# Genome and Metabolome MS-Based Mining of a Marine Strain of *Aspergillus affinis*

**DOI:** 10.3390/jof7121091

**Published:** 2021-12-18

**Authors:** Micael F. M. Gonçalves, Sandra Hilário, Marta Tacão, Yves Van de Peer, Artur Alves, Ana C. Esteves

**Affiliations:** 1 CESAM, Department of Biology, University of Aveiro, 3810-193 Aveiro, Portugal; mfmg@ua.pt (M.F.M.G.); sandra.hilario@ua.pt (S.H.); mtacao@ua.pt (M.T.); acesteves@ua.pt (A.C.E.); 2 Department of Plant Biotechnology and Bioinformatics, Ghent University, 9052 Ghent, Belgium; yves.vandepeer@psb.vib-ugent.be; 3 Center for Plant Systems Biology, VIB, 9052 Ghent, Belgium; 4 Department of Biochemistry, Genetics and Microbiology, University of Pretoria, Pretoria 0028, South Africa; 5 College of Horticulture, Academy for Advanced Interdisciplinary Studies, Nanjing Agricultural University, Nanjing 210095, China

**Keywords:** antimicrobial, anti-cancer, comparative genomics, marine fungi, metabolites, whole genome sequencing

## Abstract

*Aspergillus* section *Circumdati* encompasses several species that express both beneficial (e.g., biochemical transformation of steroids and alkaloids, enzymes and metabolites) and harmful compounds (e.g., production of ochratoxin A (OTA)). Given their relevance, it is important to analyze the genetic and metabolic diversity of the species of this section. We sequenced the genome of *Aspergillus affinis* CMG 70, isolated from sea water, and compared it with the genomes of species from section *Circumdati*, including *A. affinis*’s strain type. The *A. affinis* genome was characterized considering secondary metabolites biosynthetic gene clusters (BGCs), carbohydrate-active enzymes (CAZymes), and transporters. To uncover the biosynthetic potential of *A. affinis* CMG 70, an untargeted metabolomics (LC-MS/MS) approach was used. Cultivating the fungus in the presence and absence of sea salt showed that *A. affinis* CMG 70 metabolite profiles are salt dependent. Analyses of the methanolic crude extract revealed the presence of both unknown and well-known *Aspergillus* compounds, such as ochratoxin A, anti-viral (e.g., 3,5-Di-tert-butyl-4-hydroxybenzoic acid and epigallocatechin), anti-bacterial (e.g., 3-Hydroxybenzyl alcohol, l-pyroglutamic acid, lecanoric acid), antifungal (e.g., lpyroglutamic acid, 9,12,13-Trihydroxyoctadec-10-enoic acid, hydroxyferulic acid), and chemotherapeutic (e.g., daunomycinone, mitoxantrone) related metabolites. Comparative analysis of 17 genomes from 16 *Aspergillus* species revealed abundant CAZymes (568 per species), secondary metabolite BGCs (73 per species), and transporters (1359 per species). Some BGCs are highly conserved in this section (e.g., pyranonigrin E and UNII-YC2Q1O94PT (ACR toxin I)), while others are incomplete or completely lost among species (e.g., bikaverin and chaetoglobosins were found exclusively in series *Sclerotiorum*, while asperlactone seemed completely lost). The results of this study, including genome analysis and metabolome characterization, emphasize the molecular diversity of *A. affinis* CMG 70, as well as of other species in the section *Circumdati*.

## 1. Introduction

*Aspergillus* section *Circumdati* encompasses 27 species, many of which are economically, biotechnologically, and medically important, and having vast impacts on human and animal health [1]. *Circumdati* species are notorious for producing highly toxic fungal compounds (e.g., ochratoxin A (OTA)) [2]. In contrast, some *Circumdati* species, such as *A. ochraceus* and *A. slerotiorum*, are used for the biotransformation of steroids and alkaloids, while *A. melleus* is an important source of proteolytic enzymes [3]. The yellow aspergilli *A. affinis* was the first species of the section *Circumdati* isolated from a freshwater environment (submerged decomposing leaf litter), and is able to produce OTA [4], posing a potential danger for marine organisms and for their consumers. However, little is known about *A. affinis*‘s ecological role or biotechnological potential. Marine compounds from fungi represent the largest category of marine natural products (MNPs) [5] and are the most widely reported to exhibit a diverse source and remarkable relevant bioactivities, including antibacterial, antifungal, antiviral, anticancer, anti-inflammatory, antioxidant, and cytotoxic activities [6,7,8,9,10,11]. Recently, during a survey of marine fungi from the Portuguese coast, Gonçalves et al. [12] isolated a strain of *A. affinis* from sea water (strain CMG 70). *Aspergillus affinis* CMG 70 produces amylases, cellulases, chitinases, proteinases and xylanases, and has antimicrobial, cytotoxic and antioxidant activities [13]. The bioactivity profile of *A. affinis* CMG 70 suggests that it has the potential to represent a useful source of novel secondary metabolites. In fact, more than 30% of metabolites isolated from fungi so far are from *Aspergillus* or *Penicillium* [14].

Advances in high-throughput genome sequencing, metabolomic technologies, and bioinformatics have further enabled research on fungal biology, revealing an untapped source of (novel) biosynthetic gene clusters and compounds with a wide range of biotechnological applications [6,15,16,17]. At present, there is only one BioProject (PRJNA421325) on the whole-genome of *A. affinis* strain ATCC MYA-4773^T^ (=CBS 129190). Sixteen genomes of other species belonging to the section *Circumdati* are available at the JGI Genome Portal database.

To disclose the biotechnological potential of *A. affinis*, we sequenced and analyzed the genome of *A. affinis* CMG 70. Additionally, to assess the effect of sea salt on the metabolic output of *A. affinis* CMG 70, a metabolomic approach was used. Moreover, given the importance of the section *Circumdati*, a comparative analysis was undertaken using 16 fungal genomes of this section.

## 2. Materials and Methods

### 2.1. Culture Conditions and DNA Extraction

*Aspergillus affinis* CMG 70 was previously isolated from sea water collected at Vagueira (Portugal), during a survey of marine fungi from the Portuguese coast in 2018 [12]. Two mycelium-colonized agar plugs were inoculated in Erlenmeyer flasks containing 50 mL of Potato Dextrose Broth (Merck, Darmstadt, Germany) at 25 °C, without agitation for 7 days, in the dark. Afterwards, mycelium was filtered through sterile filter paper, and was immediately grounded in liquid nitrogen. DNA was extracted according to Pitcher et al. [18]. The quality of the DNA was assessed by agarose gel electrophoresis (0.8%). DNA purity and quantity were determined using a NanoDrop 2000 spectrophotometer (Thermo Fisher Scientific Inc., Waltham, MA, USA).

### 2.2. Genome Sequencing, Assembly, and Gene Prediction

The *A. affinis* strain CMG 70 genome was sequenced from 100 ng of genomic DNA by Genome Sequencer Illumina HiSeq (2 × 150 bp paired-end reads) with NovaSeq 6000 S2 PE150 XP platform (Eurofins, Brussels, Belgium). Adapter sequences and low-quality reads were removed from output reads using the Trimmomatic software v.0.39 [19]. Quality assessment analysis of the reads was performed with the fastQC program (Babraham, Bioinformatics, 2016). Then, the nuclear genome was assembled using SPAdes v.3.14 [20]. The QUAST web interface (http://cab.cc.spbu.ru/quast/, accessed on 10 January 2021) was used to assess the quality of the assembled genome. Gene prediction of the draft genome assembly was performed using Augustus v.3.3.3 [21] with default parameters and using *A. oryzae* gene models as training set.

### 2.3. Genome Annotation and Functional Analysis

Several complementary methodologies were used to annotate the sequences. Dispersed Repeat sequences (DRs) were identified in OmicsBox (v.1.4.12) with the Repeat Masking option (RepeatMasker v.4.0.9) [22]. Tandem Repeat sequences (TRs) were identified by Tandem Repeats Finder (TRF) (http://tandem.bu.edu/cgi-bin/trdb/trdb.exe, accessed on 5 February 2021) [23]. Analyses of noncoding RNAs, such as tRNAs were carried out using the tRNAscan-SE tool (http://lowelab.ucsc.edu/tRNAscan-SE/, accessed on 5 February 2021) with default parameters [24].

Predicted genes were functionally annotated with OmicsBox using Blast2GO [25] against NCBI’s nonredundant (Nr) database, Gene Ontology (GO), and Kyoto Encyclopedia of Genes and Genomes (KEGG) with an e-value threshold of 1 × 10^−3^. Proteins were classified using InterProScan and the Evolutionary Genealogy of Genes: Non-supervised Orthologous Groups (EggNOG), which also contains the orthologous groups from the original COG/KOG database (euKaryotic cluster of Orthologous Groups of proteins) with an e-value of 1 × 10^3^.

Carbohydrate-degrading enzymes (CAZymes) were predicted with the web-based application dbCAN (HMMs 5.0) (http://www.cazy.org/, accessed on 10 February 2021) with default settings (http://bcb.unl.edu/dbCAN2/blast.php, accessed on 10 February 2021) [26]. Fungal secreted proteins, including signal peptides, were predicted using the SignalP [27]. Transporters were identified with the BLAST analysis against the Transporter Classification (TC) Database [28], downloaded in March 2021, with an e-value threshold of 1 × 10^−5^, using the Geneious Prime v.2021.0.3 (http://www.geneious.com, accessed on 15 February 2021). The genome was also screened for the presence of Biosynthetic Gene Clusters (BGCs) using the web-based application antiSMASH v.5.0, using strictness ‘relaxed’ option for detection of well-defined and partials clusters containing the functional parts [29].

### 2.4. Comparative Analyses

The genome of *A. affinis* CMG 70 was compared to other sequenced and annotated genomes from 16 species in the section *Circumdati* (Table 1). The information available in JGI Genome Portal databases such as genome size, GC content, CAZymes, transporters, and BGCs abundance was used to evaluate genetic and metabolic diversity within the section *Circumdati*. One-way analysis of variance (ANOVA) followed by Student *t*-test (*p* < 0.05) was used to determine significant differences in CAZyme family diversity and transporters abundance between species of the three series within the section *Circumdati*. In addition, a phylogenetic analysis based on Maximum Likelihood using the sequences of the rDNA internal transcribed spacer region (ITS) and tubulin (*tub2*) of the *Aspergillus* strains was performed using MEGA7 [30]. Clade stability was assessed using a bootstrap analysis with 1000 replicates. Sequences were aligned with ClustalX version 2.1 [31] using the parameters described in Gonçalves et al. [12]. All alignments were checked and edited with the BioEdit Alignment Editor version 7.2.5 [32].

### 2.5. Small-Scale Fermentation and Extraction of Metabolites

A small-scale fermentation was carried out as described in [13]. Briefly, two plugs of mycelium-colonized agar were inoculated into 1-L Erlenmeyer flasks containing 250 mL of PDB (Merck, Germany) in two conditions: with and without 3% sea salt (Sigma-Aldrich, Darmstadt, Germany), with 4 replicates for each condition. The fungus was grown at 25 °C under stationary conditions for 14 days. Culture filtrates were obtained by filtering the mycelium through sterile filter paper. Then, the culture media was filtrated with 0.45 μm cellulose membrane (GN-6 Metricel, Pall Corporation, New York, NY, USA) followed by 0.2 μm nitrate cellulose membrane (Sartorius Stedim Biotech, Gottingen, Germany) in a vacuum system. Cultures media from the 4 replicates were pooled and lyophilized, and dried cultures media were weighed and transferred to tubes. Next, 20 mL of cold 80% MeOH (−80 °C) were added to each tube (containing 2 g of dried sample) and vortexed for 5 min. Each mixture was centrifuged at 14,000× *g* for 10 min at 4 °C to remove precipitated proteins. The supernatant was collected, and the extraction process was repeated. After extraction, the methanolic extracts were filtered using a glass microfiber filter 0.47 mm (Prat Dumas, Couze-St-Front, France), evaporated in vacuo using a rotary evaporator and lyophilized.

For LC-MS, 5 replicates of dried crude extracts (100 mg) for each condition were used. Metabolite extraction was performed by adding MeOH to each sample and vortexed for 40 min. Then, the samples were centrifuged for 5 min at 20,000× *g* and 400 μL of the methanolic fraction was vacuum dried. Afterwards, 100 μL of cyclohexane/water (1/1, *v*/*v*) was added to each sample and vortexed. Each mixture was centrifuged at 20,000× *g* for 5 min and 90 μL of the aqueous phase was filtered on a 96-filter plate and transferred to a 96-well plate. The samples were 10× diluted in water and 10 μL was analyzed by LC-MS.

### 2.6. LC-MS Data Analysis, Processing, and Visualization

UHPLC was performed on an ACQUITY UPLC I-Class system (Waters Corporation, Milford, MA, USA) consisting of a binary pump, a vacuum degasser, an autosampler, and a column oven. Chromatographic separation was carried out on an ACQUITY UPLC BEH C18 column (150 × 2.1 mm, 1.7 μm, Waters Corporation), at 40 °C. A gradient of solution A (99:1:0.1 water: acetonitrile: formic acid, pH 3) and solution B (99:1:0.1 acetonitrile: water: formic acid, pH 3) was used: 99% A for 0.1 min decreased to 50% A in 30 min, decreased to 30% in 5 min, decreased to 0% in 2 min. The flow rate was set to 0.35 mL min^−1^, and the injection volume was 10 μL. The UHPLC system was coupled to a Vion IMS QTOF hybrid mass spectrometer (Waters Corporation). The LockSpray ion source was operated in negative electrospray ionization mode under the following specific conditions: capillary voltage, 2.5 kV; reference capillary voltage, 3 kV; cone voltage, 40 V; source offset, 50 V; source temperature, 120 °C; desolvation gas temperature, 600 °C; desolvation gas flow, 800 L h^−1^; and cone gas flow, 50 L h^−1^. Mass range was set from 50 to 1000 Da. The collision energy for full HDMSe was set at 6 eV (low energy) and ramped from 20 to 70 eV (high energy), intelligent data capture intensity threshold was set at 5. Nitrogen (greater than 99.5%) was employed as desolvation and cone gas. Leucin-enkephalin (250 pg μL^−1^ in water: acetonitrile 1:1 [*v*/*v*], with 0.1% formic acid) was used for the lock mass calibration, with scanning every 2 min at a scan time of 0.1 s. Profile data were recorded through a UNIFI Scientific Information System (Waters Corporation). Data processing was performed with Progenesis QI software v.2.4 (Waters Corporation). To understand *A. affinis* metabolome dynamics in response to sea salt, an IQR (interquartile range) filtering was applied due to the large number of significant ions, resulting in a selection of a set of 2500 ions for the data modeling. Principal Component Analysis (PCA), heatmaps, and *t*-test on log-transformed and pareto-scaled (normalized) of the filtered ions were generated and analyzed using online MetaboAnalyst v.4.0 software [33]. Computed *p*-values were adjusted using the Benjamin-Hochberg False Discovery Rate (FDR) correction. Ions having an FDR < 0.01 and a log_2_ fold change (FC) > 2 or <−2 were considered differently expressed. For identification purposes, the fragmentation data (ESI negative) of the significant ions were selected and matched against in-house library and 44 external spectral libraries (https://mona.fiehnlab.ucdavis.edu/, accessed on 3 December 2021), using MSsearch software v.2.6. For each ion, the best hit was based on a matching precursor ion (*m/z* < 10 ppm difference) and matching fragments (<50 ppm accuracy), generating five common fragments, including the precursor *m/z*. For each hit, the name of the matching compound followed by the collision energy used, the parent ion as a nominal mass, the chemical formula, a matching factor (MF), a reverse matching factor (RMF), and the name of the library found were obtained (File S1). File S1 contains some positive ionizations, but only ions in negative mode were considered for identification. Thus, annotation was conducted at level 2 of the Metabolomics Standards Initiative (MSI).

## 3. Results and Discussion

### 3.1. Sequencing, Assembly Data and Genomic Characteristics

General data related to the draft genome of *A. affinis* strain CMG 70 is presented in Table 2. Briefly, the CMG 70 genome size was estimated at 37.6 Mp, assembled in 421 contigs, with 11,763 predicted coding sequences from which 13.7% encode for hypothetical proteins, and a GC content of 50.21%. The *A. affinis* CMG 70 genome size is larger (0.8%), has a slightly higher GC content (0.2%) and has 5.2% fewer genes than ATCC MYA-4773^T^.

### 3.2. Repetitive Sequences and Prediction of tRNAs

Repetitive sequences are classified in Dispersed Repeats (DRs) and Tandem Repeats (TRs). The total length of the 12,411 DRs in the *A. affinis* CMG 70 genome amounts to 570,106 bp, covering 1.52% of the genome. With respect to the TRs, there are 4491 sequences with a total length of 262,036 bp covering 0.70% of the genome. 251 tRNAs were also predicted, with a total length of 22,032 bp covering 0.06% of the genome (Table 3). Among the tRNAs, 11 are possible pseudogenes and the remaining 240 anti-codon tRNAs correspond to the 20 common amino acid codons.

### 3.3. Gene Annotation

**Figure 1:**
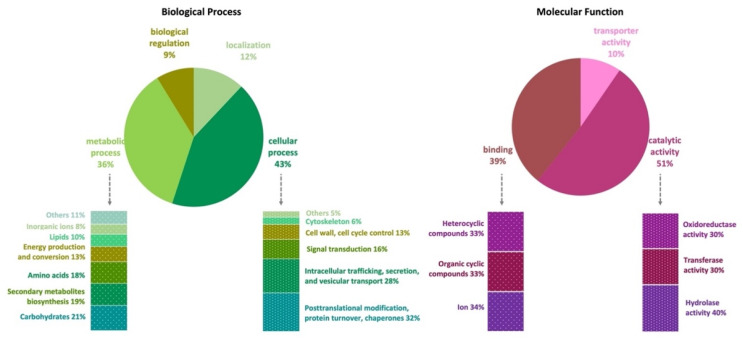
Gene Ontology (GO) functional annotation (pie charts) and EggNOG functional classification (bars charts) of the *Aspergillus affinis* CMG 70 genome.

The genome of *A. affinis* CMG 70 has 11,584 genes annotated according to the NCBI’s nonredundant protein (Nr), UniProt/Swiss-Prot, EggNOG, KEGG, GO, and Pfam databases (Table S1). There are 10,726 (91.2%) cellular proteins and approximately 1037 secreted proteins (8.8%) (Tables S1 and S3). The number of predicted proteins of CMG 70 is similar to that of ATCC MYA-4773^T^, as well as to what has been reported for other *Aspergillus* species, which vary from 9078 in *A. coremiiformis* to 14,216 in *A. transmontanensis* [34]. Functional analysis (GO, Biological Processes) showed that most genes are involved in cellular (43%) and metabolic process (36%), cellular localization (12%), and biological regulation (9%) (Figure 1, Table S1). Genes classified within the “cellular process” category were mainly classified as being involved in posttranslational modification, protein turnover, chaperones (32%); intracellular trafficking, secretion, and vesicular transport (28%); signal transduction (16%); cell wall and cell cycle control (13%); cytoskeleton (6%); and others (5%), which include defense mechanisms and extracellular structures. Within the “metabolic process” category, *A. affinis* genes are involved in the metabolism and transport of carbohydrates (21%), amino acids (18%), lipids (10%) and inorganic ions (8%); in the biosynthesis of secondary metabolites (19%); and in energy production and conversion (13%). In GO, Molecular Functions, genes are involved in catalytic (51%), binding (39%), and transport (10%) activities (Figure 1, Table S1). These values are in agreement with what has been described in the literature for fungi.

### 3.4. Carbohydrate-Active Enzymes (CAZymes)

There are 566 genes encoding putative CAZymes, from which 295 carry signal peptides, that were annotated using the HMMER database (Table S2). Among these genes, 279 encode for glycoside hydrolases (GH), 22 for carbohydrate binding modules (CBM), 96 for glycosyltransferases (GT), 107 for oxidoreductases (AA), 39 for carbohydrate esterases (CE), and 23 for pectate lyases (PL) comprising 146 distinct CAZymes families. The main GH family includes β-glucosidades (GH3), chitinases (GH18), cellulases (GH5), β-xylosidades (GH43), polygalaturonases (GH28), and amylases (GH13). Regarding GT, UDP-glucuronosyltransferase (GT1), cellulose/chitin synthases (GT2), and xylanase (GT90) were the most abundant. Carbohydrate binding modules 67, which is a lrhamnose-binding present in pectin and hemicellulose [35] and CBM20 associated with starch binding [36], were the most CBM abundant. All these enzymes have an important role in the degradation of polysaccharides, such as fucoidan, chitin, pectin, hemicellulose, and starch [37]. This may reveal a certain adaptation for the fungus to obtain carbon sources from different marine substrates, such as the algal fucoidan, pectin, cellulose, and chitin present in some algae and crab and shrimp shells. Glucooligosaccharide/chitooligosaccharide oxidases (AA7), cellobiose dehydrogenase (AA3), and copper-dependent lytic polysaccharide monooxygenases (AA9), which belong to auxiliary activity (AA) family were the most predominant. Carbohydrate esterase families are classified in 18 sub-families and catalyze the de-O or de-N-acylation of substituted saccharides. In *A. affinis*, 11 CEs are present with CE4, the most abundant. CE4 participates in the deacetylation of polysaccharides, such as xylan, chitin, and peptidoglycan [38]. Enzymes acting in the deacetylation of peptidoglycan may be involved in the degradation of bacterial cell wall, being attractive for drug design with potential application in biomedical industry. *Aspergillus affinis* genome encodes PL genes such as pectate lyase (PL1) and rhamnogalacturonan endolyase (PL4). This family is known to be involved in the breakdown of pectin that is synthesized in abundance by terrestrial plants but is not known as a marine polysaccharide [39]. However, pectin-like polysaccharides have been reported in red and green algae, microalgae and in seagrasses [40].

### 3.5. Transporter Proteins

We observed transporters from all protein classification (TC) classes: channels and pores (TC 1), electrochemical potential-driven transporters (TC 2), primary active transporters (TC 3), group translocators (TC 4), transmembrane electron carriers (TC 5), accessory factors involved in transport (TC 8), and incompletely characterized transport systems (TC 9). There were 3005 predicted genes annotated as transporters against TC database, accounting for 25.6% of the total predicted genes for *A. affinis* (Table 4 and Table S4). Genes from the TC 2 class are the most abundant transporters of *A. affinis* CMG 70 genome (32.7%), followed by genes from the classes TC 1 (19.6%), TC 9 (16.1%), and TC 3 (15.3%). The *A. affinis* genome encodes transporters involved in the transport of zinc, sugar/H+, florfenicol, pantothenate, and various MFS (Major Facilitator Superfamily) transporters. It is known that MFS transporters in fungi play an important role in multidrug resistance [41] and are required for fungal growth under stress conditions [42]. Furthermore, we identified several genes coding for glycerol, inositol, sodium, and chloride transporters. By increasing these compounds’ production and accumulation and others such as erythritol, arabitol, xylitol, mannitol, mycosporines and nitrogen-containing compounds (e.g., glycine, betaine, and free amino acids), the cell is able to maintain a positive turgor pressure [43]. Two mechanisms explain how fungi tolerate high salinity levels: their high affinity transport systems and osmoregulatory capacity. Marine fungi produce and accumulate specific solutes that allow them to function in saltwater [44]. For example, in the halophyte *Mesembryanthemum crystallinum,* the myo-inositol and its transporters play a major role in the tolerance to salt stress [45]. Moreover, Kogej et al. [43] and Plemenitaš et al. [46] also showed the production of glycerol, erythritol, arabitol, and mannitol and the involvement of alkali metal transporters (K^+^/Na^+^) by the halophilic fungus *Hortaea werneckii* as osmoadaptation. All four polyols have also been detected in *A. flavus* and *A. parasiticus* in response to osmotic stress [47]. We annotated many genes involved in glycerol, mannitol, inositol, trehalose, sorbitol, glycine, and betaine biosynthetic process, suggesting that *A. affinis* has adaptability mechanisms to thrive in saltwater. Furthermore, genes essential for the MAPK high osmolarity (*Sln1-Ypd1-Ssk1-Ssk2-Pbs2-Hog1* and *Sho1-Cdc42-Ste20(or Cla4)-Ste11-Pbs2-Hog1*) and cell wall stress cascades (*Wsc1-Rom2-Rho1-Pkc1-Bck1-Mkk1-Slt2*) were identified, resulting in glycerol accumulation to reduce the osmotic pressure and in cell wall remodeling. Gladfelter et al. [48] suggests that the high-osmolarity-glycerol signaling pathway seems in part to be linked to the water balance, cell stability and turgor in fungi. Also, transporters associated to ionic homeostasis (TC 1) encoding for calcium channels, nucleoporins, and heat shock 70 proteins’ transporters were also detected, allowing rapid changes in the cell physiology of *A. affinis*.

### 3.6. Biosynthetic Gene Clusters

Seventy-two biosynthetic gene clusters (BGCs) involved in the secondary metabolism of *A. affinis* CMG 70 were predicted (Table S5). Biosynthetic gene clusters encode a form of machinery that produce bioactive compounds. In addition to biosynthetic genes, BGC typically include genes for expression control, self-resistance, and export of the compounds they encode [49].

The BGCs identified encode 7 terpenes, 5 indoles, 23 t1PKs (type 1 polyketide synthases), 8 NRPS (non-ribosomal peptide synthase), 7 NRPS-t1PKs, 14 NRPS-like, 3 NRPS-like-t1PKs, 1 NRPS-like-indole, 3 NRPS-indole, and 1 betalactone. From the BGCs identified, 9 BGCs have 100% similarity with known BGCs, such as asperlactone (anti-fungal), serinocyclin A (anti-insect), UNII-YC2Q1O94PT (ACR toxin I), pyranonigrin E (antimicrobial), biotin (vitamin), clavaric acid (antitumor), pseurotin (antibacterial), 6-methylsalicyclic acid (mammal-toxic) and AbT1 (anti-fungal). Other BGCs, such as cluster 33, shared 75% gene similarity with nidulanin A BGC, cluster 61 is likely to be an ochratoxin A BGC (60% of genes show similarity), and cluster 41 acts as aspergillic acid coding BGC (57% of genes show similarity). Cluster 14, 22 and 34 have 50% similarity with notoamide A, hexadehydroastechrome and asperphenamate, respectively. Other genes probably involved in BGC of squalestatin S1, ankaflavin, patulin, shearinine D, NG-391 and ochrindole A, were also detected.

### 3.7. Phylogenetic Analyses

**Figure 2:**
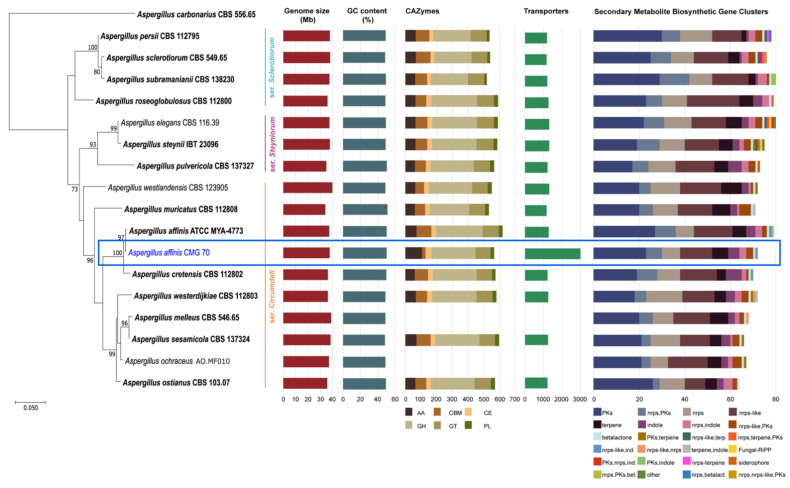
Phylogenetic relationship of 16 *Aspergillus* species from the section *Circumdati* based on ITS and *tub2* sequence data and inferred using the Maximum Likelihood method under Tamura 3-parameter model. The tree is drawn to scale, with branch lengths measured in the number of substitutions per site and rooted to *Aspergillus carbonarius* (CBS 556.65). Bootstrap values (≥70%) are shown at the nodes. Ex-type strains are in bold and the strain under study is in blue. The number of carbohydrate-active enzymes, transporters and biosynthetic gene clusters are indicated using bar graphs.

Recently, Houbraken et al. [50] created three series within the section *Circumdati* to distinguish the species, namely *Circumdati*, *Sclerotiorum*, and *Steyniorum*. As can be seen in Figure 2, *A. affinis* CMG 70 groups in the same clade as the type species of *A. affinis* strain ATCC MYA-4773 (=CBS 129190), which belongs to ser. *Circumdati*. This series forms a sister clade with ser. *Steyniorum*, which is phylogenetic related with ser. *Sclerotiorum*.

### 3.8. Comparative Analyses

#### 3.8.1. General Features

The analysis showed that the genomic features of *A. affinis* strain CMG 70 and of ATCC MYA-4773^T^ are similar, regarding genome size, GC content and number of predicted genes. The average size of all genomes from the section *Circumdati* is 37.08 Mb. *Aspergillus muricatus* CBS 112808 has the smallest genome, i.e., 1.5% smaller than *A. westlandensis* CBS 123905. Moreover, *Circumdati* species genomes have a moderate GC content around 49.37% (Figure 2, Table S6).

#### 3.8.2. CAZymes

Differences in the number of CAZymes between both strains of *A. affinis* were observed with more 8.6% CAZymes in ATCC MYA-4773^T^ (Figure 2, Table S7). In addition, some CAZymes appeared to be strain-specific, such as those from families AA2, CBM46, CBM67, CBM87, CE2, CE18, GH146, GH 109, and GT109 for strain CMG 70, while AA5, CBM1, CBM13, CBM18, CBM 32, CBM35, CBM48, CBM50, GH23, GH74, GH115, GH133, GT31, and GT41 for strain ATCC MYA-4773^T^ (Table S7). These differences may be related to the availability and type of carbohydrates in the environment where the strains where isolated from.

**Figure 3:**
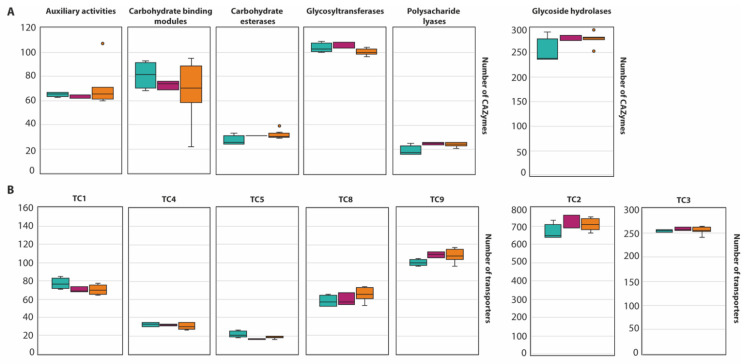
Boxplot representing the diversity of CAZyme family (**A**) and the abundance of transporters (**B**) in the section *Circumdati*: series *Sclerotiorum* (blue), series *Steyniorum* (pink) and series *Circumdati* (orange). In the boxplot, the midline represents the median and the upper and lower limit of the box represents the third and first quartile. One way analysis of variance (ANOVA) followed by Student *t*-test was used. No significant differences (*p* > 0.05) were observed.

An average of 568 CAZymes per species was predicted (Figure 2, with the exception of *A. melleus* CBS 546.65 and *A. ochraceus* AO.MF010 since no information is available at the Mycocosm portal). This is similar to what has been reported for 23 species of the section *Flavi* (598/species) [34]. Within the section *Circumdati*, there is a clear difference between the type and number of CAZymes among the three series of this section (Figure 3A). Within this section, the ser. *Steyniorum* showed the lower abundance of CAZymes, while series *Sclerotiorum* showed the highest. Carbohydrate esterases, GT, PL and GH were more prevalent in ser. *Sclerotiorum*, in opposition to ser. *Circumdati* in which AA and CBM are more prevalent.

#### 3.8.3. Transporter Proteins

The genome of *A. affinis* CMG 70 contains 57.2% more transporters compared with ATCC MYA-4773^T^ (Figure 2, Table S6). The highest number of transporters predicted in *A. affinis* CMG 70 might be associated with to the salinity control, since this strain was isolated from a marine environment.

A total of 20,387 transporters were predicted for the 15 *Circumdati* strains analyzed (approximately 1360 transporters per strain) (Table S6, Figure 2). The number of predicted transporters is similar in most species. Exceptions are *A. melleus* CBS 546.65 and *A. ochraceus* AO.MF010 with no annotated transporters and *A. affinis* CMG 70, with 2.2 times the number of transporters than the rest of the genomes analyzed. TC2 transporters’ family is the most represented in all *Circumdati* species although slightly more abundant in ser. *Steyniorum* (Figure 3B). The second most abundant class in the section *Circumdati* was the TC 3. According to our analyses, there is no difference in the distribution of this transporter family among the three series of this section (Figure 3B). No variation was also observed for TC 1, TC 4 and TC 5. In contrast, TC 8 and TC 9 are the most prevalent in the ser. *Circumdati*, followed by ser. *Steyniorum* and ser. *Sclerotiorum* (Figure 3B).

#### 3.8.4. BGCs

**Figure 4:**
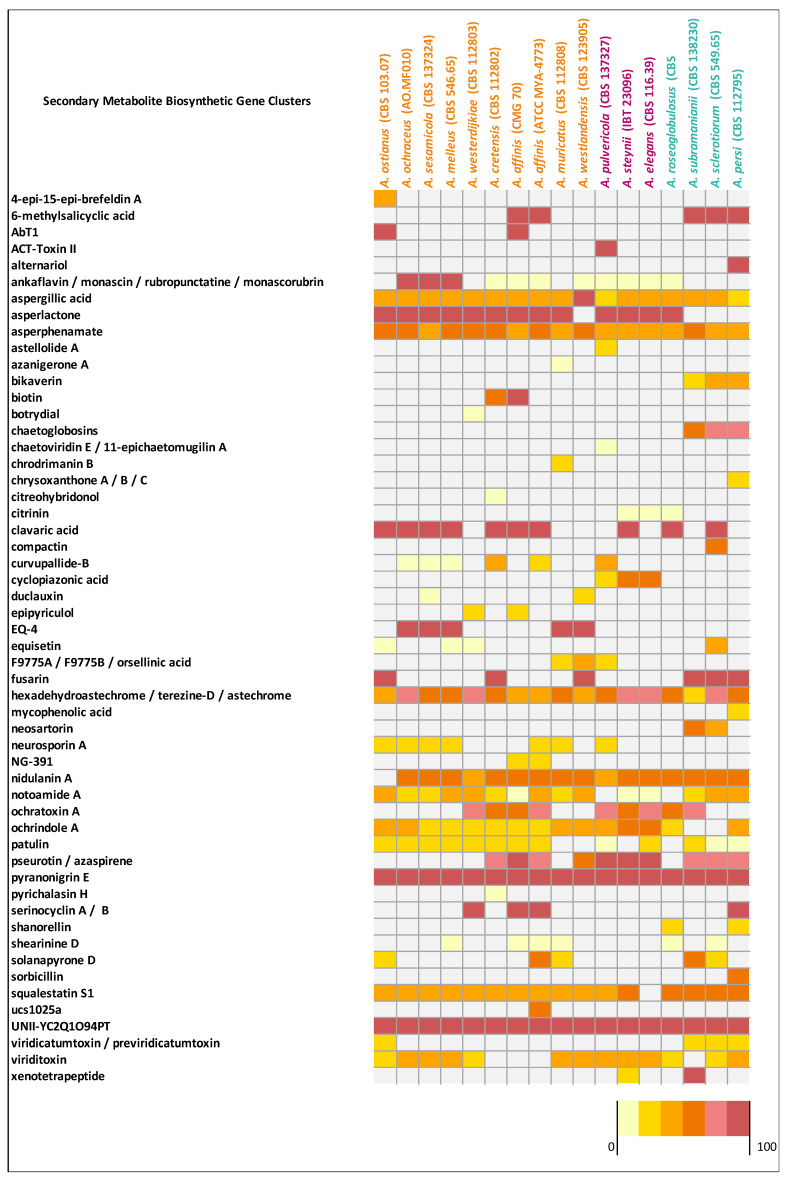
Matrix indicating the similarity of secondary metabolite gene clusters of *Circumdati* genomes (series *Sclerotiorum* in blue, series *Steyniorum* in pink, and series *Circumdati* in orange) in relation to known clusters from the antiSMASH. The color key is given as a percentage.

*Aspergillus affinis* CMG 70 contains 8.9% fewer BGCs than *A. affinis* ATCC MYA-4773^T^ (Figure 2, Table S6). Overall, genomes of *Aspergillus* species from the section *Circumdati* are rich in gene clusters involved in the synthesis of secondary metabolites (average 73/species). Type 1 polyketide synthases were the most abundant type of gene clusters, followed by NRPS and NRPS-like, PKs-NRPS hybrid clusters, terpenes, and indoles (Figure 2). *Aspergillus elegans* and *A. subramanianii* have the highest number of BGCs (80), while *A. ostianus* has the lowest (64). Figure 4 shows the list and similarity of known secondary metabolite BGCs of *Circumdati* species genomes. The pyranonigrin E and UNII-YC2Q1O94PT (ACR toxin I) BGCs were detected in all genomes with 100% similarity showing a high degree of conservation in this section. Pyranonigrin E is a PKs-NRPS hybrid metabolite from *A. niger* isolated from a marine source. Pyranonigrins are of considerable interest as potent antioxidants [51]. ACR toxin I is responsible for brown spot of rough lemon disease by the rough lemon pathotype of *Alternaria alternata* [52]. This suggests that *Circumdati* species may be able to also cause lemon leaf spot disease, but more studies are needed to understand the effect of this toxin in other plants.

Additionally, aspergillic acid, asperphenamate, and hexadehydroastechrome/terezine-D/astechrome BGCs were detected in all genomes but with similarity above 28%, 50%, and 37%, respectively, suggesting that some genes may be partially incomplete or lost. Nidulanin A, sequalestatin S1, notoamide A and ochrindole A were also detected in all genomes with exception of *A. ostianus*, *A. elegans*, *A. pulvericola*/*A*. *roseoglobulosus*, and *A. subramanianii*/*A. sclerotiorum* respectively (Figure 4). Interestingly, bikaverin and chaetoglobosins BGCs were detected exclusively in *Aspergillus* series *Sclerotiorum* (with the exception of *A. roseoglobulosus*). On the other hand, asperlactone BGC were detected in all species of series *Circumdati* and *Steyniorum* and curiously only in *A. roseoglobulosus*, which belong to series *Sclerotiorum* (Figure 4). Asperlactone belongs to methylsalicylic acid (MSA) type polyketide group and is produced by *A. westerdijkiae*. It has been reported that asperlactone has strong antibacterial and antifungal activities [53,54].

Aspergillic acid is a hydroxamic acid-containing pyrazinone isolated from *A. flavus* that exhibits antibiotic properties and toxicity for mammals [55]. Lebar et al. [56] reported that *Circumdati* species do not produce aspergillic acid, but neoaspergillic acid and its hydroxylated analog neohydroxyaspergillic acid, indicating that the cluster responsible for these is a homolog of aspergillic acid BGC. This six-gene cluster is constituted by *AsaA* (ankyrin domain protein), *AsaB* (GA4 desaturase family protein), *AsaC* (NRPS-like), *AsaD* (cytochrome P450 oxidoreductase), *AsaR* (C6 transcription factor) and *AsaE* (MFS transporter). This gene architecture was found in both strains of *A. affinis*, with the *AsaR* gene incomplete. However, the C6 transcription factor is not essential for the synthesis of aspergillic acid and its derivatives [56].

Nidulanin A is a cyclic tetrapeptide isolated from *A. nidulans*. The nidulanin A gene cluster is conserved in all *Aspergillus* and *Penicillium* spp. and its biological functions are not yet known [57]. Recently, Raffa and Keller [58] mentioned that this compound is being tested for antimicrobial or virulence-related properties. The presence of the four genes encoding nidulanin A (MFS and ABC multidrug transporter, NRPS and conserved hypothetical protein) was observed in both strains of *A. affinis.* Although we did not detect nidulanin in its metabolome, we cannot overrule the hypothesis of nidulanin being produced—or another very similar compound—by *Circumdati* species.

**Figure 5:**
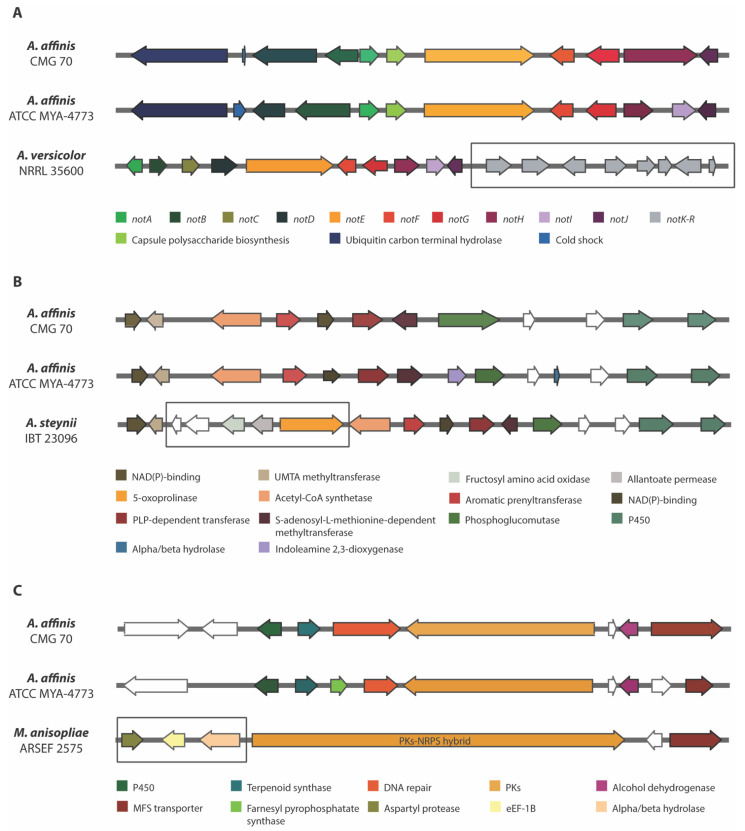
Comparison of three biosynthetic gene regions in *Aspergillus affinis* strains CMG 70 and ATCC MYA-4773^T^ with: (**A**) Notoamide BGC of *Aspergillus versicolor* NRRL 35600; (**B**) Ochrindole A BGC of *Aspergillus steynii* IBT 23096; and (**C**) NG-391 BGC of *Metarhizium anisopliae* ARSEF 2575. The genes encoding hypothetical proteins are represented as white arrows. The square box shows the missing genes in relation to the *A. affinis* cluster.

Notoamides are alkaloids with the pyranoindole ring common to stephacidins (antitumor alkaloids) found in *A. ochraceus* and in several members of the paraherquamide family. These prenylated indole alkaloids were obtained and characterized from a culture of a marine *Aspergillus* sp. isolated from the mussel *Mytilus edulis* [59]. Currently, there is no well-known property or function for notoamides, although some studies showed cytotoxicity against tumor cell lines, insecticidal, antibiotic and antiparasitic activities [60,61,62]. The genetic architecture of a notoamide BGC comprises 18 genes (*notA–R*). It was not possible to detect *notK–R* genes in both strains of *A. affinis* (Figure 5A). The cluster is identical only in 10 *not* genes (*notA–J*) and the pattern of the exon/intron arrangement in the corresponding genes is also highly similar between strains, including the 2 genes that were not described in the *not* gene cluster—the cold shock protein and the ubiquitin carbon terminal hydrolase genes. Li et al. [63] stated that the sequence similarity from *notK* to *notR* is quite reduced, and that the gene architecture differs drastically, suggesting that the previously assigned *not* gene cluster probably ends at *notJ* and the other *not* genes are unlikely involved in notoamide biosynthesis. Since *notK–R* genes were not also detected and considering the hypothesis of the *notK–R* not being involved in notoamide synthesis, it is possible that *Circumdati* species produce notoamide or a notoamide related compound.

Ochratoxin A (OTA) is a problematic toxic metabolite that is widely distributed in food products, such as cereals, rice, soya, coffee, cocoa, beans, peas, peanuts, fresh grapes, and dry fruits, posing risks to human and animal health [2]. It was first reported in *A. ochraceus*, but many other *Aspergillus* and *Penicillium* species and other molds have been reported as OTA-producing species. Recently, Gil-Serna et al. [2] showed that the genomic regions that encode for OTA widely differ in *Circumdati* species. Some species, including *A. affinis*, contain a potentially functional OTA biosynthetic cluster suggesting that these species have the potential to synthetize the toxin, and others contain partial regions which might be related to their inability to produce OTA. In *A. affinis* CMG 70 we found the cluster region containing five genes known to be involved in OTA biosynthesis: halogenase, bZIP transcription factor, cytochrome P450 monooxygenase, NRPS, and a PKs. In fact, when we analyzed the dried crude extracts of *A. affinis* CMG 70 (see Section 3.9), ochratoxin A was detected as one of the most expressed compounds.

Ochrindoles (A–D) are prenylated bis-indolyl benzoid/quinone and ochrindole A is the most common of the four ochrindole compounds known [64]. Ochrindoles are known for their anti-insect properties, making ochrindoles (or derivatives)-producing species, of interest for the pesticide industry. Kjærbølling et al. [34] already identified candidate-genes for the ochrindole cluster in *A. steynii*, a member of the section *Circumdati*. Both strains of *A. affinis* shared 12 genes within ochrindole A BGC which is comprised of 17 genes (Figure 5B). The lack of the five genes (hypothetical protein, fructosyl amino acid oxidase, allantoate permease and 5-oxoprolinase coding genes) might (or not!) compromise the synthesis of ochrindole A. A deeper investigation on this subject is needed.

Patulin is a carcinogenic mycotoxin produced by several species found in fruit and vegetable-based products, posing a serious health risk to consumers [65]. Patulin production has been doubtfully reported in several species, including some *Penicillium* and *Aspergillus* spp., such as *A. ochraceus* [66]. Confirmed and efficient production of patulin has been found only in *A. clavatus*, *A. giganteus* and *A. longivesica* (section *Clavati*). The biosynthesis of patulin and its gene cluster are well known. We identified 3 of the 15 *pat* genes: *patC* (MFS transporter), *patD* (dehydrogenase) and *patE* (oxidoreductase), in both strains of *A. affinis*, suggesting that this species and probably all the others *Circumdati* species do not produce patulin. In fact, we did not detect patulin in the extracts of *A. affinis*. Nielsen et al. [67] showed that although *Penicillium roqueforti* has most of the *pat* genes needed for production of patulin, some genes are lacking and therefore it is unable to produce it.

Squalestatin S1 (also known as zaragozic acid) is a potent inhibitor of squalene synthase, an important enzyme for sterol biosynthesis [68]. Squalestatin S1 exhibits antifungal activity [69] and was found in some ascomycetes [70,71]. More recently the squalestatin S1 producing BGC from *Aspergillus* sp. Z5 was reported in *Paecilomyces penicillatus* [72] and halophilic marine fungus *Eurotium rubrum* [73]. The cluster of both *A. affinis* strains shared three out of four genes of squalestatin S1 BGC: the core enzyme farnesyl-diphosphate farnesyltransferase (squalene synthase), a DnaJ domain protein and other one conserved hypothetical protein.

When observing the predicted BGCs of the two strains of *A. affinis* (CMG 70 and ATCC MYA-4773^T^) (Figure 4) we were able to detect some differences in the diversity of the BGCs present: the AbT1, biotin and epipyriculol BGCs were detected only in CMG 70, while curvupallide-B, neurosporin A, solanapyrone D and ucs1025a in ATCC MYA-4773^T^. Furthermore, we found that the NG-391 BGC was exclusive of *A. affinis* suggesting that this cluster region is species specific. NG-391 was firstly identified in an insect pathogen *Metarhizium robertsii* [74] with similar structure to the mutagenic and carcinogenic mycotoxin fusarin C [75]. However, Donzelli et al. [74] reported that NG-391 does not contribute significantly to *M. robertsii* virulence. Recently, Kato et al. [76] isolated a lucilactaene compound from *Fusarium* sp. RK97-94 which is structurally related to NG-391. The same authors reported that lucilactaene and NG-391 do not have the 7-methyl group present in fusarins and show antimalarial activity and moderate growth inhibitory activity against cancer cells. With a similar core biosynthetic gene and the MFS transporter, the NG-391 cluster in *A. affinis* also has a cytochrome P450, a terpenoid synthase, a DNA repair protein, an alcohol dehydrogenase, and a farnesyl pyrophosphate synthase (Figure 5C).

### 3.9. Metabolome Analysis

We profiled the metabolomes of *A. affinis* CMG 70 grown with and without sea salt. Quintuplicate profiles were combined for each condition for comparative analysis. The full list of ions is given in Table S8. Despite the presence of unknown compounds, the major classes identified were polyketides, phenolic compounds, terpenes, amino acids, drugs, mycotoxins, carbohydrates, carboxylic acids, fatty acids, alkaloids, and indoles.

**Figure 6:**
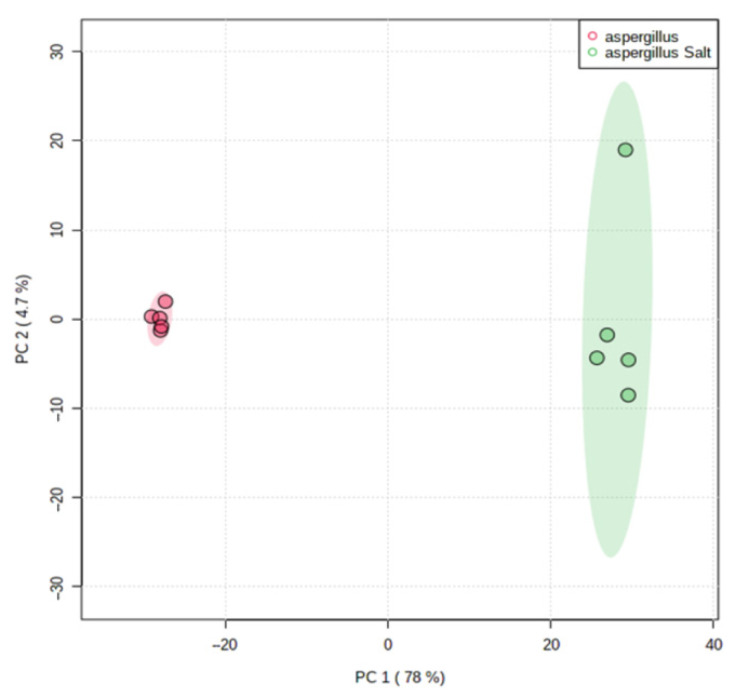
Principal Component Analysis (PCA) scores plot of salted and non-salted extracts of *Aspergillus affinis* CMG 70. Green represents salted extracts and in red non-salted extracts.

The scores of PCA on all filtered ions clearly revealed dissimilarities in the metabolome of the salted and non-salted extracts of *A. affinis* (Figure 6). These results show that *A. affinis* produces different compounds in response to osmotic stress and may adapt to salinity oscillations.

**Figure 7:**
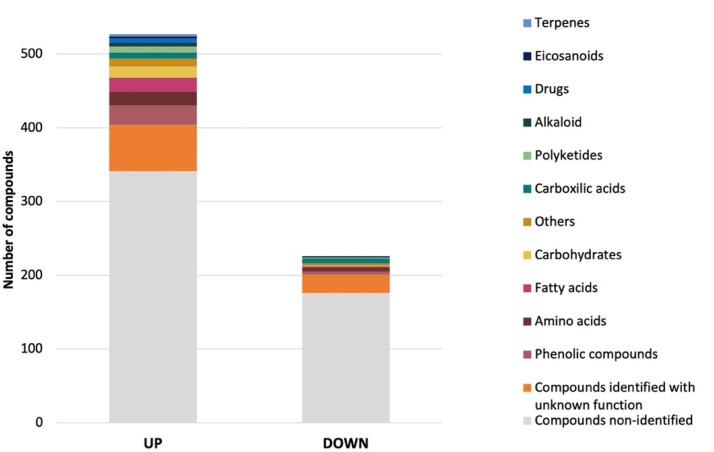
Structural classification of up and down regulated (*p* < 0.01) metabolites produced by *Aspergillus affinis* CMG 70, grown in the presence of sea salt, in comparison to *A. affinis* CMG 70, grown in the absence of sea salt.

Subsequently, statistical testing on the filtered ions was conducted using a *t*-test. Computed *p*-values were adjusted using the false discovery rate (FDR) correction. 749 ions (FDR < 0.01 and a log_2_ fold change (FC) > 2 or <−2) were significantly more (523) or less (226) abundant when the fungus was grown with sea salt (Table S9, Figure 7). Due to the lack of information, many of the predicted compounds of *A. affinis* CMG 70 remain unidentified. From the identified compounds, the most abundant in both conditions were, for example, ochratoxin A, daunomycinone, maltose, maltotriose, laminaritetraose, 3,5-Di-tert-butyl-4-hydroxybenzoic acid, methyldopa, 2-hydroxypentyl glucosinolate, 2-Chloro-N6-cyclopentyladenosine, N-Fructosyl pyroglutamate, and lecanoric acid. Gonçalves et al. [13] reported different biological activities of *A. affinis* CMG 70 under salted and non-salted conditions, suggesting that salt induces an alteration to the metabolic profile. Compounds such as 19R-Hydroxy-PGF2alpha, catechin, ferulic acid sulfate, 9S,11R-Dihydroxy-15-oxo-13E-prostenoic acid, N-Caffeoyl-O-methyltyramine, 7-(2-Cyclopentylidenehydrazinyl)-7-oxoheptanoic acid, and d-myo-Inositol-1,3,4,5,6-pentaphosphate, and hydroxyferulic acid were most abundant under salted conditions, while 4-O-β-Galactopyranosyl-d-mannopyranose, lactobionic acid, (−)-Gallocatechin 3-gallate, quercetin-3-O-xyloside, 1,6-Anhydro-β-d-glucose, kelampayoside A, (+)-Fluprostenol, maltotetraose, isoreserpin, daunomycinone, (−)-Catechin gallate, aleuritic acid, maltose, palatinose, maltotriose, and laminaritetraose under non-salted conditions. As demonstrated by Overy et al. [77] media supplemented with sea salt applies a selective pressure on the metabolic profile of the fungus, regardless of the marine or terrestrial origin of the isolates. It is noteworthy that salt induces an increase of lipids, peptides, polyketides, and phenolic compounds in *A. affinis*. This suggests possible physiological mechanisms due to the accumulation of osmolytes and mechanical strengthening of the cells to adapt and tolerate different salinity levels. At this point, it should be stressed that, as stated by Drabinska et al. [78], the presence of salt changes the nature of the molecular interactions between compounds. It might affect the quality of the extraction and induce the decrease of the intensity of the detected ions. Though some differences might be due to the presence of salts and not to differential expression by the fungus grown in the presence of salt, the water–cyclohexane extraction step should ensure that the amount of salt in the sample is reduced to a non-significant level.

Analysis of *A. affinis* extracts by LC-MS proved effective in detecting bioactive compounds that have been reported for their multiple activities, such as anti-bacterial, anti-fungal, anti-viral, anti-cancer, anti-inflammatory, and pesticides (Table 5). Despite significant efforts, new drugs are required to combat the increase in drug-resistance and the emergence of new viral infections. In this regard, we identified 3,5-Di-tert-butyl-4-hydroxybenzoic acid, that has been reported recently as a precursor of anti-viral compounds [79]. In addition, epigallocatechin, which has been associated with anti-viral properties [80], was also found in our crude extracts. To the best of our knowledge, this is the first report of these bioactive compounds in a fungus.

The dissemination of antibiotic resistance in clinical and non-clinical environments is a serious, difficult to control problem, and a risk to public health [81]. Therefore, the discovery and production of new anti-bacterial compounds is crucial and constitutes a breakthrough for medicine. In the present study, we identified anti-bacterial compounds, such as 3-Hydroxybenzyl alcohol, lPyroglutamic acid, and lecanoric acid. 3-Hydroxybenzyl alcohol was detected for the first time in *A. nidulans* isolated from a forest soil sample (India) [82]. We also identified genes involved in the biosynthesis of carbapenem, streptomycin, novobiocin, penicillin, and cephalosporin (Table S1). Apart from anti-viral and anti-bacterial compounds, we also detected antifungal compounds, such as 9,12,13-Trihydroxyoctadec-10-enoic, hydroxyferulic acid, lpyroglutamic acid, lecanoric acid, scopoletin and 4,6-dihydroxy-4-(hydroxymethyl)-3,4a,8,8-tetramethyl-5,6,7,8a-tetrahydronaphthalen-1-one. This last compound was also identified in the marine-derived fungus *A. insuetus*, which was isolated from the Mediterranean sponge *Psammocinia* sp. [83] and in *Pleosporales* sp. from marine sediments of Bohai Sea [84]. Furthermore, lecanoric acid has been detected in a marine strain of *A. versicolor* [85].

Although marine fungi are less explored compared to terrestrial fungi, some marine species have yielded a wide range of diverse compounds with anti-cancer properties [7]. In this context, some known chemotherapeutic metabolites, such as daunomycinone and mitoxantrone, were also found in the crude extract of *A. affinis*. To our knowledge, this is first report of these compounds in fungi.

**Table 5:** Metabolites with biotechnological potential of *Aspergillus affinis* CMG70 belonging to various chemical classes and related functions. Metabolites were annotated at MSI-level 2. *m/z*—ratio mass/charge detected; Rt—retention time (min). PubChem was used for class identification.

Putative Metabolite	Molecular Formula	*m/z*	Rt	Adduct	Class	Function
3-Hydroxybenzyl alcohol	C_7_H_9_O_2_	123.0445	9.22	[M–H]^–^	Benzyl alcohol	Anti-bacterial [82]
9,12,13-Trihydroxyoctadec-10-enoic acid	C_18_H_34_O_5_	329.2324	23.40	[M–H]^–^	Fatty Acid	Anti-fungal [86]
Aleuritic acid	C_16_H_32_O_5_	303.2169	19.84	[M–H]^–^	Fatty Acid	Main component of shellac, a natural resin with applications in food, pharmaceutics and coatings [87]
3,5-Di-tert-butyl-4-hydroxybenzoic acid	C_15_H_22_O_3_	249.1489	25.17	[M–H]^–^	Phenolic Compound	Antioxidant and anti-inflammatory activities. Also, it is used as a precursor to anti-viral compounds and to cyclooxygenase inhibitors [79,88]
Carbidopa	C_10_H_14_N_2_O_4_	193.0498	10.04	[M–H-H_4_N_2_]^–^	Catecholamine	Used in Parkinson’s disease treatment [89]
Catechin	C_15_H_14_O_6_	289.0013	5.34	[M–H]^–^	Phenol	Used as carbon source for growth [90]
4,6-dihydroxy-4-(hydroxymethyl)-3,4a,8,8-tetramethyl-5,6,7,8a-tetrahydronaphthalen-1-one	C_15_H_24_O_4_	267.1589	17.50	[M–H]^–^	Naphthalene	Anti-fungal [83,84]
Daunomycinone	C_21_H_18_O_8_	379.0825	1.05	[M–H-H_2_O]^–^	Naphthacene	Antibiotic with anti-cancer activity [91]
Epigallocatechin	C_15_H_14_O_7_	611.1352	2.55	[2M–H]^–^	Phenol	Anti-viral, antimicrobial, antitoxin and antitumor [80]
Folinic acid	C_20_H_23_N_7_O_7_	472.1561	4.14	[M–H]^–^	Polyketide	Used in combination with other chemotherapy drugs [92]
Hydroxyferulic acid	C_20_H_10_O_5_	209.0443	8.77	[M–H]^–^	Coumaric Acid	Anti-fungal, involved in lignin biosynthesis [93]
Guanosine	C_10_H_13_N_5_O_5_	282.0838	2.27	[M–H]^–^	Nucleoside	Antioxidant, neuroprotective, cardiotonic and immuno-modulatory properties [94]
Inosine	C_10_H_12_N_4_O_5_	267.0719	6.31	[M–H]^–^	Nucleoside	Antioxidant, neuroprotective, cardiotonic and immuno-modulatory properties [94]
Isofraxidin	C_11_H_10_O_5_	221.0444	10.81	[M–H]^–^	Coumarin	Antioxidant, anti-malarial and neuprotective [95]
L-Pyroglutamic acid	C_5_H_7_NO_3_	257.0768	1.80	[2M–H]^–^	Imino Acid	Anti-fungal and anti-bacterial [96]
Lecanoric acid	C_16_H_14_O_7_	167.0343	9.21	[M–H-C_8_H_6_O_3_]^–^	Polyphenol	Anti-bacterial, anti-fungal, anthelmintic and antioxidant properties [97,98]
Mitoxantrone	C_22_H_28_N_4_O_6_	443.1945	5.66	[M–H]^–^	Anthraquinone	Anti-cancer [99]
Ochratoxin A	C_20_H_18_ClNO_6_	402.0746	27.01	[M–H]^–^	Carboxylic Acid	Mycotoxin, nephrotoxic, immunotoxic, carcinogenic and teratogenic [100]
Saccharopine	C_11_H_20_N_2_O_6_	275.1239	2.03	[M–H]^–^	Amino Acid	Plays a role in the metabolism of lysine and swainsonine, which is a potential chemotherapy drug [101]
Scopoletin	C_10_H_8_O_4_	191.0336	14.95	[M–H]^–^	Coumarin	Anti-fungal [102]

## 4. Conclusions

This study discloses the genome sequence of *A. affinis* CMG 70 and analyses the biosynthetic potential among *Aspergillus* species from the section *Circumdati*. Overall, the present study has illustrated high similarity in genome size, GC content and transporters. Furthermore, we have also shown that members of the section *Circumdati* are a rich source of CAZymes, with different abundances between the three series of this section. We have shown that the pyranonigrin E and UNII-YC2Q1O94PT (ACR toxin I) BGCs are highly conserved in all genomes of the section *Circumdati*. Moreover, we also observed that some BGCs that are incomplete or truncated. In addition, the asperlactone cluster was detected only in series *Circumdati* and *Steyniorum* while it seemed to be completely lost in series *Sclerotiorum*. Contrarily, the bikaverin and chaetoglobosins clusters were found exclusively in *Sclerotiorum*.

The *A. affinis* CMG 70 genome has some clusters, transporters and CAZymes’ genes that appear to be strain-specific. These features might be related to fungal adaptation to the marine environment, maintaining osmotic potential through: (1) the production and accumulation of specific solutes (osmolytes) that allow them to function in saltwater; (2) increase of transporters that allow ion exchange; (3) activation of signaling pathways allowing the water balance, cell stability, and positive turgor; (4) high affinity CAZymes to marine polysaccharides enabling the efficient degradation of the available carbon sources in the marine food web. Combining genome analysis with metabolites profiling showed a variety of gene components and secondary metabolites. Additionally, efforts should also be taken to determine the properties of both known and especially unknown molecules to unravel its promising potential. We cannot rule out that many of these molecules may play an important role in the fungus’ osmoregulatory capacity to thrive in the marine environment. Moreover, different fermentation culture conditions should be used to amplify the production of specific compounds, evidencing the remarkable plasticity of fungal secondary metabolism.

## Figures and Tables

**Table 1:** List of *Aspergillus* species and strains used in this study. Accessions numbers of ITS and *tub2* are provided.

Species	Strain	Host/Substrate	ITS	*tub2*
*Aspergillus affinis*	CMG 70	Sea water	MZ230522	MZ254672
*Aspergillus affinis*	ATCC MYA-4773	Leaf Litter	MN431360	GU721092
*Aspergillus crcarbonarius*	CBS 556.65	Paper	EF661204	EF661099
*Aspergillus cretensis*	CBS 112802	Soil	FJ491572	EF661332
*Aspergillus elegans*	CBS 116.39	Japanese bread	EF661414	EF661349
*Aspergillus melleus*	CBS 546.65	Soil	EF661425	EF661326
*Aspergillus muricatus*	CBS 112808	Soil	EF661434	EF661356
*Aspergillus ochraceus*	AO.MF010	Soil	Genome	Genome
*Aspergillus ostianus*	CBS 103.07	Unknown	EF661421	EF661324
*Aspergillus persi*	CBS 112795	Toenail of patient	FJ491580	AY819988
*Aspergillus pulvericola*	CBS 137327	Indoor house dust	KJ775440	KJ775055
*Aspergillus roseoglobulosus*	CBS 112800	Decaying leaves	FJ491583	AY819984
*Aspergillus sclerotiorum*	CBS 549.65	Apple	EF661400	EF661337
*Aspergillus sesamicola*	CBS 137324	Sesame seed	KJ775437	KJ775063
*Aspergillus steynii*	IBT 23096	Green coffee bean	EF661416	EF661347
*Aspergillus subramanianii*	CBS 138230	Shelled brazil nuts	EF661403	EF661339
*Aspergillus westerdijkiae*	CBS 112803	Plant	EF661427	EF661329
*Aspergillus westlandensis*	CBS 123905	Air sample	KJ775433	KJ775065

**Table 2:** General statistics of the *Aspergillus affinis* CMG 70 genome assembly, and gene prediction.

	General Features
Genome assembled	37.6 Mb
Number of contigs (>500 bp)	421
Largest contig length	919,884 bp
N50	216,796 bp
N75	126,688 bp
L50	51 bp
L75	106 bp
GC content	50.21%
Number of predicted genes	11,763
Total length of predicted genes	18,519,399 bp
Average length of predicted genes	1574 bp
Total length of predicted genes/Genome assembled	49.3%
Average of exons per gene	3
Average of introns per gene	2

**Table 3:** Statistical results of repetitive sequences and noncoding RNAs for the *Aspergillus affinis* CMG 70 genome. SINEs: short interspersed nuclear elements; LINEs: long interspersed nuclear elements; LTRs: long terminal repeats.

Type		Number	Total Length (bp)	Percentage in Genome (%)
Interspersed repeat	SINEs	7	460	0.0012
	LINEs	69	4979	0.0133
	LTRs	124	30,681	0.0817
	DNA transposons	93	9220	0.0245
	Rolling-circles	0	0	0
	Unclassified	17	1433	0.0038
	Small RNA	124	13,588	0.0362
	Satellites	60	4618	0.0123
	Simple repeats	9755	391,258	1.0415
	Low complexicity	2162	113,869	0.3031
	Total	12,411	570,106	1.5176
Tandem repeat		4491	262,036	0.6975
tRNAs		251	22,032	0.0586

**Table 4:** Genes predicted to code for transporters in the genome of *Aspergillus affinis* CMG 70.

Transporter Class	Number of Genes (n)
Channels and pores (TC 1)	586
Electrochemical potential-driven transporters (TC 2)	983
Primary active transporters (TC 3)	460
Group translocators (TC 4)	109
Transmembrane electron carriers (TC 5)	42
Accessory factors involved in transport (TC 8)	342
Incompletely characterized transport systems (TC 9)	483
Total	3005

## Data Availability

This Whole-Genome Shotgun project has been deposited in the GenBank database under the accession number JAGXNN000000000. The genome raw sequencing data and the assembly reported in this paper is associated with NCBI BioProject: PRJNA723818 and BioSample: SAMN18830867 within GenBank. The SRA accession number is SRR14465410. Data generated or analyzed during this study are included in this published article and its supplementary information files.

## Supplementary Materials

The following are available online at https://www.mdpi.com/article/10.3390/jof7121091/s1, Table S1: Gene annotation, Table S2: Carbohydrate active enzymes prediction, Table S3: Secreted proteins, Table S4: Transporter’s prediction, Table S5: Biosynthetic Gene Clusters, Table S6: Summary of genomic features of *Circumdati* genomes, Table S7: Comparison of CAZymes families between *A. affinis* CMG 70 and ATCC MYA-4773, Table S8: Full list of compounds, Table S9: List of the significantly differential compounds, File S1: matched spectral library compounds.
