# Supplementary material for: Genome and Metabolome MS-Based Mining of a Marine Strain of Aspergillus affinis
Source: J Fungi (Basel). 2021 Dec 18;7(12):1091. doi: 10.3390/jof7121091 (PMC8709101; doi:10.3390/jof7121091)

Unknown: Unknown (0.79_556.8902m/z)
Compound in Library Factor = 0

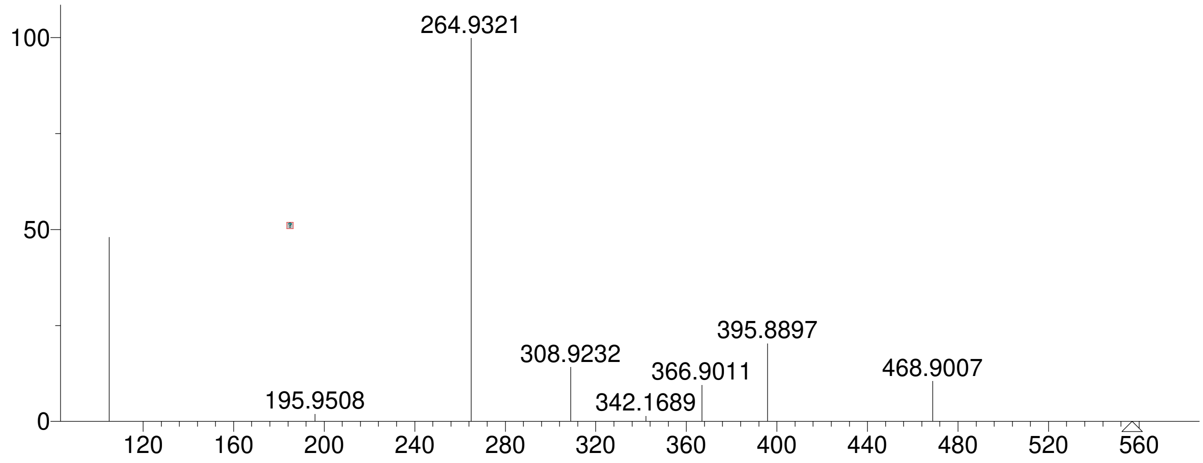

Hit 1 : Benzamide, 5-bromo-2-[(2-chloro-4-iodophenyl)amino]-N-(cyclopropylmethoxy)-3,4-difluoro- [M+H]+ IT 3
C17H13BrClF2IN2O2; MF: 0; RMF: 17; Prob 100.0%; CAS: 212631-67-9; Lib: nist_msms; ID: 372907.

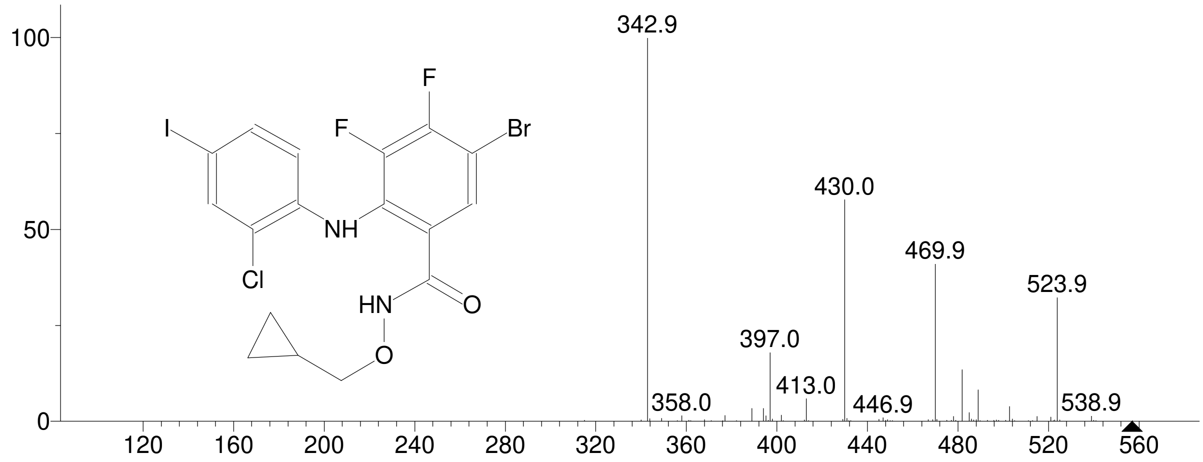

** Search Report Page 1 of 1 **

Unknown: Unknown (0.80_262.9280m/z)
Compound in Library Factor = 0

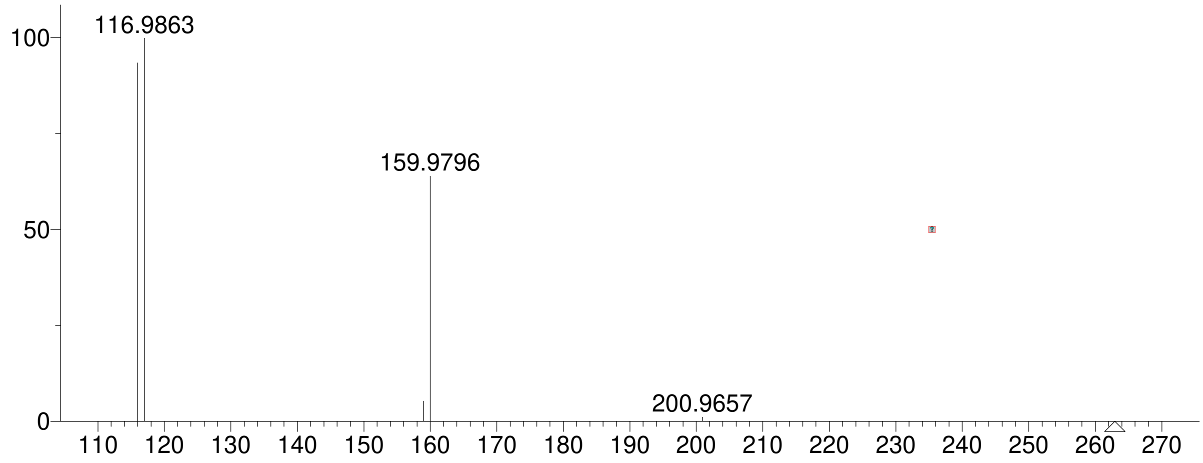

Unknown: Unknown (0.80_442.9105m/z)
Compound in Library Factor = 0

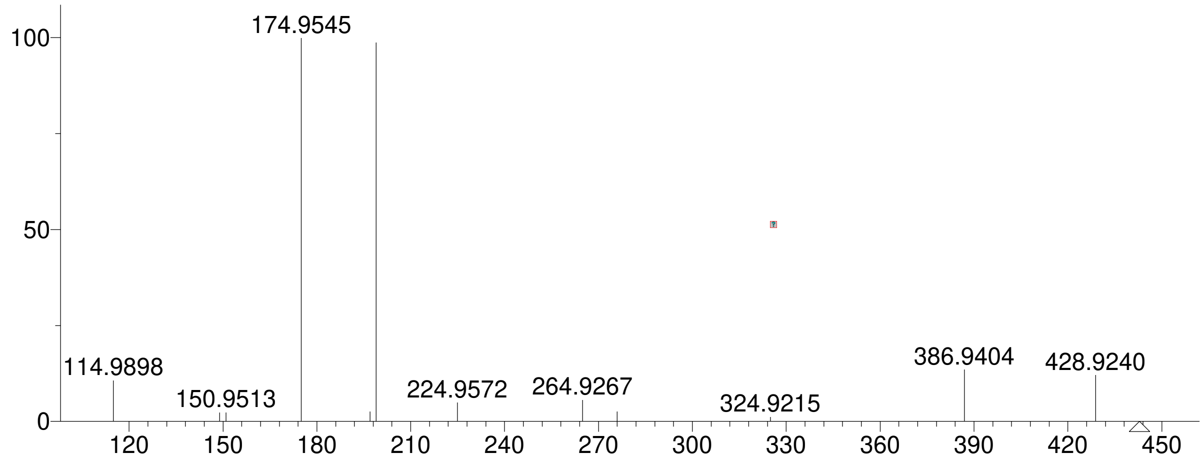

Unknown: Unknown (0.82_270.9416m/z)
Compound in Library Factor = -3273

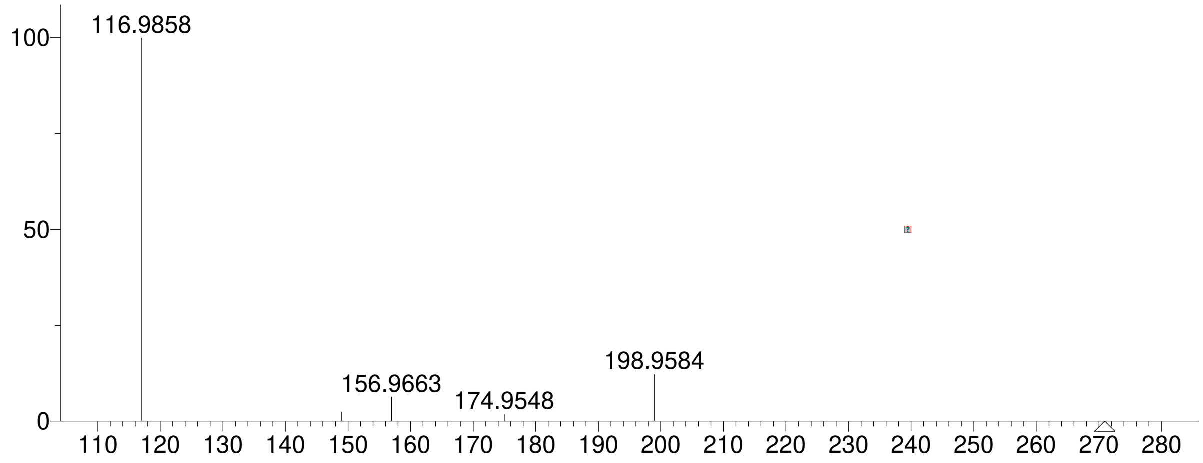

Hit 1 : Tienilic acid [M-H-C2H2O2]- HCD 78V P=270.9
C13H8Cl2O4S; MF: 15; RMF: 478; Prob 100.0%; CAS: 40180-04-9; Lib: nist_msms; ID: 429843.

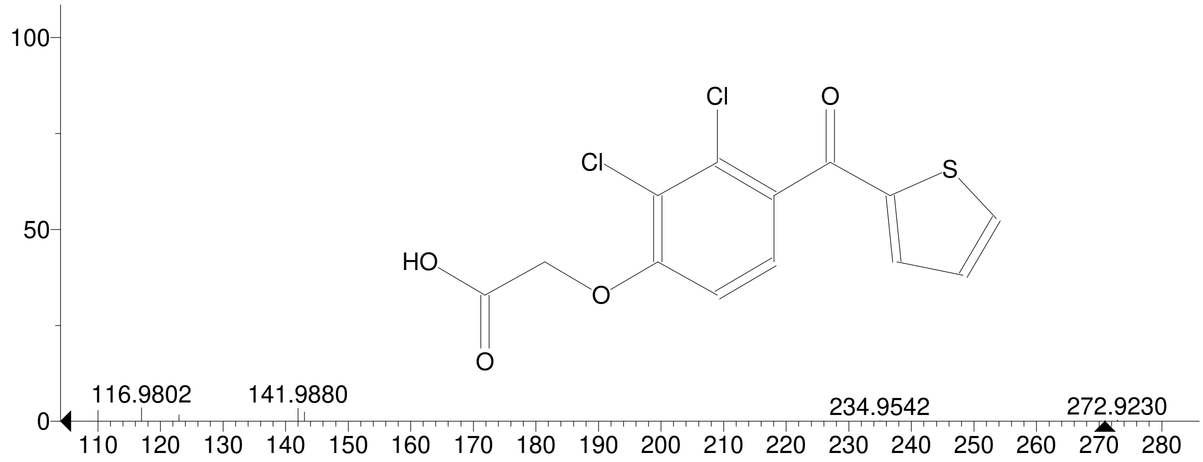

Unknown: Unknown (0.82_426.9167m/z)
Compound in Library Factor = 0

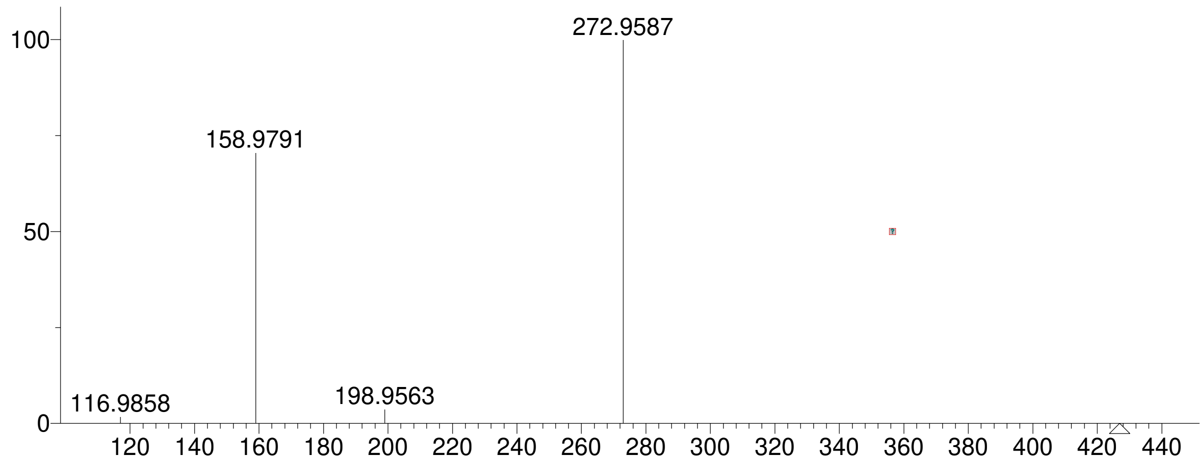

Unknown: Unknown (0.82_558.8905m/z)
Compound in Library Factor = 0

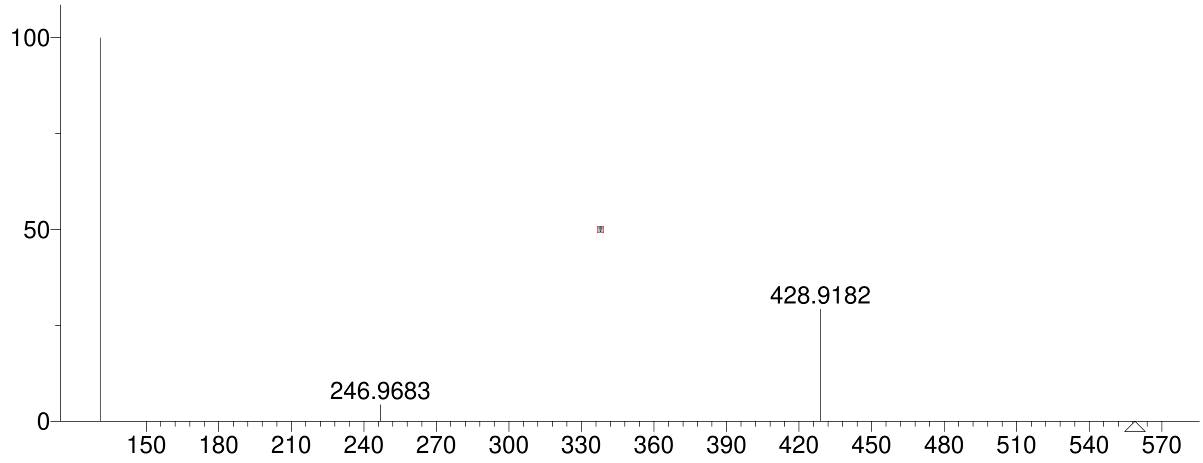

Unknown: Unknown (0.82_730.8721m/z)
Compound in Library Factor = -1848

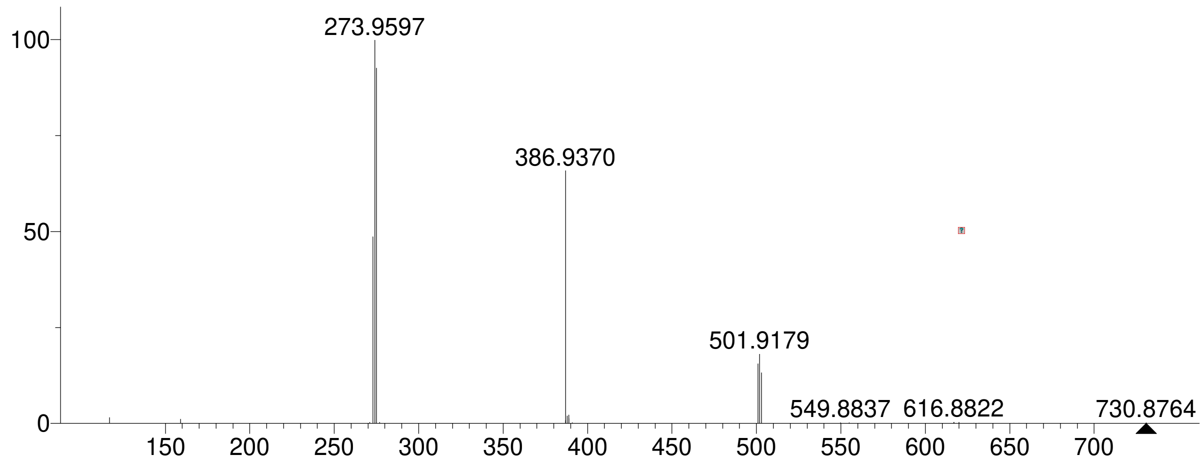

Hit 1 : Proll, DAla12 Angiotensin (I) [M-2H]2- HCD 26eV P=730.9
C70H101N19O16; MF: 33; RMF: 585; Prob 100.0%; Lib: nist_msms2; ID: 76615.

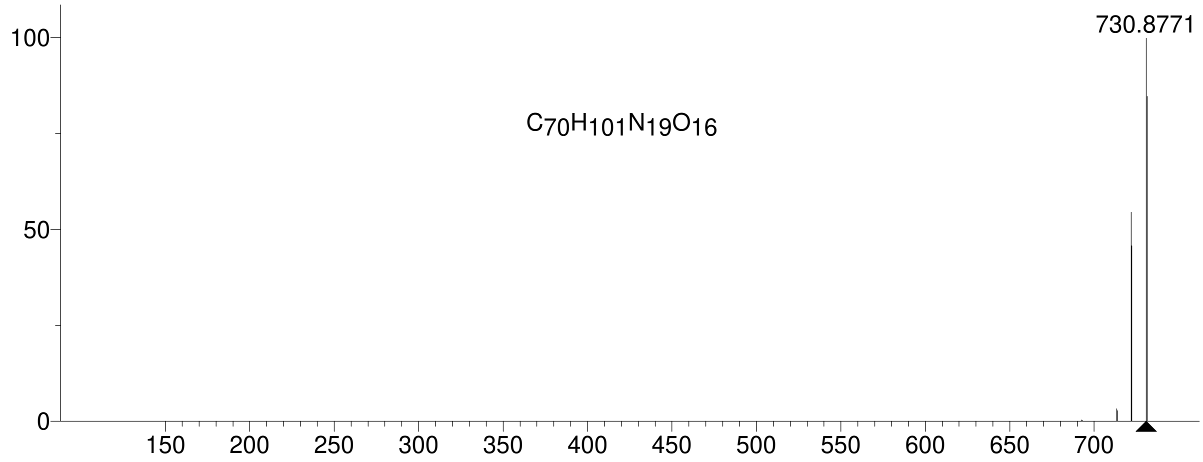

Unknown: Unknown (0.83_728.8746m/z)
Compound in Library Factor = 0

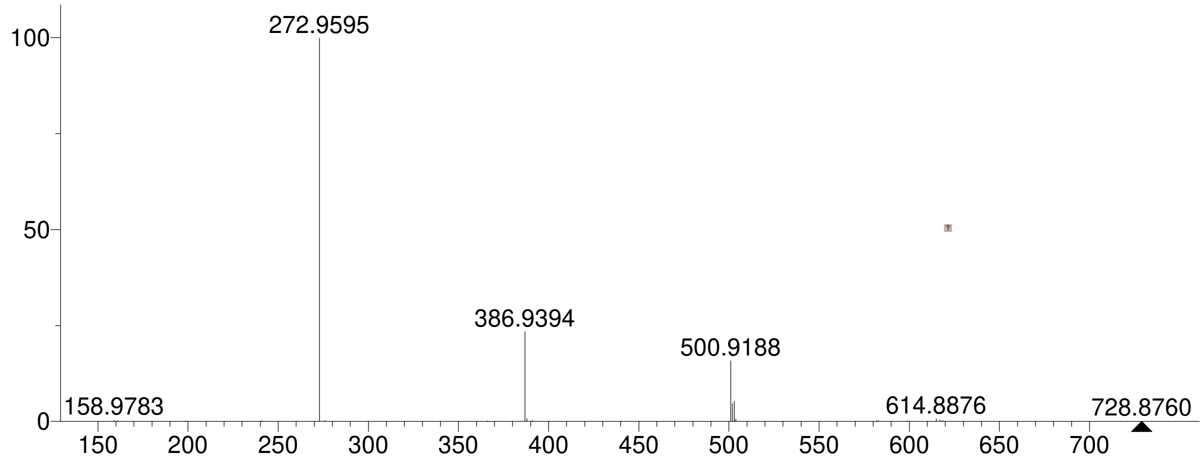

Hit 1 : CART (1-39), human, rat [M+6H]6+ QTOF 2V P=728.9
C188H303N57O63; MF: 0; RMF: 14; Prob 100.0%; Lib: nist_msms2; ID: 35836.

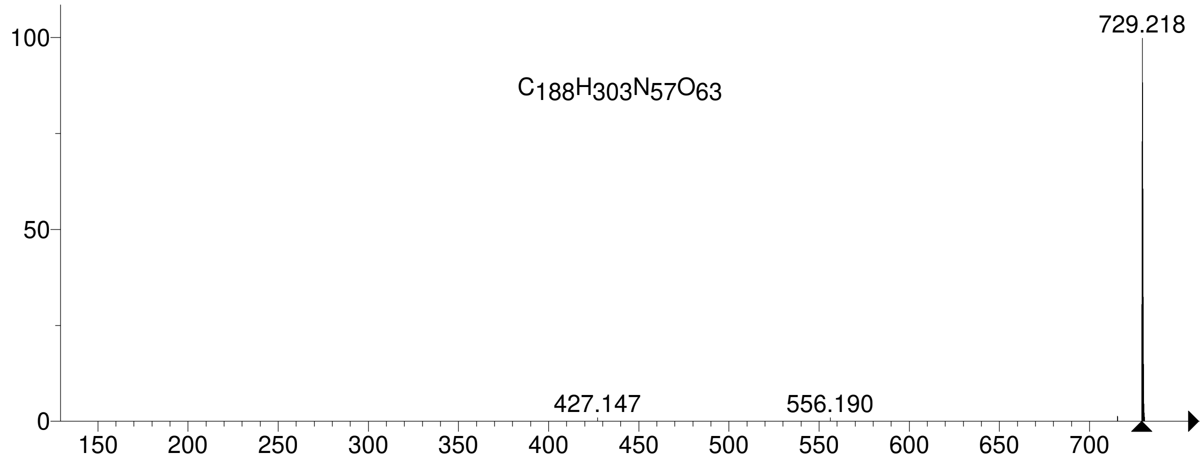

** Search Report Page 1 of 1 **

Unknown: Unknown (0.84_402.9143m/z)
Compound in Library Factor = 0

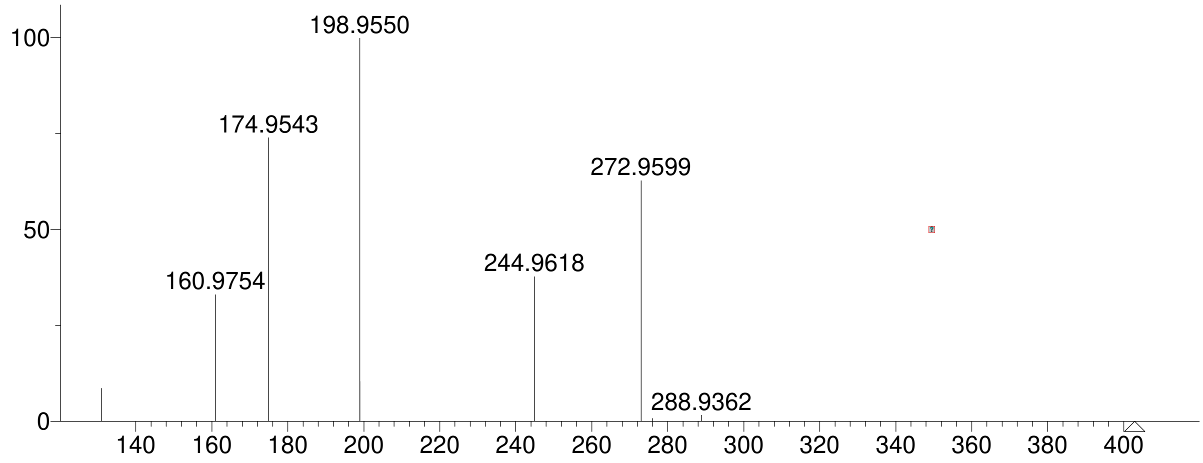

Unknown: Unknown (0.86_586.9023m/z)
Compound in Library Factor = 0

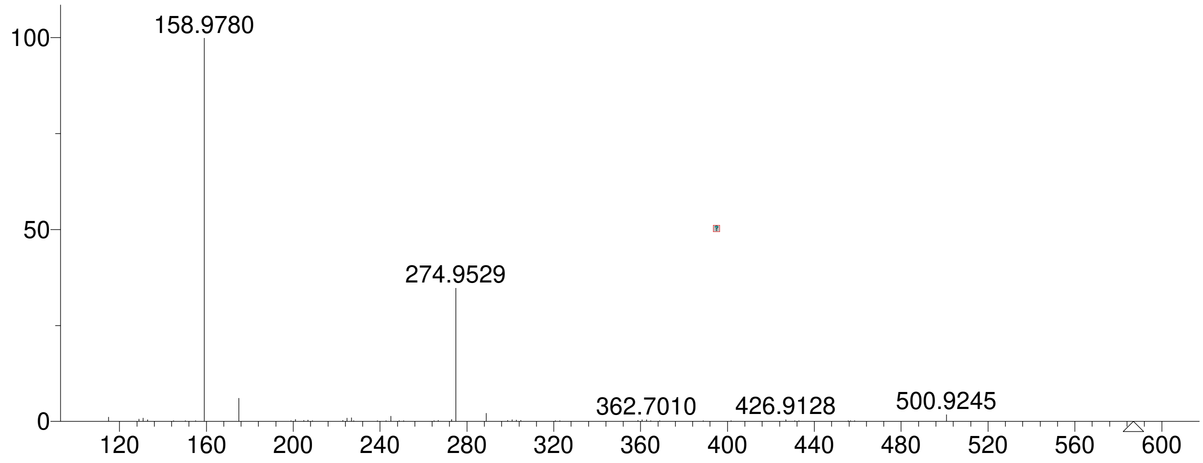

Unknown: Unknown (0.86_731.8780m/z)
Compound in Library Factor = 0

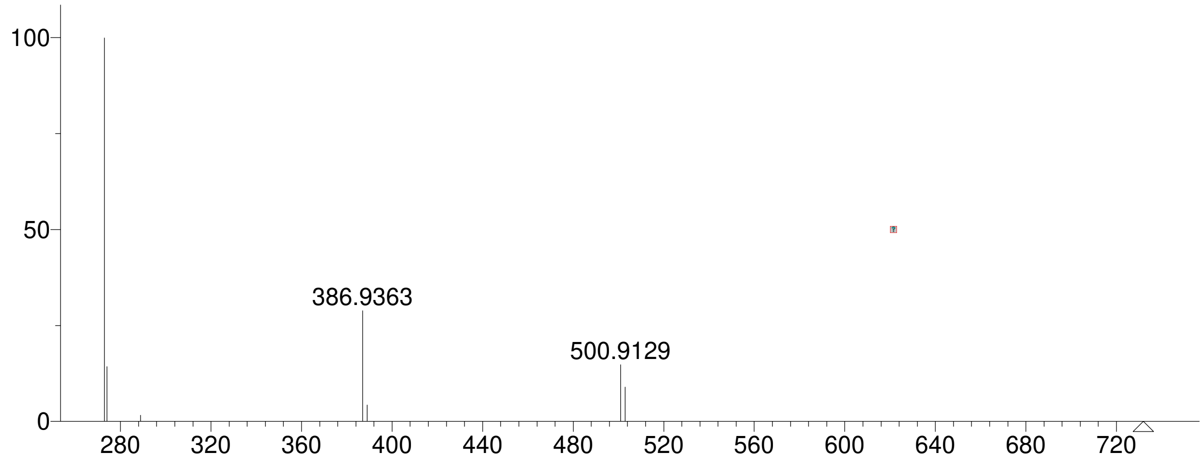

** Search Report Page 1 of 1 **

Unknown: Unknown (0.87_617.8931m/z)
Compound in Library Factor = 0

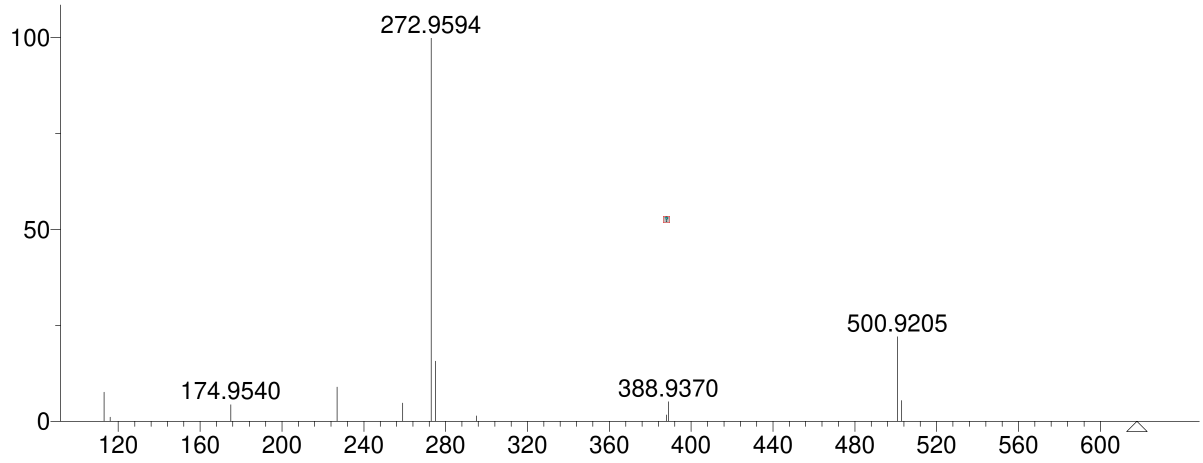

Unknown: Unknown (0.89_294.9504m/z)
Compound in Library Factor = 0

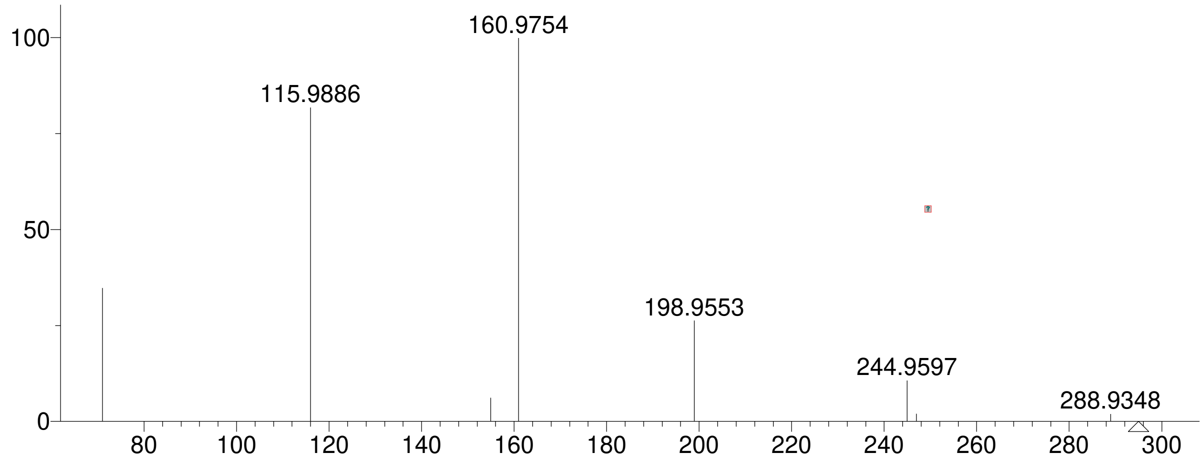

Unknown: Unknown (0.89_358.9415m/z)
Compound in Library Factor = 0

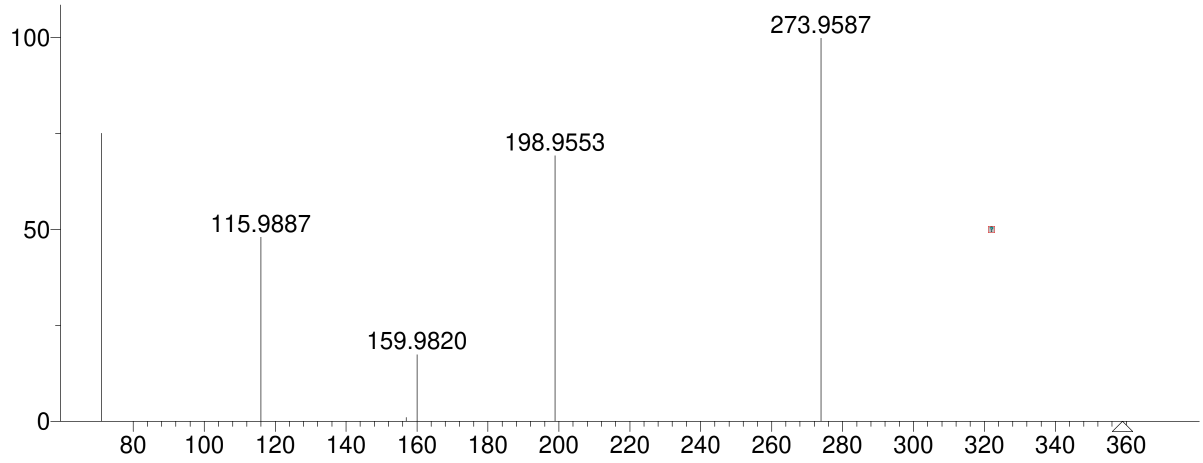

** Search Report Page 1 of 1 **

Unknown: Unknown (0.89_362.9376m/z)
Compound in Library Factor = 0

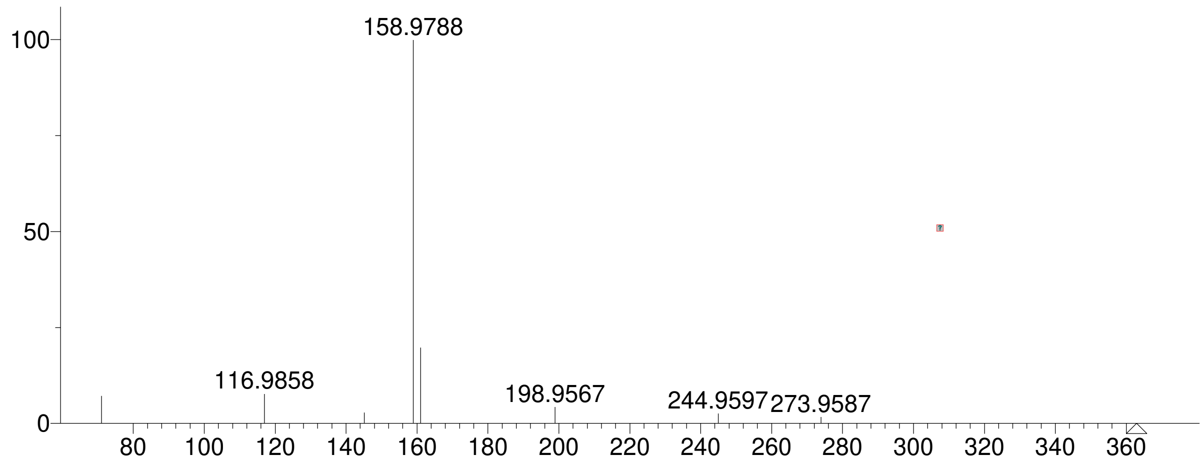

Unknown: Unknown (0.89_540.8949m/z)
Compound in Library Factor = 0

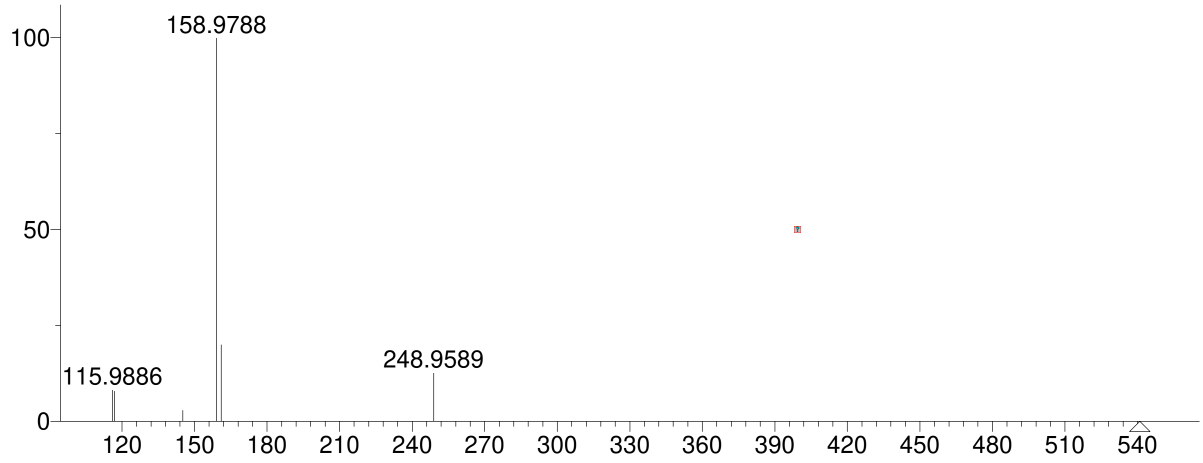

Unknown: Unknown (0.89_542.8965m/z)
Compound in Library Factor = 0

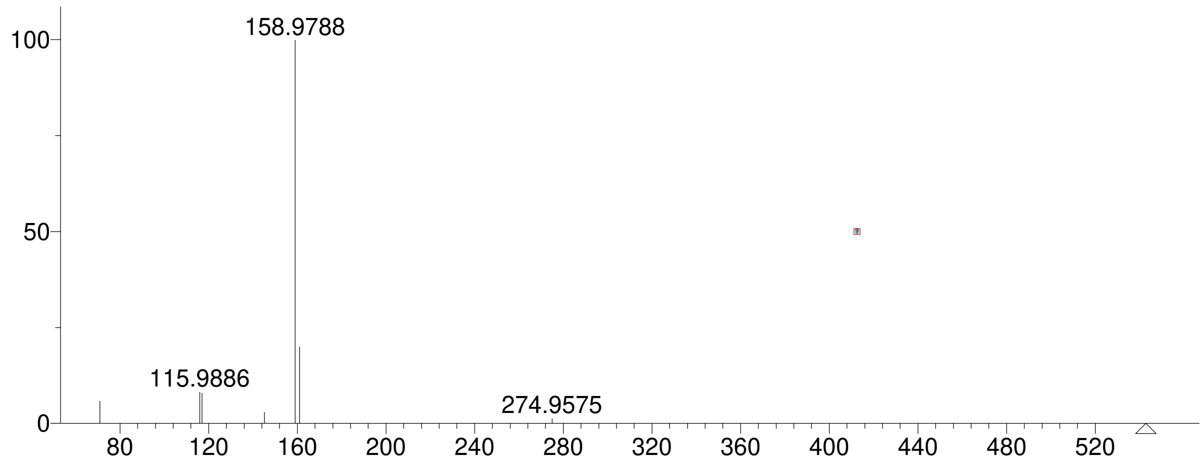

Unknown: Unknown (0.89_616.8975m/z)
Compound in Library Factor = 0

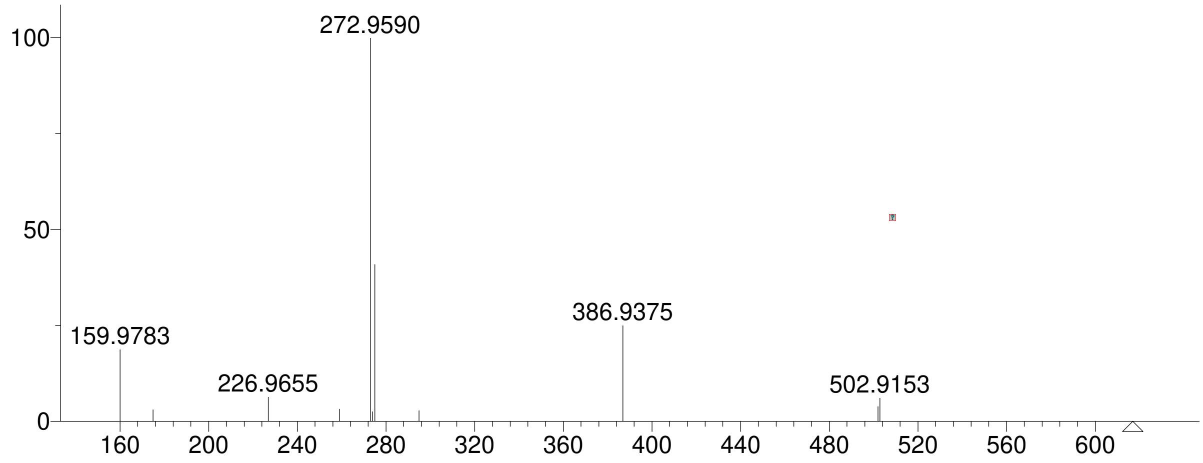

Unknown: Unknown (0.89_728.8746m/z)
Compound in Library Factor = 0

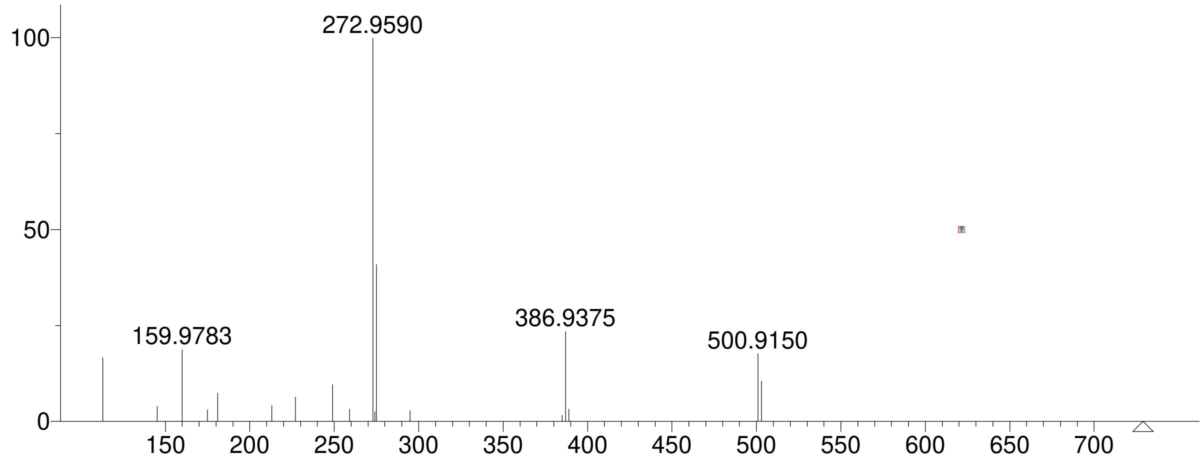

Hit 1 : CART (1-39), human, rat [M+6H]6+ QTOF 45V P=728.9
C188H303N57O63; MF: 0; RMF: 18; Prob 100.0%; Lib: nist_msms2; ID: 35851.

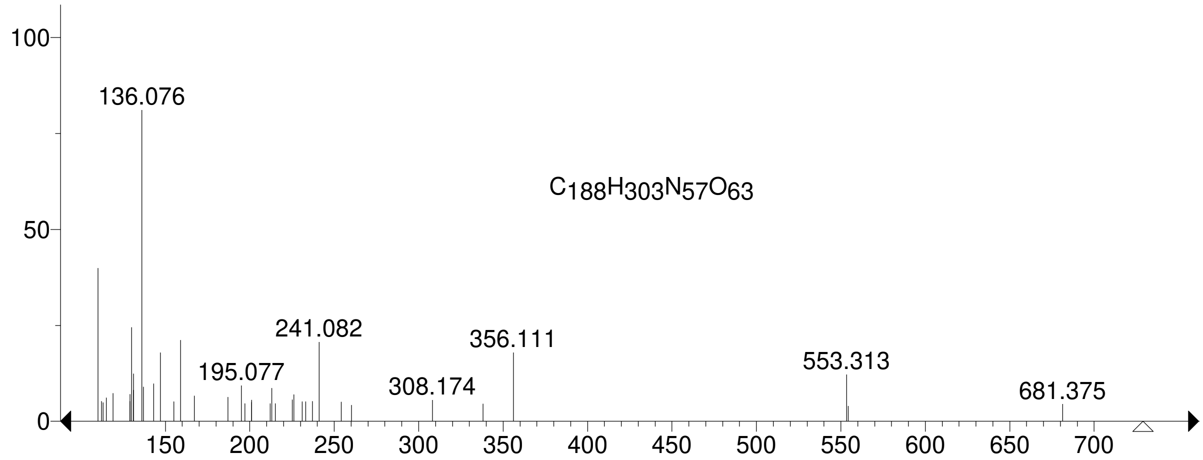

Unknown: Unknown (0.89_730.8761m/z)
Compound in Library Factor = 0

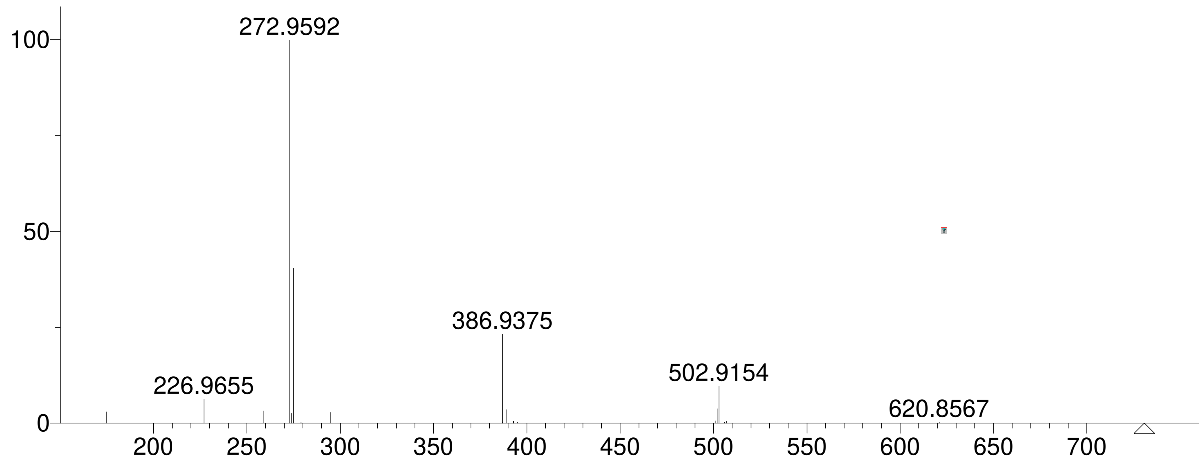

Hit 1 : ProII, DAla12 Angiotensin (I) [M-2H]2- HCD 49eV P=730.9
C70H101N19O16; MF: 0; RMF: 1; Prob 100.0%; Lib: nist_msms2; ID: 76619.

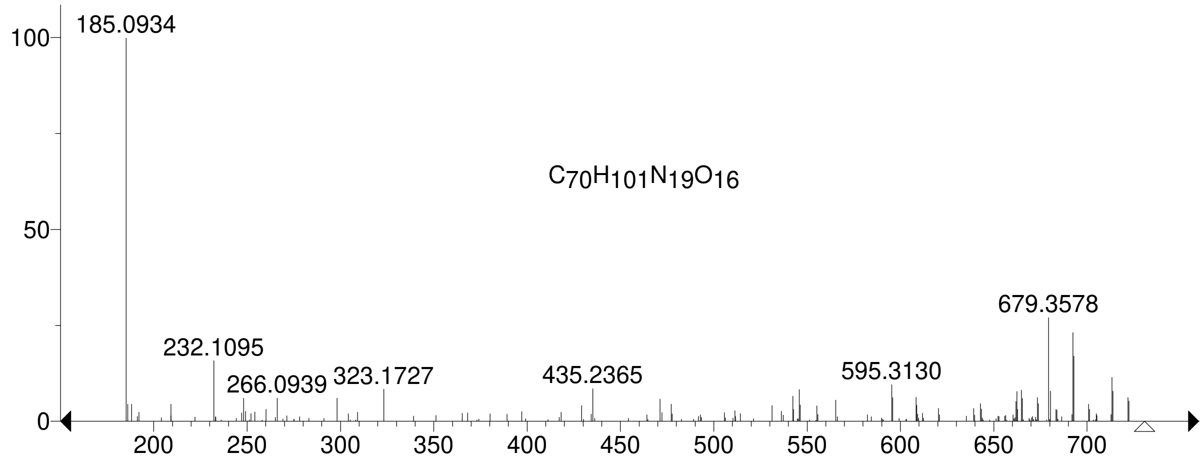

Unknown: Unknown (0.90_386.9380m/z)
Compound in Library Factor = 0

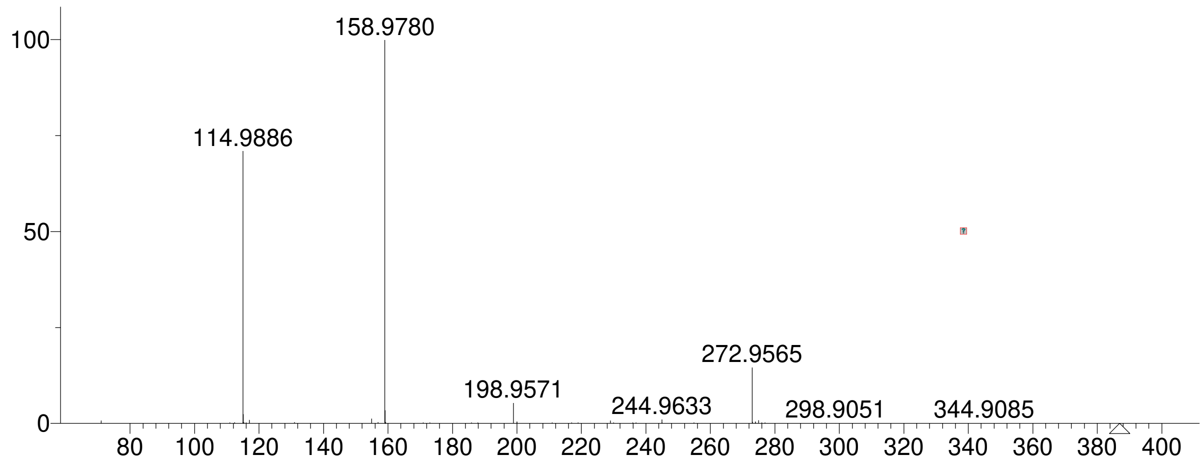

Unknown: Unknown (0.90_427.9170m/z)
Compound in Library Factor = 0

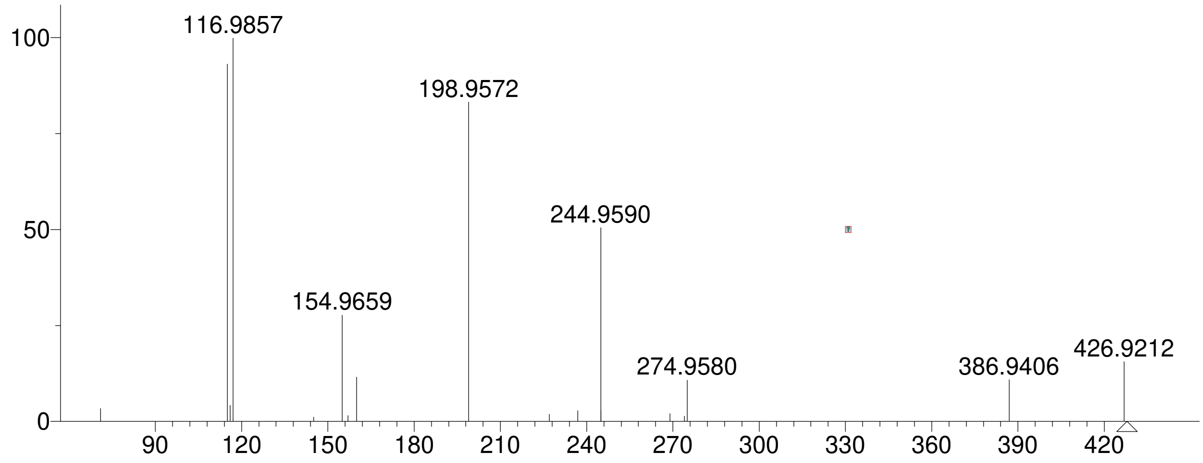

** Search Report Page 1 of 1 **

Unknown: Unknown (0.90_500.9186m/z)
Compound in Library Factor = 0

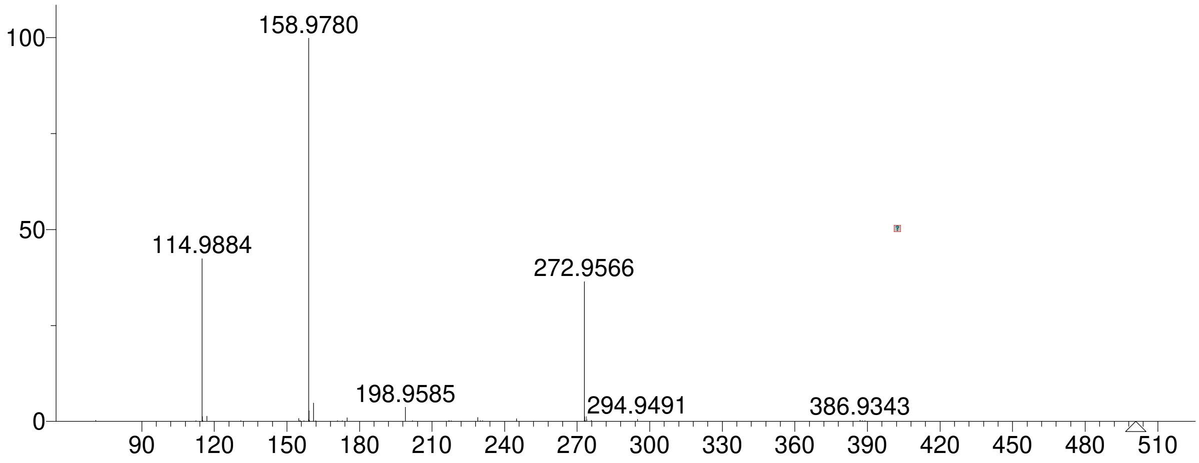

** Search Report Page 1 of 1 **

Unknown: Unknown (0.90_588.8990m/z)
Compound in Library Factor = 0

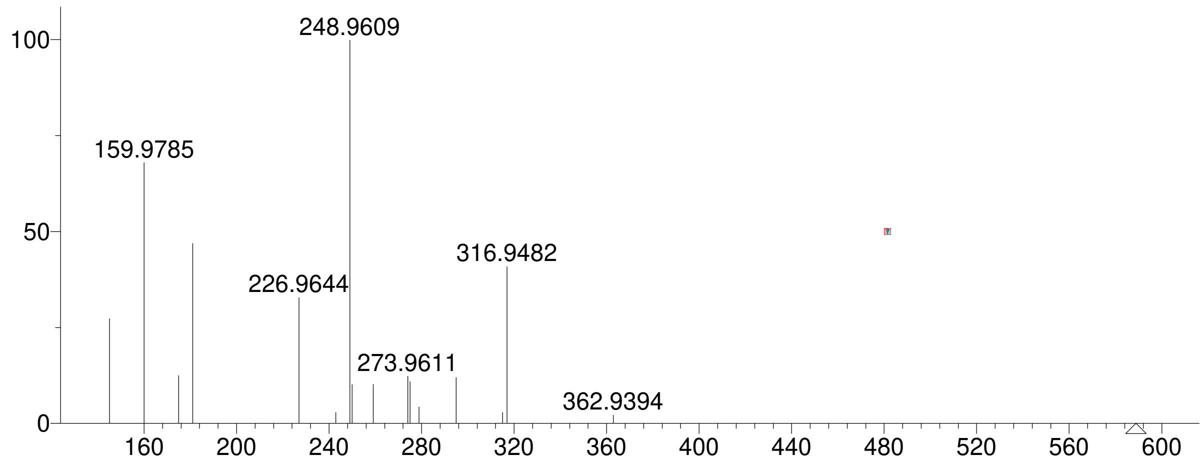

Unknown: Unknown (0.90_614.8971m/z)
Compound in Library Factor = 0

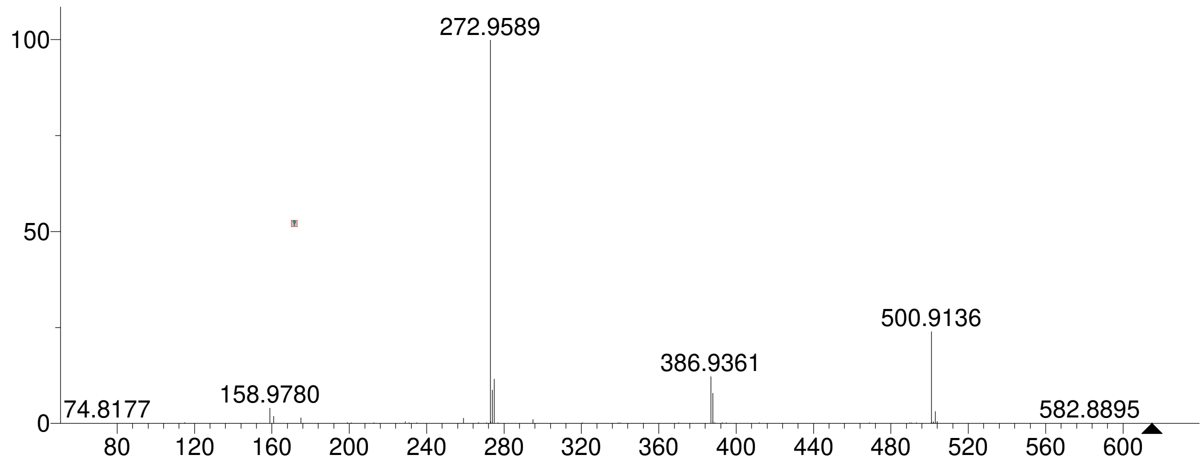

Unknown: Unknown (0.90_700.8833m/z)
Compound in Library Factor = 0

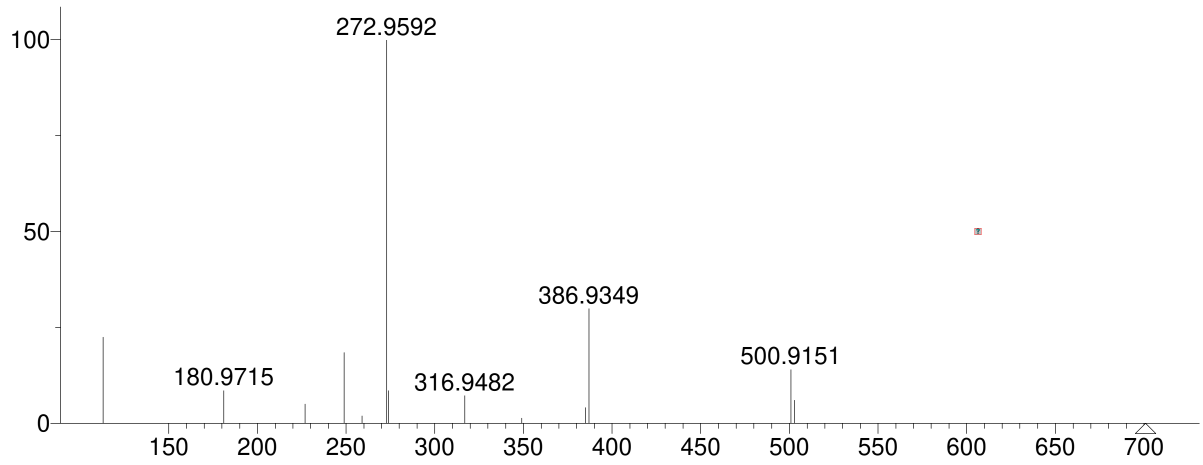

Unknown: Unknown (0.91_472.9235m/z)
Compound in Library Factor = 0

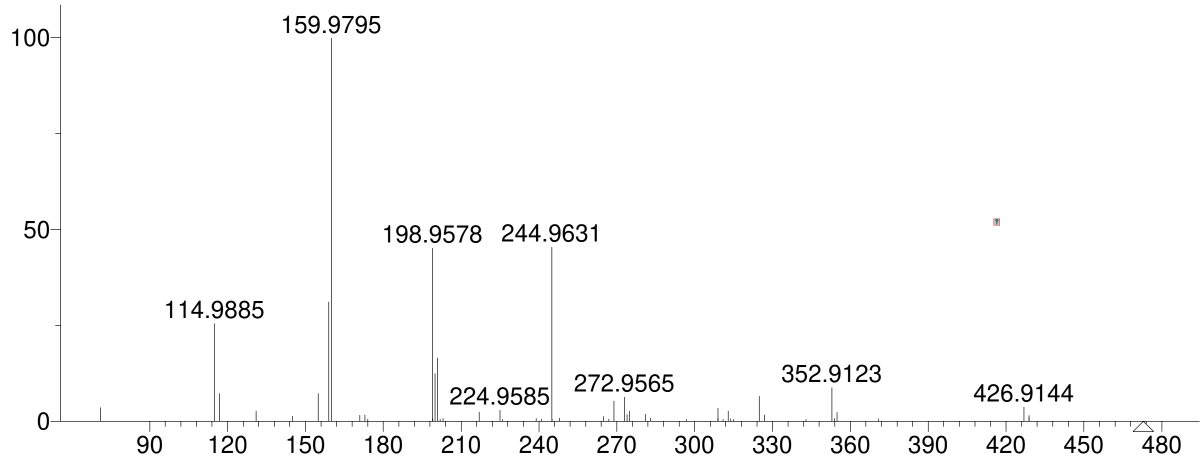

Unknown: Unknown (0.91_540.8995m/z)
Compound in Library Factor = 0

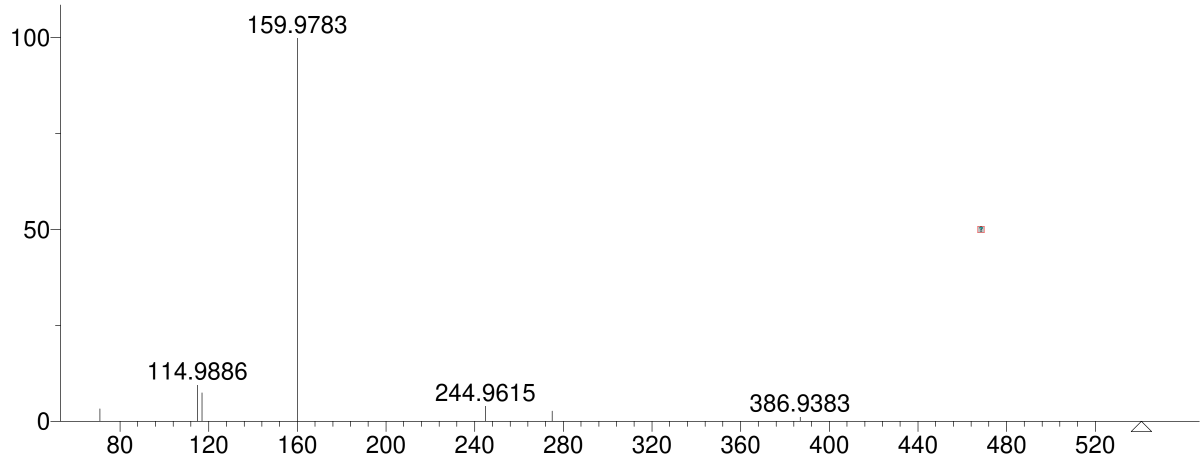

Unknown: Unknown (0.92_244.9629m/z)
Compound in Library Factor = 0

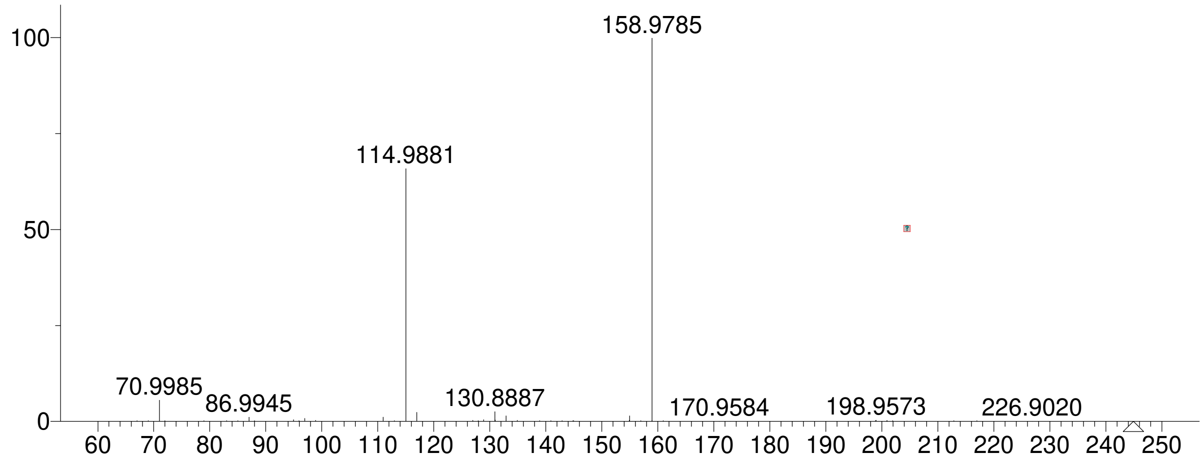

Unknown: Unknown (0.92_272.9581m/z)
Compound in Library Factor = 0

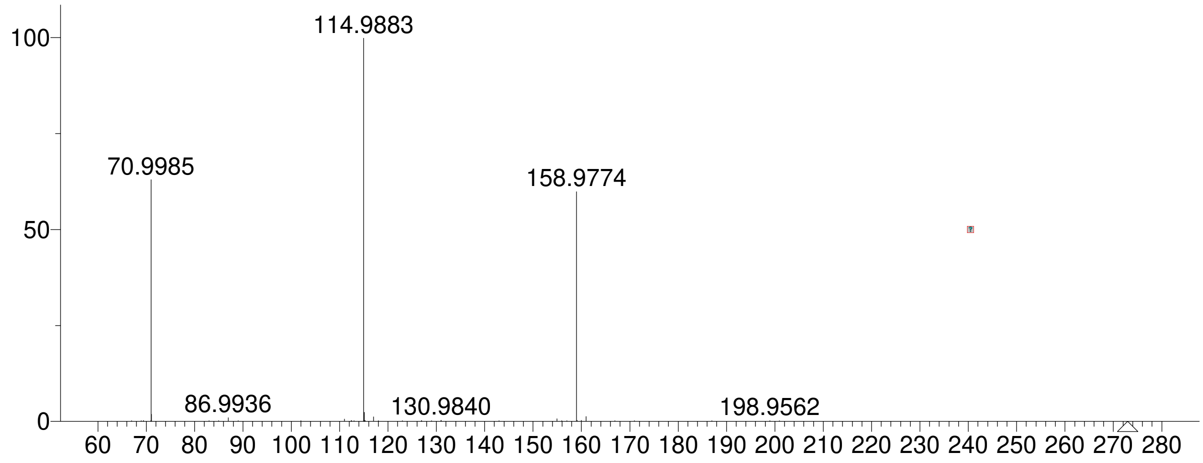

Unknown: Unknown (0.92_273.1131m/z)
Compound in Library Factor = 0

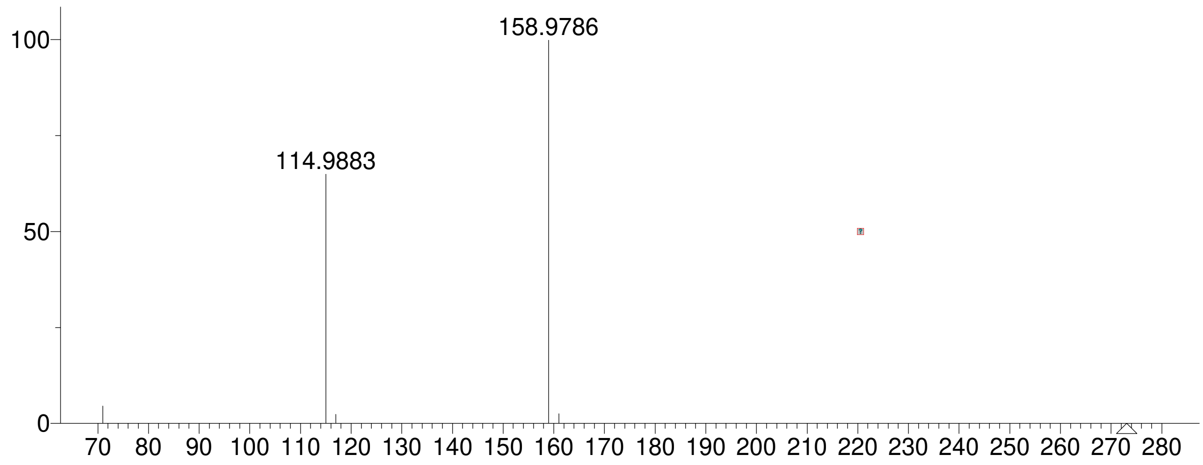

Hit 1 : ANGOLENSIN (R) [M+H]+ 65HCD P=273.1
C16H16O4; MF: 0; RMF: 1; Prob 100.0%; Lib: mona-export-vf-npl_qexactive; ID: 2856.

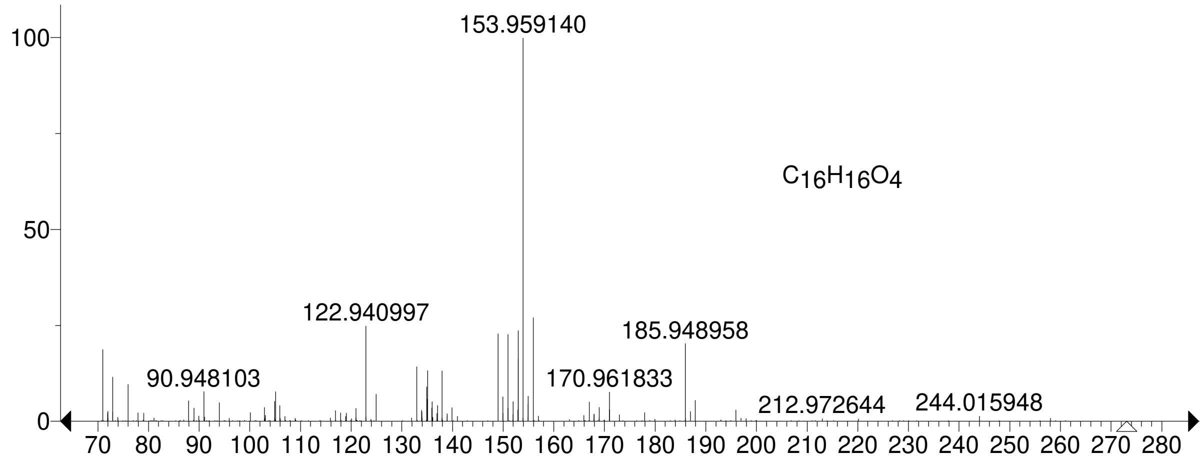

** Search Report Page 1 of 1 **

Unknown: Unknown (0.92_520.9080m/z)
Compound in Library Factor = 0

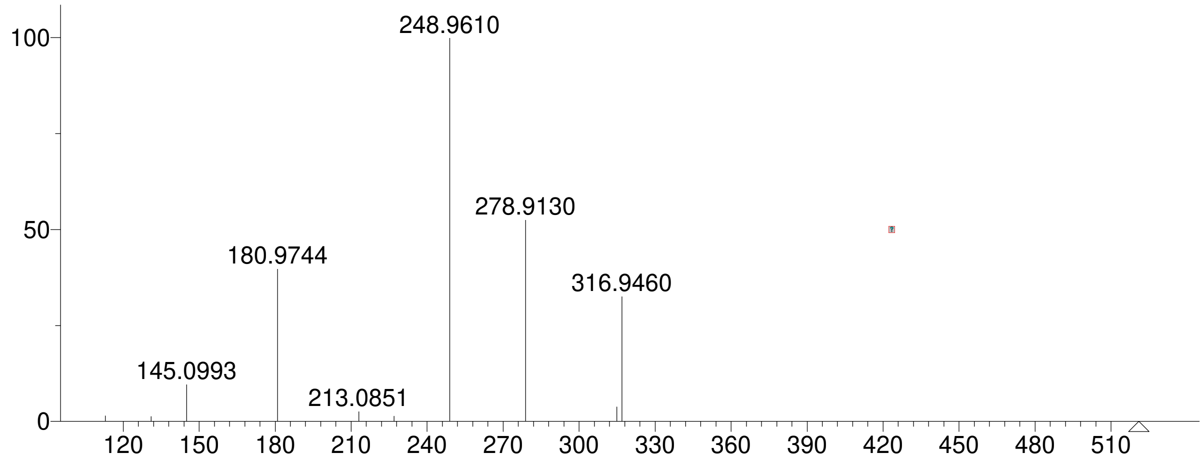

Unknown: Unknown (0.93_158.9779m/z)
Compound in Library Factor = 0

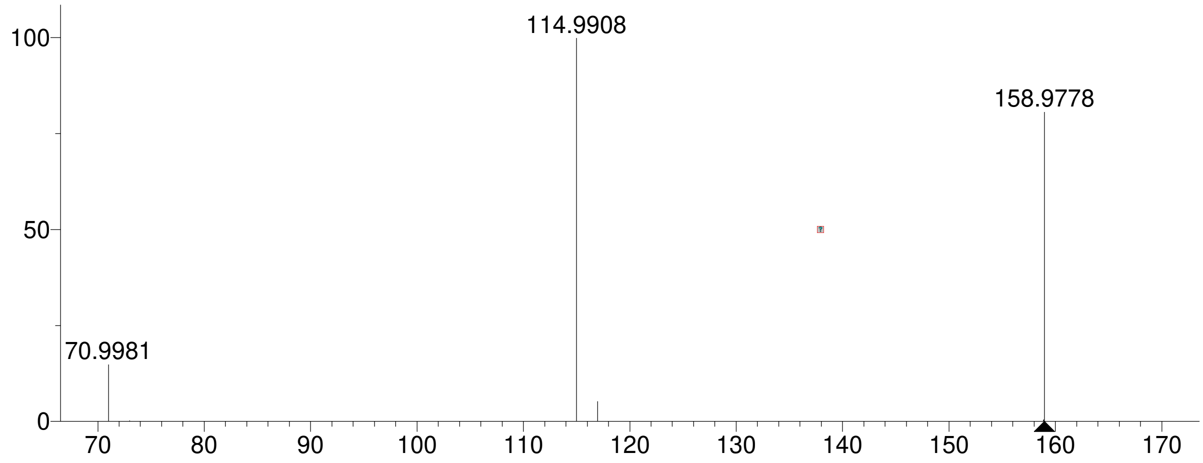

** Search Report Page 1 of 1 **

Unknown: Unknown (0.93_164.9337m/z)
Compound in Library Factor = 0

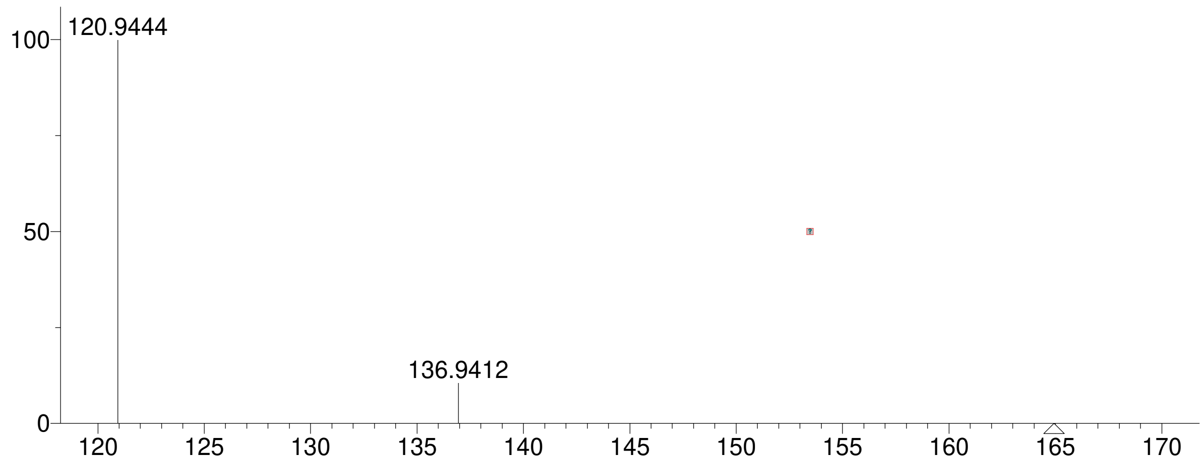

** Search Report Page 1 of 1 **

Unknown: Unknown (0.93_248.9603m/z)
Compound in Library Factor = 0

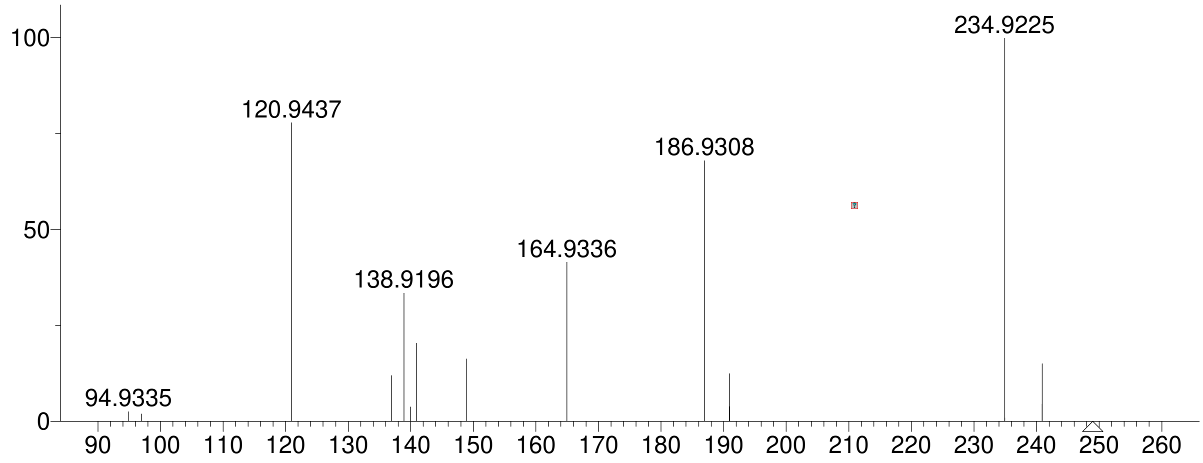

** Search Report Page 1 of 1 **

Unknown: Unknown (0.93_278.9142m/z)
Compound in Library Factor = 0

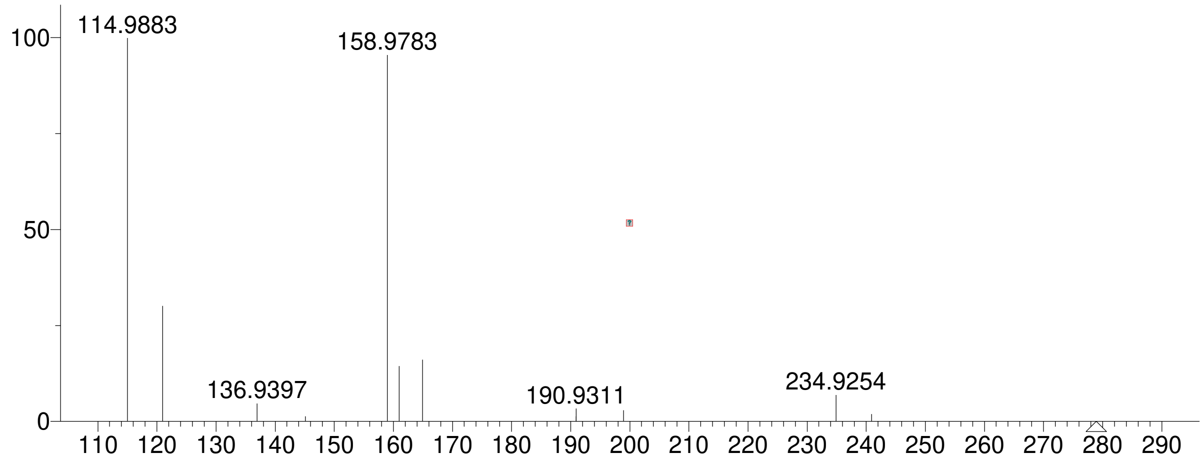

Unknown: Unknown (0.93_316.9473m/z)
Compound in Library Factor = 0

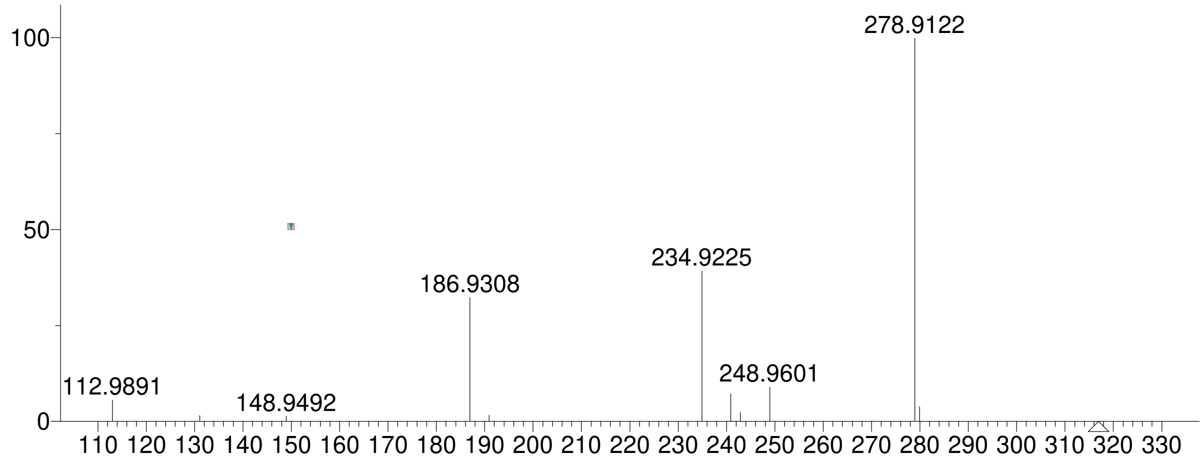

** Search Report Page 1 of 1 **

Unknown: Unknown (0.93_384.9351m/z)
Compound in Library Factor = 0

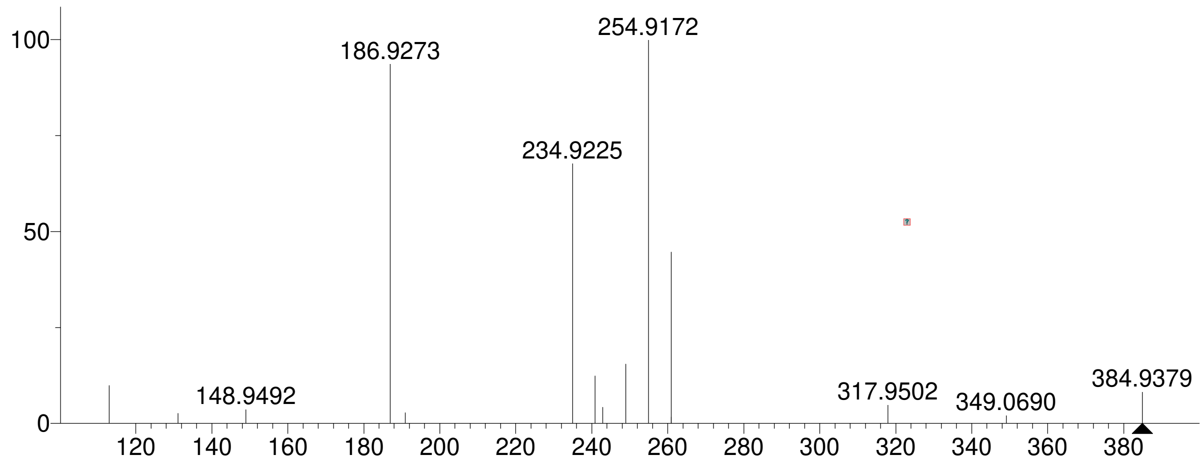

Hit 1 : Chlorfluazuron [M+H-C7H5F2ON+2i]+ HCD 30V P=384.9
C20H9Cl3F5N3O3; MF: 0; RMF: 87; Prob 100.0%; CAS: 71422-67-8; Lib: nist_msms; ID: 508071.

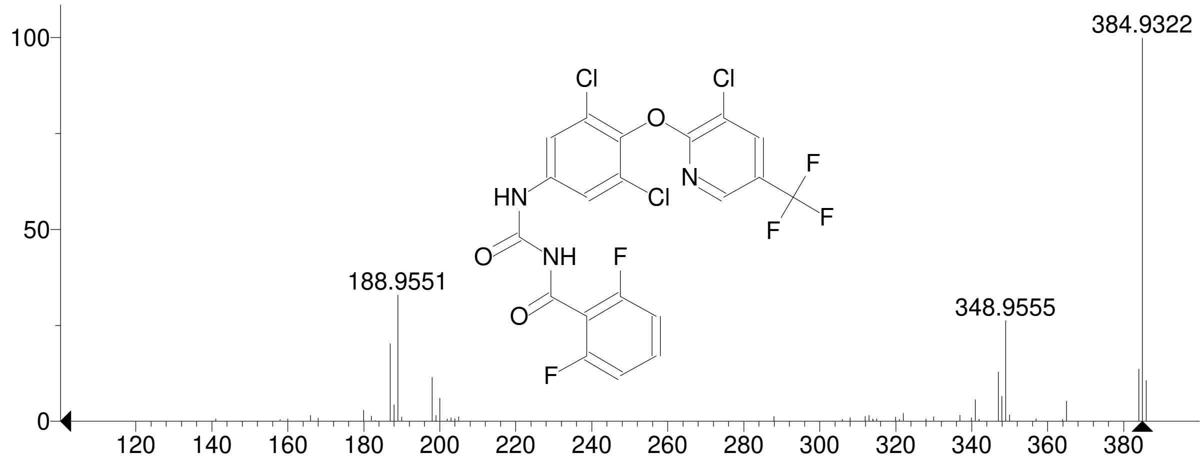

Unknown: Unknown (0.93_392.8935m/z)
Compound in Library Factor = 0

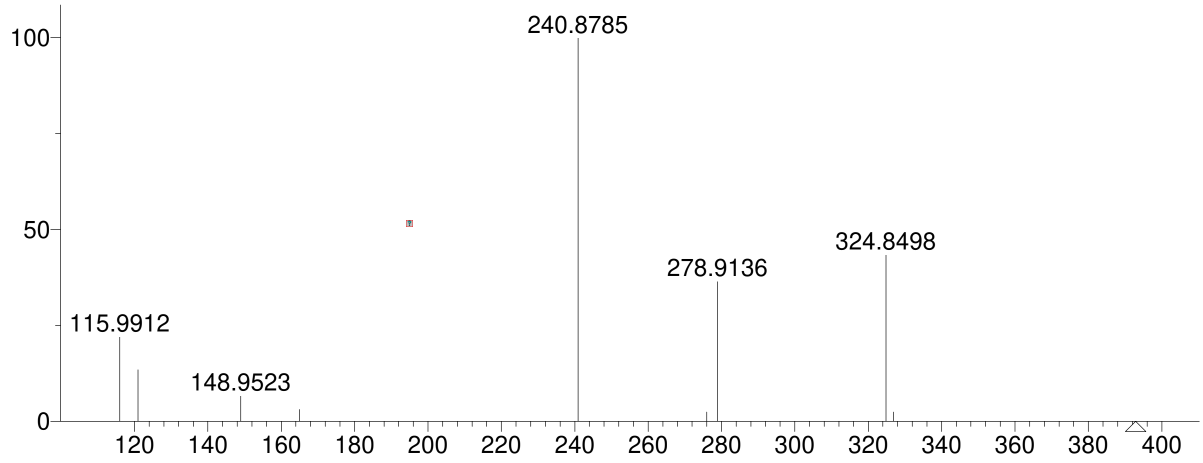

Unknown: Unknown (0.93_452.9216m/z)
Compound in Library Factor = 0

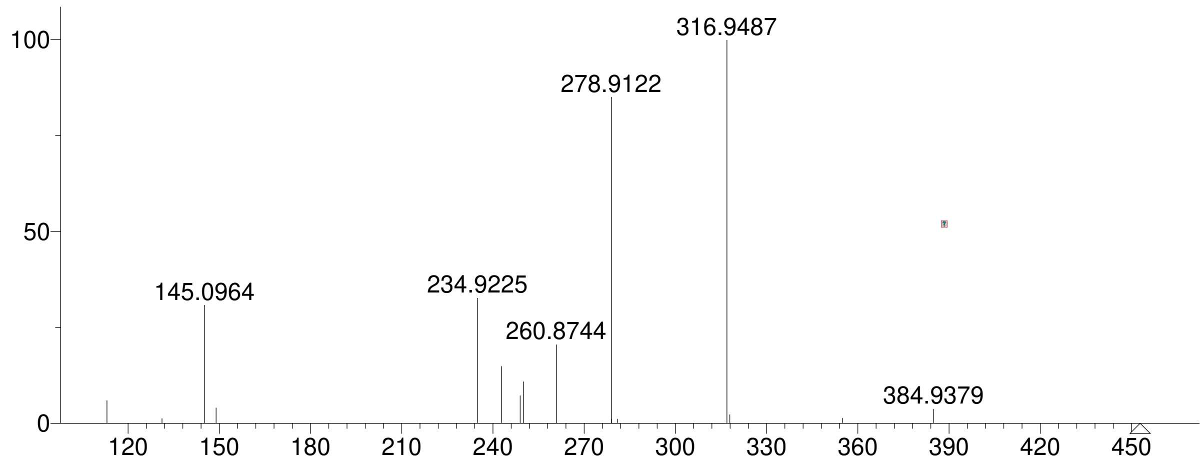

Unknown: Unknown (0.93_506.8754m/z)
Compound in Library Factor = 0

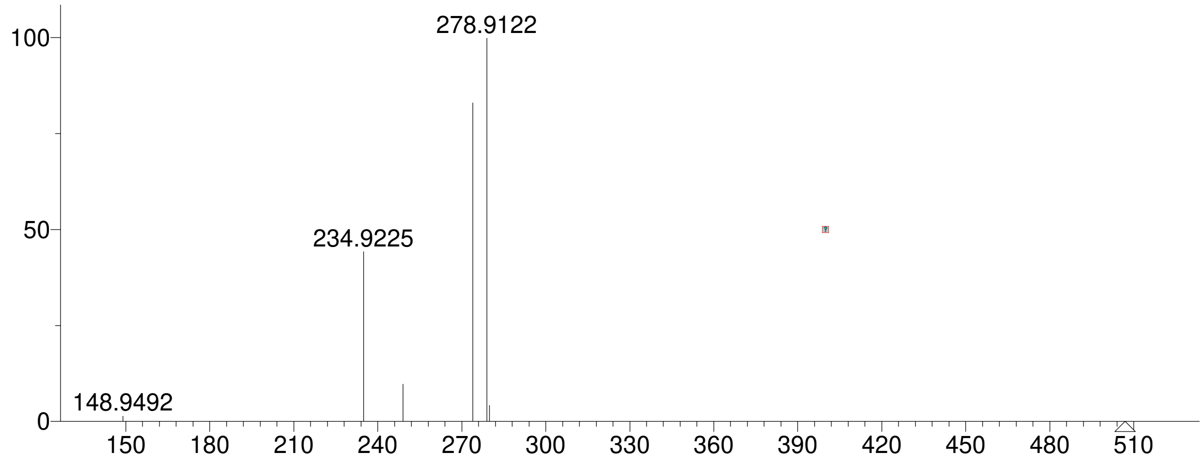

Unknown: Unknown (0.93_508.8731m/z)
Compound in Library Factor = 0

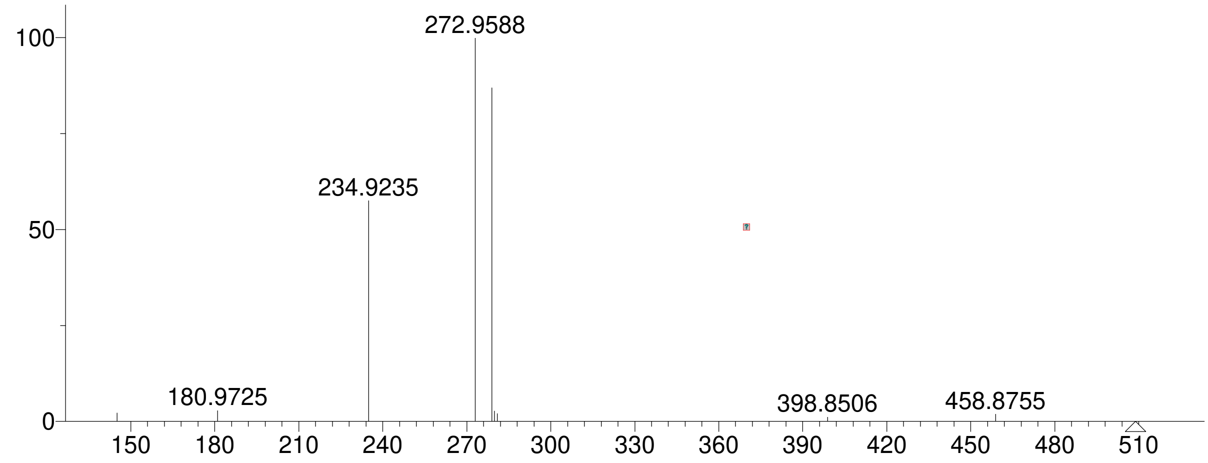

Hit 1 : 3,3'-Diiodo-L-thyronine [M+H-NH3]+ HCD 61V P=508.9
C15H13I2NO4; MF: 0; RMF: 119; Prob 100.0%; CAS: 70-40-6; Lib: nist_msms; ID: 187453.

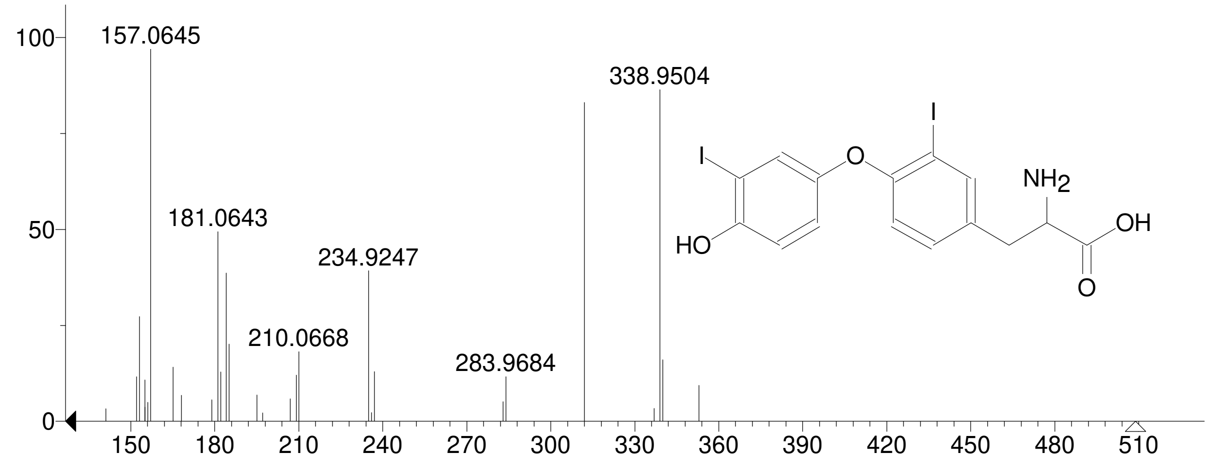

** Search Report Page 1 of 1 **

Unknown: Unknown (0.93_604.8696m/z)
Compound in Library Factor = 0

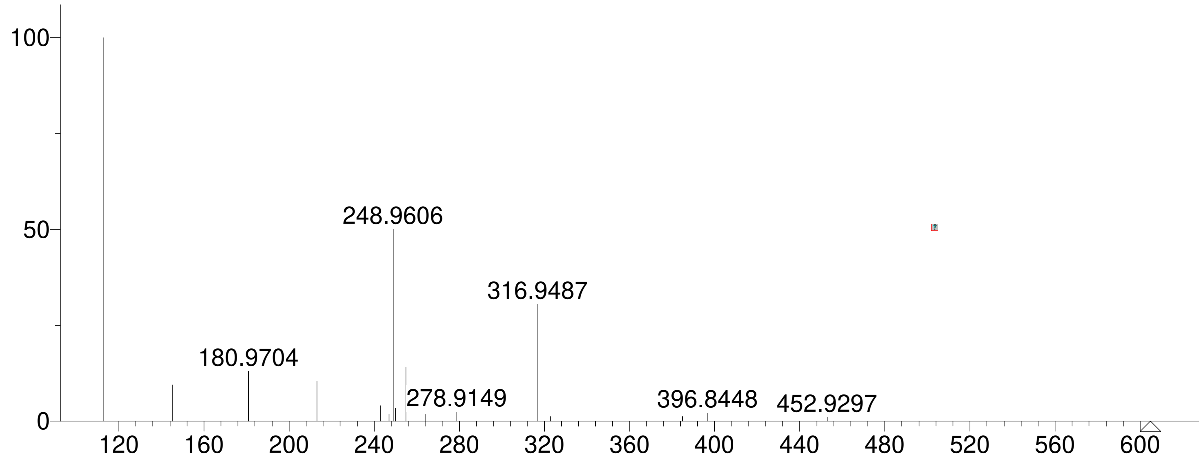

Unknown: Unknown (0.93_656.8838m/z)
Compound in Library Factor = 0

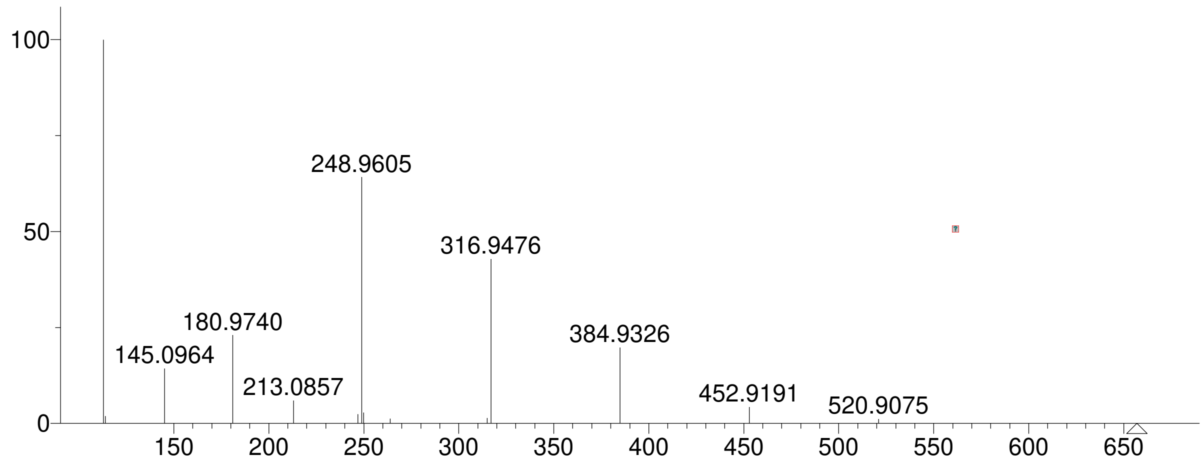

Unknown: Unknown (0.93_672.8563m/z)
Compound in Library Factor = 0

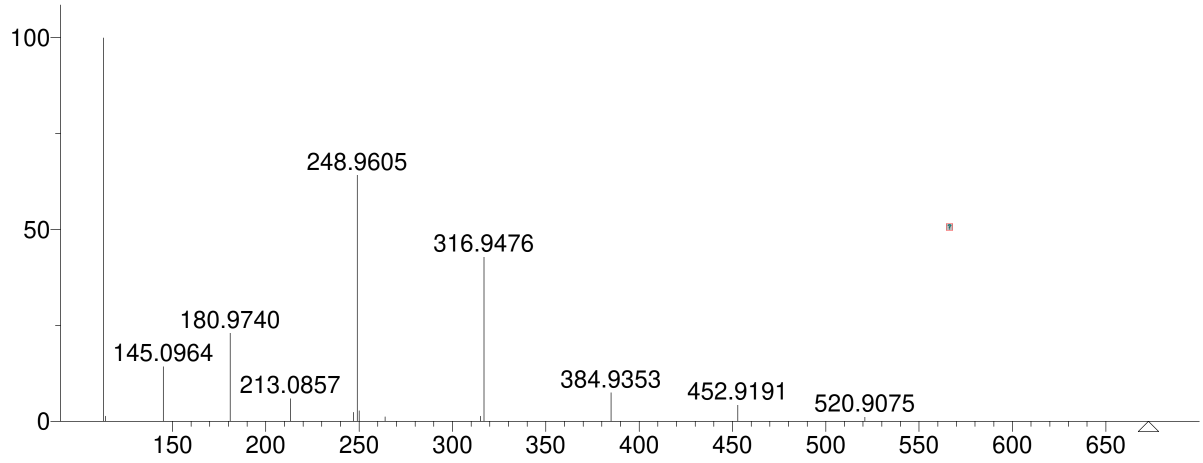

Unknown: Unknown (0.93_756.8200m/z)
Compound in Library Factor = 0

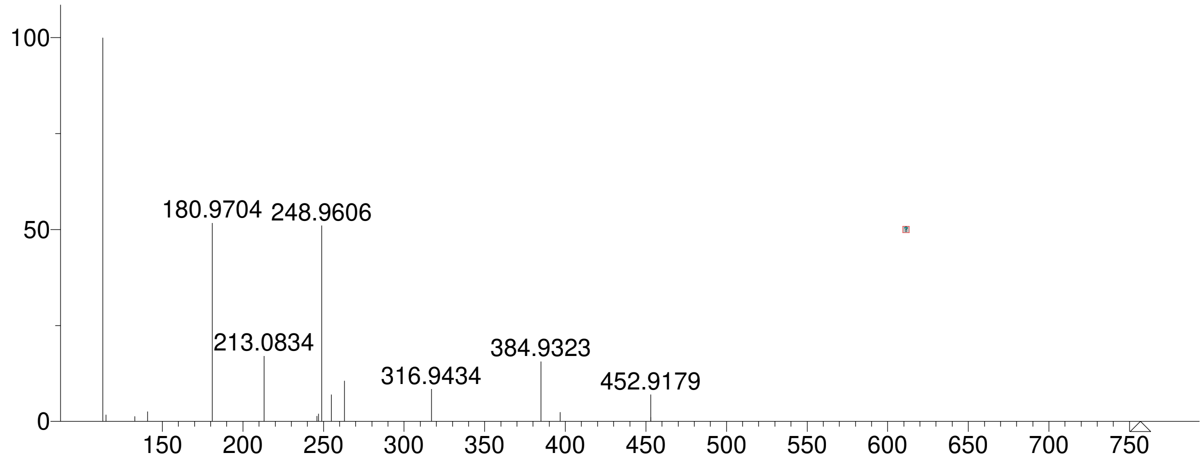

Unknown: Unknown (0.94_252.9003m/z)
Compound in Library Factor = 0

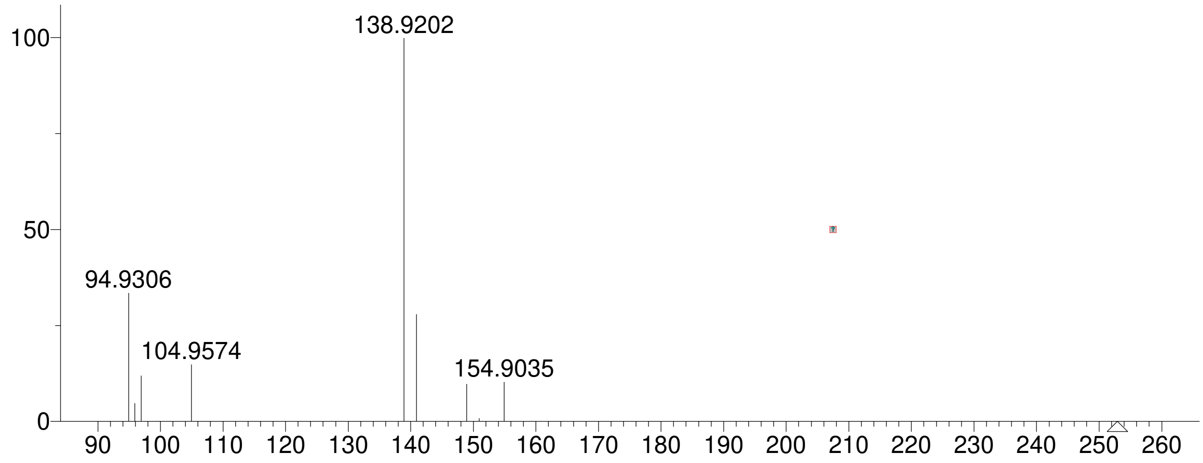

Hit 1 : Sulprofos [M+H]+=>294.9=>252.9 IT 35% P=252.9
C12H19O2PS3; MF: 0; RMF: 8; Prob 33.3%; CAS: 35400-43-2; Lib: nist_msms; ID: 129596.

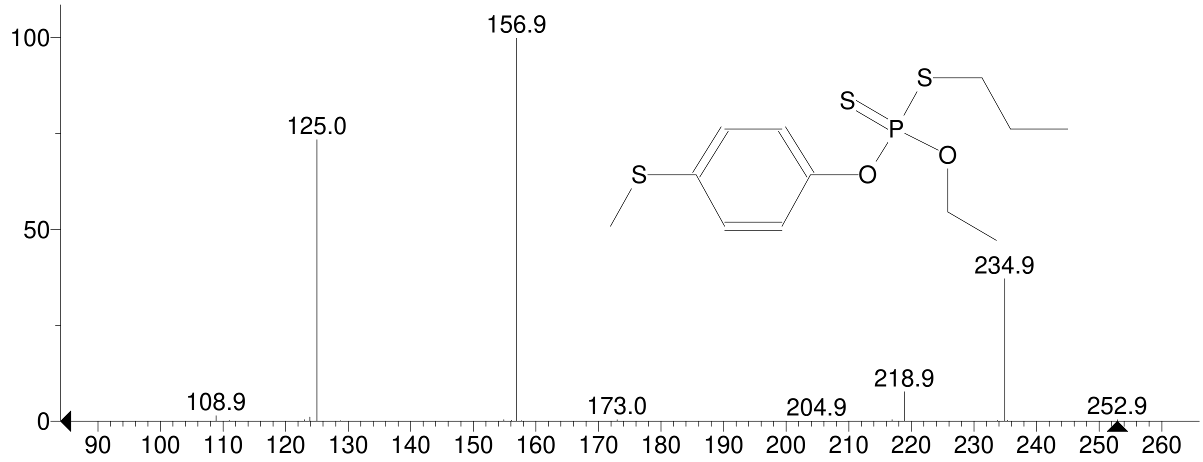

Hit 2 : Fensulfothion [M+H]+=>252.9 IT 35% P=252.9
C11H17O4PS2; MF: 0; RMF: 8; Prob 33.3%; CAS: 115-90-2; Lib: nist_msms; ID: 483029.

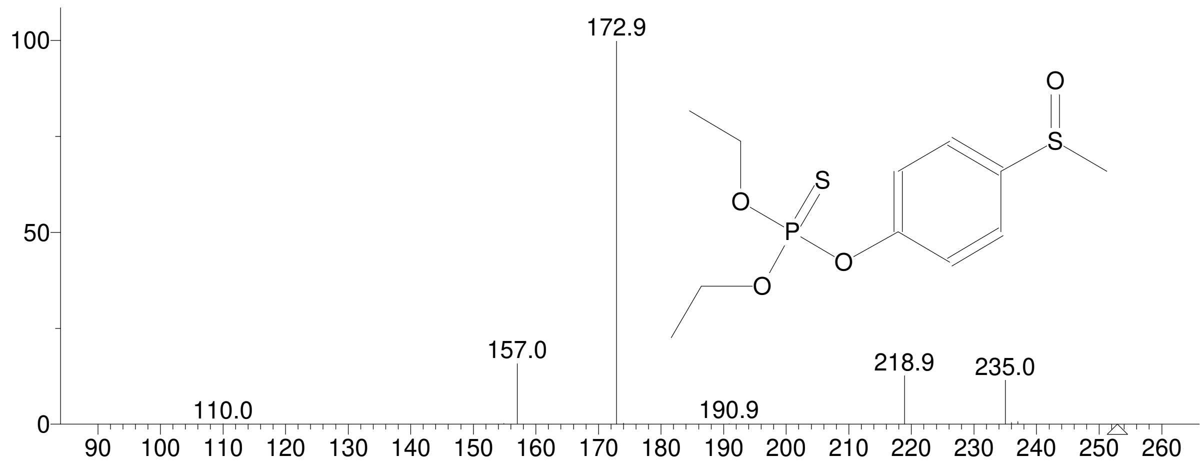

Hit 3 : Dihydroergotamine [M+H-H2O]+=>252.9 IT 35% P=252.9
C33H37N5O5; MF: 0; RMF: 5; Prob 33.3%; CAS: 511-12-6; Lib: nist_msms; ID: 140025.

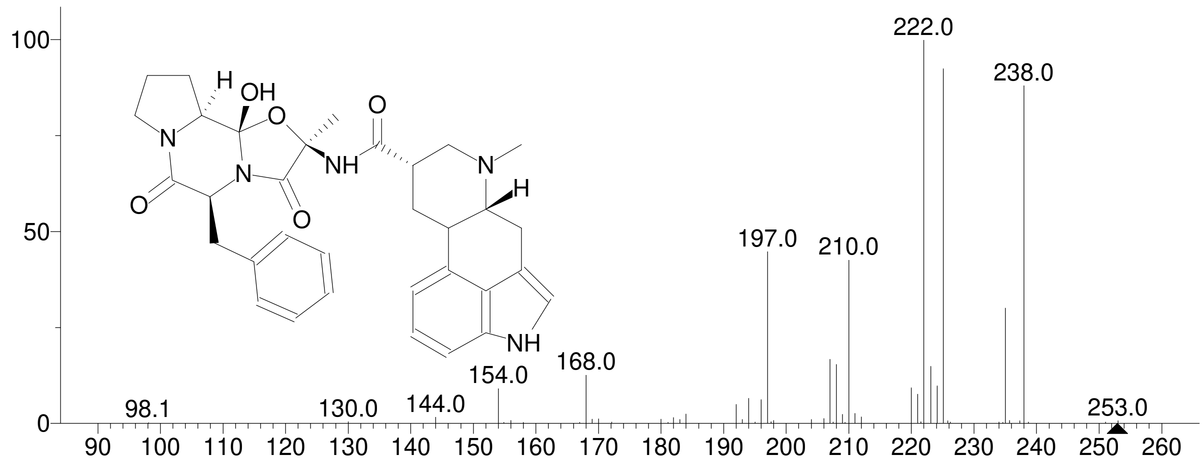

** Search Report Page 1 of 1 **

Unknown: Unknown (0.94_254.8974m/z)
Compound in Library Factor = 0

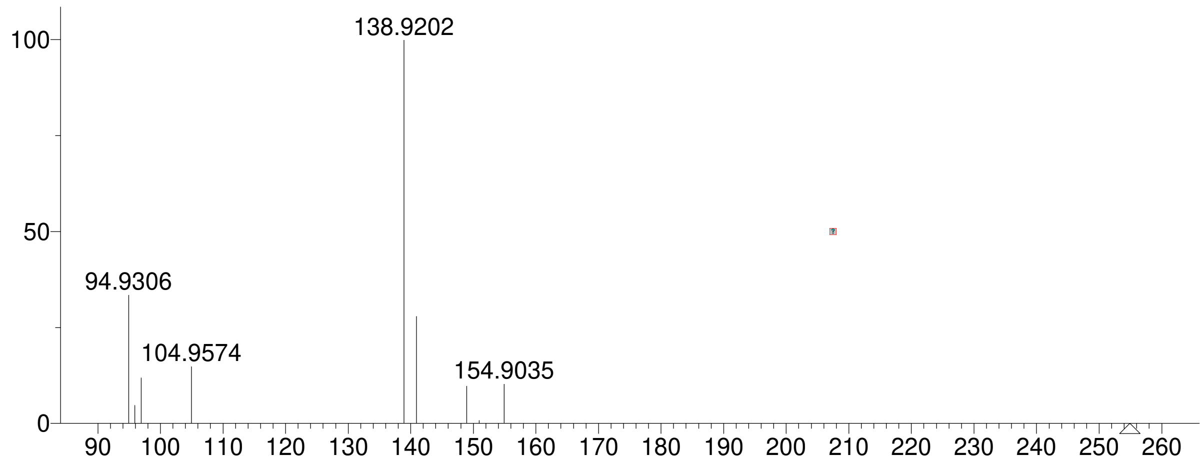

Unknown: Unknown (0.94_262.9292m/z)
Compound in Library Factor = 0

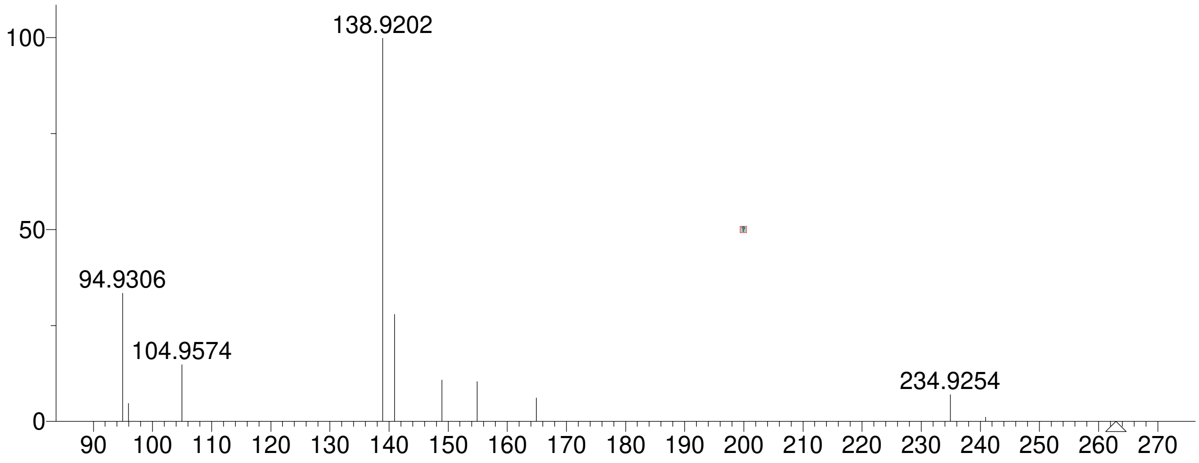

Unknown: Unknown (0.94_264.9263m/z)
Compound in Library Factor = 0

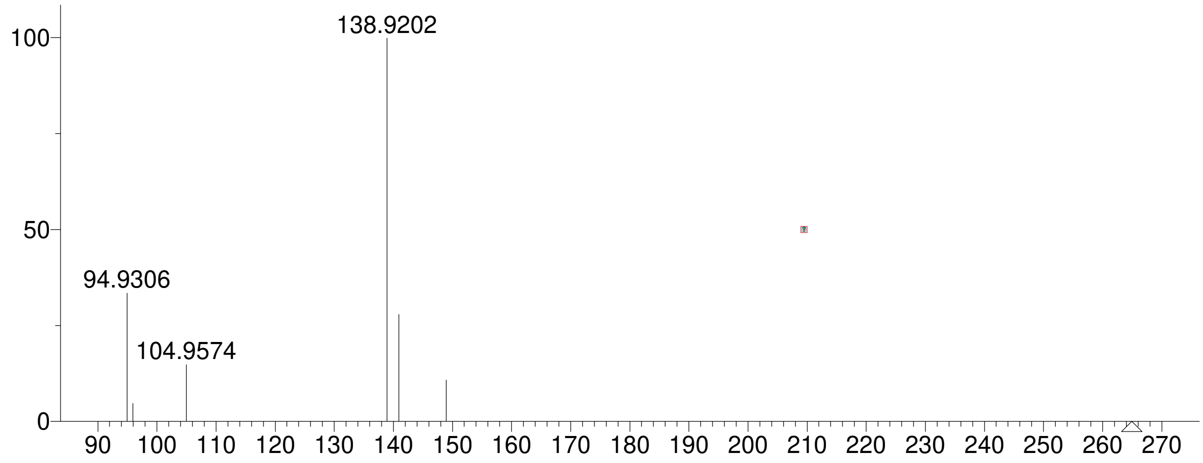

** Search Report Page 1 of 1 **

Unknown: Unknown (0.94_376.9076m/z)
Compound in Library Factor = 0

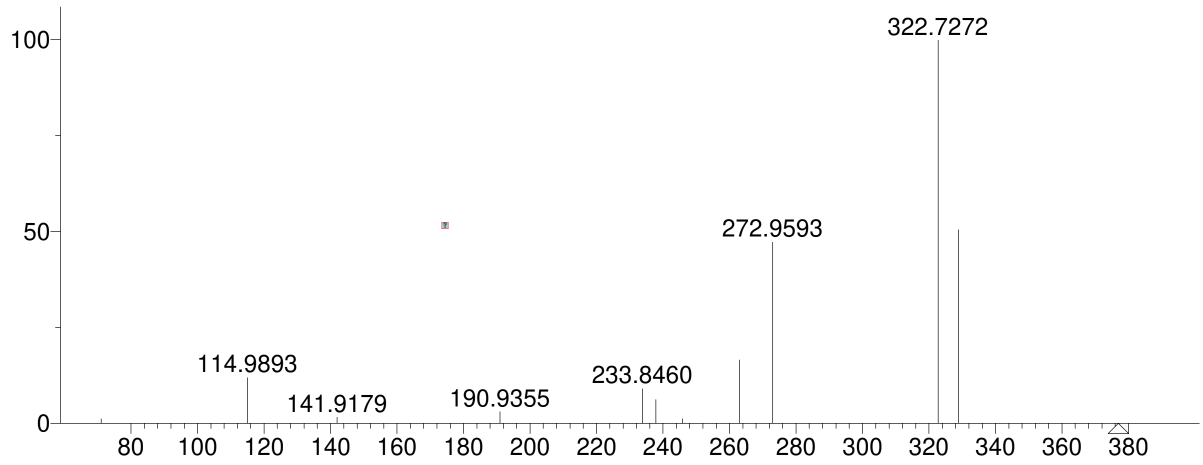

Unknown: Unknown (0.94_378.9052m/z)
Compound in Library Factor = 0

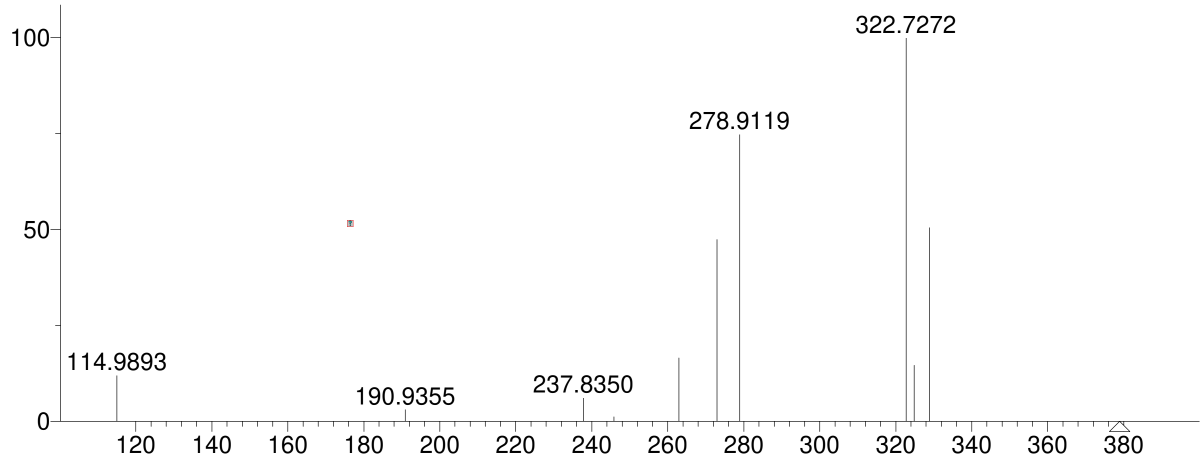

Unknown: Unknown (0.94_468.8940m/z)
Compound in Library Factor = 0

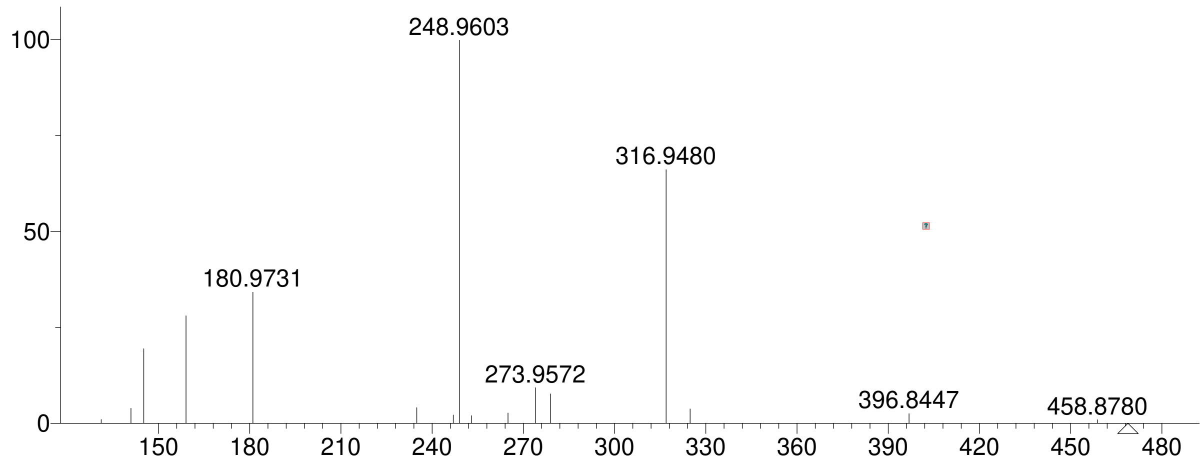

** Search Report Page 1 of 1 **

Unknown: Unknown (0.94_480.8596m/z)
Compound in Library Factor = 0

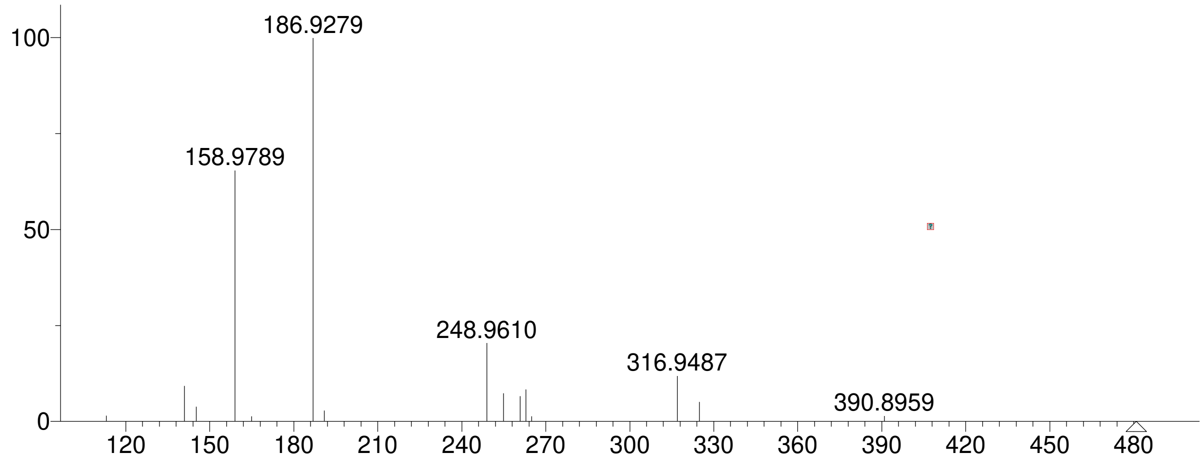

** Search Report Page 1 of 1 **

Unknown: Unknown (0.94_490.8903m/z)
Compound in Library Factor = 0

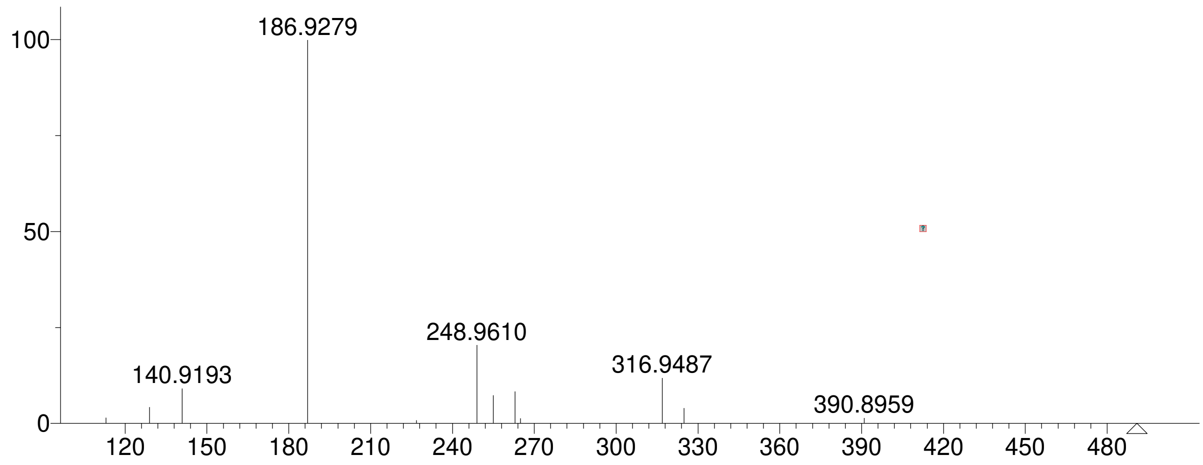

** Search Report Page 1 of 1 **

Unknown: Unknown (0.94_492.8871m/z)
Compound in Library Factor = 0

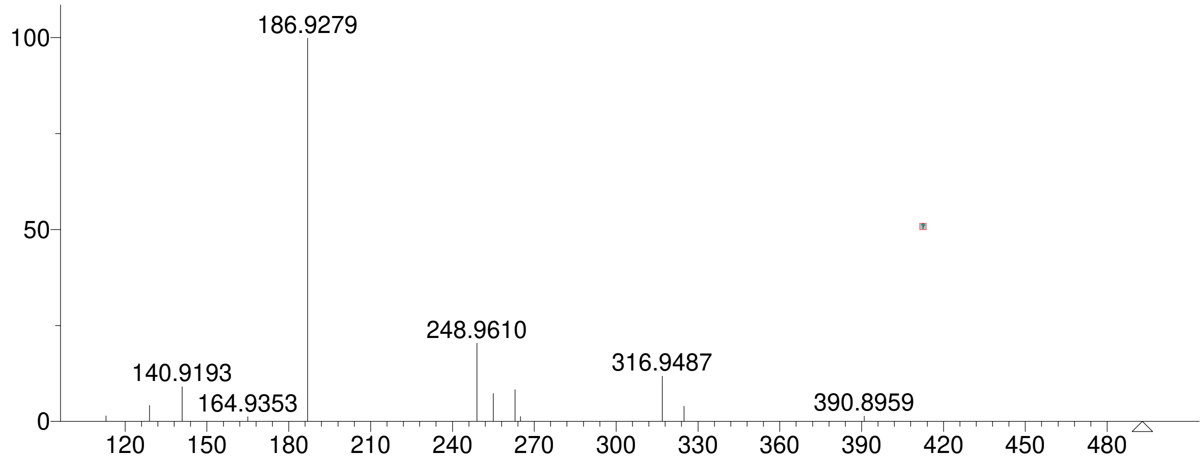

** Search Report Page 1 of 1 **

Unknown: Unknown (0.94_740.8447m/z)
Compound in Library Factor = 0

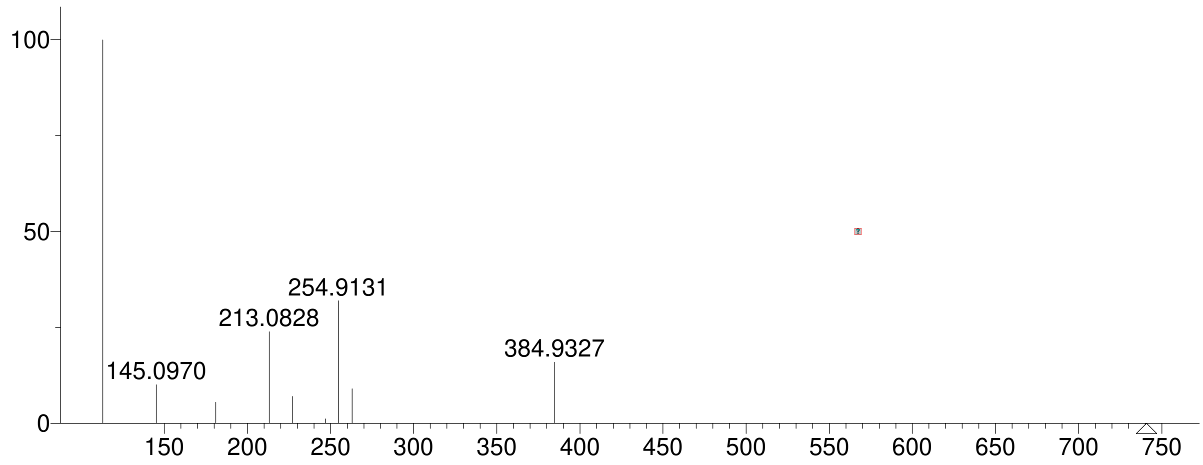

Unknown: Unknown (0.94_808.8385m/z)
Compound in Library Factor = 0

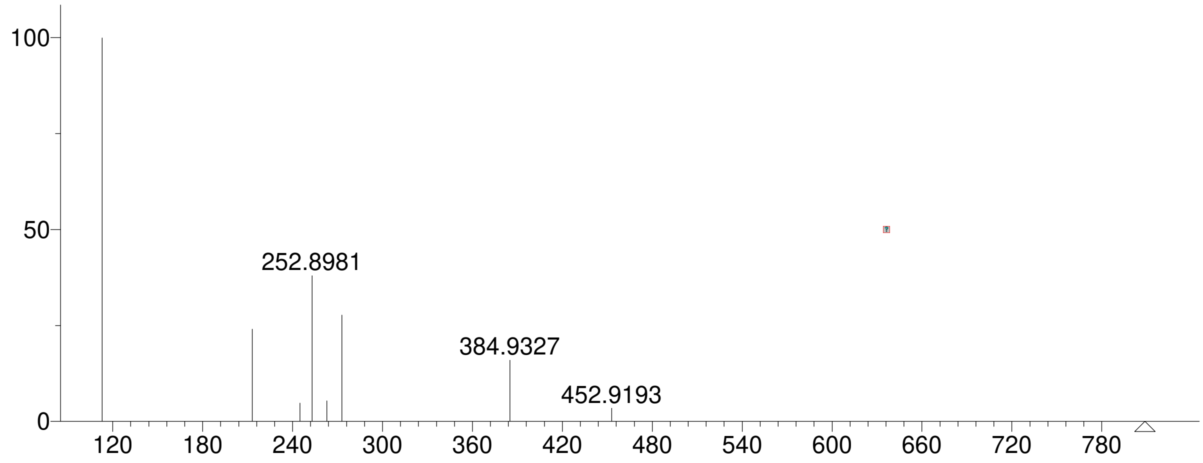

** Search Report Page 1 of 1 **

Unknown: Unknown (0.95_242.8716m/z)
Compound in Library Factor = 0

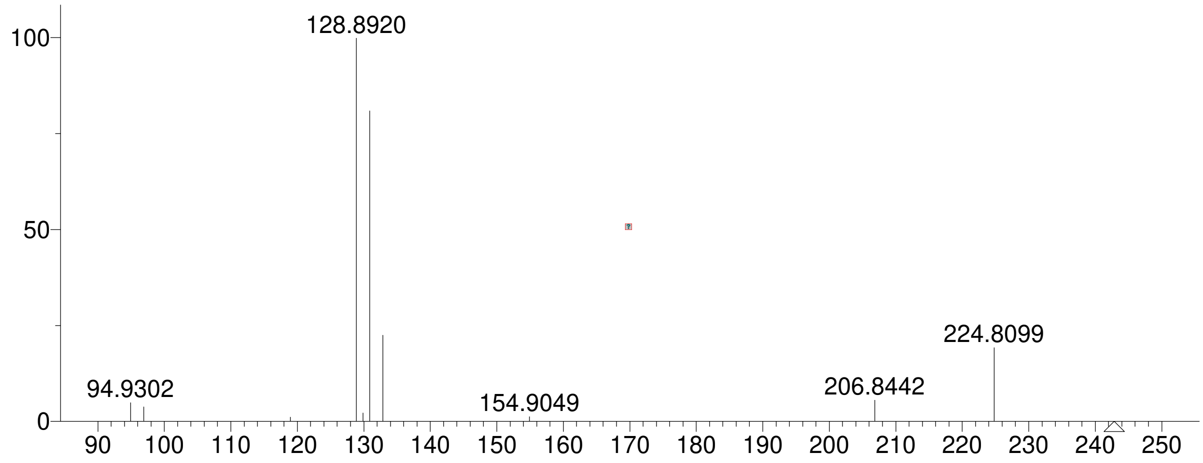

Unknown: Unknown (0.95_244.8687m/z)
Compound in Library Factor = 0

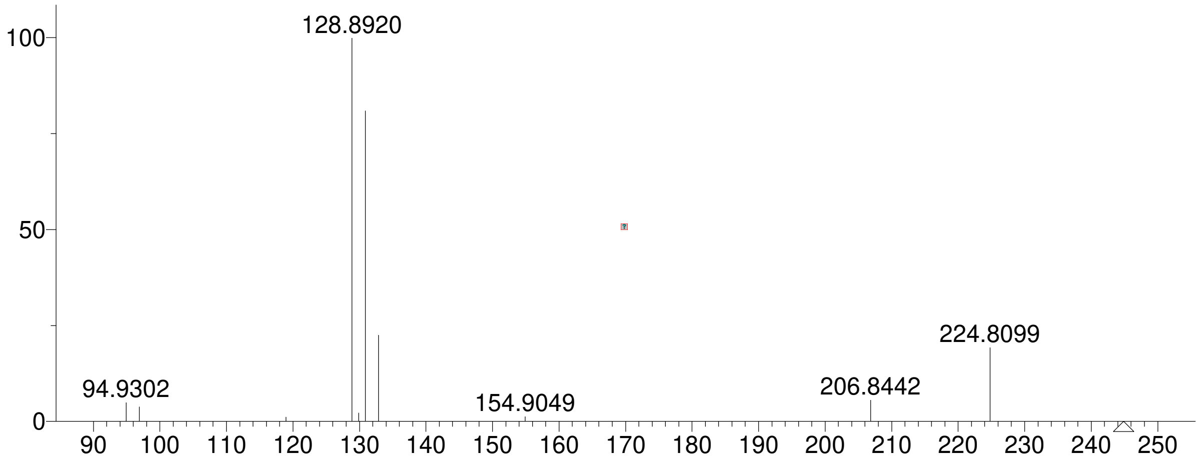

** Search Report Page 1 of 1 **

Unknown: Unknown (0.95_246.8658m/z)
Compound in Library Factor = 0

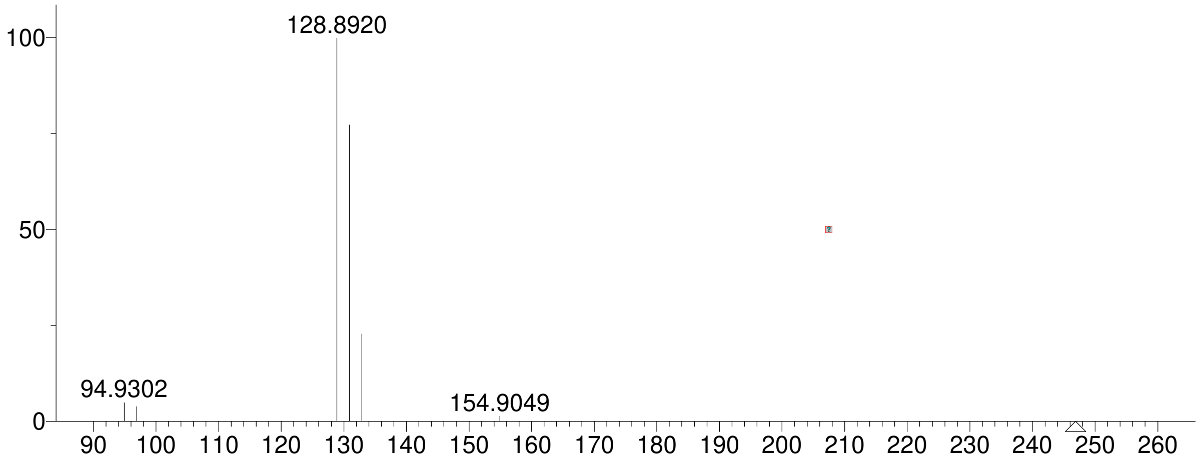

Unknown: Unknown (0.95_312.7927m/z)
Compound in Library Factor = 0

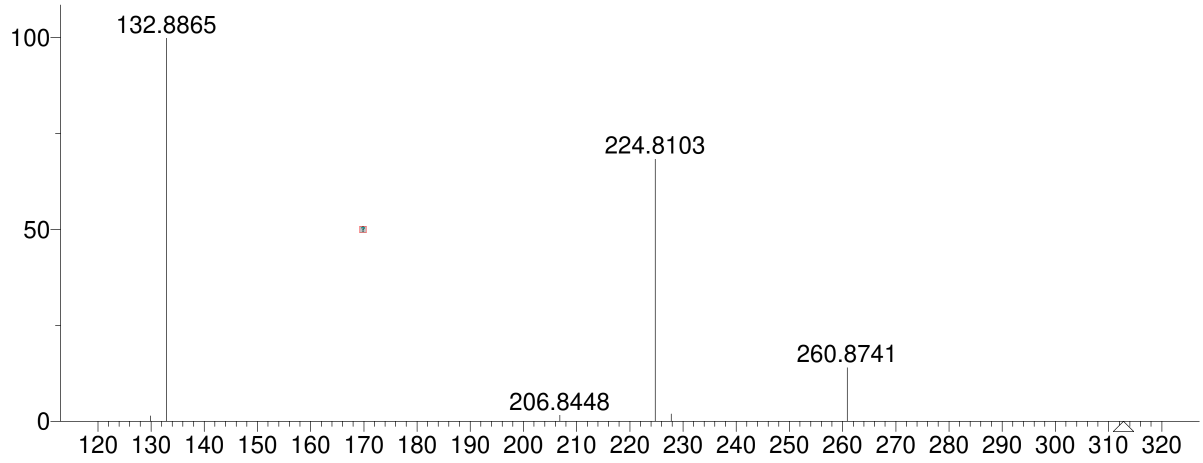

Unknown: Unknown (0.95_366.8805m/z)
Compound in Library Factor = 0

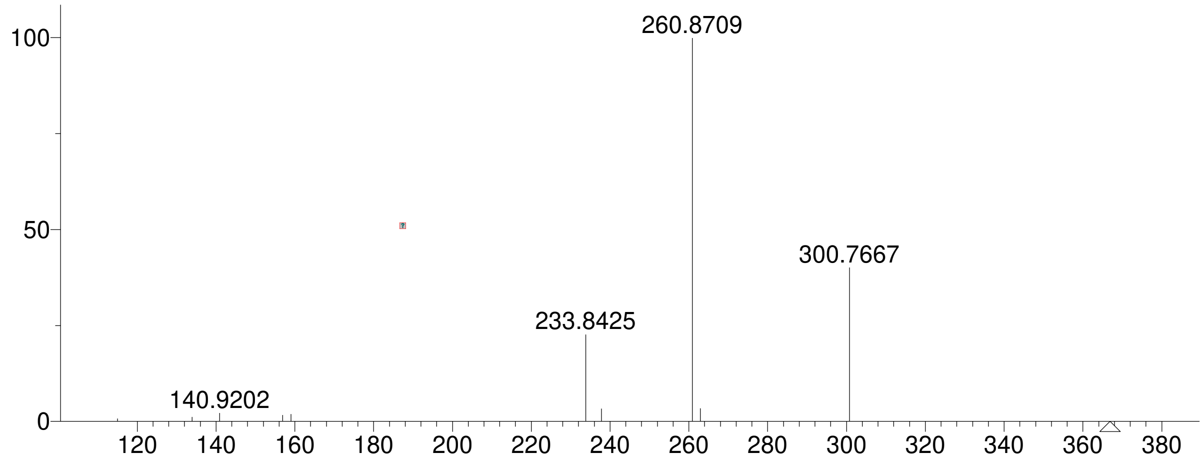

Unknown: Unknown (0.95_368.8744m/z)
Compound in Library Factor = 0

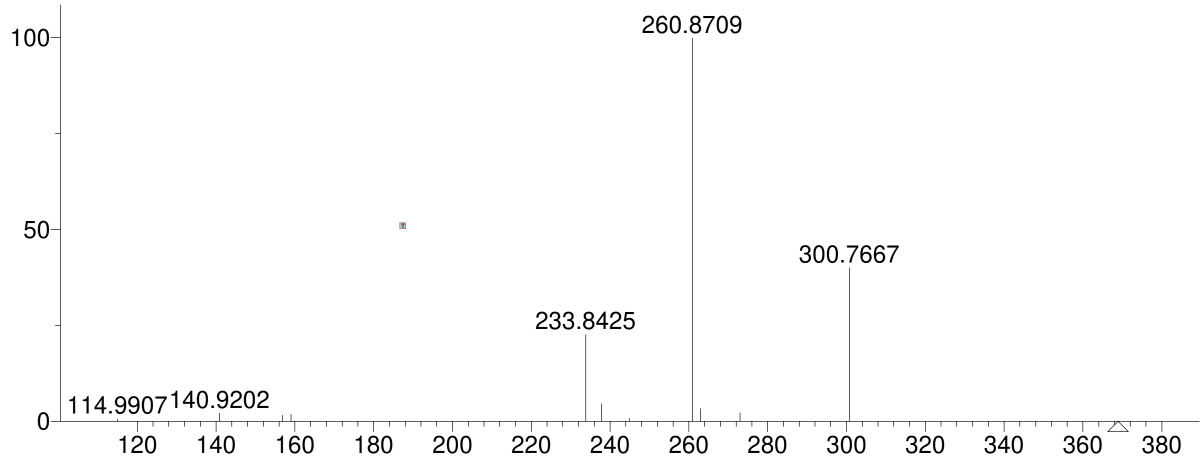

** Search Report Page 1 of 1 **

Unknown: Unknown (0.95_422.6849m/z)
Compound in Library Factor = 0

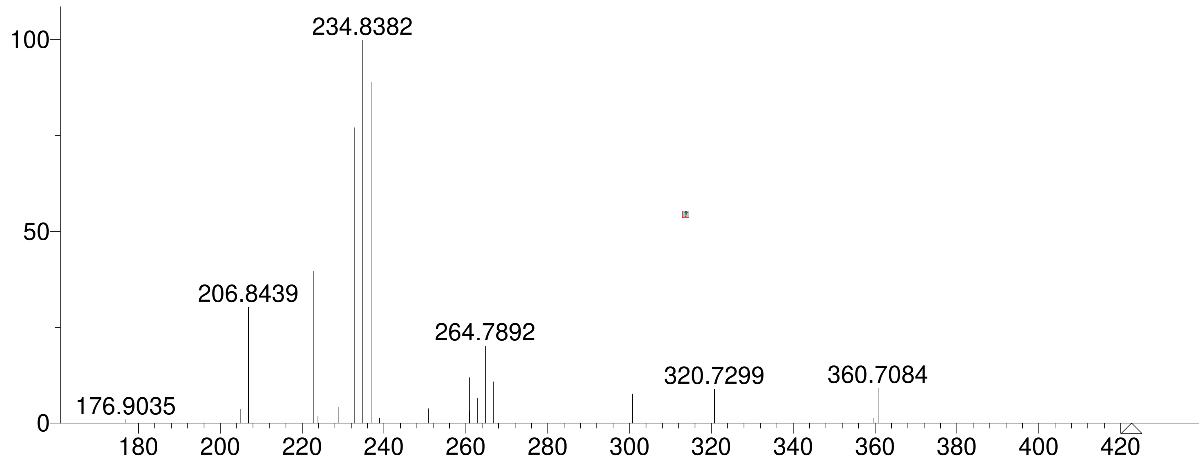

** Search Report Page 1 of 1 **

Unknown: Unknown (0.95_432.7146m/z)
Compound in Library Factor = 0

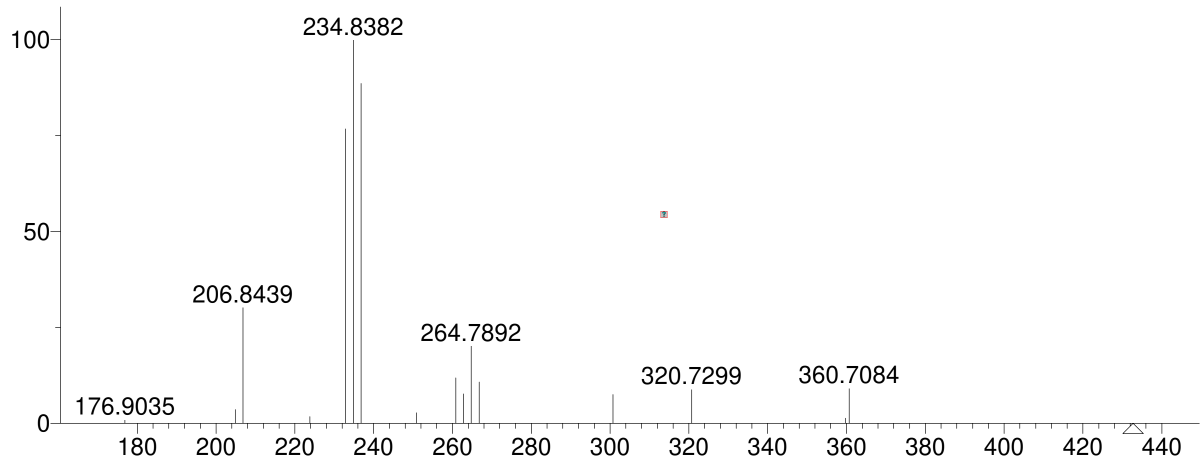

** Search Report Page 1 of 1 **

Unknown: Unknown (0.95_436.7099m/z)
Compound in Library Factor = 0

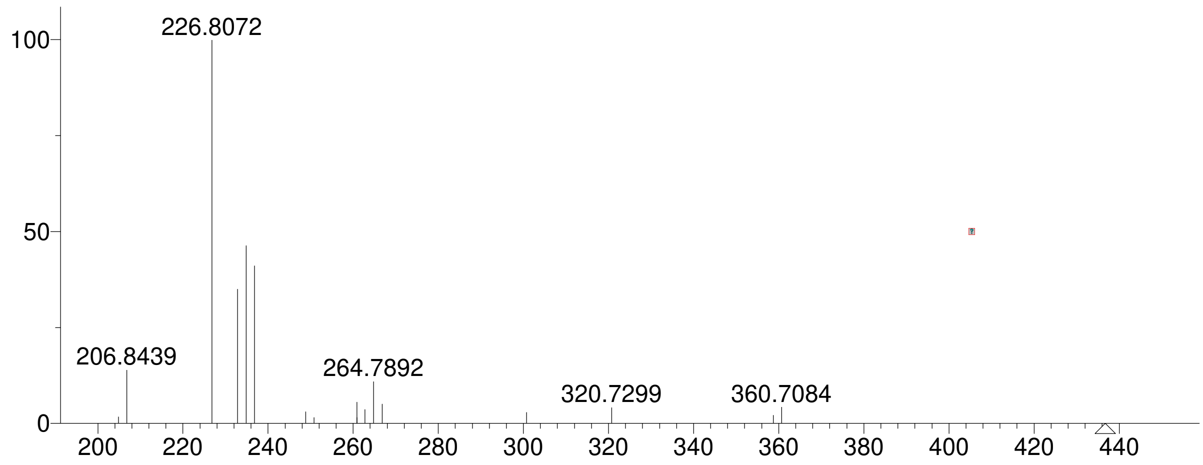

Unknown: Unknown (0.95_440.7449m/z)
Compound in Library Factor = 0

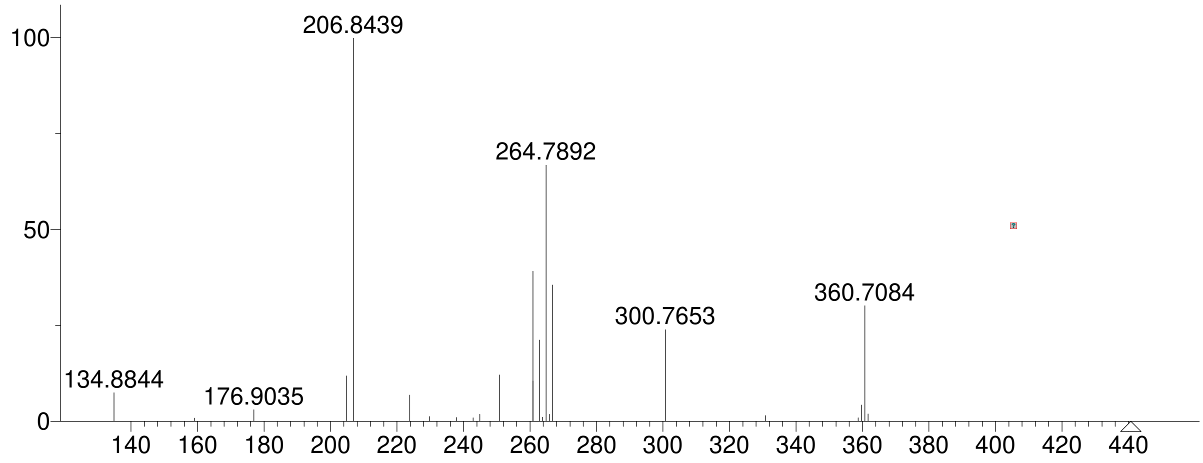

** Search Report Page 1 of 1 **

Unknown: Unknown (0.95_442.7419m/z)
Compound in Library Factor = 0

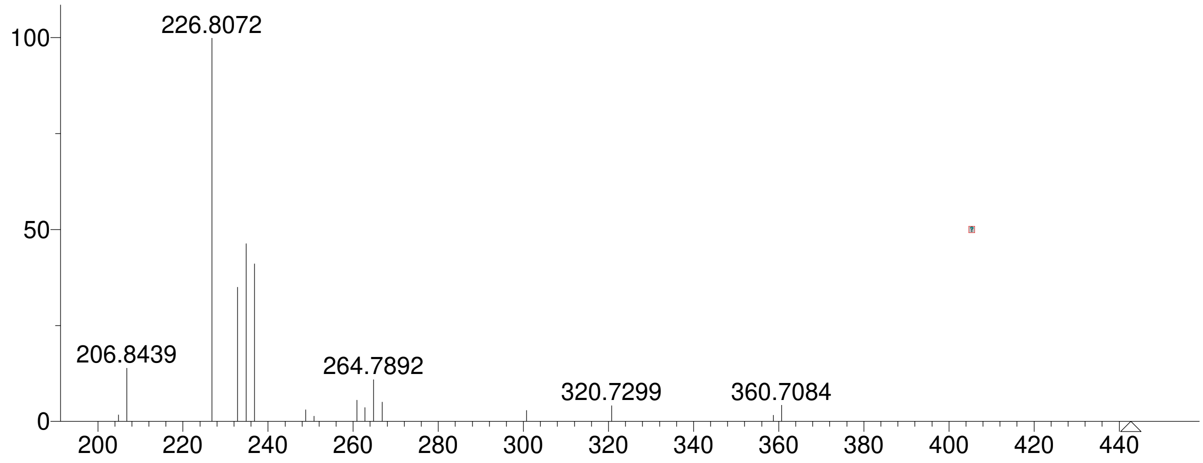

Unknown: Unknown (0.95_444.7400m/z)
Compound in Library Factor = 0

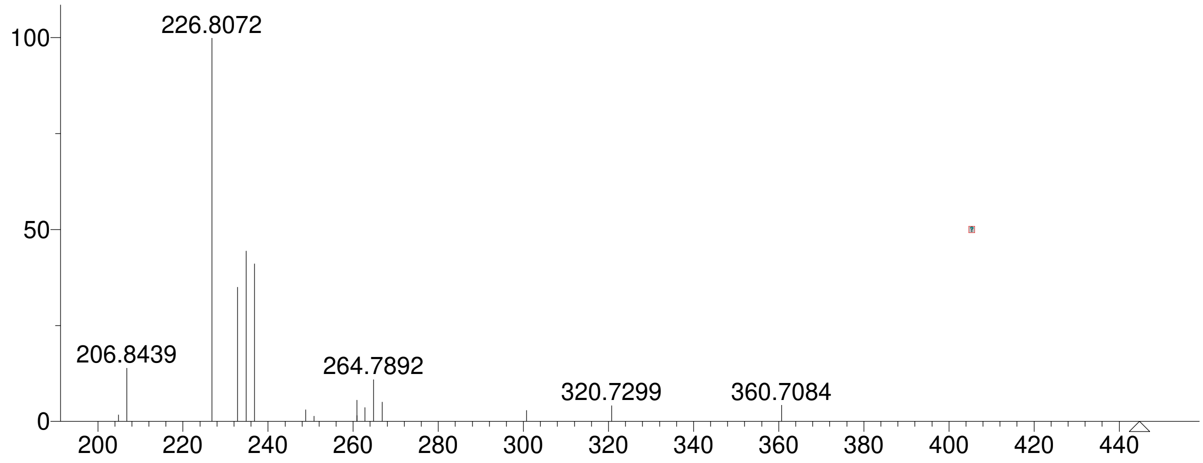

Unknown: Unknown (0.95_446.7360m/z)
Compound in Library Factor = 0

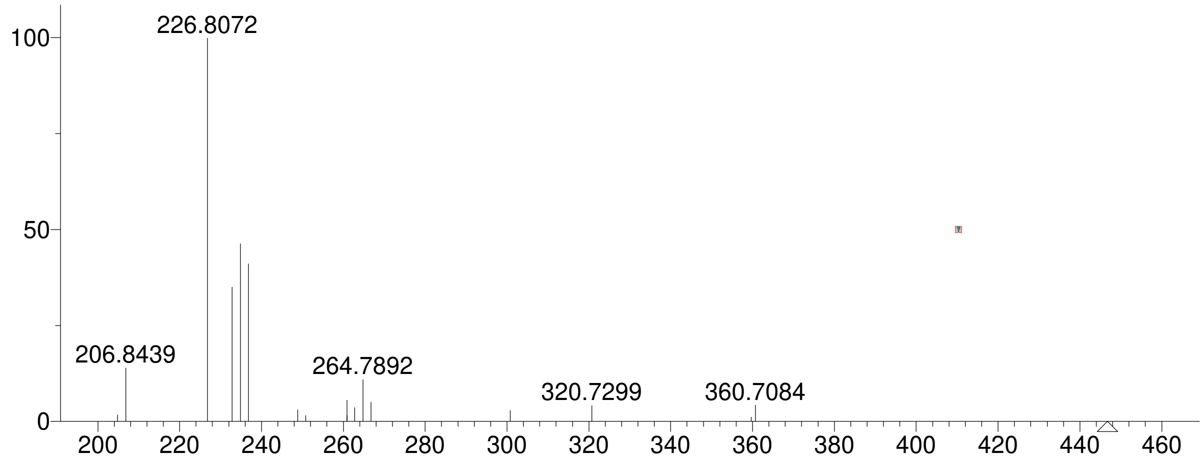

Unknown: Unknown (0.95_452.7717m/z)
Compound in Library Factor = 0

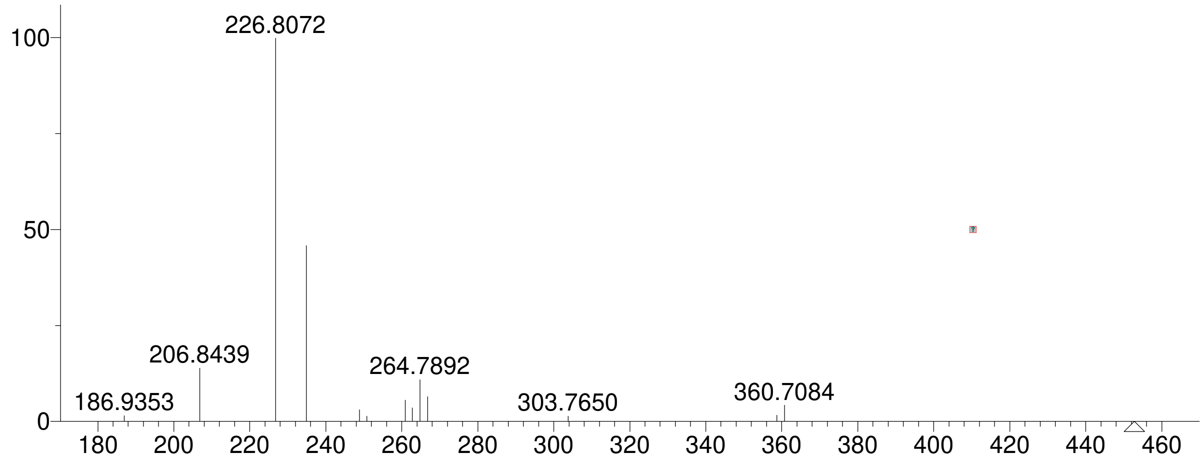

Unknown: Unknown (0.95_462.8012m/z)
Compound in Library Factor = 0

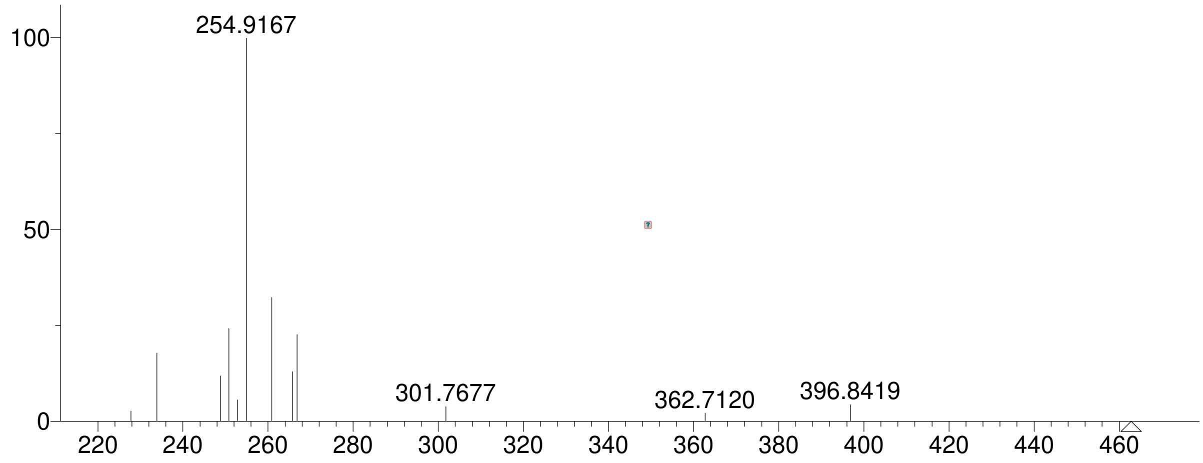

Hit 1 : D-myo-Inositol-1,3,4,5,6-pentaphosphate [M-H]-=>480.9=>462.8 IT 35% P=462.8
C6H17O21P5; MF: 0; RMF: 49; Prob 50.0%; CAS: 20298-95-7; Lib: nist_msms; ID: 191370.

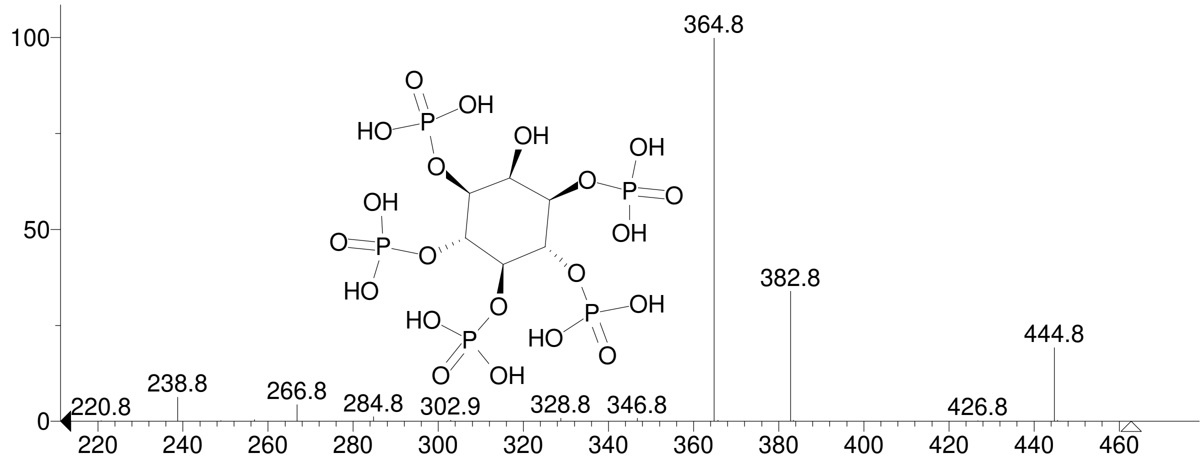

Hit 2 : D-myo-Inositol-1,2,4,5,6-pentaphosphate [M-H]-=>480.9=>462.8 IT 35% P=462.8
C6H17O21P5; MF: 0; RMF: 48; Prob 50.0%; CAS: 26326-86-3; Lib: nist_msms; ID: 192861.

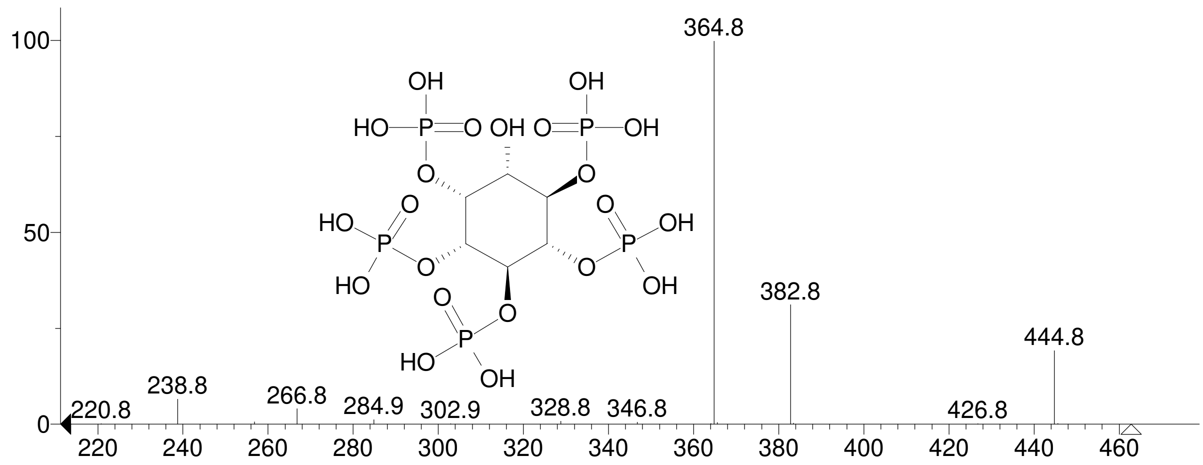

** Search Report Page 1 of 1 **

Unknown: Unknown (0.96_222.8133m/z)
Compound in Library Factor = 0

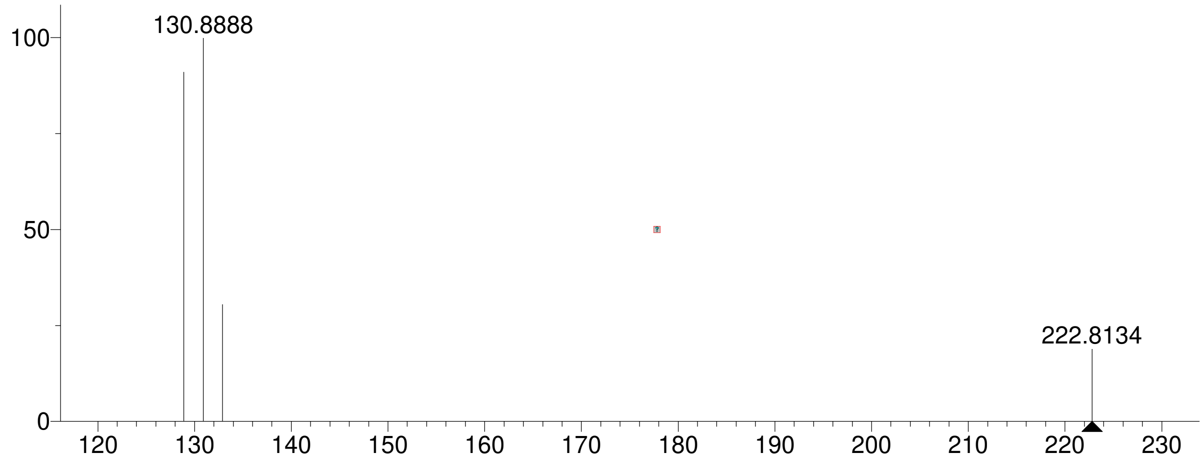

Unknown: Unknown (0.96_232.8426m/z)
Compound in Library Factor = 0

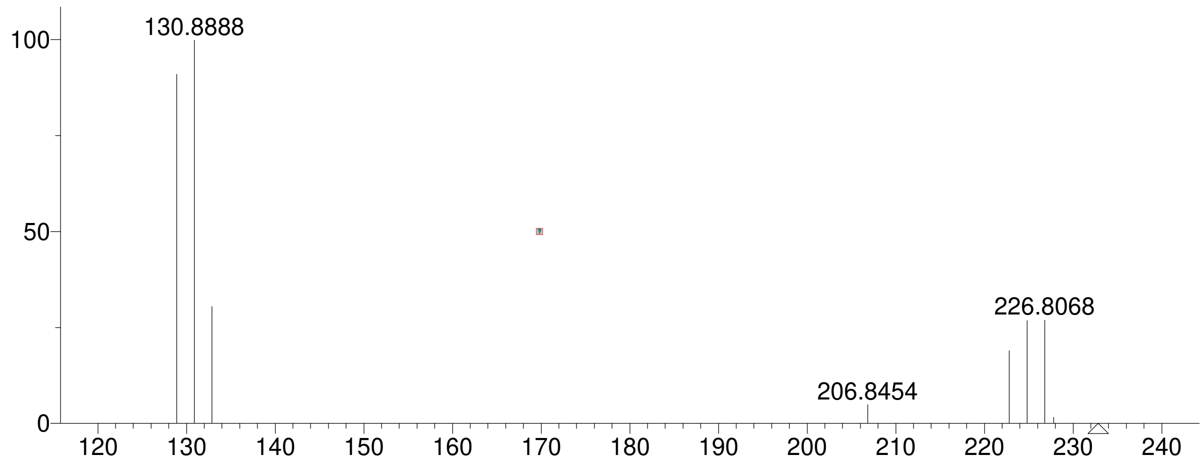

Unknown: Unknown (0.96_234.8398m/z)
Compound in Library Factor = 0

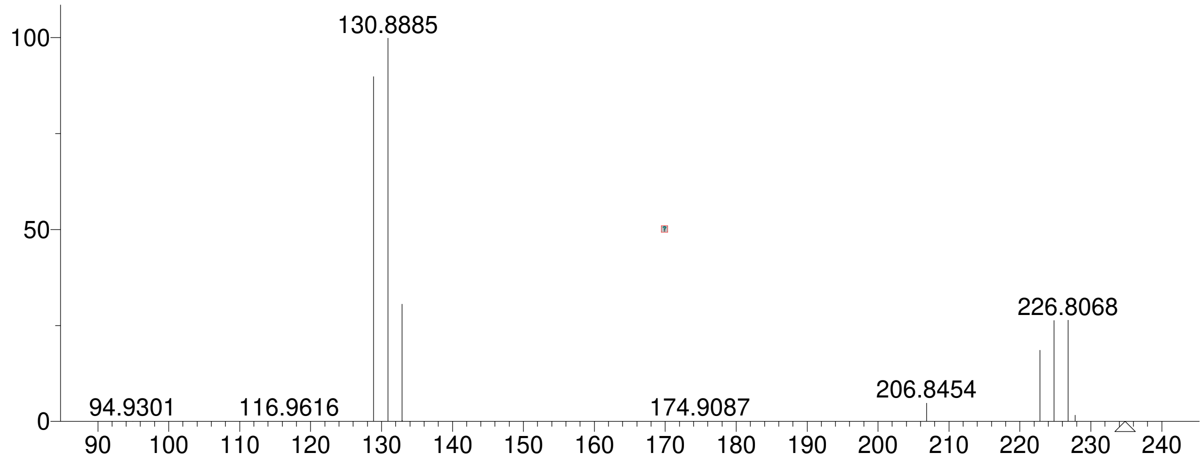

** Search Report Page 1 of 1 **

Unknown: Unknown (0.96_236.8370m/z)
Compound in Library Factor = 0

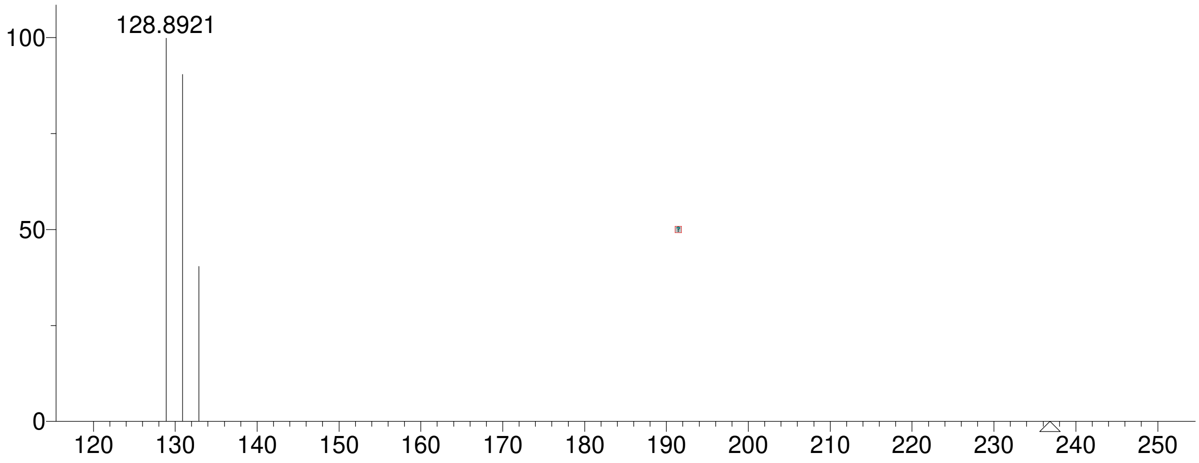

** Search Report Page 1 of 1 **

Unknown: Unknown (0.96_238.8339m/z)
Compound in Library Factor = 0

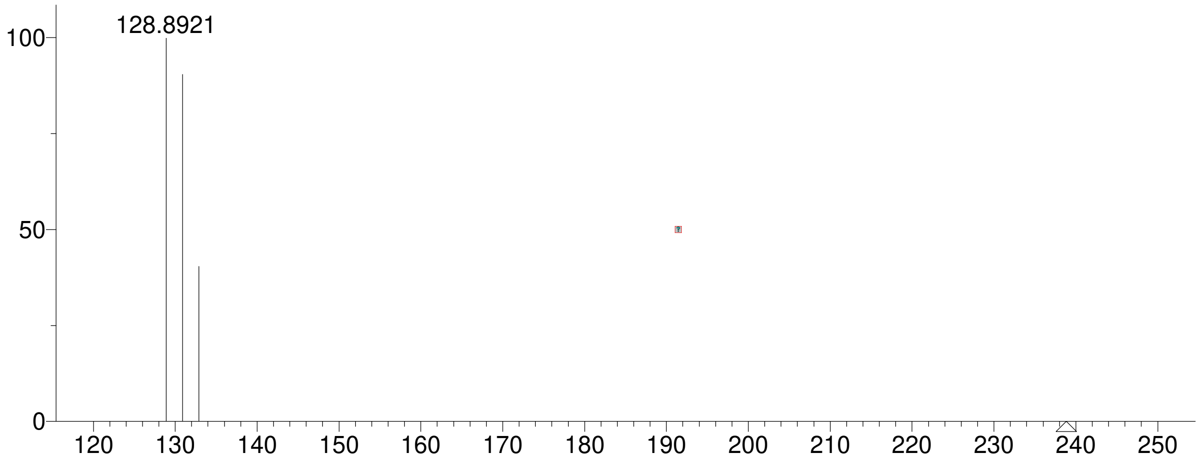

Unknown: Unknown (0.96_298.7686m/z)
Compound in Library Factor = 0

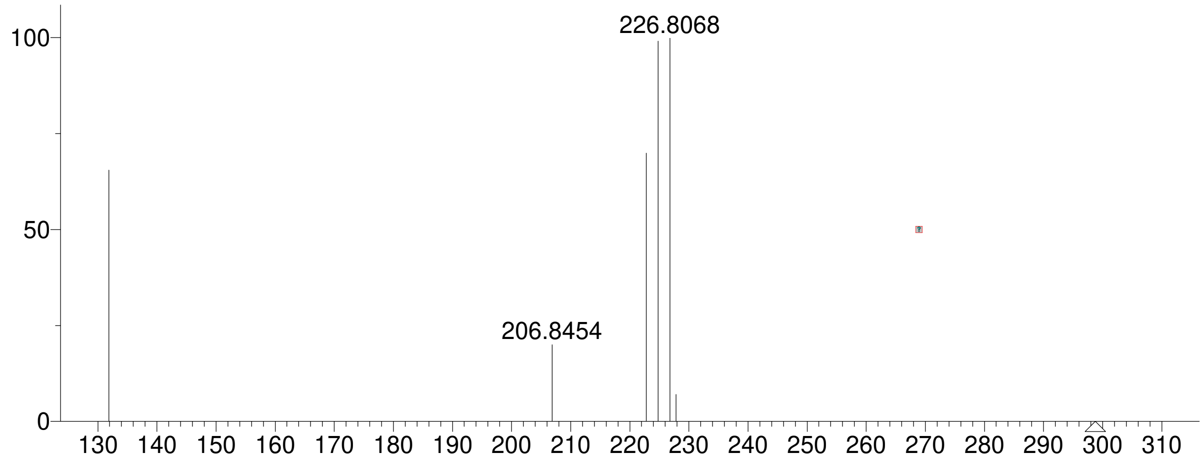

Unknown: Unknown (0.96_300.7672m/z)
Compound in Library Factor = 0

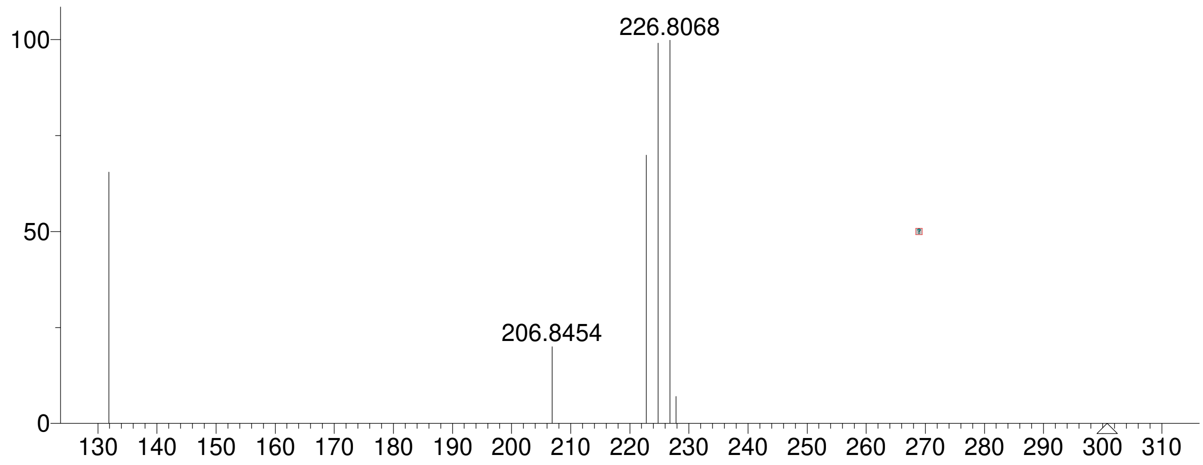

** Search Report Page 1 of 1 **

Unknown: Unknown (0.96_302.7646m/z)
Compound in Library Factor = 0

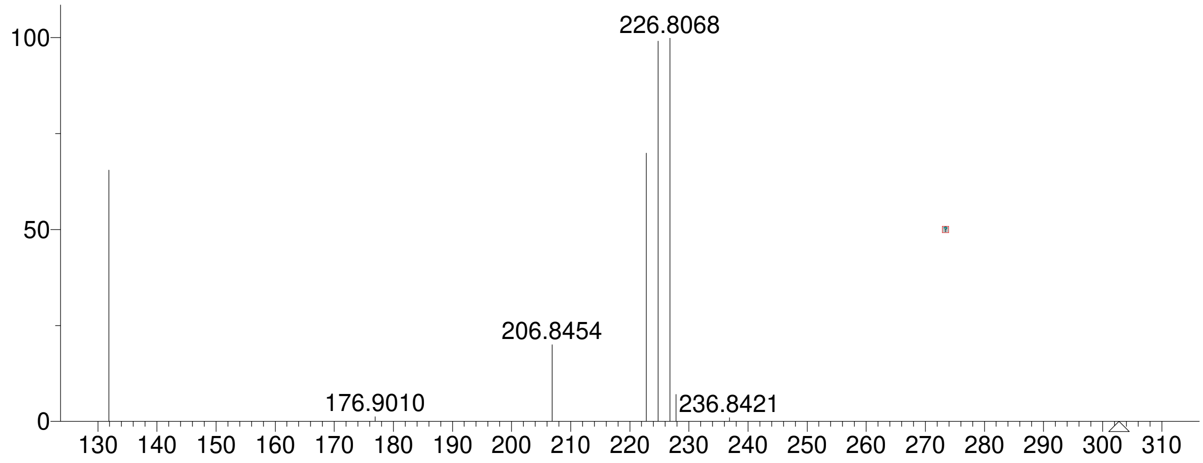

** Search Report Page 1 of 1 **

Unknown: Unknown (0.96_304.7606m/z)
Compound in Library Factor = 0

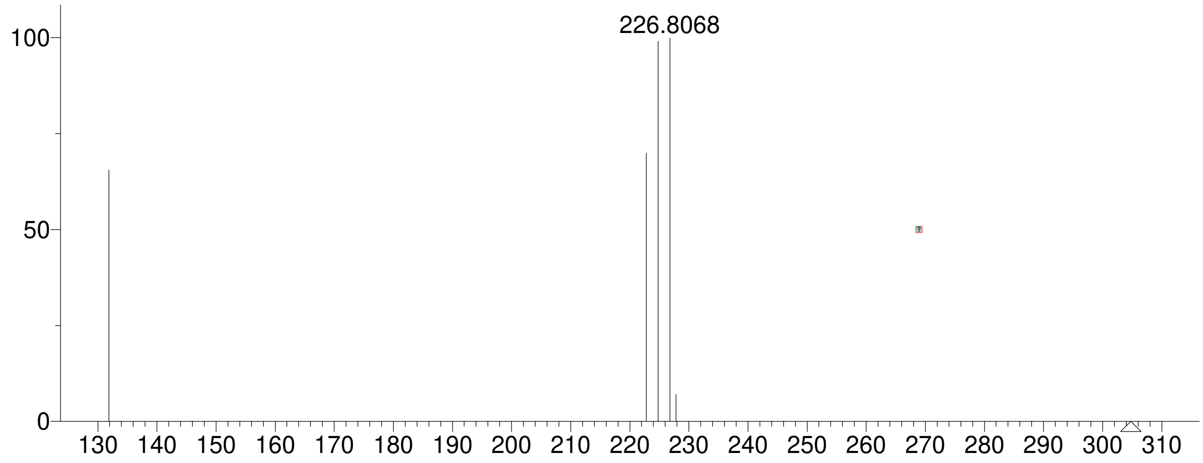

Unknown: Unknown (0.96_310.7953m/z)
Compound in Library Factor = 0

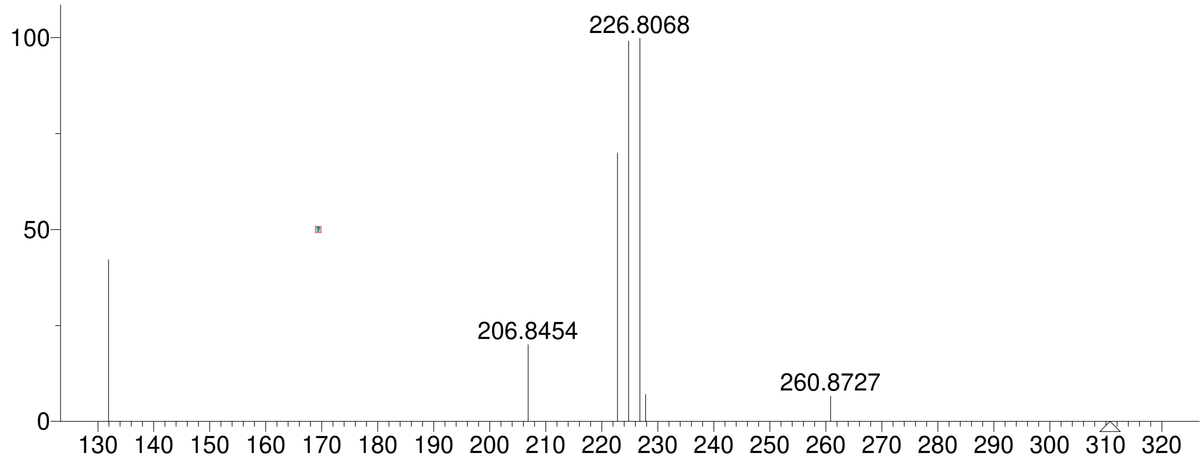

Unknown: Unknown (0.96_316.7359m/z)
Compound in Library Factor = 0

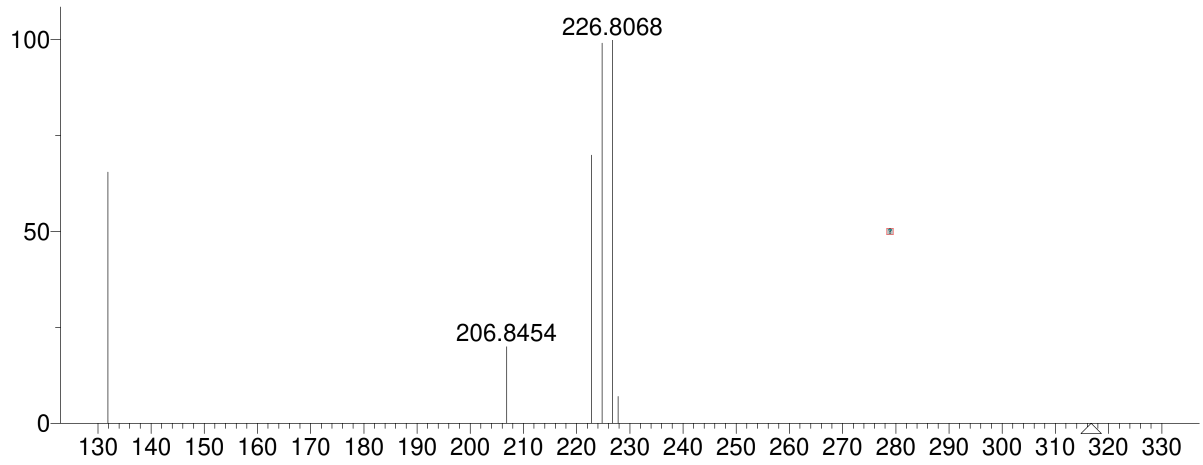

** Search Report Page 1 of 1 **

Unknown: Unknown (0.96_318.7335m/z)
Compound in Library Factor = 0

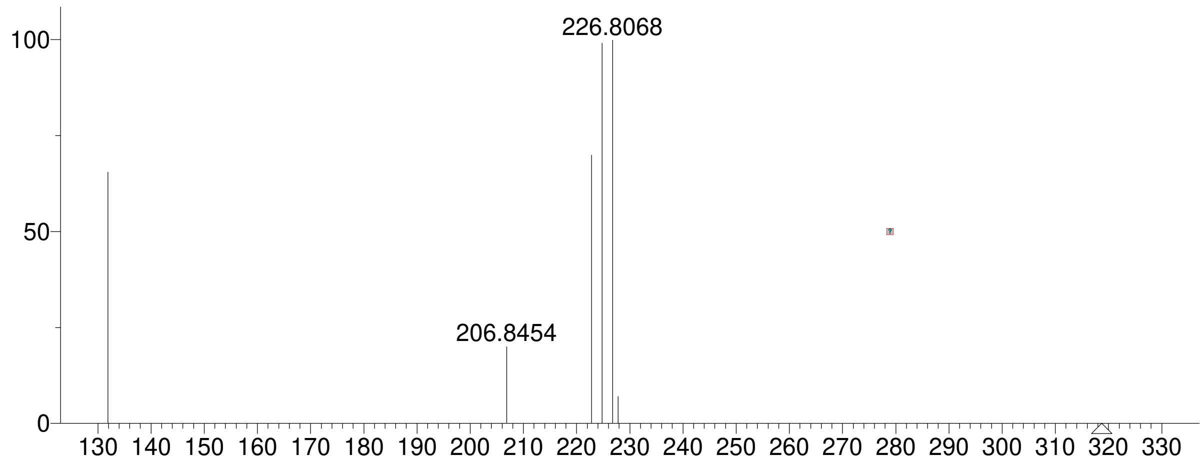

Unknown: Unknown (0.96_320.7309m/z)
Compound in Library Factor = 0

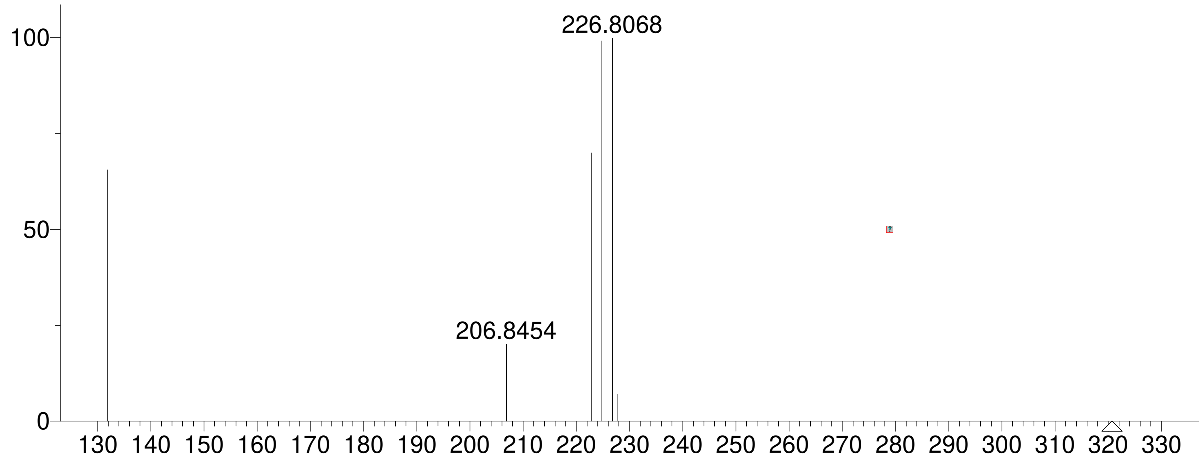

Unknown: Unknown (0.96_322.7282m/z)
Compound in Library Factor = 0

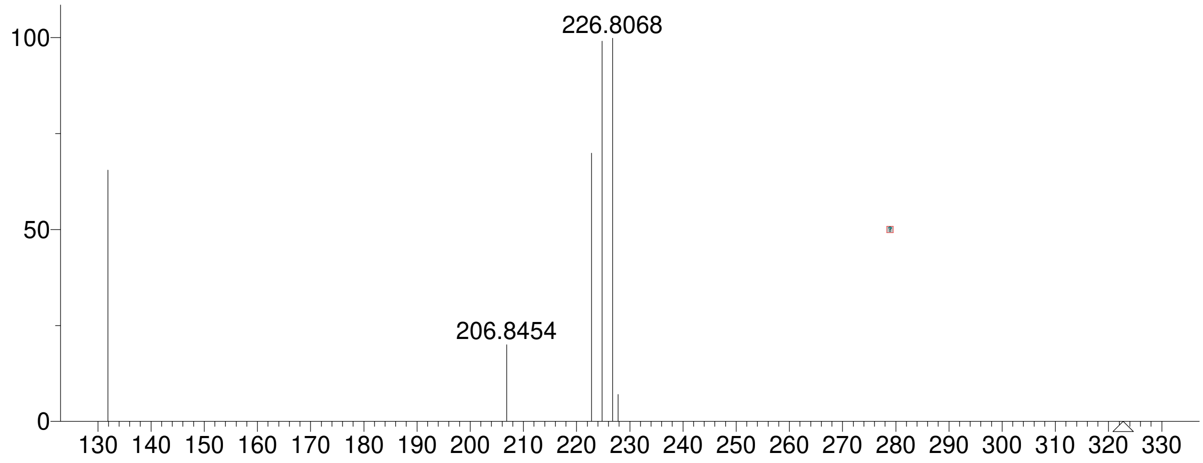

Unknown: Unknown (0.96_324.7246m/z)
Compound in Library Factor = 0

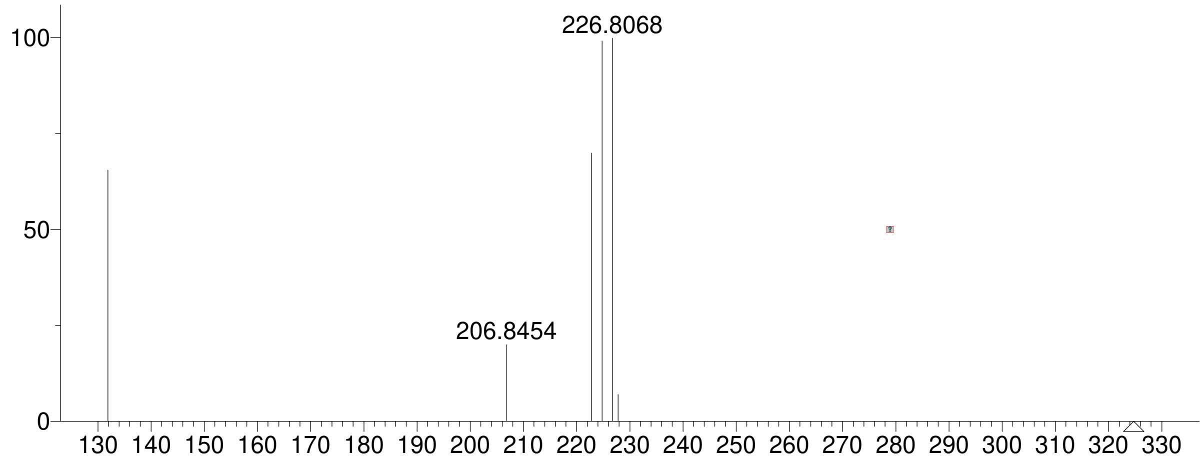

** Search Report Page 1 of 1 **

Unknown: Unknown (0.96_328.7616m/z)
Compound in Library Factor = 0

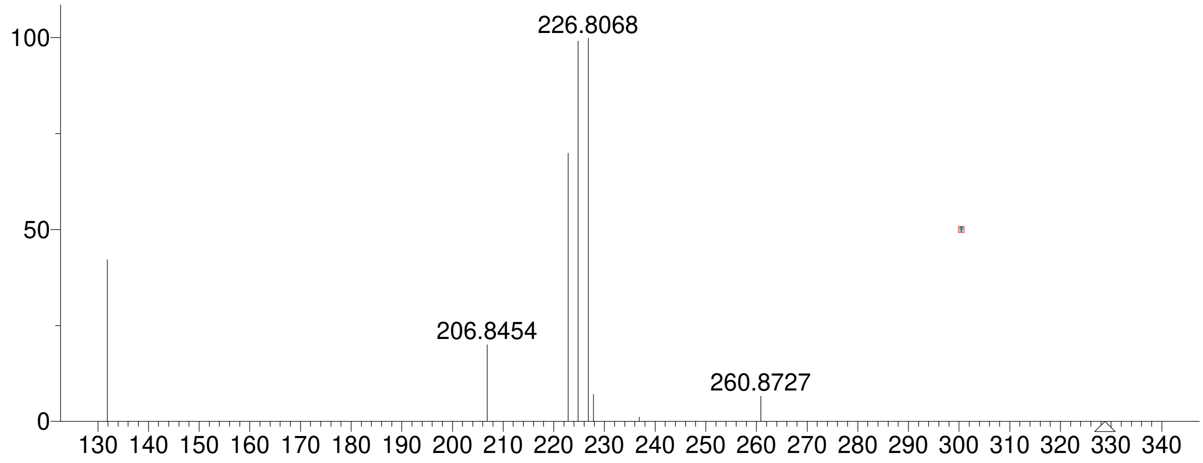

Unknown: Unknown (0.96_330.7581m/z)
Compound in Library Factor = 0

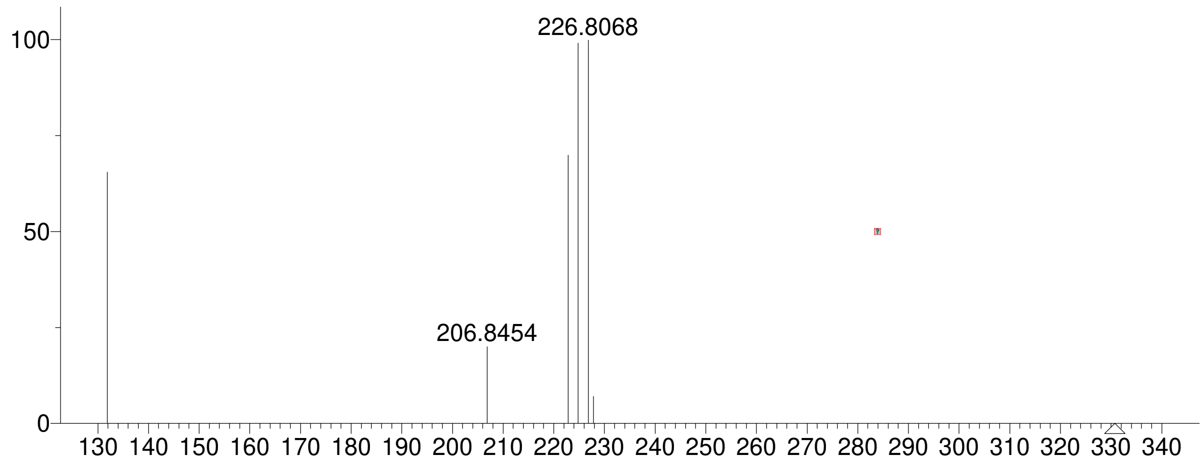

** Search Report Page 1 of 1 **

Unknown: Unknown (0.96_332.7563m/z)
Compound in Library Factor = 0

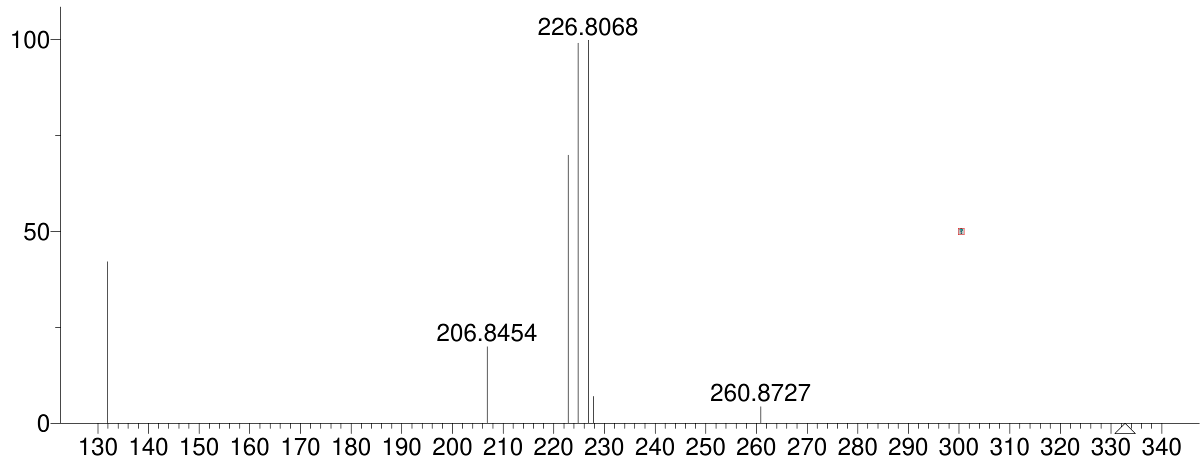

** Search Report Page 1 of 1 **

Unknown: Unknown (0.96_338.7903m/z)
Compound in Library Factor = 0

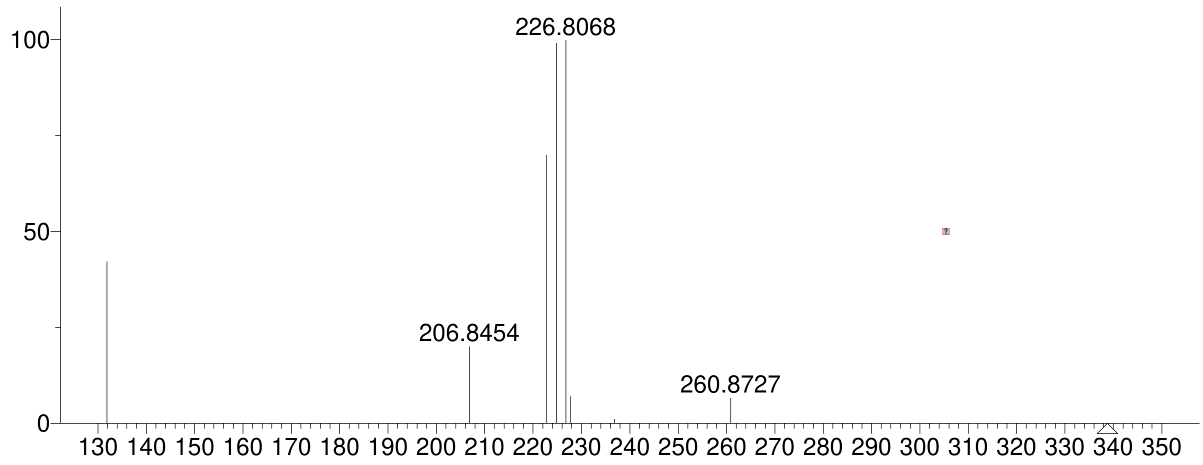

Unknown: Unknown (0.96_406.7143m/z)
Compound in Library Factor = 0

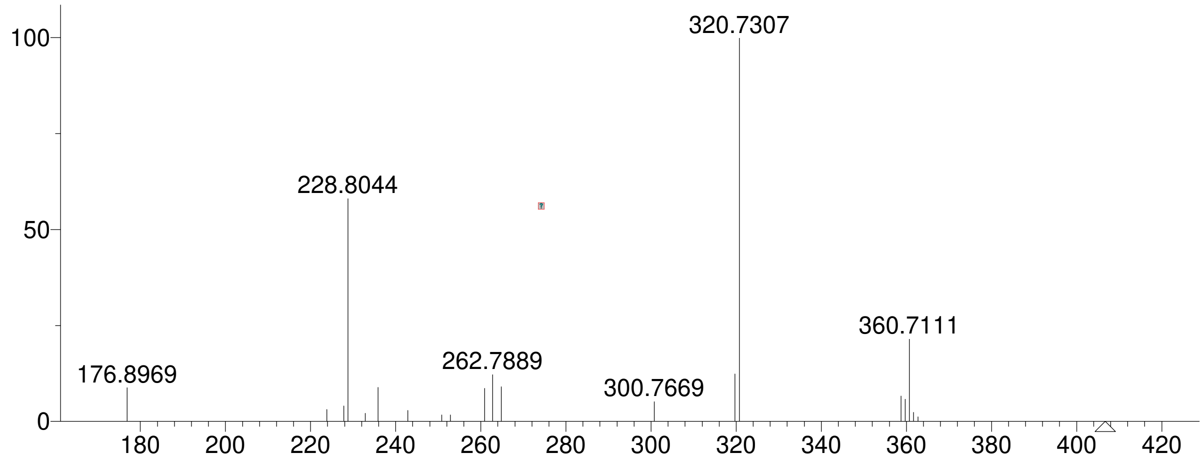

Hit 1 : Bombesin (8-14) [M+2H]2+ QQQ 12V P=406.7
C38H57N11O7S; MF: 0; RMF: 2; Prob 50.0%; CAS: 55749-98-9; Lib: nist_msms2; ID: 21234.

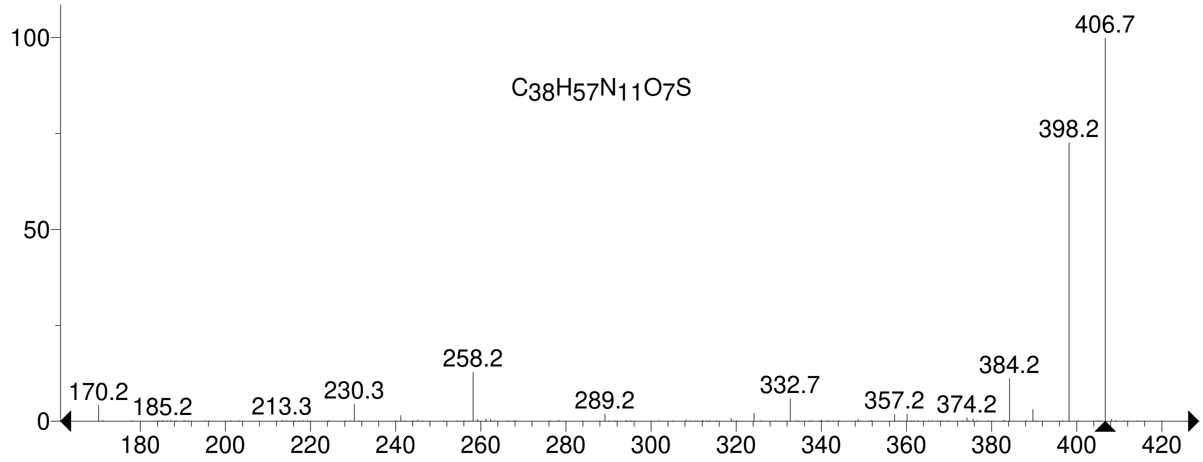

Hit 2 : GTP-Binding Protein Fragment, G alpha [M+4H]4+ QQQ 13V P=406.7
C66H118N20O23S2; MF: 0; RMF: 1; Prob 50.0%; Lib: nist_msms2; ID: 3203.

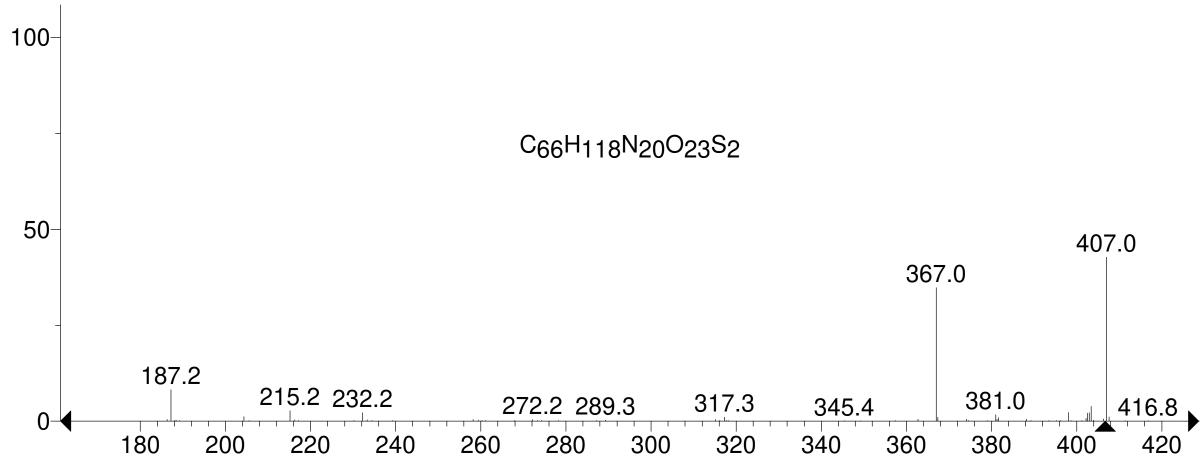

Unknown: Unknown (0.96_424.6829m/z)
Compound in Library Factor = 0

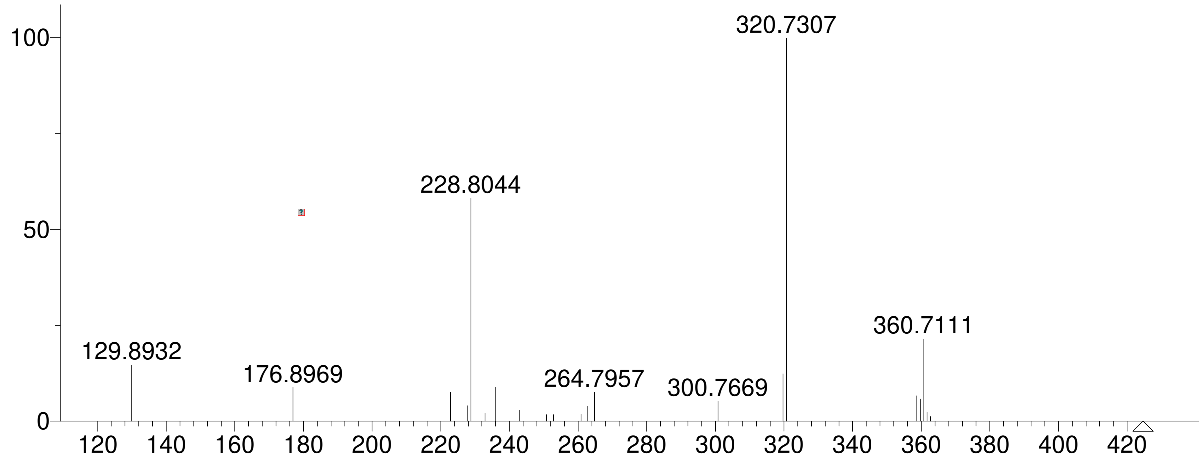

** Search Report Page 1 of 1 **

Unknown: Unknown (0.96_426.6807m/z)
Compound in Library Factor = 0

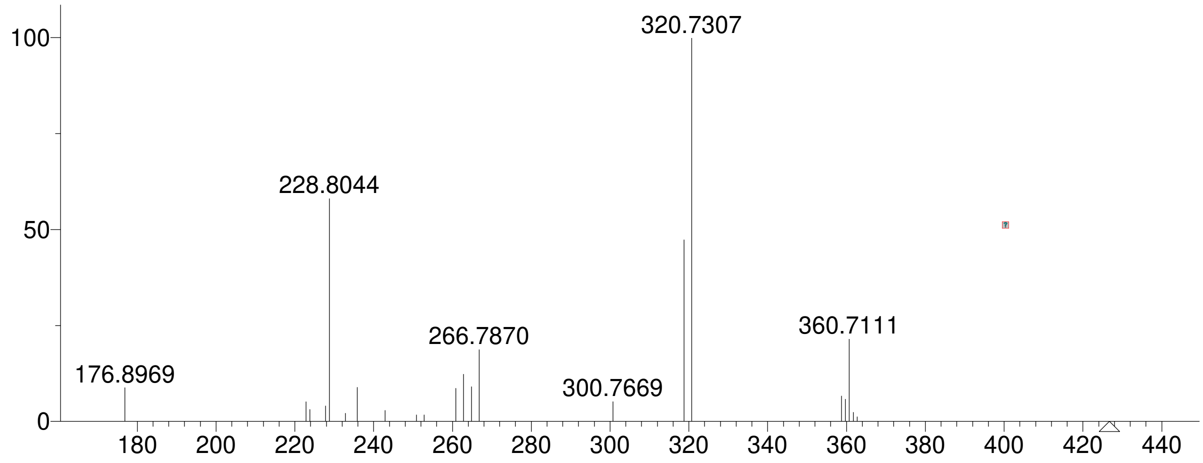

Unknown: Unknown (0.96_434.7099m/z)
Compound in Library Factor = 0

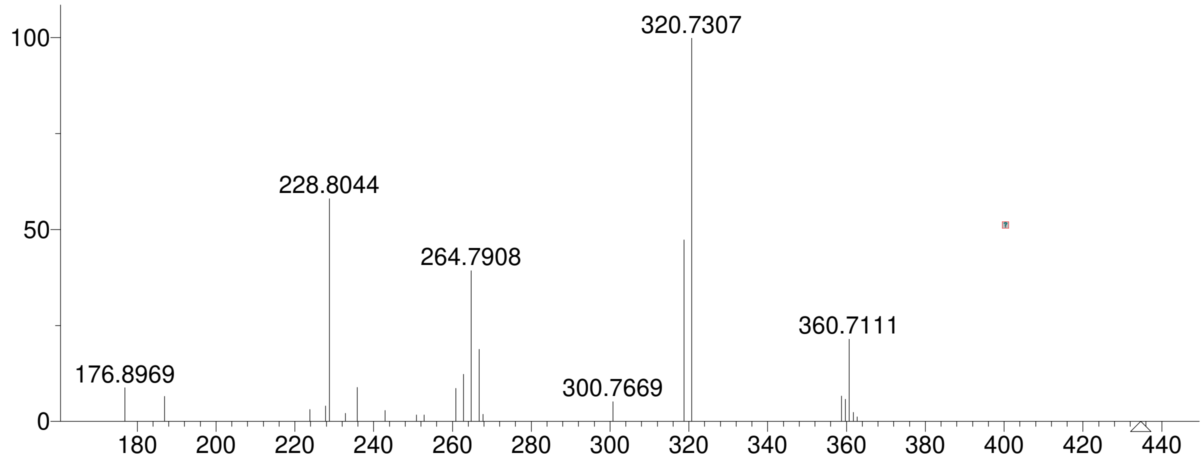

Unknown: Unknown (0.97_128.8915m/z)
Compound in Library Factor = 0

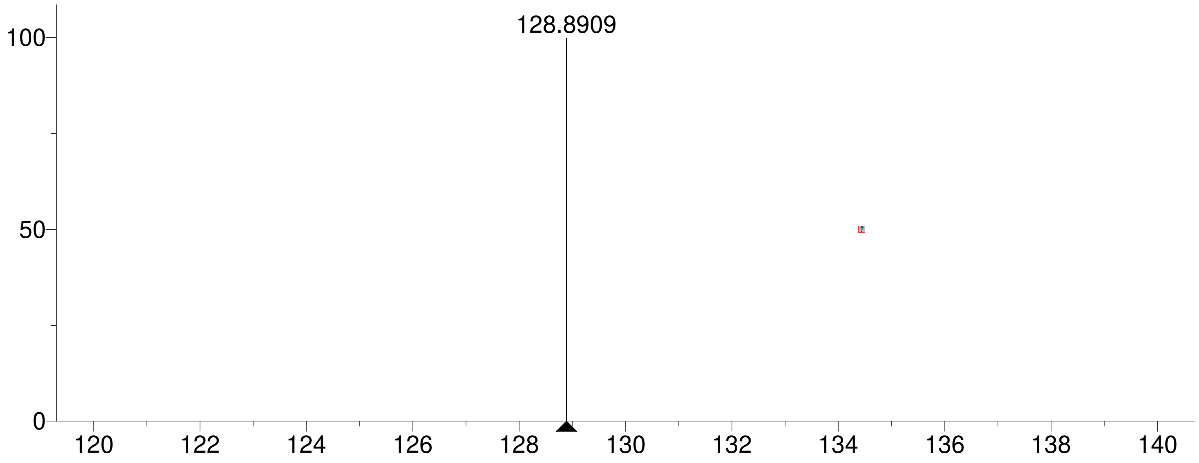

Unknown: Unknown (0.97_130.8886m/z)
Compound in Library Factor = 0

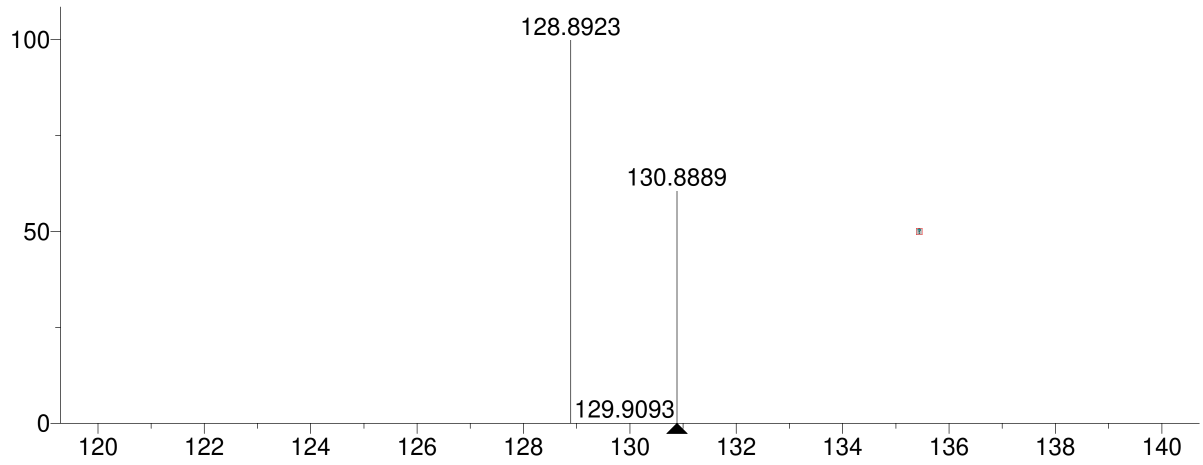

** Search Report Page 1 of 1 **

Unknown: Unknown (0.97_132.8857m/z)
Compound in Library Factor = 0

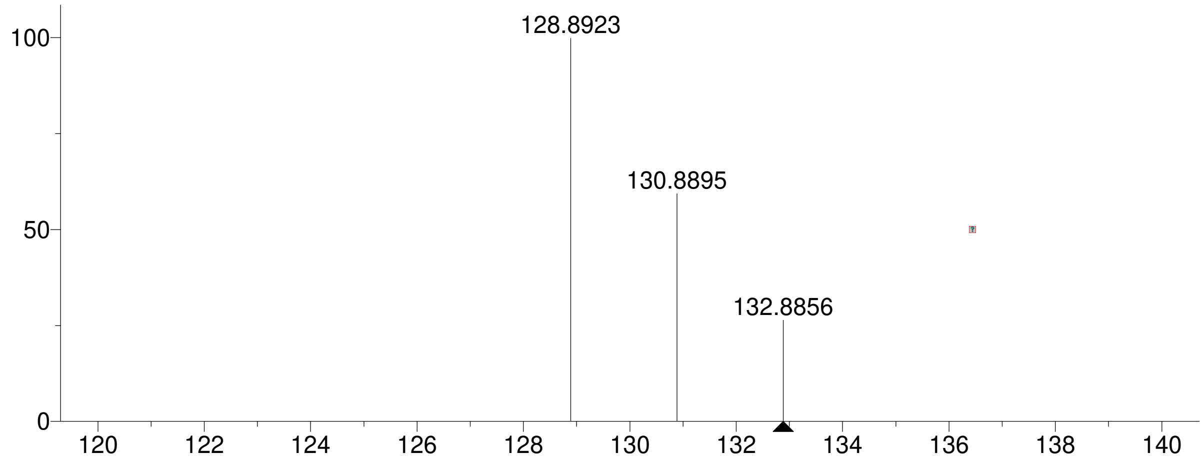

** Search Report Page 1 of 1 **

Unknown: Unknown (0.97_224.8108m/z)
Compound in Library Factor = 0

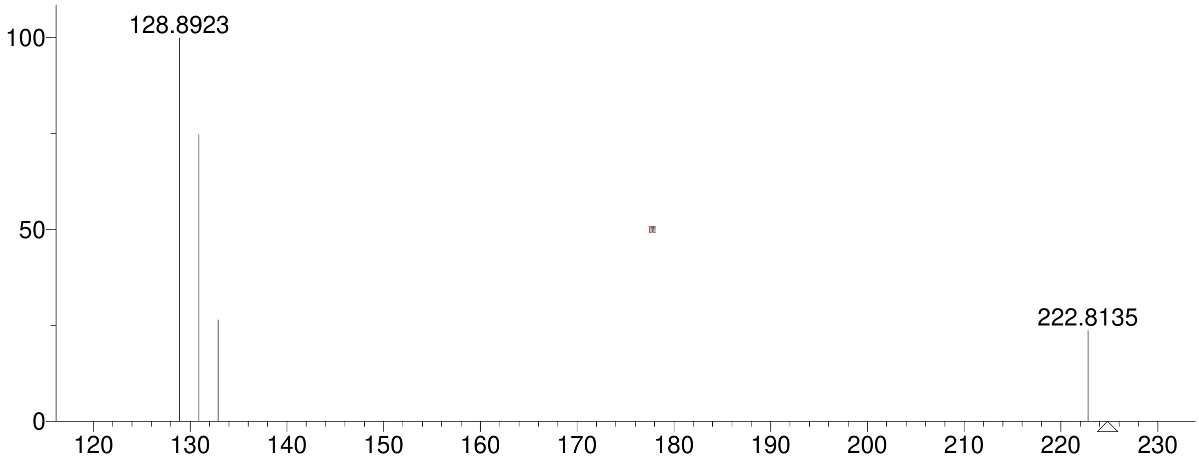

** Search Report Page 1 of 1 **

Unknown: Unknown (0.97_226.8079m/z)
Compound in Library Factor = 0

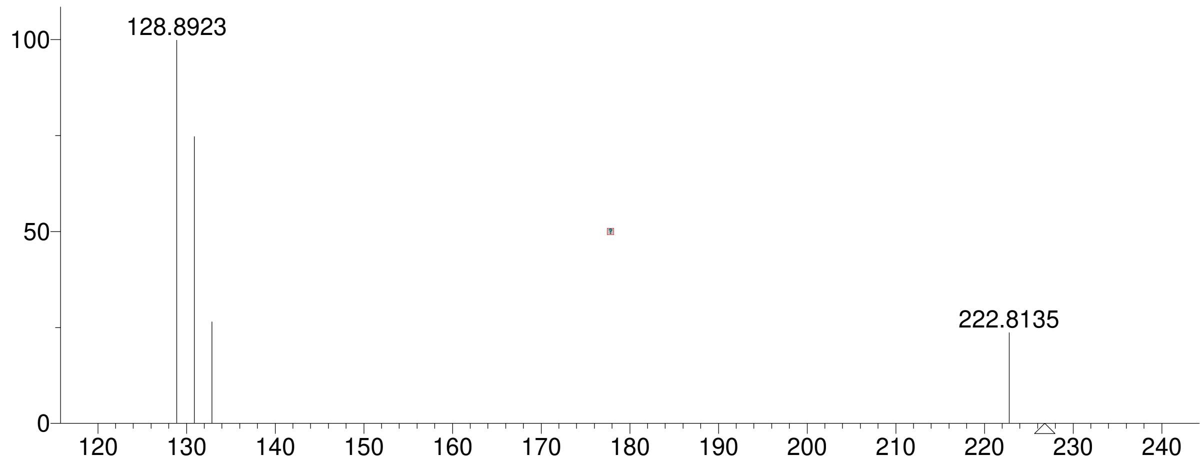

Unknown: Unknown (0.97_228.8049m/z)
Compound in Library Factor = 0

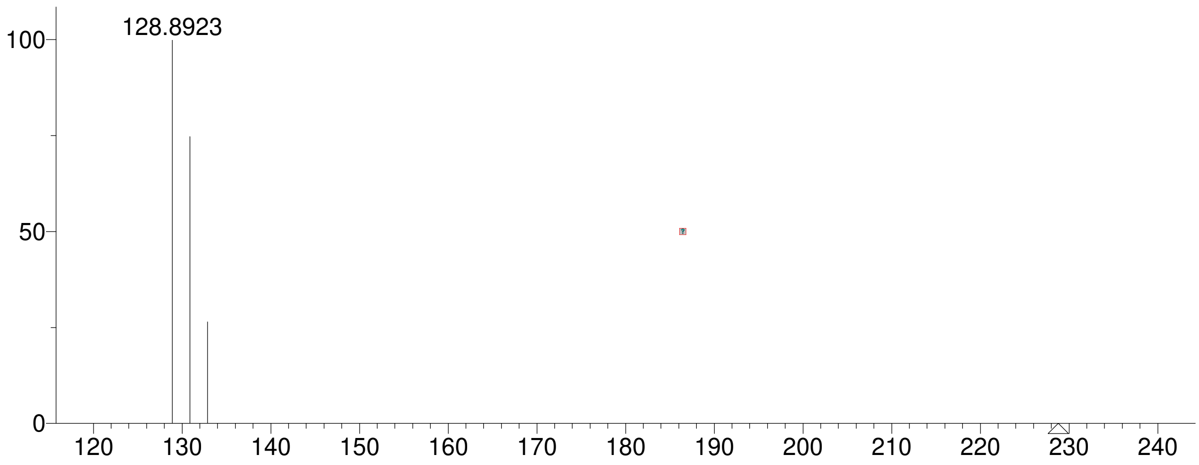

Unknown: Unknown (0.97_282.7680m/z)
Compound in Library Factor = 0

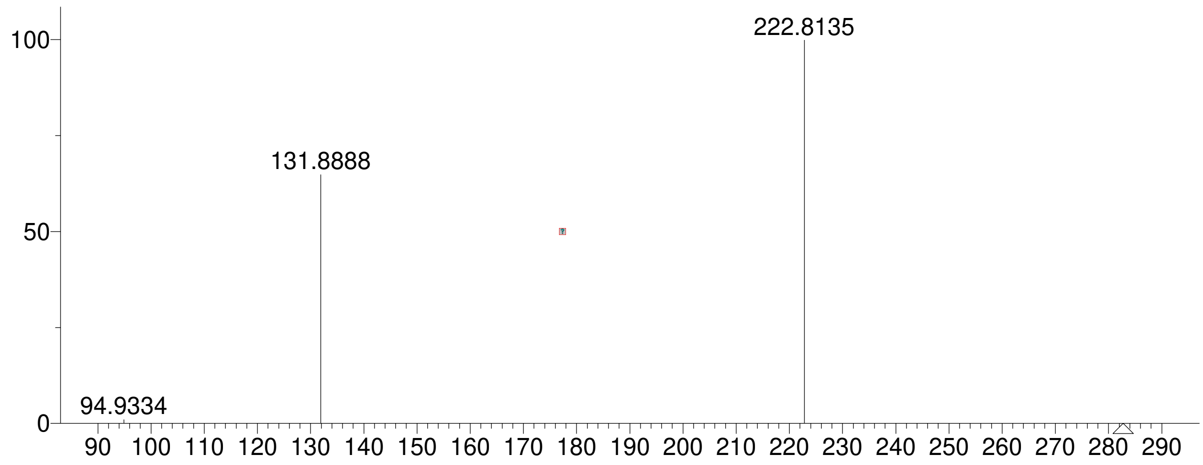

Unknown: Unknown (0.97_284.7658m/z)
Compound in Library Factor = 0

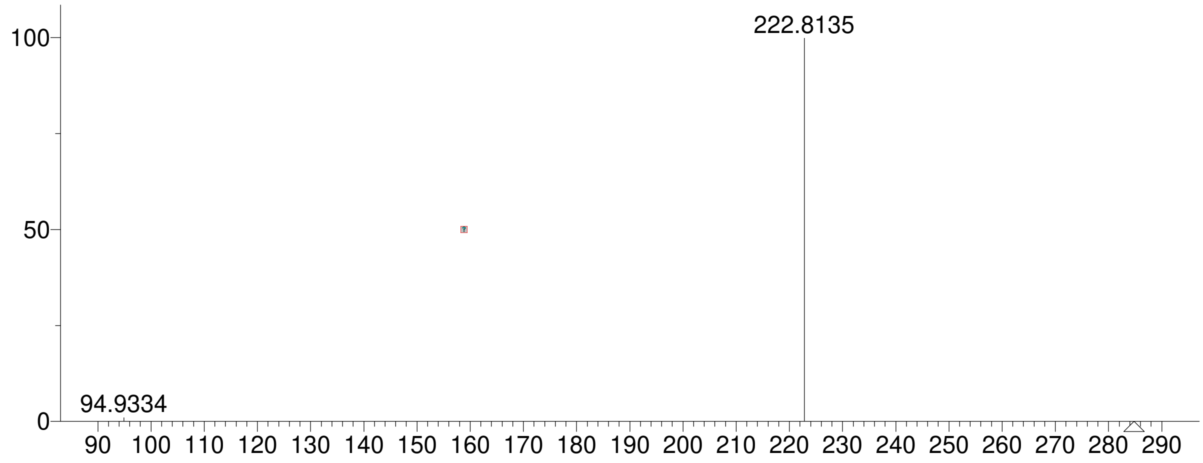

** Search Report Page 1 of 1 **

Unknown: Unknown (1.01_355.0870m/z)
Compound in Library Factor = 0

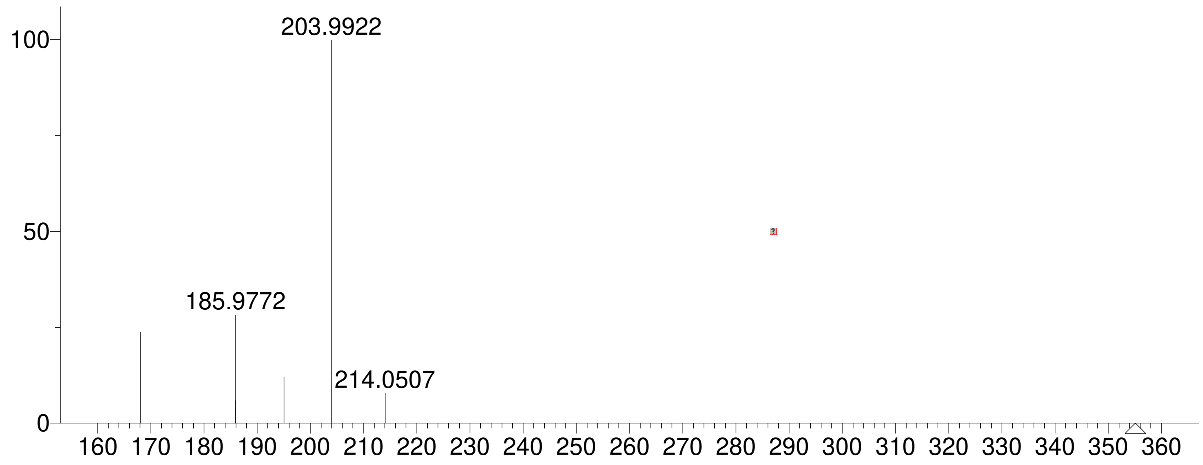

Unknown: Unknown (1.01_357.1016m/z)
Compound in Library Factor = -1309

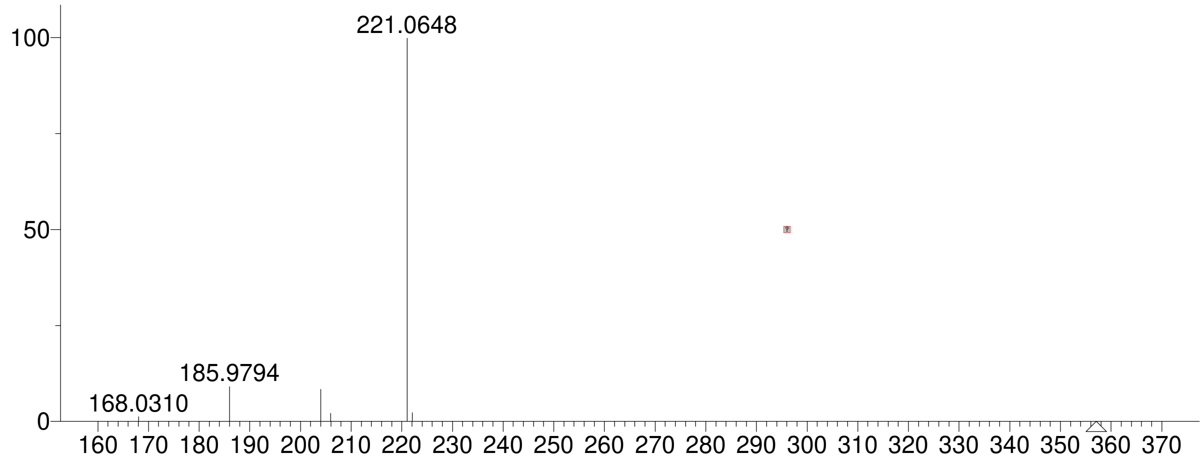

Hit 1 : Lactobionic acid [M-H]- HCD 46V P=357.1
C12H22O12; MF: 61; RMF: 582; Prob 46.3%; CAS: 96-82-2; Lib: nist_msms; ID: 407492.

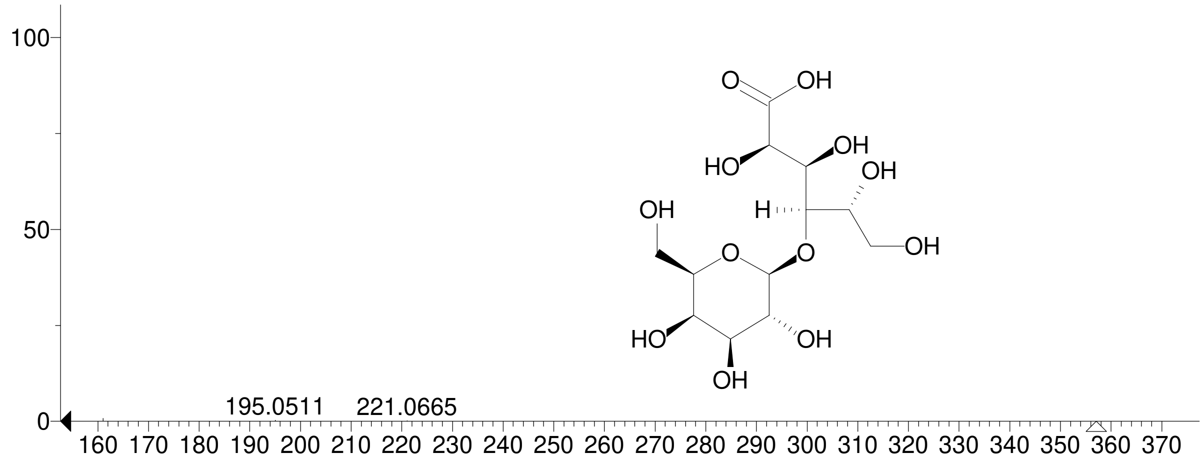

Hit 2 : N-(sec-Butyl)-N-(4-(sec-butyl(trifluoroacetyl)amino)phenyl-2,2,2-trifluoroacetamide [M+H-C4H8]+ HCD 9[illegible]
C18H22F6N2O2; MF: 3; RMF: 82; Prob 8.52%; CAS: 883829-91-2; Lib: nist_msms; ID: 199683.

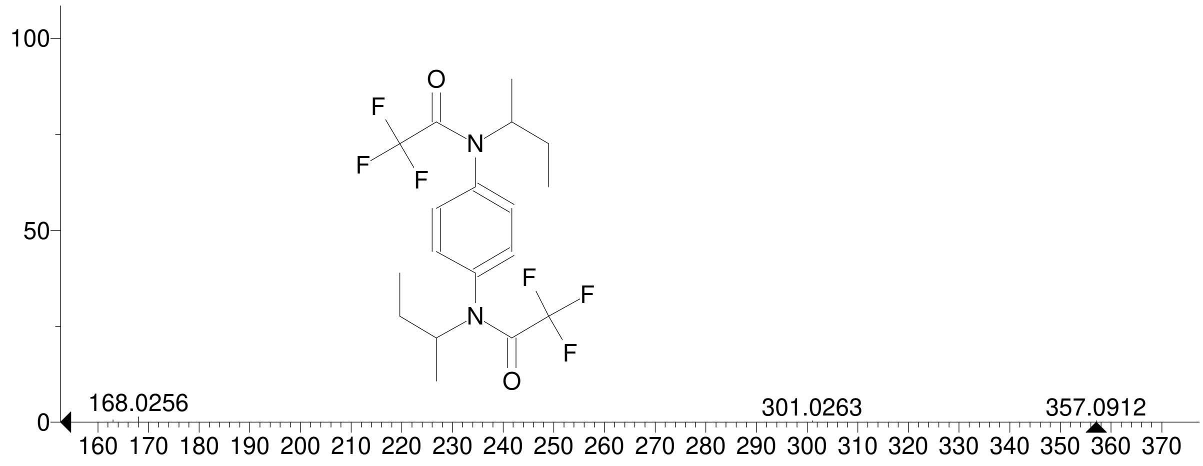

Hit 3 : 3'-α-Sialyl-N-acetyllactosamine [M+H+K]2+ HCD 19V P=357.1
C25H42N2O19; MF: 0; RMF: 31; Prob 7.53%; CAS: 81693-22-3; Lib: nist_msms; ID: 182060.

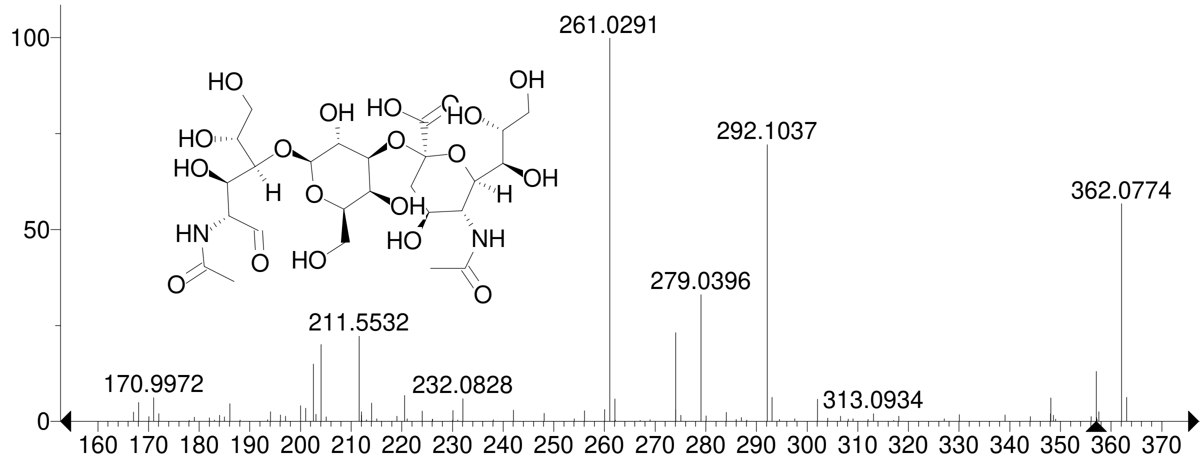

Hit 4 : Trandolapril [M+H]+=>357.1 IT 35% P=357.1
C24H34N2O5; MF: 0; RMF: 27; Prob 7.53%; CAS: 87679-37-6; Lib: nist_msms; ID: 116282.

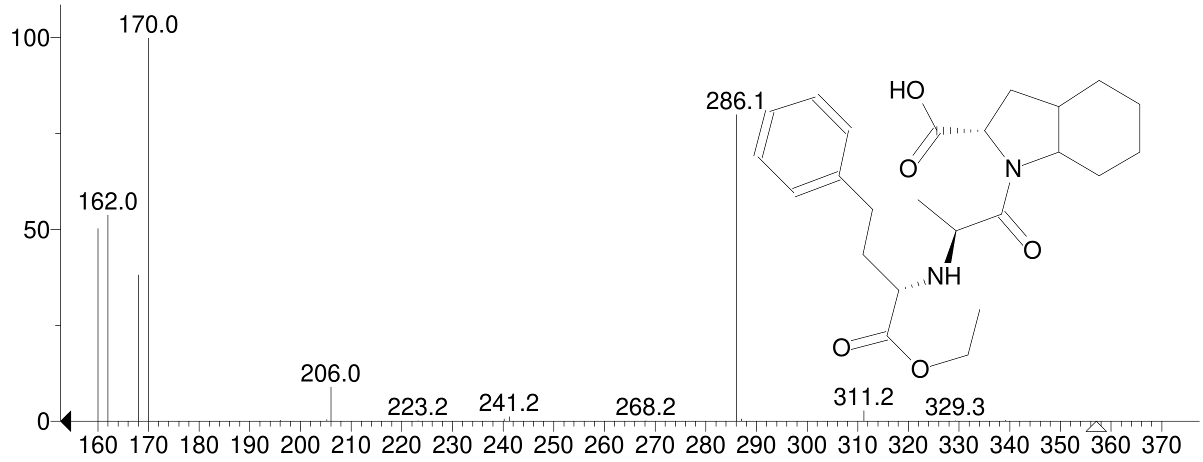

Hit 5 : 6'-Sialyl-N-acetyllactosamine [M+H+K]2+ HCD 22V P=357.1
C25H42N2O19; MF: 0; RMF: 25; Prob 7.53%; CAS: 78969-47-8; Lib: nist_msms; ID: 181459.

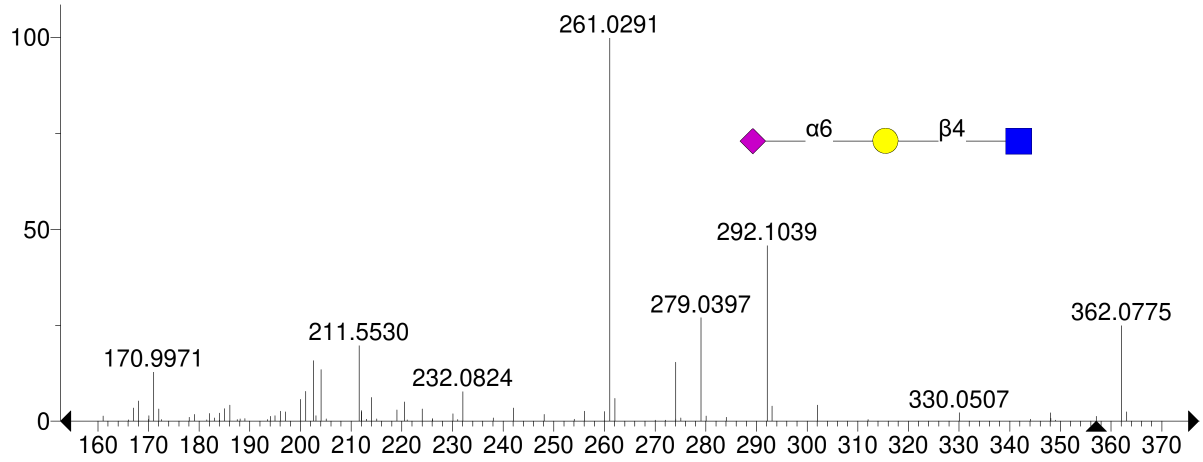

Unknown: Unknown (1.01_393.0793m/z)
Compound in Library Factor = 0

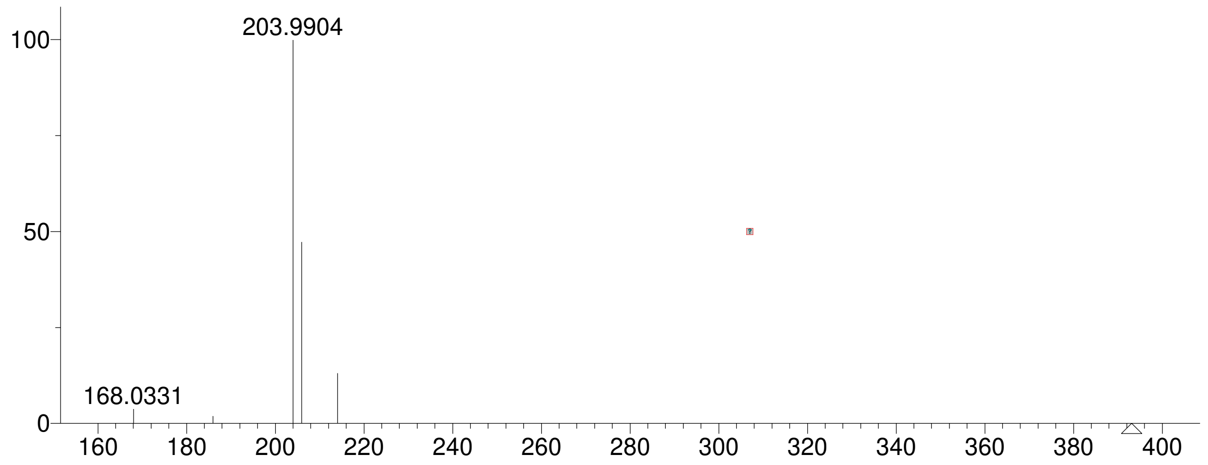

Hit 1 : fraxin [M+Na]+ 65HCD P=393.1
C16H18O10; MF: 0; RMF: 47; Prob 50.0%; Lib: mona anthocyanidin-o-glycosides; ID: 3225.

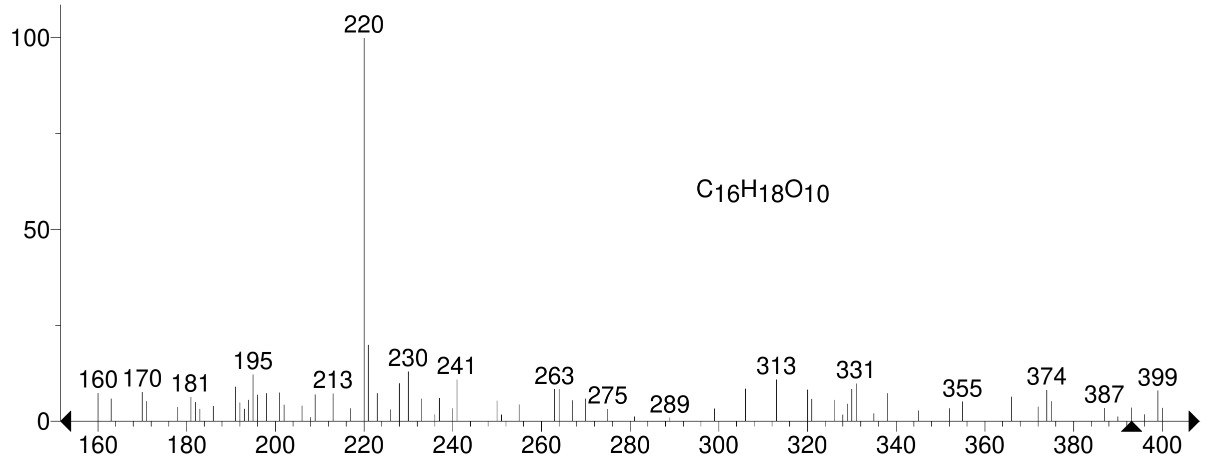

Hit 2 : 8-hydroxy-6-methoxy-7-[(2S,3R,4S,5S,6R)-3,4,5-trihydroxy-6-(hydroxymethyl)oxan-2-yl]oxychromen-2-one
C16H18O10; MF: 0; RMF: 19; Prob 50.0%; Lib: mona anthocyanidin-o-glycosides; ID: 3231.

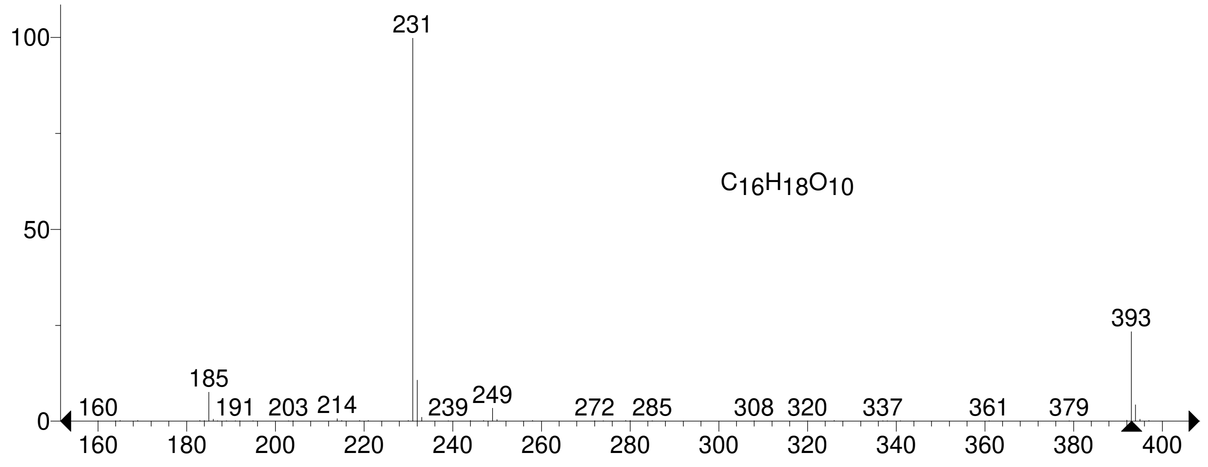

Unknown: Unknown (1.03_377.1966m/z)
Compound in Library Factor = -2176

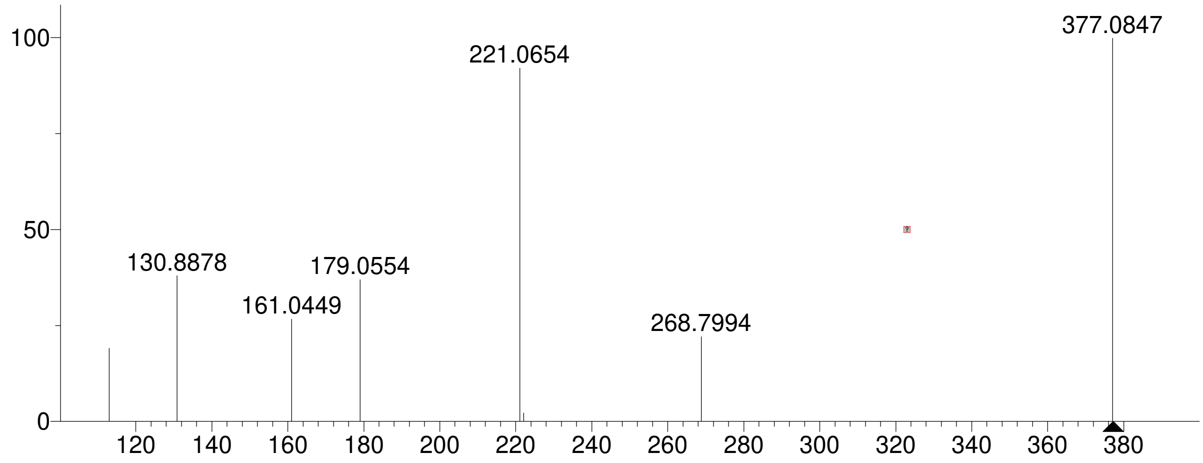

Hit 1 : Ala-Met-Arg [M+H]+ QQQ 10V P=377.2
C14H28N6O4S; MF: 61; RMF: 556; Prob 38.3%; Lib: nist_msms; ID: 41633.

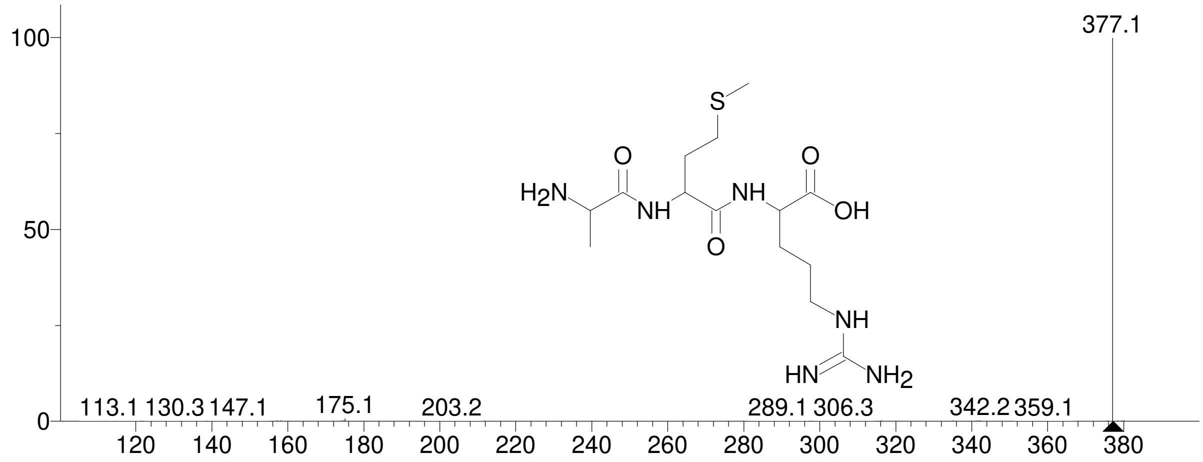

Hit 2 : Tetrahydrocurcumin [M+Na]+=>377.2 IT 35% P=377.2
C21H24O6; MF: 4; RMF: 202; Prob 7.17%; CAS: 36062-04-1; Lib: nist_msms; ID: 136919.

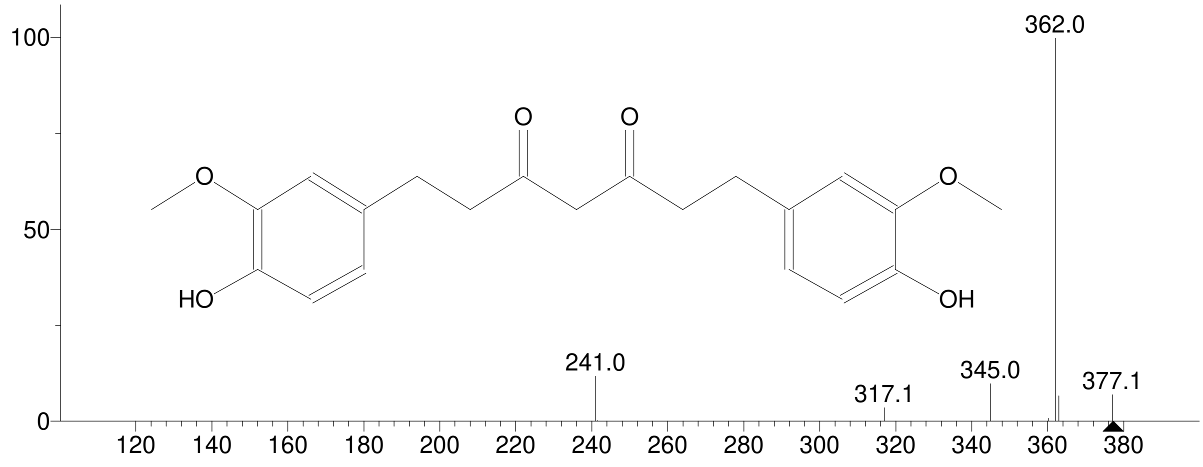

Hit 3 : 17-Trifluoromethylphenyl-13,14-dihydrotrinorprostaglandin F2α [M-H-H2O]-=>421.1=>377.2 IT 35% P=3
C24H33F3O5; MF: 0; RMF: 74; Prob 6.06%; CAS: 294856-01-2; Lib: nist_msms; ID: 93998.

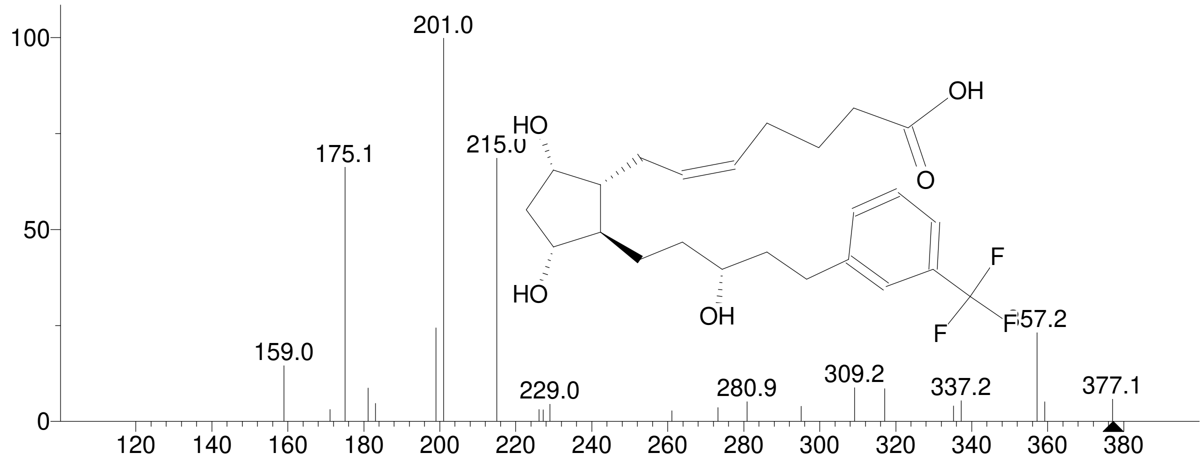

Hit 4 : Sildenafil [M+H]+=>377.2 IT 35% P=377.2
C22H30N6O4S; MF: 0; RMF: 63; Prob 6.06%; CAS: 139755-83-2; Lib: nist_msms; ID: 105397.

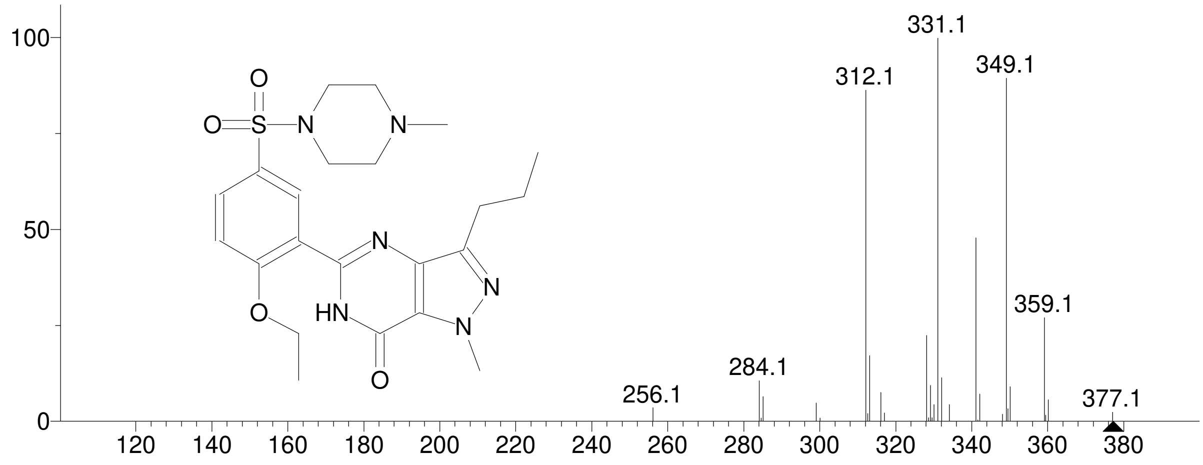

Hit 5 : Bisphenol A bis(2,3-dihydroxypropyl) ether [M+H]+ IT 35% P=377.2
C21H28O6; MF: 0; RMF: 51; Prob 6.06%; CAS: 5581-32-8; Lib: nist_msms; ID: 138122.

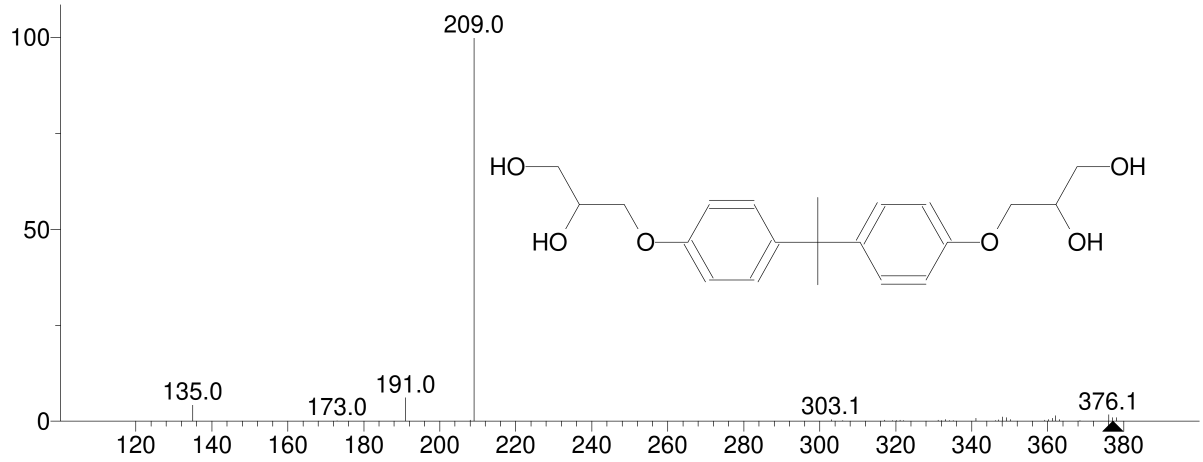

Unknown: Unknown (1.03_377.6459m/z)
Compound in Library Factor = 0

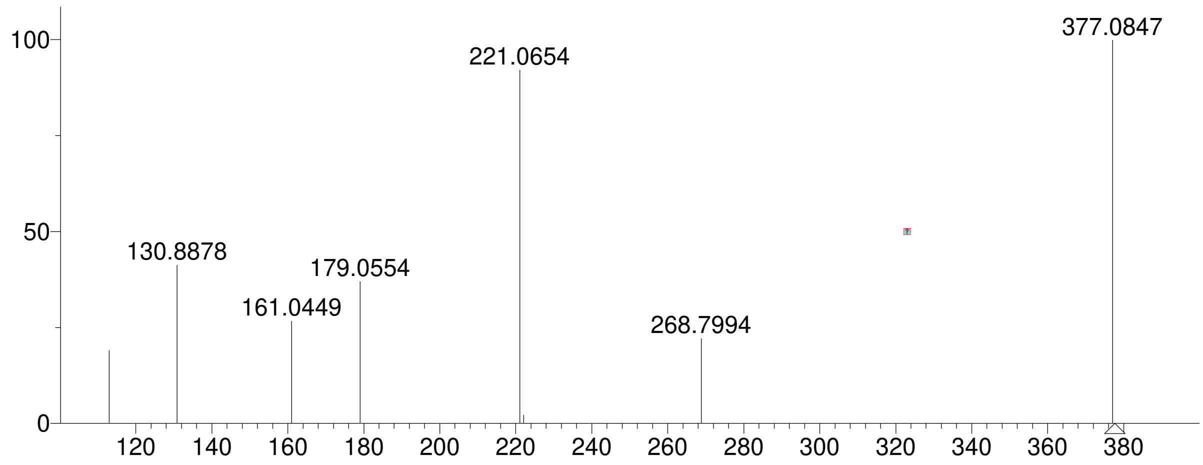

Unknown: Unknown (1.03_379.2651m/z)
Compound in Library Factor = 0

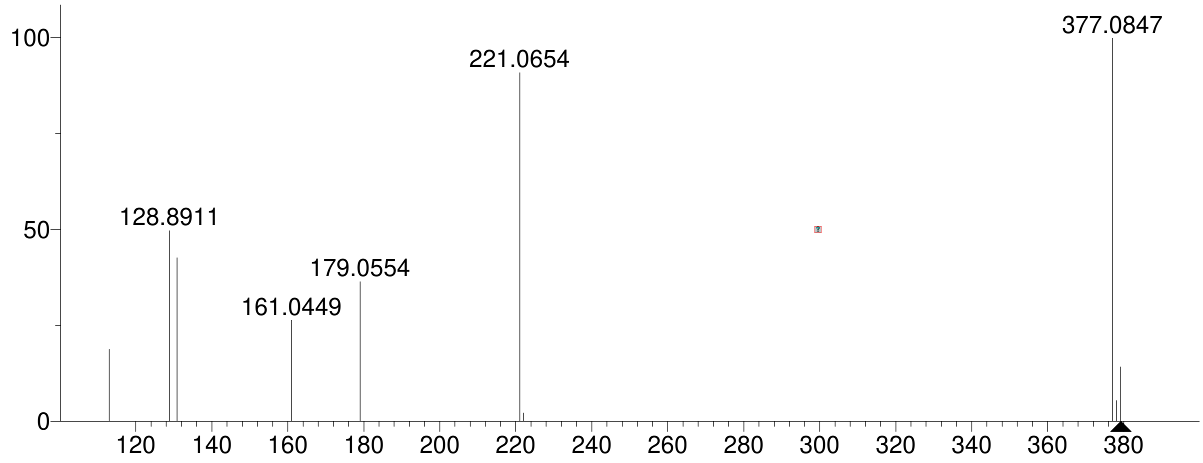

** Search Report Page 1 of 1 **

Unknown: Unknown (1.03_421.0325m/z)
Compound in Library Factor = 0

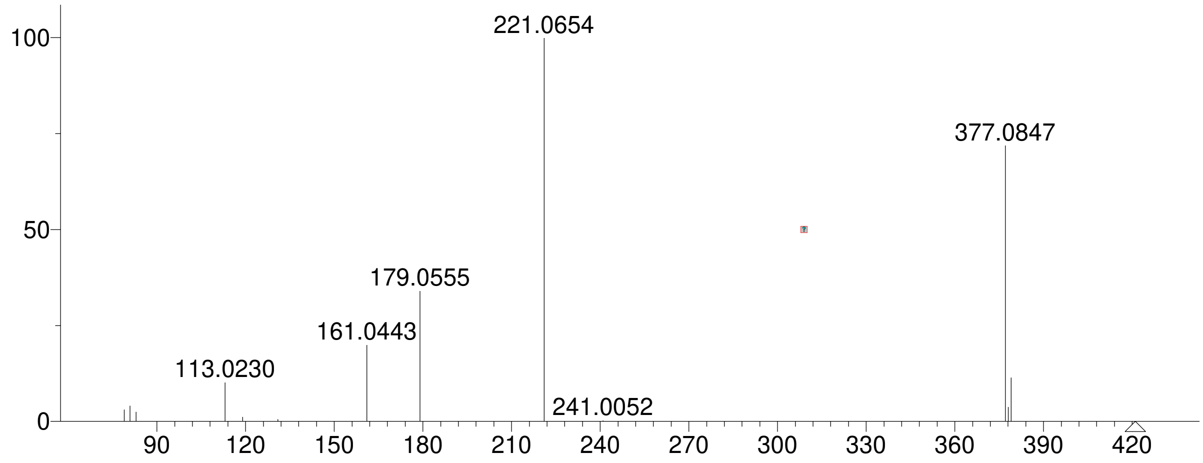

** Search Report Page 1 of 1 **

Unknown: Unknown (1.03_423.0317m/z)
Compound in Library Factor = 0

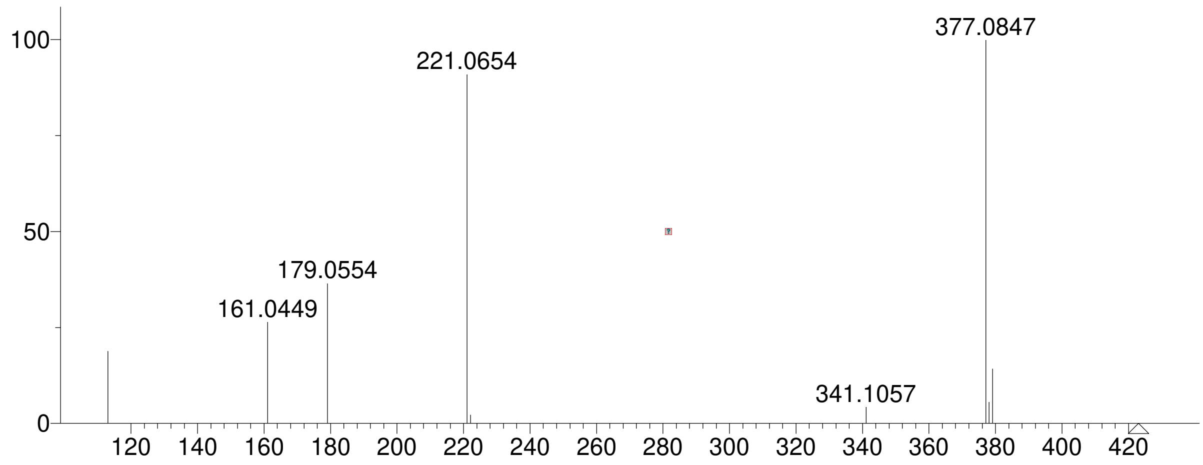

Hit 1 : Chloramphenicol succinate [M+H]+ IT 35% P=423
C15H16Cl2N2O8; MF: 0; RMF: 15; Prob 100.0%; CAS: 3544-94-3; Lib: nist_msms; ID: 152600.

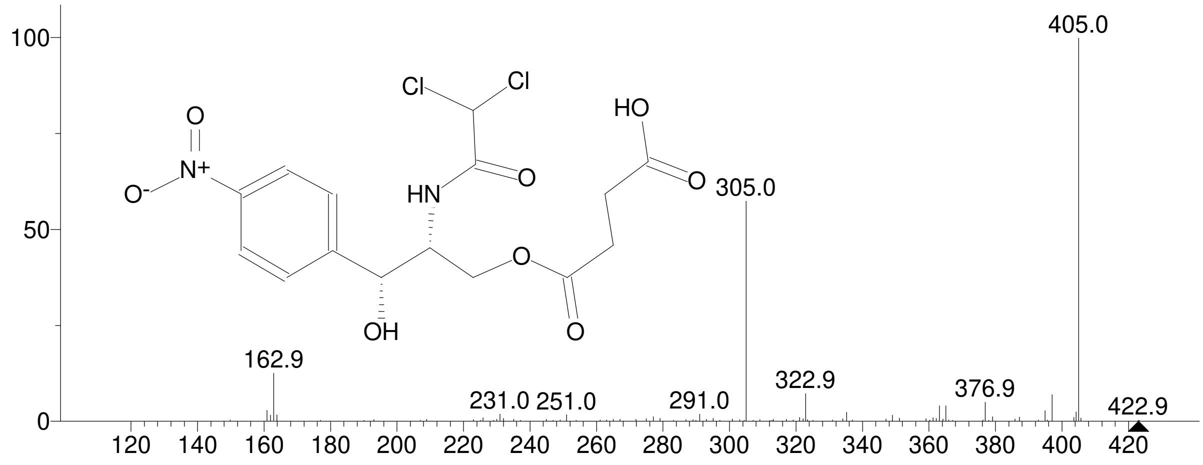

Unknown: Unknown (1.04_161.0445m/z)
Compound in Library Factor = -1453

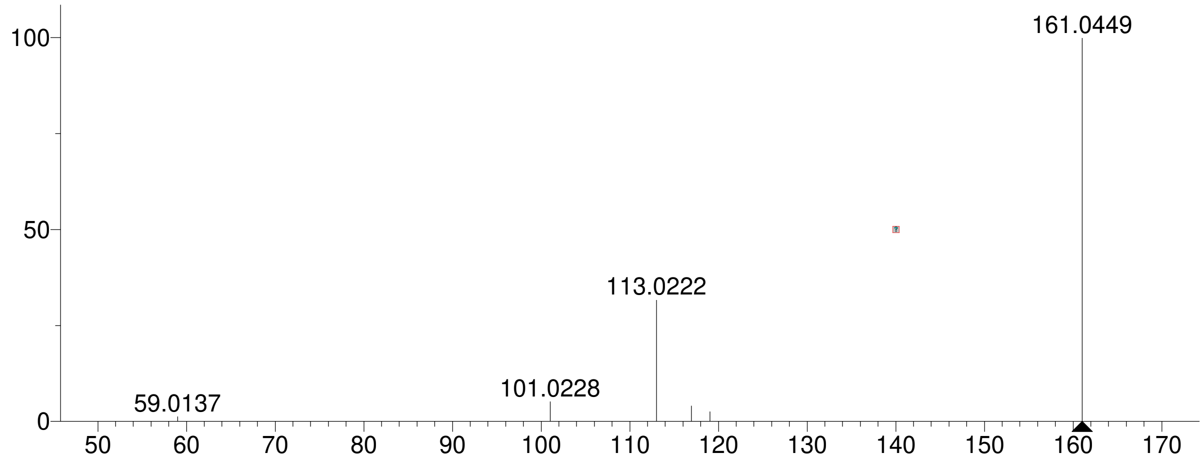

Hit 1 : 1,6-Anhydro-β-D-glucose [M-H]- HCD 7V P=161
C6H10O5; MF: 232; RMF: 830; Prob 73.7%; CAS: 498-07-7; Lib: nist_msms; ID: 289434.

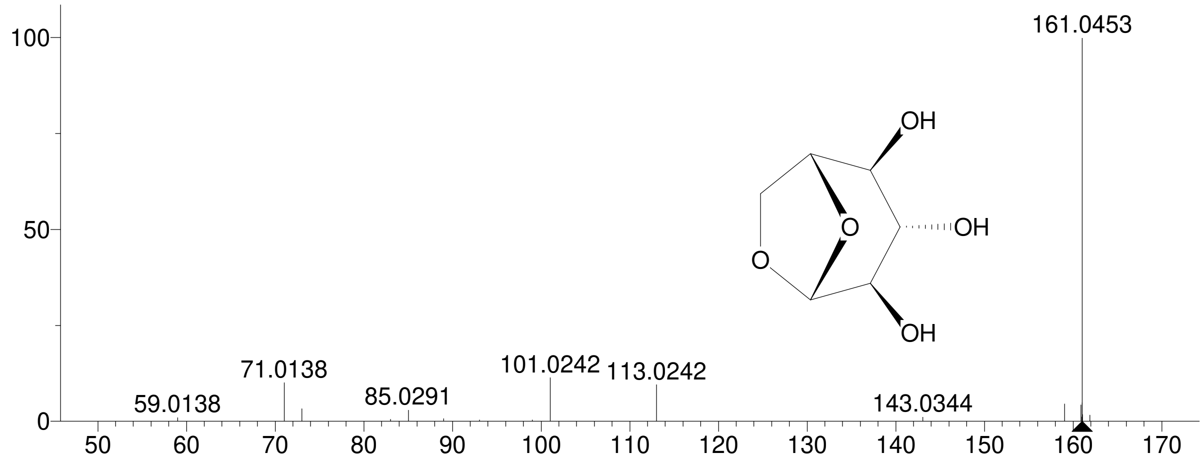

Hit 2 : D-Psicose [M-H-H2O]- HCD 11V P=161
C6H12O6; MF: 195; RMF: 834; Prob 17.9%; CAS: 551-68-8; Lib: nist_msms; ID: 499897.

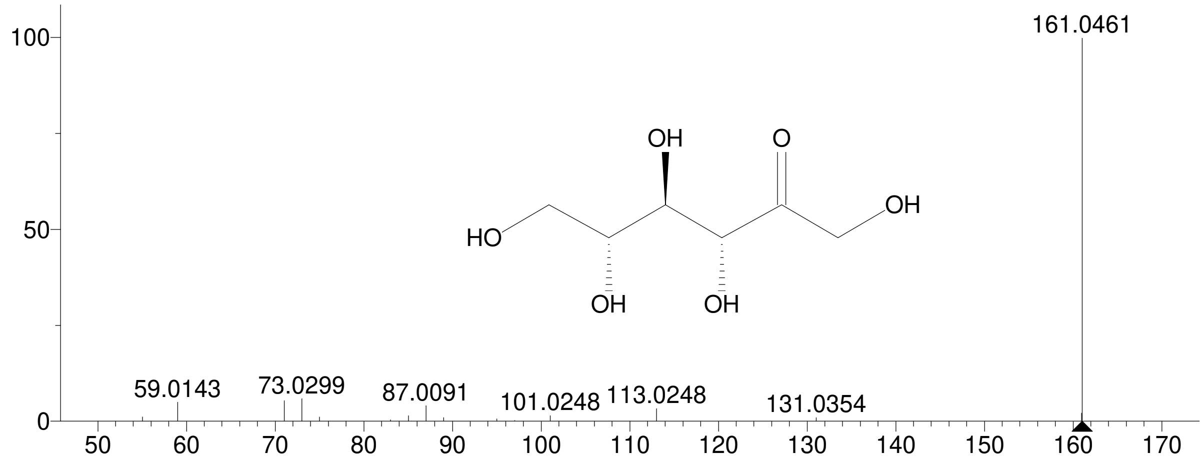

Hit 3 : Hydroxymethylglutaric acid; PlaSMA ID-124 [M-H]- P=161
C6H10O5; MF: 151; RMF: 728; Prob 3.89%; Lib: biomsms-neg-plasma; ID: 461.

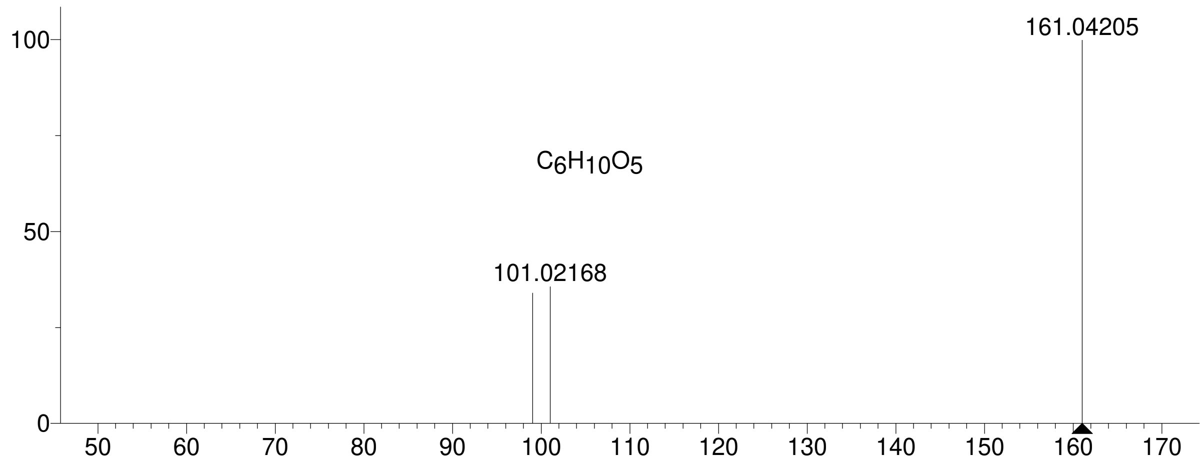

Hit 4 : 3-Hydroxy-3-methylglutaric acid [M-H]- HCD 14V P=161
C6H10O5; MF: 108; RMF: 484; Prob 0.85%; CAS: 503-49-1; Lib: nist_msms; ID: 313312.

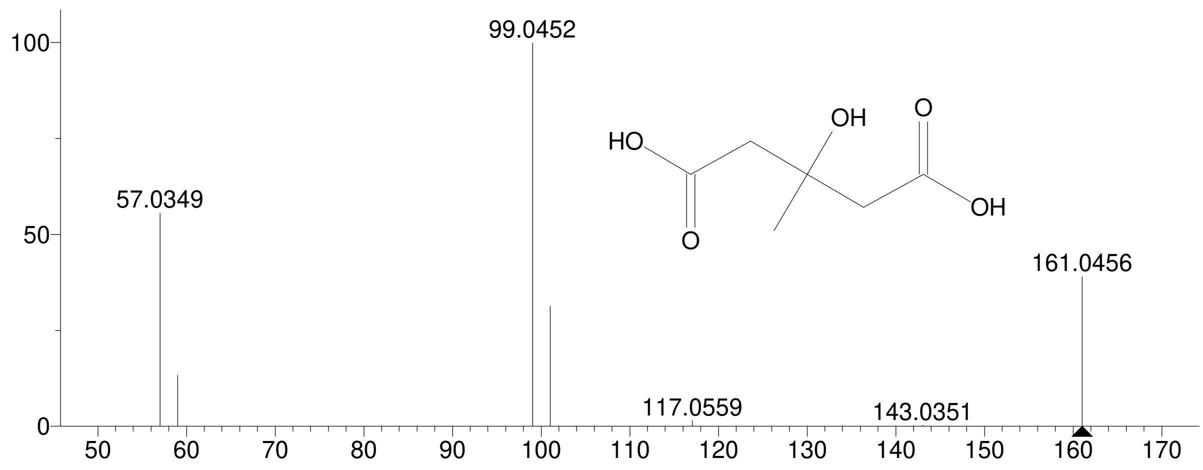

Hit 5 : D-(+)-Cellobiose [M-H-C6H12O6]- HCD 8V P=161
C12H22O11; MF: 102; RMF: 545; Prob 0.67%; CAS: 528-50-7; Lib: nist_msms; ID: 478378.

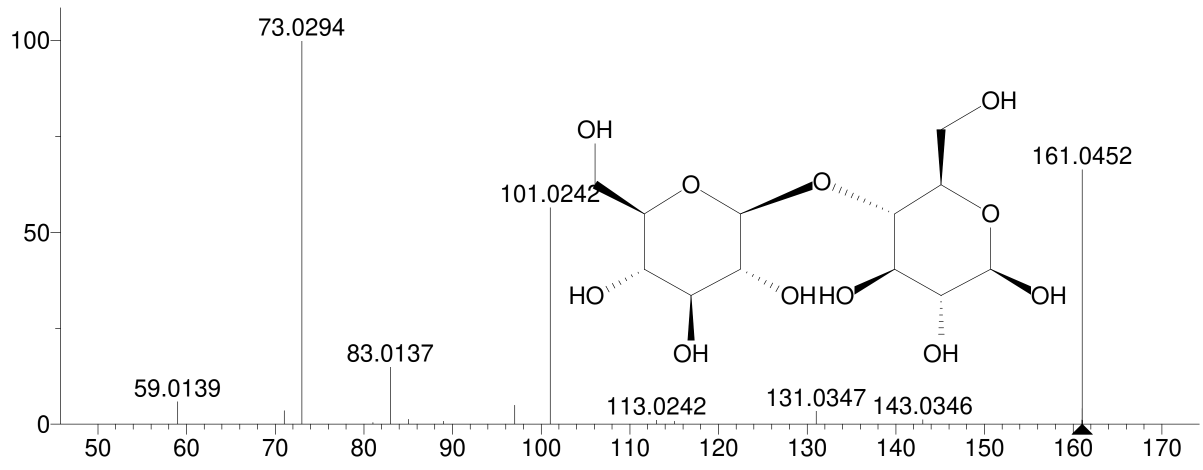

Unknown: Unknown (1.04_375.0682m/z)
Compound in Library Factor = -3273

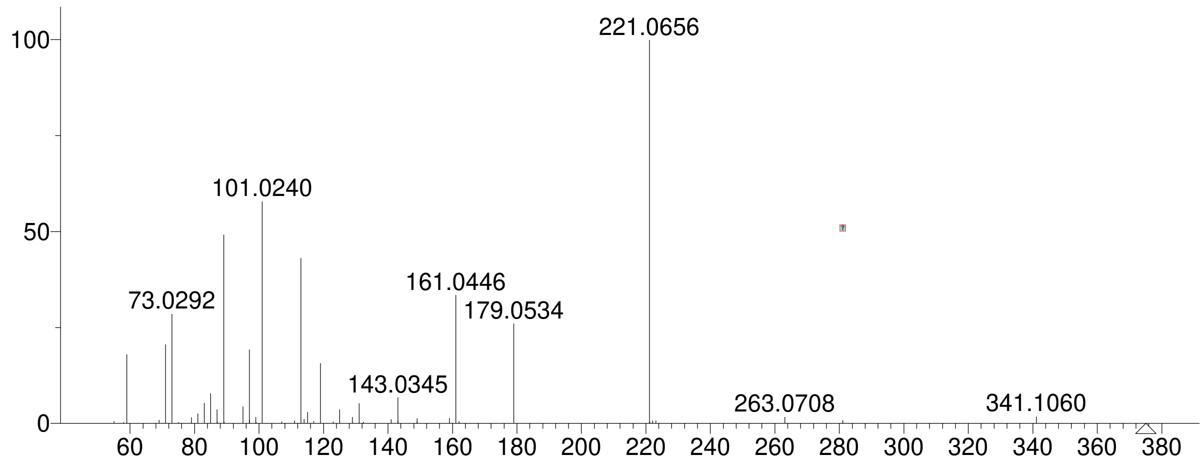

Hit 1 : LPI 2:0; [M-H]- [M-H]- P=375.1
C11H21O12P; MF: 2; RMF: 117; Prob 52.0%; Lib: msdial-insilicomsms-lipids-neg; ID: 311142.

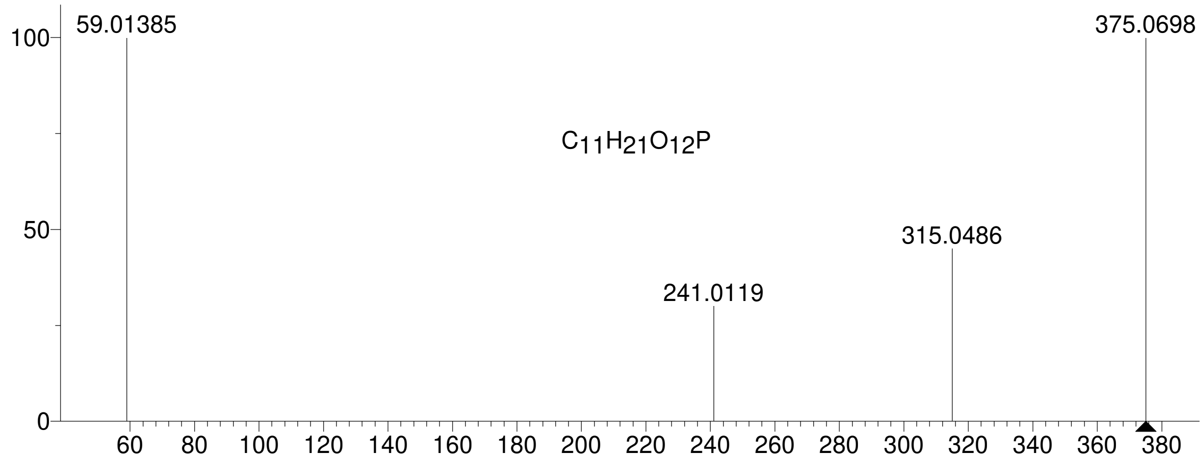

Hit 2 : Mefenpyr-diethyl [M+H+2i]+ HCD 97V P=375.1
C16H18Cl2N2O4; MF: 0; RMF: 4; Prob 48.0%; CAS: 135590-91-9; Lib: nist_msms; ID: 512691.

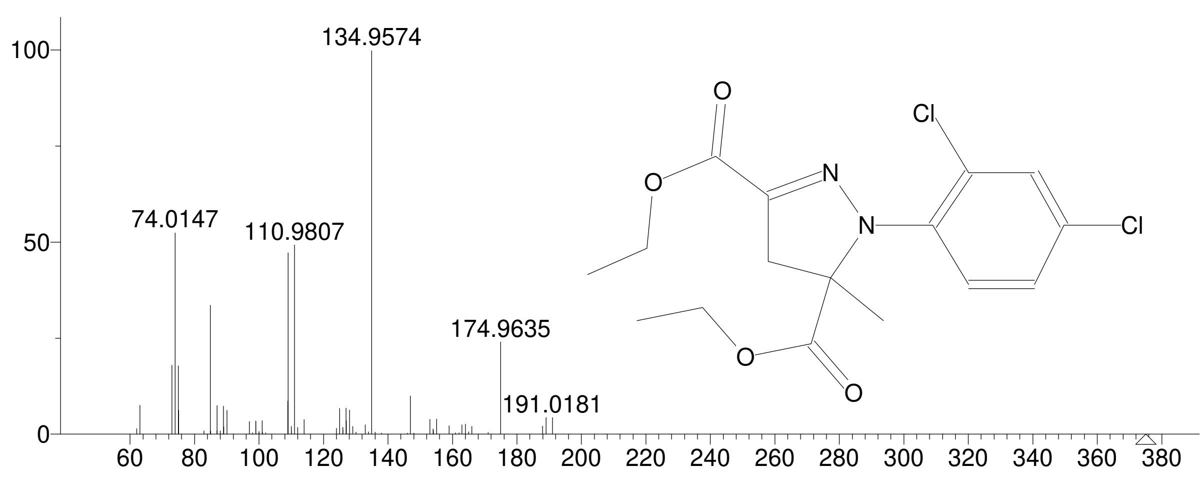

Unknown: Unknown (1.04_377.0851m/z)
Compound in Library Factor = 1485

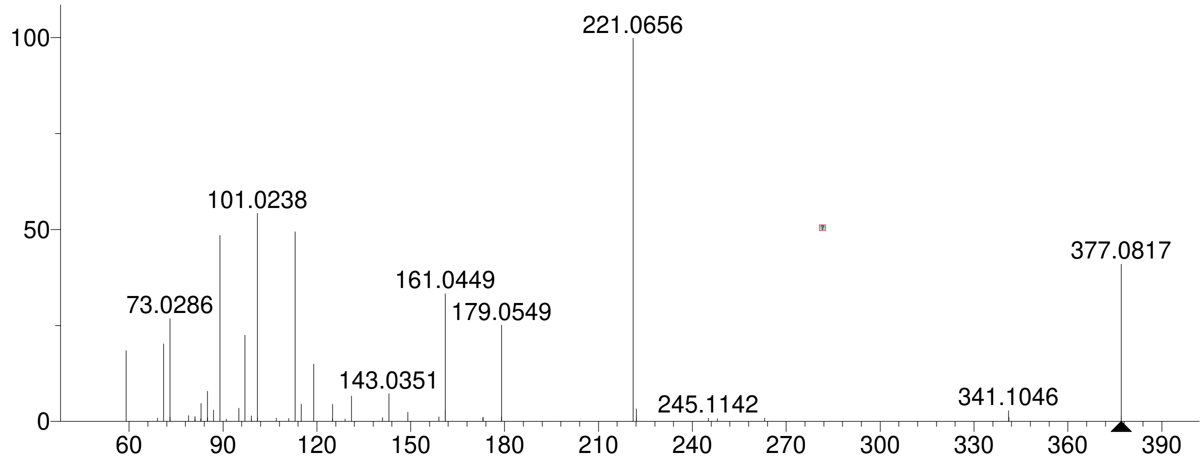

Hit 1 : Lactose [M+Cl]- HCD 33V P=377.1
C12H22O11; MF: 905; RMF: 828; Prob 26.1%; CAS: 63-42-3; Lib: nist_msms; ID: 500030.

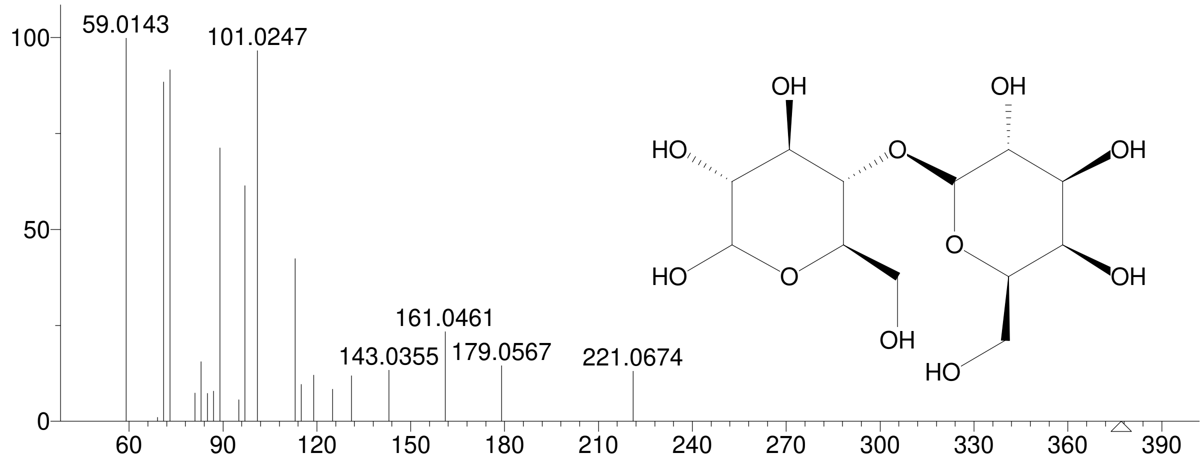

Hit 2 : Maltose [M+Cl]- HCD 26V P=377.1
C12H22O11; MF: 904; RMF: 916; Prob 25.1%; CAS: 69-79-4; Lib: nist_msms; ID: 479066.

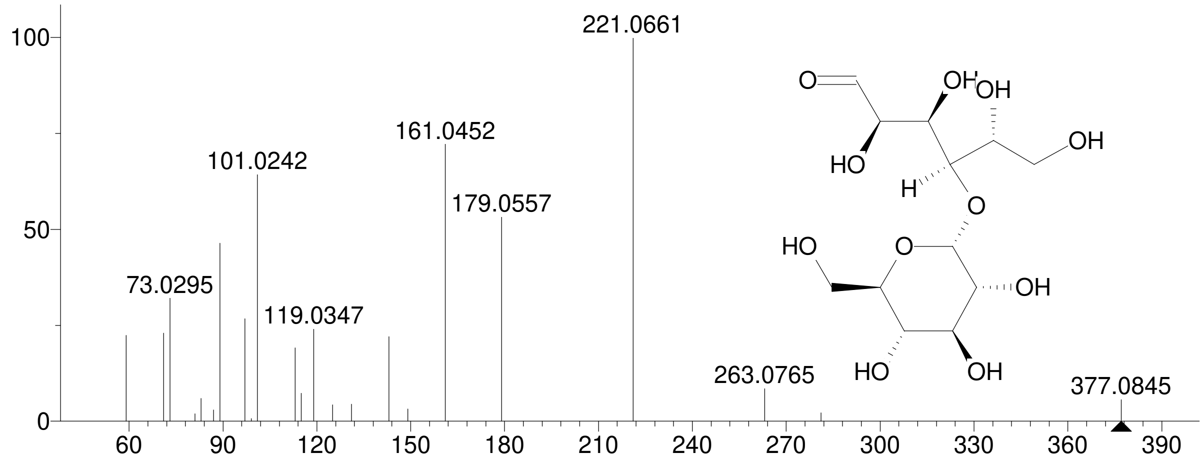

Hit 3 : D-(+)-Cellobiose [M+Cl]- HCD 33V P=377.1
C12H22O11; MF: 897; RMF: 864; Prob 19.2%; CAS: 528-50-7; Lib: nist_msms; ID: 478427.

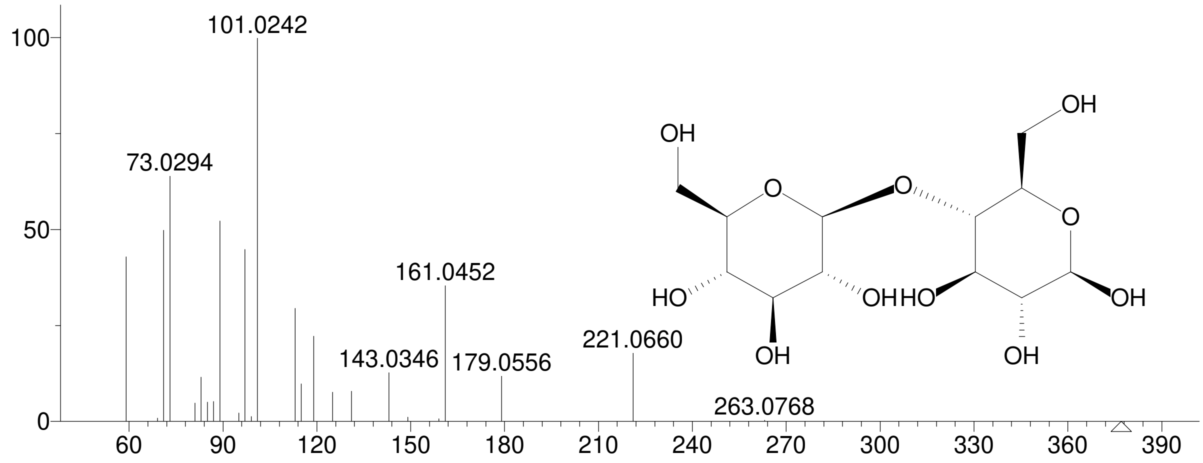

Hit 4 : Isomaltose [M+Cl]- HCD 26V P=377.1
C12H22O11; MF: 869; RMF: 885; Prob 5.54%; CAS: 499-40-1; Lib: nist_msms; ID: 499828.

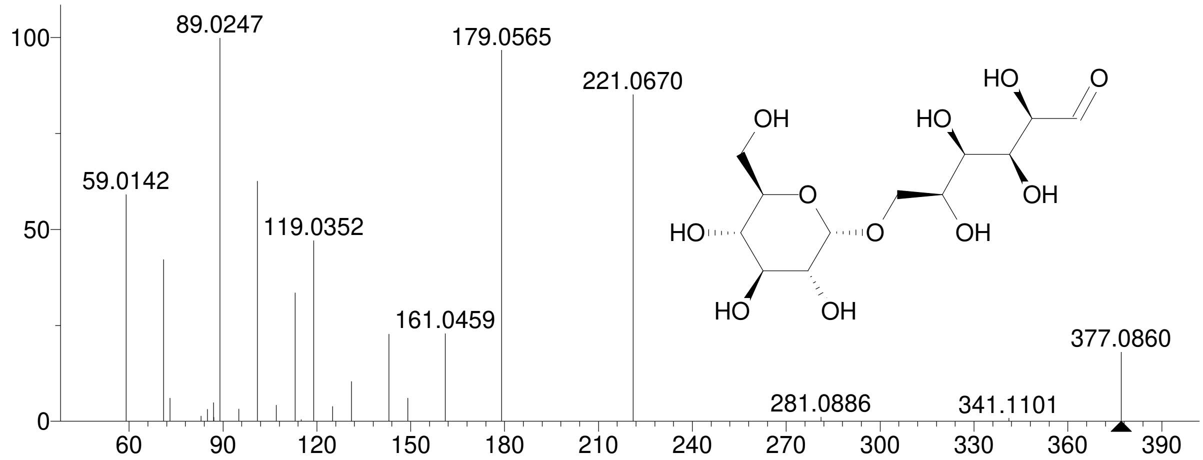

Hit 5 : β-Gentiobiose [M+Cl]- HCD 26V P=377.1
C12H22O11; MF: 867; RMF: 878; Prob 5.11%; CAS: 5996-00-9; Lib: nist_msms; ID: 477704.

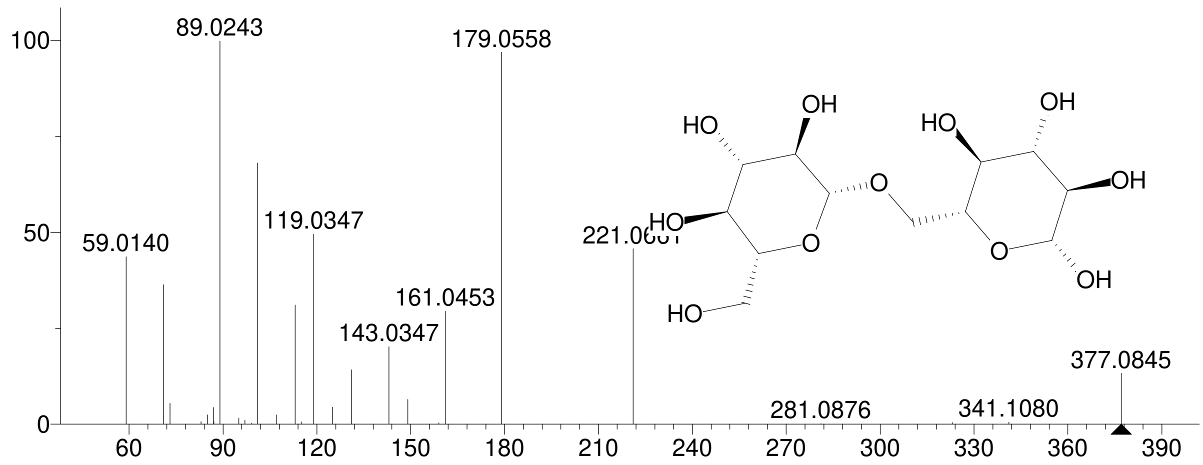

Unknown: Unknown (1.05_179.0551m/z)
Compound in Library Factor = 0

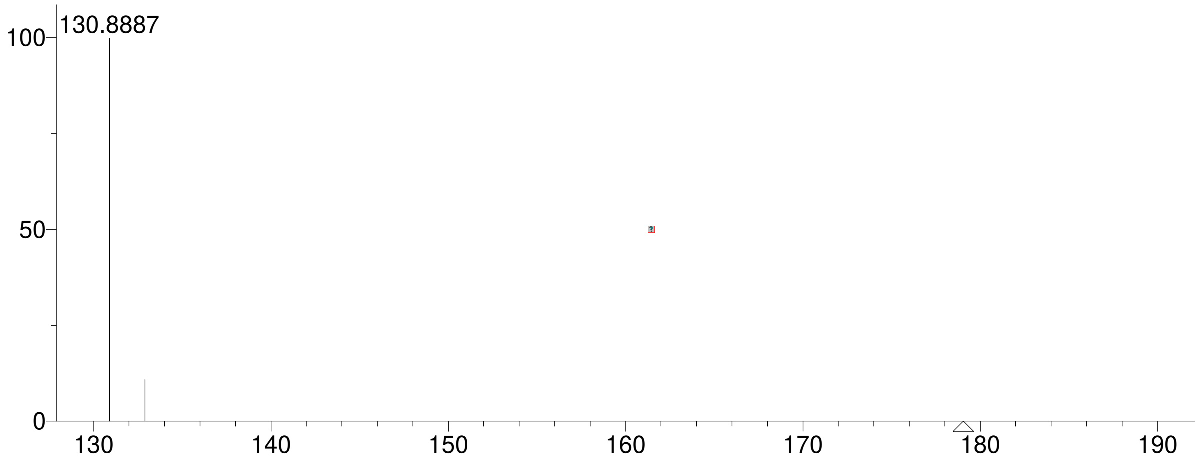

** Search Report Page 1 of 1 **

Unknown: Unknown (1.05_237.0028m/z)
Compound in Library Factor = 0

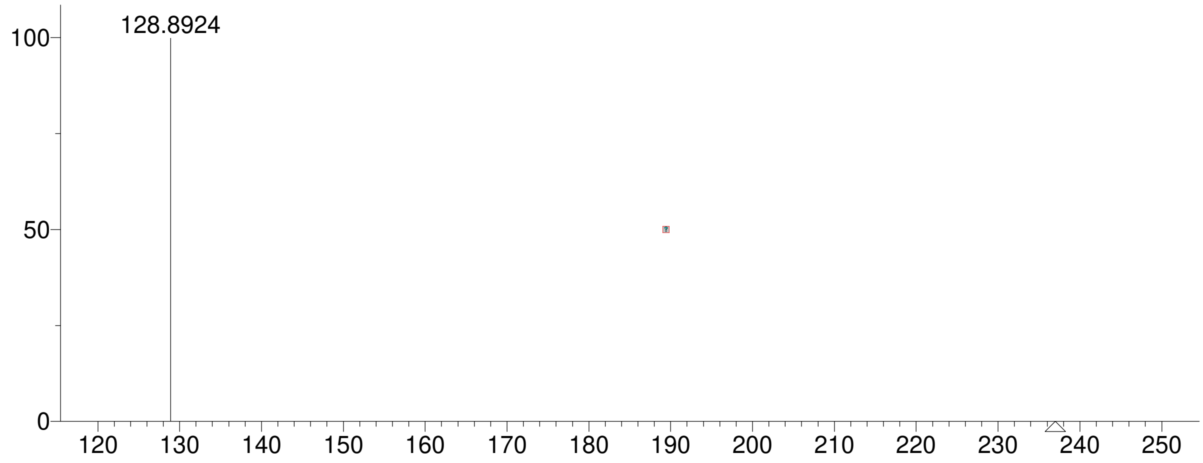

Unknown: Unknown (1.05_341.1078m/z)
Compound in Library Factor = -459

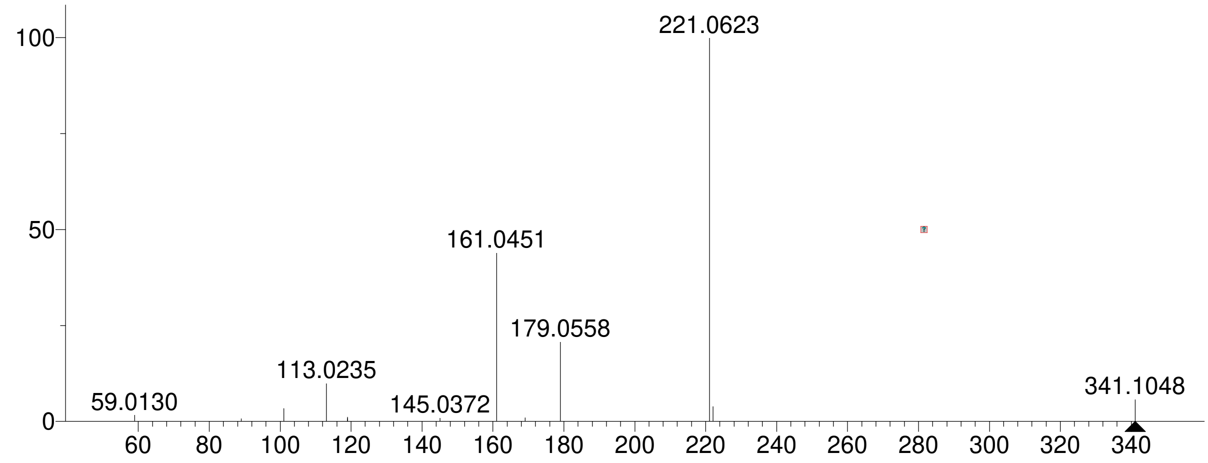

Hit 1 : Palatinose [M-H]- 5-60V P=341.1
C12H22O11; MF: 493; RMF: 637; Prob 31.2%; Lib: mona-export-lc-ms-ms_negative_mode; ID: 8796.

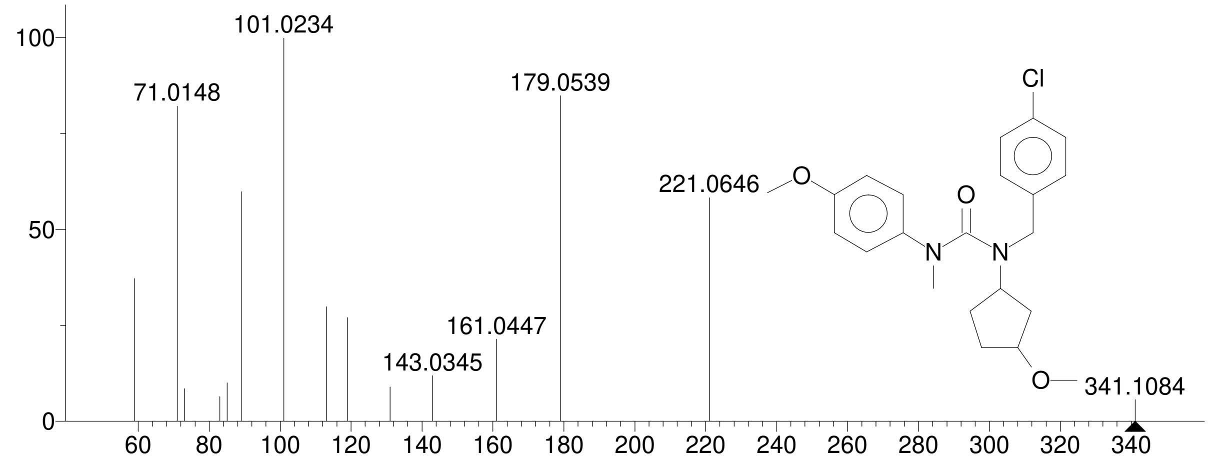

Hit 2 : Palatinose Monohydrate [M-H]- QTOF 5-45V P=341.1
C12H22O11; MF: 493; RMF: 637; Prob 31.2%; Lib: mona-export-lc-ms-ms_negative_mode; ID: 11710.

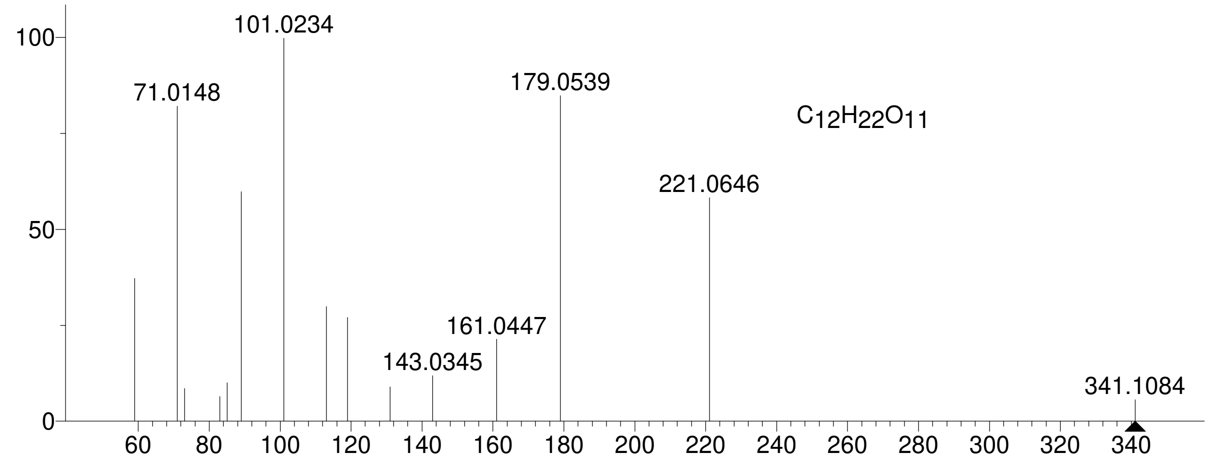

Hit 3 : Palatinose; LC-ESI-QTOF; MS2; CE [M-H]- P=341.1
C12H22O11; MF: 492; RMF: 637; Prob 30.0%; Lib: msms-neg-massbank; ID: 4681.

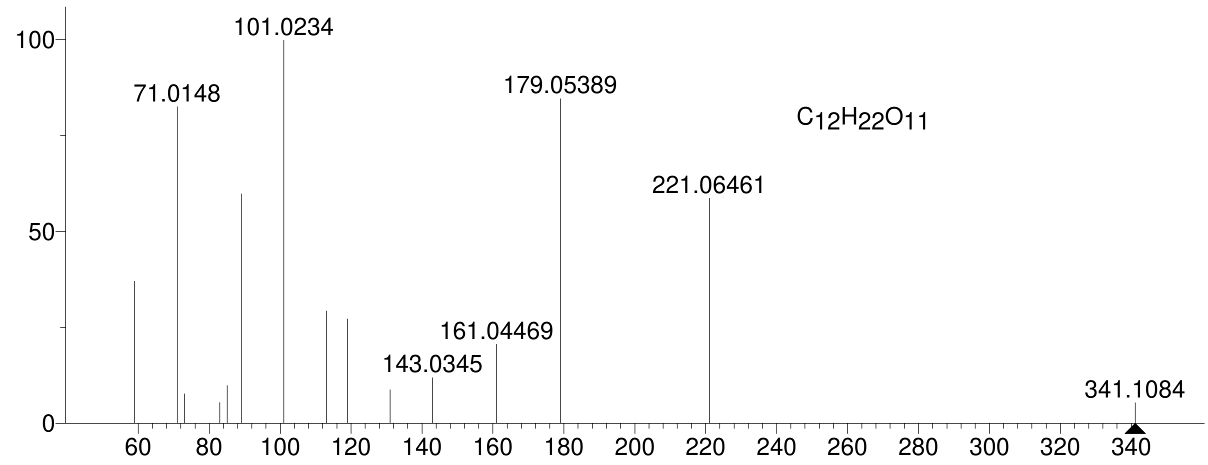

Hit 4 : 4-O-β-Galactopyranosyl-D-mannopyranose [M-H]- HCD 17V P=341.1
C12H22O11; MF: 381; RMF: 515; Prob 1.89%; CAS: 20869-27-6; Lib: nist_msms; ID: 479256.

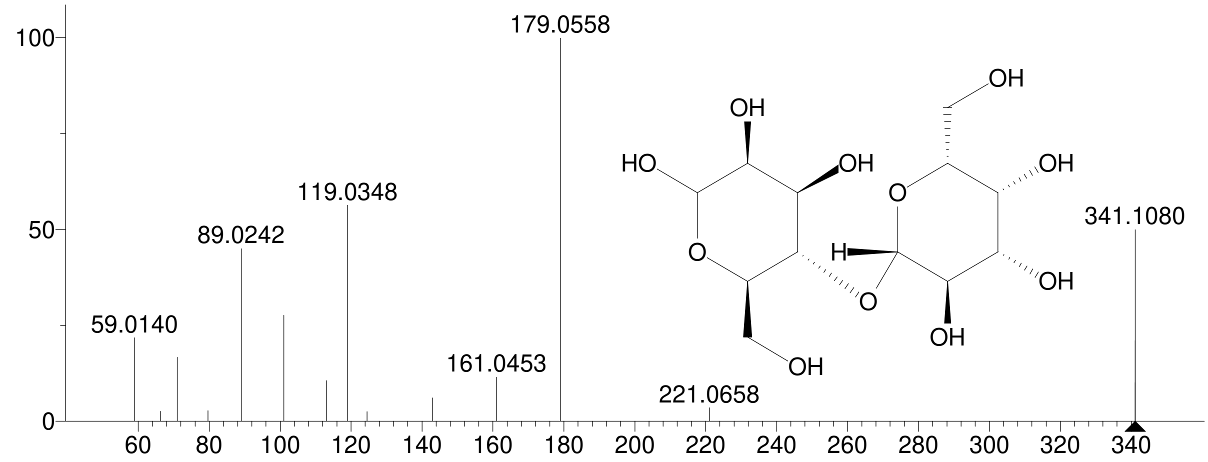

Hit 5 : Turanose [M-H]- 10 P=341.1
C12H22O11; MF: 375; RMF: 521; Prob 1.49%; Lib: msms neg mona_ipb_gnps_plasma; ID: 204.

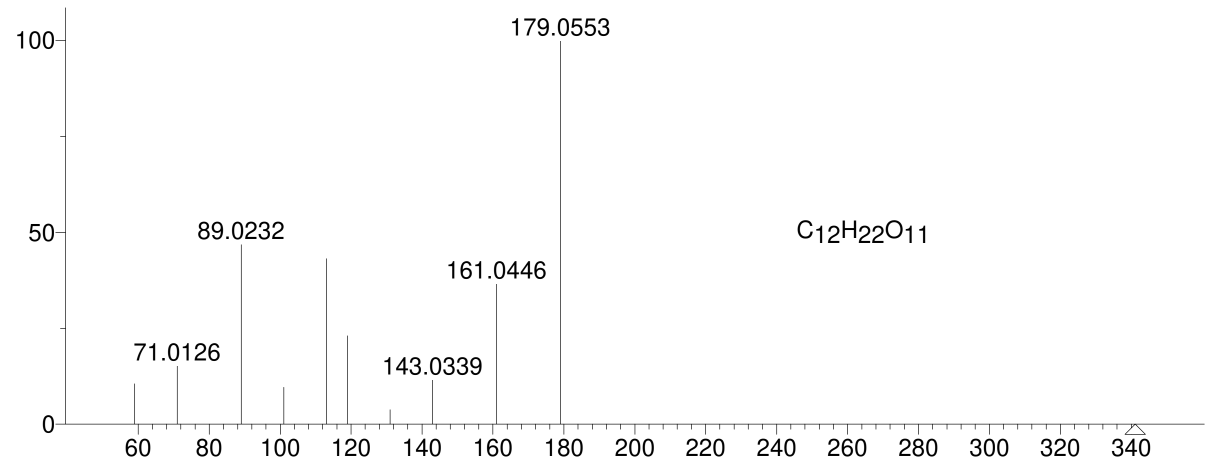

Unknown: Unknown (1.05_379.0825m/z)
Compound in Library Factor = -3273

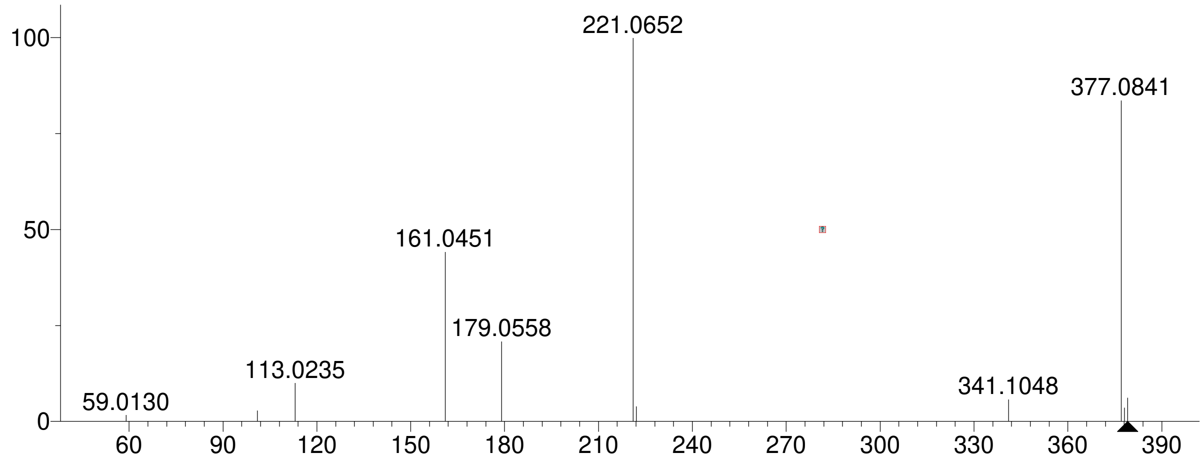

Hit 1 : Doxorubicin [M+H-C6H15NO4]+ HCD 7V P=379.1
C27H29NO11; MF: 17; RMF: 237; Prob 15.8%; CAS: 23214-92-8; Lib: nist_msms; ID: 345480.

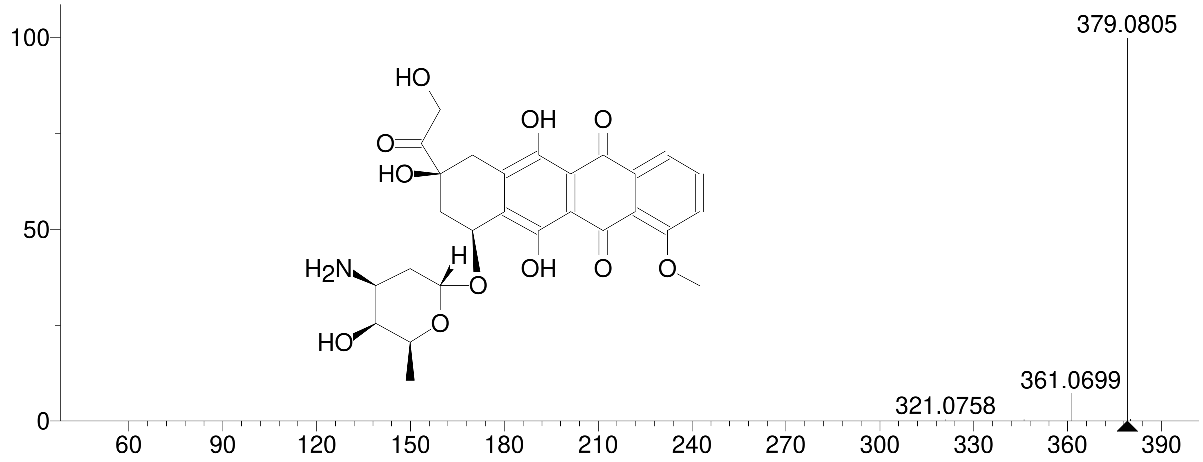

Hit 2 : Doxorubicinone [M+H-2H2O]+ HCD 7V P=379.1
C21H18O9; MF: 13; RMF: 243; Prob 13.4%; CAS: 24385-10-2; Lib: nist_msms; ID: 437347.

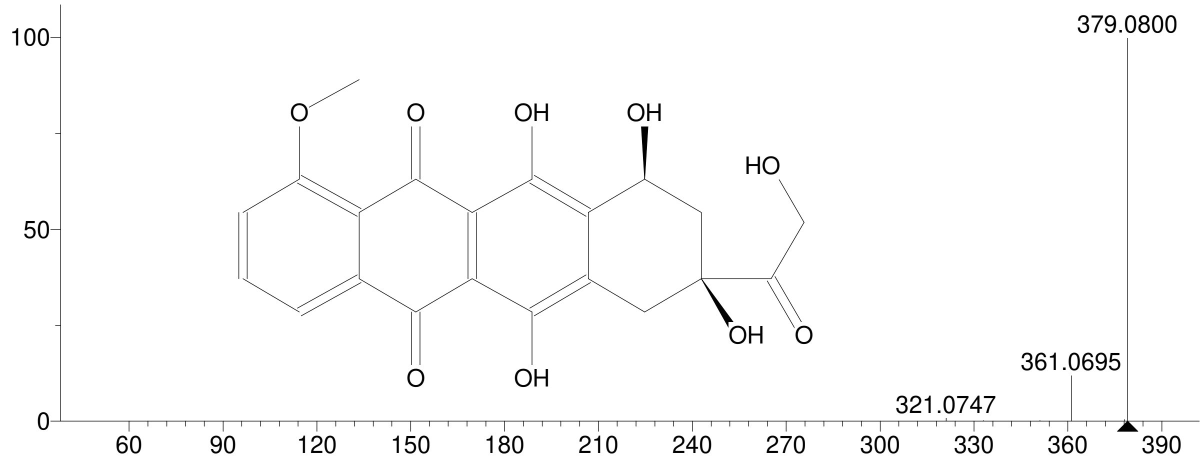

Hit 3 : Daunomycinone [M-H-H2O]- HCD 11V P=379.1
C21H18O8; MF: 13; RMF: 181; Prob 13.4%; CAS: 21794-55-8; Lib: nist_msms; ID: 450630.

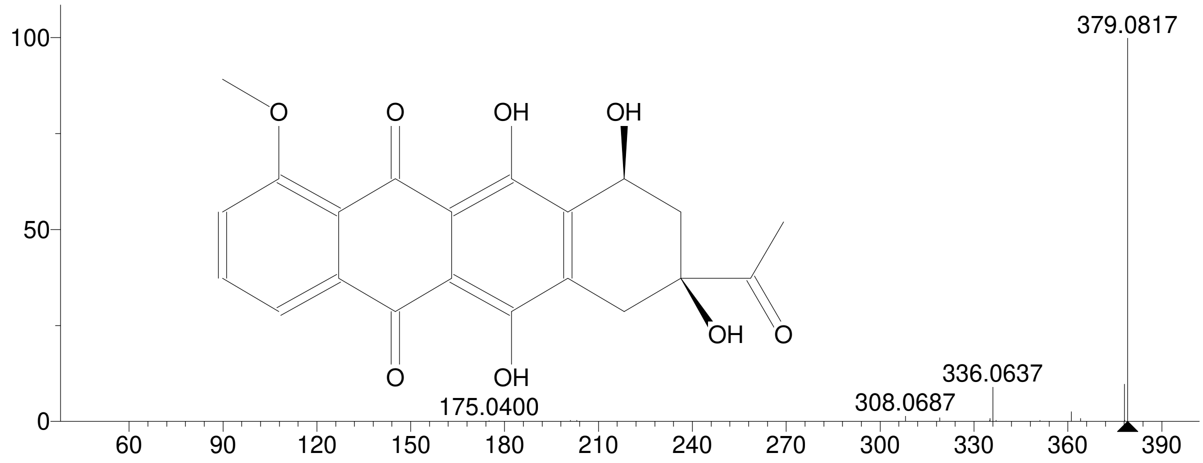

Hit 4 : Daunorubicin [M-H-C6H13O3N]- HCD 18V P=379.1
C27H29NO10; MF: 12; RMF: 151; Prob 12.8%; CAS: 20830-81-3; Lib: nist_msms; ID: 283928.

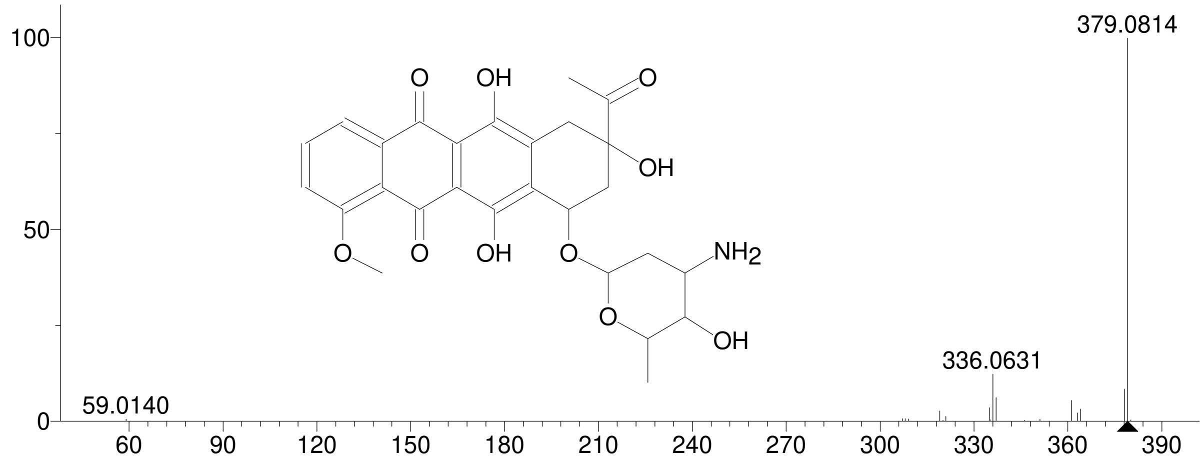

Hit 5 : Taiwanin E methyl ether [M+H]+ HCD 18V P=379.1
C21H14O7; MF: 4; RMF: 212; Prob 9.57%; CAS: 30403-00-0; Lib: nist_msms; ID: 411853.

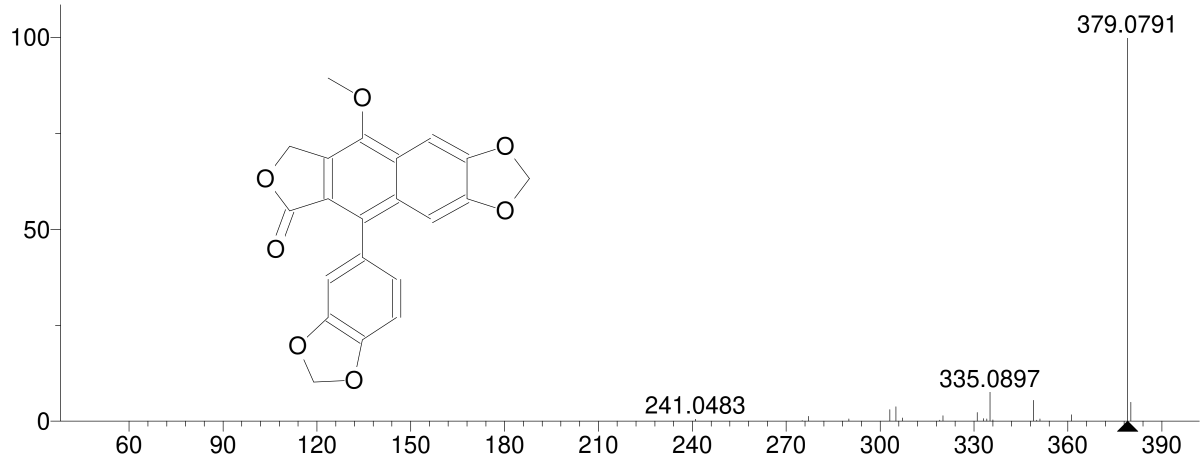

Unknown: Unknown (1.05_521.1709m/z)
Compound in Library Factor = 0

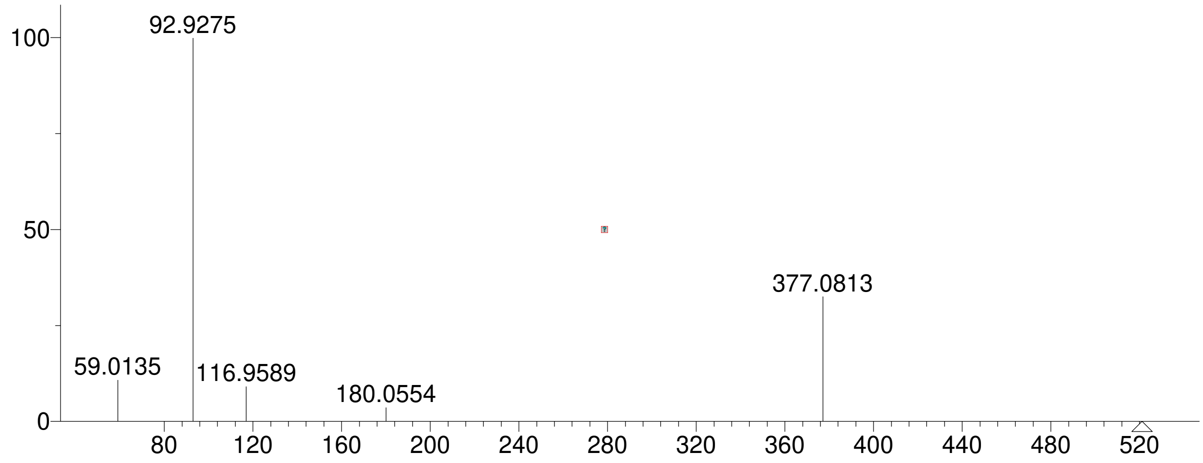

** Search Report Page 1 of 1 **

Unknown: Unknown (1.07_599.0931m/z)
Compound in Library Factor = 0

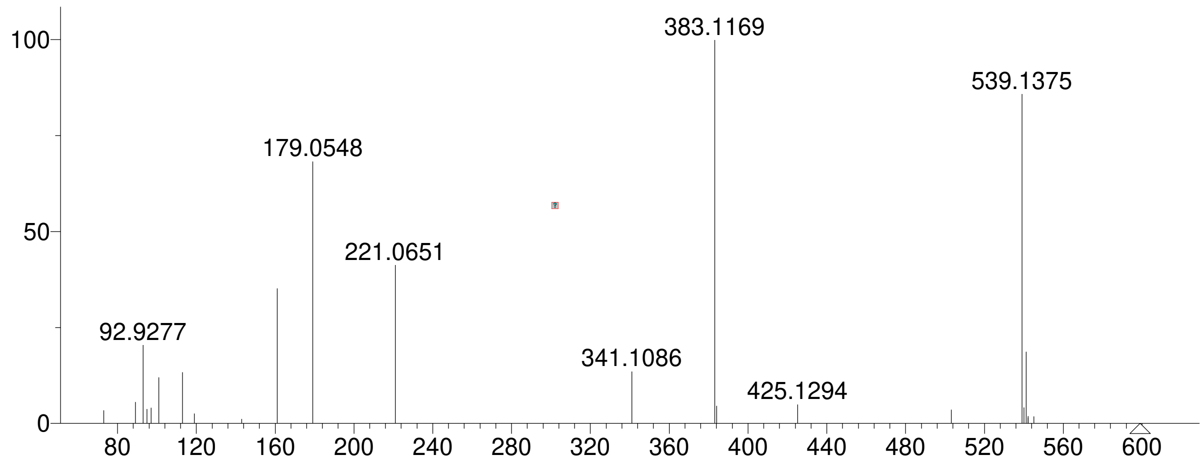

Unknown: Unknown (1.07_655.0537m/z)
Compound in Library Factor = 0

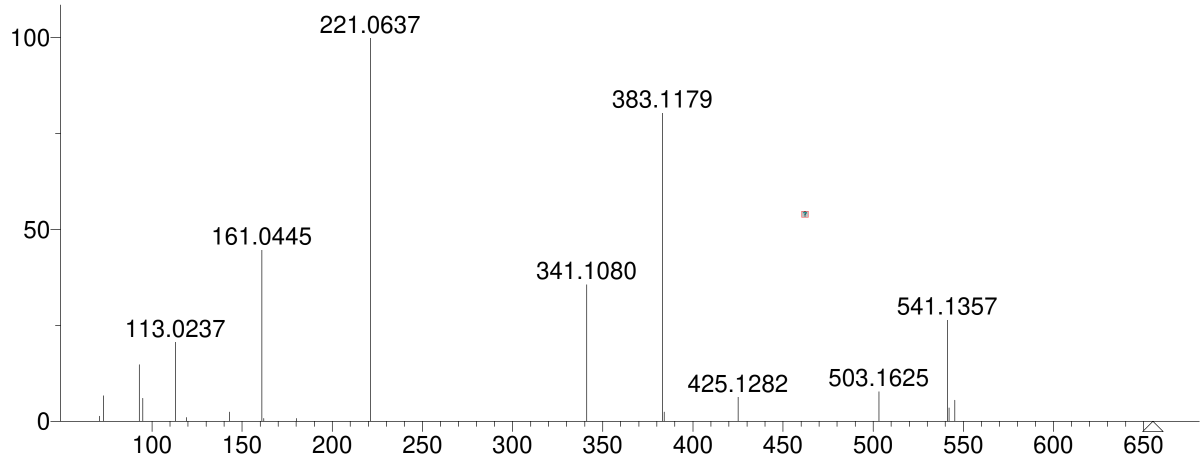

** Search Report Page 1 of 1 **

Unknown: Unknown (1.07_657.0484m/z)
Compound in Library Factor = 0

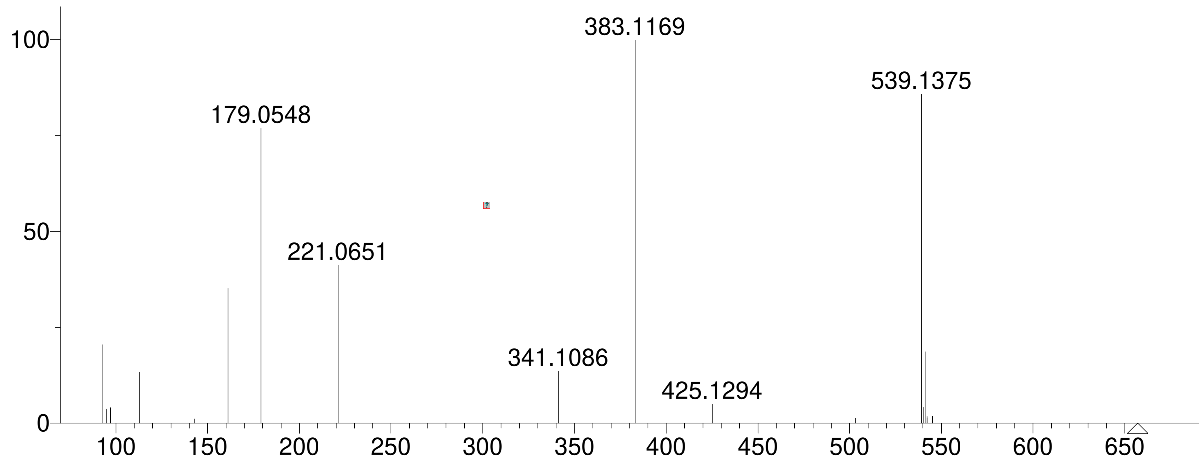

Unknown: Unknown (1.08_383.1176m/z)
Compound in Library Factor = -1523

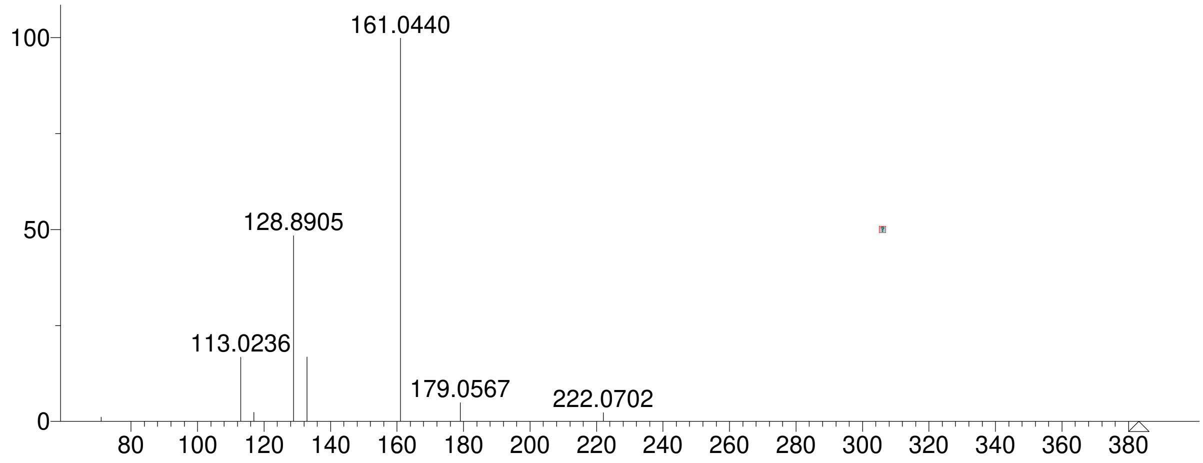

Hit 1 : Maltotetraose [M-H-C10H18O9]- IT-FT 35% P=383.1
C24H42O21; MF: 48; RMF: 376; Prob 38.8%; CAS: 34612-38-9; Lib: nist_msms; ID: 499732.

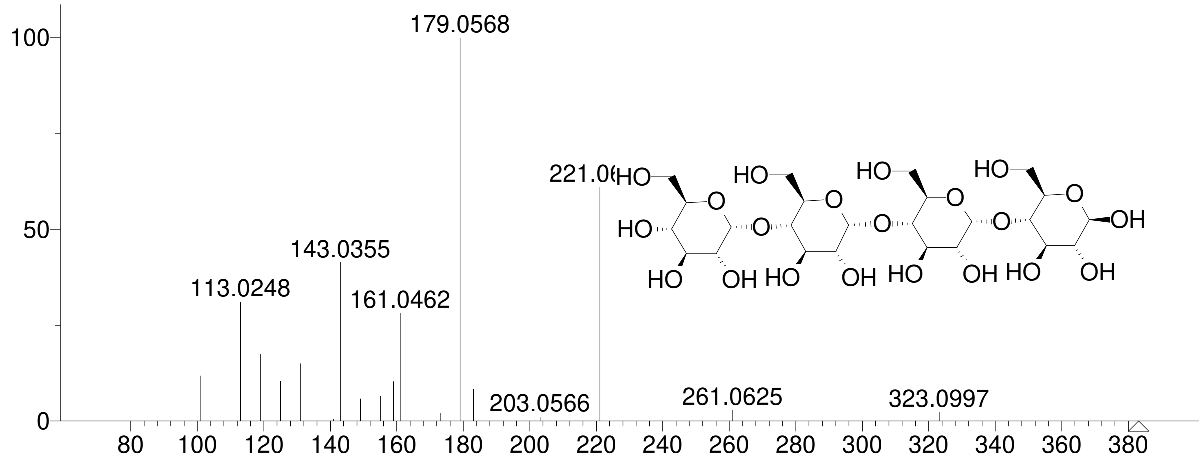

Hit 2 : Sedoheptulosan [2M-H]- HCD 61V P=383.1
C7H12O6; MF: 2; RMF: 103; Prob 8.20%; CAS: 469-90-9; Lib: nist_msms; ID: 143898.

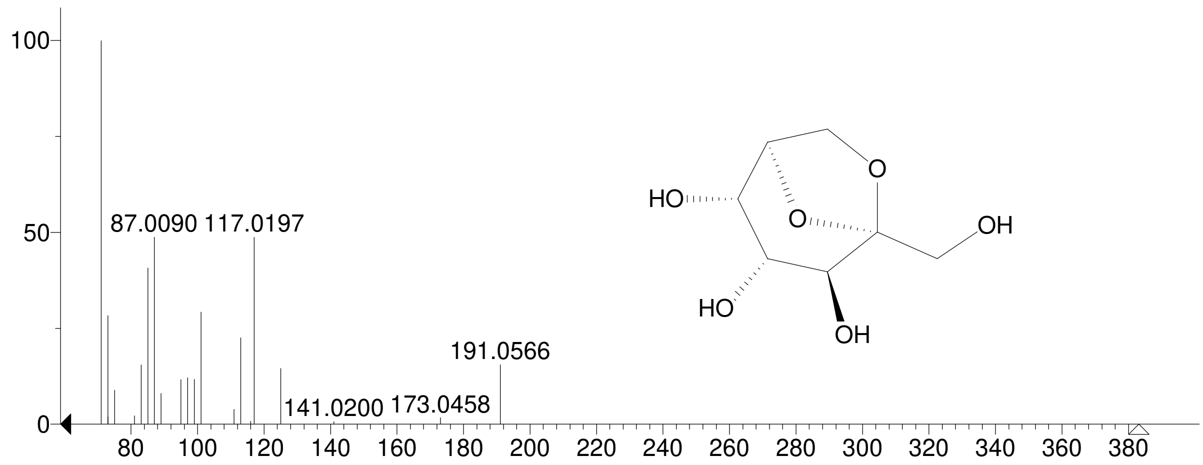

Hit 3 : (-)-Quinic acid [2M-H]- HCD 61V P=383.1
C7H12O6; MF: 0; RMF: 28; Prob 7.56%; CAS: 77-95-2; Lib: nist_msms; ID: 288459.

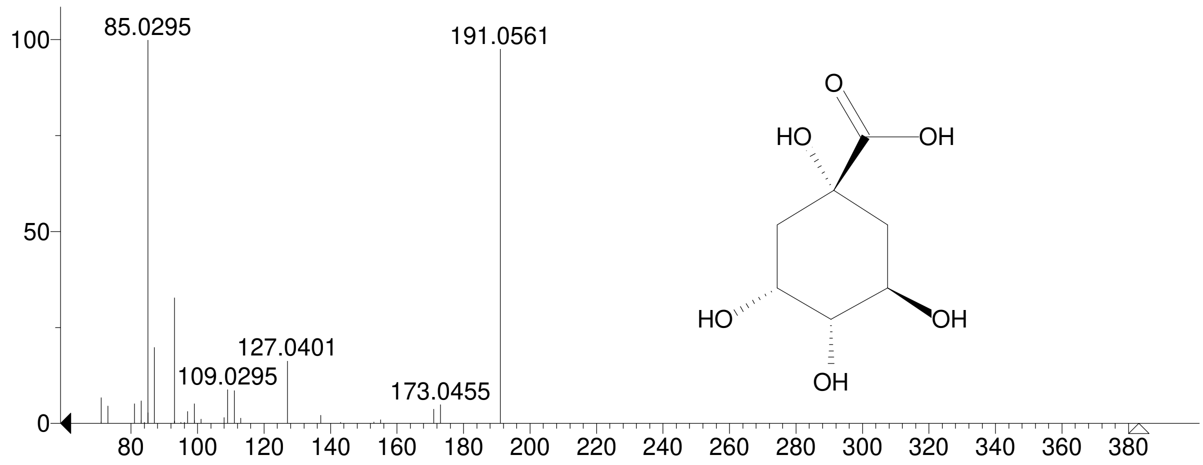

Hit 4 : S-(5'-ADENOSYL)-L-HOMOCYSTEINE [M-H]- P=383.1
C14H20N6O5S; MF: 0; RMF: 16; Prob 7.56%; Lib: mona-export-gnps; ID: 5663.

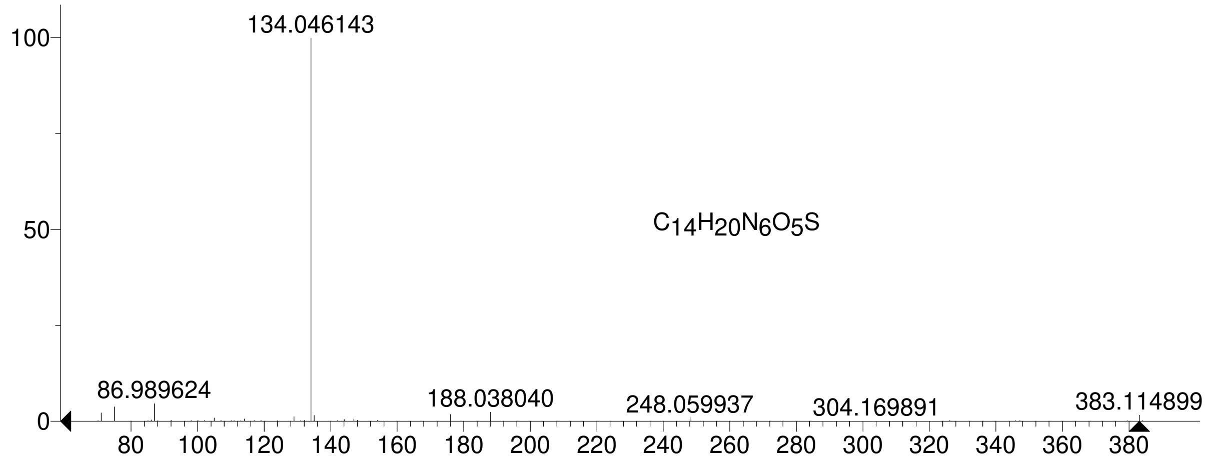

Hit 5 : S-(5#39;-ADENOSYL)-L-HOMOCYSTEINE
C14H20N6O5S; MF: 0; RMF: 15; Prob 7.56%; Lib: embl neg mcg; ID: 301.

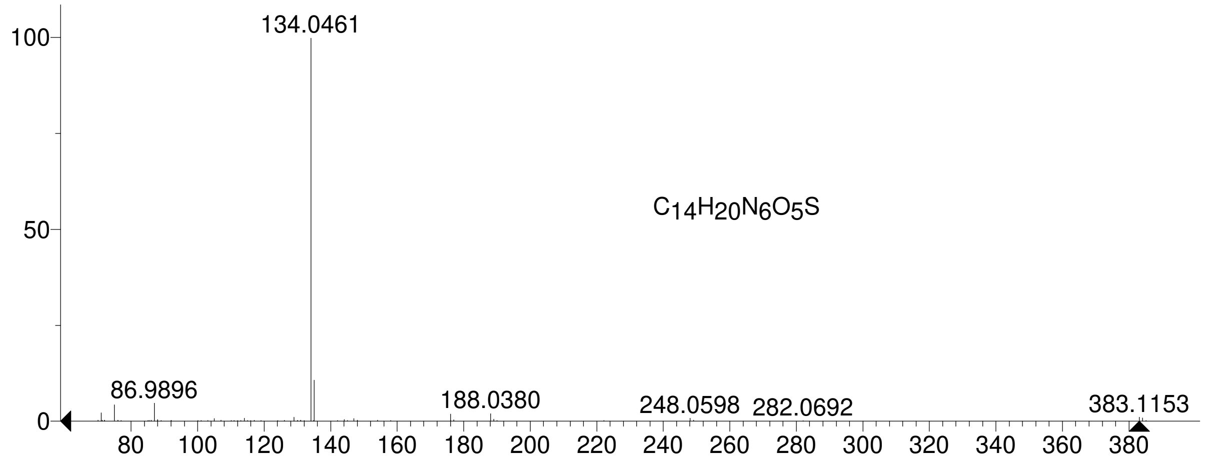

** Search Report Page 1 of 1 **

Unknown: Unknown (1.08_537.1213m/z)
Compound in Library Factor = 0

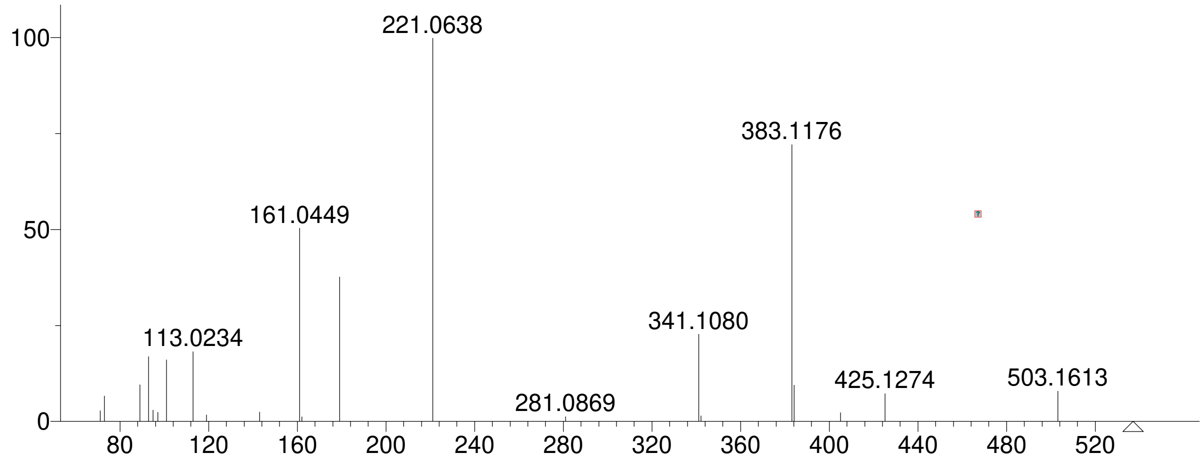

Hit 1 : 6-Oxomometasone furoate [M+H+2i]+ IT-FT 35% P=537.1
C27H28Cl2O7; MF: 0; RMF: 109; Prob 100.0%; CAS: 1305334-30-8; Lib: nist_msms; ID: 517601.

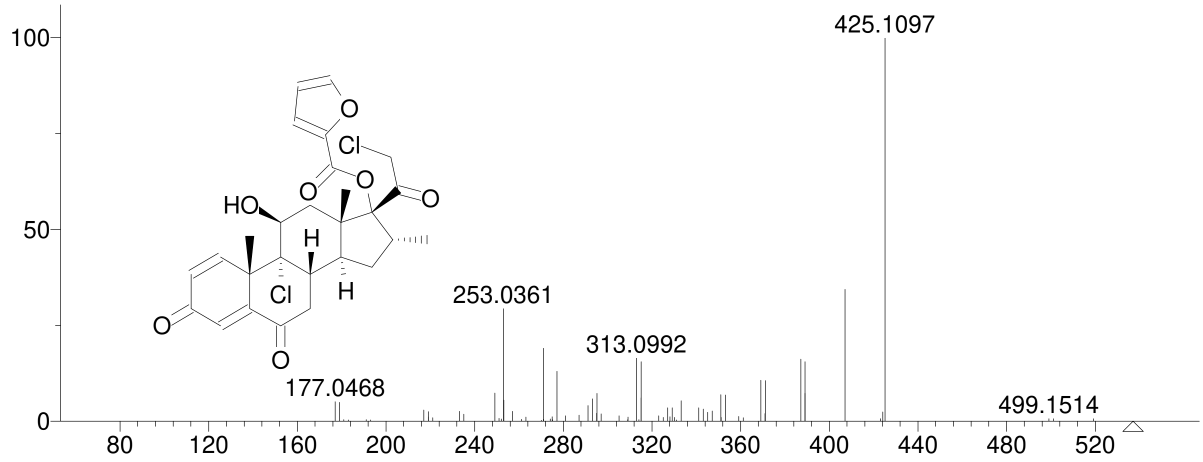

Unknown: Unknown (1.08_539.1376m/z)
Compound in Library Factor = 845

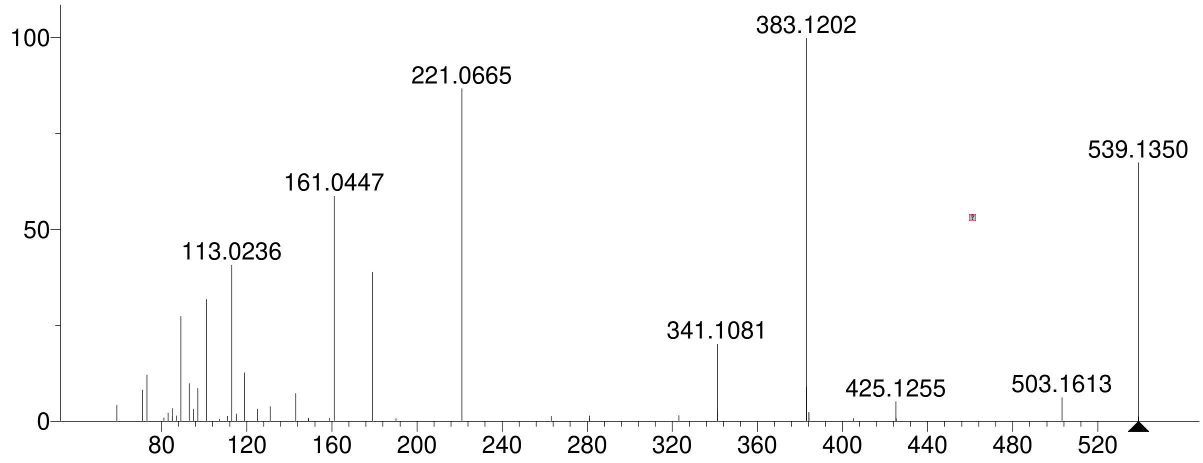

Hit 1 : Maltotriose [M+Cl]- HCD 37V P=539.1
C18H32O16; MF: 866; RMF: 842; Prob 82.2%; CAS: 1109-28-0; Lib: nist_msms; ID: 478094.

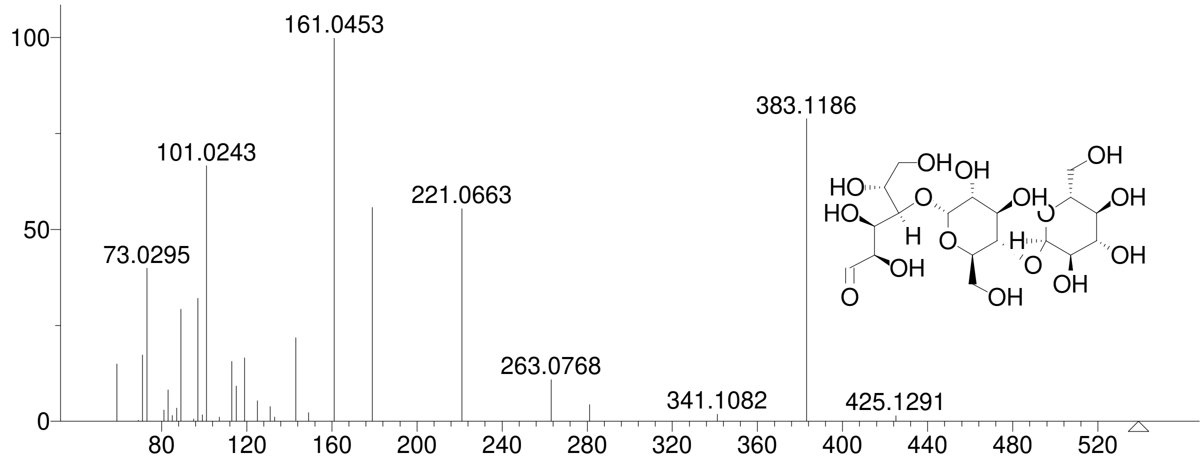

Hit 2 : Manninotriose [M+Cl]- HCD 32V P=539.1
C18H32O16; MF: 804; RMF: 836; Prob 14.0%; CAS: 13382-86-0; Lib: nist_msms; ID: 531273.

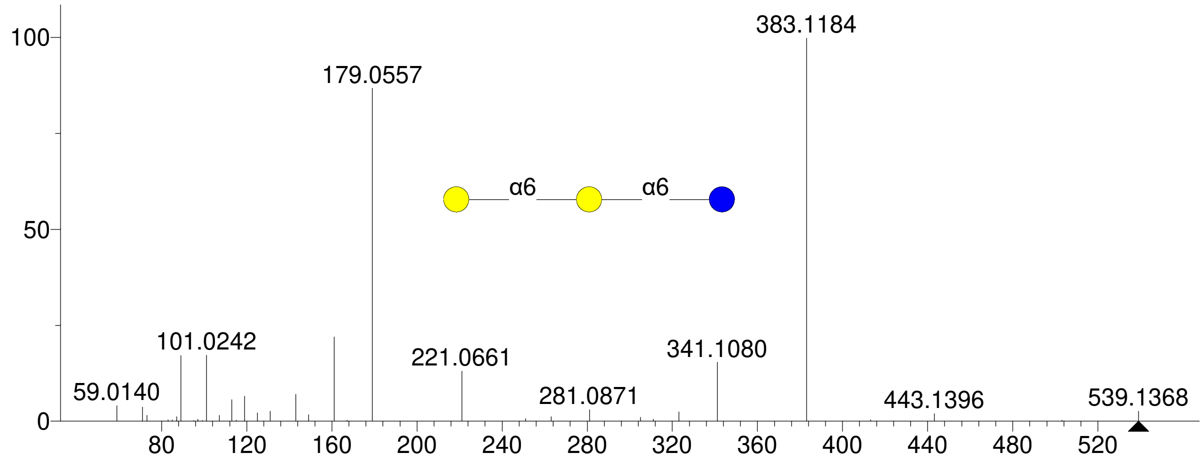

Hit 3 : Erlose [M+Cl]- HCD 37V P=539.1
C18H32O16; MF: 706; RMF: 742; Prob 1.08%; CAS: 13101-54-7; Lib: nist_msms; ID: 531028.

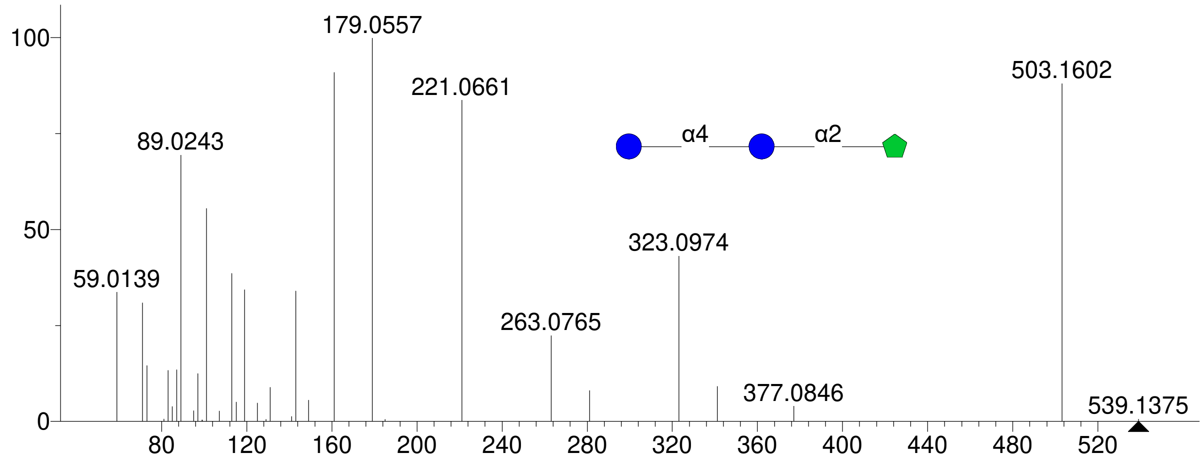

Hit 4 : 3α,4β-Galactotriose [M+Cl]- HCD 26V P=539.1
C18H32O16; MF: 704; RMF: 735; Prob 0.99%; CAS: 56038-36-9; Lib: nist_msms; ID: 527588.

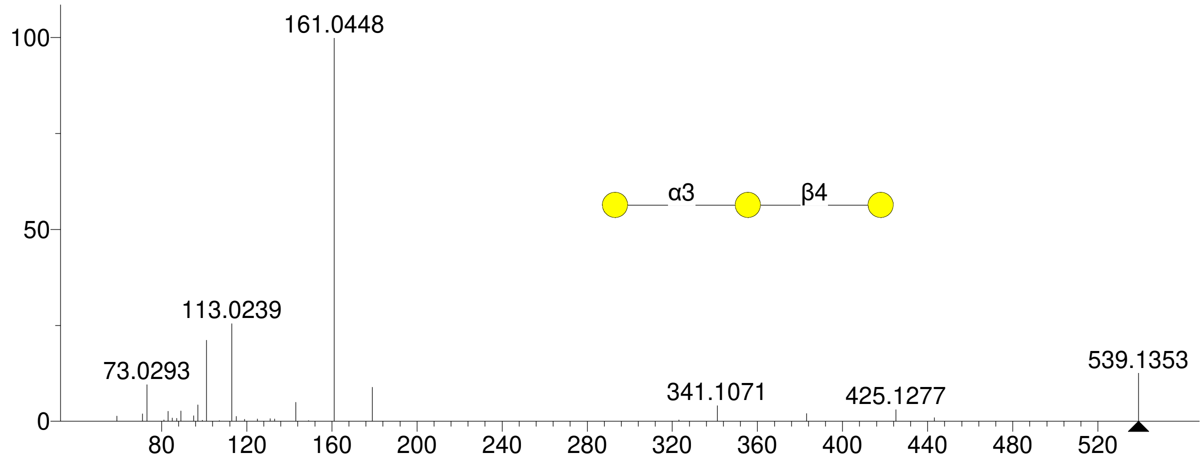

Hit 5 : 3'-Galactosyllactose [M+Cl]- HCD 26V P=539.1
C18H32O16; MF: 703; RMF: 740; Prob 0.96%; CAS: 32694-82-9; Lib: nist_msms; ID: 533312.

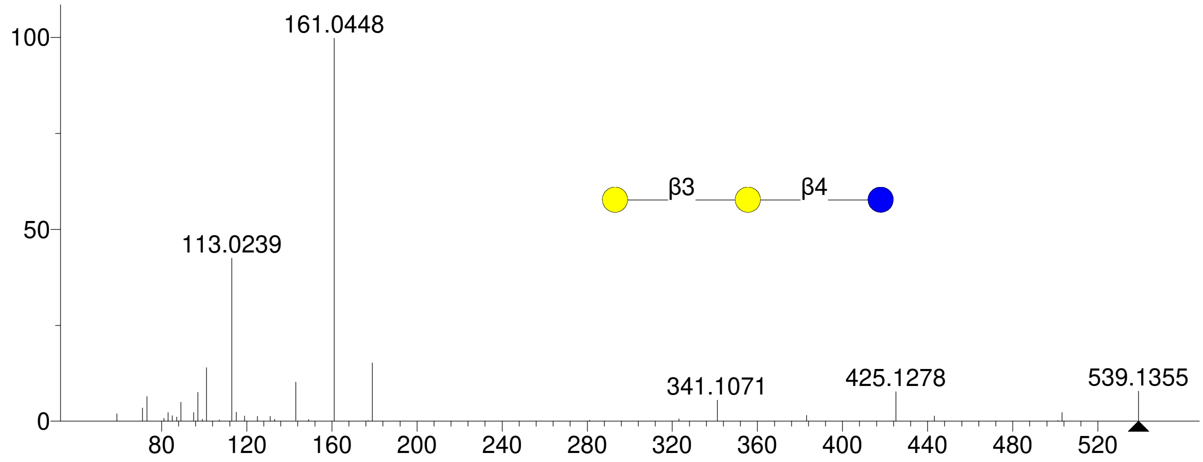

Unknown: Unknown (1.08_539.3557m/z)
Compound in Library Factor = 0

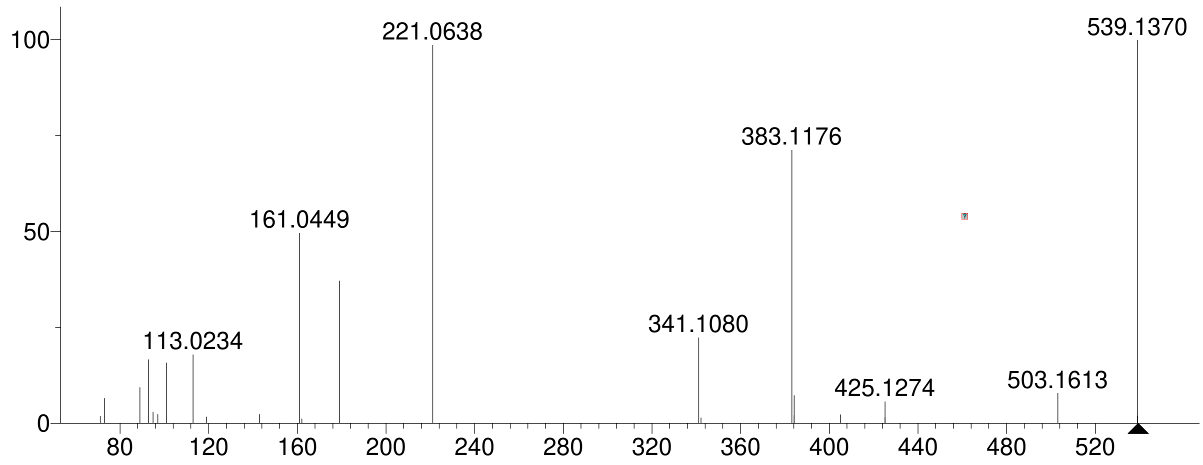

Unknown: Unknown (1.08_541.1356m/z)
Compound in Library Factor = 0

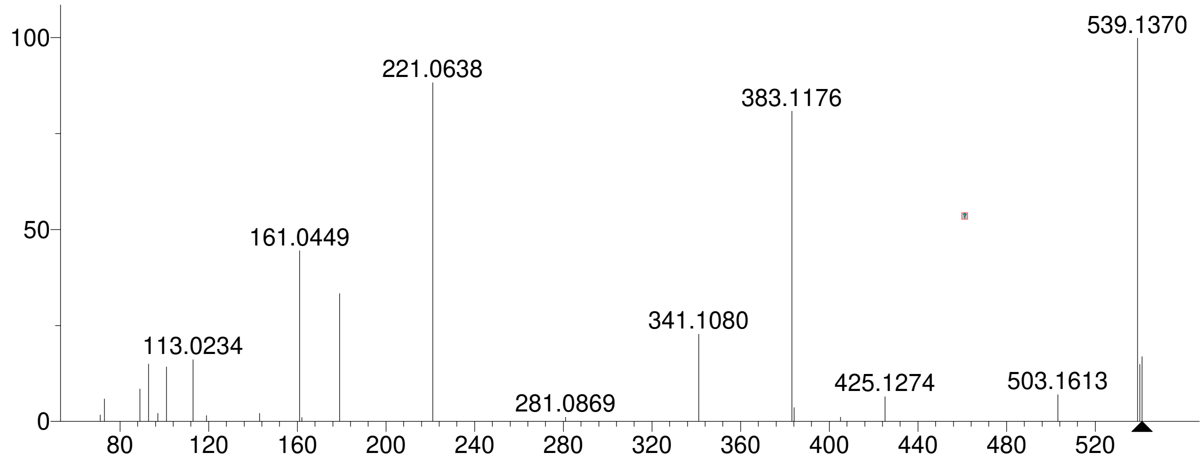

Unknown: Unknown (1.08_613.0692m/z)
Compound in Library Factor = 0

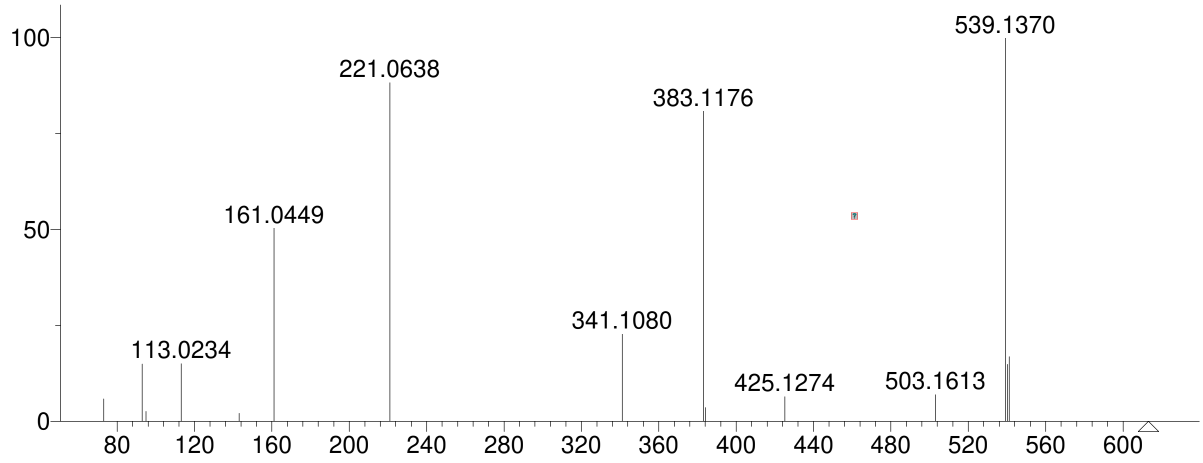

Unknown: Unknown (1.08_683.2246m/z)
Compound in Library Factor = -1848

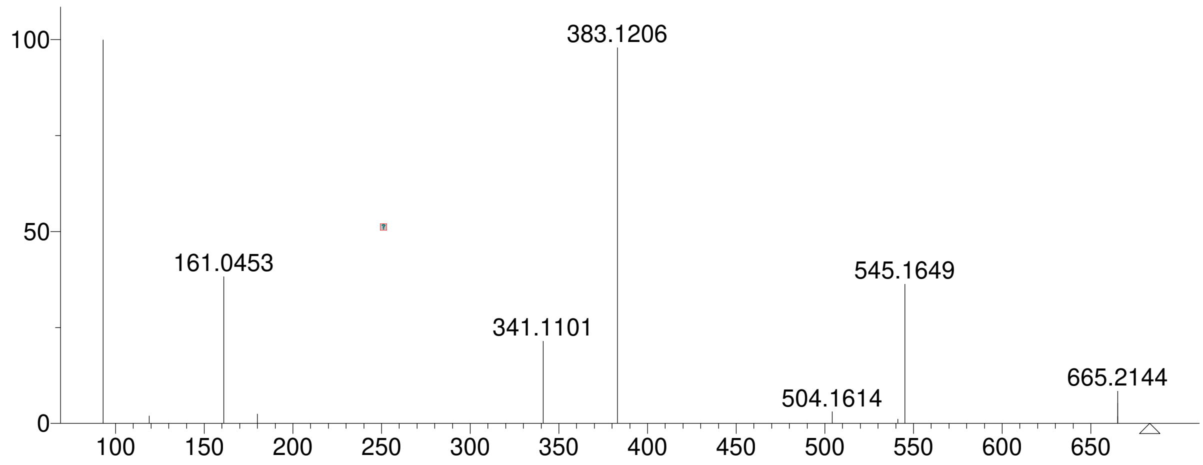

Hit 1 : Lactose [2M-H]- HCD 10V P=683.2
C12H22O11; MF: 128; RMF: 464; Prob 23.6%; CAS: 63-42-3; Lib: nist_msms; ID: 500011.

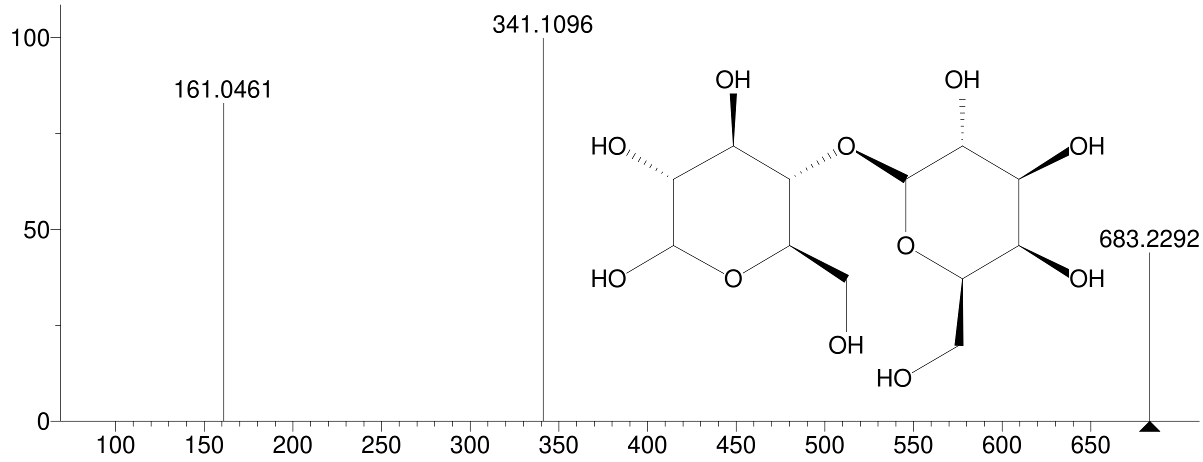

Hit 2 : 4-O-β-Galactopyranosyl-D-mannopyranose [2M-H]- HCD 10V P=683.2
C12H22O11; MF: 124; RMF: 441; Prob 19.9%; CAS: 20869-27-6; Lib: nist_msms; ID: 500321.

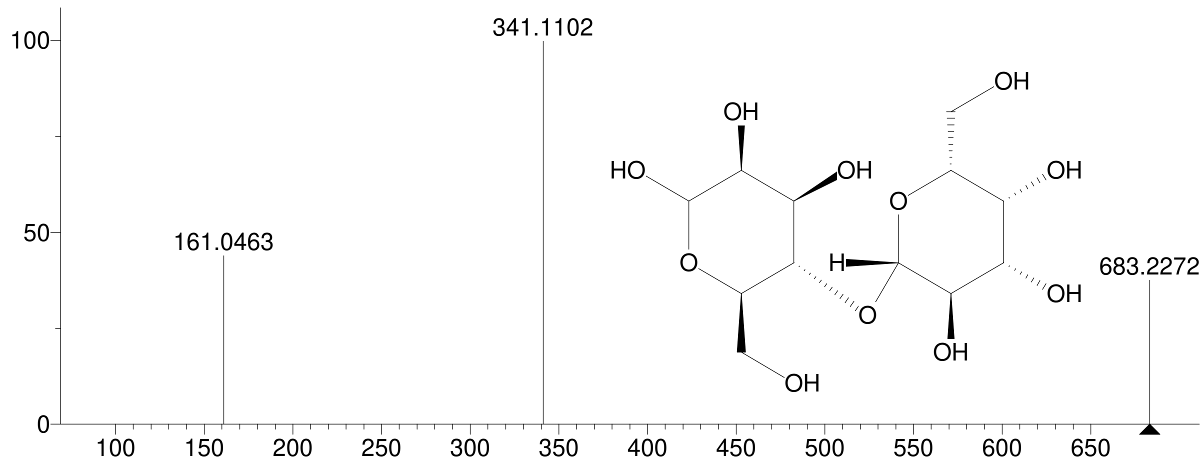

Hit 3 : 4α-Mannobiose [2M-H]- HCD 20V P=683.2
C12H22O11; MF: 120; RMF: 479; Prob 16.8%; CAS: 35438-40-5; Lib: nist_msms; ID: 534314.

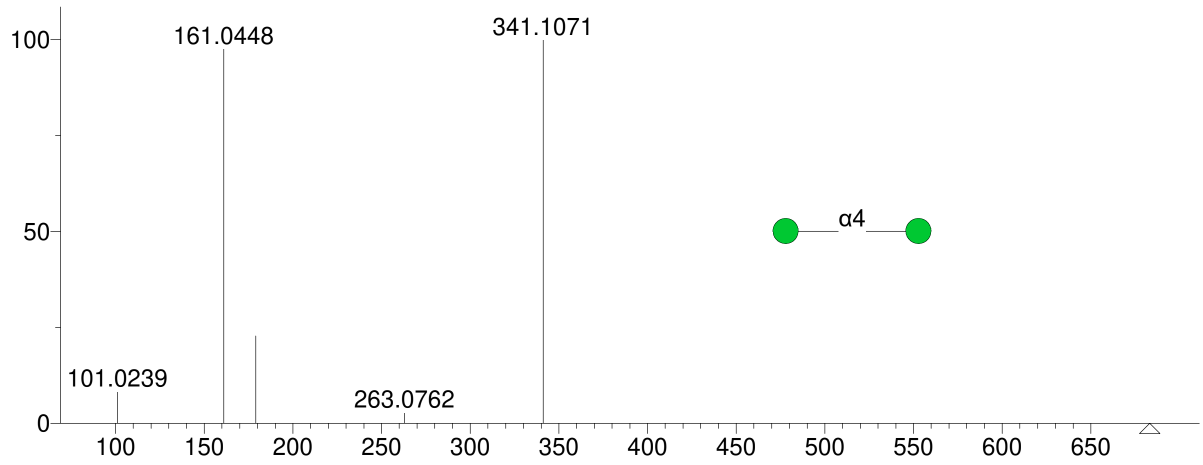

Hit 4 : Lactulose [2M-H]- HCD 13V P=683.2
C12H22O11; MF: 104; RMF: 457; Prob 9.69%; CAS: 4618-18-2; Lib: nist_msms; ID: 477980.

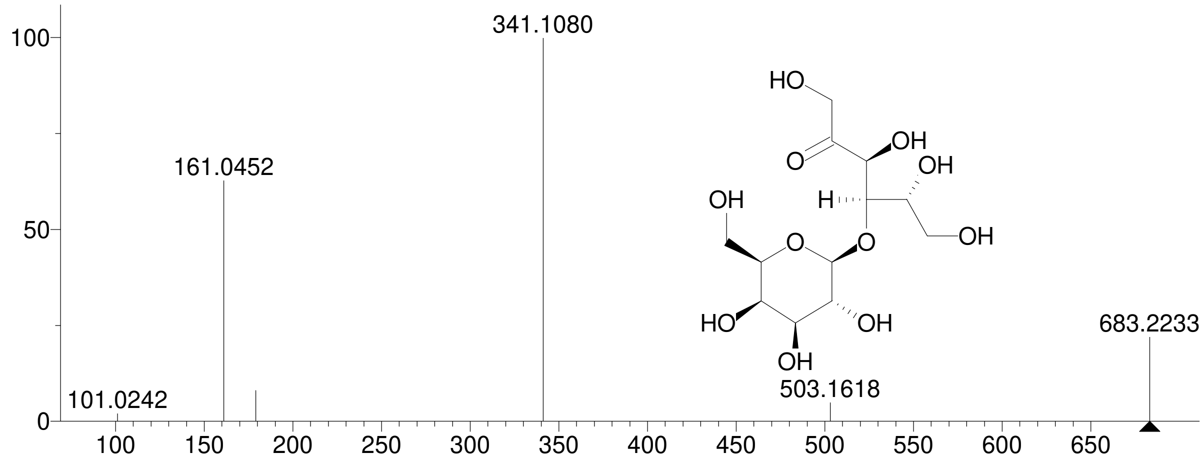

Hit 5 : Galactinol [2M-H]- HCD 27V P=683.2
C12H22O11; MF: 101; RMF: 283; Prob 8.56%; CAS: 3687-64-7; Lib: nist_msms; ID: 477813.

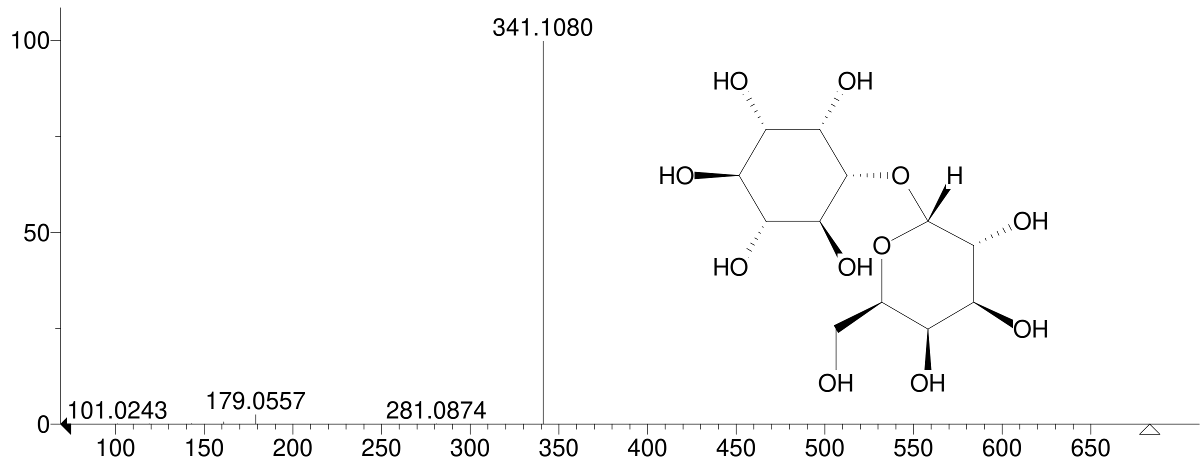

** Search Report Page 1 of 1 **

Unknown: Unknown (1.09_541.3534m/z)
Compound in Library Factor = 0

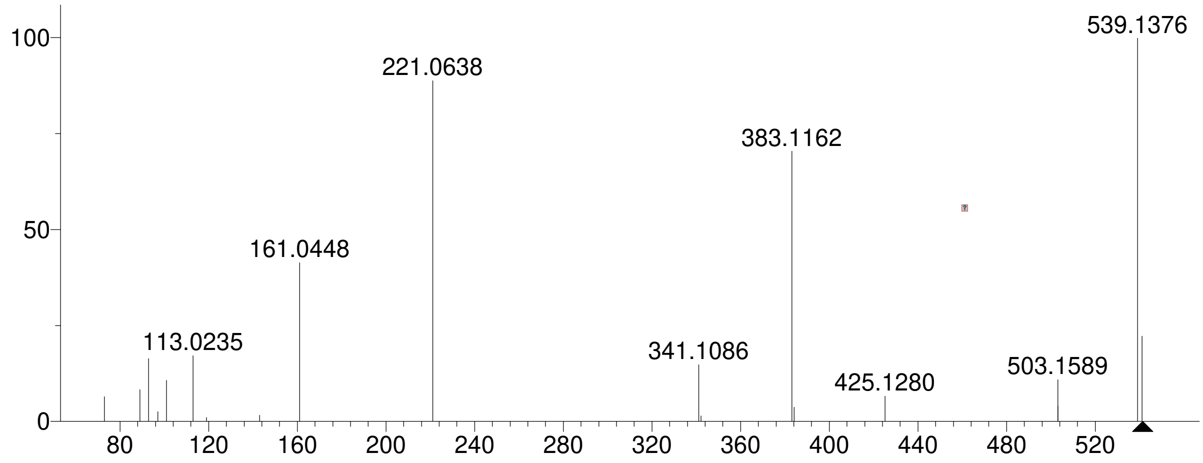

Hit 1 : Leptomycin B [M+H]+ HCD 32V P=541.4
C33H48O6; MF: 0; RMF: 8; Prob 100.0%; CAS: 87081-35-4; Lib: nist_msms; ID: 118131.

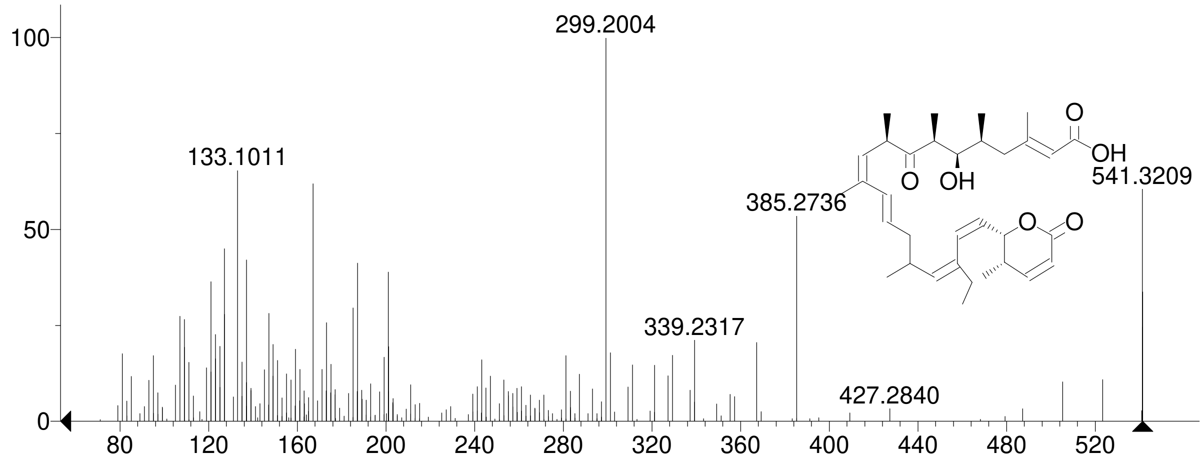

Unknown: Unknown (1.09_597.0958m/z)
Compound in Library Factor = 0

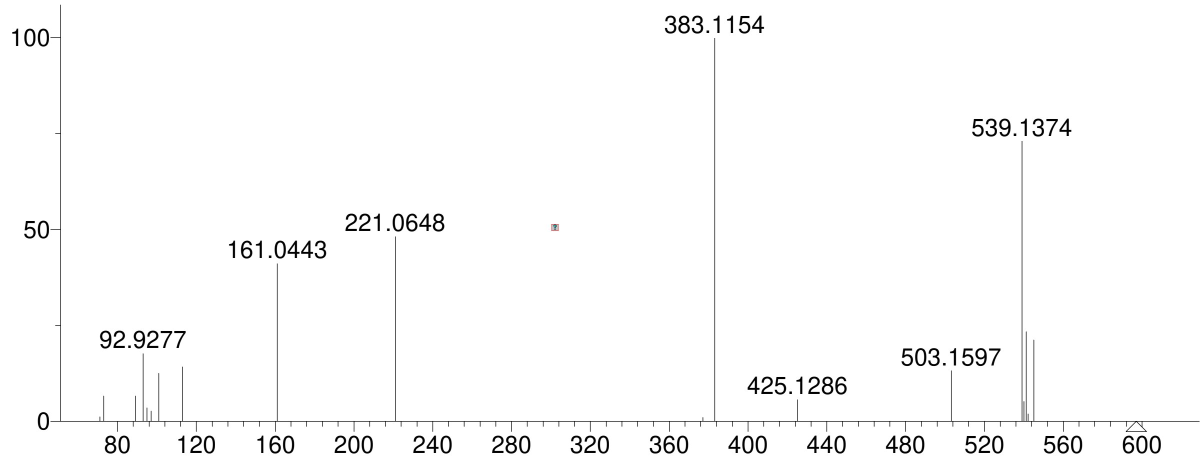

Hit 1 : 3-[5,7-dihydroxy-4-(4-hydroxyphenyl)-2-oxo-3,4-dihydrochromen-3-yl]-5,6,7,8-tetrahydroxy-4-(4-hydroxyphe
C30H22O12; MF: 0; RMF: 20; Prob 100.0%; Lib: mona-export-vf-npl_qexactive; ID: 25167.

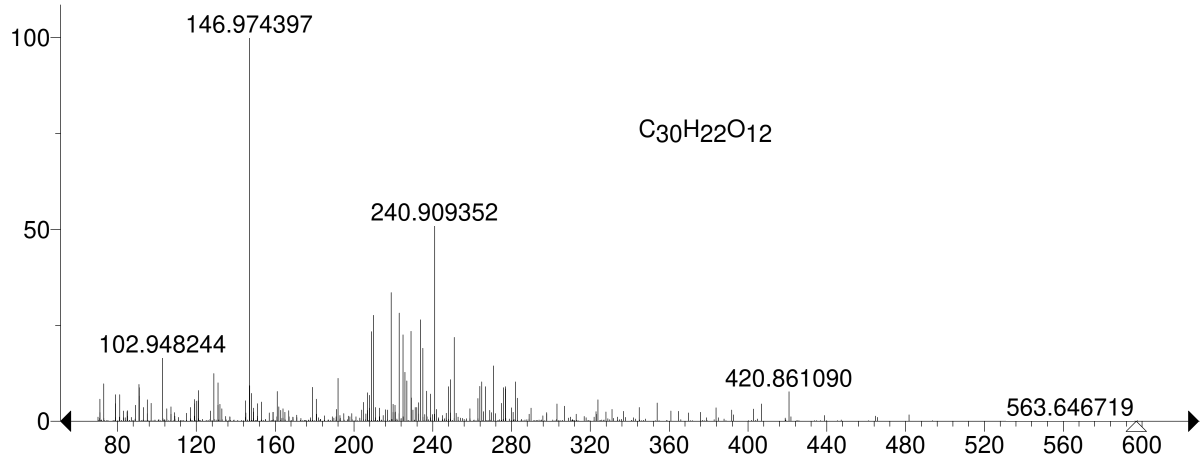

** Search Report Page 1 of 1 **

Unknown: Unknown (1.10_631.0759m/z)
Compound in Library Factor = 0

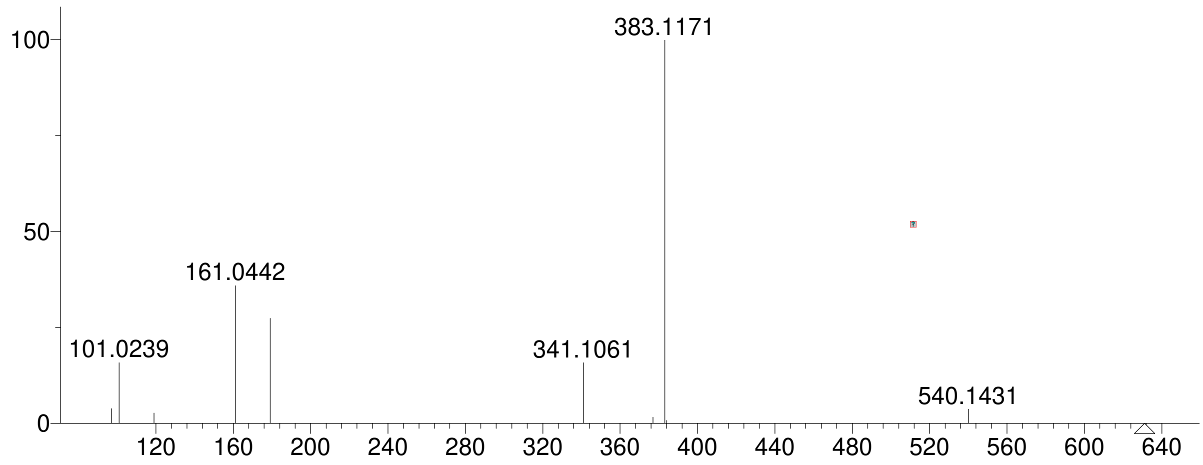

Unknown: Unknown (1.10_701.1903m/z)
Compound in Library Factor = -359

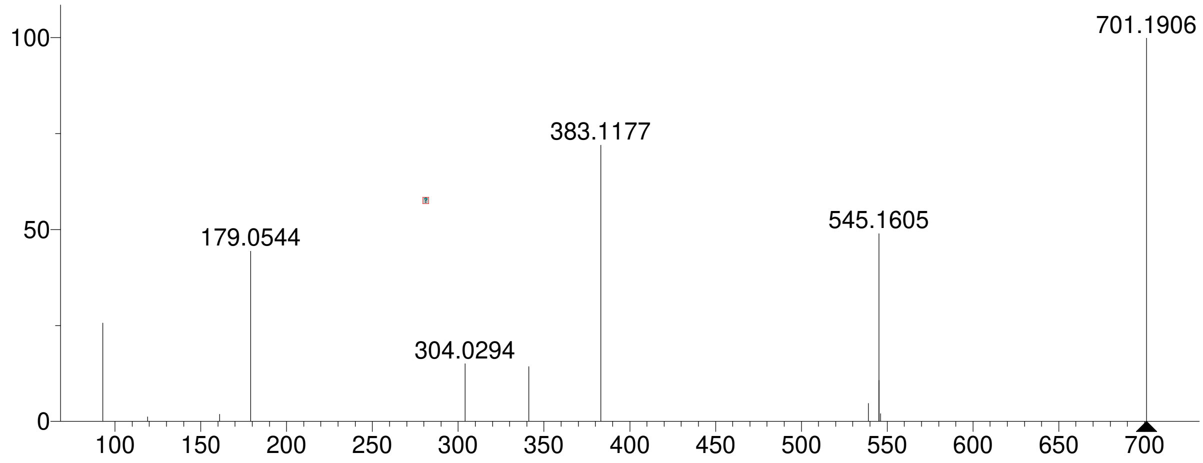

Hit 1 : Laminaritetraose [M+Cl]- HCD 10V P=701.2
C24H42O21; MF: 332; RMF: 957; Prob 81.1%; CAS: 26212-72-6; Lib: nist_msms; ID: 531112.

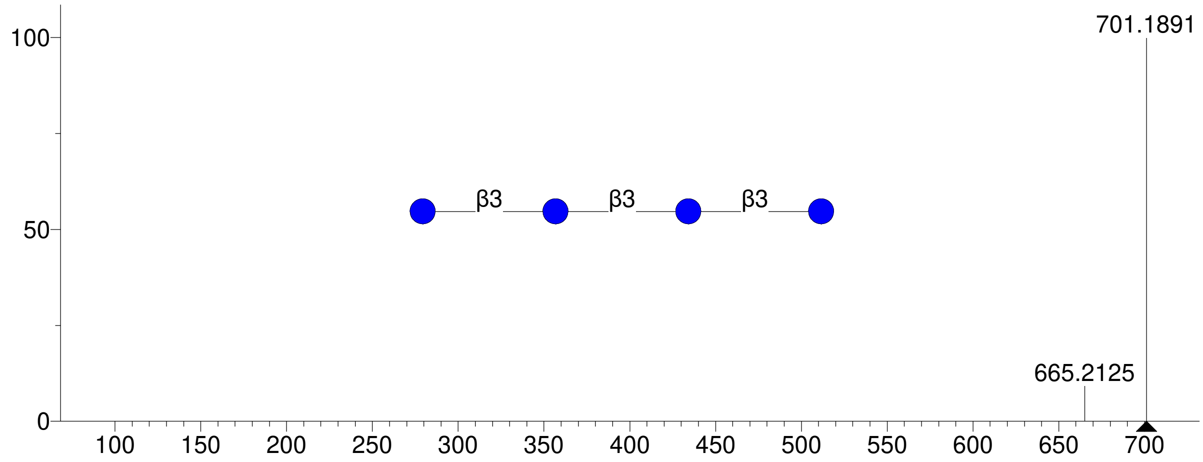

Hit 2 : Maltotetraose [M+Cl]- HCD 31V P=701.2
C24H42O21; MF: 286; RMF: 621; Prob 17.1%; CAS: 34612-38-9; Lib: nist_msms; ID: 286899.

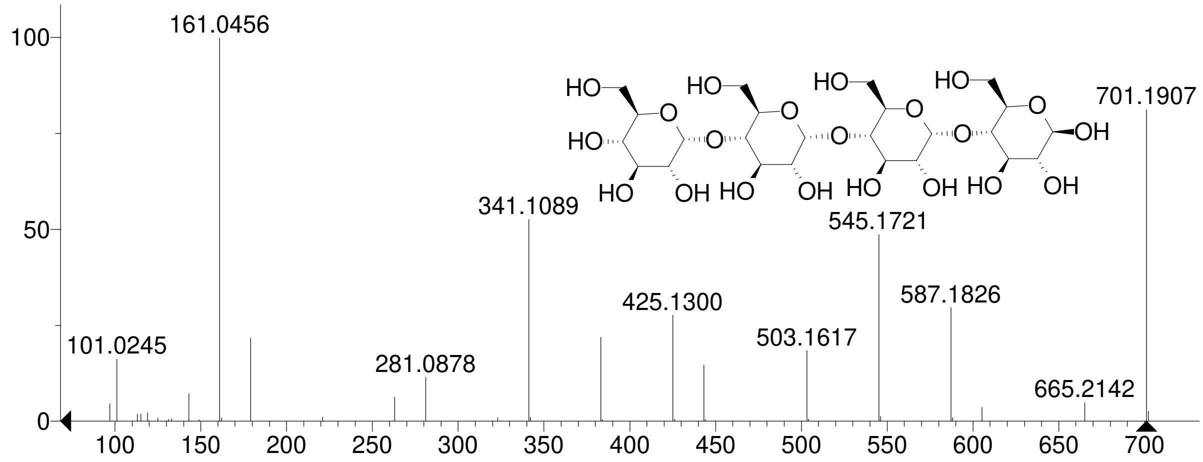

Hit 3 : Cellotetraose [M+Cl]- HCD 28V P=701.2
C24H42O21; MF: 180; RMF: 437; Prob 1.15%; CAS: 38819-01-1; Lib: nist_msms; ID: 500365.

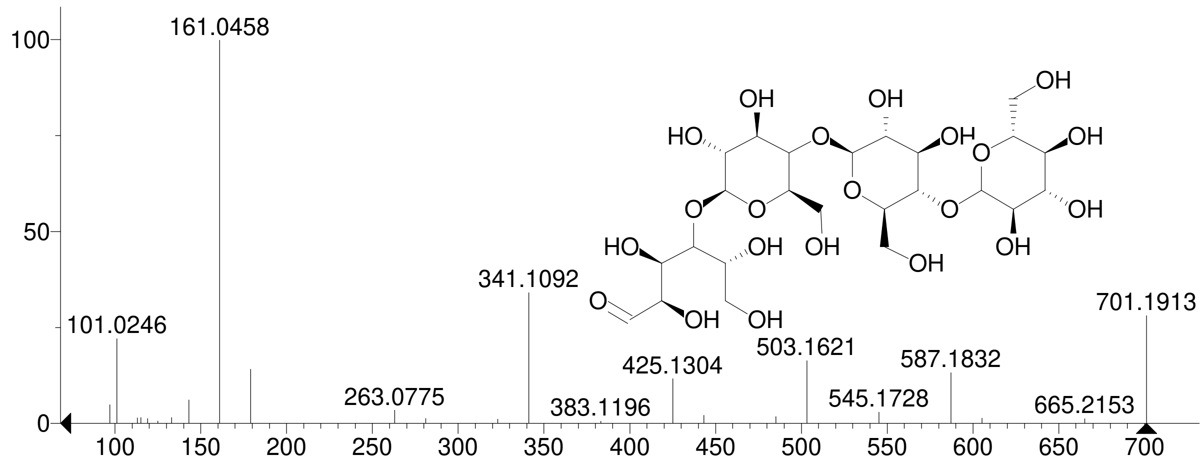

Hit 4 : 3α,4β,3α-Galactotetraose [M+Cl]- HCD 21V P=701.2
C24H42O21; MF: 162; RMF: 554; Prob 0.59%; CAS: 56038-38-1; Lib: nist_msms; ID: 530953.

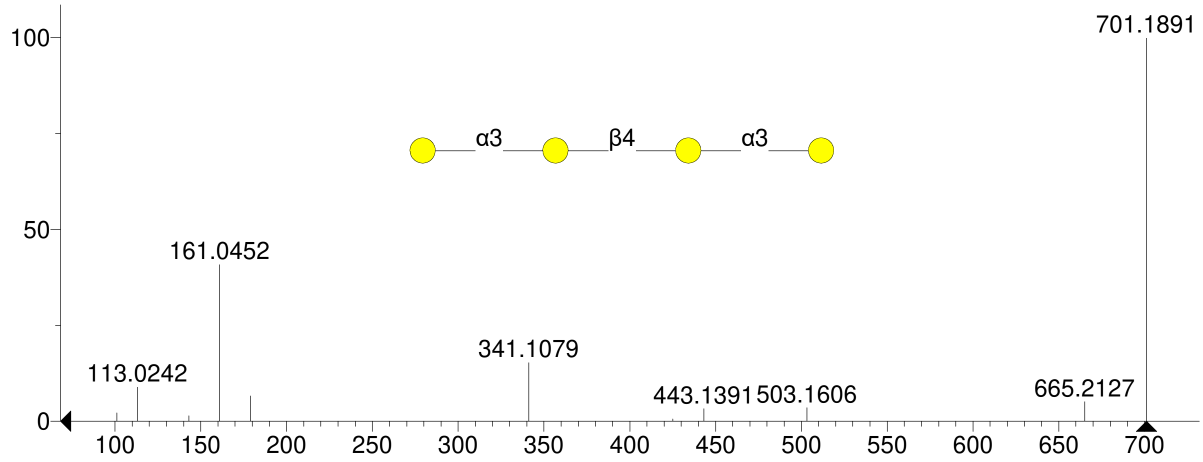

** Search Report Page 1 of 1 **

Unknown: Unknown (1.11_759.1491m/z)
Compound in Library Factor = 0

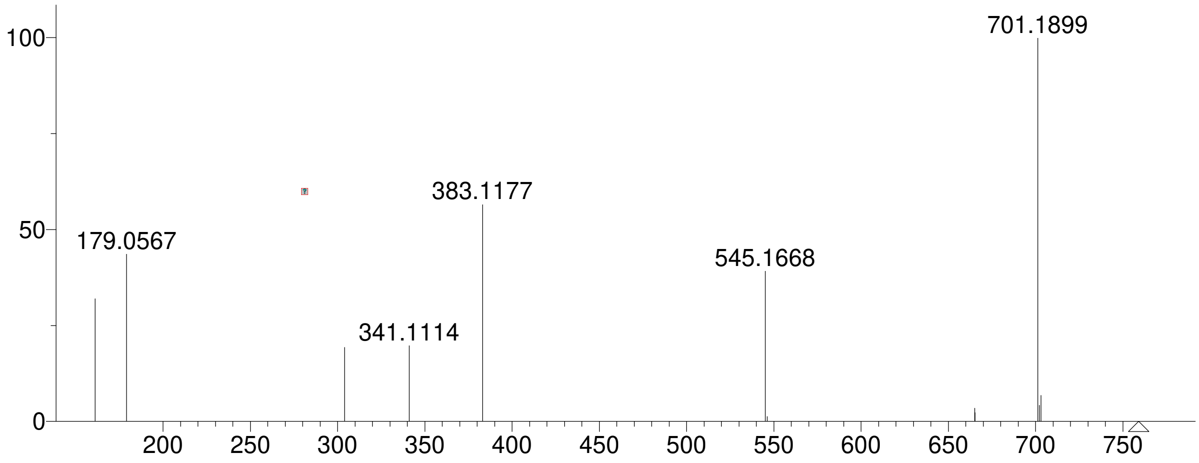

** Search Report Page 1 of 1 **

Unknown: Unknown (1.12_160.8408m/z)
Compound in Library Factor = 0

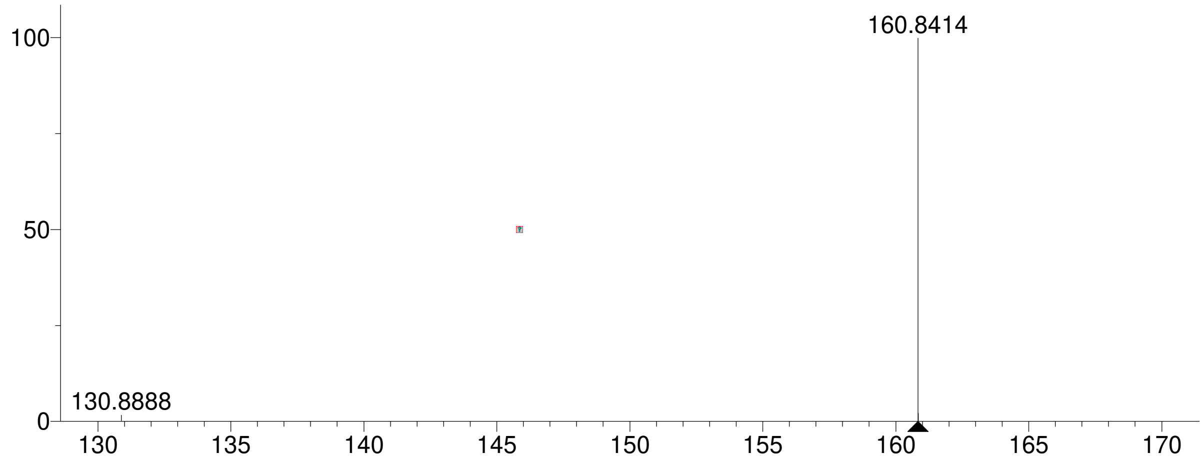

** Search Report Page 1 of 1 **

Unknown: Unknown (1.13_225.0054m/z)
Compound in Library Factor = 0

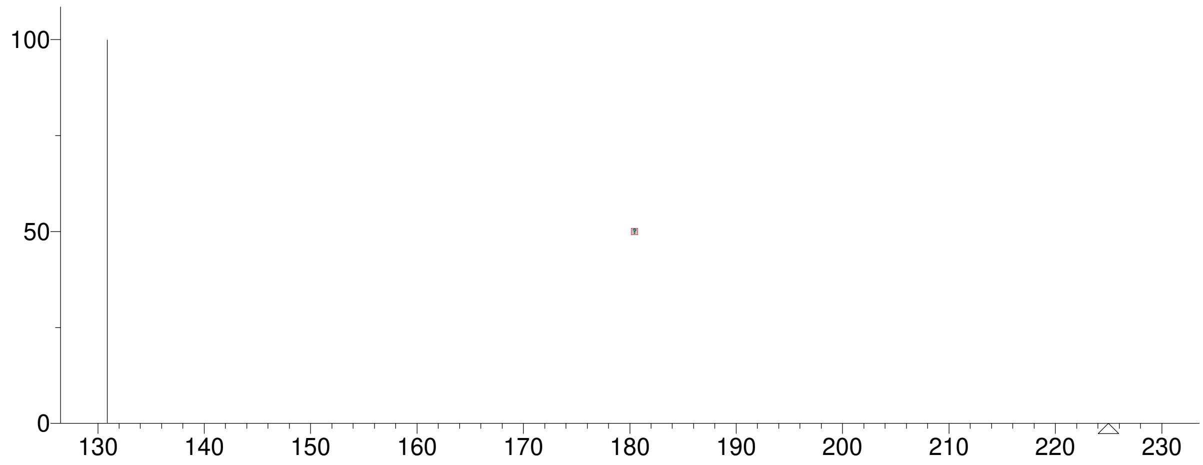

Unknown: Unknown (1.13_449.1062m/z)
Compound in Library Factor = 0

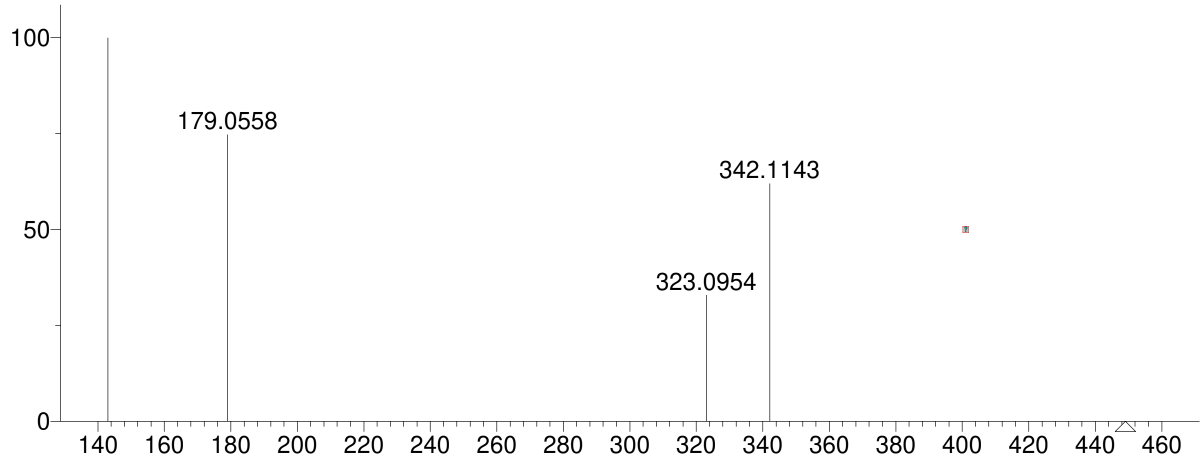

Hit 1 : Orientin [M+H]+ HCD 116V P=449.1
C21H20O11; MF: 0; RMF: 75; Prob 5.88%; CAS: 28608-75-5; Lib: nist_msms; ID: 390755.

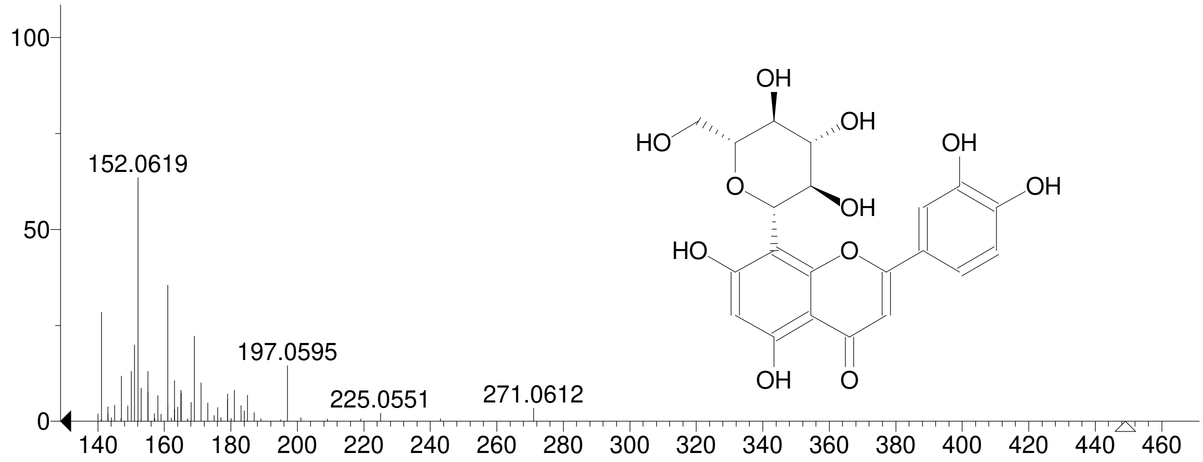

Hit 2 : Maritimein [M+H]+ HCD 67V P=449.1
C21H20O11; MF: 0; RMF: 41; Prob 5.88%; CAS: 490-54-0; Lib: nist_msms; ID: 494967.

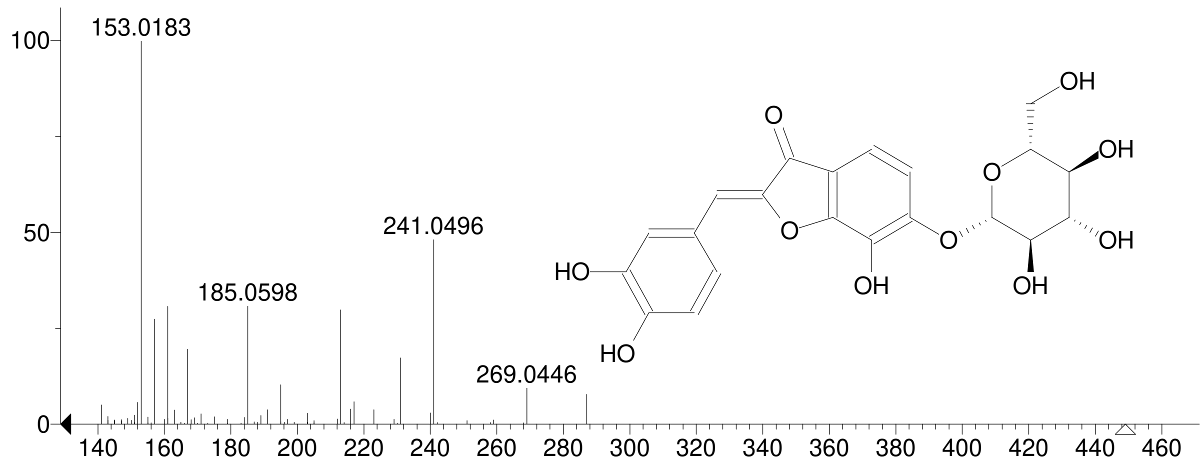

Hit 3 : luteolin-7-glucoside [M+H]+ 45HCD P=449.1
C21H20O11; MF: 0; RMF: 31; Prob 5.88%; Lib: anthocyanins mona; ID: 8111.

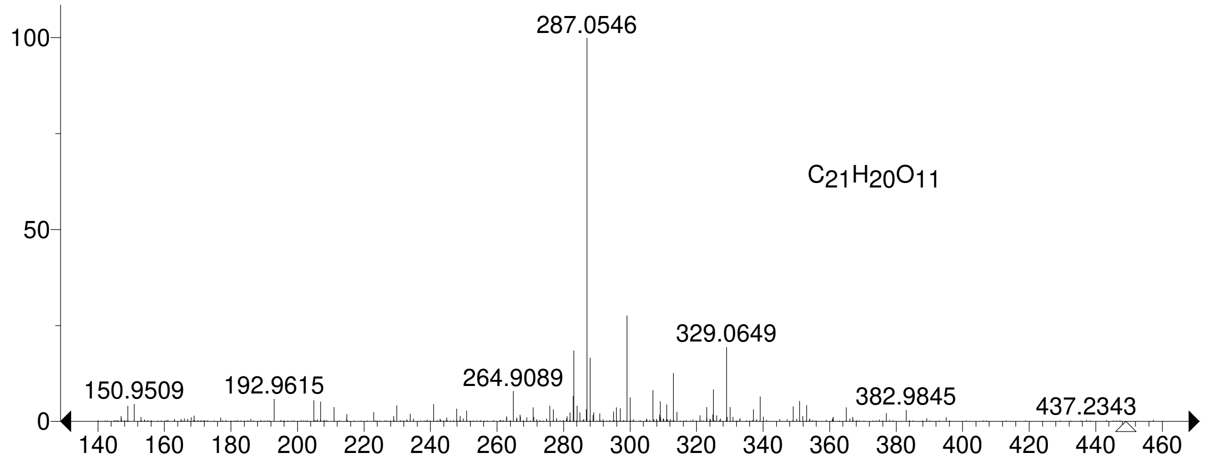

Hit 4 : Luteolin-8-C-glucoside [M+H]+ 50eV P=449.1
C21H20O11; MF: 0; RMF: 27; Prob 5.88%; Lib: anthocyanins mona; ID: 1993.

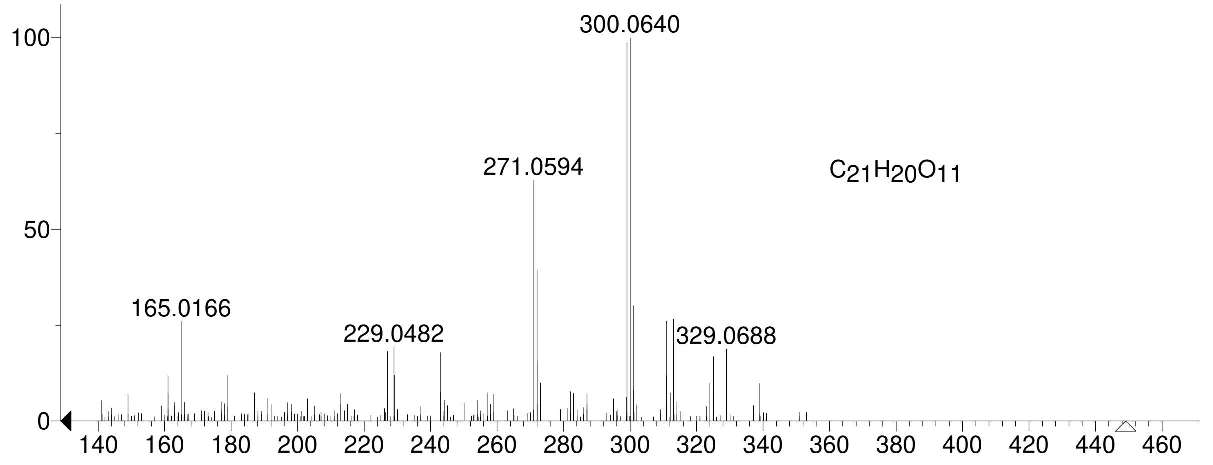

Hit 5 : NP-000286(7) [M+H]+ 30eV P=449.1
C21H20O11; MF: 0; RMF: 23; Prob 5.88%; Lib: anthocyanins mona; ID: 2947.

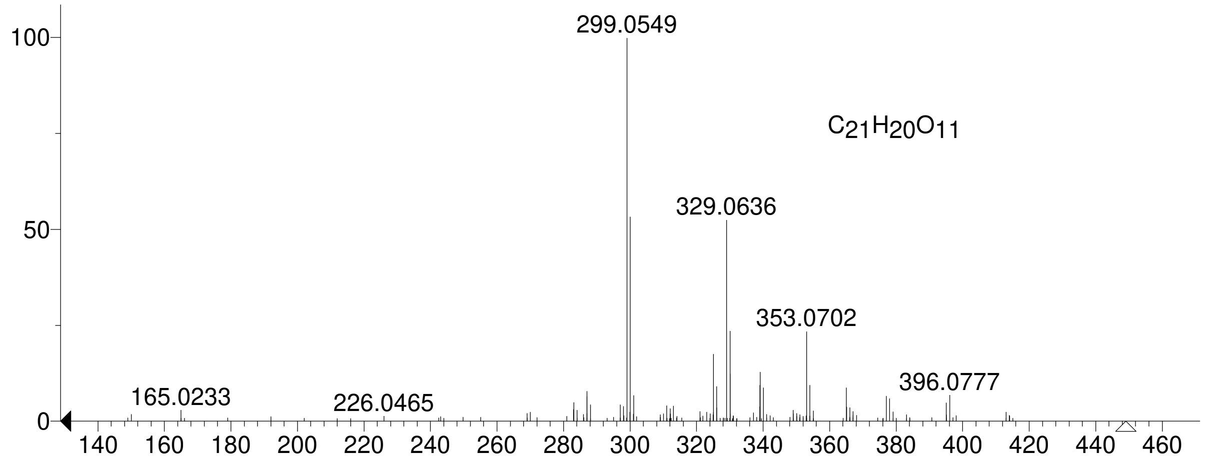

Unknown: Unknown (1.13_509.1248m/z)
Compound in Library Factor = 0

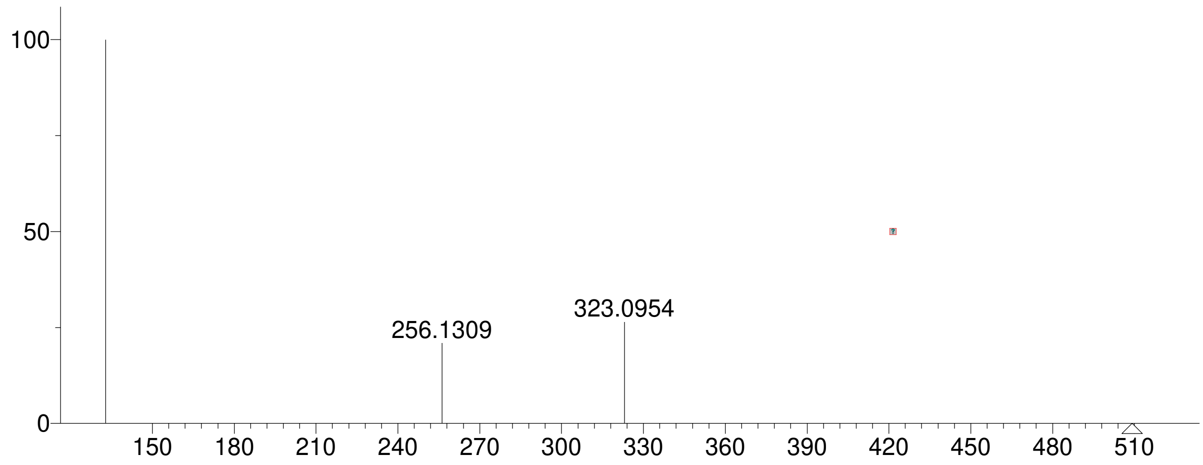

Unknown: Unknown (1.13_863.2428m/z)
Compound in Library Factor = 0

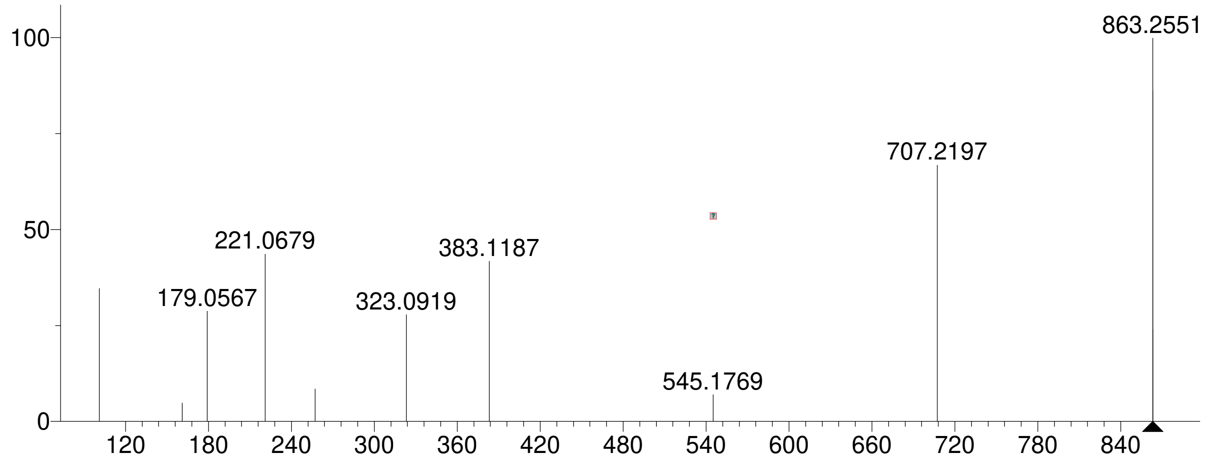

Hit 1 : [Phe10] β-Amyloid (1-40) [M+5H]5+ QTOF 3V P=863.2
C194H295N53O57S; MF: 0; RMF: 117; Prob 50.0%; Lib: nist_msms2; ID: 36926.

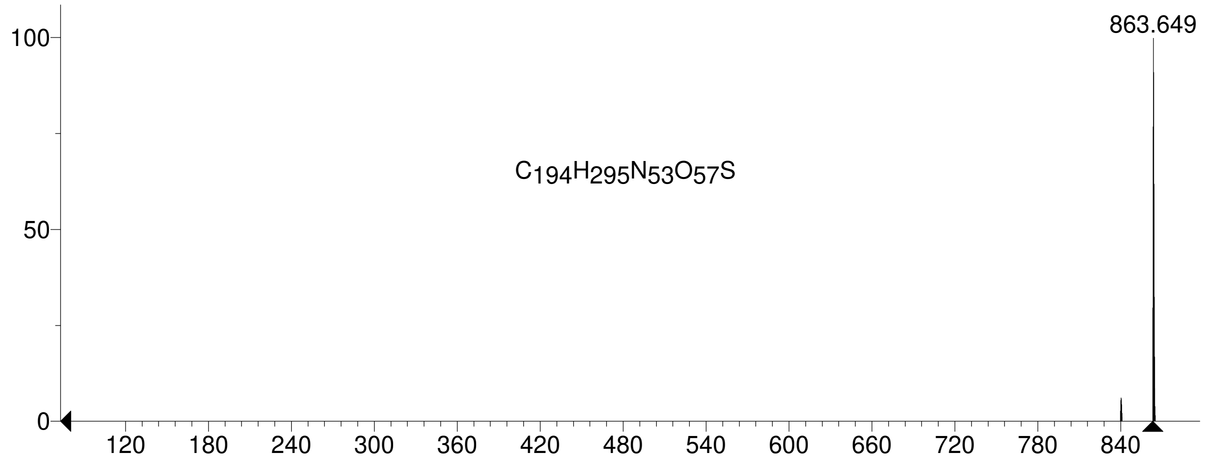

Hit 2 : Genistin [2M-H]- 35% P=863.2
C21H20O10; MF: 0; RMF: 29; Prob 50.0%; Lib: mona-export-itree; ID: 3973.

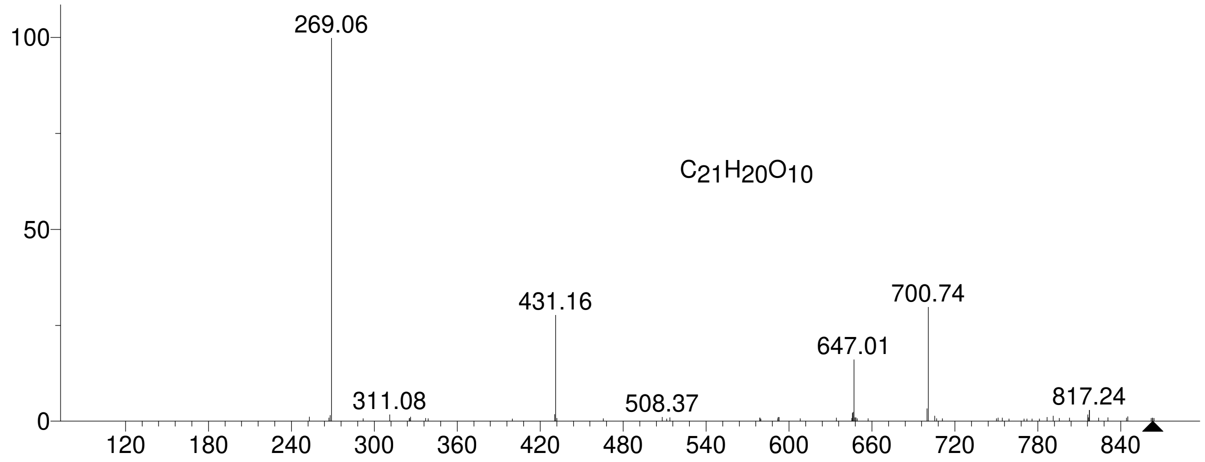

Unknown: Unknown (1.14_521.1258m/z)
Compound in Library Factor = 0

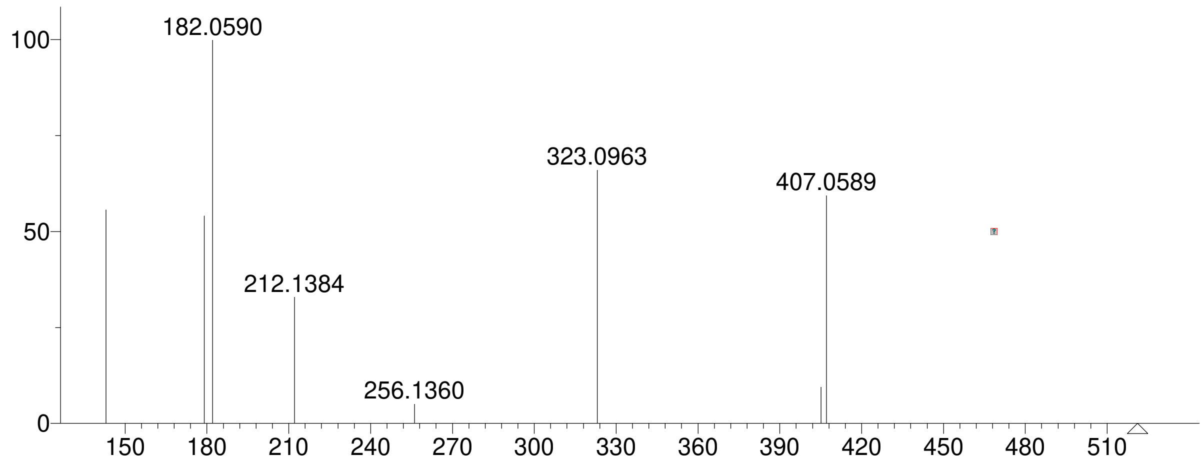

Hit 1 : verproside [M+Na]+ 65HCD P=521.1
C22H26O13; MF: 0; RMF: 14; Prob 33.3%; Lib: mona-export-vf-npl_qexactive; ID: 11988.

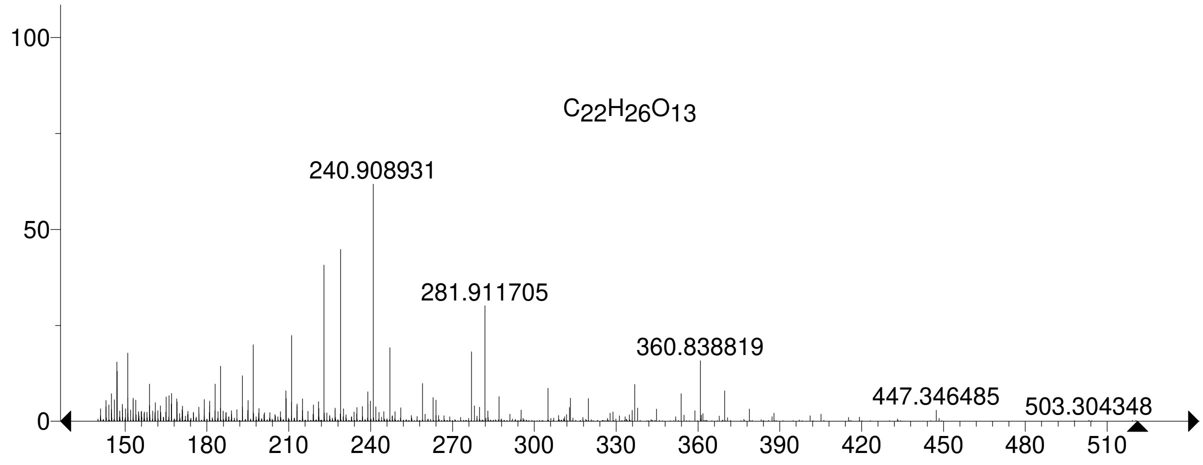

Hit 2 : Deacetoxy(7)-7-Oxokhivorinic Acid [M+H]+ 35% P=521.1
C27H36O10; MF: 0; RMF: 4; Prob 33.3%; Lib: mona-export-itree; ID: 1410.

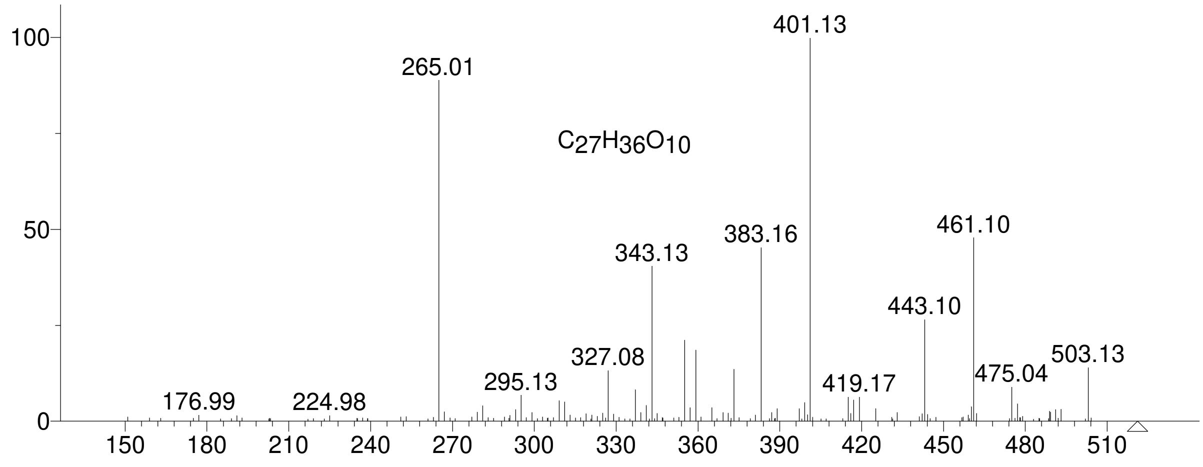

Hit 3 : 5-hydroxy-3-(5-hydroxy-2,4-dimethoxyphenyl)-6-methoxy-7-[3,4,5-trihydroxy-6-(hydroxymethyl)oxan-2-yl]ox
C24H26O13; MF: 0; RMF: 1; Prob 33.3%; Lib: anthocyanins mona; ID: 9905.

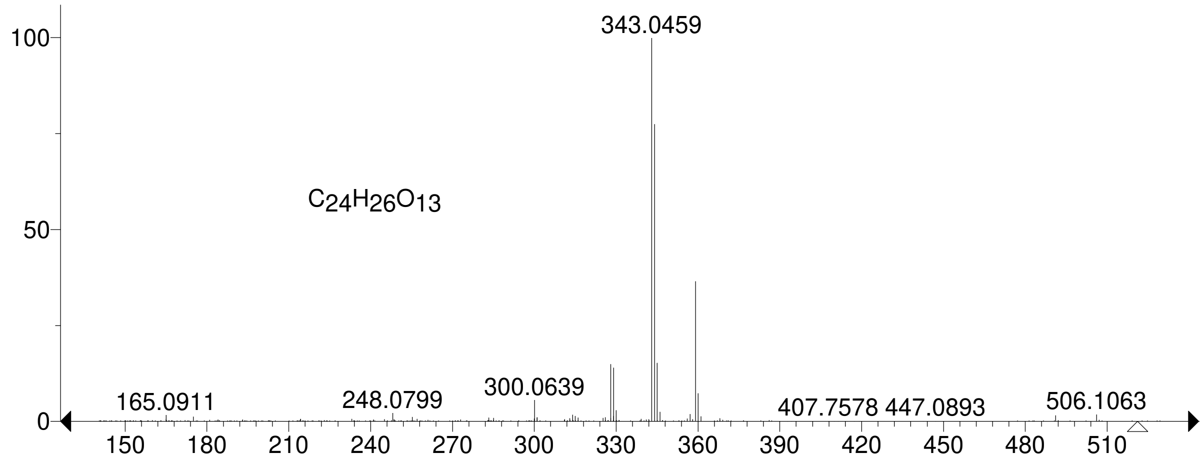

Unknown: Unknown (1.14_760.2978m/z)
Compound in Library Factor = 0

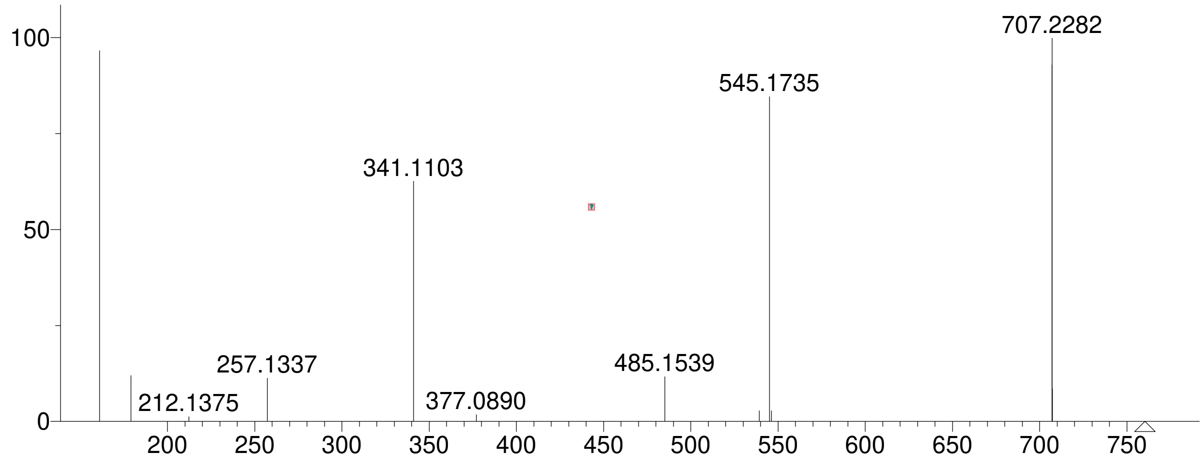

Unknown: Unknown (1.15_359.0715m/z)
Compound in Library Factor = 0

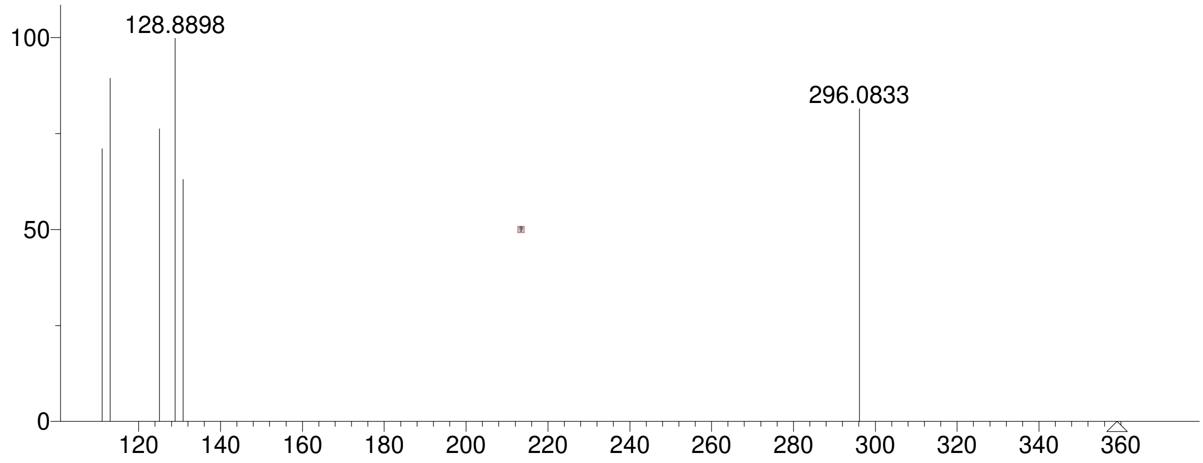

Hit 1 : 2-(4a,9,10a-trihydroxy-1-methyl-5,10-dioxo-3,4-dihydro-1H-benzo[g]isochromen-3-yl)acetic acid [M+Na]+
C16H16O8; MF: 0; RMF: 4; Prob 100.0%; Lib: mona-export-vf-npl_qexactive; ID: 19106.

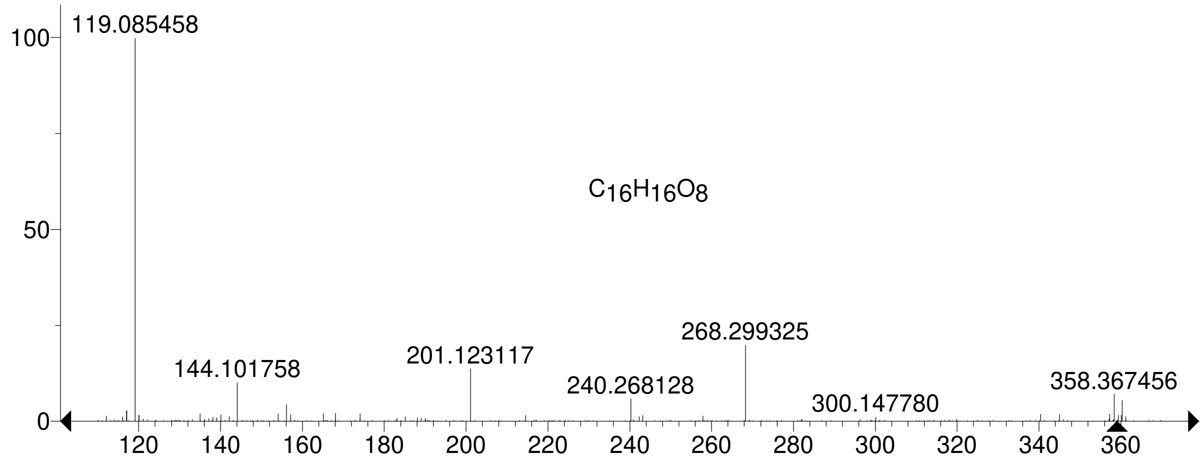

Unknown: Unknown (1.15_449.1038m/z)
Compound in Library Factor = -3273

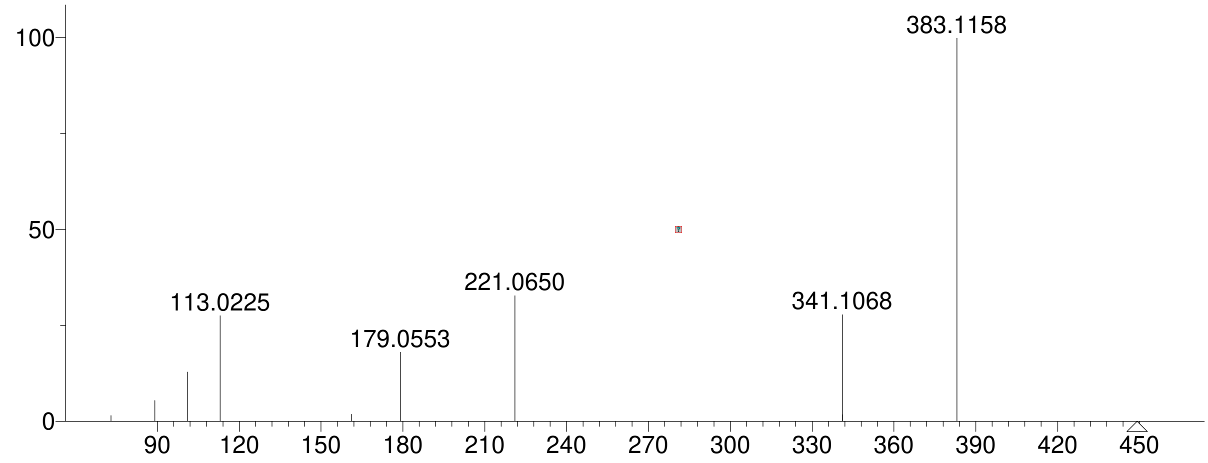

Hit 1 : Orientin [M+H]+ IT 35% P=449.1
C21H20O11; MF: 4; RMF: 263; Prob 3.04%; CAS: 28608-75-5; Lib: nist_msms; ID: 390757.

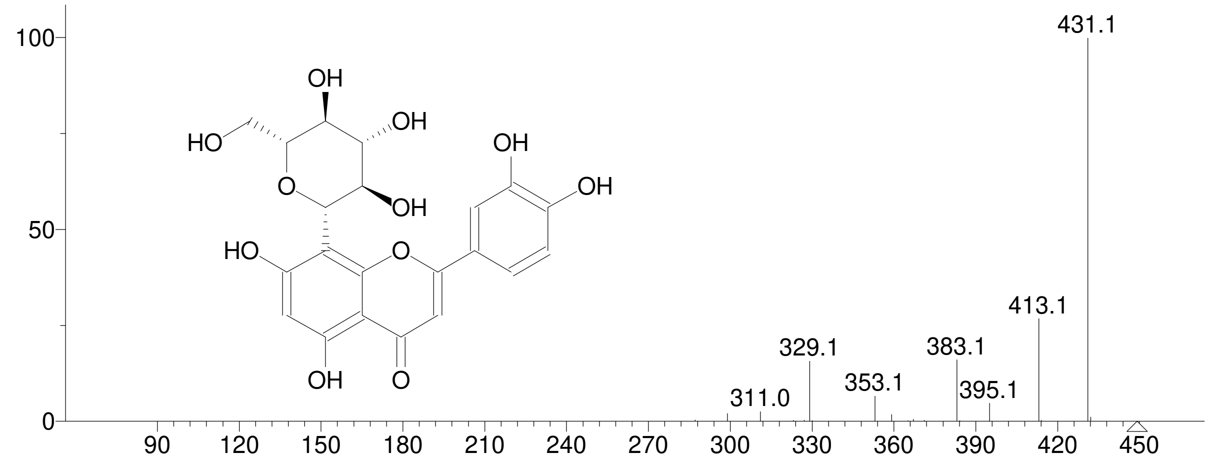

Hit 2 : Datiscin [M+H]+=>449.1 IT 35% P=449.1
C27H30O15; MF: 3; RMF: 231; Prob 2.93%; CAS: 16310-92-2; Lib: nist_msms; ID: 458608.

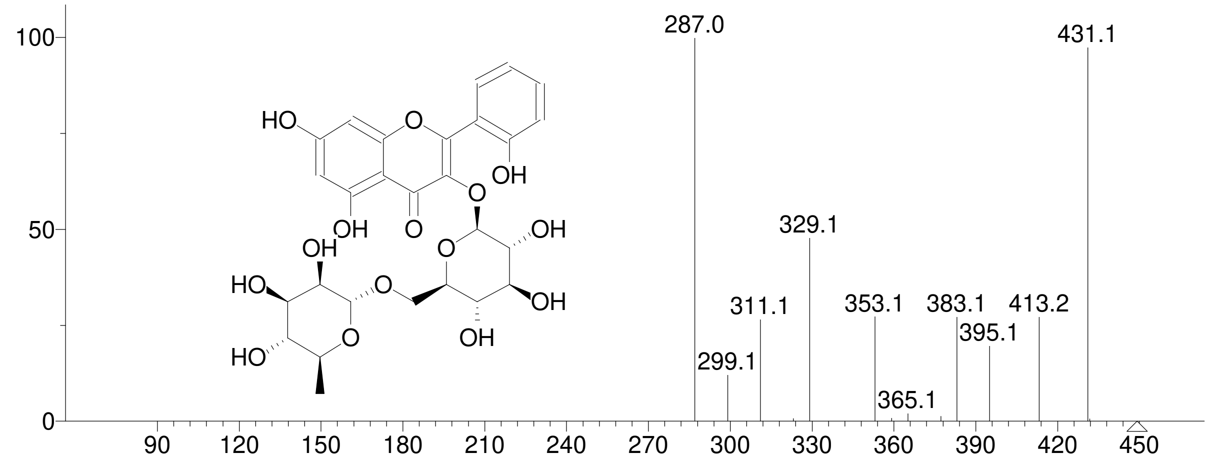

Hit 3 : Astragalin [M+H]+ IT 35% P=449.1
C21H20O11; MF: 2; RMF: 168; Prob 2.81%; CAS: 480-10-4; Lib: nist_msms; ID: 72607.

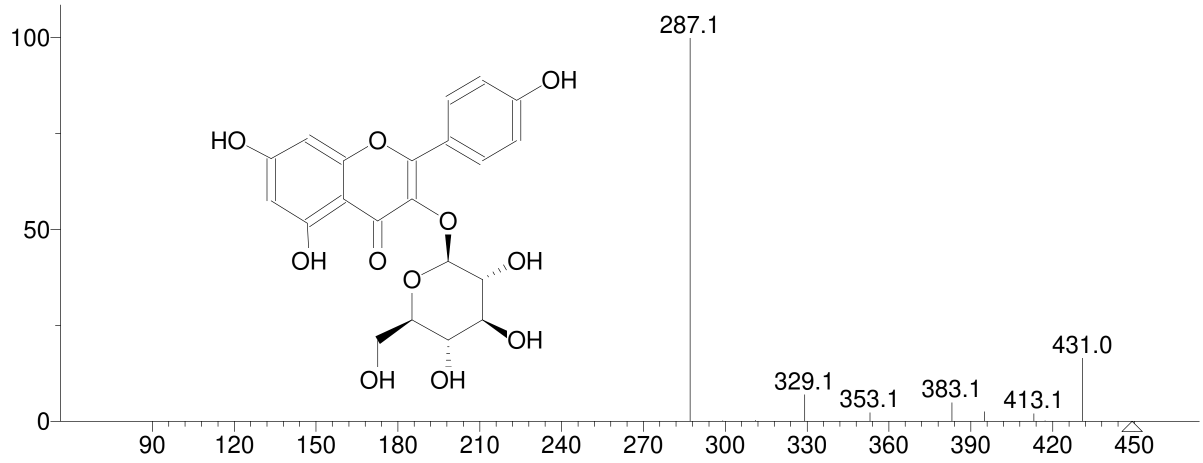

Hit 4 : Naringenin-4'-O-β-D-glucuronide [M+H]+ HCD 35V P=449.1
C21H20O11; MF: 1; RMF: 83; Prob 2.70%; CAS: 158196-35-1; Lib: nist_msms; ID: 96166.

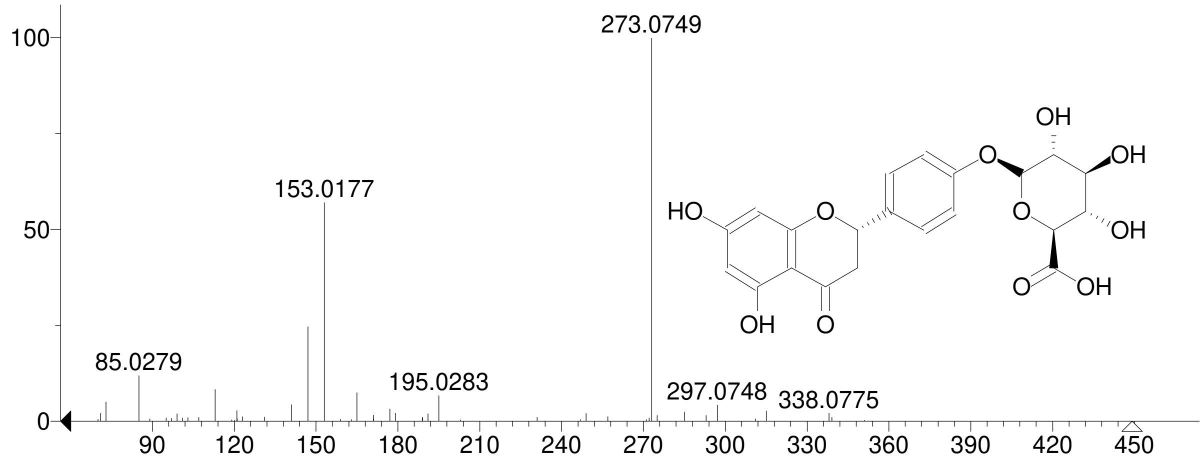

Hit 5 : 6-Oxomometasone furoate [M+H-H2O]+=>449.1 IT 35% P=449.1
C27H28Cl2O7; MF: 0; RMF: 98; Prob 2.60%; CAS: 1305334-30-8; Lib: nist_msms; ID: 465469.

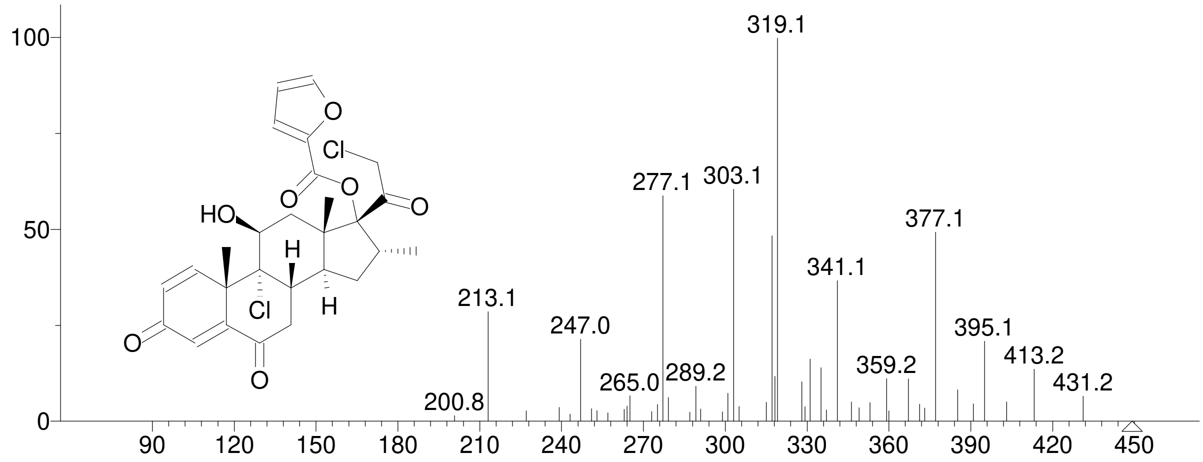

Unknown: Unknown (1.15_598.2470m/z)
Compound in Library Factor = 0

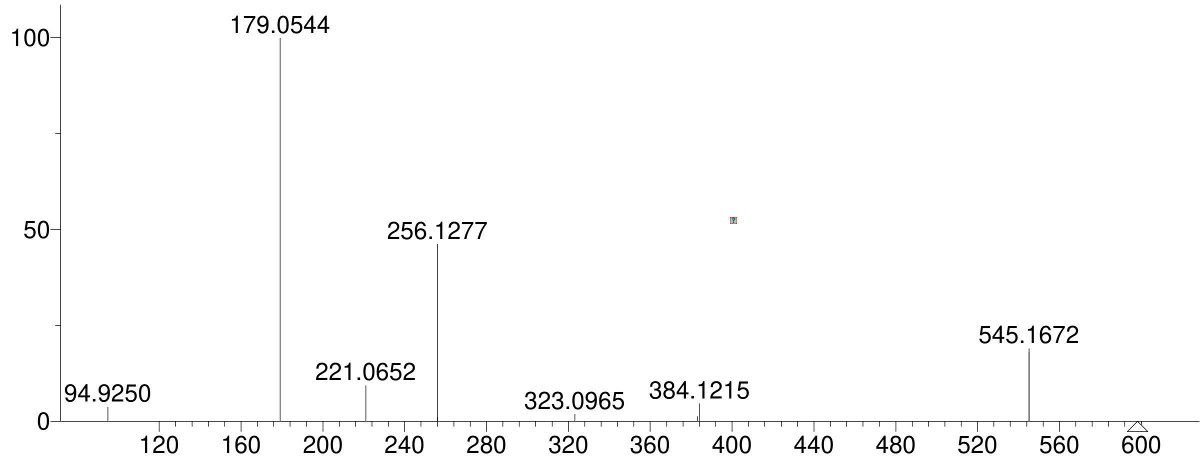

Hit 1 : NCGC00169670-02!(2S,3R,4S,5S,6R)-2-[4-[(3S,3aR,6S,6aR)-3-(4-hydroxy-3,5-dimethoxyphenyl)-1,3,3a,4
C28H36O13; MF: 0; RMF: 11; Prob 100.0%; Lib: mona-export-gnps; ID: 8125.

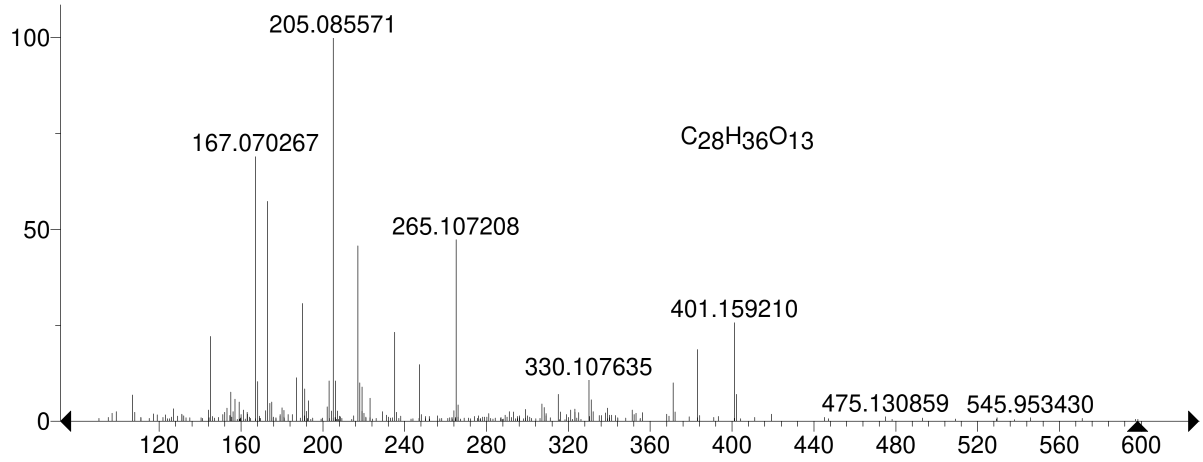

** Search Report Page 1 of 1 **

Unknown: Unknown (1.25_225.0057m/z)
Compound in Library Factor = -2632

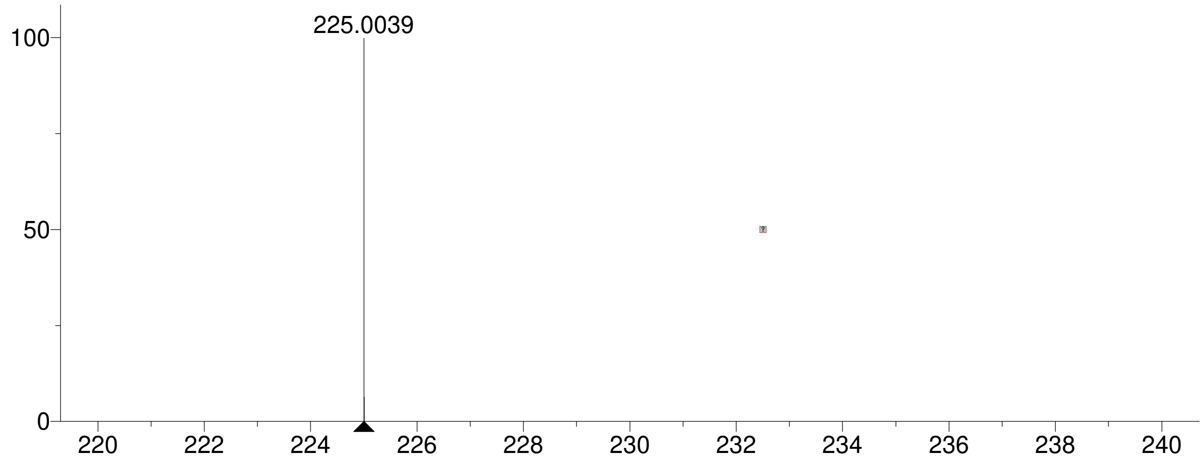

Hit 1 : 2-[[(Guanidino) (imino)methyl]sulfanyl]ethanesulfonic Acid [M-H]- P=225
C4H10N4O3S2; MF: 351; RMF: 970; Prob 50.0%; Lib: mona-export-gnps; ID: 2950.

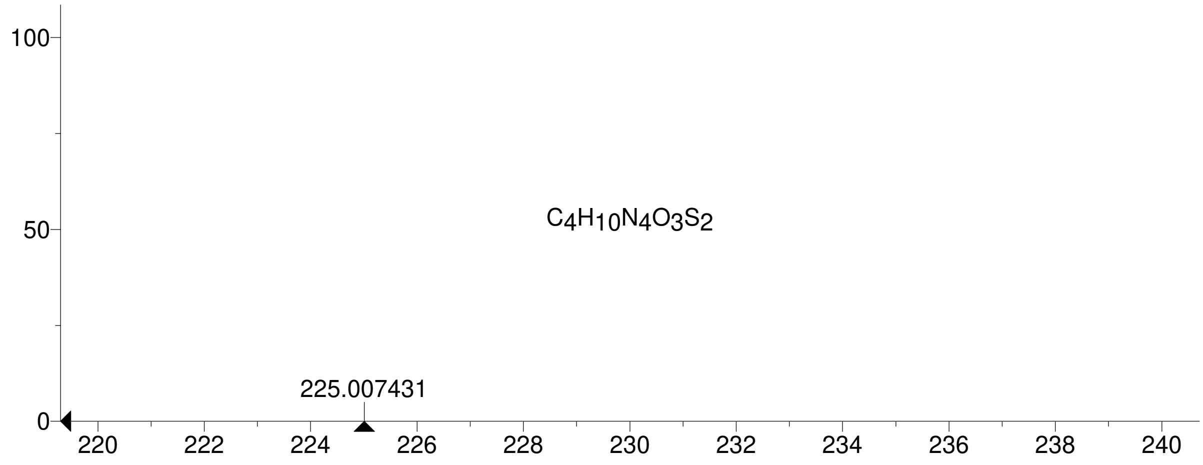

Hit 2 : 2,4,5-Phenoltricarboxylic acid [M-H]- QQQ 20V P=225
C9H6O7; MF: 351; RMF: 970; Prob 50.0%; CAS: 4961-03-9; Lib: nist_msms; ID: 1650.

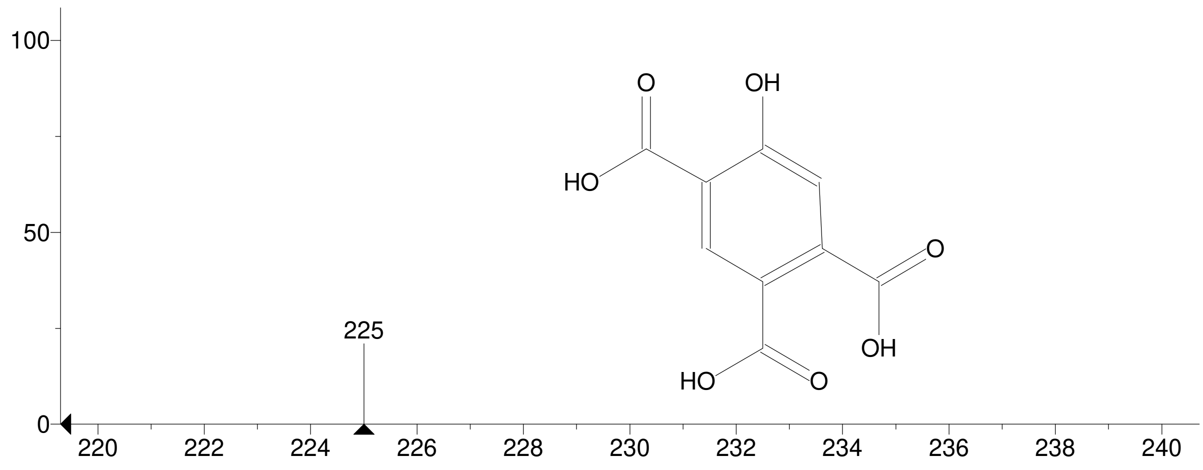

Unknown: Unknown (1.25_359.0730m/z)
Compound in Library Factor = 0

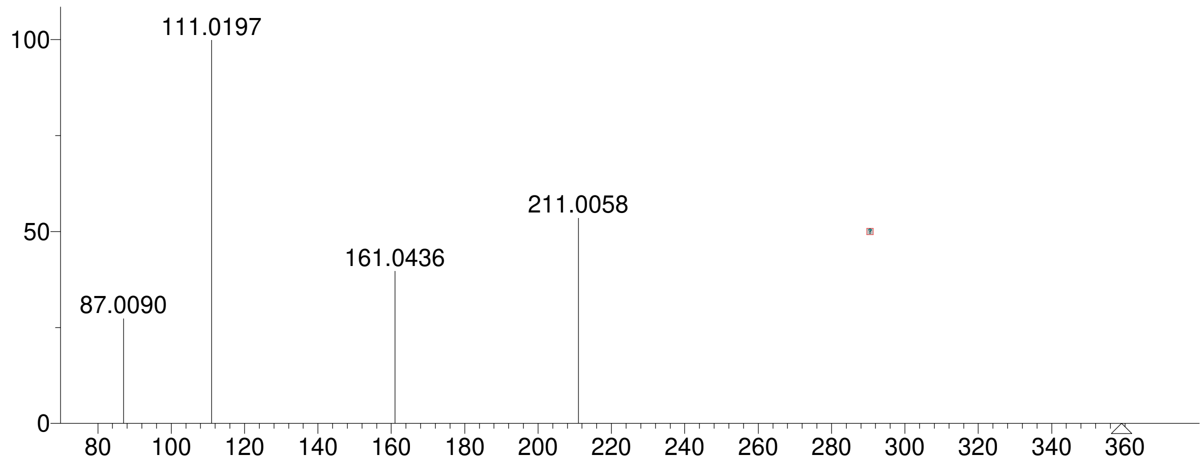

Hit 1 : 1-(3,4-dihydroxyphenyl)-6,7-dihydroxy-1,2-dihydronaphthalene-2,3-dicarboxylic acid [M+H]+ 65HCD P=3
C18H14O8; MF: 0; RMF: 6; Prob 100.0%; Lib: mona-export-vf-npl_qexactive; ID: 24906.

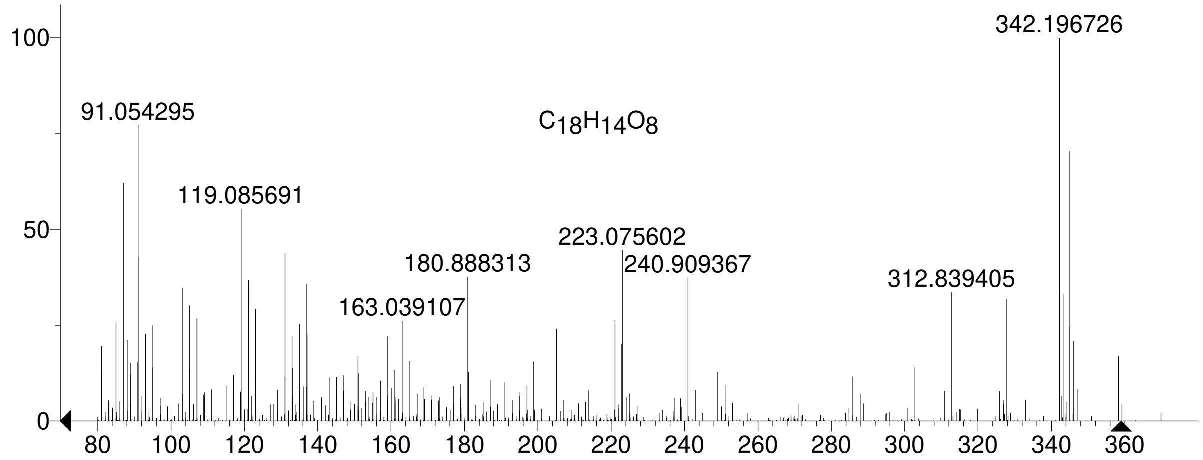

Unknown: Unknown (1.25_863.2129m/z)
Compound in Library Factor = 0

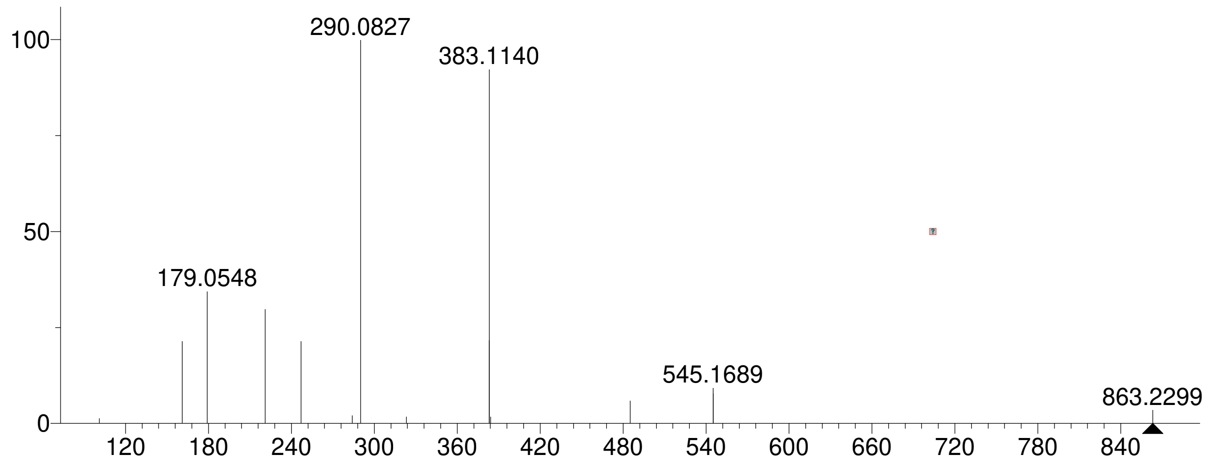

Hit 1 : Unknown (carbon number 42); PlaSMA ID-2121 [M-H]- P=863.2
C42H40O20; MF: 0; RMF: 9; Prob 100.0%; Lib: biomsms-neg-plasma; ID: 1652.

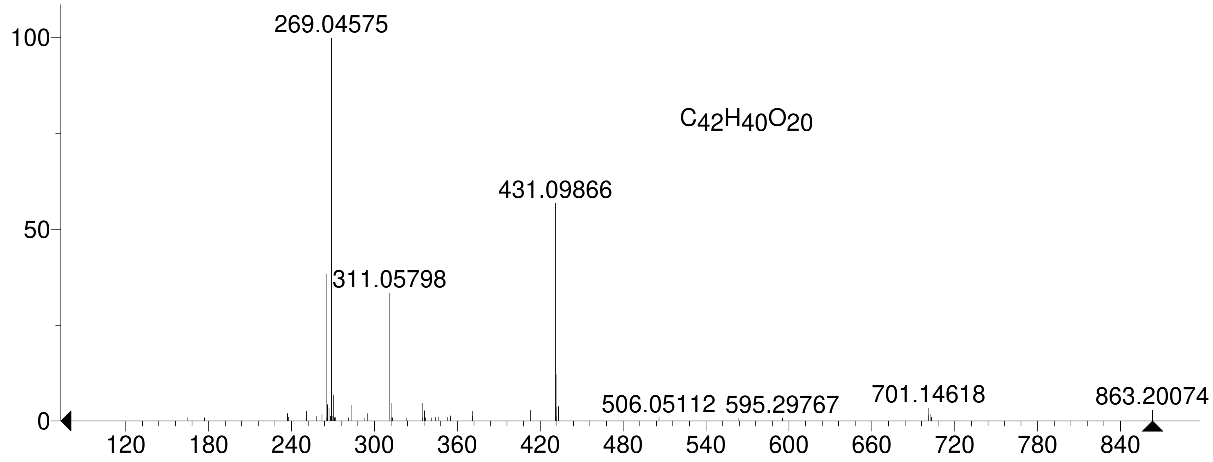

** Search Report Page 1 of 1 **

Unknown: Unknown (1.26_195.8101m/z)
Compound in Library Factor = 0

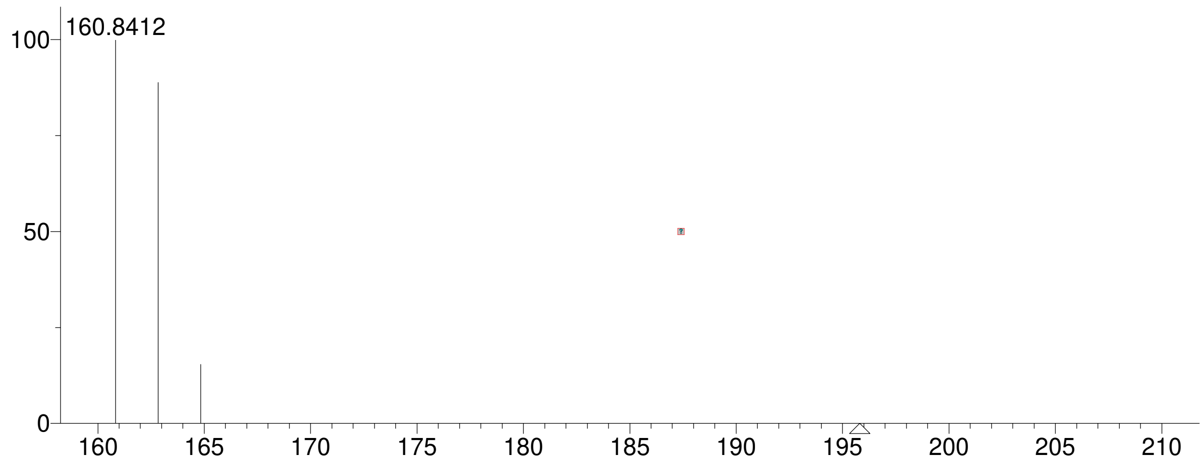

** Search Report Page 1 of 1 **

Unknown: Unknown (1.26_197.8070m/z)
Compound in Library Factor = 0

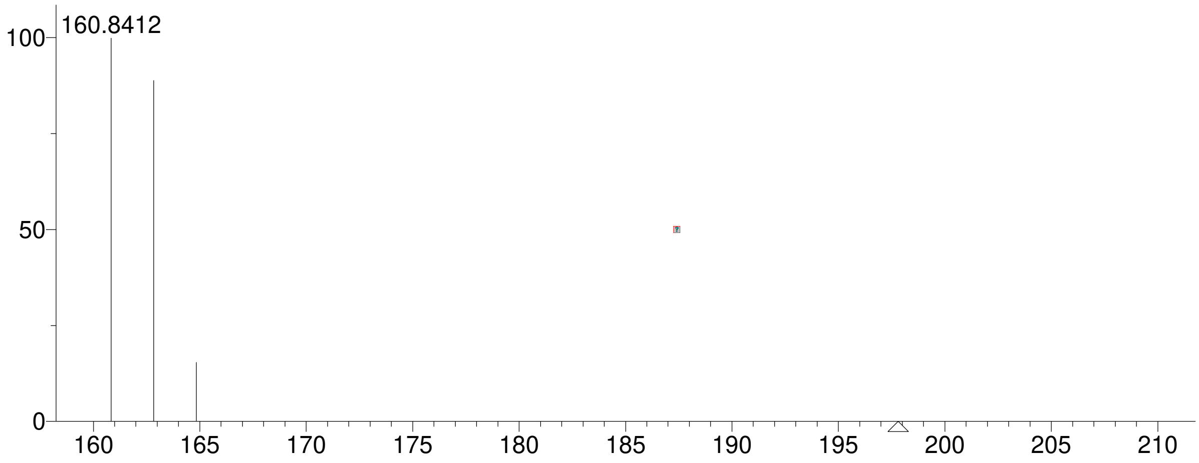

Unknown: Unknown (1.29_272.0761m/z)
Compound in Library Factor = -2176

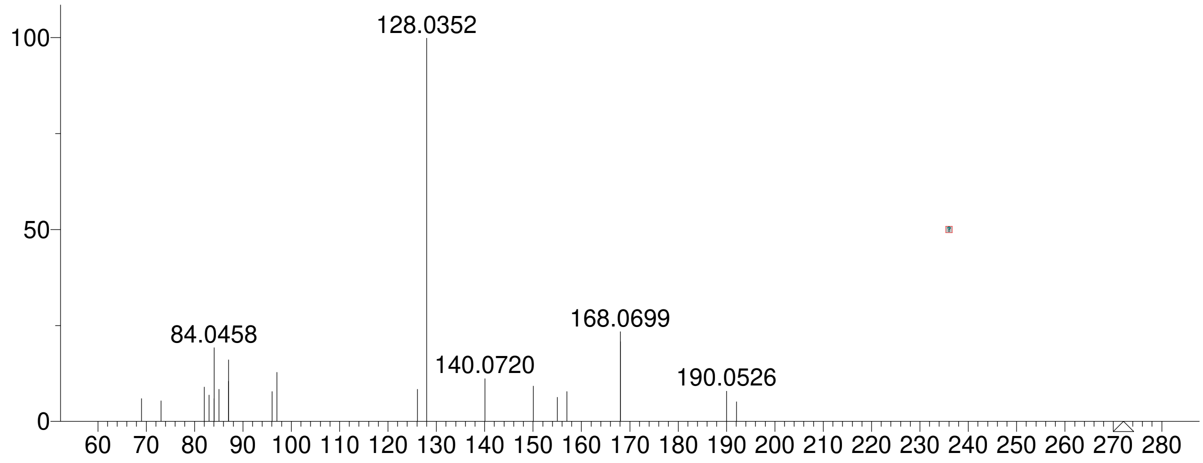

Hit 1 : N-Glycolylneuraminic acid [M+H-3H2O]+ HCD 32V P=272.1
C11H19NO10; MF: 23; RMF: 203; Prob 73.2%; CAS: 1113-83-3; Lib: nist_msms; ID: 307281.

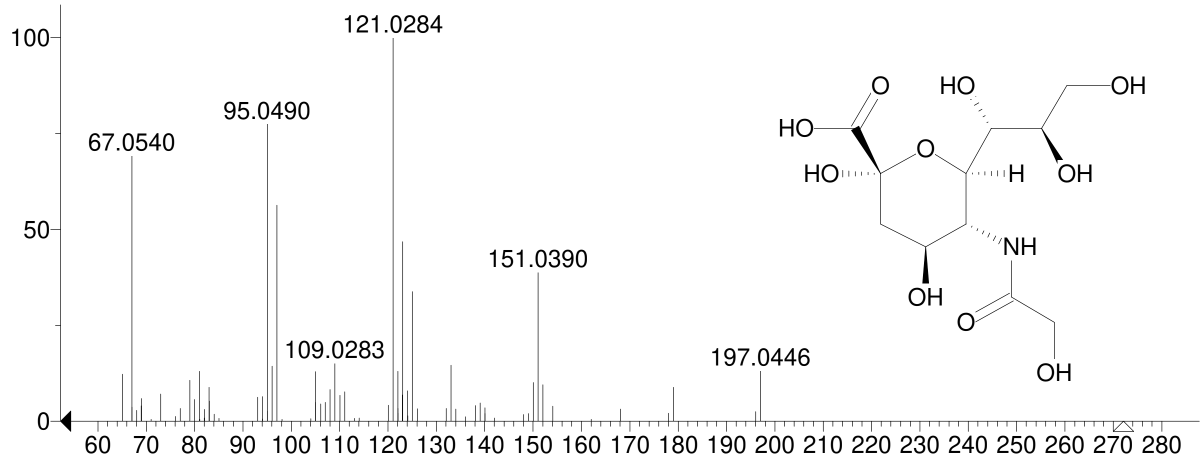

Hit 2 : NCGC00380622-01!3,5-dihydroxy-7-methoxy-6-methyl-1-oxo-3H-2-benzofuran-4-carboxylic acid [M+NH4
C11H10O7; MF: 0; RMF: 25; Prob 26.8%; Lib: mona-export-gnps; ID: 8673.

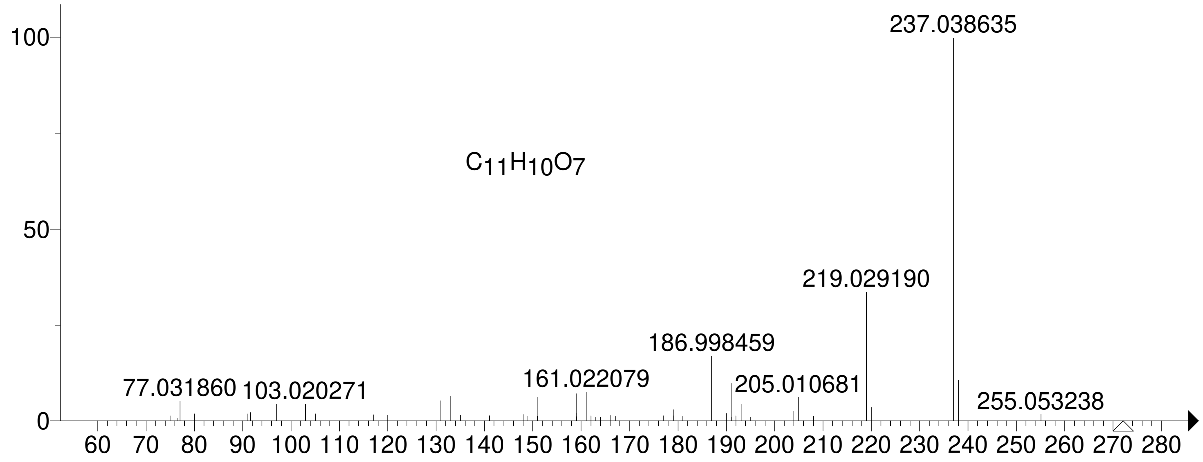

Unknown: Unknown (1.29_440.1766m/z)
Compound in Library Factor = 0

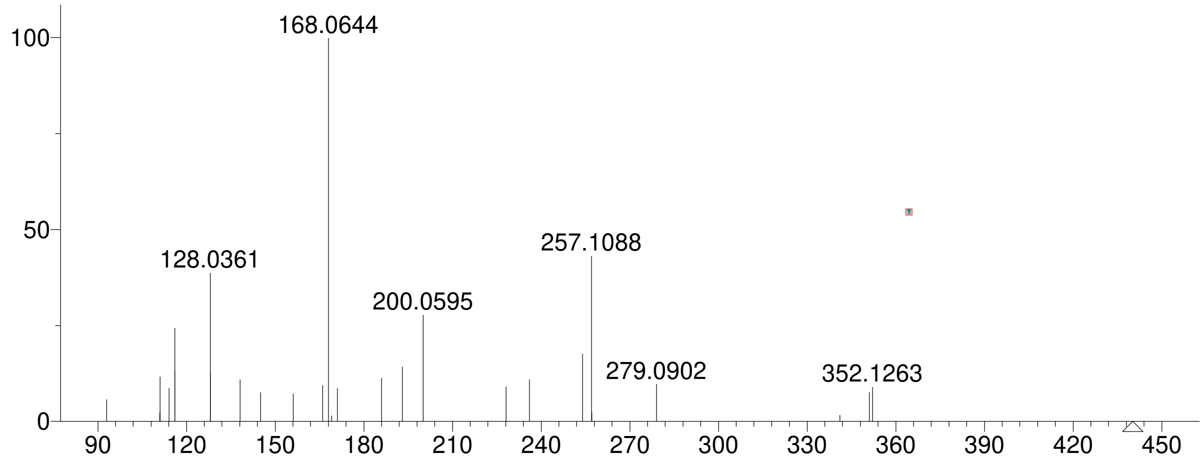

Hit 1 : Quinadoline B_130007 [M+H]+ P=440.2
C25H21N5O3; MF: 0; RMF: 24; Prob 100.0%; Lib: mona-export-gnps; ID: 4995.

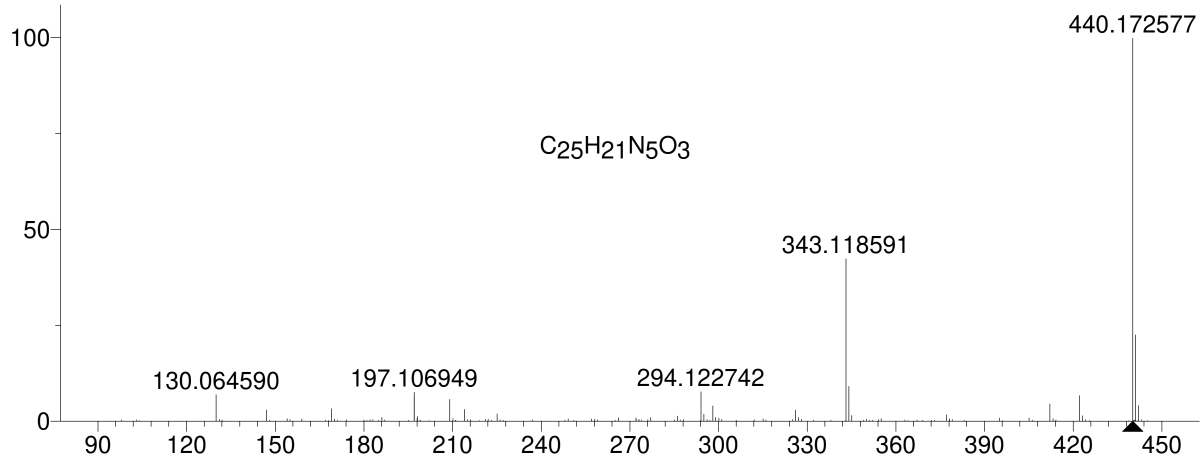

** Search Report Page 1 of 1 **

Unknown: Unknown (1.30_393.1491m/z)
Compound in Library Factor = 0

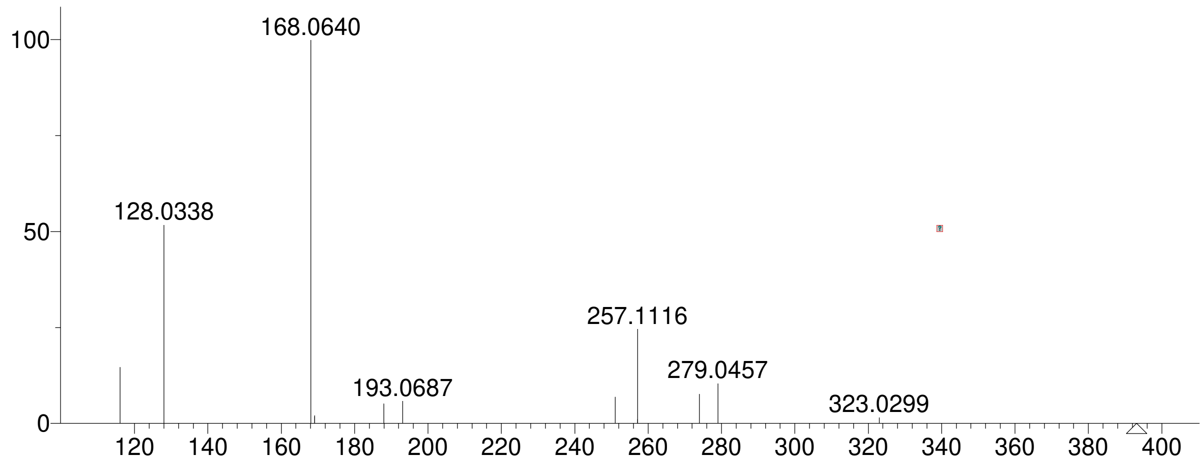

Unknown: Unknown (1.31_200.0550m/z)
Compound in Library Factor = -3273

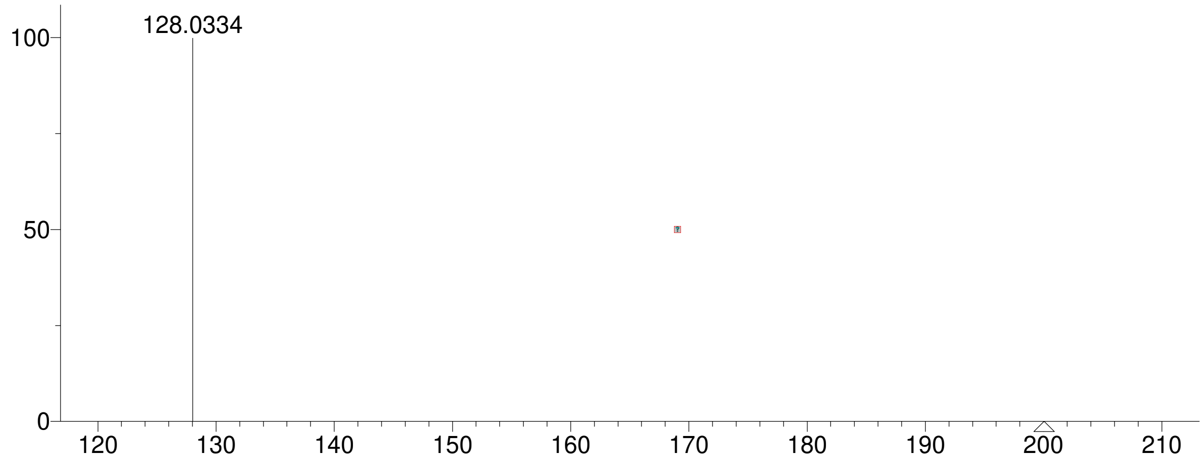

Hit 1 : O-Succinyl-L-homoserine [M-H-H2O]- HCD 26V P=200.1
C8H13NO6; MF: 2; RMF: 190; Prob 100.0%; CAS: 1492-23-5; Lib: nist_msms; ID: 125941.

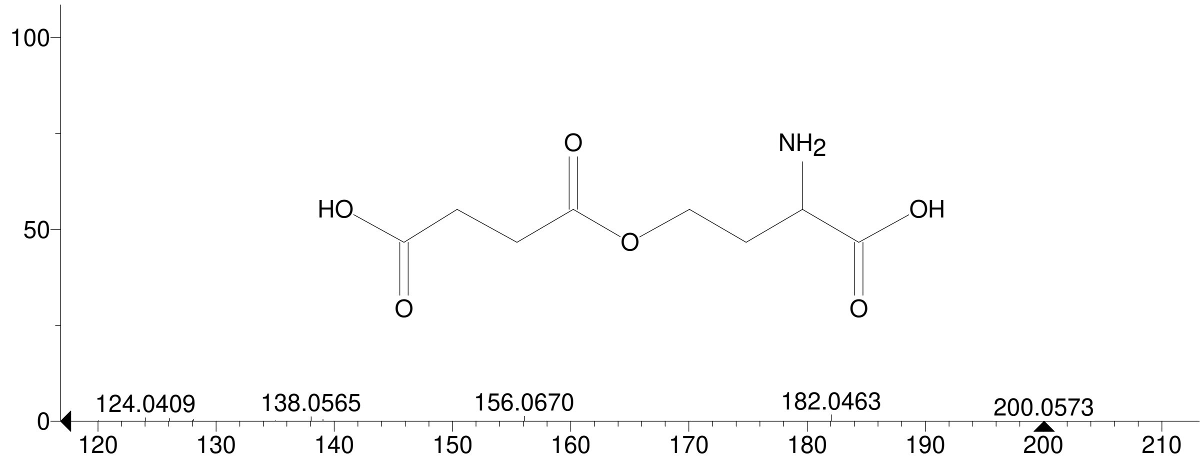

Unknown: Unknown (1.31_257.1122m/z)
Compound in Library Factor = -3273

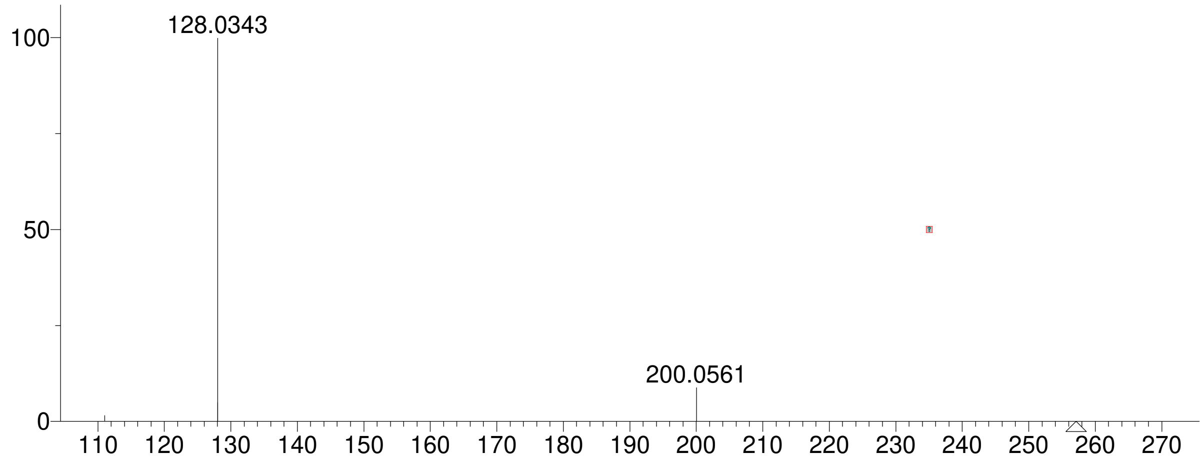

Hit 1 : L-Saccharopine [M-H-H2O]- HCD 46V P=257.1
C11H20N2O6; MF: 6; RMF: 405; Prob 100.0%; CAS: 997-68-2; Lib: nist_msms; ID: 125641.

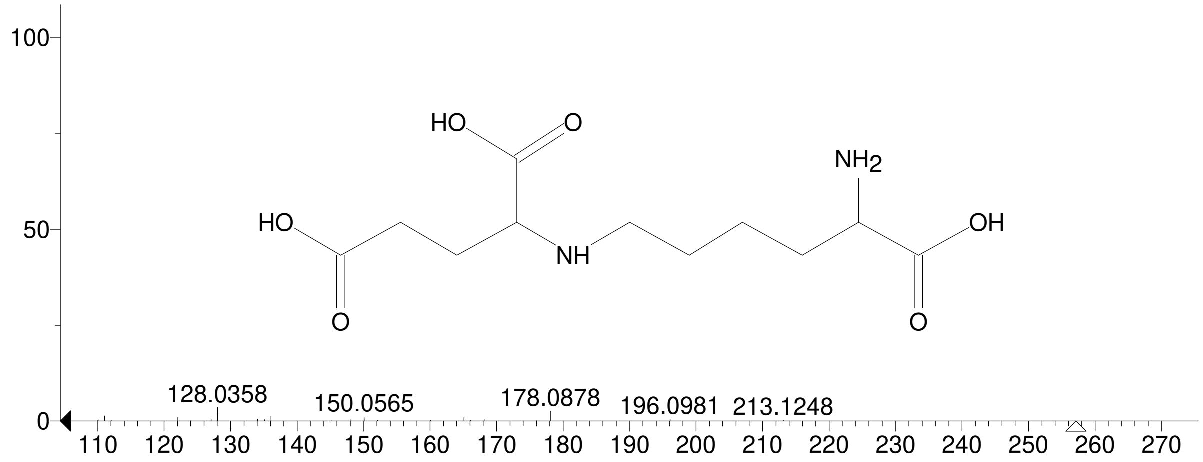

Unknown: Unknown (1.31_261.0699m/z)
Compound in Library Factor = 0

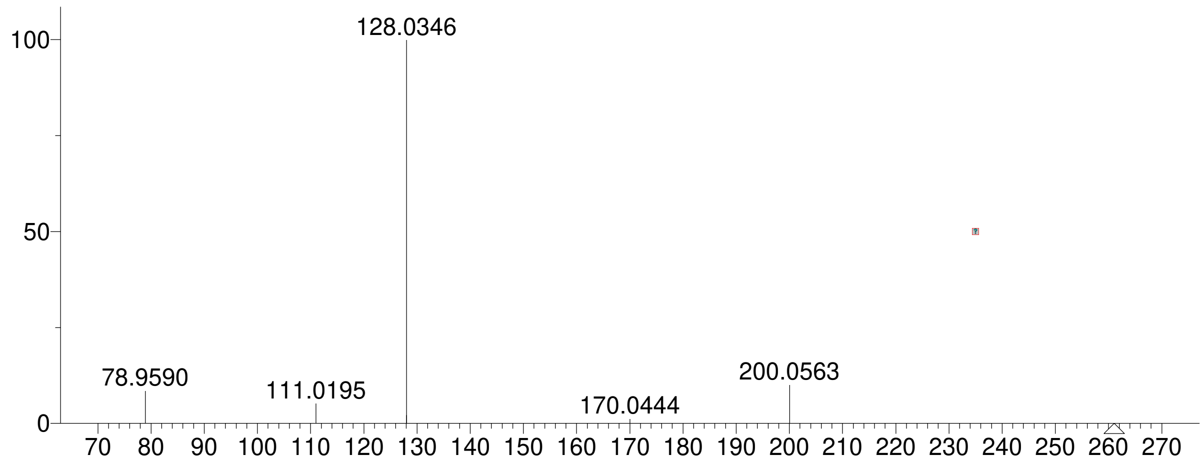

Unknown: Unknown (1.31_290.0872m/z)
Compound in Library Factor = -321

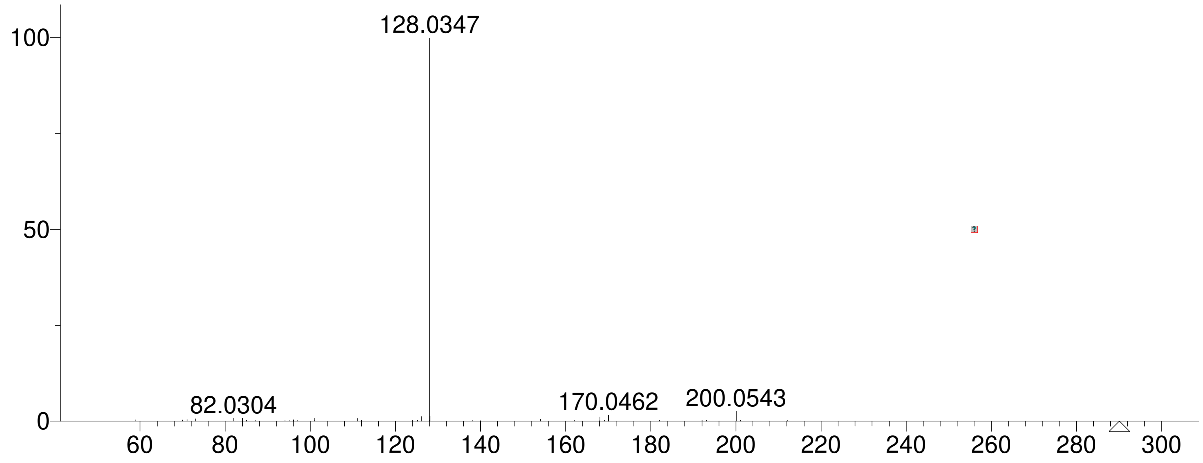

Hit 1 : N-Fructosyl pyroglutamate; PlaSMA ID-534 [M-H]- P=290.1
C11H17NO8; MF: 284; RMF: 801; Prob 98.4%; Lib: biomsms-neg-plasma; ID: 480.

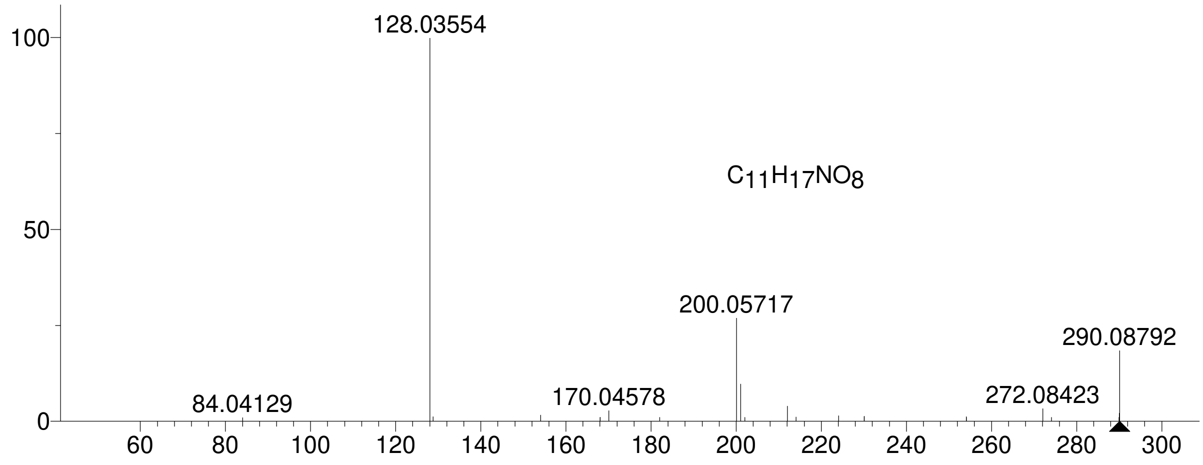

Hit 2 : (-)-N-Acetylneuraminic acid [M-H-H2O]- IT-FT 35% P=290.1
C11H19NO9; MF: 81; RMF: 140; Prob 0.98%; CAS: 131-48-6; Lib: nist_msms; ID: 479648.

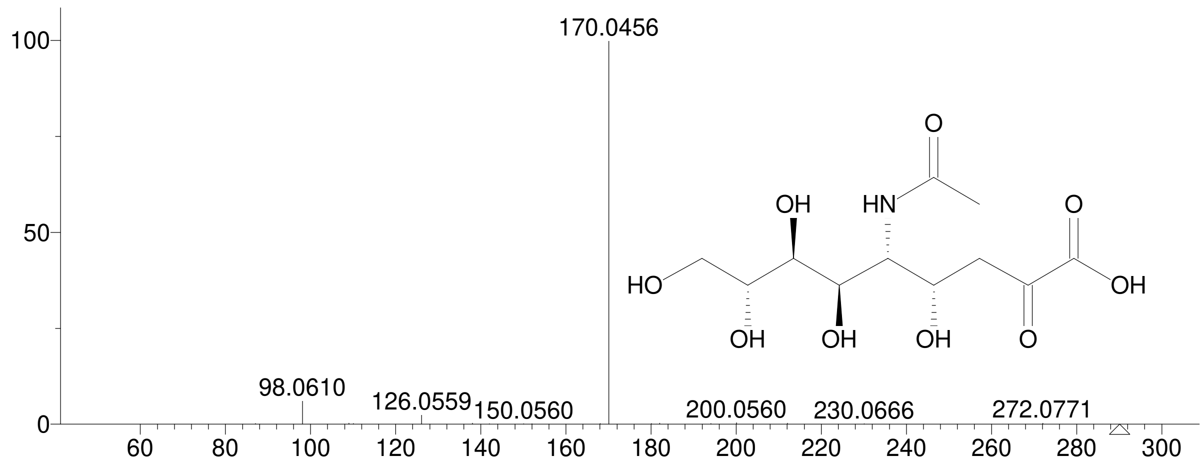

Hit 3 : N-Glycolylneuraminic acid [M+H-2H2O]+ HCD 29V P=290.1
C11H19NO10; MF: 47; RMF: 178; Prob 0.25%; CAS: 1113-83-3; Lib: nist_msms; ID: 307260.

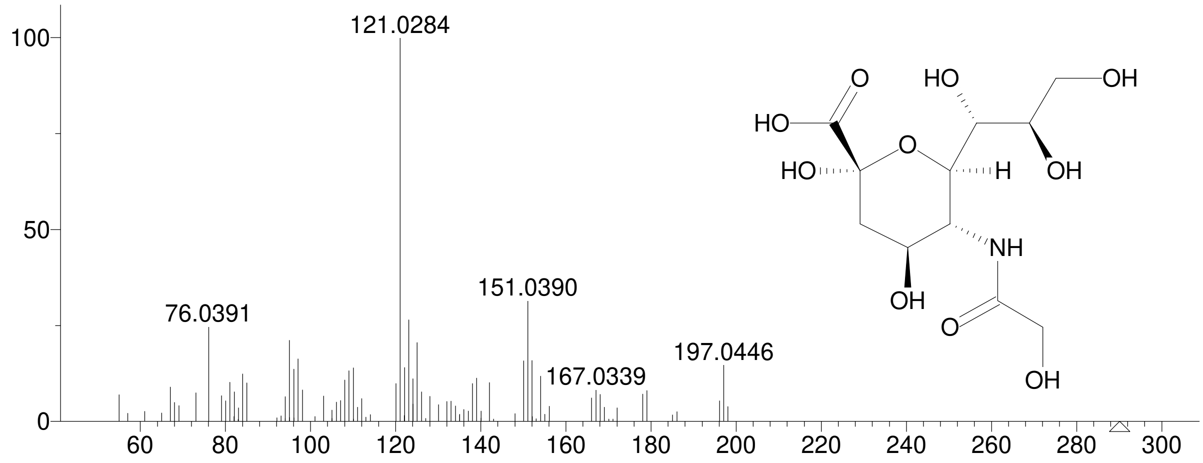

Hit 4 : 3'-α-Sialyl-N-acetyllactosamine [M-H-C14H25O11N]- IT-FT 35% P=290.1
C25H42N2O19; MF: 44; RMF: 140; Prob 0.22%; CAS: 81693-22-3; Lib: nist_msms; ID: 211511.

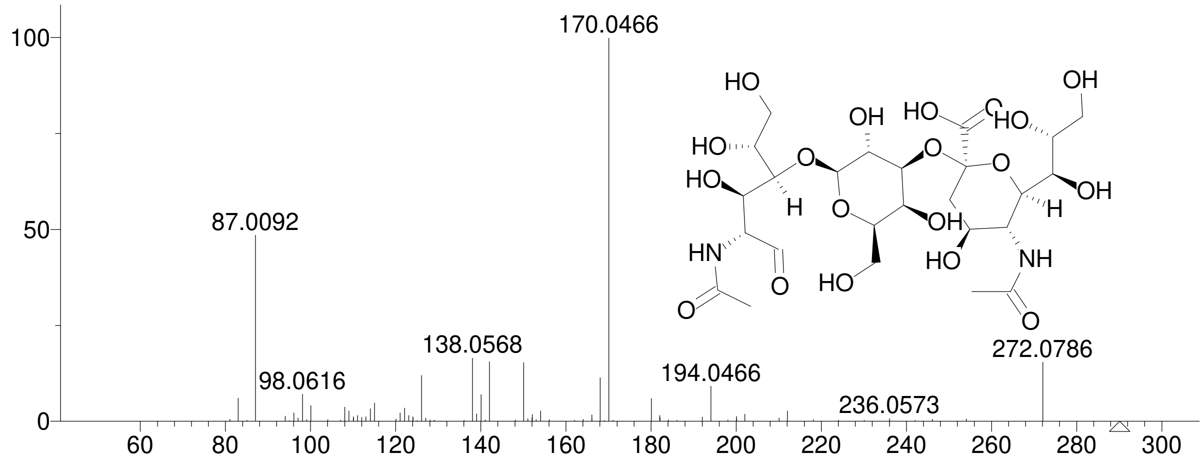

Hit 5 : 2'-Deoxyguanosine [M+Na]+ HCD 43V P=290.1
C10H13N5O4; MF: 0; RMF: 23; Prob 0.04%; CAS: 961-07-9; Lib: nist_msms; ID: 303937.

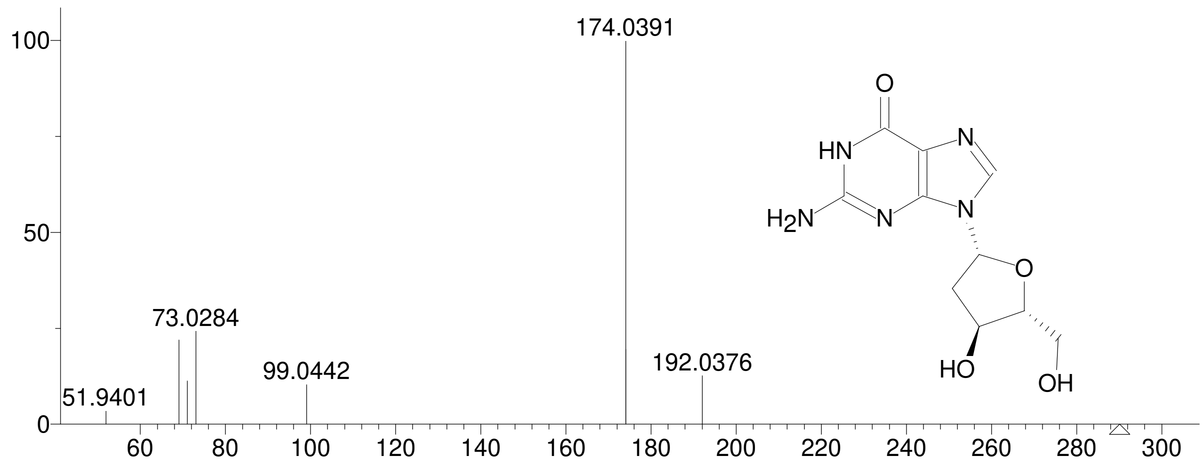

Unknown: Unknown (1.31_323.0278m/z)
Compound in Library Factor = -404

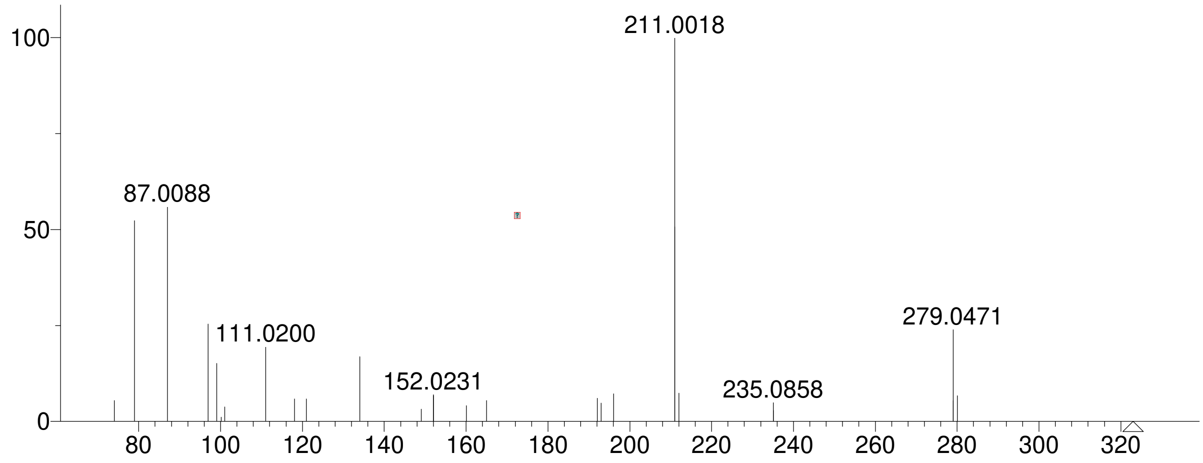

Hit 1 : 1-β-D-Arabinofuranosyluracil 5'-monophosphate [M-H]- HCD 35V P=323
C9H13N2O9P; MF: 283; RMF: 526; Prob 42.9%; CAS: 18354-06-8; Lib: nist_msms; ID: 447572.

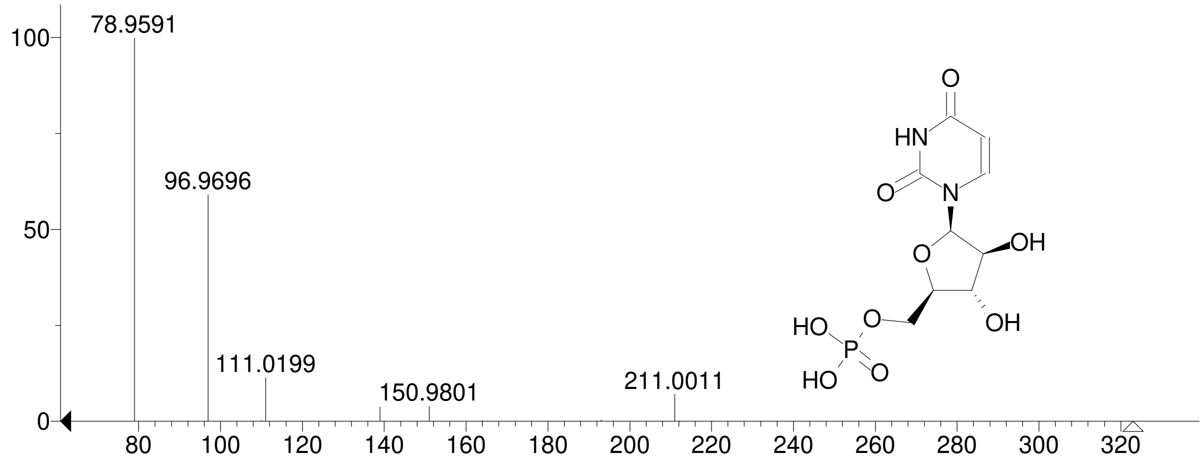

Hit 2 : Uridine 5'-diphosphoglucuronic acid [M-H-C6H9O9P]- HCD 41V P=323
C15H22N2O18P2; MF: 263; RMF: 476; Prob 19.6%; CAS: 2616-64-0; Lib: nist_msms; ID: 486425.

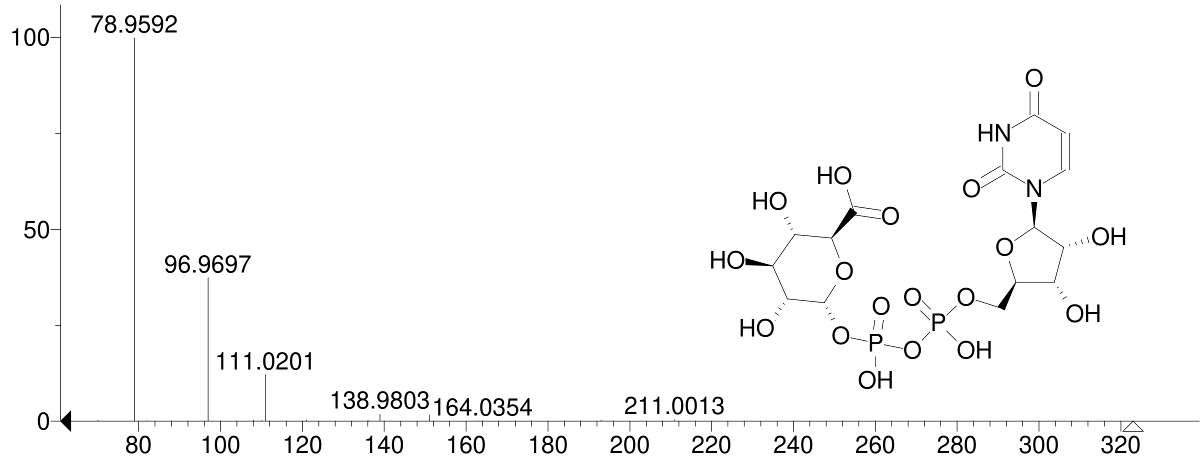

Hit 3 : URIDINE-5-MONOPHOSPHATE
C9H13N2O9P; MF: 257; RMF: 520; Prob 15.4%; Lib: embl neg mcg; ID: 631.

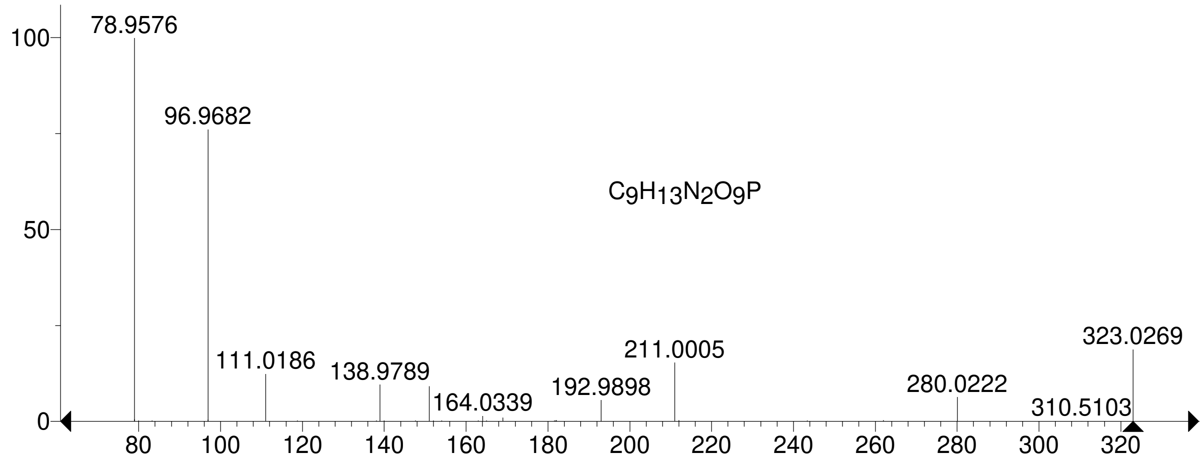

Hit 4 : Uridine 5'-monophosphate [M-H]- HCD 41V P=323
C9H13N2O9P; MF: 256; RMF: 466; Prob 14.8%; CAS: 58-97-9; Lib: nist_msms; ID: 126179.

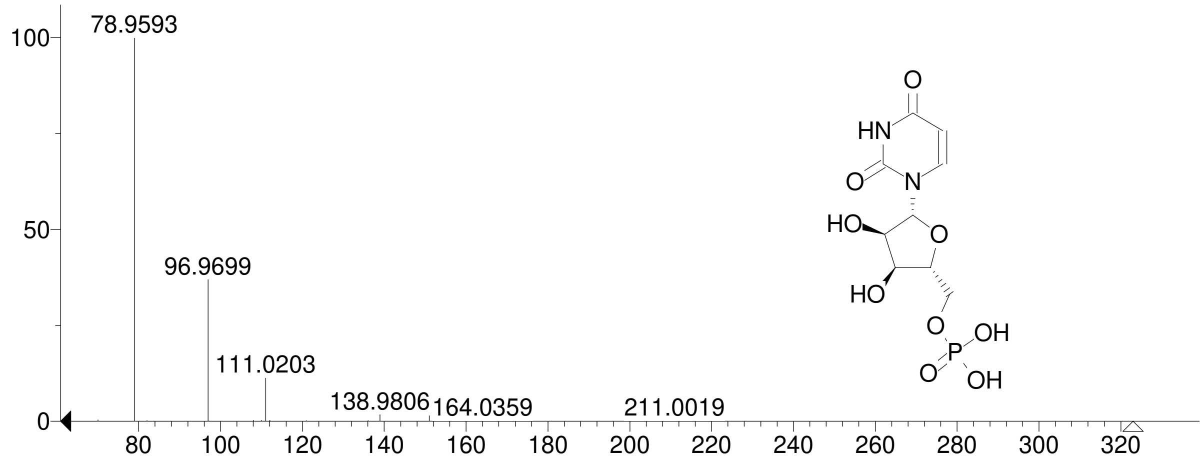

Hit 5 : URIDINE-5-MONOPHOSPHATE [M-H]- P=323
C9H13N2O9P; MF: 237; RMF: 513; Prob 7.17%; Lib: mona-export-gnps; ID: 5550.

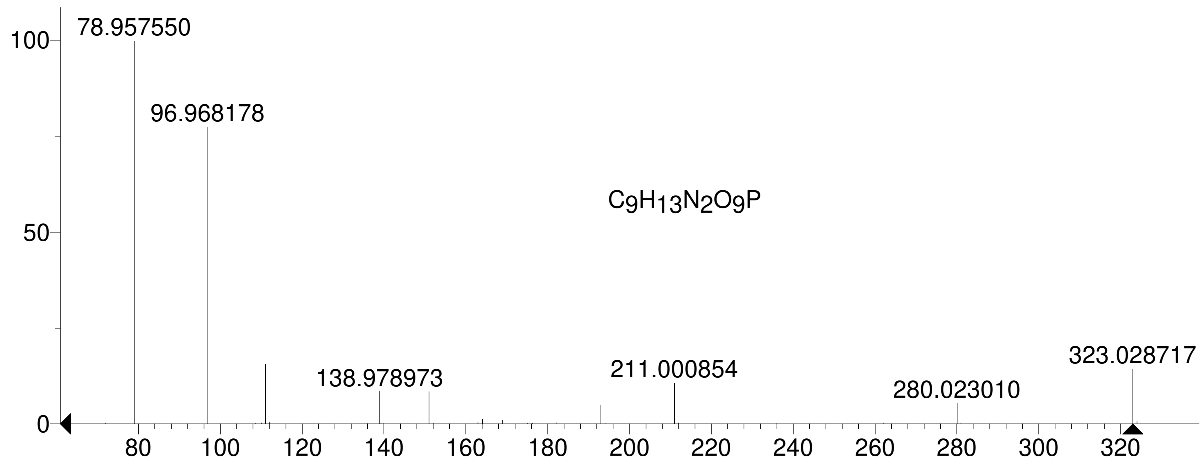

Unknown: Unknown (1.31_419.1612m/z)
Compound in Library Factor = 0

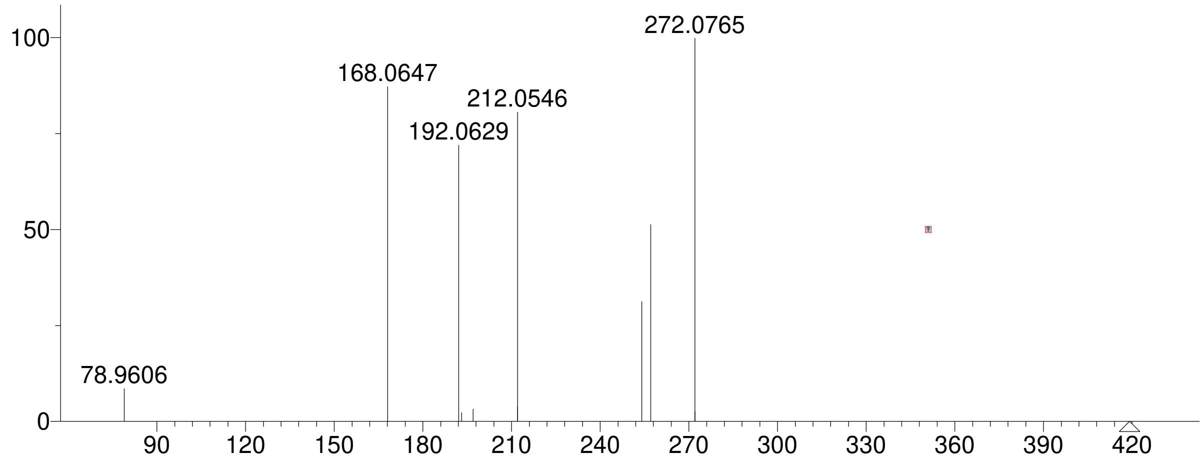

Hit 1 : NCGC00380324-01_C23H29ClO5_Benzaldehyde, 3-chloro-4,6-dihydroxy-2-methyl-5-[(2E,6E)-3-methyl-7-
C23H29ClO5; MF: 0; RMF: 17; Prob 100.0%; Lib: msms-neg-gnps; ID: 1853.

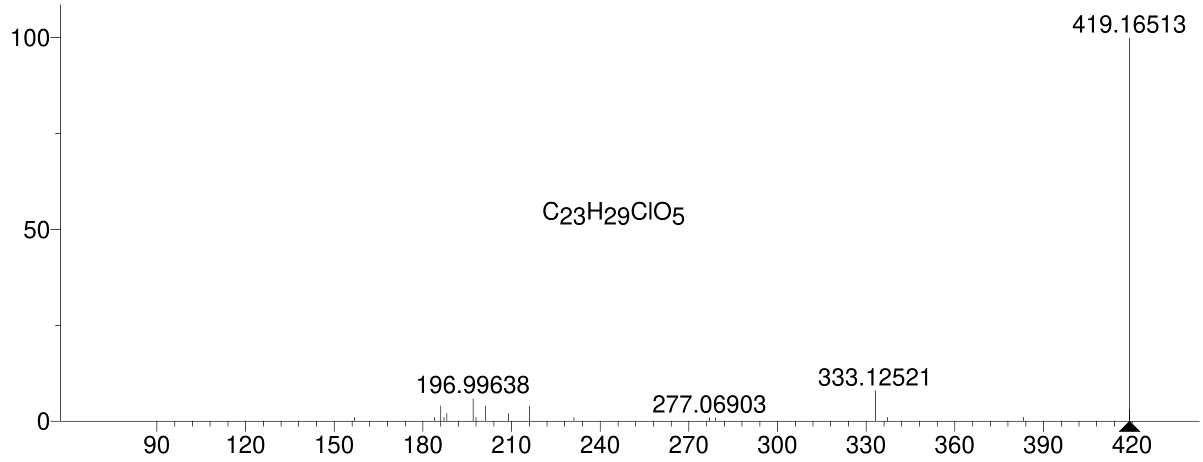

Unknown: Unknown (1.31_440.1719m/z)
Compound in Library Factor = 0

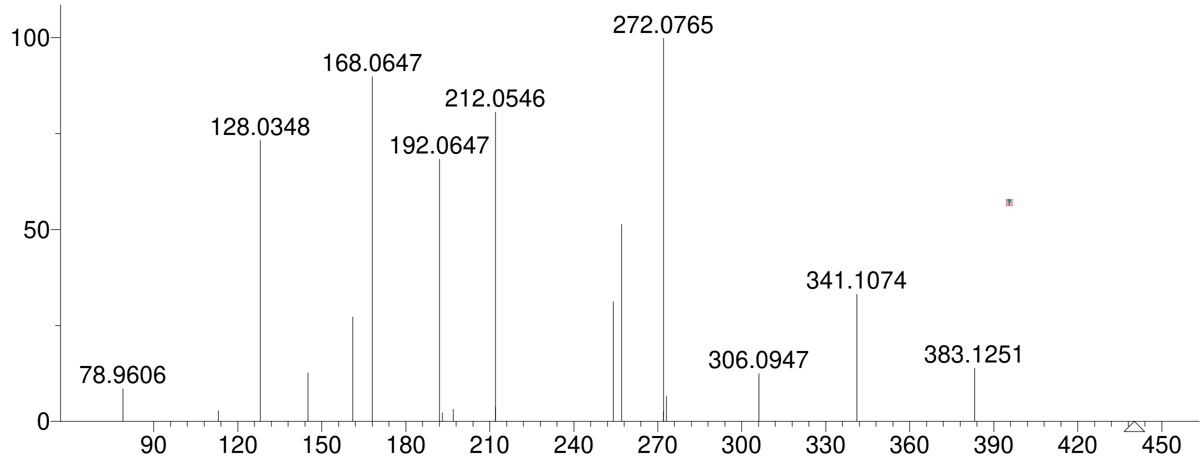

Hit 1 : Quinadoline B_130007 [M+H]+ P=440.2
C25H21N5O3; MF: 0; RMF: 35; Prob 25.0%; Lib: mona-export-gnps; ID: 4995.

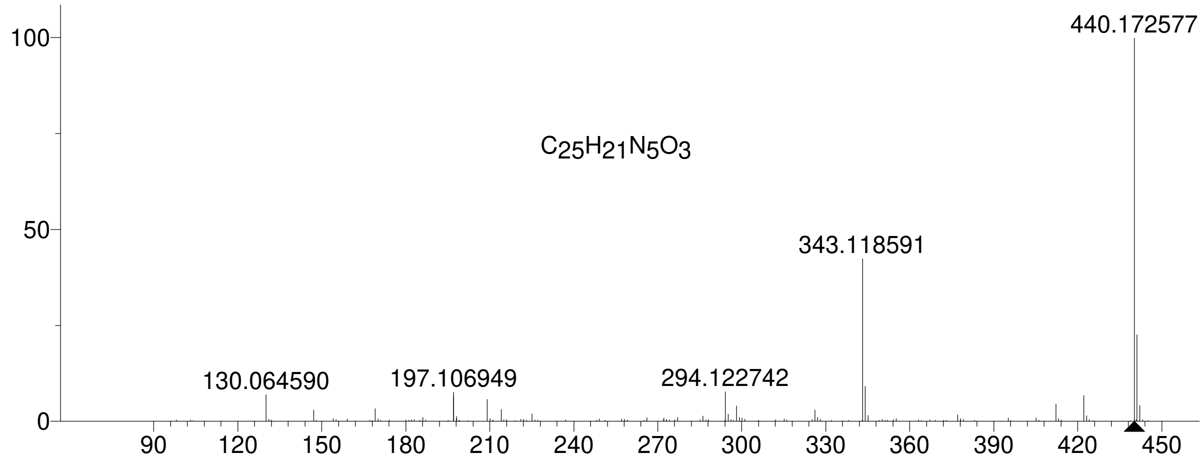

Hit 2 : _130033 [M+Na]+ P=440.2
C22H27NO7; MF: 0; RMF: 26; Prob 25.0%; Lib: mona-export-gnps; ID: 5009.

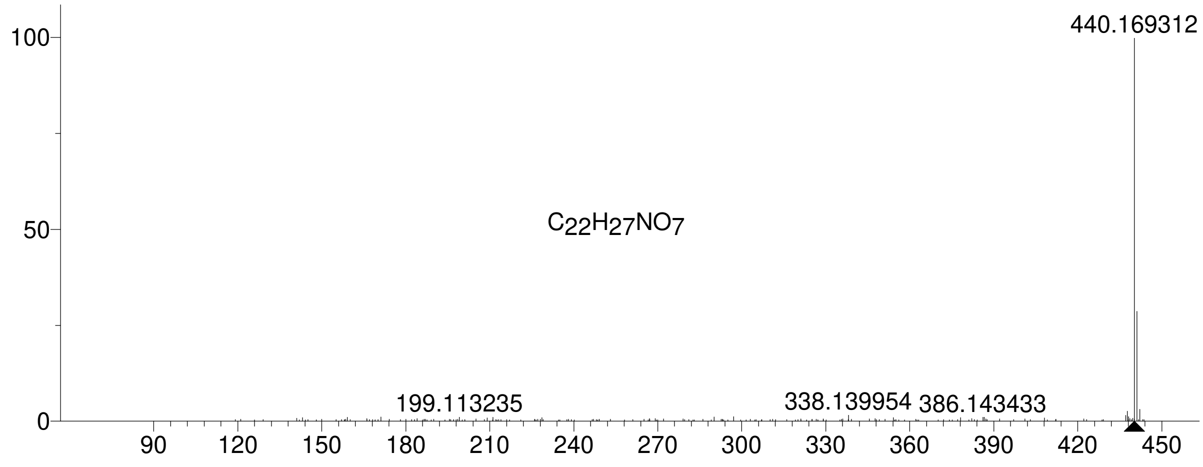

Hit 3 : Platensimycin [M-H]- HCD 57V P=440.2
C24H27NO7; MF: 0; RMF: 5; Prob 25.0%; CAS: 835876-32-9; Lib: nist_msms; ID: 265342.

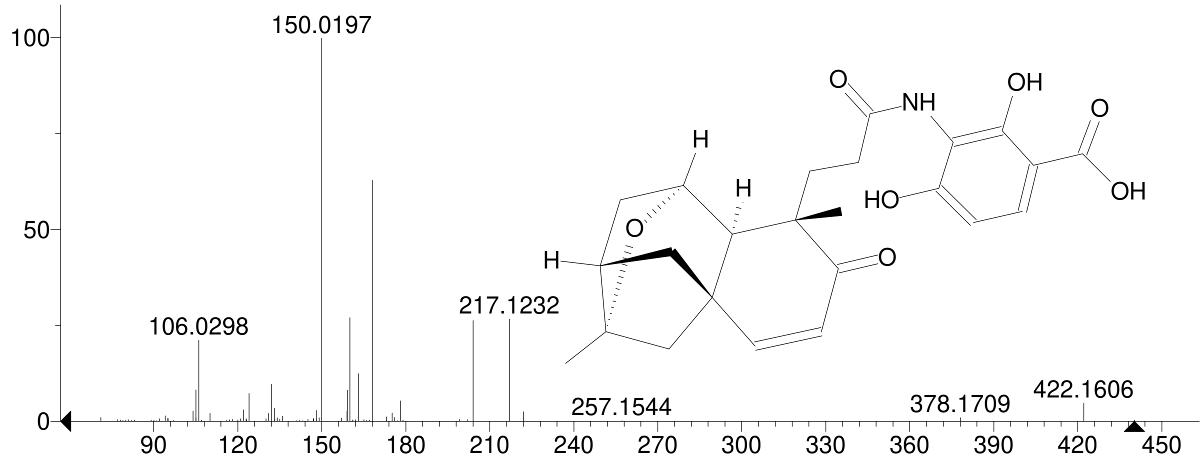

Hit 4 : NCGC00380314-01_C22H27NO7_3,5,7,9-Undecatetraenoic acid, 2-ethylidene-11-[4-hydroxy-4-(2-hydroxy
C22H27NO7; MF: 0; RMF: 4; Prob 25.0%; Lib: mona-export-gnps; ID: 12420.

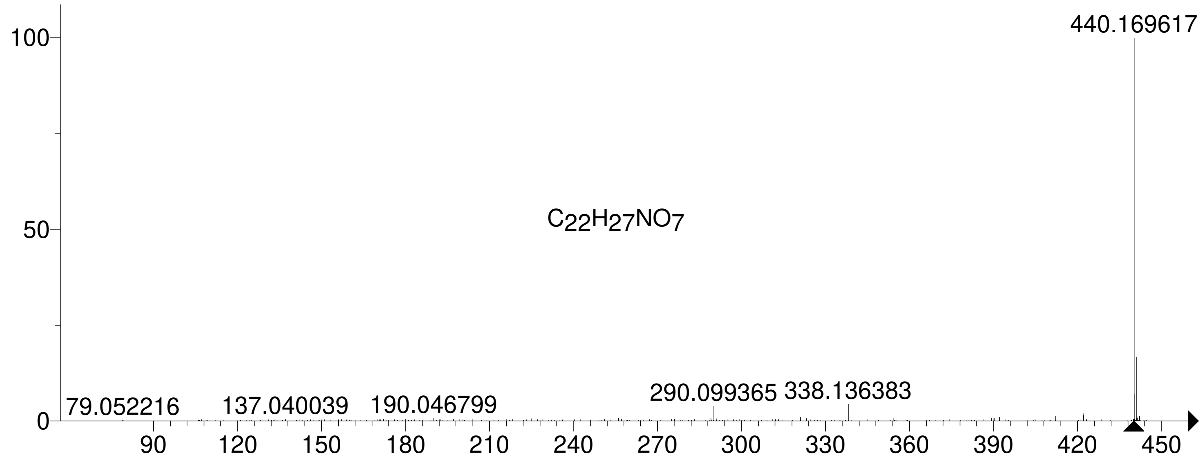

Unknown: Unknown (1.31_452.1399m/z)
Compound in Library Factor = 0

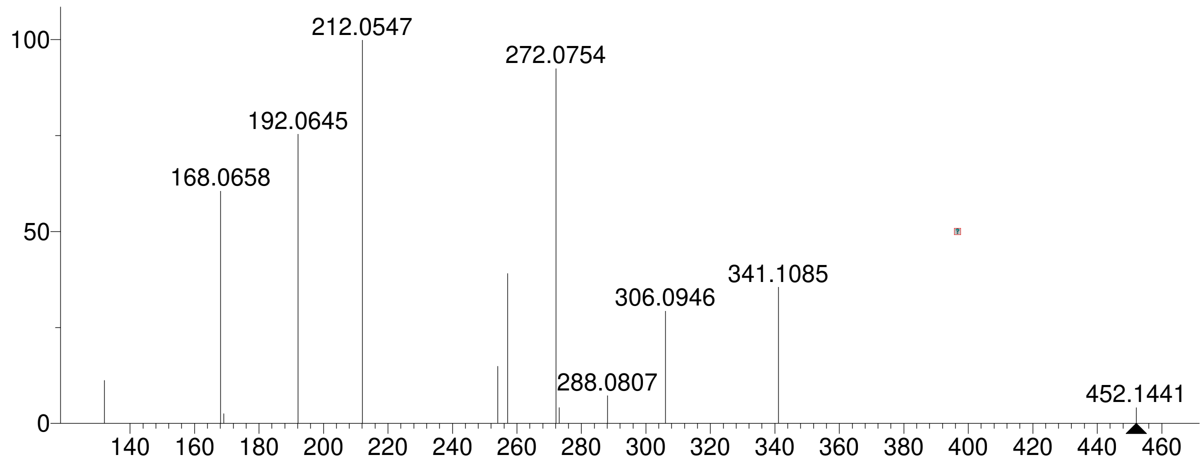

Unknown: Unknown (1.32_191.0186m/z)
Compound in Library Factor = -1042

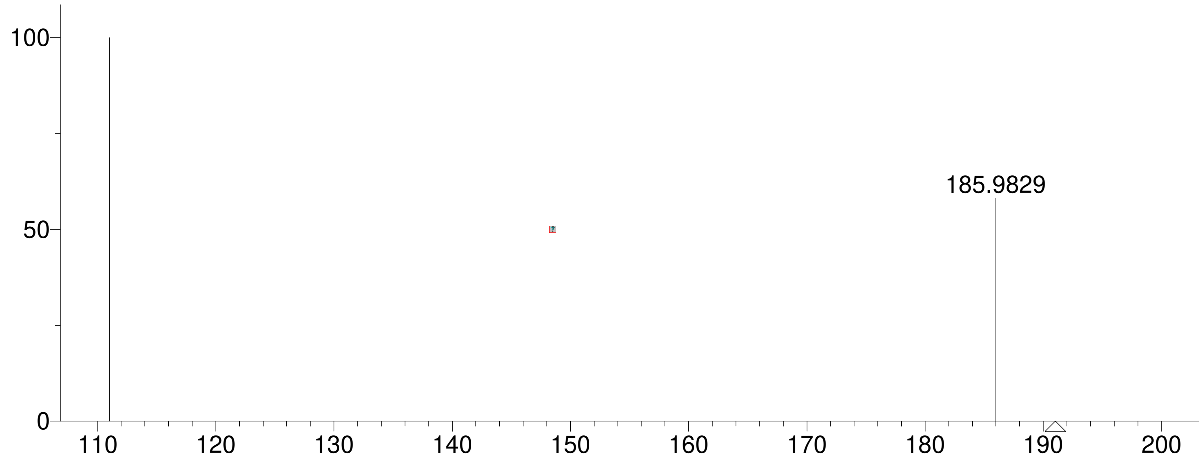

Hit 1 : Citric acid [M-H]- 20 P=191
C6H8O7; MF: 273; RMF: 795; Prob 90.7%; Lib: msms neg mona_ipb_gnps_plasma; ID: 1053.

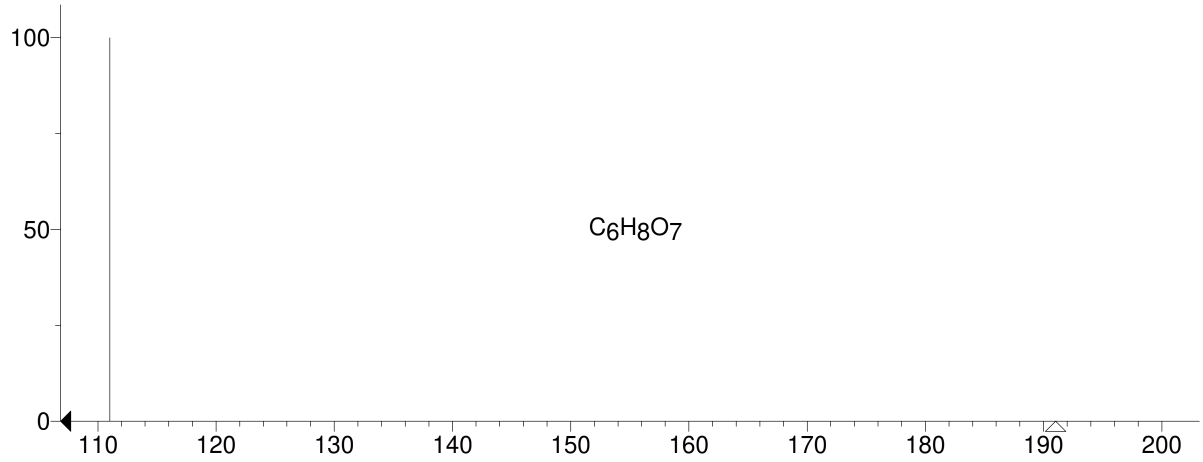

Hit 2 : Mucic acid [M-H-H2O]- HCD 30V P=191
C6H10O8; MF: 152; RMF: 764; Prob 4.62%; CAS: 526-99-8; Lib: nist_msms; ID: 303359.

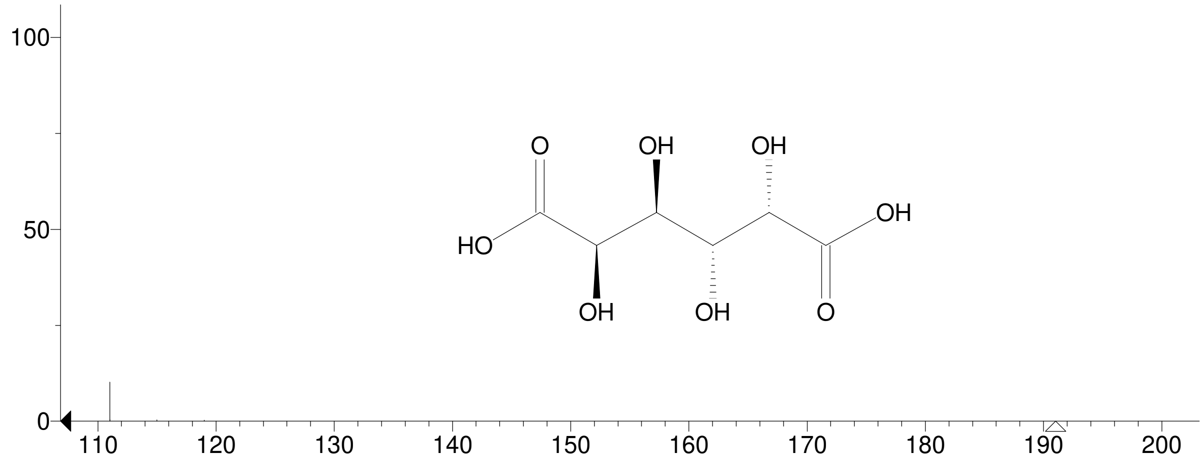

Hit 3 : citric acid [M-H]- 15 P=191
C6H8O7; MF: 117; RMF: 765; Prob 1.16%; Lib: msms neg mona_ipb_gnps_plasma; ID: 1145.

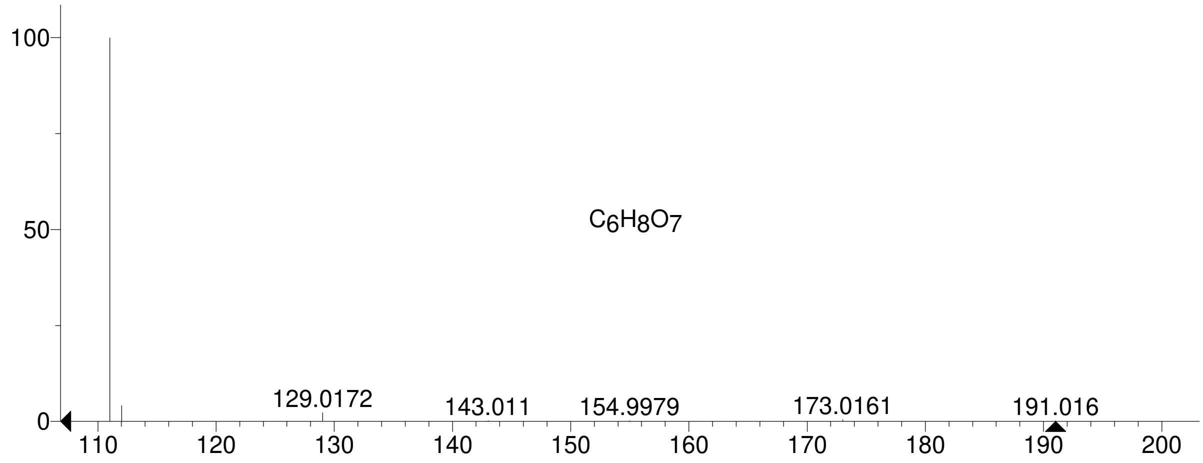

Hit 4 : Citric acid (Not validated, isomer of 228); PlaSMA ID-227 [M-H]- P=191
C6H8O7; MF: 110; RMF: 729; Prob 0.88%; Lib: biomsms-neg-plasma; ID: 463.

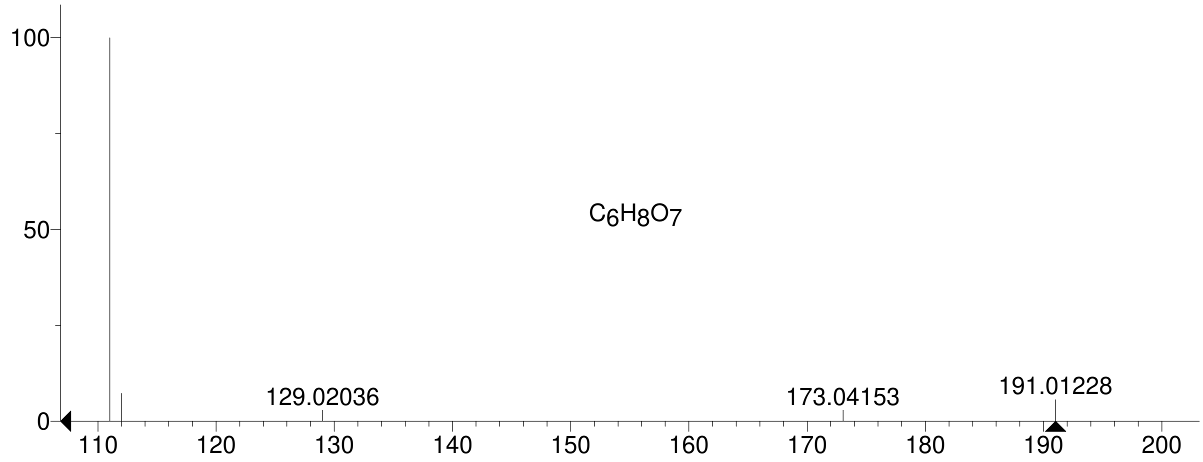

Hit 5 : CITRATE [M-H]- P=191
C6H8O7; MF: 101; RMF: 711; Prob 0.64%; Lib: msms-neg-gnps; ID: 159.

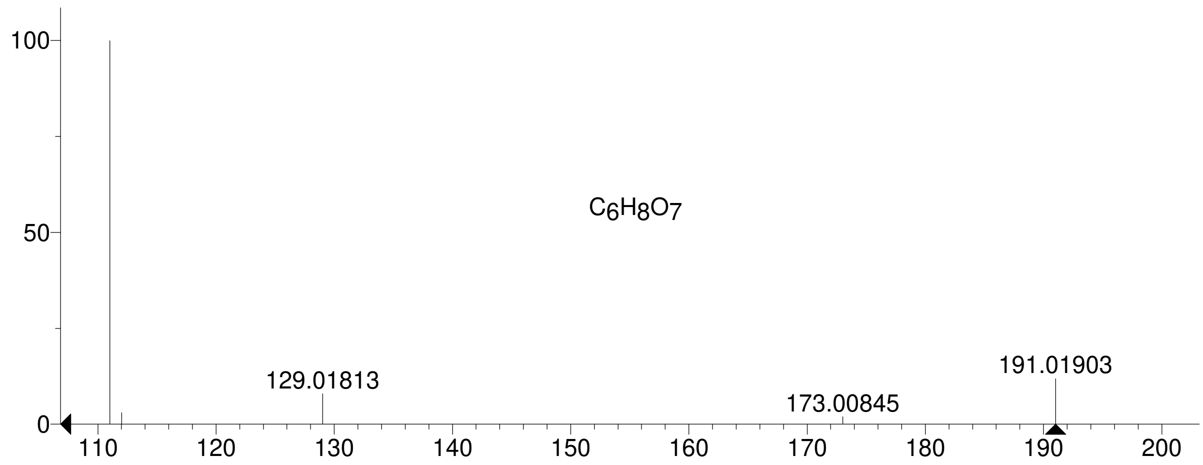

Unknown: Unknown (1.32_323.0968m/z)
Compound in Library Factor = 0

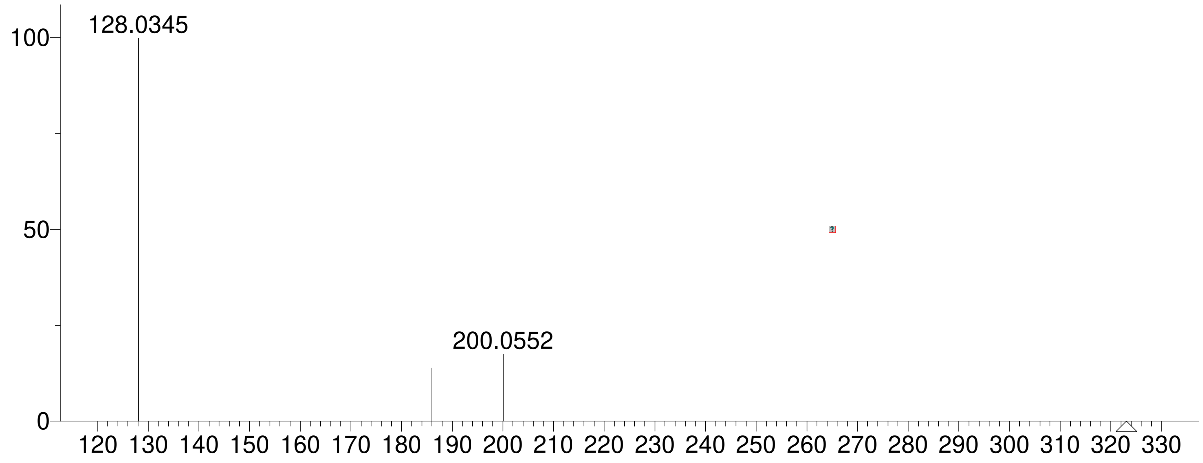

Hit 1 : Dinitramine [M+H]+ HCD 103V P=323.1
C11H13F3N4O4; MF: 0; RMF: 158; Prob 100.0%; CAS: 29091-05-2; Lib: nist_msms; ID: 100130.

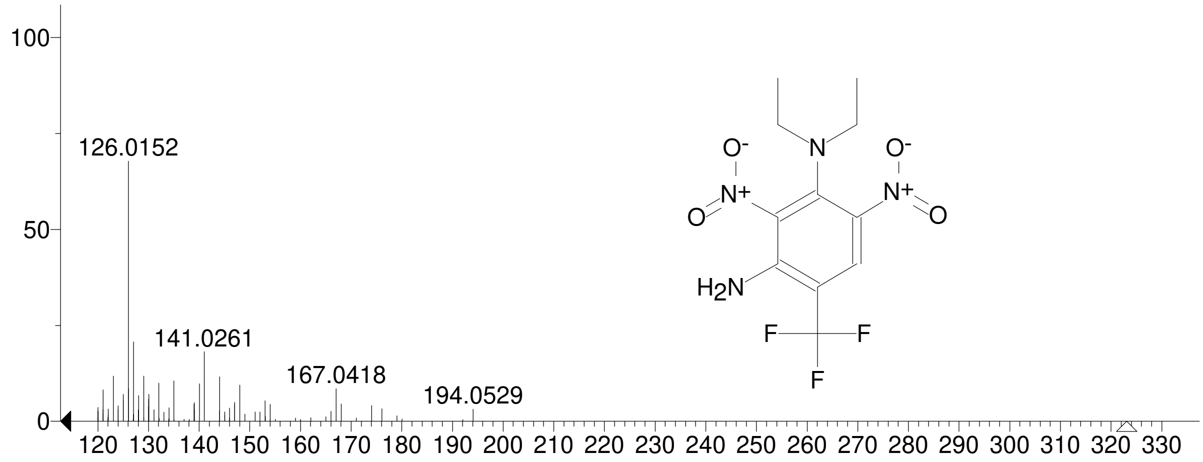

Unknown: Unknown (1.33_476.1509m/z)
Compound in Library Factor = 0

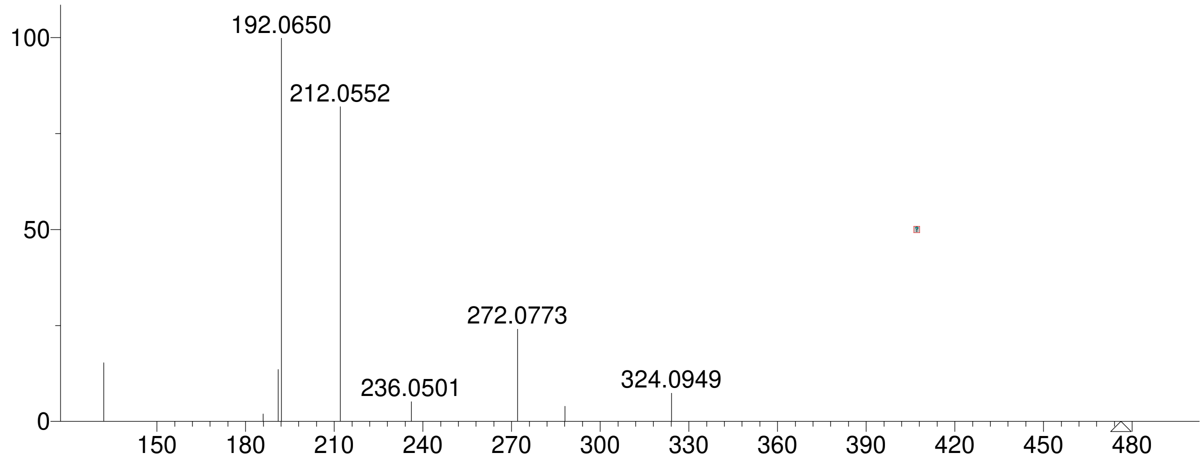

Unknown: Unknown (1.35_275.0871m/z)
Compound in Library Factor = 0

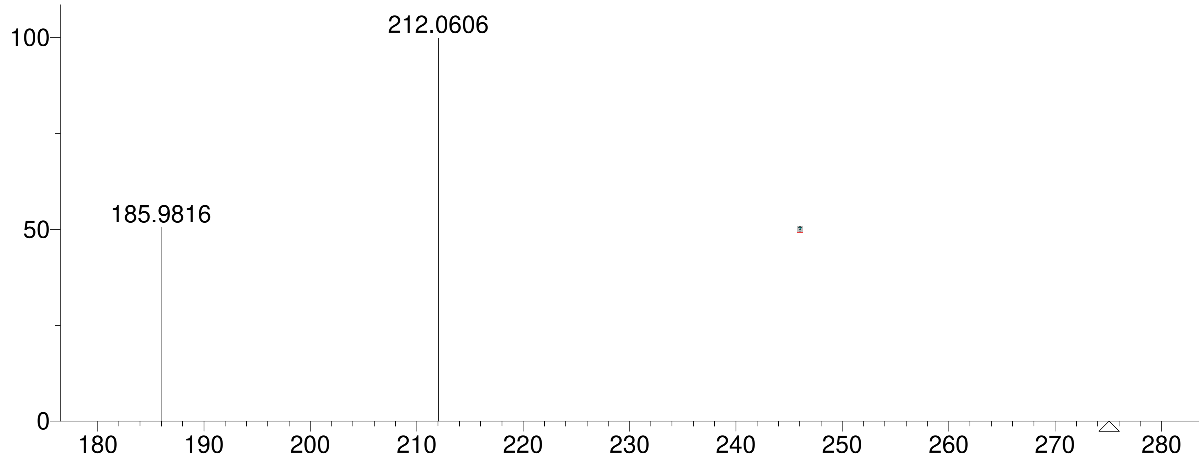

Hit 1 : NCGC00380856-01_C13H16O5_8-Methyl-3,4,6,10b-tetrahydropyrano[3,2-c]isochromene-4a,7,9(2H)-triol
C13H16O5; MF: 0; RMF: 122; Prob 100.0%; Lib: mona-export-gnps; ID: 14363.

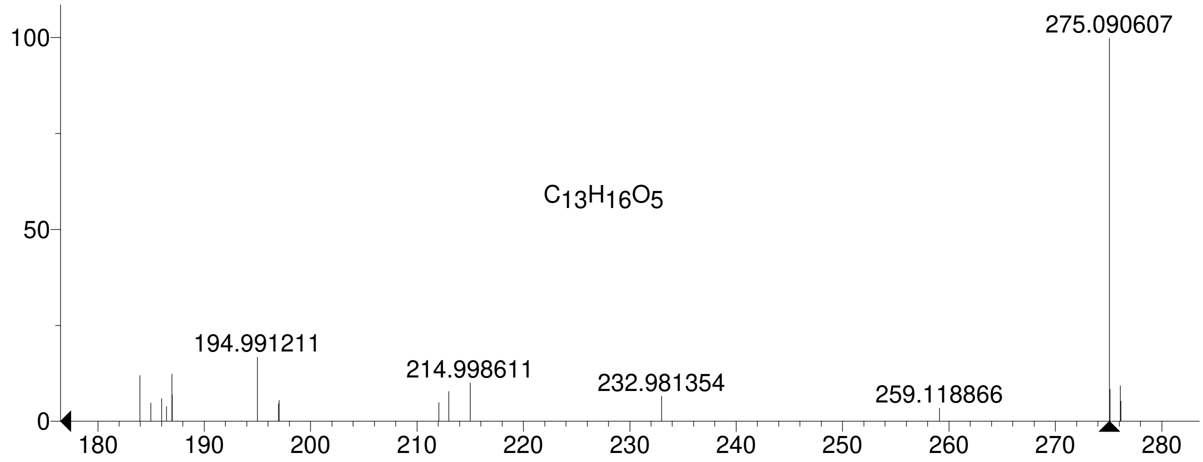

Unknown: Unknown (1.35_476.1506m/z)
Compound in Library Factor = 0

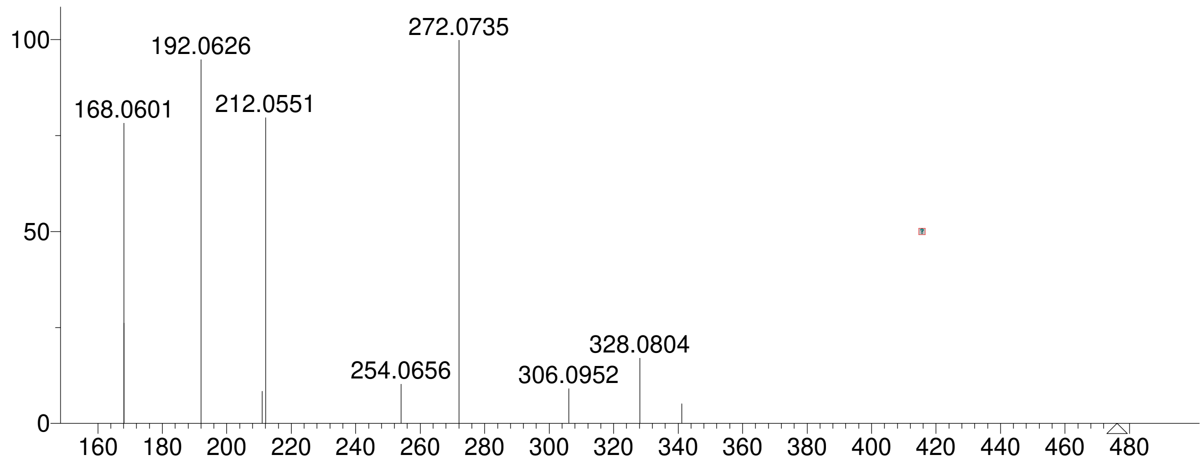

Unknown: Unknown (1.35_863.2438m/z)
Compound in Library Factor = -3273

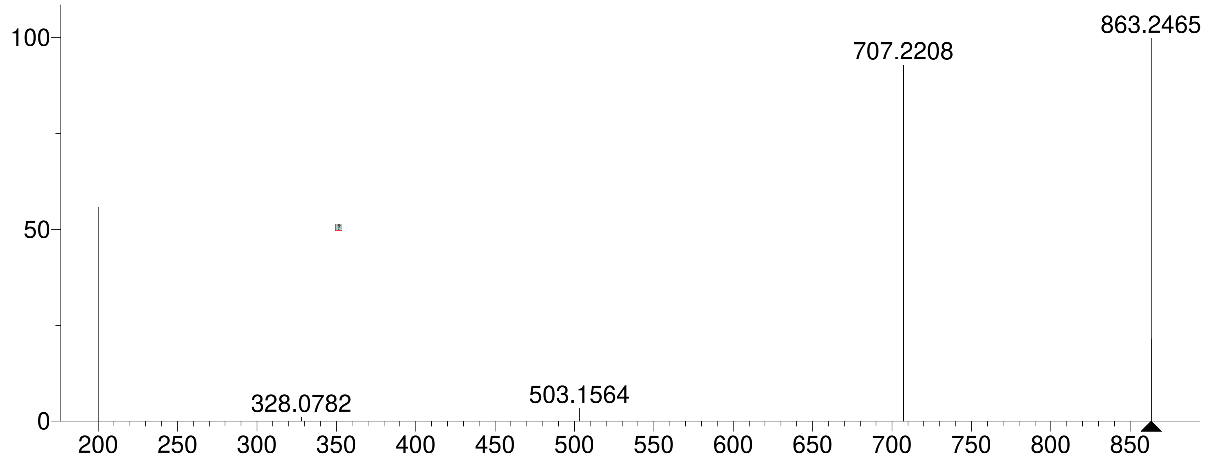

Hit 1 : [Phe10] β-Amyloid (1-40) [M+5H]5+ QTOF 3V P=863.2
C194H295N53O57S; MF: 1; RMF: 152; Prob 51.0%; Lib: nist_msms2; ID: 36926.

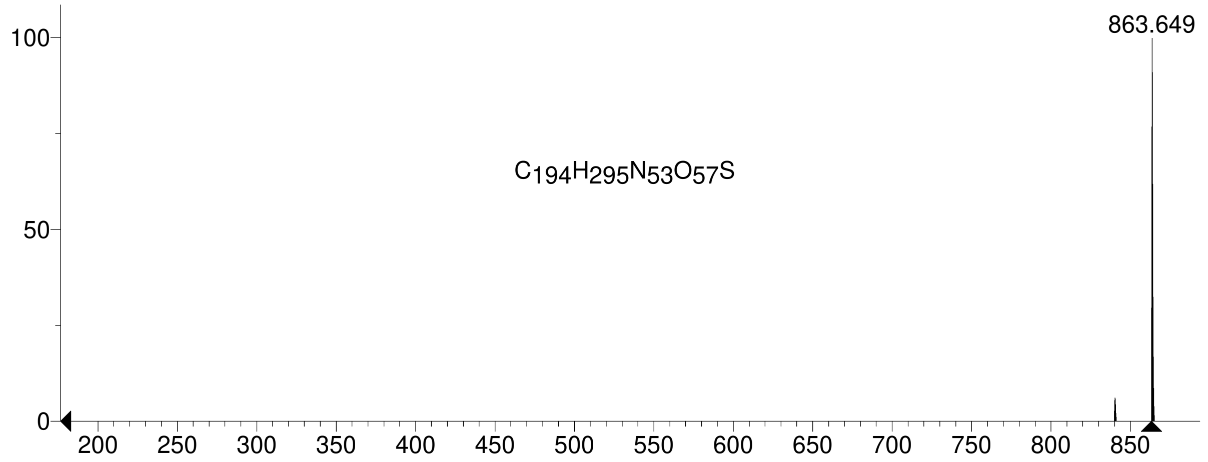

Hit 2 : Genistin [2M-H]- 35% P=863.2
C21H20O10; MF: 0; RMF: 37; Prob 49.0%; Lib: mona-export-itree; ID: 3973.

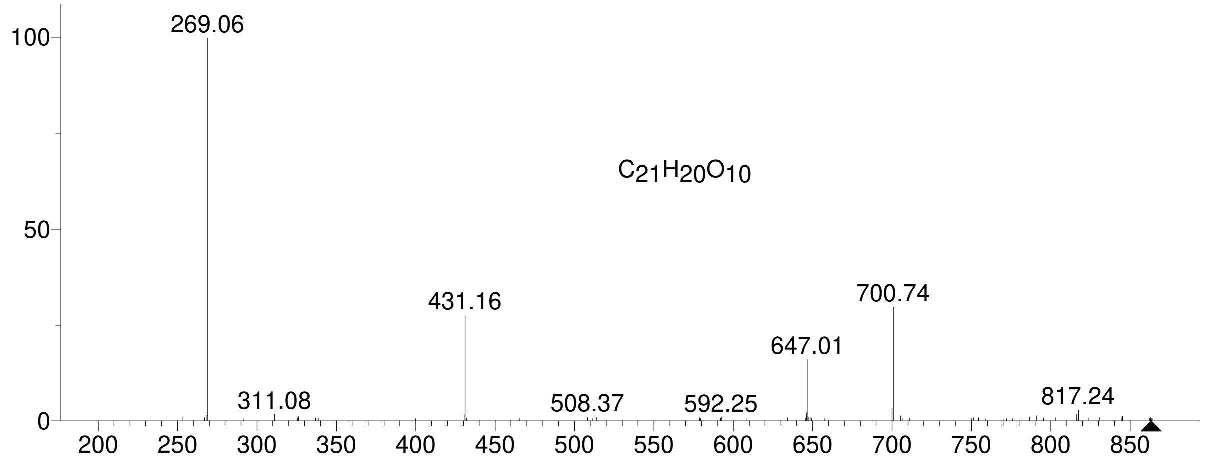

** Search Report Page 1 of 1 **

Unknown: Unknown (1.37_541.1368m/z)
Compound in Library Factor = 0

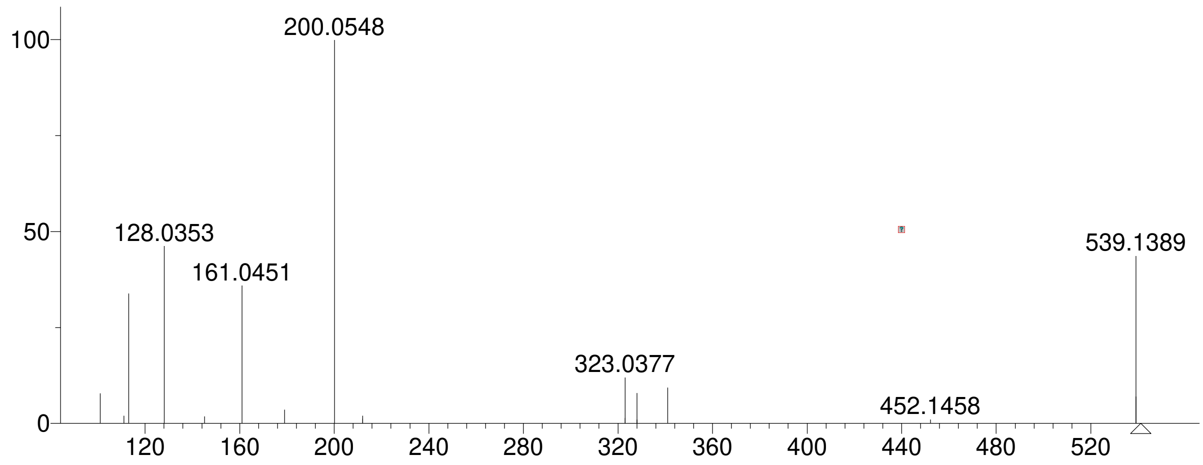

Unknown: Unknown (1.37_601.1302m/z)
Compound in Library Factor = -3273

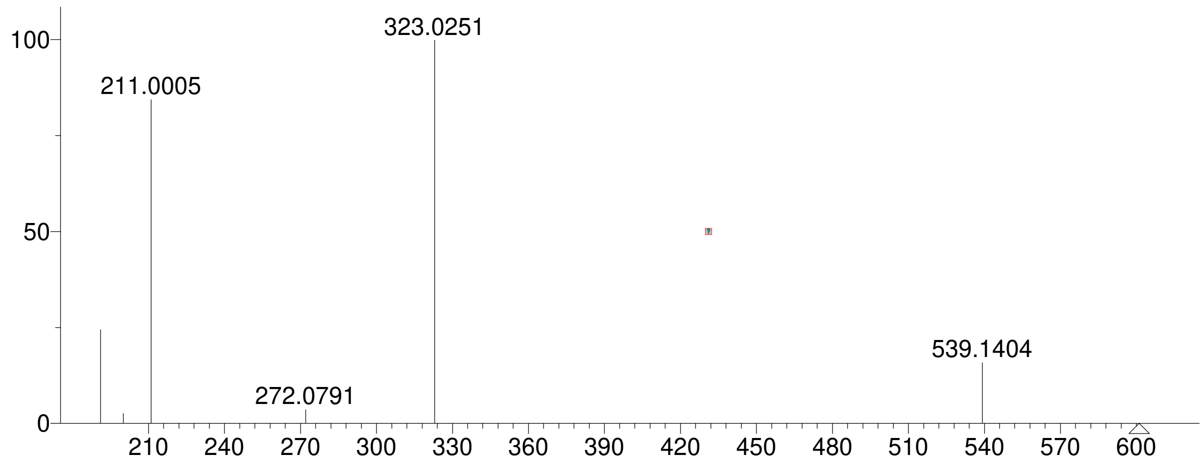

Hit 1 : 5-hydroxy-3-[(2S,3R,4S,5S)-4-hydroxy-5-(hydroxymethyl)-3-[(2S,3R,4R,5R,6S)-3,4,5-trihydroxy-6-methylox
C27H30O14; MF: 4; RMF: 386; Prob 37.2%; Lib: mona-export-itree; ID: 3497.

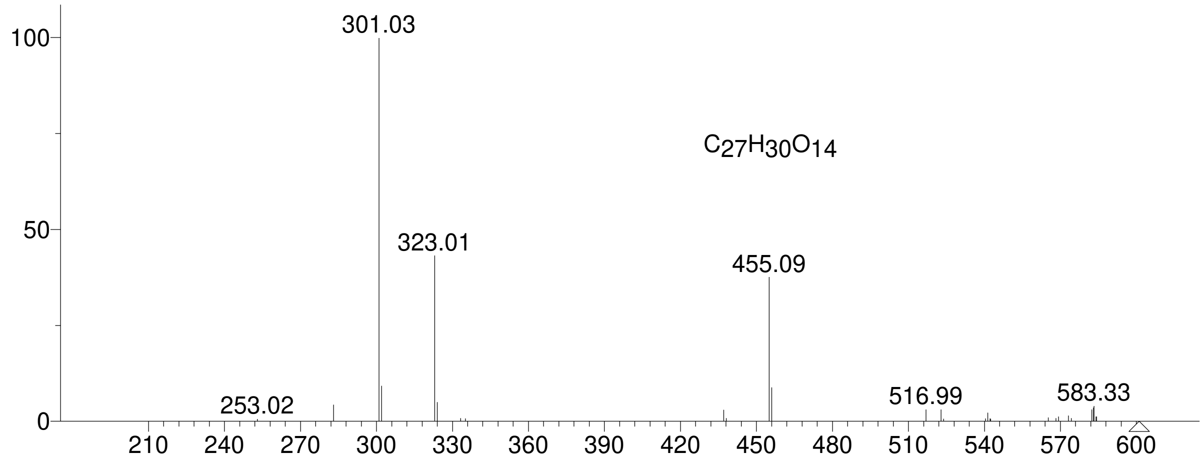

Hit 2 : 6-[4,5-dihydroxy-6-methyl-3-[(2S,3R,4R,5R,6S)-3,4,5-trihydroxy-6-methyloxan-2-yl]oxyoxan-2-yl]-5,7-dihyd
C27H30O13; MF: 0; RMF: 14; Prob 31.4%; Lib: anthocyanins mona; ID: 5070.

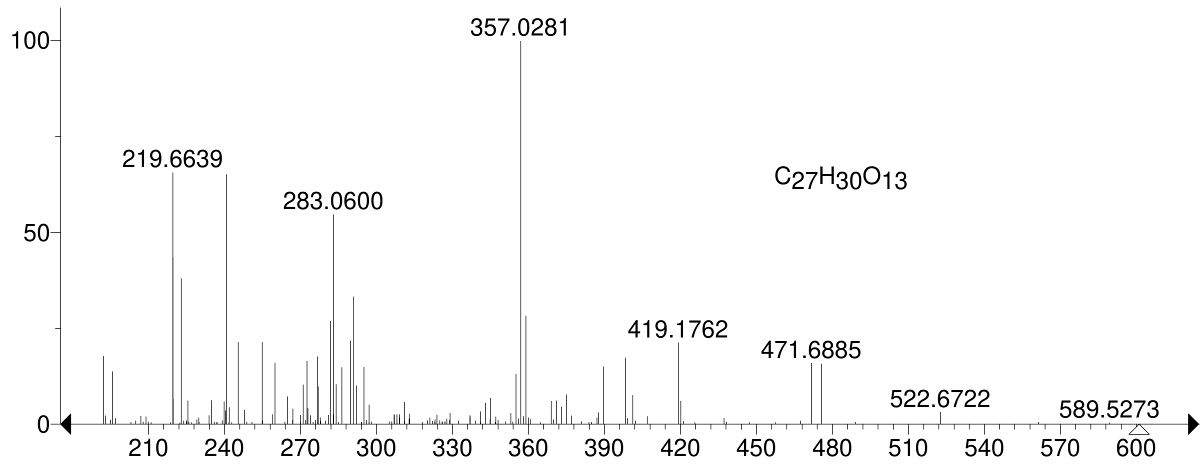

Hit 3 : 7-[3-[(2R,3R,4R)-3,4-dihydroxy-4-(hydroxymethyl)oxolan-2-yl]oxy-4,5-dihydroxy-6-(hydroxymethyl)oxan-2-y
C27H30O13; MF: 0; RMF: 2; Prob 31.4%; Lib: mona-export-vf-npl_qexactive; ID: 17025.

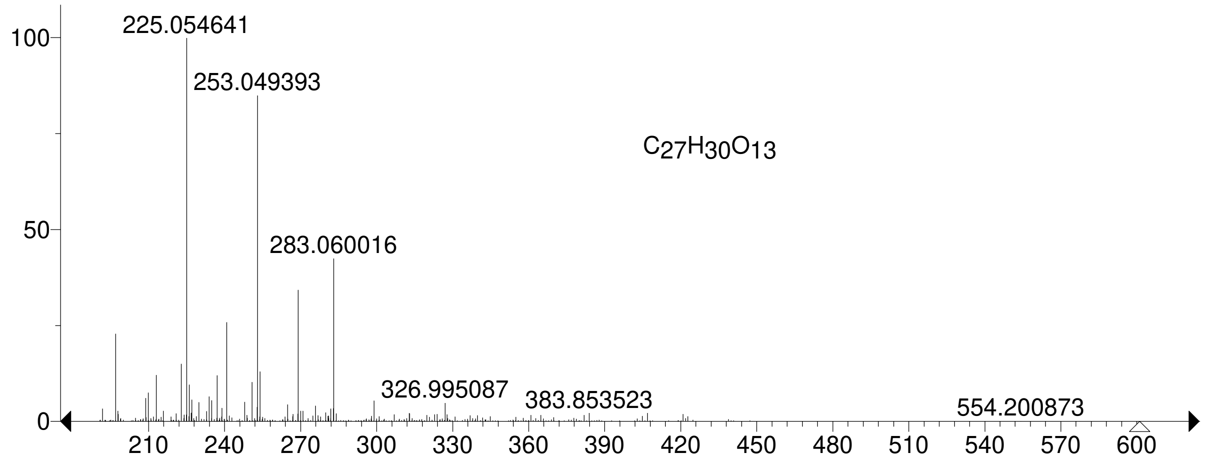

Unknown: Unknown (1.40_521.1263m/z)
Compound in Library Factor = 0

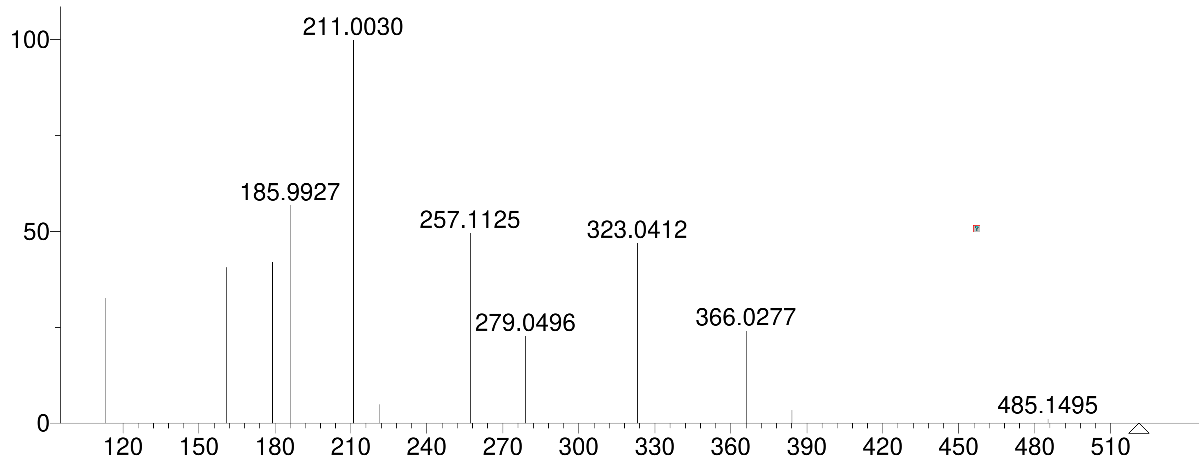

Hit 1 : Iridin [M-H]- 65HCD P=521.1
C24H26O13; MF: 0; RMF: 37; Prob 14.3%; Lib: mona anthocyanidin-o-glycosides; ID: 4756.

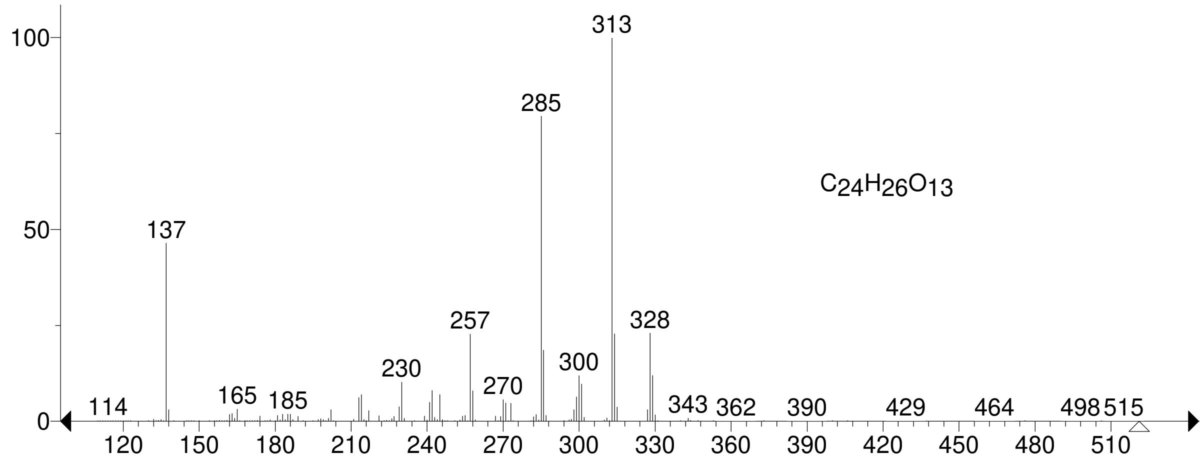

Hit 2 : verproside [M+Na]+ 35HCD P=521.1
C22H26O13; MF: 0; RMF: 37; Prob 14.3%; Lib: mona-export-vf-npl_qexactive; ID: 11986.

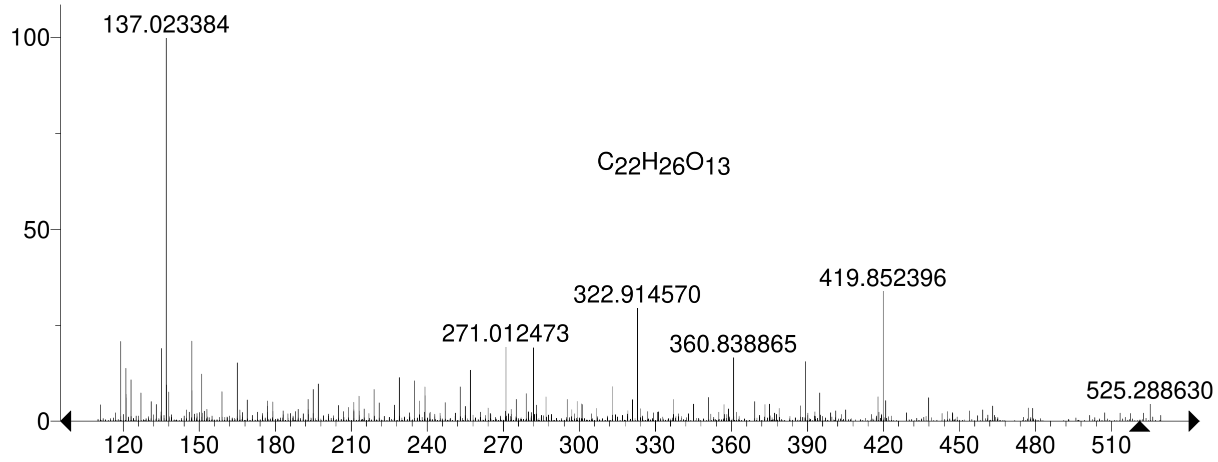

Hit 3 : 5-hydroxy-3-(5-hydroxy-2,4-dimethoxyphenyl)-6-methoxy-7-[3,4,5-trihydroxy-6-(hydroxymethyl)oxan-2-yl]ox
C24H26O13; MF: 0; RMF: 30; Prob 14.3%; Lib: mona anthocyanidin-o-glycosides; ID: 9906.

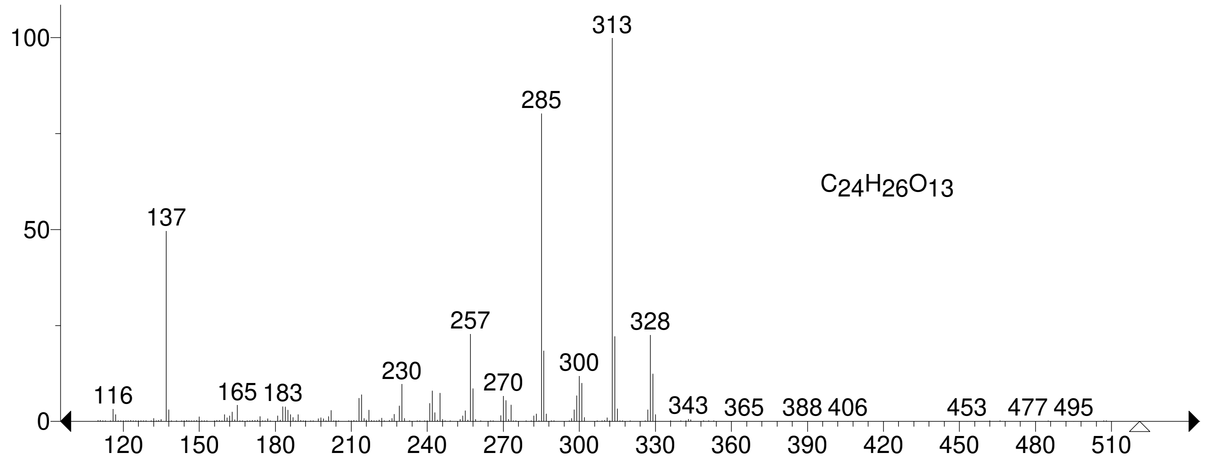

Hit 4 : NCGC00347847-02_C22H26O13_(1aS,1bS,2S,5aR,6S,6aS)-2-(beta-D-Glucopyranosyloxy)-1a-(hydroxym
C22H26O13; MF: 0; RMF: 22; Prob 14.3%; Lib: mona-export-gnps; ID: 10988.

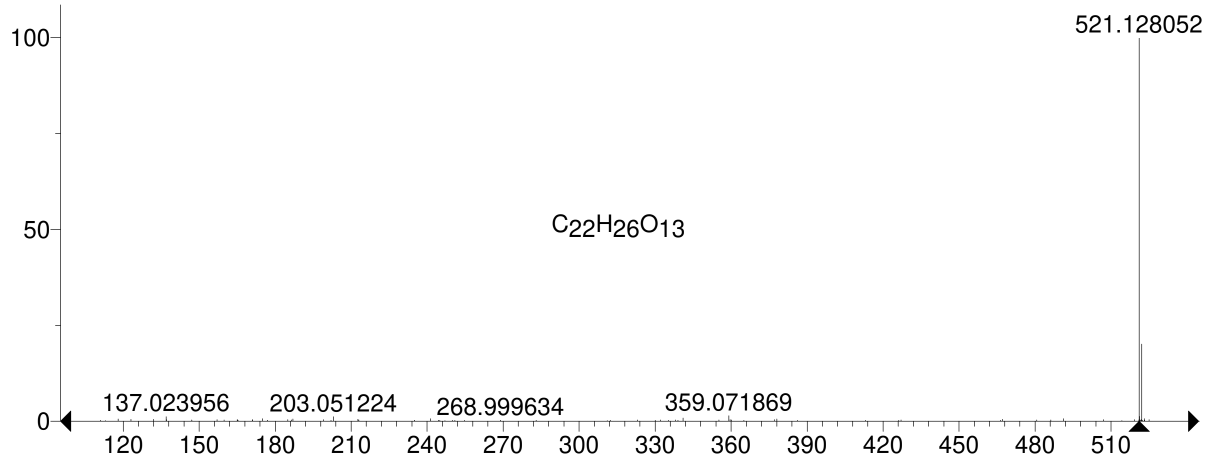

Hit 5 : [2-(3,4-dihydroxyphenyl)-5-hydroxy-7-methoxy-4-oxo-3-[(2S,3R,4R,5R,6S)-3,4,5-trihydroxy-6-methyloxan-2
C24H24O13; MF: 0; RMF: 20; Prob 14.3%; Lib: mona anthocyanidin-o-glycosides; ID: 9408.

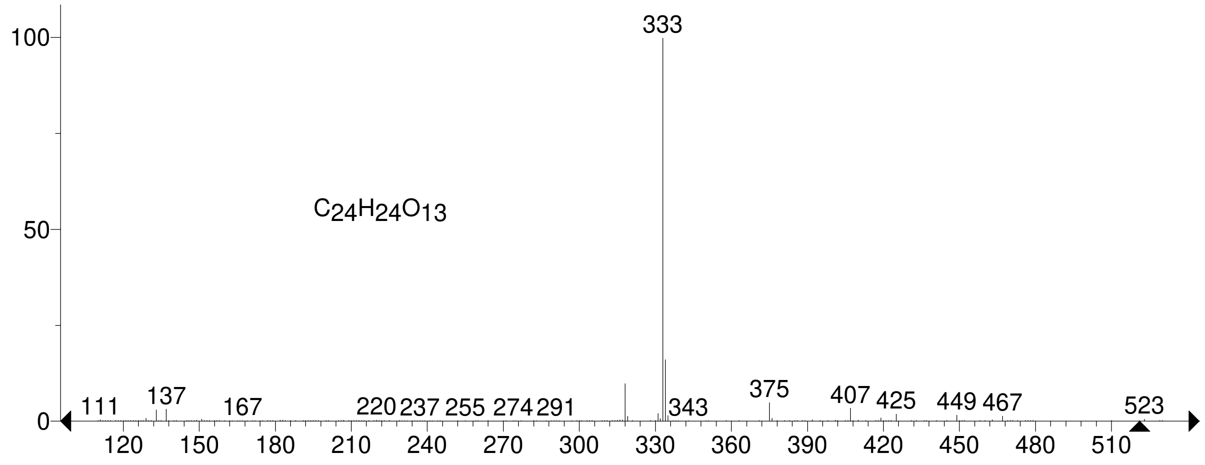

Unknown: Unknown (1.42_470.1496m/z)
Compound in Library Factor = 0

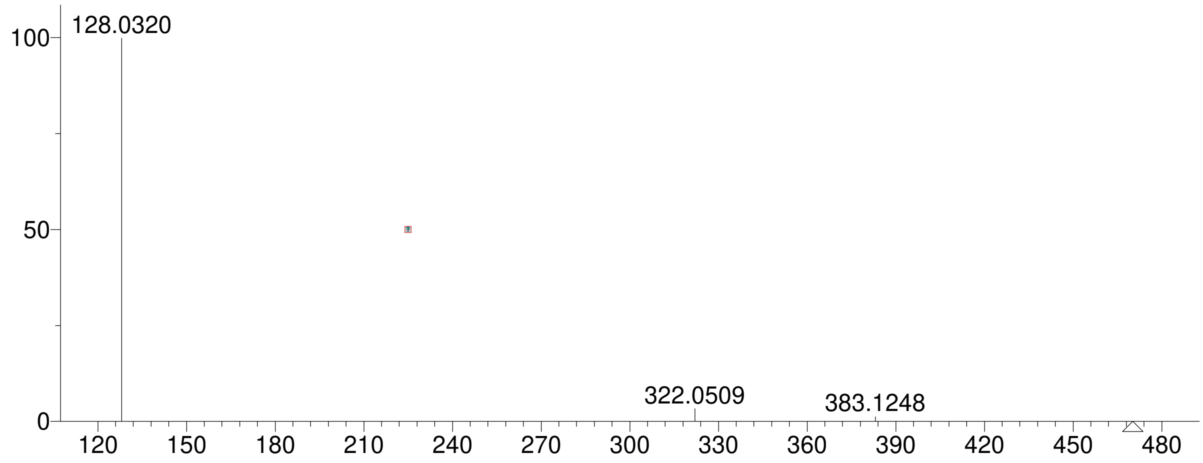

Hit 1 : 6-Sialylgalactose [M-H]- HCD 42V P=470.2
C17H29NO14; MF: 0; RMF: 32; Prob 100.0%; CAS: 35259-23-5; Lib: nist_msms; ID: 532909.

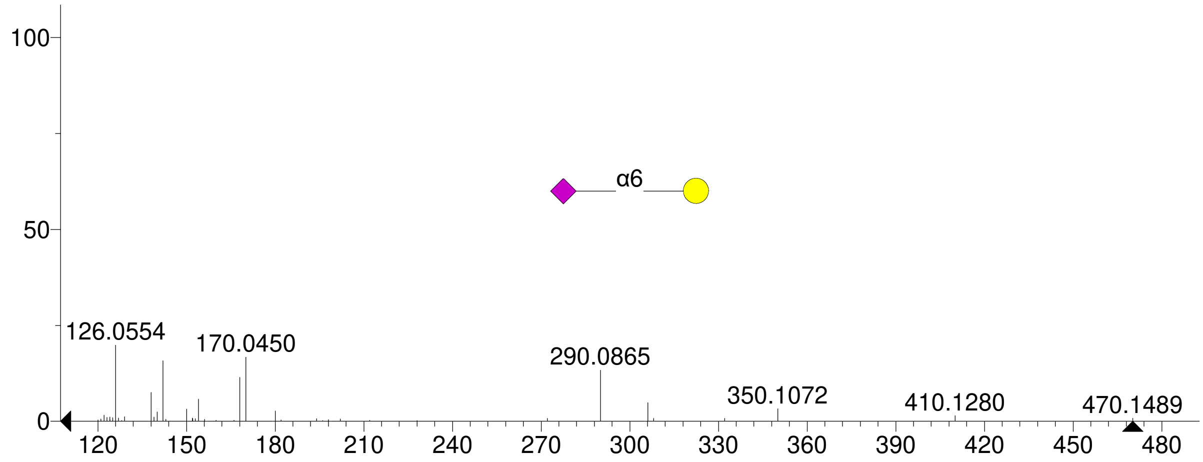

Unknown: Unknown (1.42_539.1376m/z)
Compound in Library Factor = 586

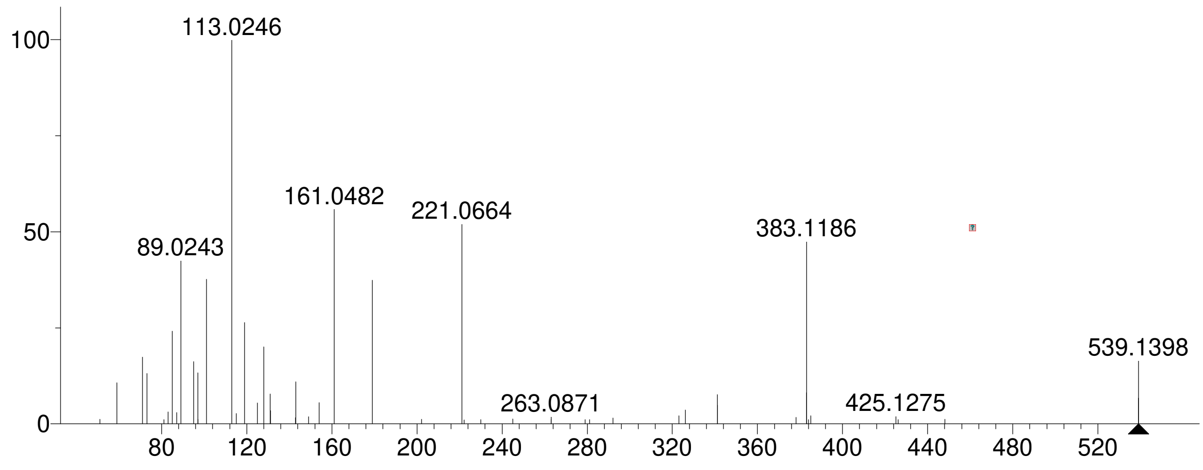

Hit 1 : Maltotriose [M+Cl]- HCD 37V P=539.1
C18H32O16; MF: 811; RMF: 837; Prob 56.0%; CAS: 1109-28-0; Lib: nist_msms; ID: 478094.

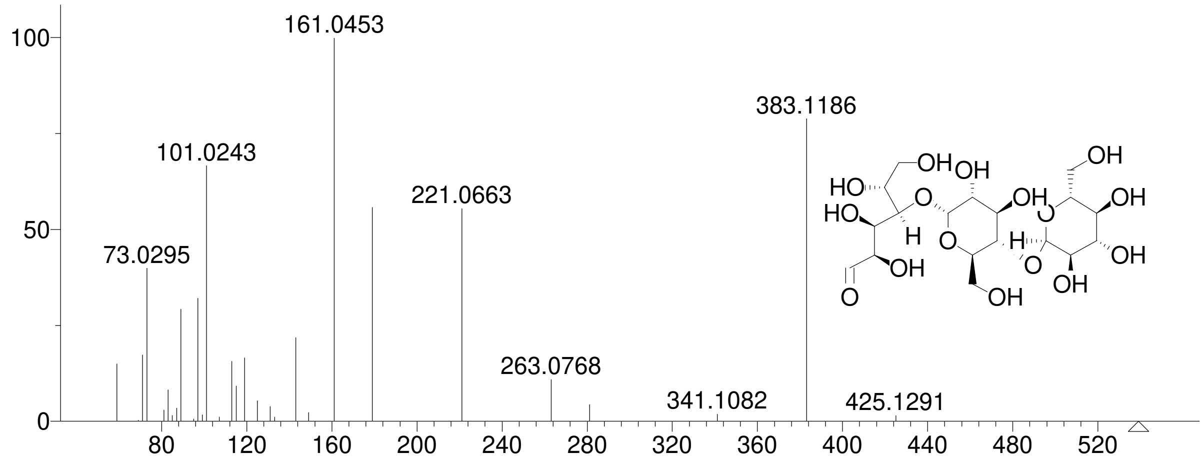

Hit 2 : 3'-Galactosyllactose [M+Cl]- HCD 37V P=539.1
C18H32O16; MF: 779; RMF: 824; Prob 15.0%; CAS: 32694-82-9; Lib: nist_msms; ID: 533314.

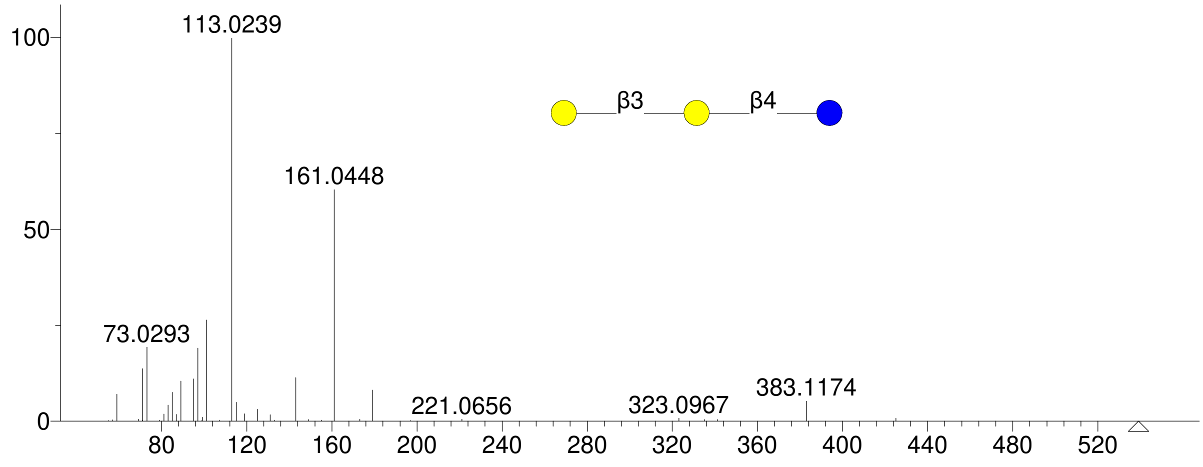

Hit 3 : Manninotriose [M+Cl]- HCD 37V P=539.1
C18H32O16; MF: 767; RMF: 803; Prob 9.96%; CAS: 13382-86-0; Lib: nist_msms; ID: 531274.

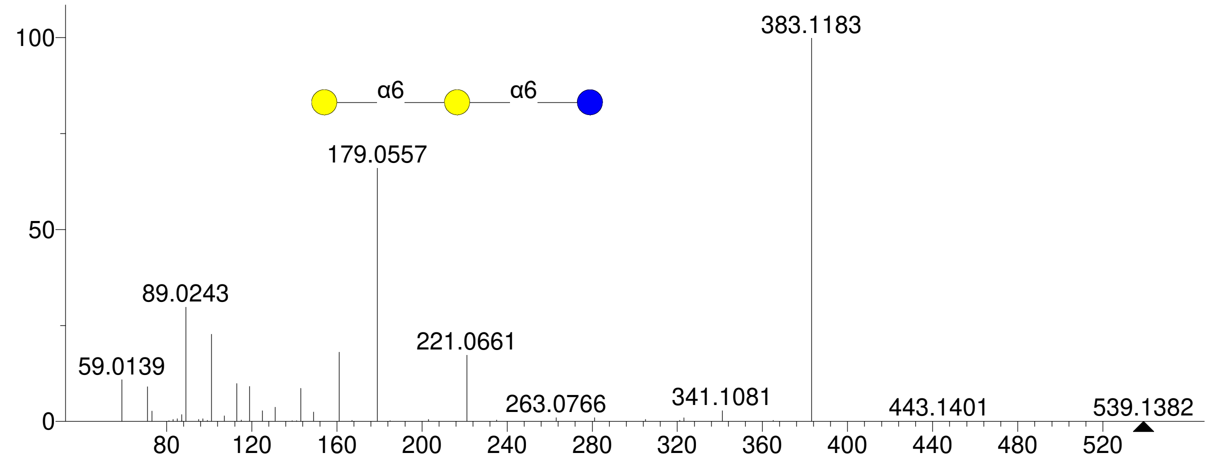

Hit 4 : Erlose [M+Cl]- HCD 48V P=539.1
C18H32O16; MF: 753; RMF: 812; Prob 6.23%; CAS: 13101-54-7; Lib: nist_msms; ID: 531029.

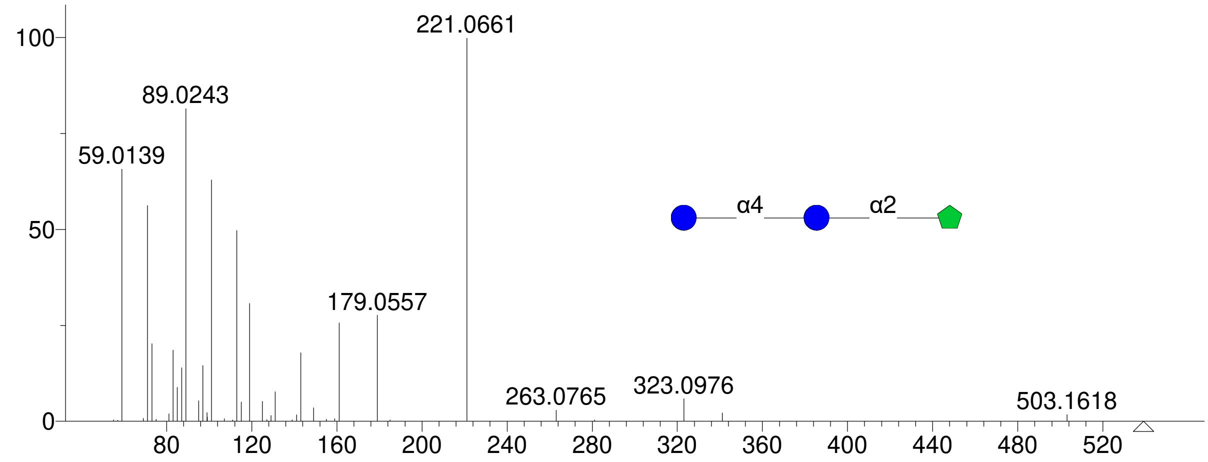

Hit 5 : 3α,4β-Galactotriose [M+Cl]- HCD 32V P=539.1
C18H32O16; MF: 751; RMF: 784; Prob 5.75%; CAS: 56038-36-9; Lib: nist_msms; ID: 527589.

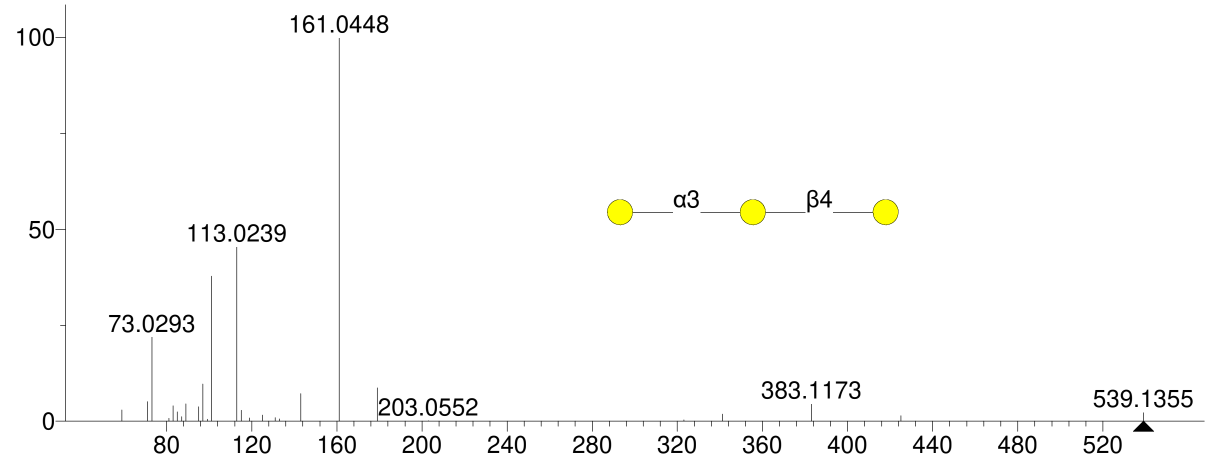

Unknown: Unknown (1.43_509.1258m/z)
Compound in Library Factor = 0

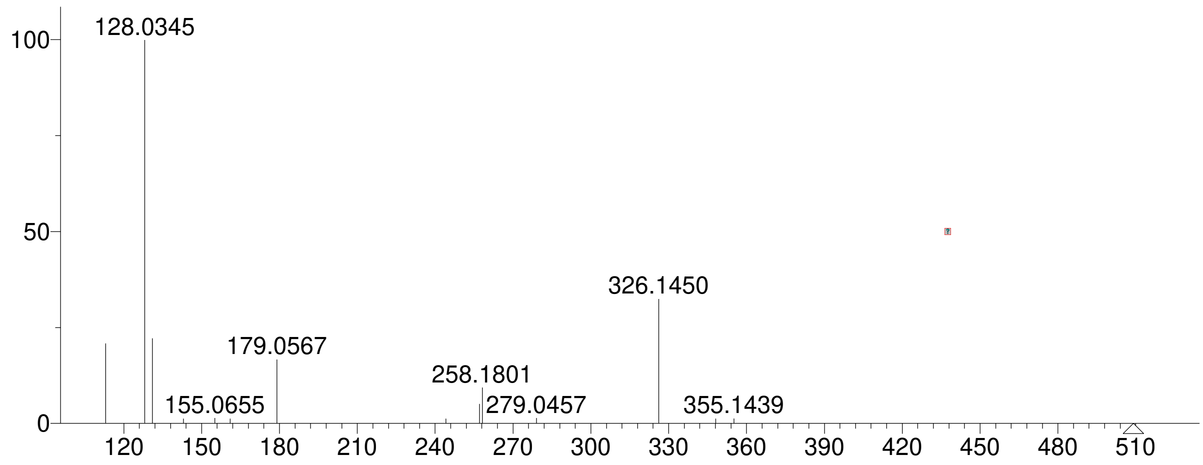

Hit 1 : Syringetin-3-O-glucoside [M+H]+ 10eV P=509.1
C23H24O13; MF: 0; RMF: 37; Prob 25.0%; Lib: anthocyanins mona; ID: 908.

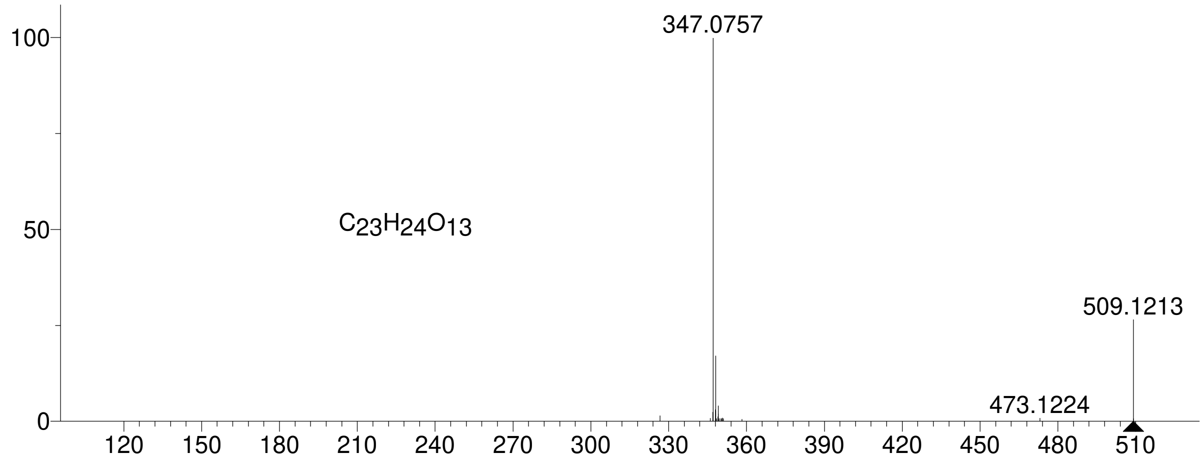

Hit 2 : NCGC00380543-01![(2R,3S,4S,5R,6R)-6-[(5,8-dihydroxy-4-oxo-2,3-dihydro-1H-naphthalen-1-yl)oxy]-3,4,5
C23H24O13; MF: 0; RMF: 19; Prob 25.0%; Lib: mona-export-gnps; ID: 6437.

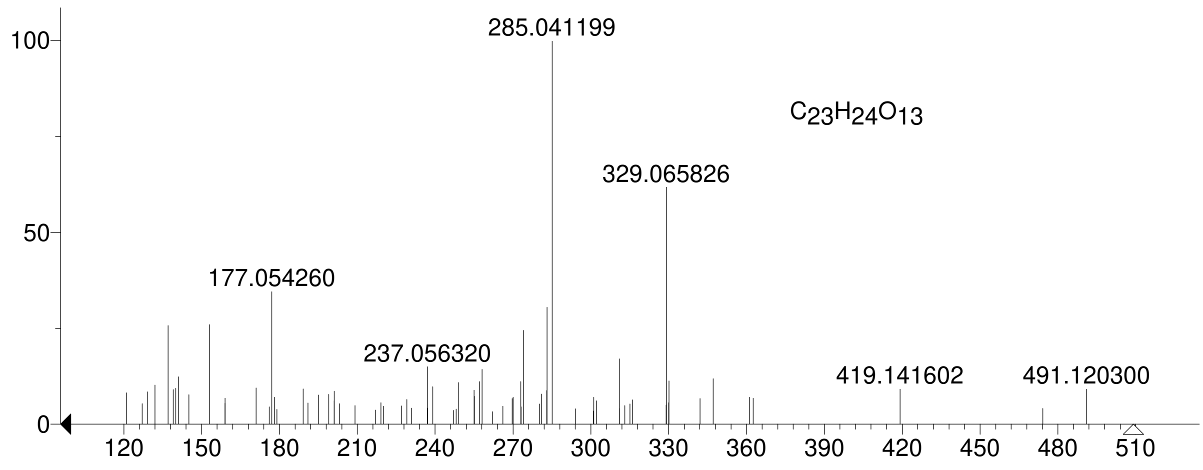

Hit 3 : 5-(1,2,3,4,5,6-hexahydroxyhexoxy)-6-hydroxy-3-methoxy-9-methyl-4,5-dihydro-3H-pyrano[3,4-h][1]benzoxe
C21H26O13; MF: 0; RMF: 19; Prob 25.0%; Lib: mona-export-vf-npl_qexactive; ID: 17685.

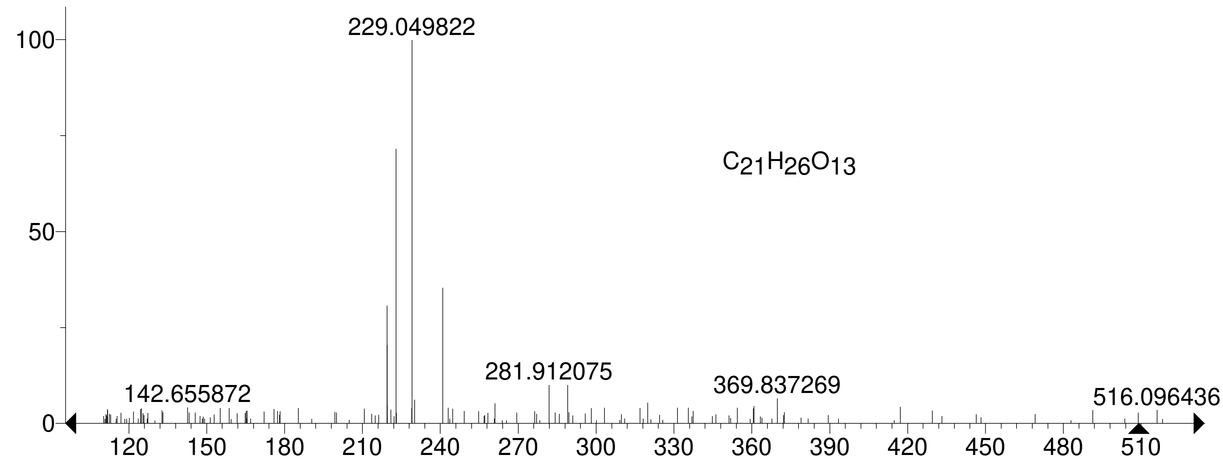

Hit 4 : Malvidin 3,5-diglucoside cation [Cat-2H-C6H8O4]- HCD 35V P=509.1
C29H35O17; MF: 0; RMF: 6; Prob 25.0%; CAS: 47863-30-9; Lib: nist_msms; ID: 503071.

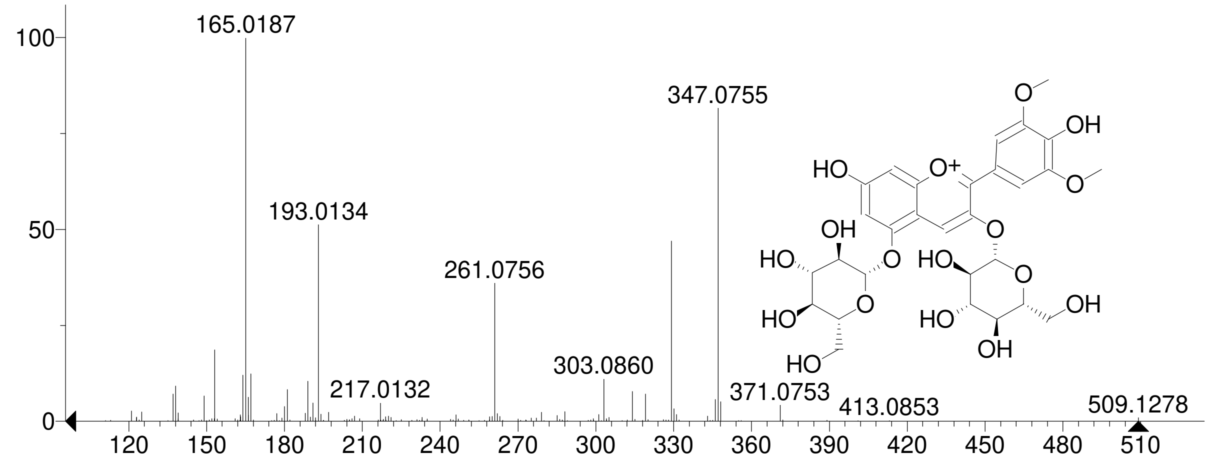

** Search Report Page 1 of 1 **

Unknown: Unknown (1.43_632.2061m/z)
Compound in Library Factor = 0

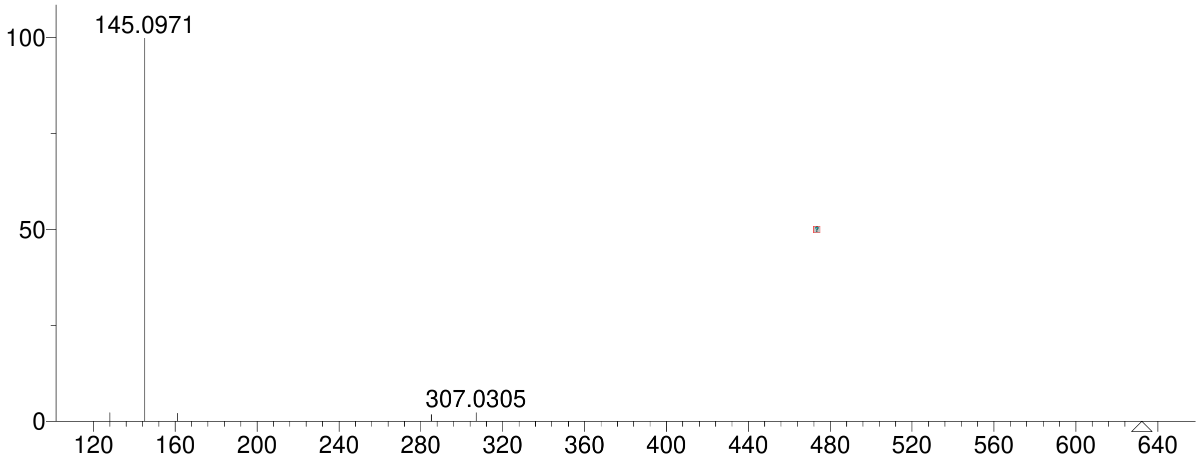

Hit 1 : 6'-Sialyllactose [M-H]- HCD 69V P=632.2
C23H39NO19; MF: 0; RMF: 22; Prob 50.0%; CAS: 74609-39-5; Lib: nist_msms; ID: 531430.

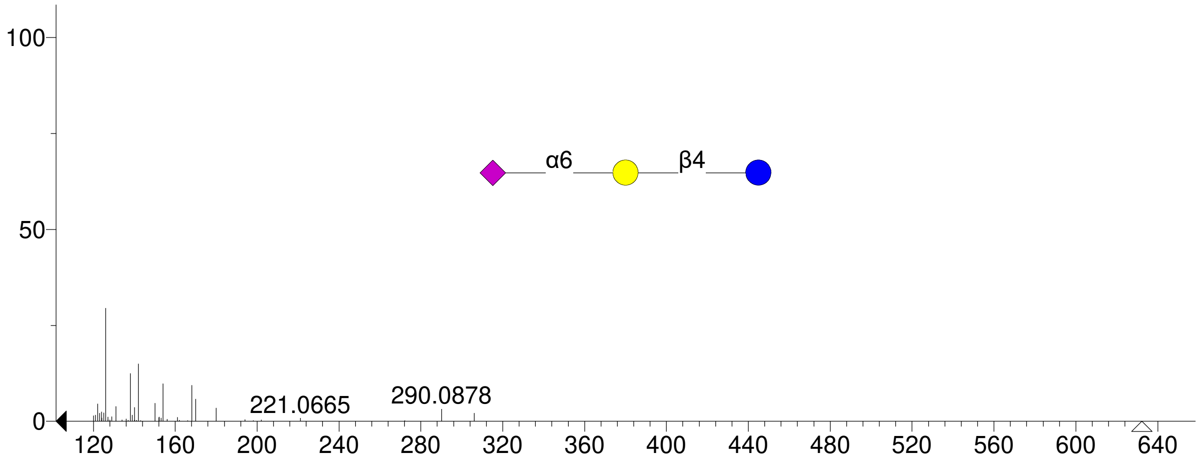

Hit 2 : 3'-Sialyllactose [M-H]- HCD 69V P=632.2
C23H39NO19; MF: 0; RMF: 18; Prob 50.0%; CAS: 35890-38-1; Lib: nist_msms; ID: 530597.

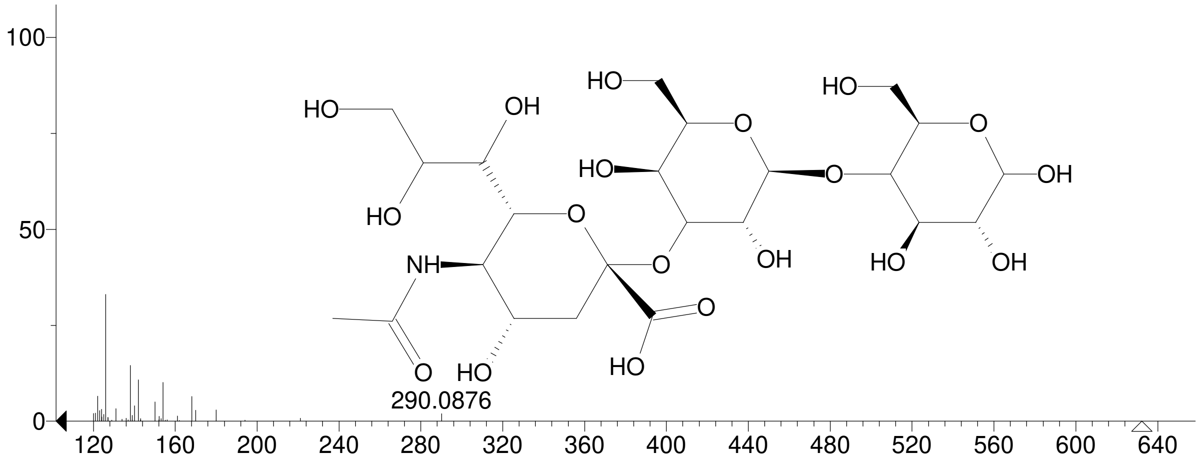

Unknown: Unknown (1.44_284.1341m/z)
Compound in Library Factor = 0

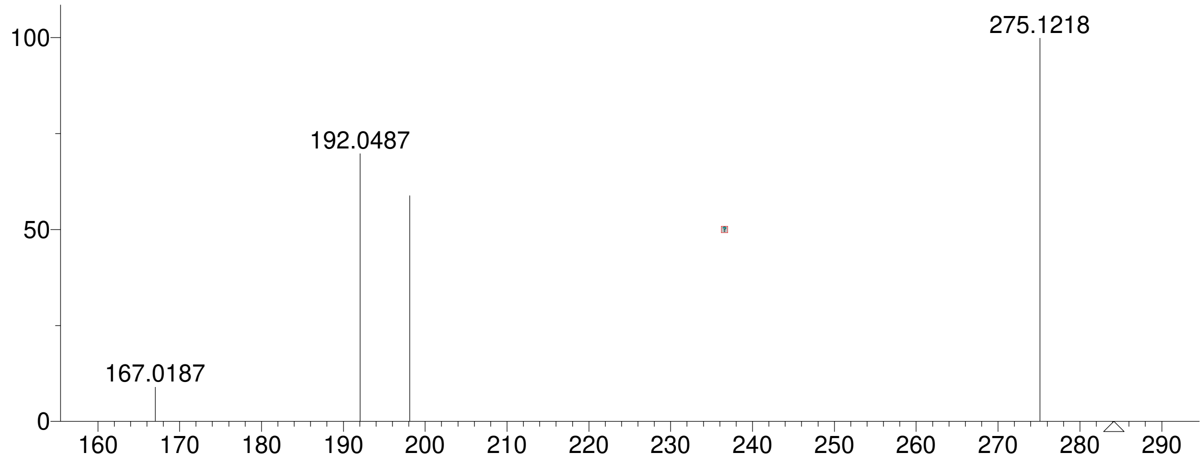

Unknown: Unknown (1.44_365.1550m/z)
Compound in Library Factor = 0

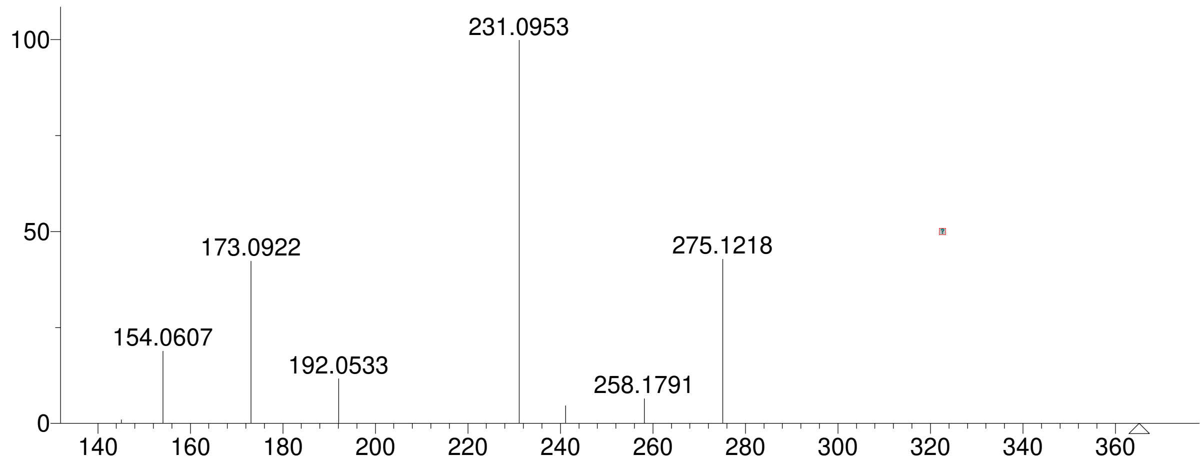

Hit 1 : MMV659004 [M+H]+ 20-30-40%) P=365.2
C21H21ClN4; MF: 0; RMF: 2; Prob 100.0%; Lib: mona-export-pathogen_box; ID: 281.

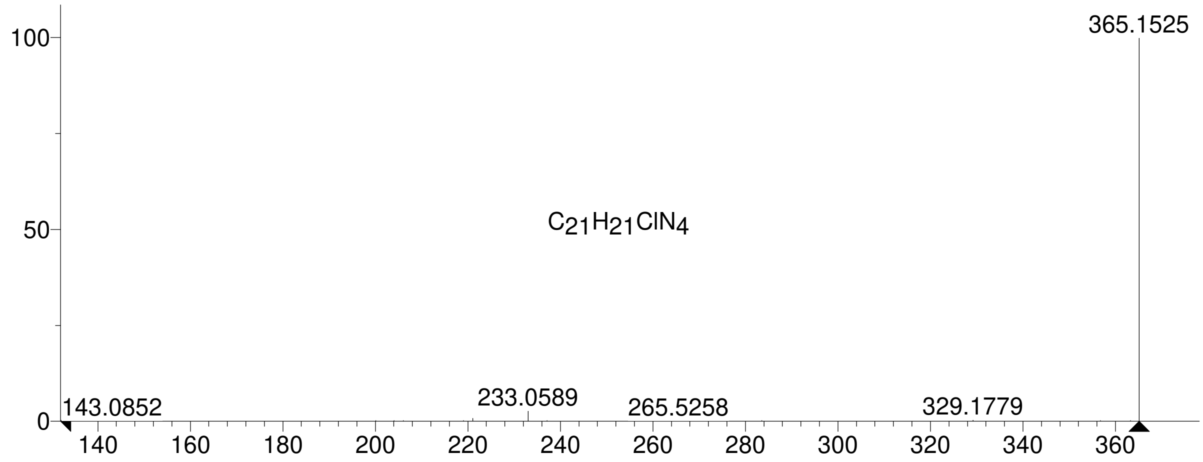

** Search Report Page 1 of 1 **

Unknown: Unknown (1.44_420.2343m/z)
Compound in Library Factor = 0

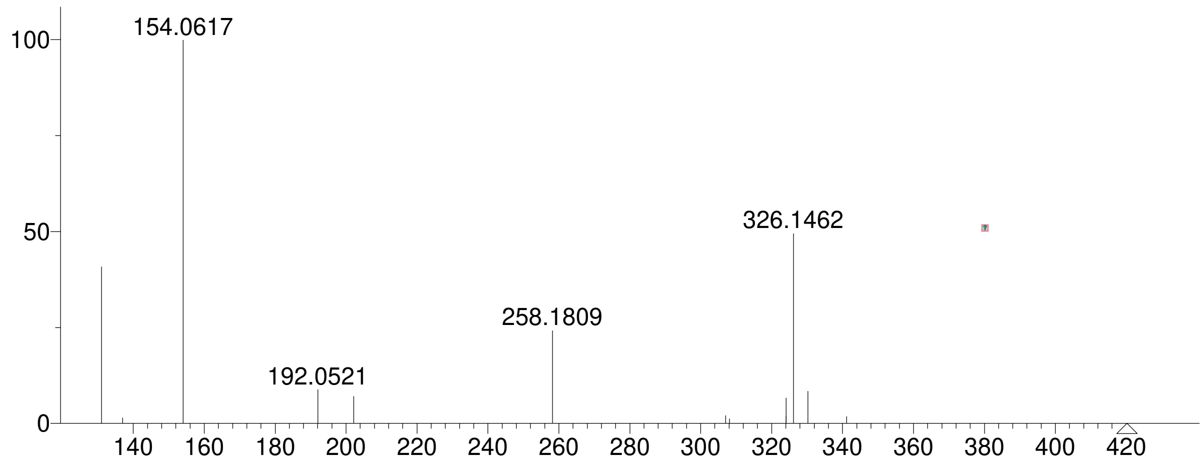

Hit 1 : TNF-α (71-82), human [M+3H]3+ HCD 35eV P=420.2
C51H91N19O18; MF: 0; RMF: 8; Prob 100.0%; Lib: nist_msms2; ID: 49961.

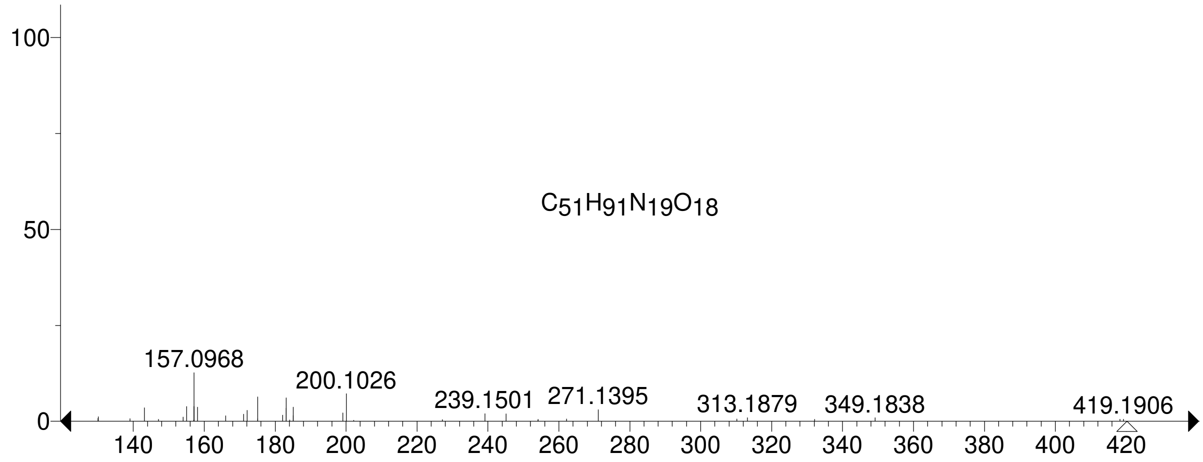

Unknown: Unknown (1.44_496.2041m/z)
Compound in Library Factor = 0

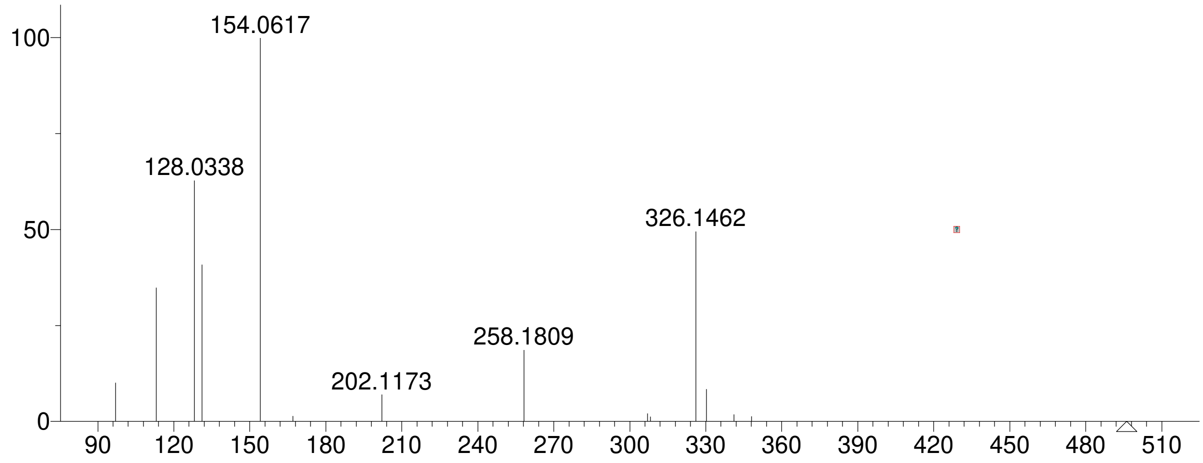

Hit 1 : (2R,3S,4S,5R,6S)-2-[(3,4,5-trihydroxyoxan-2-yl)oxymethyl]-6-(3,4,5-trimethoxyphenoxy)oxane-3,4,5-triol [M
C20H30O13; MF: 0; RMF: 192; Prob 25.0%; Lib: mona-export-vf-npl_qexactive; ID: 9258.

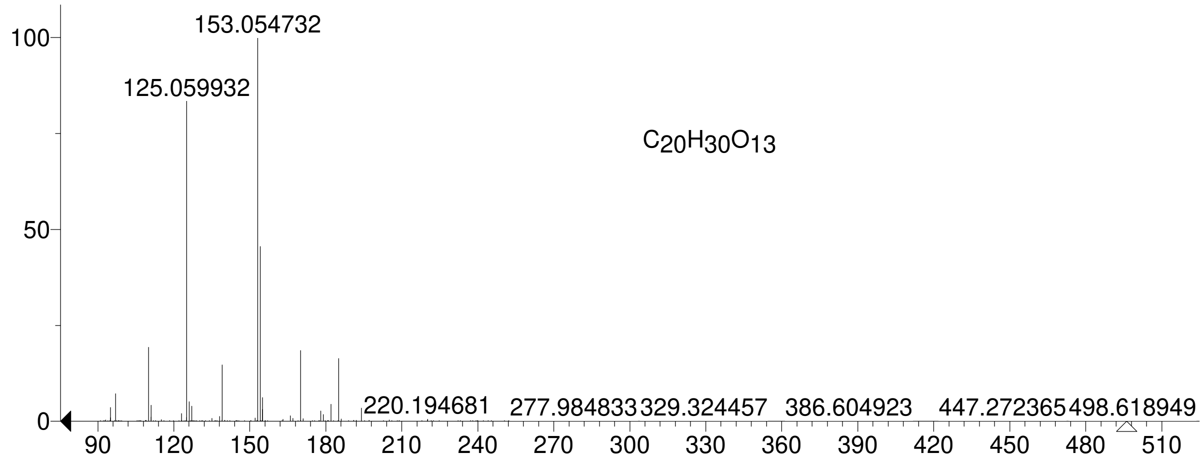

Hit 2 : (2R,3S,4S,5R,6S)-2-[[(2S,3R,4R)-3,4-dihydroxy-4-(hydroxymethyl)oxolan-2-yl]oxymethyl]-6-(3,4,5-trimetho
C20H30O13; MF: 0; RMF: 179; Prob 25.0%; Lib: mona-export-vf-npl_qexactive; ID: 9783.

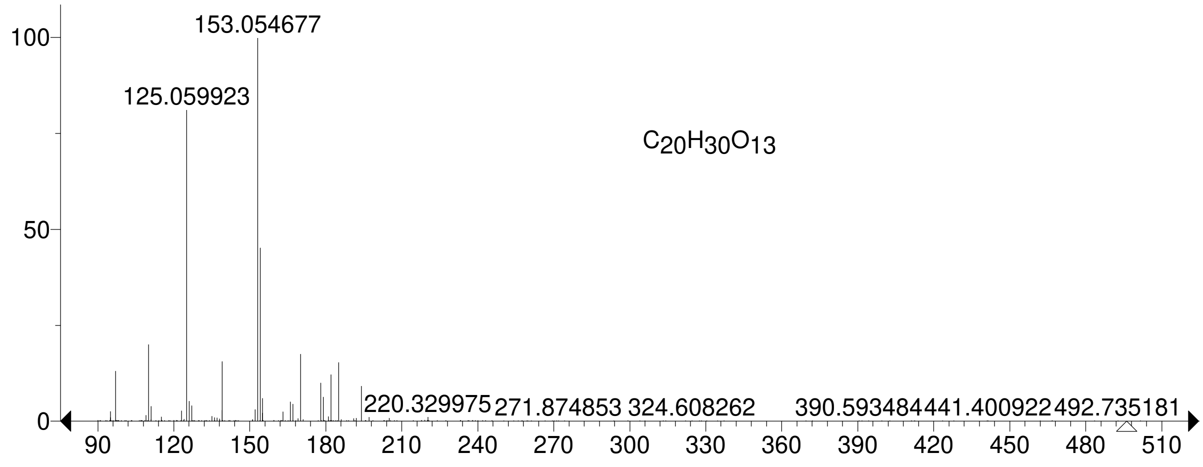

Hit 3 : NCGC00180025-03!(2R,3S,4S,5R,6S)-2-[[(2S,3R,4R)-3,4-dihydroxy-4-(hydroxymethyl)oxolan-2-yl]oxymetl
C20H30O13; MF: 0; RMF: 140; Prob 25.0%; Lib: mona-export-gnps; ID: 8565.

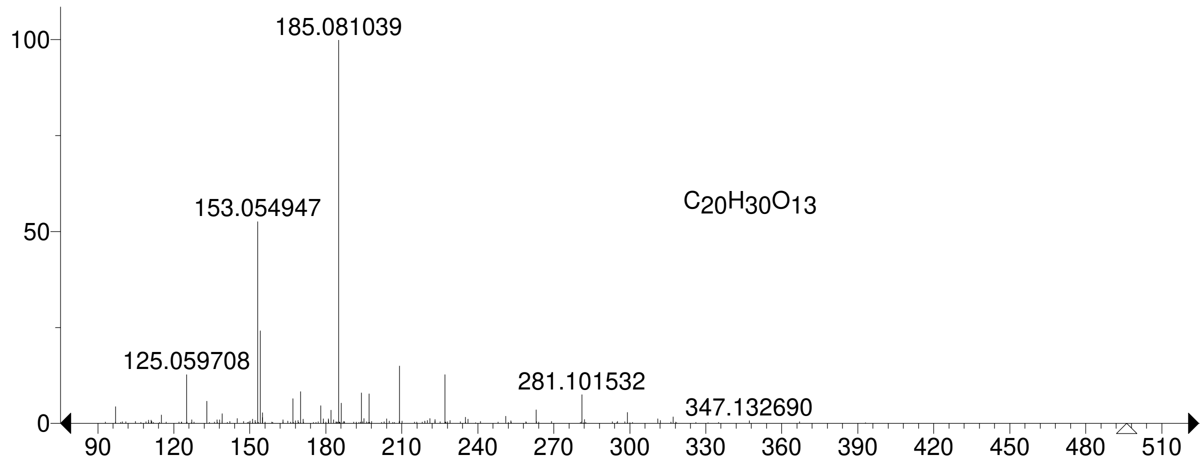

Hit 4 : NCGC00180666-02!(2R,3S,4S,5R,6S)-2-[(3,4,5-trihydroxyoxan-2-yl)oxymethyl]-6-(3,4,5-trimethoxyphenox
C20H30O13; MF: 0; RMF: 136; Prob 25.0%; Lib: mona-export-gnps; ID: 8997.

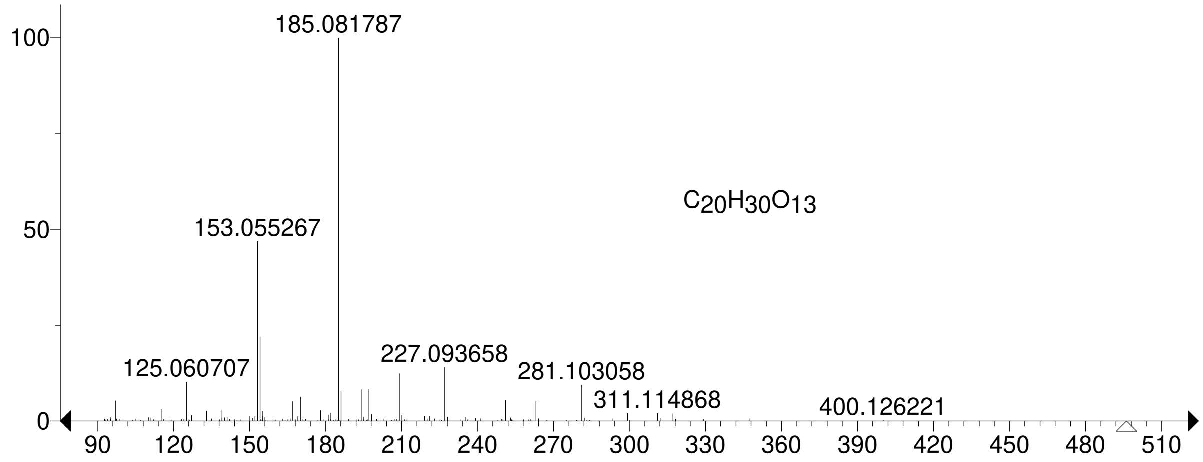

Unknown: Unknown (1.44_521.1262m/z)
Compound in Library Factor = 0

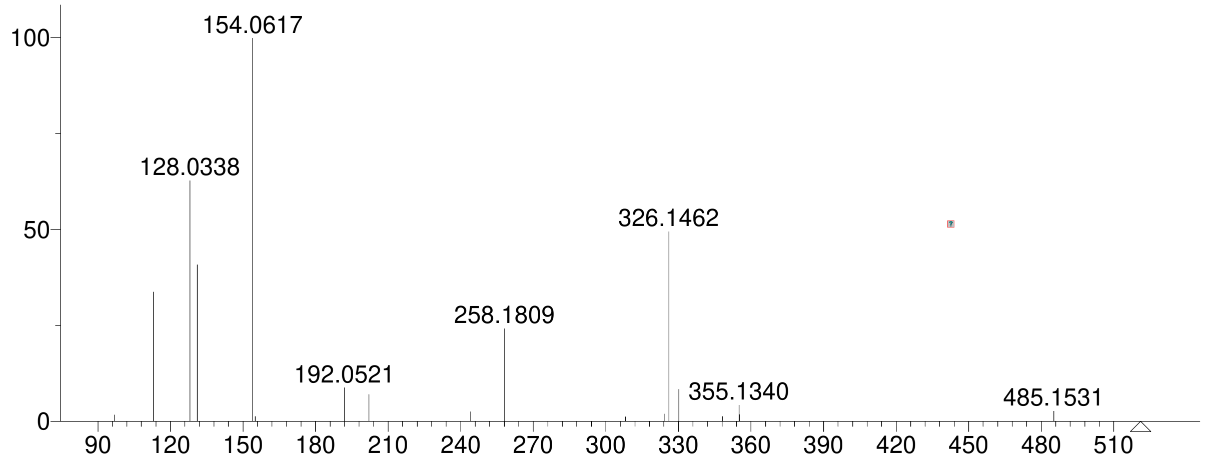

Hit 1 : verproside [M+Na]+ 65HCD P=521.1
C22H26O13; MF: 0; RMF: 24; Prob 12.5%; Lib: mona-export-vf-npl_qexactive; ID: 11988.

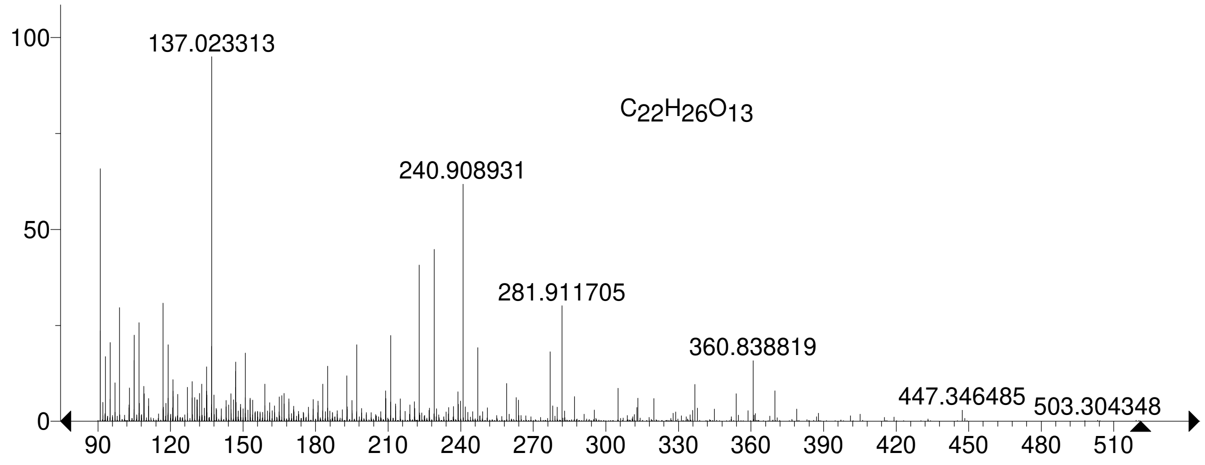

Hit 2 : NCGC00347847-02_C22H26O13_(1aS,1bS,2S,5aR,6S,6aS)-2-(beta-D-Glucopyranosyloxy)-1a-(hydroxym
C22H26O13; MF: 0; RMF: 9; Prob 12.5%; Lib: mona-export-gnps; ID: 10988.

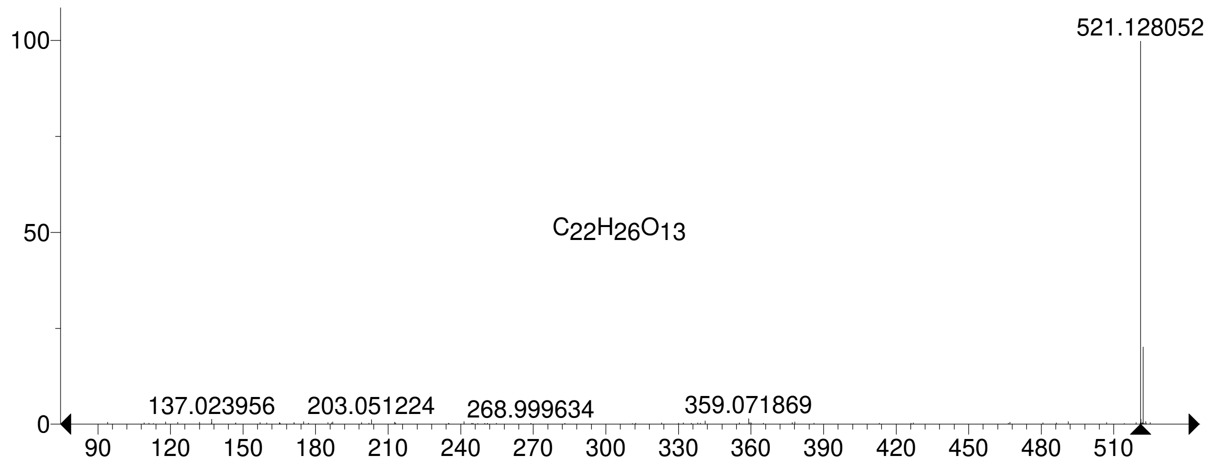

Hit 3 : 5-hydroxy-3-(5-hydroxy-2,4-dimethoxyphenyl)-6-methoxy-7-[3,4,5-trihydroxy-6-(hydroxymethyl)oxan-2-yl]ox
C24H26O13; MF: 0; RMF: 7; Prob 12.5%; Lib: anthocyanins mona; ID: 9905.

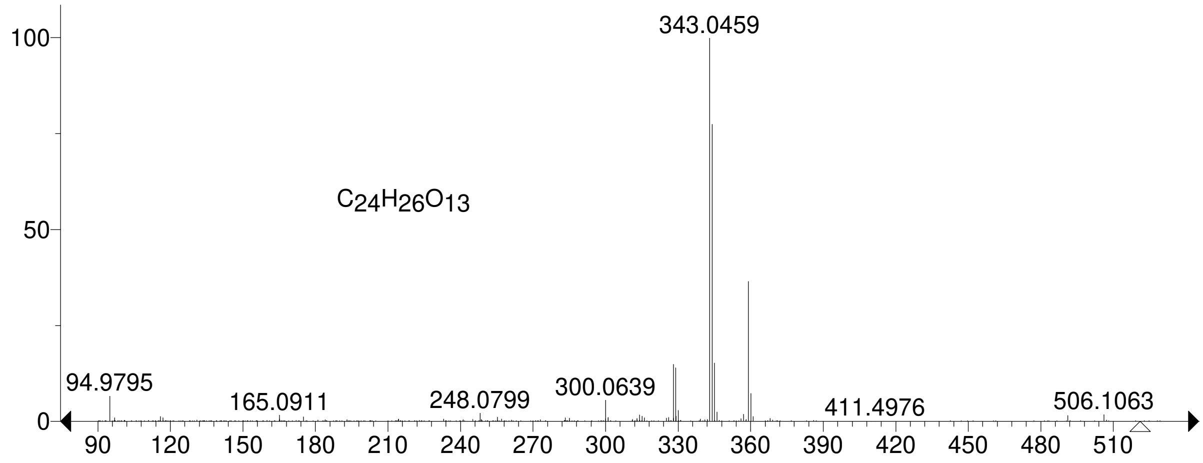

Hit 4 : C24H26O13; PlaSMA ID-1450 [M-H]- P=521.1
C24H26O13; MF: 0; RMF: 6; Prob 12.5%; Lib: biomsms-neg-plasma; ID: 886.

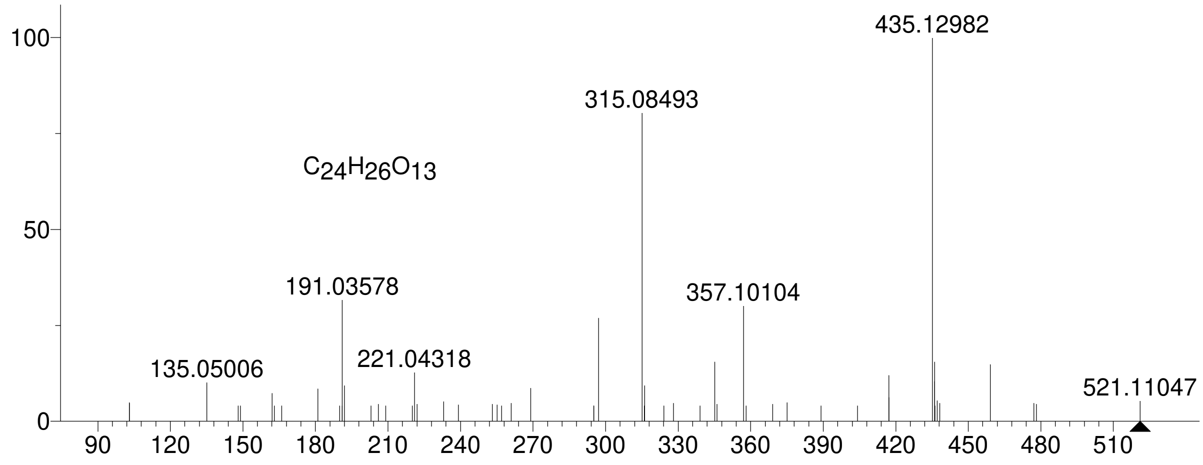

Hit 5 : salviaflaside [M-H]- 35HCD P=521.1
C24H26O13; MF: 0; RMF: 5; Prob 12.5%; Lib: mona-export-lc-ms-ms_negative_mode; ID: 22547.

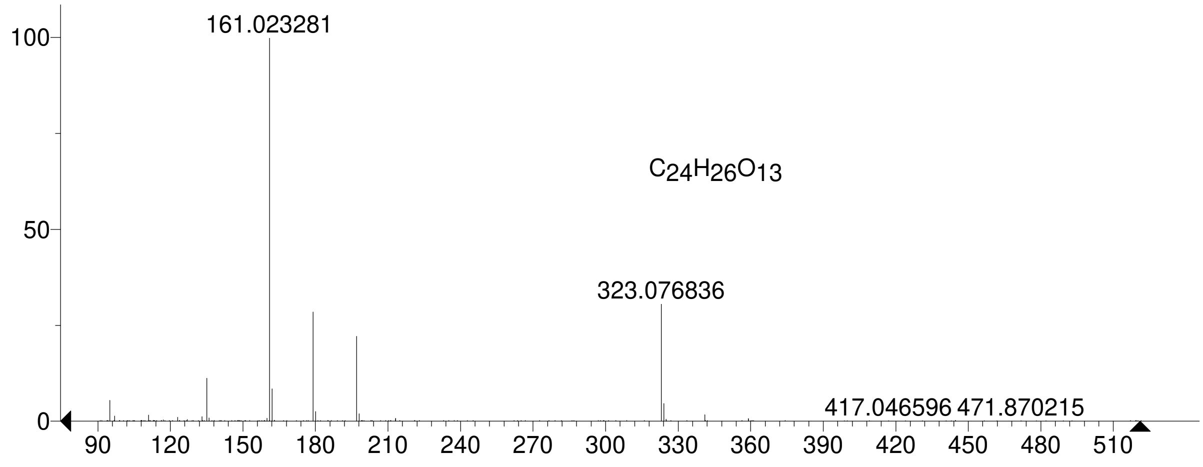

Unknown: Unknown (1.44_713.1909m/z)
Compound in Library Factor = -3273

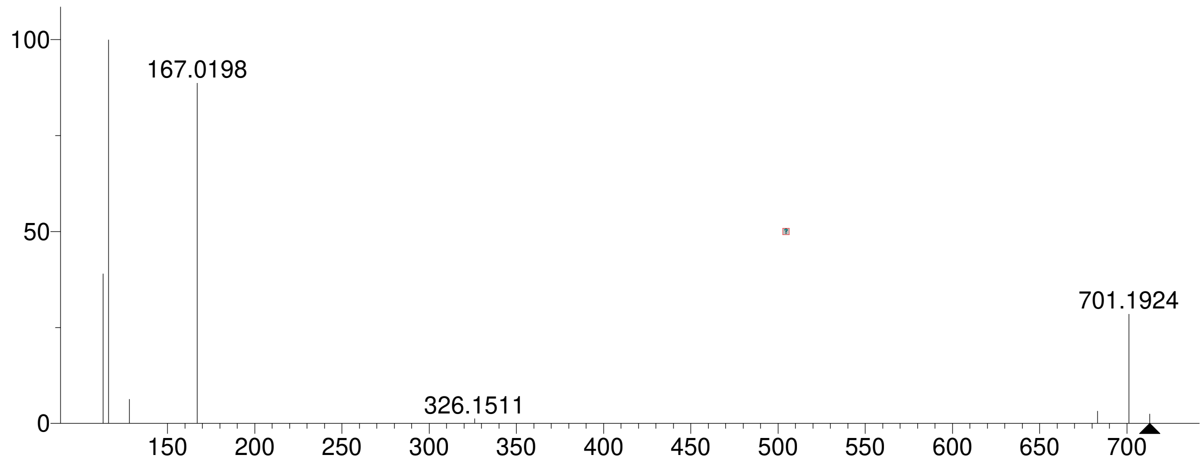

Hit 1 : Sulindac [2M+H]+ HCD 7V P=713.2
C20H17FO3S; MF: 19; RMF: 228; Prob 100.0%; CAS: 38194-50-2; Lib: nist_msms; ID: 316024.

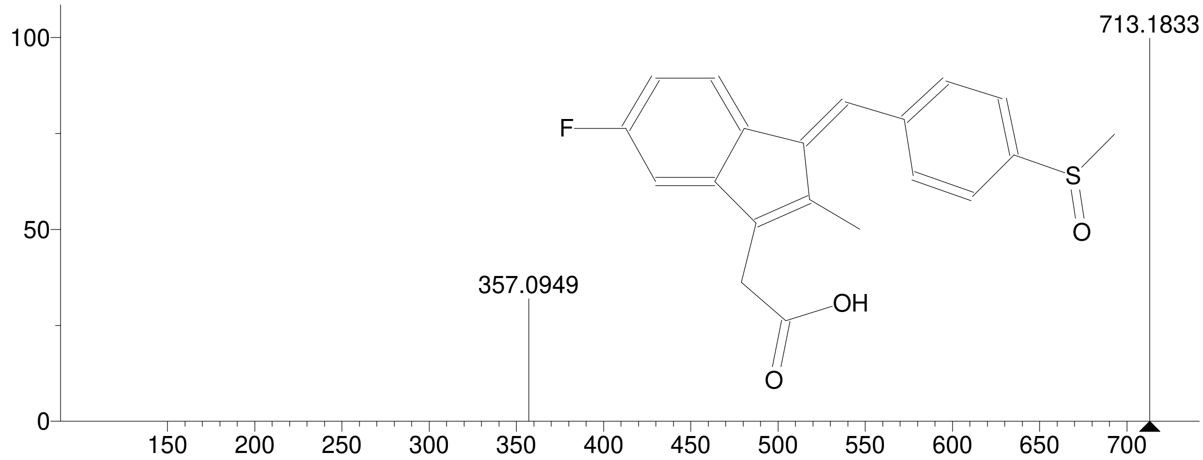

** Search Report Page 1 of 1 **

Unknown: Unknown (1.44_989.3218m/z)
Compound in Library Factor = 0

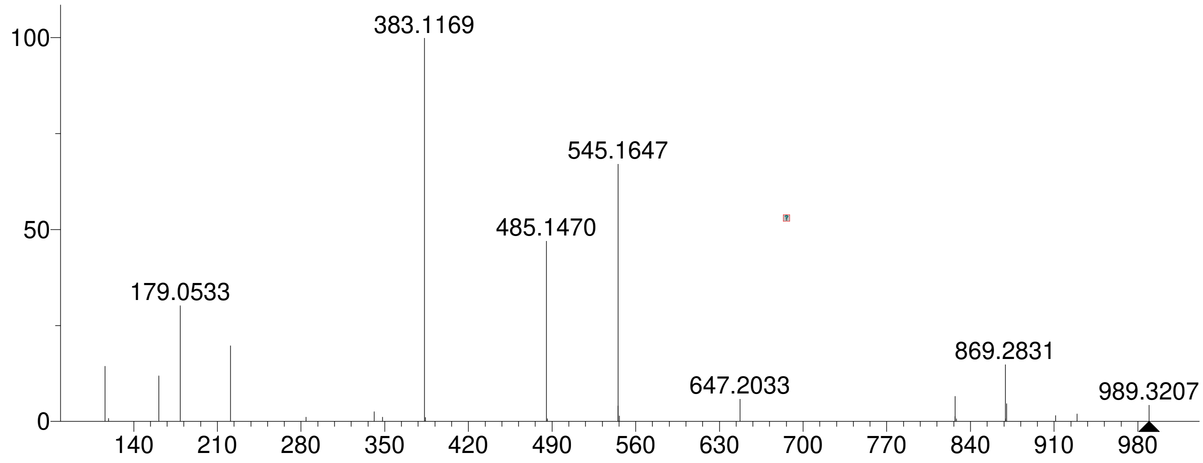

Unknown: Unknown (1.45_245.1134m/z)
Compound in Library Factor = -1615

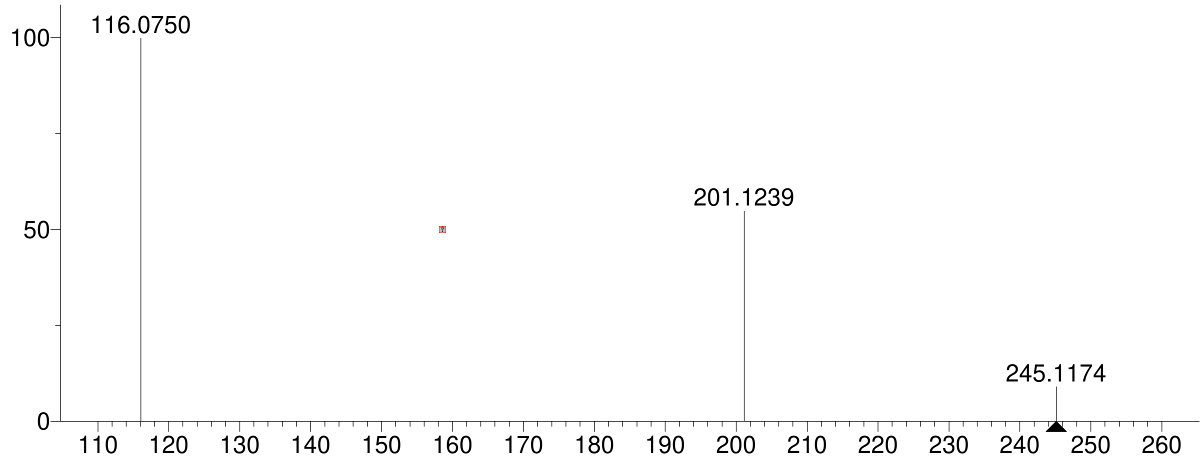

Hit 1 : Val-Glu [M-H]- HCD 14eV P=245.1
C10H18N2O5; MF: 290; RMF: 671; Prob 40.7%; Lib: nist_msms; ID: 571078.

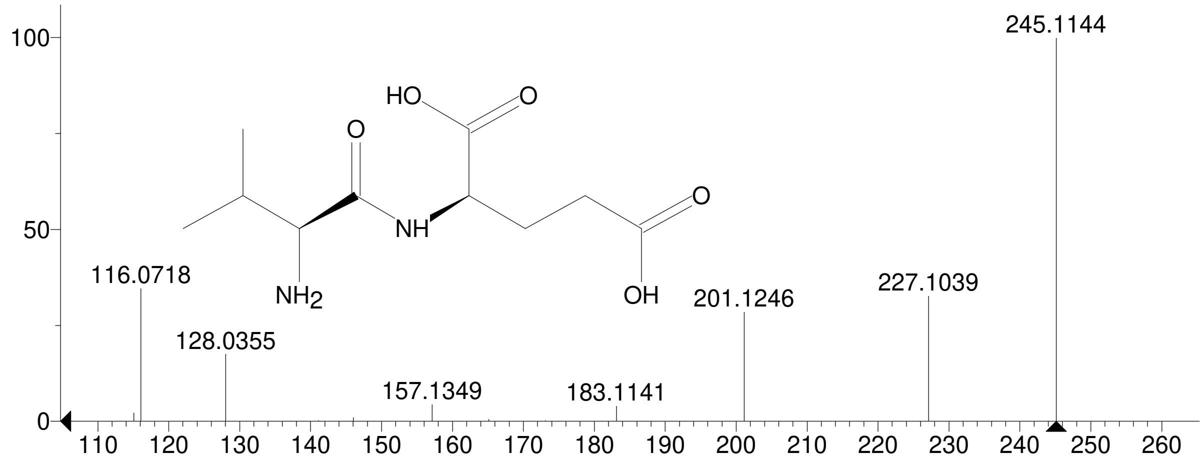

Hit 2 : Val-Glu [M-H]- HCD 14eV P=245.1
C10H18N2O5; MF: 290; RMF: 671; Prob 40.7%; CAS: 3062-07-5; Lib: nist_msms2; ID: 84811.

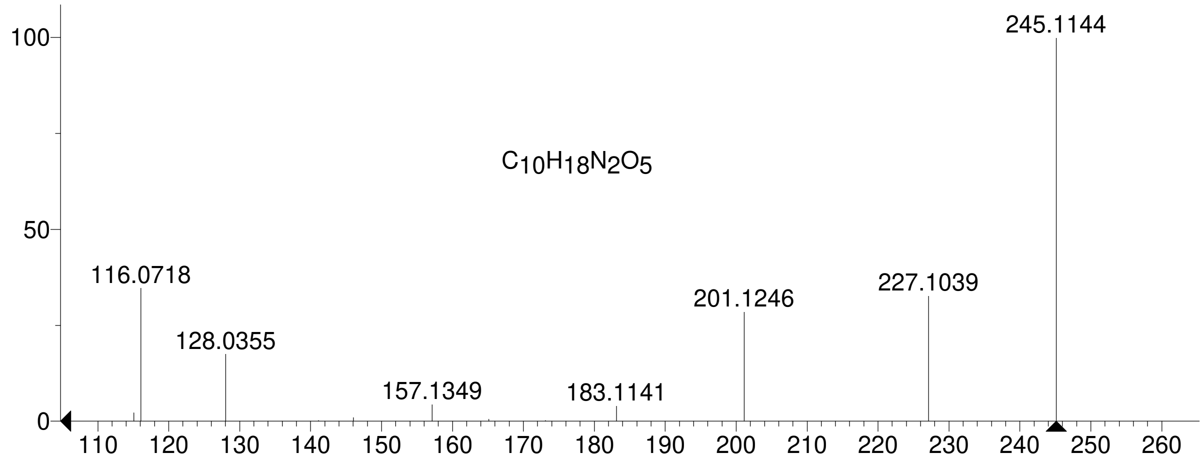

Hit 3 : Glu-Pro [M+H]+ QTOF 30V P=245.1
C10H16N2O5; MF: 265; RMF: 780; Prob 12.4%; Lib: nist_msms; ID: 47863.

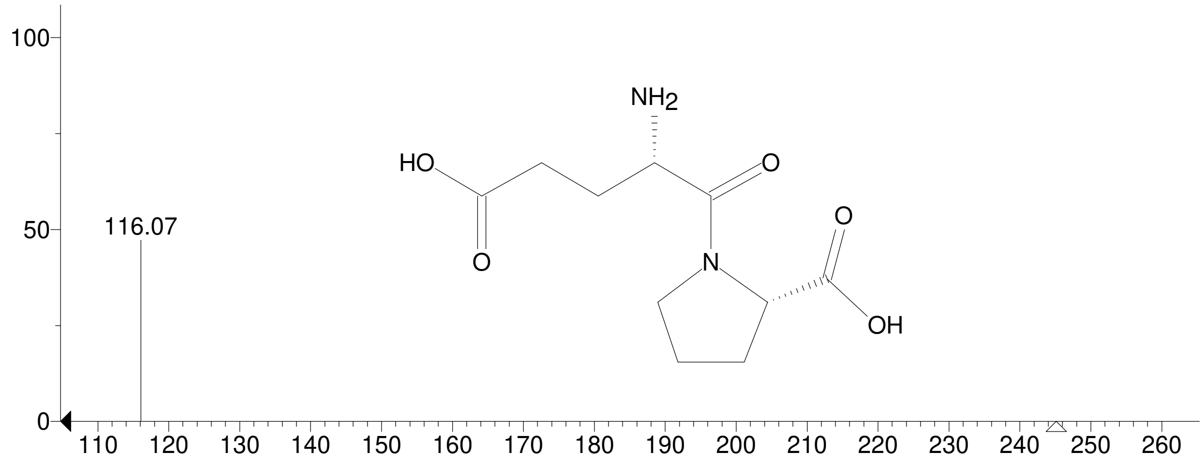

Hit 4 : Glu-Val [M-H]- HCD 12eV P=245.1
C10H18N2O5; MF: 210; RMF: 551; Prob 2.41%; Lib: nist_msms; ID: 567387.

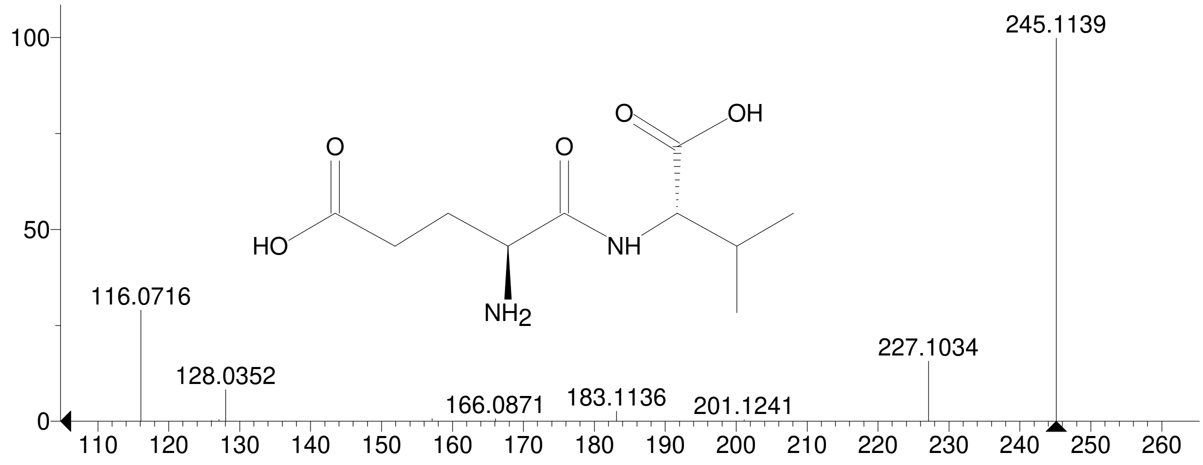

Hit 5 : Glu-Val [M-H]- HCD 12eV P=245.1
C10H18N2O5; MF: 210; RMF: 551; Prob 2.41%; CAS: 5879-06-1; Lib: nist_msms2; ID: 70244.

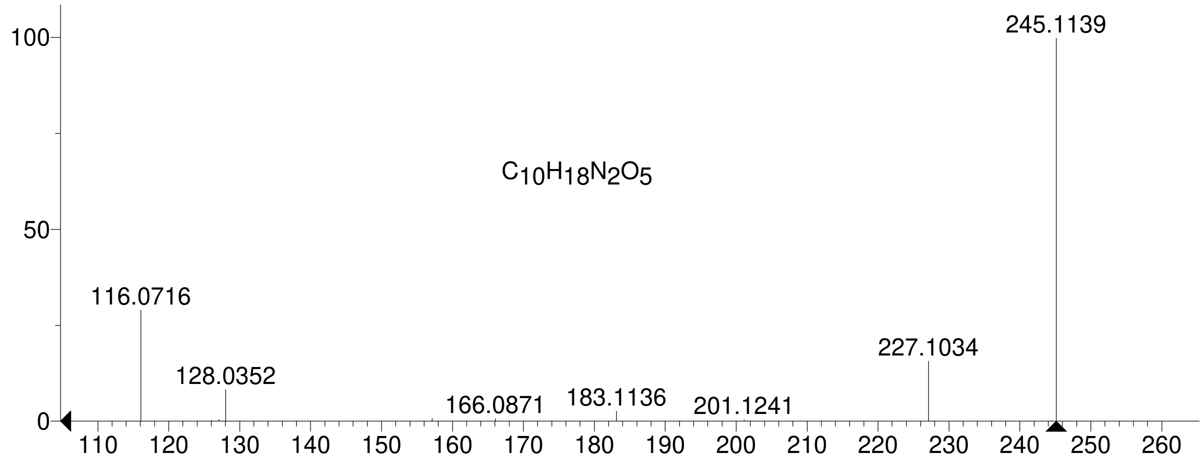

** Search Report Page 1 of 1 **

Unknown: Unknown (1.45_582.2860m/z)
Compound in Library Factor = 0

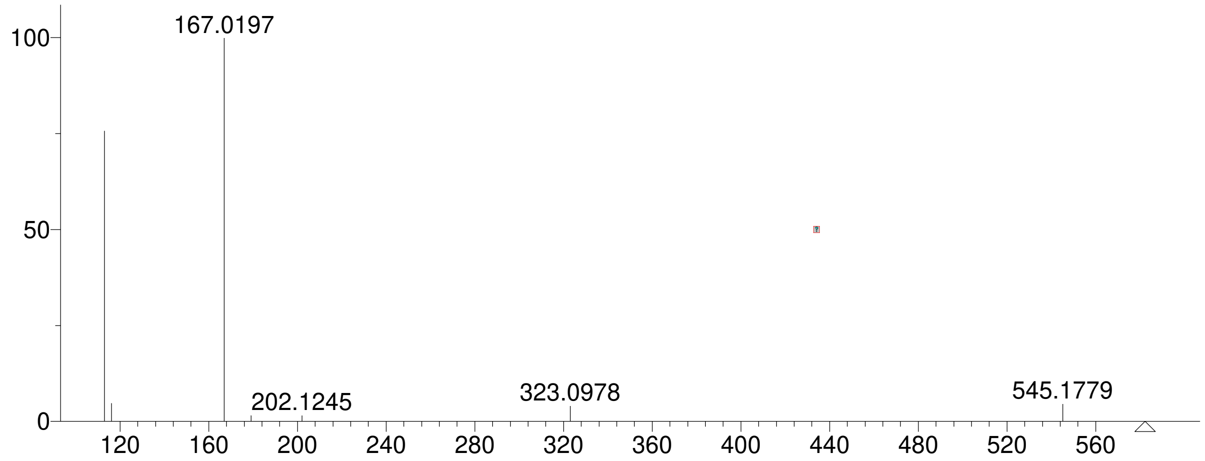

Hit 1 : Dauricine [M+H-C2H5N]+ HCD 186V P=582.3
C38H44N2O6; MF: 0; RMF: 9; Prob 100.0%; CAS: 524-17-4; Lib: nist_msms; ID: 236090.

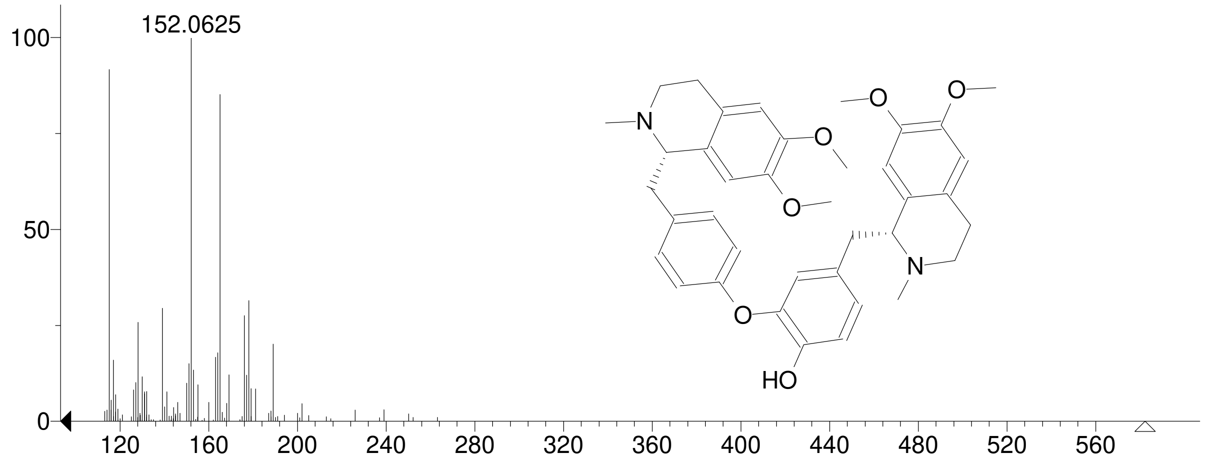

Unknown: Unknown (1.45_665.1693m/z)
Compound in Library Factor = 0

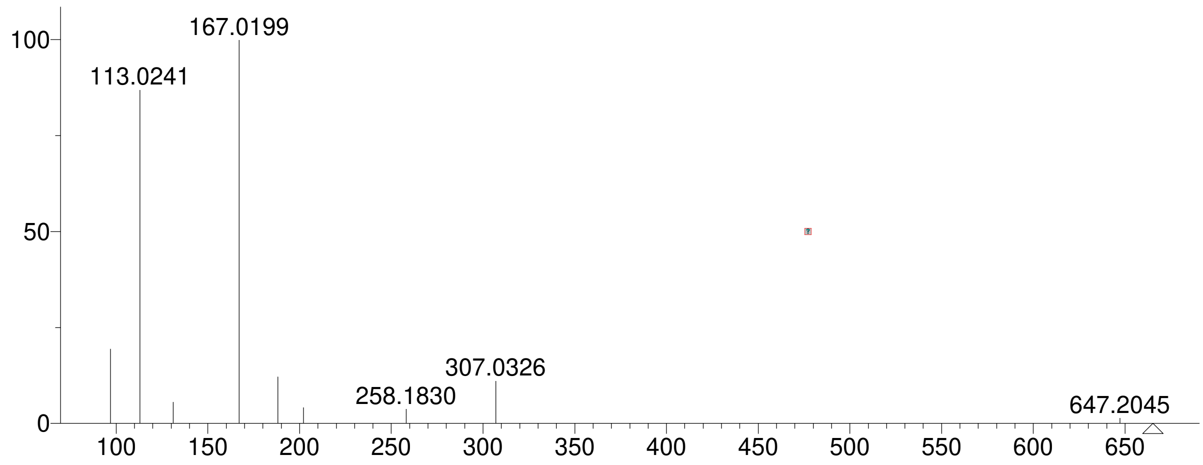

Hit 1 : [(2S,3R,4S,5S,6R)-3,4,5-trihydroxy-6-(hydroxymethyl)oxan-2-yl] (1S,4aR,6aS,6bR,9S,10R,11R,12aR)-1,10
C36H58O11; MF: 0; RMF: 85; Prob 50.0%; Lib: mona-export-itree; ID: 3655.

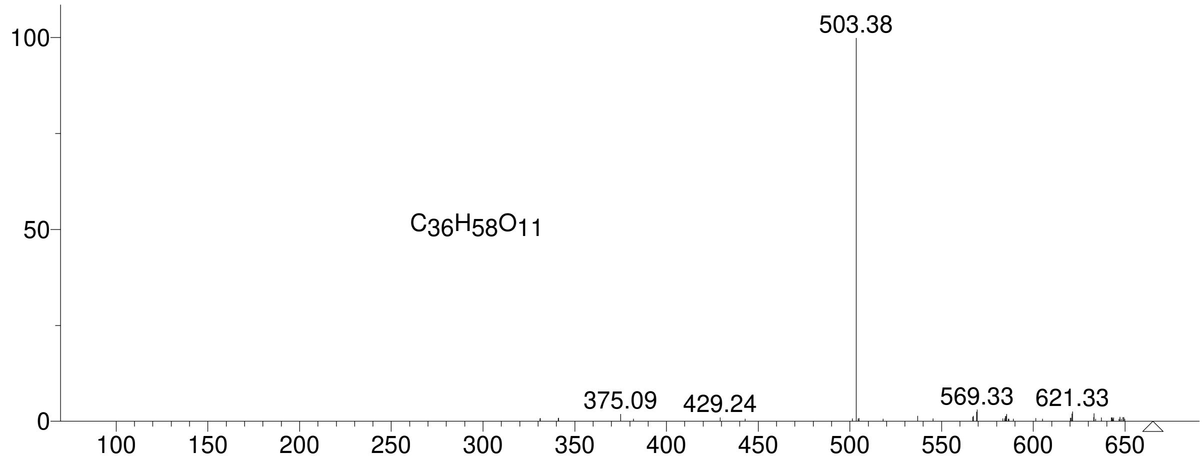

Hit 2 : Gox(8-O-4)feruloyl malate + hexose 2 (LigD) [M-H]- P=665.2
C30H34O17; MF: 0; RMF: 30; Prob 50.0%; Lib: phytofrag2018_final_060219; ID: 549.

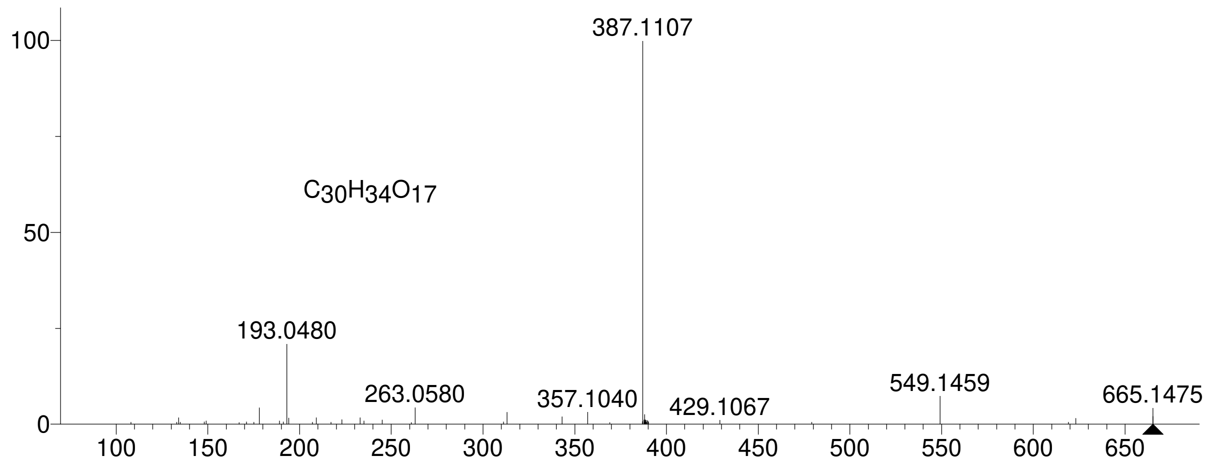

Unknown: Unknown (1.45_683.1796m/z)
Compound in Library Factor = 0

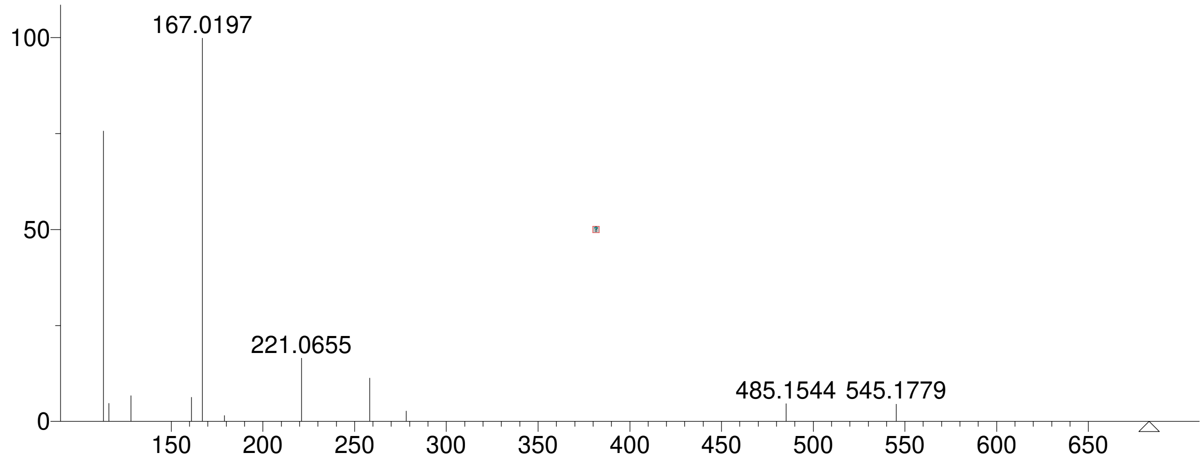

Hit 1 : [(2R,3S,4S,5R,6S)-3,4-dihydroxy-6-[3,7,8-trihydroxy-2-(4-hydroxy-3-methoxyphenyl)-4-oxochromen-5-yl]ox
C30H34O18; MF: 0; RMF: 3; Prob 100.0%; Lib: anthocyanins mona; ID: 3950.

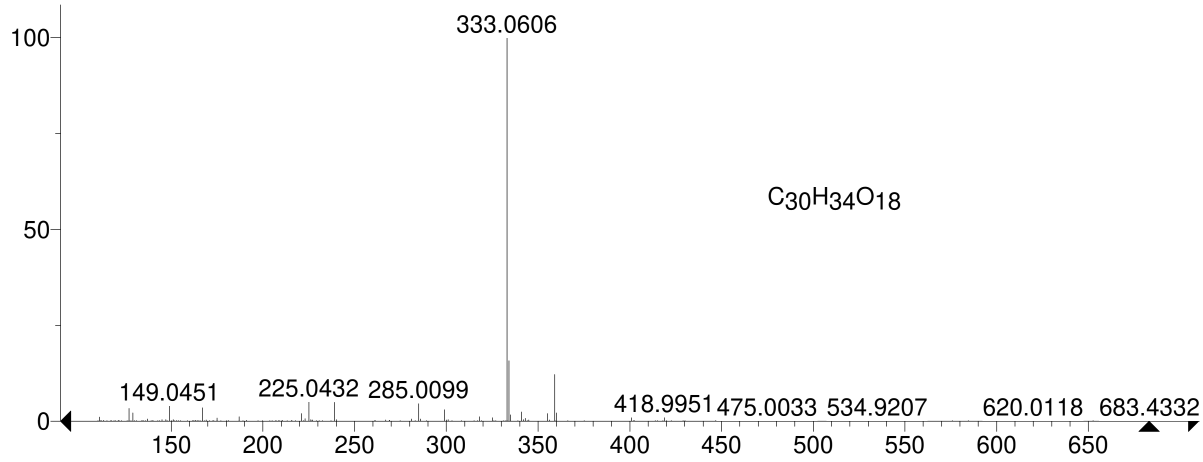

Unknown: Unknown (1.45_702.1941m/z)
Compound in Library Factor = 0

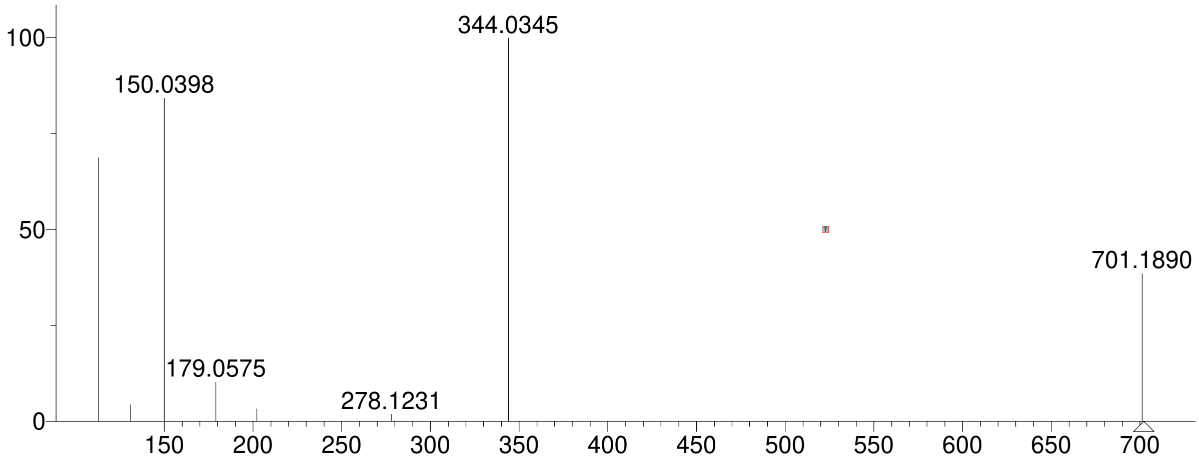

Unknown: Unknown (1.45_782.2947m/z)
Compound in Library Factor = 0

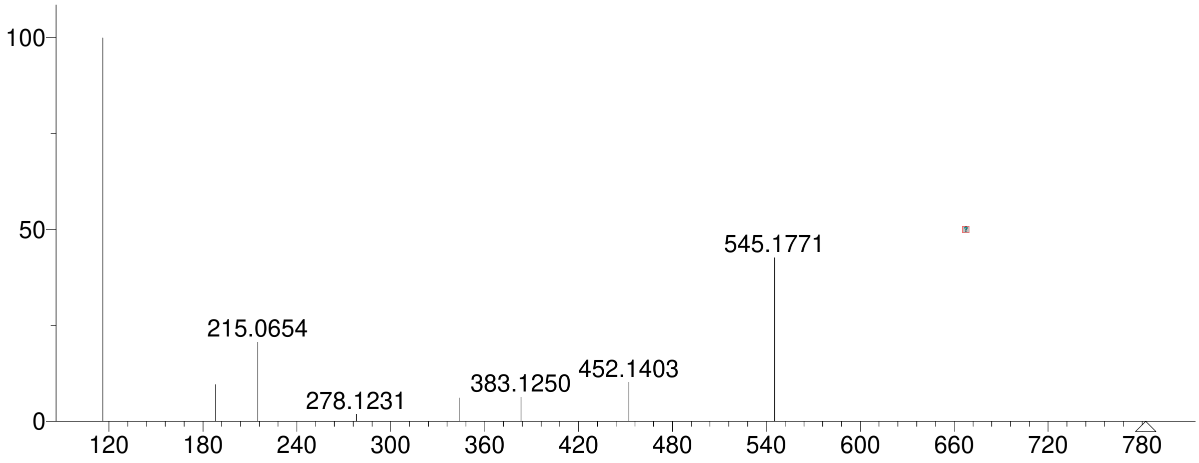

** Search Report Page 1 of 1 **

Unknown: Unknown (1.49_611.0428m/z)
Compound in Library Factor = 0

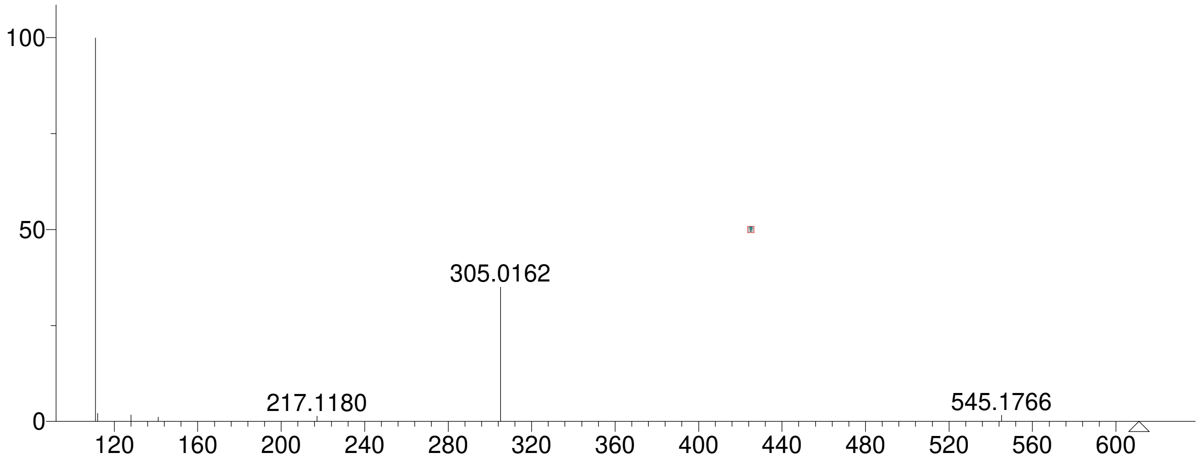

Unknown: Unknown (1.50_239.1384m/z)
Compound in Library Factor = 0

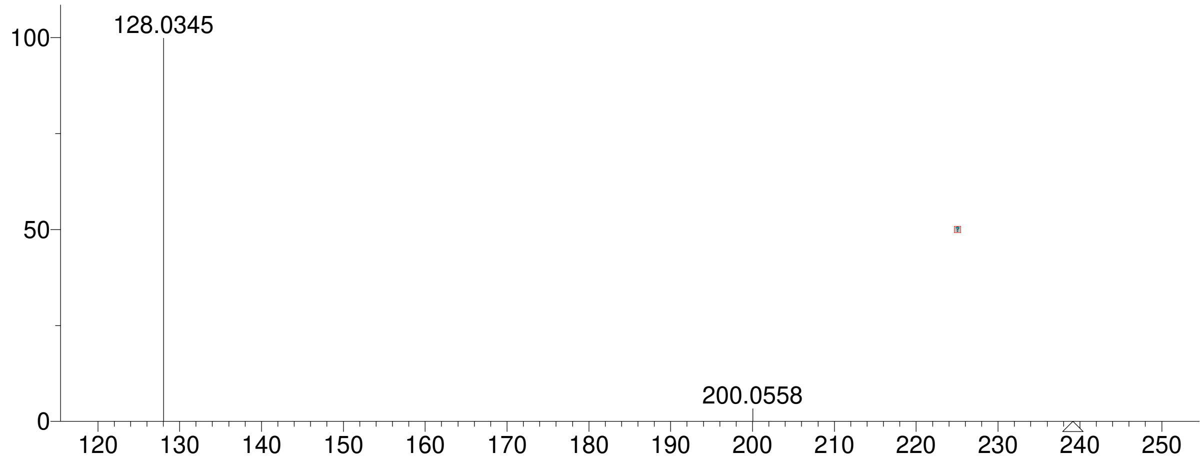

Unknown: Unknown (1.50_290.0872m/z)
Compound in Library Factor = -2176

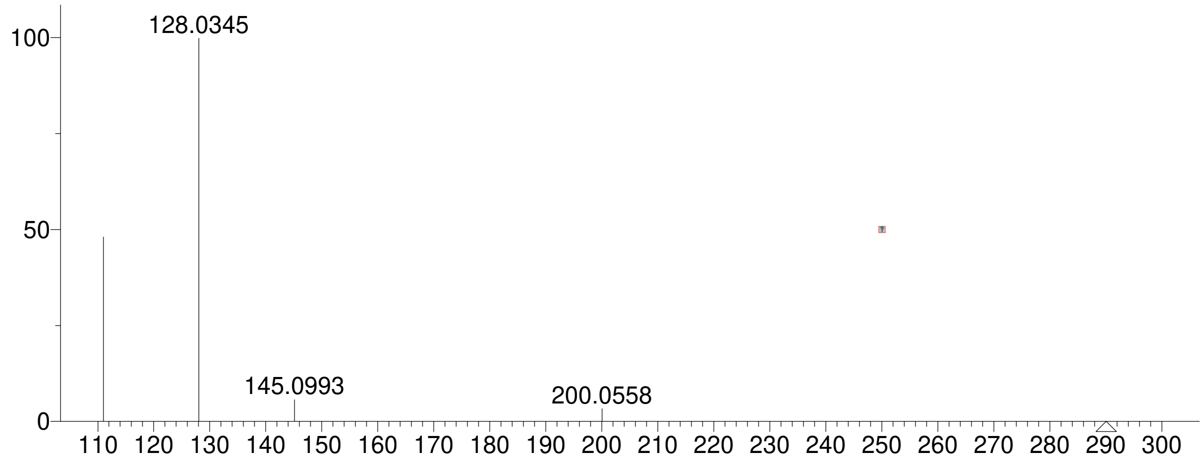

Hit 1 : N-Fructosyl pyroglutamate; PlaSMA ID-534 [M-H]- P=290.1
C11H17NO8; MF: 24; RMF: 645; Prob 49.9%; Lib: biomsms-neg-plasma; ID: 480.

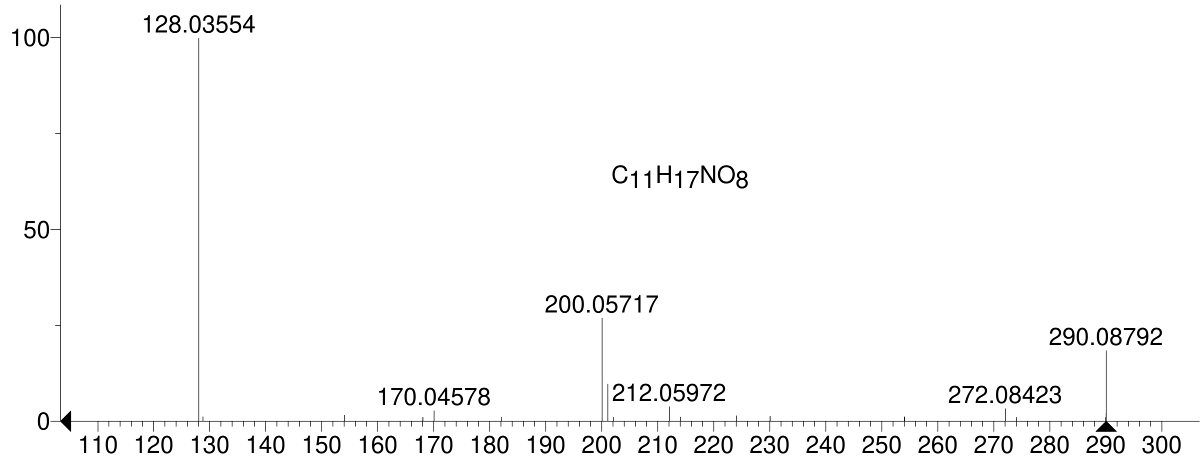

Hit 2 : N-Glycolylneuraminic acid [M+H-2H2O]+ HCD 34V P=290.1
C11H19NO10; MF: 0; RMF: 100; Prob 16.7%; CAS: 1113-83-3; Lib: nist_msms; ID: 307261.

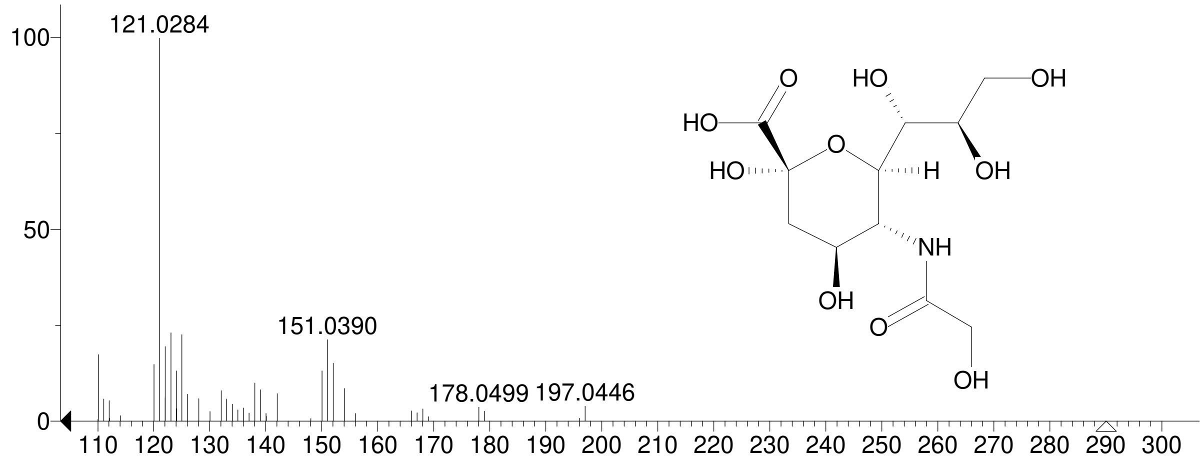

Hit 3 : (-)-N-Acetylneuraminic acid [M-H-H2O]- HCD 26V P=290.1
C11H19NO9; MF: 0; RMF: 31; Prob 16.7%; CAS: 131-48-6; Lib: nist_msms; ID: 479638.

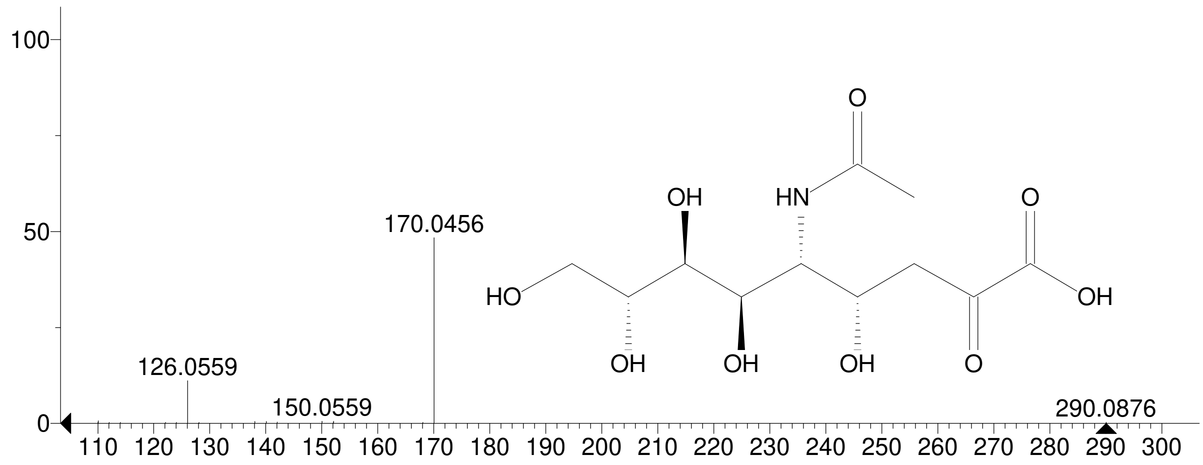

Hit 4 : 3'-α-Sialyl-N-acetyllactosamine [M-H-C14H25O11N]- IT-FT 35% P=290.1
C25H42N2O19; MF: 0; RMF: 6; Prob 16.7%; CAS: 81693-22-3; Lib: nist_msms; ID: 211511.

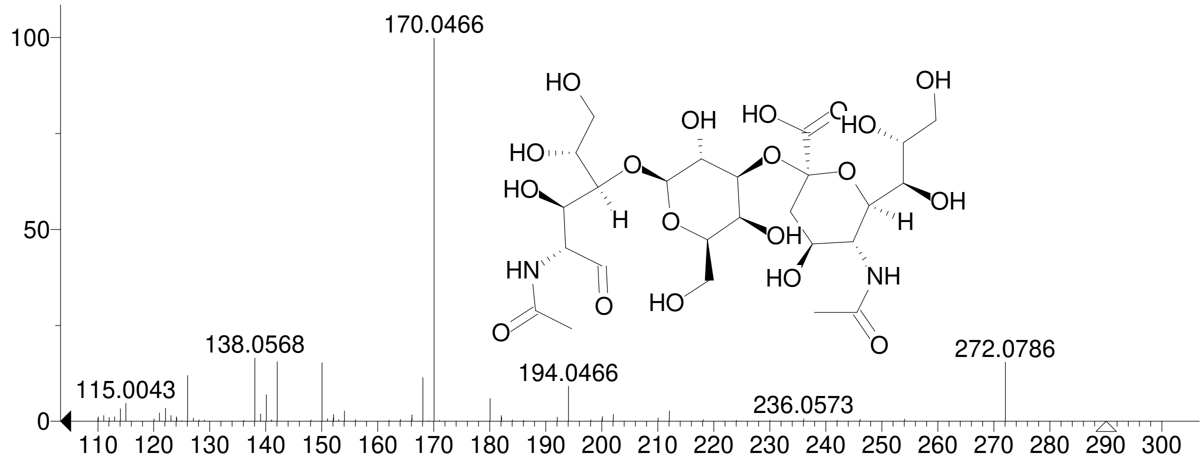

Unknown: Unknown (1.50_305.0171m/z)
Compound in Library Factor = 0

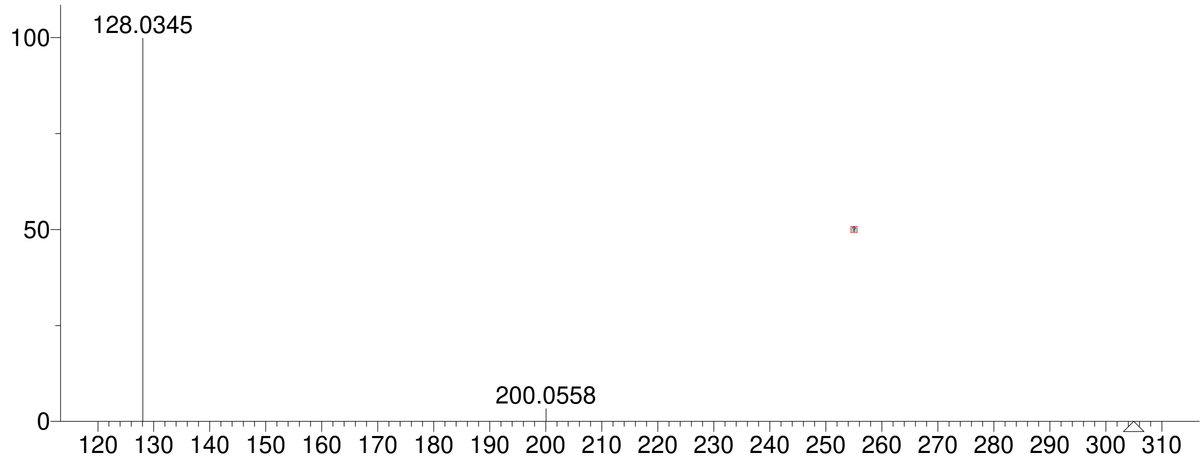

Unknown: Unknown (1.50_809.1880m/z)
Compound in Library Factor = 0

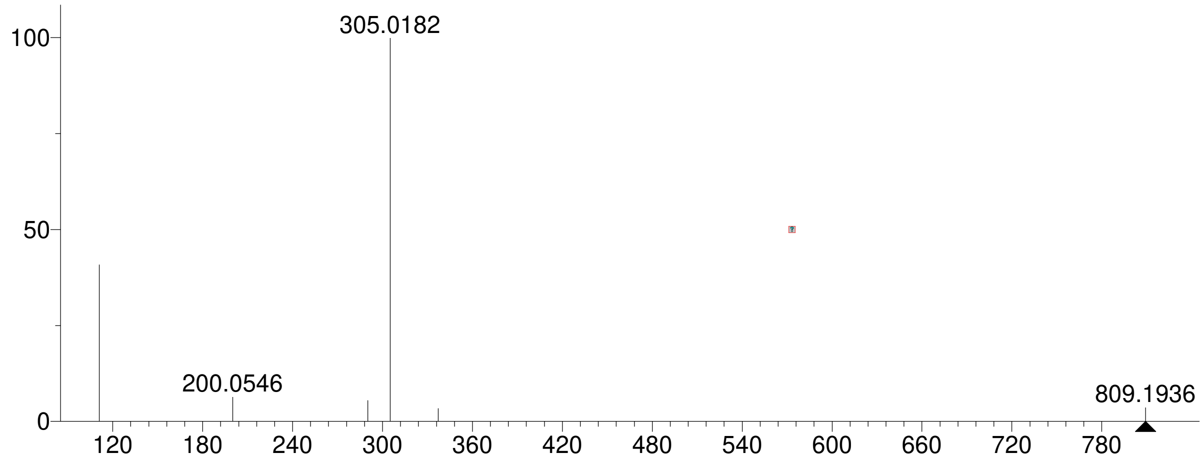

Hit 1 : Xanthorhamnin [M+K]+ 25HCD P=809.2
C34H42O20; MF: 0; RMF: 91; Prob 50.0%; Lib: mona-export-vf-npl_qexactive; ID: 15430.

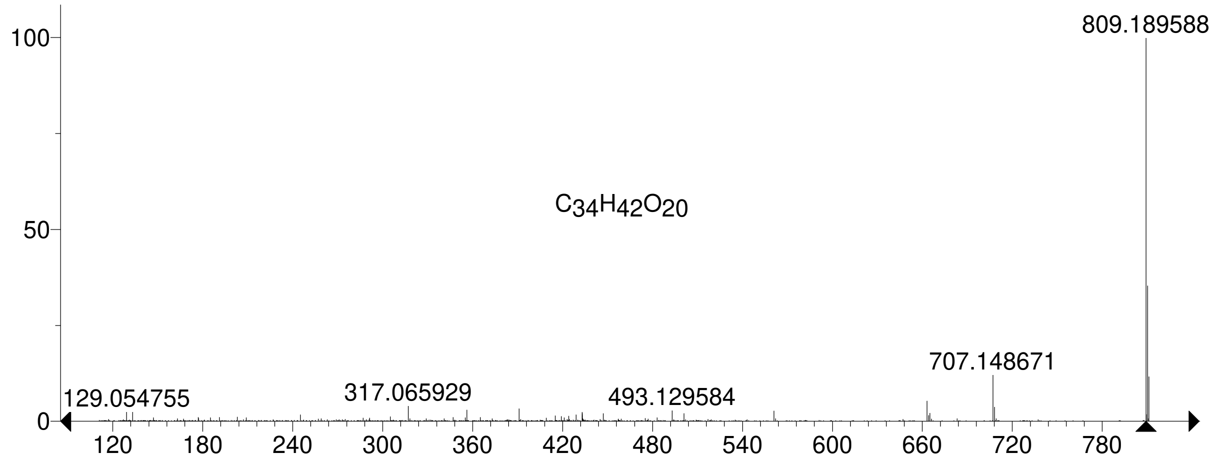

Hit 2 : 3-[(2S,3R,4S,5S,6R)-4,5-dihydroxy-3-[(2R,3R,4R,5R,6S)-3,4,5-trihydroxy-6-methyloxan-2-yl]oxy-6-[[(2R,3F
C34H42O20; MF: 0; RMF: 3; Prob 50.0%; Lib: anthocyanins mona; ID: 7686.

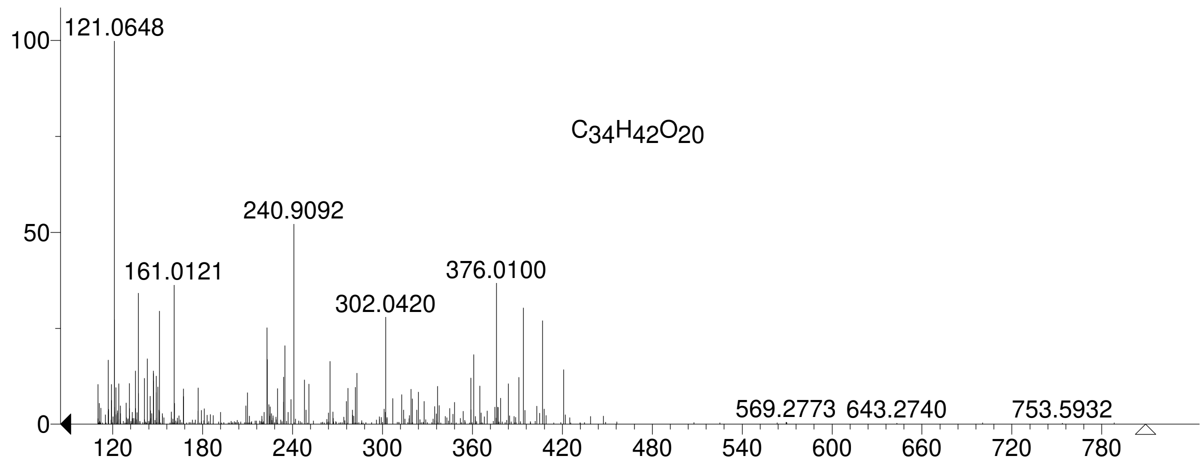

Unknown: Unknown (1.51_633.0265m/z)
Compound in Library Factor = 0

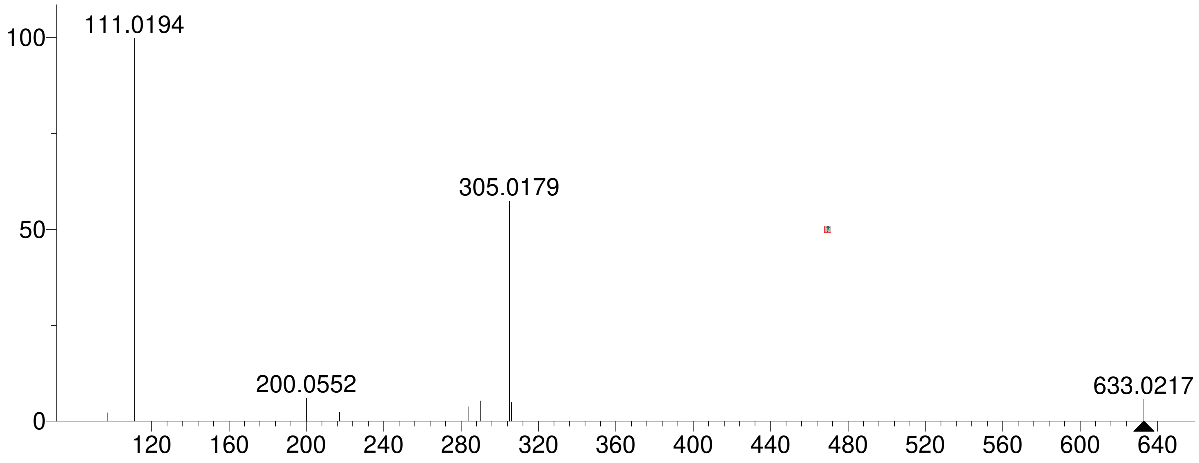

Unknown: Unknown (1.51_647.1335m/z)
Compound in Library Factor = 0

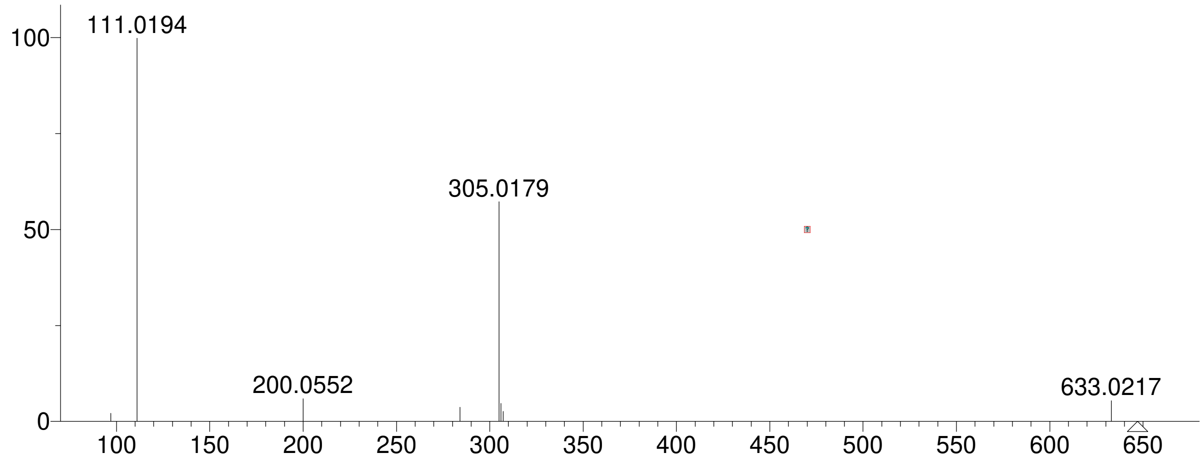

Hit 1 : Diosmin [M+K]+ 65HCD P=647.1
C28H32O15; MF: 0; RMF: 4; Prob 50.0%; Lib: mona anthocyanidin-o-glycosides; ID: 2932.

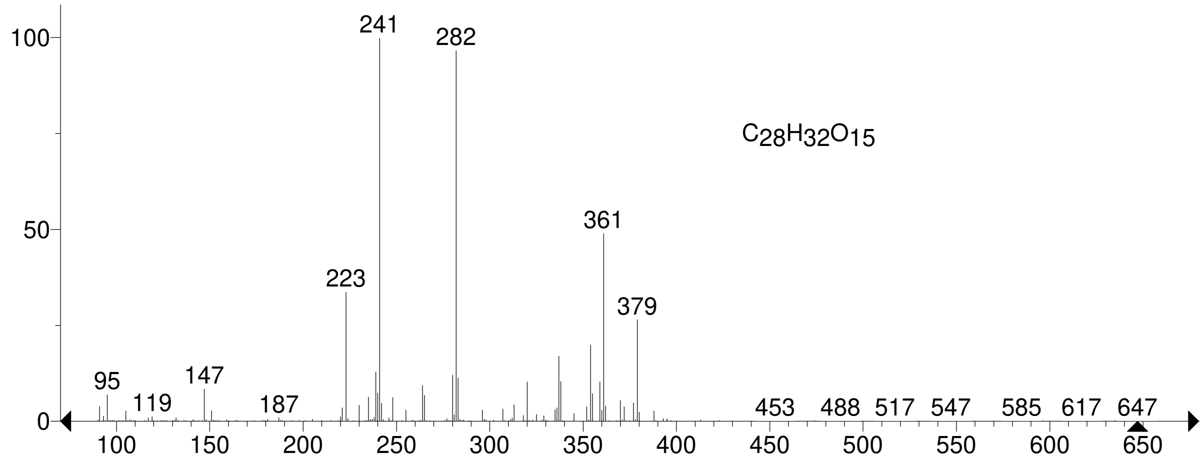

Hit 2 : 5-hydroxy-2-(4-methoxyphenyl)-3-[(2S,3R,4S,5S,6R)-3,4,5-trihydroxy-6-(hydroxymethyl)oxan-2-yl]oxy-7-[(2
C28H32O15; MF: 0; RMF: 3; Prob 50.0%; Lib: mona anthocyanidin-o-glycosides; ID: 8667.

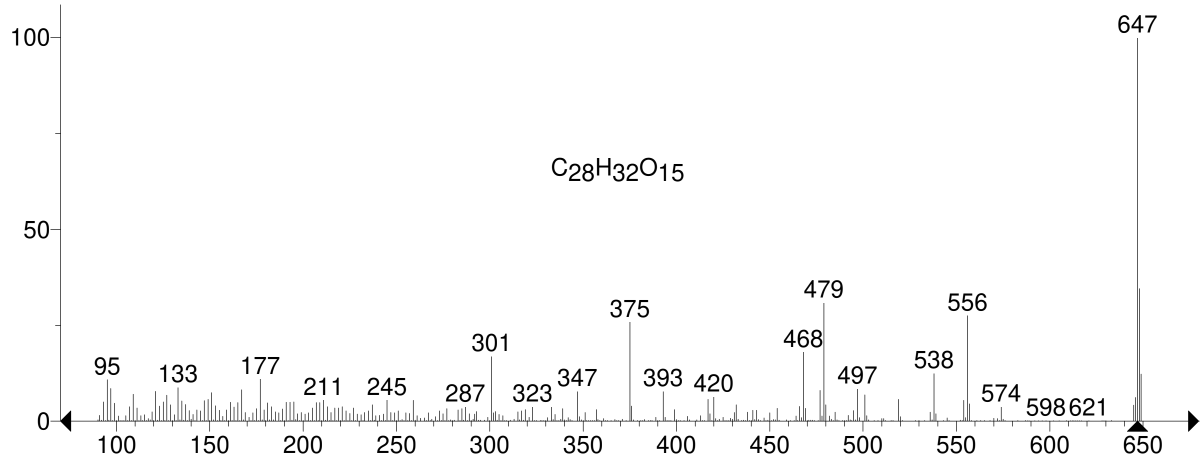

Unknown: Unknown (1.51_746.2012m/z)
Compound in Library Factor = 0

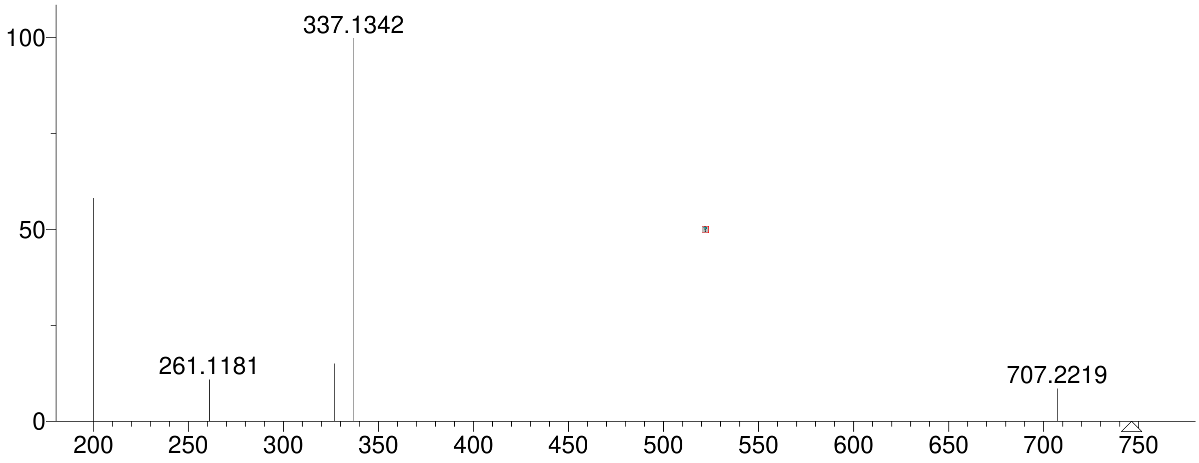

** Search Report Page 1 of 1 **

Unknown: Unknown (1.51_971.2417m/z)
Compound in Library Factor = 0

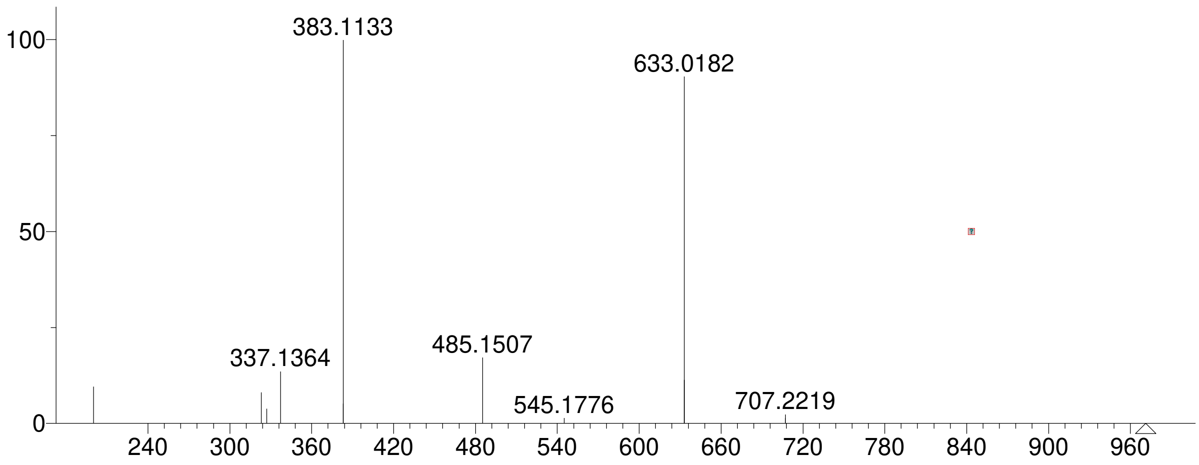

Unknown: Unknown (1.53_305.0857m/z)
Compound in Library Factor = -3273

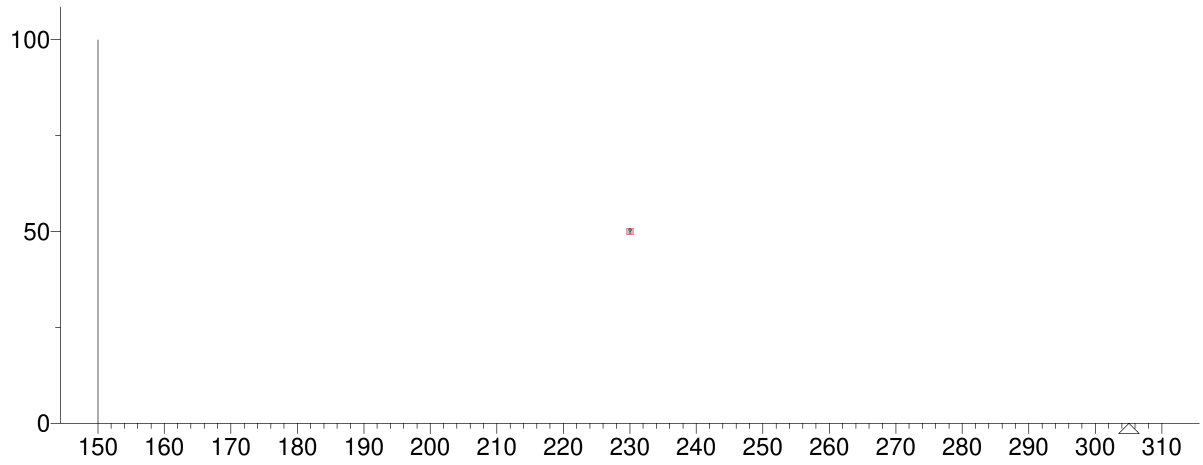

Hit 1 : 2-Hydroxyethylflurazepam [M+H-CO]+ HCD 97V P=305.1
C17H14ClFN2O2; MF: 1; RMF: 226; Prob 51.0%; CAS: 20971-53-3; Lib: nist_msms; ID: 195581.

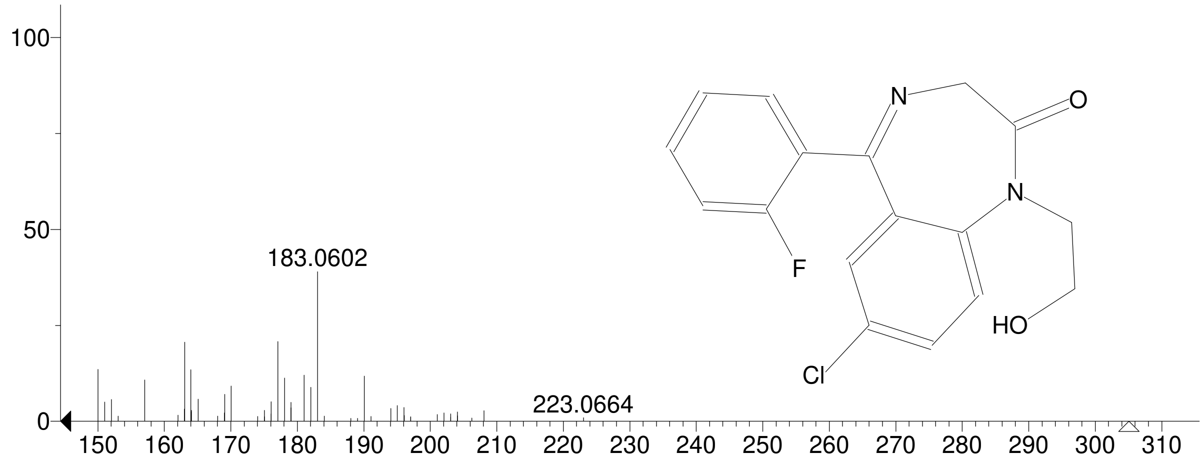

Hit 2 : Dinitramine [M+H-H2O]+ HCD 54V P=305.1
C11H13F3N4O4; MF: 0; RMF: 38; Prob 49.0%; CAS: 29091-05-2; Lib: nist_msms; ID: 100145.

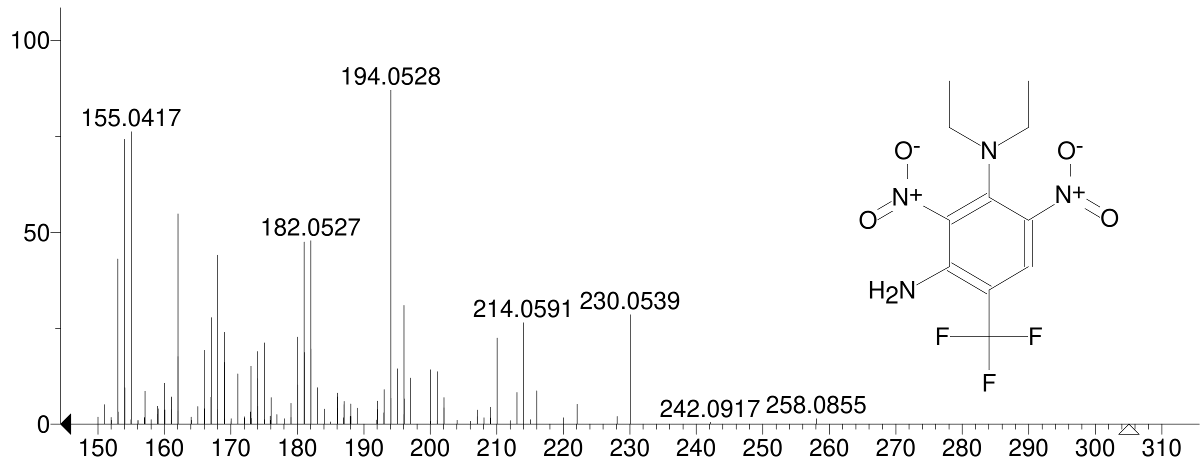

** Search Report Page 1 of 1 **

Unknown: Unknown (1.53_379.1704m/z)
Compound in Library Factor = 0

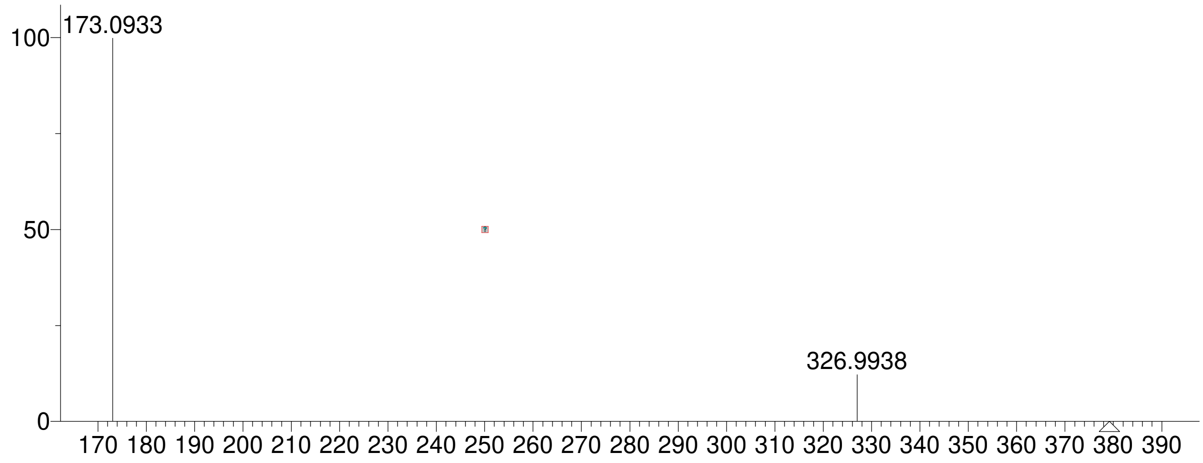

Unknown: Unknown (1.53_440.1761m/z)
Compound in Library Factor = 0

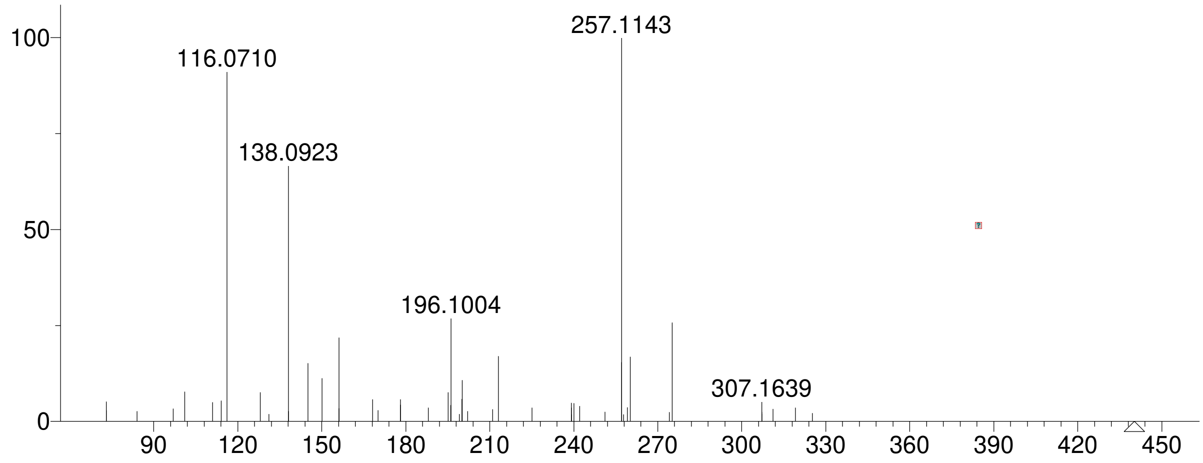

Hit 1 : Quinadoline B_130007 [M+H]+ P=440.2
C25H21N5O3; MF: 0; RMF: 20; Prob 100.0%; Lib: mona-export-gnps; ID: 4995.

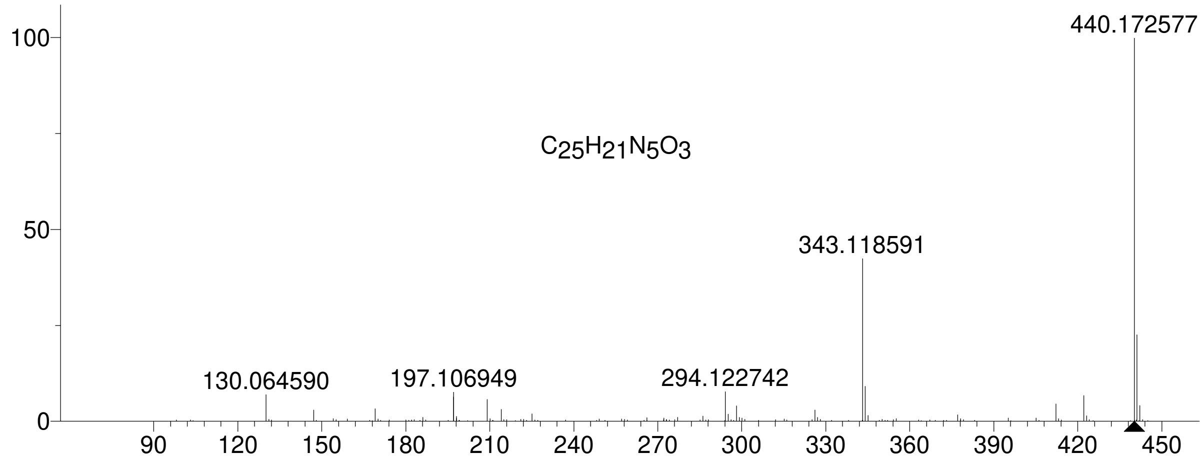

** Search Report Page 1 of 1 **

Unknown: Unknown (1.55_452.1404m/z)
Compound in Library Factor = 0

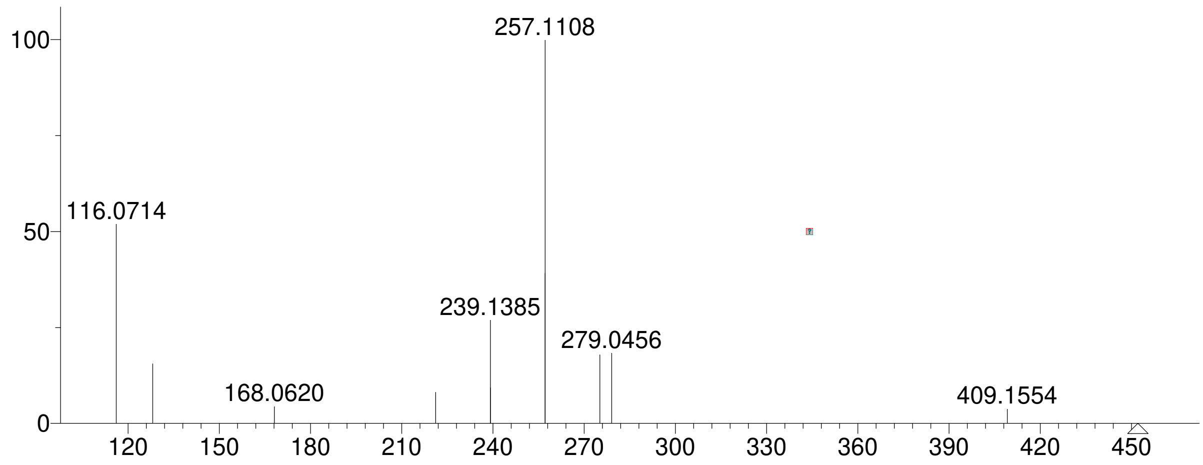

Unknown: Unknown (1.55_477.2077m/z)
Compound in Library Factor = 0

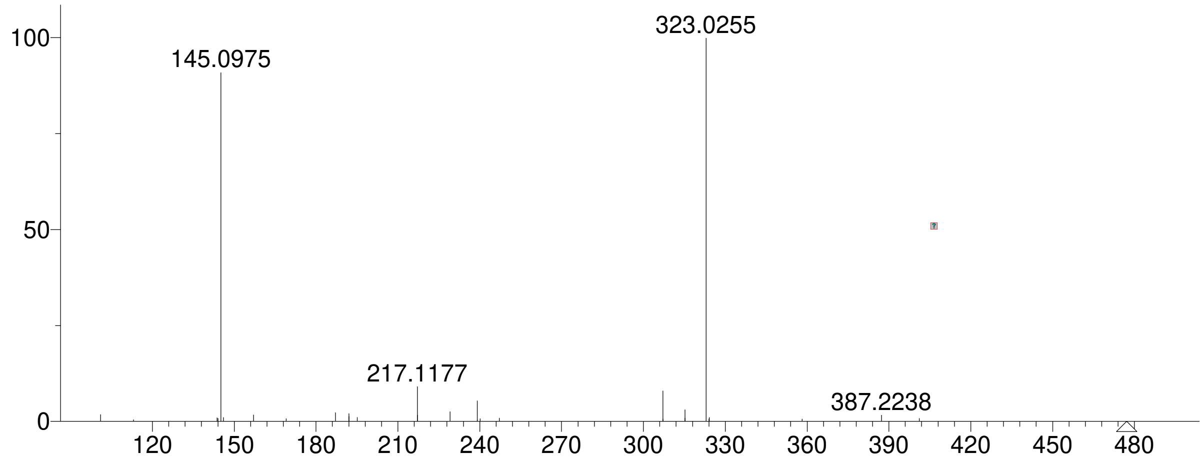

Hit 1 : 4-(2-Hydroxyethyl)piperazine-1-ethanesulfonic acid [2M+H]+ HCD 86V P=477.2
C8H18N2O4S; MF: 0; RMF: 28; Prob 33.3%; CAS: 7365-45-9; Lib: nist_msms; ID: 58517.

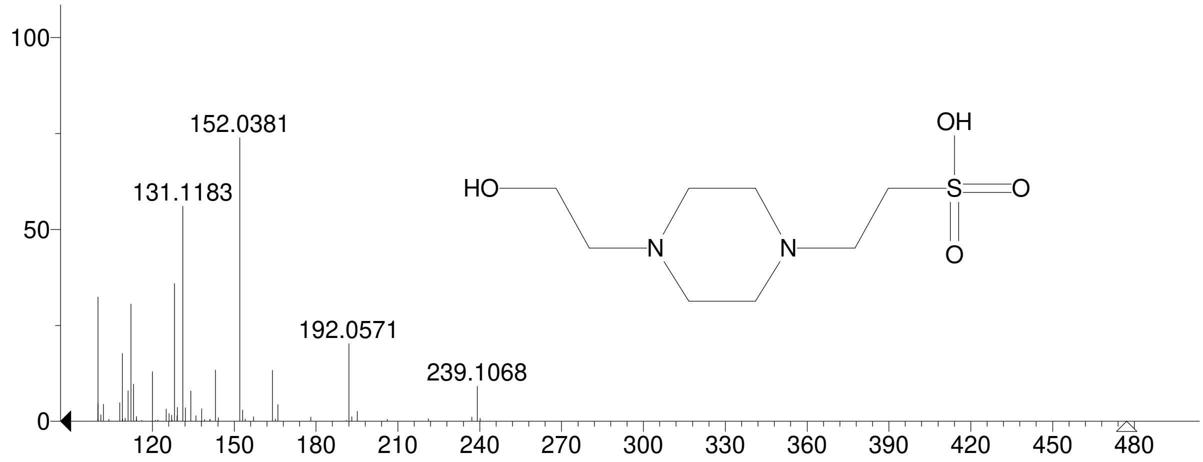

Hit 2 : 5-O-methyllicoricidin [M+K]+ 65HCD P=477.2
C27H34O5; MF: 0; RMF: 16; Prob 33.3%; Lib: mona-export-vf-npl_qexactive; ID: 966.

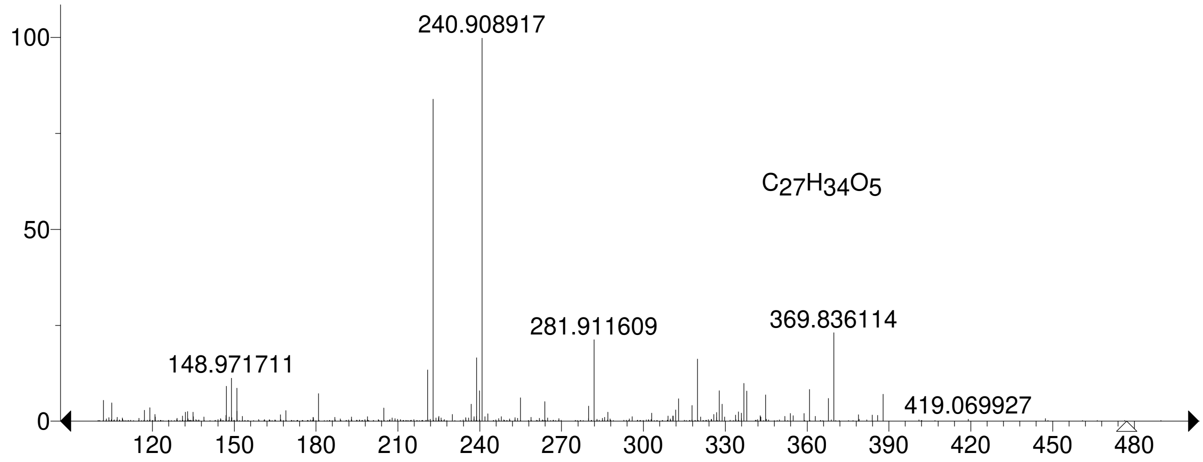

Hit 3 : 1,7-bis(4-hydroxyphenyl)-5-[(2R,3R,4S,5S,6R)-3,4,5-trihydroxy-6-(hydroxymethyl)oxan-2-yl]oxyheptan-3-or
C25H32O9; MF: 0; RMF: 3; Prob 33.3%; Lib: mona-export-vf-npl_qexactive; ID: 7568.

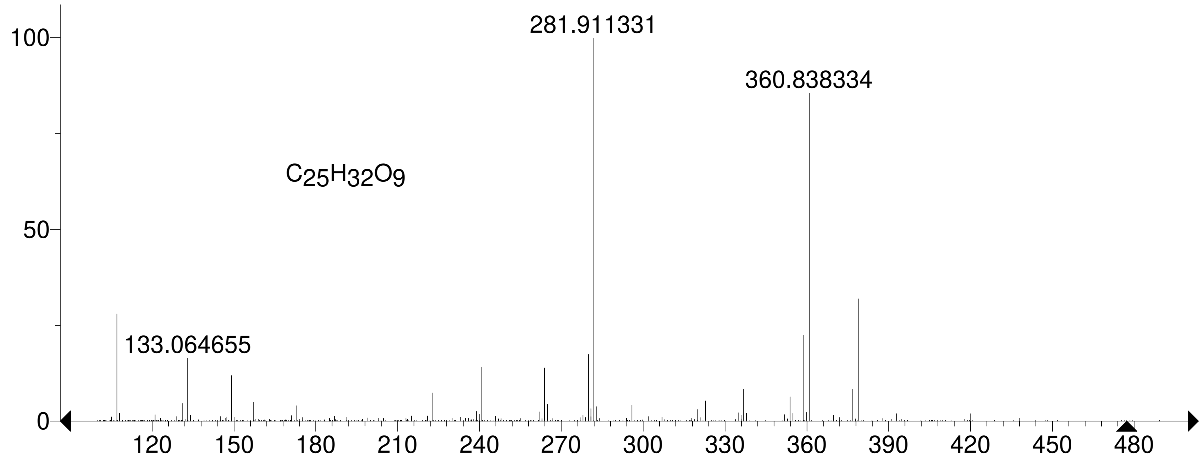

Unknown: Unknown (1.56_191.0185m/z)
Compound in Library Factor = -1224

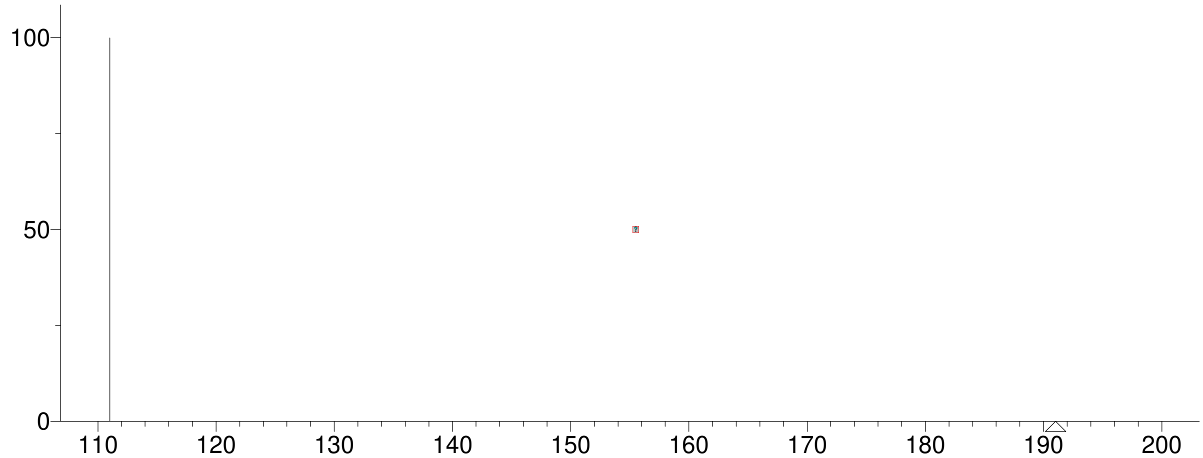

Hit 1 : Citric acid [M-H]- 20 P=191
C6H8O7; MF: 364; RMF: 999; Prob 74.1%; Lib: msms neg mona_ipb_gnps_plasma; ID: 1053.

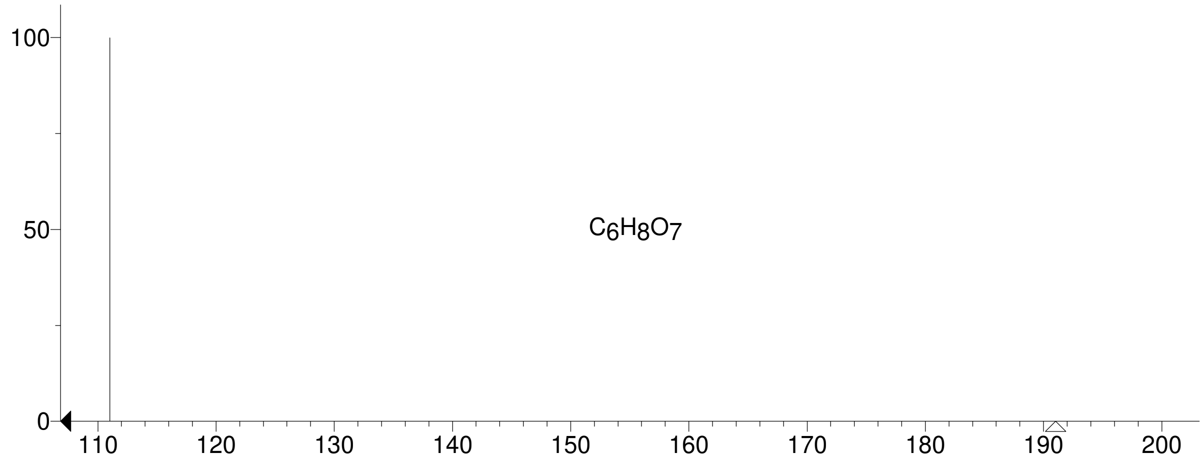

Hit 2 : Mucic acid [M-H-H2O]- HCD 30V P=191
C6H10O8; MF: 318; RMF: 961; Prob 15.6%; CAS: 526-99-8; Lib: nist_msms; ID: 303359.

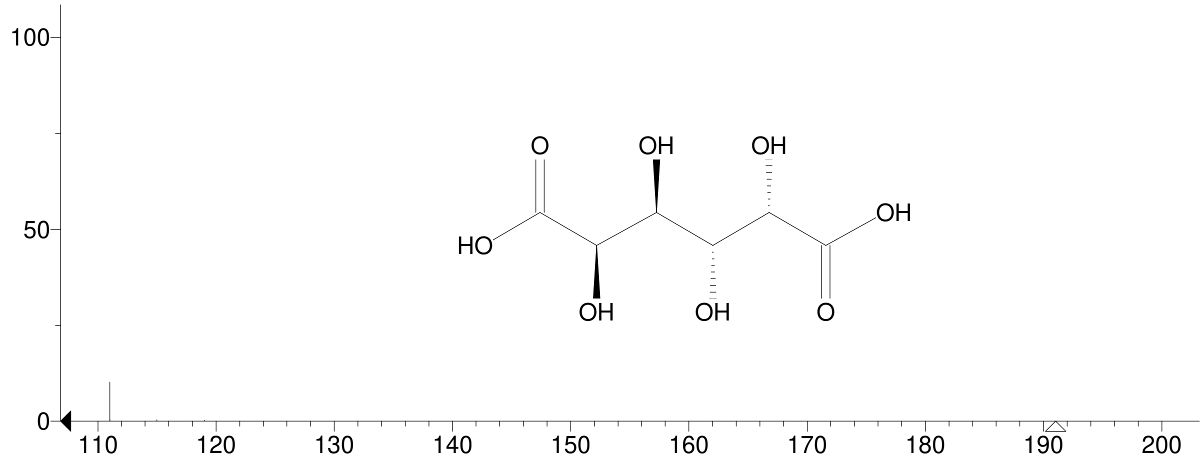

Hit 3 : citric acid [M-H]- 15 P=191
C6H8O7; MF: 299; RMF: 963; Prob 7.59%; Lib: msms neg mona_ipb_gnps_plasma; ID: 1145.

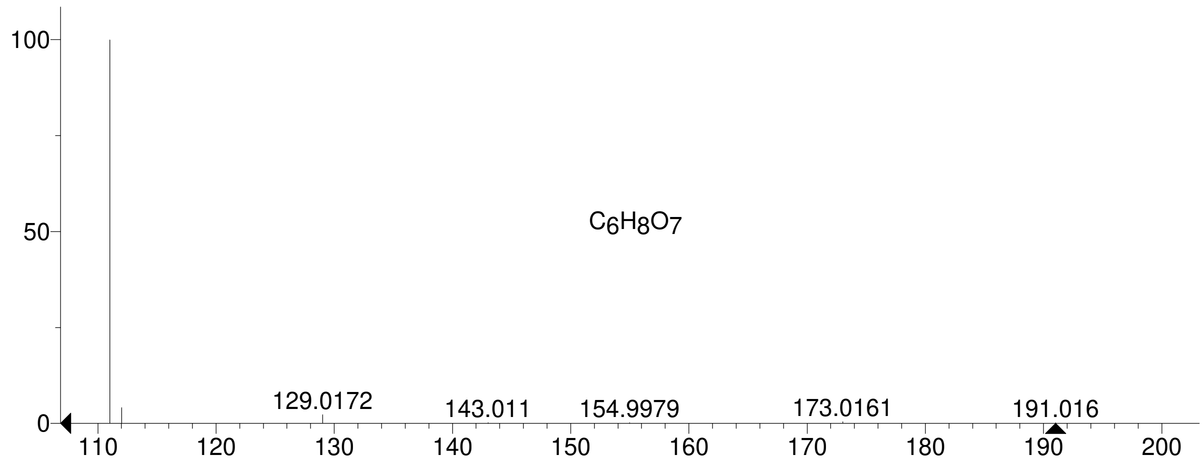

Hit 4 : Citric acid (Not validated, isomer of 228); PlaSMA ID-227 [M-H]- P=191
C6H8O7; MF: 250; RMF: 917; Prob 1.56%; Lib: biomsms-neg-plasma; ID: 463.

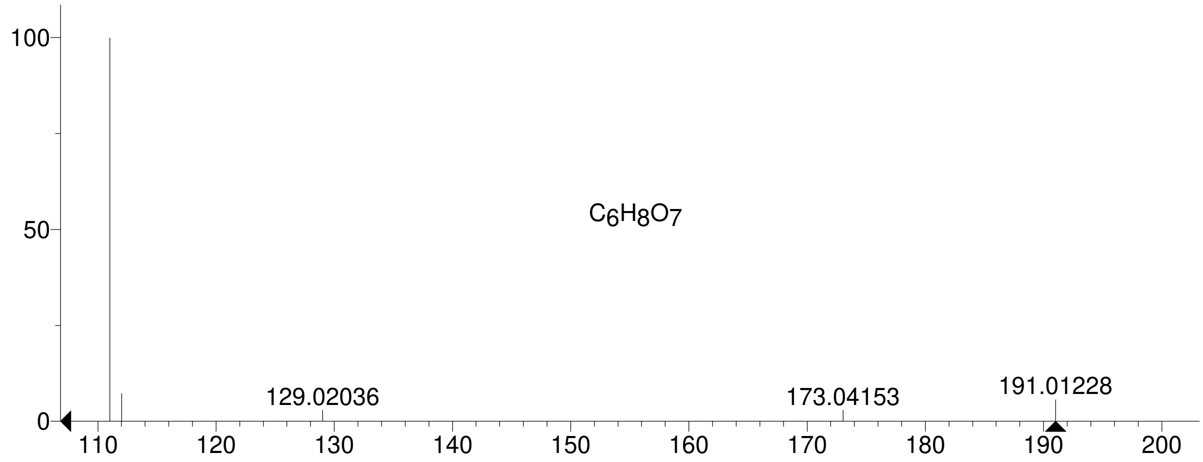

Hit 5 : CITRATE [M-H]- P=191
C6H8O7; MF: 225; RMF: 894; Prob 0.47%; Lib: msms-neg-gnps; ID: 159.

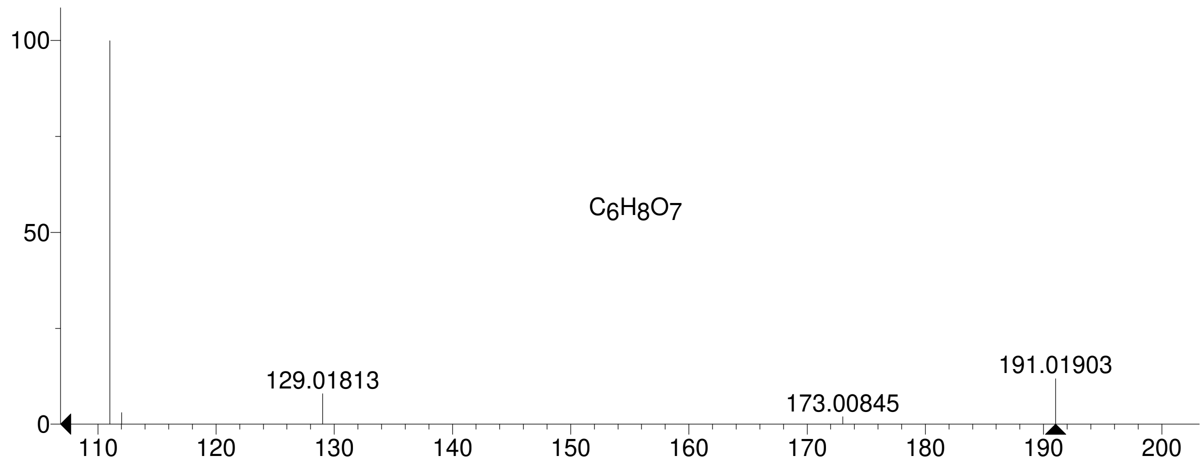

Unknown: Unknown (1.57_323.0278m/z)
Compound in Library Factor = -227

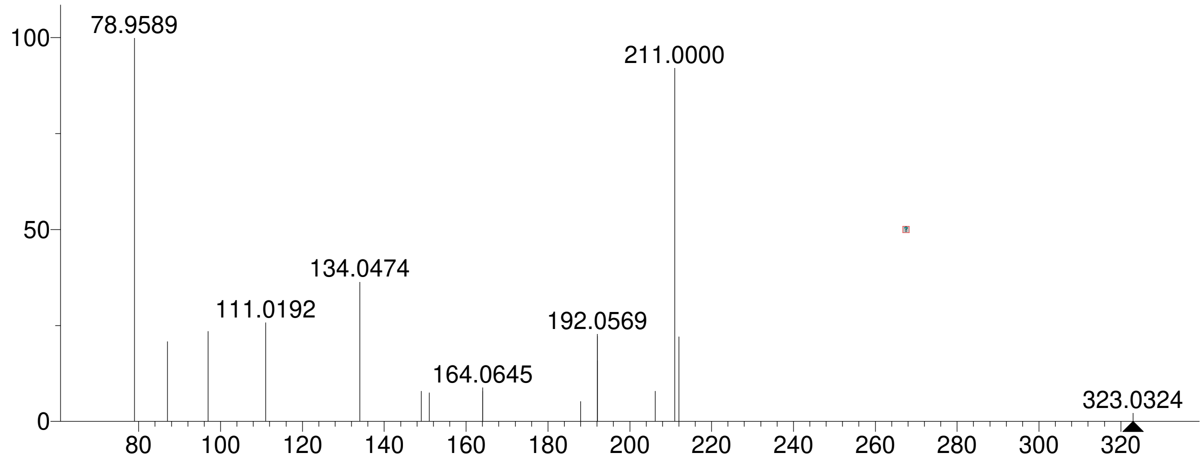

Hit 1 : 1-β-D-Arabinofuranosyluracil 5'-monophosphate [M-H]- HCD 29V P=323
C9H13N2O9P; MF: 536; RMF: 695; Prob 87.7%; CAS: 18354-06-8; Lib: nist_msms; ID: 447571.

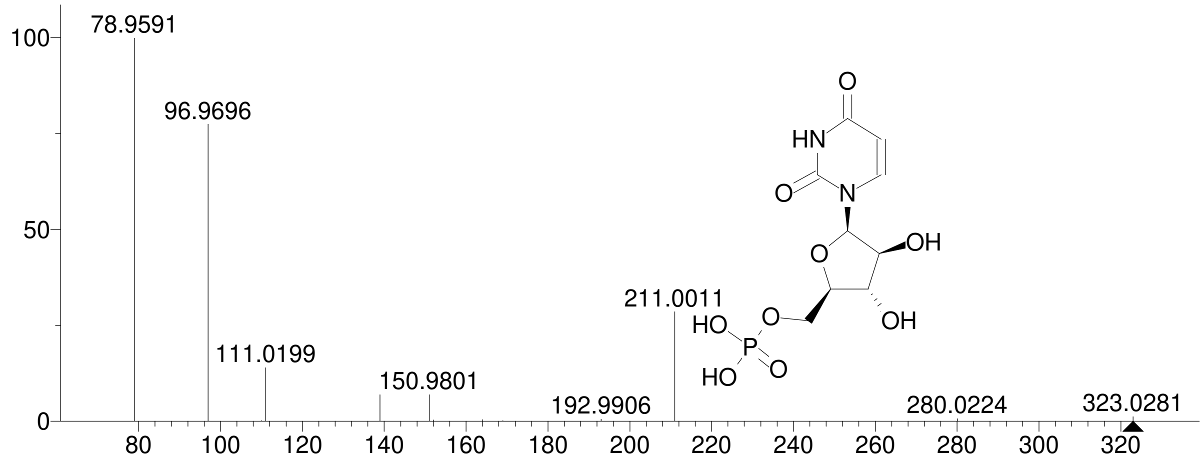

Hit 2 : Uridine 5'-monophosphate [M-H]- 20-30-40%) P=323
C9H13N2O9P; MF: 437; RMF: 644; Prob 6.64%; Lib: mona-export-fiehn_hilic; ID: 1559.

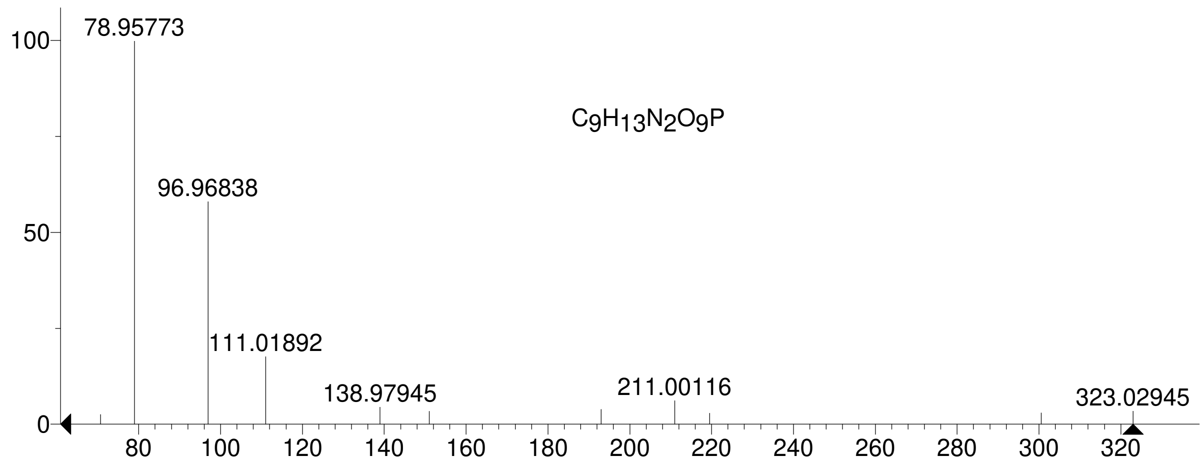

Hit 3 : URIDINE-5-MONOPHOSPHATE
C9H13N2O9P; MF: 422; RMF: 644; Prob 4.02%; Lib: embl neg mcg; ID: 631.

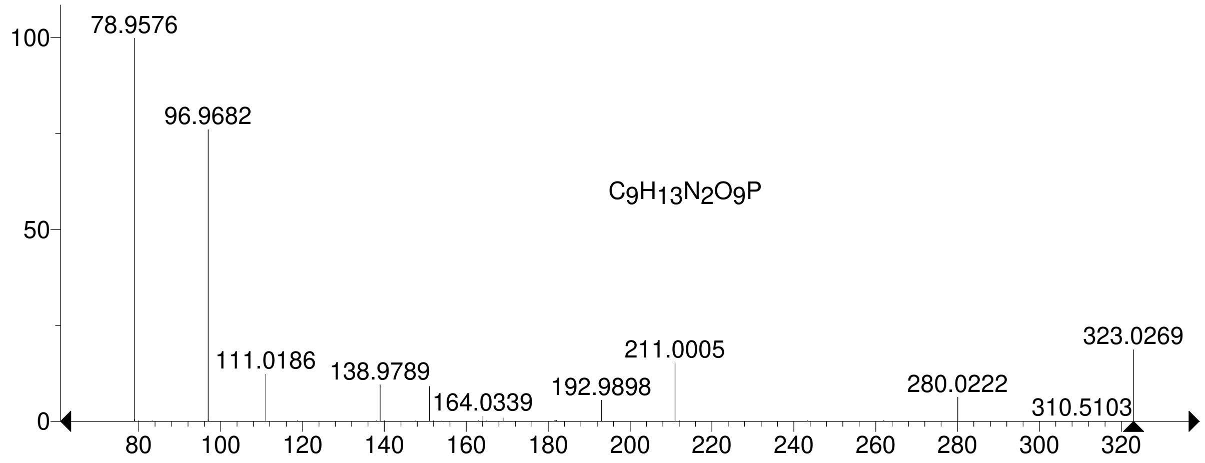

Hit 4 : URIDINE-5-MONOPHOSPHATE [M-H]- P=323
C9H13N2O9P; MF: 395; RMF: 640; Prob 1.18%; Lib: mona-export-gnps; ID: 5550.

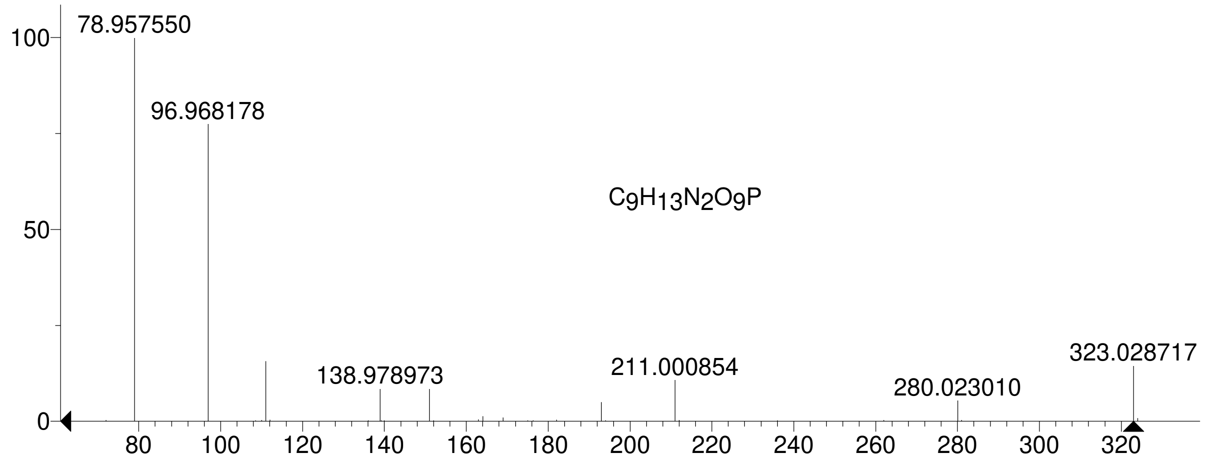

Hit 5 : Uridine 5'-diphosphoglucuronic acid [M-H-C6H9O9P]- HCD 29V P=323
C15H22N2O18P2; MF: 372; RMF: 635; Prob 0.43%; CAS: 2616-64-0; Lib: nist_msms; ID: 486423.

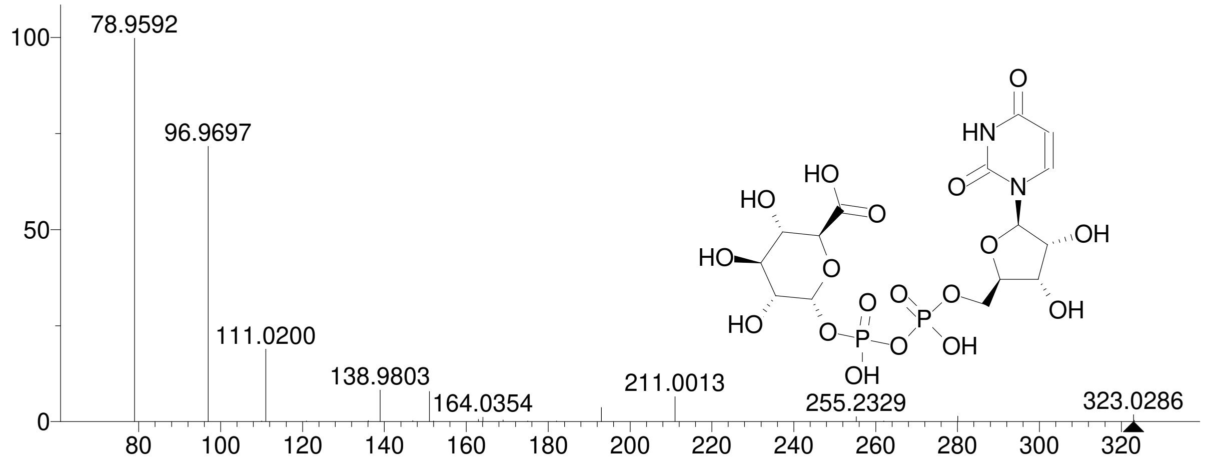

Unknown: Unknown (1.57_470.1501m/z)
Compound in Library Factor = 0

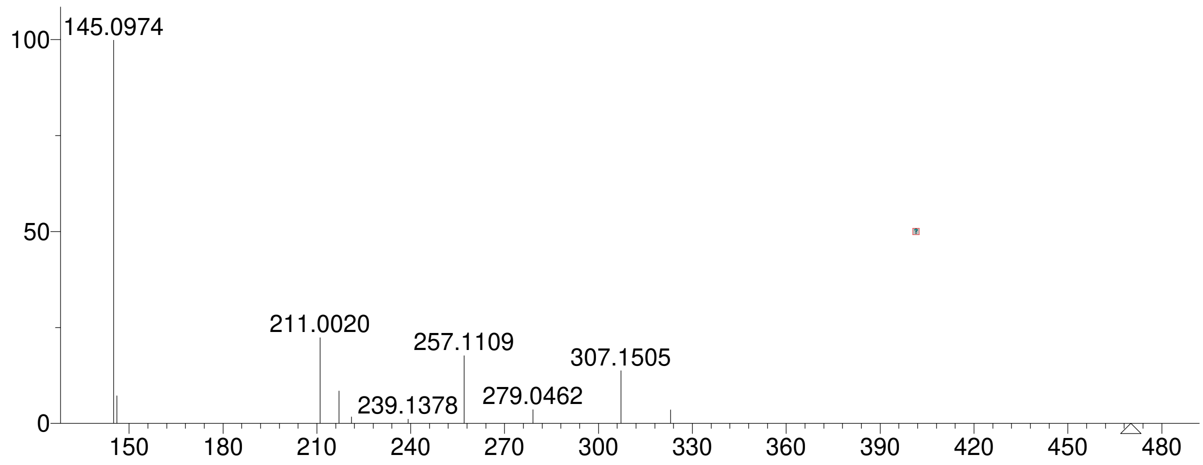

Unknown: Unknown (1.57_629.0541m/z)
Compound in Library Factor = 0

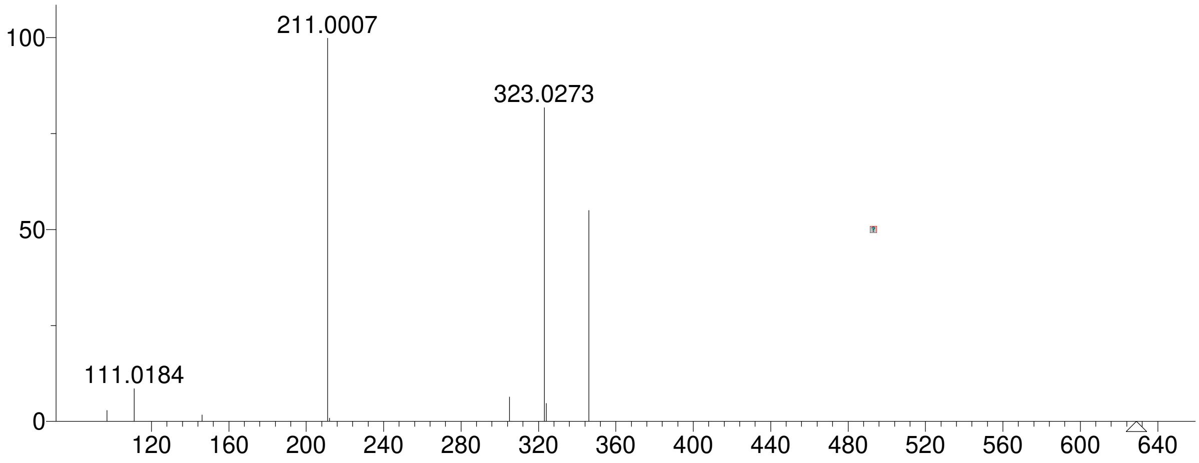

** Search Report Page 1 of 1 **

Unknown: Unknown (1.57_647.0643m/z)
Compound in Library Factor = -496

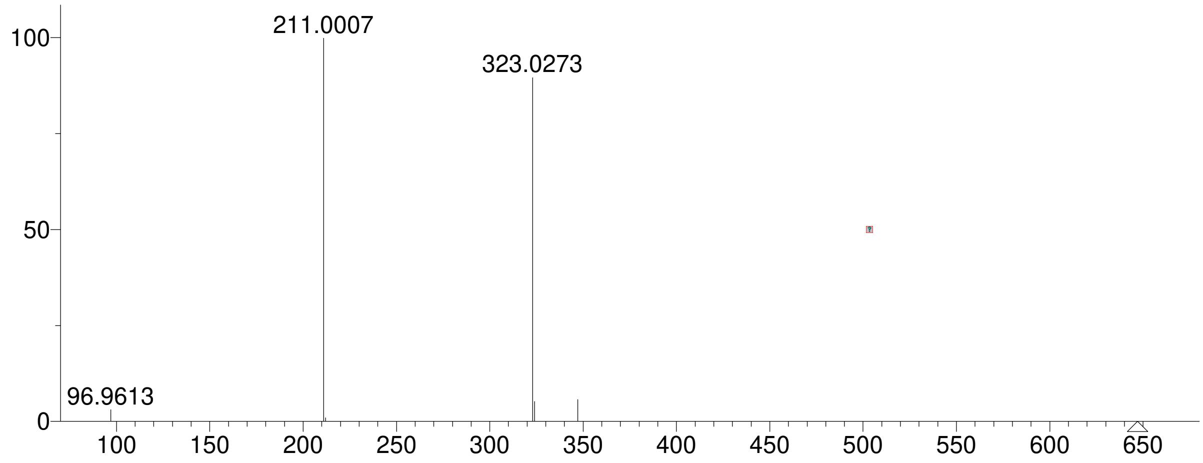

Hit 1 : 1-β-D-Arabinofuranosyluracil 5'-monophosphate [2M-H]- HCD 38V P=647.1
C9H13N2O9P; MF: 385; RMF: 813; Prob 94.9%; CAS: 18354-06-8; Lib: nist_msms; ID: 447597.

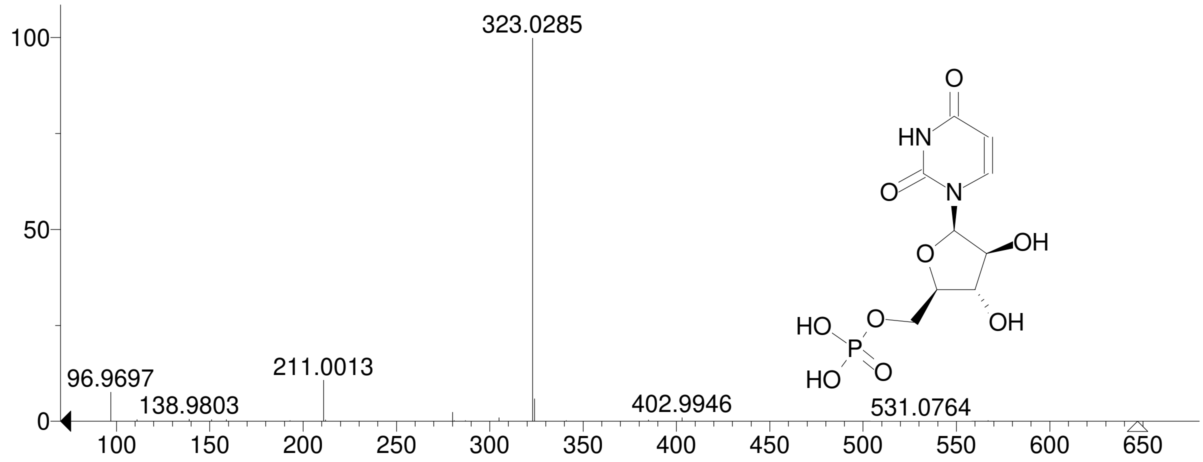

Hit 2 : Uridine 5'-monophosphate [2M-H]- HCD 20V P=647.1
C9H13N2O9P; MF: 266; RMF: 897; Prob 5.12%; CAS: 58-97-9; Lib: nist_msms; ID: 126136.

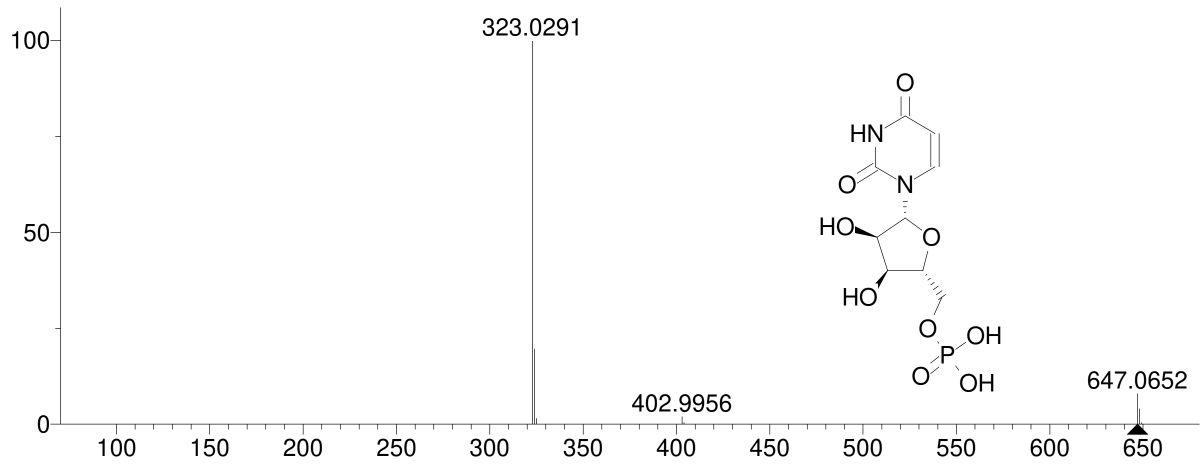

** Search Report Page 1 of 1 **

Unknown: Unknown (1.57_670.0902m/z)
Compound in Library Factor = 0

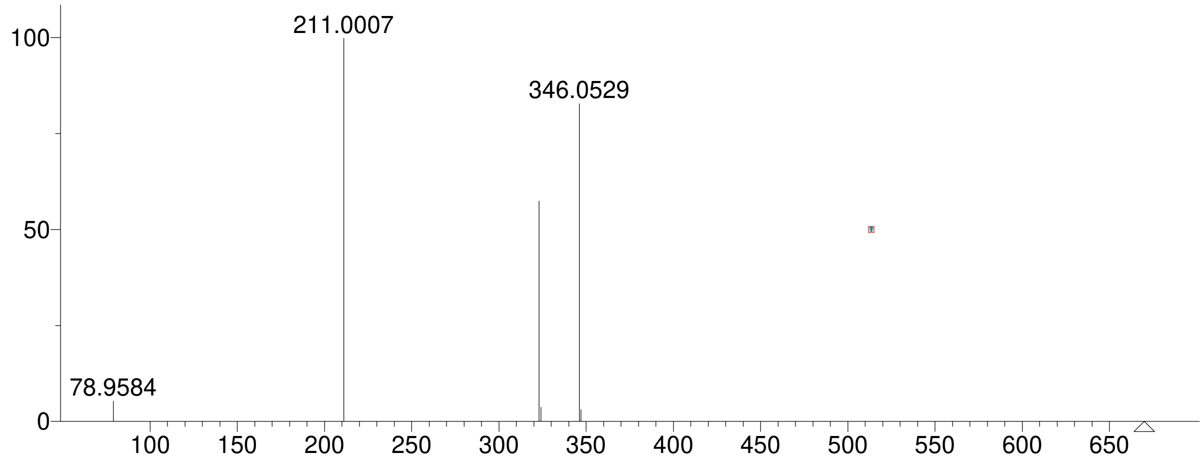

Unknown: Unknown (1.59_346.0549m/z)
Compound in Library Factor = -1014

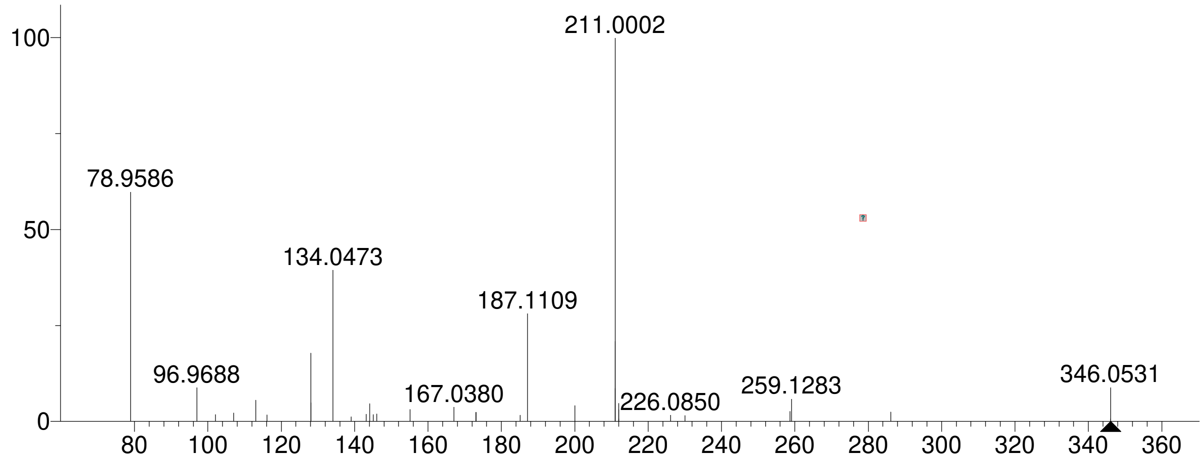

Hit 1 : Adenosine 2'-monophosphate [M-H]- HCD 24V P=346.1
C10H14N5O7P; MF: 558; RMF: 766; Prob 33.1%; CAS: 130-49-4; Lib: nist_msms; ID: 356679.

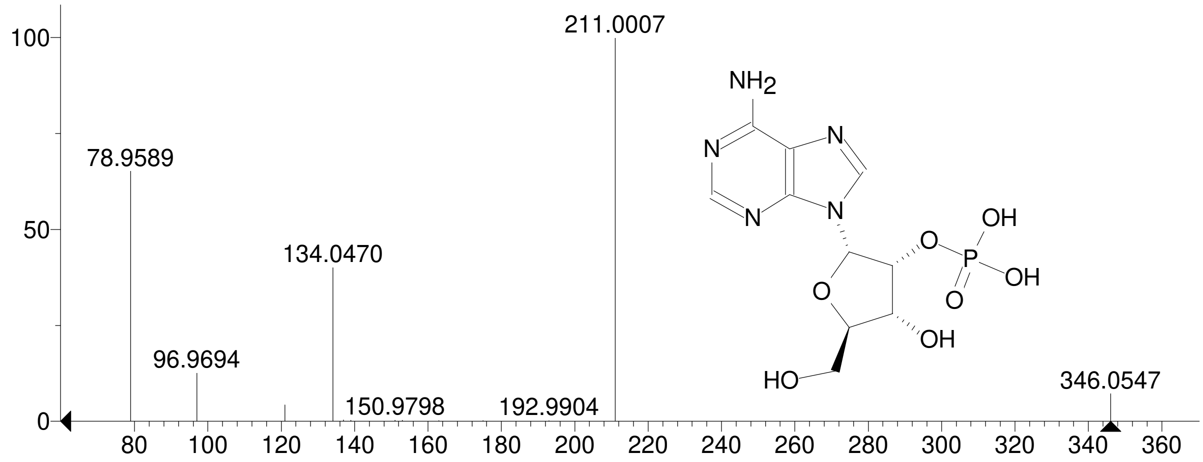

Hit 2 : Adenosine 3'-monophosphate [M-H]- HCD 31V P=346.1
C10H14N5O7P; MF: 554; RMF: 694; Prob 28.0%; CAS: 84-21-9; Lib: nist_msms; ID: 223902.

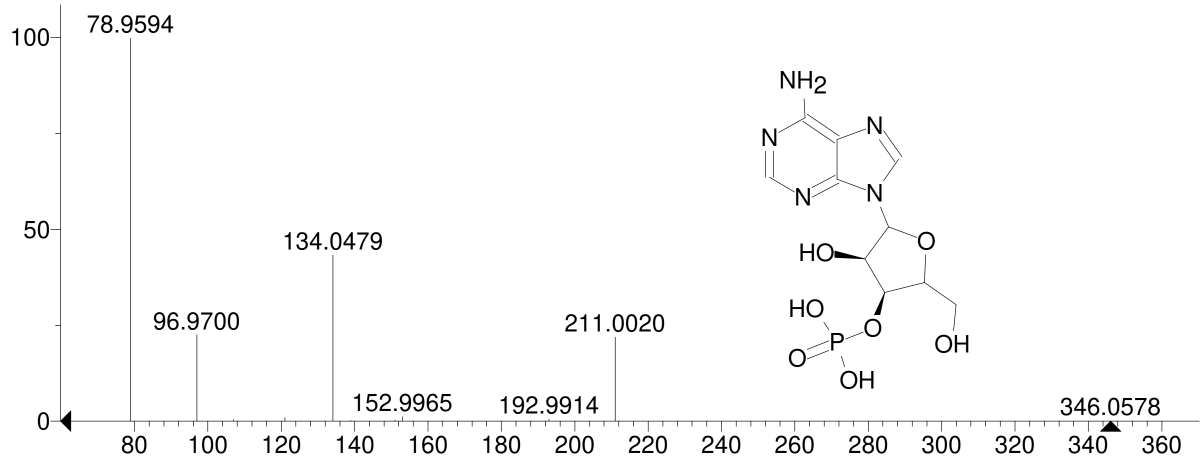

Hit 3 : Adenosine monophosphate [M-H]- HCD 31V P=346.1
C10H14N5O7P; MF: 544; RMF: 688; Prob 19.8%; Lib: nist_msms; ID: 207321.

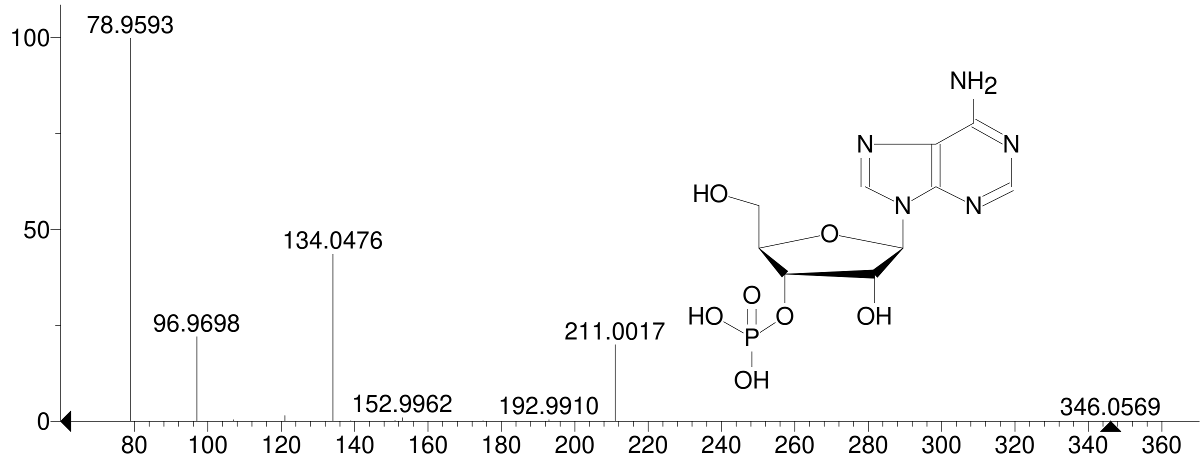

Hit 4 : Adenosine 2',3'-cyclic monophosphate [M+OH]- HCD 25V P=346.1
C10H12N5O6P; MF: 541; RMF: 744; Prob 17.5%; CAS: 634-01-5; Lib: nist_msms; ID: 207422.

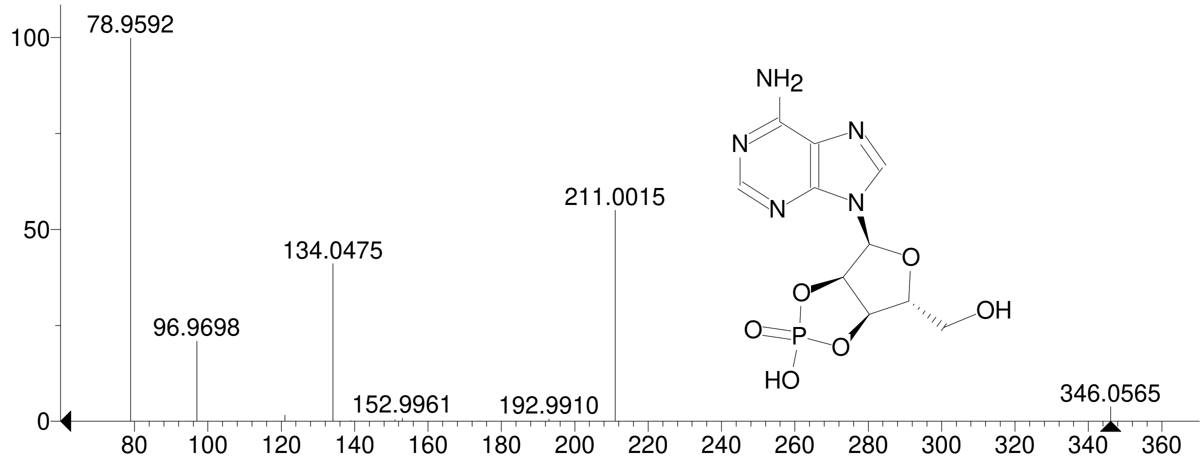

Hit 5 : Adenosine 5'-phosphosulfate [M-H-O3S]- HCD 24V P=346.1
C10H14N5O10PS; MF: 391; RMF: 599; Prob 0.48%; CAS: 485-84-7; Lib: nist_msms; ID: 392128.

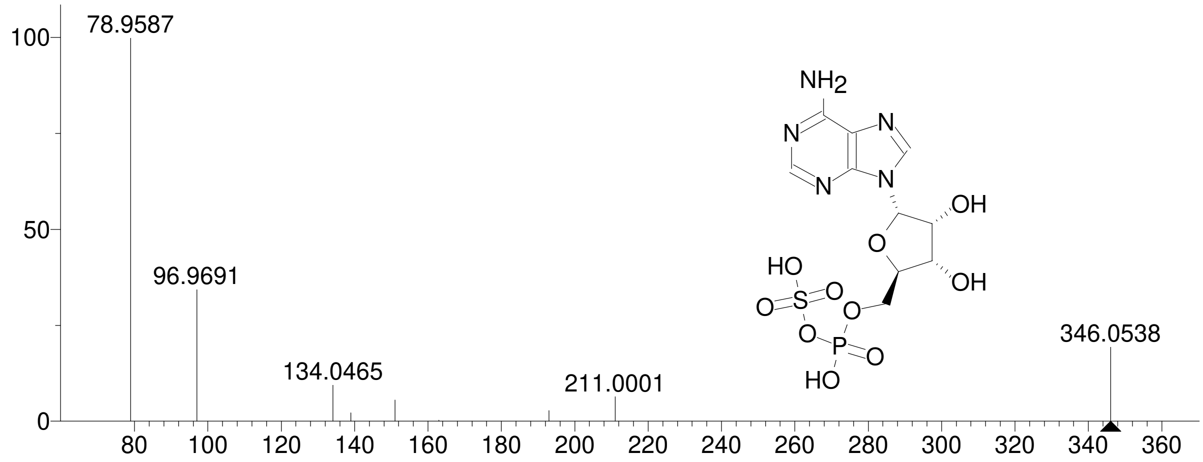

** Search Report Page 1 of 1 **

Unknown: Unknown (1.63_200.0551m/z)
Compound in Library Factor = -3273

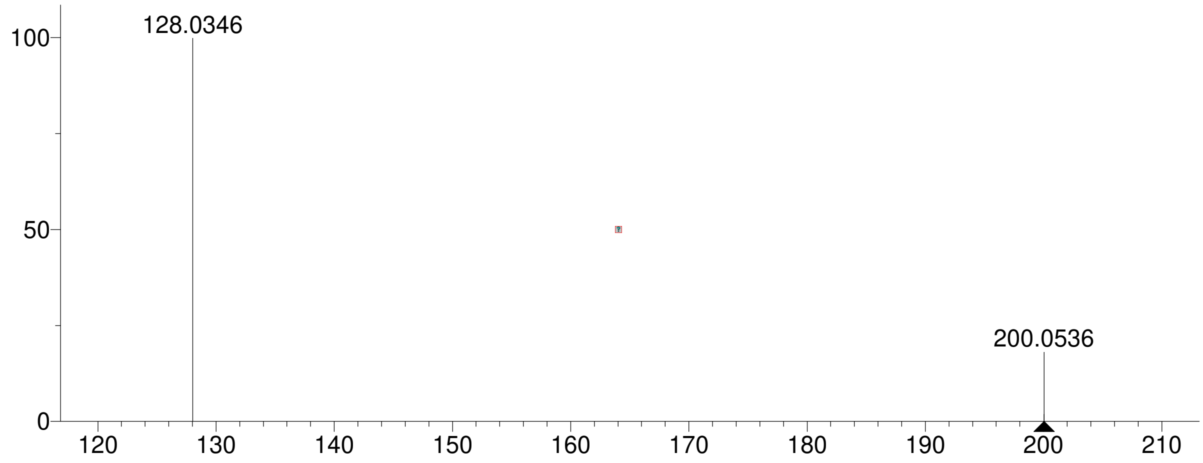

Hit 1 : O-Succinyl-L-homoserine [M-H-H2O]- HCD 6V P=200.1
C8H13NO6; MF: 19; RMF: 352; Prob 100.0%; CAS: 1492-23-5; Lib: nist_msms; ID: 125934.

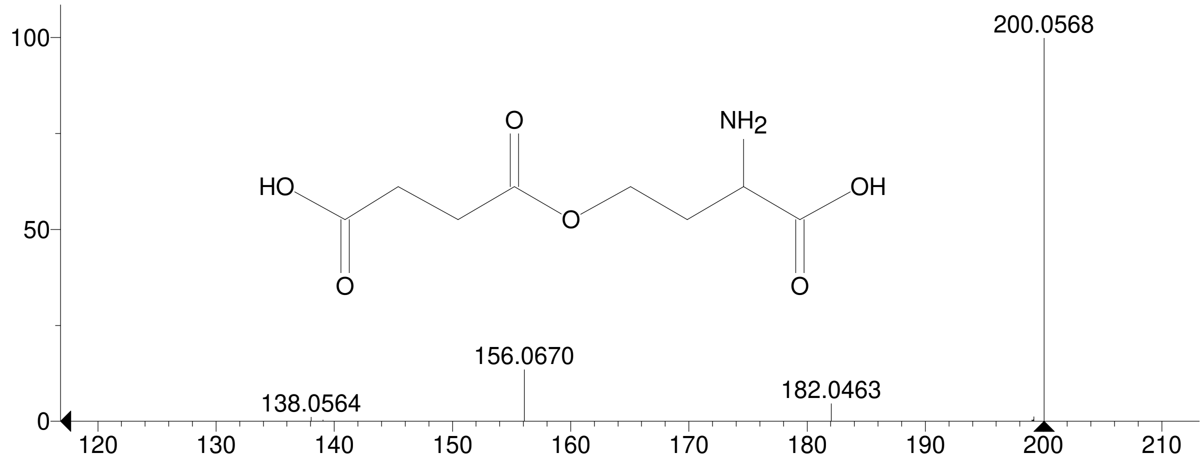

Unknown: Unknown (1.63_735.2330m/z)
Compound in Library Factor = 0

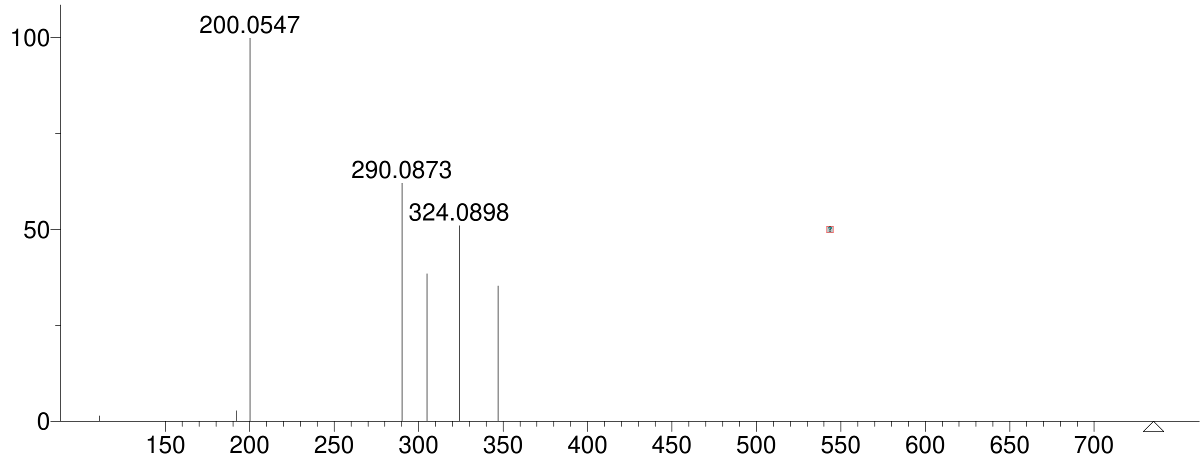

** Search Report Page 1 of 1 **

Unknown: Unknown (1.65_272.0755m/z)
Compound in Library Factor = 0

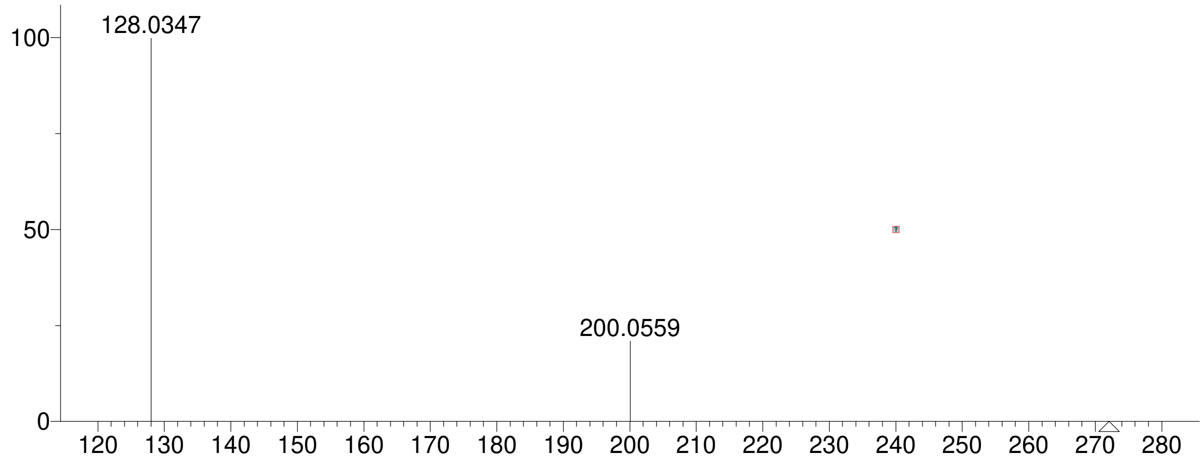

Hit 1 : N-Glycolylneuraminic acid [M+H-3H2O]+ HCD 19V P=272.1
C11H19NO10; MF: 0; RMF: 44; Prob 100.0%; CAS: 1113-83-3; Lib: nist_msms; ID: 307277.

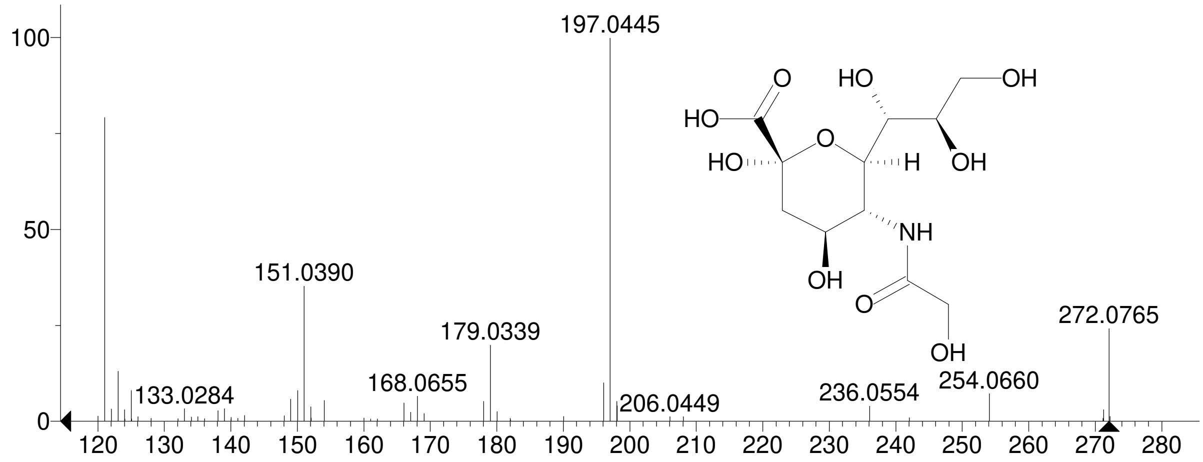

Unknown: Unknown (1.65_290.0871m/z)
Compound in Library Factor = -642

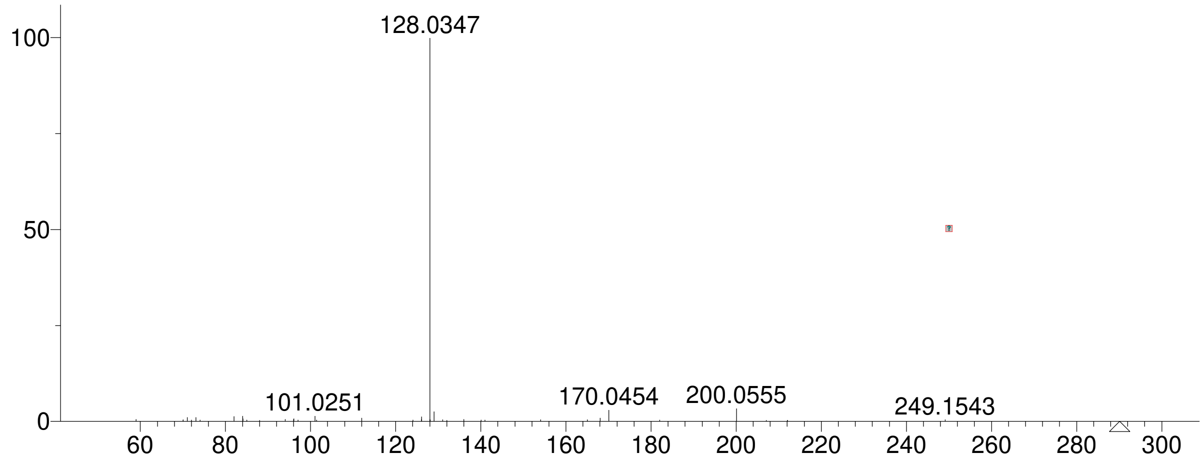

Hit 1 : N-Fructosyl pyroglutamate; PlaSMA ID-534 [M-H]- P=290.1
C11H17NO8; MF: 200; RMF: 778; Prob 92.4%; Lib: biomsms-neg-plasma; ID: 480.

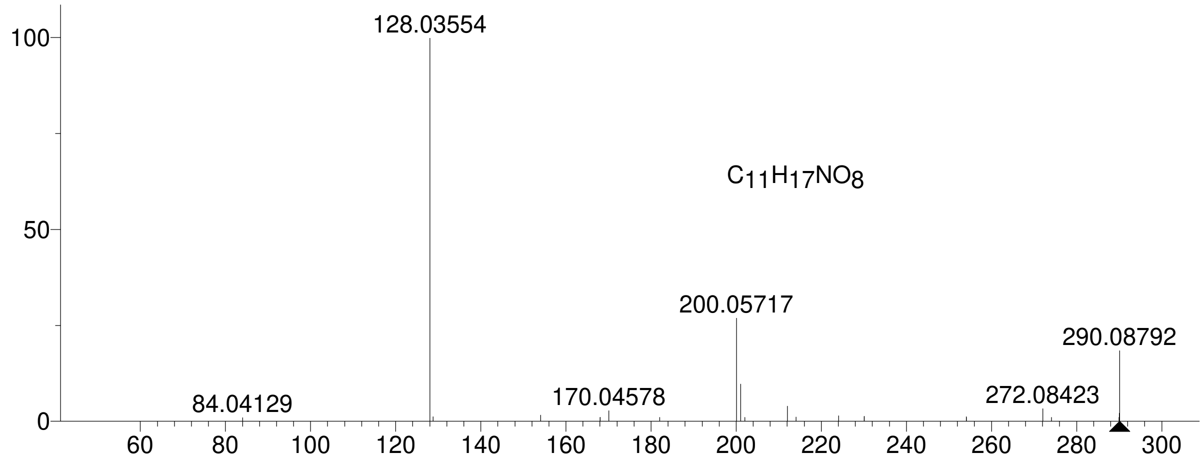

Hit 2 : (-)-N-Acetylneuraminic acid [M-H-H2O]- IT-FT 35% P=290.1
C11H19NO9; MF: 82; RMF: 179; Prob 5.12%; CAS: 131-48-6; Lib: nist_msms; ID: 479648.

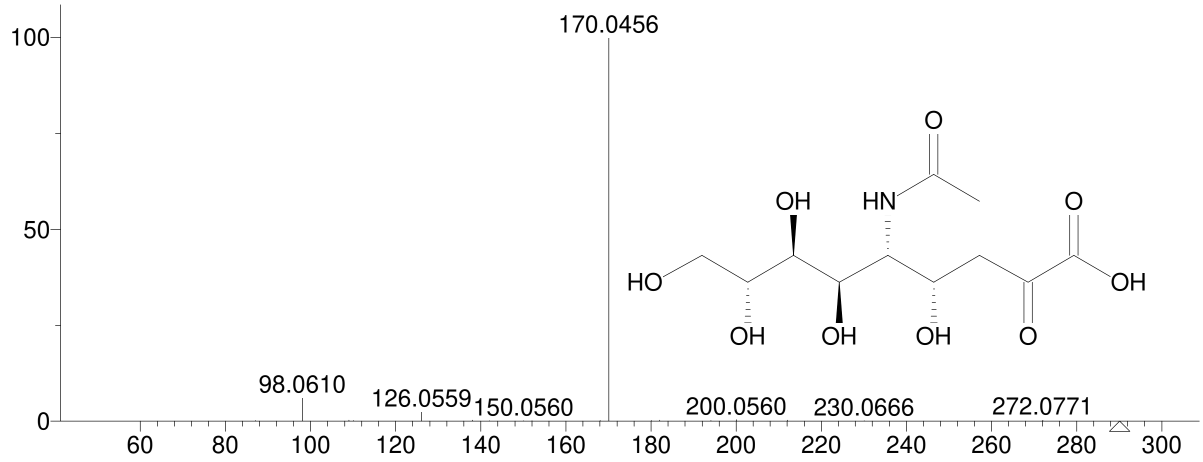

Hit 3 : N-Glycolylneuraminic acid [M+H-2H2O]+ HCD 26V P=290.1
C11H19NO10; MF: 30; RMF: 177; Prob 1.02%; CAS: 1113-83-3; Lib: nist_msms; ID: 307259.

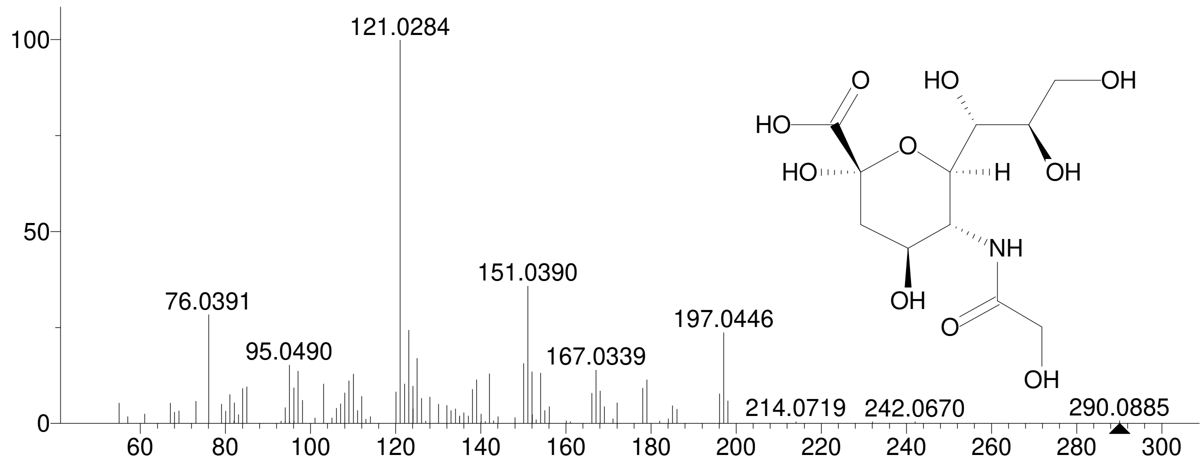

Hit 4 : 3'-α-Sialyl-N-acetyllactosamine [M-H-C14H25O11N]- HCD 46V P=290.1
C25H42N2O19; MF: 28; RMF: 149; Prob 0.94%; CAS: 81693-22-3; Lib: nist_msms; ID: 211510.

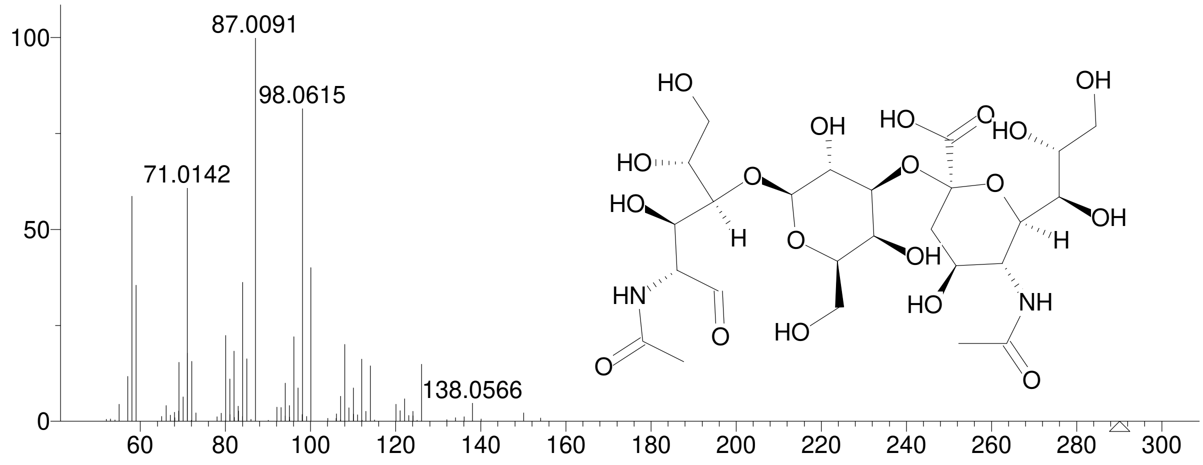

Hit 5 : 2'-Deoxyguanosine [M+Na]+ HCD 43V P=290.1
C10H13N5O4; MF: 0; RMF: 30; Prob 0.27%; CAS: 961-07-9; Lib: nist_msms; ID: 303937.

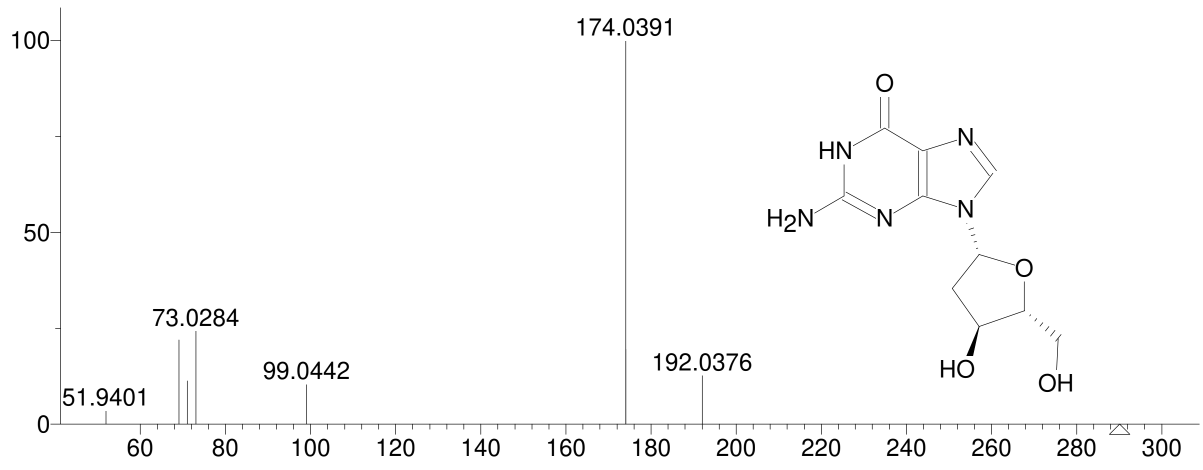

Unknown: Unknown (1.65_524.1030m/z)
Compound in Library Factor = 0

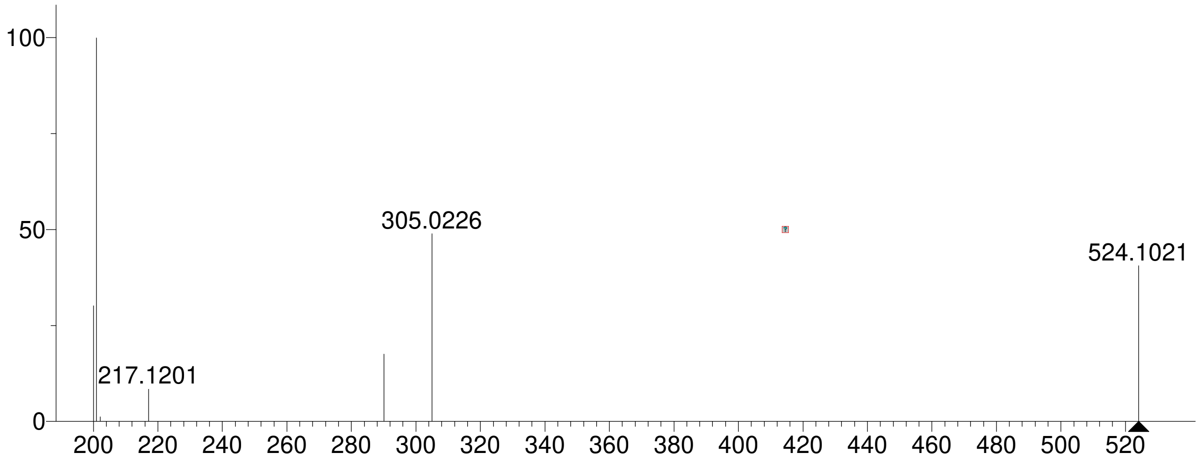

Unknown: Unknown (1.65_603.1567m/z)
Compound in Library Factor = 0

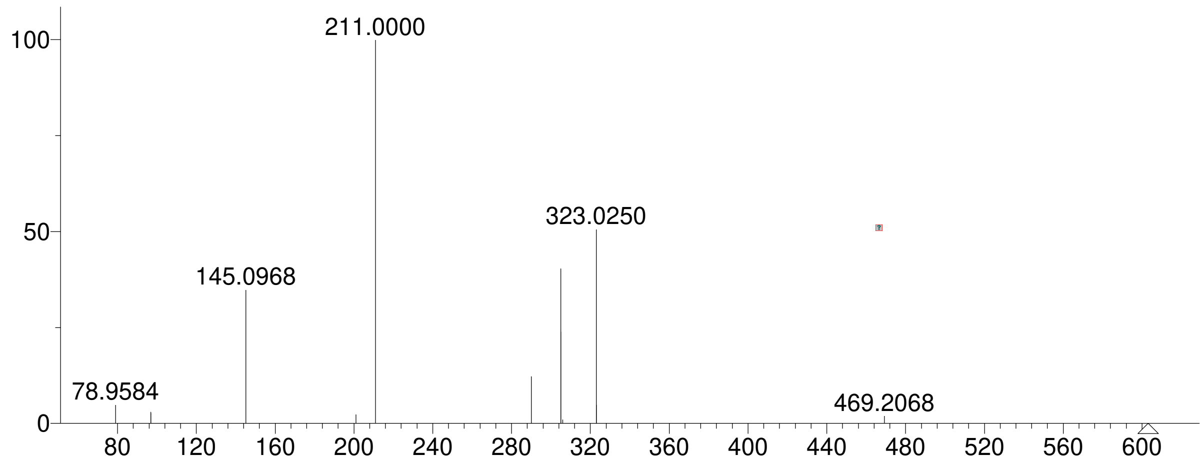

Unknown: Unknown (1.65_639.2600m/z)
Compound in Library Factor = 0

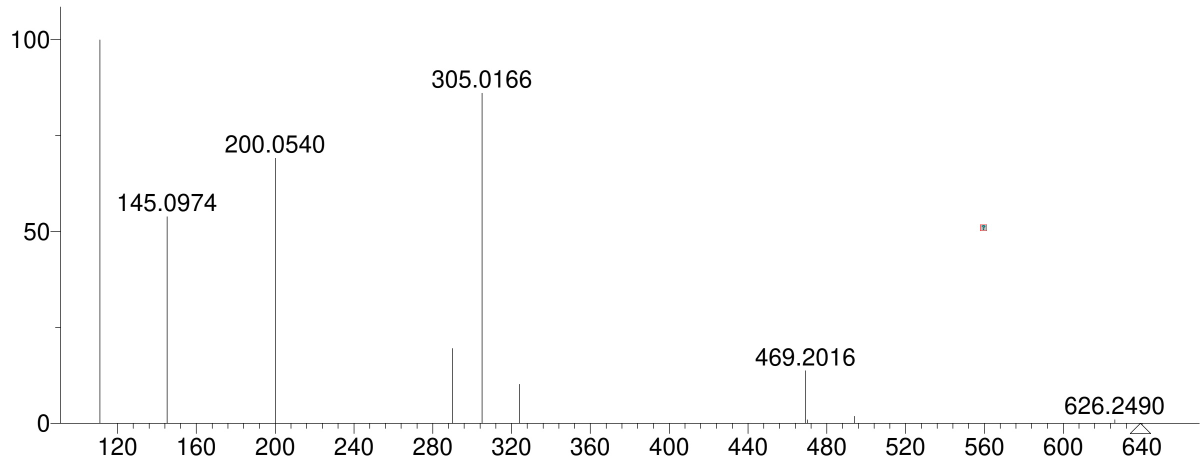

Hit 1 : Caspase 8 Substrate 1, chromogenic [M+H]+ QTOF 45V P=639.3
C27H38N6O12; MF: 0; RMF: 44; Prob 100.0%; Lib: nist_msms2; ID: 39470.

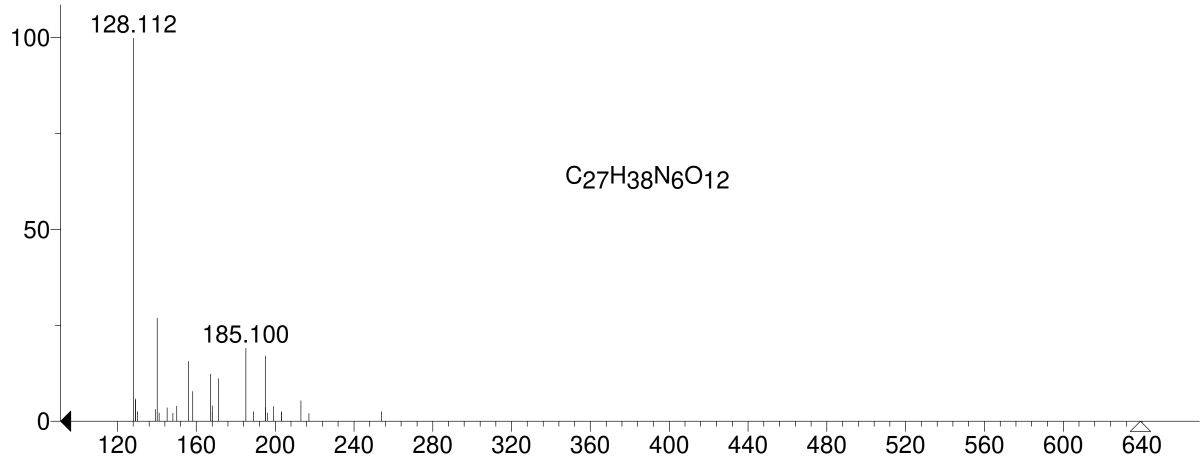

Unknown: Unknown (1.65_796.2970m/z)
Compound in Library Factor = 0

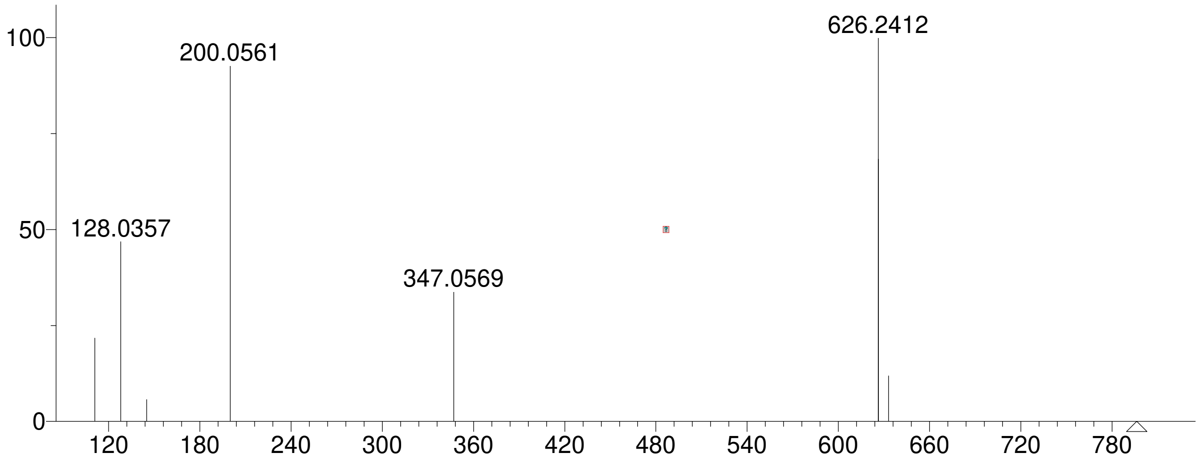

Unknown: Unknown (1.66_603.1666m/z)
Compound in Library Factor = 0

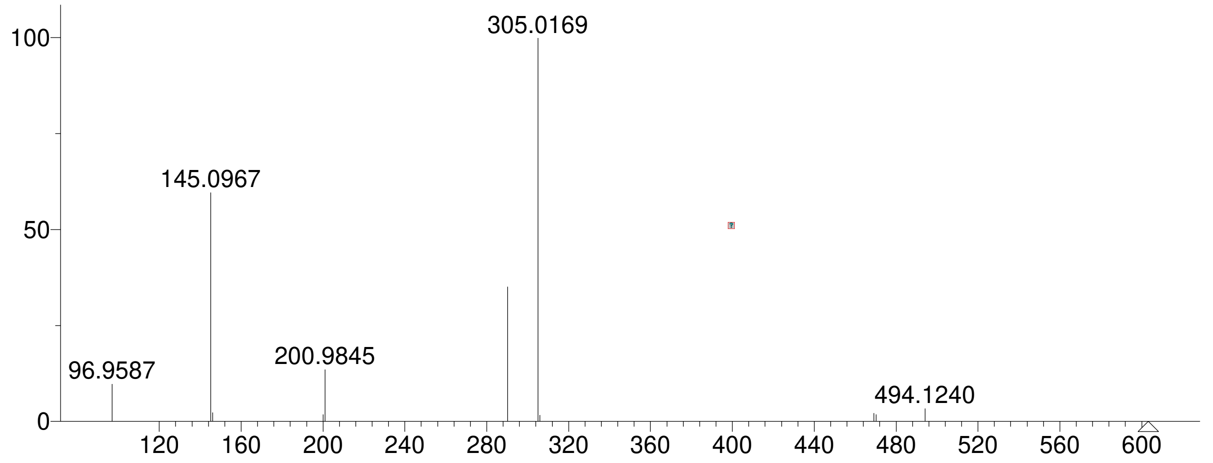

Hit 1 : [3,4,5-trihydroxy-6-[4-hydroxy-2-[(1,2,3,6-tetrahydroxycyclohexanecarbonyl)oxymethyl]phenoxy]oxan-2-yl]m
C27H32O14; MF: 0; RMF: 31; Prob 25.0%; Lib: mona-export-vf-npl_qexactive; ID: 24535.

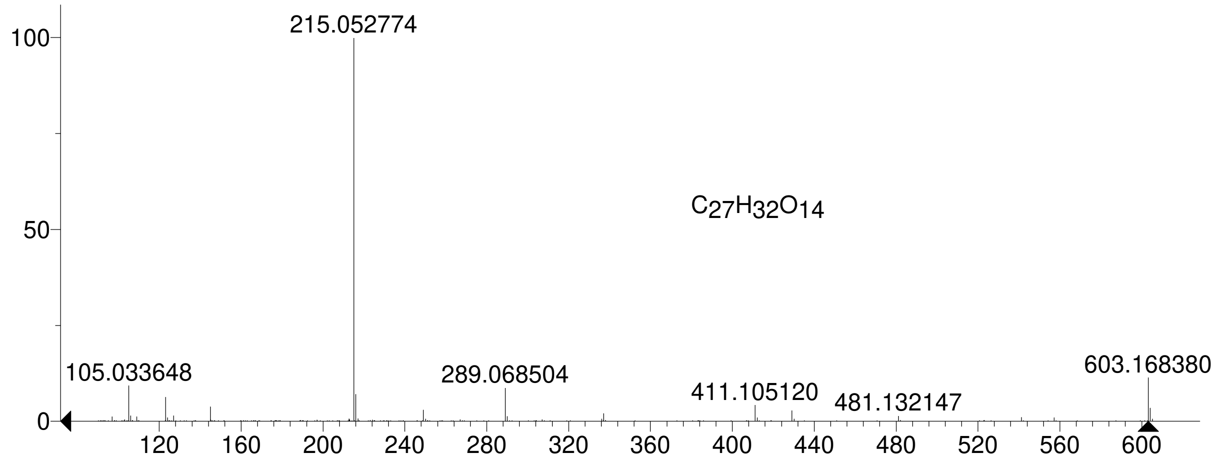

Hit 2 : naringin [M+Na]+ 45HCD P=603.2
C27H32O14; MF: 0; RMF: 24; Prob 25.0%; Lib: anthocyanins mona; ID: 7409.

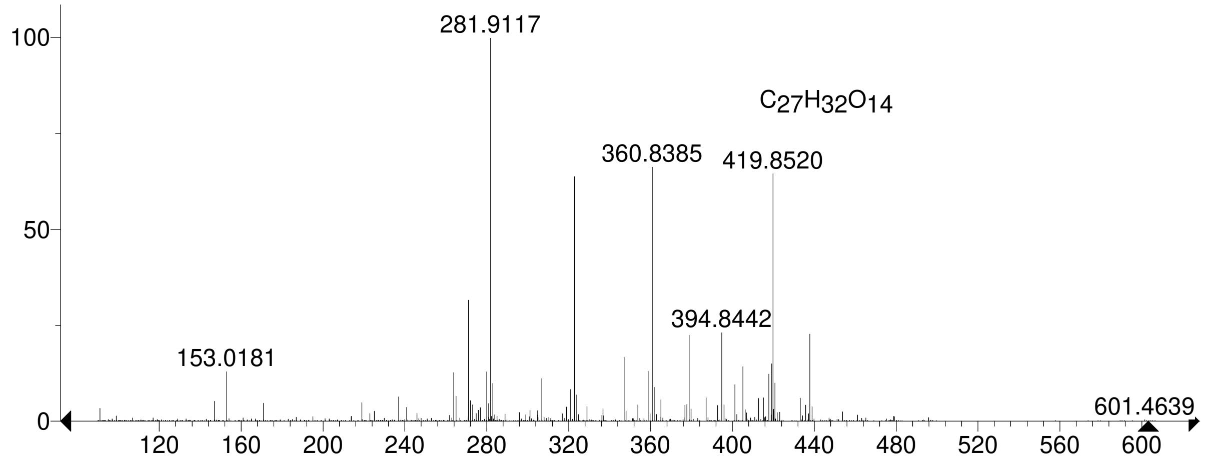

Hit 3 : NCGC00385466-01_C27H32O14_(1S,4aS,6S,7aS)-6-Hydroxy-1-({6-O-[(2E)-3-(4-hydroxy-3,5-dimethoxyp
C27H32O14; MF: 0; RMF: 17; Prob 25.0%; Lib: mona-export-gnps; ID: 10919.

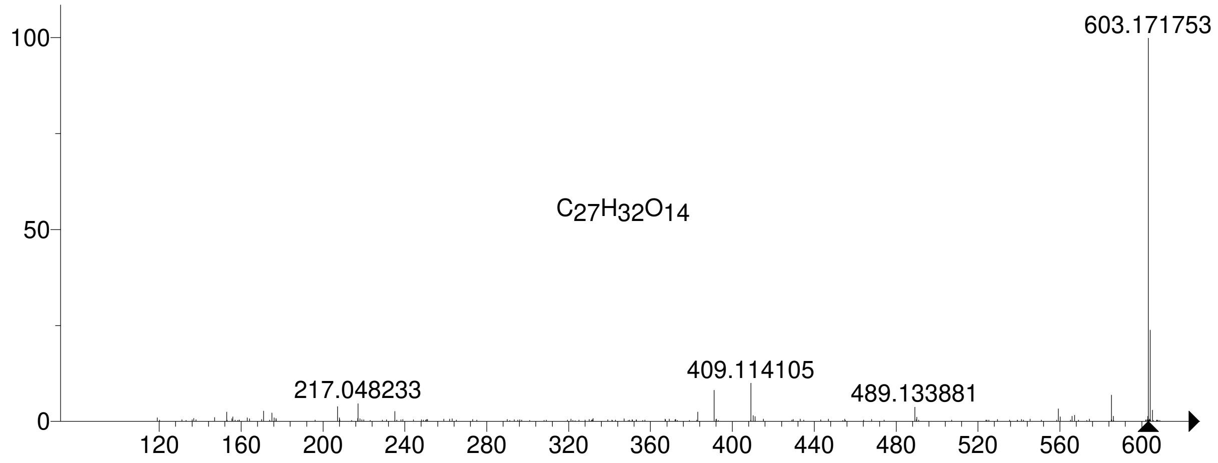

Hit 4 : 7-[4,5-dihydroxy-6-(hydroxymethyl)-3-[(2S,3R,4R,5R,6S)-3,4,5-trihydroxy-6-methyloxan-2-yl]oxyoxan-2-yl]c
C27H32O14; MF: 0; RMF: 3; Prob 25.0%; Lib: anthocyanins mona; ID: 4728.

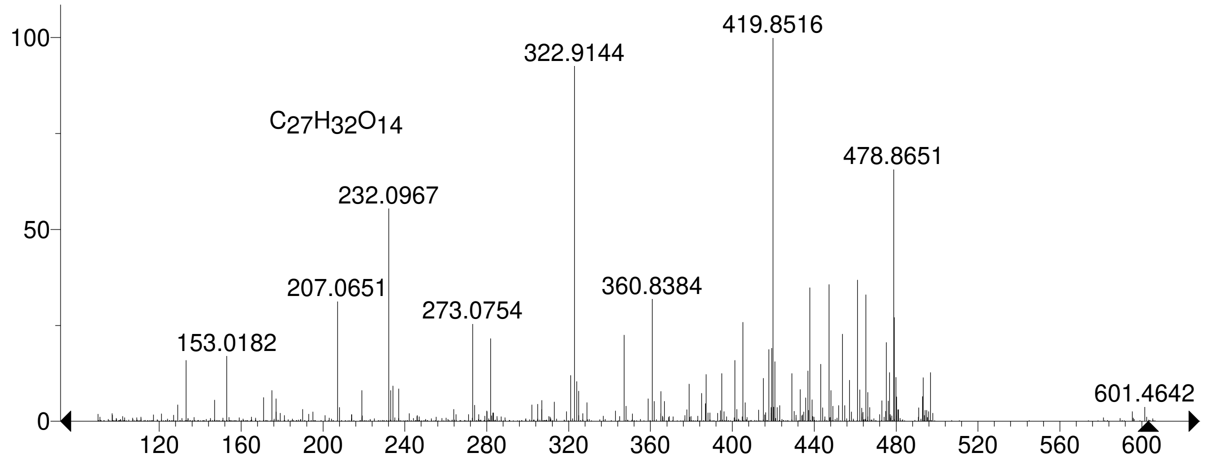

Unknown: Unknown (1.68_323.0969m/z)
Compound in Library Factor = -3273

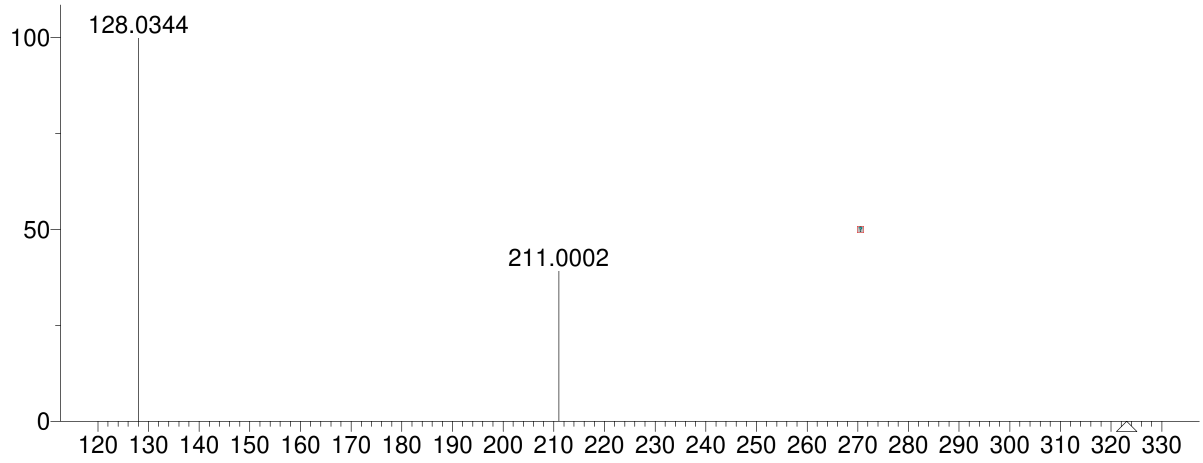

Hit 1 : 1-Piperidinecarboxylic acid, 4-[6-[4-[(methoxycarbonyl)amino]phenyl]-4-(4-morpholinyl)-1H-pyrazolo[3,4-d]
C24H29N7O5; MF: 1; RMF: 136; Prob 7.97%; CAS: 1062169-56-5; Lib: nist_msms; ID: 265735.

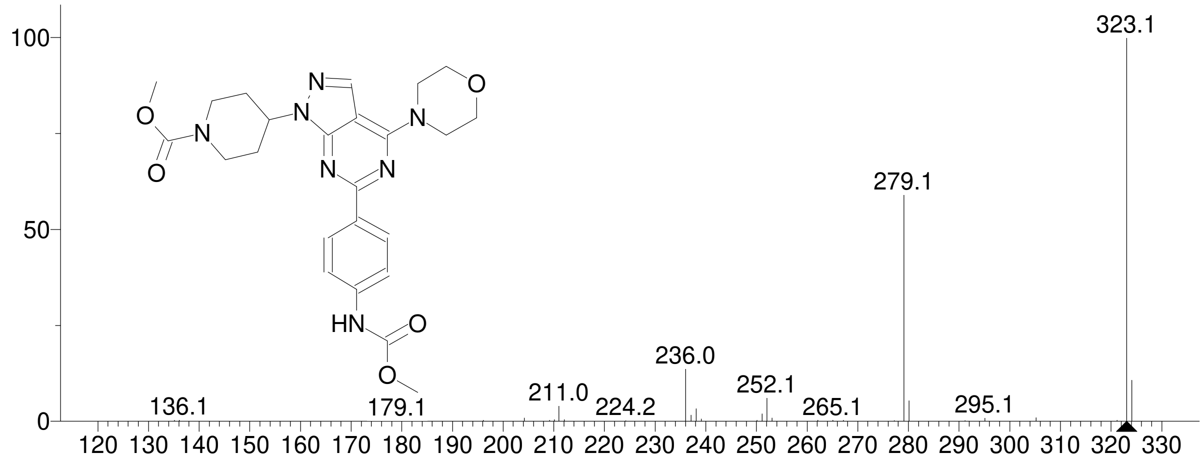

Hit 2 : Dinitramine [M+H]+ HCD 103V P=323.1
C11H13F3N4O4; MF: 0; RMF: 153; Prob 7.66%; CAS: 29091-05-2; Lib: nist_msms; ID: 100130.

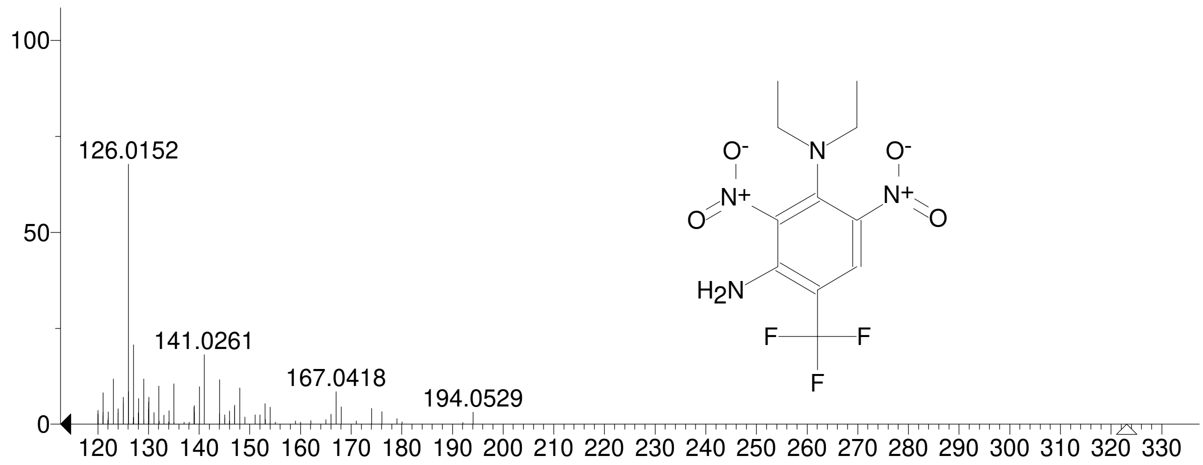

Hit 3 : Fluorometholone [M+H-H2O]+=>341.1=>323.1 IT 35% P=323.1
C22H29FO4; MF: 0; RMF: 123; Prob 7.66%; CAS: 426-13-1; Lib: nist_msms; ID: 119128.

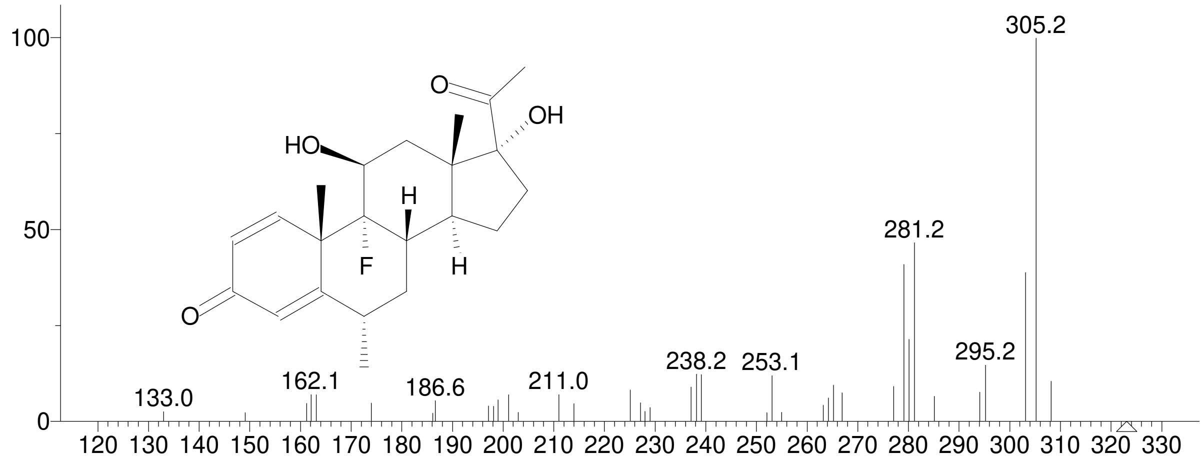

Hit 4 : Indanofan [M+H]+=>323.1 IT 35% P=323.1
C20H17ClO3; MF: 0; RMF: 86; Prob 7.66%; CAS: 133220-30-1; Lib: nist_msms; ID: 477459.

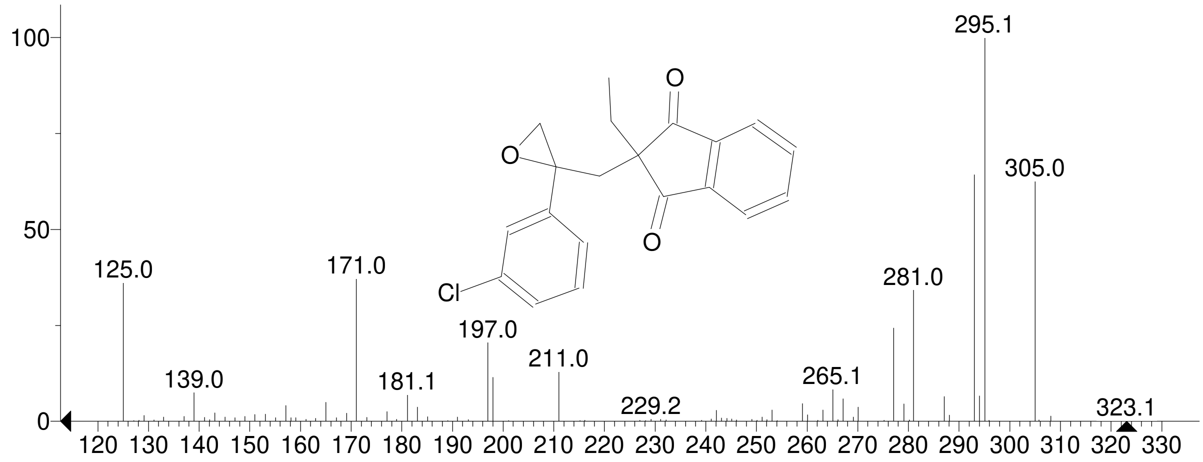

Hit 5 : Prednisone [M+H-H2O]+=>323.1 IT 35% P=323.1
C21H26O5; MF: 0; RMF: 81; Prob 7.66%; CAS: 53-03-2; Lib: nist_msms; ID: 99548.

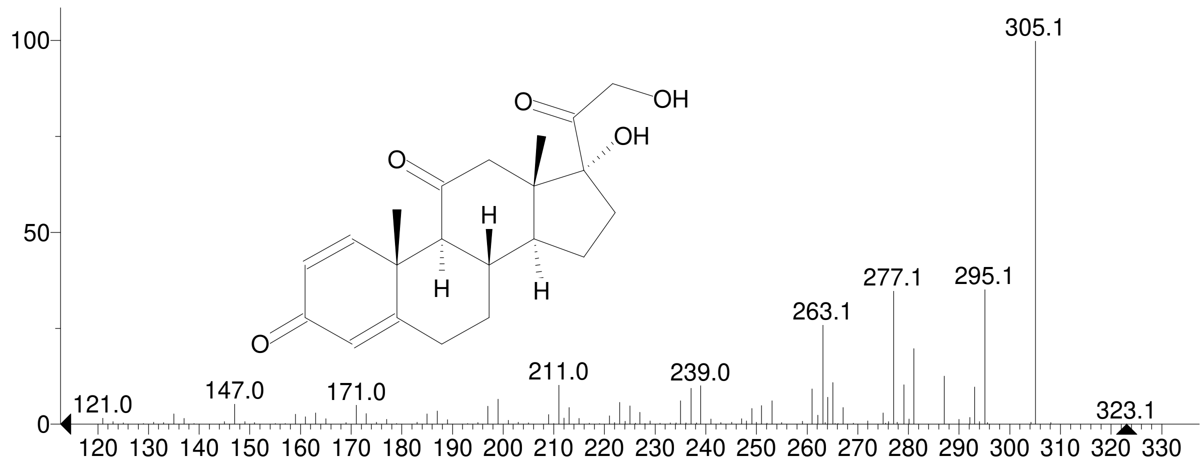

Unknown: Unknown (1.69_288.1184m/z)
Compound in Library Factor = -3273

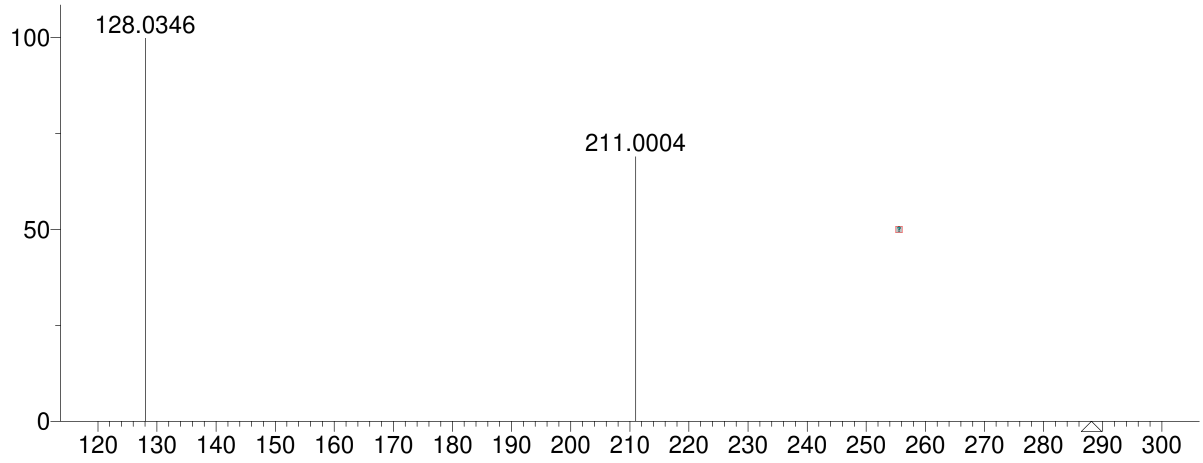

Hit 1 : OPHTHALMIC ACID [M-H]- P=288.1
C11H19N3O6; MF: 2; RMF: 252; Prob 34.7%; Lib: msms-neg-gnps; ID: 328.

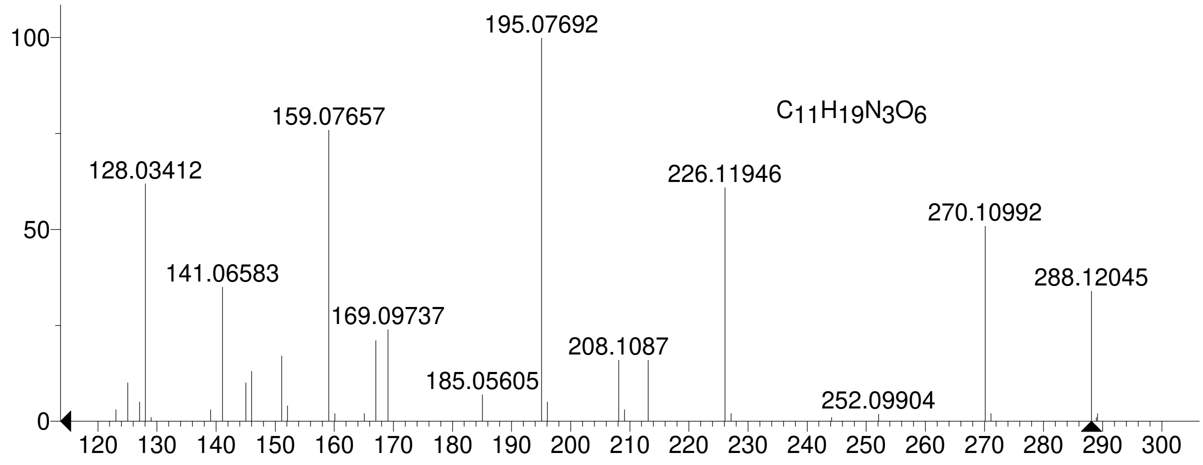

Hit 2 : OPHTHALMIC ACID
C11H19N3O6; MF: 1; RMF: 250; Prob 33.3%; Lib: embl neg mcg; ID: 381.

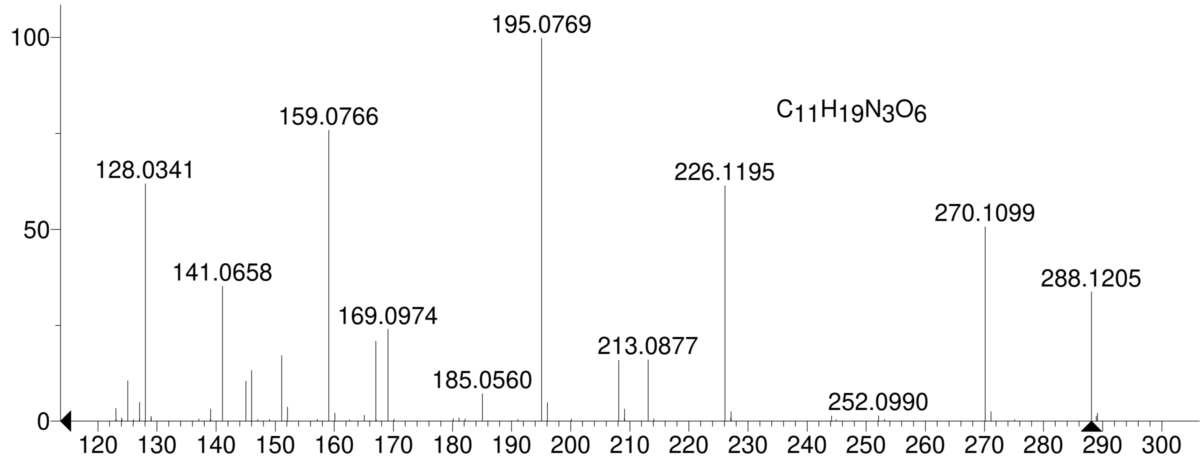

Hit 3 : ANISOMYCIN [M+Na]+ 65HCD P=288.1
C14H19NO4; MF: 0; RMF: 24; Prob 32.0%; Lib: mona-export-vf-npl_qexactive; ID: 27597.

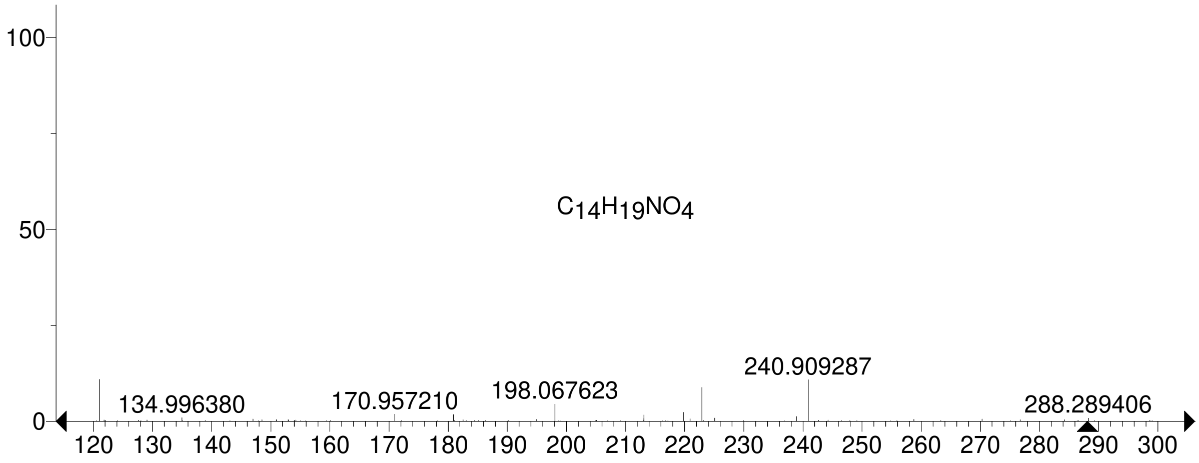

Unknown: Unknown (1.69_407.1662m/z)
Compound in Library Factor = -1848

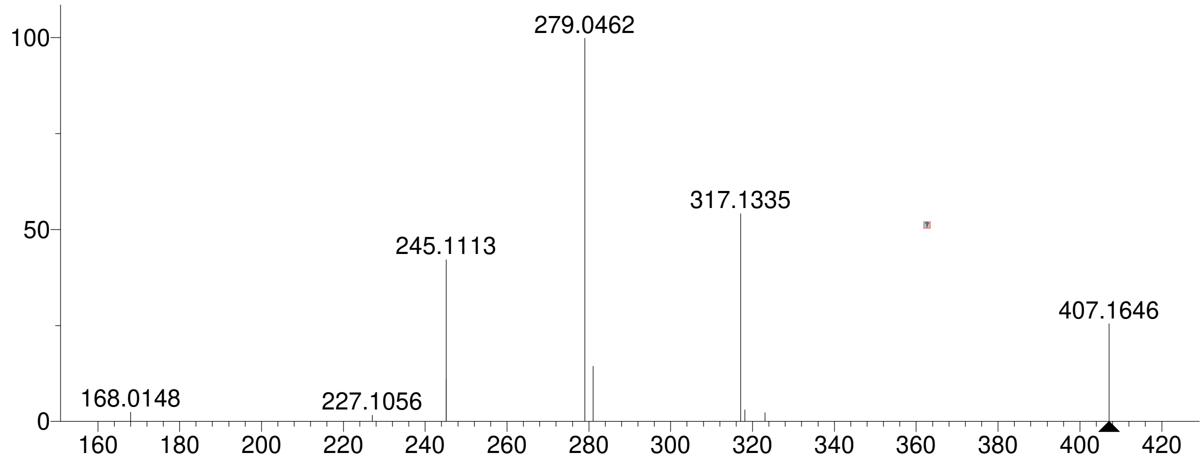

Hit 1 : (2R,3R,4S,6R)-2-((5-hydroxy-2,2-dimethyl-3,4-dihydro-2H-benzo[h]chromen-6-yl)oxy)-6-(hydroxymethyl)tet
C21H26O8; MF: 52; RMF: 363; Prob 46.6%; Lib: mona-export-gnps; ID: 2855.

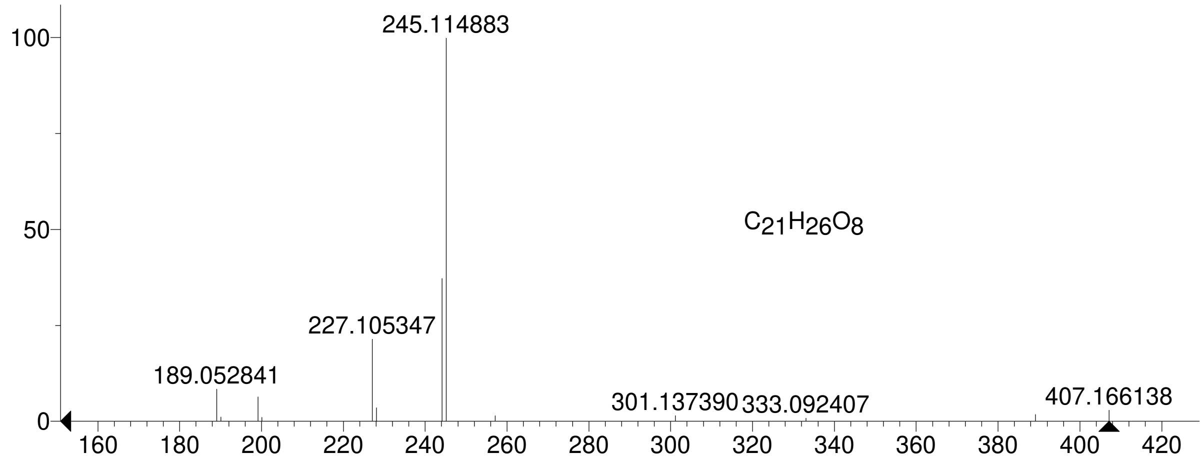

Hit 2 : Artesunate [M+Na]+ HCD 7V P=407.2
C19H28O8; MF: 22; RMF: 301; Prob 13.0%; CAS: 88495-63-0; Lib: nist_msms; ID: 119192.

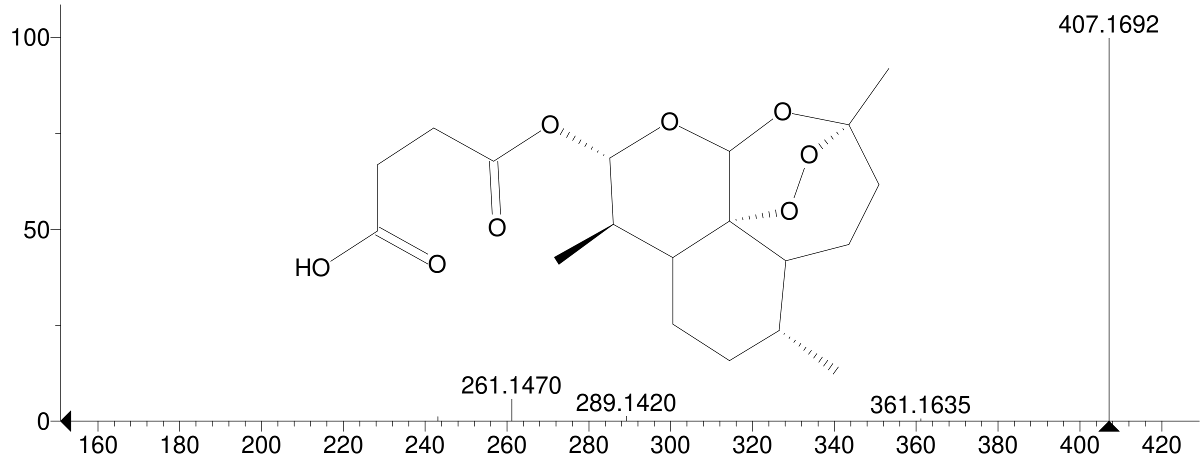

Hit 3 : N,N'-Diacetylchitobiose [M+H-H2O]+ HCD 8V P=407.2
C16H28N2O11; MF: 9; RMF: 251; Prob 8.37%; CAS: 35061-50-8; Lib: nist_msms; ID: 486176.

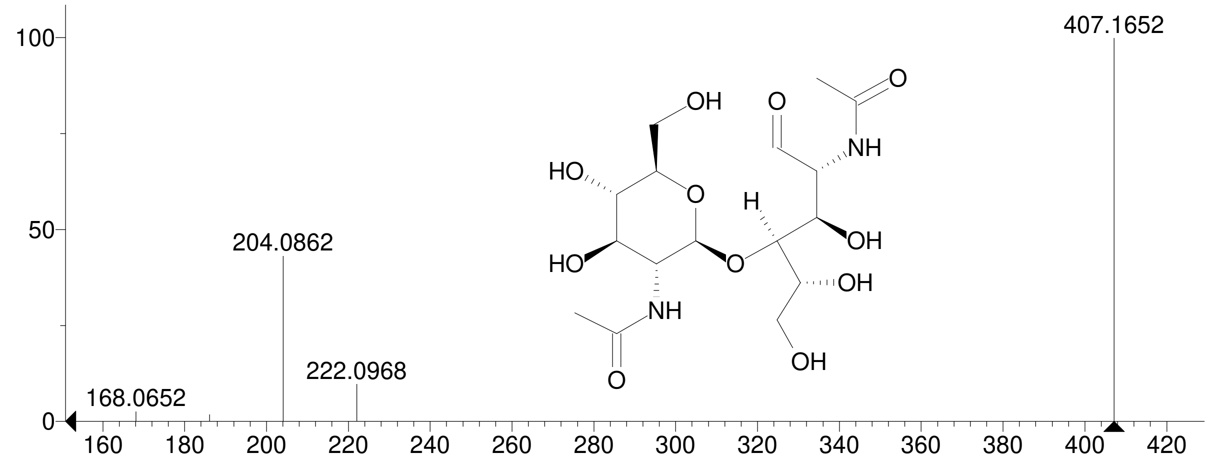

Hit 4 : (2R,3R,4S,6R)-2-((10-hydroxy-2,2-dimethyl-3,4-dihydro-2H-benzo[g]chromen-5-yl)oxy)-6-(hydroxymethyl)t
C21H26O8; MF: 8; RMF: 226; Prob 8.04%; Lib: mona-export-gnps; ID: 2848.

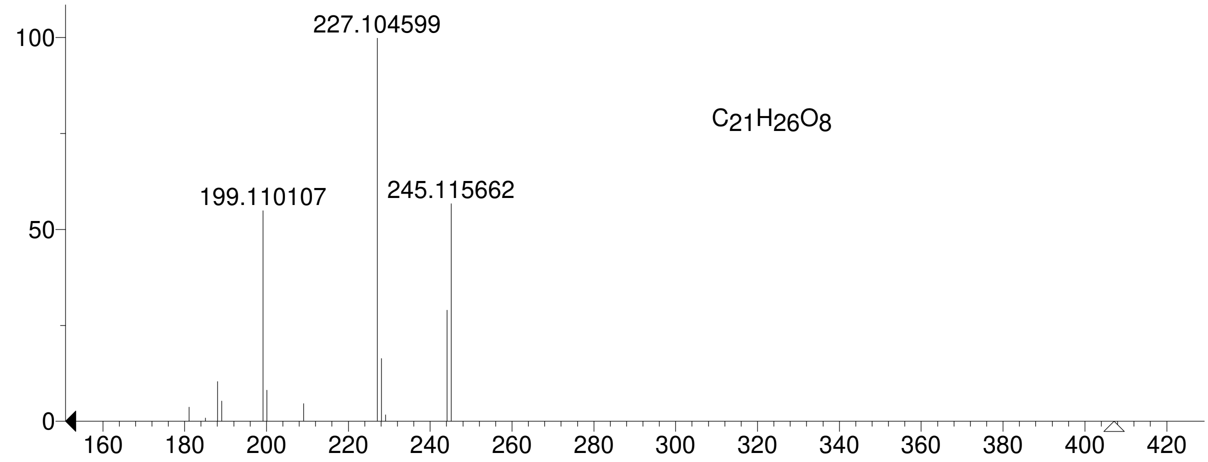

Hit 5 : Beclomethasone [M-H]- 20-30-40%) P=407.2
C22H29ClO5; MF: 3; RMF: 201; Prob 6.48%; Lib: mona-export-fiehn_hilic; ID: 1084.

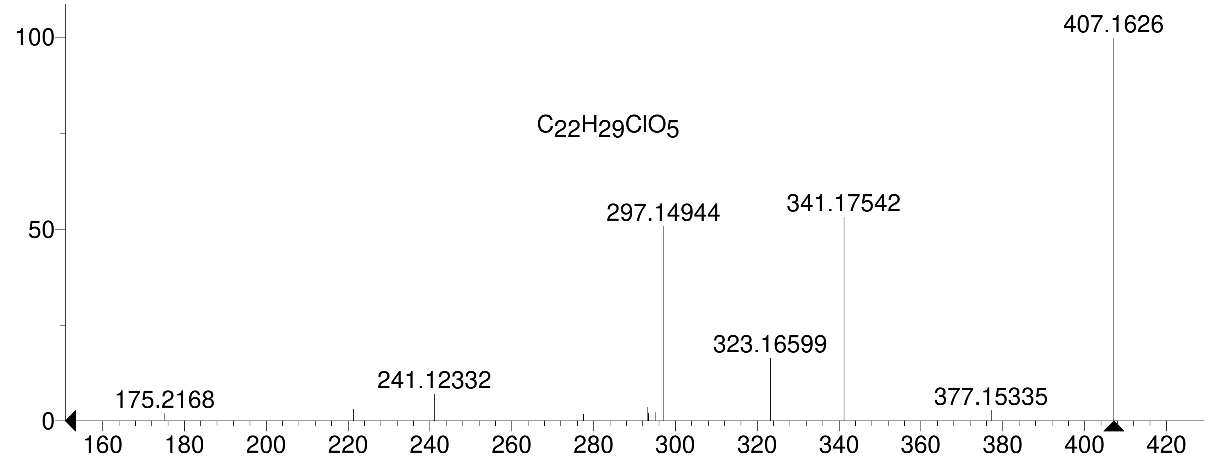

** Search Report Page 1 of 1 **

Unknown: Unknown (1.70_470.1495m/z)
Compound in Library Factor = 0

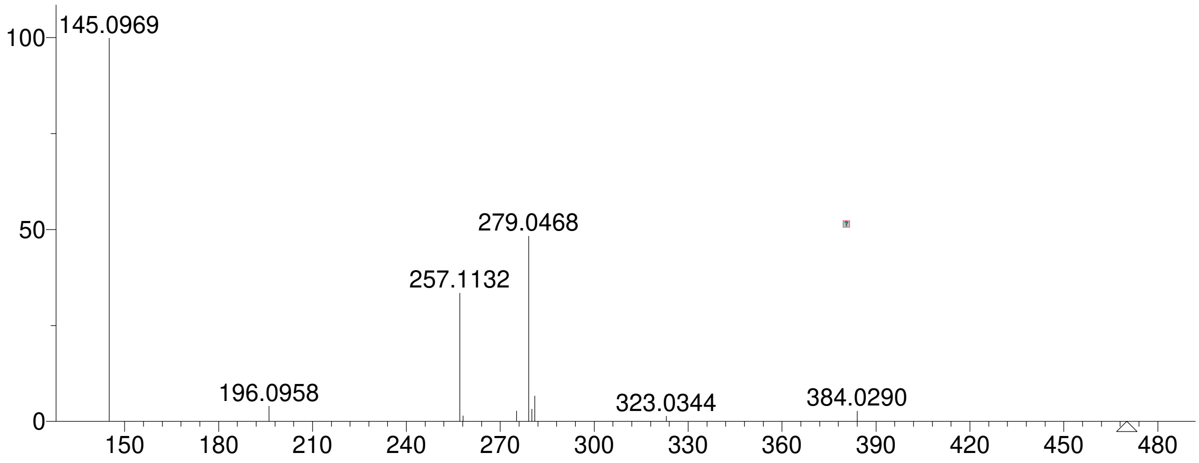

Unknown: Unknown (1.71_487.2020m/z)
Compound in Library Factor = 0

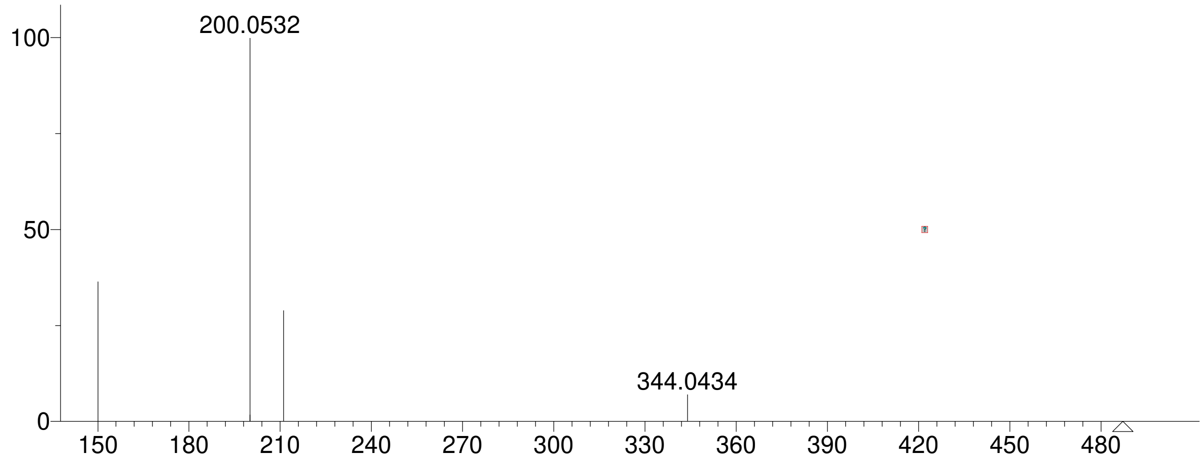

Unknown: Unknown (1.71_825.2510m/z)
Compound in Library Factor = 0

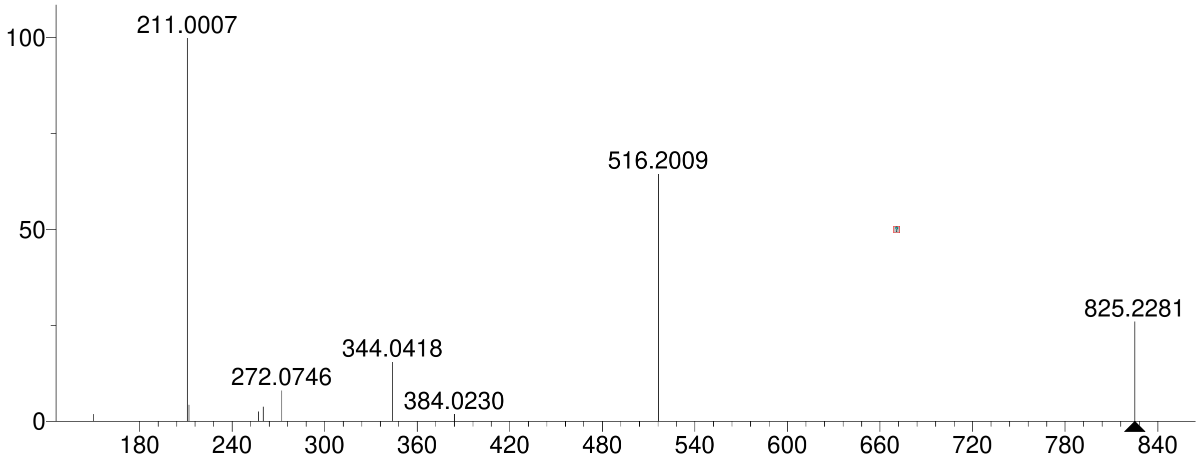

** Search Report Page 1 of 1 **

Unknown: Unknown (1.71_856.3216m/z)
Compound in Library Factor = 0

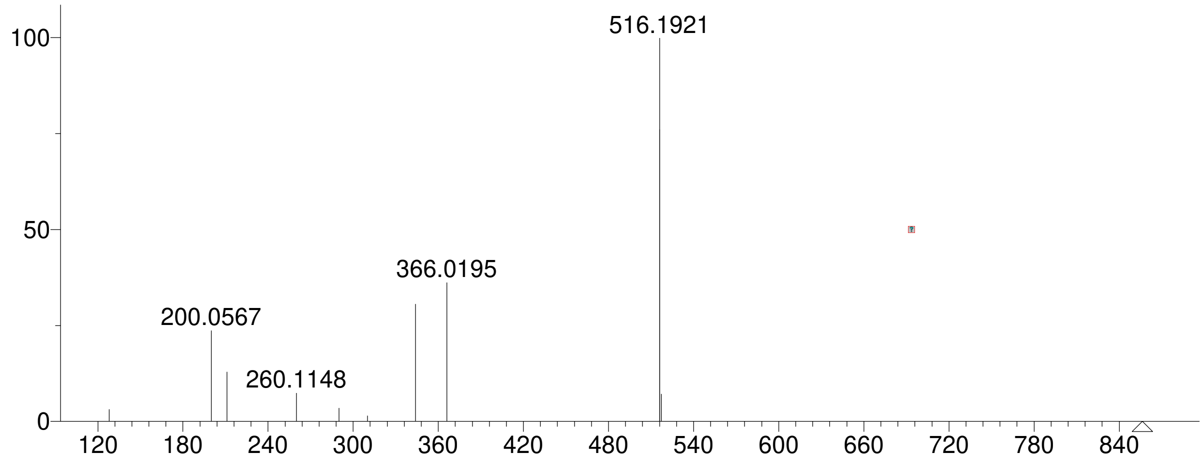

Unknown: Unknown (1.72_408.0872m/z)
Compound in Library Factor = 0

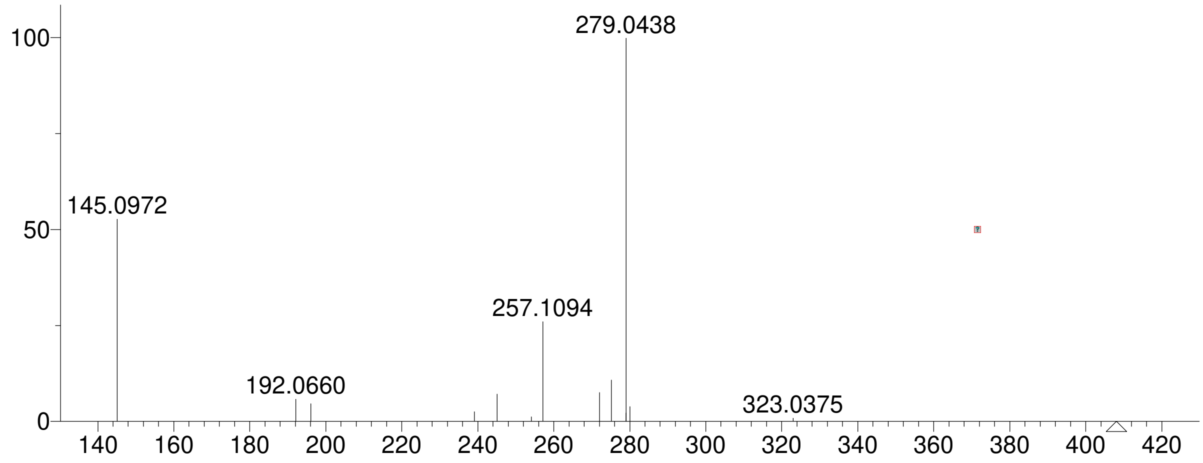

Unknown: Unknown (1.74_433.1089m/z)
Compound in Library Factor = -3273

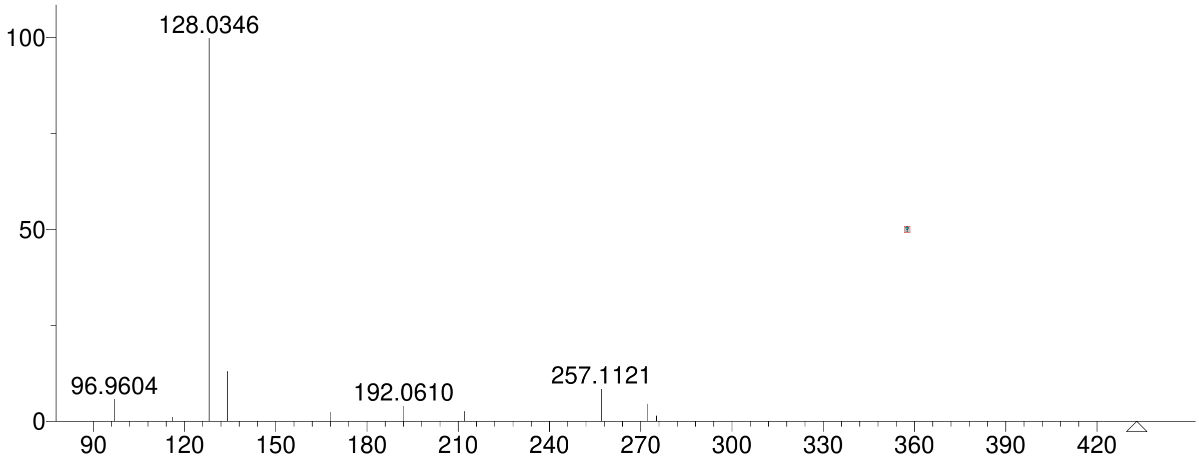

Hit 1 : Pelargonidin 3-glucoside cation [Cat]+ QTOF 23V P=433.1
C21H21O10; MF: 18; RMF: 318; Prob 5.33%; CAS: 47684-27-5; Lib: nist_msms; ID: 71646.

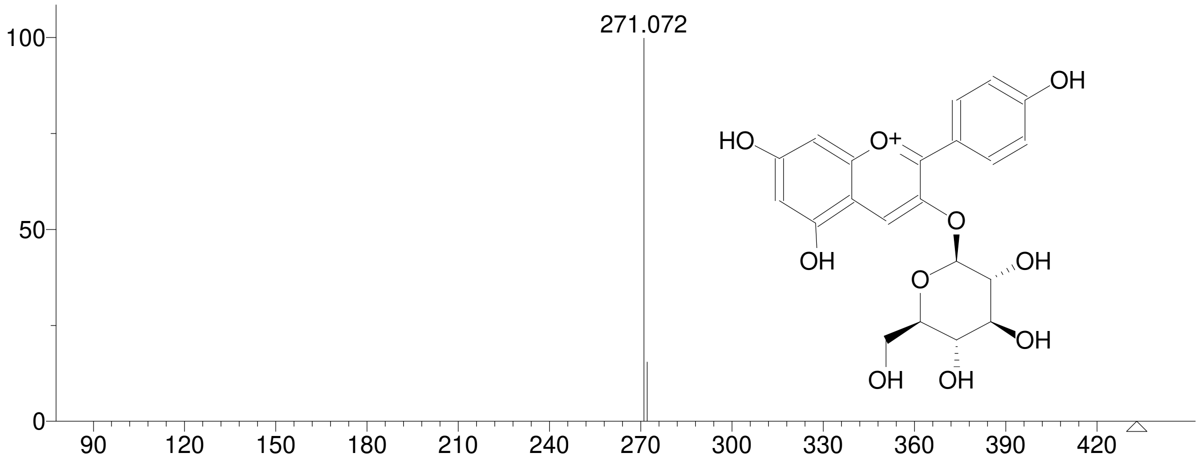

Hit 2 : Apigenin-7-O-glucoside [M+H]+ 30eV P=433.1
C21H20O10; MF: 15; RMF: 342; Prob 4.71%; Lib: anthocyanins mona; ID: 770.

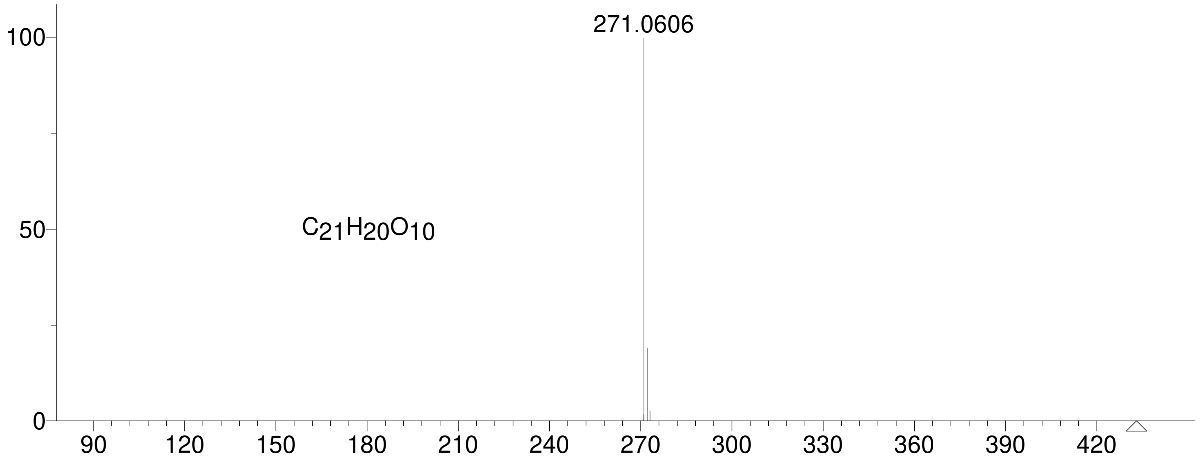

Hit 3 : Pelargonidin-3-O-glucoside [M]+ 30eV P=433.1
C21H21O10; MF: 13; RMF: 341; Prob 4.35%; Lib: anthocyanins mona; ID: 75.

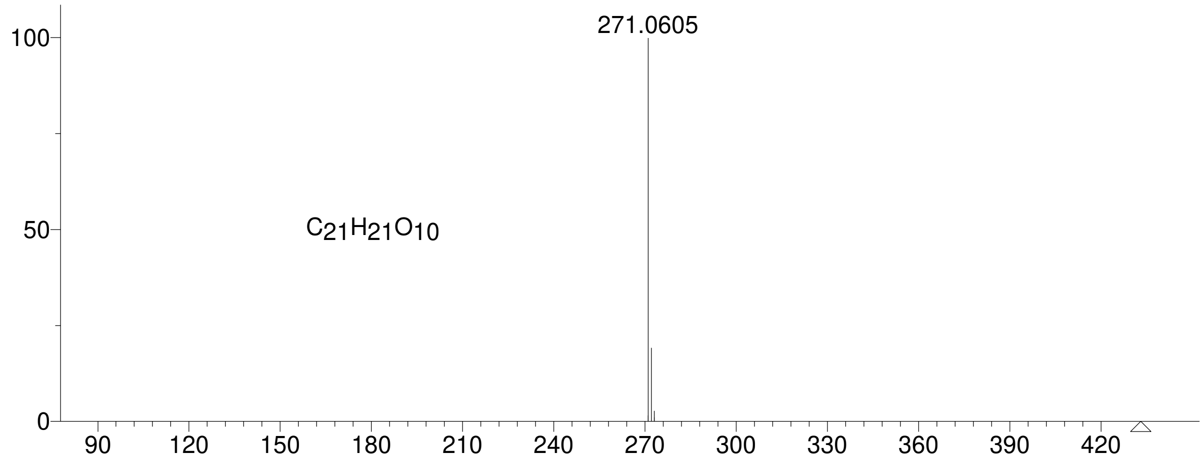

Hit 4 : Prunin [M-H]- P=433.1
C21H22O10; MF: 2; RMF: 166; Prob 2.98%; Lib: mona-export-lc-ms-ms_negative_mode; ID: 9036.

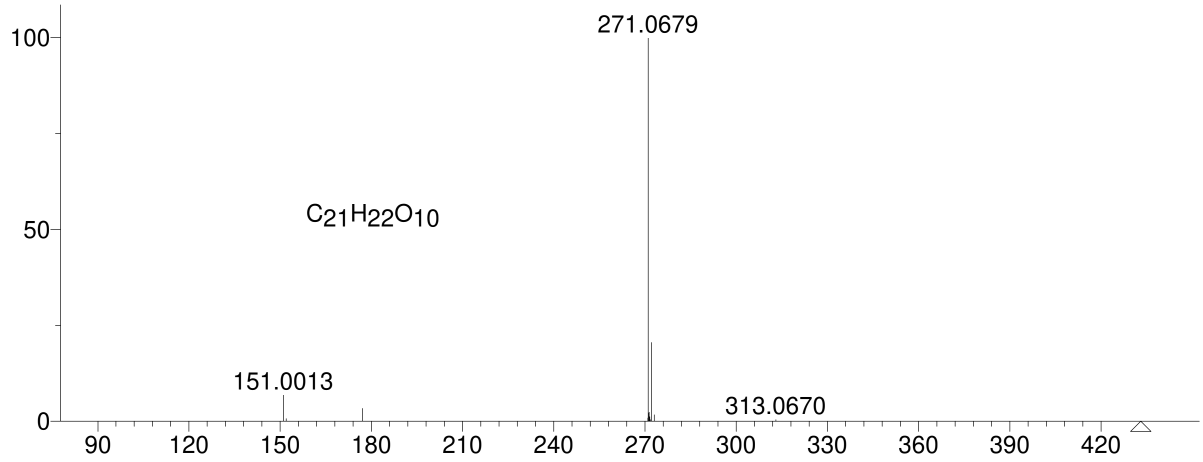

Hit 5 : α-Cypermethrin [M+NH4]+ HCD 64V P=433.1
C22H19Cl2NO3; MF: 0; RMF: 106; Prob 2.75%; CAS: 67375-30-8; Lib: nist_msms; ID: 474529.

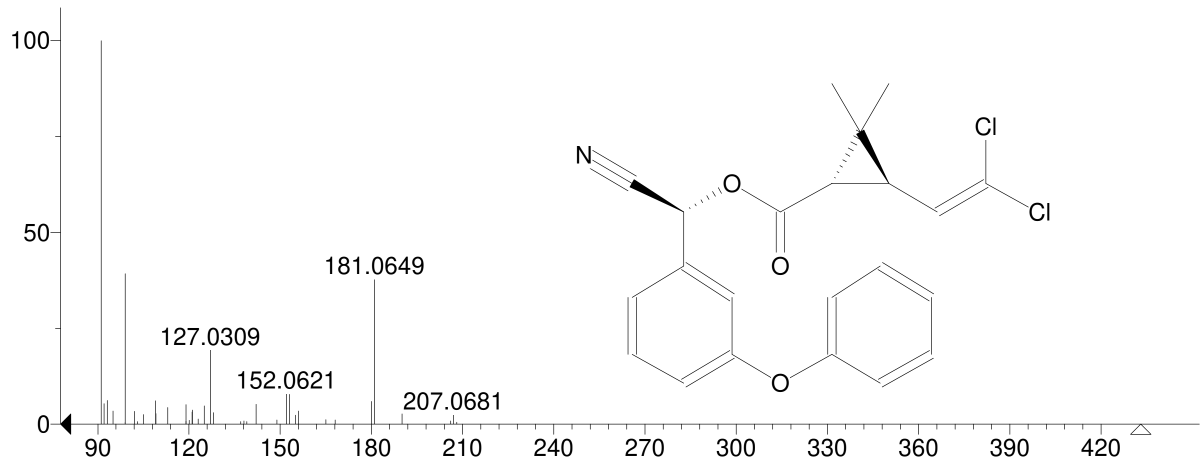

Unknown: Unknown (1.76_275.0865m/z)
Compound in Library Factor = 0

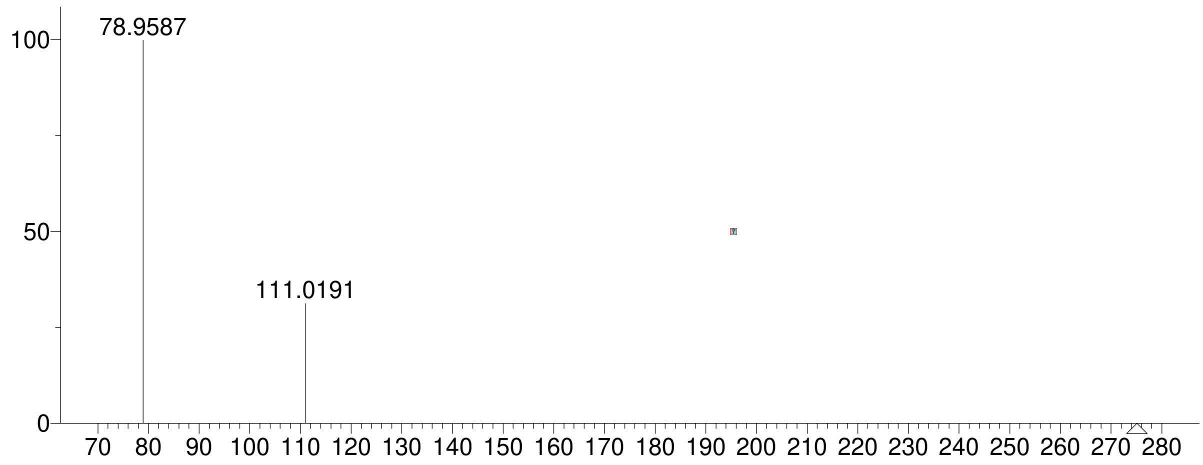

** Search Report Page 1 of 1 **

Unknown: Unknown (1.77_257.1132m/z)
Compound in Library Factor = -1523

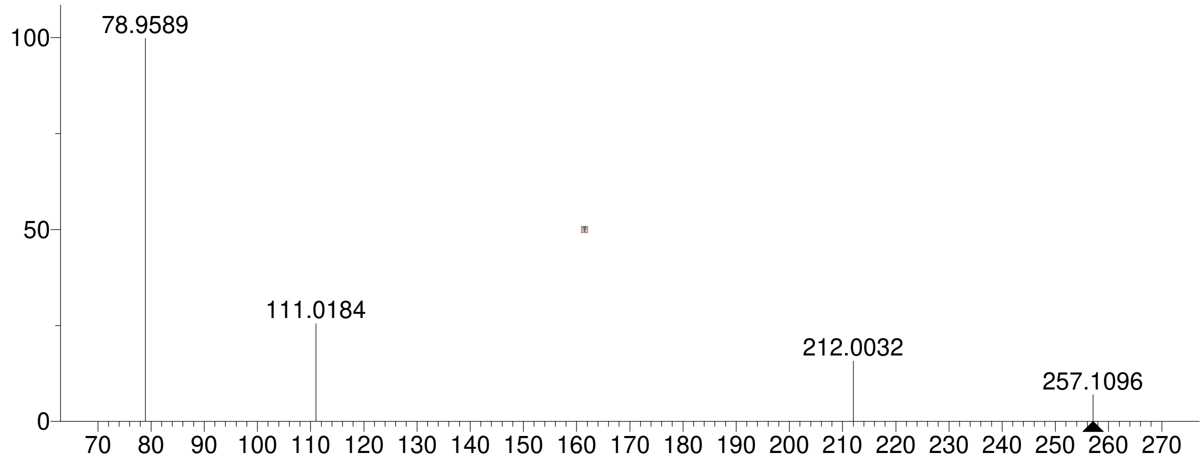

Hit 1 : α-Cyclohexylmandelic acid [M+Na]+ HCD 1V P=257.1
C14H18O3; MF: 65; RMF: 520; Prob 82.2%; CAS: 4335-77-7; Lib: nist_msms; ID: 492969.

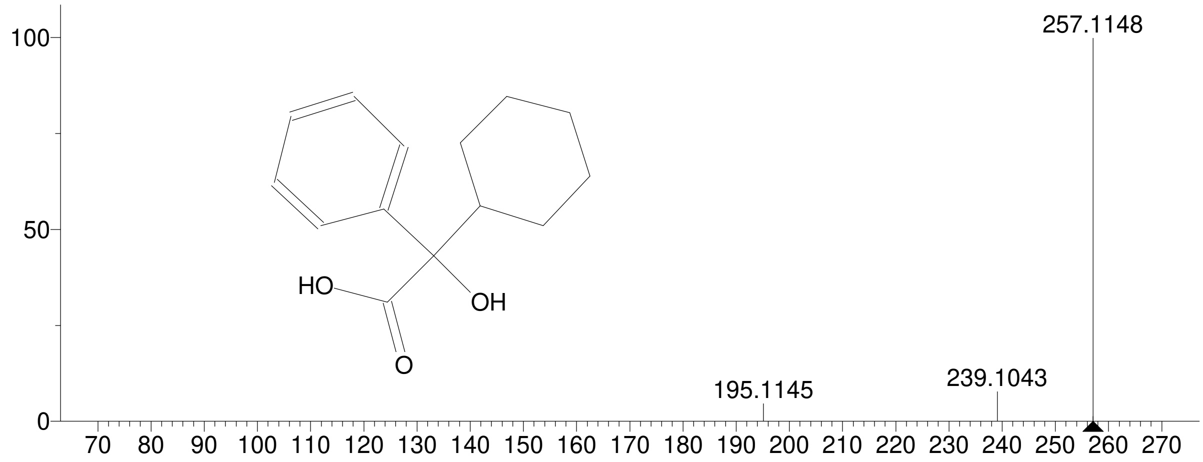

Hit 2 : L-Saccharopine [M-H-H2O]- HCD 16V P=257.1
C11H20N2O6; MF: 21; RMF: 365; Prob 17.8%; CAS: 997-68-2; Lib: nist_msms; ID: 125635.

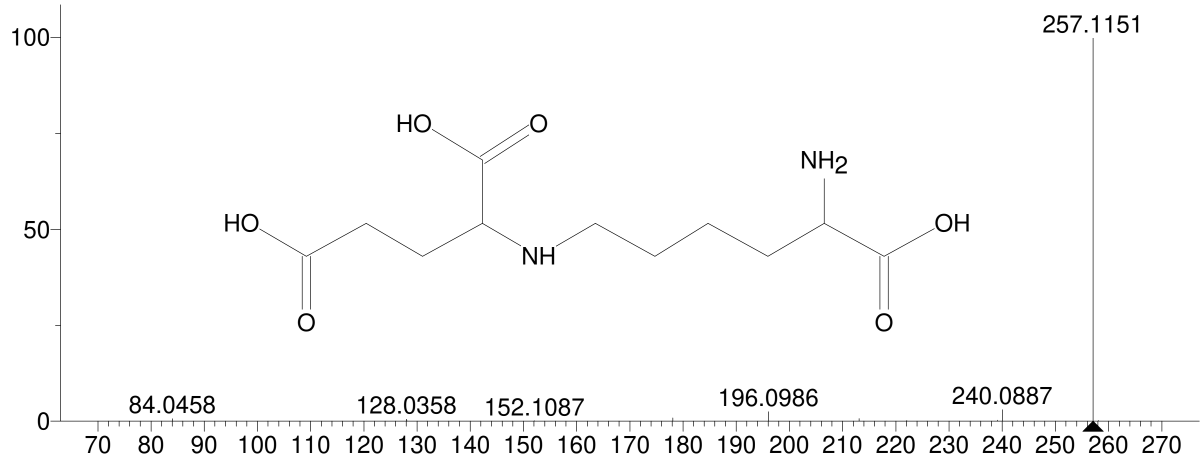

Unknown: Unknown (1.77_323.0280m/z)
Compound in Library Factor = -691

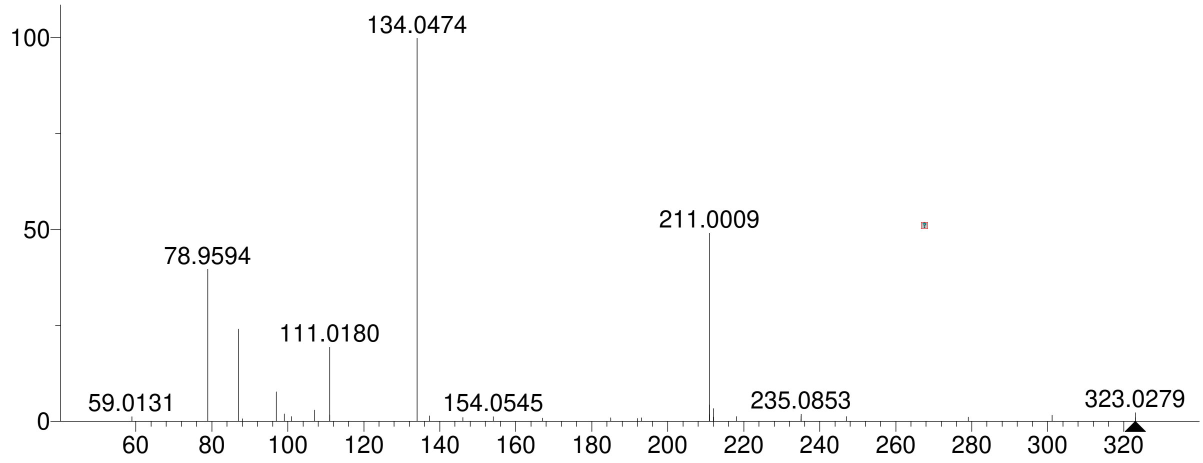

Hit 1 : 1-β-D-Arabinofuranosyluracil 5'-monophosphate [M-H]- HCD 29V P=323
C9H13N2O9P; MF: 303; RMF: 557; Prob 64.3%; CAS: 18354-06-8; Lib: nist_msms; ID: 447571.

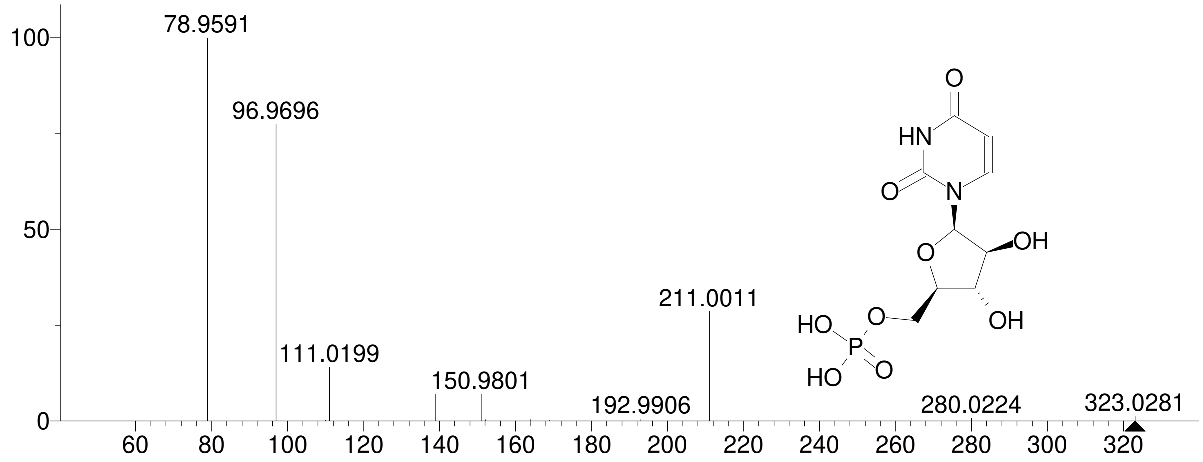

Hit 2 : Uridine 5'-monophosphate [M-H]- 20-30-40%) P=323
C9H13N2O9P; MF: 262; RMF: 514; Prob 14.7%; Lib: mona-export-fiehn_hilic; ID: 1559.

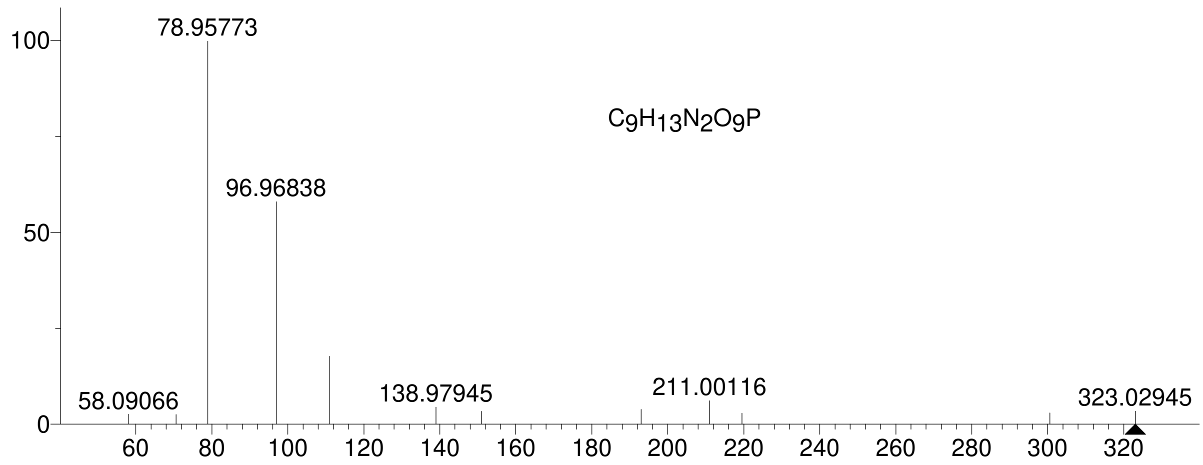

Hit 3 : URIDINE-5-MONOPHOSPHATE
C9H13N2O9P; MF: 248; RMF: 513; Prob 9.18%; Lib: embl neg mcg; ID: 631.

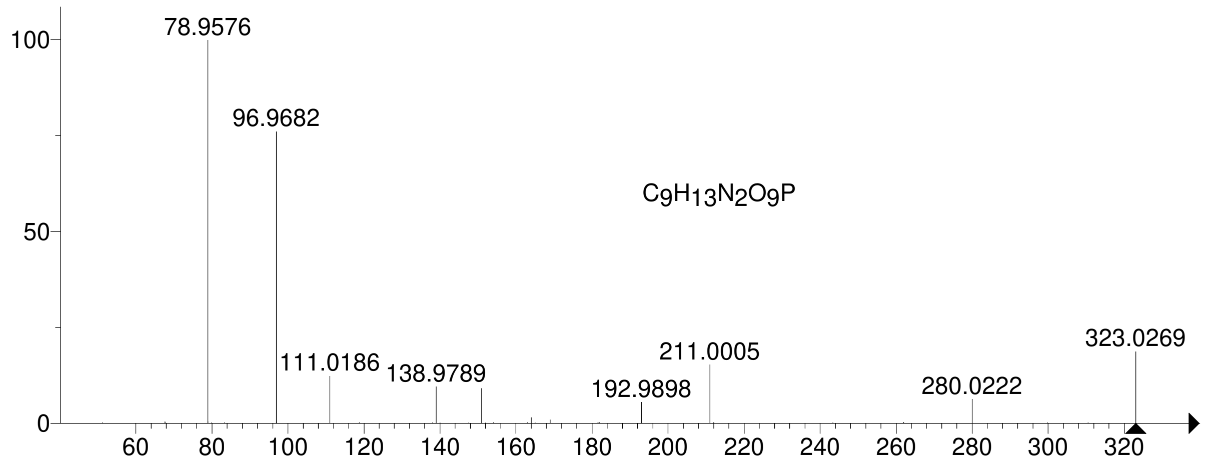

Hit 4 : URIDINE-5-MONOPHOSPHATE [M-H]- P=323
C9H13N2O9P; MF: 239; RMF: 508; Prob 6.66%; Lib: mona-export-gnps; ID: 5550.

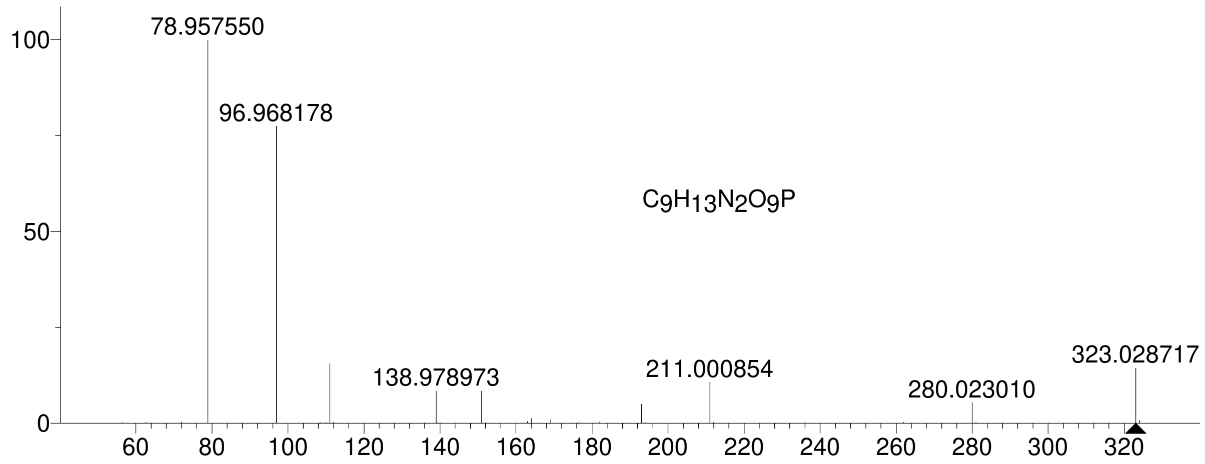

Hit 5 : Uridine 5'-diphosphoglucuronic acid [M-H-C6H9O9P]- HCD 22V P=323
C15H22N2O18P2; MF: 229; RMF: 510; Prob 4.70%; CAS: 2616-64-0; Lib: nist_msms; ID: 486422.

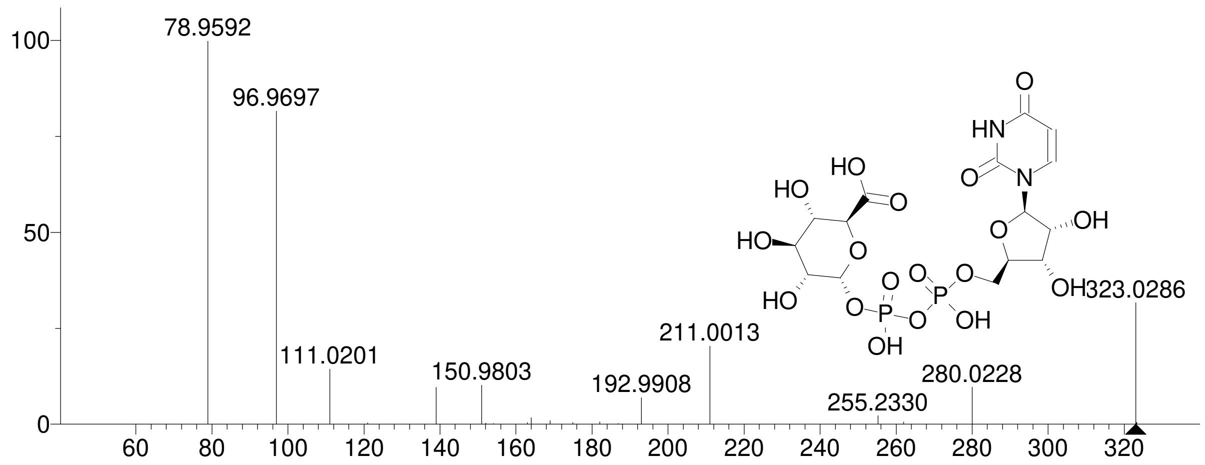

Unknown: Unknown (1.78_384.1511m/z)
Compound in Library Factor = -2176

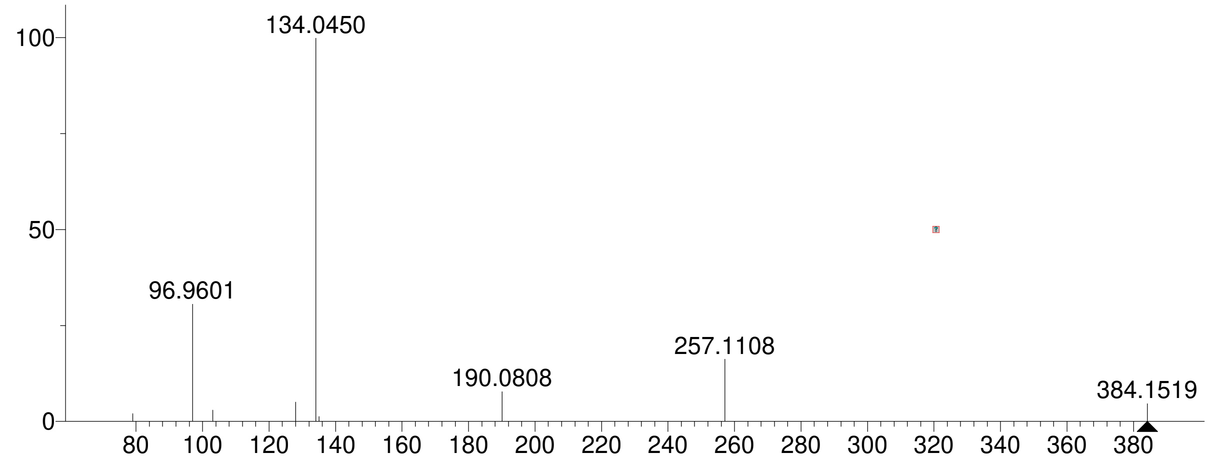

Hit 1 : 2-Acetamido-2-deoxy-3-O-(β-D-galactopyranosyl)-D-glucopyranose [M+H]+ HCD 1V P=384.2
C14H25NO11; MF: 75; RMF: 467; Prob 61.8%; CAS: 489-52-1; Lib: nist_msms; ID: 534139.

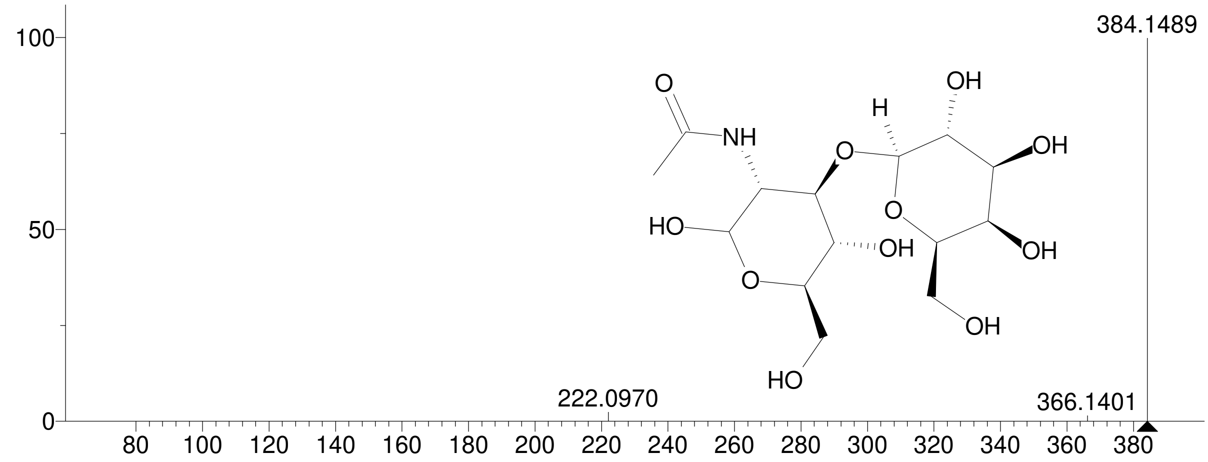

Hit 2 : N-Acetyl-D-lactosamine [M+H]+ HCD 3V P=384.2
C14H25NO11; MF: 48; RMF: 441; Prob 18.2%; CAS: 32181-59-2; Lib: nist_msms; ID: 478584.

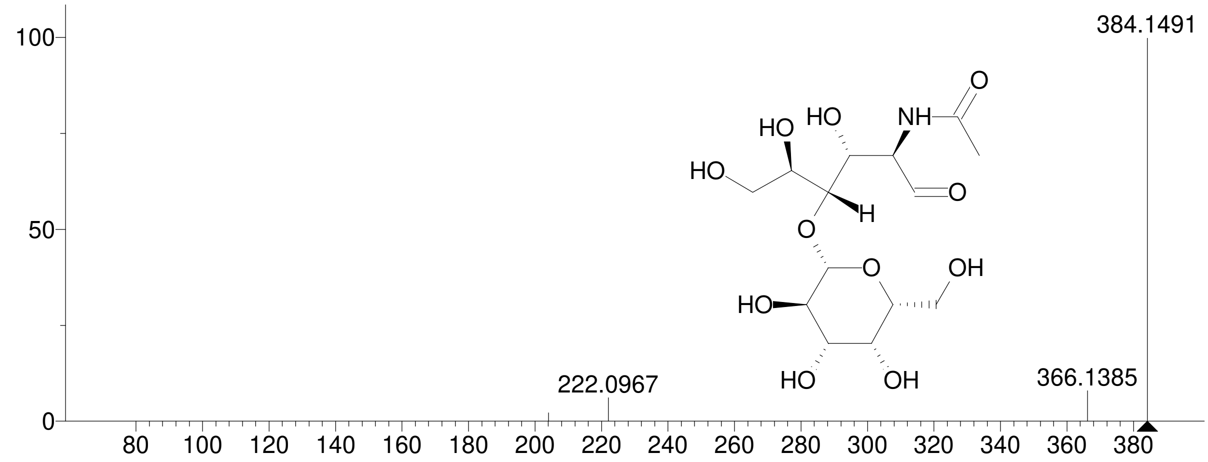

Hit 3 : Tolfenpyrad [M+H]+ HCD 17V P=384.1
C21H22ClN3O2; MF: 28; RMF: 372; Prob 8.28%; CAS: 129558-76-5; Lib: nist_msms; ID: 541788.

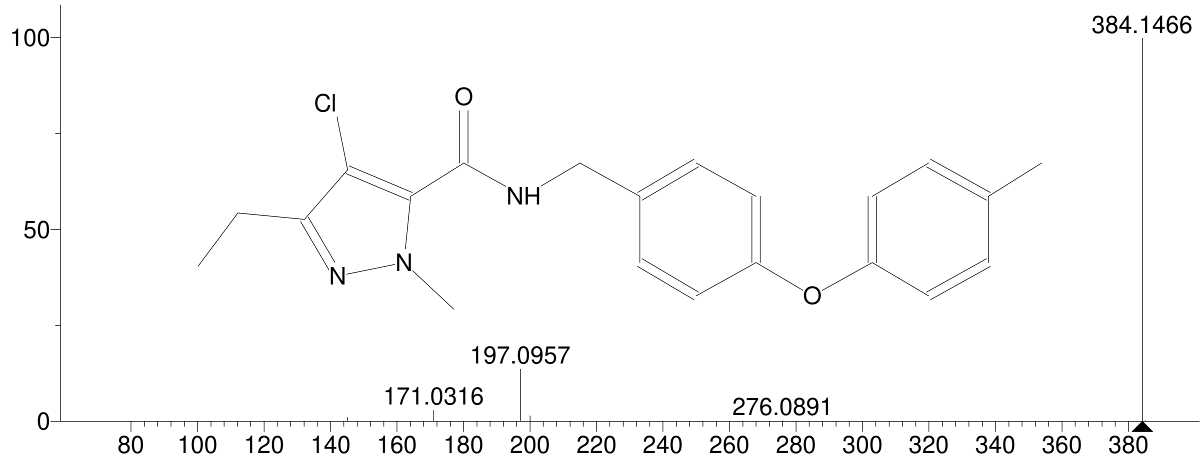

Hit 4 : 3'-α-Sialyl-N-acetyllactosamine [M+H-C11H17O8N]+ HCD 7V P=384.2
C25H42N2O19; MF: 3; RMF: 138; Prob 2.52%; CAS: 81693-22-3; Lib: nist_msms; ID: 211442.

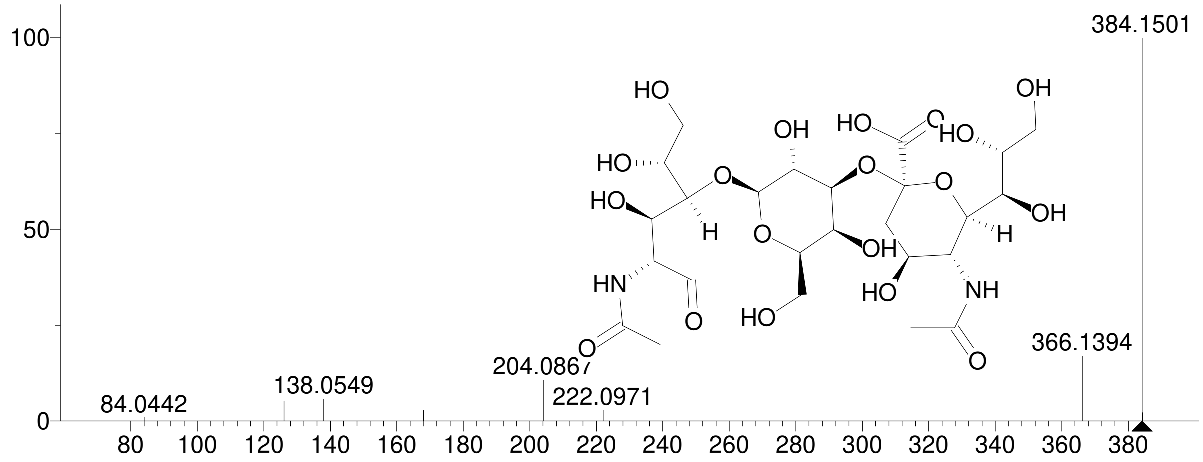

Hit 5 : Lewis X trisaccharide [M+H-C6H10O4]+ HCD 7V P=384.2
C20H35NO15; MF: 2; RMF: 132; Prob 2.42%; CAS: 71208-06-5; Lib: nist_msms; ID: 211230.

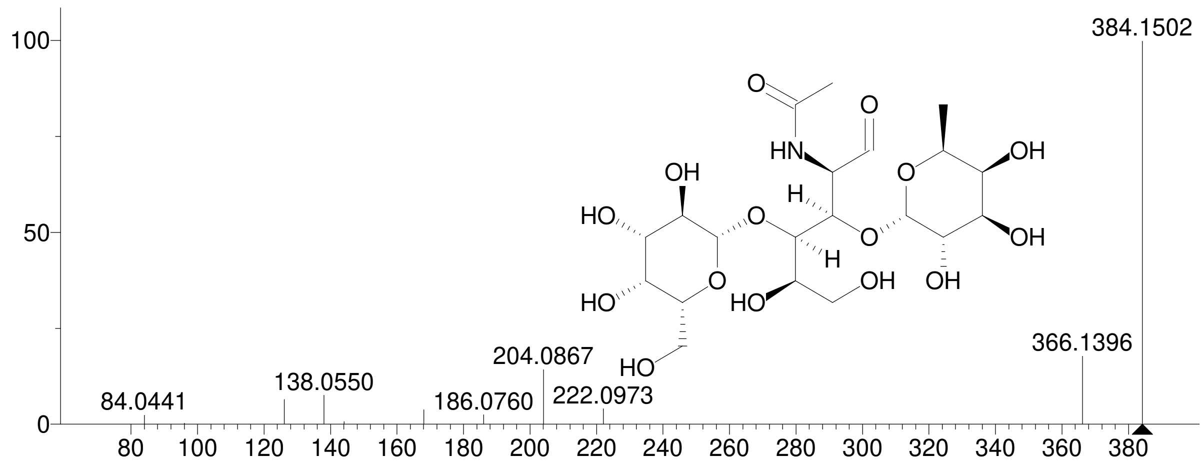

Unknown: Unknown (1.79_383.1136m/z)
Compound in Library Factor = -542

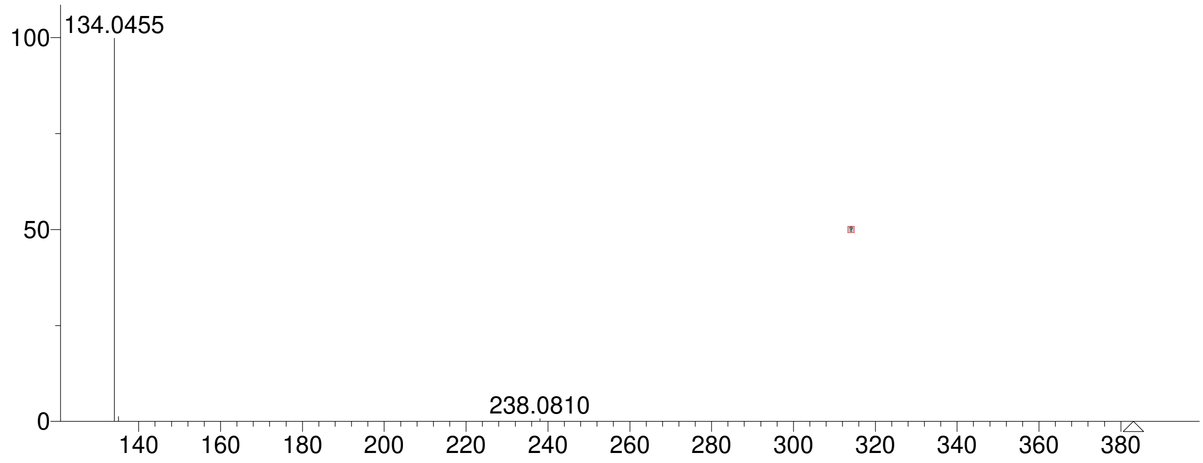

Hit 1 : S-Adenosylhomocysteine [M-H]- 40 P=383.1
C14H20N6O5S; MF: 359; RMF: 990; Prob 88.6%; Lib: msms neg mona_ipb_gnps_plasma; ID: 657.

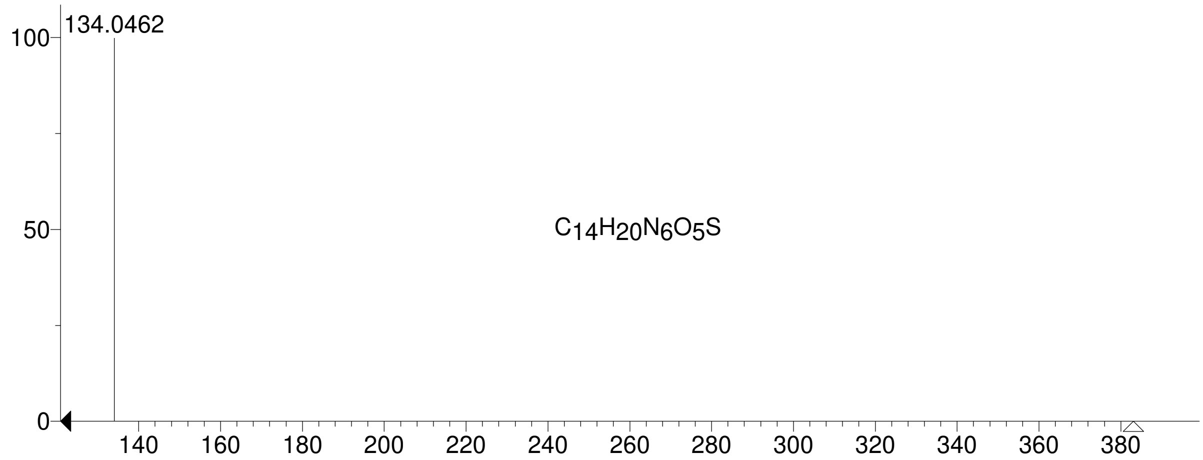

Hit 2 : S-Adenosyl-L-homocysteine [M-H]- 5-60V P=383.1
C14H20N6O5S; MF: 238; RMF: 877; Prob 4.52%; Lib: mona-export-lc-ms-ms_negative_mode; ID: 8476.

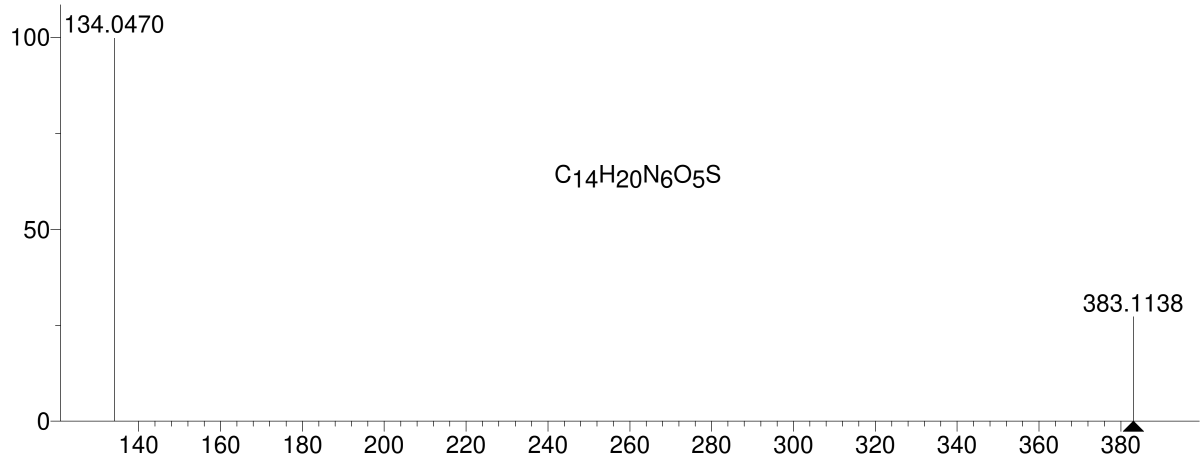

Hit 3 : S-Adenosyl-L-homocysteine; LC-ESI-QTOF; MS2; CE [M-H]- P=383.1
C14H20N6O5S; MF: 238; RMF: 877; Prob 4.52%; Lib: msms-neg-massbank; ID: 4374.

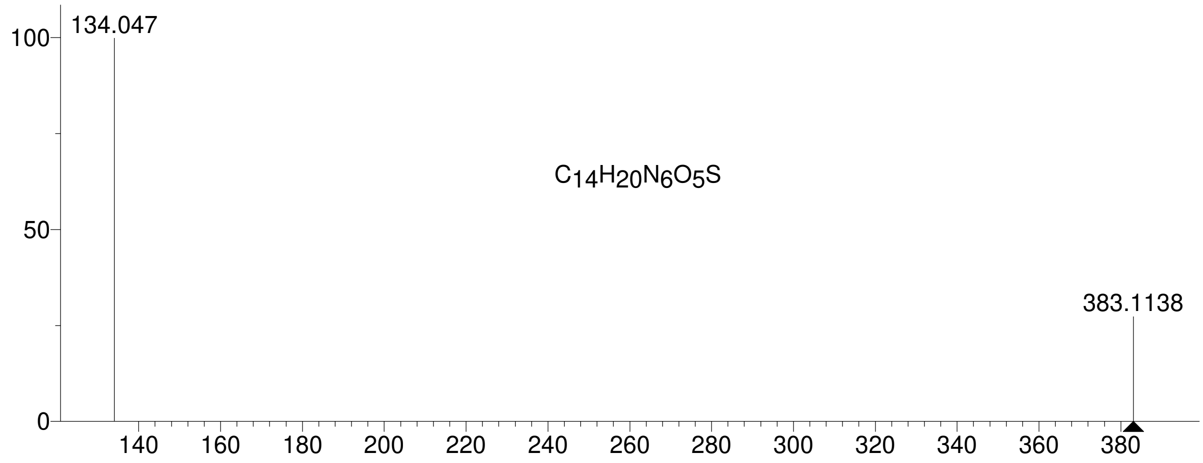

Hit 4 : S-(5#39;-ADENOSYL)-L-HOMOCYSTEINE
C14H20N6O5S; MF: 185; RMF: 936; Prob 0.89%; Lib: embl neg mcg; ID: 301.

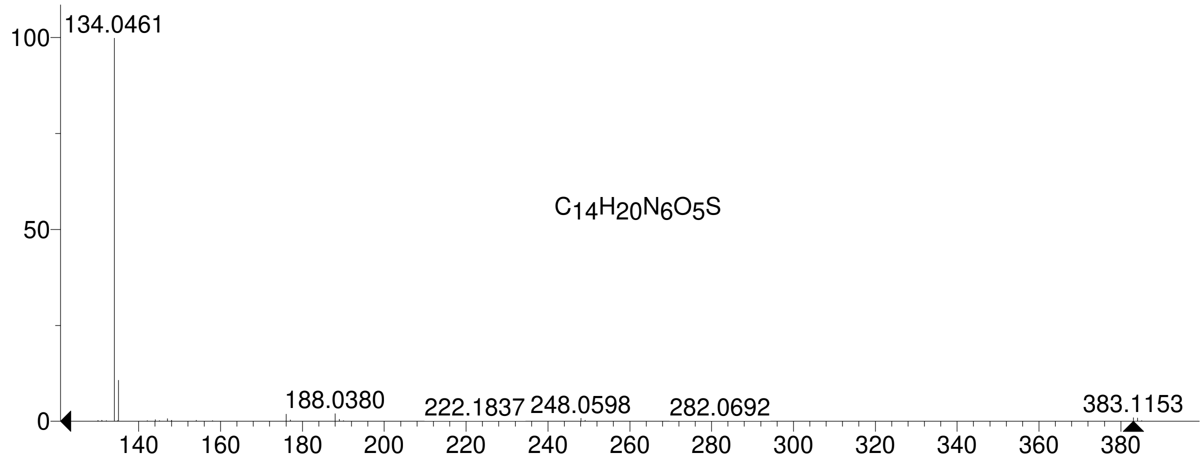

Hit 5 : S-Adenosyl-homocysteine [M-H]- 20-30-40%) P=383.1
C14H20N6O5S; MF: 182; RMF: 861; Prob 0.79%; Lib: msms-neg-fiehnhilic; ID: 626.

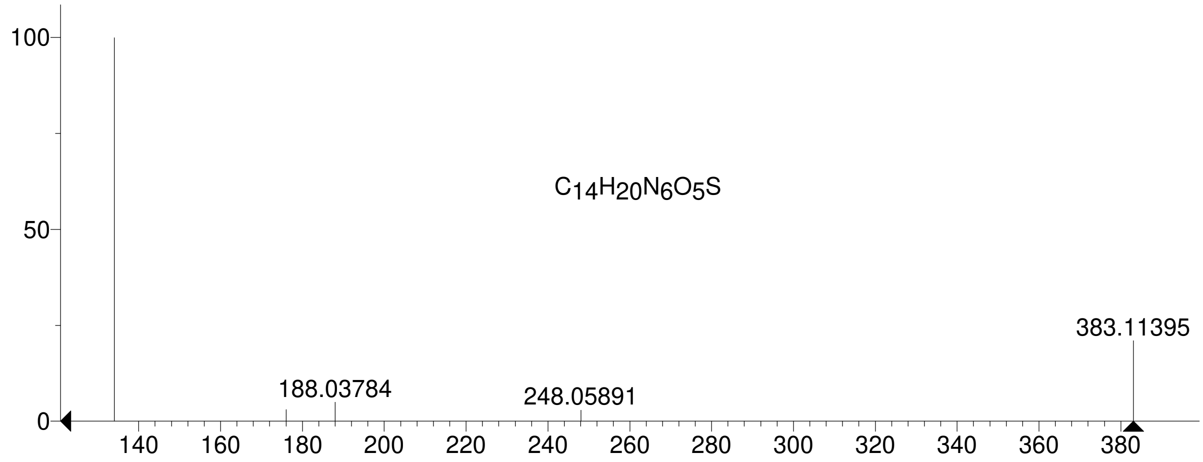

Unknown: Unknown (1.79_476.1513m/z)
Compound in Library Factor = 0

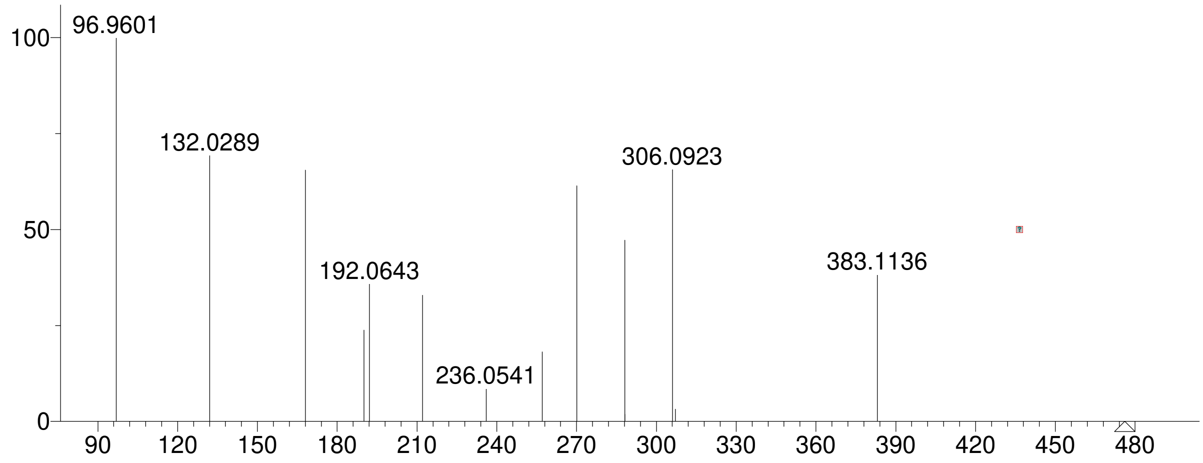

Hit 1 : NCGC00381039-01_C22H25NO8_8-Benzoyl-2-[(3Z)-1,2-dihydroxy-3-hexen-1-yl]-9-hydroxy-8-methoxy-3-r
C22H25NO8; MF: 0; RMF: 106; Prob 50.0%; Lib: mona-export-gnps; ID: 13610.

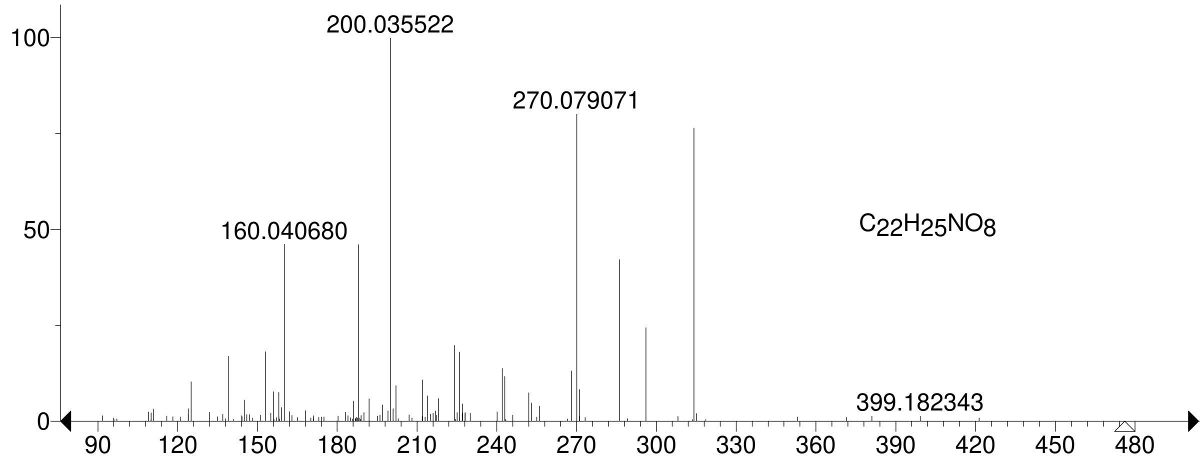

Hit 2 : NCGC00380982-01_C22H25NO8_8-Benzoyl-2-[(3E)-1,2-dihydroxy-3-hexen-1-yl]-9-hydroxy-8-methoxy-3-r
C22H25NO8; MF: 0; RMF: 94; Prob 50.0%; Lib: mona-export-gnps; ID: 12862.

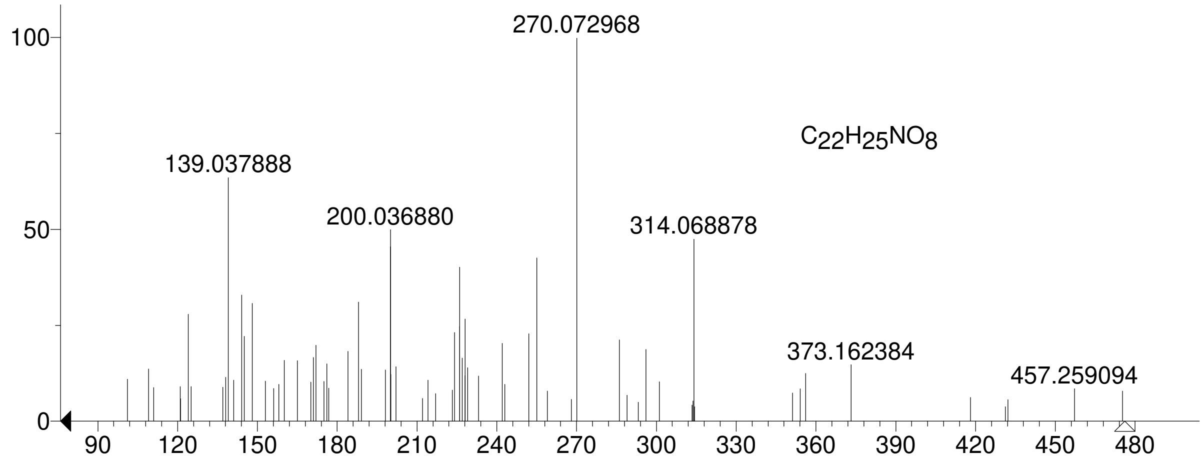

Unknown: Unknown (1.80_257.0768m/z)
Compound in Library Factor = -198

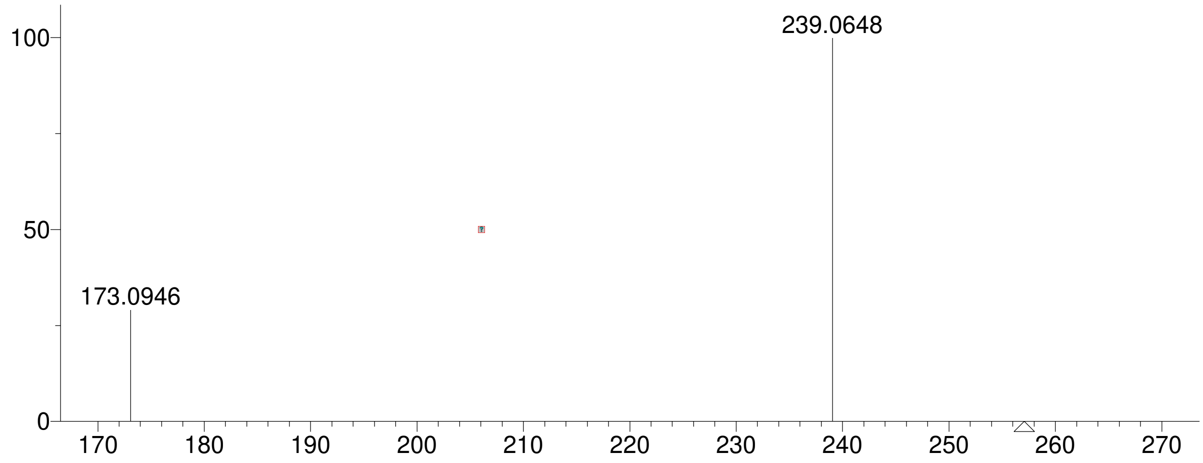

Hit 1 : L-Pyroglutamic acid [2M-H]- IT-FT 35% P=257.1
C5H7NO3; MF: 312; RMF: 880; Prob 99.0%; CAS: 98-79-3; Lib: nist_msms; ID: 296512.

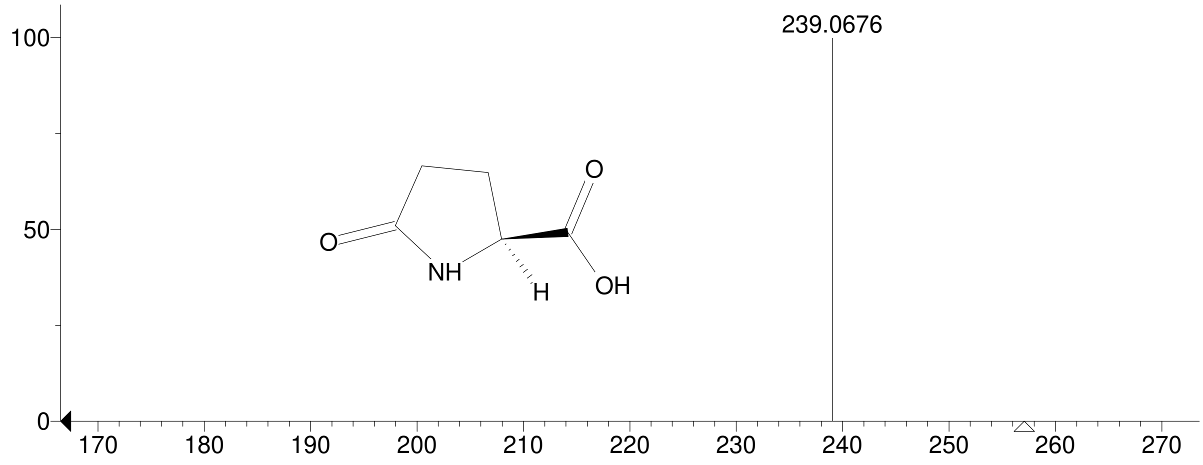

Hit 2 : L-γ-Glutamyl-L-glutamic acid [M-H-H2O]- HCD 16V P=257.1
C10H16N2O7; MF: 25; RMF: 558; Prob 0.99%; CAS: 1116-22-9; Lib: nist_msms; ID: 137327.

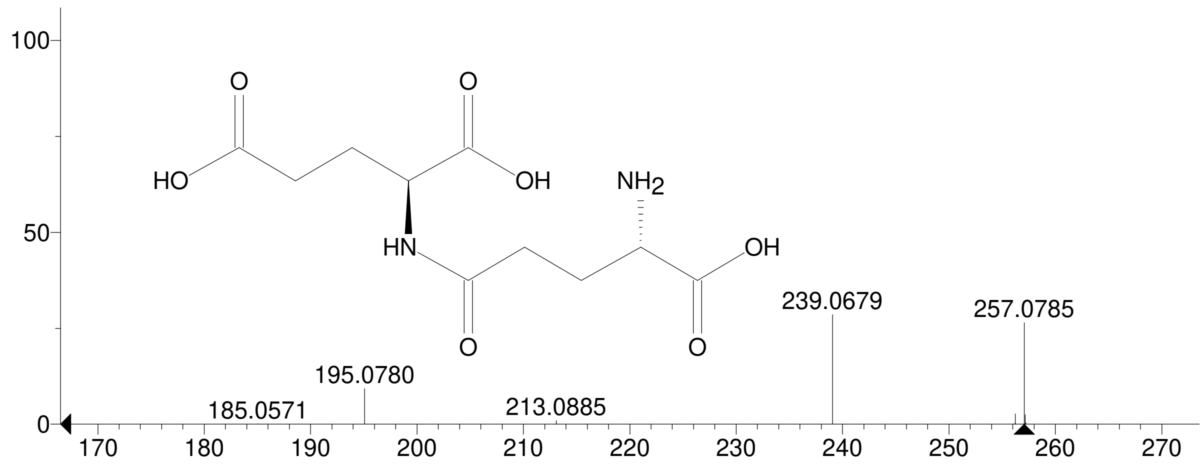

Unknown: Unknown (1.80_332.1452m/z)
Compound in Library Factor = 0

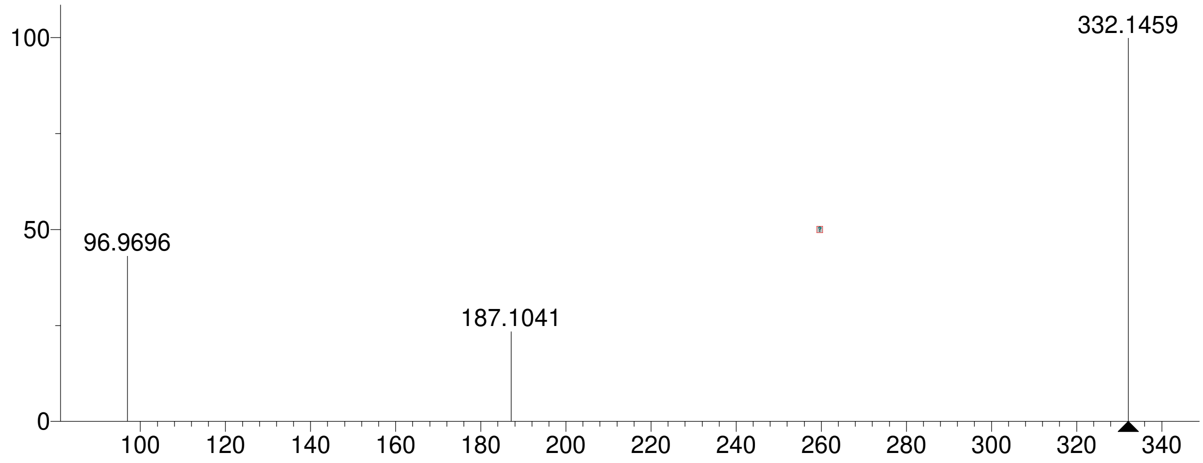

Unknown: Unknown (1.80_419.1277m/z)
Compound in Library Factor = 0

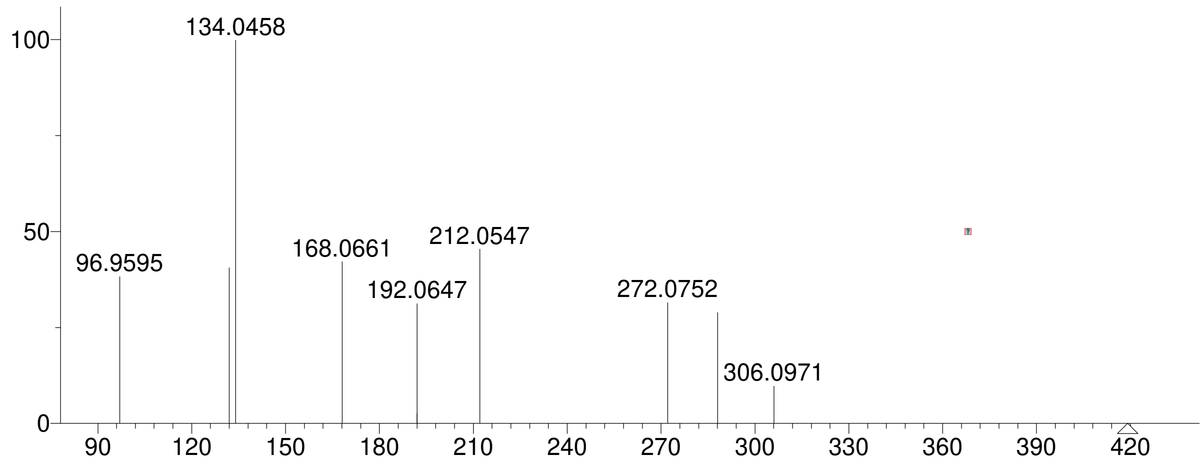

Unknown: Unknown (1.82_420.2337m/z)
Compound in Library Factor = 0

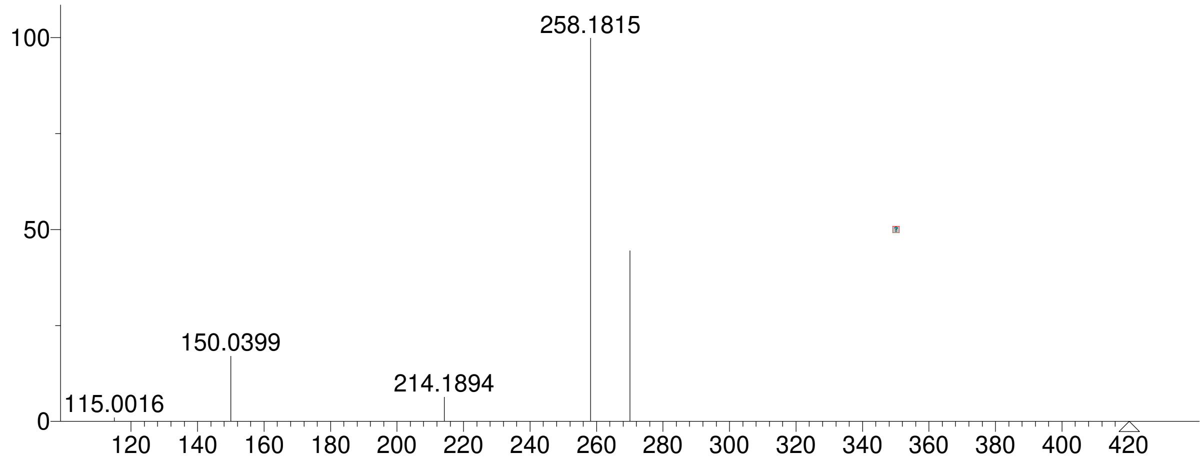

Unknown: Unknown (1.86_275.0511m/z)
Compound in Library Factor = 0

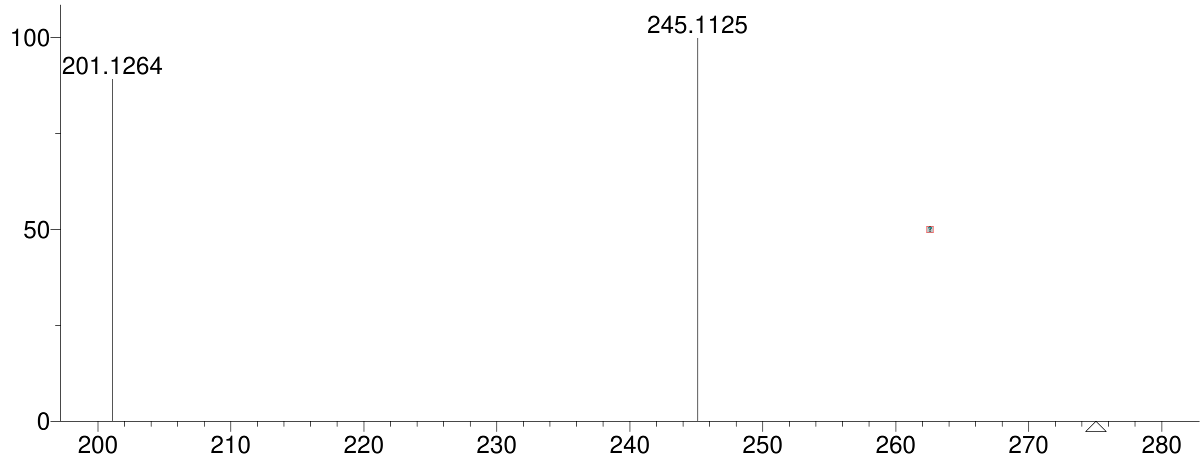

Hit 1 : 1,2,3-Benzenetricarboxylic acid, trimethyl ester [M+Na]+ IT 35% P=275.1
C12H12O6; MF: 0; RMF: 97; Prob 100.0%; CAS: 2672-57-3; Lib: nist_msms; ID: 306560.

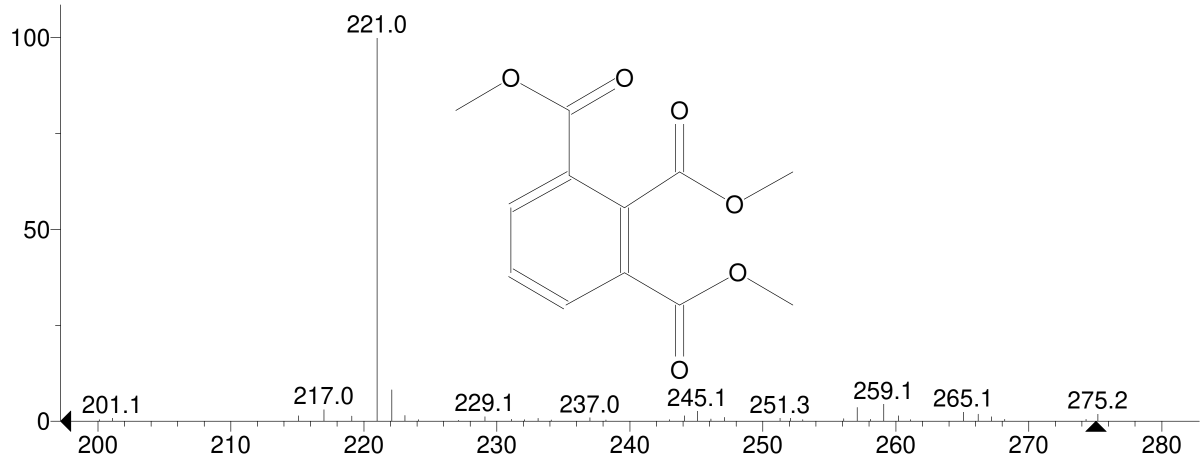

** Search Report Page 1 of 1 **

Unknown: Unknown (1.86_476.1511m/z)
Compound in Library Factor = 0

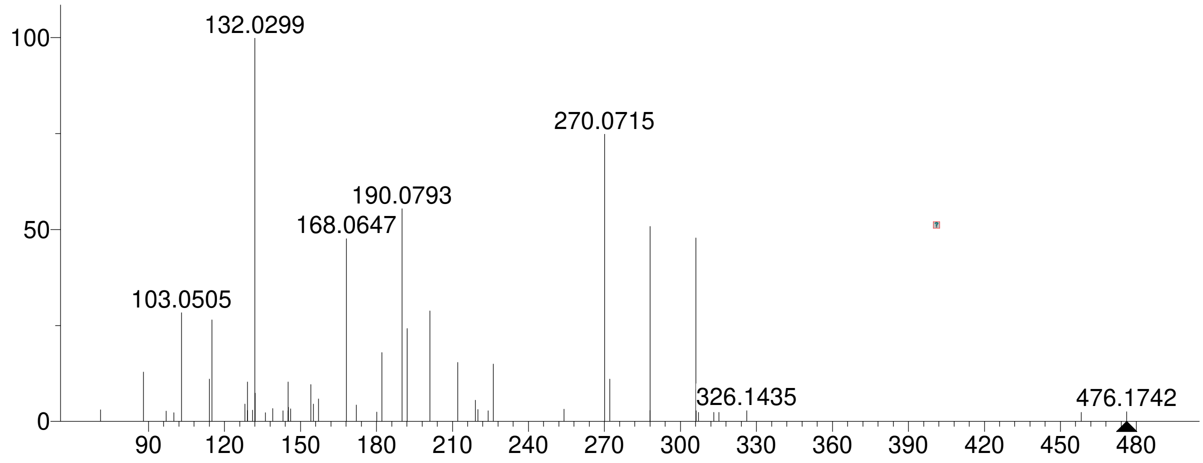

Unknown: Unknown (1.87_243.0614m/z)
Compound in Library Factor = -2387

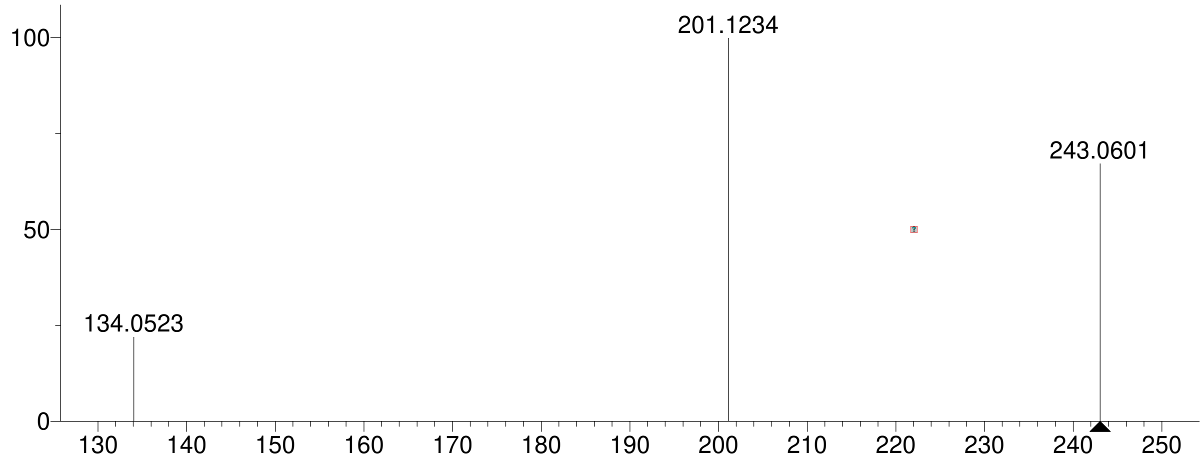

Hit 1 : Prophos [M+H]+ HCD 7V P=243.1
C8H19O2PS2; MF: 34; RMF: 508; Prob 21.1%; CAS: 13194-48-4; Lib: nist_msms; ID: 489673.

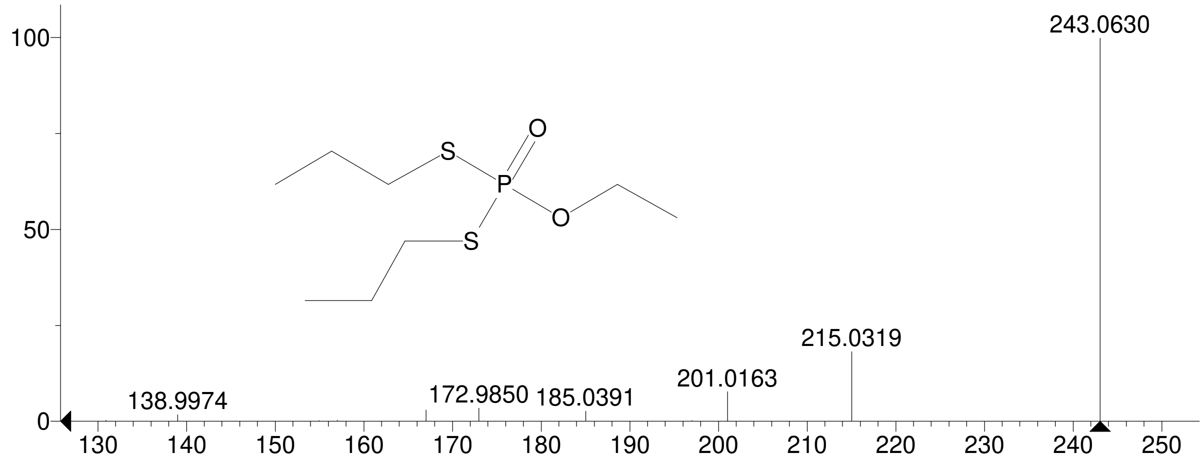

Hit 2 : Uridine [M-H]- 5-60V P=243.1
C9H12N2O6; MF: 19; RMF: 394; Prob 12.8%; Lib: mona-export-lc-ms-ms_negative_mode; ID: 8487.

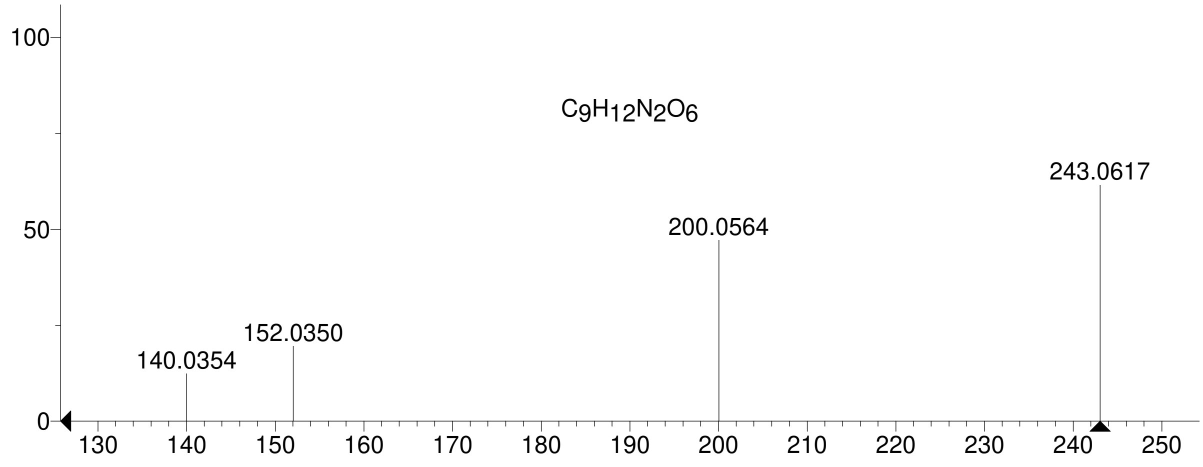

Hit 3 : Uridine; LC-ESI-QTOF; MS2; CE [M-H]- P=243.1
C9H12N2O6; MF: 19; RMF: 394; Prob 12.8%; Lib: msms-neg-massbank; ID: 4384.

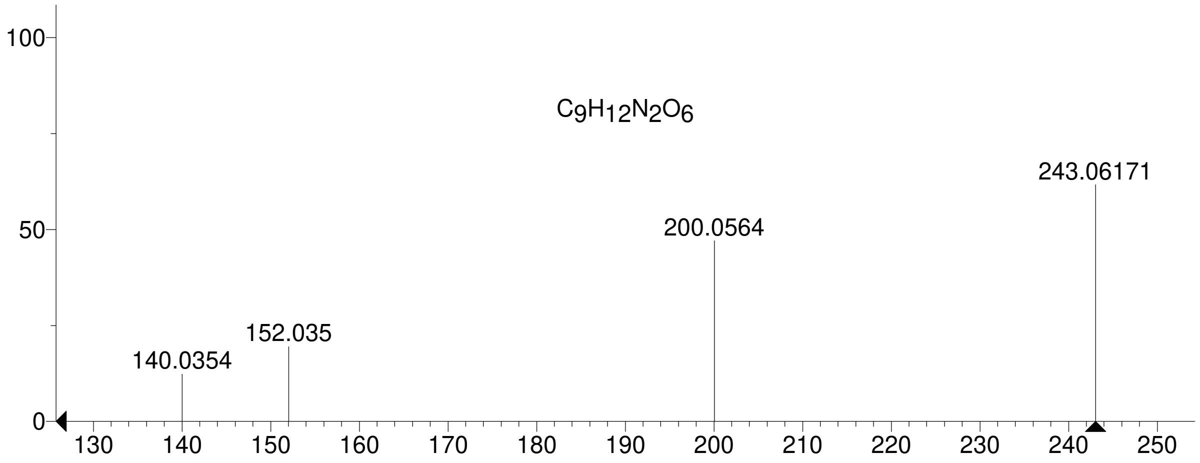

Hit 4 : Uridine (Not validated); PlaSMA ID-371 [M-H]- P=243.1
C9H12N2O6; MF: 8; RMF: 341; Prob 8.78%; Lib: biomsms-neg-plasma; ID: 471.

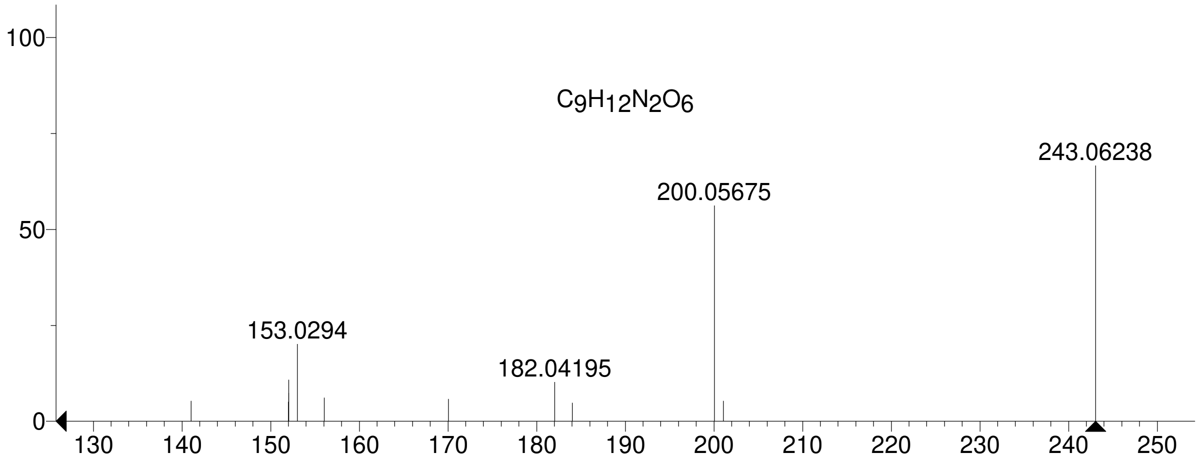

Hit 5 : uridine [M-H]- 50% (P=243.1
C9H12N2O6; MF: 7; RMF: 346; Prob 8.44%; Lib: mona-export-lc-ms-ms_negative_mode; ID: 7486.

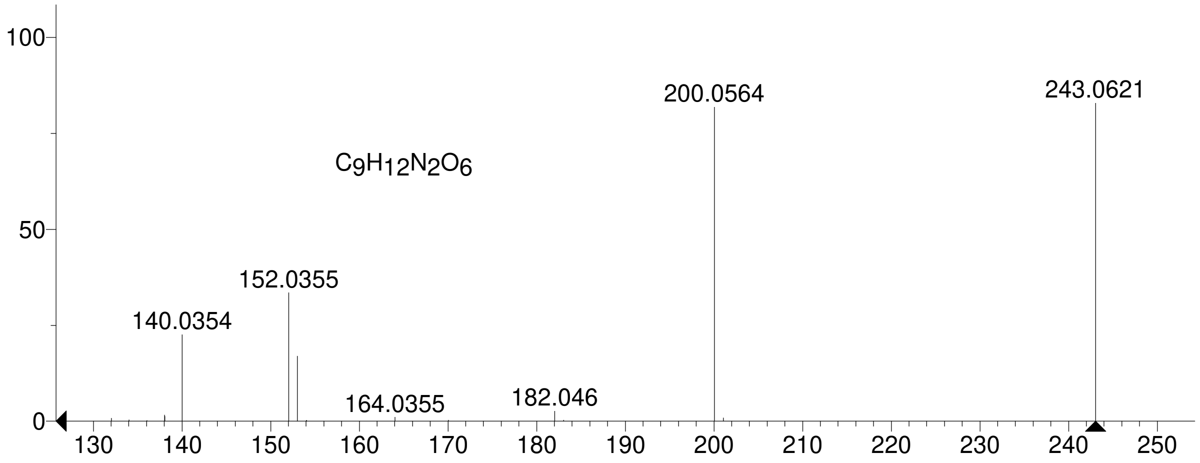

Unknown: Unknown (1.88_245.1134m/z)
Compound in Library Factor = -2387

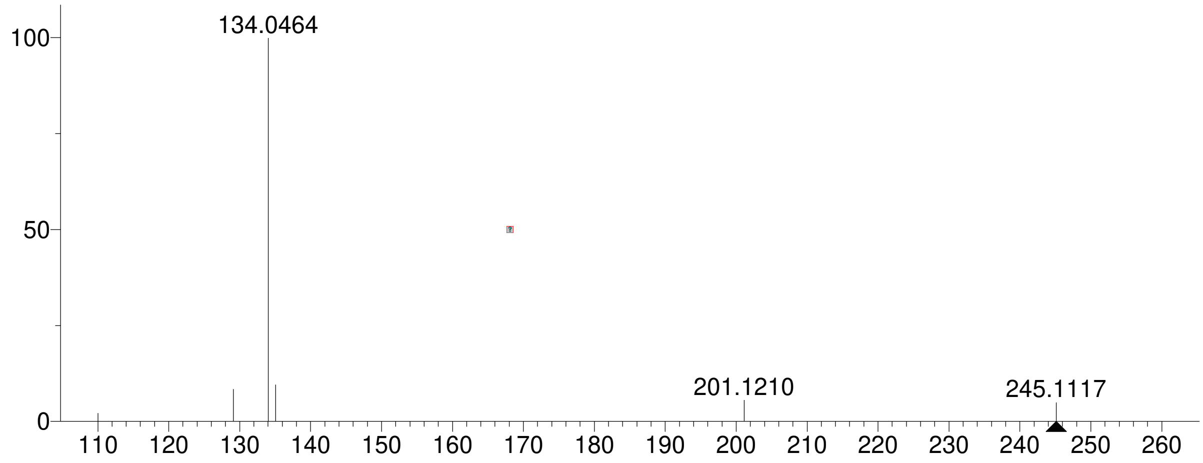

Hit 1 : Ile-Asp [M-H]- HCD 9eV P=245.1
C10H18N2O5; MF: 75; RMF: 247; Prob 17.9%; Lib: nist_msms; ID: 570699.

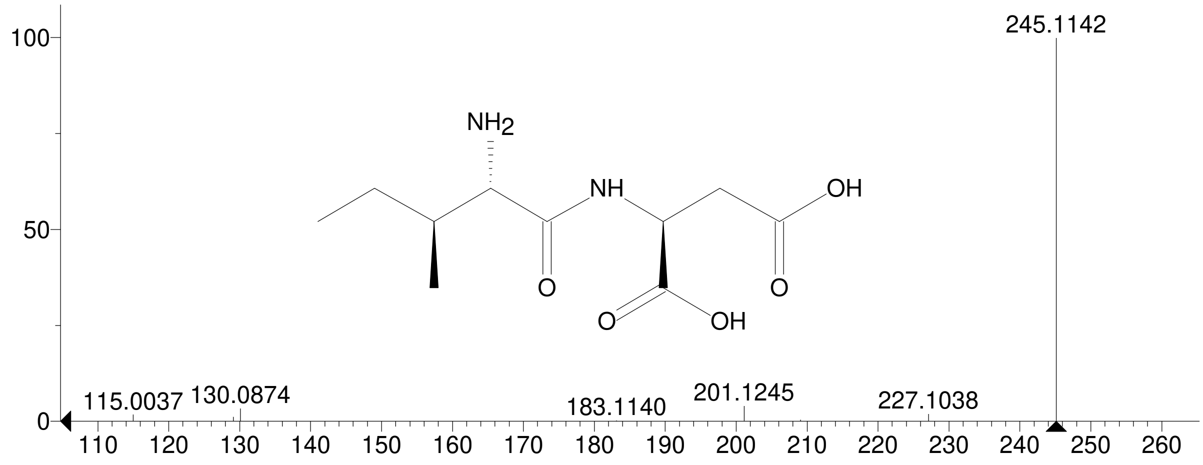

Hit 2 : Ile-Asp [M-H]- HCD 9eV P=245.1
C10H18N2O5; MF: 75; RMF: 247; Prob 17.9%; CAS: 54532-76-2; Lib: nist_msms2; ID: 84432.

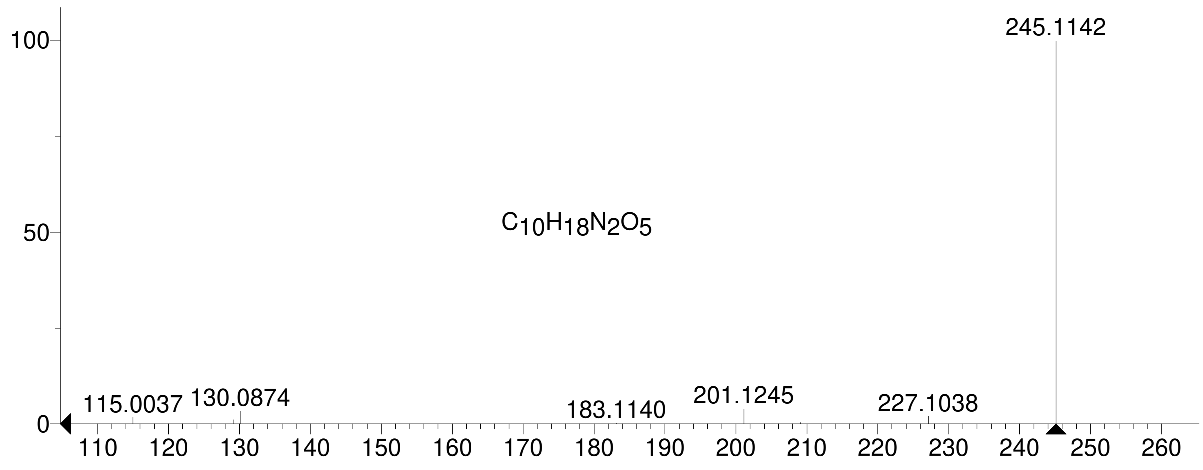

Hit 3 : Leu-Asp [M-H]- HCD 9eV P=245.1
C10H18N2O5; MF: 72; RMF: 245; Prob 15.8%; Lib: nist_msms; ID: 570768.

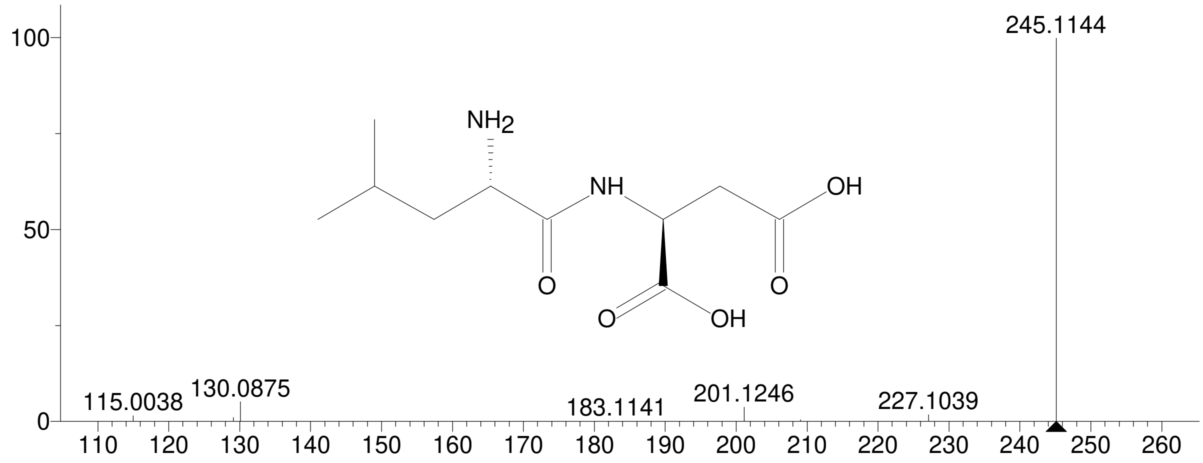

Hit 4 : Leu-Asp [M-H]- HCD 9eV P=245.1
C10H18N2O5; MF: 72; RMF: 245; Prob 15.8%; CAS: 32949-40-9; Lib: nist_msms2; ID: 84501.

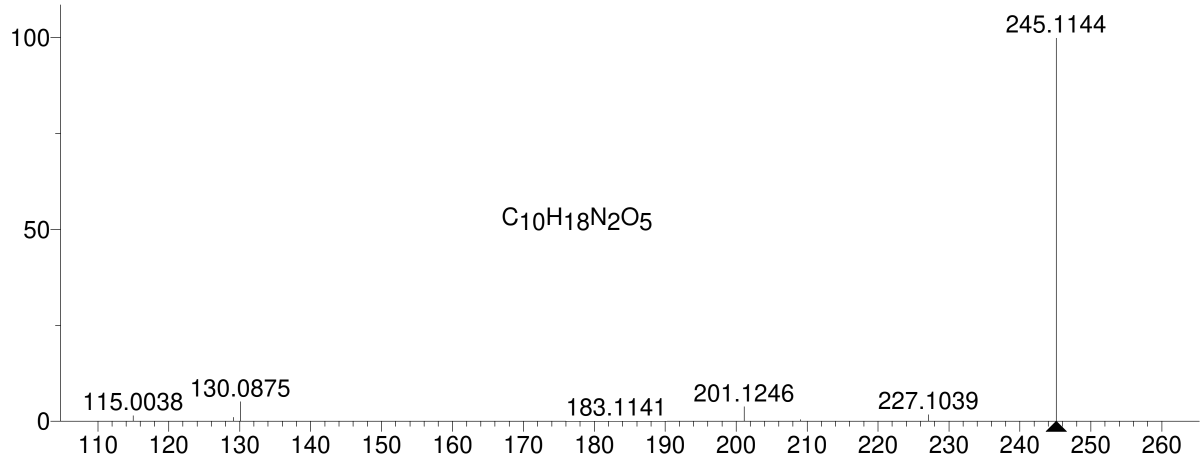

Hit 5 : Glu-Pro [M+H]+ QQQ 1V P=245.1
C10H16N2O5; MF: 23; RMF: 194; Prob 3.24%; Lib: nist_msms; ID: 37429.

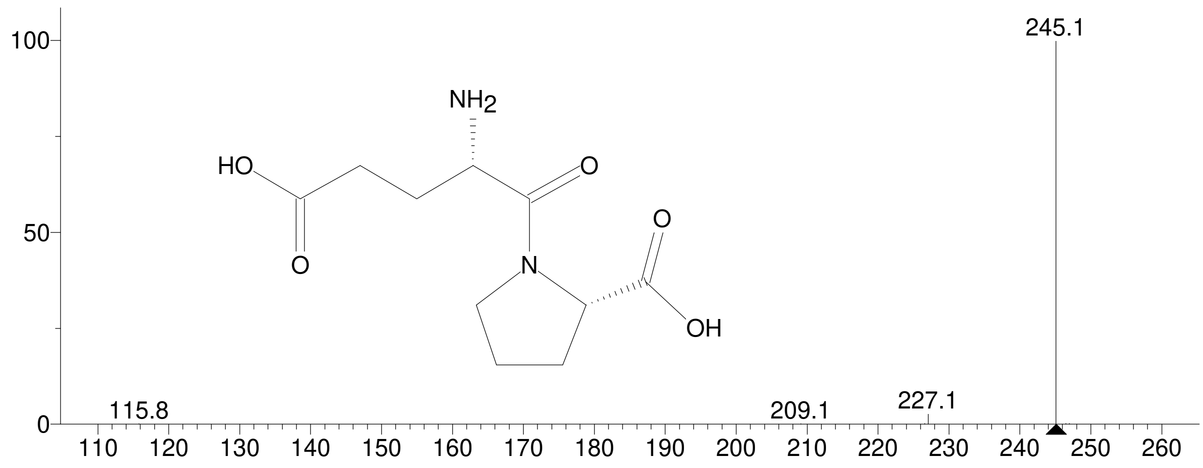

** Search Report Page 1 of 1 **

Unknown: Unknown (1.88_279.0380m/z)
Compound in Library Factor = 0

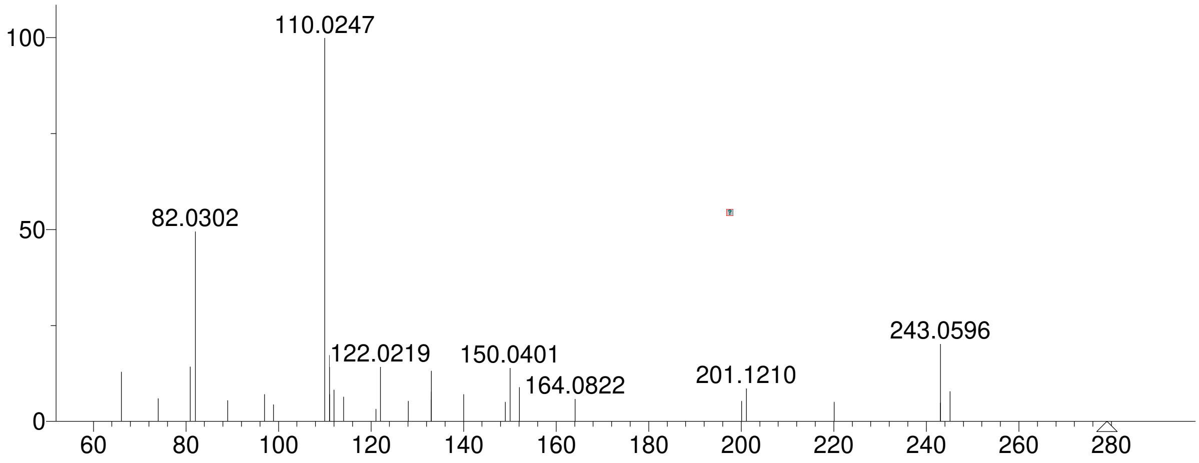

Unknown: Unknown (1.88_419.1652m/z)
Compound in Library Factor = 0

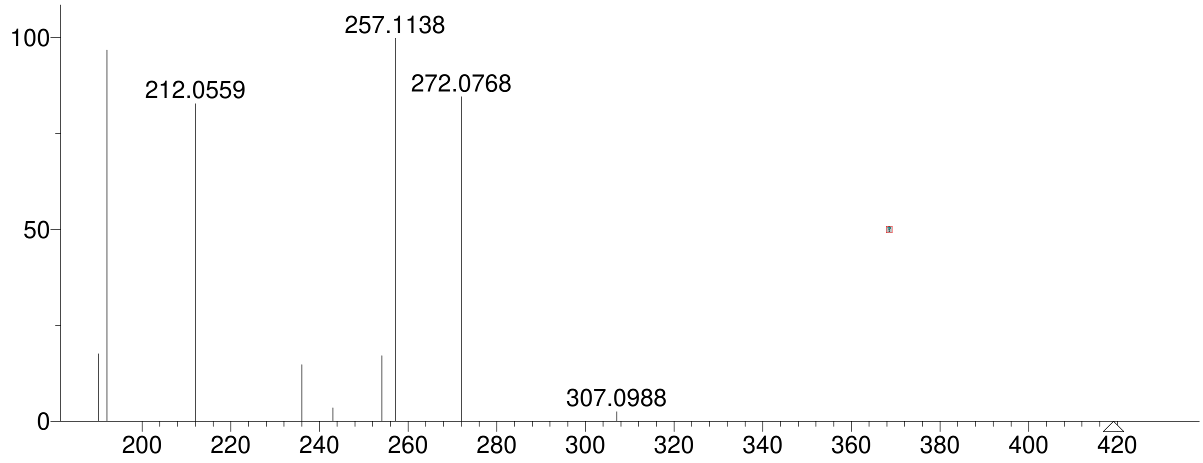

Hit 1 : (2S,3S,9R,12R,13R,14S,15R,16R,17S)-3,12,13,15,16-pentahydroxy-2,6,14,17-tetramethyl-10-oxatetracyc
C20H28O8; MF: 0; RMF: 17; Prob 50.0%; Lib: mona-export-vf-npl_qexactive; ID: 23233.

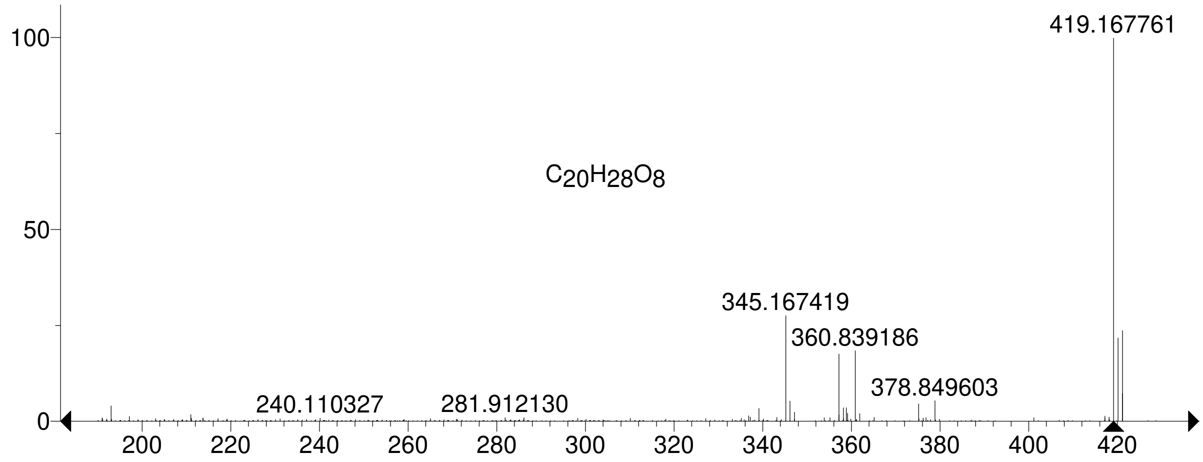

Hit 2 : Glp-His-Pro-Gly [M-H]- IT-FT 35% P=419.2
C18H24N6O6; MF: 0; RMF: 7; Prob 50.0%; Lib: nist_msms2; ID: 74068.

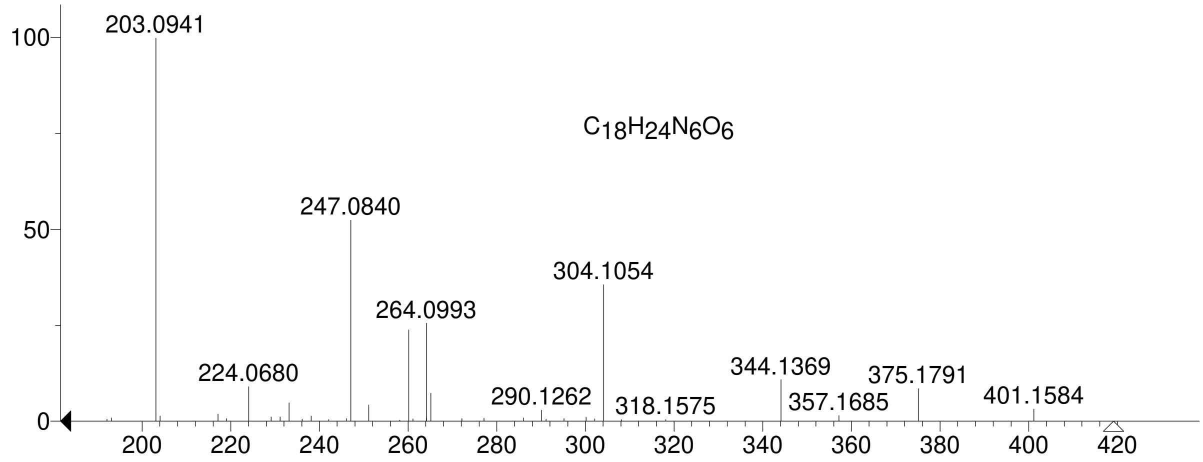

Unknown: Unknown (1.88_424.1348m/z)
Compound in Library Factor = 0

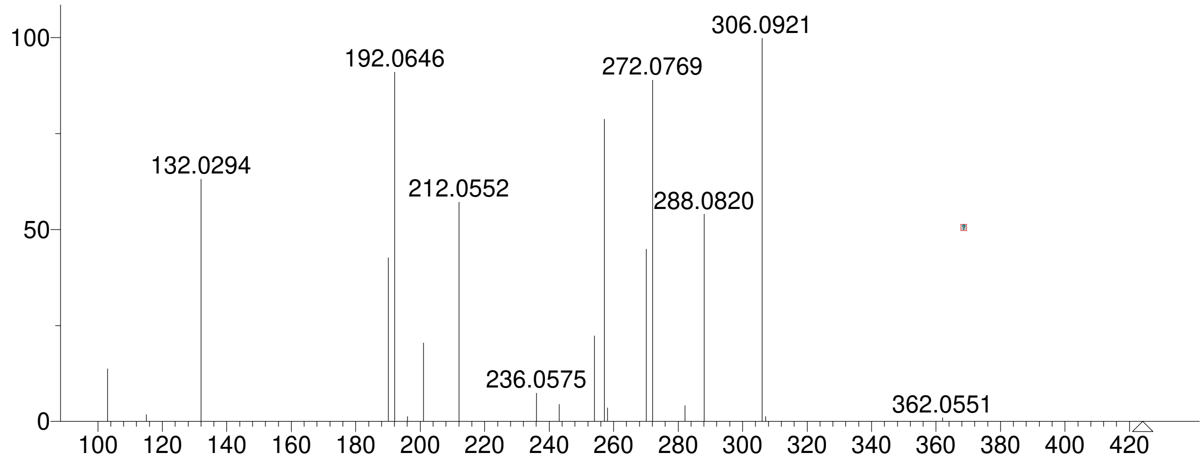

Hit 1 : Flumiclorac-pentyl [M+H]+ HCD 38V P=424.1
C21H23ClFNO5; MF: 0; RMF: 35; Prob 100.0%; CAS: 87546-18-7; Lib: nist_msms; ID: 483534.

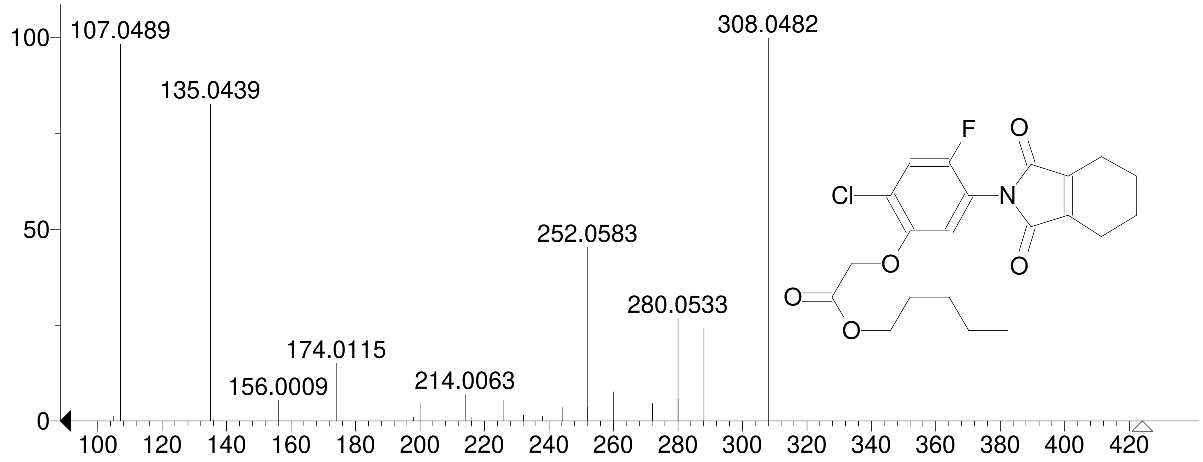

Unknown: Unknown (1.88_529.0628m/z)
Compound in Library Factor = 0

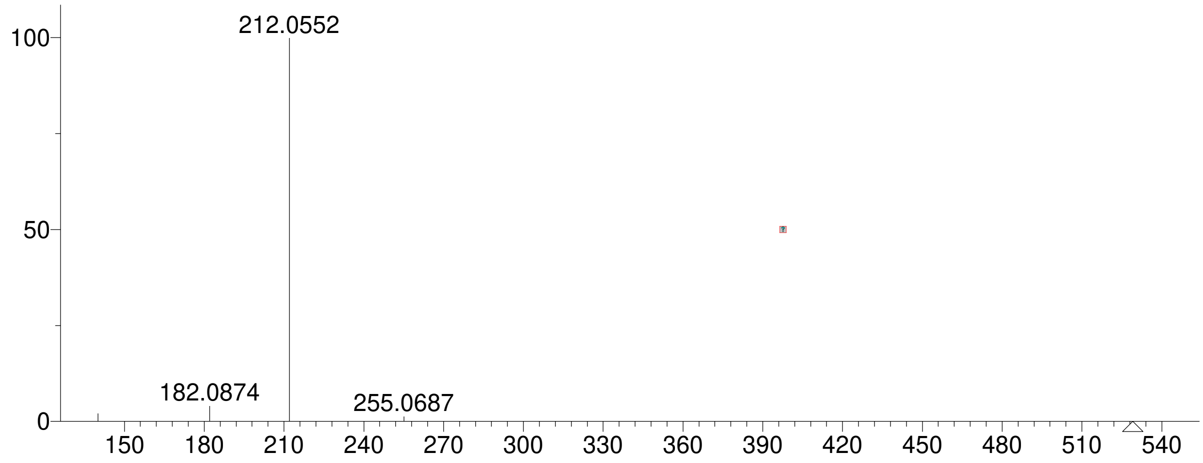

Unknown: Unknown (1.89_259.0541m/z)
Compound in Library Factor = 0

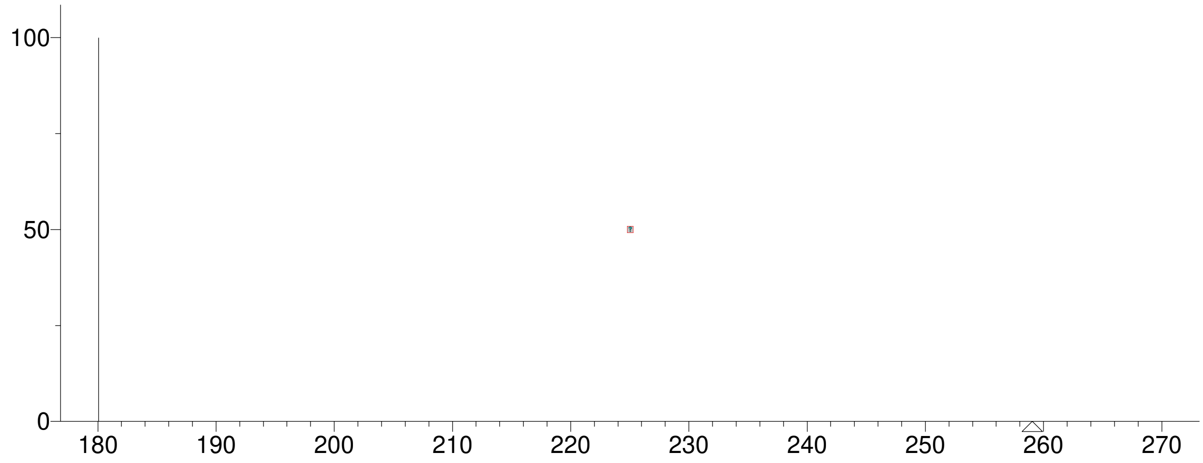

Unknown: Unknown (1.89_328.0442m/z)
Compound in Library Factor = -517

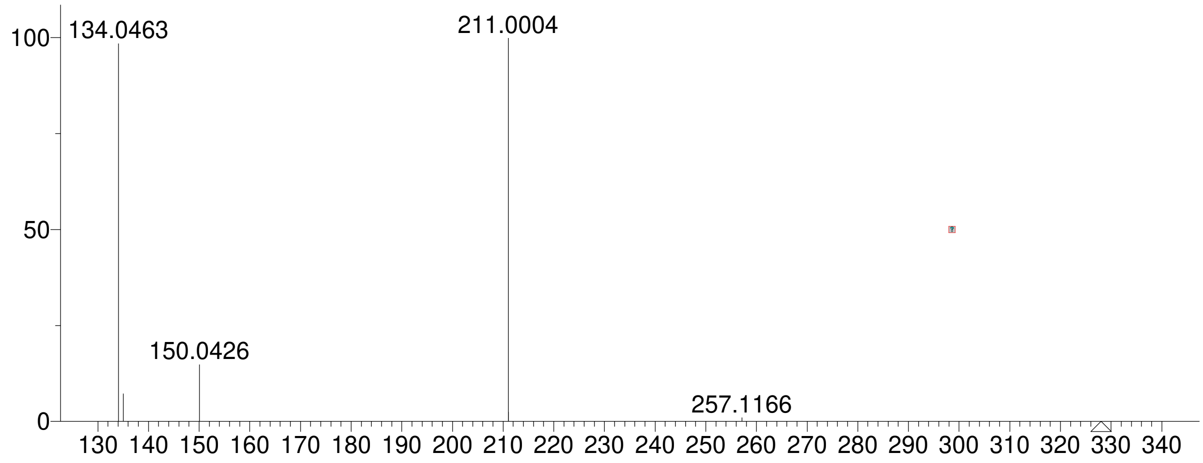

Hit 1 : Adenosine 2',3'-cyclic monophosphate [M-H]- HCD 52V P=328
C10H12N5O6P; MF: 209; RMF: 663; Prob 80.9%; CAS: 634-01-5; Lib: nist_msms; ID: 207400.

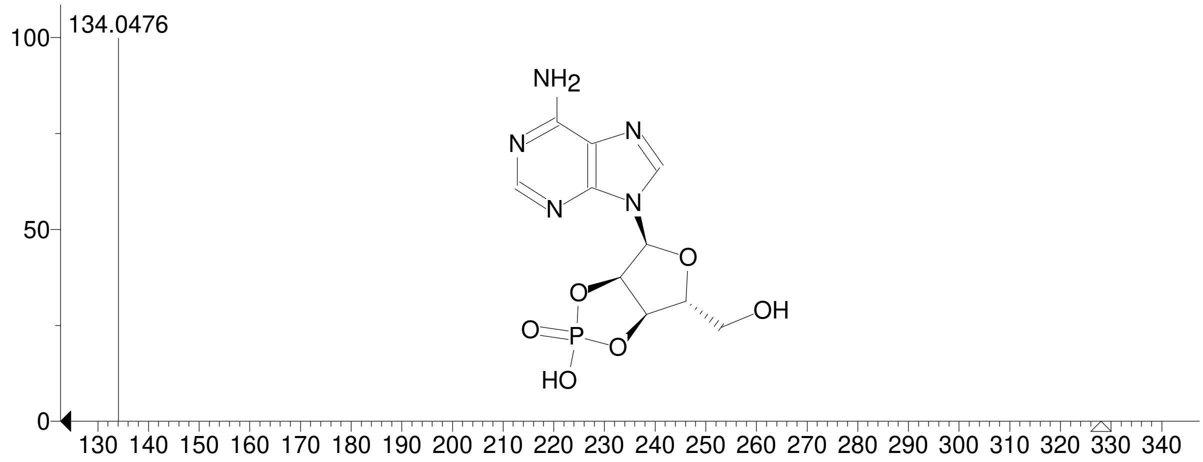

Hit 2 : Adenosine 3',5'-cyclic monophosphate [M-H]- HCD 59V P=328
C10H12N5O6P; MF: 162; RMF: 661; Prob 16.9%; CAS: 60-92-4; Lib: nist_msms; ID: 130593.

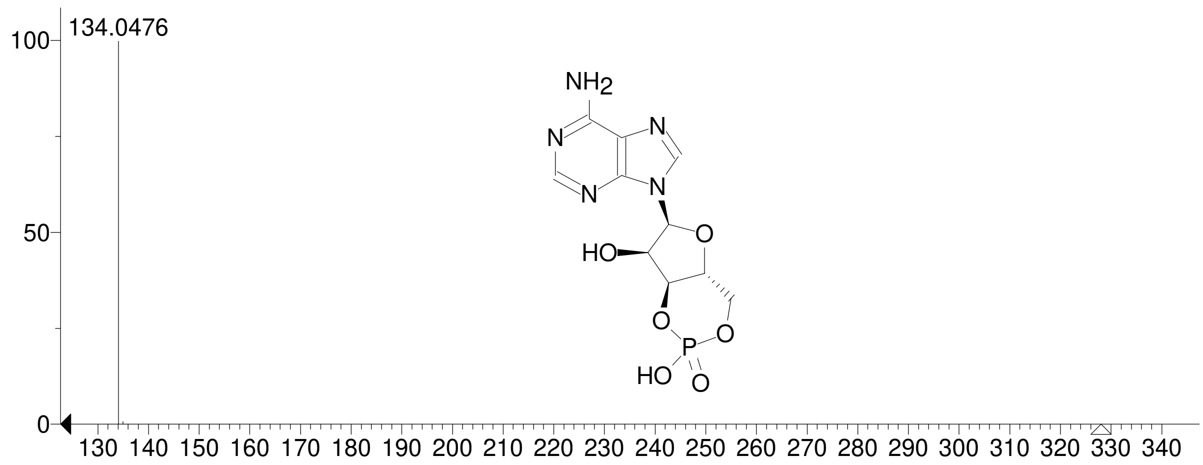

Hit 3 : cAMP [M-H]- P=328
C10H12N5O6P; MF: 36; RMF: 574; Prob 0.75%; Lib: msms neg mona_ipb_gnps_plasma; ID: 1463.

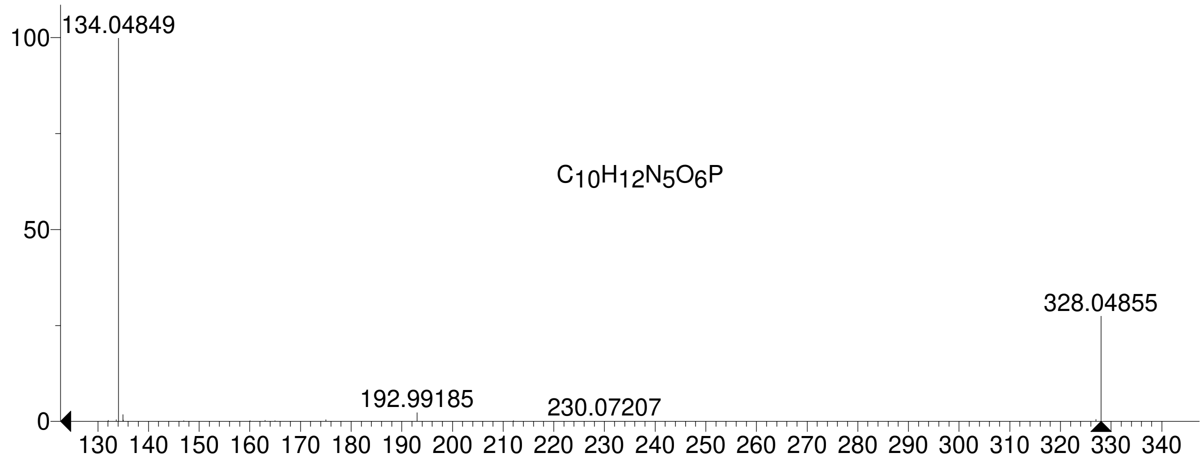

Hit 4 : ADENOSINE 3',5'-CYCLIC MONOPHOSPHATE [M-H]- P=328
C10H12N5O6P; MF: 14; RMF: 570; Prob 0.29%; Lib: msms-neg-gnps; ID: 274.

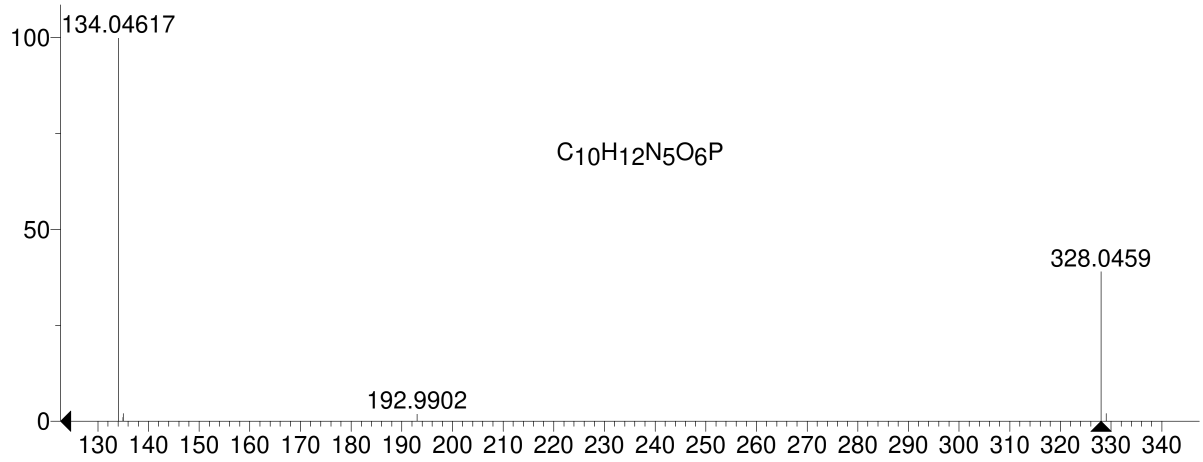

Hit 5 : 3',5'-CYCLIC AMP [M-H]- P=328
C10H12N5O6P; MF: 14; RMF: 566; Prob 0.29%; Lib: msms-neg-gnps; ID: 317.

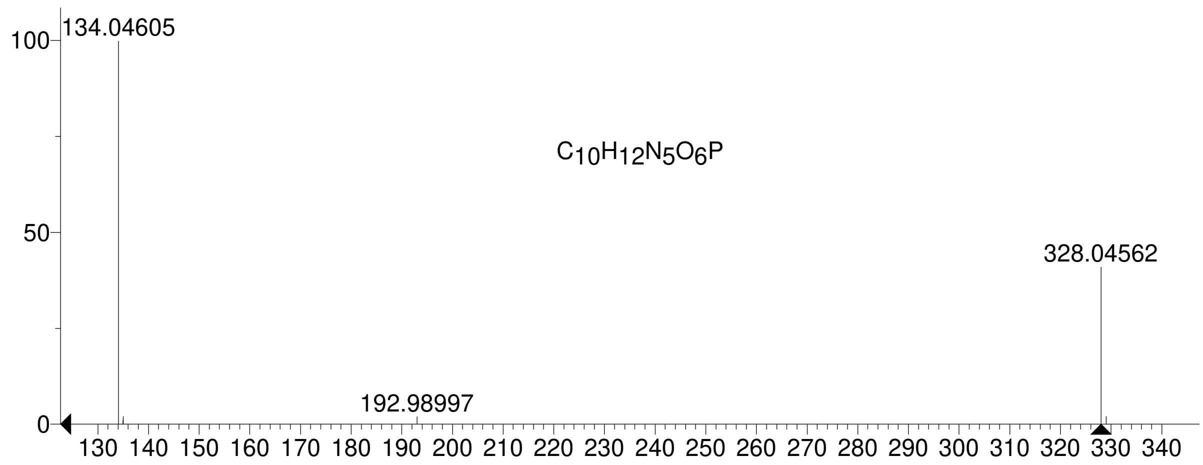

** Search Report Page 1 of 1 **

Unknown: Unknown (1.90_281.0345m/z)
Compound in Library Factor = 0

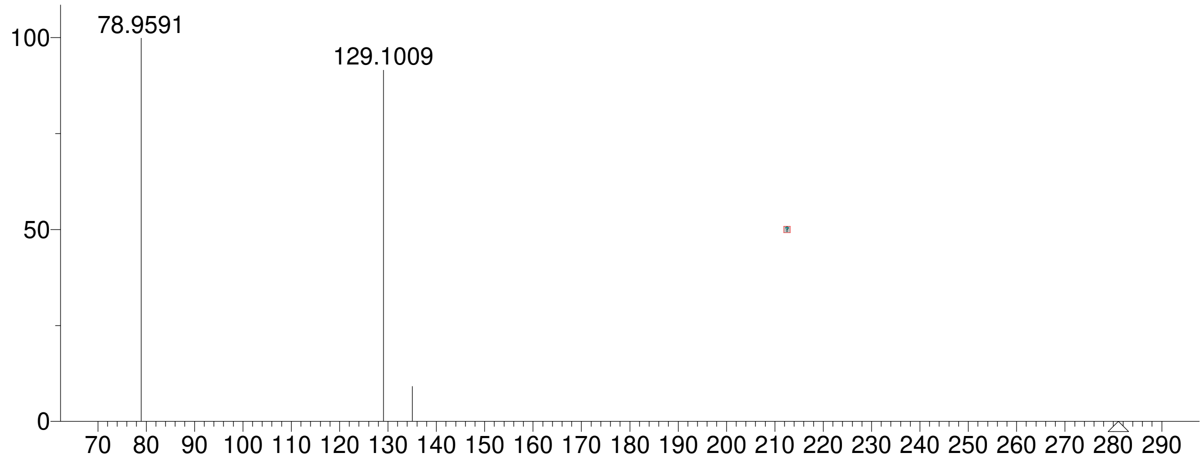

Unknown: Unknown (1.90_323.0973m/z)
Compound in Library Factor = 0

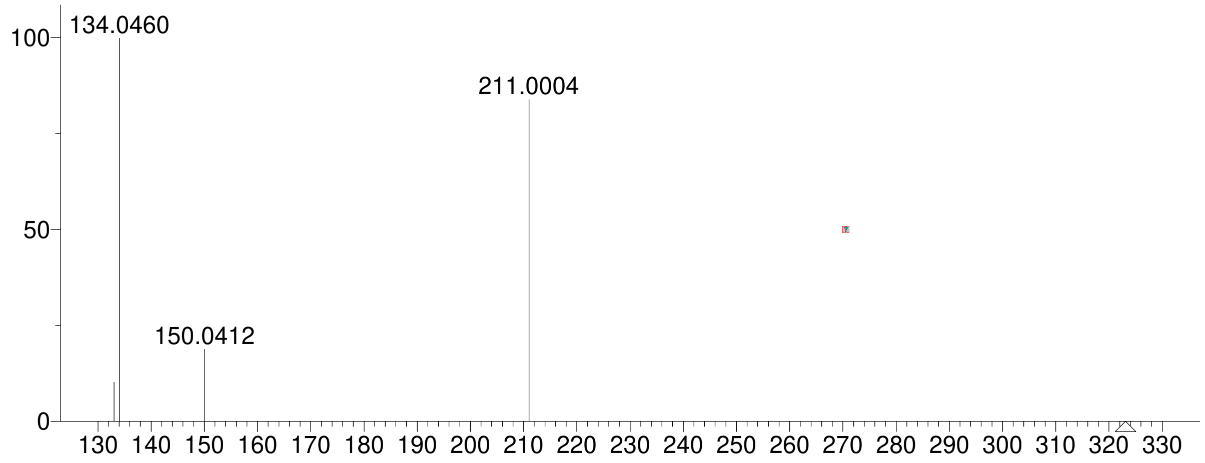

Hit 1 : 1-Piperidinecarboxylic acid, 4-[6-[4-[(methoxycarbonyl)amino]phenyl]-4-(4-morpholinyl)-1H-pyrazolo[3,4-d]|
C24H29N7O5; MF: 0; RMF: 123; Prob 7.69%; CAS: 1062169-56-5; Lib: nist_msms; ID: 265735.

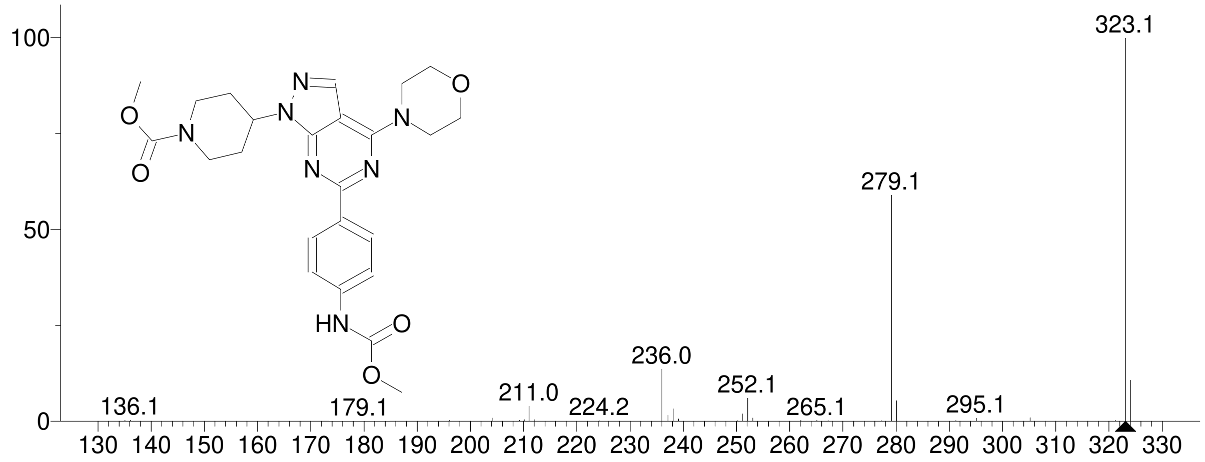

Hit 2 : Indanofan [M+H]+=>323.1 IT 35% P=323.1
C20H17ClO3; MF: 0; RMF: 102; Prob 7.69%; CAS: 133220-30-1; Lib: nist_msms; ID: 477459.

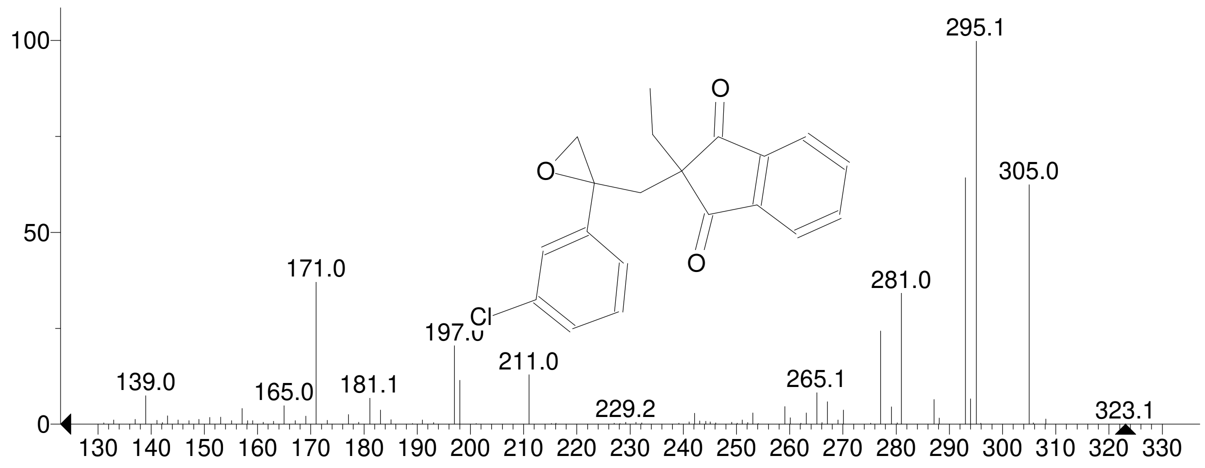

Hit 3 : Prednisone [M+H-H2O]+=>323.1 IT 35% P=323.1
C21H26O5; MF: 0; RMF: 97; Prob 7.69%; CAS: 53-03-2; Lib: nist_msms; ID: 99548.

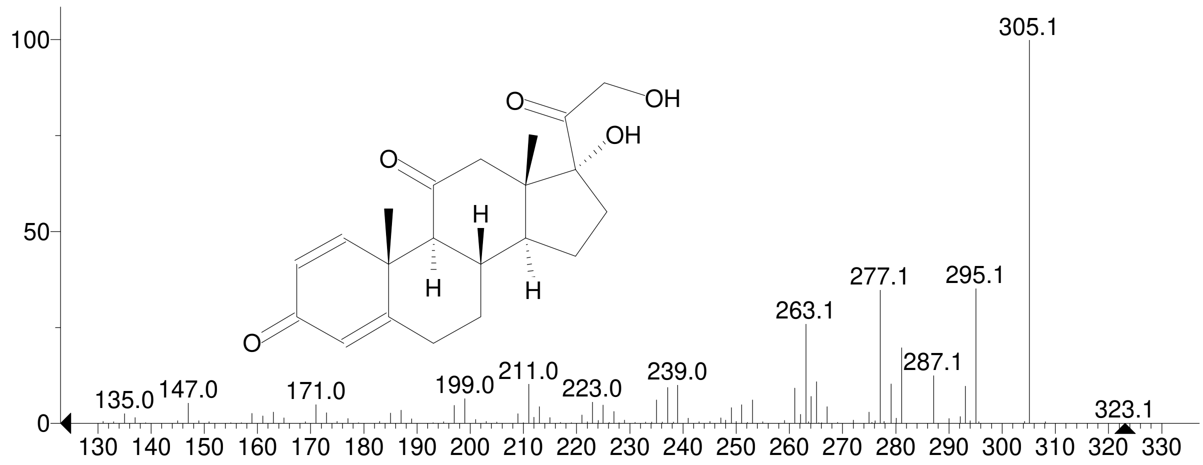

Hit 4 : Fluorometholone [M+H-H2O]+=>341.1=>323.1 IT 35% P=323.1
C22H29FO4; MF: 0; RMF: 77; Prob 7.69%; CAS: 426-13-1; Lib: nist_msms; ID: 119128.

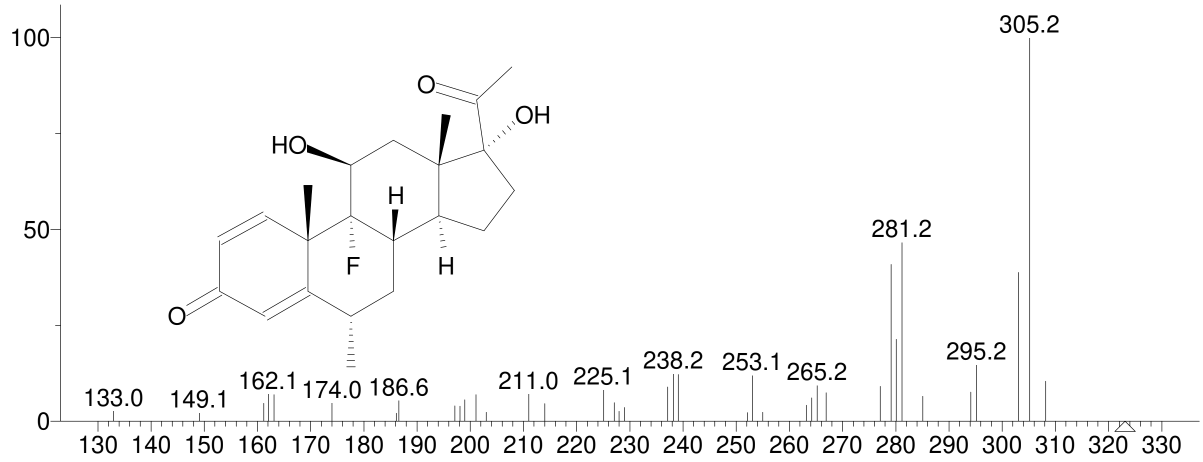

Hit 5 : Dinitramine [M+H]+ HCD 103V P=323.1
C11H13F3N4O4; MF: 0; RMF: 70; Prob 7.69%; CAS: 29091-05-2; Lib: nist_msms; ID: 100130.

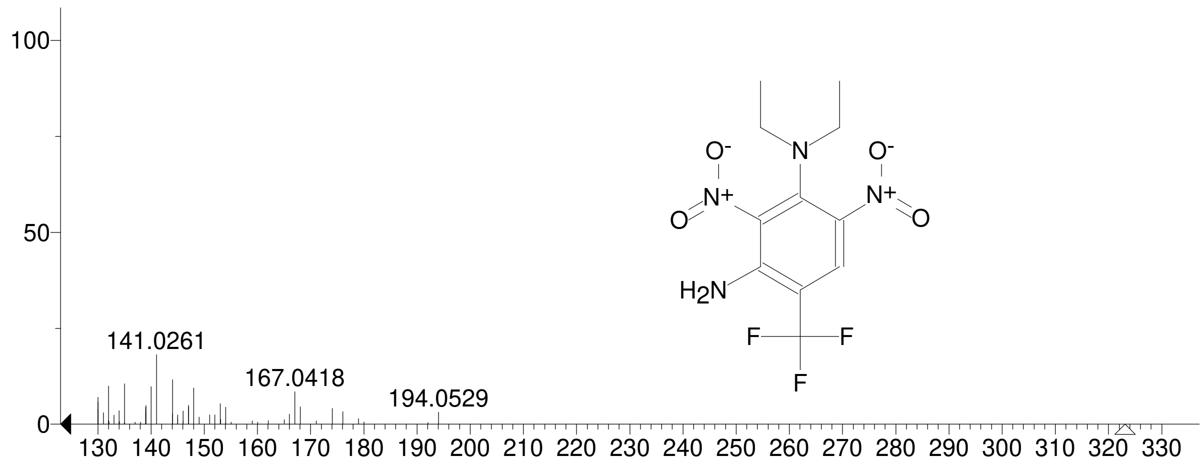

Unknown: Unknown (1.90_362.0497m/z)
Compound in Library Factor = -516

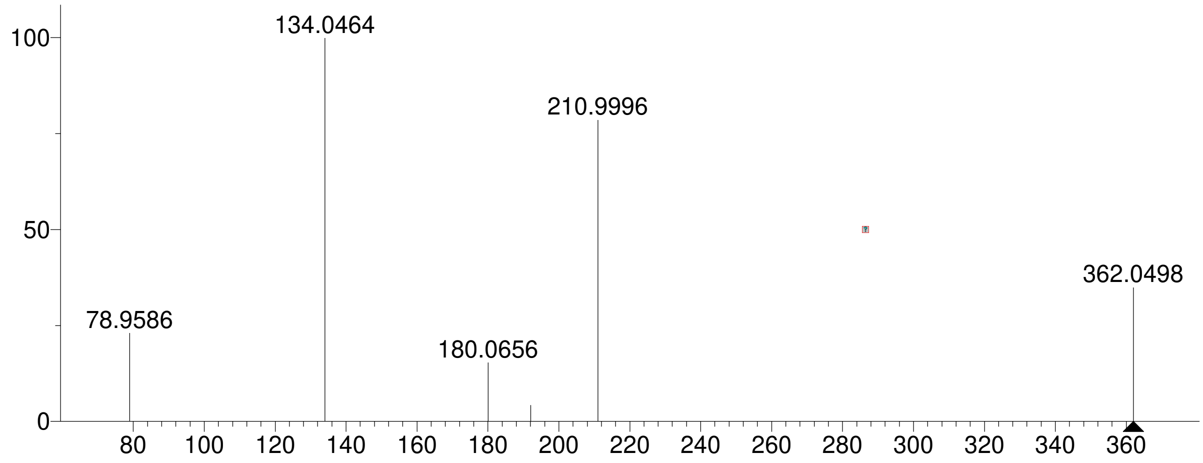

Hit 1 : Guanosine 5'-diphospho-β-L-fucose [M-H-C6H11O7P]- HCD 14V P=362.1
C16H25N5O15P2; MF: 370; RMF: 560; Prob 86.8%; CAS: 15839-70-0; Lib: nist_msms; ID: 557145.

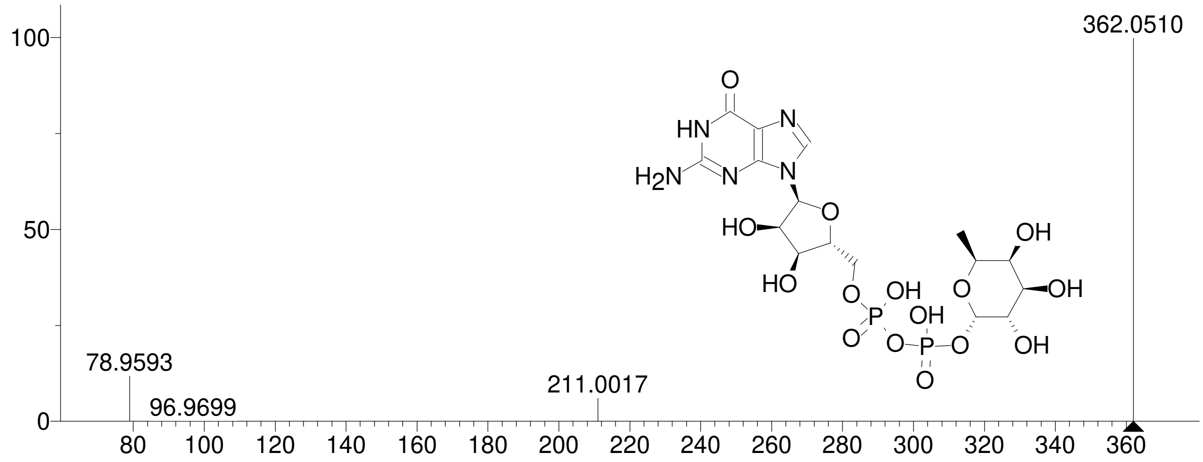

Hit 2 : Guanosine 5'-monophosphate [M-H]- HCD 14V P=362.1
C10H14N5O8P; MF: 295; RMF: 484; Prob 11.3%; CAS: 85-32-5; Lib: nist_msms; ID: 311057.

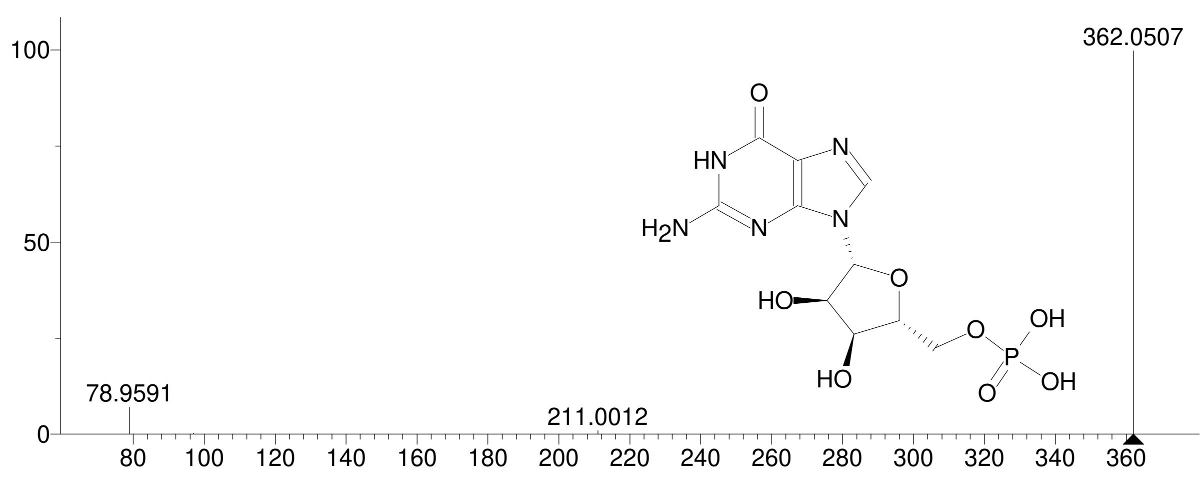

Hit 3 : Guanosine-5'-monophosphate [M-H]- 20-30-40%) P=362.1
C10H14N5O8P; MF: 229; RMF: 563; Prob 1.78%; Lib: mona-export-fiehn_hilic; ID: 1240.

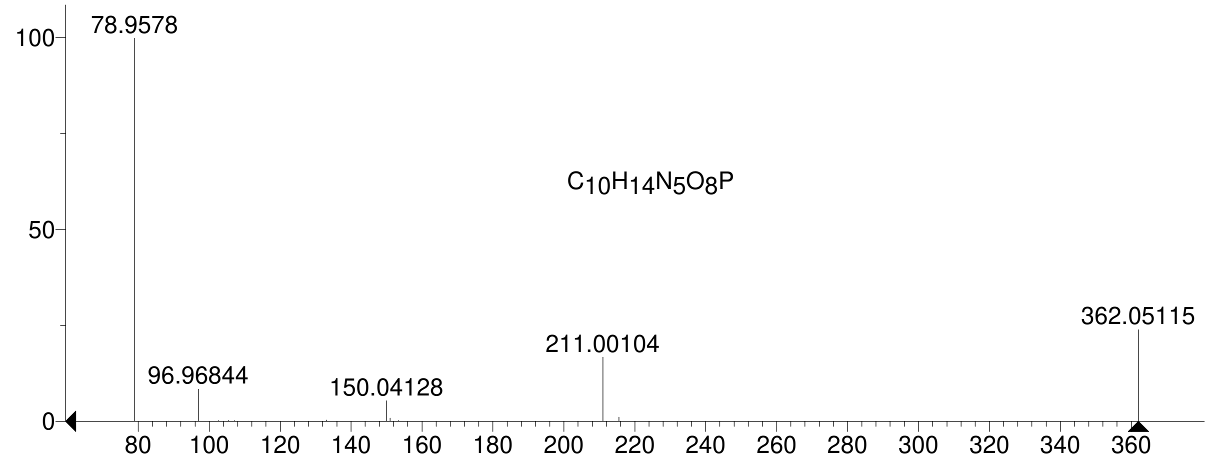

Hit 4 : GUANOSINE 5'-MONOPHOSPHATE [M-H]- P=362.1
C10H14N5O8P; MF: 112; RMF: 493; Prob 0.10%; Lib: mona-export-gnps; ID: 5674.

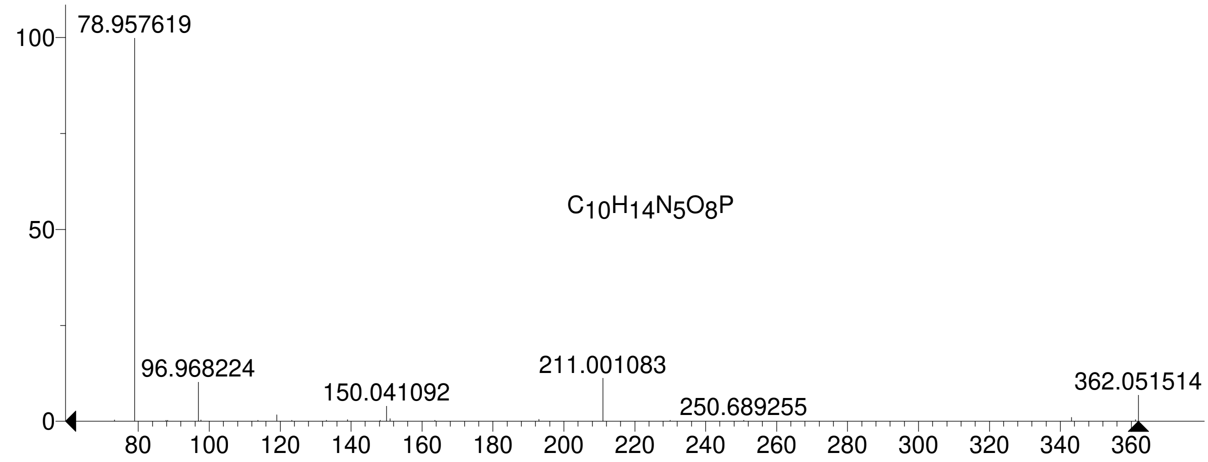

Hit 5 : Guanosine monophosphate [M-H]- 10 P=362.1
C10H14N5O8P; MF: 79; RMF: 454; Prob 0.02%; Lib: msms neg mona_ipb_gnps_plasma; ID: 592.

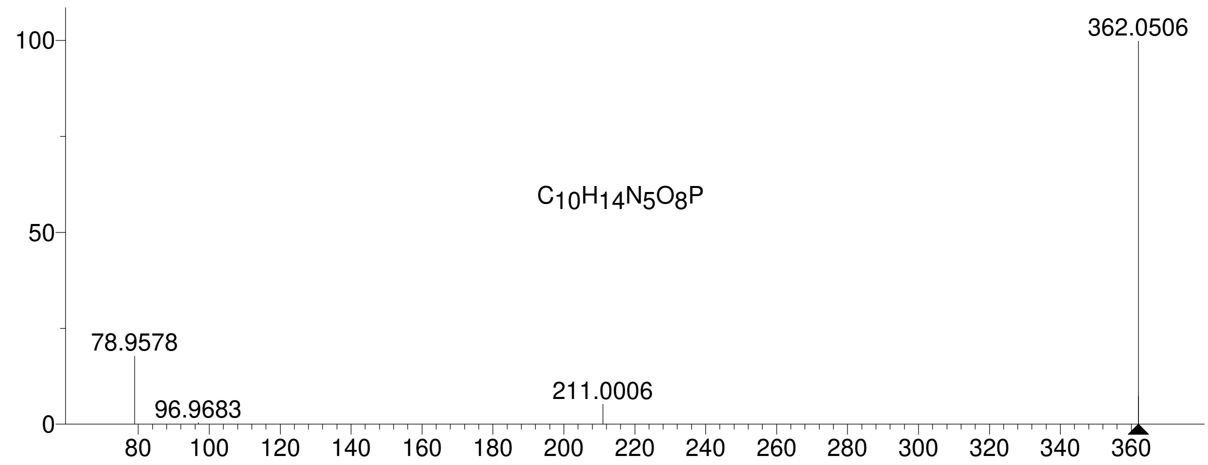

Unknown: Unknown (1.90_452.1398m/z)
Compound in Library Factor = 0

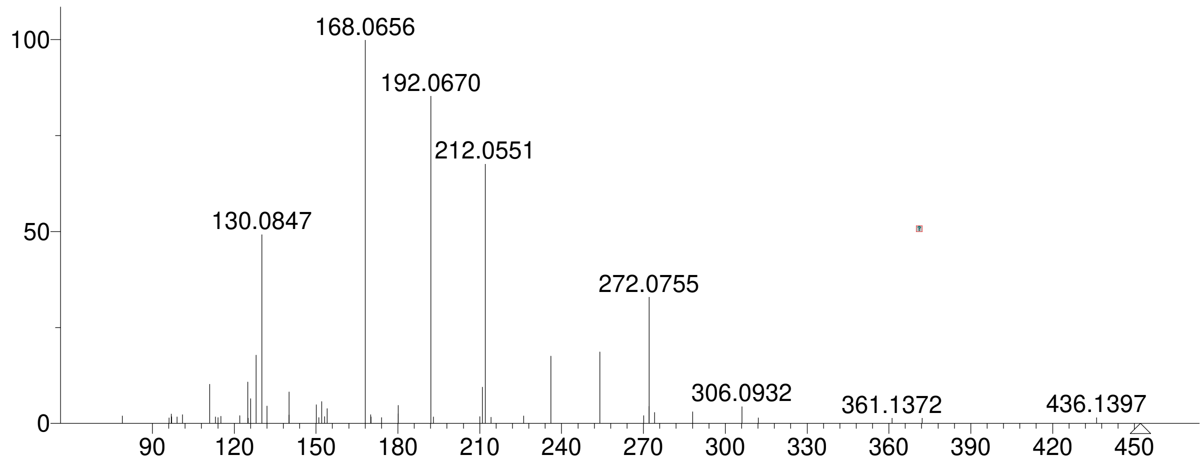

Unknown: Unknown (1.92_420.2337m/z)
Compound in Library Factor = -3273

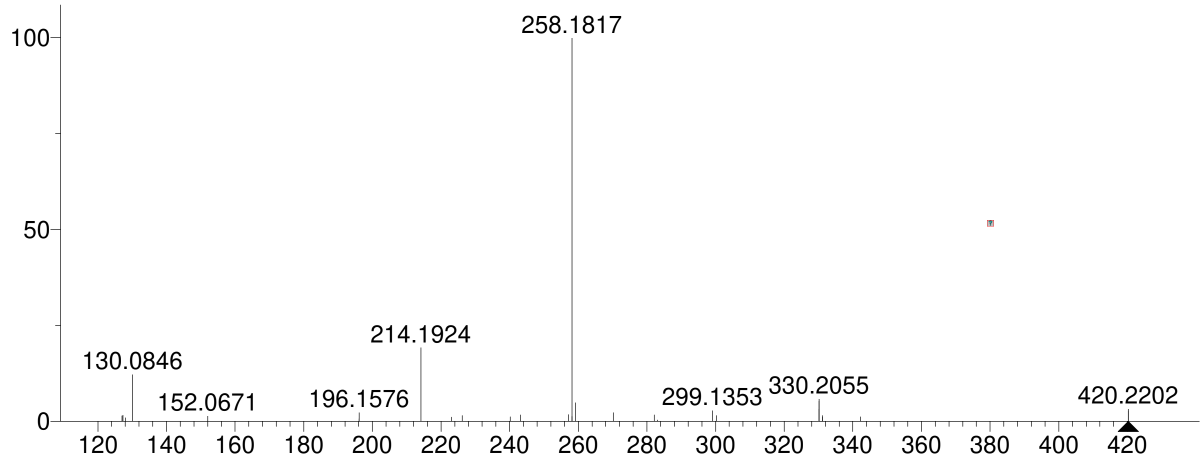

Hit 1 : TNF-α (71-82), human [M+3H]3+ HCD 10eV P=420.2
C51H91N19O18; MF: 1; RMF: 80; Prob 100.0%; Lib: nist_msms2; ID: 49954.

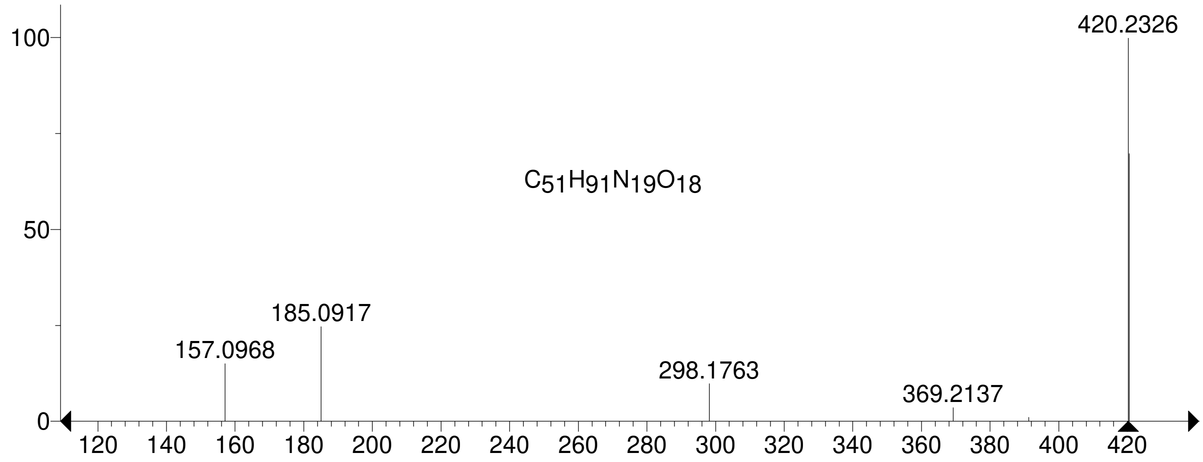

Unknown: Unknown (1.92_485.1519m/z)
Compound in Library Factor = 0

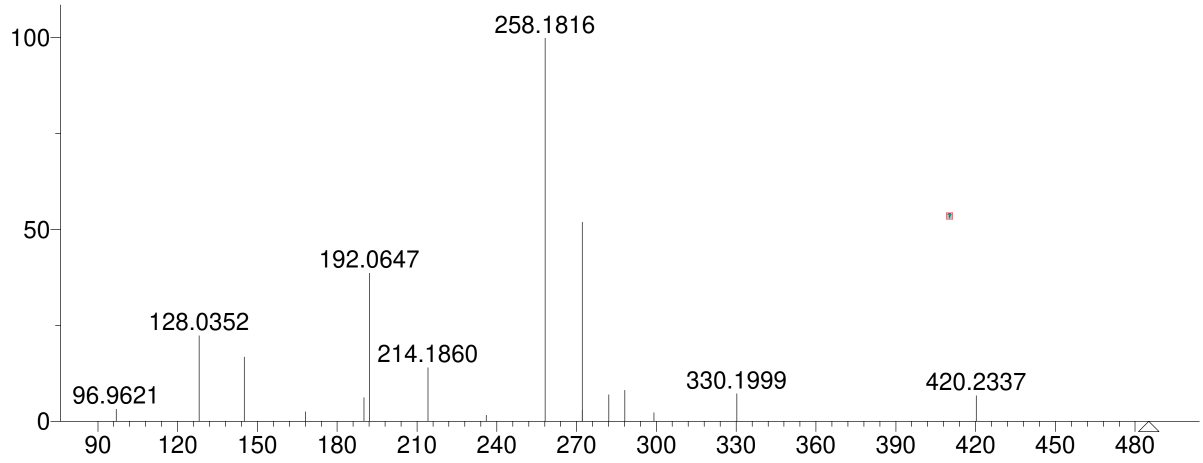

Unknown: Unknown (1.93_450.1712m/z)
Compound in Library Factor = 0

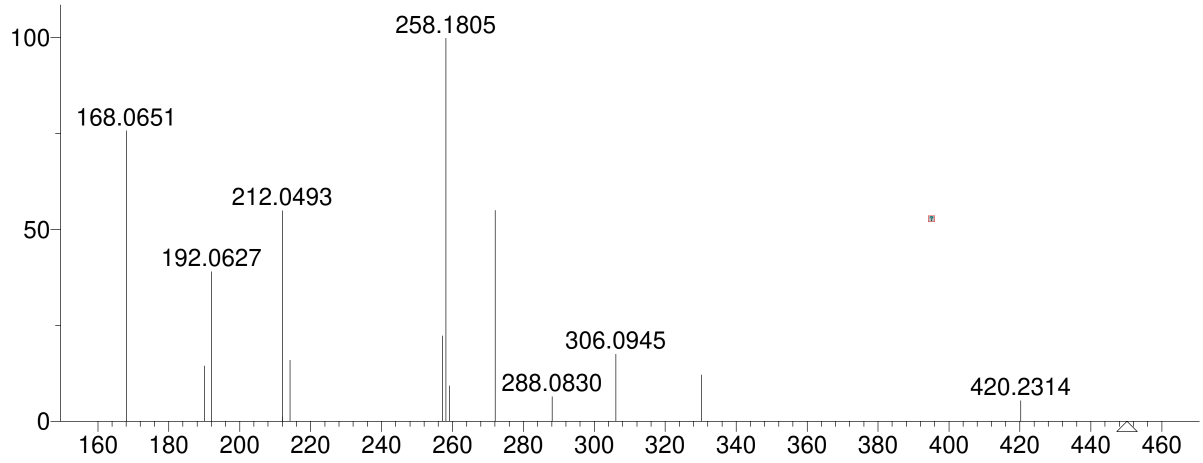

Unknown: Unknown (1.94_487.2023m/z)
Compound in Library Factor = 0

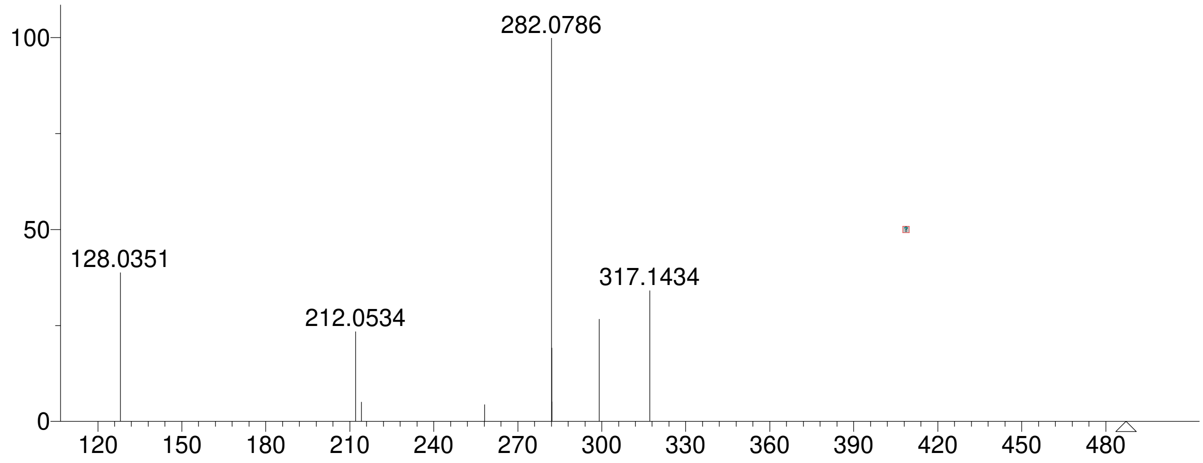

Unknown: Unknown (1.94_510.1836m/z)
Compound in Library Factor = 0

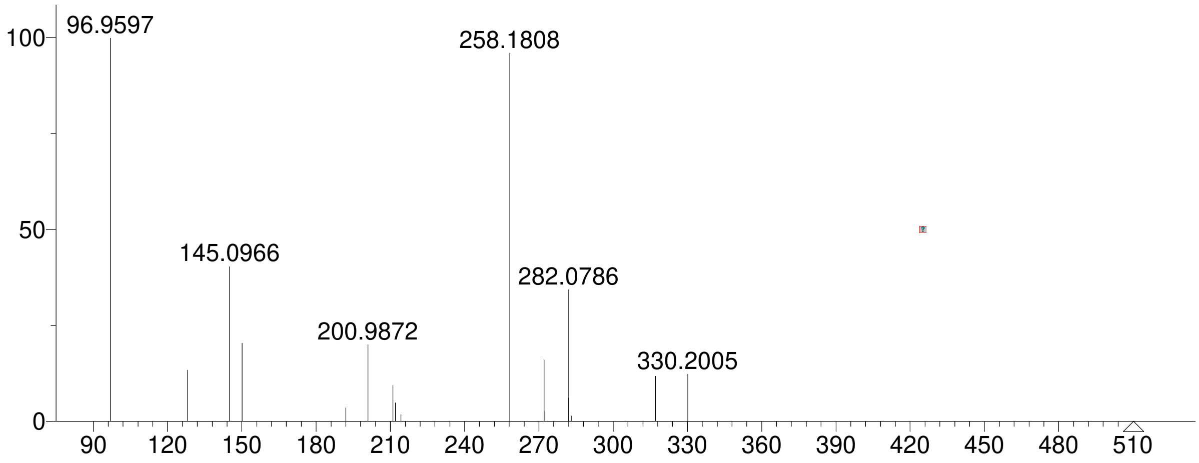

** Search Report Page 1 of 1 **

Unknown: Unknown (1.95_269.1232m/z)
Compound in Library Factor = 0

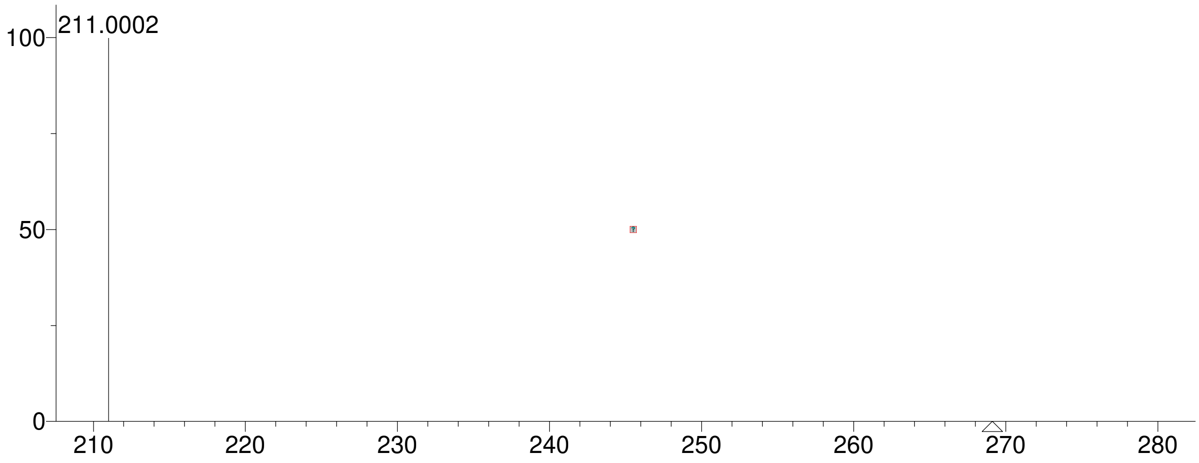

** Search Report Page 1 of 1 **

Unknown: Unknown (1.96_614.1927m/z)
Compound in Library Factor = 0

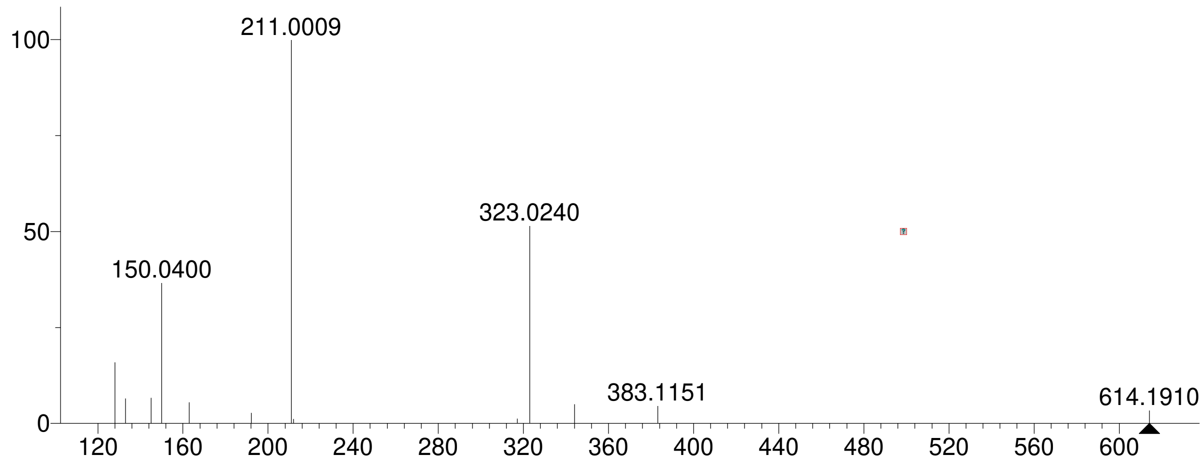

Unknown: Unknown (1.96_856.3202m/z)
Compound in Library Factor = 0

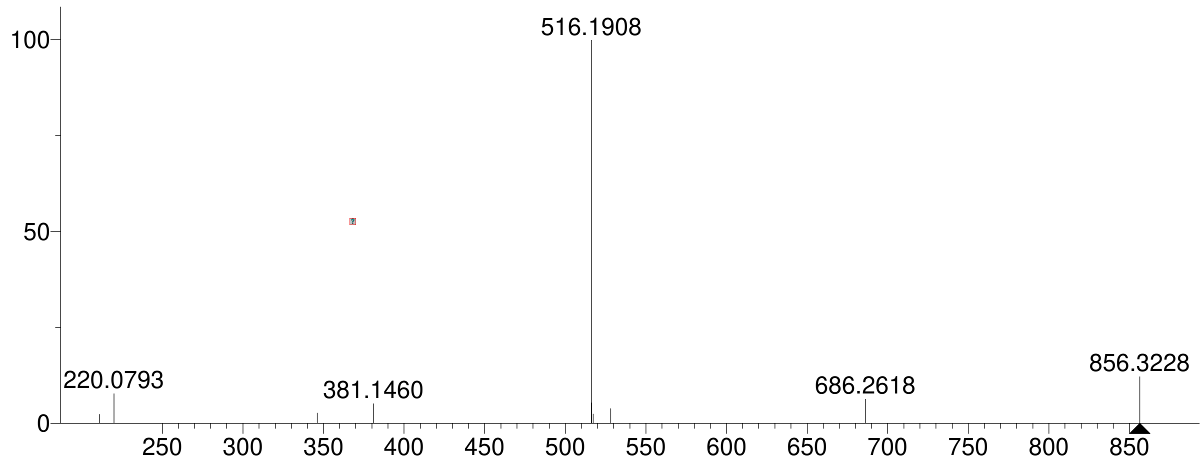

** Search Report Page 1 of 1 **

Unknown: Unknown (1.98_510.1830m/z)
Compound in Library Factor = 0

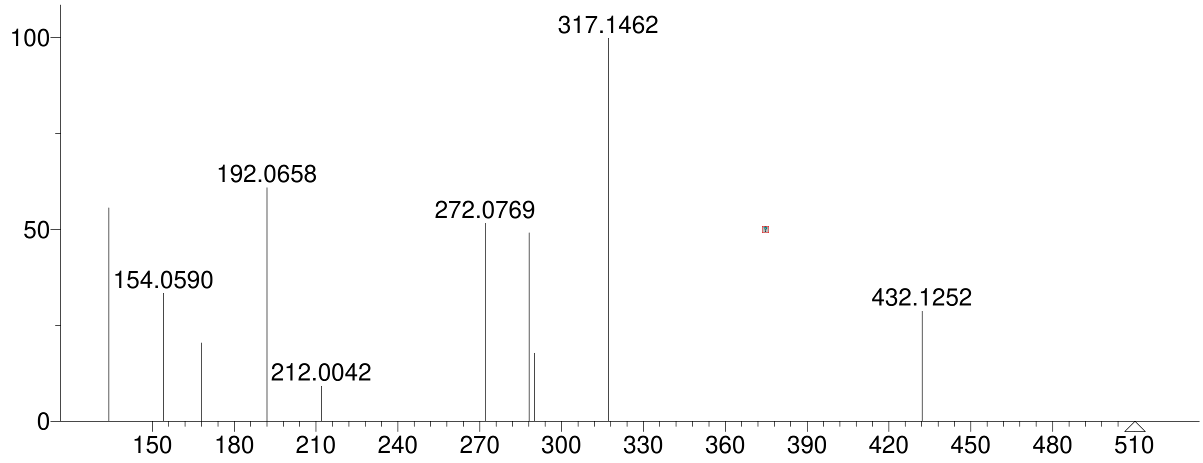

Unknown: Unknown (1.98_649.0812m/z)
Compound in Library Factor = 0

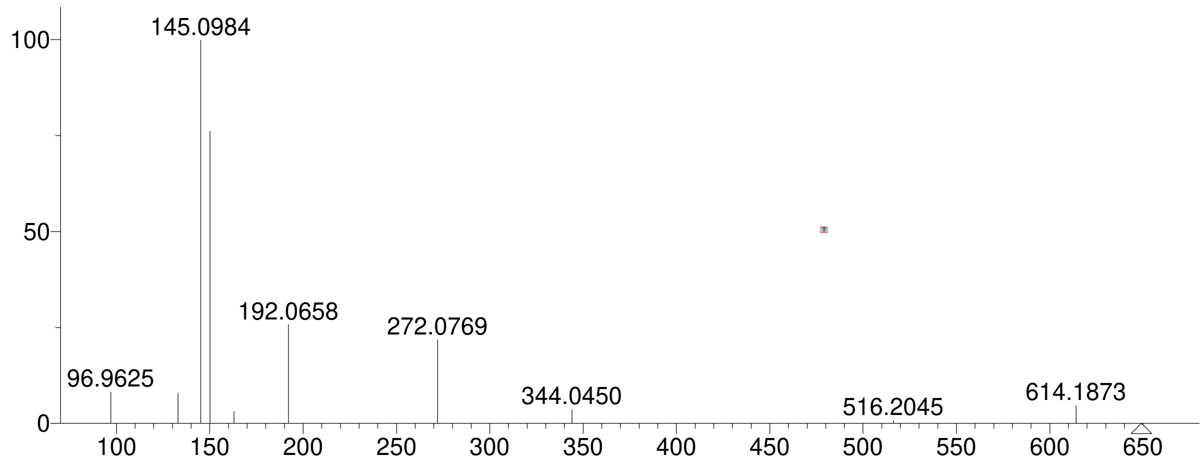

Unknown: Unknown (10.00_338.1710m/z)
Compound in Library Factor = 0

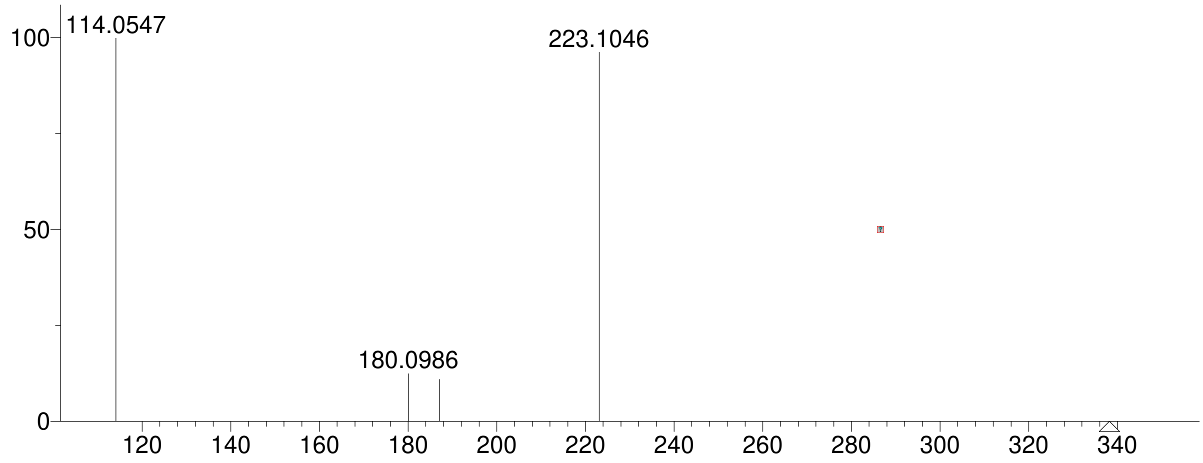

Unknown: Unknown (10.02_401.1895m/z)
Compound in Library Factor = 0

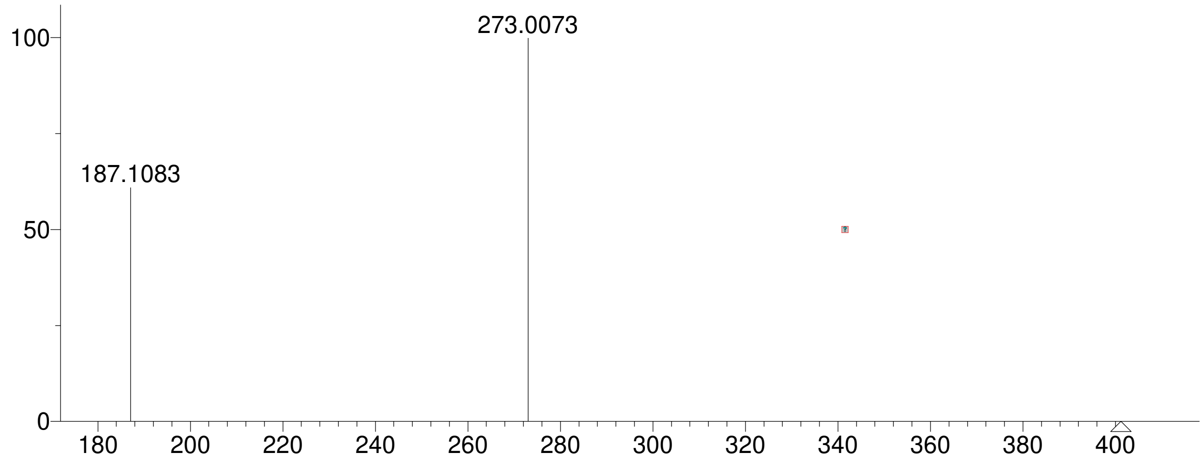

Unknown: Unknown (10.03_569.0026m/z)
Compound in Library Factor = 0

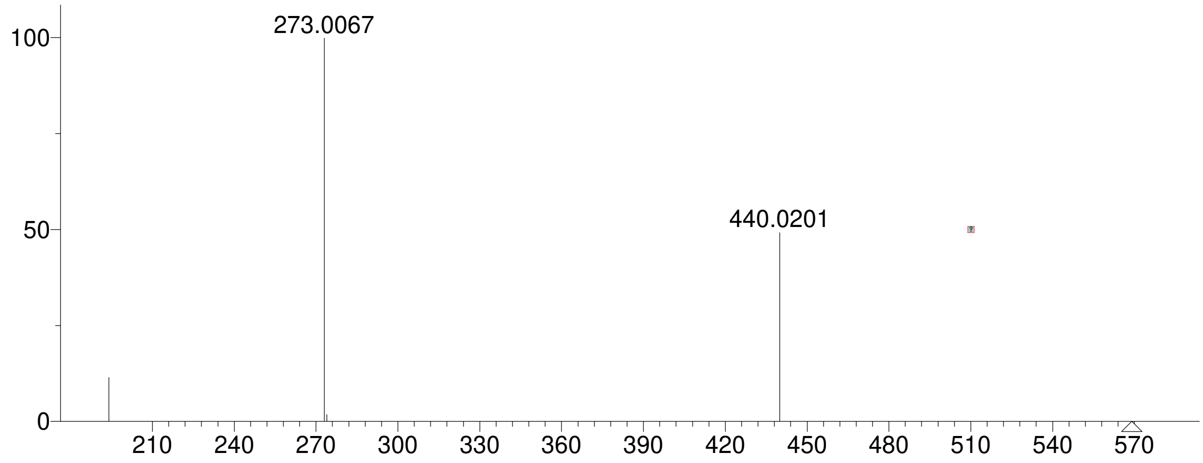

Unknown: Unknown (10.04_193.0498m/z)
Compound in Library Factor = 420

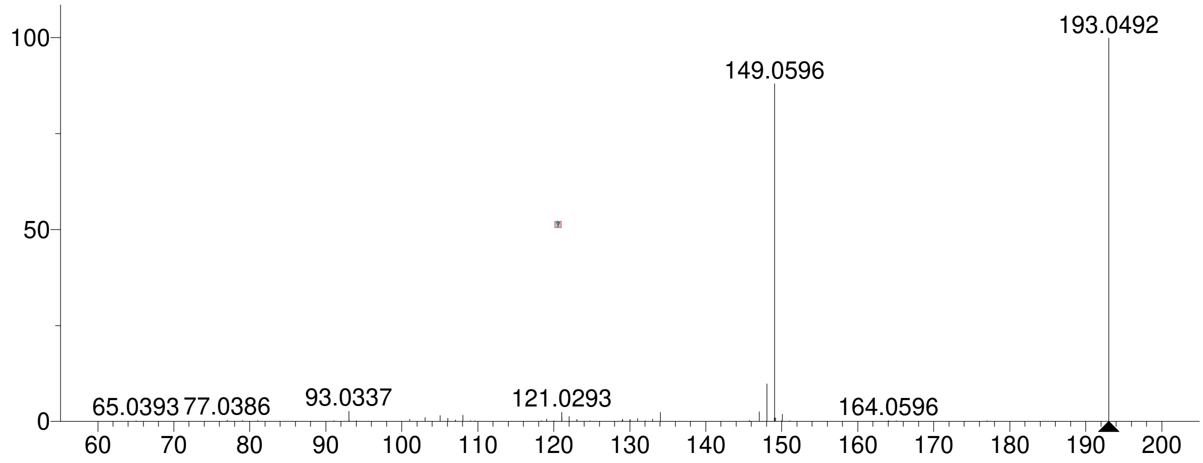

Hit 1 : Carbidopa [M-H-H4N2]- HCD 3V P=193.1
C10H14N2O4; MF: 898; RMF: 937; Prob 87.2%; CAS: 28860-95-9; Lib: nist_msms; ID: 313796.

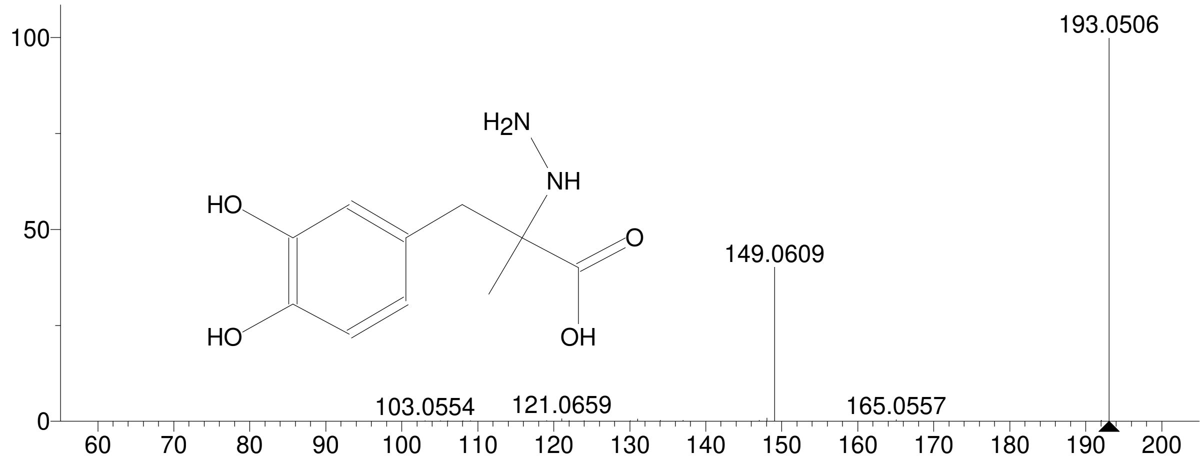

Hit 2 : Methyldopa [M-H-NH3]- HCD 14V P=193.1
C10H13NO4; MF: 827; RMF: 915; Prob 12.4%; CAS: 555-30-6; Lib: nist_msms; ID: 96914.

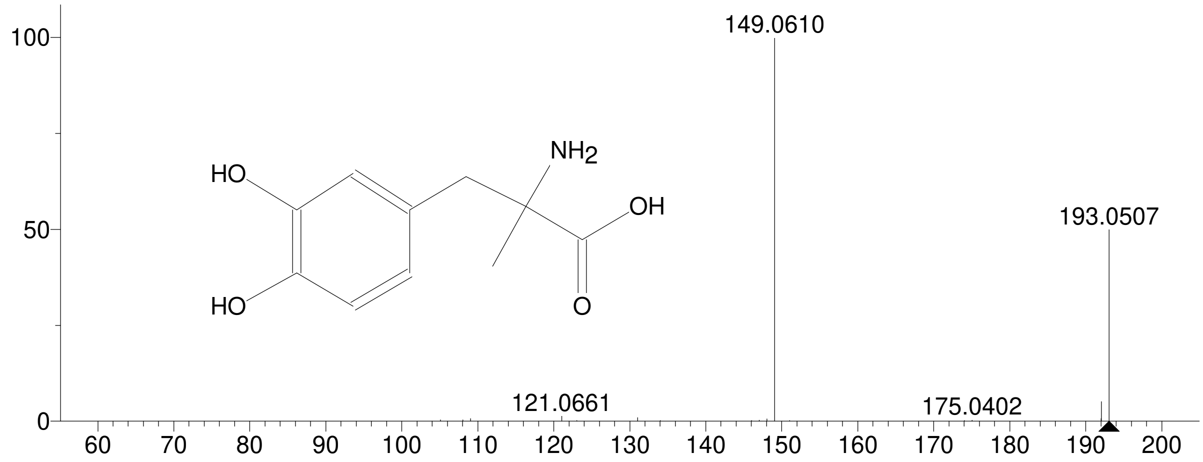

Hit 3 : Monoethyl phthalate [M-H]- HCD 7V P=193.1
C10H10O4; MF: 631; RMF: 813; Prob 0.17%; CAS: 2306-33-4; Lib: nist_msms; ID: 206557.

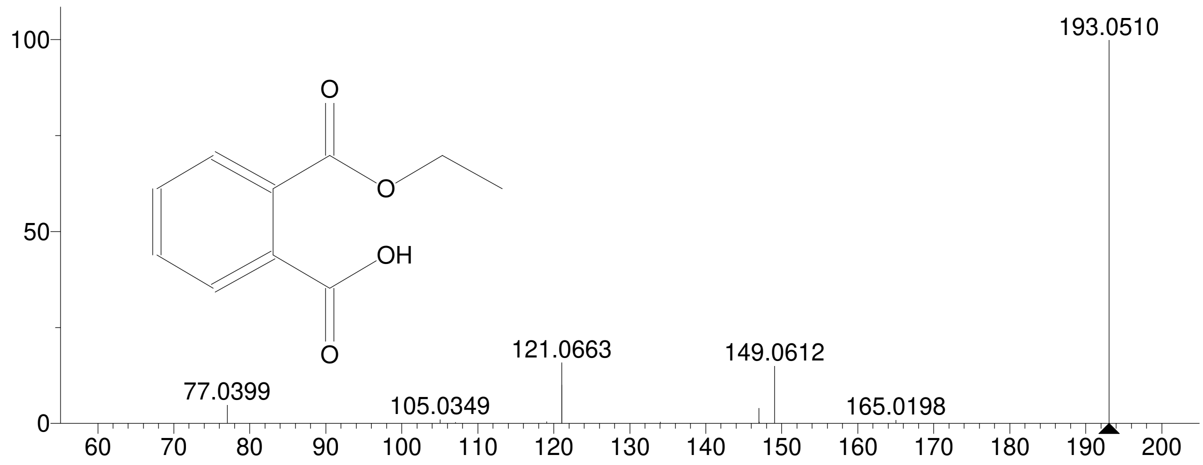

Hit 4 : Prohexadione [M-H-H2O]- HCD 11V P=193.1
C10H12O5; MF: 627; RMF: 841; Prob 0.14%; CAS: 88805-35-0; Lib: nist_msms; ID: 489541.

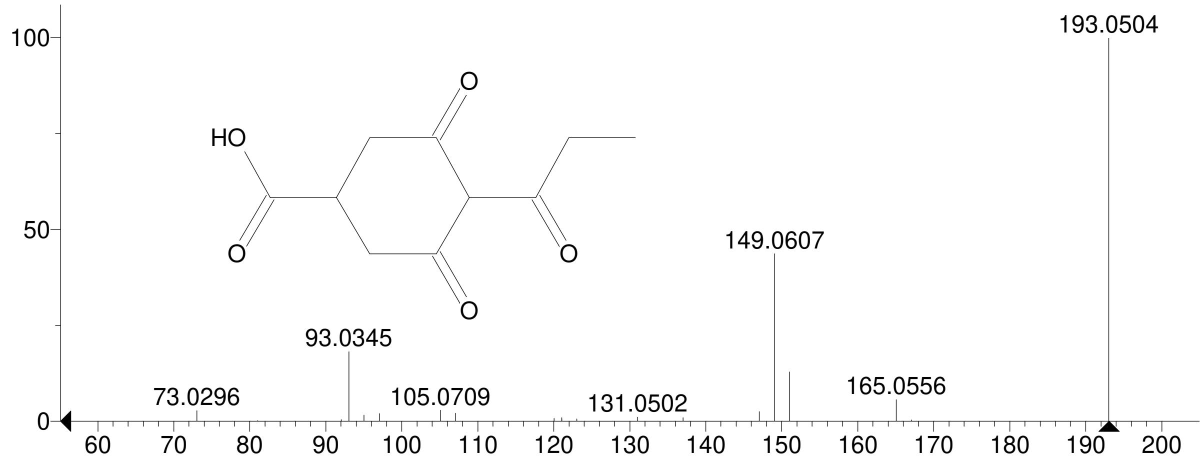

Hit 5 : trans-Ferulic acid [M-H]- HCD 7V P=193.1
C10H10O4; MF: 495; RMF: 820; Prob 0.00%; CAS: 537-98-4; Lib: nist_msms; ID: 300551.

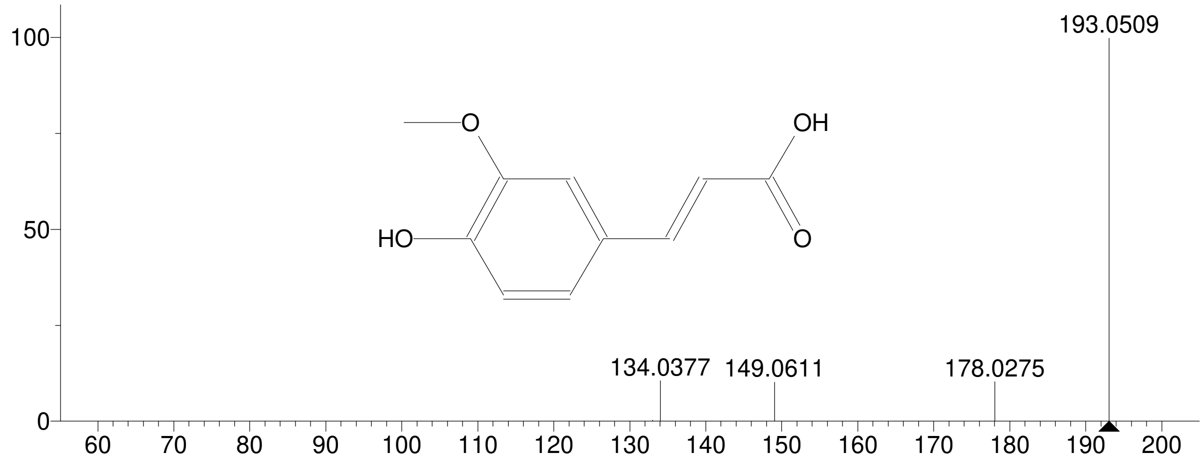

Unknown: Unknown (10.07_399.2117m/z)
Compound in Library Factor = -2176

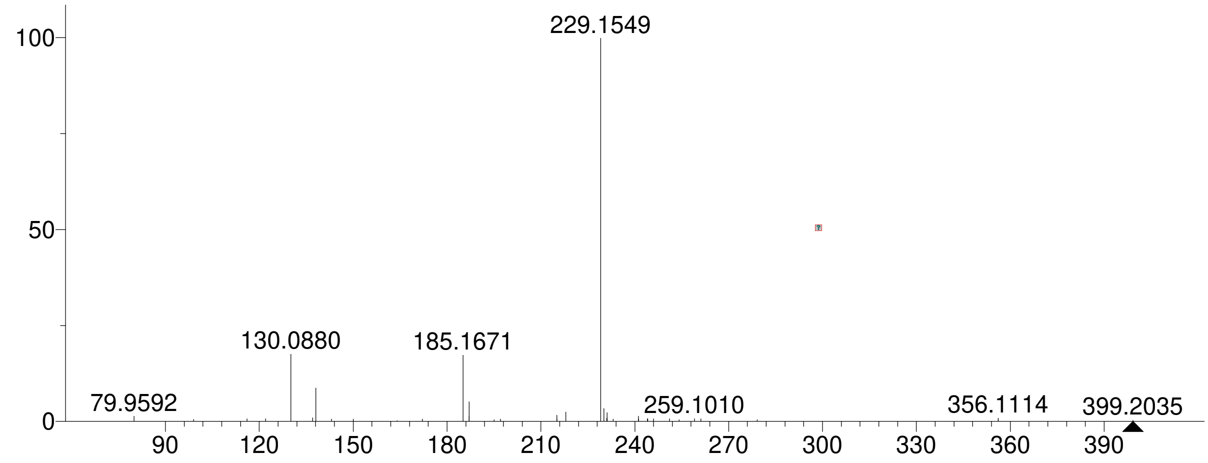

Hit 1 : Resolvin D2 [M+Na]+ HCD 15V P=399.2
C22H32O5; MF: 31; RMF: 435; Prob 38.1%; CAS: 810668-37-2; Lib: nist_msms; ID: 118154.

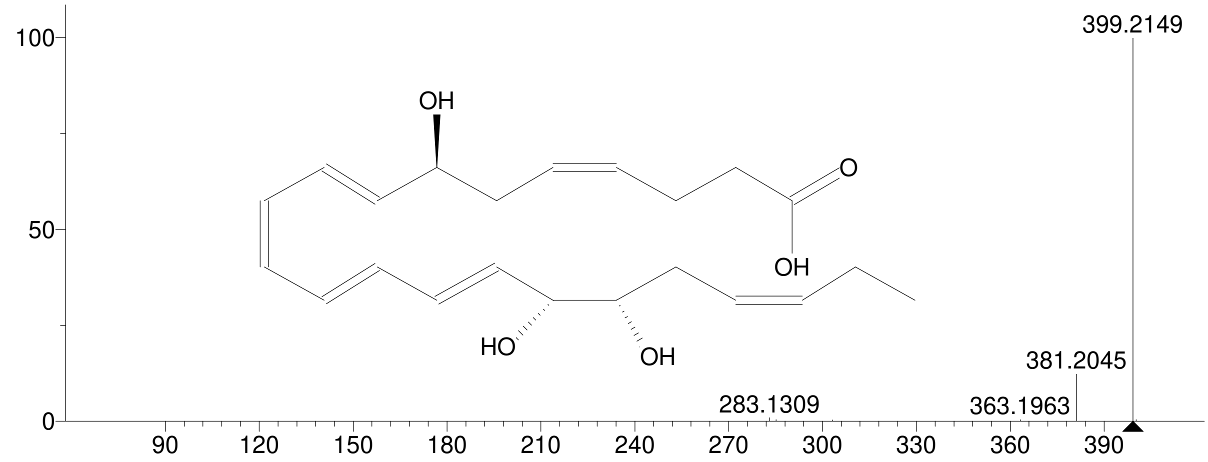

Hit 2 : N-Desmethylvinblastine [M+2H]2+ HCD 14V P=399.2
C45H56N4O9; MF: 7; RMF: 350; Prob 12.8%; CAS: 18172-50-4; Lib: nist_msms; ID: 435586.

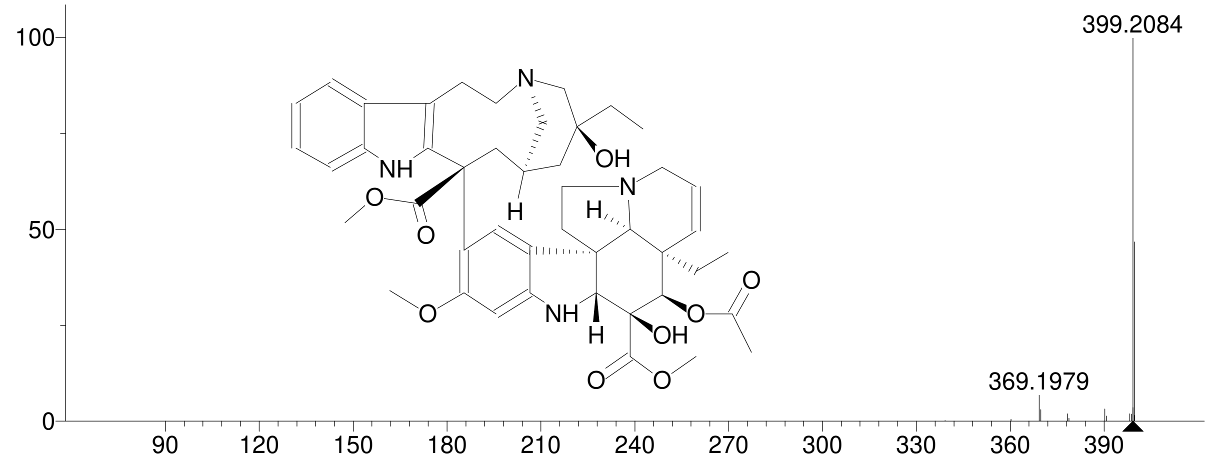

Hit 3 : 7S,8,17S-Trihydroxydocosa-4Z,9E,11E,13Z,15E,19Z-hexaenoic acid [M+Na]+ HCD 15V P=399.2
C22H32O5; MF: 1; RMF: 46; Prob 10.0%; CAS: 872993-05-0; Lib: nist_msms; ID: 118170.

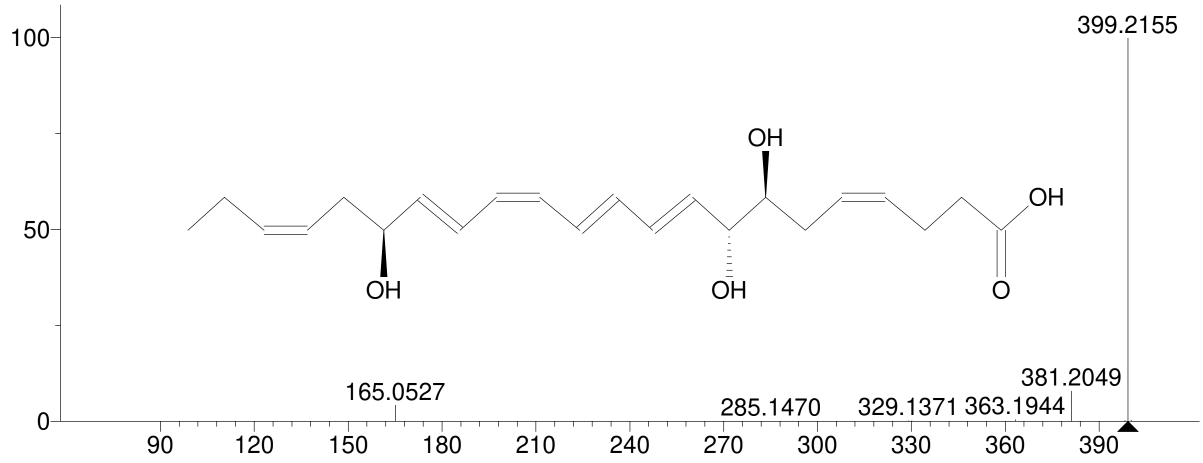

Hit 4 : NCGC00380576-01_C22H32O5_2-Pentenoic acid, 3-[(acetyloxy)methyl]-5-[(1S,4aR,8aR)-2-formyl-1,4,4a,
C22H32O5; MF: 1; RMF: 44; Prob 10.0%; Lib: mona-export-gnps; ID: 13905.

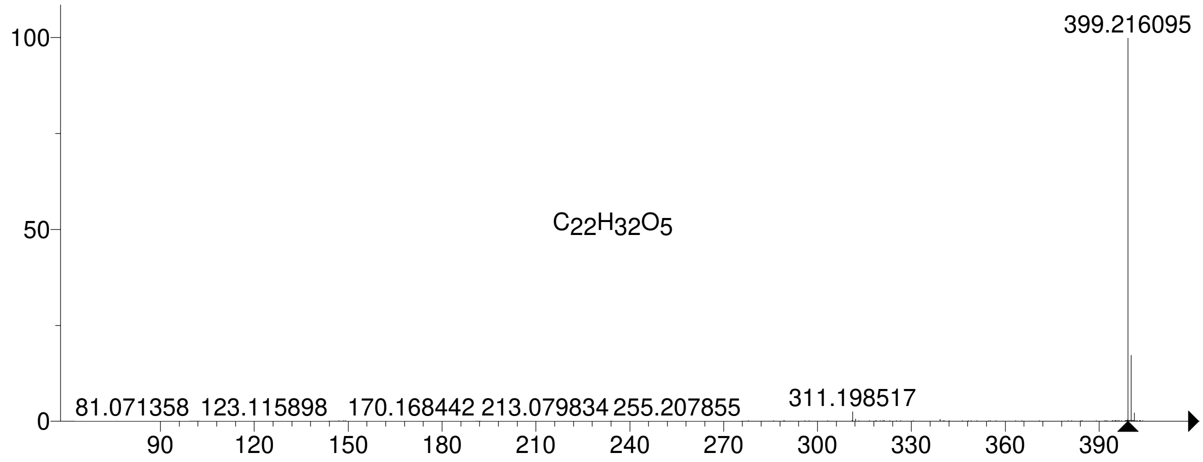

Hit 5 : His-Ser-Arg [M+H]+ QTOF 10V P=399.2
C15H26N8O5; MF: 0; RMF: 40; Prob 9.65%; Lib: nist_msms; ID: 51157.

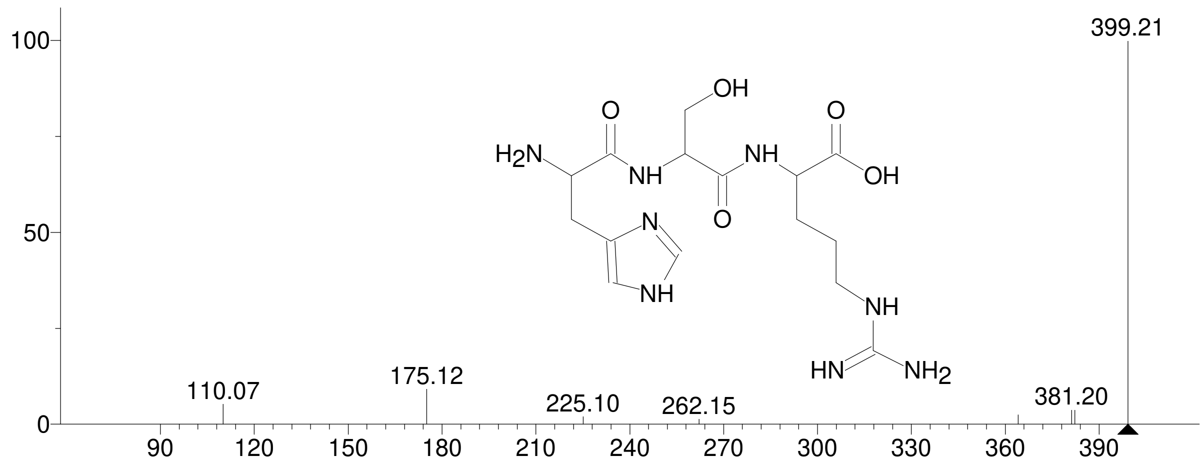

Unknown: Unknown (10.09_276.0858m/z)
Compound in Library Factor = 0

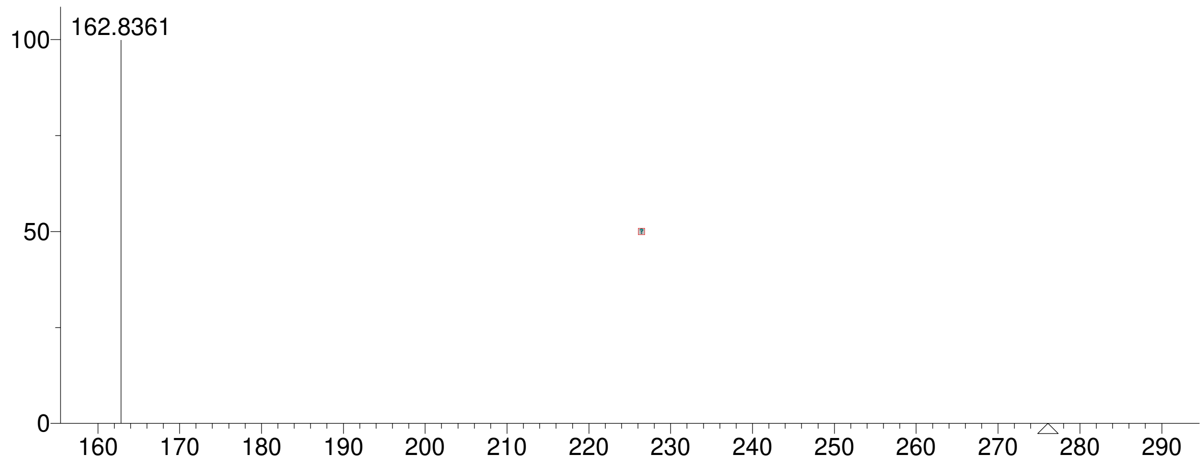

** Search Report Page 1 of 1 **

Unknown: Unknown (10.09_415.1713m/z)
Compound in Library Factor = 0

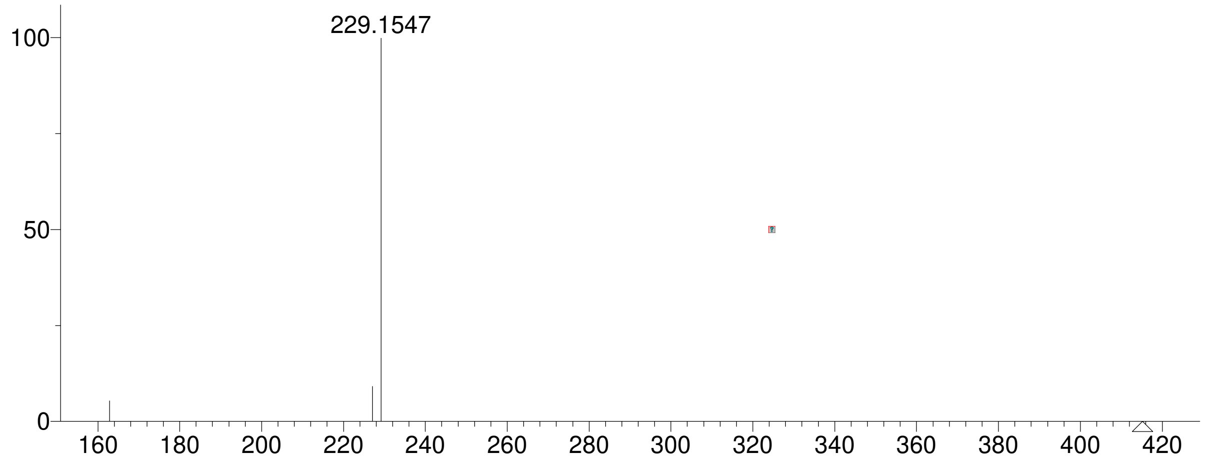

Hit 1 : 6-formyl-10-(hydroxymethyl)-5-methoxy-3-methylidene-2-oxo-2H,3H,3aH,4H,5H,8H,9H,11aH-cyclodeca[b]f
C21H28O7; MF: 0; RMF: 15; Prob 100.0%; Lib: mona-export-vf-npl_qexactive; ID: 16435.

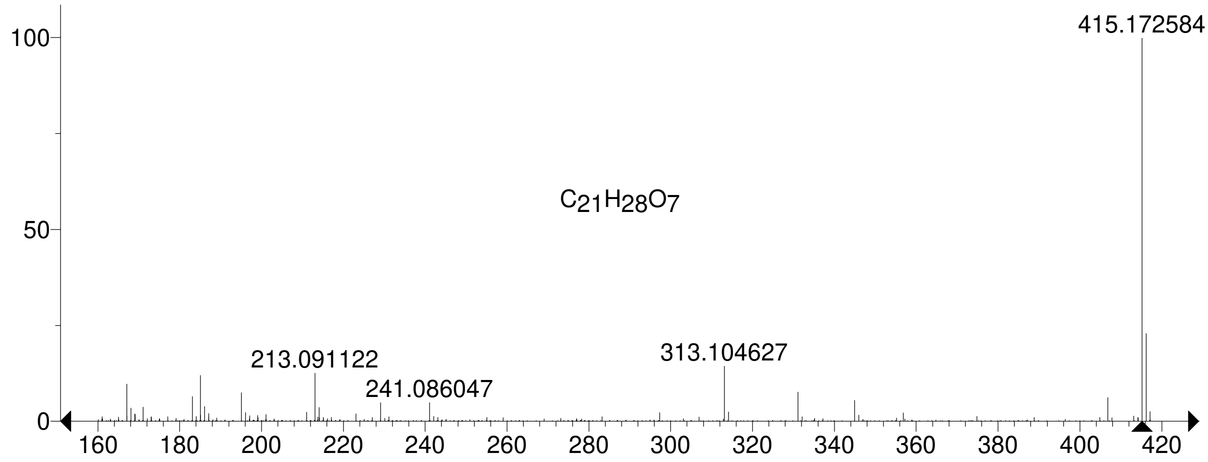

Unknown: Unknown (10.10_535.1915m/z)
Compound in Library Factor = 0

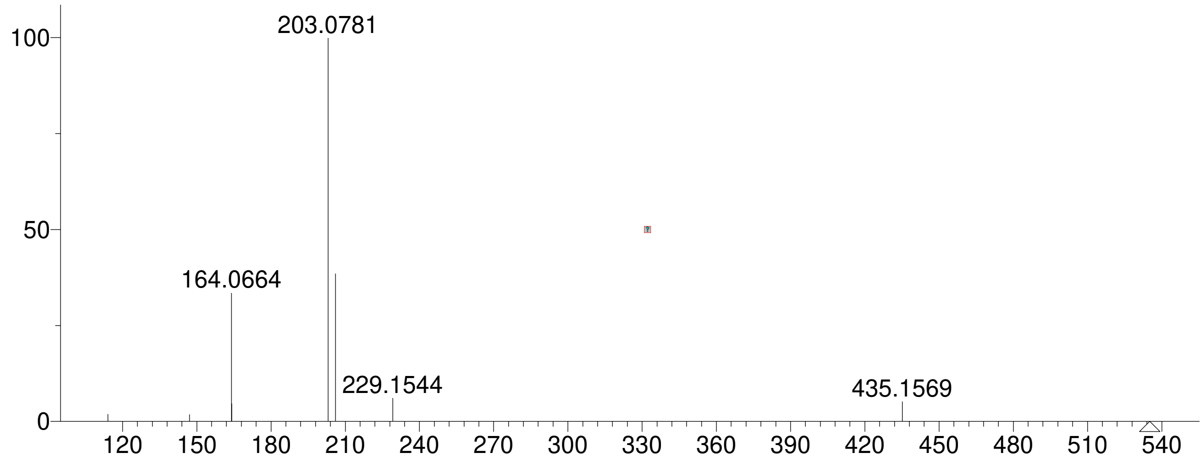

Hit 1 : NCGC00347745-02! [M-H2O+H]+ P=535.2
C30H32O10; MF: 0; RMF: 57; Prob 100.0%; Lib: mona-export-gnps; ID: 6471.

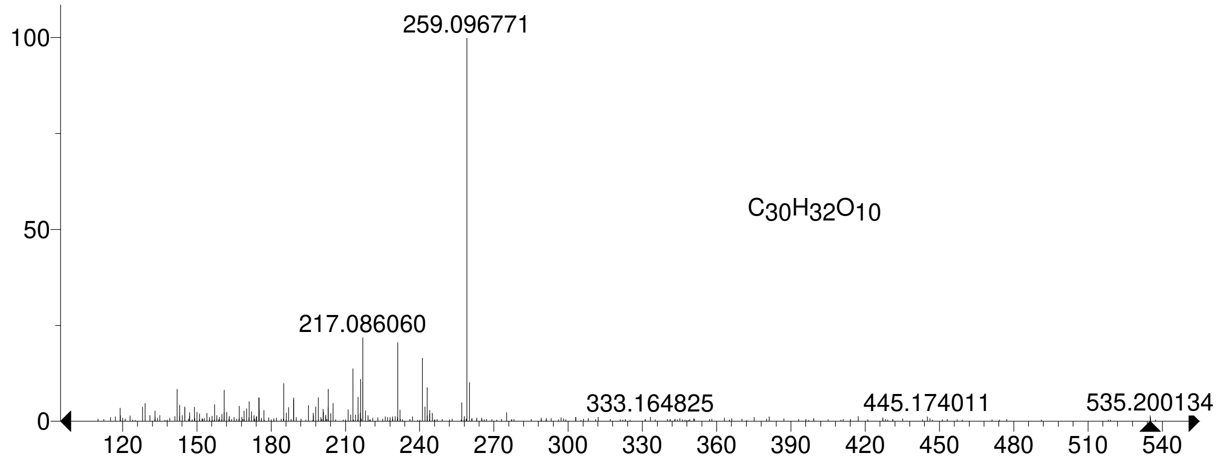

Unknown: Unknown (10.11_206.0814m/z)
Compound in Library Factor = -285

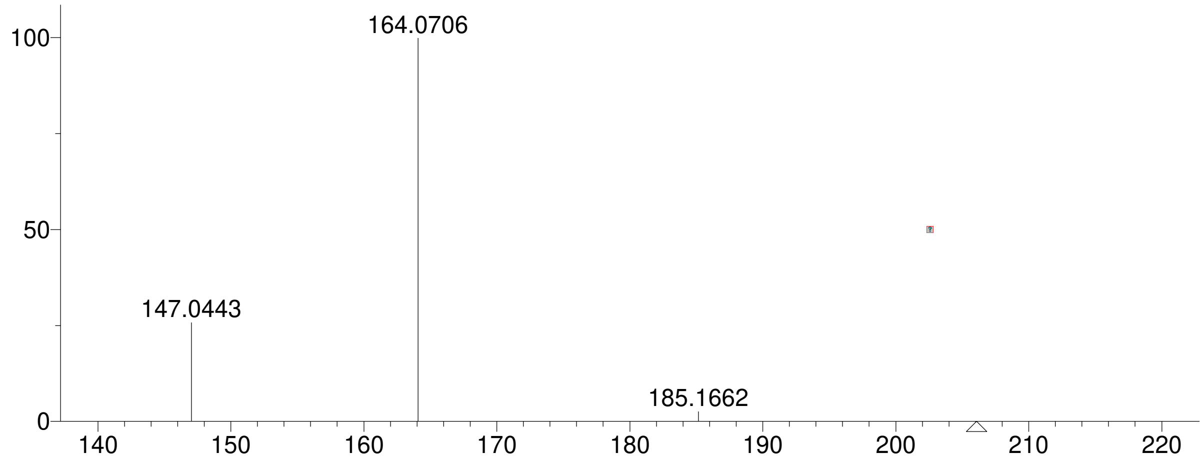

Hit 1 : N-Acetyl-L-phenylalanine [M-H]- IT-FT 35% P=206.1
C11H13NO3; MF: 559; RMF: 970; Prob 95.2%; CAS: 2018-61-3; Lib: nist_msms; ID: 290371.

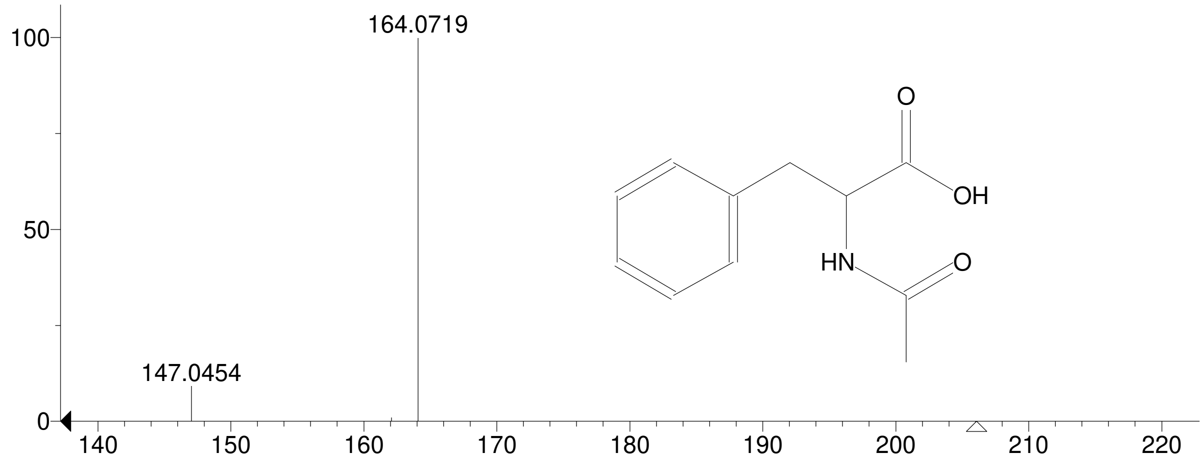

Hit 2 : N-ACETYL-L-PHENYLALANINE
C11H13NO3; MF: 425; RMF: 910; Prob 3.68%; Lib: embl neg mcg; ID: 419.

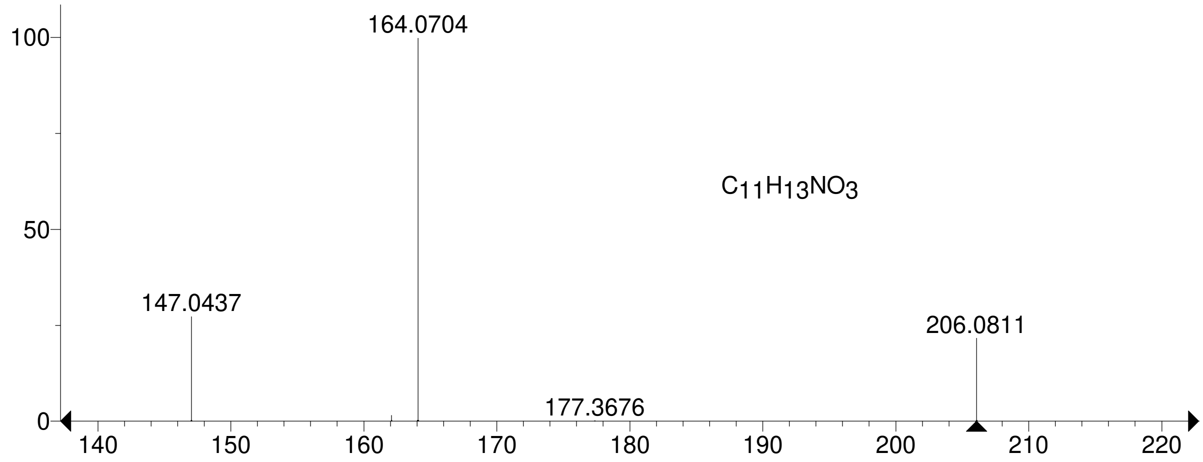

Hit 3 : N-acetylphenylalanine [M-H]- 45HCD P=206.1
C11H13NO3; MF: 372; RMF: 913; Prob 0.72%; Lib: mona-export-lc-ms-ms_negative_mode; ID: 17697.

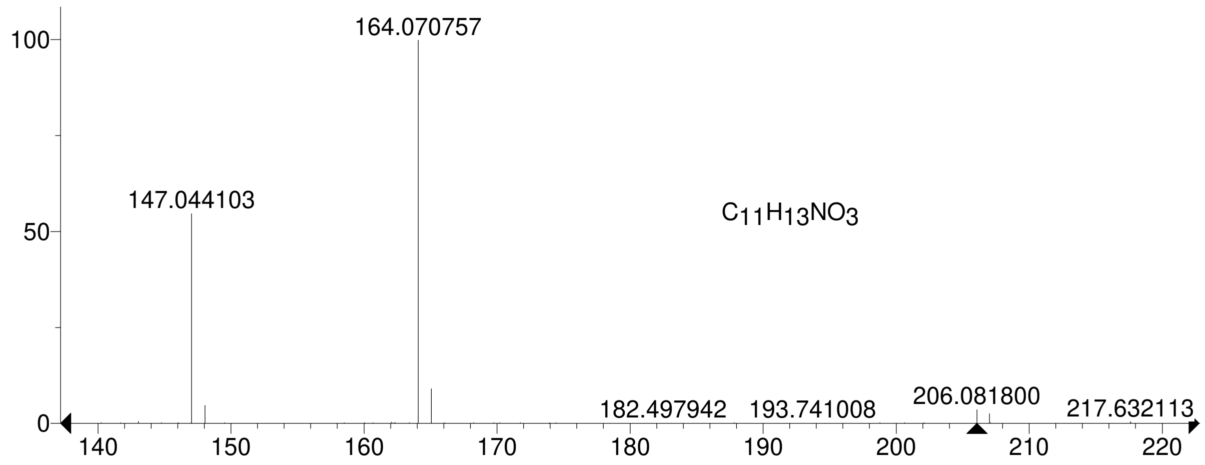

Hit 4 : N-Acetylphenylalanine [M-H]- 35eV P=206.1
C11H13NO3; MF: 354; RMF: 901; Prob 0.37%; Lib: msms-neg-fiehnhilic; ID: 1131.

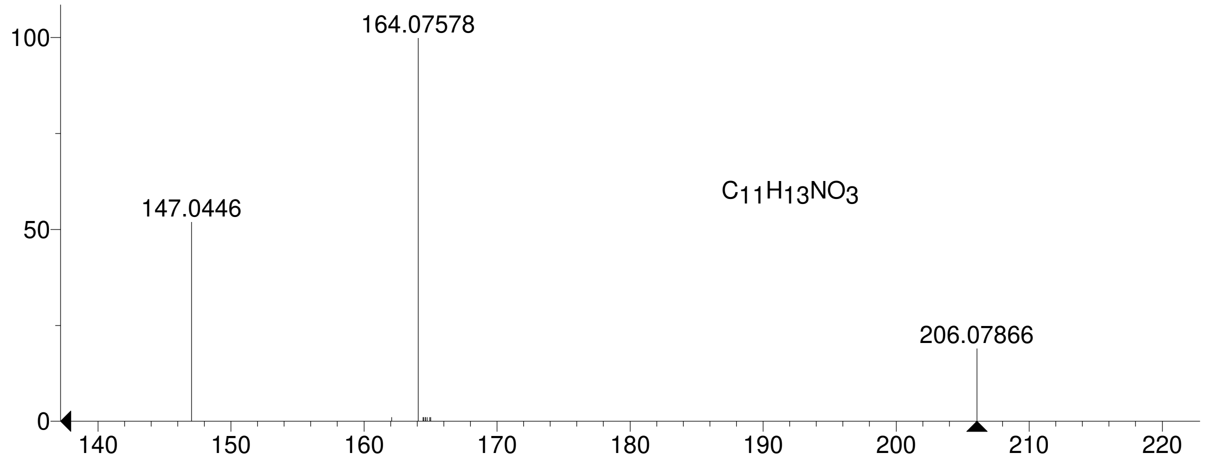

Hit 5 : N-acetylphenylalanine (CCR vessel) [M-H]- P=206.1
C11H13NO3; MF: 125; RMF: 835; Prob 0.00%; Lib: phytofrag2018_final_060219; ID: 969.

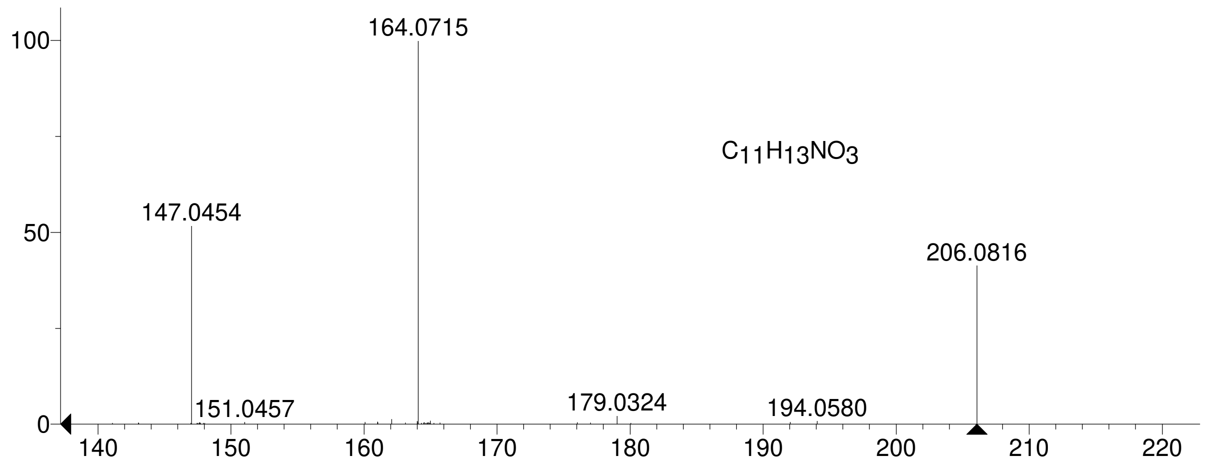

Unknown: Unknown (10.12_283.1542m/z)
Compound in Library Factor = -300

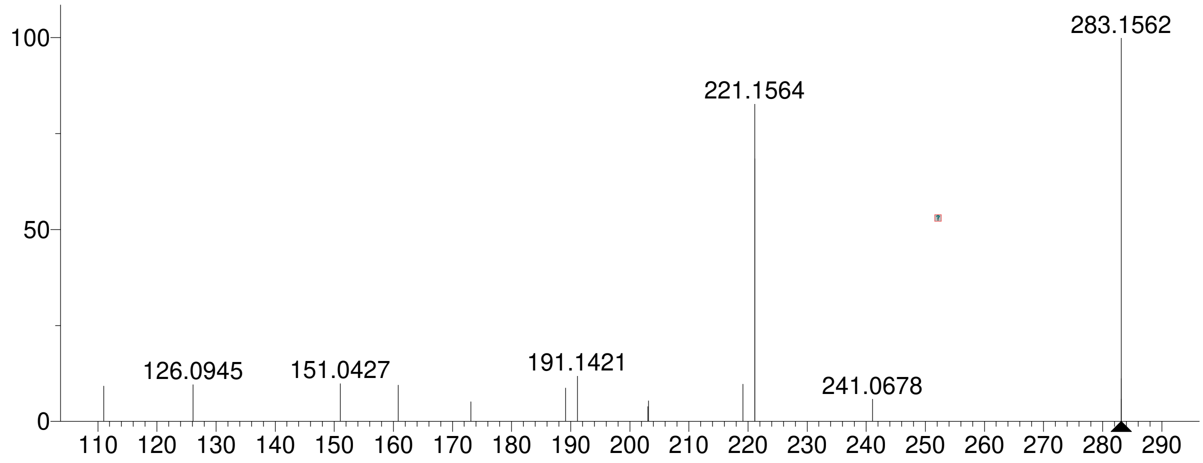

Hit 1 : Artemisinin [M+H]+ HCD 2V P=283.2
C15H22O5; MF: 246; RMF: 940; Prob 91.7%; CAS: 63968-64-9; Lib: nist_msms; ID: 118848.

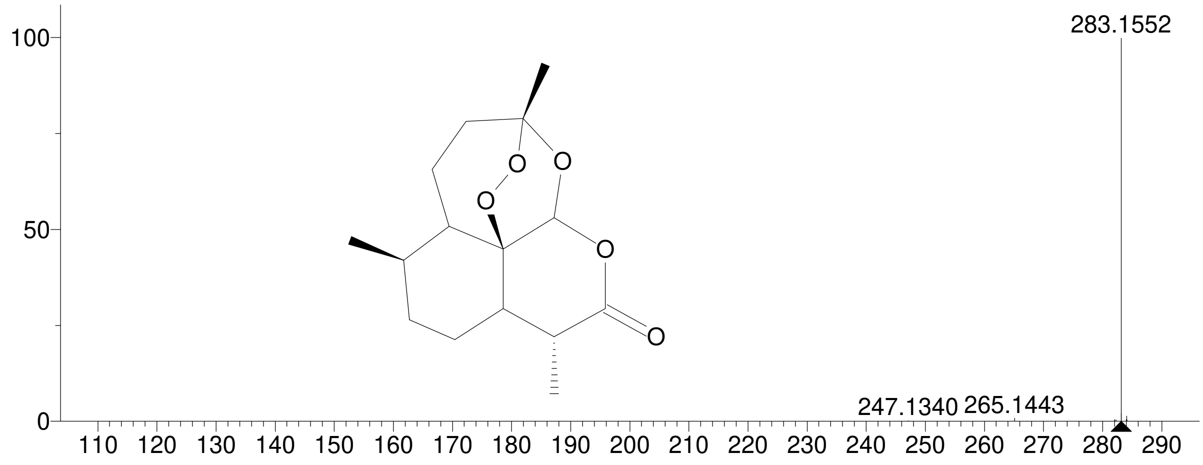

Hit 2 : isomer of dihydrophaseic acid [M+H]+ P=283.2
C15H22O5; MF: 18; RMF: 449; Prob 0.91%; Lib: mona-export-gnps; ID: 14793.

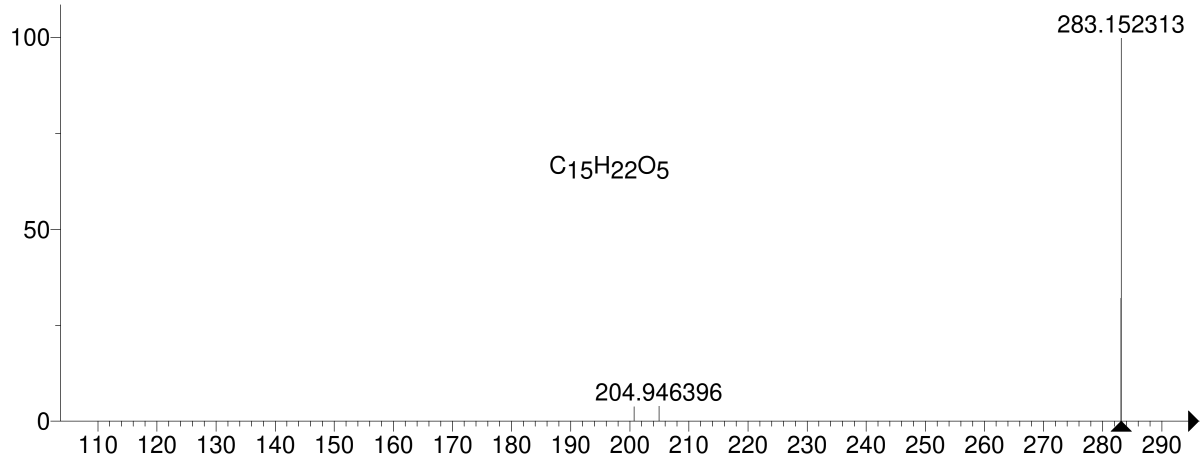

Hit 3 : Octyl gallate [M+H]+ HCD 1V P=283.2
C15H22O5; MF: 16; RMF: 511; Prob 0.84%; CAS: 1034-01-1; Lib: nist_msms; ID: 138058.

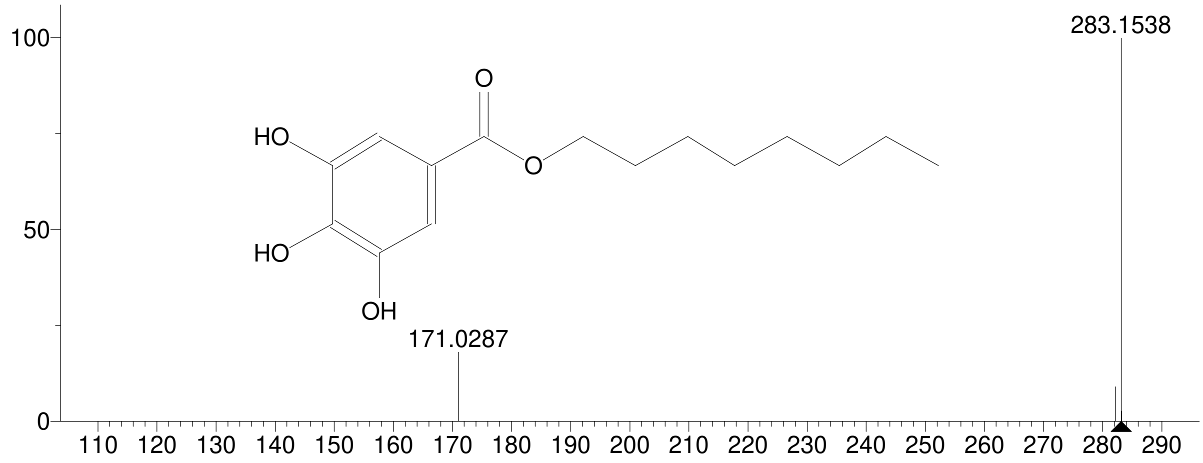

Hit 4 : (4E,6E)-2-(hydroxymethyl)-3-(3-methoxy-3-oxopropyl)deca-4,6-dienoic acid [M-H]- 35HCD P=283.2
C15H24O5; MF: 10; RMF: 140; Prob 0.66%; Lib: mona-export-lc-ms-ms_negative_mode; ID: 22052.

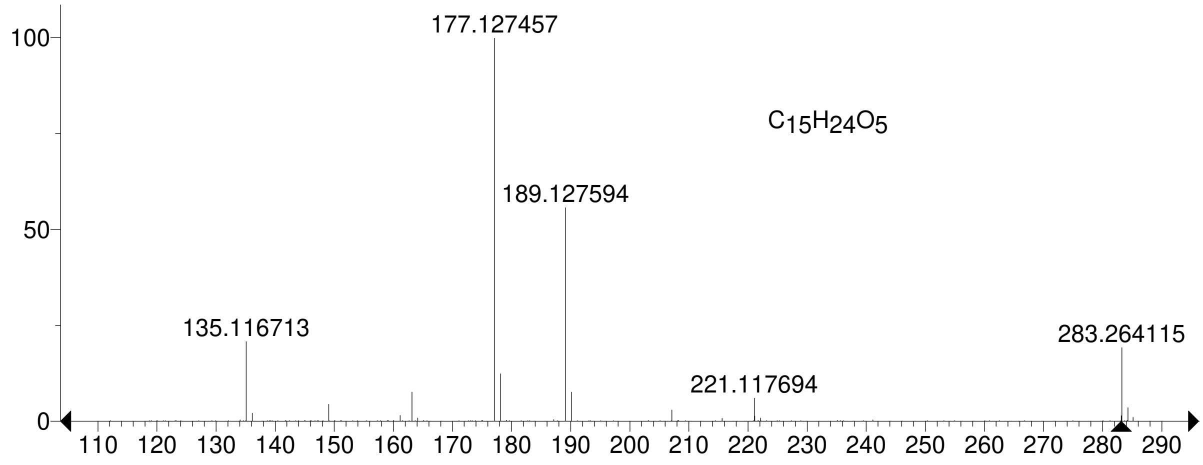

Hit 5 : 5,9-dihydroxy-7-(hydroxymethyl)-5,7-dimethyl-4,5a,6,8,8a,9-hexahydro-1H-azuleno[5,6-c]furan-3-one [M+I
C15H22O5; MF: 4; RMF: 185; Prob 0.52%; Lib: mona-export-vf-npl_qexactive; ID: 4198.

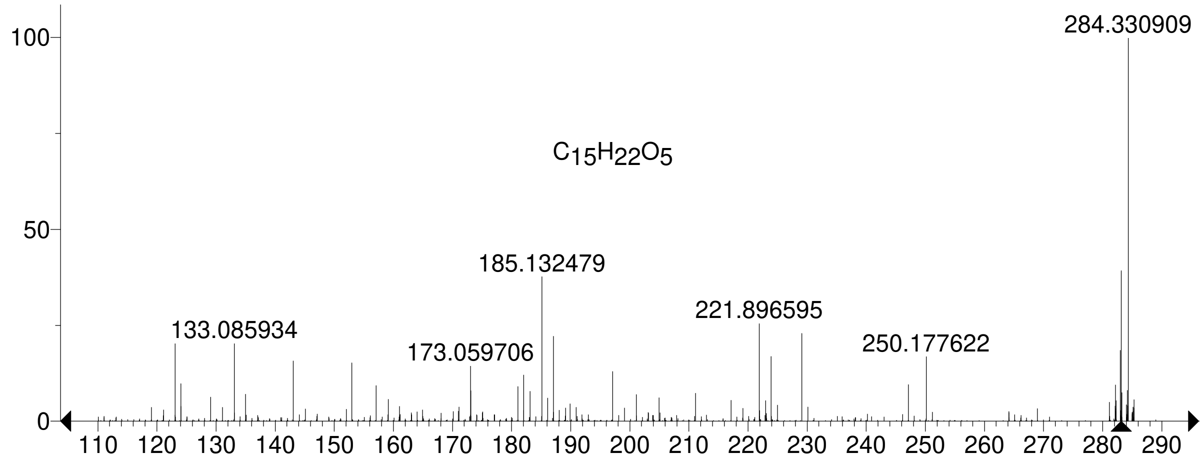

Unknown: Unknown (10.17_421.1590m/z)
Compound in Library Factor = -3273

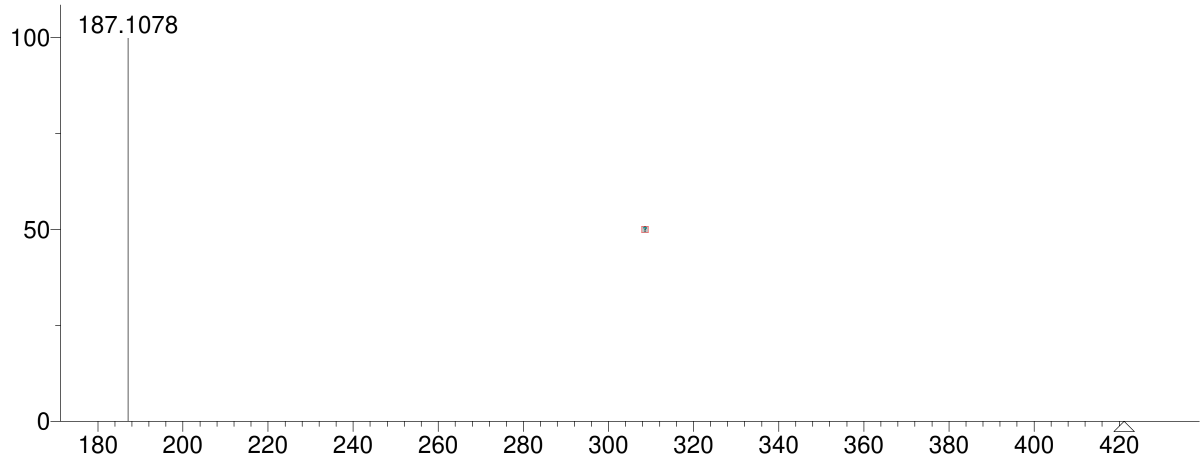

Hit 1 : 13(R),14(R)-Epoxyfluprostenol isopropyl ester [M+H-C3H12O3]+ HCD 50V P=421.2
C26H35F3O7; MF: 6; RMF: 353; Prob 55.4%; Lib: nist_msms; ID: 254116.

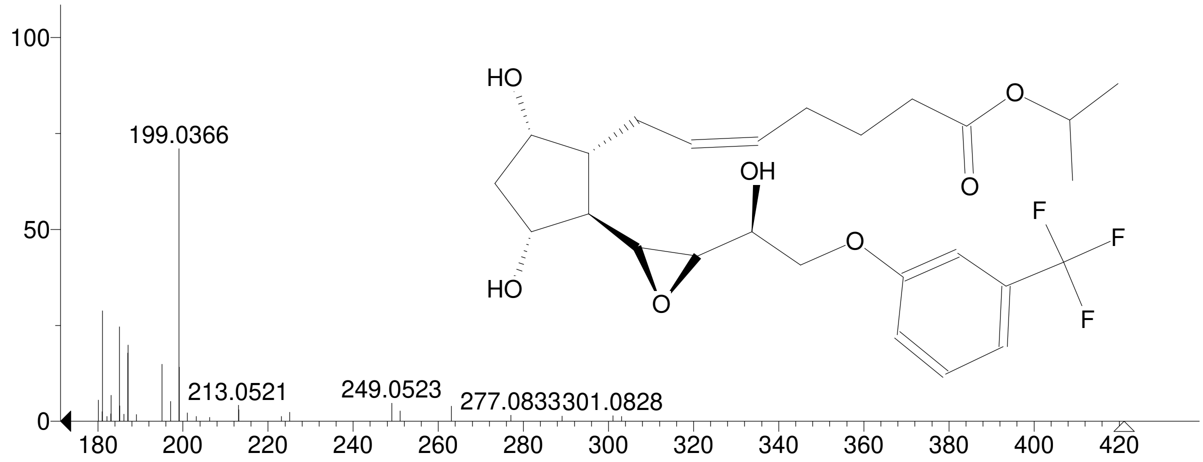

Hit 2 : Losartan [M-H]- 35eV P=421.2
C22H23ClN6O; MF: 1; RMF: 84; Prob 44.6%; Lib: msms-neg-fiehnhilic; ID: 1086.

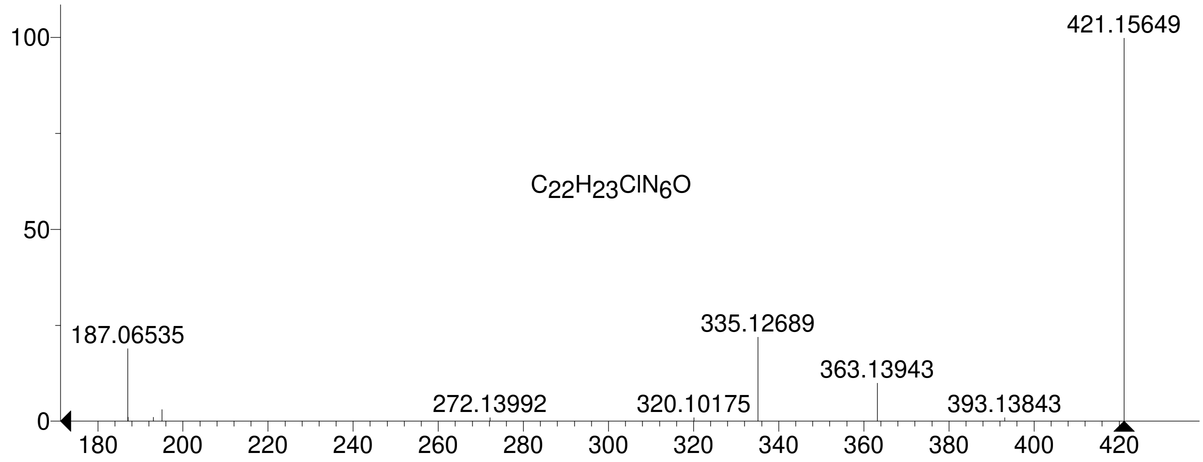

Unknown: Unknown (10.18_763.4355m/z)
Compound in Library Factor = -462

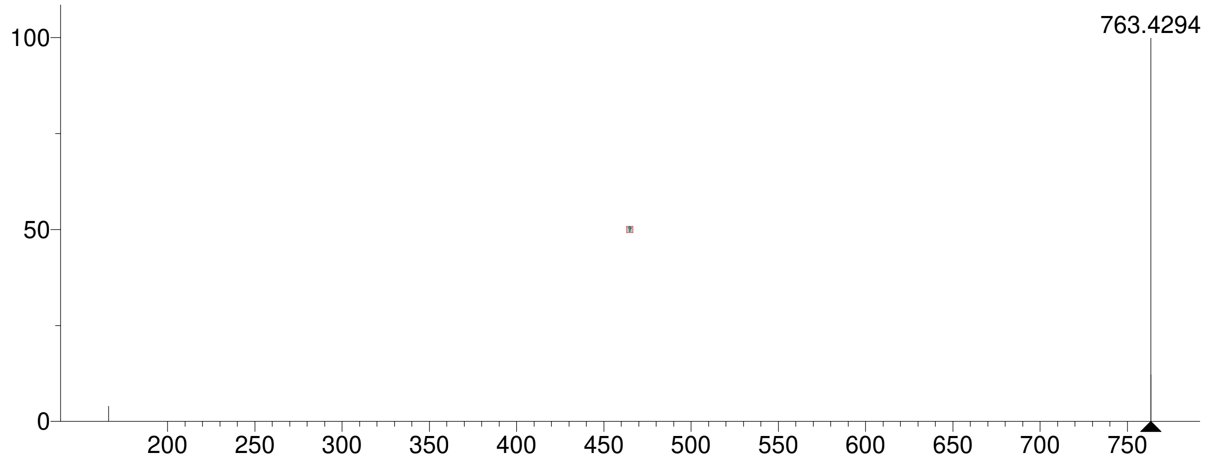

Hit 1 : MGDG 34:9 [M+Na]+ 25eV P=763.4
C43H64O10; MF: 185; RMF: 828; Prob 73.7%; Lib: mona-export-in-silico_spectra; ID: 50055.

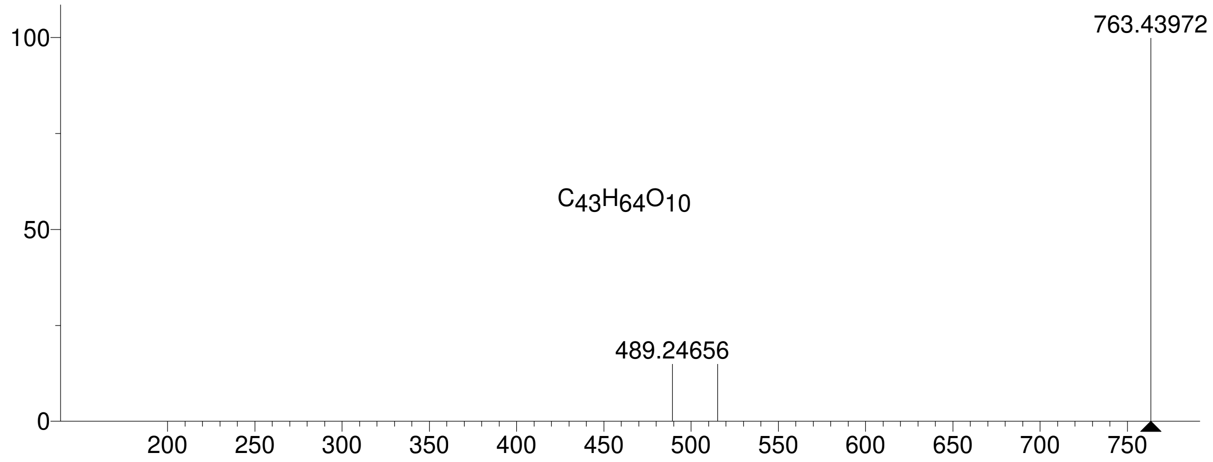

Hit 2 : PI 29:2; PI 3:0-26:2; [M-H]- [M-H]- P=763.4
C38H69O13P; MF: 44; RMF: 656; Prob 2.44%; Lib: msdial-insilicomsms-lipids-neg; ID: 326575.

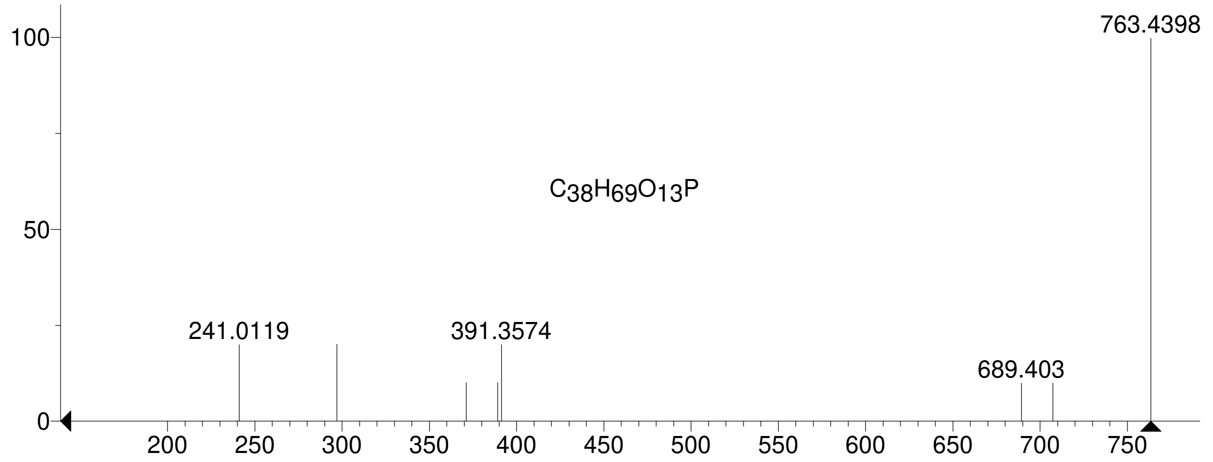

Hit 3 : PI 29:2; PI 5:0-24:2; [M-H]- [M-H]- P=763.4
C38H69O13P; MF: 44; RMF: 656; Prob 2.44%; Lib: msdial-insilicomsms-lipids-neg; ID: 326687.

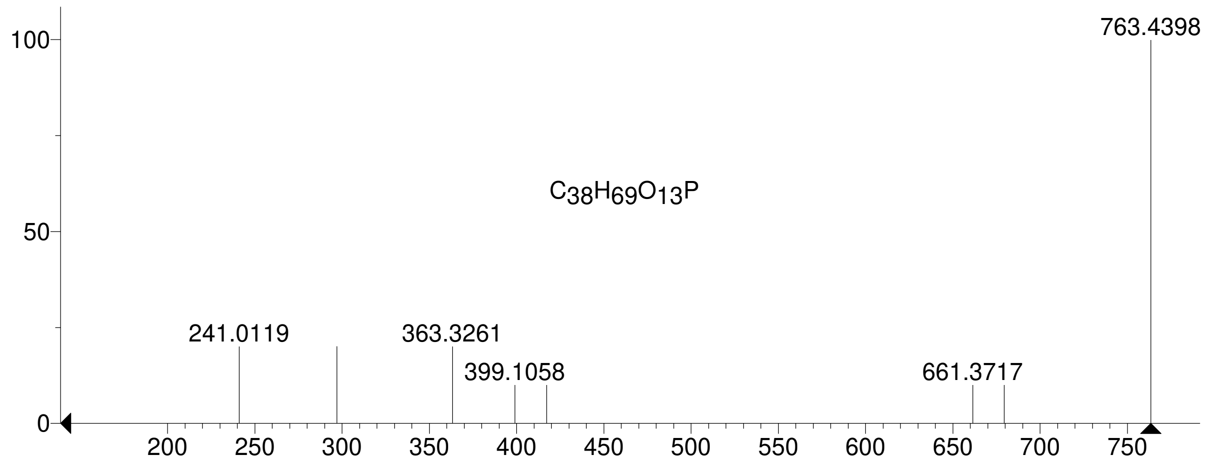

Hit 4 : PI 29:2; PI 7:0-22:2; [M-H]- [M-H]- P=763.4
C38H69O13P; MF: 44; RMF: 656; Prob 2.44%; Lib: msdial-insilicomsms-lipids-neg; ID: 326792.

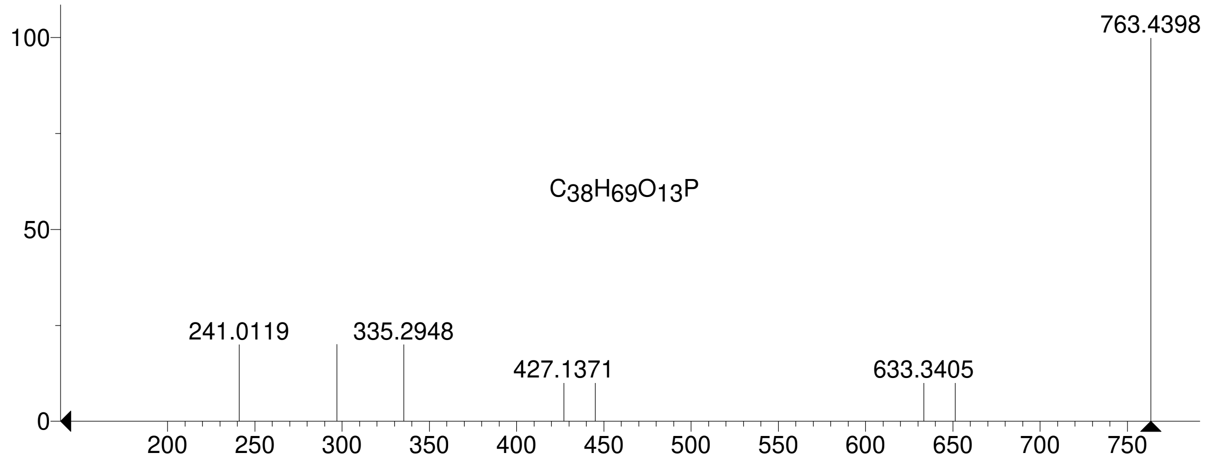

Hit 5 : PI 29:2; PI 8:0-21:2; [M-H]- [M-H]- P=763.4
C38H69O13P; MF: 44; RMF: 656; Prob 2.44%; Lib: msdial-insilicomsms-lipids-neg; ID: 326844.

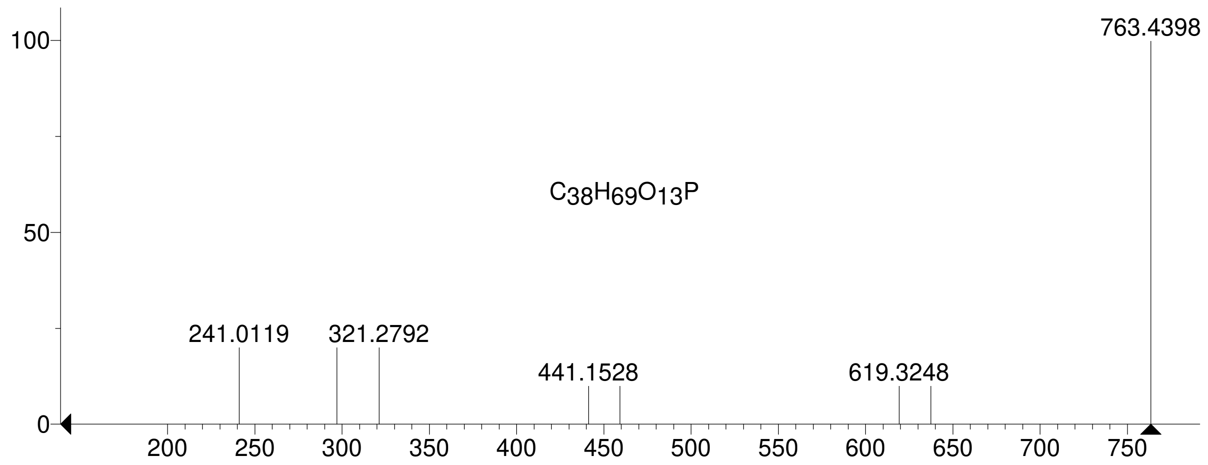

Unknown: Unknown (10.22_314.1138m/z)
Compound in Library Factor = 1575

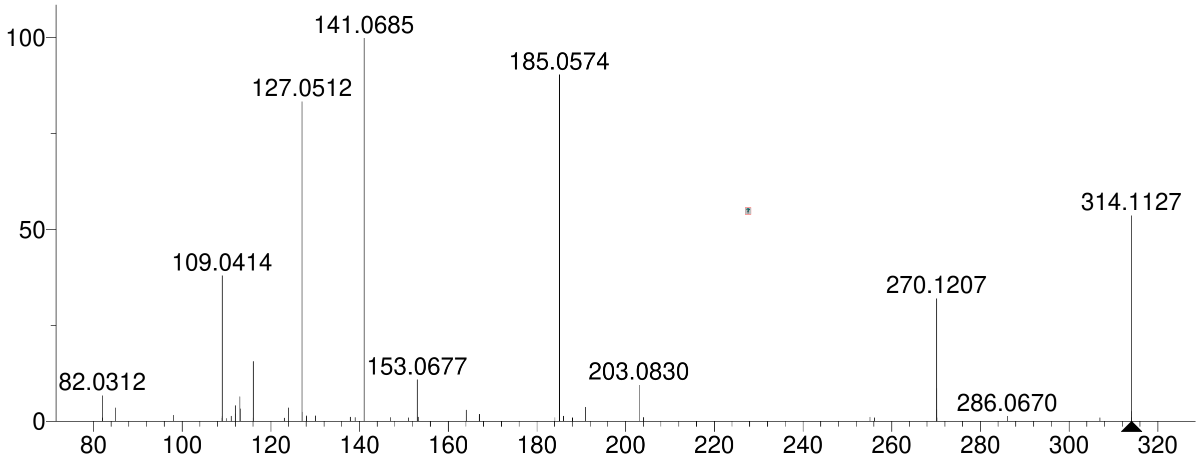

Hit 1 : PyroGlu-Trp [M-H]- HCD 23eV P=314.1
C16H17N3O4; MF: 899; RMF: 922; Prob 99.0%; CAS: 35937-24-7; Lib: nist_msms; ID: 568957.

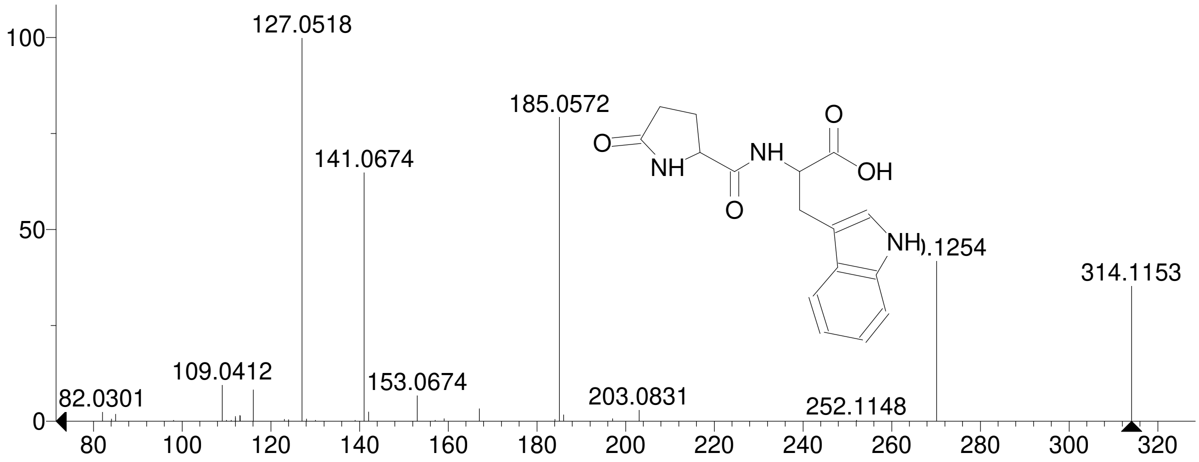

Hit 2 : Flunitrazepam [M+H]+ 20-30-40%) P=314.1
C16H12FN3O3; MF: 5; RMF: 215; Prob 0.99%; Lib: mona-export-fiehn_hilic; ID: 366.

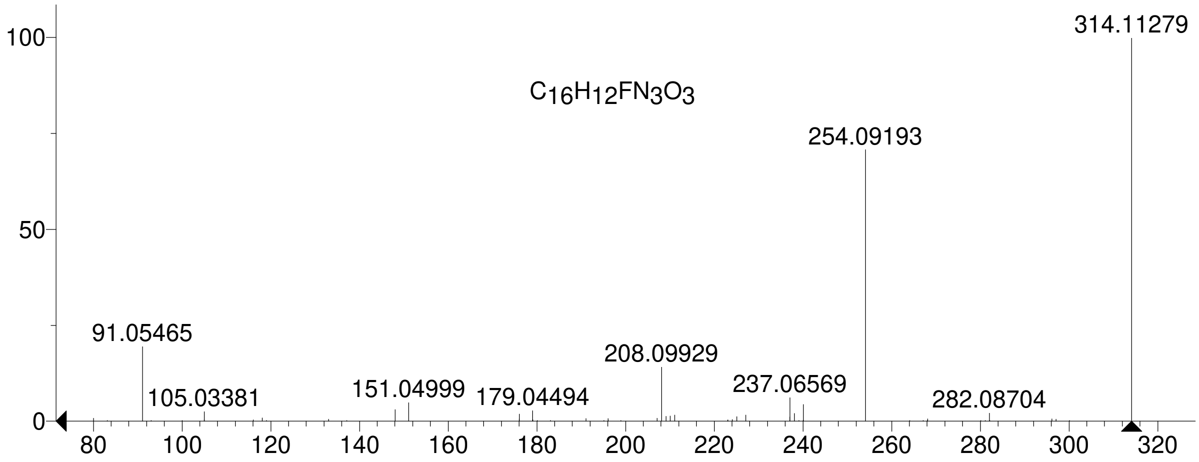

Unknown: Unknown (10.26_463.2041m/z)
Compound in Library Factor = 0

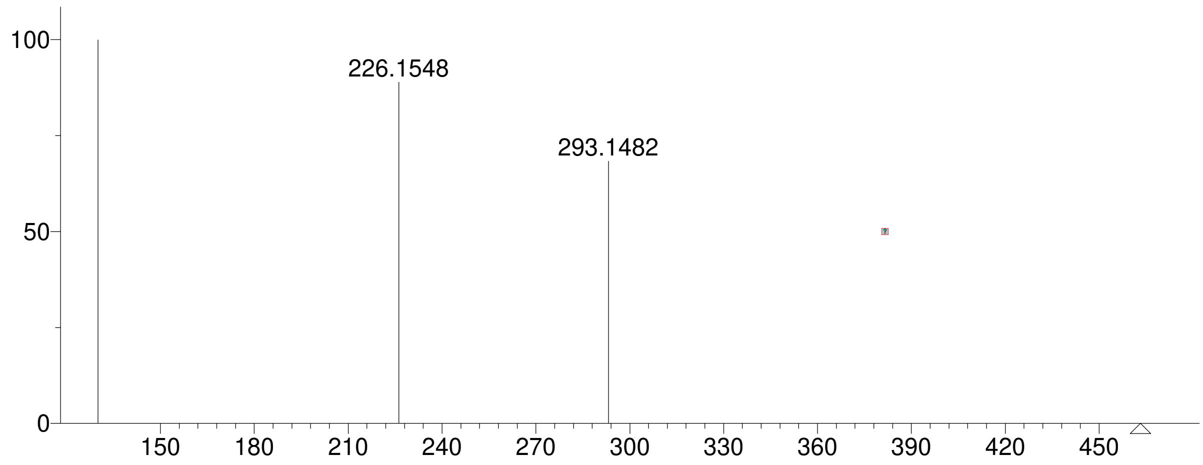

Unknown: Unknown (10.33_463.1704m/z)
Compound in Library Factor = 0

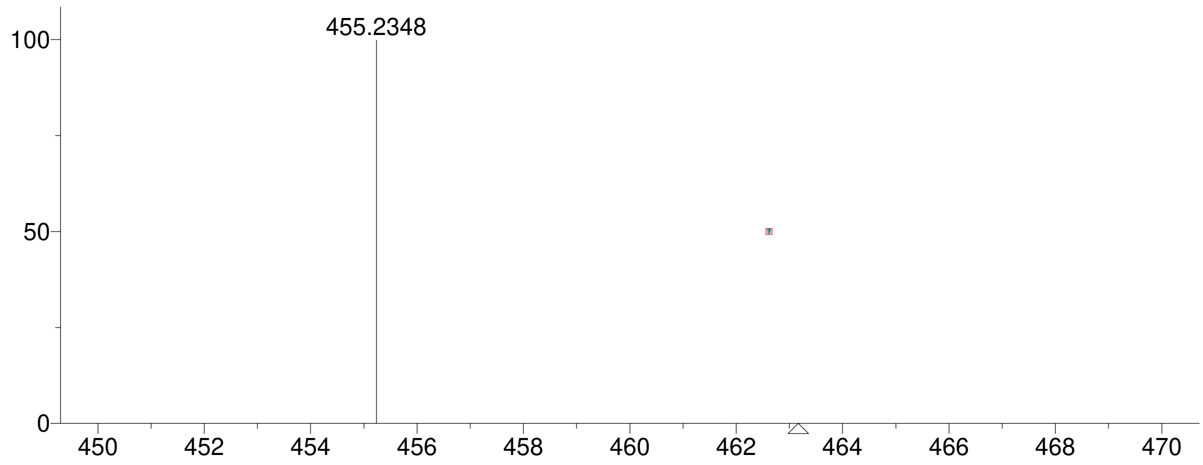

** Search Report Page 1 of 1 **

Unknown: Unknown (10.34_740.3923m/z)
Compound in Library Factor = 0

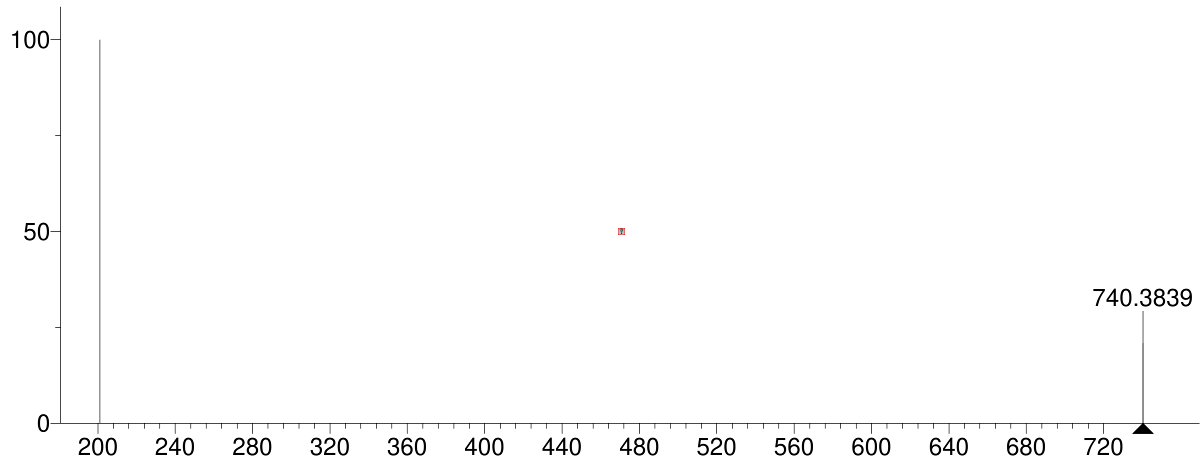

Unknown: Unknown (10.37_674.3727m/z)
Compound in Library Factor = -1848

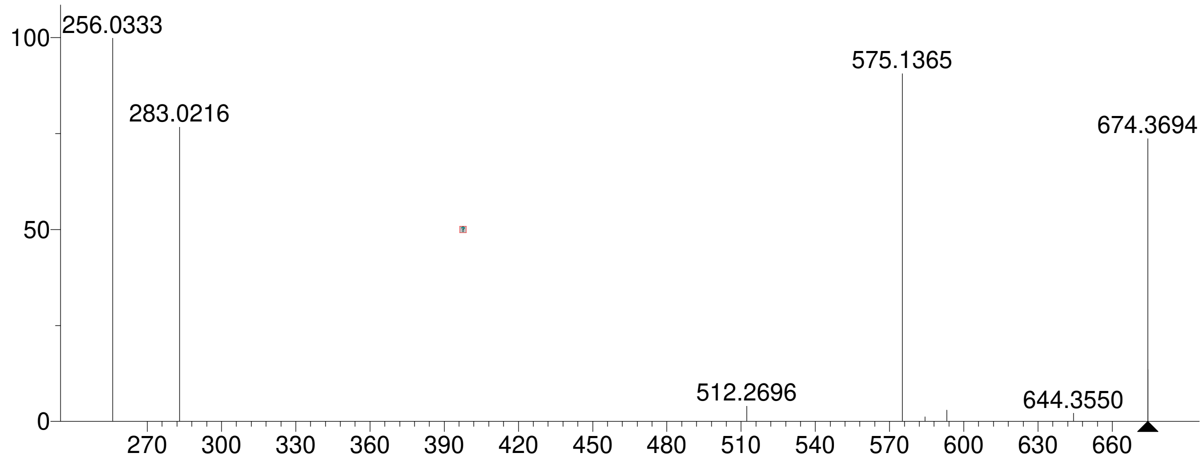

Hit 1 : Substance P reversed sequence [M+2H]2+ QQQ 10V P=674.4
C63H98N18O13S; MF: 96; RMF: 448; Prob 92.6%; Lib: nist_msms2; ID: 13465.

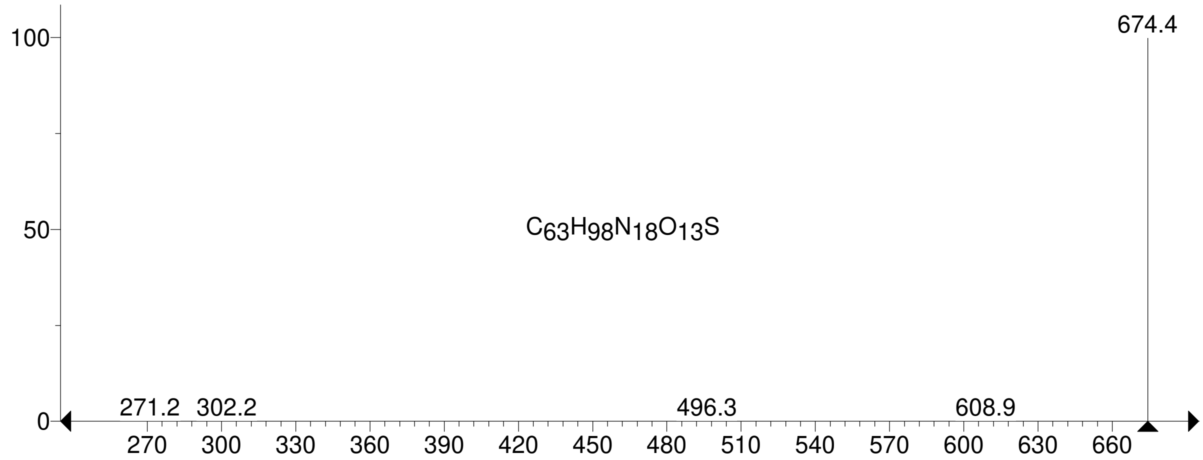

Hit 2 : Substance P [M+H]+ P=674.4
C63H98N18O13S; MF: 0; RMF: 88; Prob 7.38%; Lib: mona-export-gnps; ID: 5240.

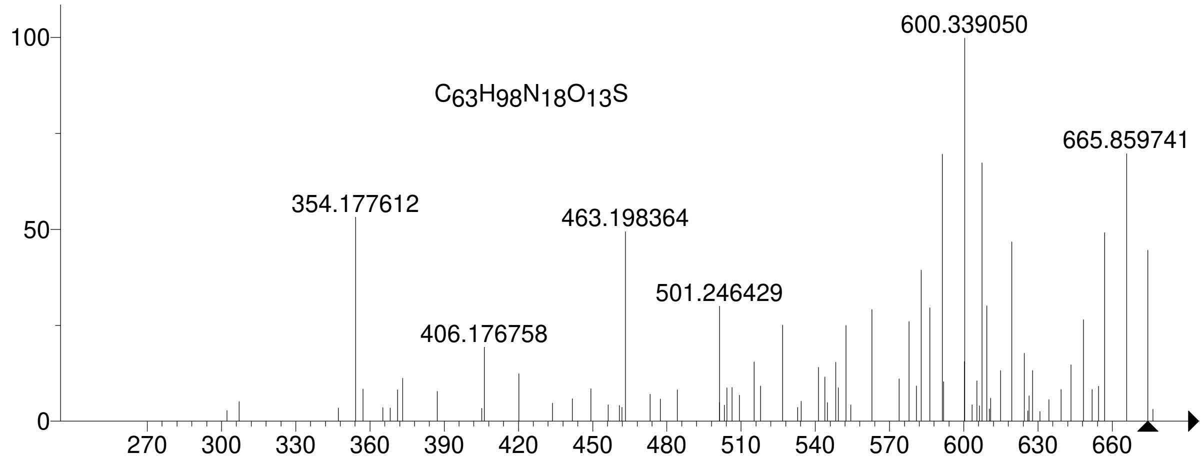

Unknown: Unknown (10.38_563.2444m/z)
Compound in Library Factor = 0

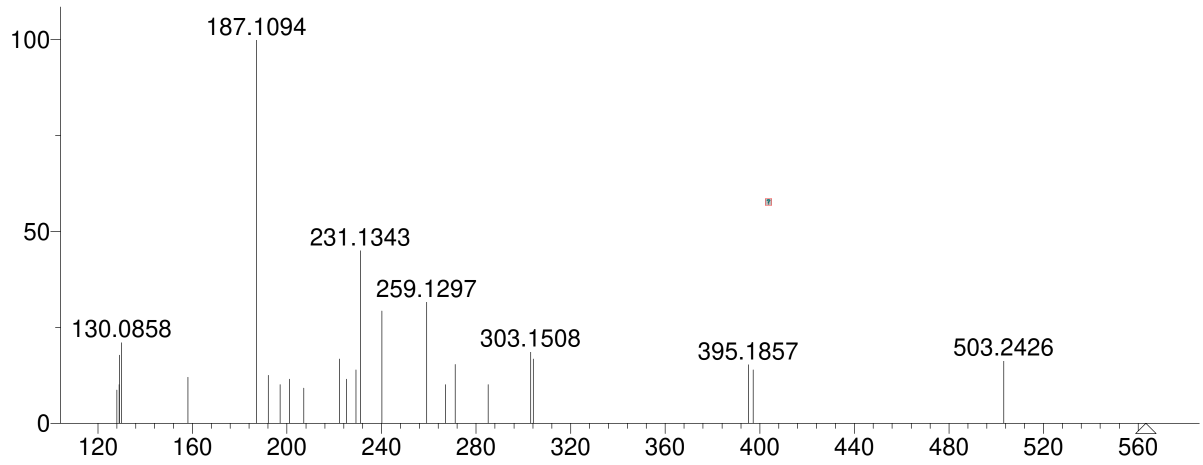

Hit 1 : Elastase Substrate IV, Colorimetric [M+H]+ QTOF 33V P=563.2
C25H34N6O9; MF: 0; RMF: 12; Prob 100.0%; Lib: nist_msms2; ID: 39070.

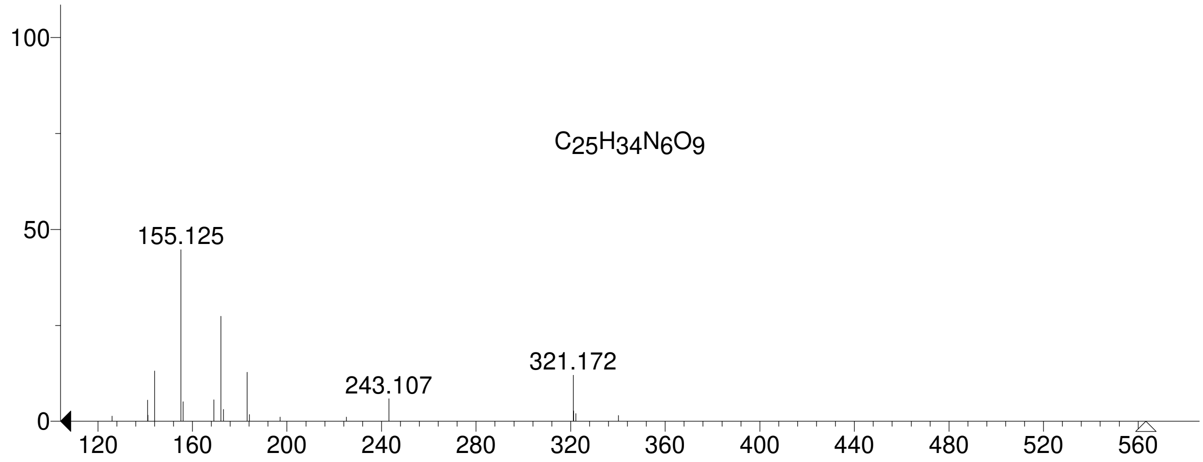

Unknown: Unknown (10.39_725.1934m/z)
Compound in Library Factor = -1194

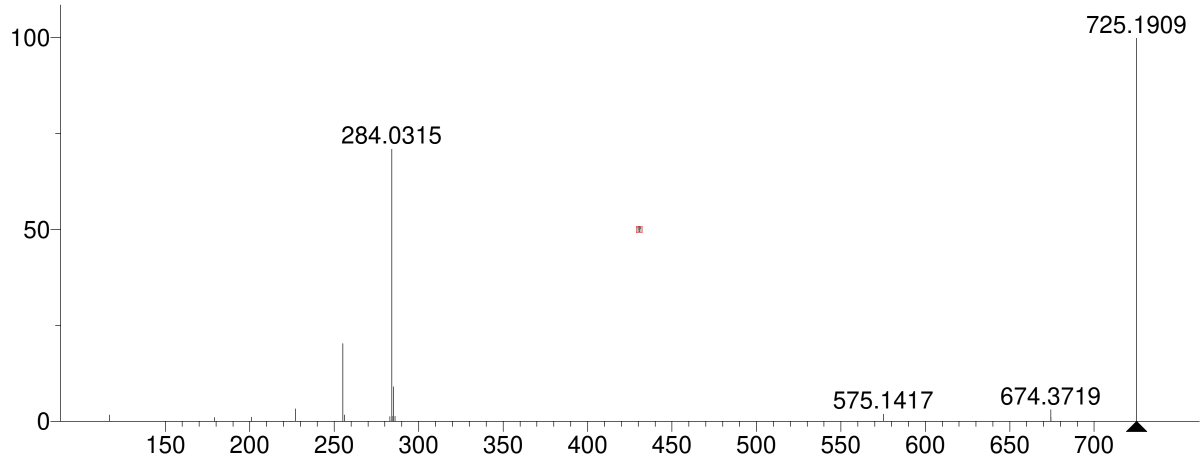

Hit 1 : NCGC00385688-01!3-[(2S,3R,4S,5S,6R)-3-[(2S,3R,4R)-3,4-dihydroxy-4-(hydroxymethyl)oxolan-2-yl]oxy-4
C32H38O19; MF: 266; RMF: 550; Prob 77.3%; Lib: msms-neg-gnps; ID: 1013.

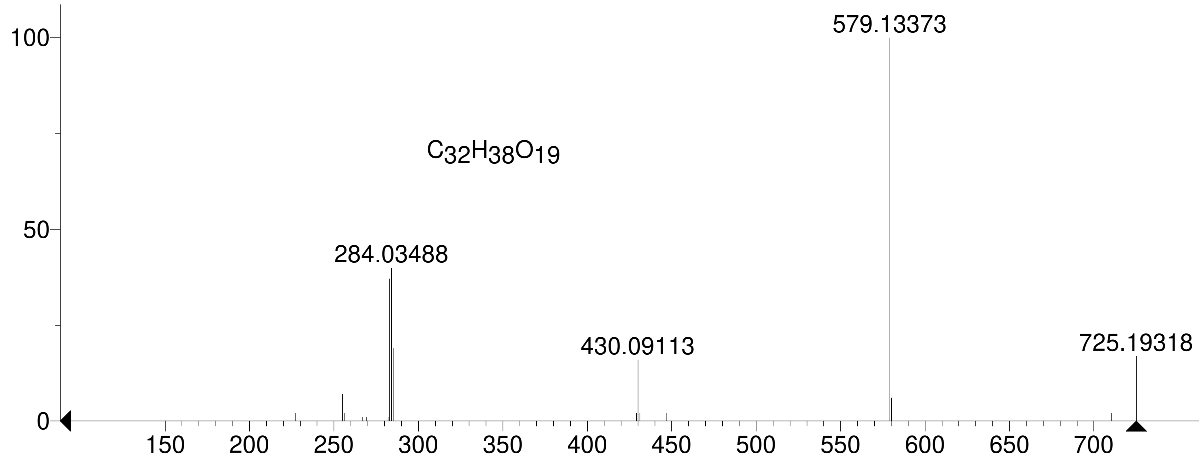

Hit 2 : Unknown (carbon number 32); PlaSMA ID-1951 [M-H]- P=725.2
C32H38O19; MF: 222; RMF: 721; Prob 16.8%; Lib: biomsms-neg-plasma; ID: 1610.

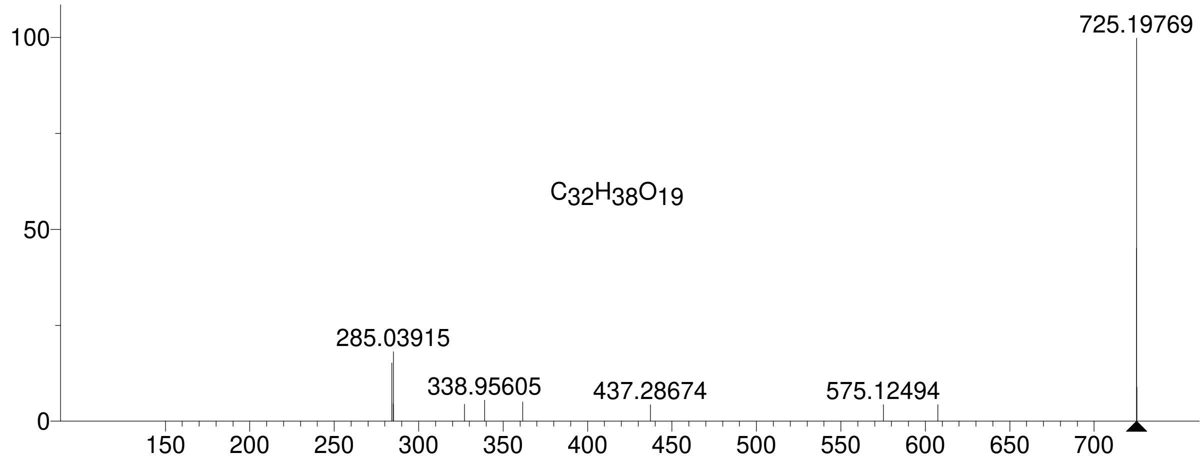

Hit 3 : NCGC00380680-01!5-hydroxy-3-[(2S,3R,4R,5S)-3-hydroxy-5-(hydroxymethyl)-4-[(2S,3R,4S,5S,6R)-3,4,5-t
C32H38O19; MF: 173; RMF: 457; Prob 3.44%; Lib: msms-neg-gnps; ID: 1331.

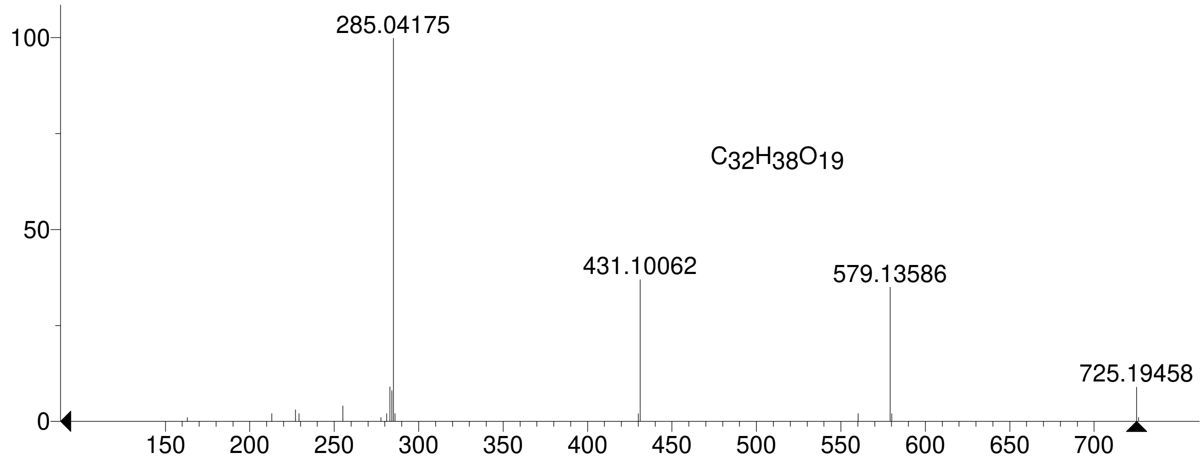

Hit 4 : 5-hydroxy-3-[(2S,3R,4R,5S)-3-hydroxy-5-(hydroxymethyl)-4-[(2S,3R,4S,5S,6R)-3,4,5-trihydroxy-6-(hydroxy
C32H38O19; MF: 161; RMF: 428; Prob 2.29%; Lib: anthocyanins mona; ID: 5277.

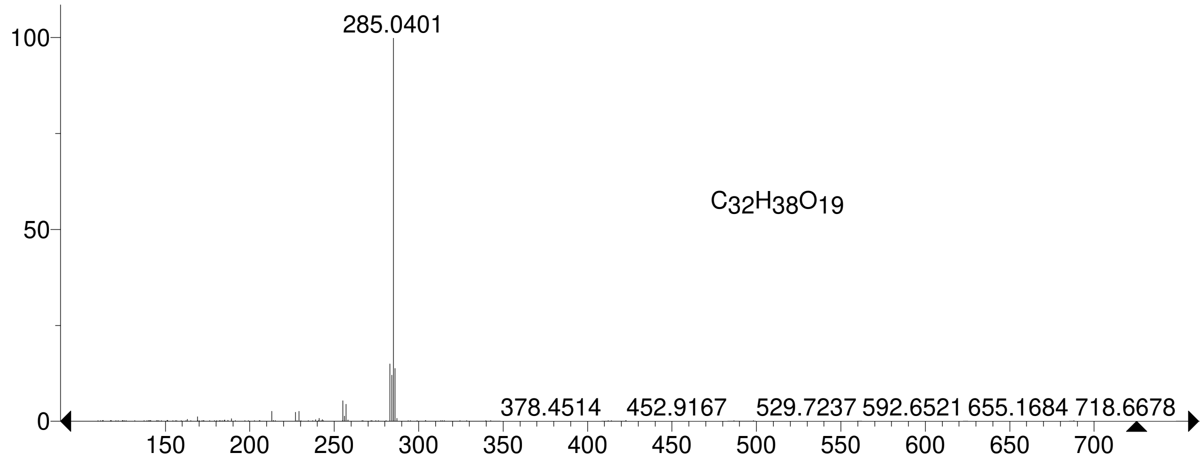

Hit 5 : apigenin 6/8C-hexoside-8/6C-pentoside + hexose (flavonoid) [M-H]- P=725.2
C32H38O19; MF: 12; RMF: 569; Prob 0.06%; Lib: phytofrag2018_final_060219; ID: 142.

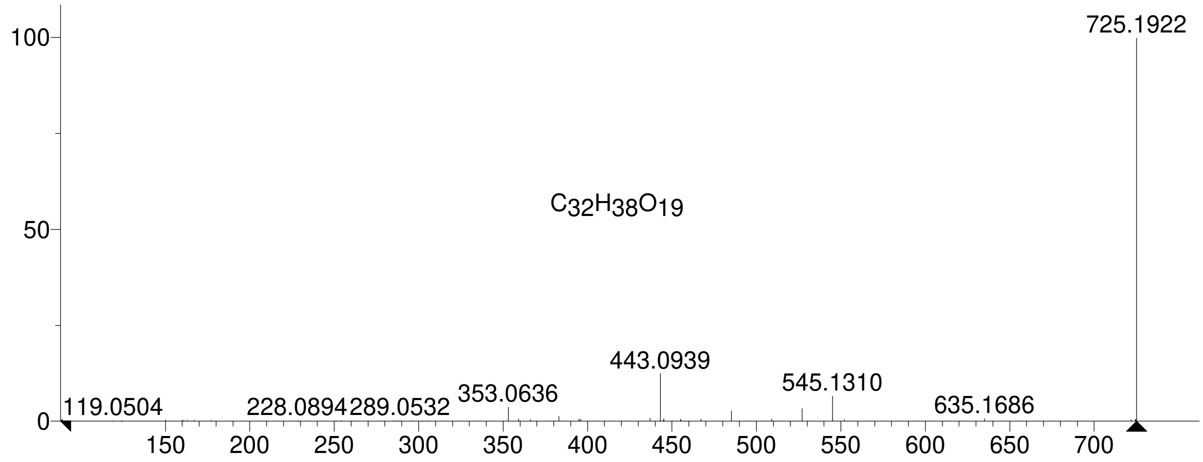

** Search Report Page 1 of 1 **

Unknown: Unknown (10.40_340.1857m/z)
Compound in Library Factor = -246

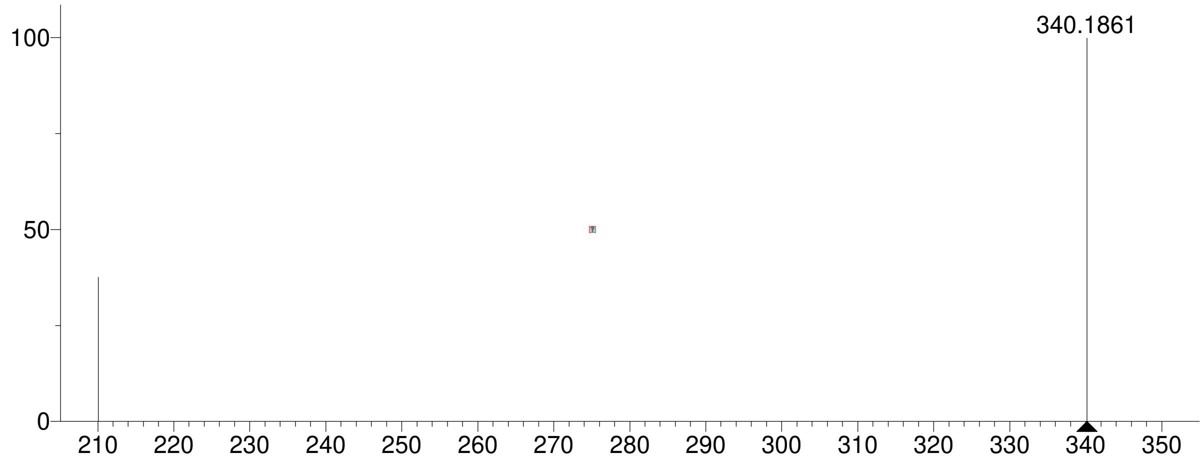

Hit 1 : Cyclohexanecarboxylic acid, 1-phenyl-, 2-(4-morpholinyl)ethyl ester [M+Na]+ HCD 13V P=340.2
C19H27NO3; MF: 266; RMF: 851; Prob 100.0%; CAS: 138847-85-5; Lib: nist_msms; ID: 423006.

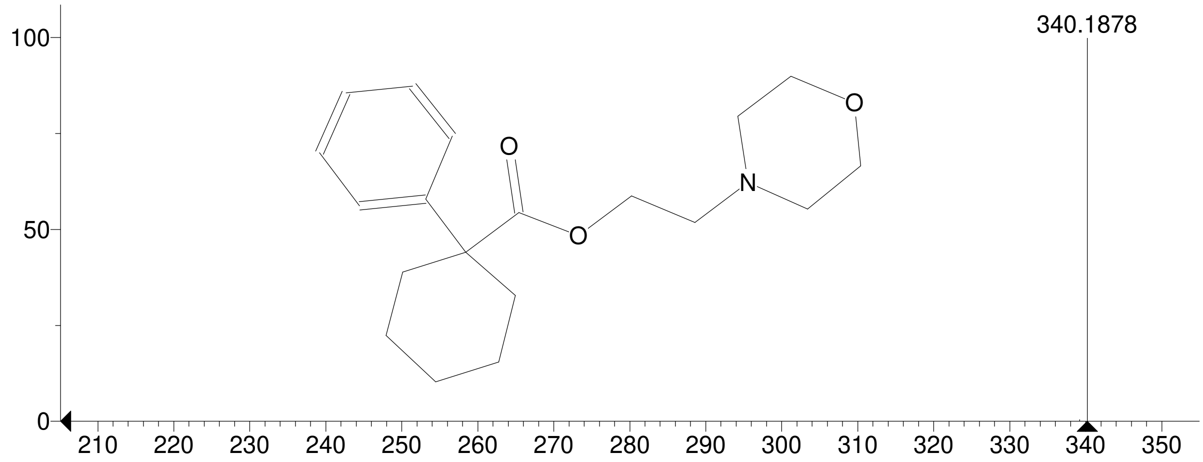

Unknown: Unknown (10.41_401.0530m/z)
Compound in Library Factor = 0

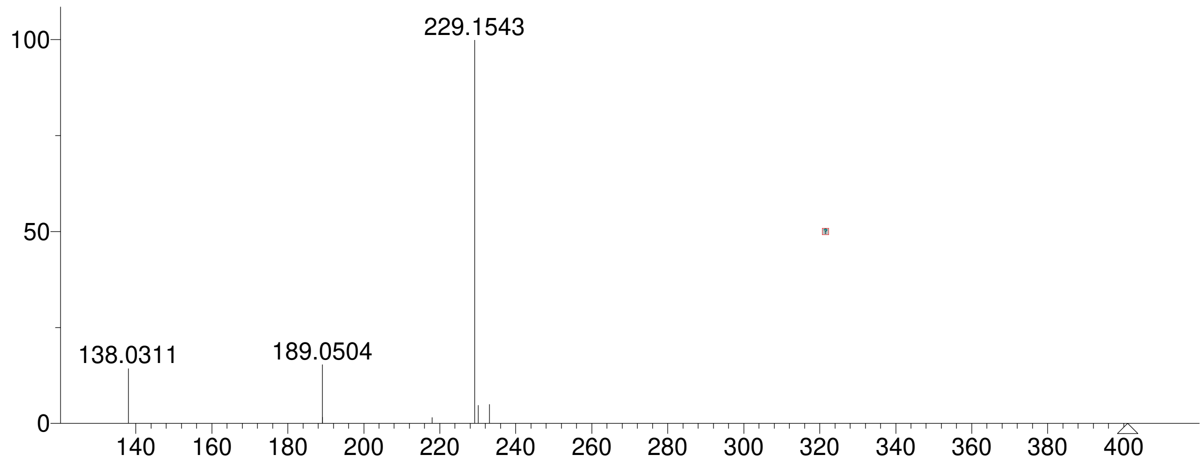

Unknown: Unknown (10.43_311.1390m/z)
Compound in Library Factor = -1848

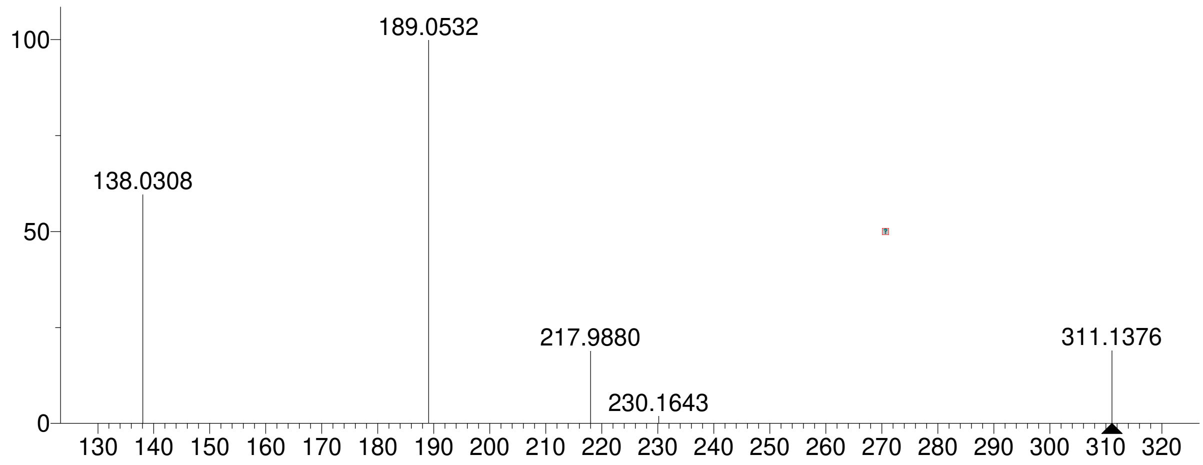

Hit 1 : Dodecyl phosphate [M-H+2Na]+ HCD 12V P=311.1
C12H27O4P; MF: 54; RMF: 308; Prob 62.4%; CAS: 2627-35-2; Lib: nist_msms; ID: 216267.

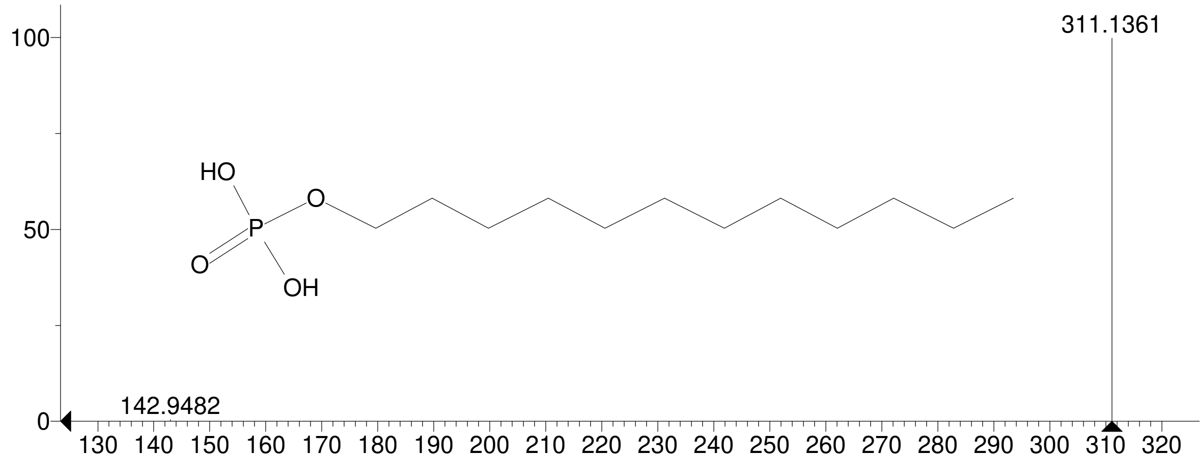

Hit 2 : 1H-Pyrrole-3-propanoic acid, 5-[(1,2-dihydro-2-oxo-3H-indol-3-ylidene)methyl]-2,4-dimethyl- [M+H]+ HCD
C18H18N2O3; MF: 24; RMF: 290; Prob 17.4%; CAS: 252916-29-3; Lib: nist_msms; ID: 419355.

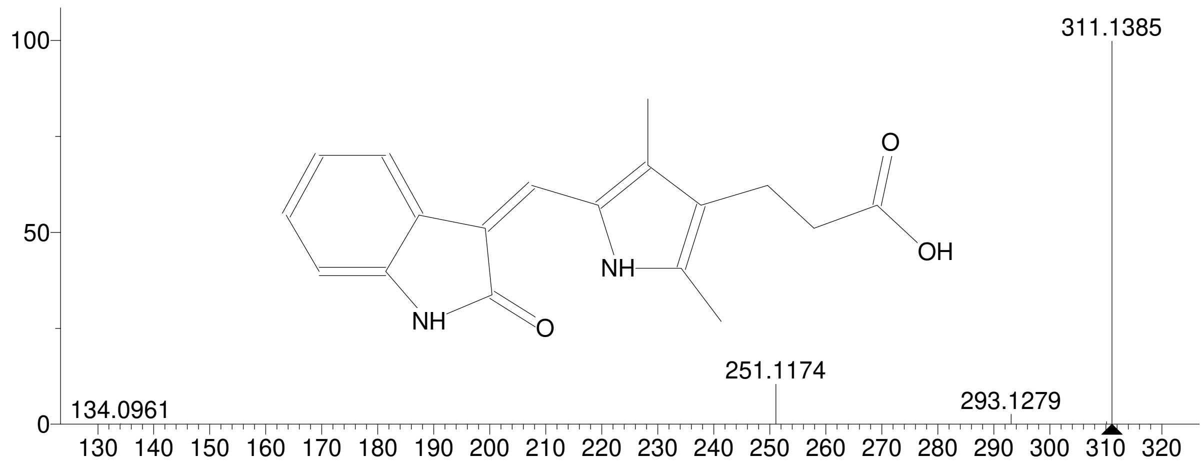

Hit 3 : Phe-Phe [M-H]- HCD 14eV P=311.1
C18H20N2O3; MF: 15; RMF: 293; Prob 12.6%; CAS: 2577-40-4; Lib: nist_msms; ID: 570068.

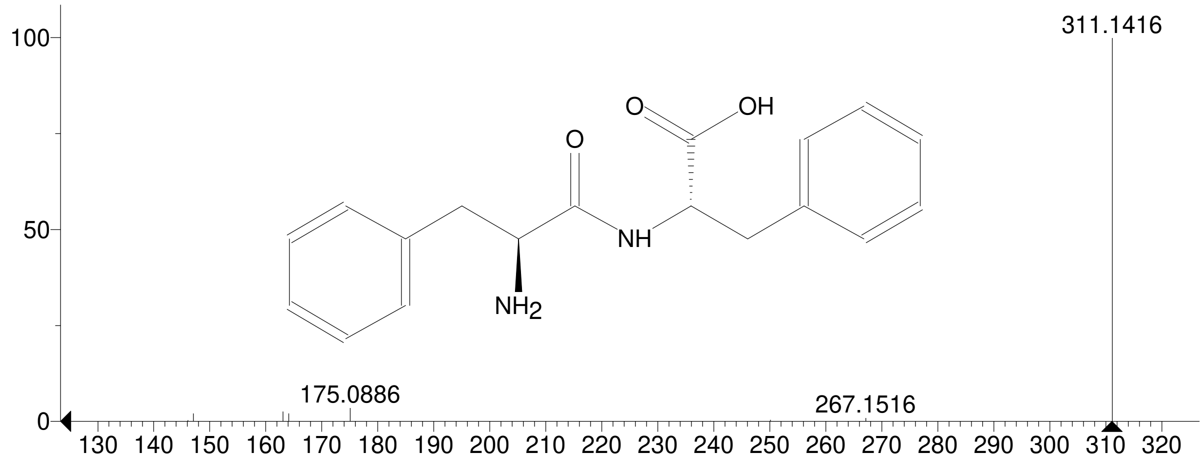

Hit 4 : Lipothiazole C [M+H]+ P=311.1
C15H22N2O3S; MF: 0; RMF: 50; Prob 7.63%; Lib: mona-export-gnps; ID: 4834.

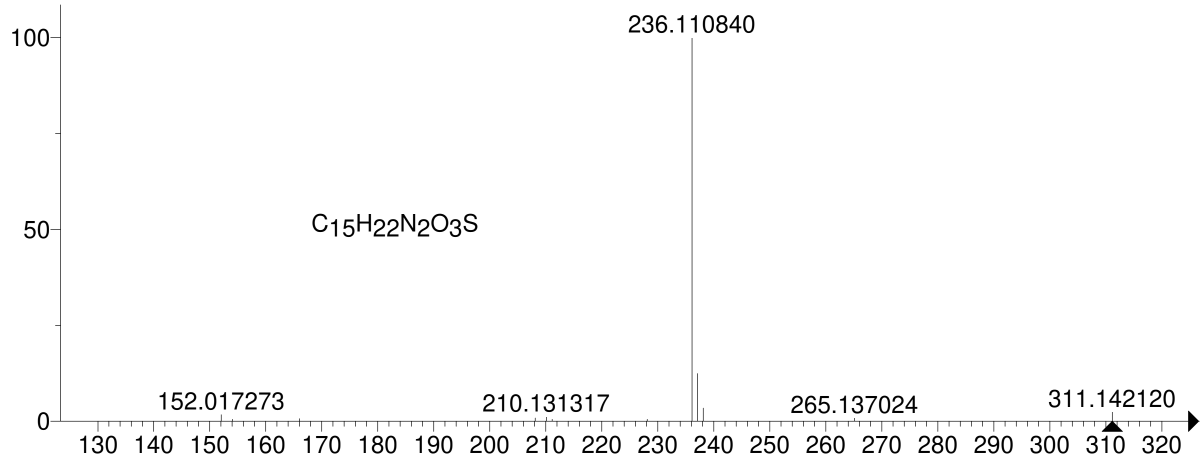

Unknown: Unknown (10.43_399.2120m/z)
Compound in Library Factor = -3273

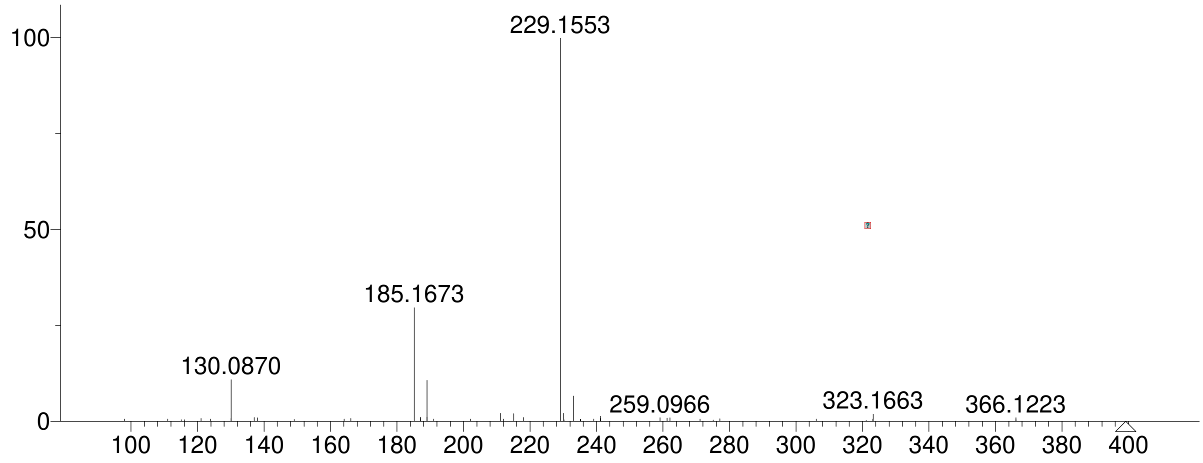

Hit 1 : His-Ser-Arg [M+H]+ QTOF 40V P=399.2
C15H26N8O5; MF: 1; RMF: 25; Prob 25.7%; Lib: nist_msms; ID: 51161.

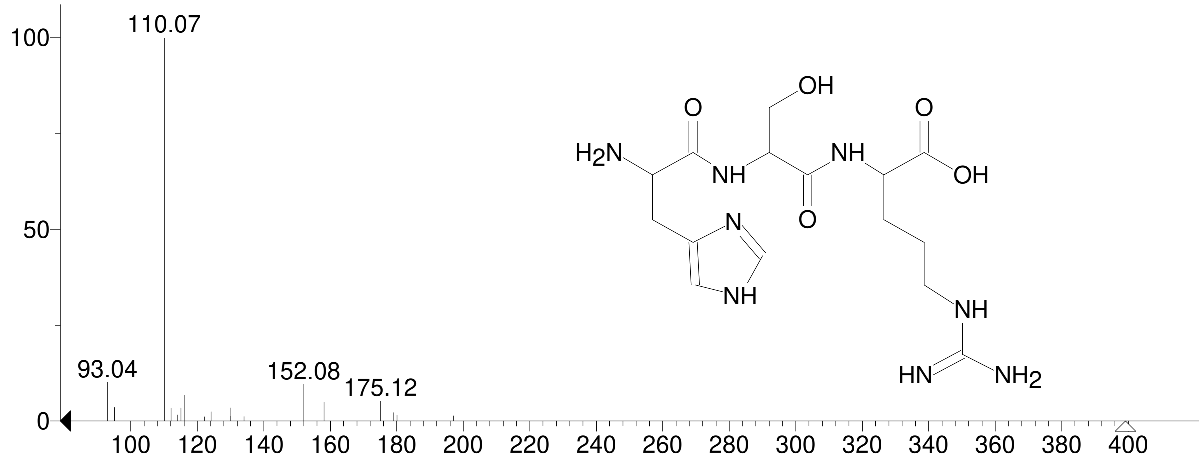

Hit 2 : N-Desmethylvinblastine [M+2H]2+ HCD 114V P=399.2
C45H56N4O9; MF: 0; RMF: 33; Prob 24.8%; CAS: 18172-50-4; Lib: nist_msms; ID: 435598.

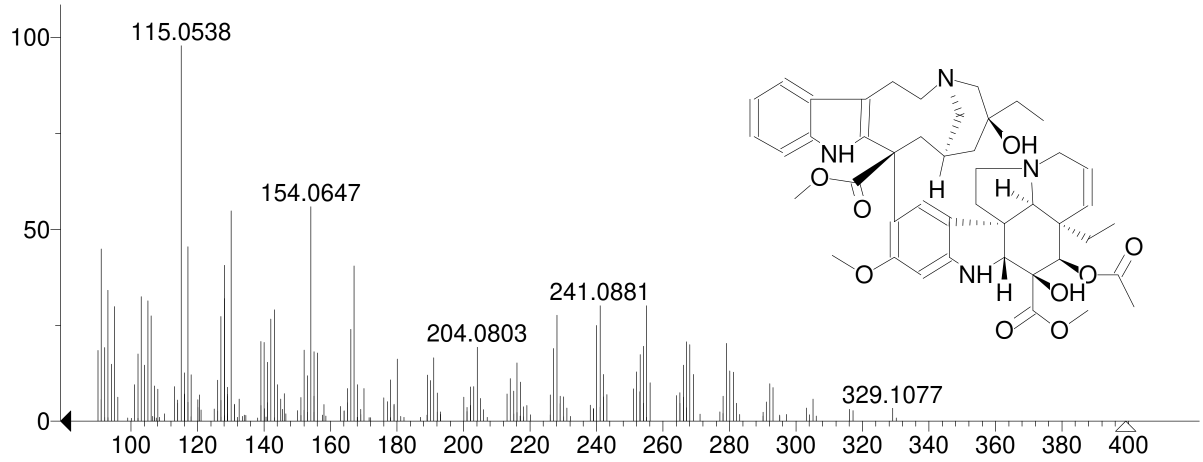

Hit 3 : Ser-His-Arg [M+H]+ QTOF 30V P=399.2
C15H26N8O5; MF: 0; RMF: 21; Prob 24.8%; Lib: nist_msms; ID: 53772.

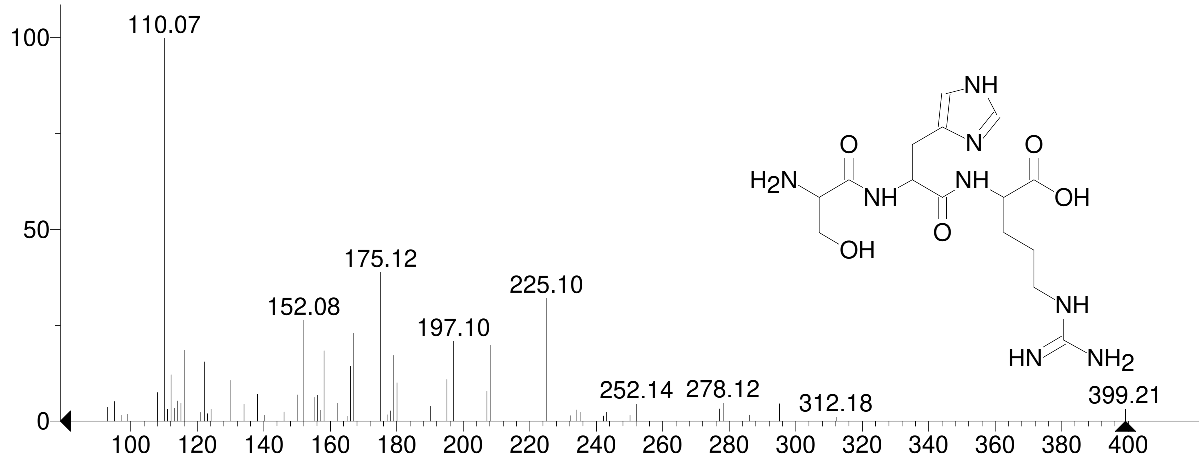

Hit 4 : NCGC00380576-01_C22H32O5_2-Pentenoic acid, 3-[(acetyloxy)methyl]-5-[(1S,4aR,8aR)-2-formyl-1,4,4a,
C22H32O5; MF: 0; RMF: 2; Prob 24.8%; Lib: mona-export-gnps; ID: 13905.

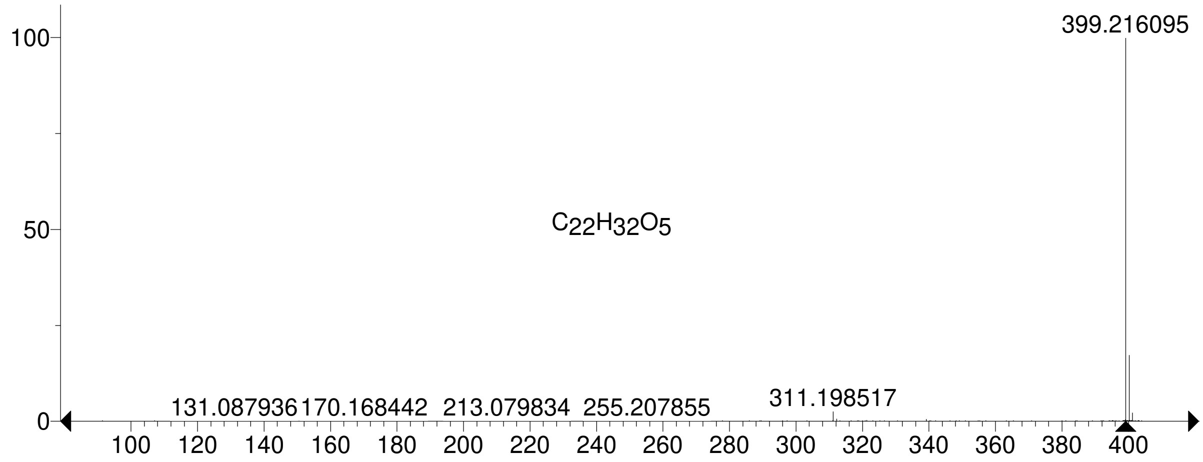

Unknown: Unknown (10.44_419.0640m/z)
Compound in Library Factor = -3273

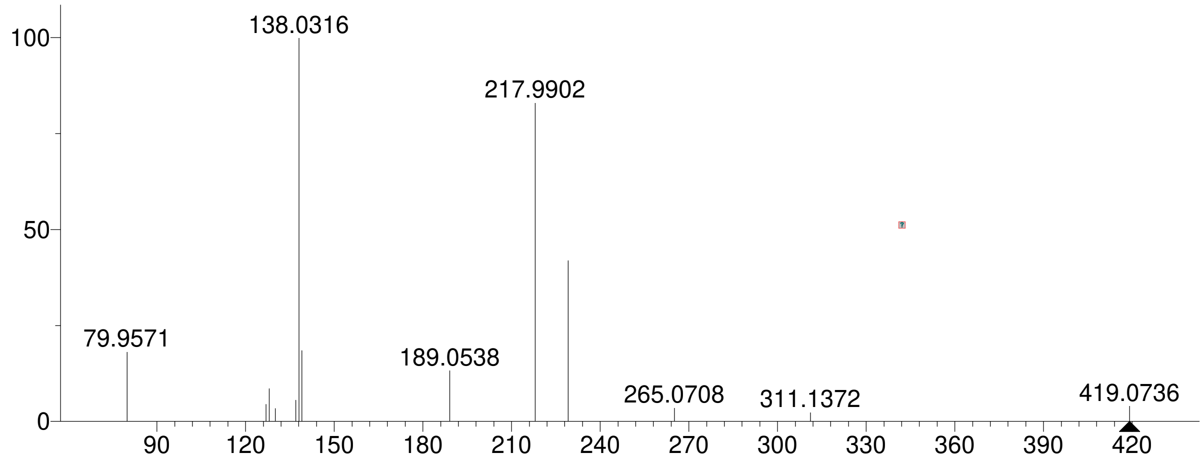

Hit 1 : D-Saccharic acid [2M-H]- HCD 2V P=419.1
C6H10O8; MF: 10; RMF: 156; Prob 57.9%; CAS: 87-73-0; Lib: nist_msms; ID: 303445.

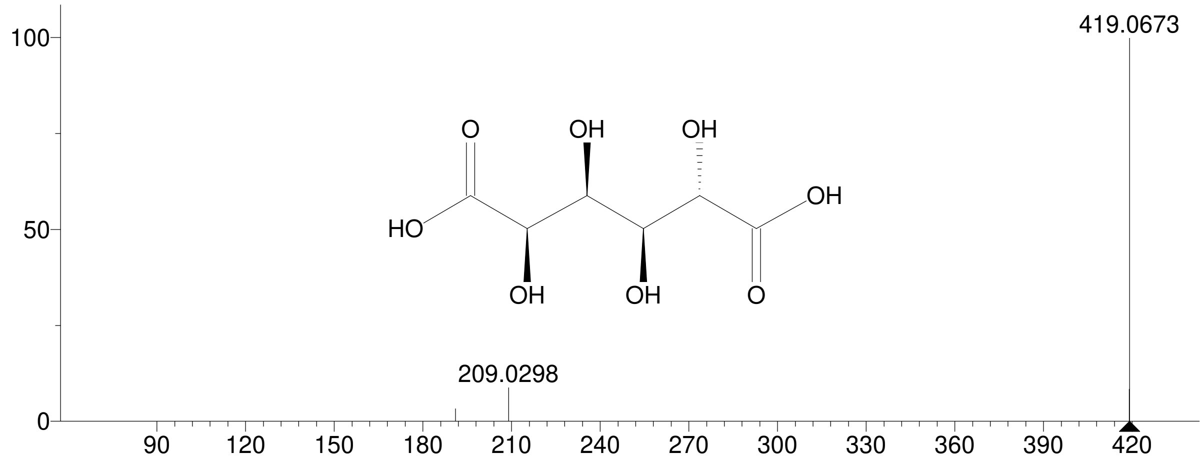

Hit 2 : Tefluthrin [M+H]+ HCD 8V P=419.1
C17H14ClF7O2; MF: 1; RMF: 100; Prob 42.1%; CAS: 79538-32-2; Lib: nist_msms; ID: 474592.

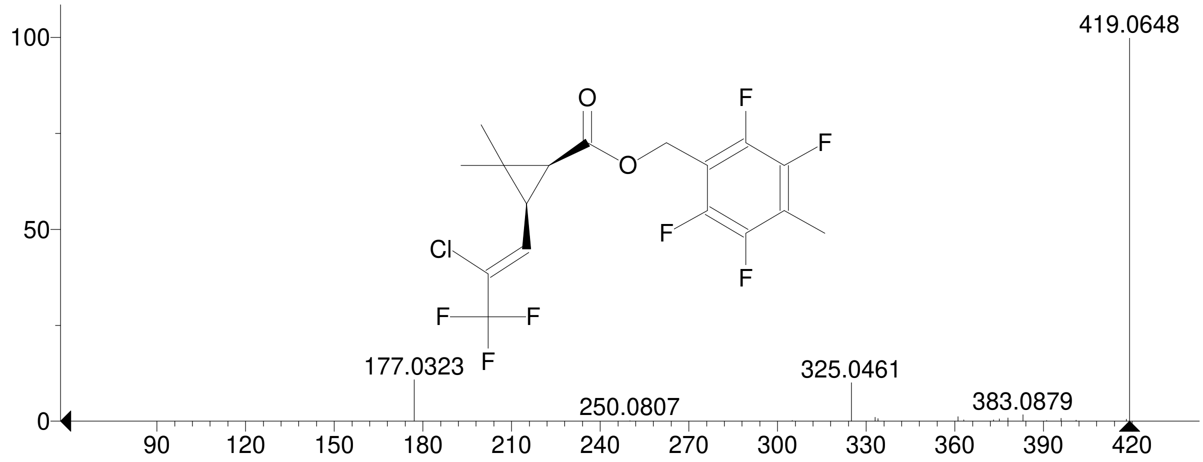

Unknown: Unknown (10.44_435.1747m/z)
Compound in Library Factor = 0

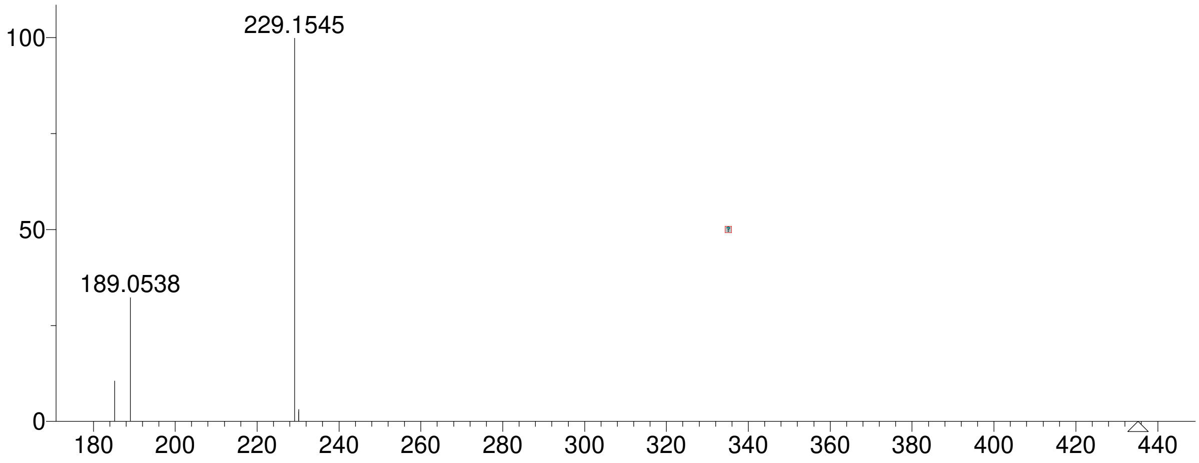

Hit 1 : 2,4-dihydroxy-5-methoxy-1'',6'',8''-trimethyldispiro[bis(oxolane)-3,2':5',7''-[3]oxatricyclo[6.3.1.0a^'',A^'1A^'2]c
C21H32O7; MF: 0; RMF: 78; Prob 100.0%; Lib: mona-export-vf-npl_qexactive; ID: 10625.

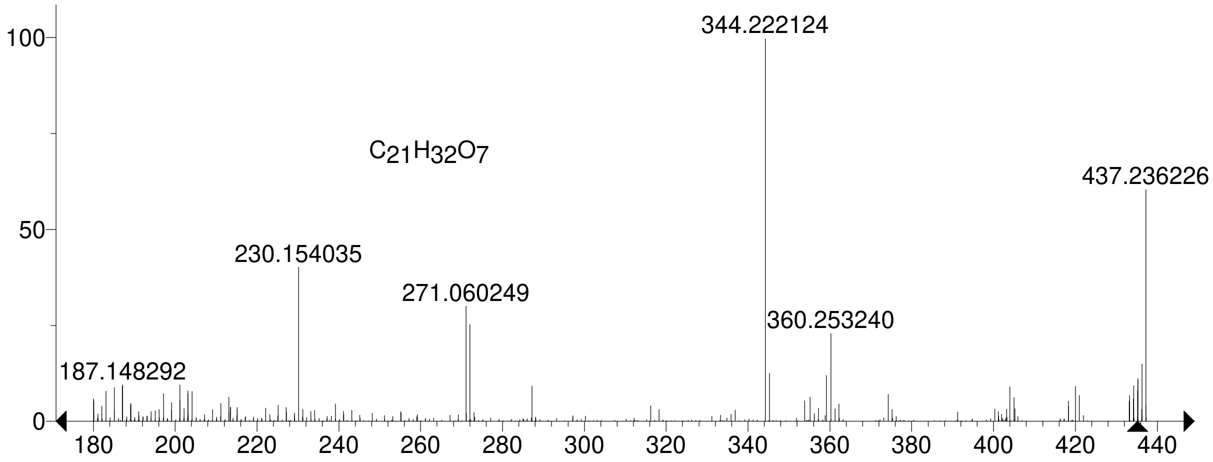

Unknown: Unknown (10.45_443.1559m/z)
Compound in Library Factor = 0

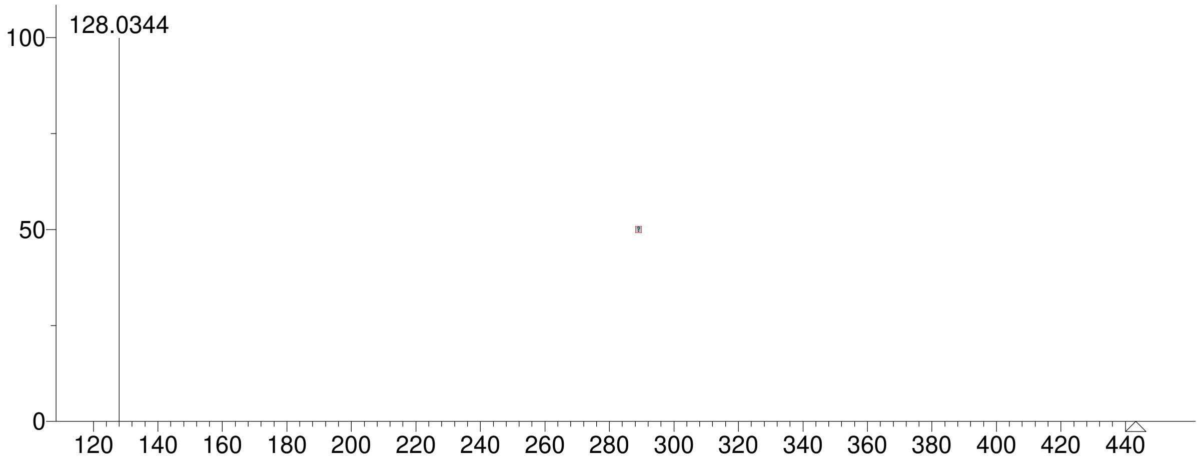

Unknown: Unknown (10.47_307.0484m/z)
Compound in Library Factor = -885

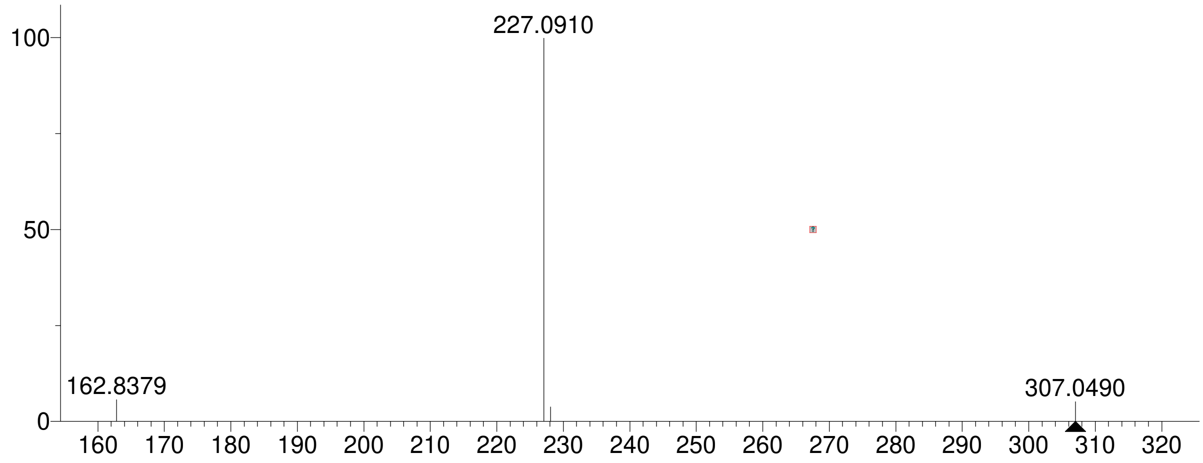

Hit 1 : 2''-Galloylhyperin [M-2H]2- HCD 6V P=307
C28H24O16; MF: 143; RMF: 839; Prob 89.4%; CAS: 53209-27-1; Lib: nist_msms; ID: 241484.

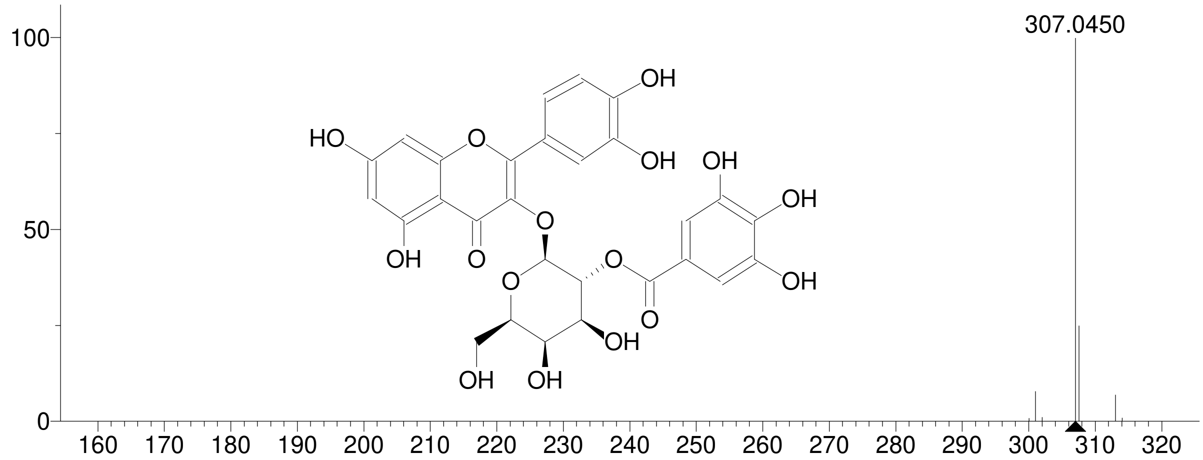

Hit 2 : 3,5-Dihydroxybenzoic acid [2M-H]- HCD 1V P=307
C7H6O4; MF: 54; RMF: 212; Prob 7.72%; CAS: 99-10-5; Lib: nist_msms; ID: 297168.

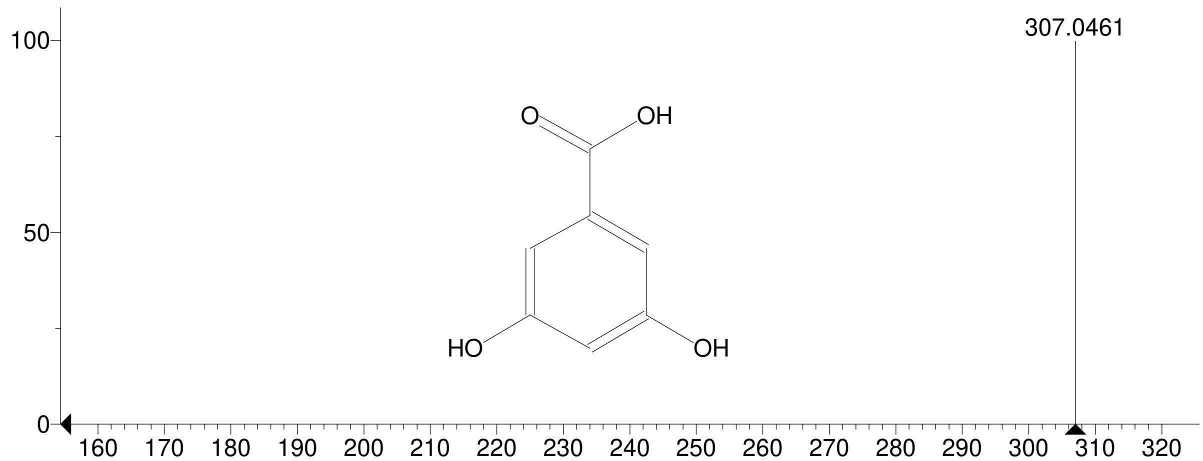

Hit 3 : Taurolidine [M+Na]+ HCD 6V P=307.1
C7H16N4O4S2; MF: 16; RMF: 202; Prob 1.84%; CAS: 19388-87-5; Lib: nist_msms; ID: 548490.

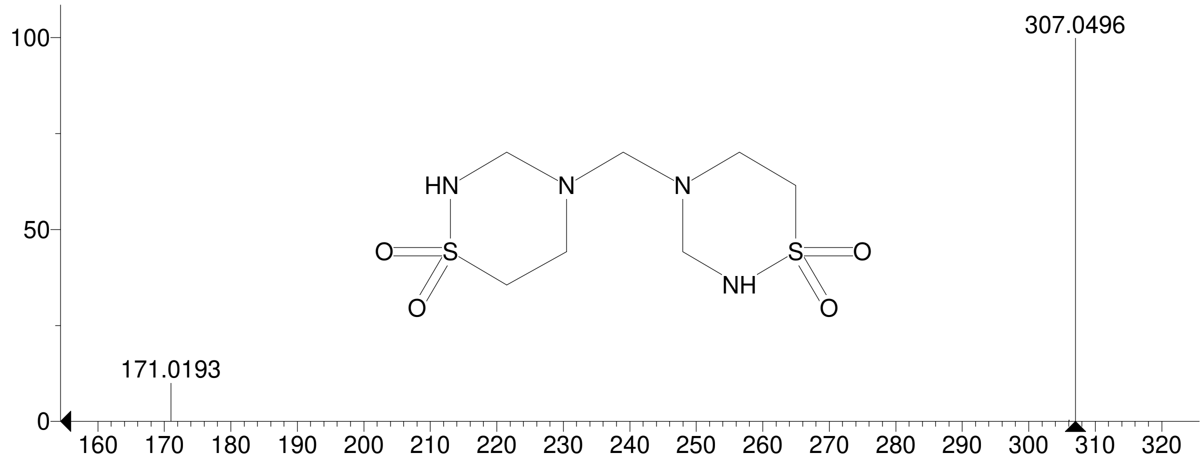

Hit 4 : methyl 1-oxo-4-(1H-pyrrol-2-yl)-2H-isoquinoline-3-carboxylate [M+K]+ 35HCD P=307
C15H12N2O3; MF: 0; RMF: 9; Prob 1.06%; Lib: mona-export-vf-npl_qexactive; ID: 19435.

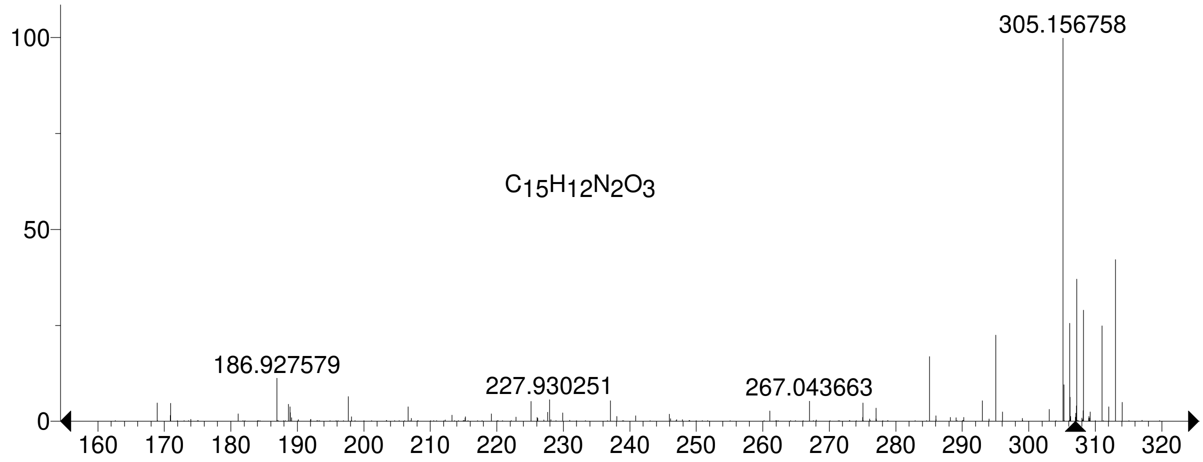

** Search Report Page 1 of 1 **

Unknown: Unknown (10.48_387.0741m/z)
Compound in Library Factor = -2176

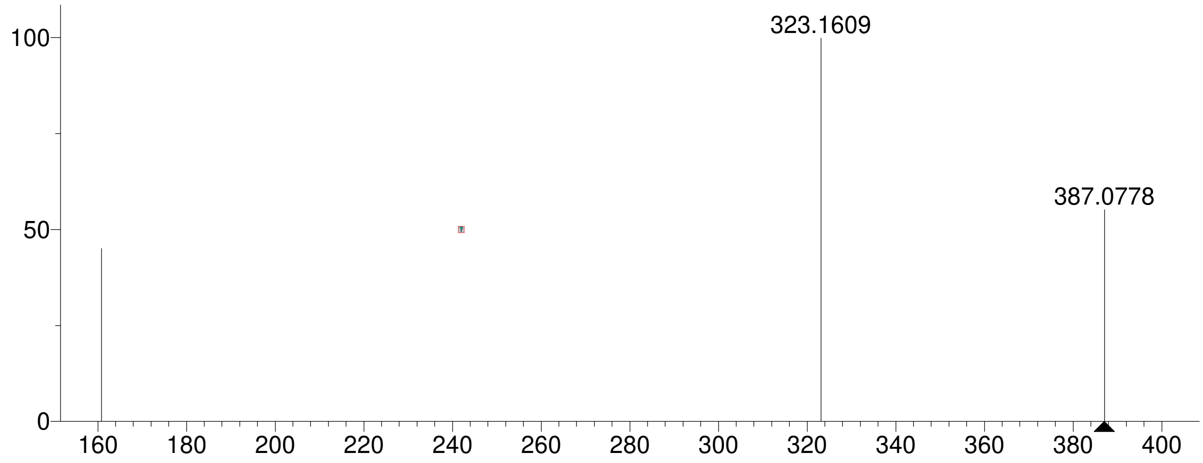

Hit 1 : Sulfometuron methyl [M+Na]+ HCD 7V P=387.1
C15H16N4O5S; MF: 50; RMF: 503; Prob 77.3%; CAS: 74222-97-2; Lib: nist_msms; ID: 490853.

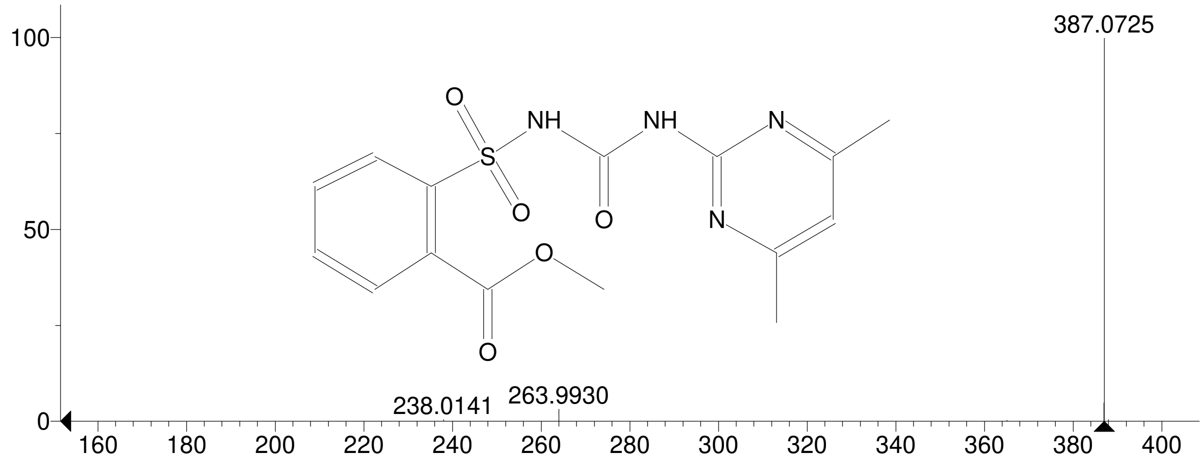

Hit 2 : 3-Isoxazolecarboxamide, 5-(5-chloro-2,4-dihydroxyphenyl)-N-ethyl-4-(4-methoxyphenyl)- [M-H]- HCD 11\
C19H17ClN2O5; MF: 23; RMF: 480; Prob 22.7%; CAS: 747413-08-7; Lib: nist_msms; ID: 274834.

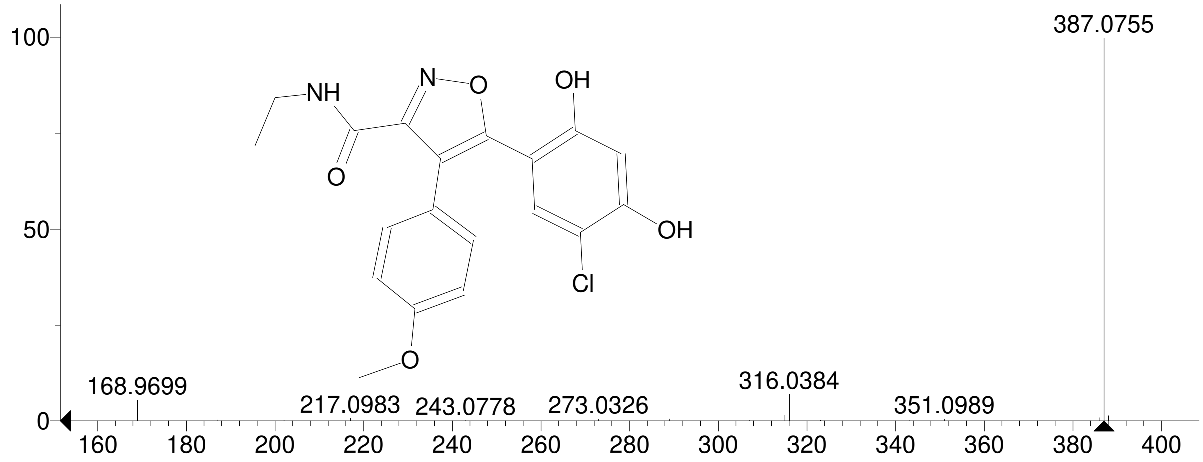

Unknown: Unknown (10.48_749.3129m/z)
Compound in Library Factor = 0

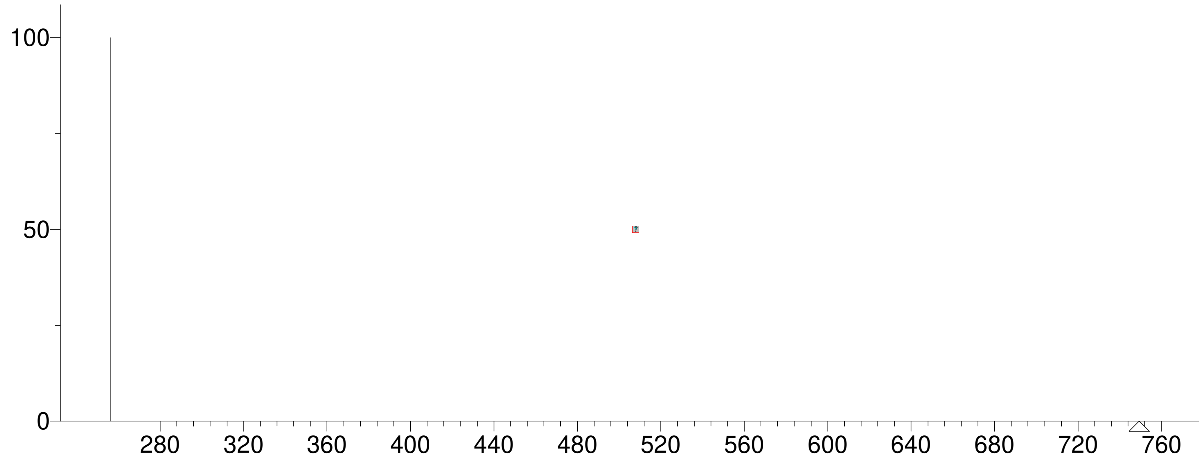

Unknown: Unknown (10.49_298.1278m/z)
Compound in Library Factor = 0

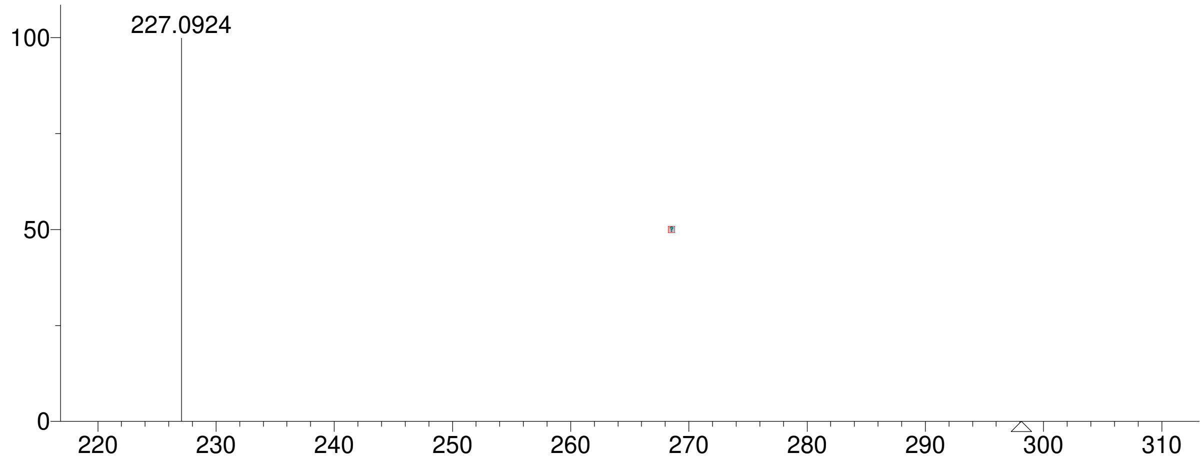

Hit 1 : (3,4,5-trihydroxy-6-methyloxan-2-yl) 2-(methylamino)benzoate [M+H]+ 35HCD P=298.1
C14H19NO6; MF: 0; RMF: 16; Prob 100.0%; Lib: mona-export-vf-npl_qexactive; ID: 19189.

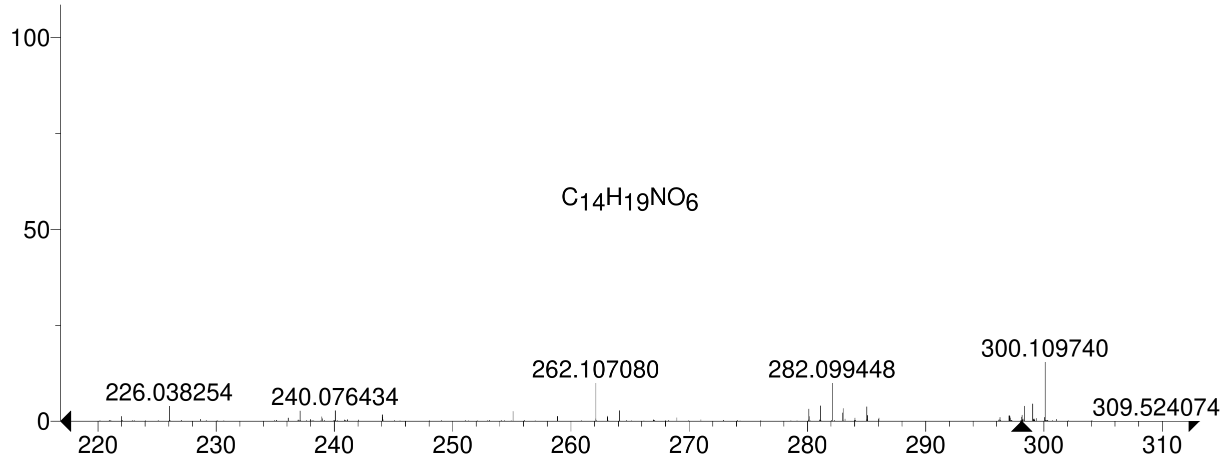

Unknown: Unknown (10.49_358.1971m/z)
Compound in Library Factor = 0

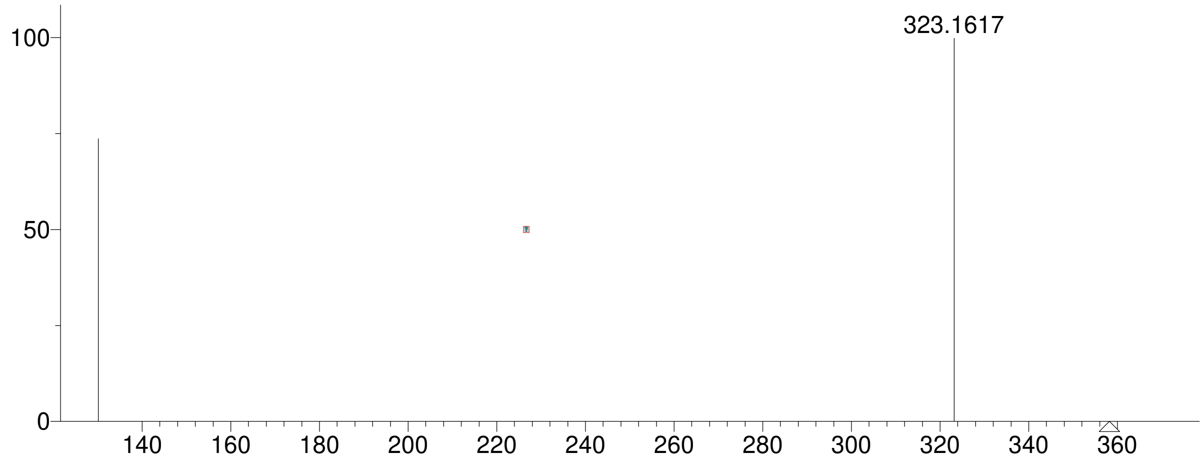

Unknown: Unknown (10.51_528.2541m/z)
Compound in Library Factor = 0

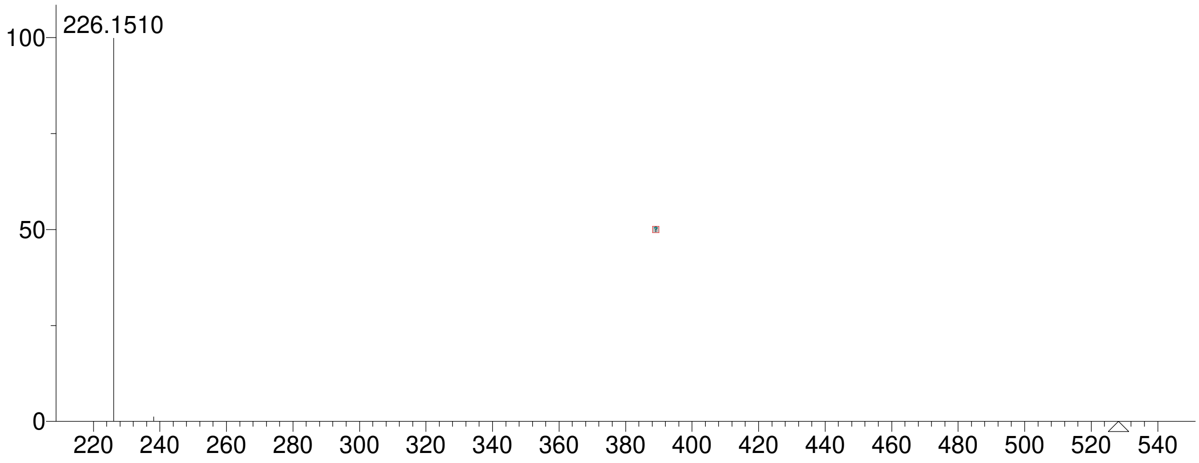

Unknown: Unknown (10.54_338.1711m/z)
Compound in Library Factor = -3273

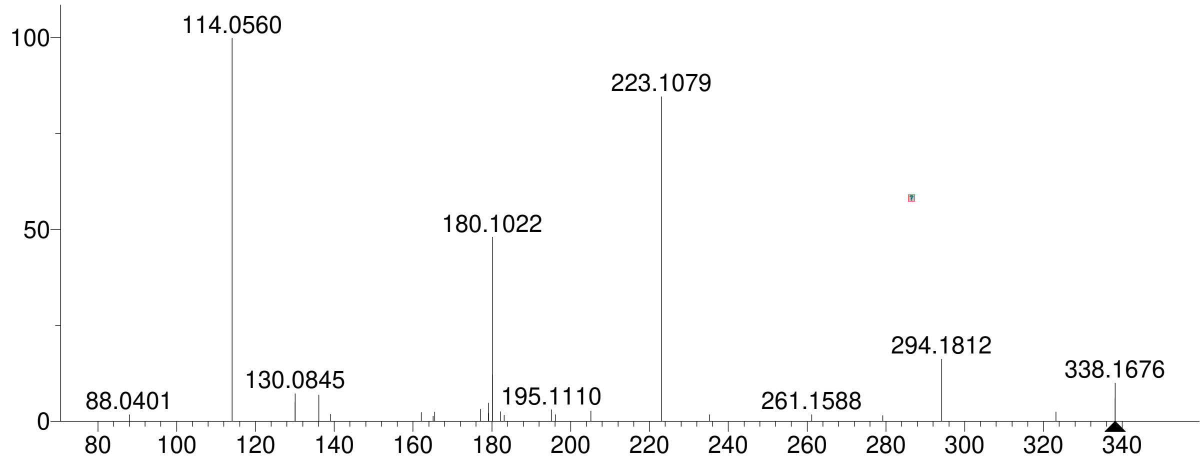

Hit 1 : DTyr-Val-Gly [M+H]+ HCD 6eV P=338.2
C16H23N3O5; MF: 6; RMF: 154; Prob 50.0%; CAS: 86030-52-6; Lib: nist_msms; ID: 564526.

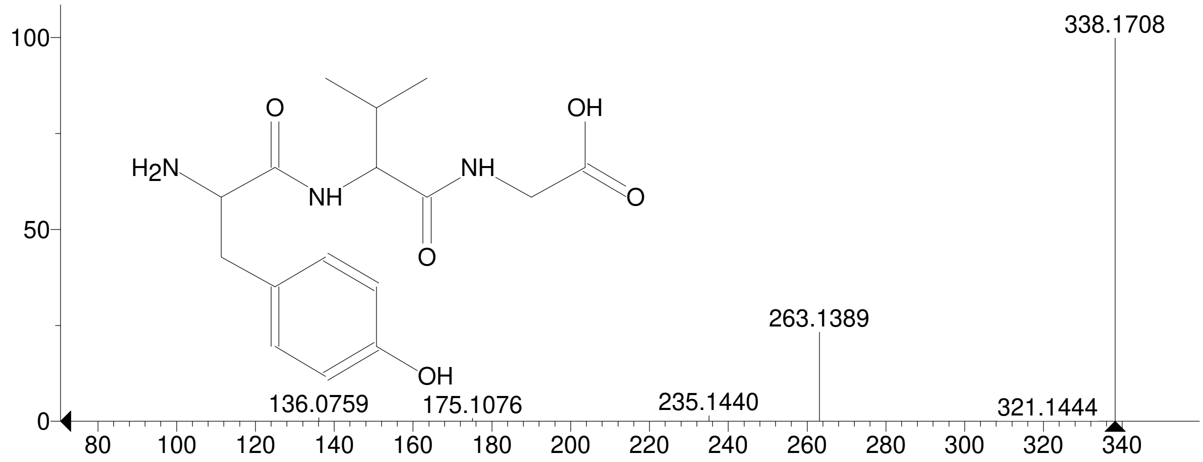

Hit 2 : DTyr-Val-Gly [M+H]+ HCD 6eV P=338.2
C16H23N3O5; MF: 6; RMF: 154; Prob 50.0%; Lib: nist_msms2; ID: 56843.

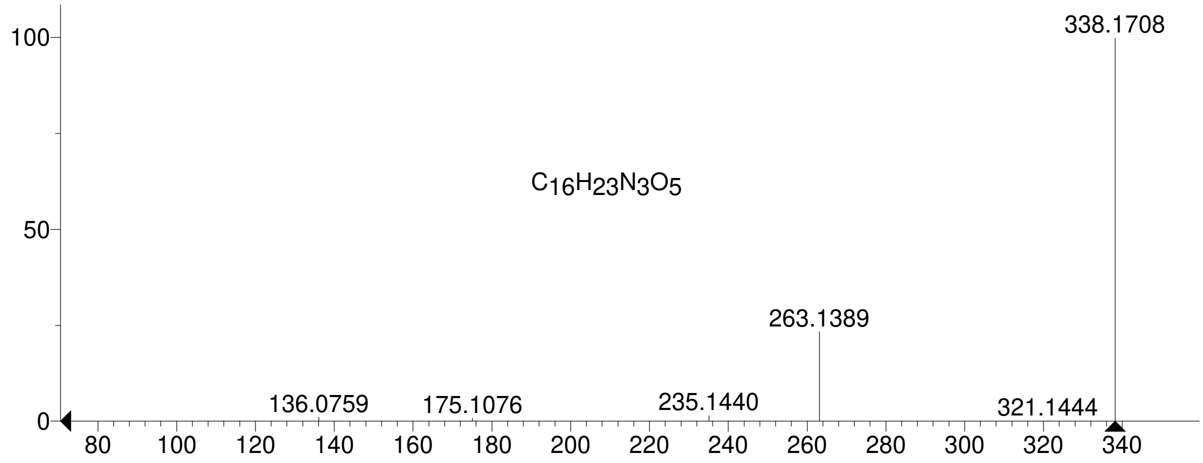

Unknown: Unknown (10.56_249.0751m/z)
Compound in Library Factor = -295

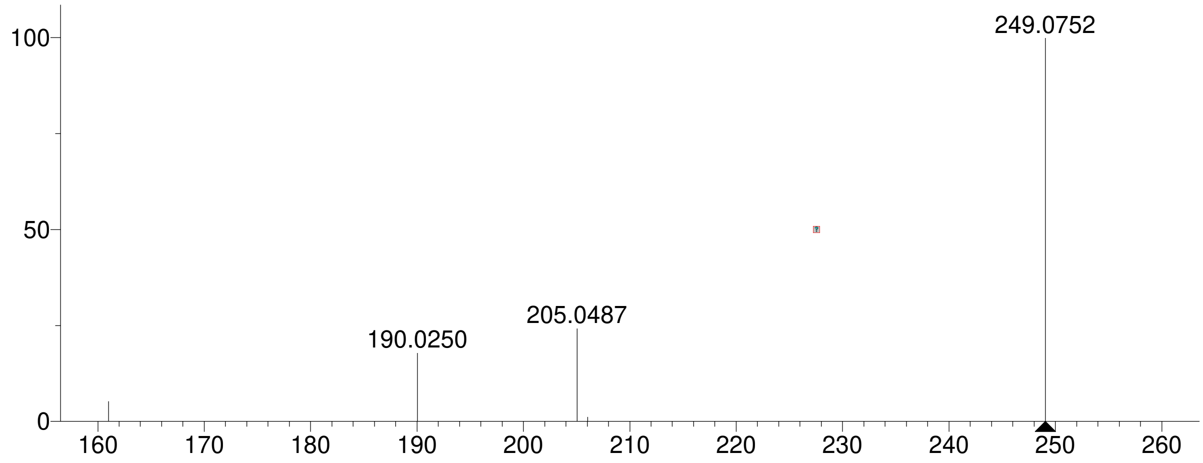

Hit 1 : Genipin [M+Na]+ HCD 2V P=249.1
C11H14O5; MF: 362; RMF: 997; Prob 98.5%; CAS: 6902-77-8; Lib: nist_msms; ID: 163060.

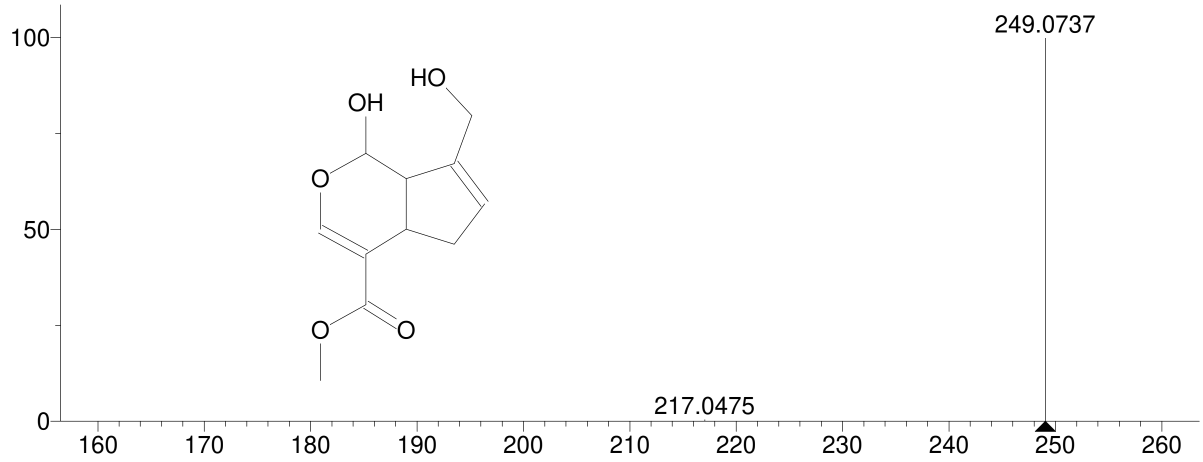

Hit 2 : Ethyl 8-methoxycoumarin-3-carboxylate [M+H]+ HCD 4V P=249.1
C13H12O5; MF: 159; RMF: 779; Prob 0.98%; CAS: 1729-02-8; Lib: nist_msms; ID: 111106.

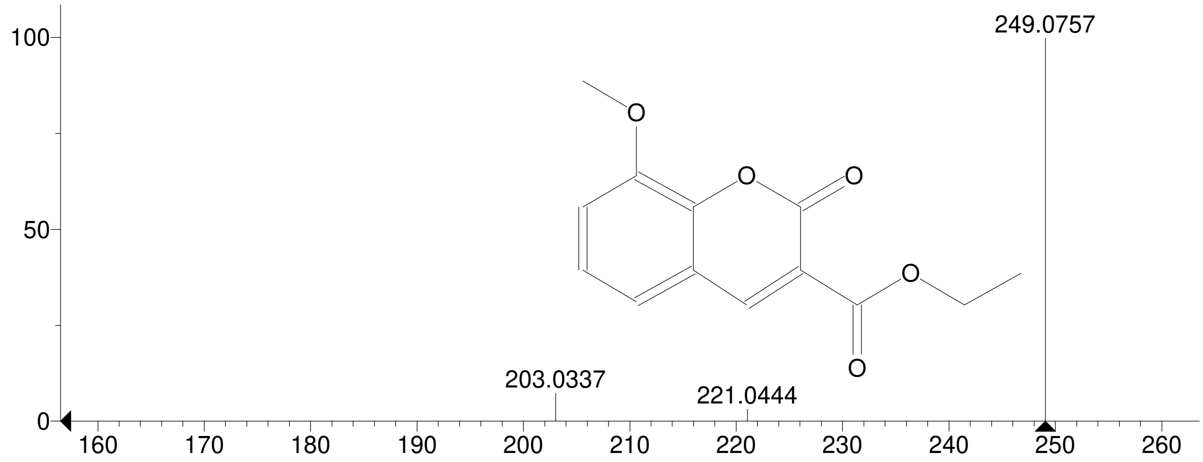

Hit 3 : Methyl 7-methoxycoumarin-4-acetate [M+H]+ HCD 14V P=249.1
C13H12O5; MF: 113; RMF: 778; Prob 0.20%; CAS: 62935-73-3; Lib: nist_msms; ID: 111496.

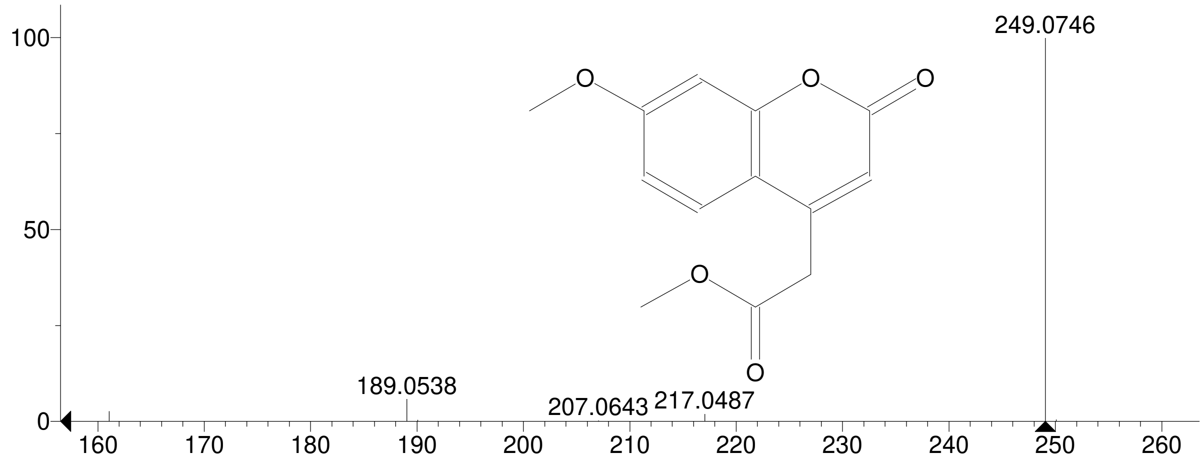

Hit 4 : 8-Acetyl-6,7-dimethoxycoumarin [M+H]+ HCD 9V P=249.1
C13H12O5; MF: 101; RMF: 760; Prob 0.13%; CAS: 263271-48-3; Lib: nist_msms; ID: 483878.

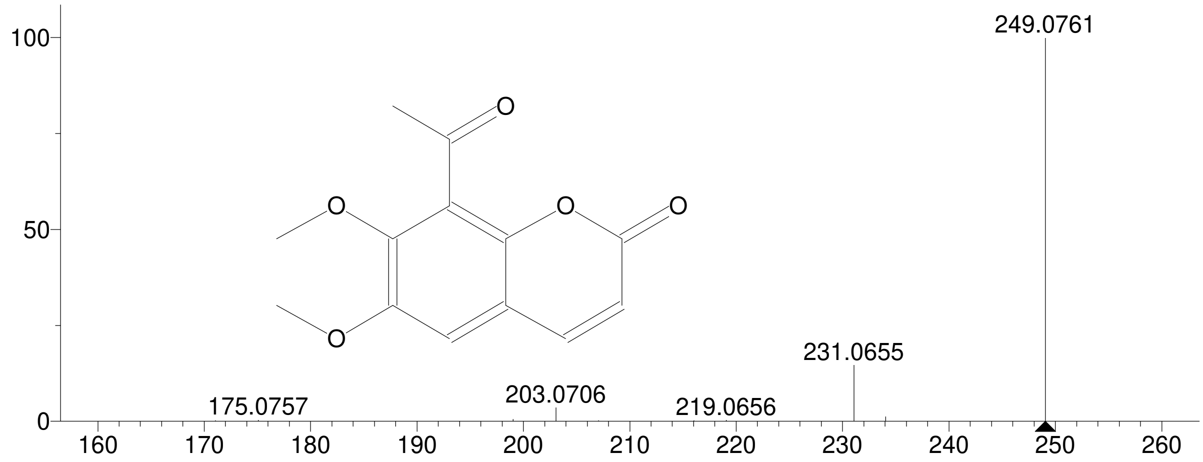

Hit 5 : p-Tolyl-β-D-glucuronide [M+H-2H2O]+ HCD 4V P=249.1
C13H16O7; MF: 82; RMF: 763; Prob 0.06%; CAS: 17680-99-8; Lib: nist_msms; ID: 299068.

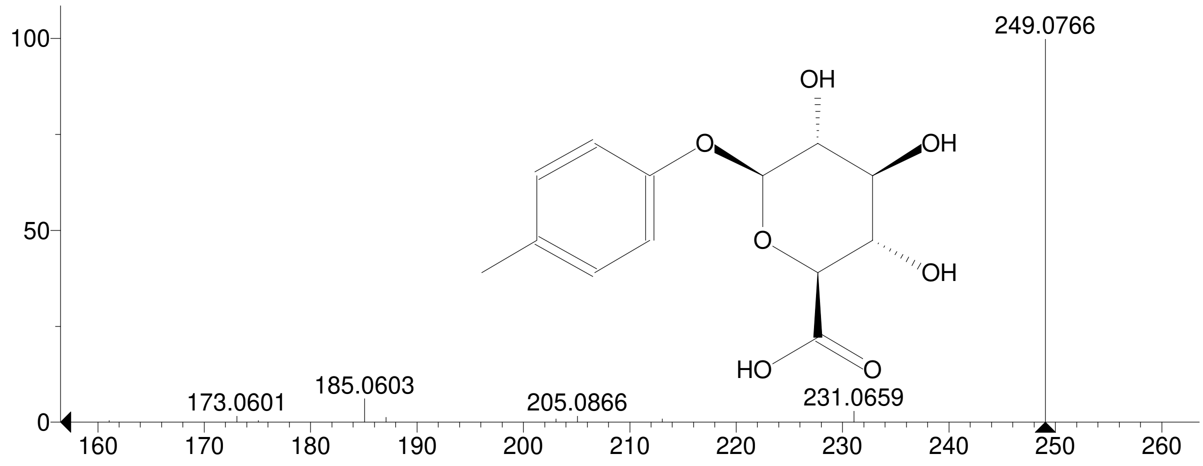

** Search Report Page 1 of 1 **

Unknown: Unknown (10.56_329.0326m/z)
Compound in Library Factor = 0

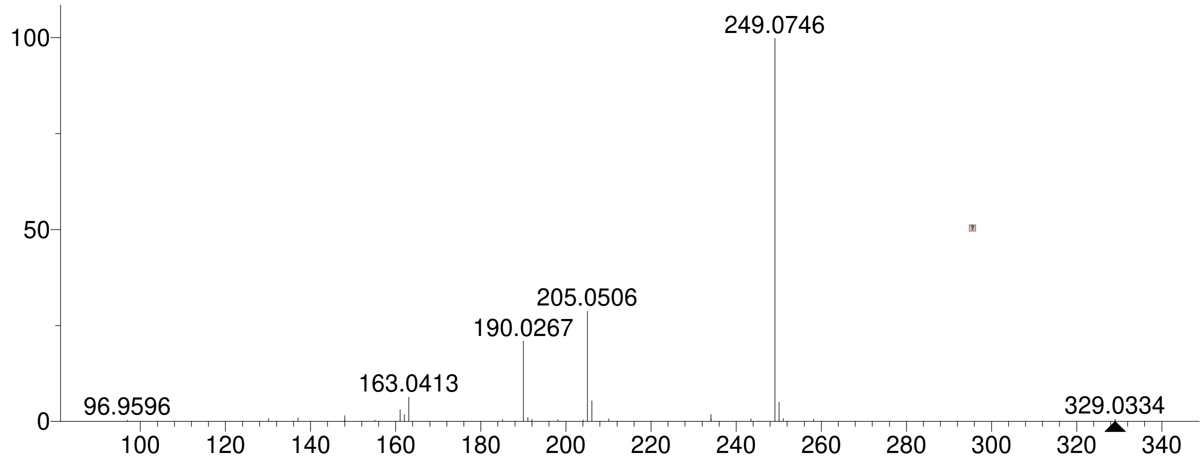

Unknown: Unknown (10.56_417.1856m/z)
Compound in Library Factor = 0

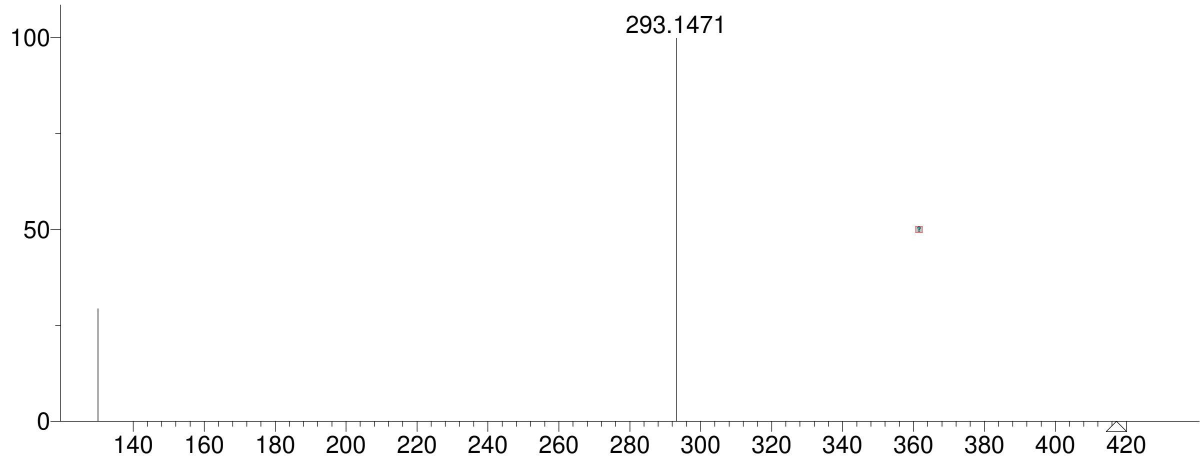

Hit 1 : Cyproterone acetate [M+H]+ IT-FT 35% P=417.2
C24H29ClO4; MF: 0; RMF: 40; Prob 50.0%; CAS: 427-51-0; Lib: nist_msms; ID: 152587.

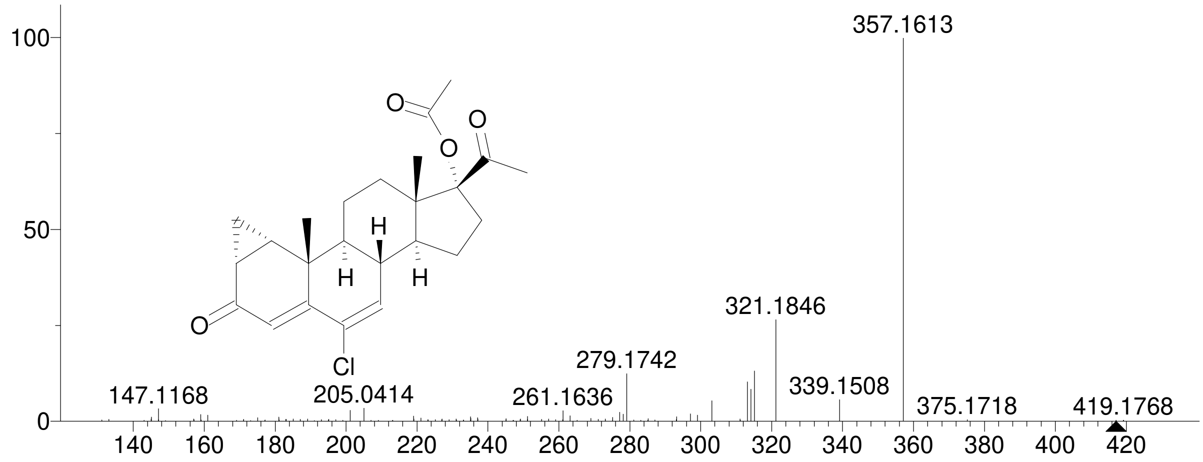

Hit 2 : MLS000859908-01!Cyproterone acetate [M+H]+ P=417.2
C24H29ClO4; MF: 0; RMF: 12; Prob 50.0%; Lib: mona-export-gnps; ID: 3413.

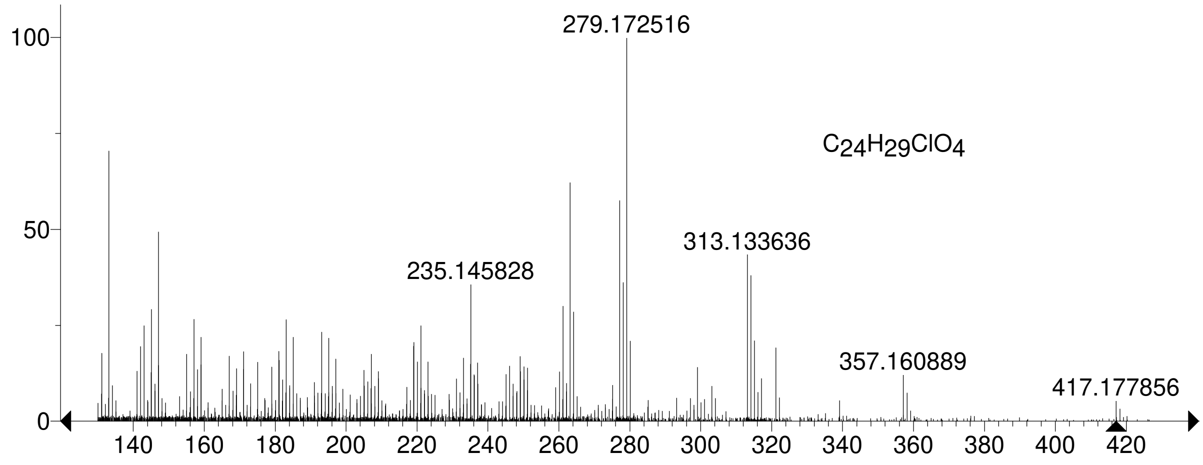

Unknown: Unknown (10.57_387.9970m/z)
Compound in Library Factor = 0

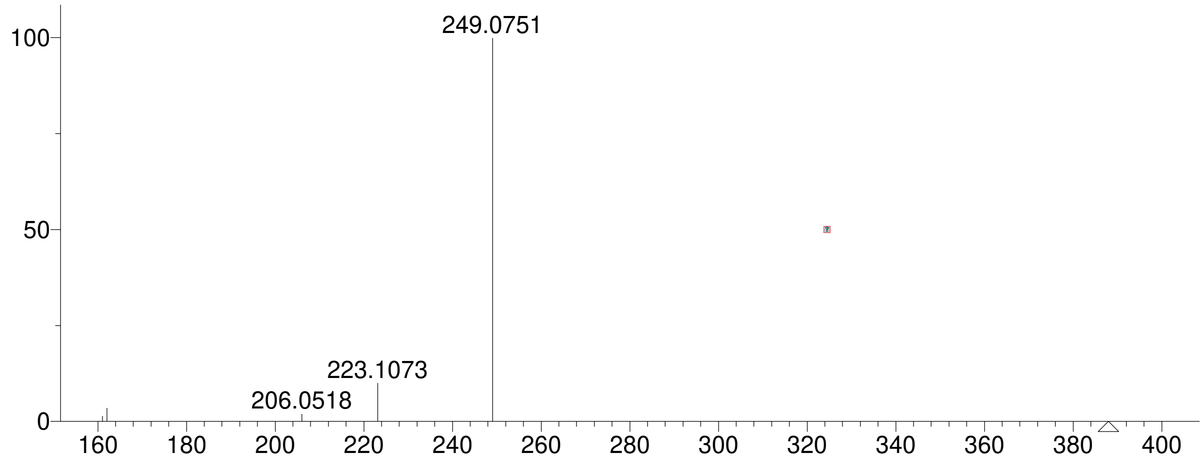

** Search Report Page 1 of 1 **

Unknown: Unknown (10.57_463.2061m/z)
Compound in Library Factor = 0

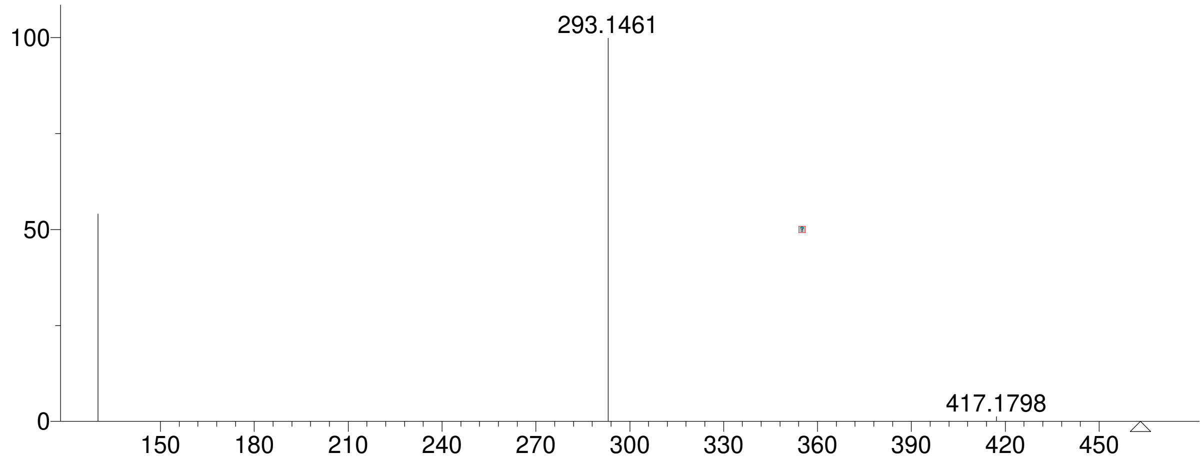

Hit 1 : 13(R),14(R)-Epoxyfluprostenol isopropyl ester [M+H-3H2O]+ IT 35% P=463.2
C26H35F3O7; MF: 0; RMF: 2; Prob 100.0%; Lib: nist_msms; ID: 254087.

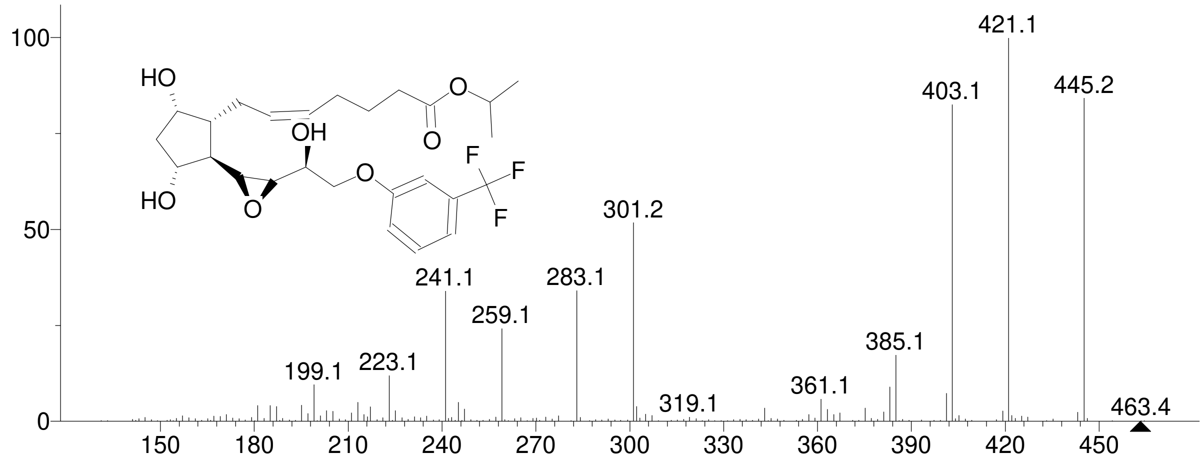

Unknown: Unknown (10.60_863.2076m/z)
Compound in Library Factor = 0

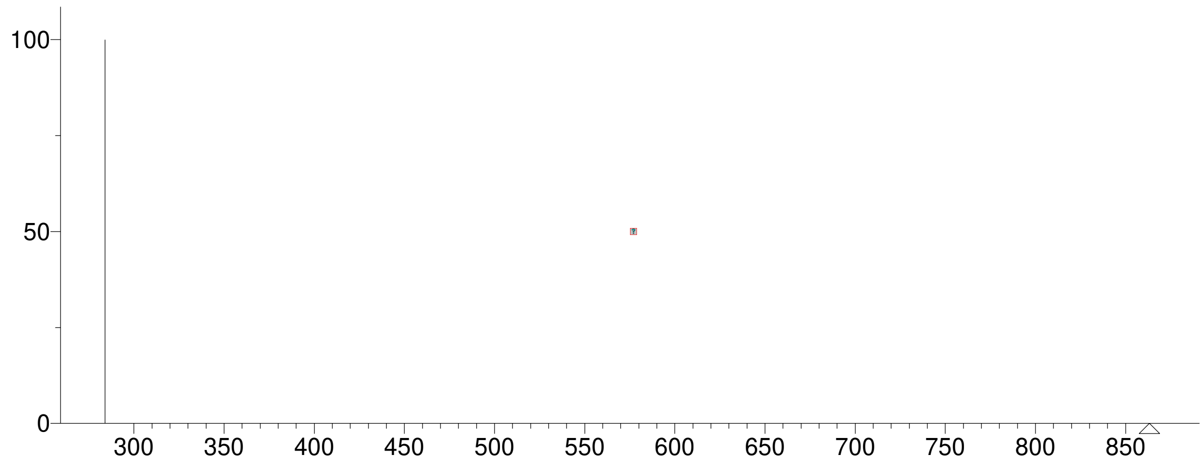

Unknown: Unknown (10.62_450.1742m/z)
Compound in Library Factor = 0

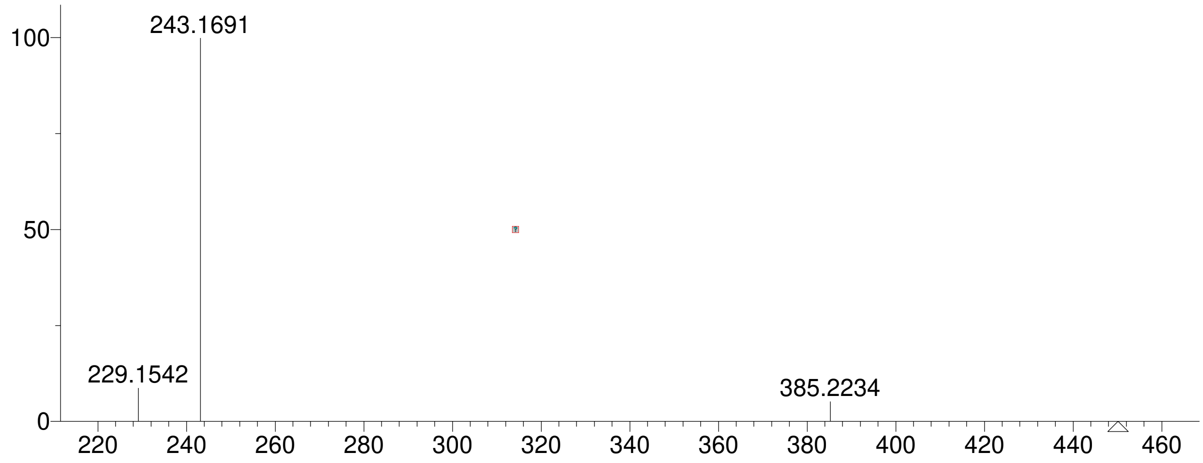

Unknown: Unknown (10.65_413.2277m/z)
Compound in Library Factor = -1309

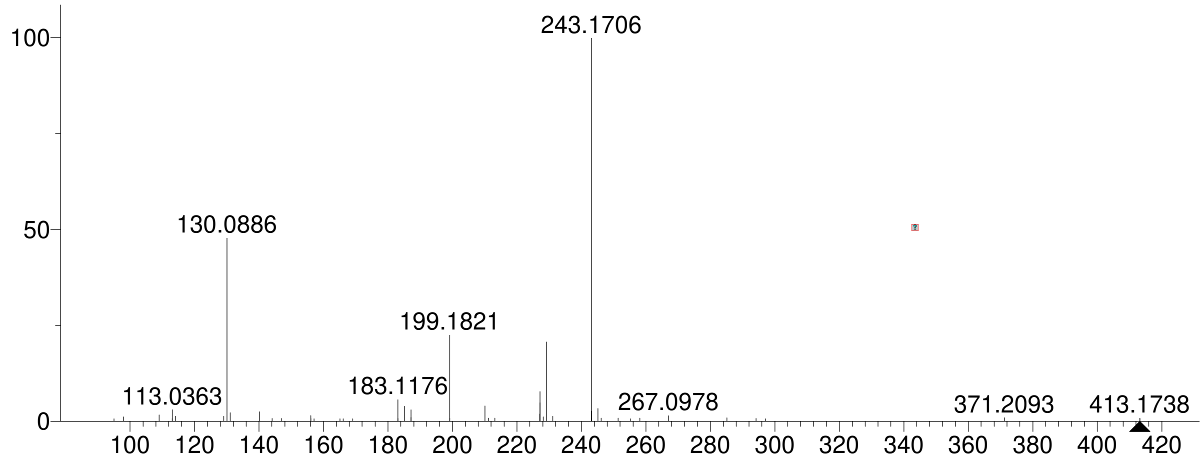

Hit 1 : Mevastatin [M+Na]+ HCD 20V P=413.2
C23H34O5; MF: 55; RMF: 419; Prob 36.9%; CAS: 73573-88-3; Lib: nist_msms; ID: 165105.

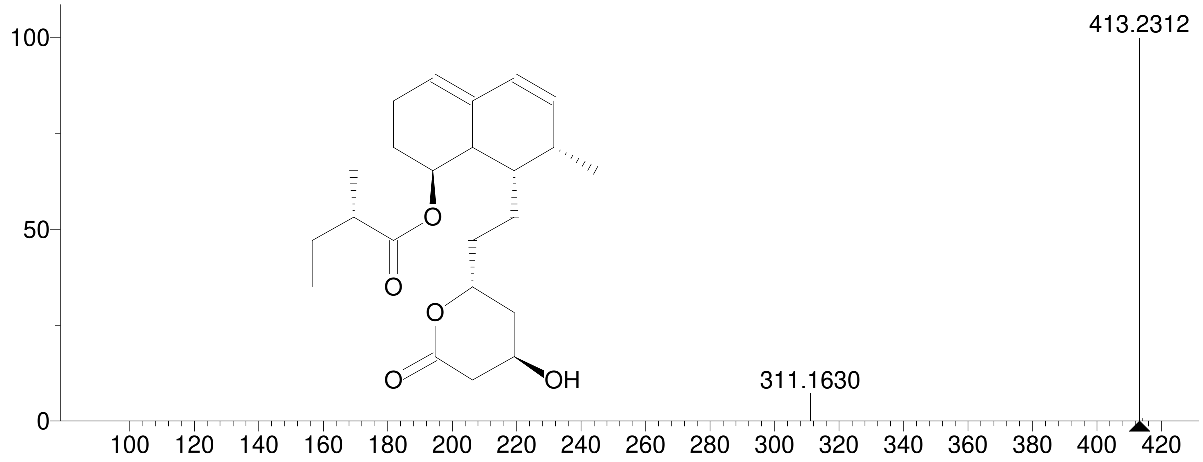

Hit 2 : Resolvin D2 methyl ester [M+Na]+ HCD 37V P=413.2
C23H34O5; MF: 1; RMF: 47; Prob 7.22%; CAS: 810668-53-2; Lib: nist_msms; ID: 378655.

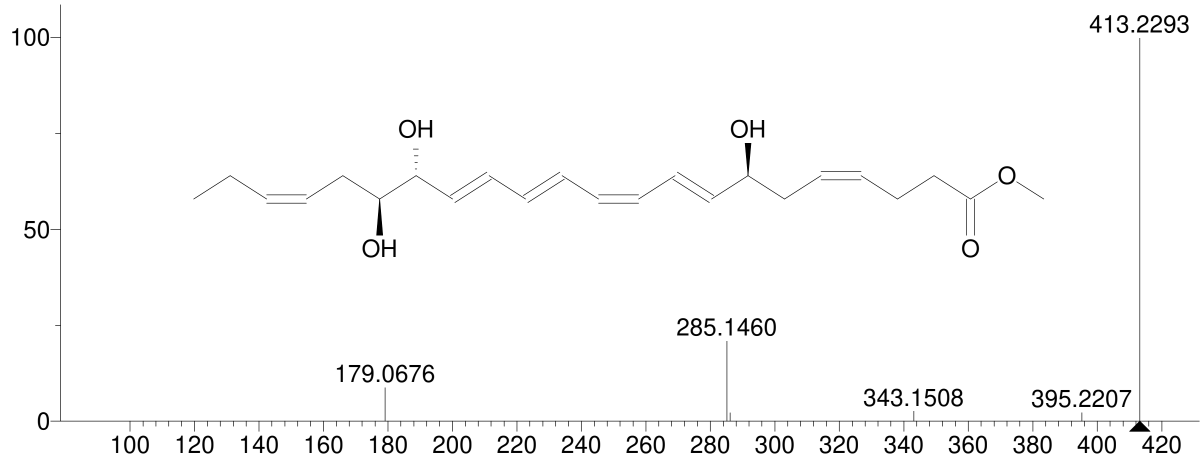

Hit 3 : Tussilagone [M+Na]+ HCD 33V P=413.2
C23H34O5; MF: 1; RMF: 36; Prob 7.22%; CAS: 104012-37-5; Lib: nist_msms; ID: 425581.

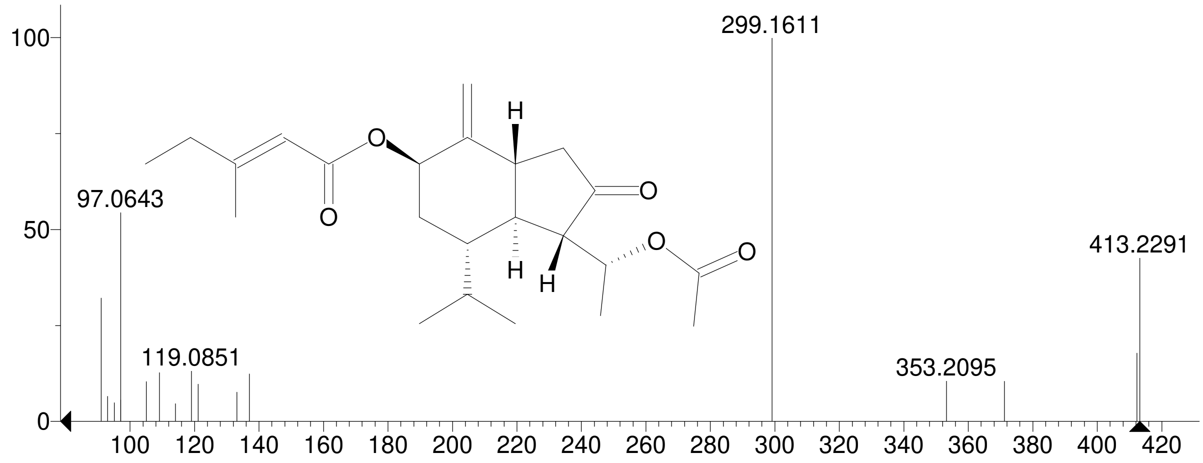

Hit 4 : 17(R)-Resolvin D1 methyl ester [M+Na]+ HCD 37V P=413.2
C23H34O5; MF: 0; RMF: 44; Prob 6.94%; CAS: 937738-64-2; Lib: nist_msms; ID: 378599.

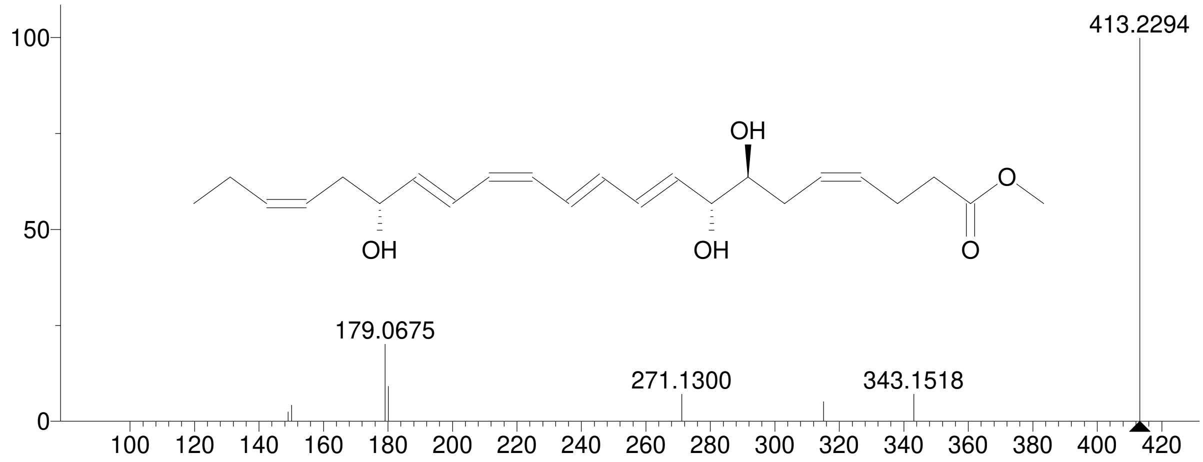

Hit 5 : Thr-His-Arg [M+H]+ QTOF 10V P=413.2
C16H28N8O5; MF: 0; RMF: 43; Prob 6.94%; Lib: nist_msms; ID: 54061.

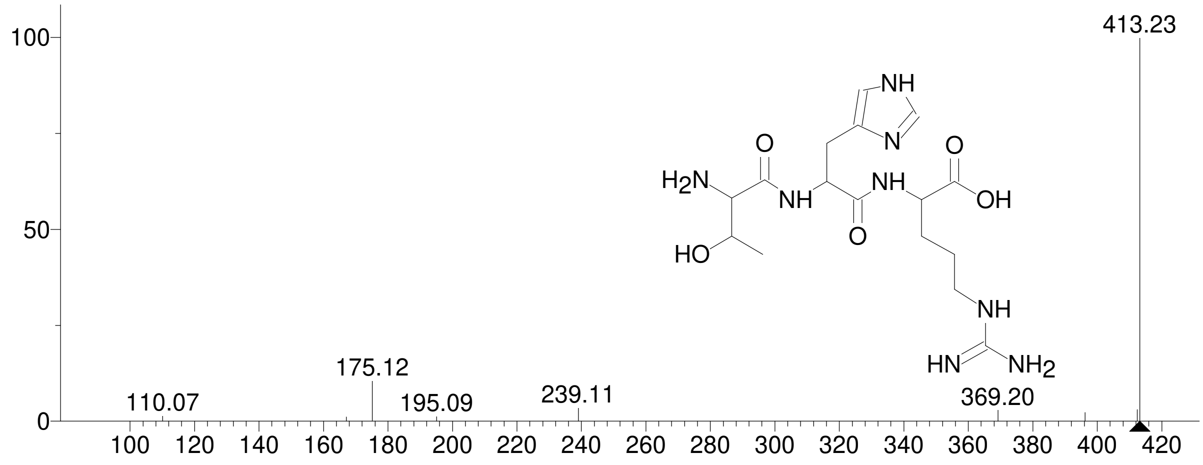

** Search Report Page 1 of 1 **

Unknown: Unknown (10.65_463.1713m/z)
Compound in Library Factor = 0

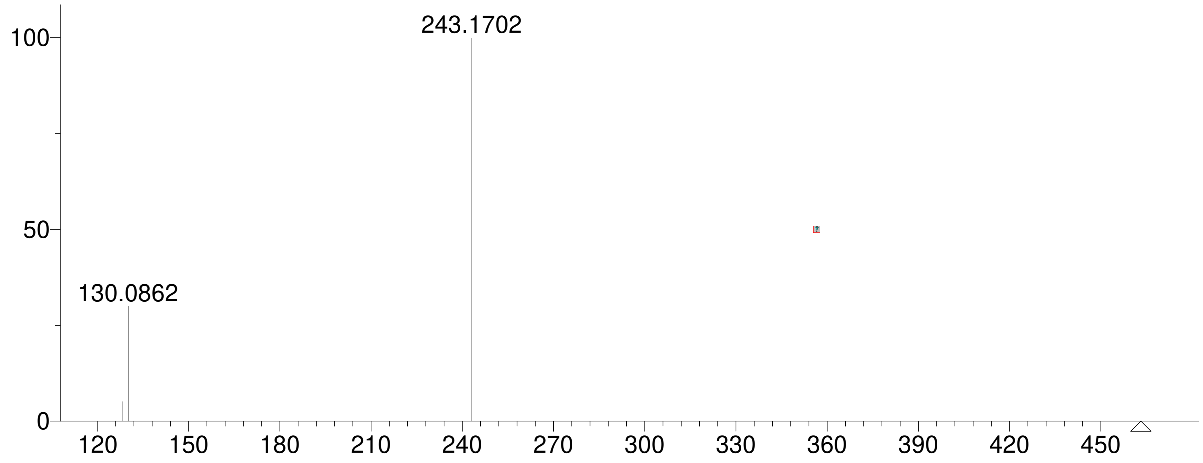

Unknown: Unknown (10.67_415.1703m/z)
Compound in Library Factor = 0

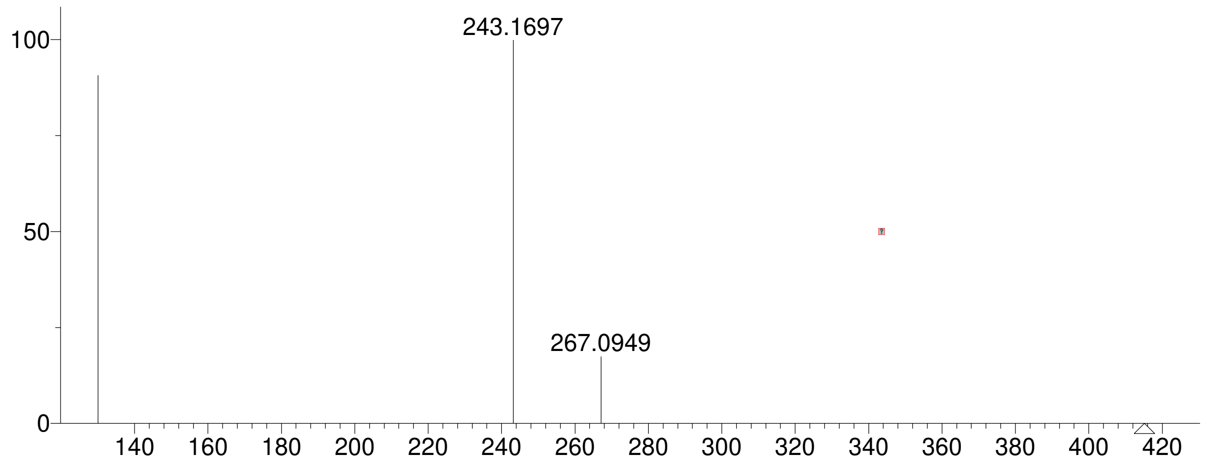

Hit 1 : NCGC00347525-02!2-amino-5-[2-[[2,3-dihydroxy-2-(1-hydroxyethyl)butanoyl]oxymethyl]-4-hydroxyanilino]-
C18H26N2O9; MF: 0; RMF: 6; Prob 50.0%; Lib: mona-export-gnps; ID: 6270.

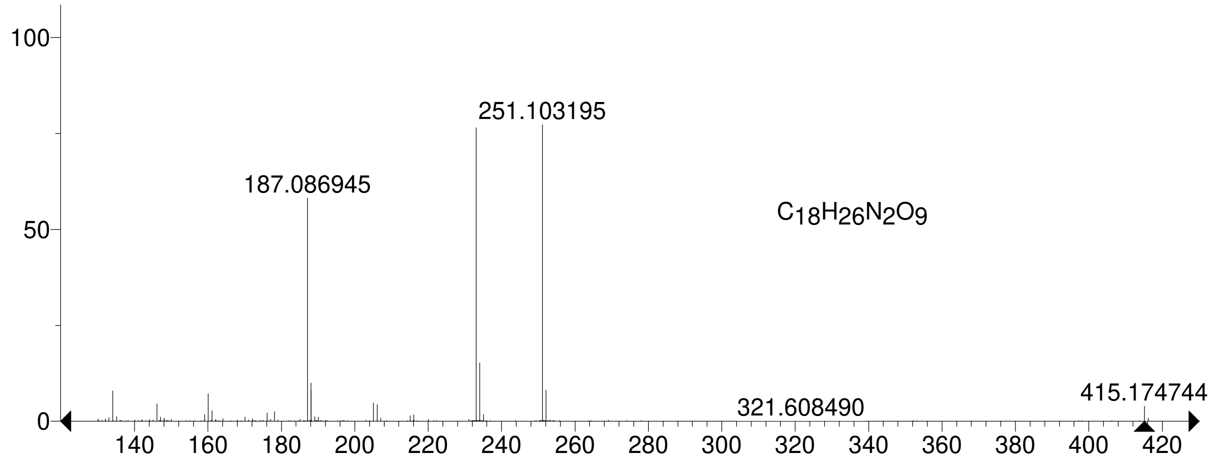

Hit 2 : 6-formyl-10-(hydroxymethyl)-5-methoxy-3-methylidene-2-oxo-2H,3H,3aH,4H,5H,8H,9H,11aH-cyclodeca[b]f
C21H28O7; MF: 0; RMF: 1; Prob 50.0%; Lib: mona-export-vf-npl_qexactive; ID: 16435.

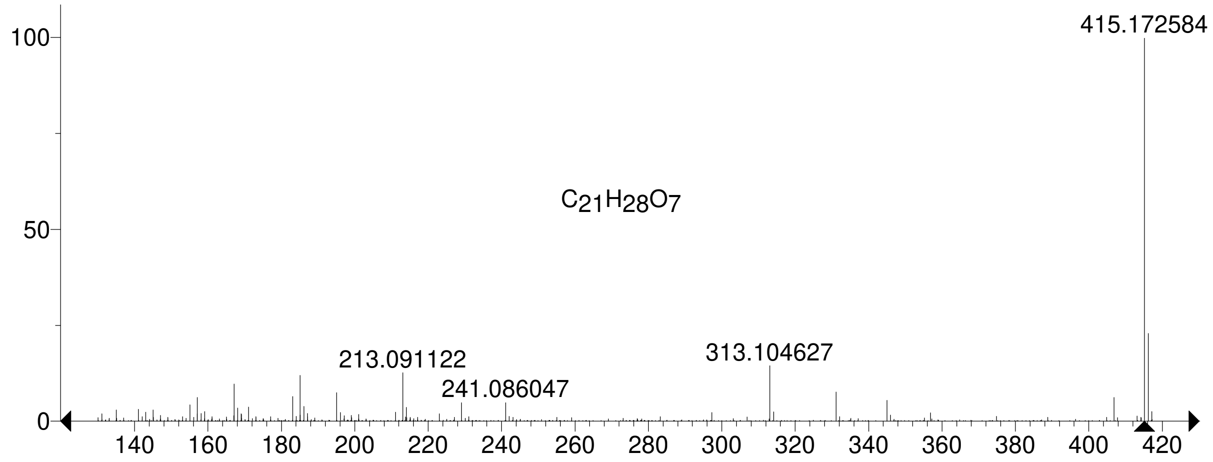

Unknown: Unknown (10.68_358.1965m/z)
Compound in Library Factor = 0

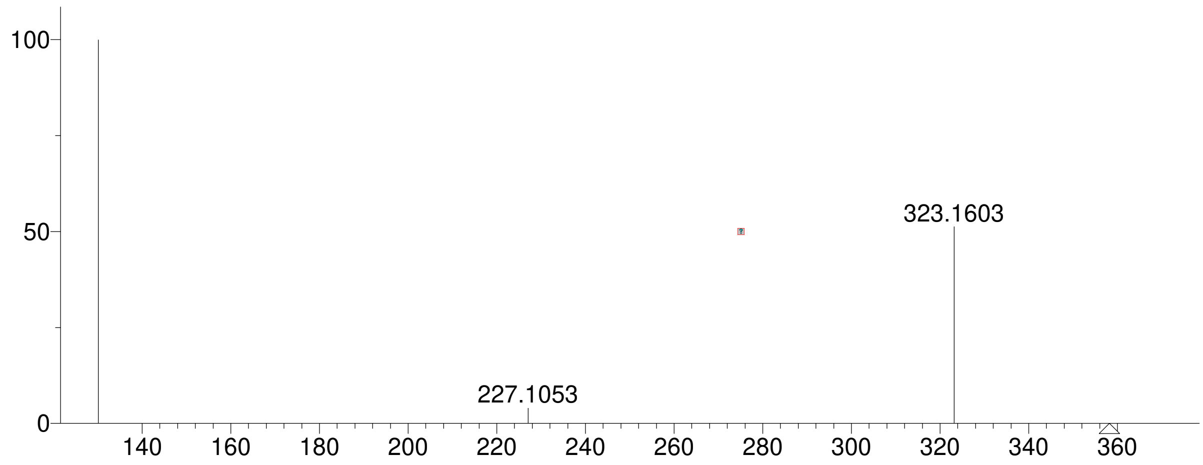

Hit 1 : Nobiletin [M+H]+=>373.2=>358.2 IT 35% P=358.2
C21H22O8; MF: 0; RMF: 7; Prob 50.0%; CAS: 478-01-3; Lib: nist_msms; ID: 178354.

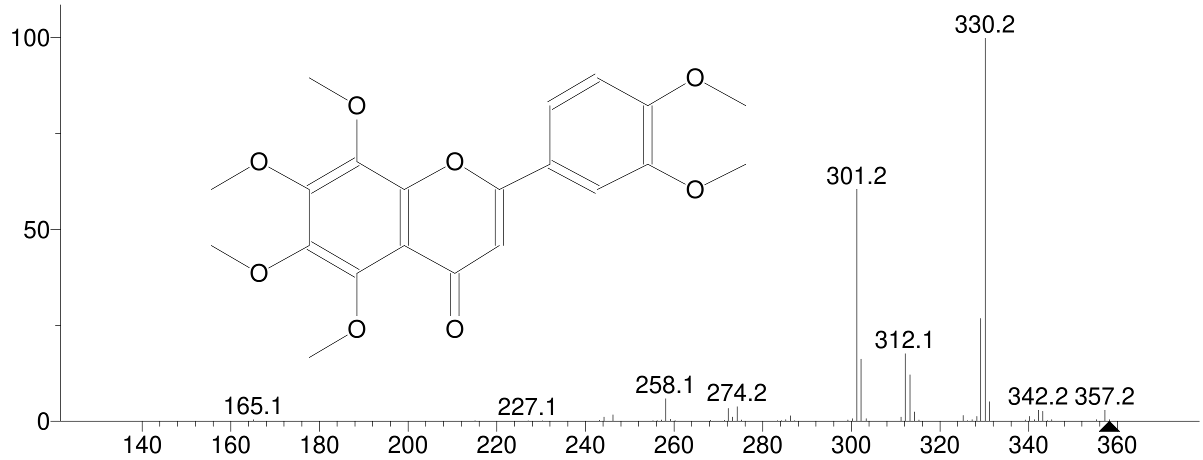

Hit 2 : cis-Moxifloxacin acyl-β-D-glucuronide [M+H-C6H8O6]+=>358.2 IT 35% P=358.2
C27H32FN3O10; MF: 0; RMF: 3; Prob 50.0%; CAS: 733002-61-4; Lib: nist_msms; ID: 463735.

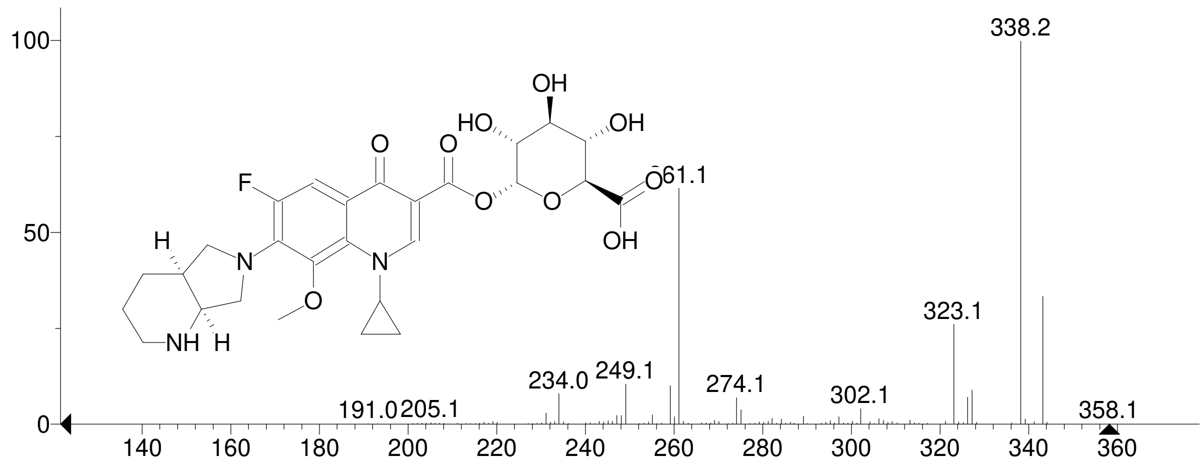

** Search Report Page 1 of 1 **

Unknown: Unknown (10.73_247.0275m/z)
Compound in Library Factor = 0

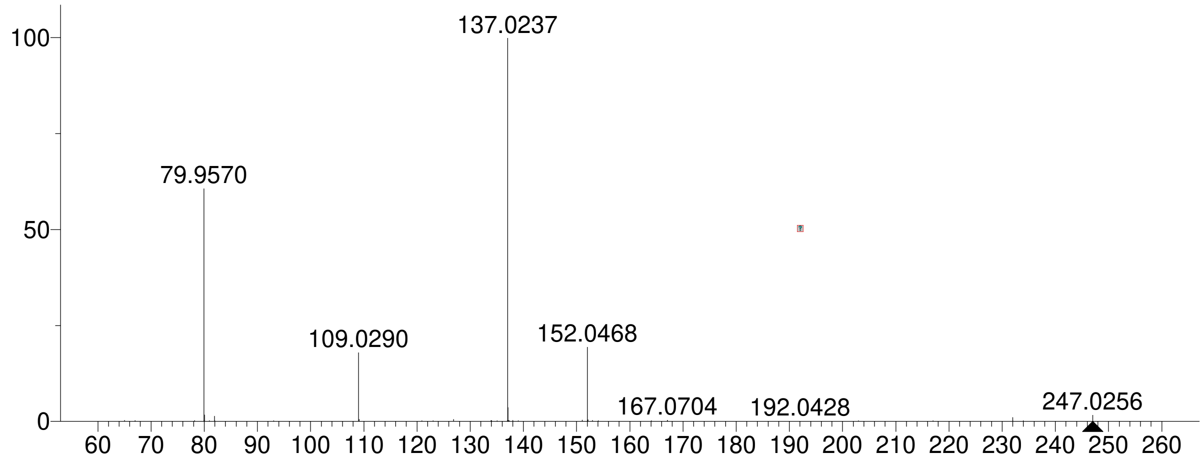

Unknown: Unknown (10.73_263.0216m/z)
Compound in Library Factor = 0

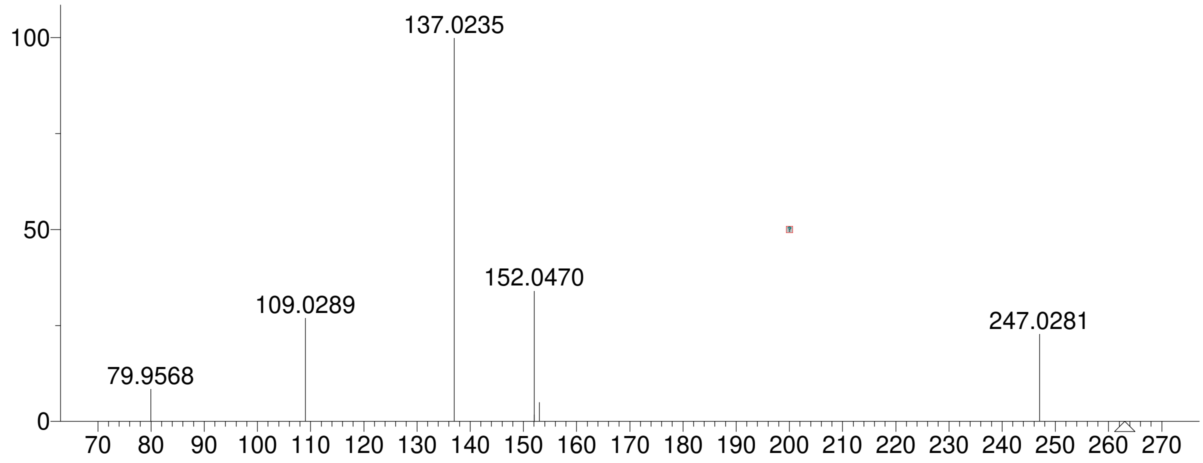

Hit 1 : 3-Methoxy-4-hydroxyphenylglycol sulfate [M-H]- QTOF 20V P=263
C9H12O7S; MF: 0; RMF: 49; Prob 100.0%; CAS: 3415-67-6; Lib: nist_msms; ID: 29981.

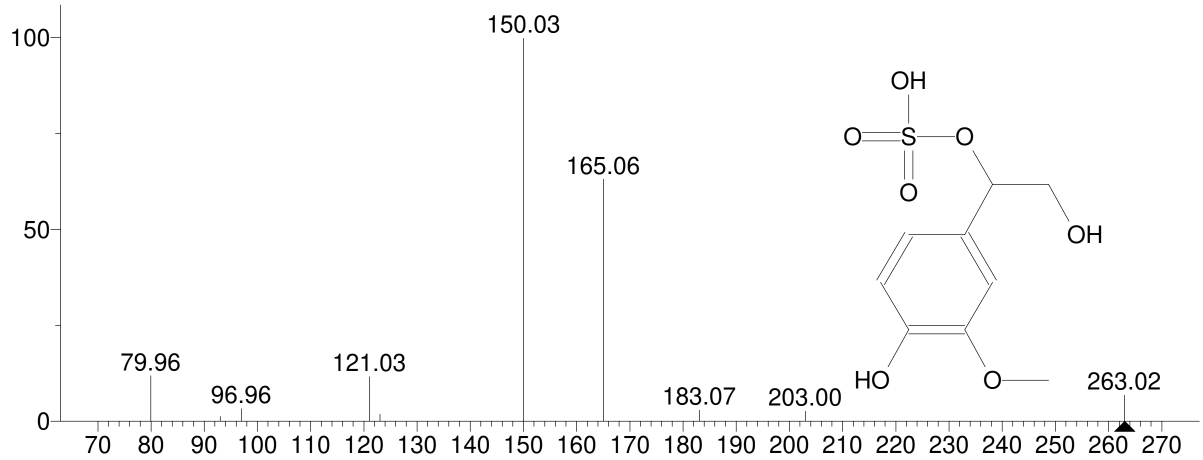

Unknown: Unknown (10.74_247.1755m/z)
Compound in Library Factor = 0

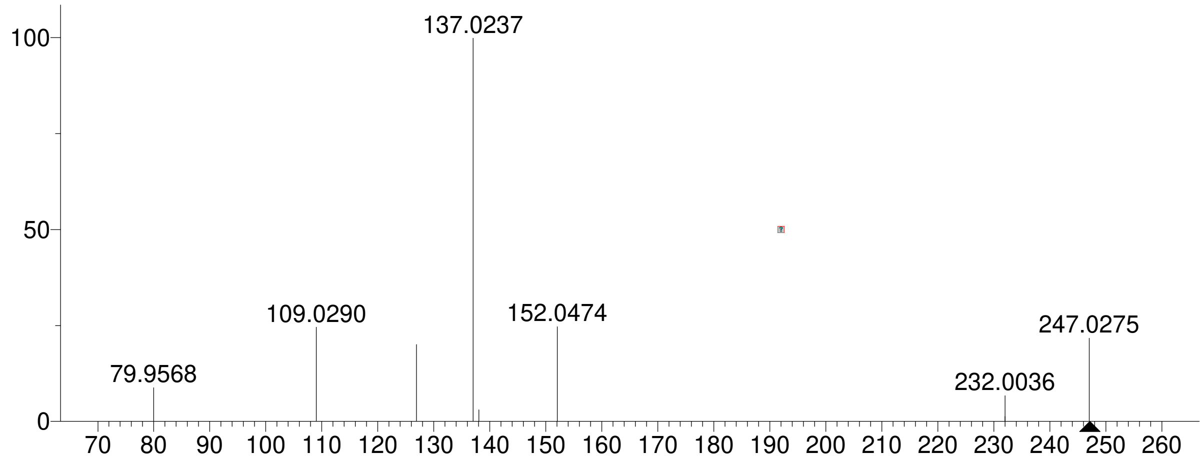

Unknown: Unknown (10.75_306.9464m/z)
Compound in Library Factor = -3273

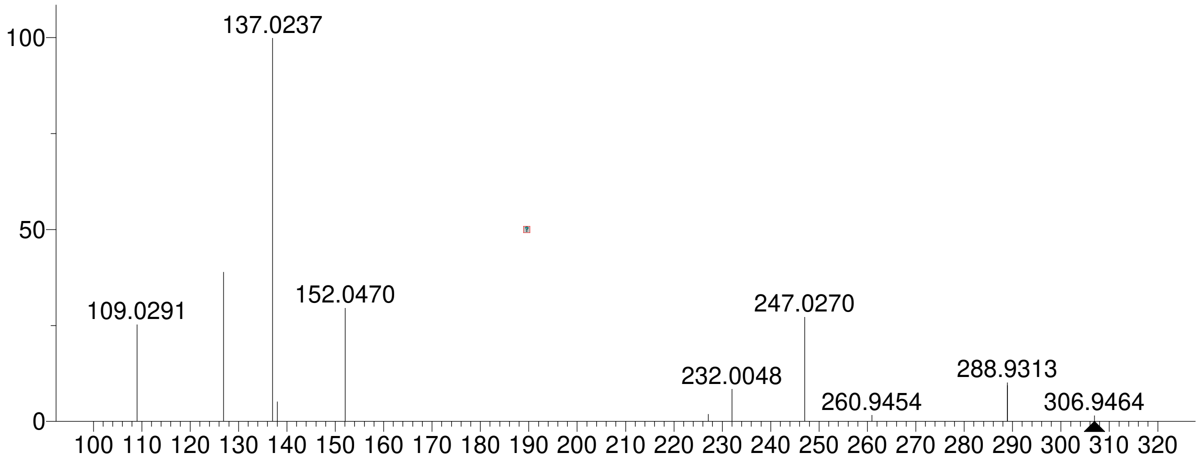

Hit 1 : 5'-Iodoresiniferatoxin [M-H-C28H30O5]- HCD 6V P=306.9
C37H39IO9; MF: 49; RMF: 346; Prob 100.0%; CAS: 535974-91-5; Lib: nist_msms; ID: 357926.

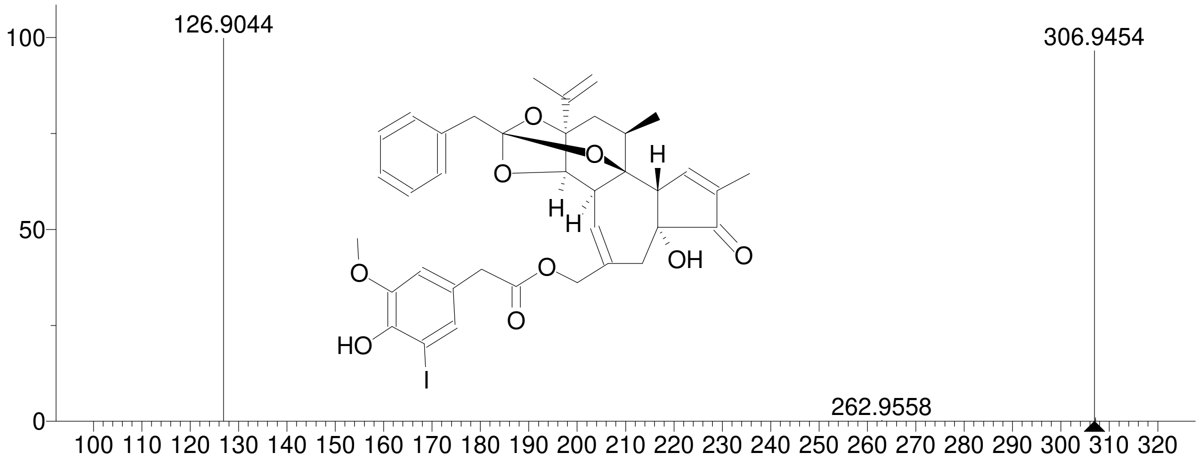

** Search Report Page 1 of 1 **

Unknown: Unknown (10.75_347.9724m/z)
Compound in Library Factor = 0

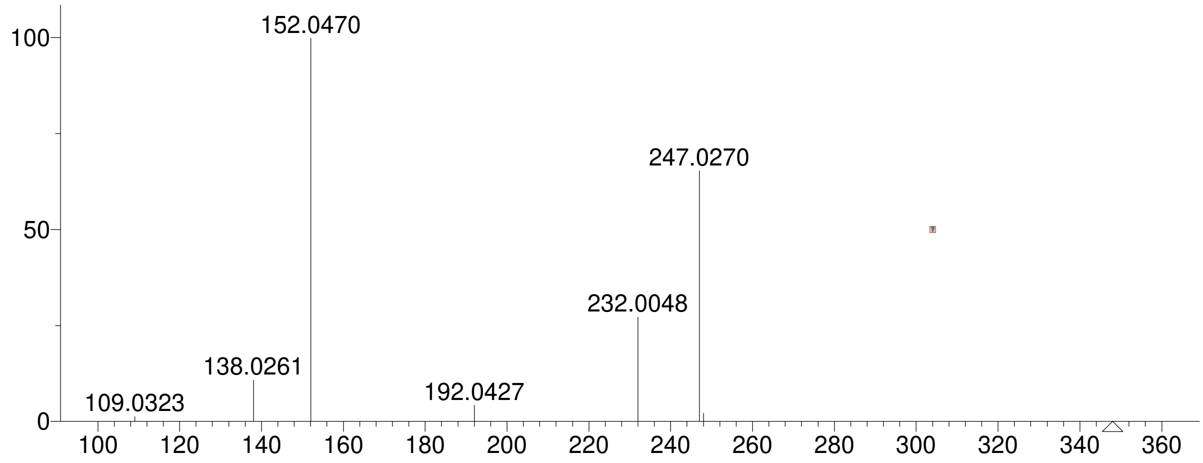

Unknown: Unknown (10.81_221.0444m/z)
Compound in Library Factor = -199

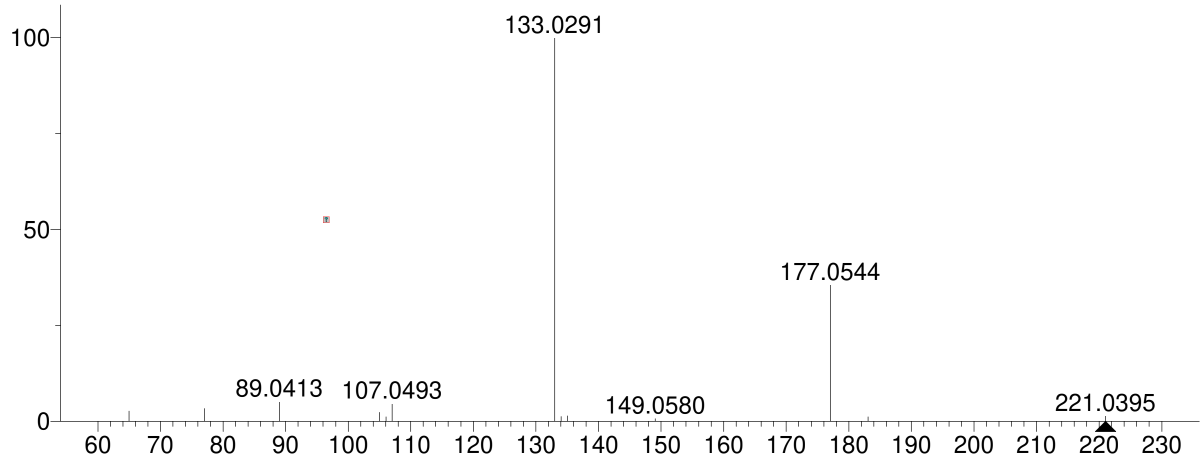

Hit 1 : 8-Methoxycoumarin-3-carboxylic acid [M+H]+ HCD 0V P=221
C11H8O5; MF: 352; RMF: 982; Prob 56.6%; CAS: 2555-20-6; Lib: nist_msms; ID: 178797.

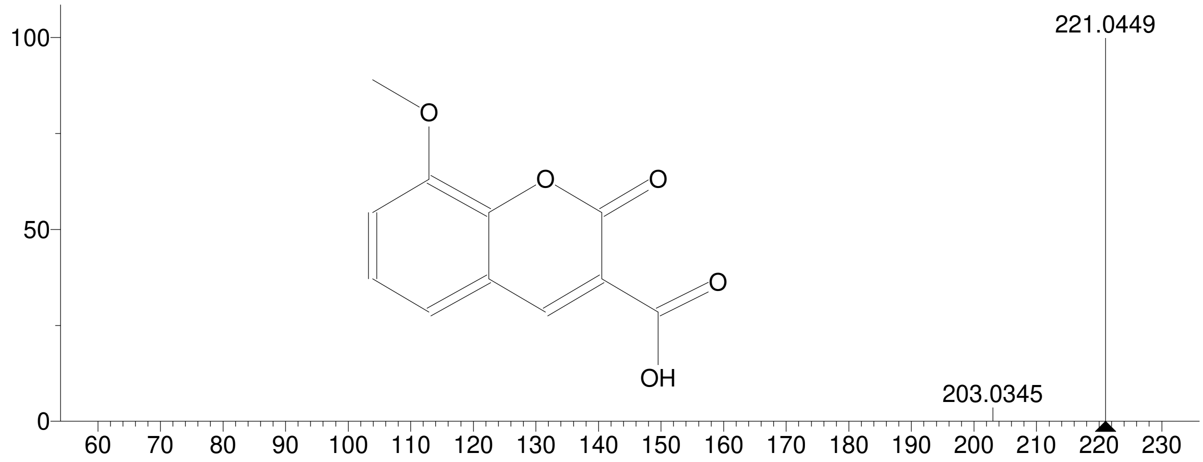

Hit 2 : Isofraxidin [M-H]- HCD 6V P=221
C11H10O5; MF: 344; RMF: 971; Prob 42.3%; CAS: 486-21-5; Lib: nist_msms; ID: 466238.

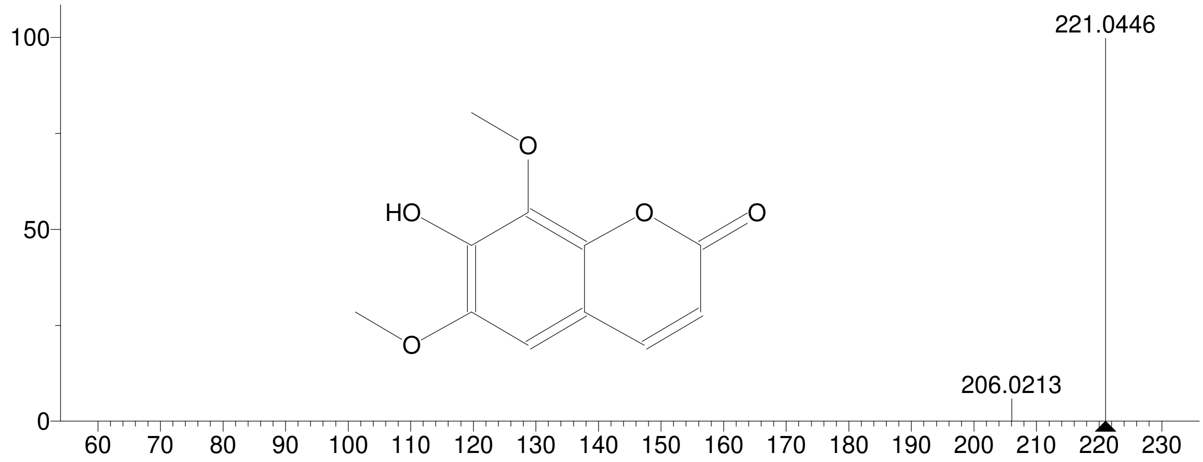

Hit 3 : 7-Hydroxycoumarin-4-acetic acid [M+H]+ HCD 11V P=221
C11H8O5; MF: 53; RMF: 249; Prob 0.42%; CAS: 6950-82-9; Lib: nist_msms; ID: 177551.

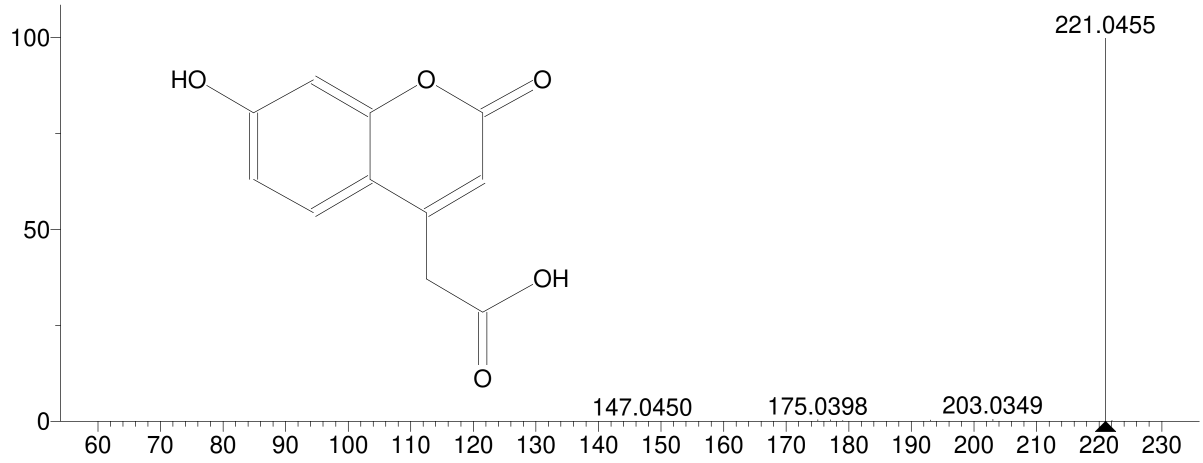

Hit 4 : Unknown (carbon number 11); PlaSMA ID-316 [M-H]- P=221
C11H10O5; MF: 13; RMF: 131; Prob 0.09%; Lib: biomsms-neg-plasma; ID: 1061.

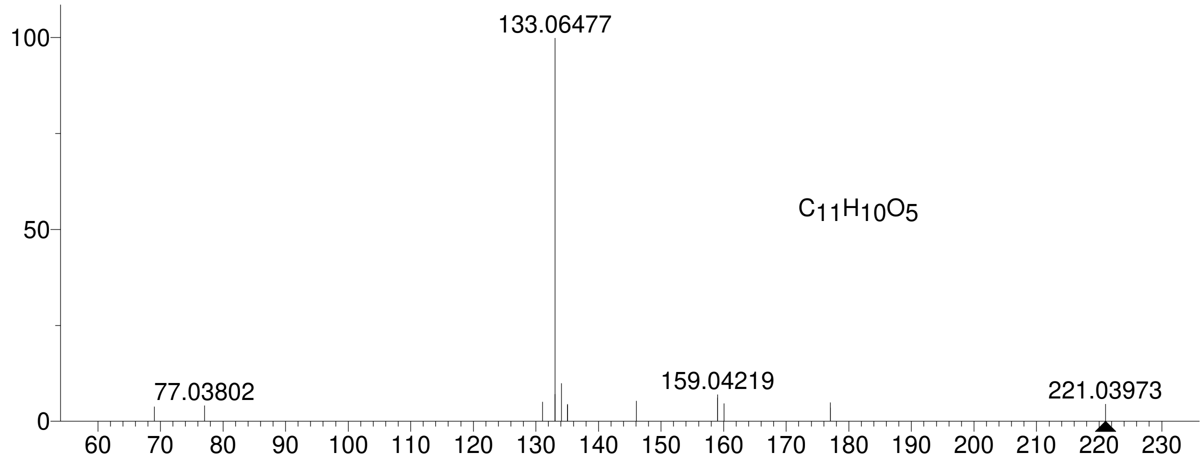

Hit 5 : Umckalin [M-H]- HCD 8V P=221
C11H10O5; MF: 5; RMF: 91; Prob 0.07%; CAS: 43053-62-9; Lib: nist_msms; ID: 234879.

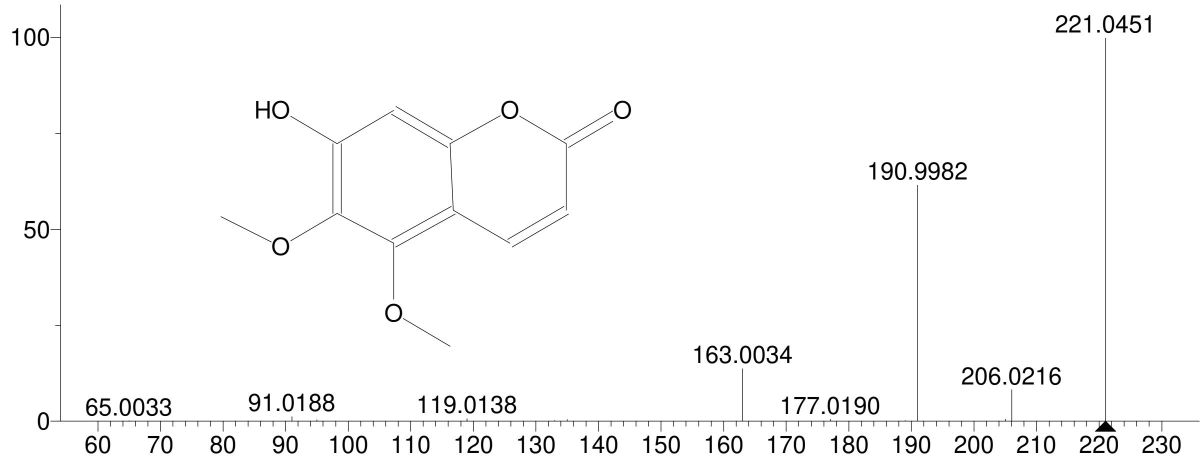

** Search Report Page 1 of 1 **

Unknown: Unknown (10.82_534.2222m/z)
Compound in Library Factor = 0

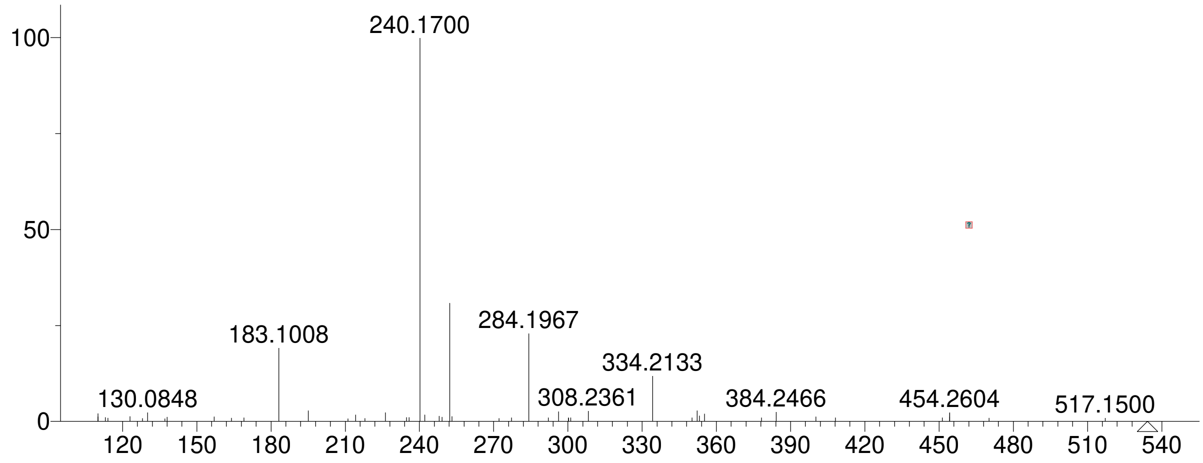

Unknown: Unknown (10.83_401.0536m/z)
Compound in Library Factor = 0

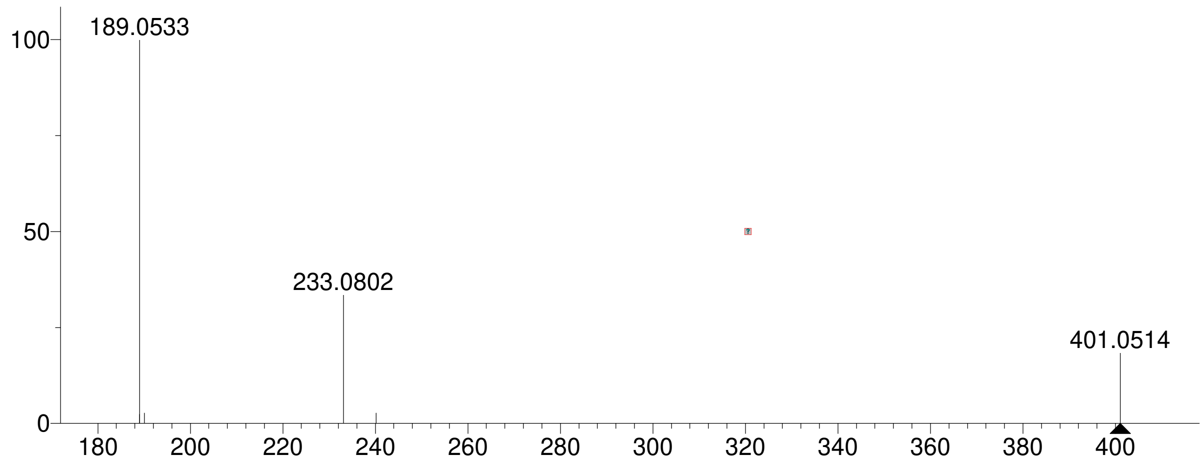

** Search Report Page 1 of 1 **

Unknown: Unknown (10.83_454.2543m/z)
Compound in Library Factor = 0

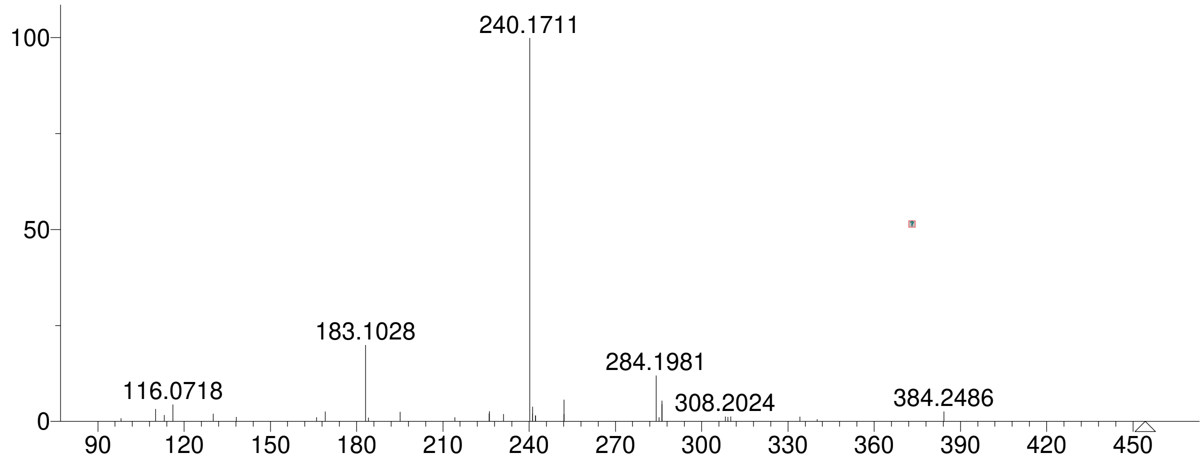

Unknown: Unknown (10.84_373.1386m/z)
Compound in Library Factor = 0

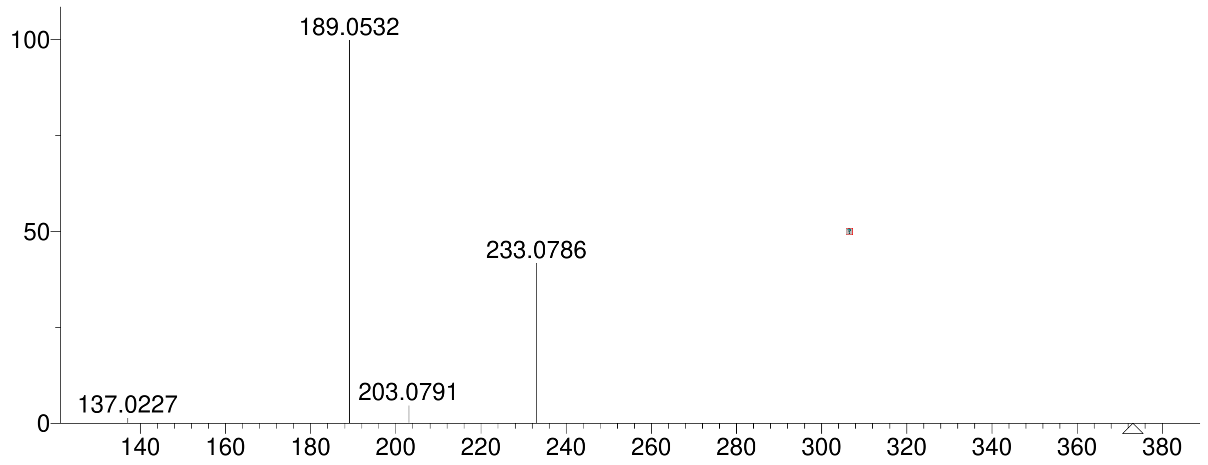

Hit 1 : [(1S,3aR,5R,5aR,8aR,9S,9aR)-1,5,8a-trimethyl-2,8-dioxo-3a,4,5,5a,9,9a-hexahydro-1H-azuleno[6,5-b]fura
C19H26O5; MF: 0; RMF: 18; Prob 100.0%; Lib: mona-export-vf-npl_qexactive; ID: 7291.

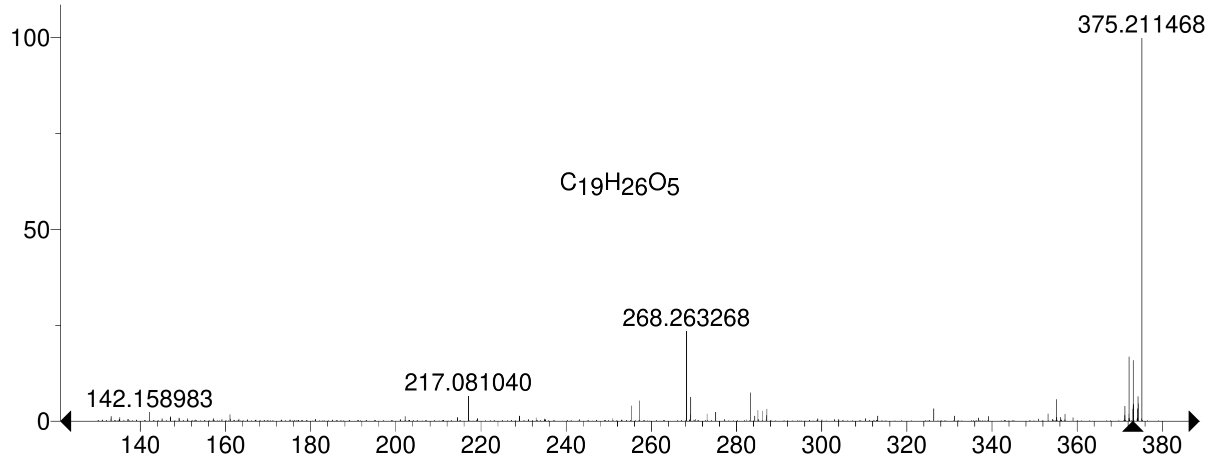

** Search Report Page 1 of 1 **

Unknown: Unknown (10.86_463.2059m/z)
Compound in Library Factor = 0

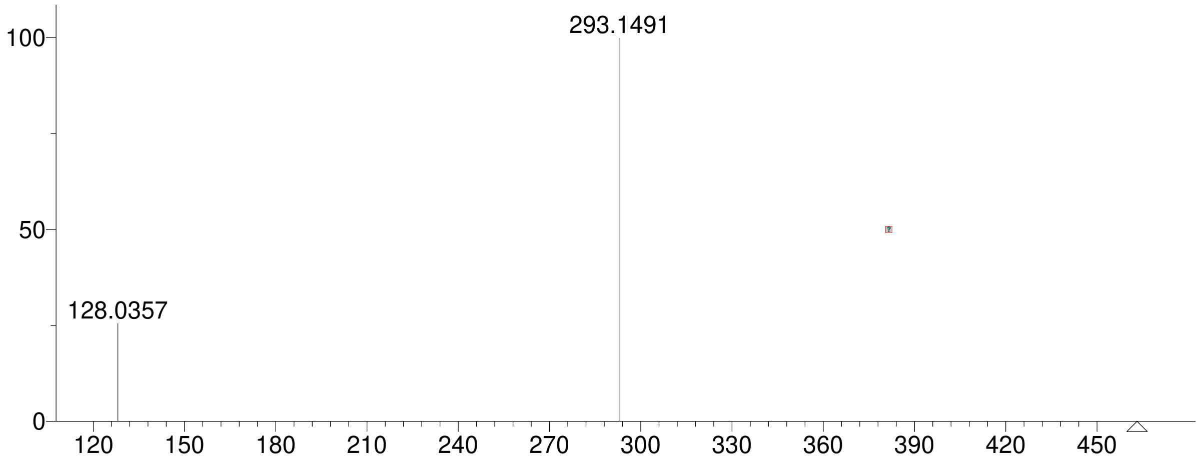

Unknown: Unknown (10.87_528.2524m/z)
Compound in Library Factor = 0

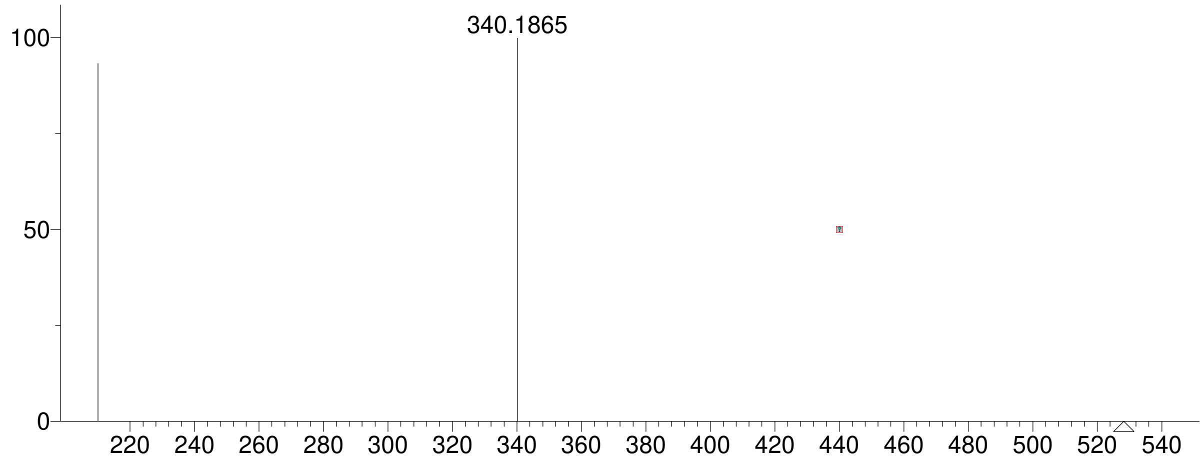

Unknown: Unknown (10.89_449.1583m/z)
Compound in Library Factor = 0

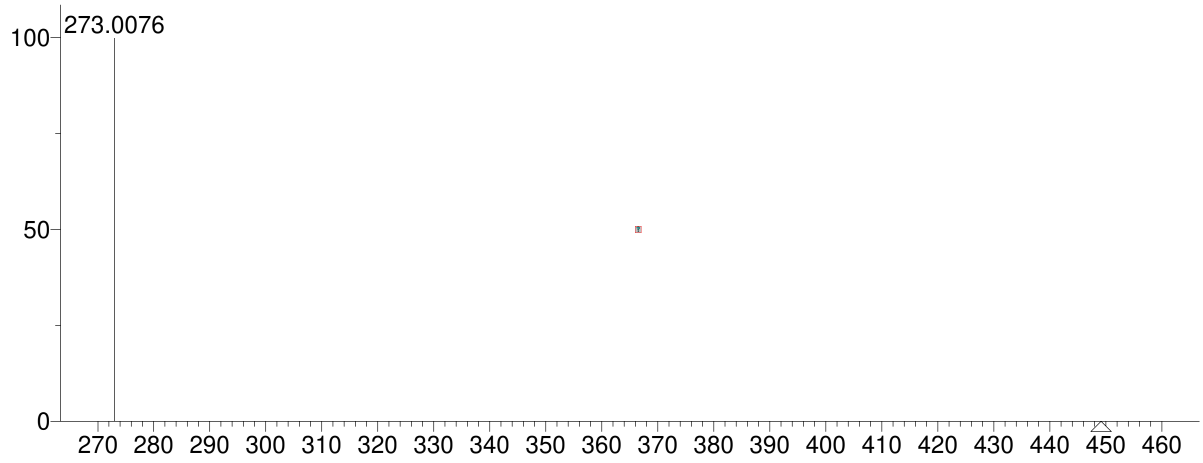

Unknown: Unknown (10.92_753.4147m/z)
Compound in Library Factor = -246

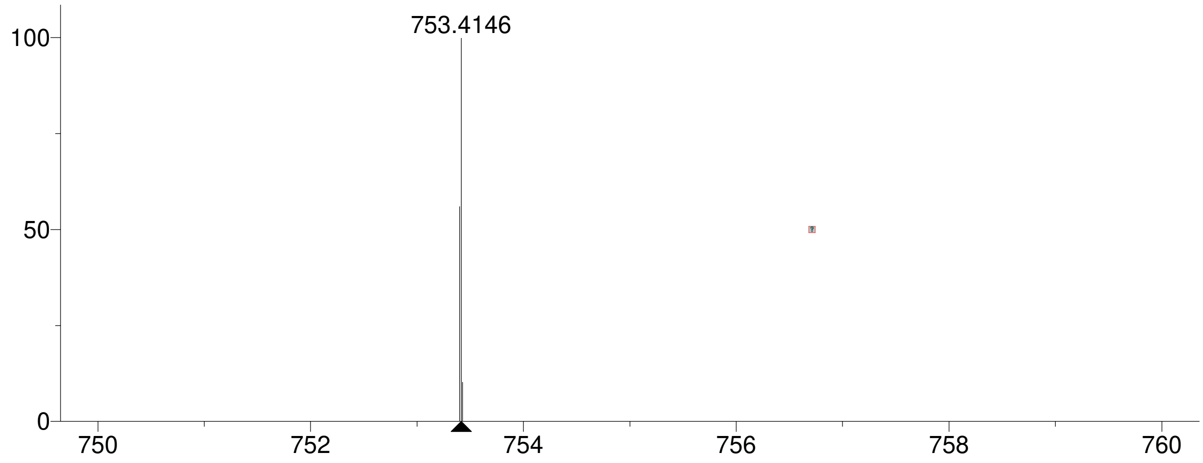

Hit 1 : GlcADG 34:9; GlcADG 16:4-18:5; [M-H]- [M-H]- P=753.4
C43H62O11; MF: 263; RMF: 775; Prob 100.0%; Lib: msdial-insilicomsms-lipids-neg; ID: 275003.

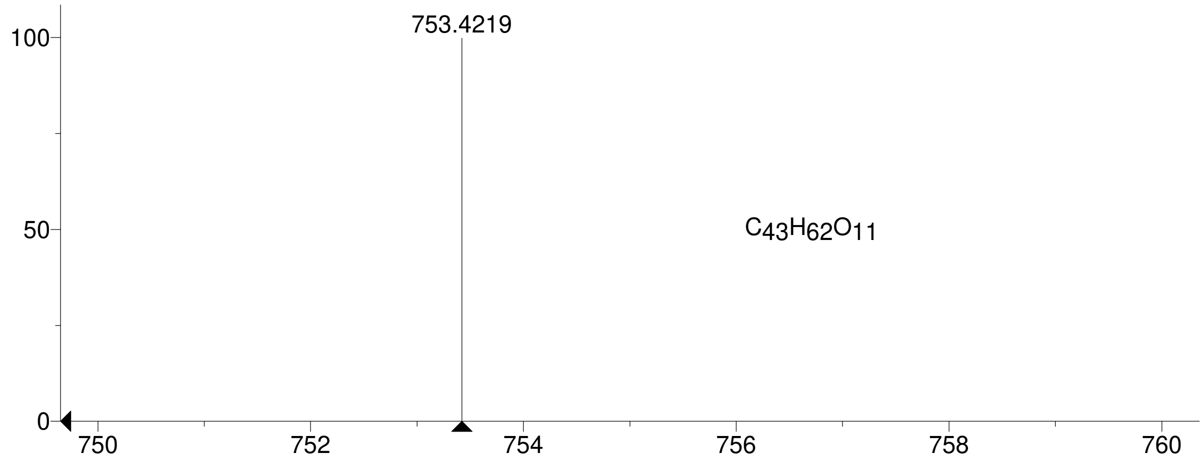

Unknown: Unknown (10.93_273.4840m/z)
Compound in Library Factor = 0

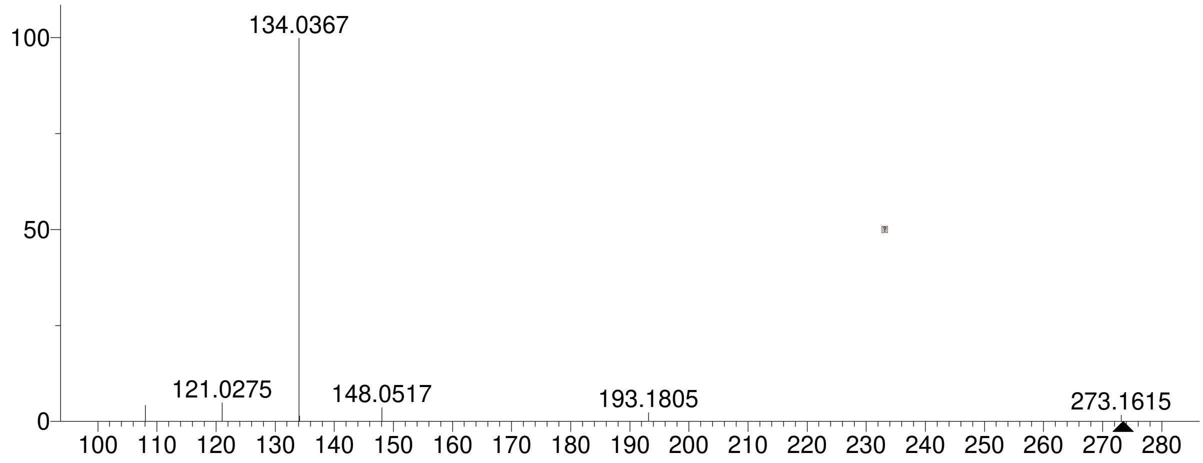

Unknown: Unknown (10.94_569.0031m/z)
Compound in Library Factor = 0

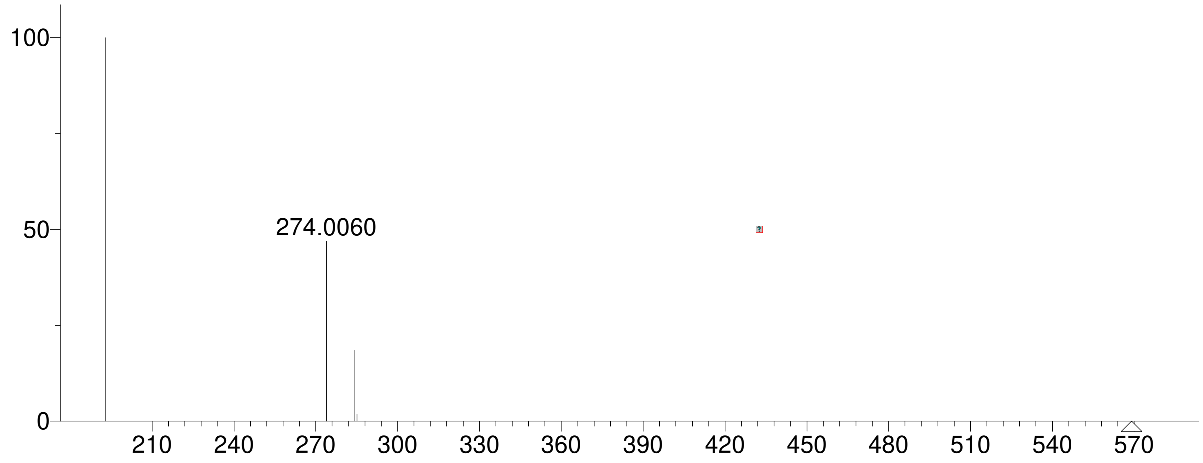

** Search Report Page 1 of 1 **

Unknown: Unknown (10.96_415.1700m/z)
Compound in Library Factor = 0

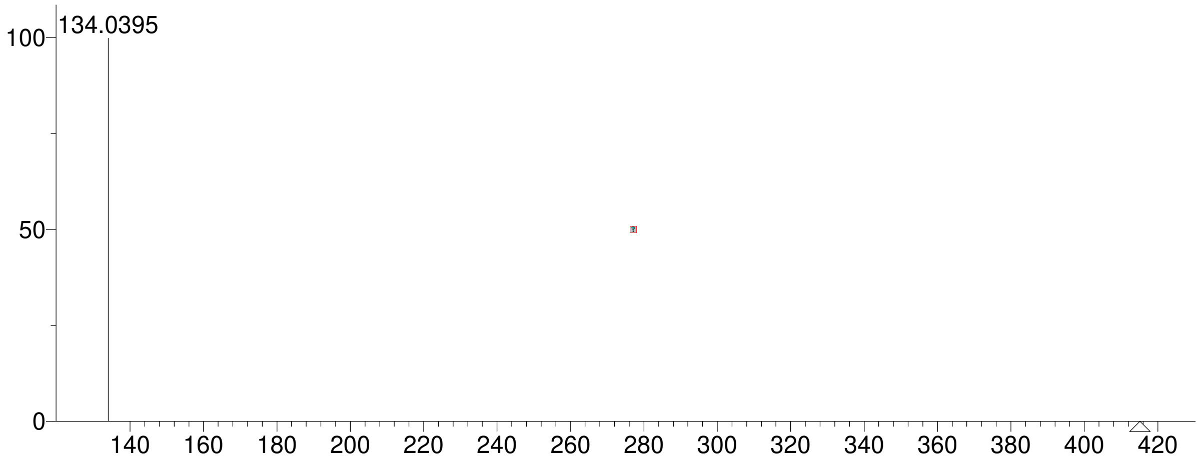

Unknown: Unknown (10.97_413.2276m/z)
Compound in Library Factor = -185

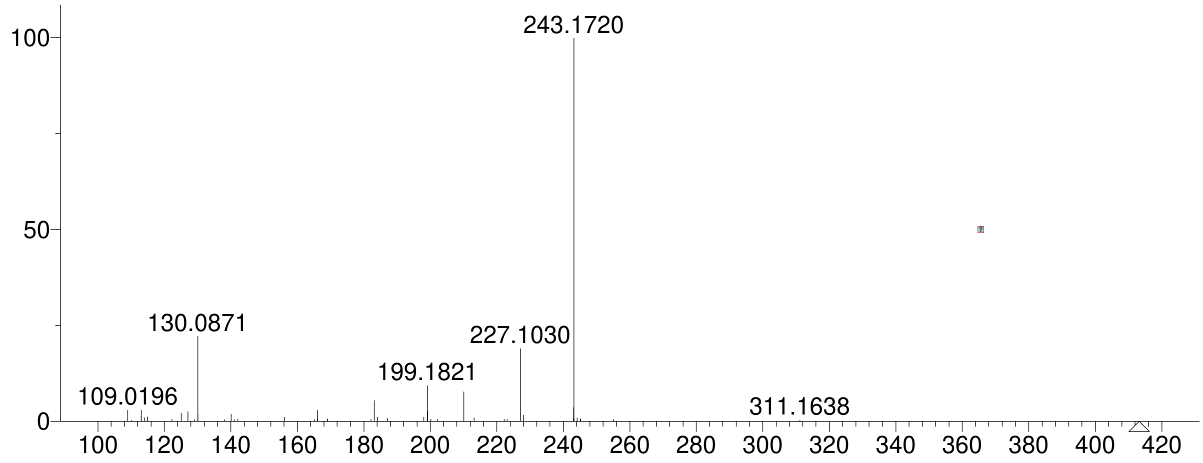

Hit 1 : Mevastatin [M+Na]+ IT-FT 35% P=413.2
C23H34O5; MF: 313; RMF: 970; Prob 93.6%; CAS: 73573-88-3; Lib: nist_msms; ID: 165122.

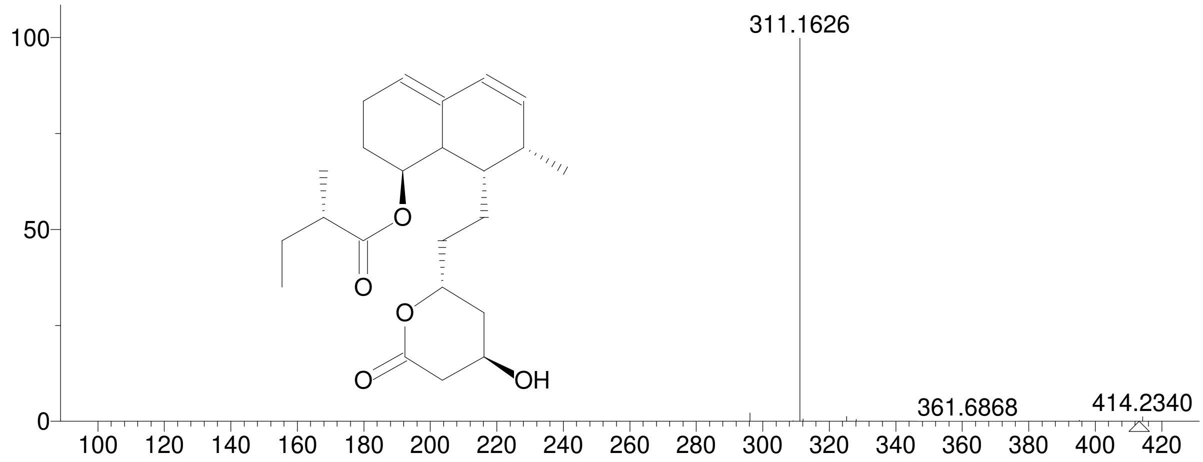

Hit 2 : His-Thr-Arg [M+H]+ QTOF 40V P=413.2
C16H28N8O5; MF: 5; RMF: 58; Prob 0.93%; Lib: nist_msms; ID: 51182.

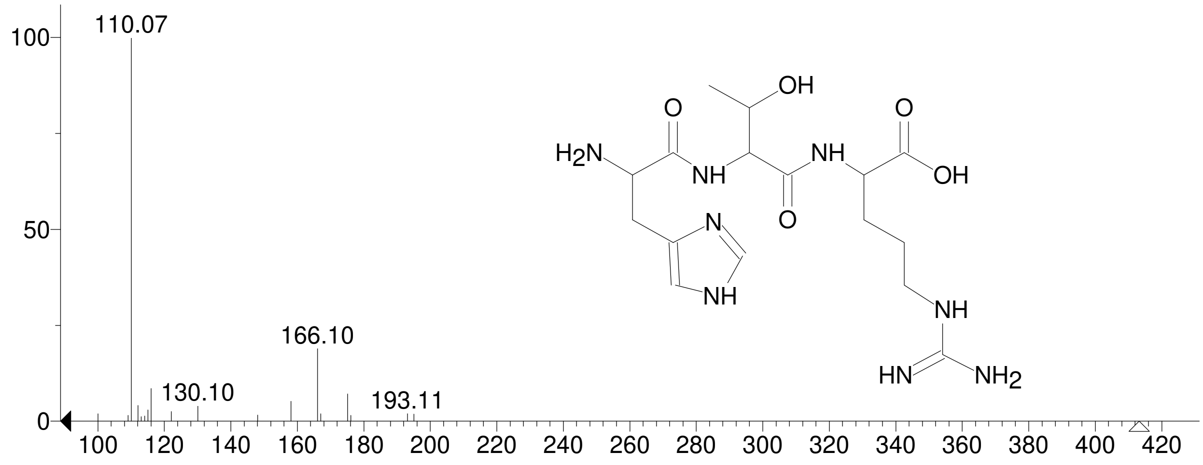

Hit 3 : 15-Deoxy-.DELTA.12,14-prostaglandin J2, 2-glycerol ester [M+Na]+ HCD 41V P=413.2
C23H34O5; MF: 3; RMF: 66; Prob 0.86%; CAS: 1176540-68-3; Lib: nist_msms; ID: 87746.

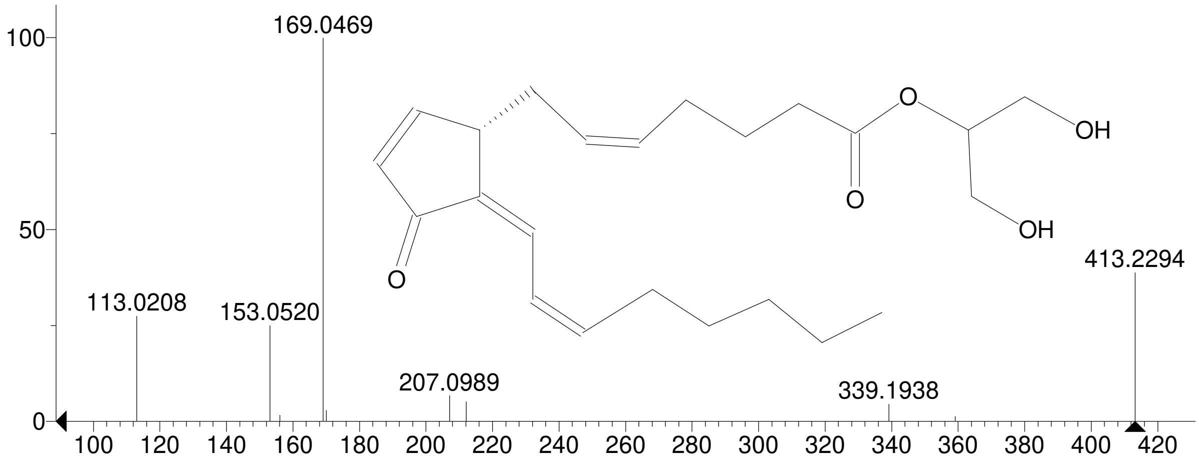

Hit 4 : Thr-His-Arg [M+H]+ QTOF 30V P=413.2
C16H28N8O5; MF: 0; RMF: 33; Prob 0.76%; Lib: nist_msms; ID: 54064.

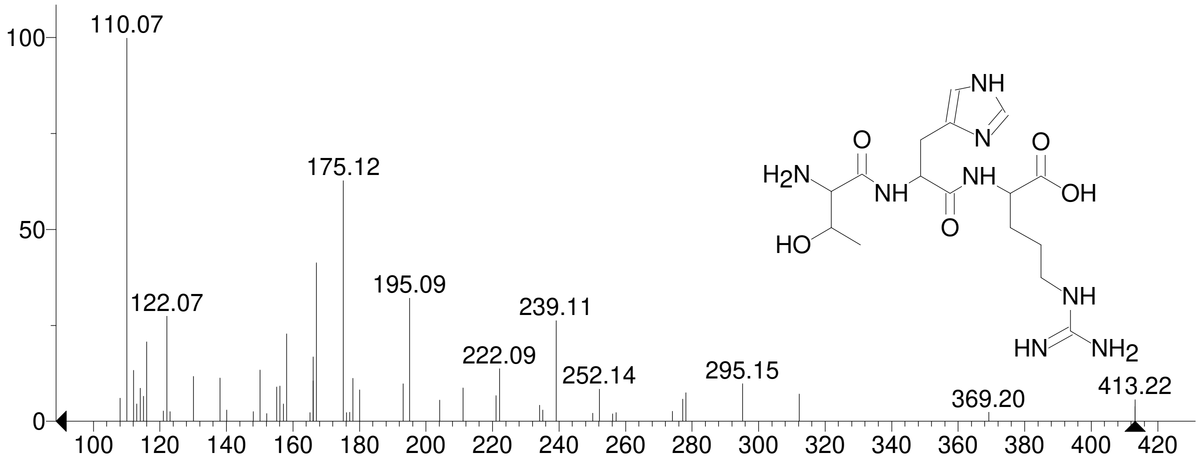

Hit 5 : Tussilagone [M+Na]+ HCD 61V P=413.2
C23H34O5; MF: 0; RMF: 11; Prob 0.76%; CAS: 104012-37-5; Lib: nist_msms; ID: 425582.

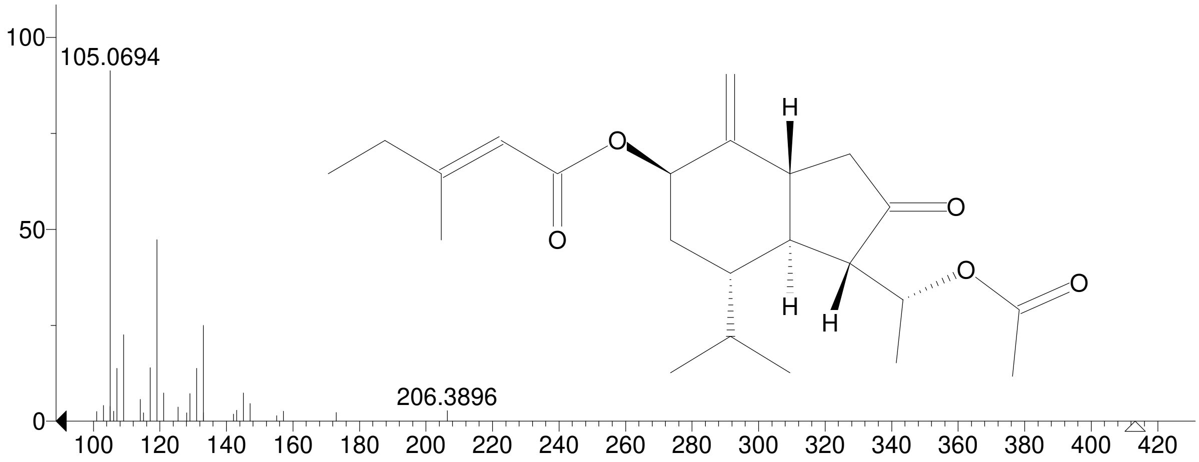

Unknown: Unknown (11.08_245.0924m/z)
Compound in Library Factor = -279

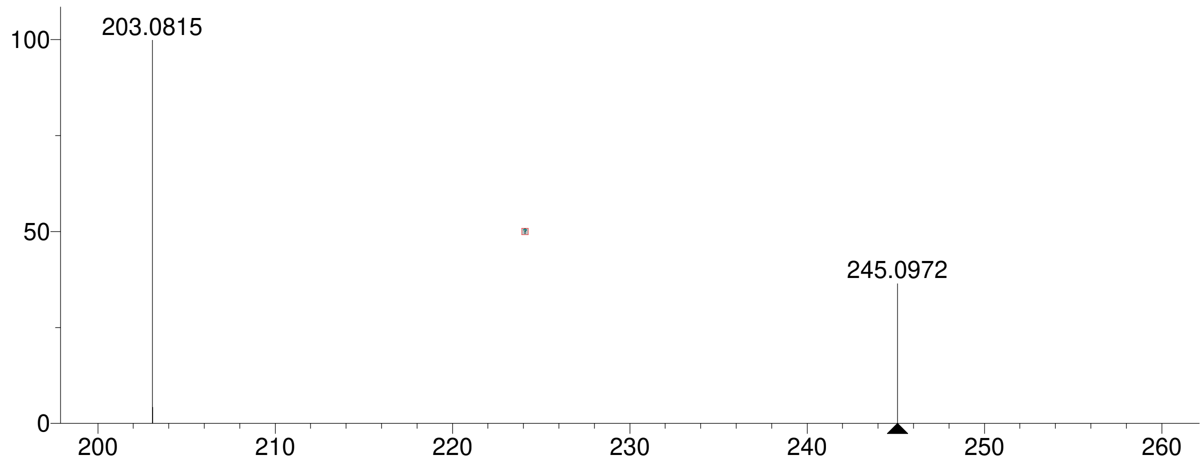

Hit 1 : N-Acetyltryptophan [M-H]- 20-30-40%) P=245.1
C13H14N2O3; MF: 581; RMF: 971; Prob 84.7%; Lib: msms-neg-fiehnhilic; ID: 488.

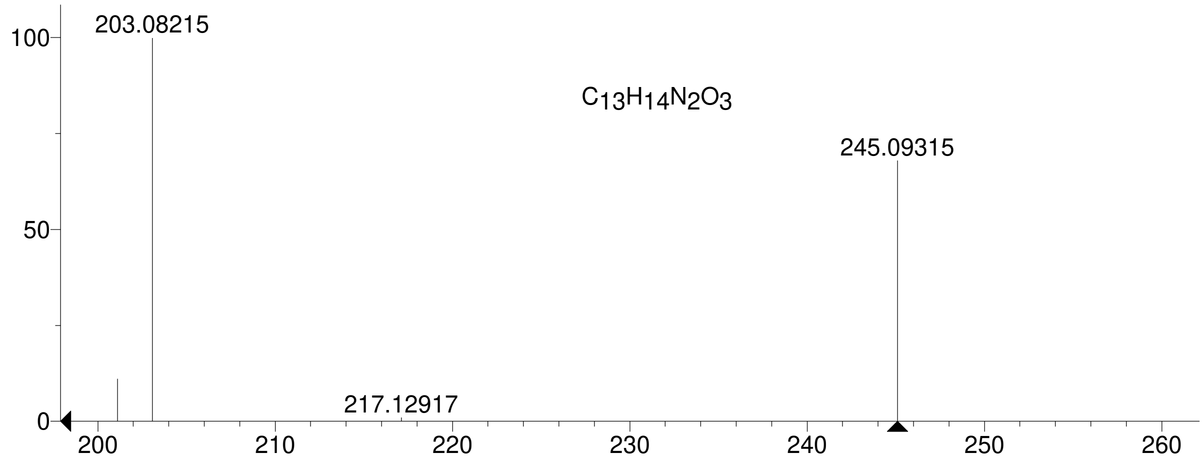

Hit 2 : N-ACETYL-D-TRYPTOPHAN
C13H14N2O3; MF: 495; RMF: 951; Prob 7.54%; Lib: embl neg mcg; ID: 175.

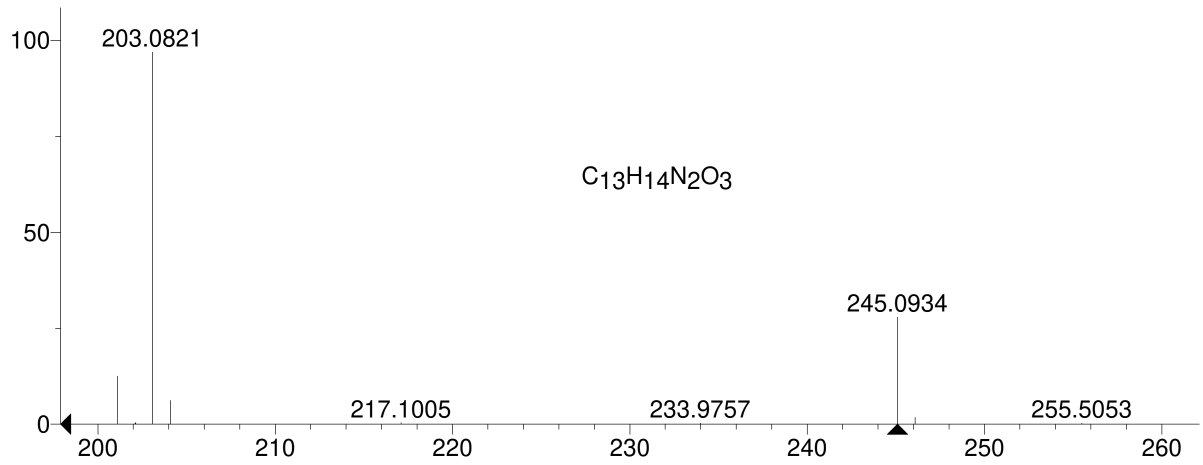

Hit 3 : N-ACETYL-D-TRYPTOPHAN [M-H]- P=245.1
C13H14N2O3; MF: 495; RMF: 951; Prob 7.54%; Lib: msms neg mona_ipb_gnps_plasma; ID: 22858.

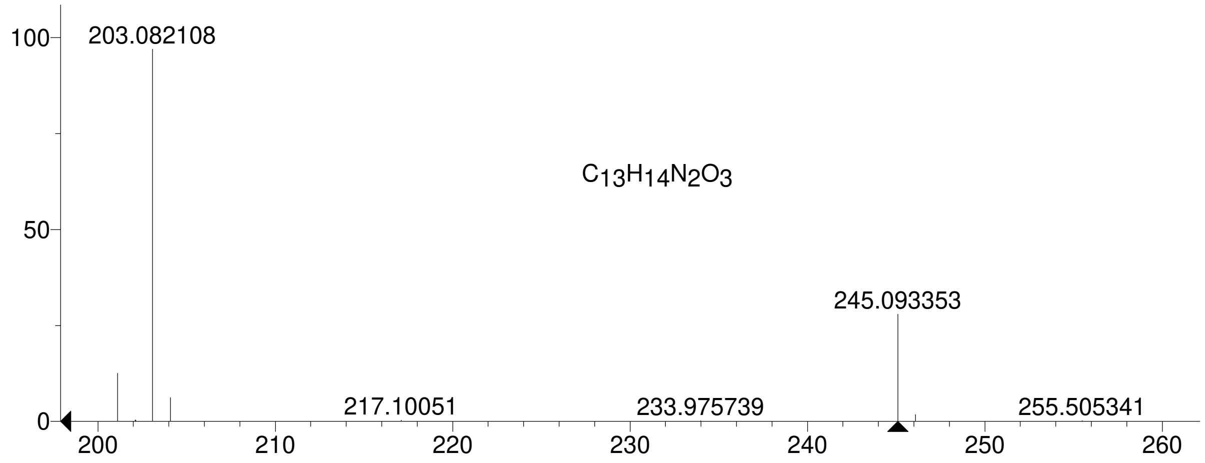

Hit 4 : N-acetyltryptophan [M-H]- 35HCD P=245.1
C13H14N2O3; MF: 343; RMF: 911; Prob 0.20%; Lib: mona-export-lc-ms-ms_negative_mode; ID: 17702.

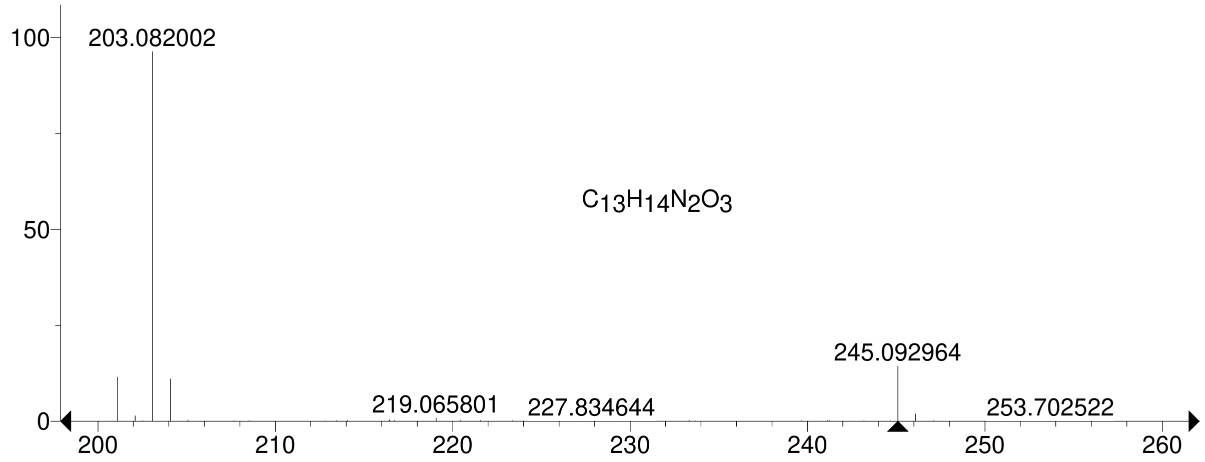

Hit 5 : Indole-3-acetyl-L-alanine [M-H]- 10 P=245.1
C13H14N2O3; MF: 156; RMF: 509; Prob 0.00%; Lib: msms neg mona_ipb_gnps_plasma; ID: 22060.

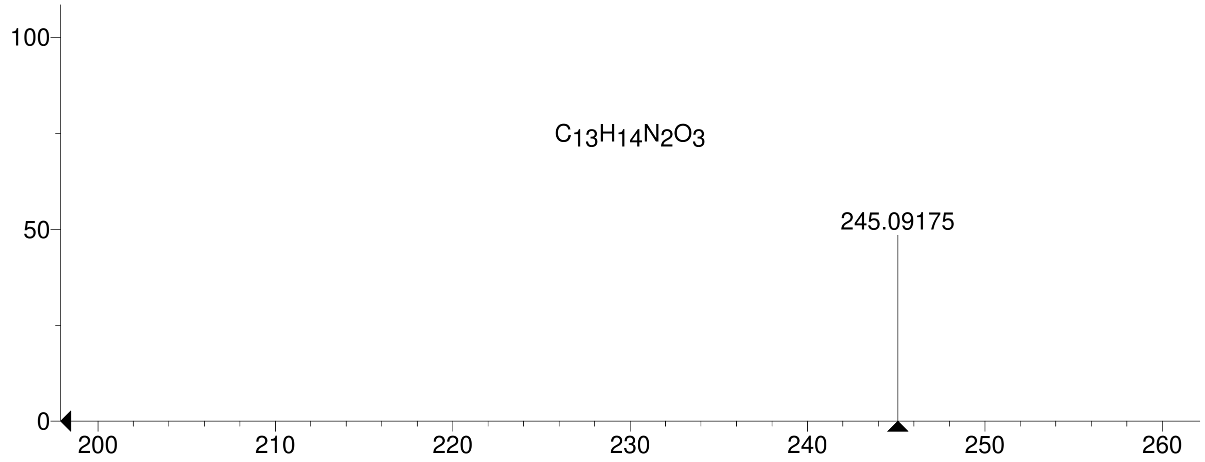

Unknown: Unknown (11.08_435.1751m/z)
Compound in Library Factor = 0

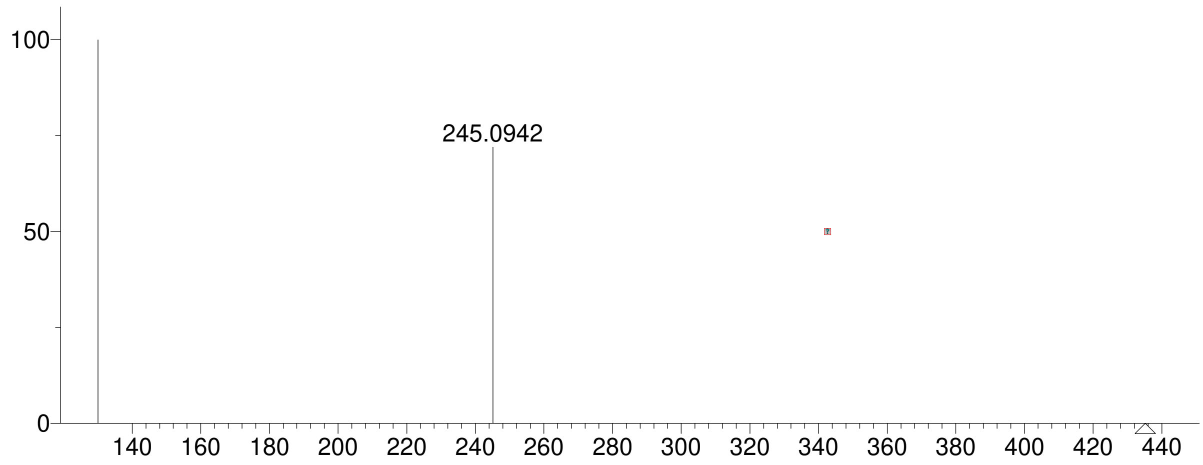

Hit 1 : 2,4-dihydroxy-5-methoxy-1'',6'',8''-trimethyldispiro[bis(oxolane)-3,2':5',7''-[3]oxatricyclo[6.3.1.0a^'',A^'1A^'2]c
C21H32O7; MF: 0; RMF: 25; Prob 100.0%; Lib: mona-export-vf-npl_qexactive; ID: 10624.

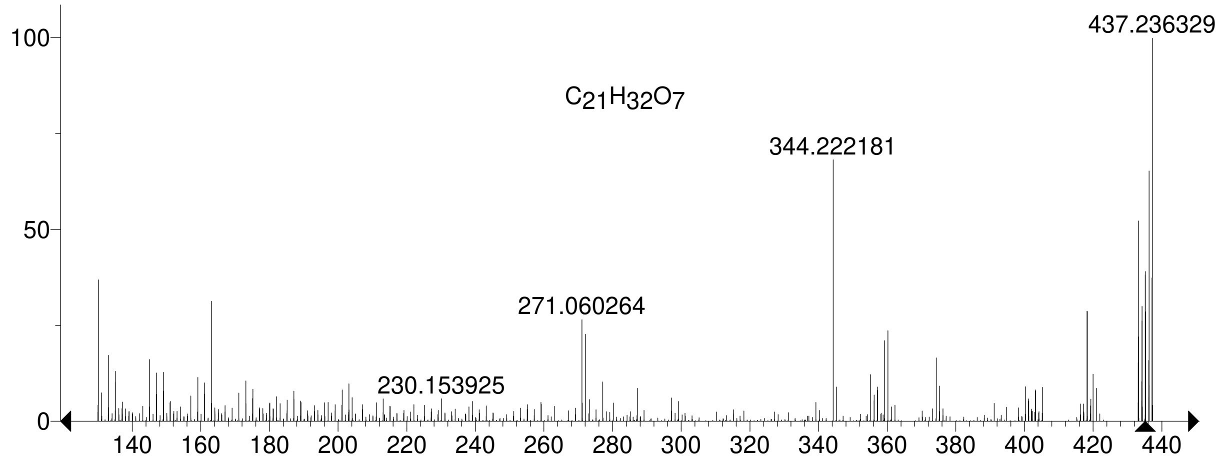

Unknown: Unknown (11.11_390.1541m/z)
Compound in Library Factor = 0

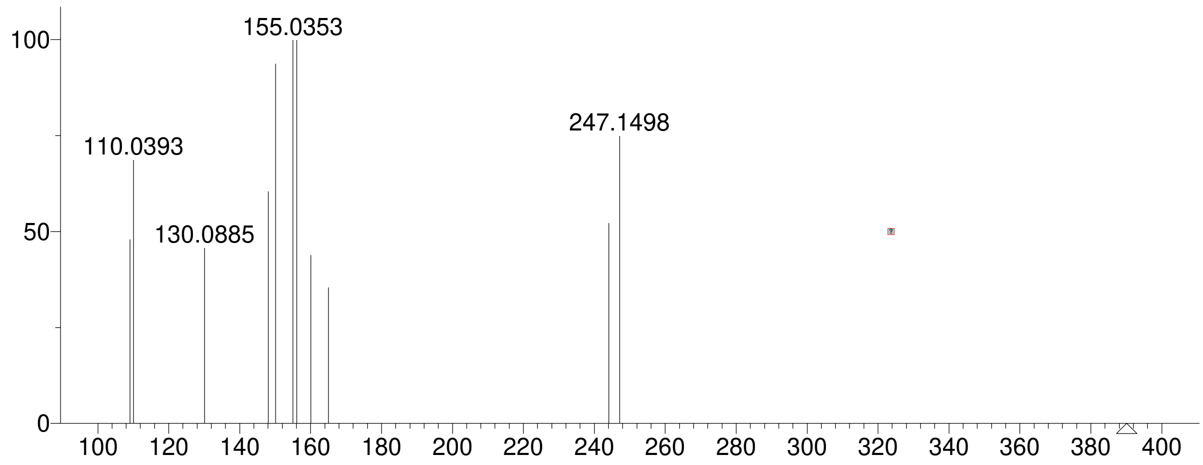

Hit 1 : [3-(4-hydroxy-3-methoxybenzoyl)-2,3-dimethyloxiran-2-yl]-(4-hydroxy-3-methoxyphenyl)methanone [M+NH
C20H20O7; MF: 0; RMF: 30; Prob 50.0%; Lib: mona-export-vf-npl_qexactive; ID: 19626.

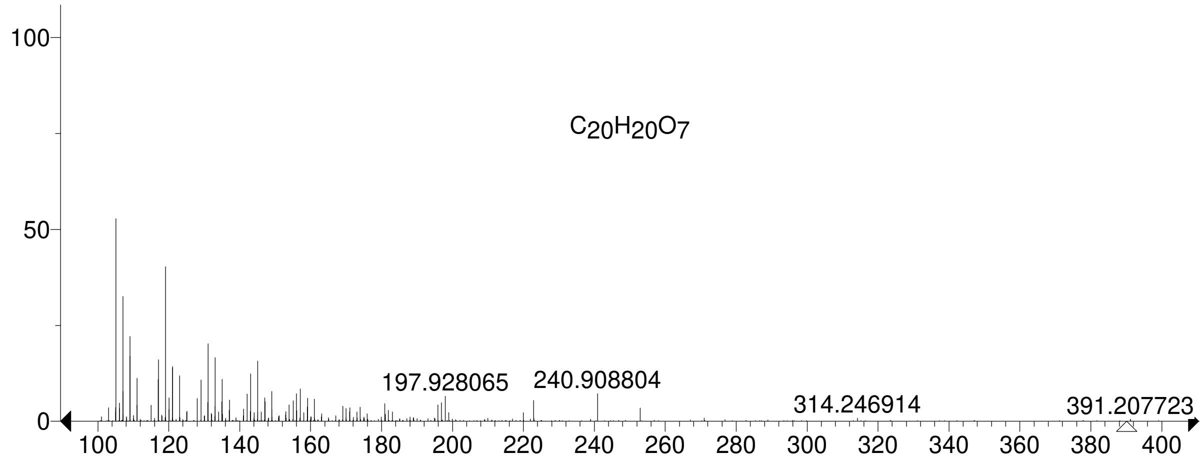

Hit 2 : (+)-Fluprostenol lactone diol [M+NH4]+ HCD 85V P=390.2
C18H19F3O5; MF: 0; RMF: 25; Prob 50.0%; CAS: 53872-60-9; Lib: nist_msms; ID: 544834.

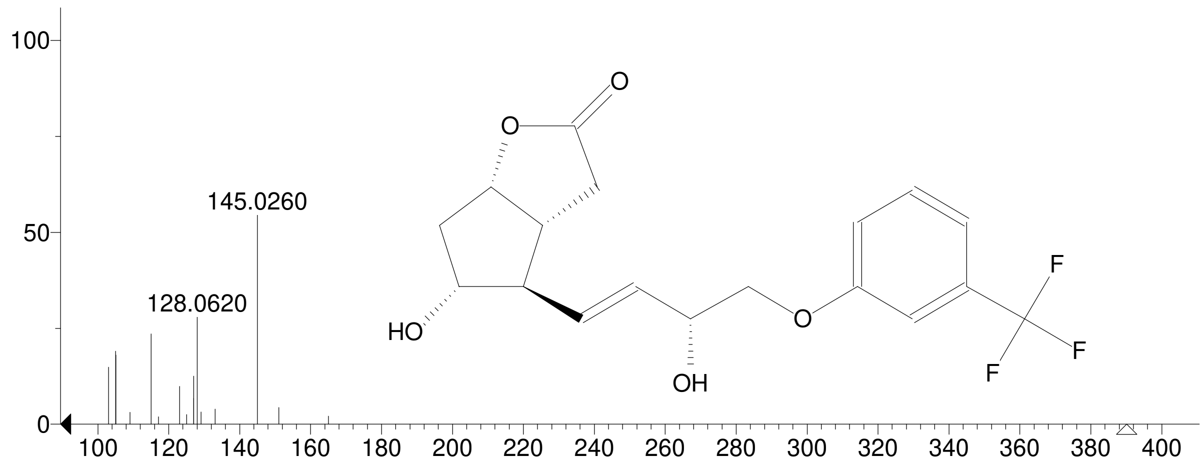

Unknown: Unknown (11.11_413.2275m/z)
Compound in Library Factor = 0

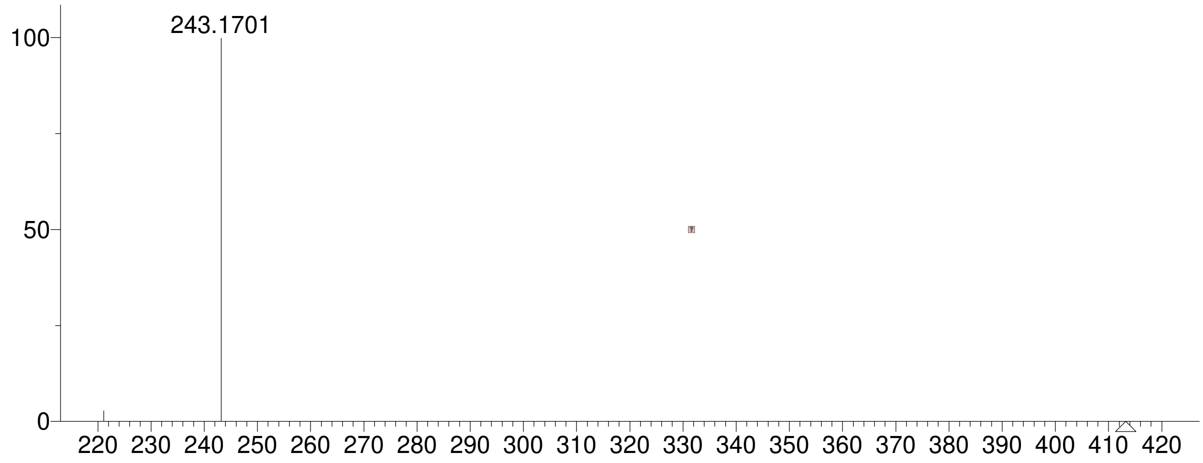

Hit 1 : His-Thr-Arg [M+H]+ QTOF 30V P=413.2
C16H28N8O5; MF: 0; RMF: 58; Prob 25.0%; Lib: nist_msms; ID: 51181.

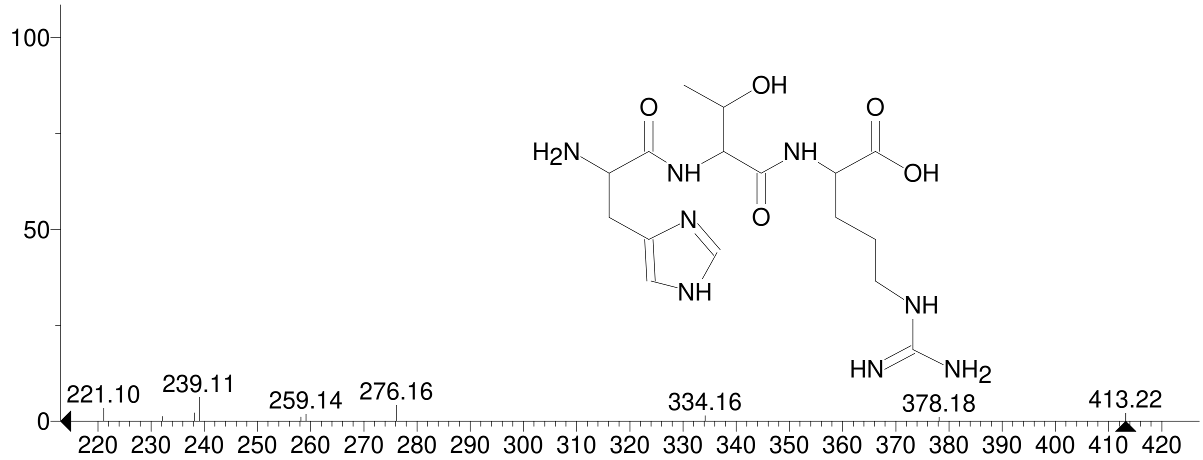

Hit 2 : Thr-His-Arg [M+H]+ QTOF 30V P=413.2
C16H28N8O5; MF: 0; RMF: 39; Prob 25.0%; Lib: nist_msms; ID: 54064.

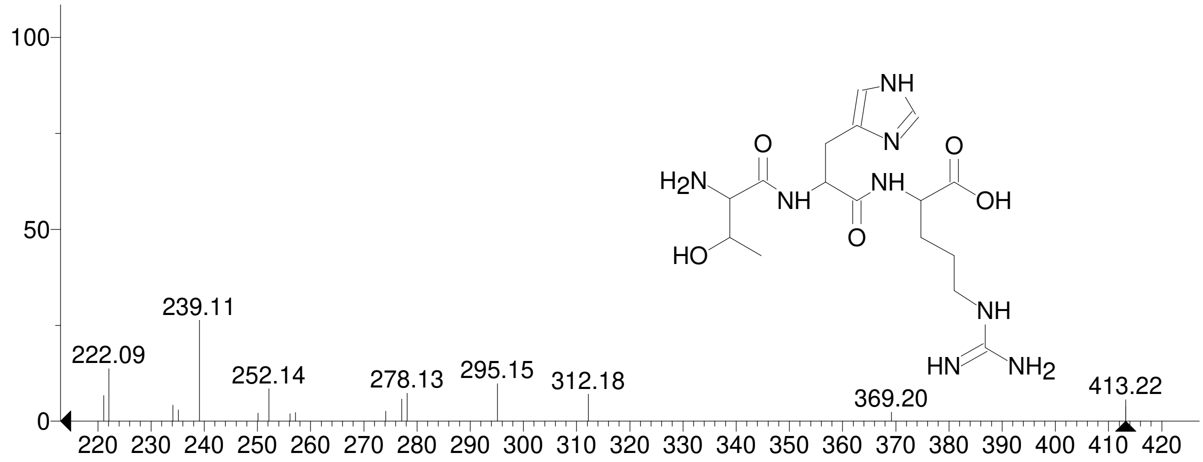

Hit 3 : 17(R)-Resolvin D1 methyl ester [M+Na]+ IT 35% P=413.2
C23H34O5; MF: 0; RMF: 4; Prob 25.0%; CAS: 937738-64-2; Lib: nist_msms; ID: 378601.

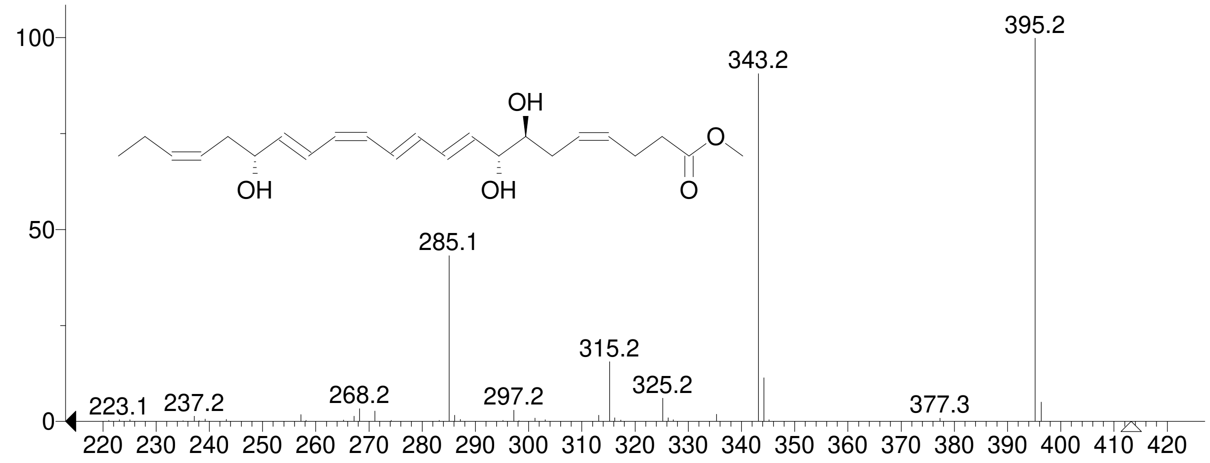

Hit 4 : Digoxigenin [M+Na]+ IT 35% P=413.2
C23H34O5; MF: 0; RMF: 3; Prob 25.0%; CAS: 1672-46-4; Lib: nist_msms; ID: 308680.

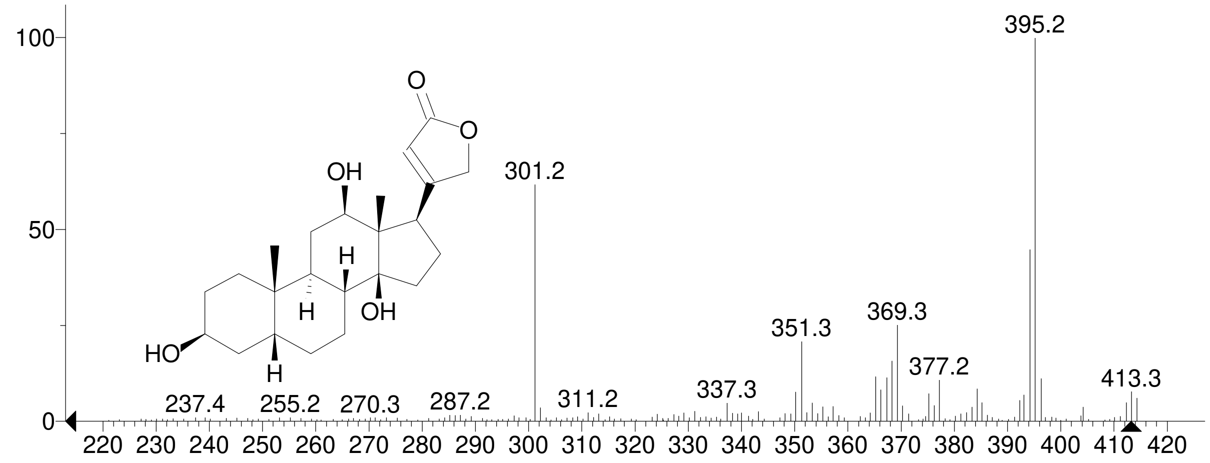

Unknown: Unknown (11.16_463.2062m/z)
Compound in Library Factor = -3273

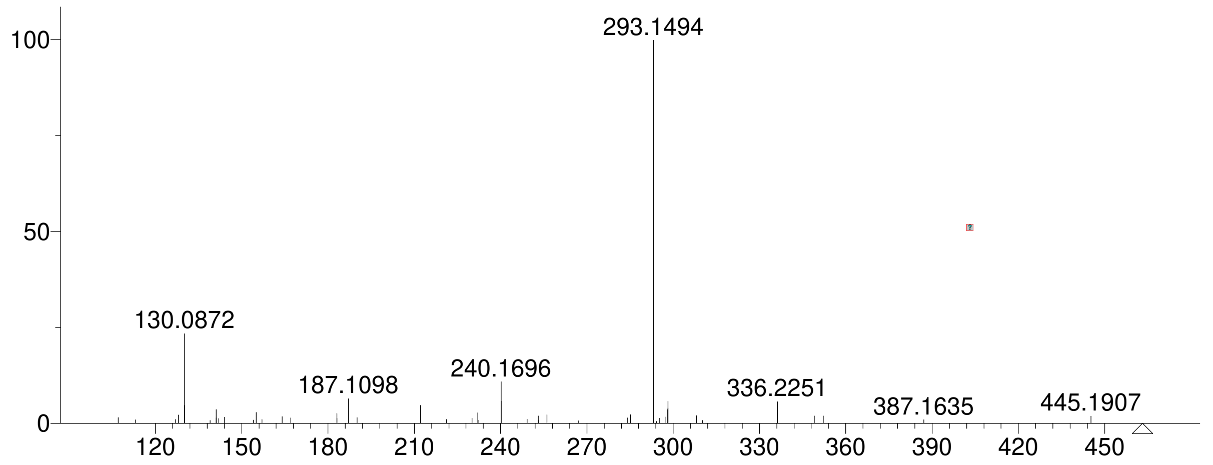

Hit 1 : 13(R),14(R)-Epoxyfluprostenol isopropyl ester [M+H-3H2O]+ HCD 83V P=463.2
C26H35F3O7; MF: 8; RMF: 95; Prob 100.0%; Lib: nist_msms; ID: 254084.

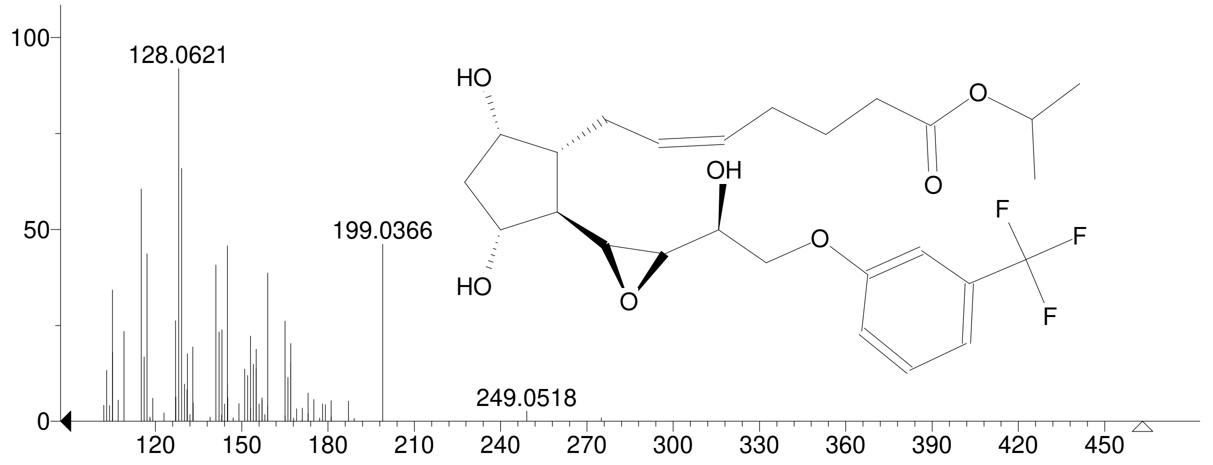

Unknown: Unknown (11.28_413.2277m/z)
Compound in Library Factor = 0

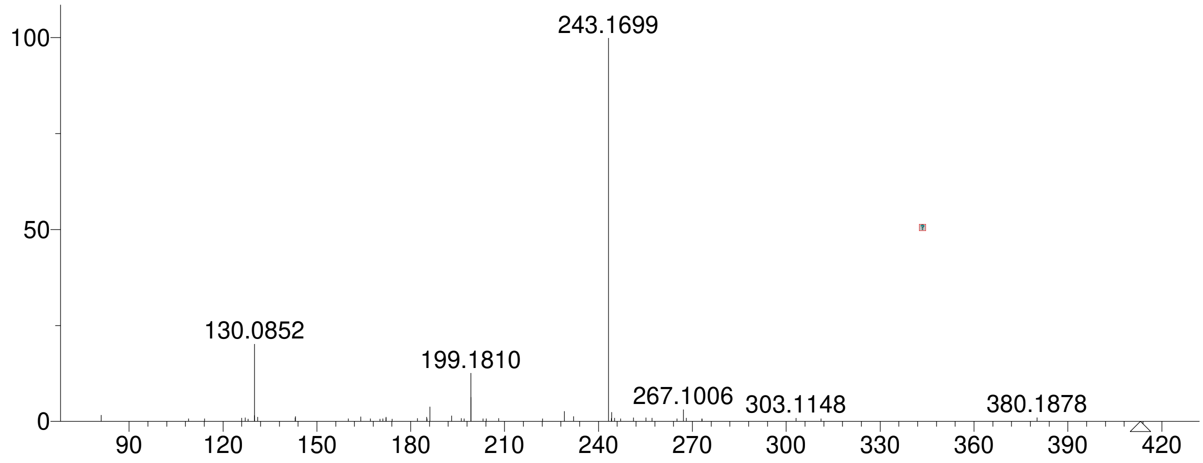

Hit 1 : Mevastatin [M+Na]+ HCD 161V P=413.2
C23H34O5; MF: 0; RMF: 17; Prob 16.7%; CAS: 73573-88-3; Lib: nist_msms; ID: 165121.

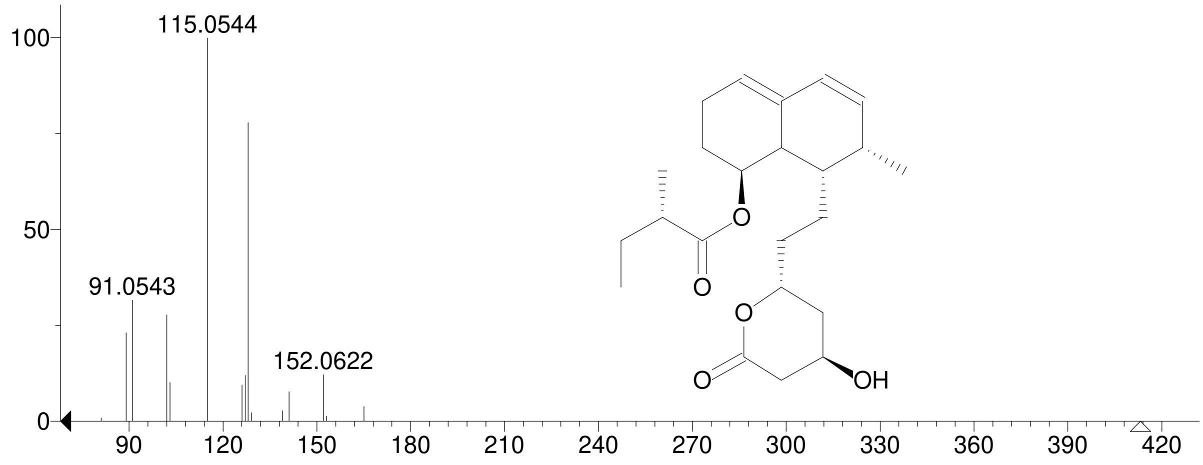

Hit 2 : Digoxigenin [M+Na]+ IT 35% P=413.2
C23H34O5; MF: 0; RMF: 16; Prob 16.7%; CAS: 1672-46-4; Lib: nist_msms; ID: 308680.

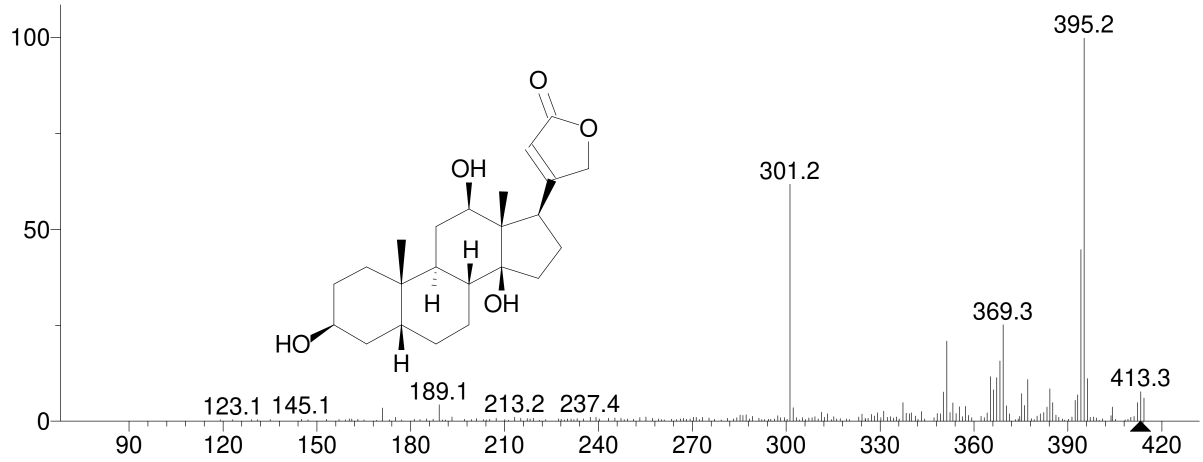

Hit 3 : Thr-His-Arg [M+H]+ QTOF 20V P=413.2
C16H28N8O5; MF: 0; RMF: 8; Prob 16.7%; Lib: nist_msms; ID: 54063.

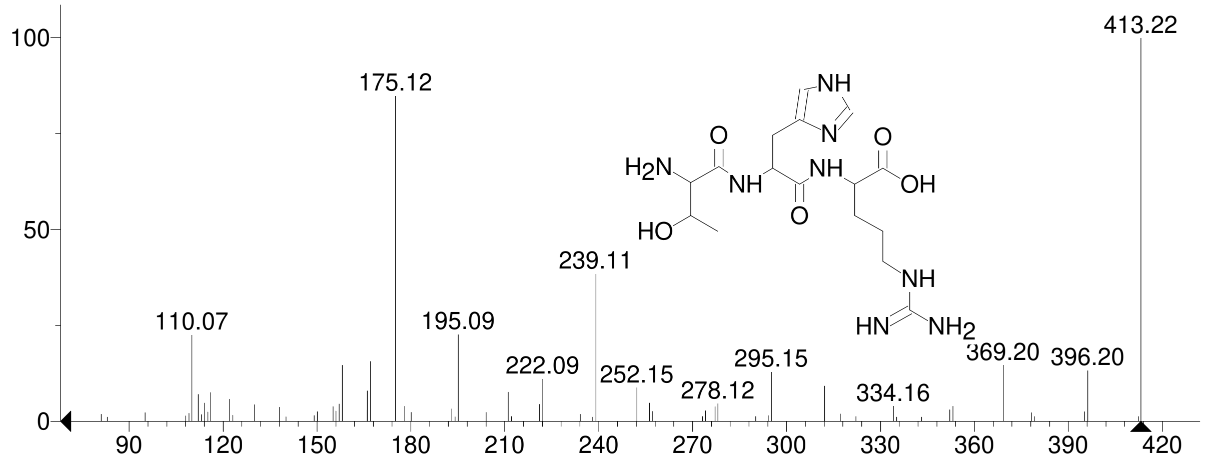

Hit 4 : Tussilagone [M+Na]+ HCD 33V P=413.2
C23H34O5; MF: 0; RMF: 6; Prob 16.7%; CAS: 104012-37-5; Lib: nist_msms; ID: 425581.

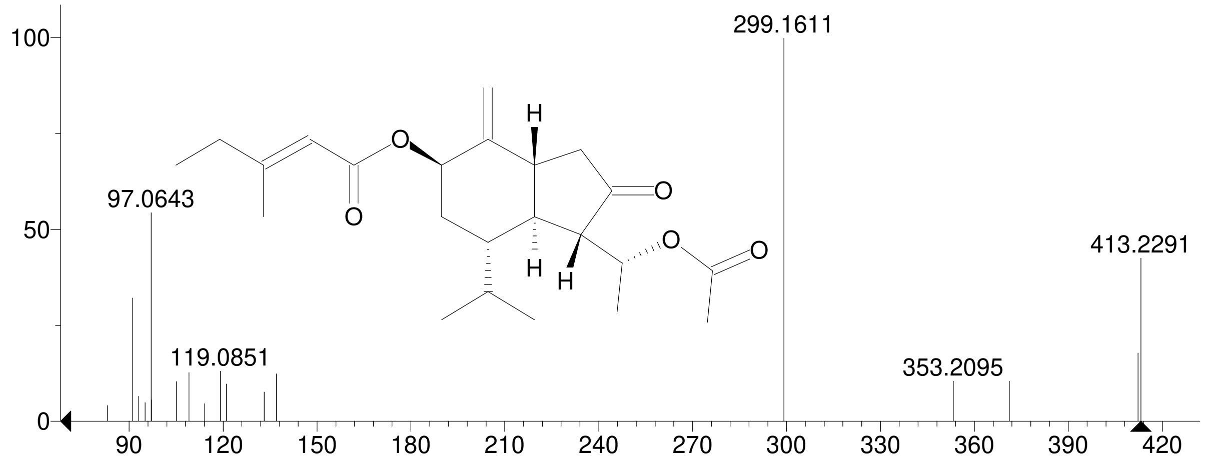

Hit 5 : 17(R)-Resolvin D1 methyl ester [M+Na]+ IT 35% P=413.2
C23H34O5; MF: 0; RMF: 2; Prob 16.7%; CAS: 937738-64-2; Lib: nist_msms; ID: 378601.

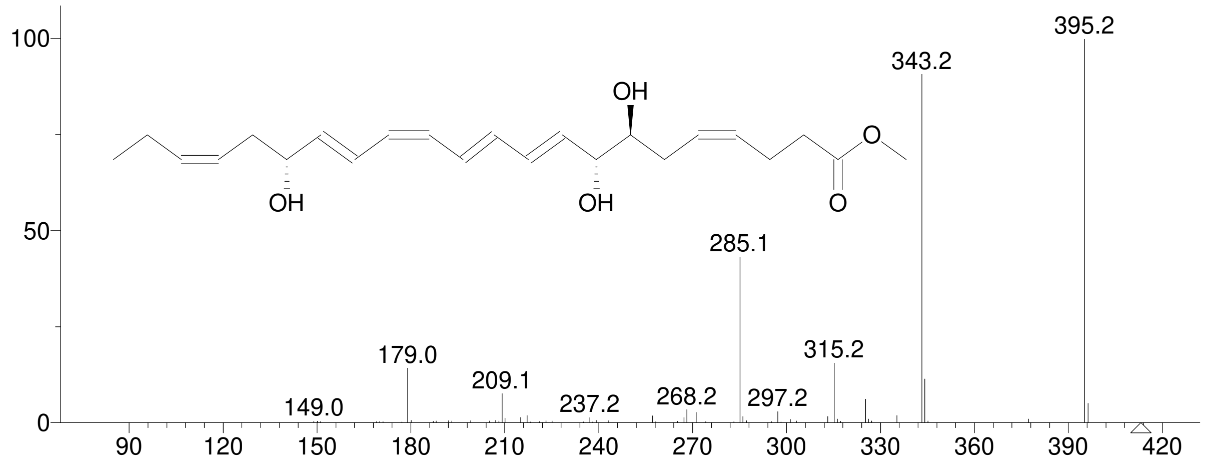

Unknown: Unknown (11.29_437.1910m/z)
Compound in Library Factor = 0

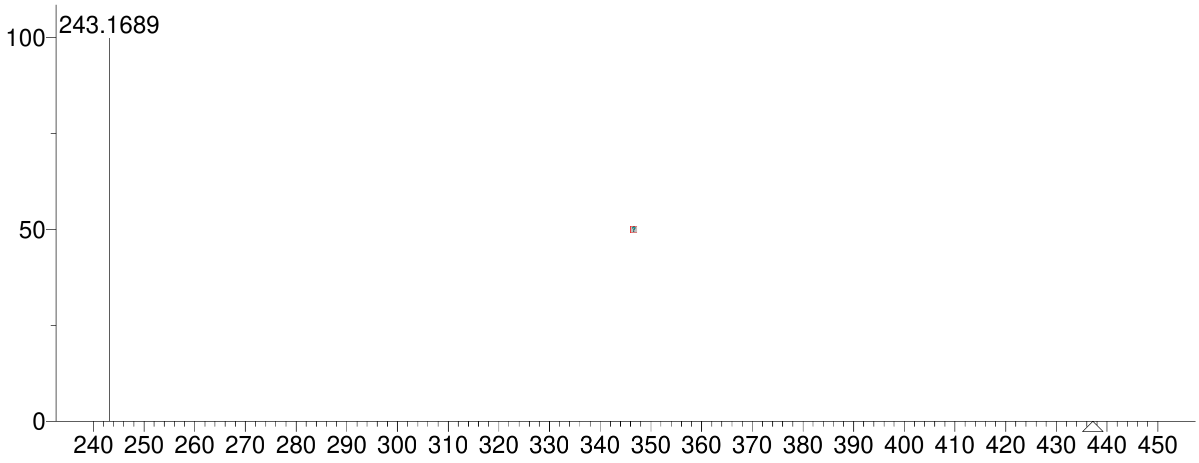

Unknown: Unknown (11.30_787.3152m/z)
Compound in Library Factor = 0

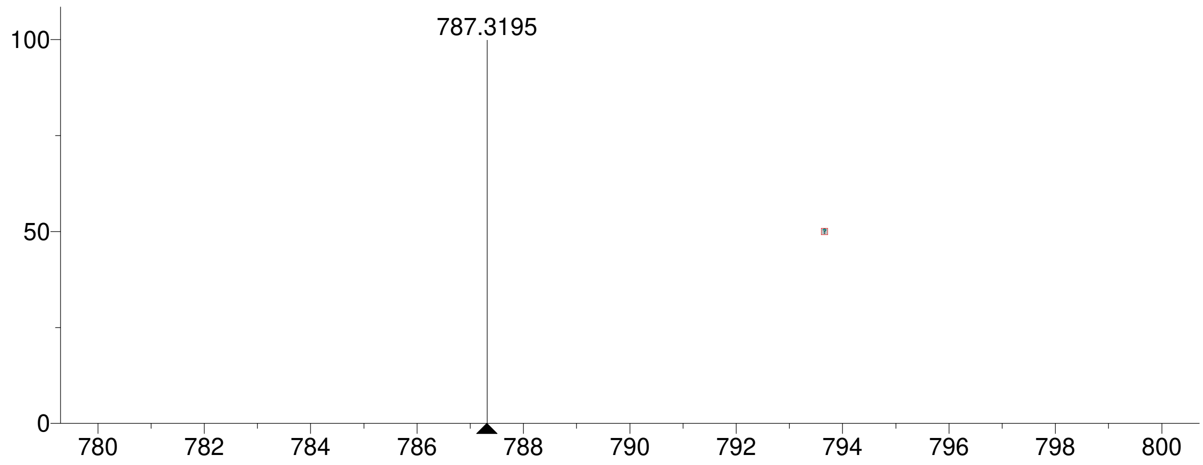

** Search Report Page 1 of 1 **

Unknown: Unknown (11.31_373.0223m/z)
Compound in Library Factor = -3273

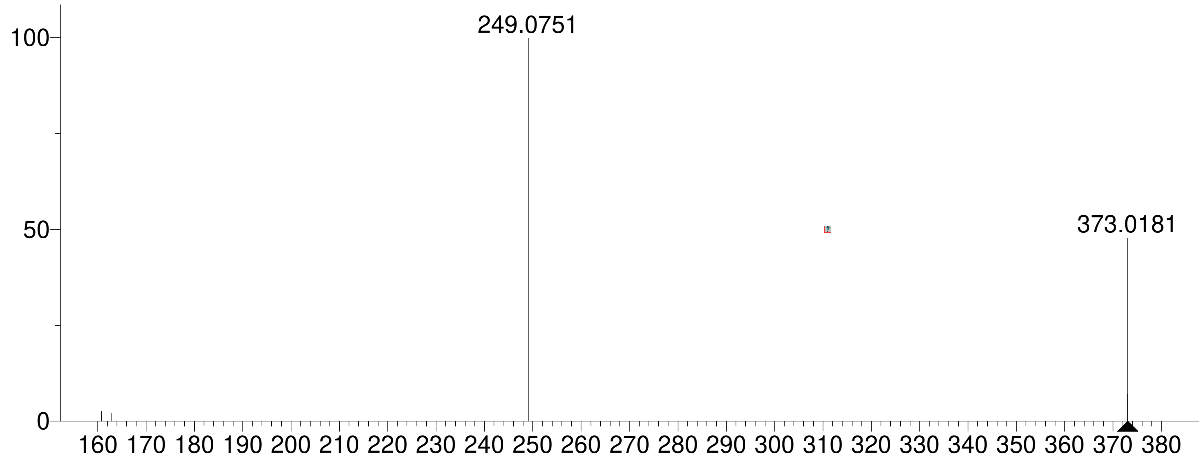

Hit 1 : Transfluthrin [M+H+2i]+ HCD 13V P=373
C15H12Cl2F4O2; MF: 2; RMF: 346; Prob 100.0%; CAS: 118712-89-3; Lib: nist_msms; ID: 521809.

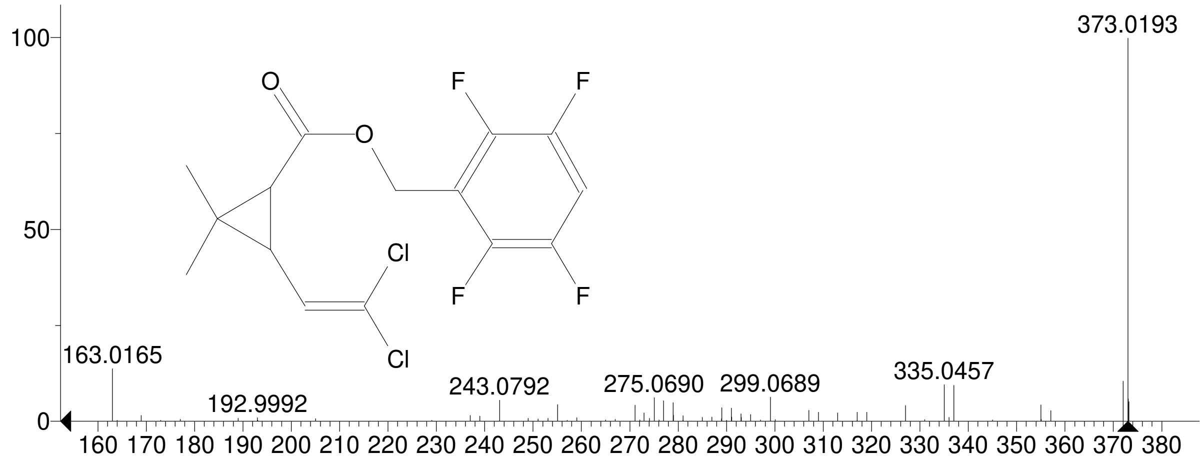

Unknown: Unknown (11.40_413.2280m/z)
Compound in Library Factor = 0

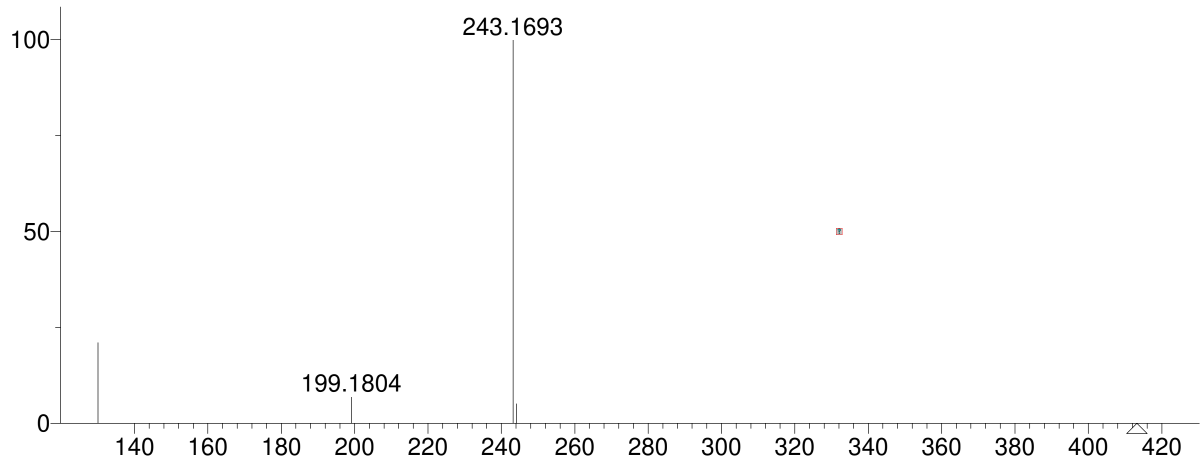

Unknown: Unknown (11.41_789.3679m/z)
Compound in Library Factor = 0

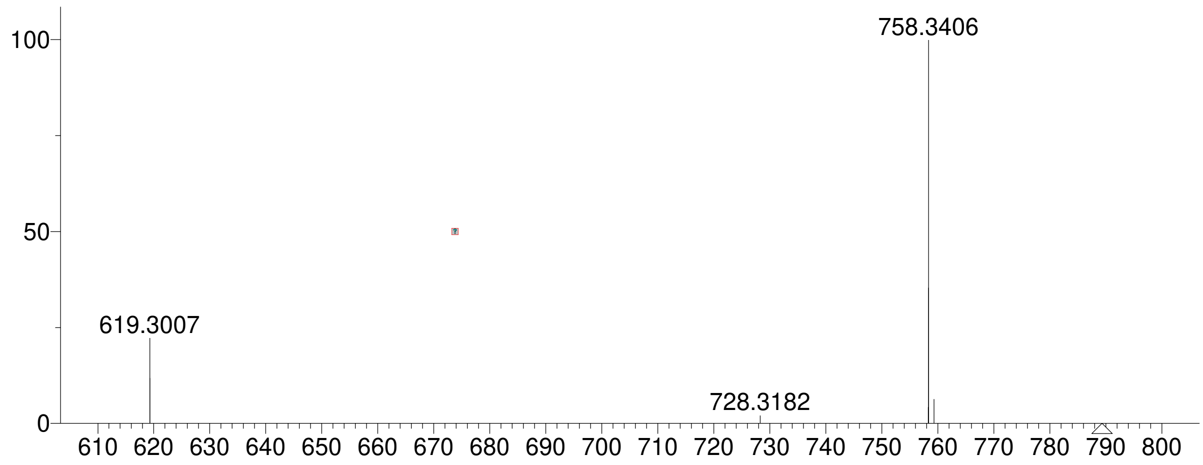

** Search Report Page 1 of 1 **

Unknown: Unknown (11.42_417.1751m/z)
Compound in Library Factor = 0

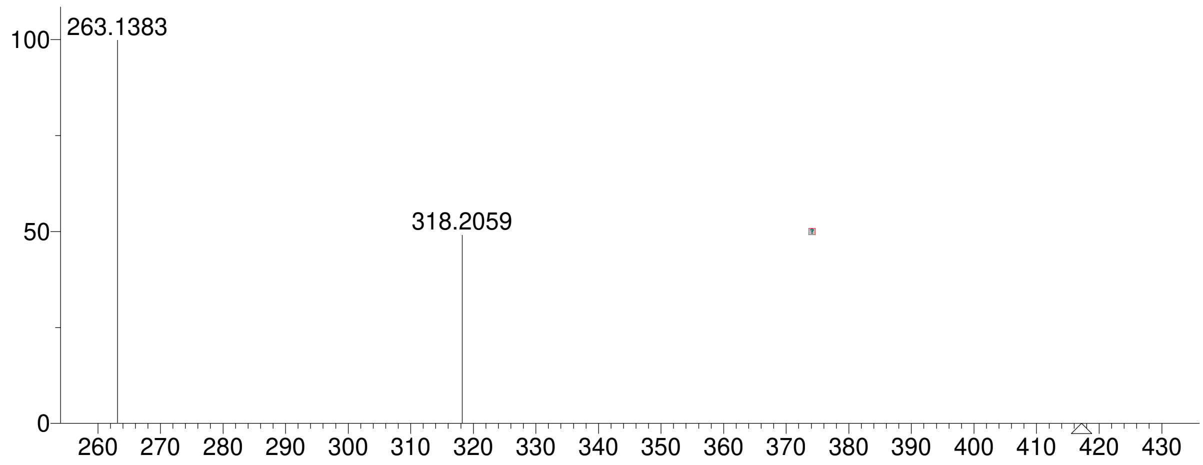

Unknown: Unknown (11.42_433.1952m/z)
Compound in Library Factor = 0

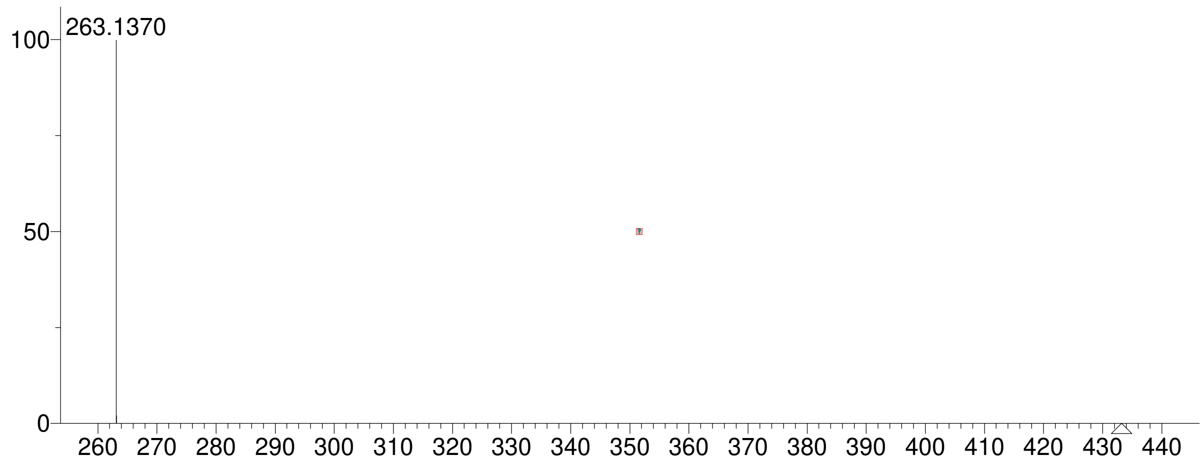

Unknown: Unknown (11.42_601.9156m/z)
Compound in Library Factor = 0

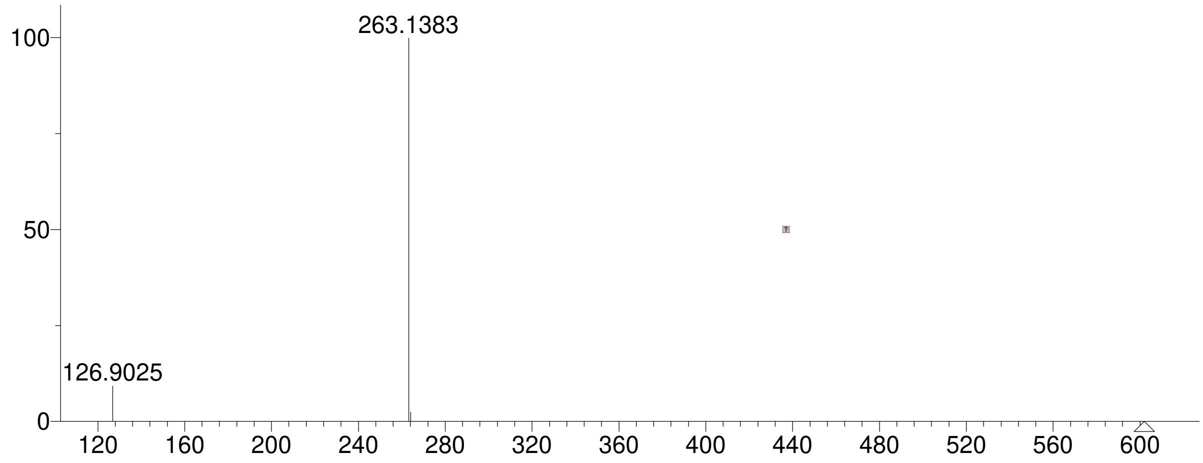

** Search Report Page 1 of 1 **

Unknown: Unknown (11.43_500.2240m/z)
Compound in Library Factor = 0

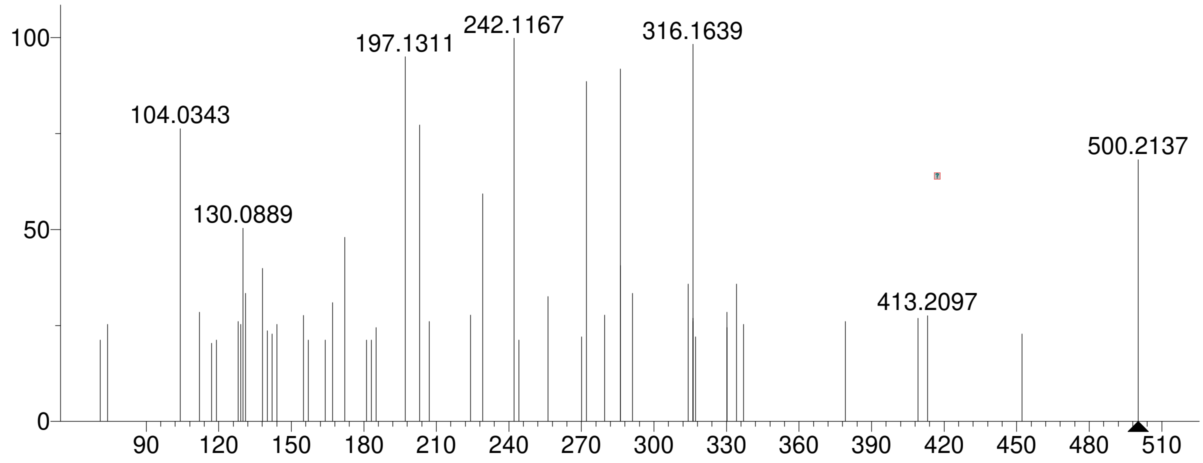

** Search Report Page 1 of 1 **

Unknown: Unknown (11.43_758.3476m/z)
Compound in Library Factor = 0

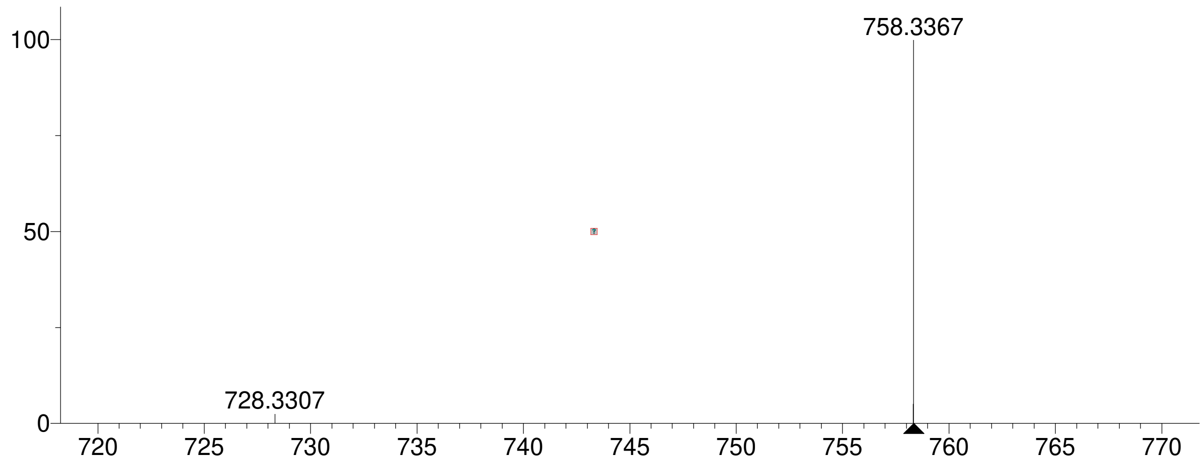

Unknown: Unknown (11.45_275.0546m/z)
Compound in Library Factor = 100

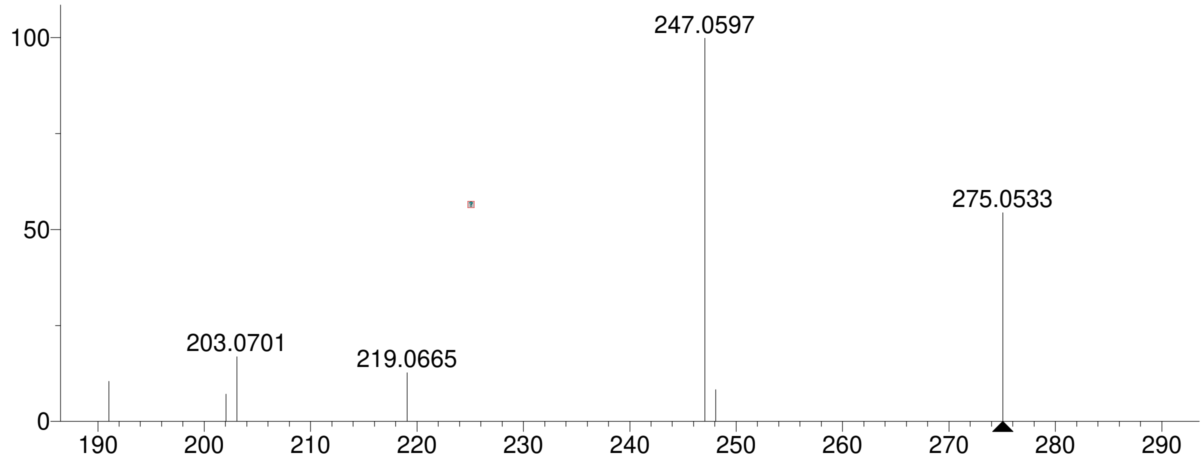

Hit 1 : Dihydromyricetin [M+H-CH2O2]+ HCD 13V P=275.1
C15H12O8; MF: 473; RMF: 765; Prob 97.7%; CAS: 27200-12-0; Lib: nist_msms; ID: 385122.

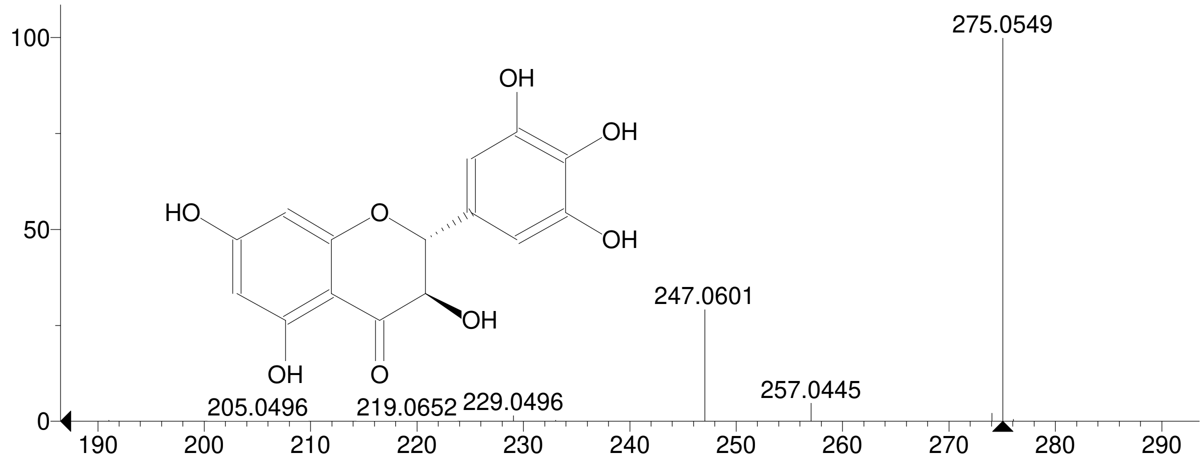

Hit 2 : 1,2,3-Benzenetricarboxylic acid, trimethyl ester [M+Na]+ HCD 16V P=275.1
C12H12O6; MF: 60; RMF: 493; Prob 0.97%; CAS: 2672-57-3; Lib: nist_msms; ID: 306556.

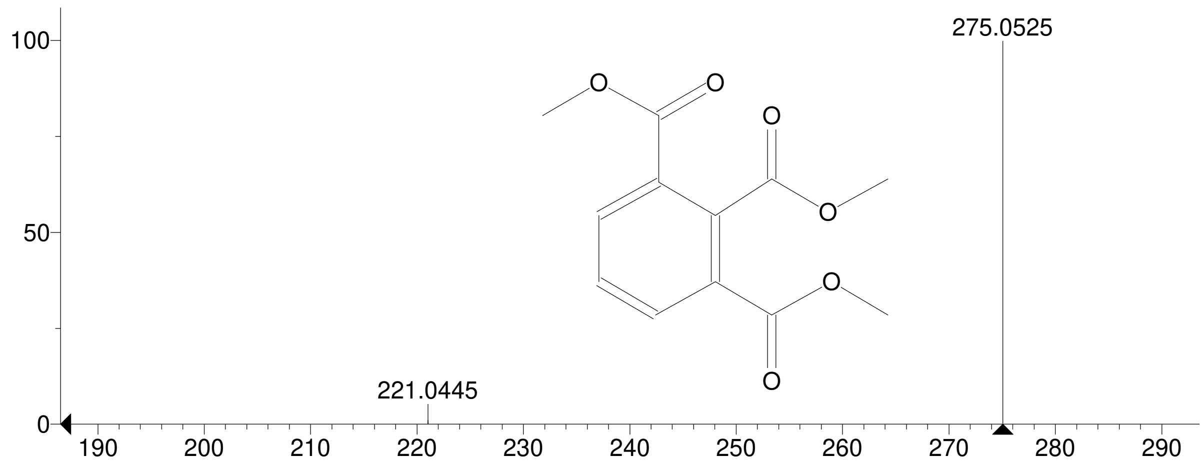

Hit 3 : Terbufos sulfone [M+H-CH2S]+ HCD 5V P=275.1
C9H21O4PS3; MF: 43; RMF: 461; Prob 0.53%; CAS: 56070-16-7; Lib: nist_msms; ID: 496673.

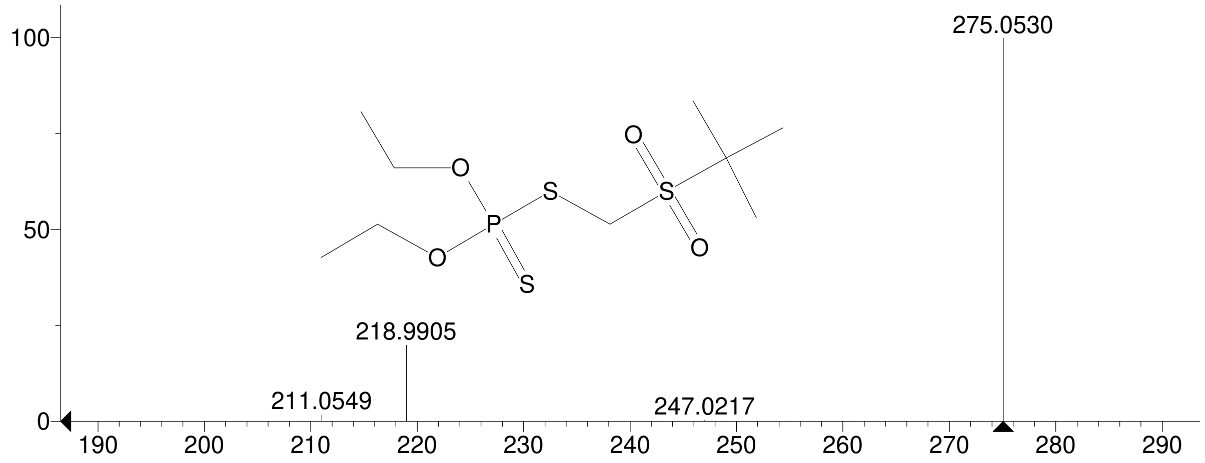

Hit 4 : NCGC00381160-01_C14H12O6_Spiro[cyclopent-4-ene-1,1'(3'H)-isobenzofuran]-3,3'-dione, 2,4'-dihydroxy
C14H12O6; MF: 31; RMF: 376; Prob 0.35%; Lib: mona-export-gnps; ID: 12305.

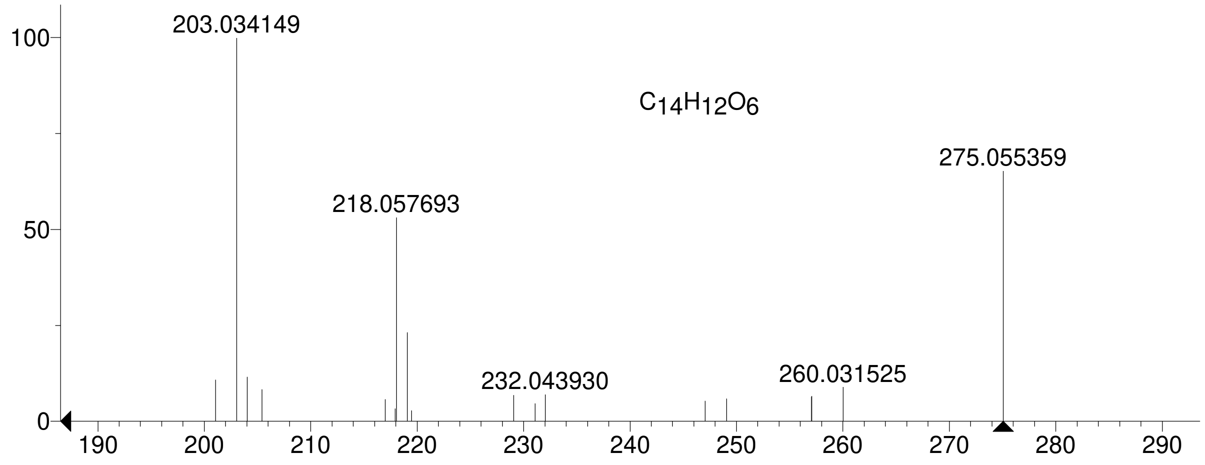

Hit 5 : NCGC00381290-01!2,3,6,8-tetrahydroxy-1-methylxanthen-9-one [M+H]+ P=275.1
C14H10O6; MF: 29; RMF: 392; Prob 0.32%; Lib: mona-export-gnps; ID: 7277.

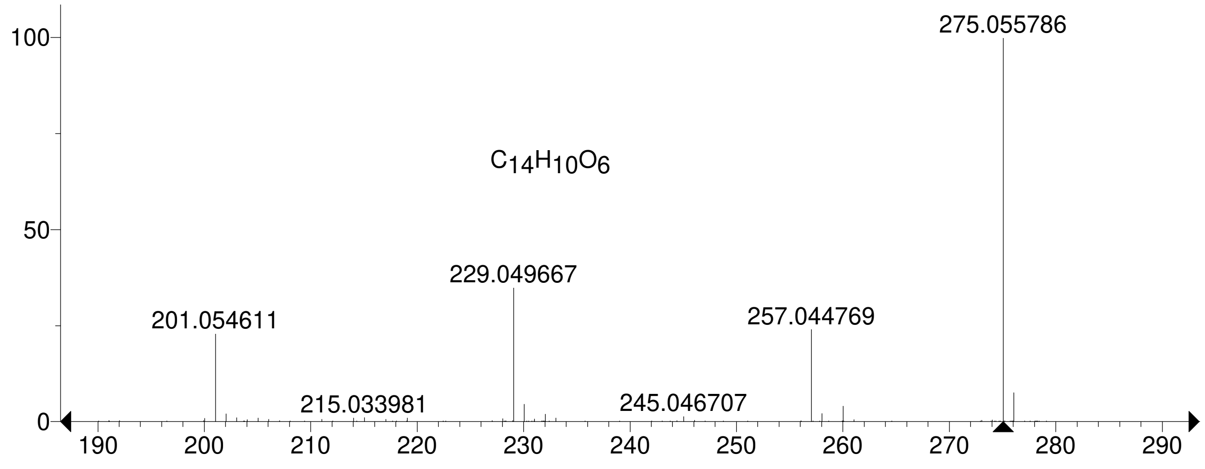

** Search Report Page 1 of 1 **

Unknown: Unknown (11.45_355.0120m/z)
Compound in Library Factor = 0

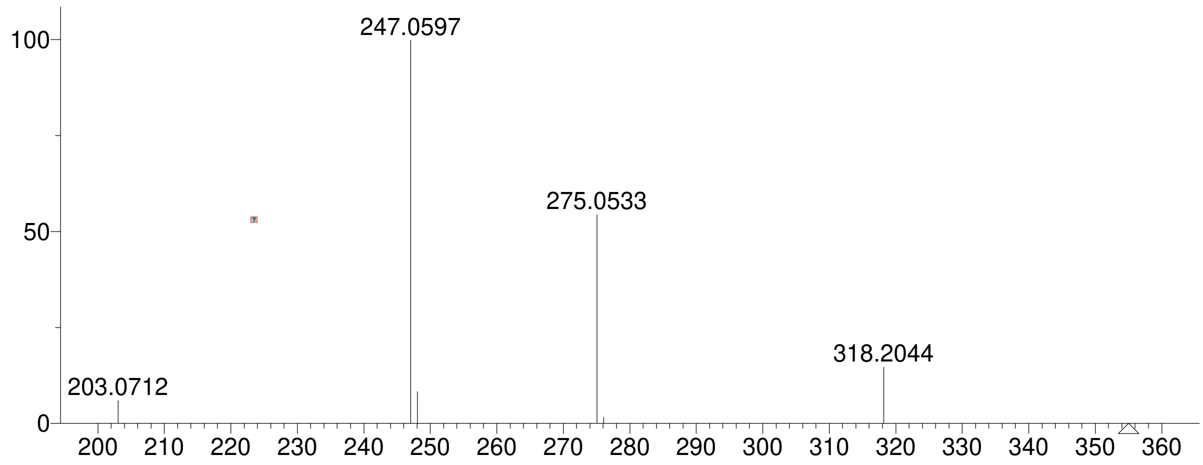

Hit 1 : 3-(3,3-dichloro-2-hydroxypropyl)-6,8-dimethoxyisochromen-1-one [M+Na]+ 35HCD P=355
C14H14Cl2O5; MF: 0; RMF: 7; Prob 100.0%; Lib: mona-export-vf-npl_qexactive; ID: 18490.

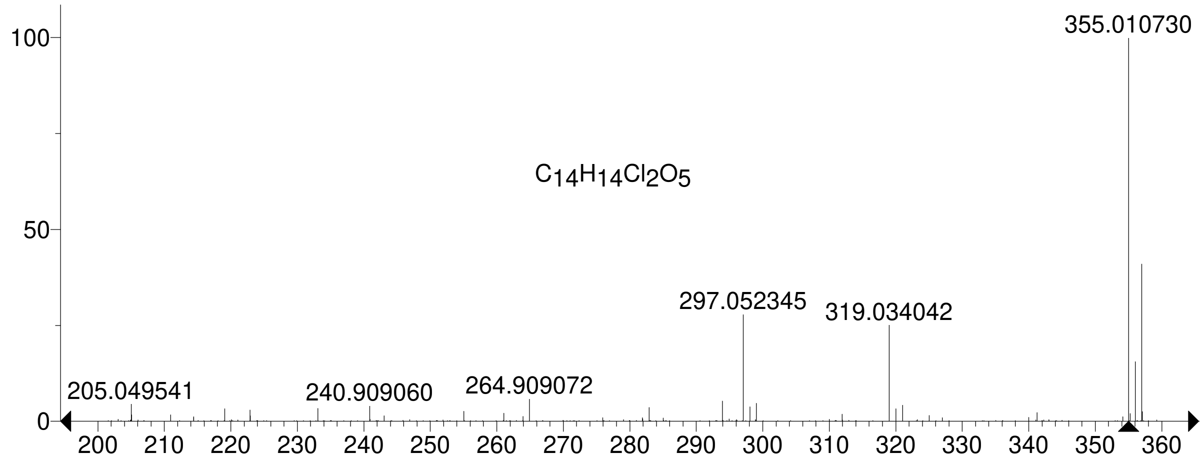

Unknown: Unknown (11.46_318.2056m/z)
Compound in Library Factor = -166

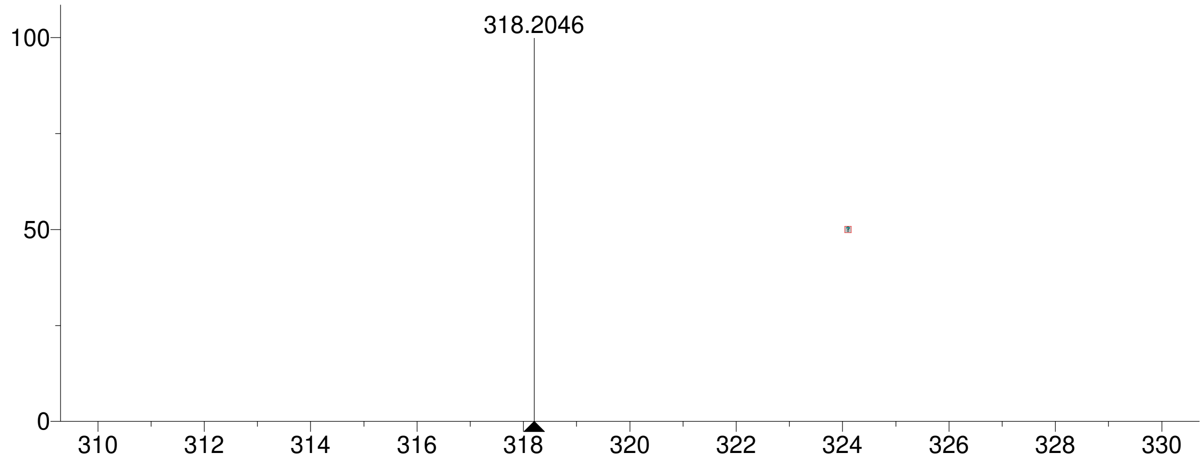

Hit 1 : Nateglinide [M+H]+ 20-30-40%) P=318.2
C19H27NO3; MF: 364; RMF: 999; Prob 33.2%; Lib: mona-export-fiehn_hilic; ID: 603.

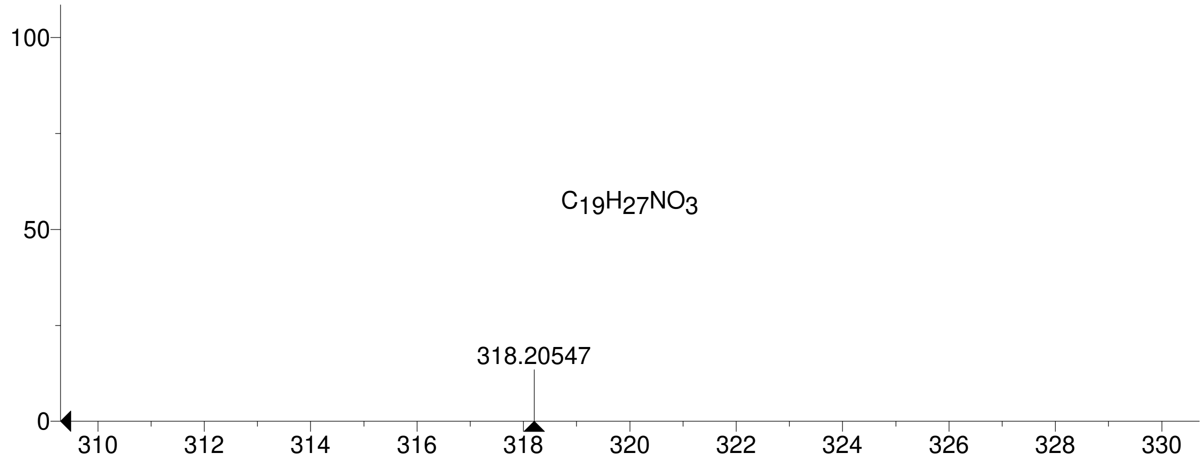

Hit 2 : Cyclohexanecarboxylic acid, 1-phenyl-, 2-(4-morpholinyl)ethyl ester [M+H]+ HCD 25V P=318.2
C19H27NO3; MF: 364; RMF: 999; Prob 33.2%; CAS: 138847-85-5; Lib: nist_msms; ID: 422975.

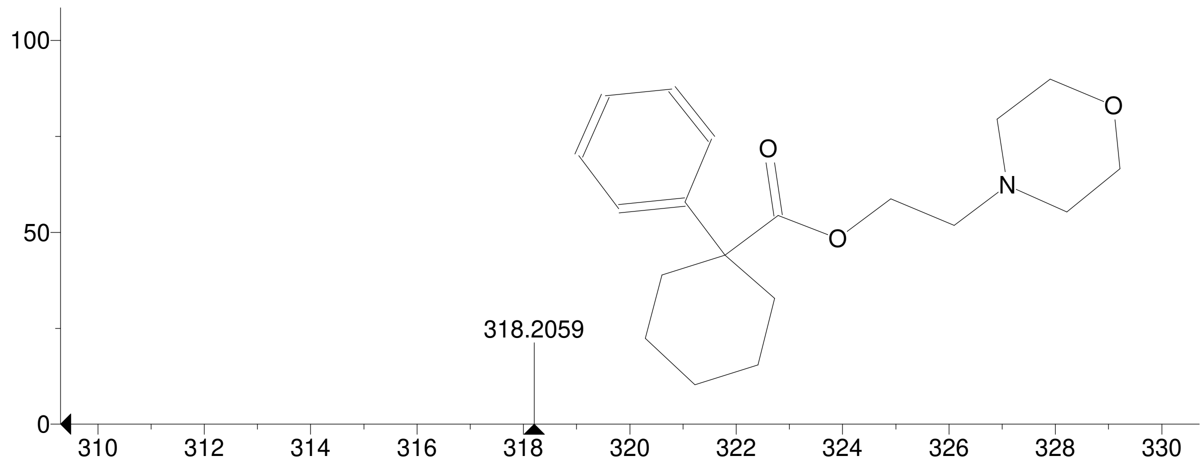

Hit 3 : Tetrabenazine [M+H]+ HCD 41V P=318.2
C19H27NO3; MF: 364; RMF: 999; Prob 33.2%; CAS: 58-46-8; Lib: nist_msms; ID: 122078.

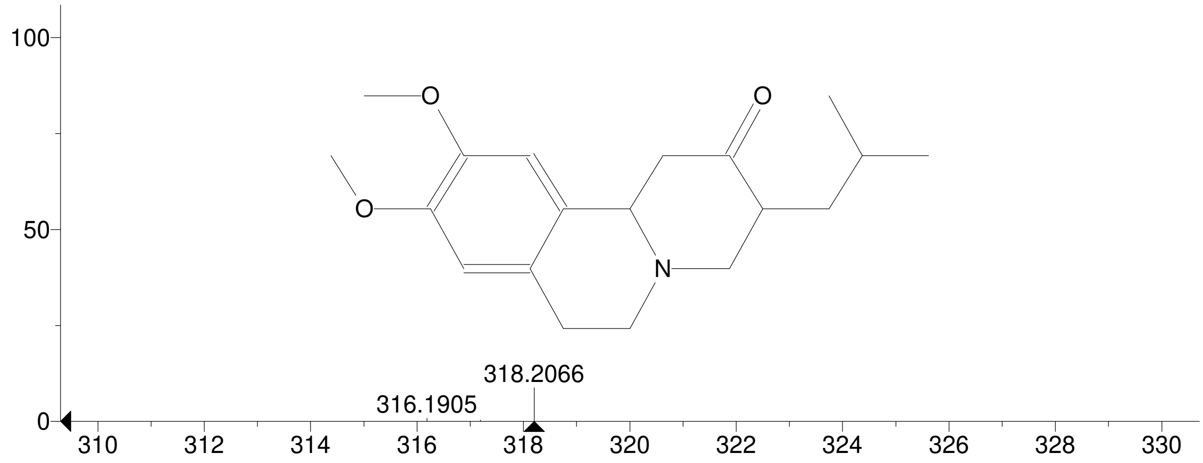

Hit 4 : Nateglinide (Starlix) [M+H]+ P=318.2
C19H27NO3; MF: 32; RMF: 723; Prob 0.33%; Lib: mona-export-gnps; ID: 815.

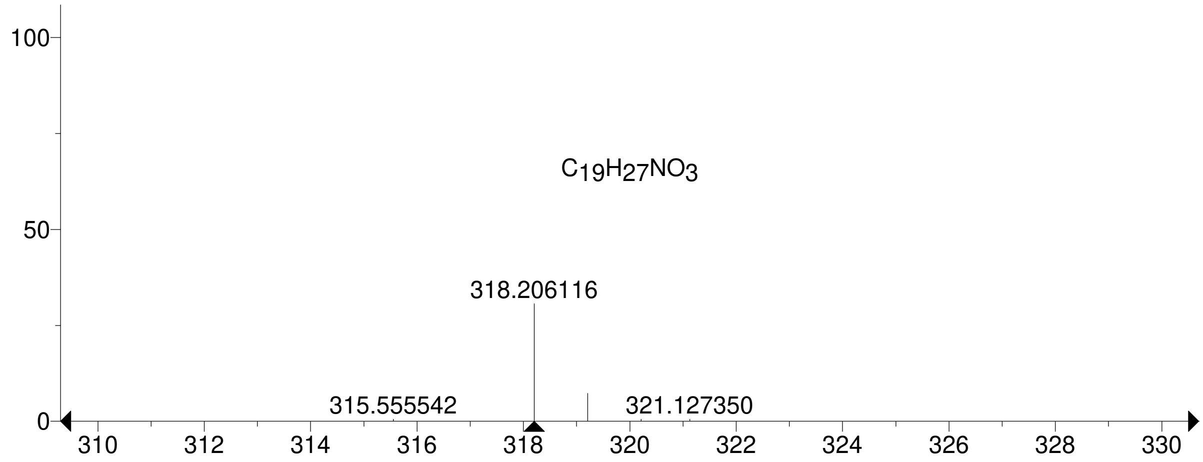

** Search Report Page 1 of 1 **

Unknown: Unknown (11.50_412.9334m/z)
Compound in Library Factor = 0

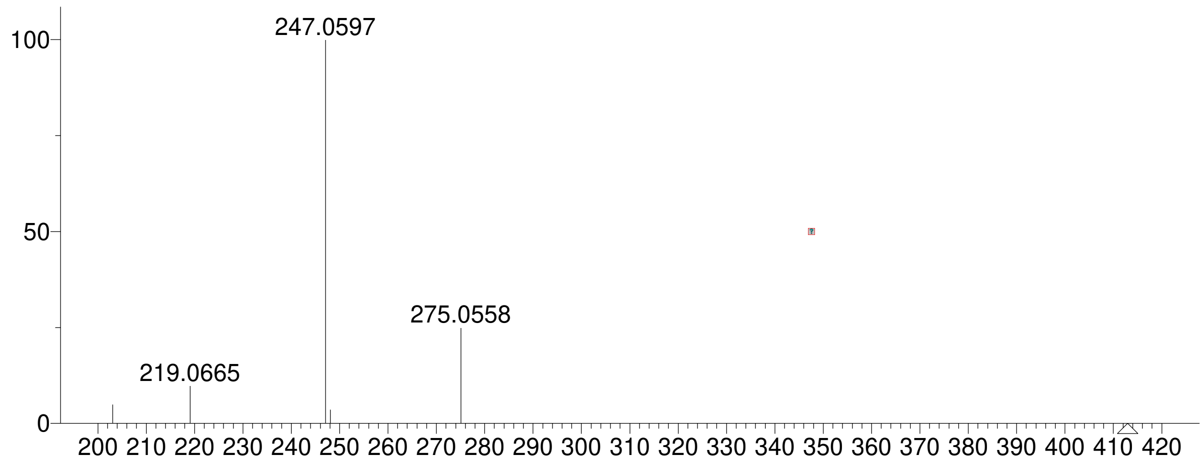

Unknown: Unknown (11.52_525.2663m/z)
Compound in Library Factor = -3273

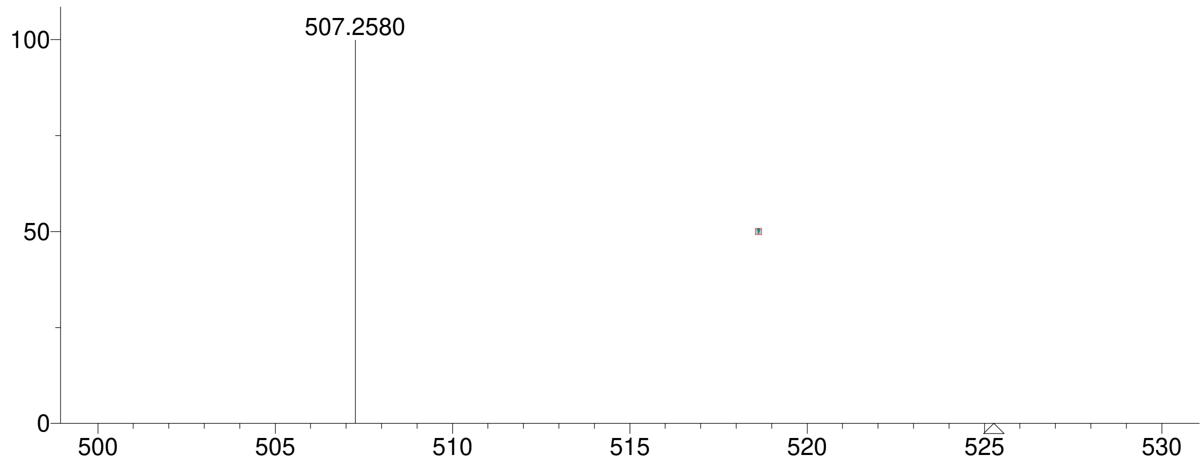

Hit 1 : FMRF Amide-Like Peptide I, lobster [M-2H]2- IT-FT 35% P=525.3
C47H72N16O12; MF: 1; RMF: 192; Prob 100.0%; Lib: nist_msms2; ID: 79293.

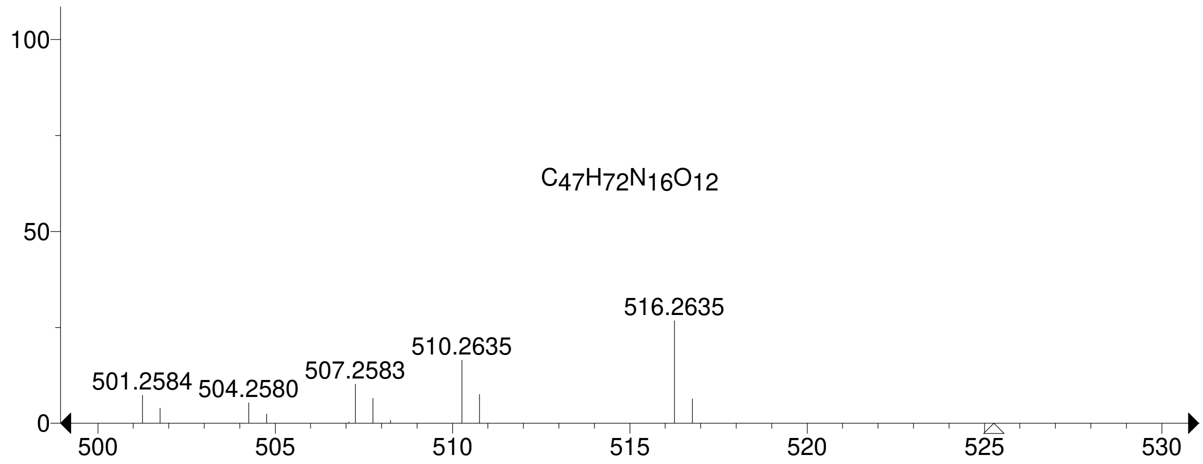

Unknown: Unknown (11.56_348.1435m/z)
Compound in Library Factor = 0

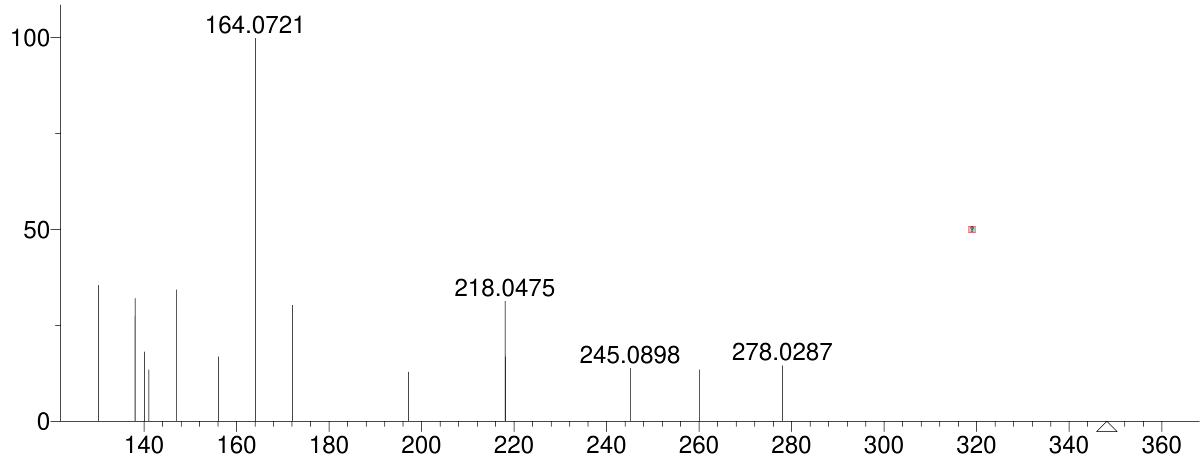

Hit 1 : 3-Pyridinecarboxamide, 6-amino-N-[3-[4-(4-morpholinyl)pyrido[3',2':4,5]furo[3,2-d]pyrimidin-2-yl]phenyl]- [N
C25H21N7O3; MF: 0; RMF: 71; Prob 14.3%; CAS: 371942-69-7; Lib: nist_msms; ID: 262104.

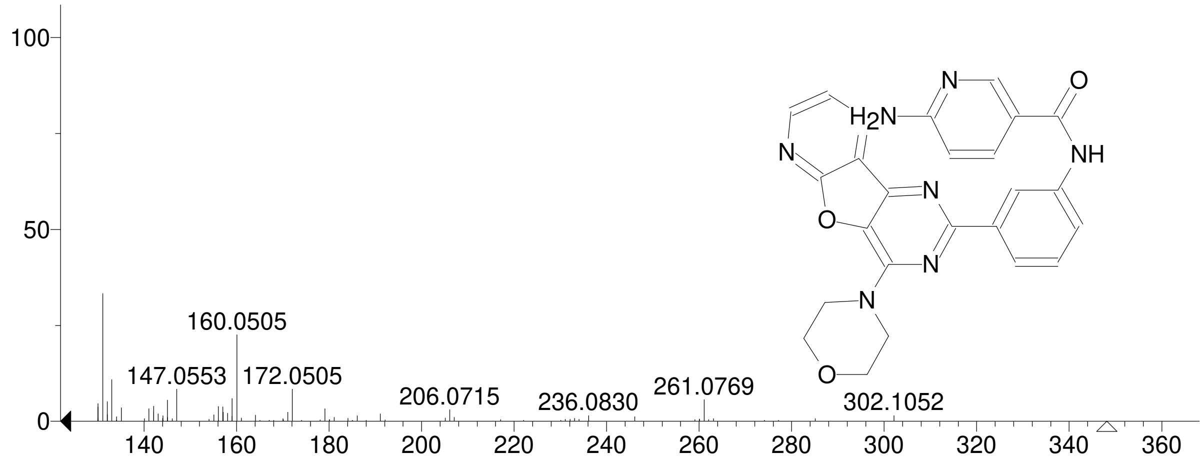

Hit 2 : 4,5-dihydroxy-4,5,6-trimethyl-2,8-dioxa-13-azatricyclo[8.5.1.0A^'1A^'3,A^'1a^!P]hexadec-10-ene-3,7-dione
C16H23NO6; MF: 0; RMF: 40; Prob 14.3%; Lib: mona-export-vf-npl_qexactive; ID: 24611.

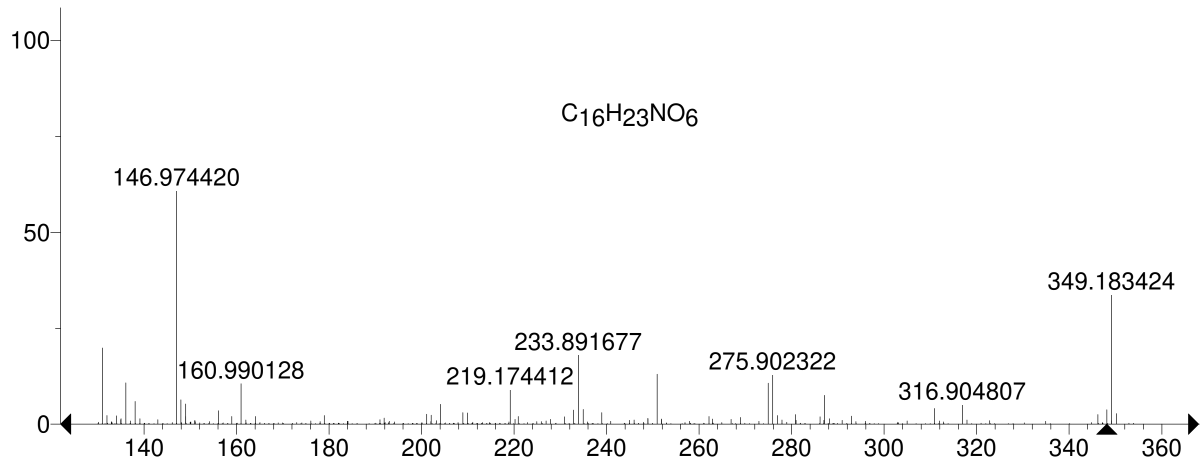

Hit 3 : Nafamostat mesylate [M+H]+ P=348.1
C21H25N5O8S2; MF: 0; RMF: 34; Prob 14.3%; Lib: mona-export-gnps; ID: 457.

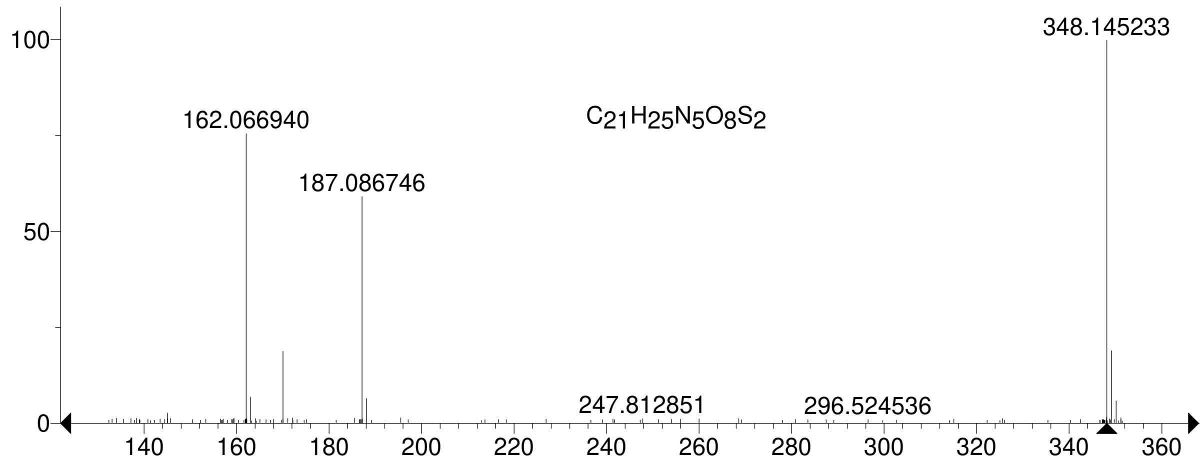

Hit 4 : Nafamostat [M+H]+ HCD 90V P=348.1
C19H17N5O2; MF: 0; RMF: 14; Prob 14.3%; CAS: 81525-10-2; Lib: nist_msms; ID: 343734.

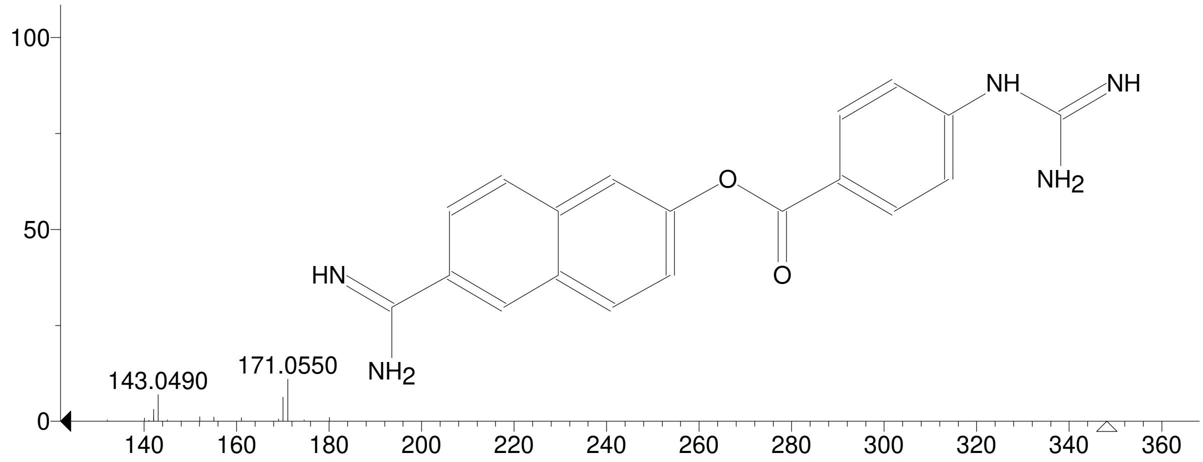

Hit 5 : His-Gly-His [M-H]- IT-FT 35% P=348.1
C14H19N7O4; MF: 0; RMF: 3; Prob 14.3%; Lib: nist_msms; ID: 574339.

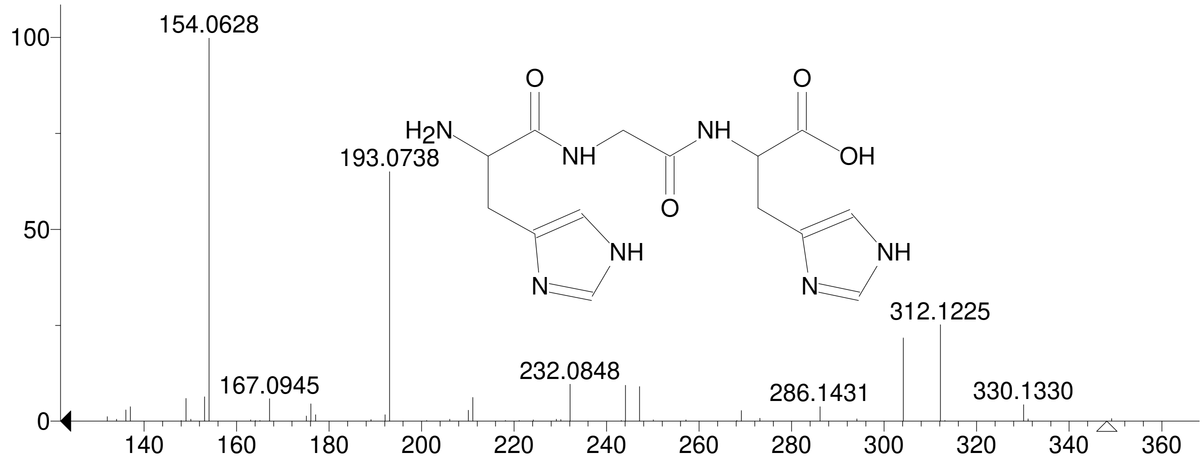

Unknown: Unknown (11.64_325.0917m/z)
Compound in Library Factor = 0

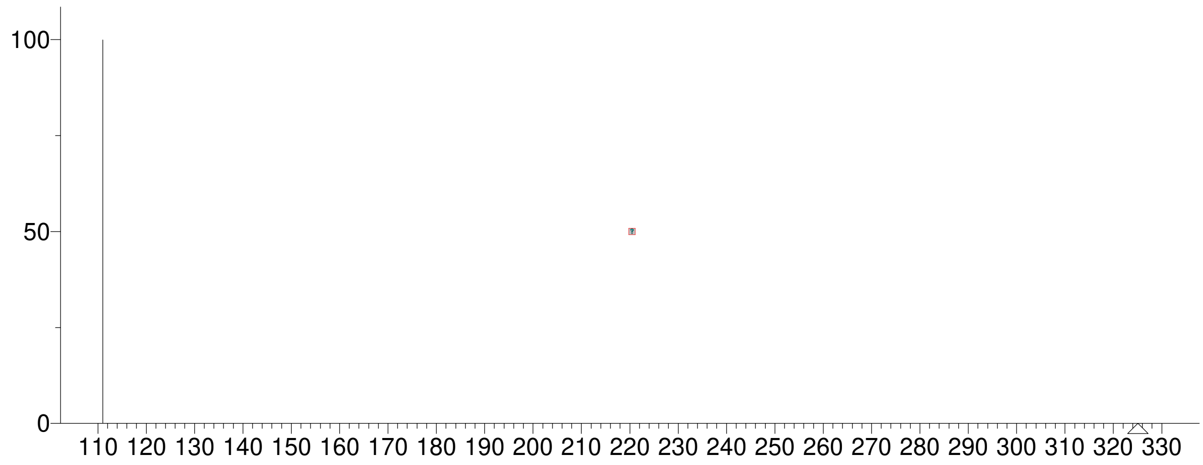

Hit 1 : 2-(3-hydroxy-5-methoxyphenoxy)-6-(hydroxymethyl)oxane-3,4,5-triol [M+Na]+ 65HCD P=325.1
C13H18O8; MF: 0; RMF: 26; Prob 50.0%; Lib: mona-export-vf-npl_qexactive; ID: 22425.

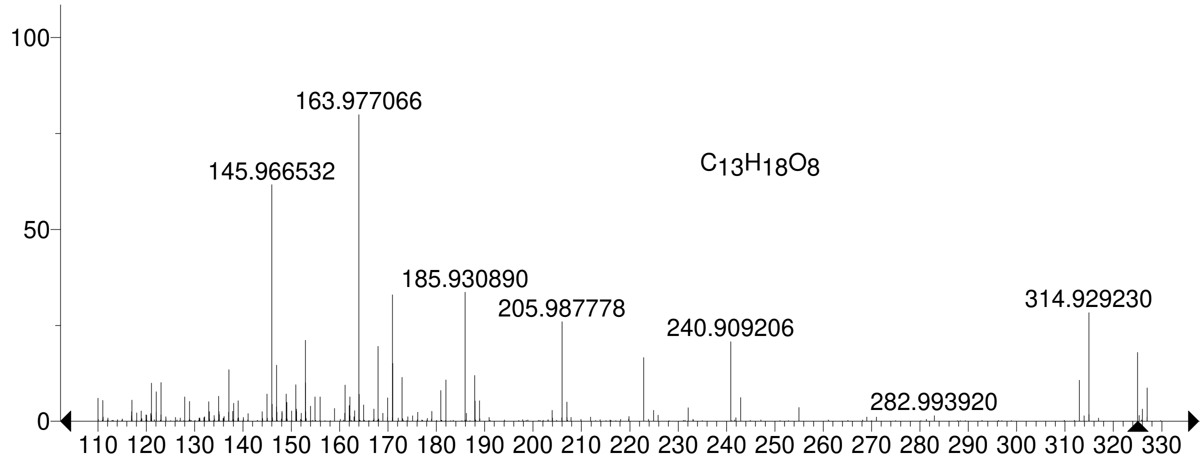

Hit 2 : (1S,4R,7S,8S,11S)-9-tert-butyl-7,9-dihydroxy-3,5,12-trioxatetracyclo[6.6.0.0A^'1,A^'1A^'1.0a^'',a^,,]tetradec
C15H18O8; MF: 0; RMF: 6; Prob 50.0%; Lib: mona-export-lc-ms-ms_negative_mode; ID: 19424.

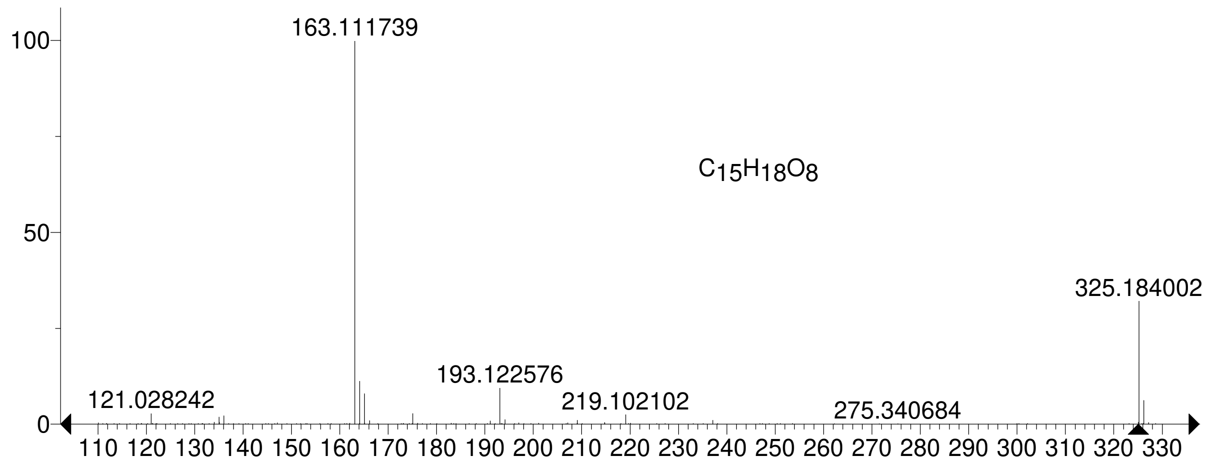

** Search Report Page 1 of 1 **

Unknown: Unknown (11.68_560.2122m/z)
Compound in Library Factor = -3273

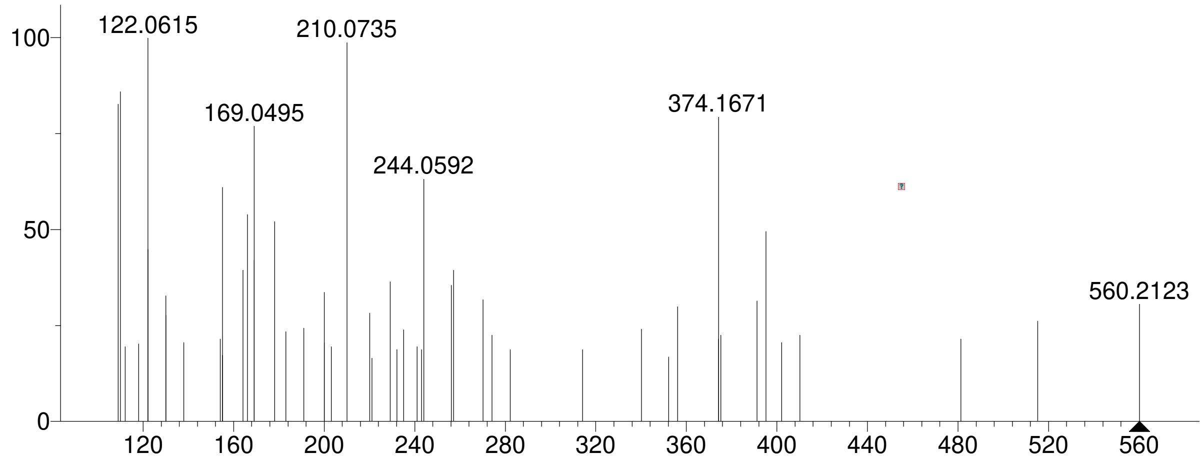

Hit 1 : 2,3,9,10,11,12-Hexahydro-10S-hydroxy-9-methyl-1-oxo-9R,12S-epoxy-1H-diindolo[1,2,3-fg:3',2',1'-kl]pyrrol
C32H31N3O5; MF: 25; RMF: 283; Prob 100.0%; CAS: 108068-98-0; Lib: nist_msms; ID: 86206.

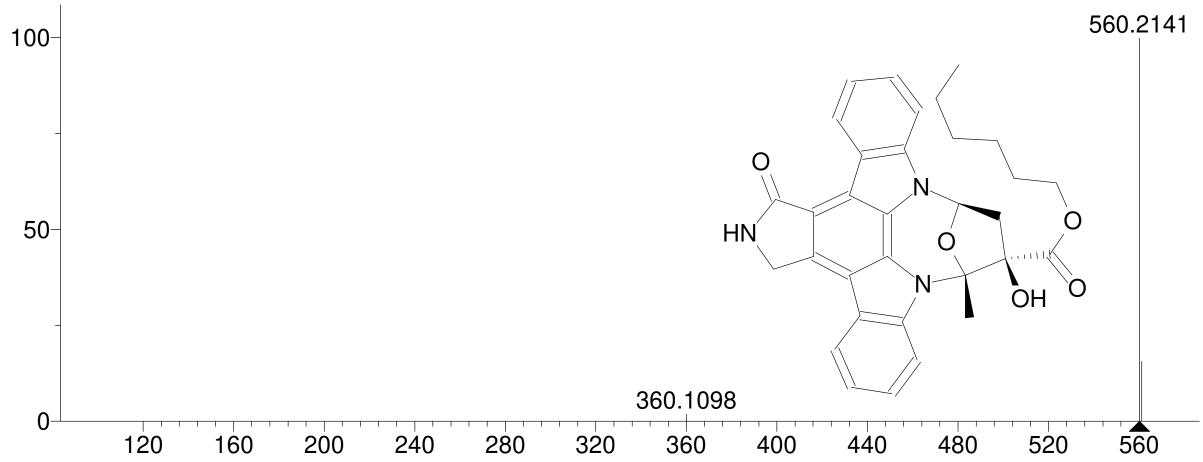

Unknown: Unknown (11.69_528.2504m/z)
Compound in Library Factor = 0

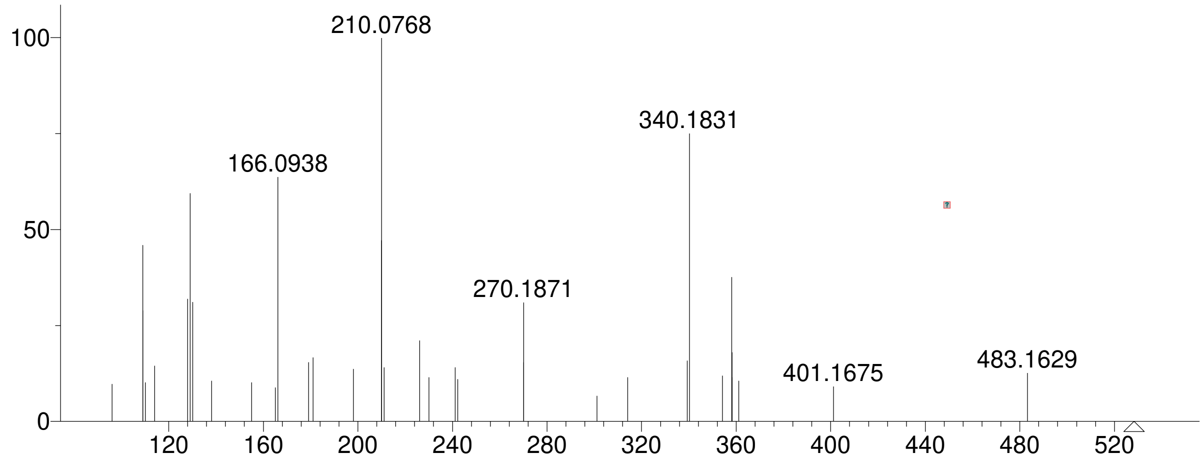

Unknown: Unknown (11.71_433.0798m/z)
Compound in Library Factor = 0

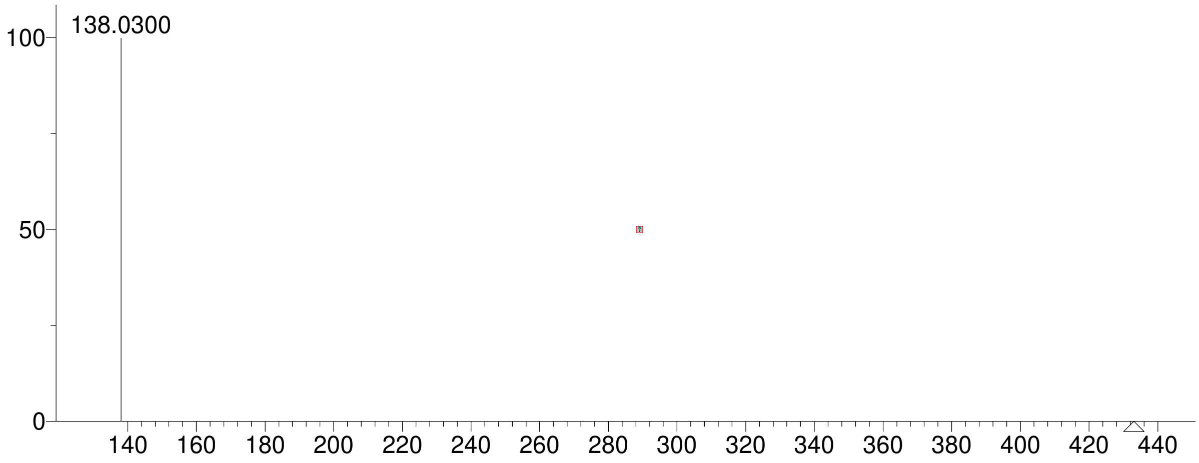

Unknown: Unknown (11.72_443.1995m/z)
Compound in Library Factor = 0

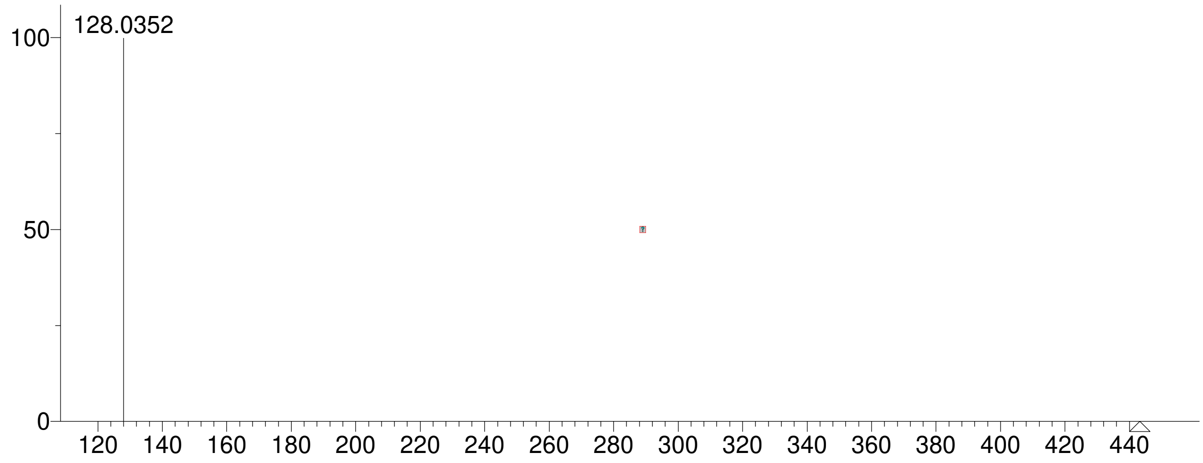

Unknown: Unknown (11.76_417.1764m/z)
Compound in Library Factor = 0

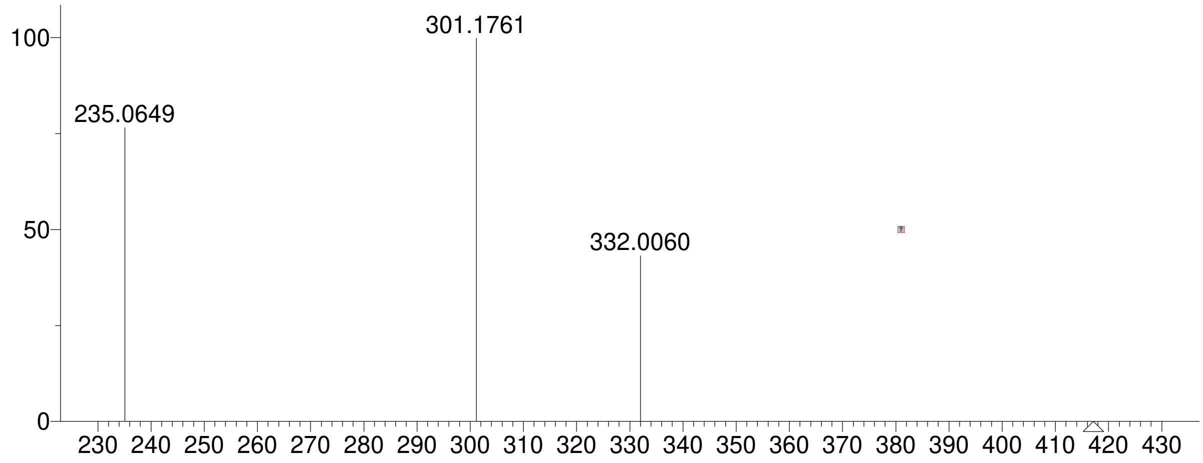

Hit 1 : (2R,3S,4S,5R,6R)-5-[(2S,3R,4R)-3,4-dihydroxy-4-(hydroxymethyl)oxolan-2-yl]oxy-2-(hydroxymethyl)-6-(2-p
C19H28O10; MF: 0; RMF: 89; Prob 33.3%; Lib: mona-export-vf-npl_qexactive; ID: 6423.

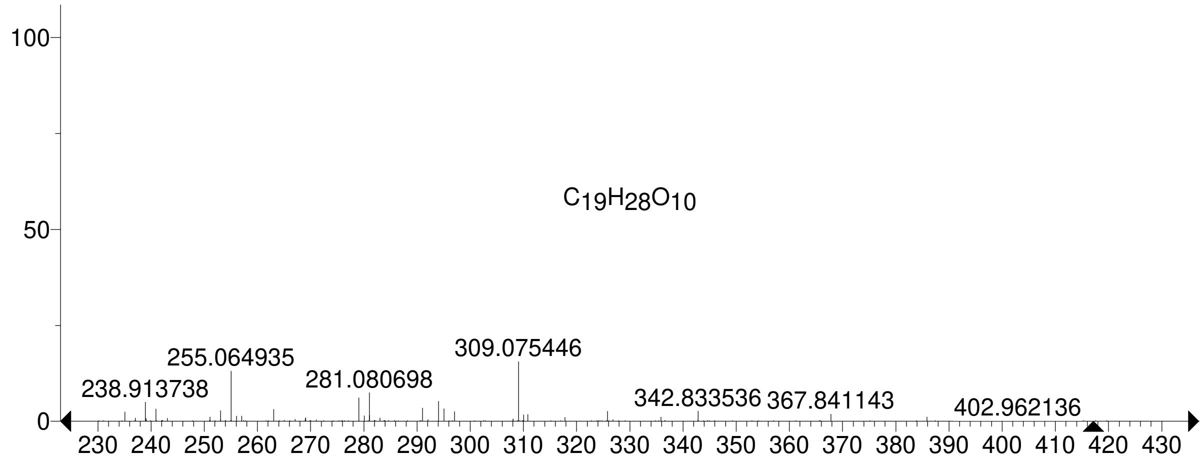

Hit 2 : (2S,3R,4S,5R)-2-[(2R,3R,4S,5S,6R)-4,5-dihydroxy-6-(hydroxymethyl)-2-(2-phenylethoxy)oxan-3-yl]oxyoxar
C19H28O10; MF: 0; RMF: 47; Prob 33.3%; Lib: mona-export-vf-npl_qexactive; ID: 8025.

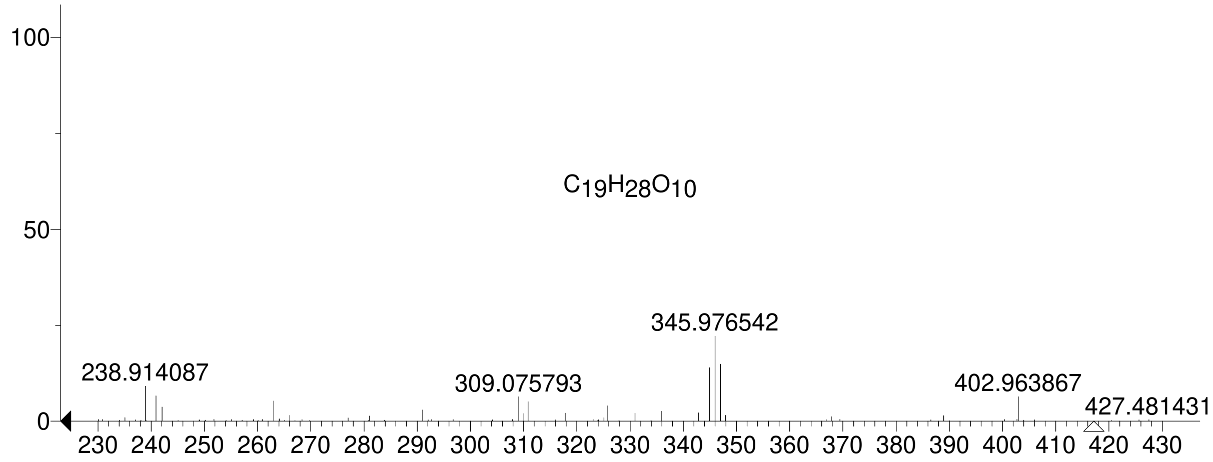

Hit 3 : (2R,3S,4S,5R,6R)-2-[[(2S,3R,4R)-3,4-dihydroxy-4-(hydroxymethyl)oxolan-2-yl]oxymethyl]-6-(2-phenylethox
C19H28O10; MF: 0; RMF: 30; Prob 33.3%; Lib: mona-export-vf-npl_qexactive; ID: 24964.

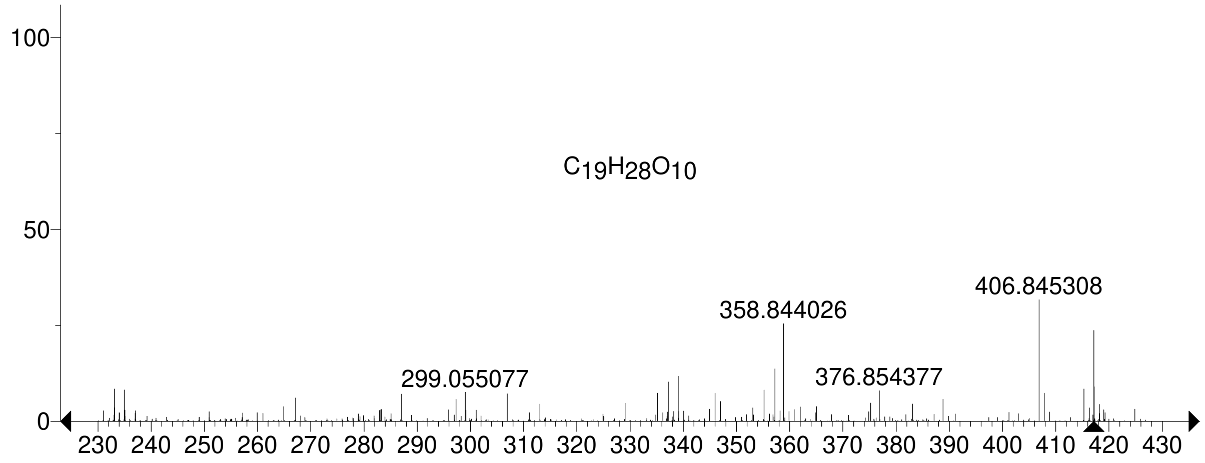

Unknown: Unknown (11.77_449.1547m/z)
Compound in Library Factor = 0

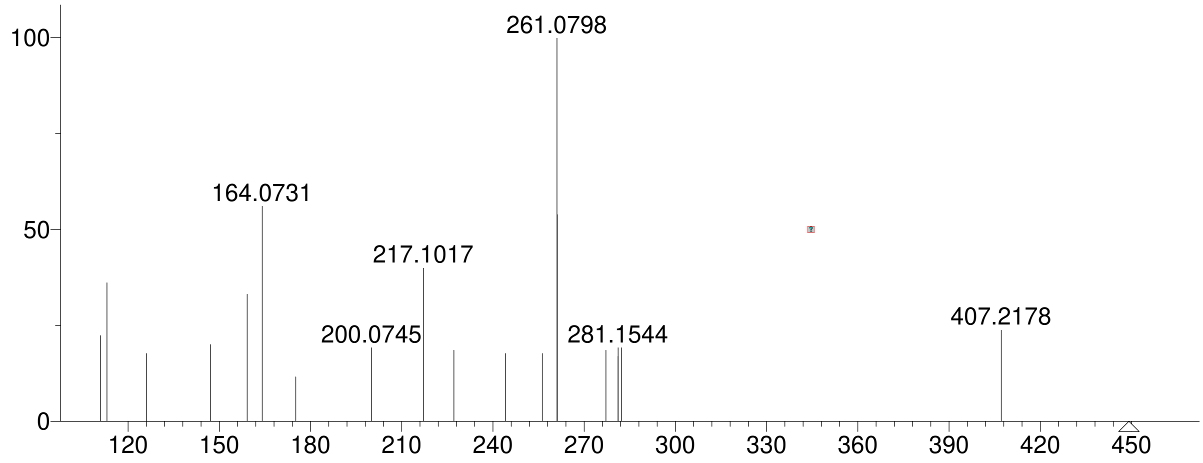

Hit 1 : [8,8-dimethyl-9-(3-methylbut-2-enoyloxy)-2-oxo-9,10-dihydropyrano[2,3-f]chromen-10-yl] 3-methylbut-2-en
C24H26O7; MF: 0; RMF: 51; Prob 25.0%; Lib: mona-export-vf-npl_qexactive; ID: 4542.

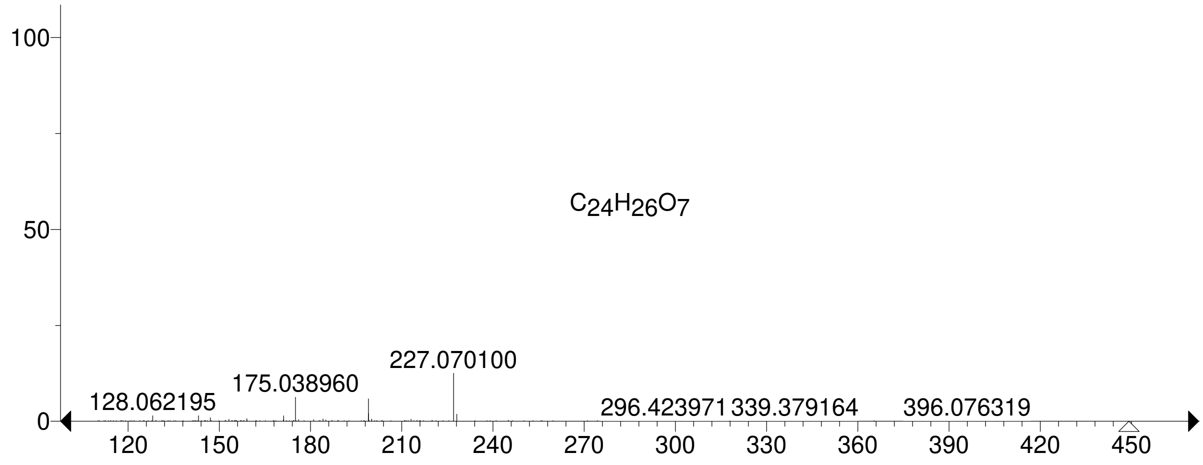

Hit 2 : NCGC00385098-01![8,8-dimethyl-9-(3-methylbut-2-enoyloxy)-2-oxo-9,10-dihydropyrano[2,3-f]chromen-10-
C24H26O7; MF: 0; RMF: 29; Prob 25.0%; Lib: mona-export-gnps; ID: 7369.

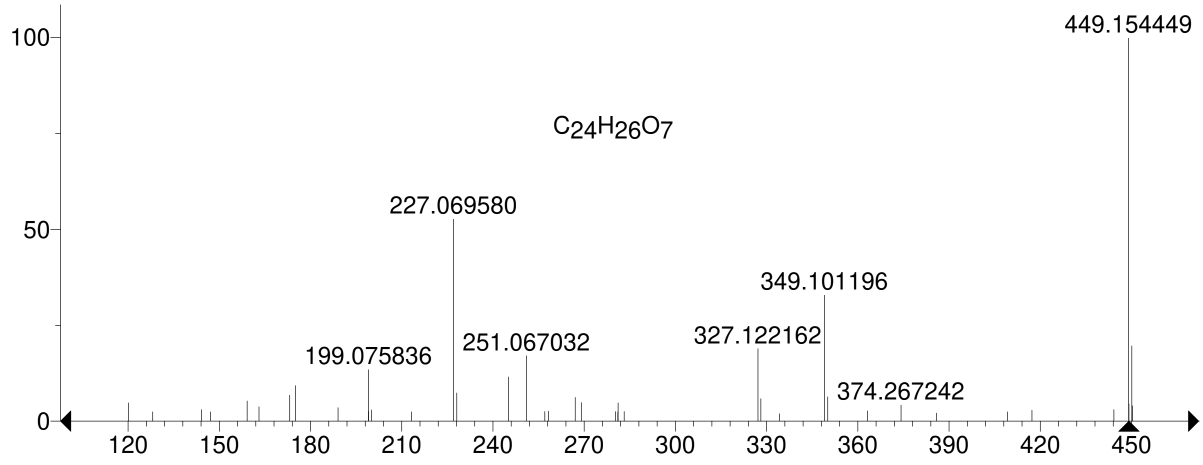

Hit 3 : Archangelicine [M+Na]+ 65HCD P=449.2
C24H26O7; MF: 0; RMF: 22; Prob 25.0%; Lib: mona-export-vf-npl_qexactive; ID: 5187.

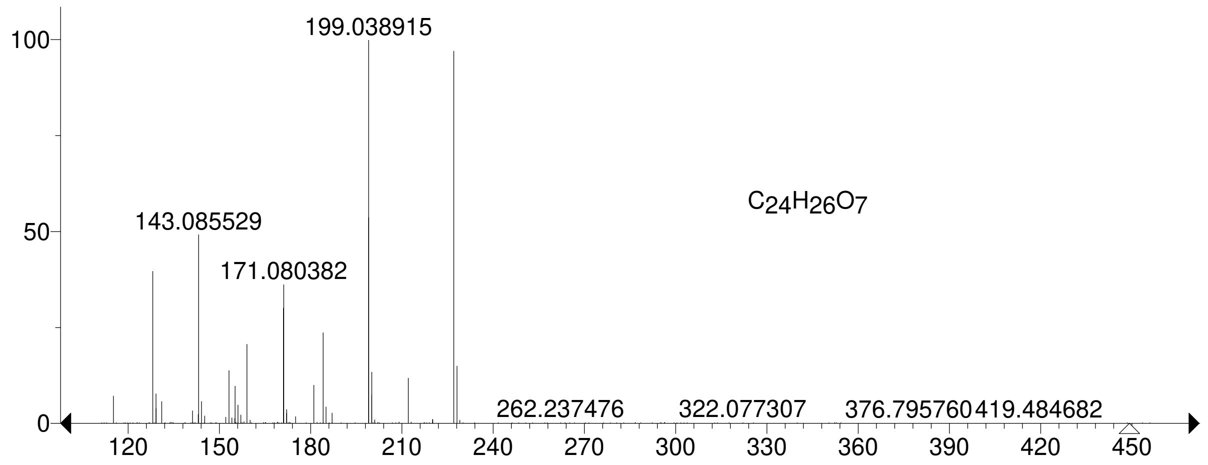

Hit 4 : NCGC00385035-01![8-[2-(3-methylbut-2-enoyloxy)propan-2-yl]-2-oxo-8,9-dihydrofuro[2,3-h]chromen-9-yl]
C24H26O7; MF: 0; RMF: 21; Prob 25.0%; Lib: mona-export-gnps; ID: 6527.

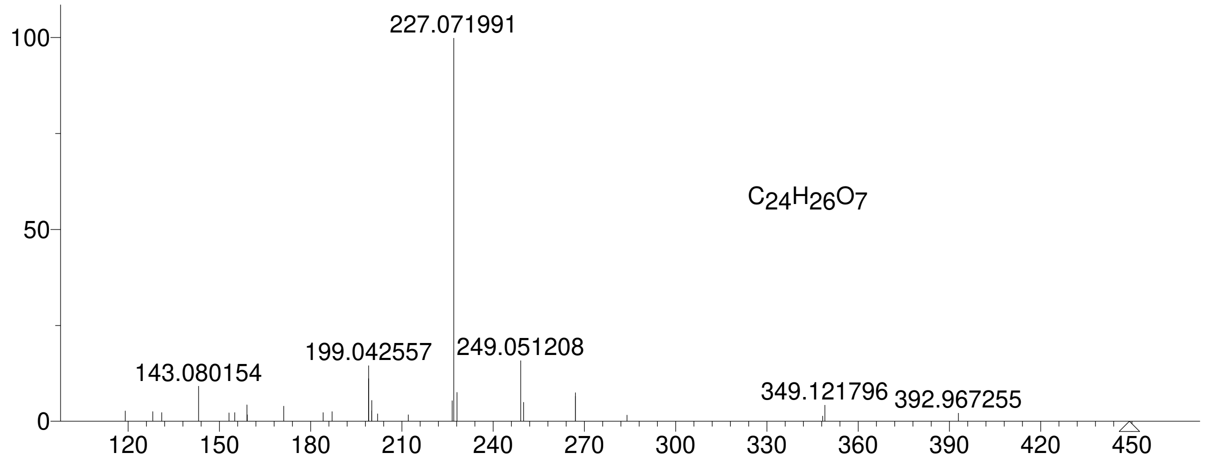

** Search Report Page 1 of 1 **

Unknown: Unknown (11.83_498.2398m/z)
Compound in Library Factor = 0

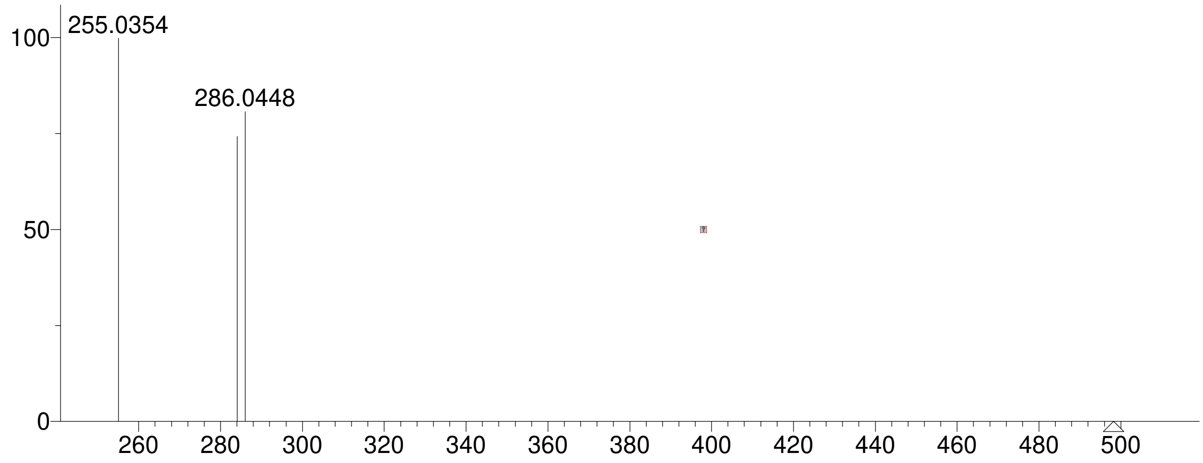

Unknown: Unknown (11.85_337.0918m/z)
Compound in Library Factor = -428

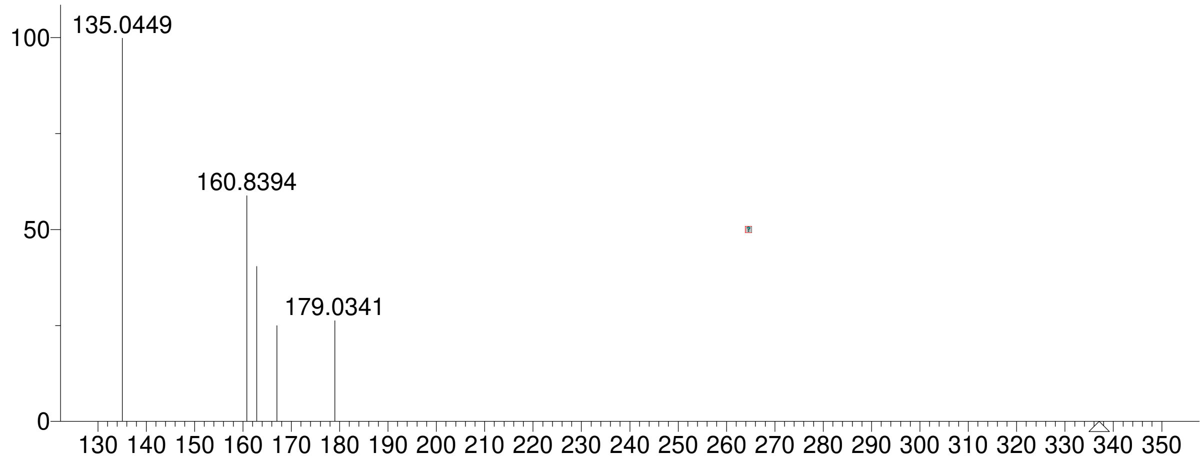

Hit 1 : Chlorogenic acid [M+H-H2O]+ HCD 87V P=337.1
C16H18O9; MF: 195; RMF: 631; Prob 93.7%; CAS: 327-97-9; Lib: nist_msms; ID: 100727.

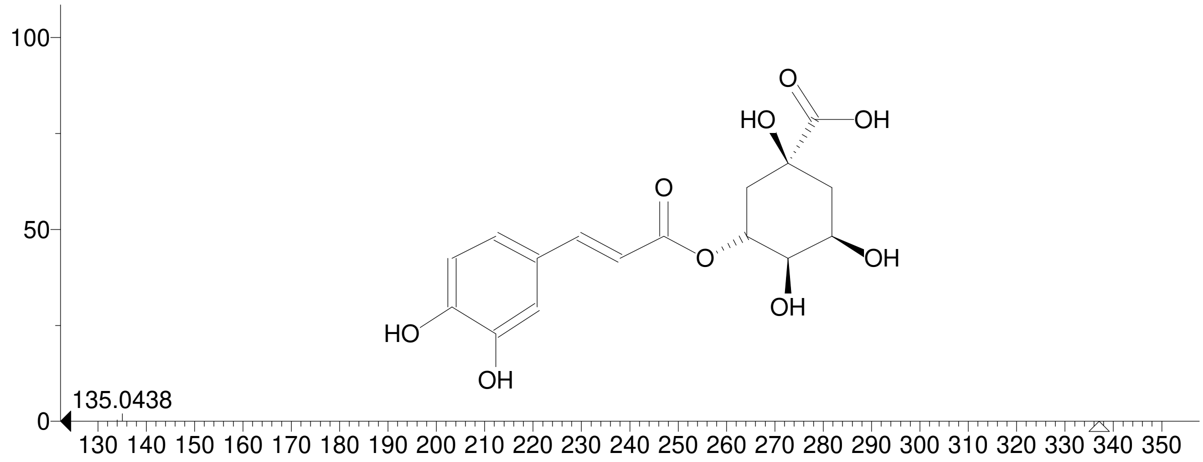

Hit 2 : Neochlorogenic acid [M+H-H2O]+ HCD 87V P=337.1
C16H18O9; MF: 32; RMF: 507; Prob 2.28%; CAS: 906-33-2; Lib: nist_msms; ID: 239450.

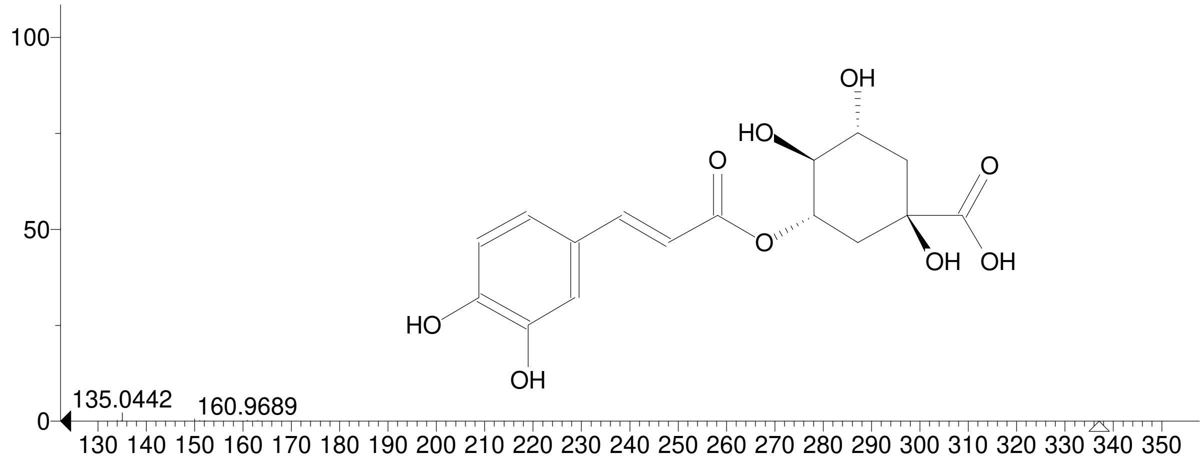

Hit 3 : NCGC00180823-02!(3R,4R,5R)-5-[(E)-3-(3,4-dihydroxyphenyl)prop-2-enoyl]oxy-3,4-dihydroxycyclohexene
C16H16O8; MF: 4; RMF: 312; Prob 0.66%; Lib: mona-export-gnps; ID: 9151.

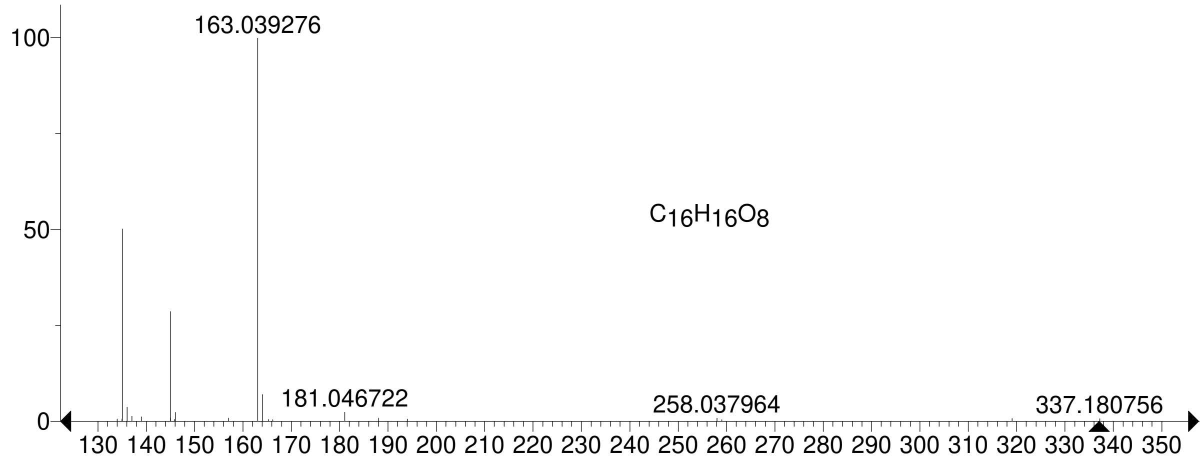

Hit 4 : NCGC00380360-01!7-methoxy-6-[(2S,3R,4S,5S,6R)-3,4,5-trihydroxy-6-(hydroxymethyl)oxan-2-yl]oxychron
C16H18O9; MF: 0; RMF: 39; Prob 0.55%; Lib: mona-export-gnps; ID: 9547.

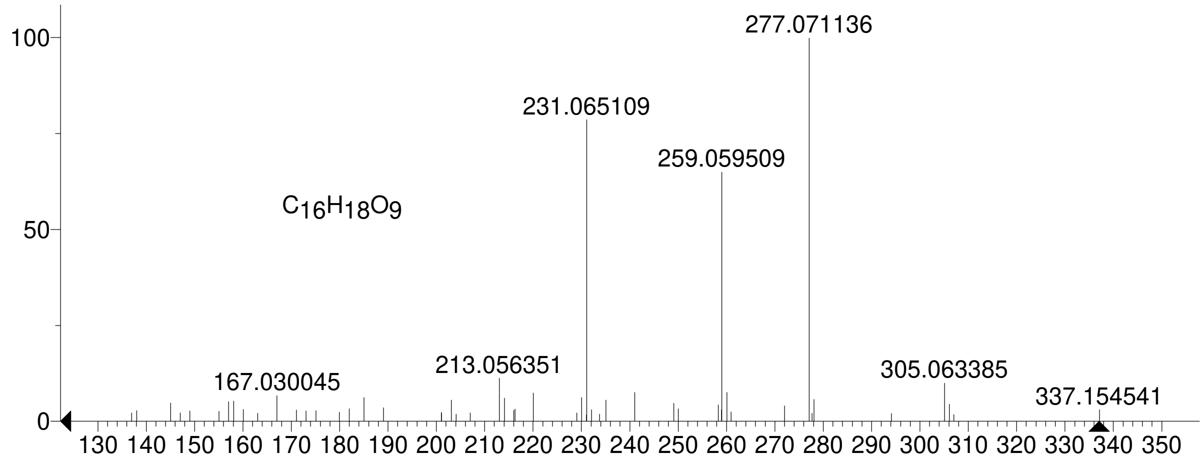

Hit 5 : 2-(4a,9,10a-trihydroxy-1-methyl-5,10-dioxo-3,4-dihydro-1H-benzo[g]isochromen-3-yl)acetic acid [M+H]+ 6
C16H16O8; MF: 0; RMF: 27; Prob 0.55%; Lib: mona-export-vf-npl_qexactive; ID: 19101.

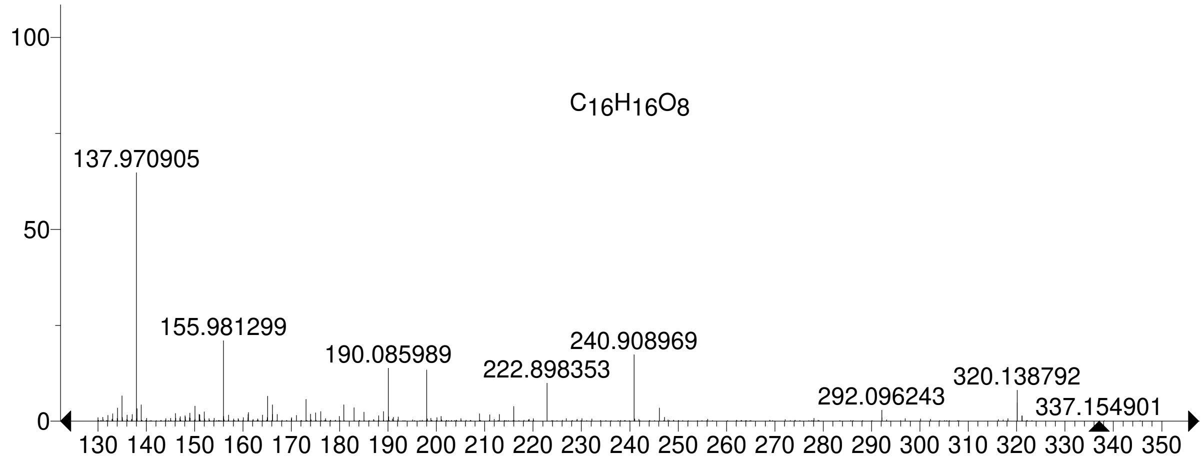

Unknown: Unknown (11.86_265.1075m/z)
Compound in Library Factor = -2387

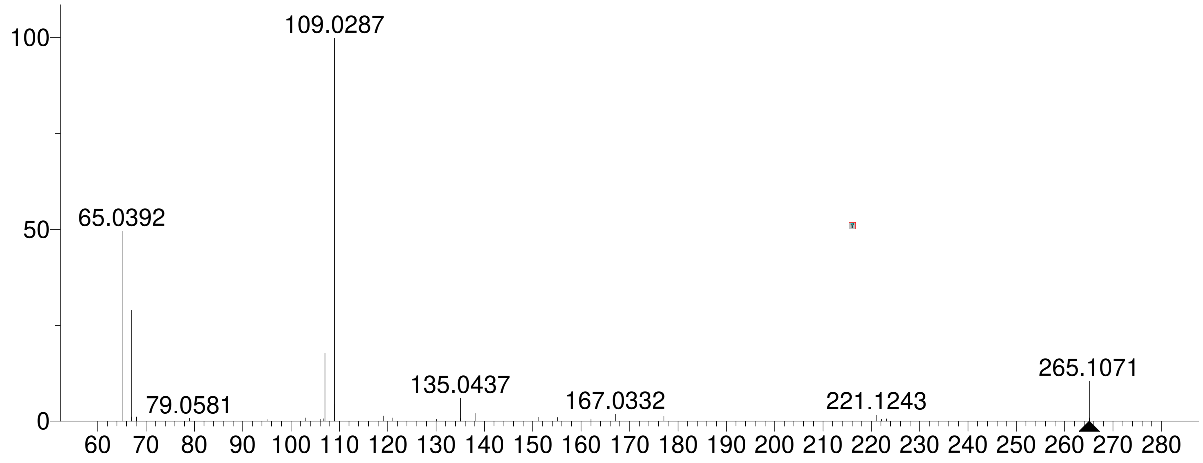

Hit 1 : Anisatin [M+H-CH4O3]+ HCD 13V P=265.1
C15H20O8; MF: 18; RMF: 201; Prob 24.0%; CAS: 5230-87-5; Lib: nist_msms; ID: 394626.

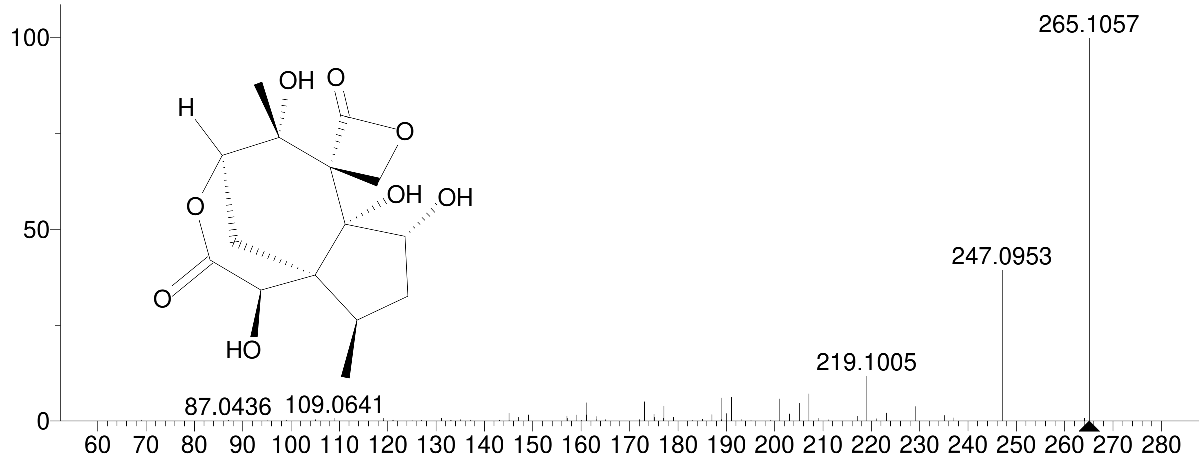

Hit 2 : 4,7,8-trimethoxy-3,5-dimethylchromen-2-one [M+H]+ 35HCD P=265.1
C14H16O5; MF: 8; RMF: 164; Prob 16.9%; Lib: mona-export-vf-npl_qexactive; ID: 23347.

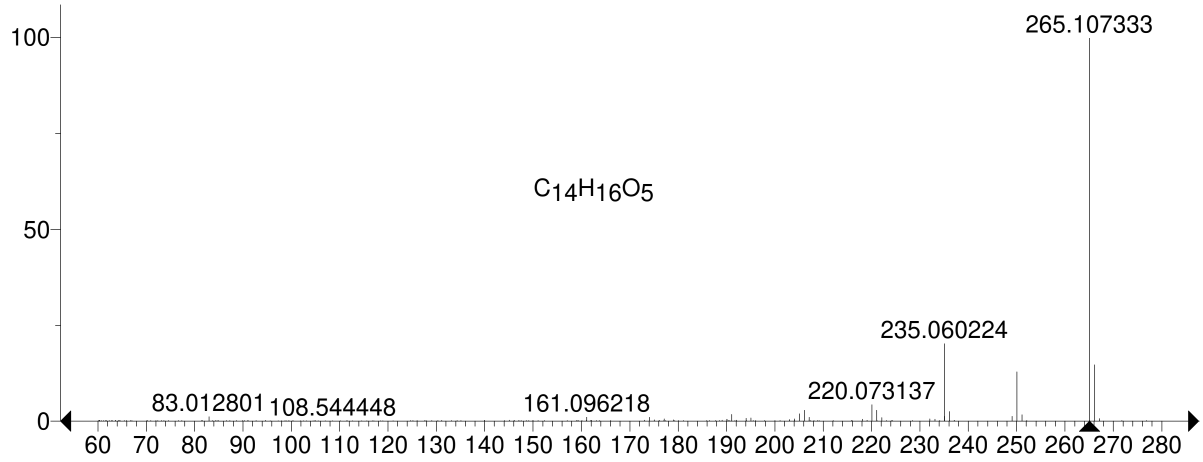

Hit 3 : NCGC00380472-01!4,7,8-trimethoxy-3,5-dimethylchromen-2-one [M+H]+ P=265.1
C14H16O5; MF: 7; RMF: 165; Prob 16.3%; Lib: mona-export-gnps; ID: 6739.

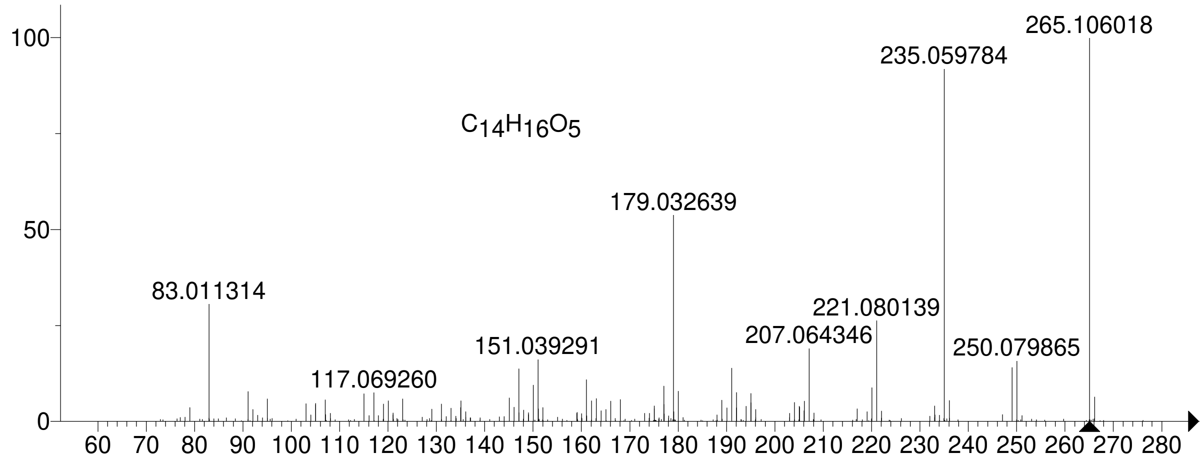

Hit 4 : NCGC00381087-01!3-(2-hydroxypropyl)-6,8-dimethoxyisochromen-1-one [M+H]+ P=265.1
C14H16O5; MF: 6; RMF: 125; Prob 15.6%; Lib: mona-export-gnps; ID: 7812.

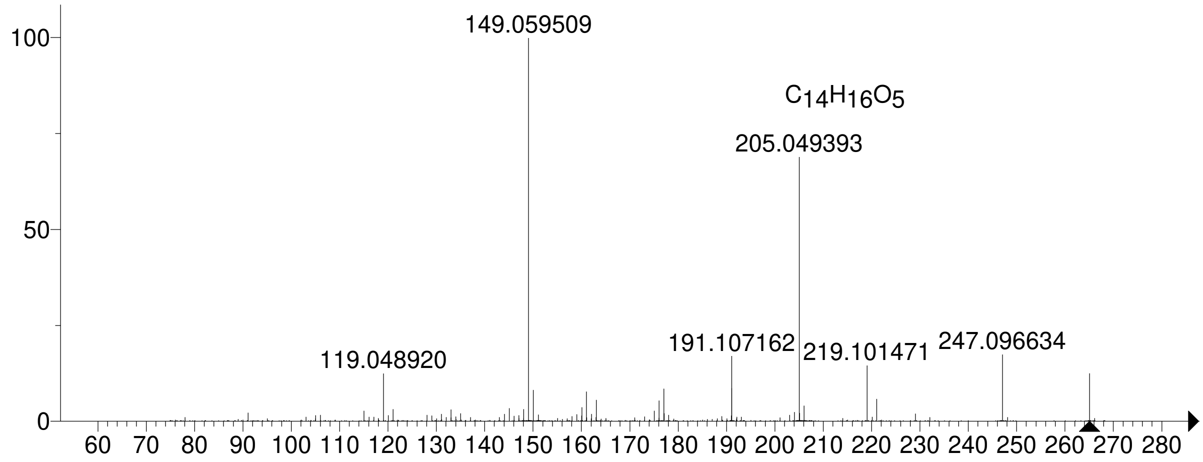

Hit 5 : (E)-5-(4-methoxy-5-methyl-6-oxopyran-2-yl)-3-methylhex-4-enoic acid [M-H]- 35HCD P=265.1
C14H18O5; MF: 5; RMF: 52; Prob 15.0%; Lib: mona-export-lc-ms-ms_negative_mode; ID: 22802.

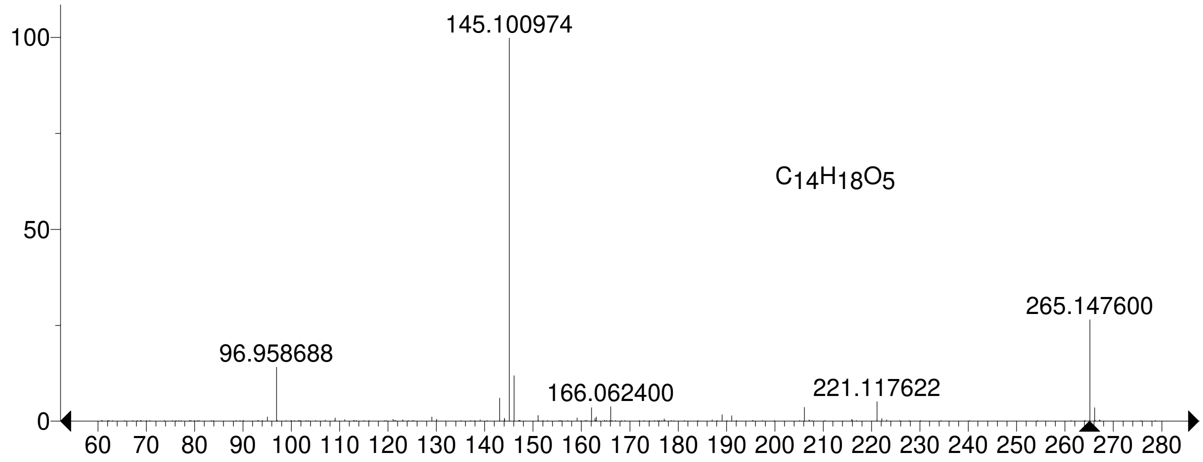

Unknown: Unknown (11.87_460.1823m/z)
Compound in Library Factor = 0

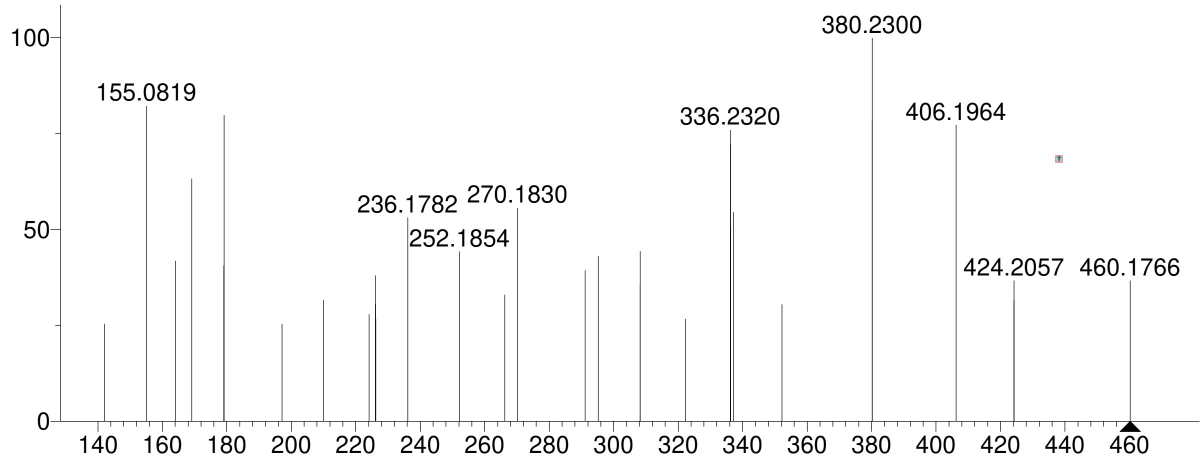

Hit 1 : α-Amanitin [M+2H]2+ HCD 12V P=460.2
C39H54N10O14S; MF: 0; RMF: 26; Prob 100.0%; CAS: 23109-05-9; Lib: nist_msms; ID: 225453.

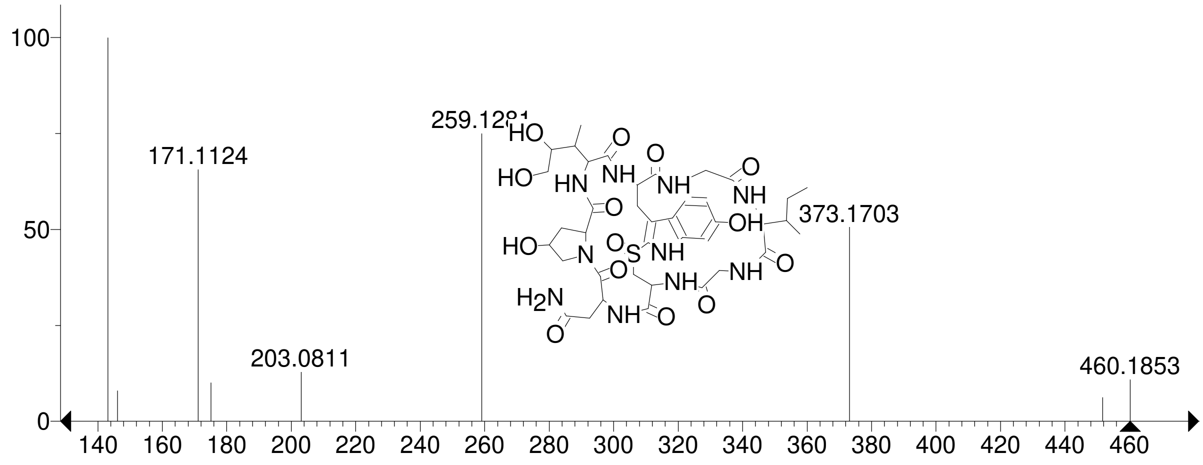

Unknown: Unknown (11.89_325.0919m/z)
Compound in Library Factor = 0

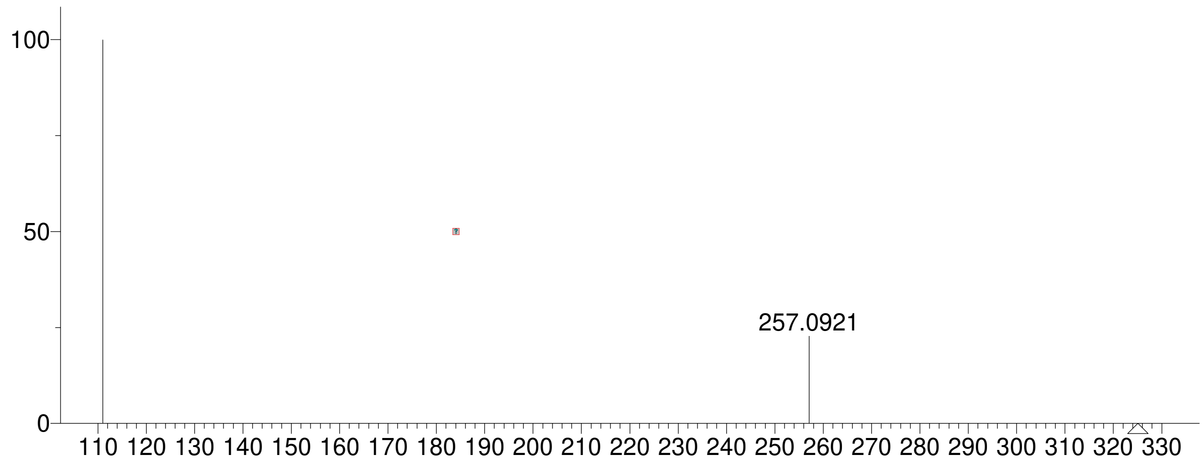

Hit 1 : 2-(3-hydroxy-5-methoxyphenoxy)-6-(hydroxymethyl)oxane-3,4,5-triol [M+Na]+ 65HCD P=325.1
C13H18O8; MF: 0; RMF: 24; Prob 50.0%; Lib: mona-export-vf-npl_qexactive; ID: 22425.

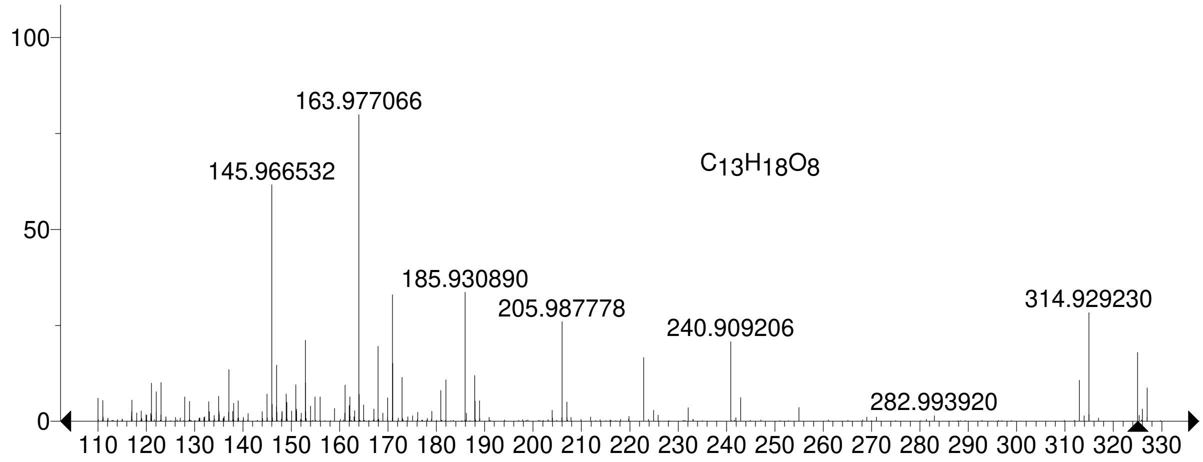

Hit 2 : (1S,4R,7S,8S,11S)-9-tert-butyl-7,9-dihydroxy-3,5,12-trioxatetracyclo[6.6.0.0A^'1,A^'1A^'1.0a^",a^,,]tetradec
C15H18O8; MF: 0; RMF: 6; Prob 50.0%; Lib: mona-export-lc-ms-ms_negative_mode; ID: 19424.

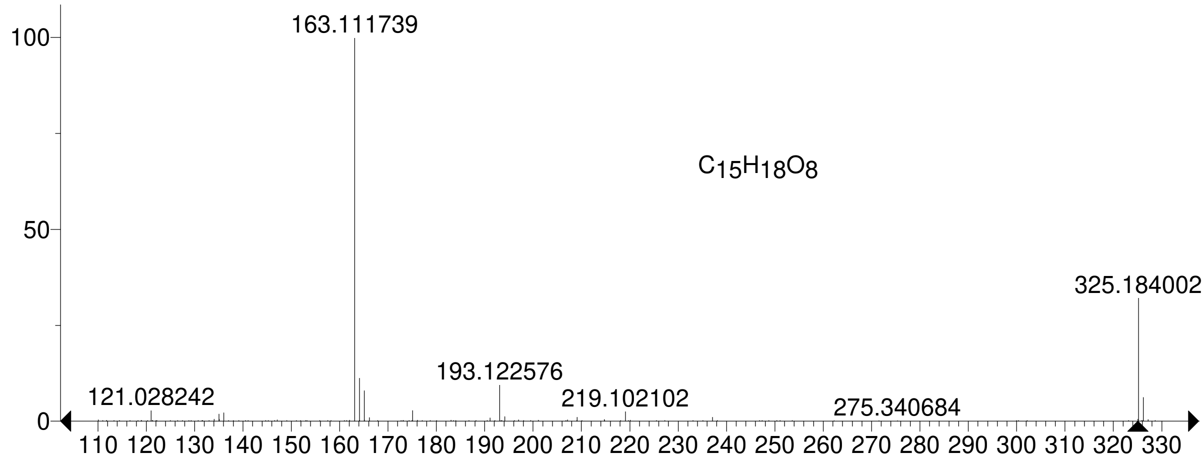

** Search Report Page 1 of 1 **

Unknown: Unknown (11.90_926.4372m/z)
Compound in Library Factor = 0

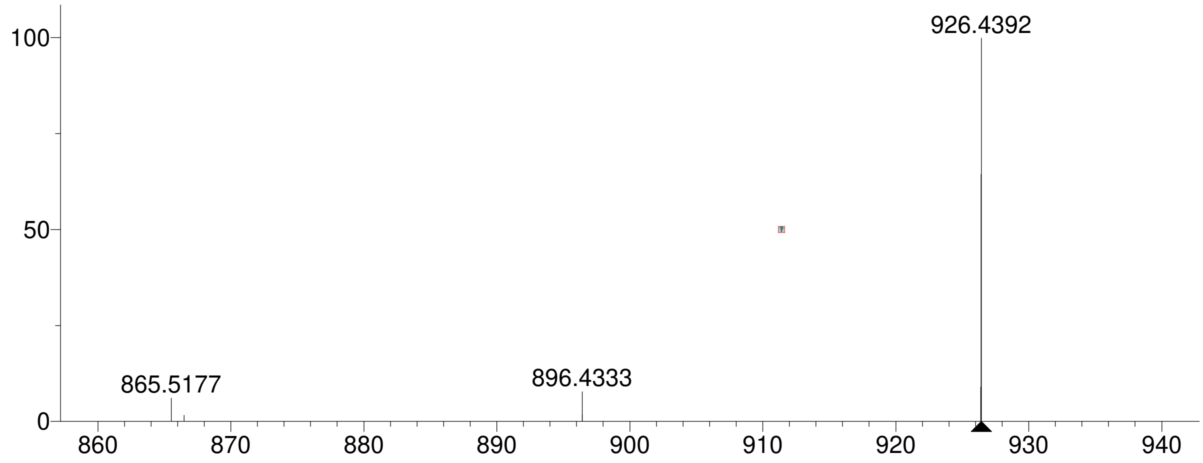

Unknown: Unknown (11.92_926.4005m/z)
Compound in Library Factor = 0

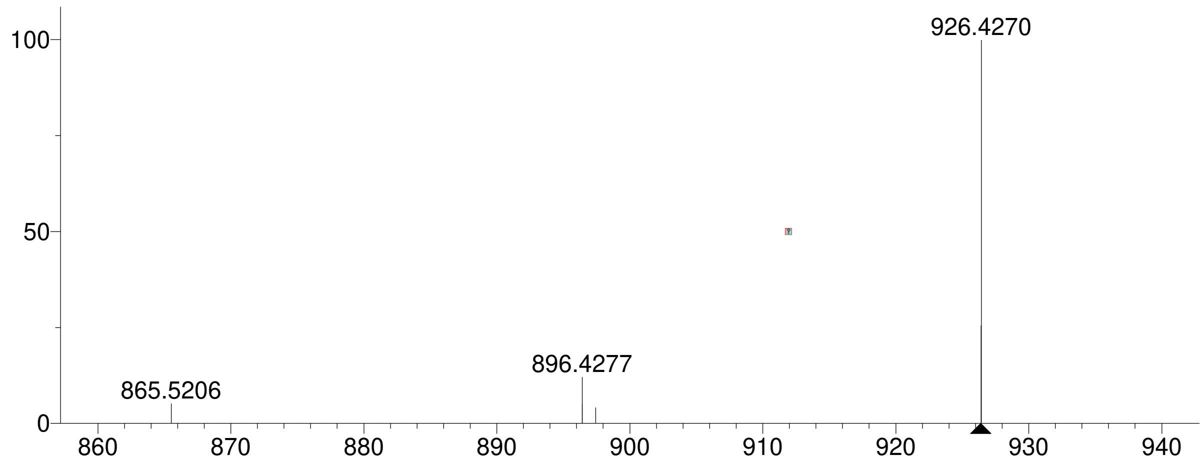

** Search Report Page 1 of 1 **

Unknown: Unknown (11.92_926.4369m/z)
Compound in Library Factor = 0

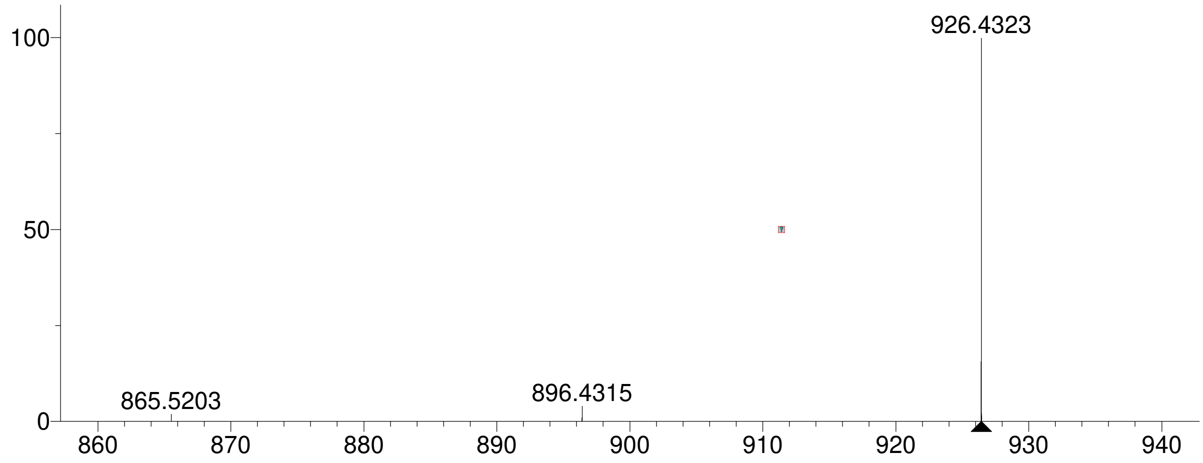

Unknown: Unknown (11.93_663.2141m/z)
Compound in Library Factor = 0

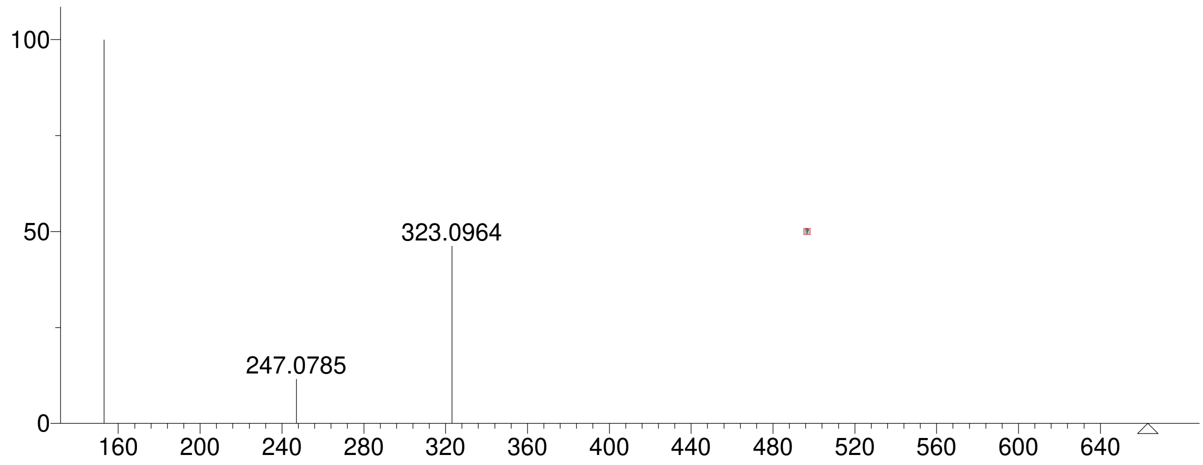

Unknown: Unknown (11.94_351.1065m/z)
Compound in Library Factor = 0

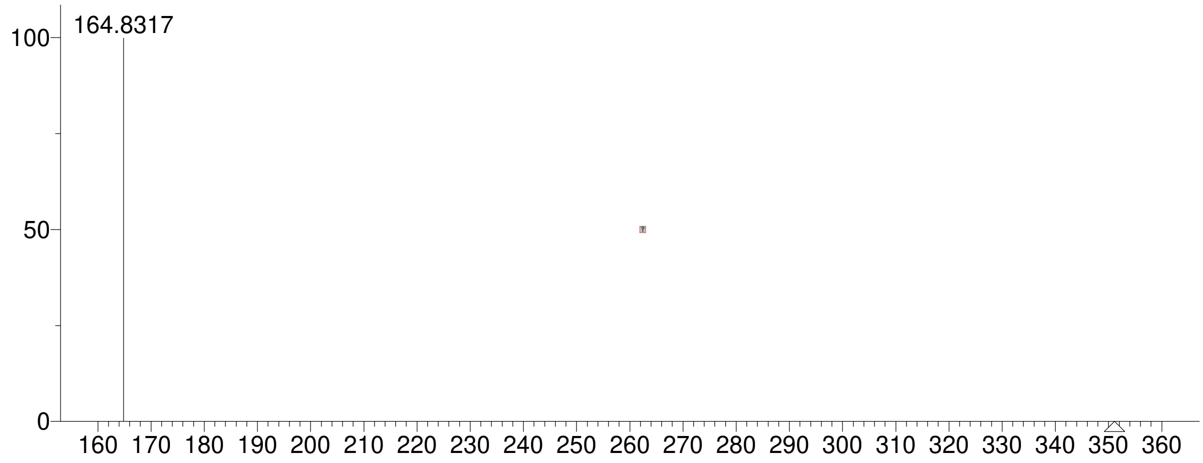

Hit 1 : 1-(4-hydroxyphenyl)-3-[(2R,3R,4S,5S,6R)-3,4,5-trihydroxy-6-(hydroxymethyl)oxan-2-yl]oxypropan-1-one [M
C15H20O8; MF: 0; RMF: 9; Prob 100.0%; Lib: anthocyanins mona; ID: 3643.

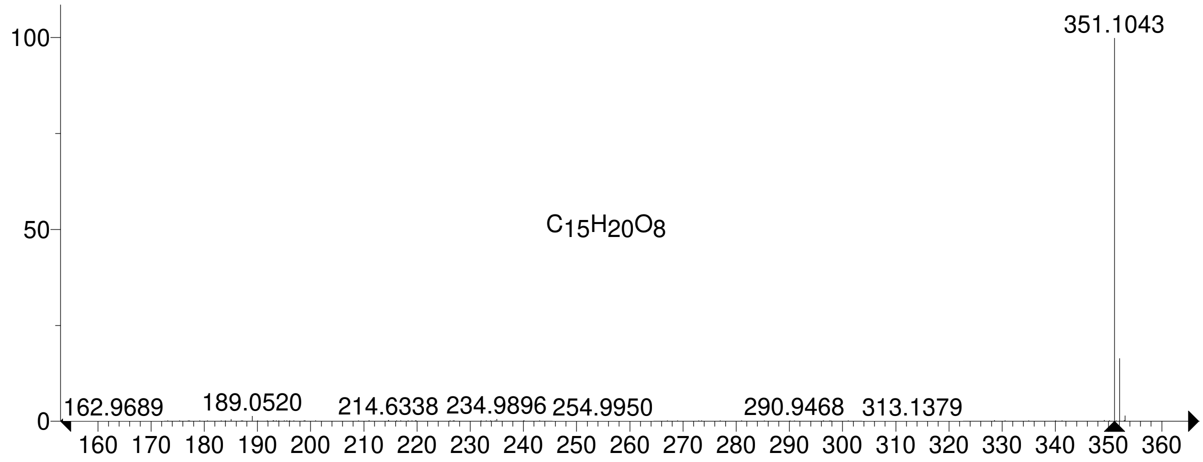

Unknown: Unknown (11.99_265.1075m/z)
Compound in Library Factor = -2387

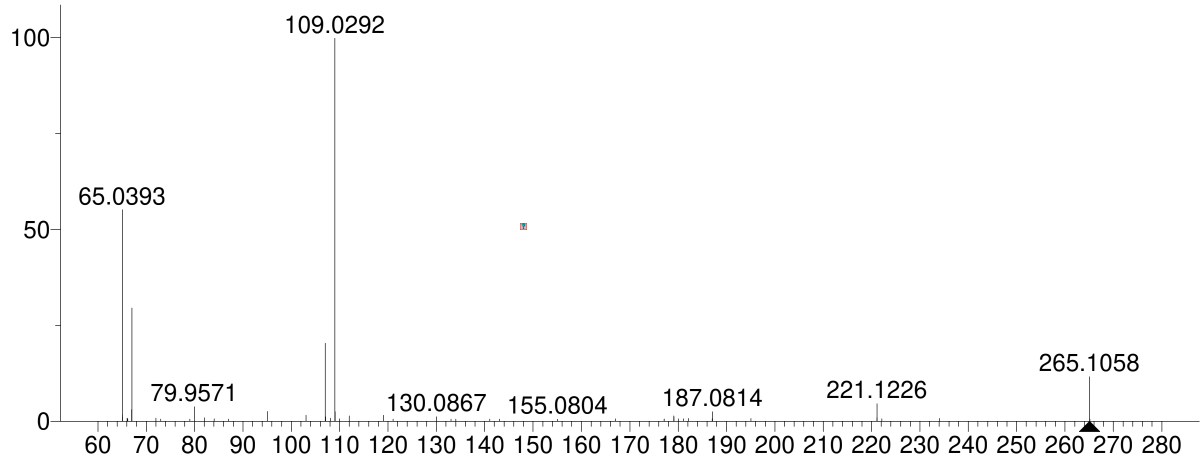

Hit 1 : Anisatin [M+H-CH4O3]+ HCD 10V P=265.1
C15H20O8; MF: 27; RMF: 477; Prob 31.5%; CAS: 5230-87-5; Lib: nist_msms; ID: 394625.

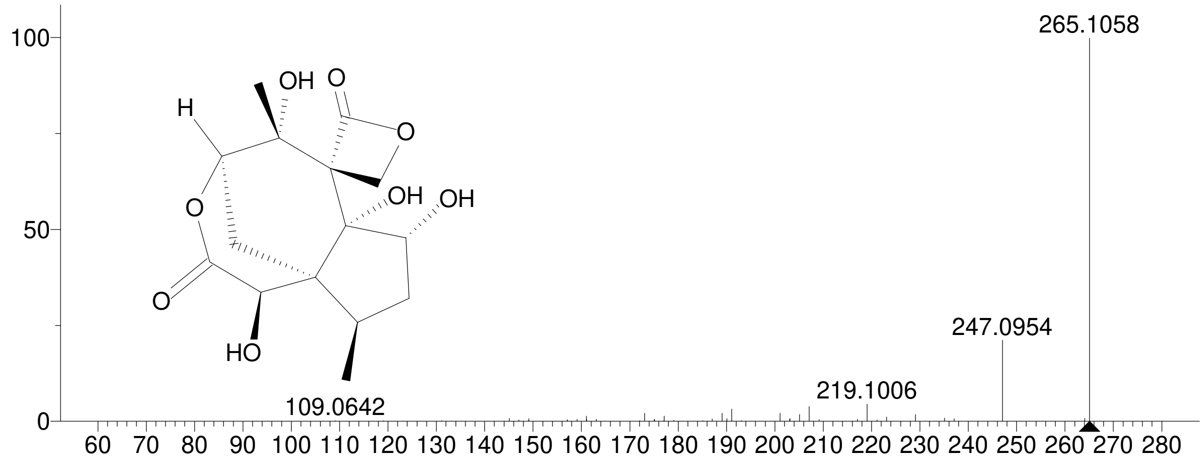

Hit 2 : NCGC00380472-01!4,7,8-trimethoxy-3,5-dimethylchromen-2-one [M+H]+ P=265.1
C14H16O5; MF: 8; RMF: 167; Prob 15.3%; Lib: mona-export-gnps; ID: 6739.

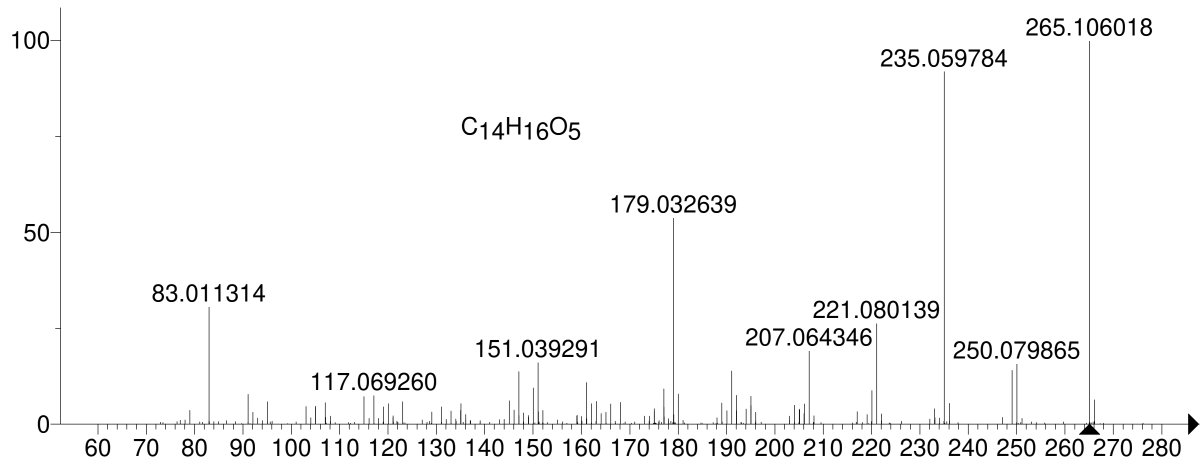

Hit 3 : 4,7,8-trimethoxy-3,5-dimethylchromen-2-one [M+H]+ 35HCD P=265.1
C14H16O5; MF: 8; RMF: 164; Prob 15.3%; Lib: mona-export-vf-npl_qexactive; ID: 23347.

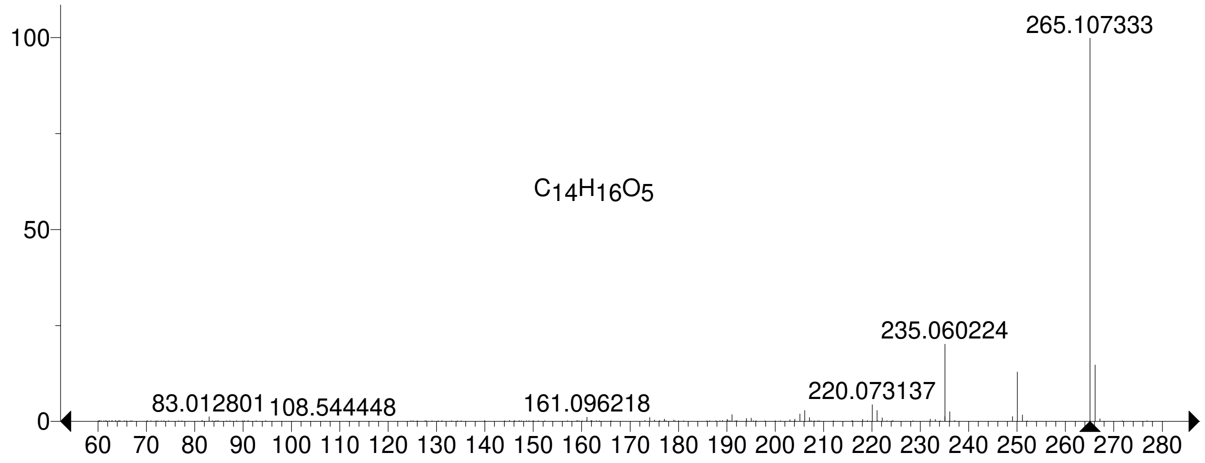

Hit 4 : NCGC00381087-01!3-(2-hydroxypropyl)-6,8-dimethoxyisochromen-1-one [M+H]+ P=265.1
C14H16O5; MF: 5; RMF: 103; Prob 13.5%; Lib: mona-export-gnps; ID: 7812.

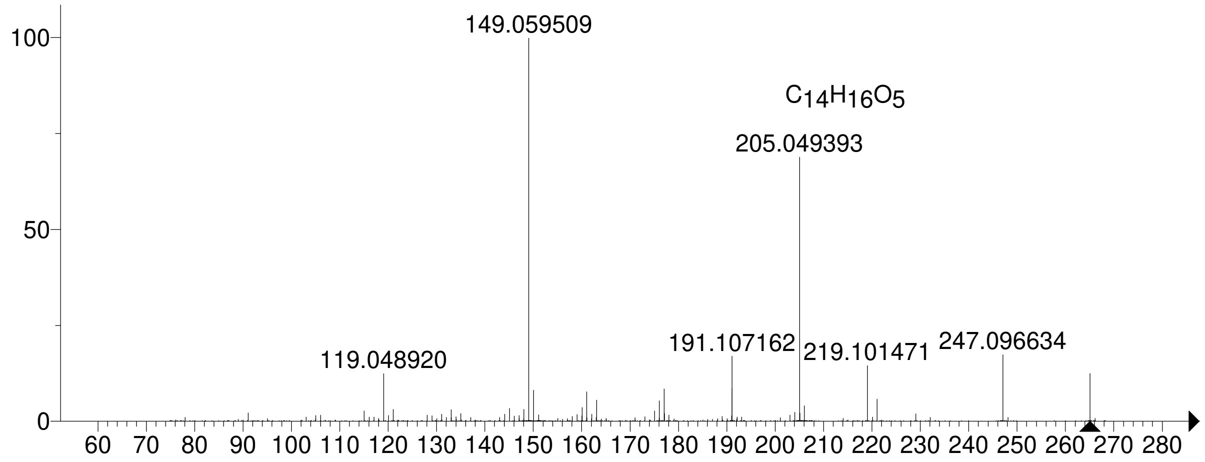

Hit 5 : (E)-5-(4-methoxy-5-methyl-6-oxopyran-2-yl)-3-methylhex-4-enoic acid [M-H]- 35HCD P=265.1
C14H18O5; MF: 5; RMF: 62; Prob 13.5%; Lib: mona-export-lc-ms-ms_negative_mode; ID: 22802.

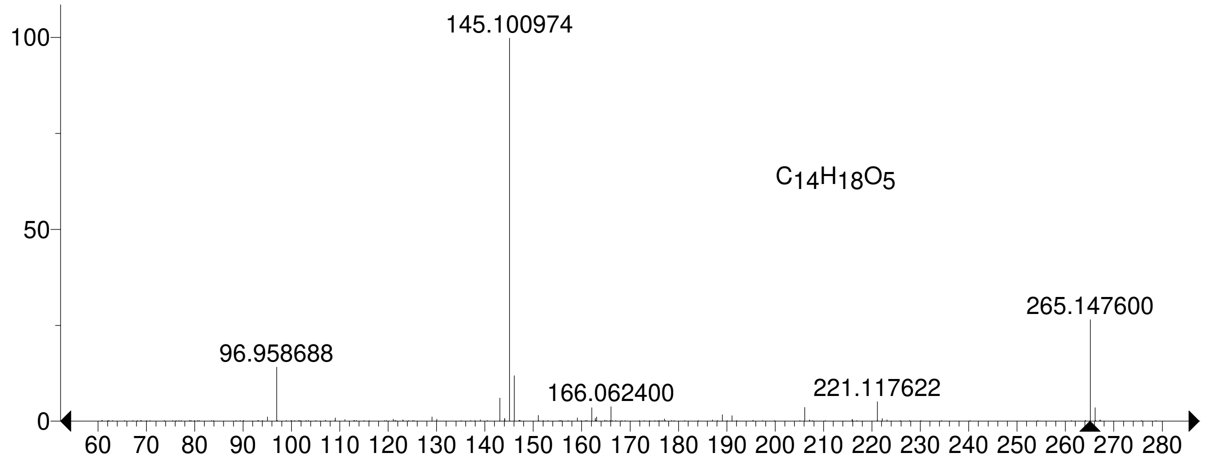

Unknown: Unknown (11.99_443.2020m/z)
Compound in Library Factor = -3273

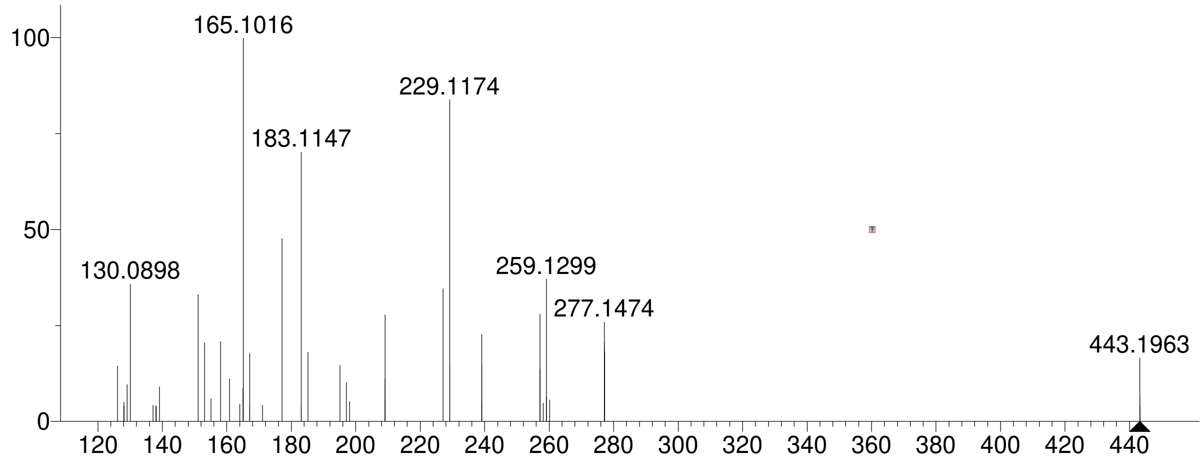

Hit 1 : Monensin [M+Na]+=>479.5=>443.2 IT 35% P=443.2
C36H62O11; MF: 6; RMF: 320; Prob 8.58%; CAS: 17090-79-8; Lib: nist_msms; ID: 145060.

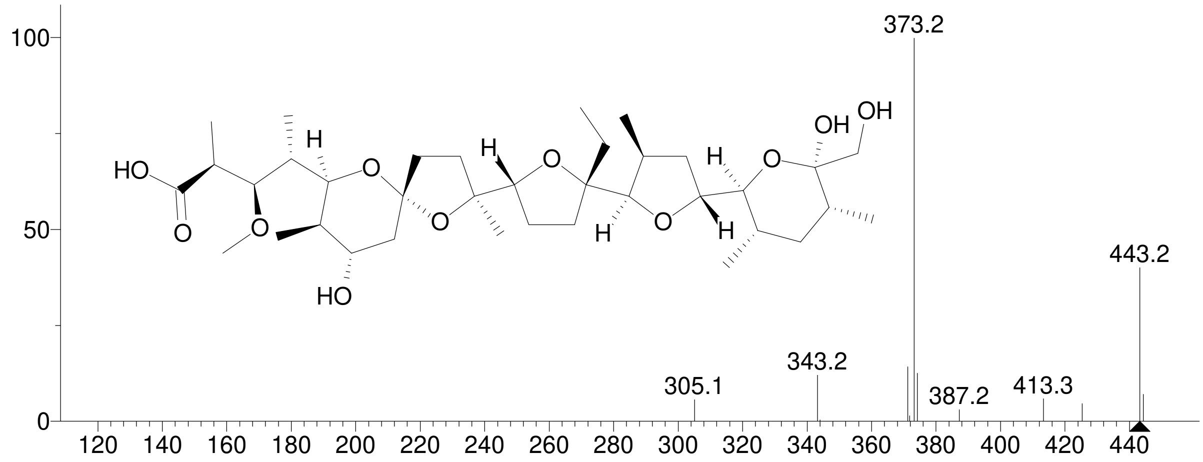

Hit 2 : Apixaban [M+H]+=>443.2 IT 35% P=443.2
C25H25N5O4; MF: 4; RMF: 80; Prob 7.92%; CAS: 503612-47-3; Lib: nist_msms; ID: 369698.

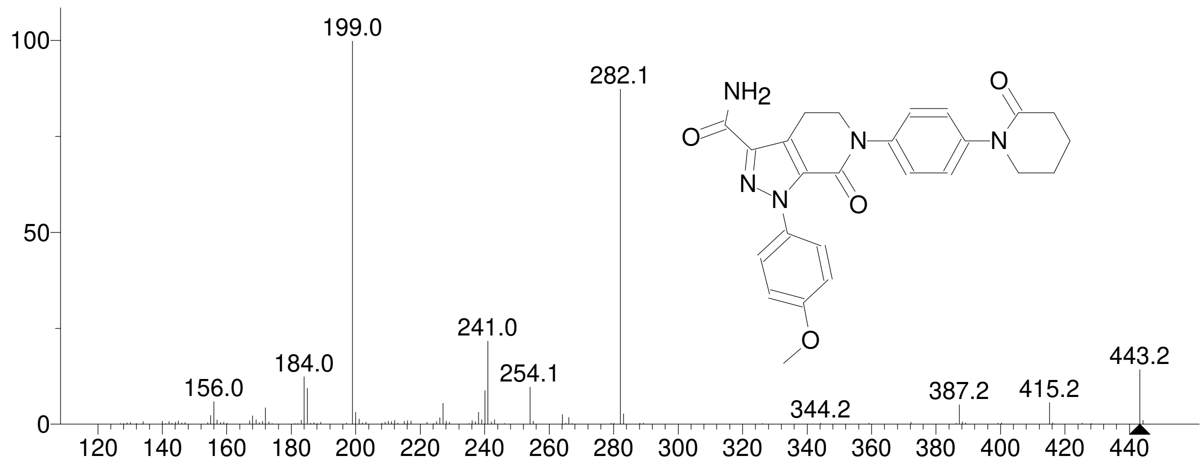

Hit 3 : NCGC00380442-01_C25H30O7_3H-5,13b-Methano-1H-furo[3',4':6,7]heptaleno[3,2-c]pyran-1,4,9(3aH,7H)
C25H30O7; MF: 3; RMF: 171; Prob 7.61%; Lib: mona-export-gnps; ID: 13756.

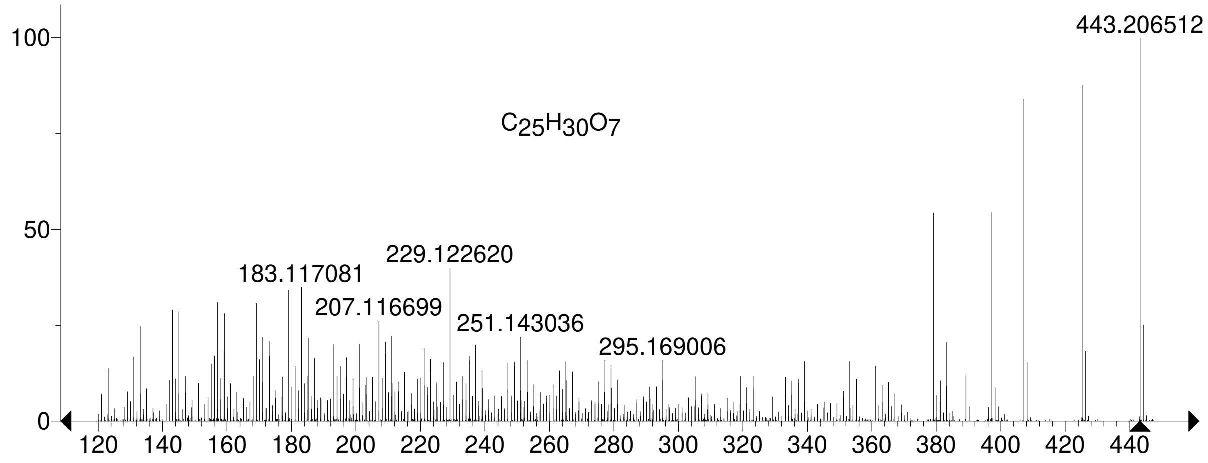

Hit 4 : Poliumoside [M-H]-=>607.3=>443.2 IT 35% P=443.2
C35H46O19; MF: 1; RMF: 86; Prob 7.02%; CAS: 94079-81-9; Lib: nist_msms; ID: 425676.

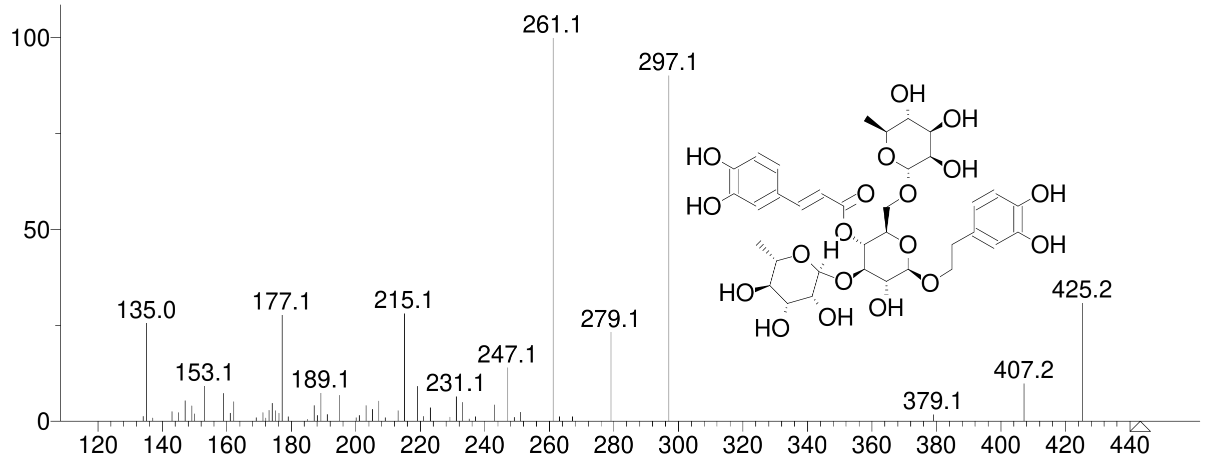

Hit 5 : NCGC00380176-01_C25H32O8_5a-Hydroxy-9-(hydroxymethyl)-13-methoxy-5,5,7a,14b-tetramethyl-1,2,5a
C25H32O8; MF: 1; RMF: 66; Prob 7.02%; Lib: mona-export-gnps; ID: 10060.

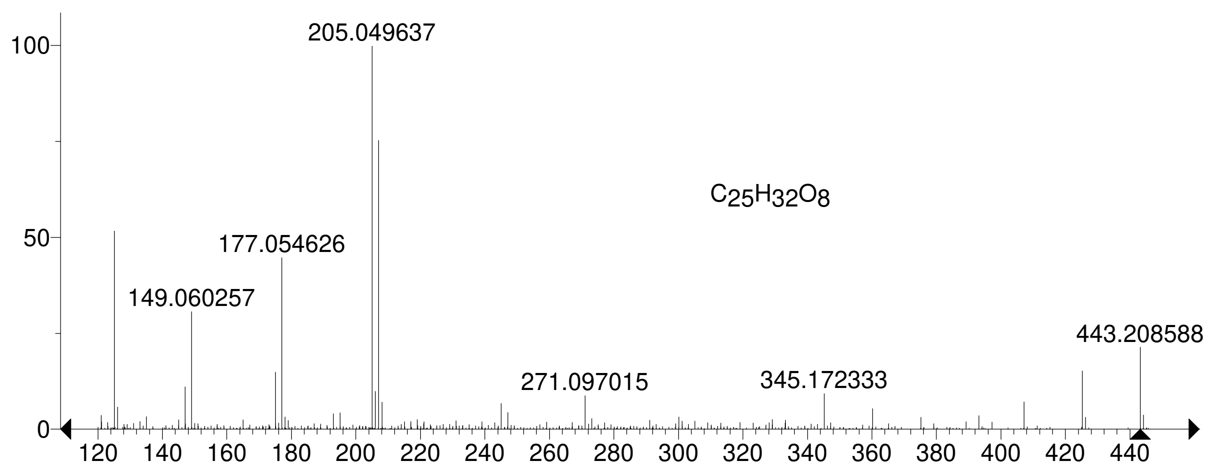

** Search Report Page 1 of 1 **

Unknown: Unknown (12.00_433.0796m/z)
Compound in Library Factor = 0

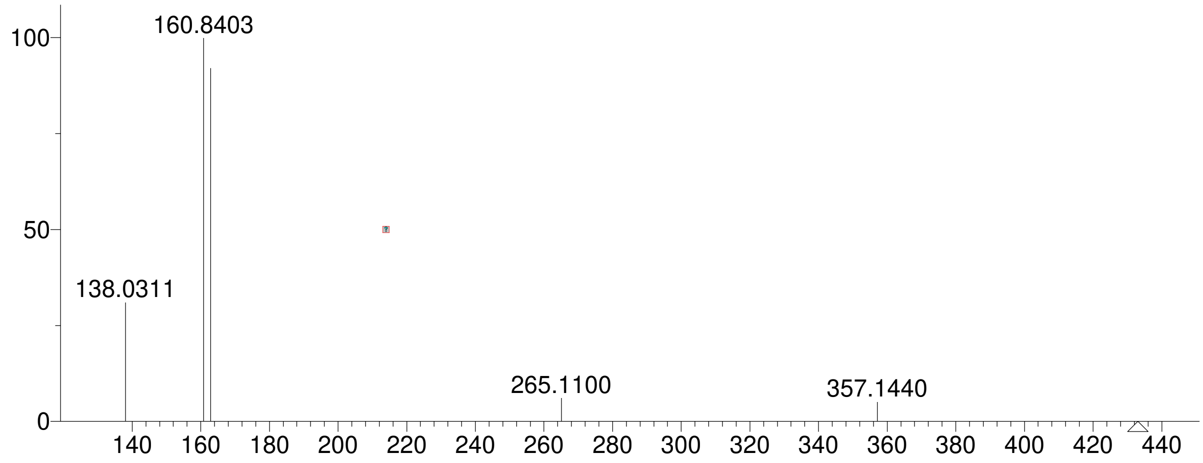

Hit 1 : avicularin [M-H]- 45HCD P=433.1
C20H18O11; MF: 0; RMF: 1; Prob 100.0%; Lib: anthocyanins mona; ID: 5373.

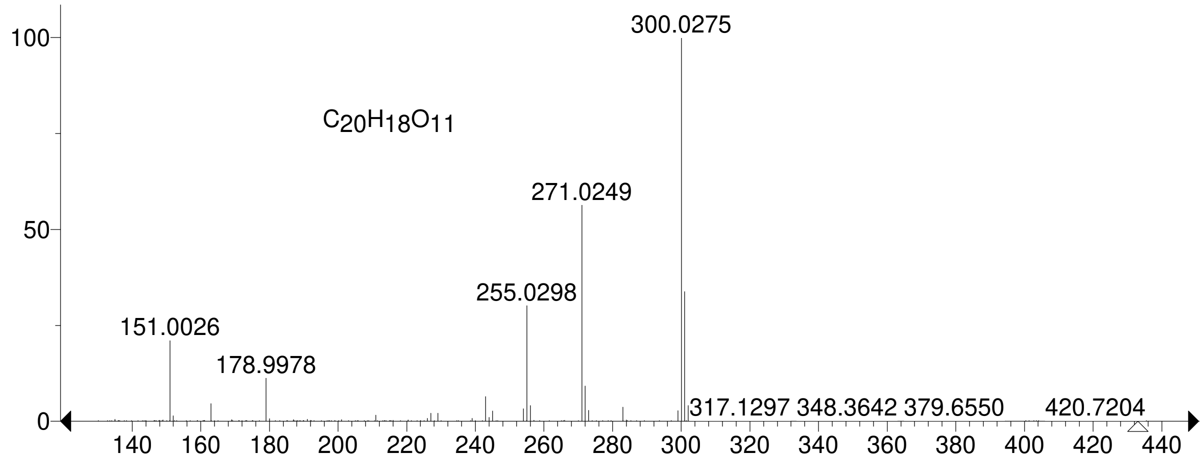

Unknown: Unknown (12.06_372.0745m/z)
Compound in Library Factor = 0

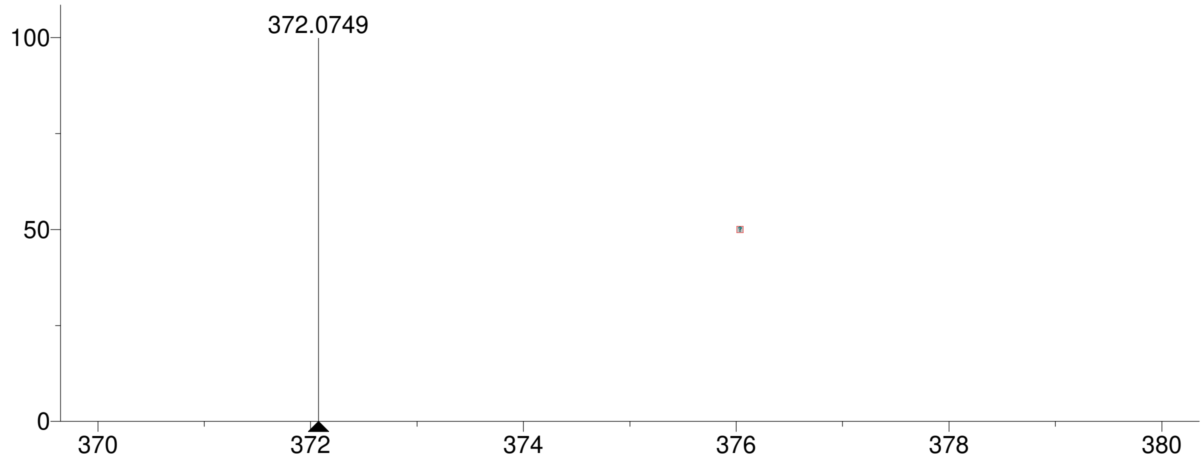

Unknown: Unknown (12.06_413.1911m/z)
Compound in Library Factor = 0

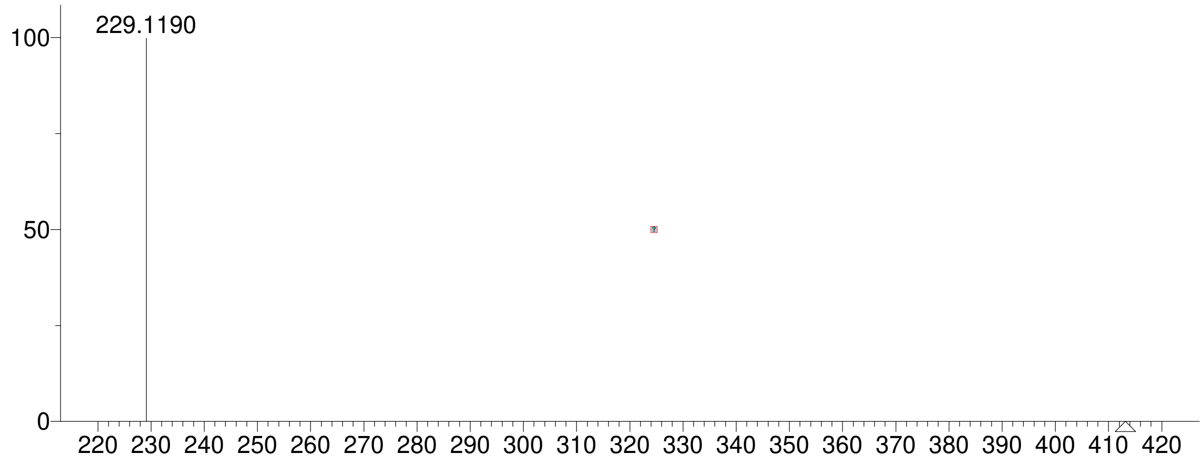

Hit 1 : 16-Phenoxytetranorprostaglandin F2α [M+Na]+ IT-FT 35% P=413.2
C22H30O6; MF: 0; RMF: 94; Prob 50.0%; CAS: 51705-19-2; Lib: nist_msms; ID: 173044.

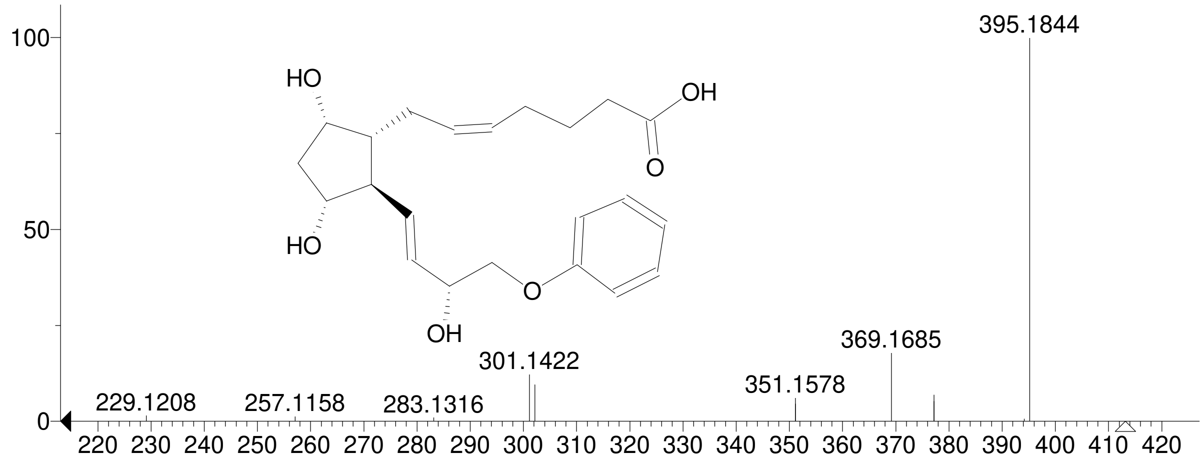

Hit 2 : (2R,3S,4S,5R,6R)-2-(hydroxymethyl)-6-[4-(4-hydroxy-2,6,6-trimethylcyclohexen-1-yl)butan-2-yloxy]oxane-3
C19H34O7; MF: 0; RMF: 27; Prob 50.0%; Lib: mona-export-vf-npl_qexactive; ID: 23533.

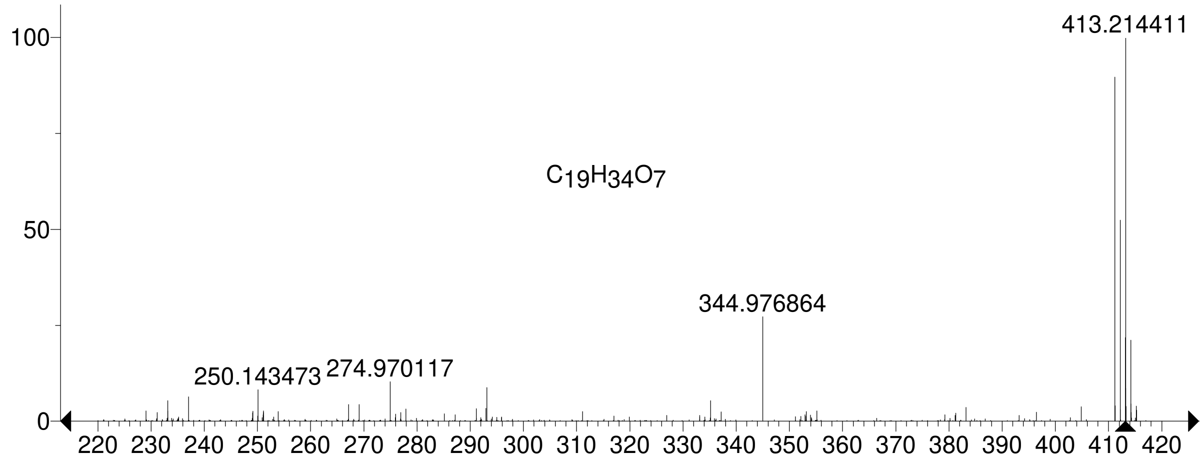

Unknown: Unknown (12.09_498.2428m/z)
Compound in Library Factor = 0

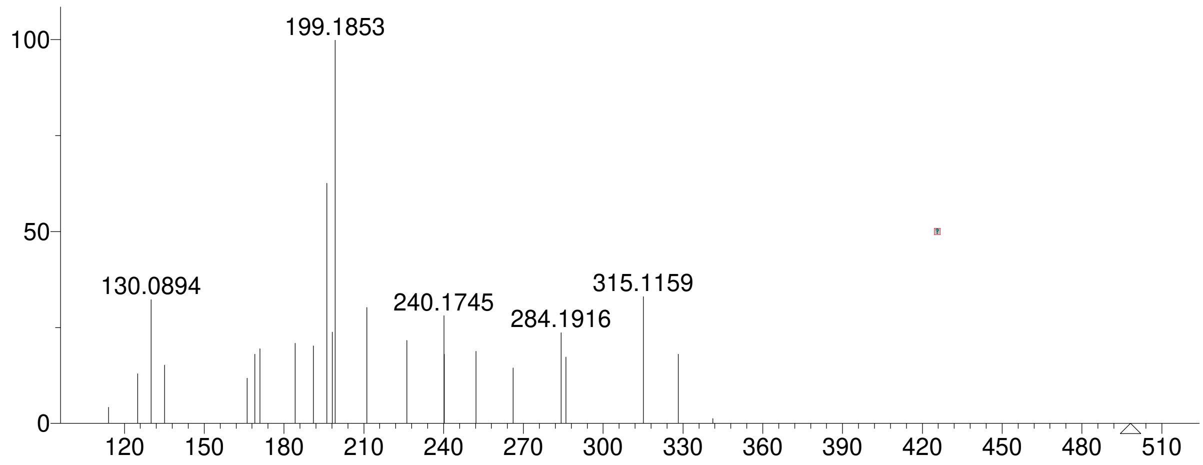

** Search Report Page 1 of 1 **

Unknown: Unknown (12.11_468.8093m/z)
Compound in Library Factor = 0

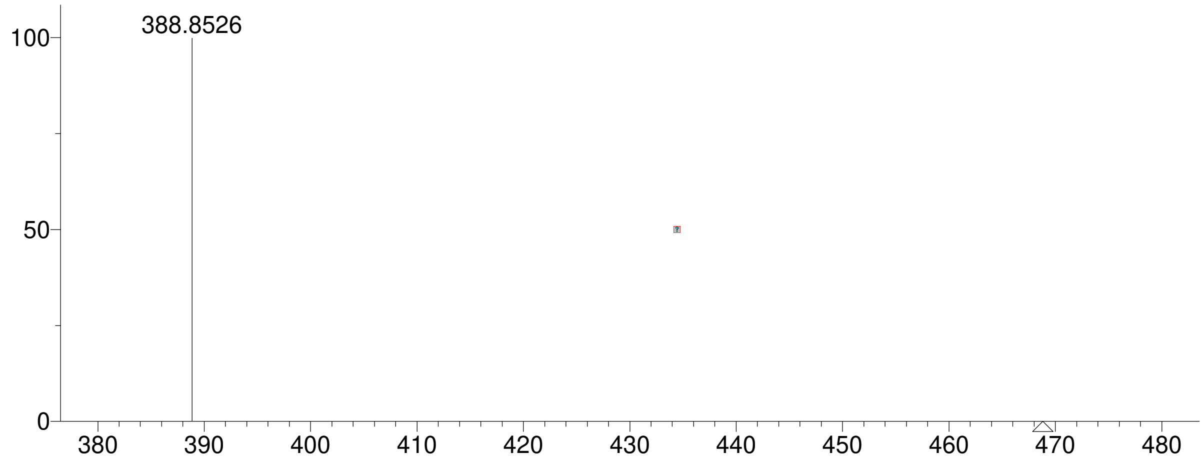

Unknown: Unknown (12.17_775.3385m/z)
Compound in Library Factor = 0

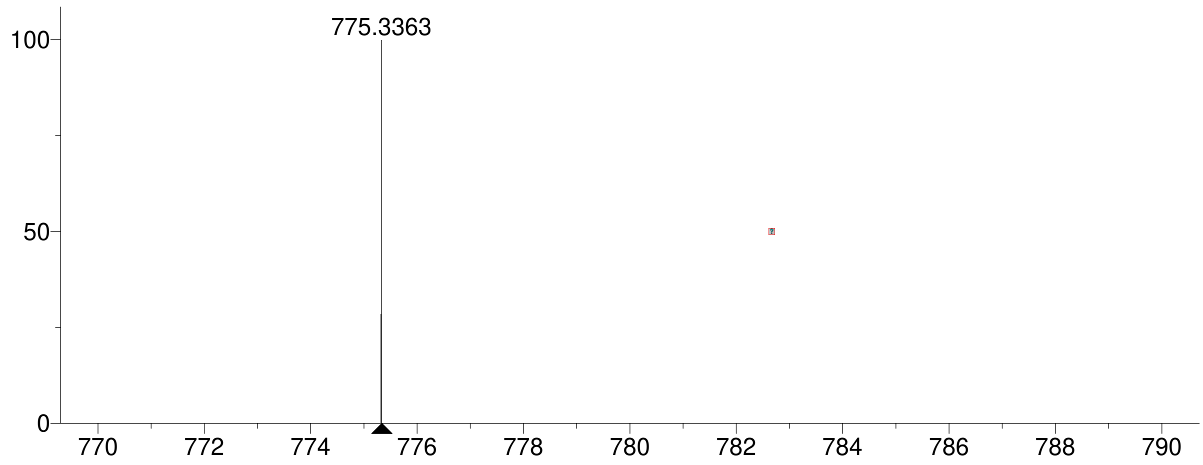

Unknown: Unknown (12.18_528.2548m/z)
Compound in Library Factor = 0

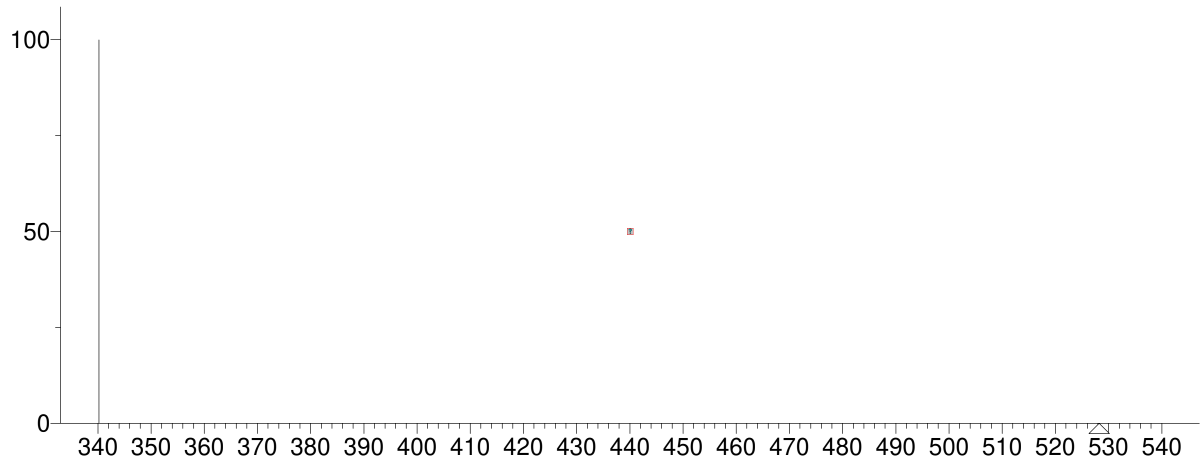

Unknown: Unknown (12.18_839.4066m/z)
Compound in Library Factor = -1090

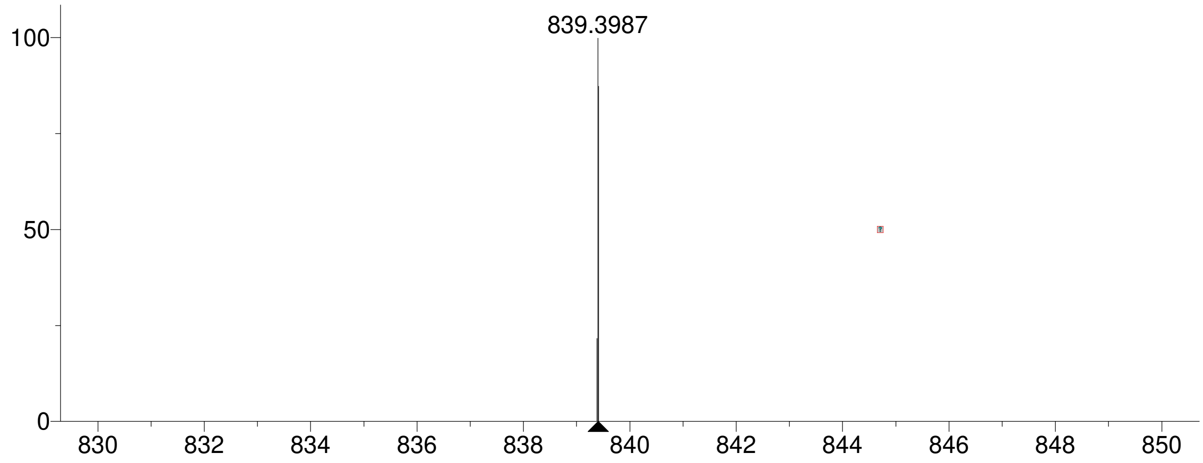

Hit 1 : Licoricesaponin G2 [M+H]+ 10eV P=839.4
C42H62O17; MF: 226; RMF: 691; Prob 32.6%; Lib: anthocyanins mona; ID: 3887.

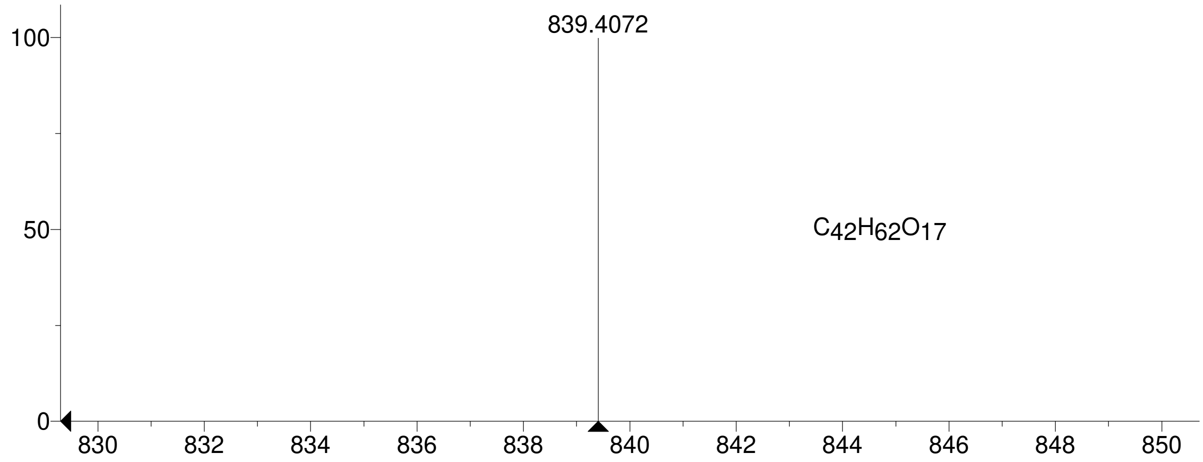

Hit 2 : Unknown (carbon number 42); PlaSMA ID-2098 [M-H]- P=839.4
C42H64O17; MF: 226; RMF: 691; Prob 32.6%; Lib: biomsms-neg-plasma; ID: 1648.

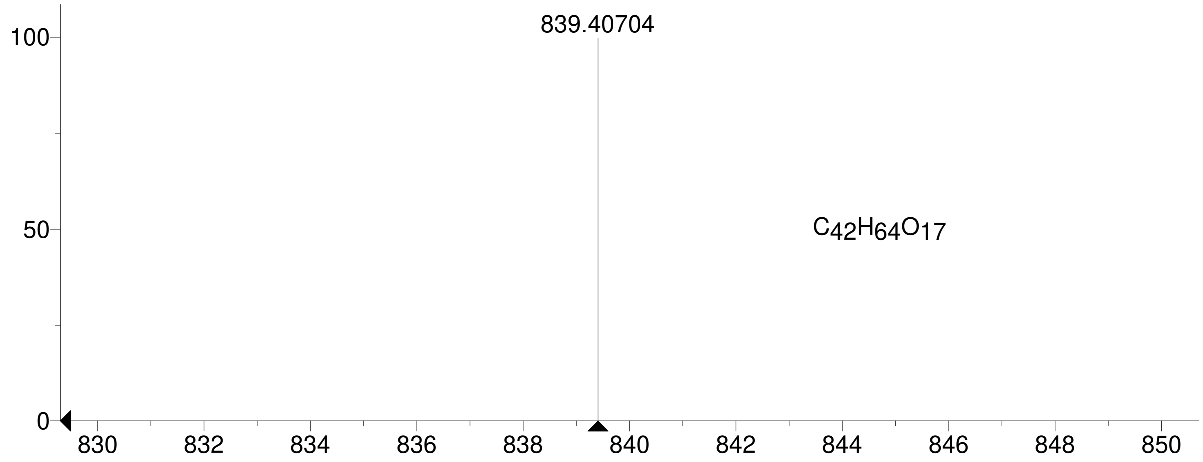

Hit 3 : DGDG 26:7 [M+FA-H]- 40eV P=839.4
C41H62O15; MF: 226; RMF: 691; Prob 32.6%; Lib: mona-export-in-silico_spectra; ID: 9785.

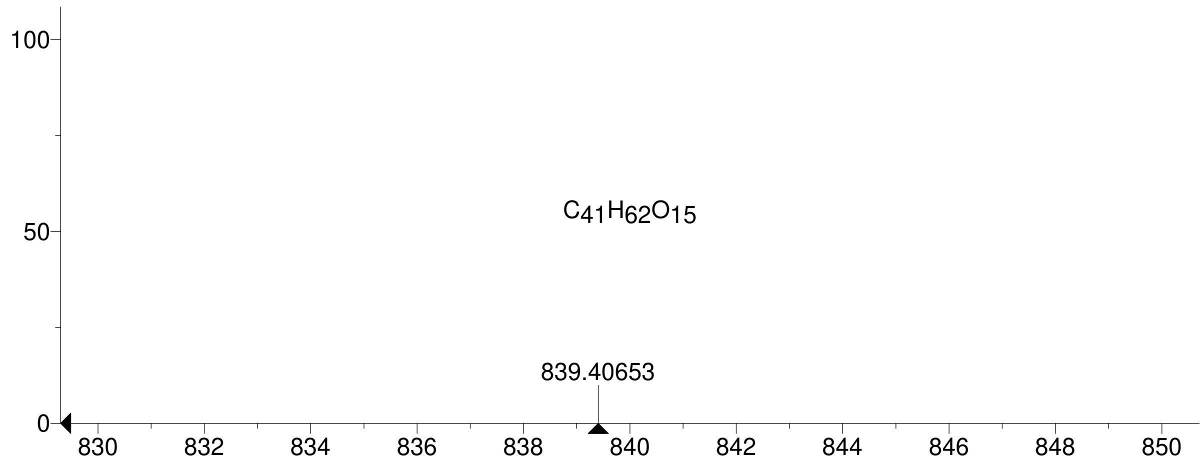

Hit 4 : NCGC00381024-01_C42H64O17_beta-D-Glucopyranose, 1-O-[(2E)-6-[[3,4-bis-O-[(2E)-6-hydroxy-2,6-dim
C42H64O17; MF: 111; RMF: 628; Prob 1.95%; Lib: msms-neg-gnps; ID: 1748.

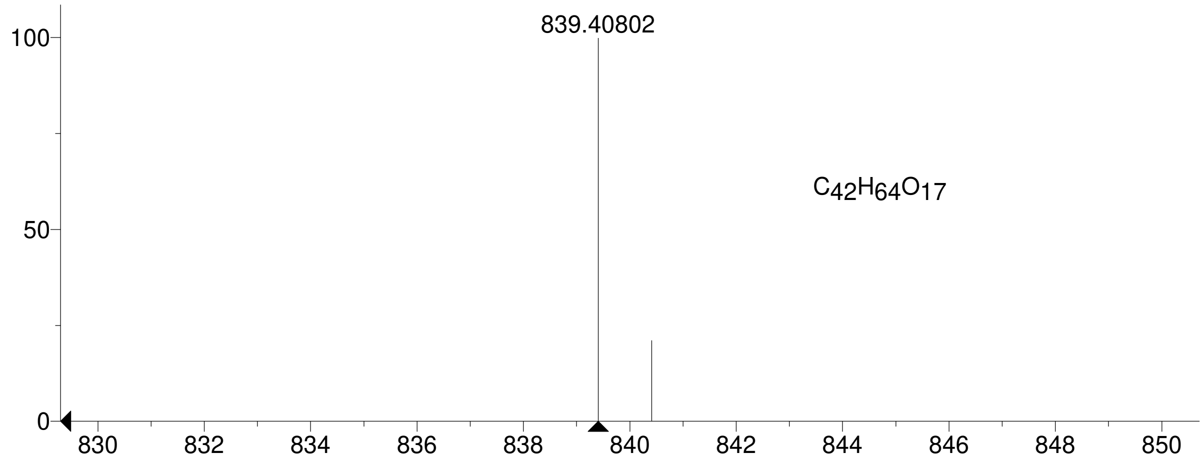

Hit 5 : 9,13-Dimethyl-17-oxo-14-(5-oxo-2,5-dihydro-3-furanyl)tetracyclo[11.3.1.01,10.04,9]heptadec-6-yl I^'2-D-glu
C42H64O17; MF: 36; RMF: 545; Prob 0.25%; Lib: mona-export-lc-ms-ms_negative_mode; ID: 21212.

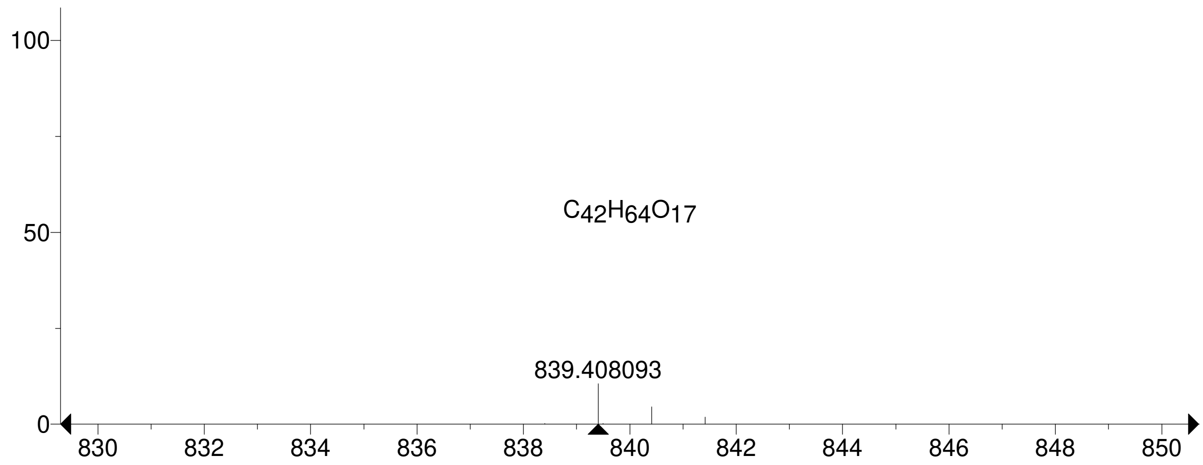

Unknown: Unknown (12.21_555.2308m/z)
Compound in Library Factor = 0

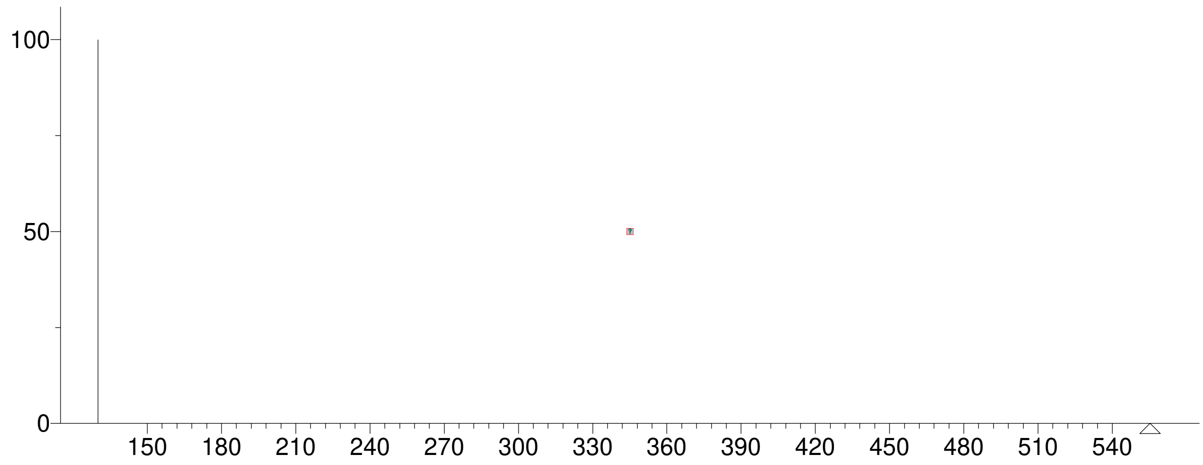

Unknown: Unknown (12.23_620.1085m/z)
Compound in Library Factor = 0

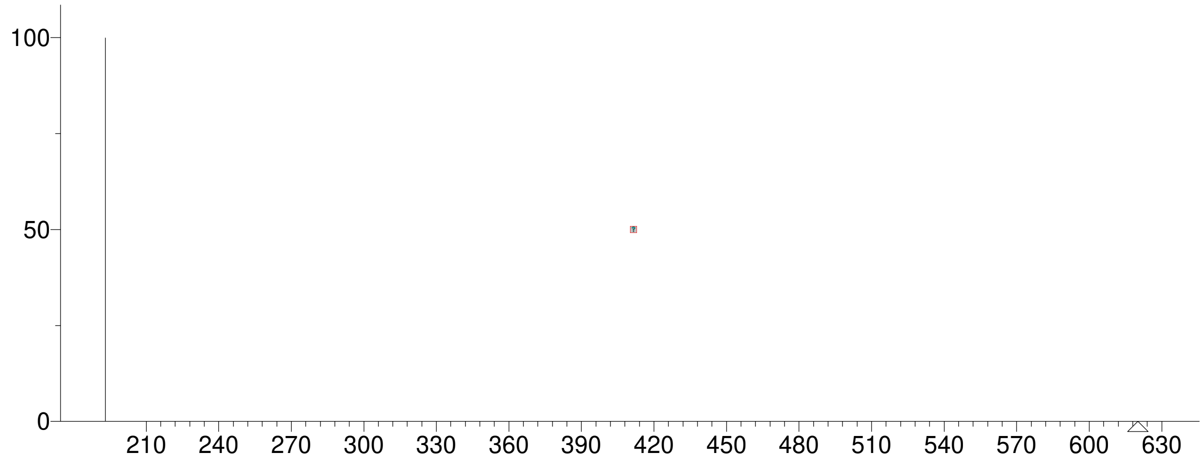

** Search Report Page 1 of 1 **

Unknown: Unknown (12.24_319.9115m/z)
Compound in Library Factor = 0

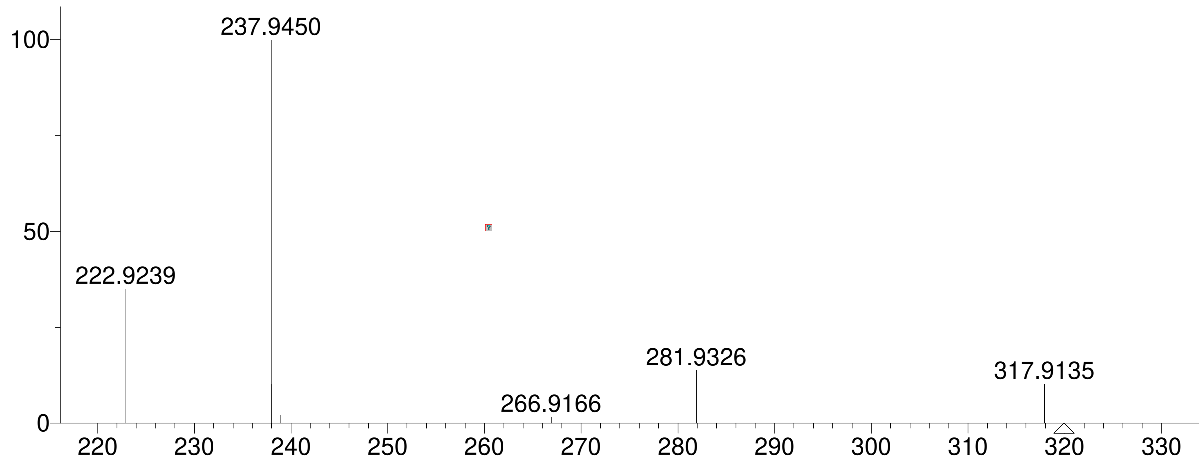

** Search Report Page 1 of 1 **

Unknown: Unknown (12.24_560.2121m/z)
Compound in Library Factor = -2860

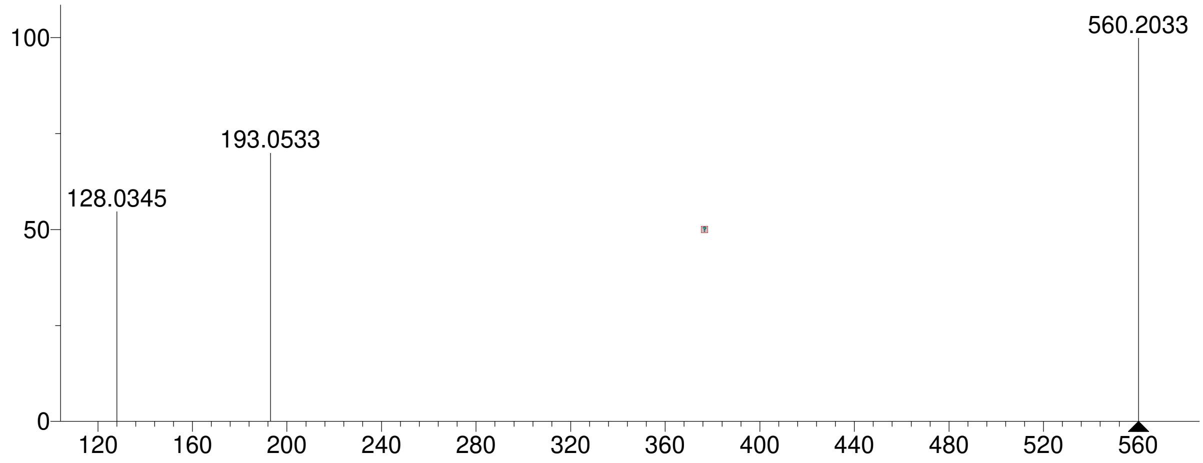

Hit 1 : 2,3,9,10,11,12-Hexahydro-10S-hydroxy-9-methyl-1-oxo-9R,12S-epoxy-1H-diindolo[1,2,3-fg:3',2',1'-kl]pyrrol
C32H31N3O5; MF: 286; RMF: 922; Prob 100.0%; CAS: 108068-98-0; Lib: nist_msms; ID: 86206.

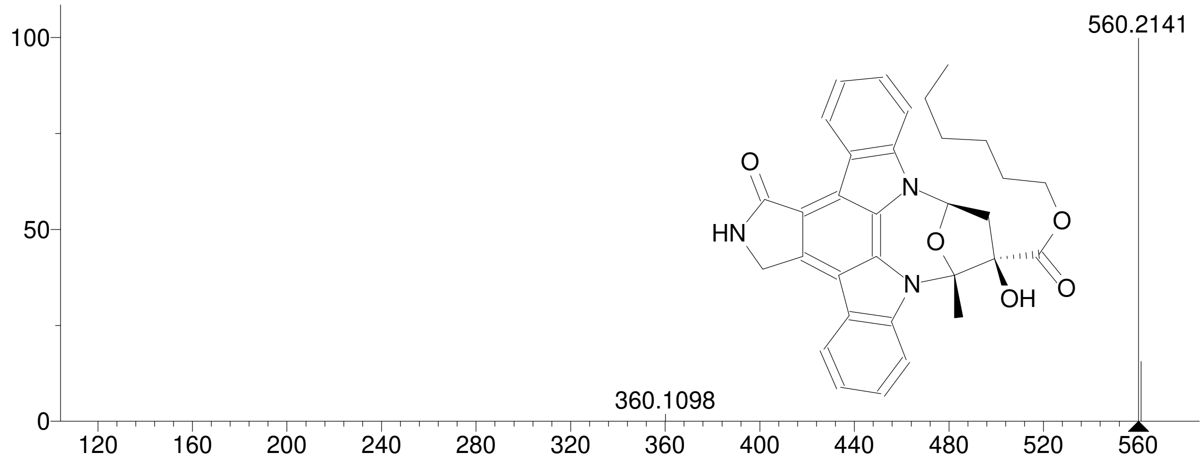

Unknown: Unknown (12.27_149.0598m/z)
Compound in Library Factor = -588

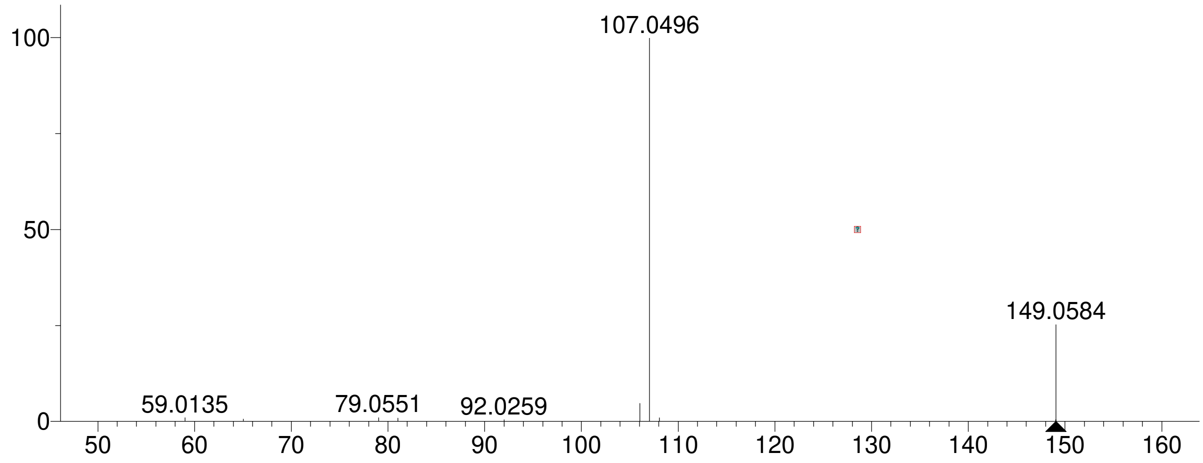

Hit 1 : 4-Chromanone [M+H]+ HCD 13V P=149.1
C9H8O2; MF: 450; RMF: 811; Prob 41.8%; CAS: 491-37-2; Lib: nist_msms; ID: 178506.

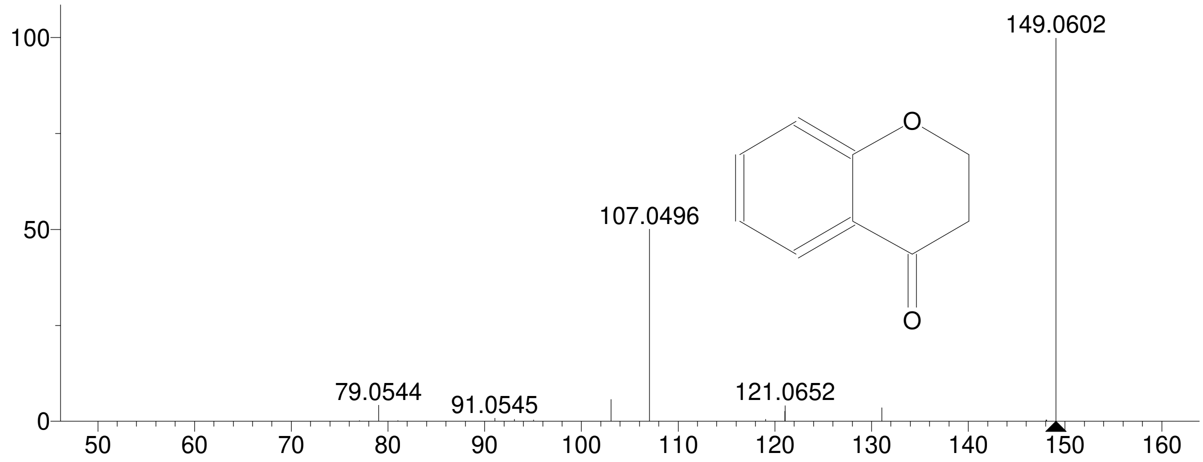

Hit 2 : 3,4-Dihydrocoumarin [M+H]+ HCD 11V P=149.1
C9H8O2; MF: 440; RMF: 794; Prob 29.5%; CAS: 119-84-6; Lib: nist_msms; ID: 111744.

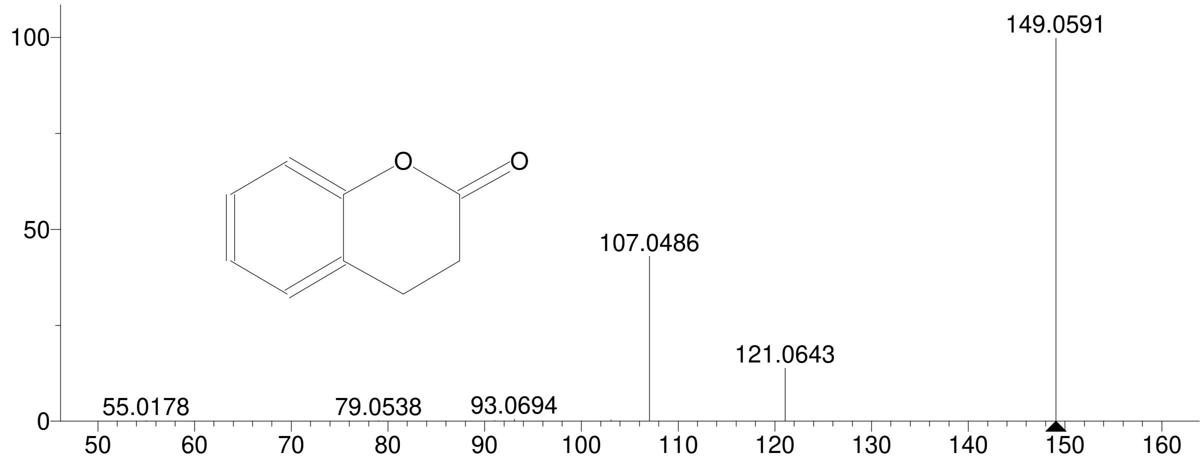

Hit 3 : 3-(2-Hydroxyphenyl)propionic acid [M+H-H2O]+ HCD 11V P=149.1
C9H10O3; MF: 430; RMF: 787; Prob 20.9%; CAS: 495-78-3; Lib: nist_msms; ID: 488257.

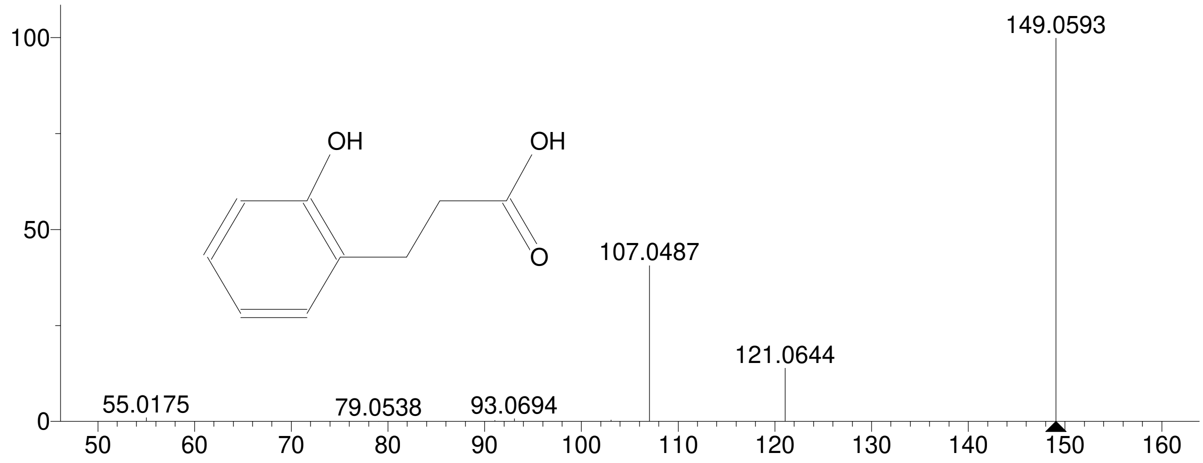

Hit 4 : 3-Phenoxypropionic acid [M+H-H2O]+ HCD 11V P=149.1
C9H10O3; MF: 405; RMF: 772; Prob 6.36%; CAS: 7170-38-9; Lib: nist_msms; ID: 149769.

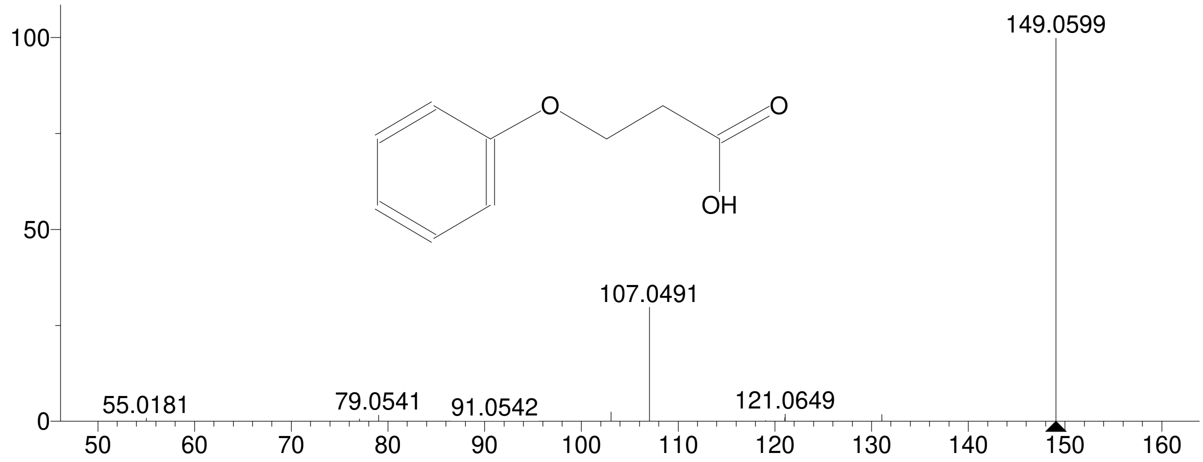

Hit 5 : Hydrocinnamic acid [M-H]- 35eV P=149.1
C9H10O2; MF: 311; RMF: 887; Prob 0.52%; Lib: mona-export-fiehn_hilic; ID: 2754.

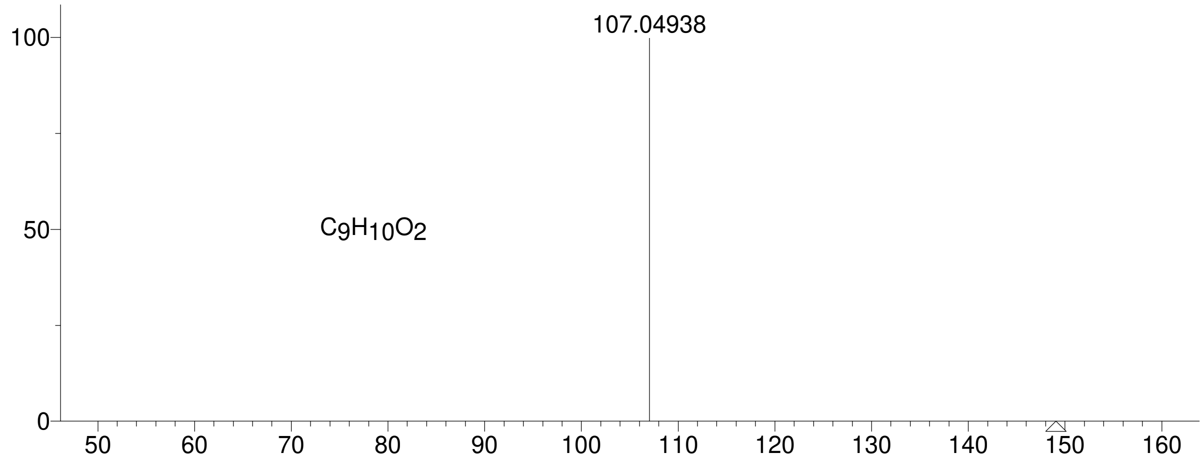

Unknown: Unknown (12.27_317.9143m/z)
Compound in Library Factor = 0

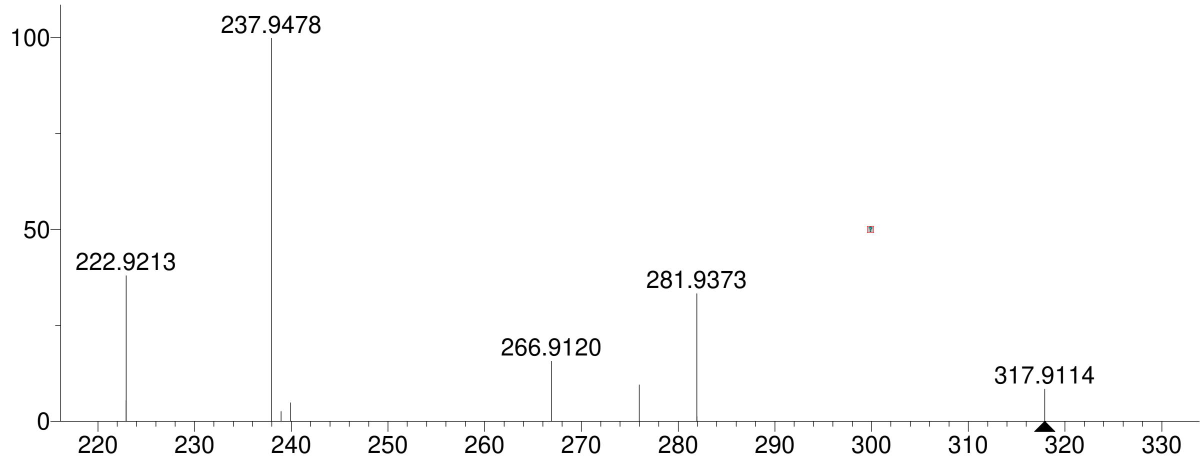

Unknown: Unknown (12.27_440.0190m/z)
Compound in Library Factor = -3273

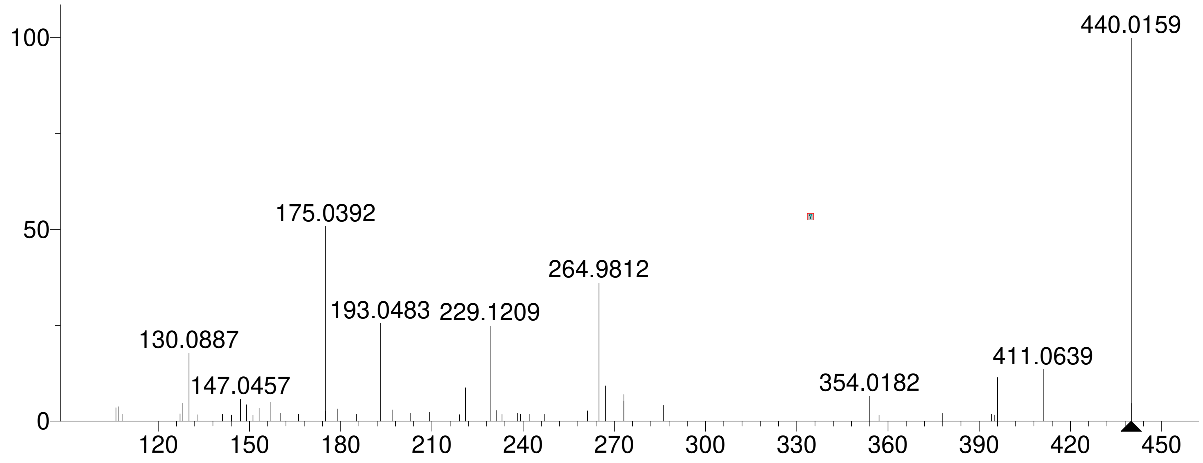

Hit 1 : Triasulfuron [M+K]+ HCD 4V P=440
C14H16ClN5O5S; MF: 77; RMF: 562; Prob 100.0%; CAS: 82097-50-5; Lib: nist_msms; ID: 541983.

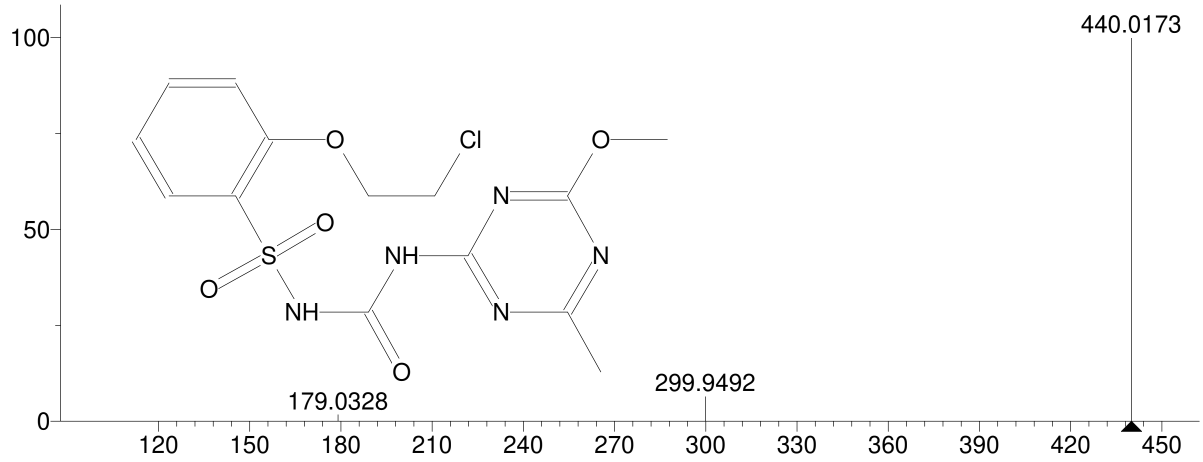

Unknown: Unknown (12.27_452.0187m/z)
Compound in Library Factor = 0

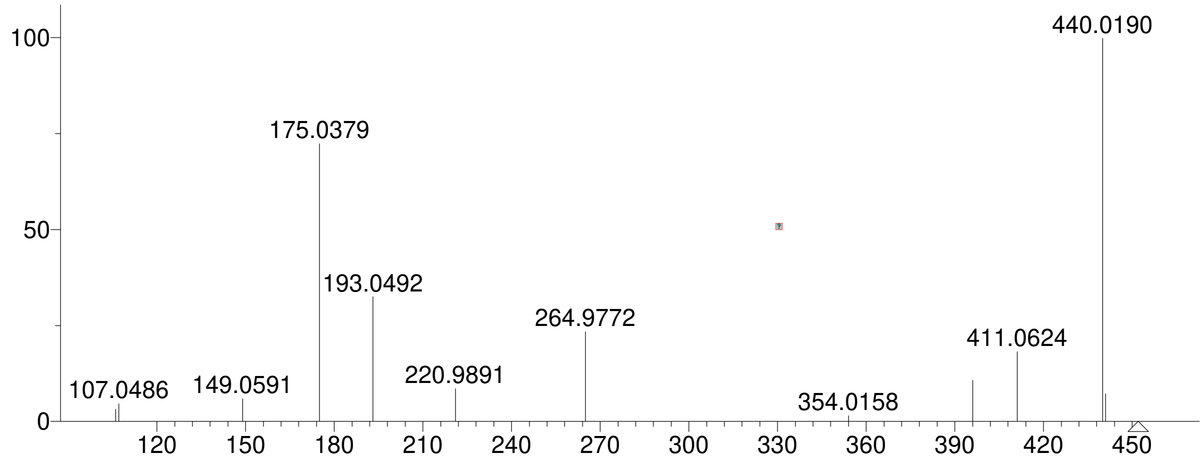

Unknown: Unknown (12.28_193.0500m/z)
Compound in Library Factor = -741

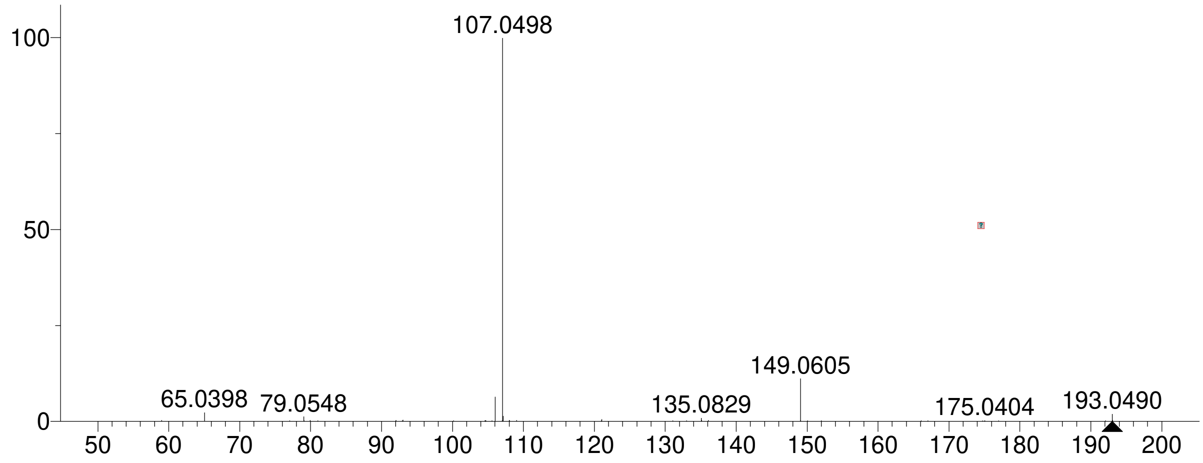

Hit 1 : Methyldopa [M-H-NH3]- HCD 9V P=193.1
C10H13NO4; MF: 338; RMF: 722; Prob 87.1%; CAS: 555-30-6; Lib: nist_msms; ID: 96911.

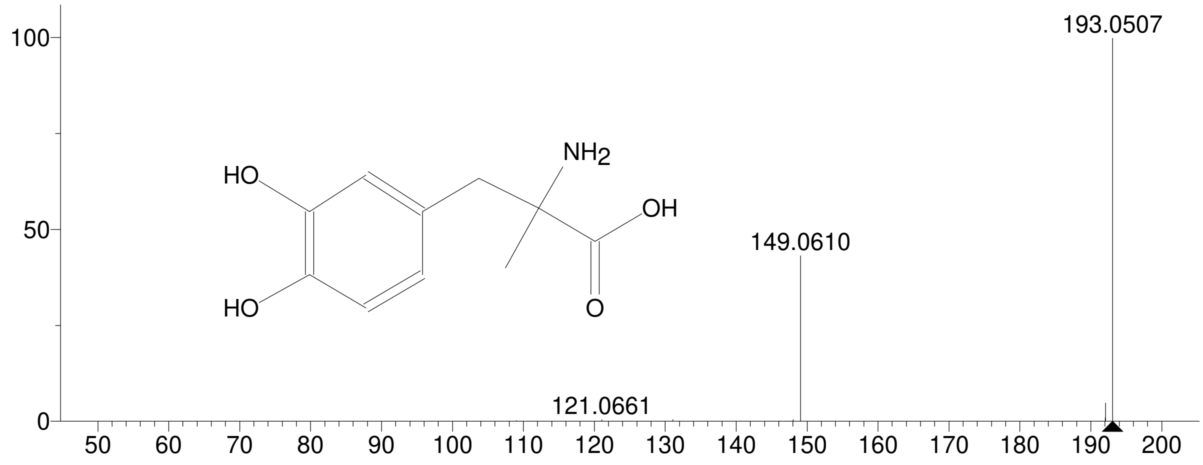

Hit 2 : Prohexadione [M-H-H2O]- HCD 17V P=193.1
C10H12O5; MF: 256; RMF: 438; Prob 8.88%; CAS: 88805-35-0; Lib: nist_msms; ID: 489543.

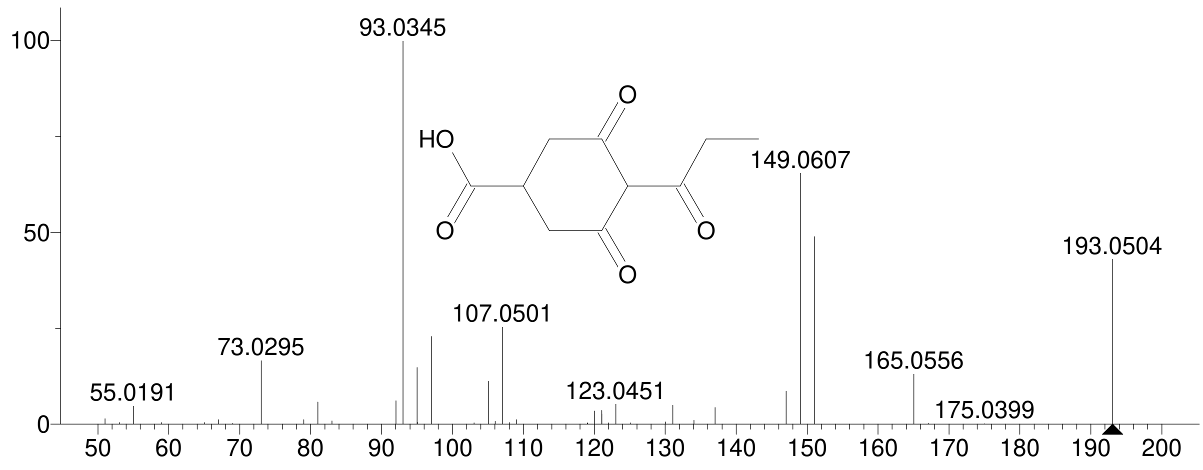

Hit 3 : trans-Ferulic acid [M-H]- HCD 8V P=193.1
C10H10O4; MF: 223; RMF: 565; Prob 2.32%; CAS: 537-98-4; Lib: nist_msms; ID: 300552.

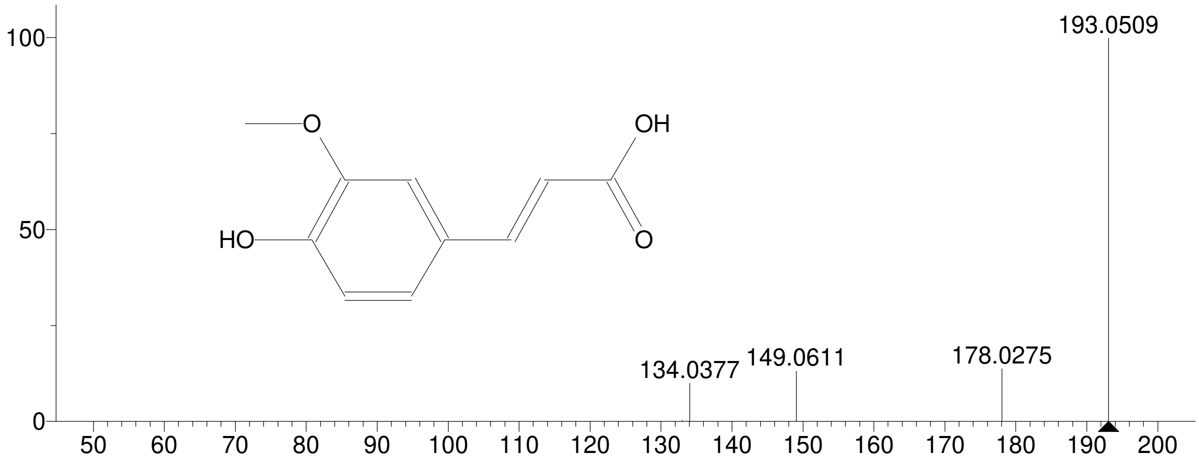

Hit 4 : Carbidopa [M-H-H4N2]- HCD 19V P=193.1
C10H14N2O4; MF: 189; RMF: 356; Prob 0.59%; CAS: 28860-95-9; Lib: nist_msms; ID: 313803.

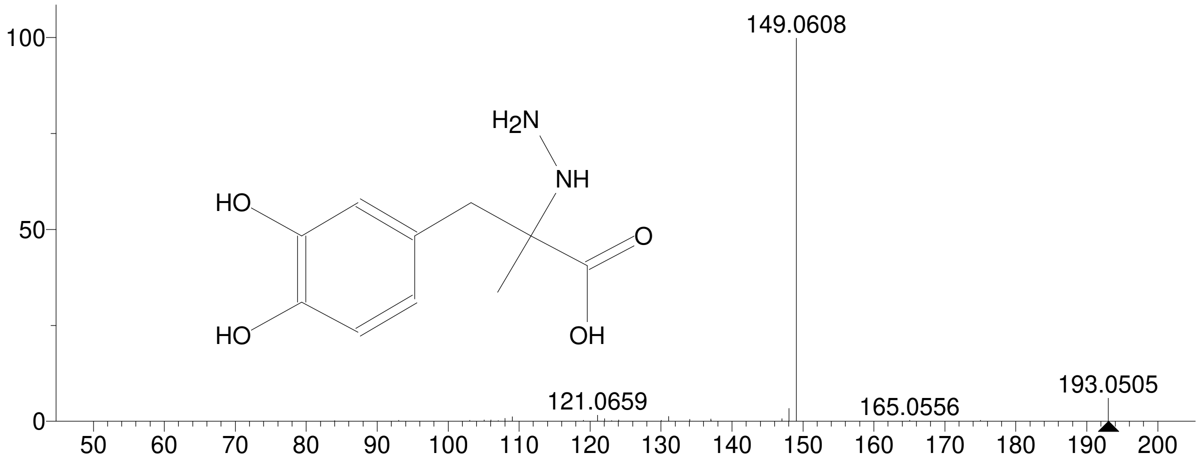

Hit 5 : Monoethyl phthalate [M-H]- 35(no P=193.1
C10H10O4; MF: 169; RMF: 643; Prob 0.27%; Lib: mona-export-lc-ms-ms_negative_mode; ID: 13731.

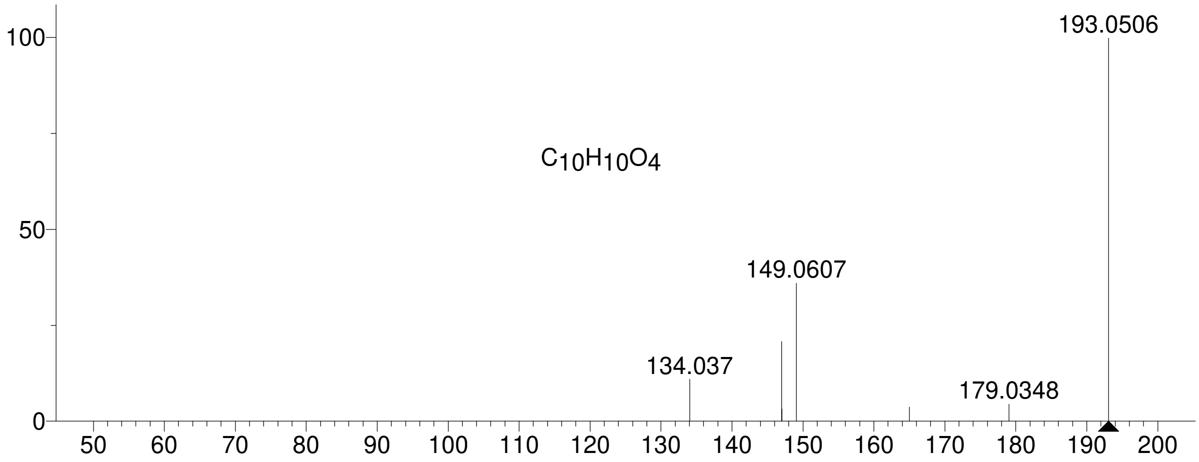

Unknown: Unknown (12.28_292.9740m/z)
Compound in Library Factor = 0

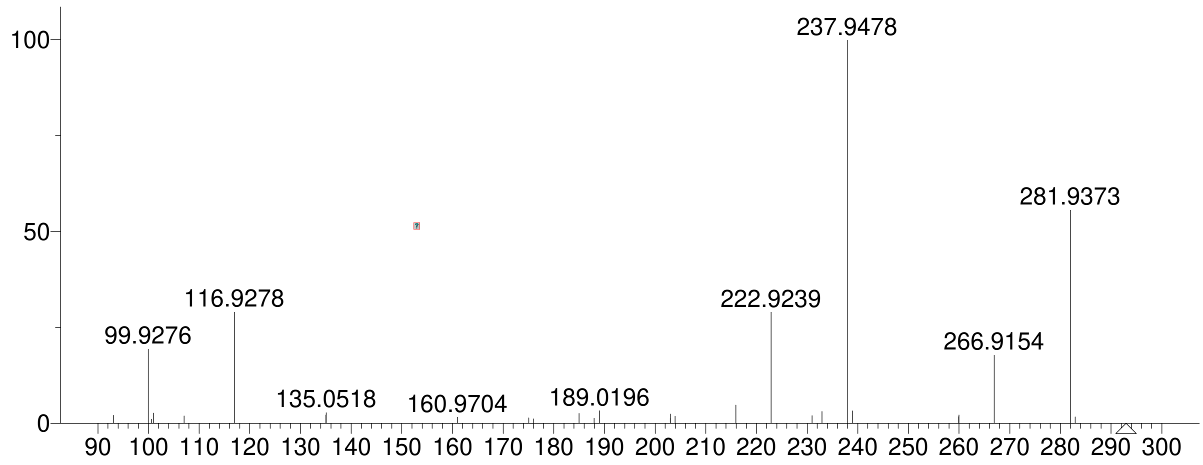

Hit 1 : Prepro-ANF (104-116), human [M+5H]5+ QQQ 11V P=293
C61H113N21O20; MF: 0; RMF: 1; Prob 100.0%; Lib: nist_msms2; ID: 18925.

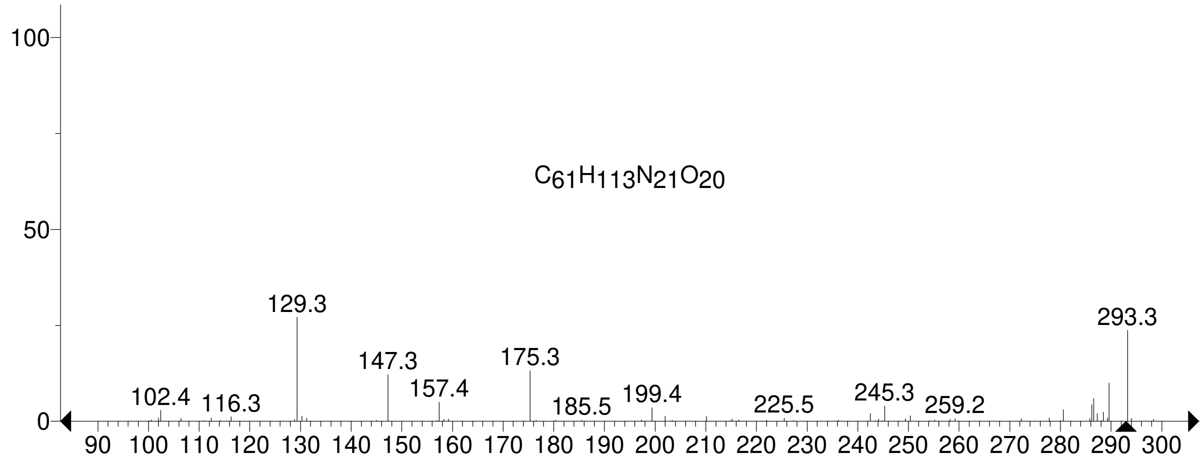

Unknown: Unknown (12.28_411.0648m/z)
Compound in Library Factor = -1848

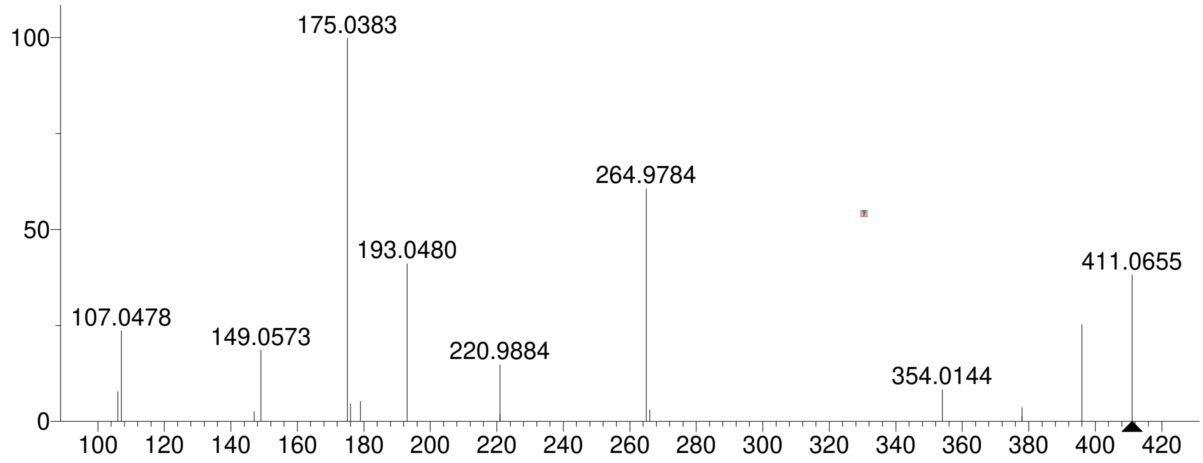

Hit 1 : Metrafenone [M+H+2i]+ HCD 4V P=411.1
C19H21BrO5; MF: 39; RMF: 430; Prob 80.9%; CAS: 220899-03-6; Lib: nist_msms; ID: 511890.

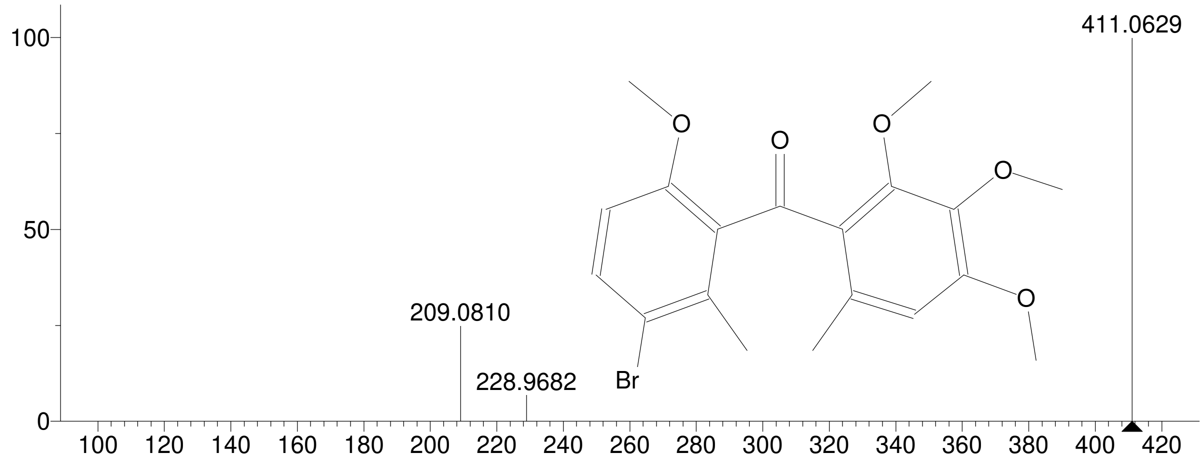

Hit 2 : (2S,3S,4S,5R,6R)-6-(3-benzoyloxy-2-hydroxypropoxy)-3,4,5-trihydroxyoxane-2-carboxylic acid [M+K]+ 65
C16H20O10; MF: 0; RMF: 14; Prob 19.1%; Lib: mona-export-vf-npl_qexactive; ID: 19893.

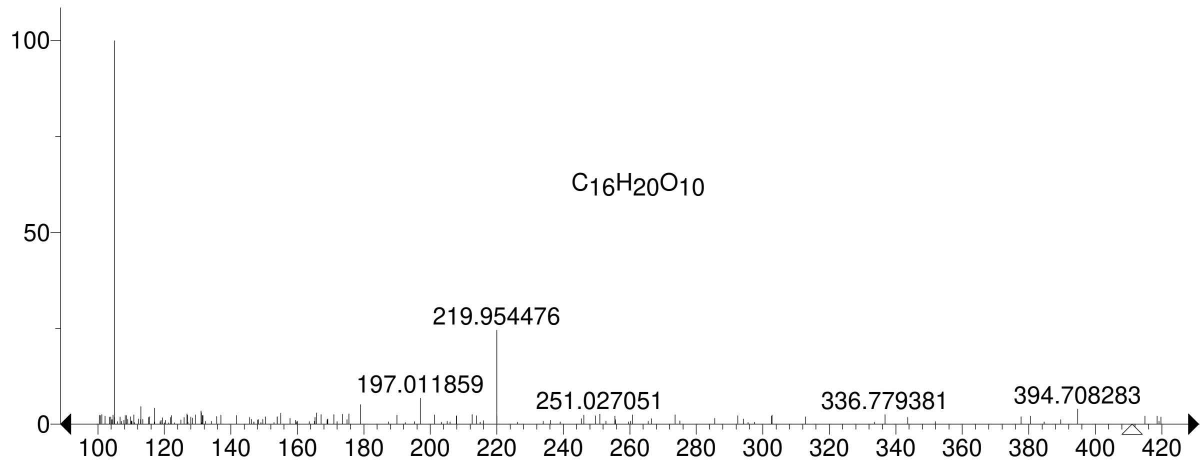

** Search Report Page 1 of 1 **

Unknown: Unknown (12.28_475.9953m/z)
Compound in Library Factor = 0

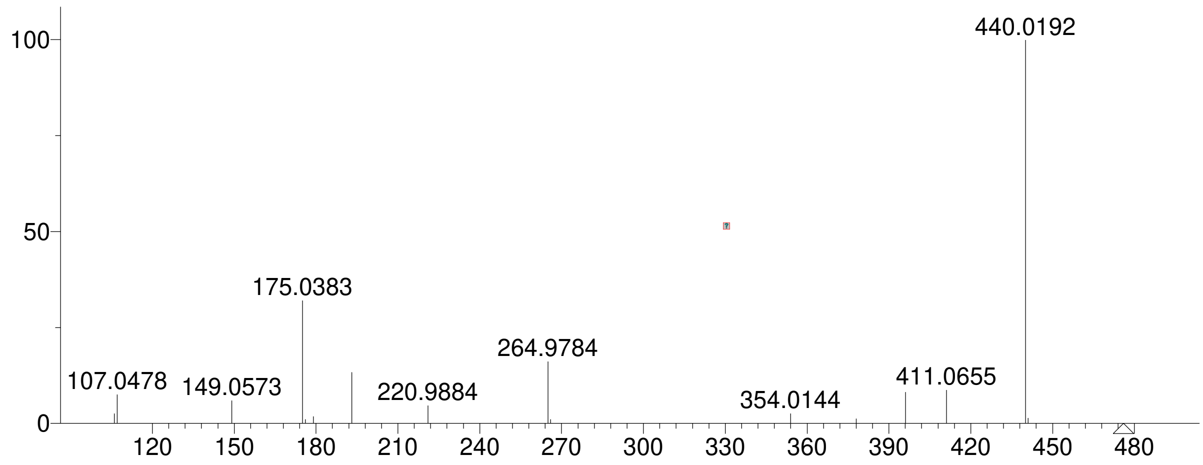

Unknown: Unknown (12.29_275.9707m/z)
Compound in Library Factor = 0

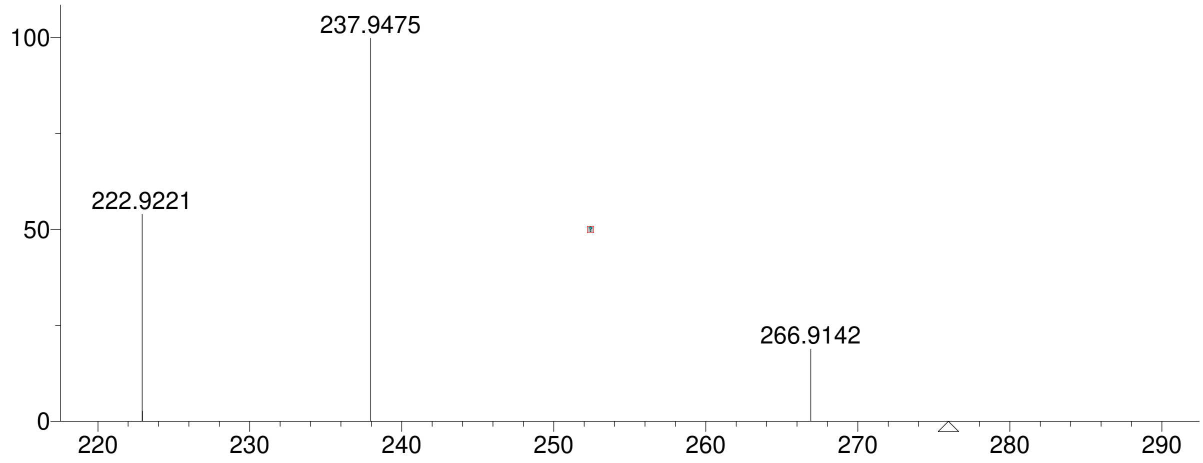

** Search Report Page 1 of 1 **

Unknown: Unknown (12.29_409.0890m/z)
Compound in Library Factor = 0

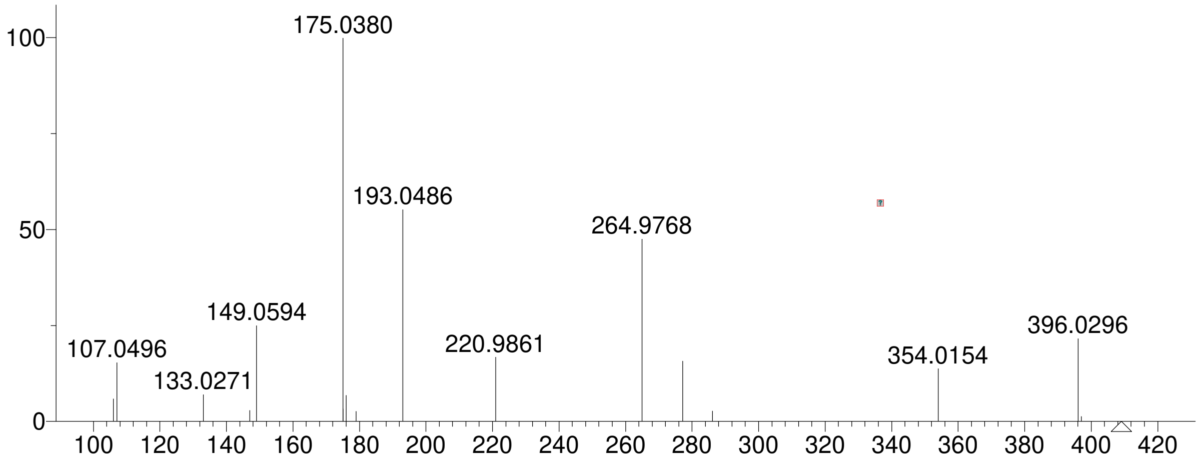

Unknown: Unknown (12.29_637.0876m/z)
Compound in Library Factor = 0

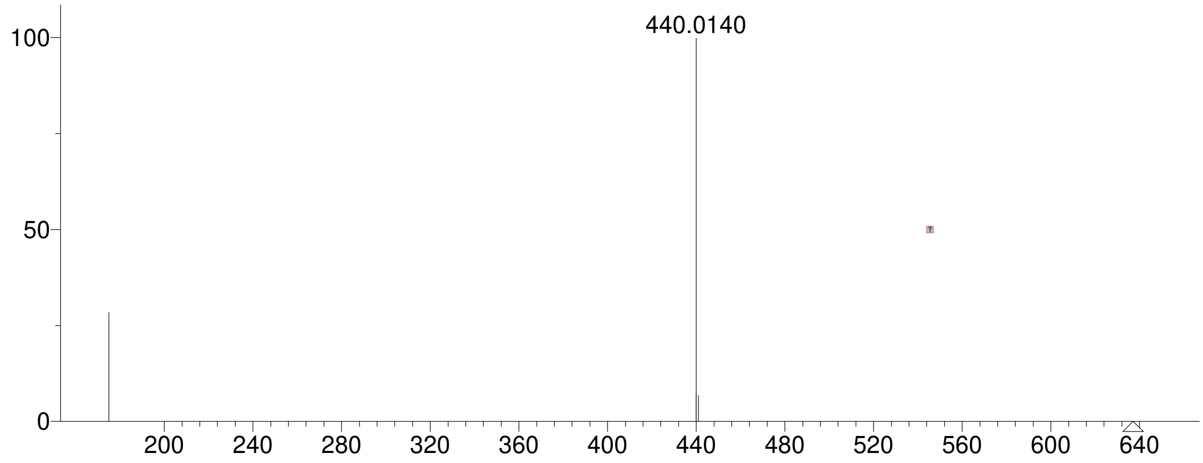

Unknown: Unknown (12.30_355.0117m/z)
Compound in Library Factor = 0

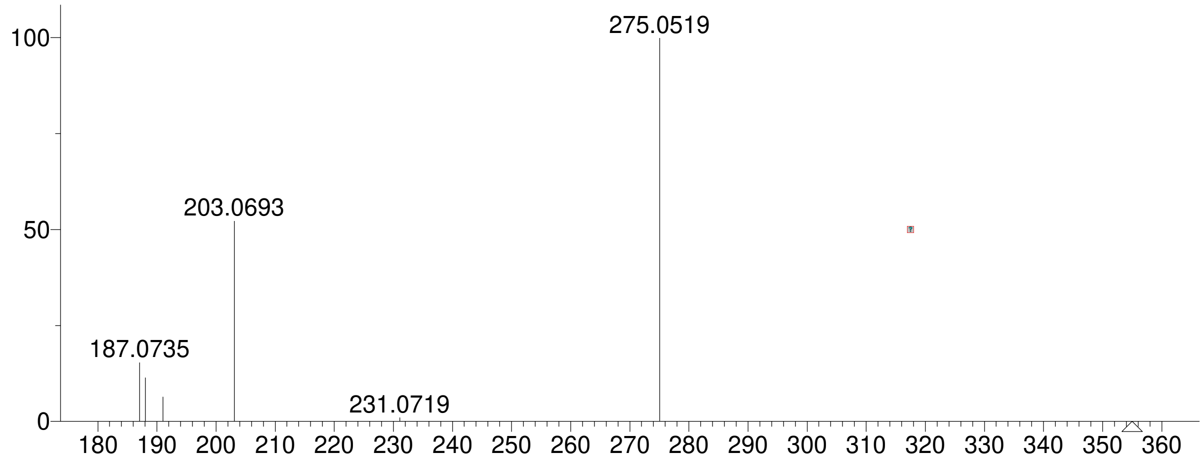

Hit 1 : 3-(3,3-dichloro-2-hydroxypropyl)-6,8-dimethoxyisochromen-1-one [M+Na]+ 35HCD P=355
C14H14Cl2O5; MF: 0; RMF: 19; Prob 100.0%; Lib: mona-export-vf-npl_qexactive; ID: 18490.

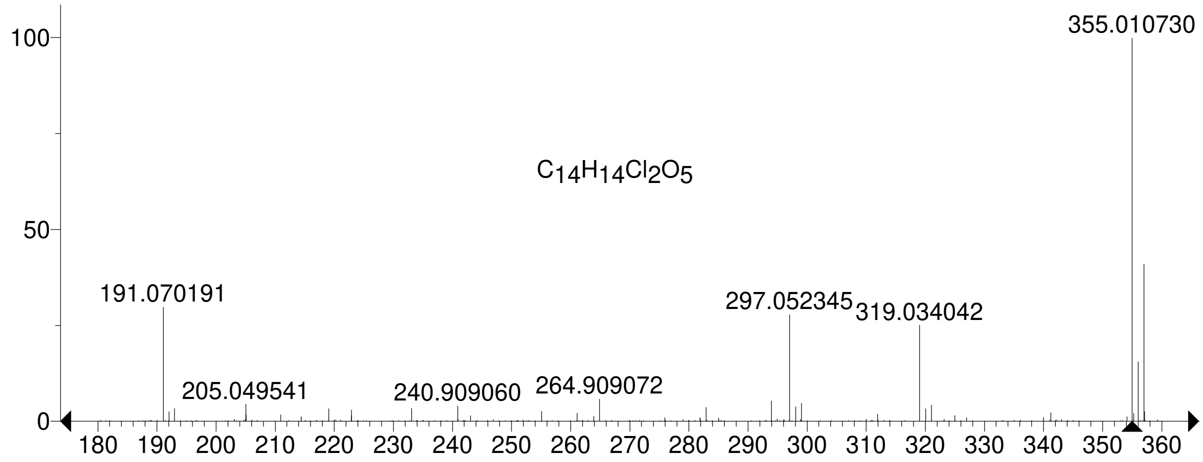

Unknown: Unknown (12.30_447.2123m/z)
Compound in Library Factor = 0

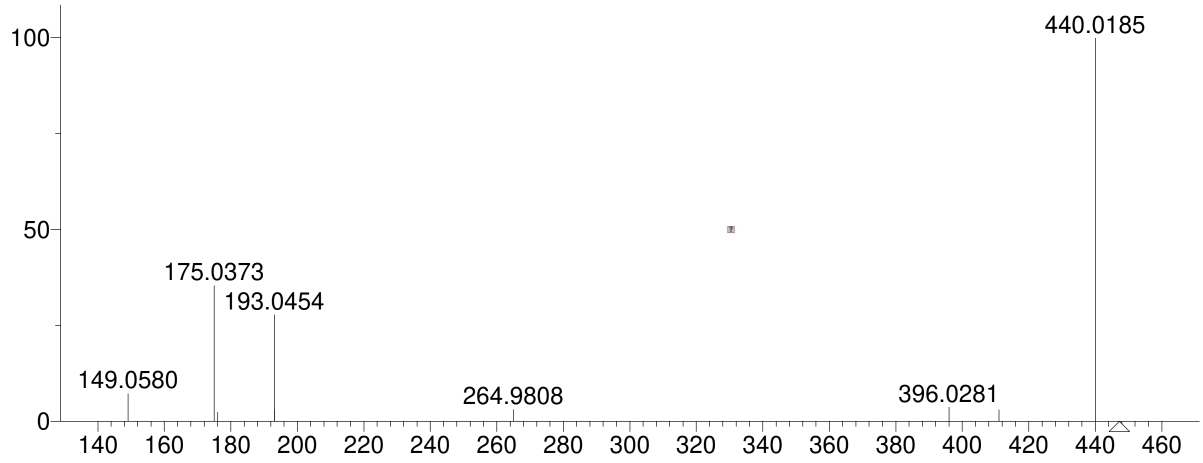

Unknown: Unknown (12.30_500.2232m/z)
Compound in Library Factor = 0

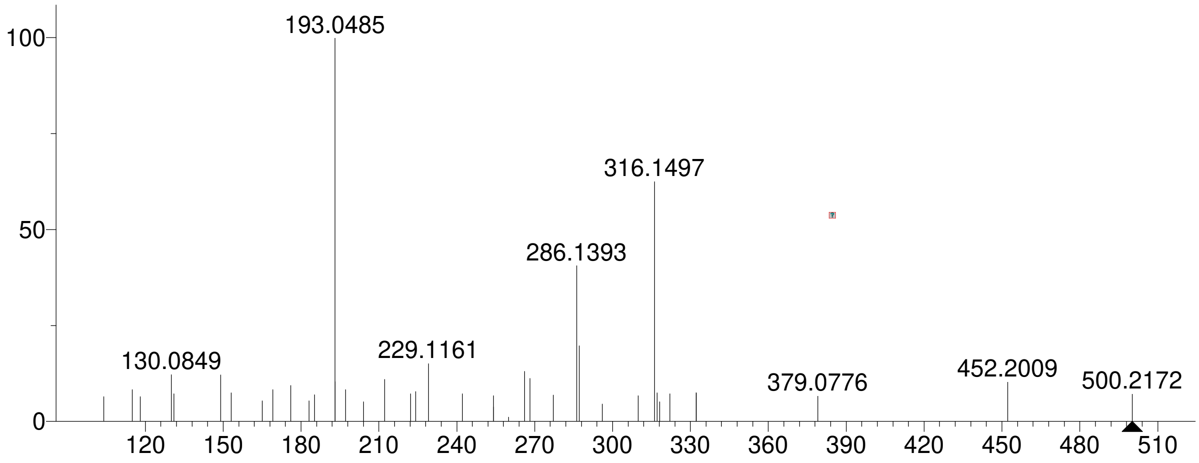

** Search Report Page 1 of 1 **

Unknown: Unknown (12.32_337.9714m/z)
Compound in Library Factor = 0

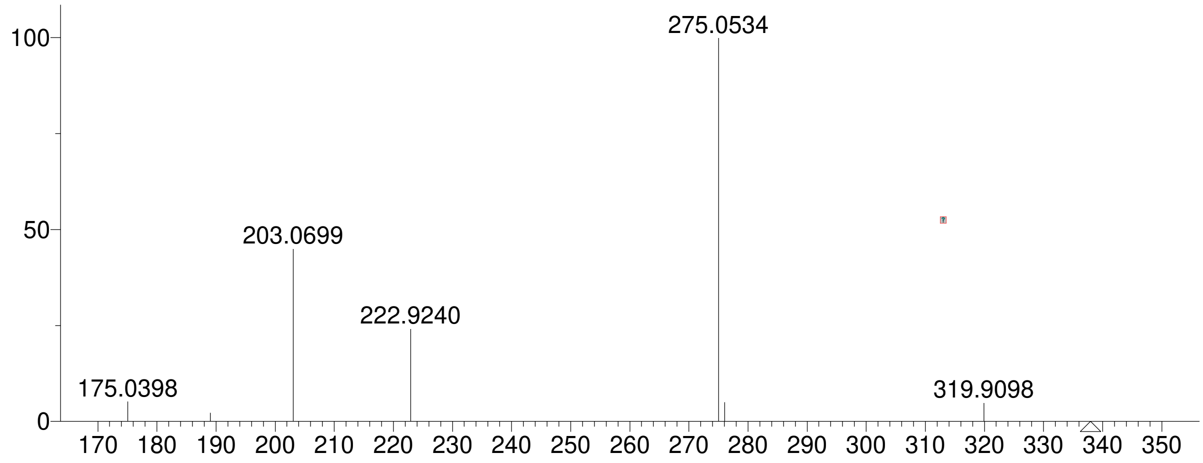

Unknown: Unknown (12.36_387.1531m/z)
Compound in Library Factor = 0

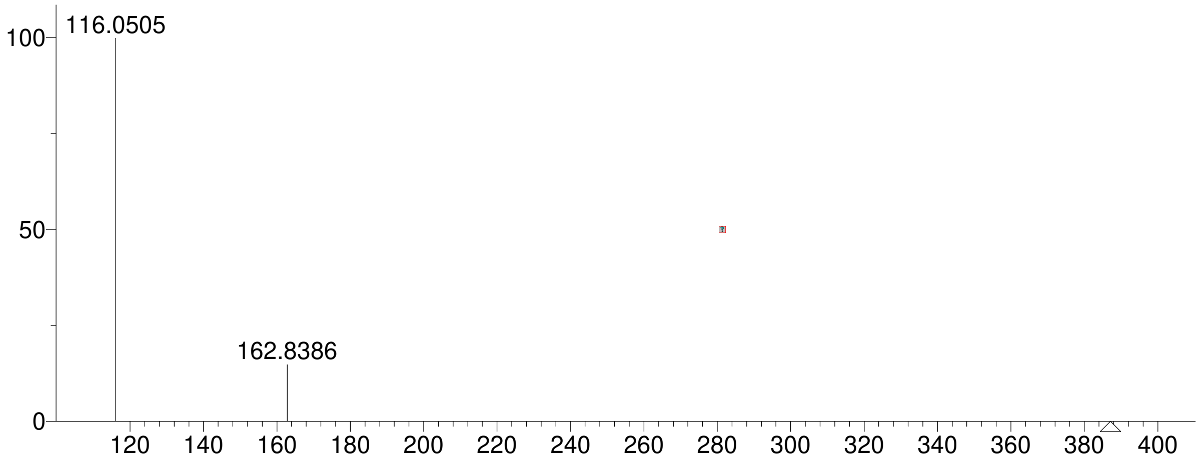

** Search Report Page 1 of 2 **

Unknown: Unknown (12.40_392.1816m/z)
Compound in Library Factor = -376

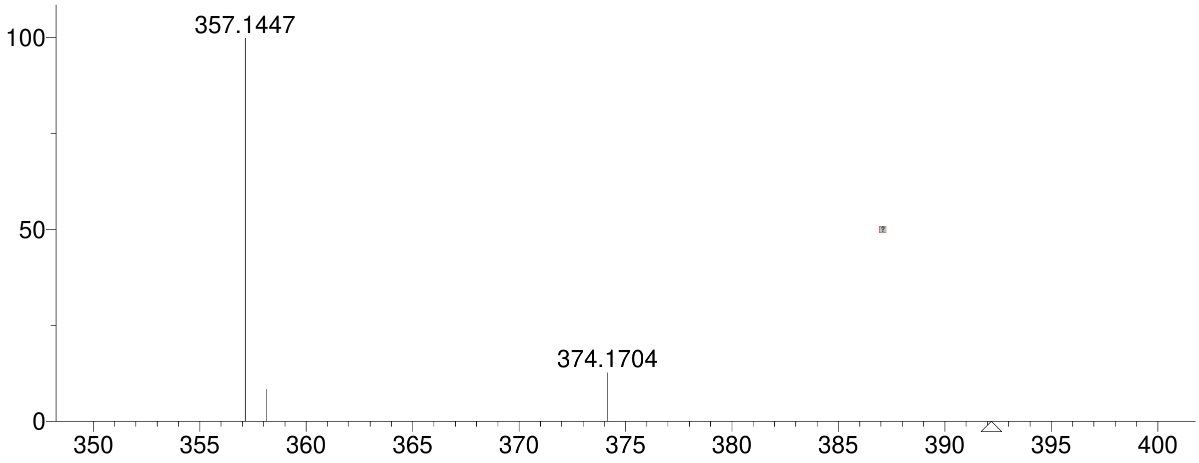

Hit 1 : Glu-Val-Phe [M-H]- IT-FT 35% P=392.2
C19H27N3O6; MF: 196; RMF: 402; Prob 96.7%; CAS: 31461-61-7; Lib: nist_msms; ID: 568009.

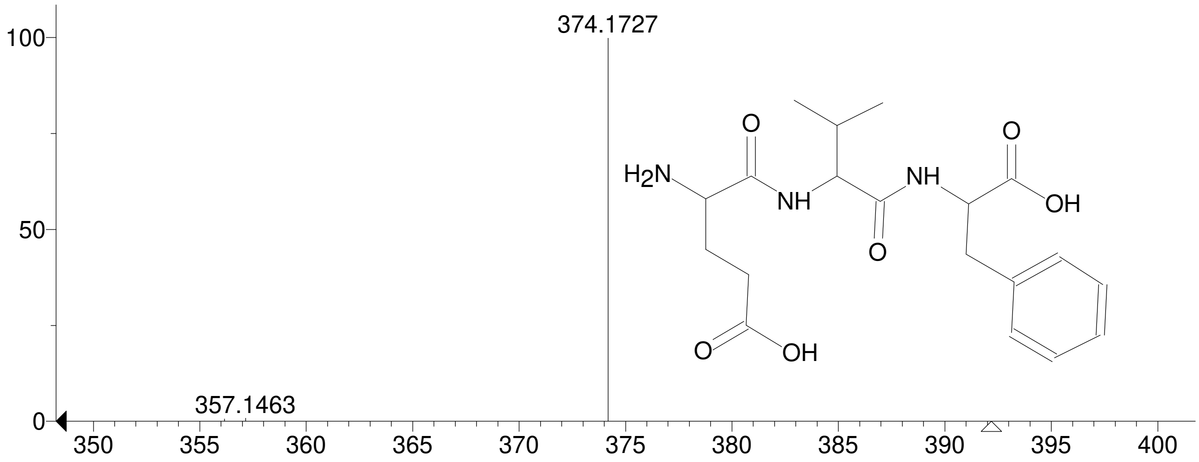

Hit 2 : lysoPE 10:0 [M+Na]+ 25eV P=392.2
C15H32NO7P; MF: 14; RMF: 229; Prob 1.66%; Lib: mona-export-in-silico_spectra; ID: 35899.

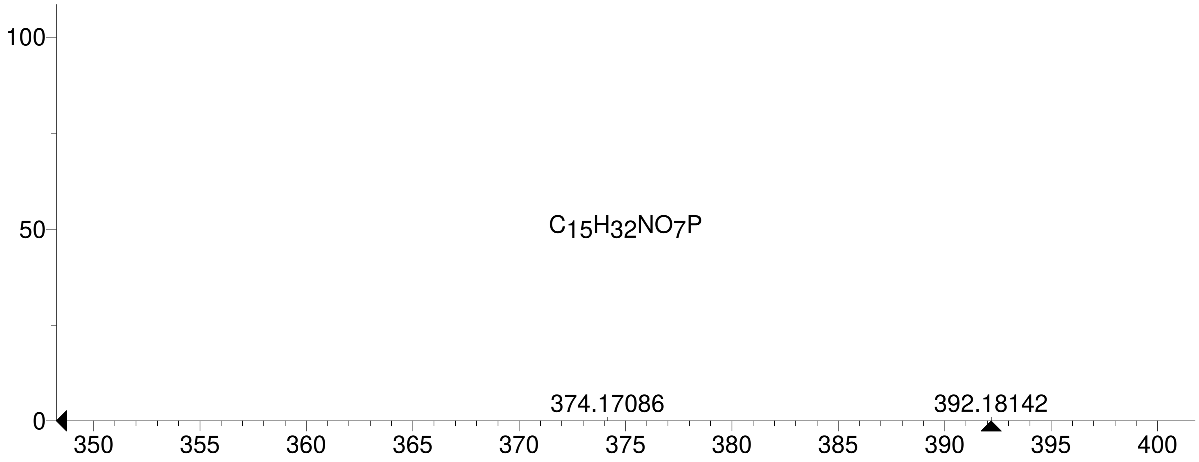

Hit 3 : Hexylglutathione [M+H]+ IT-FT 35% P=392.2
C16H29N3O6S; MF: 13; RMF: 255; Prob 1.59%; CAS: 24425-56-7; Lib: nist_msms; ID: 121555.

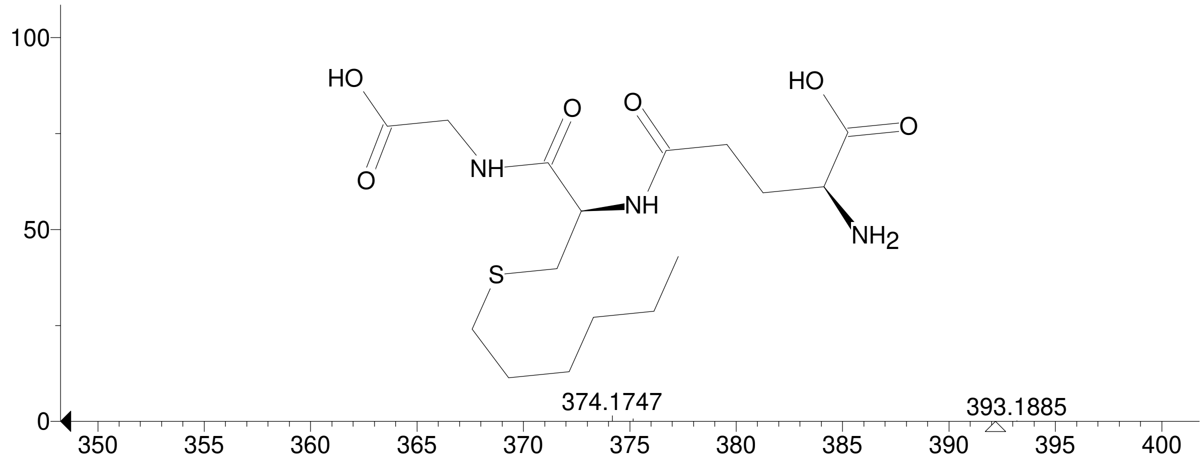

Unknown: Unknown (12.42_560.2124m/z)
Compound in Library Factor = 0

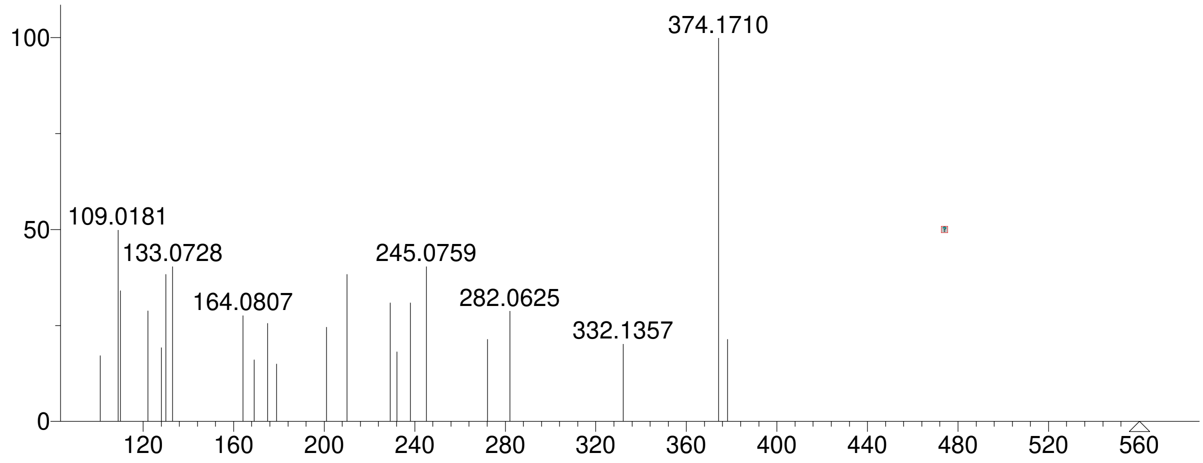

Unknown: Unknown (12.49_351.1076m/z)
Compound in Library Factor = -3273

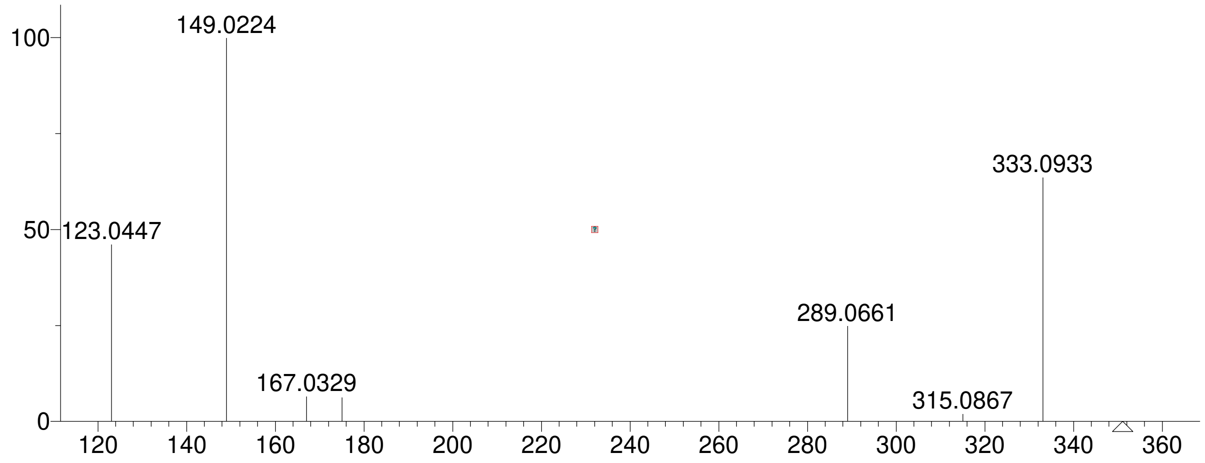

Hit 1 : Anisatin [M+Na]+ IT-FT 35% P=351.1
C15H20O8; MF: 2; RMF: 70; Prob 21.3%; CAS: 5230-87-5; Lib: nist_msms; ID: 394674.

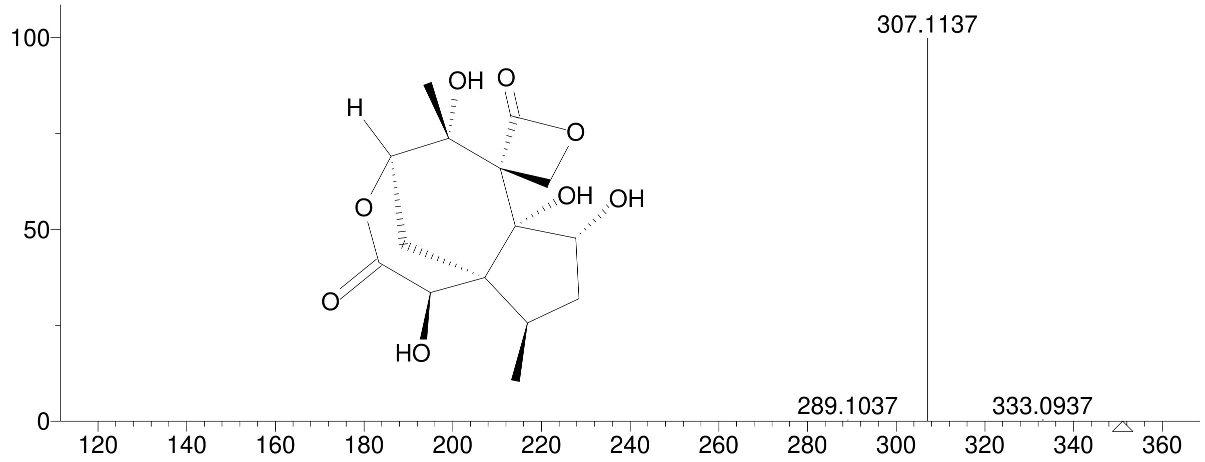

Hit 2 : Acetamide, N-[4-[5-(1,3-benzodioxol-5-yl)-3-methoxy-1H-1,2,4-triazol-1-yl]phenyl]-2-(phenylthio)- [M+H-C6
C24H20N4O4S; MF: 0; RMF: 60; Prob 19.7%; CAS: 853625-60-2; Lib: nist_msms; ID: 375962.

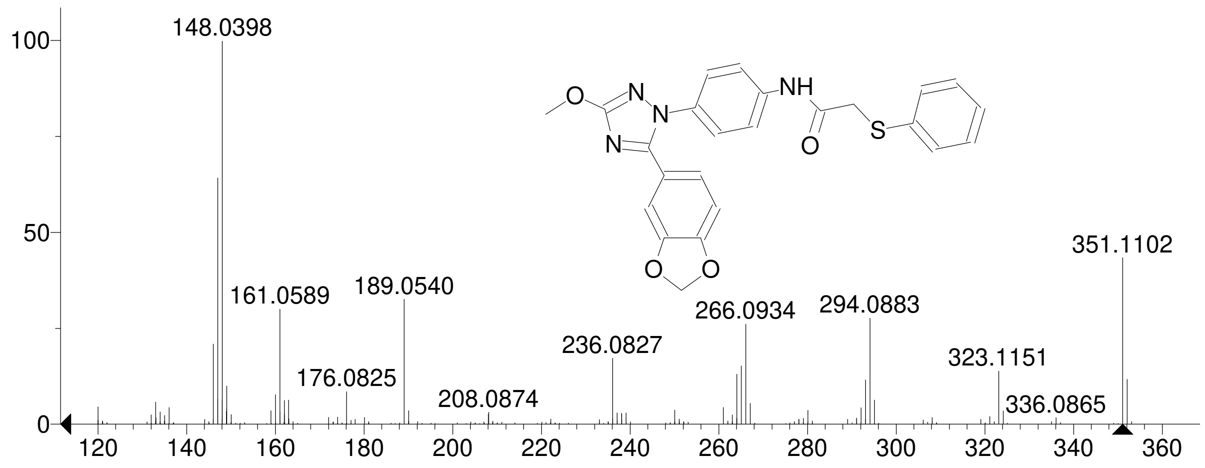

Hit 3 : methyl 2-(4a,6,10a-trihydroxy-1-methyl-5,10-dioxo-3,4-dihydro-1H-benzo[g]isochromen-3-yl)acetate [M+H]
C17H18O8; MF: 0; RMF: 26; Prob 19.7%; Lib: mona-export-vf-npl_qexactive; ID: 20964.

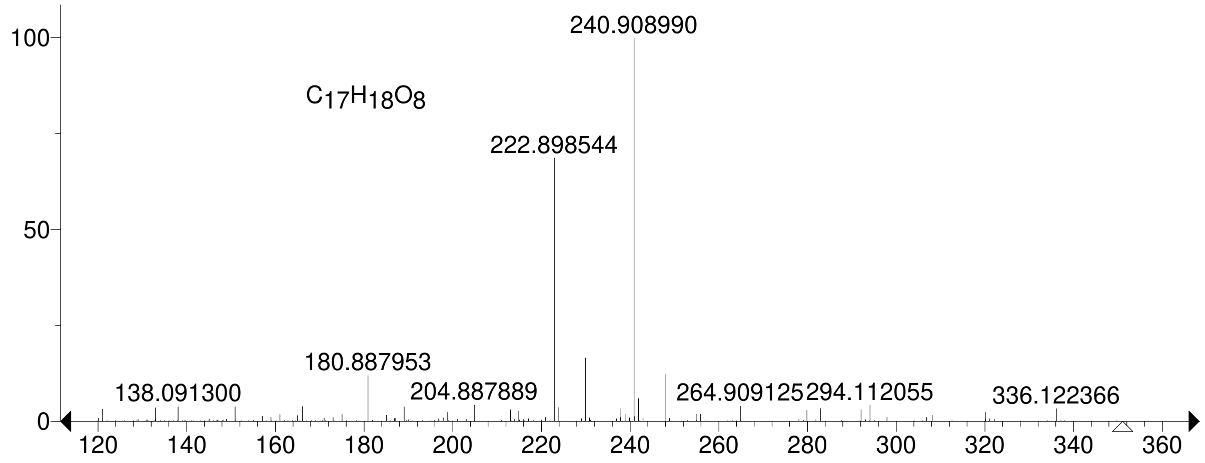

Hit 4 : N-(4-Cyanophenyl)glycine [2M-H]- IT 35% P=351.1
C9H8N2O2; MF: 0; RMF: 25; Prob 19.7%; CAS: 42288-26-6; Lib: nist_msms; ID: 202522.

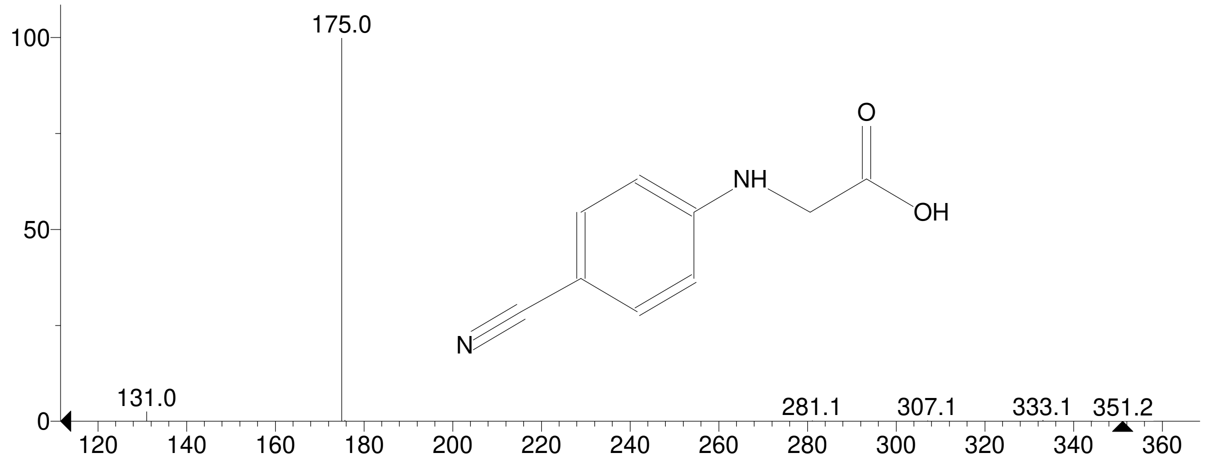

Hit 5 : 1-[2-hydroxy-6-methyl-4-[(2S,3R,4S,5S,6R)-3,4,5-trihydroxy-6-(hydroxymethyl)oxan-2-yl]oxyphenyl]ethanon
C15H20O8; MF: 0; RMF: 20; Prob 19.7%; Lib: mona-export-vf-npl_qexactive; ID: 25137.

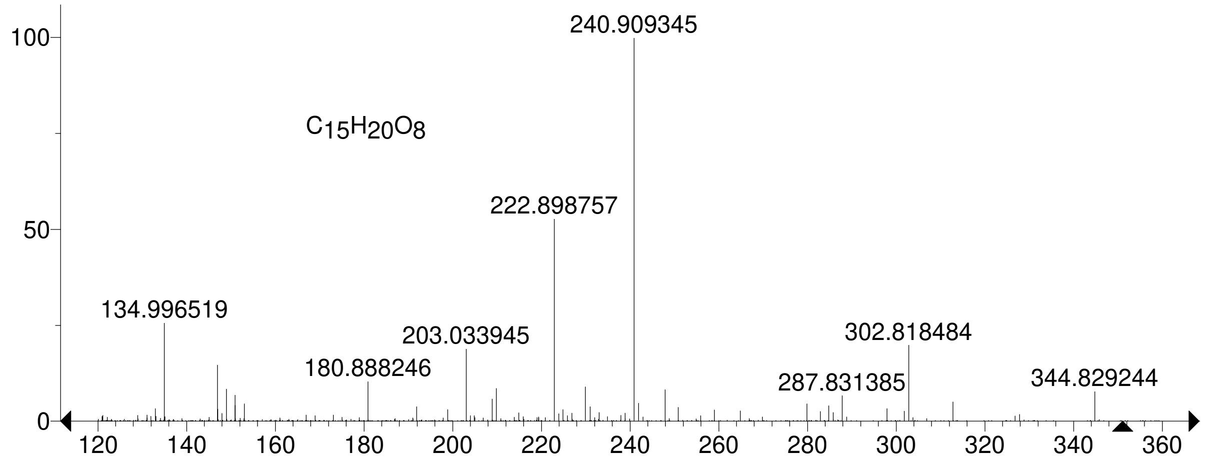

** Search Report Page 1 of 1 **

Unknown: Unknown (12.53_605.2810m/z)
Compound in Library Factor = 0

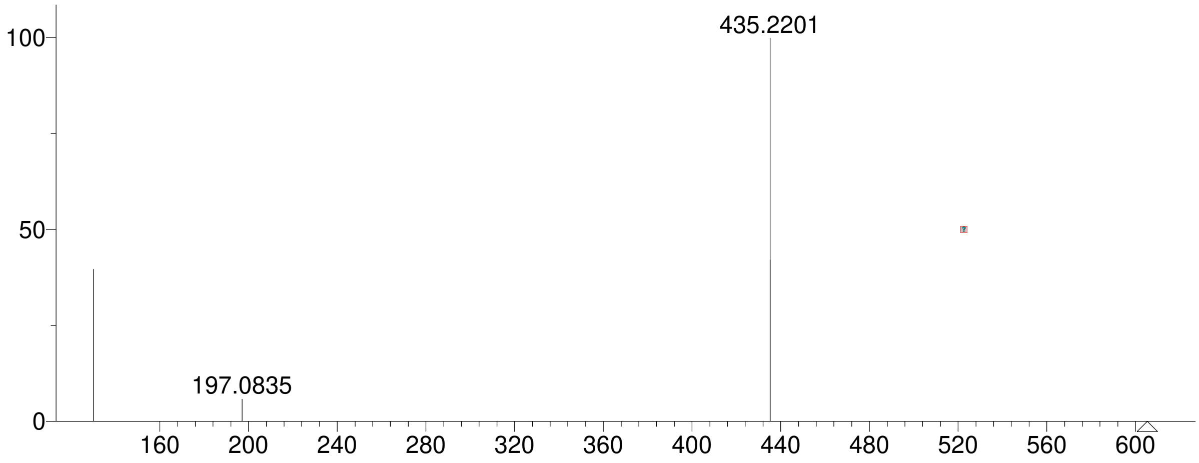

** Search Report Page 1 of 1 **

Unknown: Unknown (12.54_512.2244m/z)
Compound in Library Factor = 0

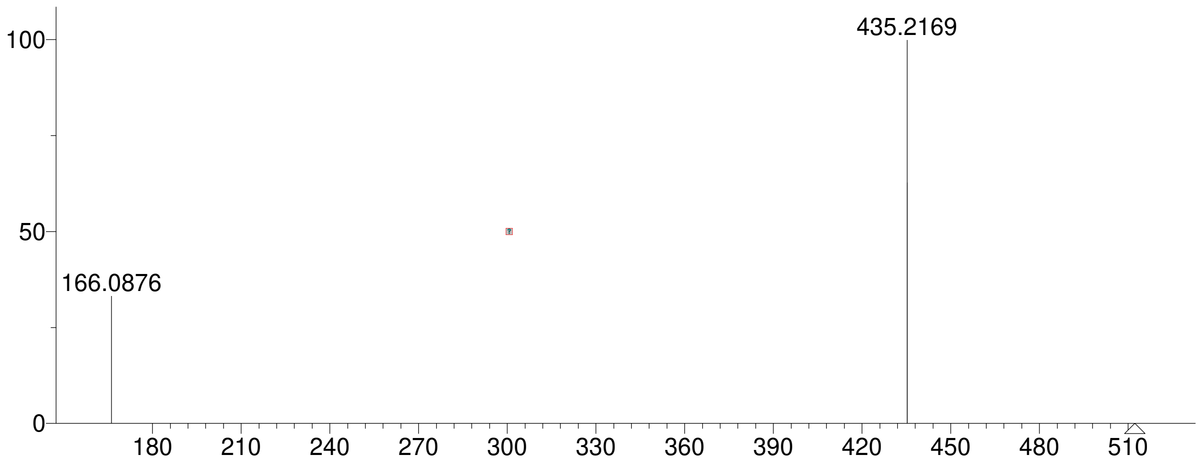

Hit 1 : 4-Heptenoic acid, 7-[(1R,2R,5S)-5-([1,1'-biphenyl]-4-ylmethoxy)-2-(4-morpholinyl)-3-oxocyclopentyl]-, (4Z)-
C29H35NO5; MF: 0; RMF: 57; Prob 100.0%; CAS: 81443-73-4; Lib: nist_msms; ID: 360435.

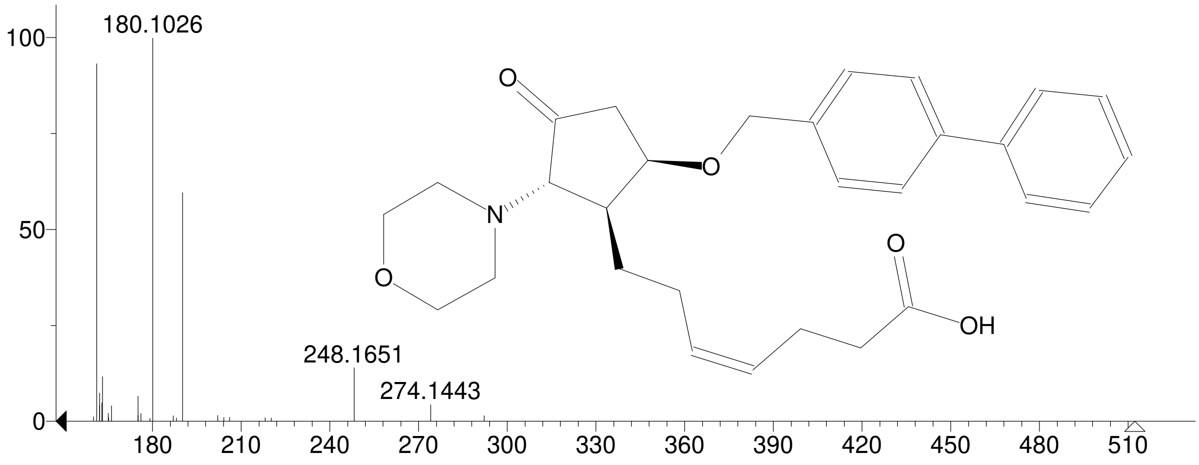

** Search Report Page 1 of 1 **

Unknown: Unknown (12.56_546.2202m/z)
Compound in Library Factor = 0

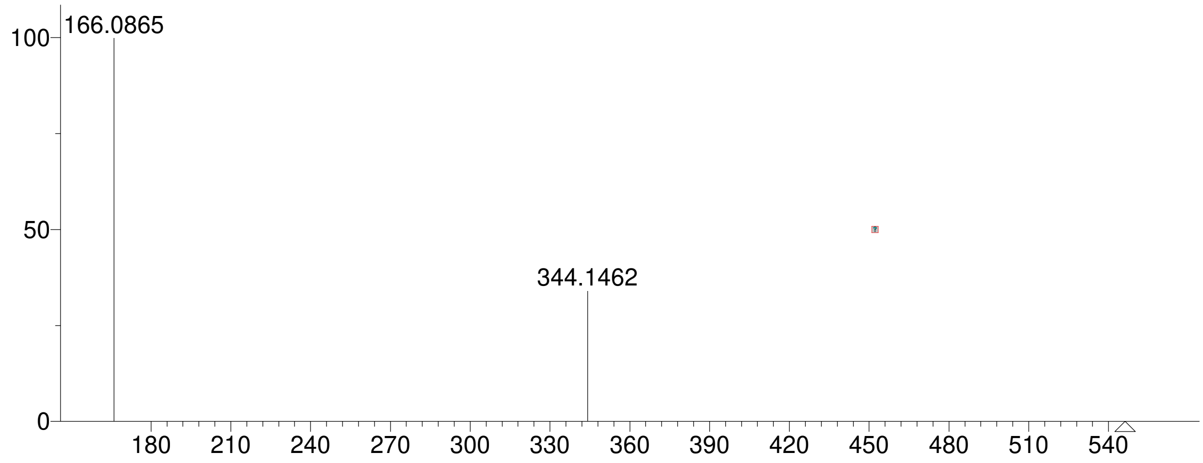

Unknown: Unknown (12.59_447.2129m/z)
Compound in Library Factor = 0

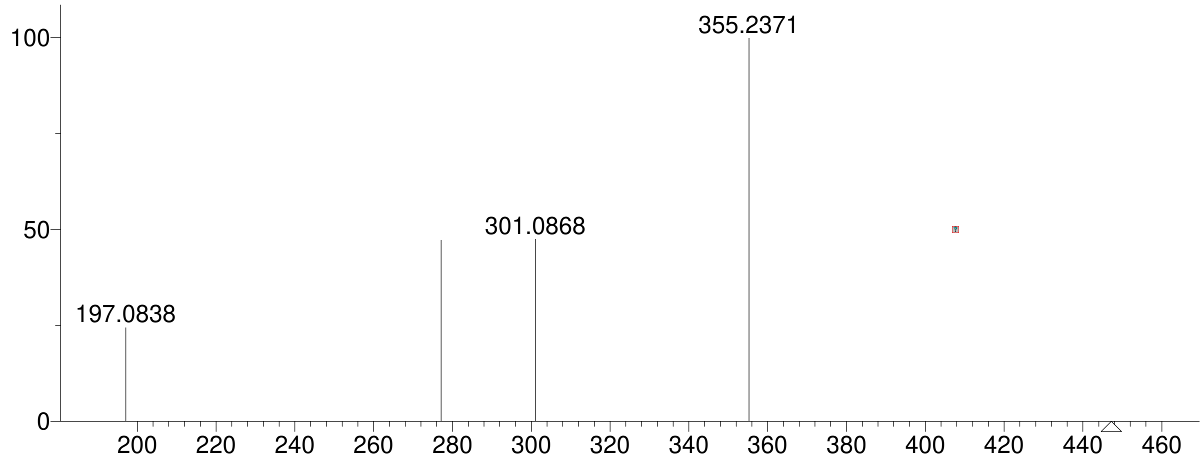

** Search Report Page 1 of 1 **

Unknown: Unknown (12.60_526.2499m/z)
Compound in Library Factor = 0

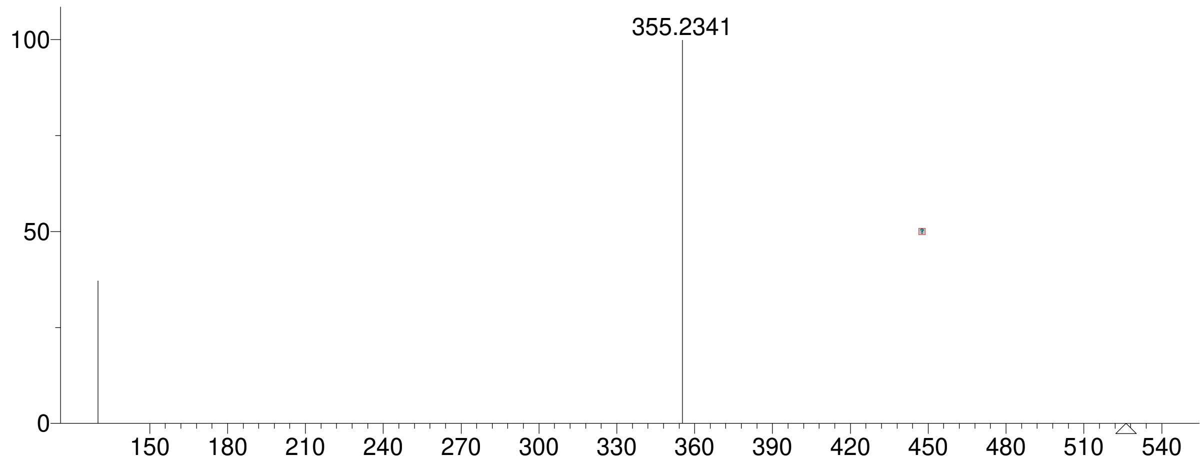

Unknown: Unknown (12.64_438.2231m/z)
Compound in Library Factor = -2387

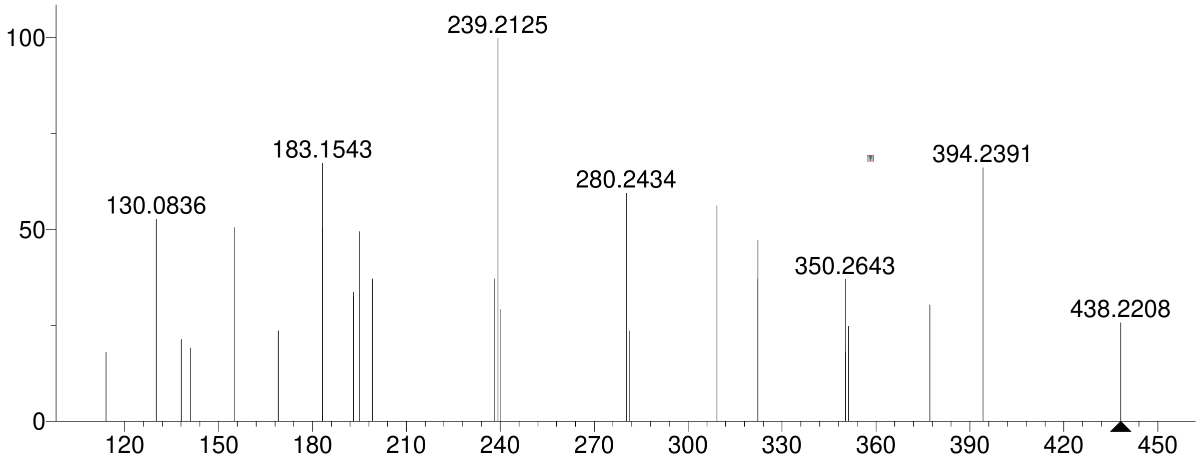

Hit 1 : Triparanol [M+H]+ QTOF 5V P=438.2
C27H32ClNO2; MF: 12; RMF: 153; Prob 19.6%; CAS: 78-41-1; Lib: nist_msms; ID: 18551.

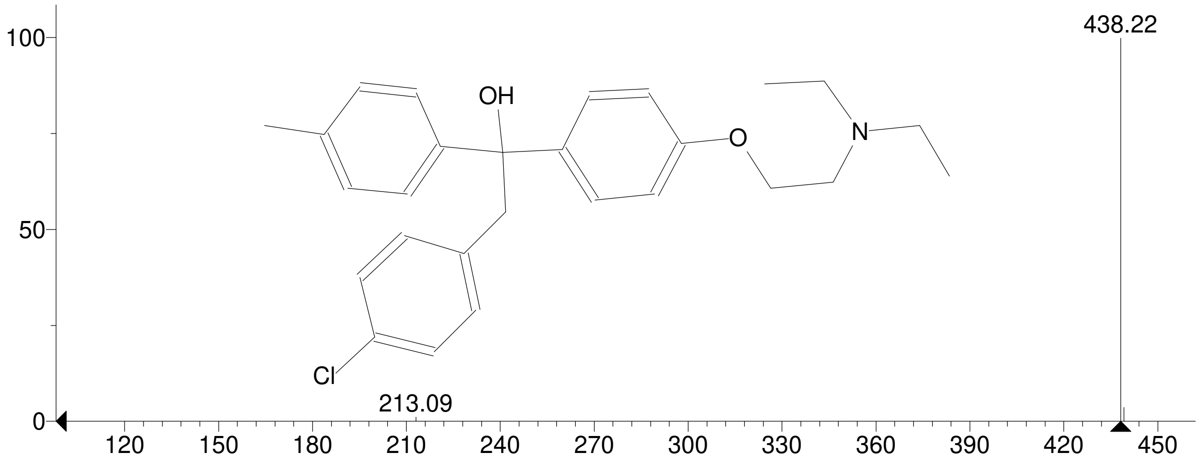

Hit 2 : PE 14:0; PE 7:0-7:0; [M-H]- [M-H]- P=438.2
C19H38NO8P; MF: 2; RMF: 96; Prob 13.8%; Lib: msdial-insilicomsms-lipids-neg; ID: 322970.

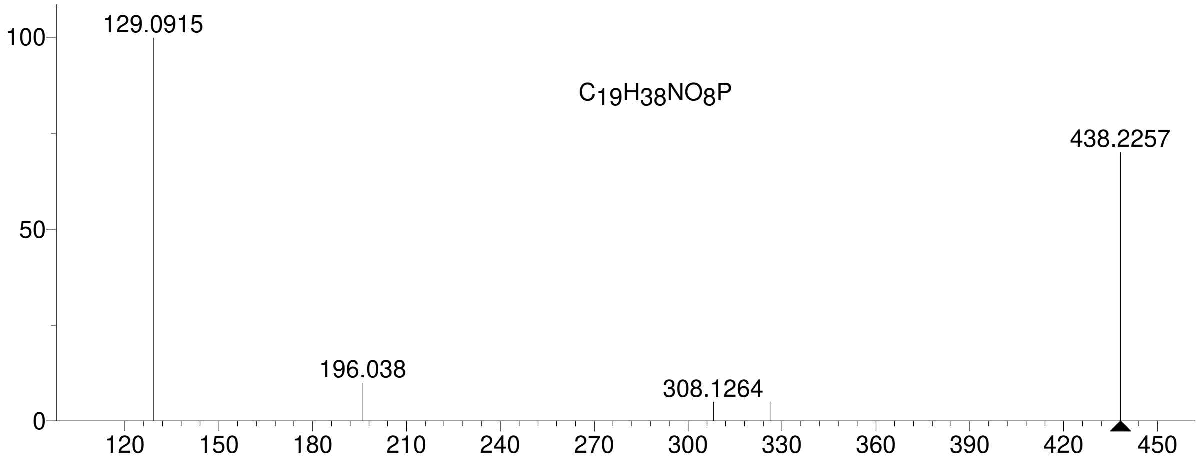

Hit 3 : PE 14:0; PE 2:0-12:0; [M-H]- [M-H]- P=438.2
C19H38NO8P; MF: 1; RMF: 92; Prob 13.3%; Lib: msdial-insilicomsms-lipids-neg; ID: 322685.

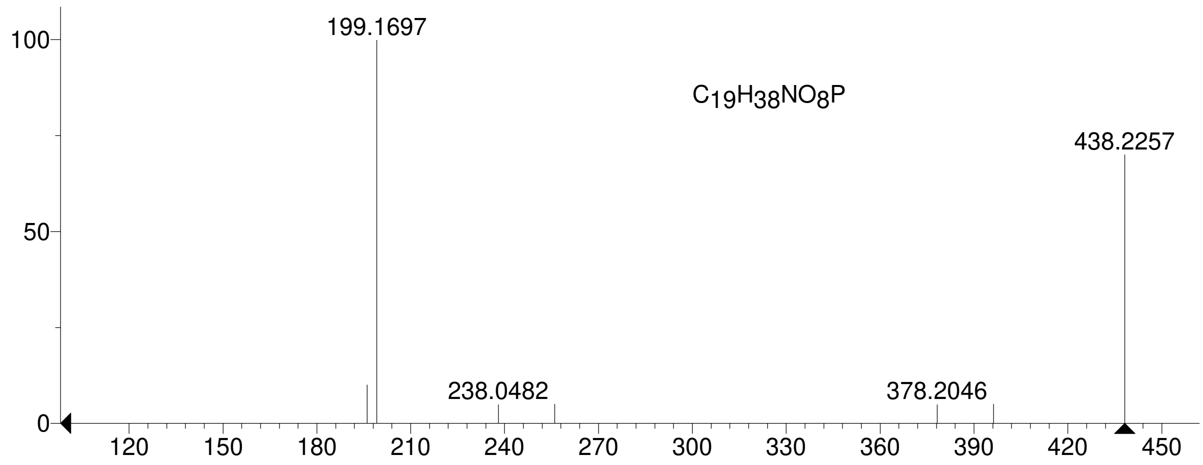

Hit 4 : PE 14:0; PE 3:0-11:0; [M-H]- [M-H]- P=438.2
C19H38NO8P; MF: 1; RMF: 92; Prob 13.3%; Lib: msdial-insilicomsms-lipids-neg; ID: 322744.

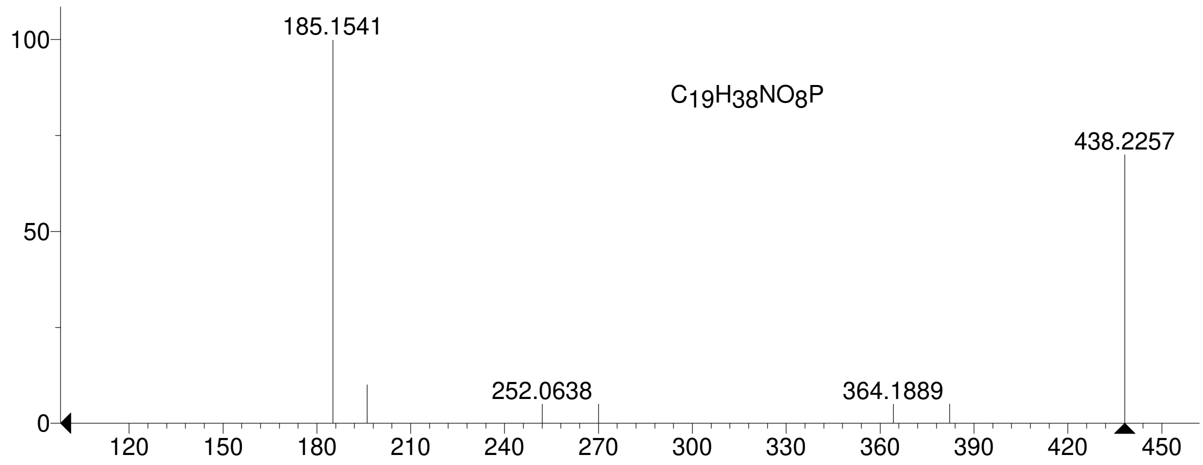

Hit 5 : PE 14:0; PE 4:0-10:0; [M-H]- [M-H]- P=438.2
C19H38NO8P; MF: 1; RMF: 92; Prob 13.3%; Lib: msdial-insilicomsms-lipids-neg; ID: 322802.

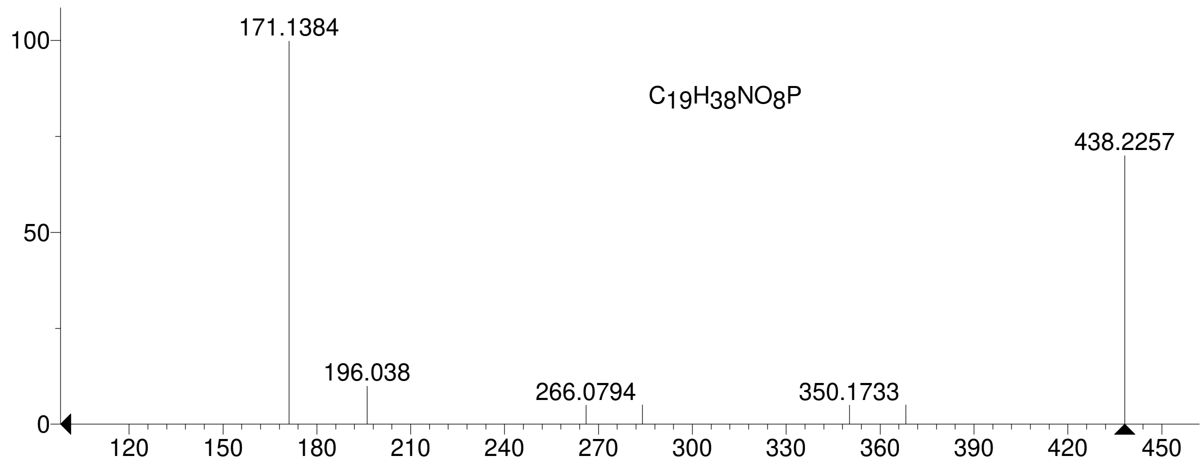

** Search Report Page 1 of 1 **

Unknown: Unknown (12.67_585.2281m/z)
Compound in Library Factor = -3273

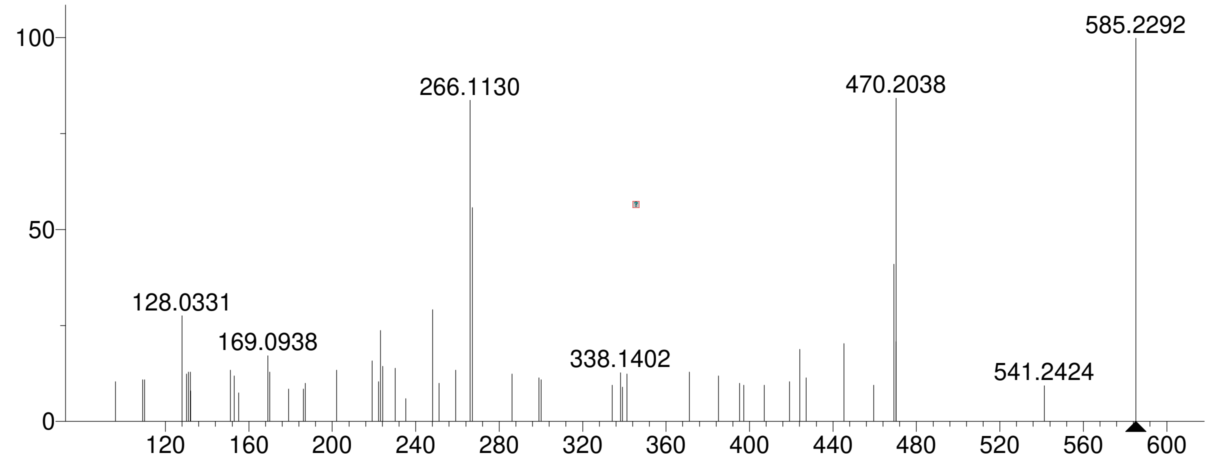

Hit 1 : Azadirachtin B [M+H-C2H6O3]+ IT 35% P=585.2
C33H42O14; MF: 2; RMF: 57; Prob 100.0%; CAS: 95507-03-2; Lib: nist_msms; ID: 223433.

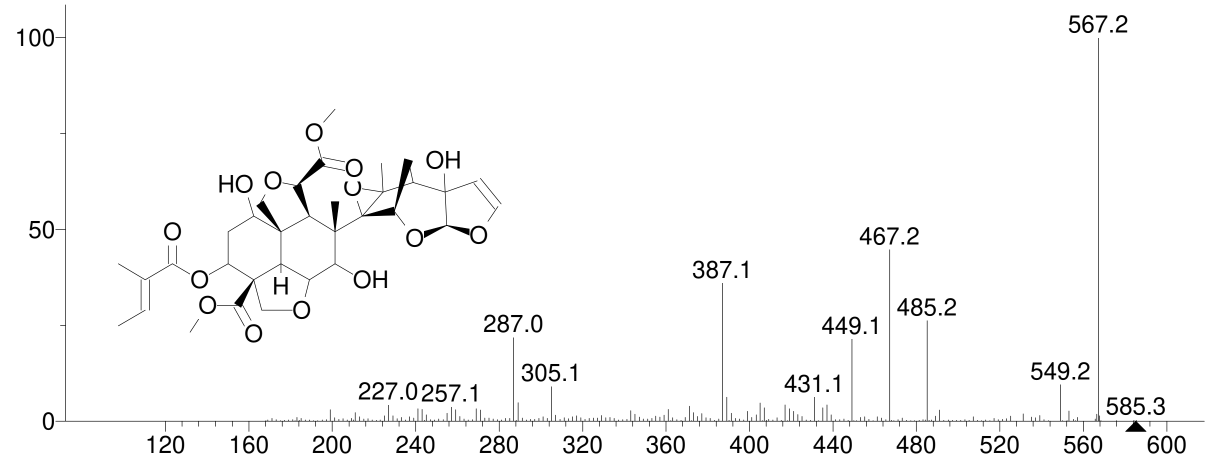

Unknown: Unknown (12.74_704.1336m/z)
Compound in Library Factor = 0

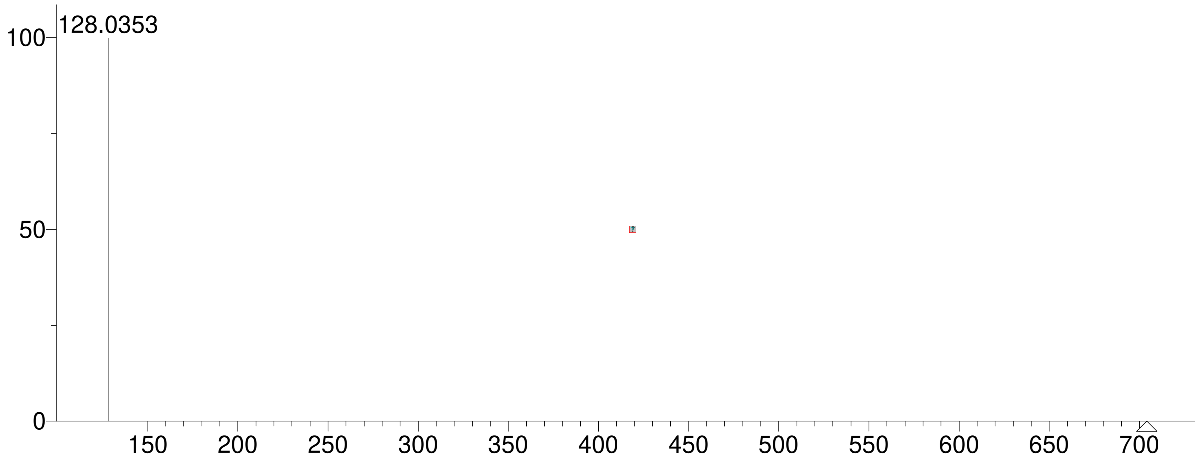

** Search Report Page 1 of 1 **

Unknown: Unknown (12.82_438.2224m/z)
Compound in Library Factor = 0

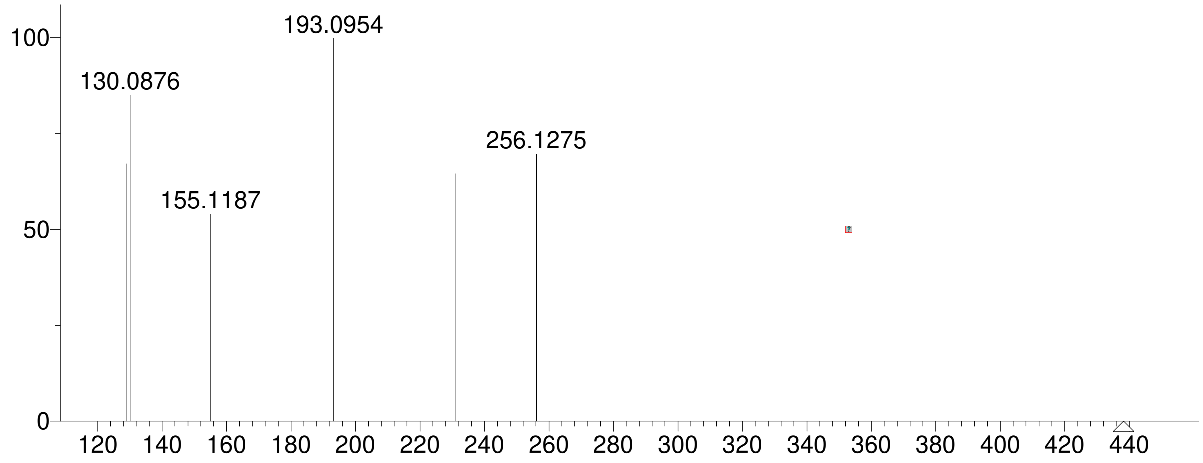

Unknown: Unknown (12.83_253.1185m/z)
Compound in Library Factor = -3273

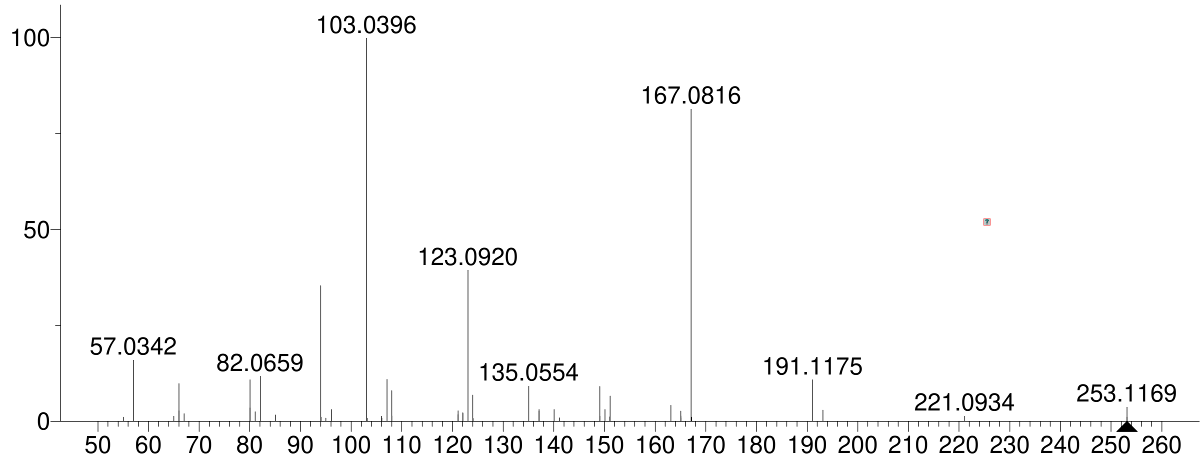

Hit 1 : Ala-Tyr [M+H]+ HCD 5eV P=253.1
C12H16N2O4; MF: 7; RMF: 151; Prob 11.8%; CAS: 3061-88-9; Lib: nist_msms2; ID: 89140.

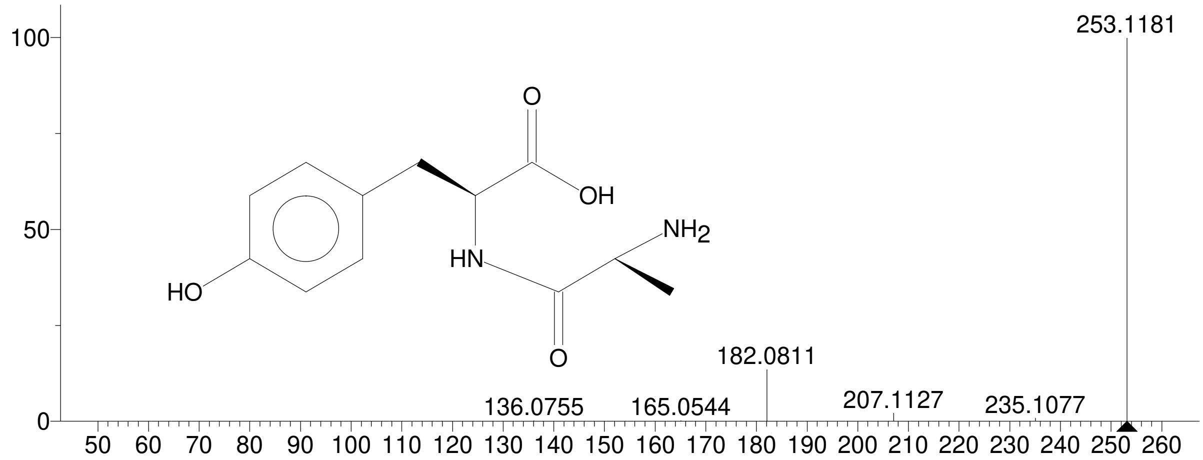

Hit 2 : Tyr-Ala [M+H]+ HCD 5eV P=253.1
C12H16N2O4; MF: 5; RMF: 96; Prob 10.9%; CAS: 730-08-5; Lib: nist_msms; ID: 564059.

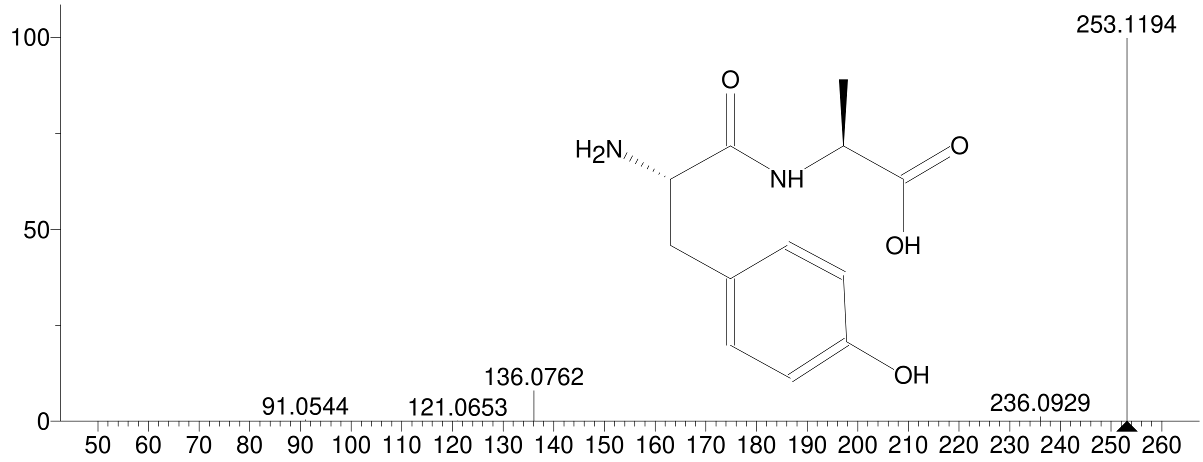

Hit 3 : Ser-Phe [M+H]+ HCD 5eV P=253.1
C12H16N2O4; MF: 4; RMF: 93; Prob 10.4%; Lib: nist_msms; ID: 571662.

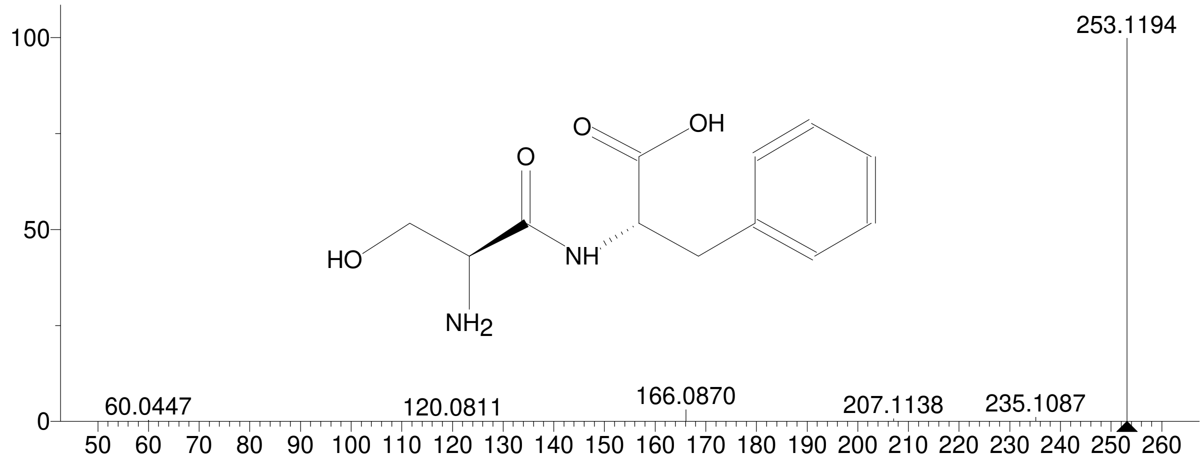

Hit 4 : Ser-Phe [M+H]+ HCD 5eV P=253.1
C12H16N2O4; MF: 4; RMF: 93; Prob 10.4%; CAS: 16875-28-8; Lib: nist_msms2; ID: 85420.

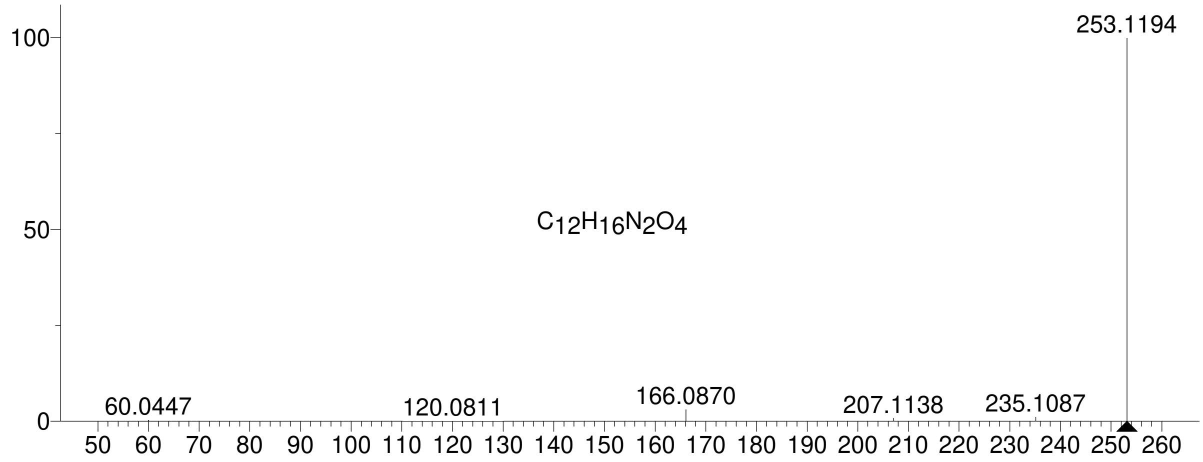

Hit 5 : Palythazine [M+H]+ P=253.1
C12H16N2O4; MF: 3; RMF: 99; Prob 10.1%; Lib: mona-export-gnps; ID: 5292.

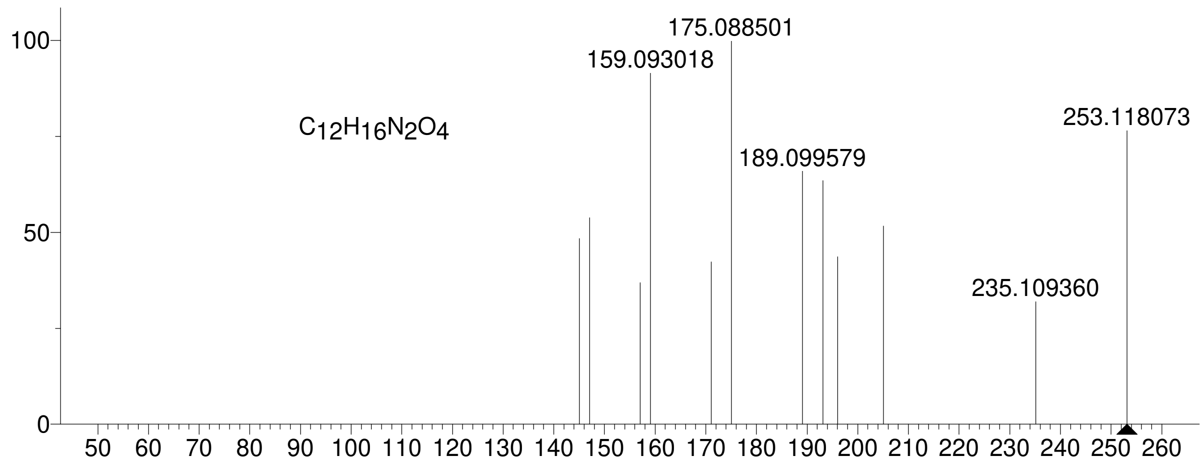

** Search Report Page 1 of 1 **

Unknown: Unknown (12.84_338.8676m/z)
Compound in Library Factor = 0

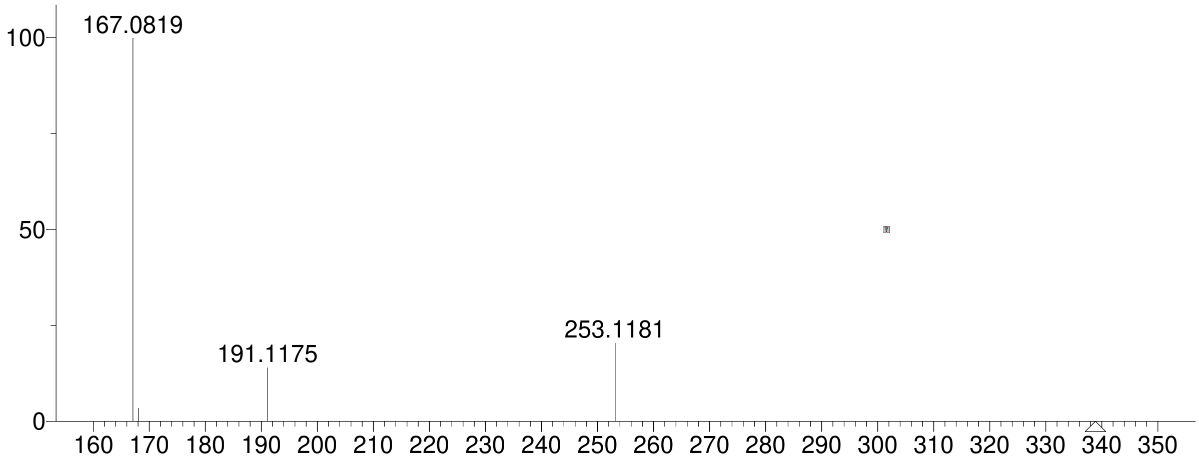

Unknown: Unknown (12.84_596.1336m/z)
Compound in Library Factor = 0

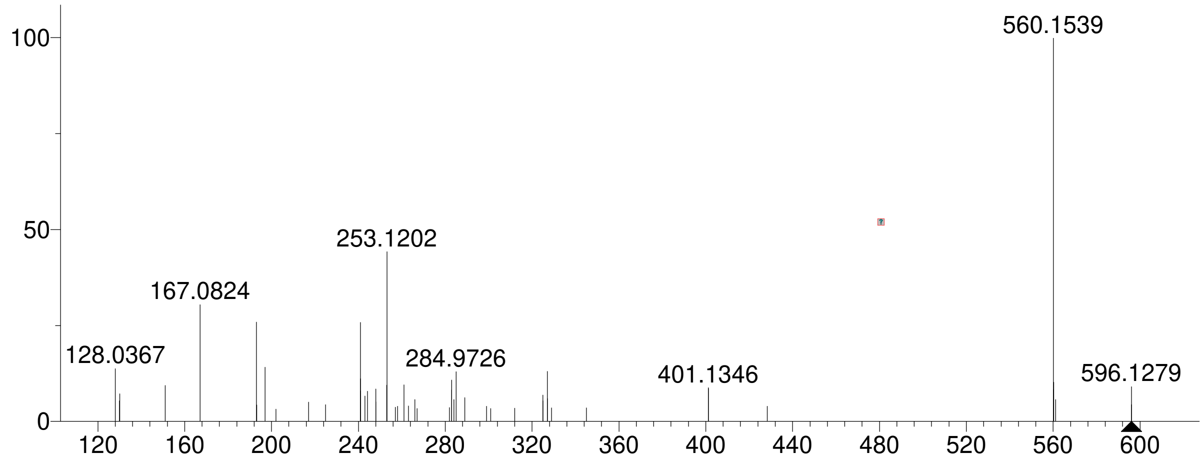

Unknown: Unknown (12.84_799.3187m/z)
Compound in Library Factor = 0

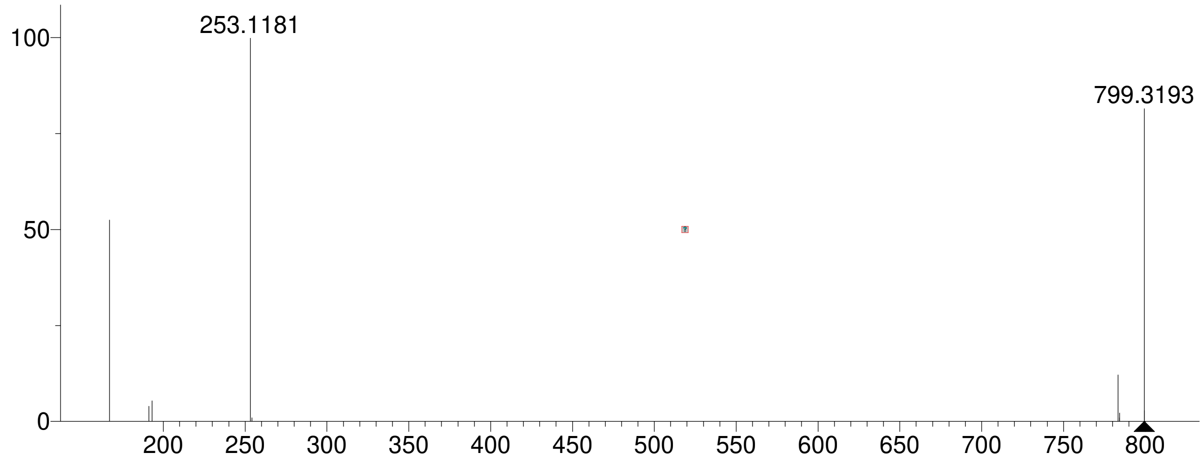

Unknown: Unknown (12.84_814.2902m/z)
Compound in Library Factor = -3273

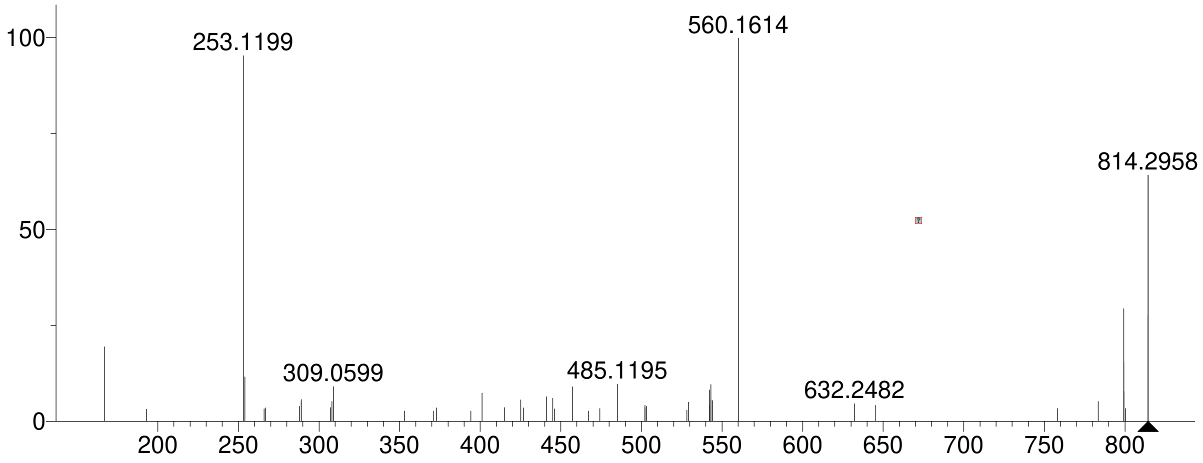

Hit 1 : NCGC00380923-01!2-[4-[4-[3,5-dihydroxy-4-[3,4,5-trihydroxy-6-(hydroxymethyl)oxan-2-yl]phenyl]-2,5-bis(4
C40H44O17; MF: 1; RMF: 71; Prob 51.0%; Lib: mona-export-gnps; ID: 7654.

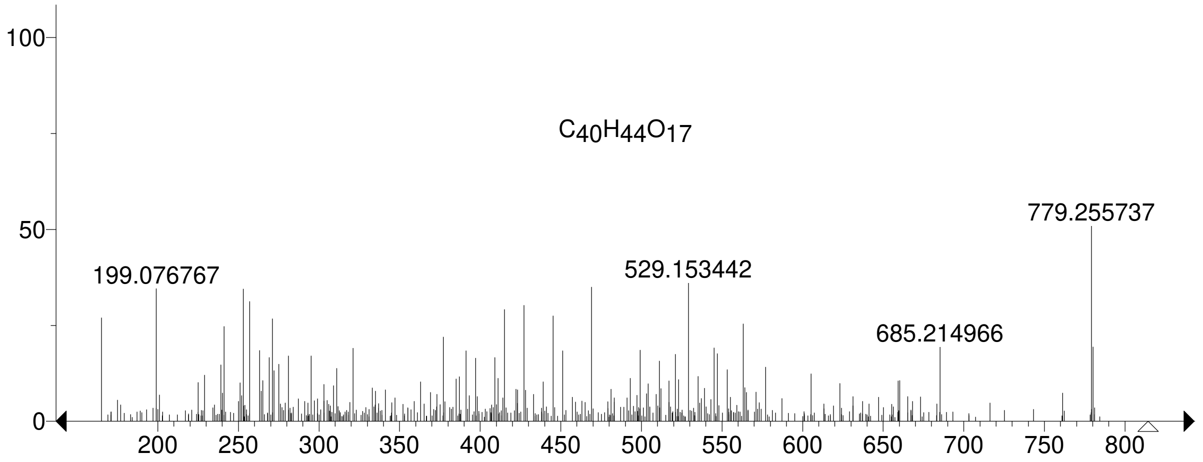

Hit 2 : 2-[4-[4-[3,5-dihydroxy-4-[3,4,5-trihydroxy-6-(hydroxymethyl)oxan-2-yl]phenyl]-2,5-bis(4-hydroxyphenyl)oxola
C40H44O17; MF: 0; RMF: 39; Prob 49.0%; Lib: anthocyanins mona; ID: 7292.

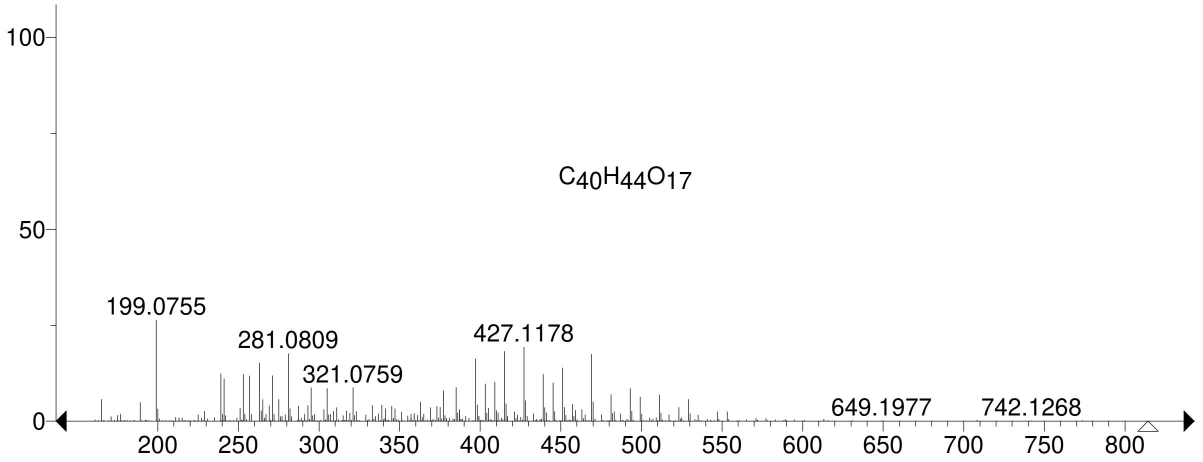

Unknown: Unknown (12.85_817.2939m/z)
Compound in Library Factor = 0

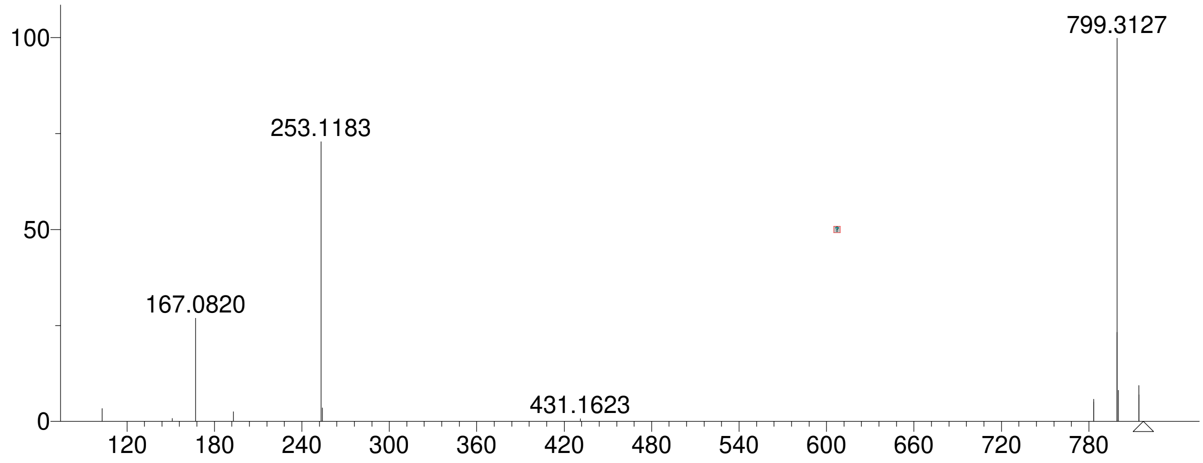

Unknown: Unknown (12.86_560.1568m/z)
Compound in Library Factor = -2176

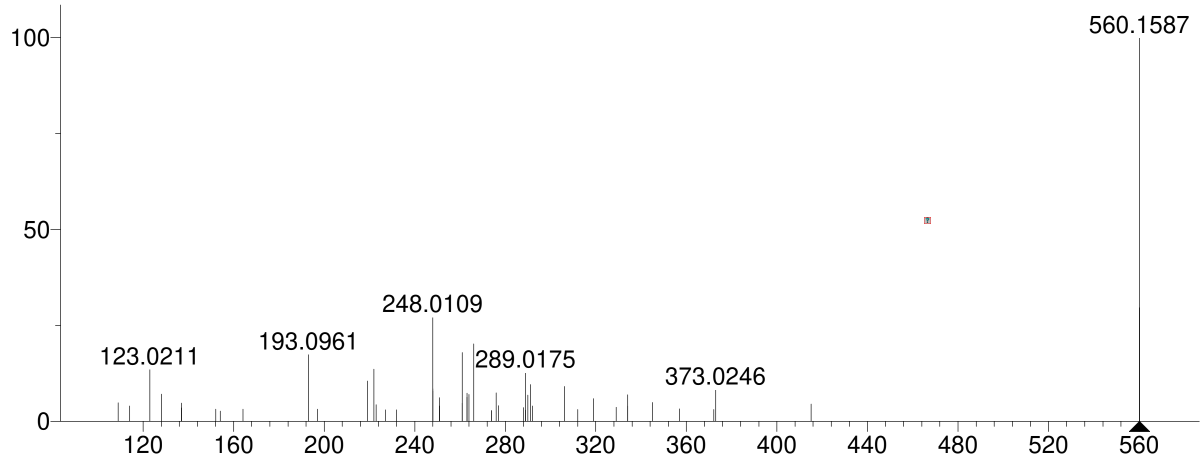

Hit 1 : Desmethylbosentan [M+Na]+ HCD 22V P=560.2
C26H27N5O6S; MF: 20; RMF: 459; Prob 30.5%; CAS: 253688-61-8; Lib: nist_msms; ID: 451527.

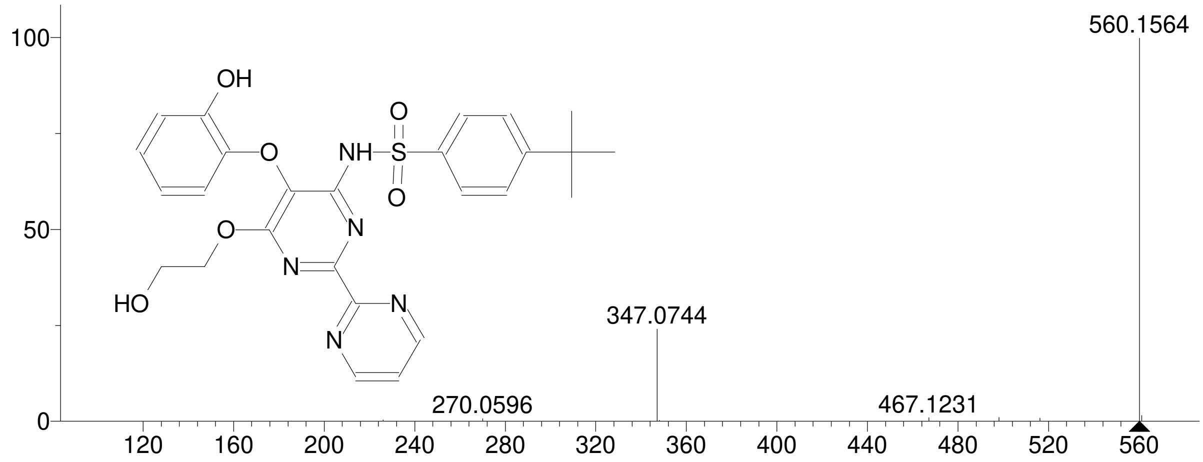

Hit 2 : (2S)-6-[(2S)-5,7-dihydroxy-2-(4-hydroxyphenyl)-4-oxo-2,3-dihydrochromen-8-yl]-5,7-dihydroxy-2-(4-hydrox
C30H22O10; MF: 0; RMF: 55; Prob 13.9%; Lib: mona-export-vf-npl_qexactive; ID: 19141.

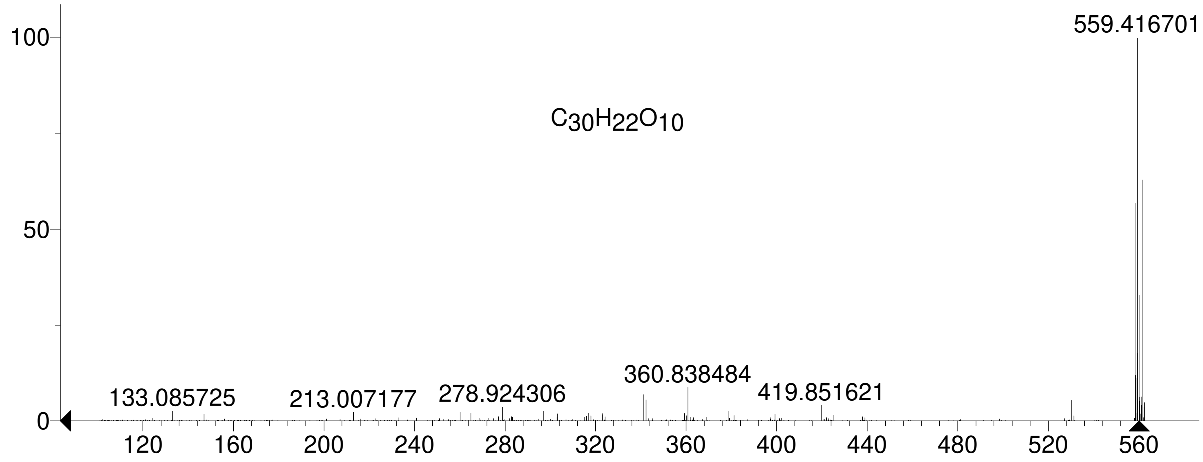

Hit 3 : (3S,4R)-3-[(3S,4R)-5,7-dihydroxy-4-(4-hydroxyphenyl)-2-oxo-3,4-dihydrochromen-3-yl]-5,7-dihydroxy-4-(4-l
C30H22O10; MF: 0; RMF: 40; Prob 13.9%; Lib: mona-export-vf-npl_qexactive; ID: 20569.

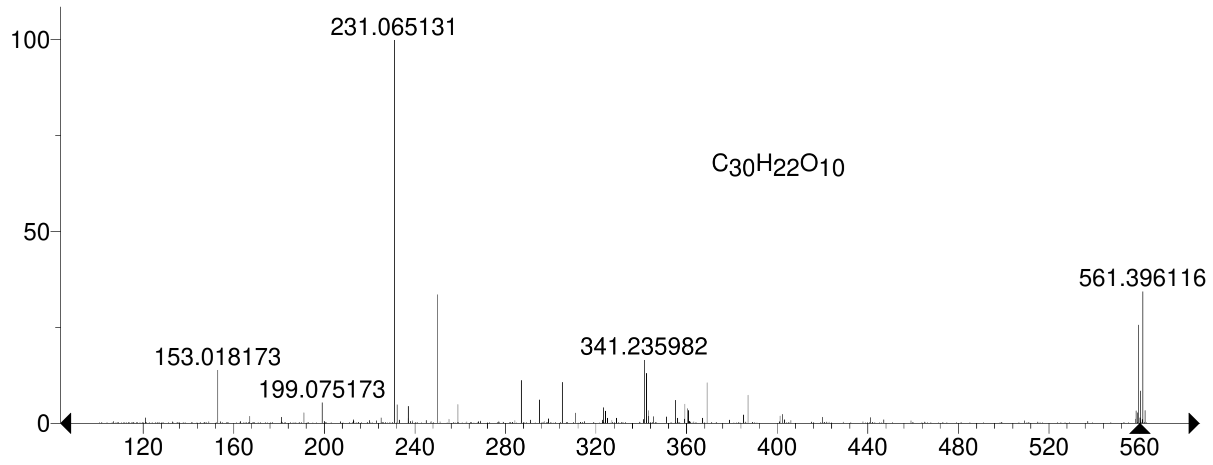

Hit 4 : 3-[1,1-bis(4-hydroxyphenyl)-3-oxo-3-(2,4,6-trihydroxyphenyl)propan-2-yl]-5,7-dihydroxychromen-4-one [M-
C30H22O10; MF: 0; RMF: 17; Prob 13.9%; Lib: mona-export-vf-npl_qexactive; ID: 22699.

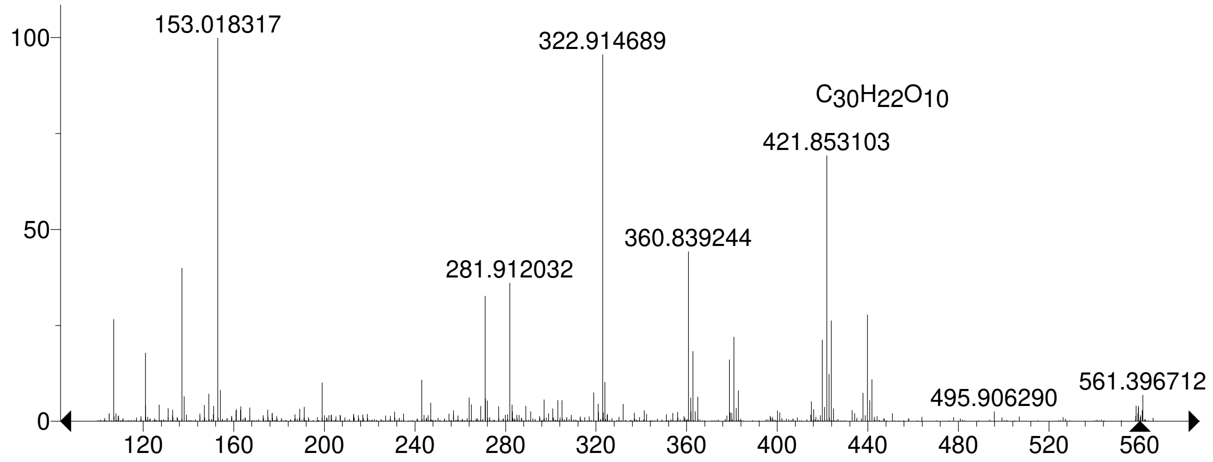

Hit 5 : NCGC00380474-01!5-hydroxy-2-(4-hydroxyphenyl)-7-[[(2R,3S)-3,5,8-trihydroxy-2-(4-hydroxyphenyl)-3,4-di
C30H22O10; MF: 0; RMF: 9; Prob 13.9%; Lib: mona-export-gnps; ID: 9344.

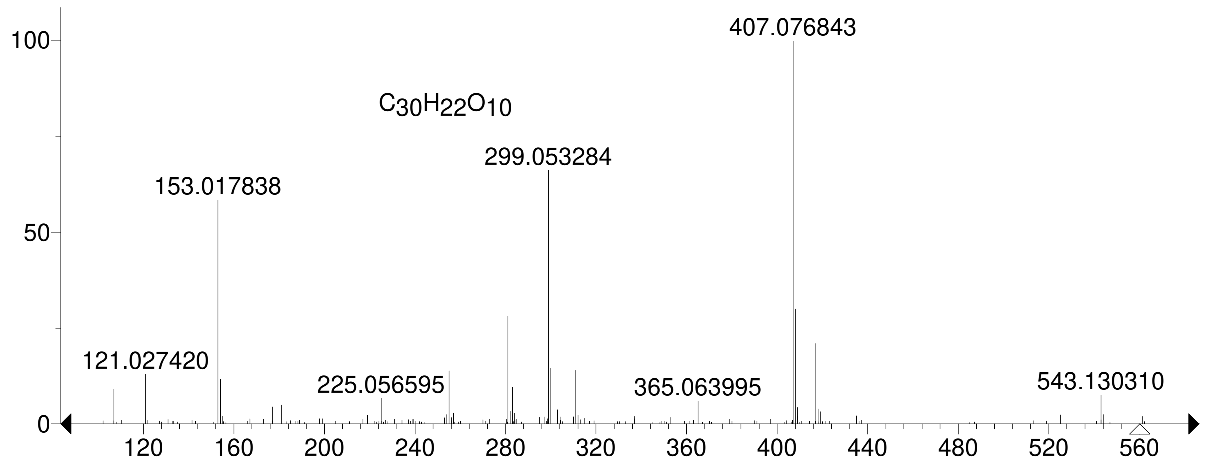

** Search Report Page 1 of 1 **

Unknown: Unknown (12.87_339.1064m/z)
Compound in Library Factor = 0

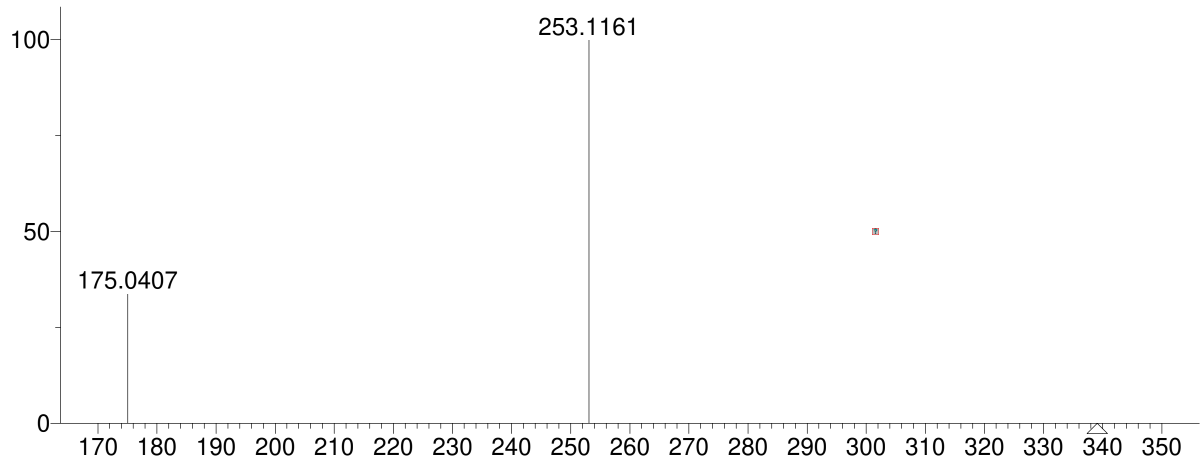

Hit 1 : NCGC00385147-01_C16H18O8_beta-D-Glucopyranoside, 4,8-dihydroxy-1-naphthalenyl [M+H]+ P=339.
C16H18O8; MF: 0; RMF: 75; Prob 50.0%; Lib: mona-export-gnps; ID: 10535.

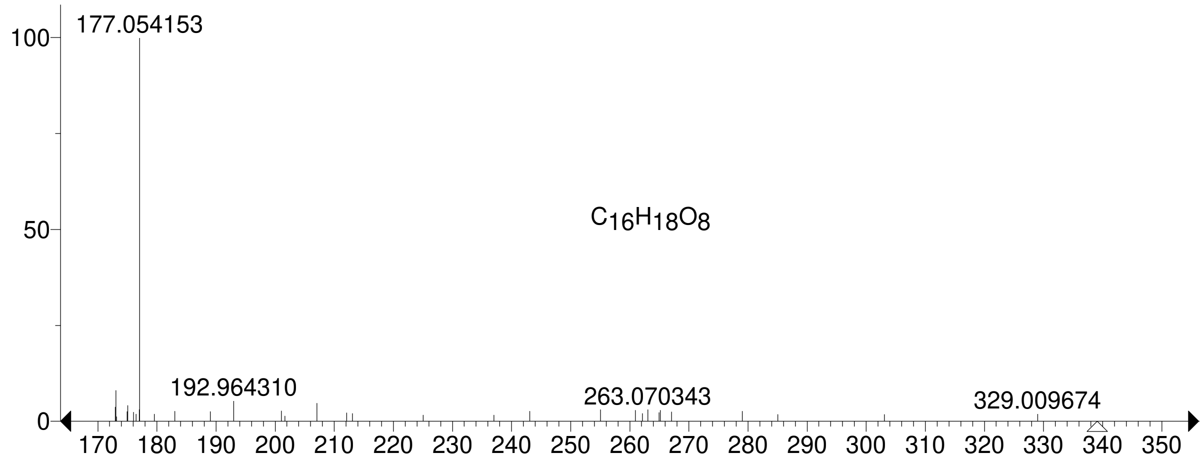

Hit 2 : (1R,3R,4S,5R)-1,3,4-trihydroxy-5-[(E)-3-(4-hydroxyphenyl)prop-2-enoyl]oxycyclohexane-1-carboxylic acid
C16H18O8; MF: 0; RMF: 20; Prob 50.0%; Lib: mona-export-vf-npl_qexactive; ID: 6316.

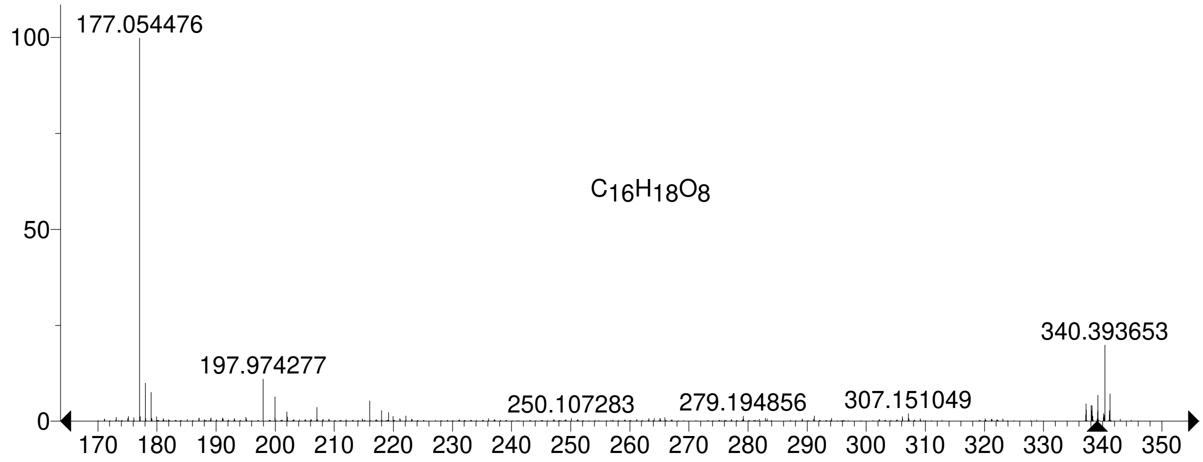

Unknown: Unknown (12.87_401.1444m/z)
Compound in Library Factor = -613

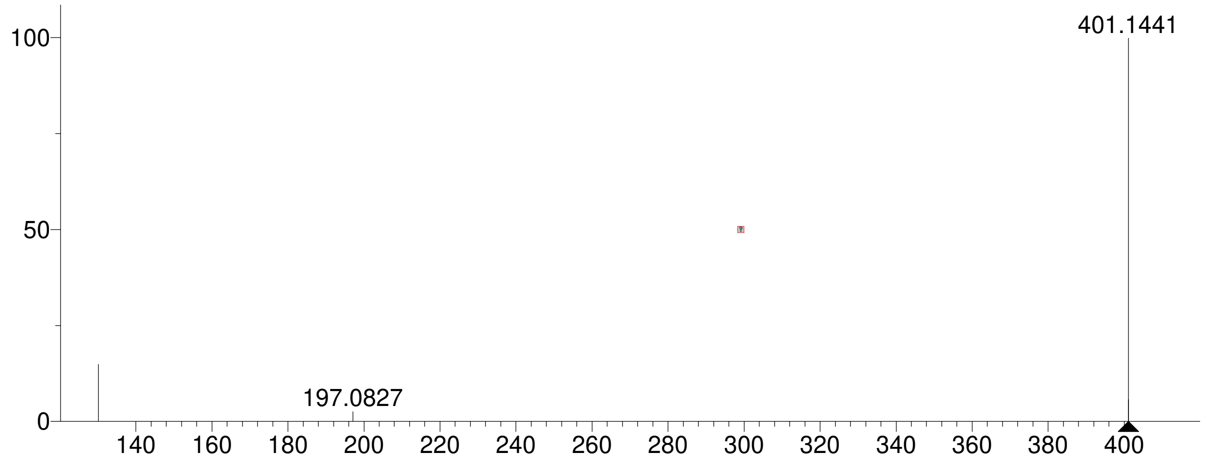

Hit 1 : Methanone, [4-amino-1-[(4-chlorophenyl)methyl]-2-methyl-5-phenyl-1H-pyrrol-3-yl]phenyl- [M+H]+ HCD
C25H21ClN2O; MF: 215; RMF: 887; Prob 91.7%; CAS: 1132922-57-6; Lib: nist_msms; ID: 415414.

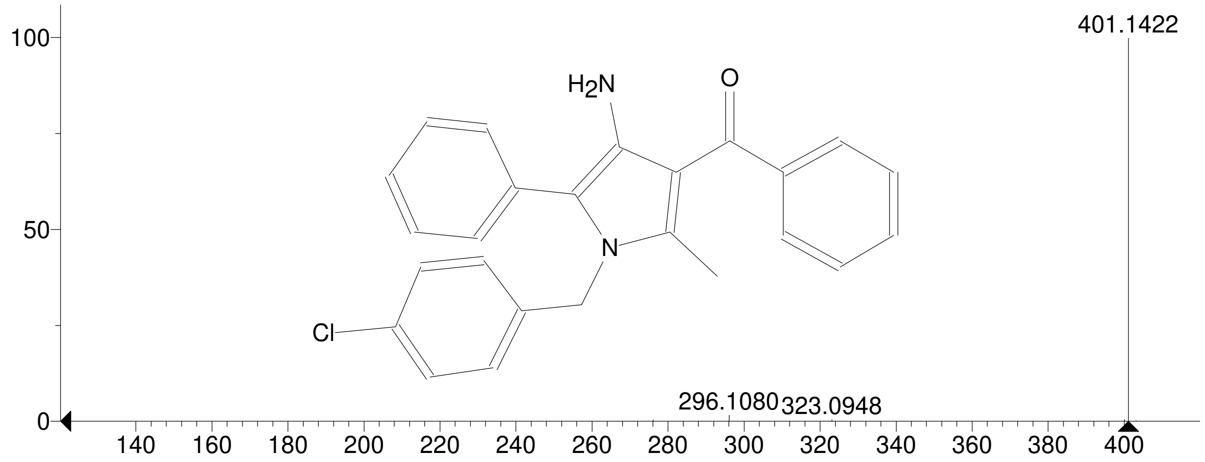

Hit 2 : cis-Clopenthixol [M+H]+ HCD 24V P=401.1
C22H25ClN2OS; MF: 100; RMF: 825; Prob 5.50%; CAS: 53772-83-1; Lib: nist_msms; ID: 135821.

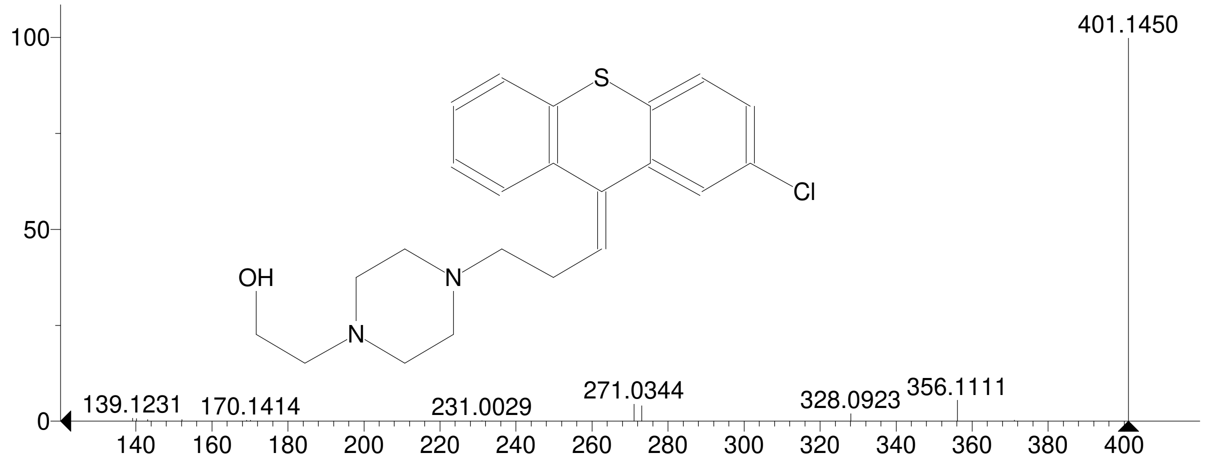

Hit 3 : Unknown (carbon number 18); PlaSMA ID-1017 [M-H]- P=401.1
C18H26O10; MF: 49; RMF: 629; Prob 1.10%; Lib: biomsms-neg-plasma; ID: 1347.

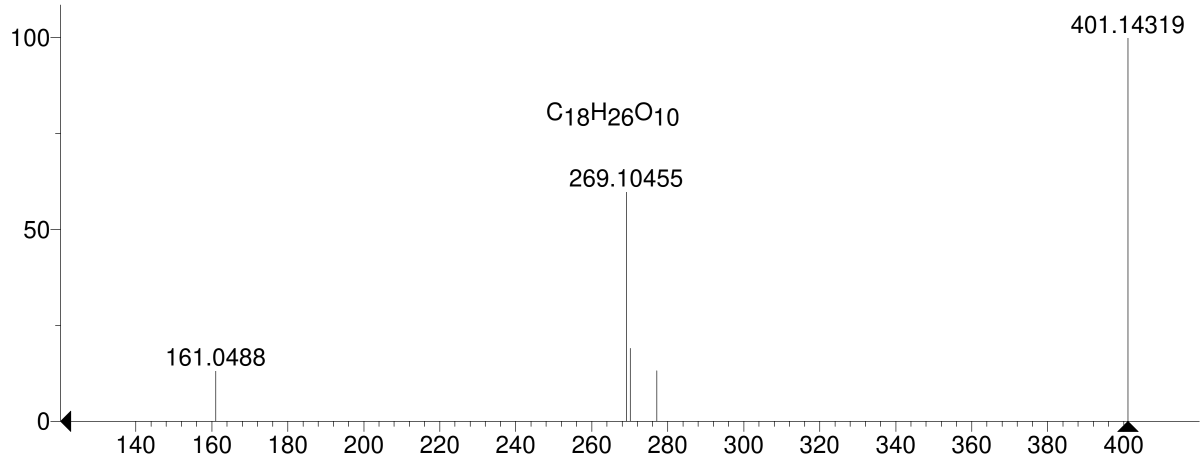

Hit 4 : Benzyl alcohol + Hex-Pen; PlaSMA ID-1015 [M-H]- P=401.1
C18H26O10; MF: 48; RMF: 668; Prob 1.06%; Lib: biomsms-neg-plasma; ID: 753.

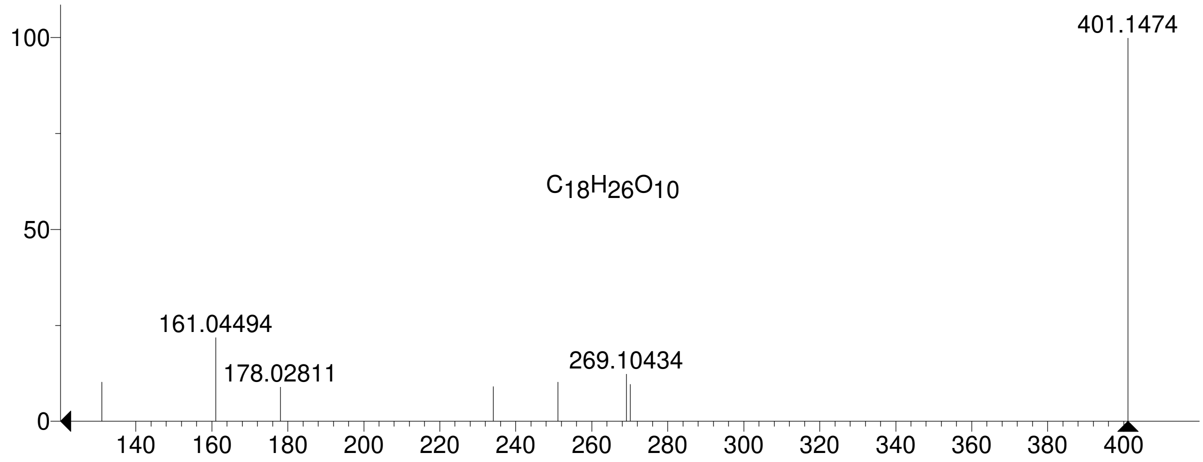

Hit 5 : Unknown (carbon number 18); PlaSMA ID-1016 [M-H]- P=401.1
C18H26O10; MF: 15; RMF: 397; Prob 0.27%; Lib: biomsms-neg-plasma; ID: 1346.

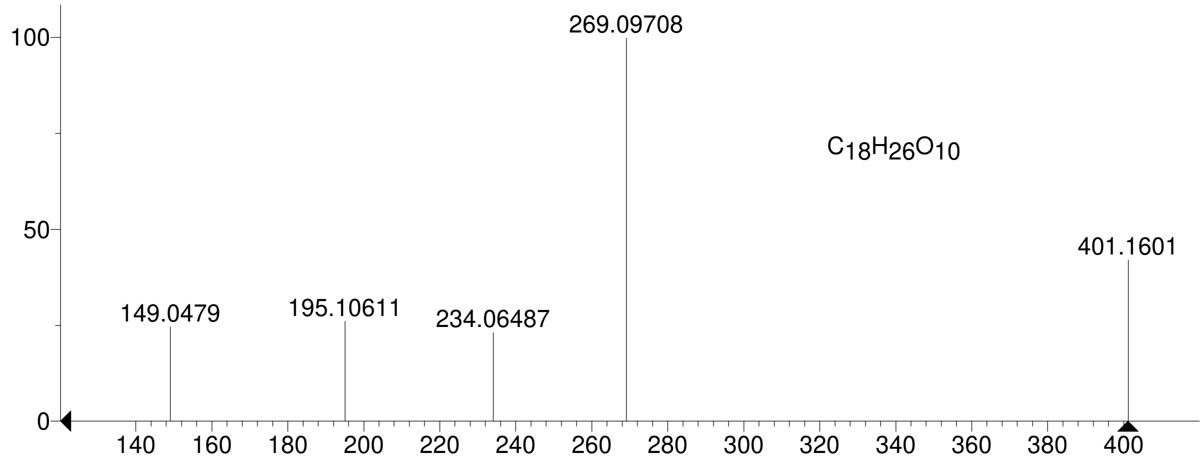

** Search Report Page 1 of 1 **

Unknown: Unknown (12.87_599.2324m/z)
Compound in Library Factor = 0

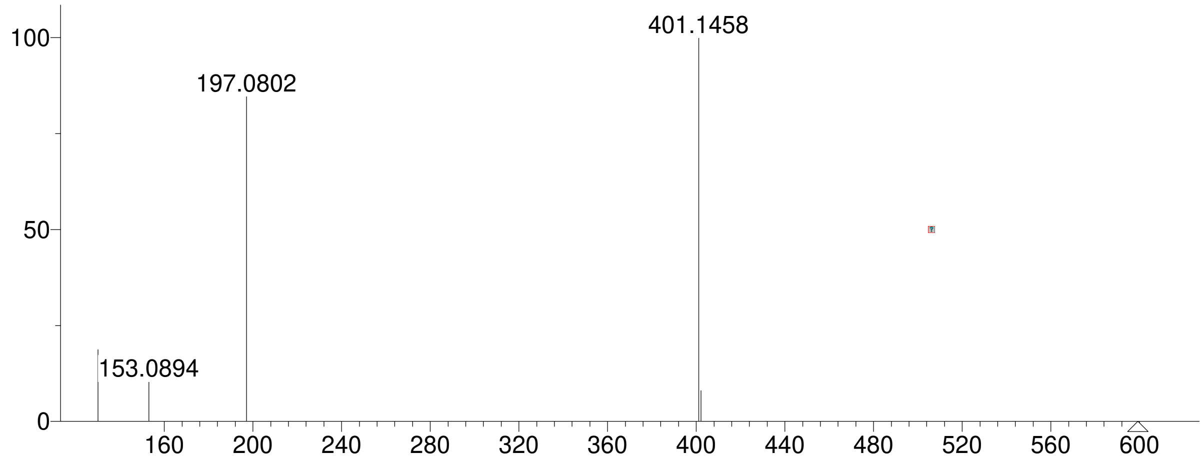

** Search Report Page 1 of 1 **

Unknown: Unknown (12.87_606.1614m/z)
Compound in Library Factor = 0

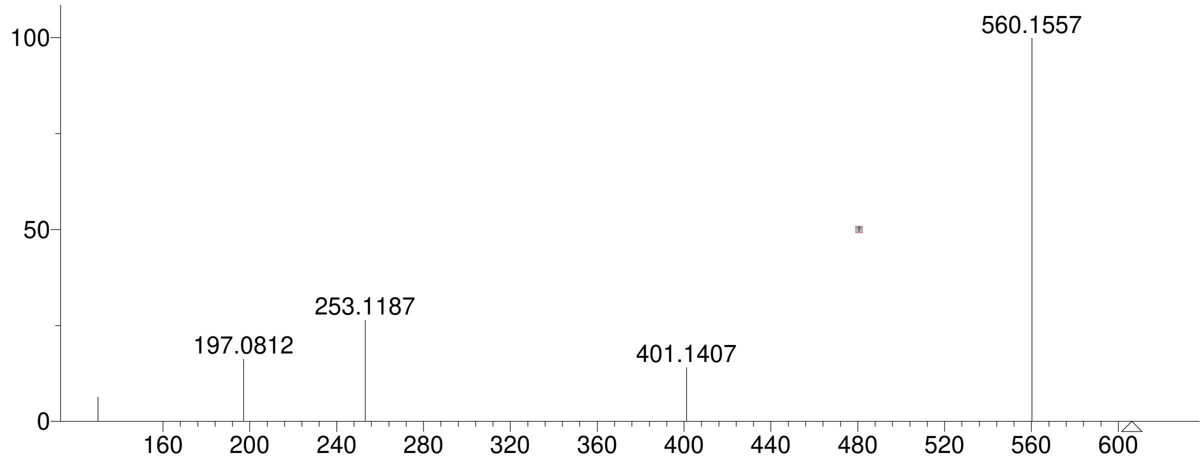

** Search Report Page 1 of 1 **

Unknown: Unknown (12.90_800.3191m/z)
Compound in Library Factor = 0

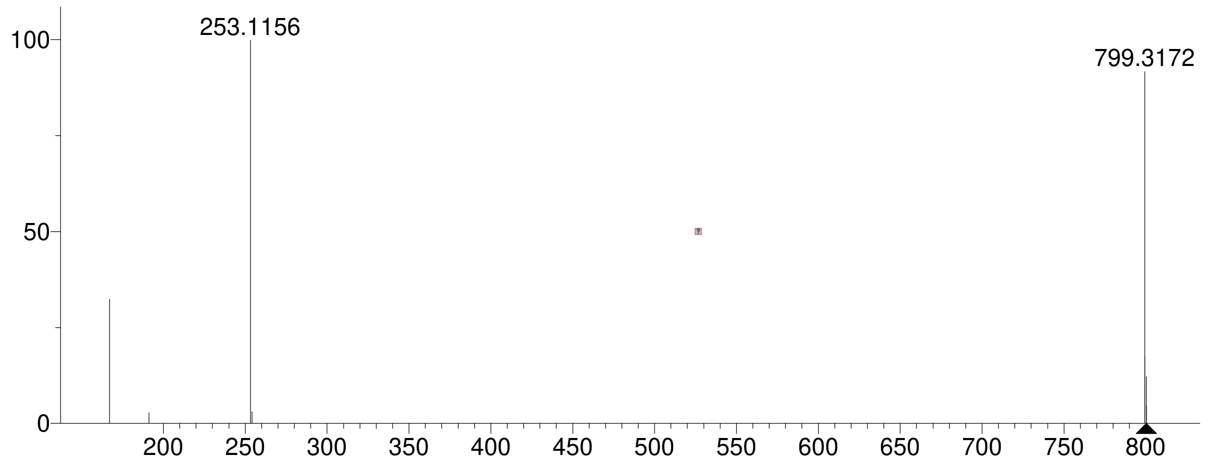

Hit 1 : NCGC00381336-01_C33H50O21_2,6-Anhydro-5-O-[4-({6-O-[4-(beta-D-glucopyranosyloxy)-2-methylenebı
C33H50O21; MF: 0; RMF: 7; Prob 100.0%; Lib: mona-export-gnps; ID: 11305.

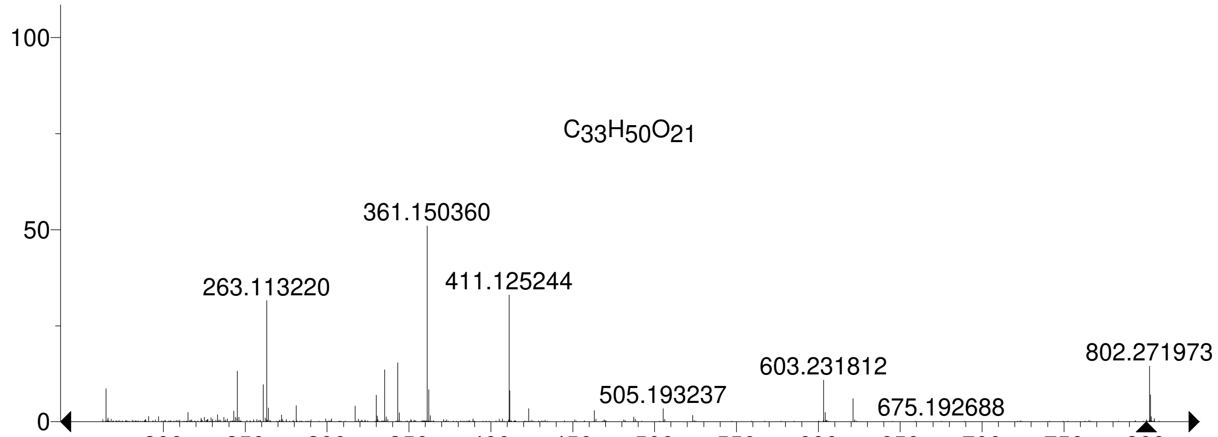

** Search Report Page 1 of 1 **

Unknown: Unknown (12.91_557.2132m/z)
Compound in Library Factor = 0

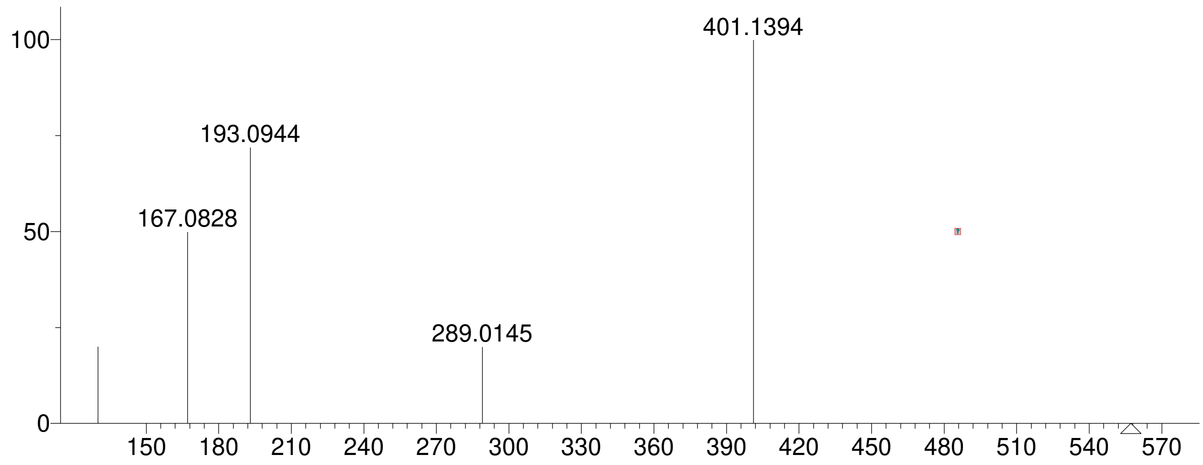

Hit 1 : (2R,3R,4S,5S,6R)-2-[(E)-3-[3,5-dimethoxy-4-[(2S,3R,4S,5S,6R)-3,4,5-trihydroxy-6-(hydroxymethyl)oxan-2-
C23H34O14; MF: 0; RMF: 31; Prob 100.0%; Lib: mona anthocyanidin-o-glycosides; ID: 2755.

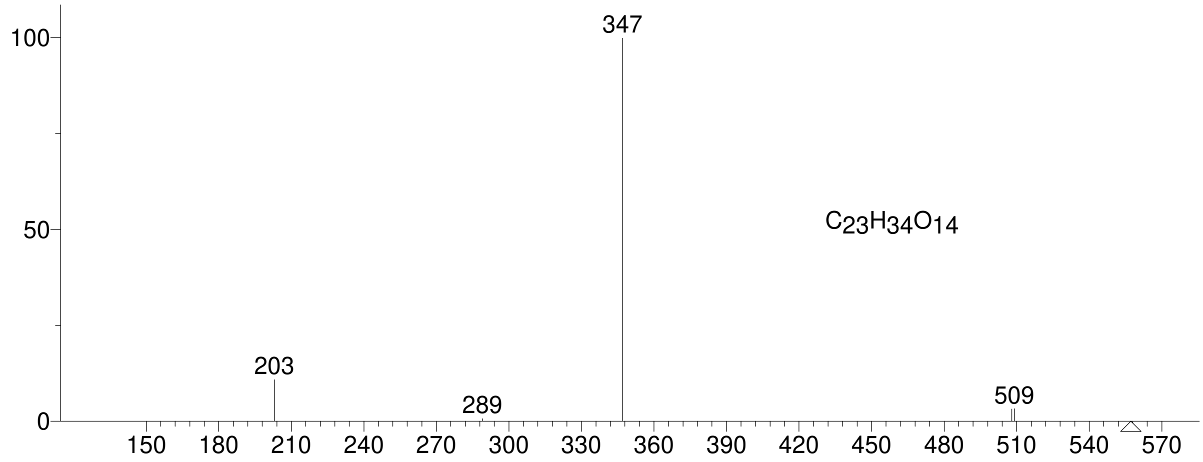

Unknown: Unknown (12.91_560.1560m/z)
Compound in Library Factor = -1072

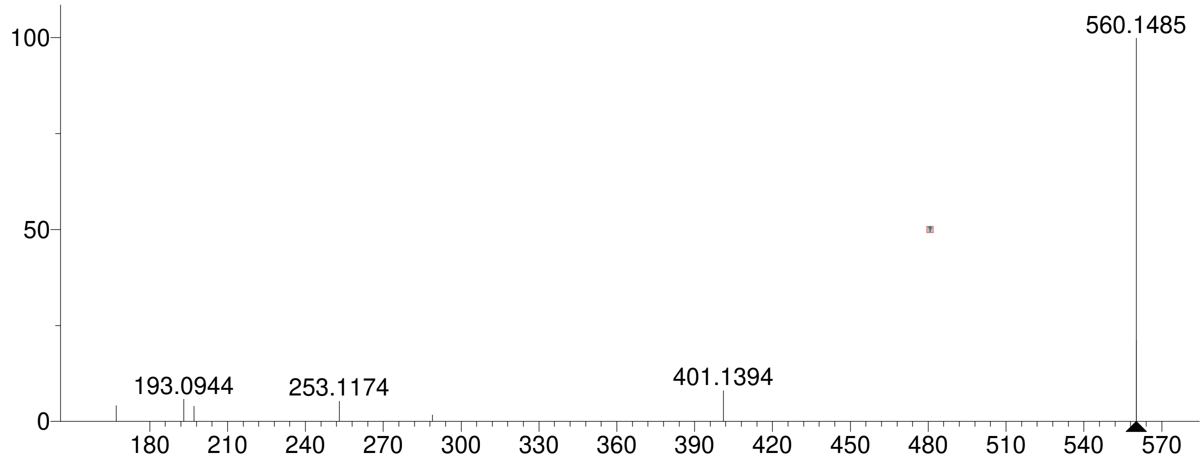

Hit 1 : Desmethylbosentan [M+Na]+ HCD 22V P=560.2
C26H27N5O6S; MF: 79; RMF: 753; Prob 74.5%; CAS: 253688-61-8; Lib: nist_msms; ID: 451527.

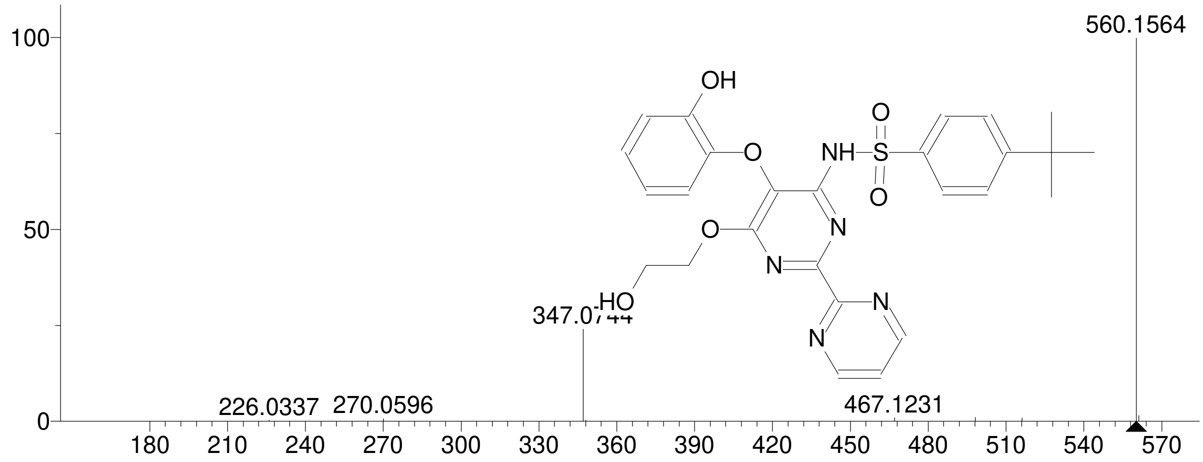

Hit 2 : (2S)-6-[(2S)-5,7-dihydroxy-2-(4-hydroxyphenyl)-4-oxo-2,3-dihydrochromen-8-yl]-5,7-dihydroxy-2-(4-hydrox
C30H22O10; MF: 0; RMF: 105; Prob 8.49%; Lib: mona-export-vf-npl_qexactive; ID: 19141.

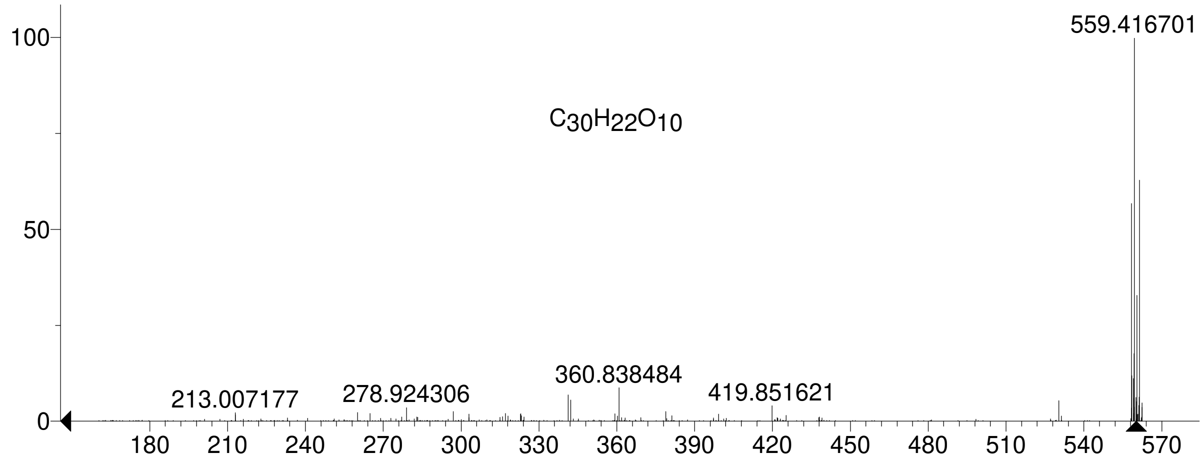

Hit 3 : (3S,4R)-3-[(3S,4R)-5,7-dihydroxy-4-(4-hydroxyphenyl)-2-oxo-3,4-dihydrochromen-3-yl]-5,7-dihydroxy-4-(4-l
C30H22O10; MF: 0; RMF: 47; Prob 8.49%; Lib: mona-export-vf-npl_qexactive; ID: 20569.

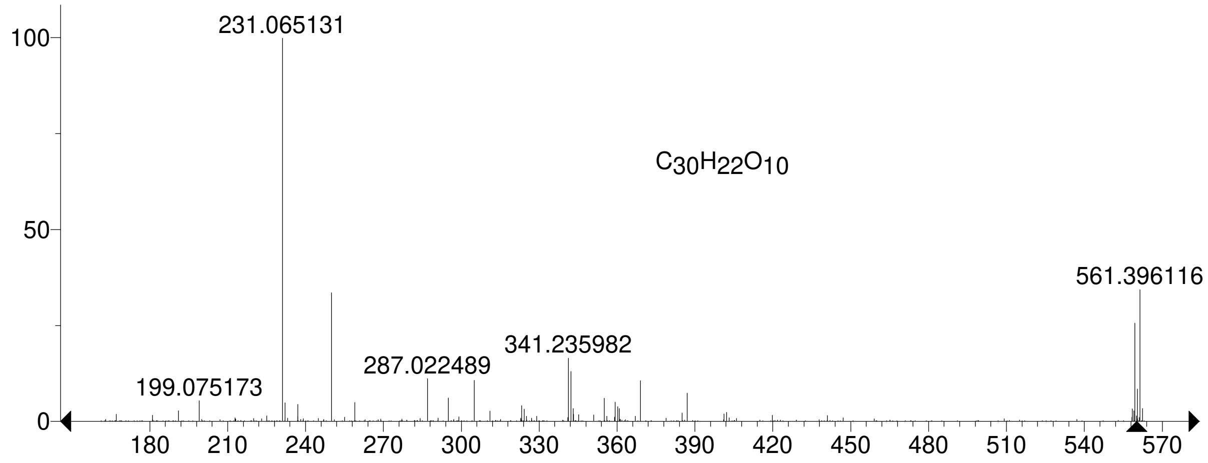

Hit 4 : 3-[1,1-bis(4-hydroxyphenyl)-3-oxo-3-(2,4,6-trihydroxyphenyl)propan-2-yl]-5,7-dihydroxychromen-4-one [M-
C30H22O10; MF: 0; RMF: 8; Prob 8.49%; Lib: mona-export-vf-npl_qexactive; ID: 22699.

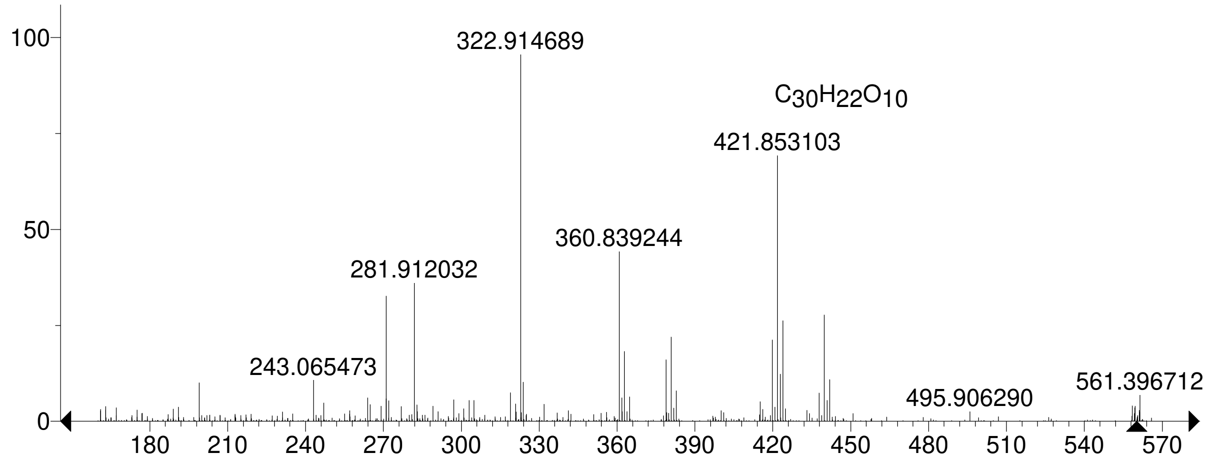

** Search Report Page 1 of 1 **

Unknown: Unknown (12.95_398.0383m/z)
Compound in Library Factor = 0

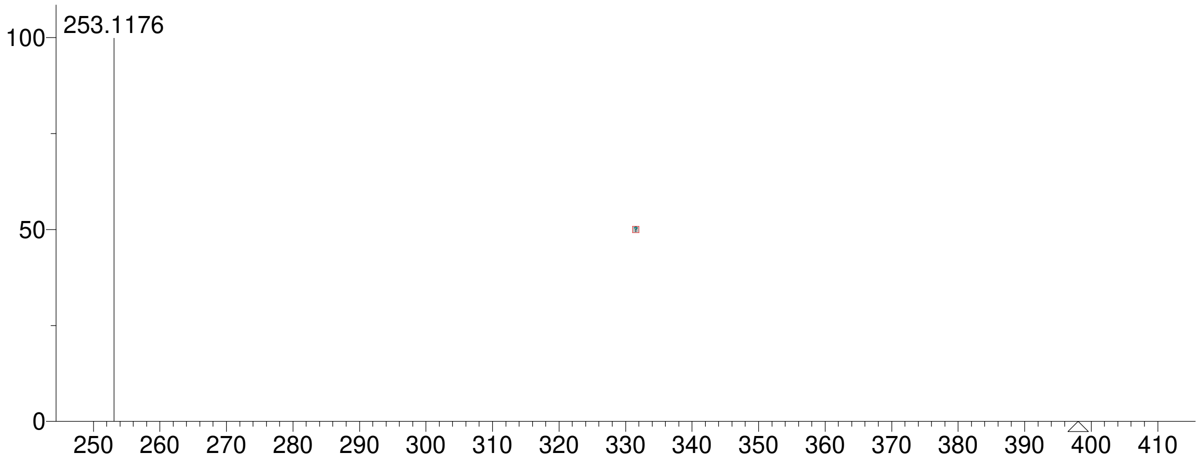

Unknown: Unknown (12.98_472.0569m/z)
Compound in Library Factor = 0

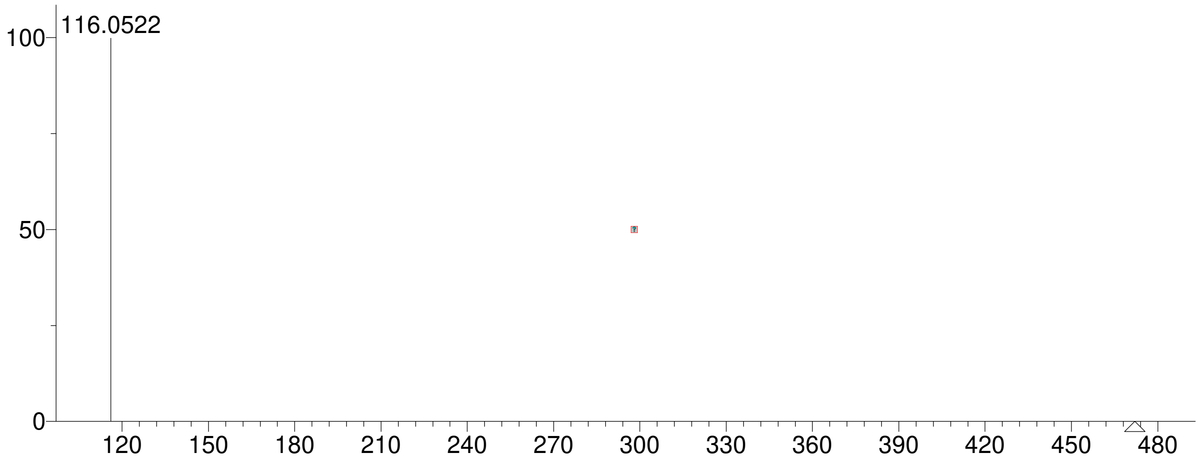

Unknown: Unknown (13.00_560.2137m/z)
Compound in Library Factor = 0

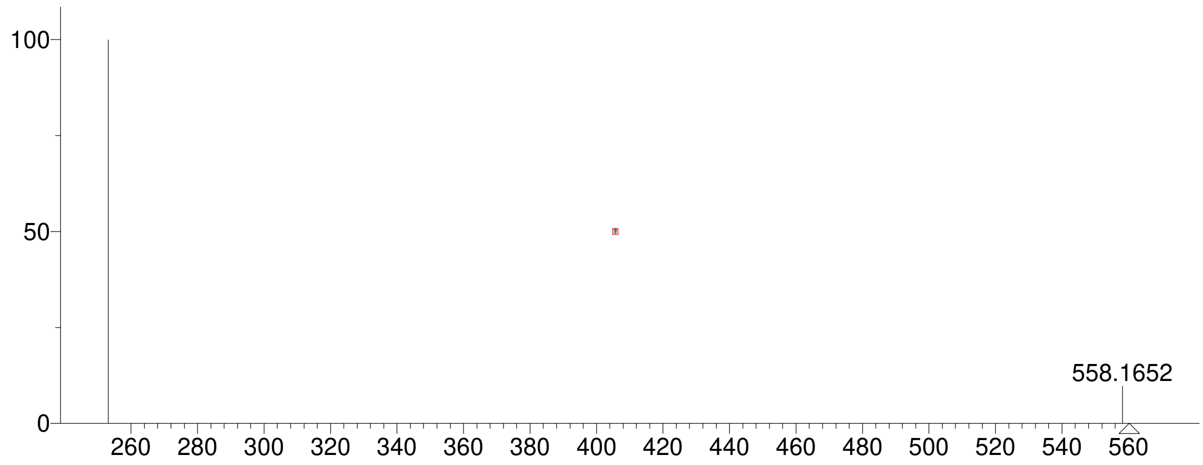

Unknown: Unknown (13.05_542.2326m/z)
Compound in Library Factor = 0

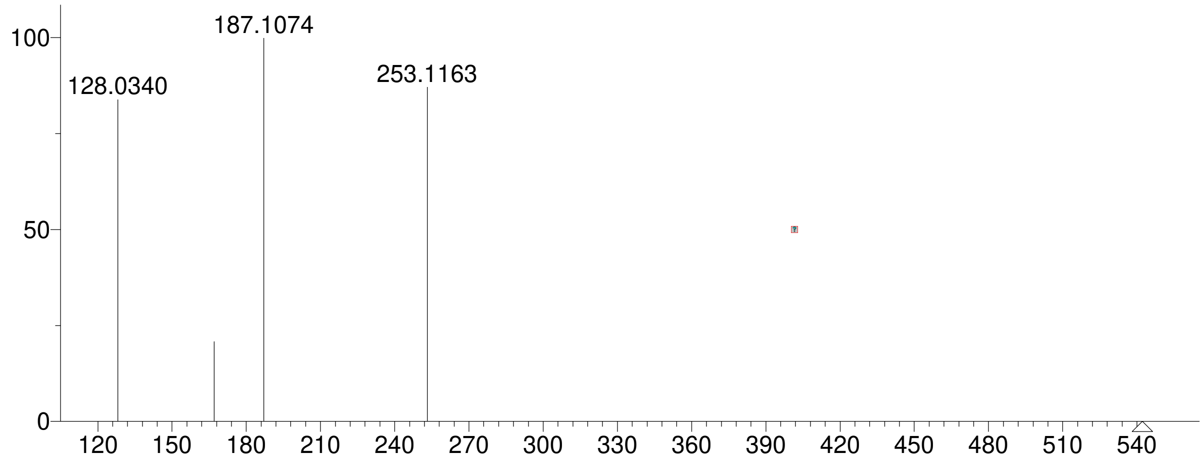

Hit 1 : NCGC00380192-01! [M+H]+ P=542.2
C29H35NO9; MF: 0; RMF: 51; Prob 100.0%; Lib: mona-export-gnps; ID: 8431.

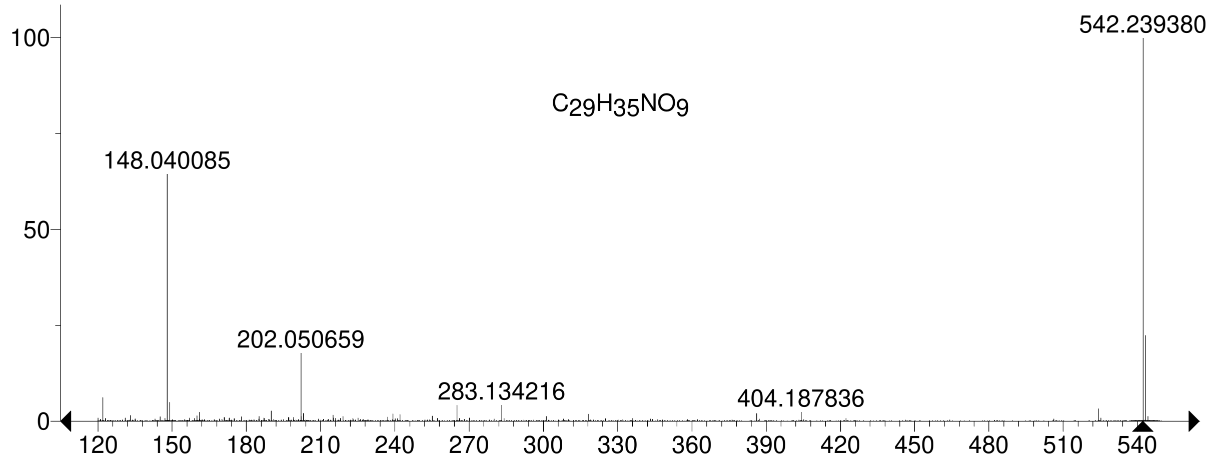

Unknown: Unknown (13.12_276.9356m/z)
Compound in Library Factor = 0

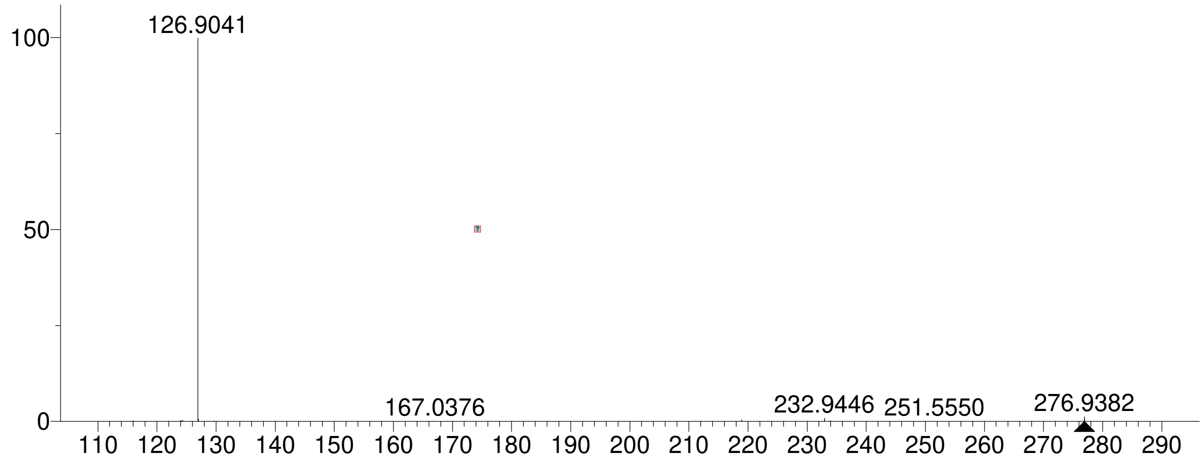

Unknown: Unknown (13.18_543.1971m/z)
Compound in Library Factor = 0

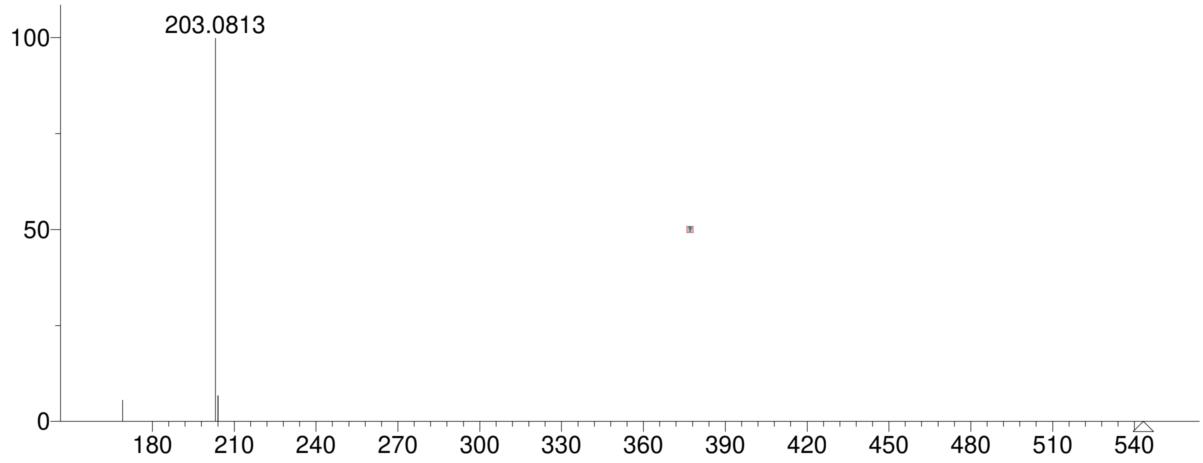

Hit 1 : NCGC00385982-01_C27H30N2O10_ [M+H]+ P=543.2
C27H30N2O10; MF: 0; RMF: 8; Prob 100.0%; Lib: mona-export-gnps; ID: 10901.

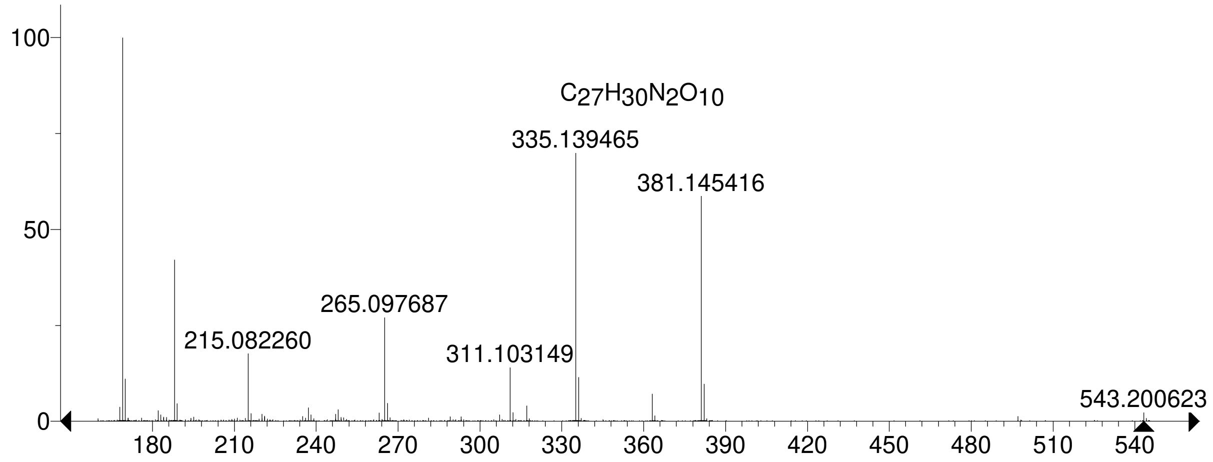

Unknown: Unknown (13.20_387.9955m/z)
Compound in Library Factor = 0

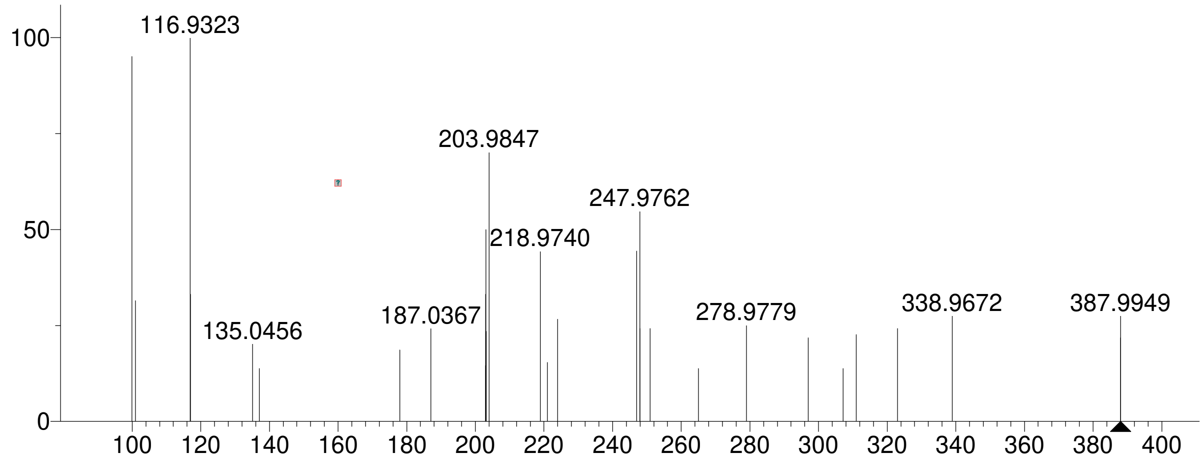

** Search Report Page 1 of 1 **

Unknown: Unknown (13.21_432.9916m/z)
Compound in Library Factor = 0

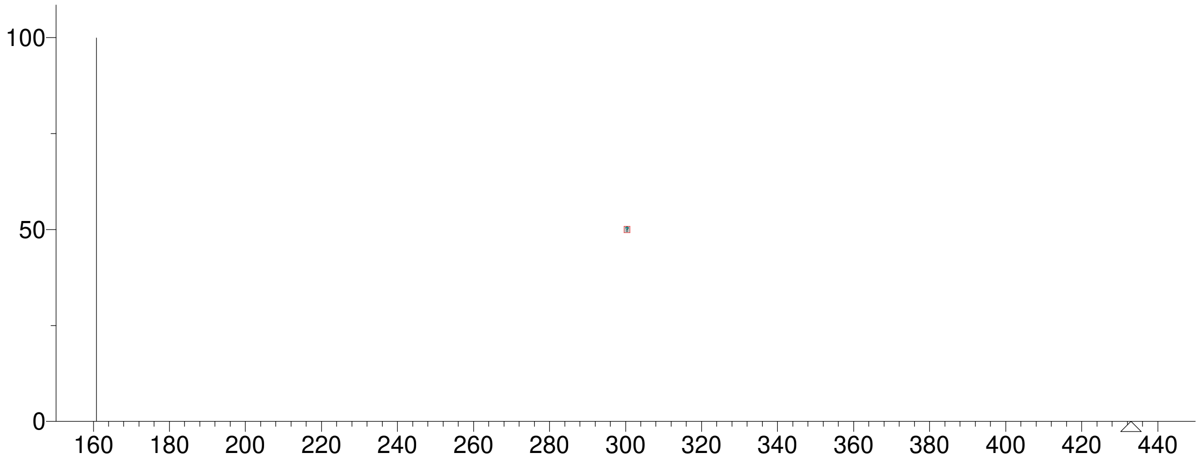

Unknown: Unknown (13.21_514.2391m/z)
Compound in Library Factor = -1142

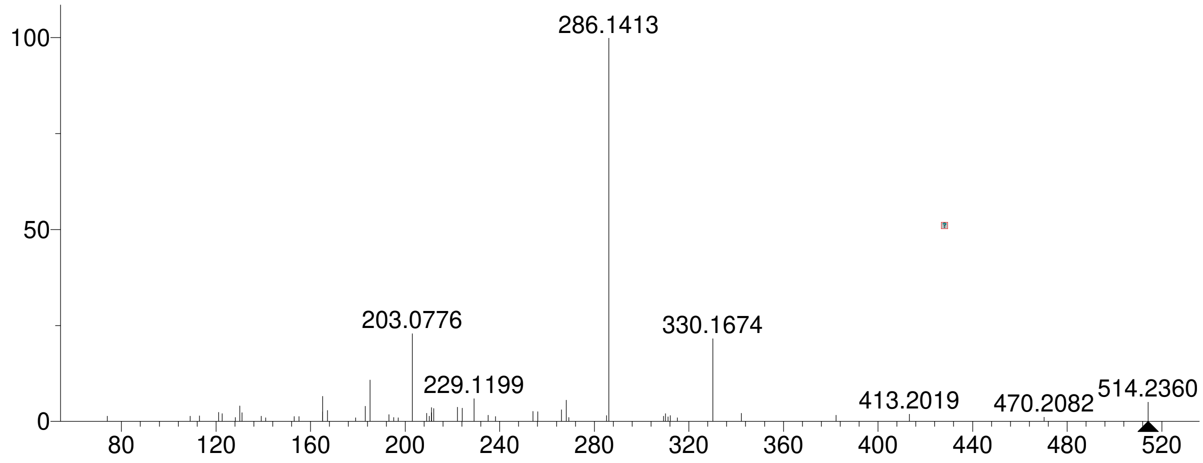

Hit 1 : Benzoic acid, 3-[[(3-carboxycyclohexyl)amino]carbonyl]-4-[3-[4-[4-(cyclohexyloxy)butoxy]phenyl]propoxy]-
C34H45NO8; MF: 82; RMF: 669; Prob 60.8%; CAS: 712313-35-4; Lib: nist_msms; ID: 190540.

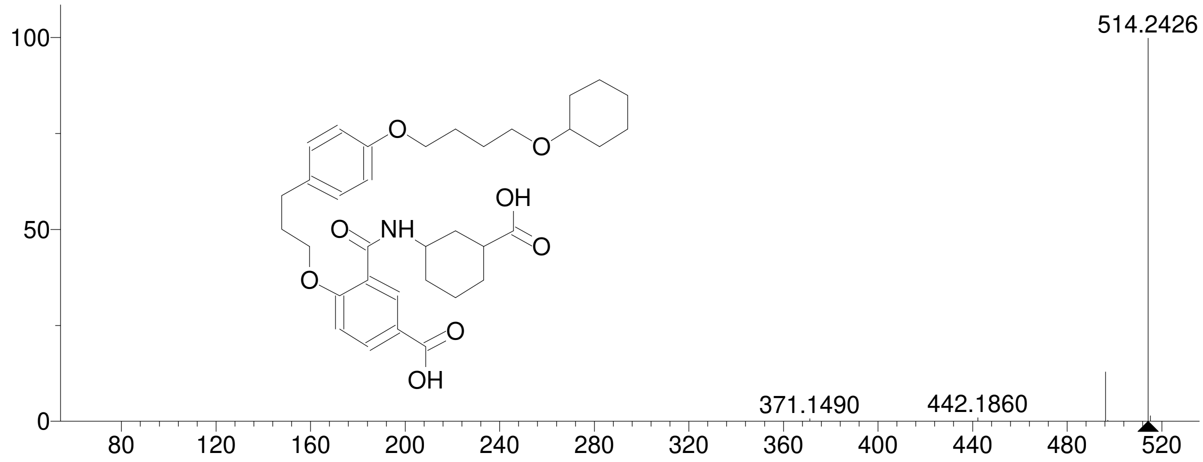

Hit 2 : 1,2-Bis(heptanoylthio) glycerophosphocholine [M+H]+ HCD 9V P=514.2
C22H44NO6PS2; MF: 14; RMF: 150; Prob 9.22%; CAS: 89019-63-6; Lib: nist_msms; ID: 171124.

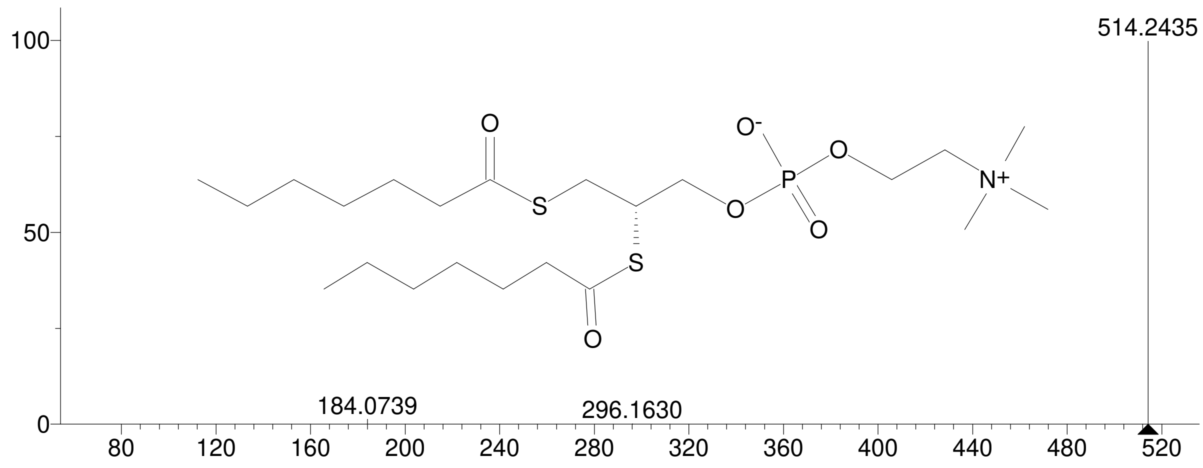

Hit 3 : Harringtonine [M+H-H2O]+ HCD 23V P=514.2
C28H37NO9; MF: 9; RMF: 152; Prob 7.43%; CAS: 26833-85-2; Lib: nist_msms; ID: 359989.

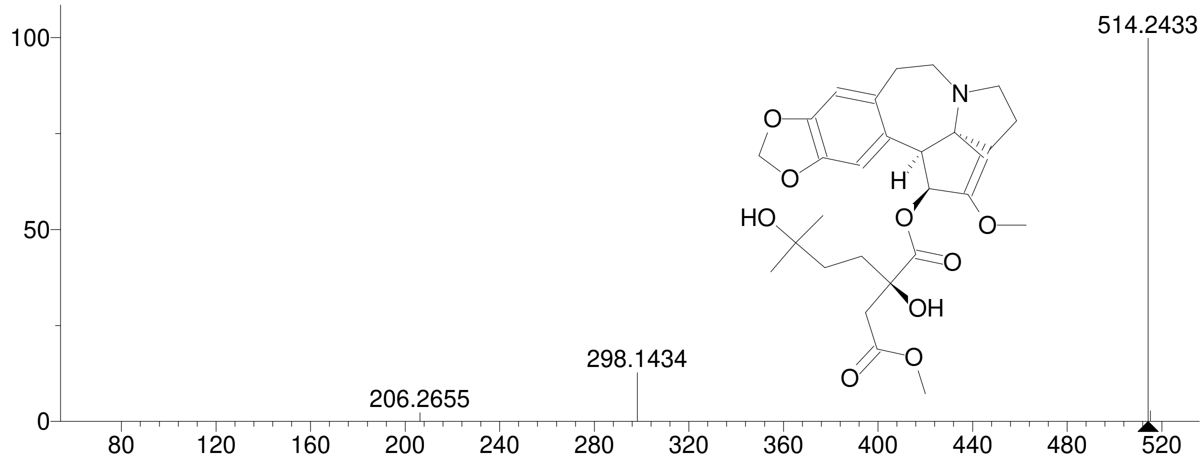

Hit 4 : (1R,4S,7R,8S)-7-[(2E,4E)-hexa-2,4-dienoyl]-3-hydroxy-8-[(2S)-3-hydroxy-2,4-dimethyl-5-oxo-2,5-dihydrofu
C28H32O8; MF: 6; RMF: 87; Prob 6.56%; Lib: mona-export-vf-npl_qexactive; ID: 21801.

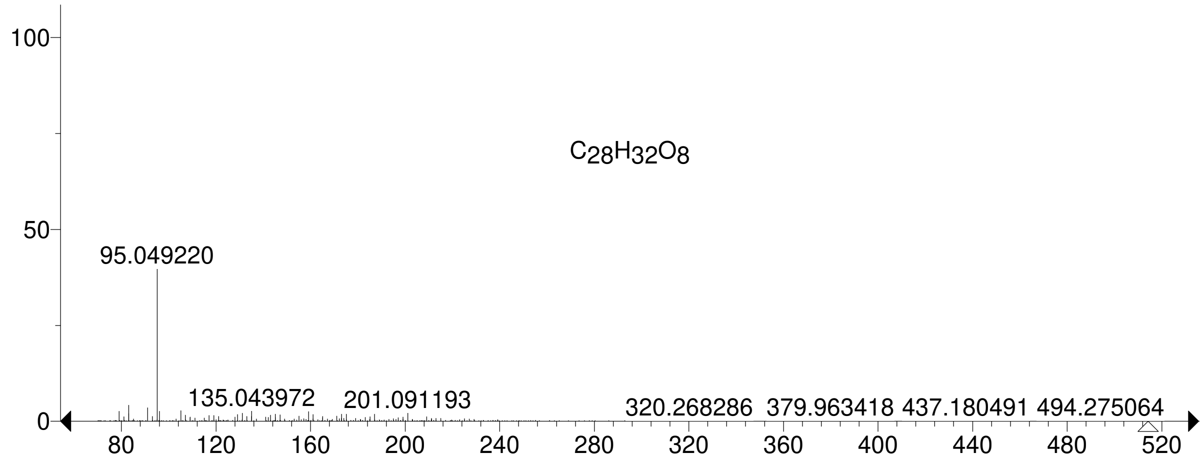

Hit 5 : Fluprostenol serinolamide [M+H-H2O]+ HCD 15V P=514.2
C26H36F3NO7; MF: 2; RMF: 116; Prob 5.54%; CAS: 1176658-85-7; Lib: nist_msms; ID: 339463.

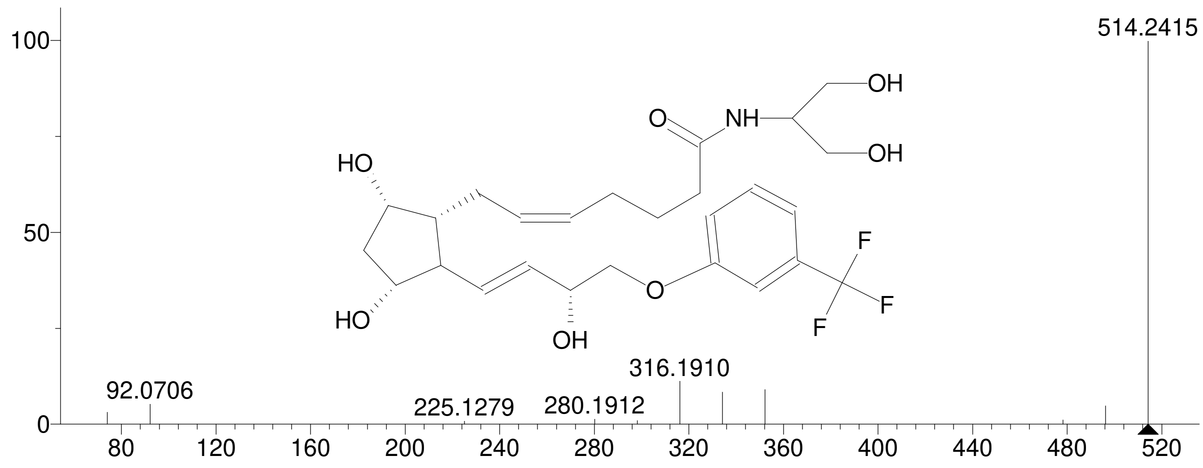

Unknown: Unknown (13.22_448.9147m/z)
Compound in Library Factor = 0

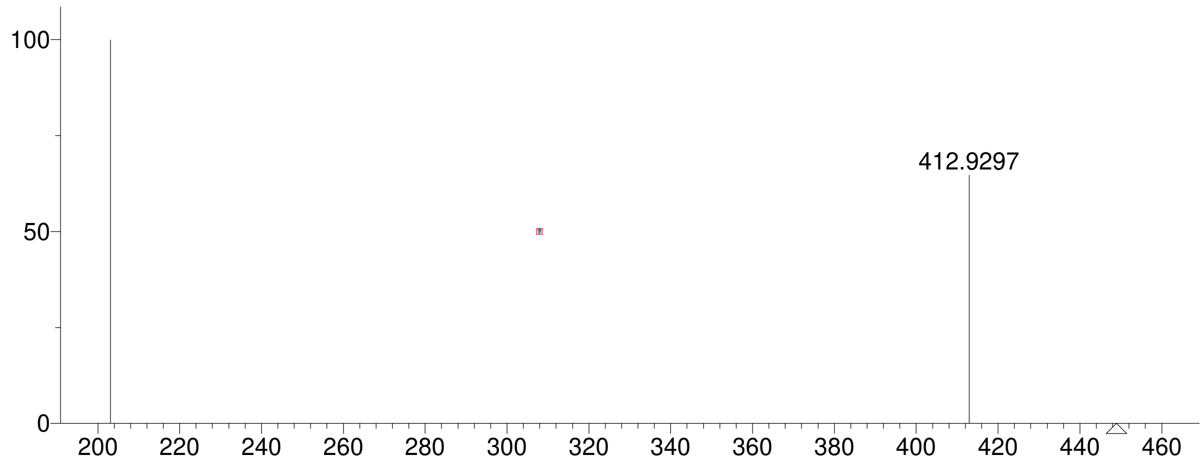

Unknown: Unknown (13.23_543.1980m/z)
Compound in Library Factor = 0

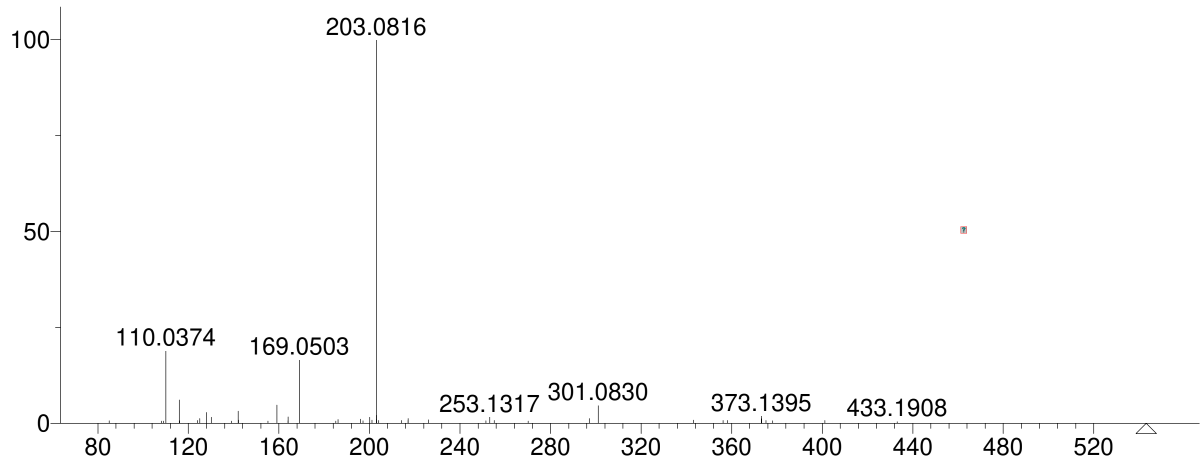

Hit 1 : NCGC00385982-01_C27H30N2O10_ [M+H]+ P=543.2
C27H30N2O10; MF: 0; RMF: 25; Prob 33.3%; Lib: mona-export-gnps; ID: 10901.

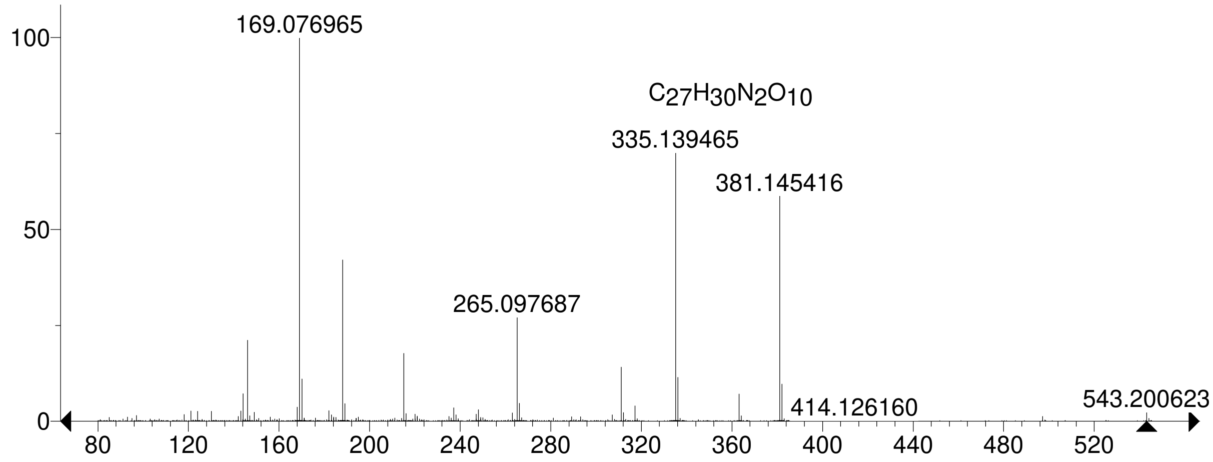

Hit 2 : 2',6'-Dihydroxy 4'-methoxydihydrochalcone [2M-H]- HCD 59V P=543.2
C16H16O4; MF: 0; RMF: 6; Prob 33.3%; CAS: 35241-55-5; Lib: nist_msms; ID: 241376.

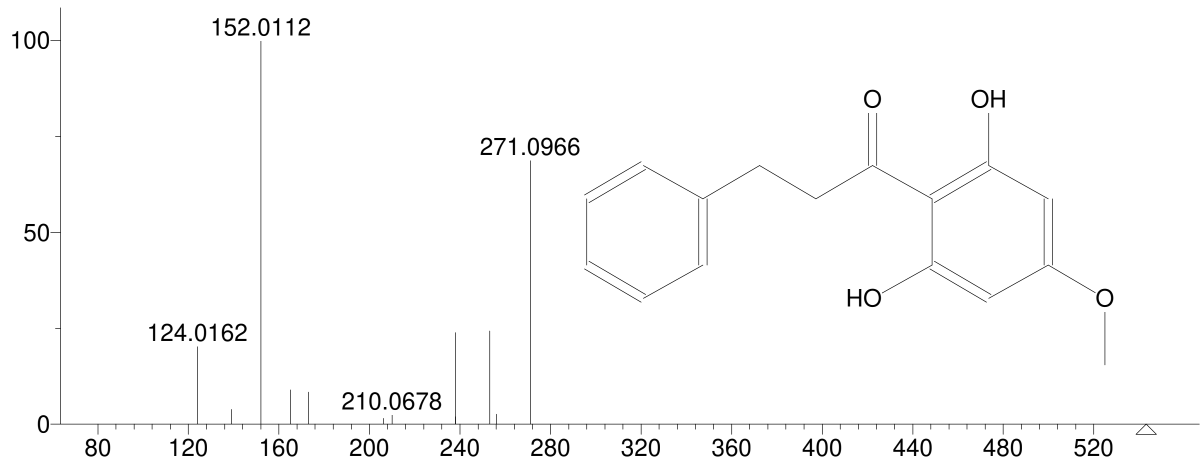

Hit 3 : Prostaglandin F2α-biotinamide [M+Na]+=>643.4=>543.2 IT 35% P=543.2
C35H60N4O6S; MF: 0; RMF: 2; Prob 33.3%; Lib: nist_msms; ID: 95551.

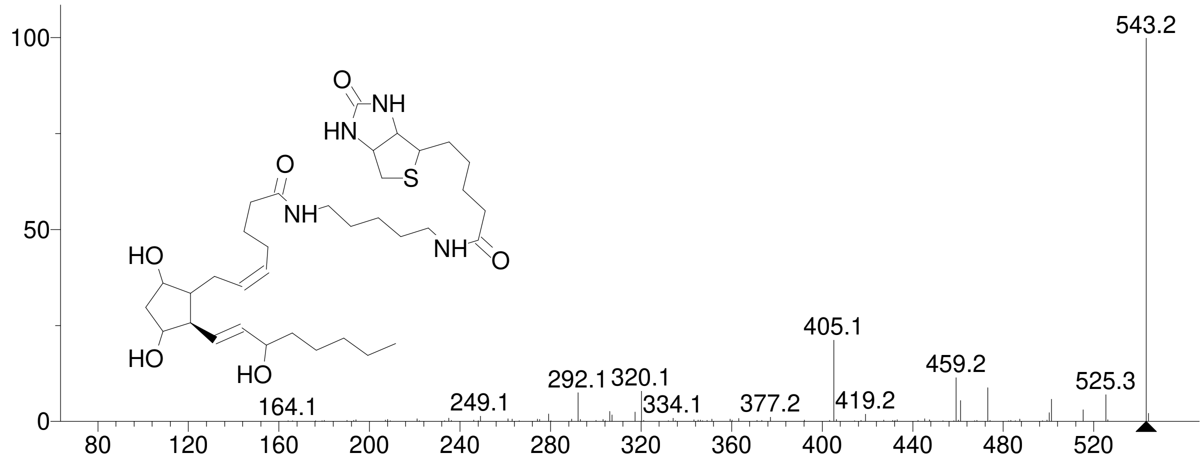

Unknown: Unknown (13.26_289.2008m/z)
Compound in Library Factor = -2176

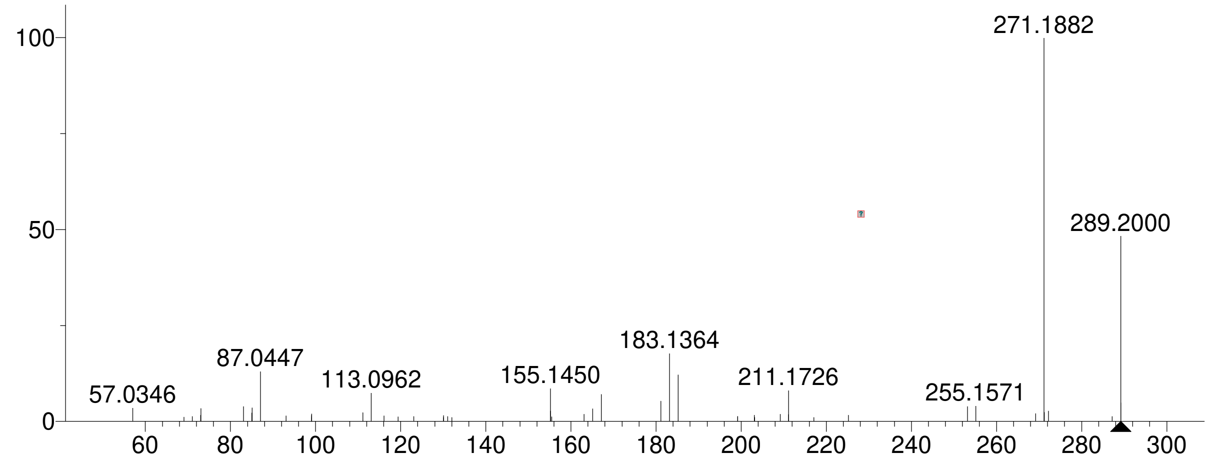

Hit 1 : 9-Oxo-11α,16R-dihydroxy-17-cyclobutyl-5Z,13E-dien-1-oic acid [M+Na]+=>289.2 IT 35% P=289.2
C23H36O5; MF: 47; RMF: 530; Prob 10.7%; CAS: 212310-16-2; Lib: nist_msms; ID: 172350.

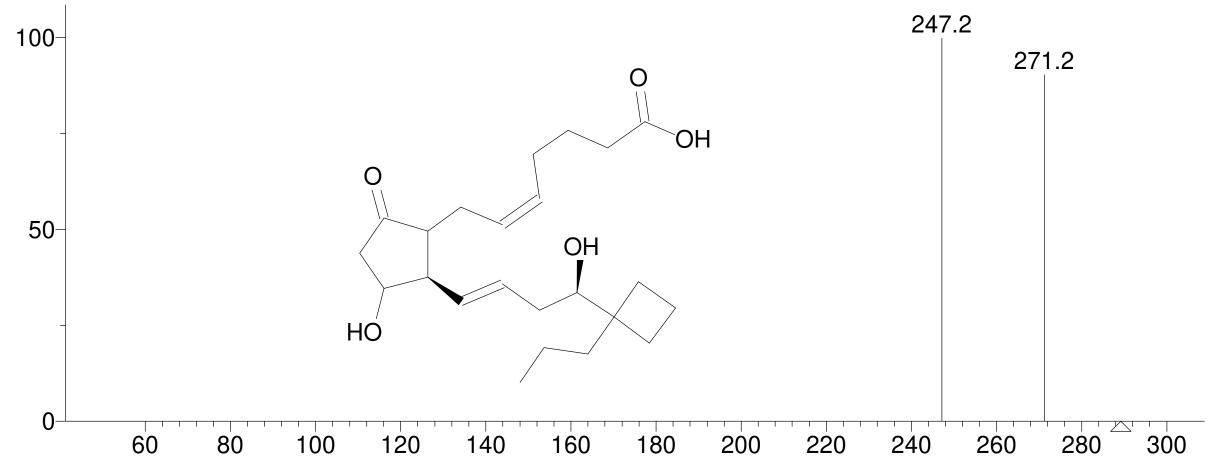

Hit 2 : Tubastatin A [M+H]+=>307.2=>289.2 IT 35% P=289.2
C20H21N3O2; MF: 22; RMF: 319; Prob 3.26%; CAS: 1252003-15-8; Lib: nist_msms; ID: 168849.

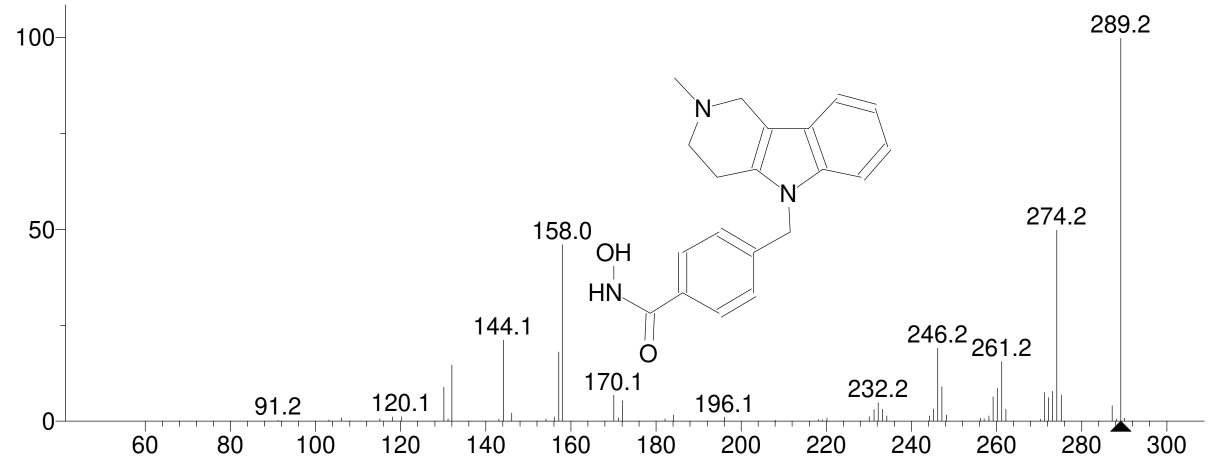

Hit 3 : 5(S),6(R),15(R)-Lipoxin A4 [M-H]-=>289.2 IT 35% P=289.2
C20H32O5; MF: 16; RMF: 388; Prob 2.56%; CAS: 171030-11-8; Lib: nist_msms; ID: 91802.

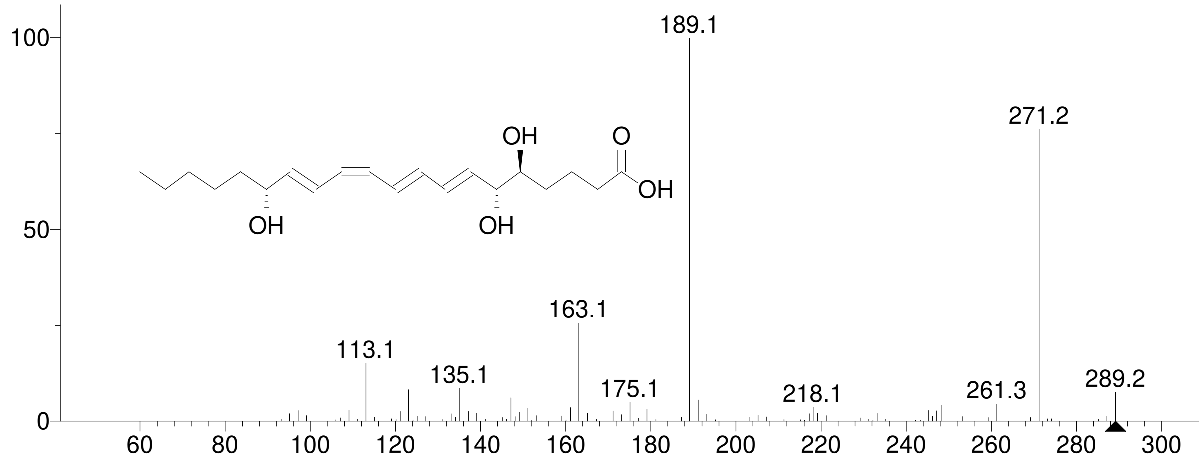

Hit 4 : 5-trans-Prostaglandin D2 [M-H-H2O]-=>289.2 IT 35% P=289.2
C20H32O5; MF: 10; RMF: 396; Prob 2.01%; Lib: nist_msms; ID: 341164.

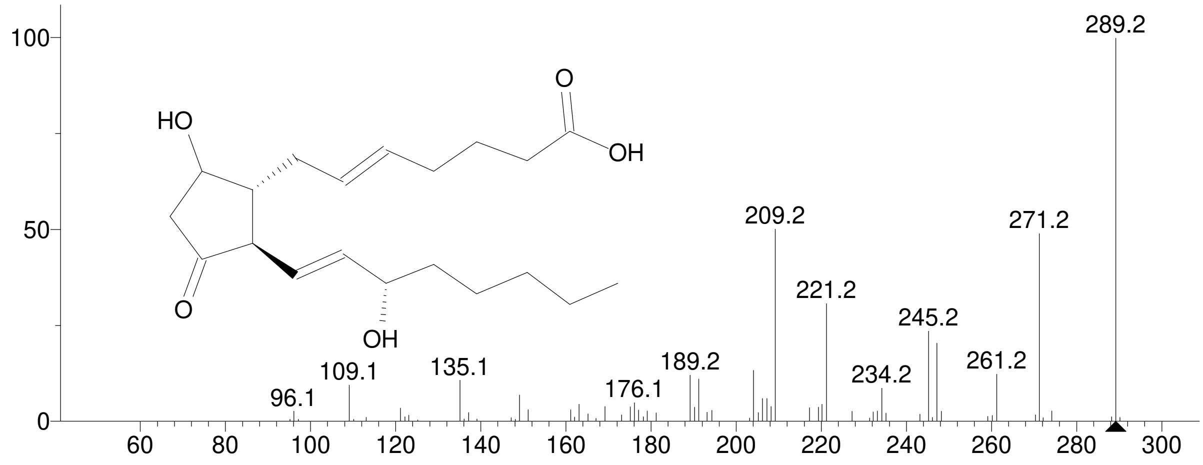

Hit 5 : 8-iso-Prostaglandin A2 [M-H]-=>289.2 IT 35% P=289.2
C20H30O4; MF: 9; RMF: 354; Prob 1.94%; CAS: 474391-66-7; Lib: nist_msms; ID: 319265.

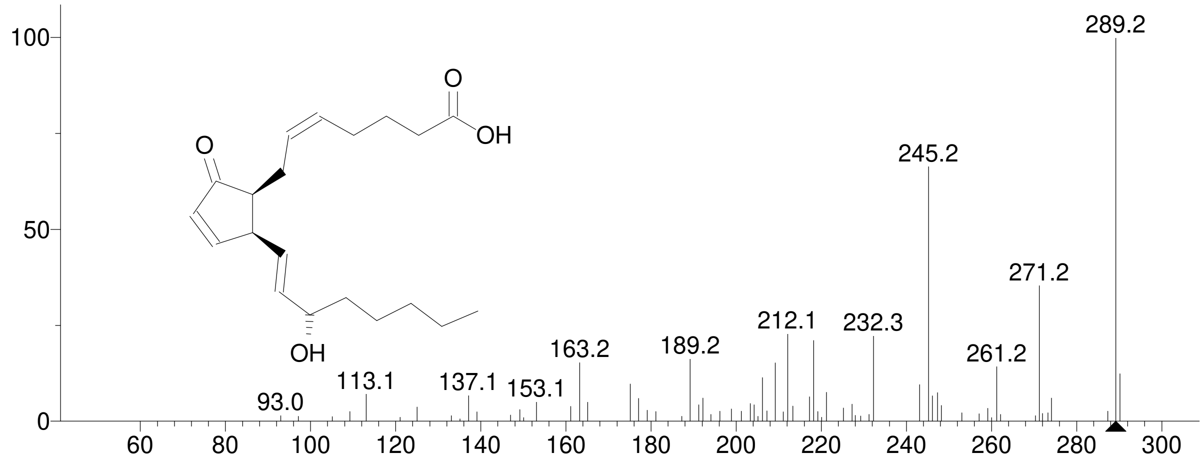

Unknown: Unknown (13.26_511.2483m/z)
Compound in Library Factor = 0

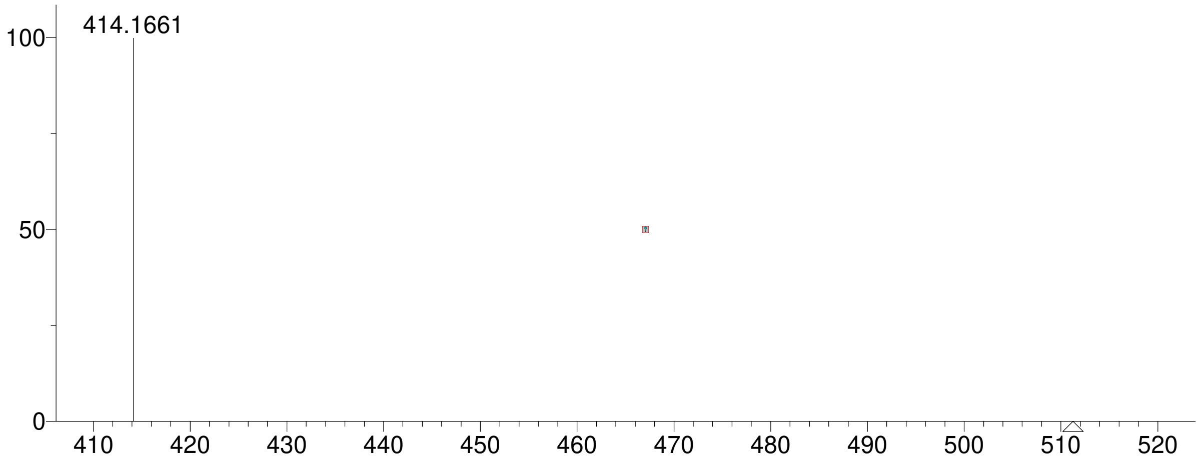

Unknown: Unknown (13.29_371.1240m/z)
Compound in Library Factor = -3273

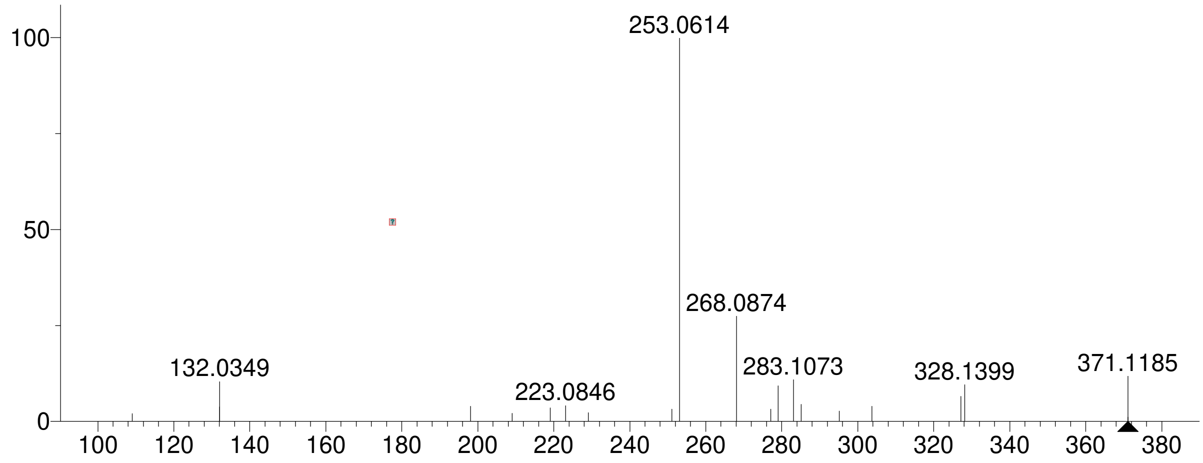

Hit 1 : 7,8-dihydroxy-3-[(1E,3E)-7-hydroxy-3,5-dimethylhepta-1,3-dienyl]-7-methyl-8H-isochromen-6-one [M+K]+
C19H24O5; MF: 1; RMF: 51; Prob 20.6%; Lib: mona-export-vf-npl_qexactive; ID: 16498.

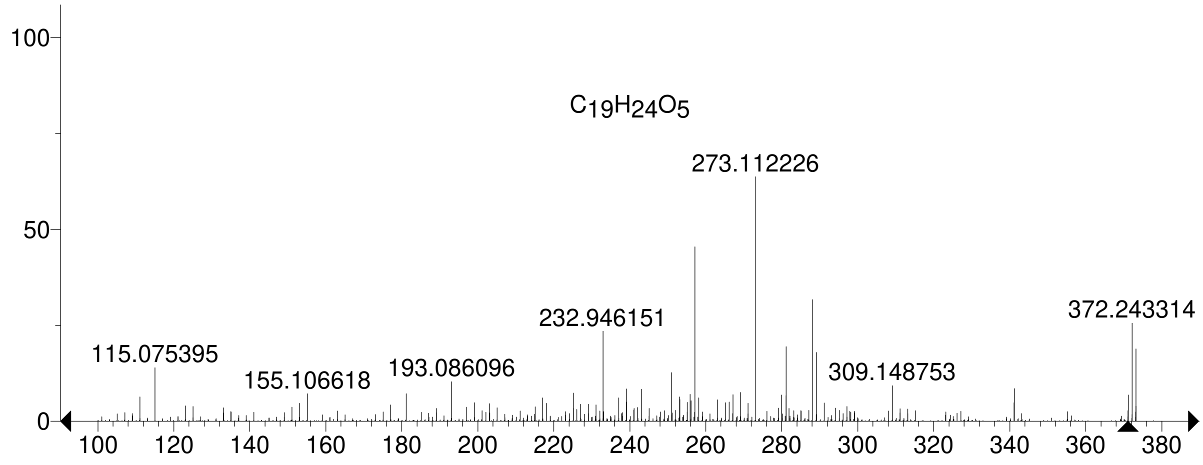

Hit 2 : G(8-O-4)5HFA'2 () [M-H]- P=371.1
C20H20O7; MF: 0; RMF: 38; Prob 19.8%; Lib: phytofrag2018_final_060219; ID: 368.

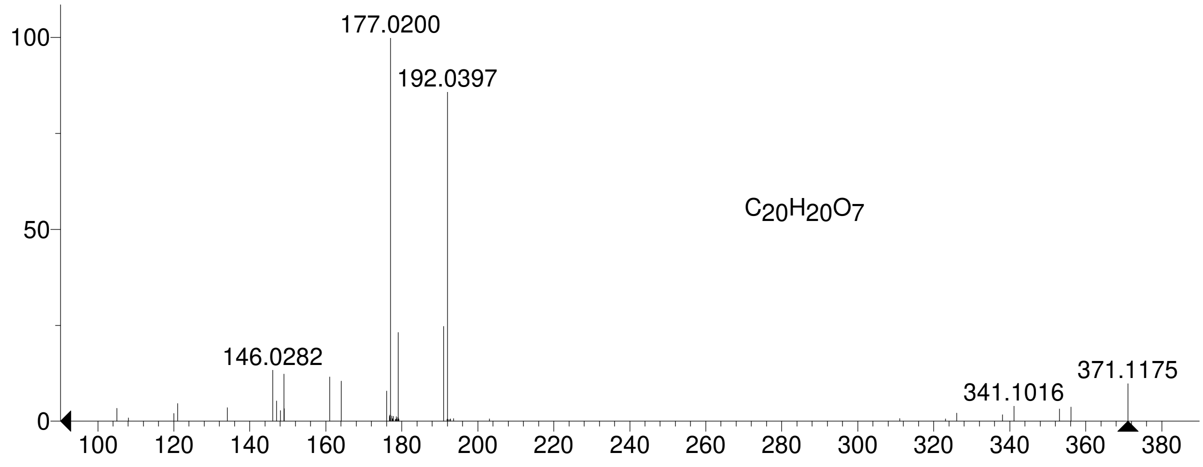

Hit 3 : Arnicolide D [M+K]+ 45HCD P=371.1
C19H24O5; MF: 0; RMF: 30; Prob 19.8%; Lib: mona-export-vf-npl_qexactive; ID: 4088.

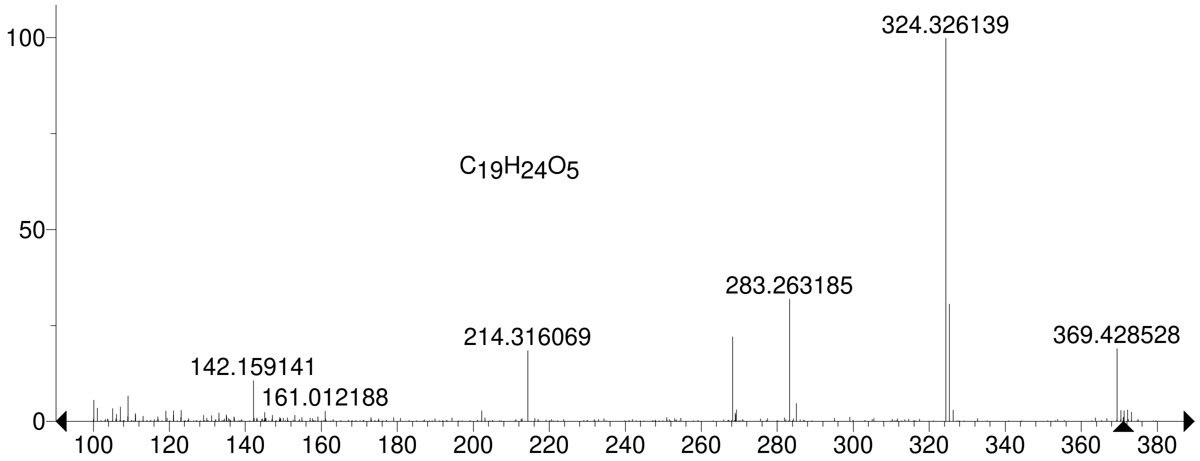

Hit 4 : 1-[2-hydroxy-4-(3-hydroxy-5-methylphenoxy)-6-methylphenyl]-3-methylbutane-2,3-diol [M+K]+ 35HCD P=
C19H24O5; MF: 0; RMF: 26; Prob 19.8%; Lib: mona-export-vf-npl_qexactive; ID: 22291.

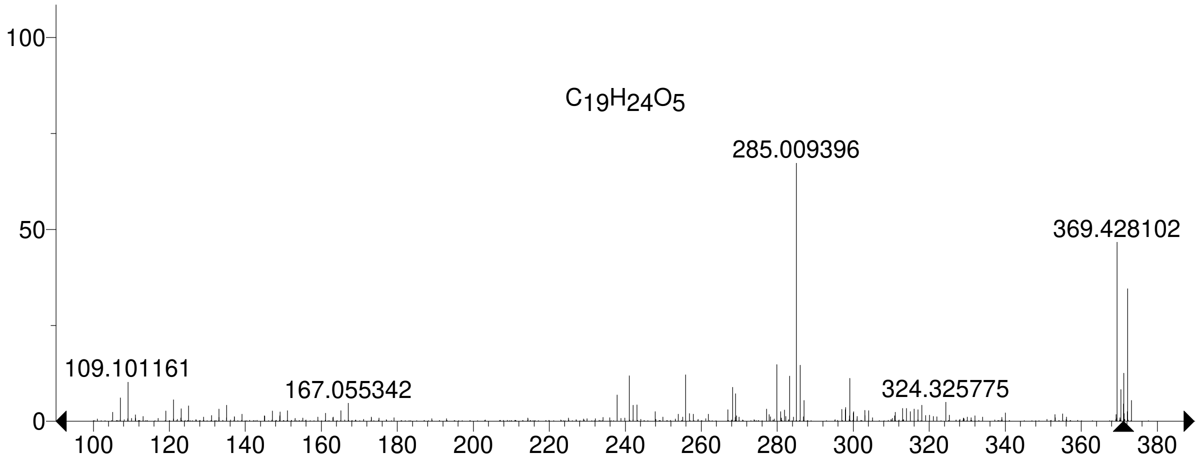

Hit 5 : methyl (2E)?3?[3?(4?hydroxy?3?methoxyphenyl)?2? (hydroxymethyl)?2,3?dihydro?1,4?benzodioxin?6? yl
C20H20O7; MF: 0; RMF: 24; Prob 19.8%; Lib: phytofrag2018_final_060219; ID: 705.

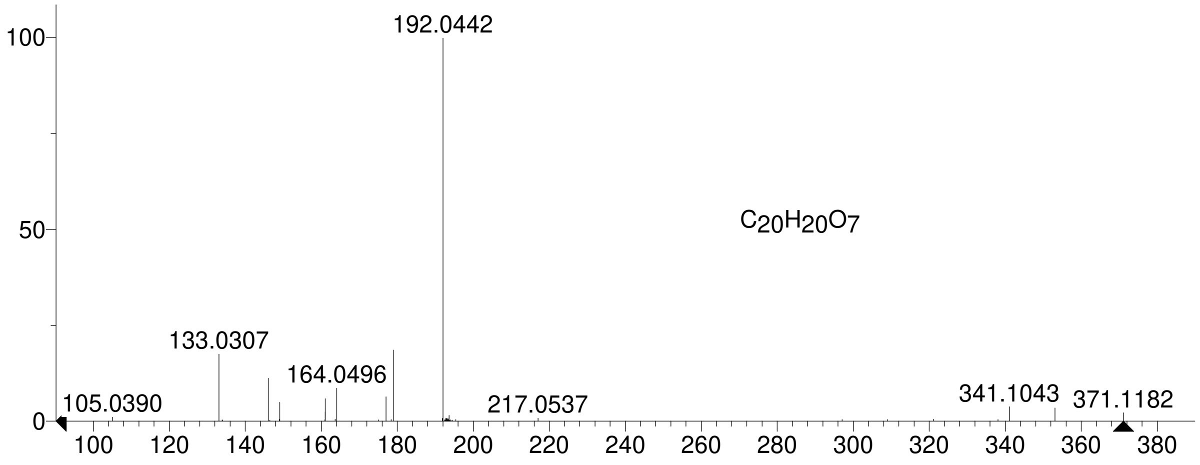

Unknown: Unknown (13.29_474.1999m/z)
Compound in Library Factor = -3273

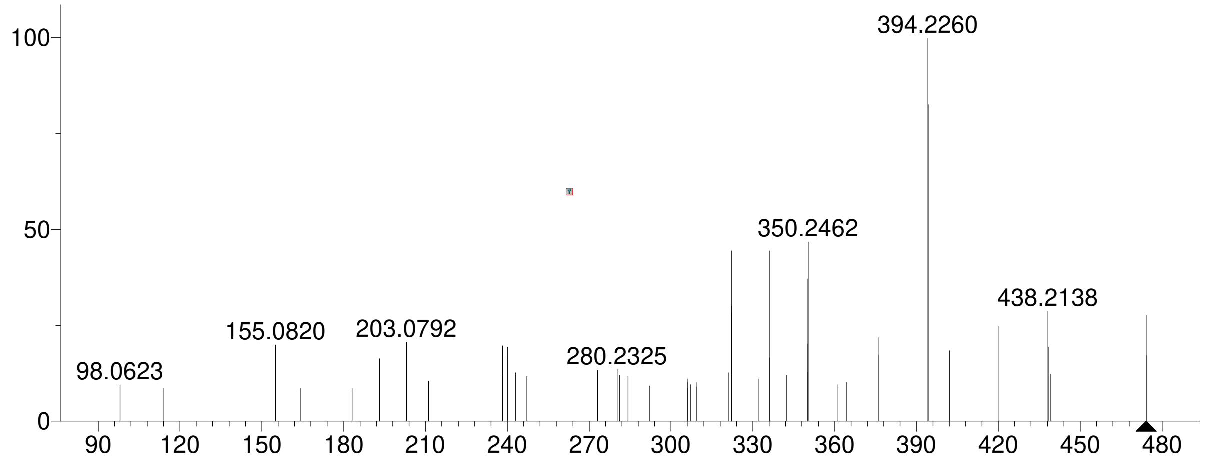

Hit 1 : Ampkinone [M-H]-=>474.2 IT 35% P=474.2
C31H23NO6; MF: 1; RMF: 69; Prob 33.8%; CAS: 1233082-79-5; Lib: nist_msms; ID: 265948.

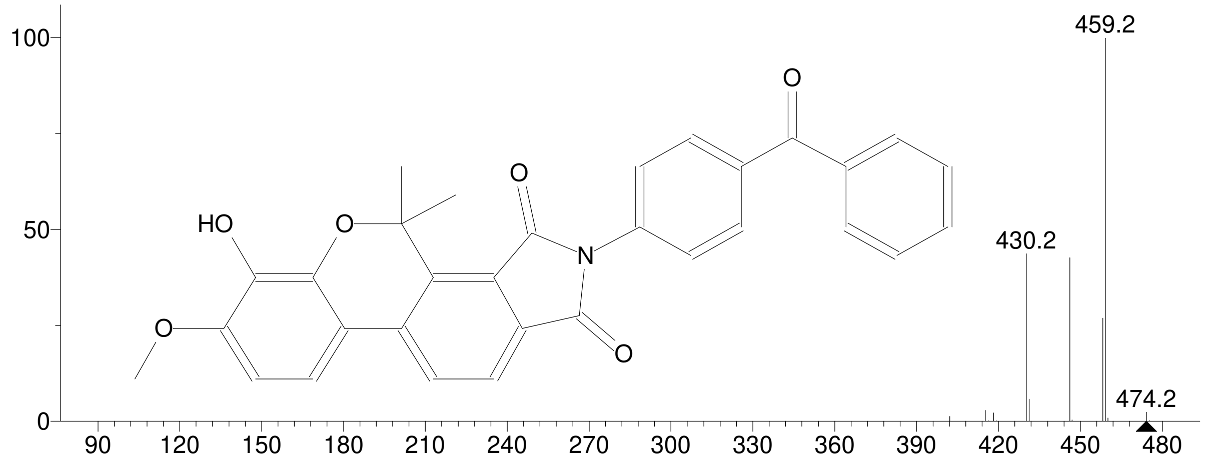

Hit 2 : 1H-Indole-3,5-dicarboxylic acid, 1-[3-[4-(decyloxy)phenoxy]-2-oxopropyl]-, 3-methyl ester [M+H-CH4O]+=>
C30H37NO7; MF: 1; RMF: 57; Prob 33.8%; CAS: 888320-29-4; Lib: nist_msms; ID: 374594.

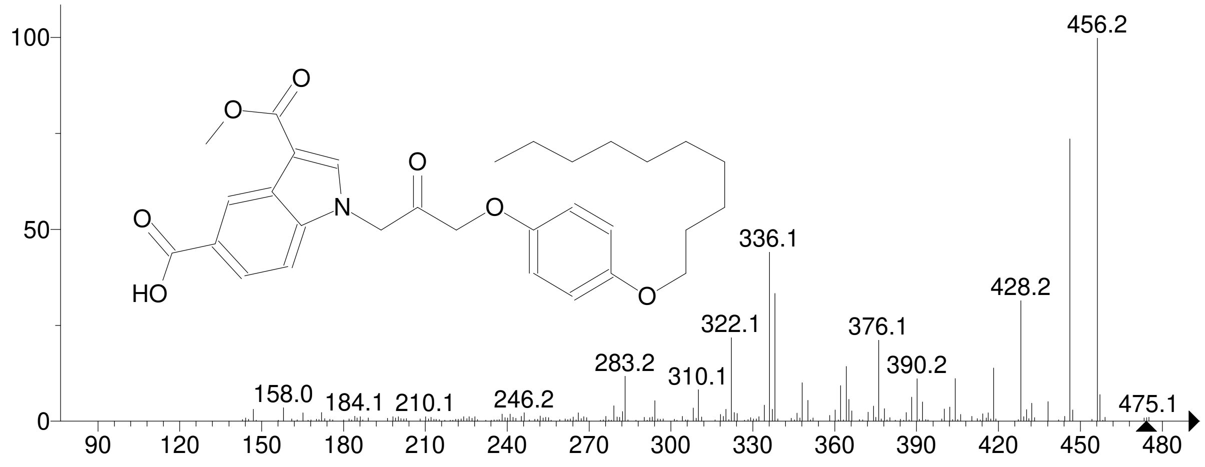

Hit 3 : NCGC00385631-01!6-[2,3-dihydroxy-3-methyl-1-[(2R,3R,4S,5S,6R)-3,4,5-trihydroxy-6-(hydroxymethyl)oxa
C21H28O11; MF: 0; RMF: 7; Prob 32.5%; Lib: mona-export-gnps; ID: 7947.

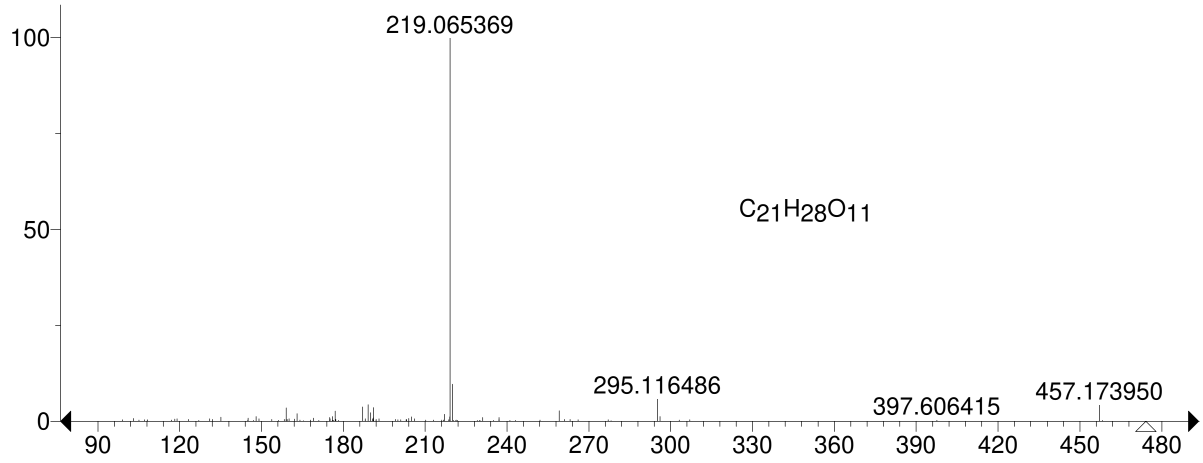

Unknown: Unknown (13.31_270.9908m/z)
Compound in Library Factor = 0

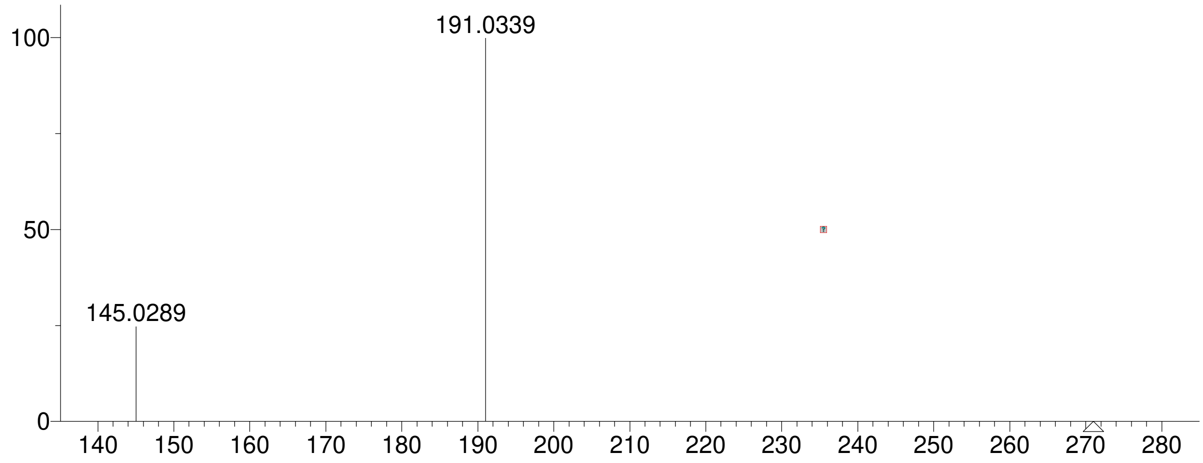

** Search Report Page 1 of 1 **

Unknown: Unknown (13.31_470.9574m/z)
Compound in Library Factor = 0

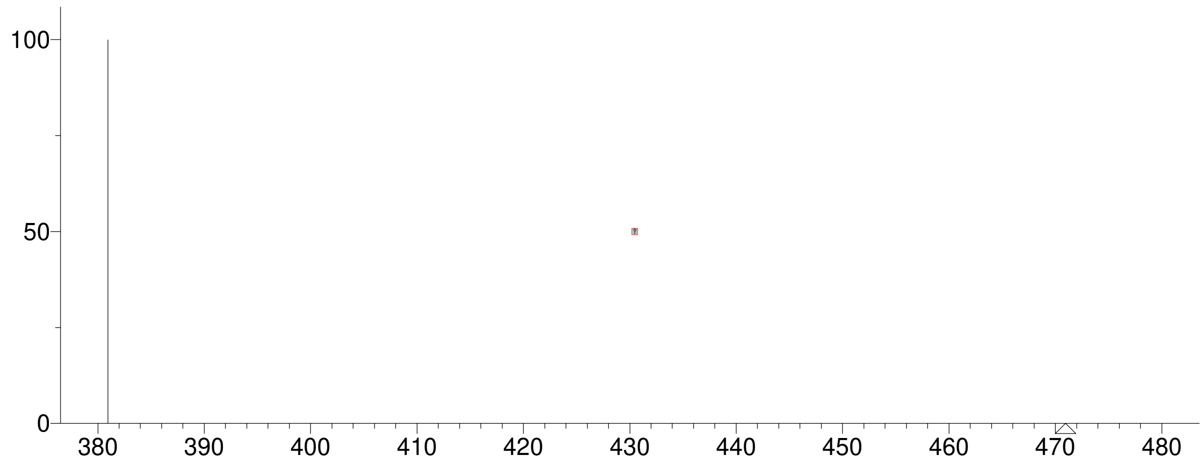

** Search Report Page 1 of 1 **

Unknown: Unknown (13.37_619.2963m/z)
Compound in Library Factor = 0

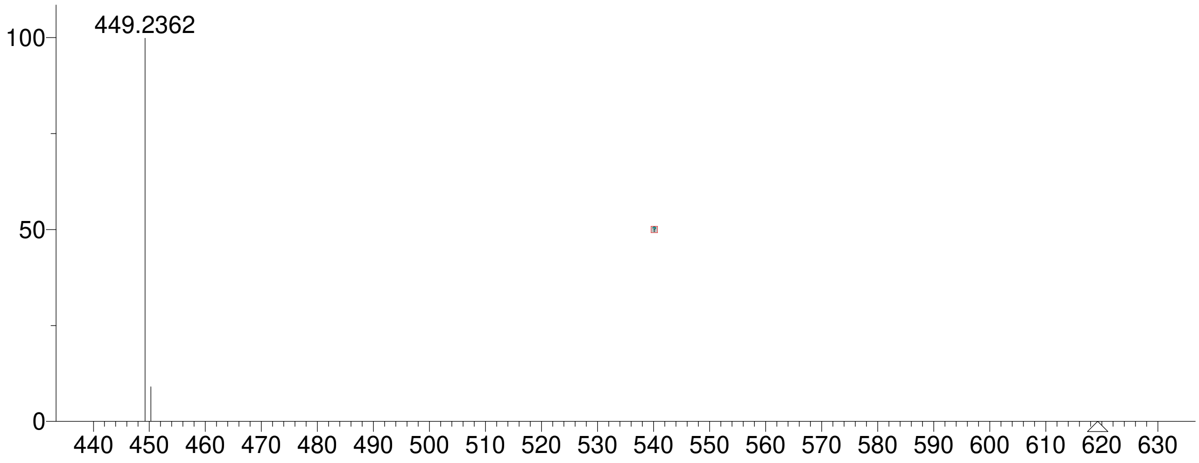

Unknown: Unknown (13.39_357.1366m/z)
Compound in Library Factor = -302

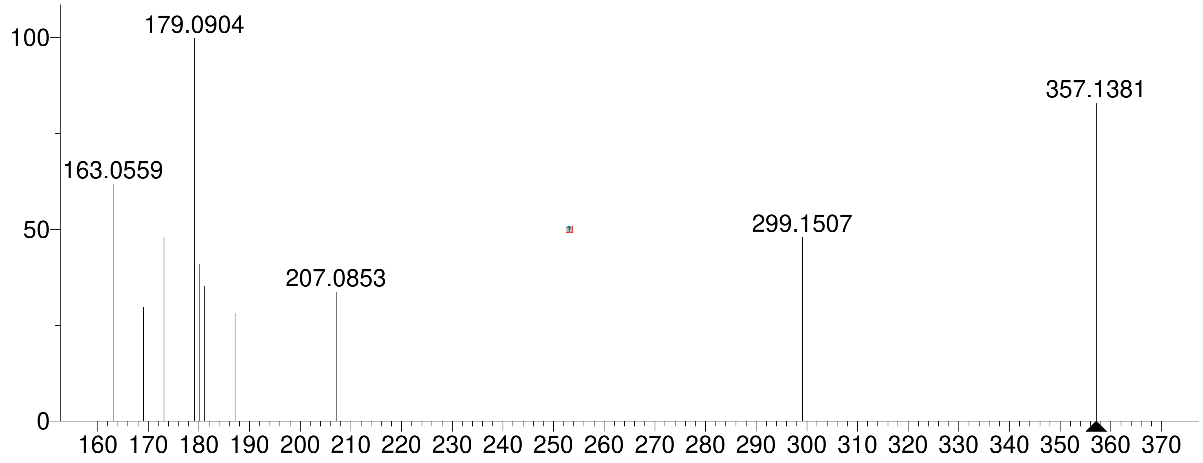

Hit 1 : Matairesinol [M-H]- 10 P=357.1
C20H22O6; MF: 362; RMF: 997; Prob 97.8%; Lib: msms neg mona_ipb_gnps_plasma; ID: 21759.

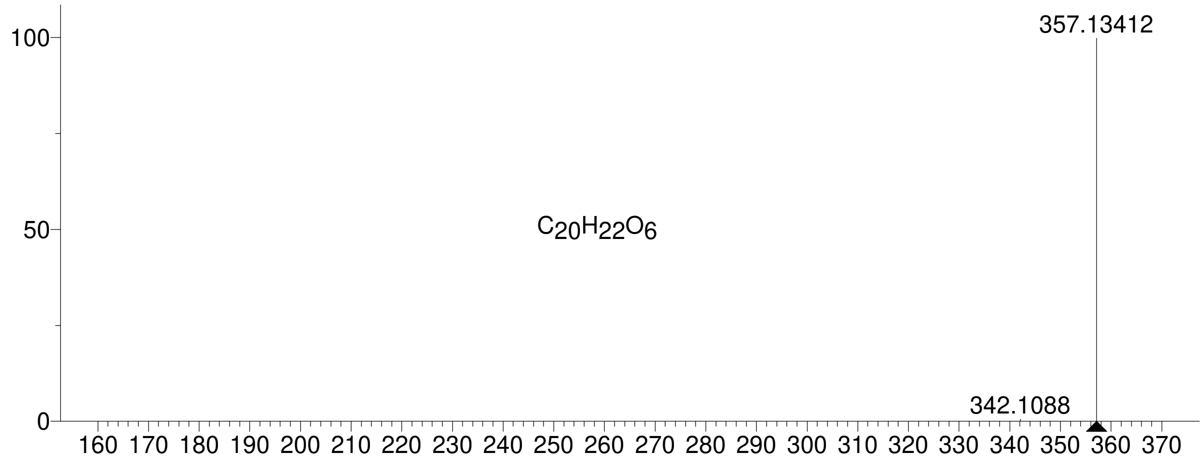

Hit 2 : Schisandrol B [M+H-C3H8O]+ HCD 14V P=357.1
C23H28O7; MF: 77; RMF: 772; Prob 0.97%; CAS: 58546-54-6; Lib: nist_msms; ID: 384792.

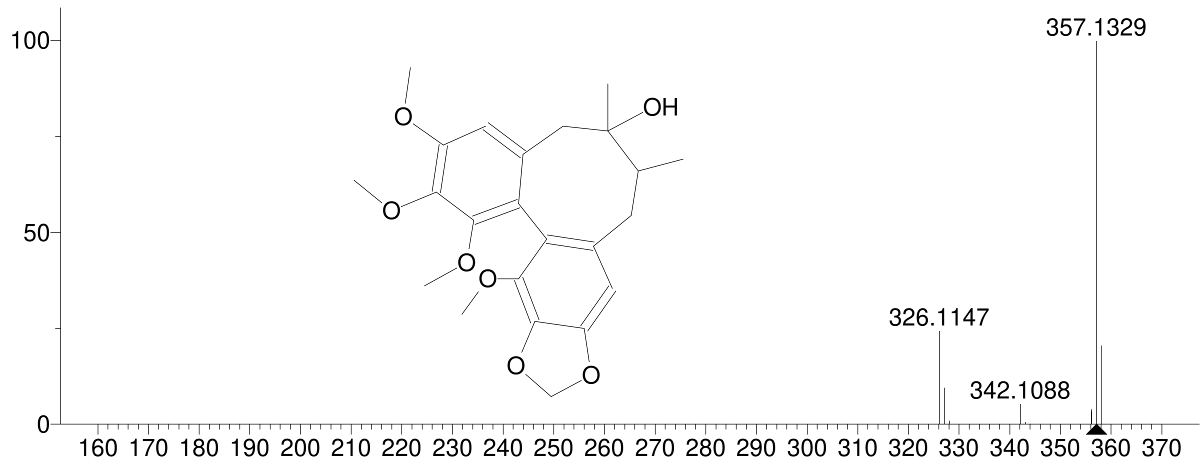

Hit 3 : (+)-Pinoresinol [M-H]- HCD 12V P=357.1
C20H22O6; MF: 51; RMF: 401; Prob 0.29%; CAS: 487-36-5; Lib: nist_msms; ID: 234442.

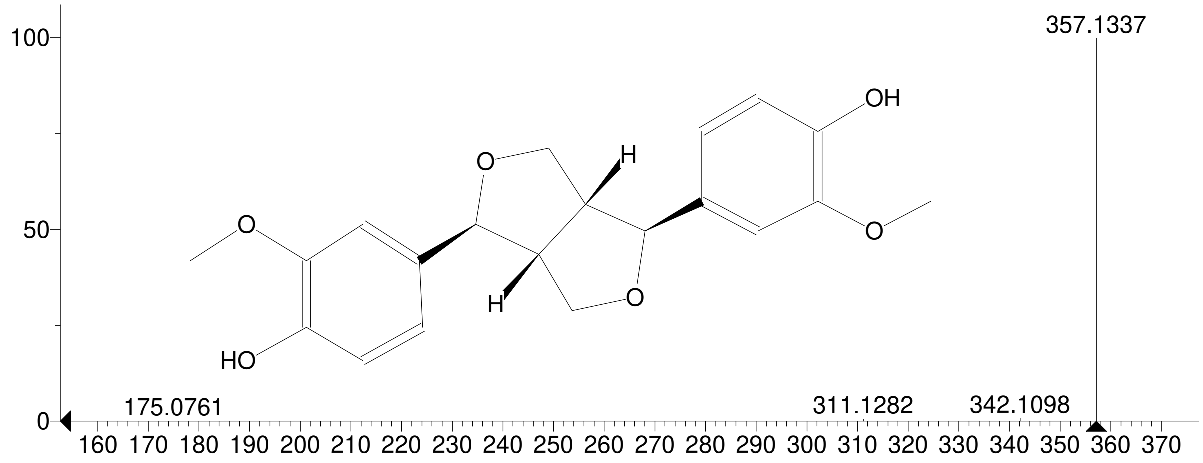

Hit 4 : Pinoresinol [M-H]- 10 P=357.1
C20H22O6; MF: 45; RMF: 388; Prob 0.23%; Lib: msms neg mona_ipb_gnps_plasma; ID: 21801.

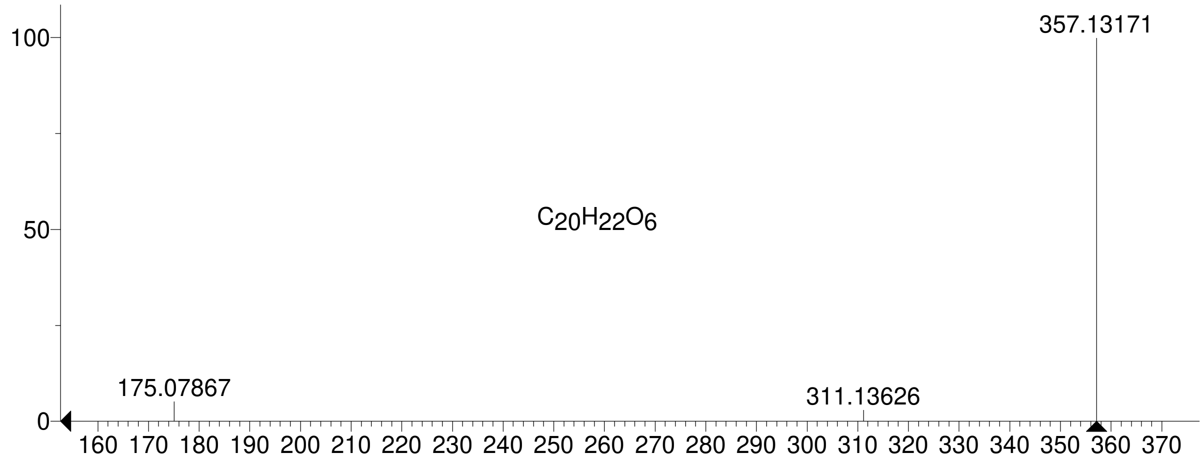

Hit 5 : (-)-Hydroxymatairesinol [M+H-H2O]+ HCD 10V P=357.1
C20H22O7; MF: 21; RMF: 384; Prob 0.07%; CAS: 81623-30-5; Lib: nist_msms; ID: 235736.

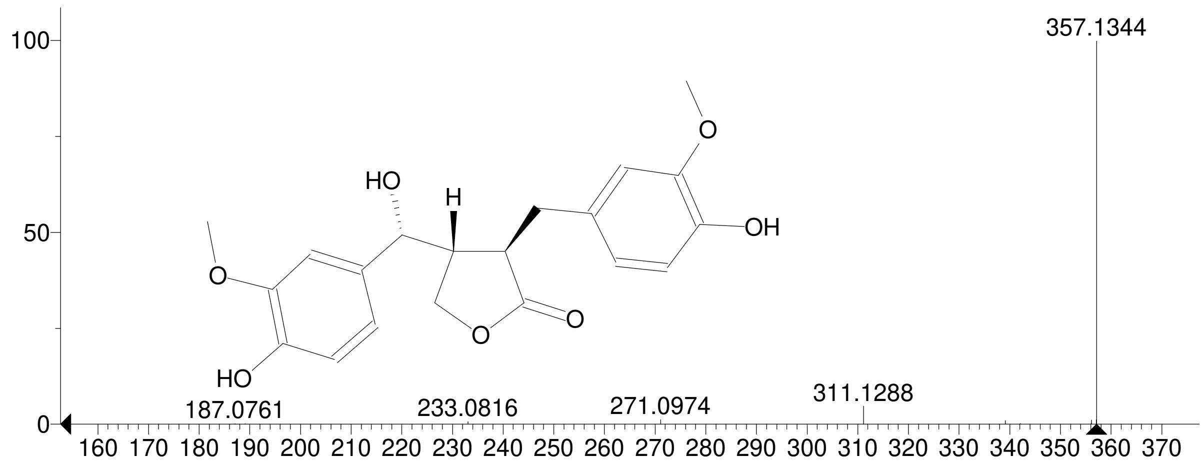

Unknown: Unknown (13.40_281.1010m/z)
Compound in Library Factor = -3273

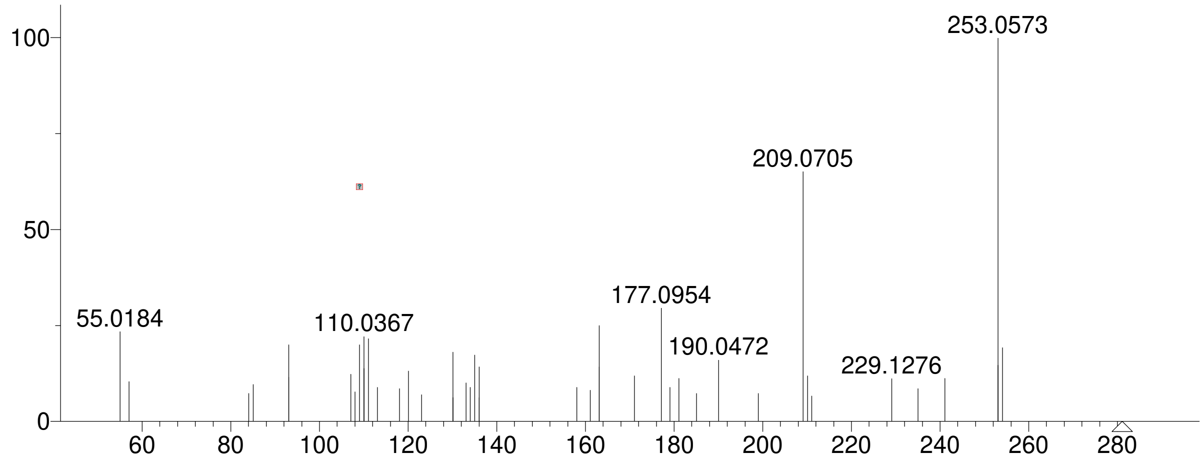

Hit 1 : methyl 3-(3,4-dihydroxy-5-phenyloxolan-2-yl)-3-hydroxypropanoate [M-H]- 65HCD P=281.1
C14H18O6; MF: 11; RMF: 160; Prob 1.85%; Lib: mona-export-lc-ms-ms_negative_mode; ID: 23317.

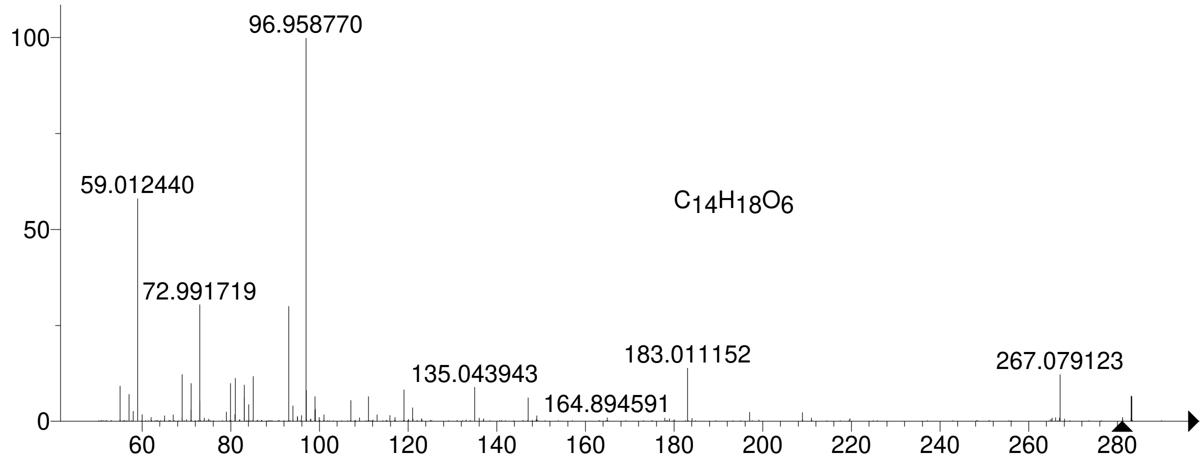

Hit 2 : 1-Palmitoyl-2-(4-ketododec-3-enedioyl)phosphatidylcholine [M+Na]+=>559.4=>281.1 IT 35% P=281.1
C36H66NO11P; MF: 0; RMF: 47; Prob 1.27%; CAS: 439904-34-4; Lib: nist_msms; ID: 94575.

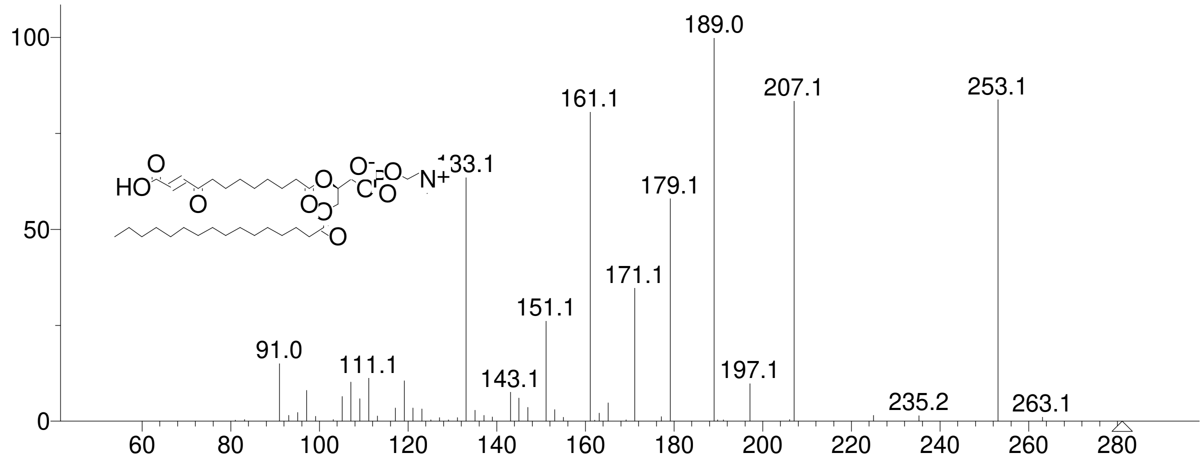

Hit 3 : N-(2,4-Dinitrophenyl)-L-leucine [M+H-HO]+ HCD 42V P=281.1
C12H15N3O6; MF: 0; RMF: 40; Prob 1.27%; CAS: 1655-57-8; Lib: nist_msms; ID: 202888.

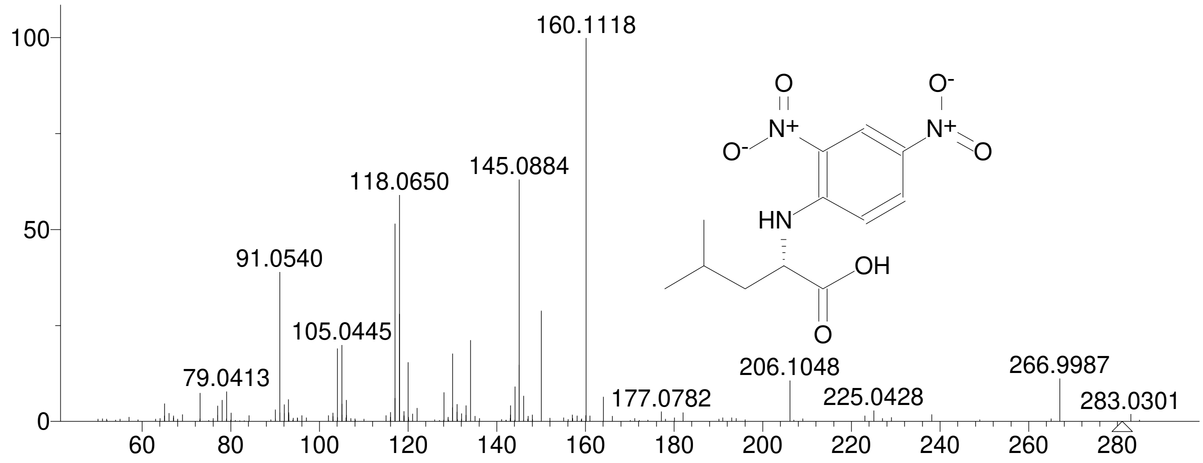

Hit 4 : Pemetrexed [M+H-C5H9NO4]+ HCD 42V P=281.1
C20H21N5O6; MF: 0; RMF: 35; Prob 1.27%; CAS: 137281-23-3; Lib: nist_msms; ID: 349969.

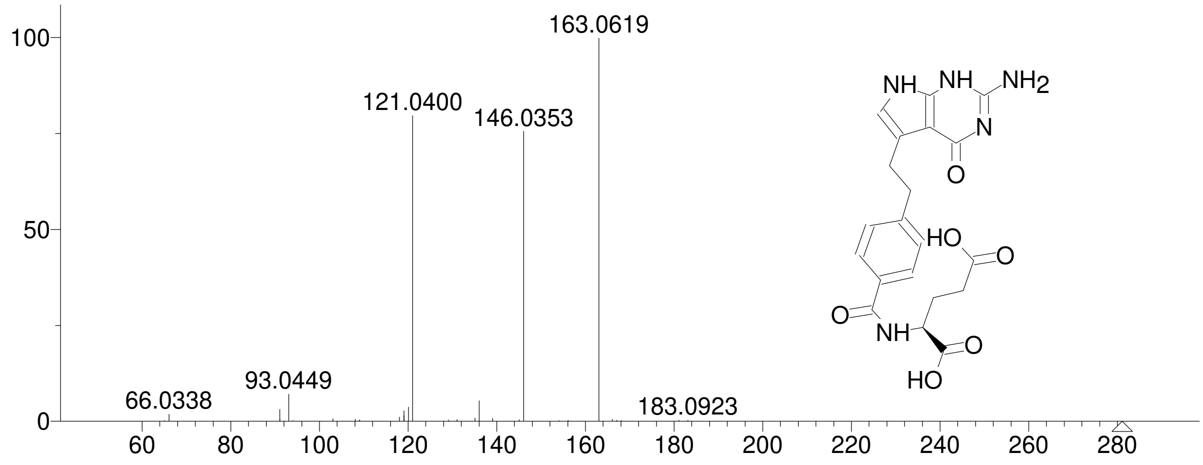

Hit 5 : Turkesterone [M+H-3H2O]+=>299.1=>281.1 IT 35% P=281.1
C27H44O8; MF: 0; RMF: 30; Prob 1.27%; CAS: 41451-87-0; Lib: nist_msms; ID: 412084.

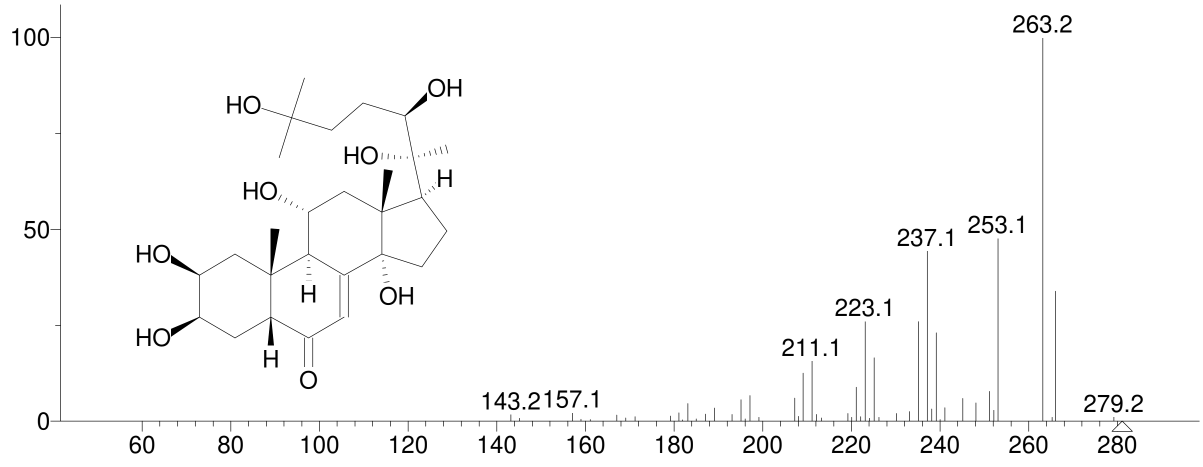

Unknown: Unknown (13.43_371.1234m/z)
Compound in Library Factor = 0

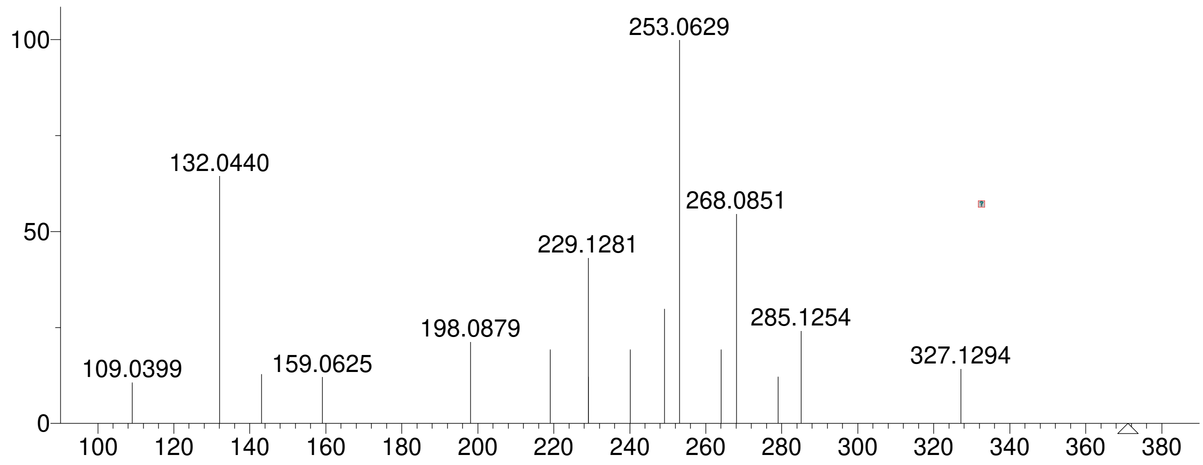

Hit 1 : 7,8-dihydroxy-3-[(1E,3E)-7-hydroxy-3,5-dimethylhepta-1,3-dienyl]-7-methyl-8H-isochromen-6-one [M+K]+
C19H24O5; MF: 0; RMF: 46; Prob 33.3%; Lib: mona-export-vf-npl_qexactive; ID: 16498.

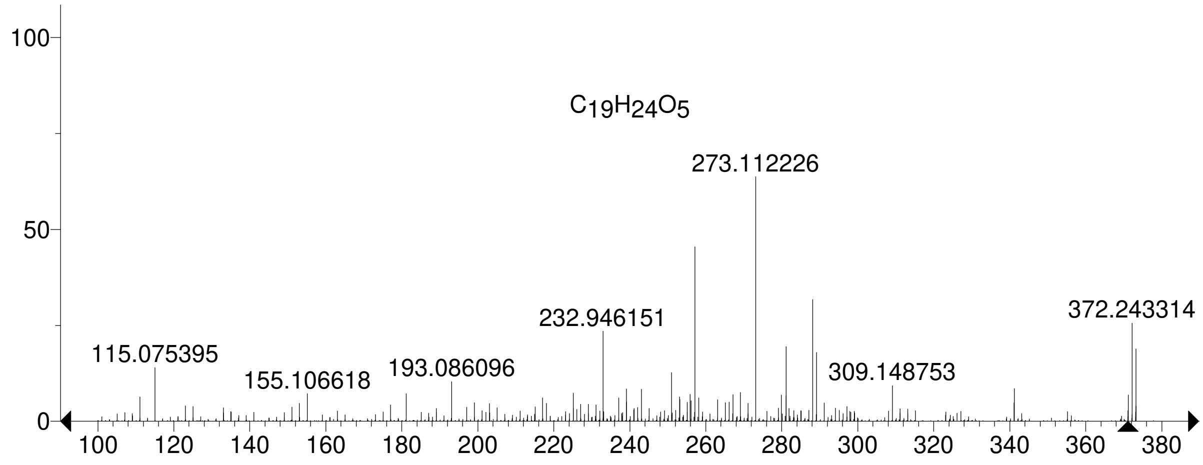

Hit 2 : Arnicolide D [M+K]+ 35HCD P=371.1
C19H24O5; MF: 0; RMF: 23; Prob 33.3%; Lib: mona-export-vf-npl_qexactive; ID: 4087.

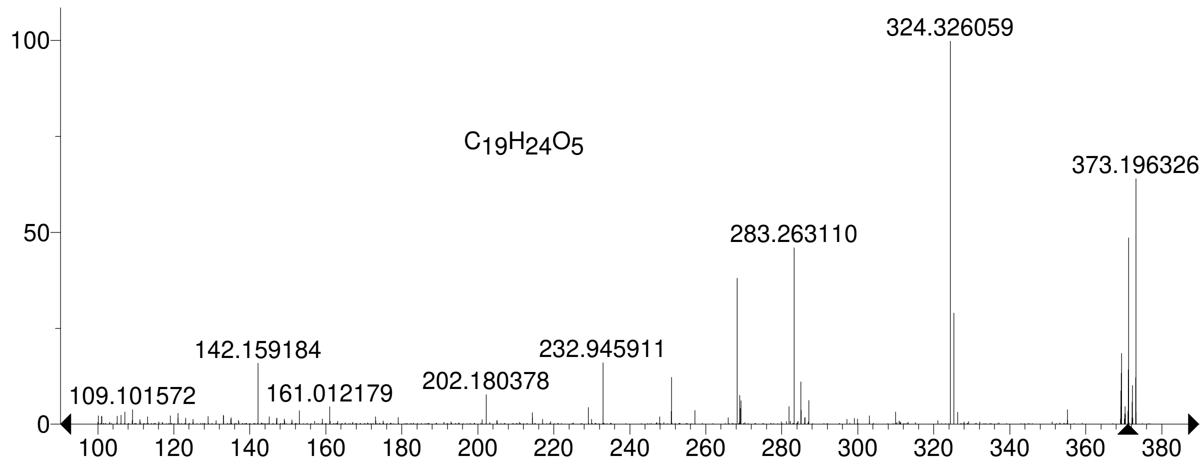

Hit 3 : 1-[2-hydroxy-4-(3-hydroxy-5-methylphenoxy)-6-methylphenyl]-3-methylbutane-2,3-diol [M+K]+ 35HCD P=
C19H24O5; MF: 0; RMF: 6; Prob 33.3%; Lib: mona-export-vf-npl_qexactive; ID: 22291.

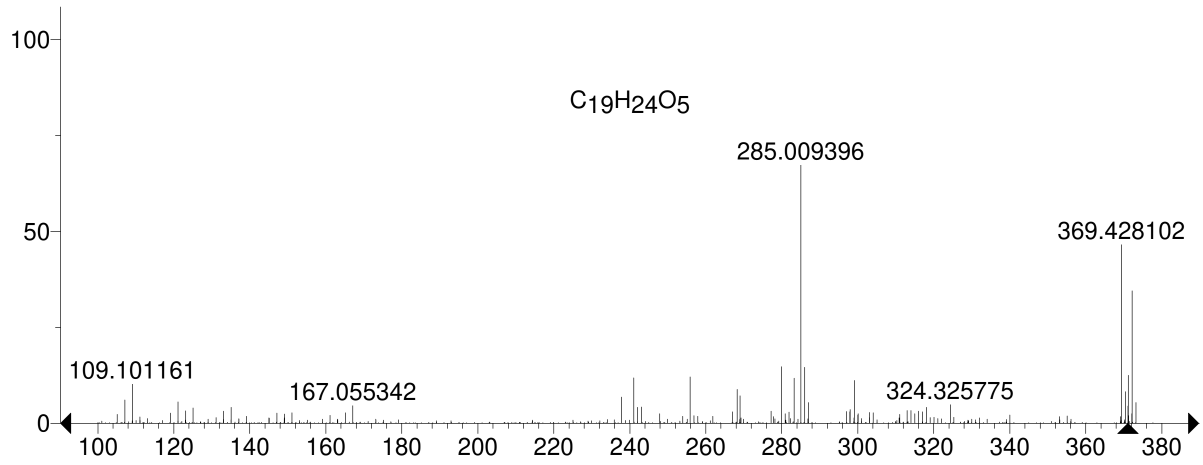

Unknown: Unknown (13.44_355.0117m/z)
Compound in Library Factor = 0

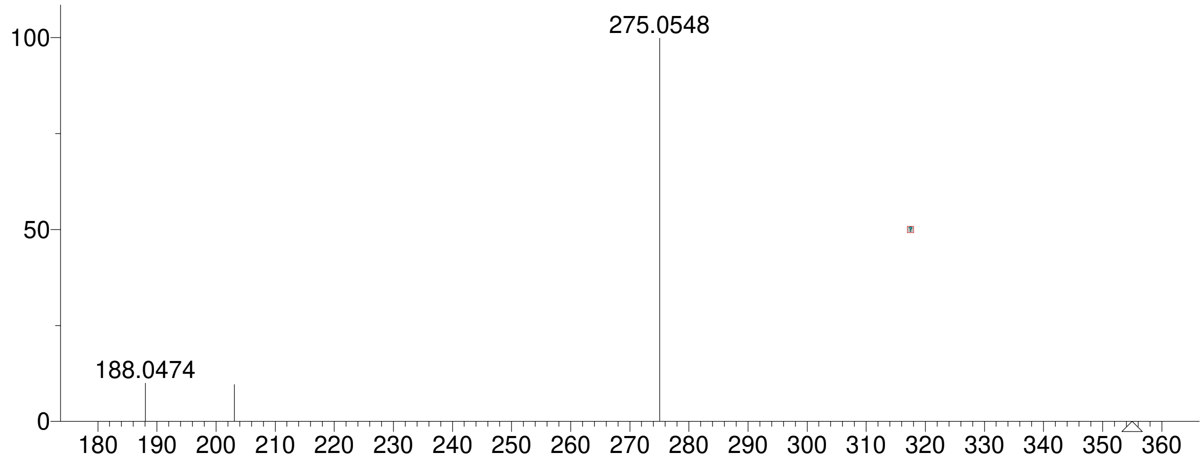

Hit 1 : 3-(3,3-dichloro-2-hydroxypropyl)-6,8-dimethoxyisochromen-1-one [M+Na]+ 35HCD P=355
C14H14Cl2O5; MF: 0; RMF: 14; Prob 100.0%; Lib: mona-export-vf-npl_qexactive; ID: 18490.

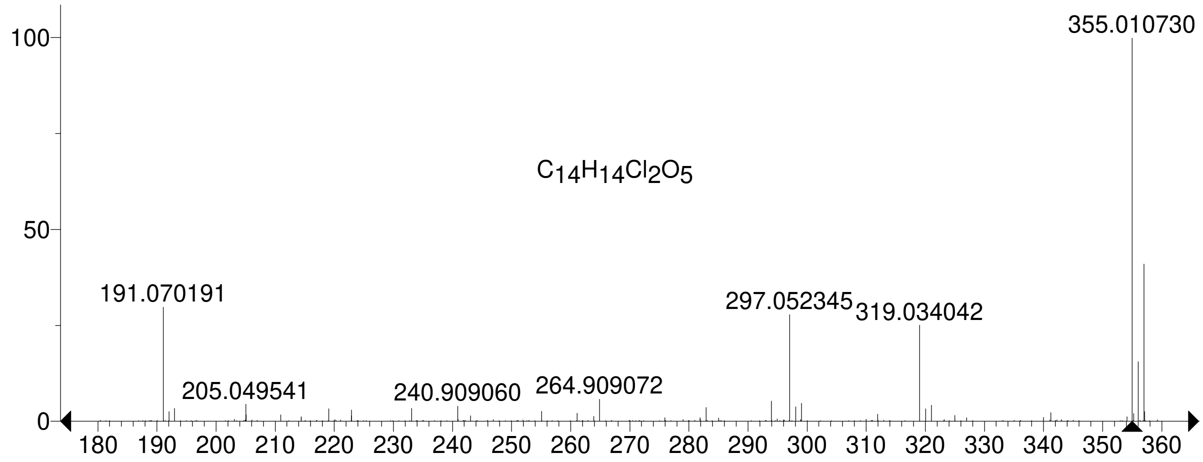

** Search Report Page 1 of 1 **

Unknown: Unknown (13.50_619.2961m/z)
Compound in Library Factor = 0

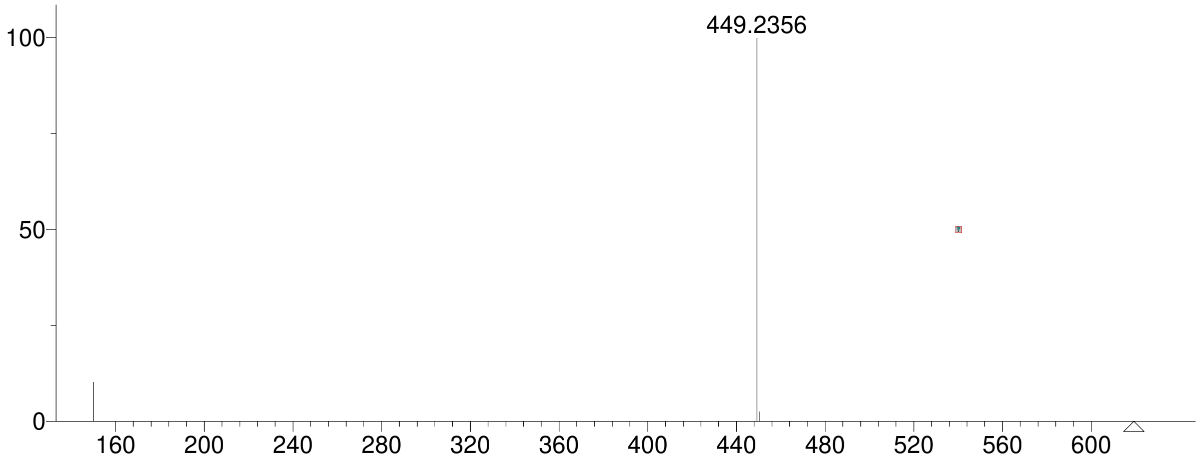

** Search Report Page 1 of 1 **

Unknown: Unknown (13.52_562.2388m/z)
Compound in Library Factor = 0

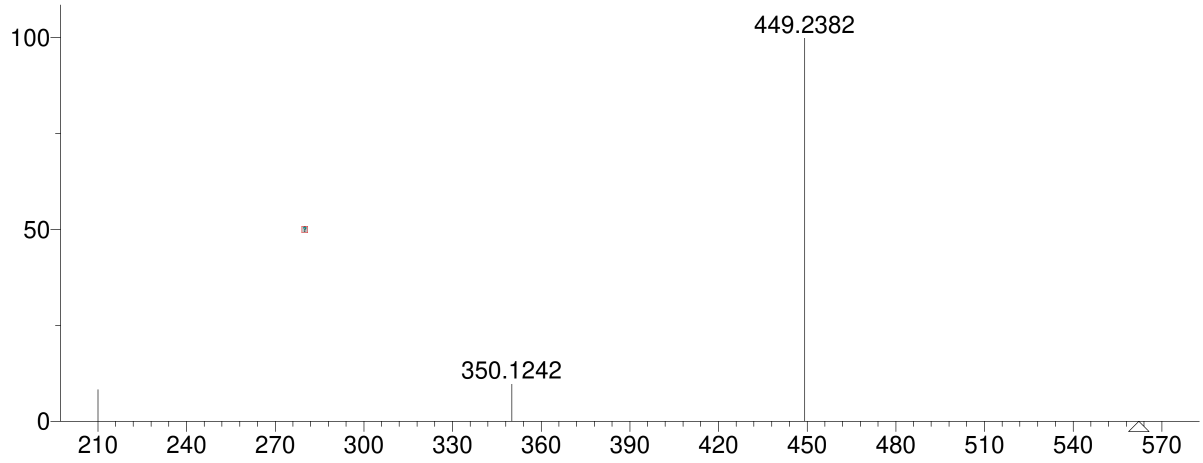

Unknown: Unknown (13.53_484.2295m/z)
Compound in Library Factor = -3273

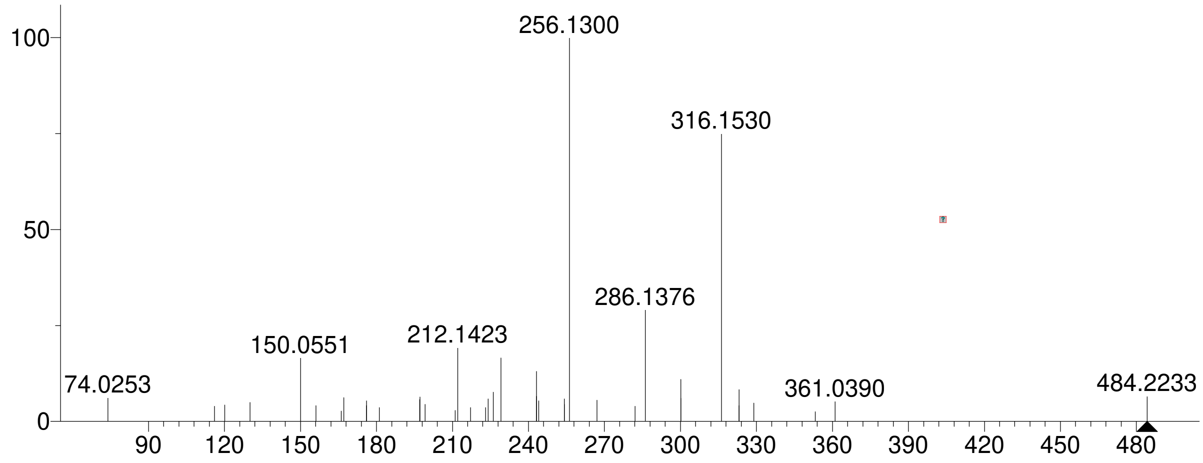

Hit 1 : PC 10:0; PC 5:0-5:0; [M+Hac-H]- [M+Hac-H]- P=484.2
C18H36NO8P; MF: 2; RMF: 65; Prob 11.3%; Lib: msdial-insilicomsms-lipids-neg; ID: 319638.

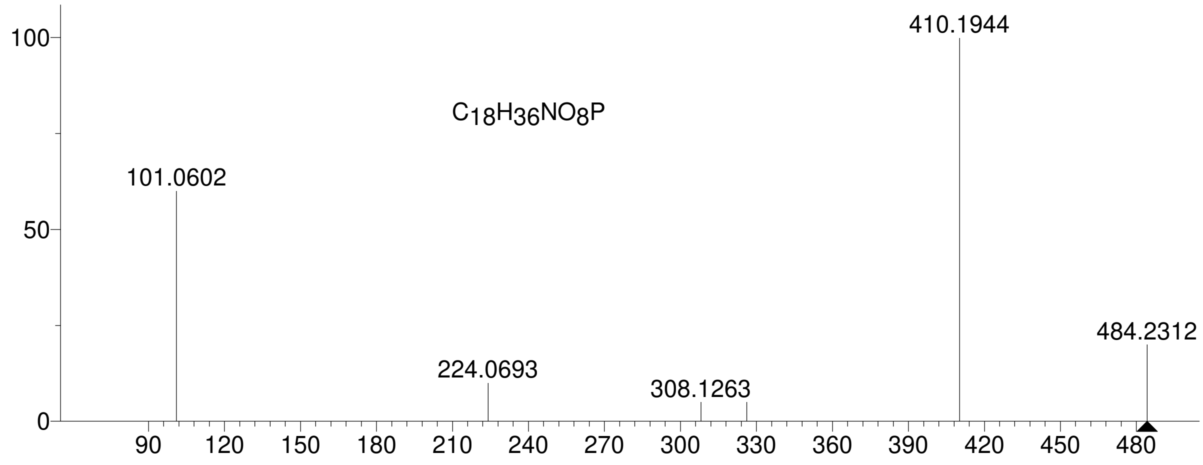

Hit 2 : PC 11:0; PC 2:0-9:0; [M+FA-H]- [M+FA-H]- P=484.2
C19H38NO8P; MF: 2; RMF: 63; Prob 11.3%; Lib: msdial-insilicomsms-lipids-neg; ID: 317574.

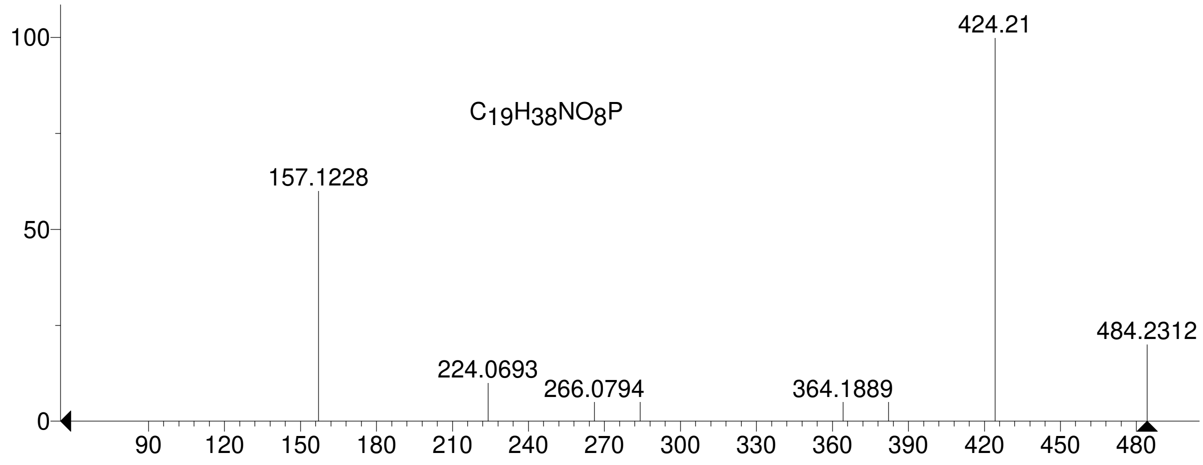

Hit 3 : PC 11:0; PC 3:0-8:0; [M+FA-H]- [M+FA-H]- P=484.2
C19H38NO8P; MF: 2; RMF: 63; Prob 11.3%; Lib: msdial-insilicomsms-lipids-neg; ID: 317633.

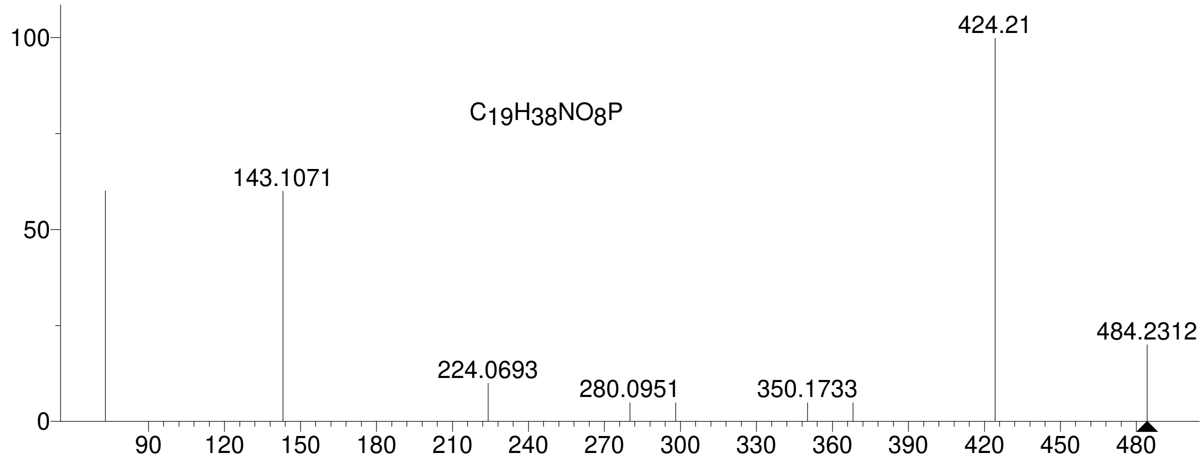

Hit 4 : PC 10:0; PC 2:0-8:0; [M+Hac-H]- [M+Hac-H]- P=484.2
C18H36NO8P; MF: 2; RMF: 63; Prob 11.3%; Lib: msdial-insilicomsms-lipids-neg; ID: 319464.

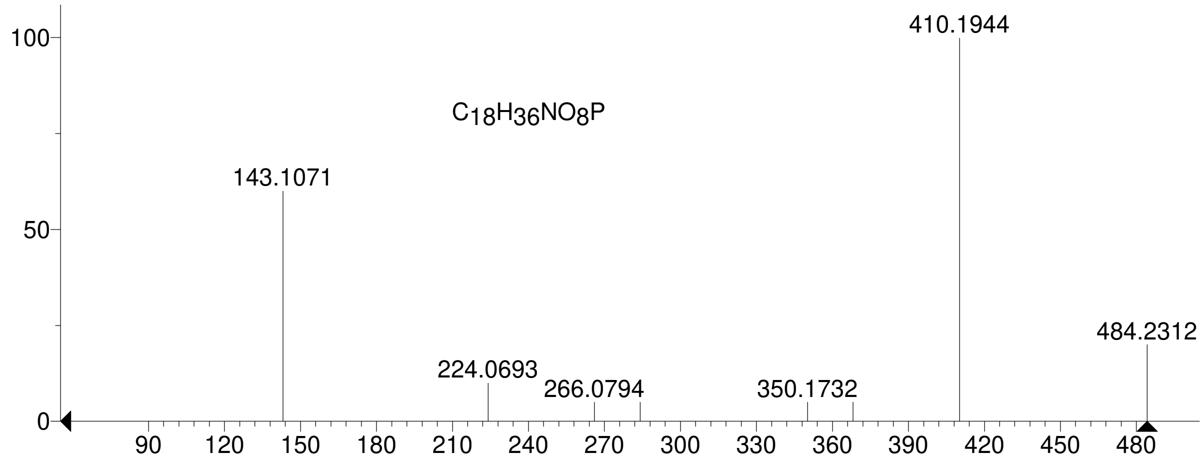

Hit 5 : PC 10:0; PC 3:0-7:0; [M+Hac-H]- [M+Hac-H]- P=484.2
C18H36NO8P; MF: 2; RMF: 63; Prob 11.3%; Lib: msdial-insilicomsms-lipids-neg; ID: 319523.

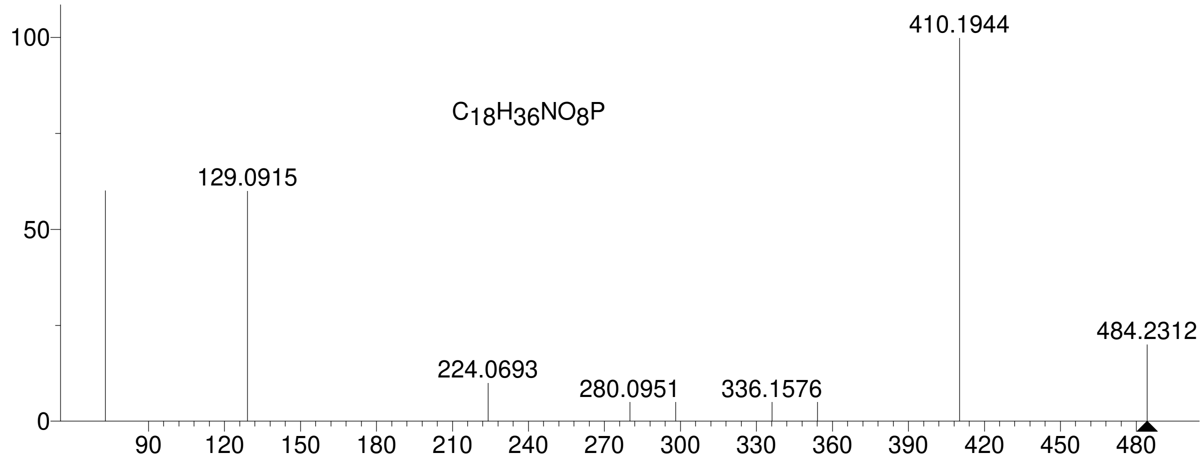

Unknown: Unknown (13.54_357.1359m/z)
Compound in Library Factor = -302

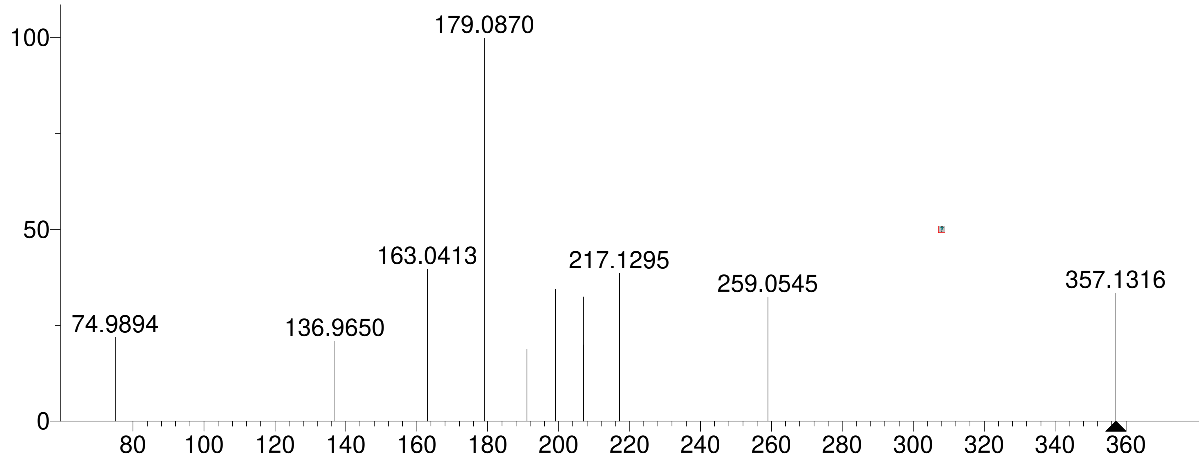

Hit 1 : Matairesinol [M-H]- 10 P=357.1
C20H22O6; MF: 362; RMF: 997; Prob 80.9%; Lib: msms neg mona_ipb_gnps_plasma; ID: 21759.

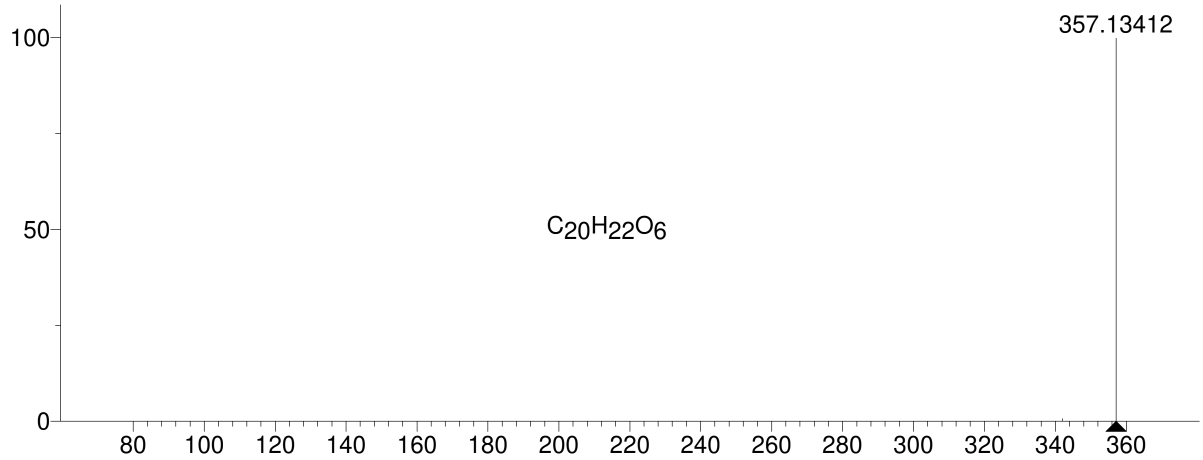

Hit 2 : Topiramate [M+NH4]+ HCD 3V P=357.1
C12H21NO8S; MF: 321; RMF: 969; Prob 18.5%; CAS: 97240-79-4; Lib: nist_msms; ID: 317944.

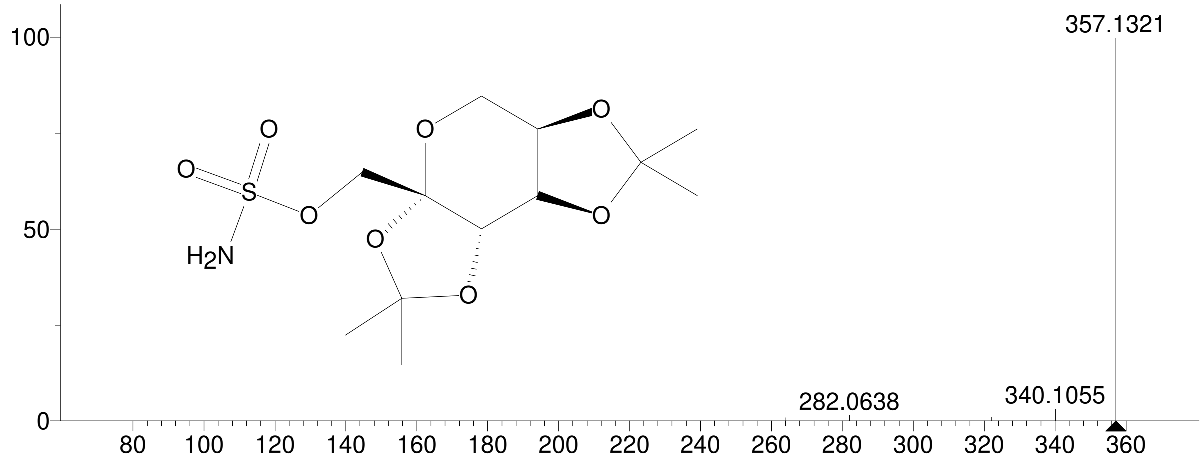

Hit 3 : Schisandrol B [M+H-C3H8O]+ HCD 14V P=357.1
C23H28O7; MF: 77; RMF: 772; Prob 0.18%; CAS: 58546-54-6; Lib: nist_msms; ID: 384792.

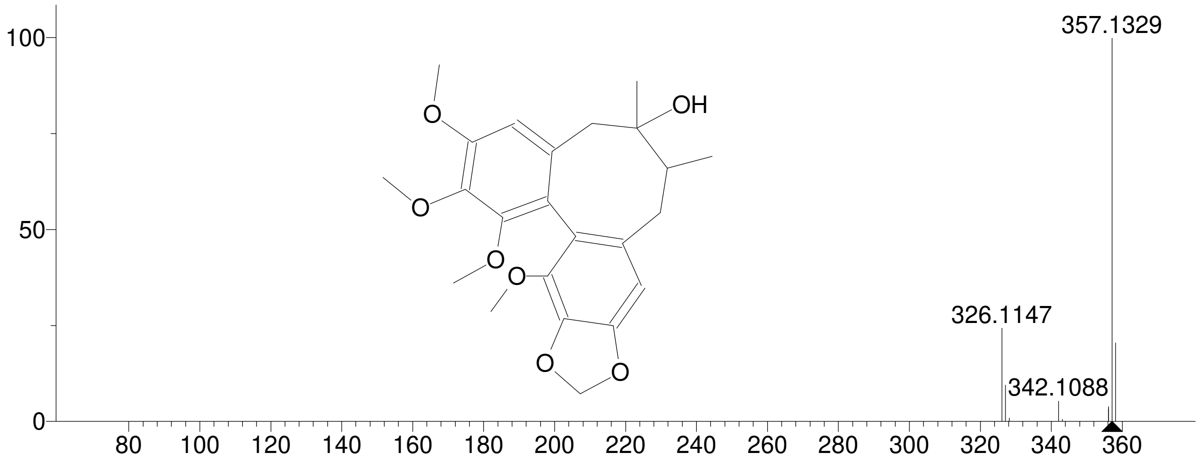

Hit 4 : (+)-Pinoresinol [M-H]- HCD 12V P=357.1
C20H22O6; MF: 20; RMF: 281; Prob 0.03%; CAS: 487-36-5; Lib: nist_msms; ID: 234442.

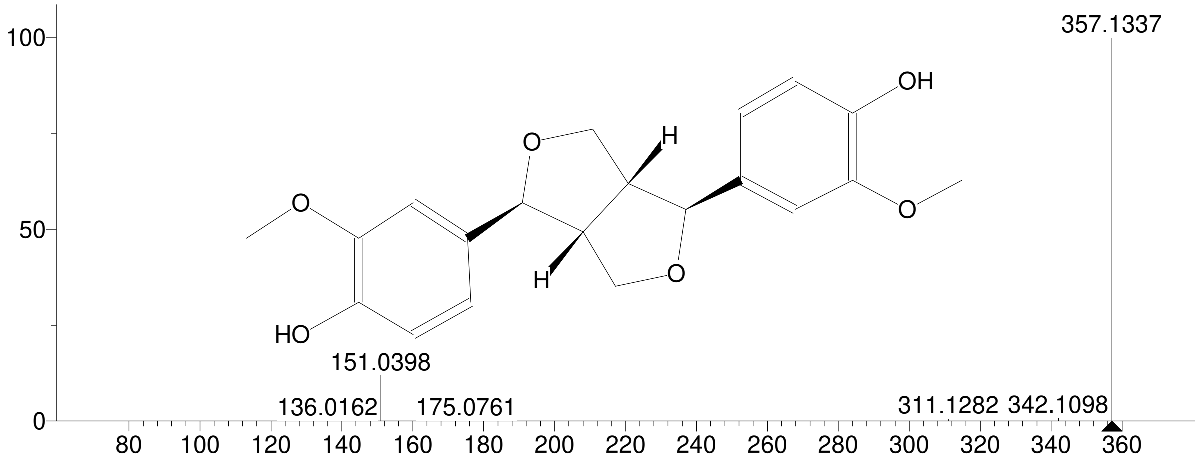

Hit 5 : Pinoresinol [M-H]- 10 P=357.1
C20H22O6; MF: 16; RMF: 272; Prob 0.02%; Lib: msms neg mona_ipb_gnps_plasma; ID: 21801.

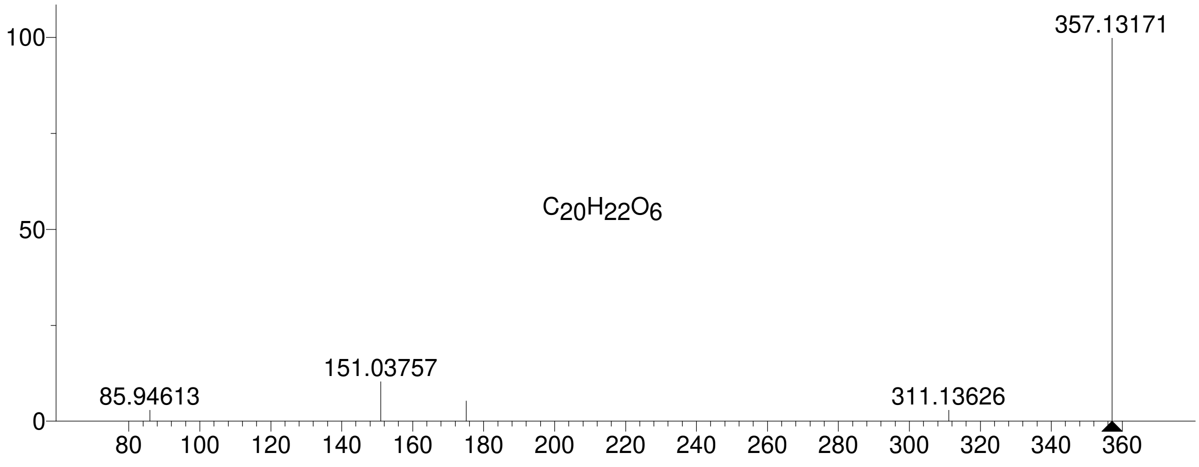

Unknown: Unknown (13.54_548.2124m/z)
Compound in Library Factor = 0

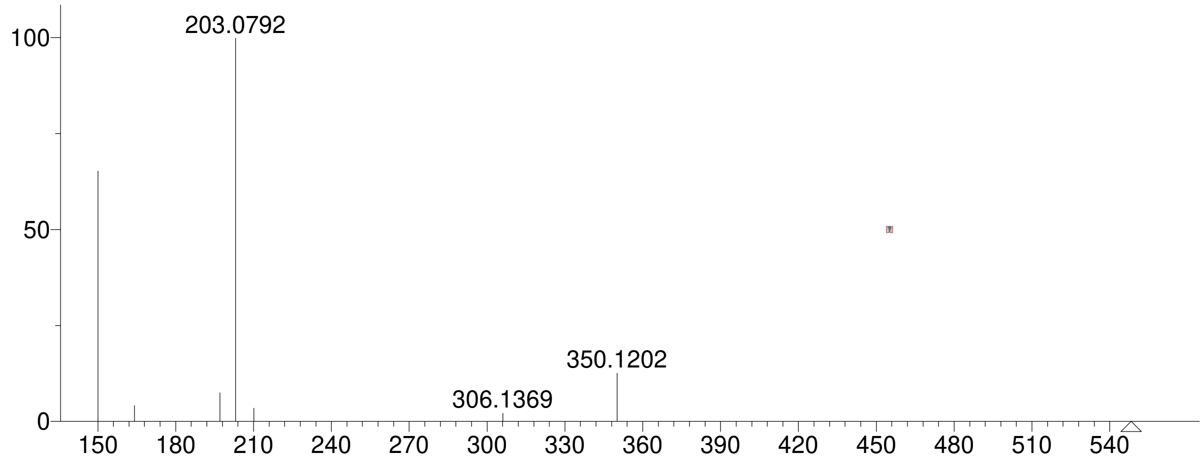

Unknown: Unknown (13.55_543.1976m/z)
Compound in Library Factor = -3273

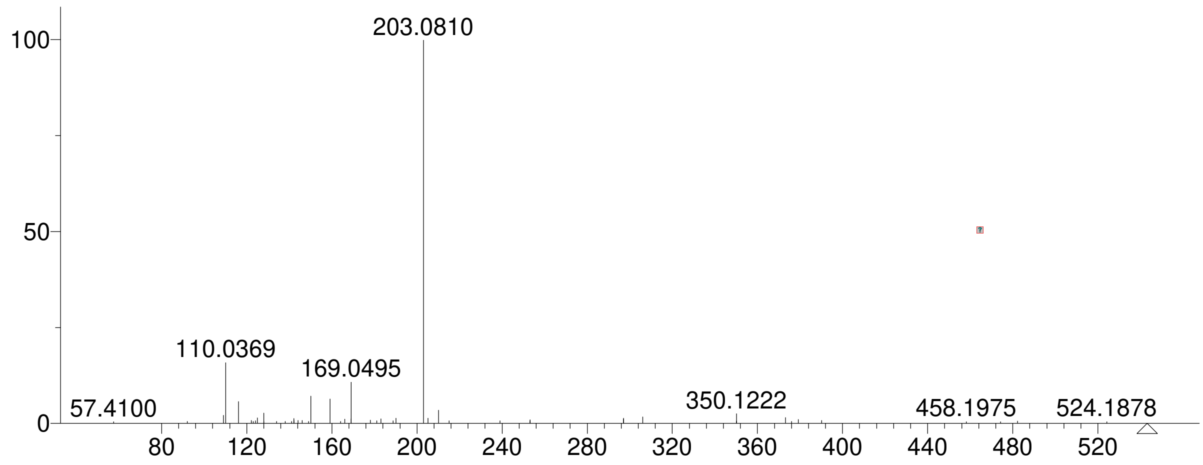

Hit 1 : NCGC00385982-01_C27H30N2O10_ [M+H]+ P=543.2
C27H30N2O10; MF: 1; RMF: 51; Prob 25.7%; Lib: mona-export-gnps; ID: 10901.

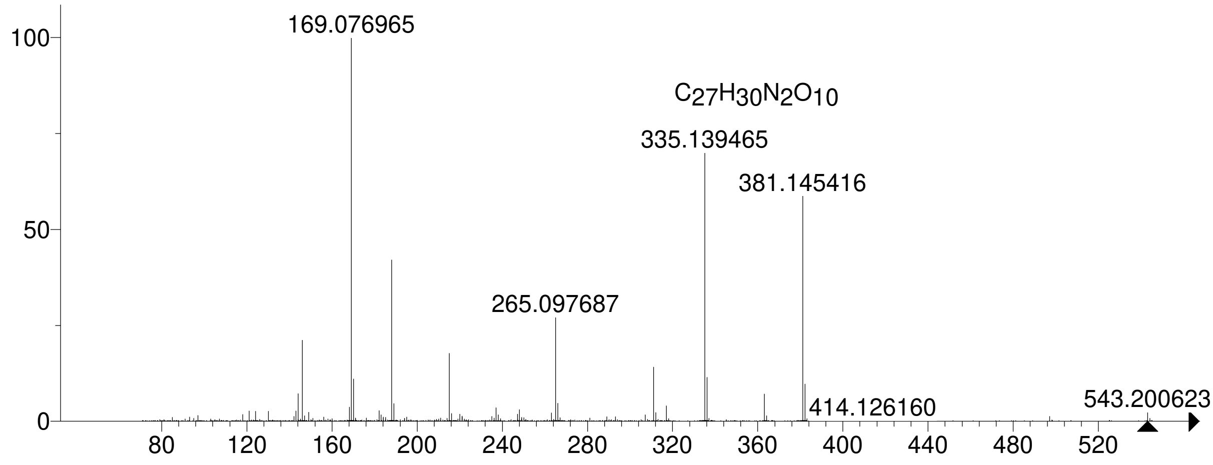

Hit 2 : 2',6'-Dihydroxy 4'-methoxydihydrochalcone [2M-H]- HCD 59V P=543.2
C16H16O4; MF: 0; RMF: 13; Prob 24.8%; CAS: 35241-55-5; Lib: nist_msms; ID: 241376.

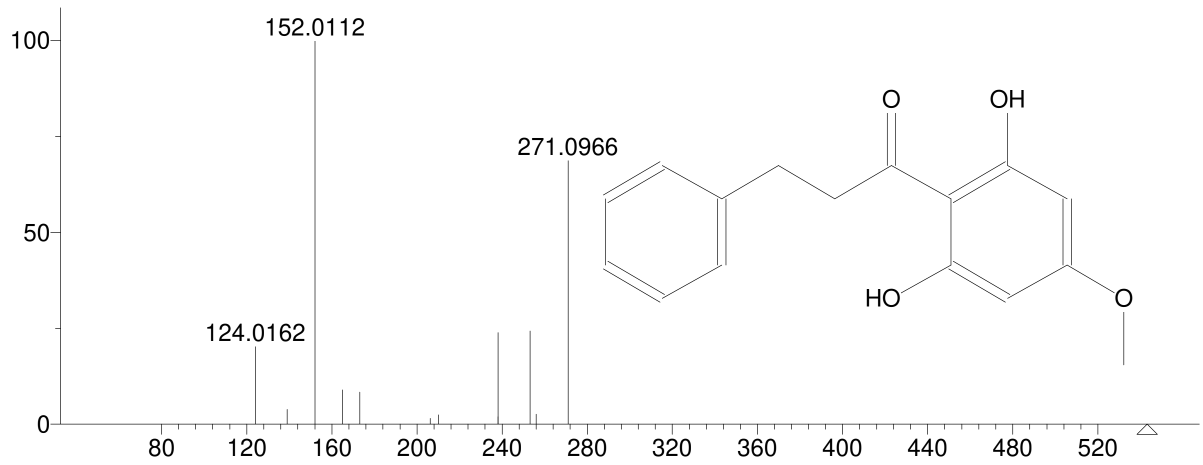

Hit 3 : 2'SLeXLeX [M+2Na]2+=>543.2 IT 35% P=543.2
C51H85N3O37; MF: 0; RMF: 2; Prob 24.8%; Lib: nist_msms; ID: 523603.

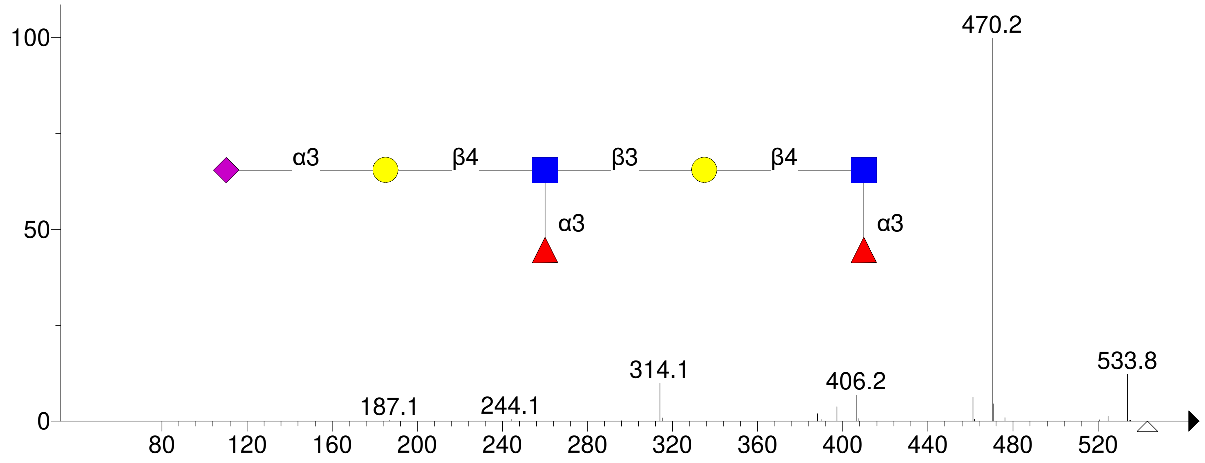

Hit 4 : Prostaglandin F2α-biotinamide [M+Na]+=>643.4=>543.2 IT 35% P=543.2
C35H60N4O6S; MF: 0; RMF: 1; Prob 24.8%; Lib: nist_msms; ID: 95551.

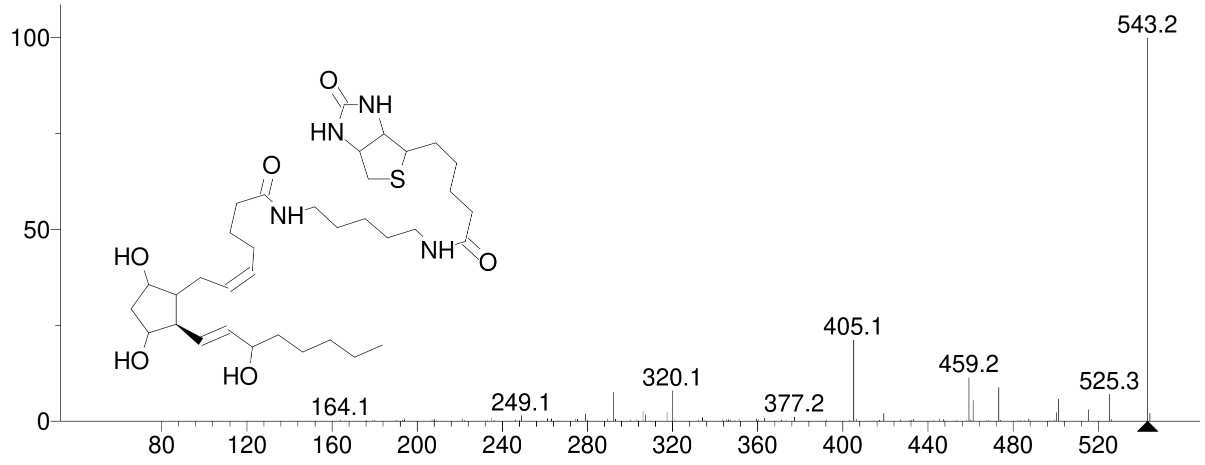

Unknown: Unknown (13.59_548.2134m/z)
Compound in Library Factor = 0

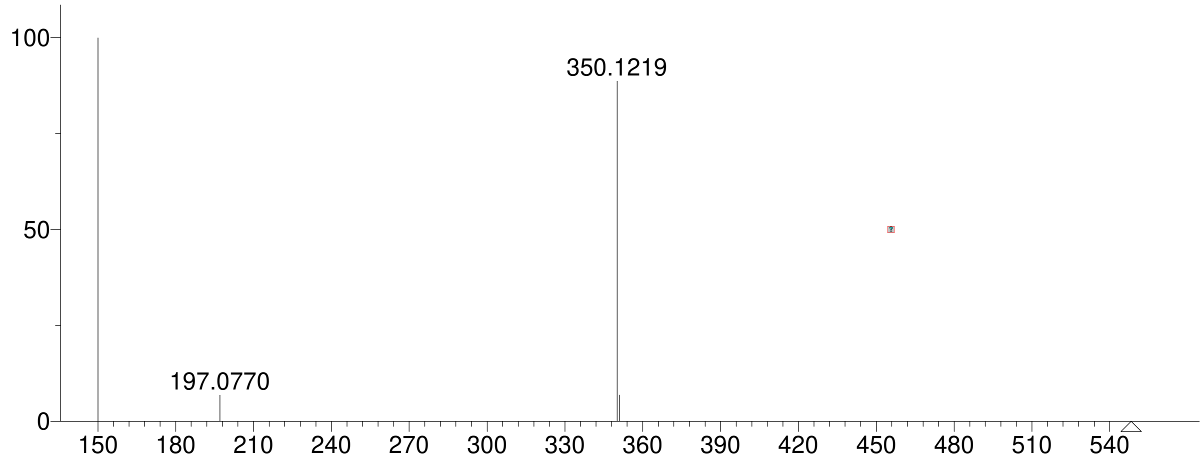

Hit 1 : [1,1'-Biphenyl]-2-carboxylic acid, 4'-[[2,3-dimethyl-5-[[[(1S)-1-(4-nitrophenyl)ethyl]amino]carbonyl]-1H-indol-
C33H29N3O5; MF: 0; RMF: 107; Prob 100.0%; CAS: 1338259-05-4; Lib: nist_msms; ID: 253410.

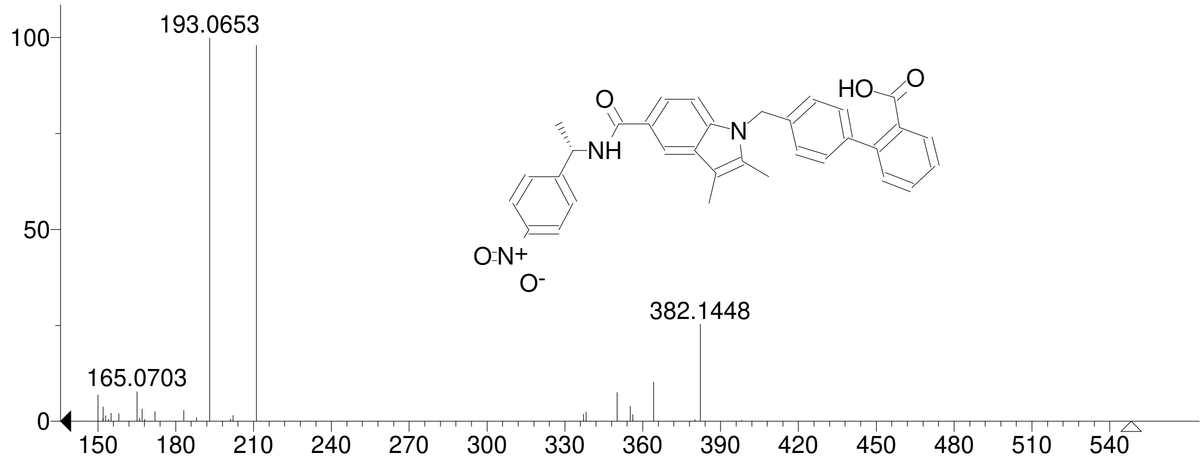

** Search Report Page 1 of 1 **

Unknown: Unknown (13.59_562.2395m/z)
Compound in Library Factor = 0

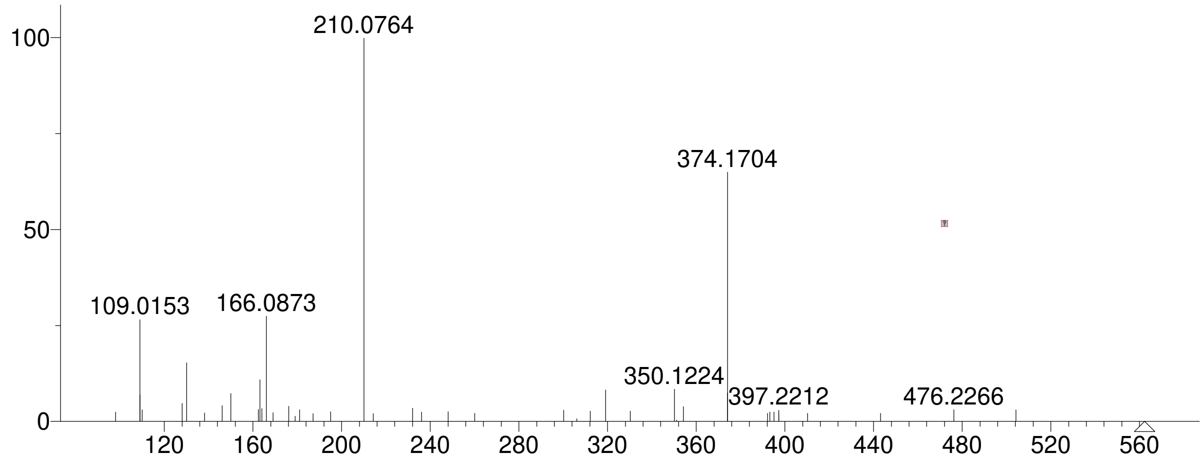

Unknown: Unknown (13.60_351.1074m/z)
Compound in Library Factor = 0

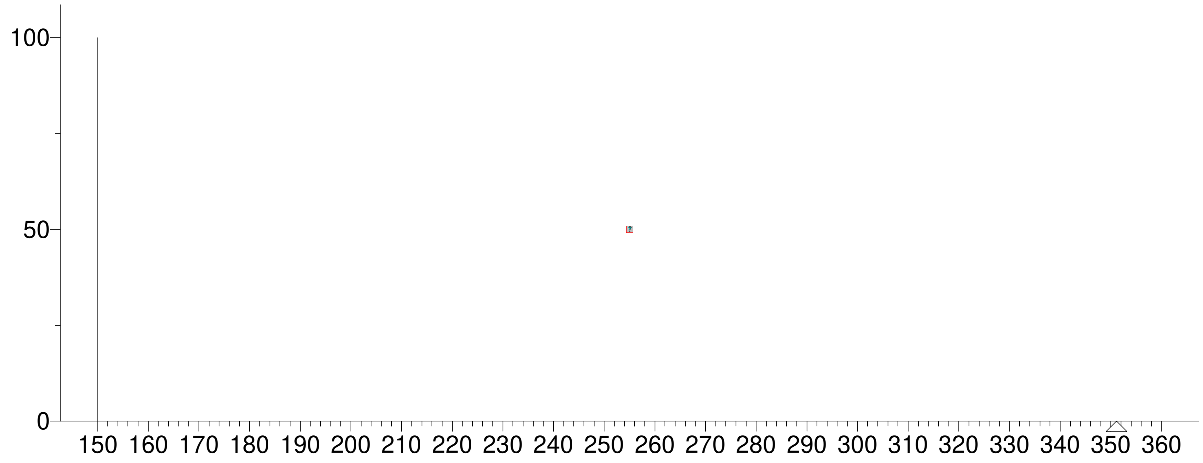

Hit 1 : Acetamide, N-[4-[5-(1,3-benzodioxol-5-yl)-3-methoxy-1H-1,2,4-triazol-1-yl]phenyl]-2-(phenylthio)- [M+H-C6
C24H20N4O4S; MF: 0; RMF: 169; Prob 100.0%; CAS: 853625-60-2; Lib: nist_msms; ID: 375967.

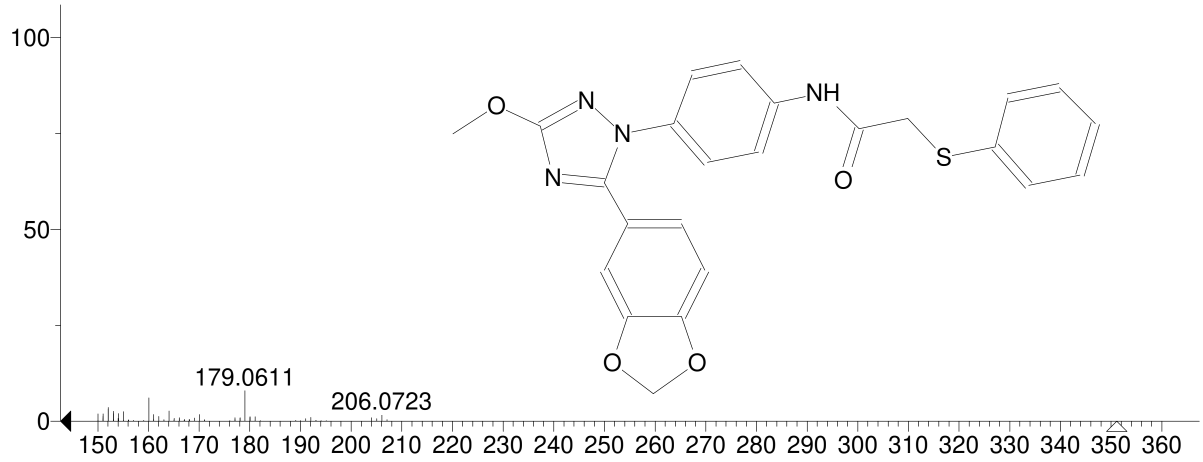

Unknown: Unknown (13.66_543.1982m/z)
Compound in Library Factor = -3273

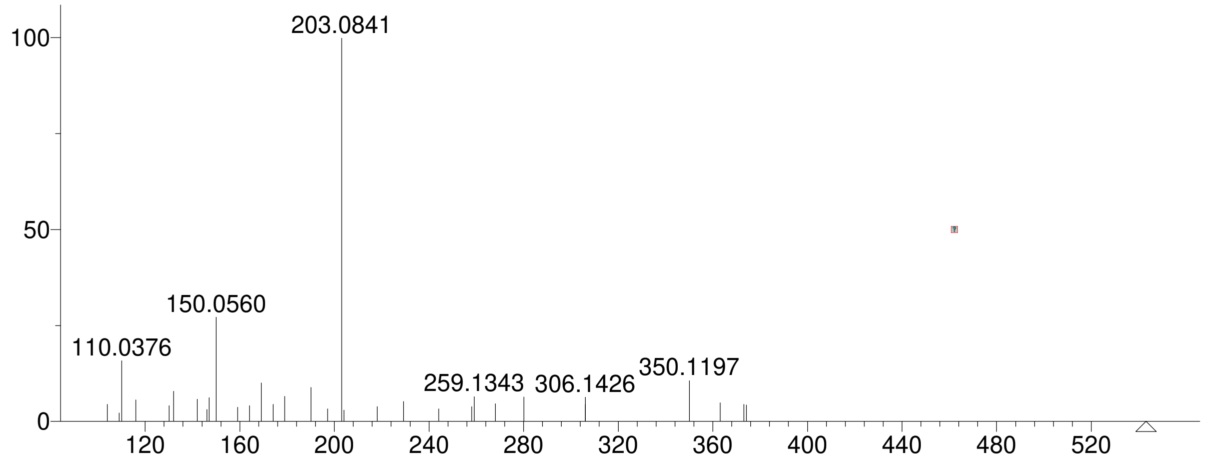

Hit 1 : NCGC00385982-01_C27H30N2O10_ [M+H]+ P=543.2
C27H30N2O10; MF: 1; RMF: 59; Prob 25.7%; Lib: mona-export-gnps; ID: 10901.

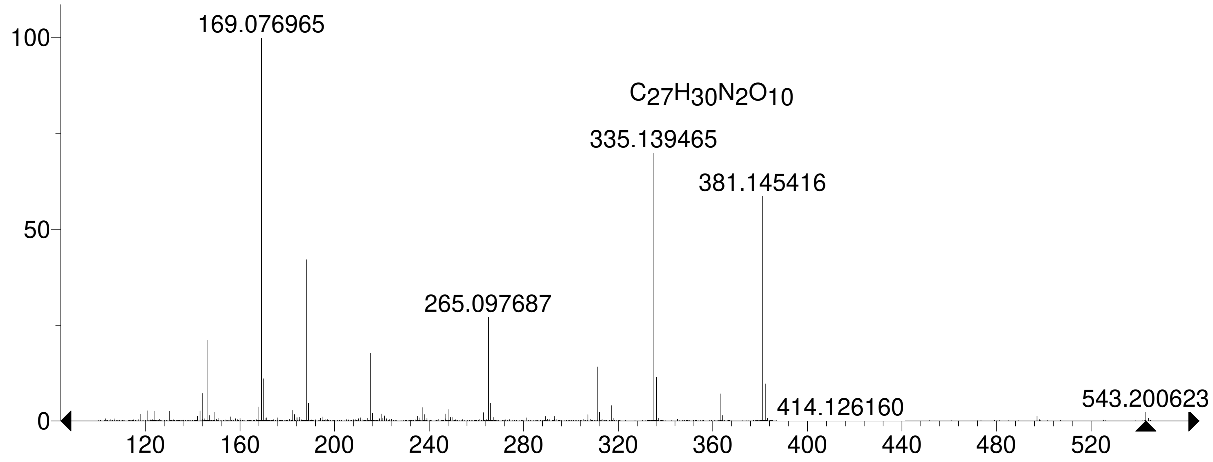

Hit 2 : Strictosamide; PlaSMA ID-1490 [M+FA-H]- P=543.2
C26H30N2O8; MF: 0; RMF: 16; Prob 24.8%; Lib: biomsms-neg-plasma; ID: 708.

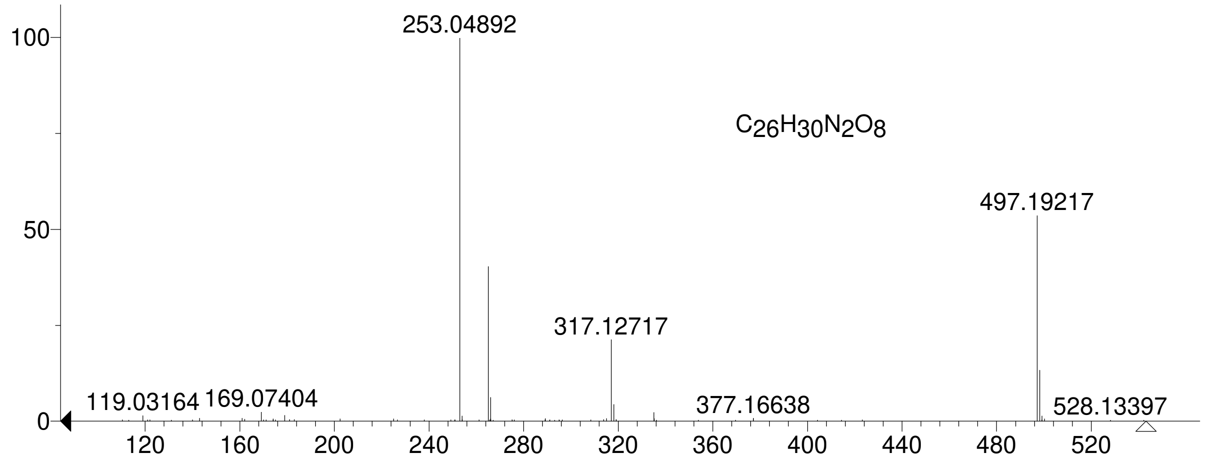

Hit 3 : Prostaglandin F2α-biotinamide [M+Na]+=>643.4=>543.2 IT 35% P=543.2
C35H60N4O6S; MF: 0; RMF: 4; Prob 24.8%; Lib: nist_msms; ID: 95551.

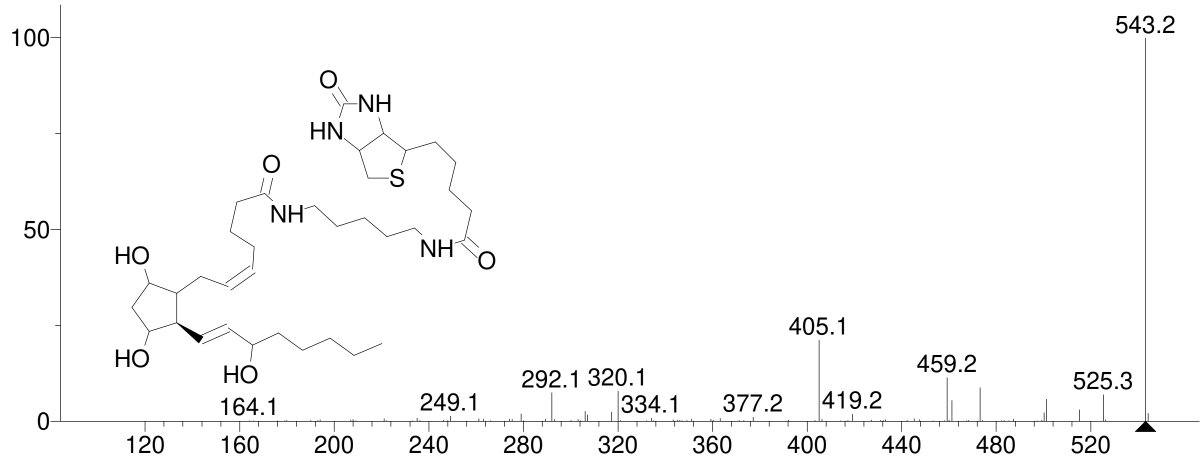

Hit 4 : Chloramphenicol palmitate [M+H]+=>543.2 IT 35% P=543.2
C27H42Cl2N2O6; MF: 0; RMF: 4; Prob 24.8%; CAS: 530-43-8; Lib: nist_msms; ID: 153359.

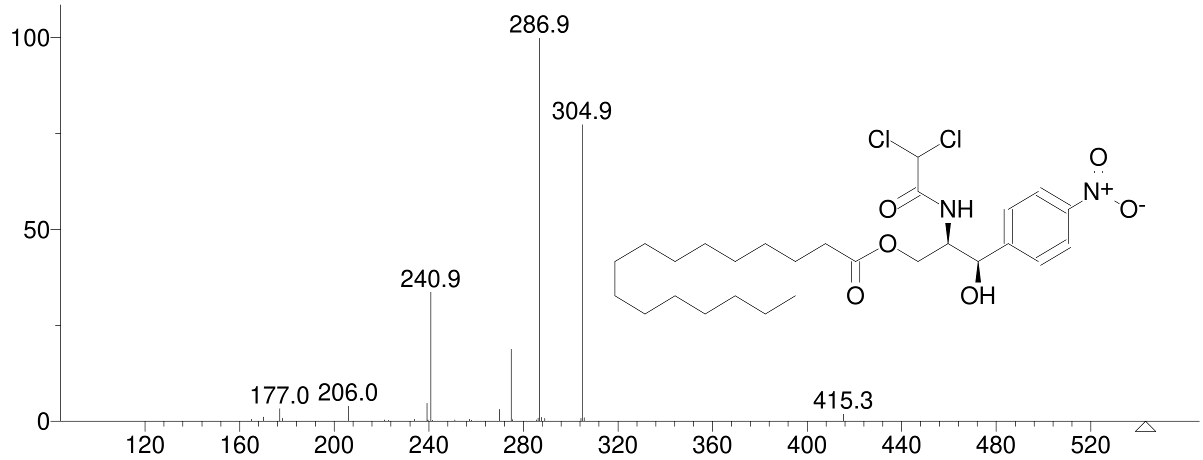

Unknown: Unknown (13.69_490.1697m/z)
Compound in Library Factor = 0

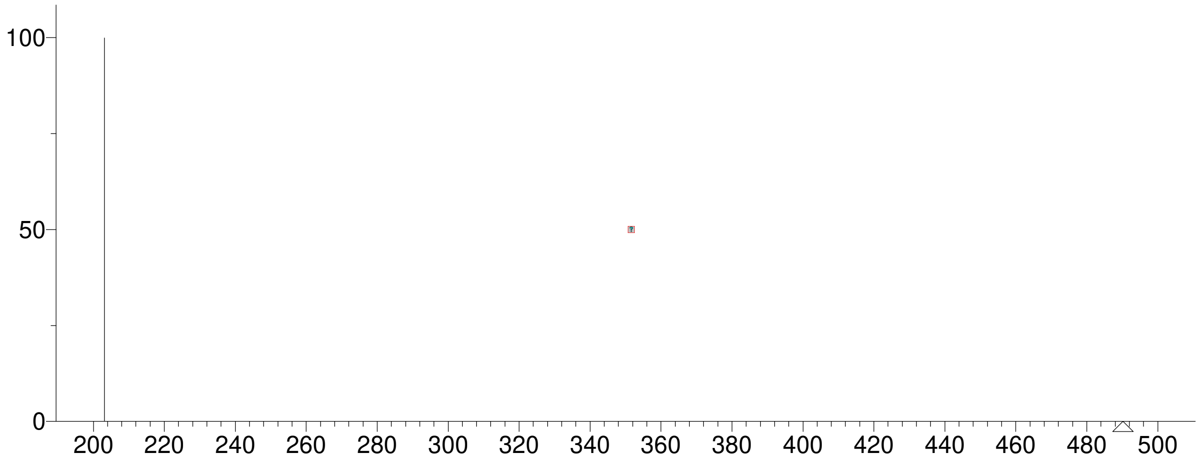

Unknown: Unknown (13.78_289.0709m/z)
Compound in Library Factor = -3273

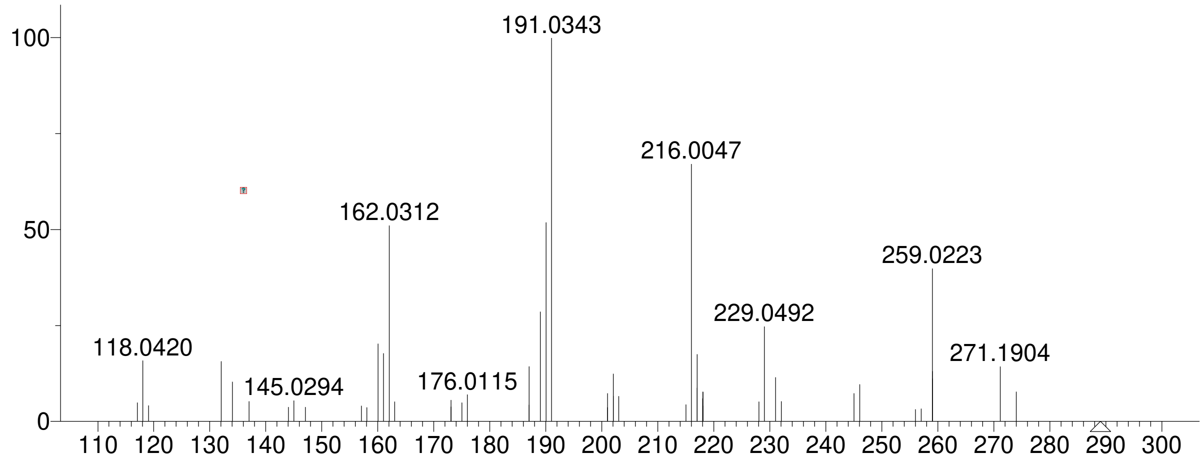

Hit 1 : (-)-Catechin gallate [M-H-C7H4O4]- HCD 92V P=289.1
C22H18O10; MF: 41; RMF: 184; Prob 5.81%; CAS: 130405-40-2; Lib: nist_msms; ID: 552328.

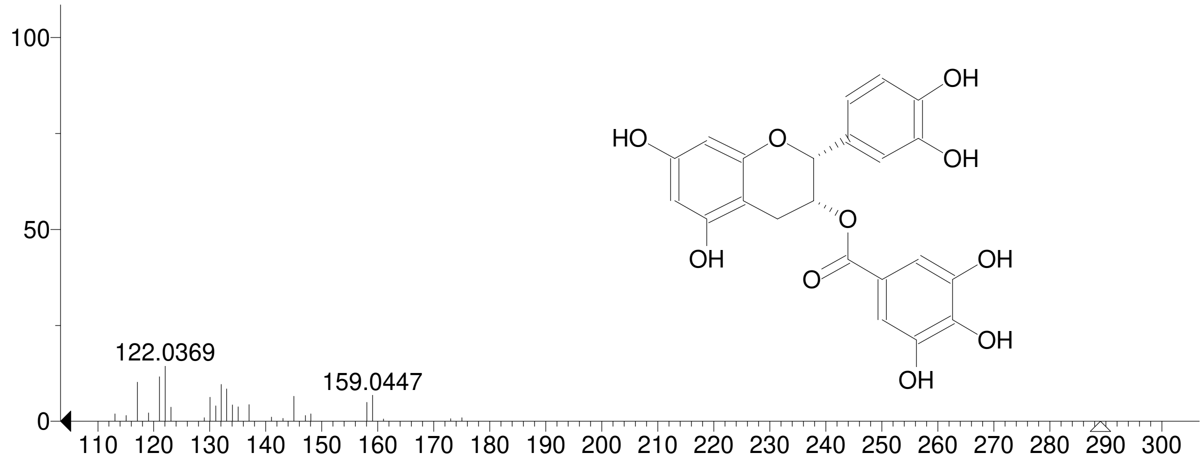

Hit 2 : NCGC00385805-01!7-methoxy-6-[(1R,4R,5S)-1-methyl-2-oxo-3,6-dioxabicyclo[3.1.0]hexan-4-yl]chromen-2
C15H12O6; MF: 34; RMF: 236; Prob 4.45%; Lib: mona-export-gnps; ID: 9447.

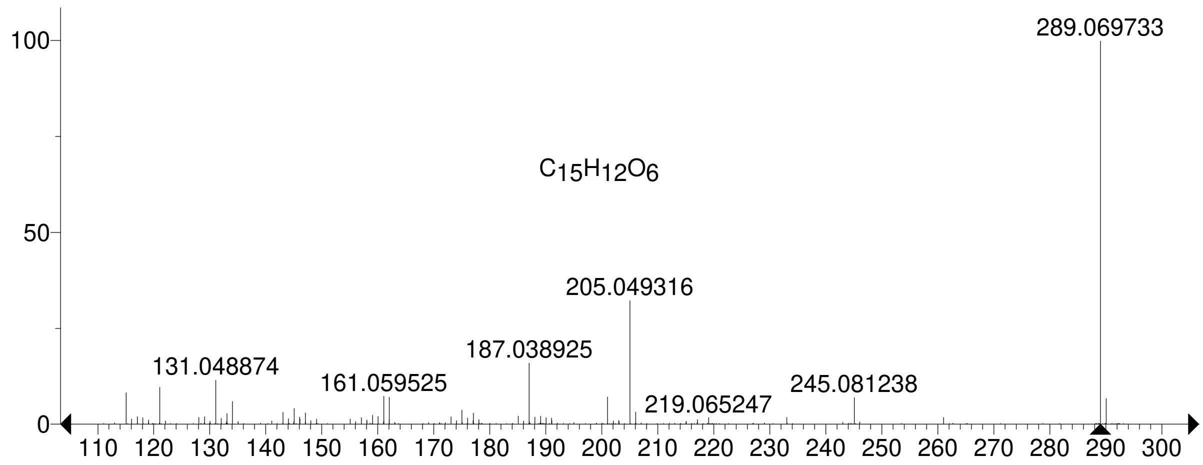

Hit 3 : (-)-Epicatechin gallate [M-H-C7H4O4]- HCD 46V P=289.1
C22H18O10; MF: 32; RMF: 201; Prob 4.10%; CAS: 1257-08-5; Lib: nist_msms; ID: 459014.

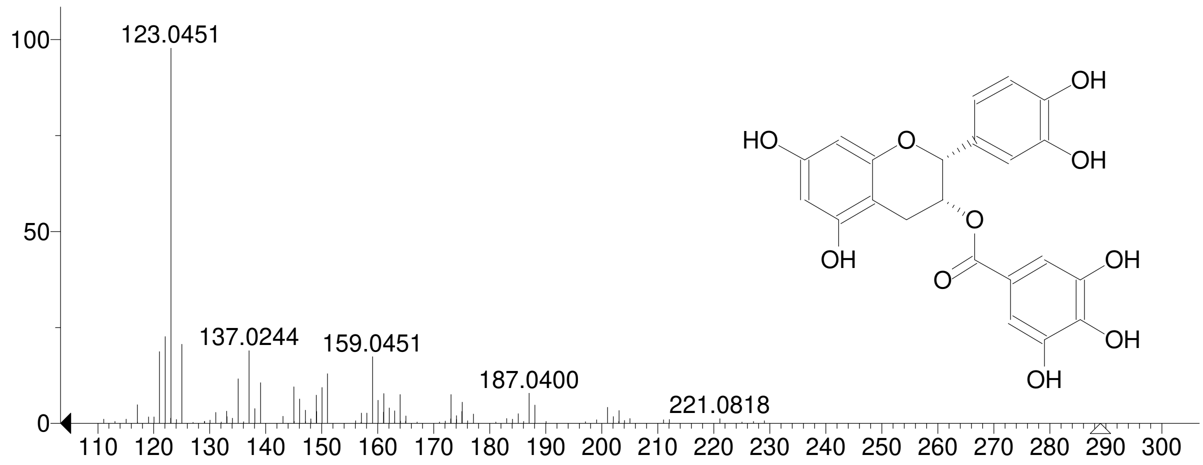

Hit 4 : Catechin [M-H]- 40 P=289.1
C15H14O6; MF: 32; RMF: 189; Prob 4.10%; Lib: mona-export-lc-ms-ms_negative_mode; ID: 27849.

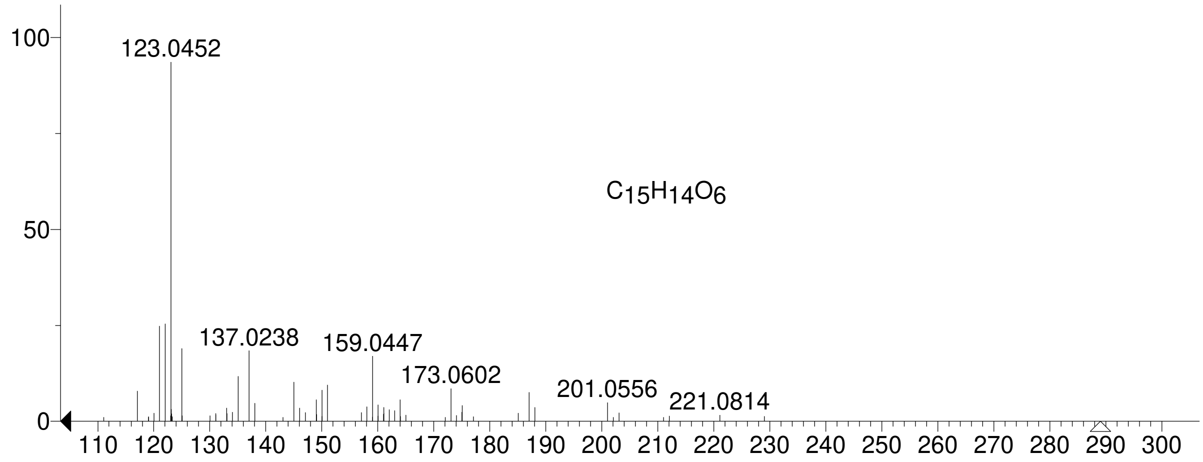

Hit 5 : Catechin; LC-ESI-QTOF; MS2; CE [M-H]- 55 P=289.1
C15H14O6; MF: 31; RMF: 175; Prob 3.94%; Lib: msms-neg-massbank; ID: 3584.

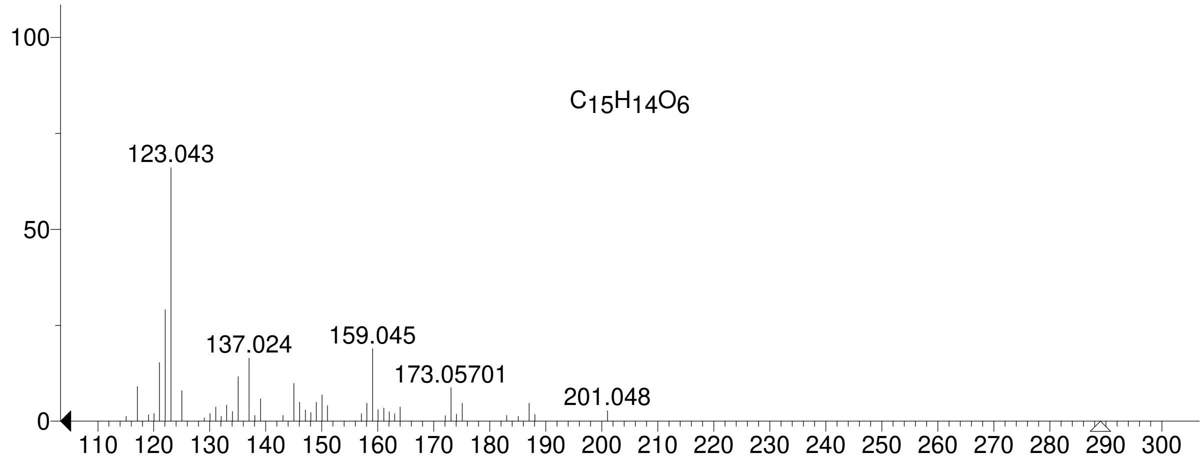

Unknown: Unknown (13.83_445.1611m/z)
Compound in Library Factor = 0

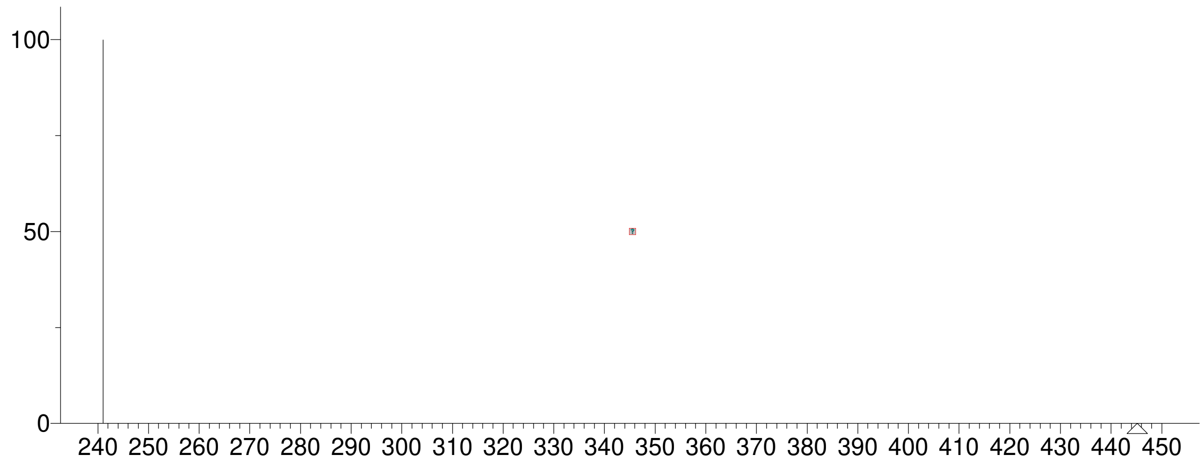

** Search Report Page 1 of 1 **

Unknown: Unknown (13.83_527.1936m/z)
Compound in Library Factor = 0

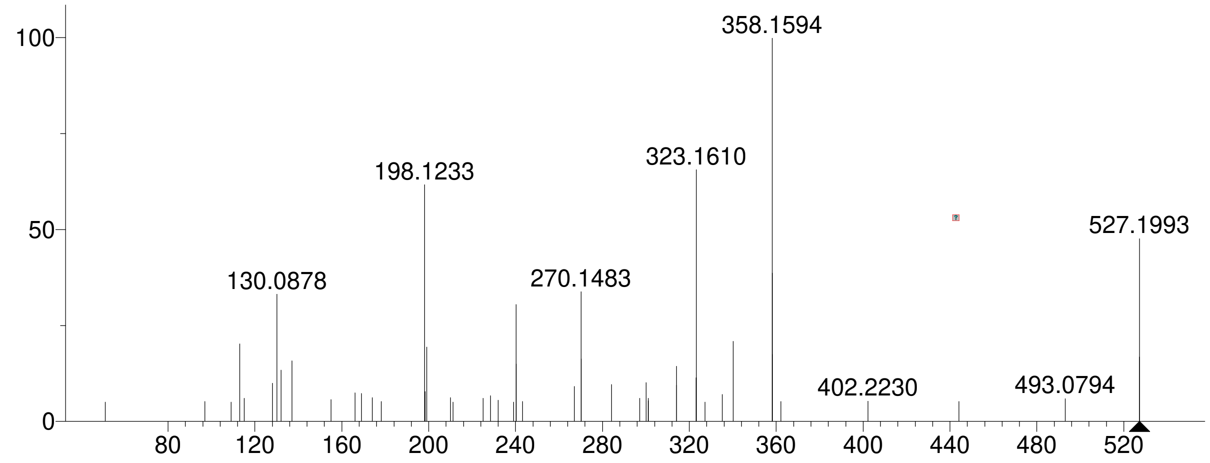

Hit 1 : (2R,7S,13R,14R,16S,19R,20S)-19-(furan-3-yl)-9,9,13,20-tetramethyl-5,12,17-trioxo-4,8,15,18-tetraoxahexa
C28H32O10; MF: 0; RMF: 31; Prob 50.0%; Lib: mona-export-lc-ms-ms_negative_mode; ID: 23168.

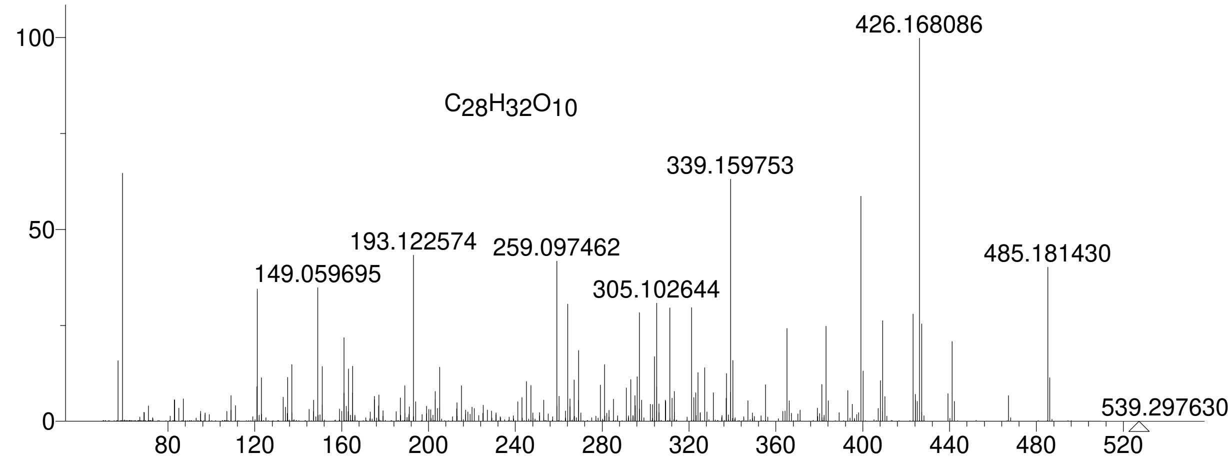

Hit 2 : Azadirachtin B [M+H-C5H12O4]+ IT 35% P=527.2
C33H42O14; MF: 0; RMF: 22; Prob 50.0%; CAS: 95507-03-2; Lib: nist_msms; ID: 223448.

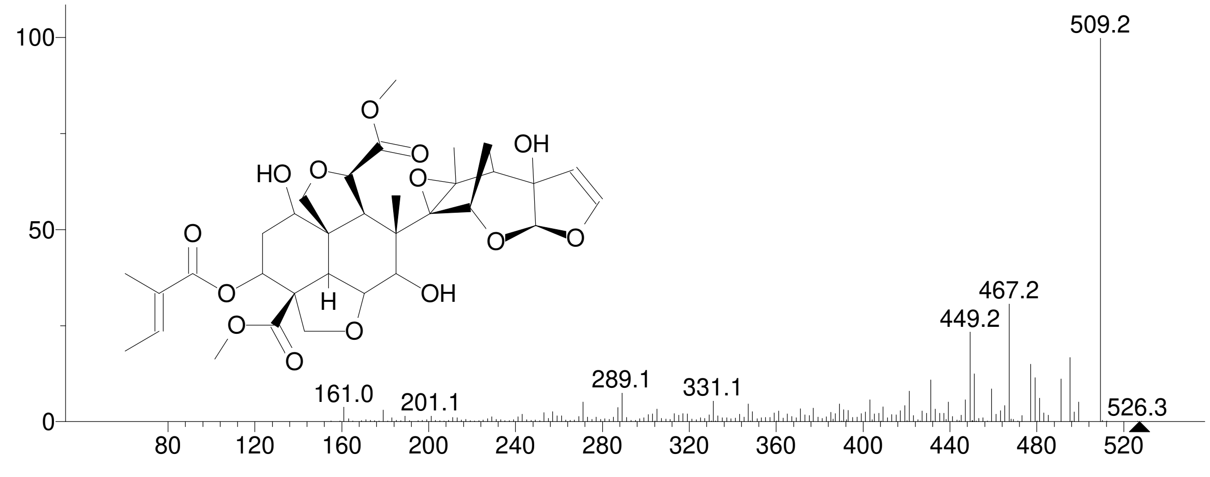

Unknown: Unknown (13.87_369.1176m/z)
Compound in Library Factor = 0

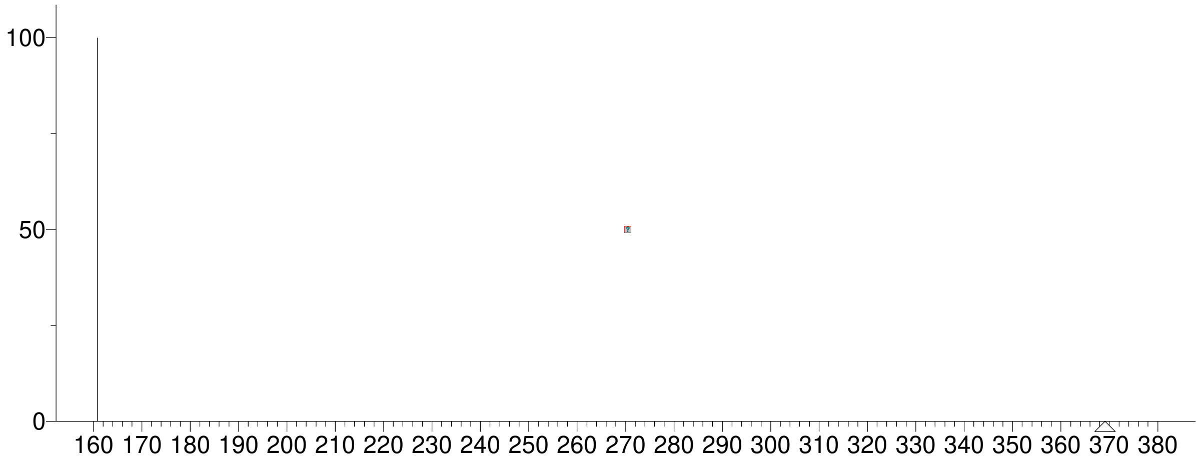

Unknown: Unknown (13.90_451.2066m/z)
Compound in Library Factor = -2176

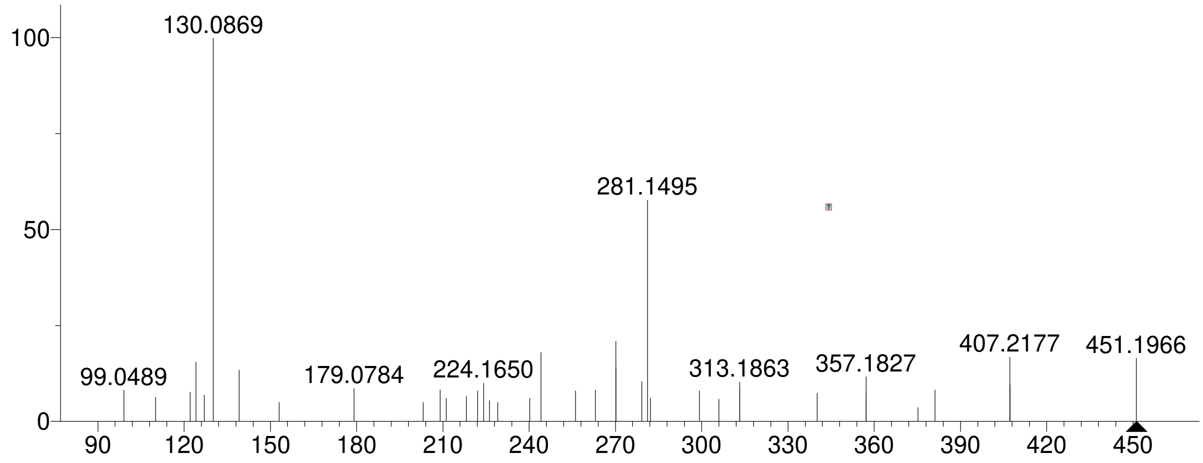

Hit 1 : Betamethasone 9,11-epoxide 17-propionate [M+Na]+ HCD 9V P=451.2
C25H32O6; MF: 37; RMF: 450; Prob 42.0%; CAS: 79578-39-5; Lib: nist_msms; ID: 448292.

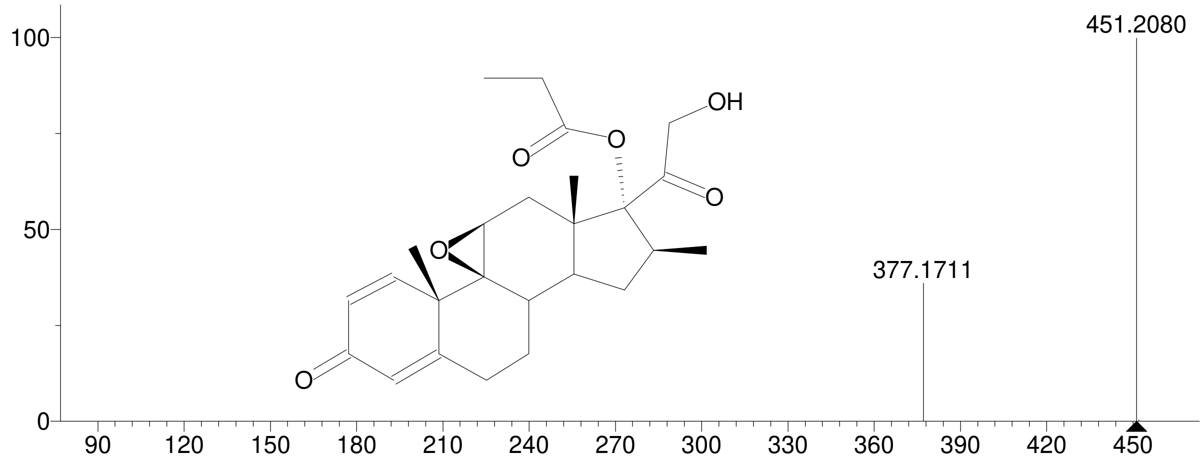

Hit 2 : L-Carnosine [2M-H]- HCD 2V P=451.2
C9H14N4O3; MF: 29; RMF: 236; Prob 31.4%; CAS: 305-84-0; Lib: nist_msms; ID: 287986.

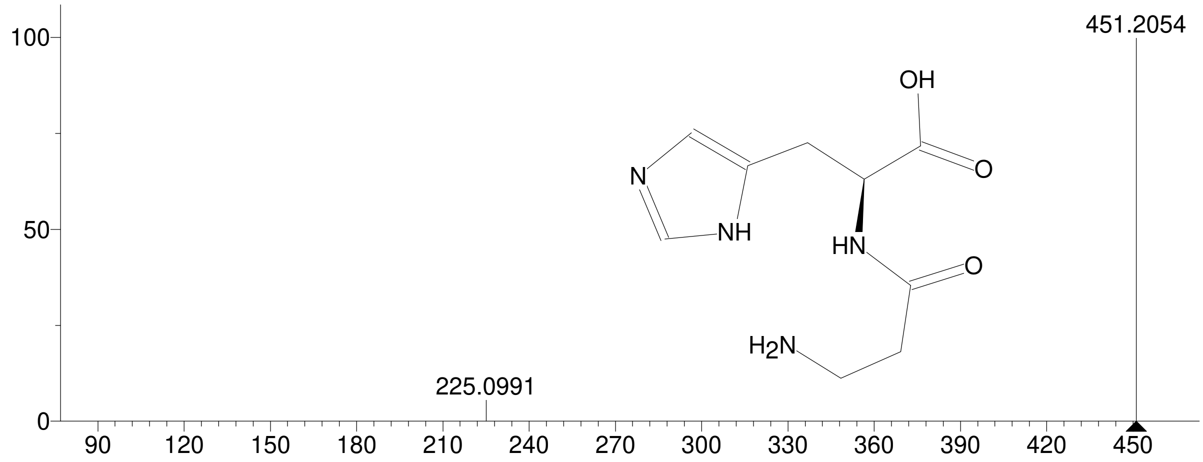

Hit 3 : Grayanotoxin I [M+K]+ 35HCD P=451.2
C22H36O7; MF: 2; RMF: 122; Prob 9.22%; Lib: mona-export-vf-npl_qexactive; ID: 21286.

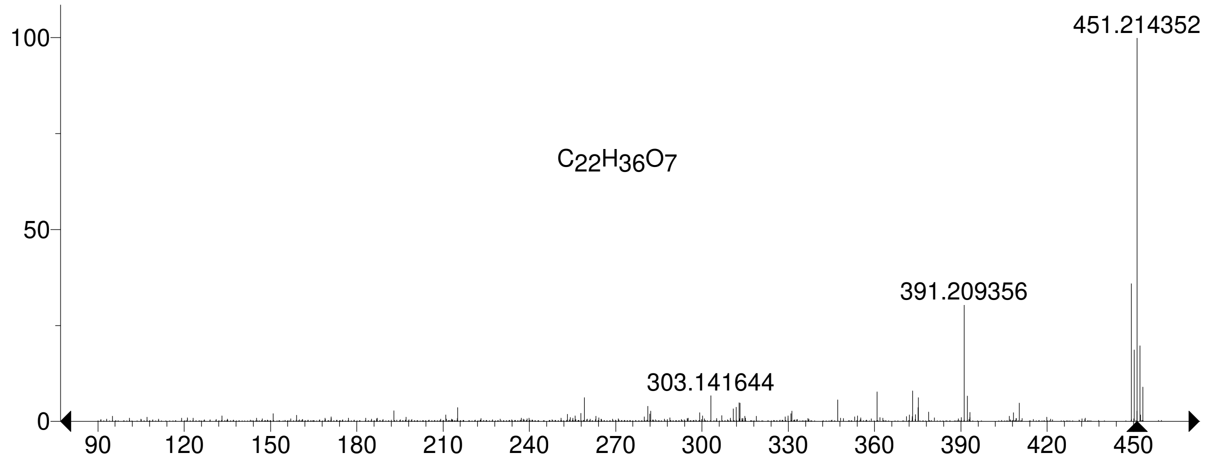

Hit 4 : 2-Naphthalenecarboxamide, N-[2-(4-oxo-1-phenyl-1,3,8-triazaspiro[4.5]dec-8-yl)ethyl]- [M+Na]+ HCD 40'
C26H28N4O2; MF: 1; RMF: 165; Prob 8.86%; CAS: 1130067-34-3; Lib: nist_msms; ID: 261179.

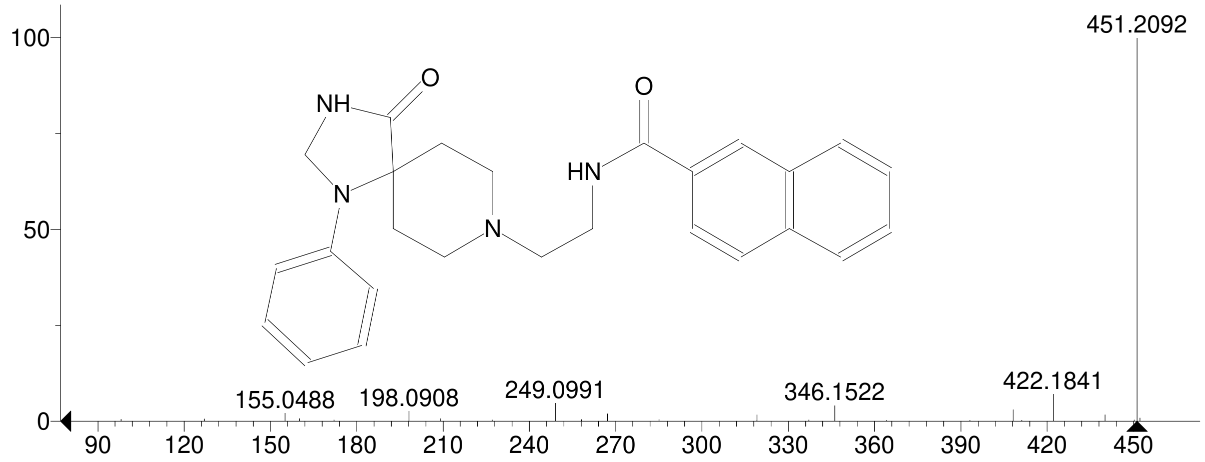

Hit 5 : Grayanotoxin I M-H 35 P=451.2
C22H36O7; MF: 0; RMF: 49; Prob 8.52%; Lib: mona-export-vf-npl_ltq; ID: 4523.

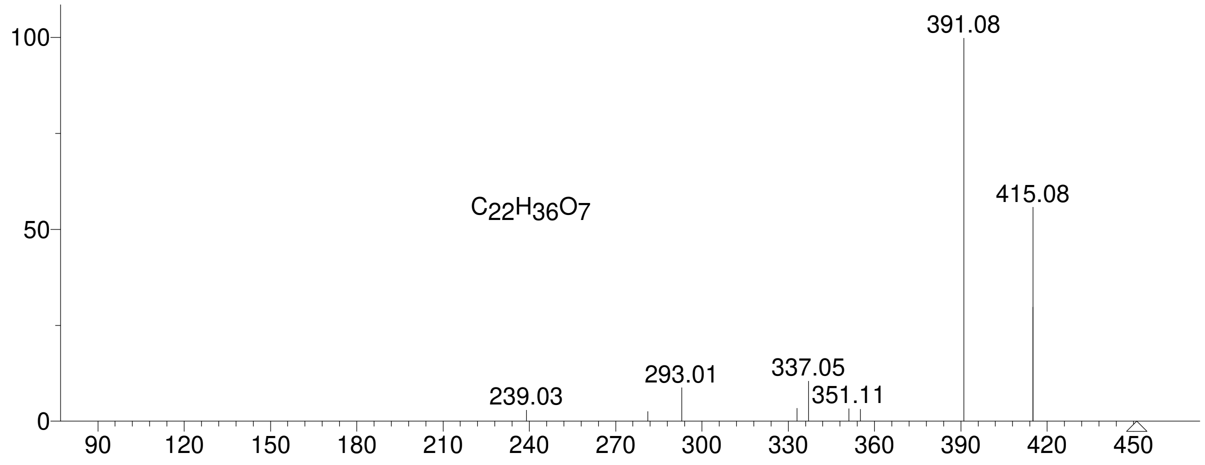

Unknown: Unknown (13.96_200.1283m/z)
Compound in Library Factor = 0

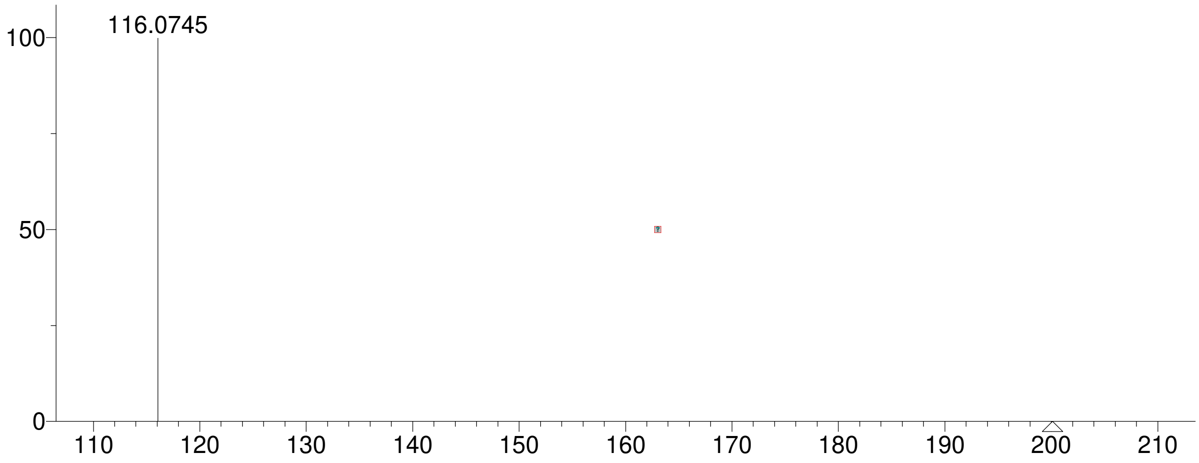

** Search Report Page 1 of 1 **

Unknown: Unknown (13.98_633.7975m/z)
Compound in Library Factor = 0

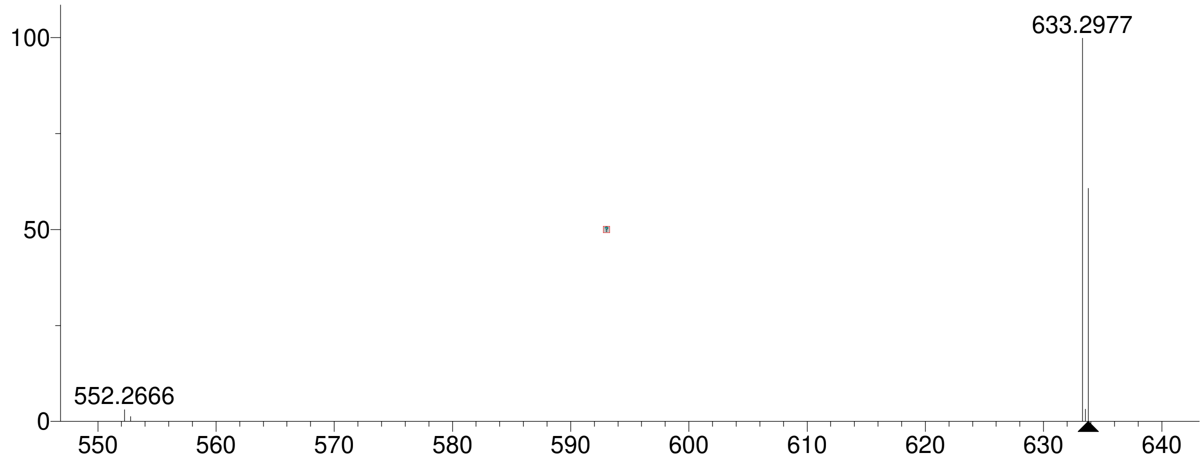

Unknown: Unknown (13.99_633.2960m/z)
Compound in Library Factor = -2387

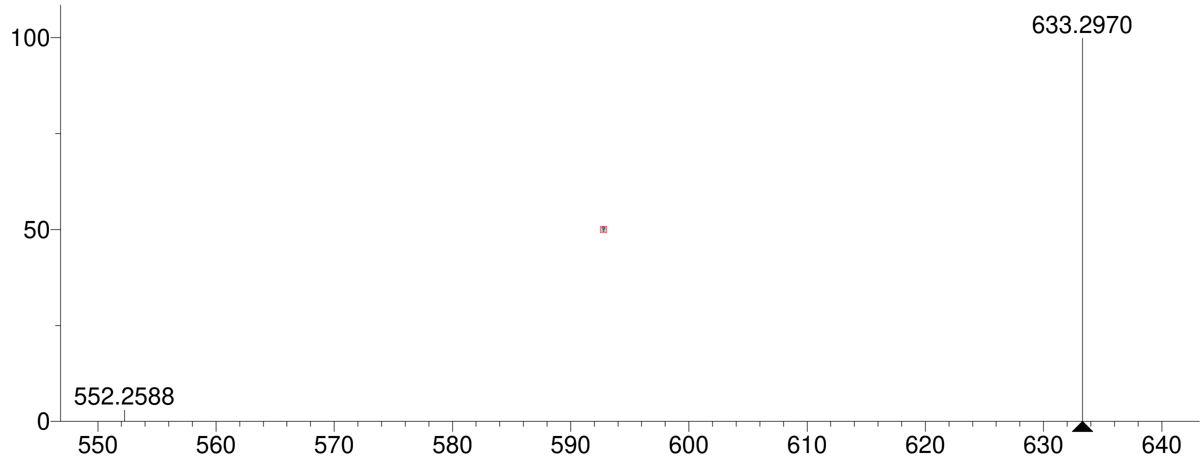

Hit 1 : Alloferon 1 [M+2H]2+ HCD 28eV P=633.3
C52H76N22O16; MF: 20; RMF: 697; Prob 100.0%; Lib: nist_msms2; ID: 49643.

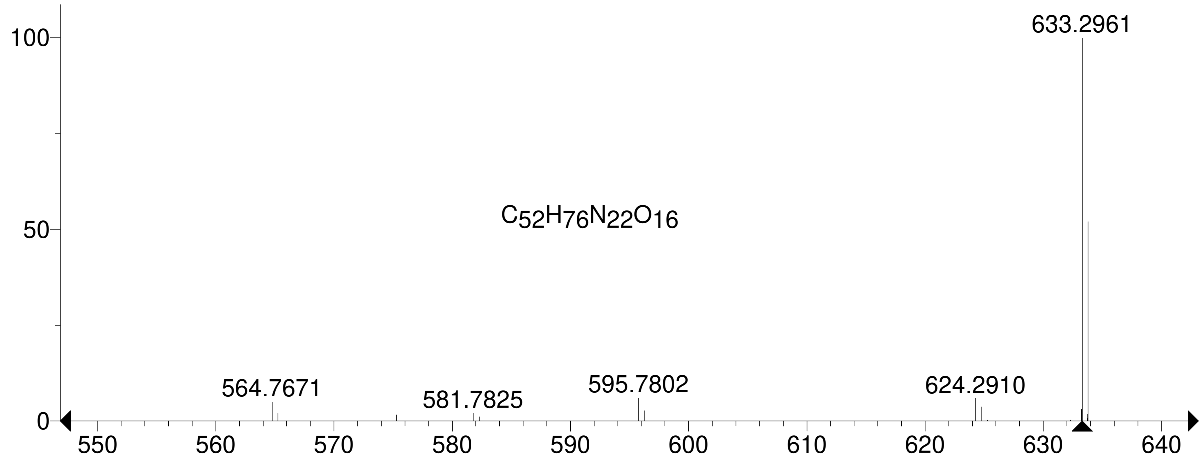

** Search Report Page 1 of 1 **

Unknown: Unknown (13.99_656.2993m/z)
Compound in Library Factor = 0

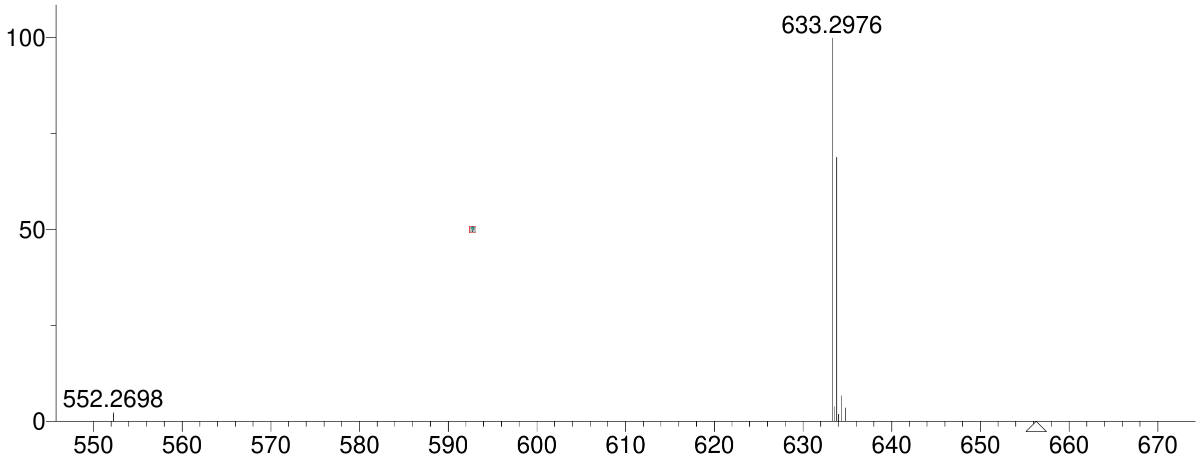

Unknown: Unknown (13.99_656.8009m/z)
Compound in Library Factor = 0

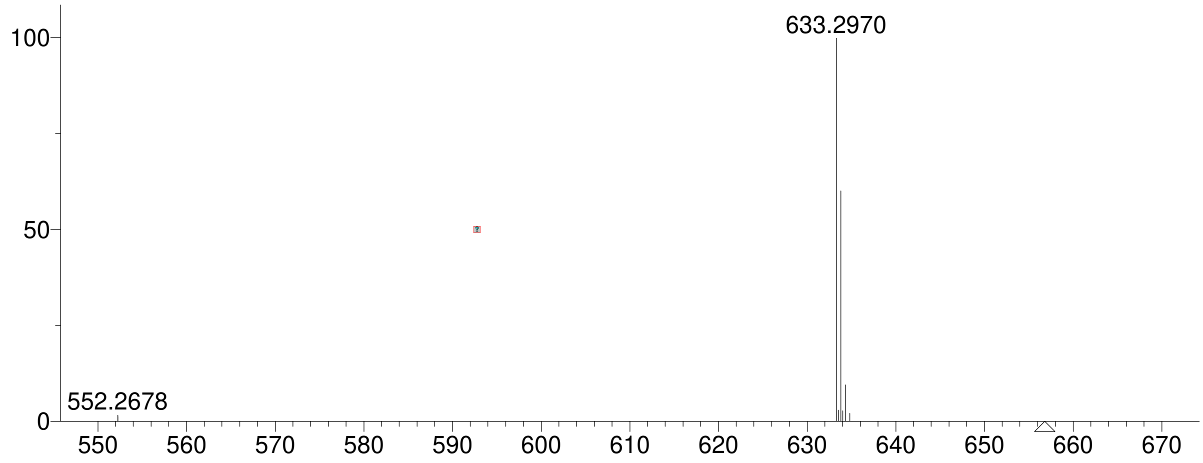

** Search Report Page 1 of 1 **

Unknown: Unknown (14.00_651.2860m/z)
Compound in Library Factor = 0

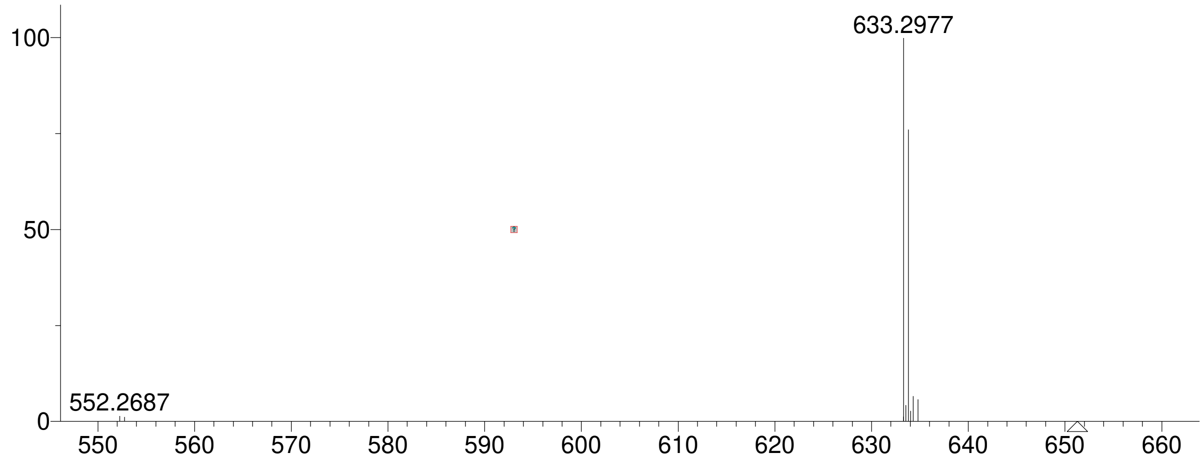

** Search Report Page 1 of 1 **

Unknown: Unknown (14.08_475.1714m/z)
Compound in Library Factor = 0

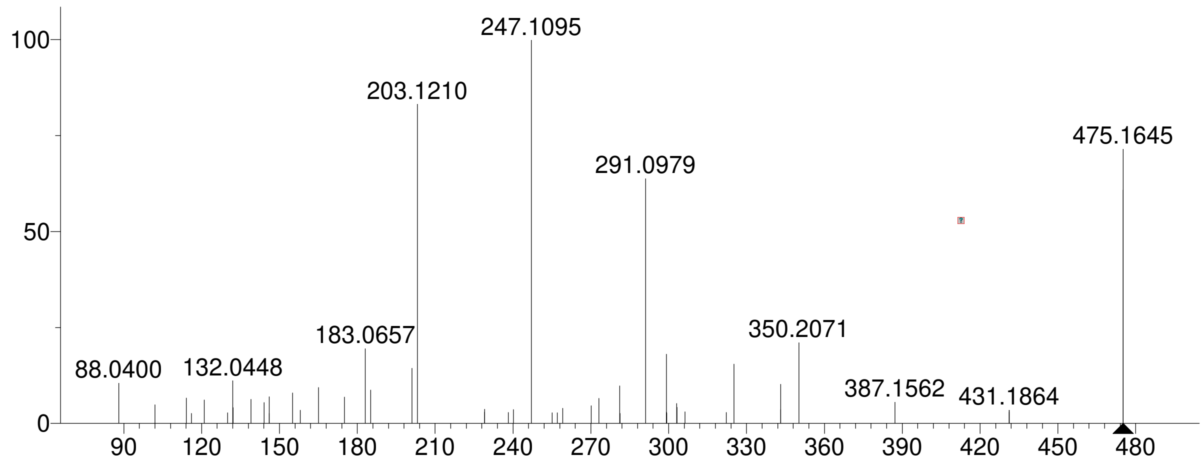

Unknown: Unknown (14.10_622.2971m/z)
Compound in Library Factor = 0

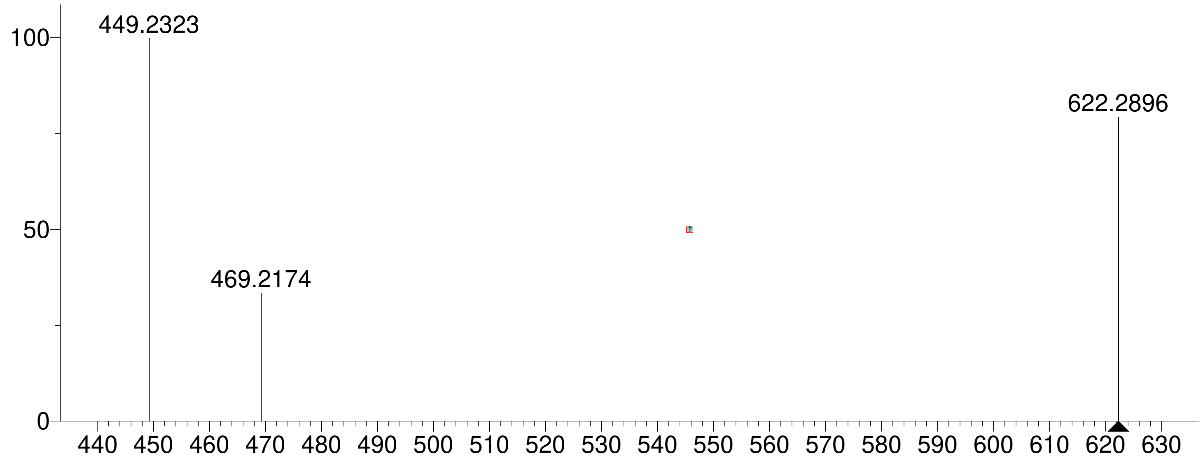

** Search Report Page 1 of 1 **

Unknown: Unknown (14.11_578.2730m/z)
Compound in Library Factor = 0

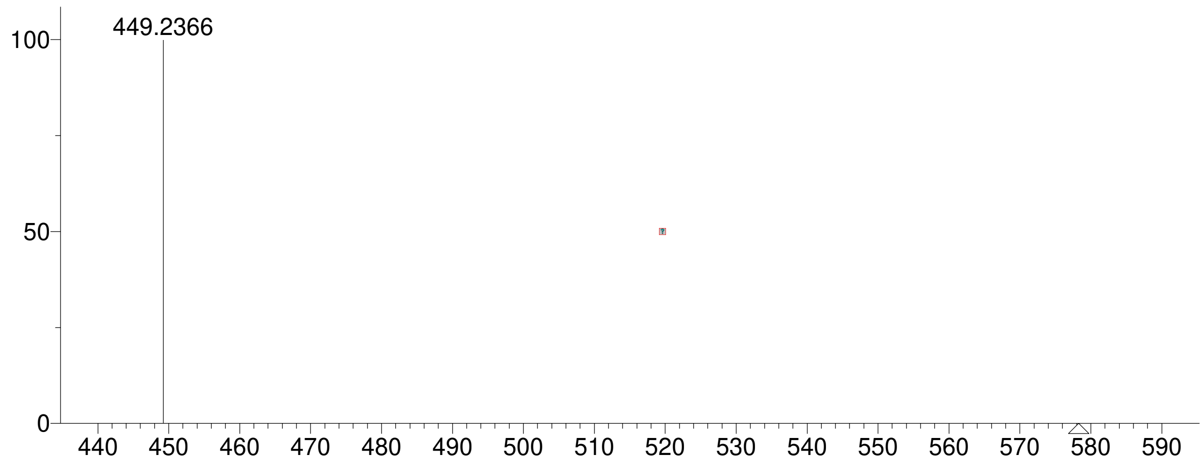

** Search Report Page 1 of 1 **

Unknown: Unknown (14.11_633.3105m/z)
Compound in Library Factor = -3273

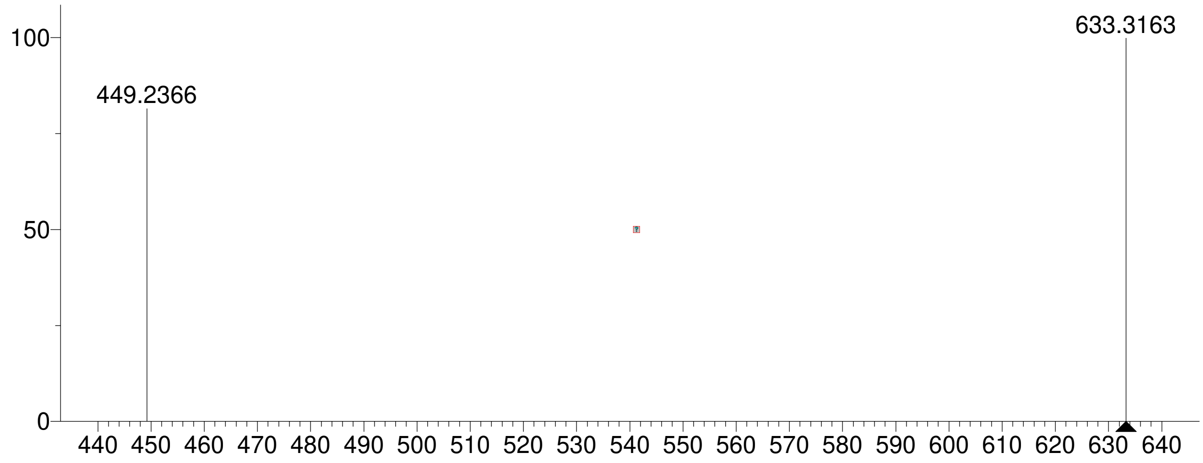

Hit 1 : [(t-Bu)DSer6, (Aza)Gly10]-LH-RH [M-2H]2- HCD 22eV P=633.3
C59H84N18O14; MF: 9; RMF: 488; Prob 100.0%; Lib: nist_msms2; ID: 80477.

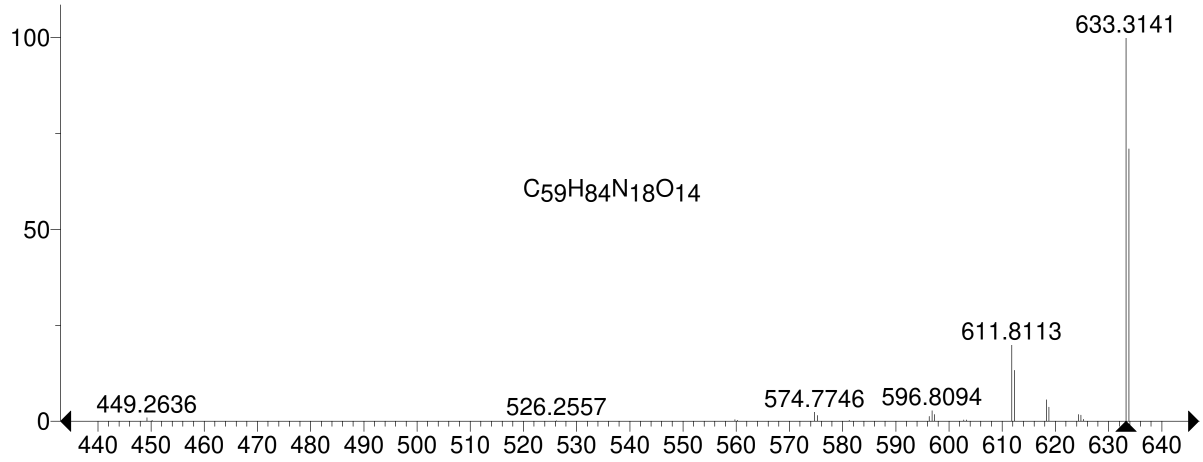

Unknown: Unknown (14.18_200.1283m/z)
Compound in Library Factor = -258

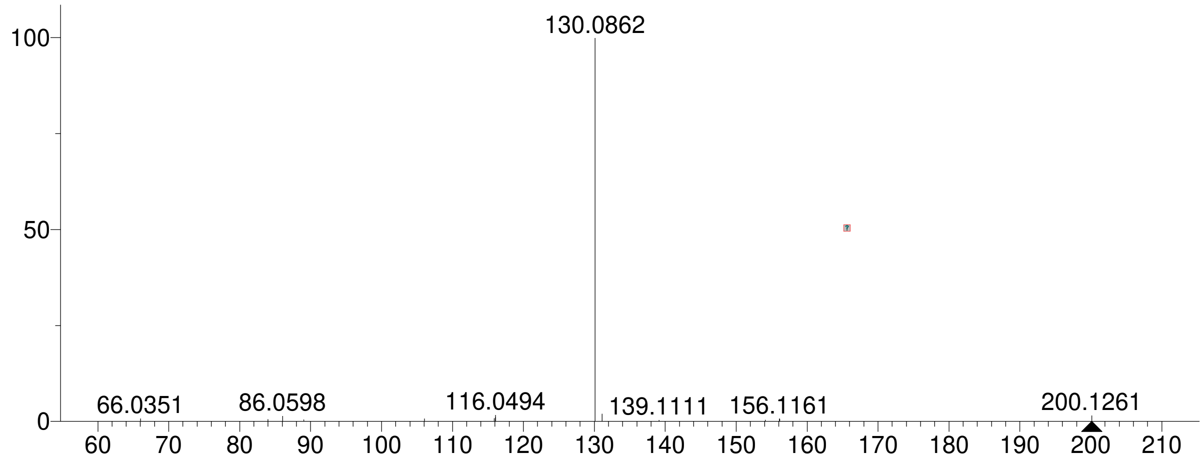

Hit 1 : Ecgonine-methyl-ester (EME) [M+H]+ 10eV P=200.1
C10H17NO3; MF: 252; RMF: 921; Prob 96.5%; Lib: mona-export-massbank; ID: 564.

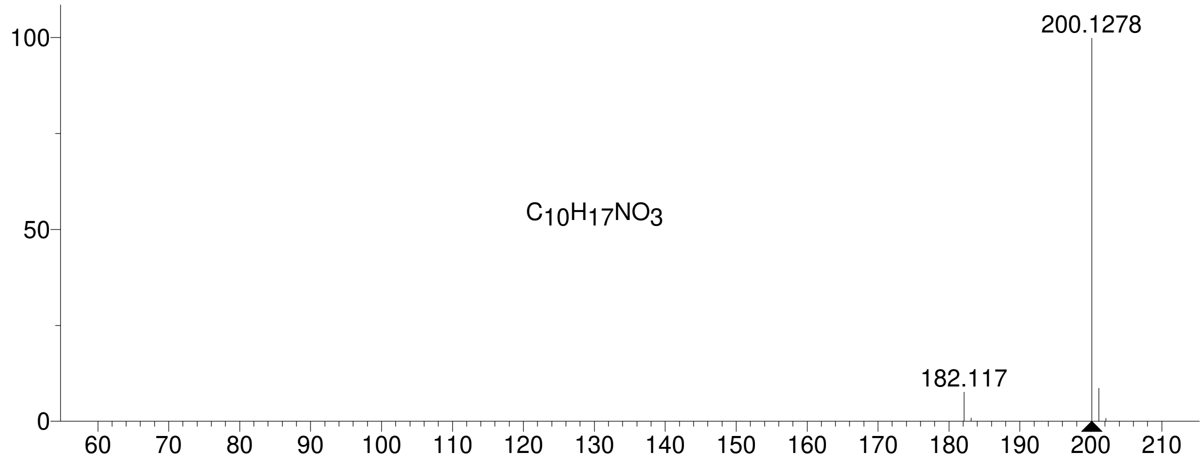

Hit 2 : N-Hexanoyl-L-homoserine lactone [M+H]+ HCD 0V P=200.1
C10H17NO3; MF: 5; RMF: 111; Prob 0.96%; CAS: 147852-83-3; Lib: nist_msms; ID: 164664.

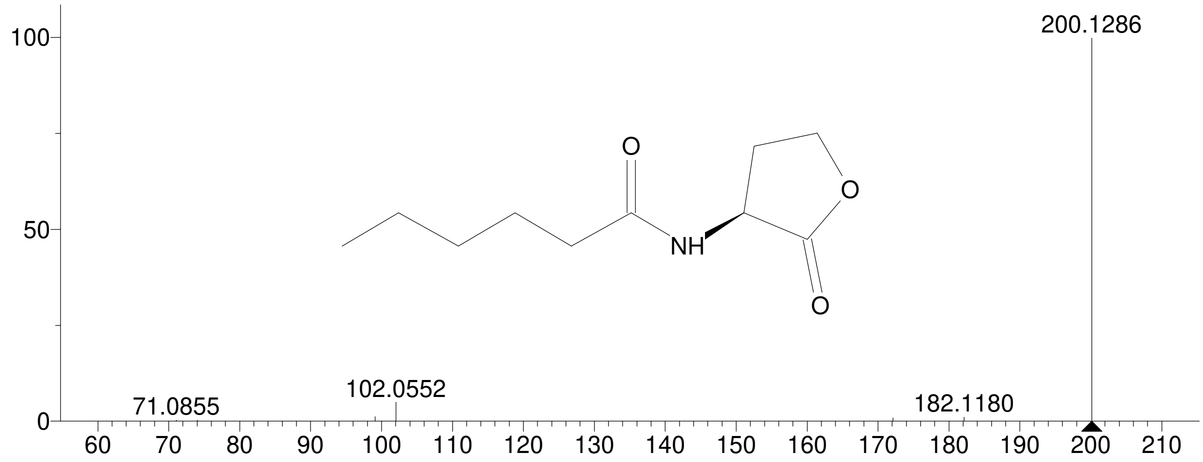

Hit 3 : N-Hexanoyl-DL-homoserine lactone [M+H]+ HCD 2V P=200.1
C10H17NO3; MF: 4; RMF: 106; Prob 0.92%; CAS: 106983-28-2; Lib: nist_msms; ID: 295469.

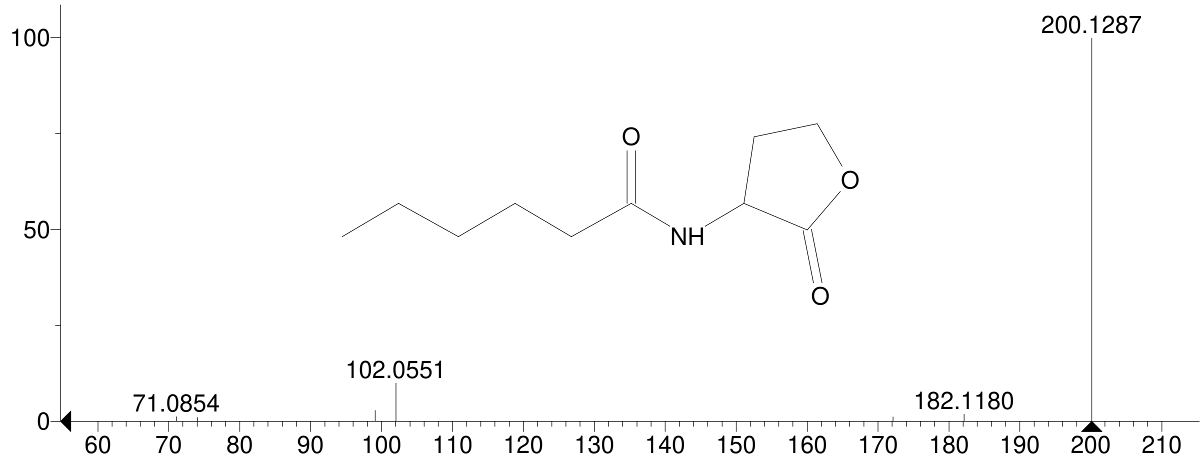

Hit 4 : Methylecgonine [M+H]+ HCD 6V P=200.1
C10H17NO3; MF: 2; RMF: 101; Prob 0.85%; CAS: 7143-09-1; Lib: nist_msms; ID: 370464.

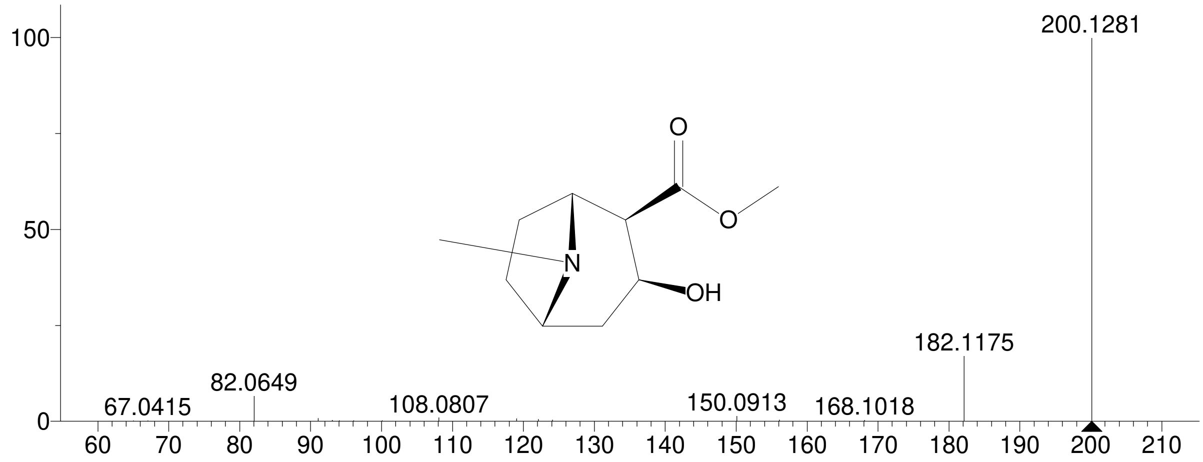

Hit 5 : Ecgonine methylester [M+H]+ 35eV P=200.1
C10H17NO3; MF: 0; RMF: 51; Prob 0.78%; Lib: mona-export-fiehn_hilic; ID: 1907.

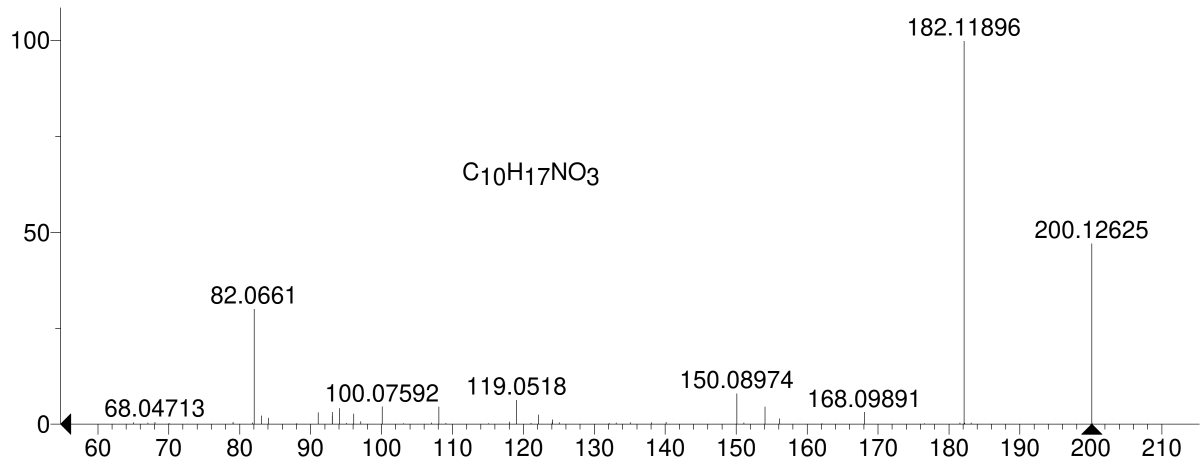

Unknown: Unknown (14.19_337.0919m/z)
Compound in Library Factor = -3273

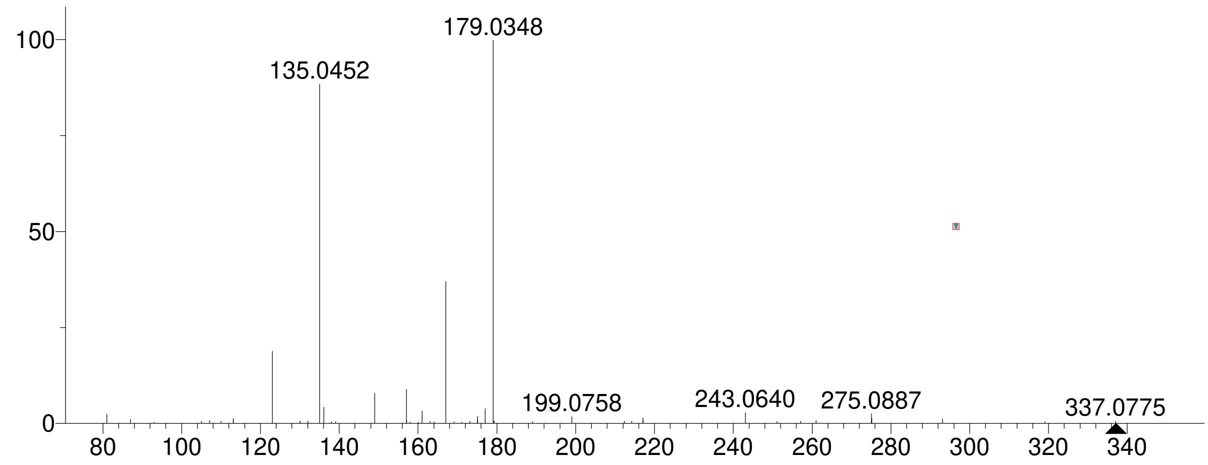

Hit 1 : Neochlorogenic acid [M+H-H2O]+ HCD 74V P=337.1
C16H18O9; MF: 18; RMF: 153; Prob 14.6%; CAS: 906-33-2; Lib: nist_msms; ID: 239449.

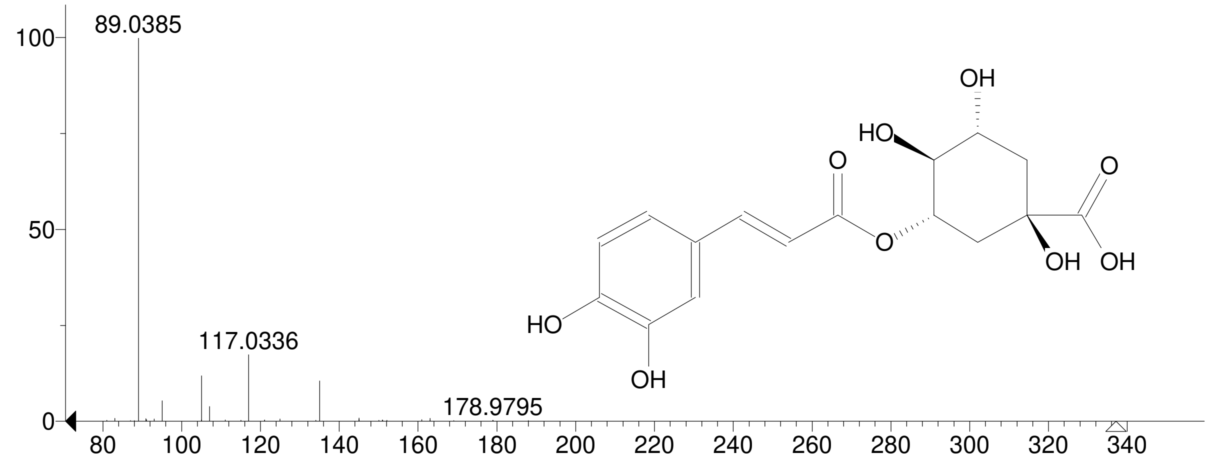

Hit 2 : Chlorogenic acid [M+H-H2O]+ HCD 33V P=337.1
C16H18O9; MF: 13; RMF: 147; Prob 11.8%; CAS: 327-97-9; Lib: nist_msms; ID: 100722.

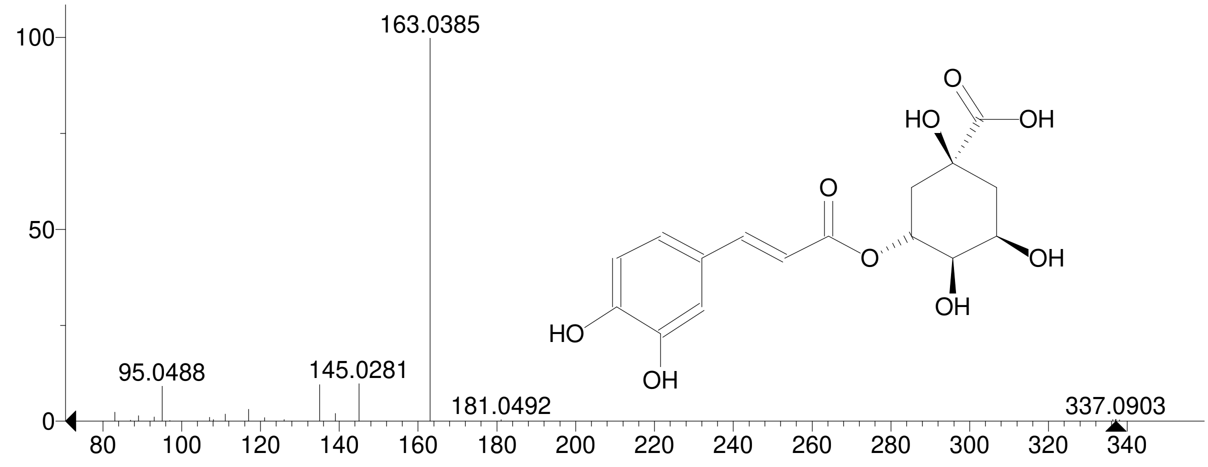

Hit 3 : NCGC00180823-02!(3R,4R,5R)-5-[(E)-3-(3,4-dihydroxyphenyl)prop-2-enoyl]oxy-3,4-dihydroxycyclohexene
C16H16O8; MF: 7; RMF: 253; Prob 9.27%; Lib: mona-export-gnps; ID: 9151.

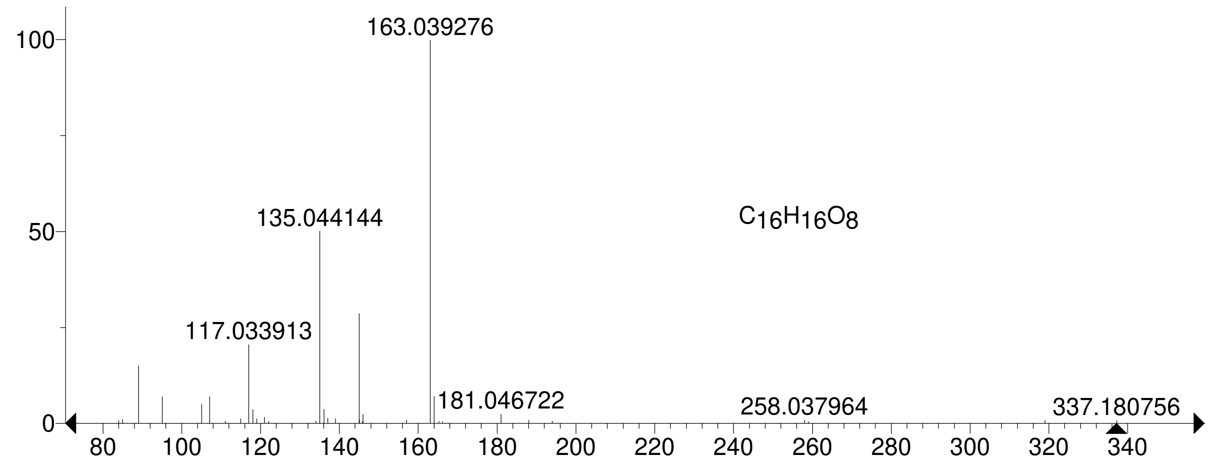

Hit 4 : NCGC00380360-01!7-methoxy-6-[(2S,3R,4S,5S,6R)-3,4,5-trihydroxy-6-(hydroxymethyl)oxan-2-yl]oxychron
C16H18O9; MF: 3; RMF: 72; Prob 7.83%; Lib: mona-export-gnps; ID: 9547.

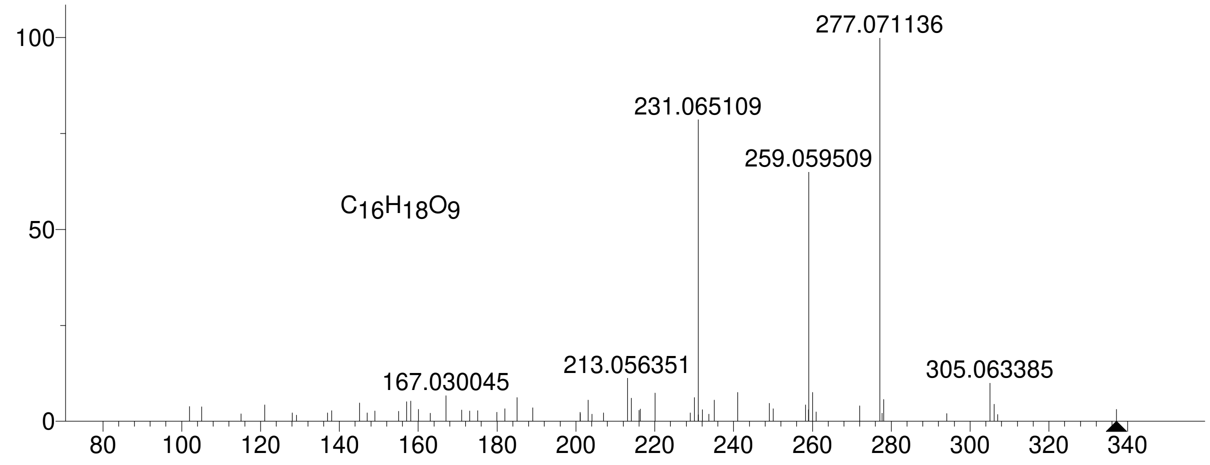

Hit 5 : 2-(4a,9,10a-trihydroxy-1-methyl-5,10-dioxo-3,4-dihydro-1H-benzo[g]isochromen-3-yl)acetic acid [M+H]+ 6
C16H16O8; MF: 1; RMF: 44; Prob 7.22%; Lib: mona-export-vf-npl_qexactive; ID: 19101.

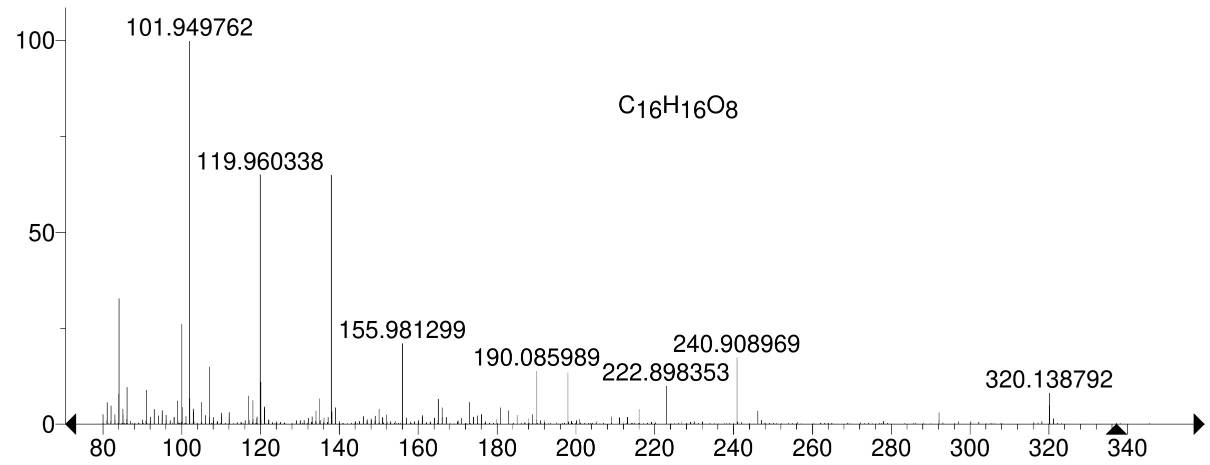

** Search Report Page 1 of 1 **

Unknown: Unknown (14.19_436.0086m/z)
Compound in Library Factor = 0

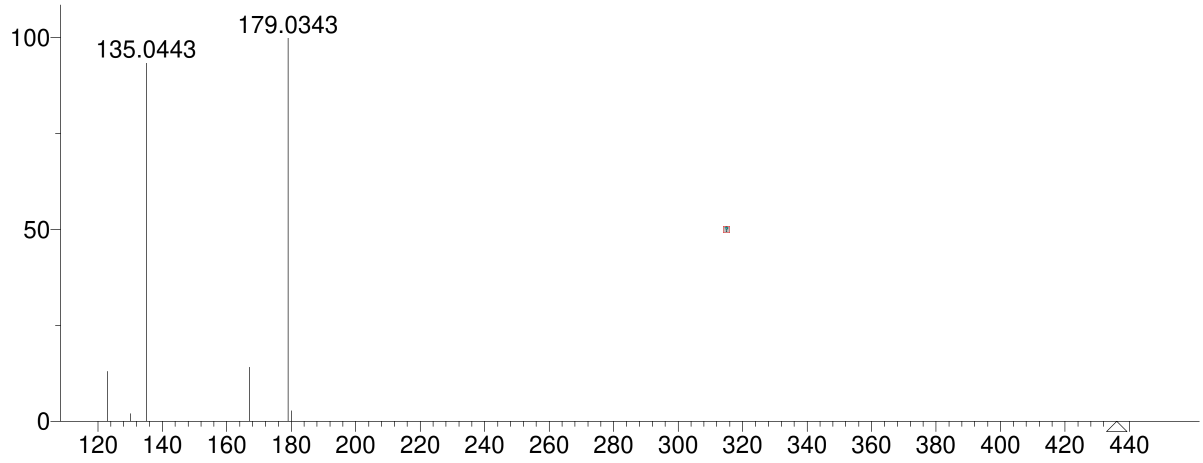

** Search Report Page 1 of 1 **

Unknown: Unknown (14.20_461.9564m/z)
Compound in Library Factor = 0

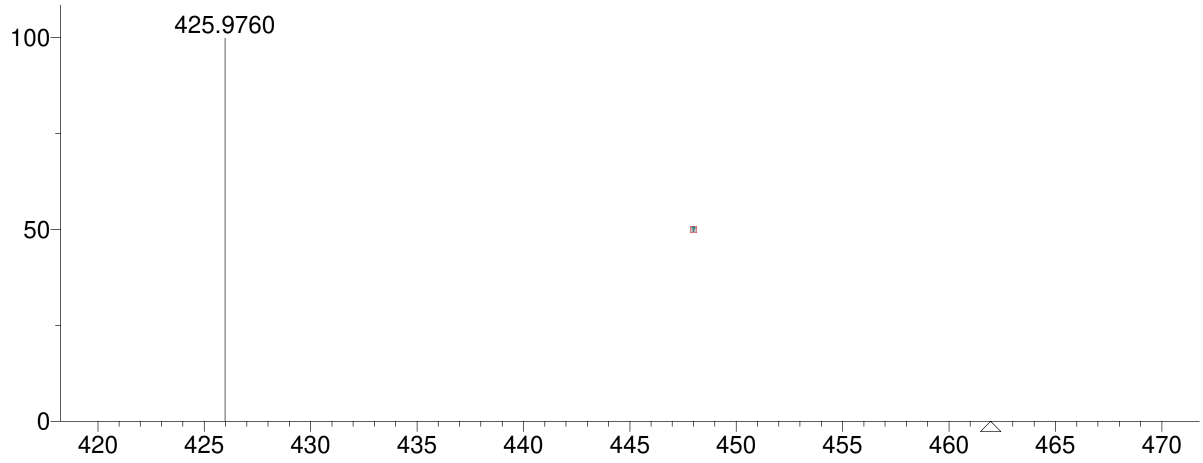

Unknown: Unknown (14.21_625.2980m/z)
Compound in Library Factor = -1309

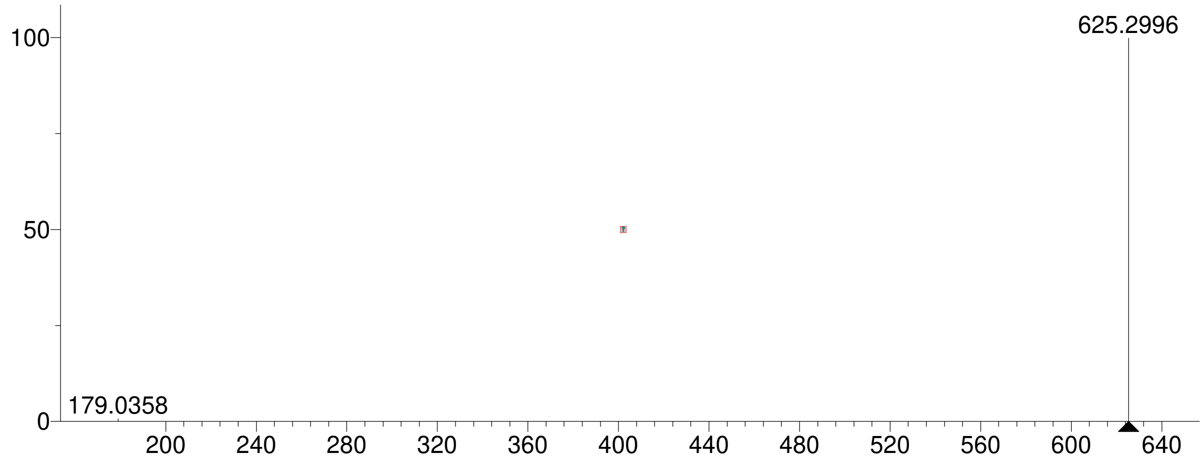

Hit 1 : PI 19:1; PI 2:0-17:1; [M-H]- [M-H]- P=625.3
C28H51O13P; MF: 57; RMF: 704; Prob 18.6%; Lib: msdial-insilicomsms-lipids-neg; ID: 326480.

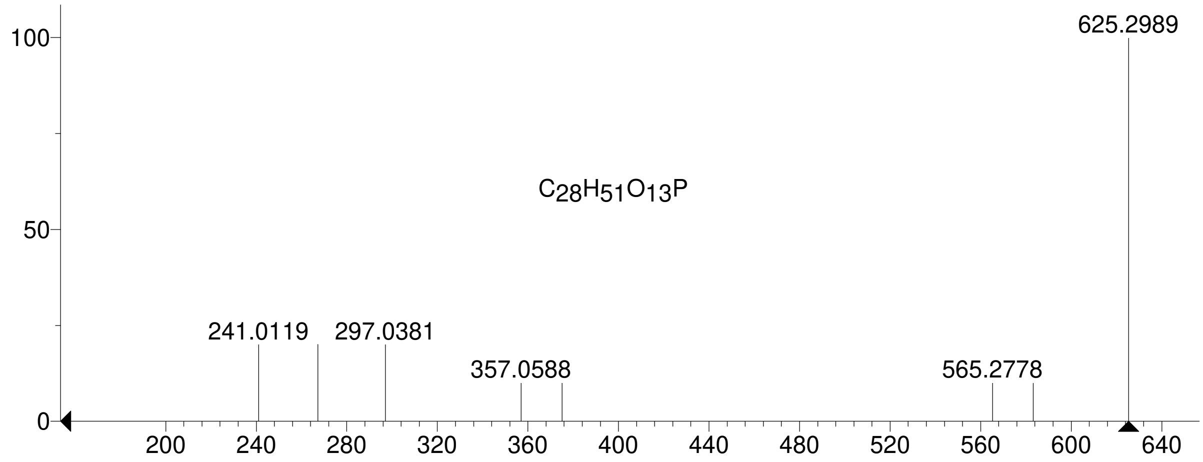

Hit 2 : PI 19:1; PI 3:0-16:1; [M-H]- [M-H]- P=625.3
C28H51O13P; MF: 57; RMF: 704; Prob 18.6%; Lib: msdial-insilicomsms-lipids-neg; ID: 326535.

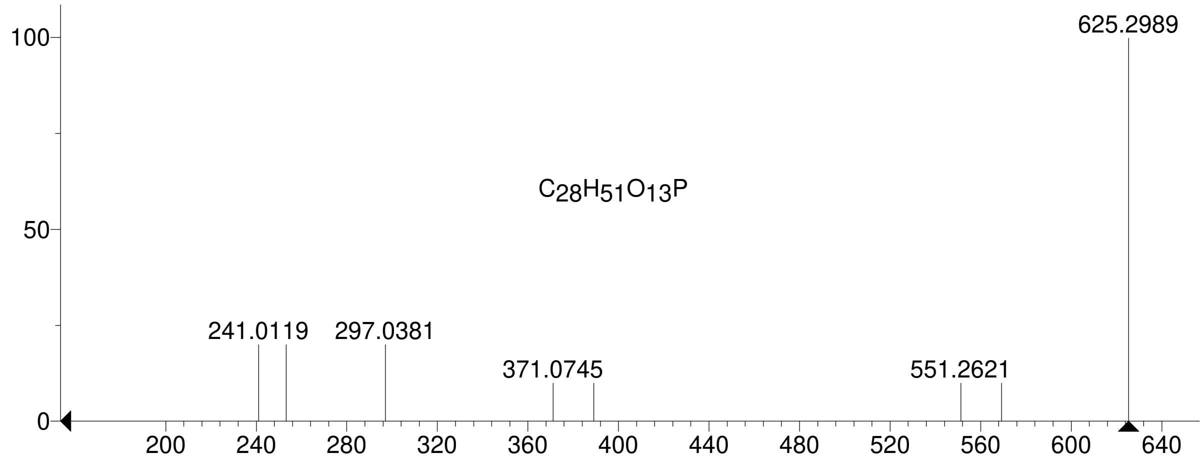

Hit 3 : PI 19:1; PI 4:0-15:1; [M-H]- [M-H]- P=625.3
C28H51O13P; MF: 57; RMF: 704; Prob 18.6%; Lib: msdial-insilicomsms-lipids-neg; ID: 326592.

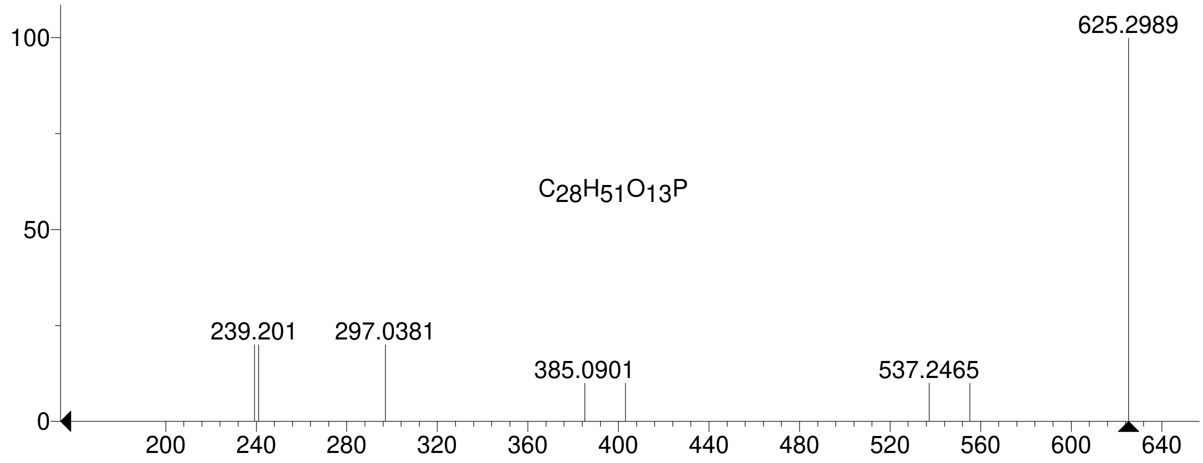

Hit 4 : PI 19:1; PI 5:0-14:1; [M-H]- [M-H]- P=625.3
C28H51O13P; MF: 57; RMF: 704; Prob 18.6%; Lib: msdial-insilicomsms-lipids-neg; ID: 326648.

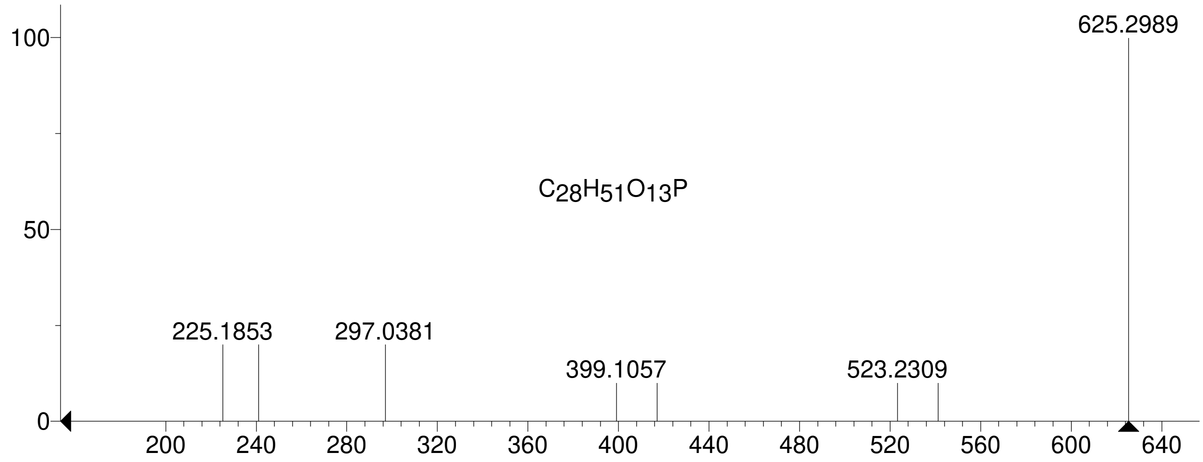

Hit 5 : PI 19:1; PI 6:0-13:1; [M-H]- [M-H]- P=625.3
C28H51O13P; MF: 57; RMF: 704; Prob 18.6%; Lib: msdial-insilicomsms-lipids-neg; ID: 326703.

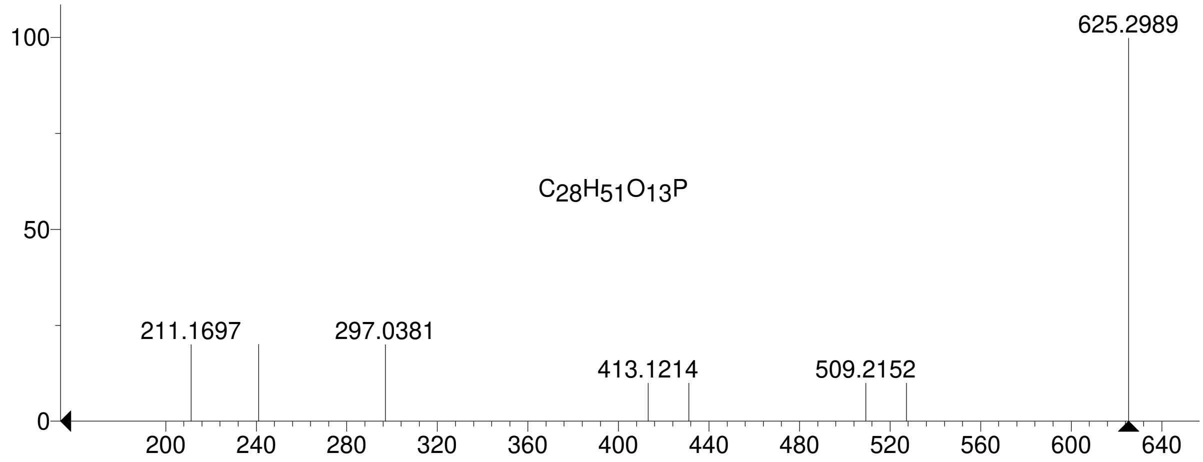

** Search Report Page 1 of 1 **

Unknown: Unknown (14.22_648.3015m/z)
Compound in Library Factor = 0

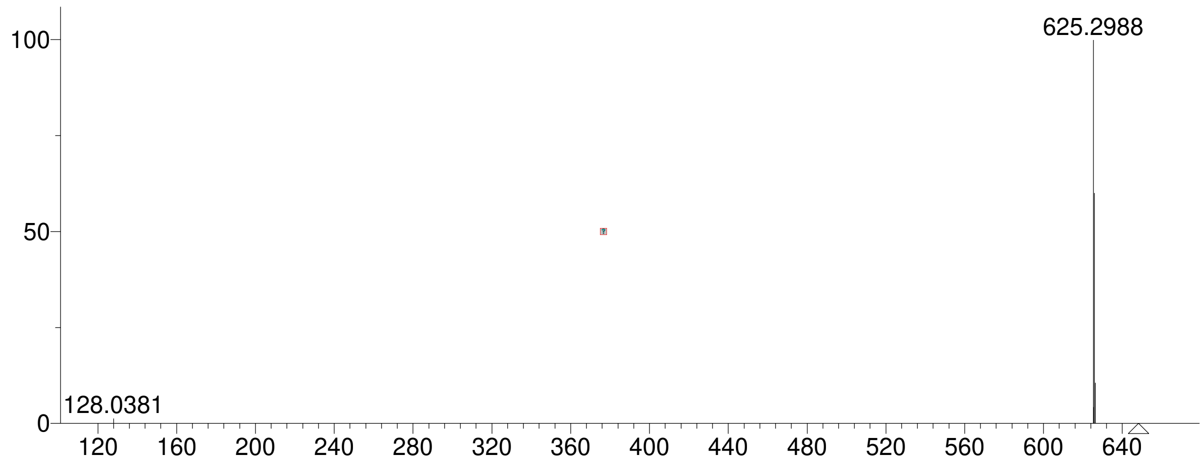

Unknown: Unknown (14.22_648.8027m/z)
Compound in Library Factor = 0

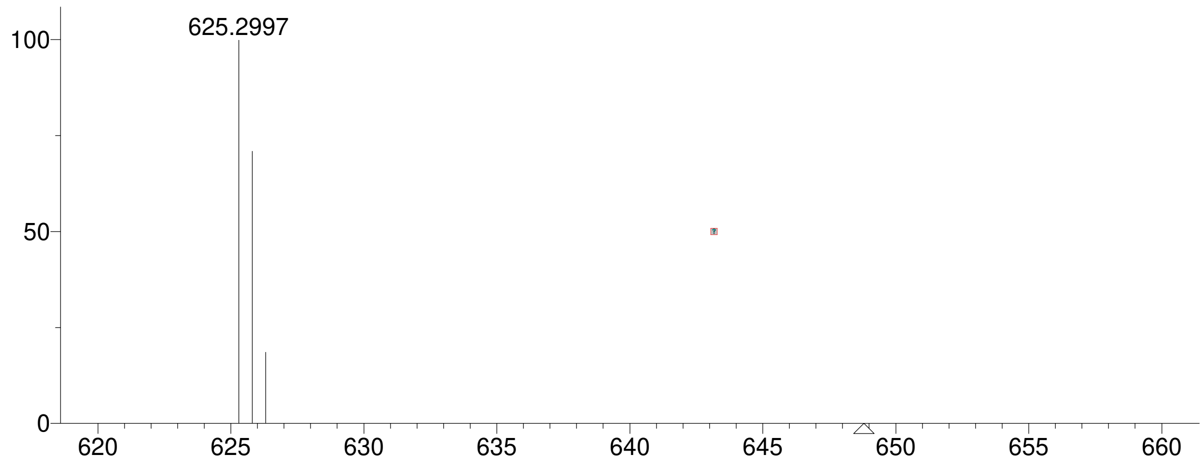

** Search Report Page 1 of 1 **

Unknown: Unknown (14.31_401.0525m/z)
Compound in Library Factor = 0

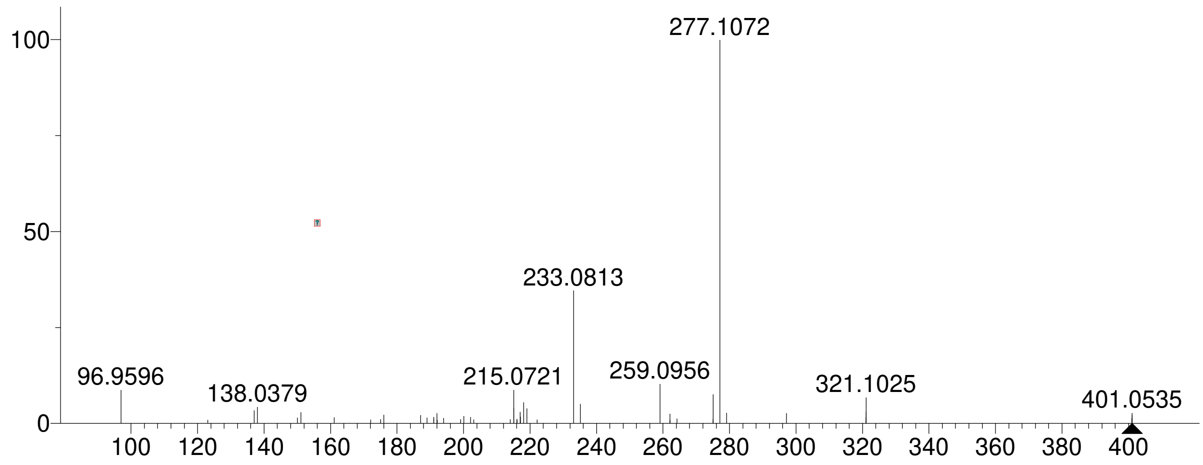

Unknown: Unknown (14.32_243.1584m/z)
Compound in Library Factor = -329

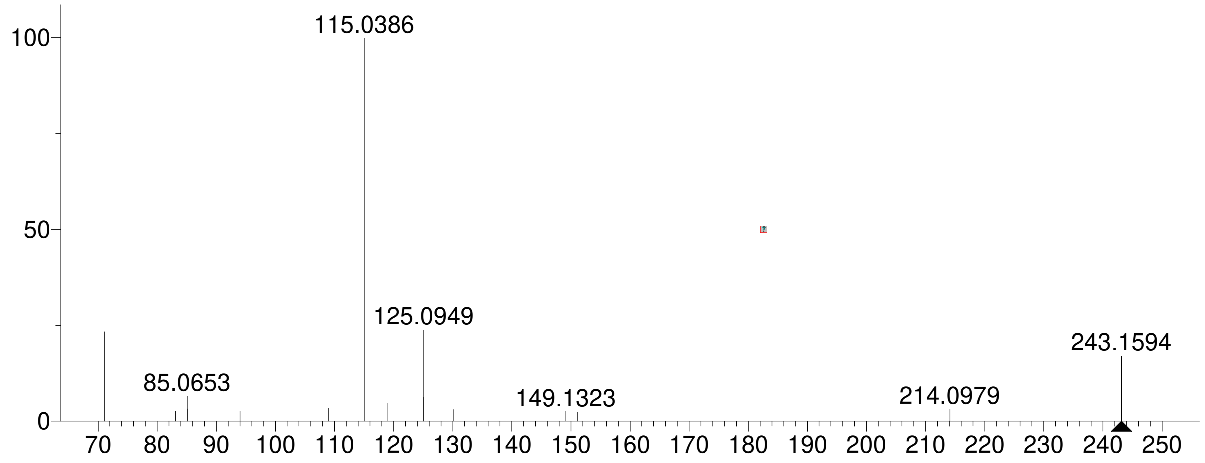

Hit 1 : 1,11-Undecanedicarboxylic acid [M-H]- HCD 14V P=243.2
C13H24O4; MF: 226; RMF: 861; Prob 98.3%; CAS: 505-52-2; Lib: nist_msms; ID: 151094.

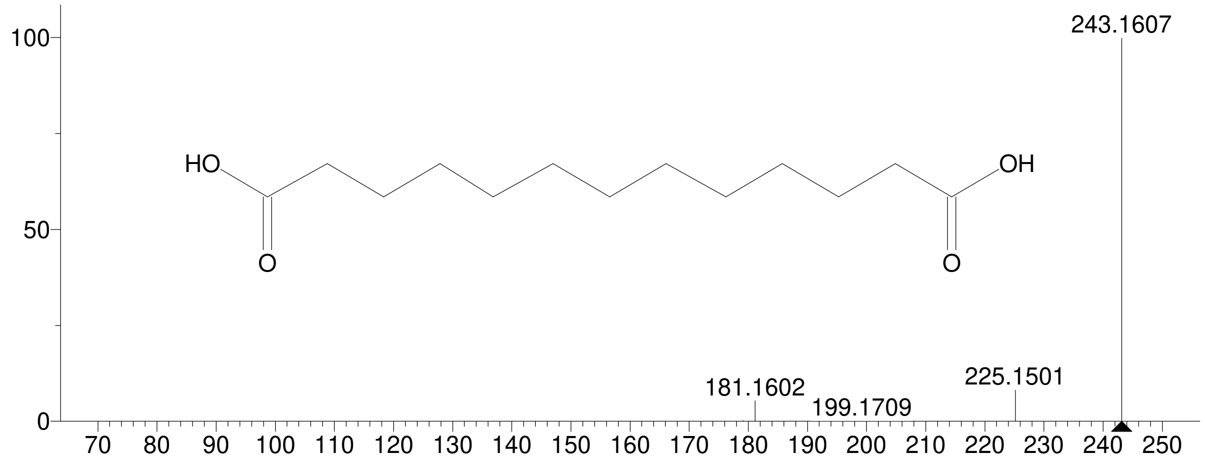

Hit 2 : Amprolium cation [Cat]+ HCD 0V P=243.2
C14H19N4; MF: 13; RMF: 273; Prob 0.98%; CAS: 13082-85-4; Lib: nist_msms; ID: 151491.

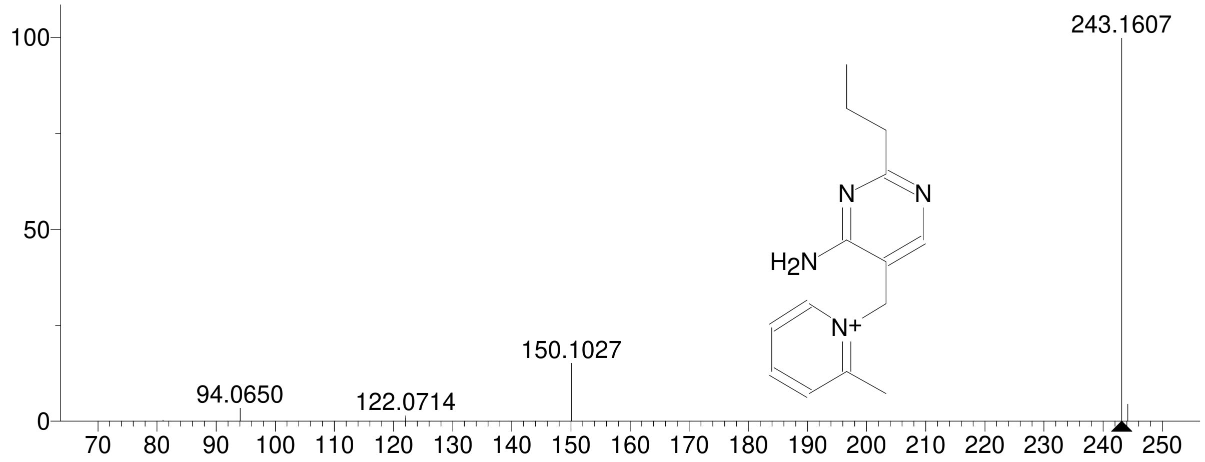

Hit 3 : (Z)-2-octylpent-2-enedioic acid [M+H]+ 35HCD P=243.2
C13H22O4; MF: 2; RMF: 129; Prob 0.67%; Lib: mona-export-vf-npl_qexactive; ID: 22777.

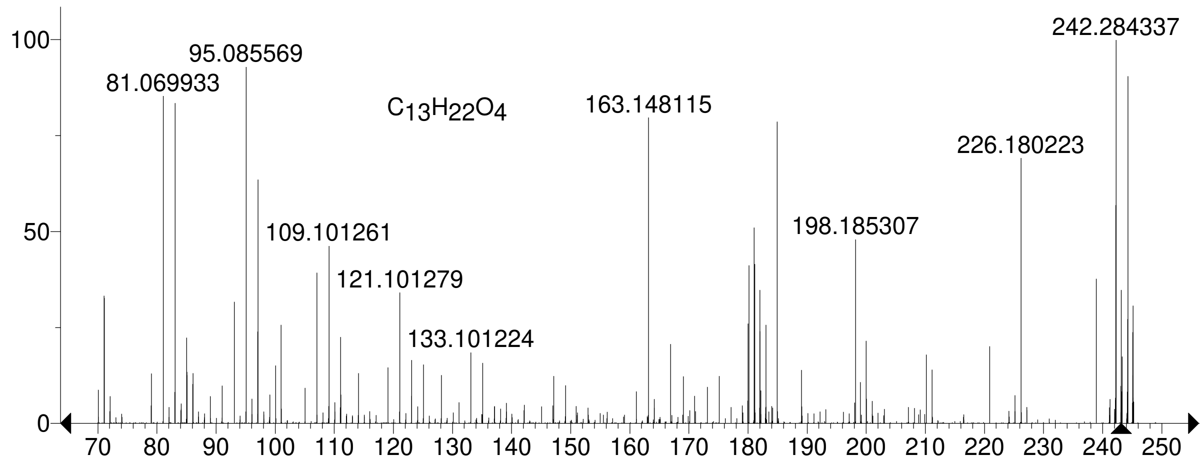

Unknown: Unknown (14.33_292.9303m/z)
Compound in Library Factor = 0

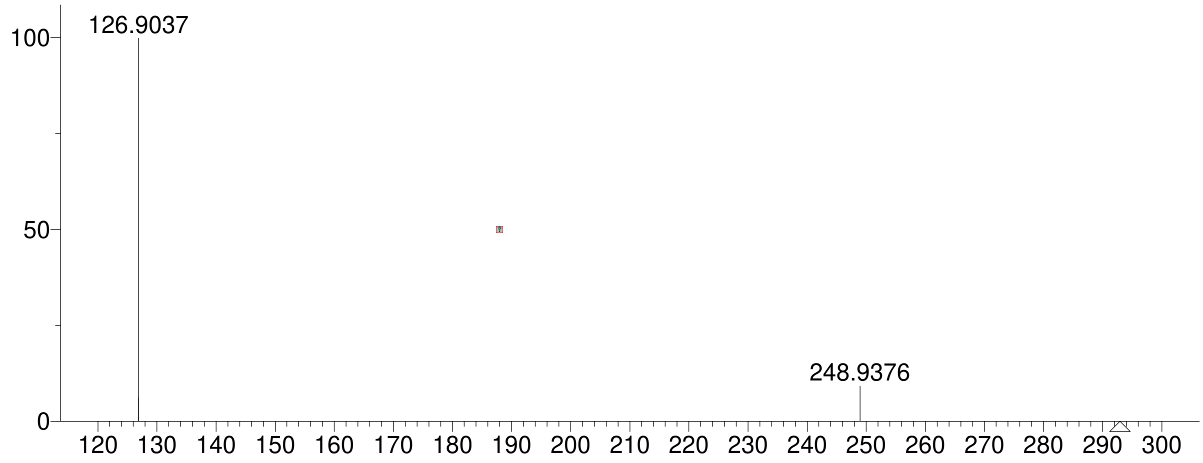

Unknown: Unknown (14.34_500.2237m/z)
Compound in Library Factor = 0

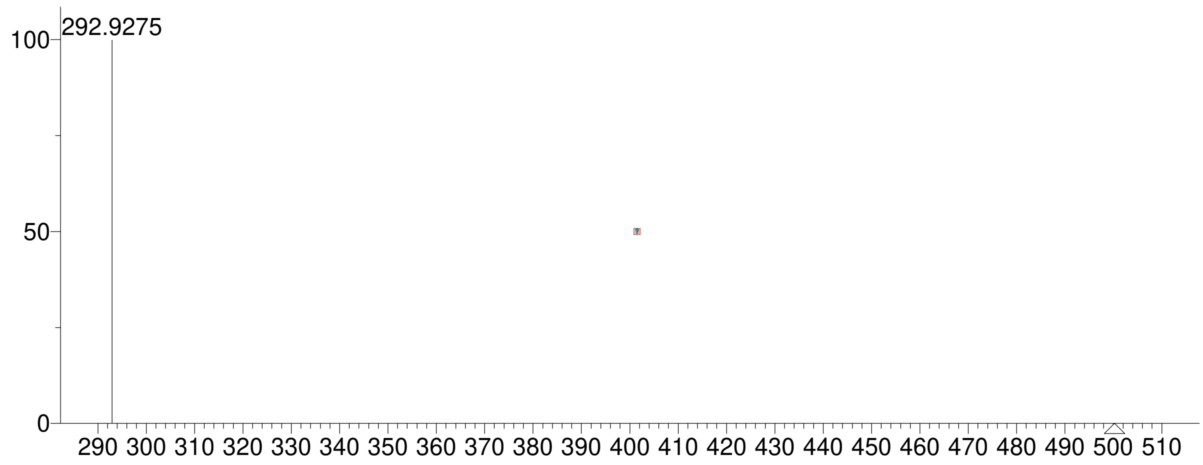

Unknown: Unknown (14.44_757.3808m/z)
Compound in Library Factor = 0

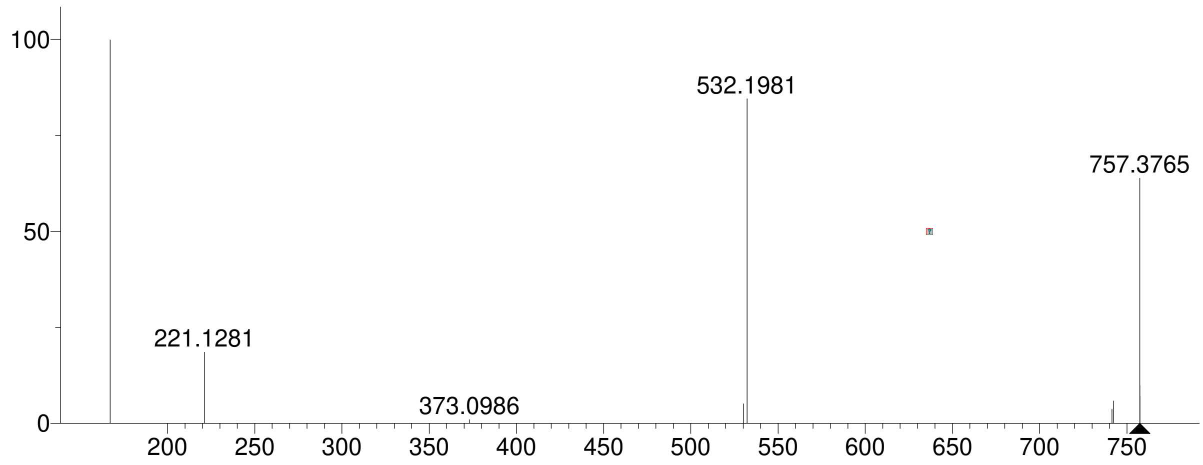

Hit 1 : ACTH (1-39), human [M+6H]6+ HCD 17eV P=757.4
C207H308N56O58S; MF: 0; RMF: 92; Prob 100.0%; CAS: 12279-41-3; Lib: nist_msms2; ID: 51586.

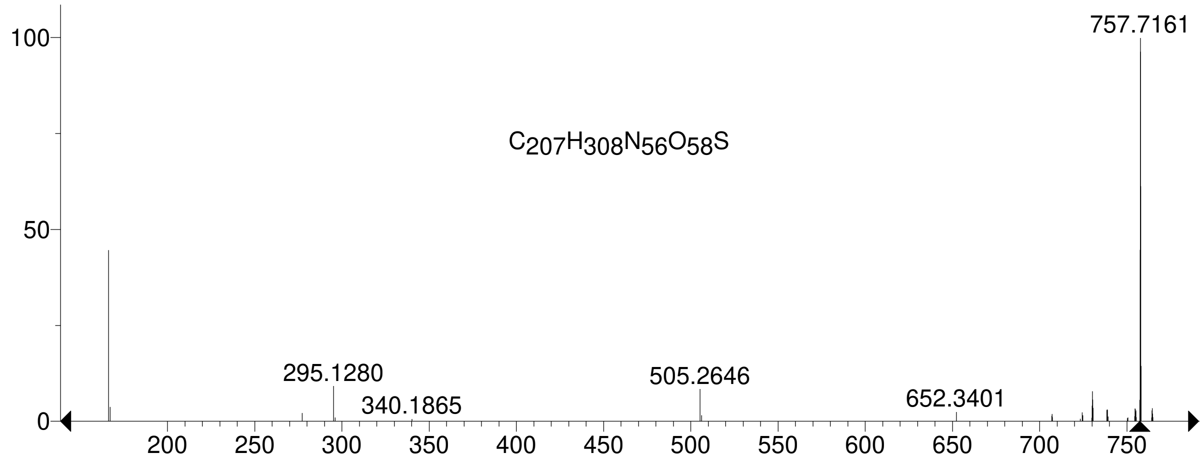

Unknown: Unknown (14.45_532.1979m/z)
Compound in Library Factor = -791

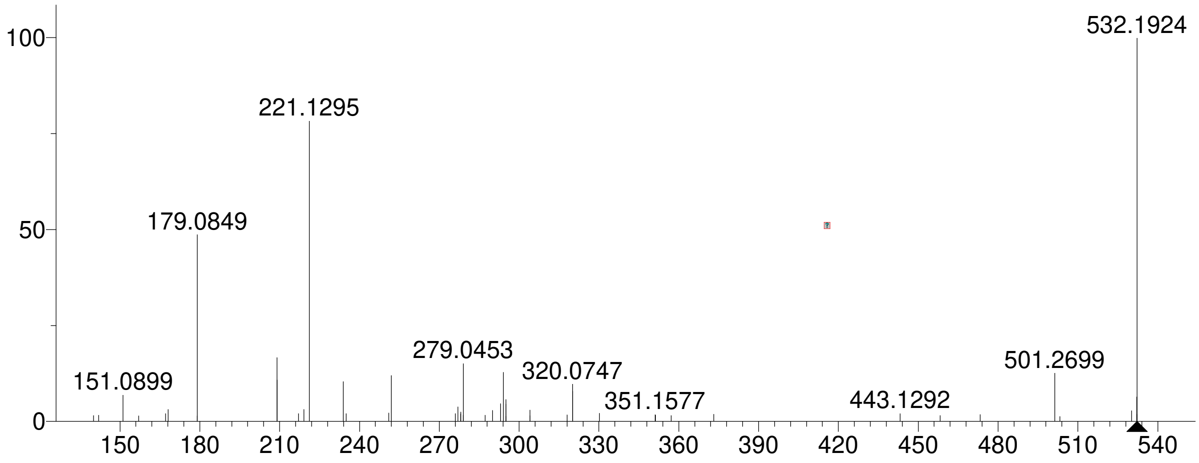

Hit 1 : LeaLeX [M+H+Na]2+ HCD 9V P=532.2
C40H68N2O29; MF: 103; RMF: 844; Prob 87.6%; Lib: nist_msms; ID: 523740.

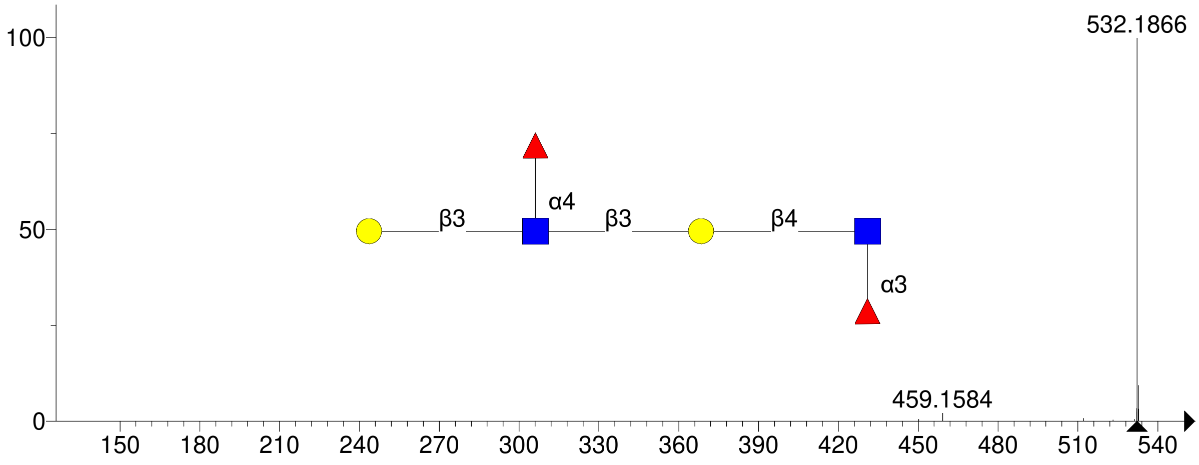

Hit 2 : [(2R,3S,4S,5R,6R)-6-[(2S,3S,4S,5R)-4-acetyloxy-3-hydroxy-2,5-bis(hydroxymethyl)oxolan-2-yl]oxy-3,4,5-tri
C23H30O13; MF: 1; RMF: 62; Prob 6.30%; Lib: mona-export-vf-npl_qexactive; ID: 12471.

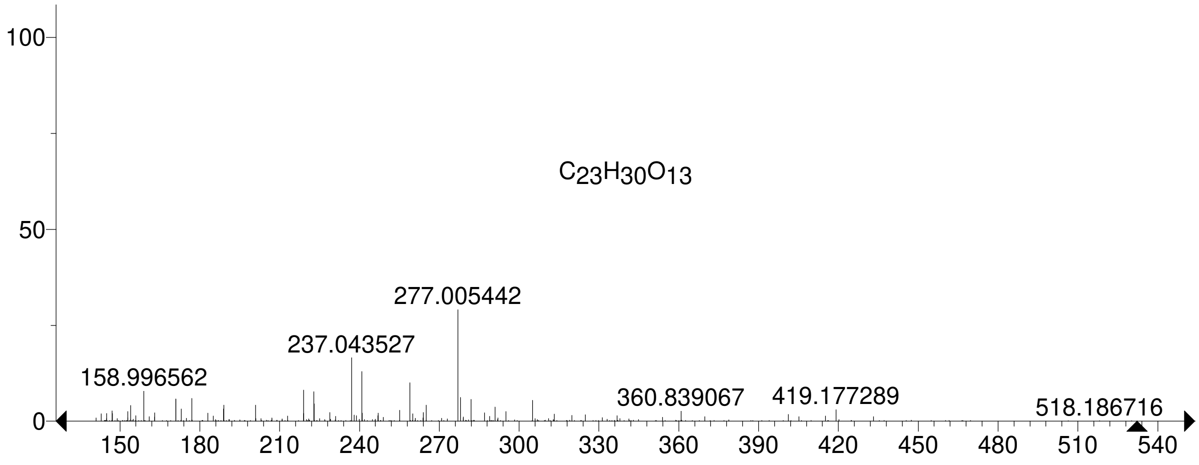

Hit 3 : NCGC00380580-01_C23H30O13_alpha-D-Glucopyranoside, 4-O-acetyl-beta-D-fructofuranosyl 6-O-[(2E)-C23H30O13; MF: 0; RMF: 55; Prob 6.05%; Lib: mona-export-gnps; ID: 13204.

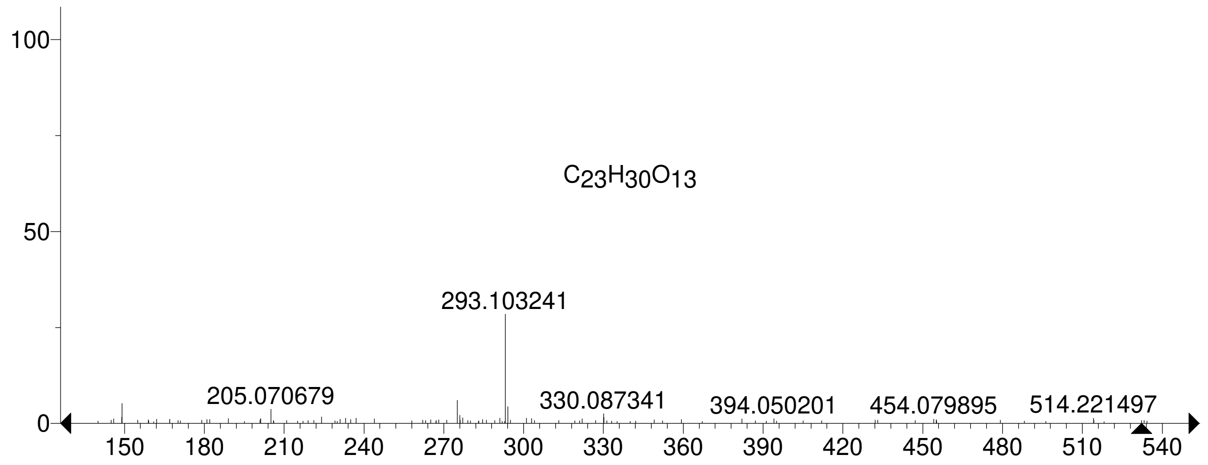

Unknown: Unknown (14.46_167.0817m/z)
Compound in Library Factor = -3273

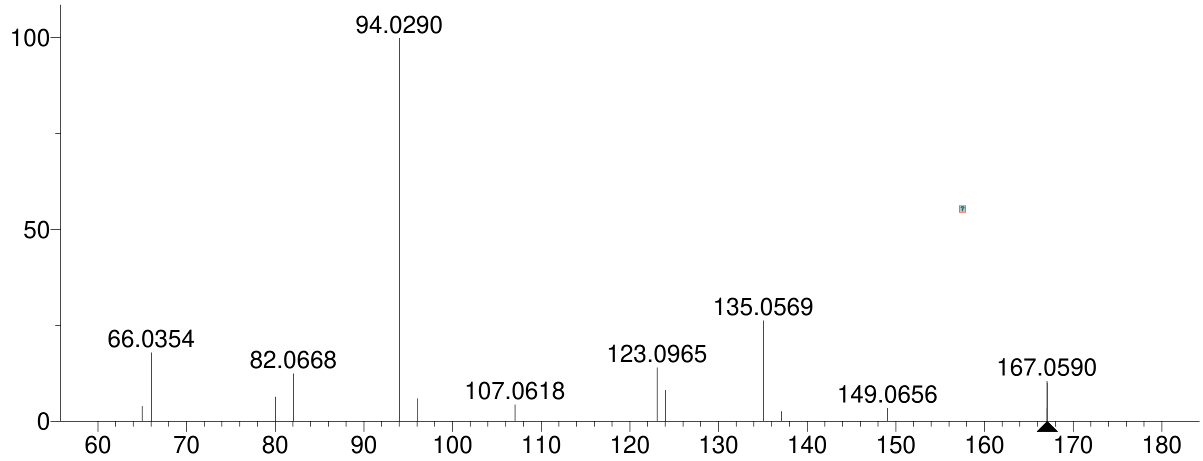

Hit 1 : Isouron [M+H-C2H7N]+ HCD 16V P=167.1
C10H17N3O2; MF: 24; RMF: 233; Prob 18.0%; CAS: 55861-78-4; Lib: nist_msms; ID: 471161.

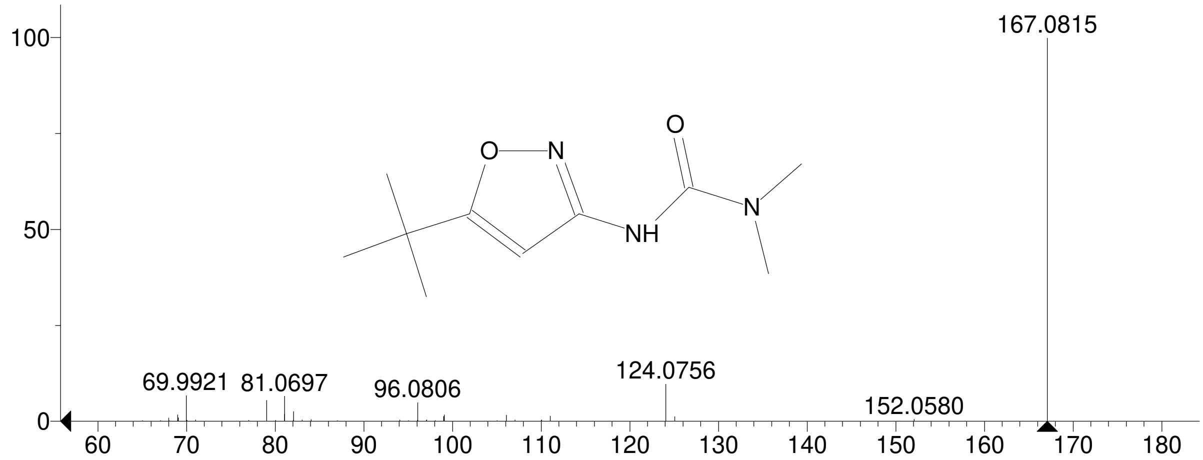

Hit 2 : Diethyl ethylphosphonate [M+H]+ HCD 1V P=167.1
C6H15O3P; MF: 17; RMF: 221; Prob 13.8%; CAS: 78-38-6; Lib: nist_msms; ID: 106000.

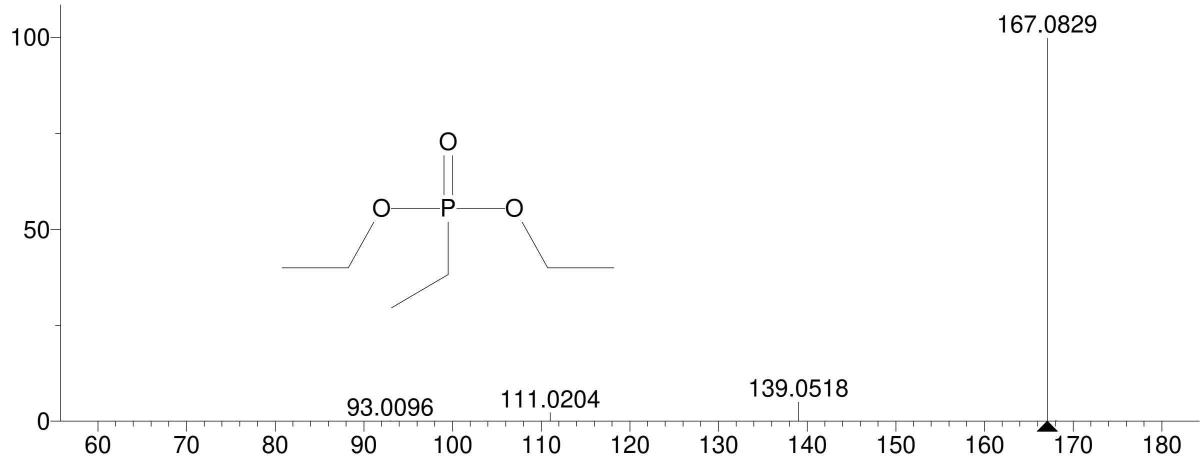

Hit 3 : PYRIDOXAMINE [M-H]- P=167.1
C8H12N2O2; MF: 16; RMF: 222; Prob 13.2%; Lib: msms-neg-gnps; ID: 278.

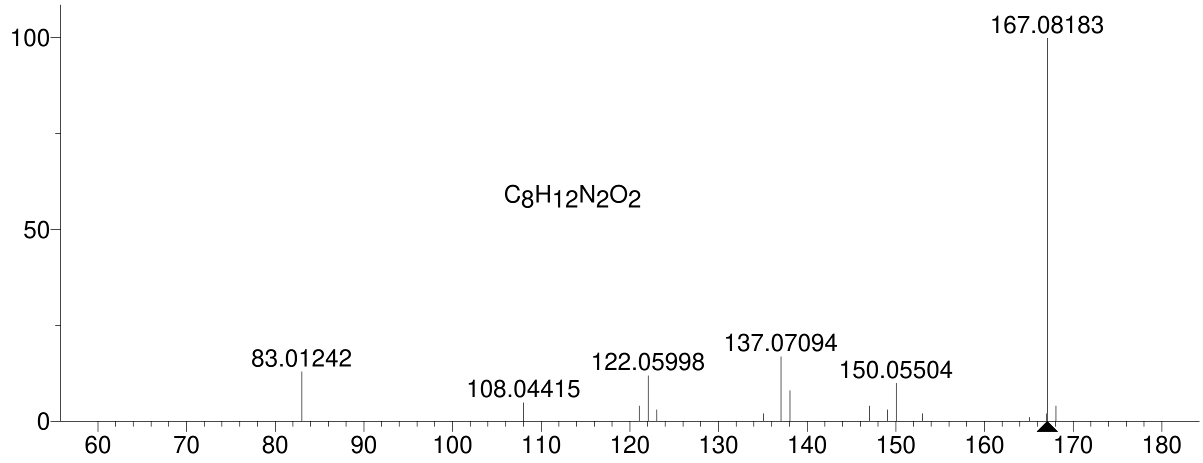

Hit 4 : Pyridoxamine; LC-ESI-QTOF; MS2; CE [M-H]- P=167.1
C8H12N2O2; MF: 11; RMF: 293; Prob 10.7%; Lib: msms-neg-massbank; ID: 4286.

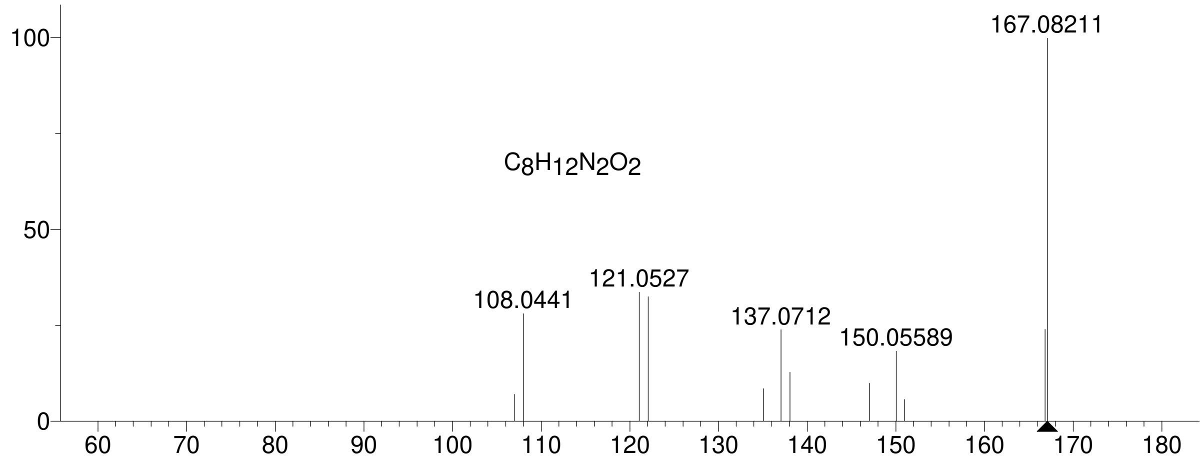

Hit 5 : Pyridoxamine [M-H]- 5-60V P=167.1
C8H12N2O2; MF: 11; RMF: 291; Prob 10.7%; Lib: mona-export-lc-ms-ms_negative_mode; ID: 8384.

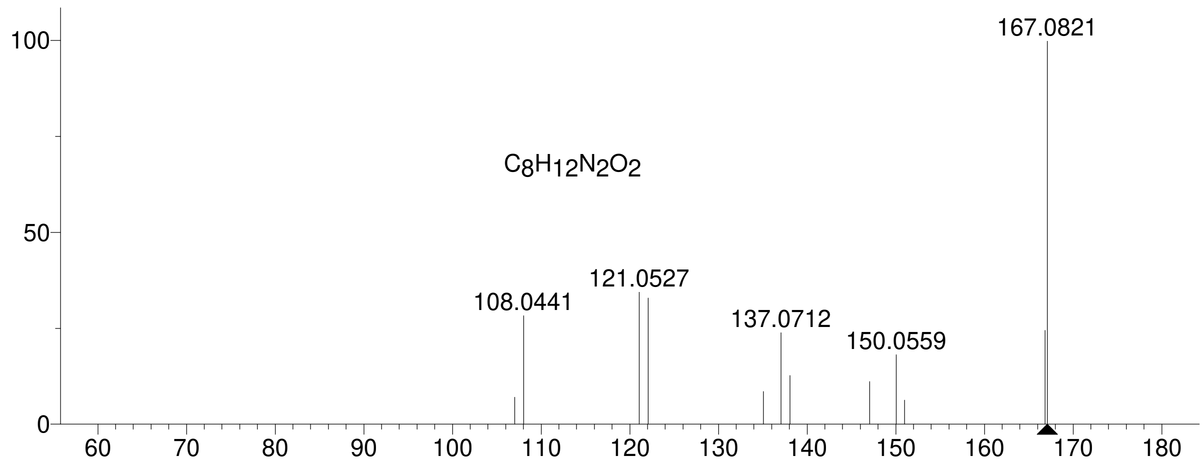

Unknown: Unknown (14.46_501.2681m/z)
Compound in Library Factor = -2387

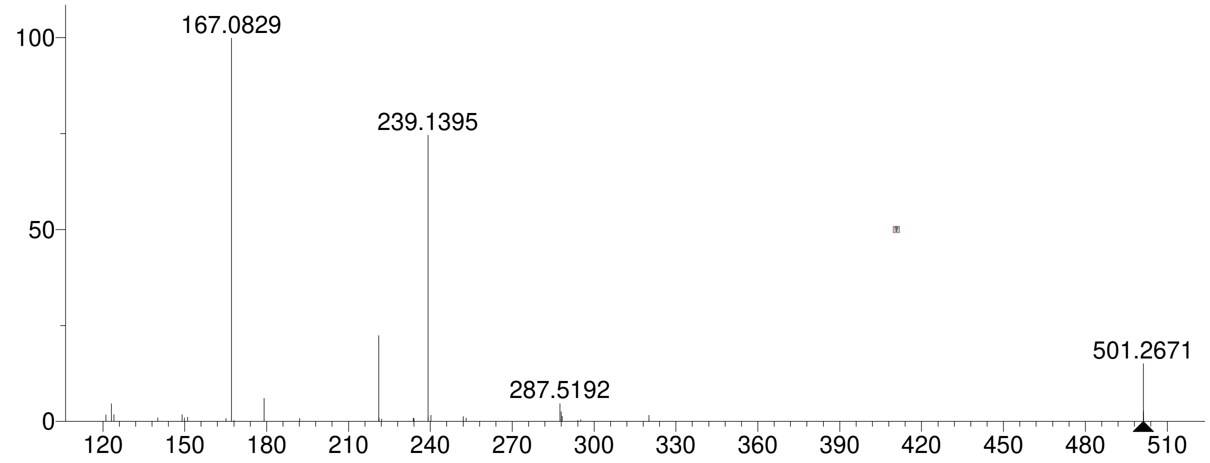

Hit 1 : Ikarugamycin [M+Na]+ HCD 35V P=501.3
C29H38N2O4; MF: 10; RMF: 292; Prob 58.6%; CAS: 36531-78-9; Lib: nist_msms; ID: 264701.

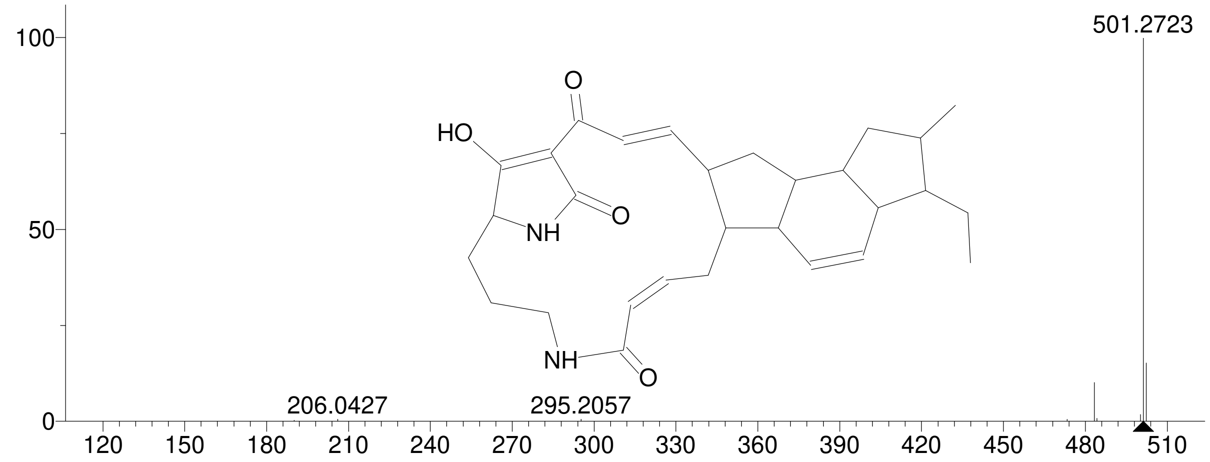

Hit 2 : 2-[(6,7-dimethoxy-3,4-dihydroisoquinolin-1-yl)methyl]-3-ethyl-9,10-dimethoxy-2,3,4,6,7,11b-hexahydro-1H-l
C29H38N2O4; MF: 0; RMF: 35; Prob 41.4%; Lib: mona-export-vf-npl_qexactive; ID: 22963.

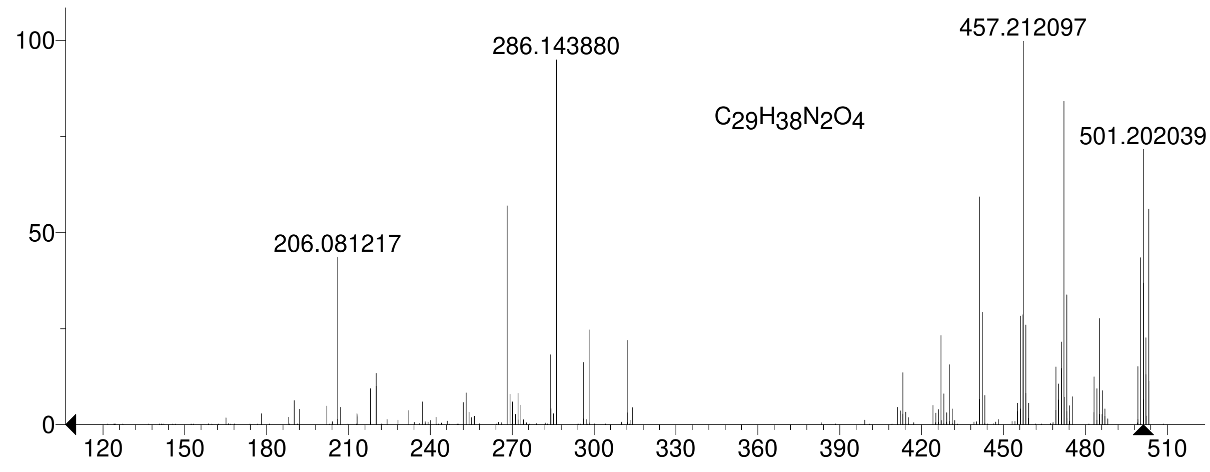

Unknown: Unknown (14.47_239.1392m/z)
Compound in Library Factor = -3273

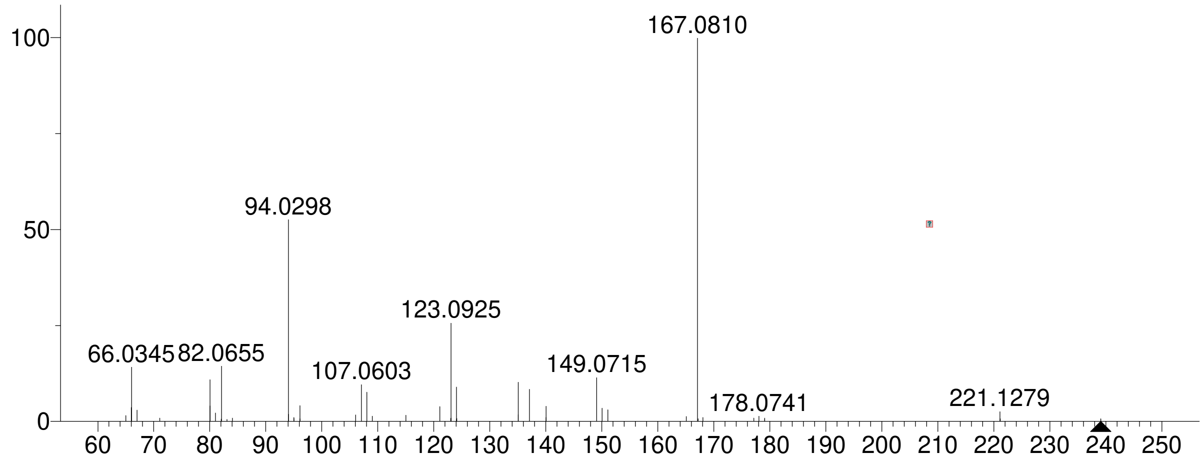

Hit 1 : Heptanedioic acid, 1-(2-cyclopentylidenehydrazide) [M-H]- HCD 14V P=239.1
C12H20N2O3; MF: 8; RMF: 59; Prob 40.0%; CAS: 1136466-93-7; Lib: nist_msms; ID: 263653.

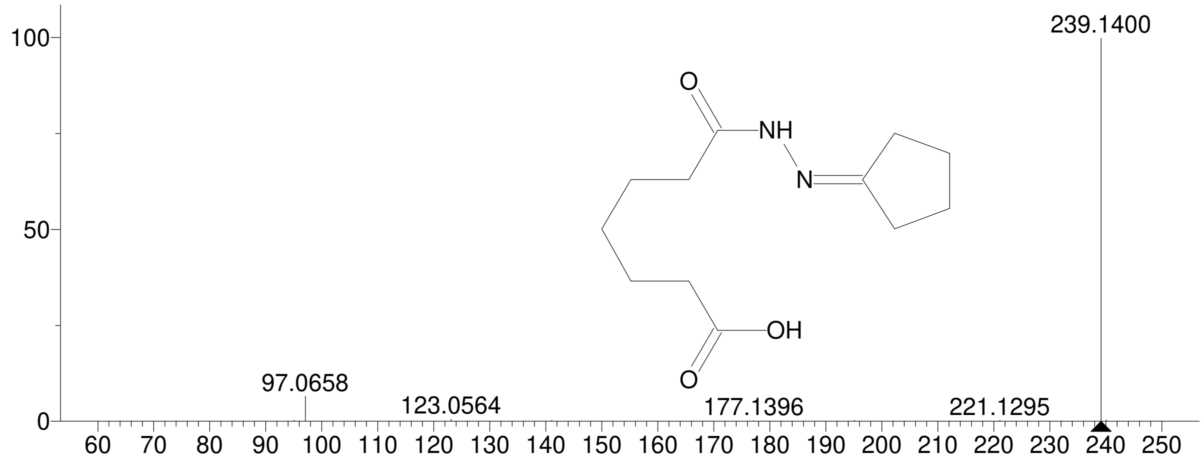

Hit 2 : Secobarbital [M+H]+ HCD 0V P=239.1
C12H18N2O3; MF: 1; RMF: 50; Prob 30.6%; CAS: 76-73-3; Lib: nist_msms; ID: 198372.

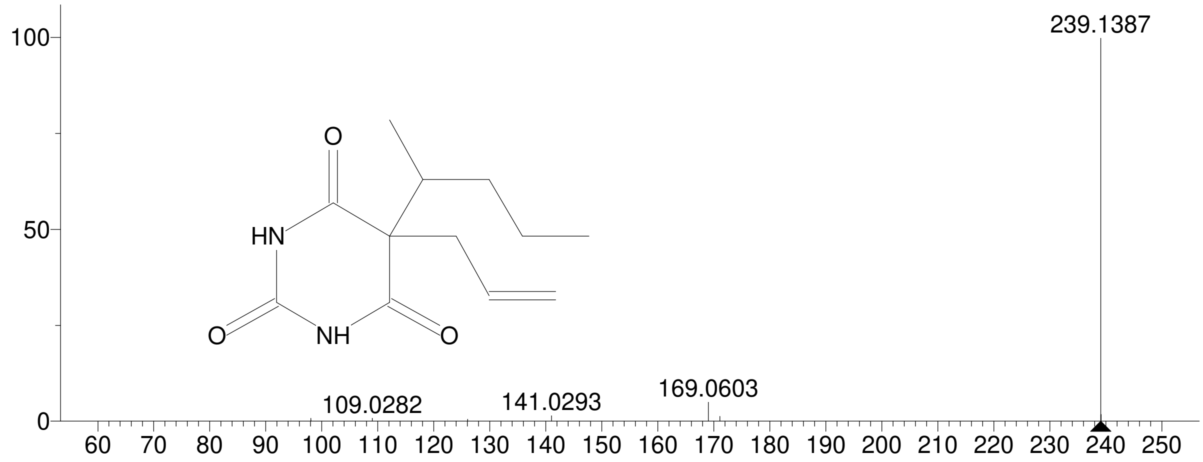

Hit 3 : CMPF [M-H]- 35% P=239.1
C12H16O5; MF: 0; RMF: 22; Prob 29.4%; Lib: mona-export-itree; ID: 3748.

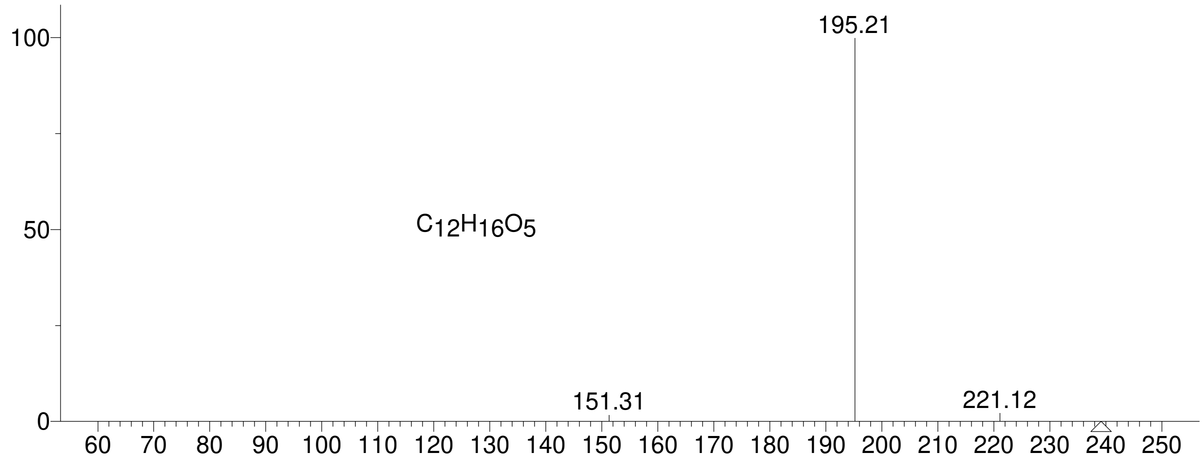

** Search Report Page 1 of 1 **

Unknown: Unknown (14.47_741.4041m/z)
Compound in Library Factor = 0

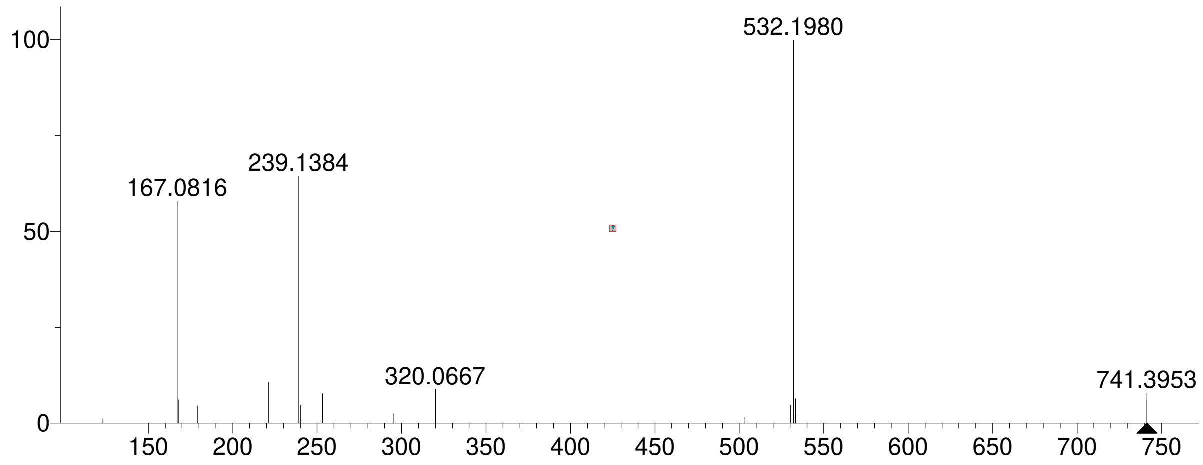

Unknown: Unknown (14.47_775.3532m/z)
Compound in Library Factor = 0

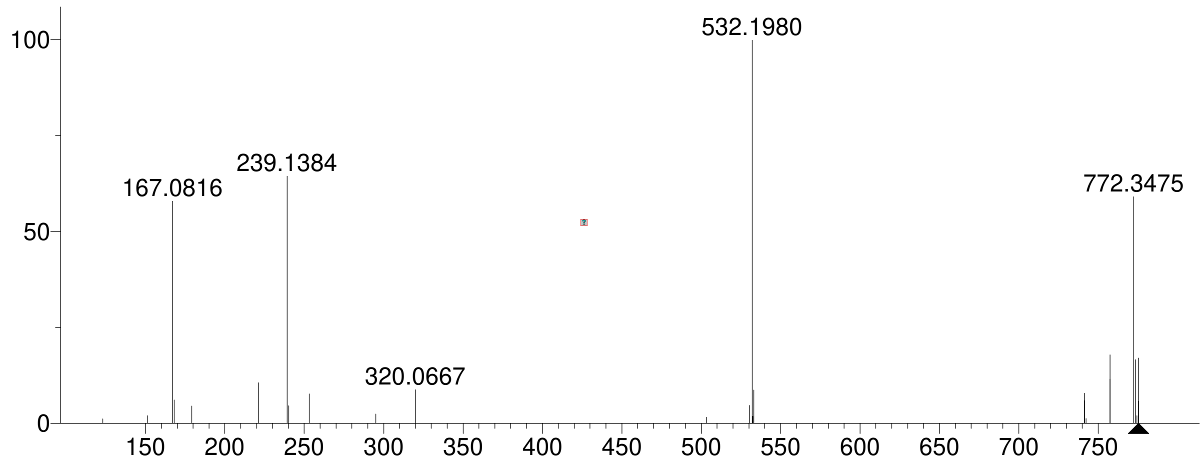

** Search Report Page 1 of 1 **

Unknown: Unknown (14.48_757.3842m/z)
Compound in Library Factor = 0

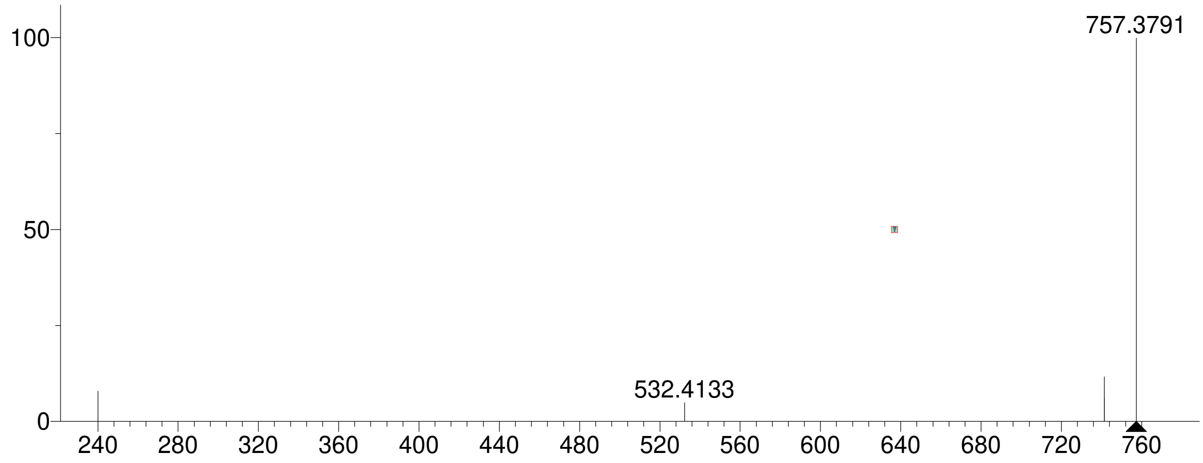

Hit 1 : ACTH (1-39), human [M+6H]6+ HCD 17eV P=757.4
C207H308N56O58S; MF: 0; RMF: 87; Prob 100.0%; CAS: 12279-41-3; Lib: nist_msms2; ID: 51586.

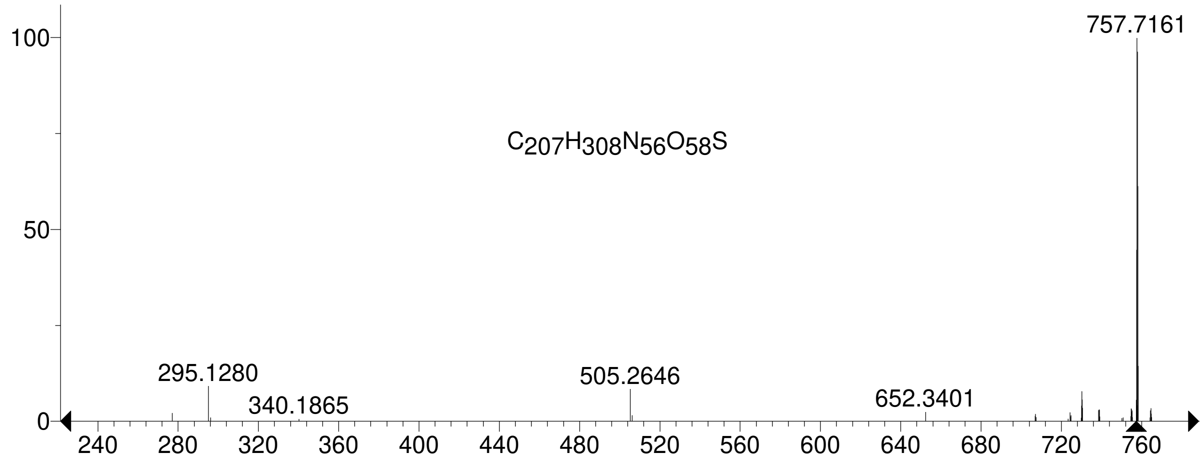

Unknown: Unknown (14.49_772.3468m/z)
Compound in Library Factor = 0

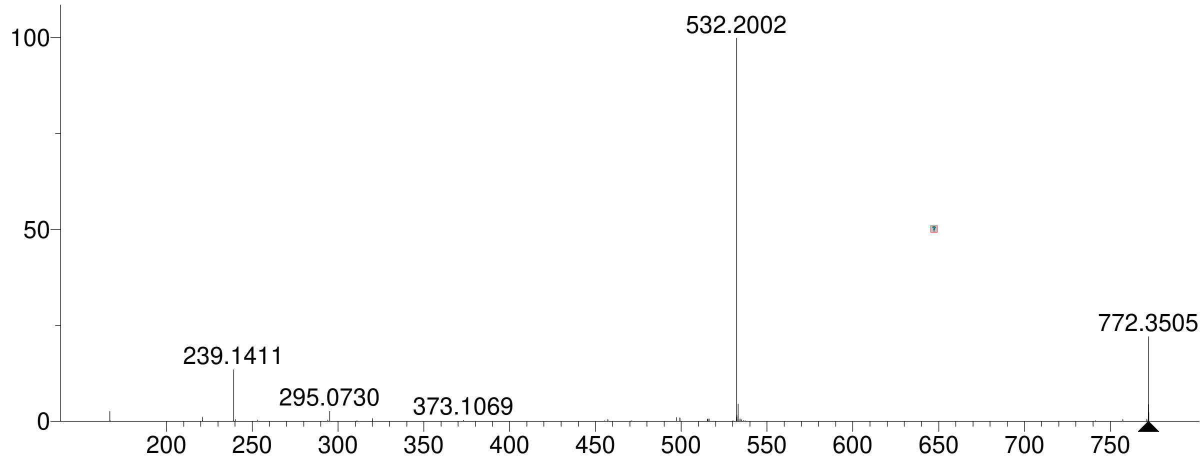

** Search Report Page 1 of 1 **

Unknown: Unknown (14.50_568.1749m/z)
Compound in Library Factor = -3273

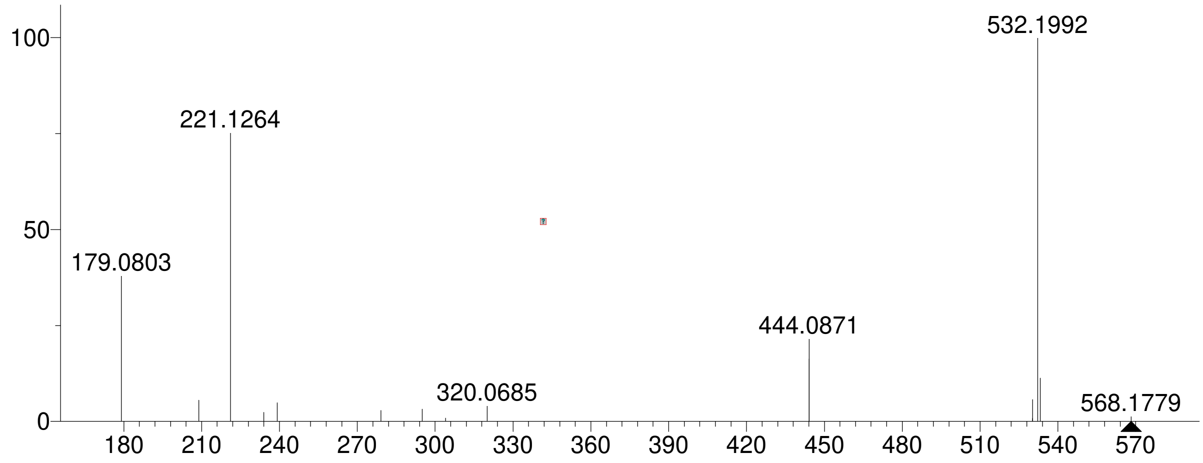

Hit 1 : 1H-Indole-5-sulfonamide, N-(3-chlorophenyl)-3-[[3,5-dimethyl-4-[(4-methyl-1-piperazinyl)carbonyl]-1H-pyrro
C28H30ClN5O4S; MF: 5; RMF: 97; Prob 100.0%; CAS: 658084-23-2; Lib: nist_msms; ID: 347758.

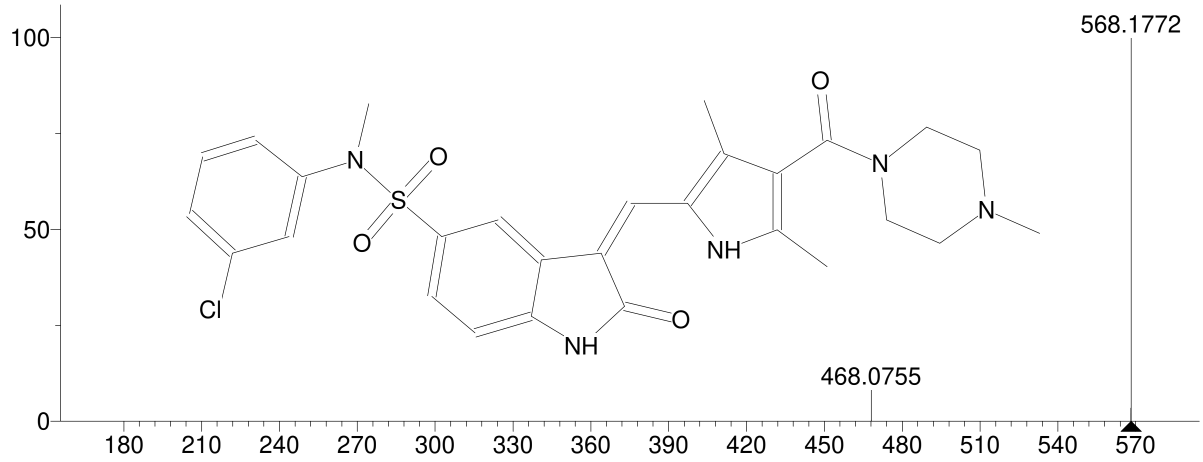

Unknown: Unknown (14.50_578.2033m/z)
Compound in Library Factor = 0

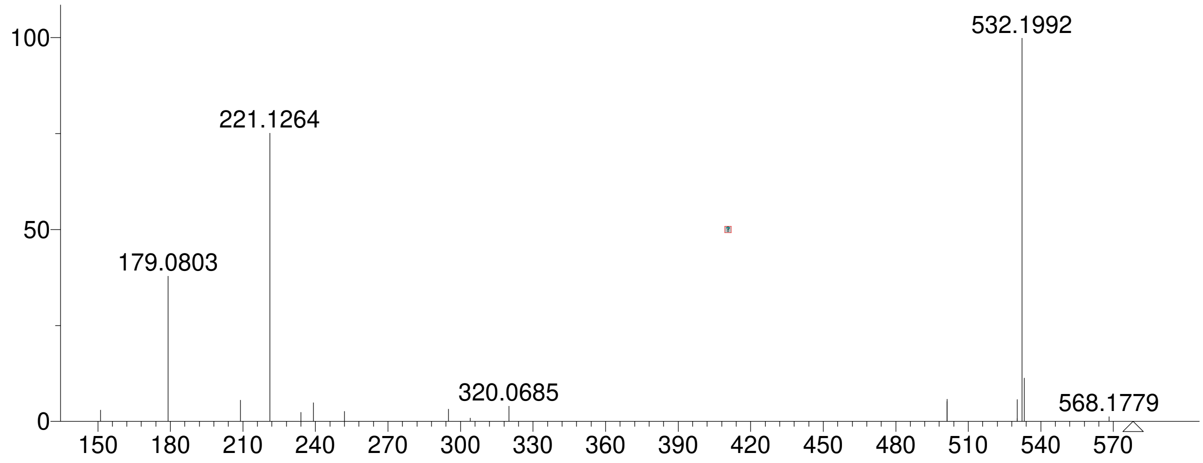

Unknown: Unknown (14.51_151.0391m/z)
Compound in Library Factor = -349

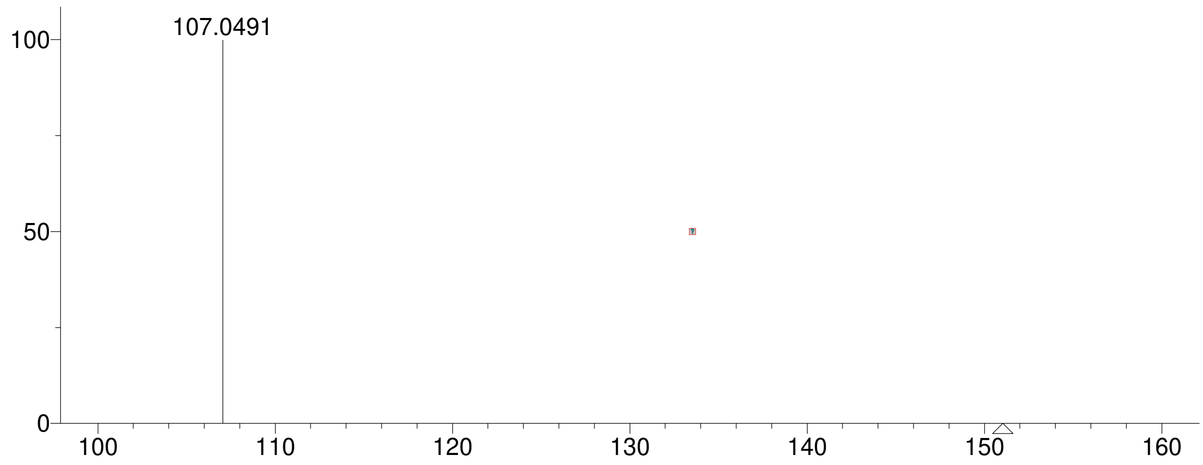

Hit 1 : p-Hydroxyphenylacetic acid [M-H]- 30 P=151
C8H8O3; MF: 364; RMF: 999; Prob 9.41%; Lib: msms neg mona_ipb_gnps_plasma; ID: 556.

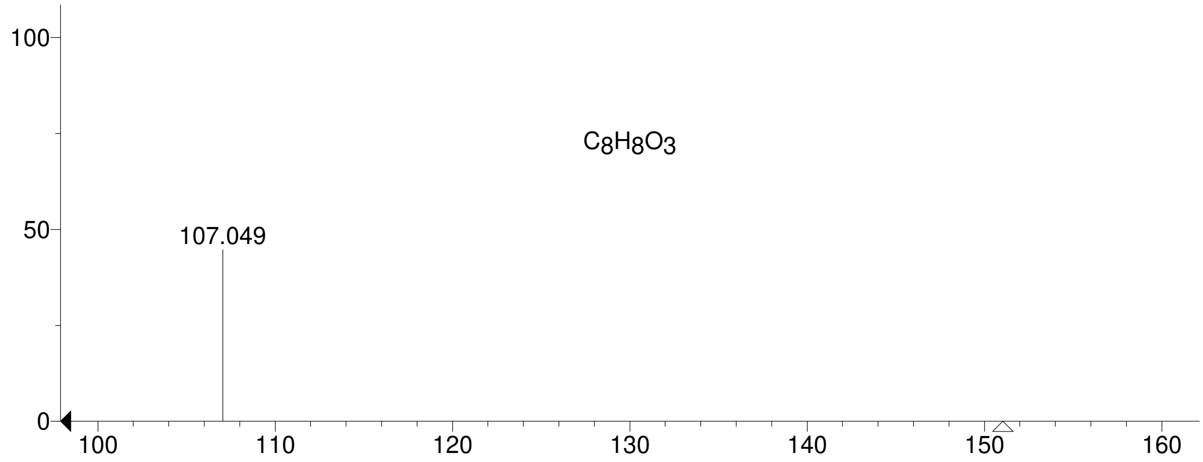

Hit 2 : 4-hydroxyphenylacetic acid [M-H]- 15 P=151
C8H8O3; MF: 364; RMF: 999; Prob 9.41%; Lib: msms neg mona_ipb_gnps_plasma; ID: 1183.

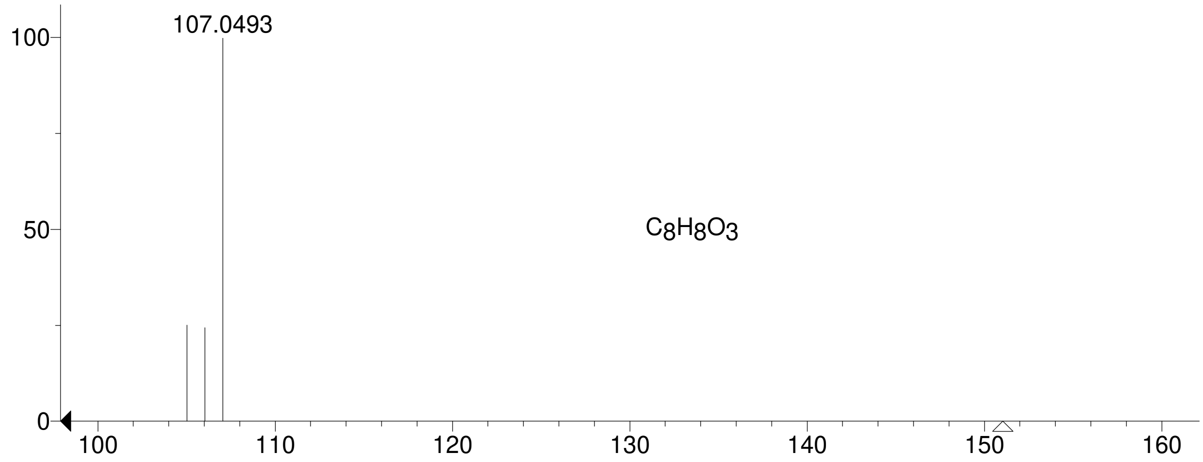

Hit 3 : 4-Hydroxyphenylacetic acid [M-H]- 35eV P=151
C8H8O3; MF: 364; RMF: 999; Prob 9.41%; Lib: msms-neg-fiehnhilic; ID: 813.

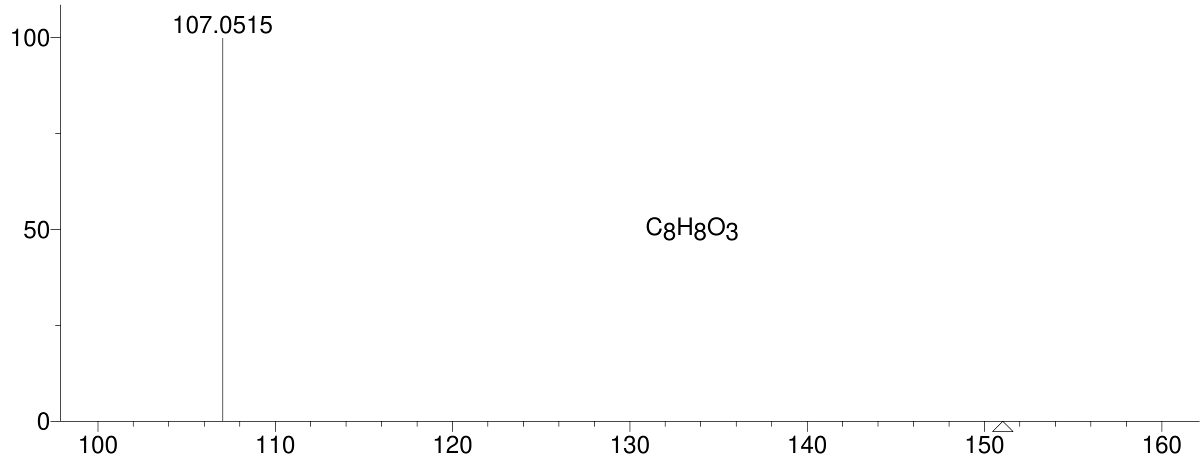

Hit 4 : 4-Hydroxy-3-methylbenzoic acid [M-H]- QTOF 20V P=151
C8H8O3; MF: 364; RMF: 999; Prob 9.41%; CAS: 499-76-3; Lib: nist_msms; ID: 29862.

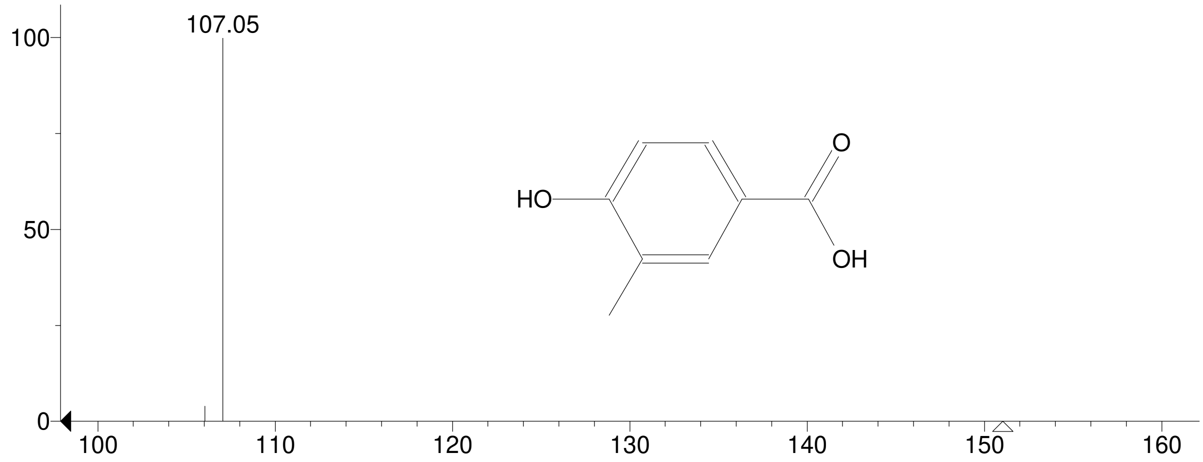

Hit 5 : 2-Methoxybenzoic acid [M-H]- HCD 33V P=151
C8H8O3; MF: 364; RMF: 999; Prob 9.41%; CAS: 579-75-9; Lib: nist_msms; ID: 554171.

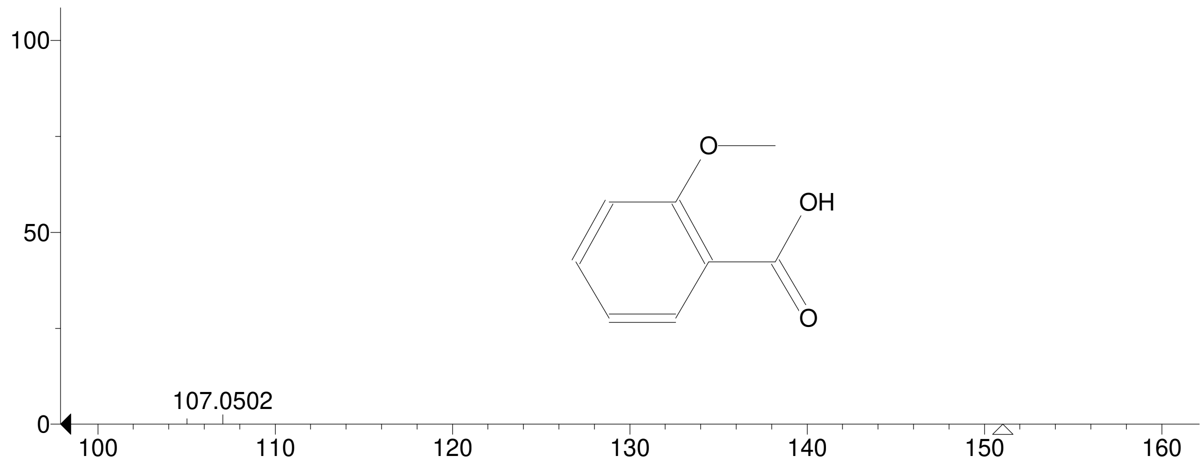

** Search Report Page 1 of 1 **

Unknown: Unknown (14.51_684.2458m/z)
Compound in Library Factor = 0

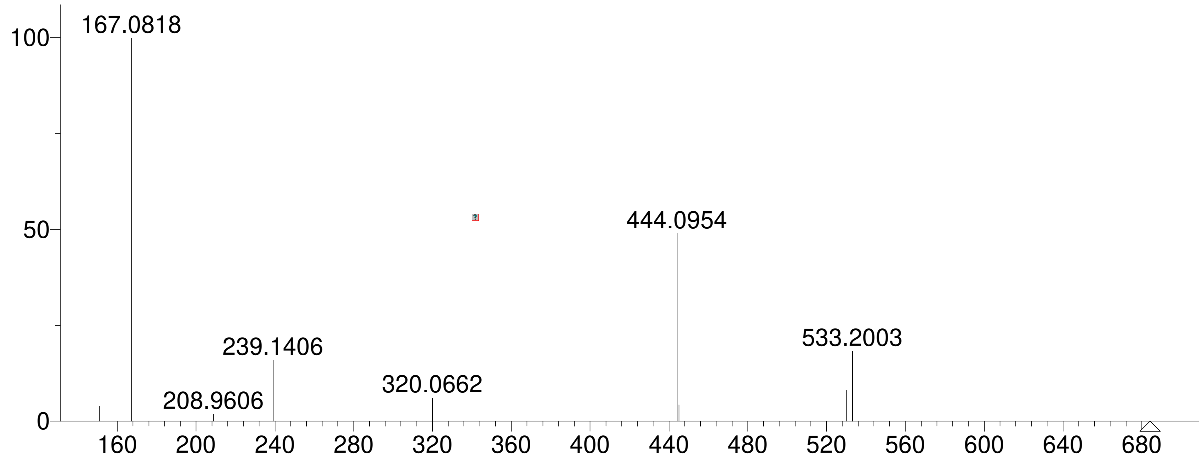

Hit 1 : 6-Acetylacteoside [M+NH4]+ IT 35% P=684.2
C31H38O16; MF: 0; RMF: 11; Prob 100.0%; CAS: 441769-43-3; Lib: nist_msms; ID: 234549.

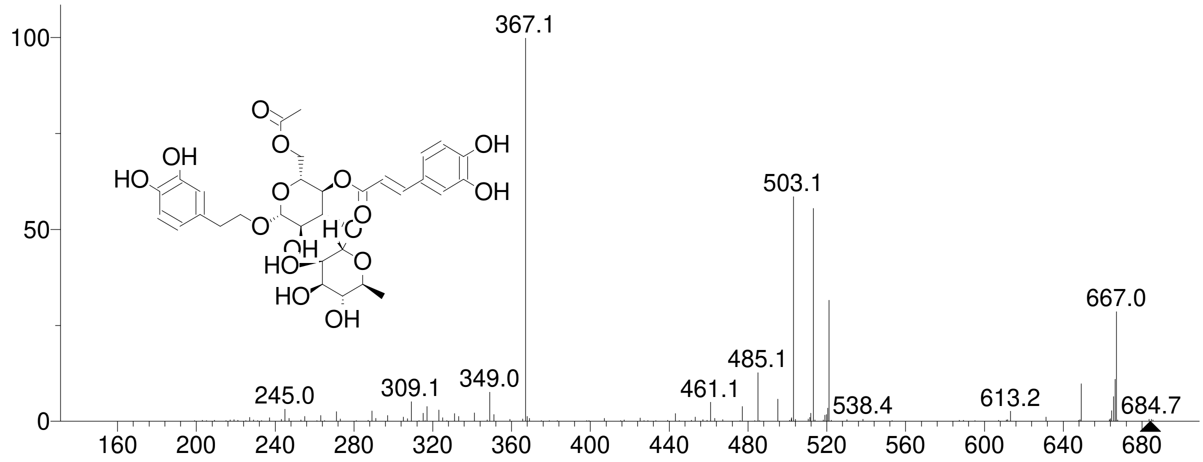

Unknown: Unknown (14.52_444.0959m/z)
Compound in Library Factor = -1072

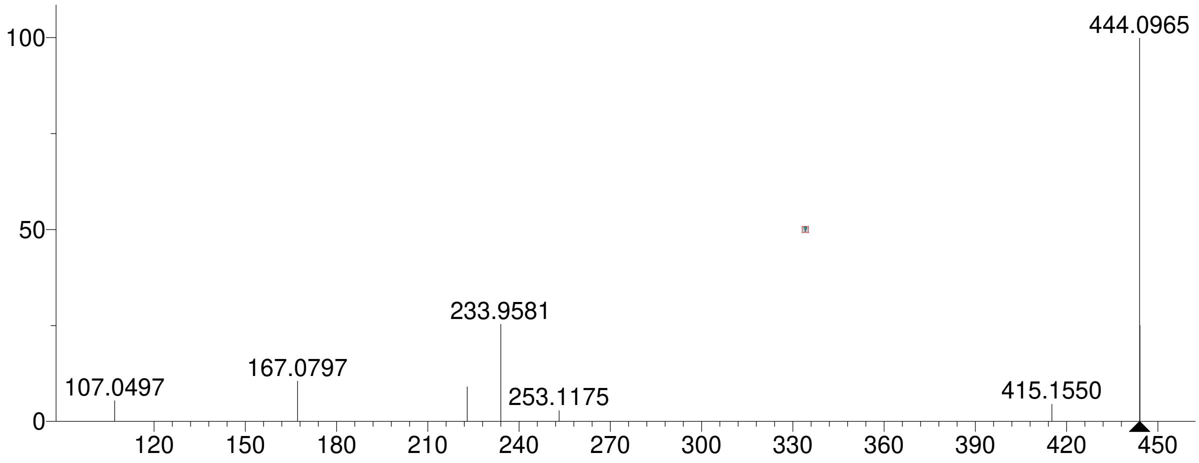

Hit 1 : Cyclosulfamuron [M+Na]+ HCD 8V P=444.1
C17H19N5O6S; MF: 75; RMF: 686; Prob 88.5%; CAS: 136849-15-5; Lib: nist_msms; ID: 294026.

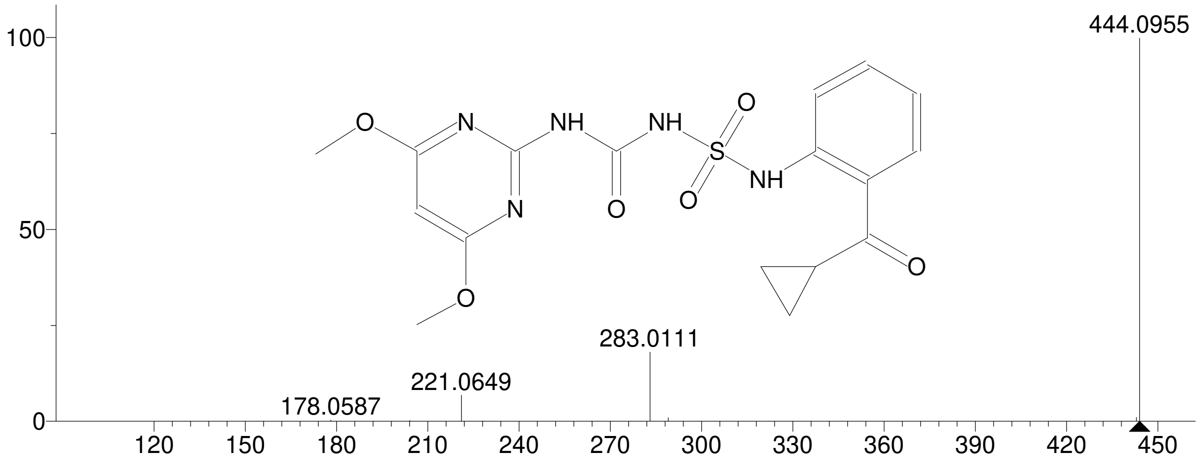

Hit 2 : Penoxsulam [M+H]+=>444.1 IT 35% P=444.1
C16H14F5N5O5S; MF: 0; RMF: 22; Prob 11.5%; CAS: 219714-96-2; Lib: nist_msms; ID: 481769.

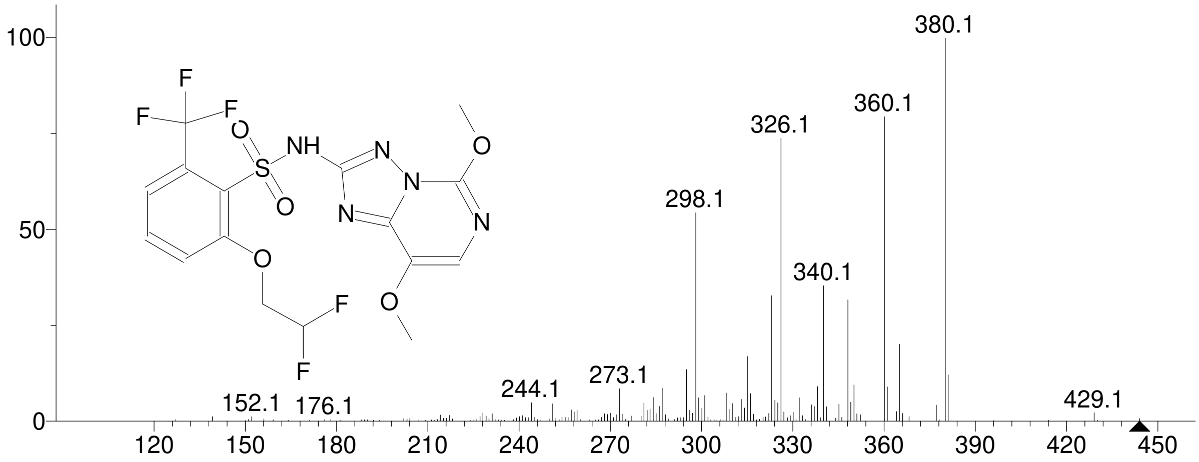

Unknown: Unknown (14.57_541.2393m/z)
Compound in Library Factor = -3273

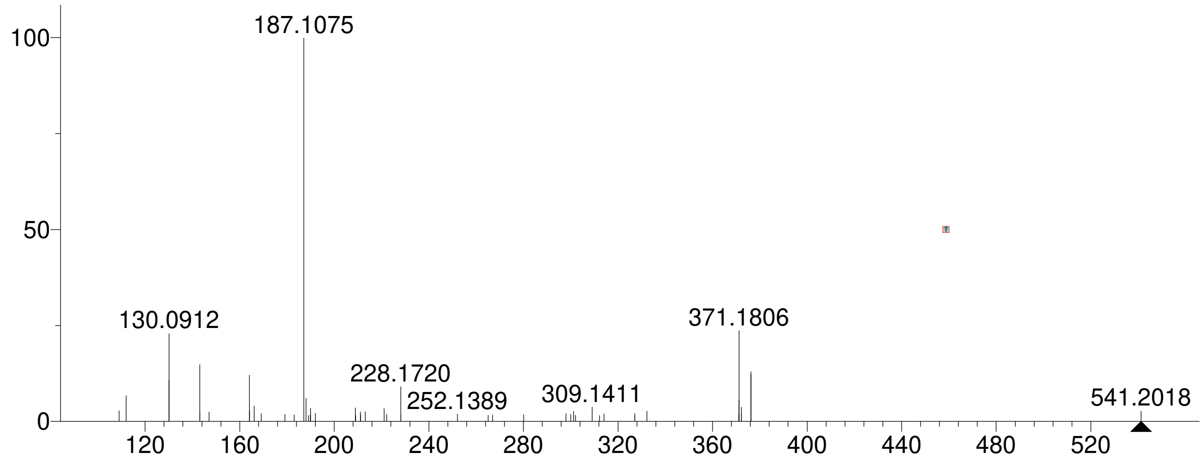

Hit 1 : methyl (1S,2R,4R,8R,9S,10R,13R,15R)-2-(acetyloxy)-13-(furan-3-yl)-9-(2-methoxy-2-oxoethyl)-4,8,10,12-t
C30H36O9; MF: 4; RMF: 152; Prob 27.6%; Lib: mona-export-vf-npl_qexactive; ID: 20633.

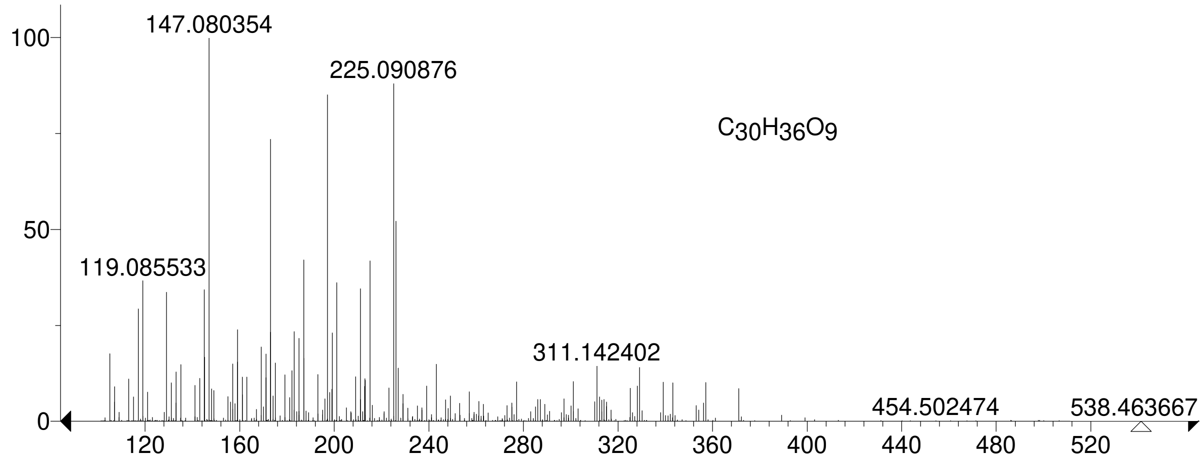

Hit 2 : NCGC00169906-03_C30H36O9_2H-Cyclopenta[b]naphtho[2,3-d]furan-10-acetic acid, 5-(acetyloxy)-2-(3-f
C30H36O9; MF: 1; RMF: 115; Prob 24.4%; Lib: mona-export-gnps; ID: 13758.

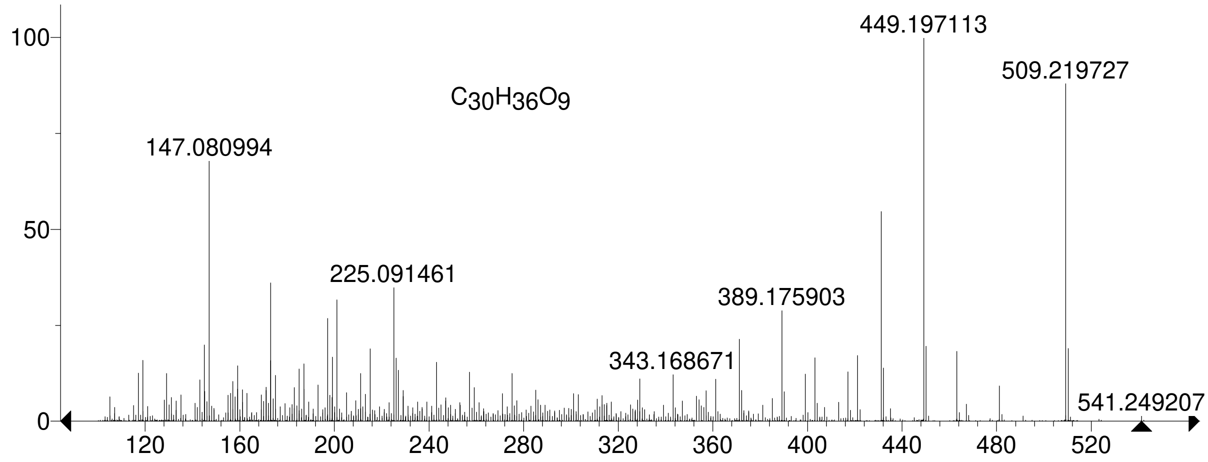

Hit 3 : 7-Deacetoxy-7-Oxokhivorin [M-H]- 35HCD P=541.2
C30H38O9; MF: 1; RMF: 55; Prob 24.4%; Lib: mona-export-lc-ms-ms_negative_mode; ID: 20717.

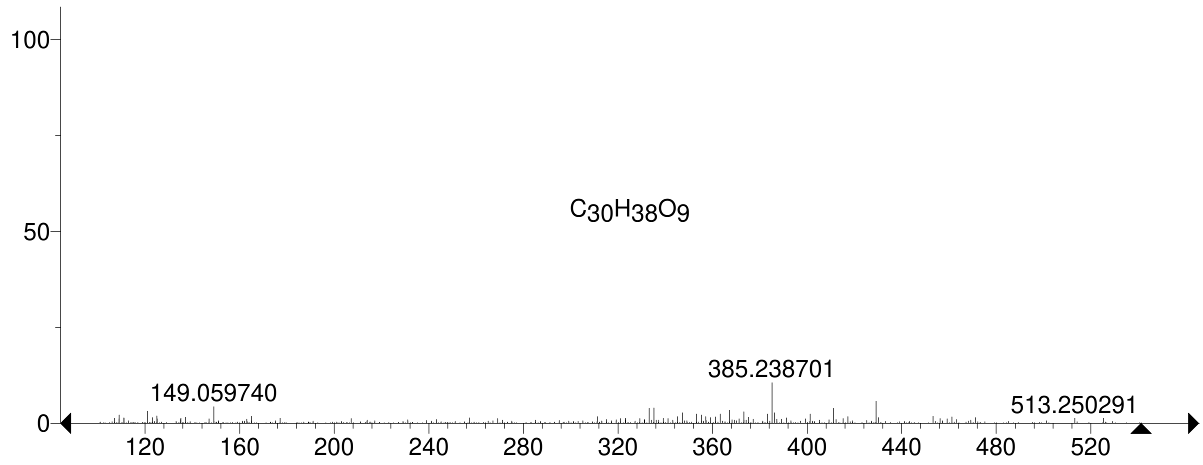

Hit 4 : Nimbin [M+H]+ IT 35% P=541.2
C30H36O9; MF: 0; RMF: 58; Prob 23.5%; CAS: 5945-86-8; Lib: nist_msms; ID: 226918.

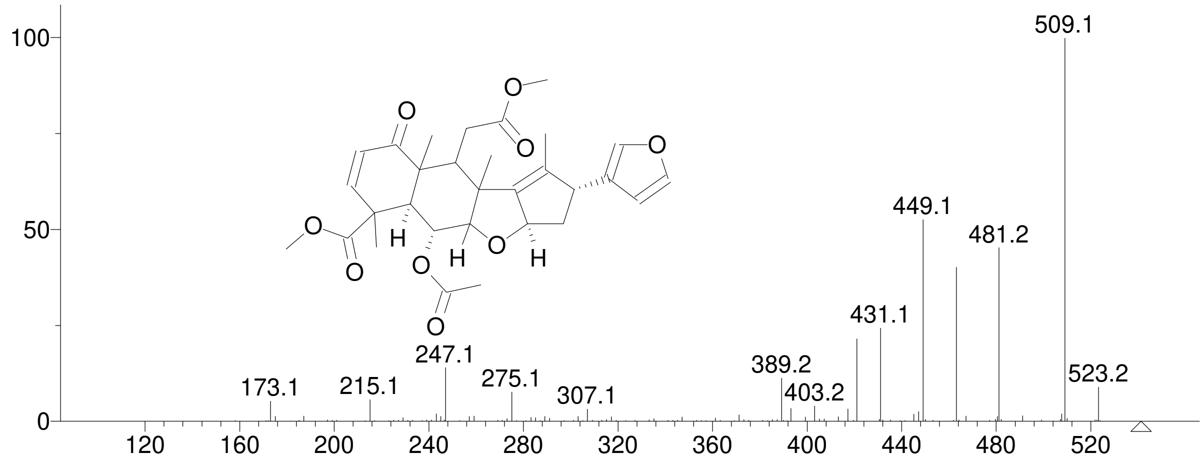

Unknown: Unknown (14.59_757.3816m/z)
Compound in Library Factor = 0

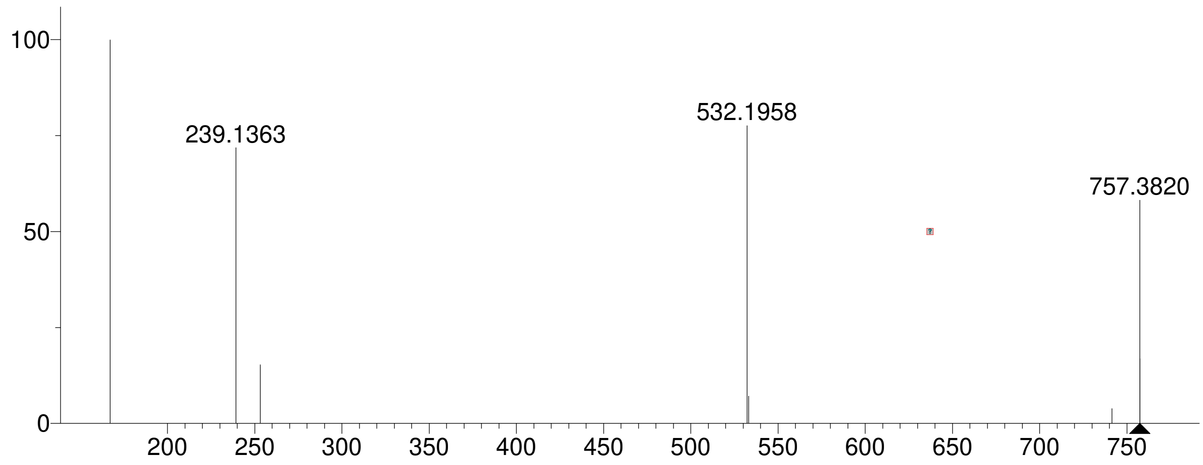

Hit 1 : ACTH (1-39), human [M+6H]6+ HCD 17eV P=757.4
C207H308N56O58S; MF: 0; RMF: 40; Prob 100.0%; CAS: 12279-41-3; Lib: nist_msms2; ID: 51586.

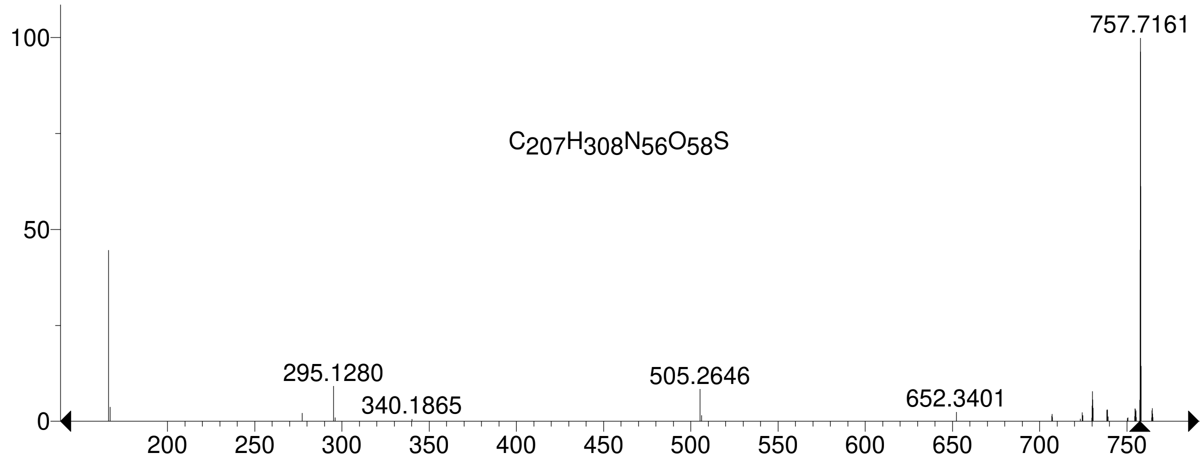

Unknown: Unknown (14.60_384.0613m/z)
Compound in Library Factor = -3273

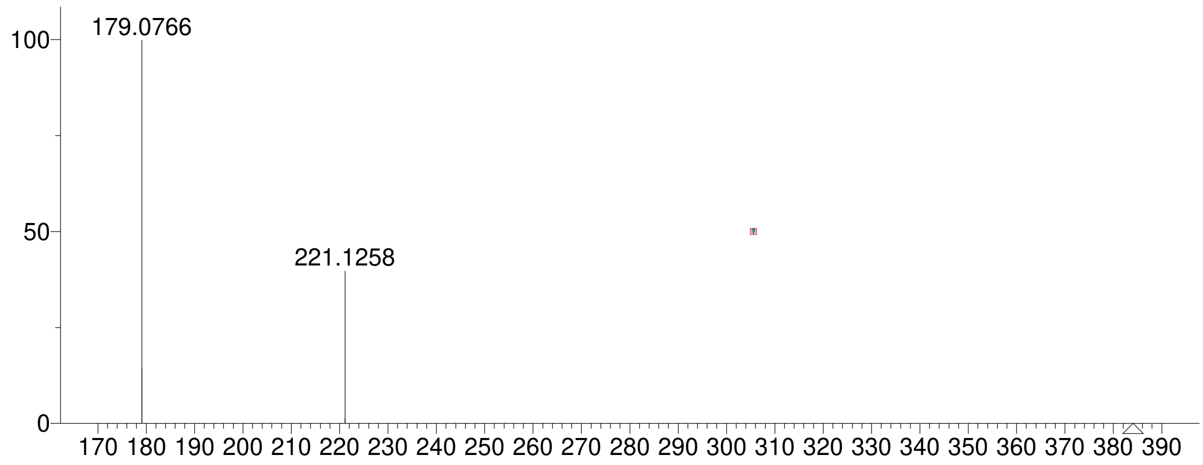

Hit 1 : Dehydrofelodipine [M+H+2i]+ HCD 122V P=384.1
C18H17Cl2NO4; MF: 3; RMF: 287; Prob 100.0%; CAS: 96382-71-7; Lib: nist_msms; ID: 517312.

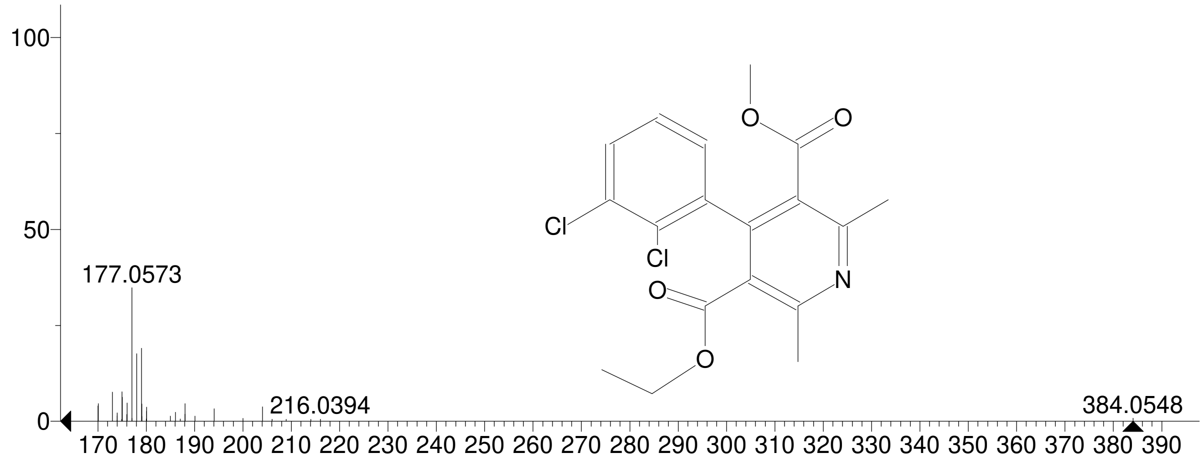

** Search Report Page 1 of 1 **

Unknown: Unknown (14.60_772.3507m/z)
Compound in Library Factor = 0

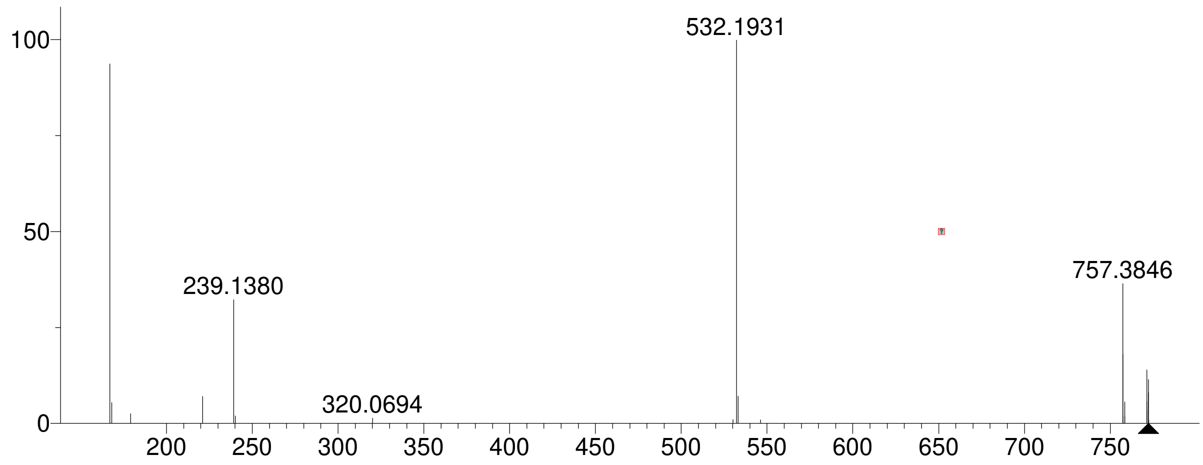

Unknown: Unknown (14.68_380.1697m/z)
Compound in Library Factor = -1142

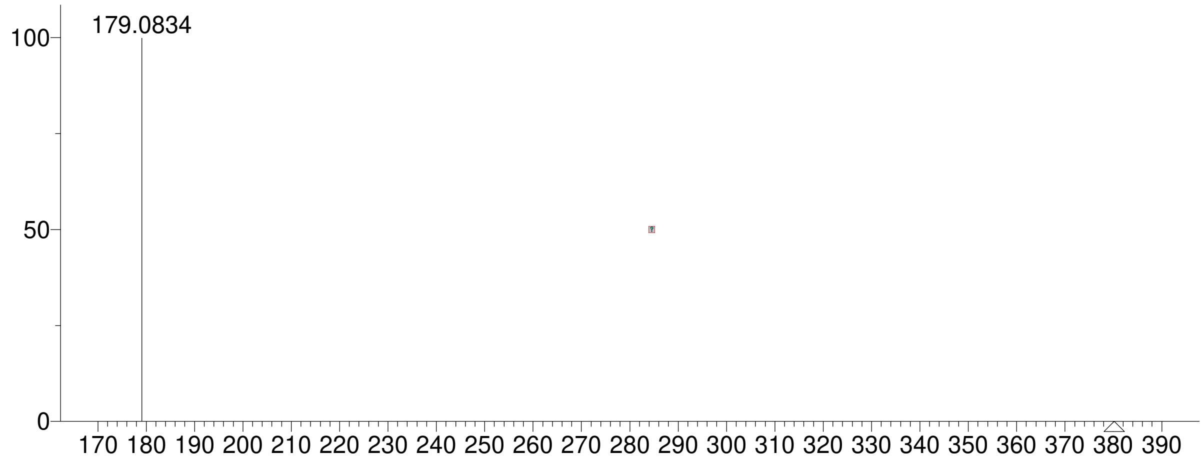

Hit 1 : Janerin [M+NH4]+ HCD 68V P=380.2
C19H22O7; MF: 62; RMF: 612; Prob 66.1%; CAS: 64205-84-1; Lib: nist_msms; ID: 225025.

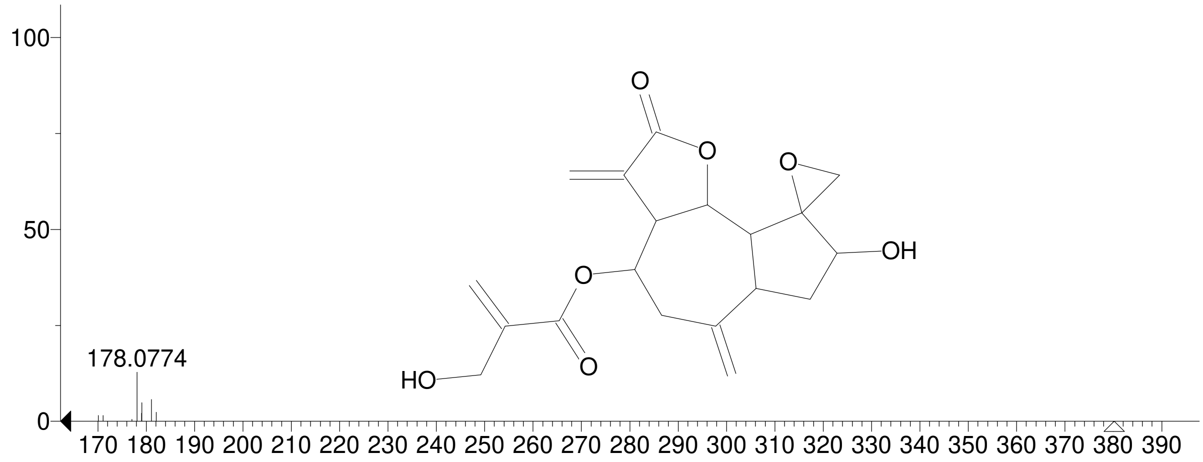

Hit 2 : [(3aS,4S,5S,6E,10Z,11aR)-6-formyl-5-hydroxy-10-(hydroxymethyl)-3-methylidene-2-oxo-3a,4,5,8,9,11a-he:
C19H22O7; MF: 0; RMF: 134; Prob 11.3%; Lib: mona-export-vf-npl_qexactive; ID: 9198.

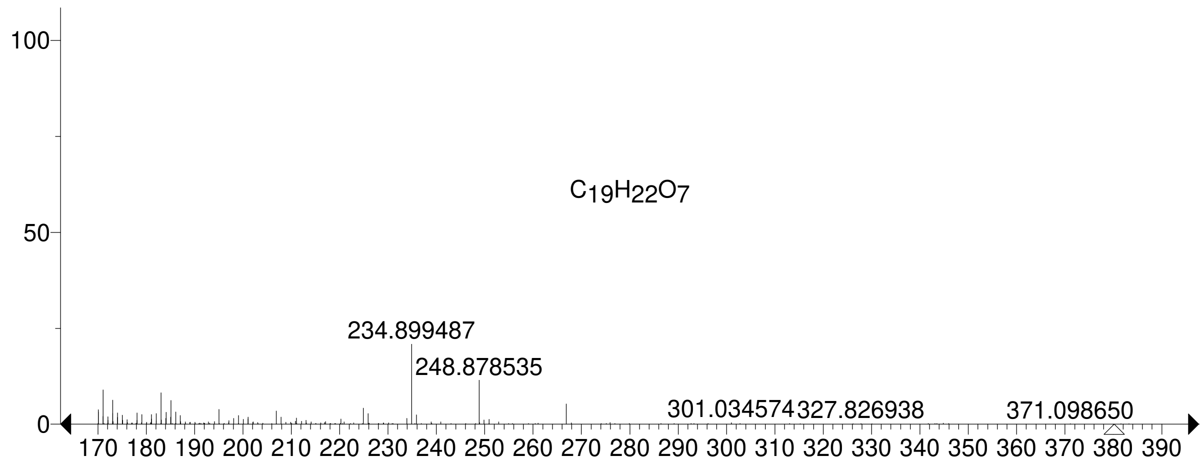

Hit 3 : Repin [M+NH4]+ IT-FT 35% P=380.2
C19H22O7; MF: 0; RMF: 47; Prob 11.3%; CAS: 11024-67-2; Lib: nist_msms; ID: 224746.

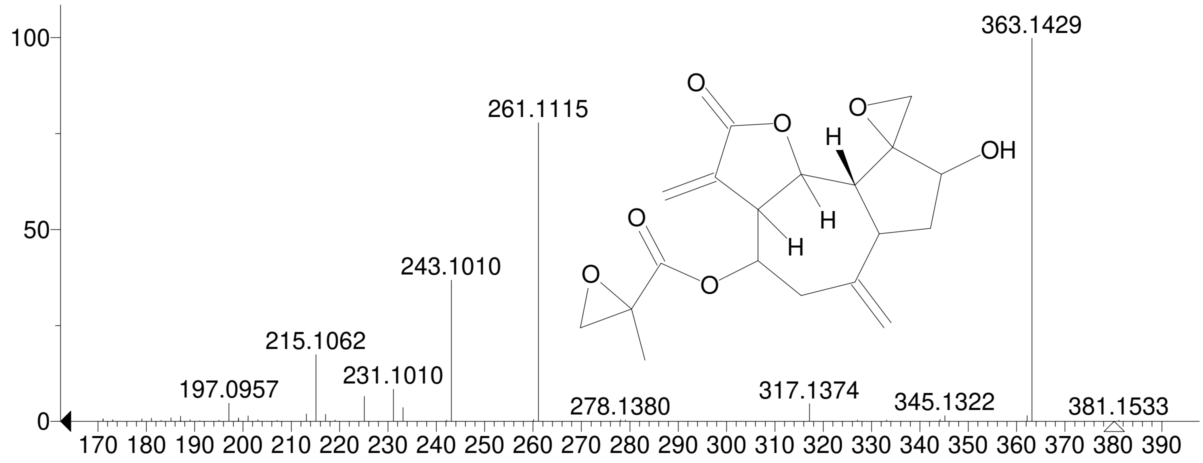

Hit 4 : Sinefungin [M-H]- IT-FT 35% P=380.2
C15H23N7O5; MF: 0; RMF: 35; Prob 11.3%; CAS: 58944-73-3; Lib: nist_msms; ID: 308141.

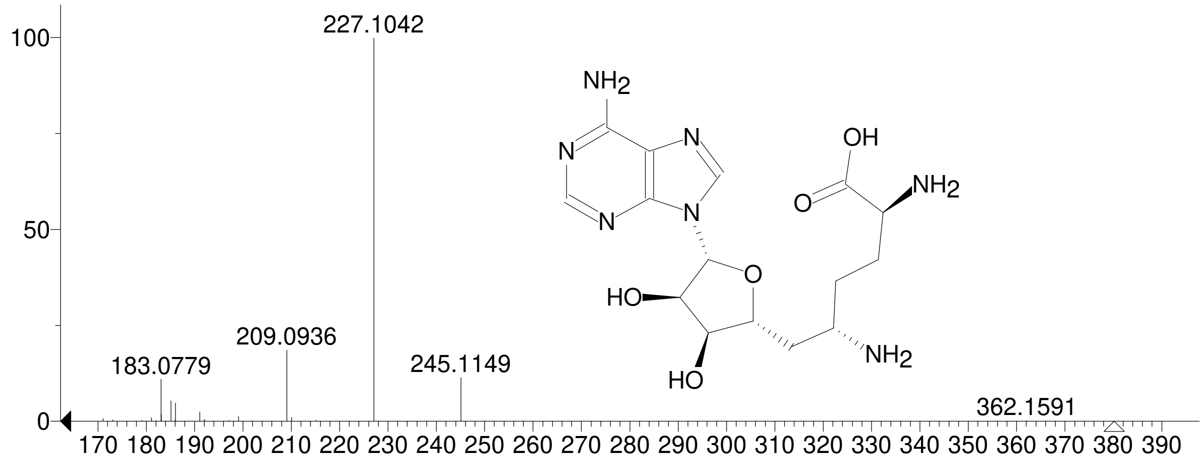

Unknown: Unknown (14.75_541.2387m/z)
Compound in Library Factor = -3273

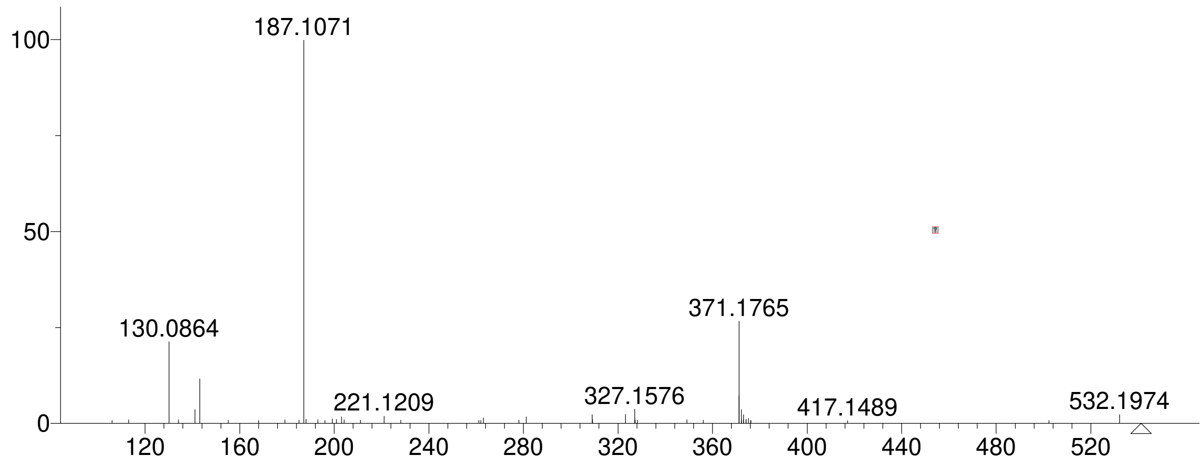

Hit 1 : methyl (1S,2R,4R,8R,9S,10R,13R,15R)-2-(acetyloxy)-13-(furan-3-yl)-9-(2-methoxy-2-oxoethyl)-4,8,10,12-t
C30H36O9; MF: 8; RMF: 198; Prob 38.0%; Lib: mona-export-vf-npl_qexactive; ID: 20632.

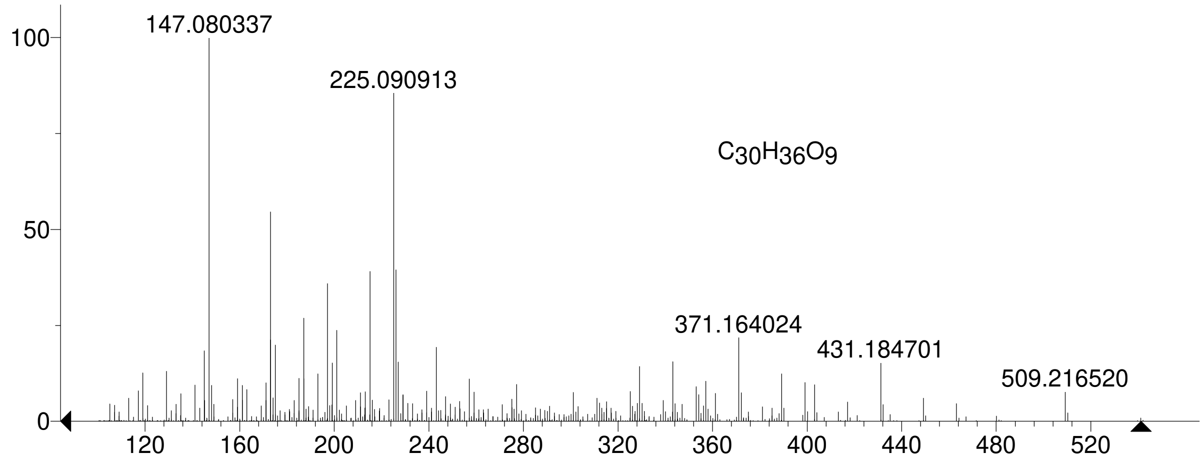

Hit 2 : NCGC00169906-03_C30H36O9_2H-Cyclopenta[b]naphtho[2,3-d]furan-10-acetic acid, 5-(acetyloxy)-2-(3-f
C30H36O9; MF: 5; RMF: 162; Prob 33.6%; Lib: mona-export-gnps; ID: 13758.

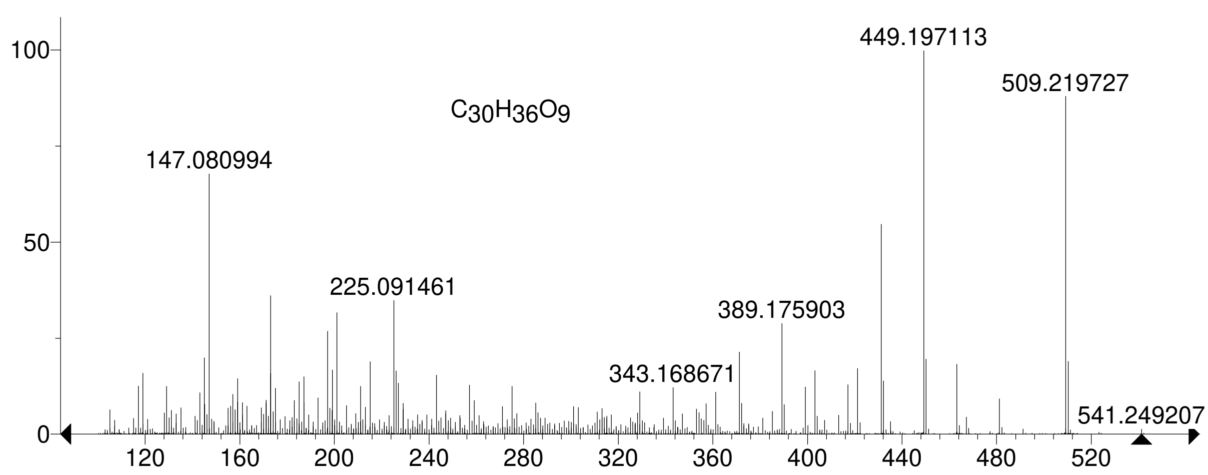

Hit 3 : Nimbin [M+H]+ IT-FT 35% P=541.2
C30H36O9; MF: 1; RMF: 63; Prob 28.4%; CAS: 5945-86-8; Lib: nist_msms; ID: 226917.

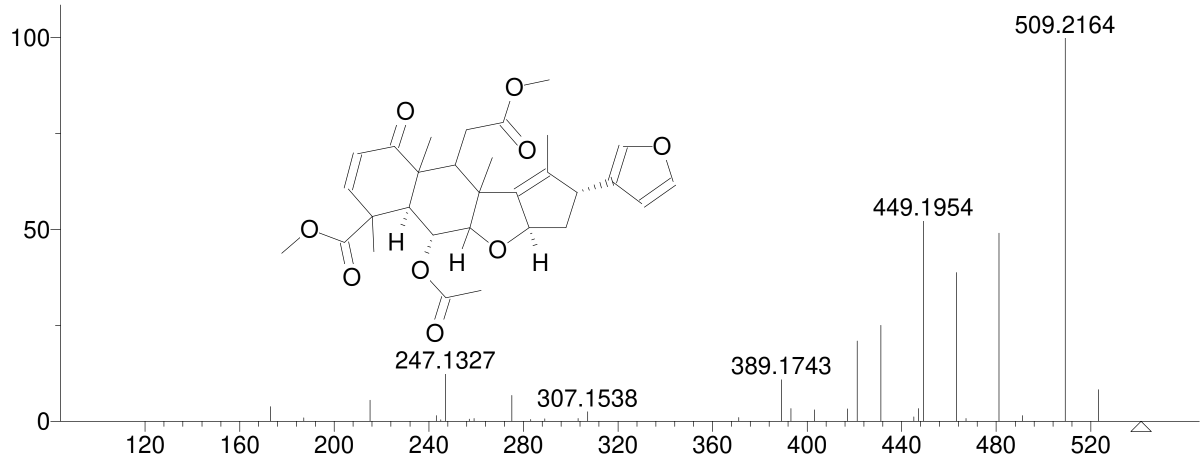

** Search Report Page 1 of 1 **

Unknown: Unknown (14.75_676.2891m/z)
Compound in Library Factor = -1072

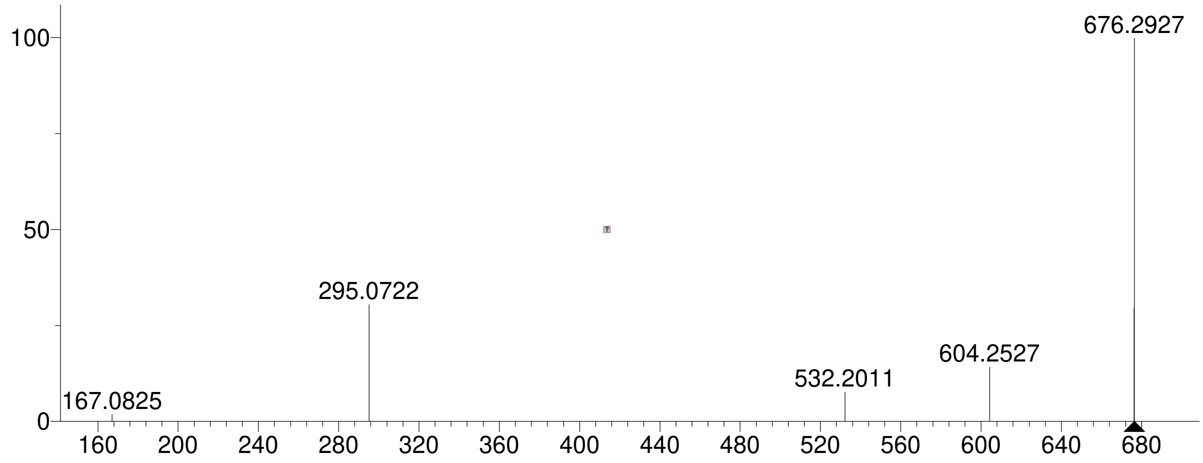

Hit 1 : Caspase-3 Precursor Protease Substrates [M+H]+ HCD 6eV P=676.3
C31H41N5O12; MF: 113; RMF: 702; Prob 100.0%; Lib: nist_msms2; ID: 54204.

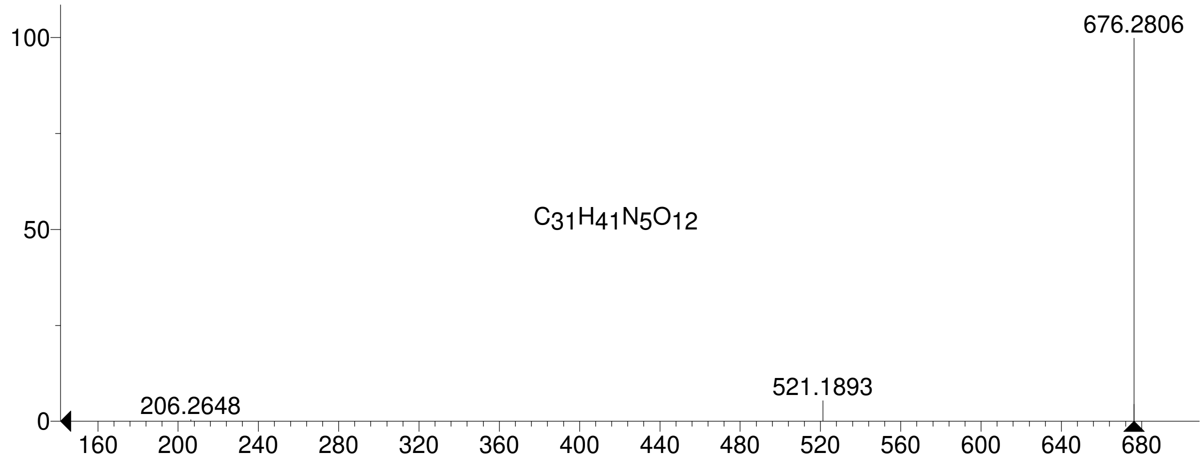

** Search Report Page 1 of 1 **

Unknown: Unknown (14.76_357.1891m/z)
Compound in Library Factor = 0

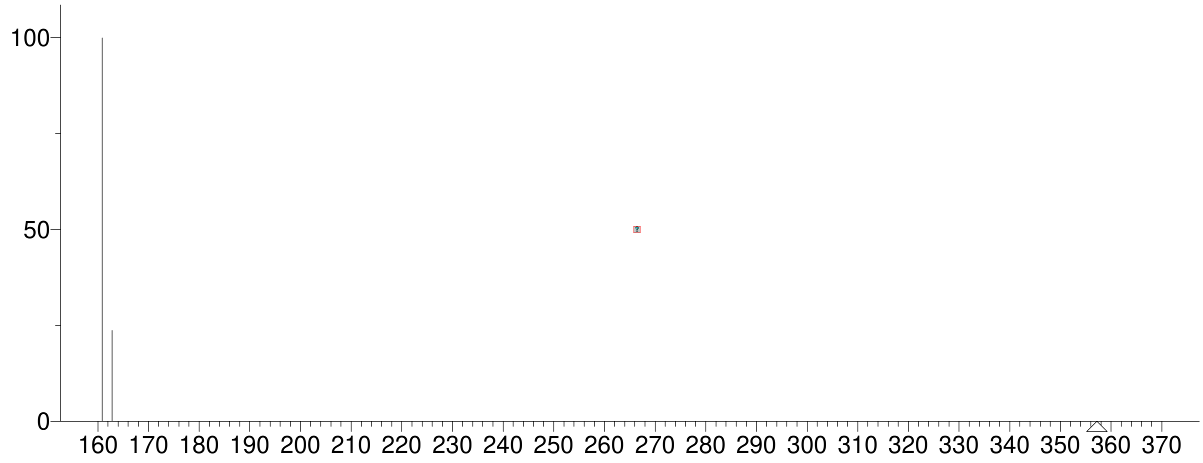

Unknown: Unknown (14.76_604.2437m/z)
Compound in Library Factor = 0

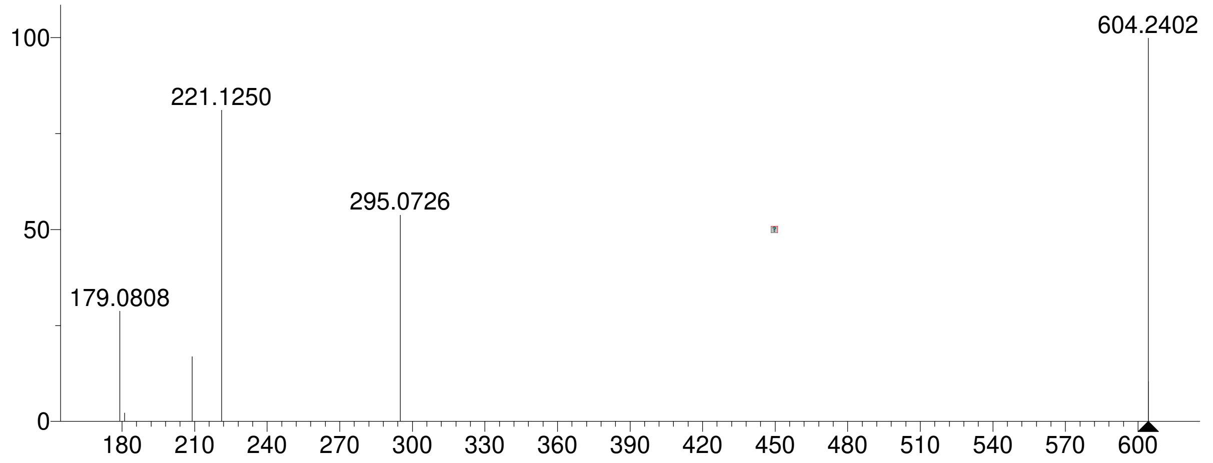

Hit 1 : NCGC00381267-01![(2S,3S,4R,5R,6R)-3,5-dihydroxy-2-methyl-6-[[(2R,3S,4S,5R,6S)-3,4,5-trihydroxy-6-[(E
C30H34O12; MF: 0; RMF: 13; Prob 50.0%; Lib: mona-export-gnps; ID: 9388.

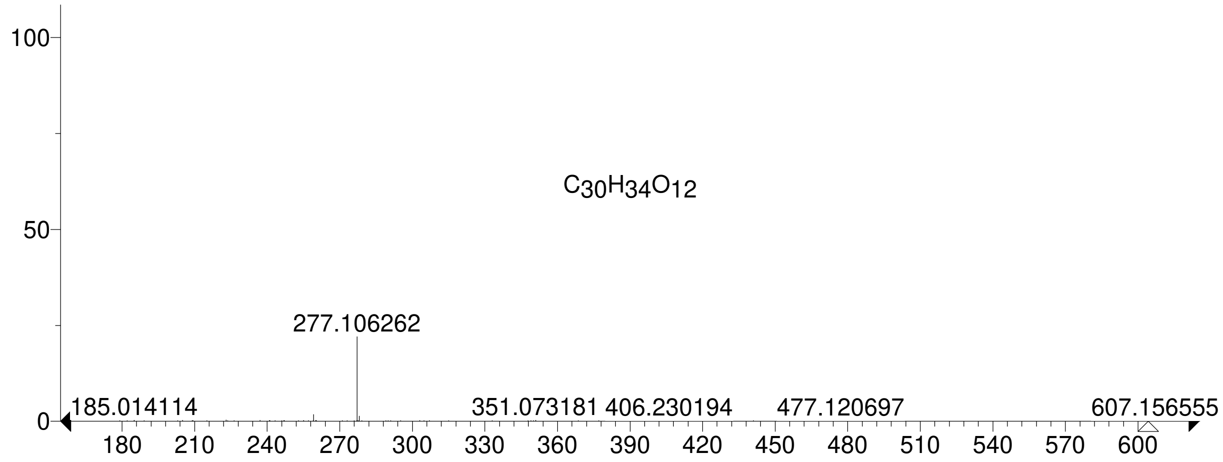

Hit 2 : NCGC00381132-01_C23H38O17_ [M+NH4]+ P=604.2
C23H38O17; MF: 0; RMF: 10; Prob 50.0%; Lib: mona-export-gnps; ID: 12990.

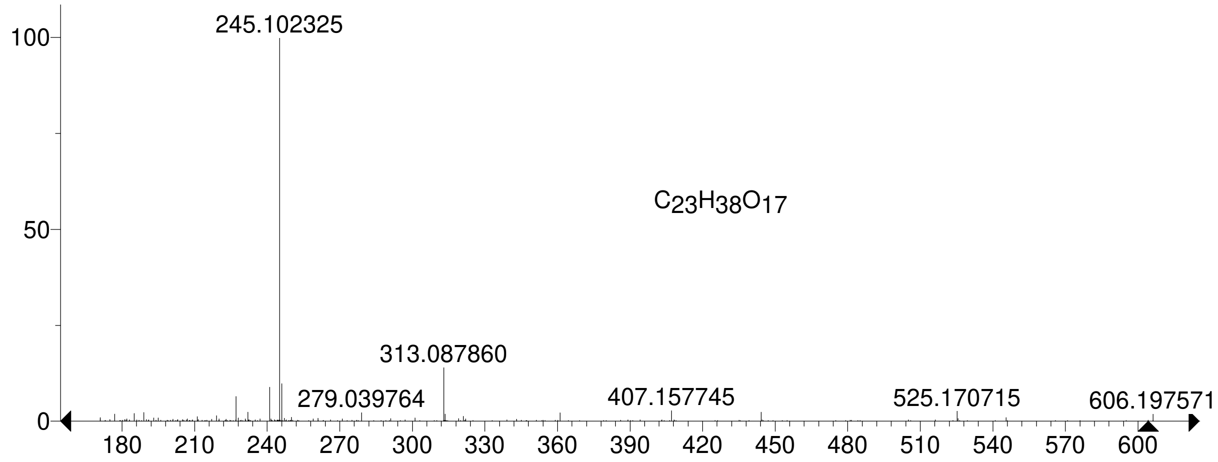

** Search Report Page 1 of 1 **

Unknown: Unknown (14.78_506.0884m/z)
Compound in Library Factor = 0

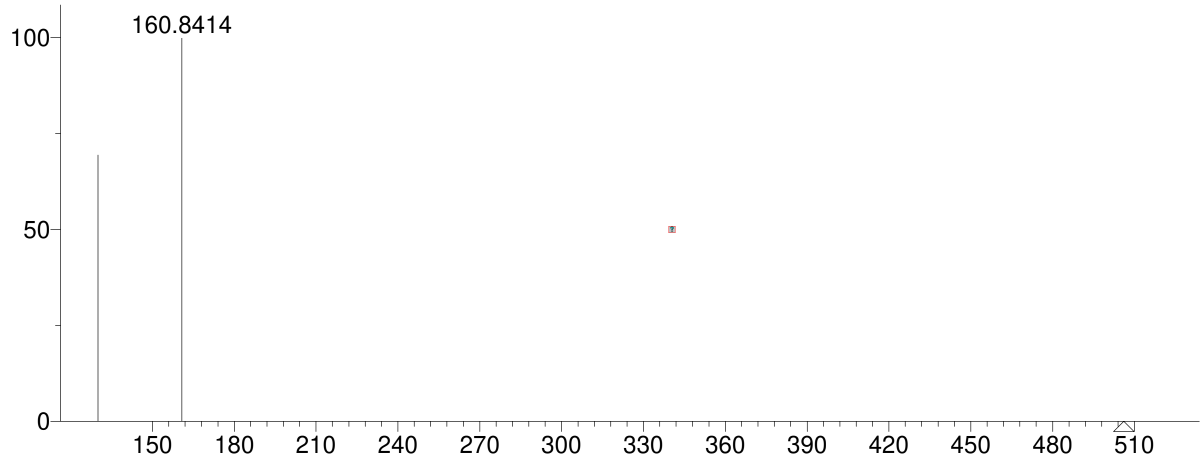

Unknown: Unknown (14.79_475.1705m/z)
Compound in Library Factor = 0

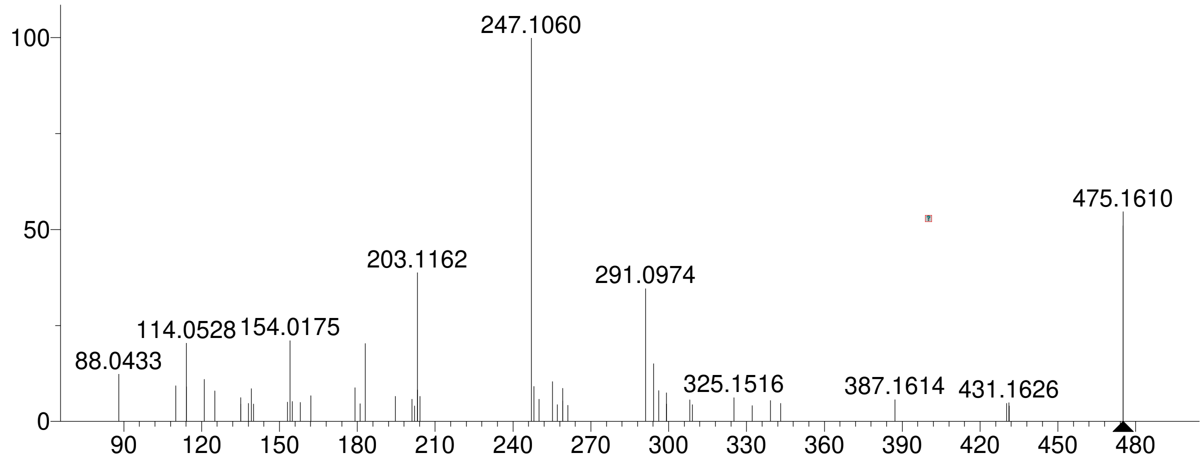

Unknown: Unknown (14.80_633.2947m/z)
Compound in Library Factor = 0

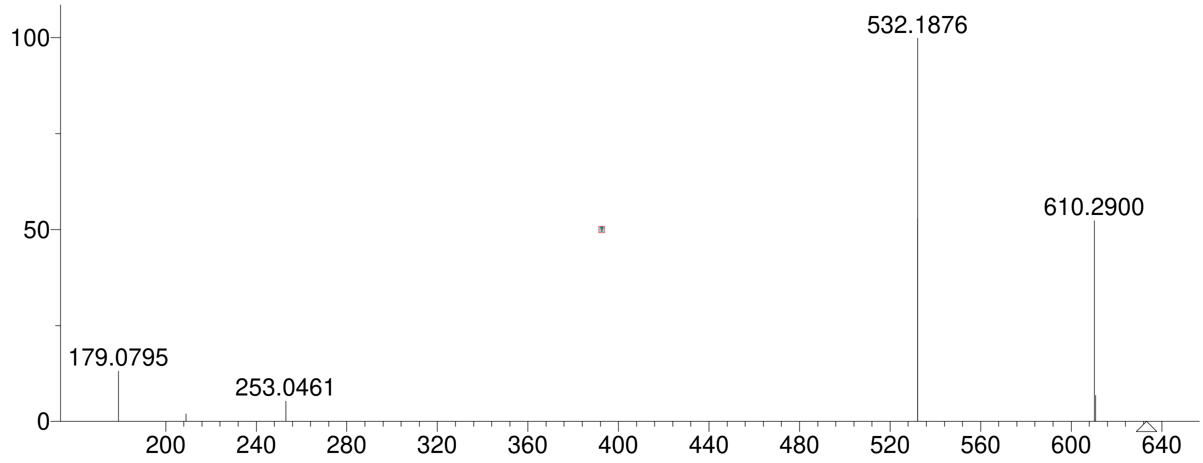

Hit 1 : Alloferon 1 [M+2H]2+ IT-FT 35% P=633.3
C52H76N22O16; MF: 0; RMF: 15; Prob 100.0%; Lib: nist_msms2; ID: 49652.

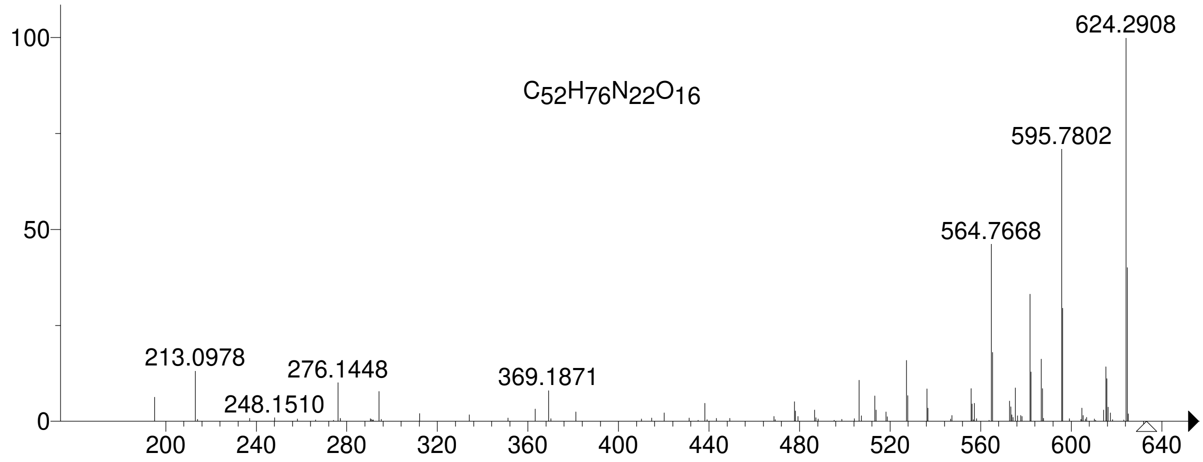

Unknown: Unknown (14.82_221.0808m/z)
Compound in Library Factor = -228

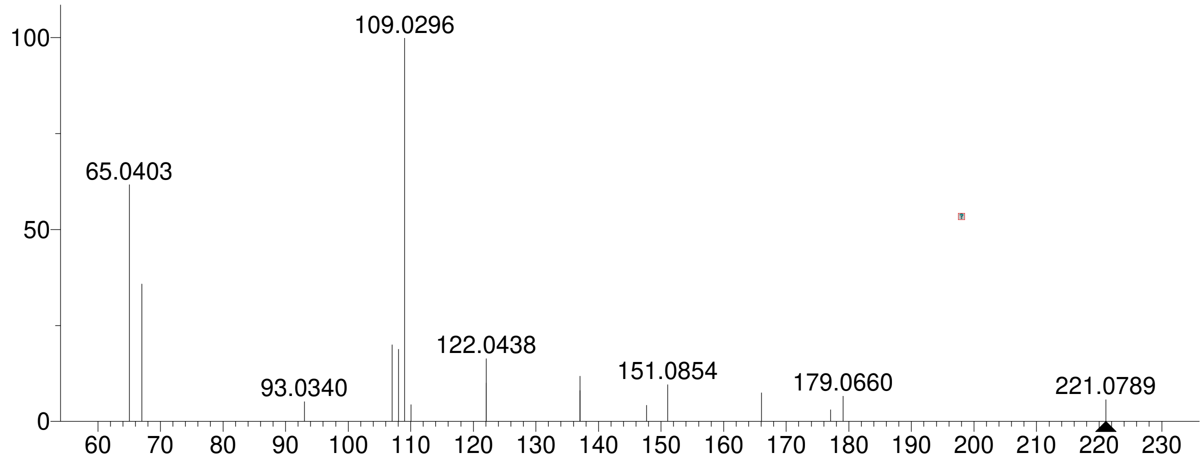

Hit 1 : 4-Ethoxy-7-methoxycoumarin [M+H]+ HCD 6V P=221.1
C12H12O4; MF: 356; RMF: 988; Prob 47.0%; CAS: 29064-32-2; Lib: nist_msms; ID: 177196.

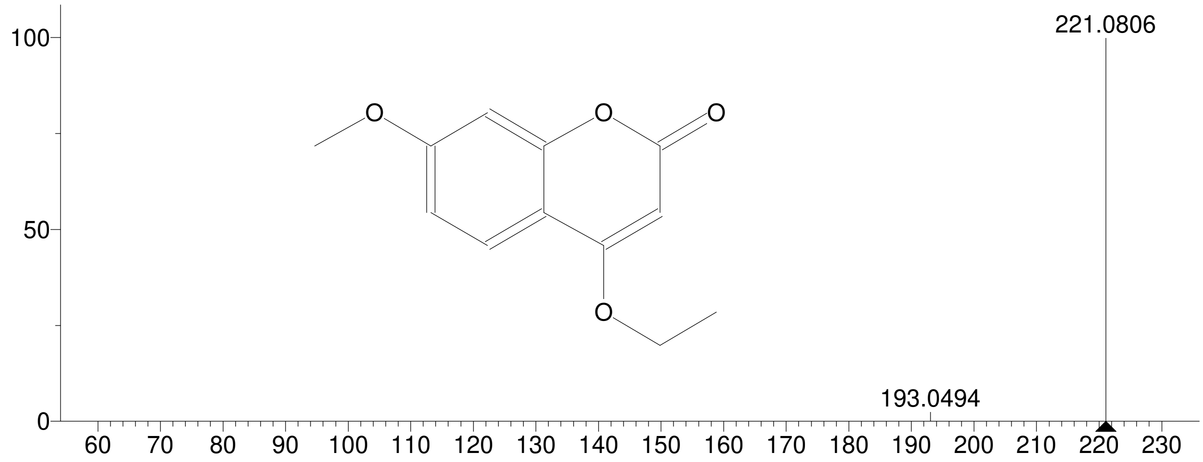

Hit 2 : 4-Ethoxy-6-methoxycoumarin [M+H]+ HCD 6V P=221.1
C12H12O4; MF: 353; RMF: 983; Prob 41.6%; CAS: 720676-54-0; Lib: nist_msms; ID: 177270.

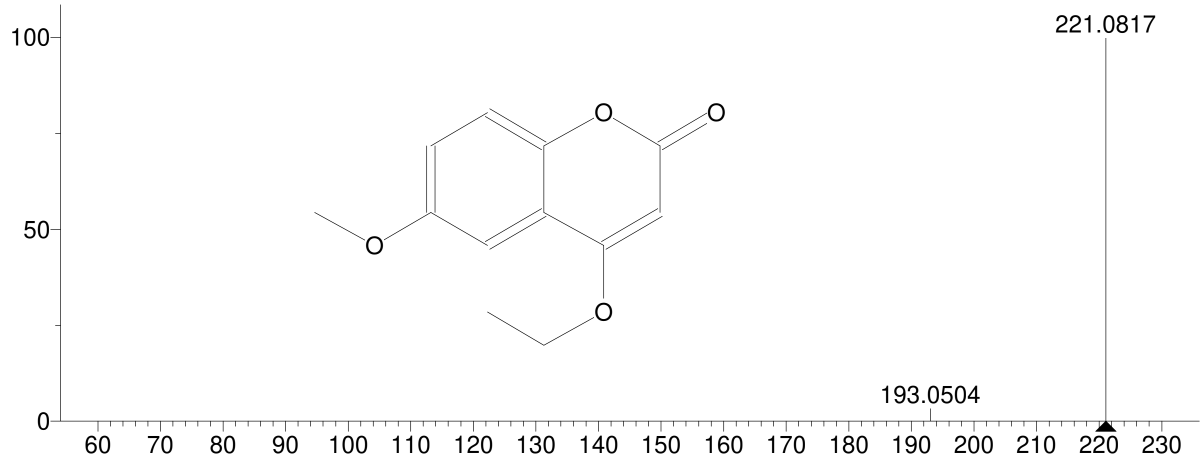

Hit 3 : Ferulic acid ethyl ester [M-H]- HCD 8V P=221.1
C12H14O4; MF: 321; RMF: 945; Prob 11.1%; CAS: 4046-02-0; Lib: nist_msms; ID: 408260.

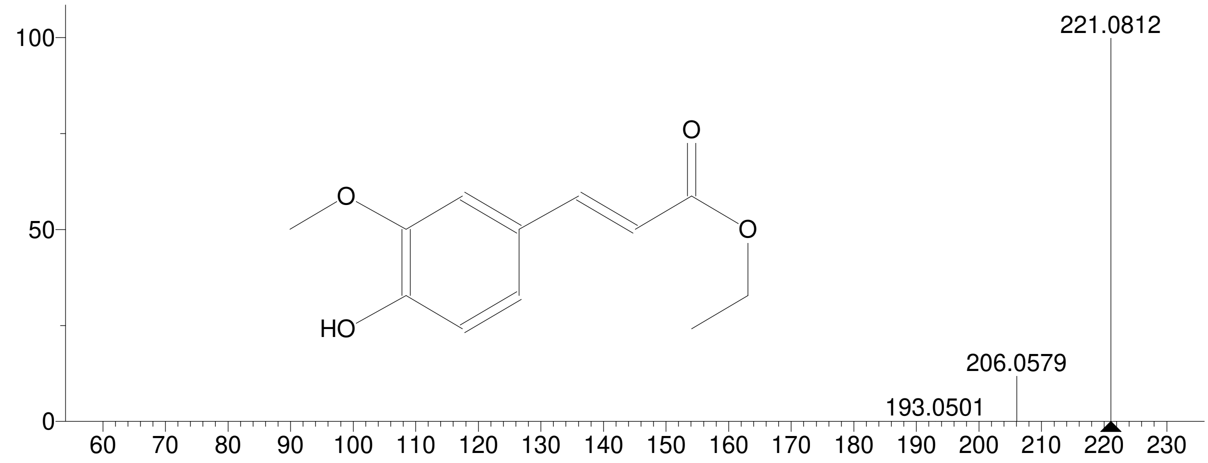

Hit 4 : Unknown (carbon number 12); PlaSMA ID-319 [M-H]- P=221.1
C12H14O4; MF: 76; RMF: 298; Prob 0.11%; Lib: biomsms-neg-plasma; ID: 1062.

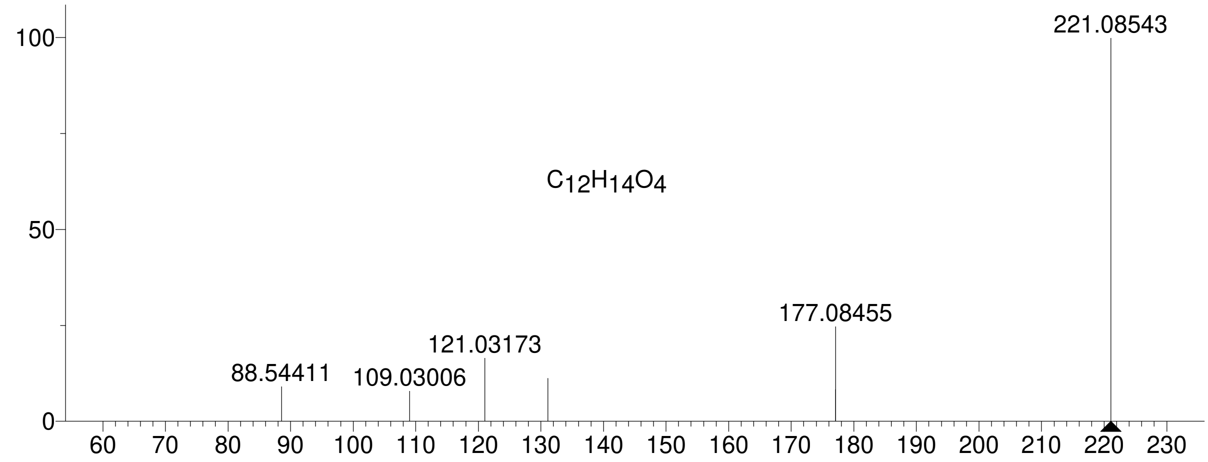

Hit 5 : 5,7-Dimethoxy-4-methylcoumarin [M+H]+ HCD 17V P=221.1
C12H12O4; MF: 20; RMF: 185; Prob 0.02%; CAS: 6093-80-7; Lib: nist_msms; ID: 110250.

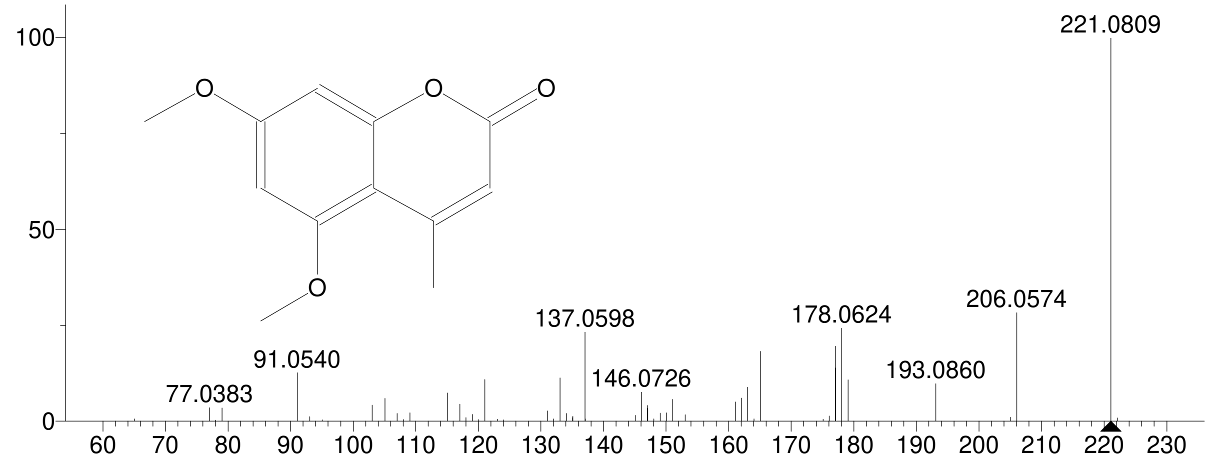

Unknown: Unknown (14.82_253.0498m/z)
Compound in Library Factor = -781

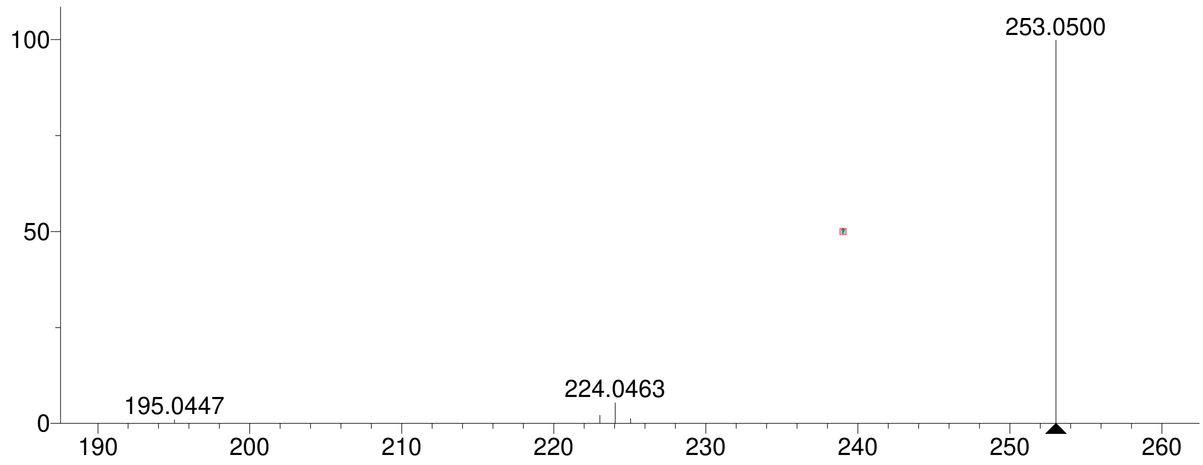

Hit 1 : Daidzein; LC-ESI-QTOF; MS2; CE [M-H]- P=253.1
C15H10O4; MF: 649; RMF: 941; Prob 77.2%; Lib: msms-neg-massbank; ID: 4125.

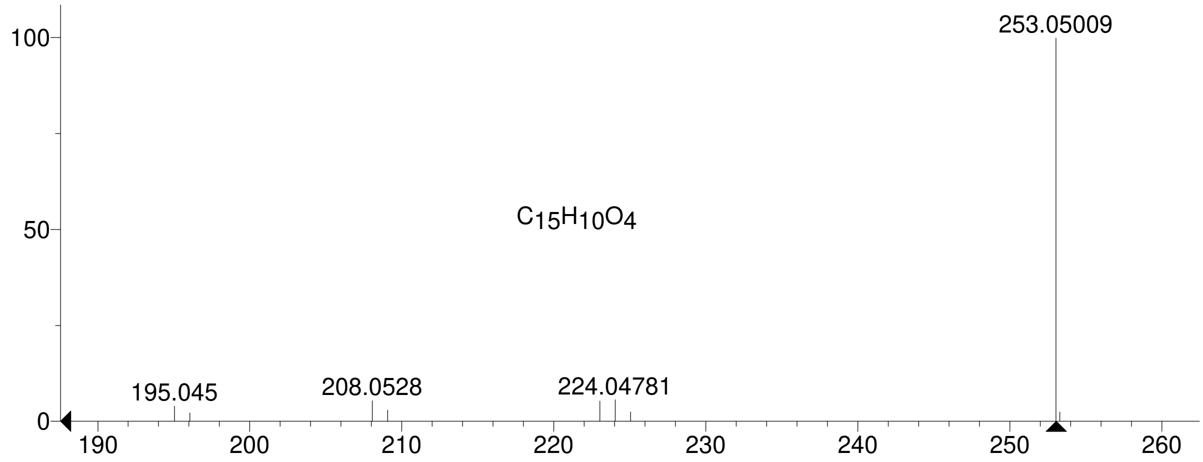

Hit 2 : Daidzein [M-H]- 10 P=253.1
C15H10O4; MF: 603; RMF: 952; Prob 16.3%; Lib: msms neg mona_ipb_gnps_plasma; ID: 20109.

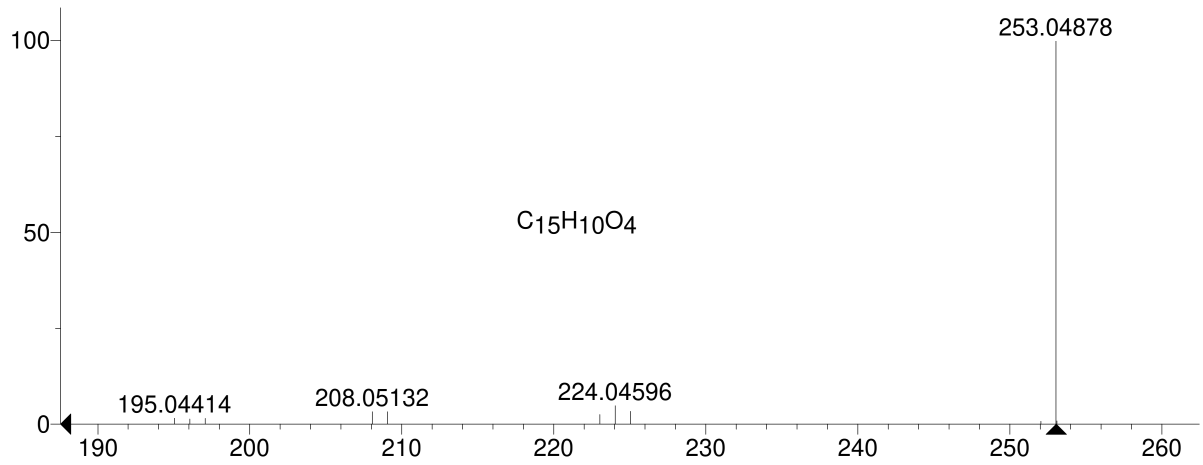

Hit 3 : 3,3'-Dihydroxyflavone [M-H]- 10ev P=253.1
C15H10O4; MF: 564; RMF: 919; Prob 3.84%; Lib: mona-export-lc-ms-ms_negative_mode; ID: 9141.

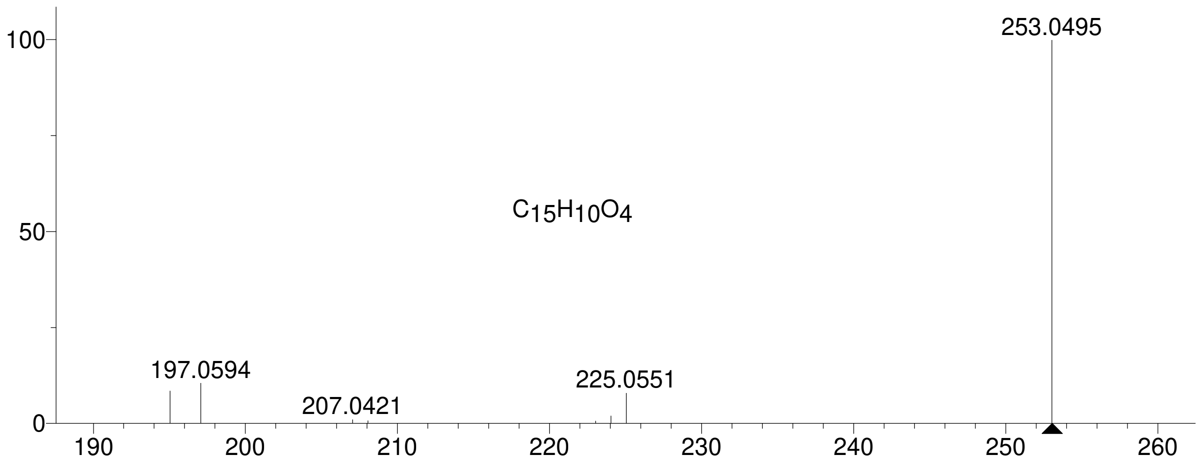

Hit 4 : Flavonol base + 1O [M-H]- 6V P=253
C15H10O4; MF: 513; RMF: 912; Prob 0.77%; Lib: anthocyanins mona; ID: 6876.

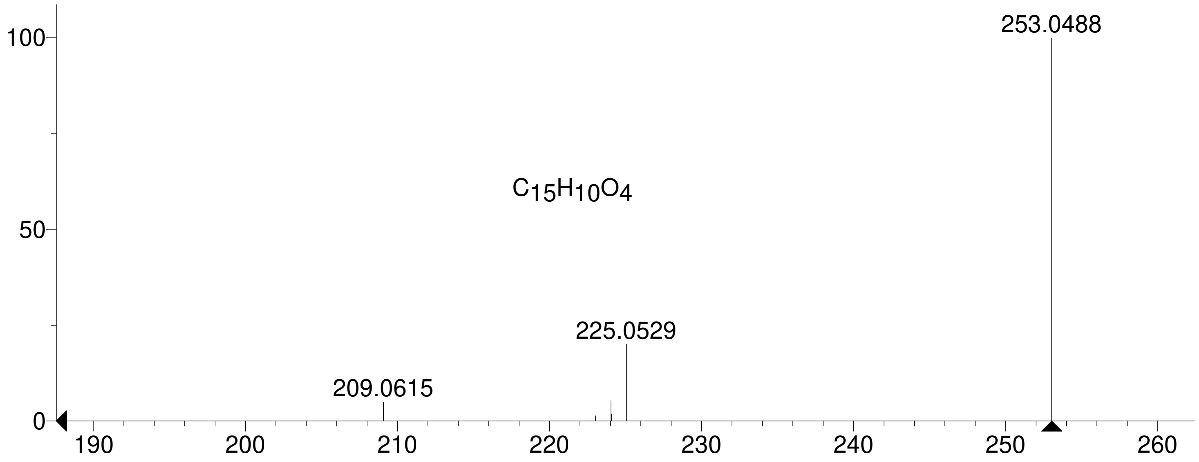

Hit 5 : Flavonol base + 1O; PlaSMA ID-410 [M-H]- P=253
C15H10O4; MF: 513; RMF: 912; Prob 0.77%; Lib: biomsms-neg-plasma; ID: 555.

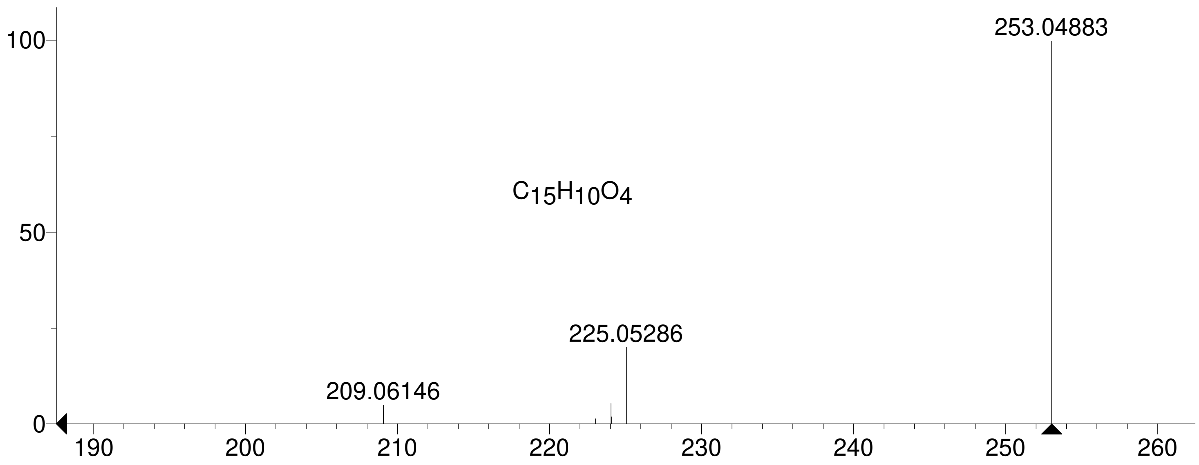

Unknown: Unknown (14.95_191.0336m/z)
Compound in Library Factor = -459

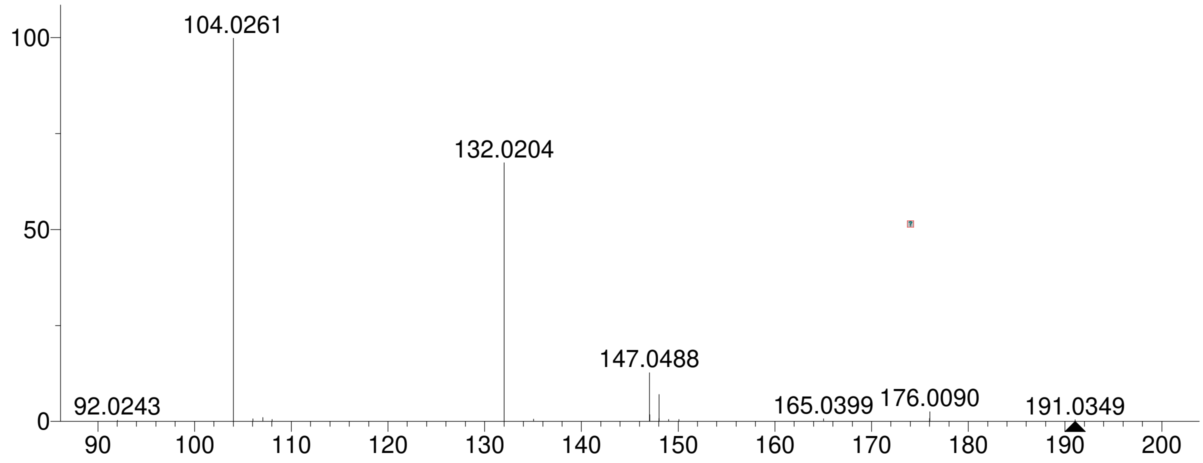

Hit 1 : Scopoletin [M-H]- HCD 9V P=191
C10H8O4; MF: 478; RMF: 785; Prob 94.0%; CAS: 92-61-5; Lib: nist_msms; ID: 552763.

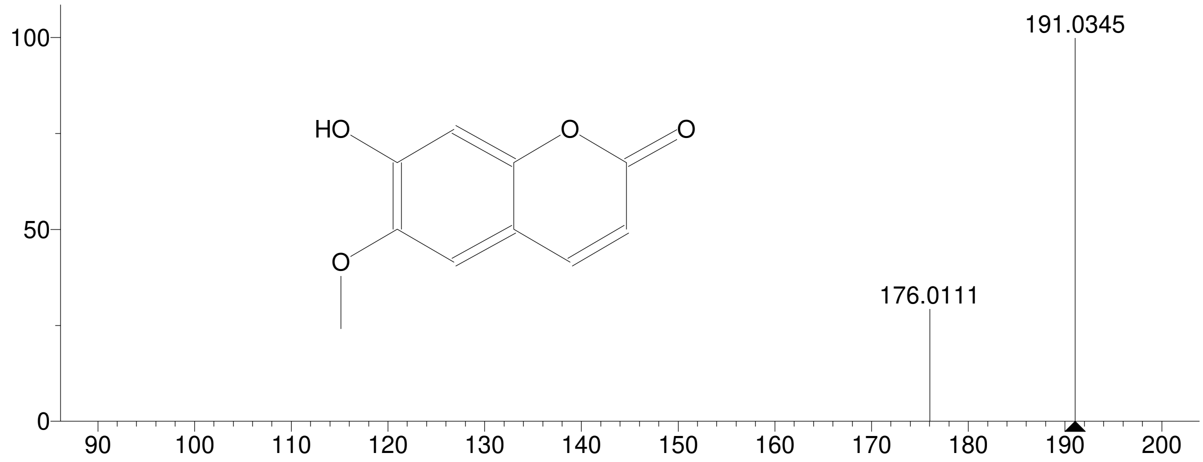

Hit 2 : 6-Hydroxy-7-methoxycoumarin [M-H]- HCD 24V P=191
C10H8O4; MF: 359; RMF: 520; Prob 5.07%; CAS: 776-86-3; Lib: nist_msms; ID: 106577.

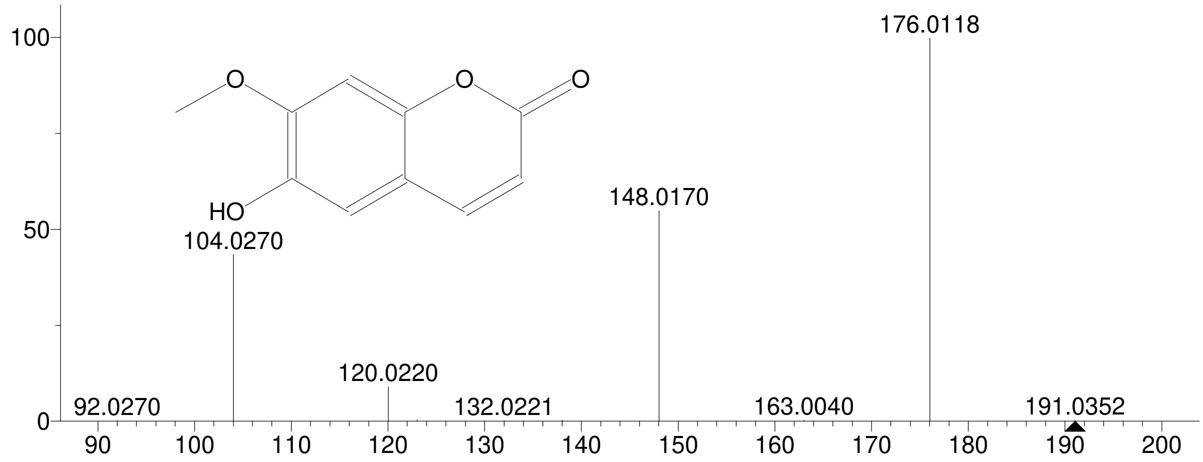

Hit 3 : Scopoletin; LC-ESI-QTOF; MS2; CE [M-H]- P=191
C10H8O4; MF: 282; RMF: 458; Prob 0.61%; Lib: msms-neg-massbank; ID: 4682.

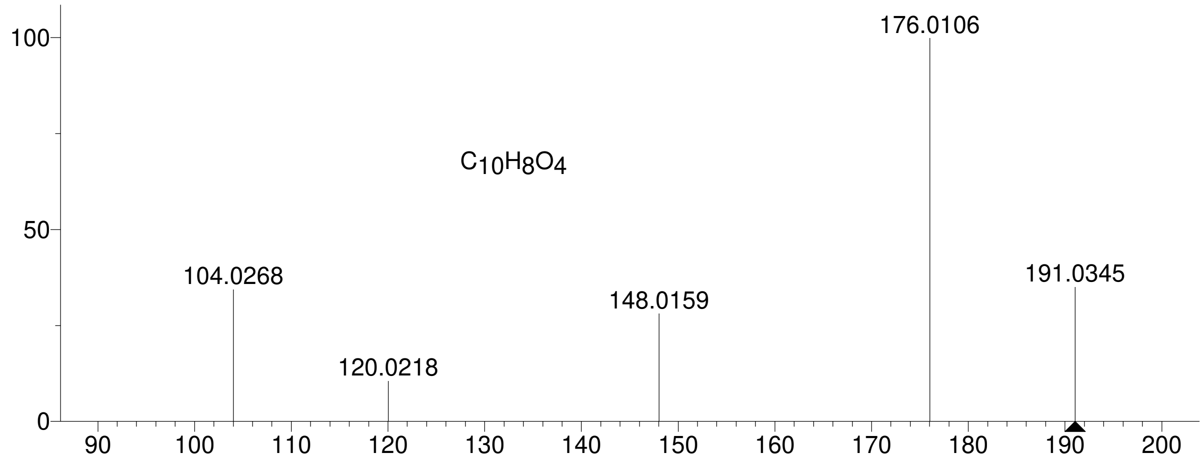

Hit 4 : Coumarin base + 1O, 1MeO; PlaSMA ID-233 [M-H]- P=191
C10H8O4; MF: 258; RMF: 798; Prob 0.20%; Lib: biomsms-neg-plasma; ID: 683.

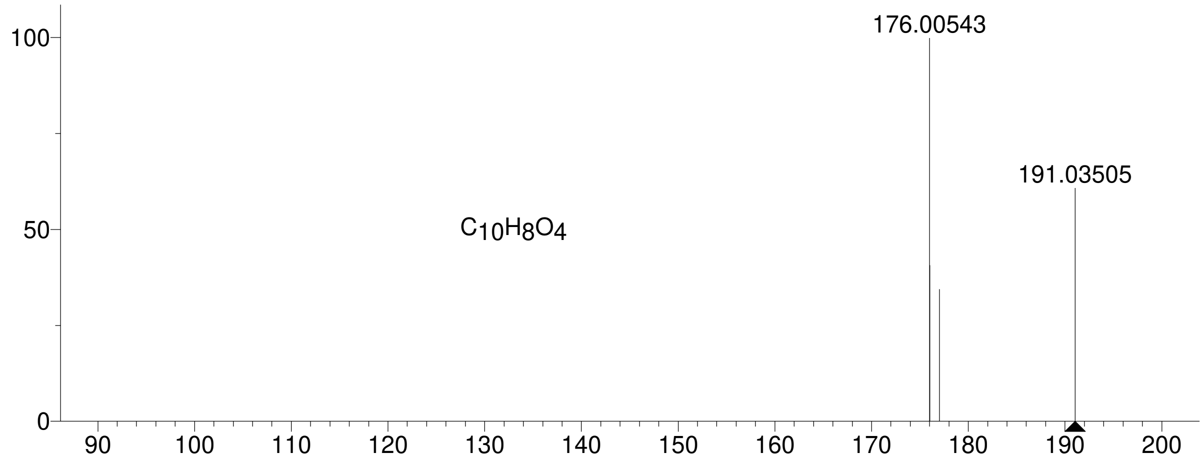

Hit 5 : 6-Methoxy-7-hydroxycoumarin [M-H]- 10 P=191
C10H8O4; MF: 223; RMF: 387; Prob 0.05%; Lib: msms neg mona_ipb_gnps_plasma; ID: 20632.

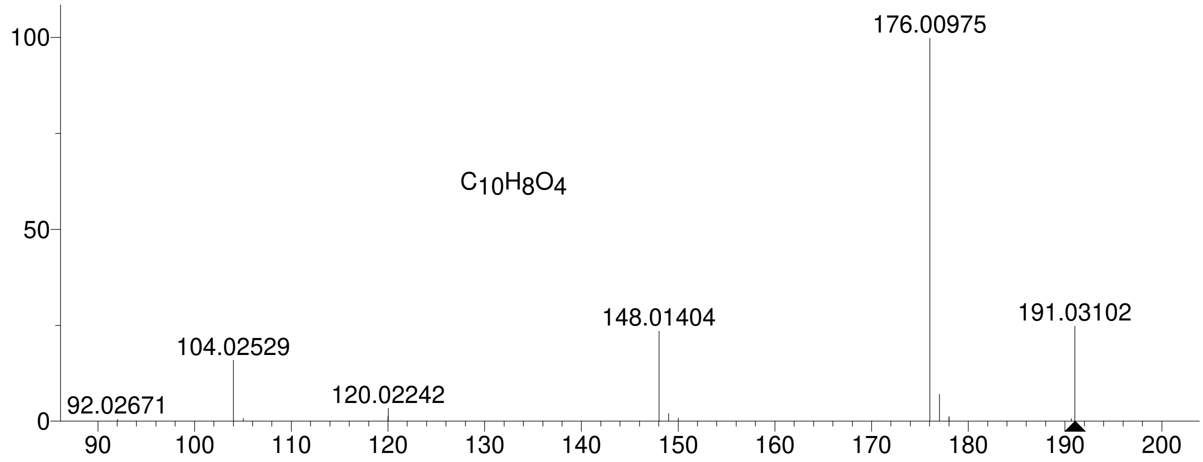

Unknown: Unknown (15.03_264.1233m/z)
Compound in Library Factor = -2387

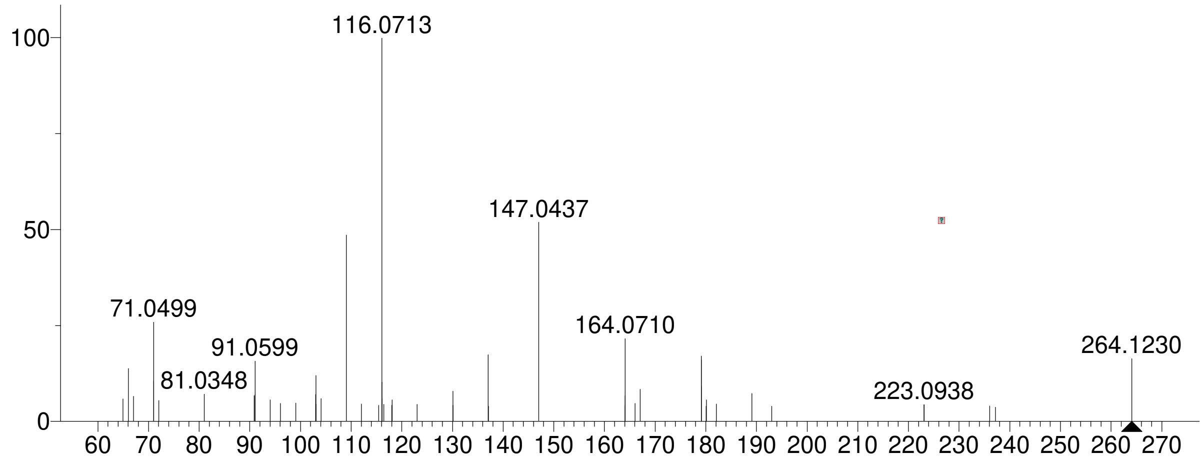

Hit 1 : N-α-(tert-Butoxycarbonyl)-L-phenylalanine [M-H]- HCD 1V P=264.1
C14H19NO4; MF: 50; RMF: 523; Prob 27.5%; CAS: 13734-34-4; Lib: nist_msms; ID: 306371.

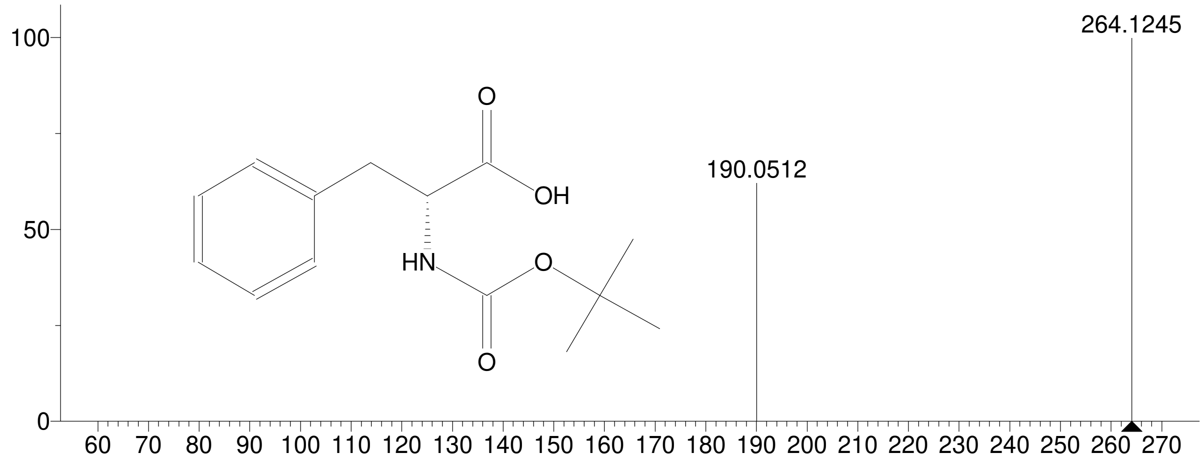

Hit 2 : 6-Benzyloxycarbonylaminohexanoic acid [M-H]- HCD 0V P=264.1
C14H19NO4; MF: 43; RMF: 323; Prob 21.1%; CAS: 1947-00-8; Lib: nist_msms; ID: 127720.

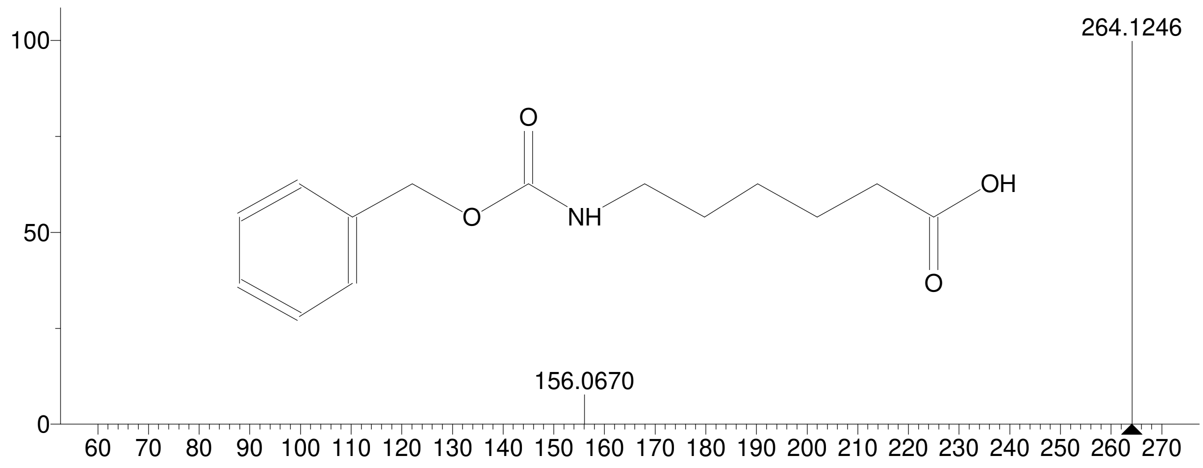

Hit 3 : Alachlor OA [M-H]- HCD 1V P=264.1
C14H19NO4; MF: 24; RMF: 317; Prob 10.2%; CAS: 171262-17-2; Lib: nist_msms; ID: 473262.

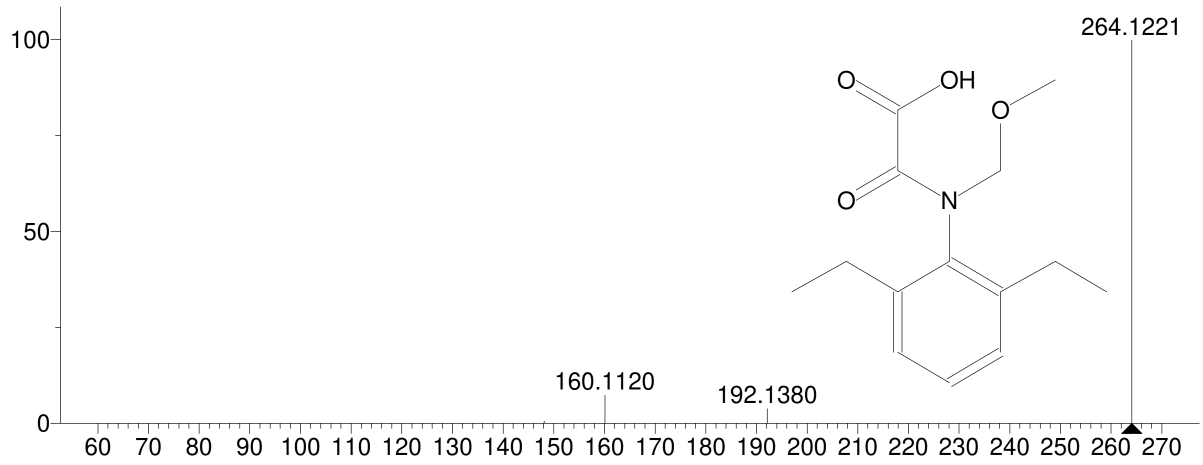

Hit 4 : Zaleplon [M+H-C2H2O]+ HCD 15V P=264.1
C17H15N5O; MF: 18; RMF: 316; Prob 8.03%; CAS: 151319-34-5; Lib: nist_msms; ID: 329246.

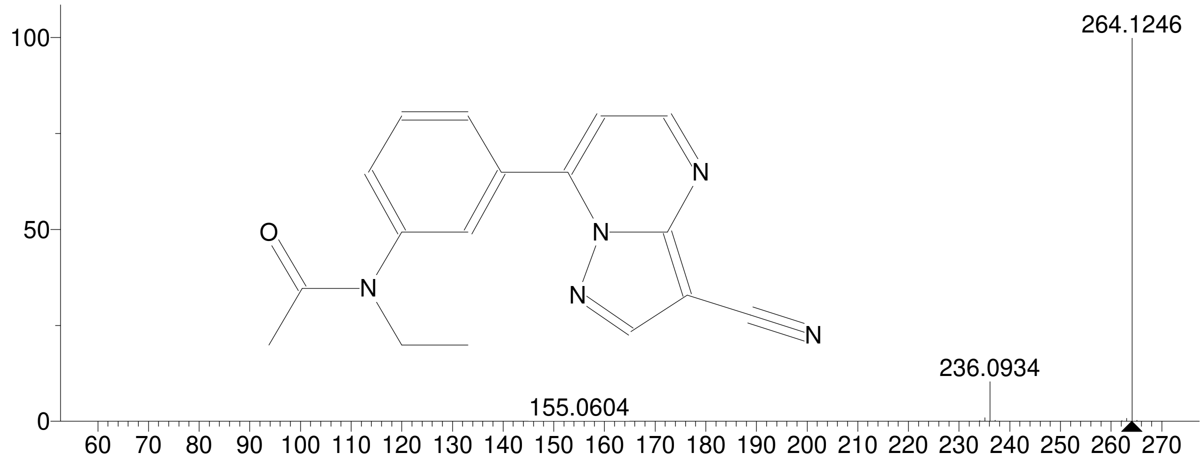

Hit 5 : CGA62826 (2-[2,6-dimethylphenyl)-methoxyacetylamino]propionic acid [M-H]- 15(no P=264.1
C14H19NO4; MF: 0; RMF: 97; Prob 4.14%; Lib: mona-export-lc-ms-ms_negative_mode; ID: 4254.

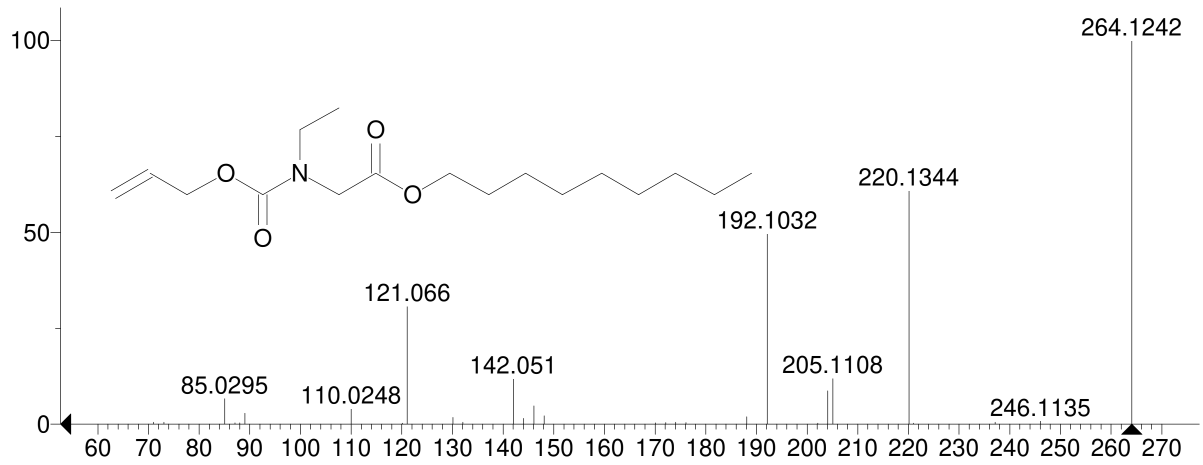

Unknown: Unknown (15.03_552.2688m/z)
Compound in Library Factor = -1848

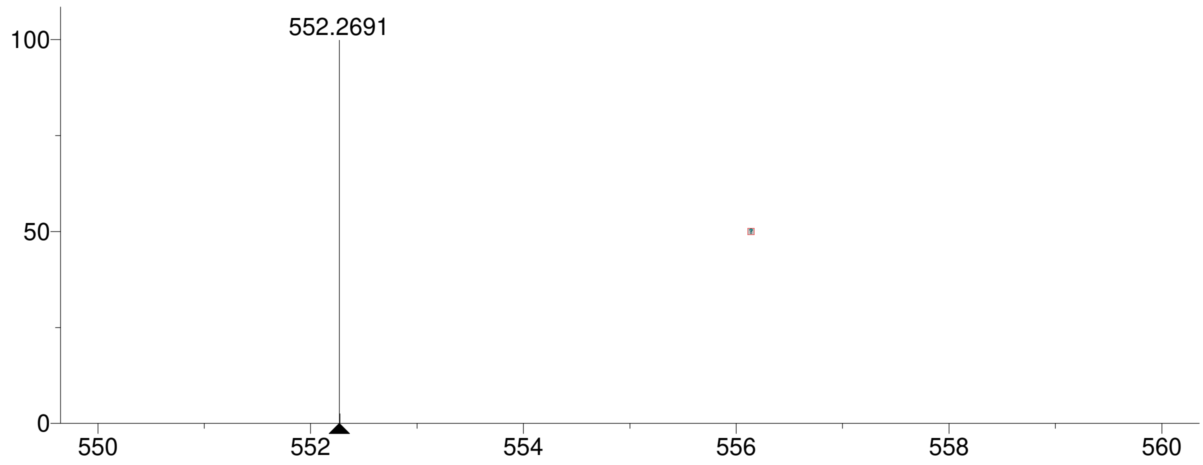

Hit 1 : Big Endothelin-2 (22-37), human [M+3H]3+ HCD 9eV P=552.3
C73H111N19O25; MF: 51; RMF: 739; Prob 100.0%; Lib: nist_msms2; ID: 61869.

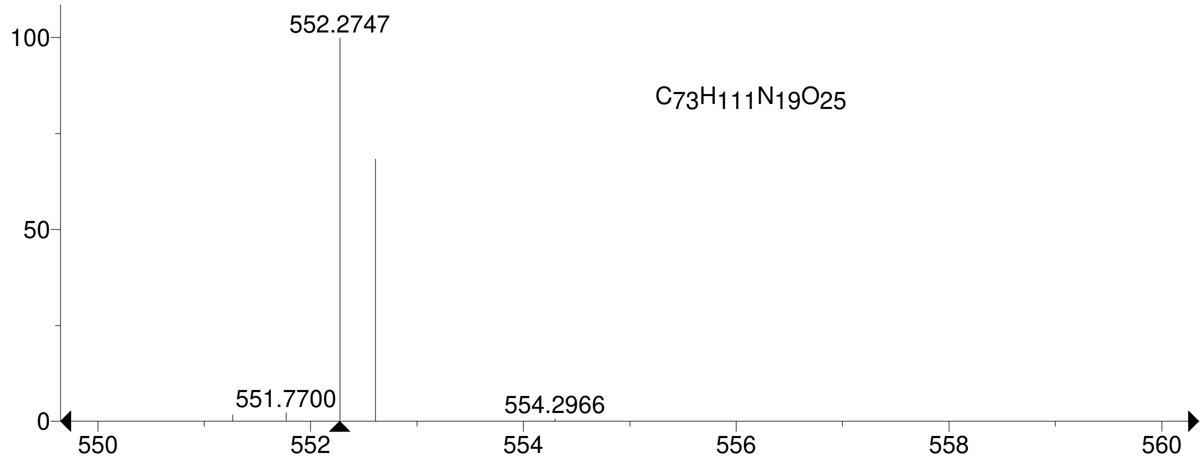

Unknown: Unknown (15.03_575.7736m/z)
Compound in Library Factor = 0

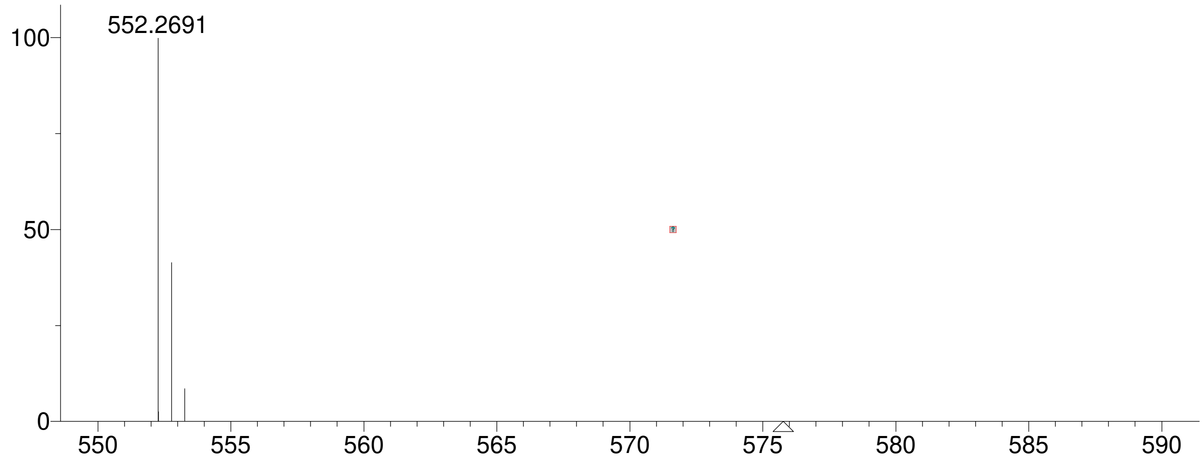

Hit 1 : [Lys8] LH-RH [M-2H]2- HCD 31eV P=575.8
C55H75N15O13; MF: 0; RMF: 74; Prob 100.0%; Lib: nist_msms2; ID: 80377.

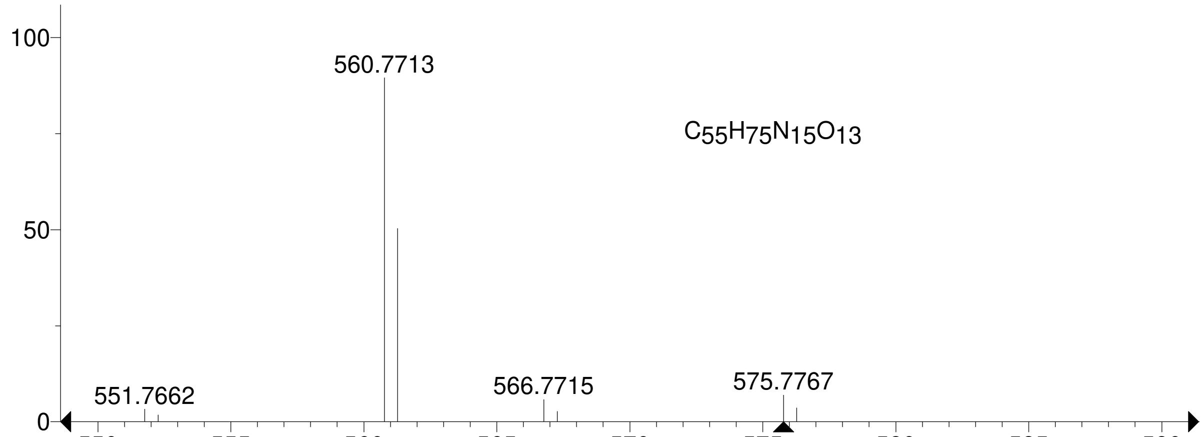

Unknown: Unknown (15.04_575.2722m/z)
Compound in Library Factor = 0

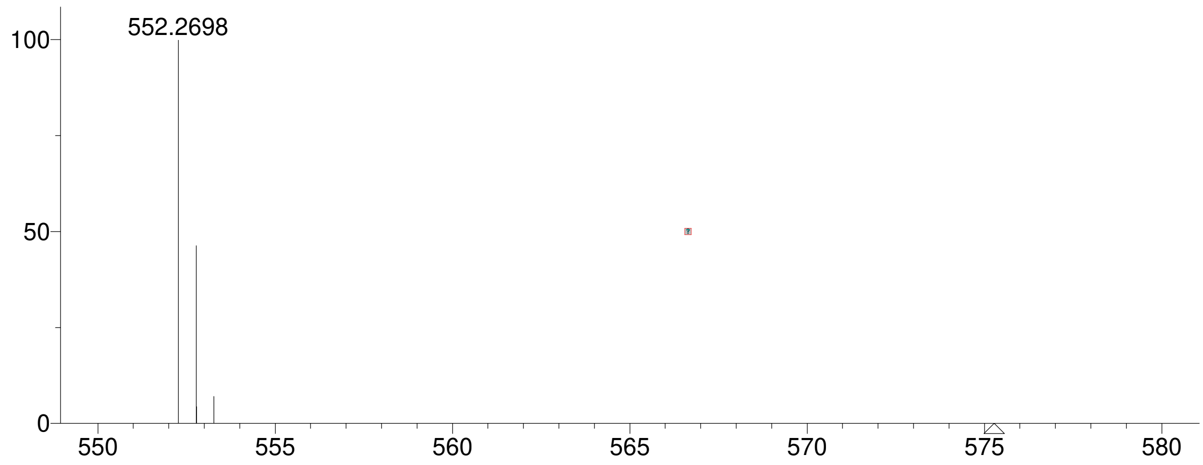

Unknown: Unknown (15.12_711.3673m/z)
Compound in Library Factor = 0

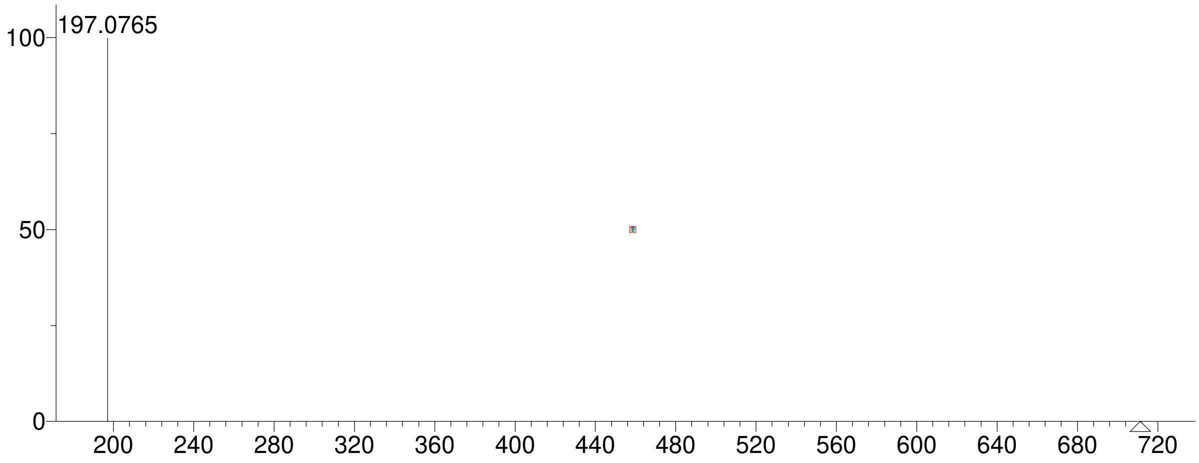

** Search Report Page 1 of 1 **

Unknown: Unknown (15.14_677.8063m/z)
Compound in Library Factor = 0

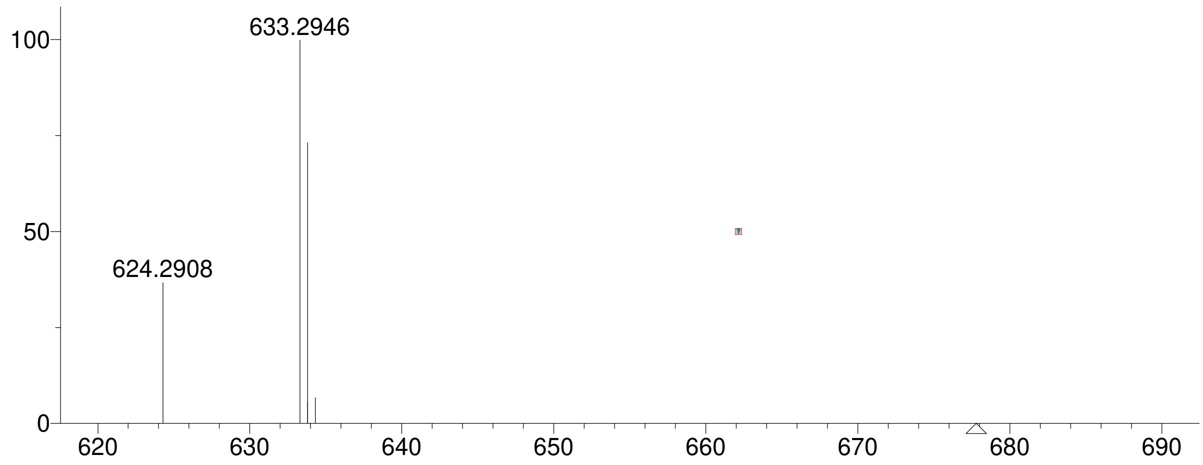

** Search Report Page 1 of 1 **

Unknown: Unknown (15.15_575.2709m/z)
Compound in Library Factor = 0

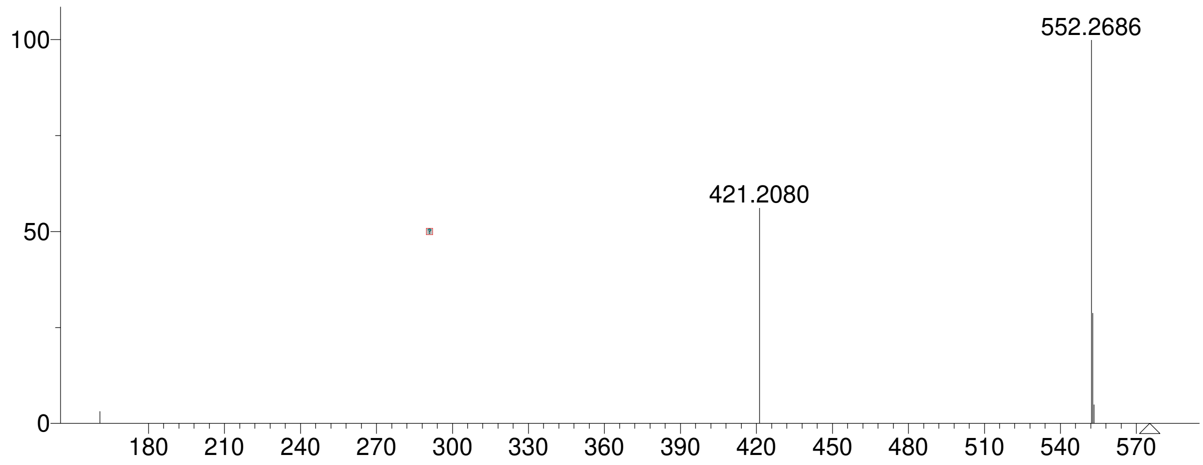

** Search Report Page 1 of 1 **

Unknown: Unknown (15.15_677.3046m/z)
Compound in Library Factor = 0

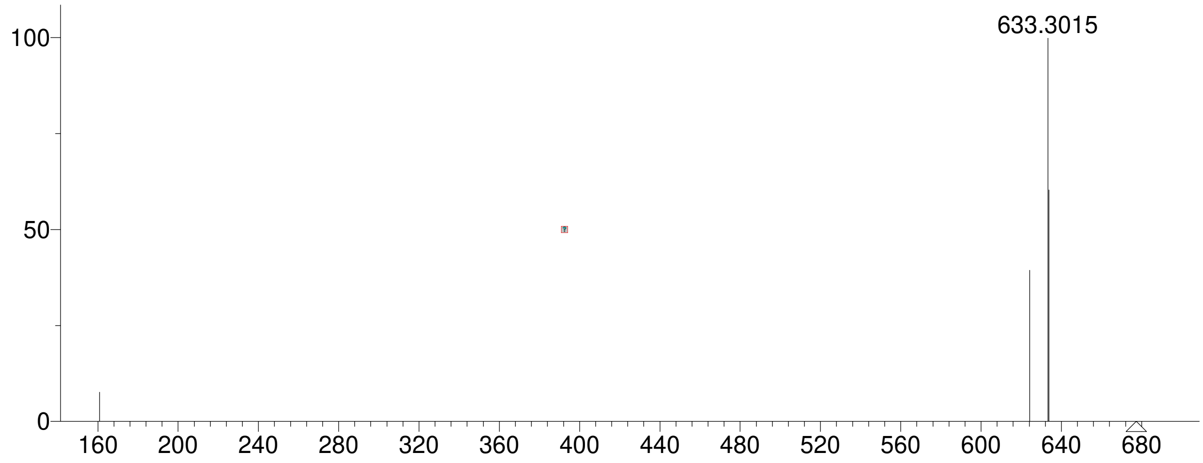

Unknown: Unknown (15.16_467.2124m/z)
Compound in Library Factor = -2176

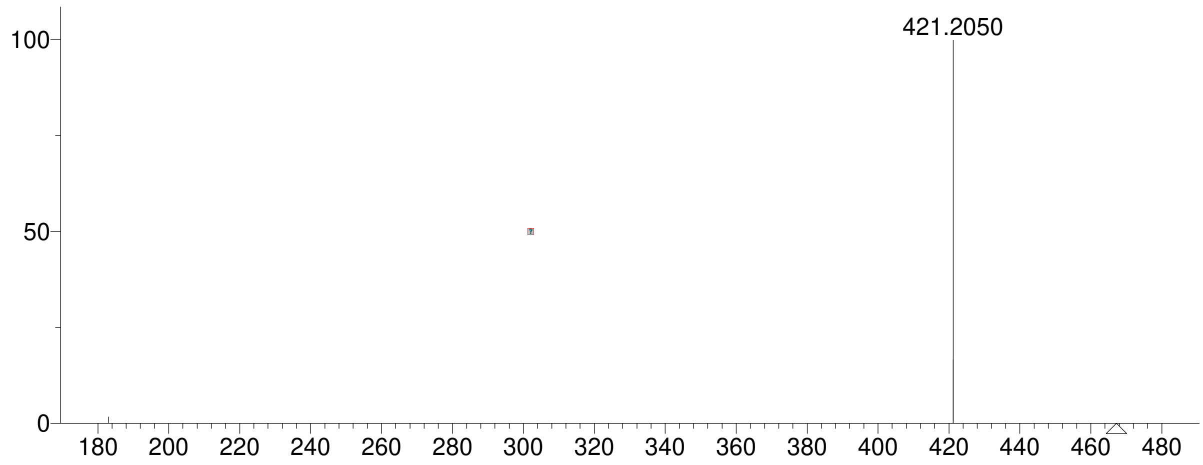

Hit 1 : NCGC00385558-01!(2R,3S,4S,5R,6R)-2-[[(2R,3R,4R)-3,4-dihydroxy-4-(hydroxymethyl)oxolan-2-yl]oxymet
C19H34O10; MF: 55; RMF: 740; Prob 46.9%; Lib: msms-neg-gnps; ID: 939.

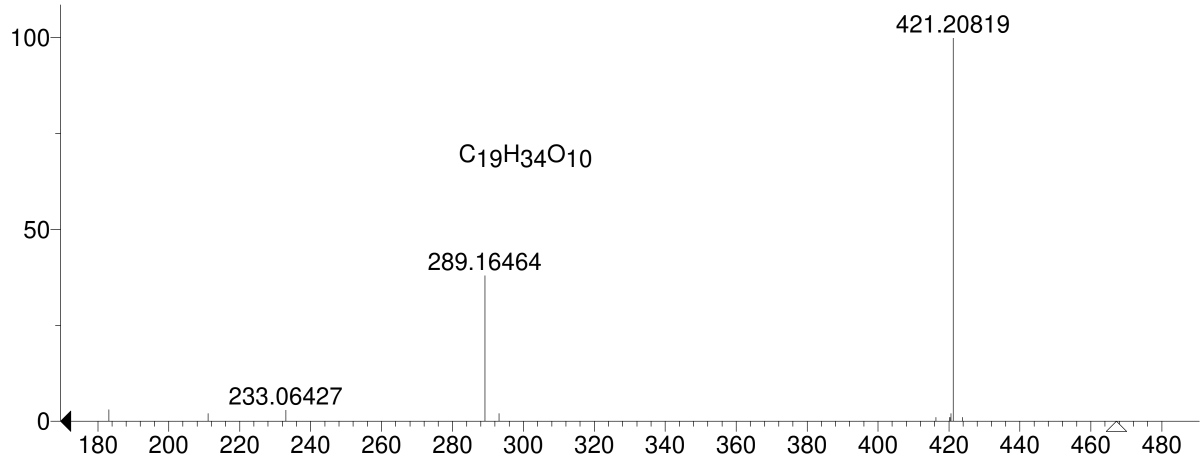

Hit 2 : MGDG 10:0; MGDG 5:0-5:0; [M+FA-H]- [M+FA-H]- P=467.2
C19H34O10; MF: 20; RMF: 411; Prob 11.8%; Lib: msdial-insilicomsms-lipids-neg; ID: 311444.

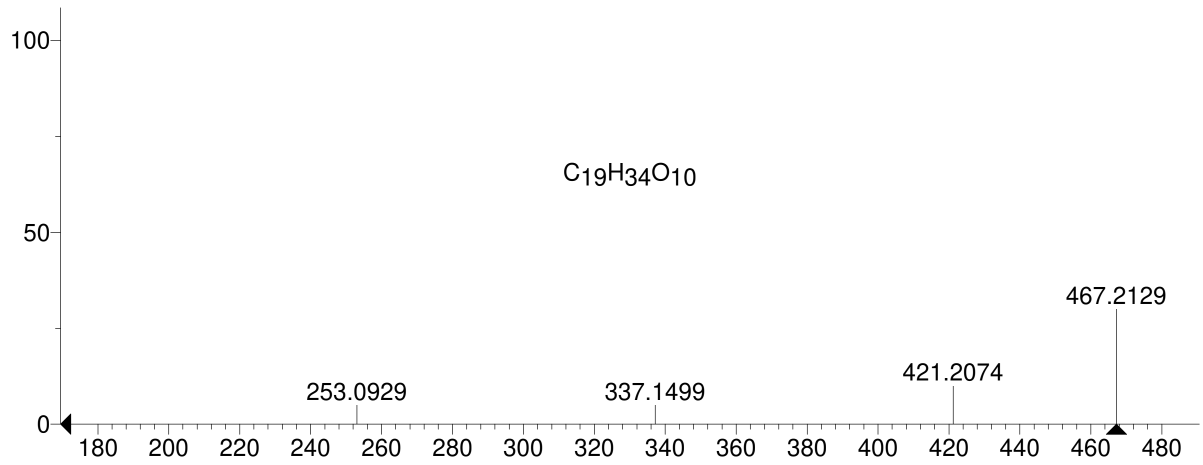

Hit 3 : NCGC00169122-02!2-oct-1-en-3-yloxy-6-[(3,4,5-trihydroxyoxan-2-yl)oxymethyl]oxane-3,4,5-triol [M+FA-H]
C19H34O10; MF: 19; RMF: 566; Prob 11.3%; Lib: mona-export-gnps; ID: 7249.

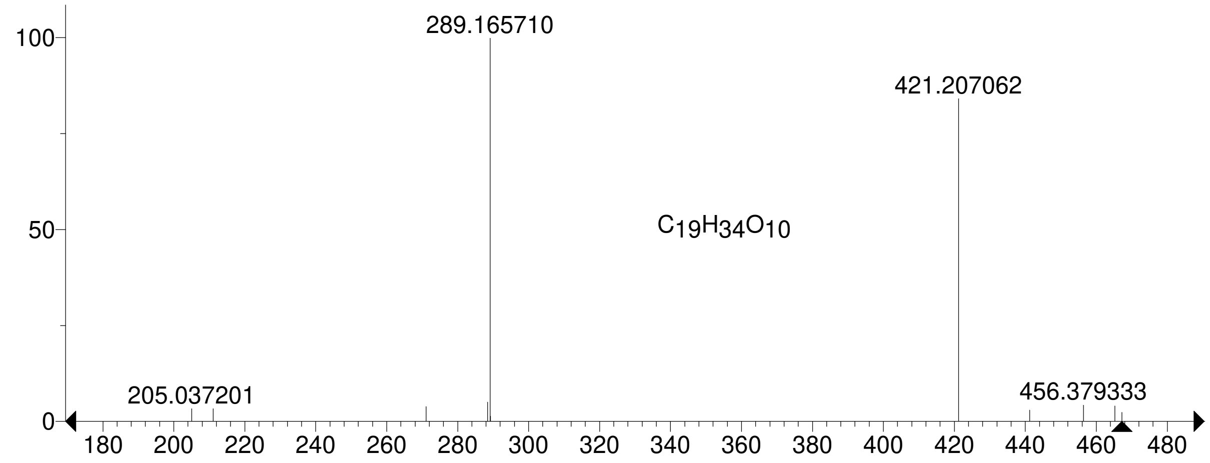

Hit 4 : MGDG 10:0; MGDG 2:0-8:0; [M+FA-H]- [M+FA-H]- P=467.2
C19H34O10; MF: 16; RMF: 392; Prob 9.99%; Lib: msdial-insilicomsms-lipids-neg; ID: 311270.

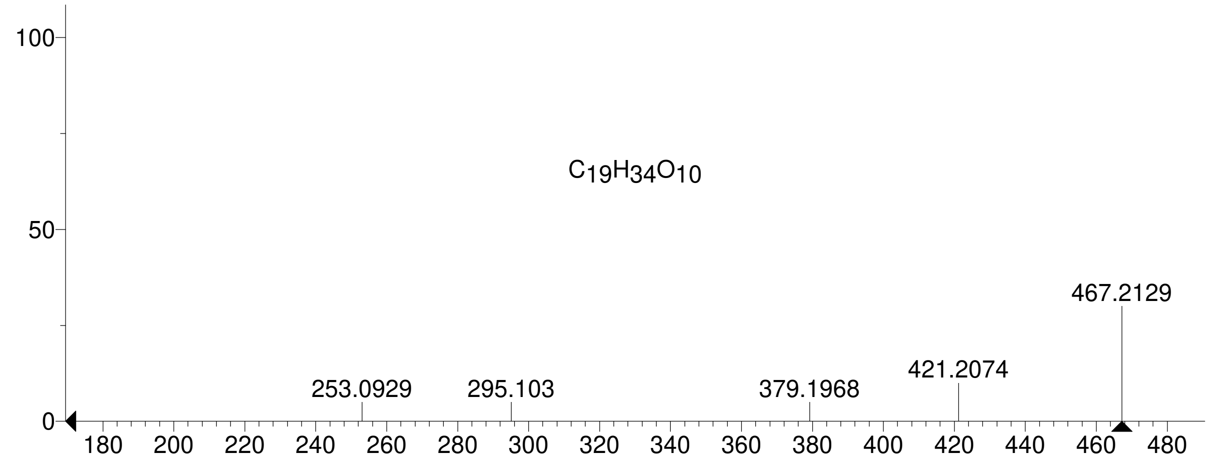

Hit 5 : MGDG 10:0; MGDG 3:0-7:0; [M+FA-H]- [M+FA-H]- P=467.2
C19H34O10; MF: 16; RMF: 392; Prob 9.99%; Lib: msdial-insilicomsms-lipids-neg; ID: 311329.

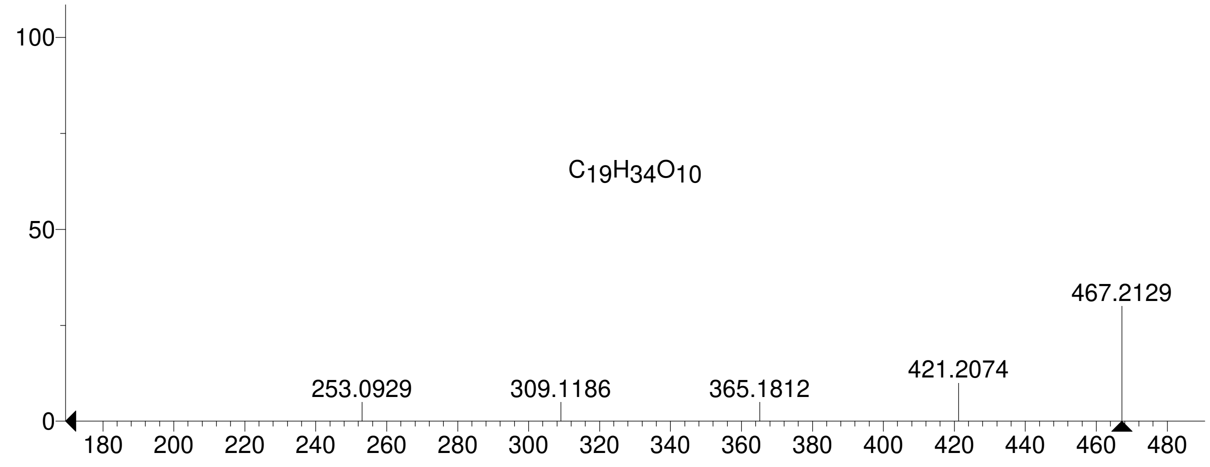

Unknown: Unknown (15.19_351.1616m/z)
Compound in Library Factor = -885

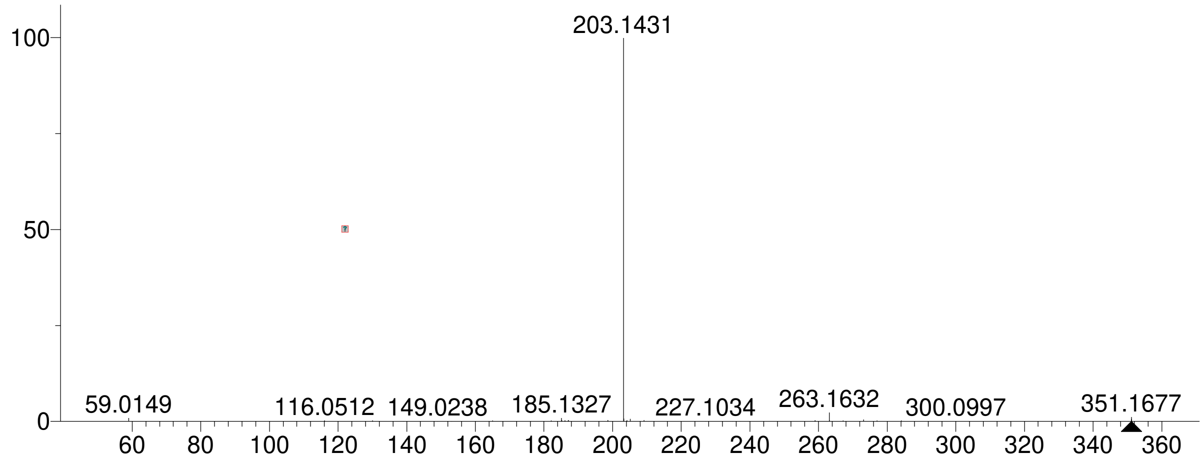

Hit 1 : Dienestrol diacetate [M+H]+ HCD 3V P=351.2
C22H22O4; MF: 88; RMF: 659; Prob 73.3%; CAS: 84-19-5; Lib: nist_msms; ID: 284291.

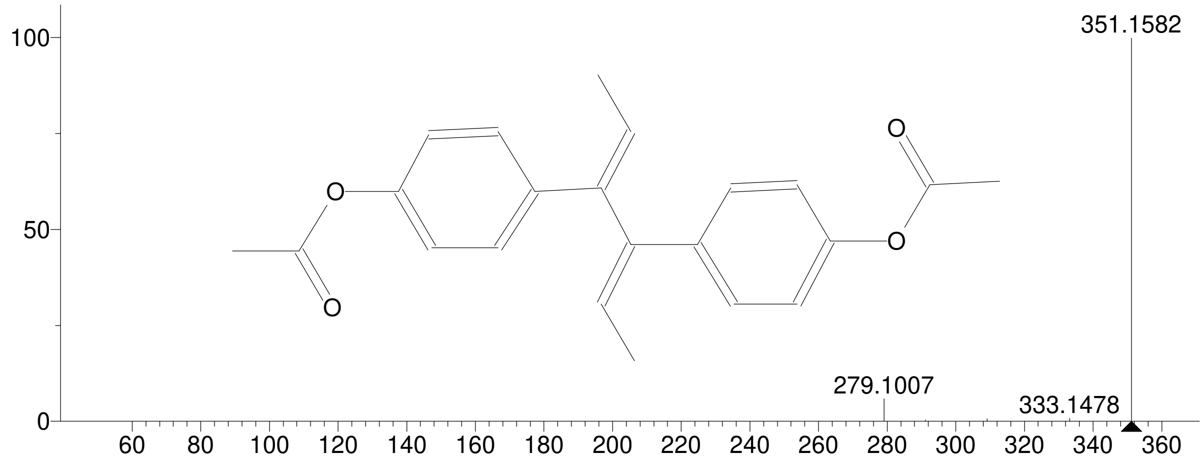

Hit 2 : 3-Dehydroepiandrosterone sulfate [M+H-H2O]+ HCD 14V P=351.2
C19H28O5S; MF: 8; RMF: 83; Prob 8.06%; CAS: 651-48-9; Lib: nist_msms; ID: 102169.

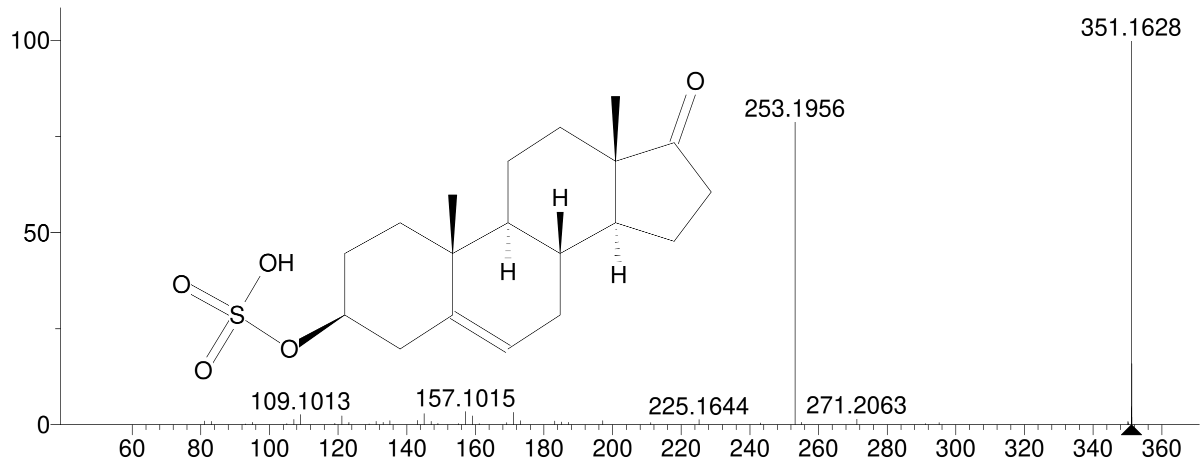

Hit 3 : 16-Phenoxytetranorprostaglandin A2 [M-H-H2O]- HCD 15V P=351.2
C22H26O5; MF: 5; RMF: 85; Prob 7.12%; CAS: 51639-10-2; Lib: nist_msms; ID: 319582.

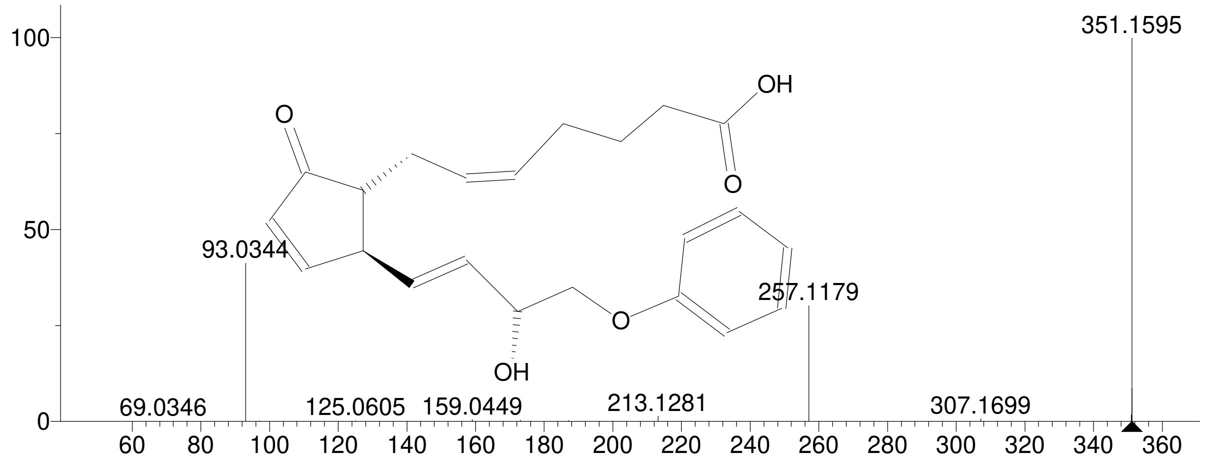

Hit 4 : (2-acetyloxy-3,6-diphenylcyclohexyl) acetate [M-H]- 35HCD P=351.2
C22H24O4; MF: 0; RMF: 15; Prob 5.74%; Lib: mona-export-lc-ms-ms_negative_mode; ID: 22883.

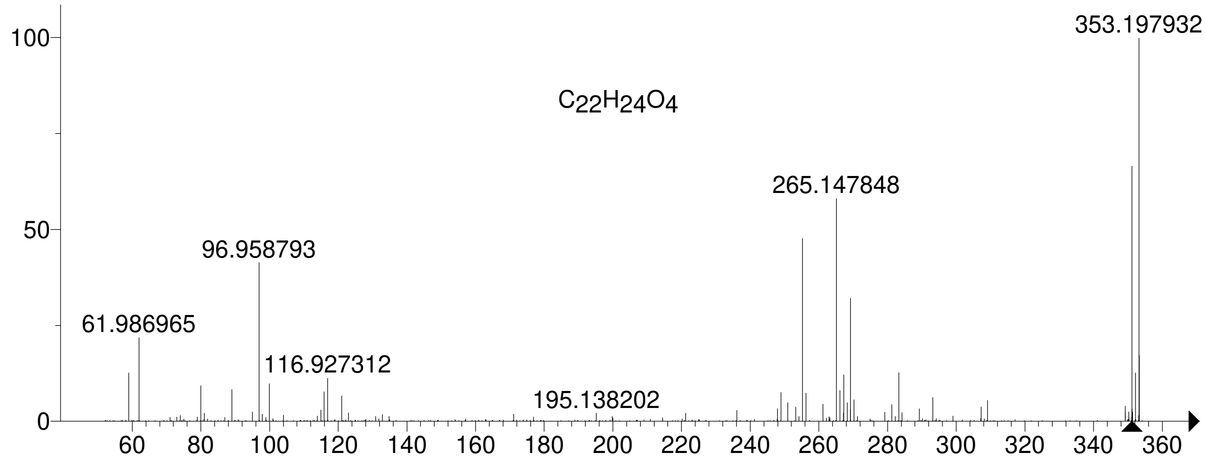

Hit 5 : candidone [M-H]- 35HCD P=351.2
C22H24O4; MF: 0; RMF: 10; Prob 5.74%; Lib: mona-export-lc-ms-ms_negative_mode; ID: 18584.

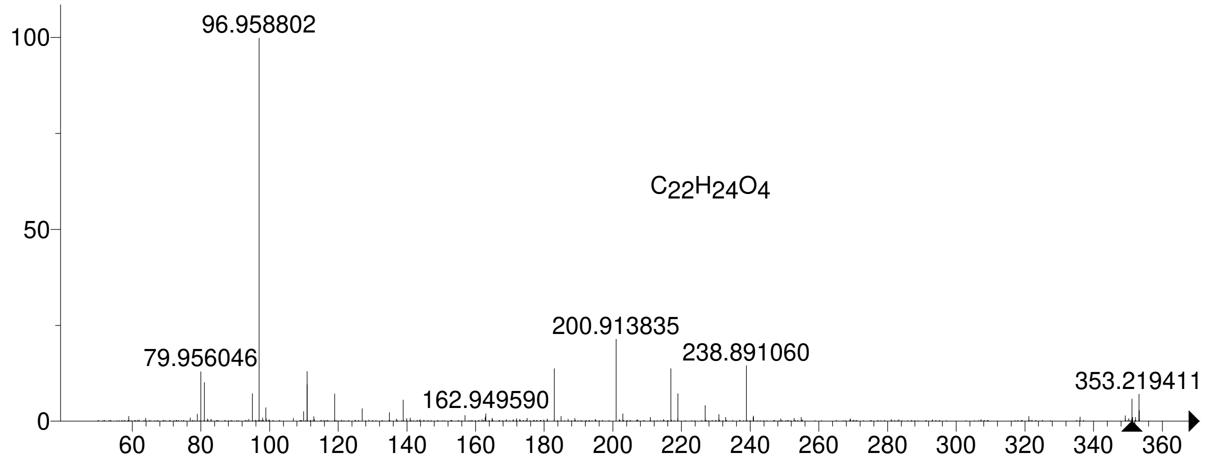

Unknown: Unknown (15.26_238.1075m/z)
Compound in Library Factor = 0

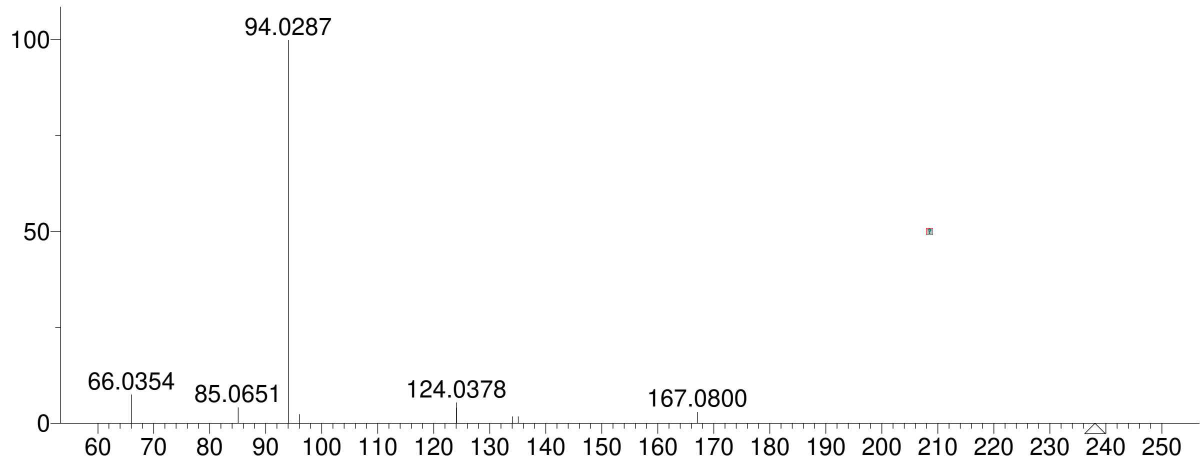

Hit 1 : Sildenafil [M+2H]2+ HCD 77V P=238.1
C22H30N6O4S; MF: 0; RMF: 61; Prob 20.0%; CAS: 139755-83-2; Lib: nist_msms; ID: 135220.

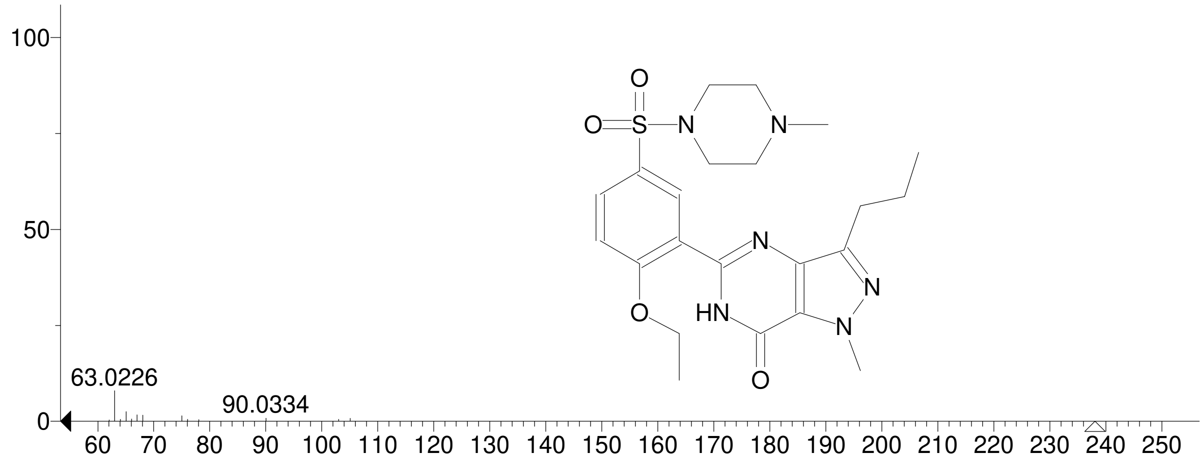

Hit 2 : 5-Methoxymethylone [M+H]+ HCD 42V P=238.1
C12H15NO4; MF: 0; RMF: 18; Prob 20.0%; Lib: nist_msms; ID: 547136.

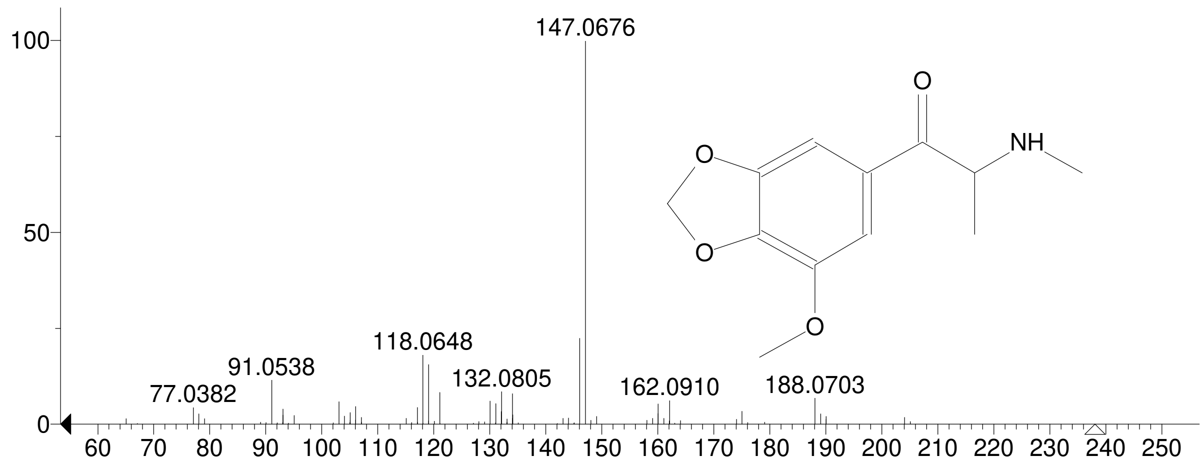

Hit 3 : NCGC00384987-01_C12H15NO4_N-Acetyl-O-methyltyrosine [M+H]+ P=238.1
C12H15NO4; MF: 0; RMF: 11; Prob 20.0%; Lib: mona-export-gnps; ID: 10703.

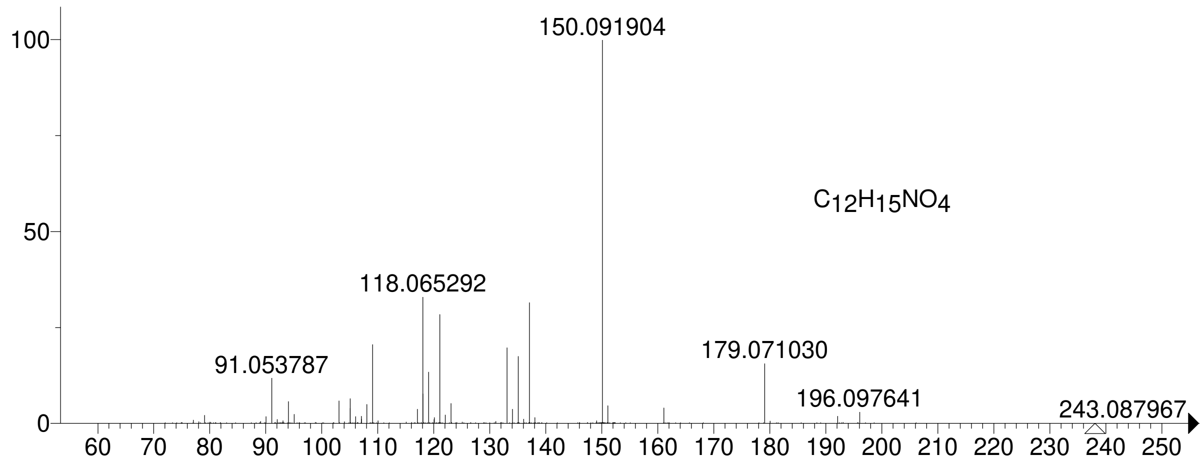

Hit 4 : Pesticide3_3-Hydroxycarbofuran_C12H15NO4_3,7-Benzofurandiol, 2,3-dihydro-2,2-dimethyl-, 7-(methylca
C12H15NO4; MF: 0; RMF: 5; Prob 20.0%; Lib: mona-export-gnps; ID: 13869.

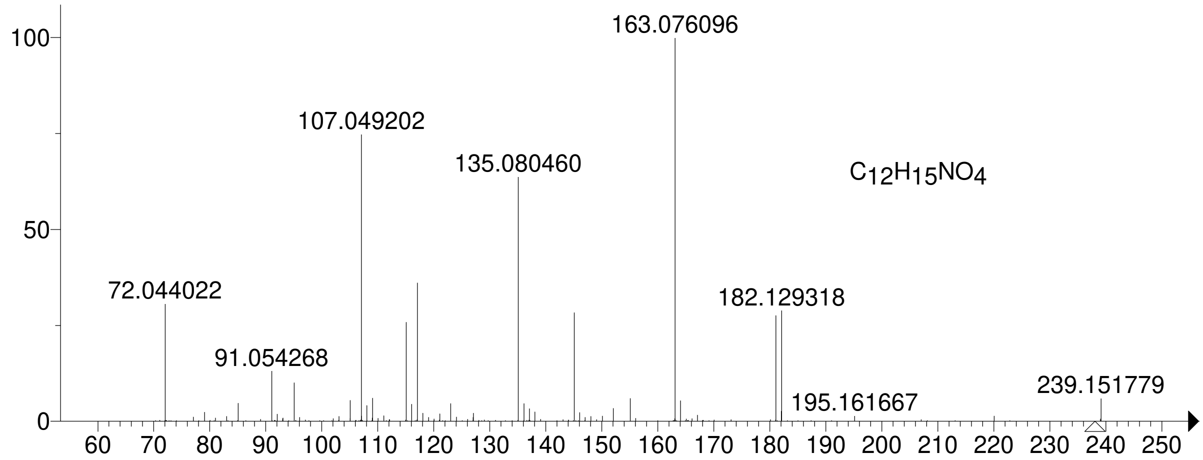

Hit 5 : N-acetyl-O-methyltyrosine [M+H]+ 45HCD P=238.1
C12H15NO4; MF: 0; RMF: 5; Prob 20.0%; Lib: mona-export-vf-npl_qexactive; ID: 1169.

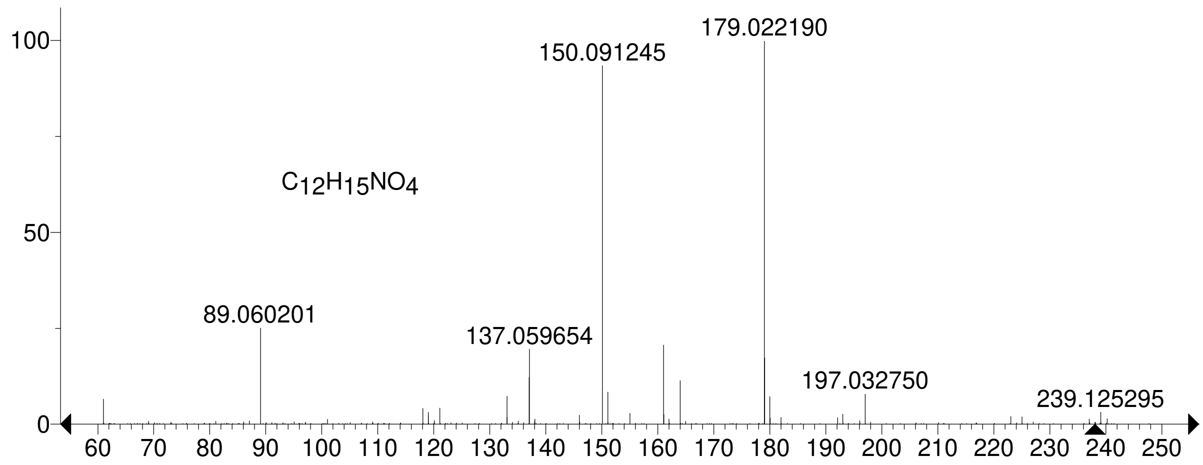

Unknown: Unknown (15.27_325.1286m/z)
Compound in Library Factor = -3273

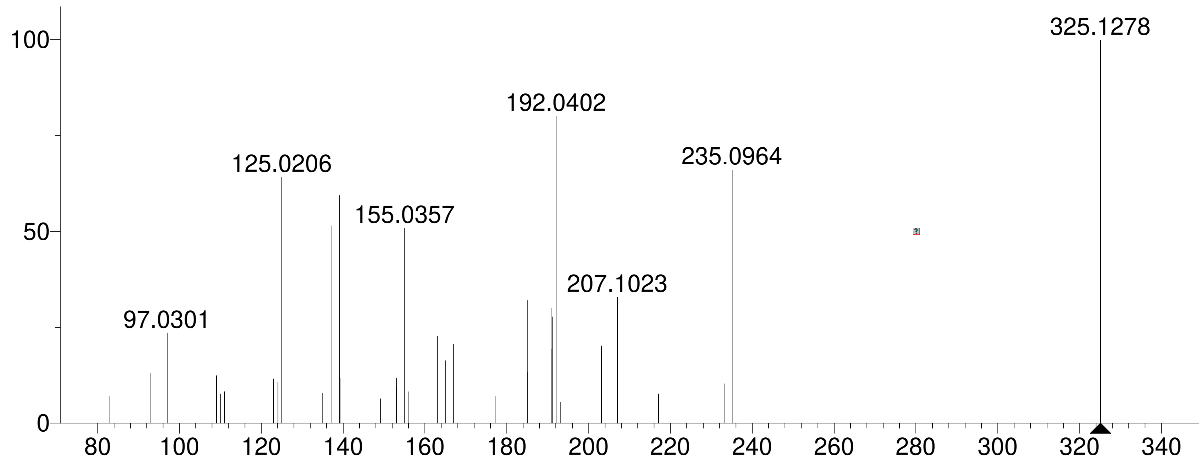

Hit 1 : Uridine 5_-monophosphate [M+H]+ P=325.1
C9H13N2O9P; MF: 9; RMF: 174; Prob 57.9%; Lib: mona-export-gnps; ID: 5232.

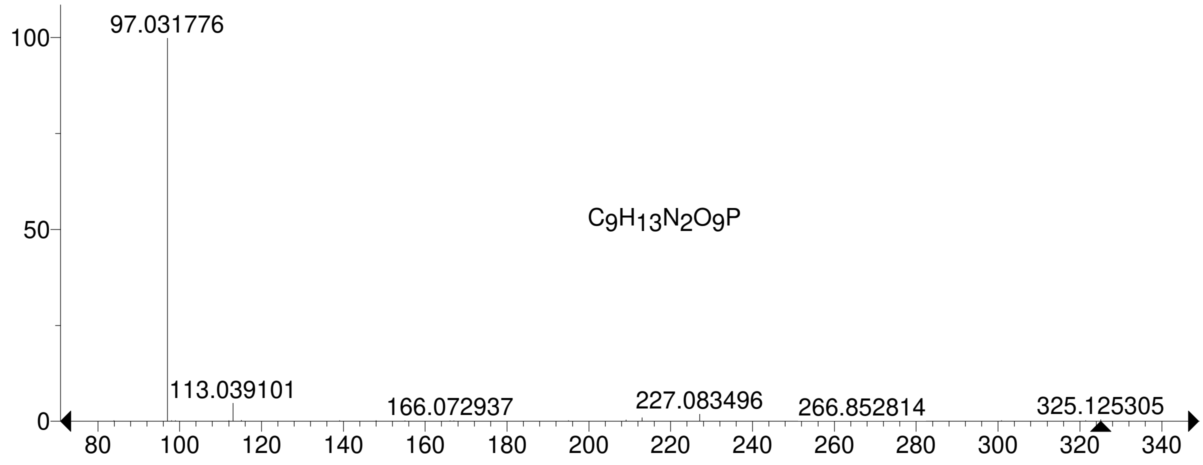

Hit 2 : [(4E)-7-acetyloxy-6-hydroxy-2-methyl-10-oxo-2,3,6,7,8,9-hexahydrooxecin-3-yl] (E)-but-2-enoate [M-H]- 3
C16H22O7; MF: 0; RMF: 1; Prob 42.1%; Lib: mona-export-lc-ms-ms_negative_mode; ID: 17951.

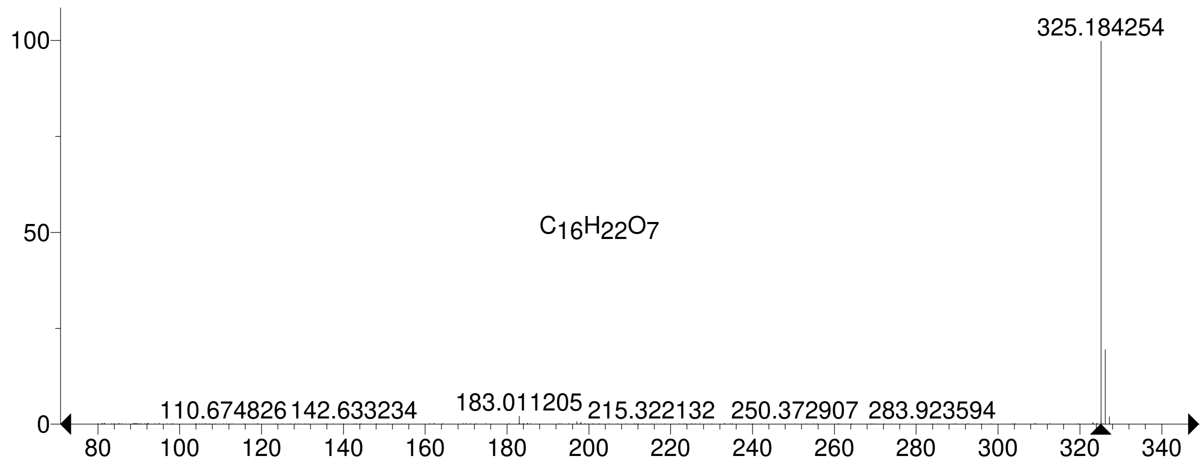

Unknown: Unknown (15.27_405.0488m/z)
Compound in Library Factor = 0

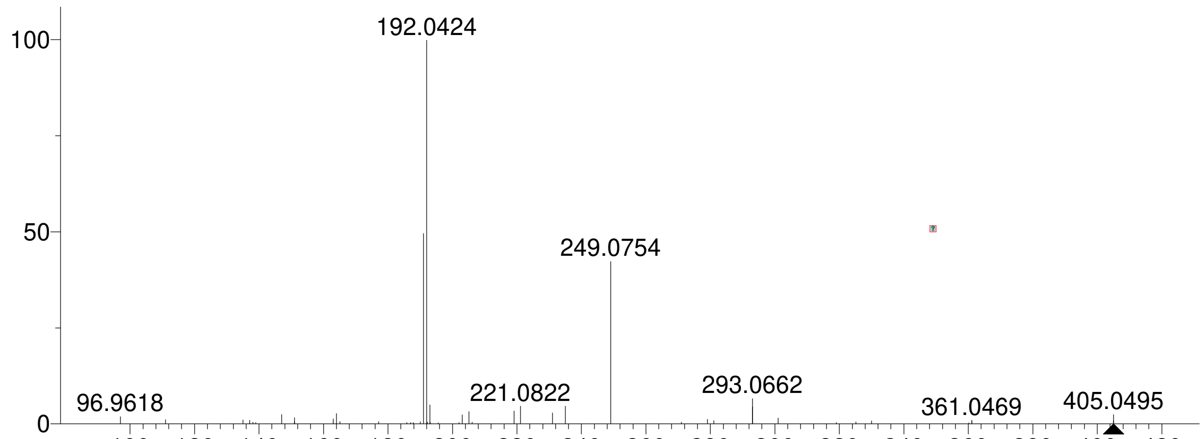

Hit 1 : [(6E,10Z)-10-(acetyloxymethyl)-6-formyl-3-methylidene-2-oxo-3a,4,5,8,9,11a-hexahydrocyclodeca[b]furan-
C22H28O7; MF: 0; RMF: 23; Prob 100.0%; Lib: mona-export-vf-npl_ltq; ID: 4842.

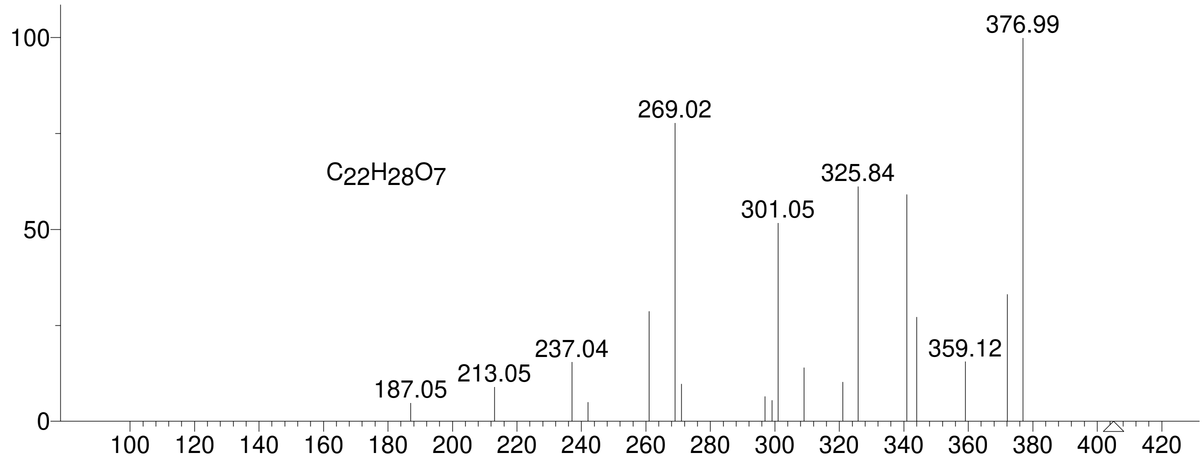

Unknown: Unknown (15.29_560.2671m/z)
Compound in Library Factor = 0

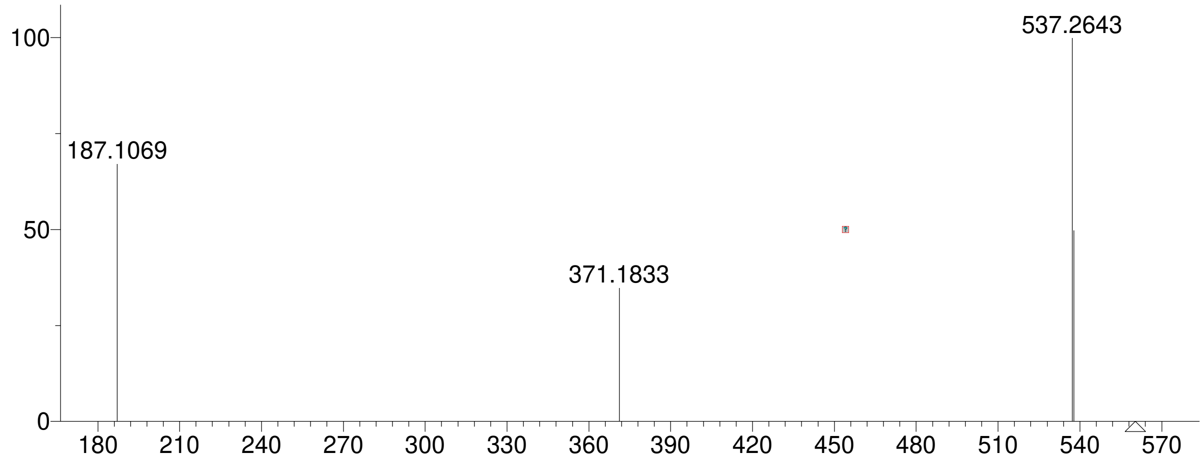

Unknown: Unknown (15.30_541.2398m/z)
Compound in Library Factor = -3273

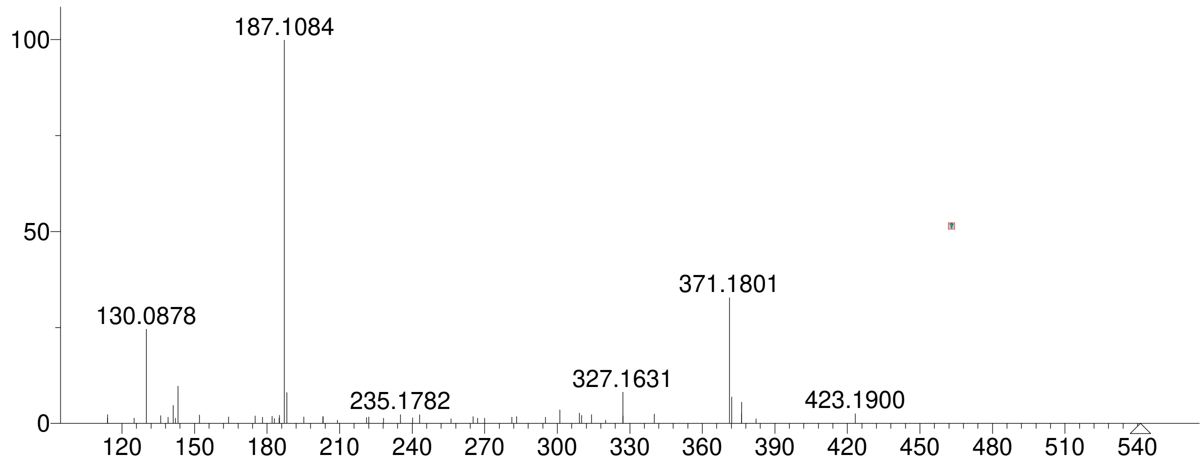

Hit 1 : methyl (1S,2R,4R,8R,9S,10R,13R,15R)-2-(acetyloxy)-13-(furan-3-yl)-9-(2-methoxy-2-oxoethyl)-4,8,10,12-t
C30H36O9; MF: 4; RMF: 171; Prob 27.0%; Lib: mona-export-vf-npl_qexactive; ID: 20632.

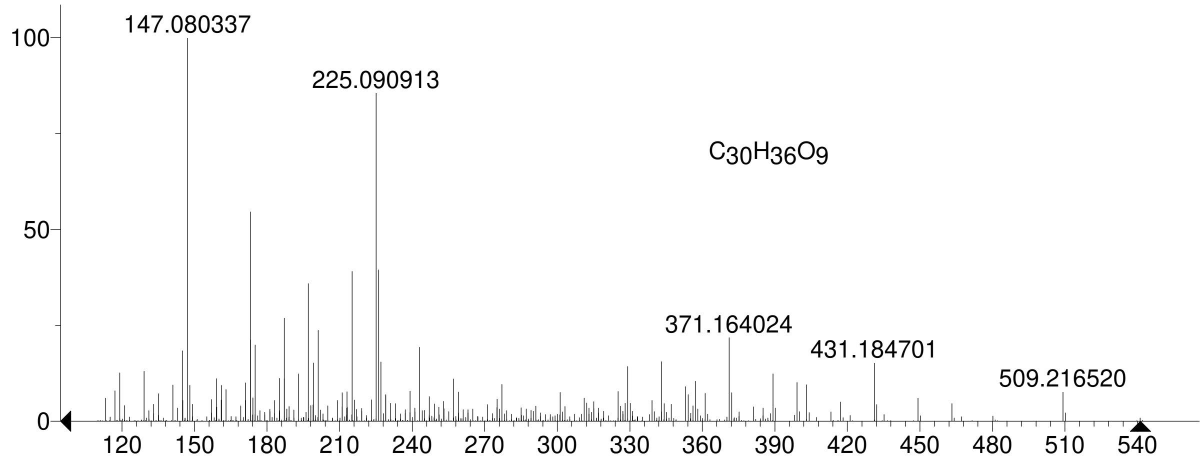

Hit 2 : NCGC00169906-03_C30H36O9_2H-Cyclopenta[b]naphtho[2,3-d]furan-10-acetic acid, 5-(acetyloxy)-2-(3-f
C30H36O9; MF: 3; RMF: 139; Prob 26.0%; Lib: mona-export-gnps; ID: 13758.

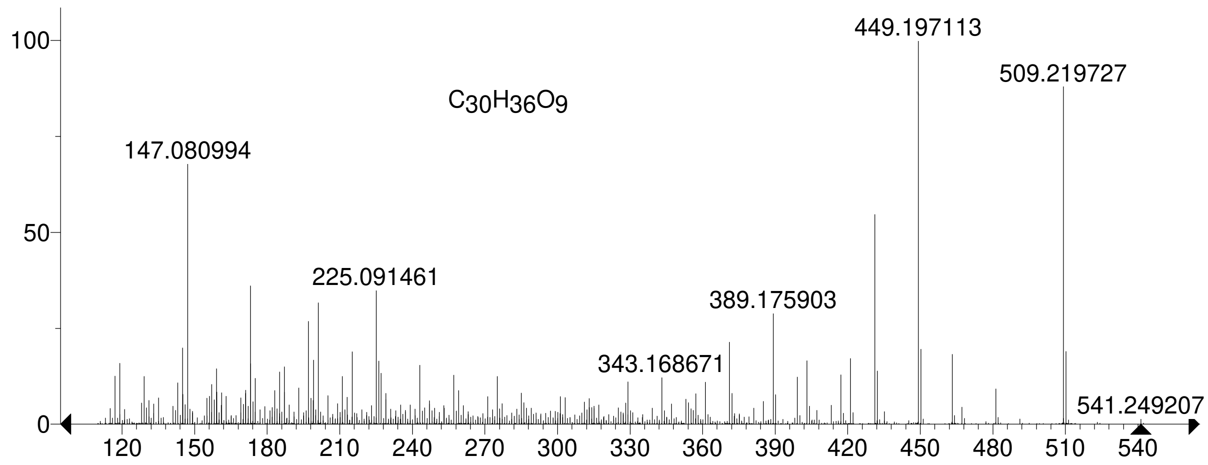

Hit 3 : 7-Deacetoxy-7-Oxokhivorin [M-H]- 35HCD P=541.2
C30H38O9; MF: 1; RMF: 60; Prob 24.0%; Lib: mona-export-lc-ms-ms_negative_mode; ID: 20717.

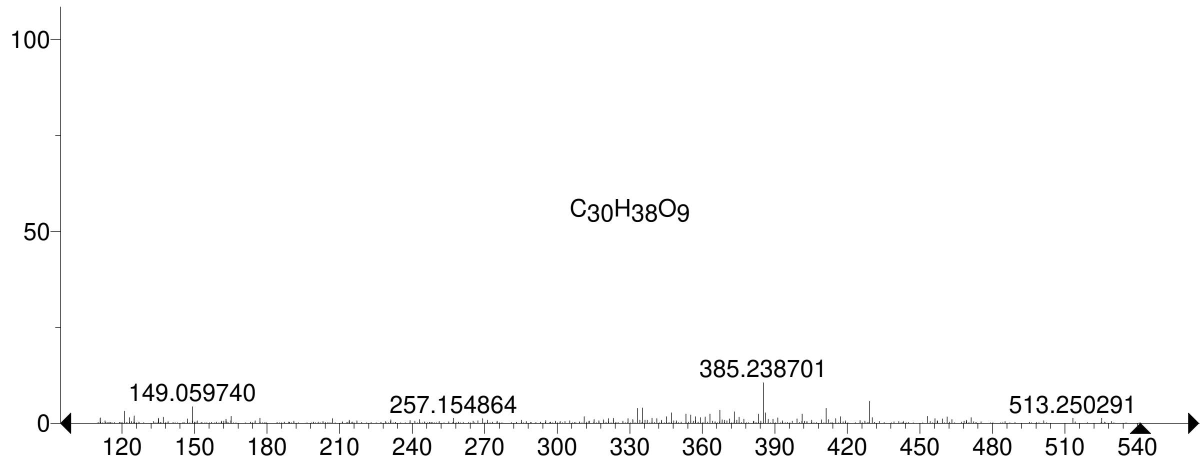

Hit 4 : Nimbin [M+H]+ IT 35% P=541.2
C30H36O9; MF: 0; RMF: 65; Prob 23.0%; CAS: 5945-86-8; Lib: nist_msms; ID: 226918.

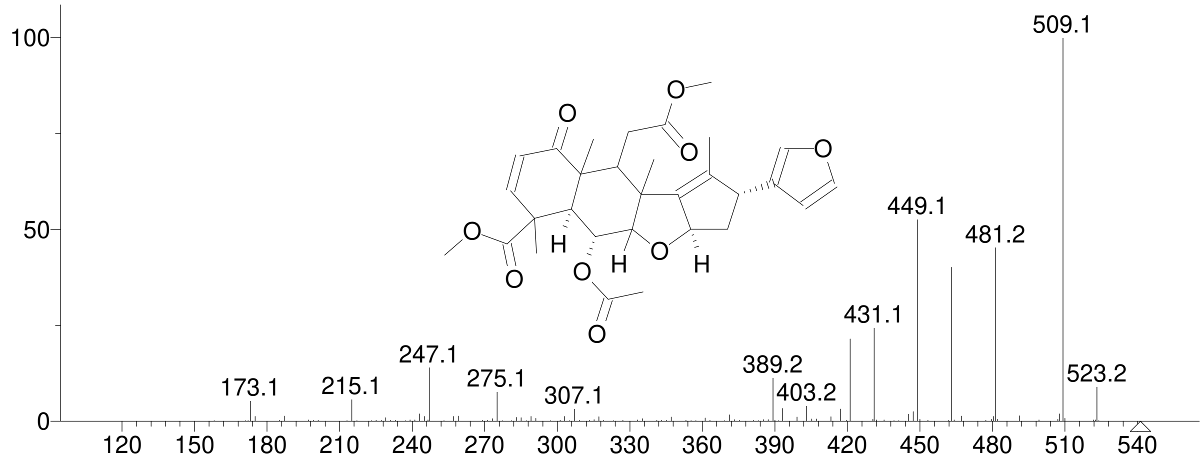

** Search Report Page 1 of 1 **

Unknown: Unknown (15.32_677.3037m/z)
Compound in Library Factor = 0

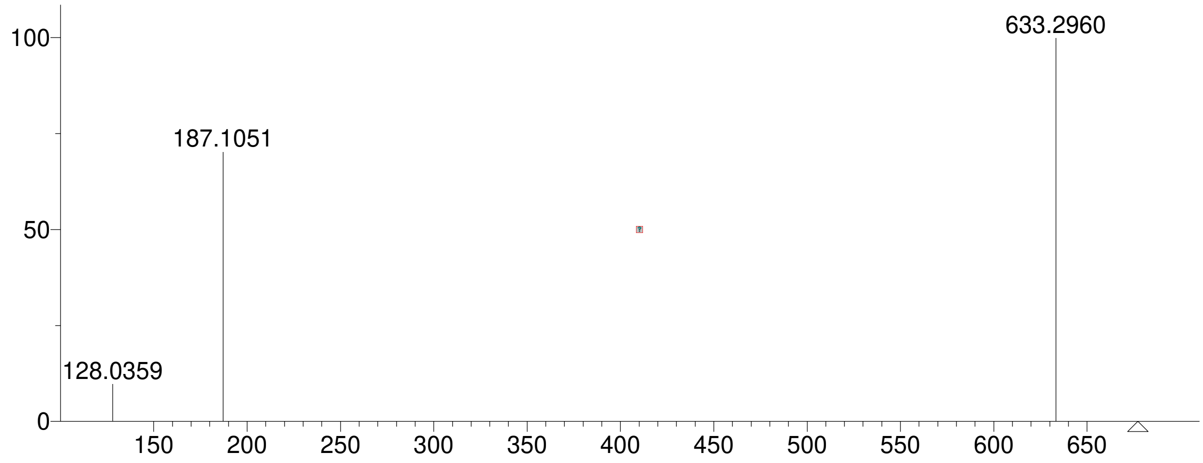

Unknown: Unknown (15.34_394.1860m/z)
Compound in Library Factor = -2387

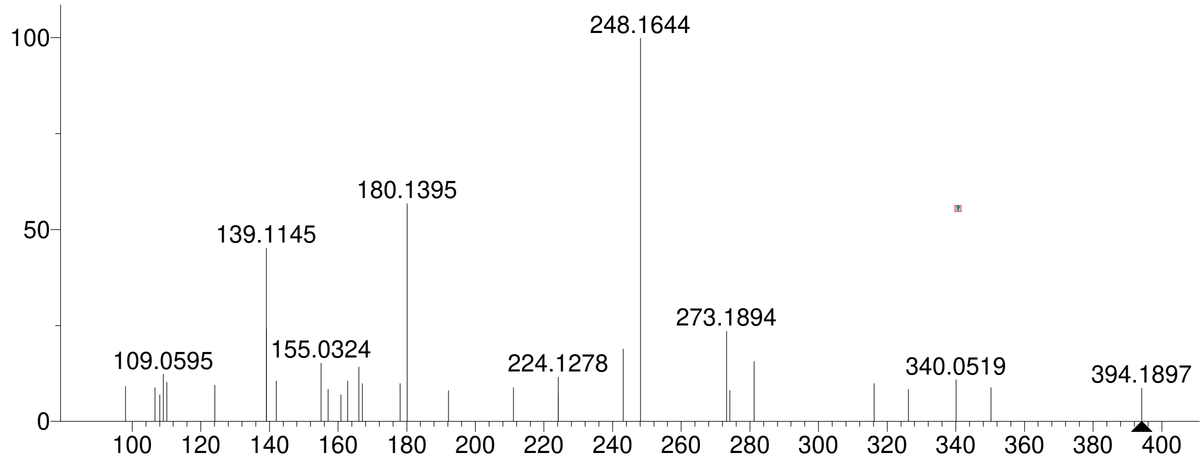

Hit 1 : 3',4',5'-Trimethoxycocaine [M+H]+ HCD 15V P=394.2
C20H27NO7; MF: 18; RMF: 177; Prob 14.7%; CAS: 156301-59-6; Lib: nist_msms; ID: 383469.

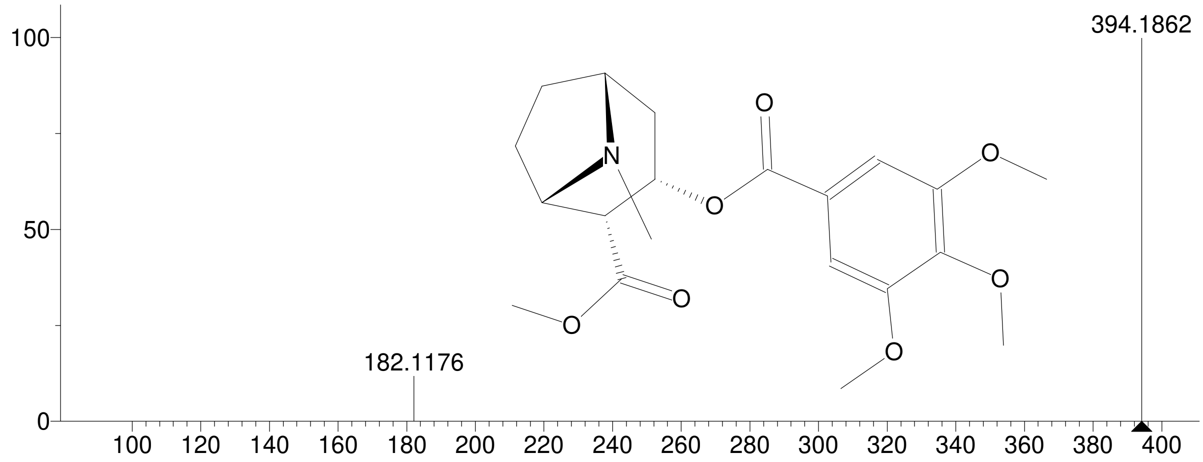

Hit 2 : [1,3-dihydroxy-1-(7-methoxy-2-oxochromen-6-yl)-3-methylbutan-2-yl] 3-methylbut-2-enoate [M+NH4]+ 35
C20H24O7; MF: 1; RMF: 60; Prob 8.03%; Lib: mona-export-vf-npl_qexactive; ID: 24781.

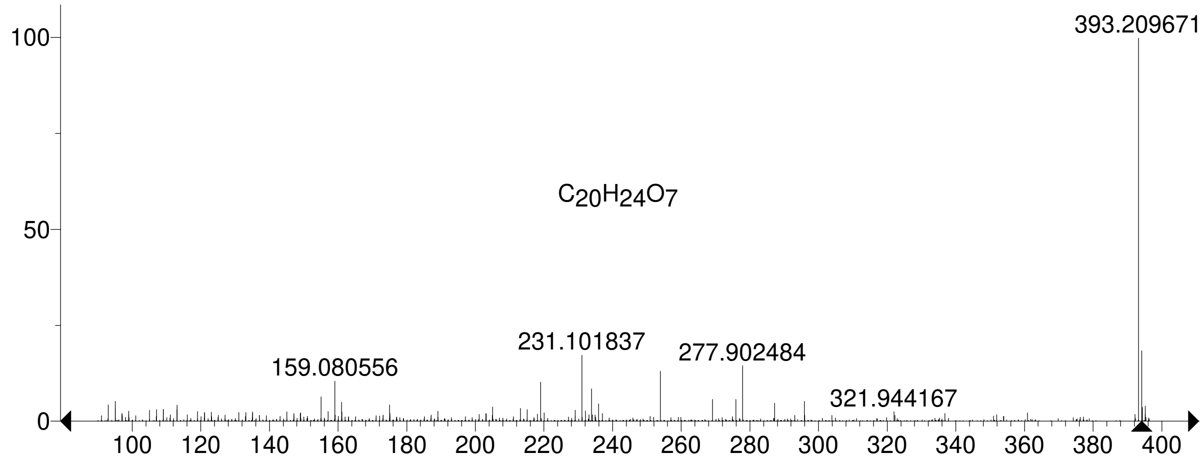

Hit 3 : rac-N,N-Didesmethyl-O-desmethylvenlafaxine glucuronide [M+H-H2O]+ HCD 11V P=394.2
C20H29NO8; MF: 0; RMF: 106; Prob 7.72%; CAS: 1799830-07-1; Lib: nist_msms; ID: 436197.

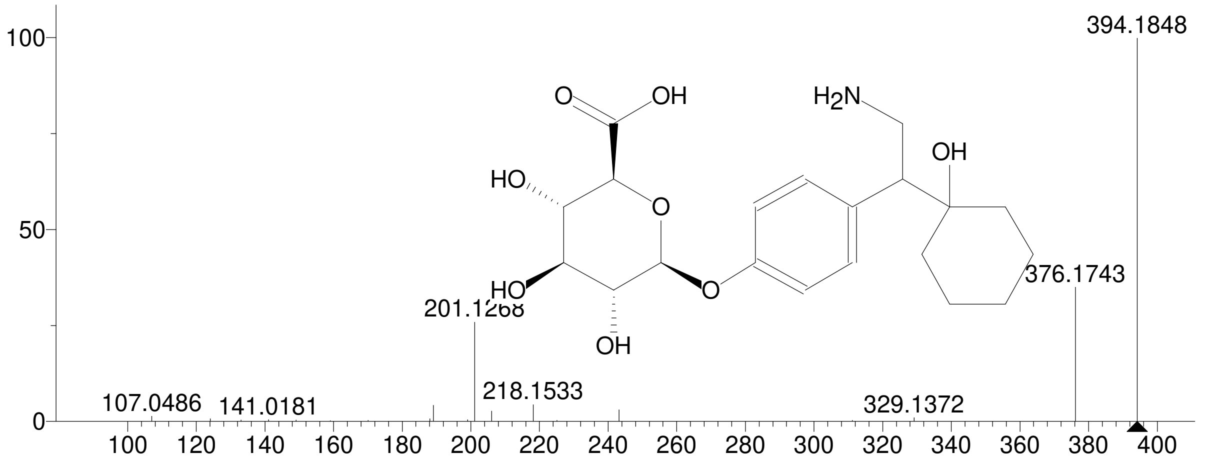

Hit 4 : NCGC00381074-01![1,3-dihydroxy-1-(7-methoxy-2-oxochromen-6-yl)-3-methylbutan-2-yl] 3-methylbut-2-er
C20H24O7; MF: 0; RMF: 44; Prob 7.72%; Lib: mona-export-gnps; ID: 8644.

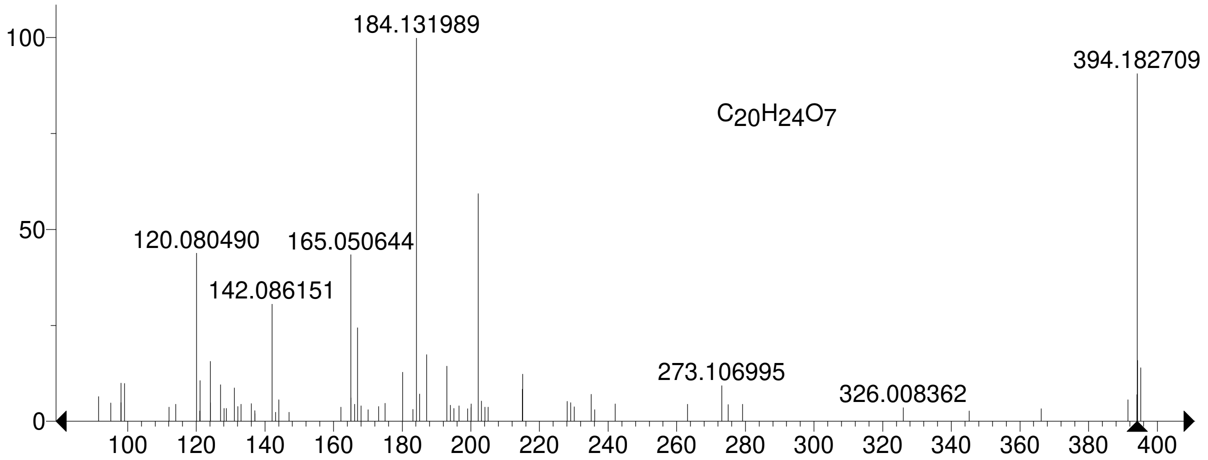

Hit 5 : 4-[4-[hydroxy-(4-hydroxy-3-methoxyphenyl)methyl]-3-(hydroxymethyl)oxolan-2-yl]-2-methoxyphenol [M+NH
C20H24O7; MF: 0; RMF: 42; Prob 7.72%; Lib: mona-export-vf-npl_qexactive; ID: 7279.

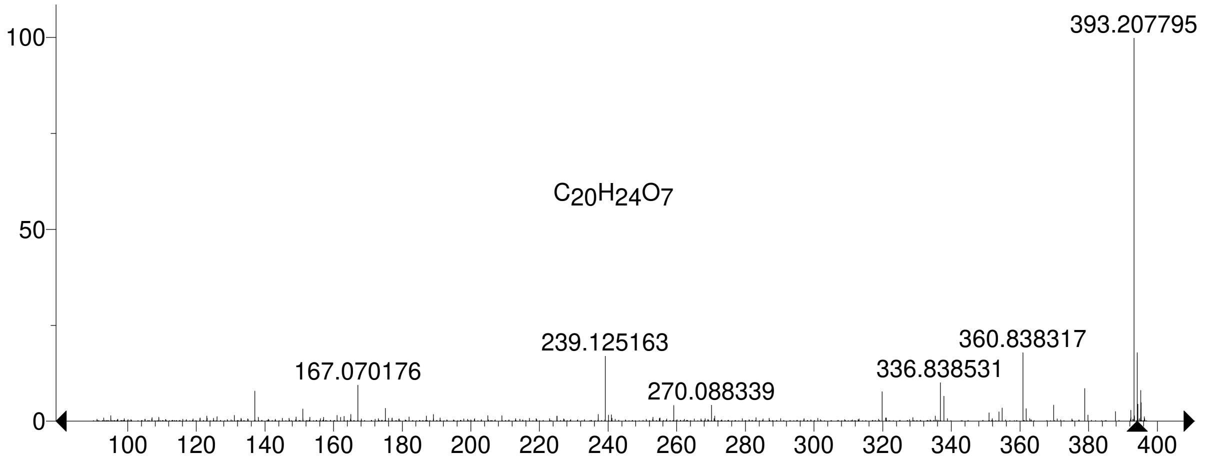

Unknown: Unknown (15.38_610.3200m/z)
Compound in Library Factor = 0

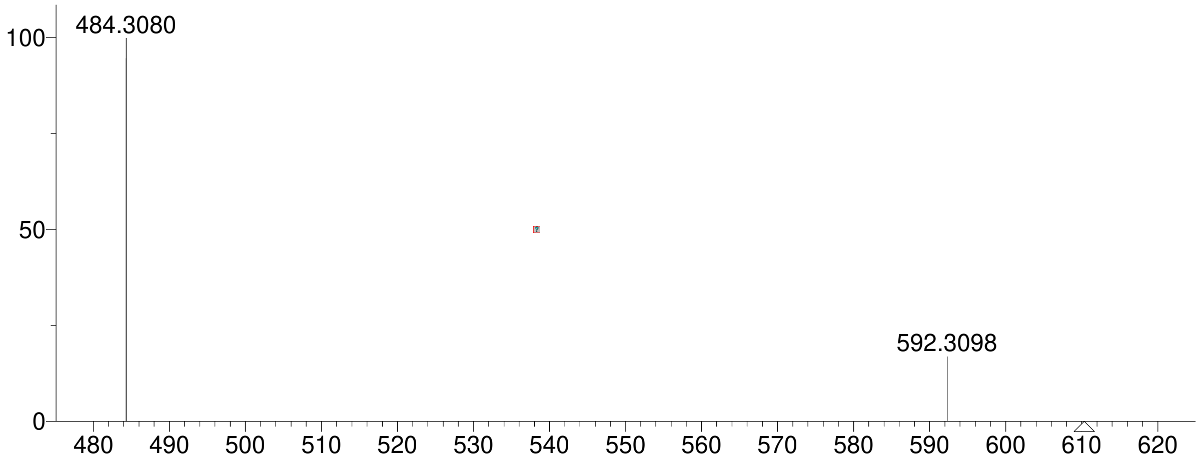

** Search Report Page 1 of 1 **

Unknown: Unknown (15.42_654.3022m/z)
Compound in Library Factor = -3273

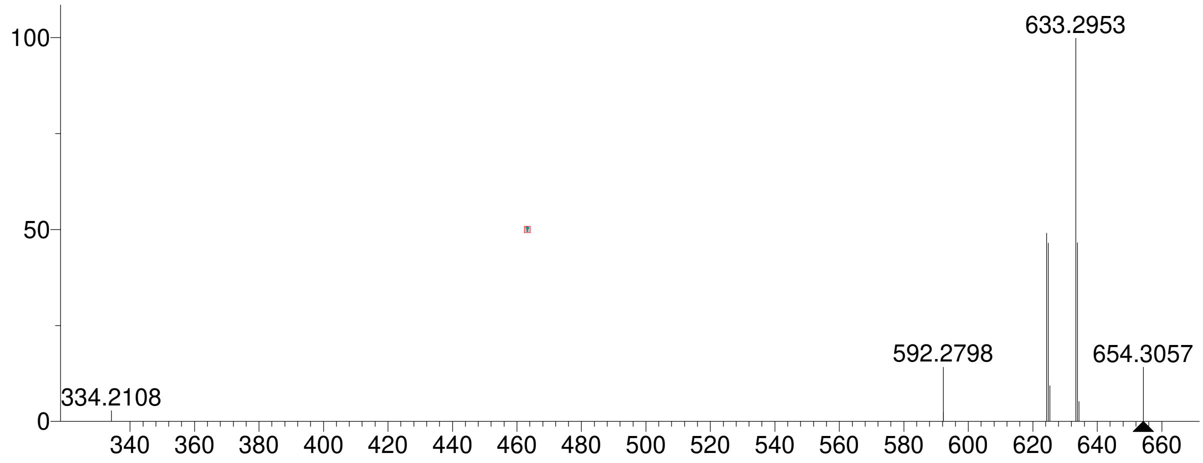

Hit 1 : [DTrp6]-LH-RH, amide [M-2H]2- HCD 20eV P=654.3
C64H82N18O13; MF: 4; RMF: 162; Prob 100.0%; Lib: nist_msms2; ID: 80487.

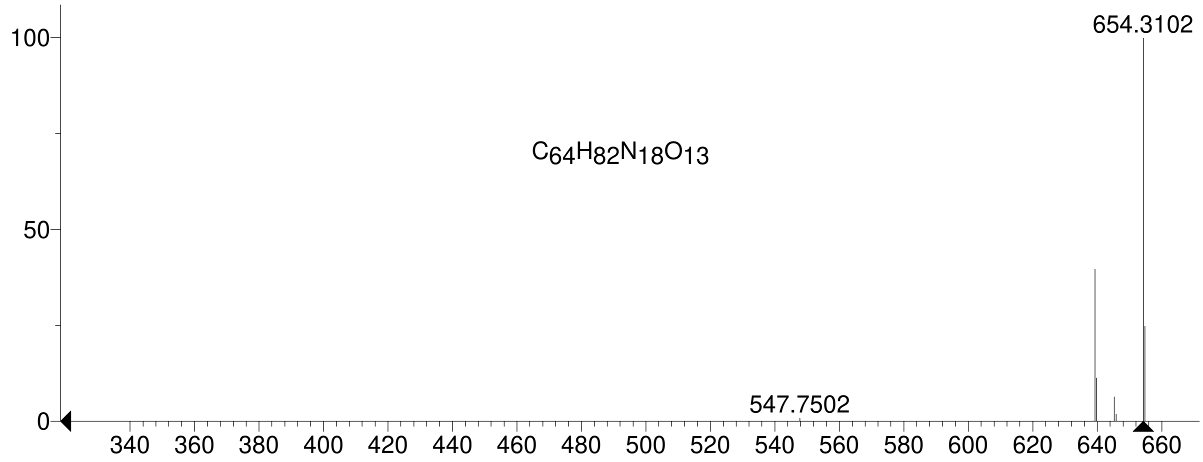

Unknown: Unknown (15.43_592.2858m/z)
Compound in Library Factor = -1309

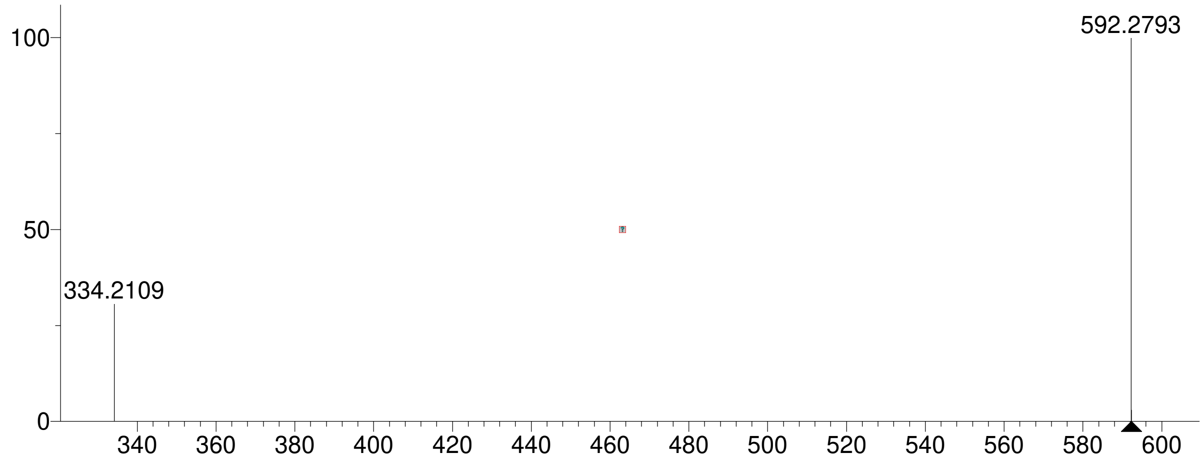

Hit 1 : LH-RH, free acid [M+2H]2+ QTOF 7V P=592.3
C55H74N16O14; MF: 56; RMF: 697; Prob 100.0%; Lib: nist_msms2; ID: 38405.

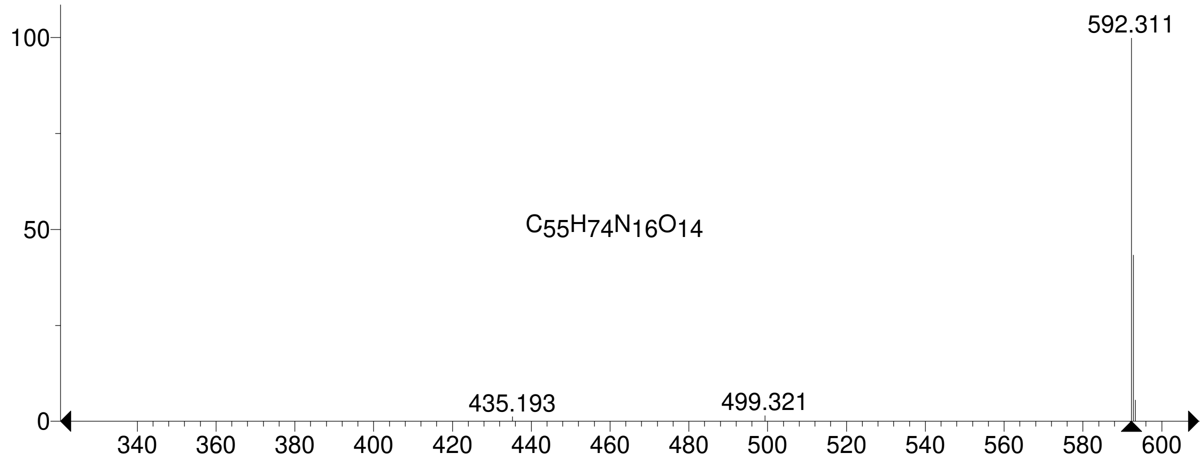

Unknown: Unknown (15.44_677.3040m/z)
Compound in Library Factor = 0

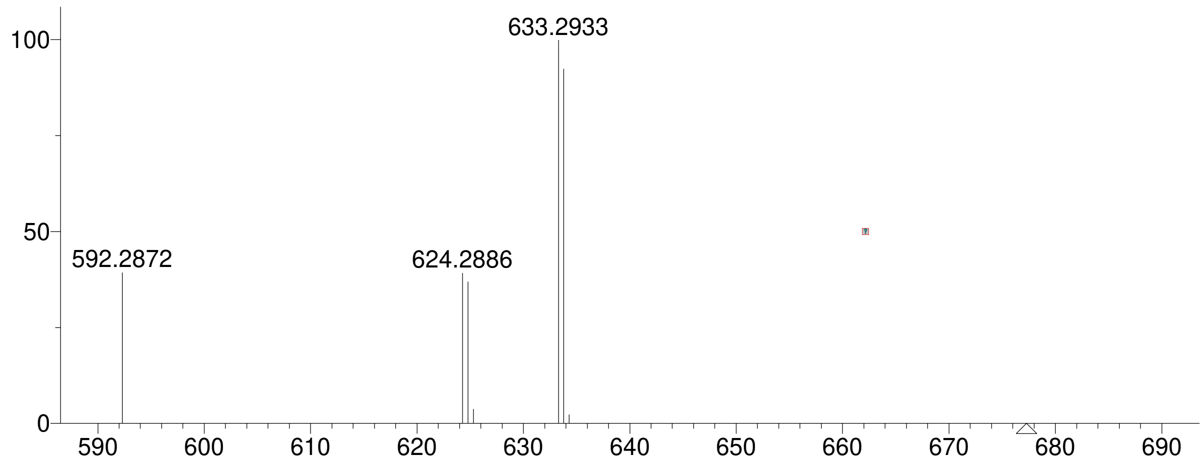

** Search Report Page 1 of 1 **

Unknown: Unknown (15.44_677.8047m/z)
Compound in Library Factor = 0

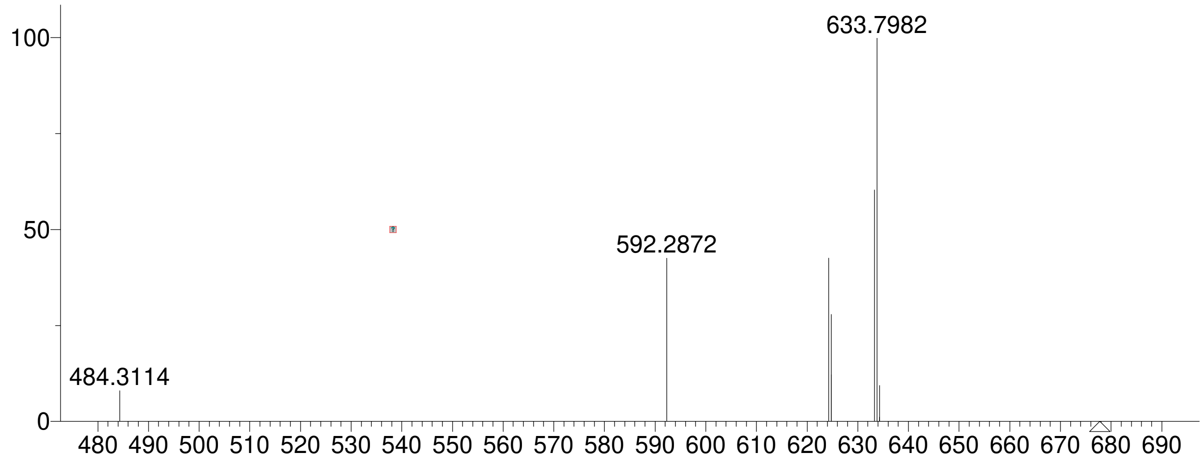

Unknown: Unknown (15.48_223.1441m/z)
Compound in Library Factor = -2387

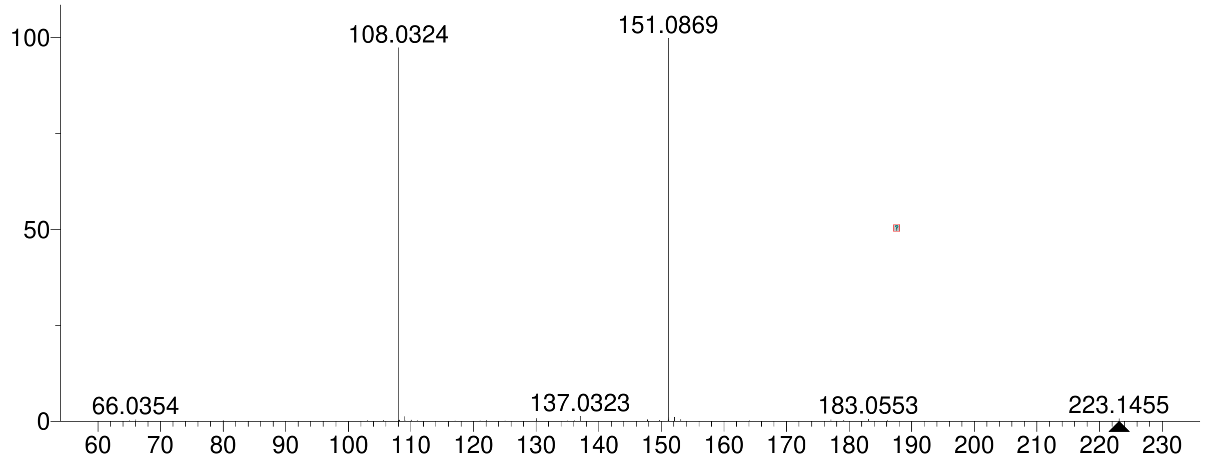

Hit 1 : Zectran [M+H]+ HCD 6V P=223.1
C12H18N2O2; MF: 18; RMF: 381; Prob 36.9%; CAS: 315-18-4; Lib: nist_msms; ID: 542422.

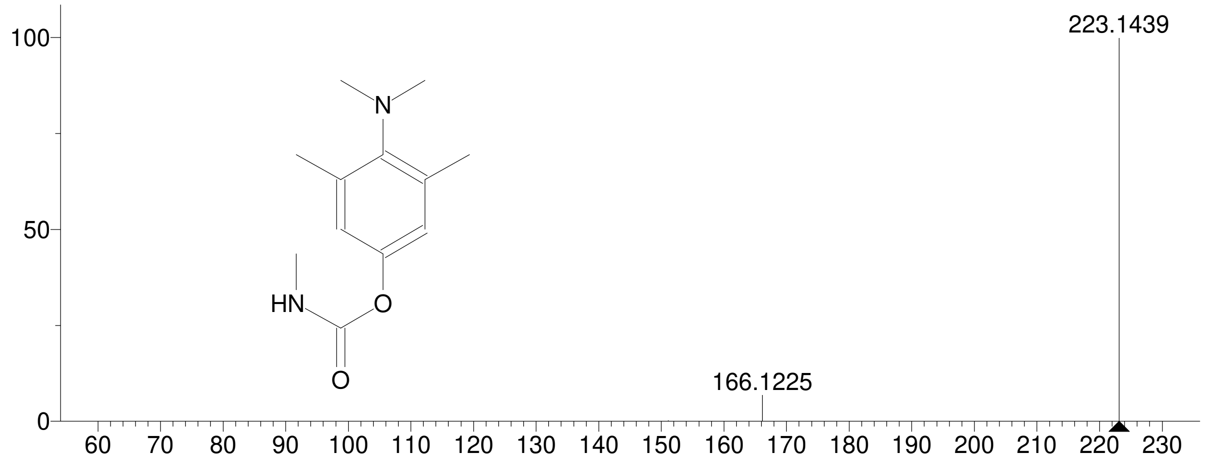

Hit 2 : Pesticide9_Mexacarbate_C12H18N2O2_4-(Dimethylamino)-3,5-dimethylphenyl methylcarbamate [M+H]+
C12H18N2O2; MF: 6; RMF: 79; Prob 24.5%; Lib: mona-export-gnps; ID: 11521.

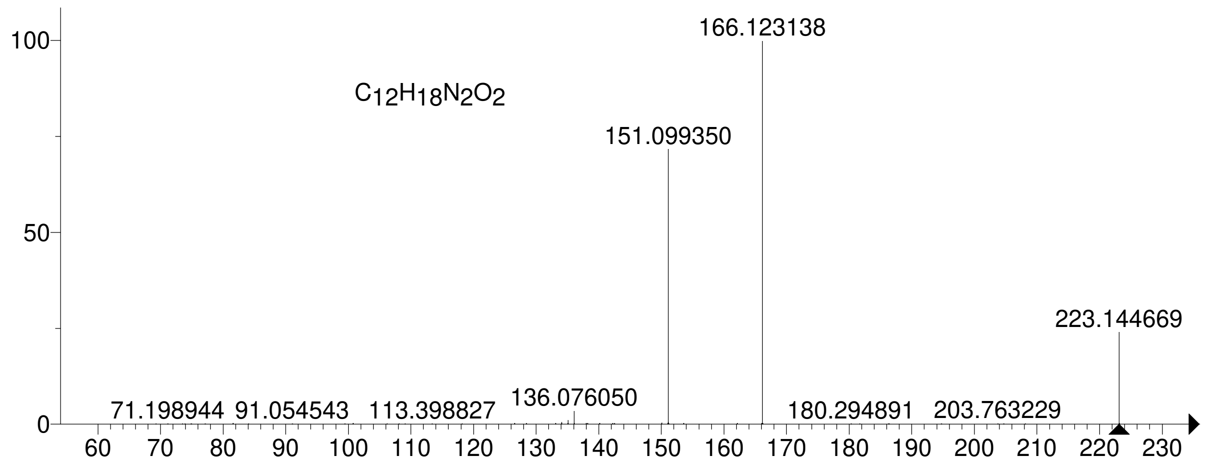

Hit 3 : Neostigmine cation [Cat]+ HCD 13V P=223.1
C12H19N2O2; MF: 0; RMF: 57; Prob 19.3%; CAS: 59-99-4; Lib: nist_msms; ID: 221803.

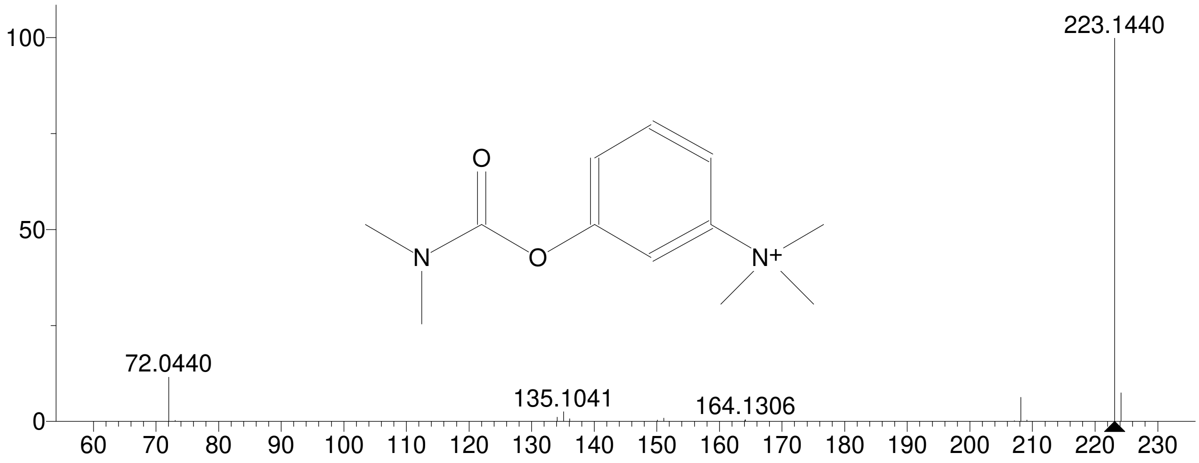

Hit 4 : Heptanedioic acid, 1-(2-cyclopentylidenehydrazide) [M+H-H2O]+ HCD 2V P=223.1
C12H20N2O3; MF: 0; RMF: 44; Prob 19.3%; CAS: 1136466-93-7; Lib: nist_msms; ID: 263606.

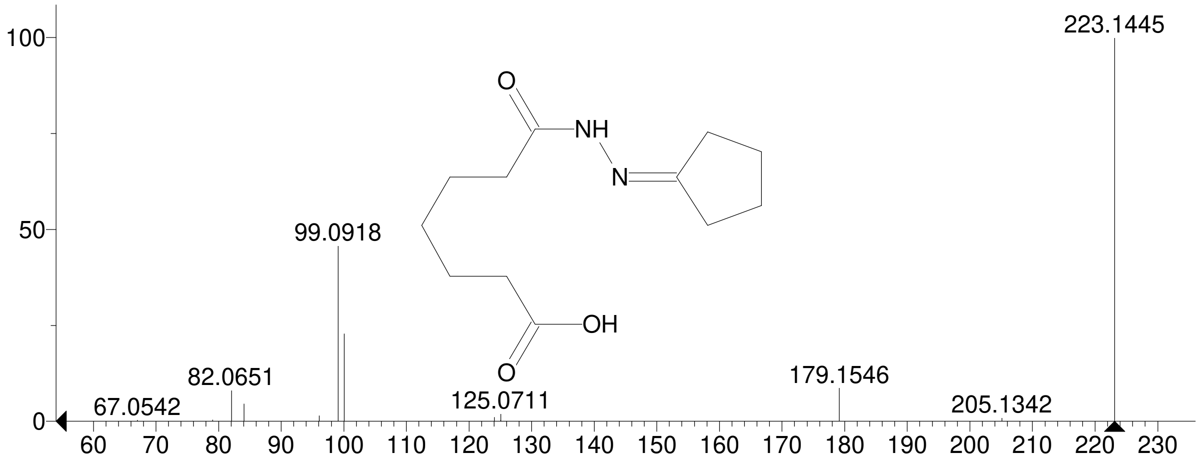

Unknown: Unknown (15.49_339.1078m/z)
Compound in Library Factor = -3273

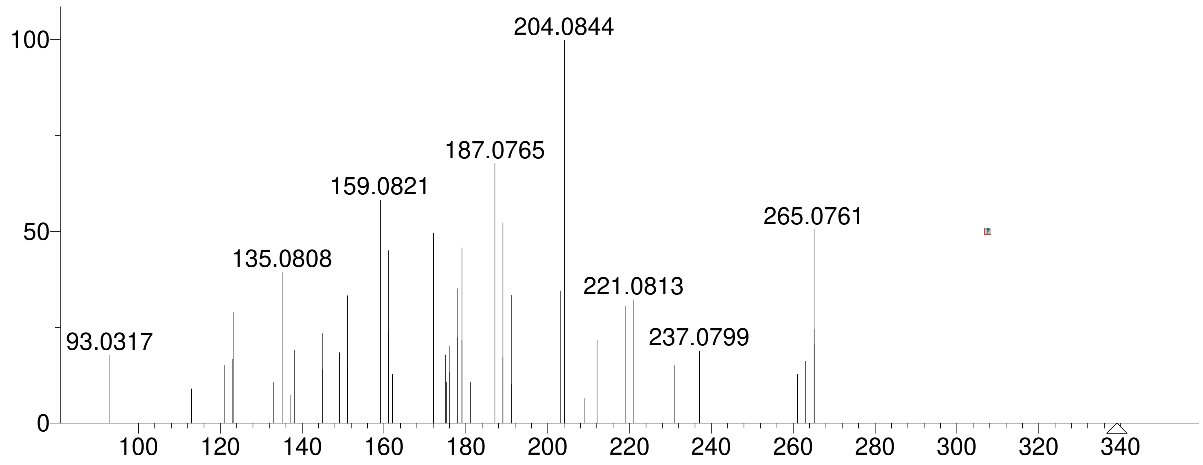

Hit 1 : gerberinside [M+H]+ 35HCD P=339.1
C16H18O8; MF: 9; RMF: 96; Prob 9.51%; Lib: mona-export-vf-npl_qexactive; ID: 17899.

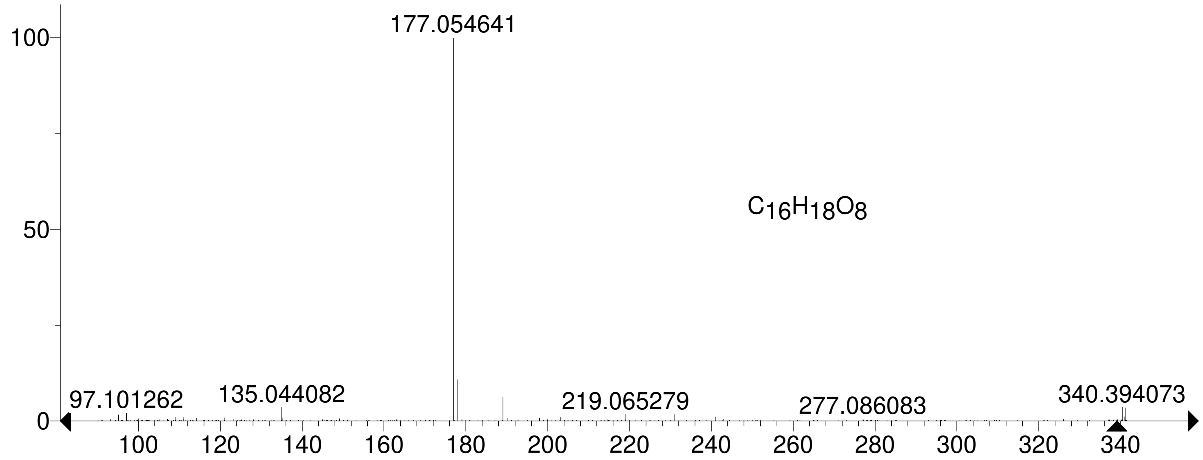

Hit 2 : (1R,3R,4S,5R)-1,3,4-trihydroxy-5-[(E)-3-(4-hydroxyphenyl)prop-2-enoyl]oxycyclohexane-1-carboxylic acid
C16H18O8; MF: 7; RMF: 98; Prob 8.77%; Lib: mona-export-vf-npl_qexactive; ID: 6317.

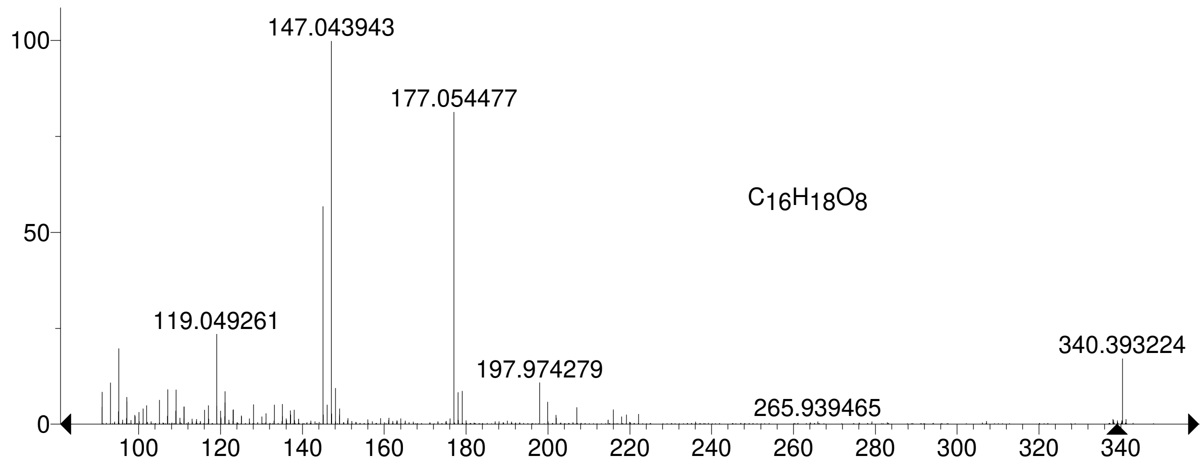

Hit 3 : NCGC00385147-01_C16H18O8_beta-D-Glucopyranoside, 4,8-dihydroxy-1-naphthalenyl [M+H]+ P=339.
C16H18O8; MF: 6; RMF: 113; Prob 8.43%; Lib: mona-export-gnps; ID: 10535.

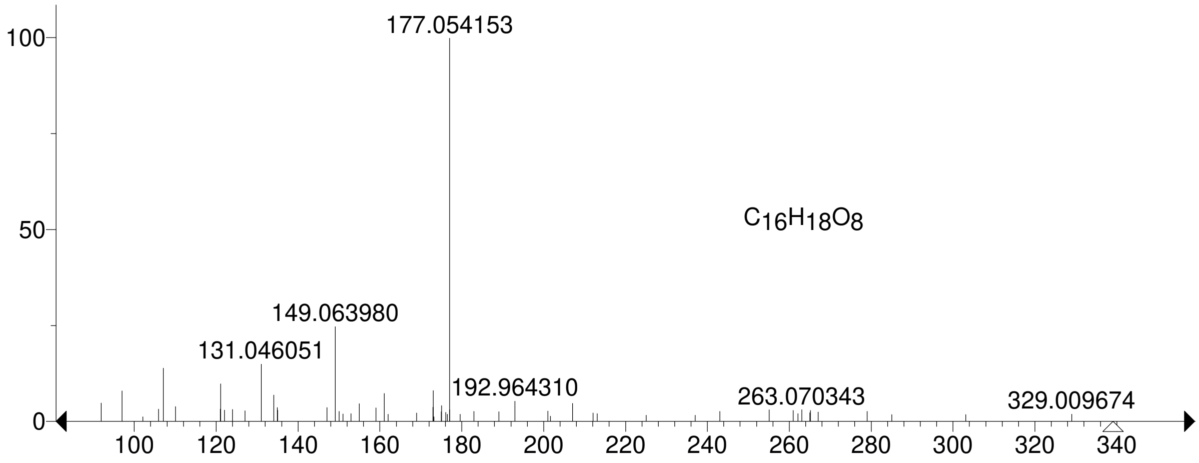

Hit 4 : 4-Methylumbelliferyl β-D-glucopyranoside [M+H]+ HCD 50V P=339.1
C16H18O8; MF: 3; RMF: 73; Prob 7.45%; CAS: 18997-57-4; Lib: nist_msms; ID: 460021.

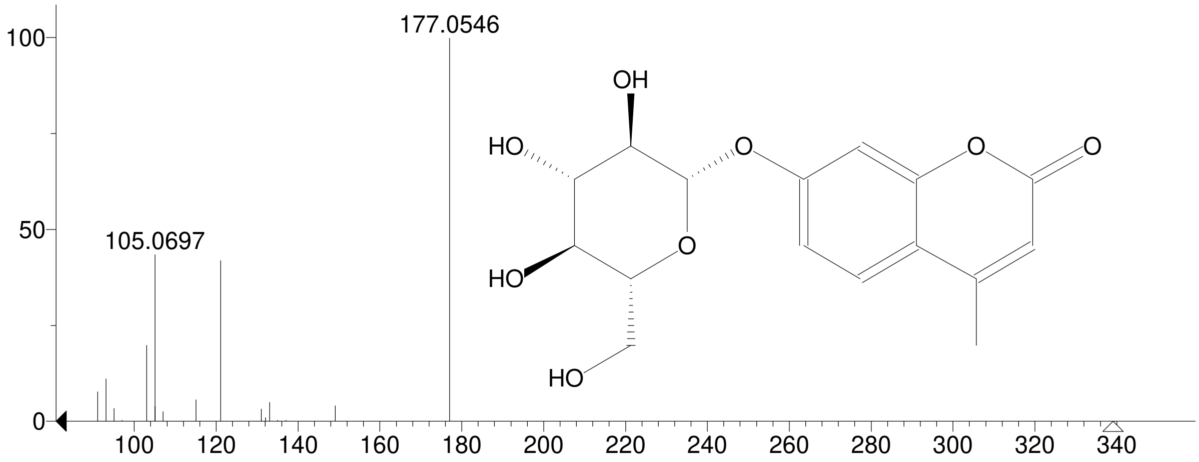

Hit 5 : 4-Demethylsimmondsin 2'-ferulate [M+H-C9H13O4N]+ HCD 50V P=339.1
C25H31NO12; MF: 0; RMF: 52; Prob 6.58%; CAS: 162290-39-3; Lib: nist_msms; ID: 217717.

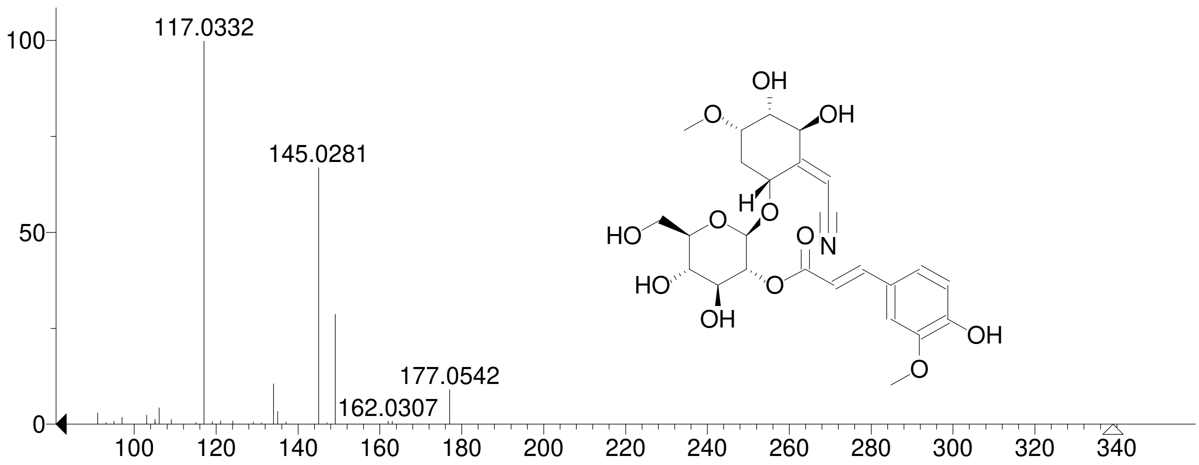

Unknown: Unknown (15.51_669.3061m/z)
Compound in Library Factor = 0

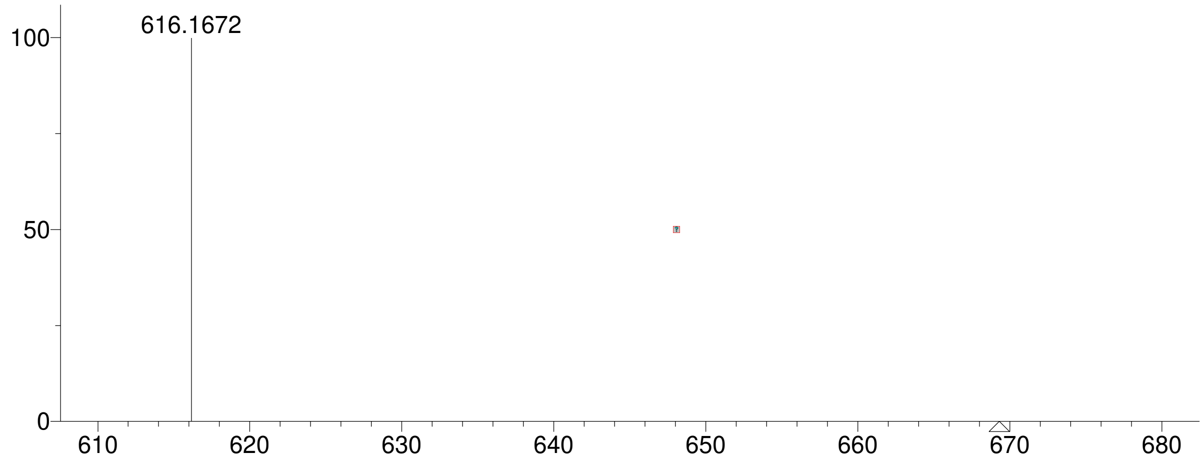

Unknown: Unknown (15.52_234.1125m/z)
Compound in Library Factor = -1309

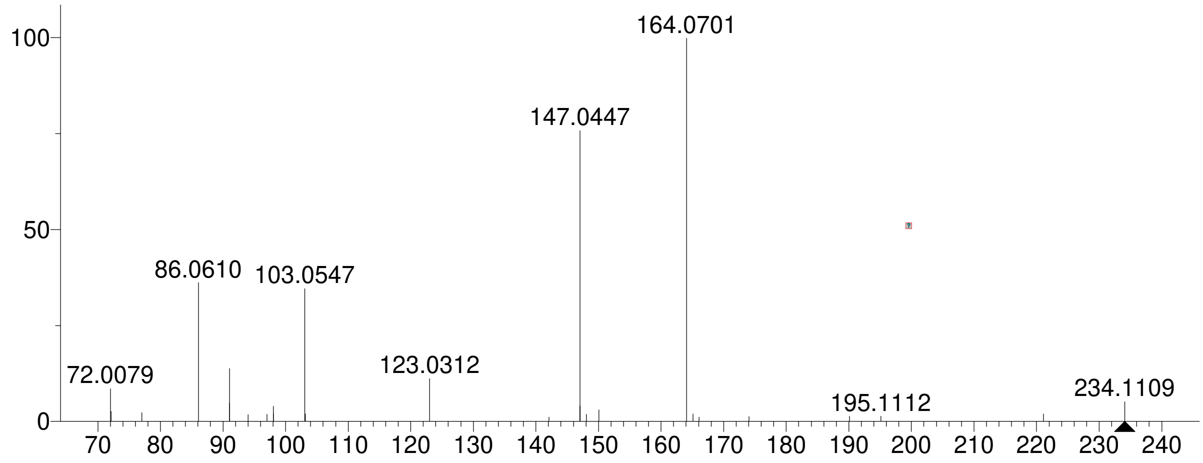

Hit 1 : Benzyl-L-glutamine methyl ester [M+H-NH3]+ HCD 11V P=234.1
C13H18N2O3; MF: 59; RMF: 235; Prob 66.3%; CAS: 727973-94-6; Lib: nist_msms; ID: 203918.

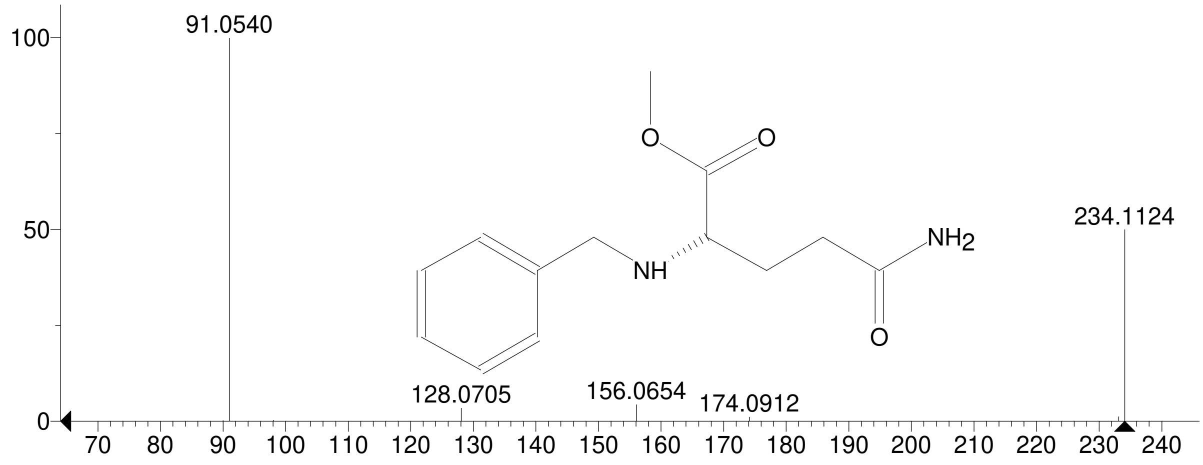

Hit 2 : Alloxydim [M+H-C4H10O2]+ HCD 35V P=234.1
C17H25NO5; MF: 8; RMF: 132; Prob 13.4%; CAS: 115263-07-5; Lib: nist_msms; ID: 543478.

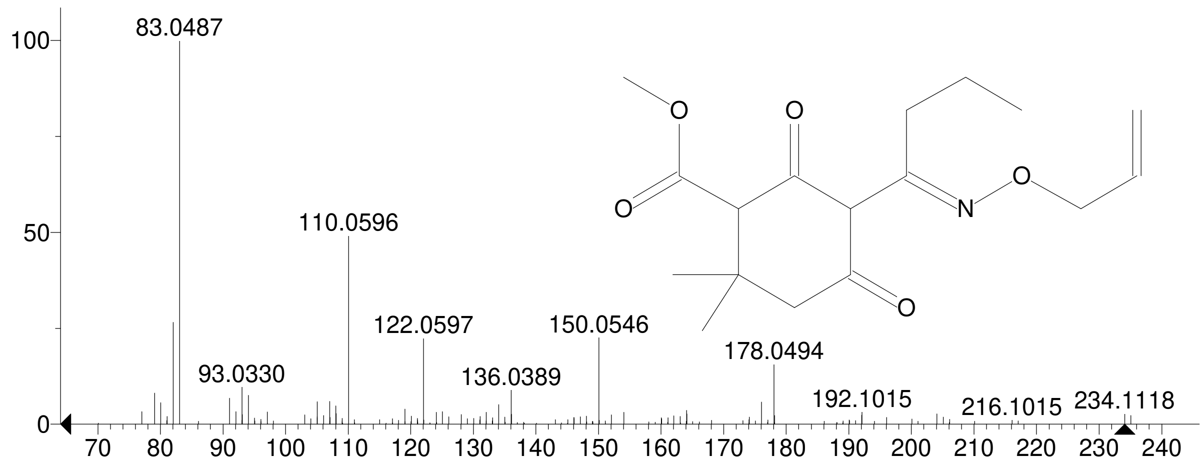

Hit 3 : Mecarbinate [M+H]+ P=234.1
C13H15NO3; MF: 3; RMF: 110; Prob 10.8%; Lib: mona-export-gnps; ID: 746.

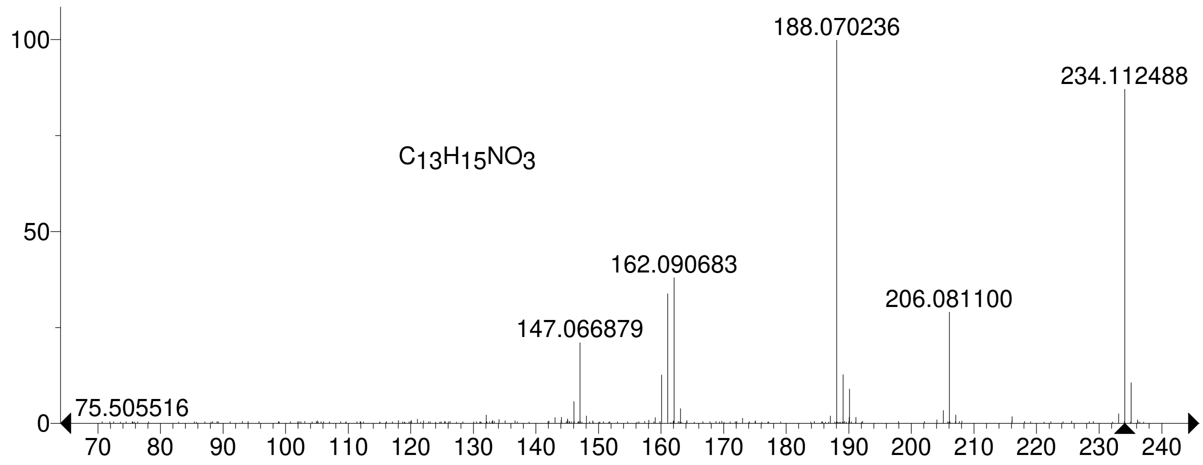

Hit 4 : Euparin [M+NH4]+ 65HCD P=234.1
C13H12O3; MF: 0; RMF: 52; Prob 9.52%; Lib: mona-export-vf-npl_qexactive; ID: 26061.

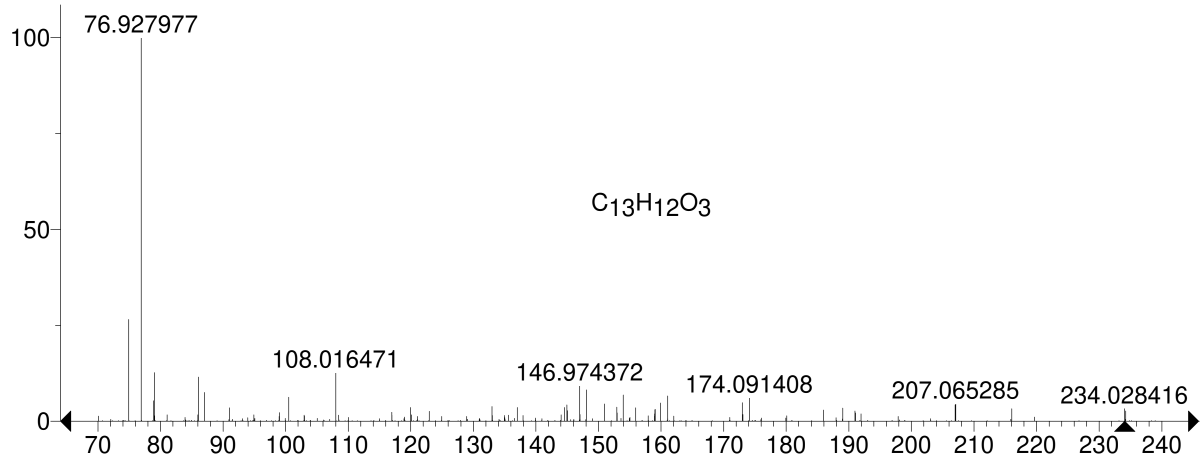

** Search Report Page 1 of 1 **

Unknown: Unknown (15.54_442.1697m/z)
Compound in Library Factor = 0

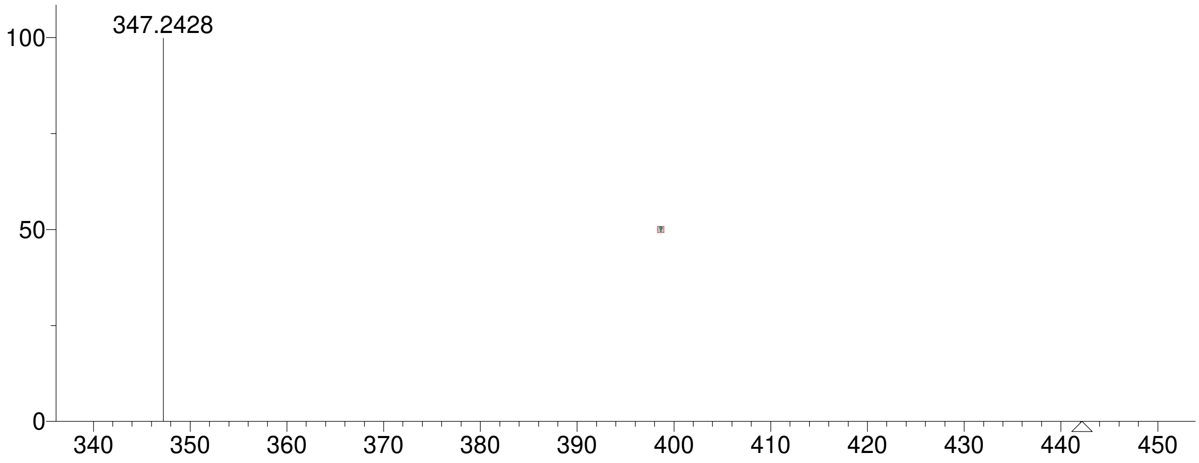

Unknown: Unknown (15.56_346.2378m/z)
Compound in Library Factor = -2632

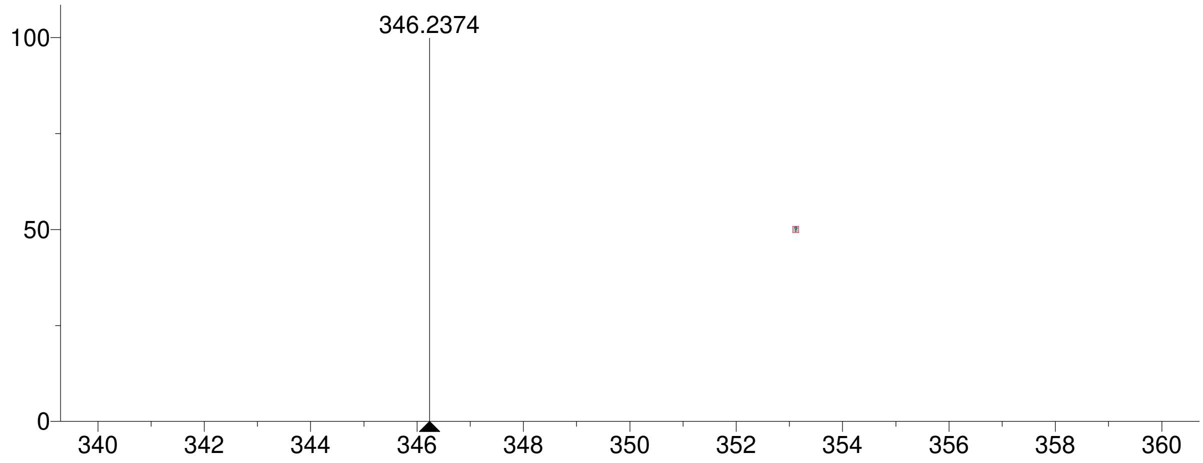

Hit 1 : 1-Pyrrolidineheptanoic acid, 2-[(1E,3S,4S)-3-hydroxy-4-methyl-1-nonen-6-yn-1-yl]-5-oxo-, (2R)- [M+H-H2C
C21H33NO4; MF: 364; RMF: 999; Prob 50.0%; CAS: 1628759-75-0; Lib: nist_msms; ID: 361796.

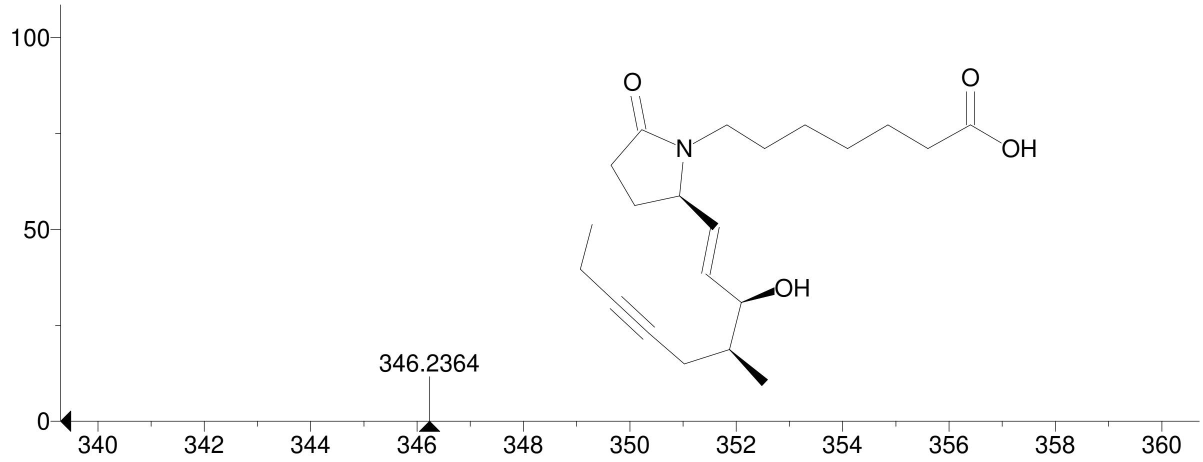

Hit 2 : Octanoic acid, 4-[(1-oxo-7-phenylheptyl)amino]-, (4R)- [M-H]- HCD 17V P=346.2
C21H33NO3; MF: 364; RMF: 999; Prob 50.0%; CAS: 1101136-50-8; Lib: nist_msms; ID: 415067.

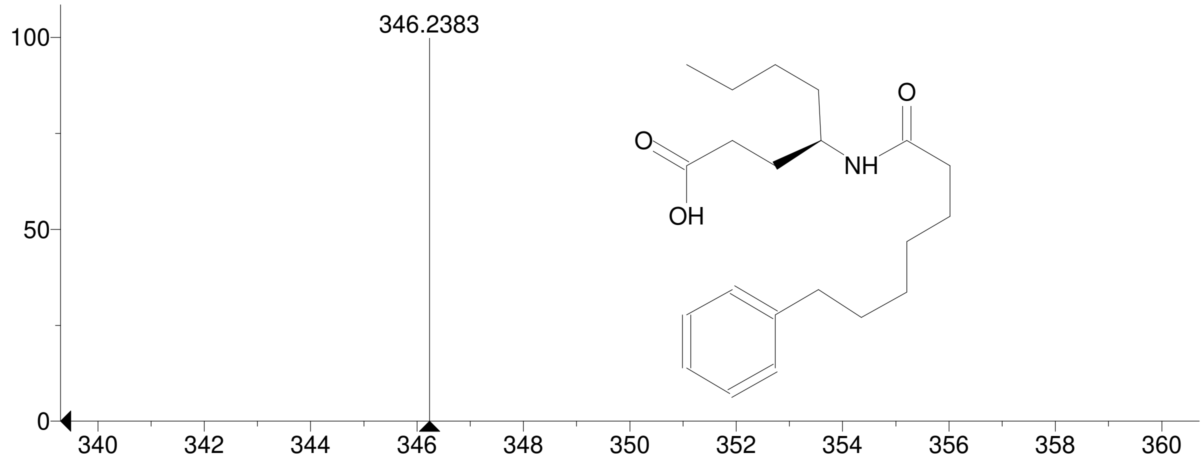

** Search Report Page 1 of 1 **

Unknown: Unknown (15.62_592.2869m/z)
Compound in Library Factor = -3273

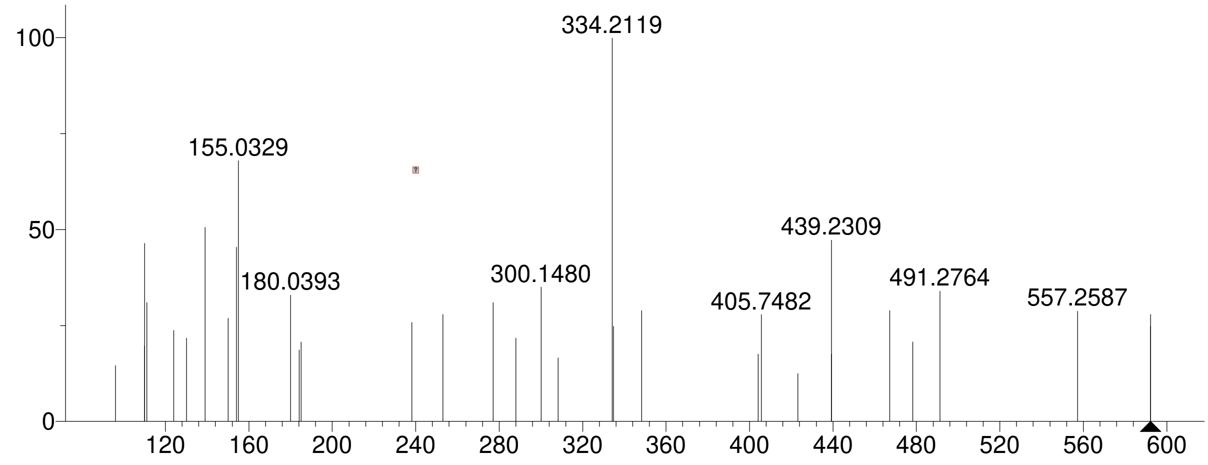

Hit 1 : Ergocristine [M+H-H2O]+ HCD 17V P=592.3
C35H39N5O5; MF: 6; RMF: 182; Prob 54.2%; CAS: 511-08-0; Lib: nist_msms; ID: 239281.

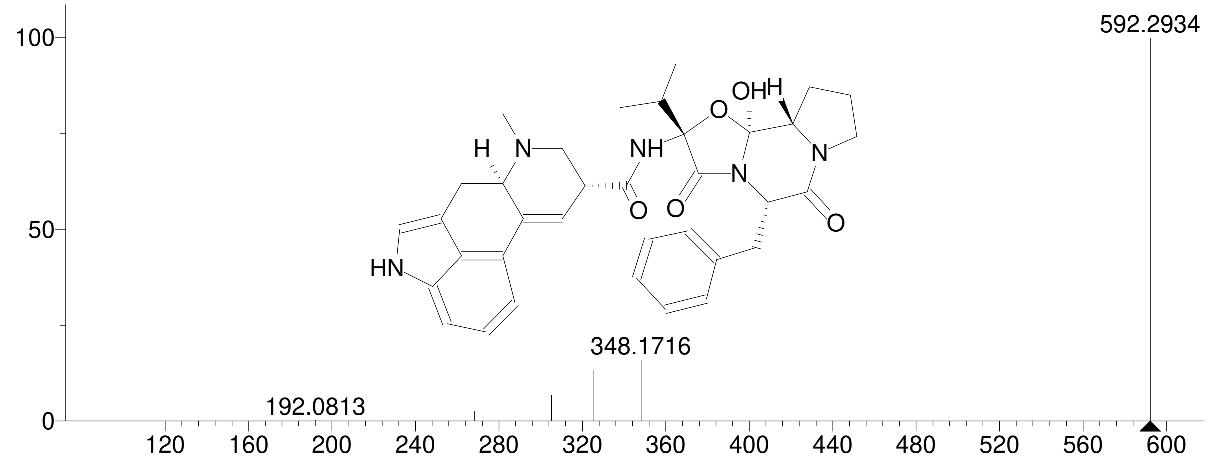

Hit 2 : LH-RH, free acid [M+2H]2+ QTOF 7V P=592.3
C55H74N16O14; MF: 2; RMF: 160; Prob 45.8%; Lib: nist_msms2; ID: 38405.

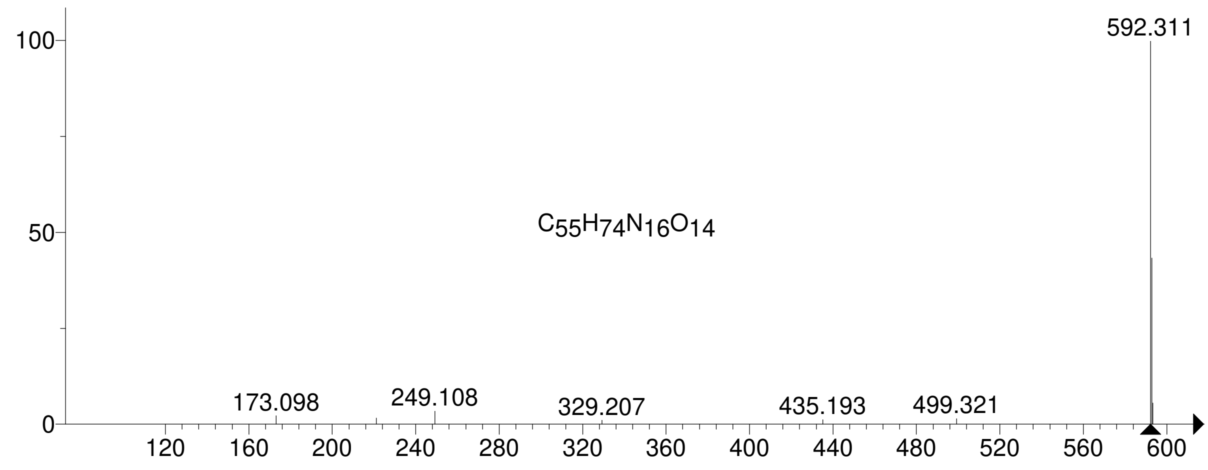

Unknown: Unknown (15.70_432.8428m/z)
Compound in Library Factor = 0

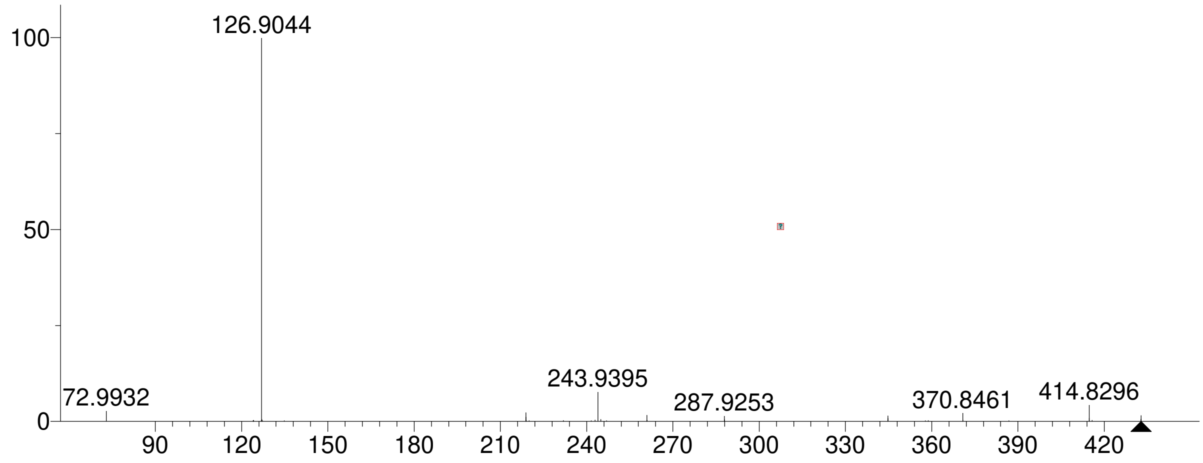

Unknown: Unknown (15.71_281.1012m/z)
Compound in Library Factor = 0

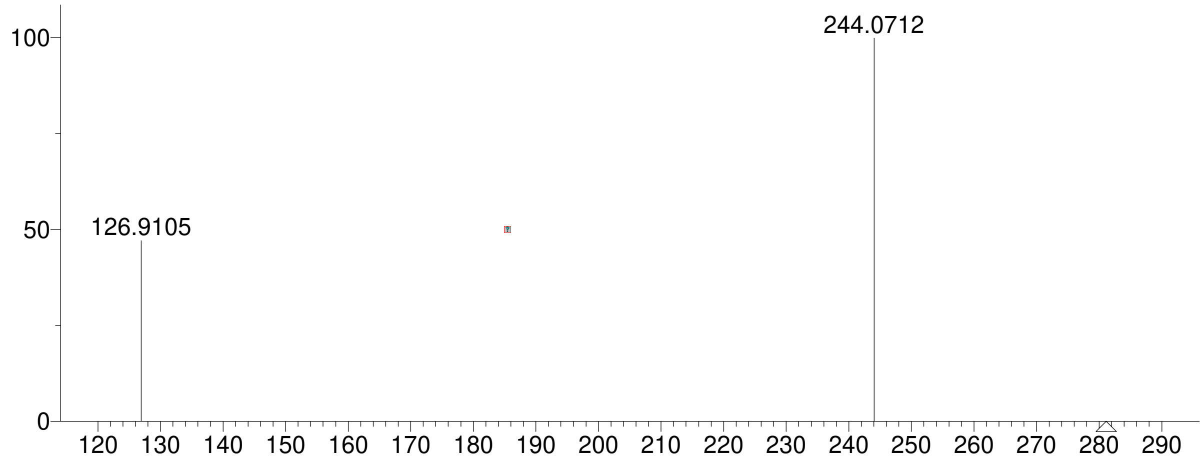

** Search Report Page 1 of 1 **

Unknown: Unknown (15.71_557.7089m/z)
Compound in Library Factor = 0

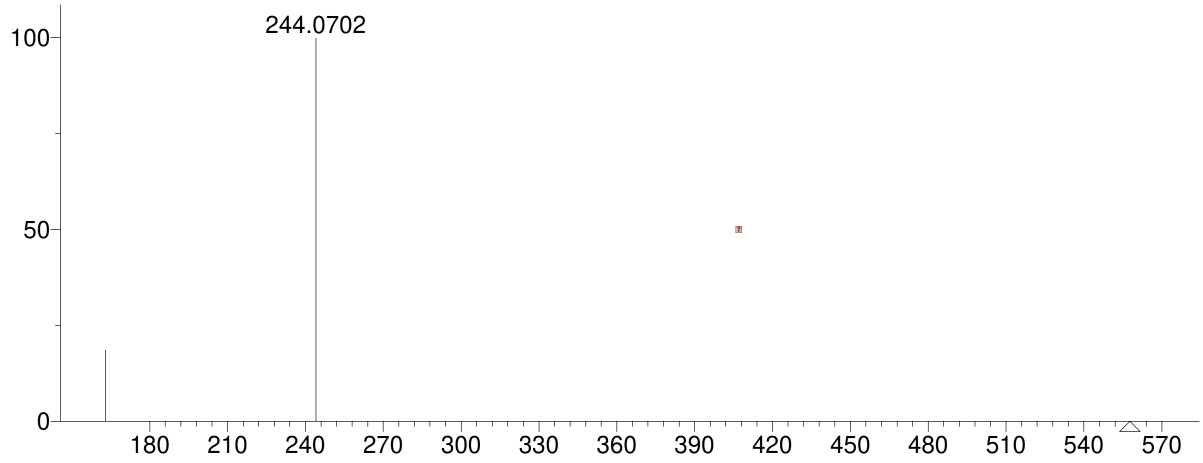

Unknown: Unknown (15.77_246.9240m/z)
Compound in Library Factor = 116

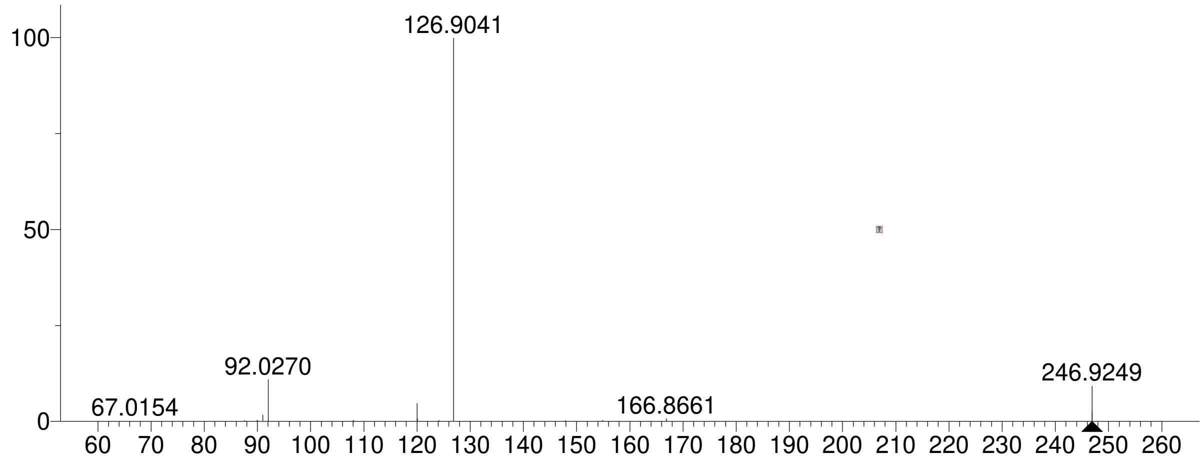

Hit 1 : 3-Iodobenzoic acid [M-H]- HCD 16V P=246.9
C7H5IO2; MF: 560; RMF: 979; Prob 99.0%; CAS: 618-51-9; Lib: nist_msms; ID: 128592.

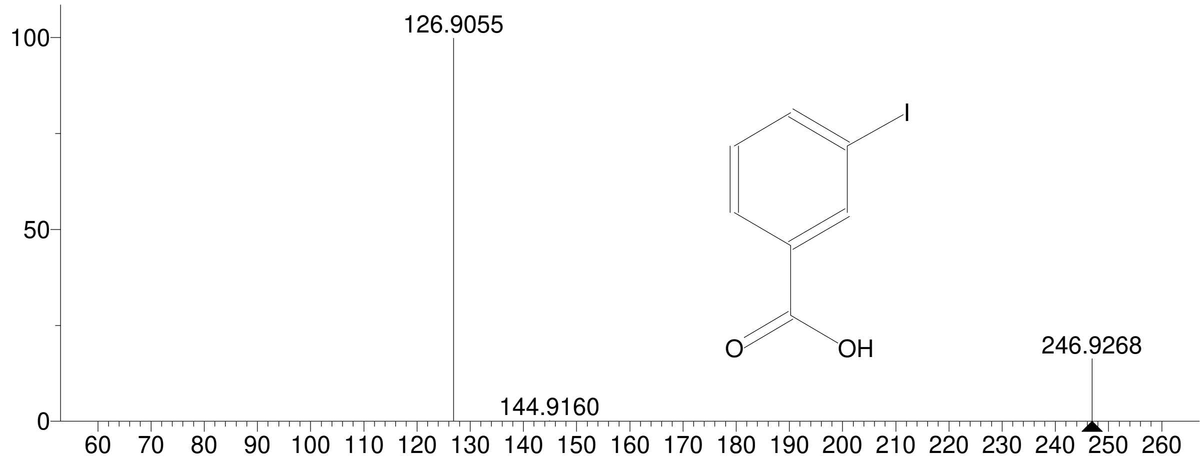

Hit 2 : Terrazole [M+H]+ HCD 4V P=246.9
C5H5Cl3N2OS; MF: 130; RMF: 785; Prob 0.98%; CAS: 2593-15-9; Lib: nist_msms; ID: 471720.

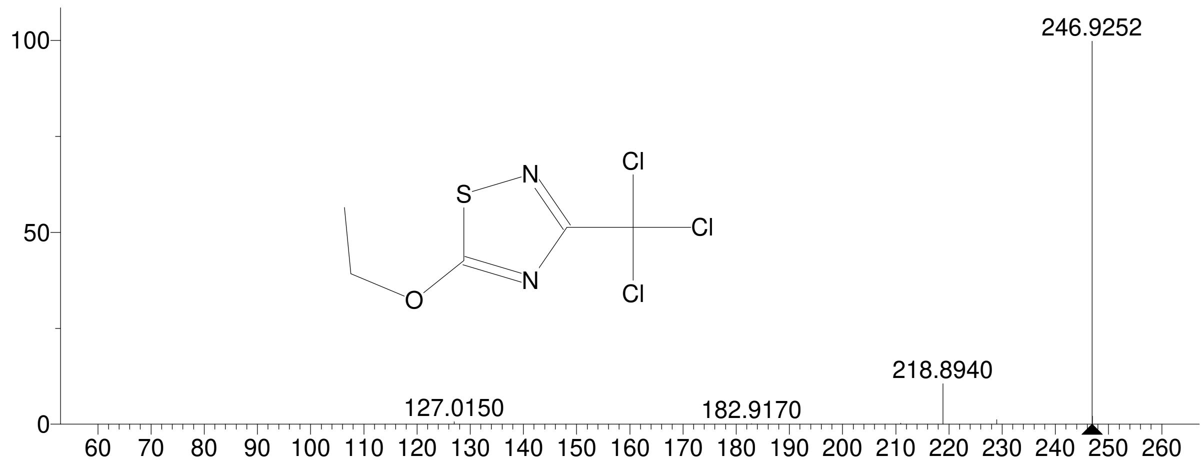

Hit 3 : p-iodobenzoic acid (Van de Wouwer et al./Van de Wouwer et al.) [M-H]- P=246.9
C7H5IO2; MF: 5; RMF: 372; Prob 0.04%; Lib: phytofrag2018_final_060219; ID: 762.

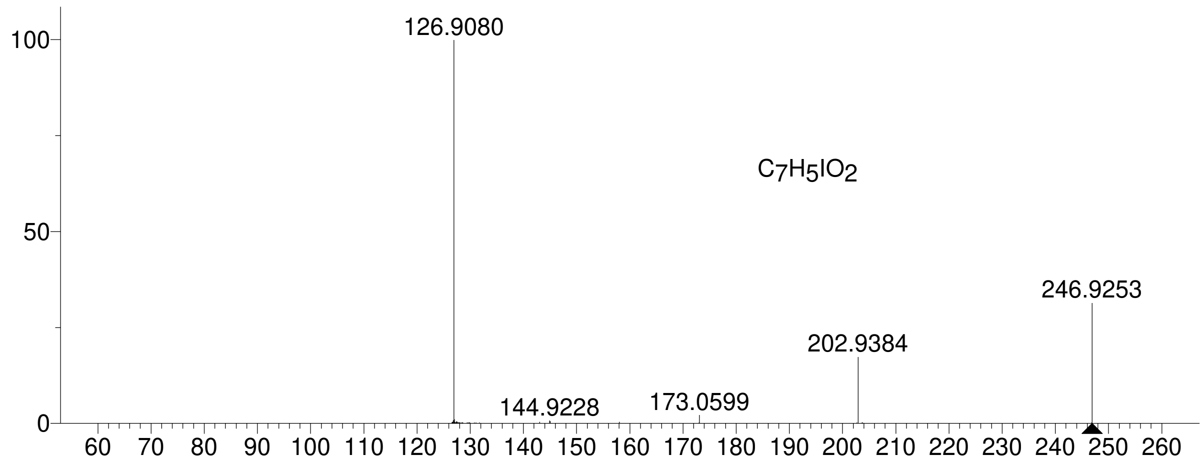

Unknown: Unknown (15.78_358.0592m/z)
Compound in Library Factor = 0

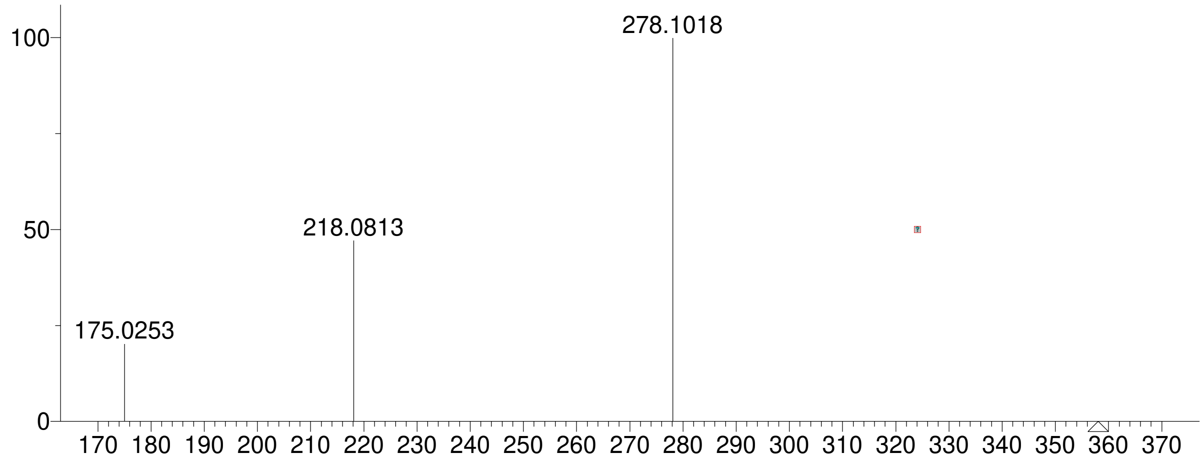

Hit 1 : Urea, N-[2-chloro-4-[(6,7-dimethoxy-4-quinazolinyl)oxy]phenyl]-N'-propyl- [M+H-C3H9N]+ HCD 66V P=3
C20H21ClN4O4; MF: 0; RMF: 25; Prob 100.0%; CAS: 286370-15-8; Lib: nist_msms; ID: 344081.

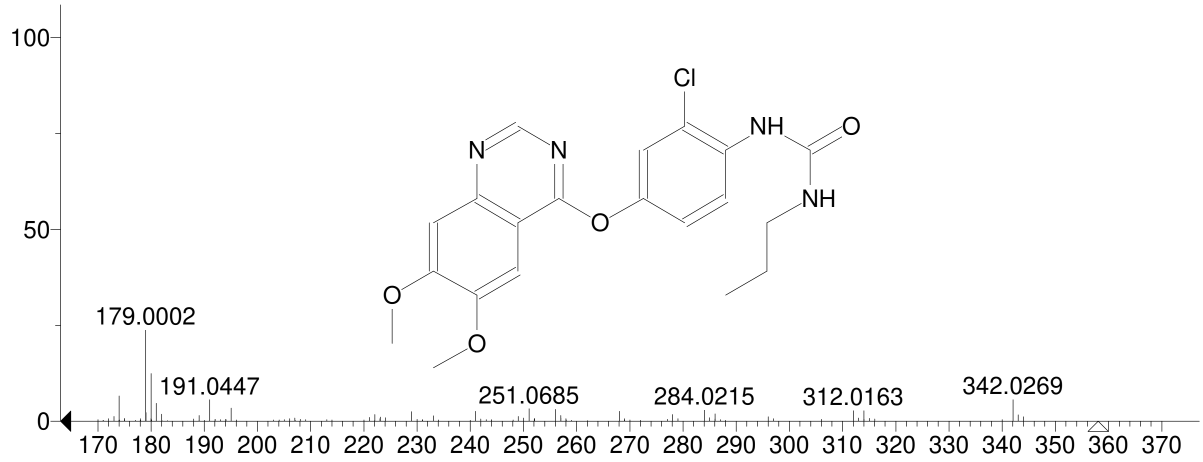

Unknown: Unknown (15.85_371.1243m/z)
Compound in Library Factor = -3273

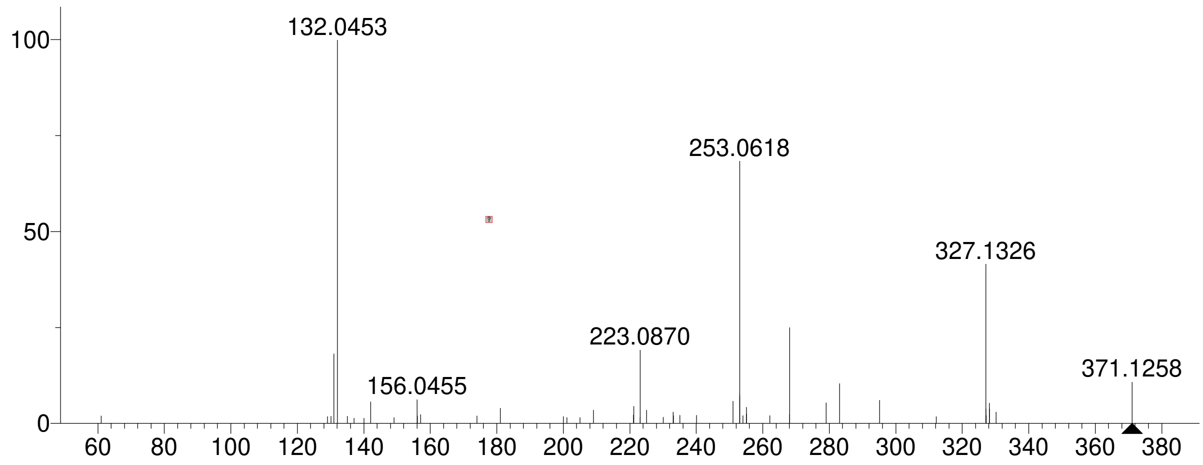

Hit 1 : 7,8-dihydroxy-3-[(1E,3E)-7-hydroxy-3,5-dimethylhepta-1,3-dienyl]-7-methyl-8H-isochromen-6-one [M+K]+
C19H24O5; MF: 2; RMF: 66; Prob 21.3%; Lib: mona-export-vf-npl_qexactive; ID: 16498.

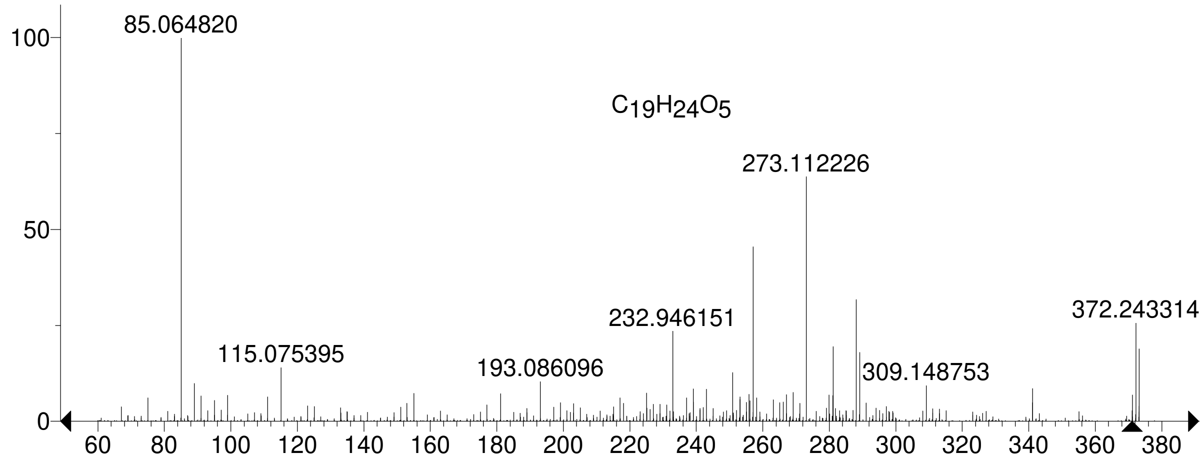

Hit 2 : G(8-O-4)5HFA'2 () [M-H]- P=371.1
C20H20O7; MF: 0; RMF: 32; Prob 19.7%; Lib: phytofrag2018_final_060219; ID: 368.

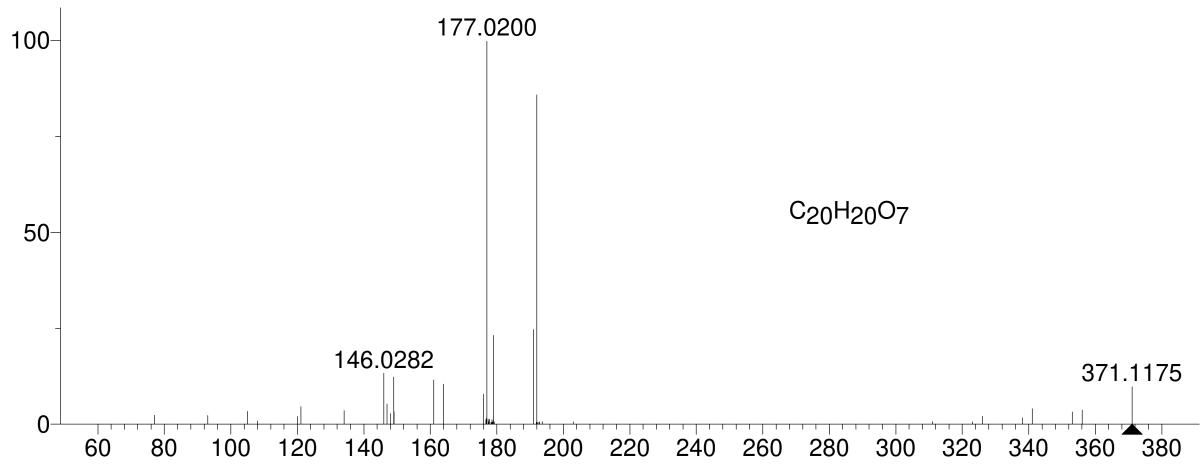

Hit 3 : methyl (2E)?3?[3?(4?hydroxy?3?methoxyphenyl)?2? (hydroxymethyl)?2,3?dihydro?1,4?benzodioxin?6? yl
C20H20O7; MF: 0; RMF: 26; Prob 19.7%; Lib: phytofrag2018_final_060219; ID: 705.

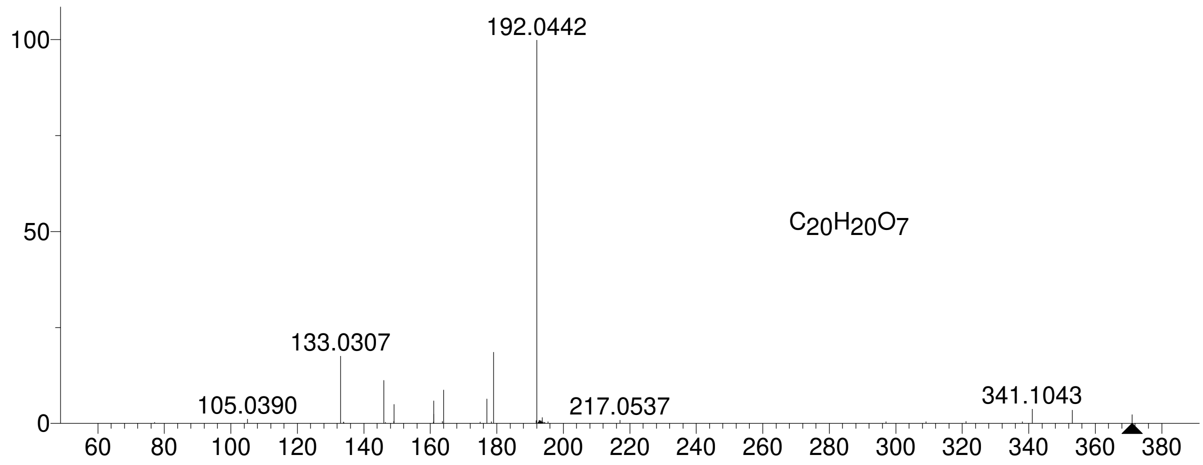

Hit 4 : 1-[2-hydroxy-4-(3-hydroxy-5-methylphenoxy)-6-methylphenyl]-3-methylbutane-2,3-diol [M+K]+ 35HCD P=
C19H24O5; MF: 0; RMF: 22; Prob 19.7%; Lib: mona-export-vf-npl_qexactive; ID: 22291.

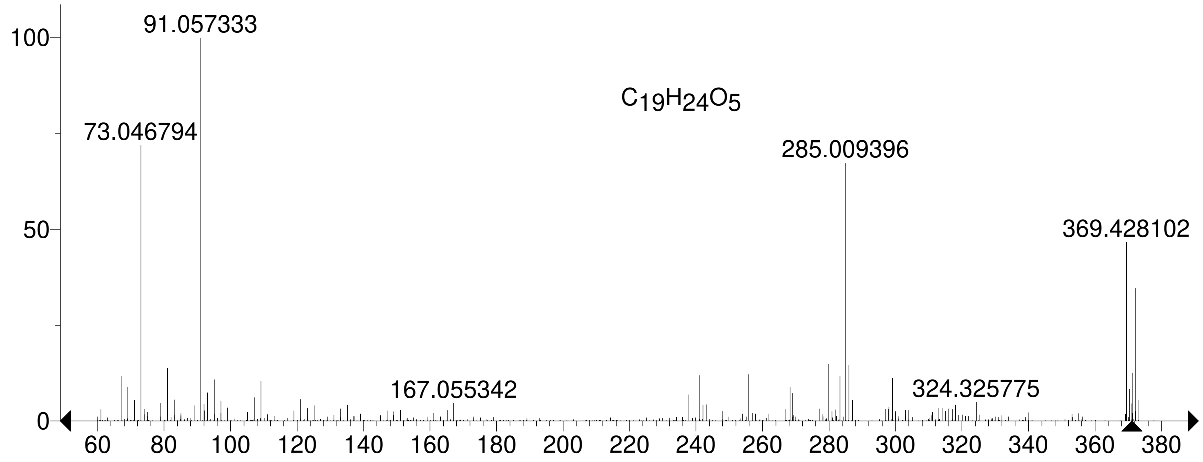

Hit 5 : Arnicolide D [M+K]+ 45HCD P=371.1
C19H24O5; MF: 0; RMF: 19; Prob 19.7%; Lib: mona-export-vf-npl_qexactive; ID: 4088.

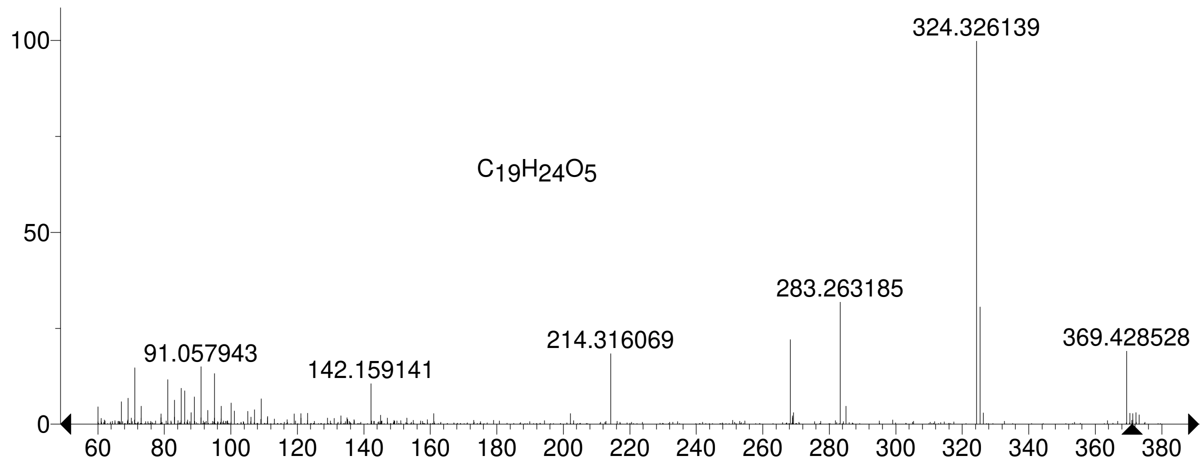

Unknown: Unknown (15.86_428.1708m/z)
Compound in Library Factor = -3273

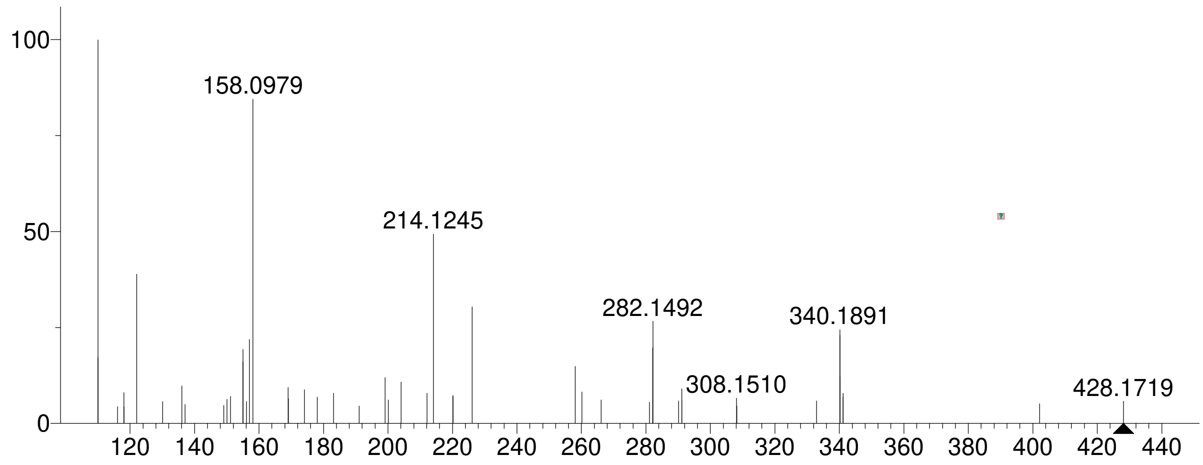

Hit 1 : (2s,6as,12as)-6a-hydroxy-8,9-dimethoxy-2-(prop-1-en-2-yl)-1,2,12,12a-tetrahydrochromeno[3,4-b]furo[2,3-
C23H22O7; MF: 1; RMF: 45; Prob 14.8%; Lib: mona-export-vf-npl_qexactive; ID: 4286.

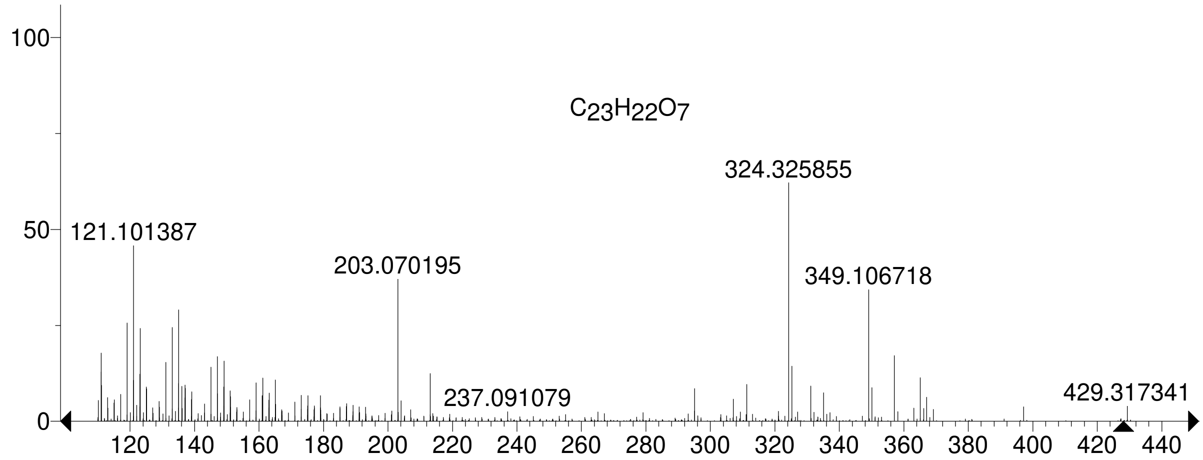

Hit 2 : tephrosin [M+NH4]+ 65HCD P=428.2
C23H22O7; MF: 0; RMF: 34; Prob 14.2%; Lib: mona-export-vf-npl_qexactive; ID: 4047.

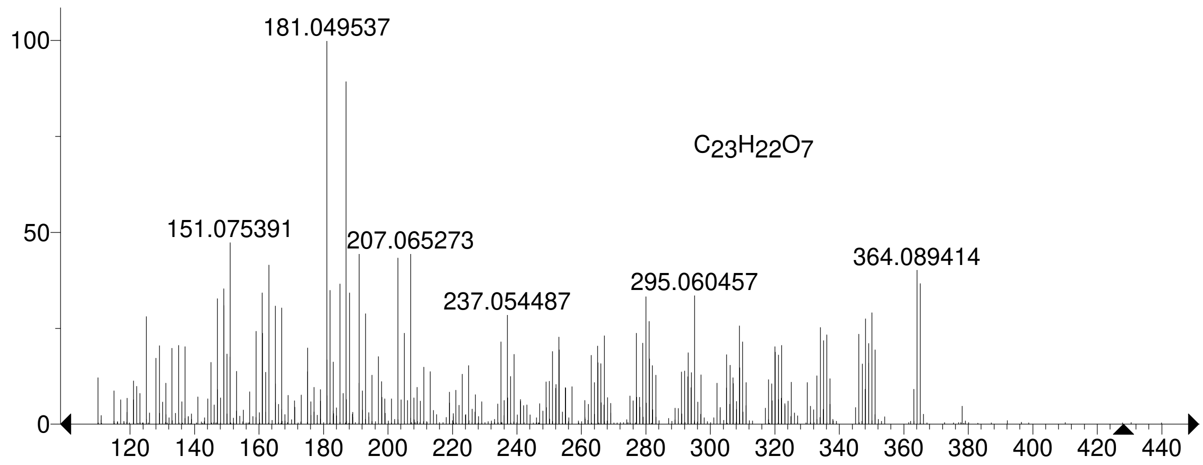

Hit 3 : PC 6:0; PC 3:0-3:0; [M+Hac-H]- [M+Hac-H]- P=428.2
C14H28NO8P; MF: 0; RMF: 33; Prob 14.2%; Lib: msdial-insilicomsms-lipids-neg; ID: 319519.

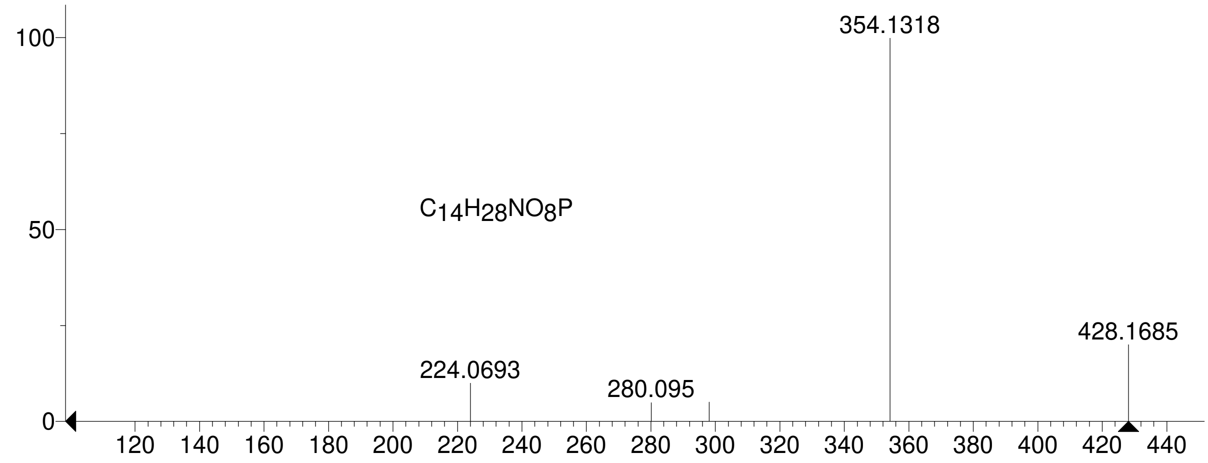

Hit 4 : PC 7:0; PC 2:0-5:0; [M+FA-H]- [M+FA-H]- P=428.2
C15H30NO8P; MF: 0; RMF: 32; Prob 14.2%; Lib: msdial-insilicomsms-lipids-neg; ID: 317570.

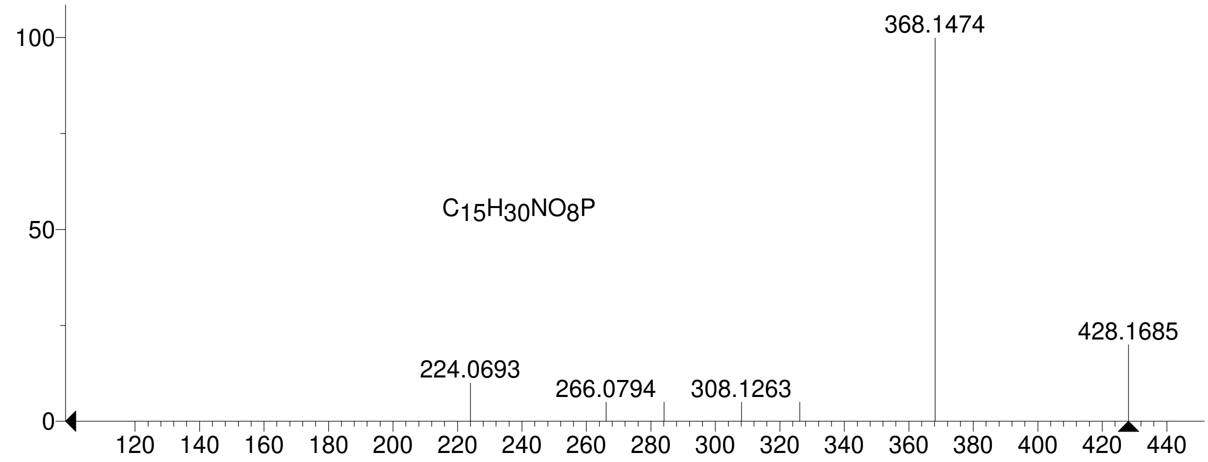

Hit 5 : PC 7:0; PC 3:0-4:0; [M+FA-H]- [M+FA-H]- P=428.2
C15H30NO8P; MF: 0; RMF: 32; Prob 14.2%; Lib: msdial-insilicomsms-lipids-neg; ID: 317629.

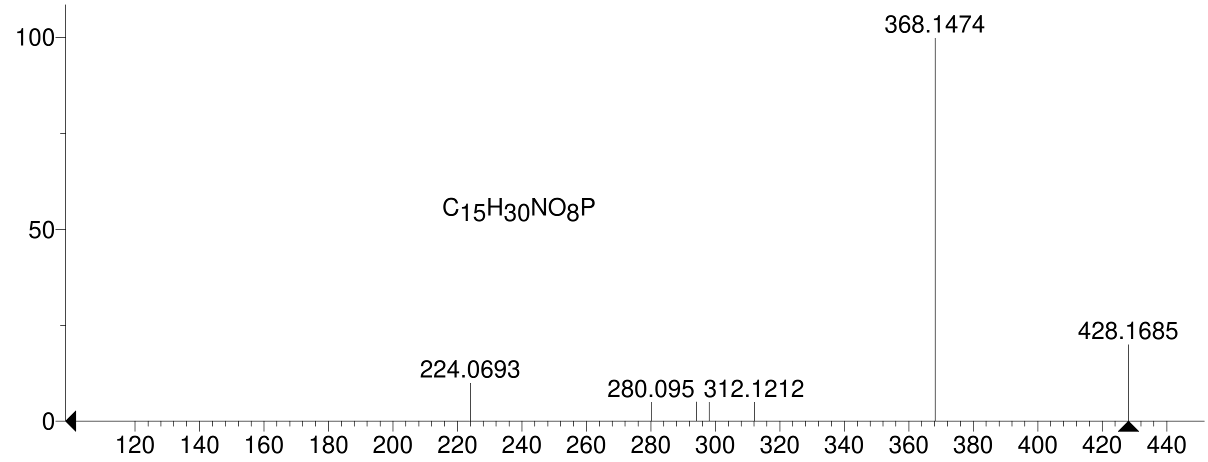

Unknown: Unknown (15.90_291.0537m/z)
Compound in Library Factor = -321

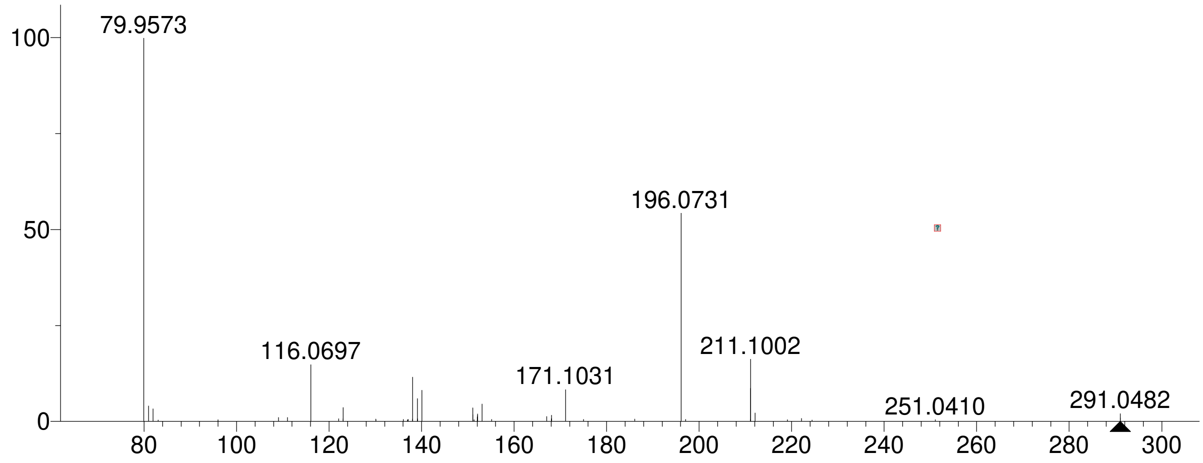

Hit 1 : N4-Acetylsulfadiazine [M-H]- 15% (P=291.1
C12H12N4O3S; MF: 294; RMF: 840; Prob 92.0%; Lib: mona-export-lc-ms-ms_negative_mode; ID: 2464.

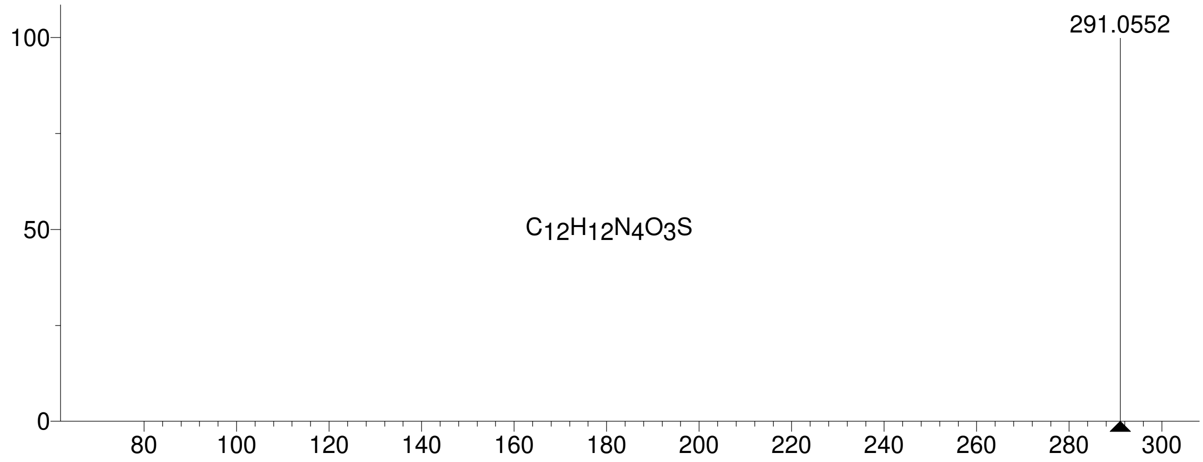

Hit 2 : N4-Acetylsulfadiazine; LC-ESI-ITFT; MS2; CE [M-H]- 15 P=291.1
C12H12N4O3S; MF: 206; RMF: 762; Prob 8.02%; Lib: msms-neg-massbank; ID: 2282.

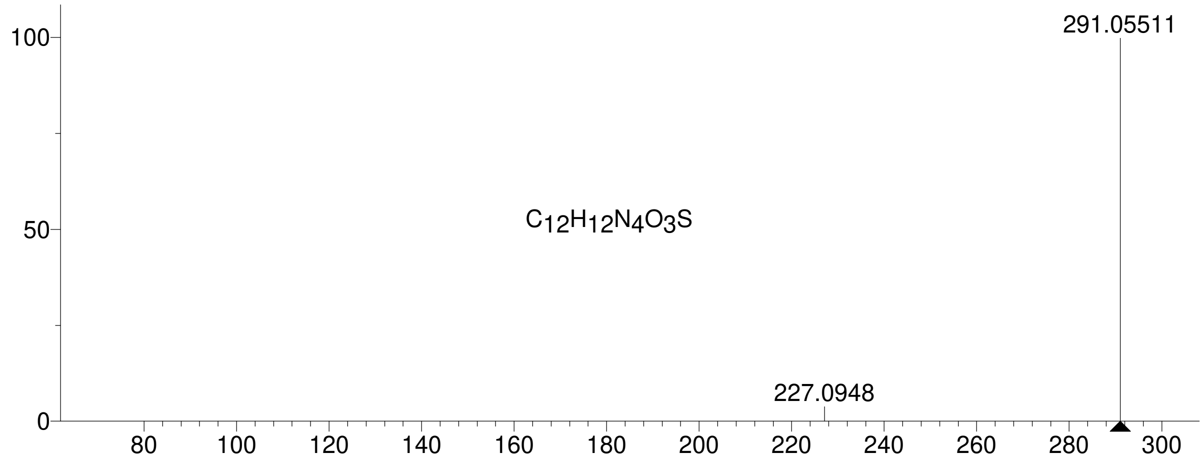

Unknown: Unknown (15.91_314.1962m/z)
Compound in Library Factor = -3273

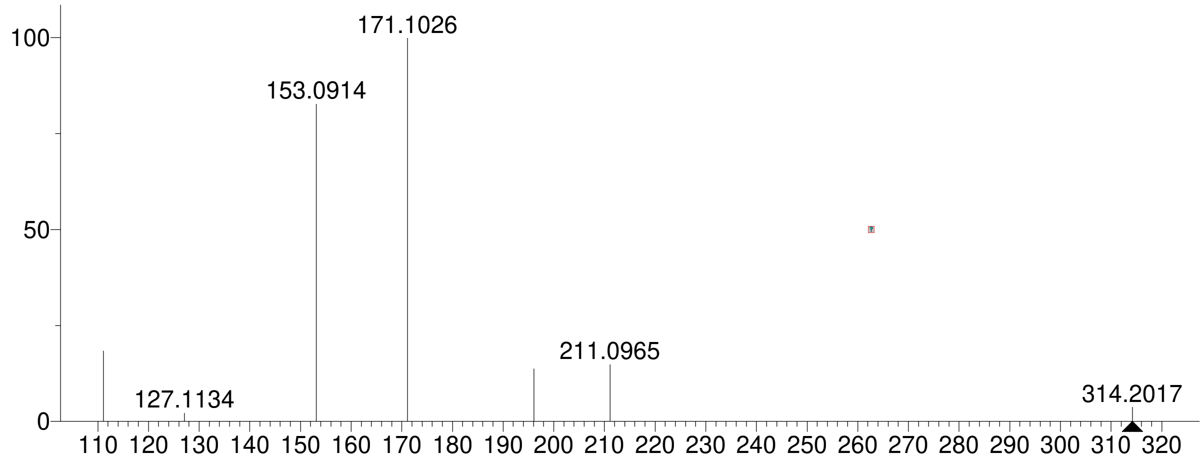

Hit 1 : Heliotrine [M+H]+ HCD 15V P=314.2
C16H27NO5; MF: 7; RMF: 120; Prob 56.6%; CAS: 303-33-3; Lib: nist_msms; ID: 236012.

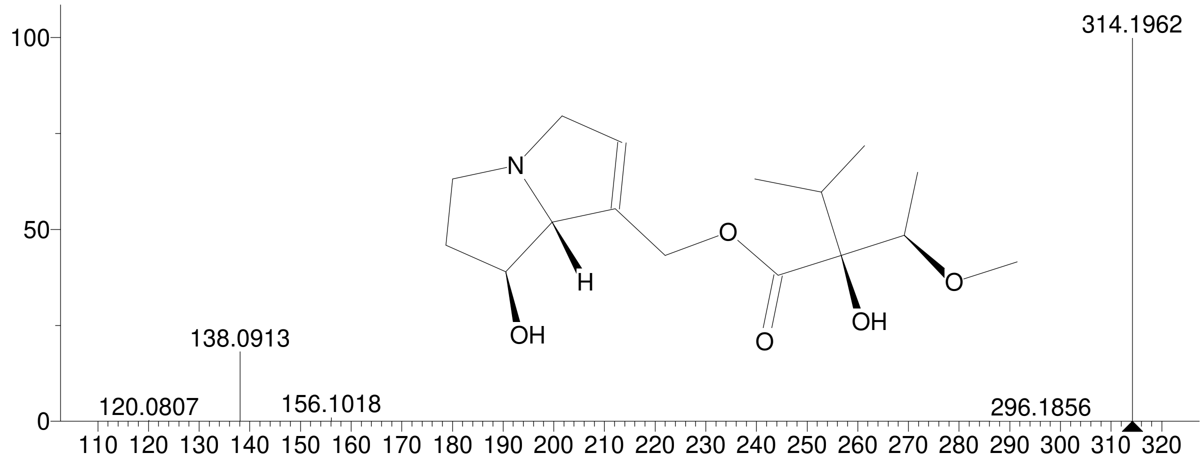

Hit 2 : NCGC00385977-01_C16H24O5_4-Hydroxy-5-methoxy-4-[2-methyl-3-(3-methyl-2-buten-1-yl)-2-oxiranyl]-1
C16H24O5; MF: 0; RMF: 70; Prob 43.4%; Lib: mona-export-gnps; ID: 12532.

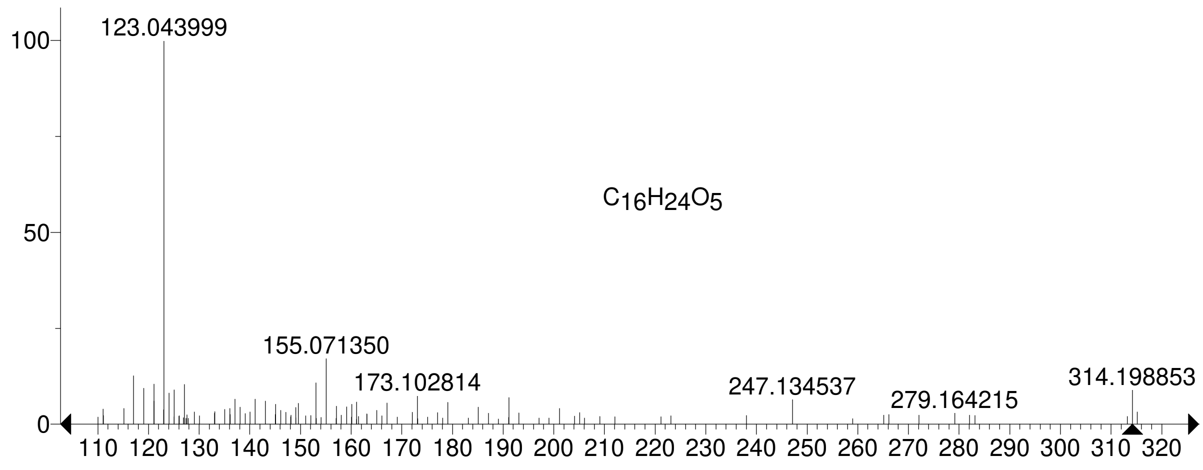

** Search Report Page 1 of 1 **

Unknown: Unknown (15.99_498.1061m/z)
Compound in Library Factor = 0

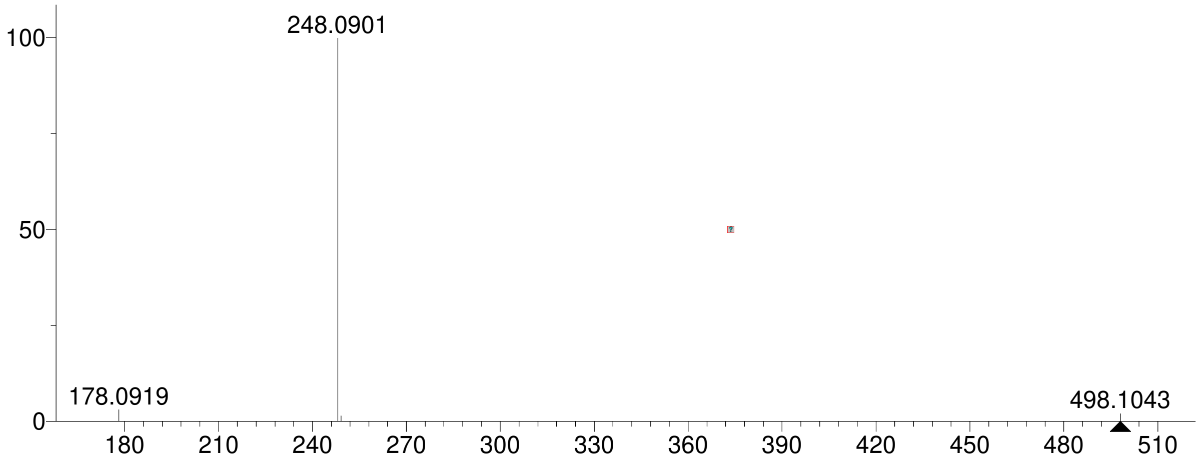

Unknown: Unknown (16.08_272.0916m/z)
Compound in Library Factor = 0

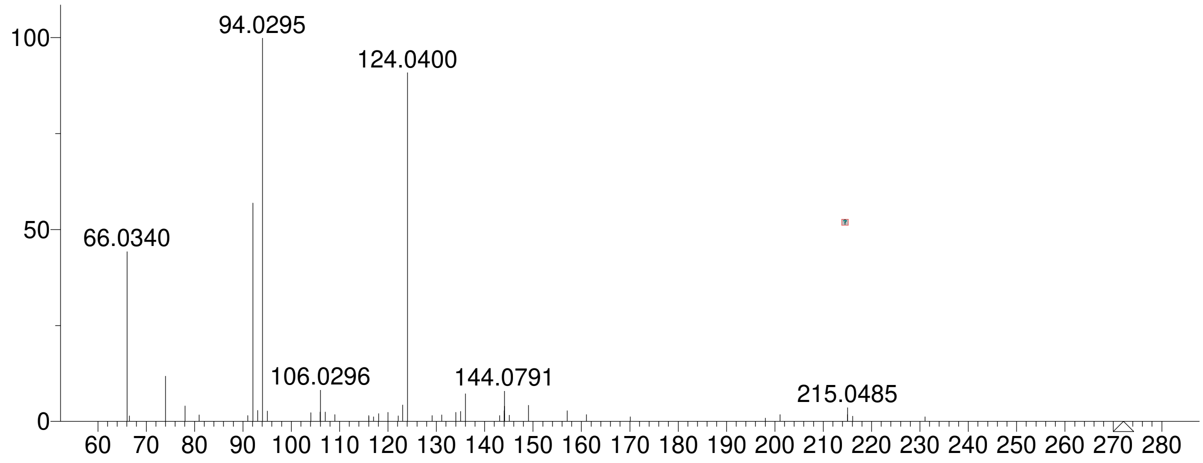

Hit 1 : 5-Hydroxymethylcytidine [M-H]- 20-30-40%) P=272.1
C10H15N3O6; MF: 0; RMF: 43; Prob 33.3%; Lib: mona-export-fiehn_hilic; ID: 1015.

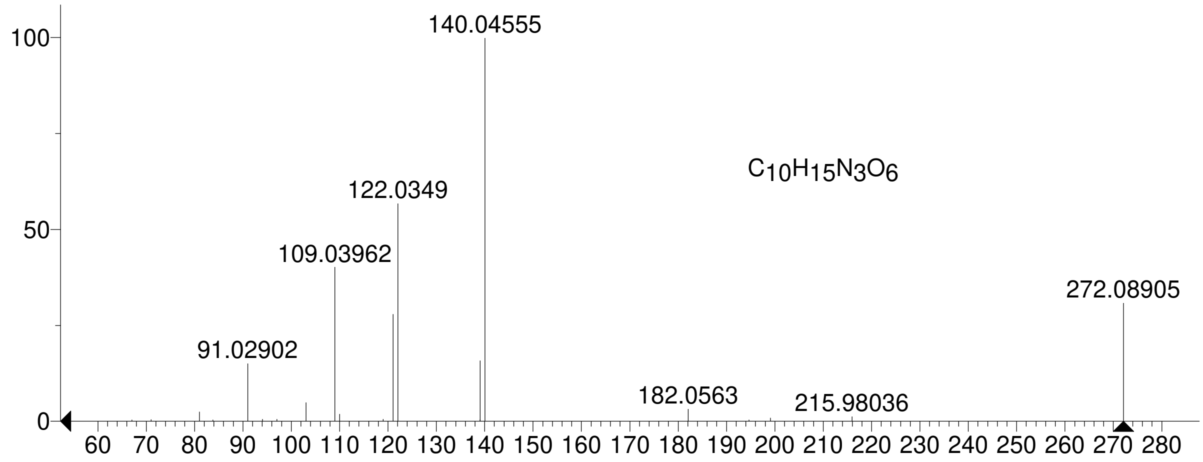

Hit 2 : L-Thyronine [M-H]- HCD 35V P=272.1
C15H15NO4; MF: 0; RMF: 25; Prob 33.3%; CAS: 1596-67-4; Lib: nist_msms; ID: 277919.

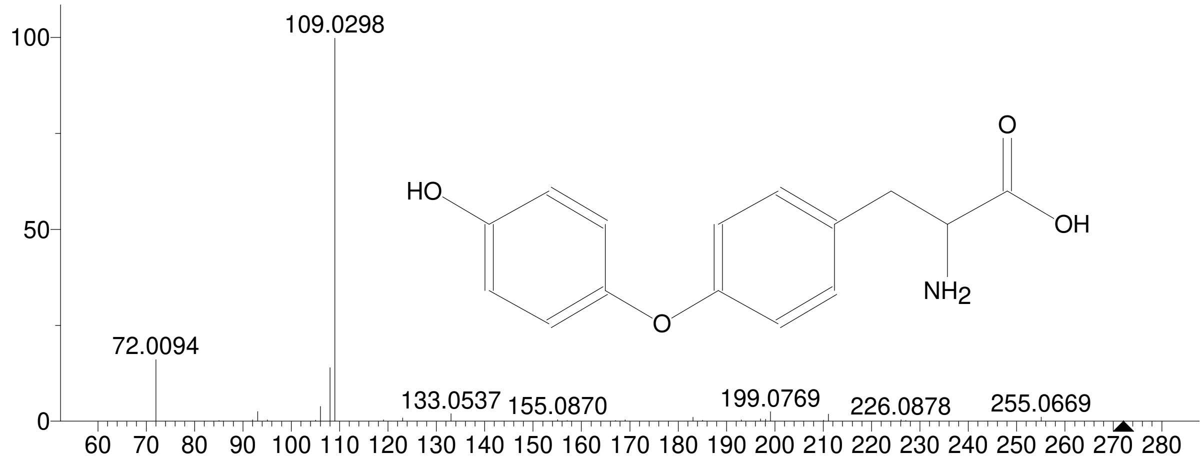

Hit 3 : rubiadin [M+NH4]+ 65HCD P=272.1
C15H10O4; MF: 0; RMF: 13; Prob 33.3%; Lib: mona-export-vf-npl_qexactive; ID: 3999.

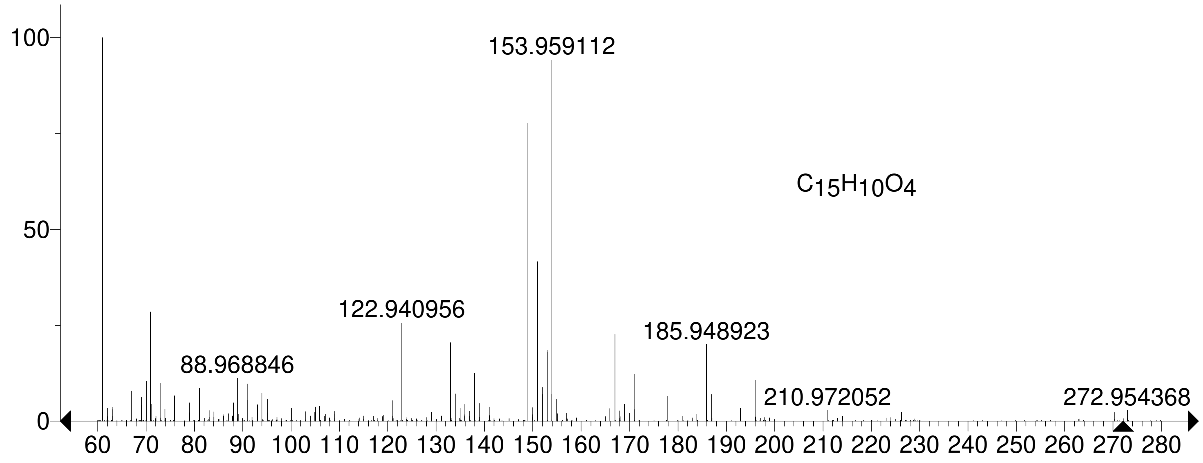

Unknown: Unknown (16.08_272.1496m/z)
Compound in Library Factor = 0

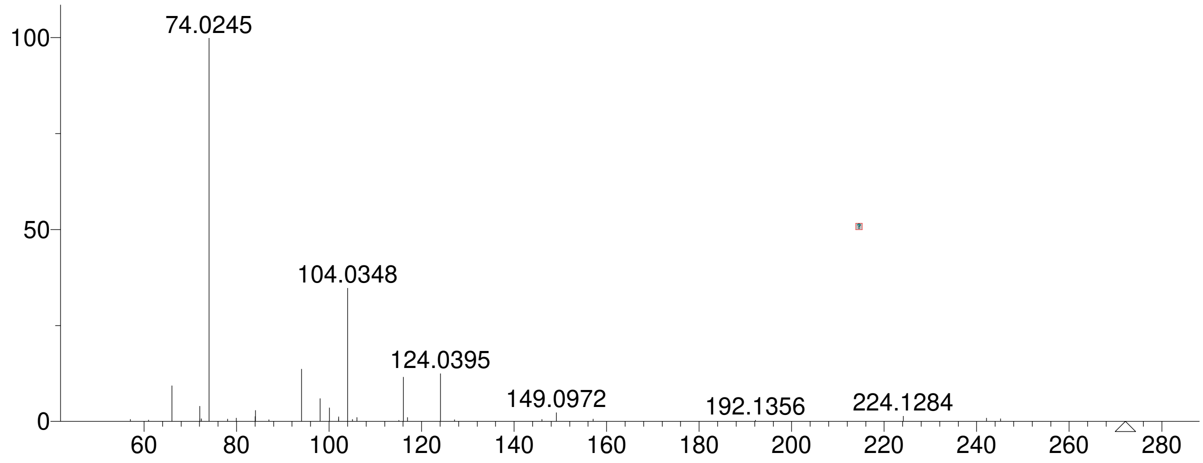

Unknown: Unknown (16.19_320.1129m/z)
Compound in Library Factor = -3273

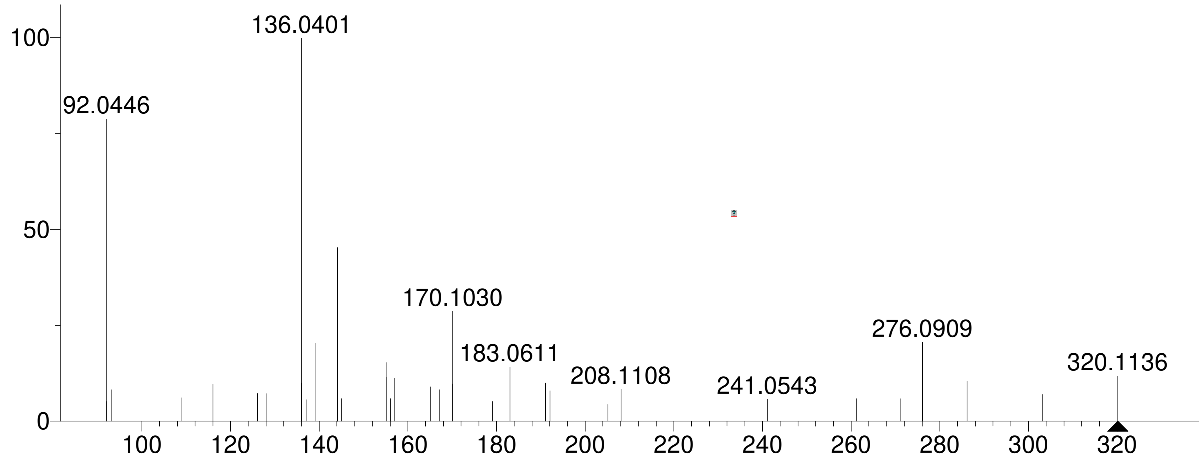

Hit 1 : (2R)-5,8-dihydroxy-2-(2-hydroxyphenyl)-7-methoxy-2,3-dihydrochromen-4-one [M+NH4]+ 65HCD P=320
C16H14O6; MF: 1; RMF: 55; Prob 34.2%; Lib: mona-export-vf-npl_qexactive; ID: 9708.

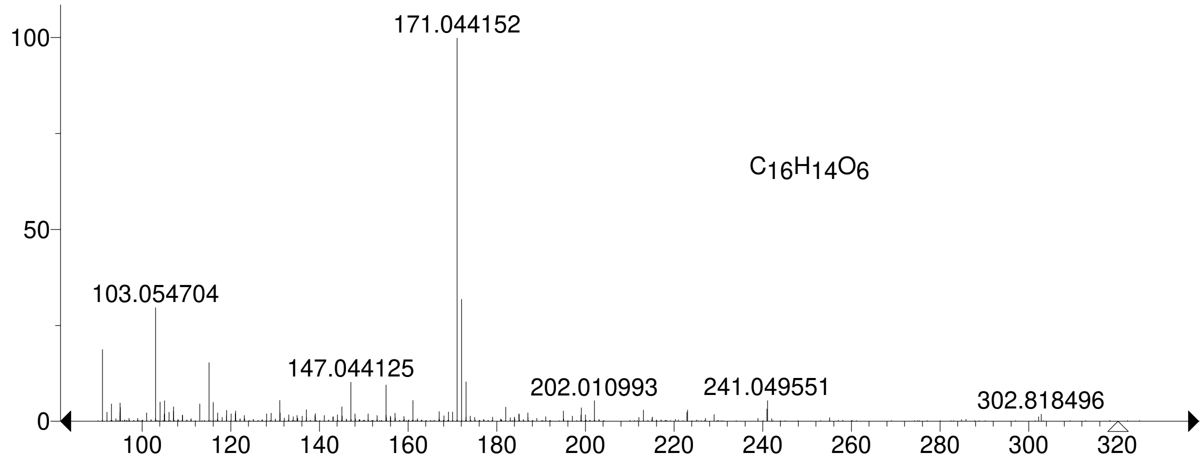

Hit 2 : (3,4,5-trihydroxy-6-methyloxan-2-yl) 2-(methylamino)benzoate [M+Na]+ 35HCD P=320.1
C14H19NO6; MF: 0; RMF: 26; Prob 32.9%; Lib: mona-export-vf-npl_qexactive; ID: 19195.

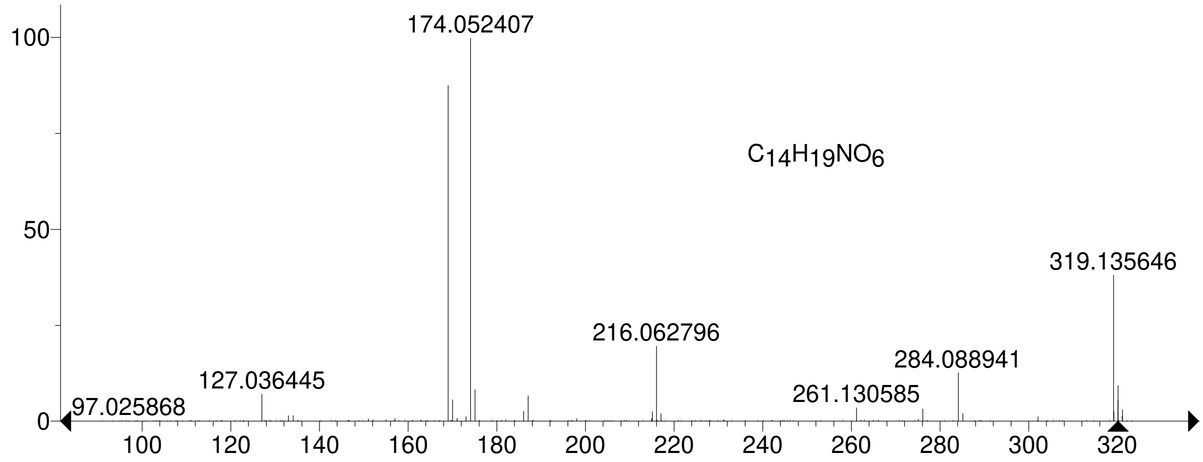

Hit 3 : Hematoxylin [M+NH4]+ 45HCD P=320.1
C16H14O6; MF: 0; RMF: 18; Prob 32.9%; Lib: mona-export-vf-npl_qexactive; ID: 17570.

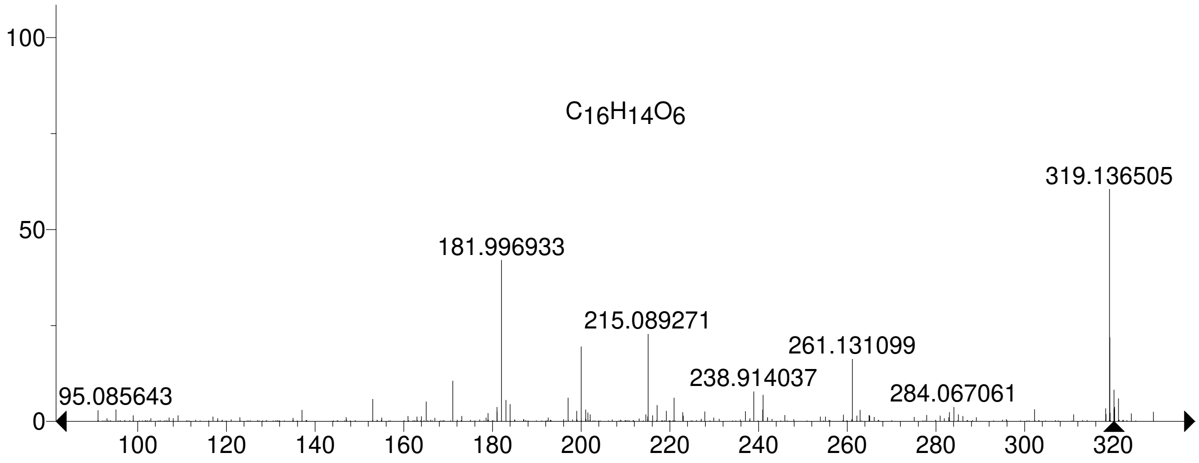

Unknown: Unknown (16.26_275.0216m/z)
Compound in Library Factor = 0

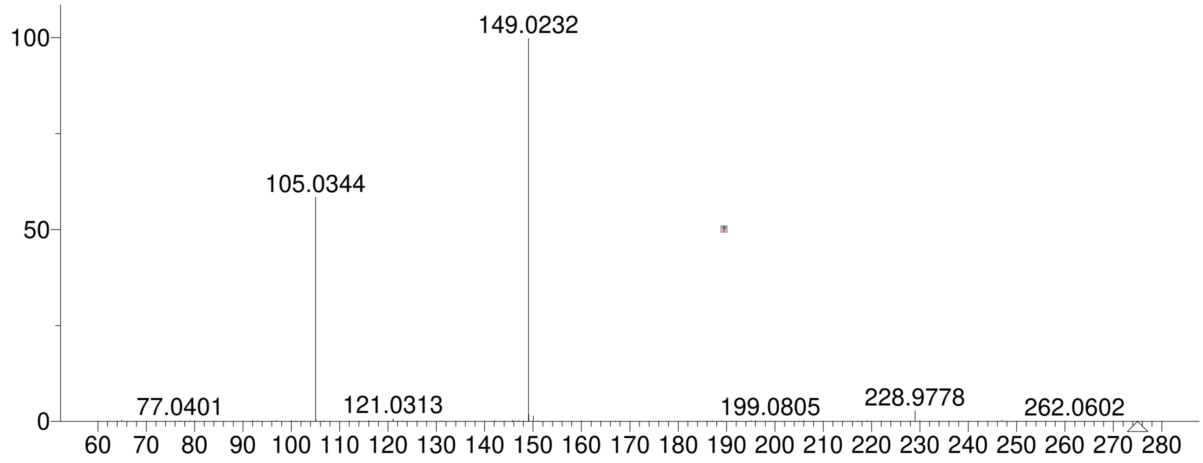

Hit 1 : 2-Thiophenemethanol, 5,5'-(2,5-furandiyl)bis- [M+H-H2O]+ HCD 60V P=275
C14H12O3S2; MF: 0; RMF: 13; Prob 100.0%; CAS: 213261-59-7; Lib: nist_msms; ID: 418063.

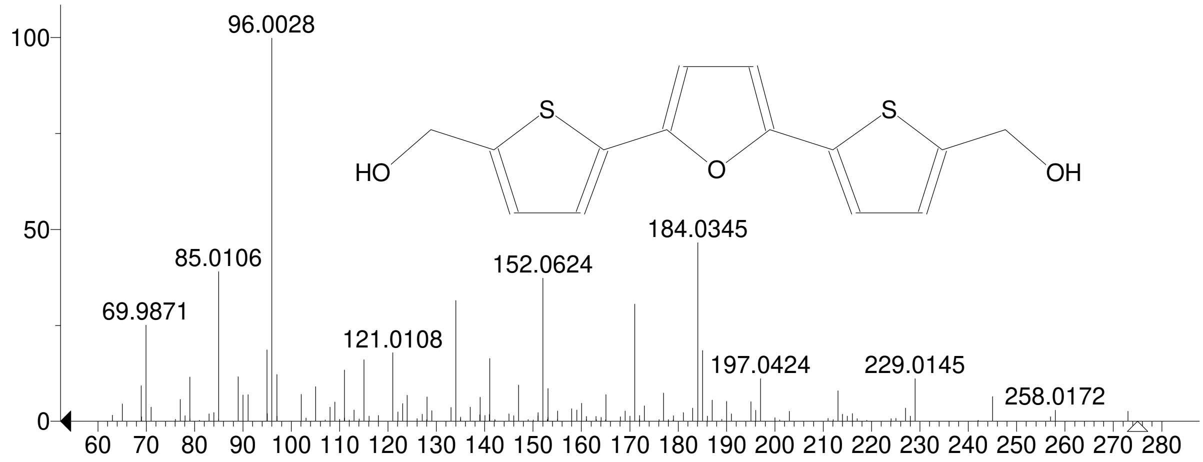

Unknown: Unknown (16.26_498.1056m/z)
Compound in Library Factor = 0

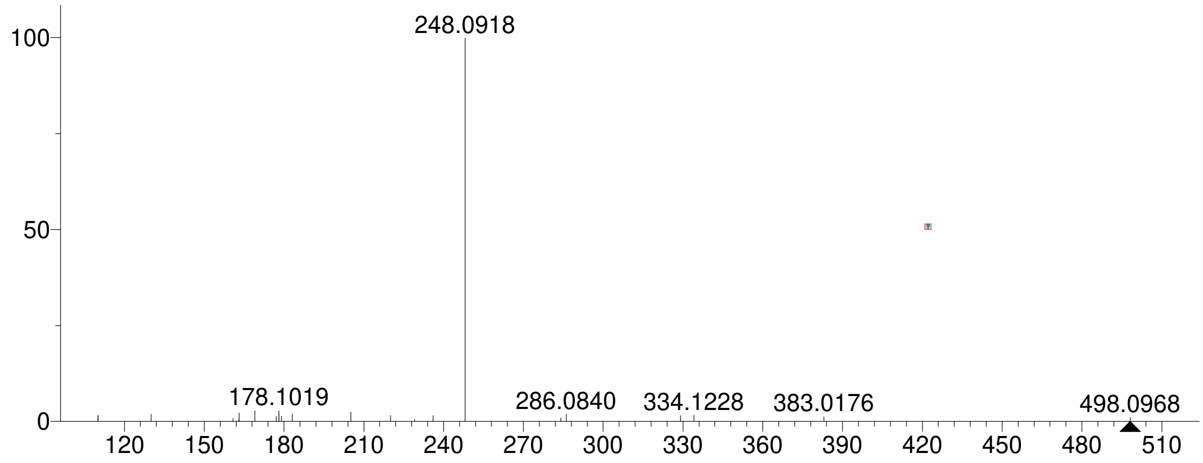

** Search Report Page 1 of 1 **

Unknown: Unknown (16.26_711.3686m/z)
Compound in Library Factor = 0

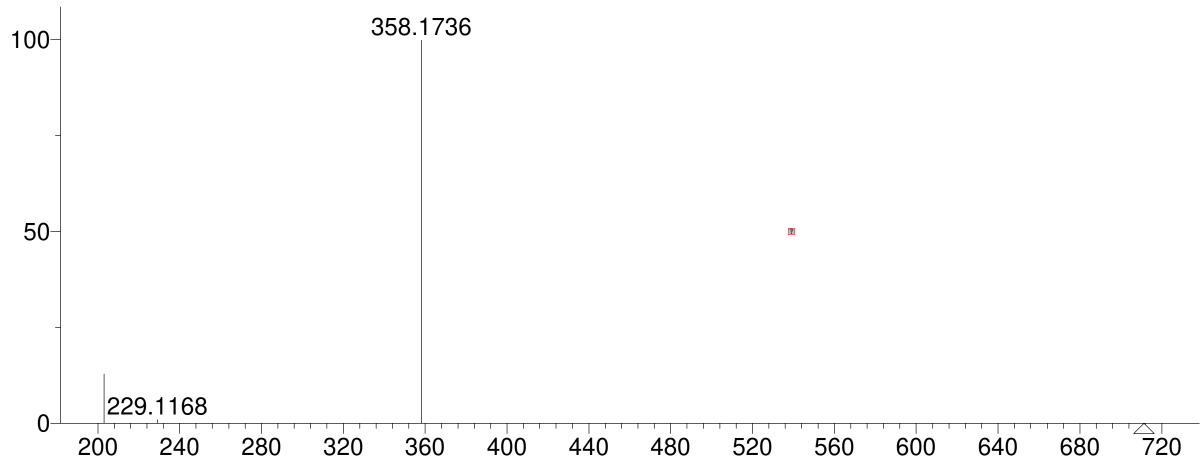

Hit 1 : Gastric Inhibitory Peptide, porcine [M+7H]7+ QTOF 30V P=711.4
C225H342N60O66S; MF: 0; RMF: 72; Prob 100.0%; CAS: 11063-17-5; Lib: nist_msms2; ID: 36869.

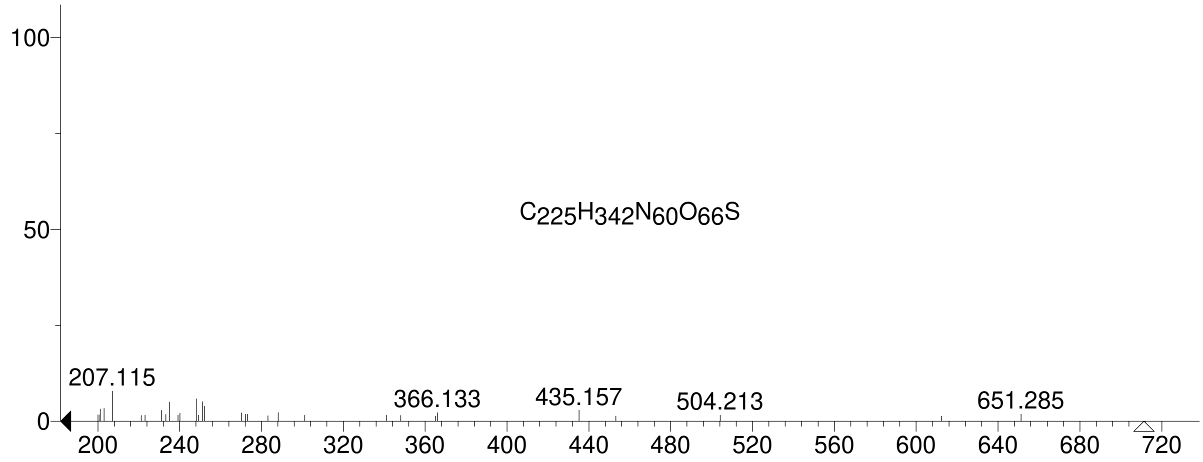

Unknown: Unknown (16.27_549.1035m/z)
Compound in Library Factor = 0

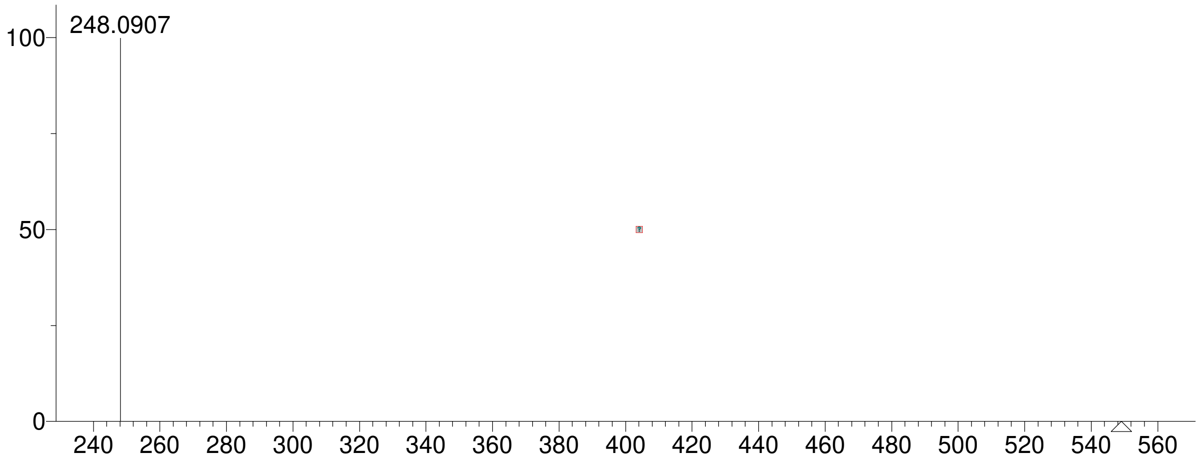

Unknown: Unknown (16.29_458.0991m/z)
Compound in Library Factor = 0

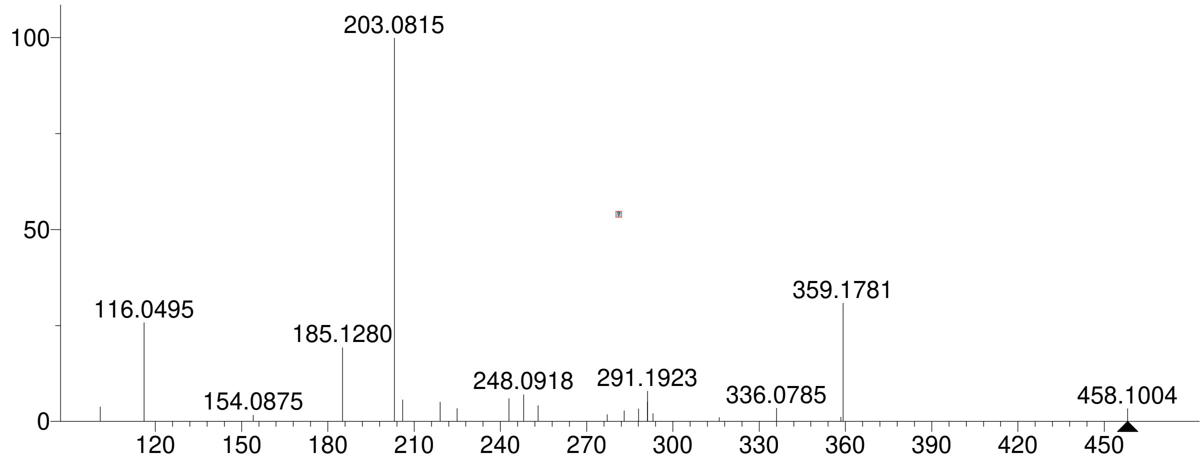

Hit 1 : Desmethylbosentan [M-H]-=>474.1=>458.1 IT 35% P=458.1
C26H27N5O6S; MF: 0; RMF: 88; Prob 25.0%; CAS: 253688-61-8; Lib: nist_msms; ID: 451556.

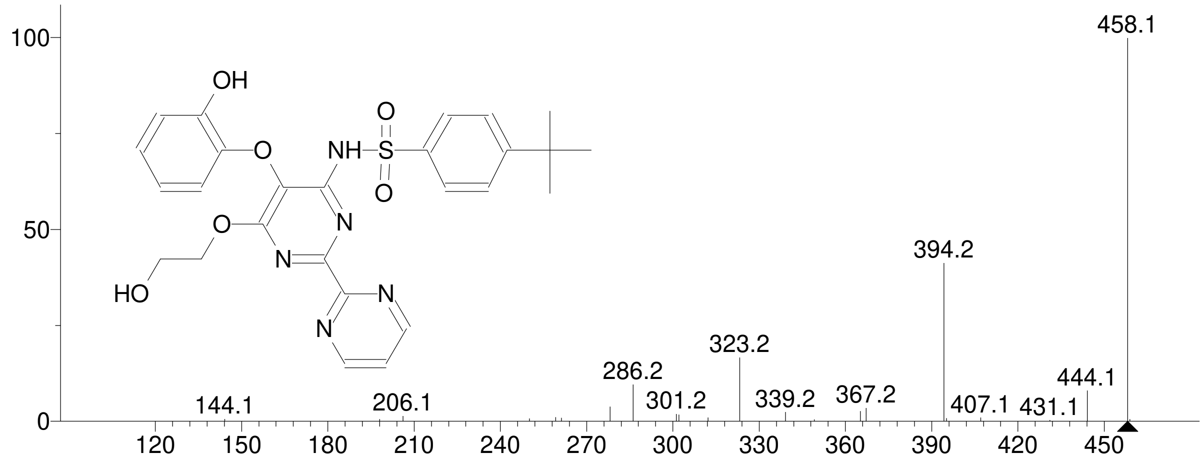

Hit 2 : Lapatinib [M+H]+=>458.1 IT 35% P=458.1
C29H26ClFN4O4S; MF: 0; RMF: 63; Prob 25.0%; CAS: 231277-92-2; Lib: nist_msms; ID: 254911.

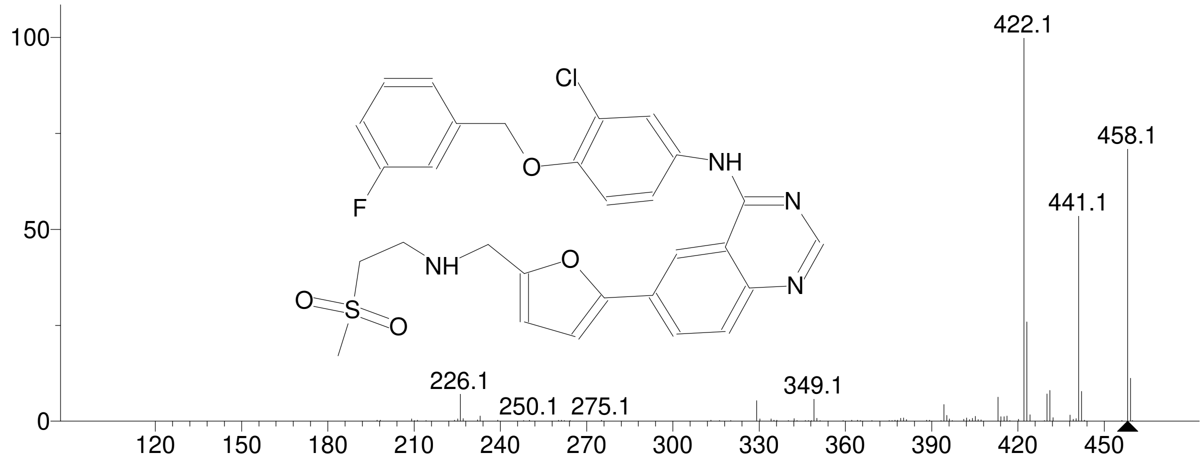

Hit 3 : Sialyl Lewis A [M+Na-H2O]+=>476.1=>458.1 IT 35% P=458.1
C31H52N2O23; MF: 0; RMF: 48; Prob 25.0%; CAS: 92448-22-1; Lib: nist_msms; ID: 533705.

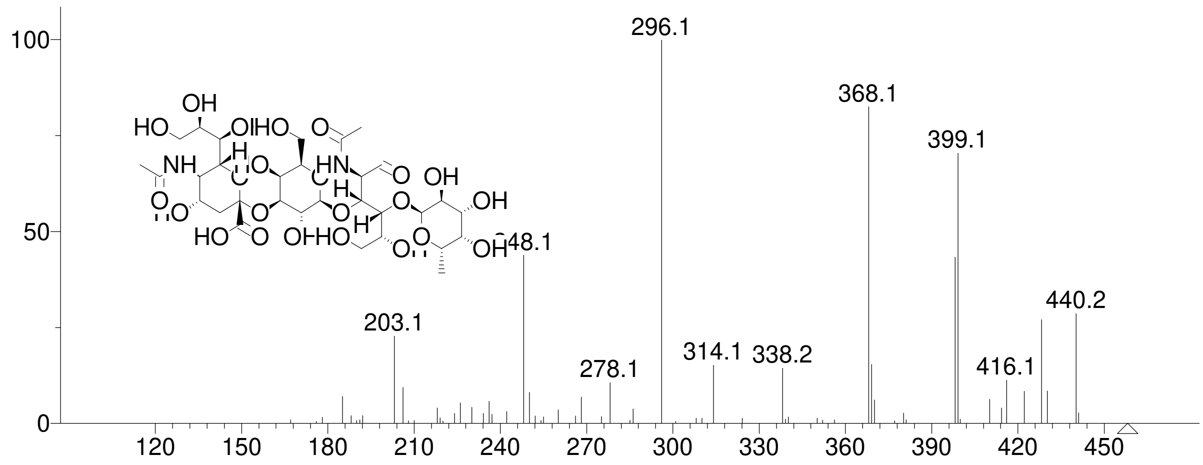

Hit 4 : Gossypol [M+H-H2O]+=>473.2=>458.1 IT 35% P=458.1
C30H30O8; MF: 0; RMF: 9; Prob 25.0%; CAS: 303-45-7; Lib: nist_msms; ID: 406557.

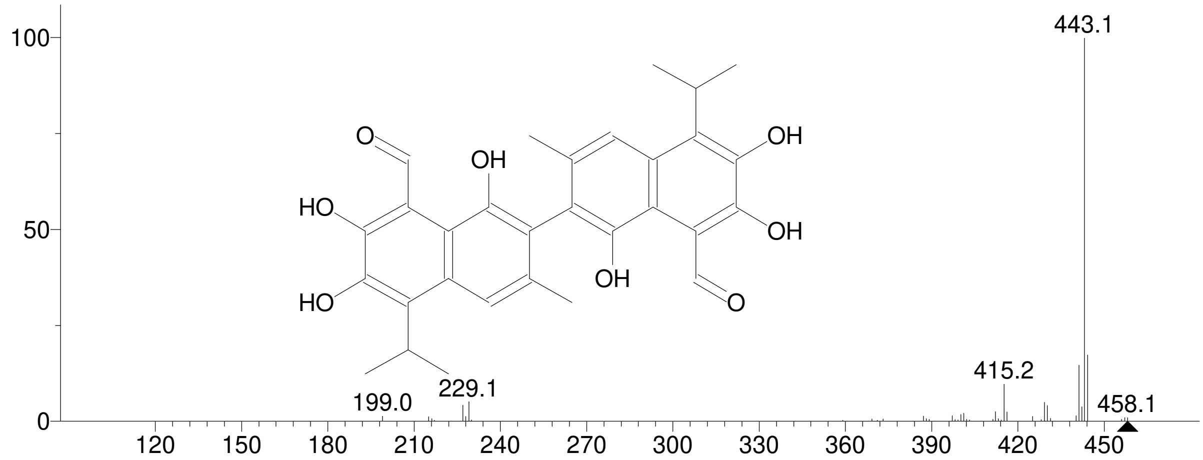

Unknown: Unknown (16.29_519.0171m/z)
Compound in Library Factor = 0

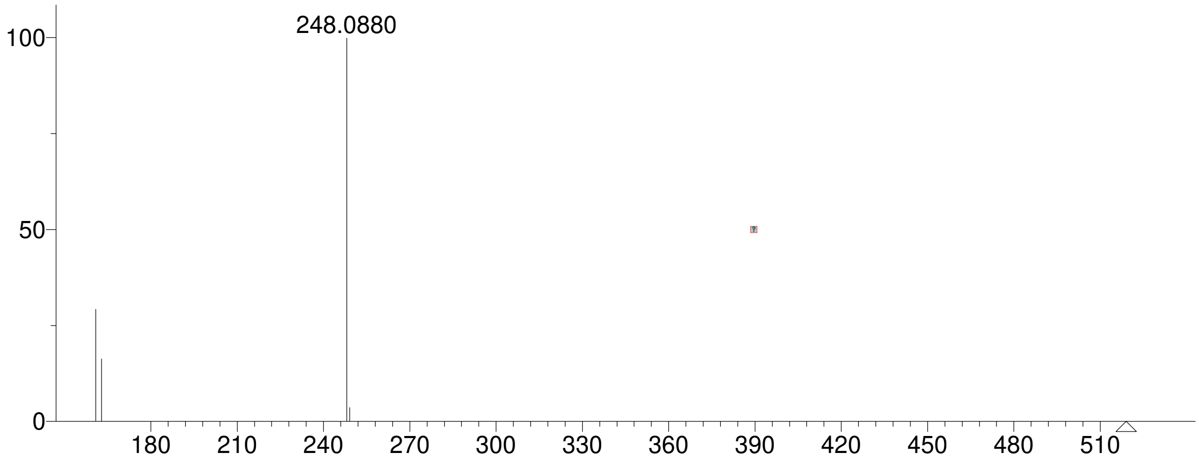

Unknown: Unknown (16.31_358.1766m/z)
Compound in Library Factor = -674

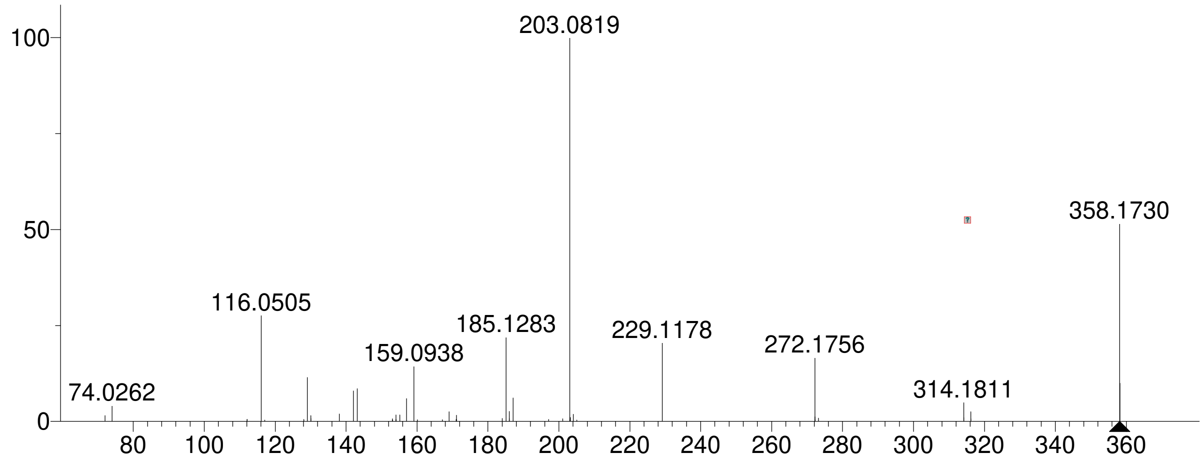

Hit 1 : 1H-Indazole-1-pentanoic acid, 3-[[[1-(aminocarbonyl)-2,2-dimethylpropyl]amino]carbonyl]- [M+H-NH3]+ H
C19H26N4O4; MF: 129; RMF: 696; Prob 91.2%; Lib: nist_msms; ID: 258958.

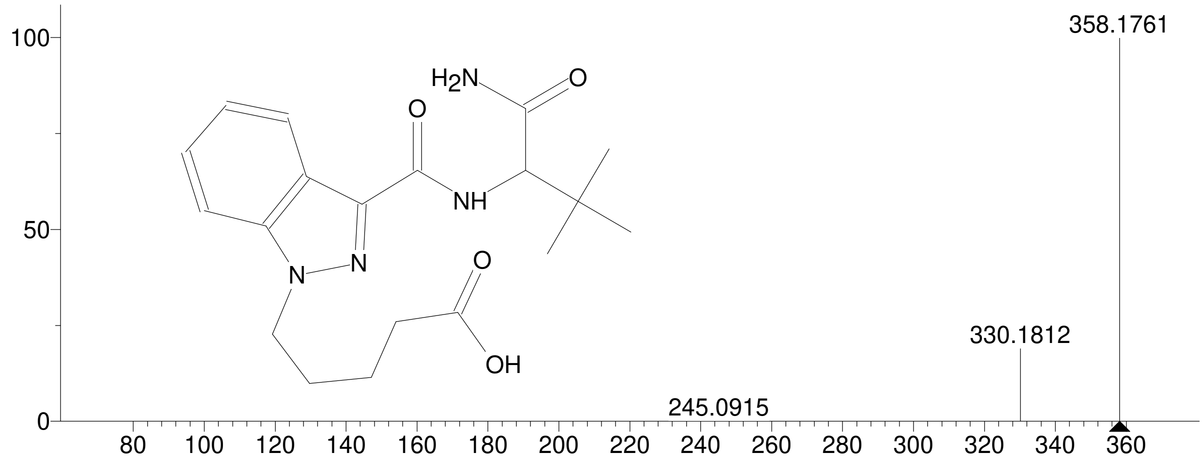

Hit 2 : NCGC00380403-01_C19H23N3O4_2H-Pyrazino[1',2':1,5]pyrrolo[2,3-b]indole-1,4(3H,5aH)-dione, 6-acetyl-
C19H23N3O4; MF: 16; RMF: 217; Prob 5.61%; Lib: mona-export-gnps; ID: 10237.

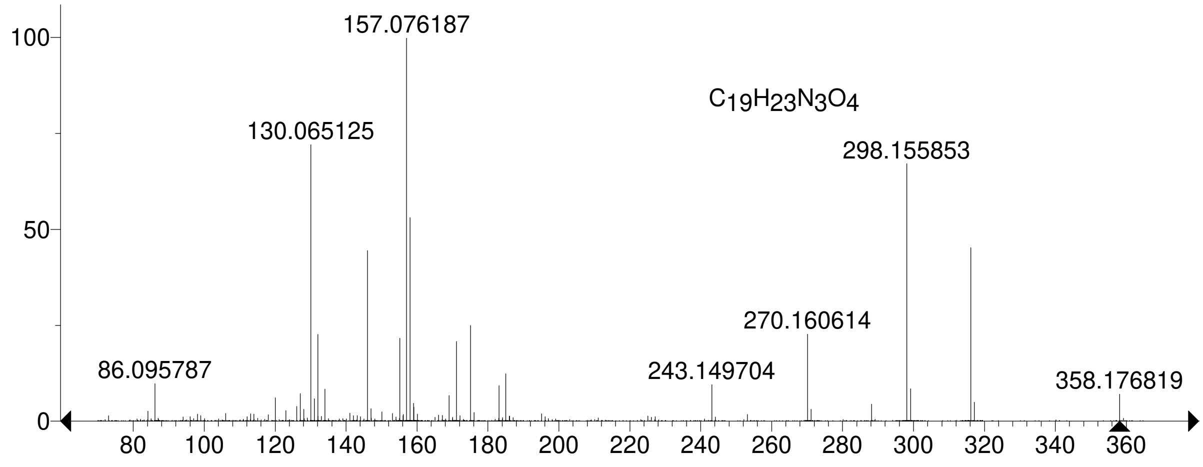

Hit 3 : AMG-073 HCl (Cinacalcet hydrochloride) [M+H]+ P=358.2
C22H23ClF3N; MF: 0; RMF: 48; Prob 3.23%; Lib: mona-export-gnps; ID: 403.

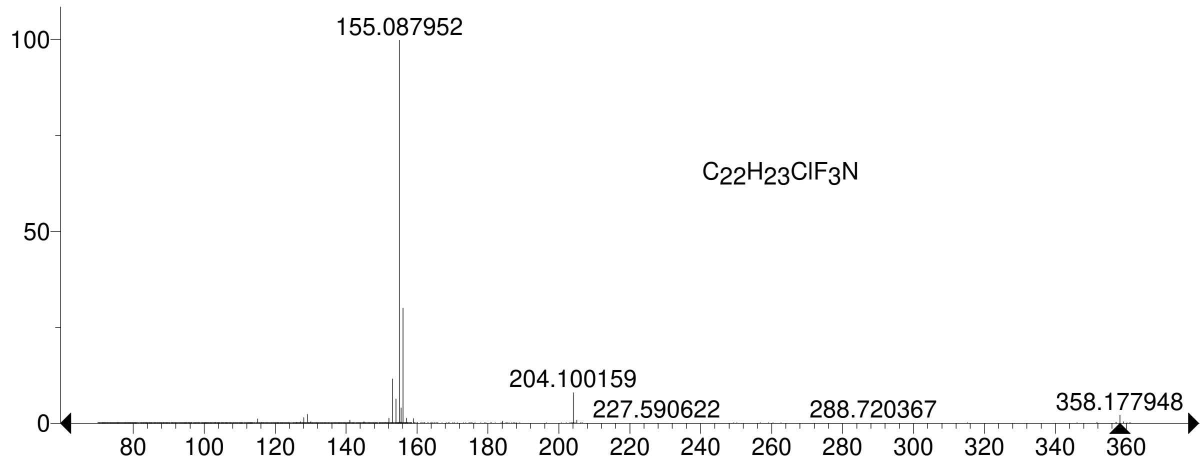

** Search Report Page 1 of 1 **

Unknown: Unknown (16.31_358.3541m/z)
Compound in Library Factor = 0

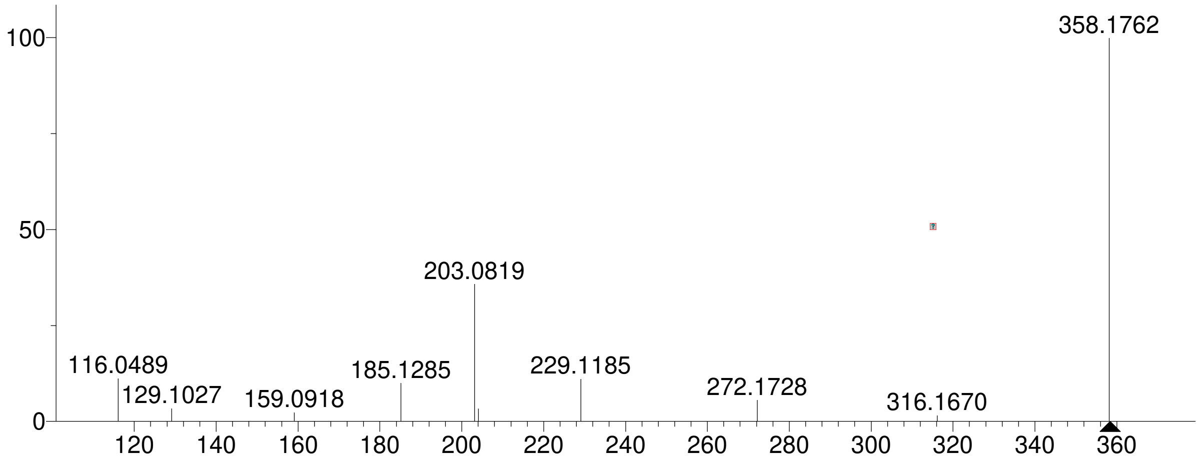

** Search Report Page 1 of 1 **

Unknown: Unknown (16.32_739.3442m/z)
Compound in Library Factor = 0

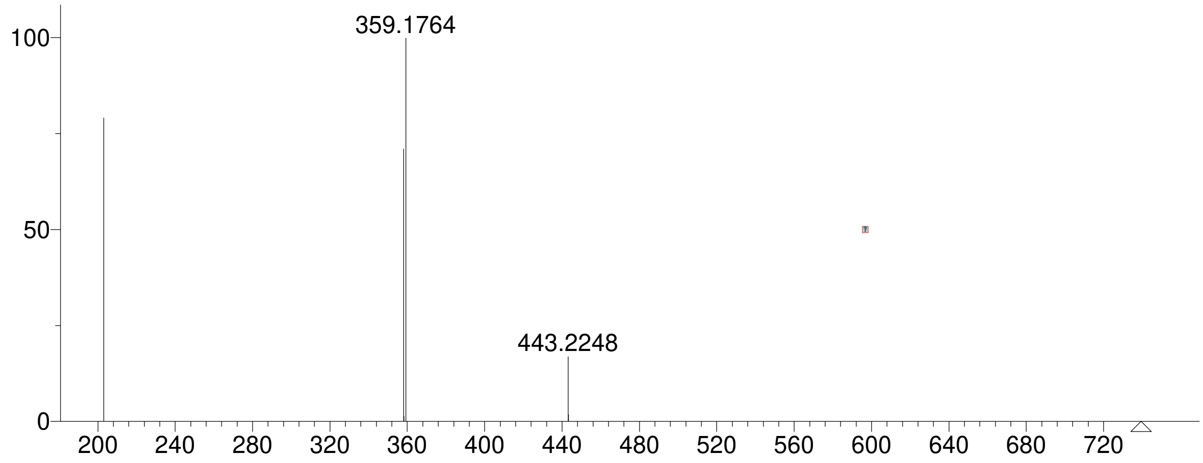

** Search Report Page 1 of 2 **

Unknown: Unknown (16.33_458.1003m/z)
Compound in Library Factor = -3273

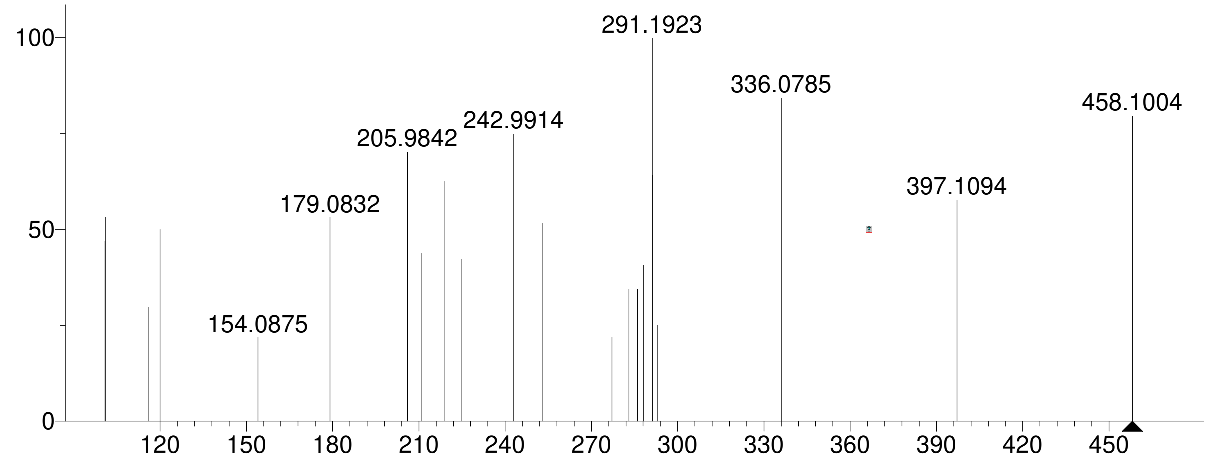

Hit 1 : Desmethylbosentan [M-H]-=>474.1=>458.1 IT 35% P=458.1
C26H27N5O6S; MF: 2; RMF: 203; Prob 35.1%; CAS: 253688-61-8; Lib: nist_msms; ID: 451556.

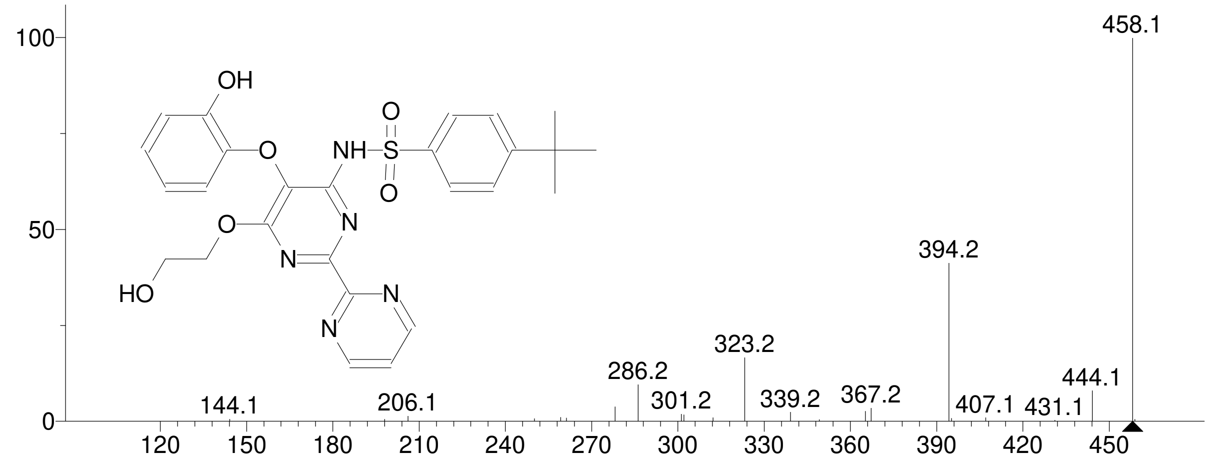

Hit 2 : Lapatinib [M+H]+=>458.1 IT 35% P=458.1
C29H26ClFN4O4S; MF: 0; RMF: 136; Prob 32.4%; CAS: 231277-92-2; Lib: nist_msms; ID: 254911.

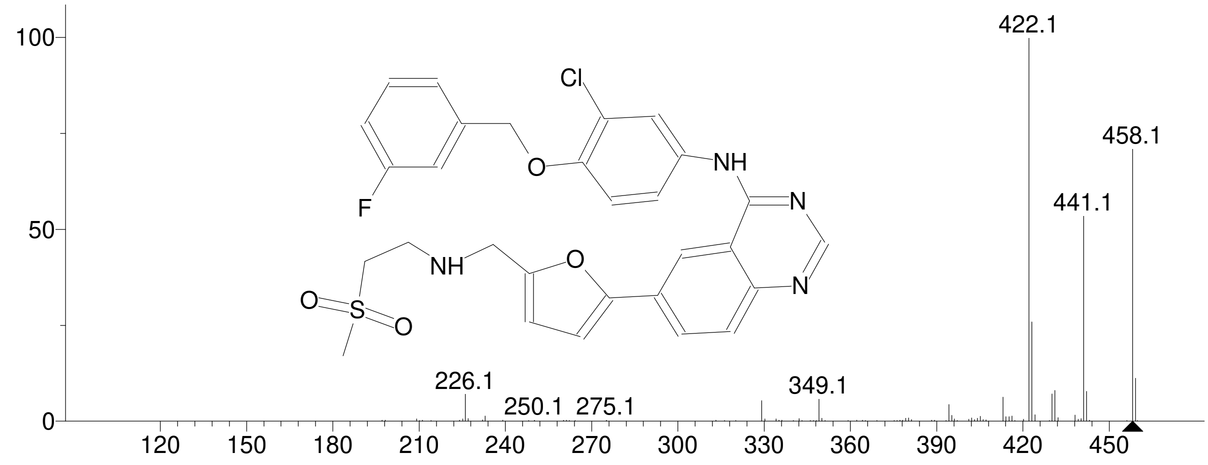

Hit 3 : Gossypol [M+H-H2O]+=>473.2=>458.1 IT 35% P=458.1
C30H30O8; MF: 0; RMF: 43; Prob 32.4%; CAS: 303-45-7; Lib: nist_msms; ID: 406557.

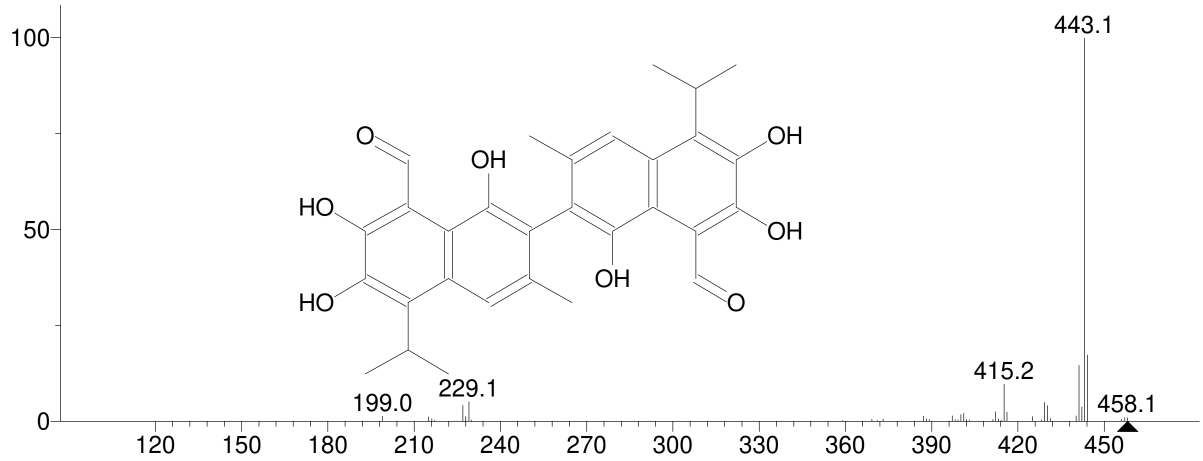

Unknown: Unknown (16.33_487.2547m/z)
Compound in Library Factor = 0

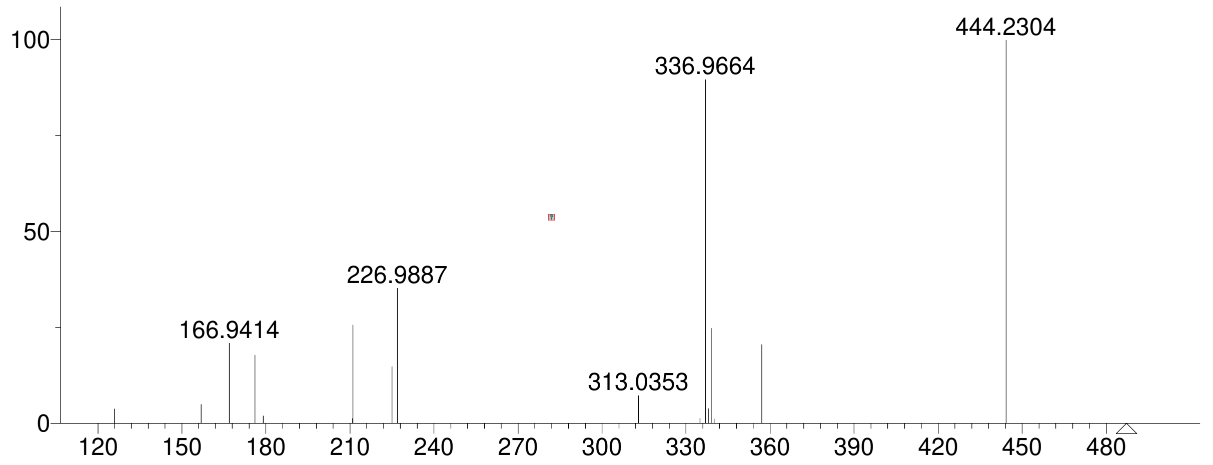

Hit 1 : 1-[[(2S,3R,11bR)-3-ethyl-9,10-dimethoxy-2,3,4,6,7,11b-hexahydro-1H-benzo[a]quinolizin-2-yl]methyl]-7-me
C28H36N2O4; MF: 0; RMF: 30; Prob 100.0%; Lib: mona-export-vf-npl_qexactive; ID: 19092.

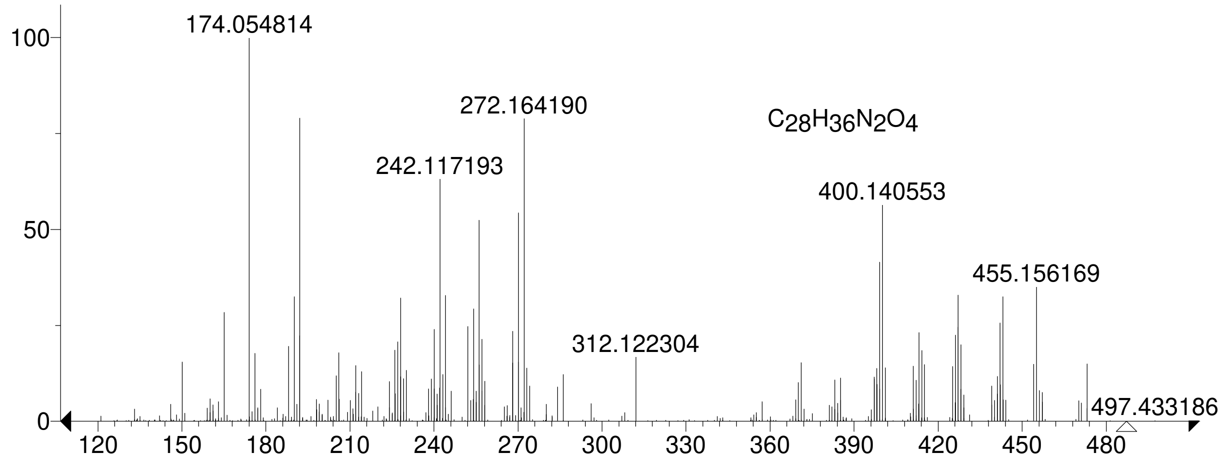

Unknown: Unknown (16.34_443.2291m/z)
Compound in Library Factor = -1523

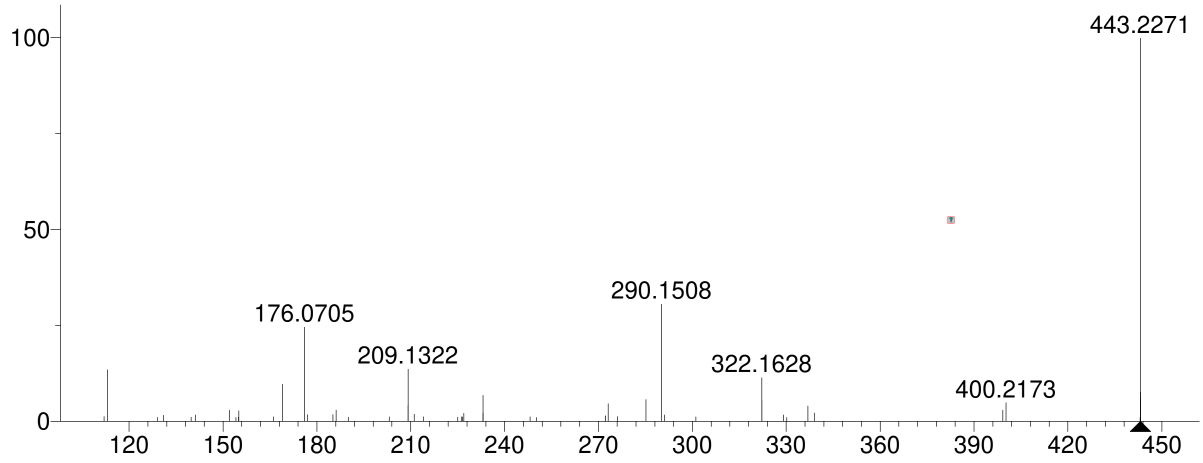

Hit 1 : Rhodamine B cation [Cat]+ IT-FT 35% P=443.2
C28H31N2O3; MF: 117; RMF: 789; Prob 72.1%; CAS: 64381-98-2; Lib: nist_msms; ID: 493932.

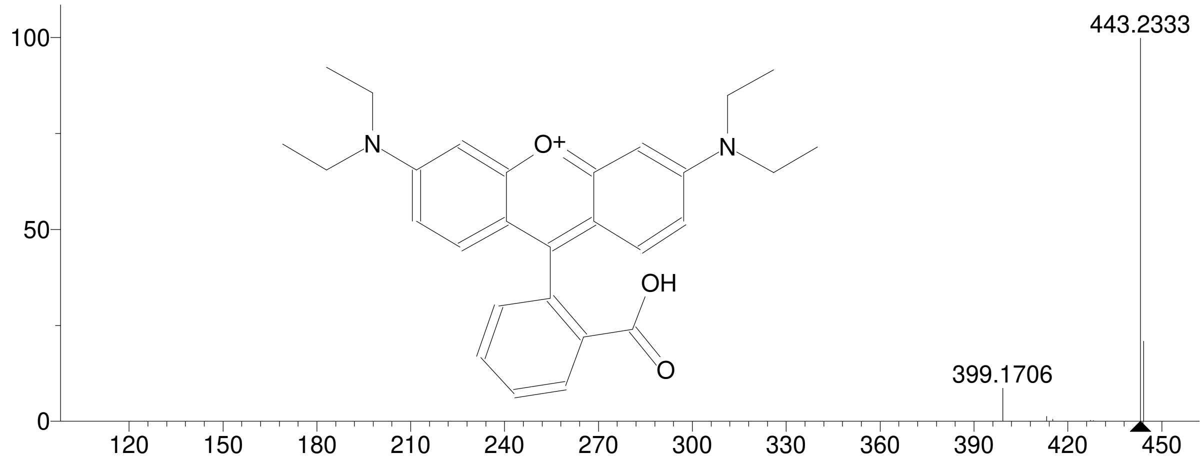

Hit 2 : L-Valinamide, N-(4-methoxy-1,4-dioxobutyl)-L-alanyl-L-alanyl-L-prolyl-N-(4-methyl-2-oxo-2H-1-benzopyran
C31H41N5O9; MF: 68; RMF: 369; Prob 14.8%; CAS: 72252-90-5; Lib: nist_msms; ID: 548181.

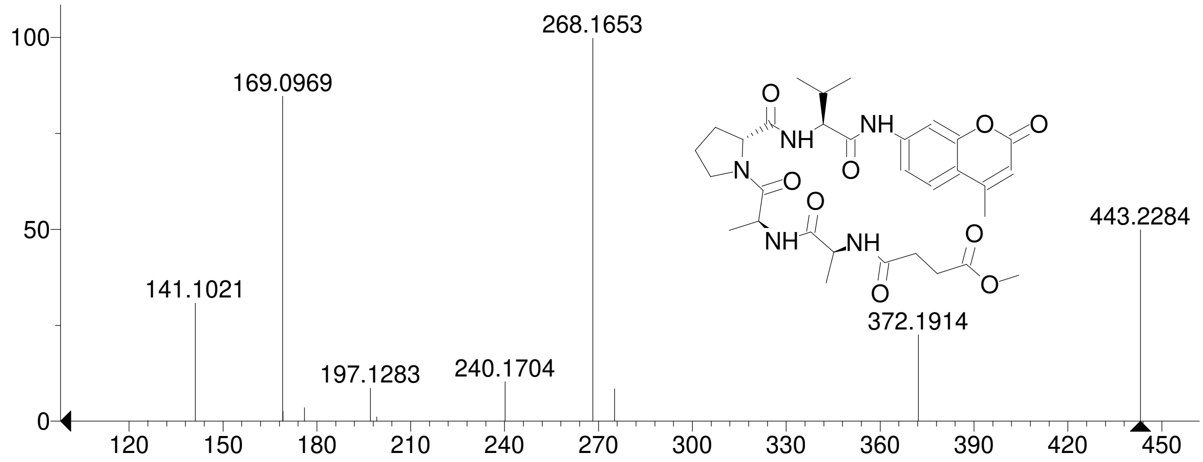

Hit 3 : Rhodamine 6G cation [Cat]+ HCD 31V P=443.2
C28H31N2O3; MF: 58; RMF: 847; Prob 10.4%; CAS: 47724-48-1; Lib: nist_msms; ID: 278957.

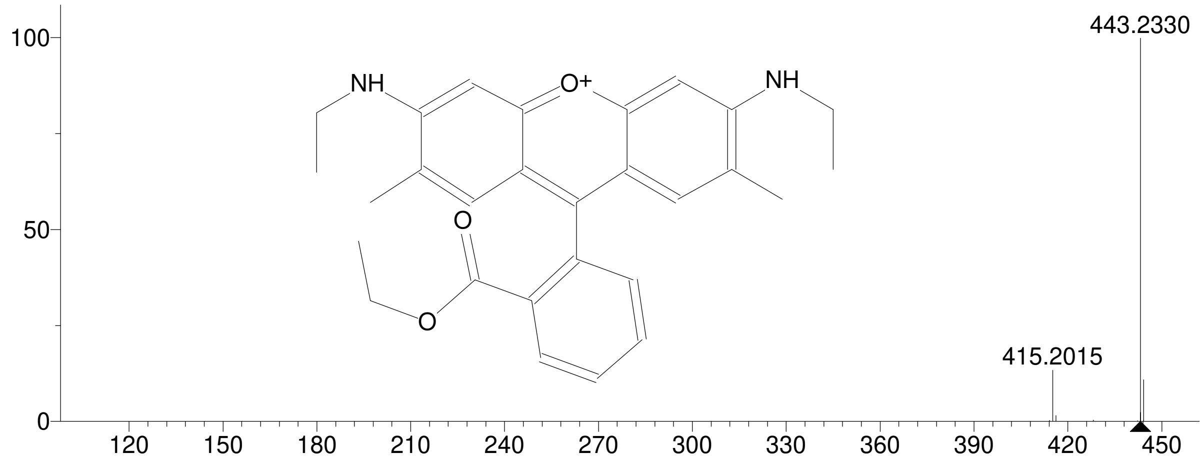

Hit 4 : O-Desmethylastemizole [M-H]- HCD 26V P=443.2
C27H29FN4O; MF: 23; RMF: 527; Prob 2.62%; CAS: 73736-50-2; Lib: nist_msms; ID: 451450.

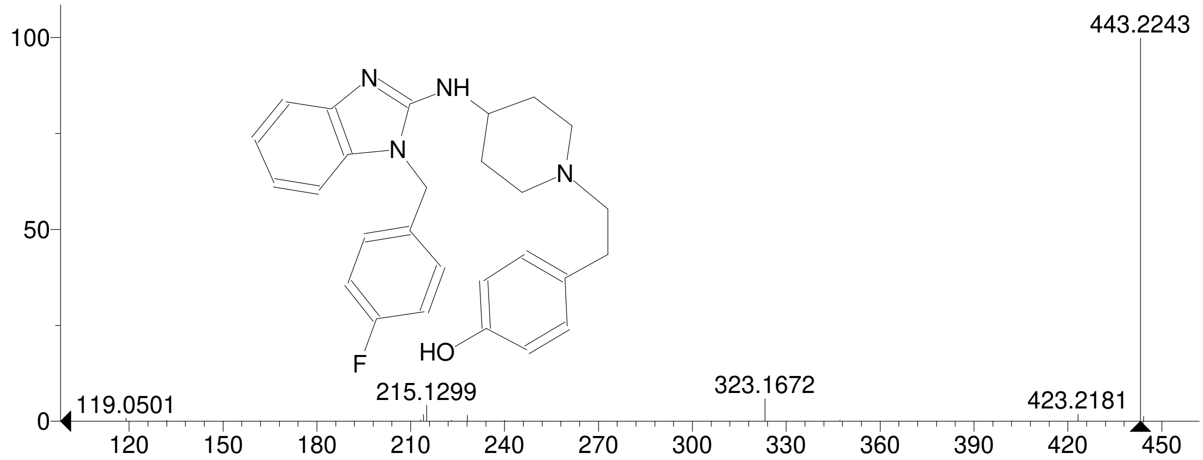

Unknown: Unknown (16.34_846.4412m/z)
Compound in Library Factor = 0

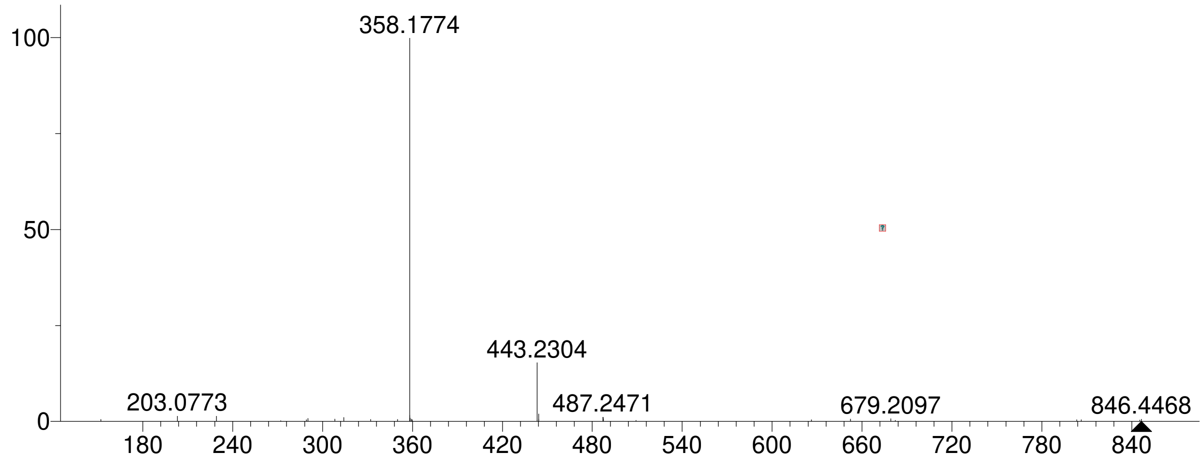

Unknown: Unknown (16.35_373.9351m/z)
Compound in Library Factor = 0

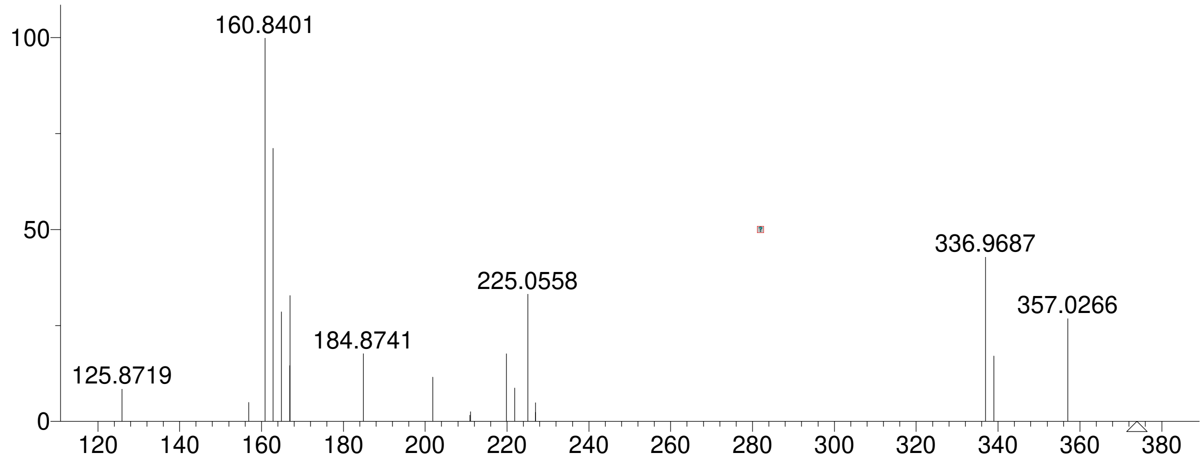

Unknown: Unknown (16.35_383.9638m/z)
Compound in Library Factor = 0

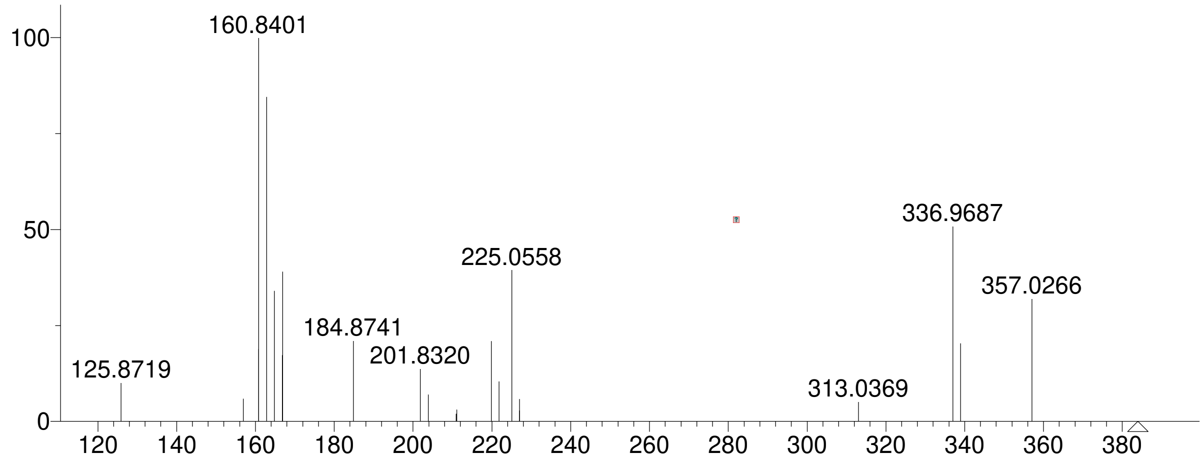

** Search Report Page 1 of 1 **

Unknown: Unknown (16.35_391.9956m/z)
Compound in Library Factor = 0

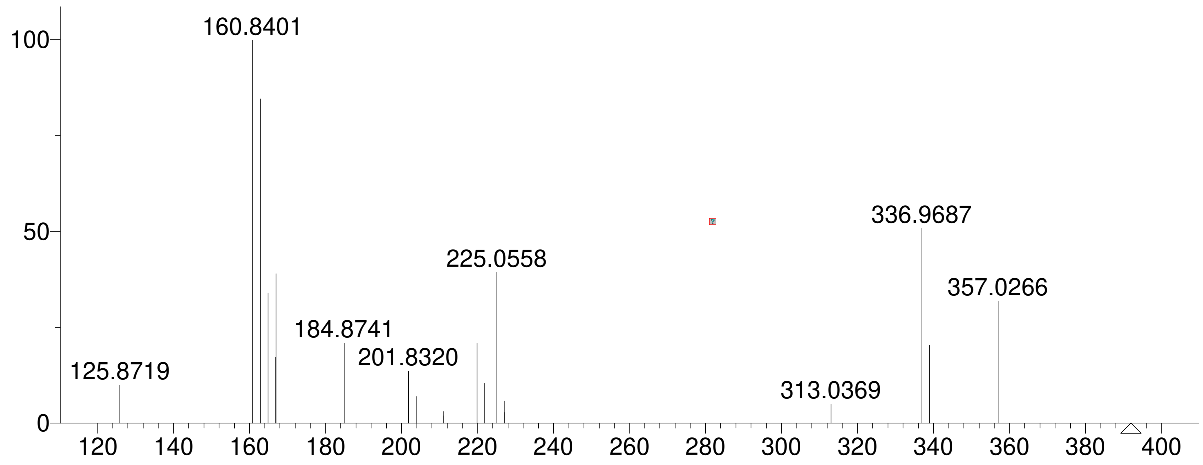

** Search Report Page 1 of 1 **

Unknown: Unknown (16.35_402.0246m/z)
Compound in Library Factor = 0

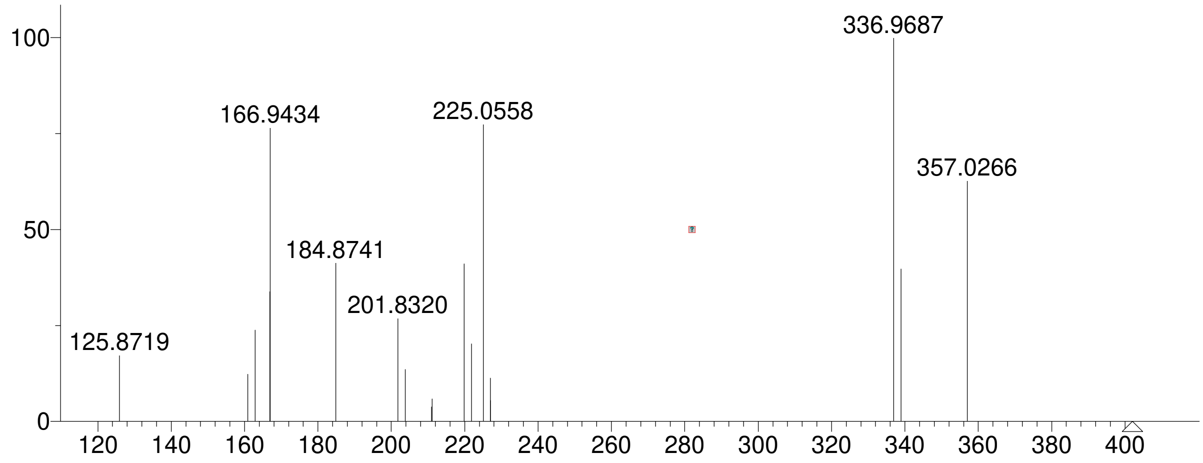

Hit 1 : Benzenesulfonamide, 4-[5-(4-chlorophenyl)-3-(trifluoromethyl)-1H-pyrazol-1-yl]- [M+H]+ HCD 60V P=40[illegible]
C16H11ClF3N3O2S; MF: 0; RMF: 7; Prob 100.0%; CAS: 170569-86-5; Lib: nist_msms; ID: 427054.

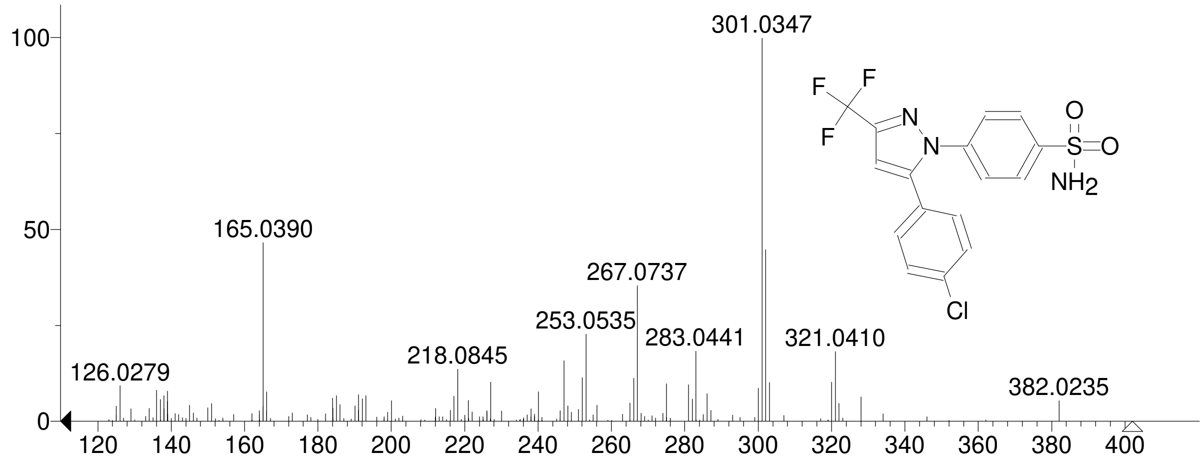

Unknown: Unknown (16.35_558.0951m/z)
Compound in Library Factor = -2387

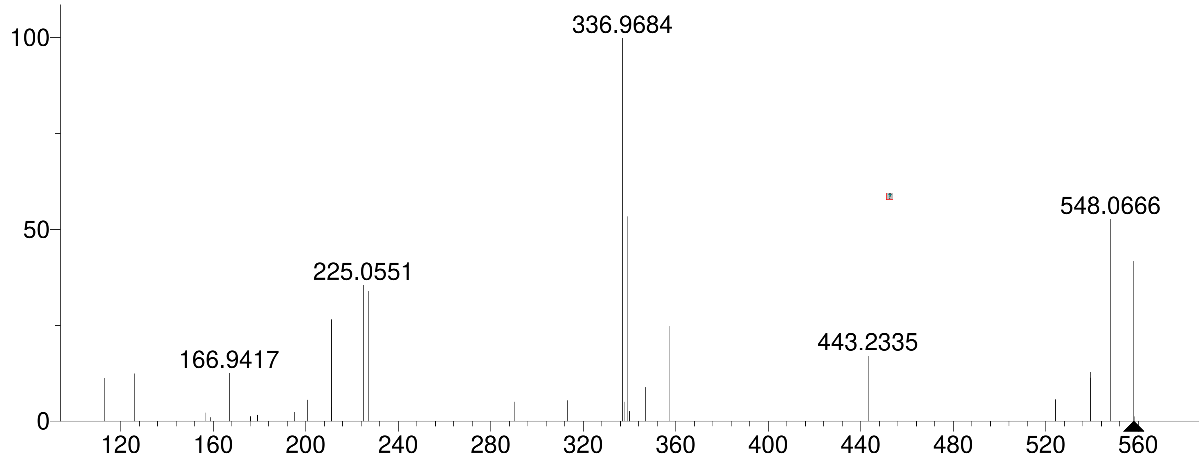

Hit 1 : Ergocryptine [in-source] QIT 25->3 P=558.1
C32H41N5O5; MF: 10; RMF: 264; Prob 100.0%; Lib: mona-export-respect; ID: 6090.

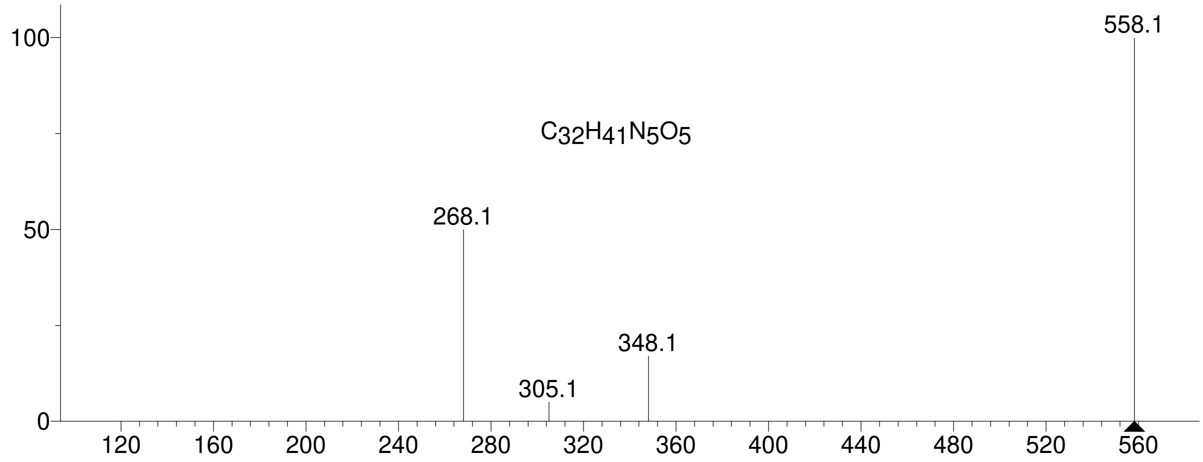

Unknown: Unknown (16.36_381.9669m/z)
Compound in Library Factor = 0

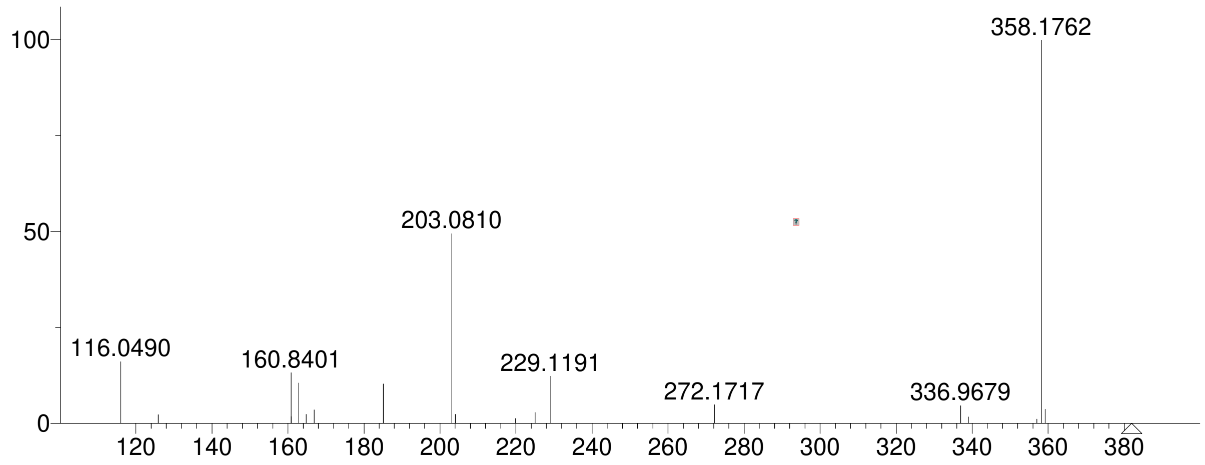

Hit 1 : 3,3'-Diiodo-L-thyronine [M+H-H3NI]+ IT-FT 35% P=382
C15H13I2NO4; MF: 0; RMF: 64; Prob 100.0%; CAS: 70-40-6; Lib: nist_msms; ID: 187503.

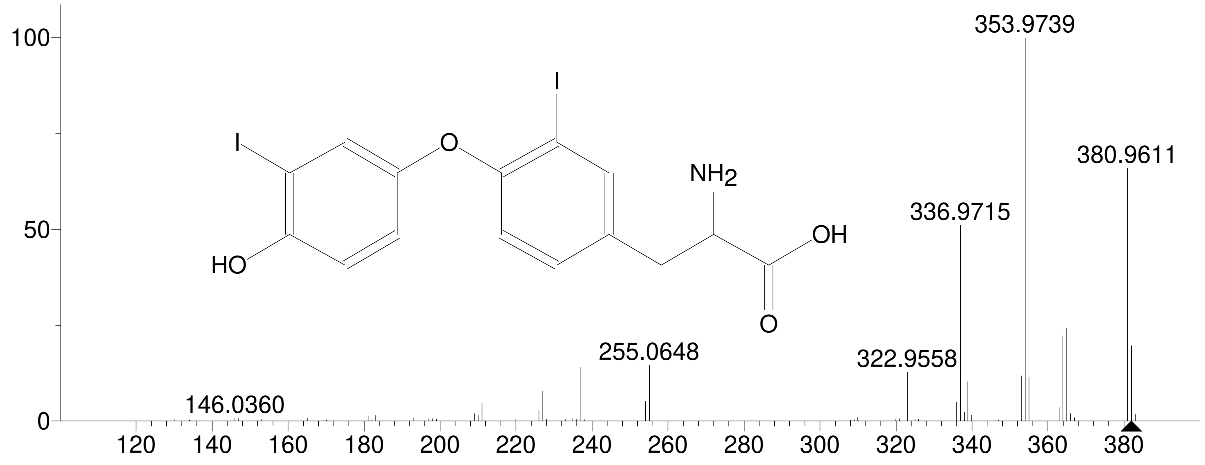

** Search Report Page 1 of 1 **

Unknown: Unknown (16.36_568.1242m/z)
Compound in Library Factor = 0

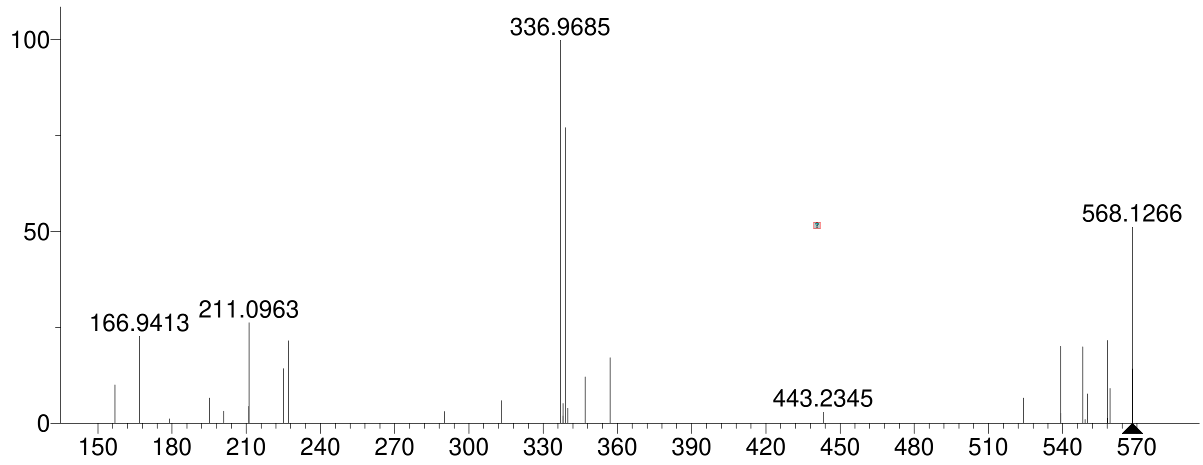

** Search Report Page 1 of 1 **

Unknown: Unknown (16.37_371.9378m/z)
Compound in Library Factor = 0

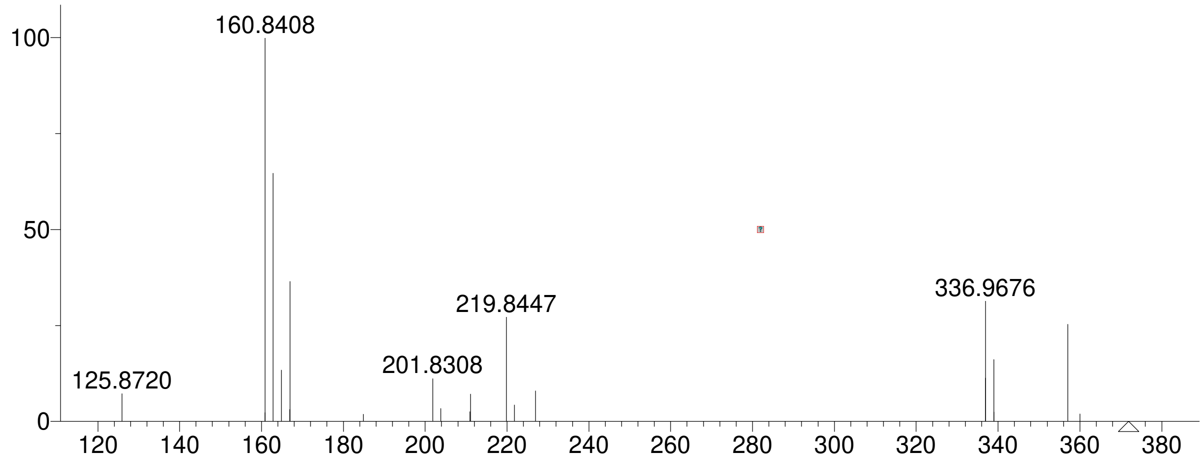

** Search Report Page 1 of 1 **

Unknown: Unknown (16.37_452.0063m/z)
Compound in Library Factor = 0

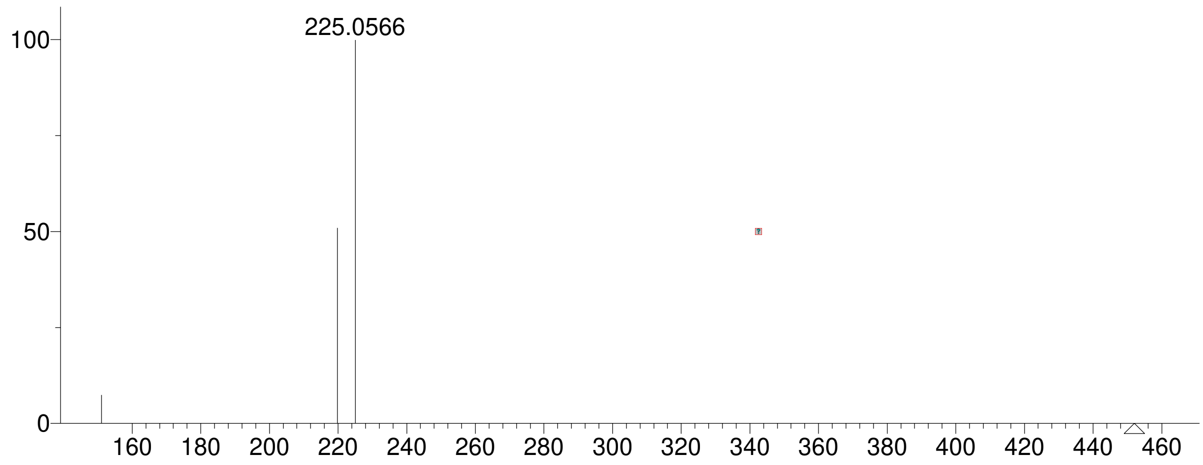

Unknown: Unknown (16.37_548.0664m/z)
Compound in Library Factor = 0

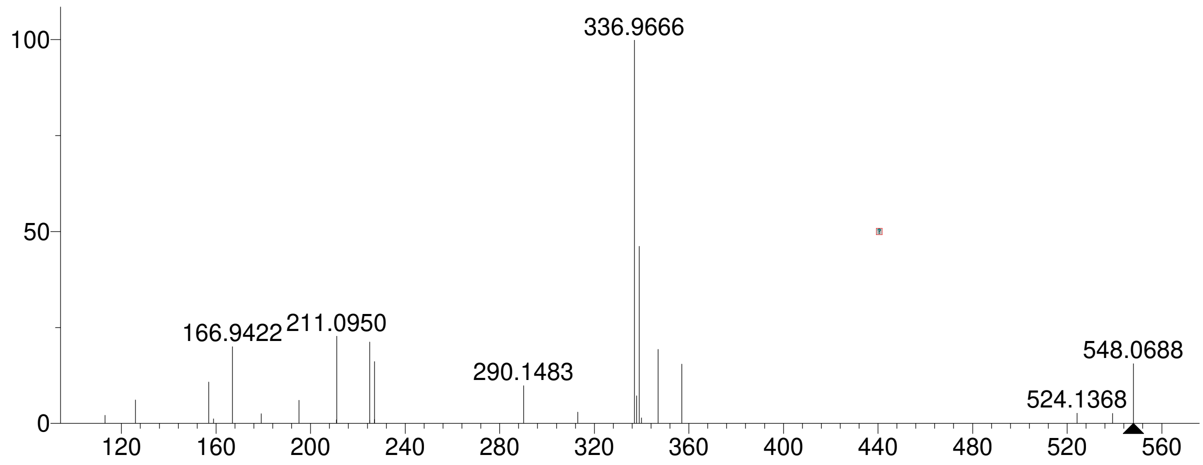

Unknown: Unknown (16.41_346.1284m/z)
Compound in Library Factor = 0

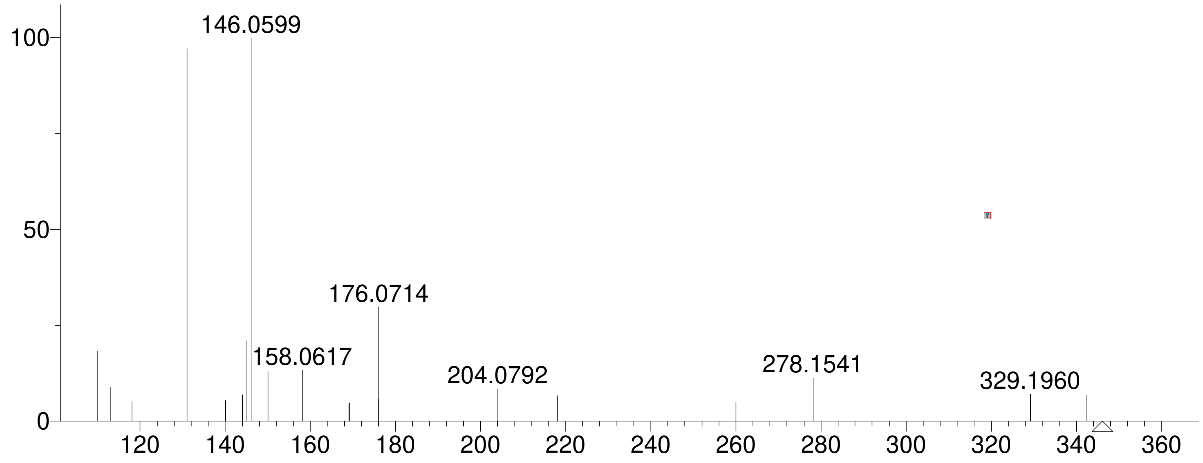

Hit 1 : Deoxyguanylic acid [M-H]- 35% P=346.1
C10H14N5O7P; MF: 0; RMF: 32; Prob 100.0%; Lib: mona-export-itree; ID: 316.

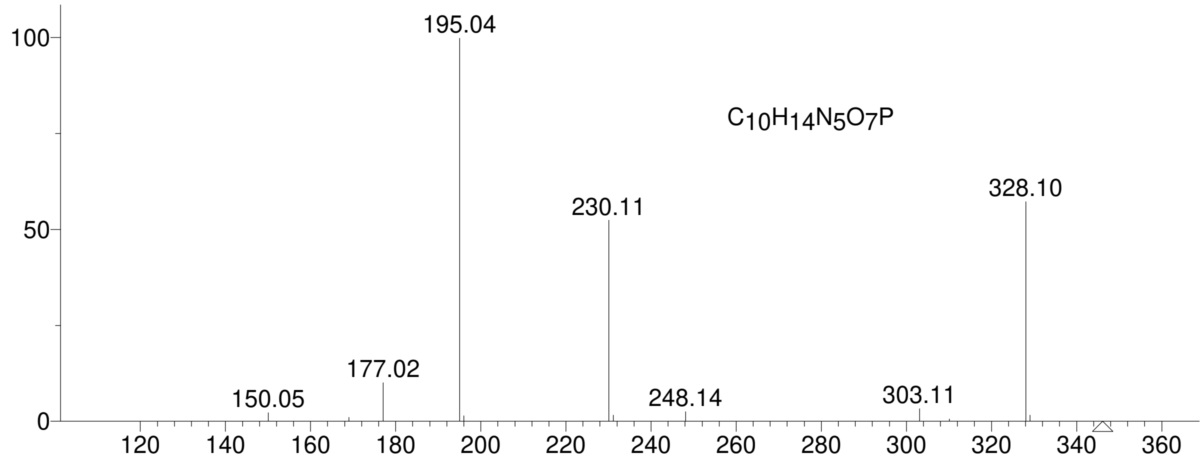

Unknown: Unknown (16.46_413.2183m/z)
Compound in Library Factor = -434

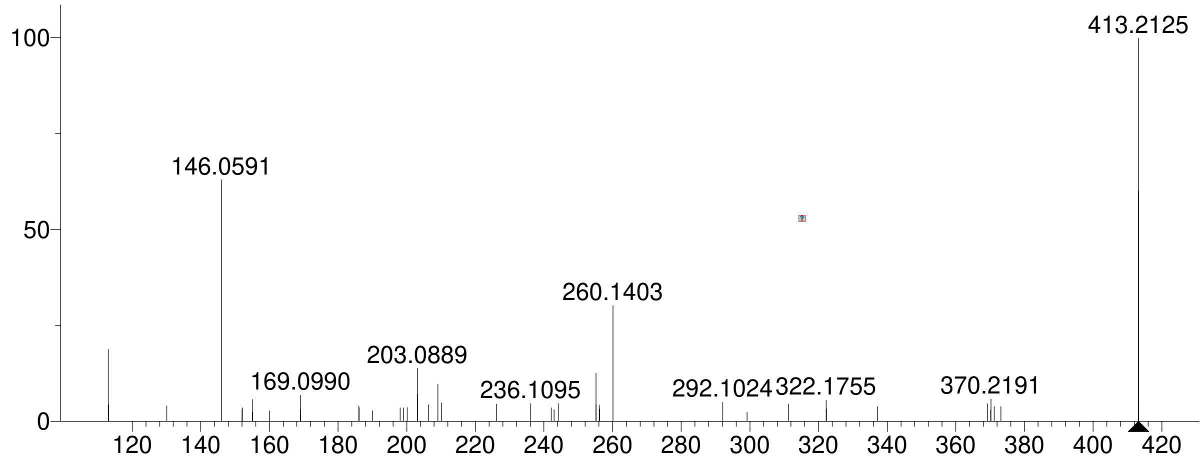

Hit 1 : Unknown (carbon number 21); PlaSMA ID-1051 [M-H]- P=413.2
C21H34O8; MF: 163; RMF: 539; Prob 87.0%; Lib: biomsms-neg-plasma; ID: 1355.

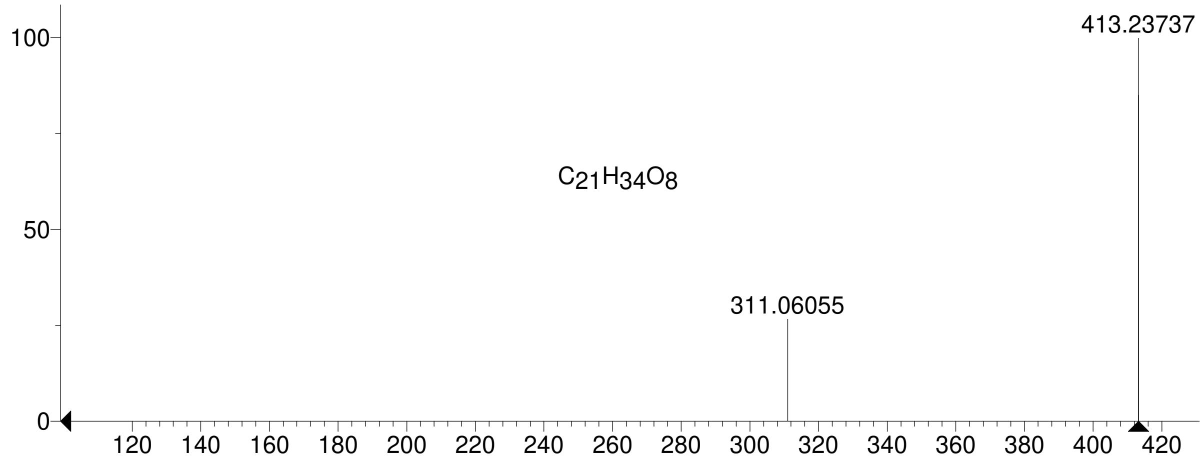

Hit 2 : L-Arginine, N2-[2-(2,2-diphenylethoxy)acetyl]- [M+H]+ HCD 28V P=413.2
C22H28N4O4; MF: 13; RMF: 253; Prob 2.43%; CAS: 259218-28-5; Lib: nist_msms; ID: 355405.

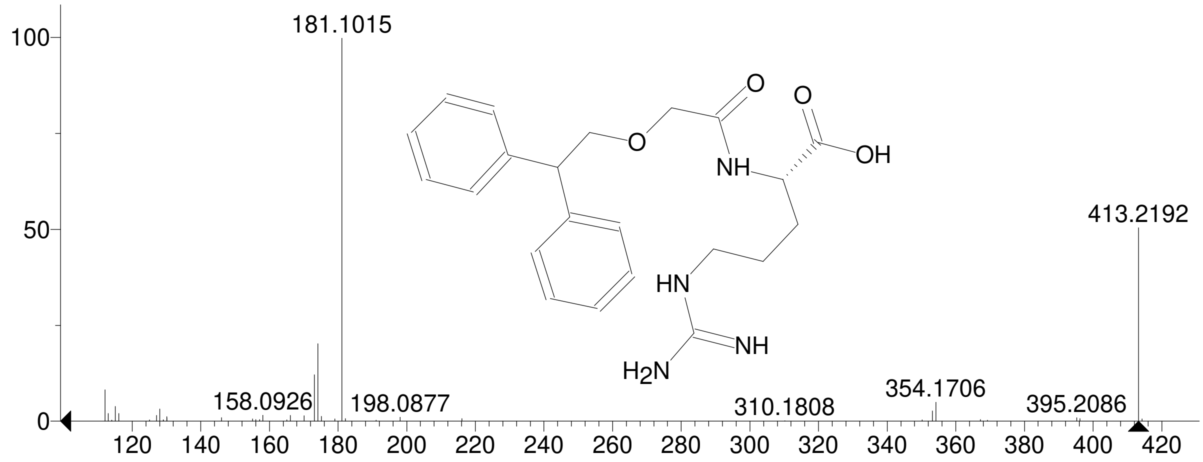

Hit 3 : His-Glu-Lys [M+H]+ QTOF 10V P=413.2
C17H28N6O6; MF: 10; RMF: 368; Prob 2.15%; Lib: nist_msms; ID: 50934.

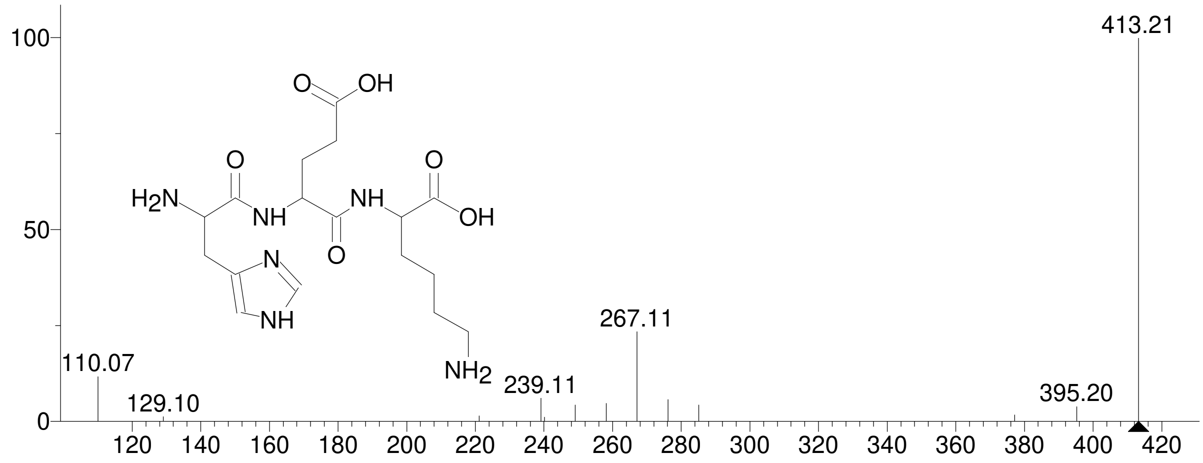

Hit 4 : Unknown (carbon number 21); PlaSMA ID-1052 [M-H]- P=413.2
C21H34O8; MF: 9; RMF: 302; Prob 2.06%; Lib: biomsms-neg-plasma; ID: 1356.

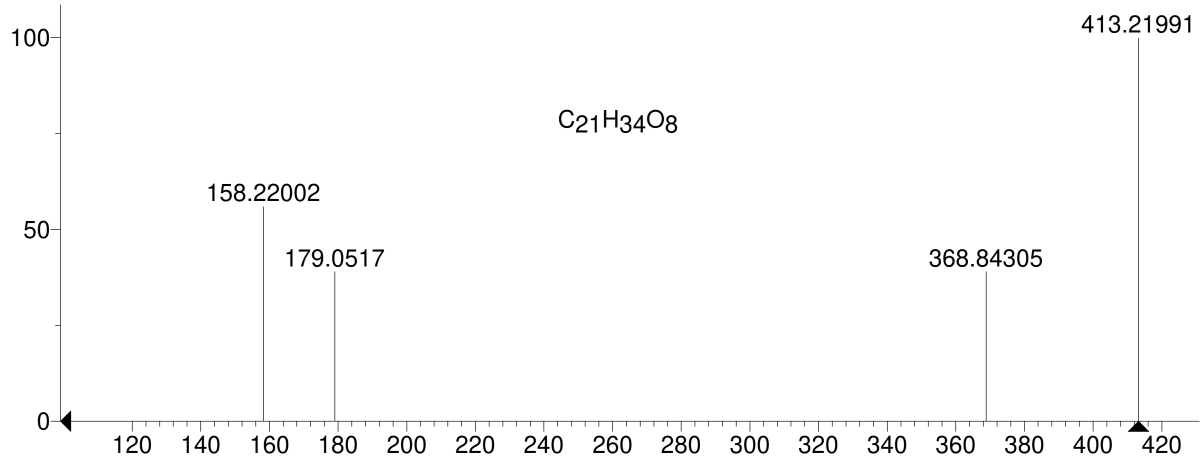

Hit 5 : (2R,3R,4S,5S,6R)-2-[6-hydroxy-3-[(E)-3-hydroxybut-1-enyl]-2,4,4-trimethylcyclohexyl]oxy-6-(hydroxymethy
C19H34O8; MF: 6; RMF: 380; Prob 1.82%; Lib: anthocyanins mona; ID: 5985.

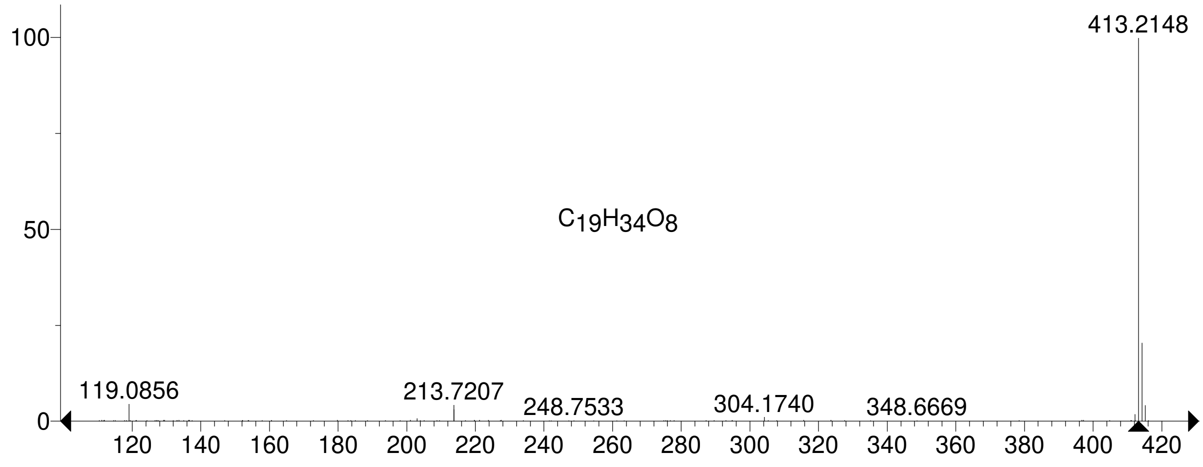

Unknown: Unknown (16.46_596.2778m/z)
Compound in Library Factor = 0

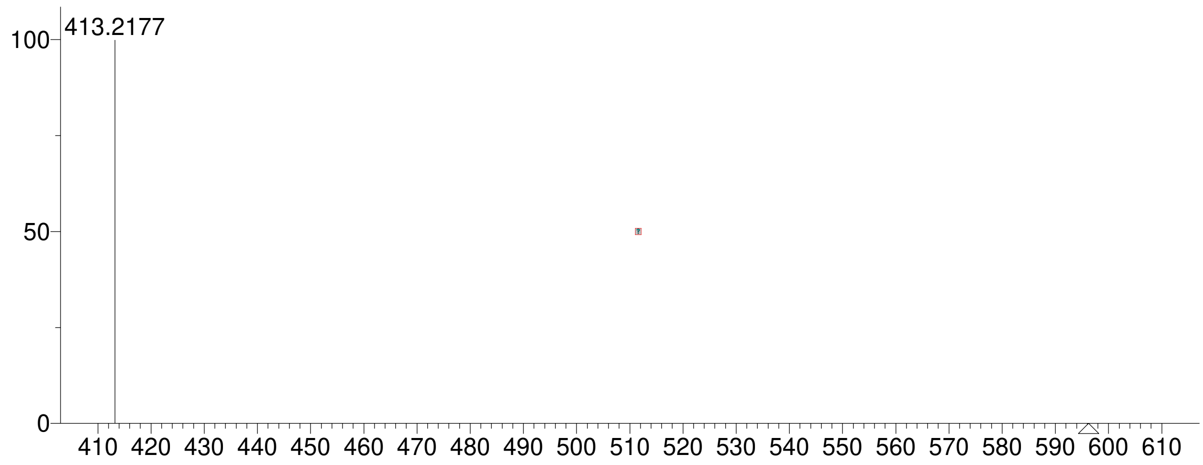

Unknown: Unknown (16.67_535.2398m/z)
Compound in Library Factor = 0

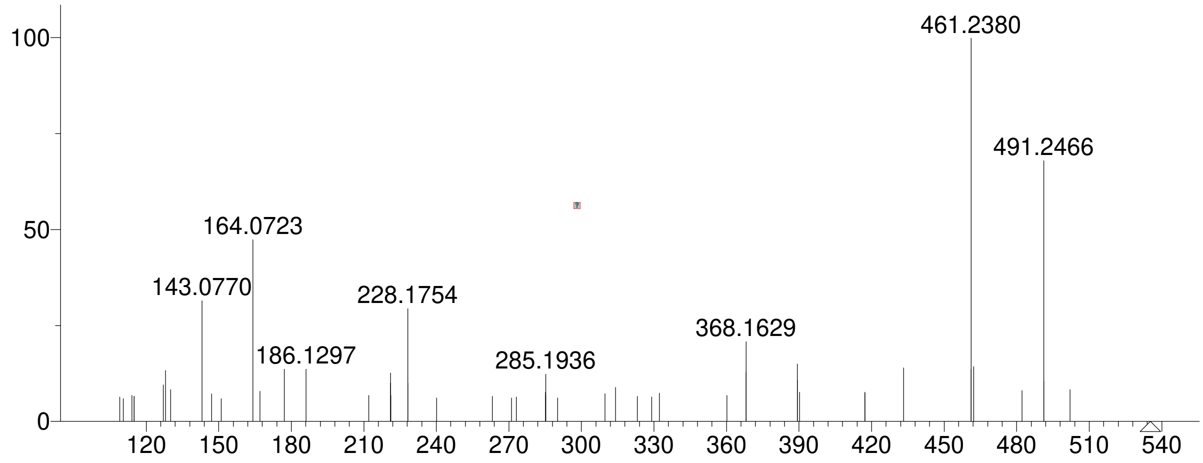

Hit 1 : 3,6-dibenzyl-9-[(2,3-diamino-1,2-dihydroindol-3-yl)methyl]-1,4,7-triazonane-2,5,8-trione [M+Na]+ 35HCD
C29H32N6O3; MF: 0; RMF: 6; Prob 100.0%; Lib: mona-export-vf-npl_qexactive; ID: 18547.

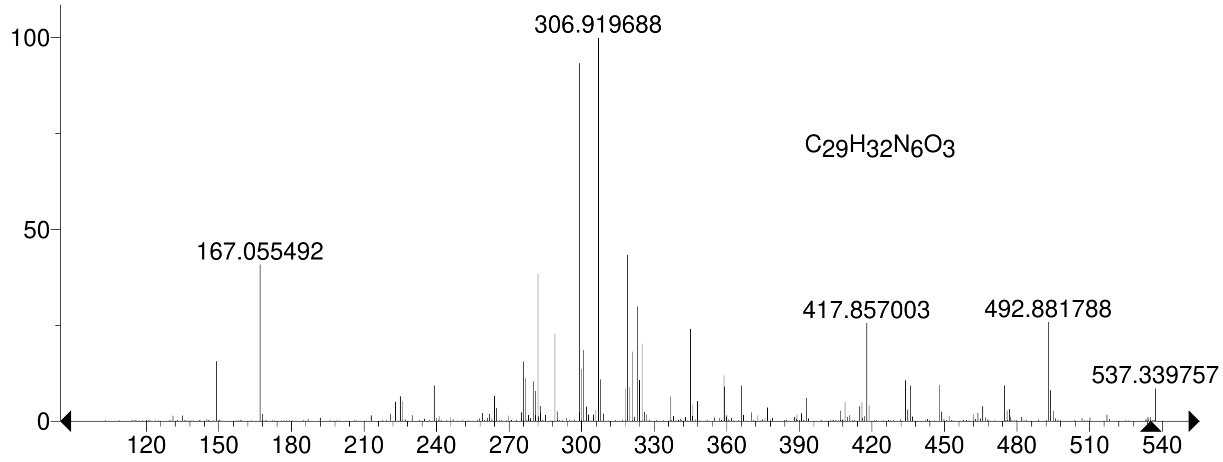

Unknown: Unknown (16.71_416.1319m/z)
Compound in Library Factor = 0

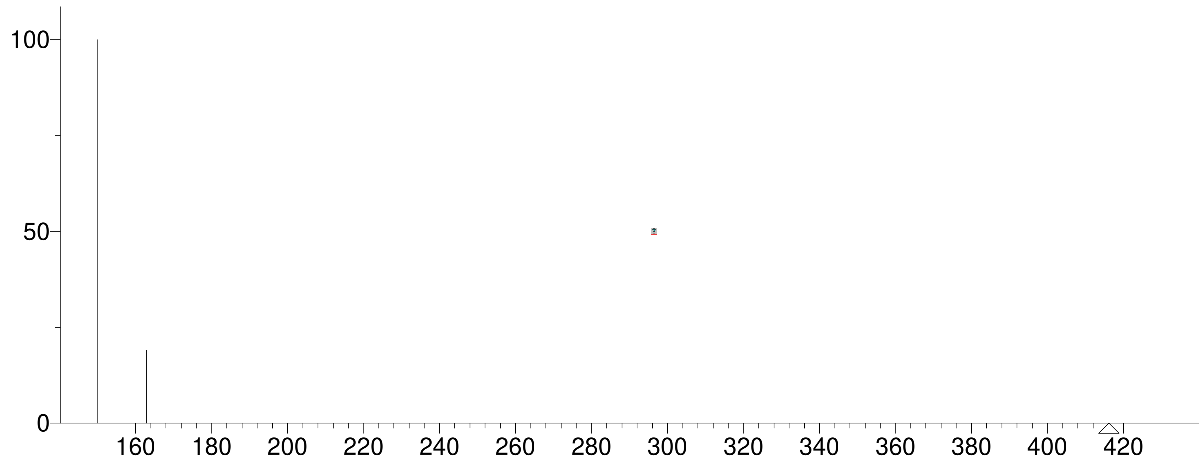

Hit 1 : NCGC00380250-01_C21H23NO8_(2E)-4-({(3S,3aR,4E,7aS)-7-Hydroxy-4-[(2E,4E)-1-hydroxy-2,4-hexadie
C21H23NO8; MF: 0; RMF: 95; Prob 50.0%; Lib: mona-export-gnps; ID: 11477.

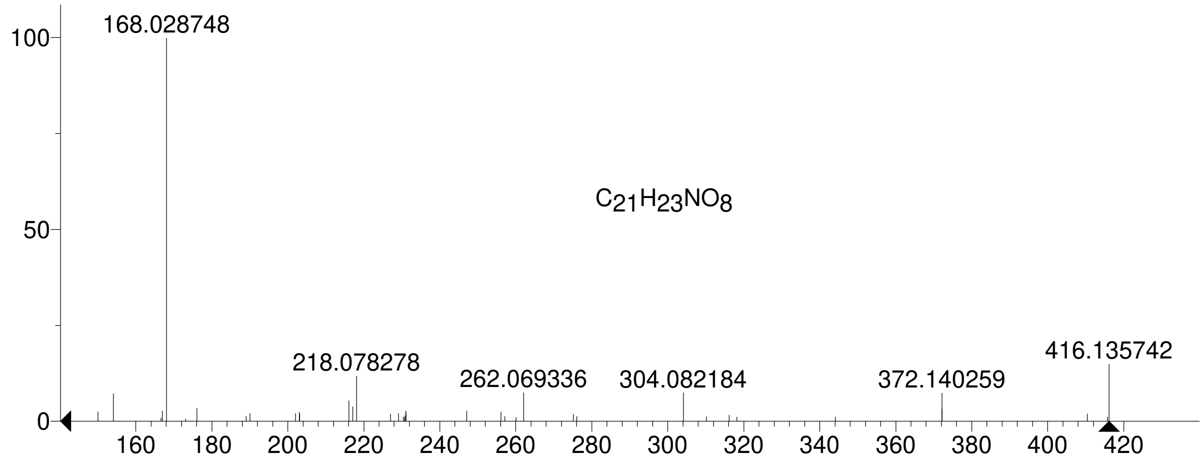

Hit 2 : Oxytetracycline [M-H-CHNO]- HCD 54V P=416.1
C22H24N2O9; MF: 0; RMF: 17; Prob 50.0%; CAS: 79-57-2; Lib: nist_msms; ID: 481718.

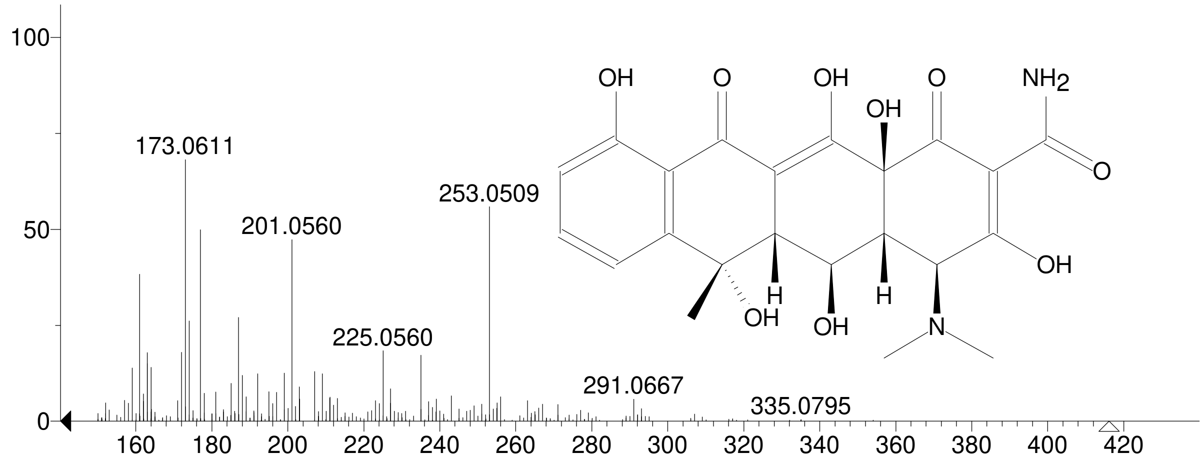

Unknown: Unknown (16.77_209.0938m/z)
Compound in Library Factor = -3273

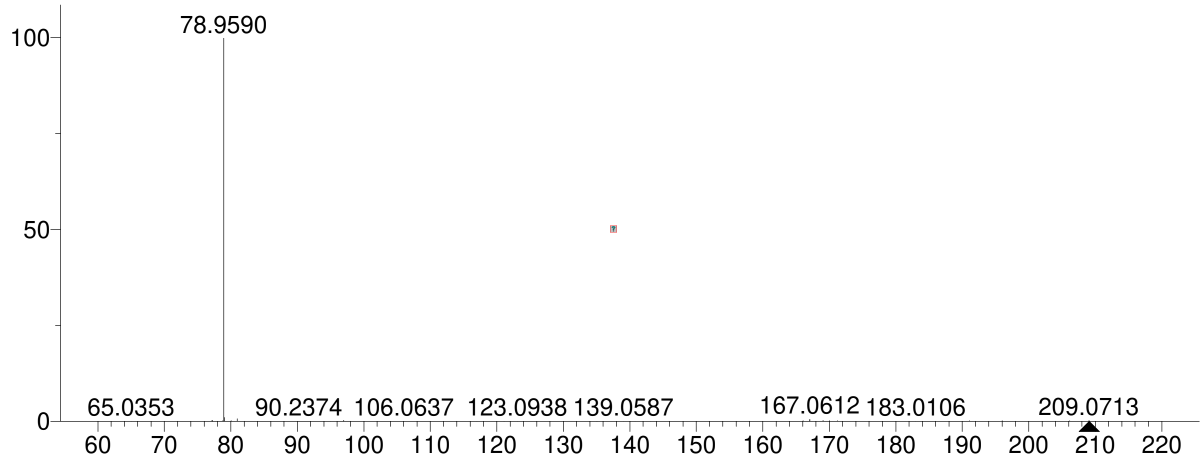

Hit 1 : Bromazepam [M+H-CBrO]+ HCD 20V P=209.1
C14H10BrN3O; MF: 6; RMF: 134; Prob 19.8%; CAS: 1812-30-2; Lib: nist_msms; ID: 194115.

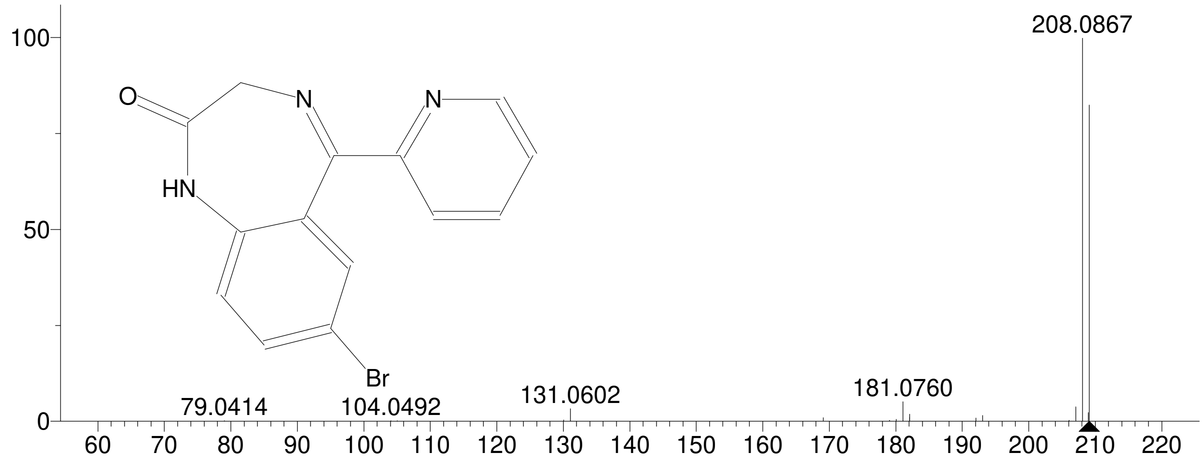

Hit 2 : Aprobarbital [M-H]- 20-30-40%) P=209.1
C10H14N2O3; MF: 3; RMF: 171; Prob 17.5%; Lib: msms-neg-fiehnhilic; ID: 198.

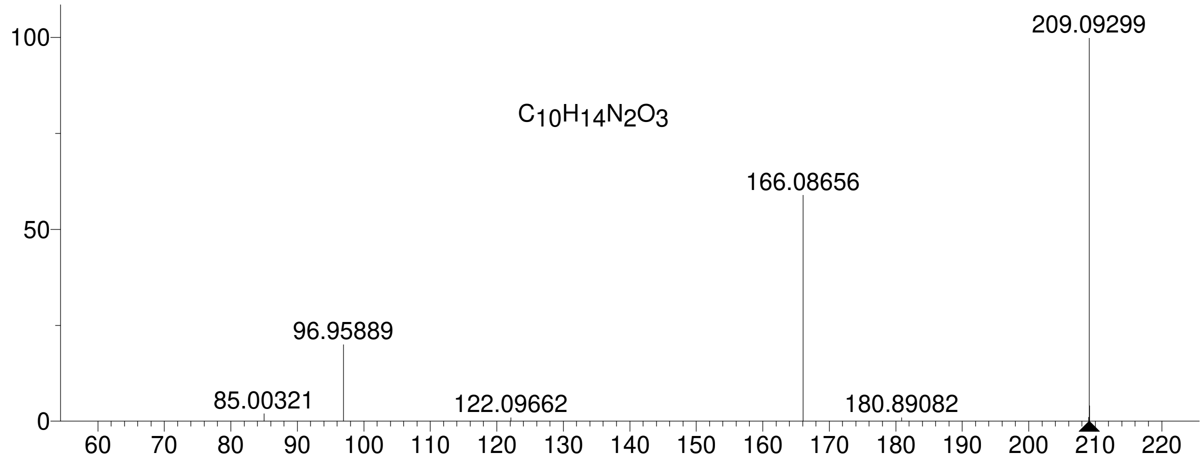

Hit 3 : Kynurenine [M+H]+ 10eV P=209.1
C10H12N2O3; MF: 1; RMF: 107; Prob 16.1%; Lib: mona-export-massbank; ID: 27196.

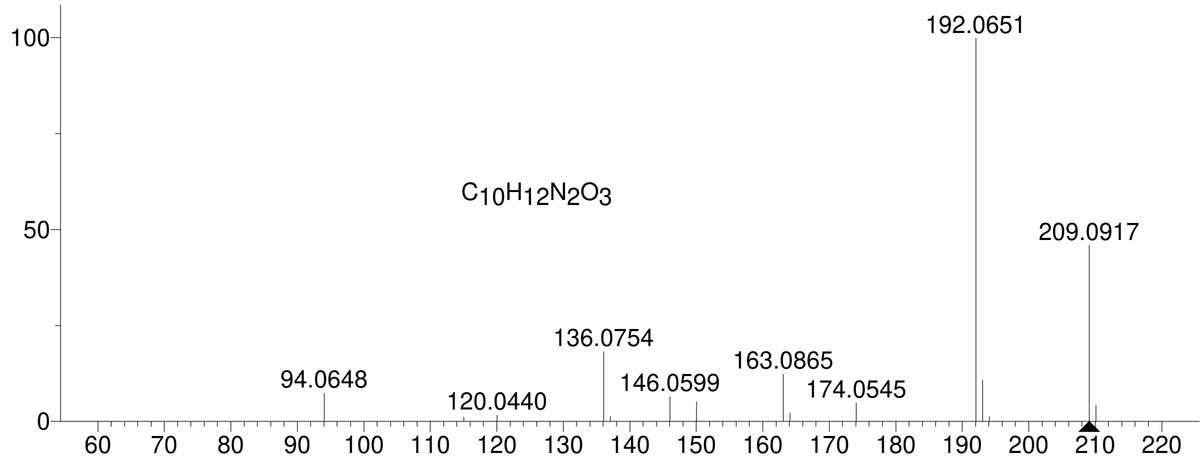

Hit 4 : L-Kynurenine [M+H]+ HCD 0V P=209.1
C10H12N2O3; MF: 0; RMF: 21; Prob 15.5%; CAS: 2922-83-0; Lib: nist_msms; ID: 288000.

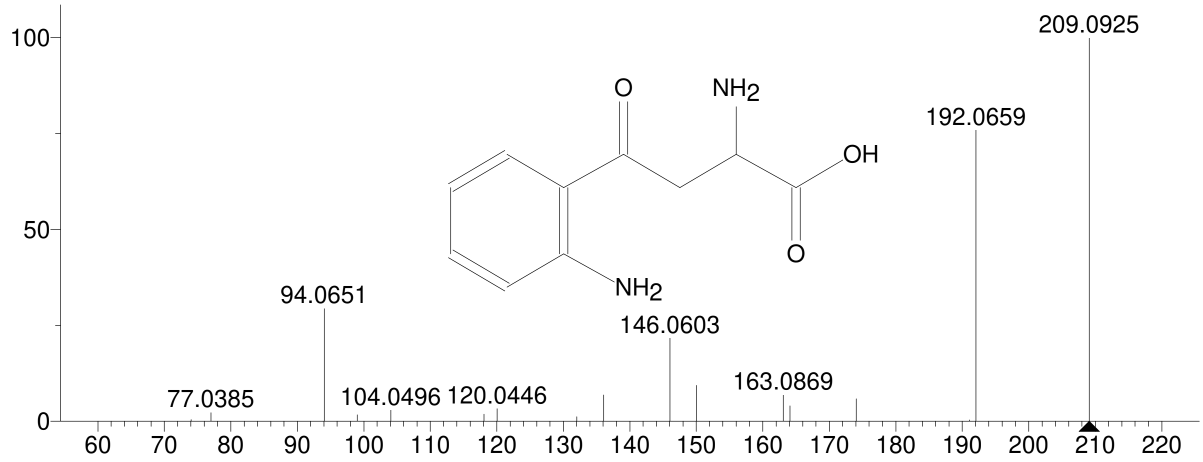

Hit 5 : L-kynurenine [M+H]+ 25HCD P=209.1
C10H12N2O3; MF: 0; RMF: 7; Prob 15.5%; Lib: mona-export-vf-npl_qexactive; ID: 154.

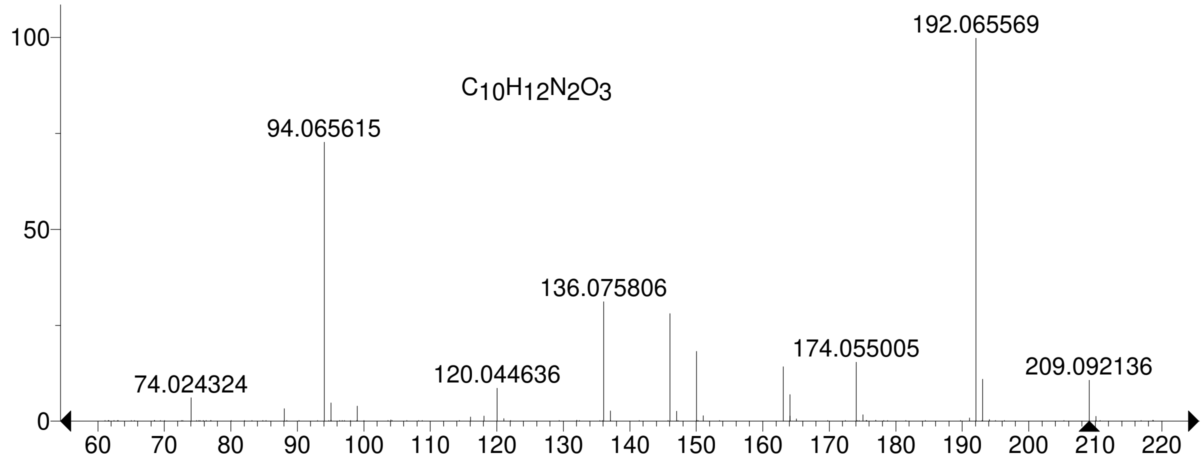

** Search Report Page 1 of 1 **

Unknown: Unknown (16.78_401.0533m/z)
Compound in Library Factor = 0

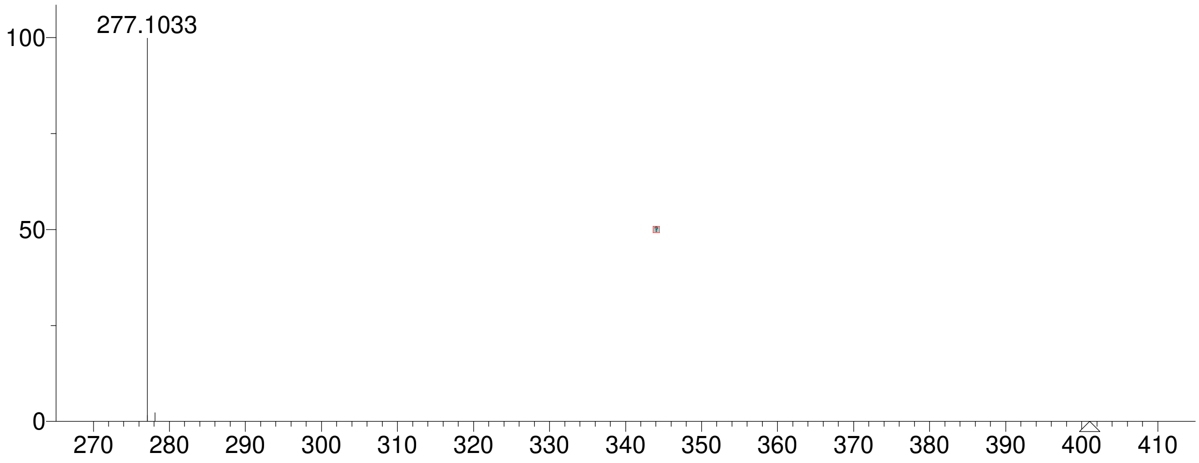

Unknown: Unknown (16.80_577.1493m/z)
Compound in Library Factor = 0

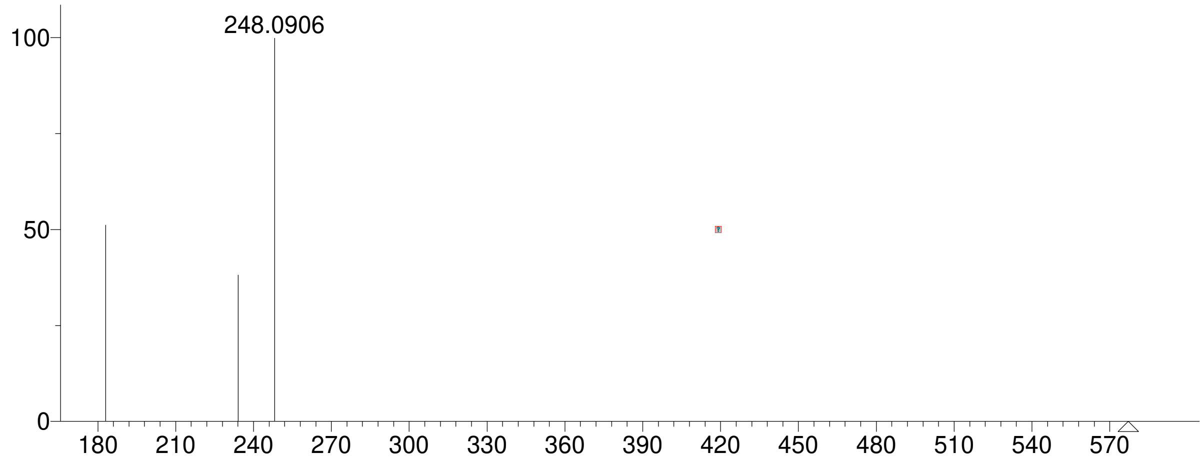

Unknown: Unknown (16.97_239.1386m/z)
Compound in Library Factor = -3273

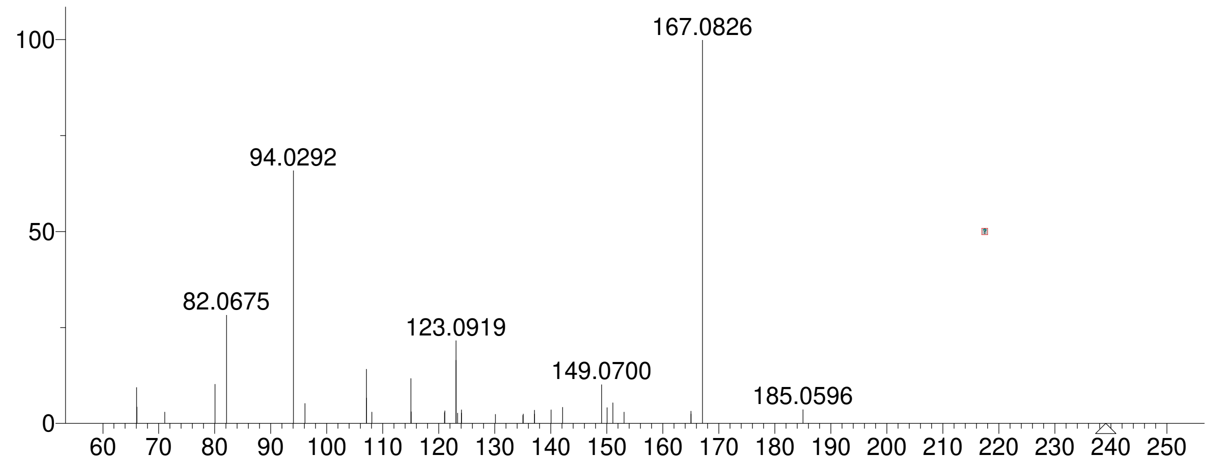

Hit 1 : Heptanedioic acid, 1-(2-cyclopentylidenehydrazide) [M-H]- HCD 45V P=239.1
C12H20N2O3; MF: 1; RMF: 30; Prob 51.0%; CAS: 1136466-93-7; Lib: nist_msms; ID: 263660.

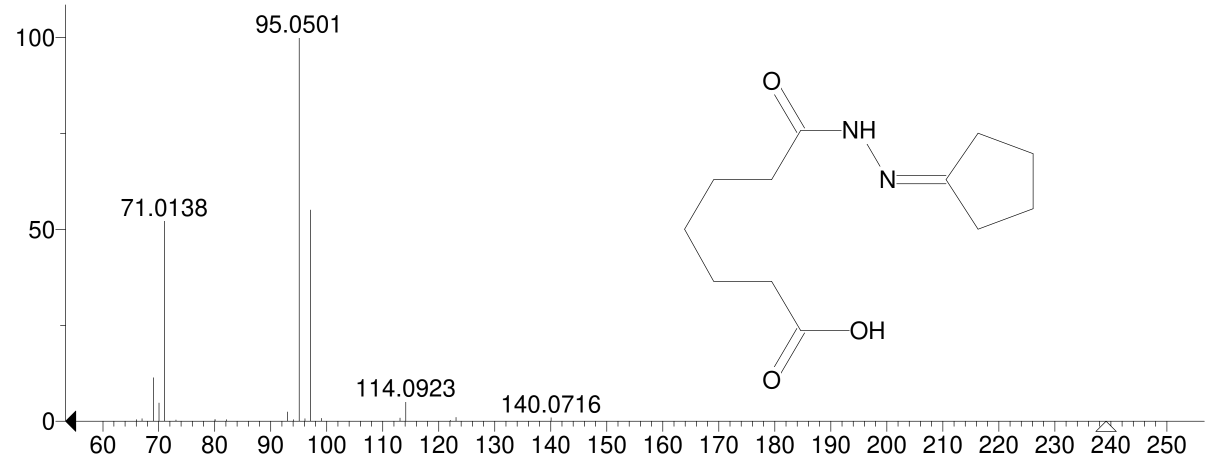

Hit 2 : Secobarbital [M+H]+ HCD 43V P=239.1
C12H18N2O3; MF: 0; RMF: 17; Prob 49.0%; CAS: 76-73-3; Lib: nist_msms; ID: 198385.

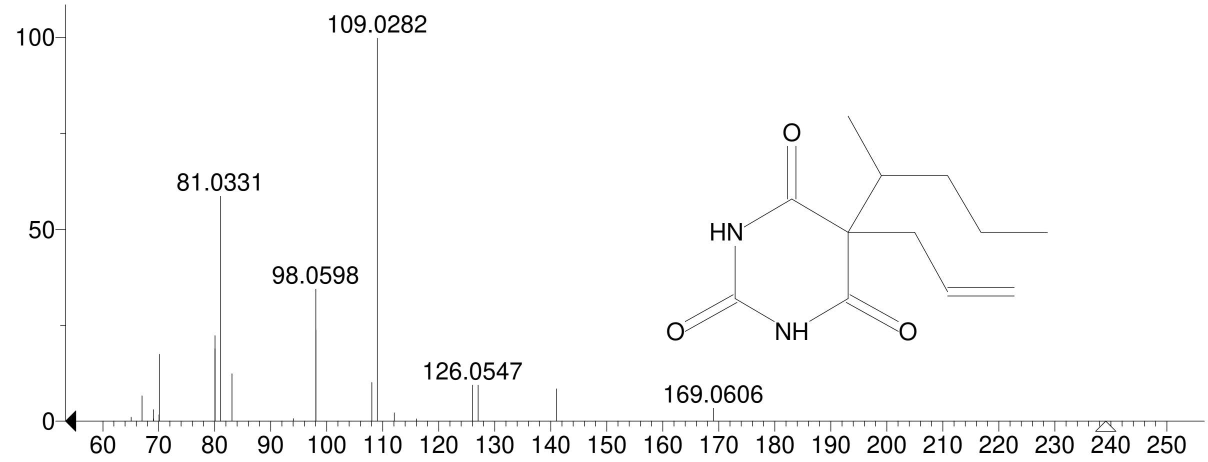

Unknown: Unknown (17.00_610.3193m/z)
Compound in Library Factor = 0

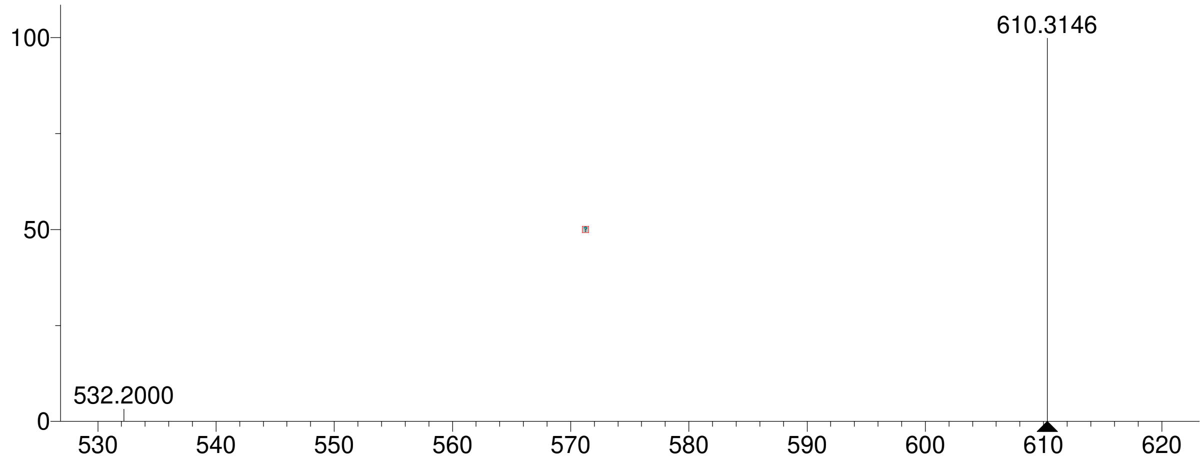

Unknown: Unknown (17.01_264.0863m/z)
Compound in Library Factor = -2387

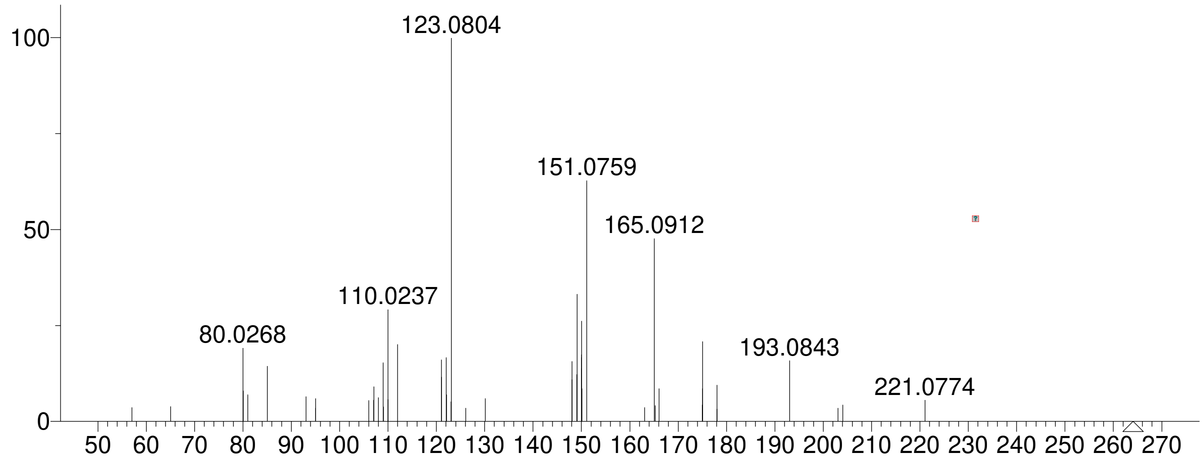

Hit 1 : 3,4-Dihydroxycinnamic acid (L-alanine methyl ester) amide [M-H]- HCD 50V P=264.1
C13H15NO5; MF: 18; RMF: 119; Prob 46.1%; CAS: 778624-05-8; Lib: nist_msms; ID: 548704.

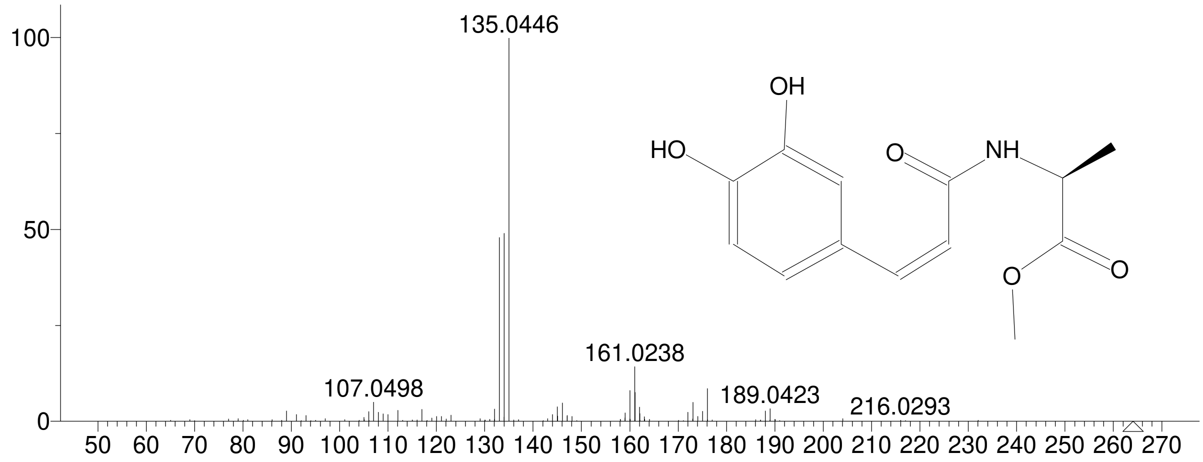

Hit 2 : 2,3',4,6-Tetrahydroxybenzophenone [M+NH4]+ 65HCD P=264.1
C13H10O5; MF: 5; RMF: 91; Prob 29.8%; Lib: mona-export-vf-npl_qexactive; ID: 4668.

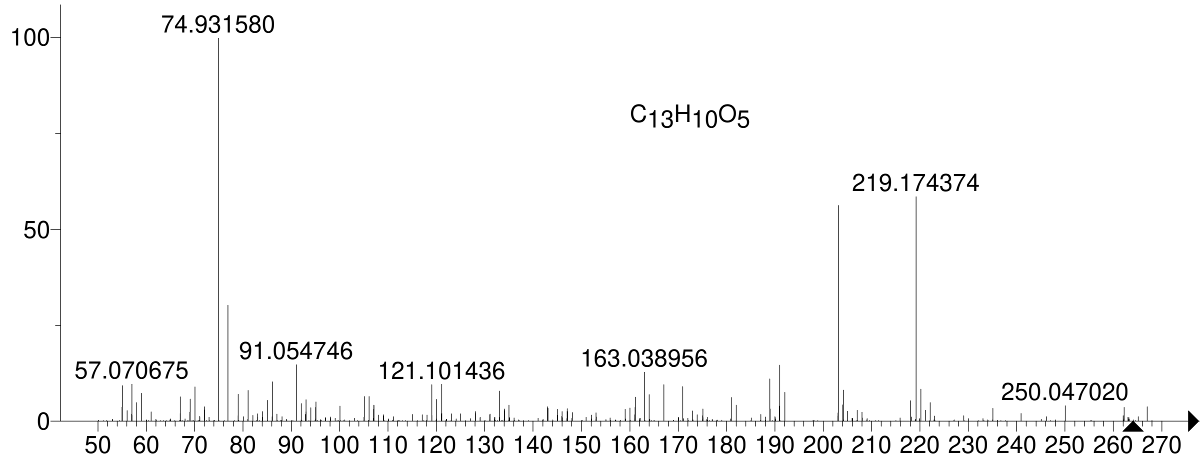

Hit 3 : NCGC00095569-05!4,9-dimethoxyfuro[3,2-g]chromen-7-one [M+NH4]+ P=264.1
C13H10O5; MF: 0; RMF: 14; Prob 24.0%; Lib: mona-export-gnps; ID: 8525.

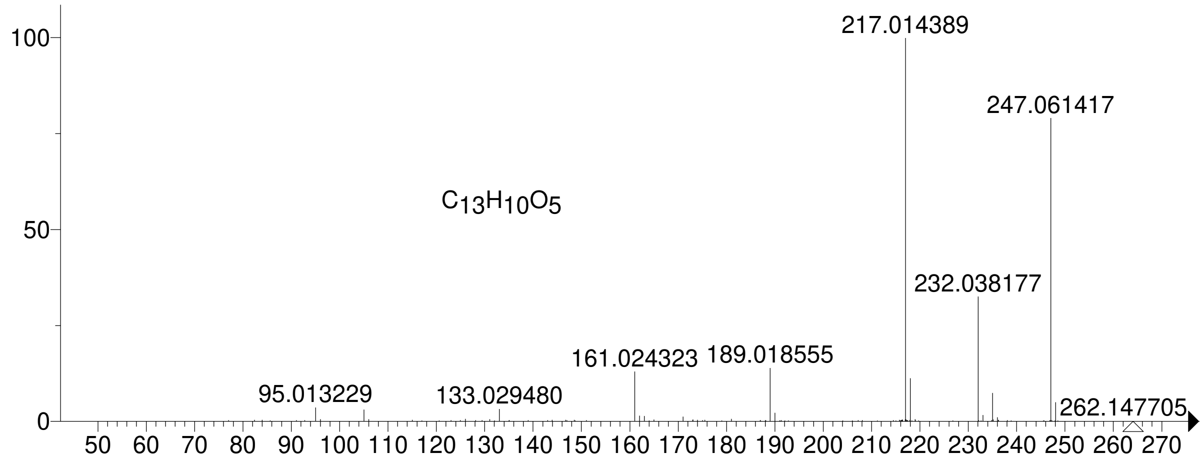

Unknown: Unknown (17.04_369.1355m/z)
Compound in Library Factor = -885

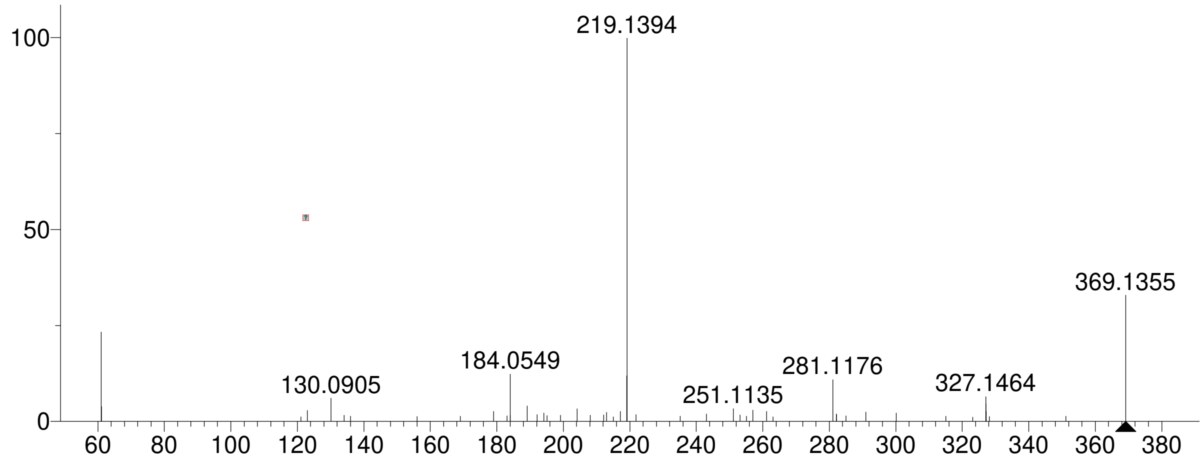

Hit 1 : Epimedin B [M+H-C17H28O13]+ HCD 18V P=369.1
C38H48O19; MF: 145; RMF: 744; Prob 20.7%; CAS: 110623-73-9; Lib: nist_msms; ID: 257878.

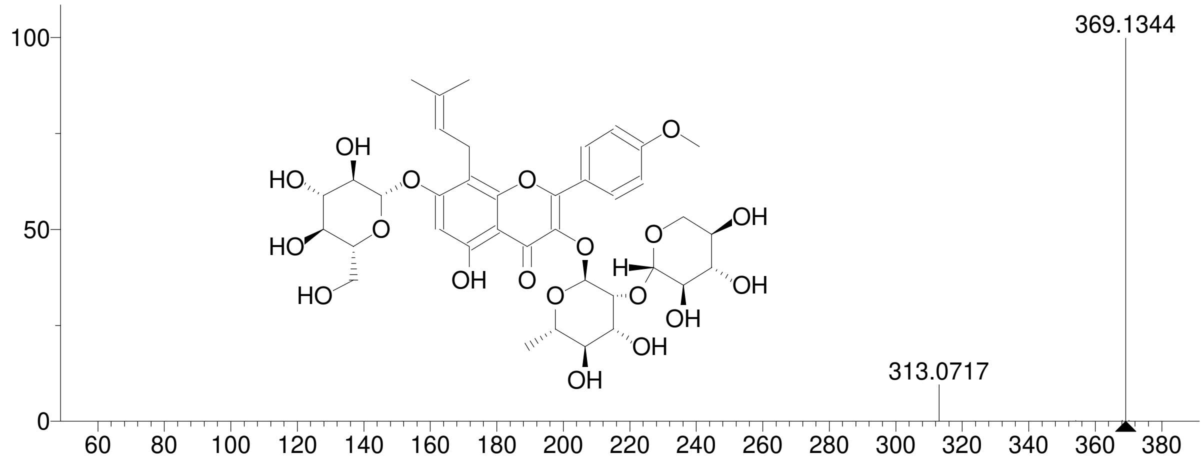

Hit 2 : Epimedin C [M+H-C18H30O13]+ HCD 18V P=369.1
C39H50O19; MF: 145; RMF: 744; Prob 20.7%; CAS: 110642-44-9; Lib: nist_msms; ID: 257938.

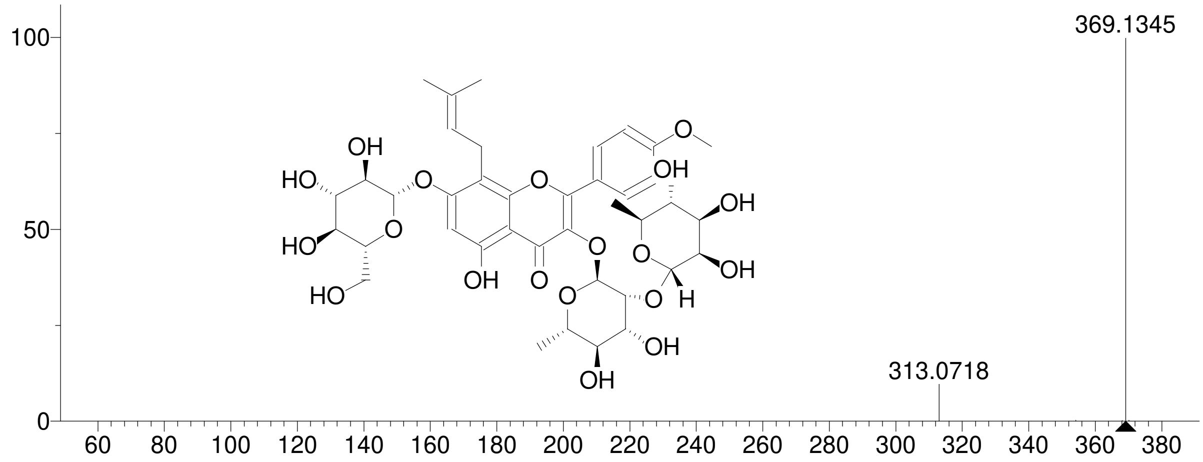

Hit 3 : Epimedin A [M+H-C18H30O14]+ HCD 18V P=369.1
C39H50O20; MF: 143; RMF: 744; Prob 19.1%; CAS: 110623-72-8; Lib: nist_msms; ID: 257761.

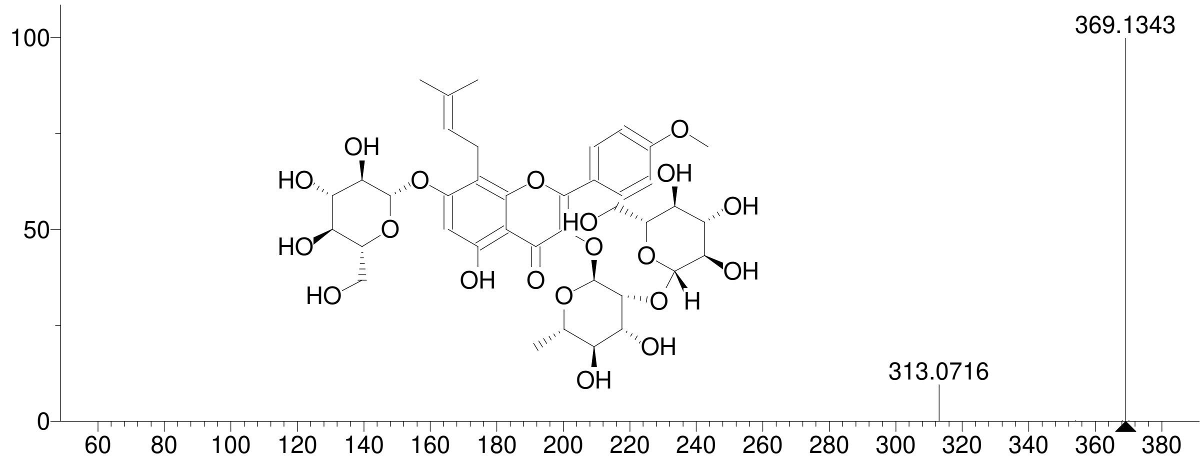

Hit 4 : Icariside I [M+H-C6H10O5]+ HCD 18V P=369.1
C27H30O11; MF: 128; RMF: 745; Prob 11.6%; CAS: 56725-99-6; Lib: nist_msms; ID: 232874.

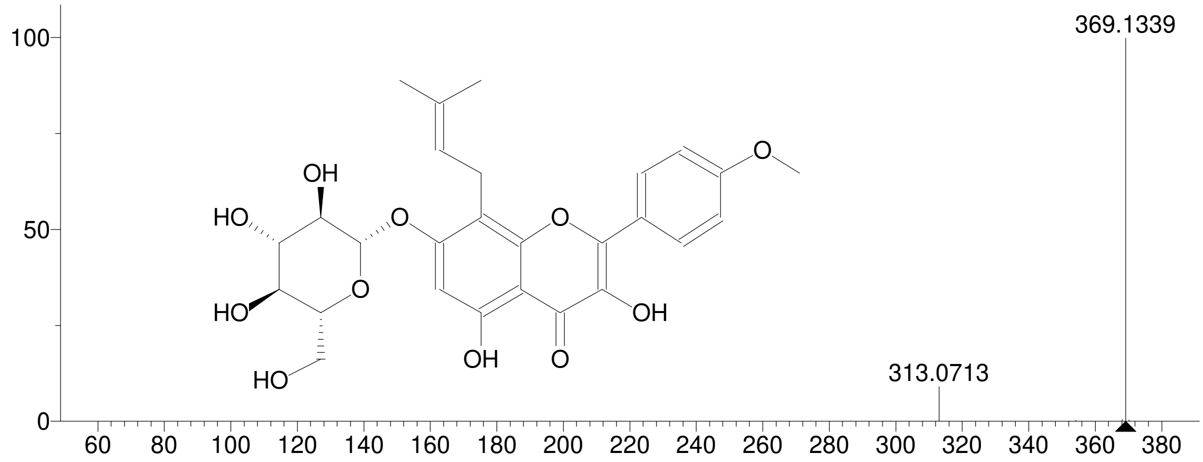

Hit 5 : Icariside II [M+H-C6H10O4]+ HCD 18V P=369.1
C27H30O10; MF: 123; RMF: 739; Prob 9.33%; CAS: 113558-15-9; Lib: nist_msms; ID: 232522.

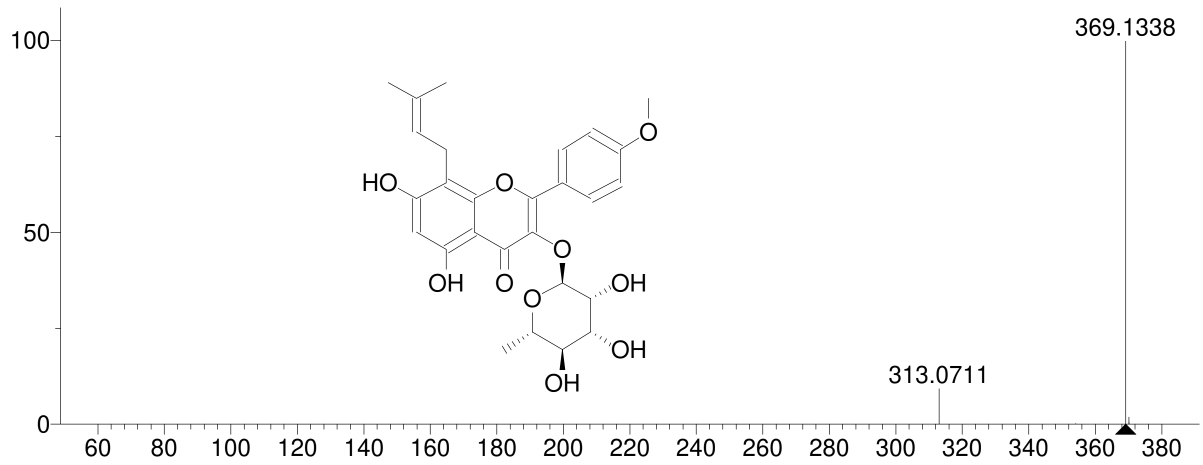

Unknown: Unknown (17.10_431.1372m/z)
Compound in Library Factor = -3273

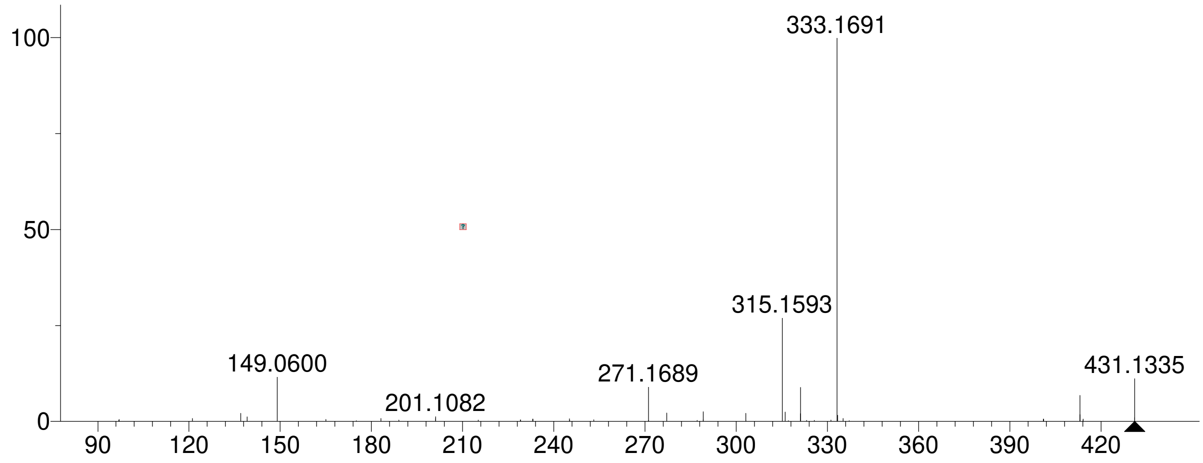

Hit 1 : Agnuside [M+H-2H2O]+ HCD 12V P=431.1
C22H26O11; MF: 14; RMF: 194; Prob 10.7%; CAS: 11027-63-7; Lib: nist_msms; ID: 389578.

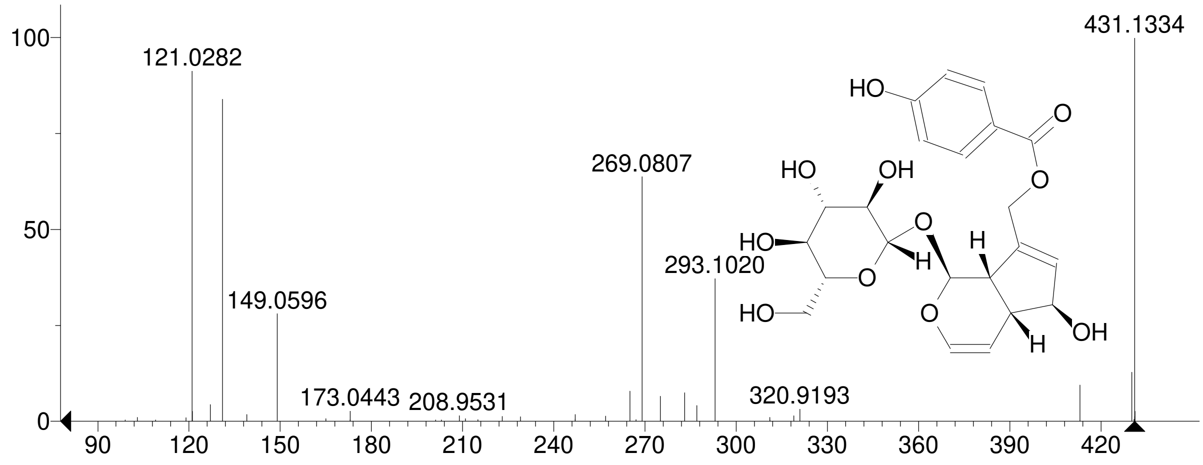

Hit 2 : Ononin [M+H]+ 15eV P=431.1
C22H22O9; MF: 7; RMF: 158; Prob 8.23%; Lib: mona-export-massbank; ID: 28099.

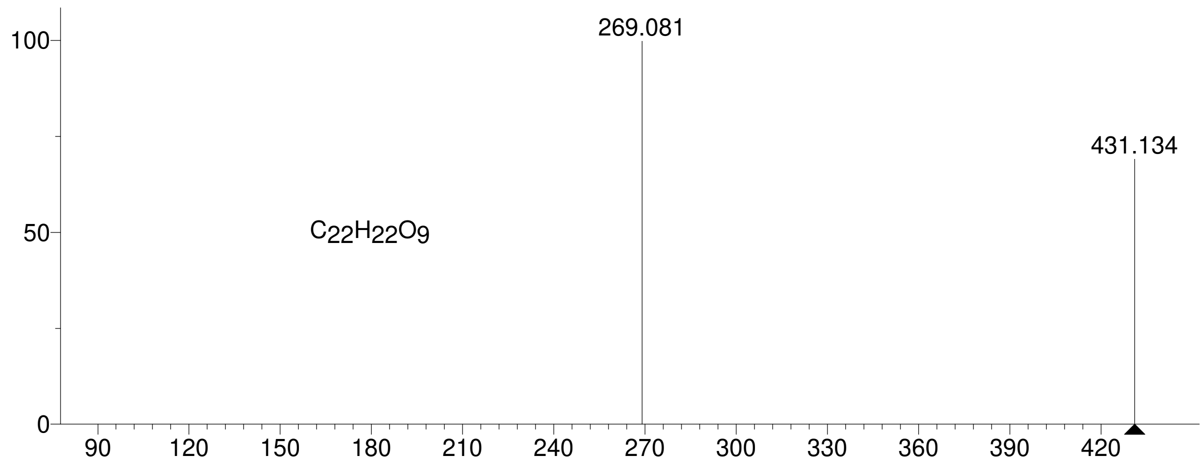

Hit 3 : Formononetin-7-O-glucoside [M+H]+ 10eV P=431.1
C22H22O9; MF: 4; RMF: 179; Prob 7.27%; Lib: anthocyanins mona; ID: 1117.

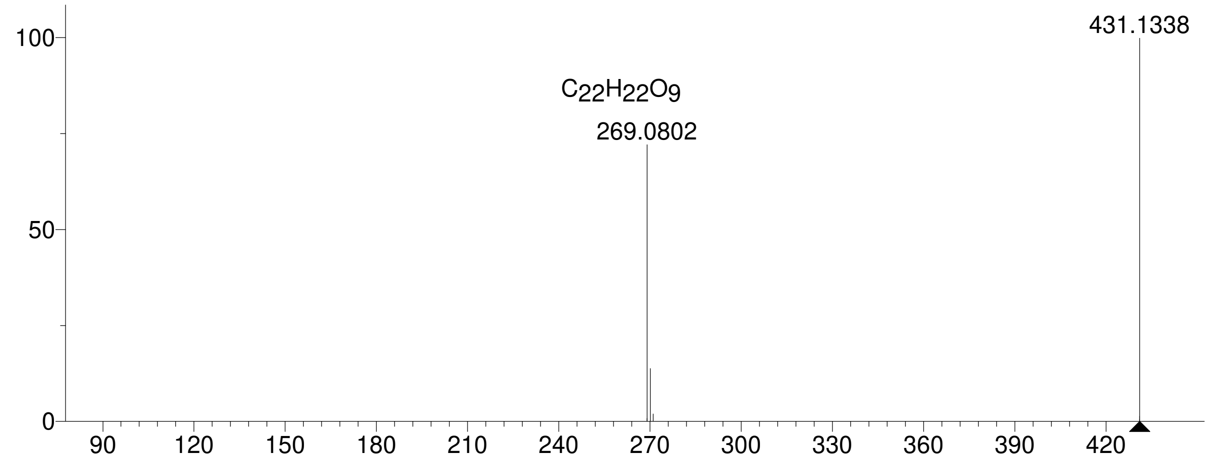

Hit 4 : populoside B2 (pubchem) [M-H]- P=431.1
C22H24O9; MF: 0; RMF: 88; Prob 6.14%; Lib: phytofrag2018_final_060219; ID: 779.

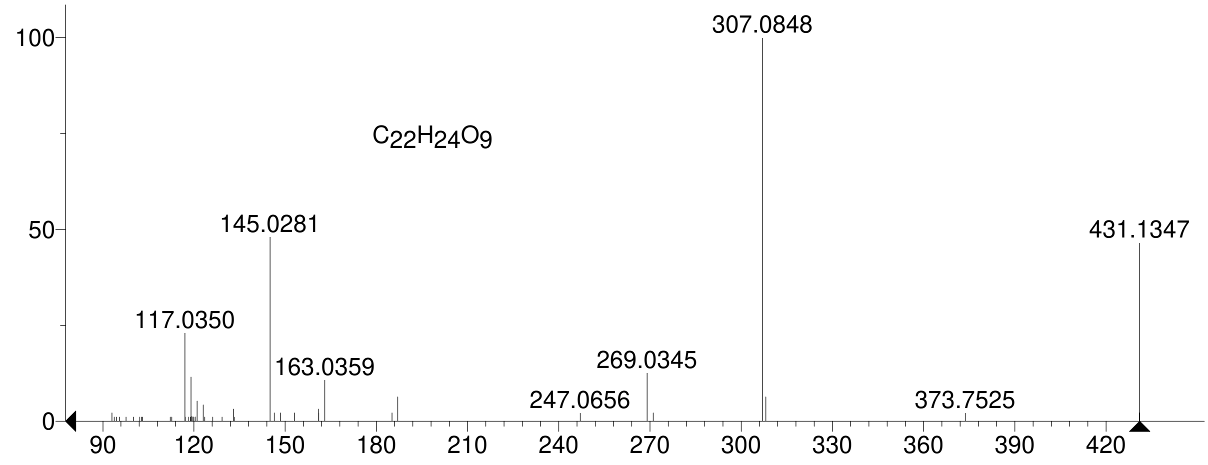

Hit 5 : populoside B4 (pubchem) [M-H]- P=431.1
C22H24O9; MF: 0; RMF: 82; Prob 6.14%; Lib: phytofrag2018_final_060219; ID: 781.

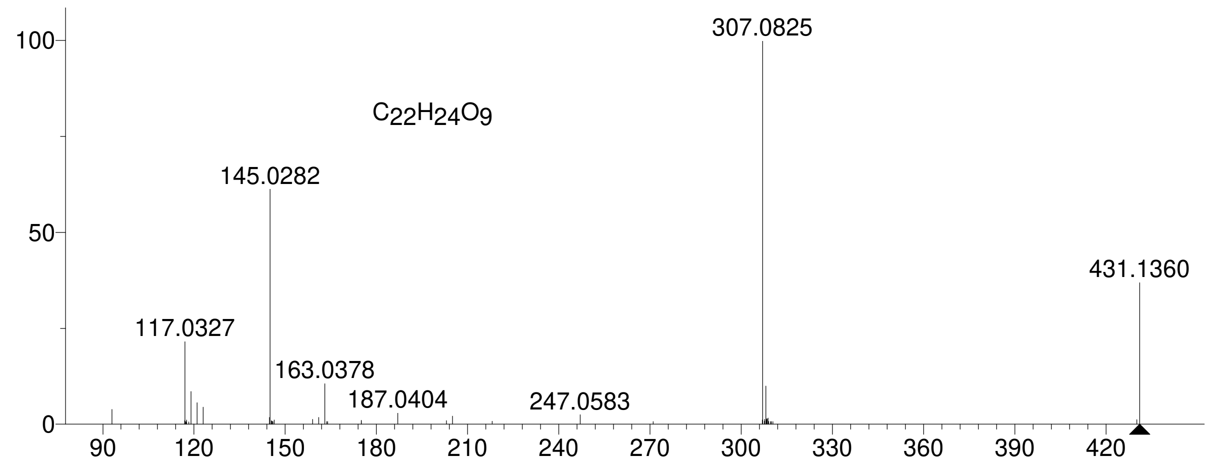

Unknown: Unknown (17.20_214.1441m/z)
Compound in Library Factor = -2387

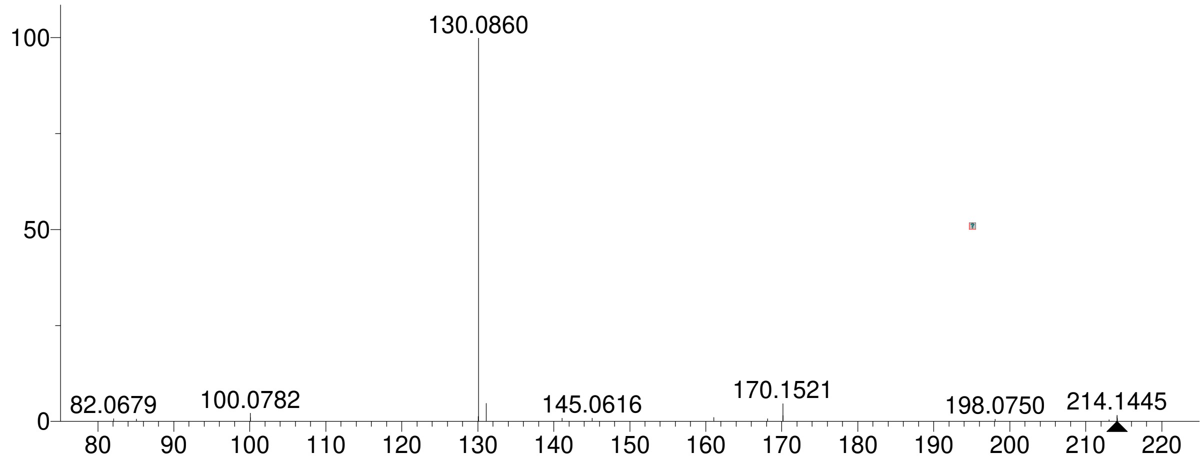

Hit 1 : Ecgonine ethyl ester [M+H]+ HCD 4V P=214.1
C11H19NO3; MF: 15; RMF: 128; Prob 43.4%; CAS: 70939-97-8; Lib: nist_msms; ID: 195186.

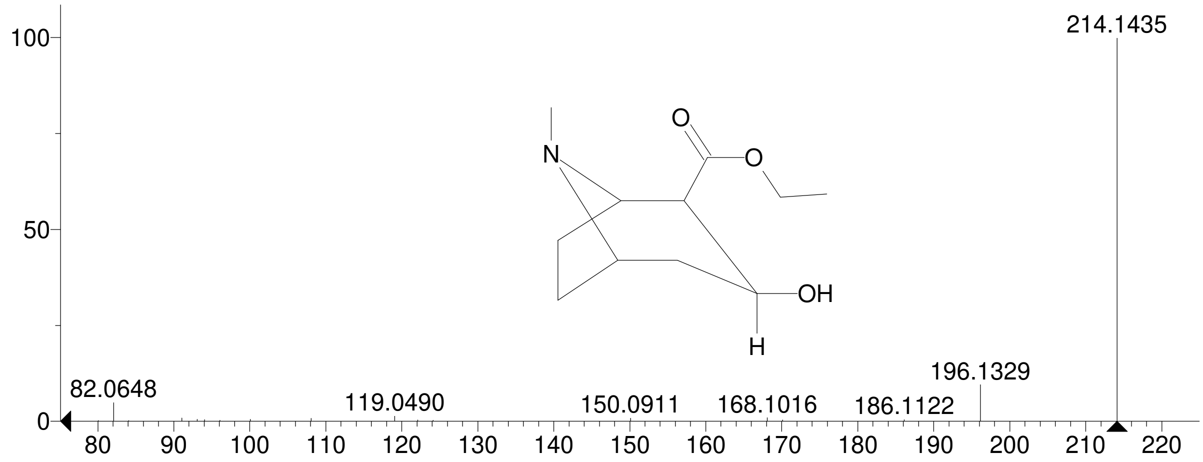

Hit 2 : N-Heptanoyl-L-homoserine lactone [M+H]+ HCD 0V P=214.1
C11H19NO3; MF: 5; RMF: 112; Prob 30.7%; CAS: 177158-20-2; Lib: nist_msms; ID: 165292.

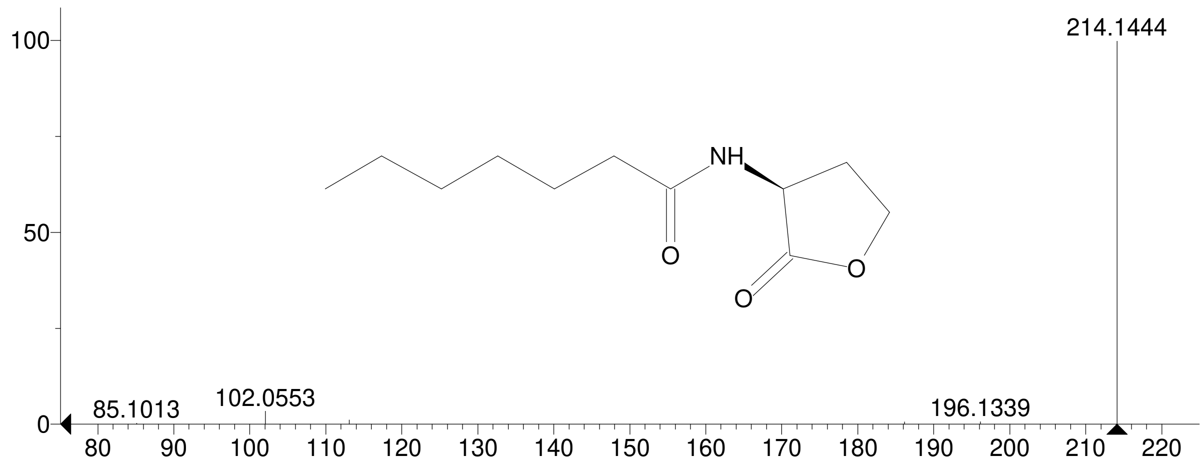

Hit 3 : N-Heptanoyl-DL-homoserine lactone [M+H]+ QTOF 3V P=214.1
C11H19NO3; MF: 1; RMF: 72; Prob 25.9%; CAS: 106983-26-0; Lib: nist_msms; ID: 23507.

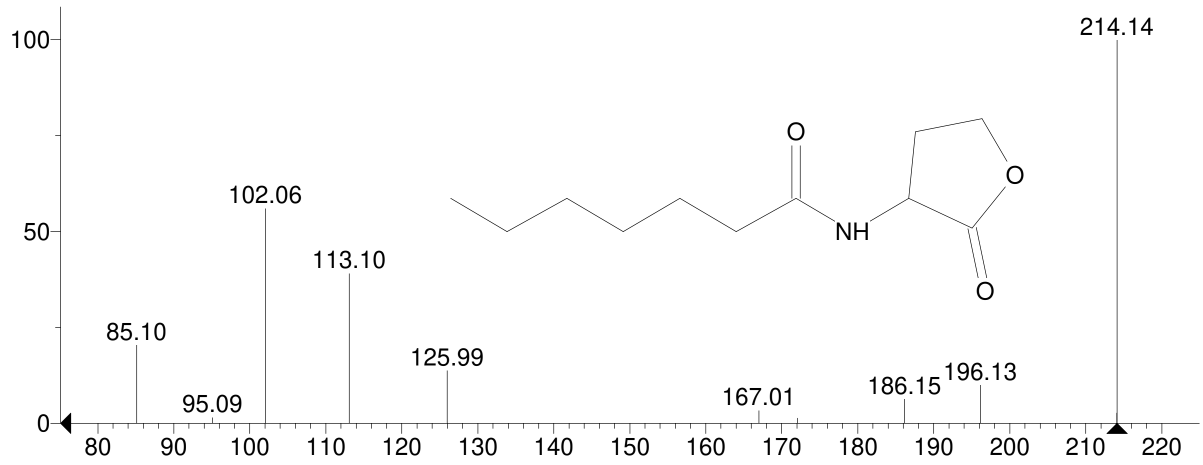

** Search Report Page 1 of 1 **

Unknown: Unknown (17.20_484.2537m/z)
Compound in Library Factor = 0

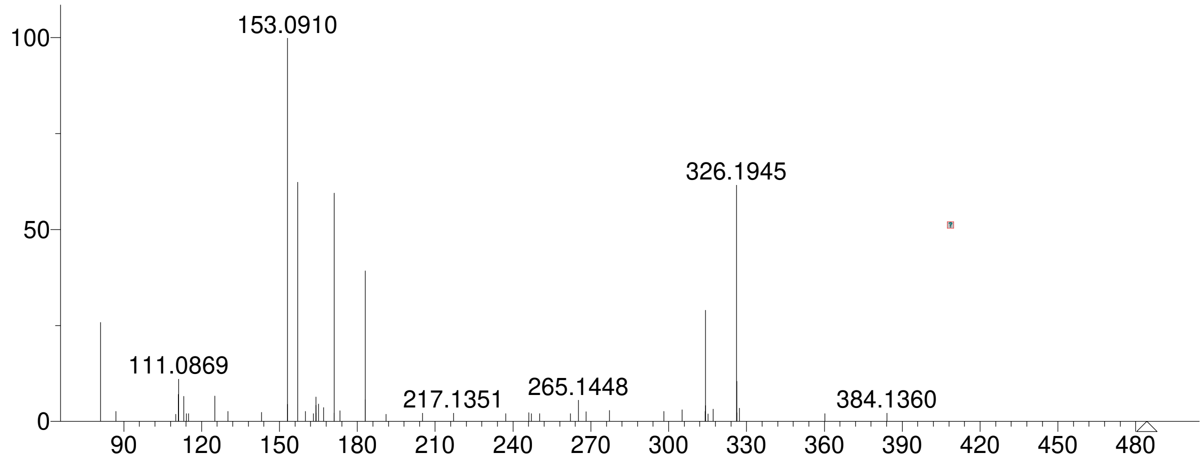

Hit 1 : 2-[2,6-bis(3-methylbut-2-enyl)-4-[(2S,3R,4S,5S,6R)-3,4,5-trihydroxy-6-(hydroxymethyl)oxan-2-yl]oxyphenyl
C24H34O9; MF: 0; RMF: 50; Prob 50.0%; Lib: mona-export-vf-npl_qexactive; ID: 21829.

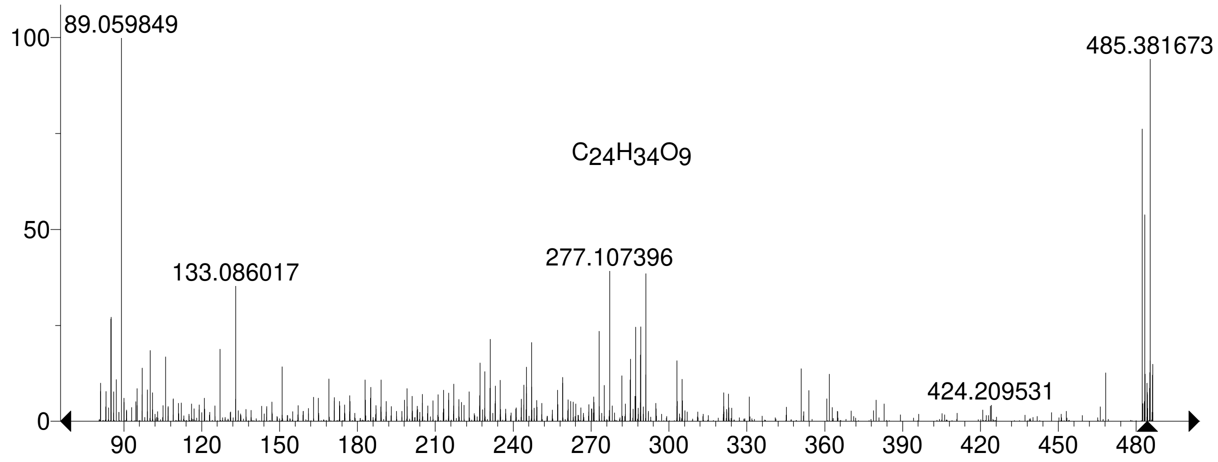

Hit 2 : NCGC00169100-02!2-[2,6-bis(3-methylbut-2-enyl)-4-[(2S,3R,4S,5S,6R)-3,4,5-trihydroxy-6-(hydroxymethyl
C24H34O9; MF: 0; RMF: 21; Prob 50.0%; Lib: mona-export-gnps; ID: 6241.

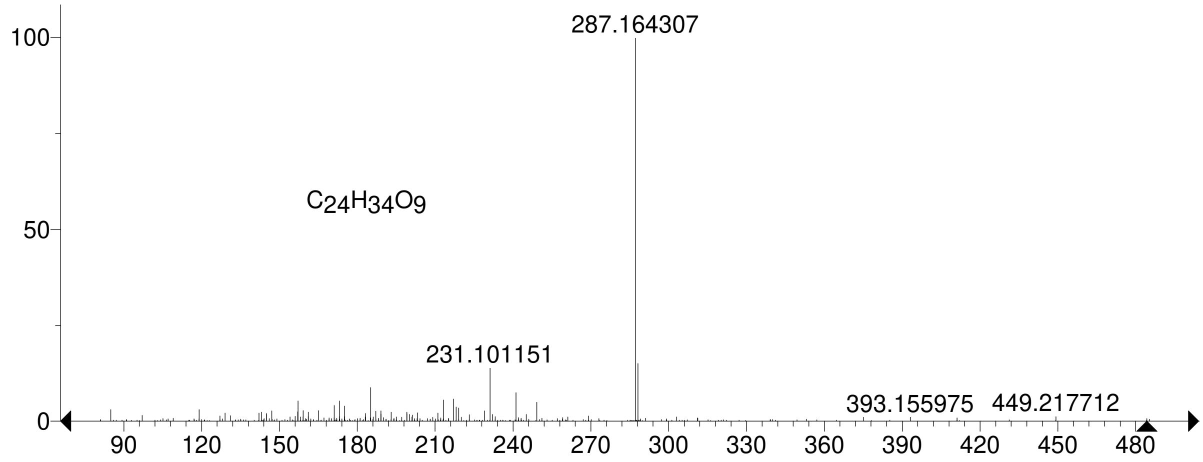

Unknown: Unknown (17.24_394.1856m/z)
Compound in Library Factor = 0

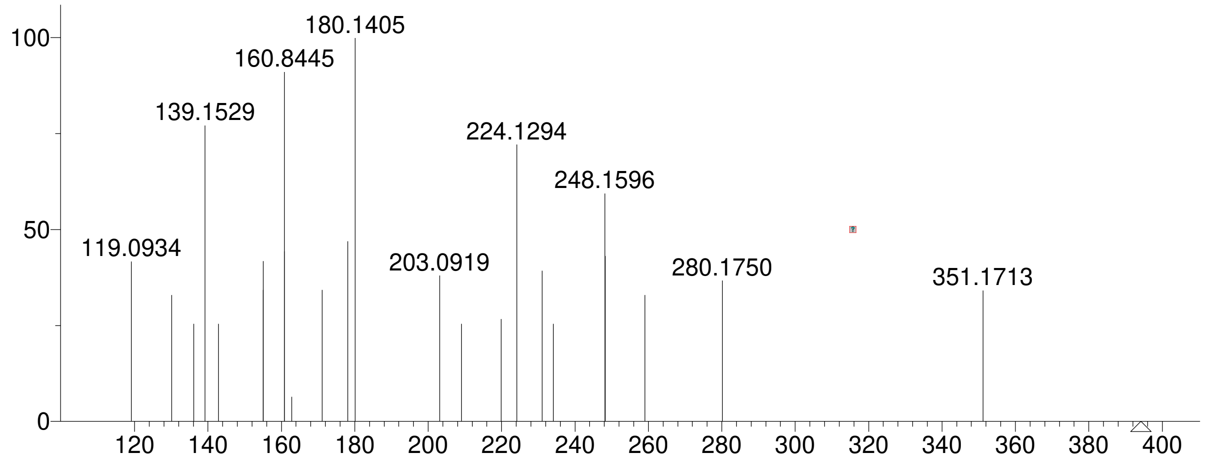

Hit 1 : NCGC00381047-01_C20H24O7_ [M+NH4]+ P=394.2
C20H24O7; MF: 0; RMF: 59; Prob 9.09%; Lib: mona-export-gnps; ID: 11699.

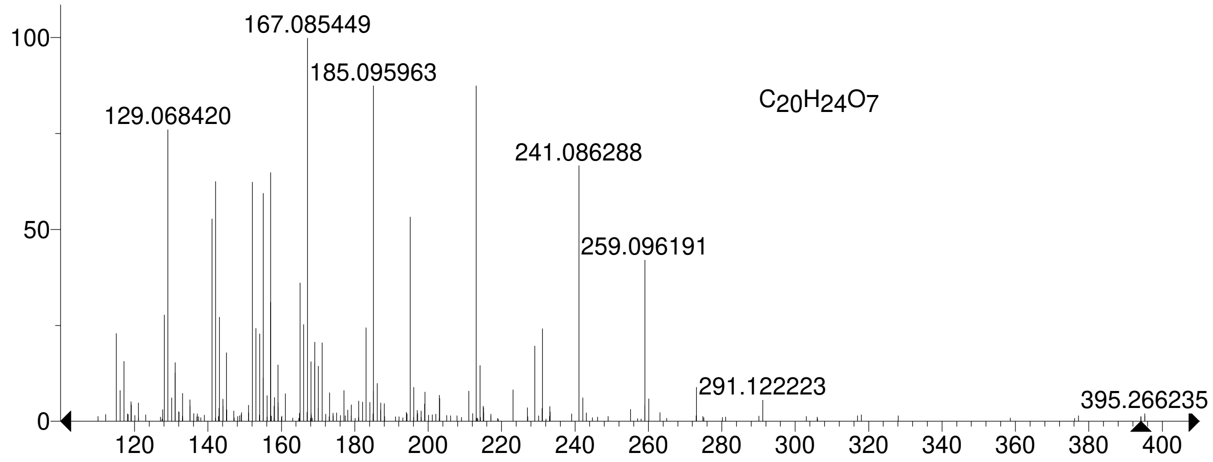

Hit 2 : [1,3-dihydroxy-1-(7-methoxy-2-oxochromen-6-yl)-3-methylbutan-2-yl] 3-methylbut-2-enoate [M+NH4]+ 35
C20H24O7; MF: 0; RMF: 37; Prob 9.09%; Lib: mona-export-vf-npl_qexactive; ID: 24781.

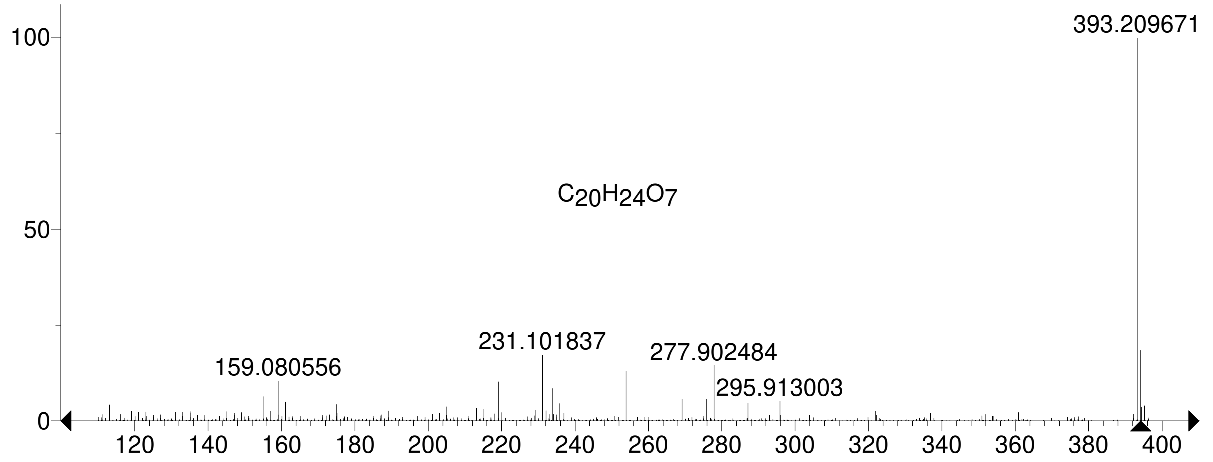

Hit 3 : [2,3-dihydroxy-1-(7-methoxy-2-oxochromen-6-yl)-3-methylbutyl] (Z)-2-methylbut-2-enoate [M+NH4]+ 35H[illegible]
C20H24O7; MF: 0; RMF: 25; Prob 9.09%; Lib: mona-export-vf-npl_qexactive; ID: 5716.

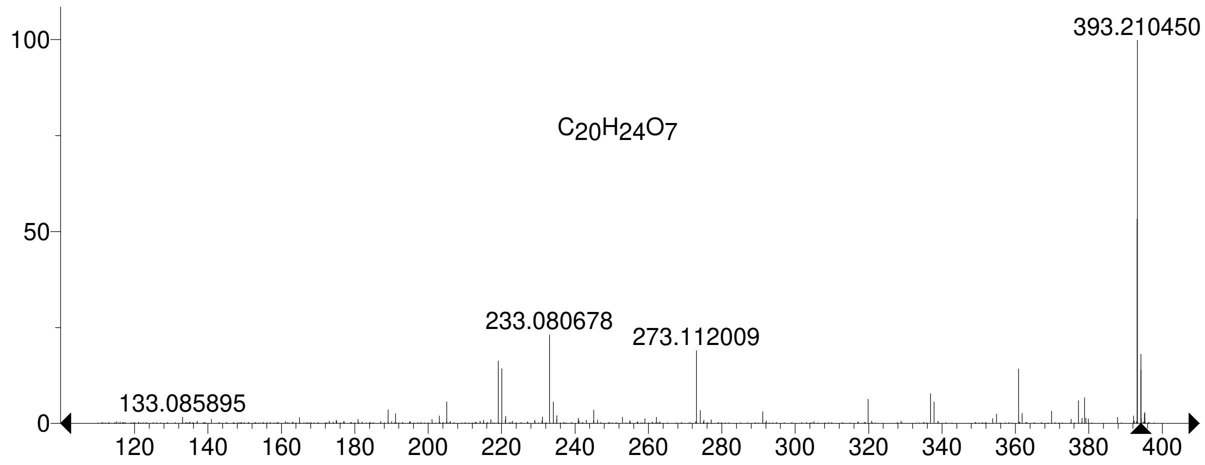

Hit 4 : NCGC00180381-03_C20H24O7_2-Butenoic acid, 2-methyl-, (1aR,1bS,2aS,4R,4aS,7aR,8S)-dodecahydro
C20H24O7; MF: 0; RMF: 23; Prob 9.09%; Lib: mona-export-gnps; ID: 10736.

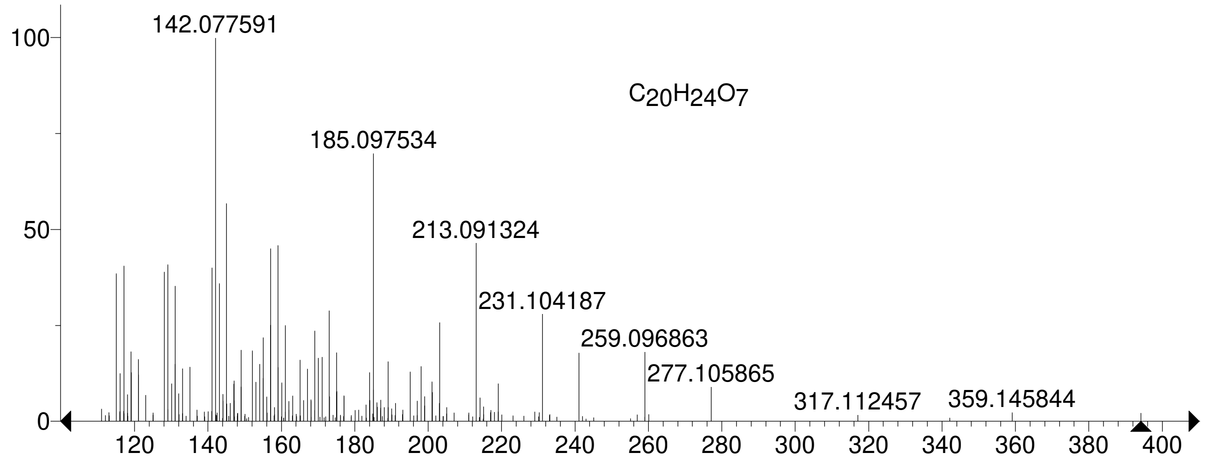

Hit 5 : [1,3-dihydroxy-1-(7-methoxy-2-oxochromen-6-yl)-3-methylbutan-2-yl] (E)-2-methylbut-2-enoate [M+NH4]+
C20H24O7; MF: 0; RMF: 22; Prob 9.09%; Lib: mona-export-vf-npl_qexactive; ID: 5462.

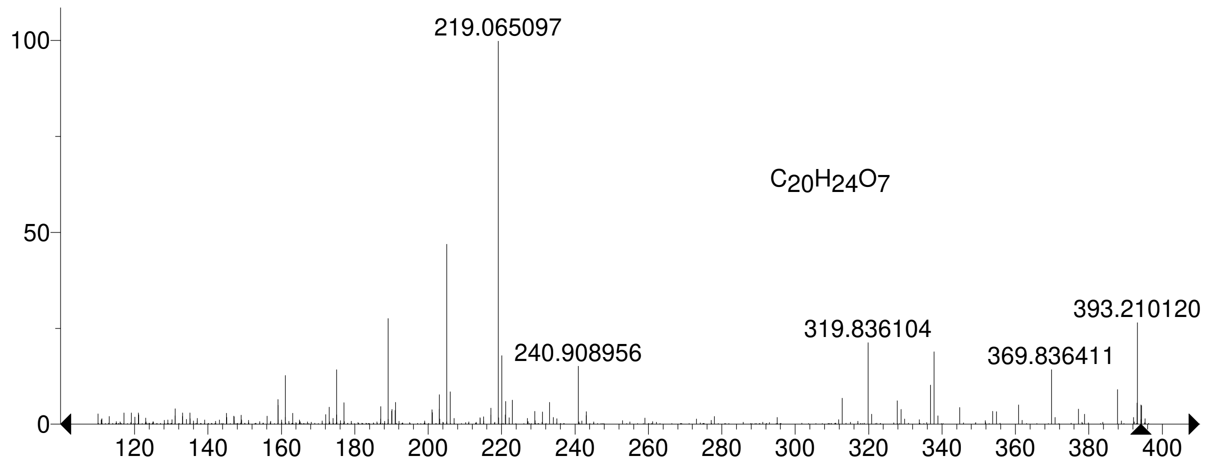

** Search Report Page 1 of 2 **

Unknown: Unknown (17.28_501.1760m/z)
Compound in Library Factor = 0

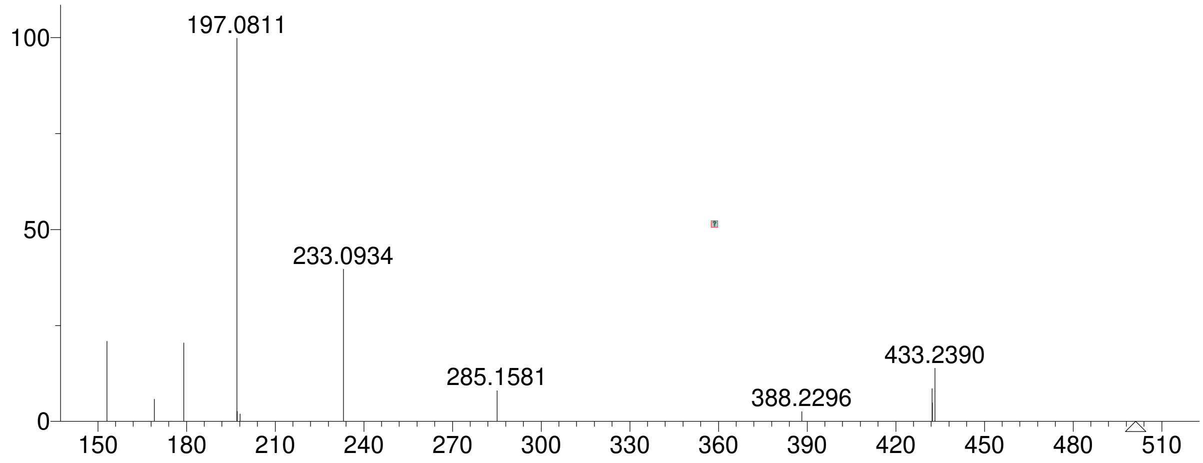

Hit 1 : (1R,4S,7S,8S,9R,12E,20R,21R,22S,23S)-7,8,21,22,23-pentahydroxy-12,16-dimethyl-3,5,10,19,24-pentao
C21H34O11; MF: 0; RMF: 60; Prob 33.3%; Lib: mona-export-vf-npl_qexactive; ID: 19953.

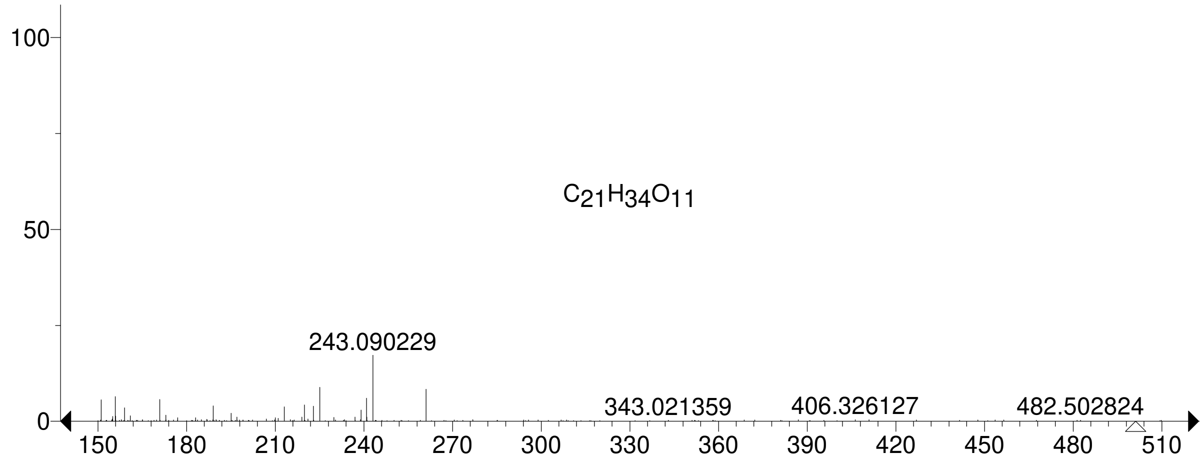

Hit 2 : (2S,3R,4S,5R)-2-[4-[7-hydroxy-3-(hydroxymethyl)-5-(3-hydroxypropyl)-2,3-dihydro-1-benzofuran-2-yl]-2-me
C24H30O10; MF: 0; RMF: 40; Prob 33.3%; Lib: mona-export-vf-npl_qexactive; ID: 1942.

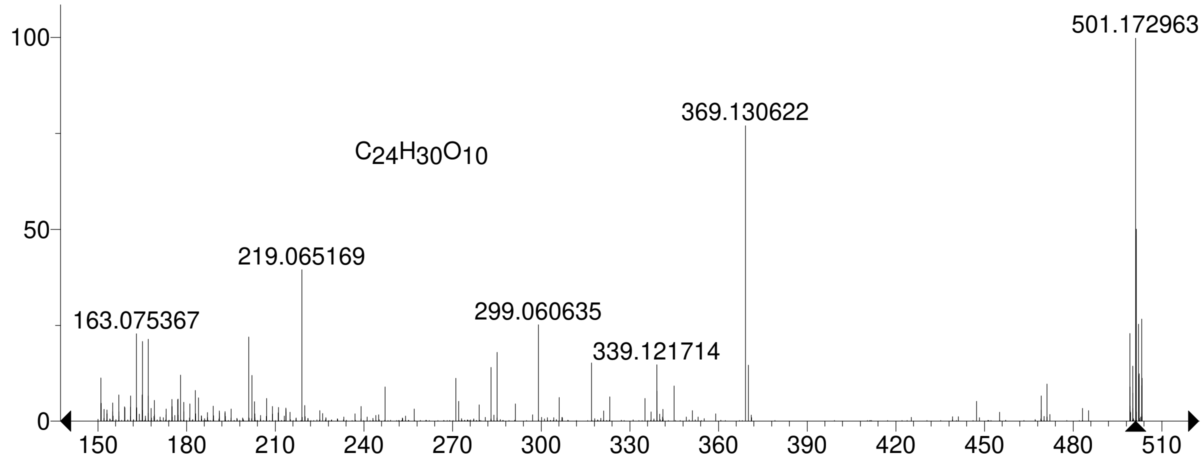

Hit 3 : (1R,4R,7S,11E,15S,19R,20R,21S,22S,24R)-7,20,21,22,24-pentahydroxy-11,15-dimethyl-3,5,9,18,23-penta
C21H34O11; MF: 0; RMF: 24; Prob 33.3%; Lib: mona-export-vf-npl_qexactive; ID: 22606.

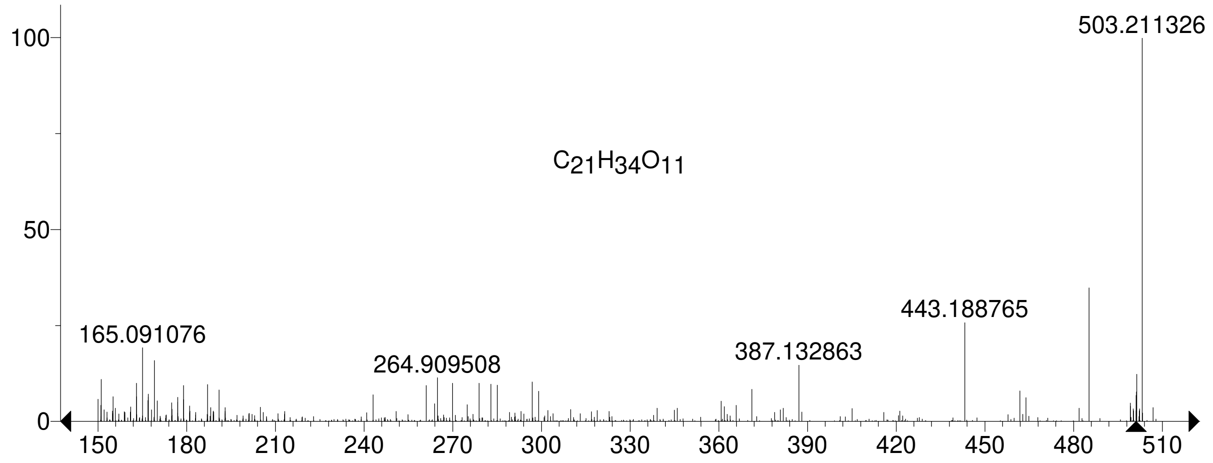

Unknown: Unknown (17.28_620.1776m/z)
Compound in Library Factor = 0

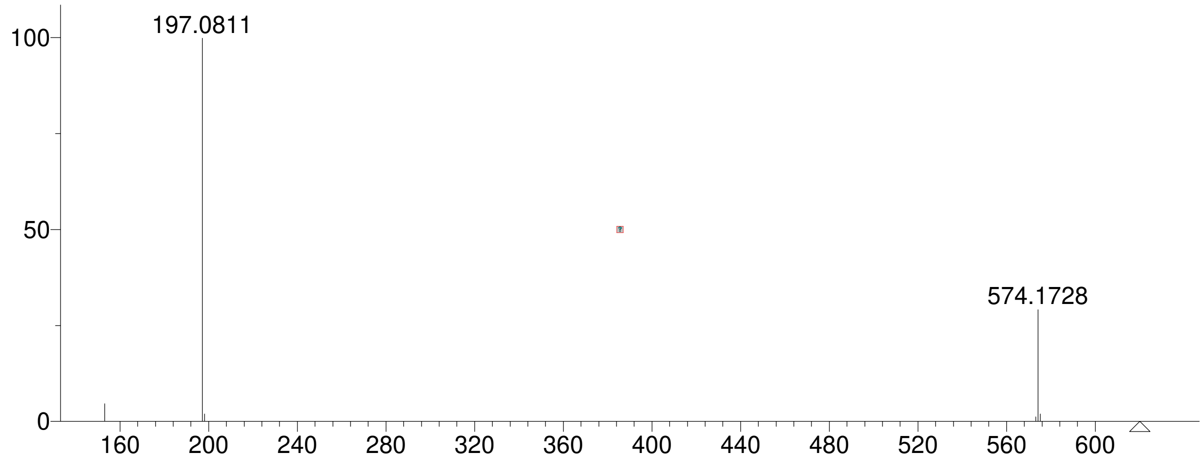

Hit 1 : BEKANAMYCIN SULFATE [M+K]+ 35% P=620.2
C18H39N5O14S; MF: 0; RMF: 2; Prob 100.0%; Lib: mona-export-itree; ID: 1511.

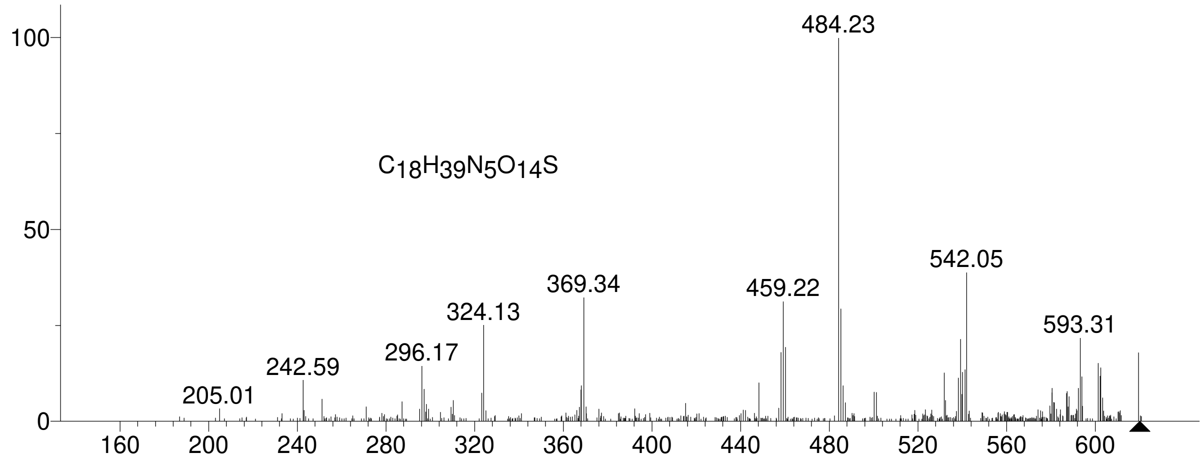

Unknown: Unknown (17.30_431.4242m/z)
Compound in Library Factor = 0

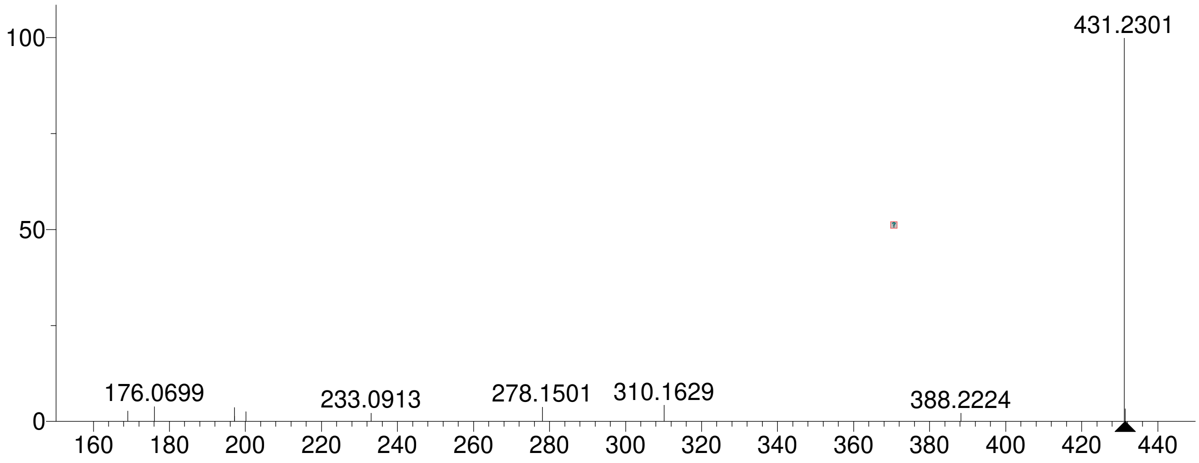

Unknown: Unknown (17.30_636.0978m/z)
Compound in Library Factor = 0

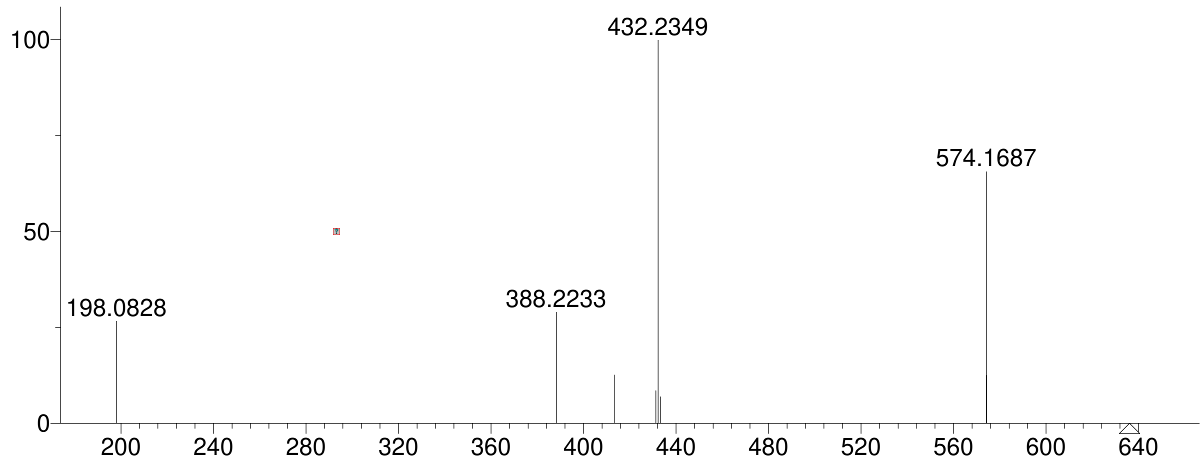

** Search Report Page 1 of 1 **

Unknown: Unknown (17.32_659.2451m/z)
Compound in Library Factor = 0

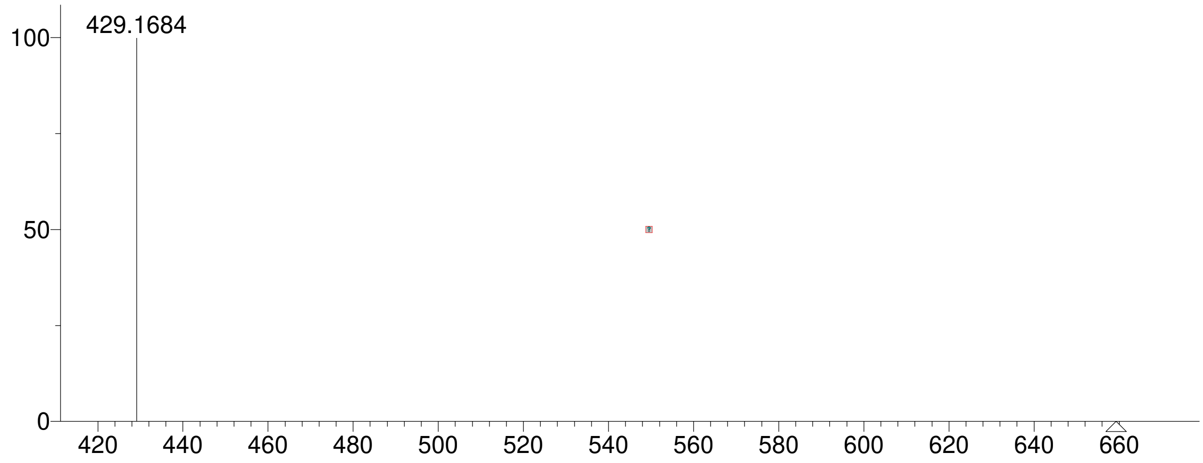

Unknown: Unknown (17.34_267.1594m/z)
Compound in Library Factor = -1309

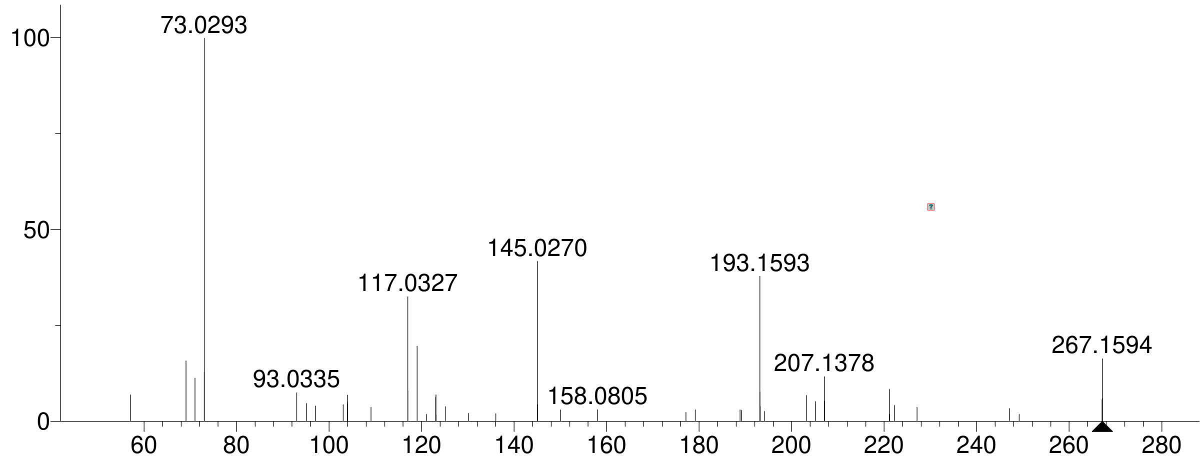

Hit 1 : Dihydroartemisinin [M+H-H2O]+ HCD 7V P=267.2
C15H24O5; MF: 67; RMF: 264; Prob 25.4%; CAS: 71939-50-9; Lib: nist_msms; ID: 121293.

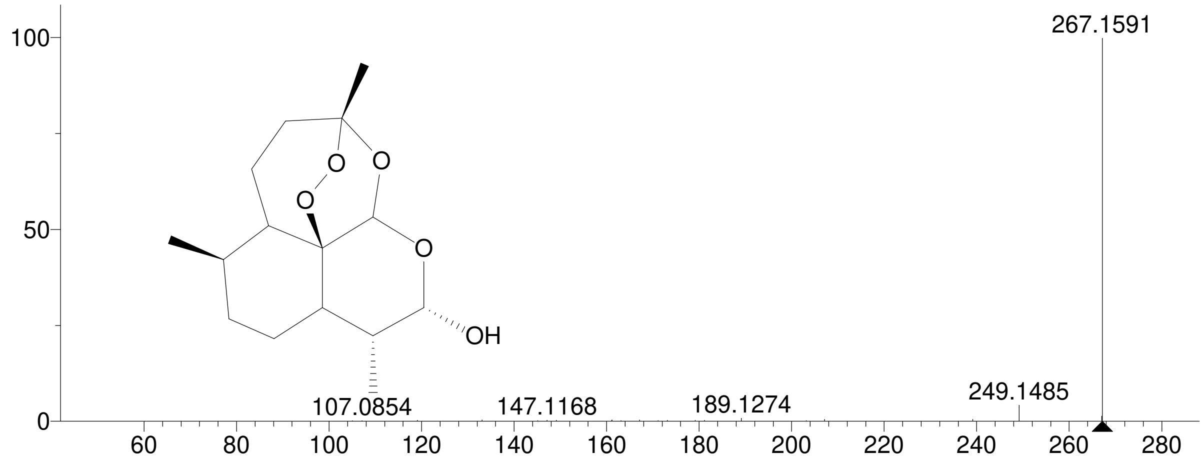

Hit 2 : NCGC00381369-01_C15H22O4_3a,7-Ethano-3aH-indene-4-carboxylic acid, octahydro-6-hydroxy-3,8,8-tri
C15H22O4; MF: 8; RMF: 137; Prob 4.59%; Lib: mona-export-gnps; ID: 10301.

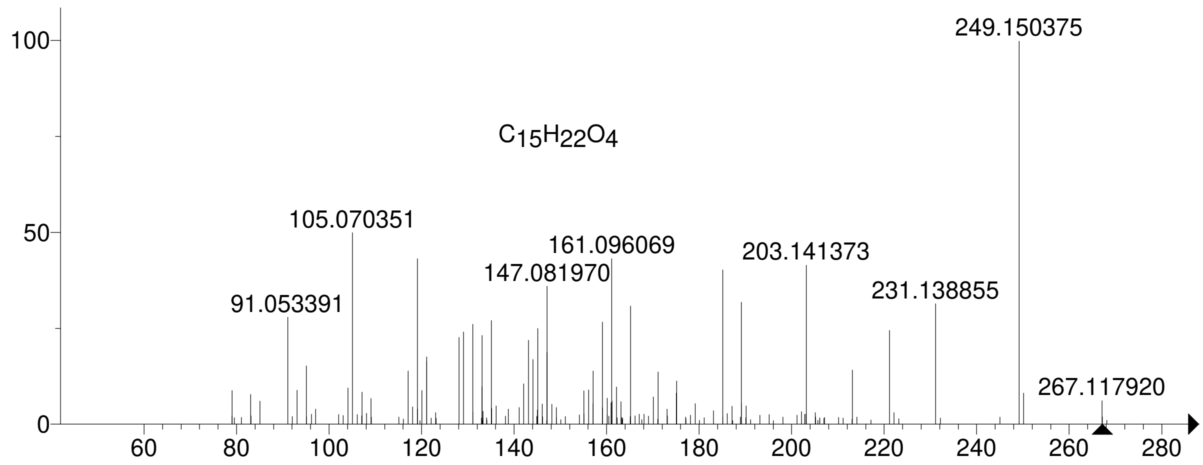

Hit 3 : NCGC00380735-01_C15H22O4_Naphtho[2,3-b]furan-2(4H)-one, 4a,5,6,7,8,8a,9,9a-octahydro-9,9a-dihyd
C15H22O4; MF: 8; RMF: 136; Prob 4.59%; Lib: mona-export-gnps; ID: 11971.

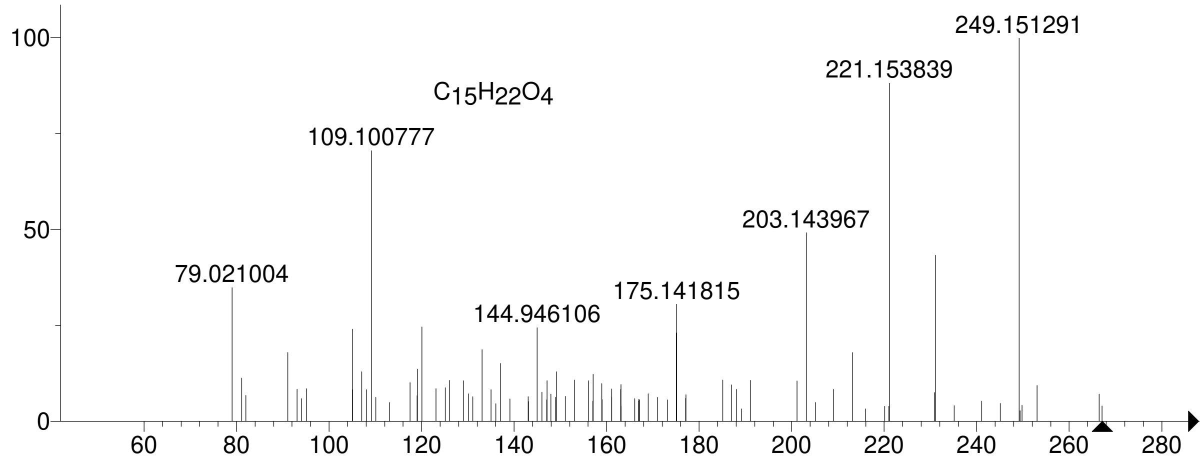

Hit 4 : NCGC00385633-01_C15H22O4_Naphtho[1,2-c]furan-3(1H)-one, 5,5a,6,7,8,9,9a,9b-octahydro-5-hydroxy-
C15H22O4; MF: 7; RMF: 140; Prob 4.41%; Lib: mona-export-gnps; ID: 13883.

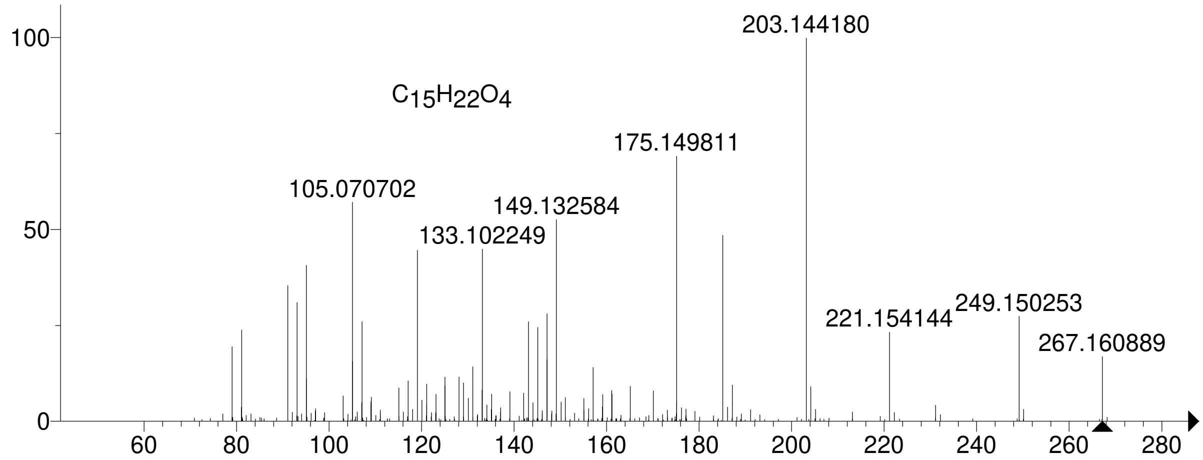

Hit 5 : 1,4-dihydroxy-6,6,9a-trimethyl-4,5,5a,7,8,9-hexahydro-1H-benzo[e][2]benzofuran-3-one [M+H]+ 35HCD I
C15H22O4; MF: 7; RMF: 136; Prob 4.41%; Lib: mona-export-vf-npl_qexactive; ID: 10777.

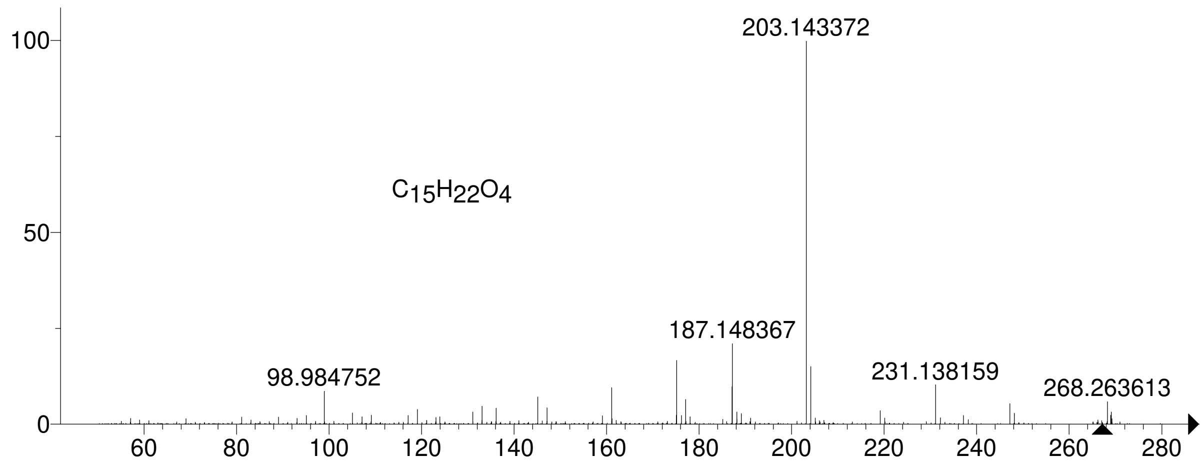

Unknown: Unknown (17.36_445.2446m/z)
Compound in Library Factor = 0

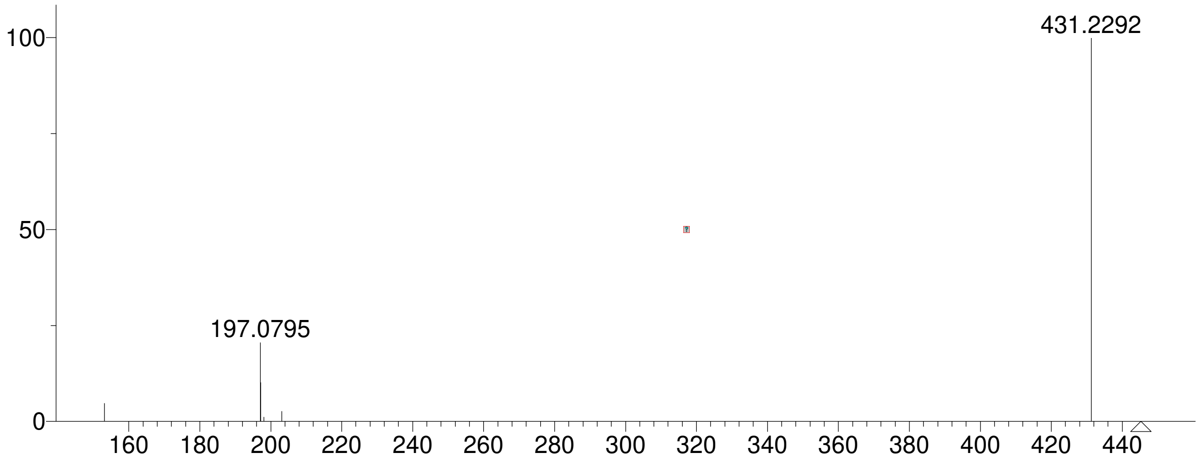

Unknown: Unknown (17.37_401.2186m/z)
Compound in Library Factor = -2387

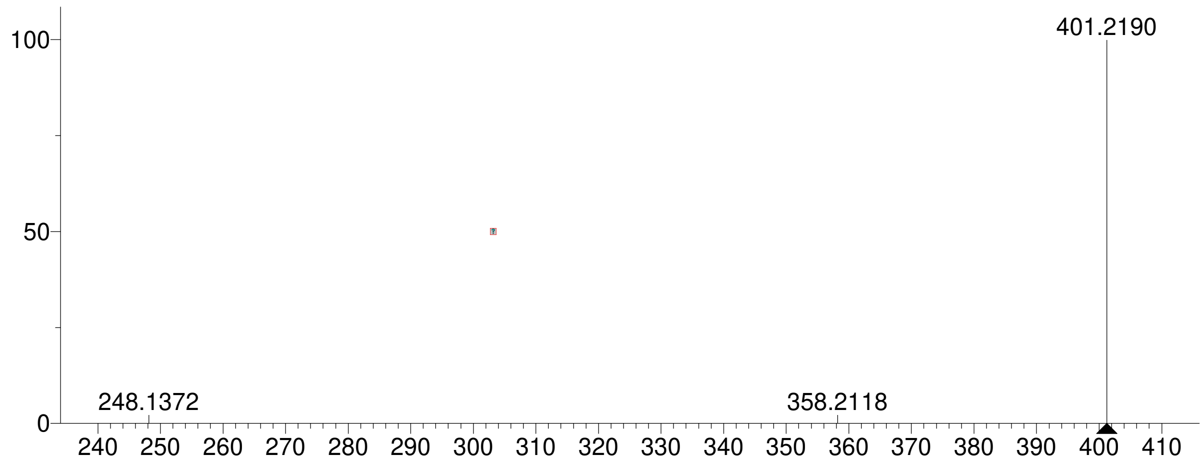

Hit 1 : GTP-Binding Protein Fragment, Gs alpha [M+5H]5+ HCD 12eV P=401.2
C85H144N30O24S; MF: 17; RMF: 540; Prob 100.0%; Lib: nist_msms2; ID: 57649.

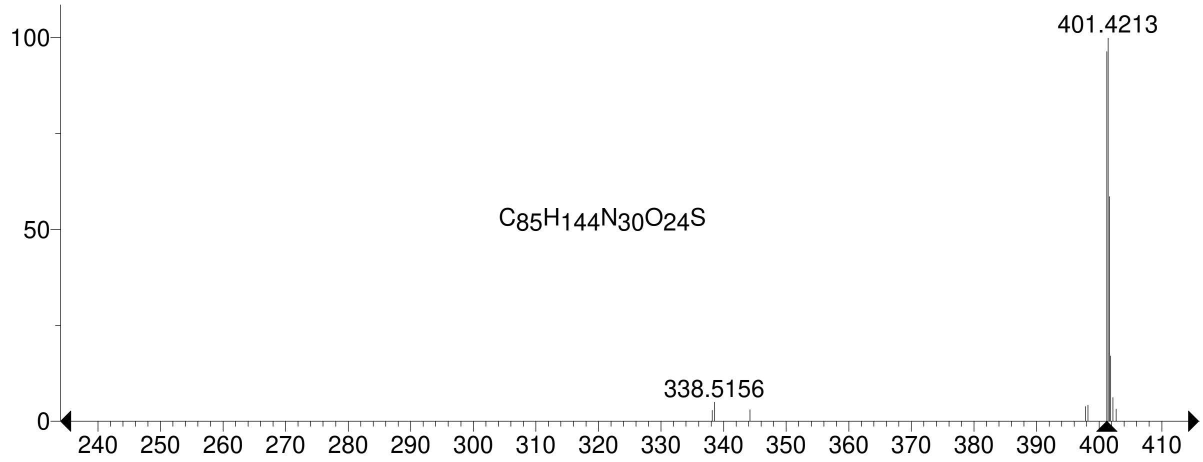

Unknown: Unknown (17.37_501.1762m/z)
Compound in Library Factor = 0

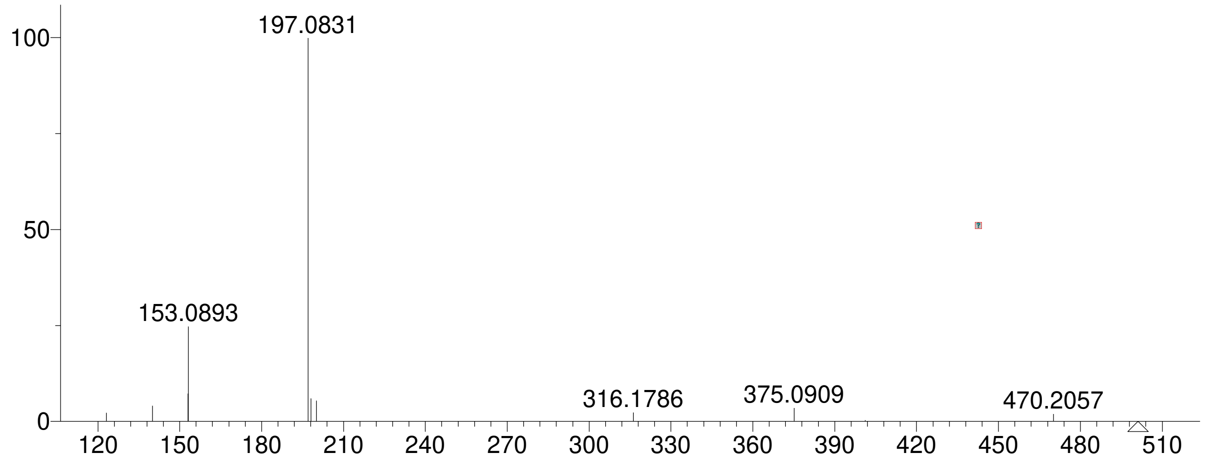

Hit 1 : (1R,4S,7S,8S,9R,12E,20R,21R,22S,23S)-7,8,21,22,23-pentahydroxy-12,16-dimethyl-3,5,10,19,24-pentaox
C21H34O11; MF: 0; RMF: 64; Prob 33.3%; Lib: mona-export-vf-npl_qexactive; ID: 19953.

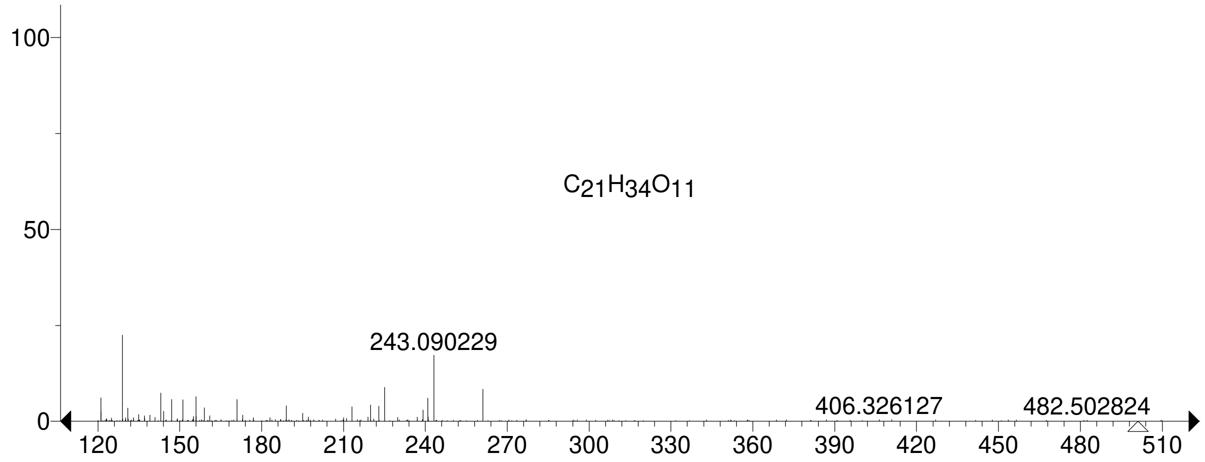

Hit 2 : (2S,3R,4S,5R)-2-[4-[7-hydroxy-3-(hydroxymethyl)-5-(3-hydroxypropyl)-2,3-dihydro-1-benzofuran-2-yl]-2-me
C24H30O10; MF: 0; RMF: 43; Prob 33.3%; Lib: mona-export-vf-npl_qexactive; ID: 1942.

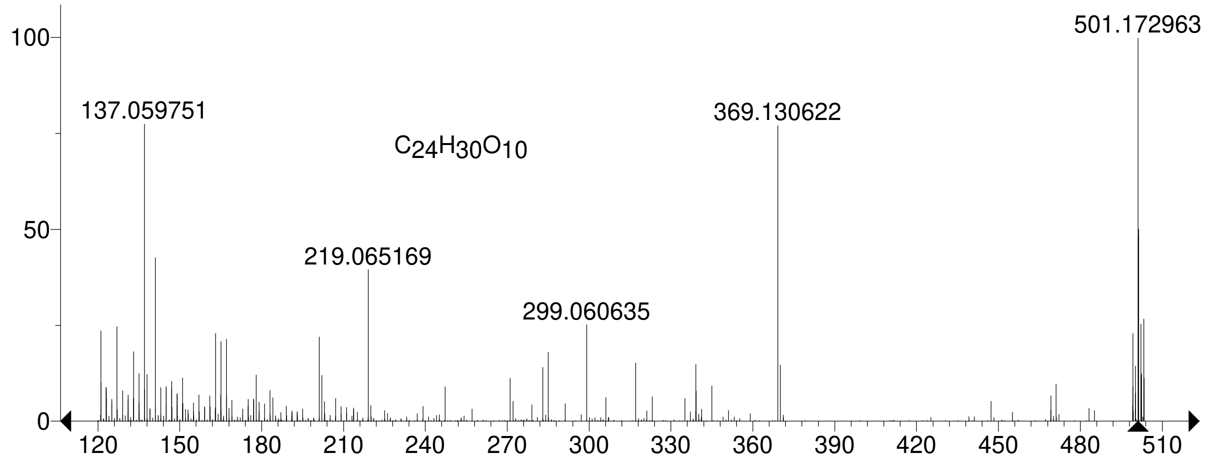

Hit 3 : (1R,4R,7S,11E,15S,19R,20R,21S,22S,24R)-7,20,21,22,24-pentahydroxy-11,15-dimethyl-3,5,9,18,23-penta
C21H34O11; MF: 0; RMF: 32; Prob 33.3%; Lib: mona-export-vf-npl_qexactive; ID: 22608.

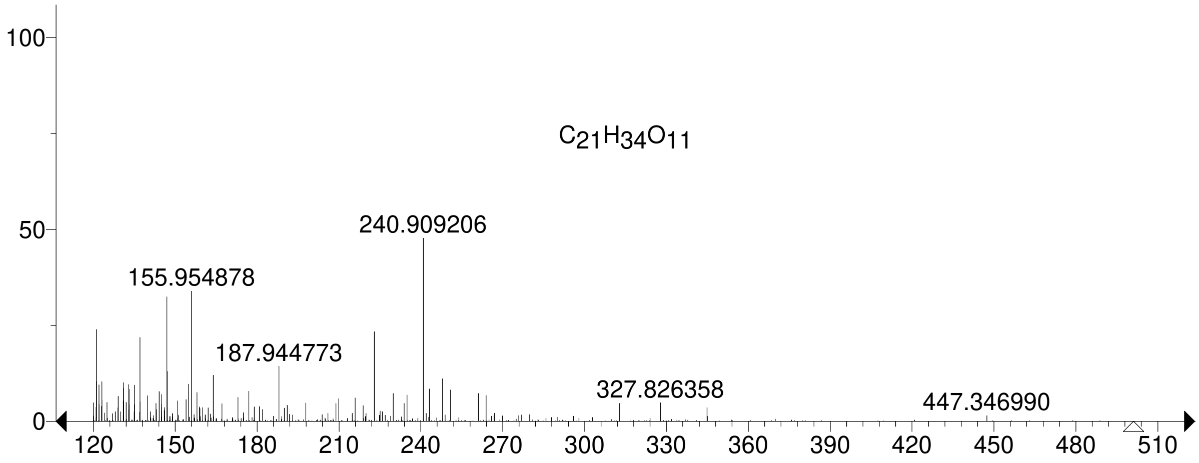

Unknown: Unknown (17.39_394.1859m/z)
Compound in Library Factor = -2387

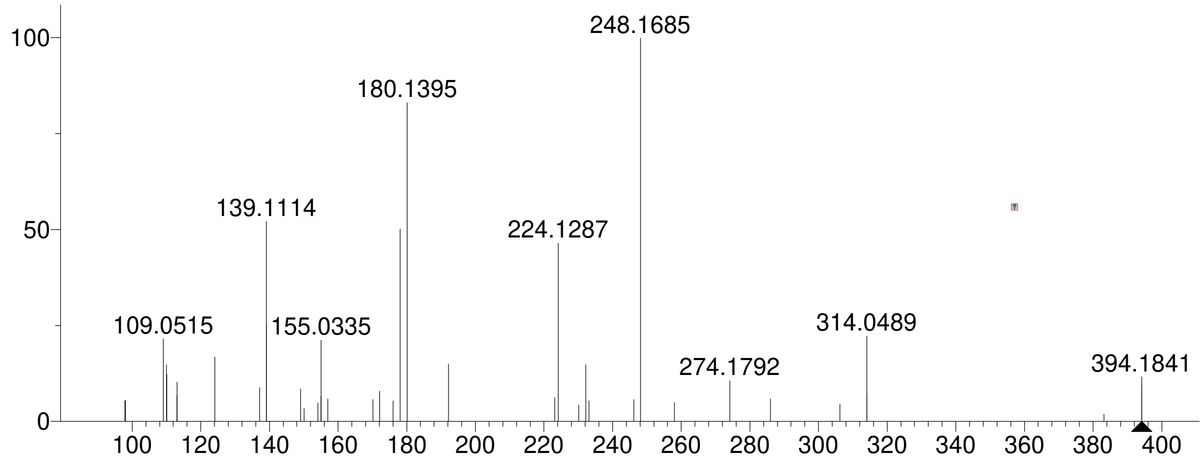

Hit 1 : 3',4',5'-Trimethoxycocaine [M+H]+ HCD 15V P=394.2
C20H27NO7; MF: 21; RMF: 194; Prob 15.5%; CAS: 156301-59-6; Lib: nist_msms; ID: 383469.

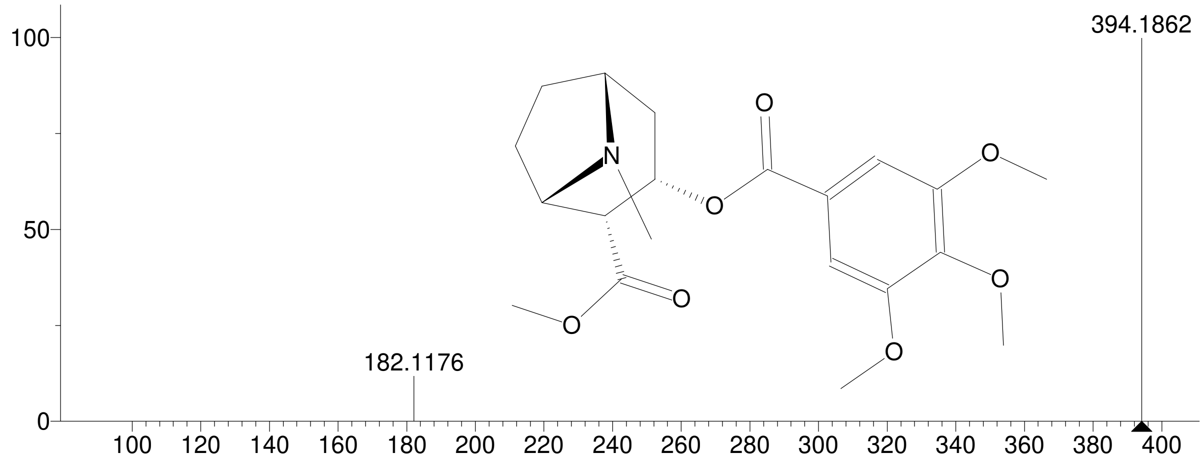

Hit 2 : [1,3-dihydroxy-1-(7-methoxy-2-oxochromen-6-yl)-3-methylbutan-2-yl] 3-methylbut-2-enoate [M+NH4]+ 35I
C20H24O7; MF: 2; RMF: 96; Prob 7.54%; Lib: mona-export-vf-npl_qexactive; ID: 24781.

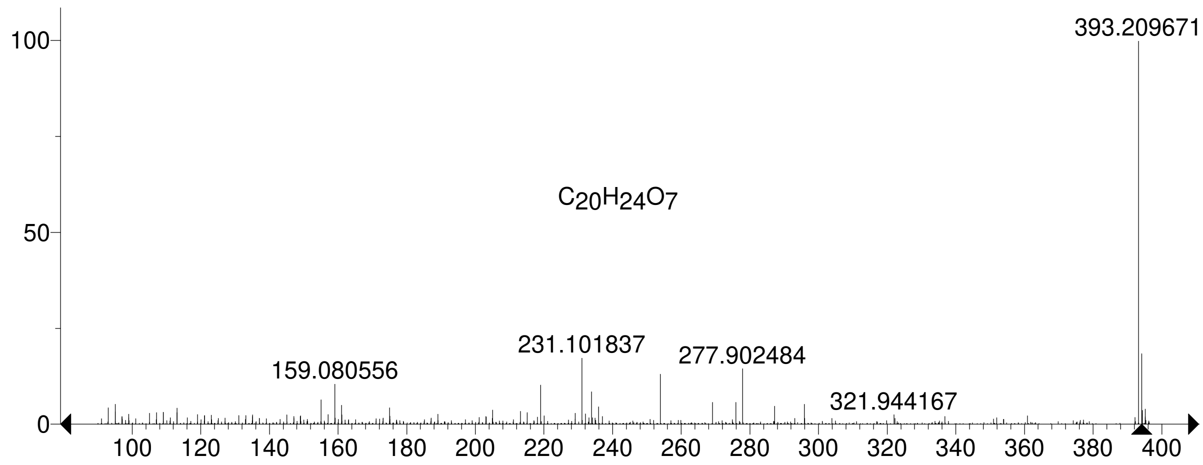

Hit 3 : [1,3-dihydroxy-1-(7-methoxy-2-oxochromen-6-yl)-3-methylbutan-2-yl] (E)-2-methylbut-2-enoate [M+NH4]+
C20H24O7; MF: 1; RMF: 61; Prob 7.24%; Lib: mona-export-vf-npl_qexactive; ID: 5461.

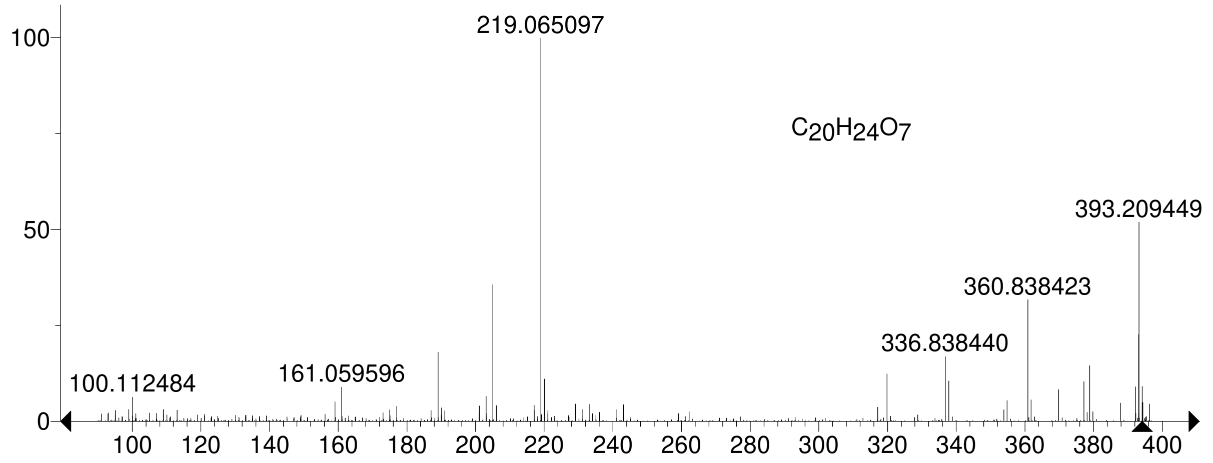

Hit 4 : rac-N,N-Didesmethyl-O-desmethylvenlafaxine glucuronide [M+H-H2O]+ HCD 11V P=394.2
C20H29NO8; MF: 0; RMF: 108; Prob 6.96%; CAS: 1799830-07-1; Lib: nist_msms; ID: 436197.

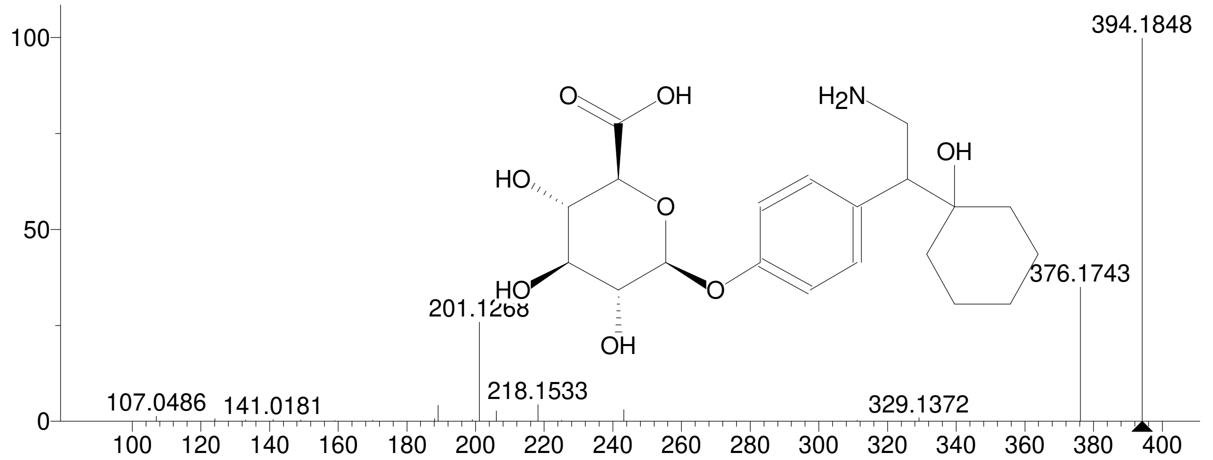

Hit 5 : NCGC00381074-01![1,3-dihydroxy-1-(7-methoxy-2-oxochromen-6-yl)-3-methylbutan-2-yl] 3-methylbut-2-er
C20H24O7; MF: 0; RMF: 57; Prob 6.96%; Lib: mona-export-gnps; ID: 8644.

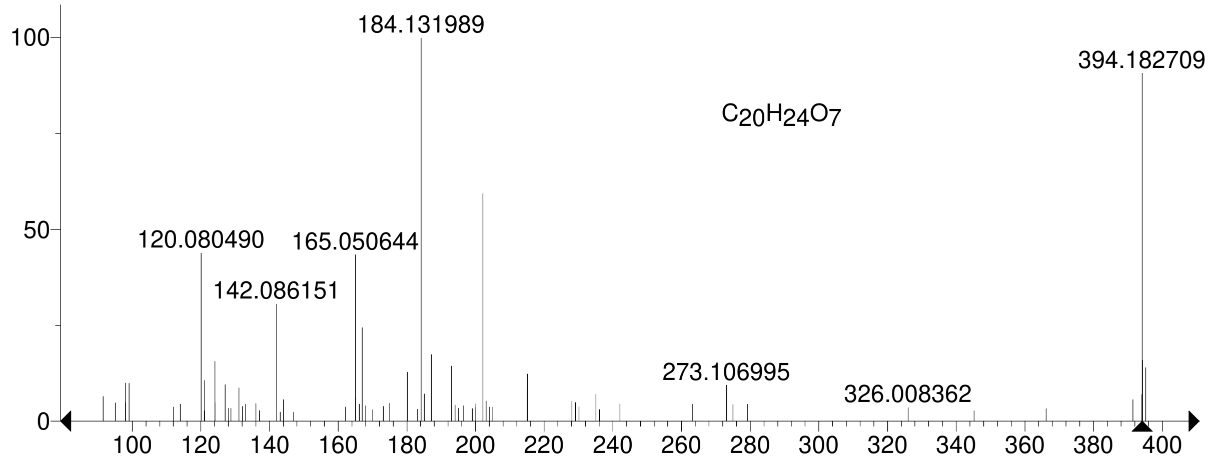

Unknown: Unknown (17.45_423.2128m/z)
Compound in Library Factor = -3273

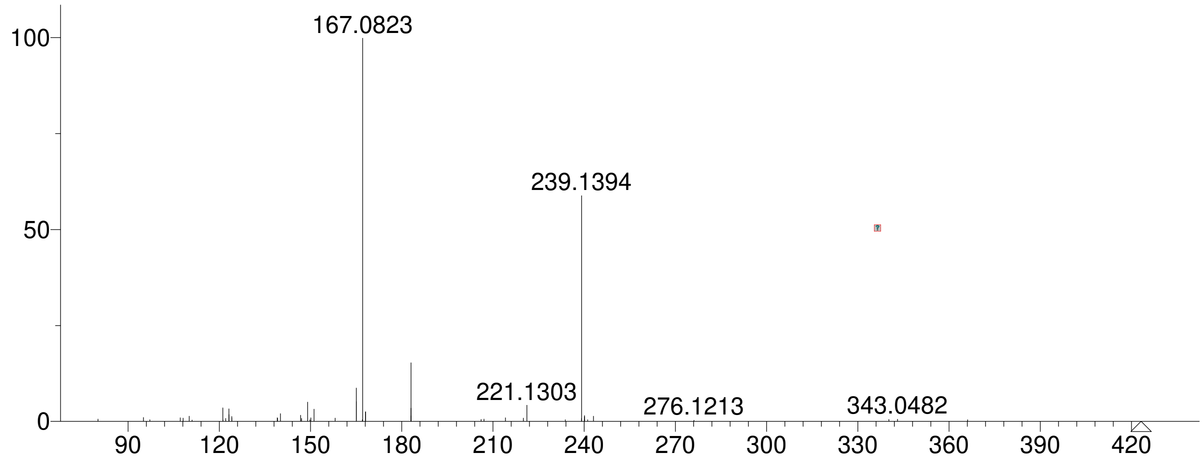

Hit 1 : Cinobufotalin [M+H-2H2O]+ HCD 50V P=423.2
C26H34O7; MF: 4; RMF: 98; Prob 22.1%; CAS: 1108-68-5; Lib: nist_msms; ID: 391346.

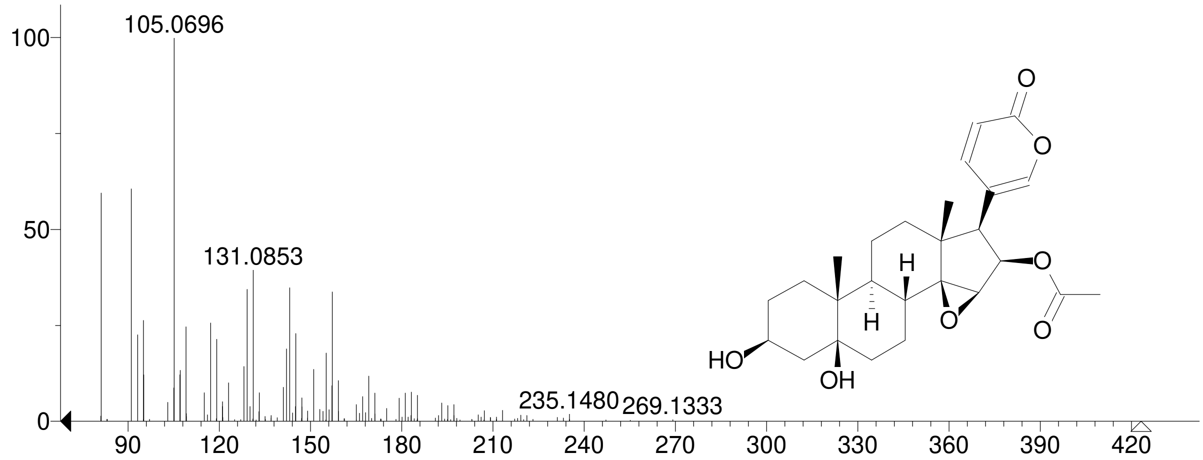

Hit 2 : [4,5-dihydroxy-3,4-bis(hydroxymethyl)-4a,8,8-trimethyl-5,6,7,8a-tetrahydro-1H-naphthalen-1-yl] hexanoate
C21H36O6; MF: 2; RMF: 84; Prob 20.4%; Lib: mona-export-vf-npl_qexactive; ID: 8154.

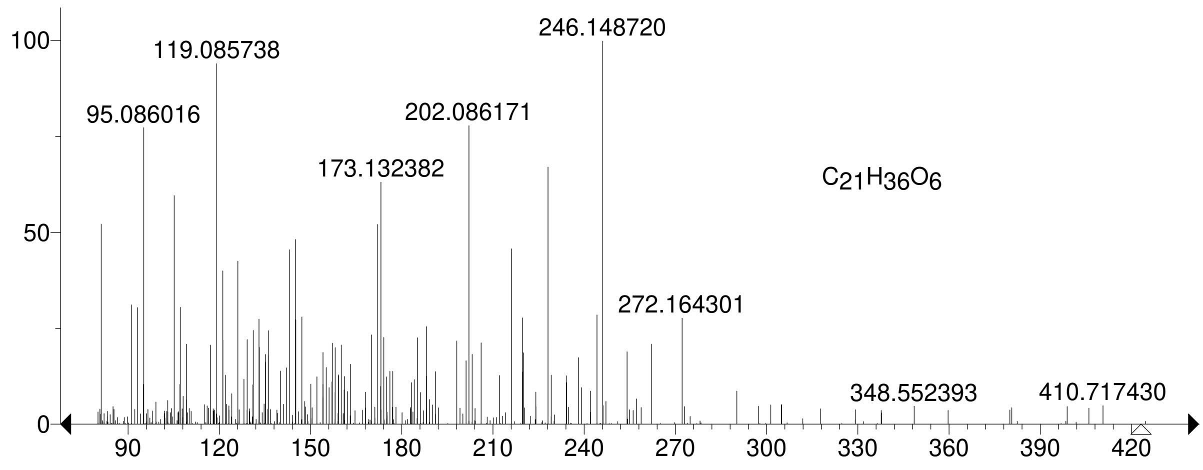

Hit 3 : 4-Methylabyssinone V [M+H]+ 65HCD P=423.2
C26H30O5; MF: 1; RMF: 40; Prob 19.6%; Lib: mona-export-vf-npl_qexactive; ID: 6033.

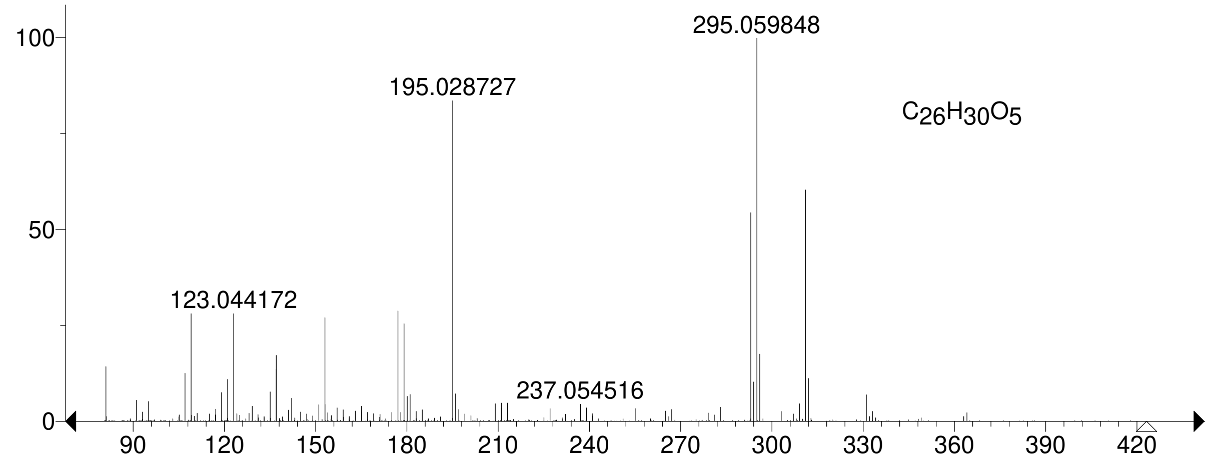

Hit 4 : NCGC00384666-01!(2S)-5,7-dihydroxy-2-[4-methoxy-3,5-bis(3-methylbut-2-enyl)phenyl]-2,3-dihydrochrom
C26H30O5; MF: 0; RMF: 38; Prob 18.9%; Lib: mona-export-gnps; ID: 7800.

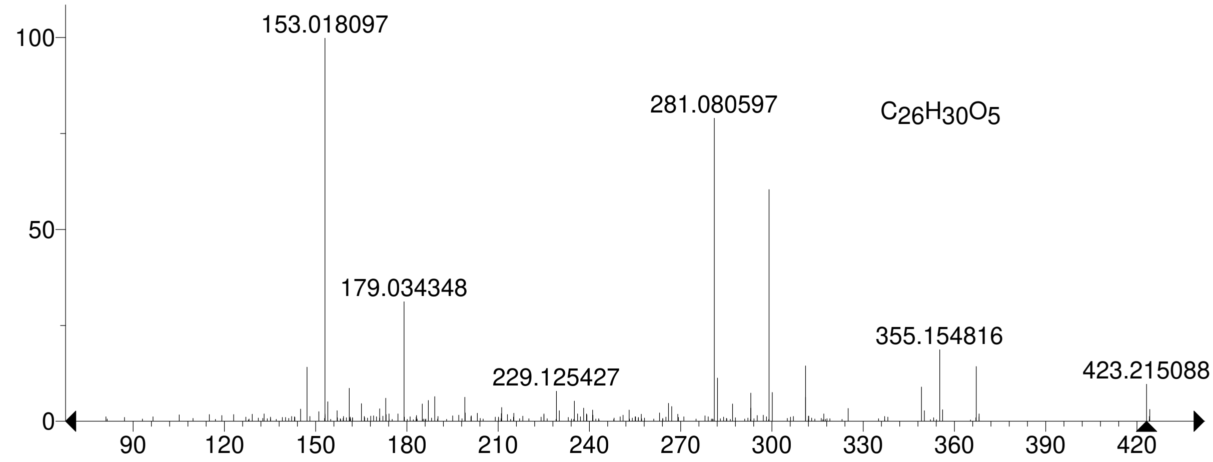

Hit 5 : PyroGlu-His-Arg [M+H]+ QTOF 20V P=423.2
C17H26N8O5; MF: 0; RMF: 12; Prob 18.9%; Lib: nist_msms; ID: 53165.

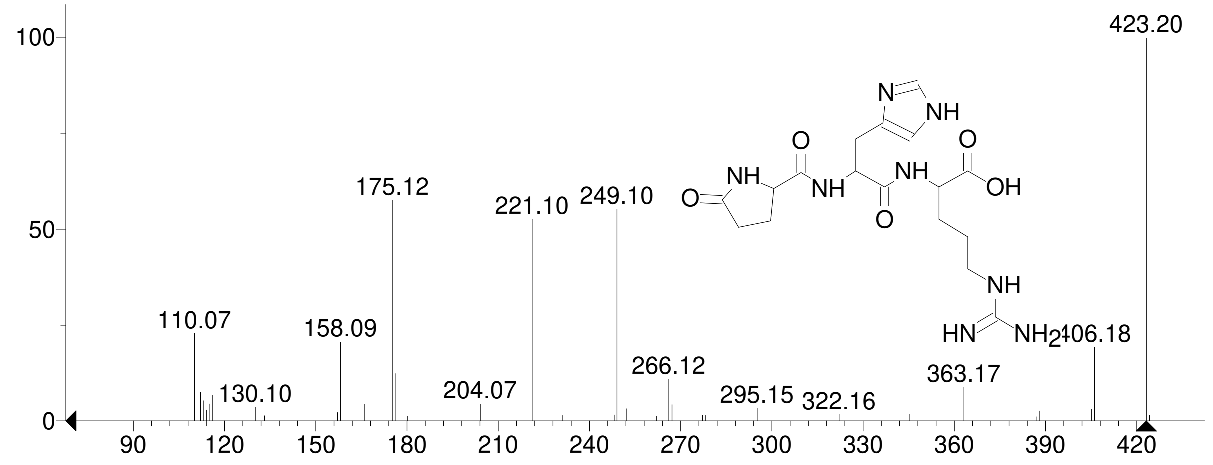

Unknown: Unknown (17.46_568.1343m/z)
Compound in Library Factor = 0

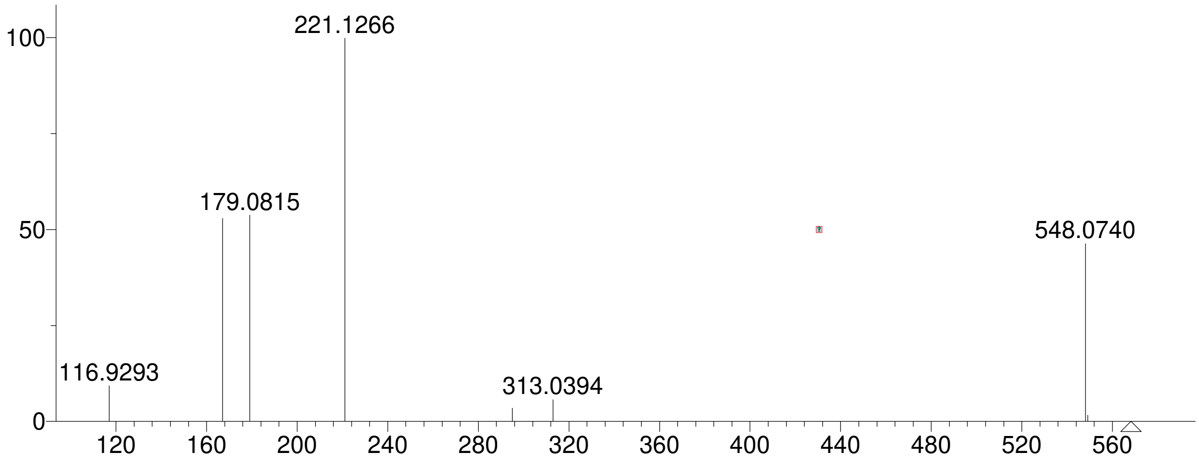

Unknown: Unknown (17.50_267.1589m/z)
Compound in Library Factor = -3273

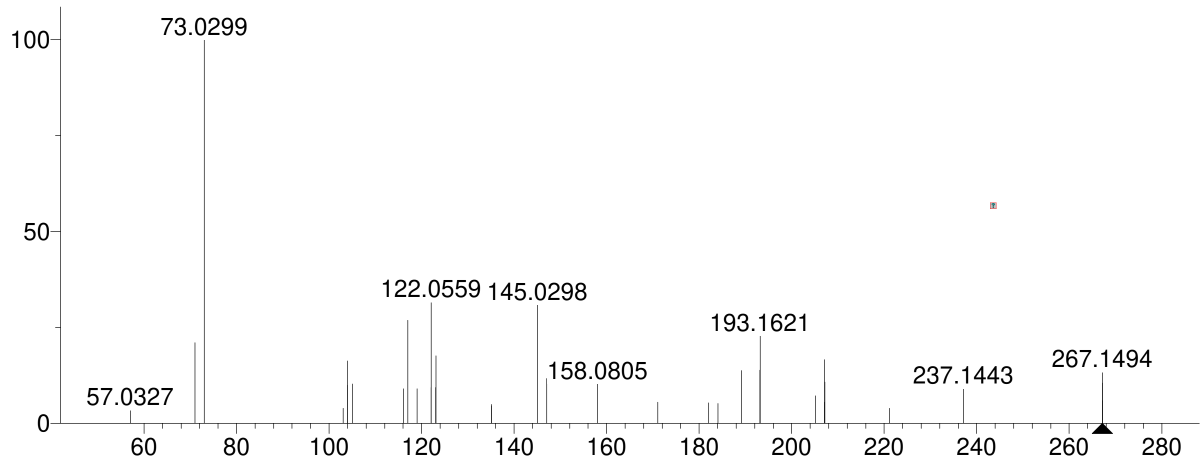

Hit 1 : Dihydroartemisinin [M+H-H2O]+ HCD 10V P=267.2
C15H24O5; MF: 9; RMF: 195; Prob 6.72%; CAS: 71939-50-9; Lib: nist_msms; ID: 121294.

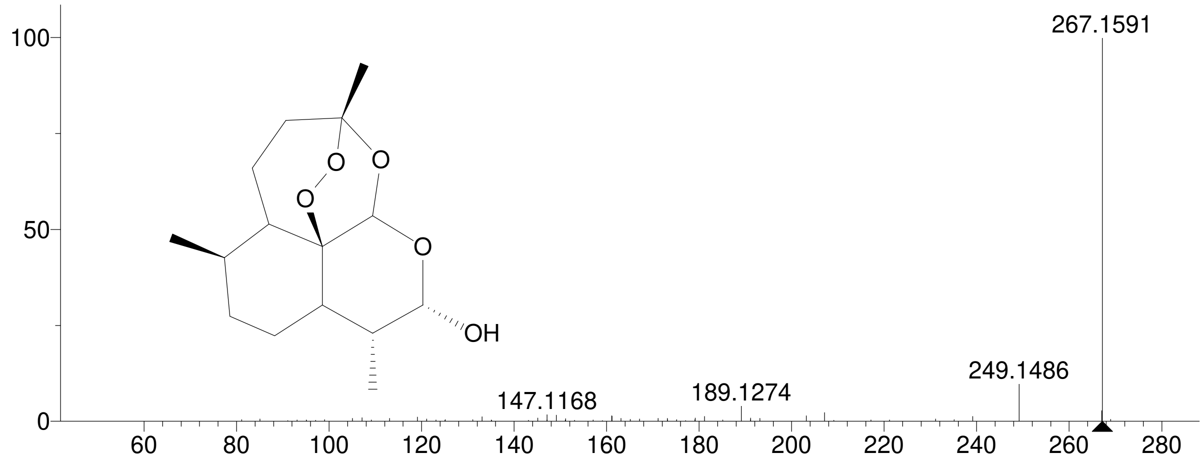

Hit 2 : 4,6-dihydroxy-4-(hydroxymethyl)-3,4a,8,8-tetramethyl-5,6,7,8a-tetrahydronaphthalen-1-one [M-H]- 35HCD
C15H24O4; MF: 4; RMF: 122; Prob 5.42%; Lib: mona-export-lc-ms-ms_negative_mode; ID: 19568.

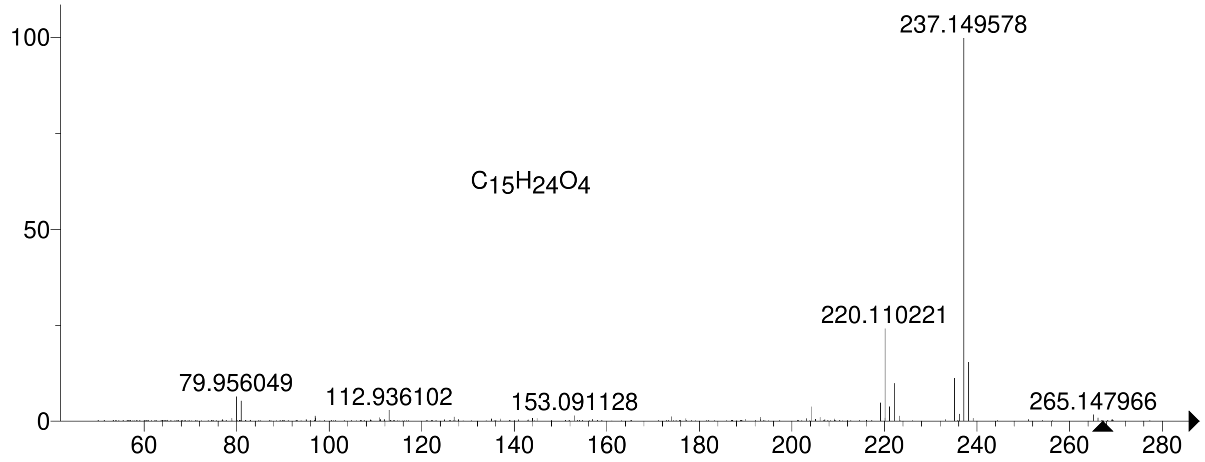

Hit 3 : NCGC00385871-01_C15H22O4_2,8-Dihydroxy-5,5,8-trimethyl-11-oxatetracyclo[7.3.1.0~1,9~.0~3,7~]tride
C15H22O4; MF: 3; RMF: 71; Prob 5.21%; Lib: mona-export-gnps; ID: 10011.

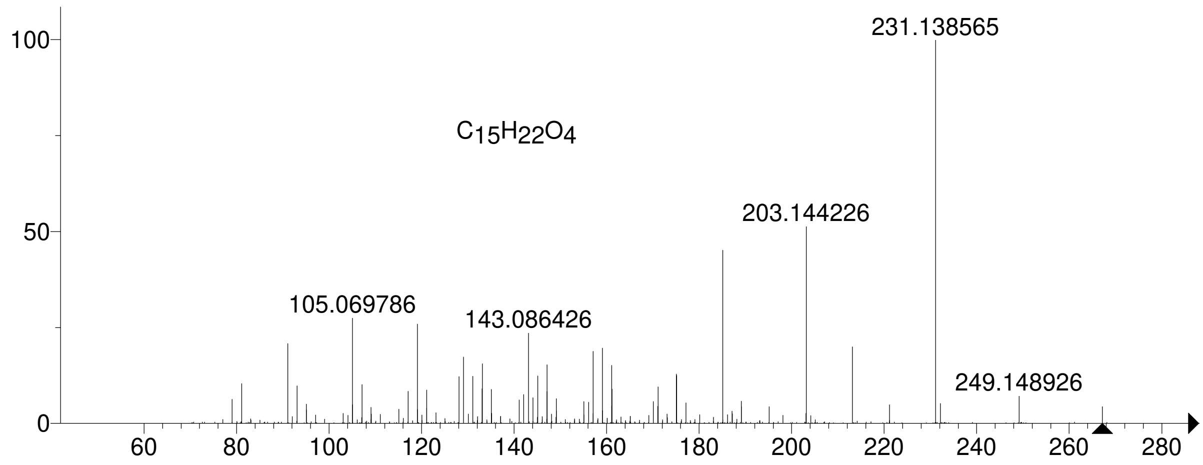

Hit 4 : NCGC00385923-01_C15H22O4_2-[(1E,3E)-1,3-Hexadien-1-yl]-5-(3-hydroxy-2-methoxypropyl)-2-methyl-3
C15H22O4; MF: 2; RMF: 94; Prob 5.00%; Lib: mona-export-gnps; ID: 14368.

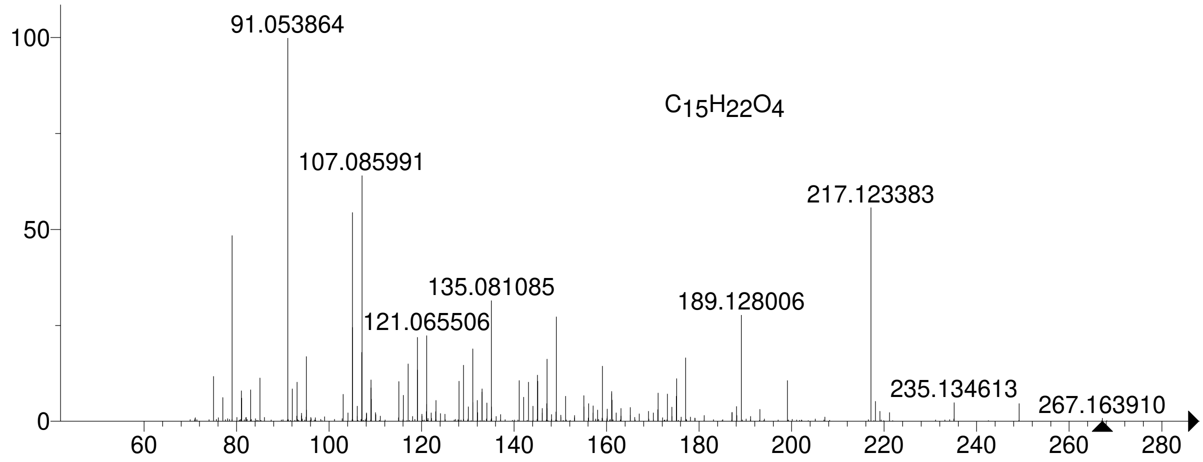

Hit 5 : (4aR,5S)-9,9a-dihydroxy-3,4a,5-trimethyl-5,6,7,8,8a,9-hexahydro-4H-benzo[f][1]benzofuran-2-one [M+H]+
C15H22O4; MF: 2; RMF: 91; Prob 5.00%; Lib: mona-export-vf-npl_qexactive; ID: 1837.

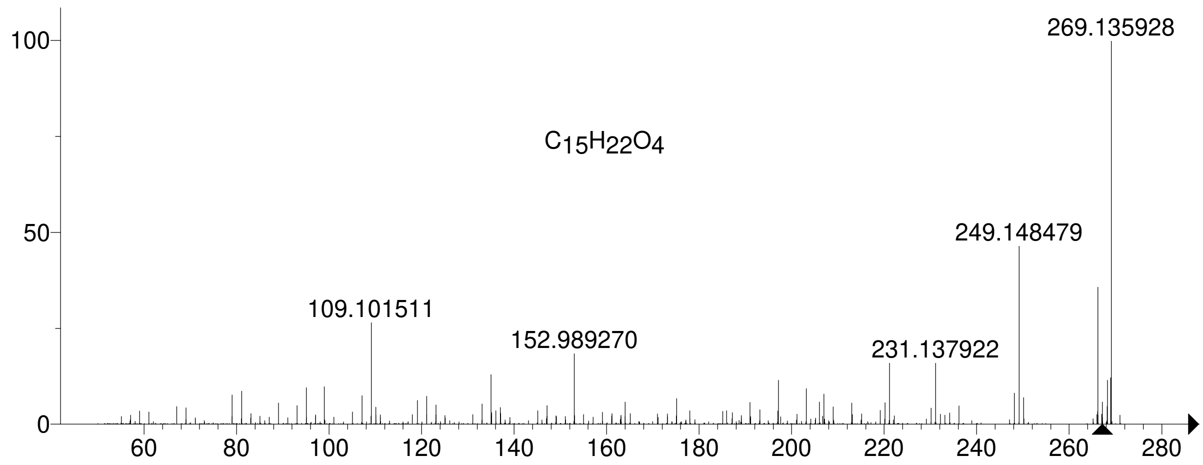

Unknown: Unknown (17.52_418.1486m/z)
Compound in Library Factor = 0

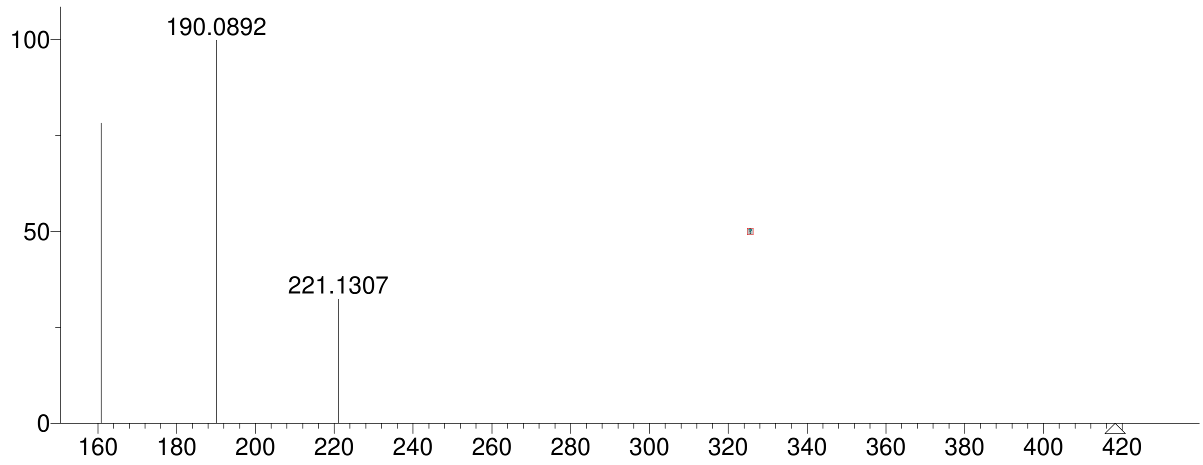

Hit 1 : NCGC00380250-01_C21H23NO8_(2E)-4-({(3S,3aR,4E,7aS)-7-Hydroxy-4-[(2E,4E)-1-hydroxy-2,4-hexadie
C21H23NO8; MF: 0; RMF: 69; Prob 50.0%; Lib: mona-export-gnps; ID: 11631.

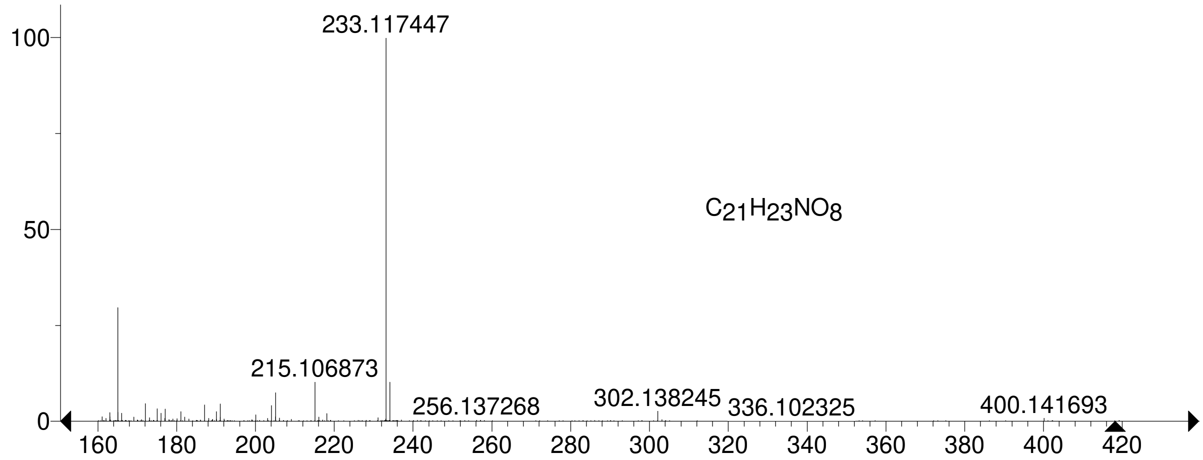

Hit 2 : 3,8-dihydroxy-7,14-dimethoxy-17-methyl-12,16,21-trioxapentacyclo[15.3.1.0A^'2,A^'1a^*u.0a^'',A^'1A^'3.0a
C21H20O8; MF: 0; RMF: 2; Prob 50.0%; Lib: mona-export-vf-npl_qexactive; ID: 17720.

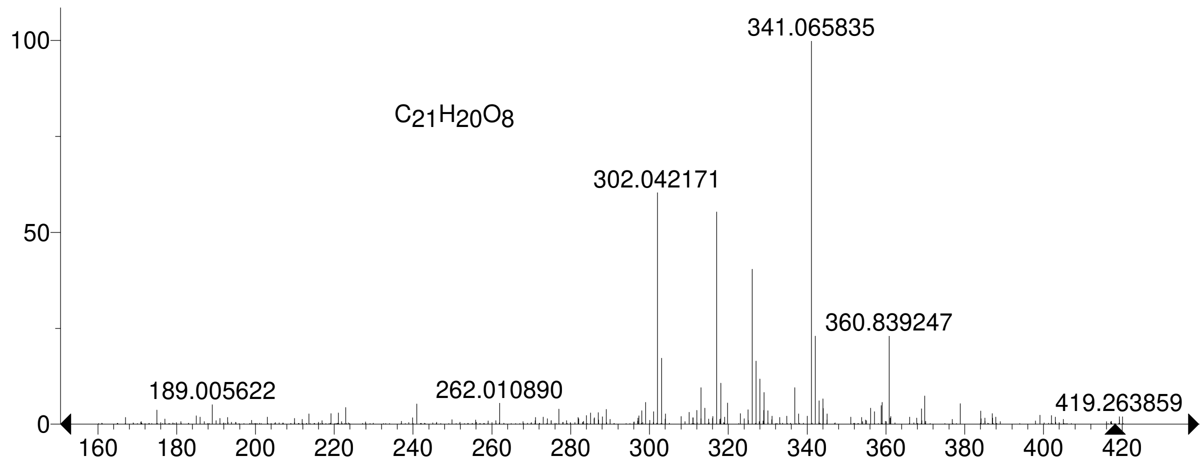

Unknown: Unknown (17.54_370.9883m/z)
Compound in Library Factor = 0

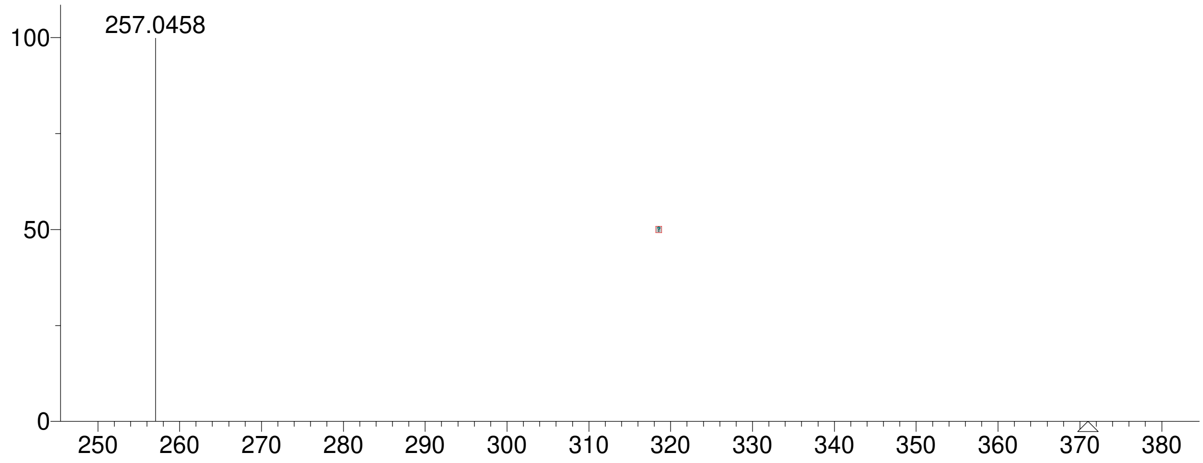

Unknown: Unknown (17.56_394.1856m/z)
Compound in Library Factor = -2387

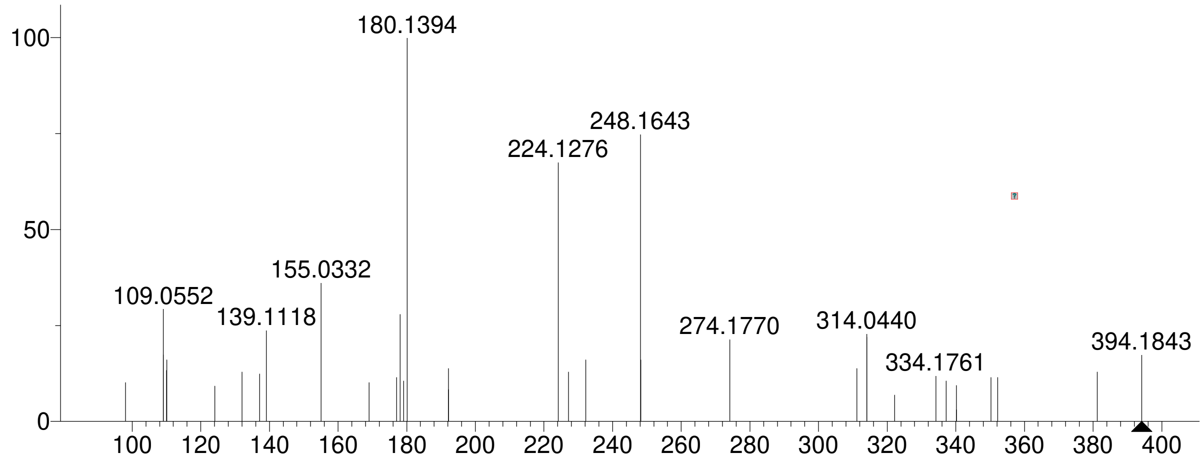

Hit 1 : 3',4',5'-Trimethoxycocaine [M+H]+ HCD 15V P=394.2
C20H27NO7; MF: 22; RMF: 201; Prob 15.3%; CAS: 156301-59-6; Lib: nist_msms; ID: 383469.

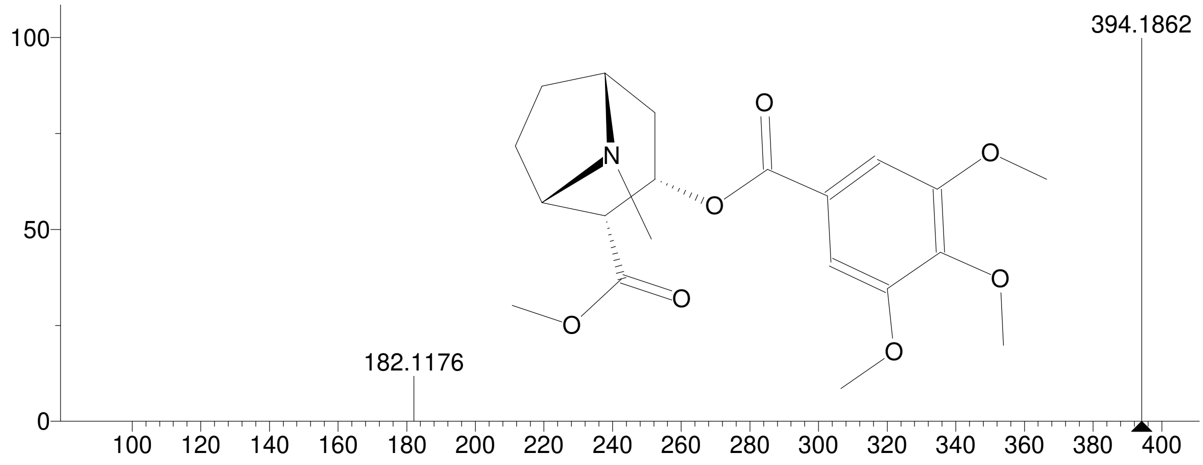

Hit 2 : rac-N,N-Didesmethyl-O-desmethylvenlafaxine glucuronide [M+H-H2O]+ HCD 11V P=394.2
C20H29NO8; MF: 6; RMF: 126; Prob 8.83%; CAS: 1799830-07-1; Lib: nist_msms; ID: 436197.

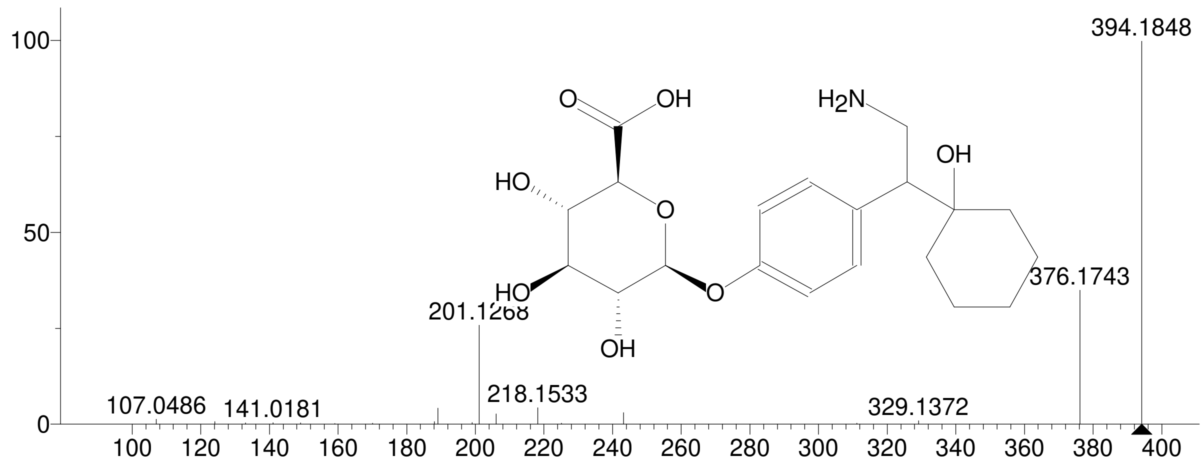

Hit 3 : [1,3-dihydroxy-1-(7-methoxy-2-oxochromen-6-yl)-3-methylbutan-2-yl] 3-methylbut-2-enoate [M+NH4]+ 45
C20H24O7; MF: 1; RMF: 63; Prob 7.11%; Lib: mona-export-vf-npl_qexactive; ID: 24782.

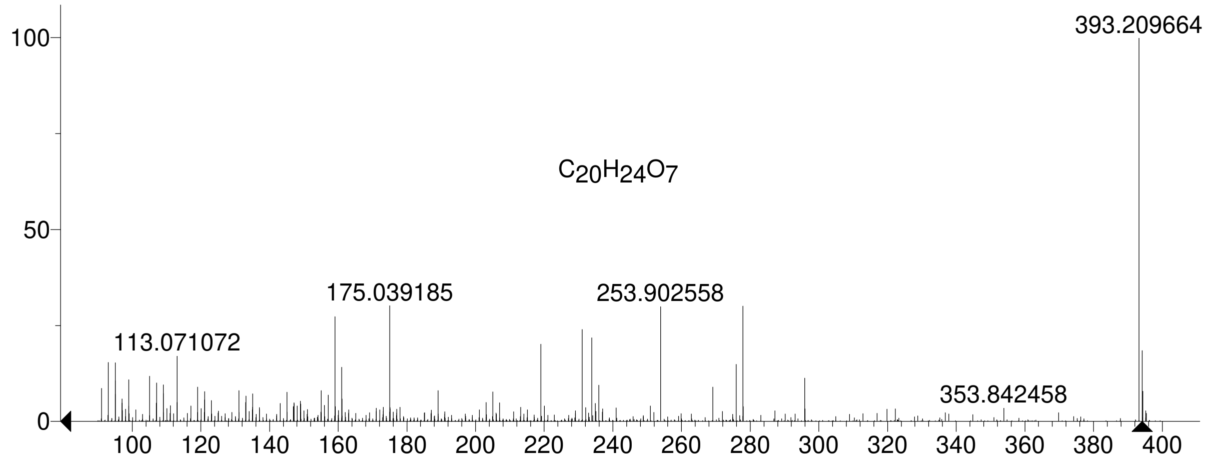

Hit 4 : [1,3-dihydroxy-1-(7-methoxy-2-oxochromen-6-yl)-3-methylbutan-2-yl] (E)-2-methylbut-2-enoate [M+NH4]+
C20H24O7; MF: 1; RMF: 49; Prob 7.11%; Lib: mona-export-vf-npl_qexactive; ID: 5461.

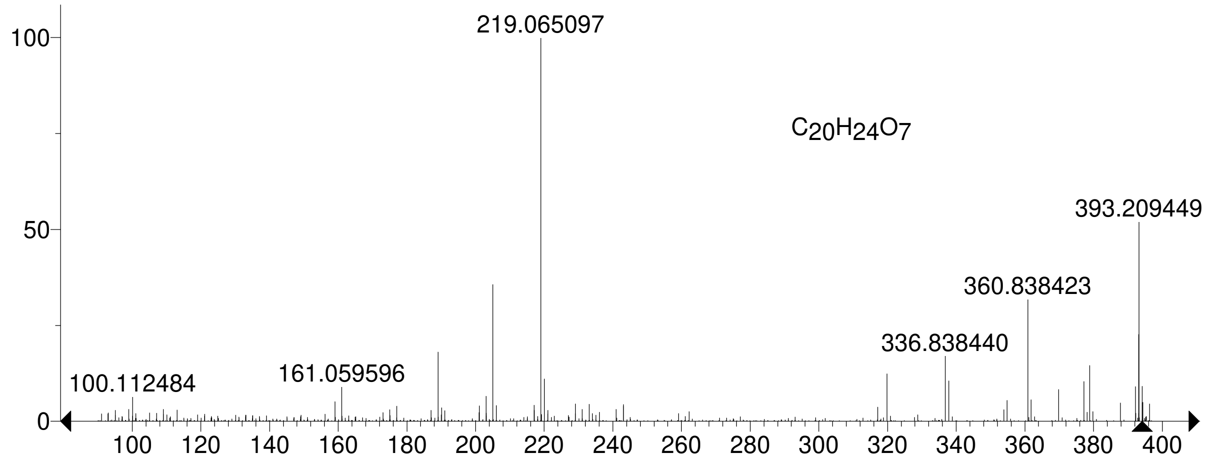

Hit 5 : NCGC00381074-01![1,3-dihydroxy-1-(7-methoxy-2-oxochromen-6-yl)-3-methylbutan-2-yl] 3-methylbut-2-er
C20H24O7; MF: 0; RMF: 59; Prob 6.84%; Lib: mona-export-gnps; ID: 8644.

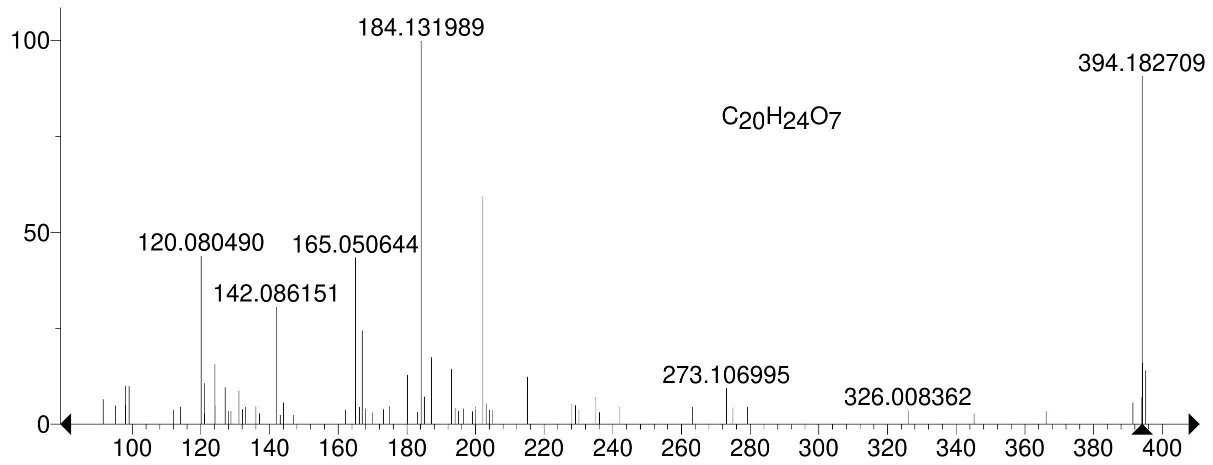

Unknown: Unknown (17.57_428.1707m/z)
Compound in Library Factor = -3273

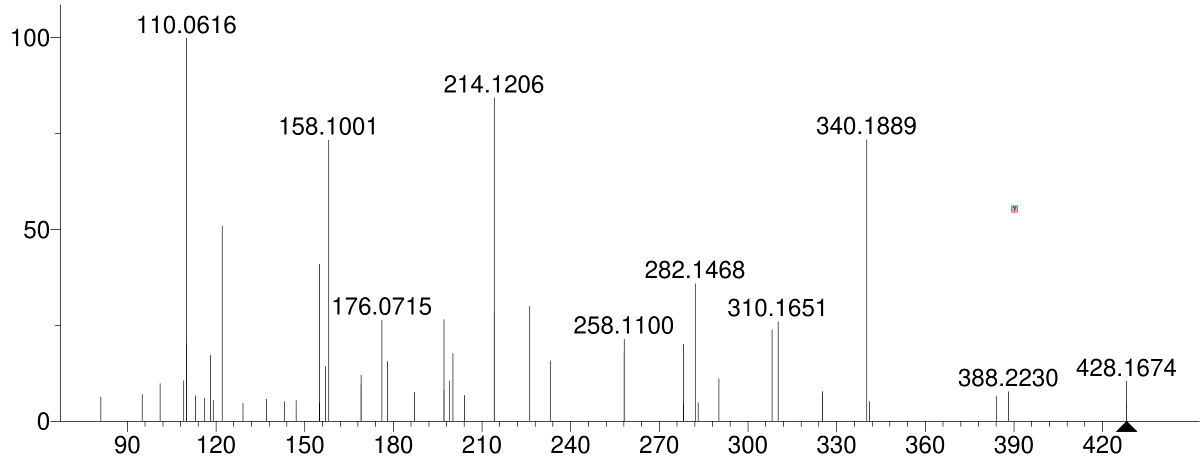

Hit 1 : (2s,6as,12as)-6a-hydroxy-8,9-dimethoxy-2-(prop-1-en-2-yl)-1,2,12,12a-tetrahydrochromeno[3,4-b]furo[2,3-
C23H22O7; MF: 2; RMF: 57; Prob 15.3%; Lib: mona-export-vf-npl_qexactive; ID: 4287.

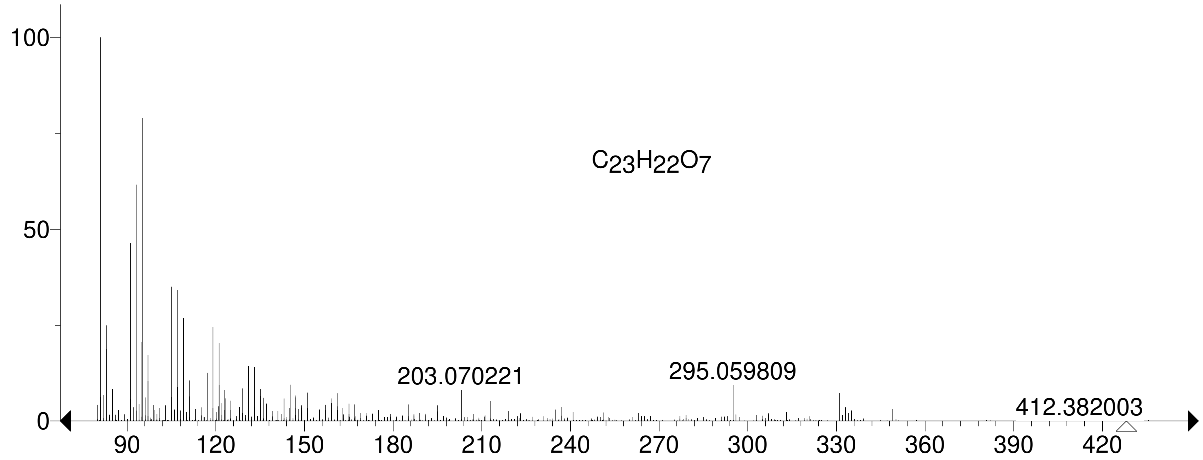

Hit 2 : PC 6:0; PC 3:0-3:0; [M+Hac-H]- [M+Hac-H]- P=428.2
C14H28NO8P; MF: 0; RMF: 39; Prob 14.1%; Lib: msdial-insilicomsms-lipids-neg; ID: 319519.

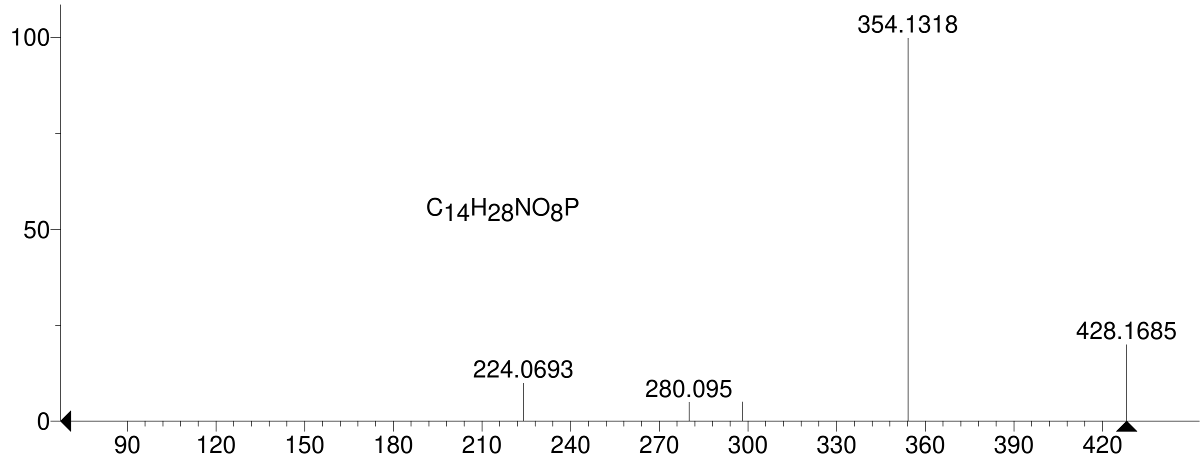

Hit 3 : NCGC00380614-01_C24H23N5O4_2H-Pyrazino[2,1-b]quinazoline-3,6(1H,4H)-dione, 1-methyl-4-[[(2S,9S,
C24H23N5O4; MF: 0; RMF: 33; Prob 14.1%; Lib: mona-export-gnps; ID: 10706.

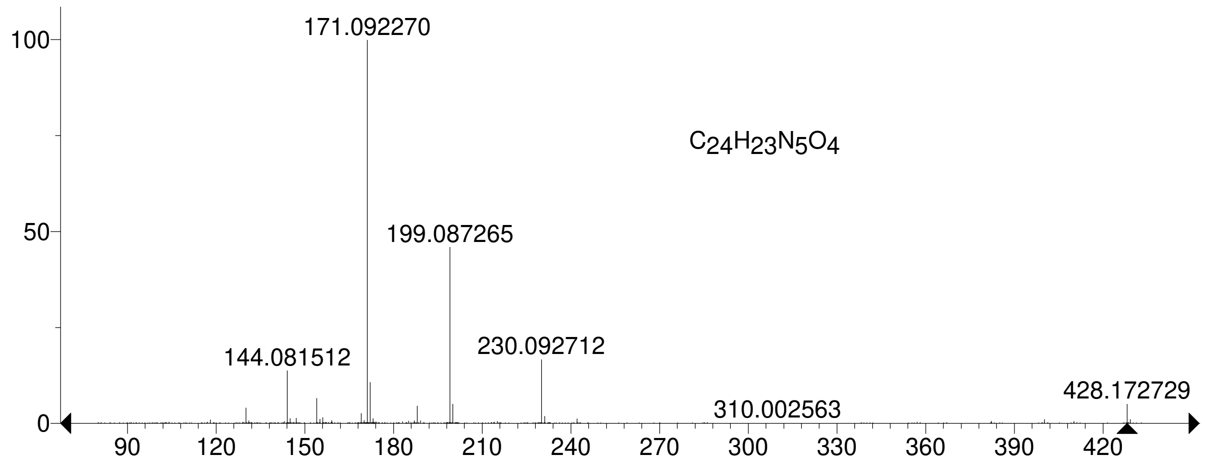

Hit 4 : tephrosin [M+NH4]+ 65HCD P=428.2
C23H22O7; MF: 0; RMF: 33; Prob 14.1%; Lib: mona-export-vf-npl_qexactive; ID: 4047.

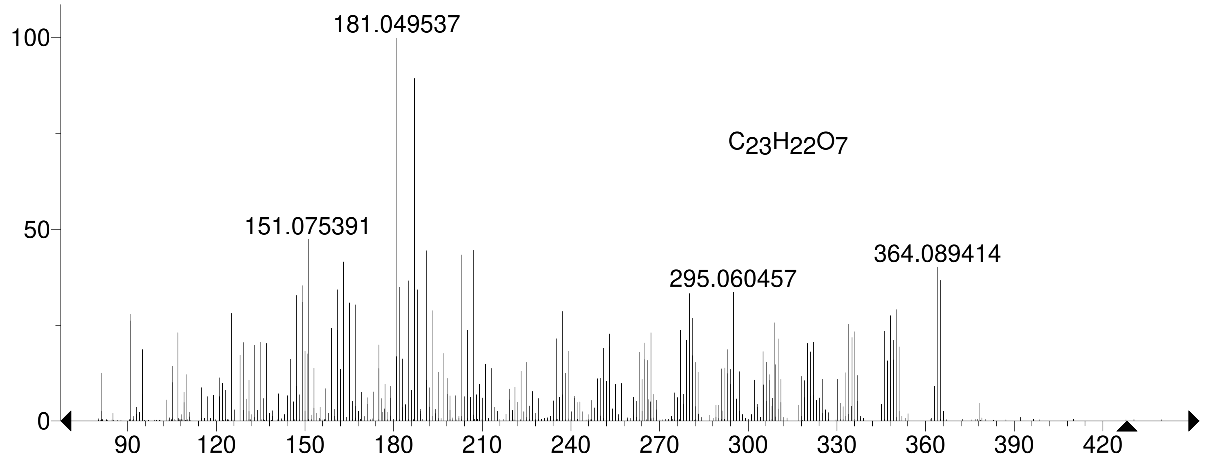

Hit 5 : PC 7:0; PC 2:0-5:0; [M+FA-H]- [M+FA-H]- P=428.2
C15H30NO8P; MF: 0; RMF: 31; Prob 14.1%; Lib: msdial-insilicomsms-lipids-neg; ID: 317570.

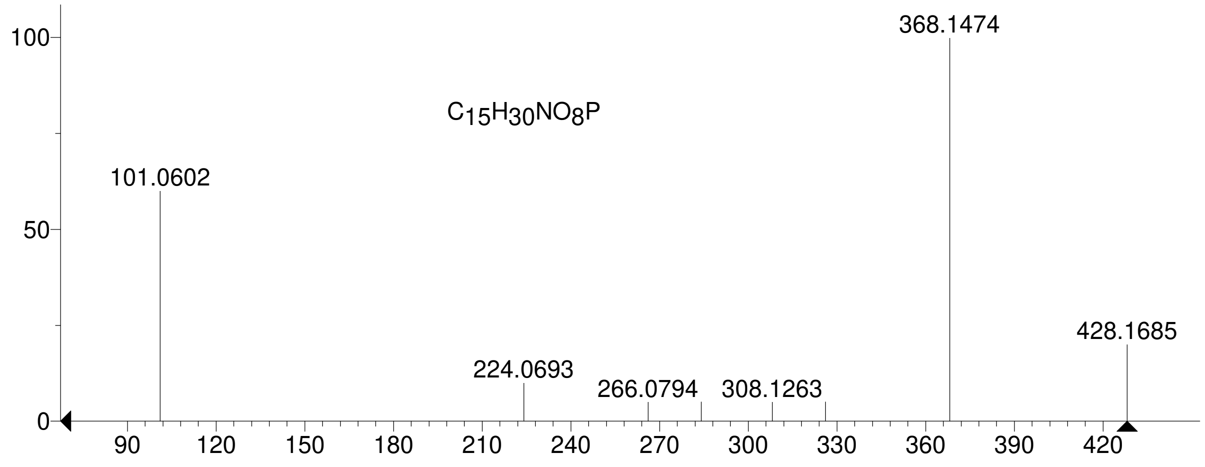

Unknown: Unknown (17.60_411.1758m/z)
Compound in Library Factor = 0

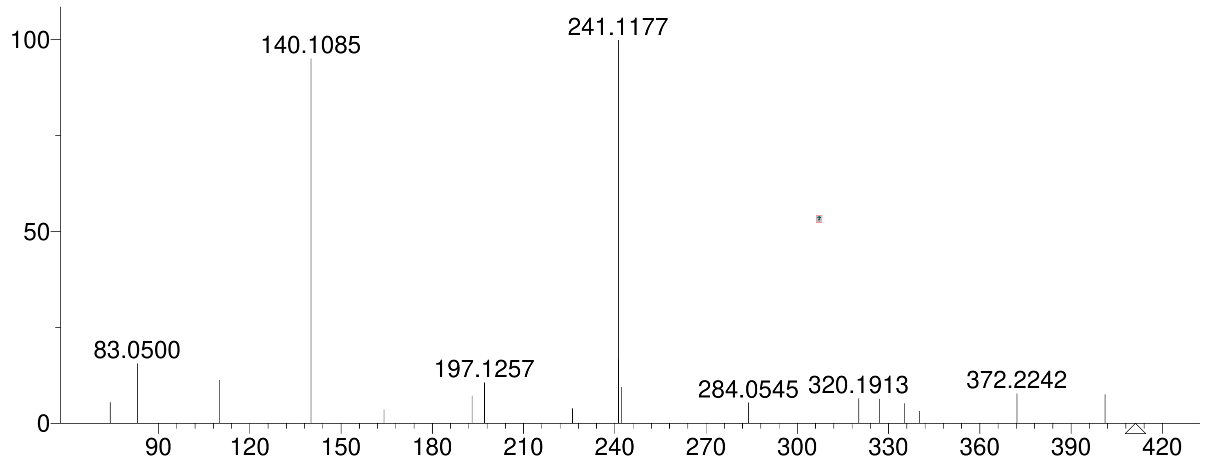

Hit 1 : Quassin [M+Na]+ 65HCD P=411.2
C22H28O6; MF: 0; RMF: 31; Prob 20.0%; Lib: mona-export-vf-npl_qexactive; ID: 26442.

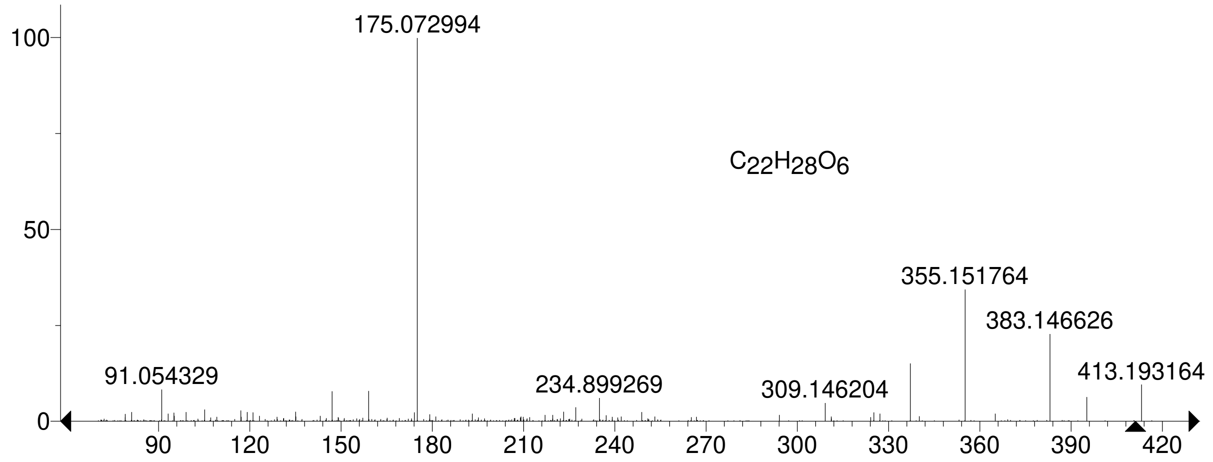

Hit 2 : 3-(5-hydroxy-2,2,7,8-tetramethyl-6-oxo-7,8-dihydropyrano[3,2-g]chromen-10-yl)hexanoic acid [M+Na]+ 3[illegible]
C22H28O6; MF: 0; RMF: 21; Prob 20.0%; Lib: mona-export-vf-npl_qexactive; ID: 982.

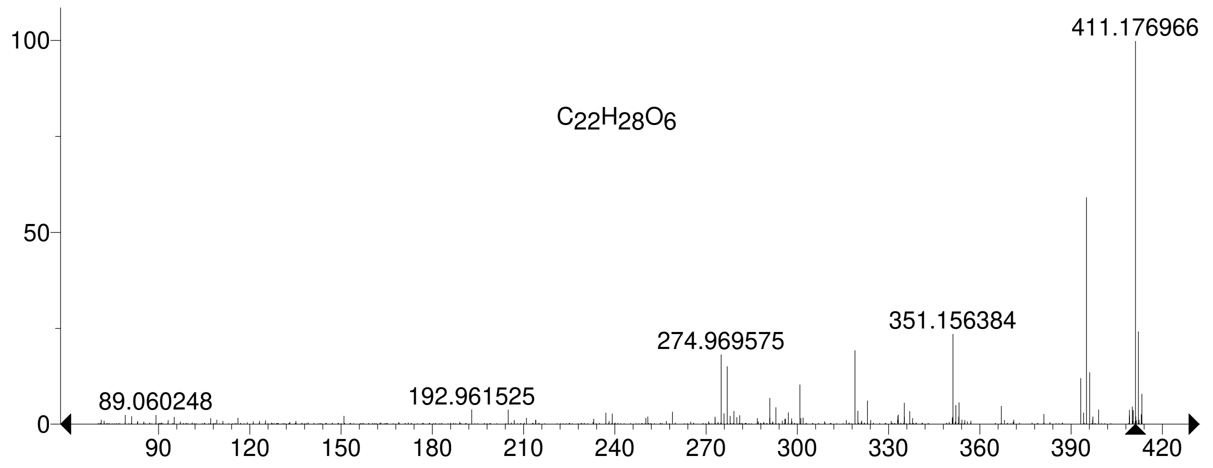

Hit 3 : [9b-hydroxy-6-(hydroxymethyl)-6,9a-dimethyl-3-oxo-1,3a,4,5,5a,7,8,9-octahydrobenzo[e][2]benzofuran-5-yl
C22H28O6; MF: 0; RMF: 15; Prob 20.0%; Lib: mona-export-vf-npl_qexactive; ID: 18077.

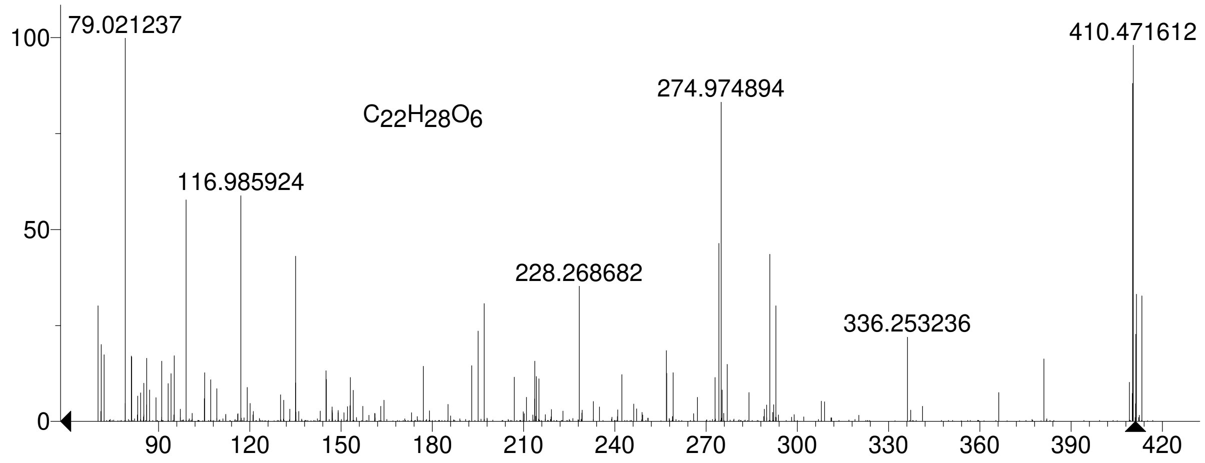

Hit 4 : (4E,8E)-10-(4-hydroxy-6-methoxy-7-methyl-3-oxo-1H-2-benzofuran-5-yl)-4,8-dimethyldeca-4,8-dienoic acic
C22H28O6; MF: 0; RMF: 14; Prob 20.0%; Lib: mona-export-vf-npl_qexactive; ID: 19695.

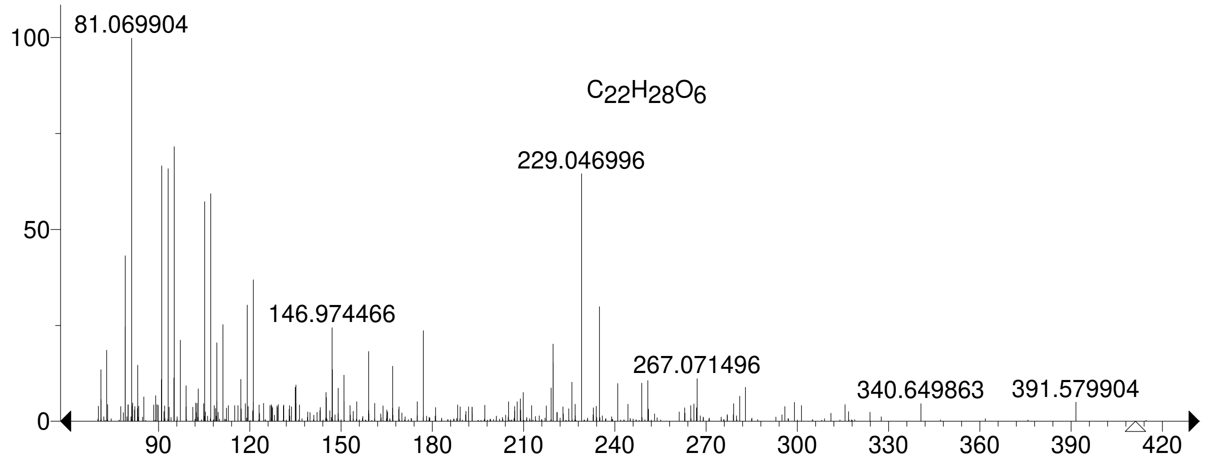

Hit 5 : NCGC00380628-01_C22H28O6_(4E,8E)-10-(4-Hydroxy-6-methoxy-7-methyl-3-oxo-1,3-dihydro-2-benzofu
C22H28O6; MF: 0; RMF: 5; Prob 20.0%; Lib: mona-export-gnps; ID: 13319.

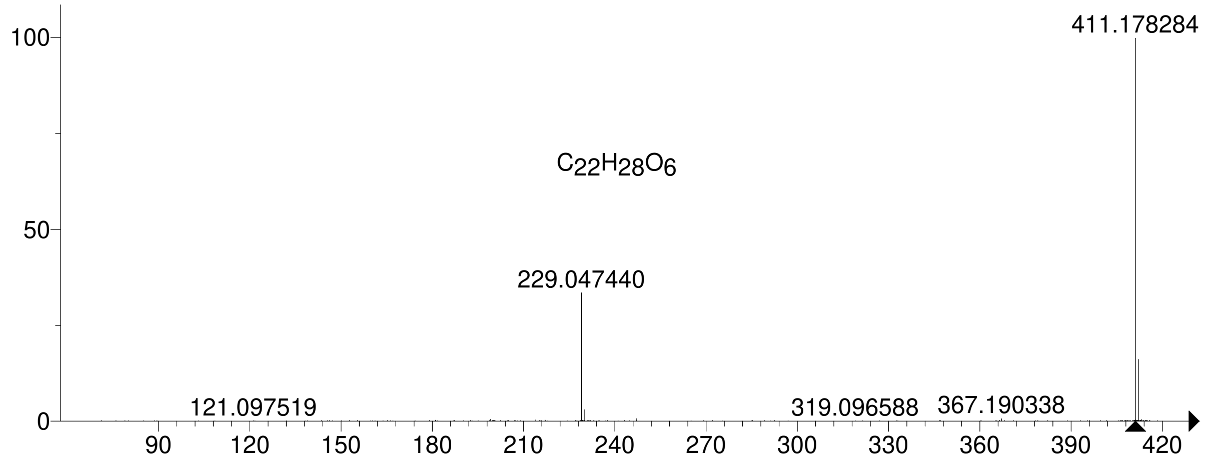

Unknown: Unknown (17.66_239.1392m/z)
Compound in Library Factor = -2387

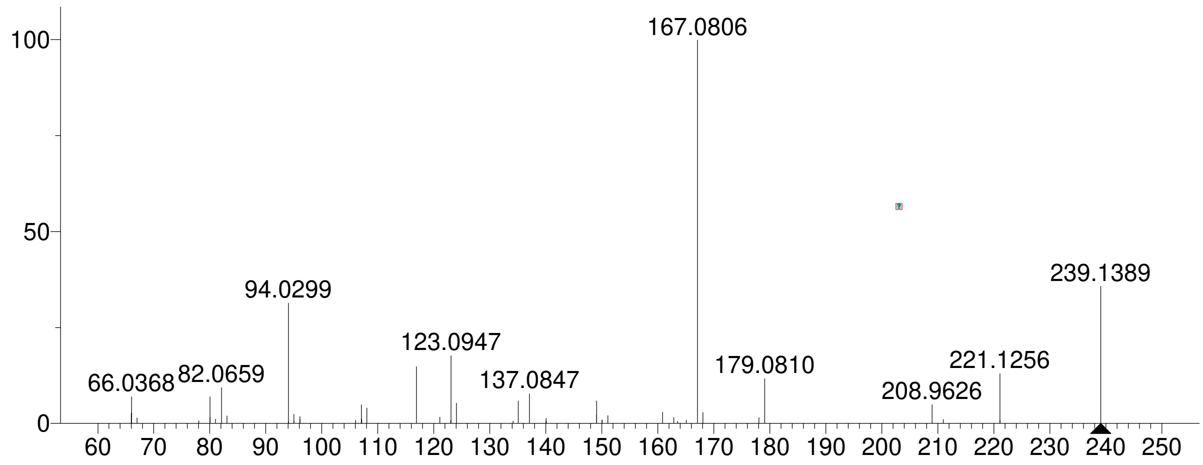

Hit 1 : Secobarbital [M+H]+ HCD 4V P=239.1
C12H18N2O3; MF: 14; RMF: 338; Prob 38.1%; CAS: 76-73-3; Lib: nist_msms; ID: 198374.

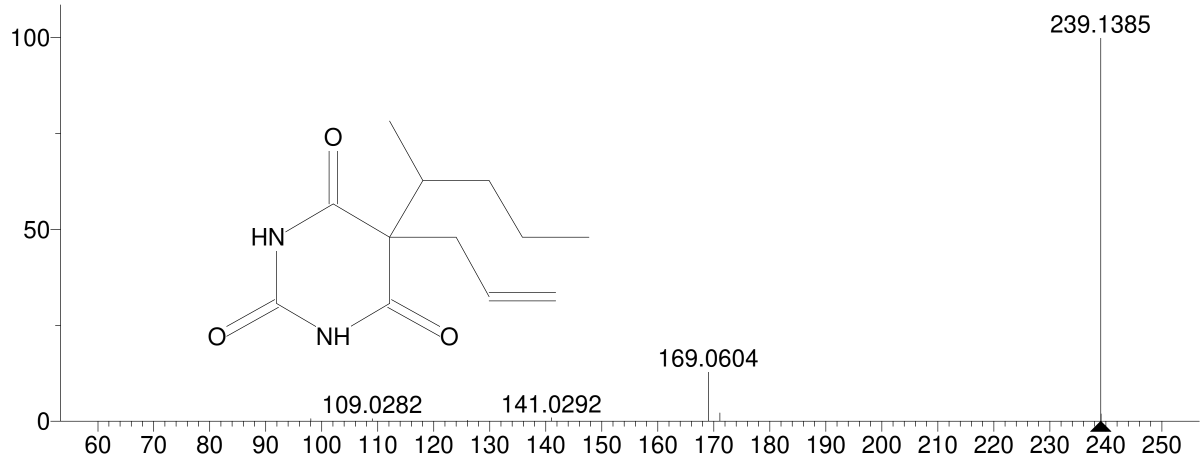

Hit 2 : Heptanedioic acid, 1-(2-cyclopentylidenehydrazide) [M-H]- HCD 21V P=239.1
C12H20N2O3; MF: 14; RMF: 245; Prob 38.1%; CAS: 1136466-93-7; Lib: nist_msms; ID: 263655.

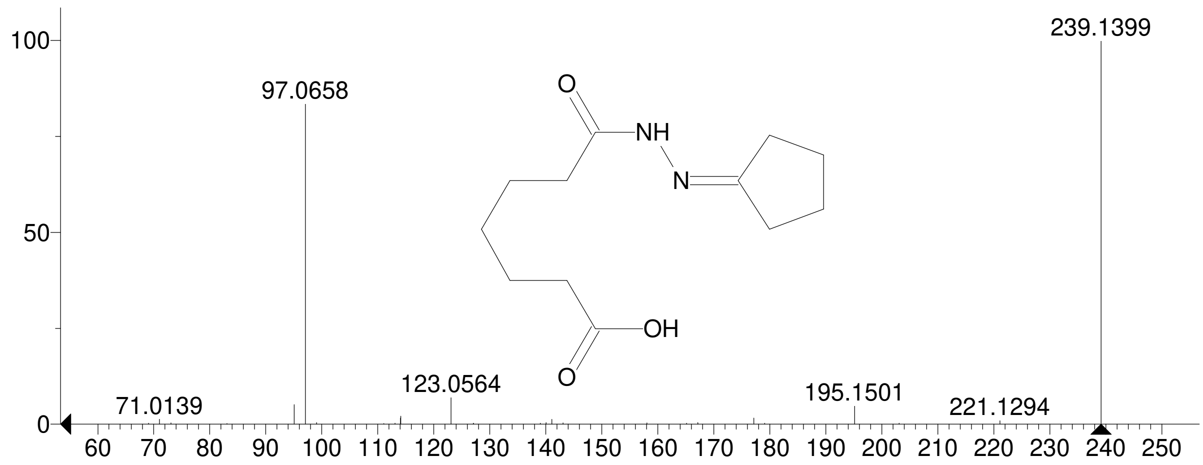

Hit 3 : CMPF [M-H]- 35% P=239.1
C12H16O5; MF: 0; RMF: 38; Prob 23.8%; Lib: mona-export-itree; ID: 3748.

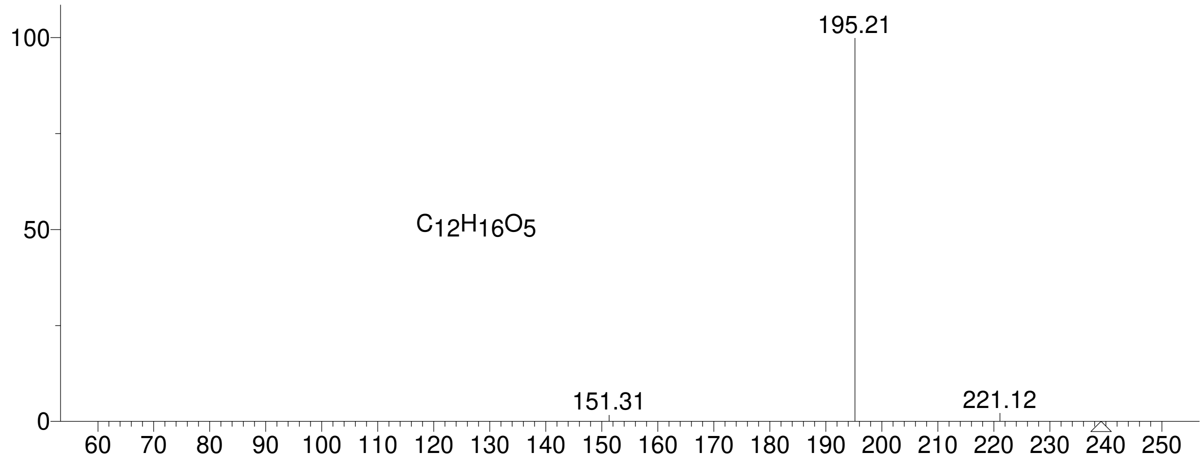

Unknown: Unknown (17.67_214.1441m/z)
Compound in Library Factor = 0

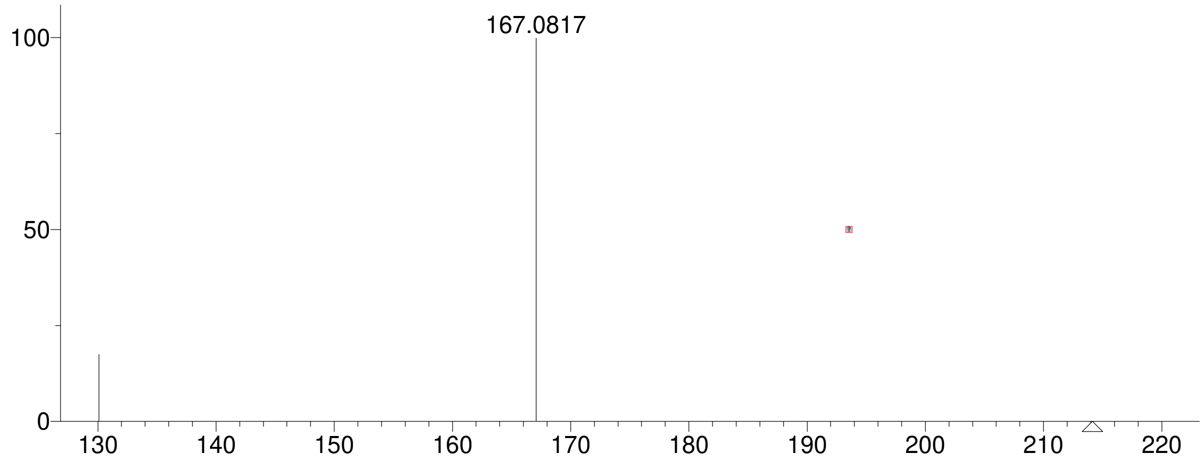

Unknown: Unknown (17.67_239.1392m/z)
Compound in Library Factor = -2387

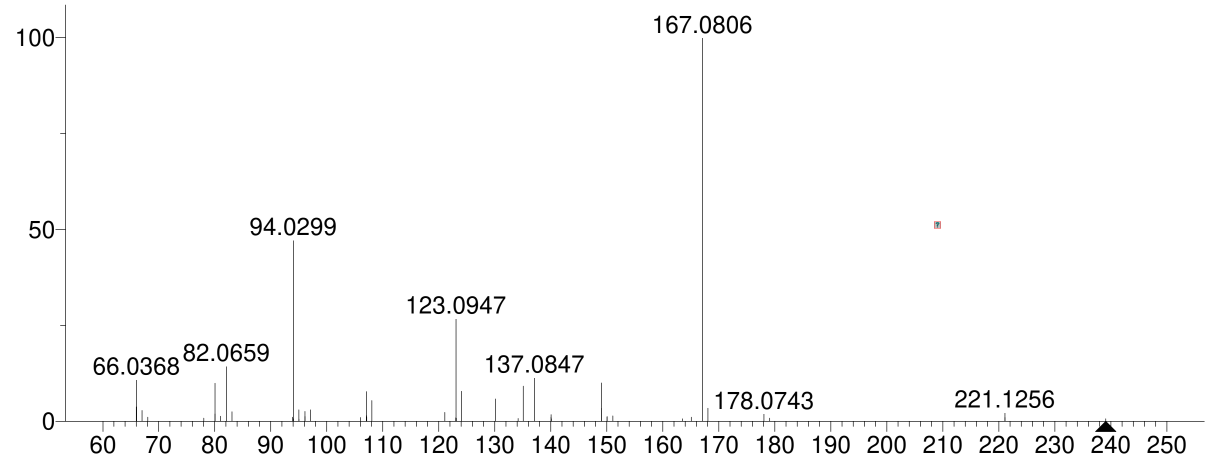

Hit 1 : Heptanedioic acid, 1-(2-cyclopentylidenehydrazide) [M-H]- HCD 26V P=239.1
C12H20N2O3; MF: 19; RMF: 109; Prob 49.7%; CAS: 1136466-93-7; Lib: nist_msms; ID: 263656.

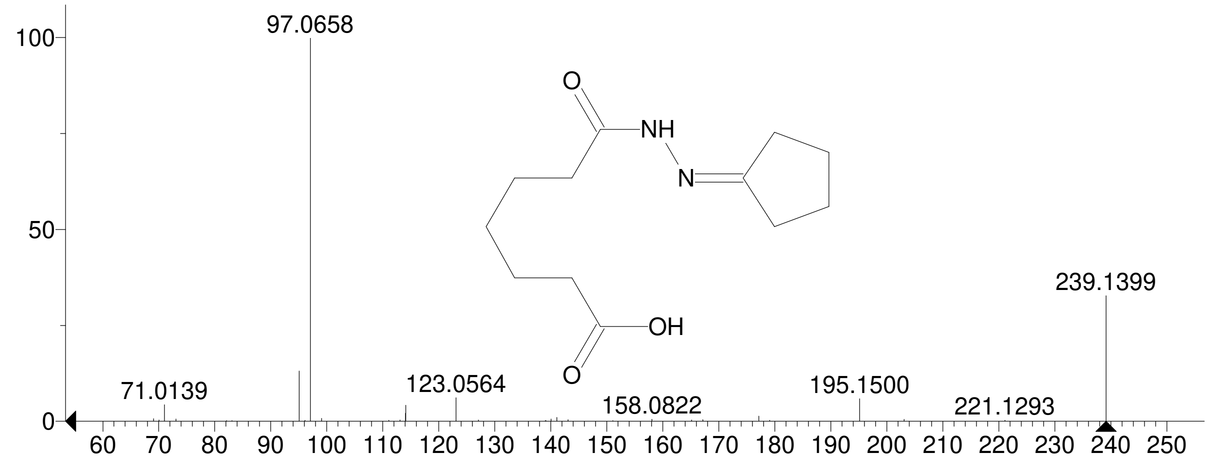

Hit 2 : Secobarbital [M+H]+ HCD 0V P=239.1
C12H18N2O3; MF: 1; RMF: 51; Prob 25.6%; CAS: 76-73-3; Lib: nist_msms; ID: 198372.

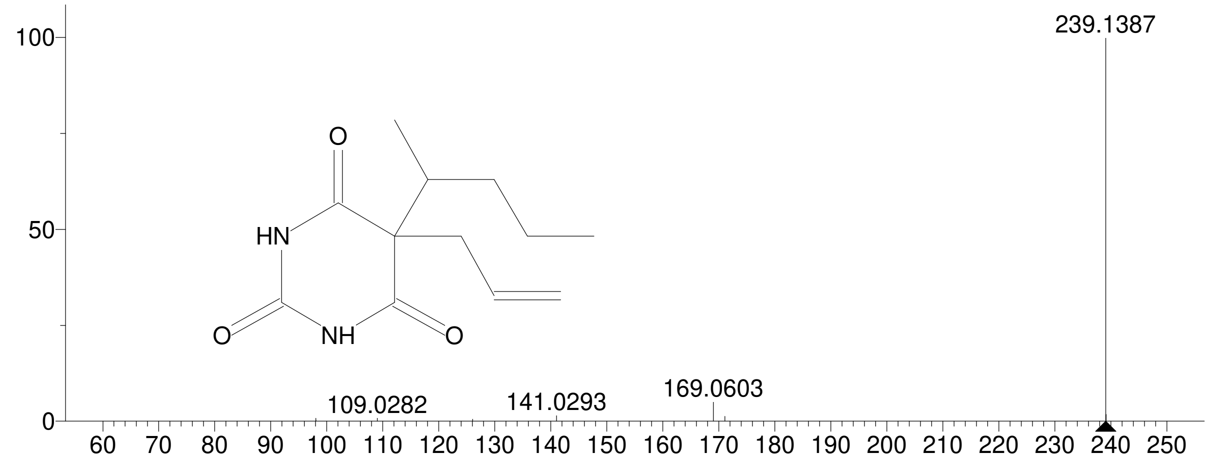

Hit 3 : CMPF [M-H]- 35% P=239.1
C12H16O5; MF: 0; RMF: 20; Prob 24.6%; Lib: mona-export-itree; ID: 3748.

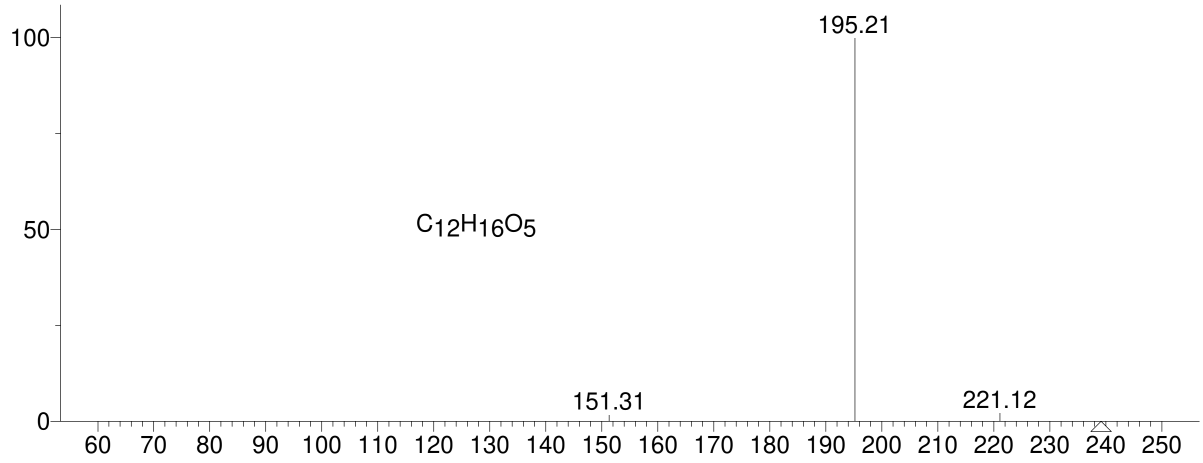

Unknown: Unknown (17.68_349.0920m/z)
Compound in Library Factor = -2387

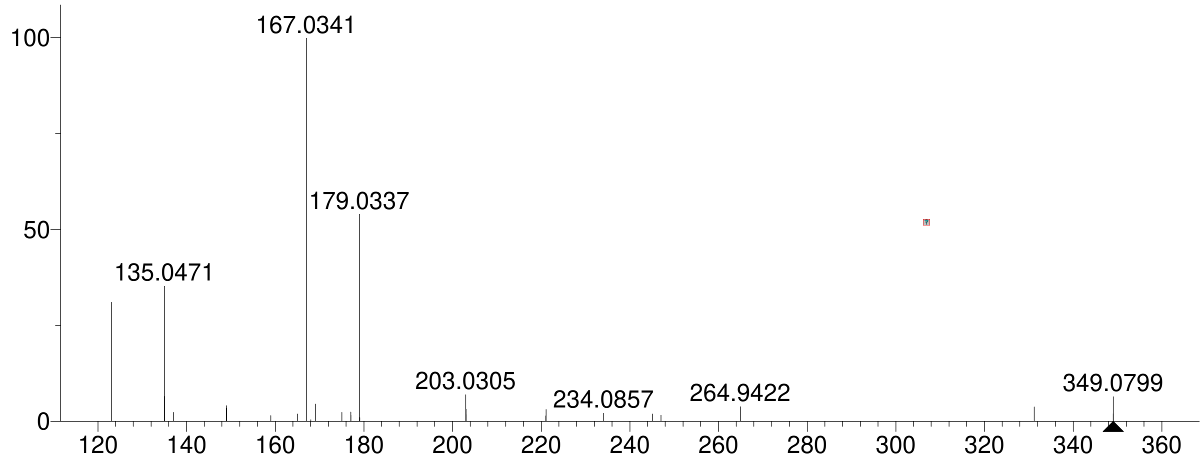

Hit 1 : Hydroxysulochrin_120241 [M+H]+ P=349.1
C17H16O8; MF: 16; RMF: 184; Prob 14.7%; Lib: mona-export-gnps; ID: 4987.

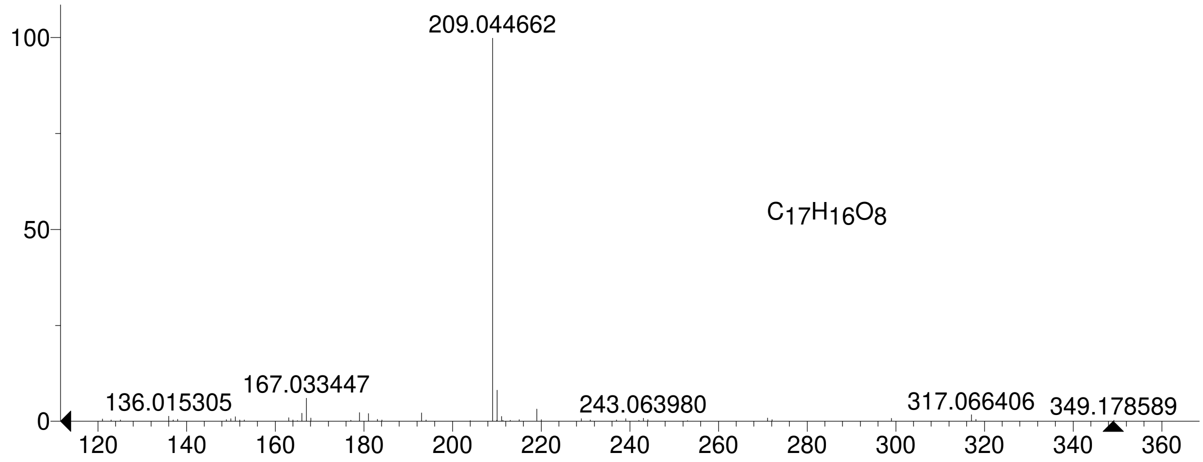

Hit 2 : methyl 2-(4a,6,10a-trihydroxy-1-methyl-5,10-dioxo-3,4-dihydro-1H-benzo[g]isochromen-3-yl)acetate [M-H]
C17H18O8; MF: 4; RMF: 80; Prob 9.75%; Lib: mona-export-lc-ms-ms_negative_mode; ID: 22564.

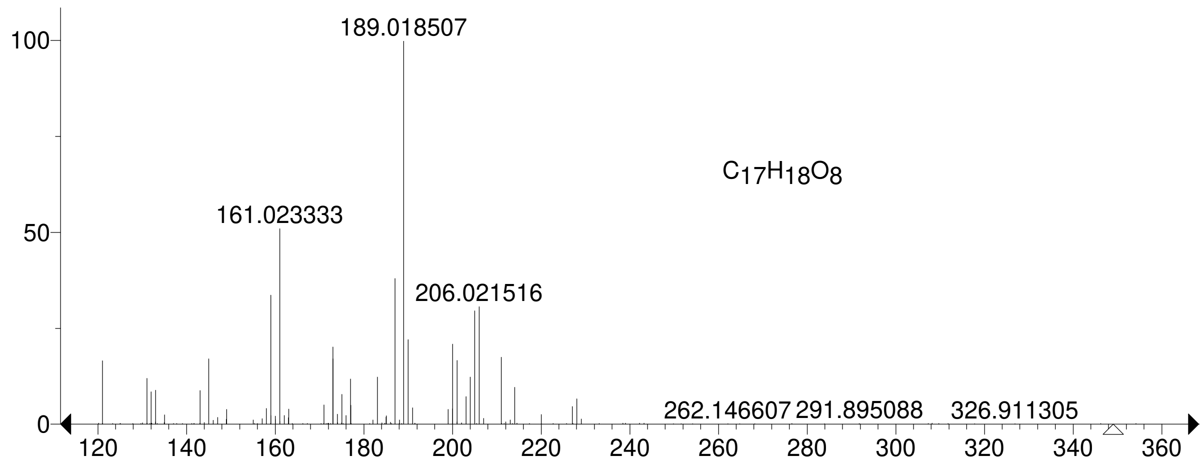

Hit 3 : (1S,4R,7S,8S,11S)-9-tert-butyl-7,9-dihydroxy-3,5,12-trioxatetracyclo[6.6.0.0A^'1,A^'1A^'1.0a^",a^,,]tetradec
C15H18O8; MF: 1; RMF: 95; Prob 8.62%; Lib: mona-export-vf-npl_qexactive; ID: 8436.

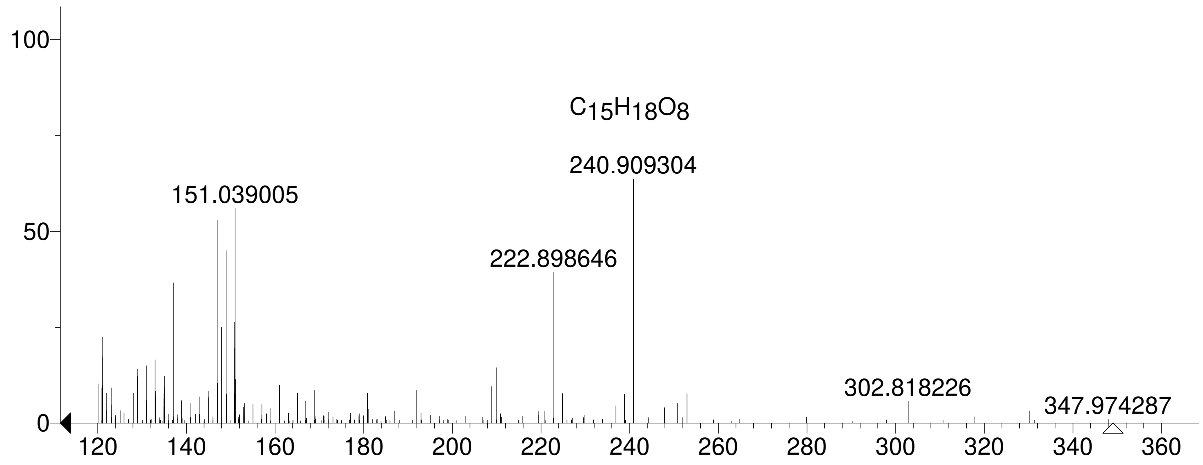

Hit 4 : (E)-3-[2-[(2S,3R,4S,5S,6R)-3,4,5-trihydroxy-6-(hydroxymethyl)oxan-2-yl]oxyphenyl]prop-2-enoic acid [M+N
C15H18O8; MF: 1; RMF: 79; Prob 8.62%; Lib: mona-export-vf-npl_qexactive; ID: 5110.

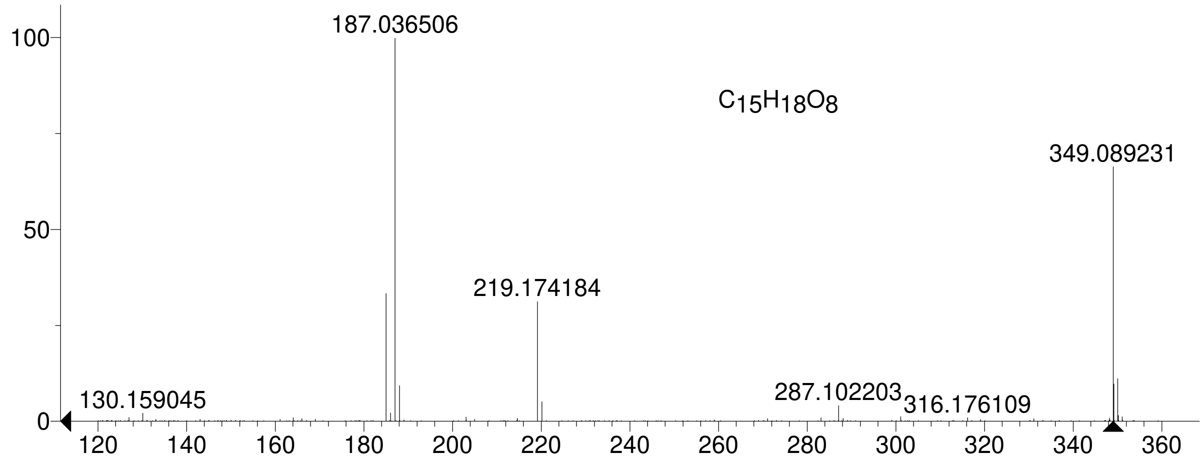

Hit 5 : NCGC00381013-01!5-hydroxy-2-(3-hydroxy-2-methoxycarbonyl-5-methylphenoxy)-3-methoxybenzoic acid
C17H16O8; MF: 1; RMF: 41; Prob 8.62%; Lib: mona-export-gnps; ID: 9371.

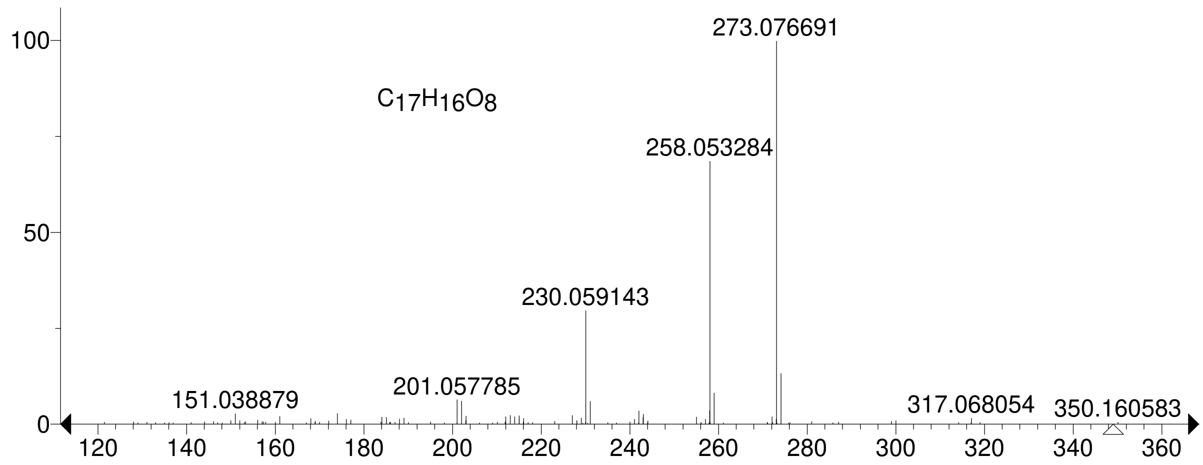

Unknown: Unknown (17.68_491.1998m/z)
Compound in Library Factor = -3273

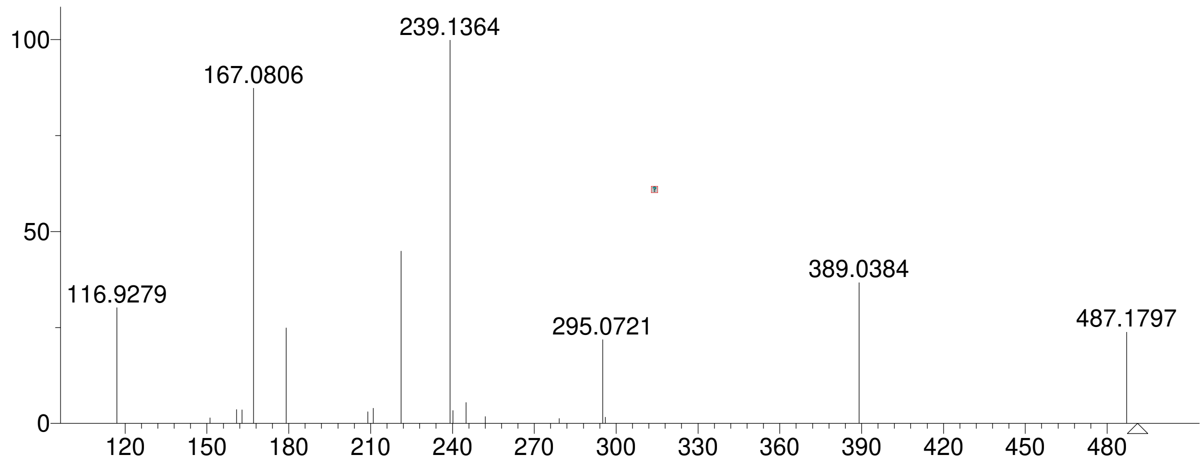

Hit 1 : [(1S,2R,4aR,8aR)-1-acetyloxy-1,4a-dimethyl-6-oxo-7-propan-2-ylidene-2,3,4,5,8,8a-hexahydronaphthalen-
C24H36O8; MF: 3; RMF: 168; Prob 53.1%; Lib: mona-export-vf-npl_qexactive; ID: 23071.

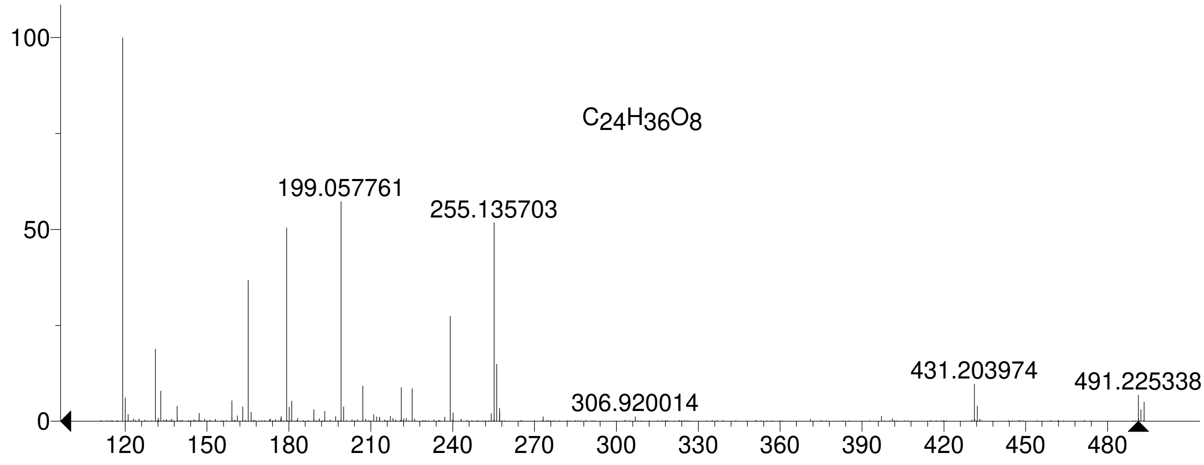

Hit 2 : Paprotrain [2M+H]+ HCD 108V P=491.2
C16H11N3; MF: 0; RMF: 79; Prob 46.9%; CAS: 57046-73-8; Lib: nist_msms; ID: 247753.

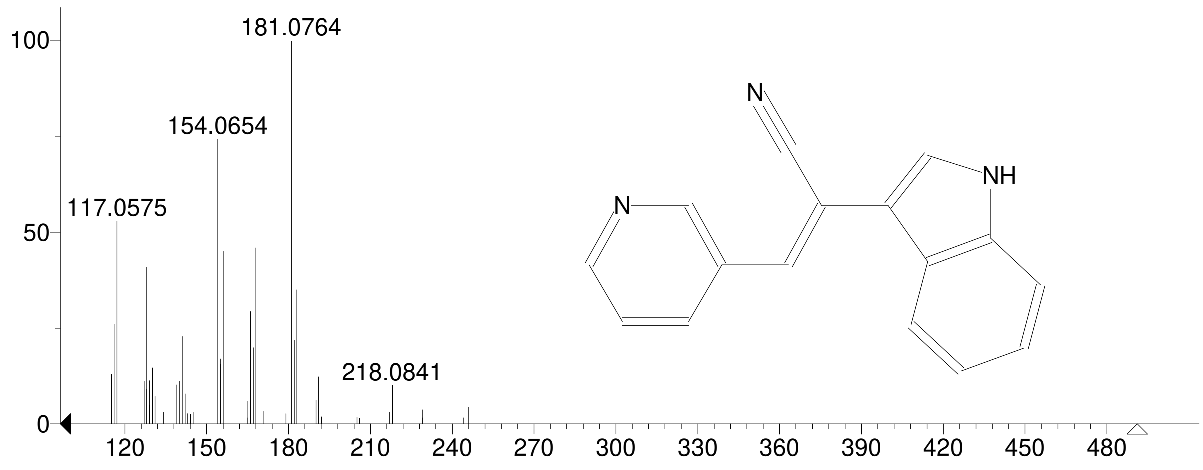

Unknown: Unknown (17.68_522.1302m/z)
Compound in Library Factor = 0

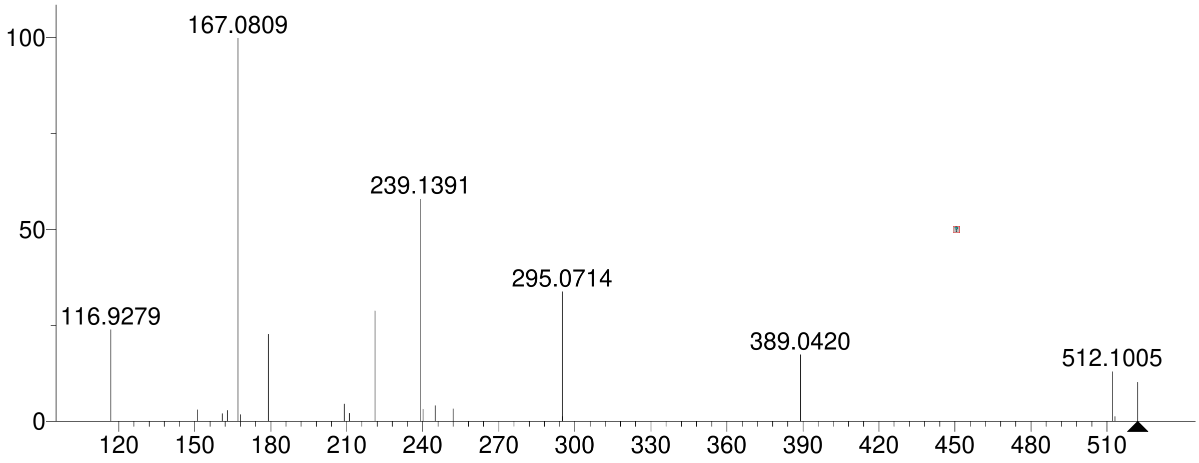

Unknown: Unknown (17.69_487.1814m/z)
Compound in Library Factor = -1142

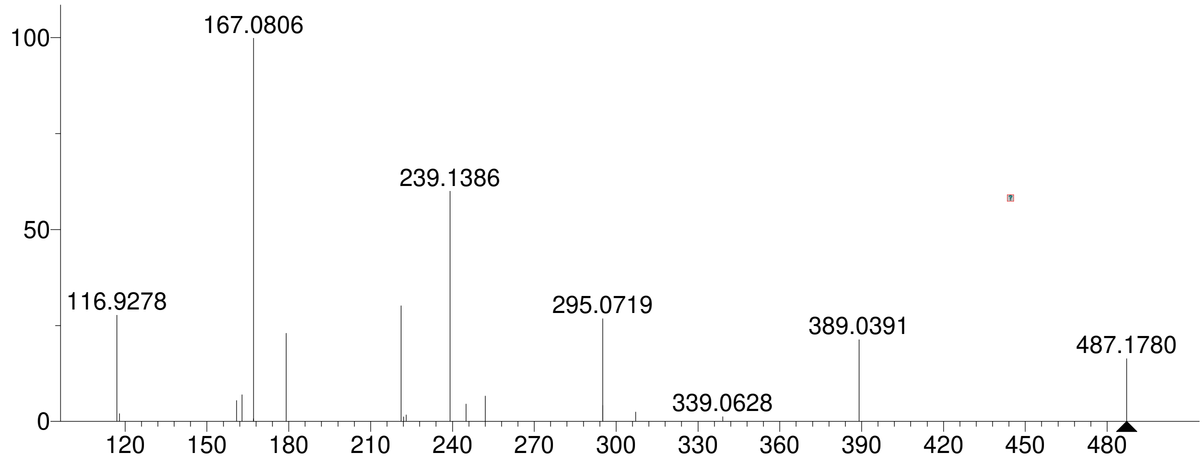

Hit 1 : Beclomethasone 21-propionate [M+Na]+ HCD 9V P=487.2
C25H33ClO6; MF: 123; RMF: 820; Prob 78.1%; CAS: 69224-79-9; Lib: nist_msms; ID: 447840.

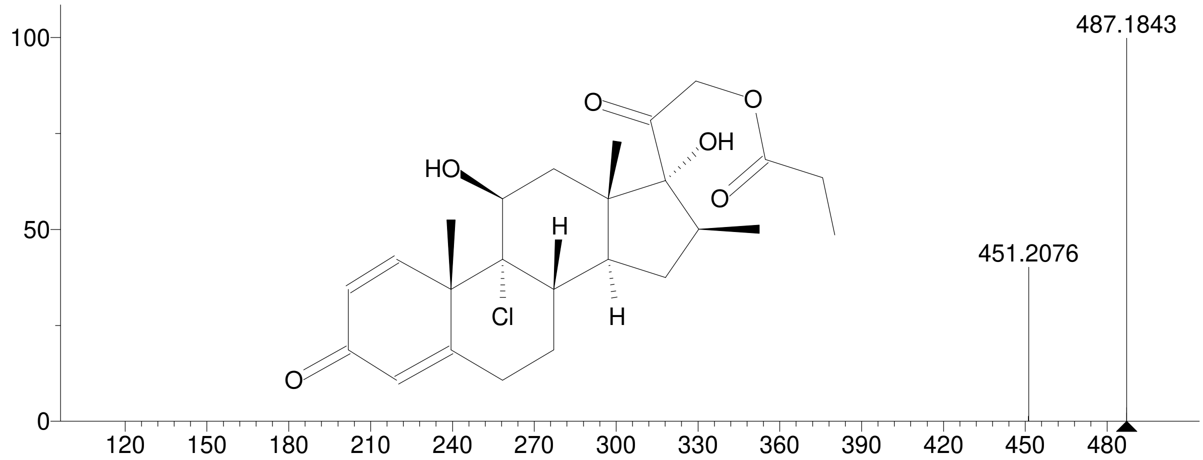

Hit 2 : Beclomethasone 17-propionate [M+Na]+ HCD 9V P=487.2
C25H33ClO6; MF: 77; RMF: 611; Prob 16.5%; CAS: 5534-18-9; Lib: nist_msms; ID: 447795.

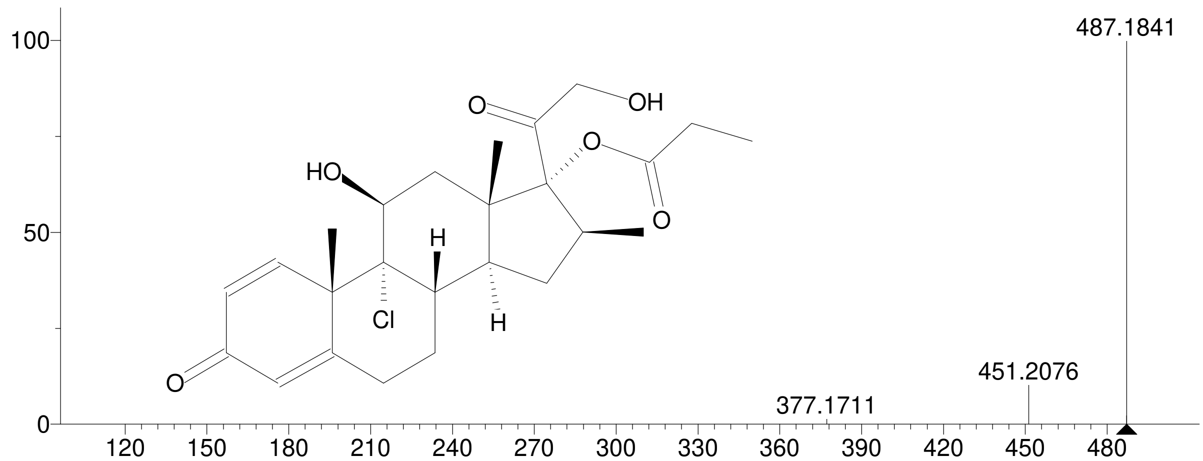

Hit 3 : Cytidine [2M+H]+ HCD 4V P=487.2
C9H13N3O5; MF: 17; RMF: 208; Prob 2.92%; CAS: 65-46-3; Lib: nist_msms; ID: 228109.

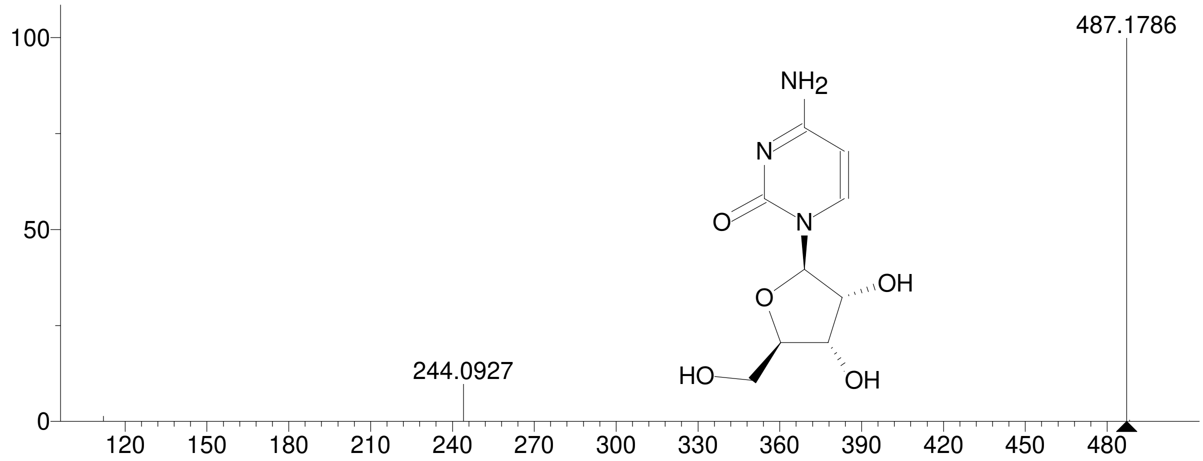

Hit 4 : Cytarabine [2M+H]+ HCD 4V P=487.2
C9H13N3O5; MF: 13; RMF: 204; Prob 2.47%; CAS: 147-94-4; Lib: nist_msms; ID: 120706.

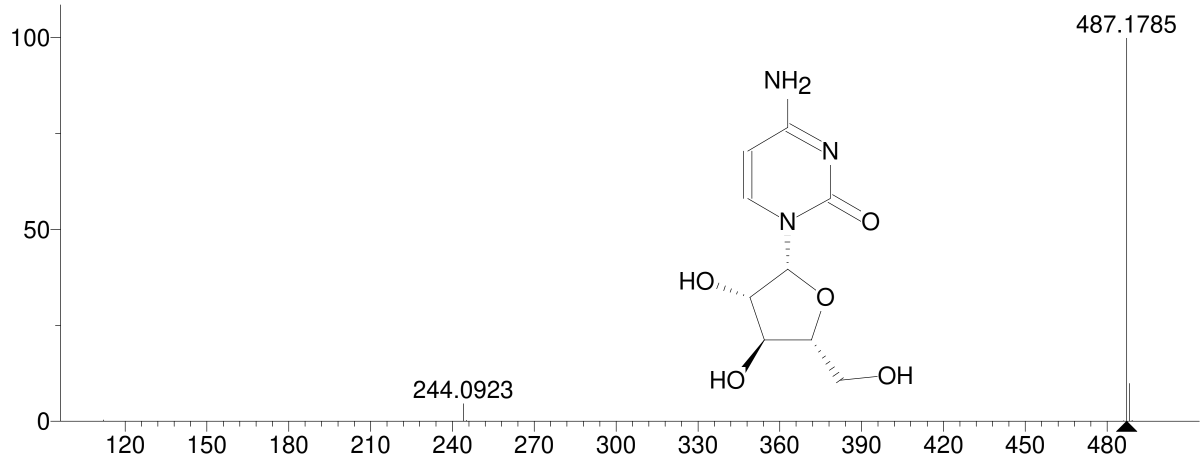

Unknown: Unknown (17.82_487.1812m/z)
Compound in Library Factor = -422

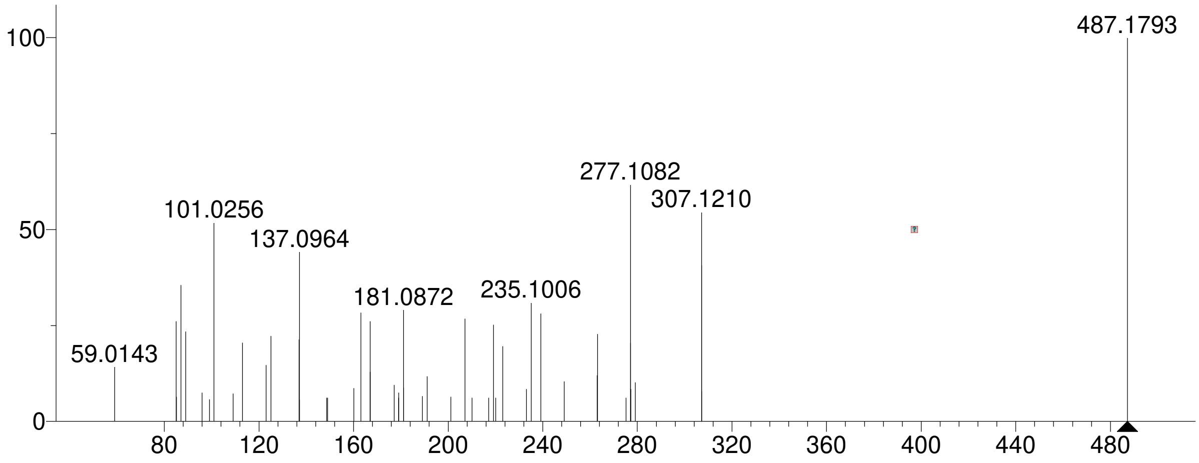

Hit 1 : Beclomethasone 17-propionate [M+Na]+ HCD 9V P=487.2
C25H33ClO6; MF: 267; RMF: 928; Prob 96.6%; CAS: 5534-18-9; Lib: nist_msms; ID: 447795.

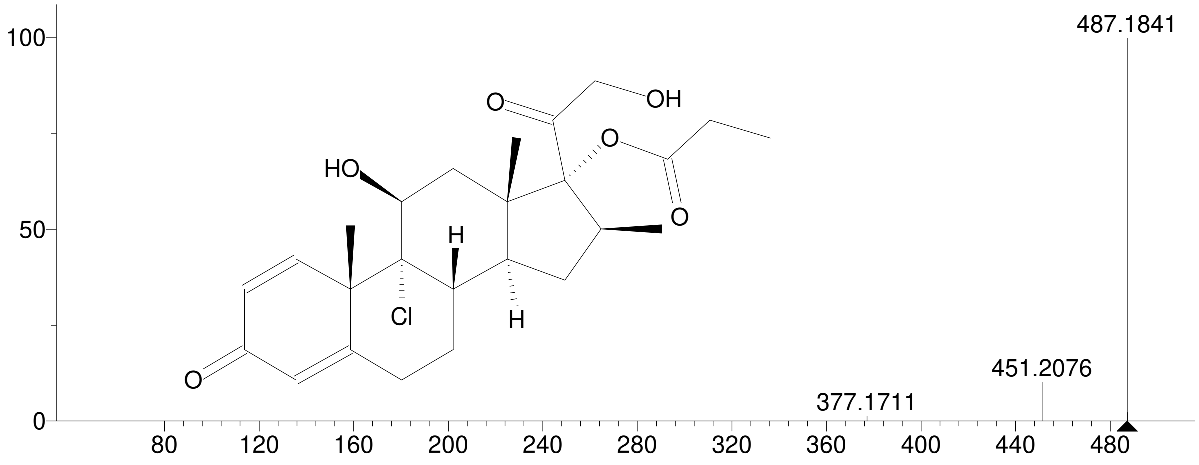

Hit 2 : Beclomethasone 21-propionate [M+Na]+ HCD 9V P=487.2
C25H33ClO6; MF: 123; RMF: 820; Prob 2.97%; CAS: 69224-79-9; Lib: nist_msms; ID: 447840.

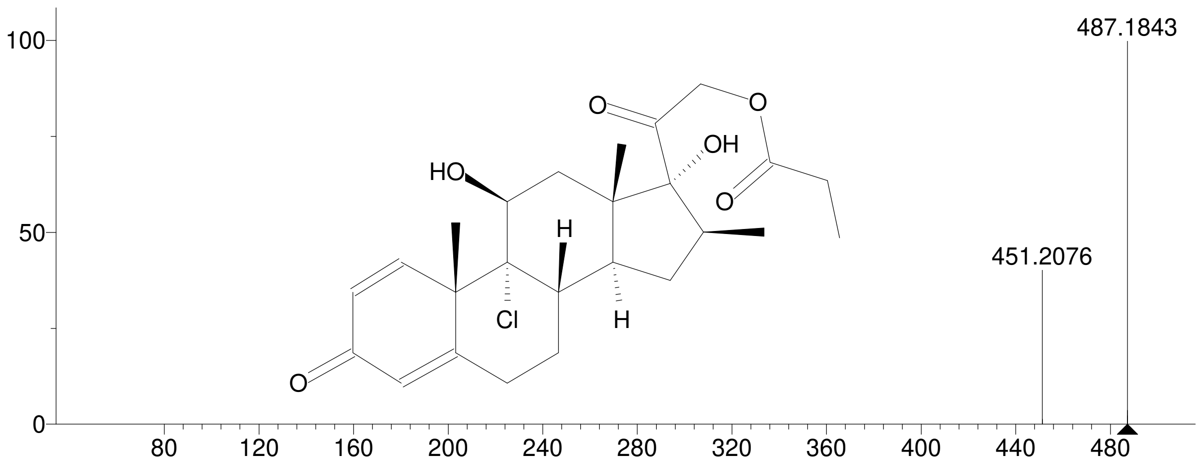

Hit 3 : Cytidine [2M+H]+ HCD 4V P=487.2
C9H13N3O5; MF: 24; RMF: 332; Prob 0.22%; CAS: 65-46-3; Lib: nist_msms; ID: 228109.

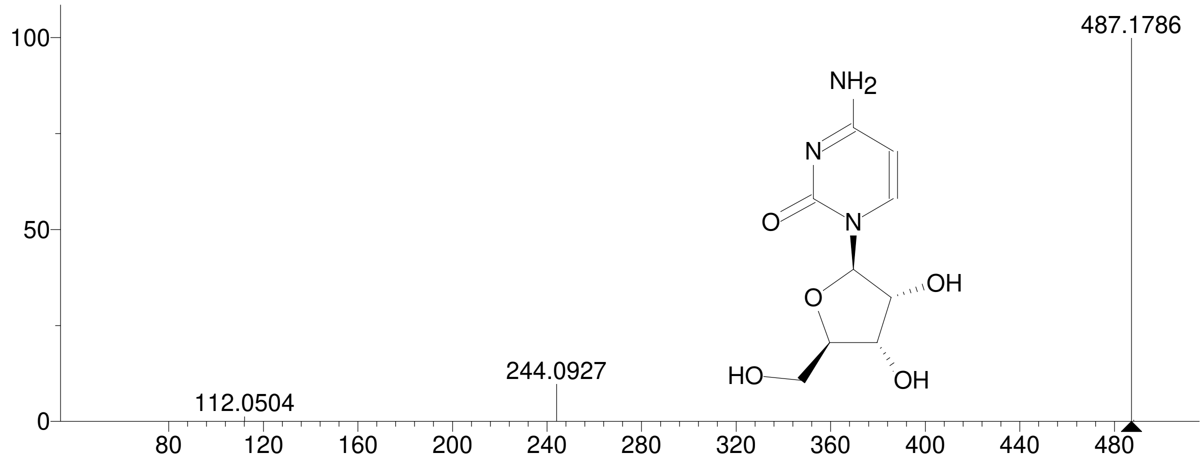

Hit 4 : Cytarabine [2M+H]+ HCD 4V P=487.2
C9H13N3O5; MF: 20; RMF: 326; Prob 0.19%; CAS: 147-94-4; Lib: nist_msms; ID: 120706.

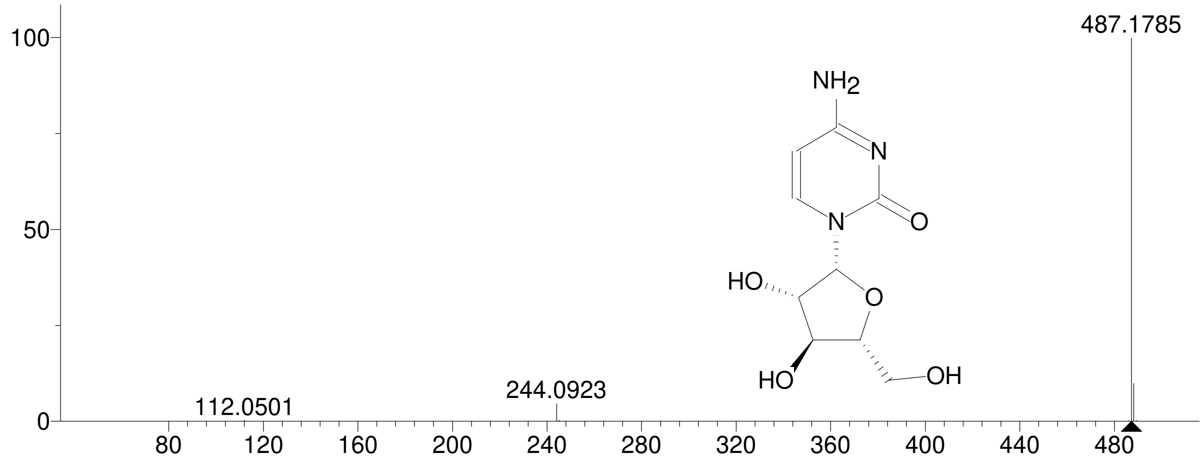

Unknown: Unknown (17.88_325.1648m/z)
Compound in Library Factor = -3273

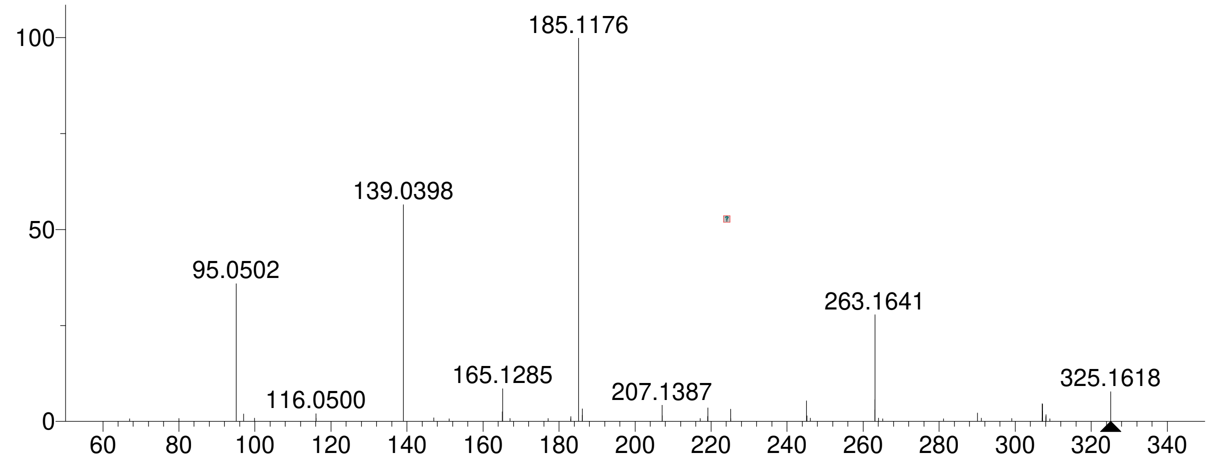

Hit 1 : Chamissonolide [M+H]+ HCD 1V P=325.2
C17H24O6; MF: 8; RMF: 160; Prob 15.5%; CAS: 78853-98-2; Lib: nist_msms; ID: 500759.

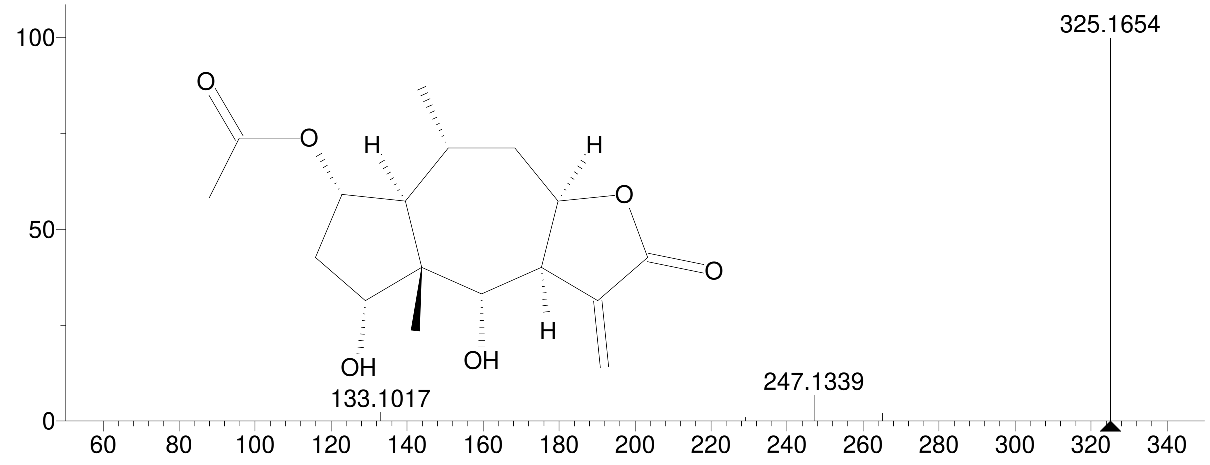

Hit 2 : Tributyrin [M+Na]+ HCD 13V P=325.2
C15H26O6; MF: 8; RMF: 148; Prob 15.5%; CAS: 60-01-5; Lib: nist_msms; ID: 144821.

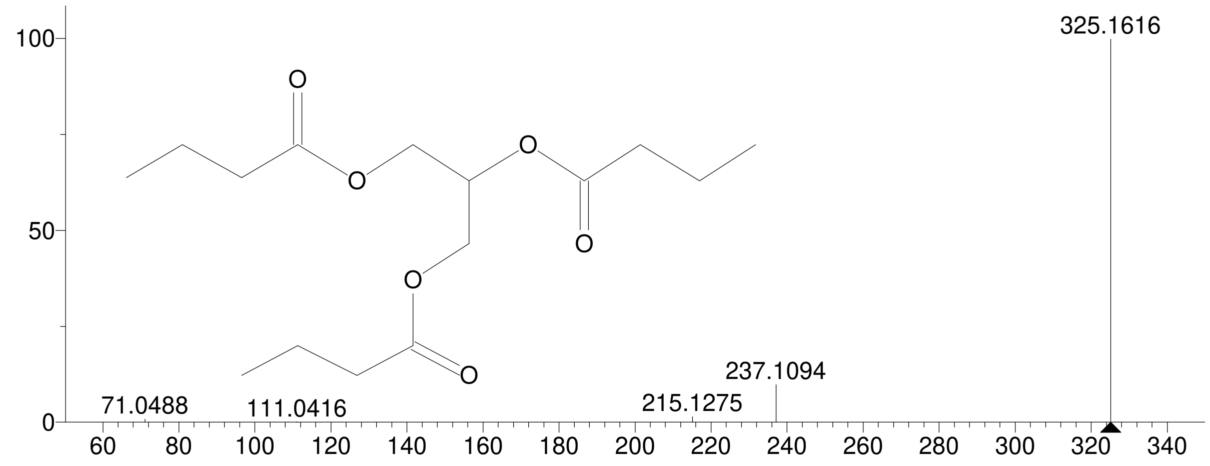

Hit 3 : MMV023985 [M+H]+ 20-30-40%) P=325.2
C18H20N4O2; MF: 3; RMF: 139; Prob 12.5%; Lib: mona-export-pathogen_box; ID: 292.

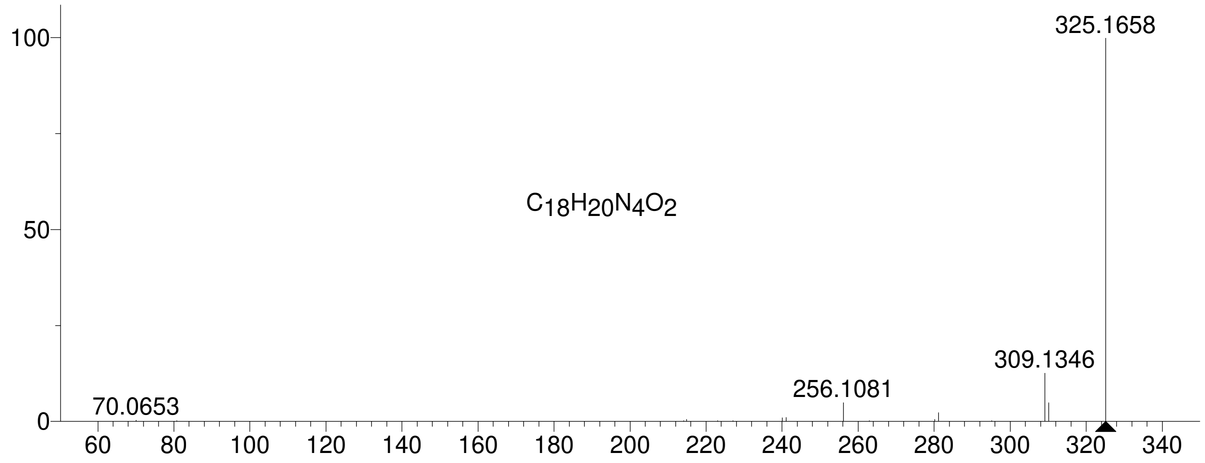

Hit 4 : 2H-Benzimidazol-2-one, 1-ethyl-1,3-dihydro- [2M+H]+ HCD 3V P=325.2
C9H10N2O; MF: 2; RMF: 92; Prob 12.0%; CAS: 10045-45-1; Lib: nist_msms; ID: 364753.

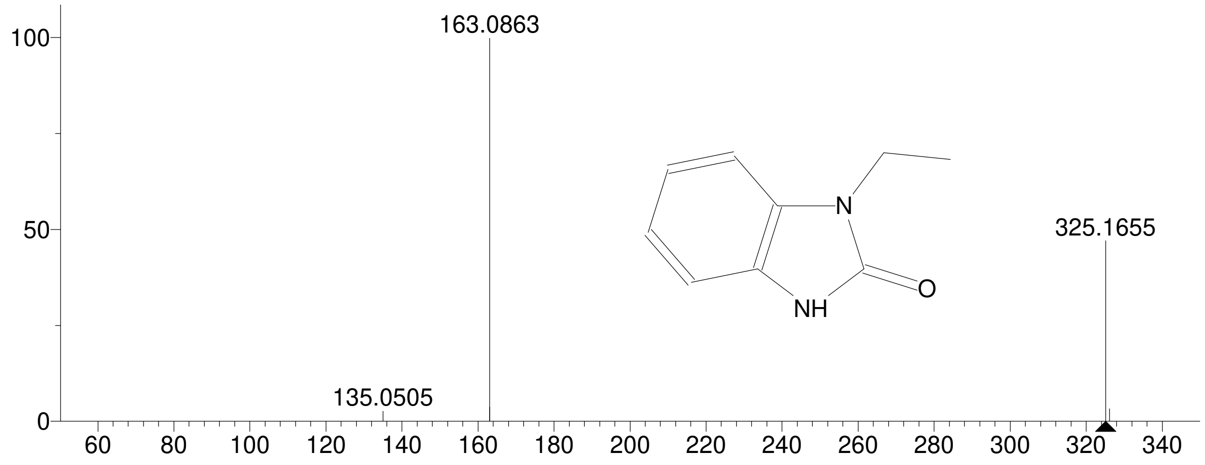

Hit 5 : NCGC00180769-03_C17H24O6_2-Butenoic acid, 2-methyl-, (1S,5R,6S)-5-(acetyloxy)-3-(hydroxymethyl)-
C17H24O6; MF: 0; RMF: 85; Prob 11.1%; Lib: mona-export-gnps; ID: 12444.

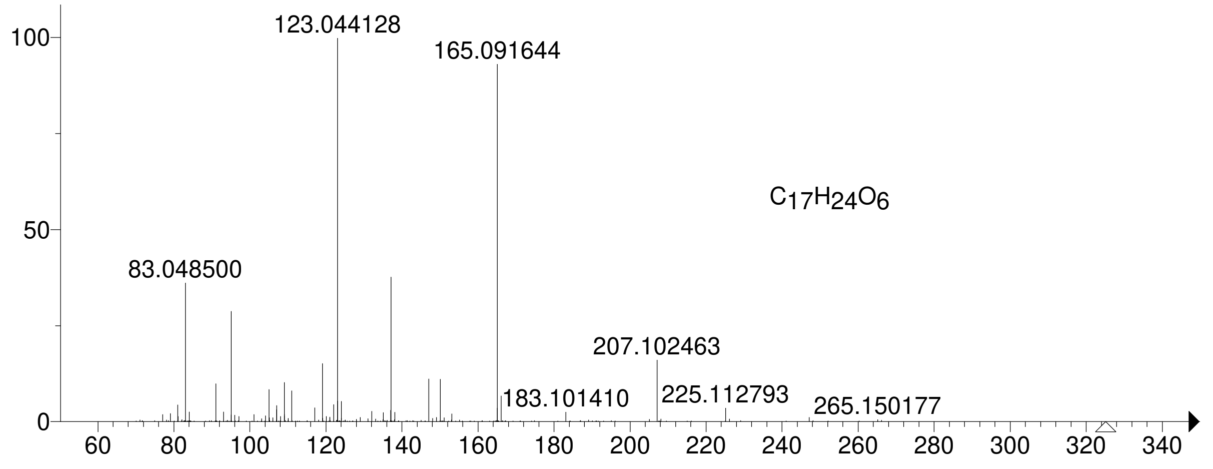

Unknown: Unknown (18.00_265.1072m/z)
Compound in Library Factor = -1848

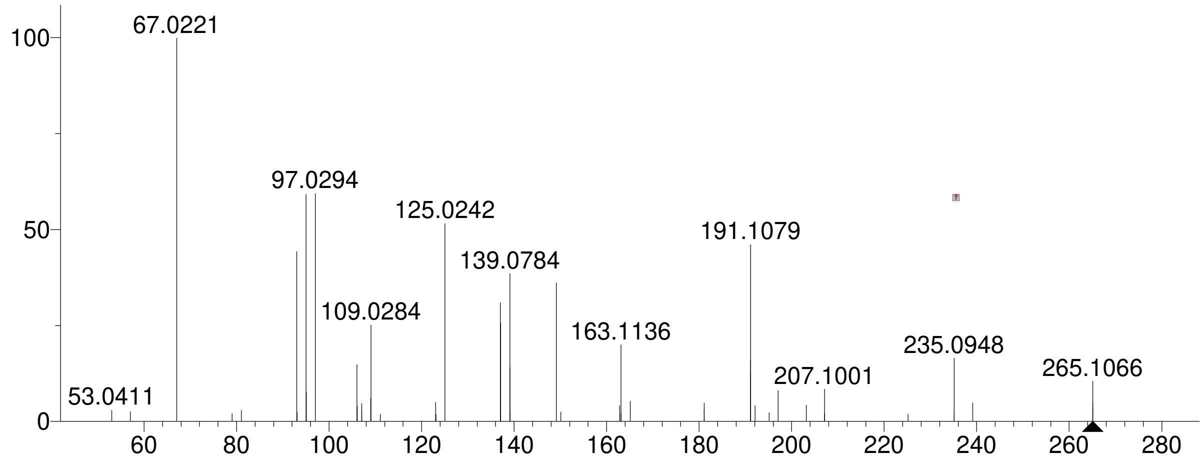

Hit 1 : Anisatin [M+H-CH4O3]+ HCD 10V P=265.1
C15H20O8; MF: 44; RMF: 244; Prob 47.6%; CAS: 5230-87-5; Lib: nist_msms; ID: 394625.

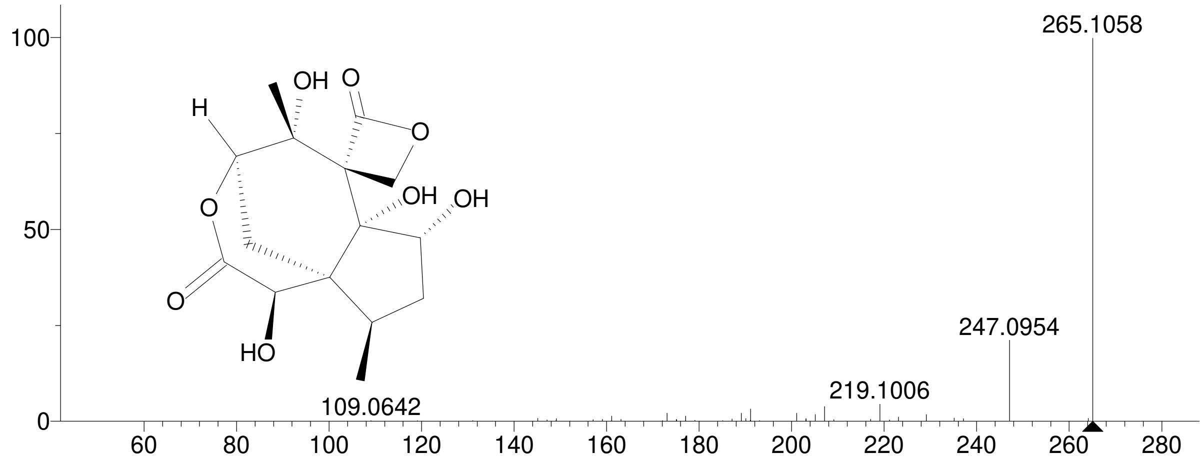

Hit 2 : D-α-Tocopherol succinate [M+H-C19H38]+ IT-FT 35% P=265.1
C33H54O5; MF: 6; RMF: 105; Prob 11.4%; CAS: 4345-03-3; Lib: nist_msms; ID: 308331.

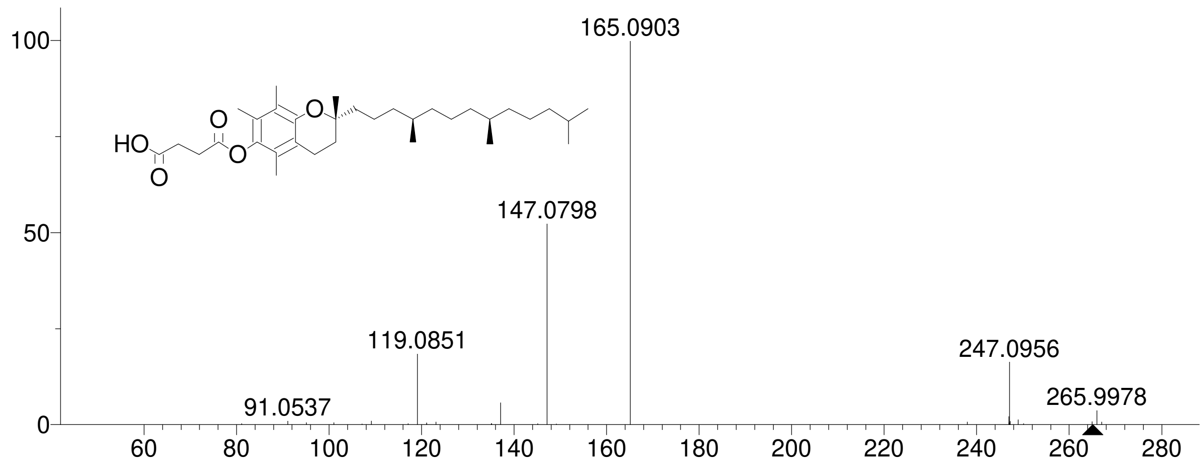

Hit 3 : 4,7,8-trimethoxy-3,5-dimethylchromen-2-one [M+H]+ 35HCD P=265.1
C14H16O5; MF: 5; RMF: 113; Prob 11.0%; Lib: mona-export-vf-npl_qexactive; ID: 23347.

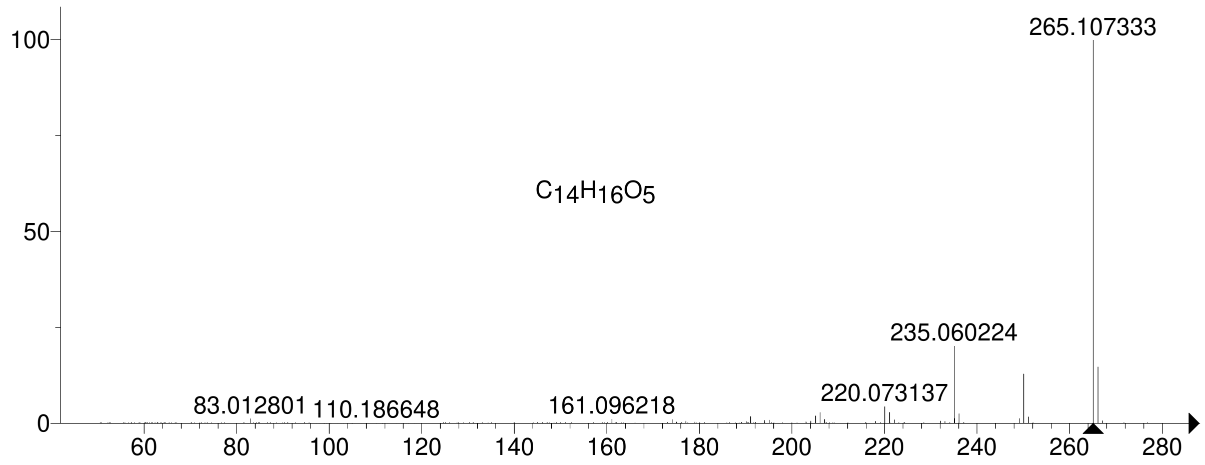

Hit 4 : NCGC00381087-01!3-(2-hydroxypropyl)-6,8-dimethoxyisochromen-1-one [M+H]+ P=265.1
C14H16O5; MF: 4; RMF: 121; Prob 10.5%; Lib: mona-export-gnps; ID: 7812.

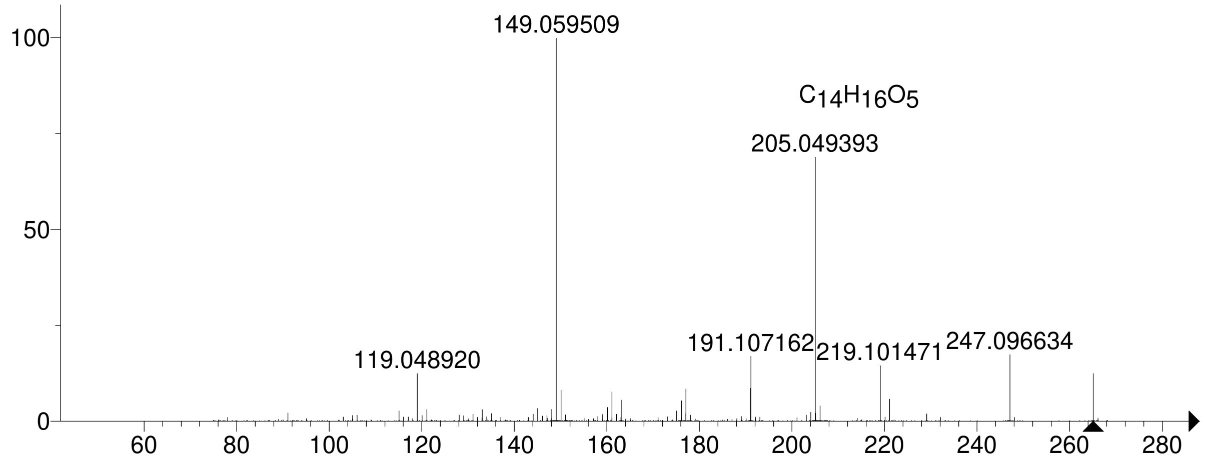

Hit 5 : (E)-5-(4-methoxy-5-methyl-6-oxopyran-2-yl)-3-methylhex-4-enoic acid [M-H]- 35HCD P=265.1
C14H18O5; MF: 3; RMF: 47; Prob 10.1%; Lib: mona-export-lc-ms-ms_negative_mode; ID: 22802.

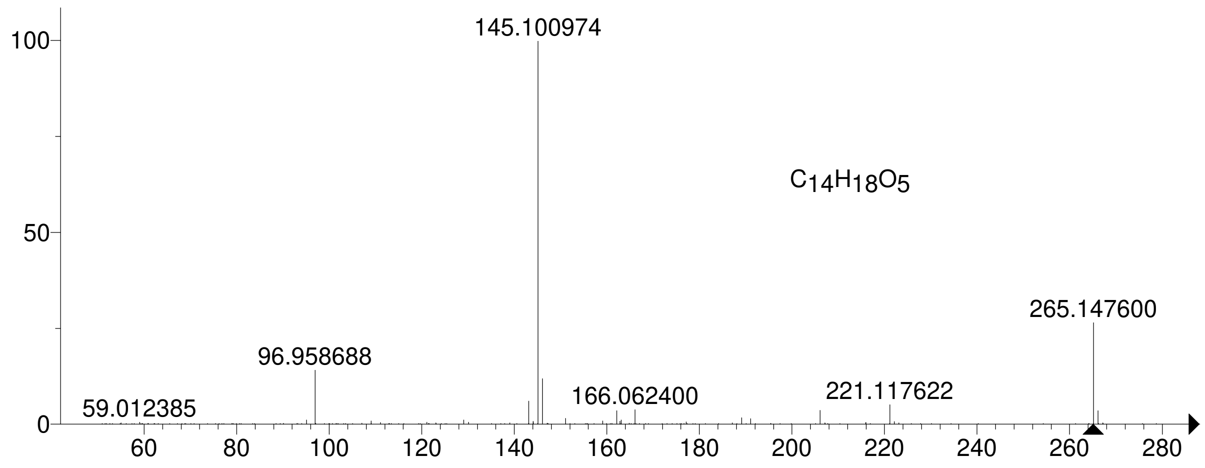

Unknown: Unknown (18.05_484.2537m/z)
Compound in Library Factor = -3273

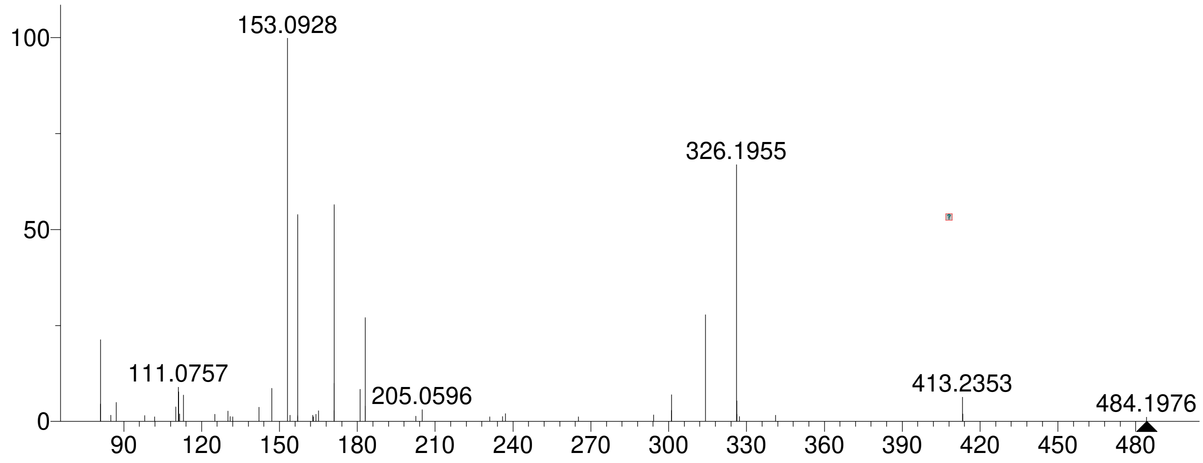

Hit 1 : 2-[2,6-bis(3-methylbut-2-enyl)-4-[(2S,3R,4S,5S,6R)-3,4,5-trihydroxy-6-(hydroxymethyl)oxan-2-yl]oxyphenyl
C24H34O9; MF: 1; RMF: 80; Prob 50.0%; Lib: mona-export-vf-npl_qexactive; ID: 21831.

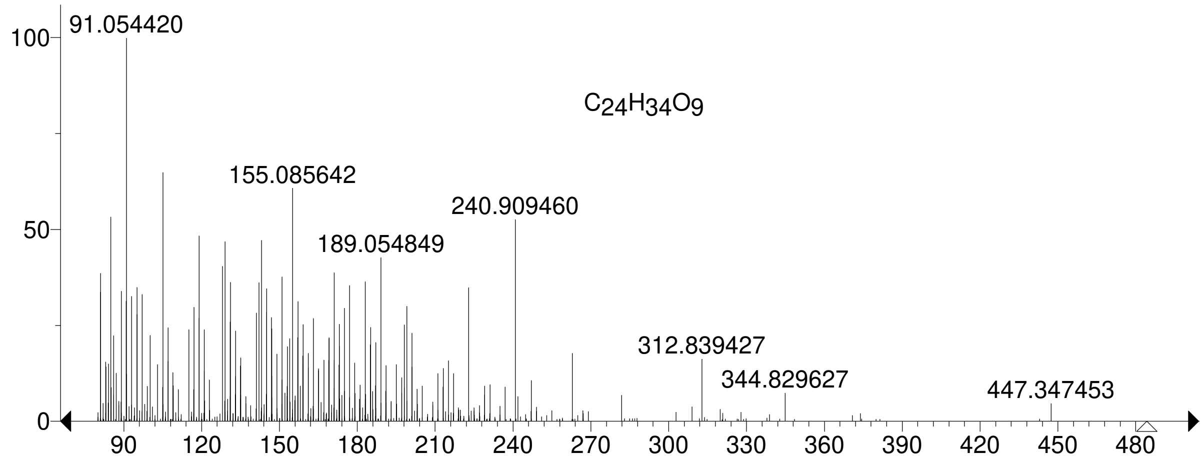

Hit 2 : NCGC00169100-02!2-[2,6-bis(3-methylbut-2-enyl)-4-[(2S,3R,4S,5S,6R)-3,4,5-trihydroxy-6-(hydroxymethyl
C24H34O9; MF: 1; RMF: 49; Prob 50.0%; Lib: mona-export-gnps; ID: 6241.

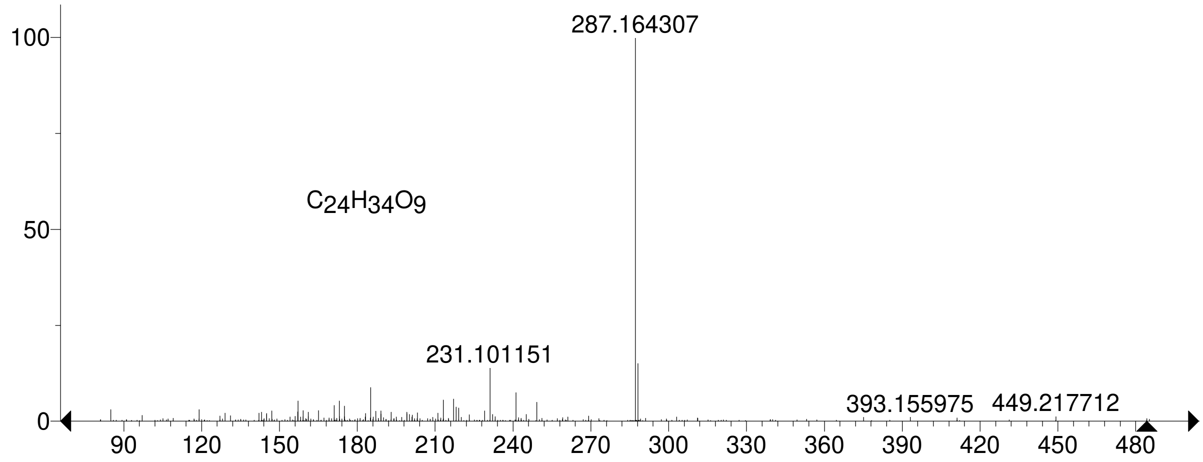

** Search Report Page 1 of 1 **

Unknown: Unknown (18.10_647.8122m/z)
Compound in Library Factor = 0

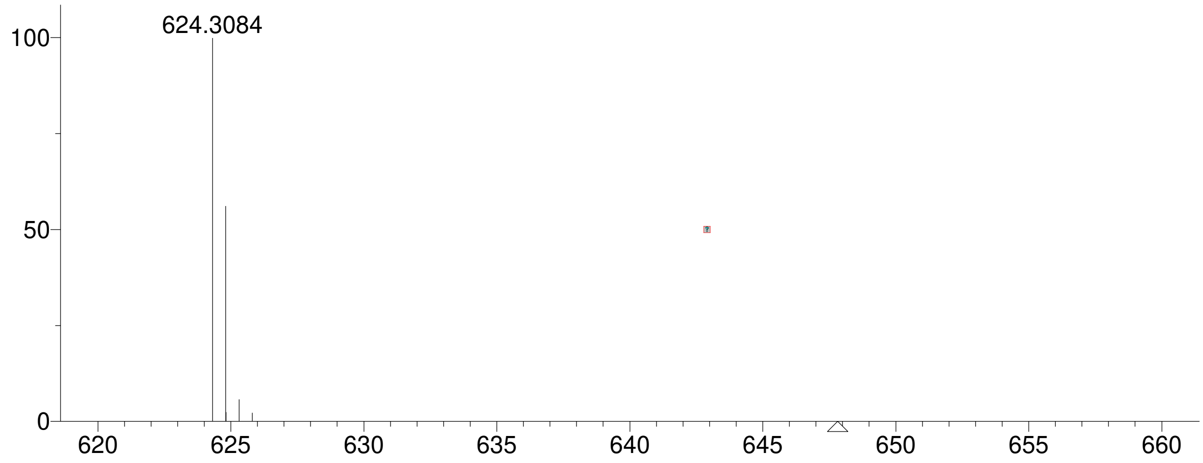

Unknown: Unknown (18.12_647.3119m/z)
Compound in Library Factor = 0

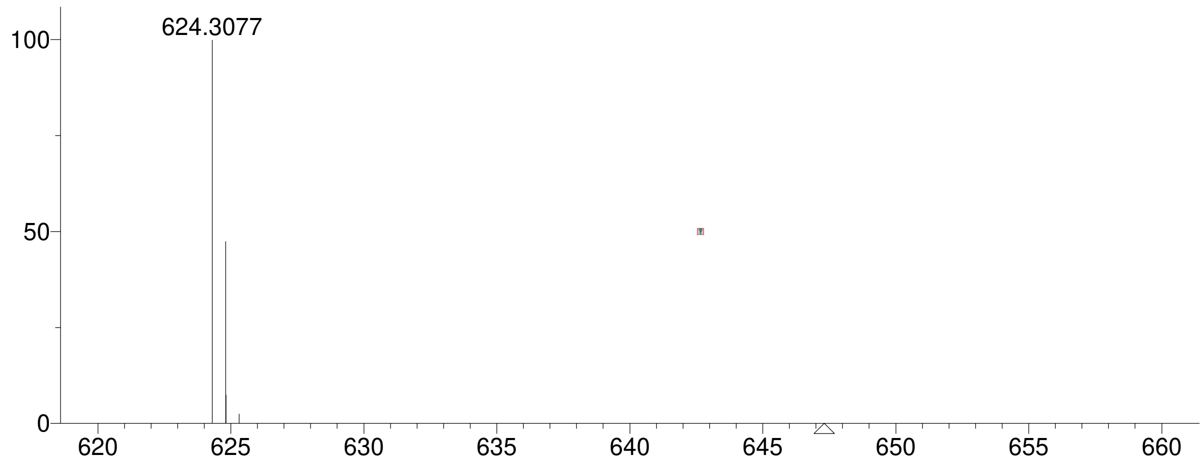

Unknown: Unknown (18.14_394.1862m/z)
Compound in Library Factor = -2176

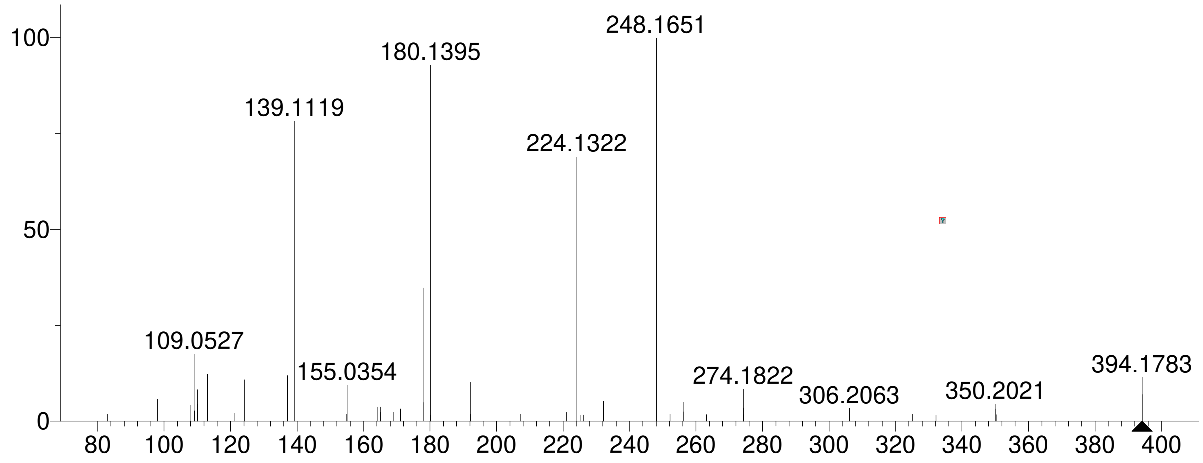

Hit 1 : 3',4',5'-Trimethoxycocaine [M+H]+ HCD 15V P=394.2
C20H27NO7; MF: 21; RMF: 199; Prob 15.9%; CAS: 156301-59-6; Lib: nist_msms; ID: 383469.

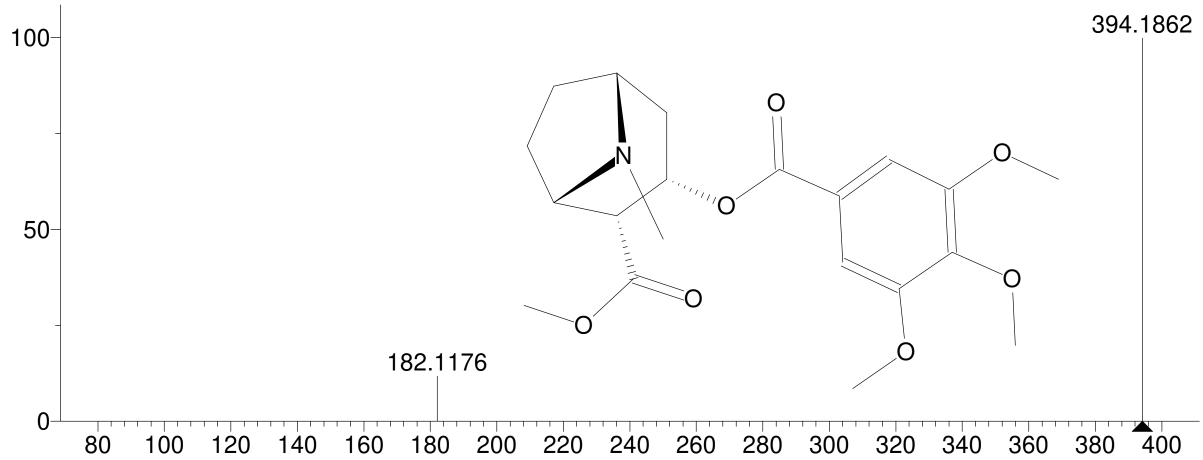

Hit 2 : [1,3-dihydroxy-1-(7-methoxy-2-oxochromen-6-yl)-3-methylbutan-2-yl] 3-methylbut-2-enoate [M+NH4]+ 35l
C20H24O7; MF: 1; RMF: 73; Prob 7.24%; Lib: mona-export-vf-npl_qexactive; ID: 24781.

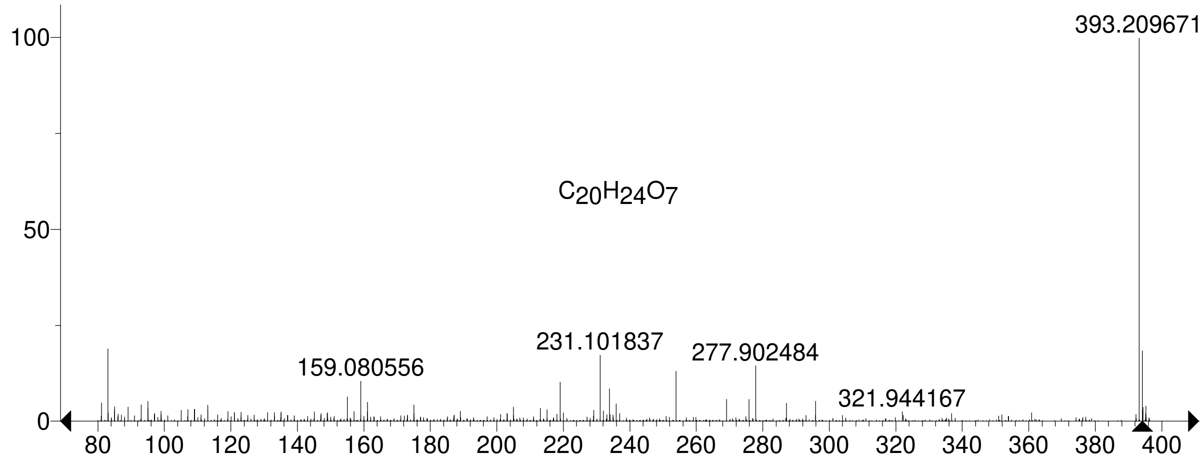

Hit 3 : [1,3-dihydroxy-1-(7-methoxy-2-oxochromen-6-yl)-3-methylbutan-2-yl] (E)-2-methylbut-2-enoate [M+NH4]+
C20H24O7; MF: 1; RMF: 58; Prob 7.24%; Lib: mona-export-vf-npl_qexactive; ID: 5461.

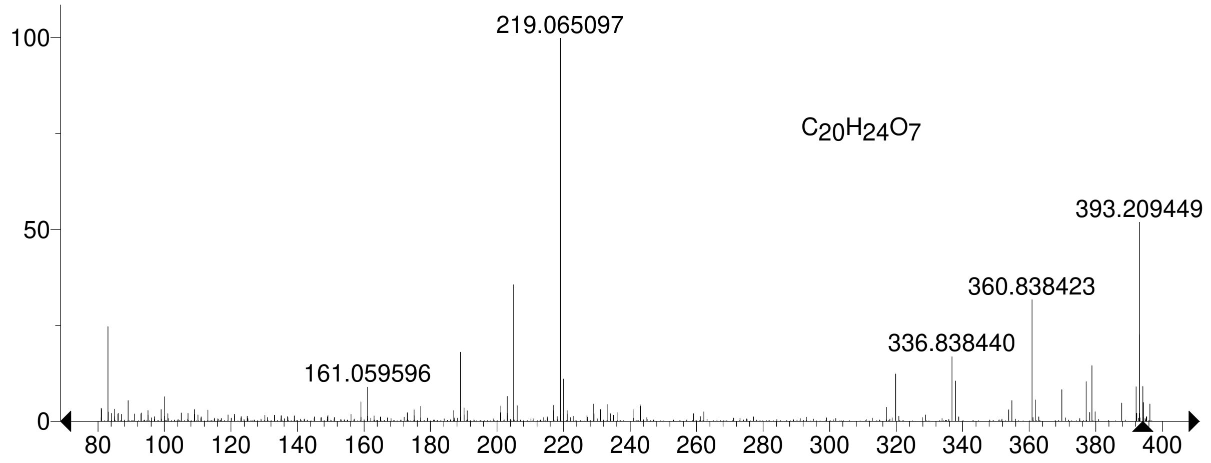

Hit 4 : rac-N,N-Didesmethyl-O-desmethylvenlafaxine glucuronide [M+H-H2O]+ HCD 11V P=394.2
C20H29NO8; MF: 0; RMF: 112; Prob 6.96%; CAS: 1799830-07-1; Lib: nist_msms; ID: 436197.

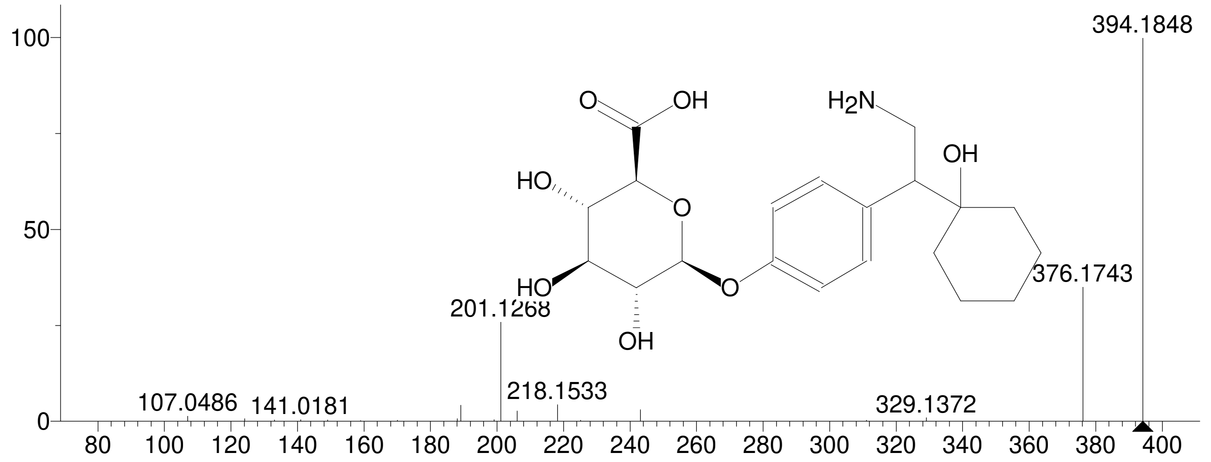

Hit 5 : NCGC00381074-01![1,3-dihydroxy-1-(7-methoxy-2-oxochromen-6-yl)-3-methylbutan-2-yl] 3-methylbut-2-er
C20H24O7; MF: 0; RMF: 54; Prob 6.96%; Lib: mona-export-gnps; ID: 8644.

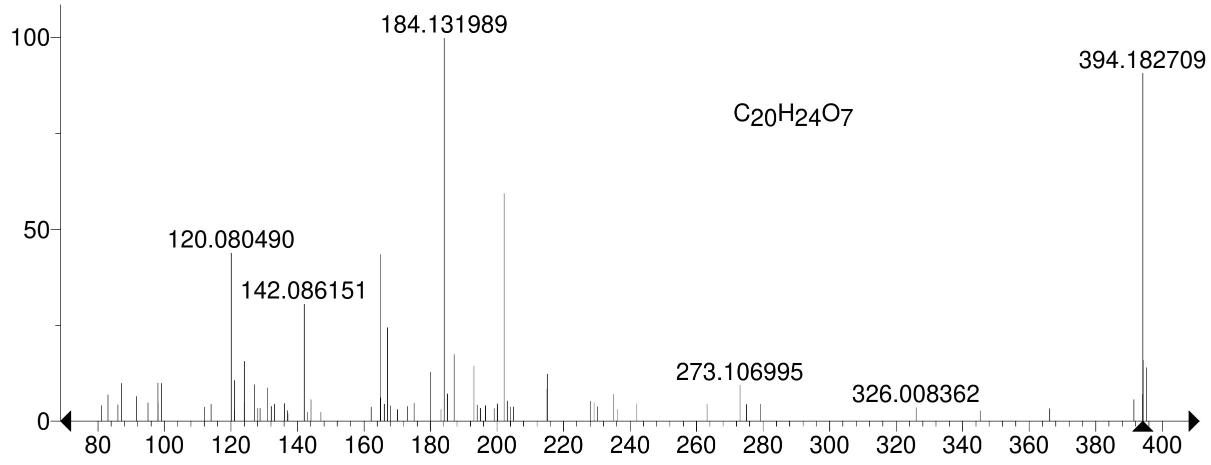

Unknown: Unknown (18.15_402.8321m/z)
Compound in Library Factor = 0

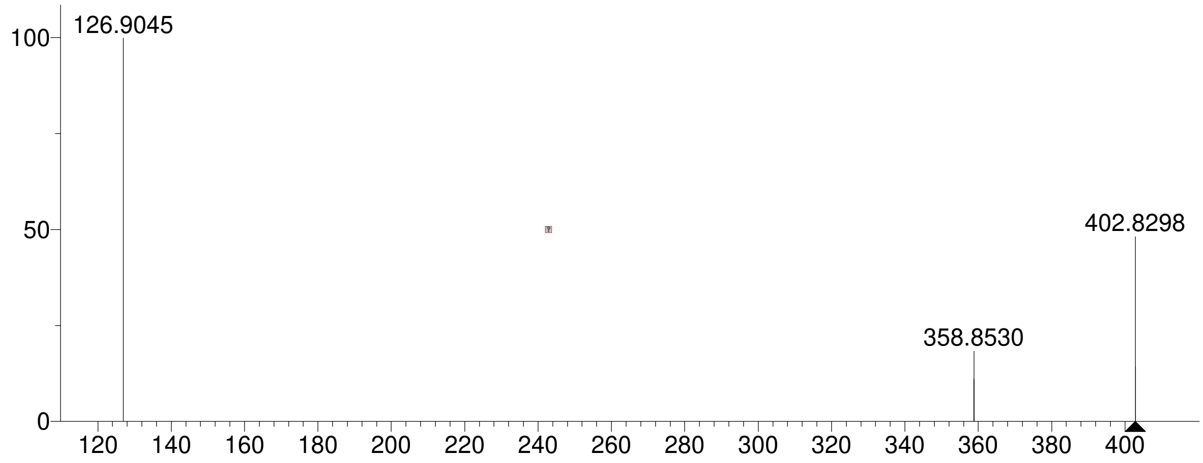

Unknown: Unknown (18.26_520.0691m/z)
Compound in Library Factor = 0

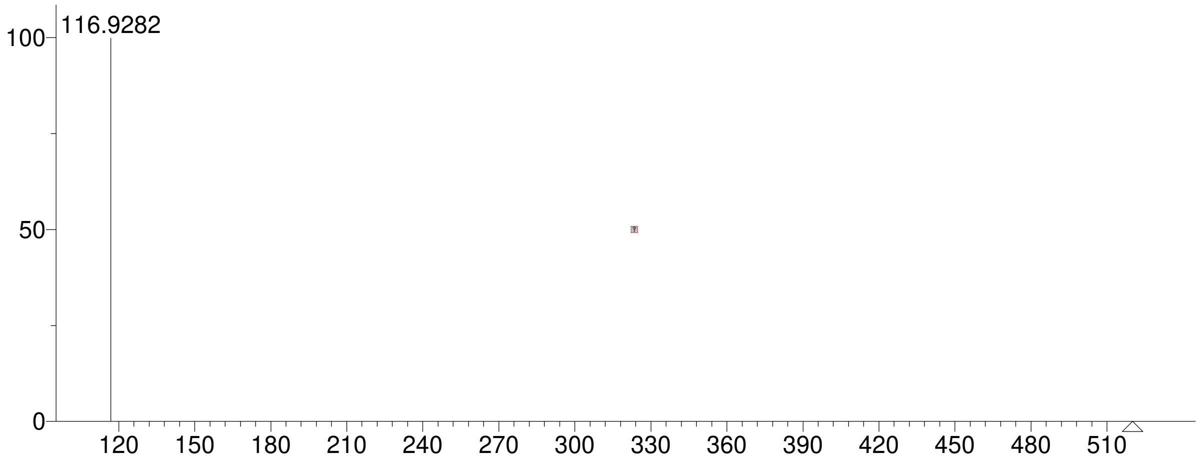

Unknown: Unknown (18.27_673.3202m/z)
Compound in Library Factor = -2387

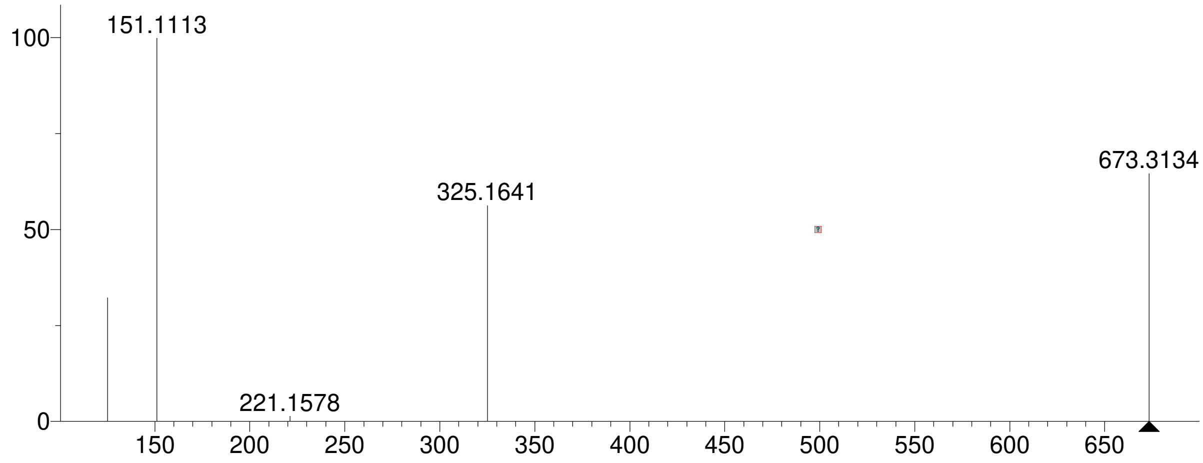

Hit 1 : Thapsigargin [M+Na]+ HCD 20V P=673.3
C34H50O12; MF: 18; RMF: 439; Prob 48.3%; CAS: 67526-95-8; Lib: nist_msms; ID: 247695.

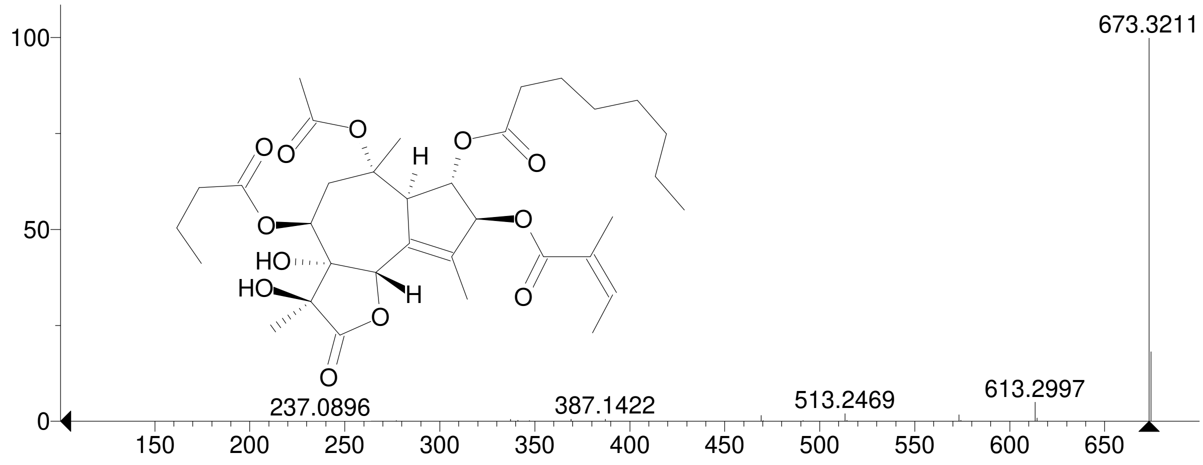

Hit 2 : 7,13,17-O-Triacetyl-5-O-(2-methylbutyryl)-3-O-propanoyl-14-oxopremyrsinol [M+Na]+ P=673.3
C34H50O12; MF: 1; RMF: 164; Prob 26.4%; Lib: mona-export-gnps; ID: 5960.

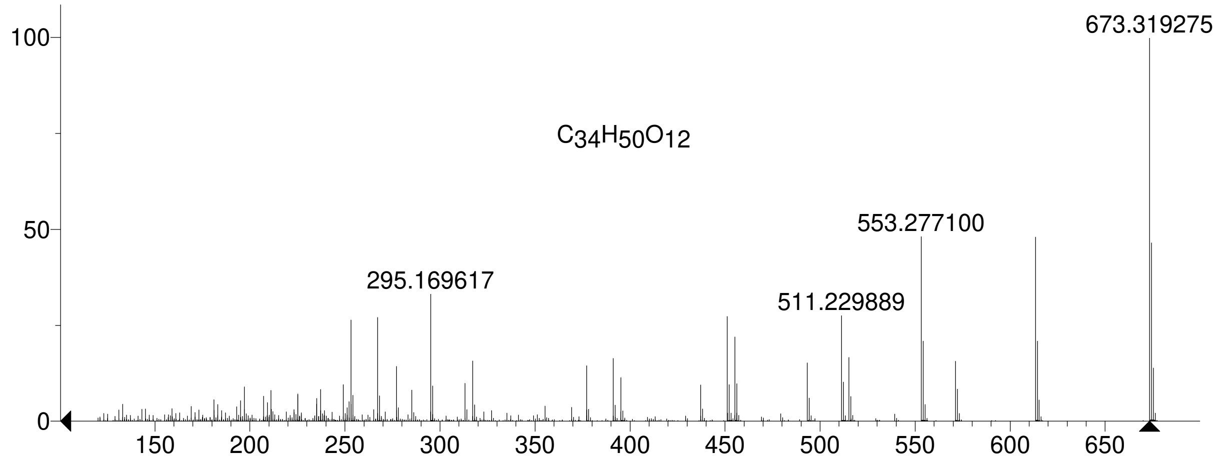

Hit 3 : [6-acetyloxy-4-butanoyloxy-3,3a-dihydroxy-3,6,9-trimethyl-8-[(E)-2-methylbut-2-enoyl]oxy-2-oxo-4,5,6a,7,8,
C34H50O12; MF: 0; RMF: 12; Prob 25.3%; Lib: mona-export-vf-npl_qexactive; ID: 18161.

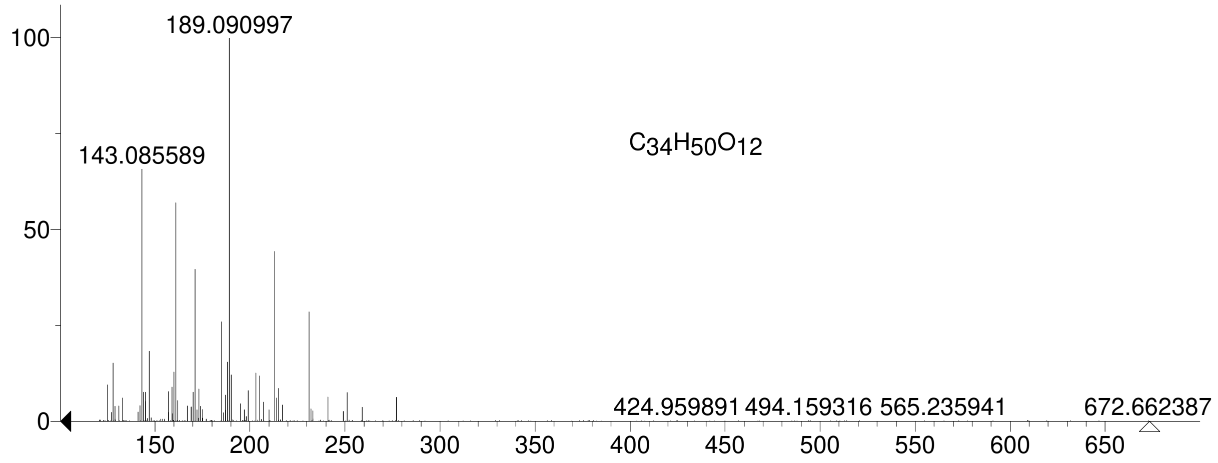

Unknown: Unknown (18.28_151.1115m/z)
Compound in Library Factor = -144

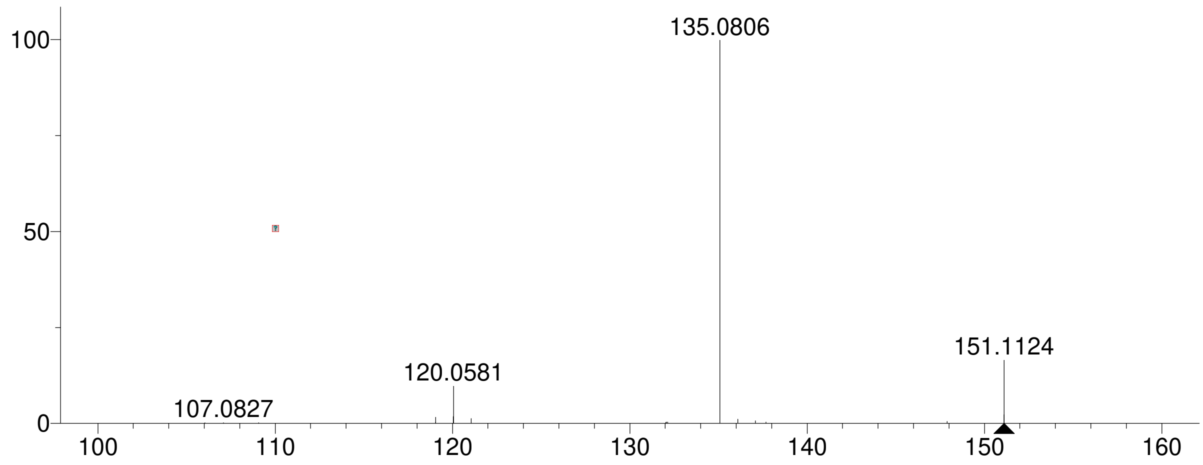

Hit 1 : 2-Furanpropanoic acid, 3-carboxy-4-methyl-5-propyl- [M-H-C2O4]- HCD 16V P=151.1
C12H16O5; MF: 454; RMF: 655; Prob 94.1%; CAS: 86879-39-2; Lib: nist_msms; ID: 428346.

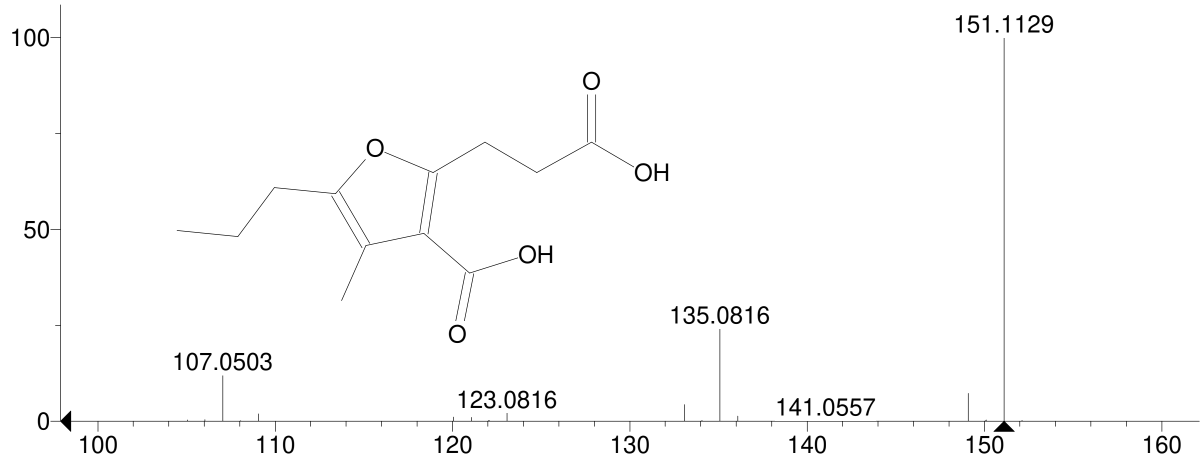

Hit 2 : L-Carvone [M+H]+ HCD 6V P=151.1
C10H14O; MF: 102; RMF: 342; Prob 0.94%; CAS: 6485-40-1; Lib: nist_msms; ID: 145173.

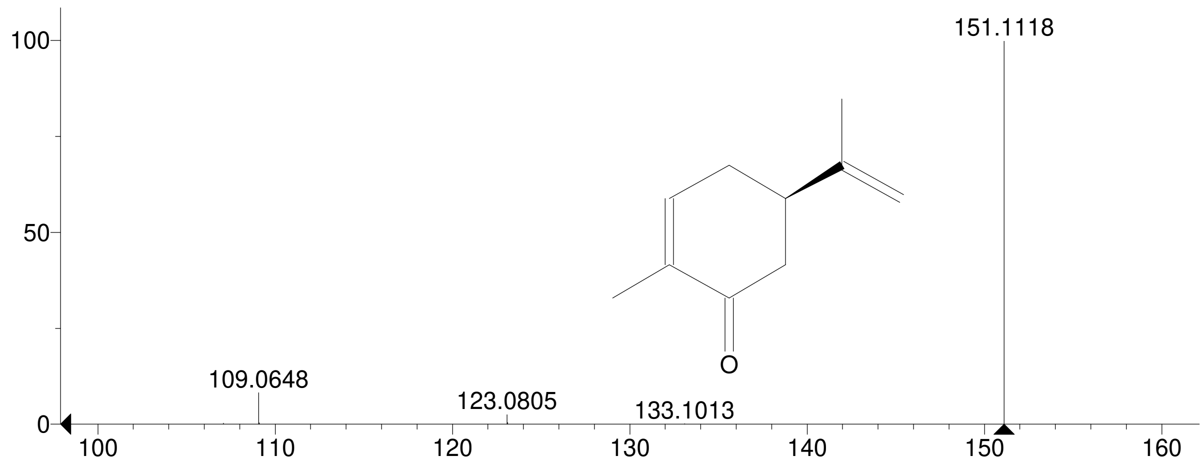

Hit 3 : (-)-Verbenone [M+H]+ HCD 3V P=151.1
C10H14O; MF: 96; RMF: 341; Prob 0.73%; CAS: 1196-01-6; Lib: nist_msms; ID: 143387.

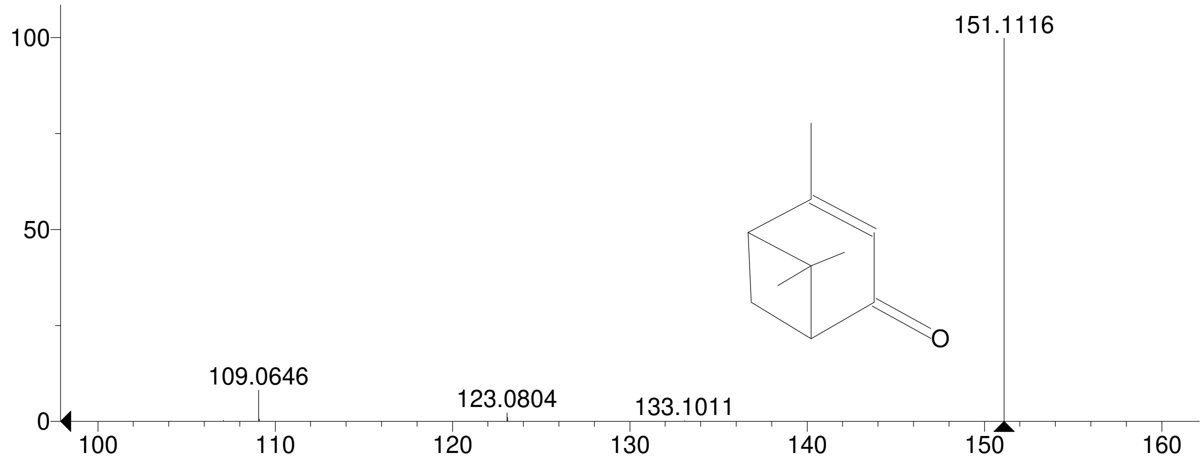

Hit 4 : 2-Adamantanone [M+H]+ HCD 18V P=151.1
C10H14O; MF: 96; RMF: 339; Prob 0.73%; CAS: 700-58-3; Lib: nist_msms; ID: 395499.

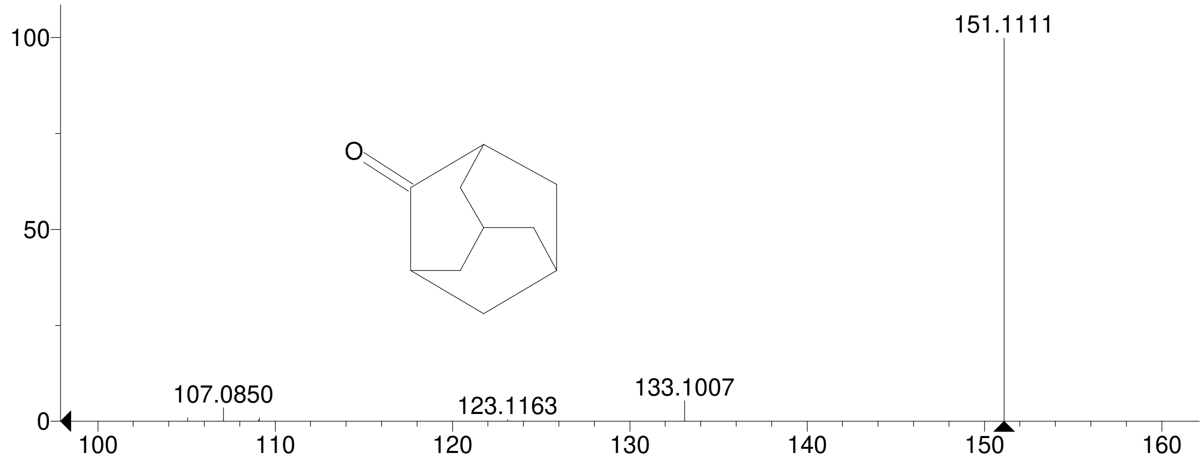

Hit 5 : (+)-Carvone [M+H]+ HCD 6V P=151.1
C10H14O; MF: 89; RMF: 336; Prob 0.56%; CAS: 2244-16-8; Lib: nist_msms; ID: 384974.

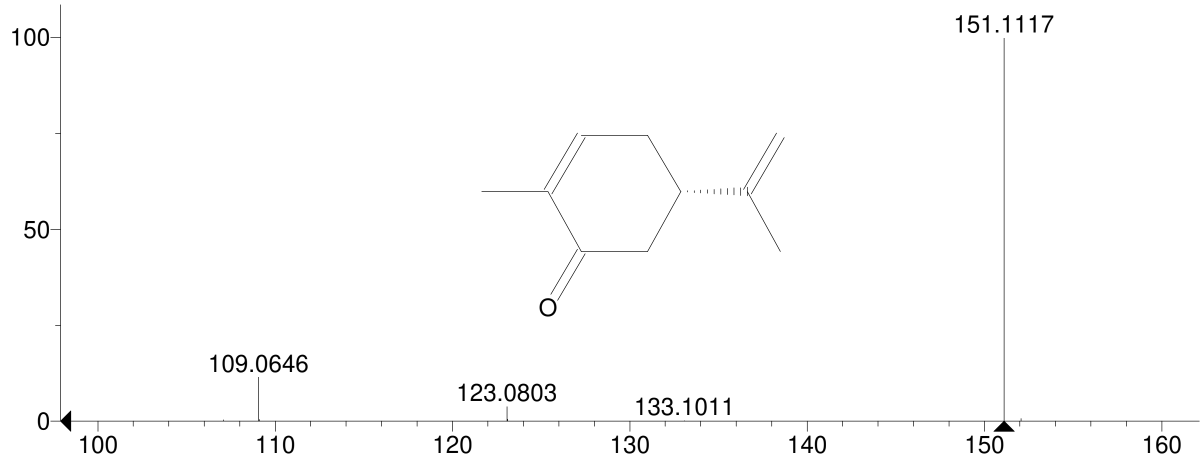

Unknown: Unknown (18.28_221.1529m/z)
Compound in Library Factor = -3273

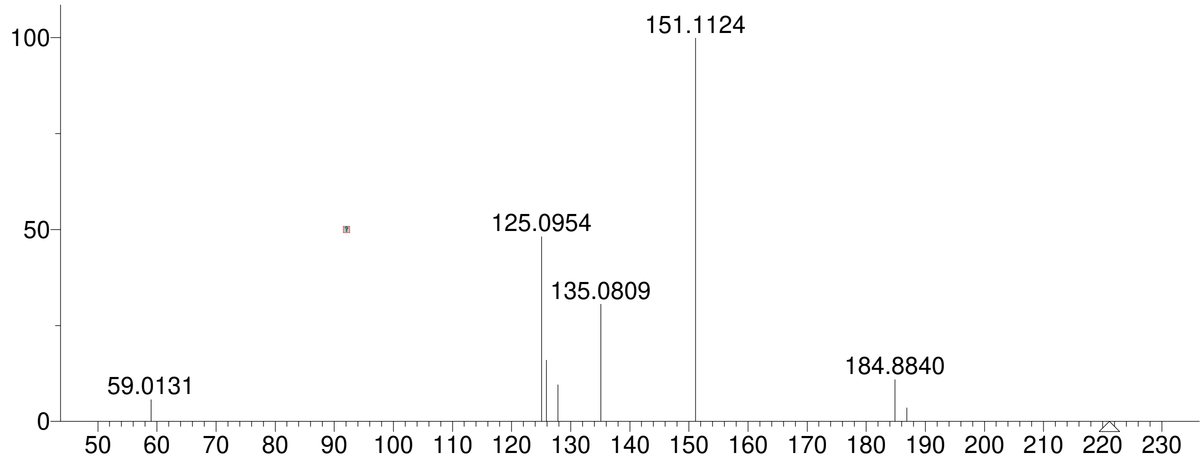

Hit 1 : 2,6-Di-tert-butylbenzoquinone [M+H]+ HCD 26V P=221.2
C14H20O2; MF: 3; RMF: 186; Prob 12.4%; CAS: 719-22-2; Lib: nist_msms; ID: 144841.

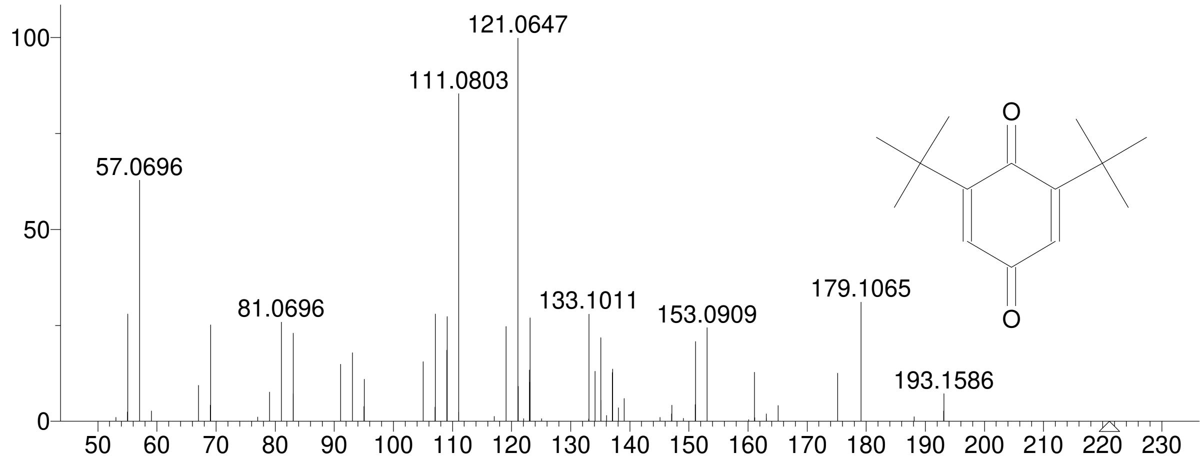

Hit 2 : 2,5-Di-tert-butyl-1,4-benzoquinone [M+H]+ HCD 26V P=221.2
C14H20O2; MF: 0; RMF: 153; Prob 10.9%; CAS: 2460-77-7; Lib: nist_msms; ID: 141999.

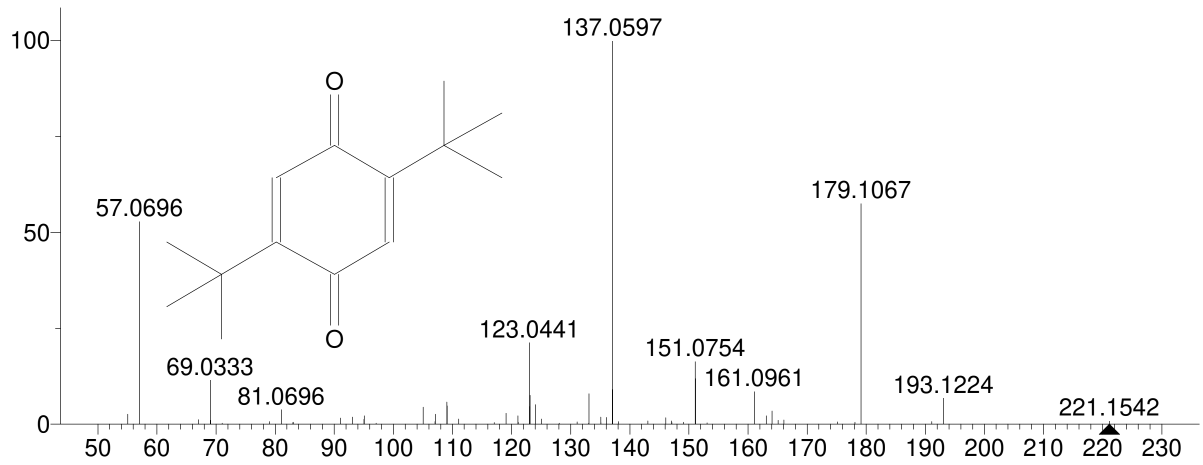

Hit 3 : 8,11-Tridecadienoic acid, 13-(3-pentyl-2-oxiranyl)-, (8Z,11Z)- [M-H-C6H12O]- HCD 19V P=221.2
C20H34O3; MF: 0; RMF: 92; Prob 10.9%; CAS: 351533-80-7; Lib: nist_msms; ID: 428462.

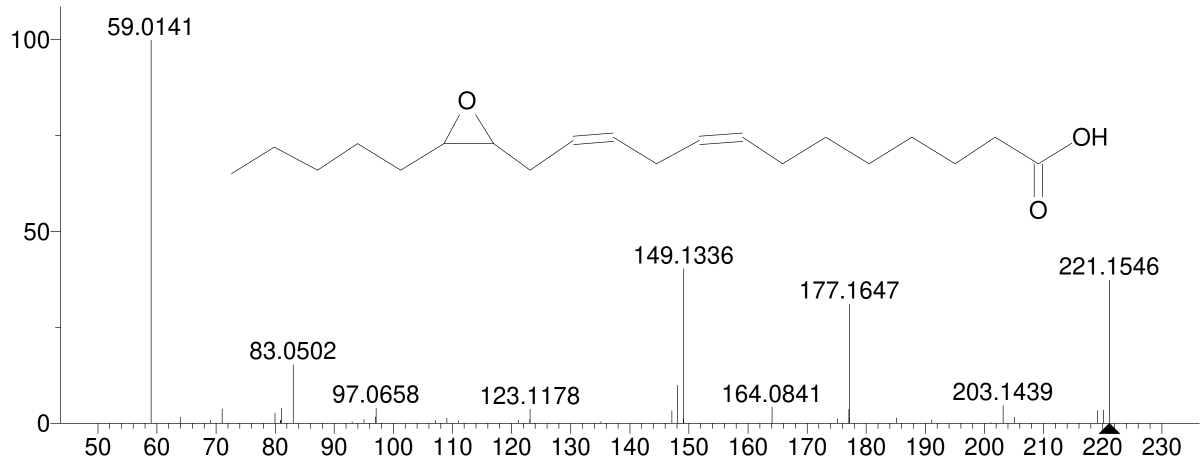

Hit 4 : NCGC00381404-01_C14H22O3_2-Naphthalenecarboxylic acid, 3,4,4a,5,6,7,8,8a-octahydro-3-hydroxy-5,5
C14H22O3; MF: 0; RMF: 60; Prob 10.9%; Lib: mona-export-gnps; ID: 13406.

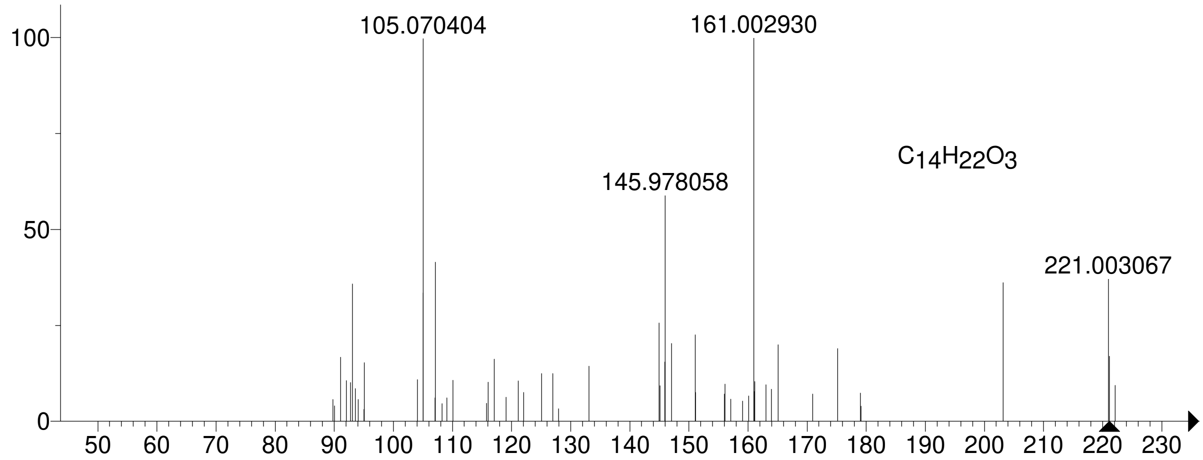

Hit 5 : Rhodinyl Acetate [M+Na]+ 35HCD P=221.2
C12H22O2; MF: 0; RMF: 33; Prob 10.9%; Lib: mona-export-vf-npl_qexactive; ID: 26275.

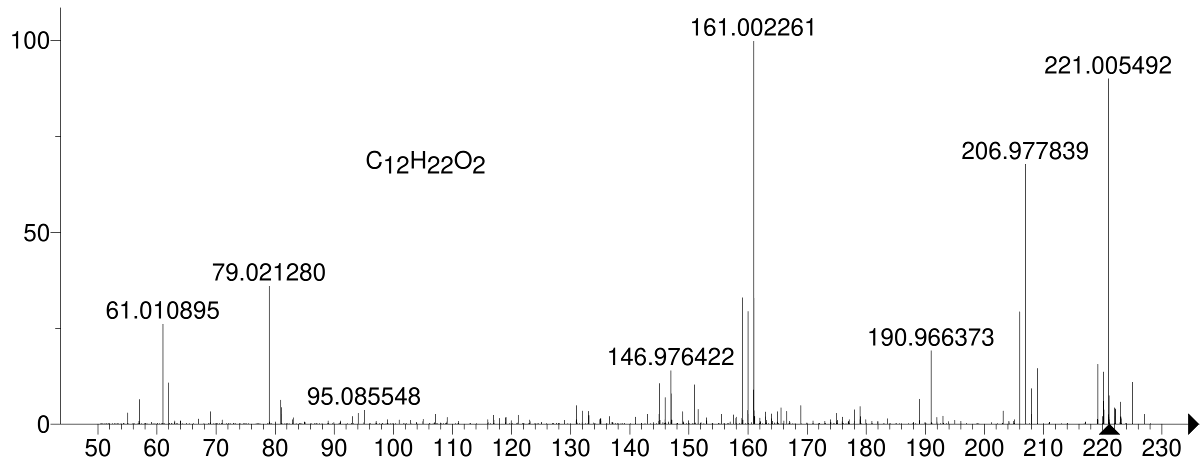

Unknown: Unknown (18.28_325.1652m/z)
Compound in Library Factor = -3273

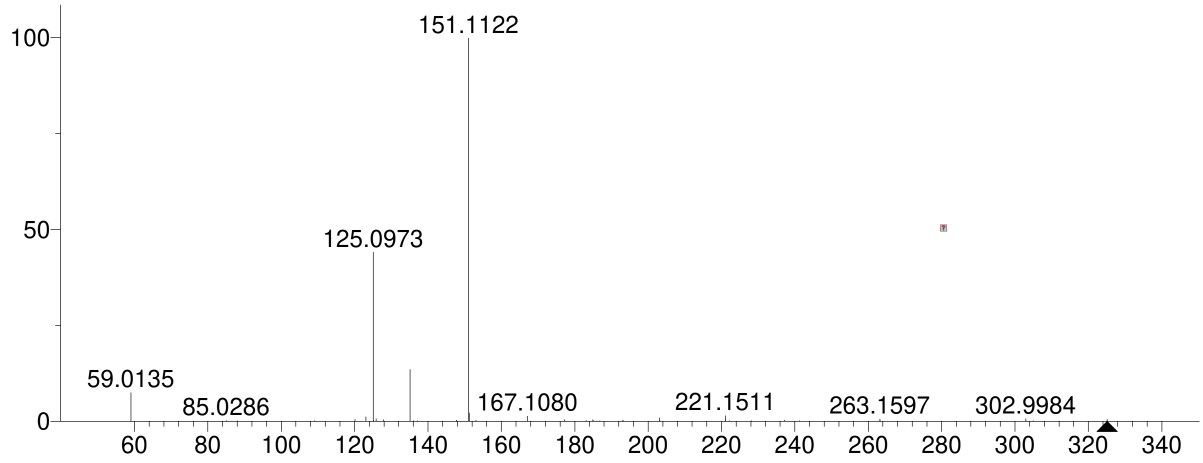

Hit 1 : Chamissonolide [M+H]+ HCD 1V P=325.2
$C_{17}H_{24}O_6$; MF: 2; RMF: 57; Prob 14.9%; CAS: 78853-98-2; Lib: nist_msms; ID: 500759.

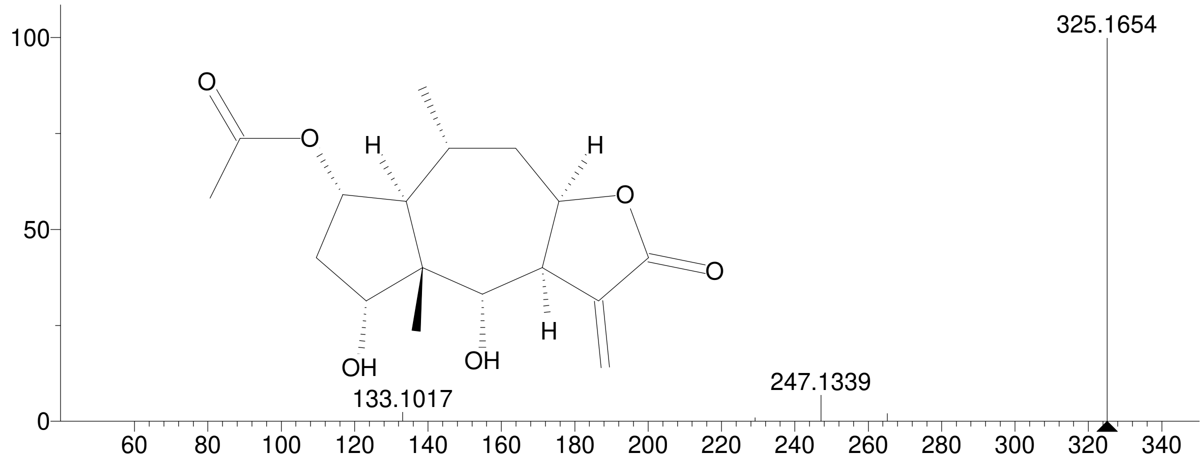

Hit 2 : Tributyrin [M+Na]+ HCD 13V P=325.2
$C_{15}H_{26}O_6$; MF: 2; RMF: 49; Prob 14.9%; CAS: 60-01-5; Lib: nist_msms; ID: 144821.

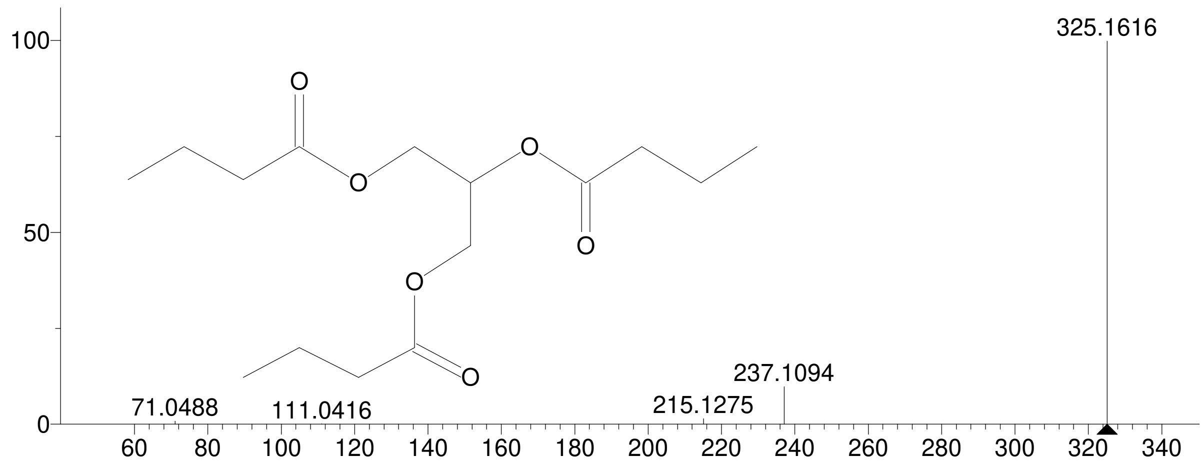

Hit 3 : NCGC00180769-03_C17H24O6_2-Butenoic acid, 2-methyl-, (1S,5R,6S)-5-(acetyloxy)-3-(hydroxymethyl)-6
C17H24O6; MF: 2; RMF: 38; Prob 14.9%; Lib: mona-export-gnps; ID: 12444.

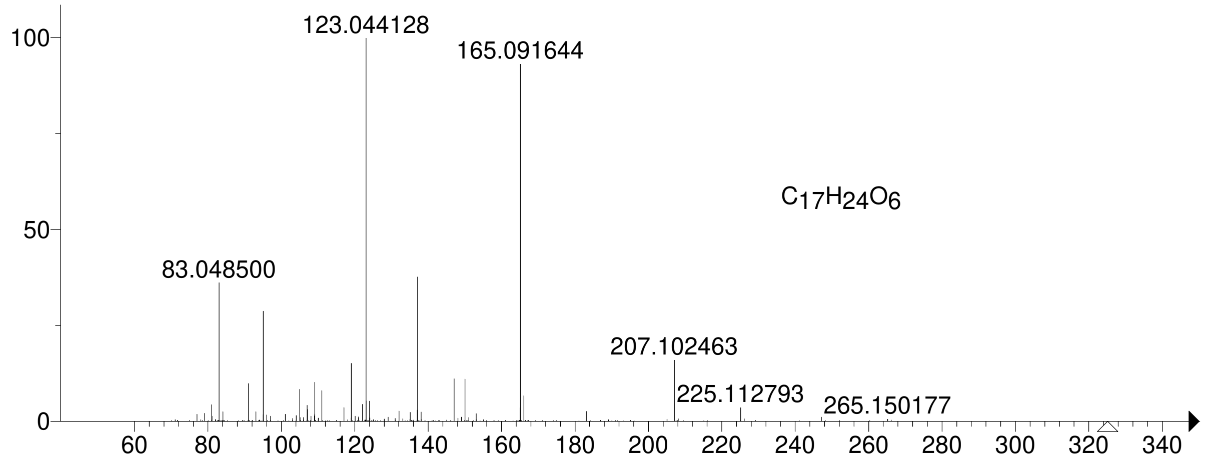

Hit 4 : 2H-Benzimidazol-2-one, 1-ethyl-1,3-dihydro- [2M+H]+ HCD 6V P=325.2
C9H10N2O; MF: 1; RMF: 55; Prob 14.3%; CAS: 10045-45-1; Lib: nist_msms; ID: 364754.

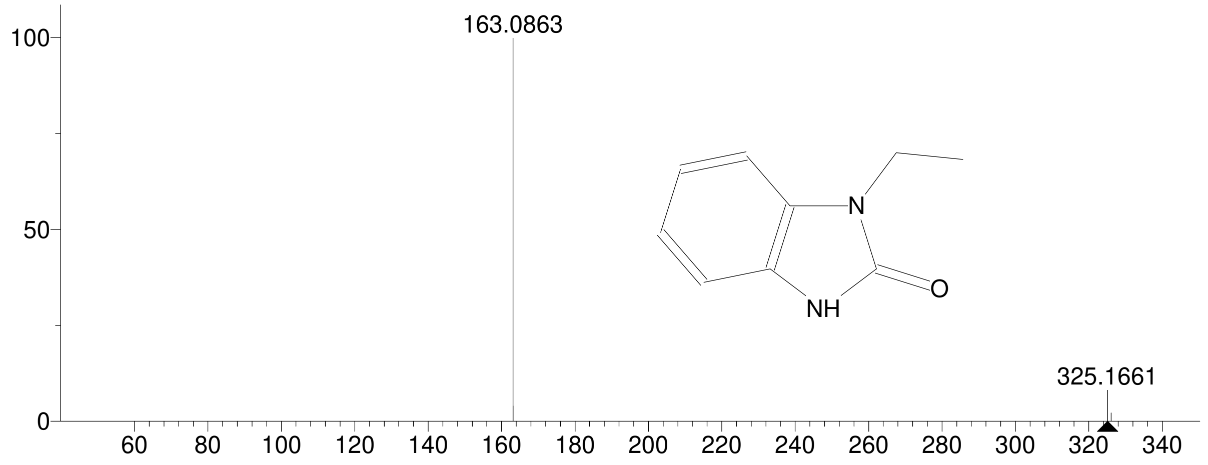

Hit 5 : MMV023985 [M+H]+ 20-30-40%) P=325.2
C18H20N4O2; MF: 0; RMF: 46; Prob 13.7%; Lib: mona-export-pathogen_box; ID: 292.

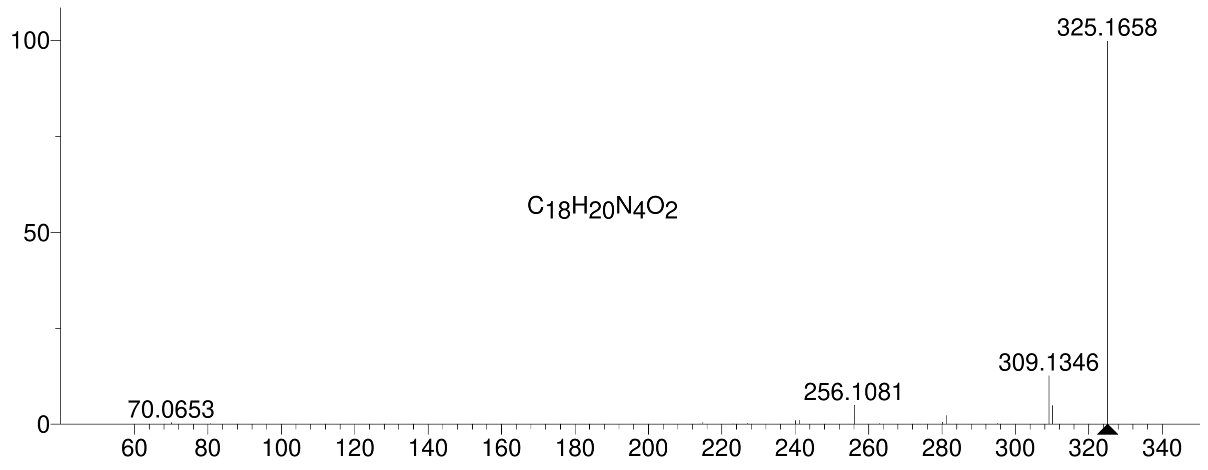

Unknown: Unknown (18.29_325.3344m/z)
Compound in Library Factor = 0

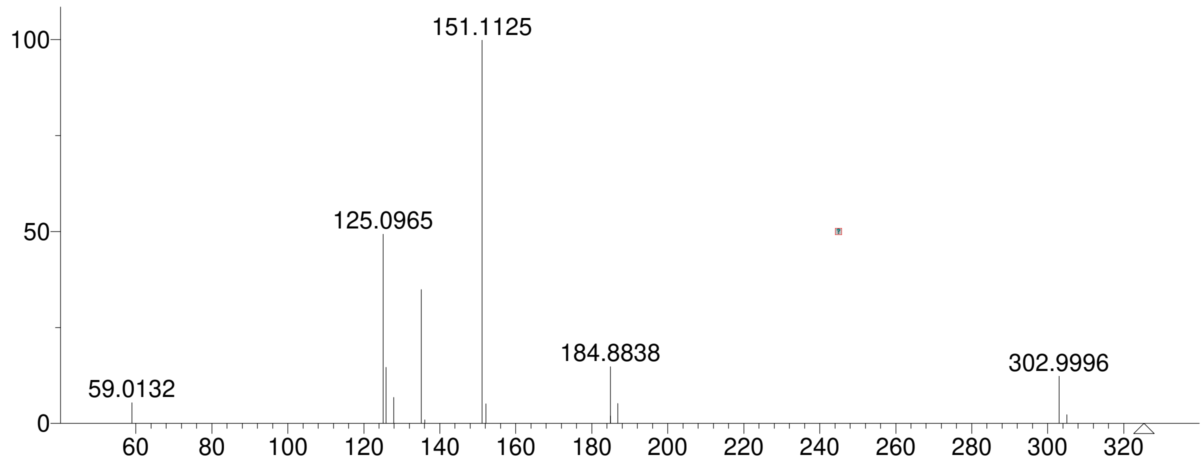

Unknown: Unknown (18.29_408.0852m/z)
Compound in Library Factor = 0

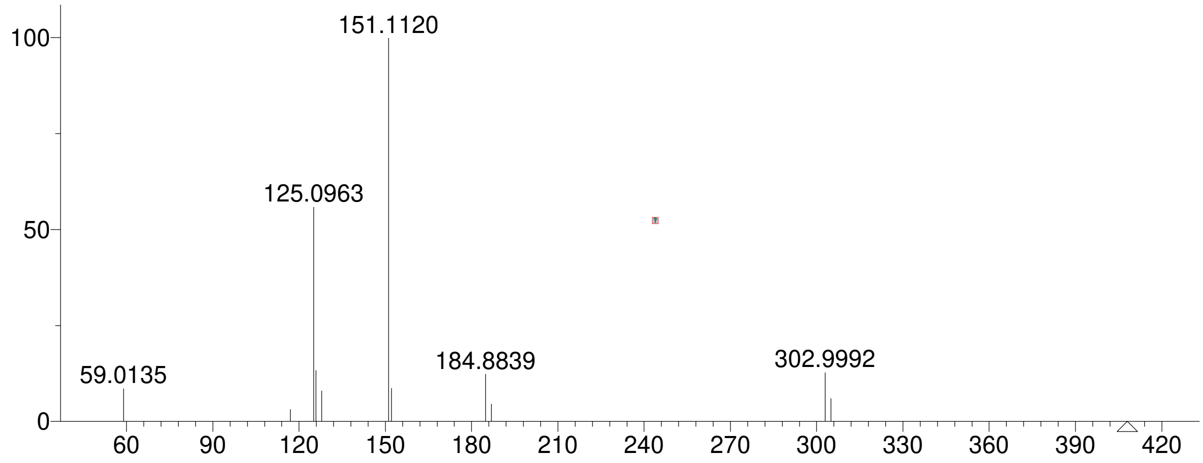

** Search Report Page 1 of 1 **

Unknown: Unknown (18.29_460.0563m/z)
Compound in Library Factor = 0

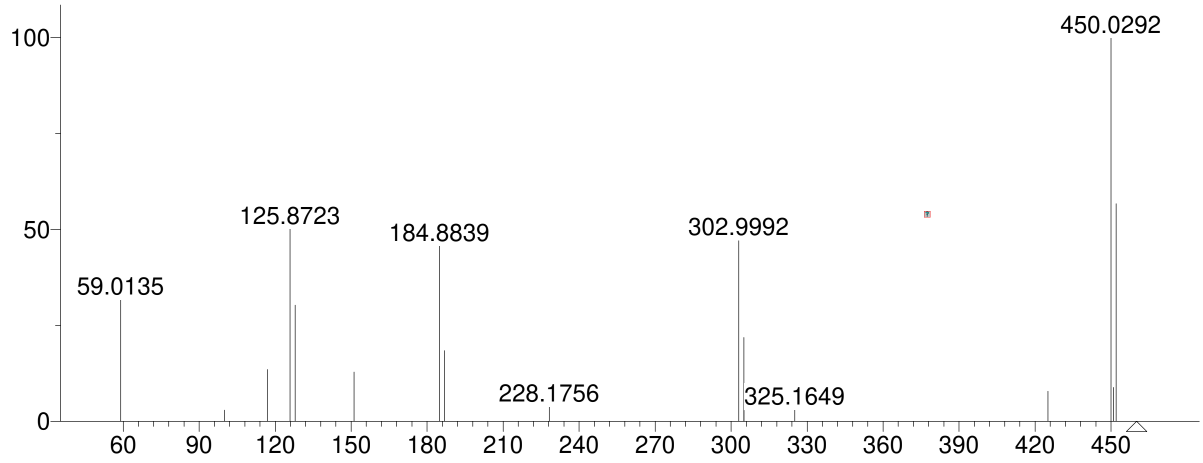

Unknown: Unknown (18.29_660.2601m/z)
Compound in Library Factor = 0

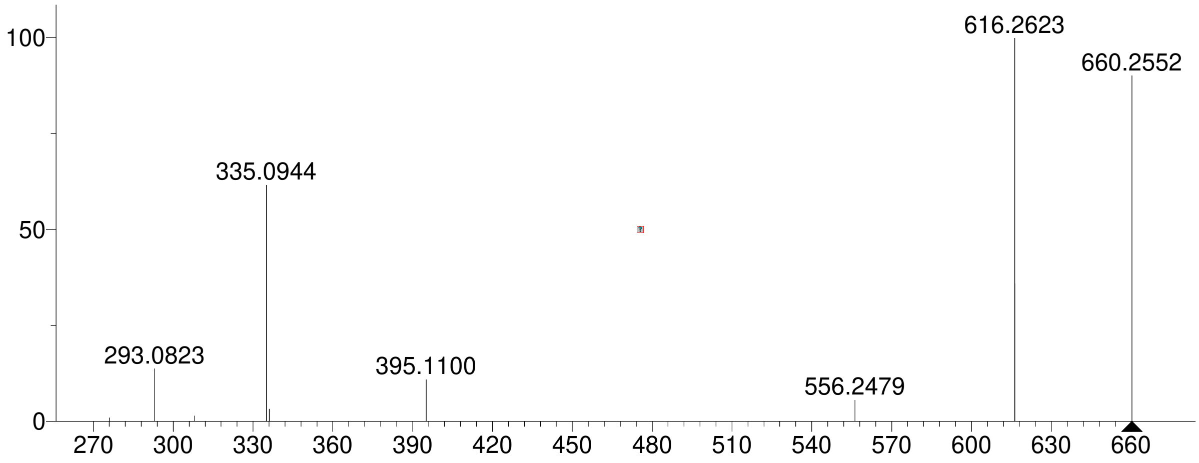

Unknown: Unknown (18.29_704.2502m/z)
Compound in Library Factor = 0

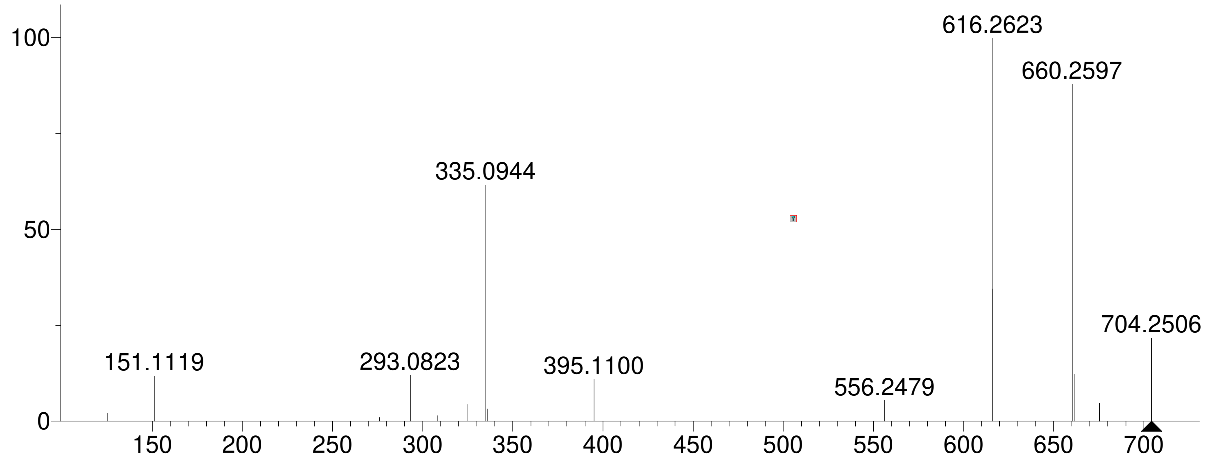

Hit 1 : DFLNHc [M-H+HCO2]2- IT 35% P=704.2
C52H88N2O39; MF: 0; RMF: 20; Prob 100.0%; Lib: nist_msms; ID: 532655.

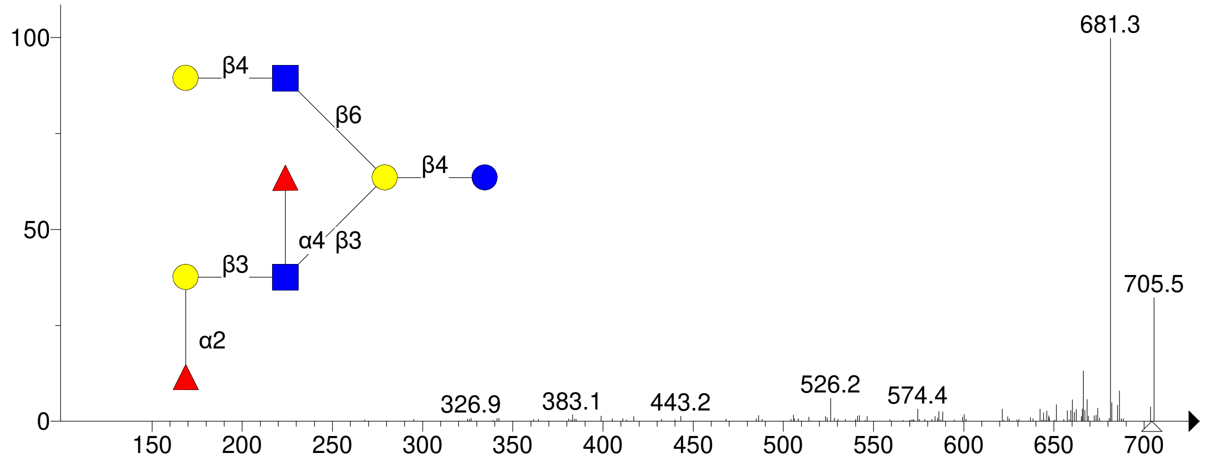

** Search Report Page 1 of 1 **

Unknown: Unknown (18.29_740.2263m/z)
Compound in Library Factor = 0

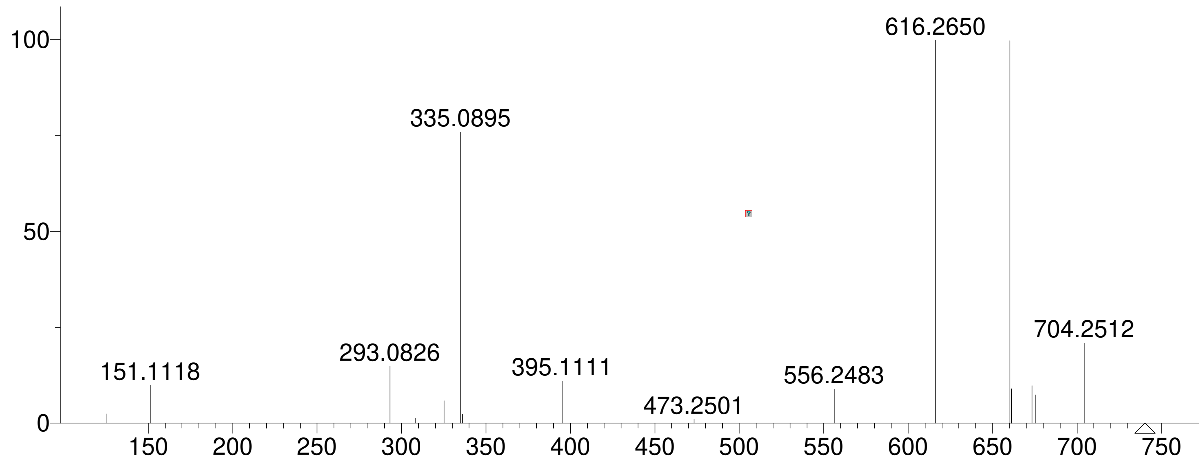

Unknown: Unknown (18.30_675.2961m/z)
Compound in Library Factor = 0

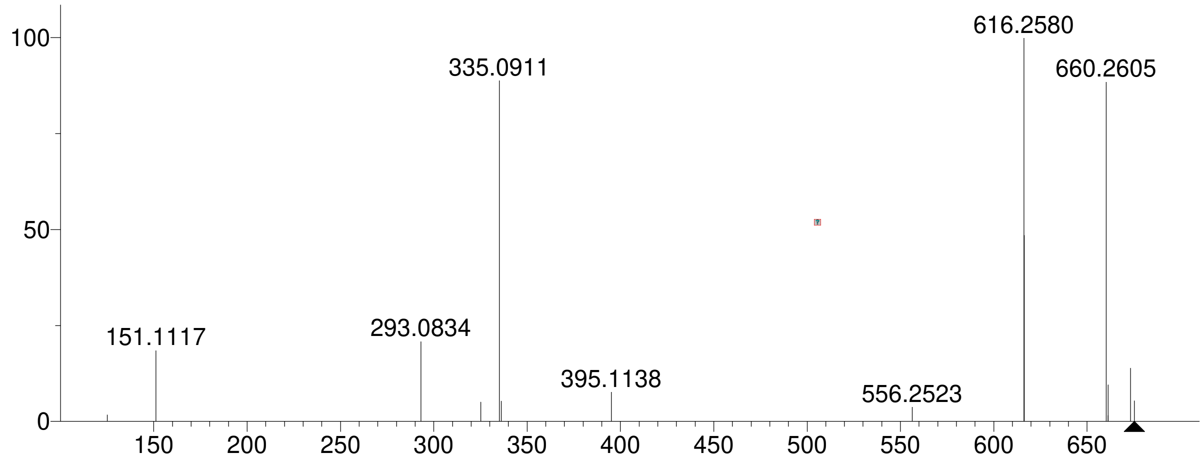

Hit 1 : ATROPINE SULFATE [M-H]- 35HCD P=675.3
C34H48N2O10S; MF: 0; RMF: 25; Prob 100.0%; Lib: mona-export-lc-ms-ms_negative_mode; ID: 23921.

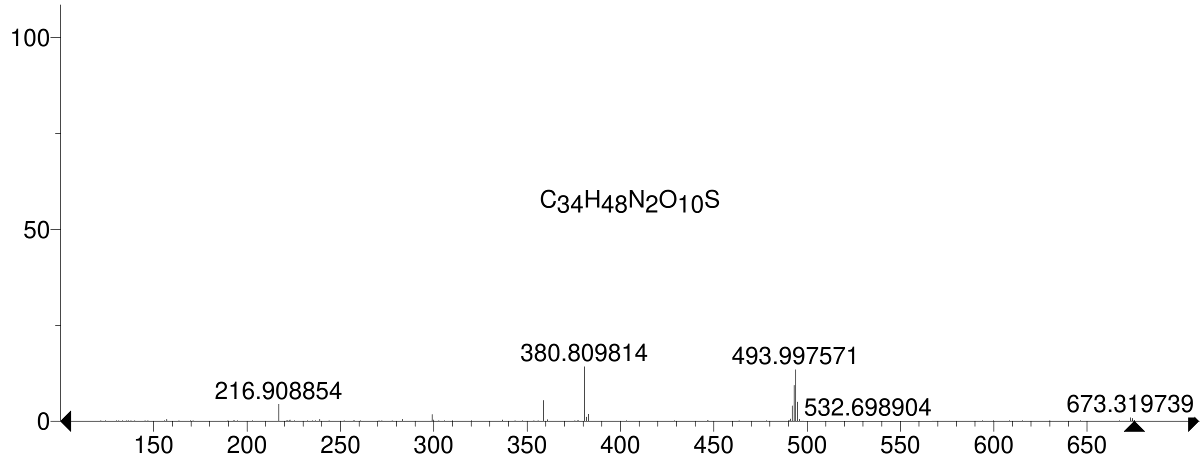

** Search Report Page 1 of 1 **

Unknown: Unknown (18.31_371.1962m/z)
Compound in Library Factor = -3273

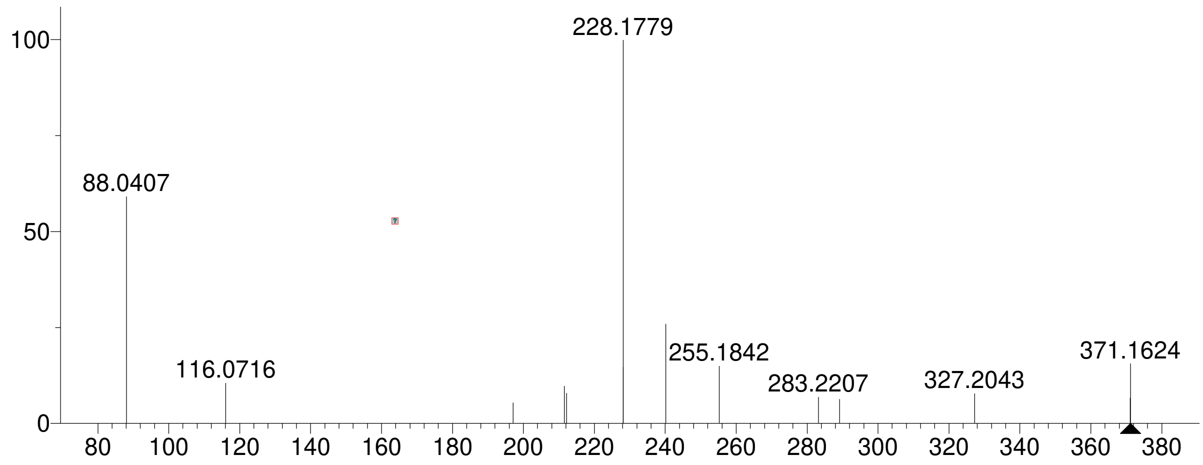

Hit 1 : N,N,Diacetyl-Lys-DAla-DAla [M-H]- HCD 36eV P=371.2
C16H28N4O6; MF: 4; RMF: 297; Prob 100.0%; Lib: nist_msms2; ID: 75619.

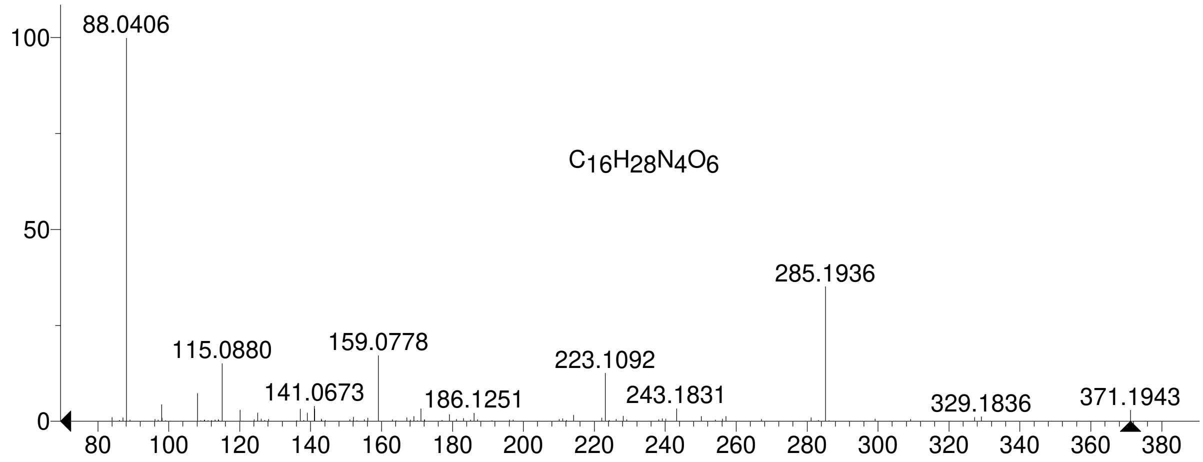

** Search Report Page 1 of 2 **

Unknown: Unknown (18.31_415.1858m/z)
Compound in Library Factor = 0

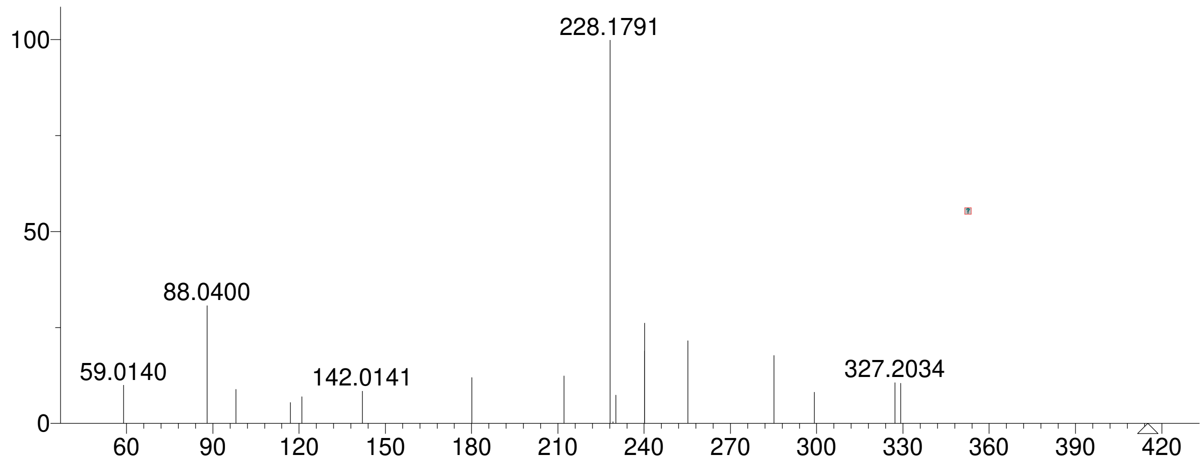

Hit 1 : Dexamethasone [M+Na]+ IT 35% P=415.2
C22H29FO5; MF: 0; RMF: 17; Prob 20.0%; CAS: 50-02-2; Lib: nist_msms; ID: 310175.

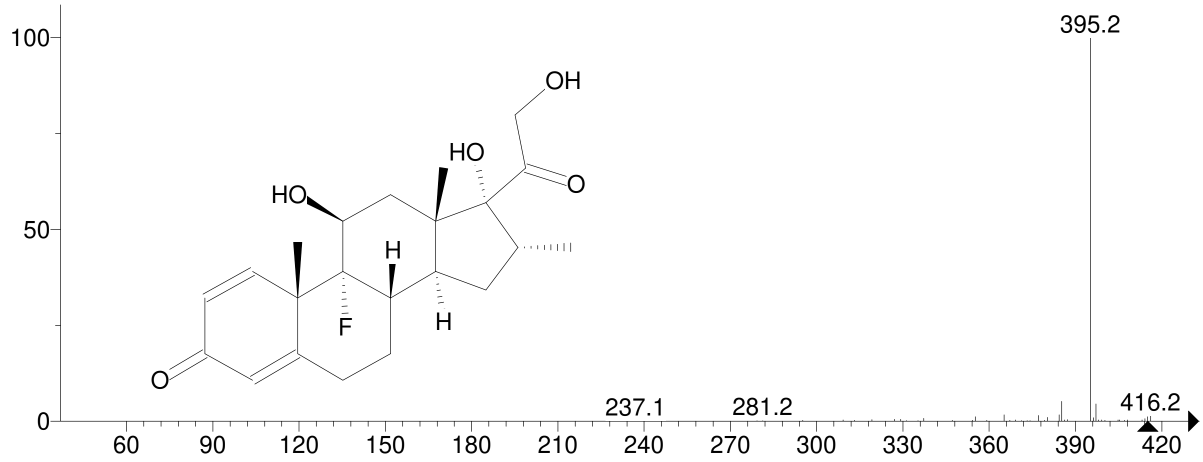

Hit 2 : His-Cys-Arg [M+H]+ IT 35% P=415.2
C15H26N8O4S; MF: 0; RMF: 16; Prob 20.0%; Lib: nist_msms; ID: 47288.

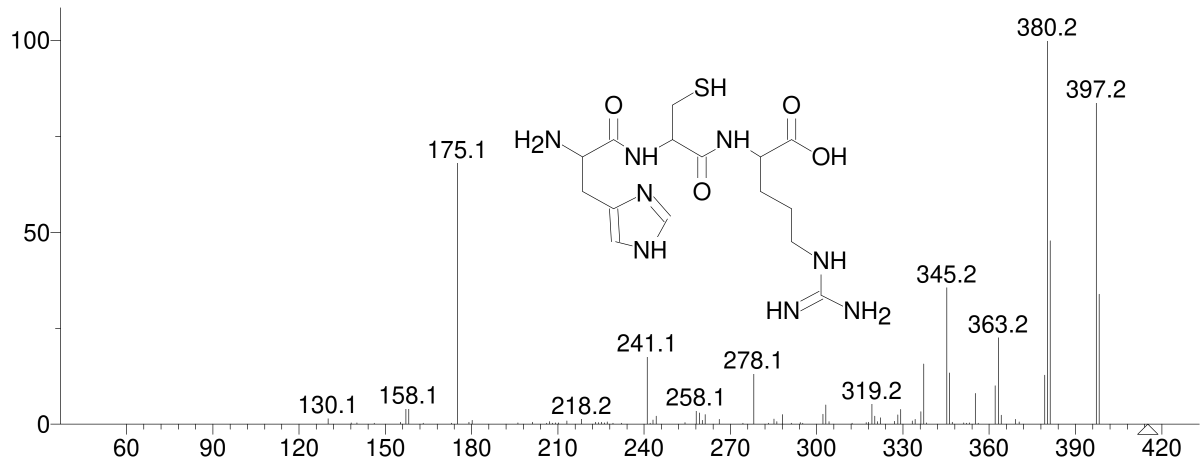

Hit 3 : Cys-His-Arg [M+H]+ QTOF 20V P=415.2
C15H26N8O4S; MF: 0; RMF: 10; Prob 20.0%; Lib: nist_msms; ID: 49596.

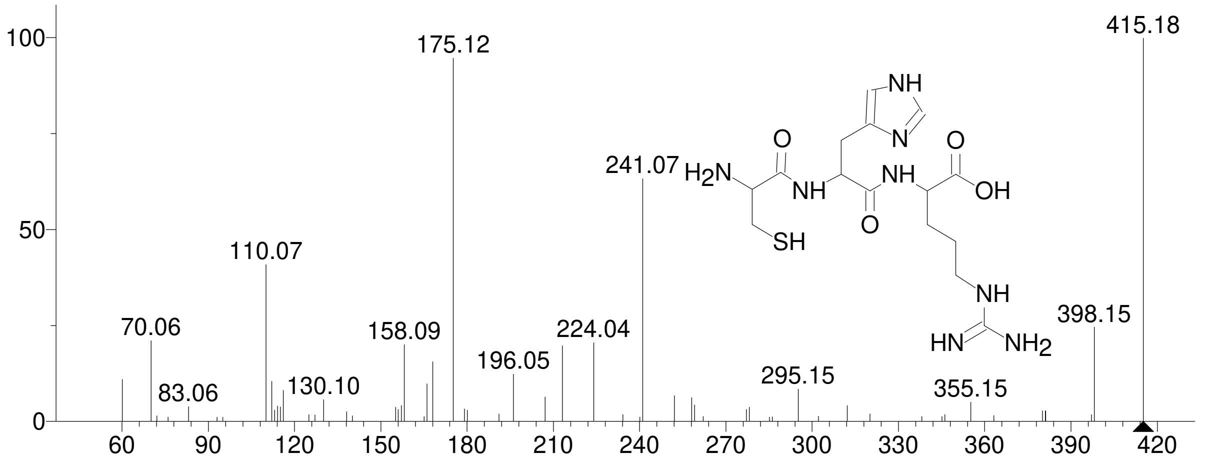

Hit 4 : Betamethasone [M+Na]+ IT 35% P=415.2
C22H29FO5; MF: 0; RMF: 9; Prob 20.0%; CAS: 378-44-9; Lib: nist_msms; ID: 409066.

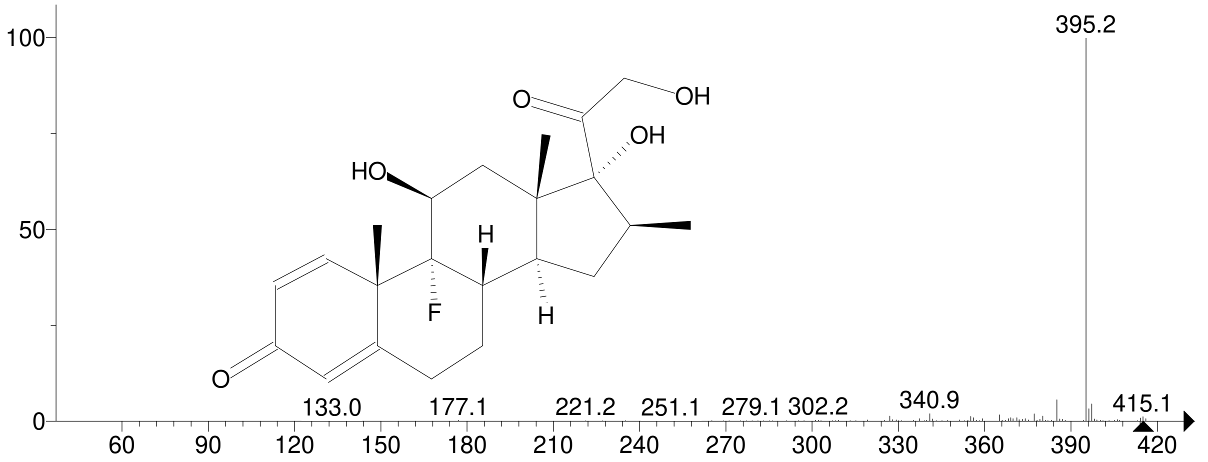

Hit 5 : 2-methoxy-3,5-dimethyl-6-[(4Z)-4-[(E)-2-methyl-3-(4-nitrophenyl)prop-2-enylidene]oxolan-2-yl]pyran-4-one
C22H23NO6; MF: 0; RMF: 3; Prob 20.0%; Lib: mona-export-vf-npl_qexactive; ID: 24481.

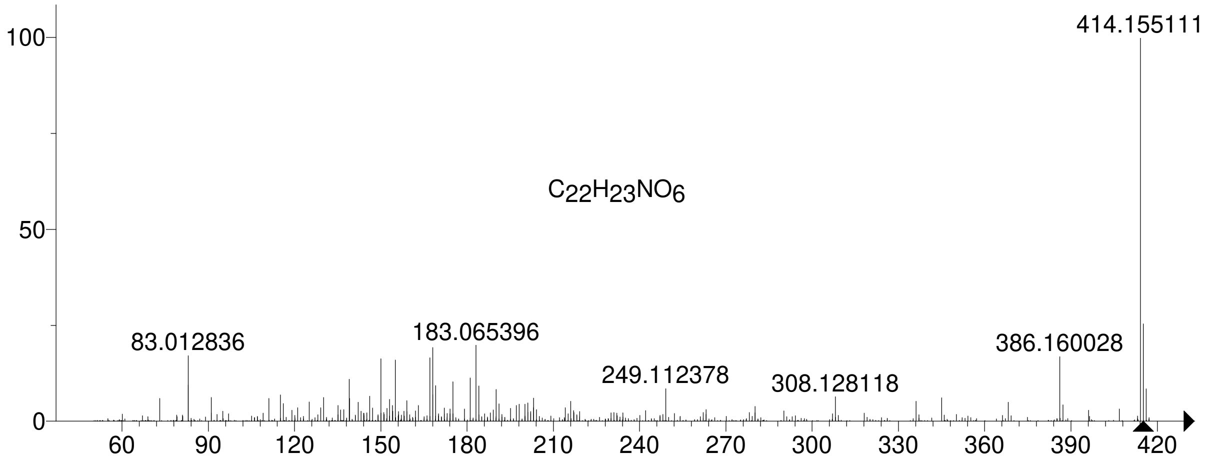

Unknown: Unknown (18.35_706.2631m/z)
Compound in Library Factor = 0

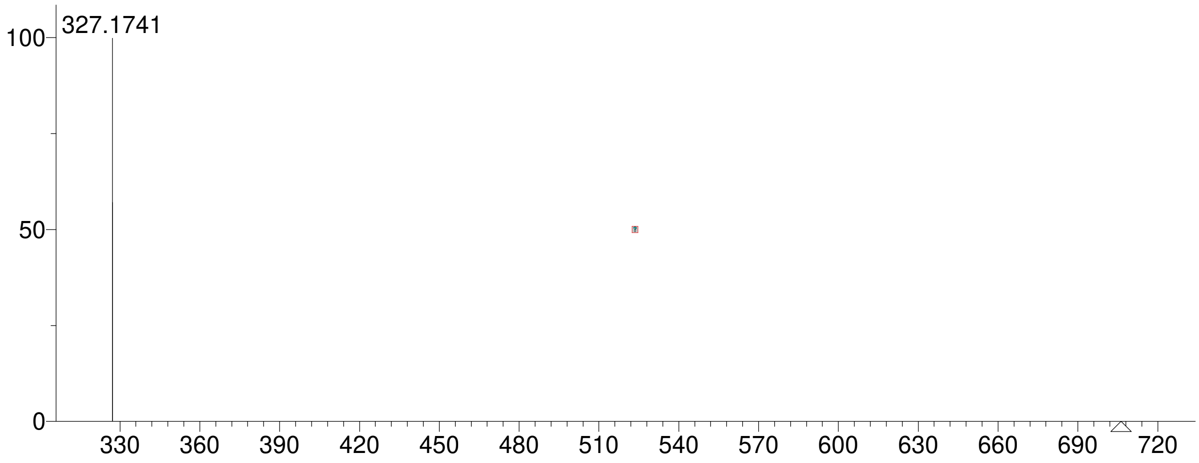

Unknown: Unknown (18.37_311.1853m/z)
Compound in Library Factor = -1848

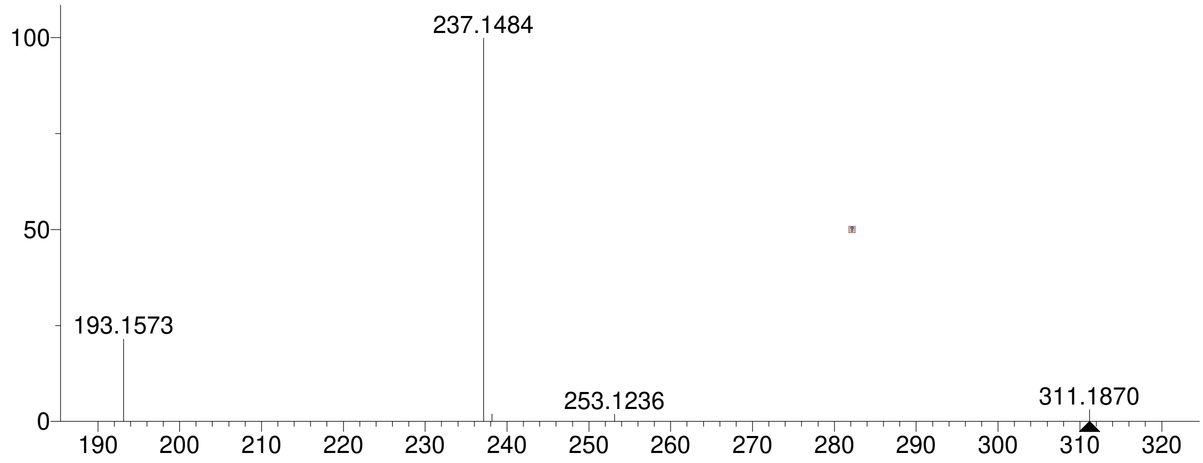

Hit 1 : 1,2-Dihexanoyl-sn-glycerol [M+Na]+ HCD 31V P=311.2
C15H28O5; MF: 37; RMF: 155; Prob 67.4%; CAS: 30403-47-5; Lib: nist_msms; ID: 87073.

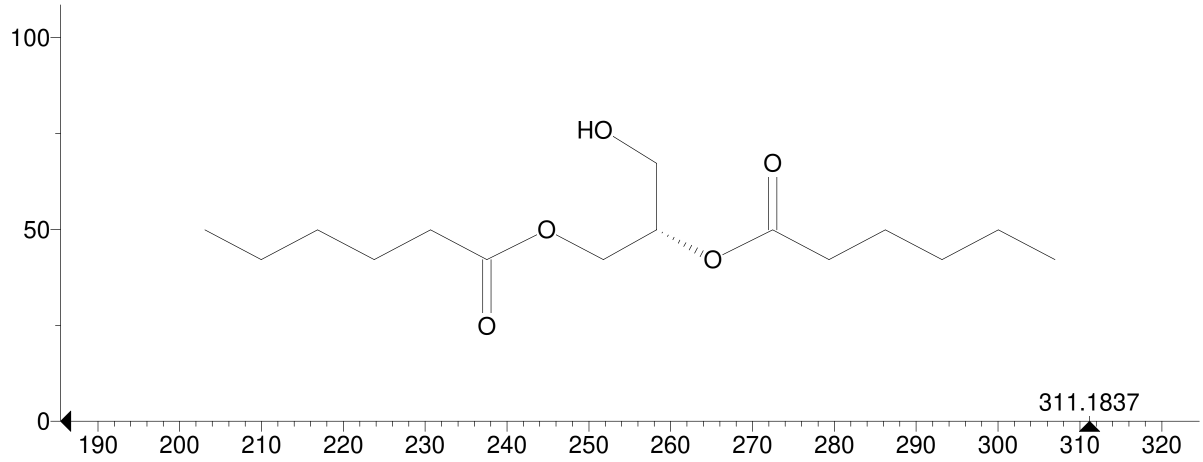

Hit 2 : Gly-Pro-Arg-Pro-amide [M+H-C5H10ON2]+ HCD 15V P=311.2
C18H32N8O4; MF: 3; RMF: 140; Prob 17.3%; CAS: 126047-75-4; Lib: nist_msms; ID: 200731.

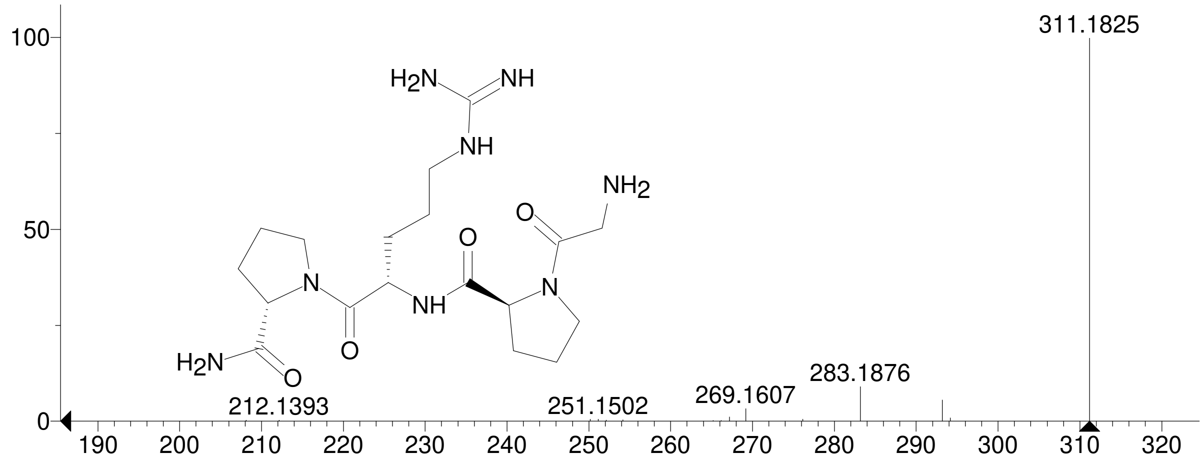

Hit 3 : 2-decyl-3-hydroxypentanedioic acid [M+Na]+ 35HCD P=311.2
C15H28O5; MF: 0; RMF: 34; Prob 15.3%; Lib: mona-export-vf-npl_qexactive; ID: 8950.

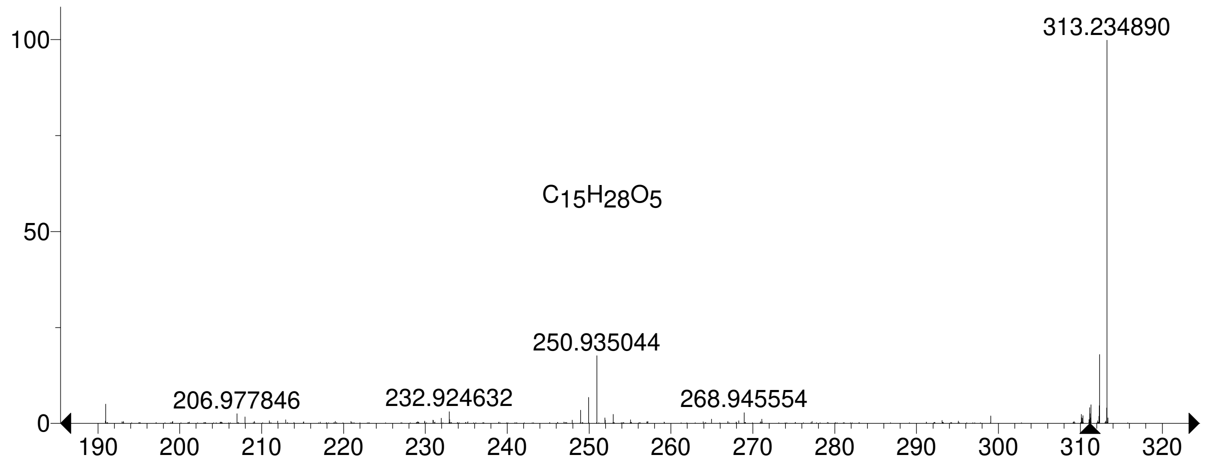

** Search Report Page 1 of 1 **

Unknown: Unknown (18.38_327.3501m/z)
Compound in Library Factor = 0

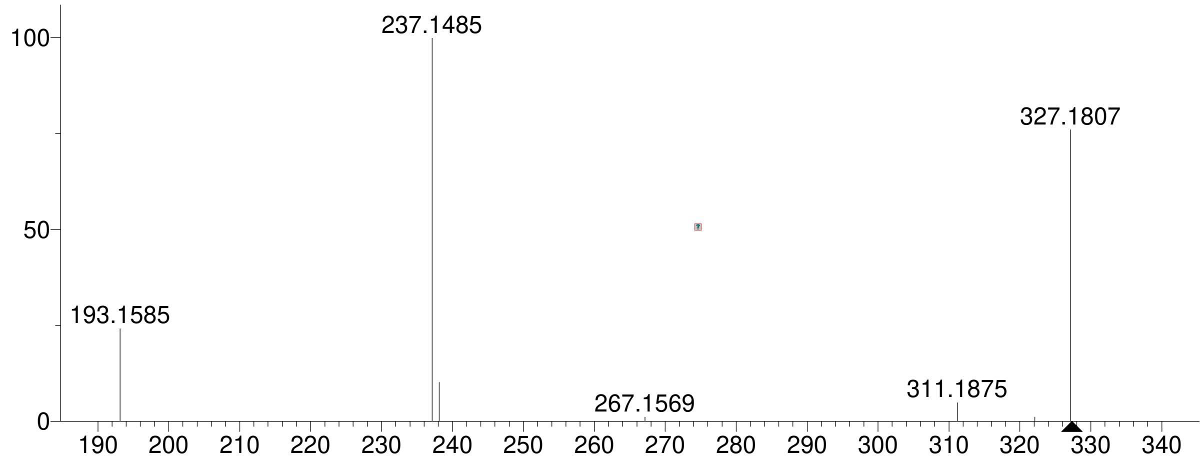

Unknown: Unknown (18.38_341.1418m/z)
Compound in Library Factor = -1523

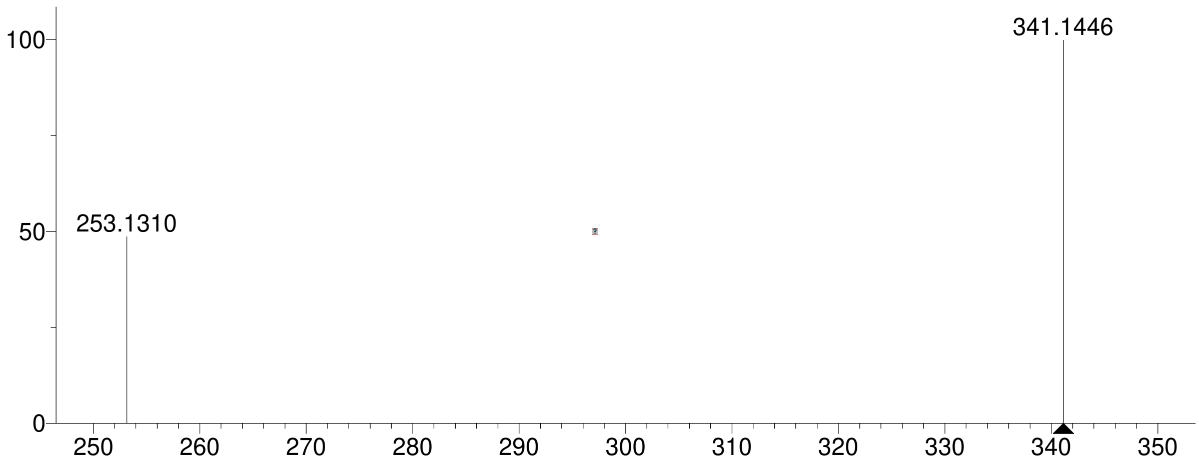

Hit 1 : Matairesinol [M+H-H2O]+ HCD 13V P=341.1
C20H22O6; MF: 130; RMF: 777; Prob 45.6%; CAS: 580-72-3; Lib: nist_msms; ID: 339853.

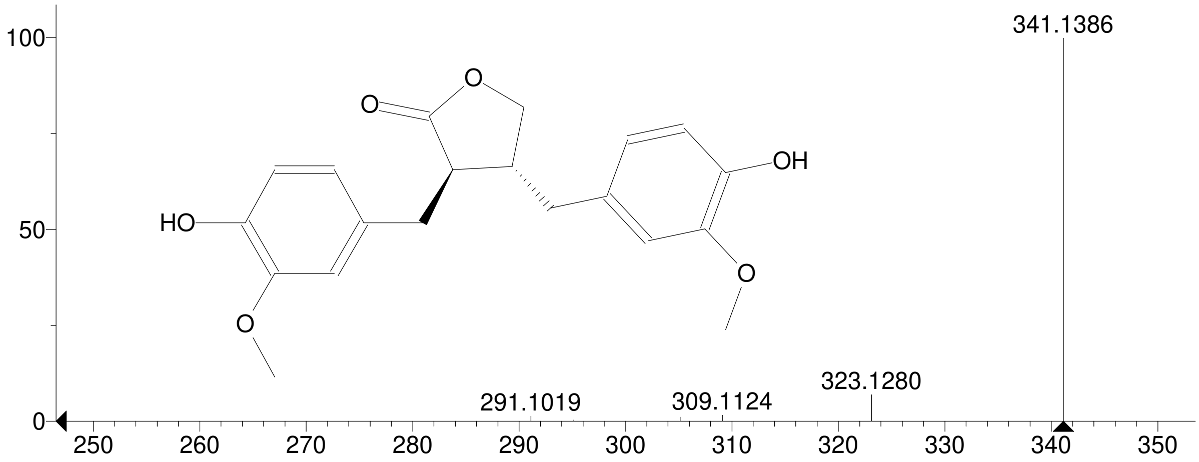

Hit 2 : 4H-Pyrrolo[3,2-c]pyridin-4-one, 1,5,6,7-tetrahydro-2-[2-(3-quinolinyl)-4-pyridinyl]- [M+H]+ HCD 34V P=34
C21H16N4O; MF: 125; RMF: 862; Prob 36.7%; CAS: 724711-21-1; Lib: nist_msms; ID: 421538.

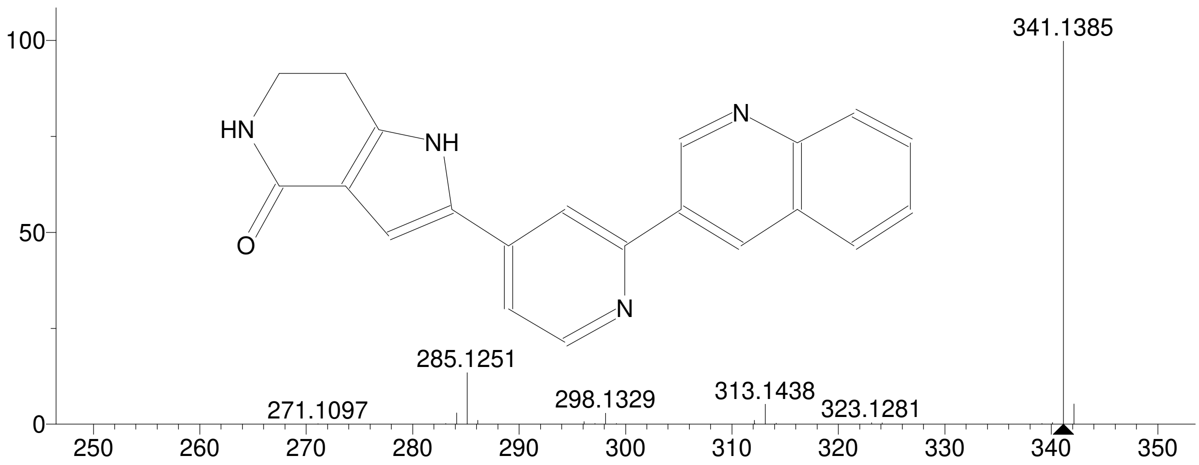

Hit 3 : 2',3,4,5,6'-Pentamethoxychalcone [M+H-H2O]+ HCD 17V P=341.1
C20H22O6; MF: 83; RMF: 718; Prob 8.24%; CAS: 944447-14-7; Lib: nist_msms; ID: 111167.

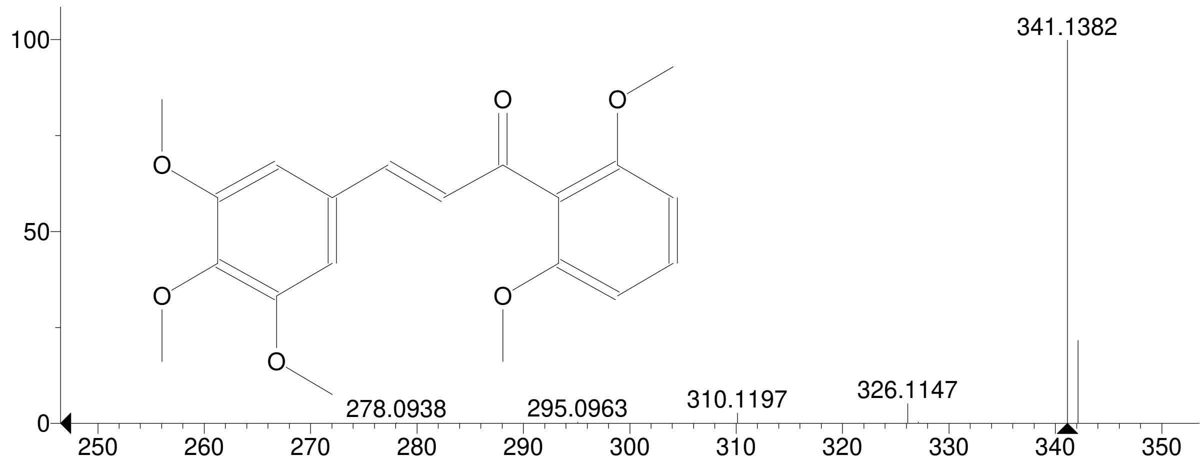

Hit 4 : (+)-Pinoresinol [M+H-H2O]+ HCD 10V P=341.1
C20H22O6; MF: 76; RMF: 741; Prob 6.31%; CAS: 487-36-5; Lib: nist_msms; ID: 234395.

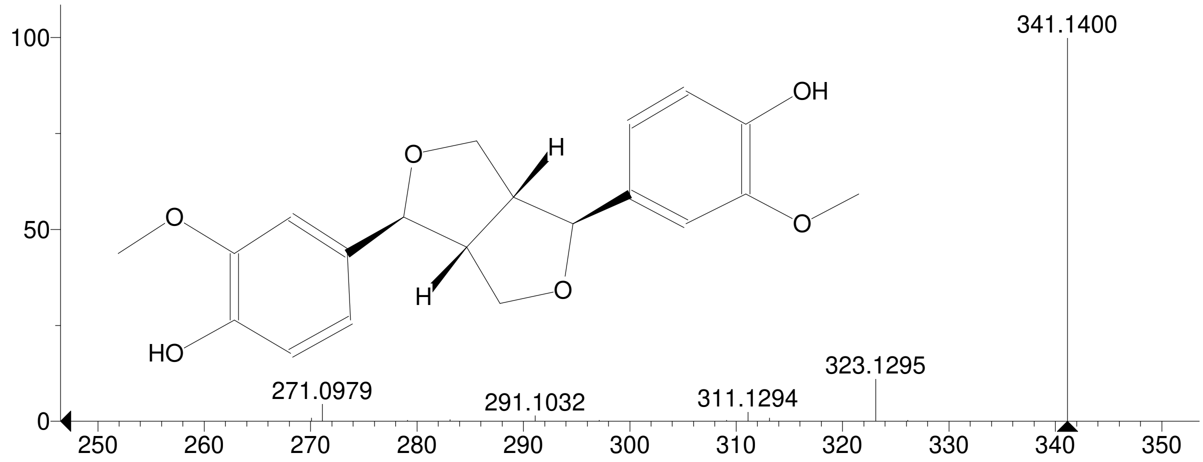

Hit 5 : 3-(3,4-Dimethoxyphenyl)-6-ethoxy-4-methylcoumarin [M+H]+ HCD 88V P=341.1
C20H20O5; MF: 43; RMF: 482; Prob 1.65%; CAS: 720674-30-6; Lib: nist_msms; ID: 109799.

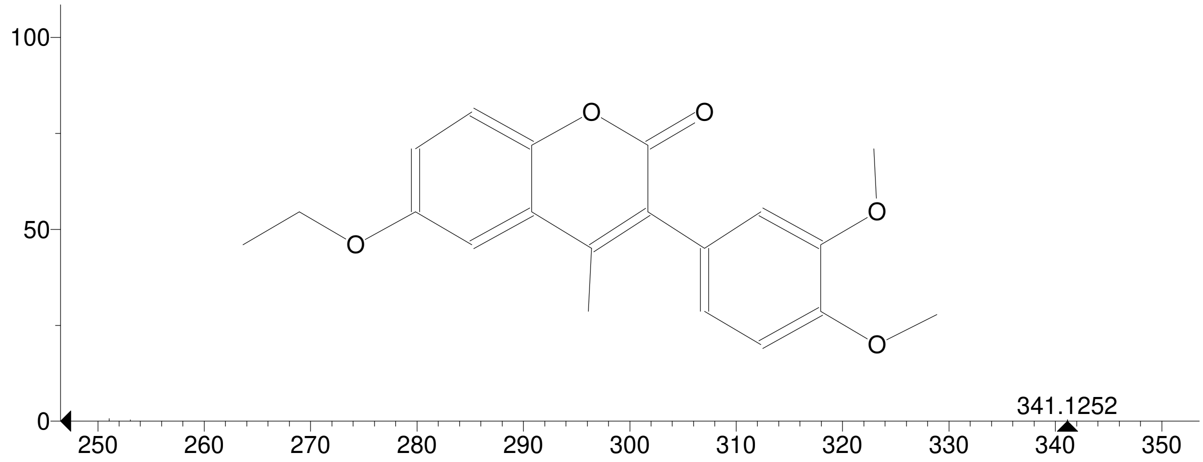

** Search Report Page 1 of 1 **

Unknown: Unknown (18.38_395.1668m/z)
Compound in Library Factor = 0

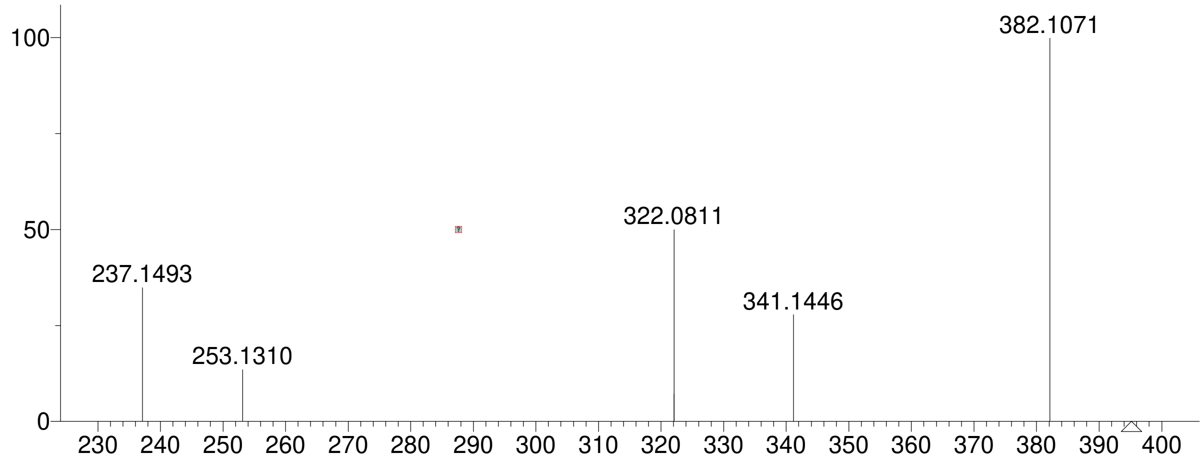

Unknown: Unknown (18.45_405.1214m/z)
Compound in Library Factor = -3273

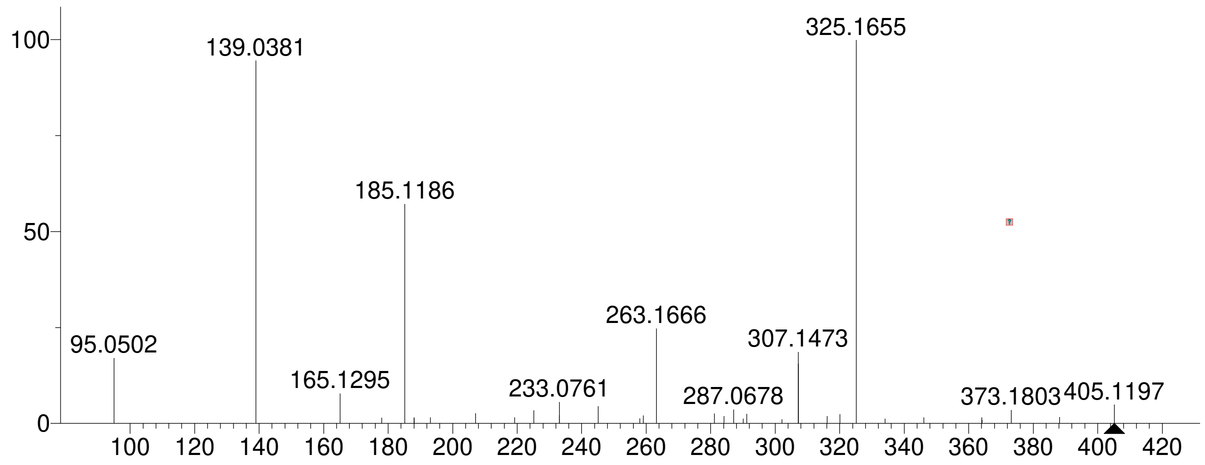

Hit 1 : 2,3,4',5-Tetrahydroxystilbene 2-glucoside [M-H]- HCD 12V P=405.1
C20H22O9; MF: 11; RMF: 143; Prob 11.8%; CAS: 82373-94-2; Lib: nist_msms; ID: 240828.

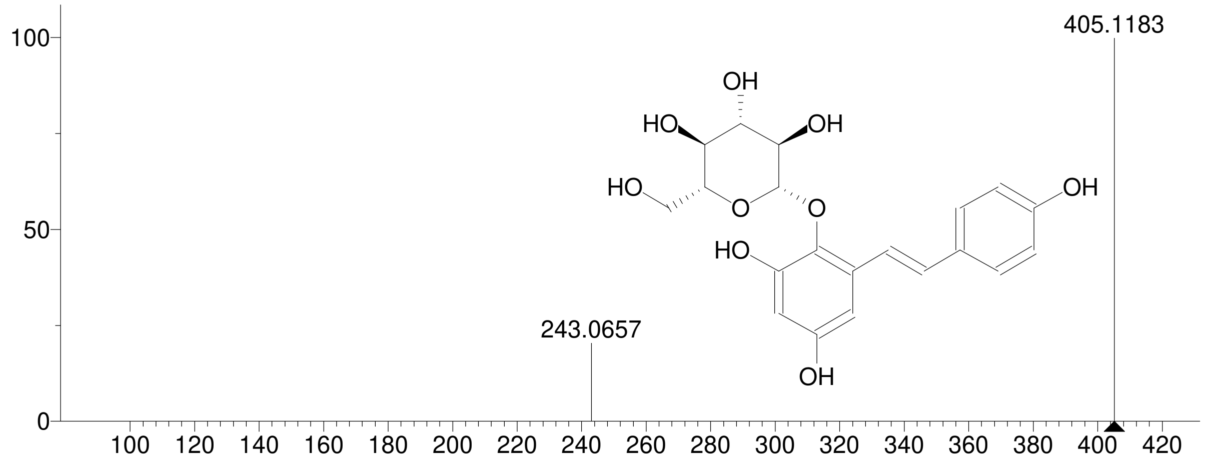

Hit 2 : 7-[3,4-dihydroxy-5-(hydroxymethyl)oxolan-2-yl]oxy-2-(4-hydroxyphenyl)-3,4-dihydro-2H-chromene-3,5-diol
C20H22O9; MF: 2; RMF: 125; Prob 8.58%; Lib: anthocyanins mona; ID: 2182.

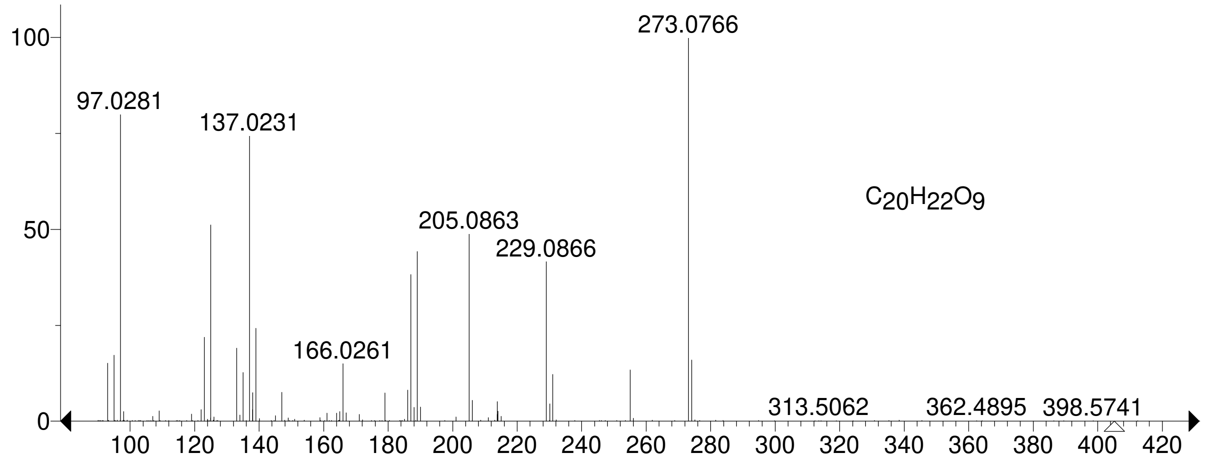

Hit 3 : Resveratrol 4'-O-D-glucuronide [M+H]+ HCD 8V P=405.1
C20H20O9; MF: 1; RMF: 99; Prob 8.24%; CAS: 387372-20-5; Lib: nist_msms; ID: 189809.

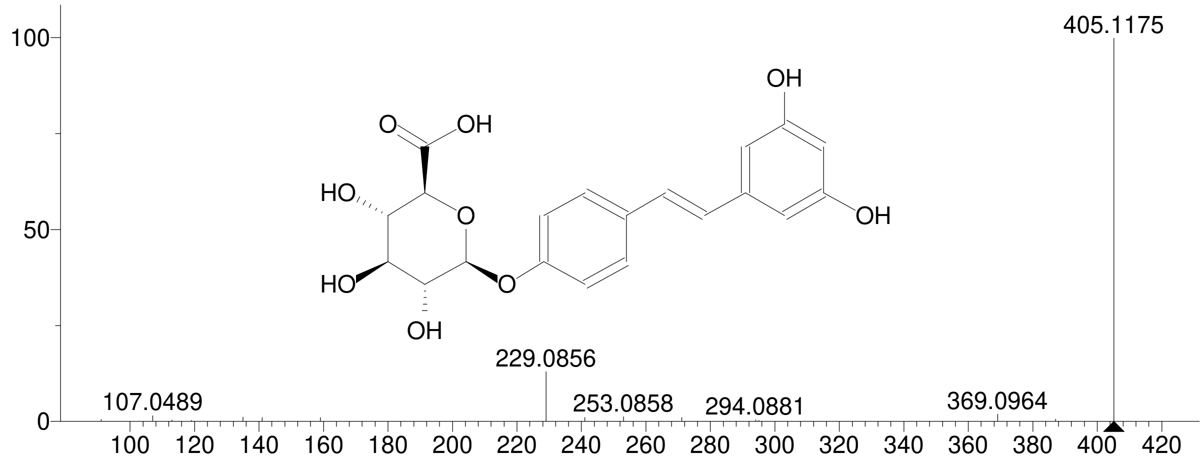

Hit 4 : Strophanthidin [M+H]+ 35% P=405.1
C23H32O6; MF: 0; RMF: 74; Prob 7.92%; Lib: mona-export-itree; ID: 988.

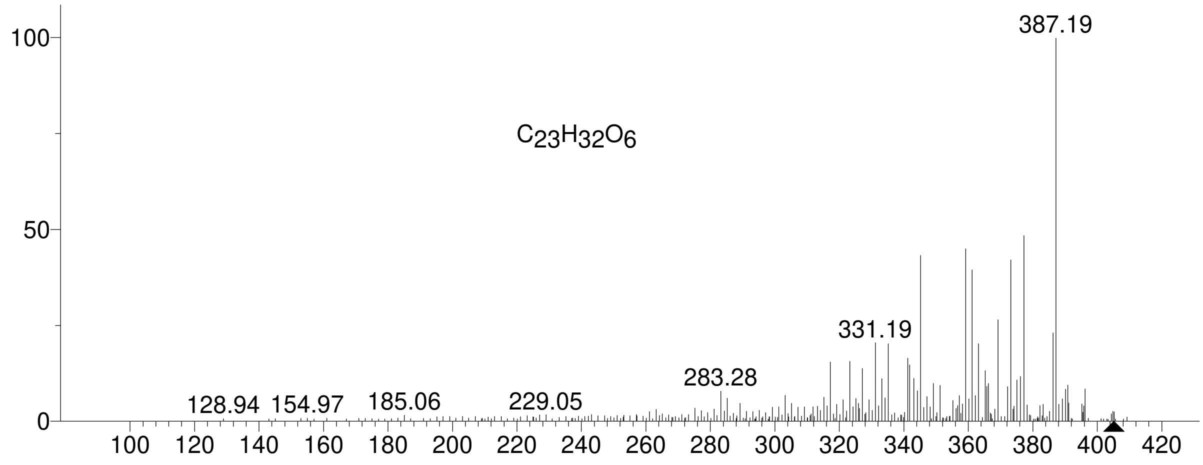

Hit 5 : 5,7-dihydroxy-3,6-dimethoxy-2-(3,4,5-trimethoxyphenyl)-4H-chromen-4-one [M+H]+ P=405.1
C20H20O9; MF: 0; RMF: 46; Prob 7.92%; Lib: mona-export-gnps; ID: 4640.

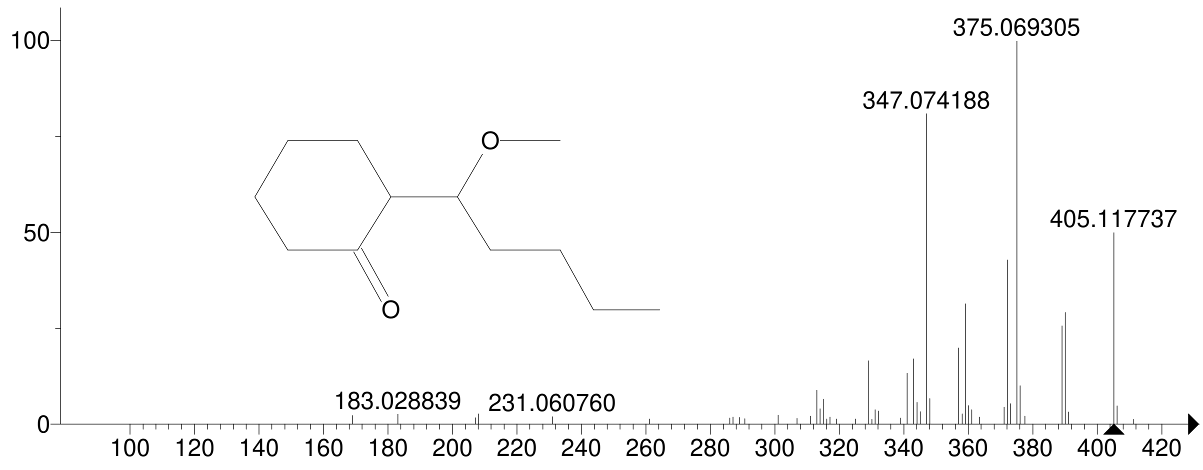

Unknown: Unknown (18.45_437.1921m/z)
Compound in Library Factor = -3273

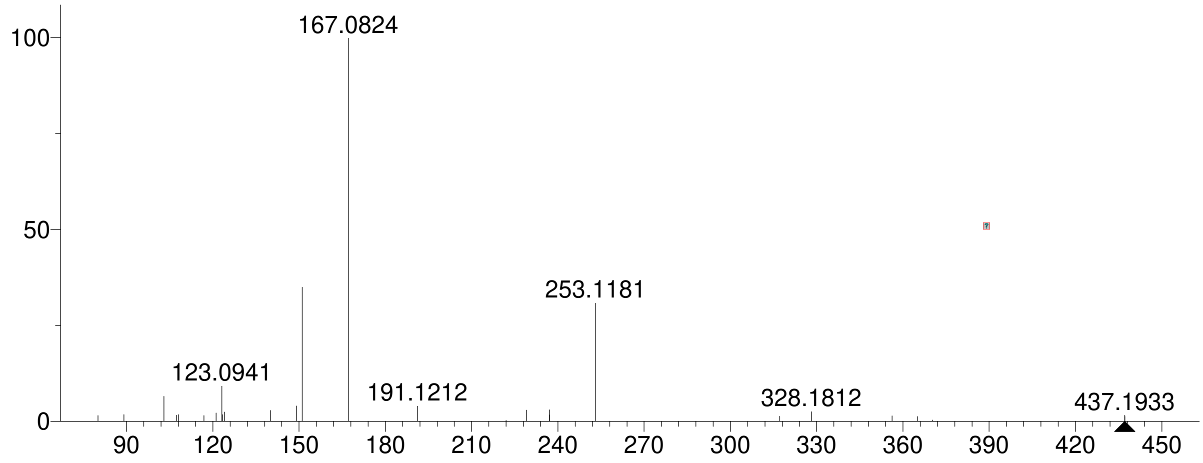

Hit 1 : Ethynylestradiol 17-β-D-glucuronide [M+H-2H2O]+ HCD 13V P=437.2
C26H32O8; MF: 5; RMF: 88; Prob 22.0%; CAS: 75803-39-3; Lib: nist_msms; ID: 438306.

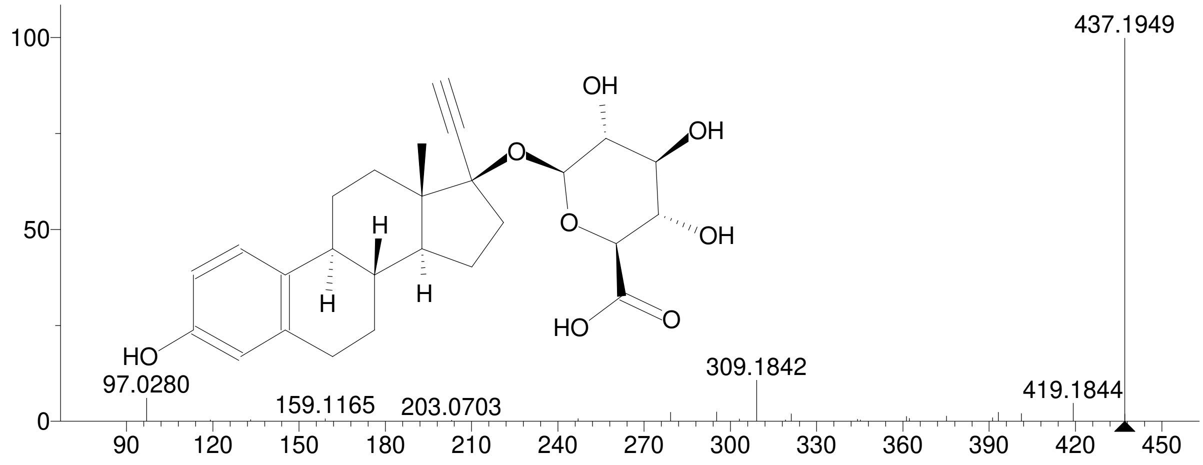

Hit 2 : Ethynylestradiol 3-β-D-glucuronide [M+H-2H2O]+ HCD 13V P=437.2
C26H32O8; MF: 5; RMF: 85; Prob 22.0%; CAS: 60134-76-1; Lib: nist_msms; ID: 438189.

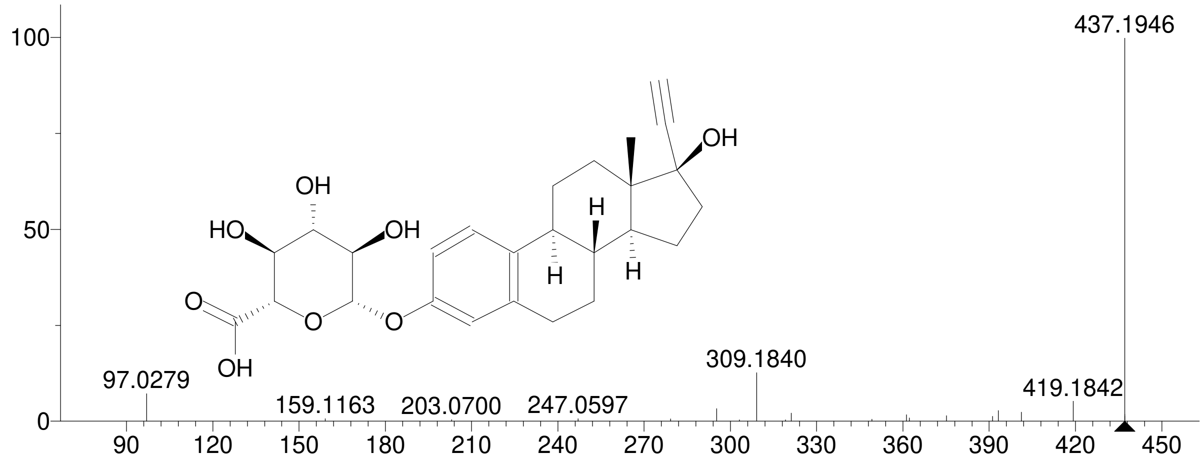

Hit 3 : 17-Trifluoromethylphenyltrinorprostaglandin F2α [M-H-H2O]- HCD 17V P=437.2
C24H31F3O5; MF: 2; RMF: 95; Prob 19.4%; CAS: 221246-34-0; Lib: nist_msms; ID: 93923.

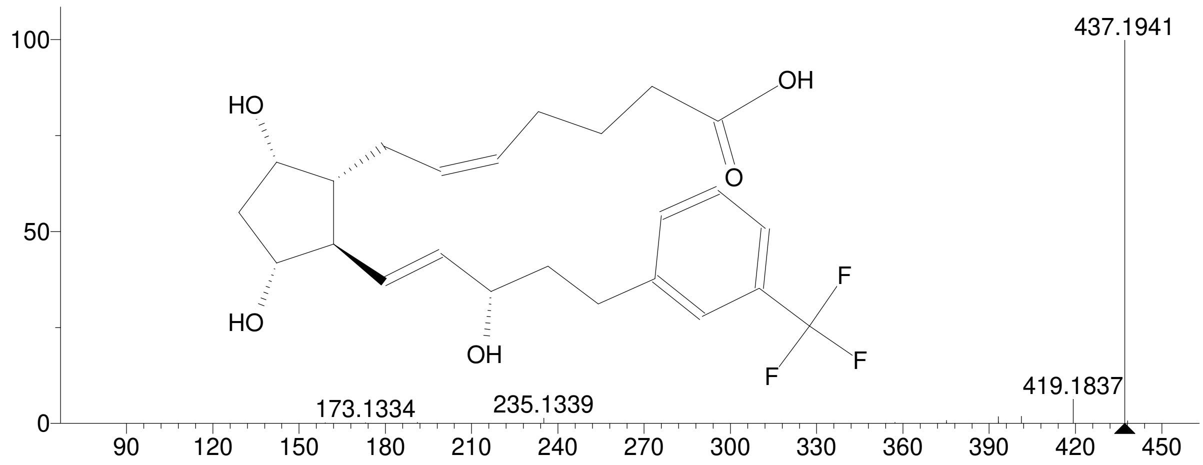

Hit 4 : NCGC00381156-01_C24H30O6_Methyl 3-[(1E,3E)-3,5-dimethyl-1,3-heptadien-1-yl]-8-hydroxy-6a,8-dimetl
C24H30O6; MF: 1; RMF: 78; Prob 18.7%; Lib: mona-export-gnps; ID: 13910.

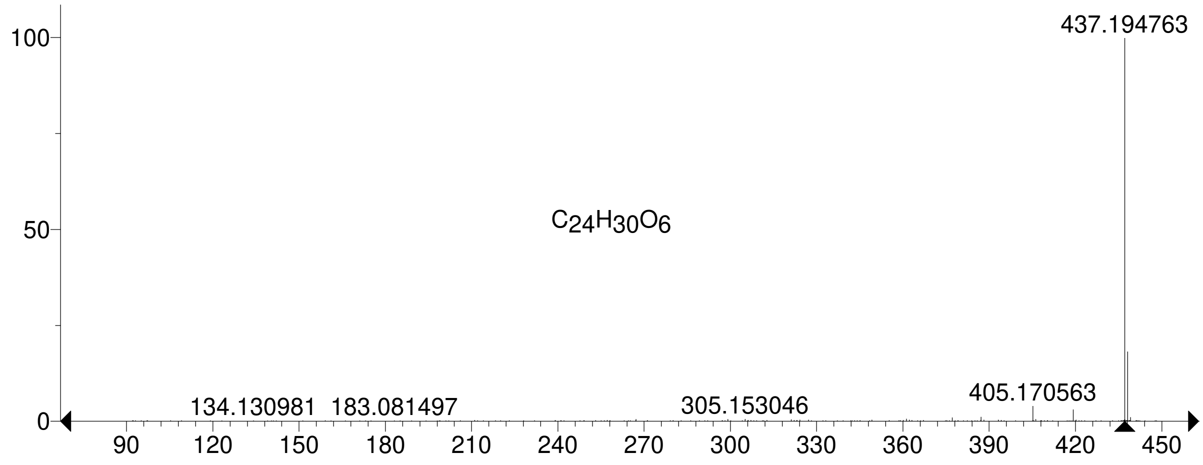

Hit 5 : NCGC00384865-01!5-hydroxy-7-[4-hydroxy-2-methoxy-3-(3-methylbut-2-enyl)phenyl]-2,2-dimethyl-7,8-dih
C26H28O6; MF: 0; RMF: 14; Prob 17.9%; Lib: mona-export-gnps; ID: 7877.

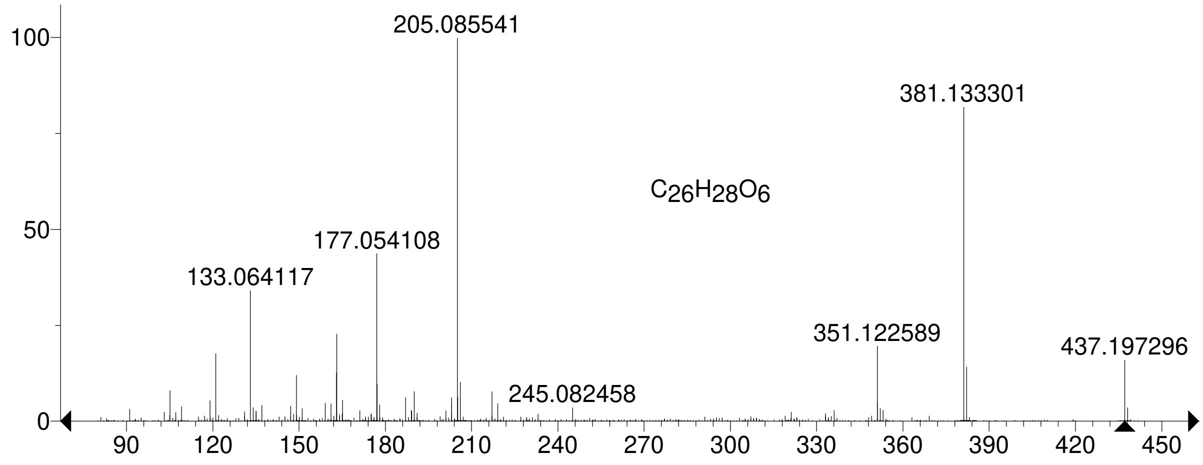

Unknown: Unknown (18.53_373.0225m/z)
Compound in Library Factor = -3273

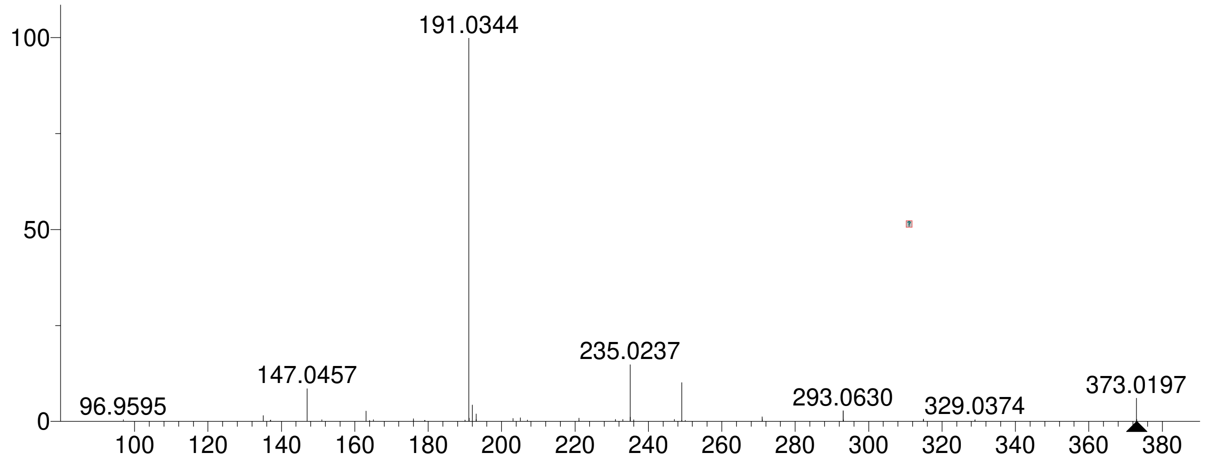

Hit 1 : Transfluthrin [M+H+2i]+ HCD 13V P=373
C15H12Cl2F4O2; MF: 2; RMF: 128; Prob 100.0%; CAS: 118712-89-3; Lib: nist_msms; ID: 521809.

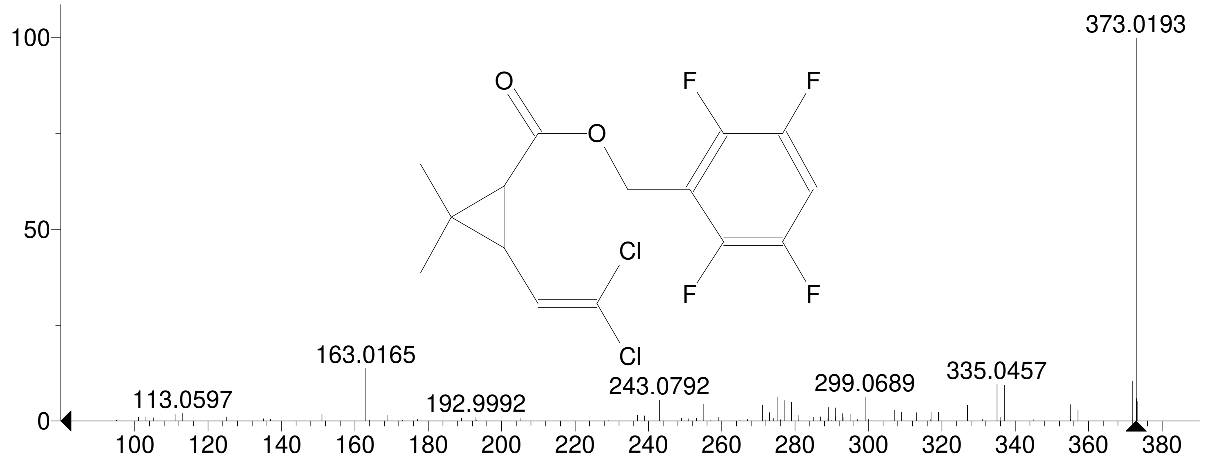

Unknown: Unknown (18.53_544.2282m/z)
Compound in Library Factor = 0

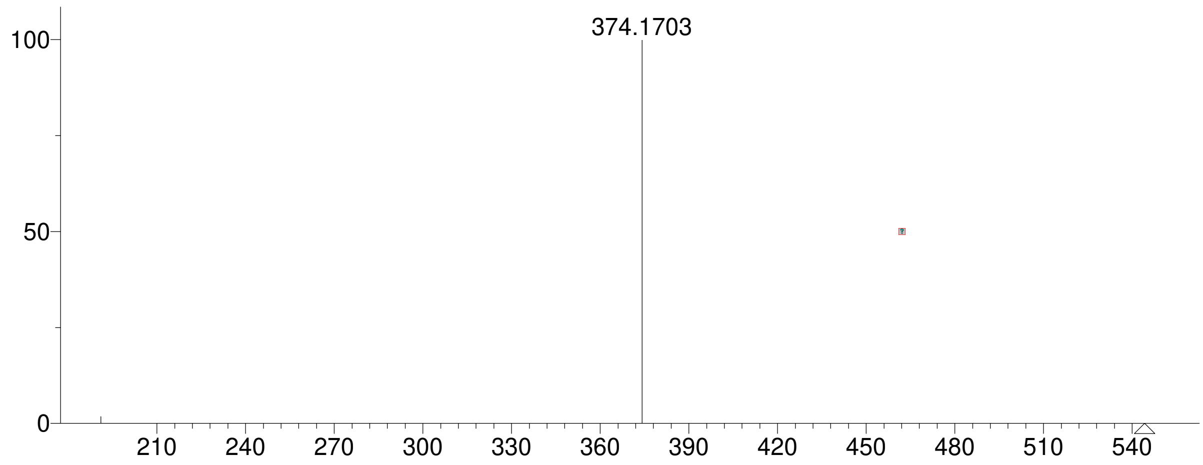

Unknown: Unknown (18.62_423.2127m/z)
Compound in Library Factor = -3273

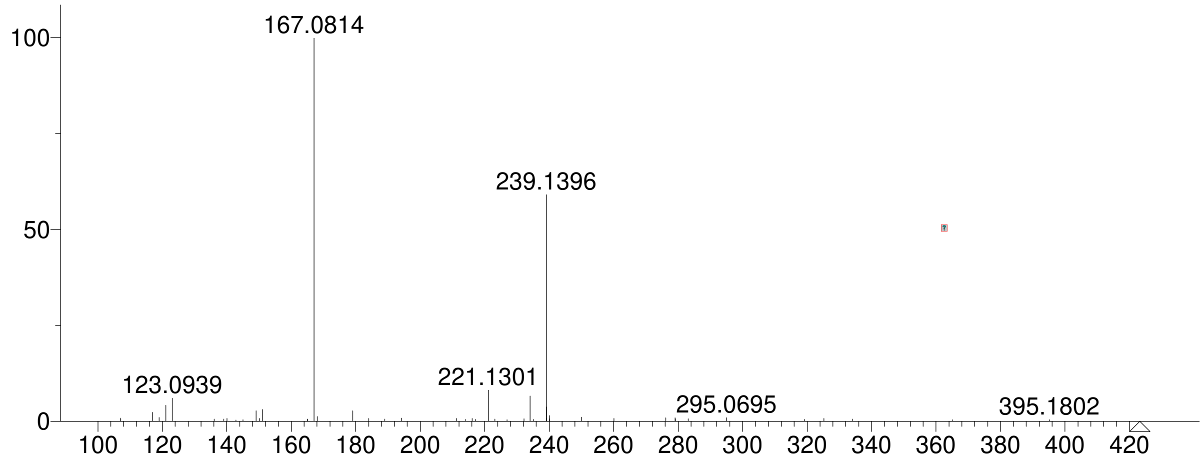

Hit 1 : Cinobufotalin [M+H-2H2O]+ HCD 63V P=423.2
C26H34O7; MF: 7; RMF: 141; Prob 20.6%; CAS: 1108-68-5; Lib: nist_msms; ID: 391347.

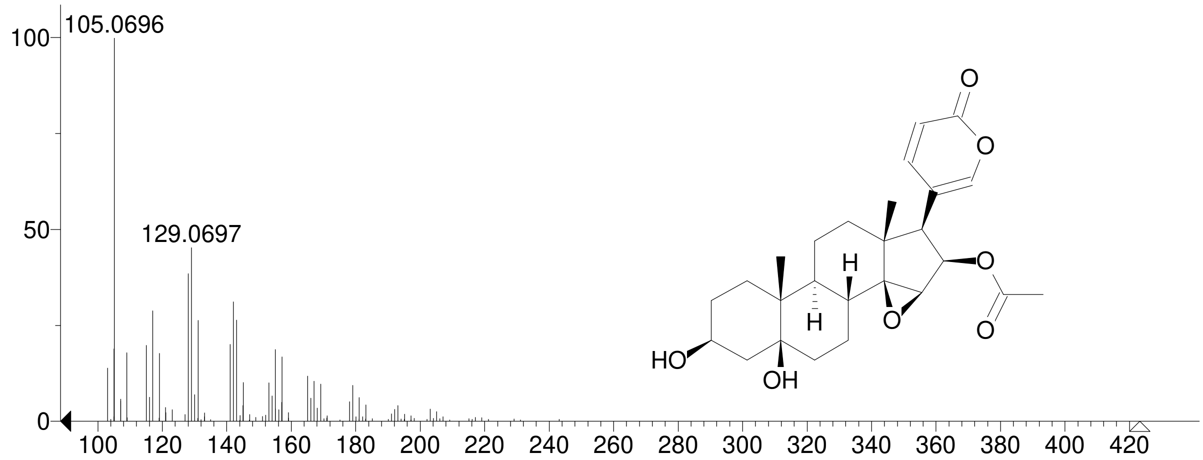

Hit 2 : [4,5-dihydroxy-3,4-bis(hydroxymethyl)-4a,8,8-trimethyl-5,6,7,8a-tetrahydro-1H-naphthalen-1-yl] hexanoate
C21H36O6; MF: 2; RMF: 85; Prob 16.6%; Lib: mona-export-vf-npl_qexactive; ID: 8154.

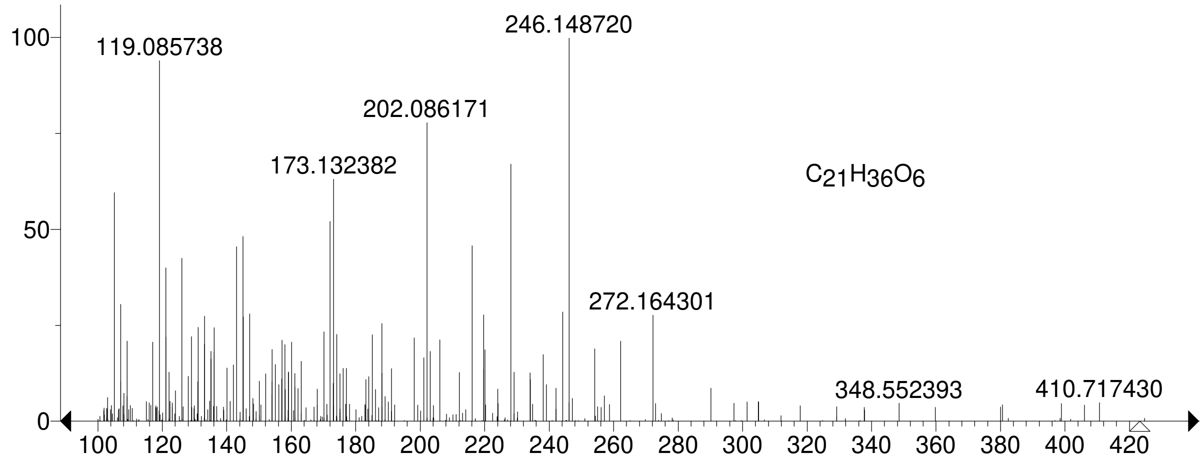

Hit 3 : 4-Methylabyssinone V [M+H]+ 65HCD P=423.2
C26H30O5; MF: 1; RMF: 58; Prob 16.0%; Lib: mona-export-vf-npl_qexactive; ID: 6033.

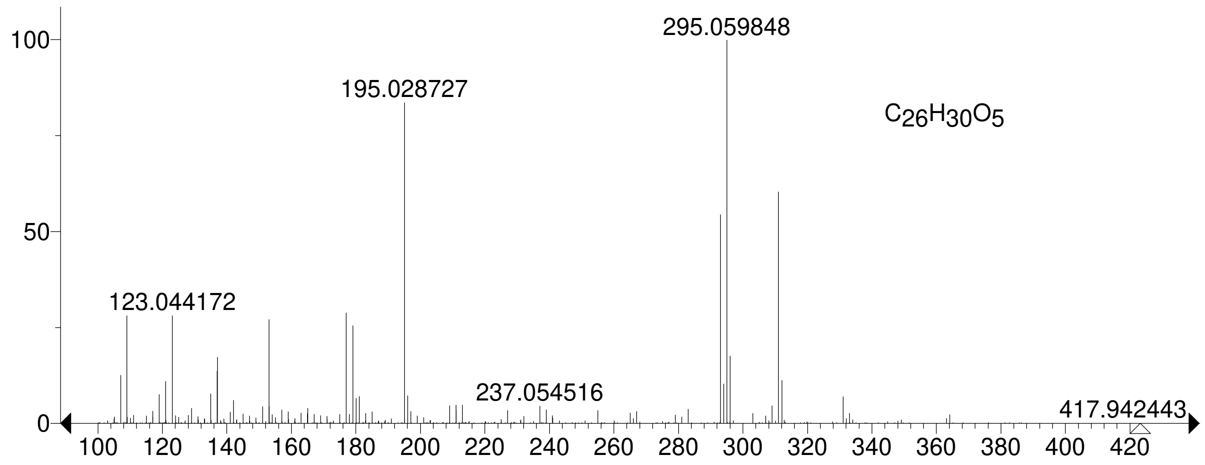

Hit 4 : NCGC00384666-01!(2S)-5,7-dihydroxy-2-[4-methoxy-3,5-bis(3-methylbut-2-enyl)phenyl]-2,3-dihydrochrom
C26H30O5; MF: 1; RMF: 43; Prob 16.0%; Lib: mona-export-gnps; ID: 7800.

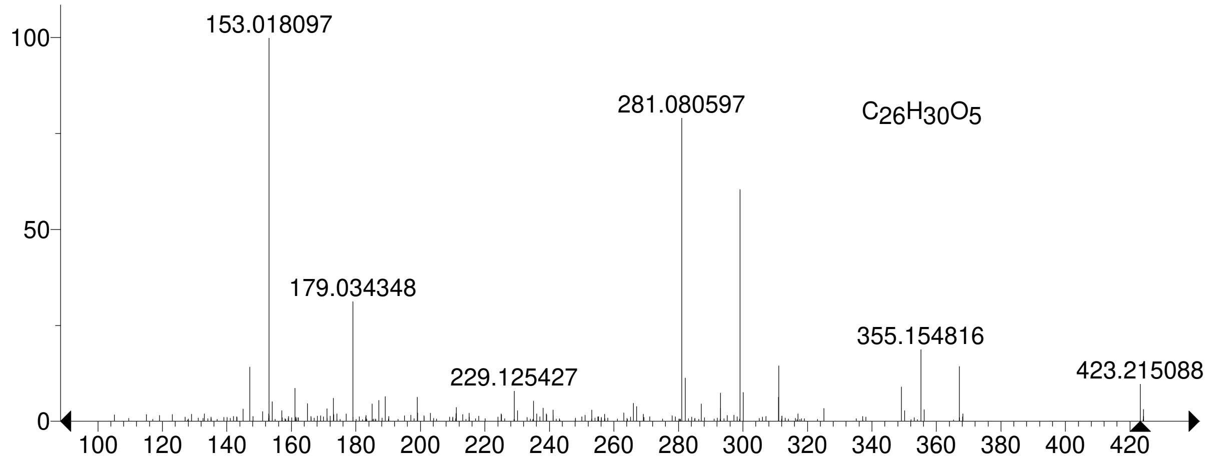

Hit 5 : PA 16:0; PA 7:0-9:0; [M-H]- [M-H]- P=423.2
C19H37O8P; MF: 0; RMF: 9; Prob 15.4%; Lib: msdial-insilicomsms-lipids-neg; ID: 315973.

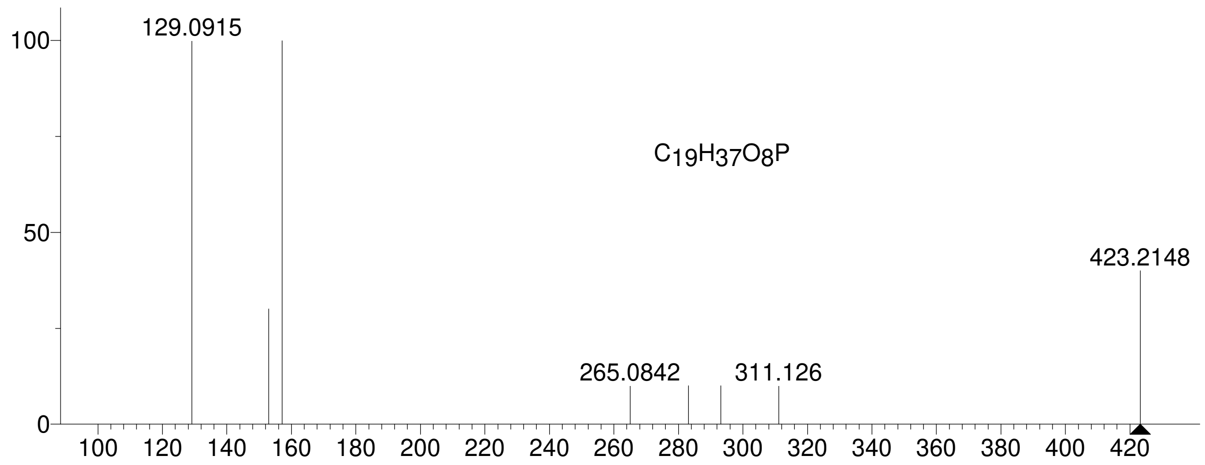

Unknown: Unknown (18.62_523.1368m/z)
Compound in Library Factor = 0

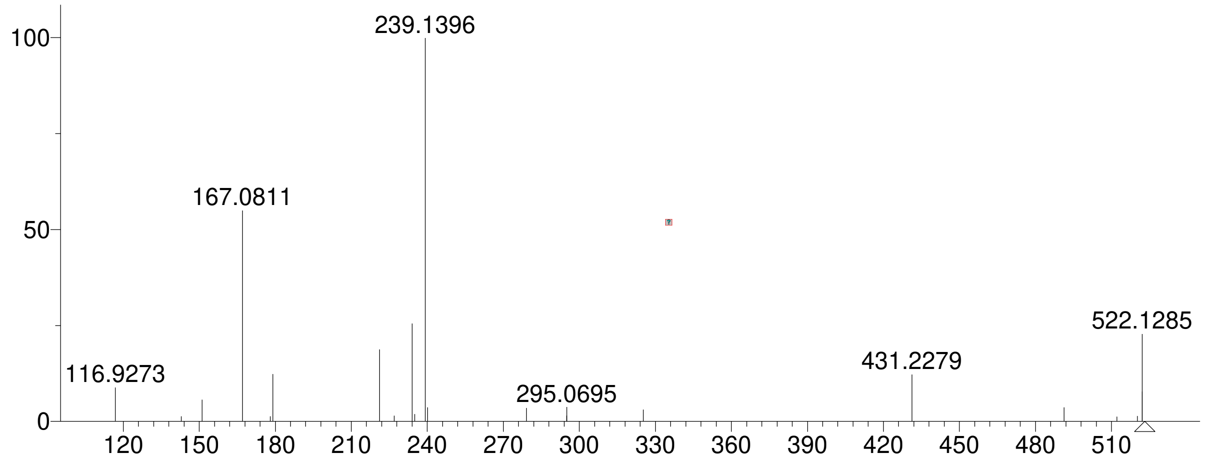

Hit 1 : (2R,7S,13R,14R,16S,19R,20S)-19-(furan-3-yl)-11-hydroxy-9,9,13,20-tetramethyl-4,8,15,18-tetraoxahexacy
C26H28O9; MF: 0; RMF: 52; Prob 50.0%; Lib: mona-export-vf-npl_qexactive; ID: 9555.

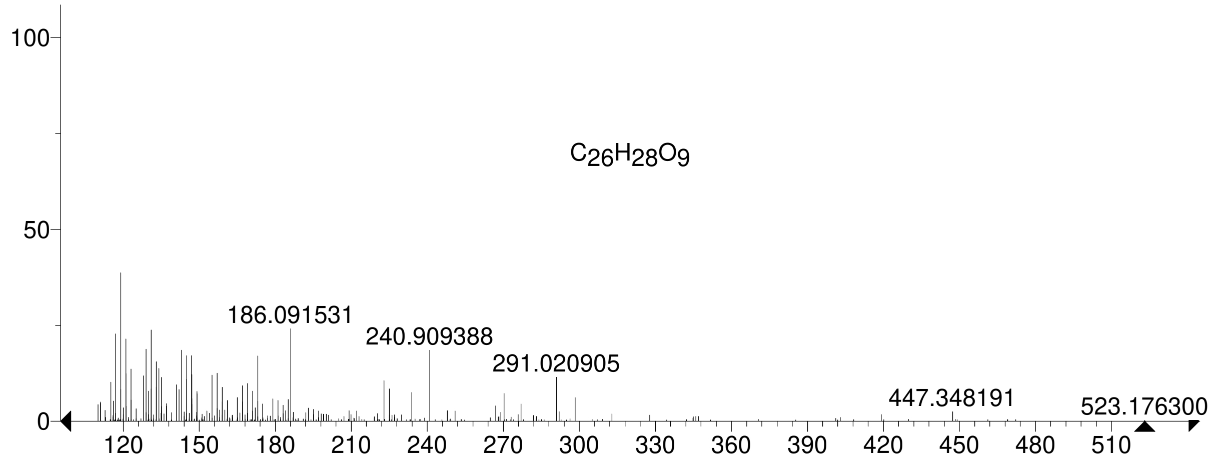

Hit 2 : [(2R,3S,4S,5R,6R)-3,4,5-trihydroxy-6-[2-(3-hydroxy-5-oxooxolan-3-yl)propoxy]oxan-2-yl]methyl (E)-3-(3,4-c
C22H28O12; MF: 0; RMF: 2; Prob 50.0%; Lib: mona-export-itree; ID: 2069.

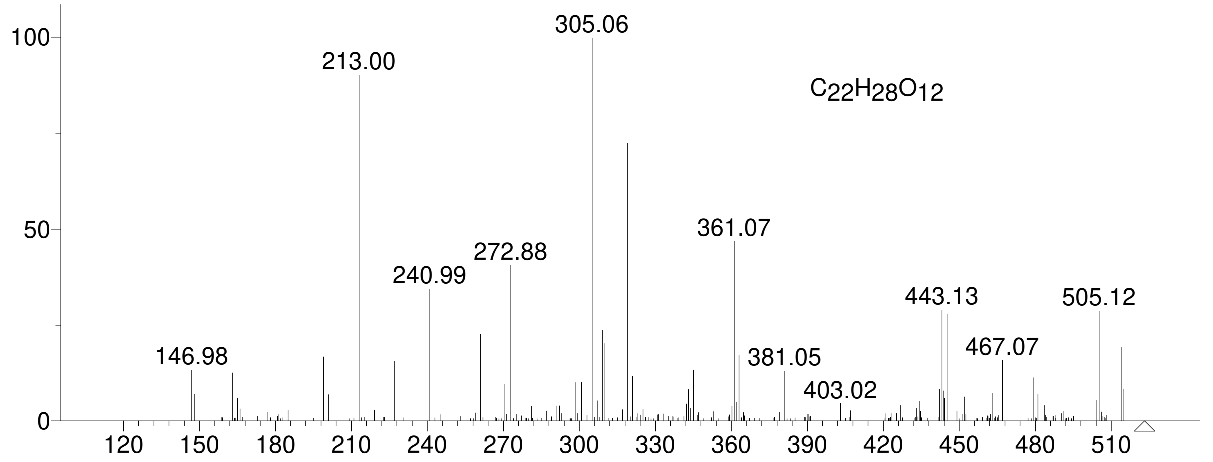

** Search Report Page 1 of 1 **

Unknown: Unknown (18.62_548.0781m/z)
Compound in Library Factor = 0

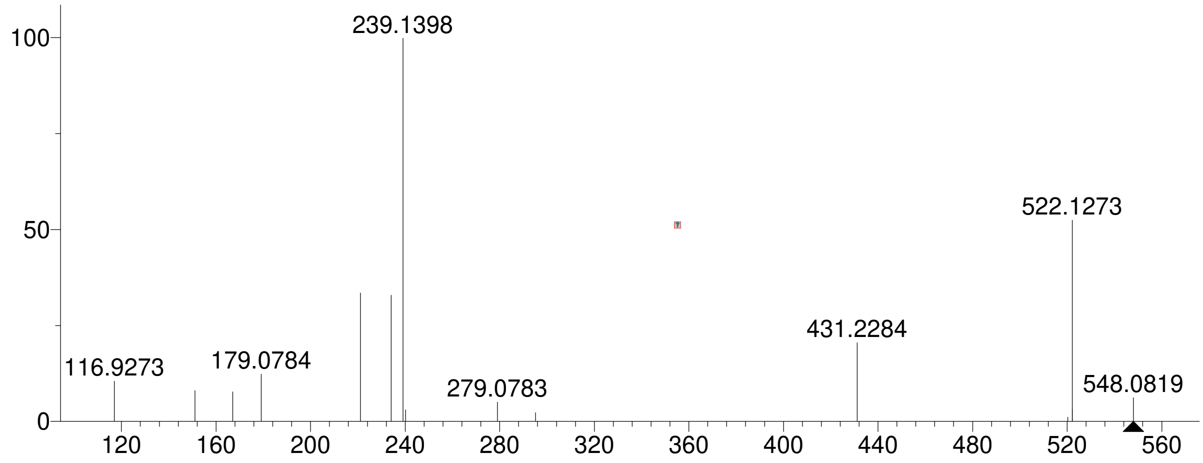

Unknown: Unknown (18.63_239.1393m/z)
Compound in Library Factor = -2387

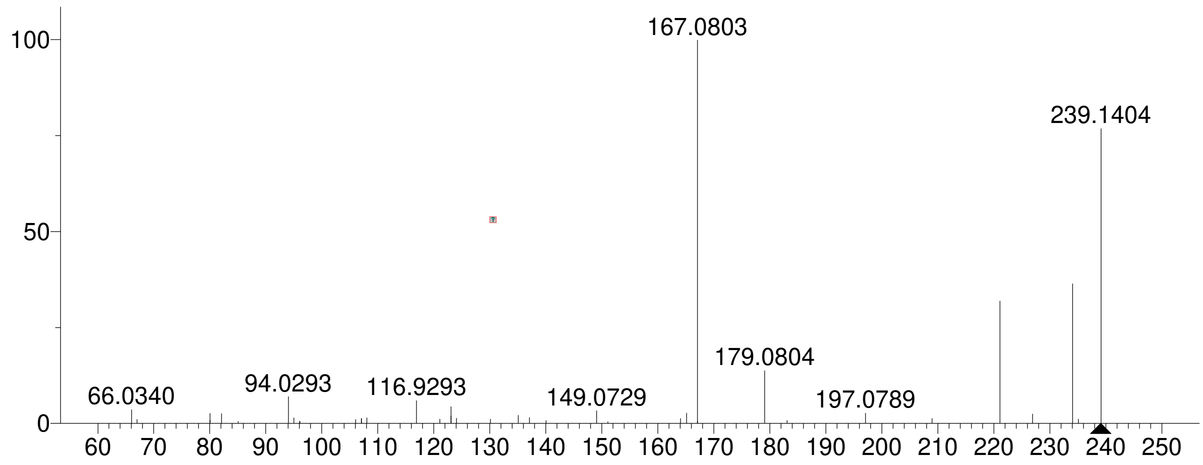

Hit 1 : Secobarbital [M+H]+ HCD 0V P=239.1
C12H18N2O3; MF: 23; RMF: 476; Prob 43.2%; CAS: 76-73-3; Lib: nist_msms; ID: 198372.

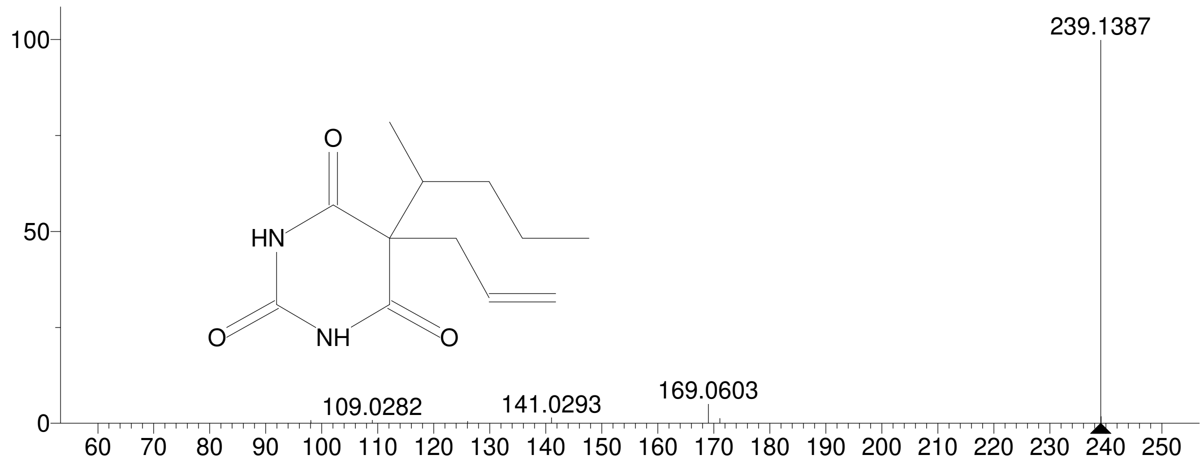

Hit 2 : Heptanedioic acid, 1-(2-cyclopentylidenehydrazide) [M-H]- HCD 14V P=239.1
C12H20N2O3; MF: 20; RMF: 502; Prob 38.2%; CAS: 1136466-93-7; Lib: nist_msms; ID: 263653.

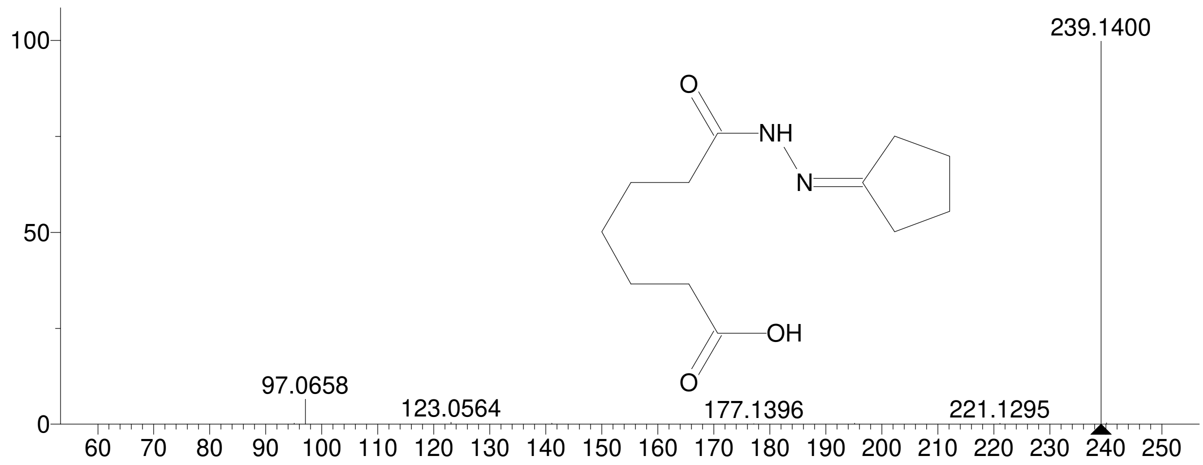

Hit 3 : CMPF [M-H]- 35% P=239.1
C12H16O5; MF: 1; RMF: 49; Prob 18.6%; Lib: mona-export-itree; ID: 3748.

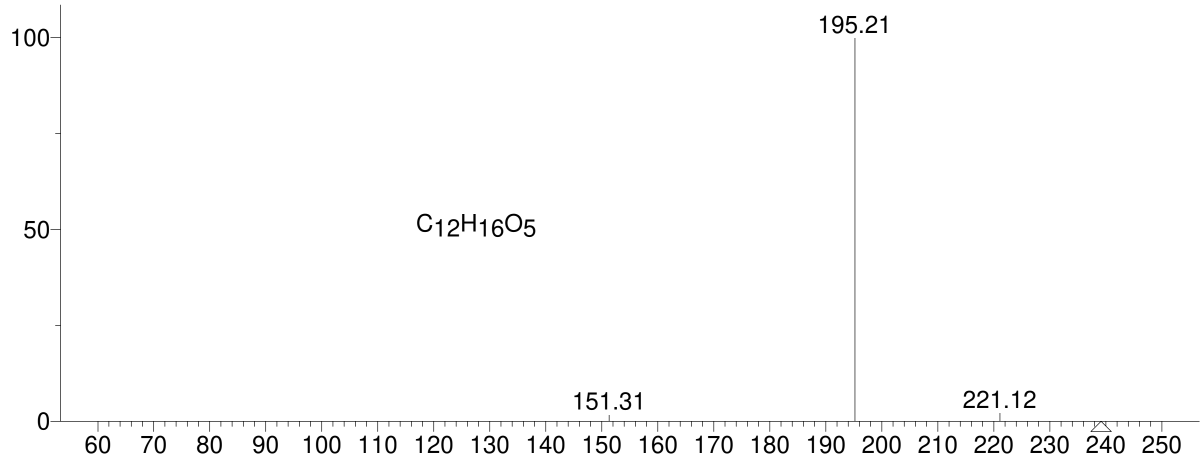

Unknown: Unknown (18.63_568.1349m/z)
Compound in Library Factor = 0

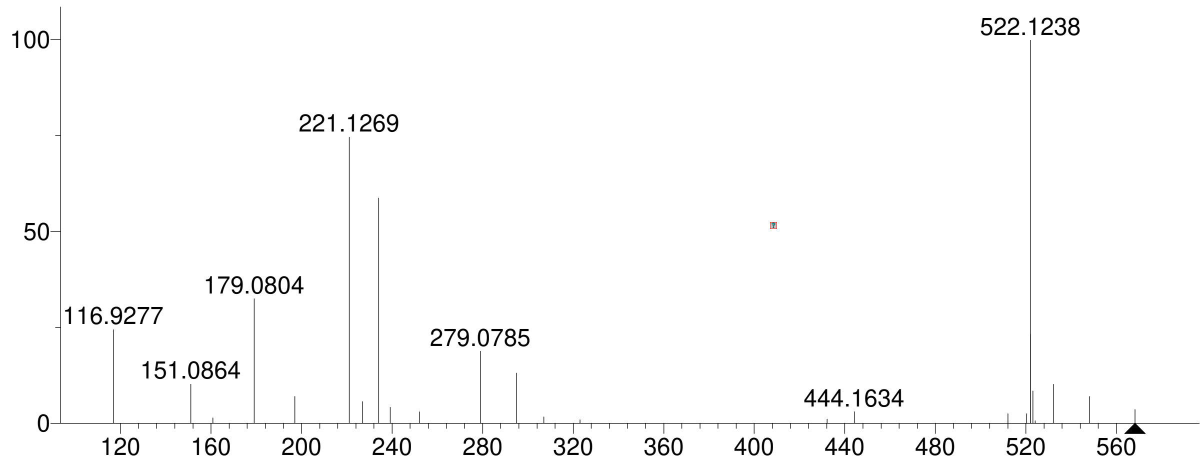

** Search Report Page 1 of 1 **

Unknown: Unknown (18.64_239.1387m/z)
Compound in Library Factor = -2387

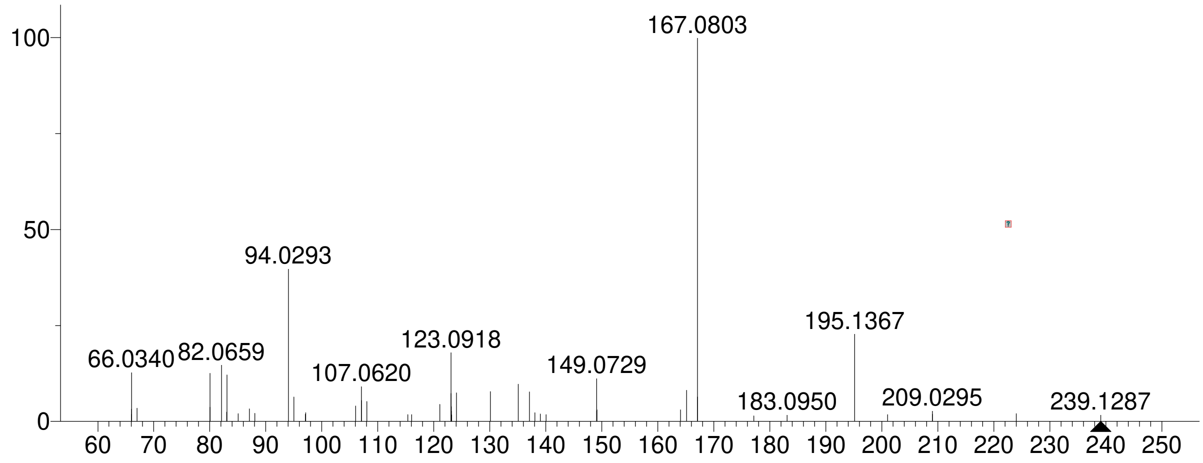

Hit 1 : Heptanedioic acid, 1-(2-cyclopentylidenehydrazide) [M-H]- HCD 21V P=239.1
C12H20N2O3; MF: 15; RMF: 97; Prob 61.5%; CAS: 1136466-93-7; Lib: nist_msms; ID: 263655.

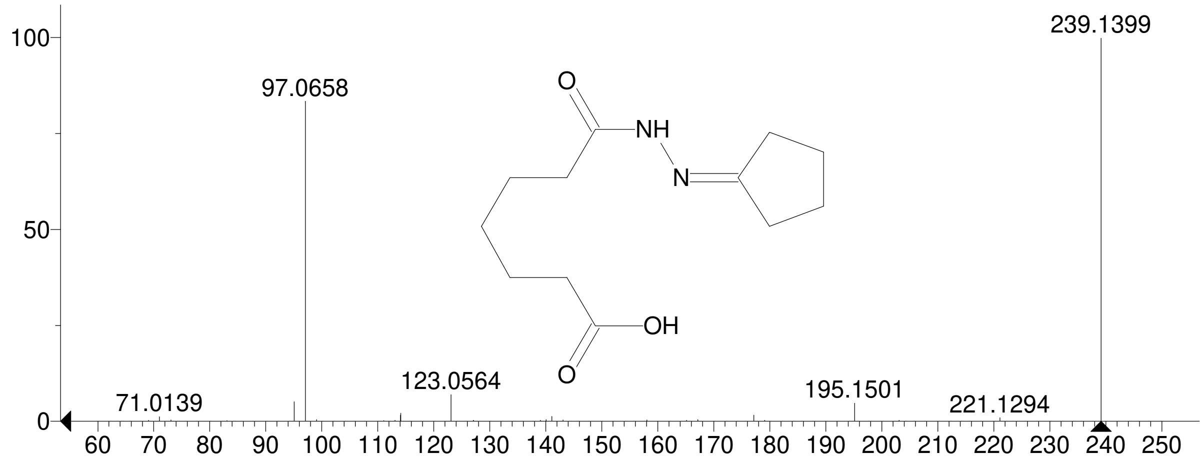

Hit 2 : Secobarbital [M+H]+ HCD 0V P=239.1
C12H18N2O3; MF: 1; RMF: 73; Prob 38.5%; CAS: 76-73-3; Lib: nist_msms; ID: 198372.

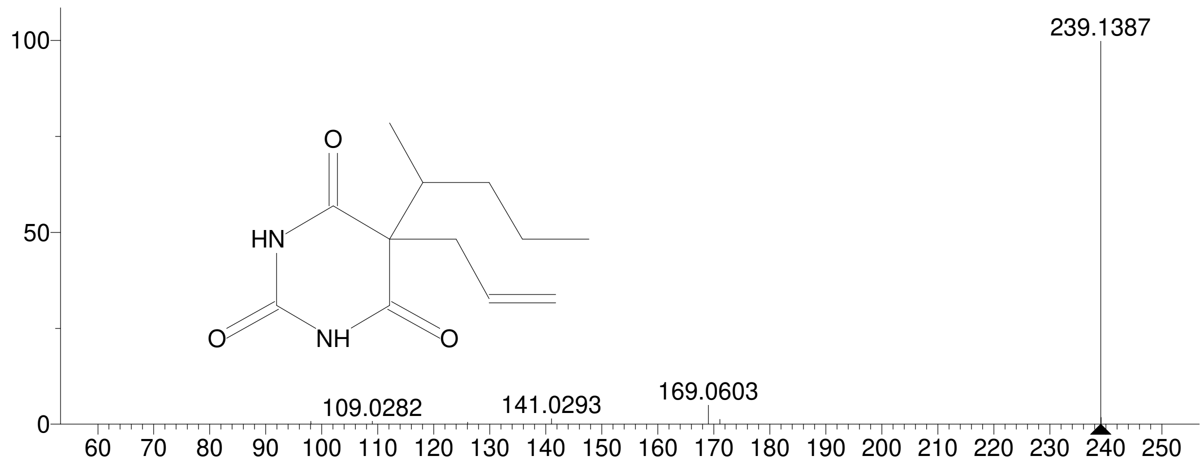

Unknown: Unknown (18.64_444.1652m/z)
Compound in Library Factor = -3273

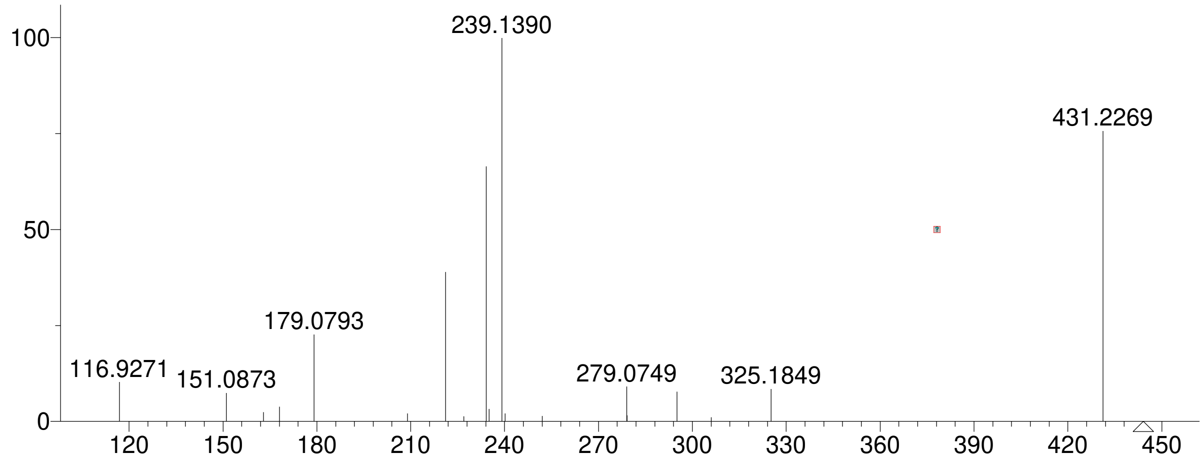

Hit 1 : dabinol [M+NH4]+ 65HCD P=444.2
C23H22O8; MF: 1; RMF: 73; Prob 14.8%; Lib: mona-export-vf-npl_qexactive; ID: 10569.

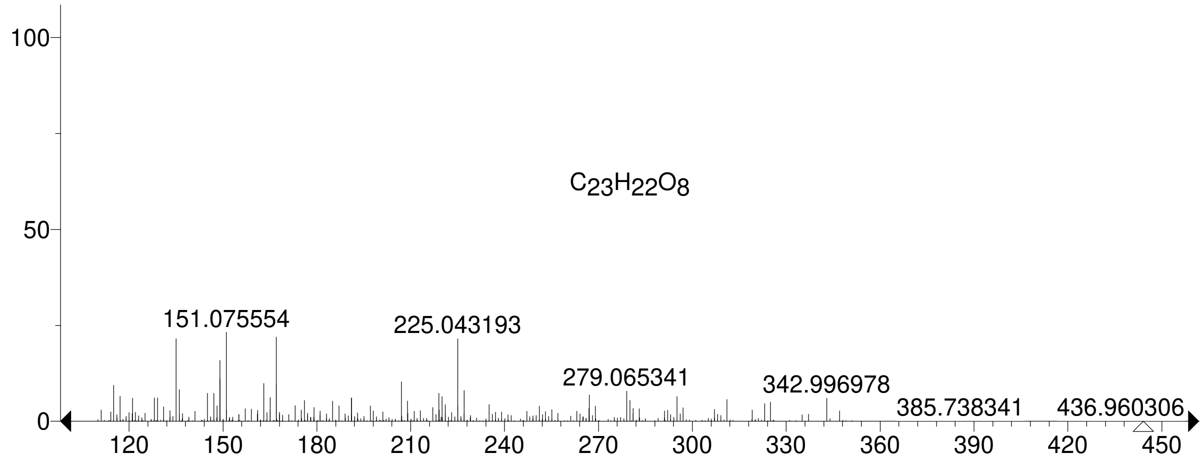

Hit 2 : Dihydrofolic acid [M+H]+ HCD 53V P=444.2
C19H21N7O6; MF: 0; RMF: 113; Prob 14.2%; CAS: 4033-27-6; Lib: nist_msms; ID: 205208.

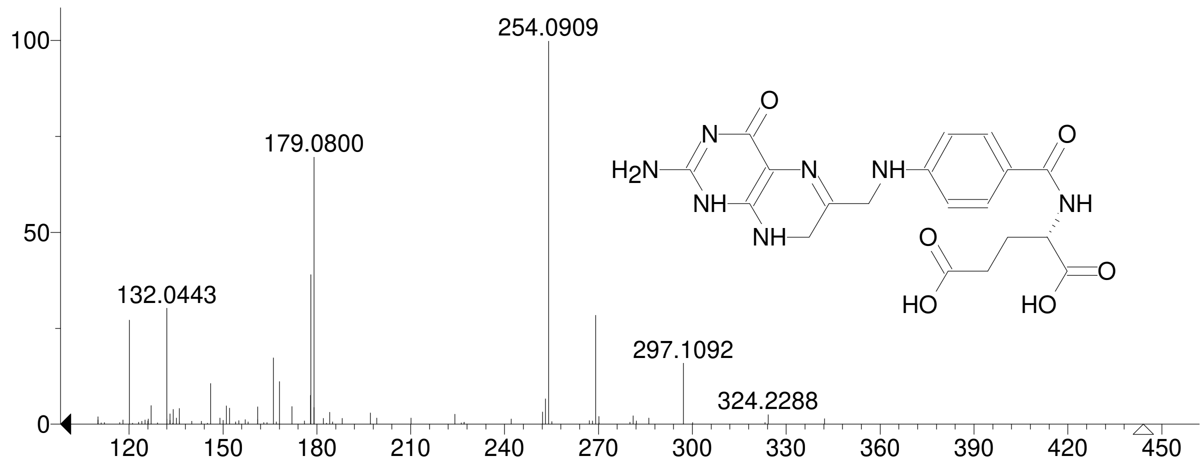

Hit 3 : 6-Hydroxysumatrol [M+NH4]+ 45HCD P=444.2
C23H22O8; MF: 0; RMF: 37; Prob 14.2%; Lib: mona-export-vf-npl_qexactive; ID: 7100.

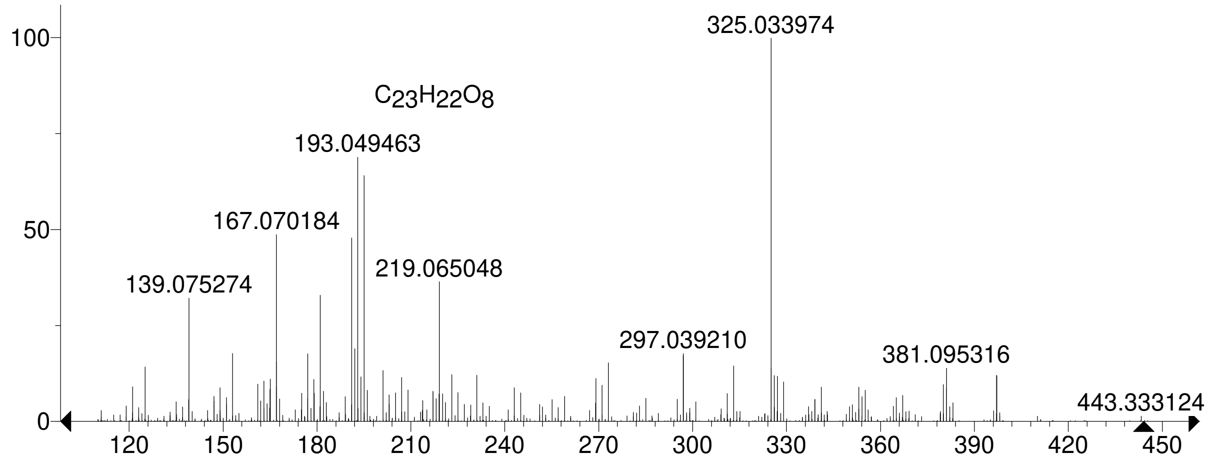

Hit 4 : Morphine-6-glucuronide [M+H-H2O]+ HCD 53V P=444.2
C23H27NO9; MF: 0; RMF: 33; Prob 14.2%; CAS: 20290-10-2; Lib: nist_msms; ID: 324980.

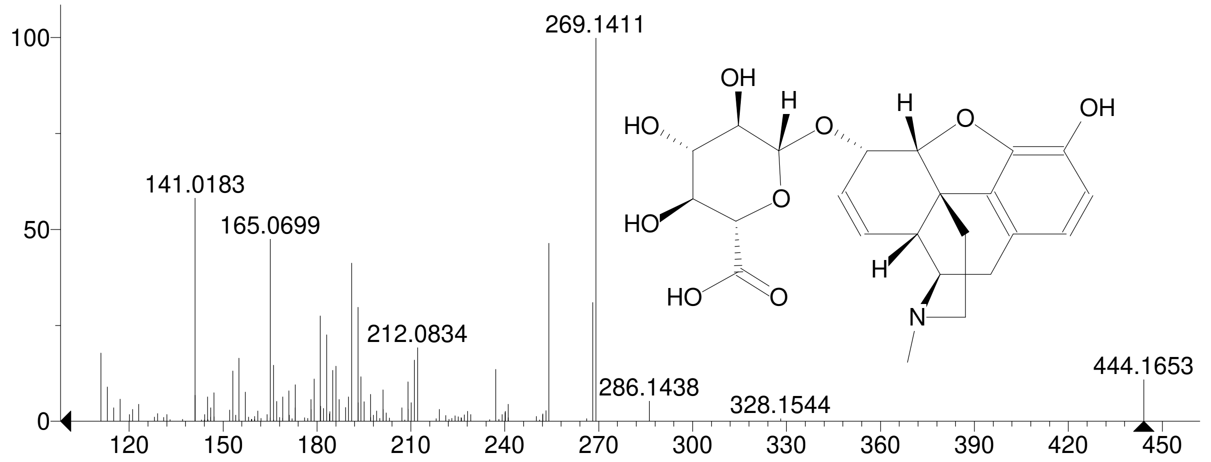

Hit 5 : NCGC00380154-01! [M+H]+ P=444.2
C24H21N5O4; MF: 0; RMF: 11; Prob 14.2%; Lib: mona-export-gnps; ID: 6697.

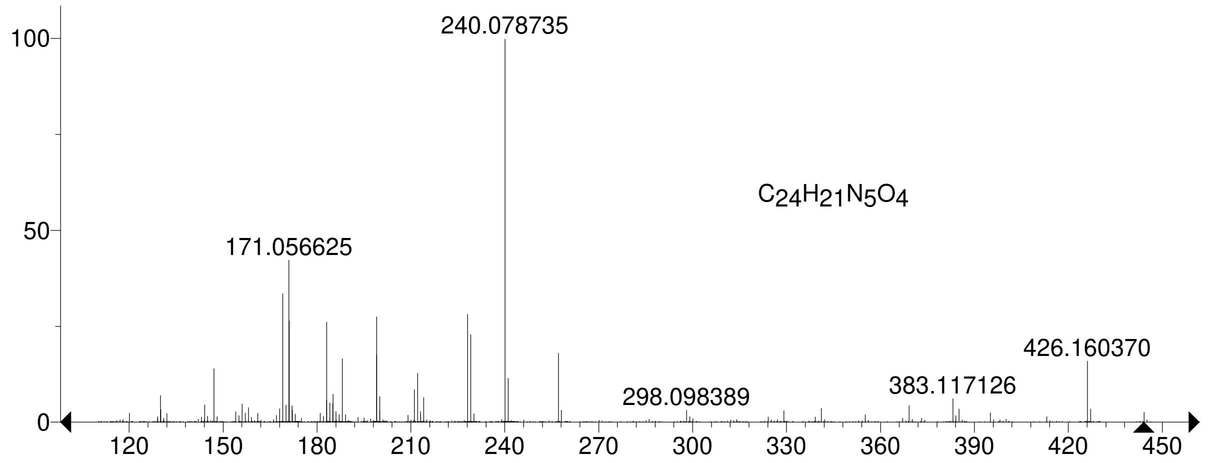

** Search Report Page 1 of 2 **

Unknown: Unknown (18.66_431.2284m/z)
Compound in Library Factor = -3273

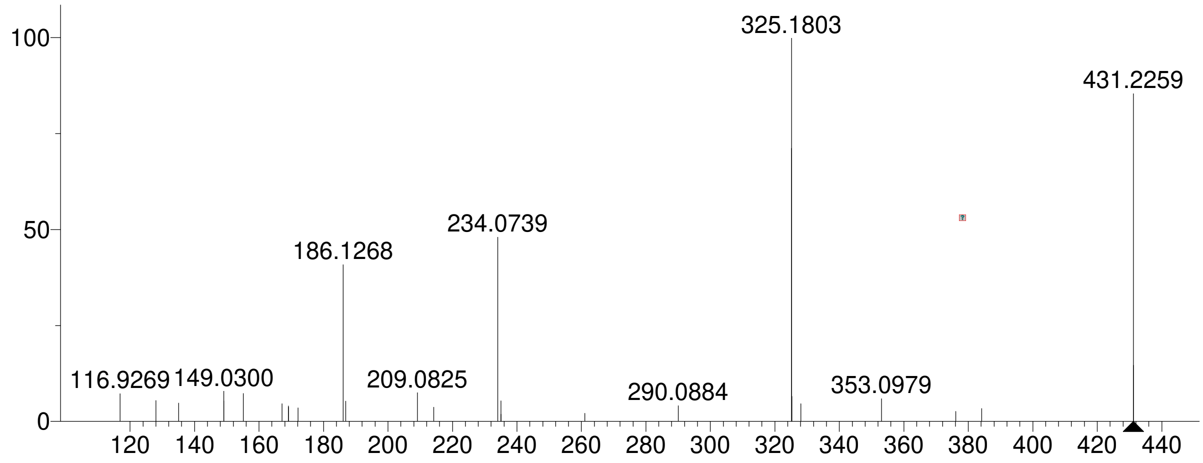

Hit 1 : 4-Piperidinecarboxylic acid, 1-[[4-(acetylamino)phenyl]methyl]-4-(2-phenylethyl)-, ethyl ester [M+Na]+ HC
C25H32N2O3; MF: 6; RMF: 330; Prob 38.1%; CAS: 1069498-96-9; Lib: nist_msms; ID: 420451.

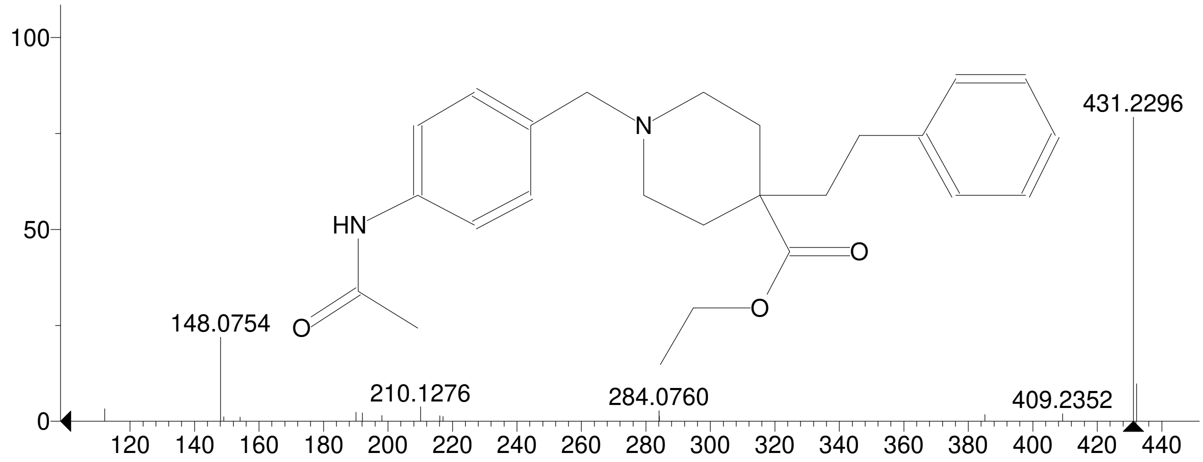

Hit 2 : Acetyl-(Lys0,Nle3)-γ2-MSH, amide [M+4H]4+ HCD 13eV P=431.2
C83H116N24O17; MF: 2; RMF: 239; Prob 32.2%; CAS: 237761-41-0; Lib: nist_msms2; ID: 49099.

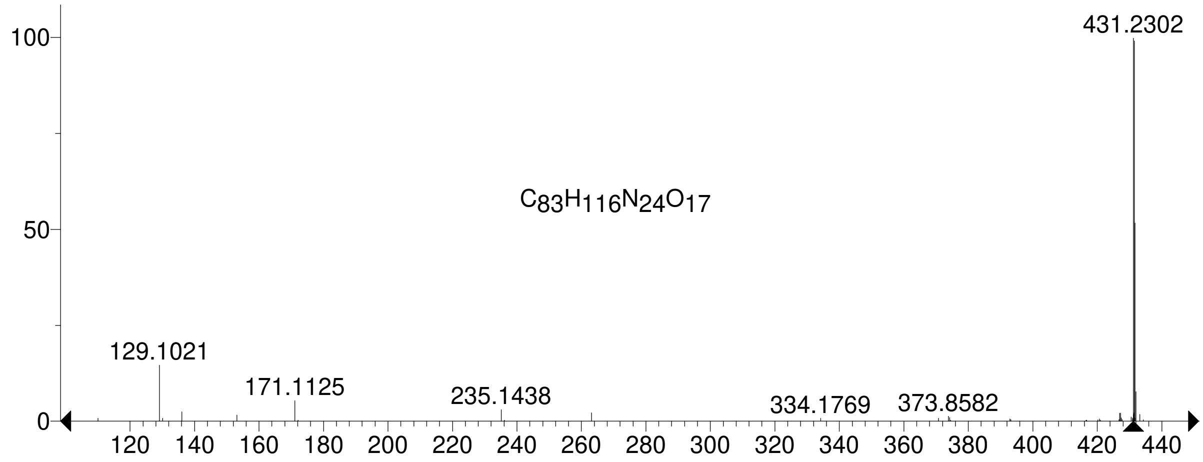

Hit 3 : NCGC00384740-01_C21H34O9_2(1H)-Azulenone, 6-(beta-D-glucopyranosyloxy)-4,5,6,7,8,8a-hexahydro-C21H34O9; MF: 0; RMF: 10; Prob 29.7%; Lib: mona-export-gnps; ID: 10841.

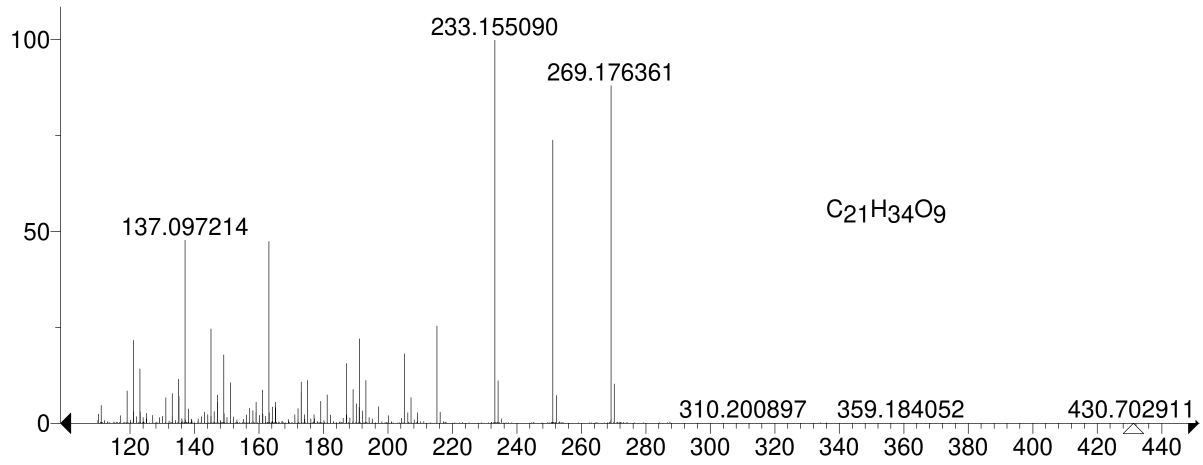

Unknown: Unknown (18.73_296.1489m/z)
Compound in Library Factor = -3273

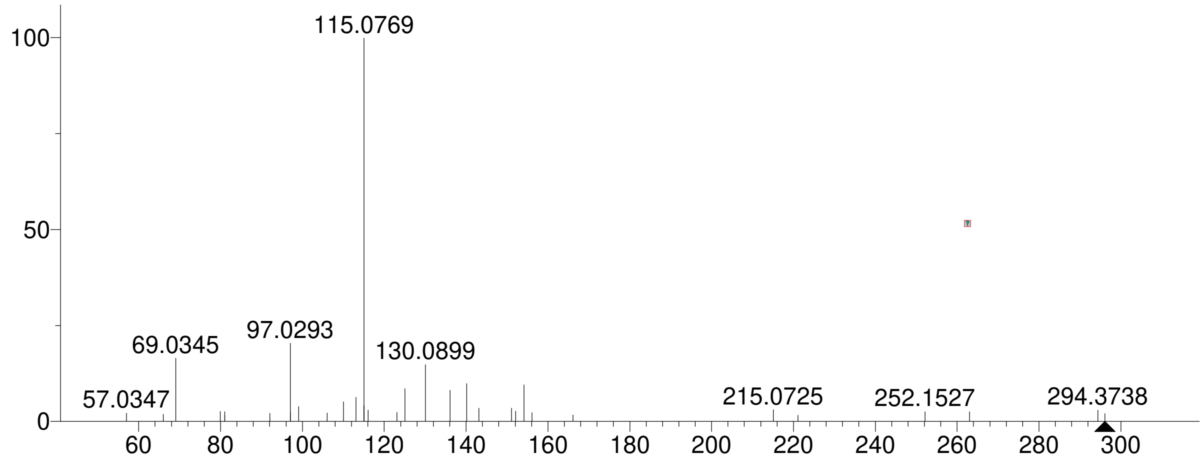

Hit 1 : [Met5,Arg6] Enkephalin-Arg [M+3H]3+ HCD 5eV P=296.1
C39H59N13O9S; MF: 2; RMF: 184; Prob 11.8%; Lib: nist_msms2; ID: 59110.

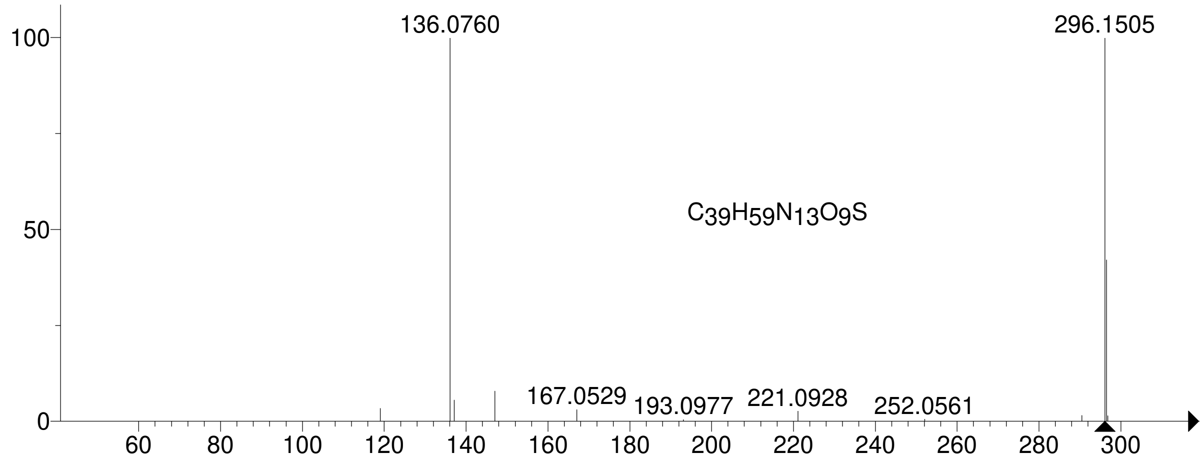

Hit 2 : (3aR,4R,6E,10Z,11aR)-4-hydroxy-10-(hydroxymethyl)-3-methylidene-2-oxo-3a,4,5,8,9,11a-hexahydrocyclo
C15H18O5; MF: 1; RMF: 53; Prob 11.4%; Lib: mona-export-vf-npl_qexactive; ID: 19378.

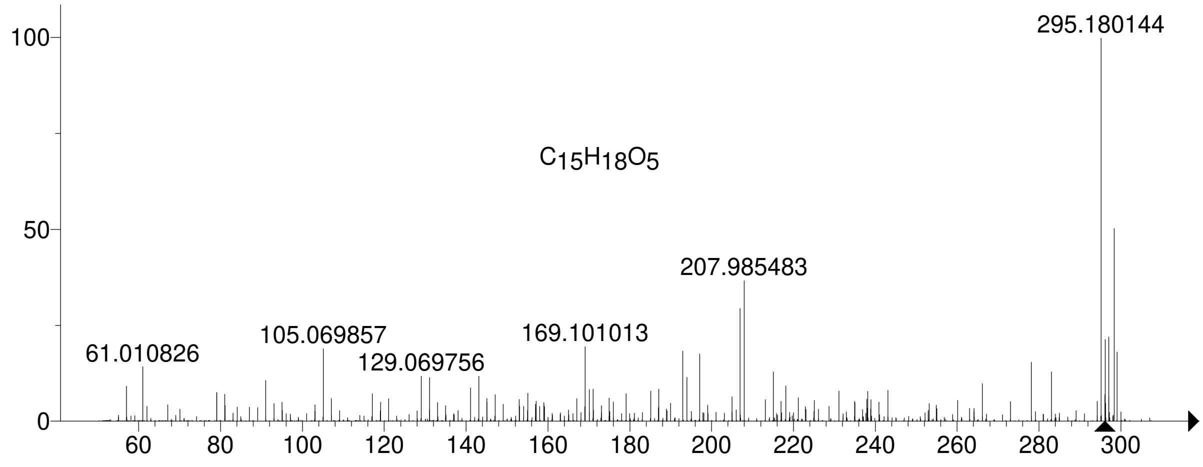

Hit 3 : (2S,3S,4S)-3,4-dihydroxy-6-methoxy-3-methyl-7-methylidene-2-pentyl-2,4-dihydropyrano[2,3-c]pyrrol-5-on
C15H23NO5; MF: 1; RMF: 49; Prob 11.4%; Lib: mona-export-lc-ms-ms_negative_mode; ID: 22789.

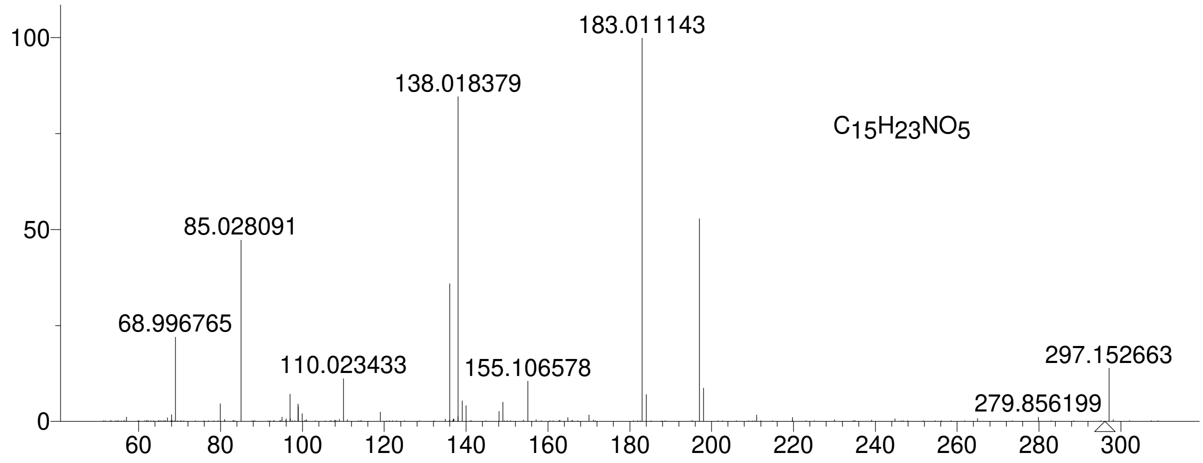

Hit 4 : NCGC00385487-01_C15H21NO5_ [M+H]+ P=296.1
C15H21NO5; MF: 0; RMF: 51; Prob 10.9%; Lib: mona-export-gnps; ID: 10113.

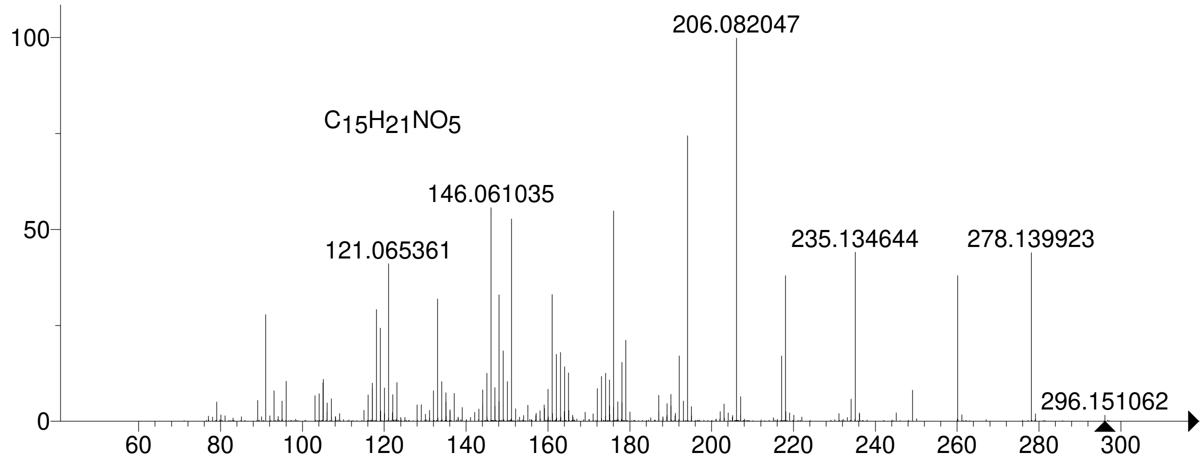

Hit 5 : Dothiepin [M+H]+ HCD 76V P=296.1
C19H21NS; MF: 0; RMF: 32; Prob 10.9%; CAS: 113-53-1; Lib: nist_msms; ID: 198884.

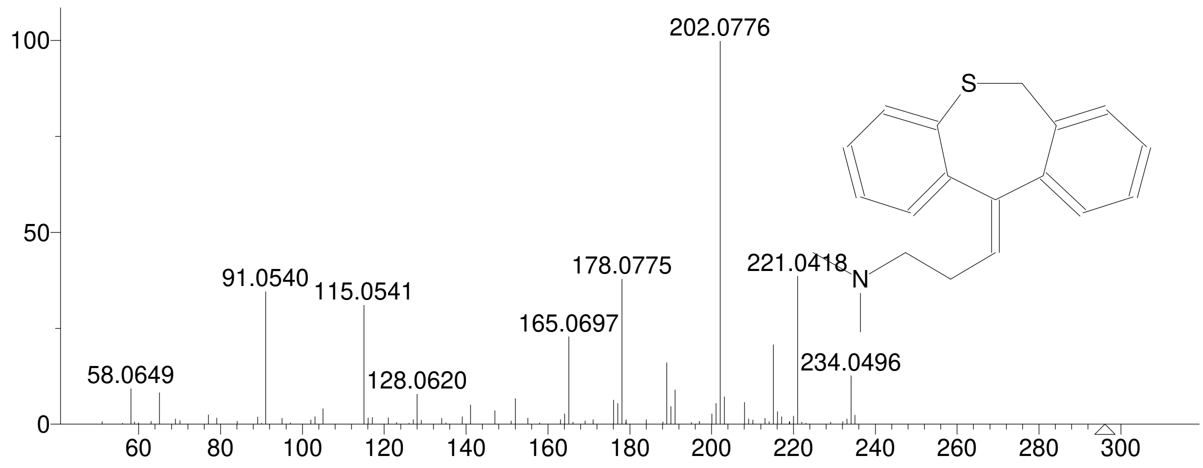

Unknown: Unknown (18.82_511.1988m/z)
Compound in Library Factor = -3273

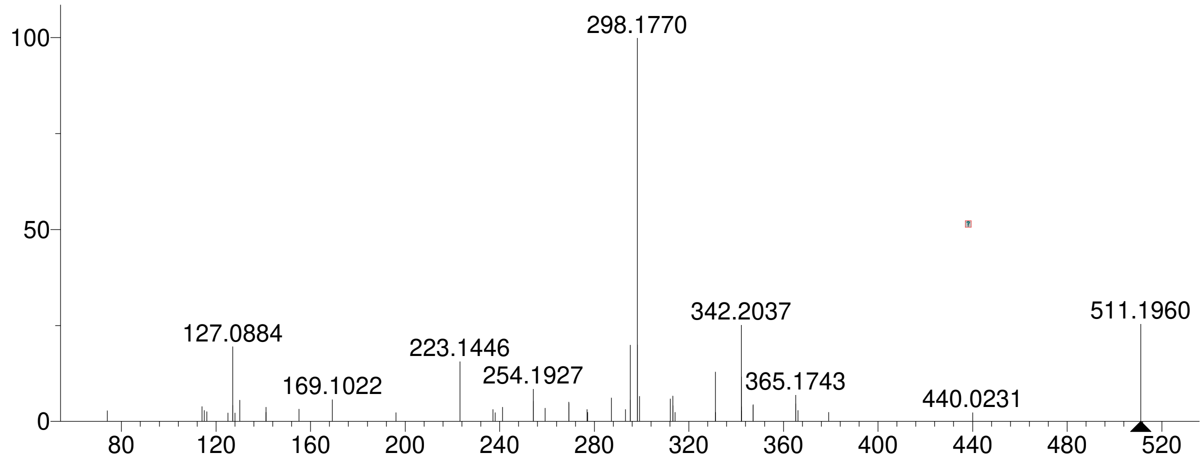

Hit 1 : Ganciclovir [2M+H]+ HCD 5V P=511.2
C9H13N5O4; MF: 6; RMF: 182; Prob 29.8%; CAS: 82410-32-0; Lib: nist_msms; ID: 263891.

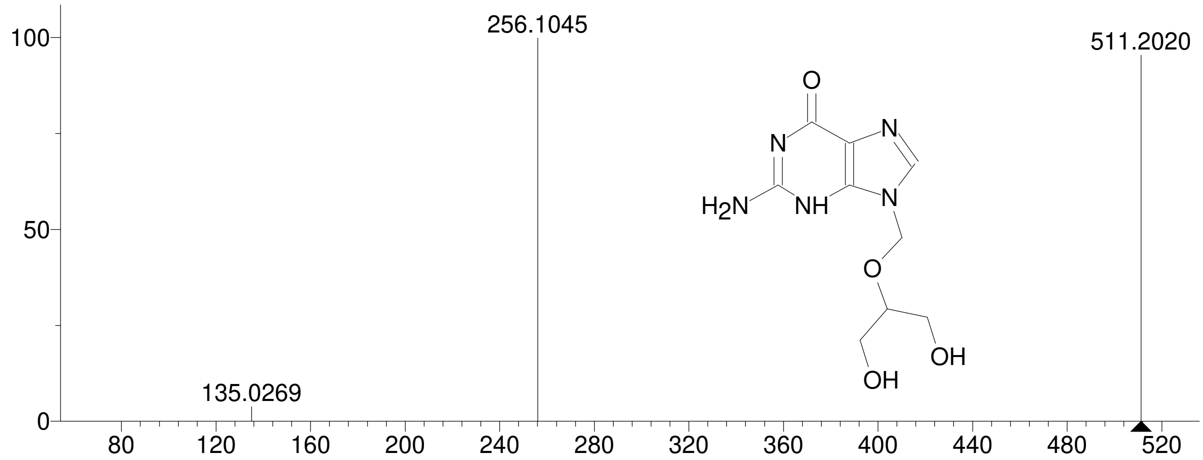

Hit 2 : NCGC00381012-01_C26H32O9_4aH-Phenanthro[2,1-c]pyran-4a-carboxylic acid, 1,3,4,4b,5,7,8,9,10,10a,
C26H32O9; MF: 0; RMF: 79; Prob 23.4%; Lib: mona-export-gnps; ID: 14259.

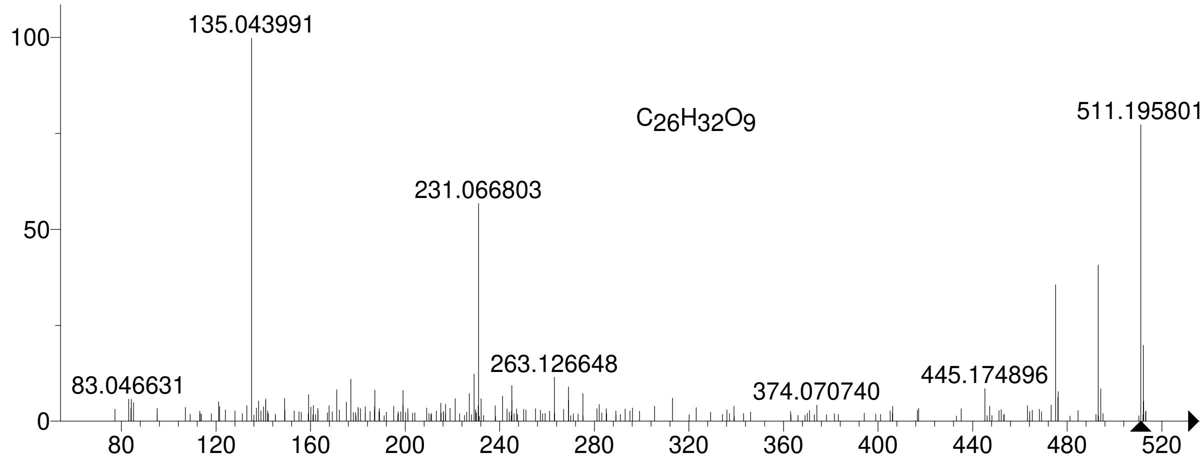

Hit 3 : Dutasteride [M+H]+=>511.2 IT 35% P=511.2
C27H30F6N2O2; MF: 0; RMF: 46; Prob 23.4%; CAS: 164656-23-9; Lib: nist_msms; ID: 356310.

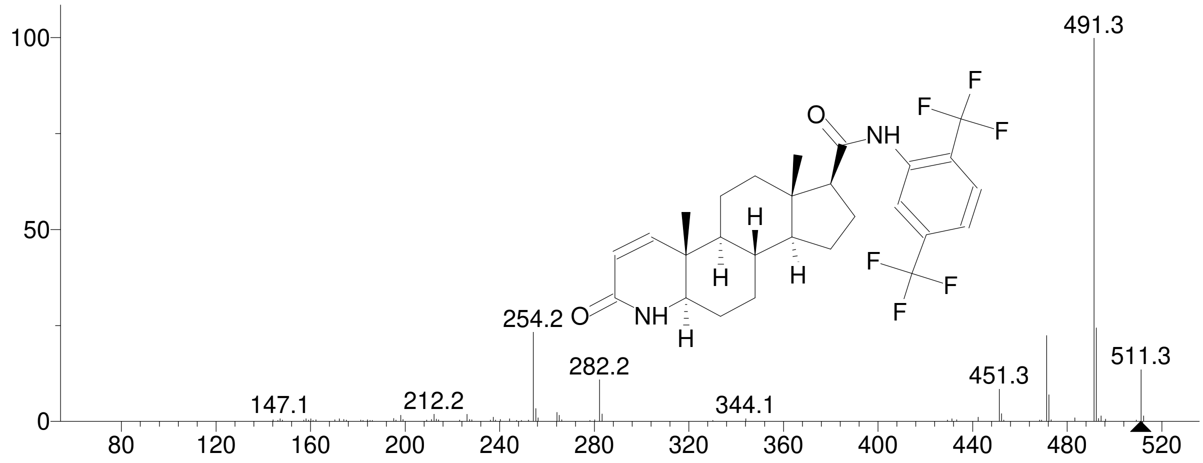

Hit 4 : Pluripotin [M+H]+=>511.2 IT 35% P=511.2
C27H25F3N8O2; MF: 0; RMF: 17; Prob 23.4%; CAS: 839707-37-8; Lib: nist_msms; ID: 156609.

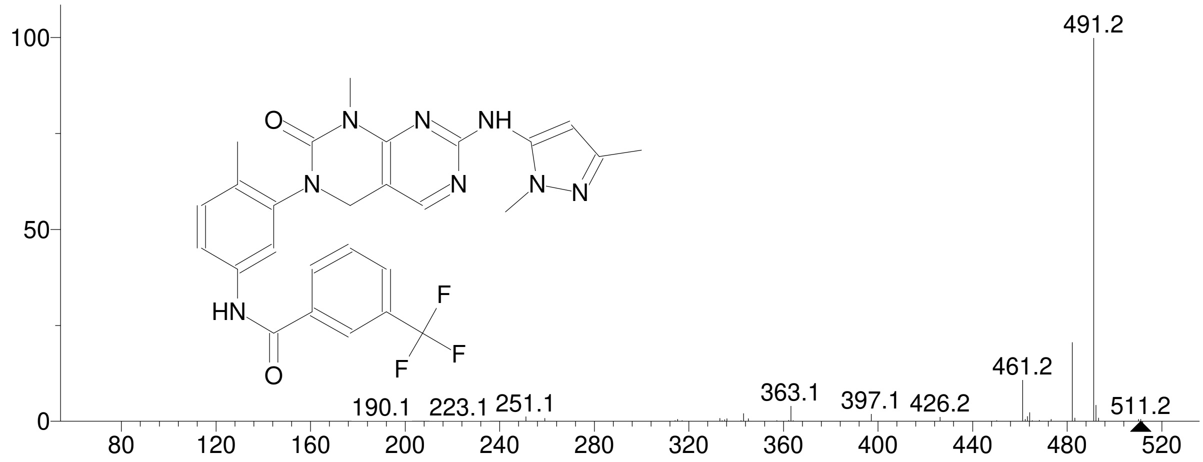

Unknown: Unknown (18.84_248.1287m/z)
Compound in Library Factor = -462

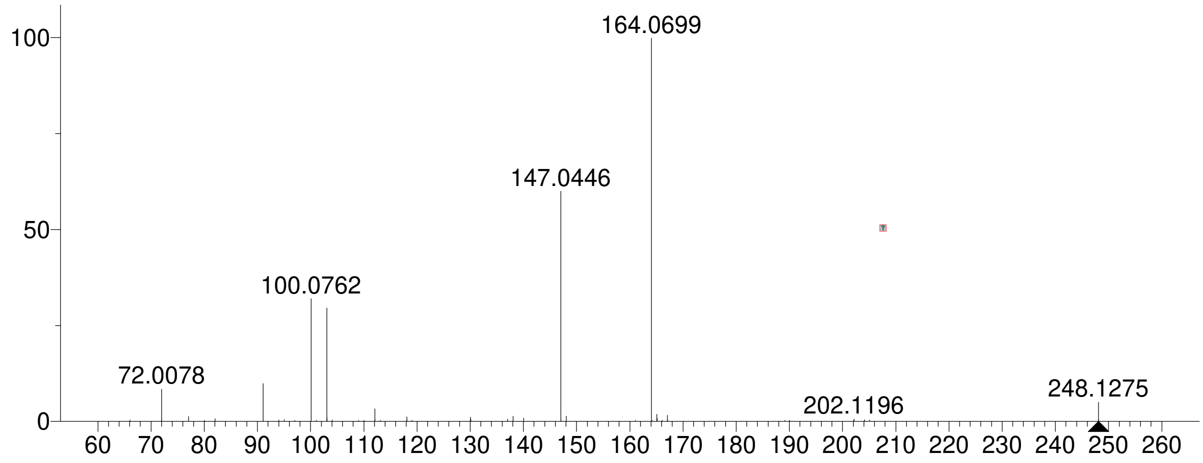

Hit 1 : Mefenoxam [M+H-CH4O]+ HCD 2V P=248.1
C15H21NO4; MF: 195; RMF: 797; Prob 94.7%; CAS: 70630-17-0; Lib: nist_msms; ID: 397662.

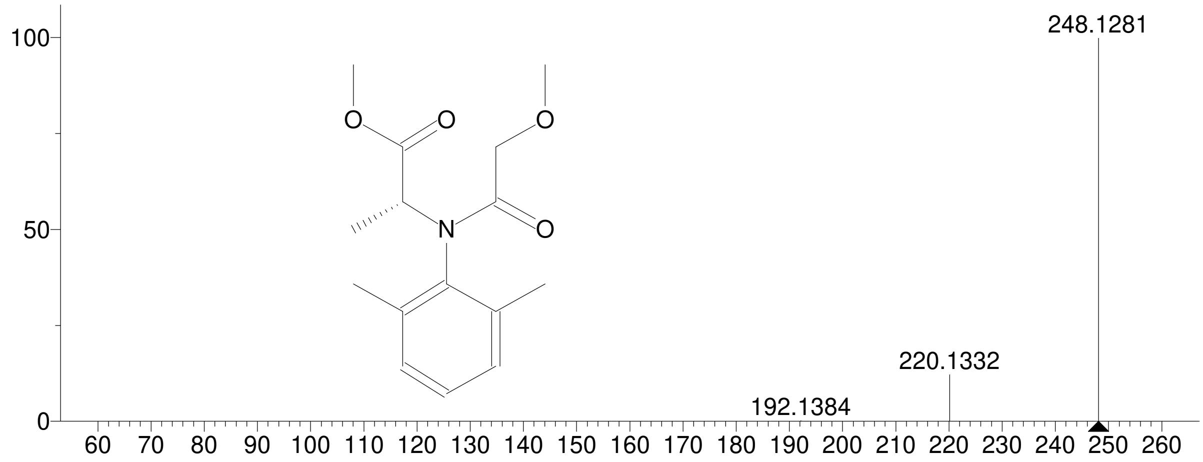

Hit 2 : 3',4'-Methylenedioxy-α-pyrrolidinopropiophenone [M+H]+ HCD 54V P=248.1
C14H17NO3; MF: 48; RMF: 260; Prob 2.76%; CAS: 783241-66-7; Lib: nist_msms; ID: 325332.

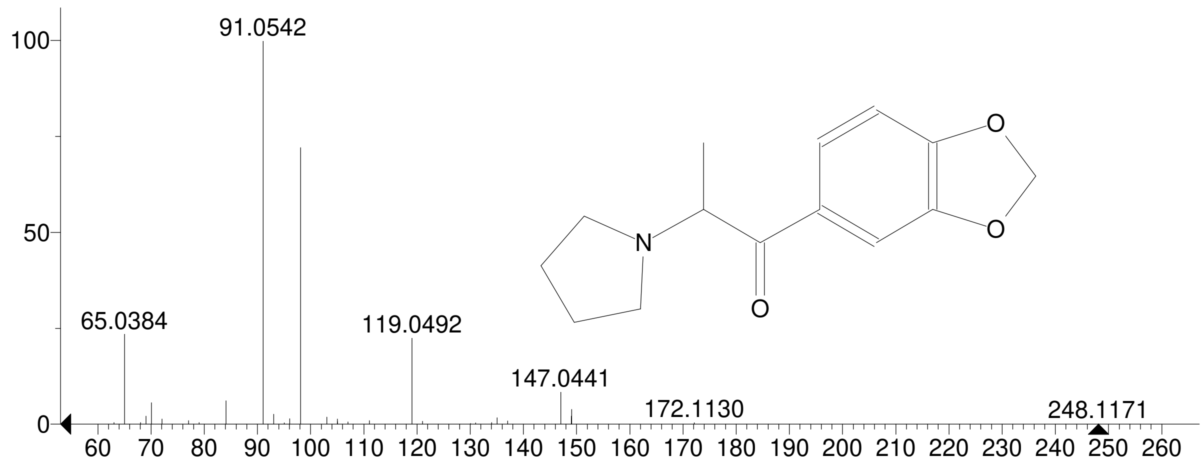

Hit 3 : Metolachlor OA [M+H-CH4O]+ HCD 79V P=248.1
C15H21NO4; MF: 30; RMF: 223; Prob 1.42%; CAS: 152019-73-3; Lib: nist_msms; ID: 473391.

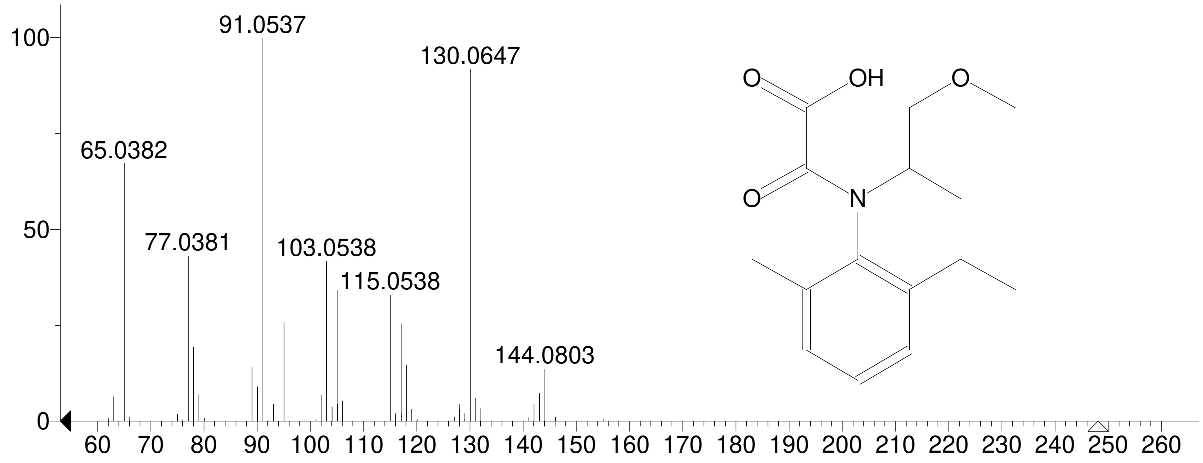

Hit 4 : 6-Benzyloxycarbonylaminohexanoic acid [M+H-H2O]+ HCD 6V P=248.1
C14H19NO4; MF: 24; RMF: 142; Prob 1.12%; CAS: 1947-00-8; Lib: nist_msms; ID: 127702.

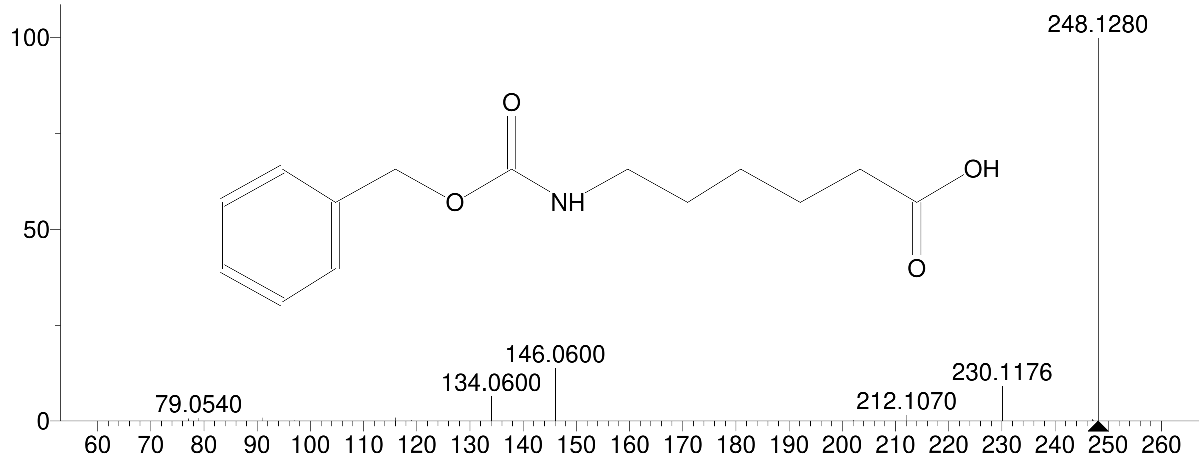

Unknown: Unknown (18.96_411.1763m/z)
Compound in Library Factor = -3273

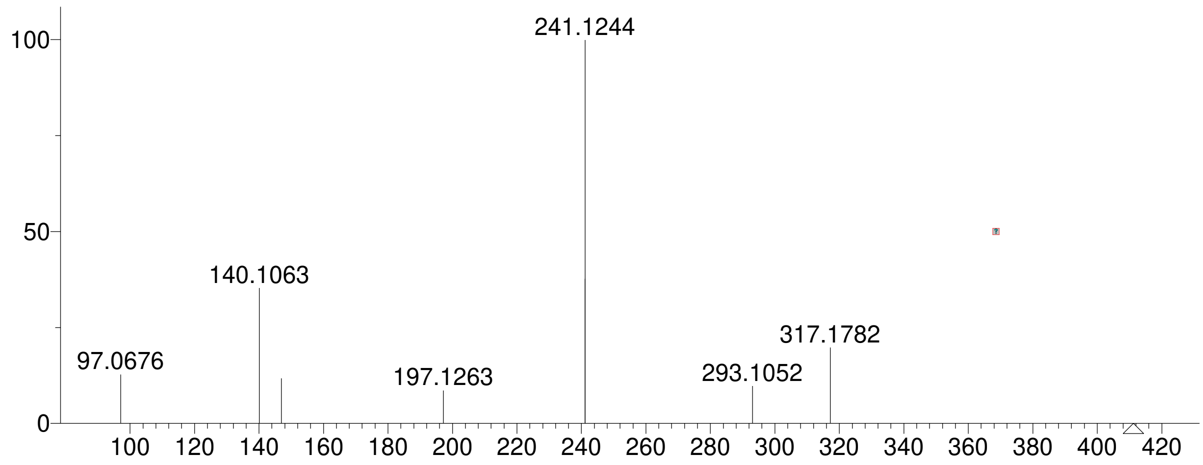

Hit 1 : 21-Carboxylic acid triamcinolone acetonide [M+H-H3FO]+ HCD 28V P=411.2
C24H29FO7; MF: 1; RMF: 93; Prob 17.2%; CAS: 53962-41-7; Lib: nist_msms; ID: 449235.

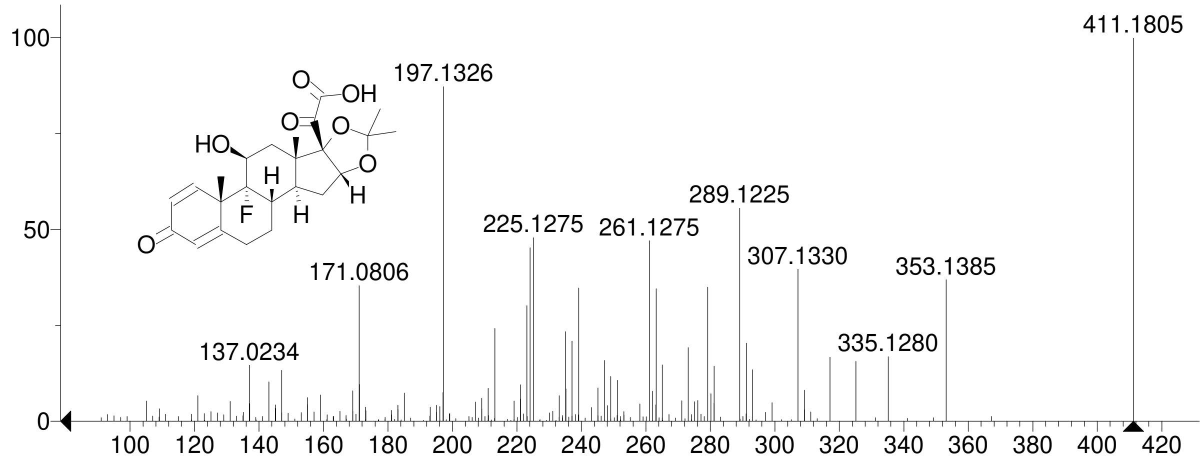

Hit 2 : (4E,8E)-10-(4-hydroxy-6-methoxy-7-methyl-3-oxo-1H-2-benzofuran-5-yl)-4,8-dimethyldeca-4,8-dienoic acid
C22H28O6; MF: 0; RMF: 31; Prob 16.6%; Lib: mona-export-vf-npl_qexactive; ID: 19695.

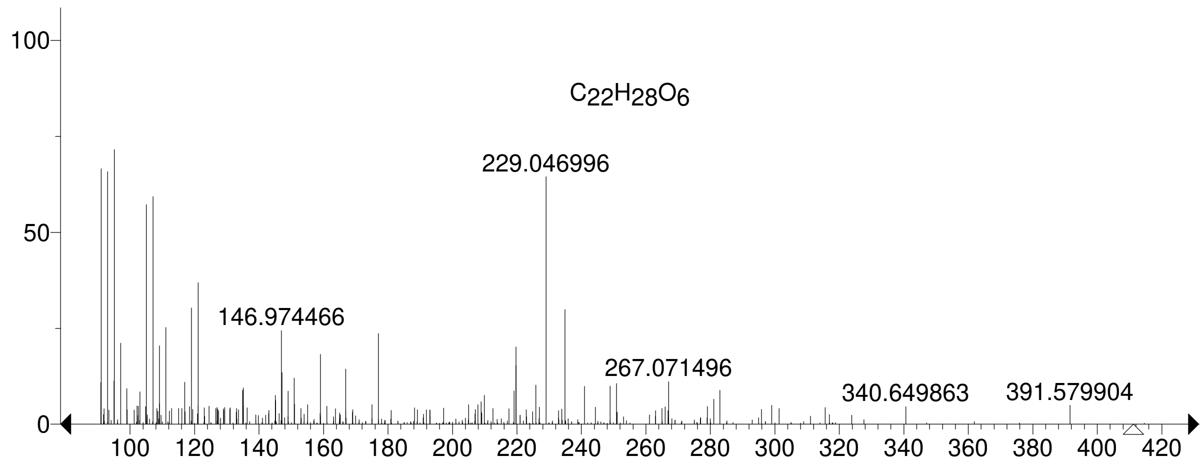

Hit 3 : Mangostine [M+H]+ QQQ 42V P=411.2
C24H26O6; MF: 0; RMF: 31; Prob 16.6%; CAS: 6147-11-1; Lib: nist_msms; ID: 79870.

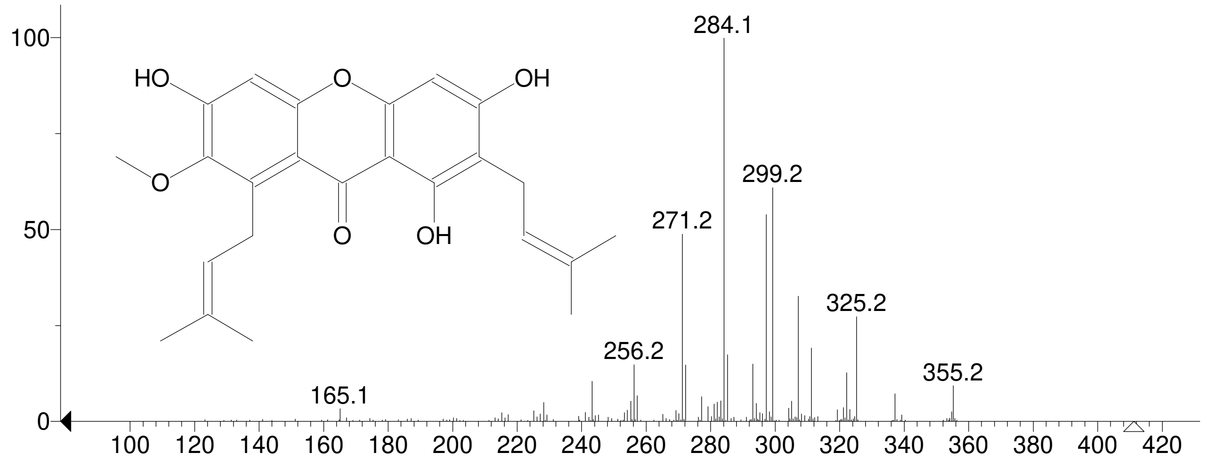

Hit 4 : Quassin [M+Na]+ 65HCD P=411.2
C22H28O6; MF: 0; RMF: 25; Prob 16.6%; Lib: mona-export-vf-npl_qexactive; ID: 26442.

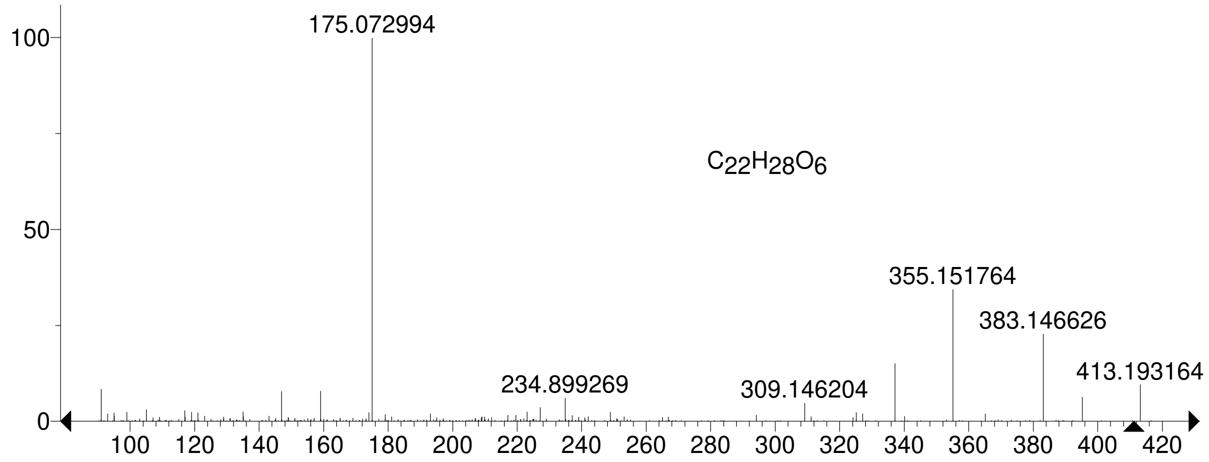

Hit 5 : 3-(5-hydroxy-2,2,7,8-tetramethyl-6-oxo-7,8-dihydropyrano[3,2-g]chromen-10-yl)hexanoic acid [M+Na]+ 45
C22H28O6; MF: 0; RMF: 11; Prob 16.6%; Lib: mona-export-vf-npl_qexactive; ID: 983.

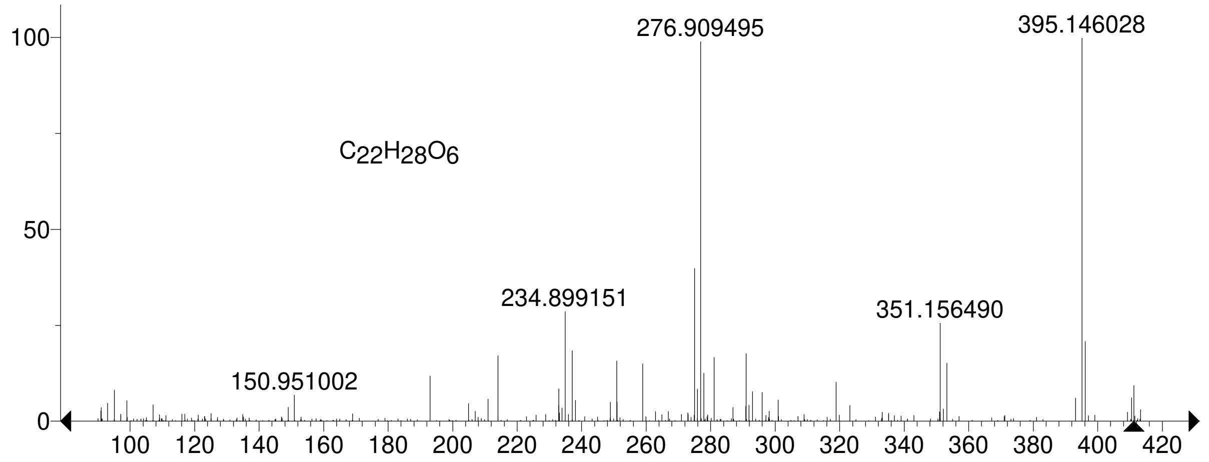

Unknown: Unknown (19.02_237.1234m/z)
Compound in Library Factor = -246

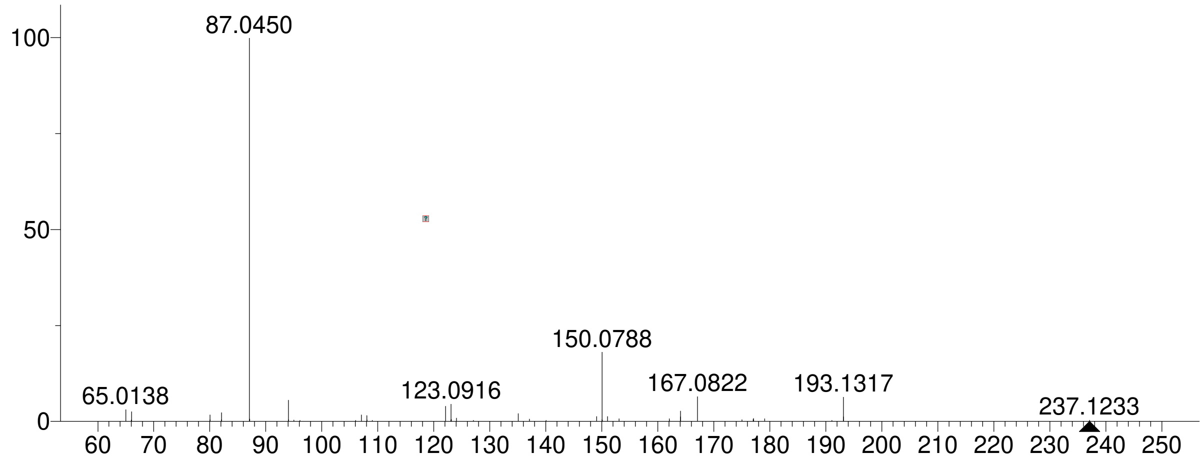

Hit 1 : Secobarbital [M-H]- 35(no P=237.1
C12H18N2O3; MF: 279; RMF: 907; Prob 93.8%; Lib: mona-export-lc-ms-ms_negative_mode; ID: 13759.

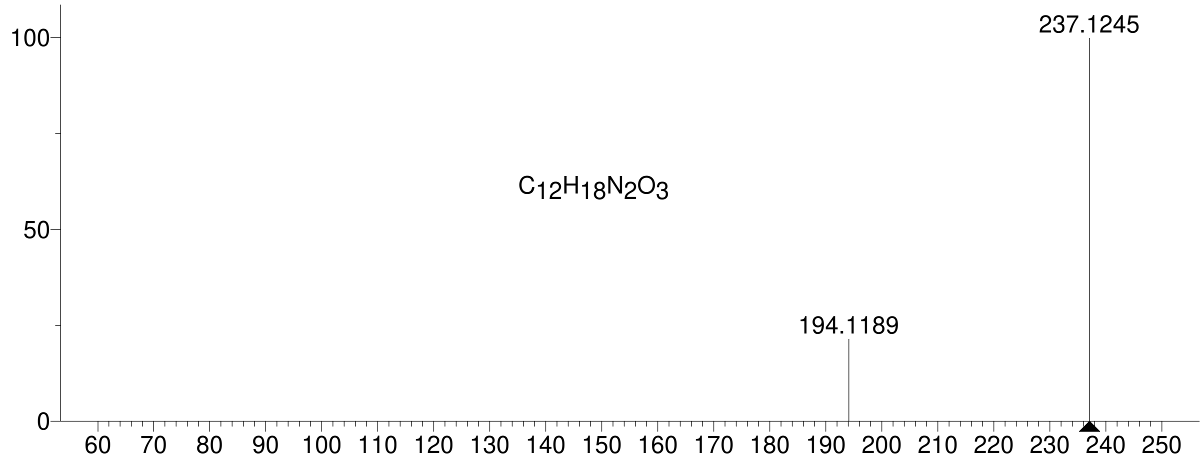

Hit 2 : Ala-Phe [M+H]+ HCD 2eV P=237.1
C12H16N2O3; MF: 10; RMF: 147; Prob 0.93%; CAS: 3061-90-3; Lib: nist_msms2; ID: 89123.

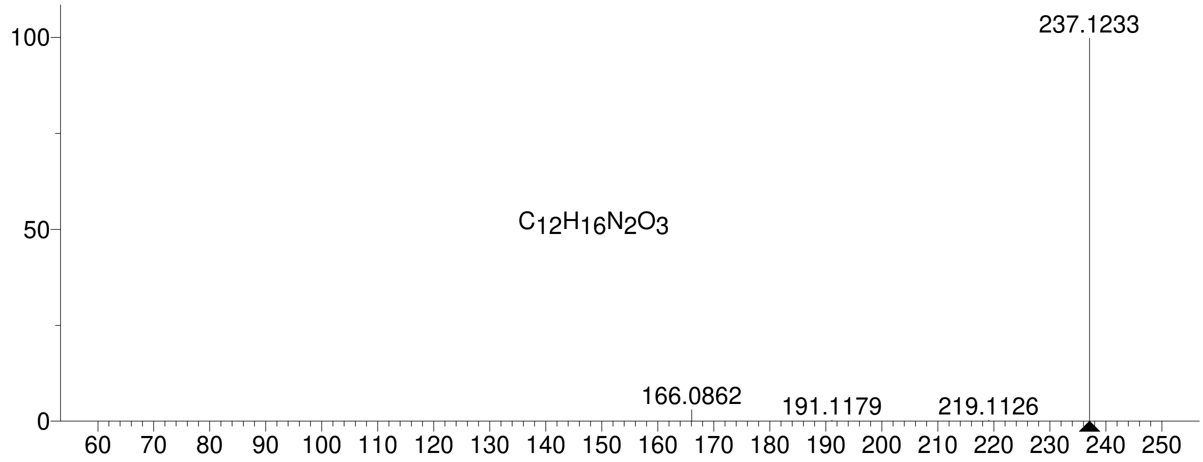

Hit 3 : Carbetamide [M+H]+ HCD 0V P=237.1
C12H16N2O3; MF: 2; RMF: 53; Prob 0.69%; CAS: 16118-49-3; Lib: nist_msms; ID: 282730.

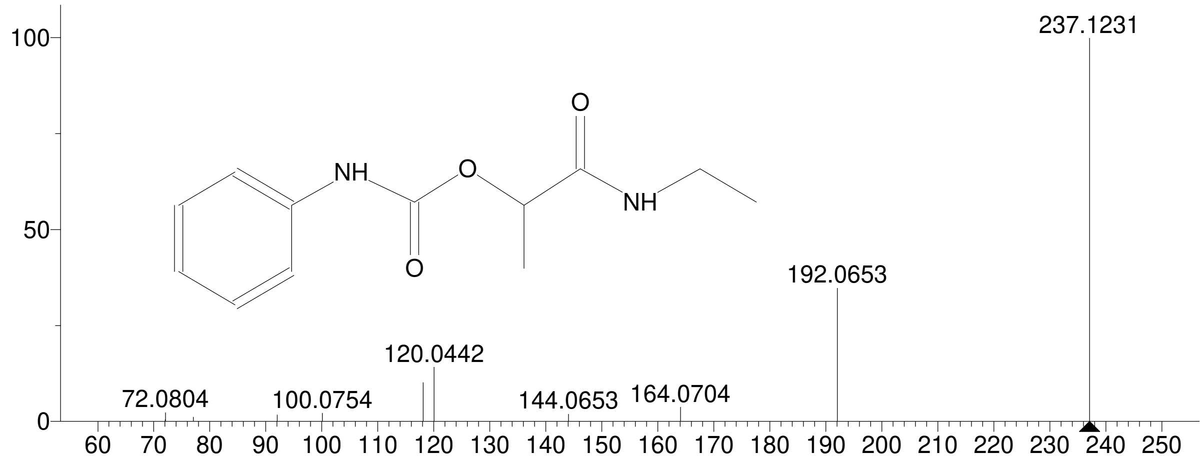

Hit 4 : Phe-Ala [M+H]+ HCD 4eV P=237.1
C12H16N2O3; MF: 1; RMF: 77; Prob 0.67%; CAS: 3918-87-4; Lib: nist_msms; ID: 571919.

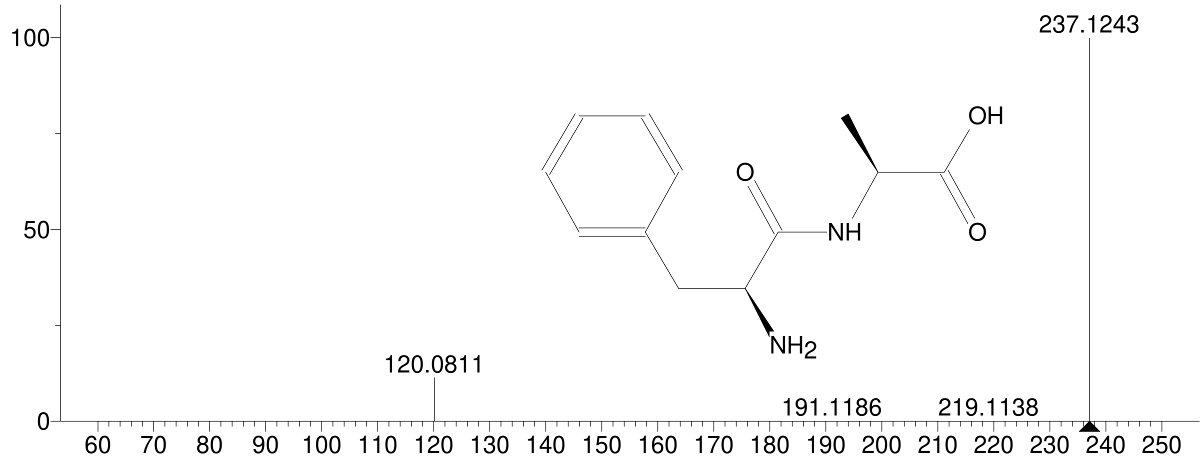

Hit 5 : Hexobarbital [M+H]+ HCD 4V P=237.1
C12H16N2O3; MF: 1; RMF: 43; Prob 0.67%; CAS: 56-29-1; Lib: nist_msms; ID: 195763.

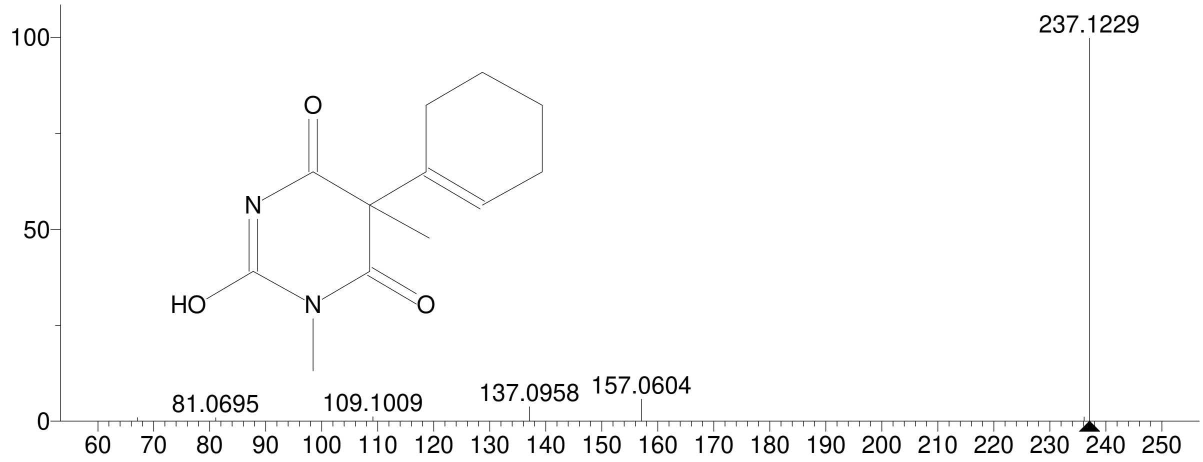

** Search Report Page 1 of 1 **

Unknown: Unknown (19.03_428.0514m/z)
Compound in Library Factor = 0

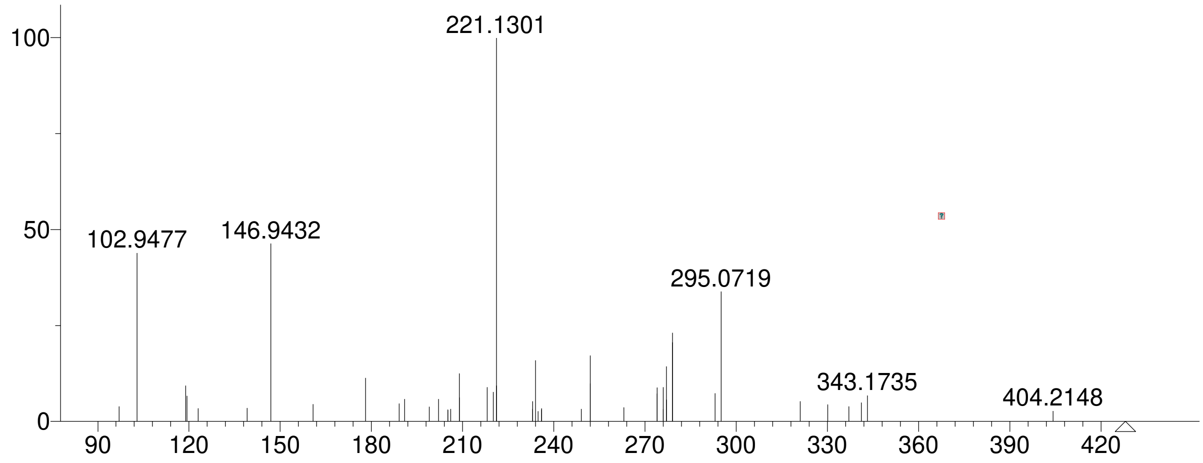

Unknown: Unknown (19.10_501.1959m/z)
Compound in Library Factor = 0

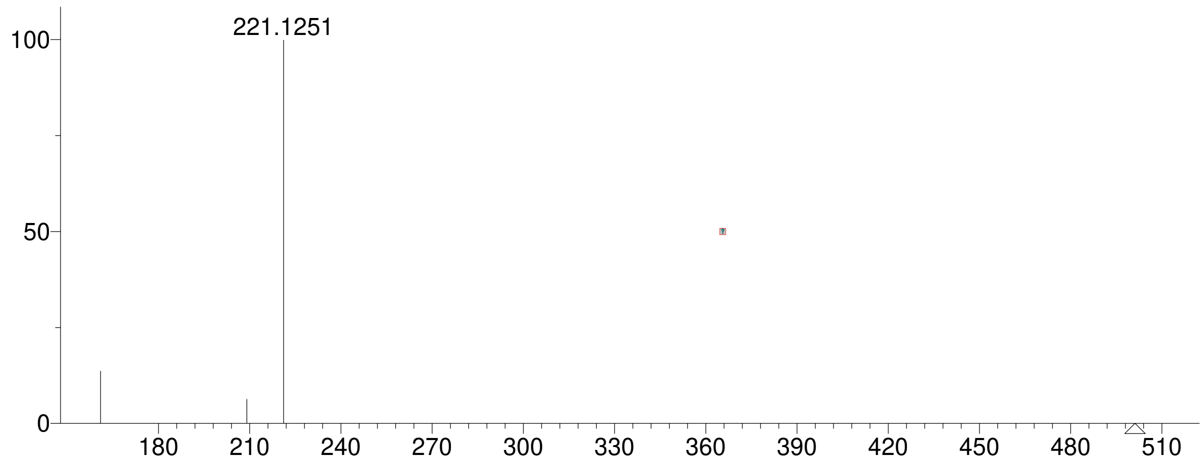

Hit 1 : "Fluticasone propionate (Flonase, Veramyst)" [M+H]+ P=501.2
C25H31F3O5S; MF: 0; RMF: 18; Prob 100.0%; Lib: mona-export-gnps; ID: 701.

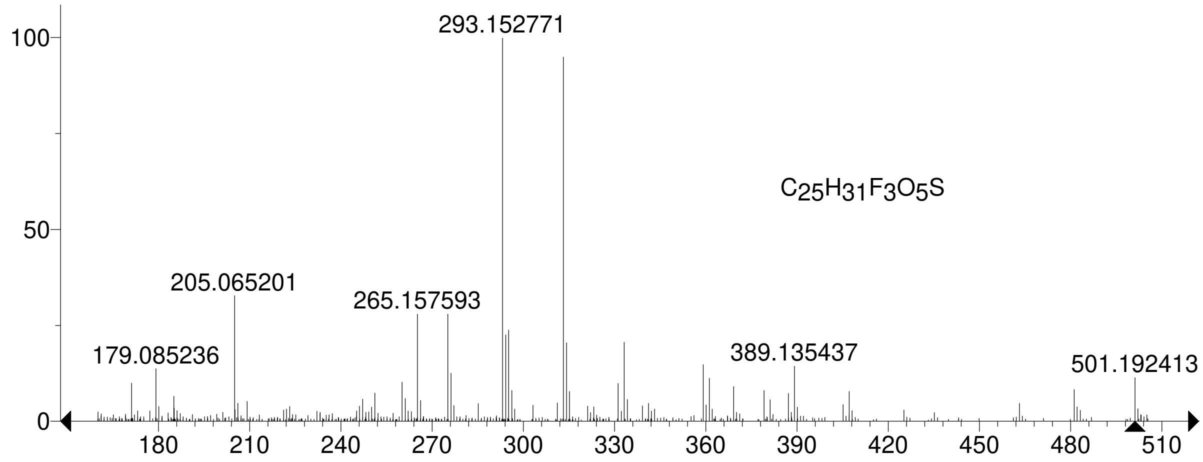

Unknown: Unknown (19.11_454.1859m/z)
Compound in Library Factor = -3273

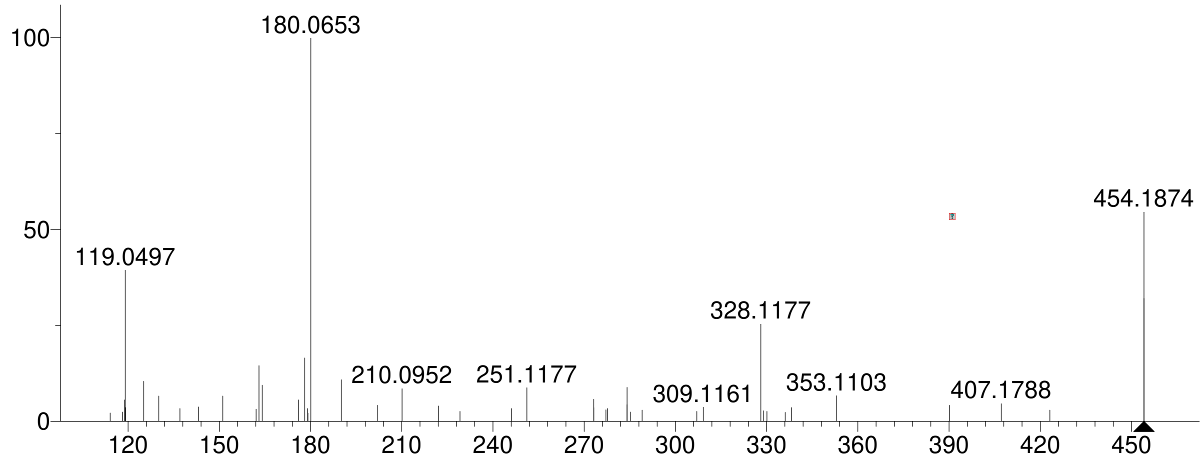

Hit 1 : PS 12:0; PS 6:0-6:0; [M-H]- [M-H]- P=454.2
C18H34NO10P; MF: 3; RMF: 154; Prob 17.2%; Lib: msdial-insilicomsms-lipids-neg; ID: 329913.

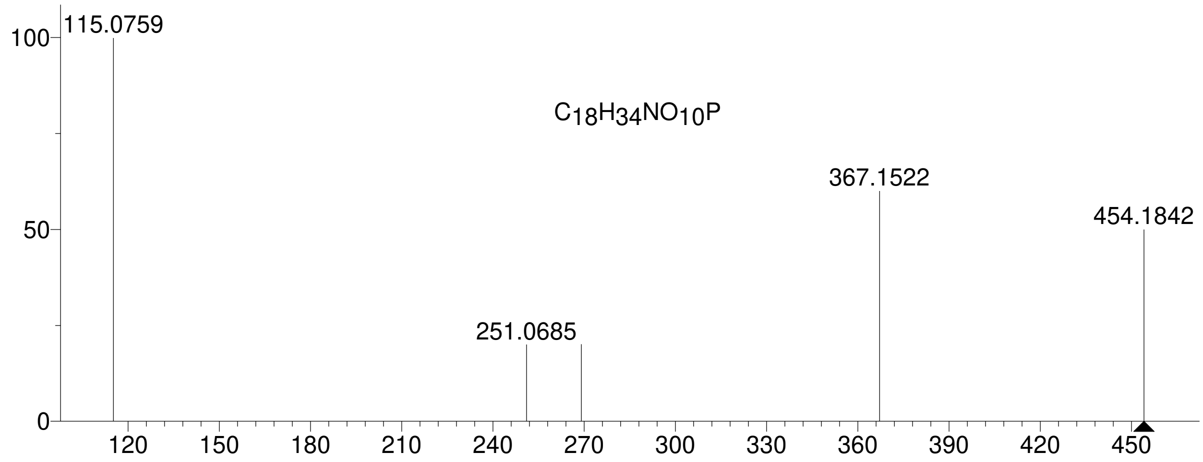

Hit 2 : PS 12:0; PS 2:0-10:0; [M-H]- [M-H]- P=454.2
C18H34NO10P; MF: 2; RMF: 142; Prob 16.6%; Lib: msdial-insilicomsms-lipids-neg; ID: 329683.

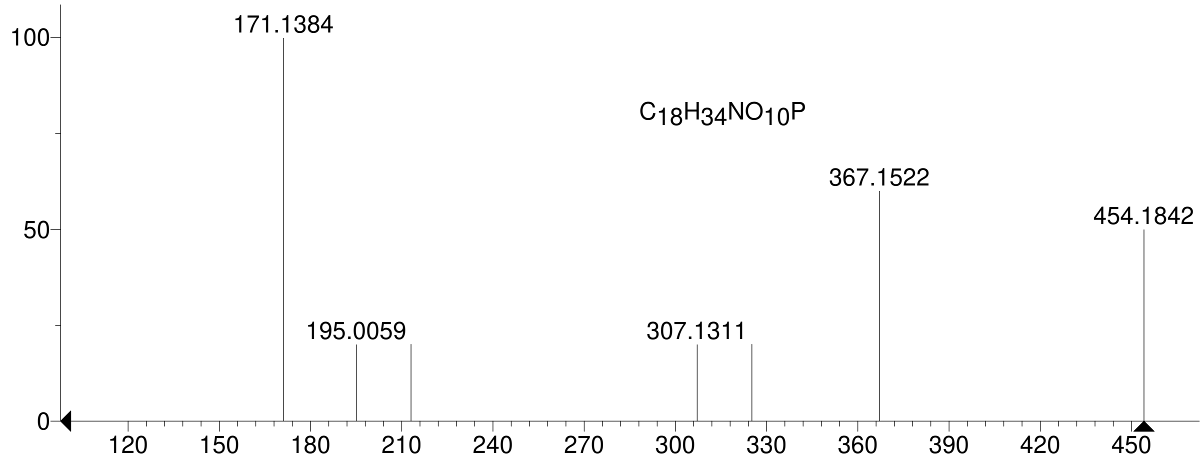

Hit 3 : PS 12:0; PS 3:0-9:0; [M-H]- [M-H]- P=454.2
C18H34NO10P; MF: 2; RMF: 142; Prob 16.6%; Lib: msdial-insilicomsms-lipids-neg; ID: 329742.

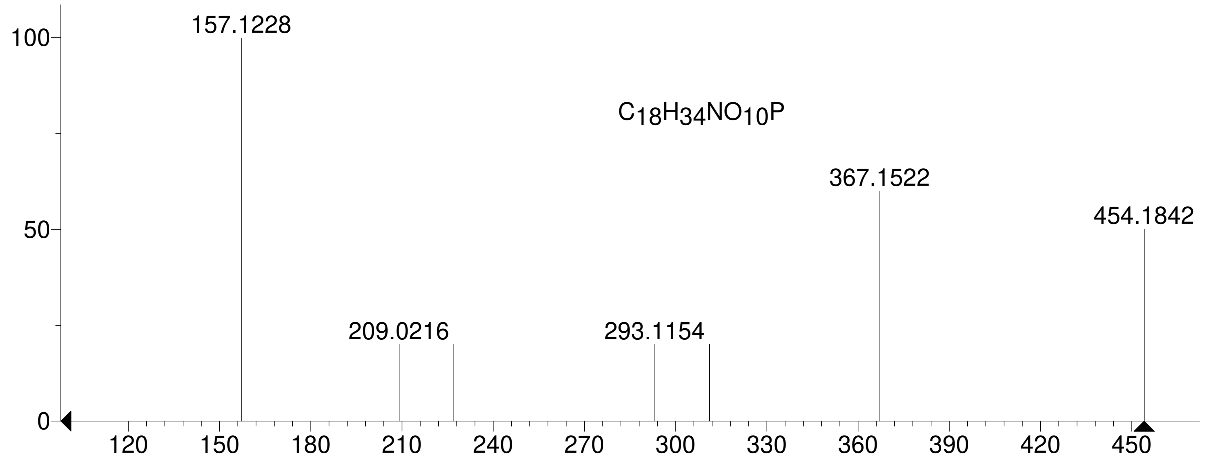

Hit 4 : PS 12:0; PS 4:0-8:0; [M-H]- [M-H]- P=454.2
C18H34NO10P; MF: 2; RMF: 142; Prob 16.6%; Lib: msdial-insilicomsms-lipids-neg; ID: 329800.

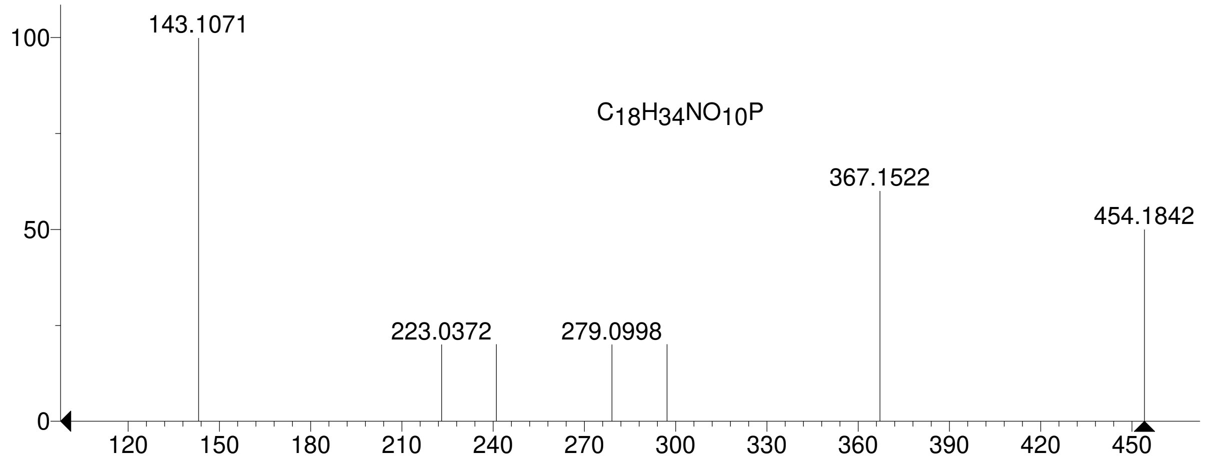

Hit 5 : PS 12:0; PS 5:0-7:0; [M-H]- [M-H]- P=454.2
C18H34NO10P; MF: 2; RMF: 142; Prob 16.6%; Lib: msdial-insilicomsms-lipids-neg; ID: 329857.

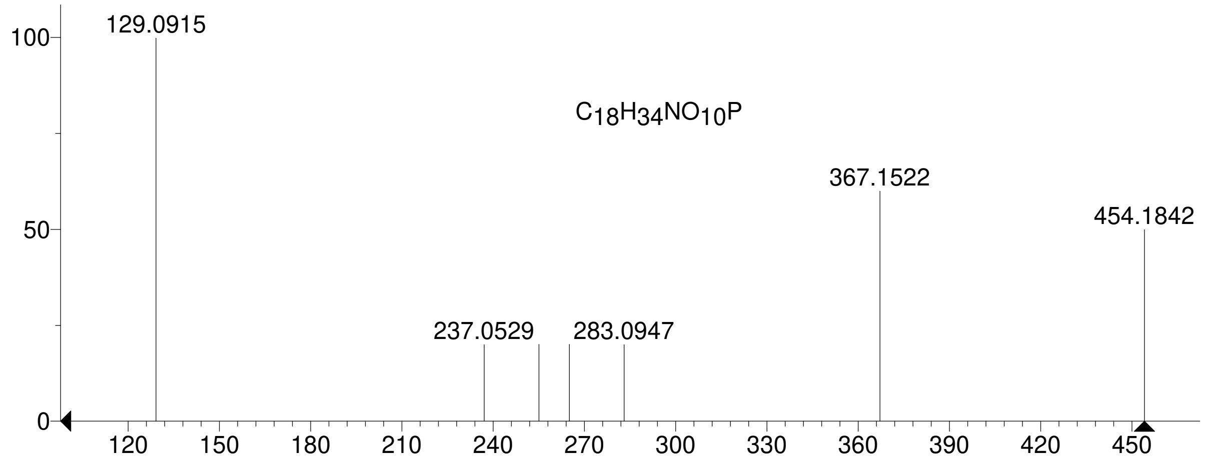

** Search Report Page 1 of 1 **

Unknown: Unknown (19.13_530.2987m/z)
Compound in Library Factor = 0

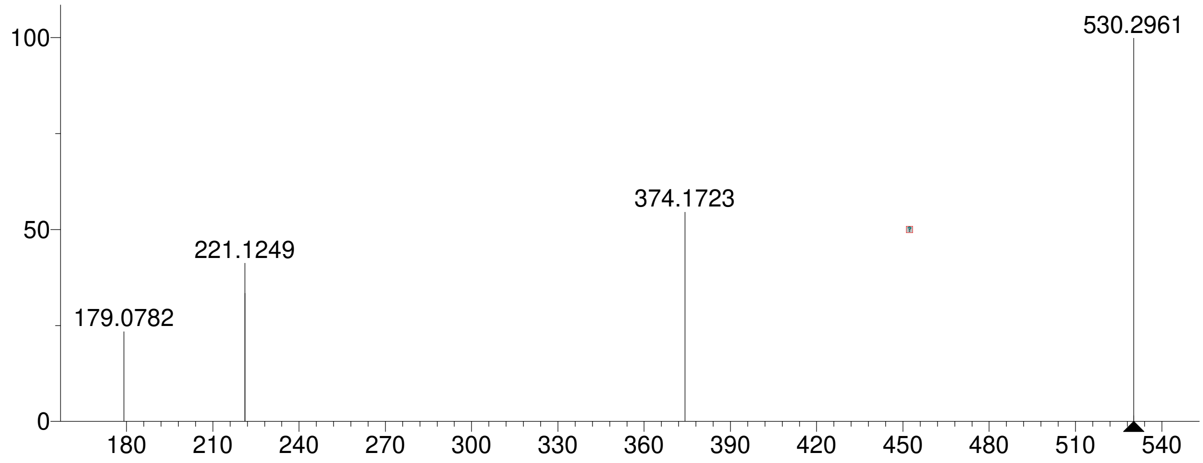

Unknown: Unknown (19.18_308.1855m/z)
Compound in Library Factor = -199

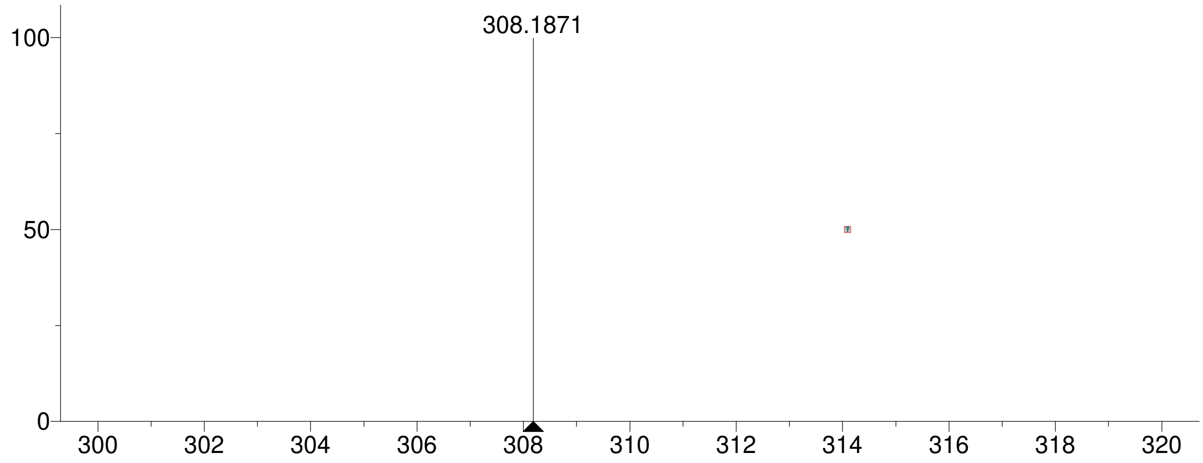

Hit 1 : Buflomedil [M+H]+ 20-30-40%) P=308.2
C17H25NO4; MF: 364; RMF: 999; Prob 49.7%; Lib: mona-export-fiehn_hilic; ID: 241.

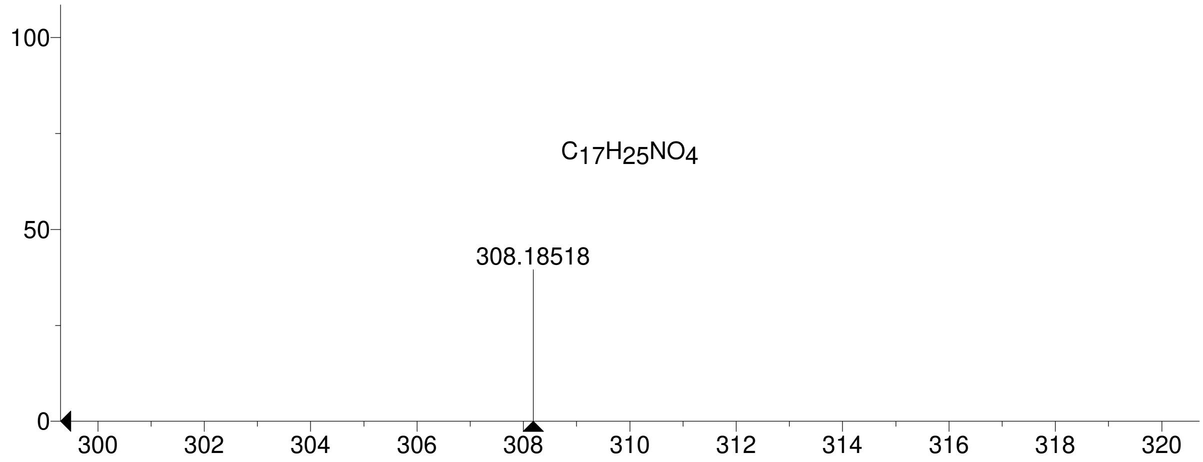

Hit 2 : Senecionine [M+H-CO]+ HCD 36V P=308.2
C18H25NO5; MF: 364; RMF: 999; Prob 49.7%; CAS: 130-01-8; Lib: nist_msms; ID: 219415.

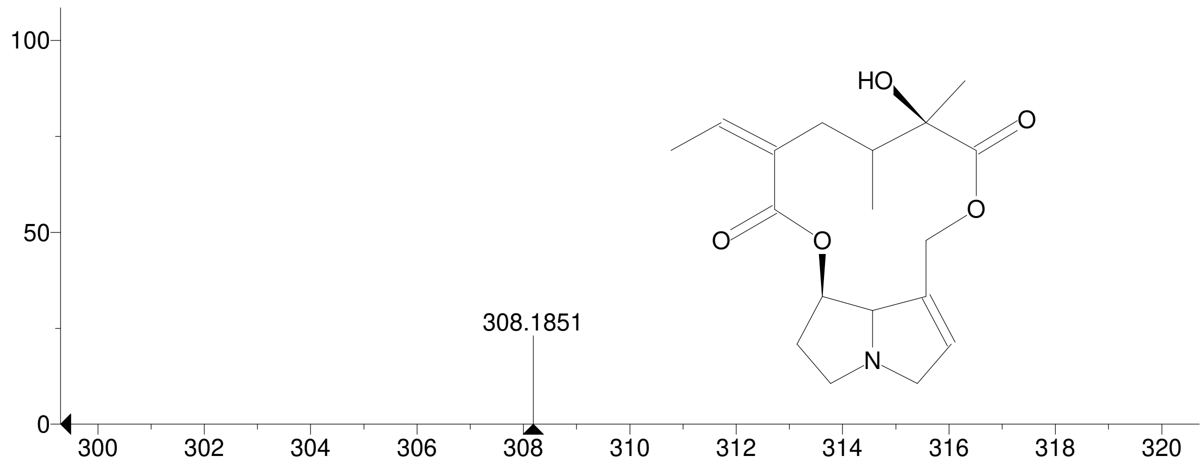

Hit 3 : Buflomedil HCl [M+H]+ P=308.2
C17H26ClNO4; MF: 87; RMF: 750; Prob 0.49%; Lib: mona-export-gnps; ID: 856.

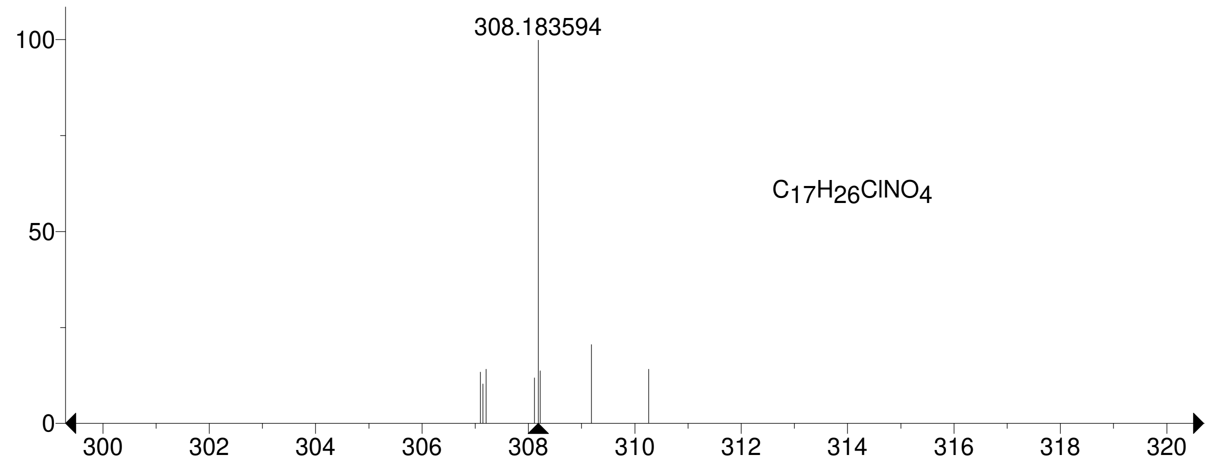

Hit 4 : Protein Kinase C (19-36) Peptide [M+7H]7+ HCD 9eV P=308.2
C93H159N35O24; MF: 4; RMF: 486; Prob 0.04%; Lib: nist_msms2; ID: 47643.

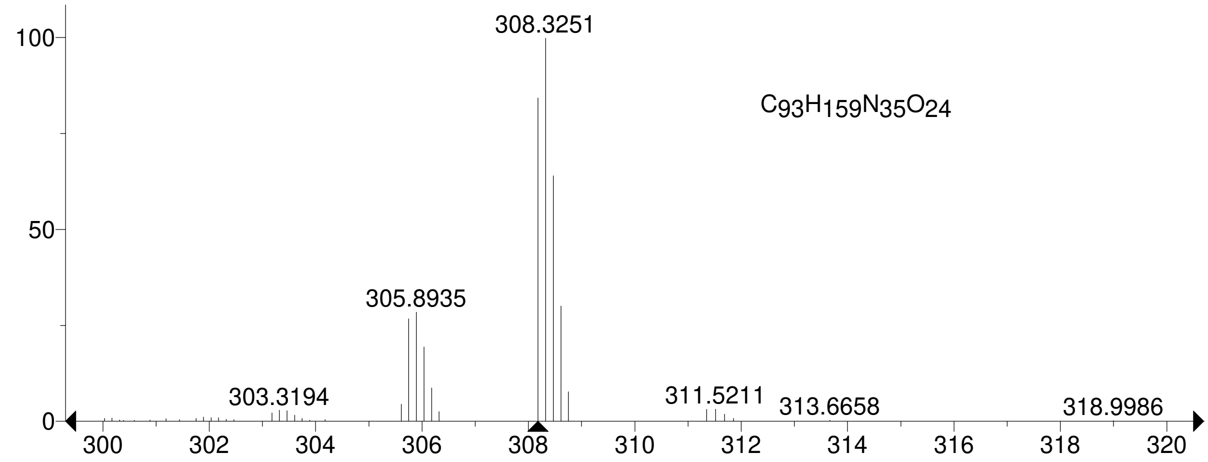

Hit 5 : Tulipinolide [M+NH4]+ 35HCD P=308.2
C17H22O4; MF: 1; RMF: 171; Prob 0.04%; Lib: mona-export-vf-npl_qexactive; ID: 5902.

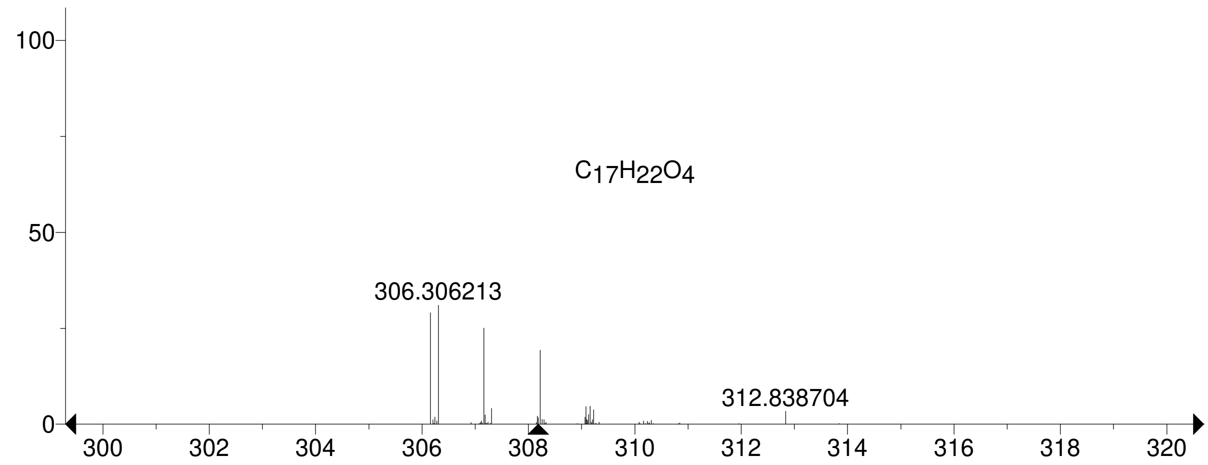

Unknown: Unknown (19.21_365.1051m/z)
Compound in Library Factor = -1523

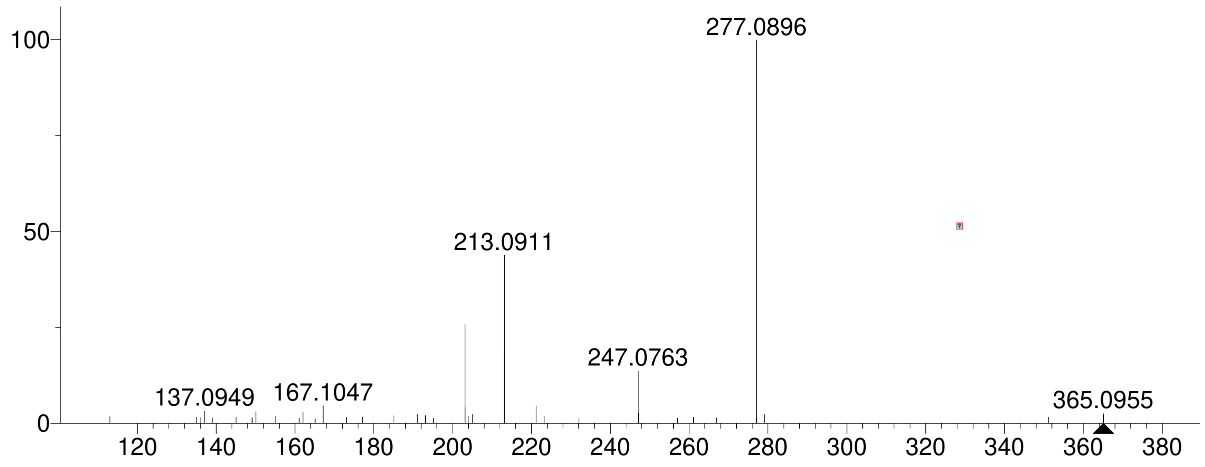

Hit 1 : Lactose [M+Na]+ QTOF 16V P=365.1
C12H22O11; MF: 123; RMF: 559; Prob 40.3%; CAS: 63-42-3; Lib: nist_msms; ID: 28960.

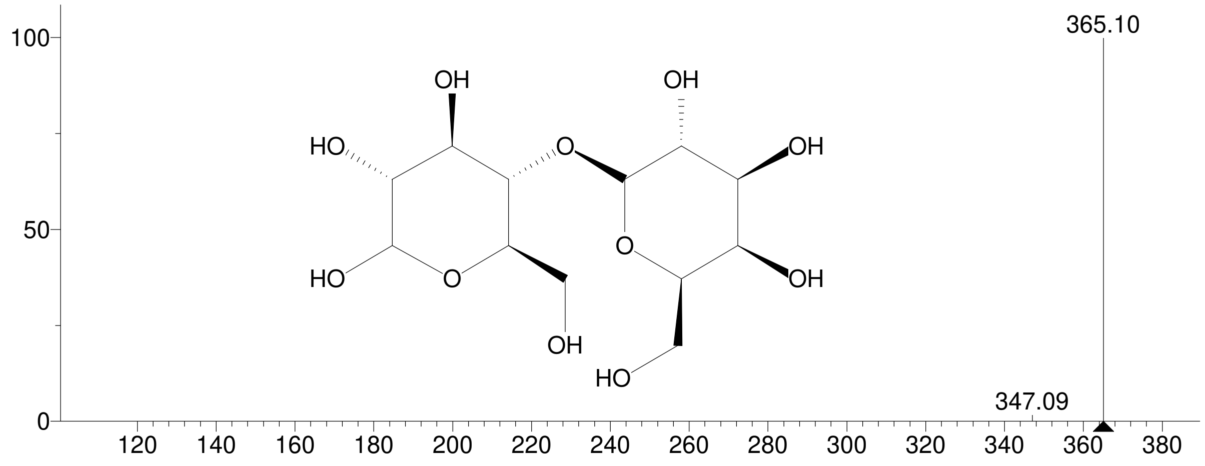

Hit 2 : Lactulose [M+Na]+ QTOF 6V P=365.1
C12H22O11; MF: 110; RMF: 547; Prob 26.0%; CAS: 4618-18-2; Lib: nist_msms; ID: 30110.

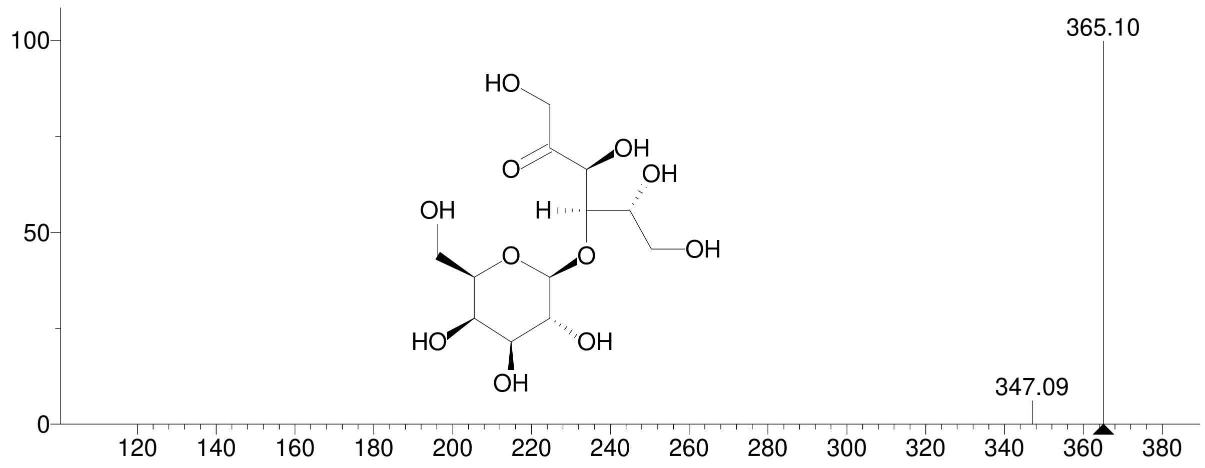

Hit 3 : 2α-Mannobiose [M+Na]+ QQQ 40V P=365.1
C12H22O11; MF: 78; RMF: 265; Prob 6.95%; CAS: 15548-39-7; Lib: nist_msms; ID: 59256.

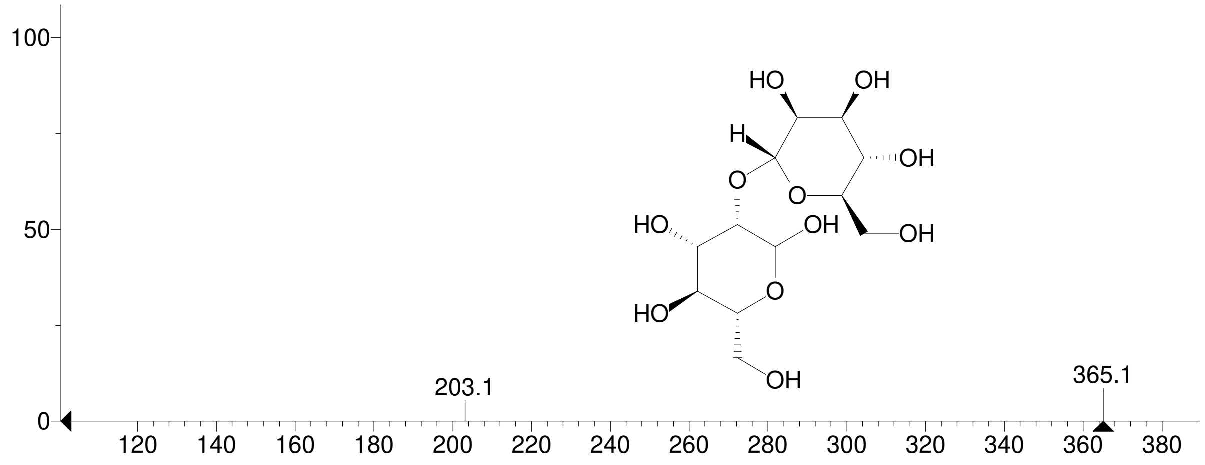

Hit 4 : Palatinose [M+Na]+ QQQ 40V P=365.1
C12H22O11; MF: 77; RMF: 281; Prob 6.68%; CAS: 13718-94-0; Lib: nist_msms; ID: 58882.

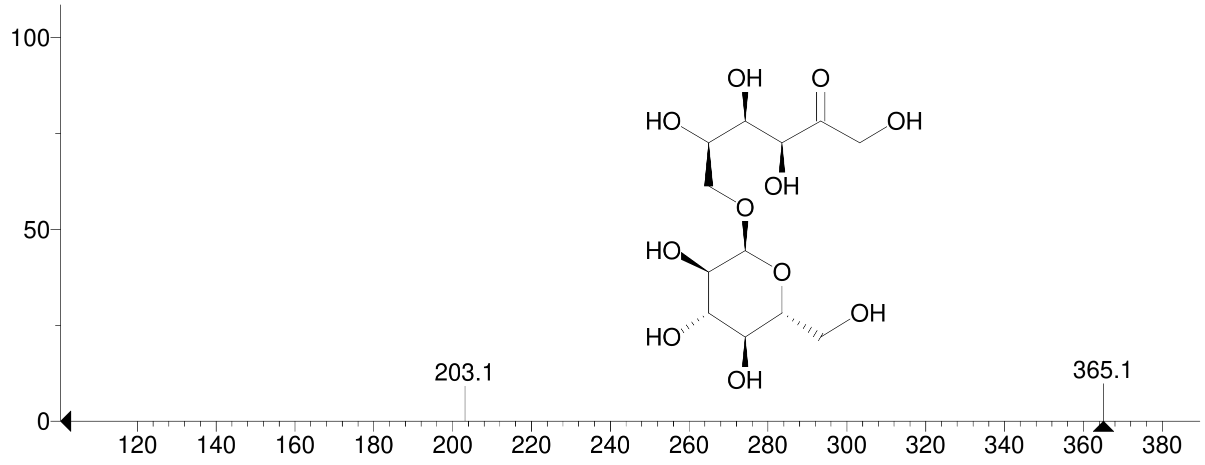

Hit 5 : D-(+)-Trehalose [M+Na]+ QQQ 36V P=365.1
C12H22O11; MF: 73; RMF: 308; Prob 5.64%; CAS: 99-20-7; Lib: nist_msms; ID: 59149.

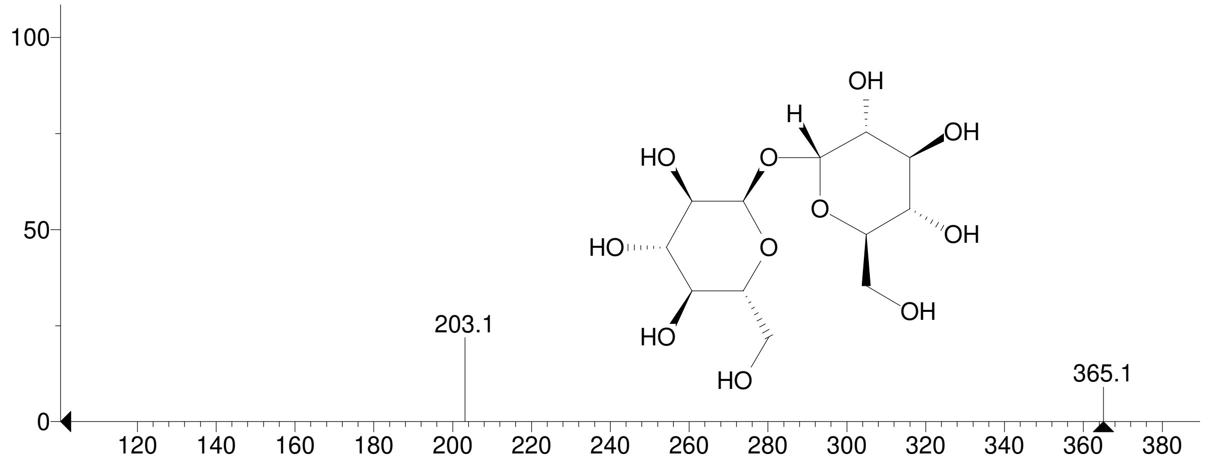

Unknown: Unknown (19.52_407.1701m/z)
Compound in Library Factor = 0

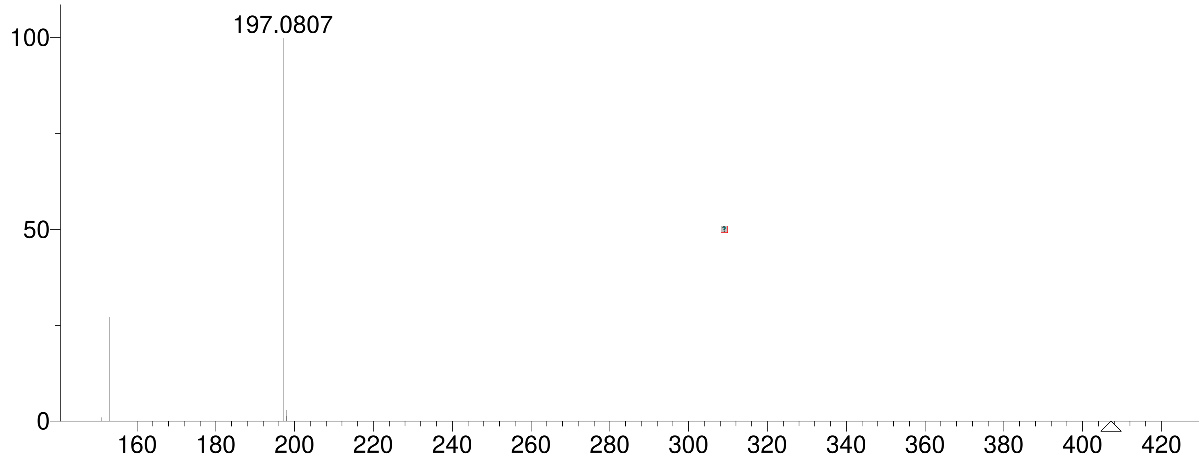

Hit 1 : [5-chloro-3-[(1E,3E)-3,5-dimethylhepta-1,3-dienyl]-7-methyl-6,8-dioxo-2H-isoquinolin-7-yl] acetate [M+NH4
C21H24ClNO4; MF: 0; RMF: 55; Prob 25.0%; Lib: mona-export-vf-npl_qexactive; ID: 25203.

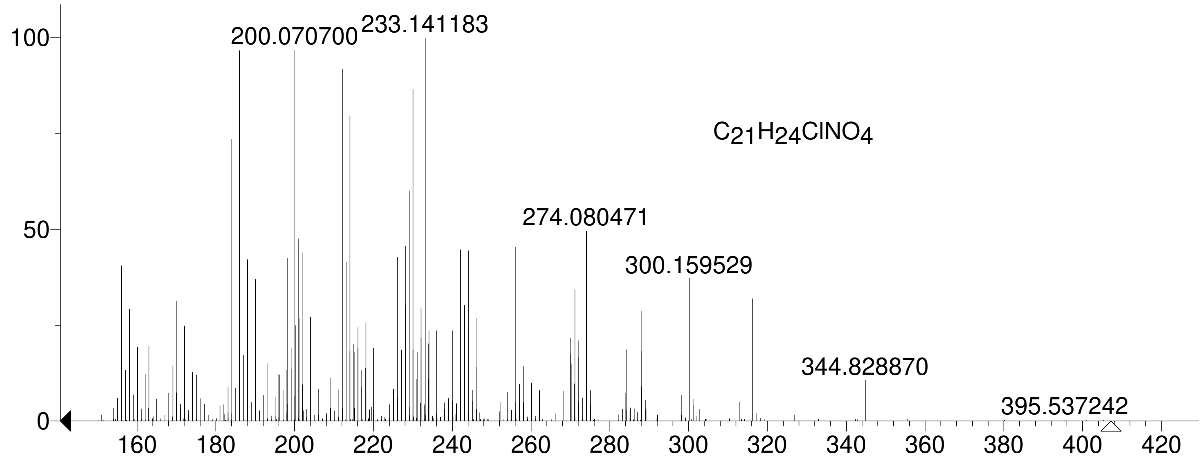

Hit 2 : Artesunate [M+Na]+ HCD 32V P=407.2
C19H28O8; MF: 0; RMF: 13; Prob 25.0%; CAS: 88495-63-0; Lib: nist_msms; ID: 119196.

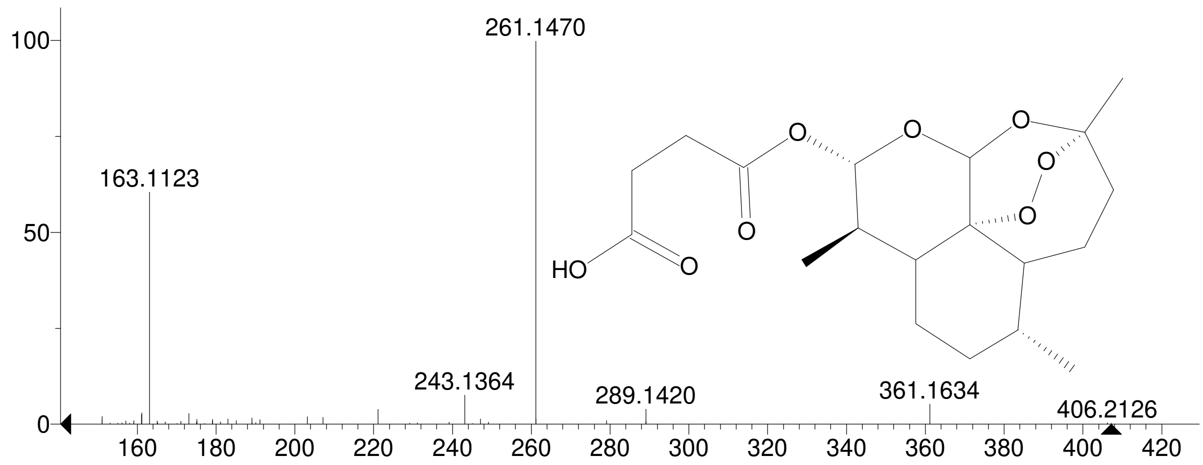

Hit 3 : Cys-Glu-Arg [M+H]+ QTOF 30V P=407.2
C14H26N6O6S; MF: 0; RMF: 12; Prob 25.0%; Lib: nist_msms; ID: 49547.

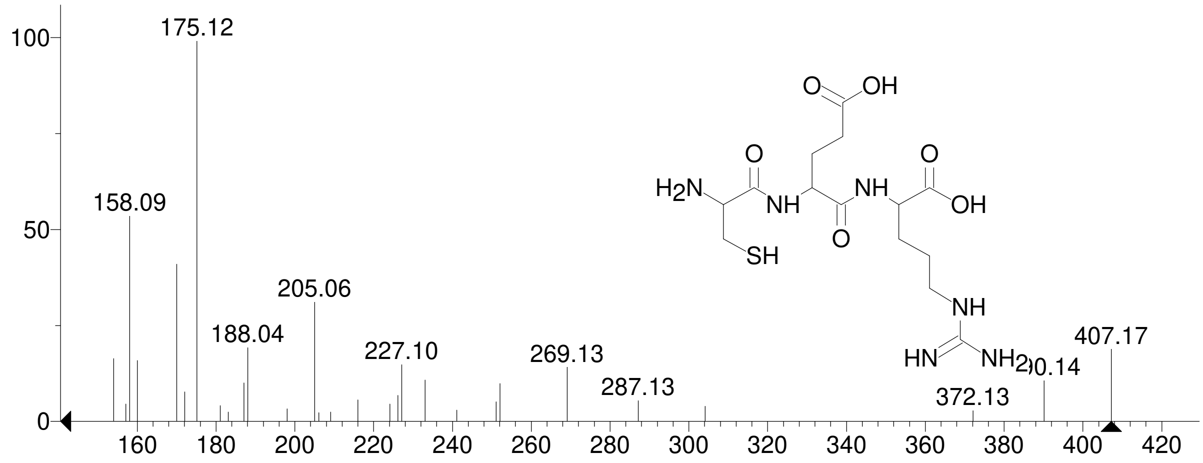

Hit 4 : Glu-Cys-Arg [M+H]+ QTOF 27V P=407.2
C14H26N6O6S; MF: 0; RMF: 10; Prob 25.0%; Lib: nist_msms; ID: 55676.

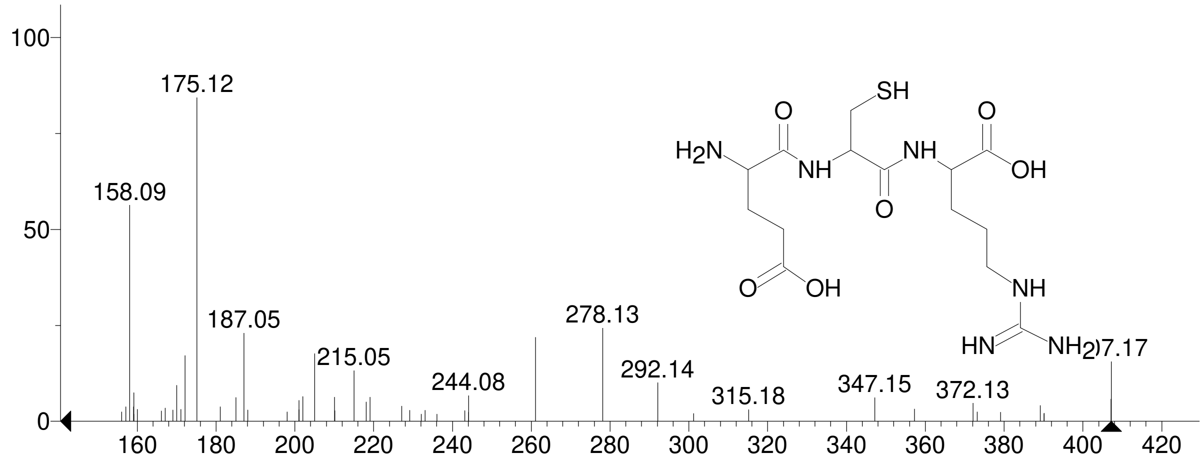

Unknown: Unknown (19.56_459.2589m/z)
Compound in Library Factor = -226

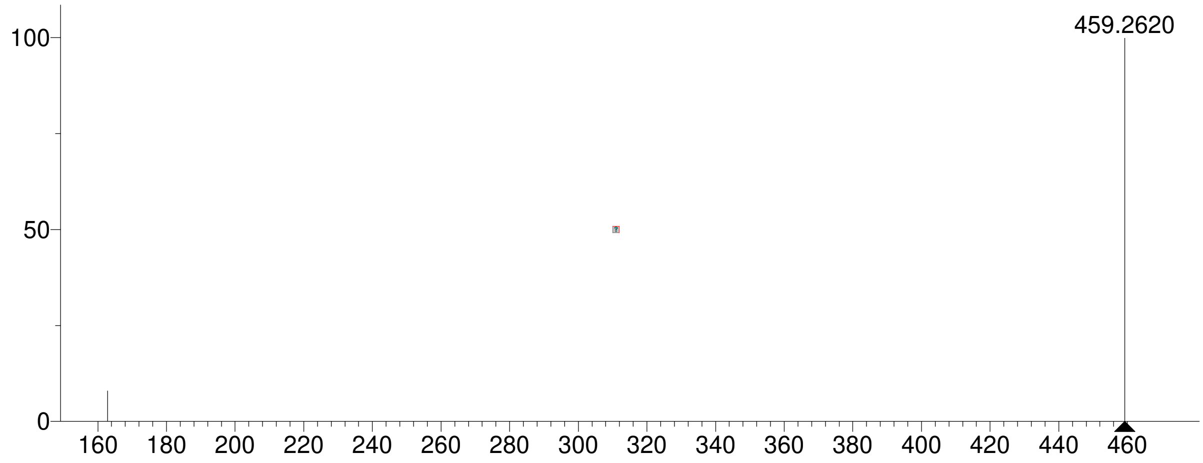

Hit 1 : β-Lipotropin (88-91) [M-H]- HCD 16eV P=459.3
C19H36N6O7; MF: 316; RMF: 950; Prob 99.0%; CAS: 72189-84-5; Lib: nist_msms2; ID: 72157.

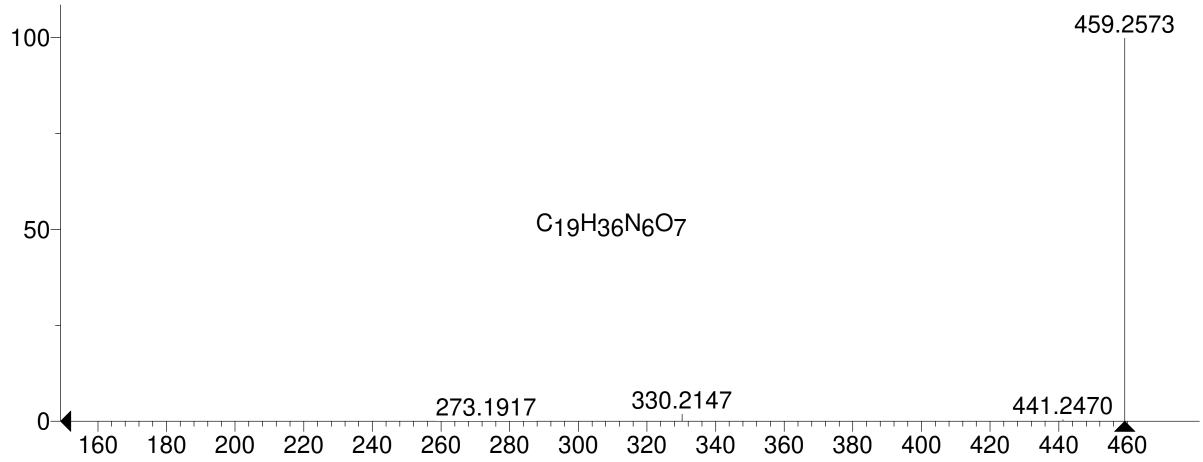

Hit 2 : Astemizole [M+H]+ HCD 24V P=459.3
C28H31FN4O; MF: 55; RMF: 753; Prob 0.99%; CAS: 68844-77-9; Lib: nist_msms; ID: 129929.

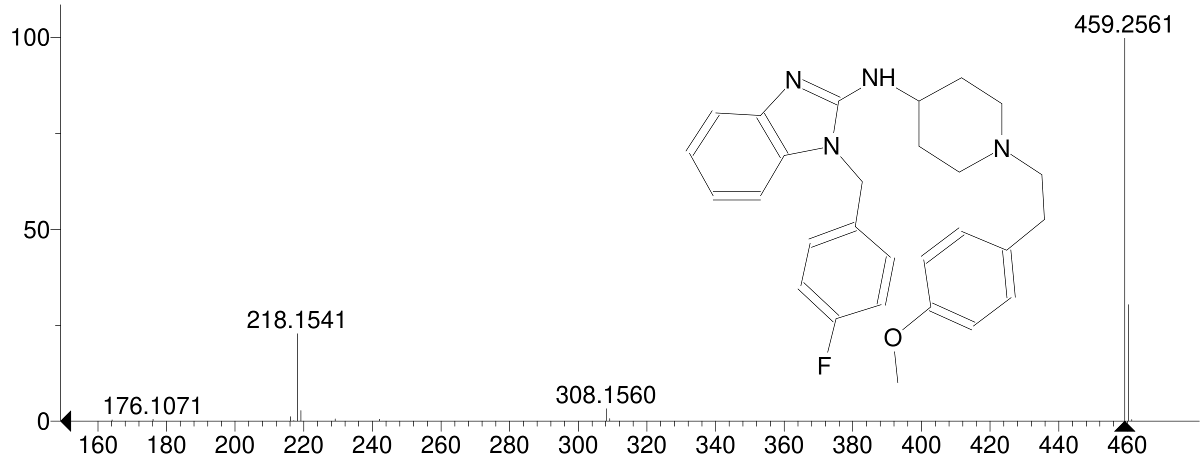

Unknown: Unknown (19.67_511.2001m/z)
Compound in Library Factor = -3273

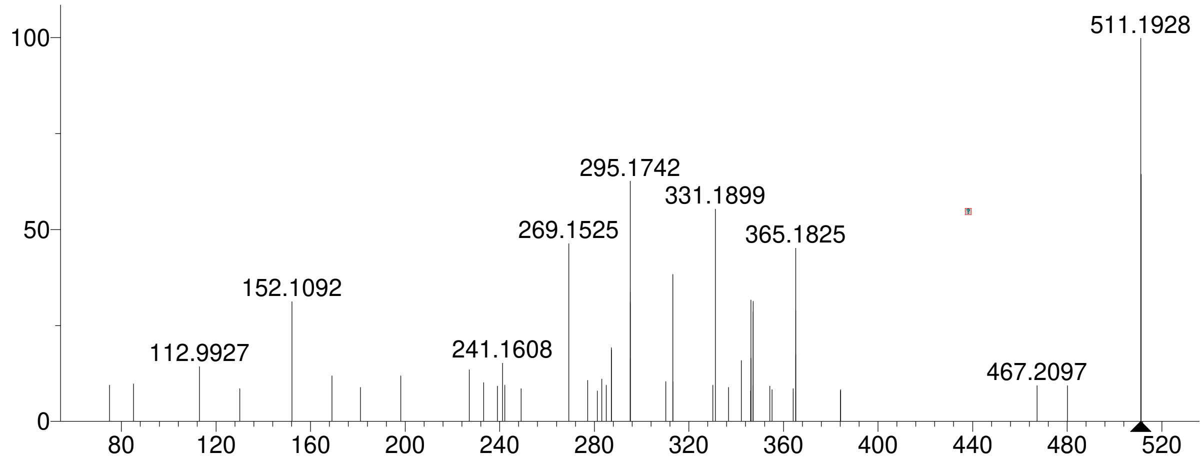

Hit 1 : Ganciclovir [2M+H]+ HCD 5V P=511.2
C9H13N5O4; MF: 8; RMF: 220; Prob 40.1%; CAS: 82410-32-0; Lib: nist_msms; ID: 263891.

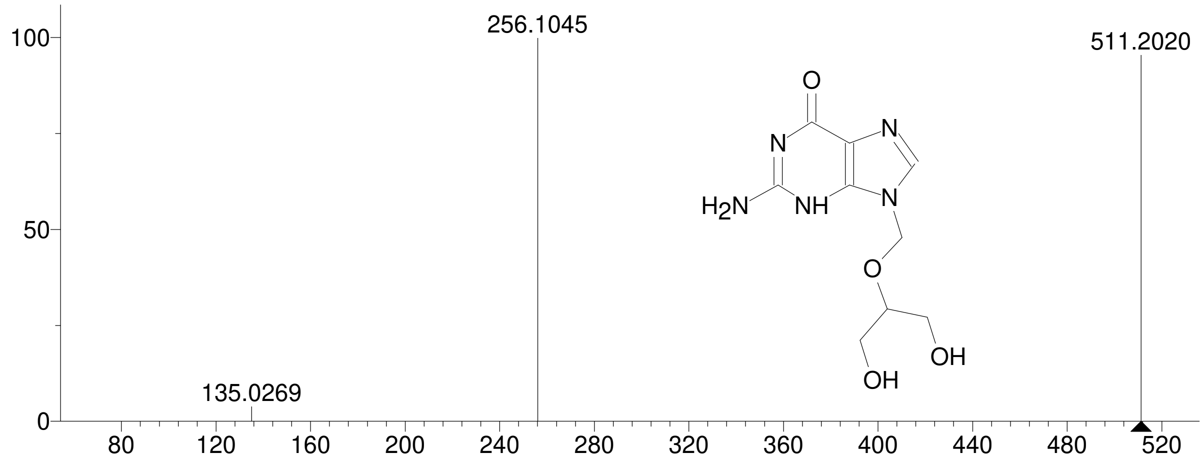

Hit 2 : Pluripotin [M+H]+=>511.2 IT 35% P=511.2
C27H25F3N8O2; MF: 0; RMF: 18; Prob 29.9%; CAS: 839707-37-8; Lib: nist_msms; ID: 156609.

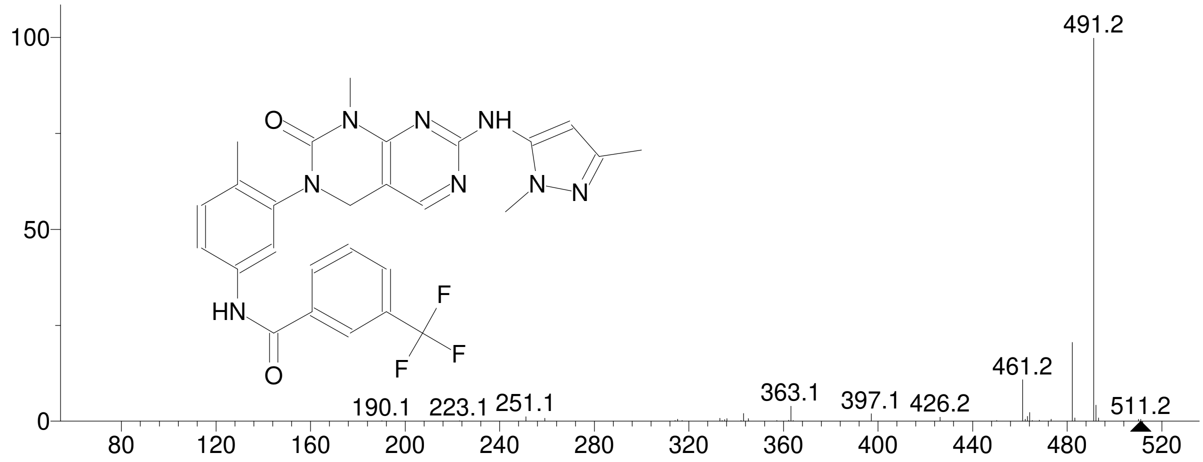

Hit 3 : 5-Heptenoic acid, 7-[(1R,2R,3R,5S)-3,5-dihydroxy-2-[(3R)-3-hydroxy-5-phenylpentyl]cyclopentyl]-, 4-(3-thic
C32H38O5S3; MF: 0; RMF: 14; Prob 29.9%; CAS: 1088434-86-9; Lib: nist_msms; ID: 262366.

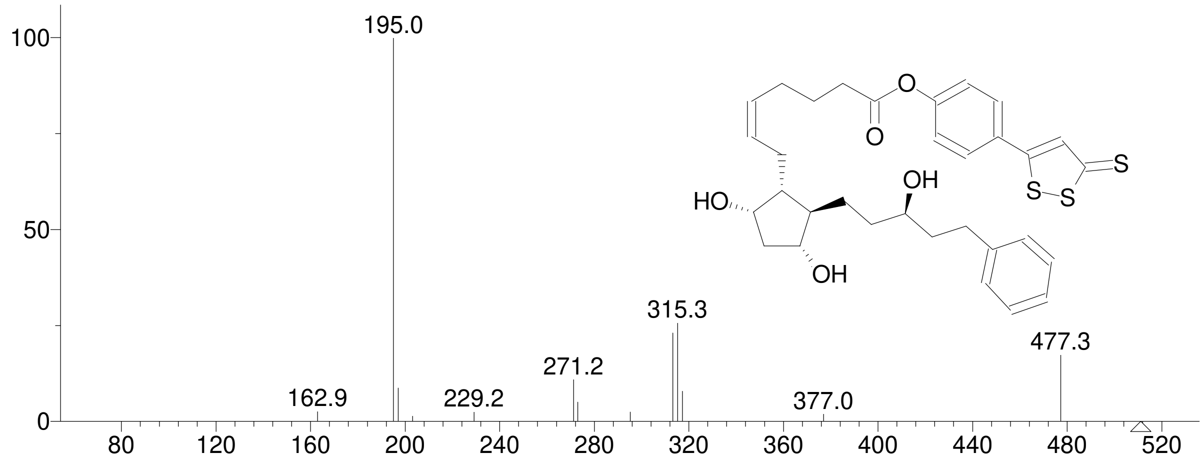

Unknown: Unknown (19.68_291.1227m/z)
Compound in Library Factor = -1523

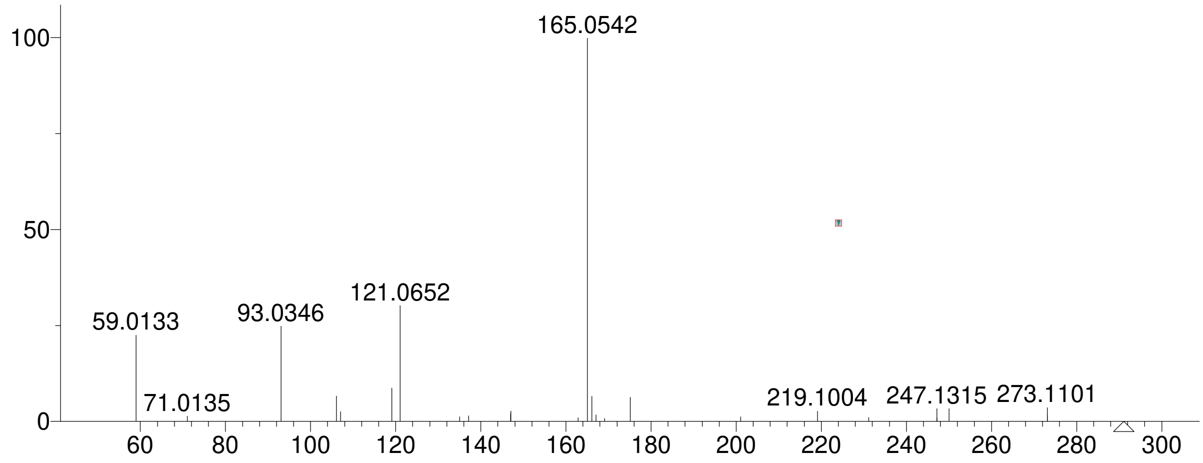

Hit 1 : 2-Cyclopentene-1-octanoic acid, 5-(2-carboxyethyl)-ε,4-dioxo-, (1S,5R)- [M-H-H2O]- IT-FT 35% P=291.1
C16H22O6; MF: 68; RMF: 256; Prob 30.5%; CAS: 52510-53-9; Lib: nist_msms; ID: 551876.

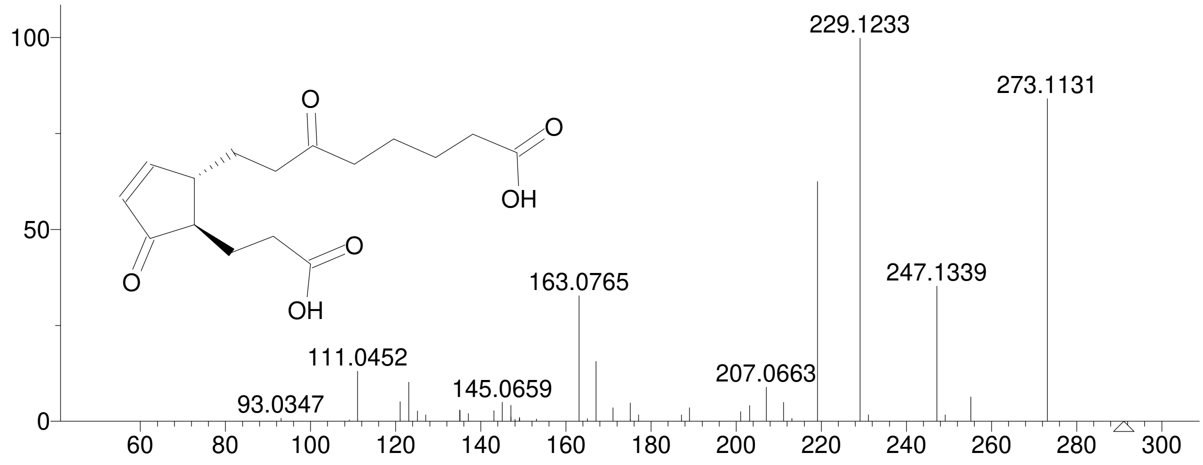

Hit 2 : Cyclopenta[b]pyran-5-octanoic acid, octahydro-ε,2,6-trioxo-, (4aR,5R,7aS)- [M-H-H2O]- HCD 32V P=29
C16H22O6; MF: 27; RMF: 217; Prob 6.95%; Lib: nist_msms; ID: 377569.

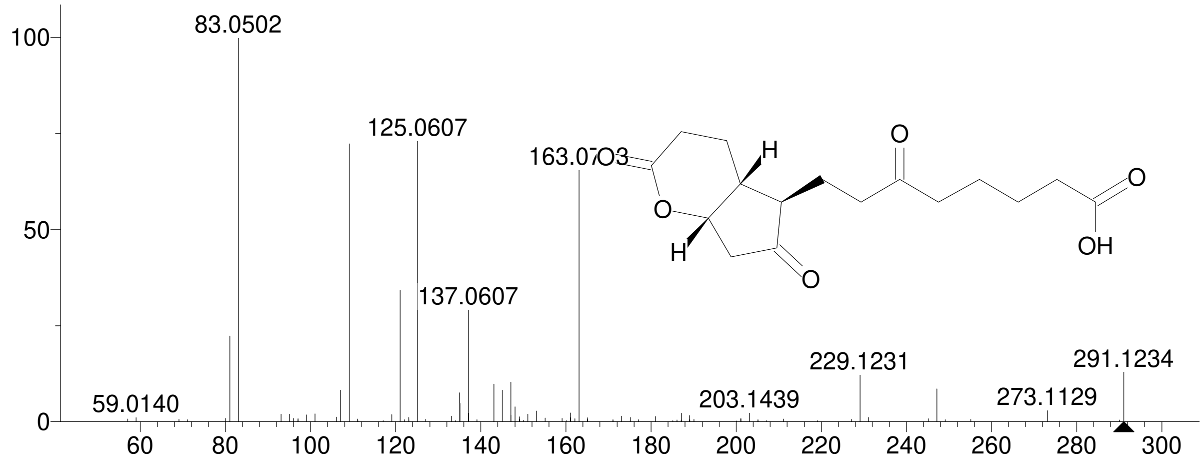

Hit 3 : Tetranorprostaglandin J2 [M-H-H2O]- HCD 26V P=291.1
C16H22O6; MF: 27; RMF: 210; Prob 6.95%; CAS: 1352751-83-7; Lib: nist_msms; ID: 377420.

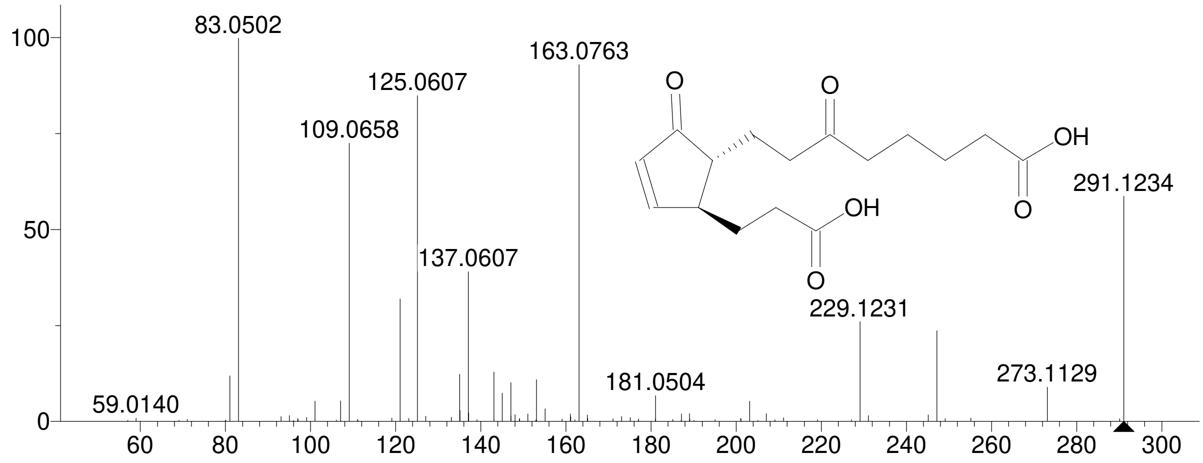

Hit 4 : 8-(2-hydroxy-3-methoxy-3-methylbutyl)-7-methoxychromen-2-one [M-H]- 35HCD P=291.1
C16H20O5; MF: 23; RMF: 242; Prob 5.87%; Lib: mona-export-lc-ms-ms_negative_mode; ID: 19661.

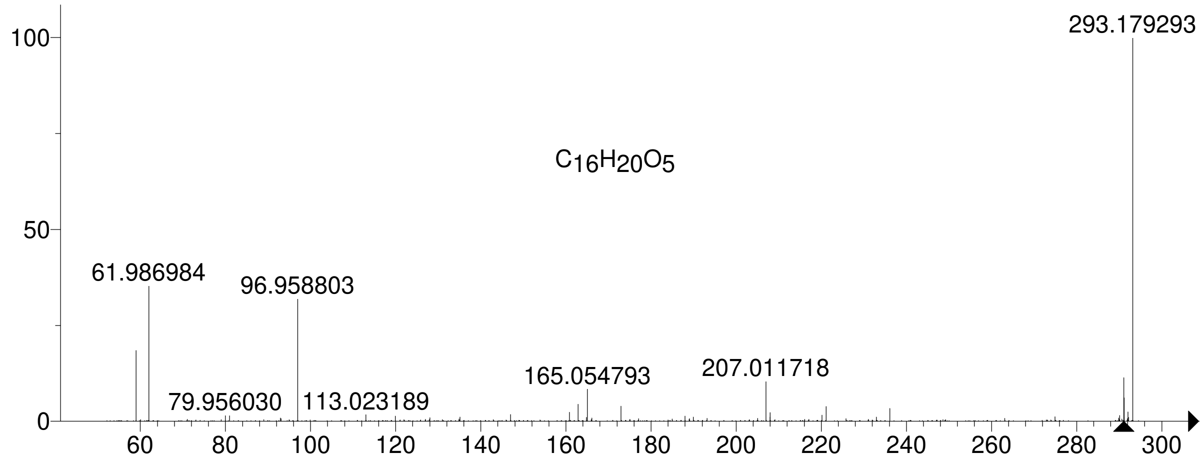

Hit 5 : 4'-O-β-D-Glucosyl-5-O-methylvisamminol [M+H-C6H10O5]+ IT 35% P=291.1
C22H28O10; MF: 10; RMF: 170; Prob 3.79%; CAS: 84272-85-5; Lib: nist_msms; ID: 236324.

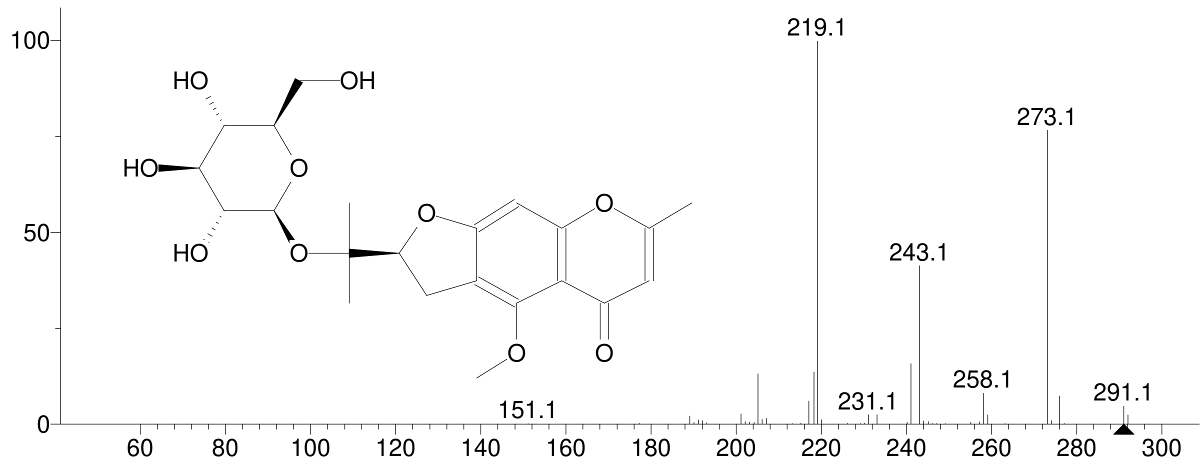

Unknown: Unknown (19.78_400.1751m/z)
Compound in Library Factor = -301

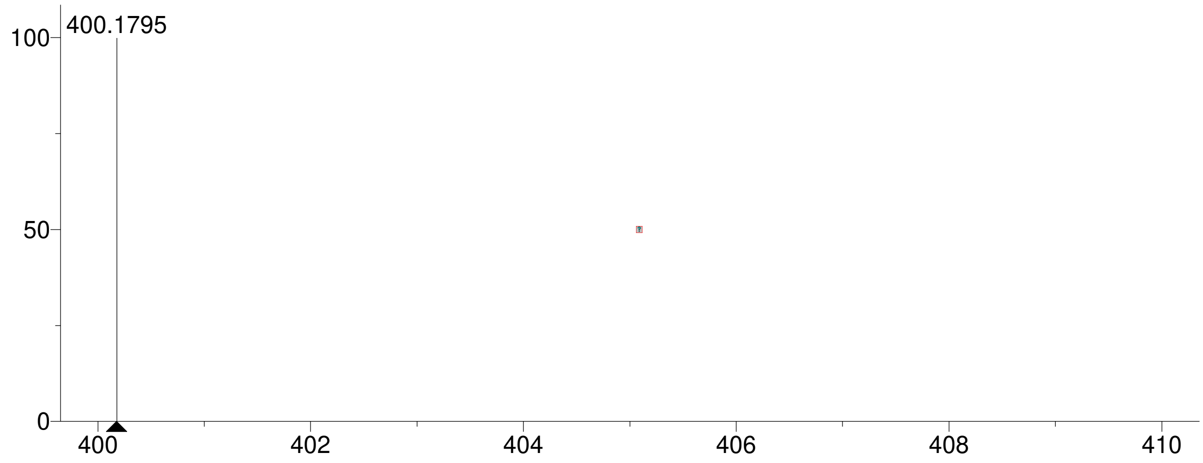

Hit 1 : LPC 6:0; [M+FA-H]- [M+FA-H]- P=400.2
C14H30NO7P; MF: 364; RMF: 999; Prob 33.1%; Lib: msdial-insilicomsms-lipids-neg; ID: 310902.

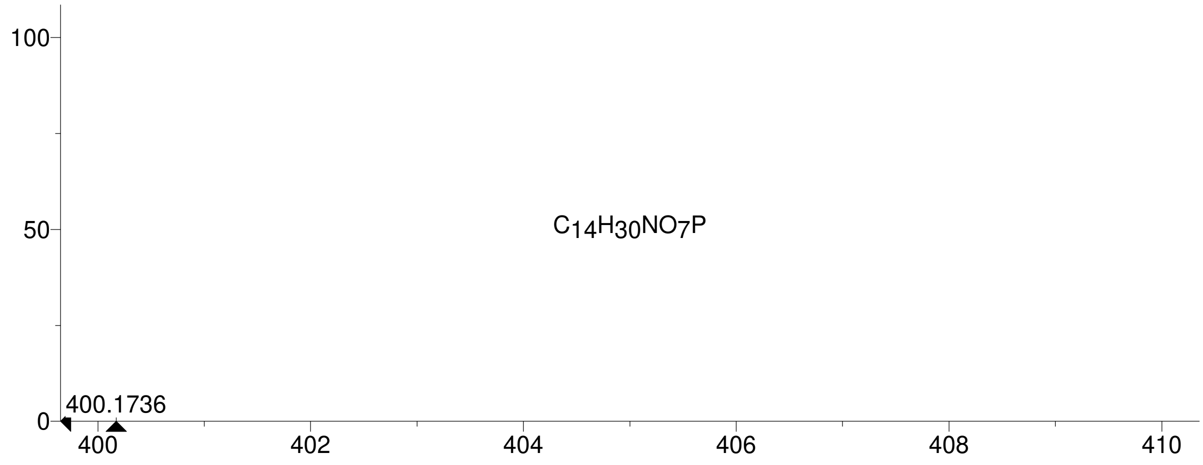

Hit 2 : LPC 5:0; [M+Hac-H]- [M+Hac-H]- P=400.2
C13H28NO7P; MF: 364; RMF: 999; Prob 33.1%; Lib: msdial-insilicomsms-lipids-neg; ID: 310962.

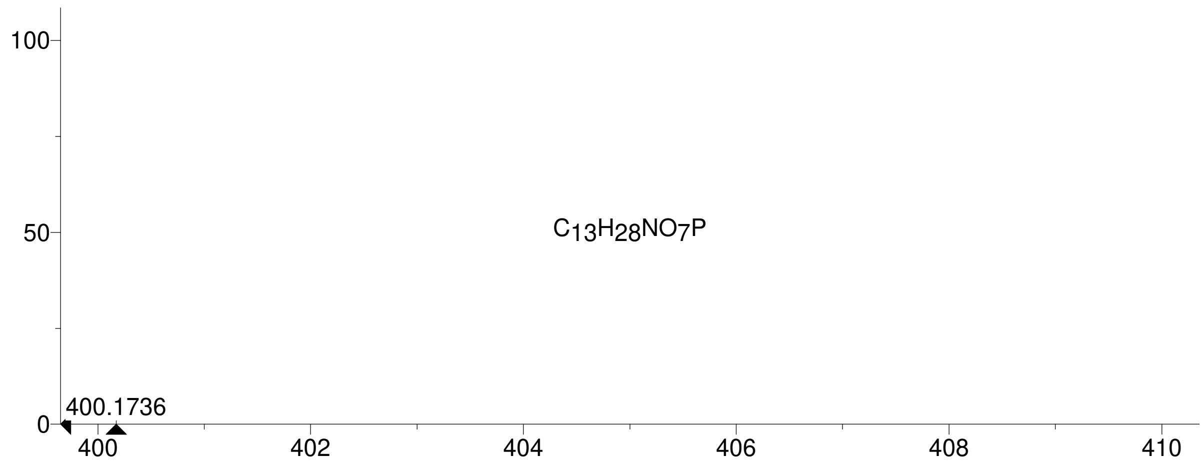

Hit 3 : Colchicine [M+H]+ HCD 40V P=400.2
C22H25NO6; MF: 364; RMF: 999; Prob 33.1%; CAS: 64-86-8; Lib: nist_msms; ID: 126601.

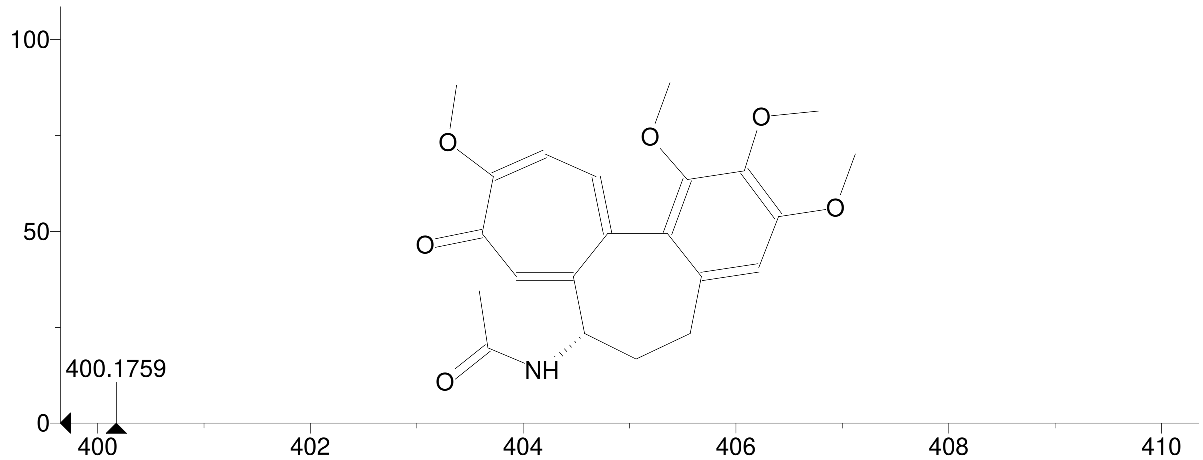

Hit 4 : NCGC00025125-21!N-[(7S)-1,2,3,10-tetramethoxy-9-oxo-6,7-dihydro-5H-benzo[a]heptalen-7-yl]acetamide
C22H25NO6; MF: 195; RMF: 918; Prob 0.74%; Lib: mona-export-gnps; ID: 6932.

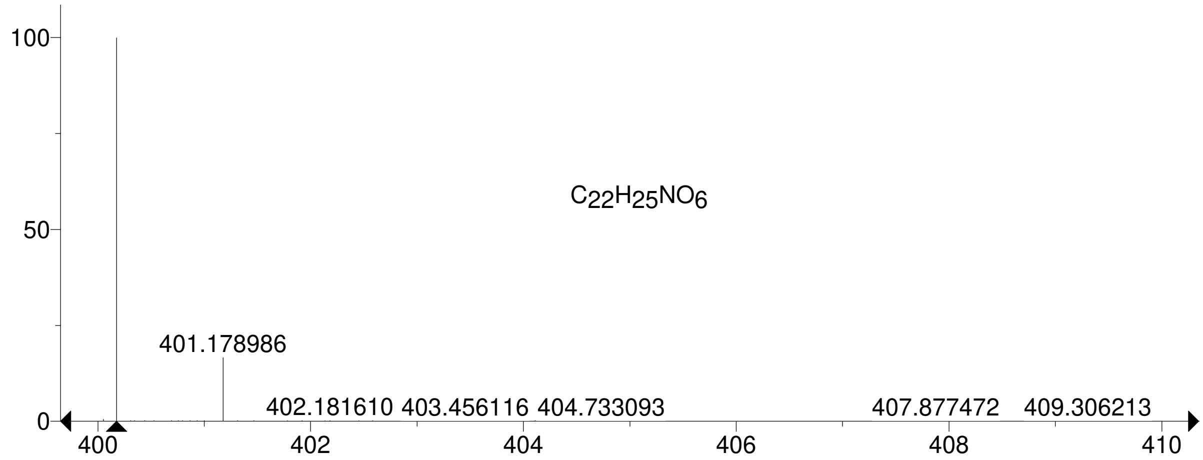

Hit 5 : NCGC00380314-01_C22H27NO7_3,5,7,9-Undecatetraenoic acid, 2-ethylidene-11-[4-hydroxy-4-(2-hydrox
C22H27NO7; MF: 2; RMF: 239; Prob 0.01%; Lib: mona-export-gnps; ID: 10356.

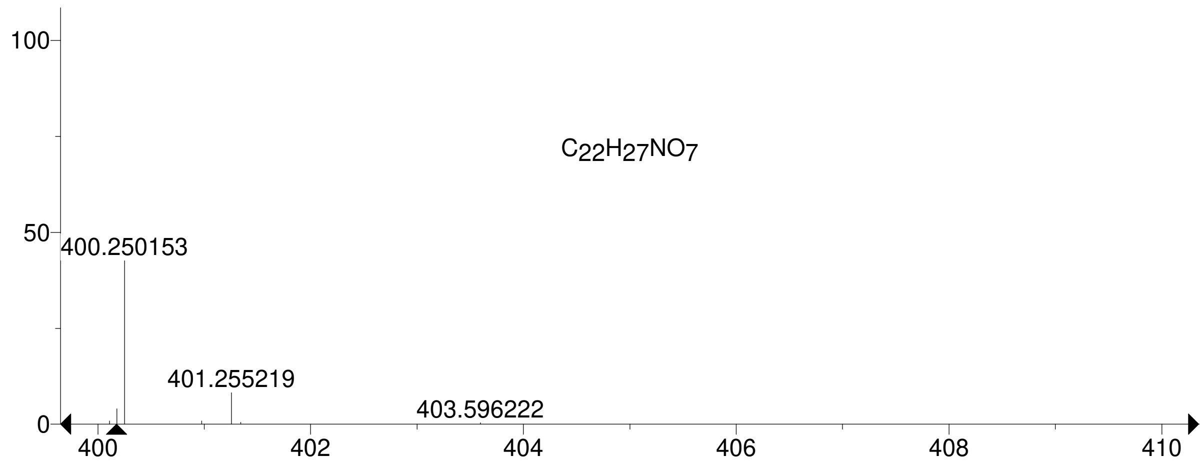

Unknown: Unknown (19.84_303.2169m/z)
Compound in Library Factor = -2176

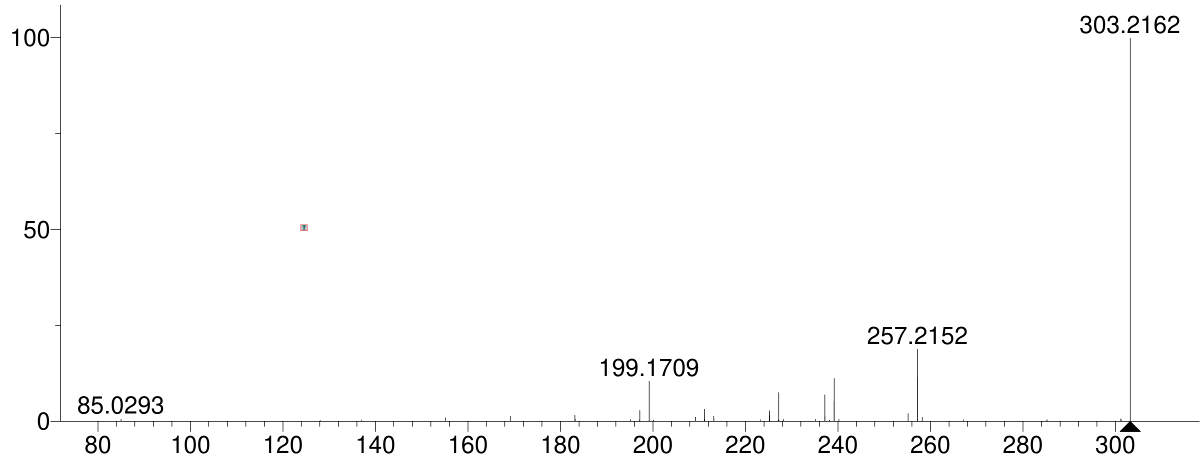

Hit 1 : Aleuritic acid [M-H]- IT-FT 35% P=303.2
C16H32O5; MF: 58; RMF: 232; Prob 55.7%; CAS: 533-87-9; Lib: nist_msms; ID: 411121.

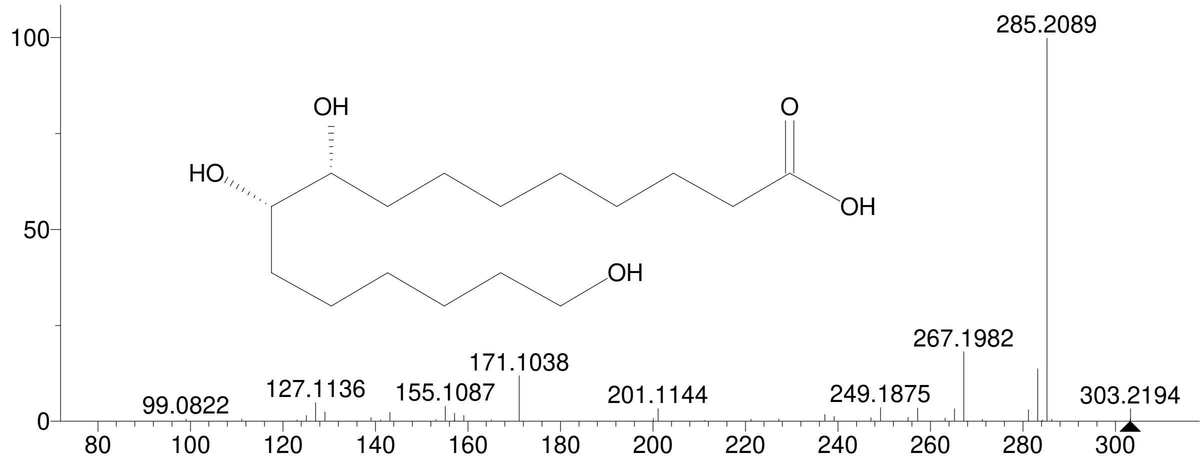

Hit 2 : Arg-Lys [M+H]+ QTOF 5V P=303.2
C12H26N6O3; MF: 30; RMF: 592; Prob 16.1%; CAS: 40968-46-5; Lib: nist_msms; ID: 55168.

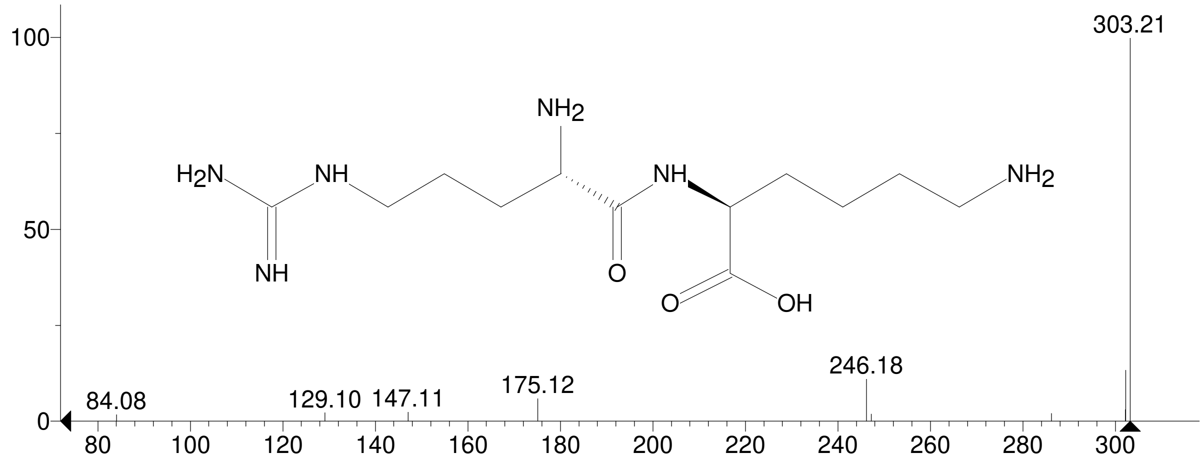

Hit 3 : Aleuretic Acid [M-H]- 35HCD P=303.2
C16H32O5; MF: 28; RMF: 609; Prob 14.9%; Lib: mona-export-lc-ms-ms_negative_mode; ID: 20798.

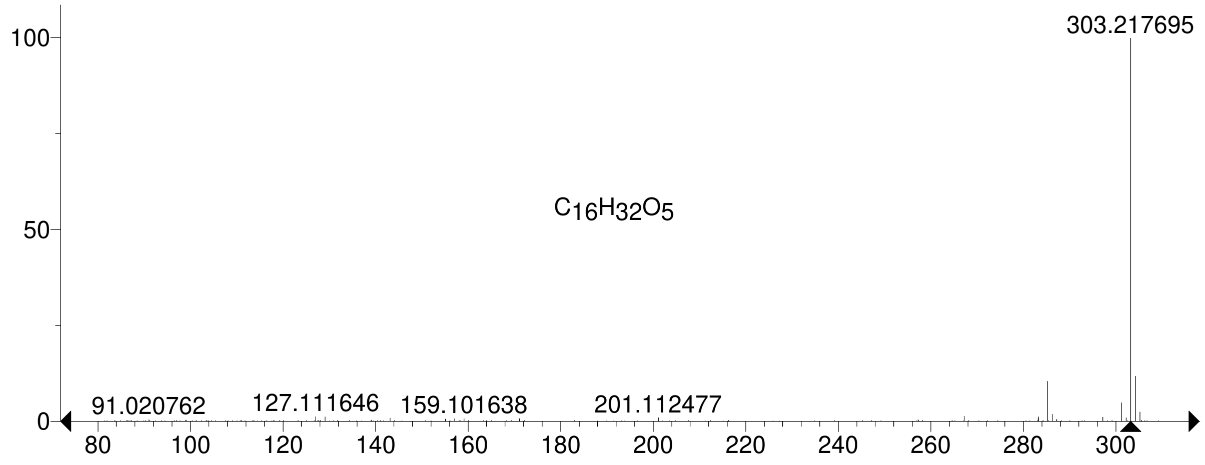

Hit 4 : Lys-Arg [M+H]+ QTOF 8V P=303.2
C12H26N6O3; MF: 9; RMF: 669; Prob 7.21%; Lib: nist_msms; ID: 48416.

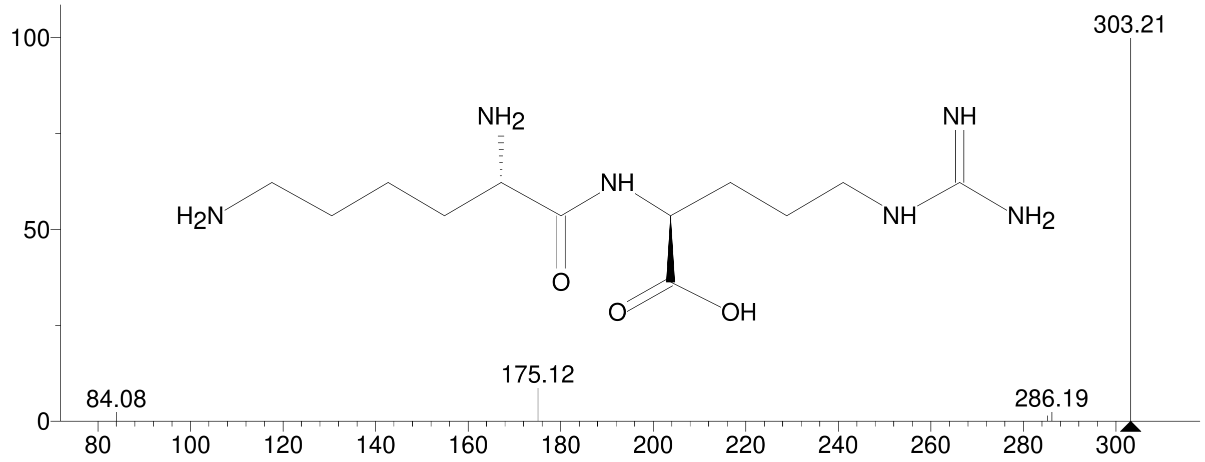

Hit 5 : Lys-Arg [M+H]+ IT-FT 35% P=303.2
C12H26N6O3; MF: 5; RMF: 119; Prob 6.09%; CAS: 29586-66-1; Lib: nist_msms2; ID: 41410.

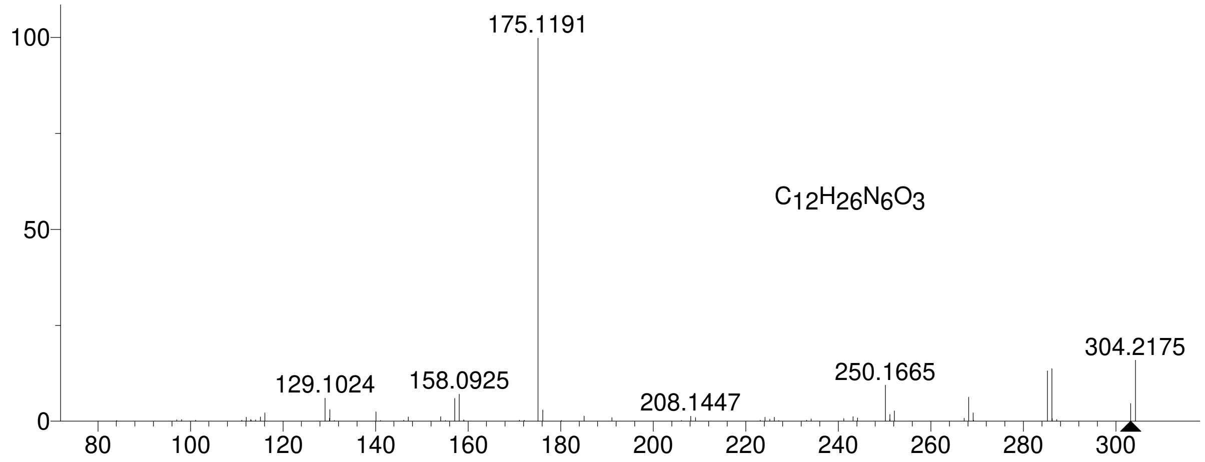

Unknown: Unknown (19.85_351.0895m/z)
Compound in Library Factor = -1142

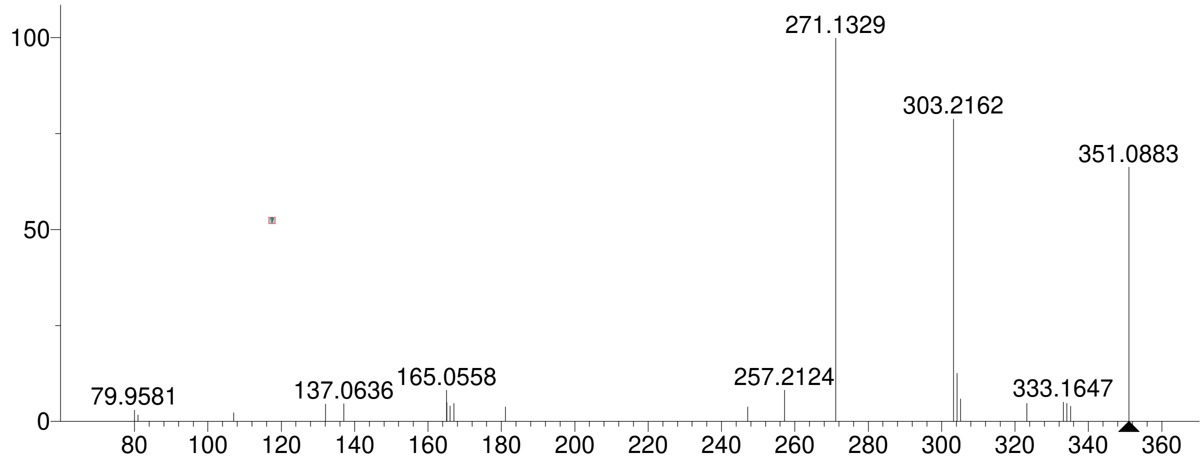

Hit 1 : Spinosine [M+H-C8H18O9]+ HCD 21V P=351.1
C28H32O15; MF: 77; RMF: 730; Prob 79.4%; CAS: 72063-39-9; Lib: nist_msms; ID: 237126.

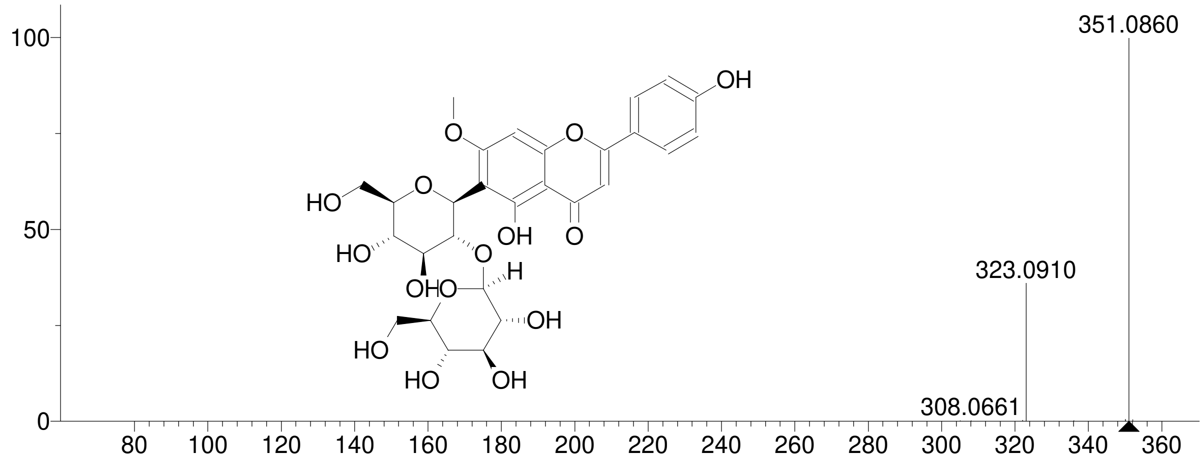

Hit 2 : NCGC00169972-02!3,6-dihydroxy-8-methoxy-3-methyl-2,4-dihydrobenzo[a]anthracene-1,7,12-trione [M-H
C20H16O6; MF: 8; RMF: 364; Prob 11.8%; Lib: mona-export-gnps; ID: 7860.

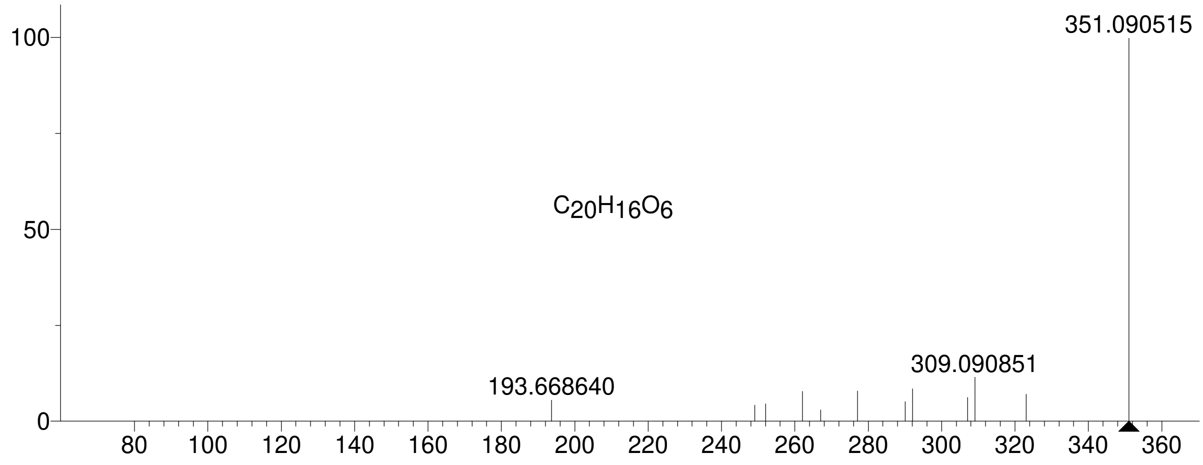

Hit 3 : 2',4',11-trioxaspiro[tricyclo[4.4.1.0A^'1,a^!P]undecane-2,3'-tricyclo[7.3.1.0a^*u,A^'1A^'3]tridecane]-1'(12'),3,
C20H16O6; MF: 0; RMF: 30; Prob 8.80%; Lib: mona-export-lc-ms-ms_negative_mode; ID: 22757.

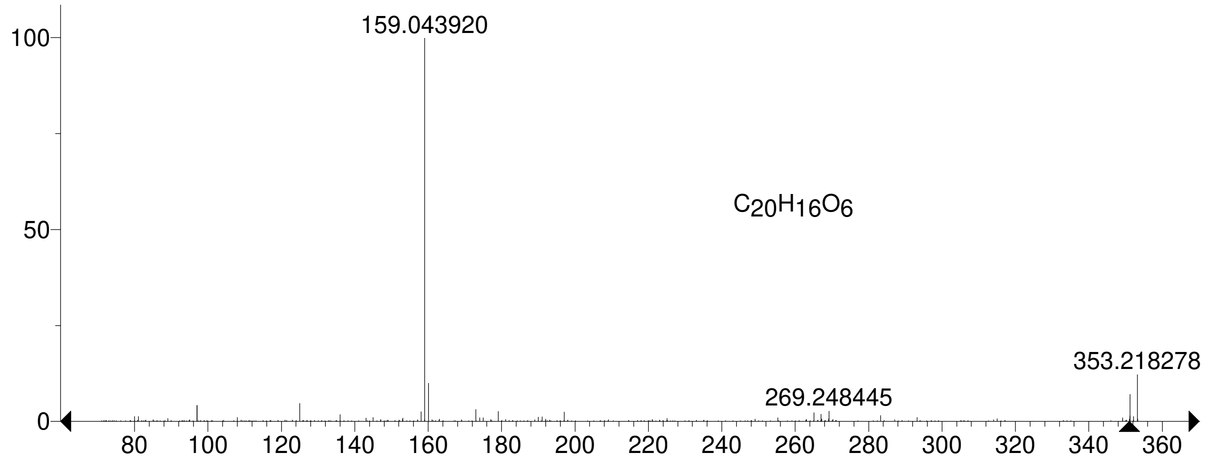

Unknown: Unknown (19.96_303.2163m/z)
Compound in Library Factor = -1848

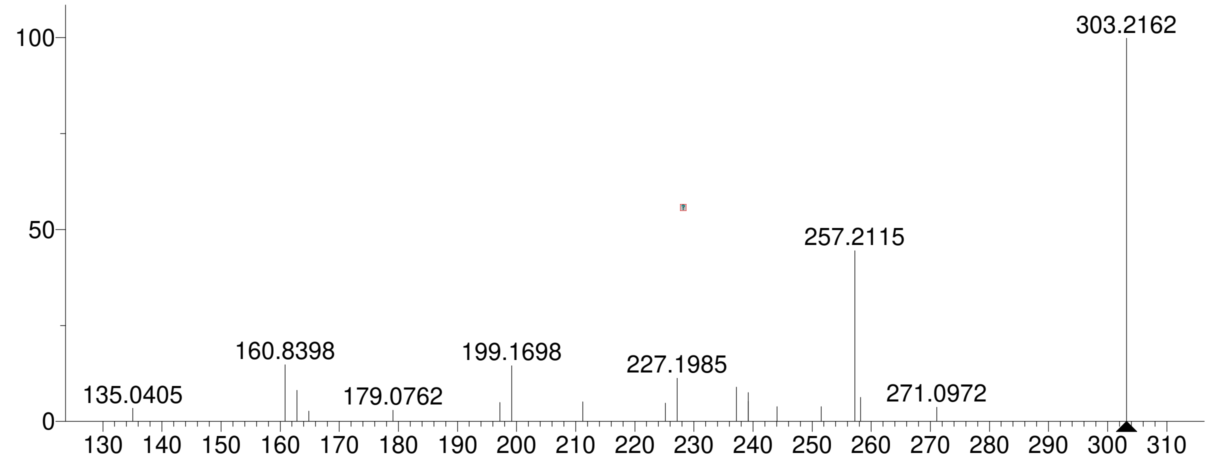

Hit 1 : Lys-Arg [M+H]+ QTOF 8V P=303.2
C12H26N6O3; MF: 71; RMF: 588; Prob 45.7%; Lib: nist_msms; ID: 48416.

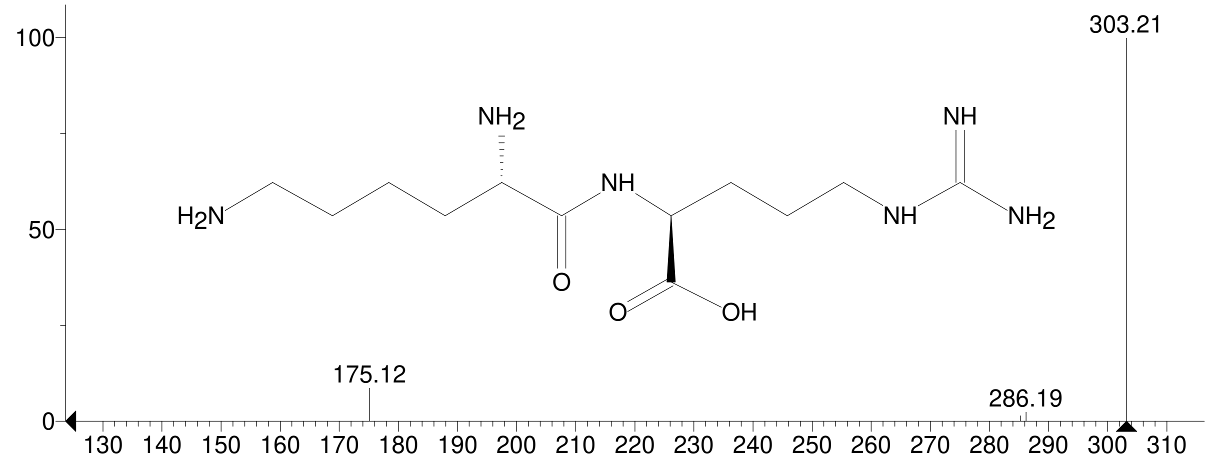

Hit 2 : Arg-Lys [M+H]+ HCD 13eV P=303.2
C12H26N6O3; MF: 64; RMF: 607; Prob 35.0%; CAS: 40968-46-5; Lib: nist_msms; ID: 565904.

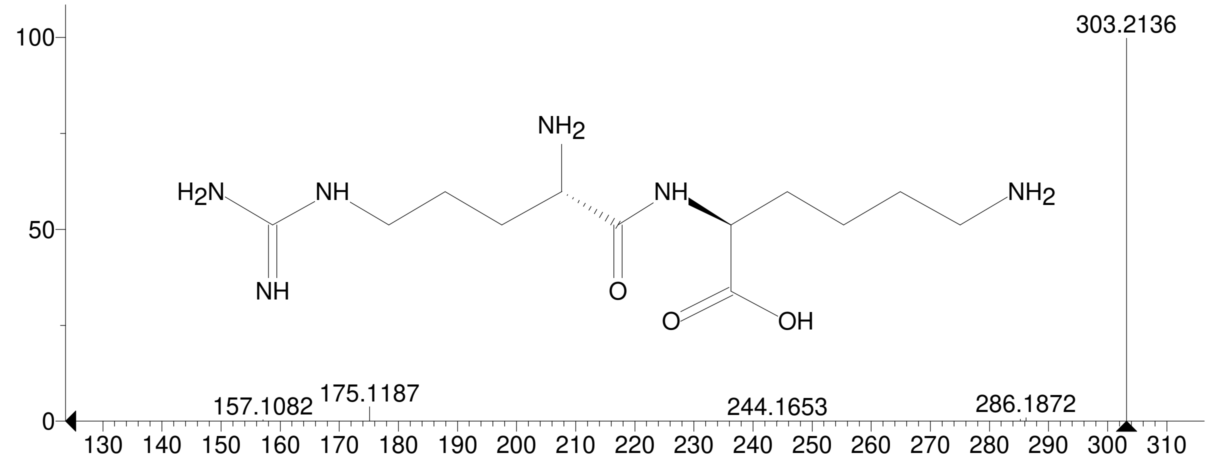

Hit 3 : Lys-Arg [M+H]+ HCD 15eV P=303.2
C12H26N6O3; MF: 32; RMF: 571; Prob 9.34%; CAS: 29586-66-1; Lib: nist_msms2; ID: 41398.

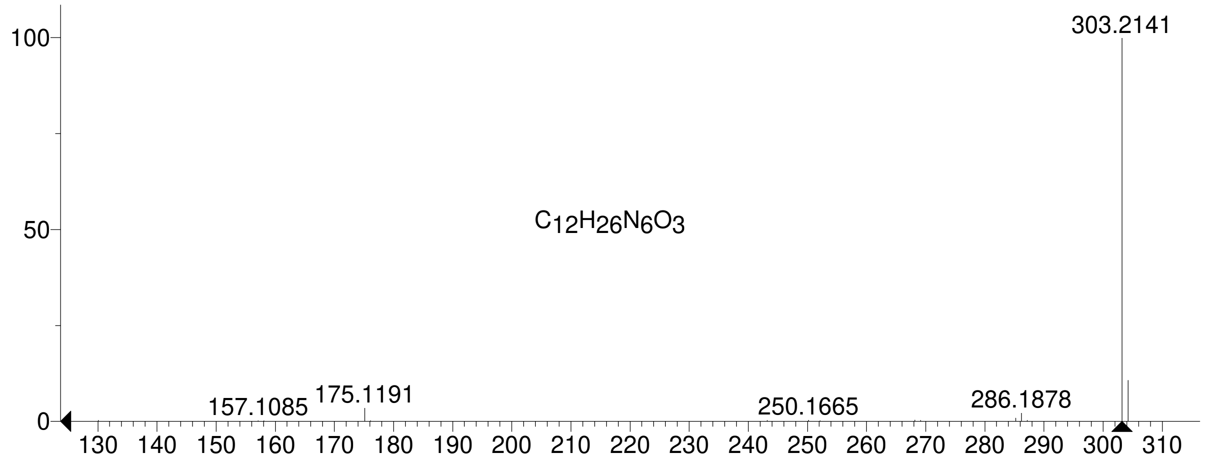

Hit 4 : Aleuritic acid [M-H]- IT-FT 35% P=303.2
C16H32O5; MF: 22; RMF: 183; Prob 6.59%; CAS: 533-87-9; Lib: nist_msms; ID: 411121.

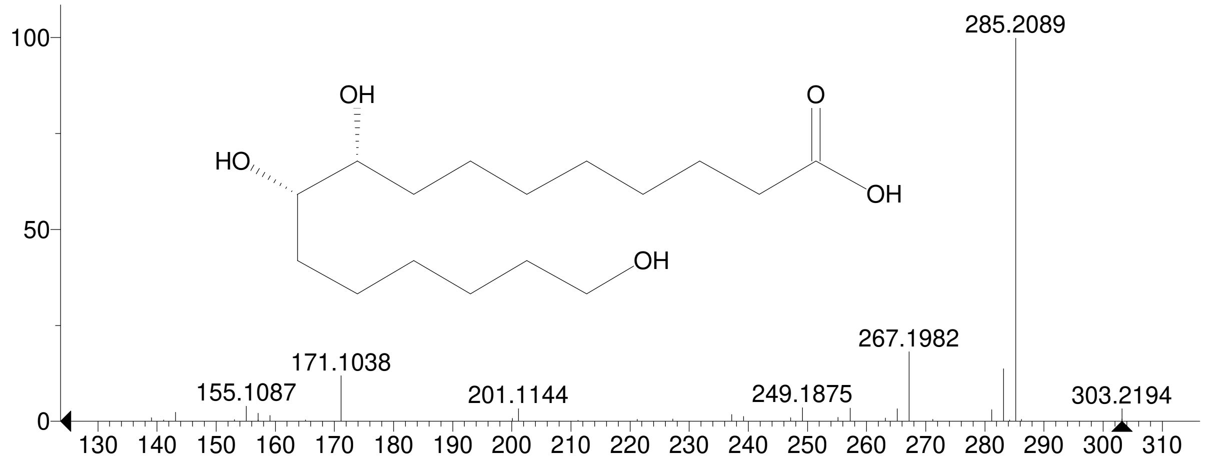

Hit 5 : Aleuretic Acid [M-H]- 45HCD P=303.2
C16H32O5; MF: 4; RMF: 419; Prob 3.40%; Lib: mona-export-lc-ms-ms_negative_mode; ID: 20799.

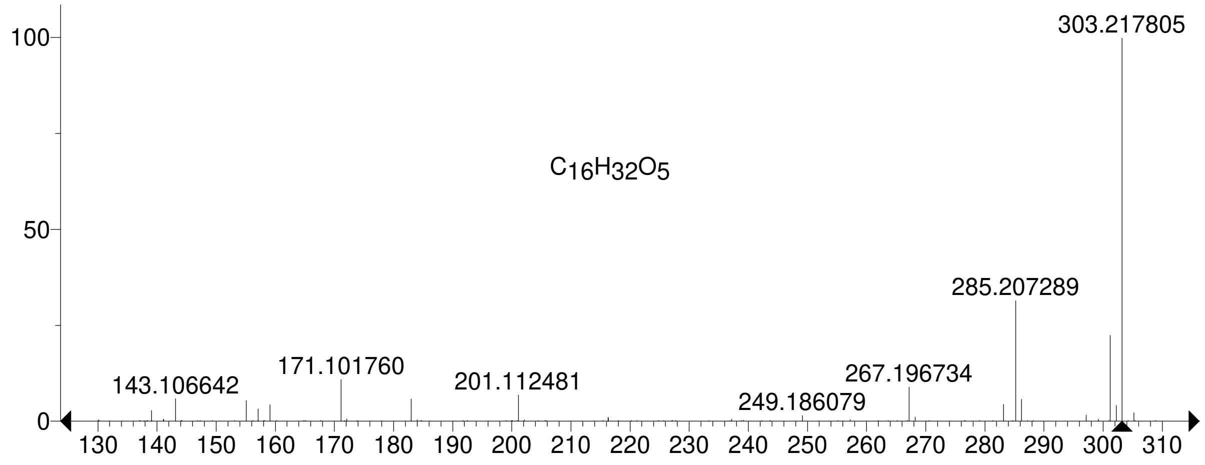

Unknown: Unknown (19.99_181.0863m/z)
Compound in Library Factor = -3273

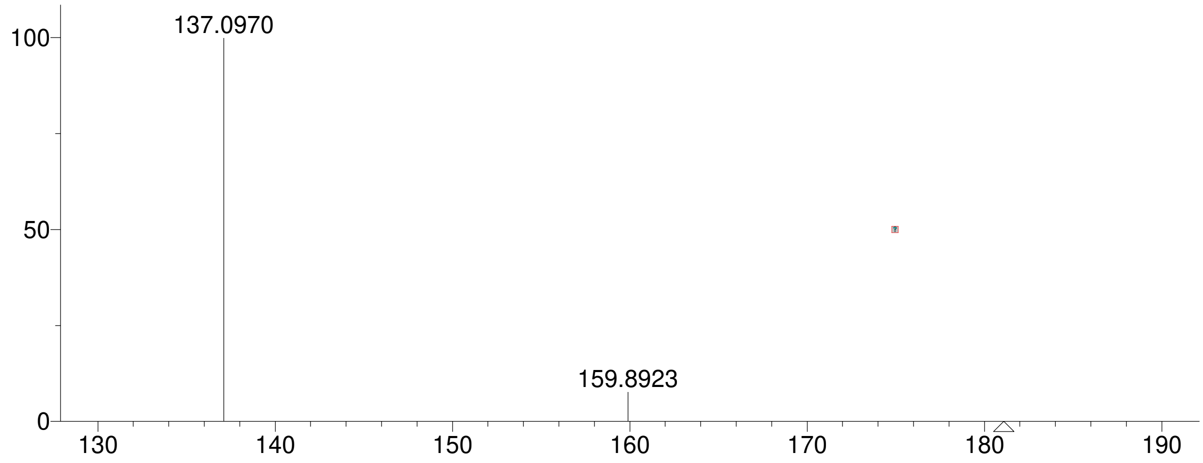

Hit 1 : Benzeneethanamine, 4-chloro-2,5-dimethoxy-N-[(3,4,5-trimethoxyphenyl)methyl]- [M+H-C10H14ClNO2]+
C20H26ClNO5; MF: 4; RMF: 251; Prob 19.1%; CAS: 1445574-98-0; Lib: nist_msms; ID: 351623.

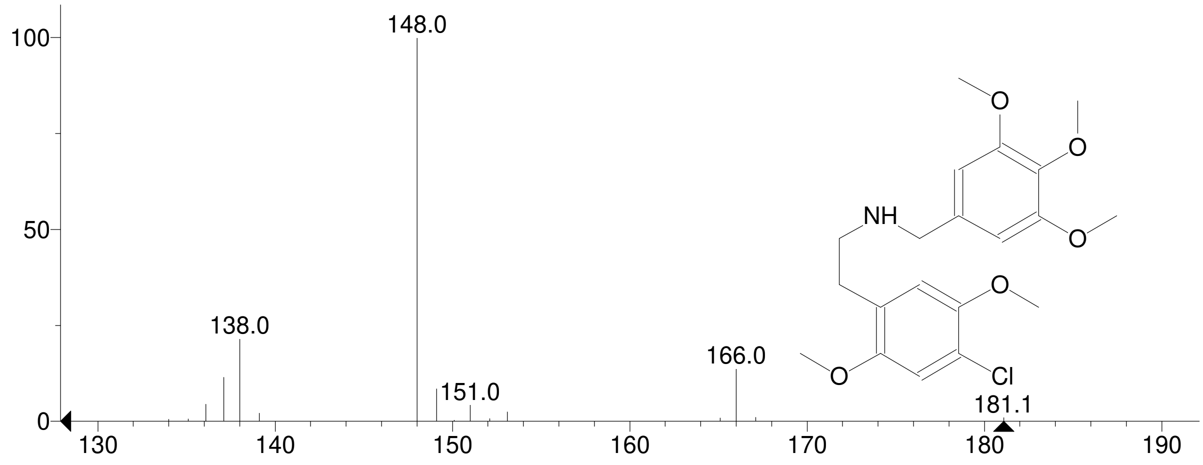

Hit 2 : 3-Oxo-1,8-octanedicarboxylic acid [M+H-2H2O]+ IT 35% P=181.1
C10H16O5; MF: 0; RMF: 67; Prob 16.2%; CAS: 114212-45-2; Lib: nist_msms; ID: 301941.

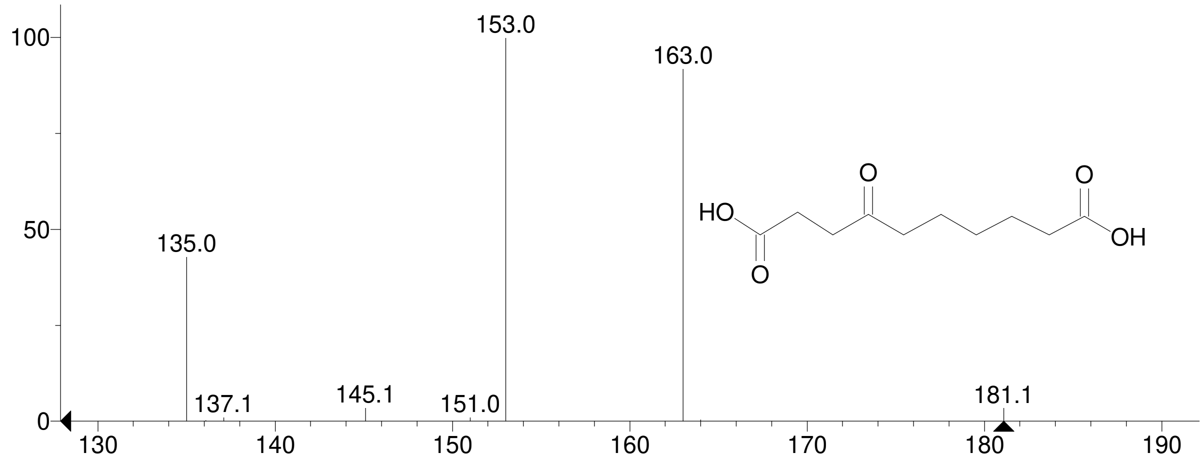

Hit 3 : β-Asarone [M+H-C2H4]+ IT 35% P=181.1
C12H16O3; MF: 0; RMF: 59; Prob 16.2%; CAS: 5273-86-9; Lib: nist_msms; ID: 234667.

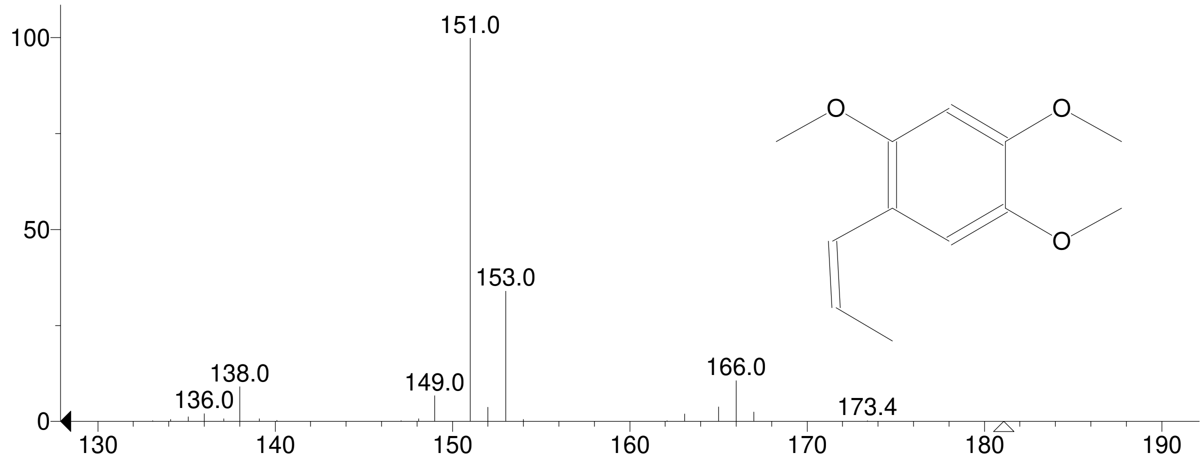

Hit 4 : Escaline [M+H-C2H7N]+ IT 35% P=181.1
C12H19NO3; MF: 0; RMF: 55; Prob 16.2%; CAS: 39201-82-6; Lib: nist_msms; ID: 364356.

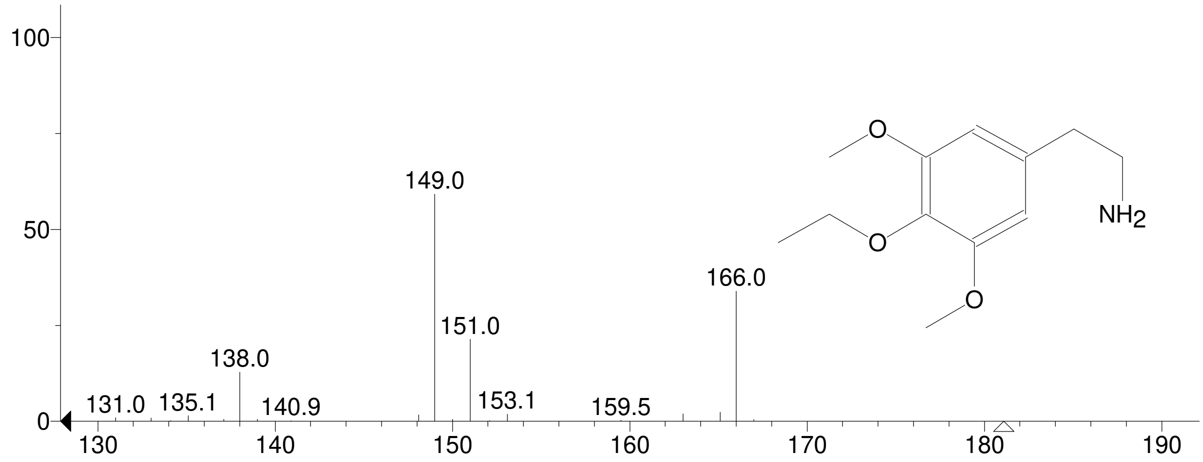

Hit 5 : α-Asarone [M+H-C2H4]+ IT 35% P=181.1
C12H16O3; MF: 0; RMF: 42; Prob 16.2%; CAS: 2883-98-9; Lib: nist_msms; ID: 234607.

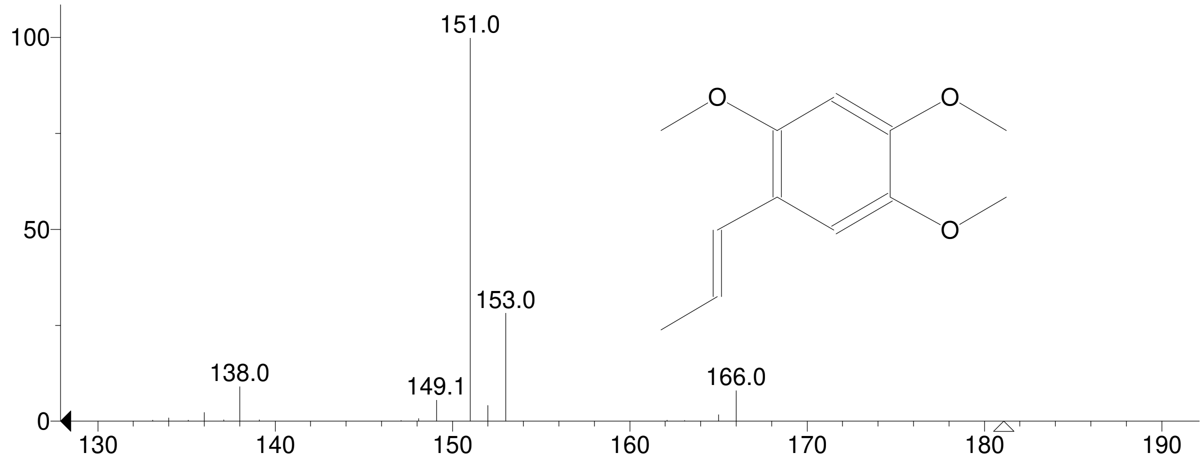

Unknown: Unknown (2.02_275.1236m/z)
Compound in Library Factor = 534

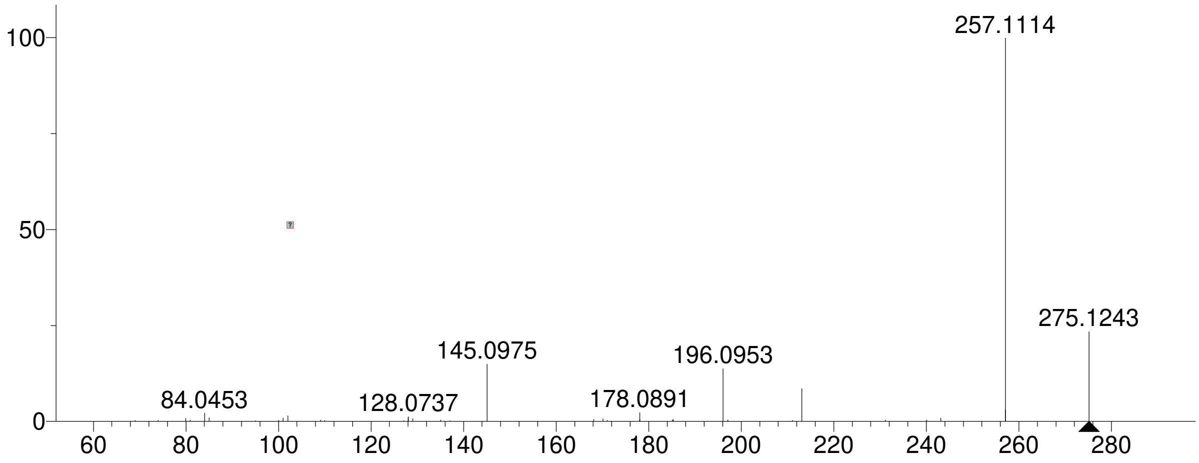

Hit 1 : L-Saccharopine [M-H]- 5-60V P=275.1
C11H20N2O6; MF: 879; RMF: 967; Prob 31.7%; Lib: mona-export-lc-ms-ms_negative_mode; ID: 8572.

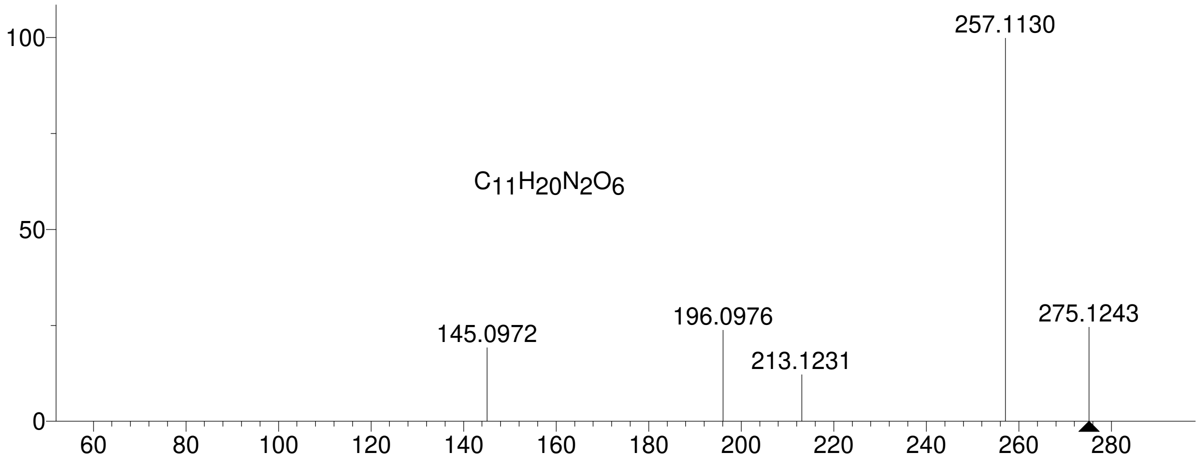

Hit 2 : L-saccharopine [M-H]- QTOF 5-45V P=275.1
C11H20N2O6; MF: 879; RMF: 967; Prob 31.7%; Lib: mona-export-lc-ms-ms_negative_mode; ID: 11459.

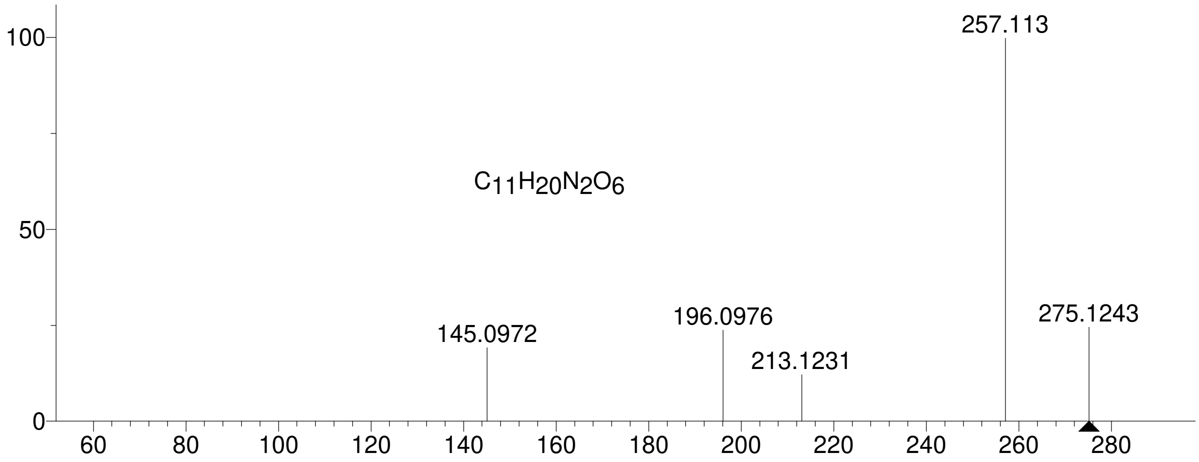

Hit 3 : L-Saccharopine; LC-ESI-QTOF; MS2; CE [M-H]- P=275.1
C11H20N2O6; MF: 879; RMF: 967; Prob 31.7%; Lib: msms-neg-massbank; ID: 4468.

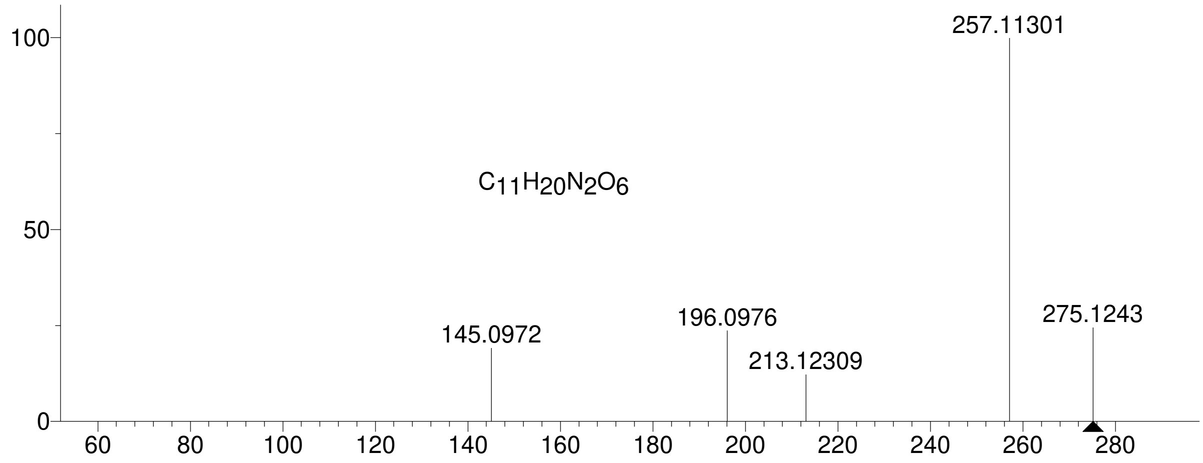

Hit 4 : Saccharopine [M-H]- 10 P=275.1
C11H20N2O6; MF: 812; RMF: 904; Prob 4.90%; Lib: msms neg mona_ipb_gnps_plasma; ID: 537.

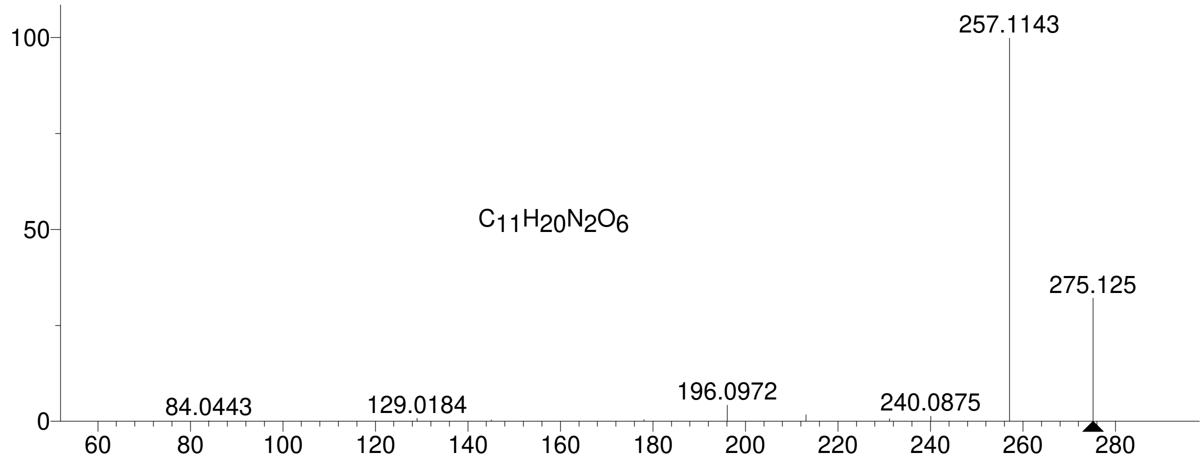

Hit 5 : NCGC00169882-02_C14H20O4_(8aR,12S,12aR)-12-Hydroxy-4-methyl-4,5,6,7,8,8a,12,12a-octahydro-2H
C14H20O4; MF: 18; RMF: 358; Prob 0.04%; Lib: mona-export-gnps; ID: 11517.

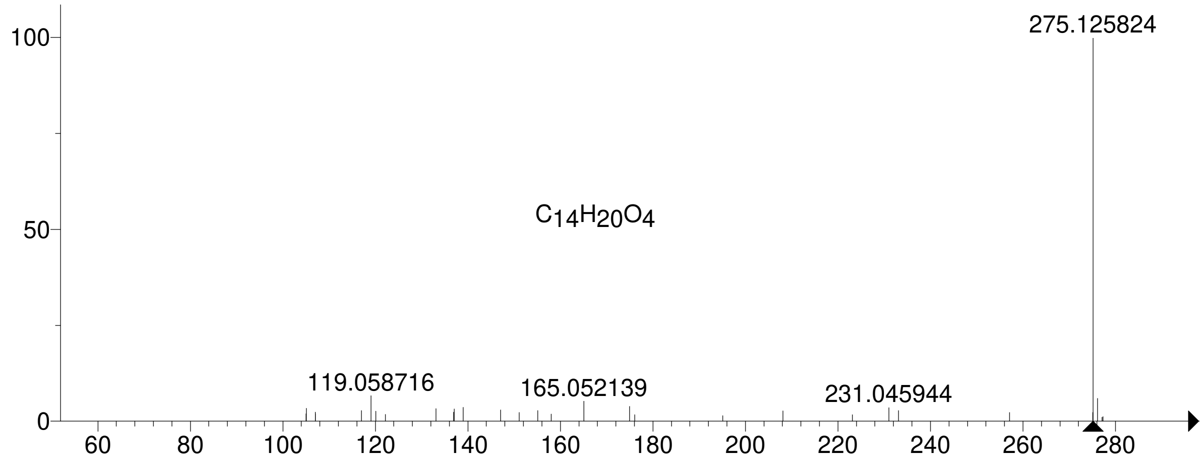

Unknown: Unknown (2.02_379.1702m/z)
Compound in Library Factor = 0

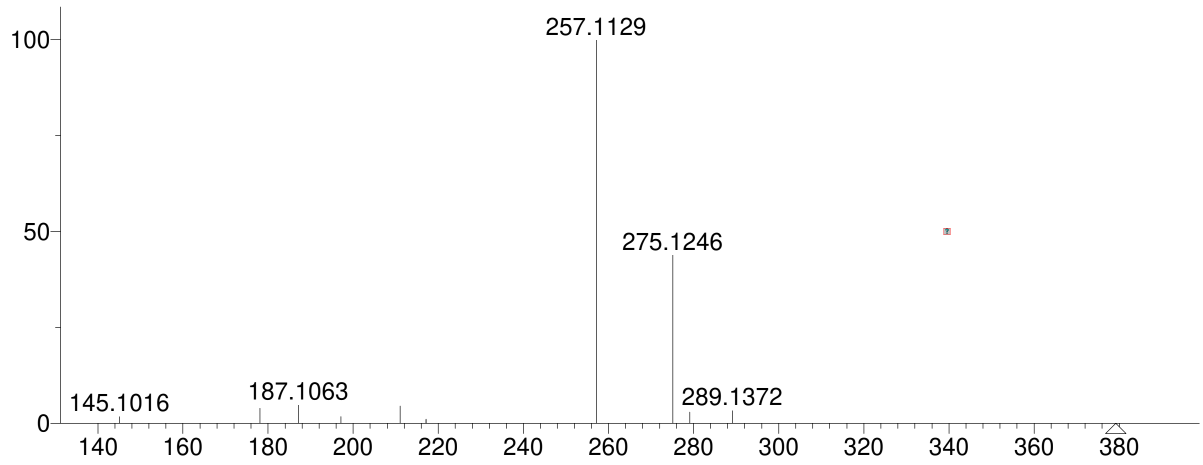

Hit 1 : [Glu1] TRH [M-H]- IT-FT 35% P=379.2
C16H24N6O5; MF: 0; RMF: 19; Prob 33.3%; CAS: 51826-17-6; Lib: nist_msms; ID: 569038.

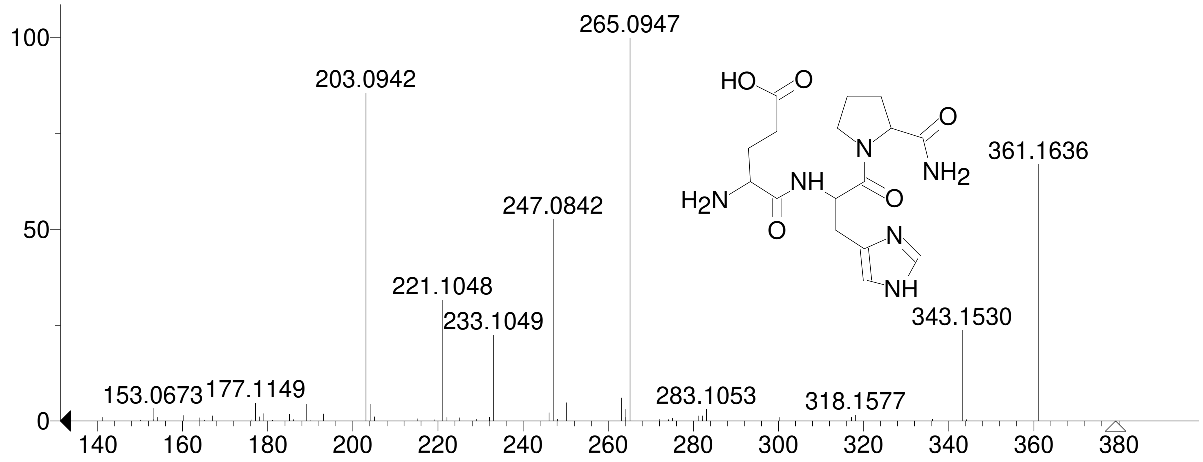

Hit 2 : [Glu1] TRH [M-H]- IT-FT 35% P=379.2
C16H24N6O5; MF: 0; RMF: 19; Prob 33.3%; Lib: nist_msms2; ID: 74030.

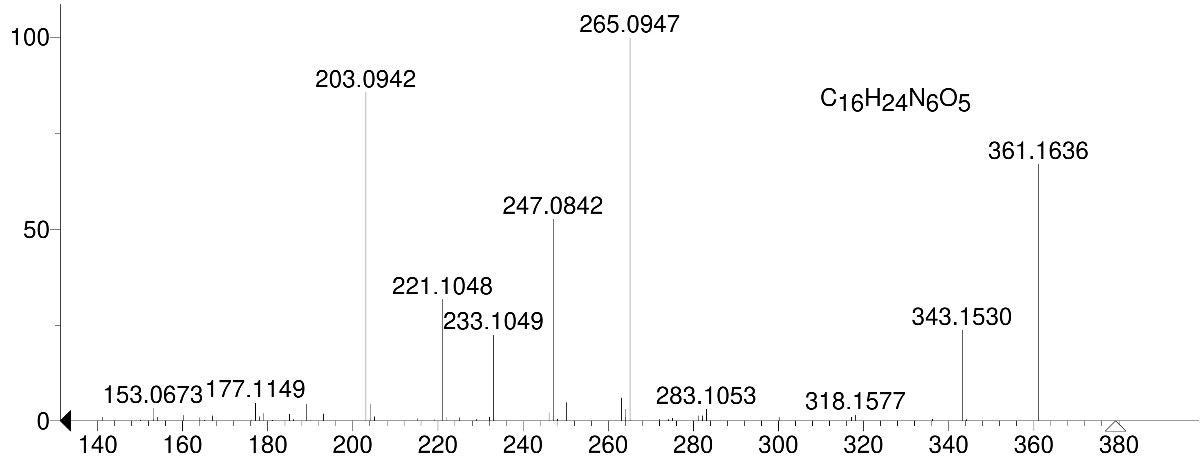

Hit 3 : 11-Nor-9-carboxy-.DELTA.9-tetrahydrocannabinol [M+Cl]- HCD 60V P=379.2
C21H28O4; MF: 0; RMF: 4; Prob 33.3%; CAS: 56354-06-4; Lib: nist_msms; ID: 187080.

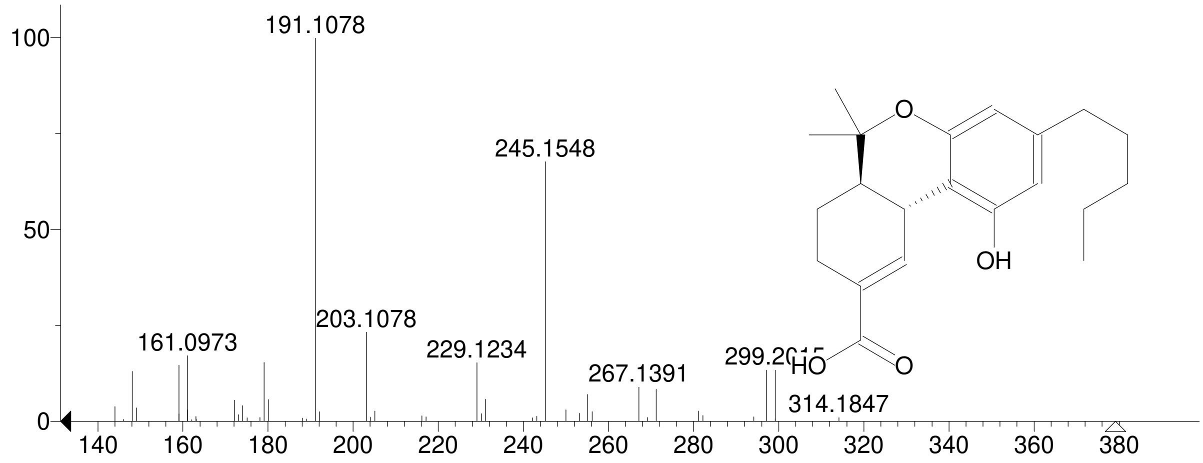

Unknown: Unknown (2.03_275.1239m/z)
Compound in Library Factor = -151

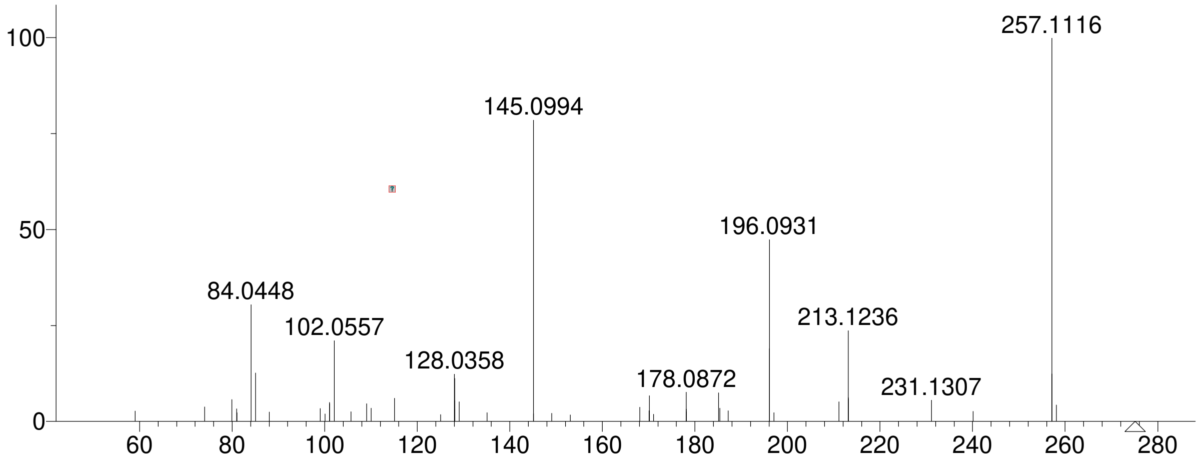

Hit 1 : Saccharopine [M-H]- 20 P=275.1
C11H20N2O6; MF: 701; RMF: 760; Prob 80.4%; Lib: msms neg mona_ipb_gnps_plasma; ID: 578.

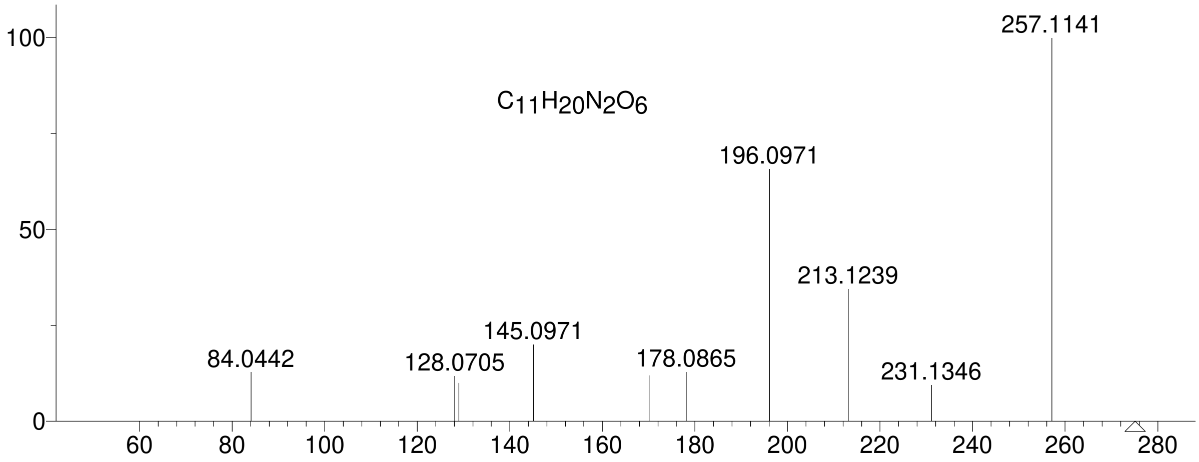

Hit 2 : L-Saccharopine [M-H]- HCD 26V P=275.1
C11H20N2O6; MF: 662; RMF: 792; Prob 19.0%; CAS: 997-68-2; Lib: nist_msms; ID: 125655.

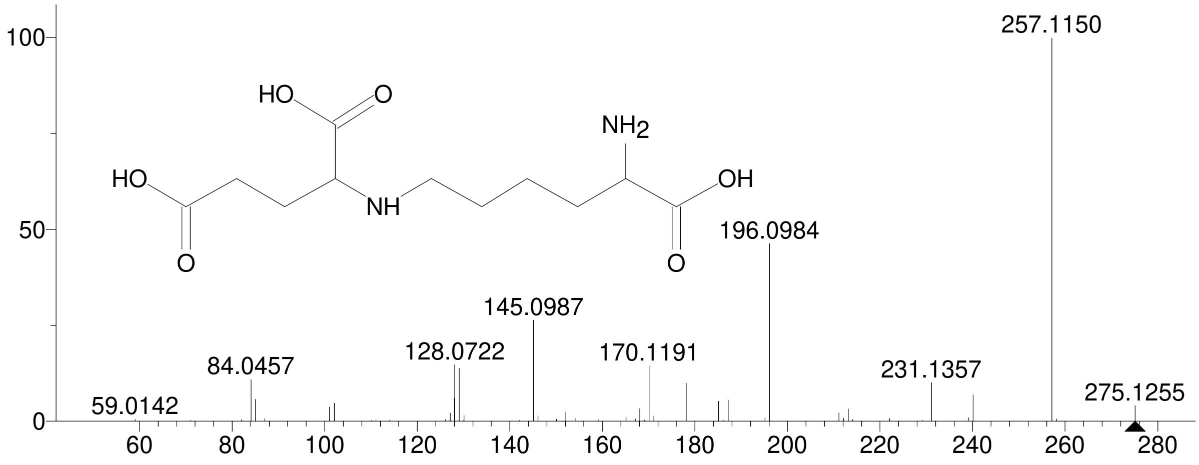

Hit 3 : L-saccharopine [M-H]- QTOF 5-45V P=275.1
C11H20N2O6; MF: 477; RMF: 745; Prob 0.30%; Lib: mona-export-lc-ms-ms_negative_mode; ID: 11459.

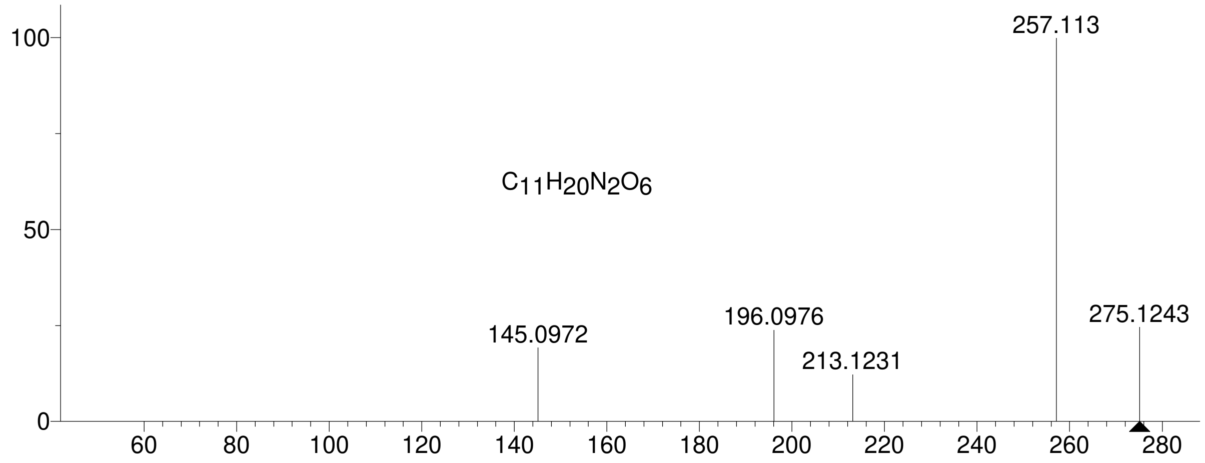

Hit 4 : L-Saccharopine; LC-ESI-QTOF; MS2; CE [M-H]- P=275.1
C11H20N2O6; MF: 477; RMF: 745; Prob 0.30%; Lib: msms-neg-massbank; ID: 4468.

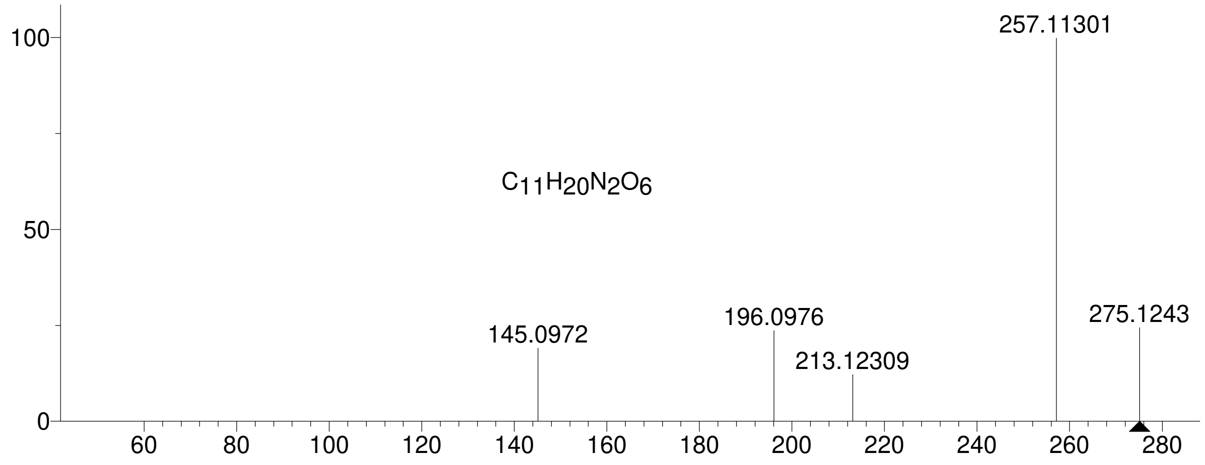

Hit 5 : NCGC00169882-02_C14H20O4_(8aR,12S,12aR)-12-Hydroxy-4-methyl-4,5,6,7,8,8a,12,12a-octahydro-2H
C14H20O4; MF: 0; RMF: 53; Prob 0.00%; Lib: mona-export-gnps; ID: 11517.

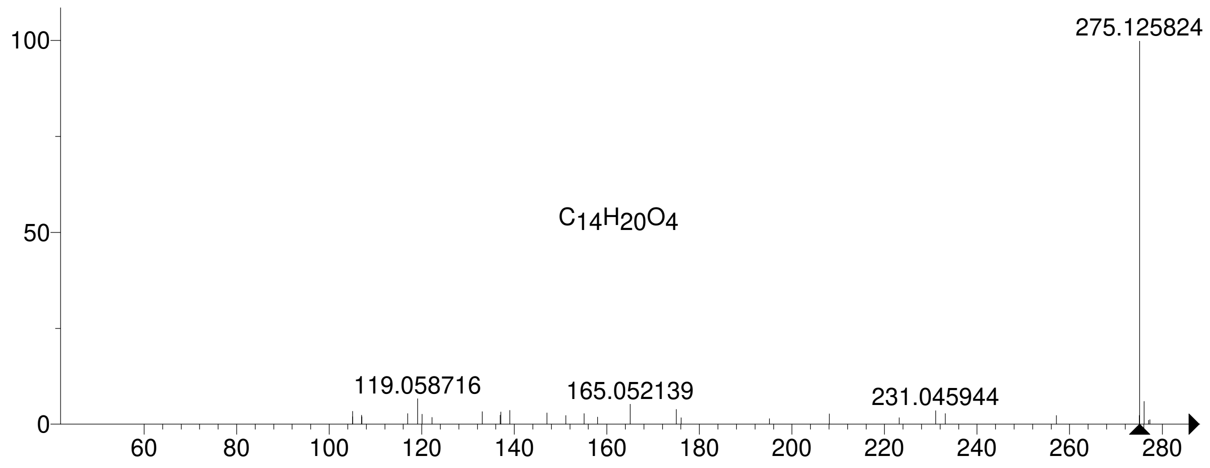

Unknown: Unknown (2.03_445.1817m/z)
Compound in Library Factor = -3273

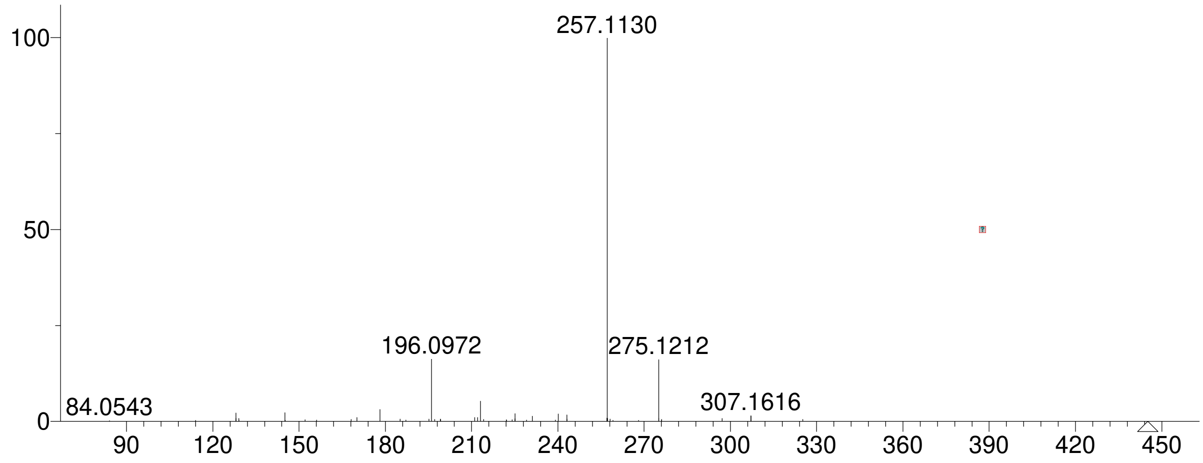

Hit 1 : Difenacoum [M+H]+ IT 35% P=445.2
C31H24O3; MF: 9; RMF: 517; Prob 21.2%; CAS: 56073-07-5; Lib: nist_msms; ID: 399396.

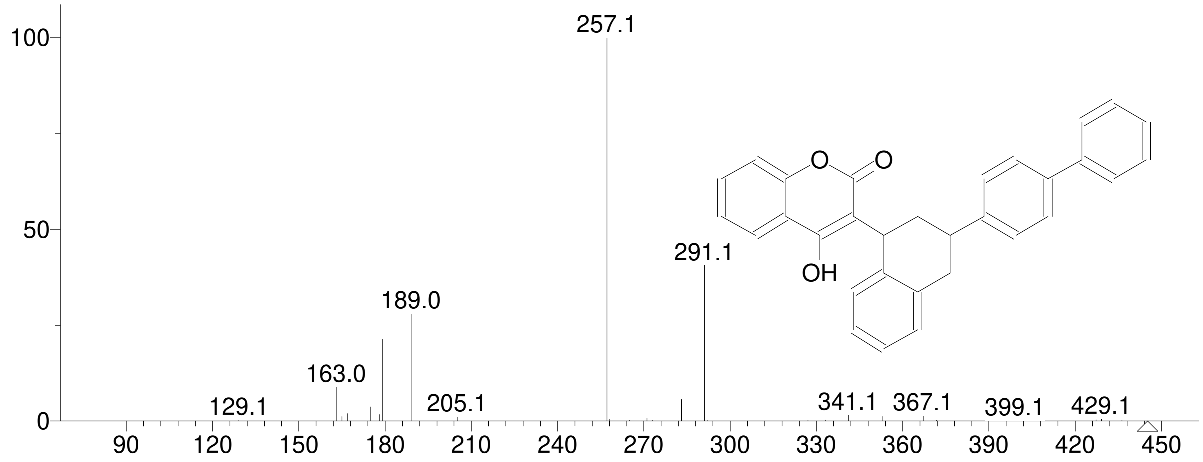

Hit 2 : 11-(hydroxymethyl)-2,6,6,14,19-pentamethyl-7,16,18-trioxapentacyclo[12.6.1.0A^'2,A^'1A^'2.0a^*u,A^'1a^'c
C24H28O8; MF: 5; RMF: 110; Prob 17.9%; Lib: mona-export-vf-npl_qexactive; ID: 21173.

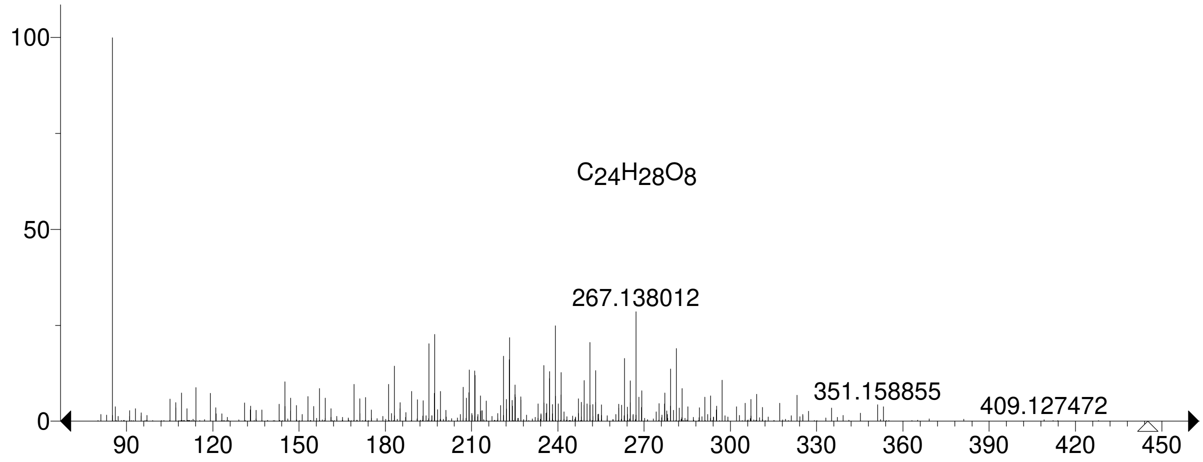

Hit 3 : NCGC00381216-01_C24H28O8_Furo[4,3,2-ij]pyrano[4',3':4,5]cyclohepta[1,2-f][2]benzopyran-1,5,9,13(2H,
C24H28O8; MF: 3; RMF: 116; Prob 16.5%; Lib: mona-export-gnps; ID: 11597.

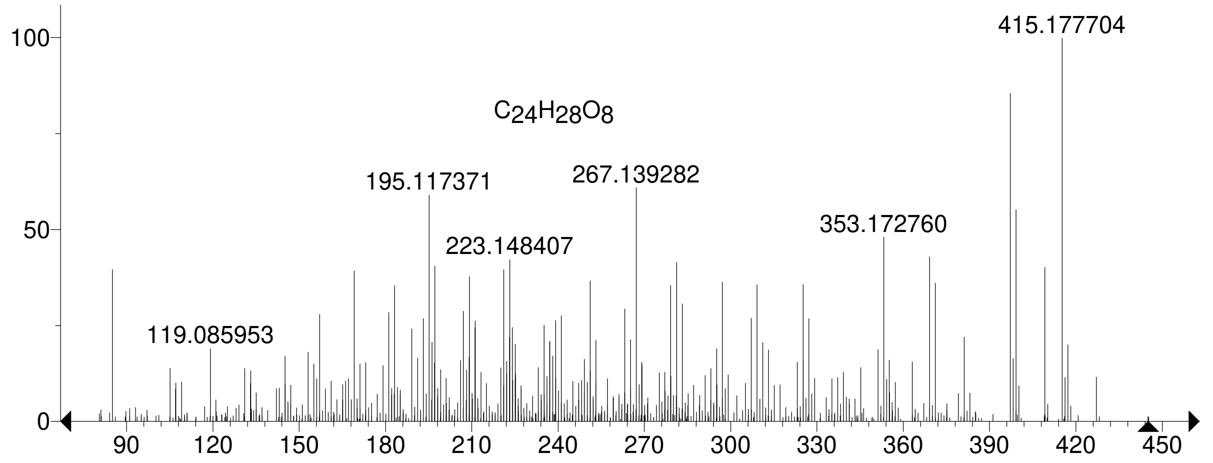

Hit 4 : [(6Z,10Z)-6-(acetyloxymethyl)-10-(hydroperoxymethyl)-3-methylidene-2-oxo-3a,4,5,8,9,11a-hexahydrocycl
C22H30O8; MF: 1; RMF: 61; Prob 15.2%; Lib: mona-export-vf-npl_qexactive; ID: 19873.

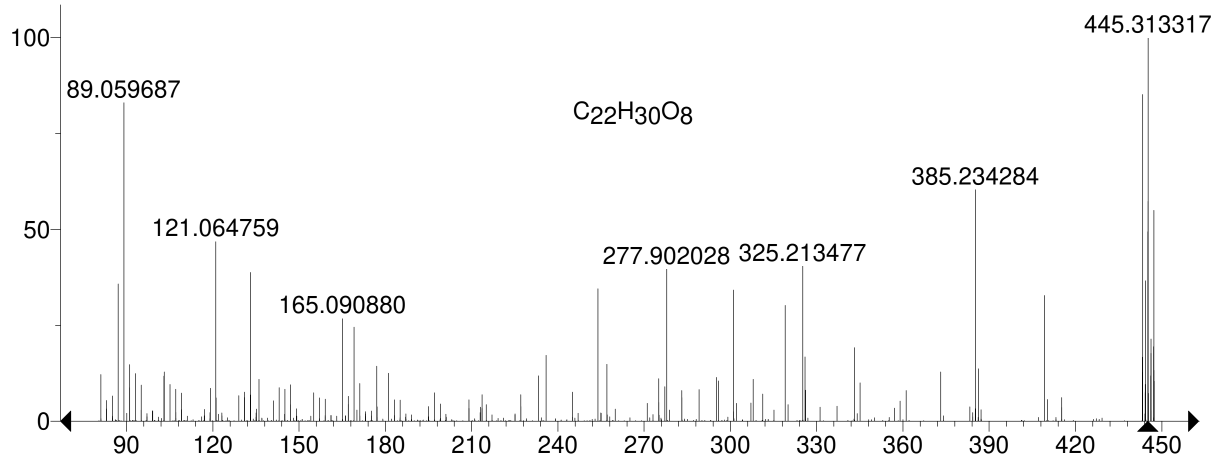

Hit 5 : NCGC00381245-01_C22H30O8_2-[6-{[(2Z)-4-Acetoxy-2-methyl-2-butenoyl]oxy}-2-hydroxy-3-(3-hydroxy-1
C22H30O8; MF: 0; RMF: 15; Prob 14.6%; Lib: mona-export-gnps; ID: 10571.

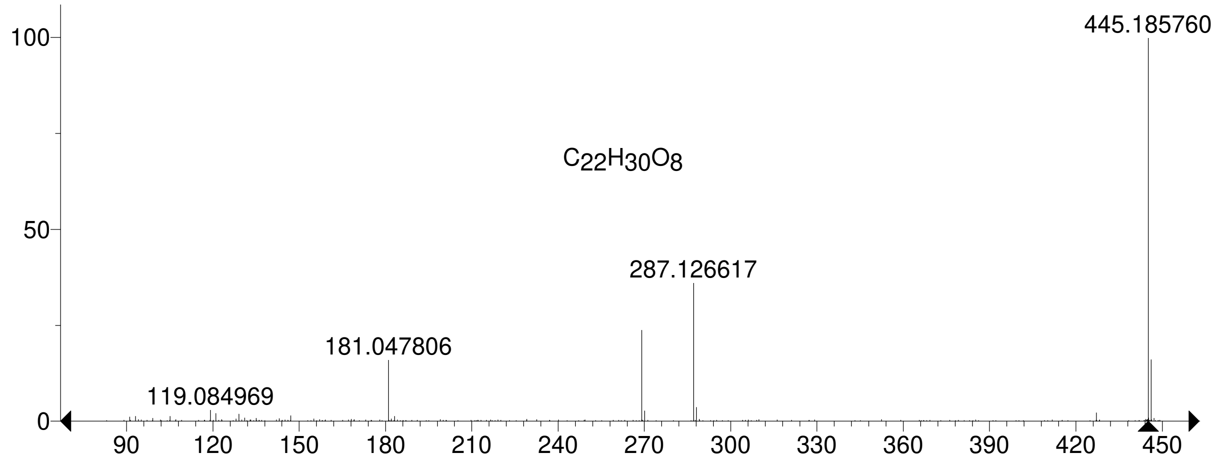

Unknown: Unknown (2.03_504.1708m/z)
Compound in Library Factor = 0

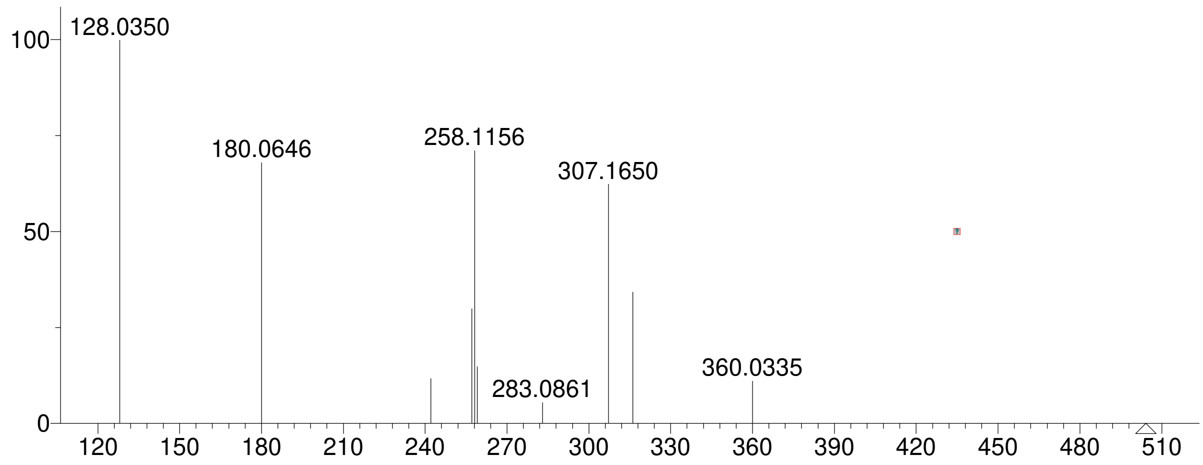

Hit 1 : 5-(1,2,3,4,5,6-hexahydroxyhexoxy)-6-hydroxy-3-methoxy-9-methyl-4,5-dihydro-3H-pyrano[3,4-h][1]benzoxe
C21H26O13; MF: 0; RMF: 7; Prob 100.0%; Lib: mona-export-vf-npl_qexactive; ID: 17689.

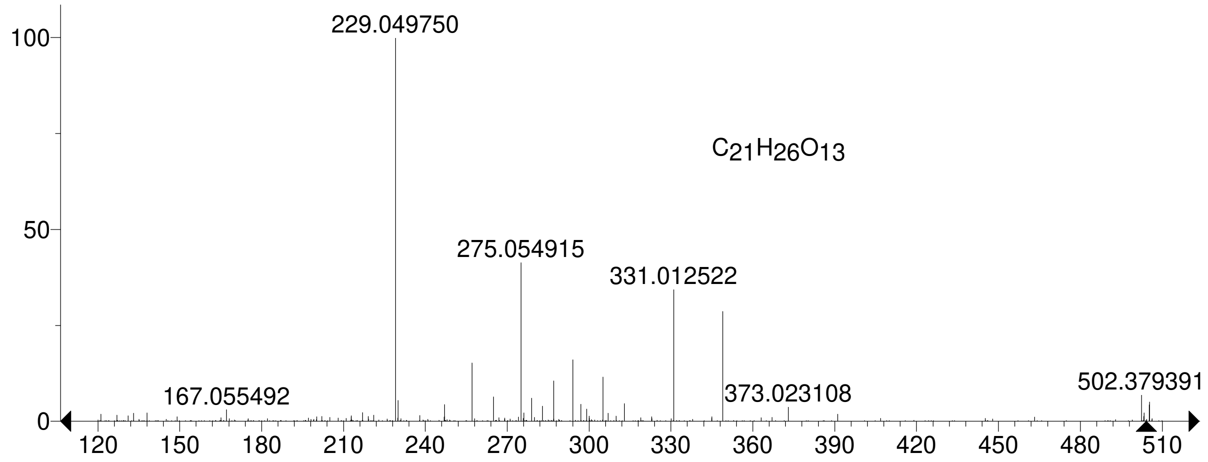

Unknown: Unknown (2.04_602.1827m/z)
Compound in Library Factor = 0

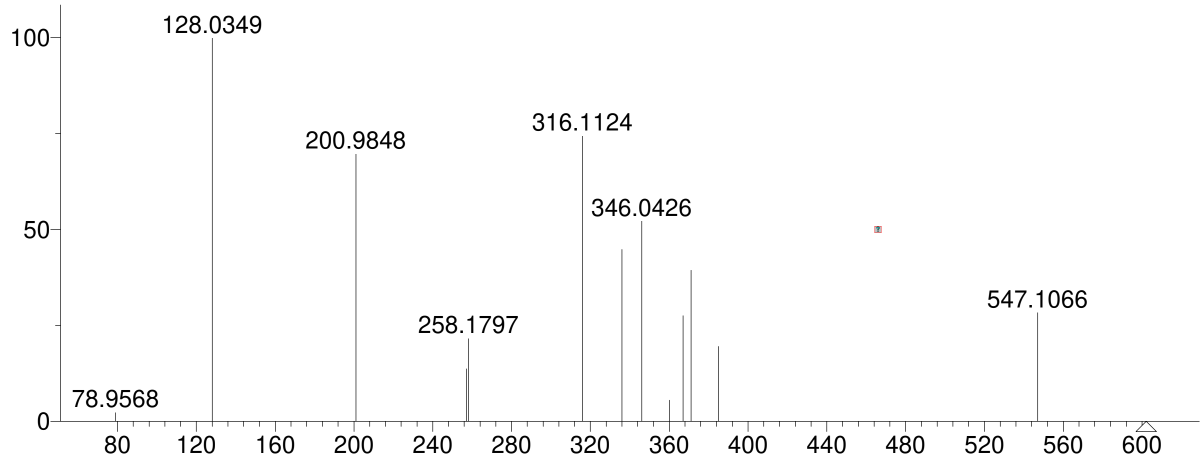

Unknown: Unknown (2.04_610.0586m/z)
Compound in Library Factor = 0

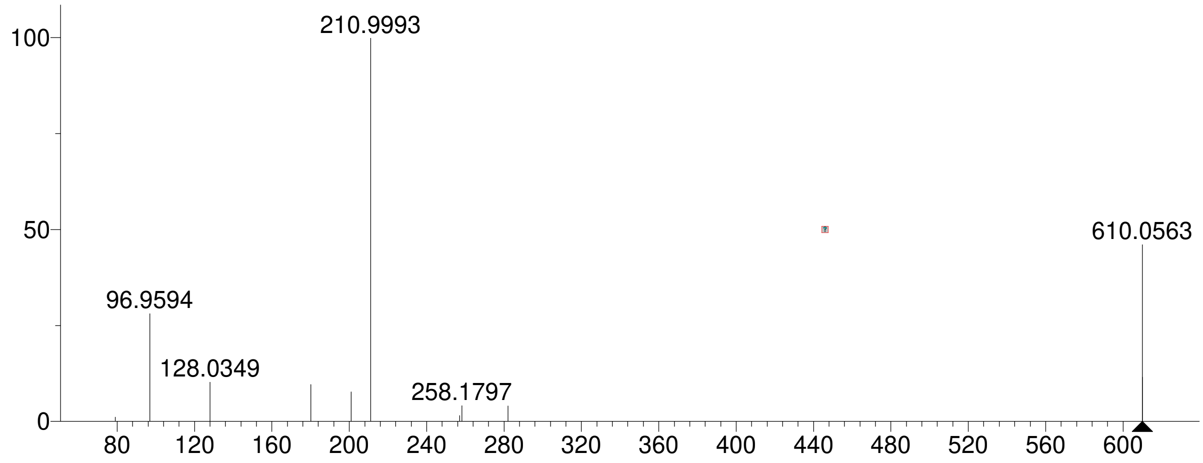

Unknown: Unknown (2.06_385.1355m/z)
Compound in Library Factor = 0

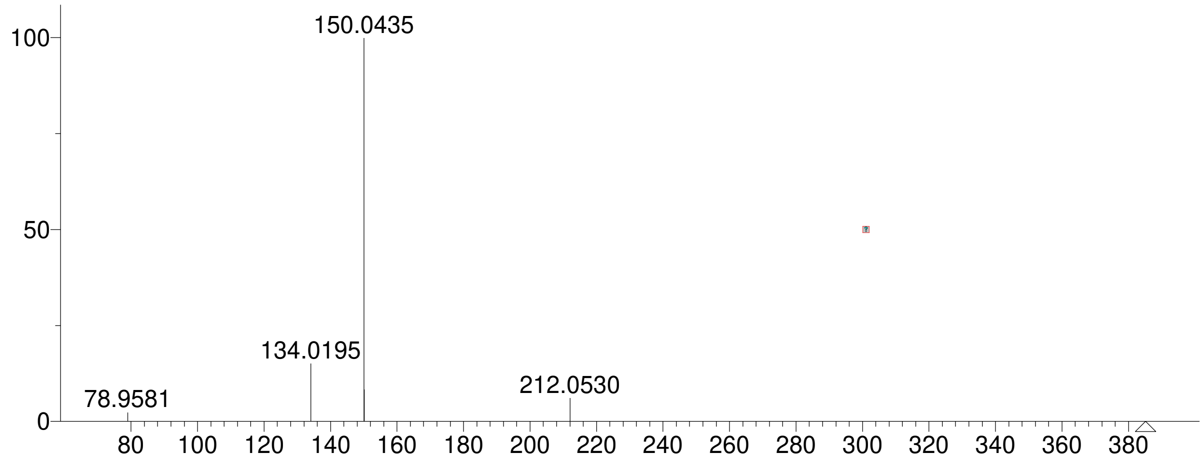

Unknown: Unknown (2.06_602.1252m/z)
Compound in Library Factor = 0

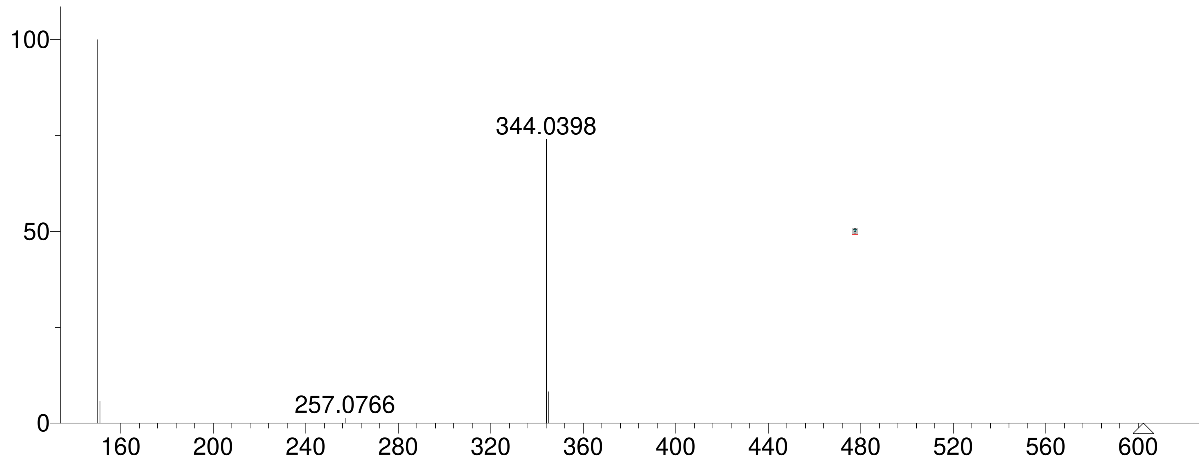

Unknown: Unknown (2.07_344.0396m/z)
Compound in Library Factor = -294

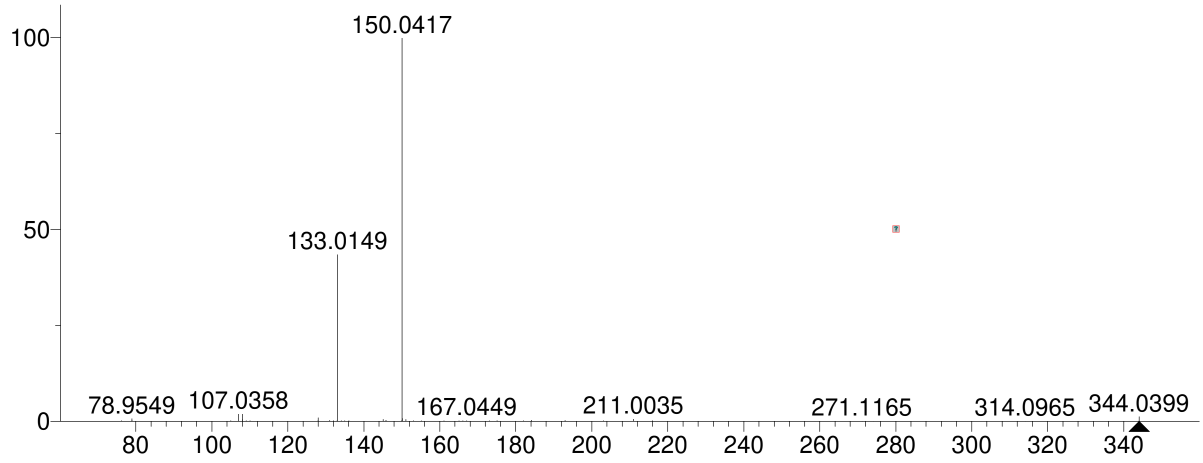

Hit 1 : cyclic gmp [M-H]- P=344
C10H12N5O7P; MF: 545; RMF: 658; Prob 45.0%; Lib: msms neg mona_ipb_gnps_plasma; ID: 1423.

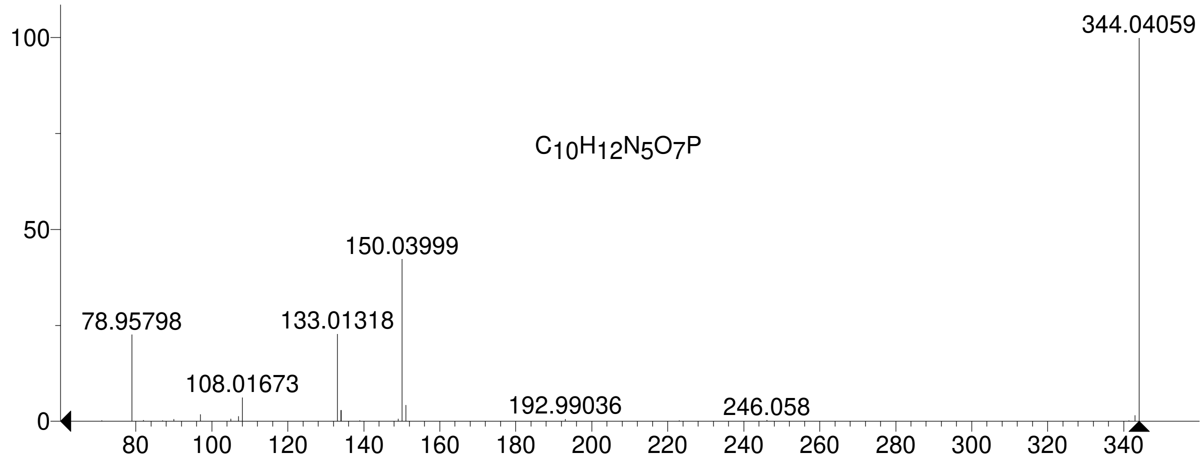

Hit 2 : Guanosine-3',5'-cyclic monophosphate [M-H]- 5-60V P=344
C10H12N5O7P; MF: 524; RMF: 757; Prob 19.2%; Lib: mona-export-lc-ms-ms_negative_mode; ID: 8435.

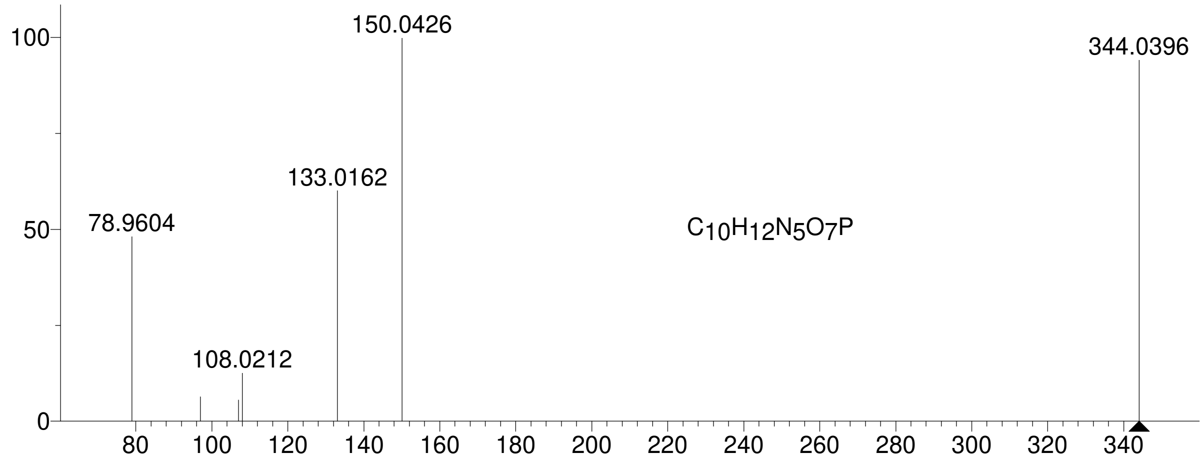

Hit 3 : Guanosine-3',5'-cyclic monophosphate; LC-ESI-QTOF; MS2; CE [M-H]- P=344
C10H12N5O7P; MF: 524; RMF: 757; Prob 19.2%; Lib: msms-neg-massbank; ID: 4337.

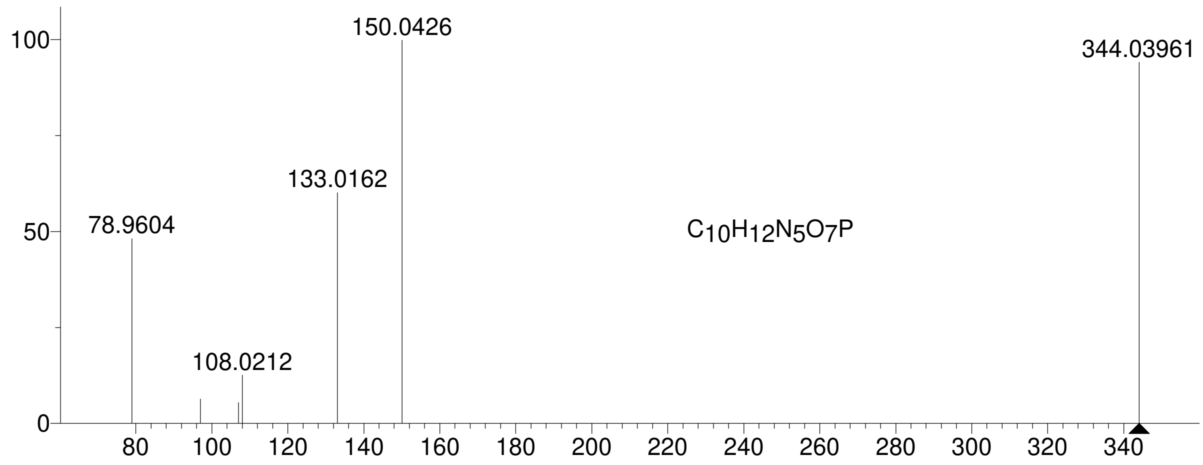

Hit 4 : 3`,5`-cyclic GMP [M-H]- 35 P=344
C10H12N5O7P; MF: 503; RMF: 717; Prob 8.15%; Lib: msms neg mona_ipb_gnps_plasma; ID: 1249.

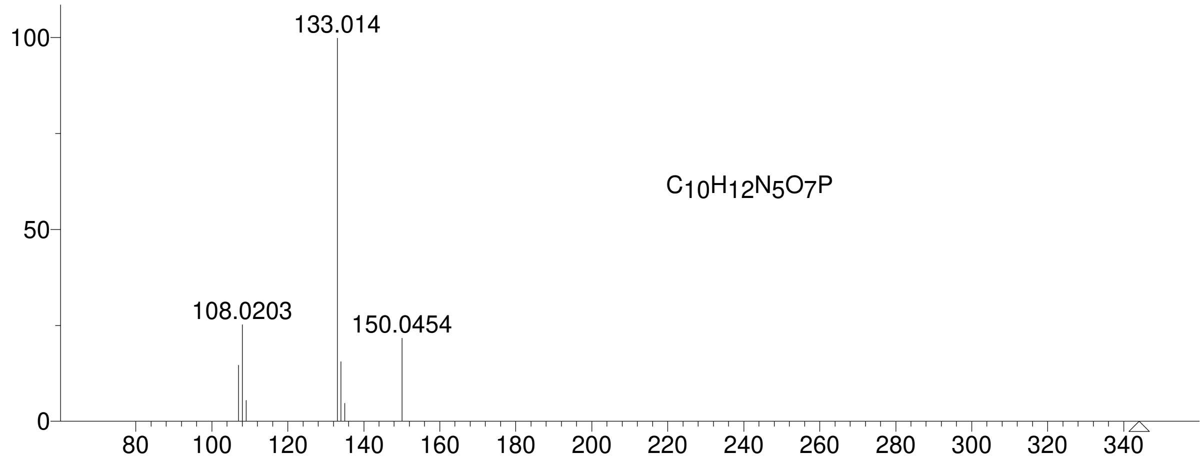

Hit 5 : Guanosine cyclic monophosphate [M-H]- 45HCD P=344
C10H12N5O7P; MF: 501; RMF: 817; Prob 7.52%; Lib: mona-export-lc-ms-ms_negative_mode; ID: 17508.

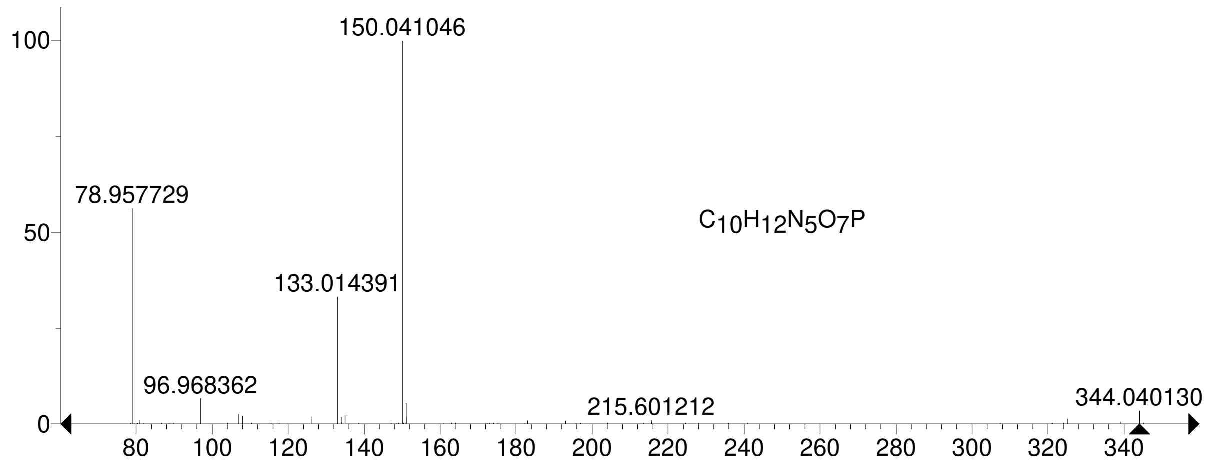

Unknown: Unknown (2.07_689.0870m/z)
Compound in Library Factor = -1679

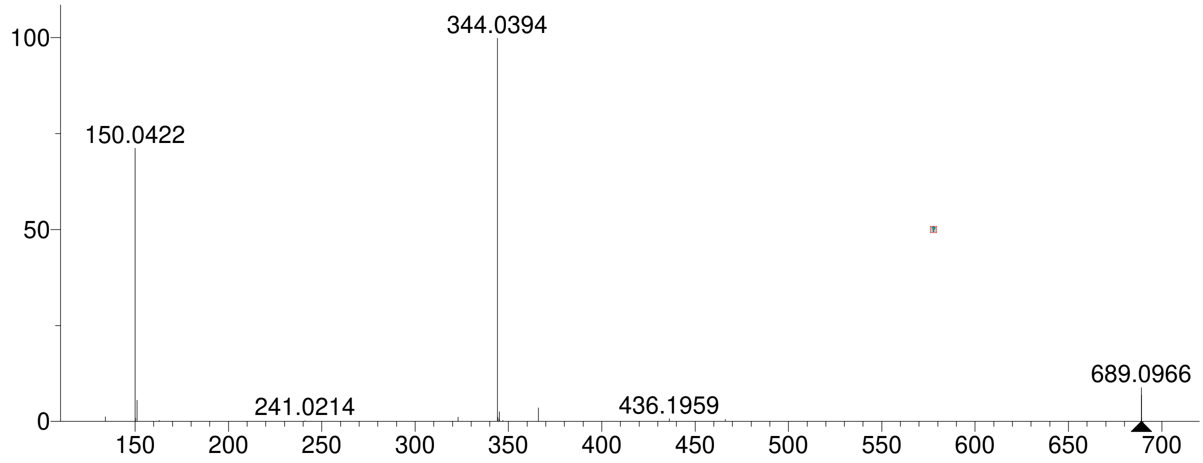

Hit 1 : Guanosine 3',5'-cyclic monophosphate [2M-H]- HCD 65V P=689.1
C10H12N5O7P; MF: 649; RMF: 884; Prob 100.0%; CAS: 7665-99-8; Lib: nist_msms; ID: 132119.

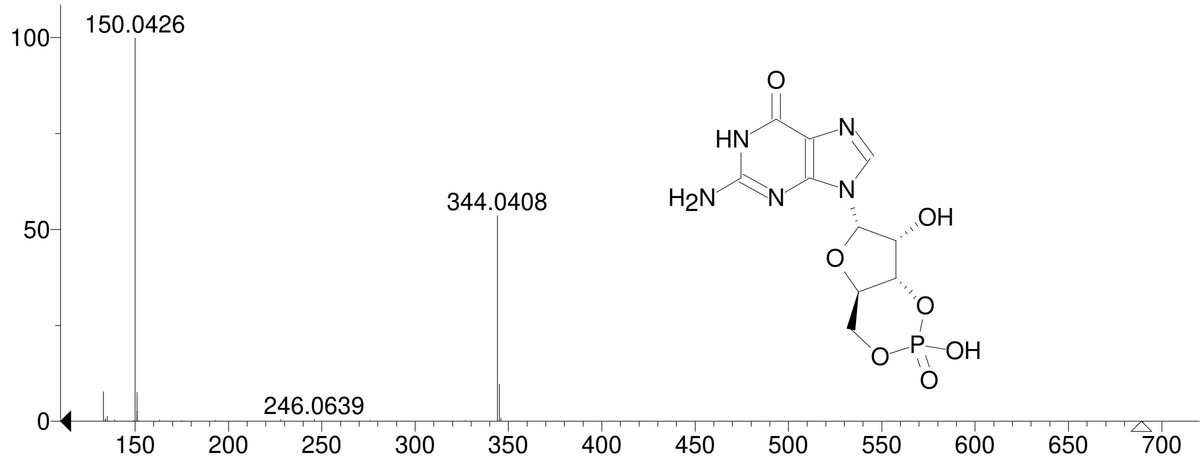

** Search Report Page 1 of 1 **

Unknown: Unknown (2.08_344.2135m/z)
Compound in Library Factor = 0

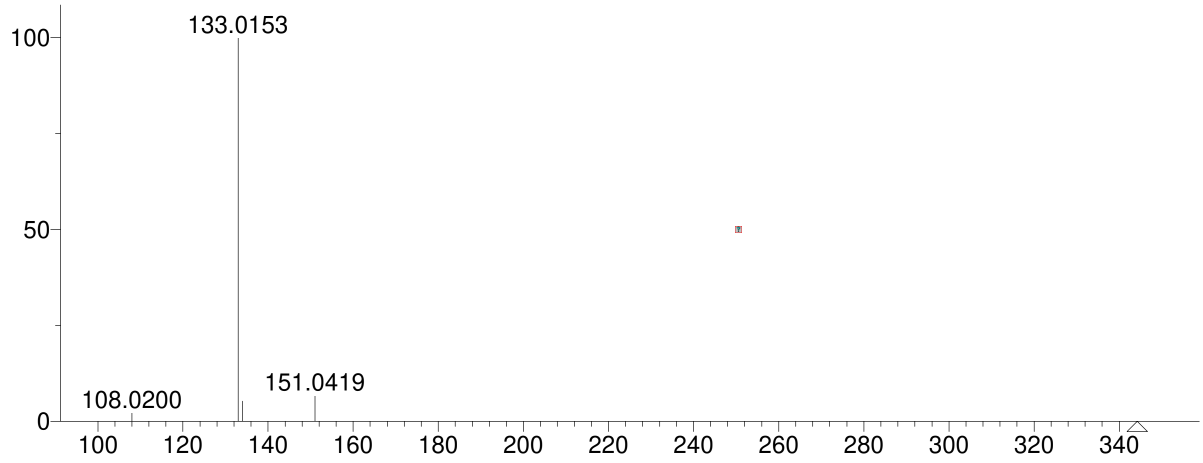

Unknown: Unknown (2.08_606.2486m/z)
Compound in Library Factor = 0

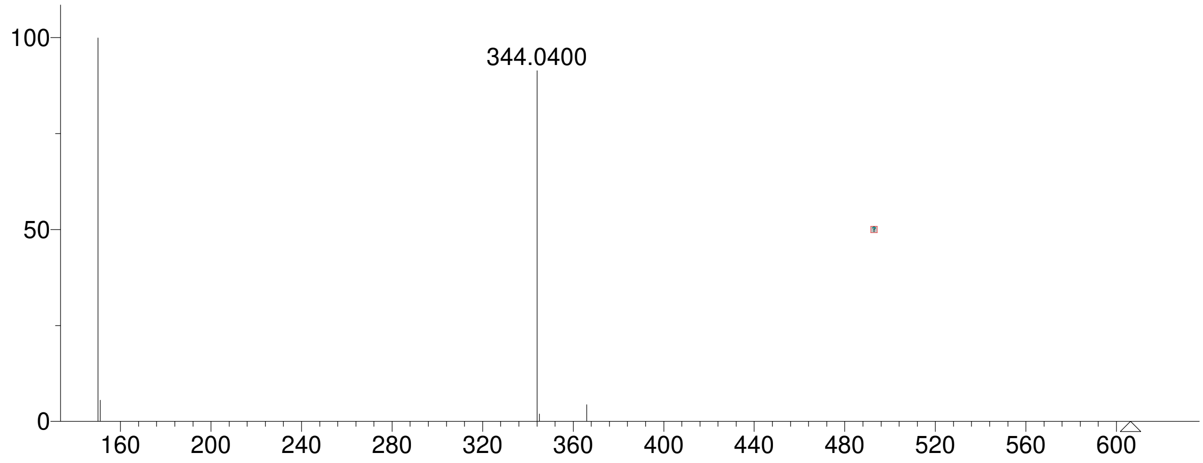

** Search Report Page 1 of 1 **

Unknown: Unknown (2.09_711.0709m/z)
Compound in Library Factor = 0

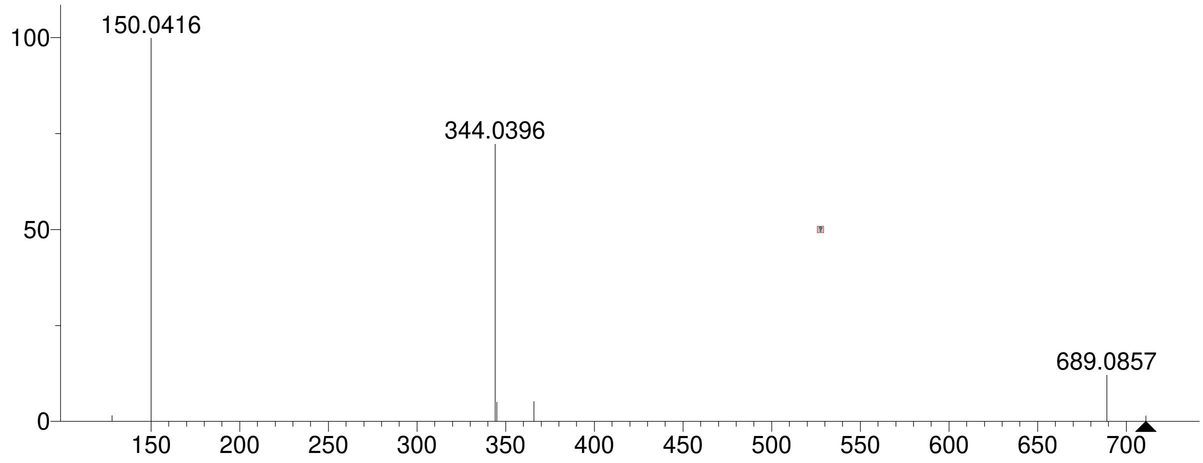

** Search Report Page 1 of 1 **

Unknown: Unknown (2.10_330.1184m/z)
Compound in Library Factor = 0

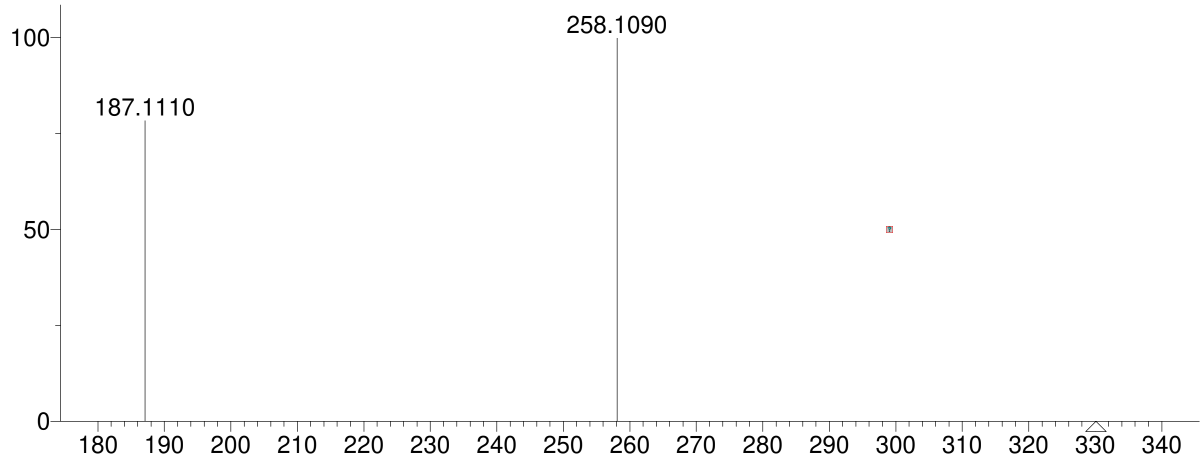

Unknown: Unknown (2.11_134.0466m/z)
Compound in Library Factor = -218

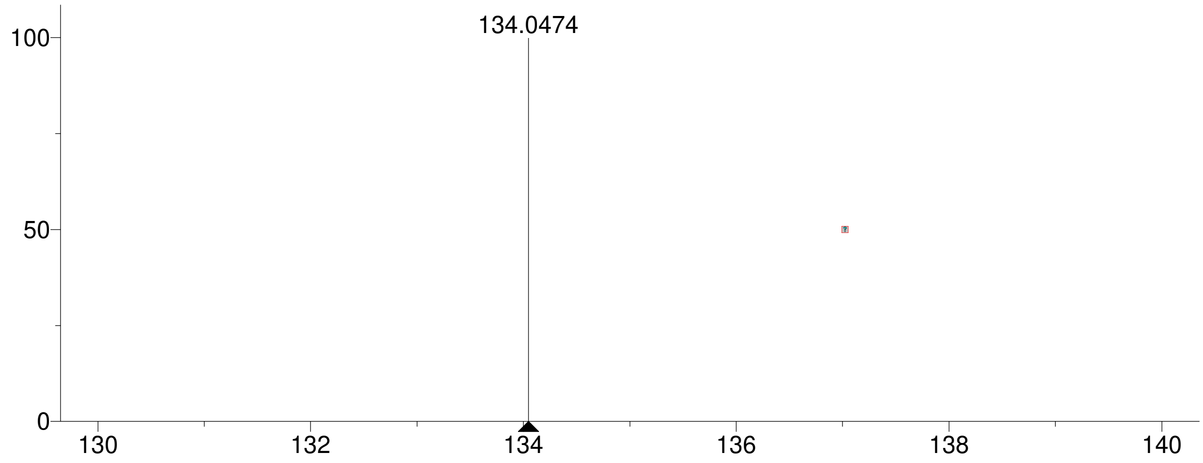

Hit 1 : Adenine [M-H]- 30V P=134
C5H5N5; MF: 364; RMF: 999; Prob 8.56%; Lib: mona-export-lc-ms-ms_negative_mode; ID: 8364.

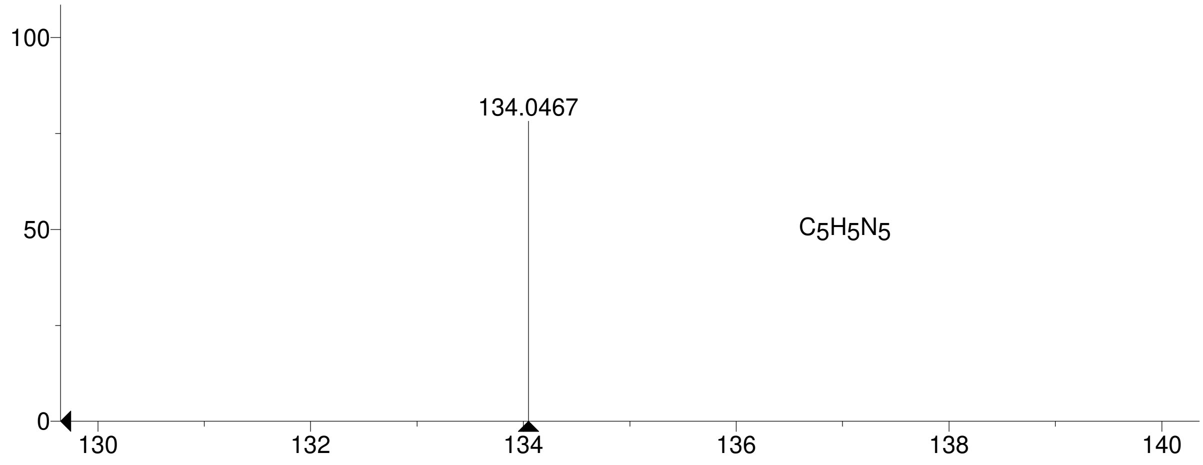

Hit 2 : C5H5N5; PlaSMA ID-63 [M-H]- P=134
C5H5N5; MF: 364; RMF: 999; Prob 8.56%; Lib: biomsms-neg-plasma; ID: 936.

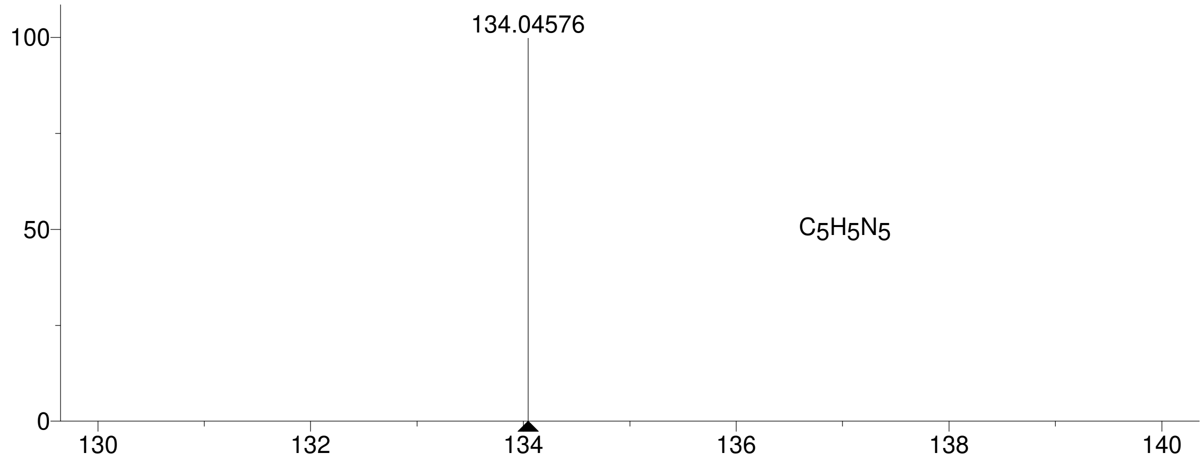

Hit 3 : adenine [M-H]- P=134
C5H5N5; MF: 364; RMF: 999; Prob 8.56%; Lib: msms neg mona_ipb_gnps_plasma; ID: 1503.

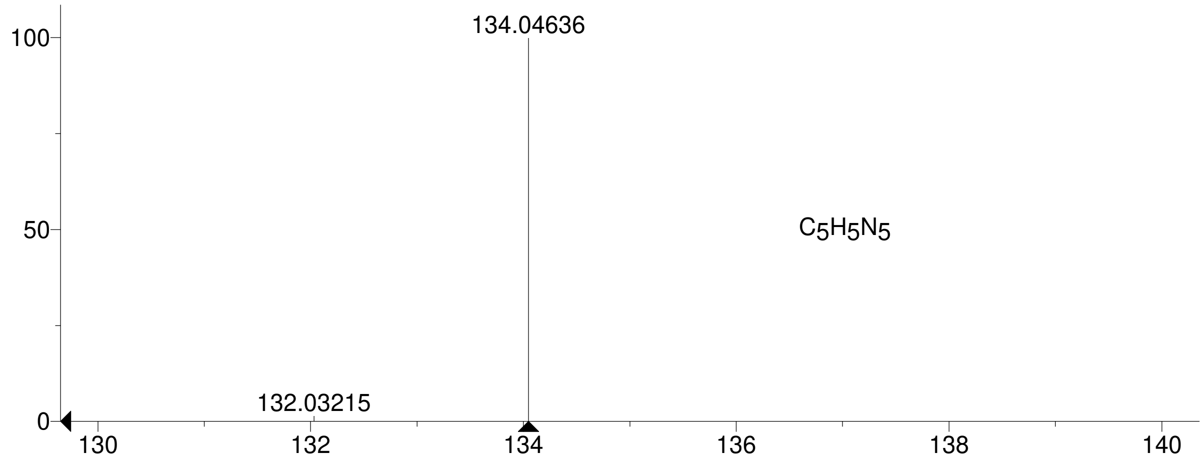

Hit 4 : Adenine; LC-ESI-QTOF; MS2; CE [M-H]- P=134
C5H5N5; MF: 364; RMF: 999; Prob 8.56%; Lib: msms-neg-massbank; ID: 4268.

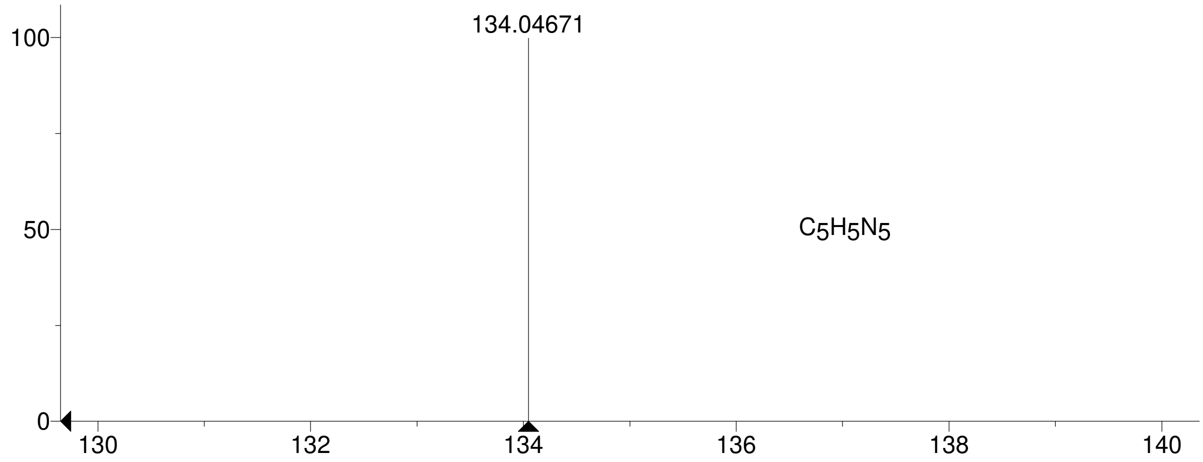

Hit 5 : 6-Chloroguanine [M+H-HCl]+ HCD 34V P=134
C5H4ClN5; MF: 364; RMF: 999; Prob 8.56%; CAS: 10310-21-1; Lib: nist_msms; ID: 285585.

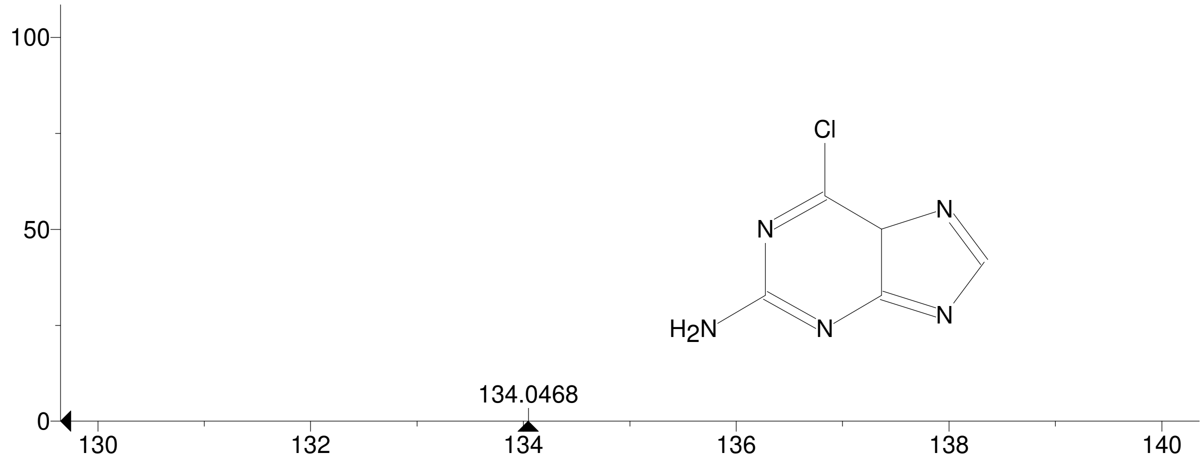

** Search Report Page 1 of 1 **

Unknown: Unknown (2.11_459.1930m/z)
Compound in Library Factor = 0

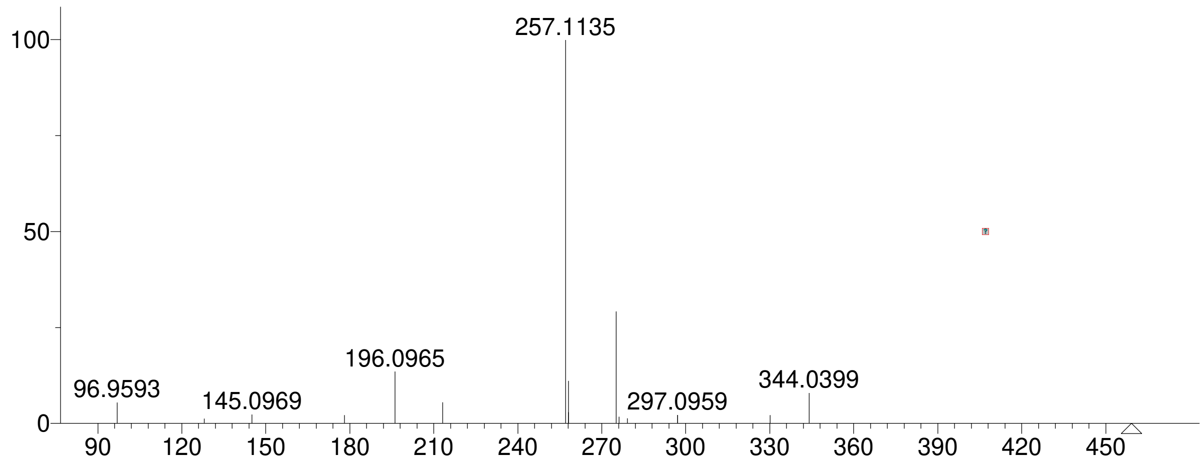

Unknown: Unknown (2.12_257.1134m/z)
Compound in Library Factor = -289

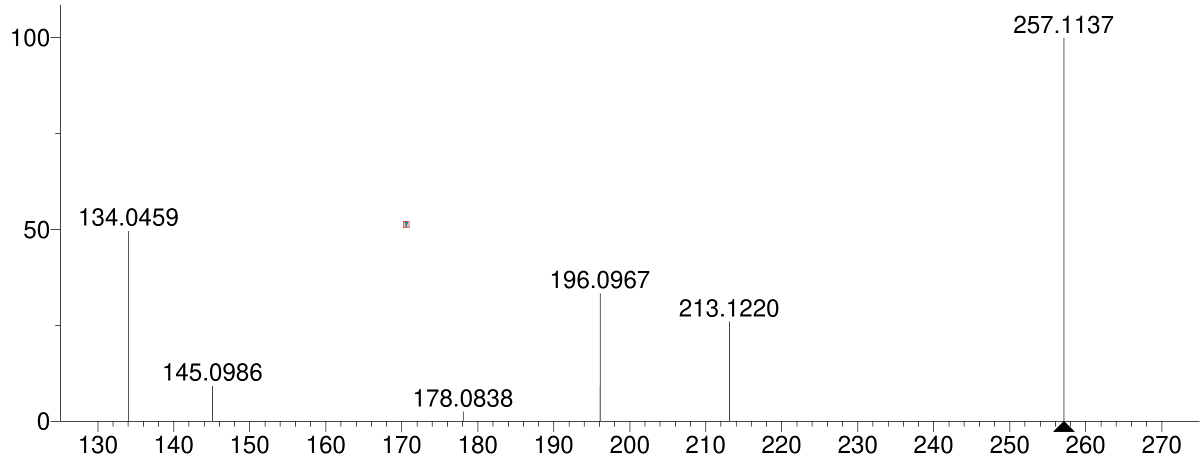

Hit 1 : L-Saccharopine [M-H-H2O]- HCD 24V P=257.1
C11H20N2O6; MF: 332; RMF: 767; Prob 99.0%; CAS: 997-68-2; Lib: nist_msms; ID: 125637.

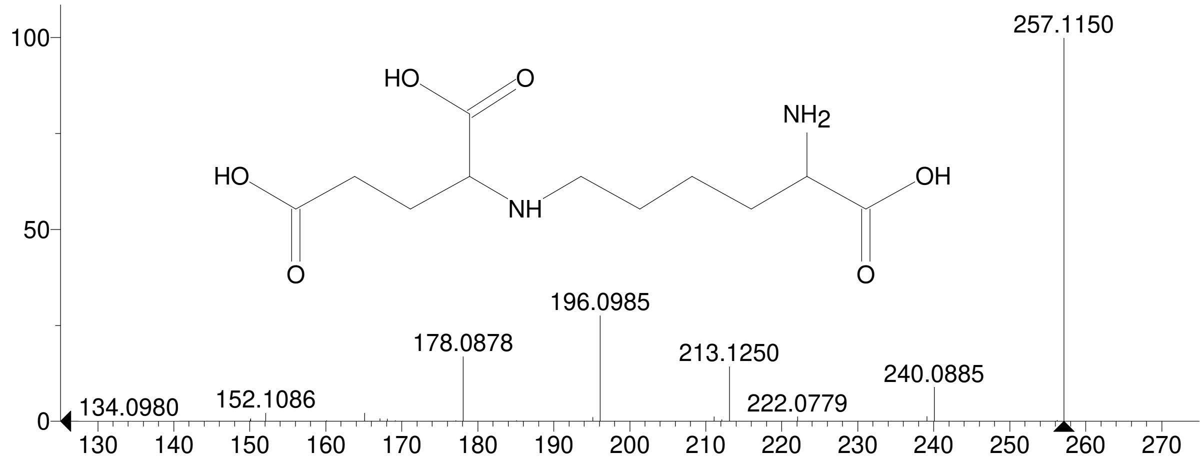

Hit 2 : α-Cyclohexylmandelic acid [M+Na]+ HCD 1V P=257.1
C14H18O3; MF: 122; RMF: 722; Prob 0.99%; CAS: 4335-77-7; Lib: nist_msms; ID: 492969.

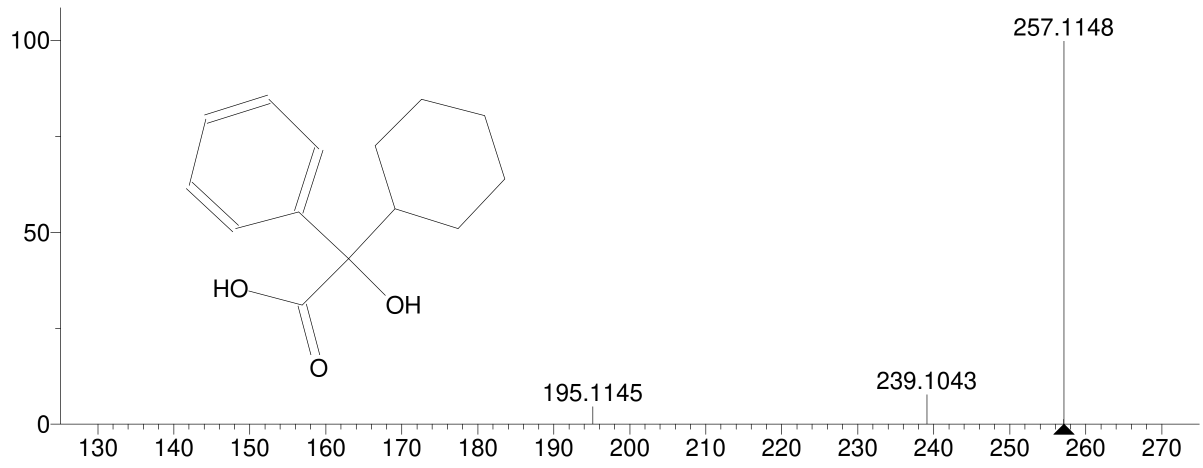

Unknown: Unknown (2.12_376.1349m/z)
Compound in Library Factor = 0

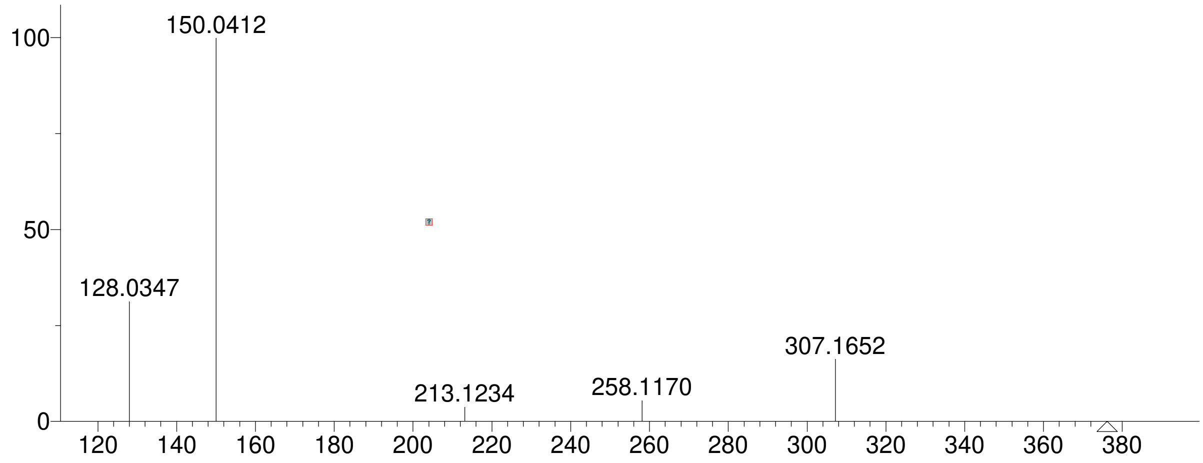

Hit 1 : Ofloxacin N-oxide [M-H]- HCD 60V P=376.1
C18H20FN3O5; MF: 0; RMF: 45; Prob 50.0%; CAS: 104721-52-0; Lib: nist_msms; ID: 464829.

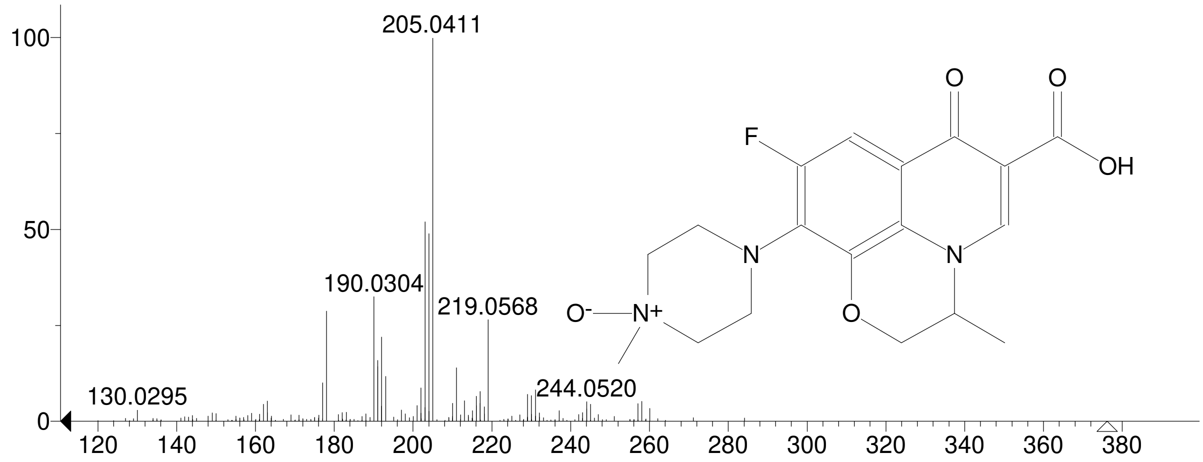

Hit 2 : NCGC00381076-01_C17H23NO7_4-[(2-{[(2-Ethyl-2,3-dihydroxybutanoyl)oxy]methyl}phenyl)amino]-4-oxob
C17H23NO7; MF: 0; RMF: 4; Prob 50.0%; Lib: mona-export-gnps; ID: 10257.

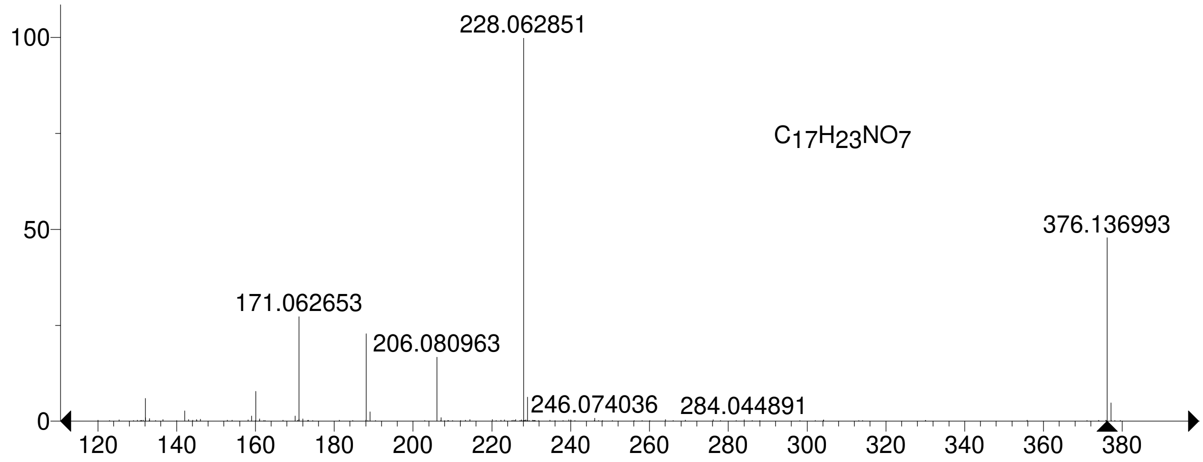

Unknown: Unknown (2.12_379.1704m/z)
Compound in Library Factor = 0

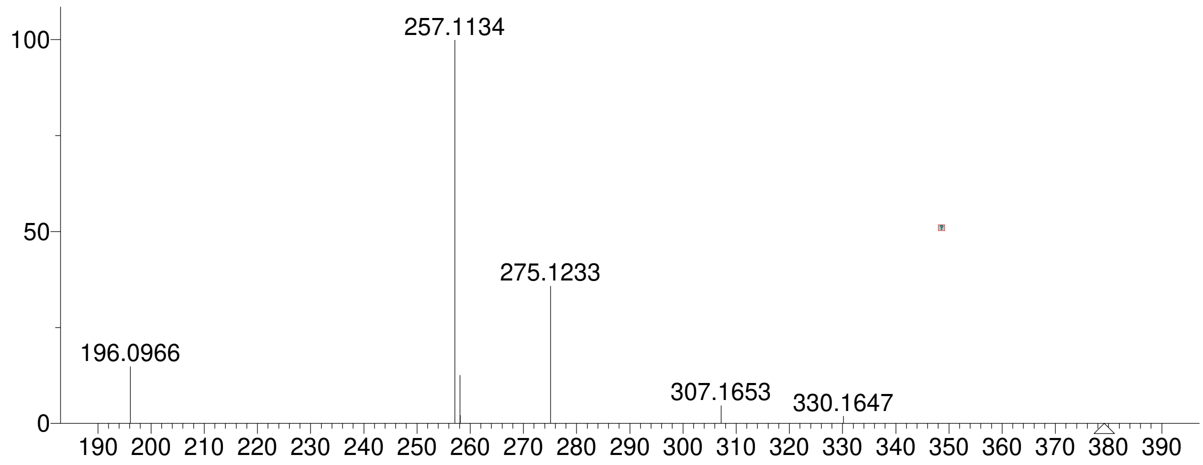

Hit 1 : [Glu1] TRH [M-H]- HCD 28eV P=379.2
C16H24N6O5; MF: 0; RMF: 19; Prob 33.3%; CAS: 51826-17-6; Lib: nist_msms; ID: 569033.

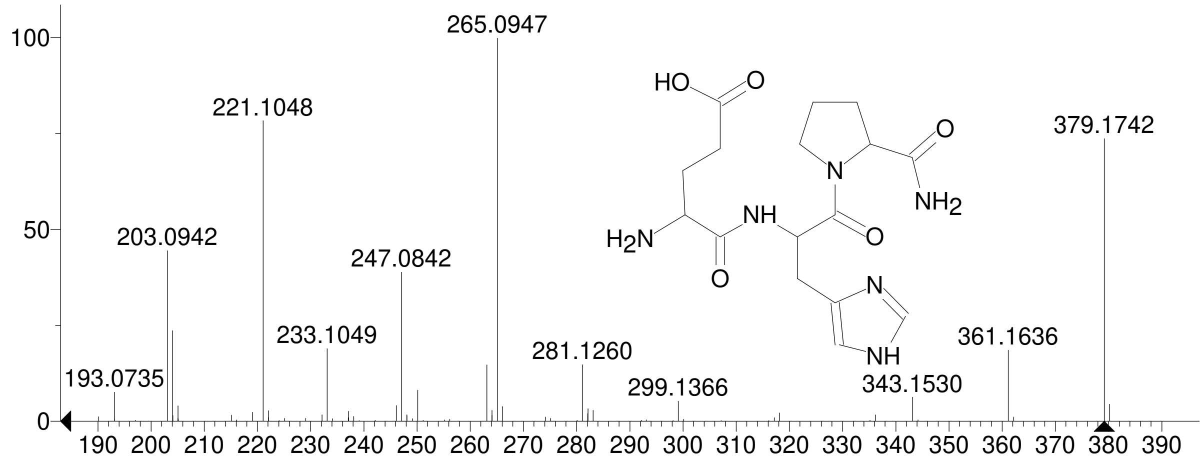

Hit 2 : [Glu1] TRH [M-H]- HCD 28eV P=379.2
C16H24N6O5; MF: 0; RMF: 19; Prob 33.3%; Lib: nist_msms2; ID: 74025.

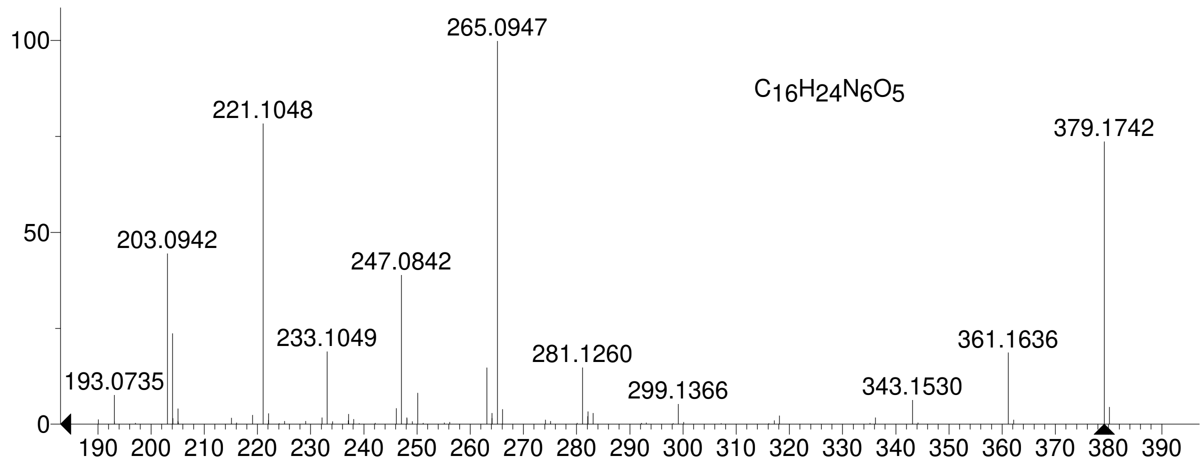

Hit 3 : Bisphenol PH [M-H]- HCD 49V P=379.2
C27H24O2; MF: 0; RMF: 17; Prob 33.3%; CAS: 24038-68-4; Lib: nist_msms; ID: 403295.

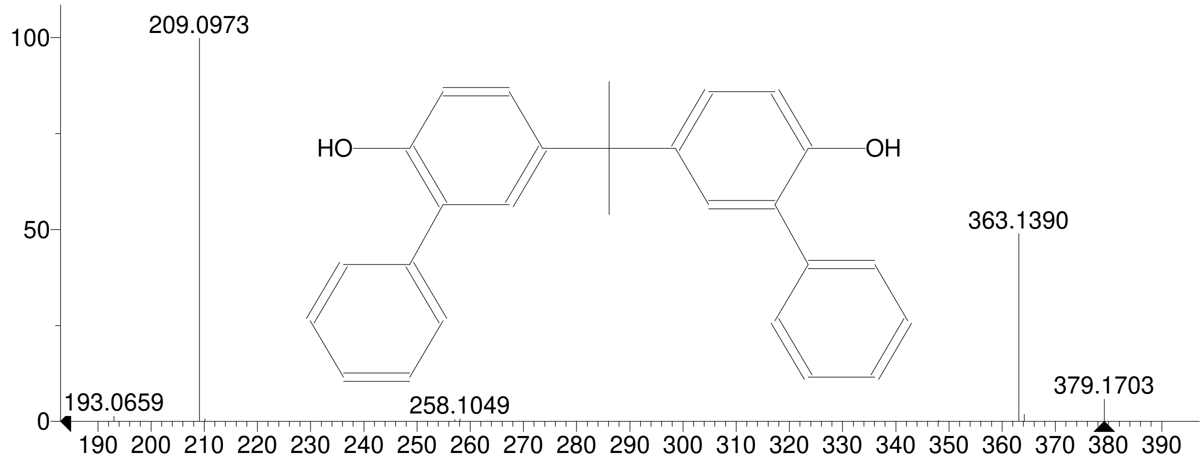

Unknown: Unknown (2.12_445.1817m/z)
Compound in Library Factor = -3273

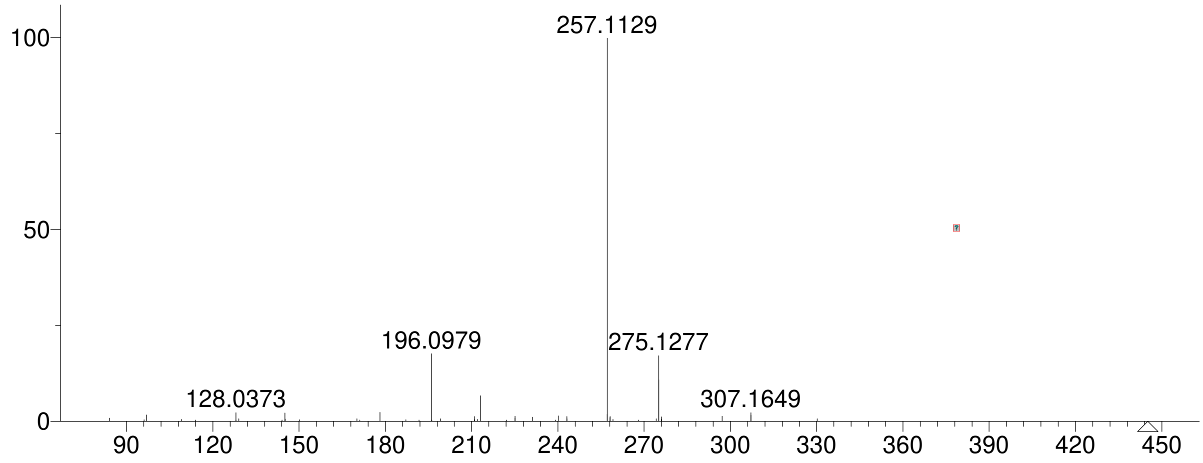

Hit 1 : Difenacoum [M+H]+ IT 35% P=445.2
C31H24O3; MF: 8; RMF: 495; Prob 20.8%; CAS: 56073-07-5; Lib: nist_msms; ID: 399396.

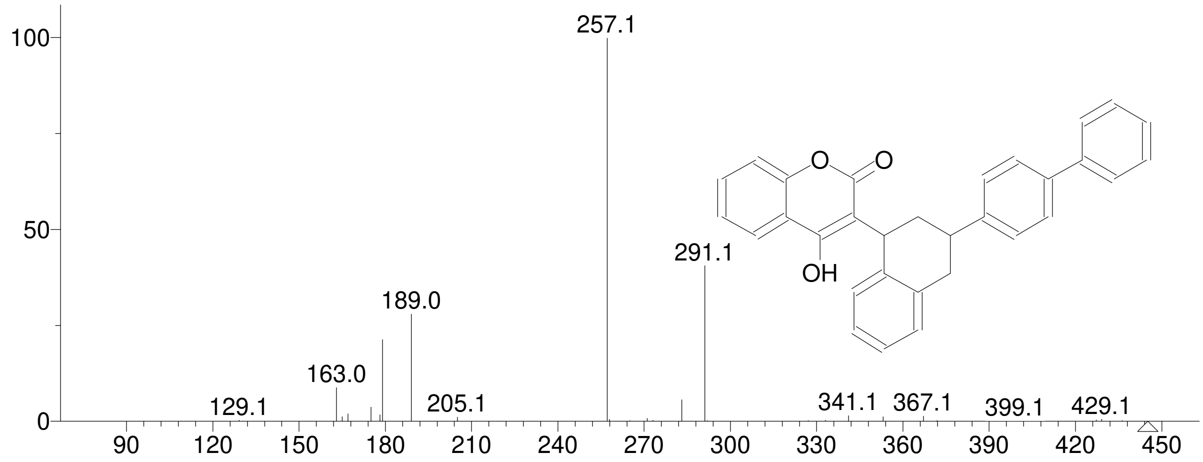

Hit 2 : 11-(hydroxymethyl)-2,6,6,14,19-pentamethyl-7,16,18-trioxapentacyclo[12.6.1.0A^'2,A^'1A^'2.0a^*u,A^'1a^'c
C24H28O8; MF: 4; RMF: 99; Prob 17.6%; Lib: mona-export-vf-npl_qexactive; ID: 21174.

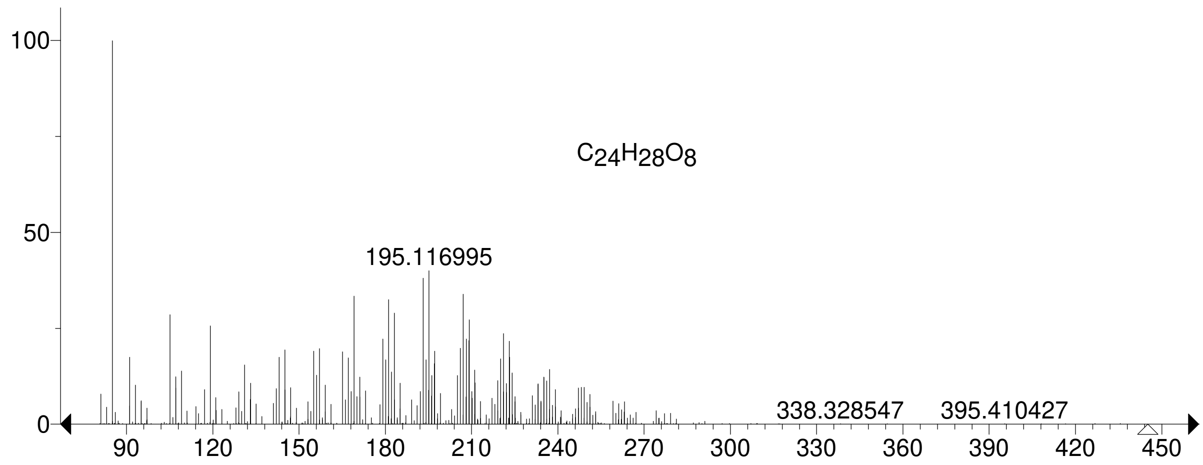

Hit 3 : NCGC00381216-01_C24H28O8_Furo[4,3,2-ij]pyrano[4',3':4,5]cyclohepta[1,2-f][2]benzopyran-1,5,9,13(2H,
C24H28O8; MF: 3; RMF: 121; Prob 16.9%; Lib: mona-export-gnps; ID: 11597.

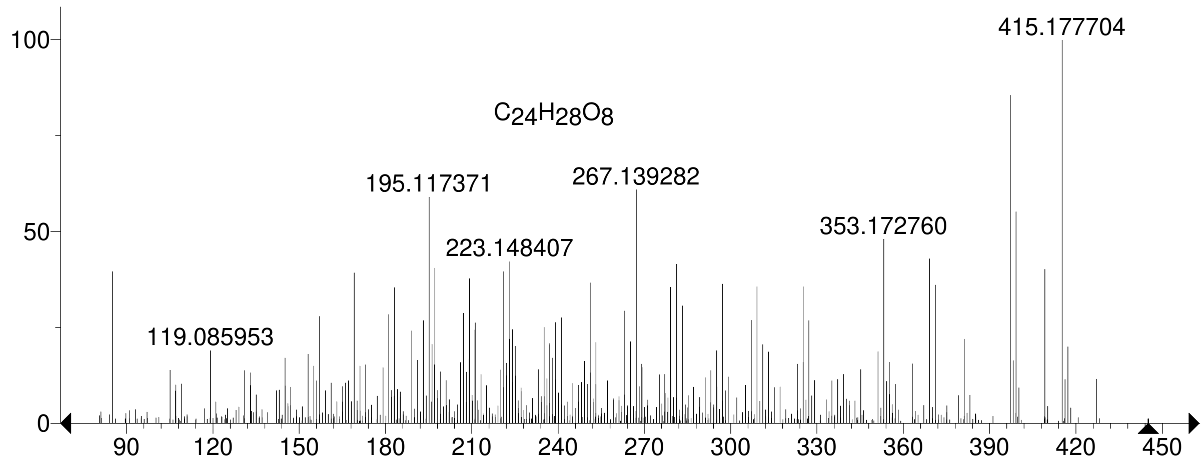

Hit 4 : [(6Z,10Z)-6-(acetyloxymethyl)-10-(hydroperoxymethyl)-3-methylidene-2-oxo-3a,4,5,8,9,11a-hexahydrocycl
C22H30O8; MF: 0; RMF: 48; Prob 14.9%; Lib: mona-export-vf-npl_qexactive; ID: 19873.

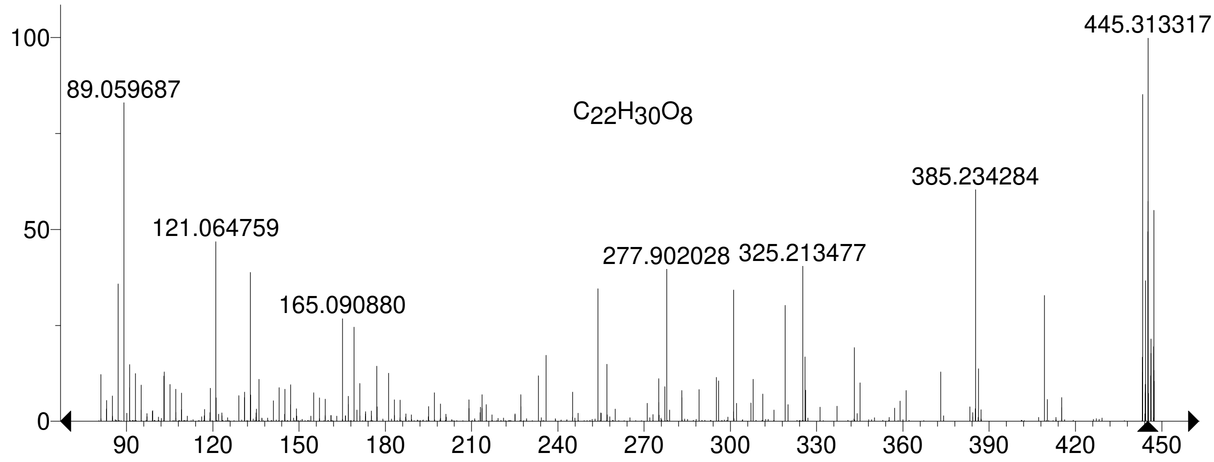

Hit 5 : NCGC00381245-01_C22H30O8_2-[6-{[(2Z)-4-Acetoxy-2-methyl-2-butenoyl]oxy}-2-hydroxy-3-(3-hydroxy-1
C22H30O8; MF: 0; RMF: 13; Prob 14.9%; Lib: mona-export-gnps; ID: 10571.

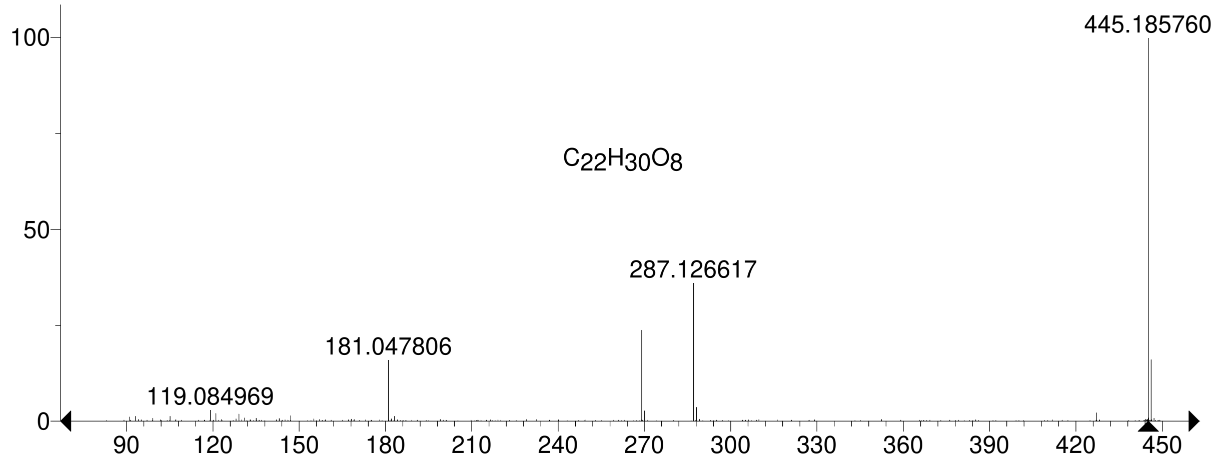

Unknown: Unknown (2.12_467.1570m/z)
Compound in Library Factor = 0

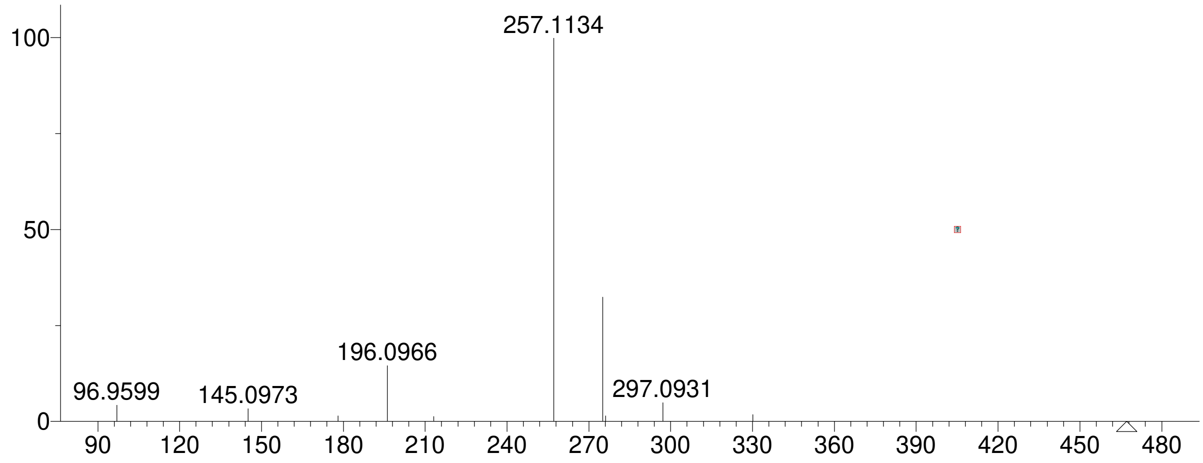

Hit 1 : agnuside [M+H]+ 45HCD P=467.2
C22H26O11; MF: 0; RMF: 11; Prob 50.0%; Lib: mona-export-vf-npl_qexactive; ID: 4715.

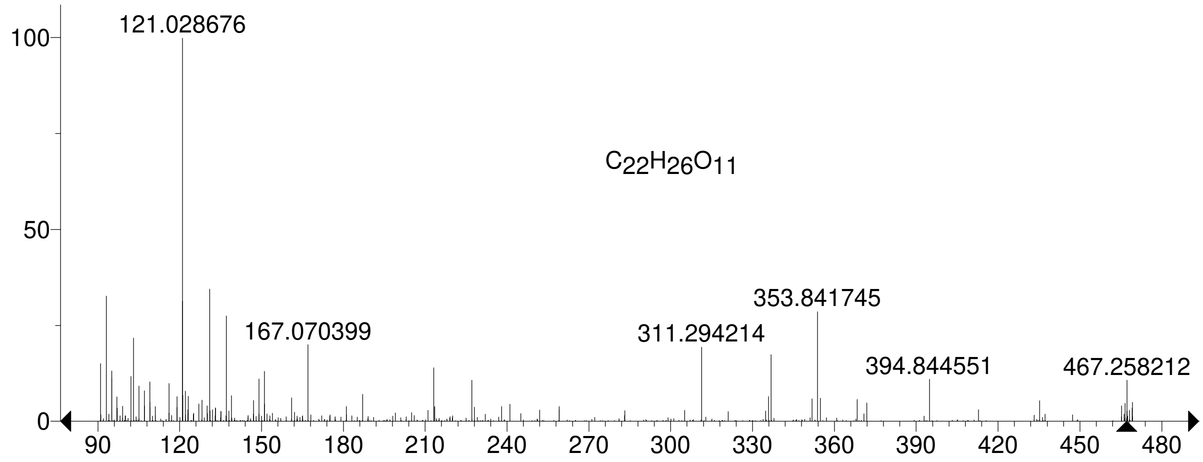

Hit 2 : [(1aS,1bS,2S,5aR,6S,6aS)-1a-(hydroxymethyl)-2-[(2S,3R,4S,5S,6R)-3,4,5-trihydroxy-6-(hydroxymethyl)oxa
C22H26O11; MF: 0; RMF: 4; Prob 50.0%; Lib: mona-export-vf-npl_qexactive; ID: 12085.

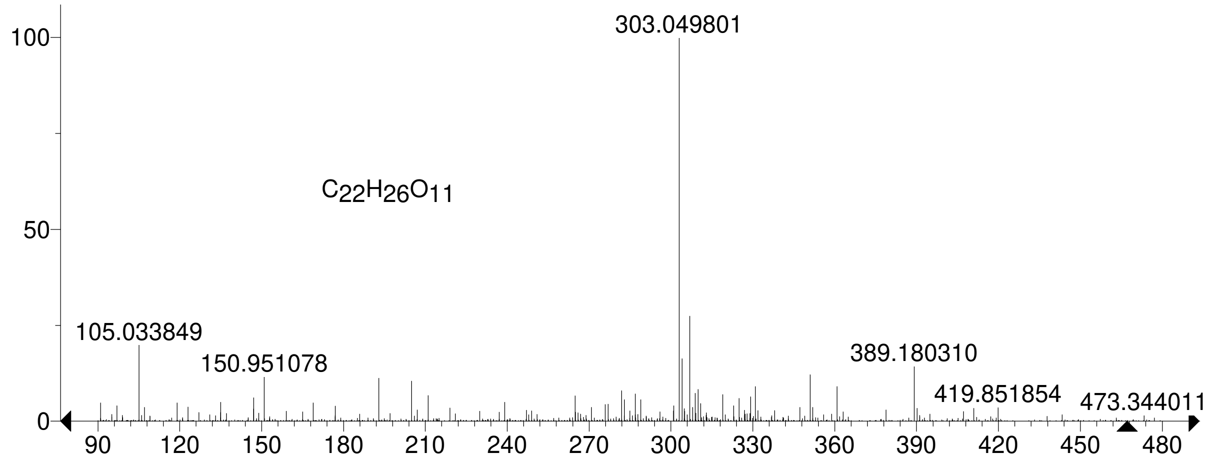

Unknown: Unknown (2.12_571.1296m/z)
Compound in Library Factor = -3273

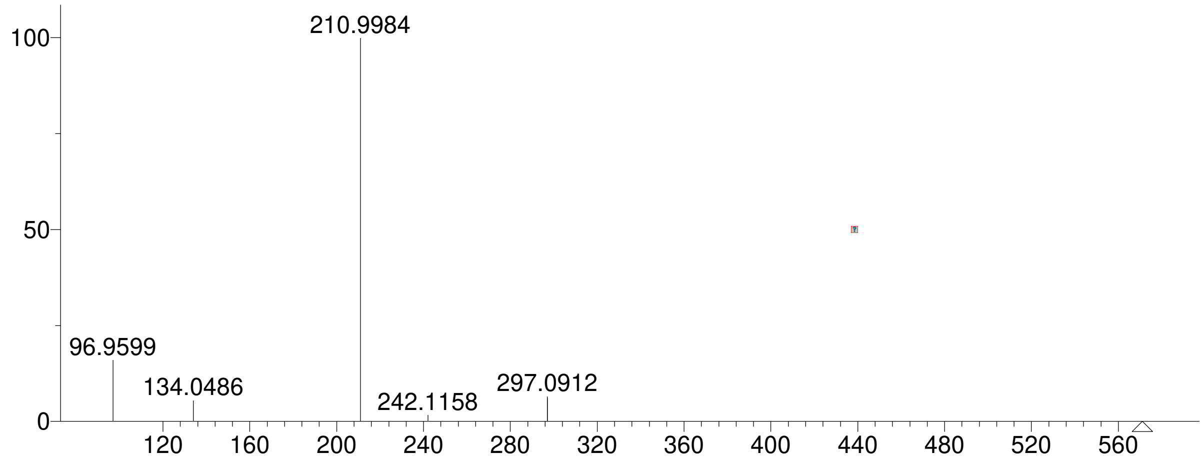

Hit 1 : Adenylyl(3'-5')cytidine [M-H]- QTOF 40V P=571.1
C19H25N8O11P; MF: 3; RMF: 175; Prob 53.1%; CAS: 2382-66-3; Lib: nist_msms; ID: 28245.

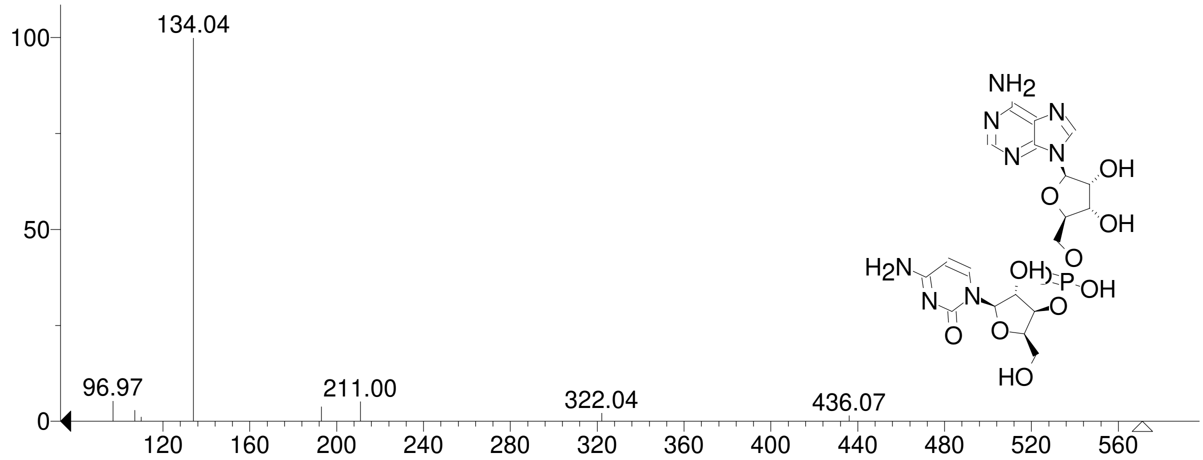

Hit 2 : methyl 8,11,15,20,22-pentahydroxy-24-methyl-13,18,27-trioxo-6-oxaheptacyclo[15.10.2.0A^'1,A^'1a^'1.0A^
C31H24O11; MF: 0; RMF: 32; Prob 46.9%; Lib: mona-export-lc-ms-ms_negative_mode; ID: 23338.

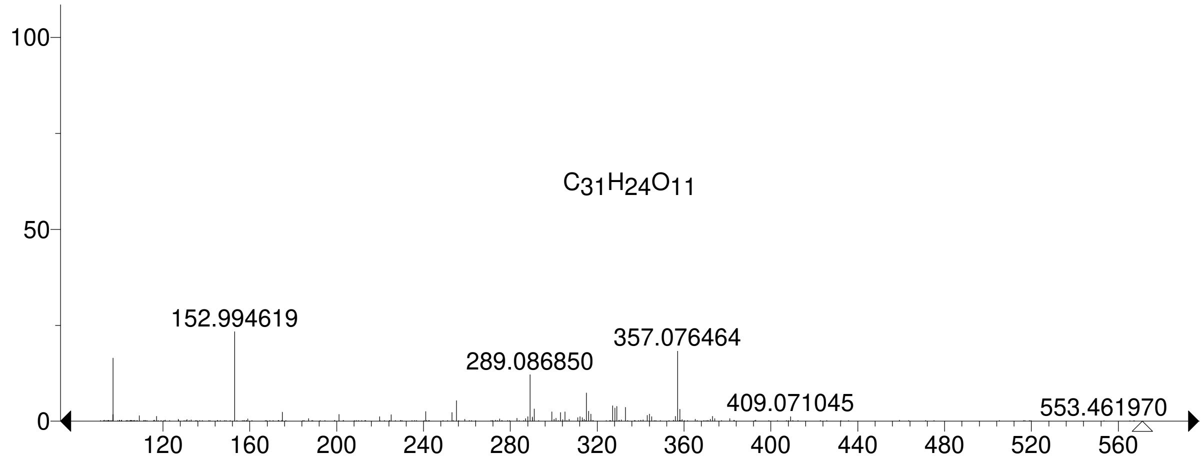

** Search Report Page 1 of 1 **

Unknown: Unknown (2.14_405.1613m/z)
Compound in Library Factor = 0

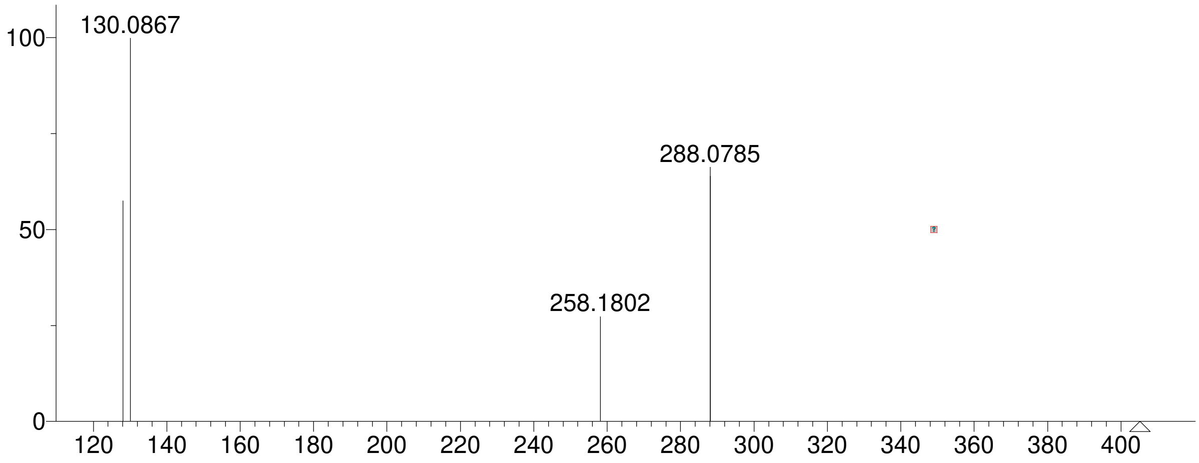

Unknown: Unknown (2.15_323.0972m/z)
Compound in Library Factor = 0

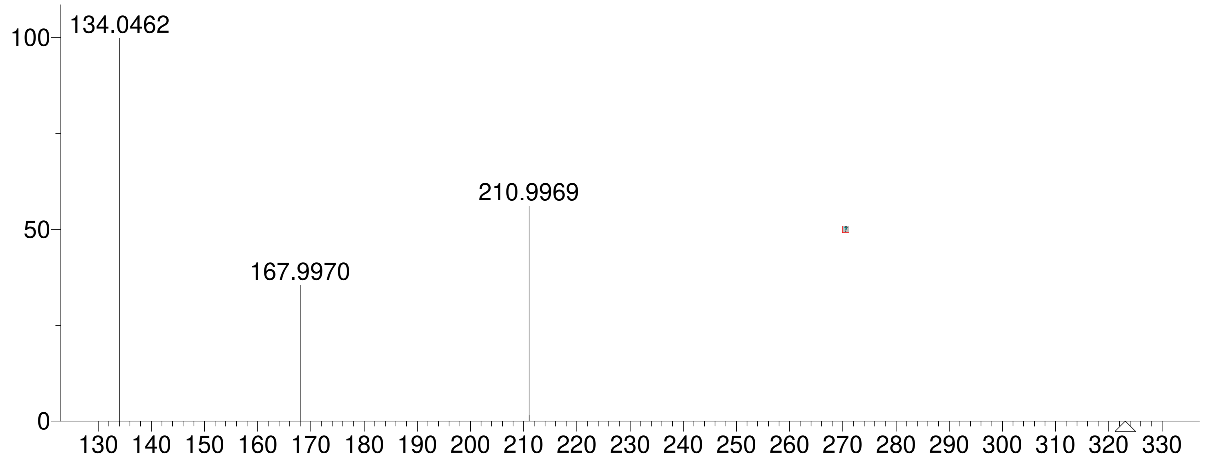

Hit 1 : 1-Piperidinecarboxylic acid, 4-[6-[4-[(methoxycarbonyl)amino]phenyl]-4-(4-morpholinyl)-1H-pyrazolo[3,4-d]|
C24H29N7O5; MF: 0; RMF: 105; Prob 7.69%; CAS: 1062169-56-5; Lib: nist_msms; ID: 265735.

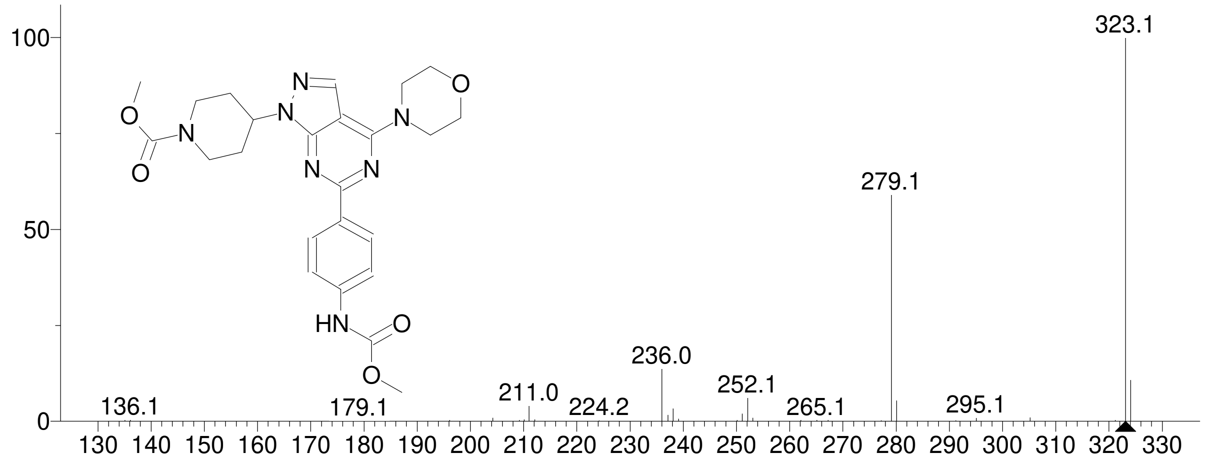

Hit 2 : Indanofan [M+H]+=>323.1 IT 35% P=323.1
C20H17ClO3; MF: 0; RMF: 88; Prob 7.69%; CAS: 133220-30-1; Lib: nist_msms; ID: 477459.

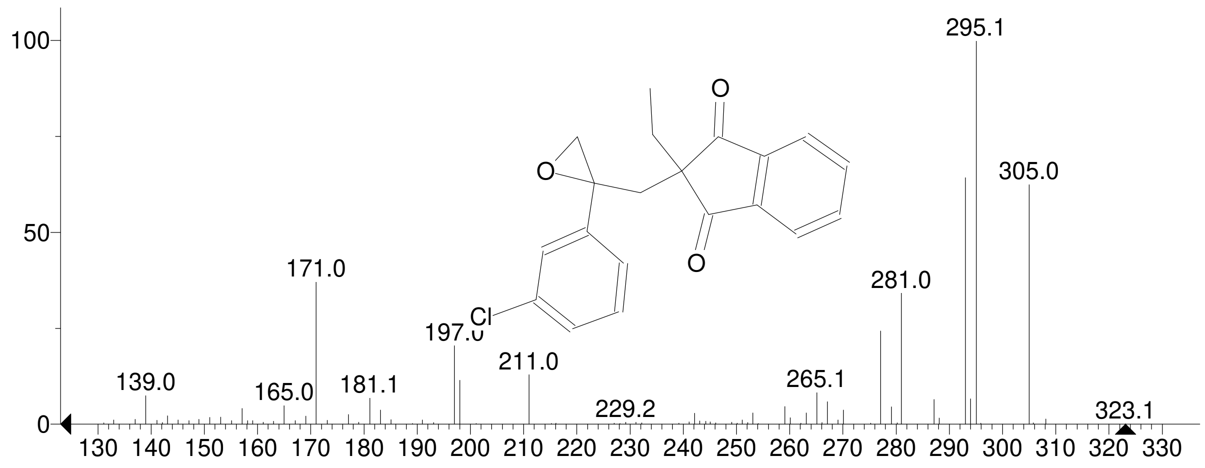

Hit 3 : Prednisone [M+H-H2O]+=>323.1 IT 35% P=323.1
C21H26O5; MF: 0; RMF: 83; Prob 7.69%; CAS: 53-03-2; Lib: nist_msms; ID: 99548.

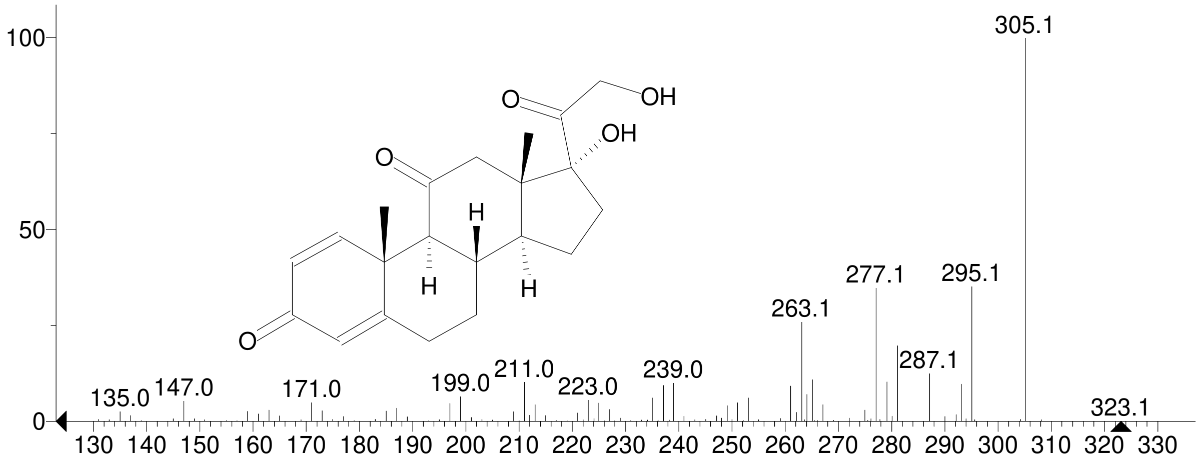

Hit 4 : Fluorometholone [M+H-H2O]+=>341.1=>323.1 IT 35% P=323.1
C22H29FO4; MF: 0; RMF: 66; Prob 7.69%; CAS: 426-13-1; Lib: nist_msms; ID: 119128.

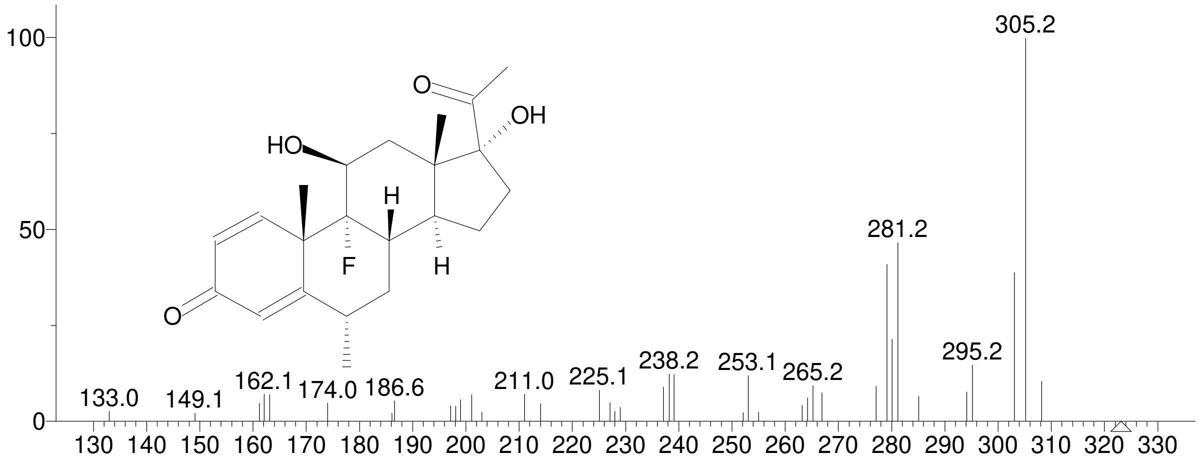

Hit 5 : Budesonide [M+H-H2O]+=>323.1 IT 35% P=323.1
C25H34O6; MF: 0; RMF: 49; Prob 7.69%; CAS: 51333-22-3; Lib: nist_msms; ID: 152692.

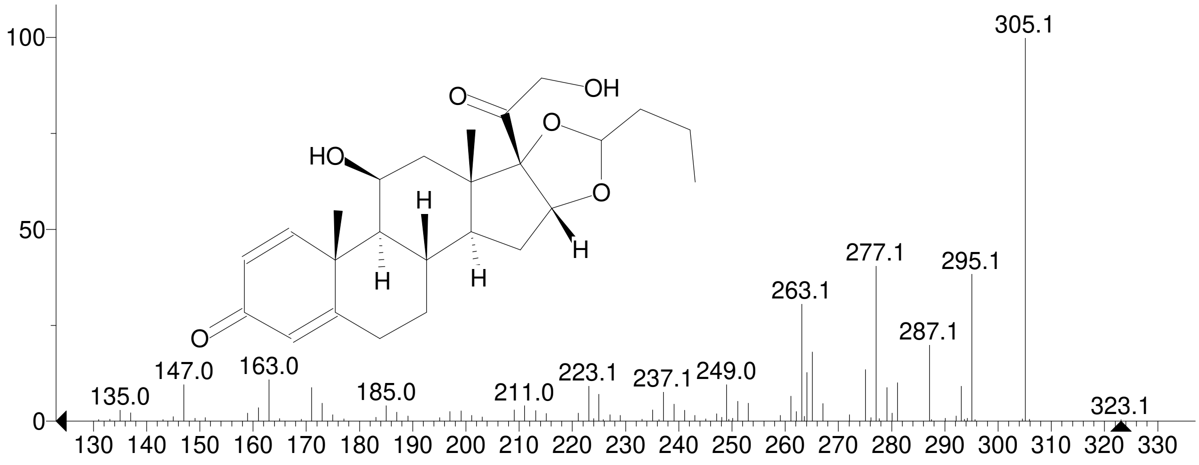

Unknown: Unknown (2.15_352.0694m/z)
Compound in Library Factor = 0

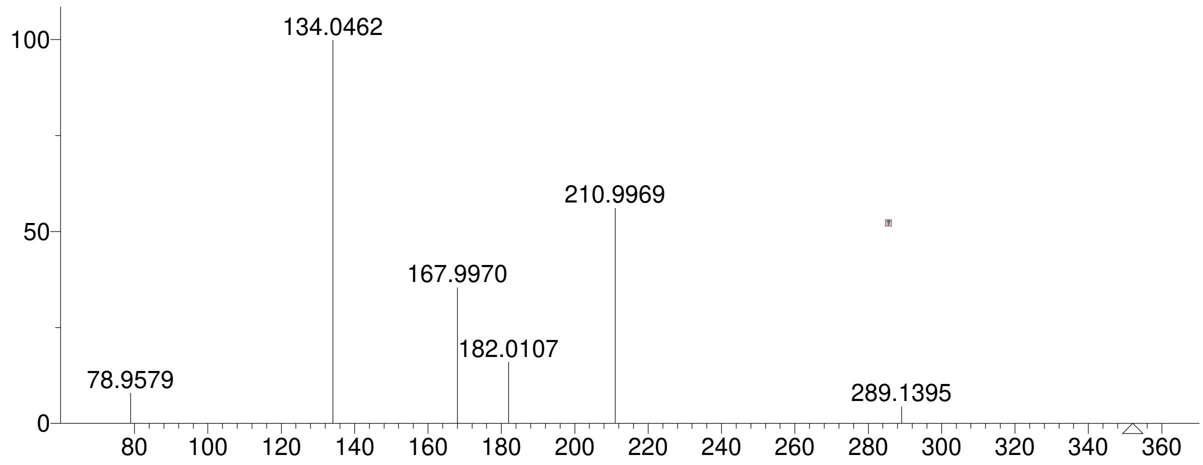

Hit 1 : Lansoprazole [M+H-H2O]+ HCD 47V P=352.1
C16H14F3N3O2S; MF: 0; RMF: 14; Prob 100.0%; CAS: 103577-45-3; Lib: nist_msms; ID: 316346.

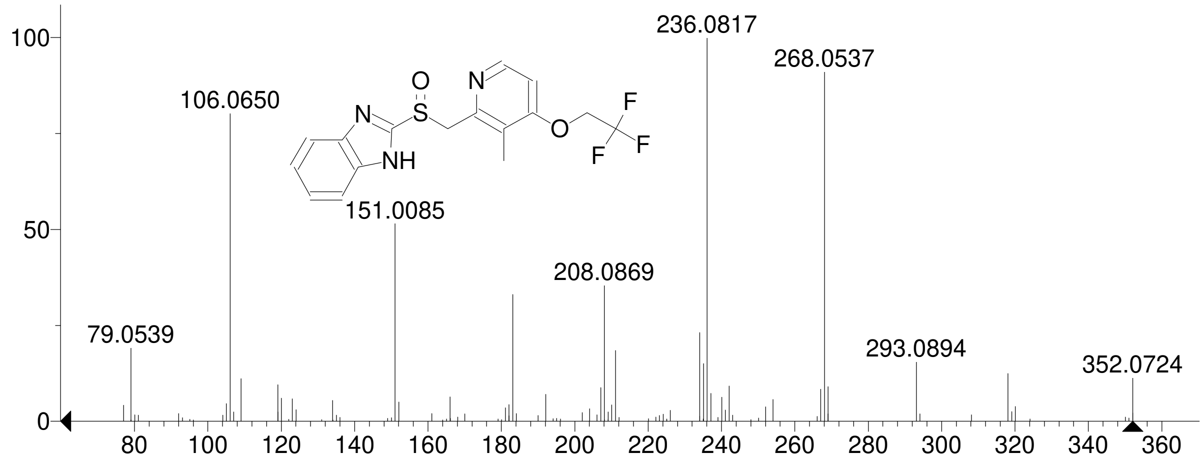

Unknown: Unknown (2.15_403.1336m/z)
Compound in Library Factor = 0

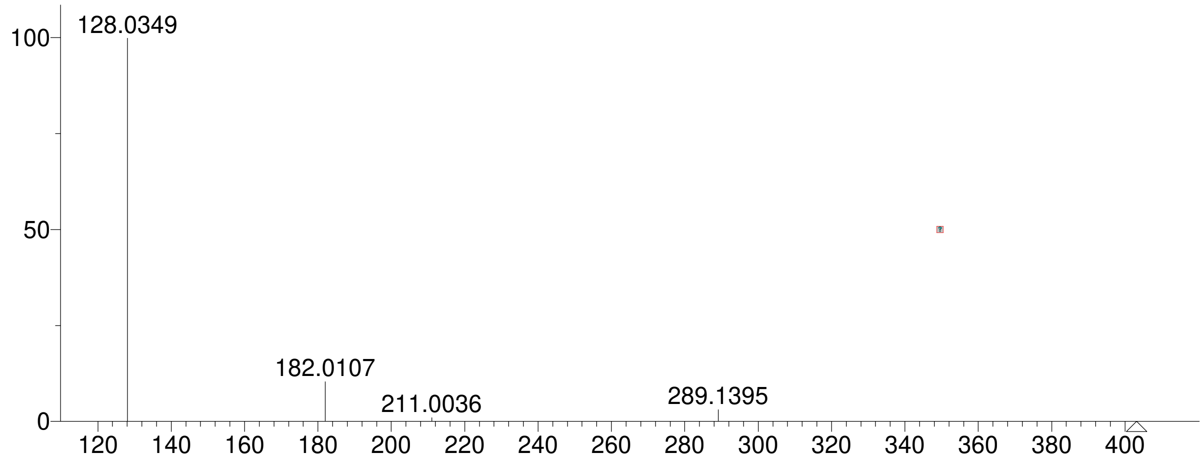

Hit 1 : Egtazic acid [M+Na]+ HCD 52V P=403.1
C14H24N2O10; MF: 0; RMF: 6; Prob 50.0%; CAS: 67-42-5; Lib: nist_msms; ID: 81815.

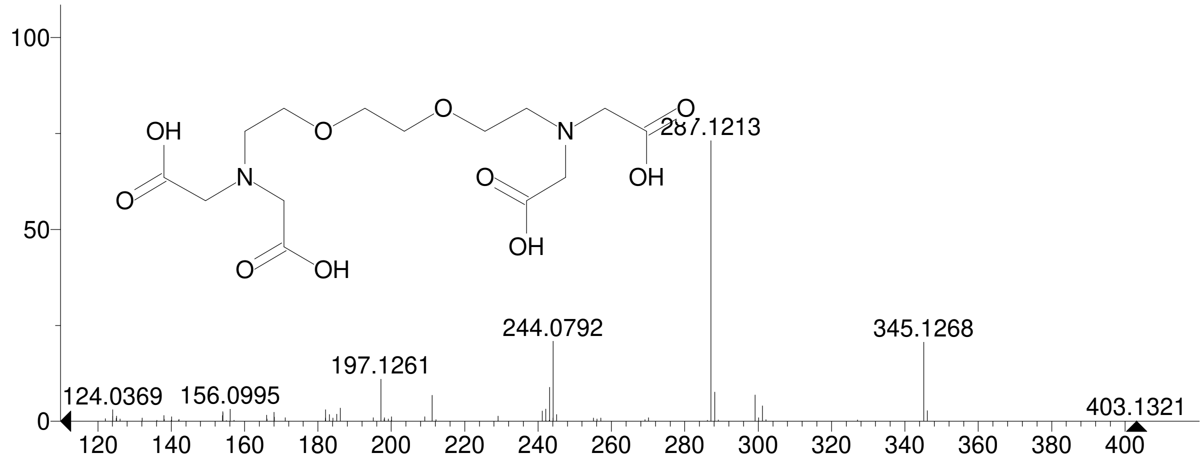

Hit 2 : methyl 2-(4,5-dihydroxy-6-methoxy-3,6-dimethyl-2-oxocyclohex-3-en-1-yl)oxy-4-hydroxy-3,6-dimethylbenzc
C19H24O8; MF: 0; RMF: 2; Prob 50.0%; Lib: mona-export-vf-npl_qexactive; ID: 23487.

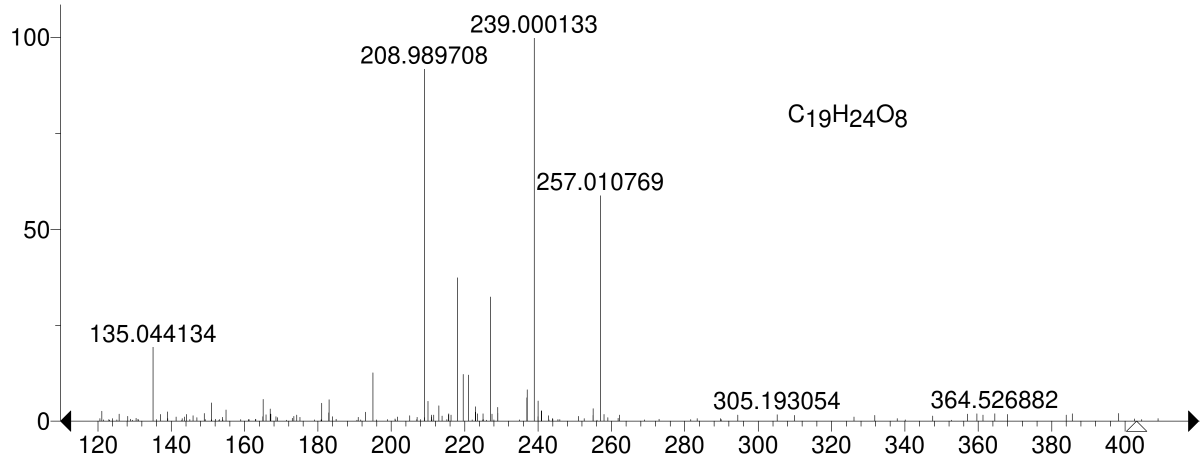

Unknown: Unknown (2.16_302.0651m/z)
Compound in Library Factor = -2387

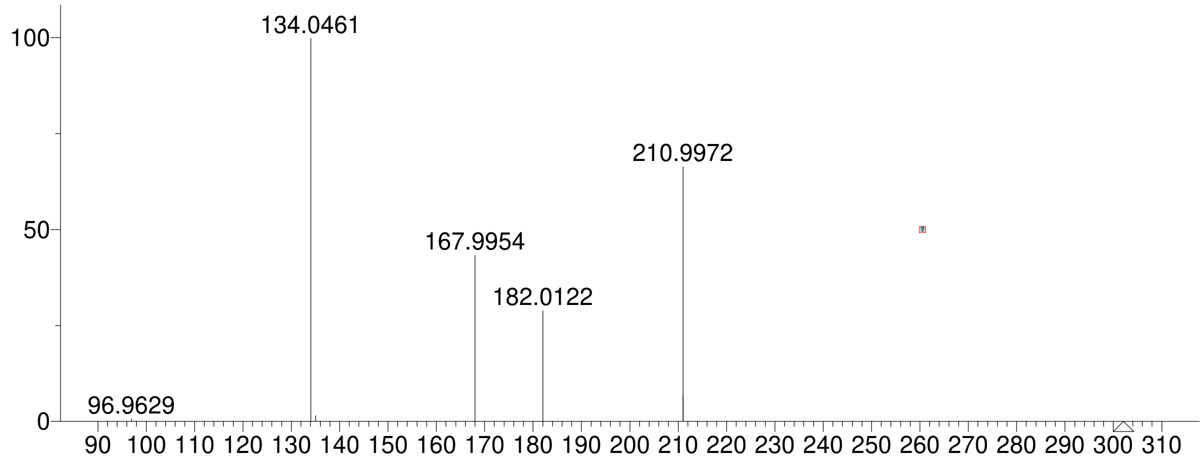

Hit 1 : 2-Chloroadenosine [M+H]+ HCD 57V P=302.1
C10H12ClN5O4; MF: 21; RMF: 506; Prob 100.0%; CAS: 146-77-0; Lib: nist_msms; ID: 116075.

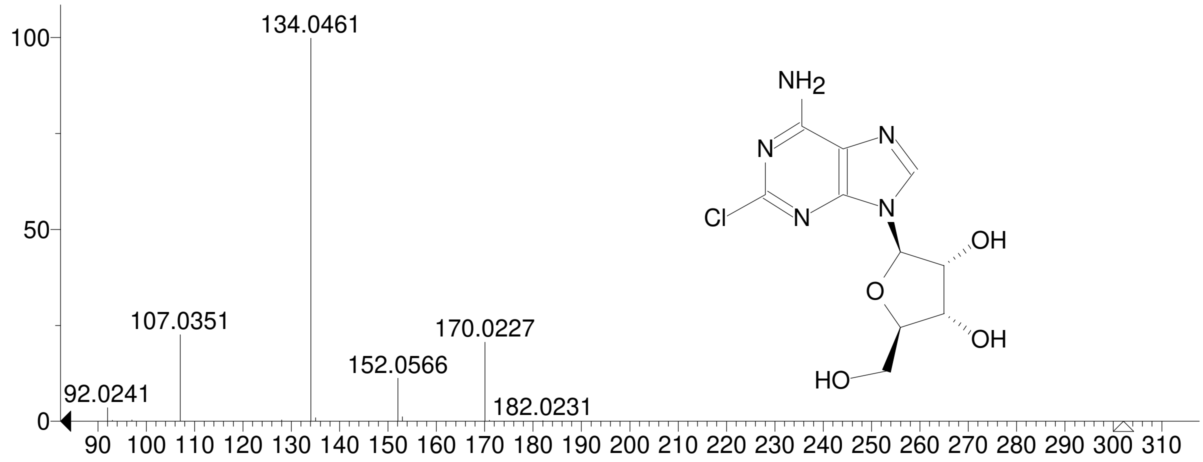

** Search Report Page 1 of 1 **

Unknown: Unknown (2.16_338.0528m/z)
Compound in Library Factor = 0

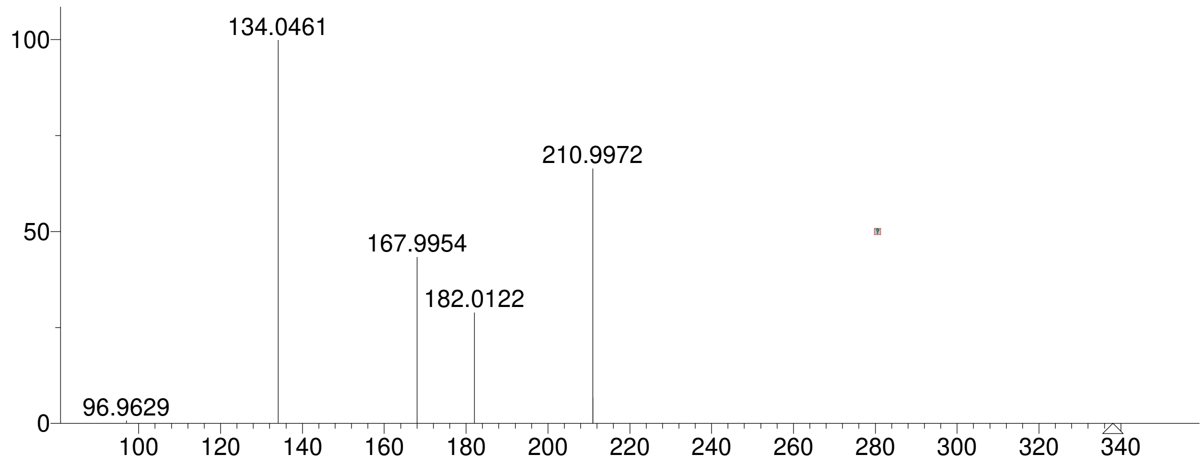

Unknown: Unknown (2.16_420.1979m/z)
Compound in Library Factor = 0

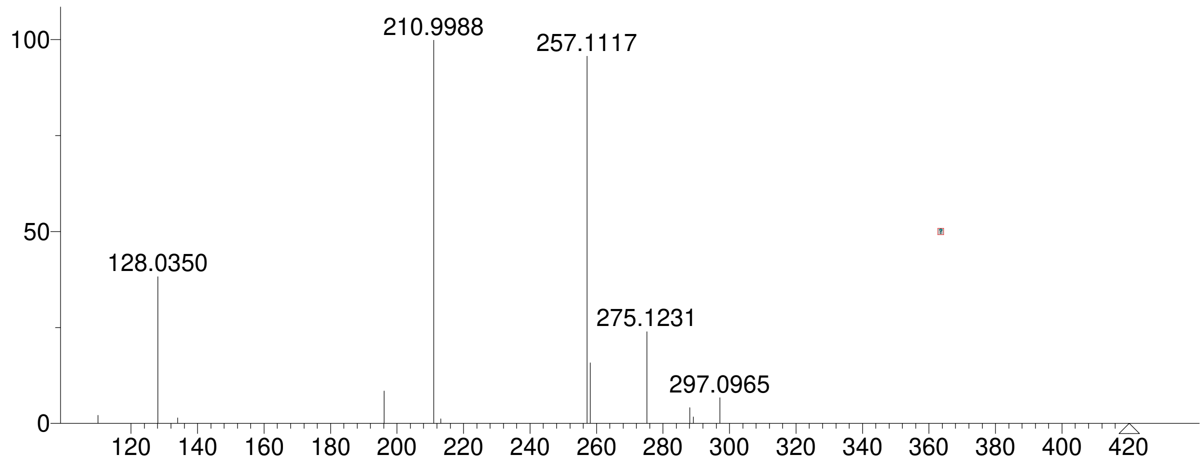

Hit 1 : (2R,3R,3aR,5R)-5,7-dimethoxy-2-(7-methoxy-1,3-benzodioxol-5-yl)-3-methyl-3a-prop-2-enyl-2,3,4,5-tetrah
C22H26O7; MF: 0; RMF: 54; Prob 14.3%; Lib: mona-export-vf-npl_qexactive; ID: 23179.

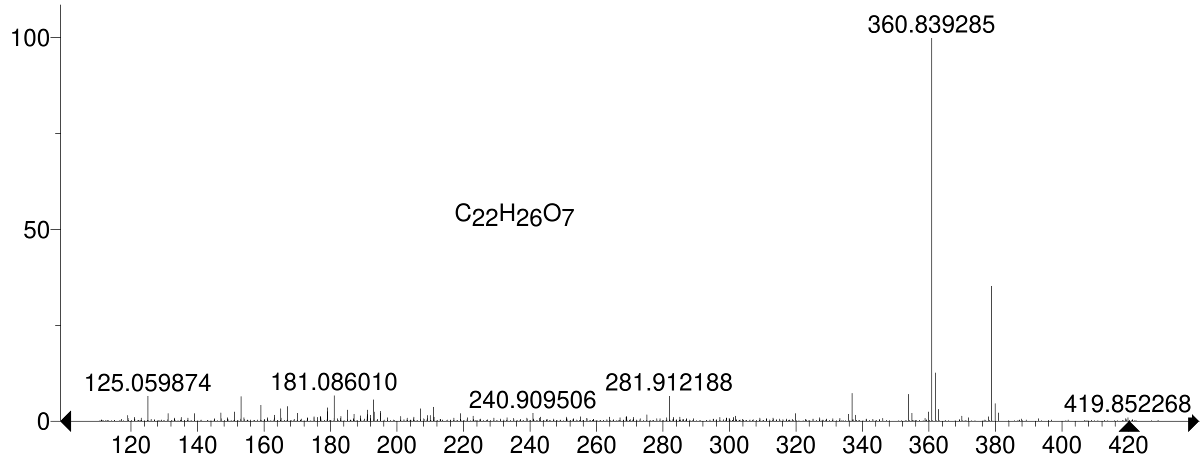

Hit 2 : Montelukast-1,2-diol [M+H-C6H10O2S]+=>420.2 IT 35% P=420.2
C35H36ClNO4S; MF: 0; RMF: 53; Prob 14.3%; CAS: 186352-97-6; Lib: nist_msms; ID: 463638.

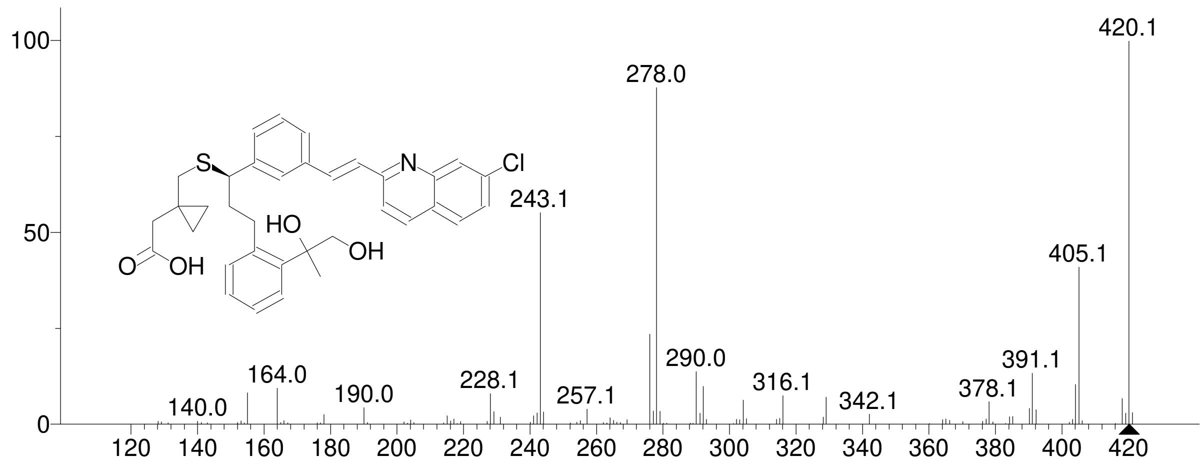

Hit 3 : NCGC00381253-01_C22H29NO7_2H-Isoindole-2-acetic acid, 7-carboxy-1,3-dihydro-6-methoxy-5-methyl-
C22H29NO7; MF: 0; RMF: 38; Prob 14.3%; Lib: mona-export-gnps; ID: 13397.

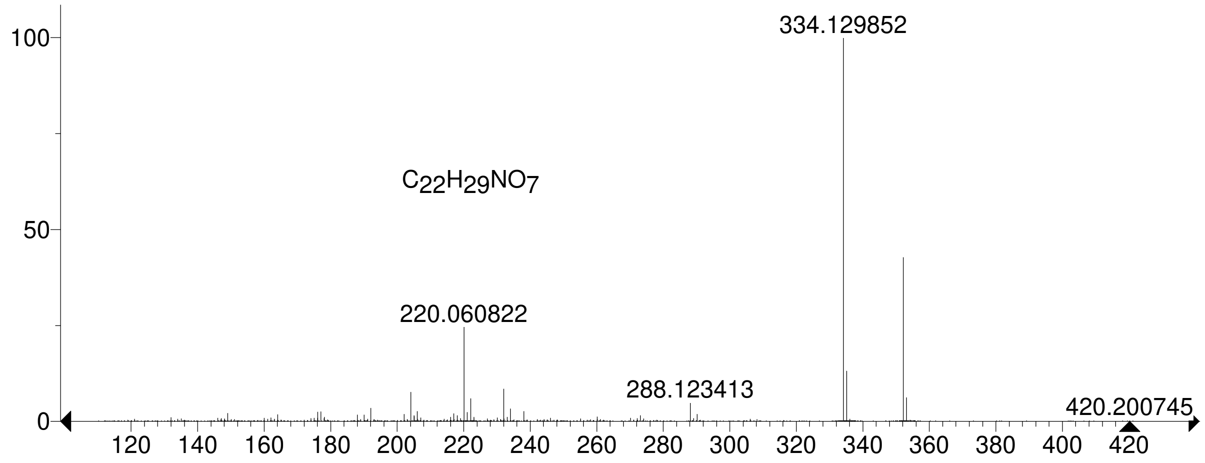

Hit 4 : [2-[2-(2-methylpropanoyloxy)propan-2-yl]-7-oxo-2,3-dihydrofuro[3,2-g]chromen-3-yl] 2-methylpropanoate [
C22H26O7; MF: 0; RMF: 29; Prob 14.3%; Lib: mona-export-vf-npl_qexactive; ID: 9047.

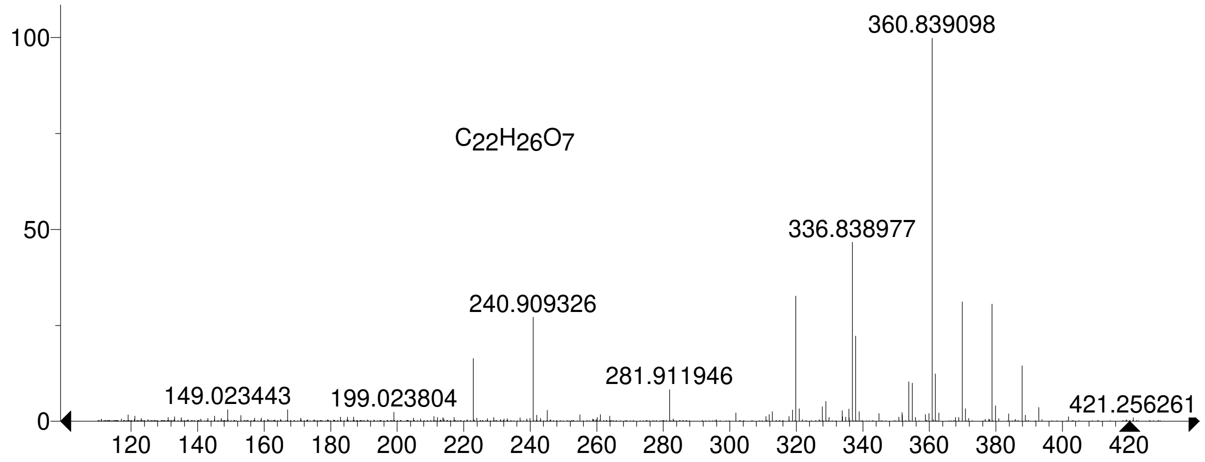

Hit 5 : 5-trans-17-Phenyltrinorprostaglandin F2α ethylamide [M+Na]+=>420.2 IT 35% P=420.2
C25H37NO4; MF: 0; RMF: 19; Prob 14.3%; CAS: 1163135-95-2; Lib: nist_msms; ID: 156516.

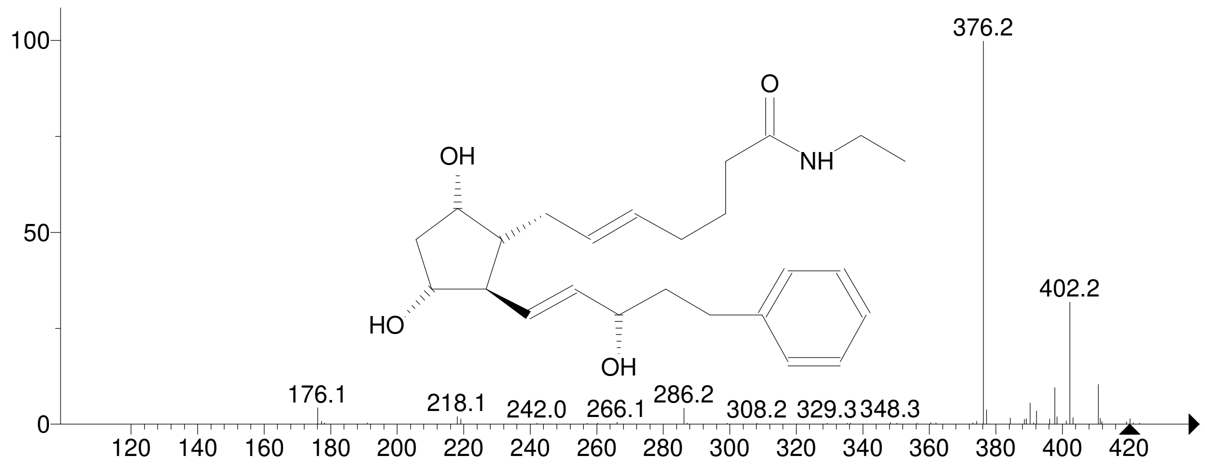

Unknown: Unknown (2.16_638.1987m/z)
Compound in Library Factor = 0

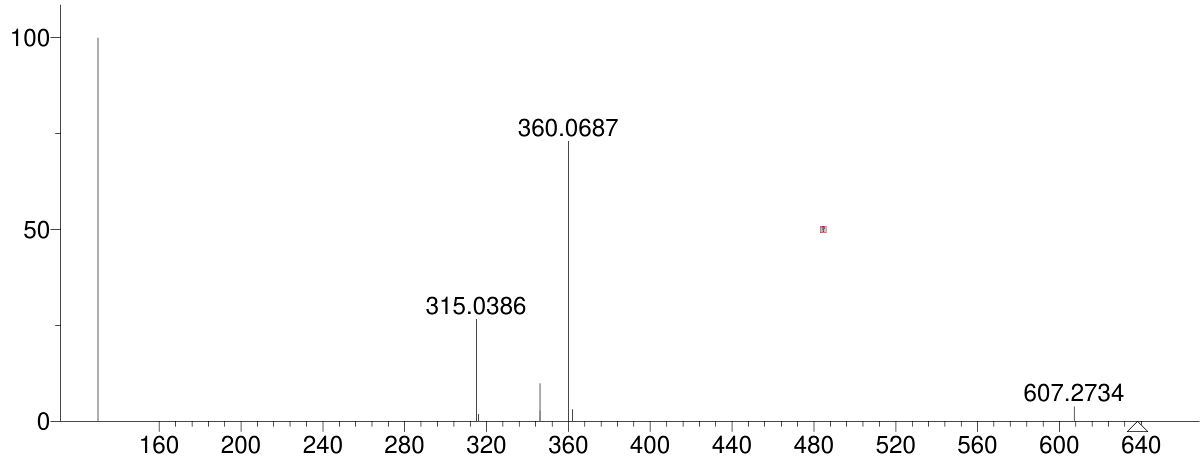

Unknown: Unknown (2.17_359.0970m/z)
Compound in Library Factor = -3273

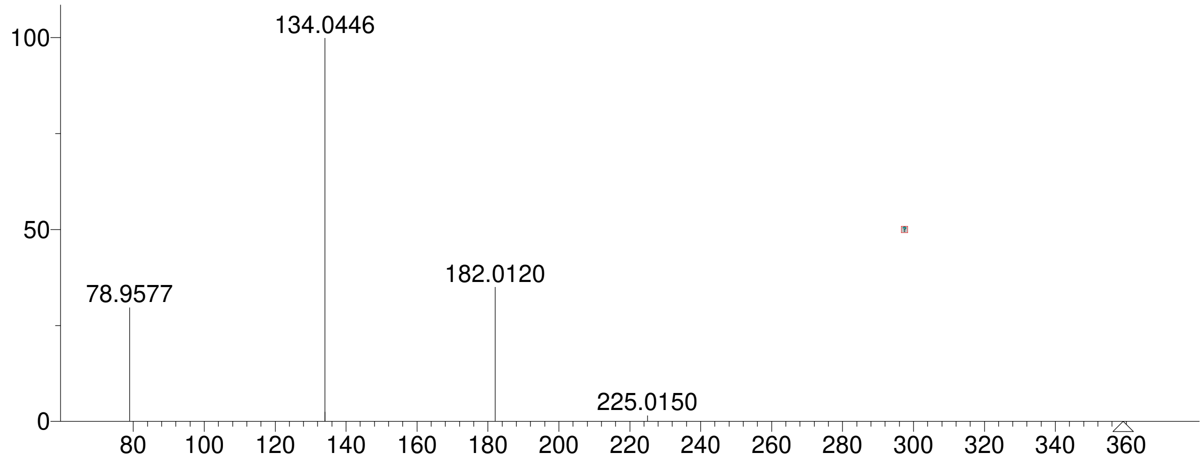

Hit 1 : Benzoic acid + 1O, 2MeO, O-Hex [M-H]- 6V P=359.1
C15H20O10; MF: 2; RMF: 157; Prob 21.0%; Lib: anthocyanins mona; ID: 7465.

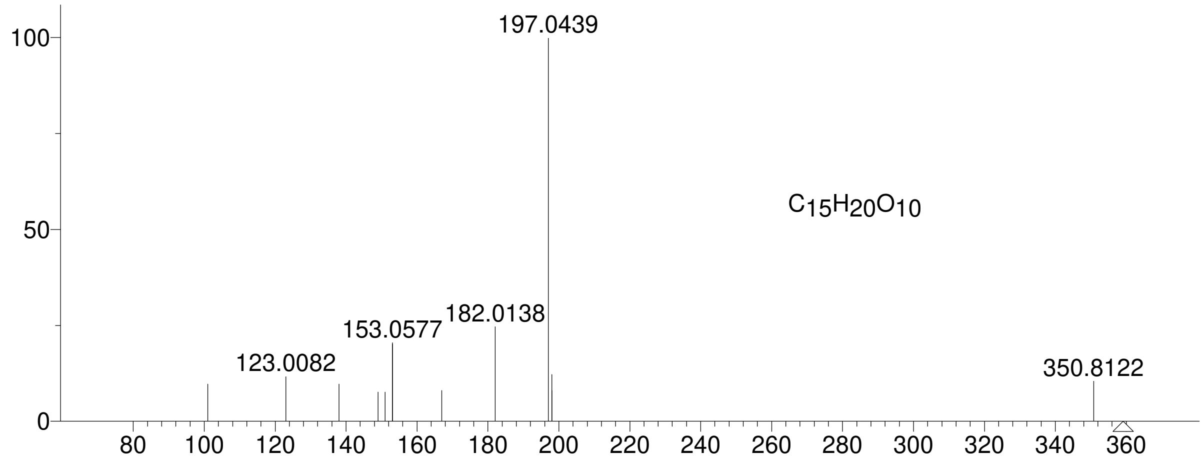

Hit 2 : Benzoic acid + 1O, 2MeO, O-Hex; PlaSMA ID-868 [M-H]- P=359.1
C15H20O10; MF: 2; RMF: 157; Prob 21.0%; Lib: biomsms-neg-plasma; ID: 397.

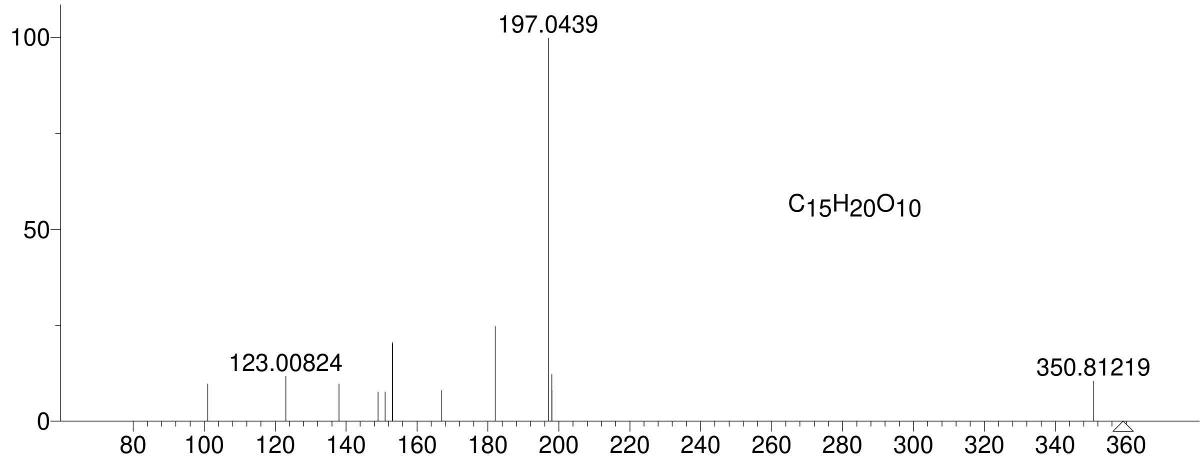

Hit 3 : Rosmarinic acid [M-H]- P=359.1
C18H16O8; MF: 0; RMF: 99; Prob 19.3%; Lib: mona-export-gnps; ID: 1168.

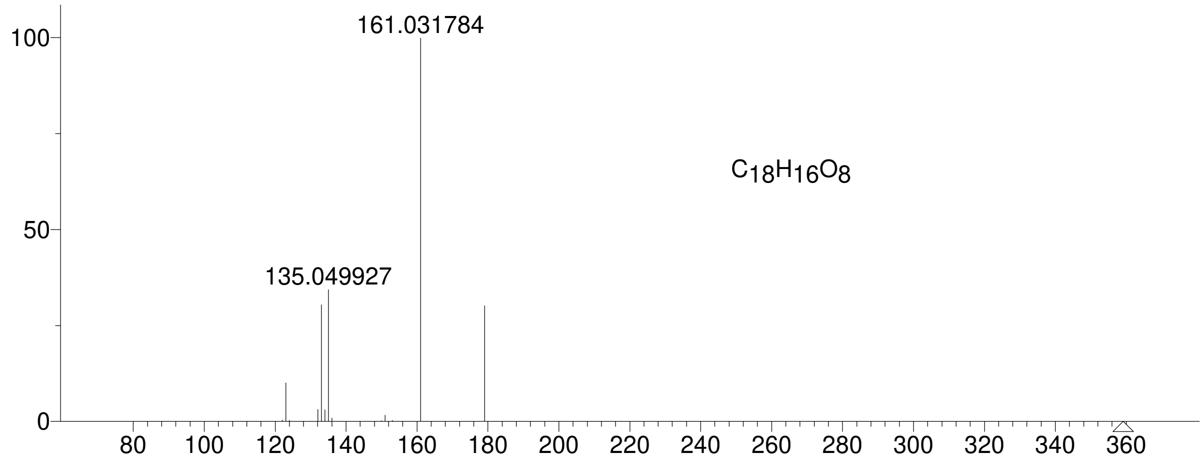

Hit 4 : C15H20O10; PlaSMA ID-869 [M-H]- P=359.1
C15H20O10; MF: 0; RMF: 80; Prob 19.3%; Lib: biomsms-neg-plasma; ID: 816.

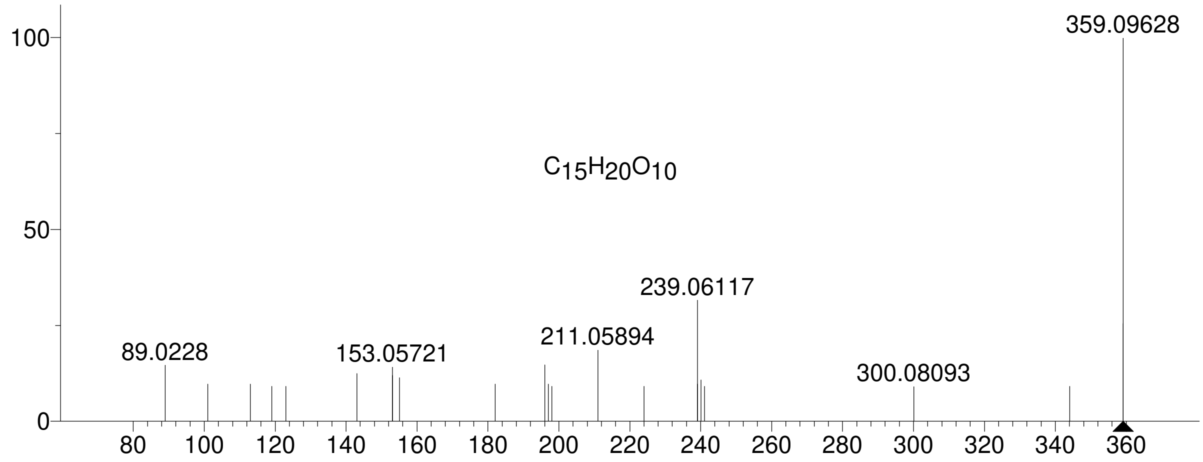

Hit 5 : syringic acid 4-O-hexoside () [M-H]- P=359.1
C15H20O10; MF: 0; RMF: 76; Prob 19.3%; Lib: phytofrag2018_final_060219; ID: 860.

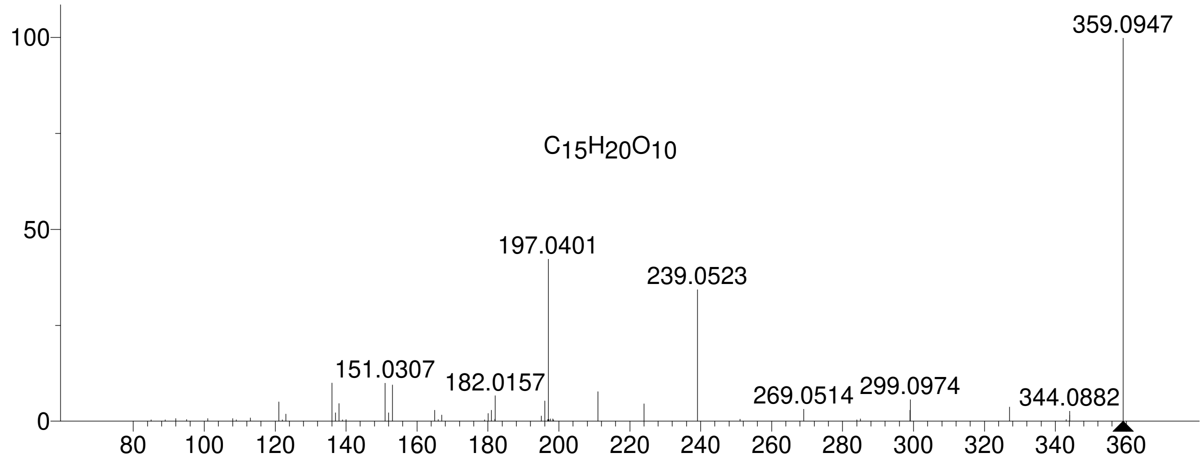

Unknown: Unknown (2.17_548.1026m/z)
Compound in Library Factor = 0

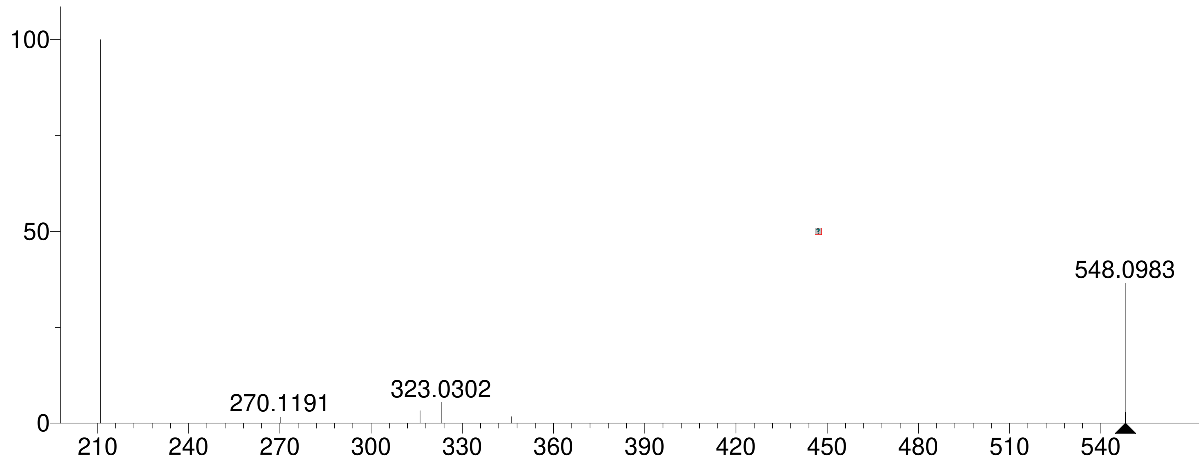

Unknown: Unknown (2.17_611.0429m/z)
Compound in Library Factor = 0

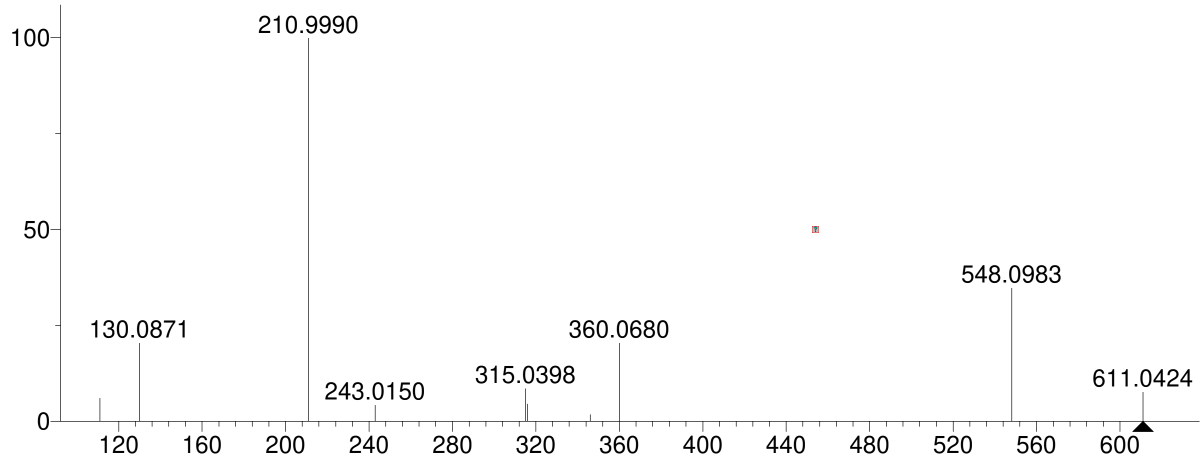

Unknown: Unknown (2.18_360.0707m/z)
Compound in Library Factor = 0

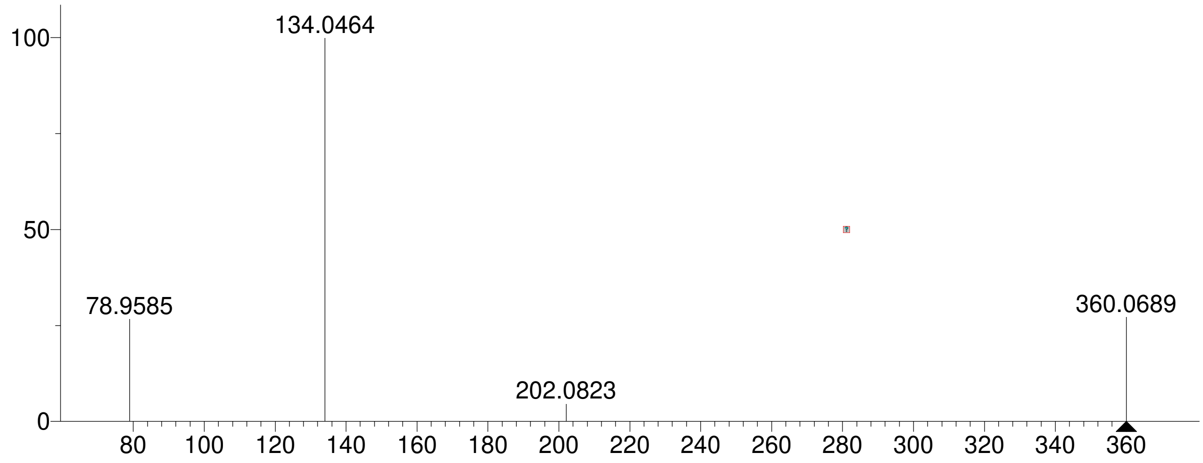

Unknown: Unknown (2.18_607.2683m/z)
Compound in Library Factor = 0

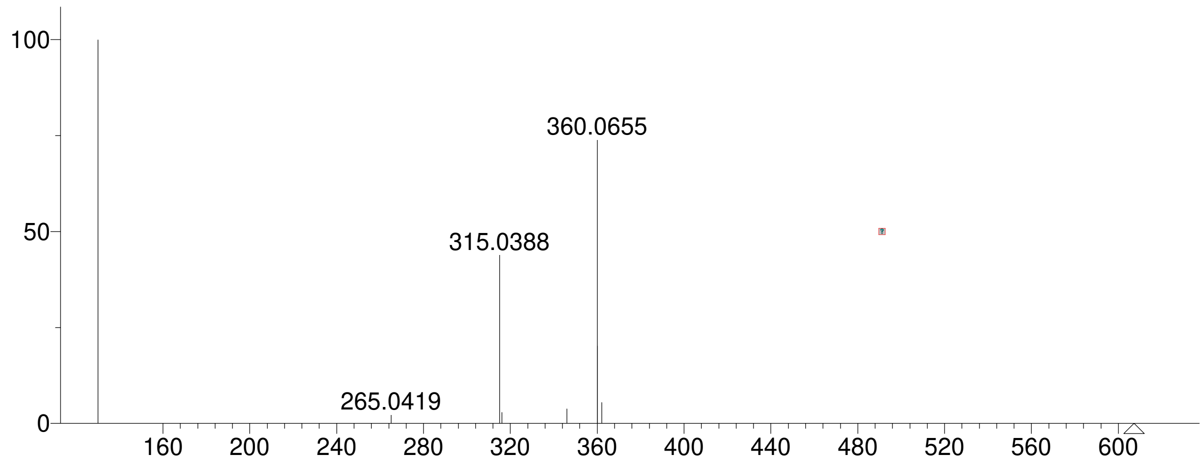

Hit 1 : Reserpine [M-H]- 38.22 P=607.3
C33H40N2O9; MF: 0; RMF: 37; Prob 50.0%; Lib: msms neg mona_ipb_gnps_plasma; ID: 1257.

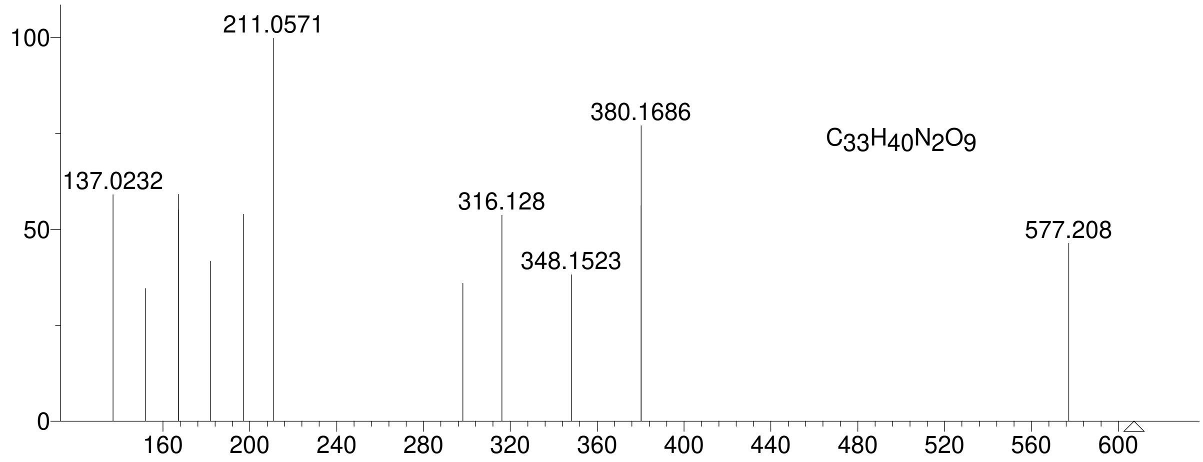

Hit 2 : 3-[(1R,3S,5S,10R,11R,13R,14S,17R)-1,5,11,14-tetrahydroxy-10-(hydroxymethyl)-13-methyl-3-[(2R,3S,4S,5
C29H44O12; MF: 0; RMF: 3; Prob 50.0%; Lib: mona-export-vf-npl_qexactive; ID: 14858.

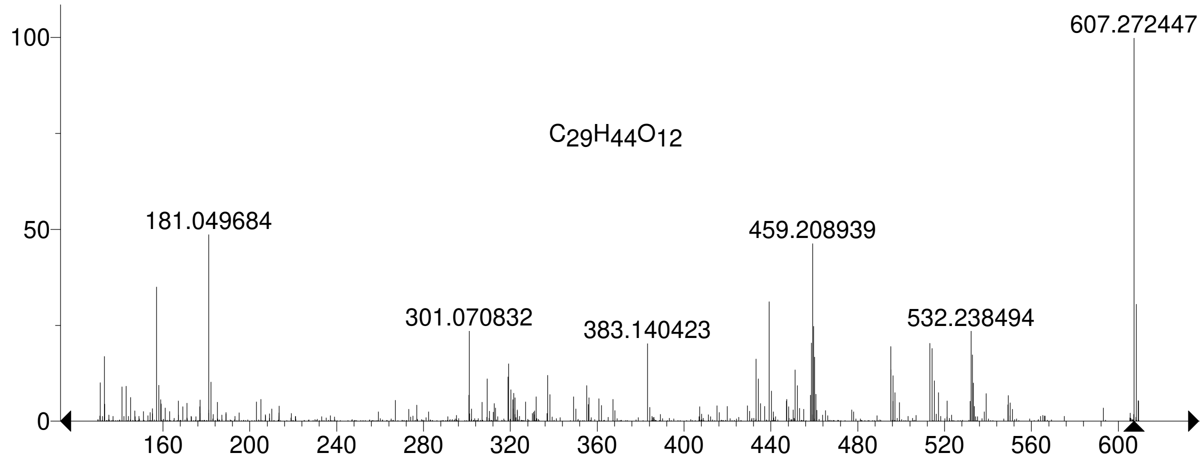

** Search Report Page 1 of 1 **

Unknown: Unknown (2.19_485.1879m/z)
Compound in Library Factor = 0

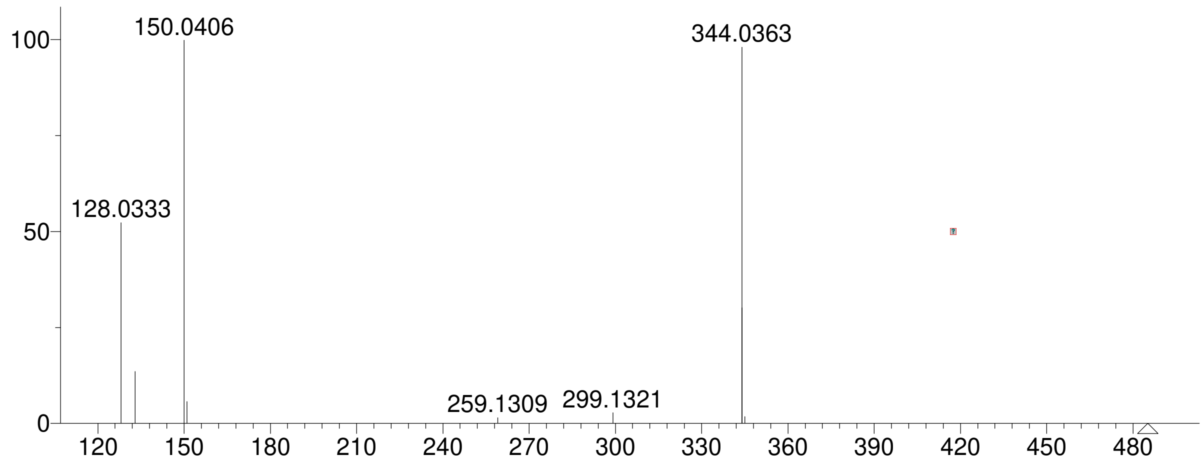

Unknown: Unknown (2.19_856.3218m/z)
Compound in Library Factor = 0

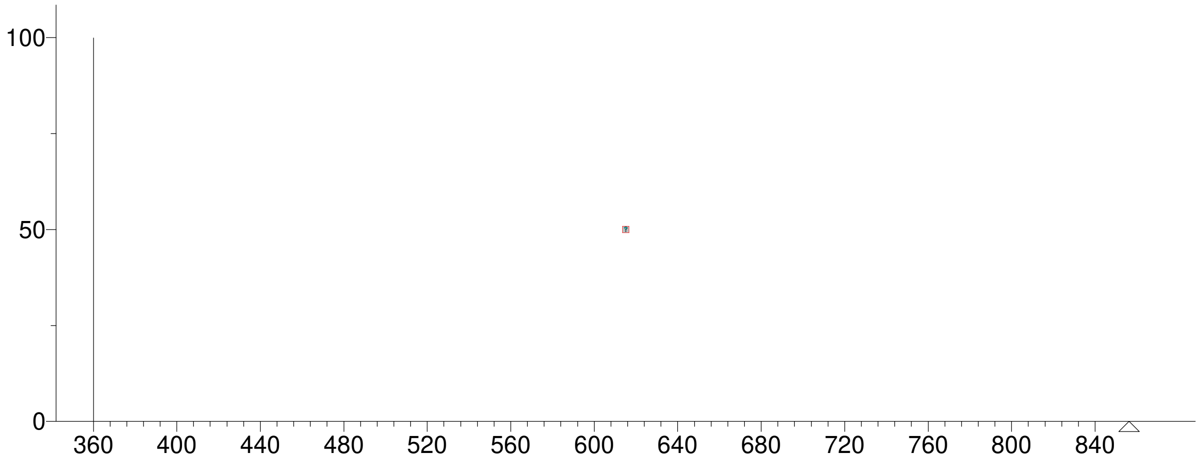

Unknown: Unknown (2.21_259.1289m/z)
Compound in Library Factor = -2176

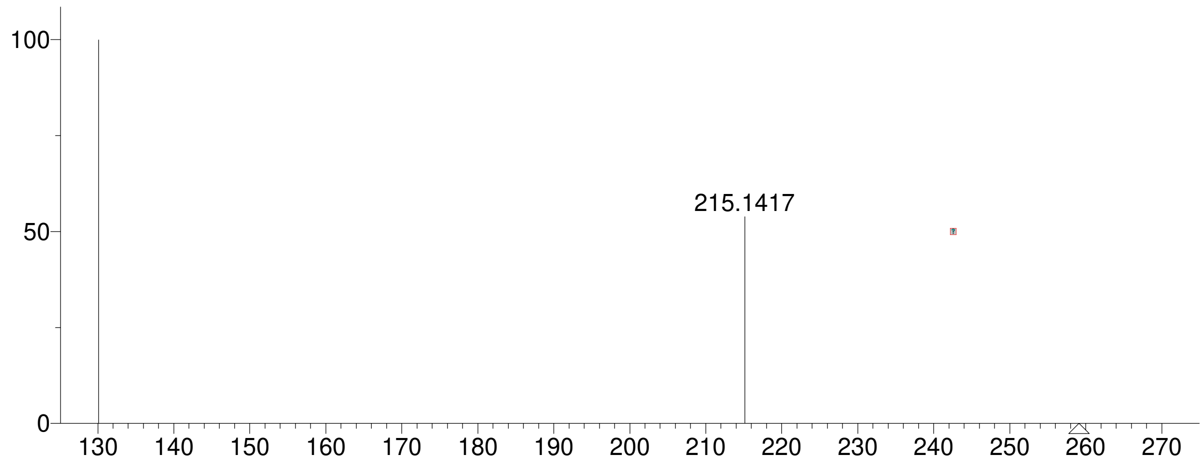

Hit 1 : Leu-Glu [M-H]- HCD 23eV P=259.1
C11H20N2O5; MF: 141; RMF: 770; Prob 22.9%; Lib: nist_msms; ID: 570784.

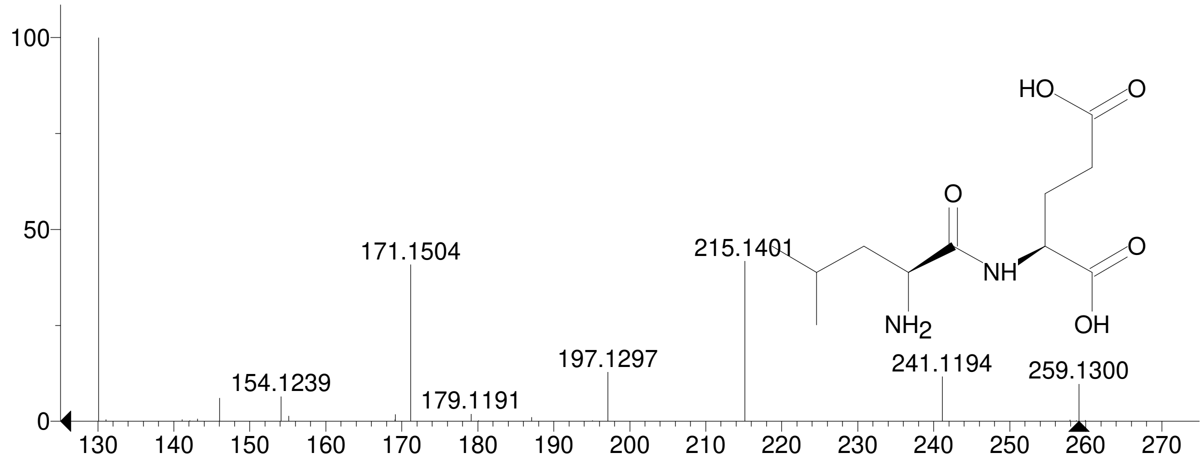

Hit 2 : Leu-Glu [M-H]- HCD 23eV P=259.1
C11H20N2O5; MF: 141; RMF: 770; Prob 22.9%; CAS: 16364-31-1; Lib: nist_msms2; ID: 84517.

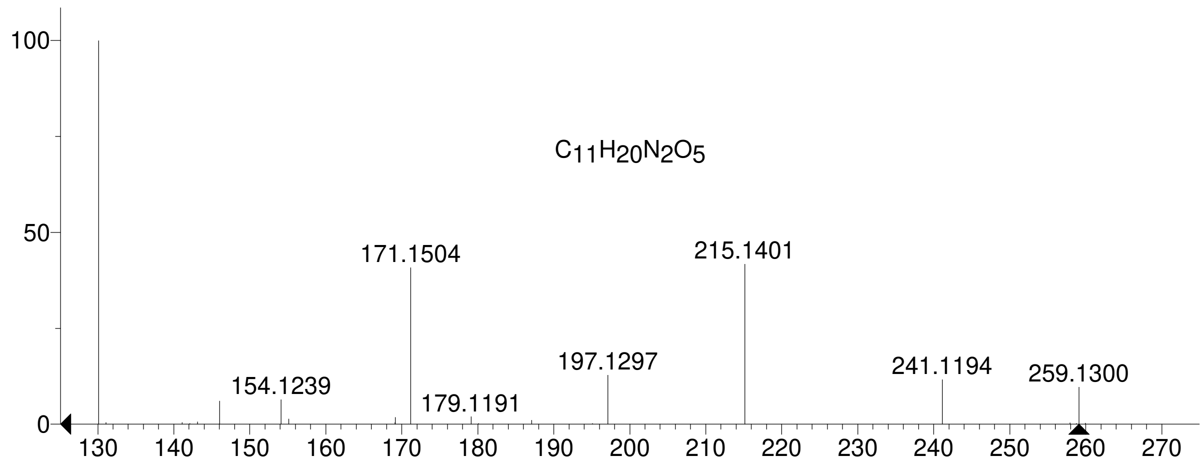

Hit 3 : Ile-Glu [M-H]- HCD 23eV P=259.1
C11H20N2O5; MF: 141; RMF: 754; Prob 22.9%; Lib: nist_msms; ID: 570716.

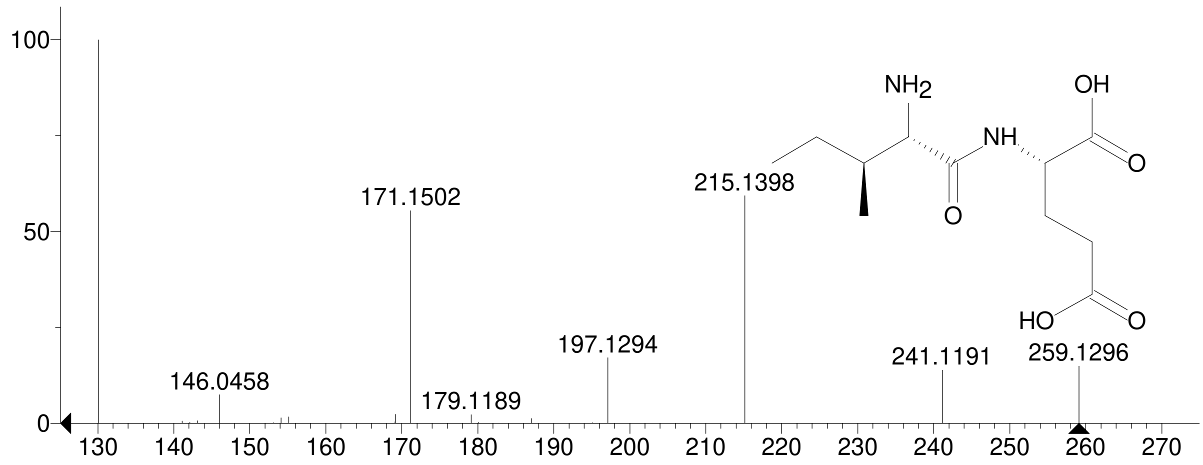

Hit 4 : Ile-Glu [M-H]- HCD 23eV P=259.1
C11H20N2O5; MF: 141; RMF: 754; Prob 22.9%; CAS: 42516-53-0; Lib: nist_msms2; ID: 84449.

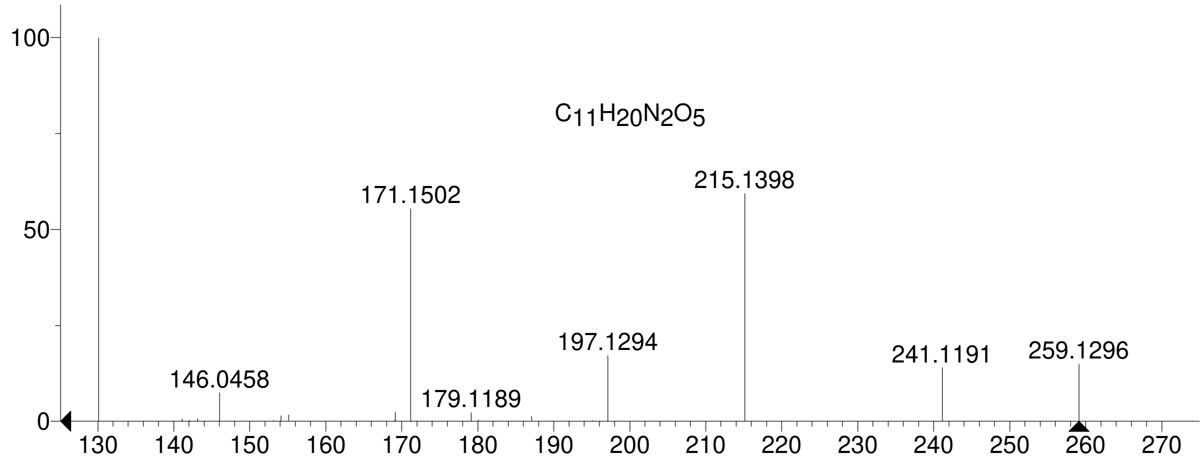

Hit 5 : Glu-Leu [M-H]- HCD 41eV P=259.1
C11H20N2O5; MF: 62; RMF: 740; Prob 2.60%; Lib: nist_msms; ID: 570065.

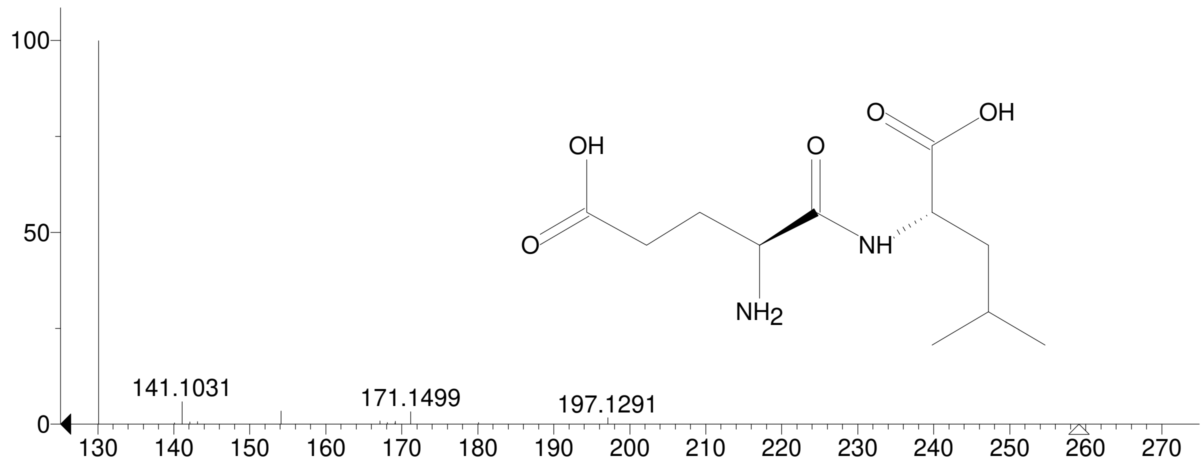

Unknown: Unknown (2.21_439.1695m/z)
Compound in Library Factor = -3273

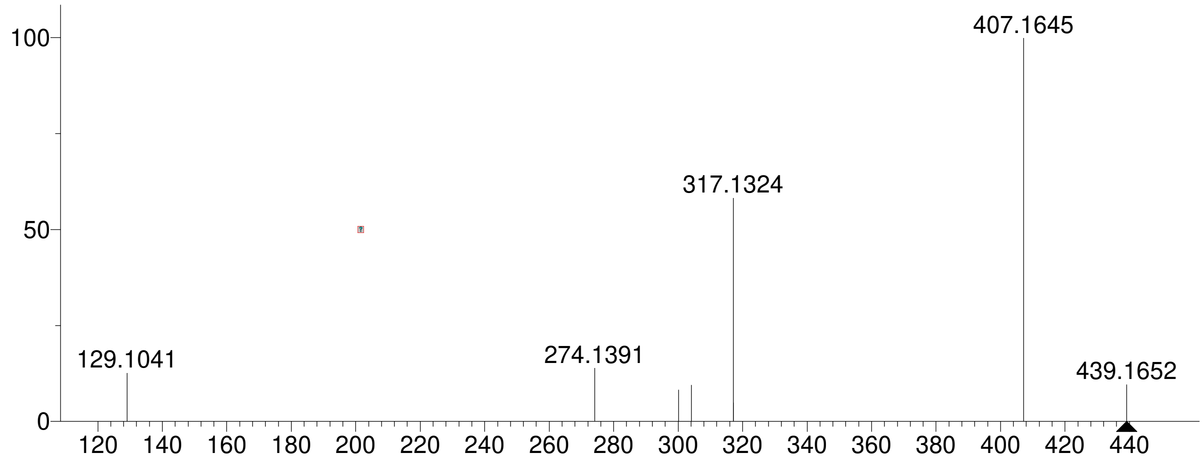

Hit 1 : (+)-Fluprostenol [M-H-H2O]- HCD 13V P=439.2
C23H29F3O6; MF: 12; RMF: 207; Prob 22.4%; CAS: 54276-17-4; Lib: nist_msms; ID: 155447.

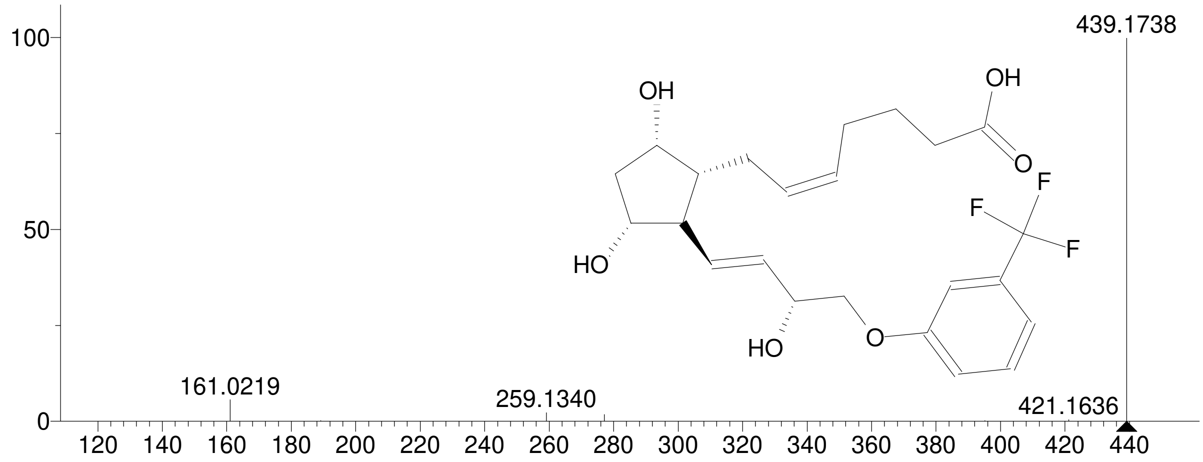

Hit 2 : 15-Ketofluprostenol [M+H-H2O]+ HCD 7V P=439.2
C23H27F3O6; MF: 9; RMF: 208; Prob 19.8%; Lib: nist_msms; ID: 155572.

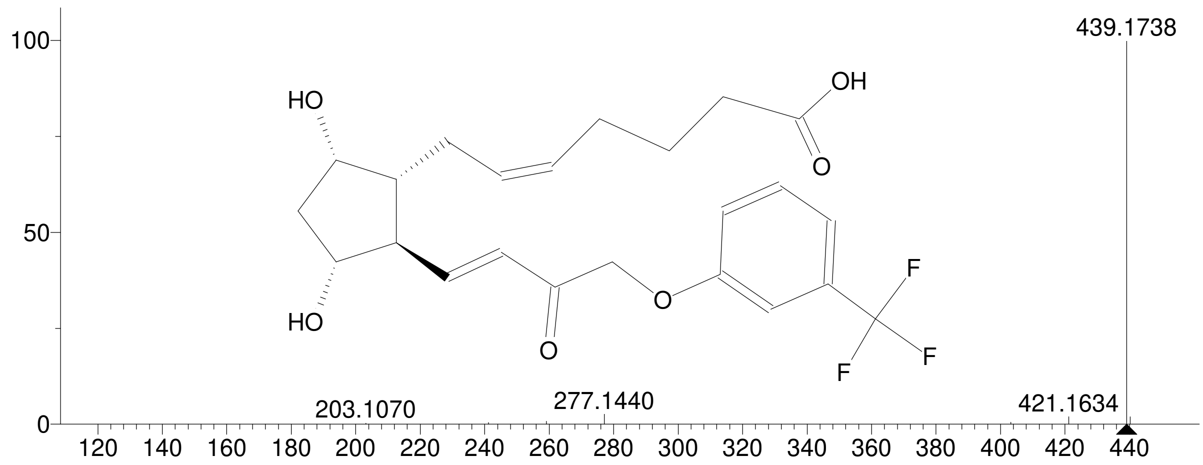

Hit 3 : 13(R),14(R)-Epoxyfluprostenol isopropyl ester [M+H-C3H10O2]+ HCD 13V P=439.2
C26H35F3O7; MF: 5; RMF: 193; Prob 16.7%; Lib: nist_msms; ID: 254094.

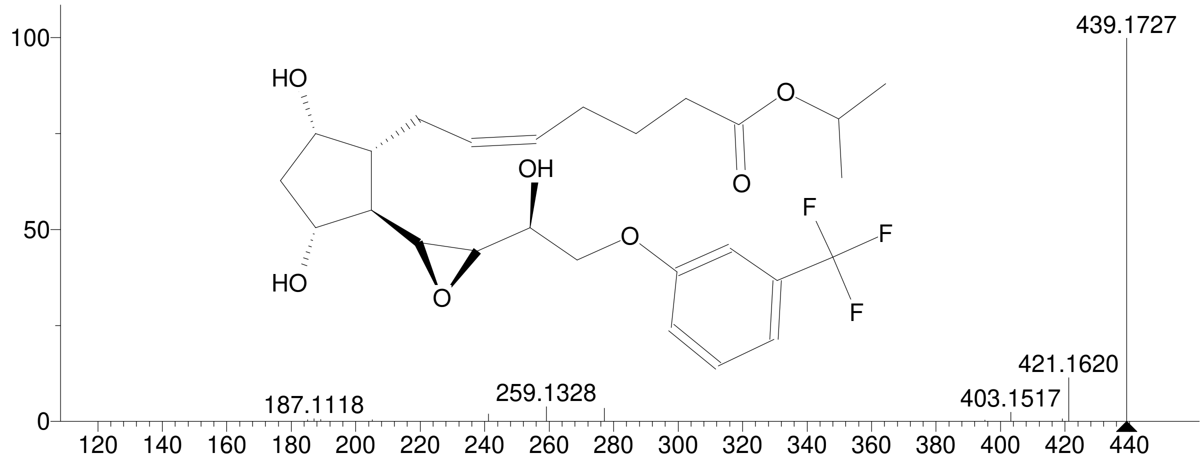

Hit 4 : NCGC00385132-01_C23H28O7_1-Phenanthrenecarboxylic acid, 10-(acetyloxy)-1,2,4a,9,10,10a-hexahydı
C23H28O7; MF: 1; RMF: 167; Prob 14.1%; Lib: mona-export-gnps; ID: 14186.

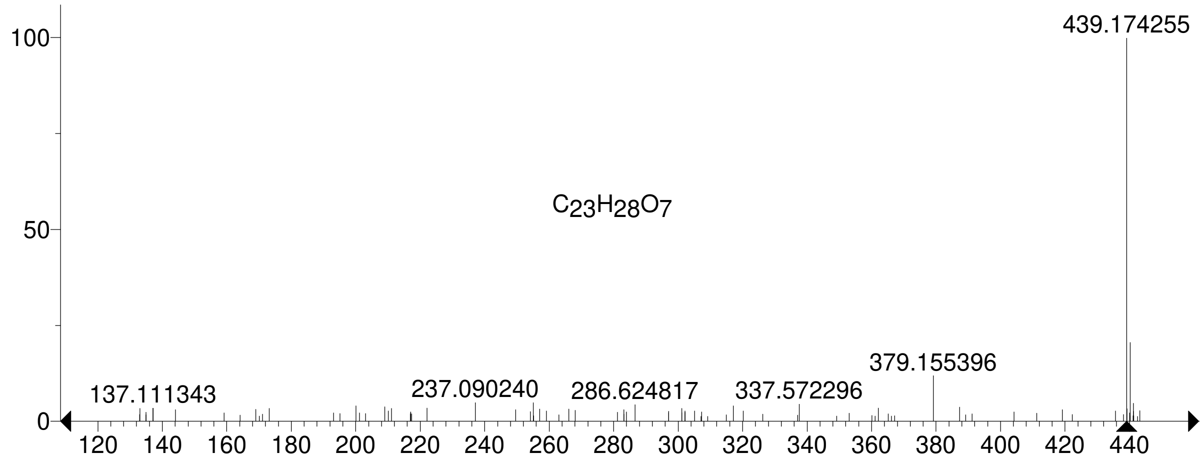

Hit 5 : NCGC00380879-01_C23H28O7_1,3-Benzodioxole, 4-methoxy-6-[(2S,3R,4R,5S)-tetrahydro-3,4-dimethyl-!
C23H28O7; MF: 0; RMF: 155; Prob 13.6%; Lib: mona-export-gnps; ID: 11784.

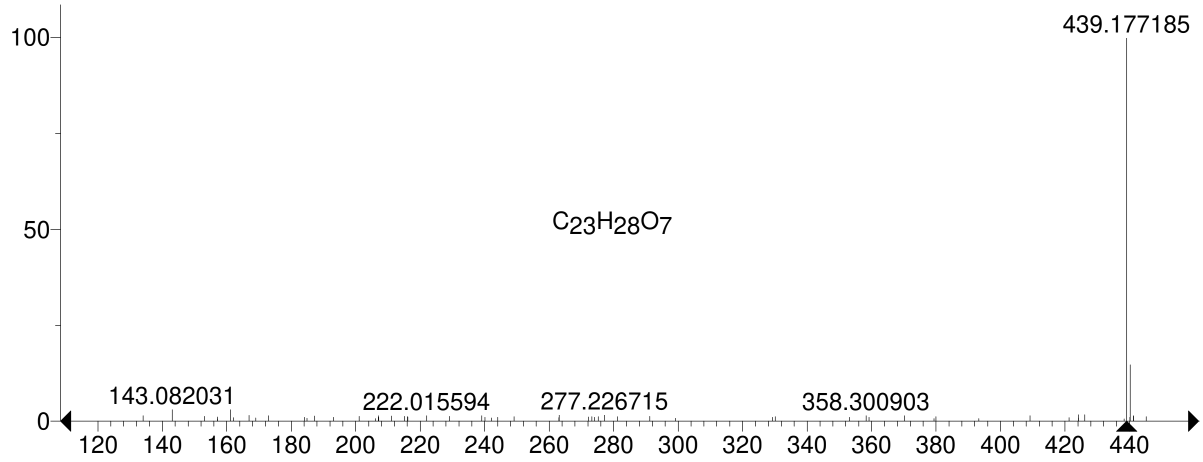

** Search Report Page 1 of 1 **

Unknown: Unknown (2.21_650.0651m/z)
Compound in Library Factor = 0

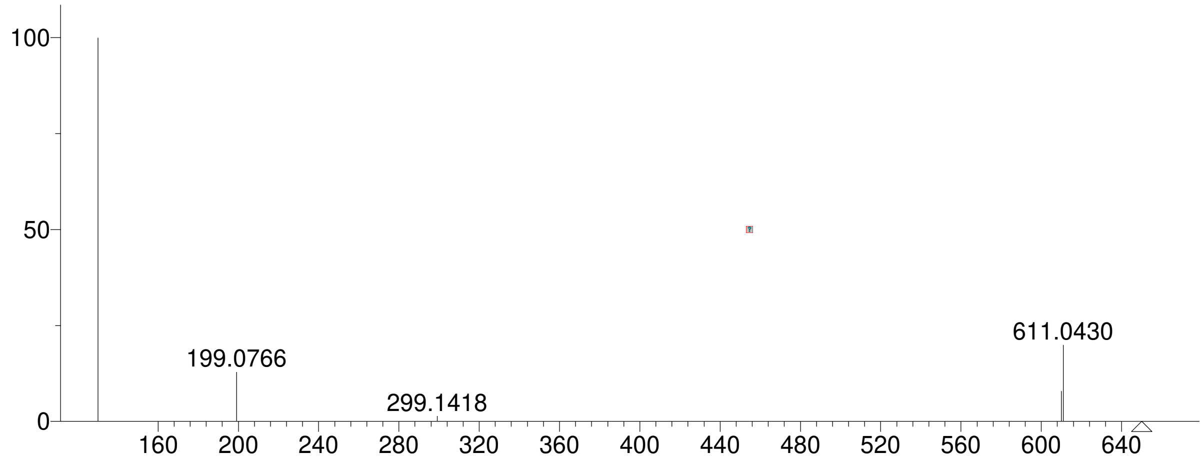

Unknown: Unknown (2.22_374.1541m/z)
Compound in Library Factor = 0

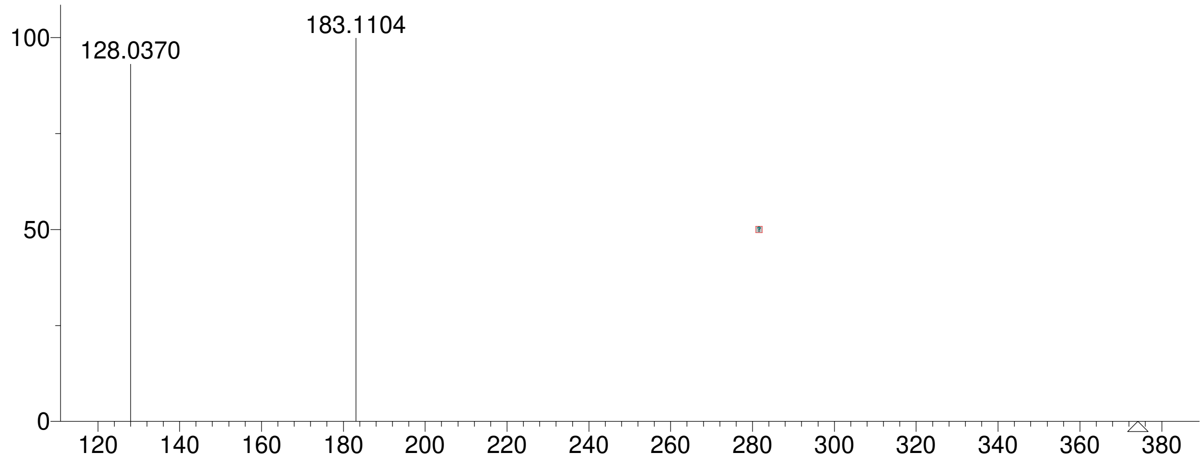

Hit 1 : Gatifloxacin [M-H]- HCD 93V P=374.2
C19H22FN3O4; MF: 0; RMF: 21; Prob 100.0%; CAS: 112811-59-3; Lib: nist_msms; ID: 357666.

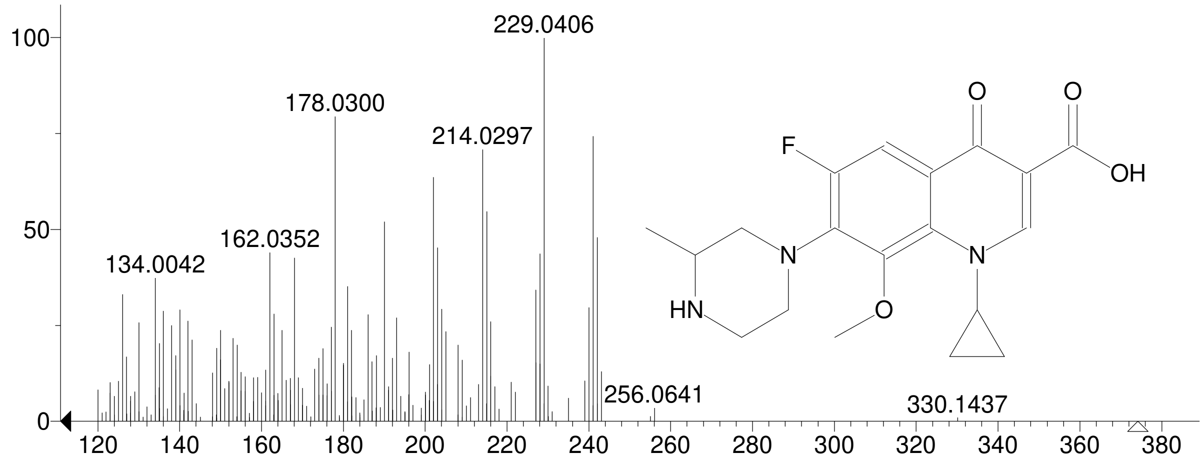

** Search Report Page 1 of 1 **

Unknown: Unknown (2.25_606.2489m/z)
Compound in Library Factor = 0

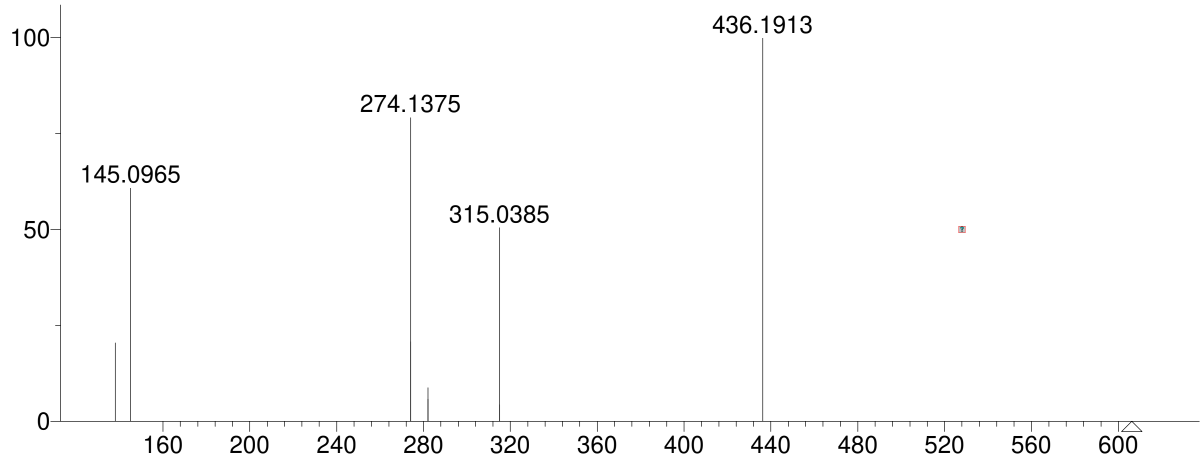

Unknown: Unknown (2.26_464.1856m/z)
Compound in Library Factor = 0

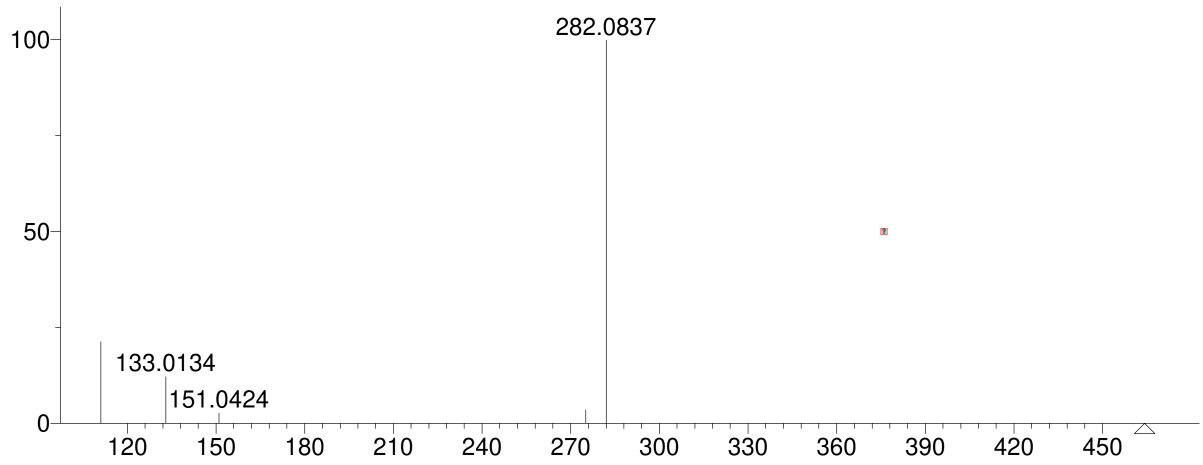

** Search Report Page 1 of 1 **

Unknown: Unknown (2.26_602.1821m/z)
Compound in Library Factor = 0

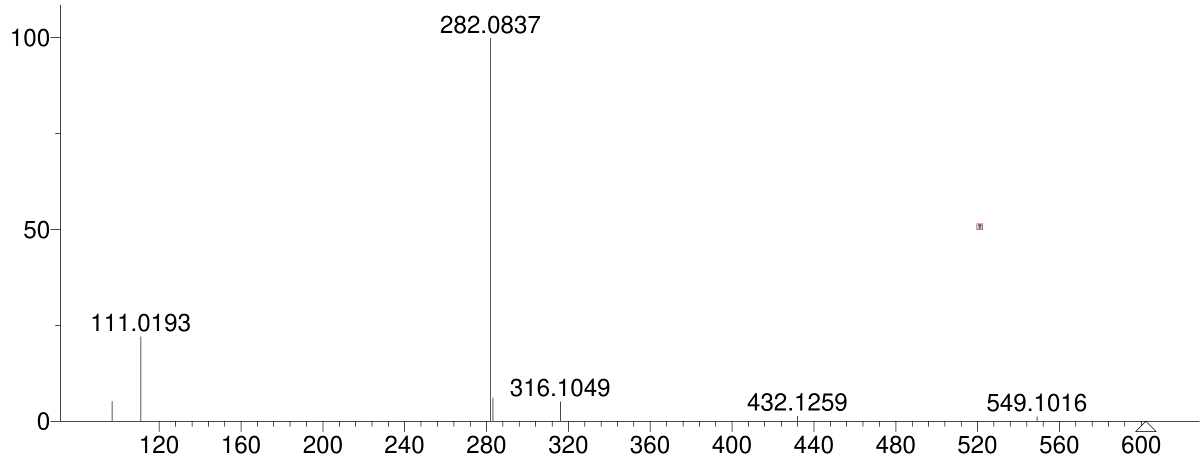

Unknown: Unknown (2.27_282.0838m/z)
Compound in Library Factor = 327

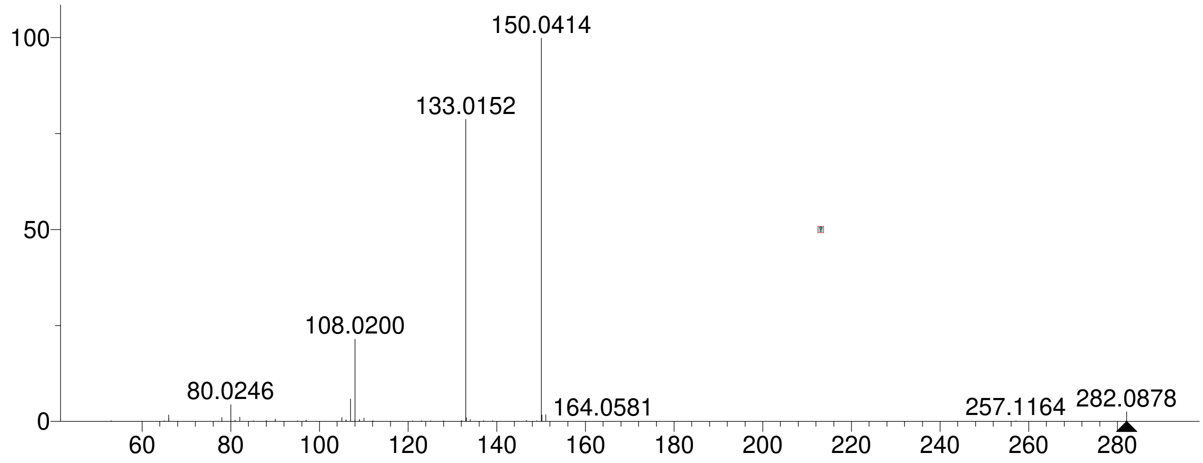

Hit 1 : Guanosine [M-H]- HCD 36V P=282.1
C10H13N5O5; MF: 909; RMF: 946; Prob 96.8%; CAS: 118-00-3; Lib: nist_msms; ID: 228186.

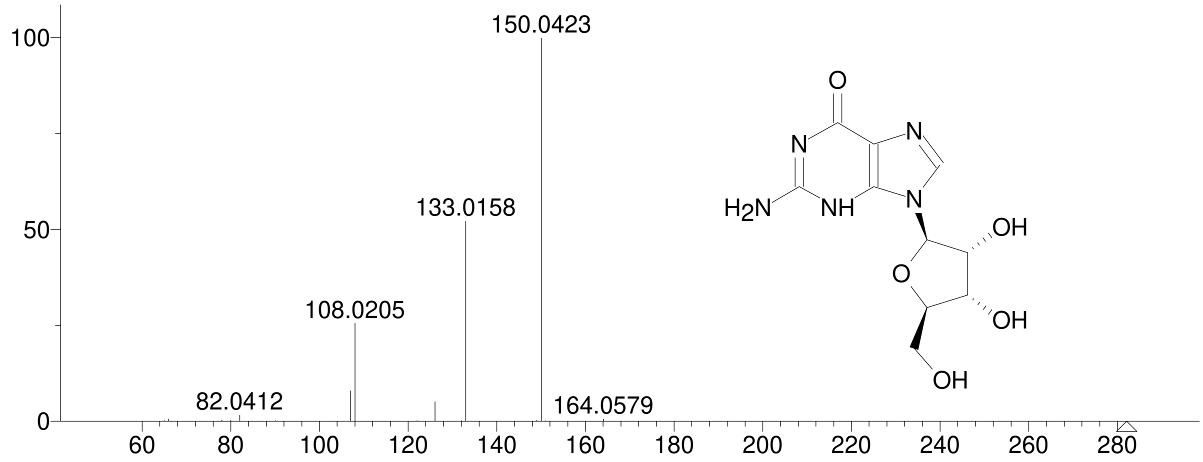

Hit 2 : Guanosine; LC-ESI-QTOF; MS2; CE [M-H]- P=282.1
C10H13N5O5; MF: 766; RMF: 919; Prob 3.05%; Lib: msms-neg-massbank; ID: 4336.

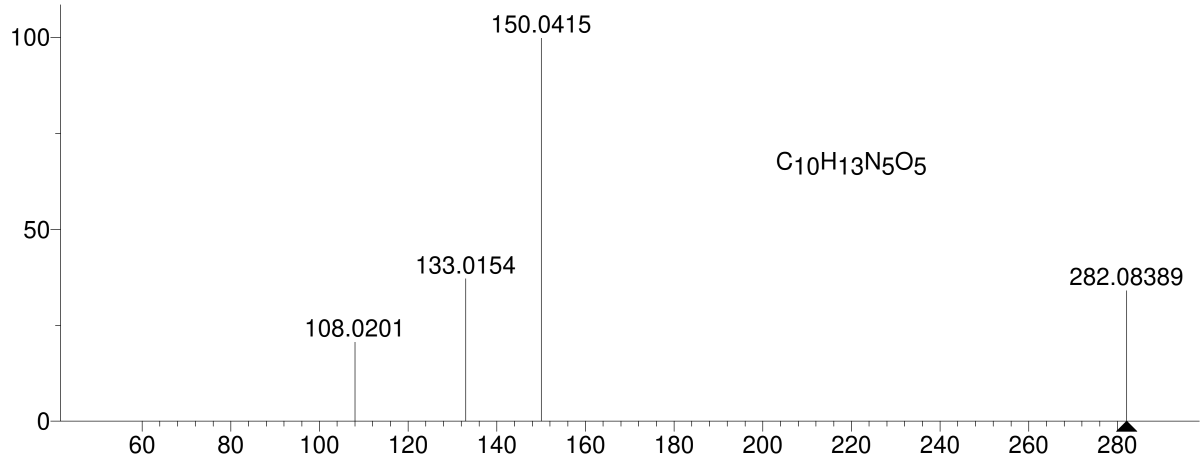

Hit 3 : Guanosine
C10H13N5O5; MF: 617; RMF: 805; Prob 0.08%; Lib: embl neg mcg; ID: 151.

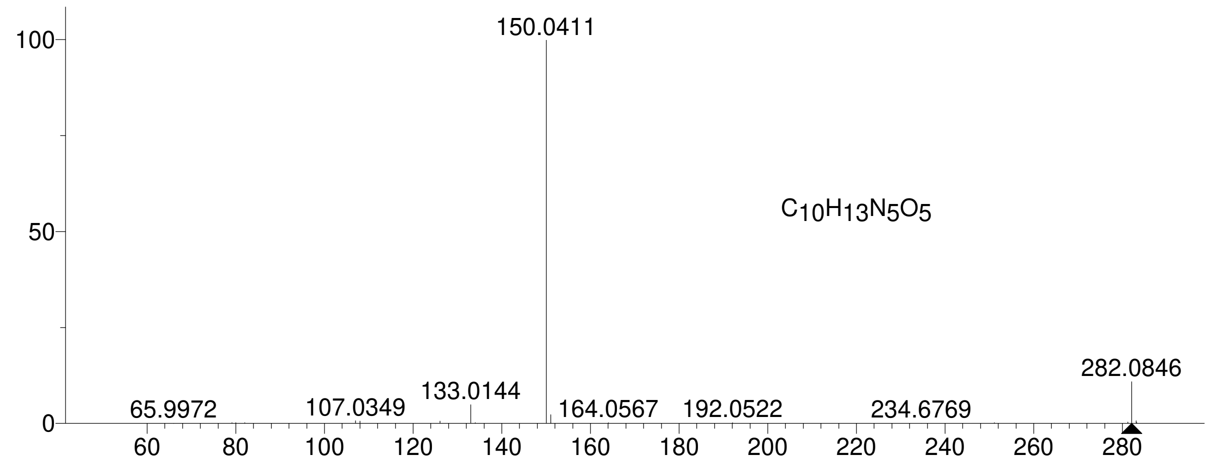

Hit 4 : GUANOSINE [M-H]- P=282.1
C10H13N5O5; MF: 617; RMF: 805; Prob 0.08%; Lib: mona-export-gnps; ID: 5564.

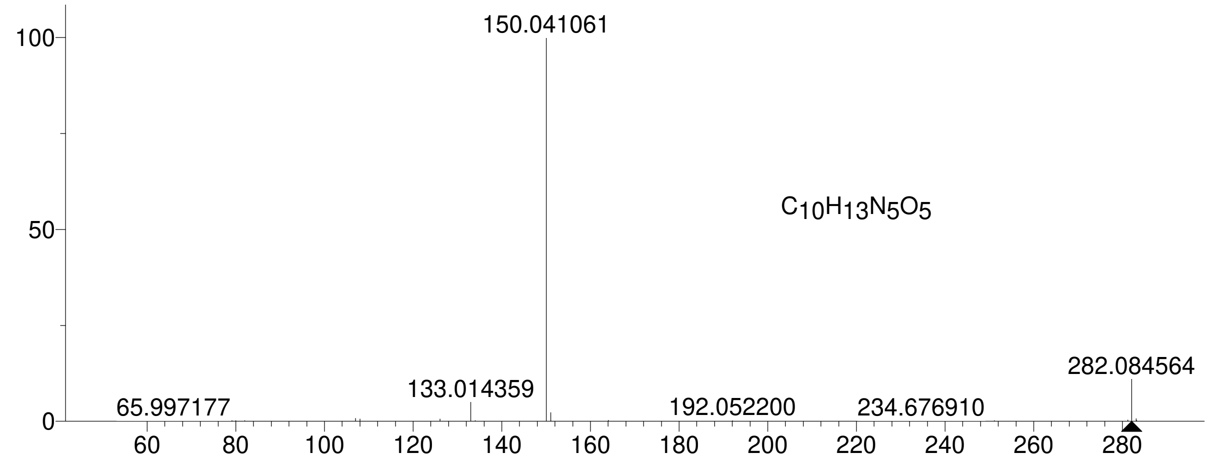

Hit 5 : Guanosine (Not validated); PlaSMA ID-499 [M-H]- P=282.1
C10H13N5O5; MF: 557; RMF: 808; Prob 0.01%; Lib: biomsms-neg-plasma; ID: 470.

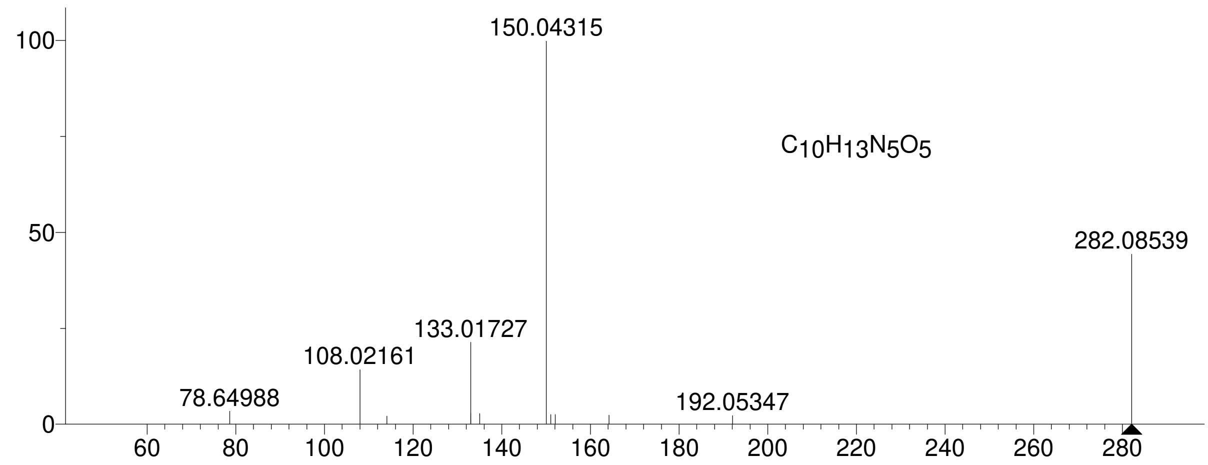

Unknown: Unknown (2.27_548.1029m/z)
Compound in Library Factor = 0

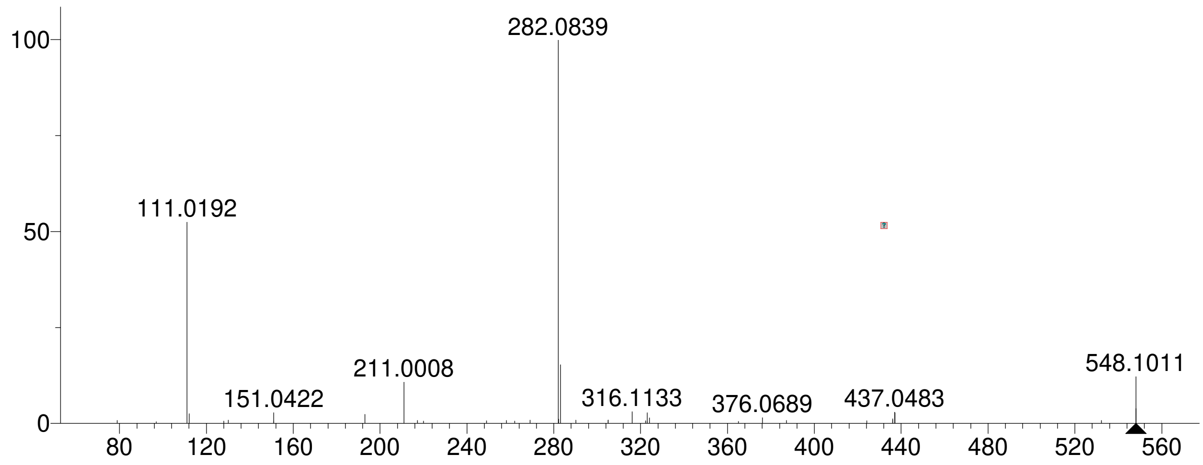

** Search Report Page 1 of 1 **

Unknown: Unknown (2.28_282.2416m/z)
Compound in Library Factor = 0

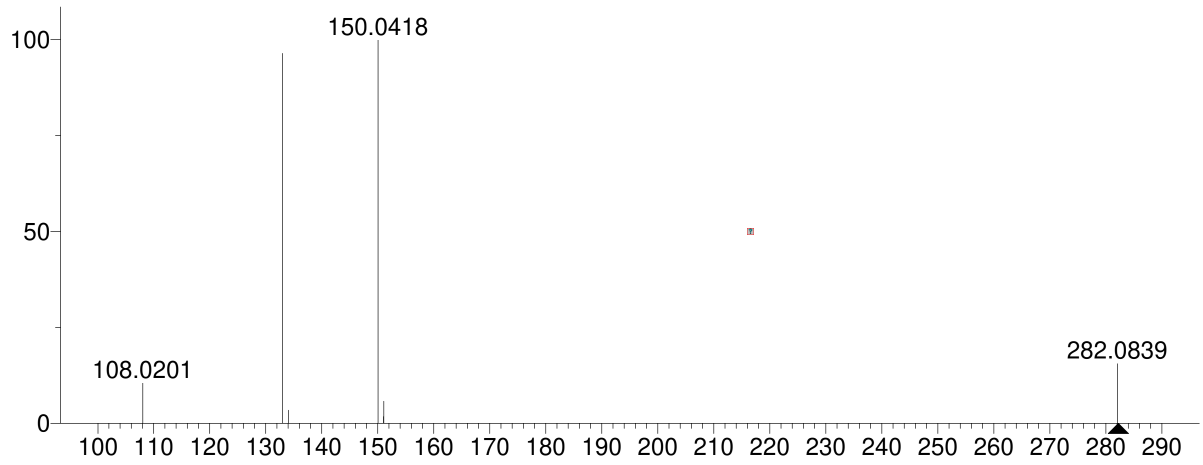

Hit 1 : MLS001146935-01!ANISOTROPINE METHYLBROMIDE [M+H]+ P=282.2
C17H32BrNO2; MF: 0; RMF: 6; Prob 100.0%; Lib: mona-export-gnps; ID: 3650.

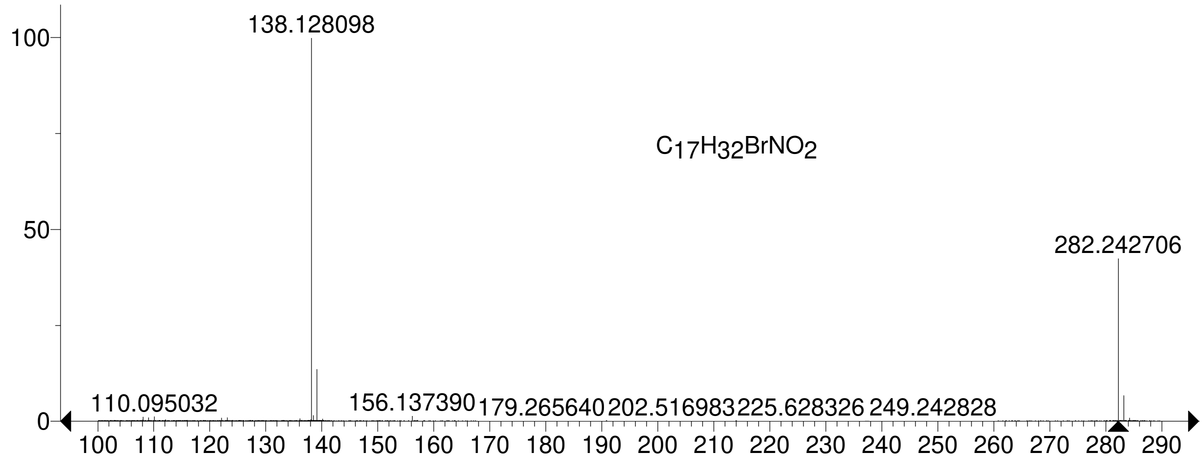

** Search Report Page 1 of 1 **

Unknown: Unknown (2.29_385.1344m/z)
Compound in Library Factor = 0

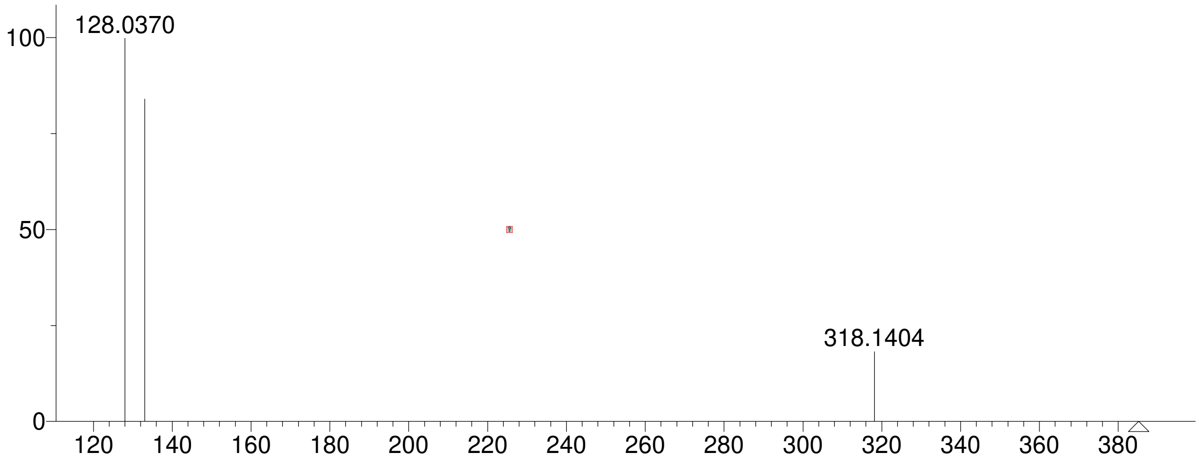

Unknown: Unknown (2.29_601.1518m/z)
Compound in Library Factor = -3273

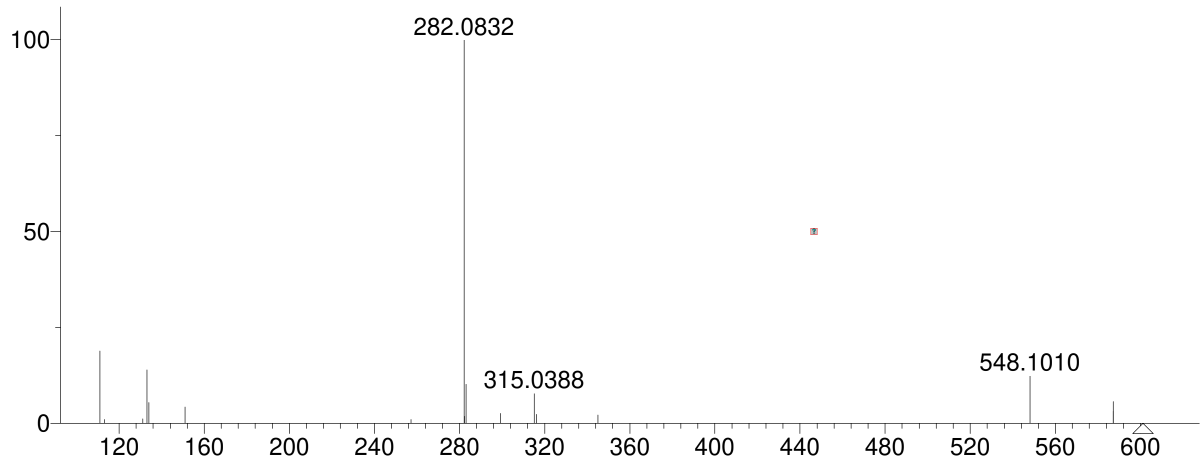

Hit 1 : Kaempferitrin [M+Na]+ HCD 90V P=601.2
C27H30O14; MF: 3; RMF: 81; Prob 22.1%; CAS: 482-38-2; Lib: nist_msms; ID: 389463.

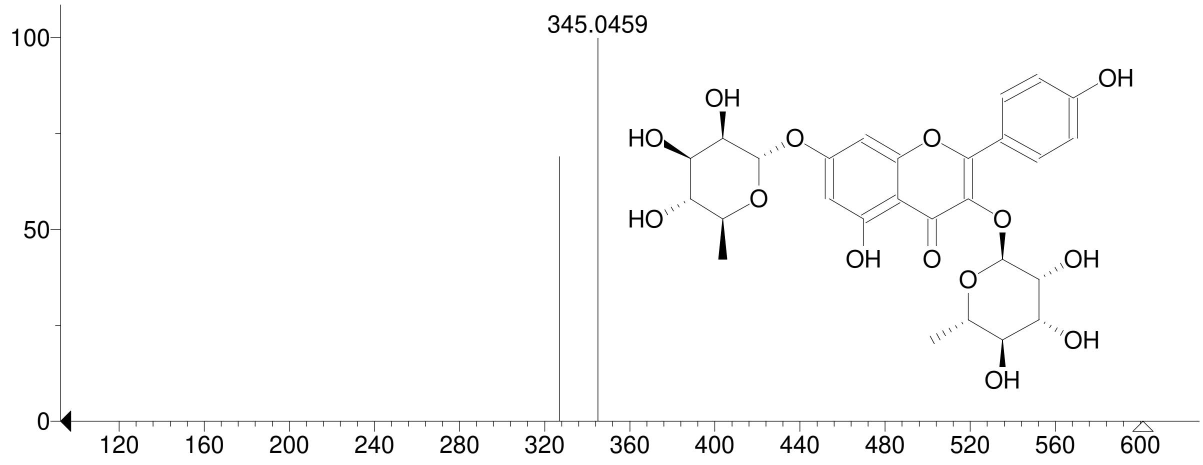

Hit 2 : lespenefril [M+Na]+ 45HCD P=601.2
C27H30O14; MF: 0; RMF: 41; Prob 19.5%; Lib: anthocyanins mona; ID: 5801.

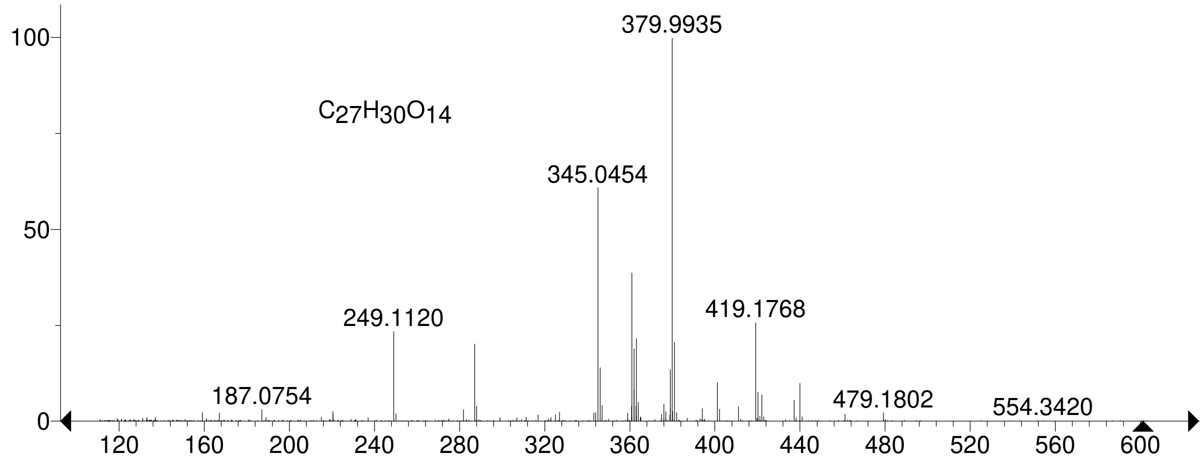

Hit 3 : 5,7-dihydroxy-2-(4-hydroxyphenyl)-6-[(2S,3R,4R,5S,6R)-3,4,5-trihydroxy-6-(hydroxymethyl)oxan-2-yl]-8-[(2
C27H30O14; MF: 0; RMF: 33; Prob 19.5%; Lib: anthocyanins mona; ID: 8004.

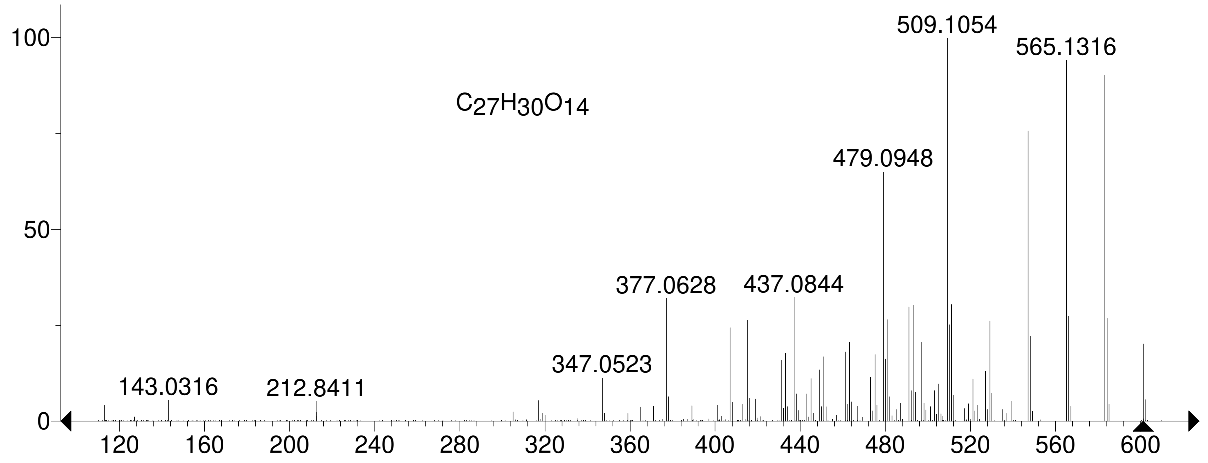

Hit 4 : 5-hydroxy-3-[(2S,3R,4S,5S)-4-hydroxy-5-(hydroxymethyl)-3-[(2S,3R,4R,5R,6S)-3,4,5-trihydroxy-6-methylo›
C27H30O14; MF: 0; RMF: 25; Prob 19.5%; Lib: anthocyanins mona; ID: 9580.

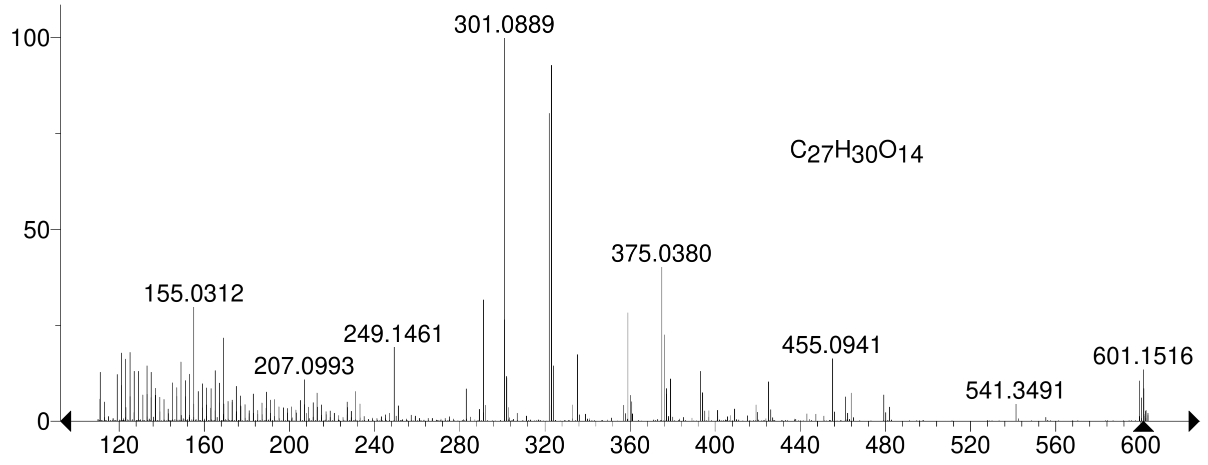

Hit 5 : 2-O-Rhamnosylvitexin [M+Na]+ 65HCD P=601.2
C27H30O14; MF: 0; RMF: 17; Prob 19.5%; Lib: anthocyanins mona; ID: 3132.

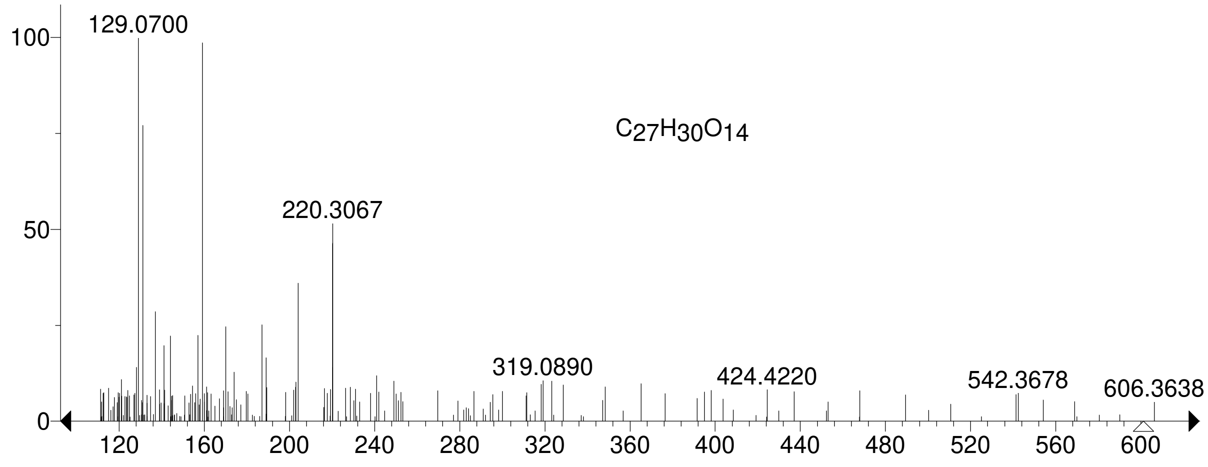

Unknown: Unknown (2.30_611.0426m/z)
Compound in Library Factor = 0

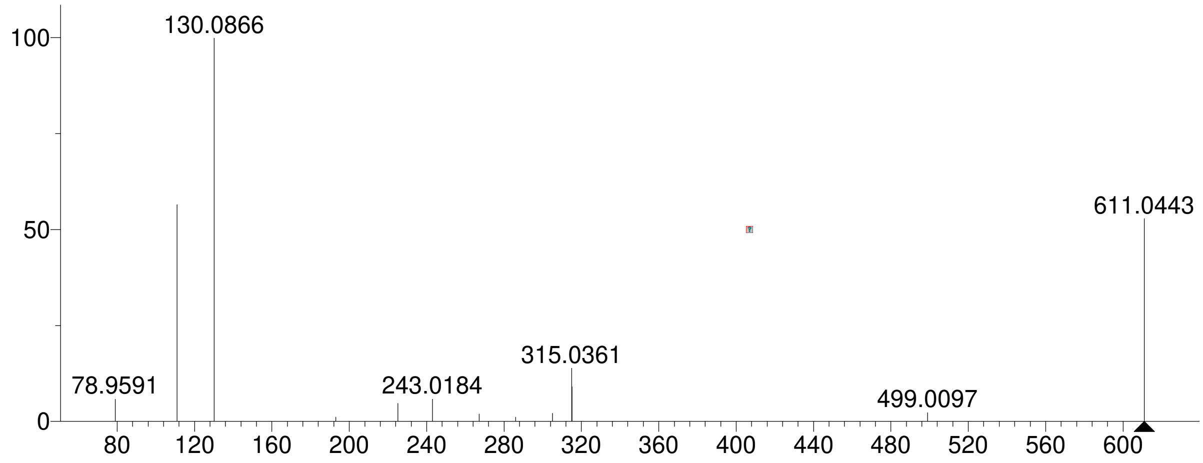

Unknown: Unknown (2.31_469.1921m/z)
Compound in Library Factor = 0

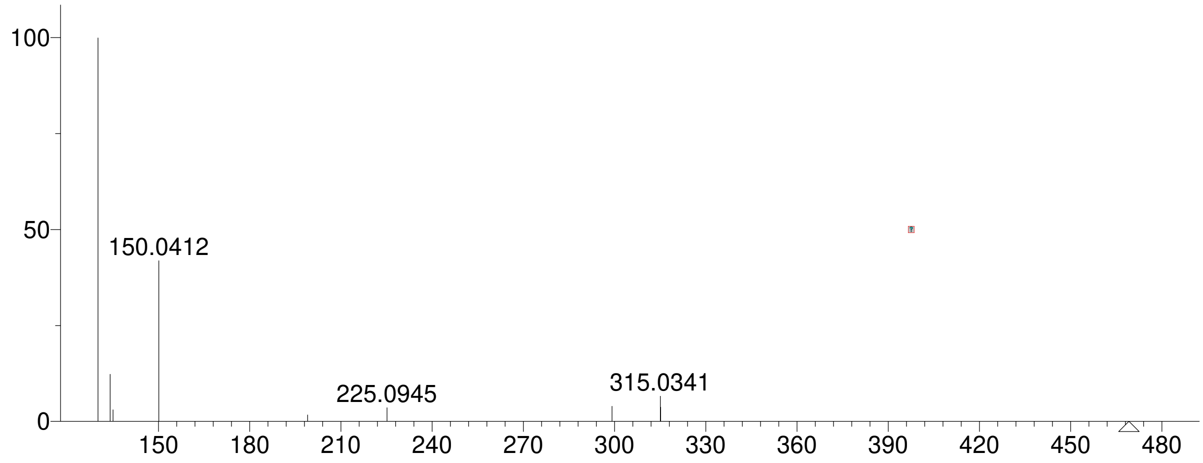

Hit 1 : Flucythrinate [M+NH4]+ HCD 121V P=469.2
C26H23F2NO4; MF: 0; RMF: 94; Prob 50.0%; CAS: 70124-77-5; Lib: nist_msms; ID: 388430.

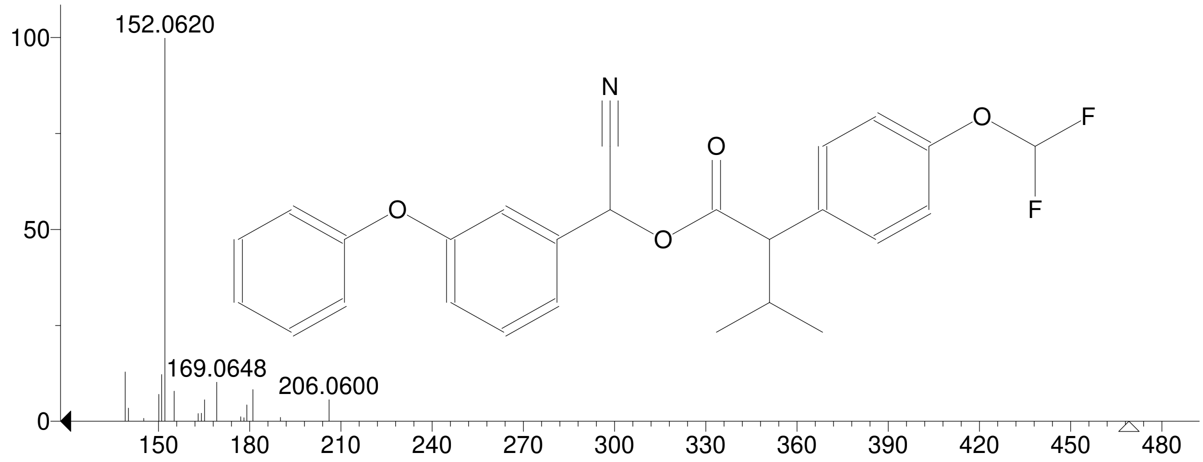

Hit 2 : Mexicanolide [M+H]+ 35% P=469.2
C27H32O7; MF: 0; RMF: 18; Prob 50.0%; Lib: mona-export-itree; ID: 713.

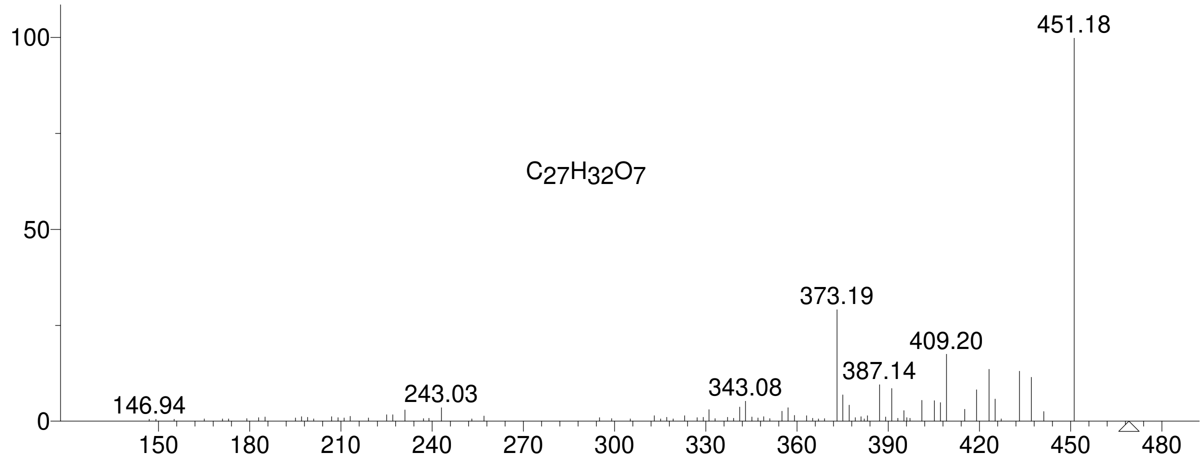

** Search Report Page 1 of 1 **

Unknown: Unknown (2.31_485.1875m/z)
Compound in Library Factor = 0

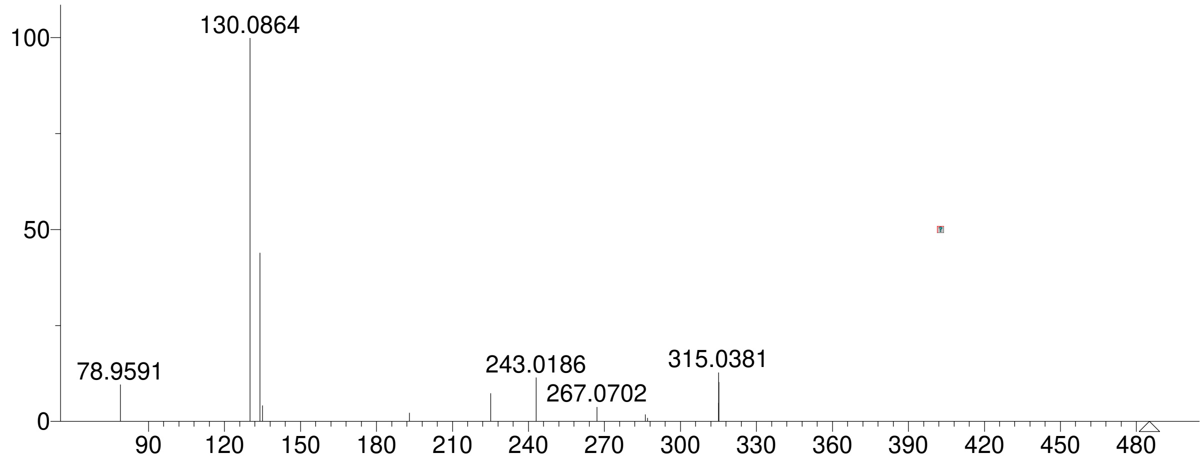

Hit 1 : Alpha-Dihydrogedunol [M-H]- 35% P=485.2
C28H38O7; MF: 0; RMF: 20; Prob 50.0%; Lib: mona-export-itree; ID: 2342.

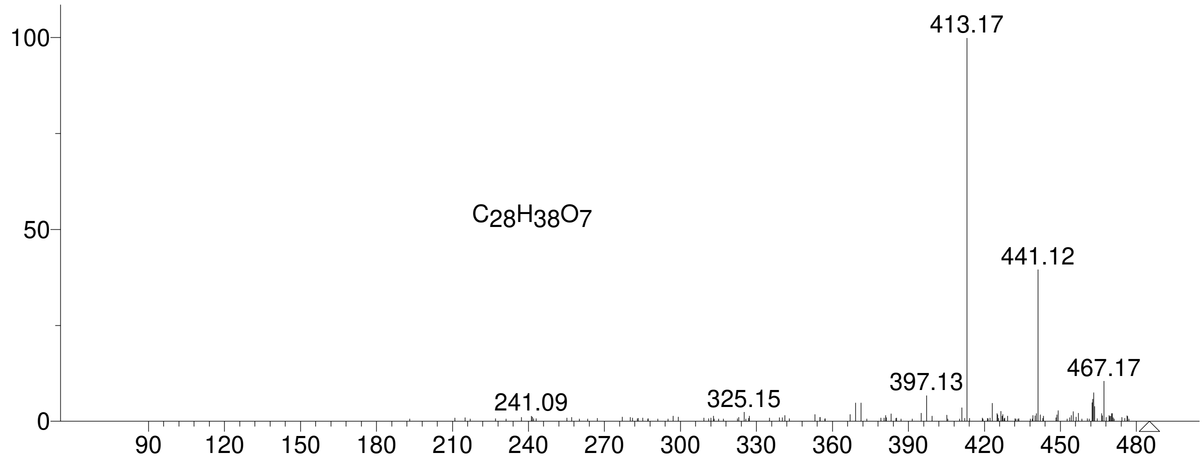

Hit 2 : Thymidine [2M+H]+ QTOF 4V P=485.2
C10H14N2O5; MF: 0; RMF: 19; Prob 50.0%; CAS: 50-89-5; Lib: nist_msms; ID: 34484.

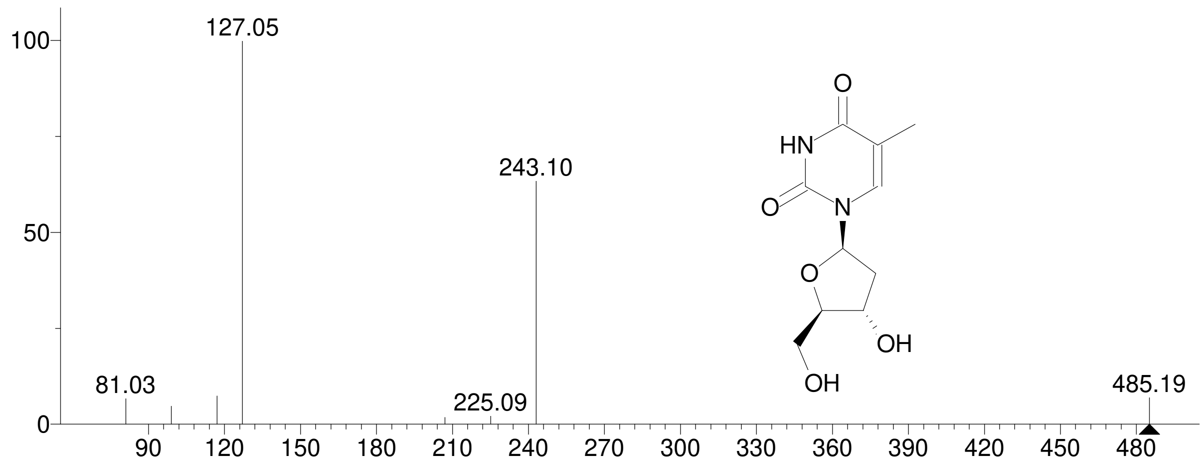

Unknown: Unknown (2.32_407.1661m/z)
Compound in Library Factor = -2387

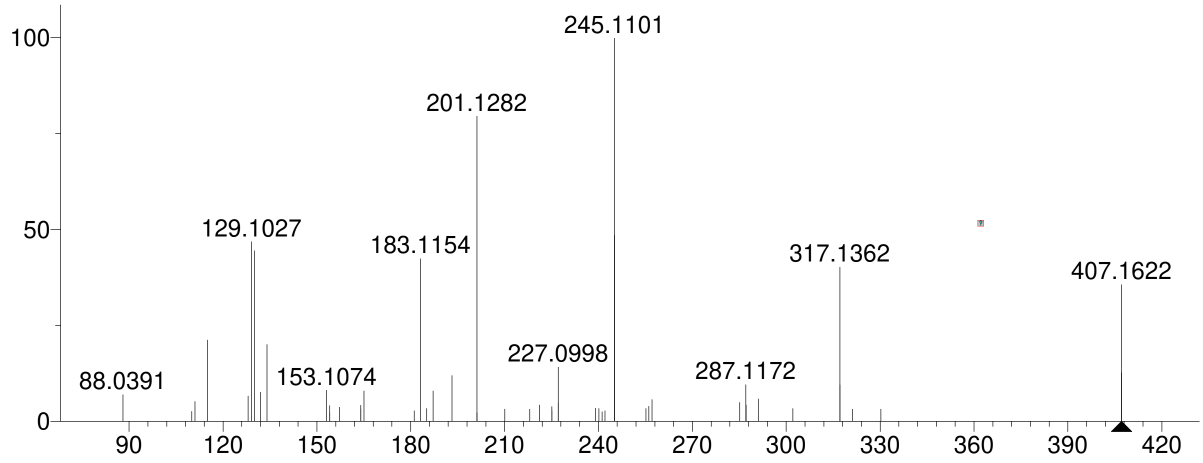

Hit 1 : (2R,3R,4S,6R)-2-((5-hydroxy-2,2-dimethyl-3,4-dihydro-2H-benzo[h]chromen-6-yl)oxy)-6-(hydroxymethyl)tet
C21H26O8; MF: 27; RMF: 365; Prob 21.8%; Lib: mona-export-gnps; ID: 2855.

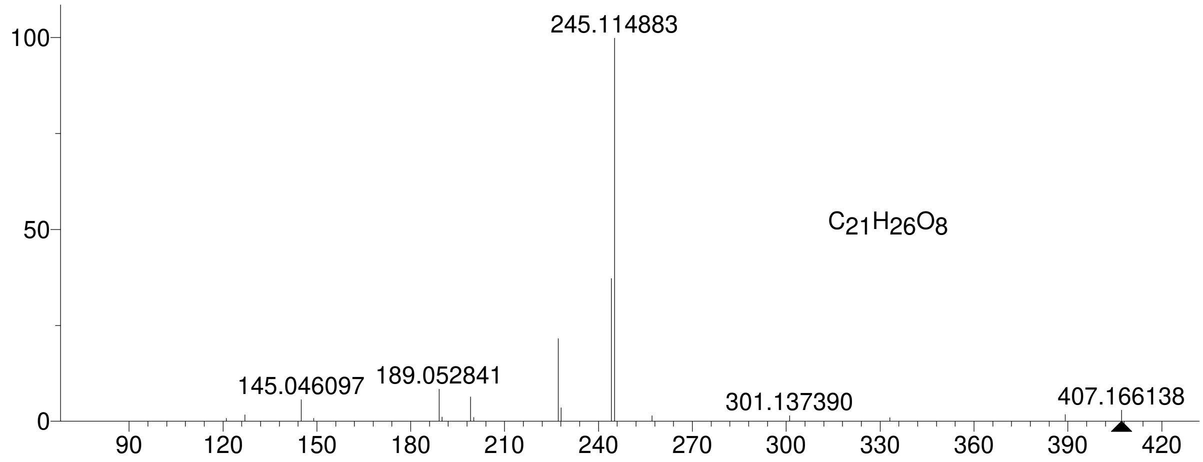

Hit 2 : (2R,3R,4S,6R)-2-((10-hydroxy-2,2-dimethyl-3,4-dihydro-2H-benzo[g]chromen-5-yl)oxy)-6-(hydroxymethyl)te
C21H26O8; MF: 15; RMF: 258; Prob 14.5%; Lib: mona-export-gnps; ID: 2848.

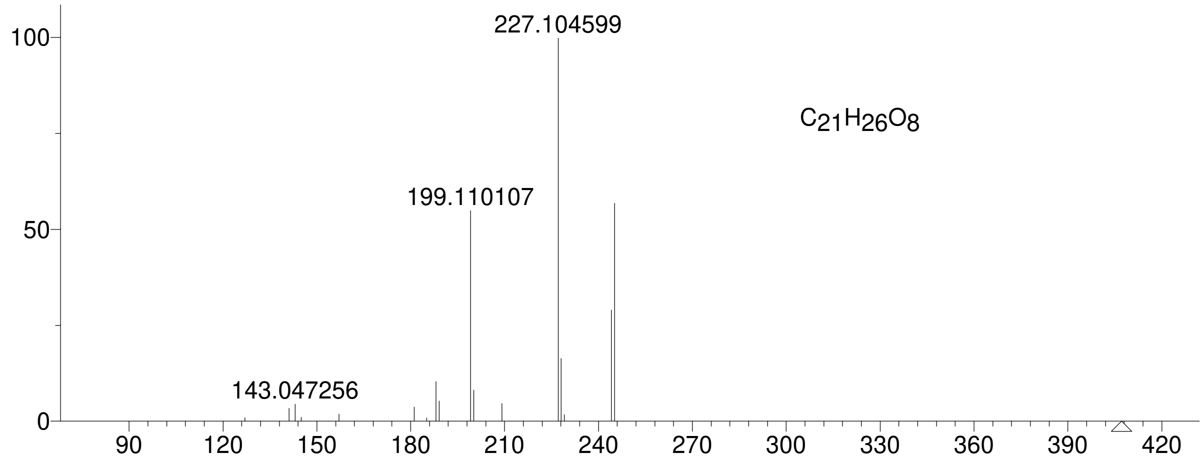

Hit 3 : Artesunate [M+Na]+ HCD 7V P=407.2
C19H28O8; MF: 12; RMF: 249; Prob 12.8%; CAS: 88495-63-0; Lib: nist_msms; ID: 119192.

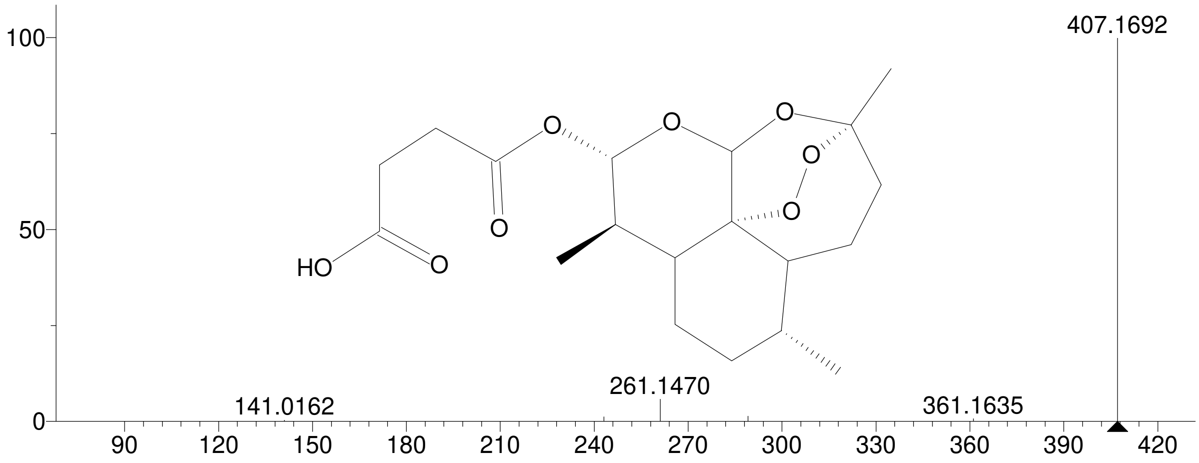

Hit 4 : N,N'-Diacetylchitobiose [M+H-H2O]+ HCD 8V P=407.2
C16H28N2O11; MF: 4; RMF: 185; Prob 9.55%; CAS: 35061-50-8; Lib: nist_msms; ID: 486176.

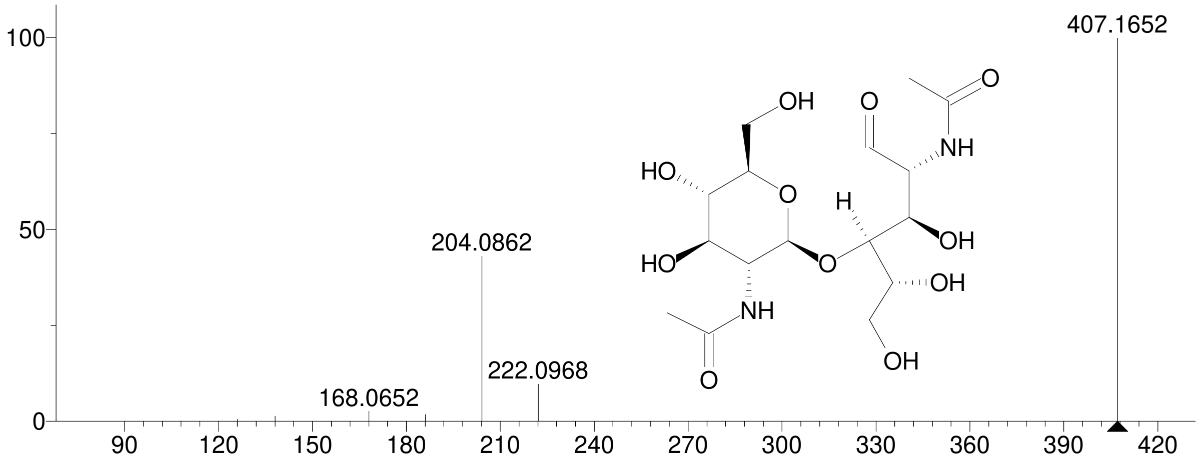

Hit 5 : 16-Phenyltetranorprostaglandin E2 [M+Cl]- HCD 36V P=407.2
C22H28O5; MF: 2; RMF: 58; Prob 8.81%; CAS: 38315-44-5; Lib: nist_msms; ID: 545300.

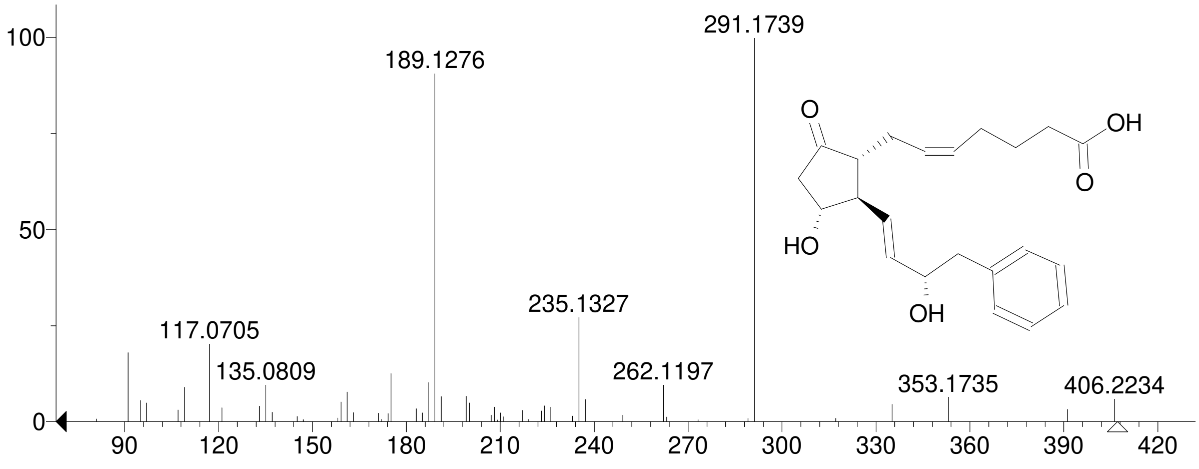

Unknown: Unknown (2.32_485.1531m/z)
Compound in Library Factor = 0

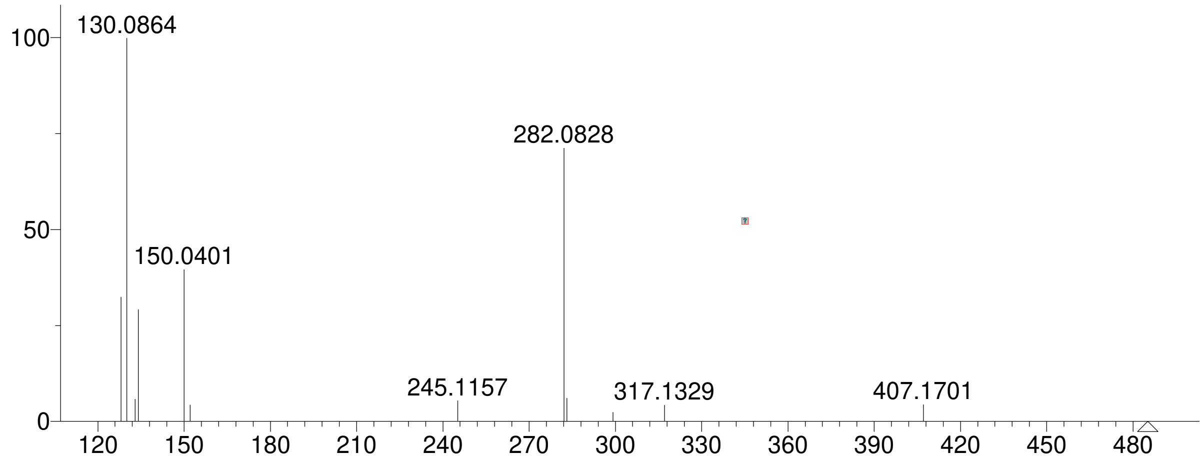

Hit 1 : epiyangambin [M+K]+ 35HCD P=485.2
C24H30O8; MF: 0; RMF: 12; Prob 100.0%; Lib: mona-export-vf-npl_qexactive; ID: 4132.

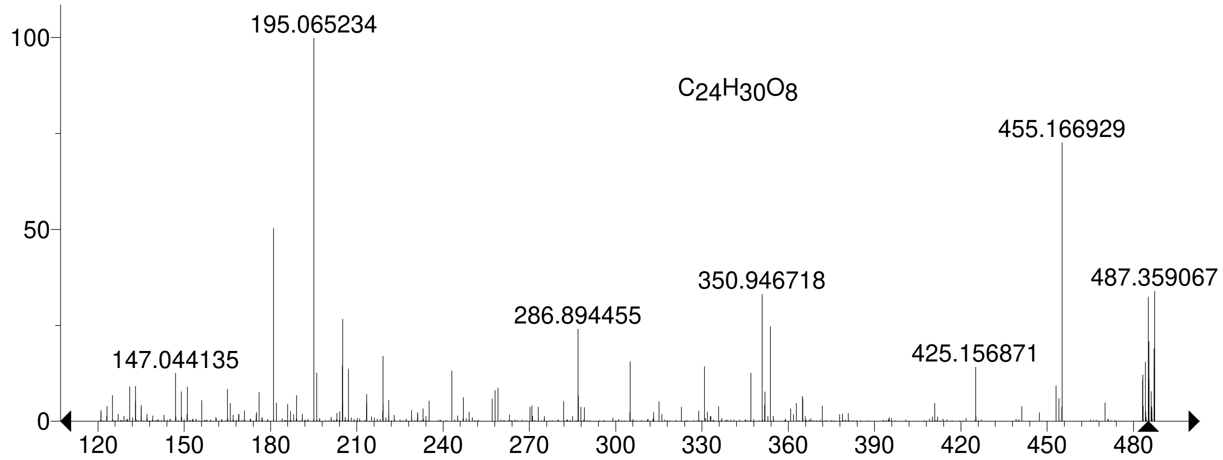

Unknown: Unknown (2.33_607.2677m/z)
Compound in Library Factor = -171

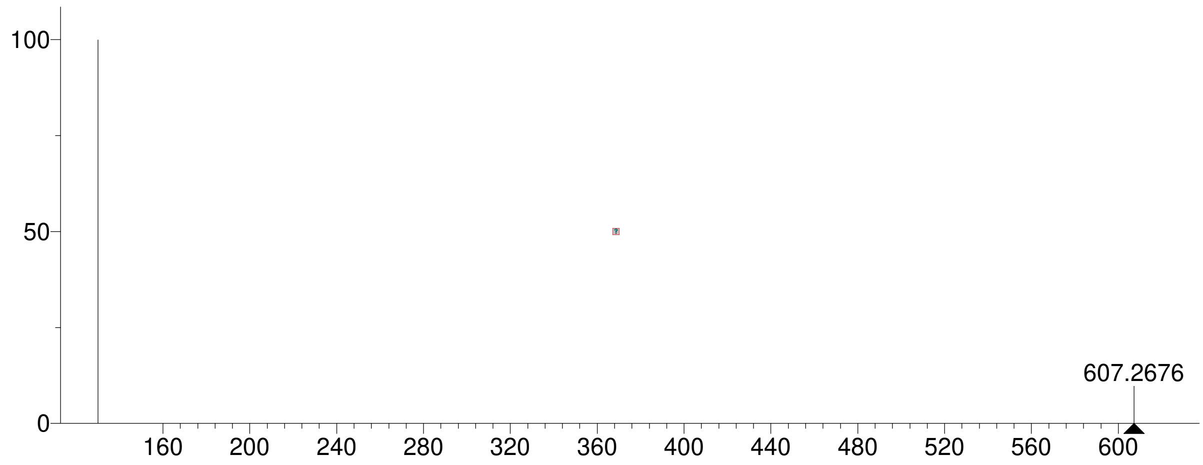

Hit 1 : Isoreserpin [M-H]- 10 P=607.3
C33H40N2O9; MF: 362; RMF: 997; Prob 97.1%; Lib: msms neg mona_ipb_gnps_plasma; ID: 18755.

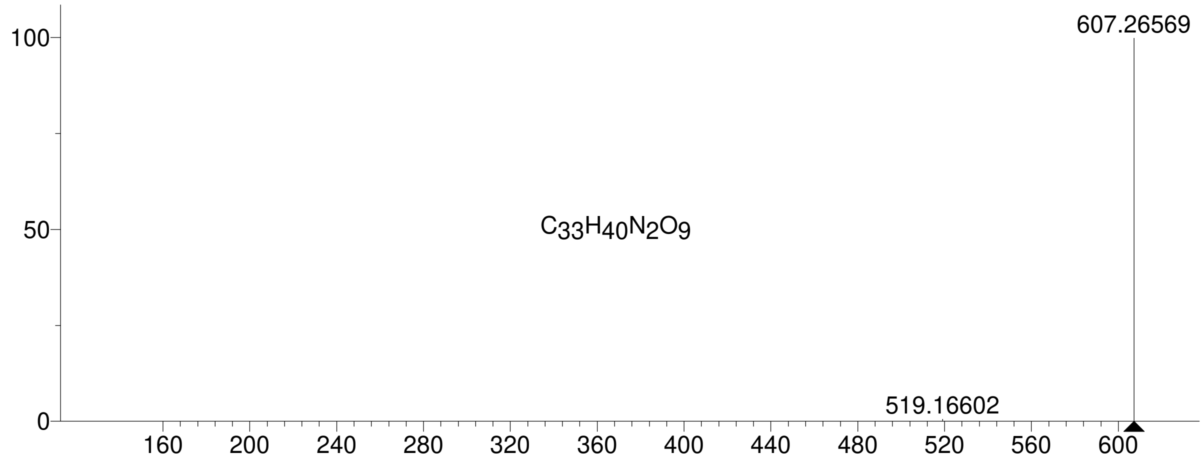

Hit 2 : Reserpine [M-H]- 10 P=607.3
C33H40N2O9; MF: 33; RMF: 830; Prob 0.97%; Lib: mona-export-lc-ms-ms_negative_mode; ID: 27799.

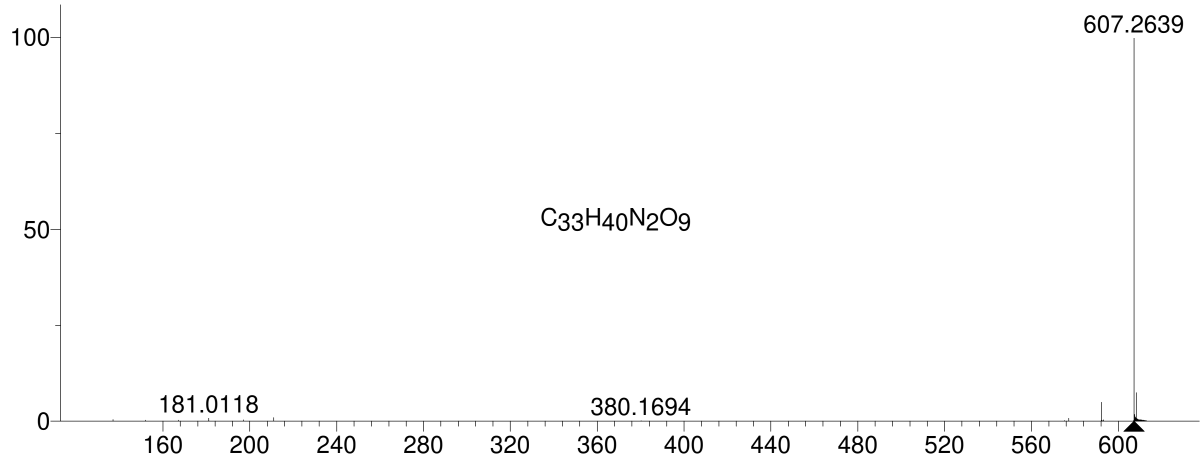

Hit 3 : Ouabain [M+Na]+ HCD 60V P=607.3
C29H44O12; MF: 18; RMF: 263; Prob 0.58%; CAS: 630-60-4; Lib: nist_msms; ID: 384921.

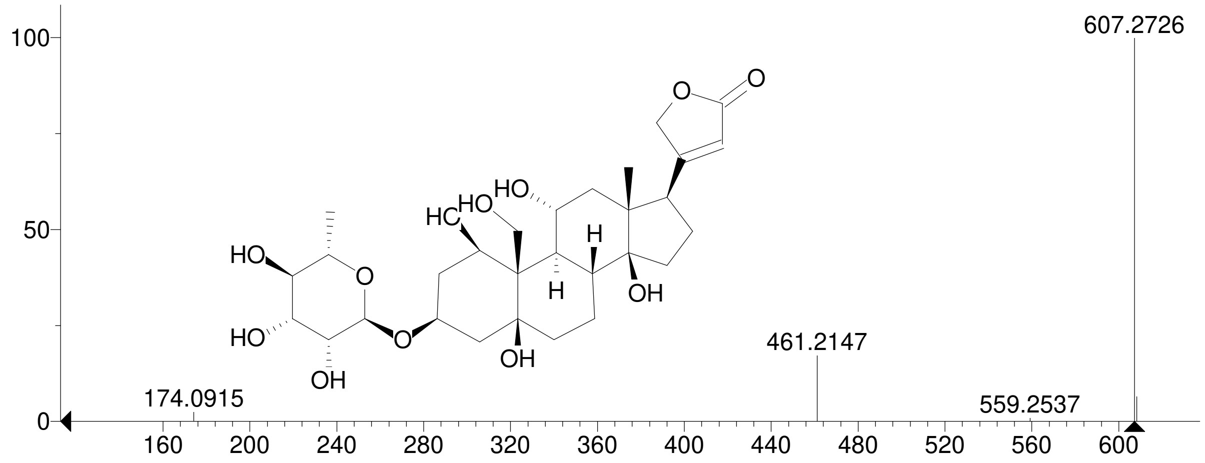

Hit 4 : NCGC00180829-02_C29H44O12_Card-20(22)-enolide, 3-[(6-deoxy-beta-D-talopyranosyl)oxy]-1,5,11,14,1
C29H44O12; MF: 3; RMF: 247; Prob 0.35%; Lib: mona-export-gnps; ID: 14671.

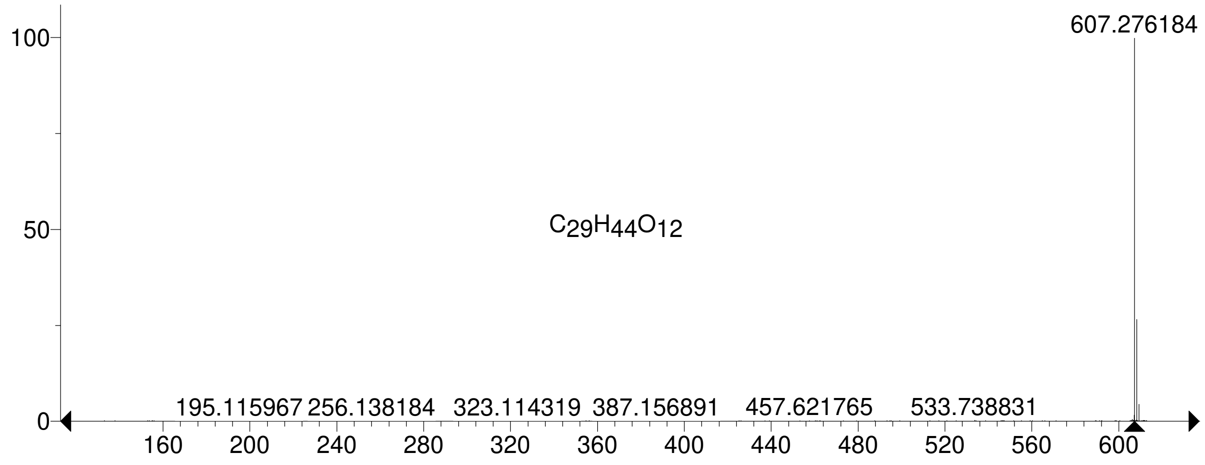

Hit 5 : OUABAIN [M+Na]+ 25HCD P=607.3
C29H44O12; MF: 3; RMF: 243; Prob 0.35%; Lib: anthocyanins mona; ID: 7849.

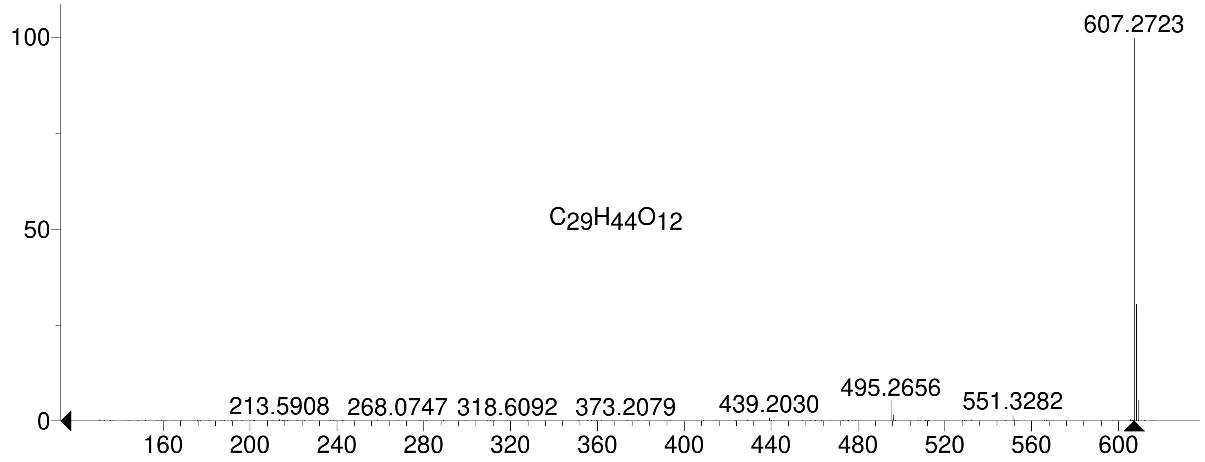

** Search Report Page 1 of 1 **

Unknown: Unknown (2.33_638.1988m/z)
Compound in Library Factor = 0

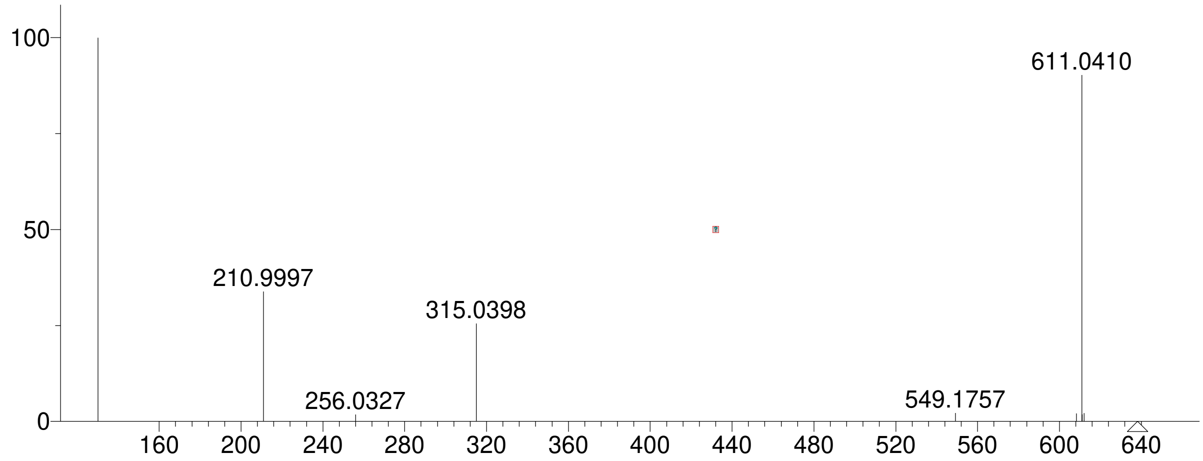

Unknown: Unknown (2.34_403.1350m/z)
Compound in Library Factor = -258

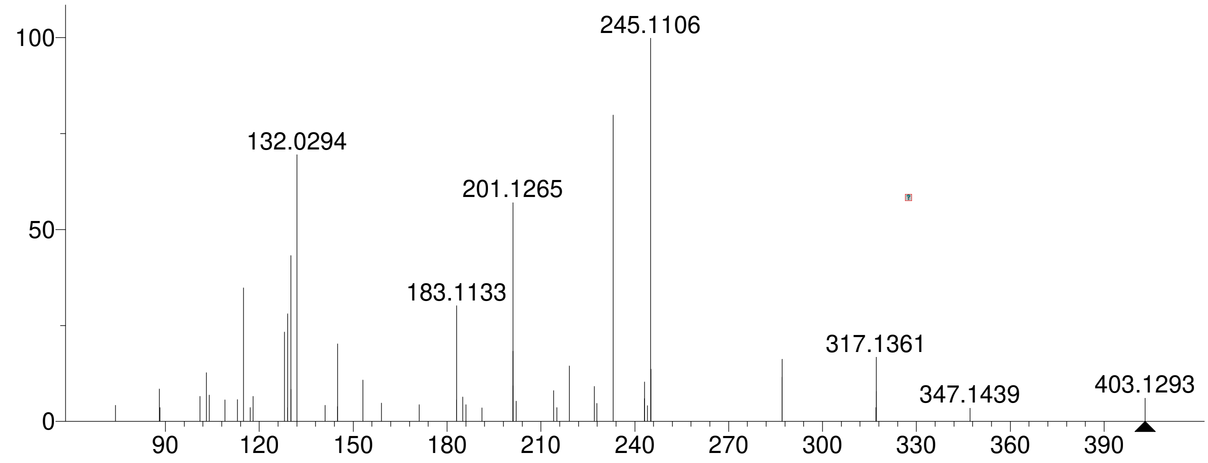

Hit 1 : Nobiletin [M+H]+ HCD 20V P=403.1
C21H22O8; MF: 269; RMF: 935; Prob 95.1%; CAS: 478-01-3; Lib: nist_msms; ID: 178331.

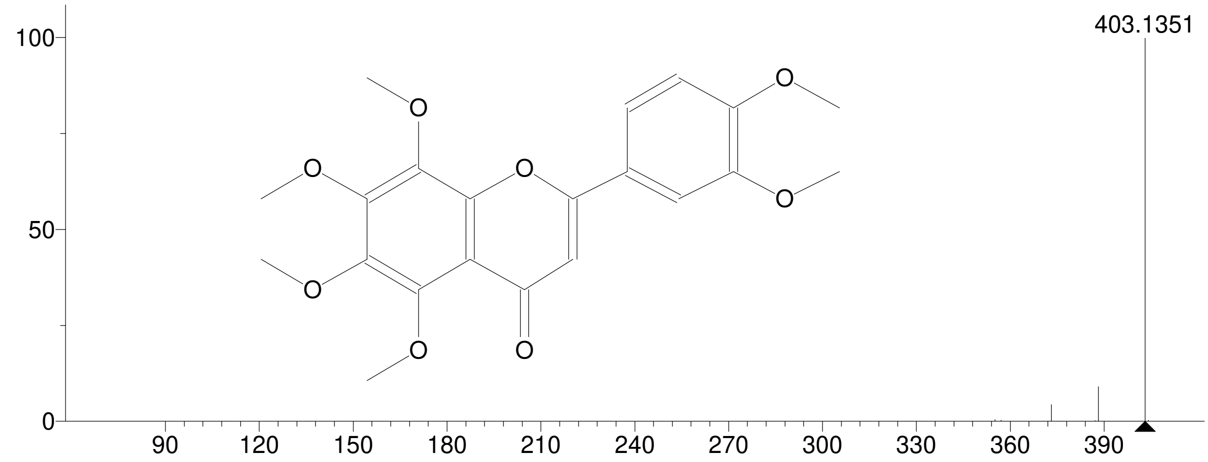

Hit 2 : 3',4',5',5,6,7-Hexamethoxyflavone [M+H]+ HCD 24V P=403.1
C21H22O8; MF: 28; RMF: 637; Prob 0.95%; CAS: 29043-07-0; Lib: nist_msms; ID: 462282.

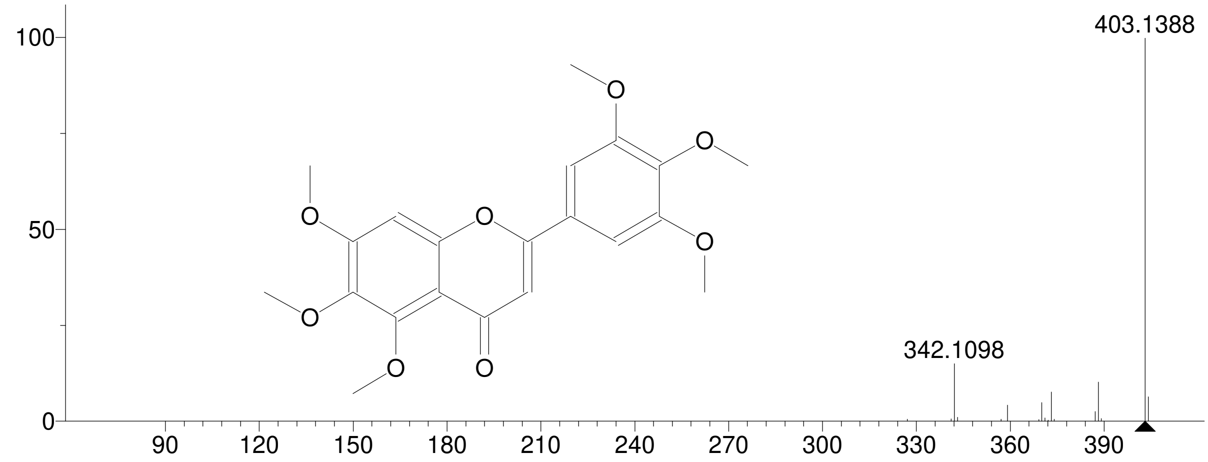

Hit 3 : Egtazic acid [M+Na]+ QTOF 15V P=403.1
C14H24N2O10; MF: 15; RMF: 101; Prob 0.61%; CAS: 67-42-5; Lib: nist_msms; ID: 21811.

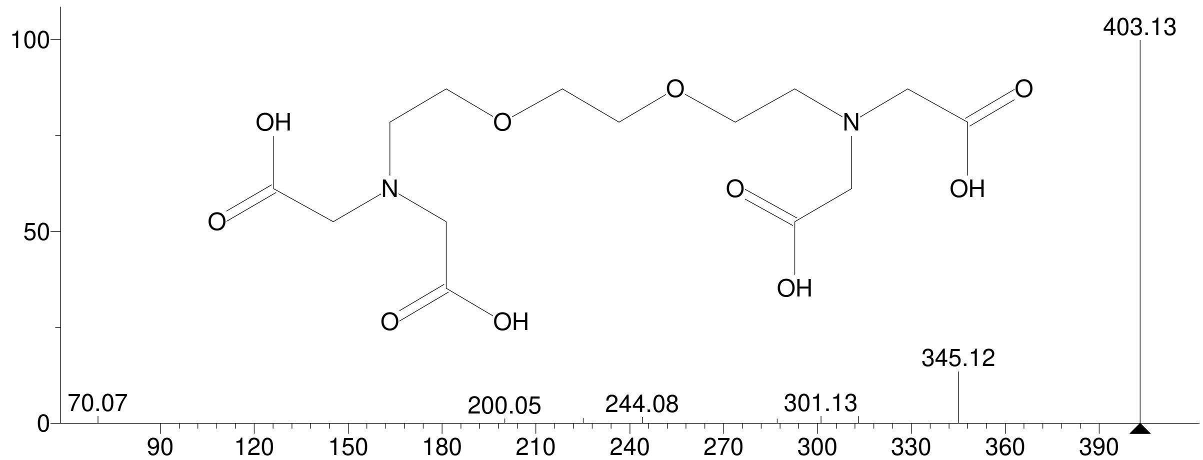

Hit 4 : Naringin dihydrochalcone [M+H-C6H12O6]+ HCD 36V P=403.1
C27H34O14; MF: 3; RMF: 56; Prob 0.40%; CAS: 18916-17-1; Lib: nist_msms; ID: 237395.

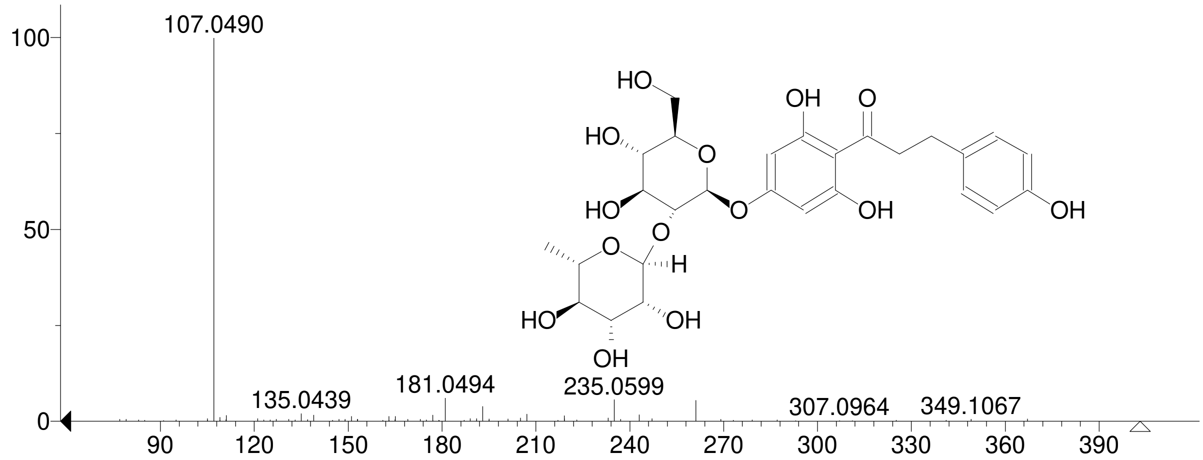

Hit 5 : NCGC00347547-02!methyl 2-(4,5-dihydroxy-6-methoxy-3,6-dimethyl-2-oxocyclohex-3-en-1-yl)oxy-4-hydro:
C19H24O8; MF: 1; RMF: 70; Prob 0.37%; Lib: mona-export-gnps; ID: 8950.

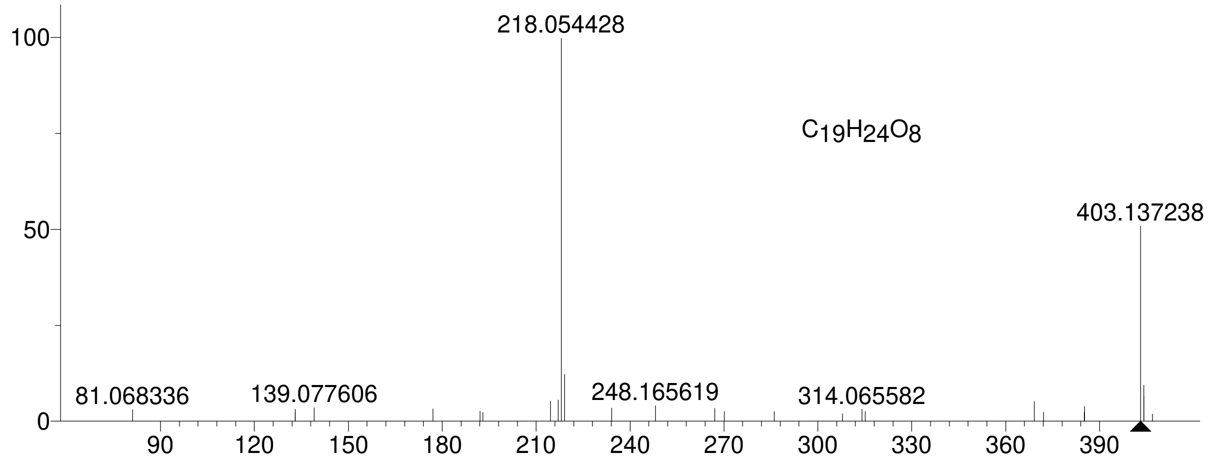

** Search Report Page 1 of 1 **

Unknown: Unknown (2.37_288.1078m/z)
Compound in Library Factor = 0

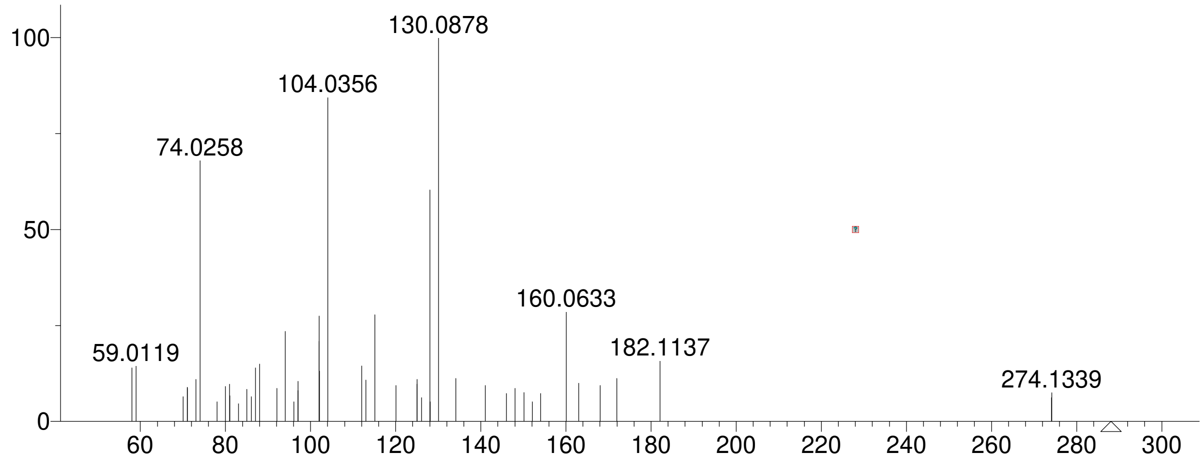

Unknown: Unknown (2.37_393.1860m/z)
Compound in Library Factor = -3273

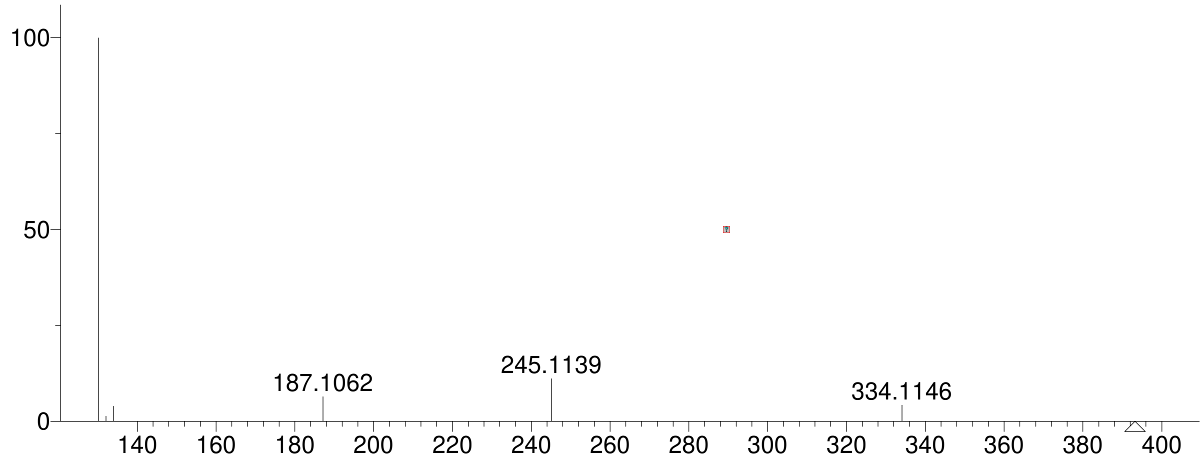

Hit 1 : Dovitinib [M+H]+ IT-FT 35% P=393.2
C21H21FN6O; MF: 1; RMF: 159; Prob 100.0%; CAS: 405169-16-6; Lib: nist_msms; ID: 381645.

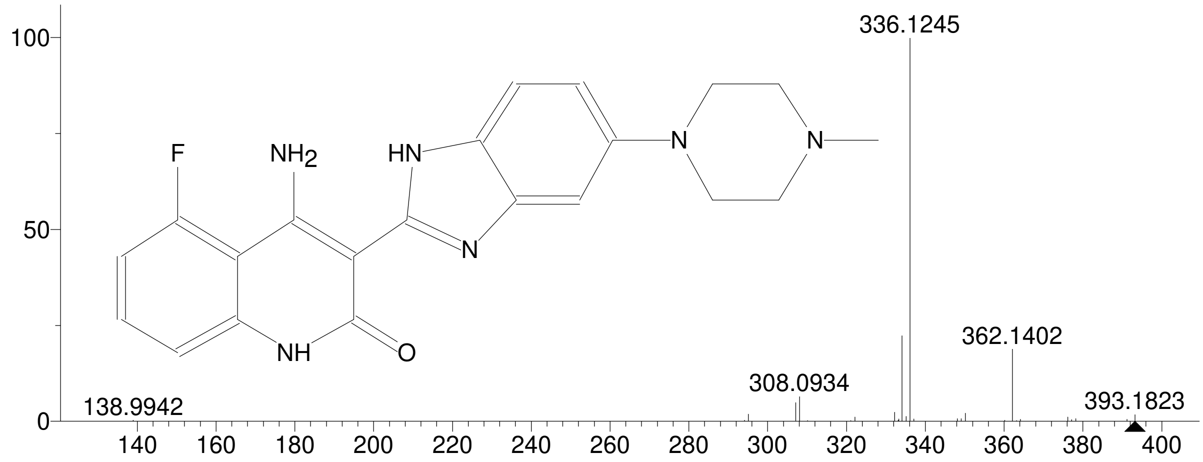

** Search Report Page 1 of 1 **

Unknown: Unknown (2.37_607.2678m/z)
Compound in Library Factor = 0

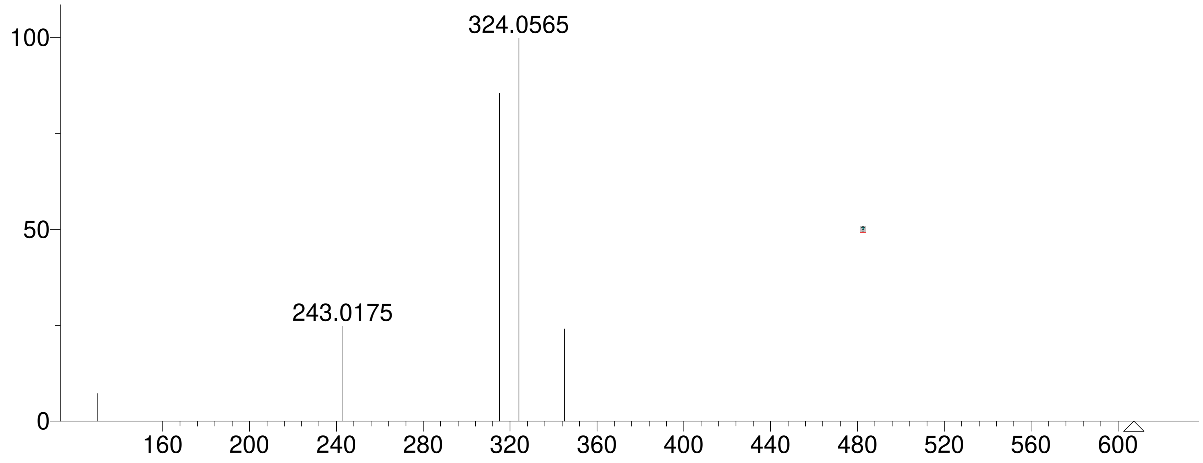

** Search Report Page 1 of 1 **

Unknown: Unknown (2.37_638.2009m/z)
Compound in Library Factor = 0

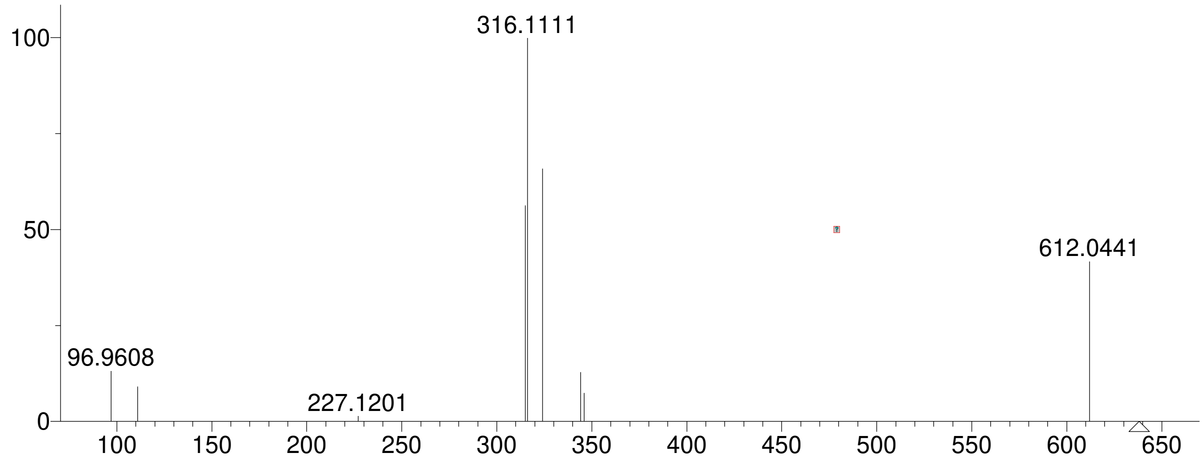

Unknown: Unknown (2.38_397.1711m/z)
Compound in Library Factor = 0

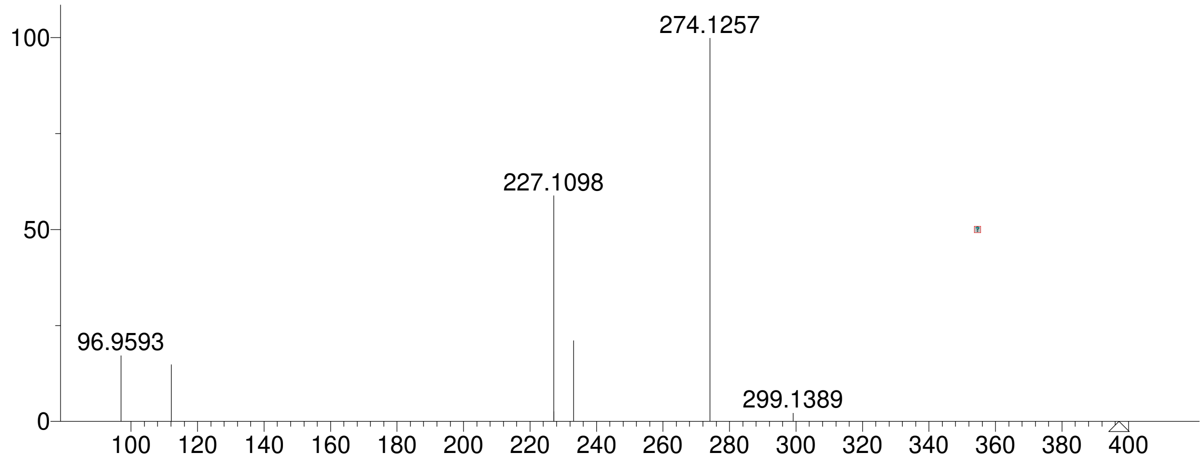

Hit 1 : Benzoic acid, 2-(diphenylphosphino)-4-[21-[(3aS,4S,6aR)-hexahydro-2-oxo-1H-thieno[3,4-d]imidazol-4-yl]-
C41H53N4O8PS; MF: 0; RMF: 36; Prob 50.0%; CAS: 608514-42-7; Lib: nist_msms; ID: 376823.

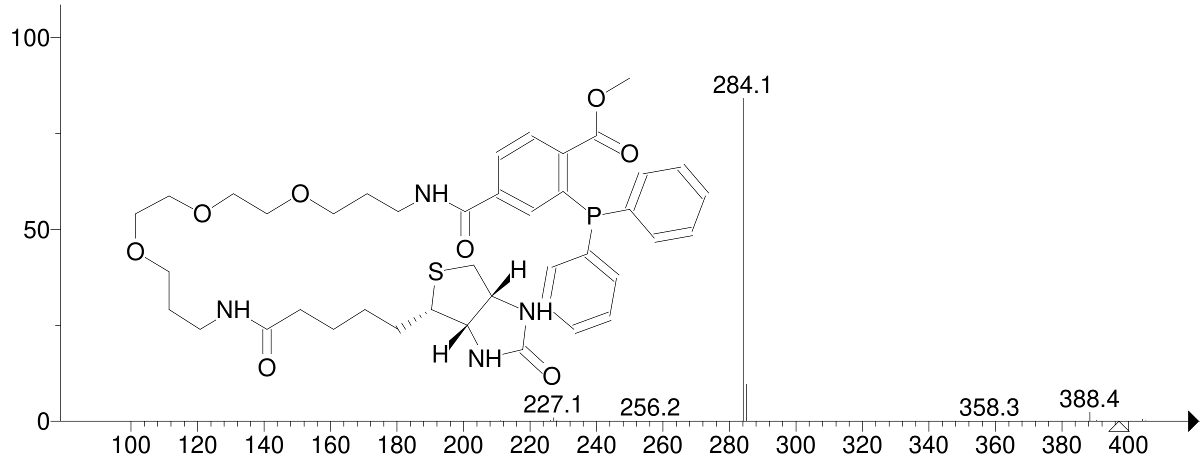

Hit 2 : Atorvastatin [M-H-C7H12O4]- HCD 111V P=397.2
C33H35FN2O5; MF: 0; RMF: 9; Prob 50.0%; CAS: 134523-00-5; Lib: nist_msms; ID: 389044.

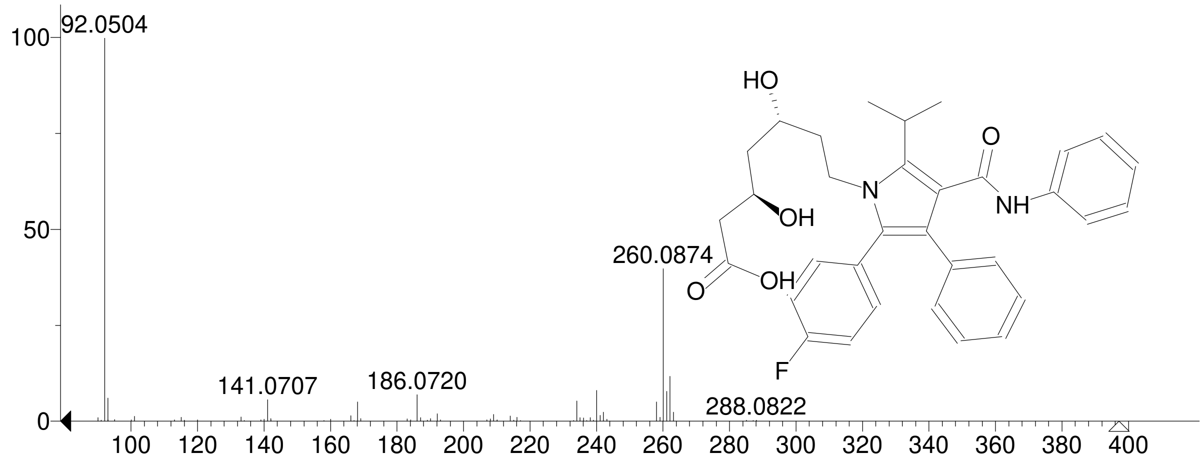

Unknown: Unknown (2.39_460.1660m/z)
Compound in Library Factor = 0

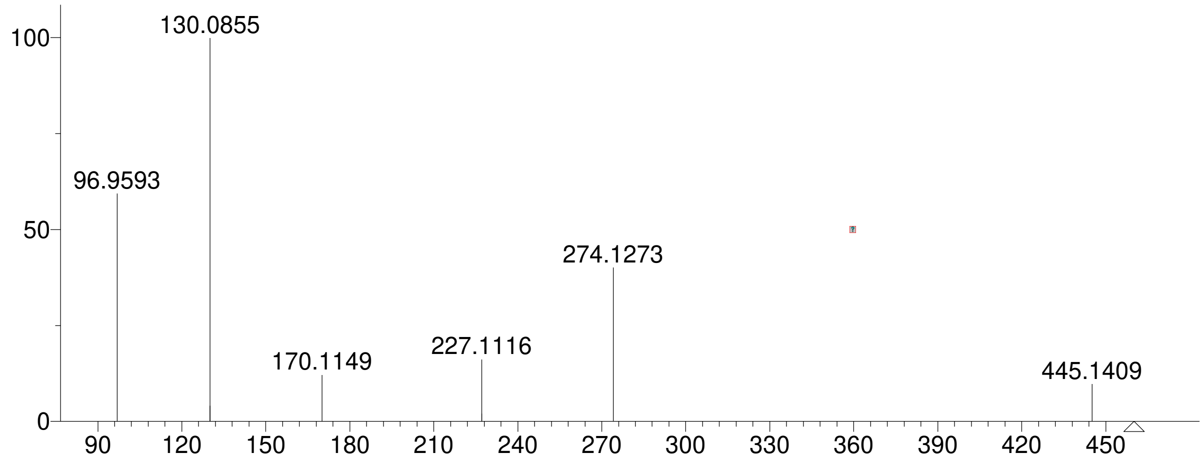

Hit 1 : Benzoic acid, 4-[[[2,4-bis(acetylamino)-6-pteridinyl]methyl]methylamino]-, ethyl ester [M+Na]+ IT-FT 35%
C21H23N7O4; MF: 0; RMF: 20; Prob 100.0%; CAS: 1330265-87-6; Lib: nist_msms; ID: 436001.

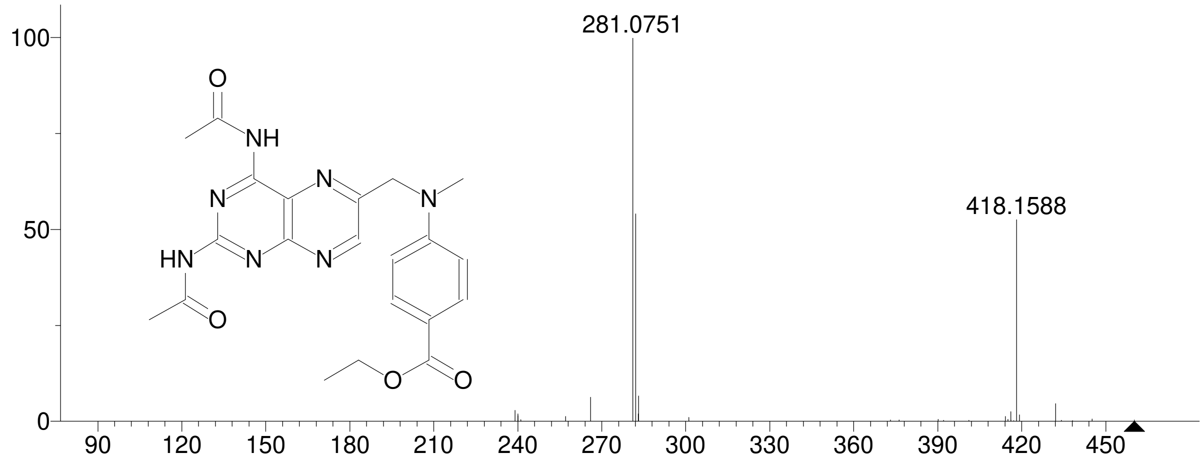

** Search Report Page 1 of 1 **

Unknown: Unknown (2.40_259.1285m/z)
Compound in Library Factor = 0

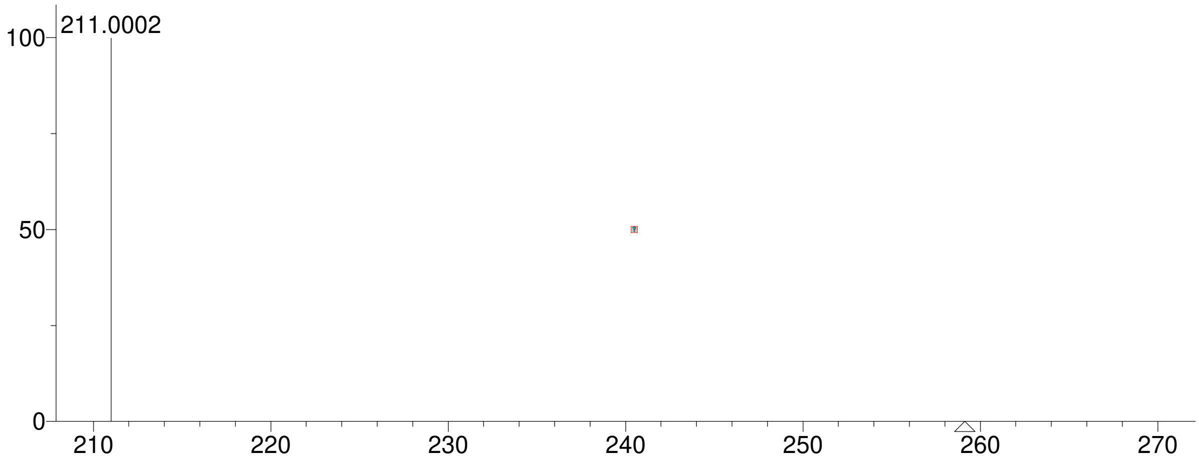

Unknown: Unknown (2.41_424.1438m/z)
Compound in Library Factor = 0

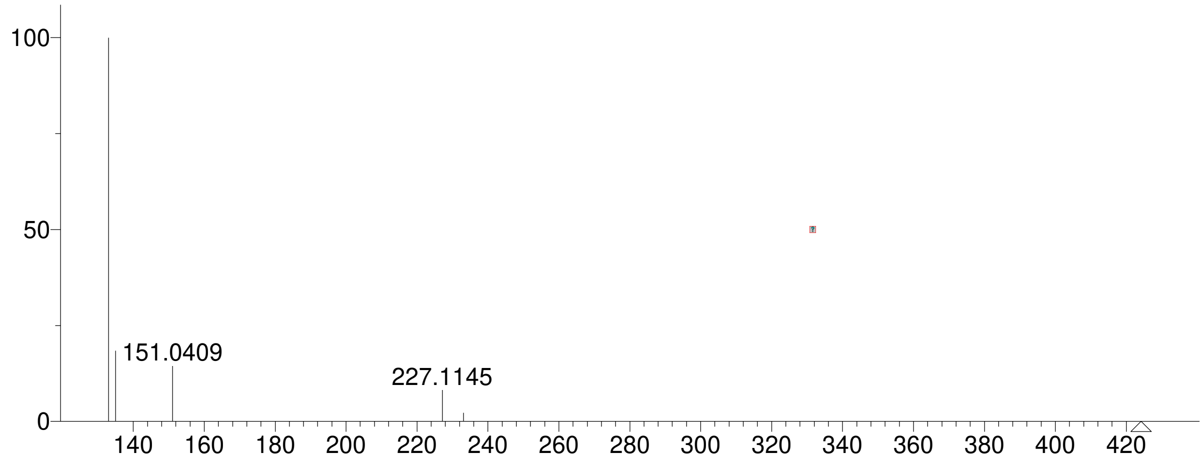

Unknown: Unknown (2.41_439.1716m/z)
Compound in Library Factor = 0

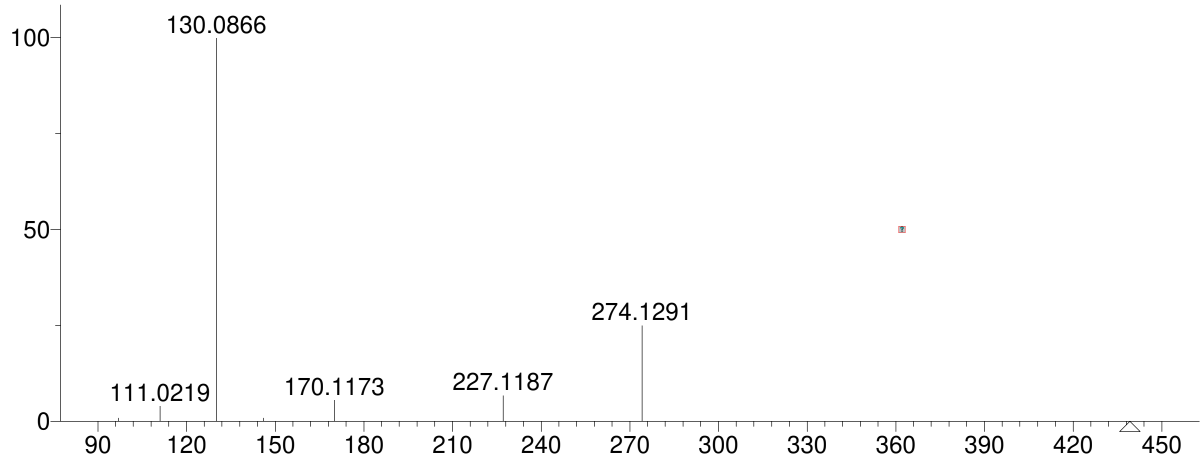

Hit 1 : 15-Ketofluprostenol [M+H-H2O]+ HCD 43V P=439.2
C23H27F3O6; MF: 0; RMF: 15; Prob 16.7%; Lib: nist_msms; ID: 155577.

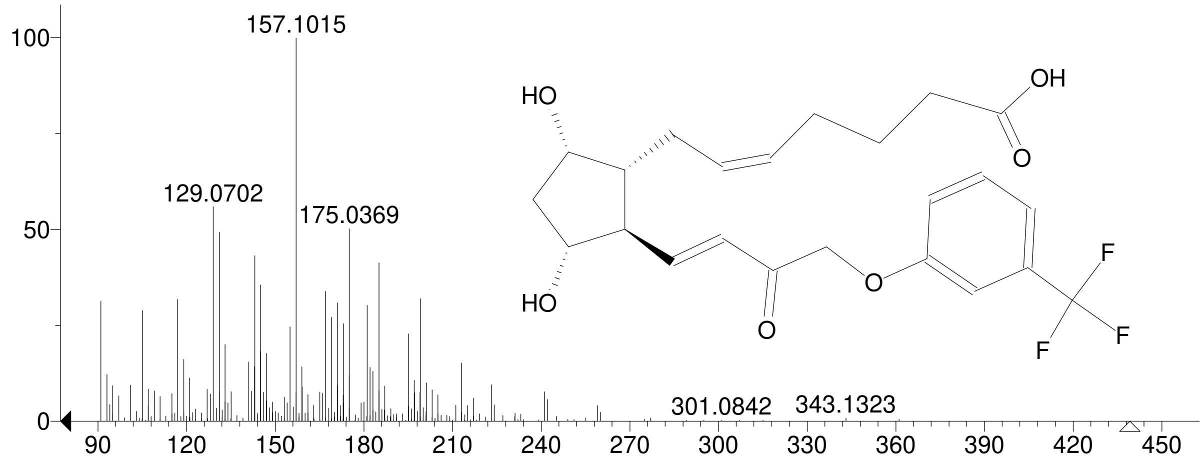

Hit 2 : methyl (1R,4aS,9S,10S)-10-acetyloxy-5,9-dihydroxy-1,4a-dimethyl-2-oxo-7-propan-2-yl-10,10a-dihydro-9H
C23H28O7; MF: 0; RMF: 13; Prob 16.7%; Lib: mona-export-vf-npl_qexactive; ID: 18731.

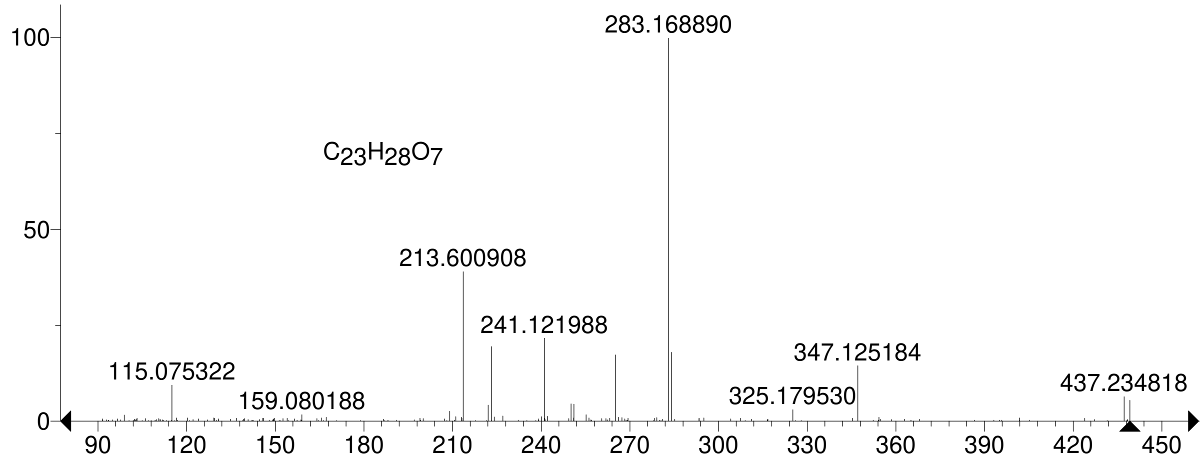

Hit 3 : methyl 2-[[4-hydroxy-3-(3-methylbut-2-enyl)phenyl]methyl]-3-(4-hydroxyphenyl)-4-methoxy-5-oxofuran-2-ca
C25H26O7; MF: 0; RMF: 11; Prob 16.7%; Lib: mona-export-vf-npl_qexactive; ID: 10071.

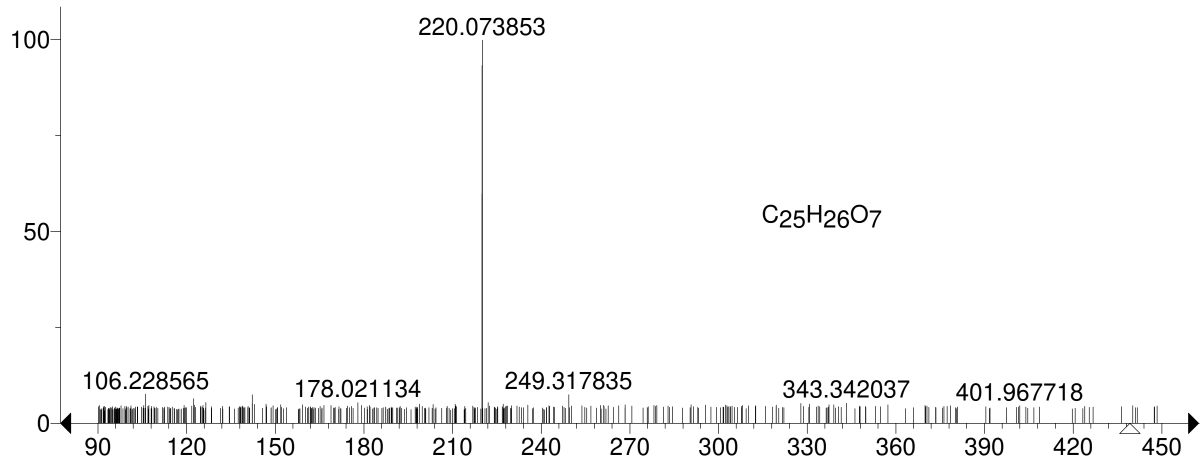

Hit 4 : (+)-Fluprostenol [M-H-H2O]- HCD 79V P=439.2
C23H29F3O6; MF: 0; RMF: 9; Prob 16.7%; CAS: 54276-17-4; Lib: nist_msms; ID: 155456.

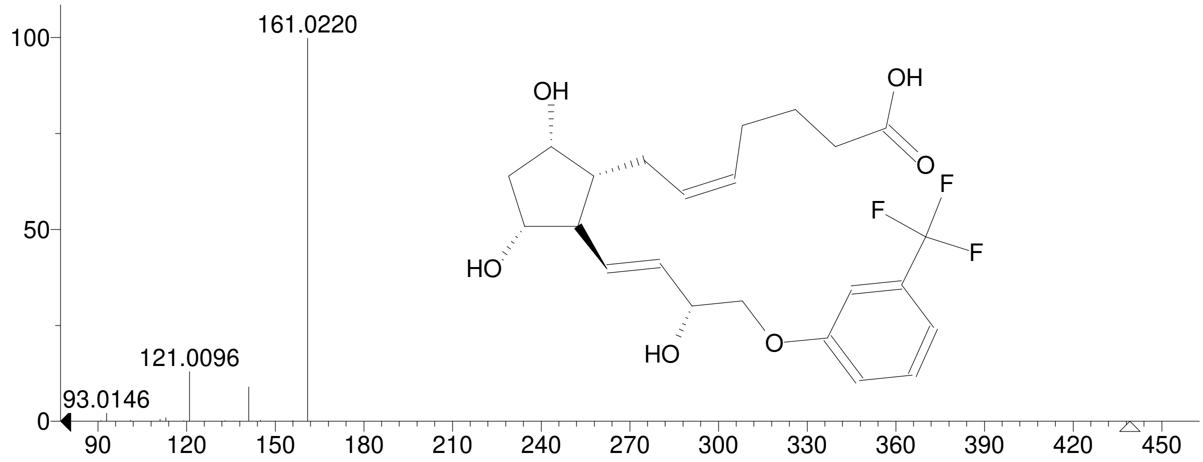

Hit 5 : Lobaric acid [M+H-H2O]+ HCD 43V P=439.2
C25H28O8; MF: 0; RMF: 5; Prob 16.7%; CAS: 522-53-2; Lib: nist_msms; ID: 223163.

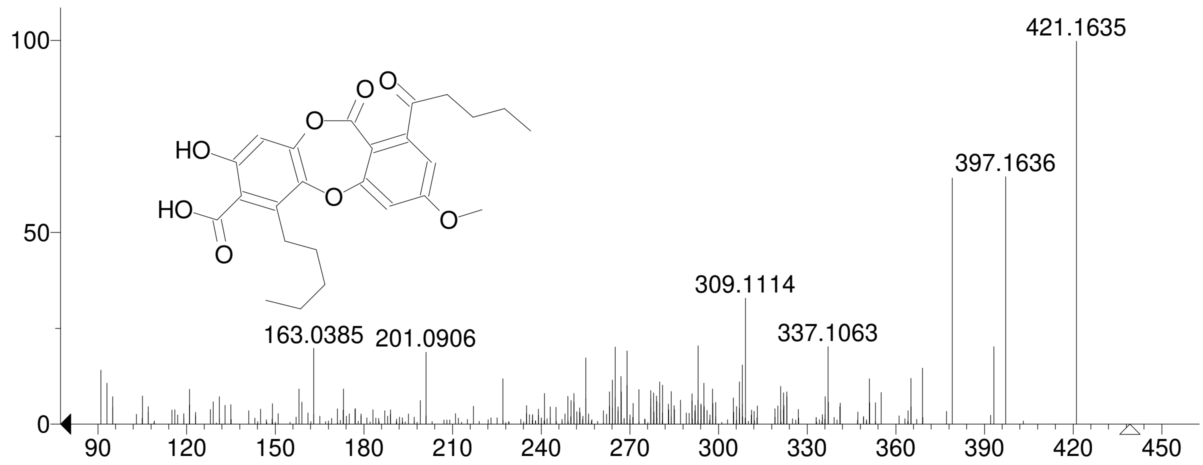

Unknown: Unknown (2.41_444.1361m/z)
Compound in Library Factor = -2387

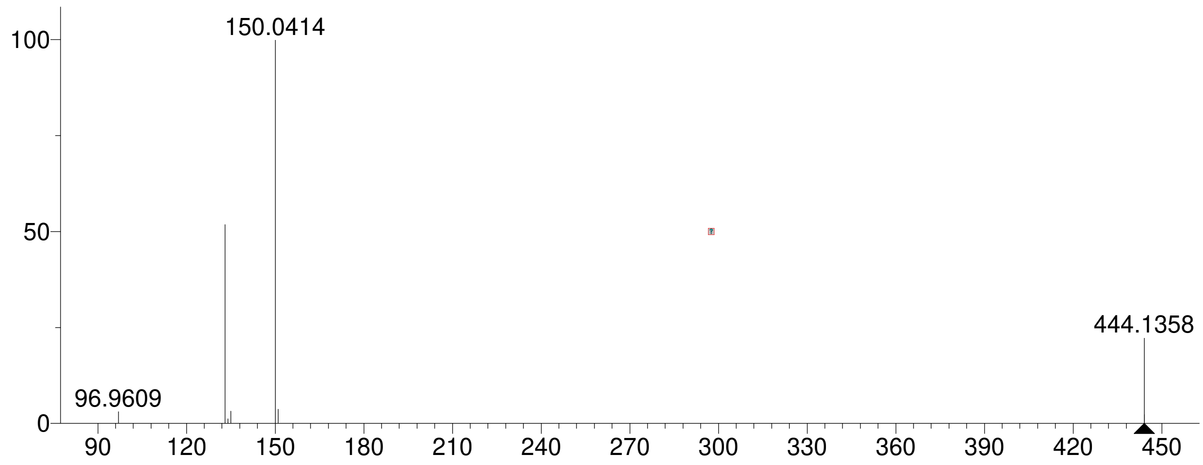

Hit 1 : Propaquizafop [M+H]+ HCD 8V P=444.1
C22H22ClN3O5; MF: 31; RMF: 334; Prob 39.5%; CAS: 111479-05-1; Lib: nist_msms; ID: 138674.

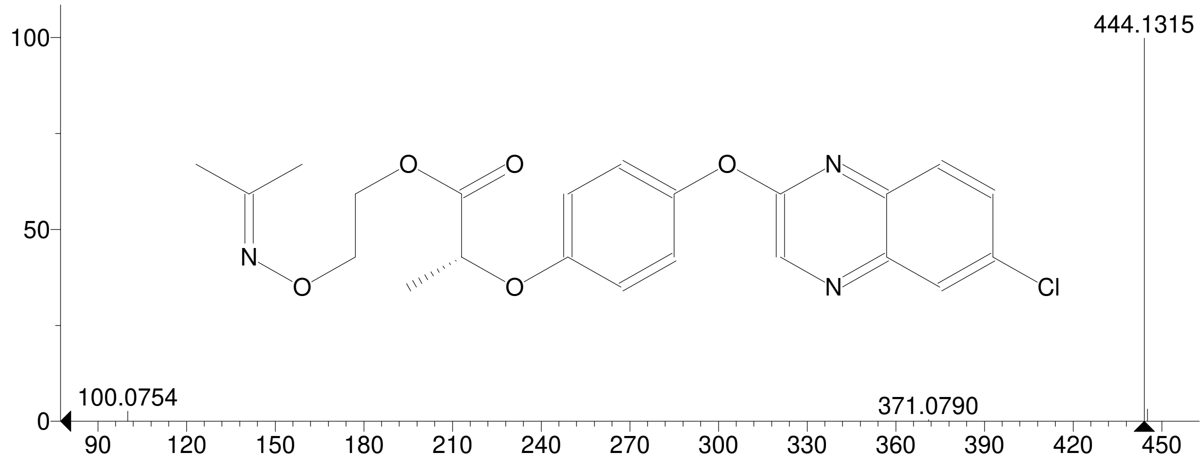

Hit 2 : N-(Deshydroxyethyl)dasatinib [M+H]+ HCD 26V P=444.1
C20H22ClN7OS; MF: 14; RMF: 320; Prob 21.5%; CAS: 910297-51-7; Lib: nist_msms; ID: 434471.

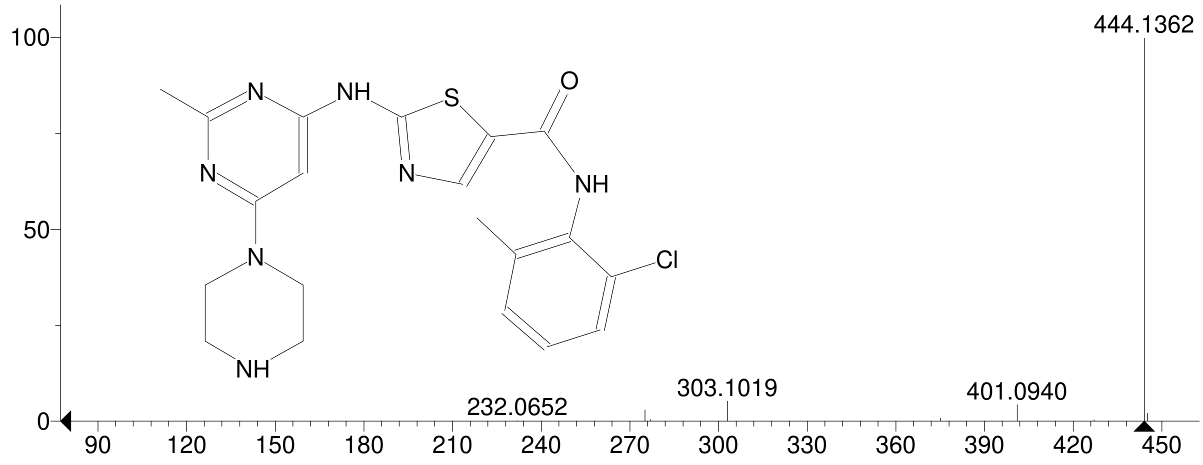

Hit 3 : 7-methyl-octyl-glucosinolate REV 1 () [M-H]- P=444.1
C16H31NO9S2; MF: 12; RMF: 331; Prob 19.9%; Lib: phytofrag2018_final_060219; ID: 109.

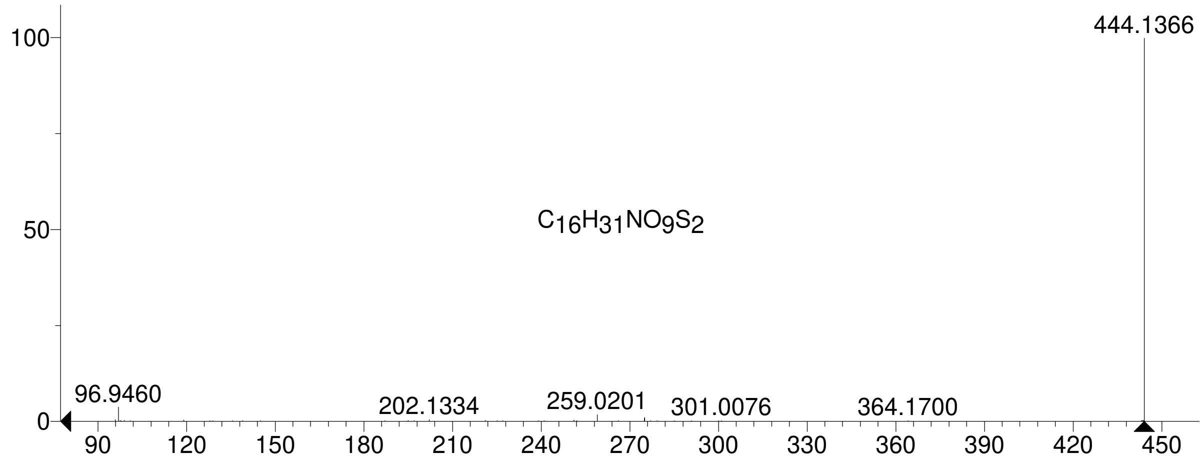

Hit 4 : 7-methyl-octyl-glucosinolate REV 2 () [M-H]- P=444.1
C16H31NO9S2; MF: 11; RMF: 330; Prob 19.1%; Lib: phytofrag2018_final_060219; ID: 110.

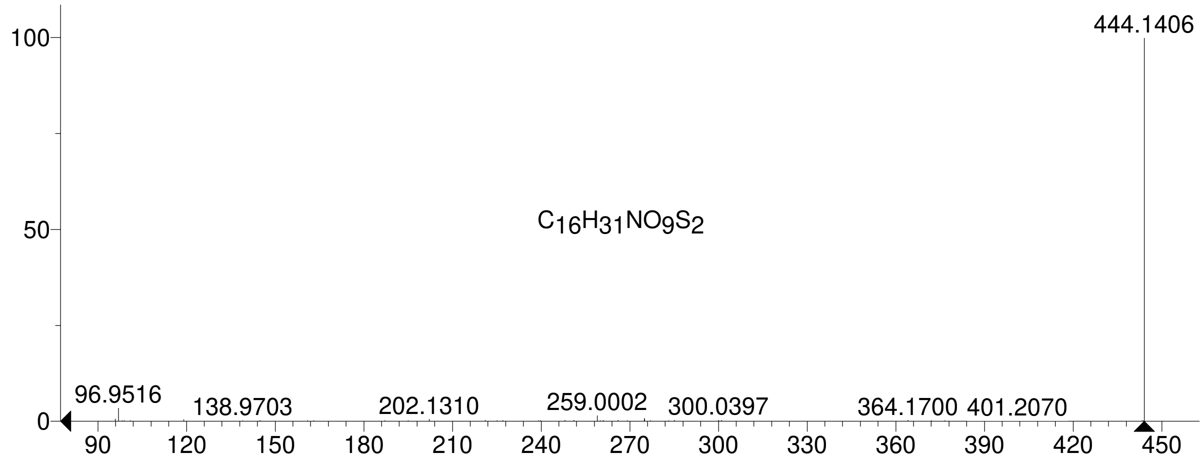

Unknown: Unknown (2.43_397.1674m/z)
Compound in Library Factor = 0

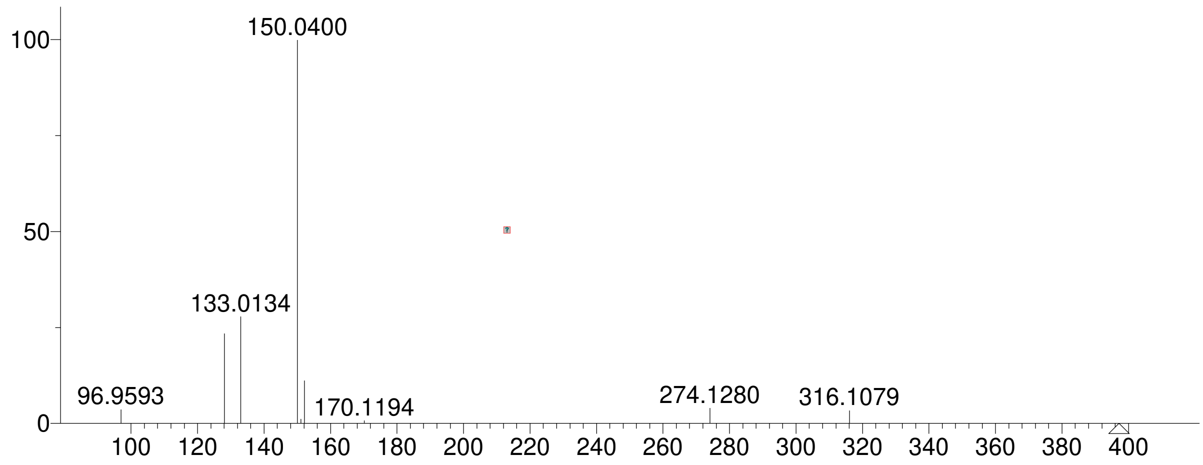

Hit 1 : Dimethoxycurcumin [M+H]+ HCD 154V P=397.2
C23H24O6; MF: 0; RMF: 164; Prob 20.0%; CAS: 160096-59-3; Lib: nist_msms; ID: 167685.

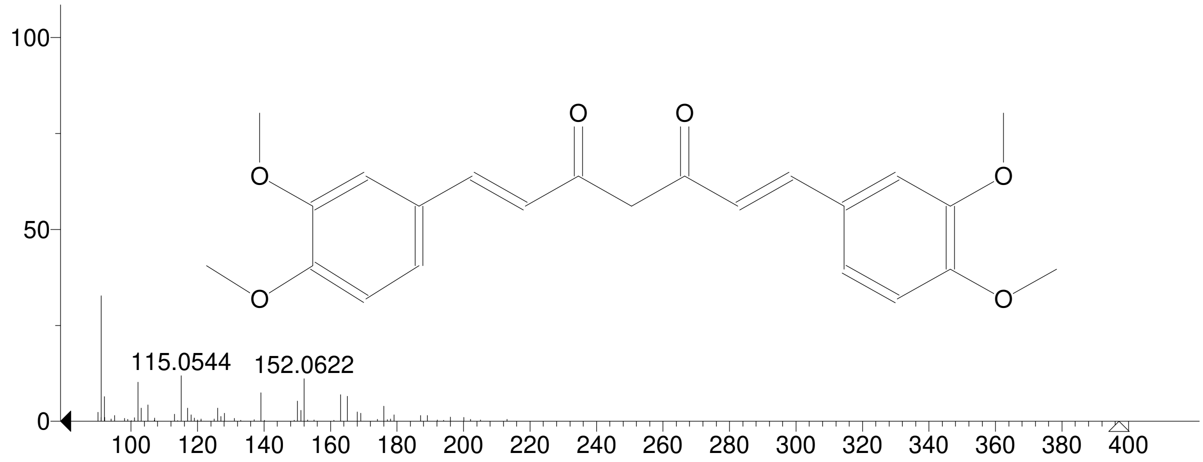

Hit 2 : Gomisin C [M+H-C7H8O3]+ HCD 127V P=397.2
C30H32O9; MF: 0; RMF: 128; Prob 20.0%; CAS: 58546-56-8; Lib: nist_msms; ID: 403958.

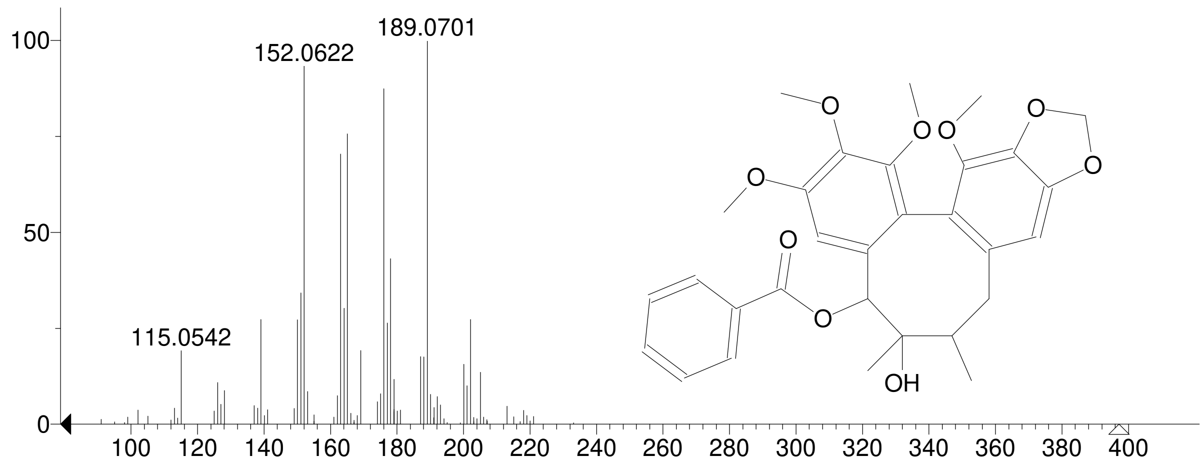

Hit 3 : 3-[1-(3-Aminopropyl)-1H-indol-3-yl]-4-(1-methyl-1H-indol-3-yl)-1H-pyrrole-2,5-dione [M-H]- HCD 51V P=
C24H22N4O2; MF: 0; RMF: 32; Prob 20.0%; CAS: 138516-31-1; Lib: nist_msms; ID: 321333.

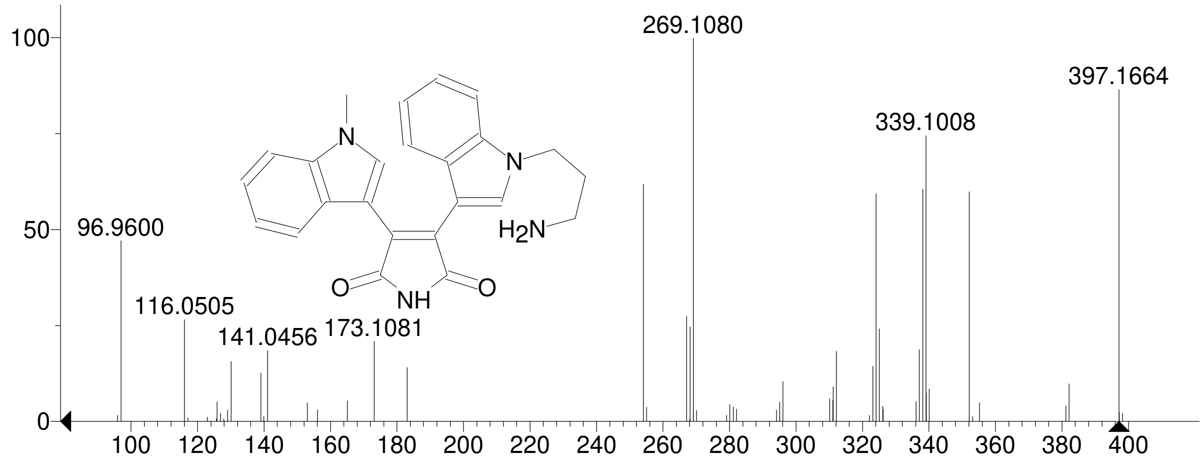

Hit 4 : Garcinone C [M+H-H2O]+ HCD 103V P=397.2
C23H26O7; MF: 0; RMF: 18; Prob 20.0%; CAS: 76996-27-5; Lib: nist_msms; ID: 389313.

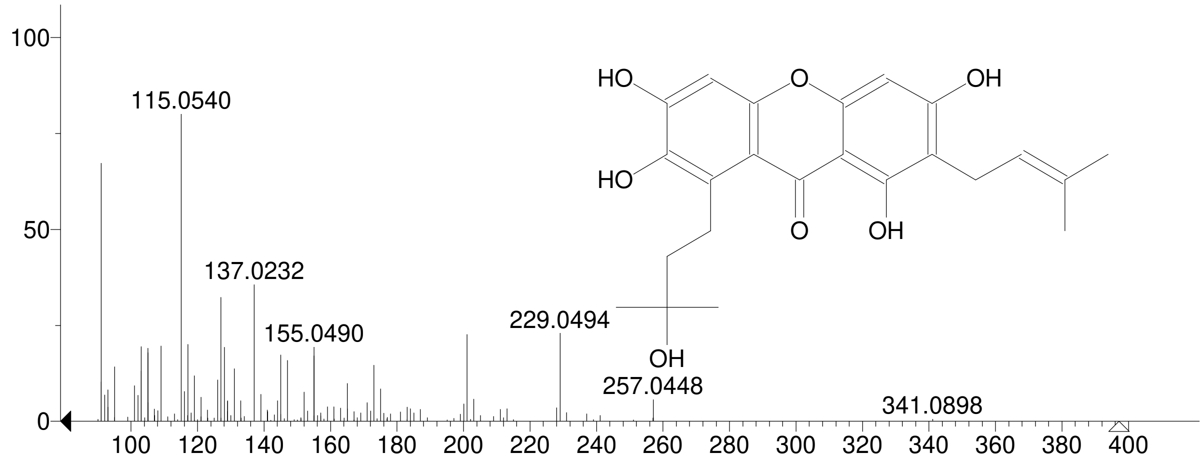

Hit 5 : 1-Naphthalenesulfonamide, N-(10-aminodecyl)-5-chloro- [M+H]+ IT 35% P=397.2
C20H29ClN2O2S; MF: 0; RMF: 9; Prob 20.0%; CAS: 80467-76-1; Lib: nist_msms; ID: 382044.

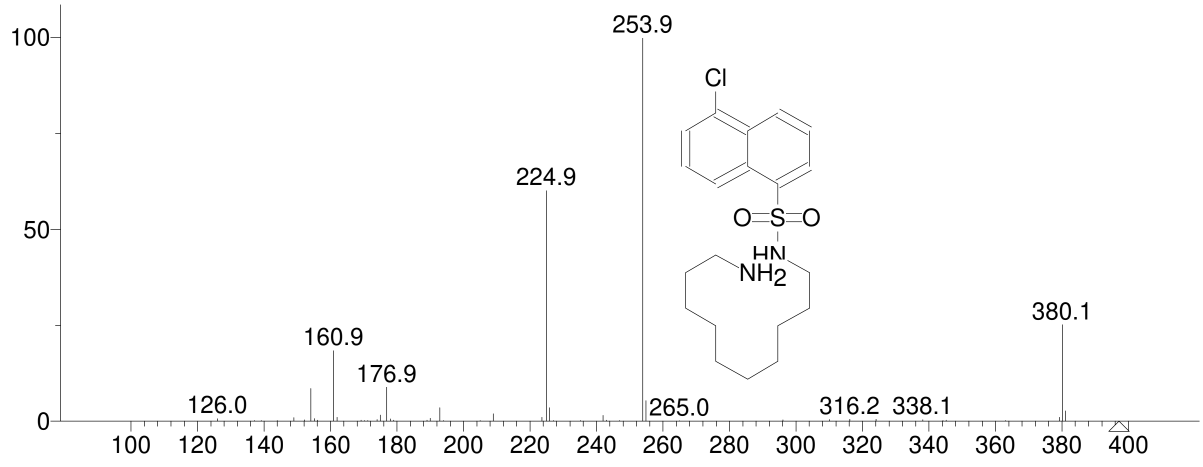

Unknown: Unknown (2.44_433.1555m/z)
Compound in Library Factor = 0

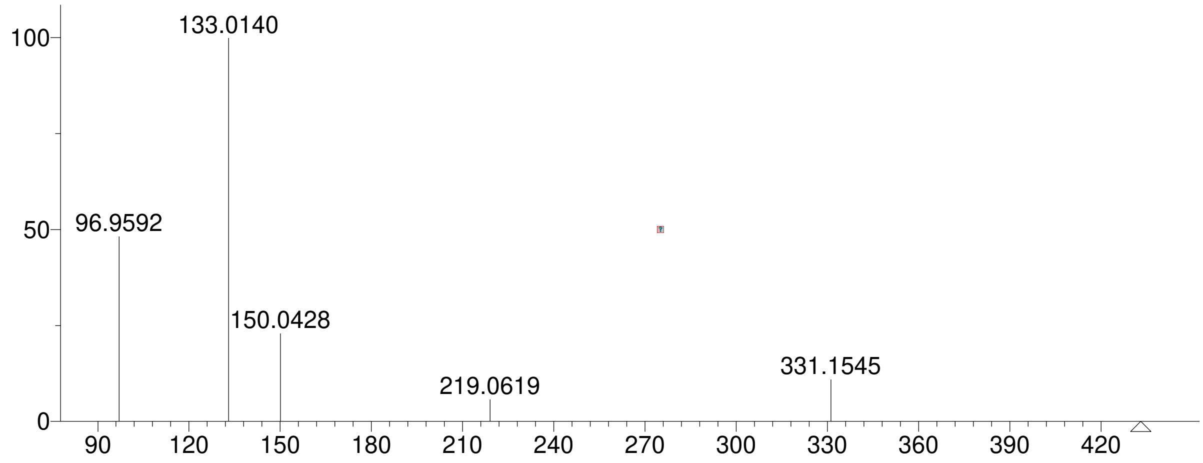

Hit 1 : dihydroferuloyk-beta-keto acid(4-O-8)G 2 (curcumin) [M-H]- P=433.2
C22H26O9; MF: 0; RMF: 56; Prob 50.0%; Lib: phytofrag2018_final_060219; ID: 1025.

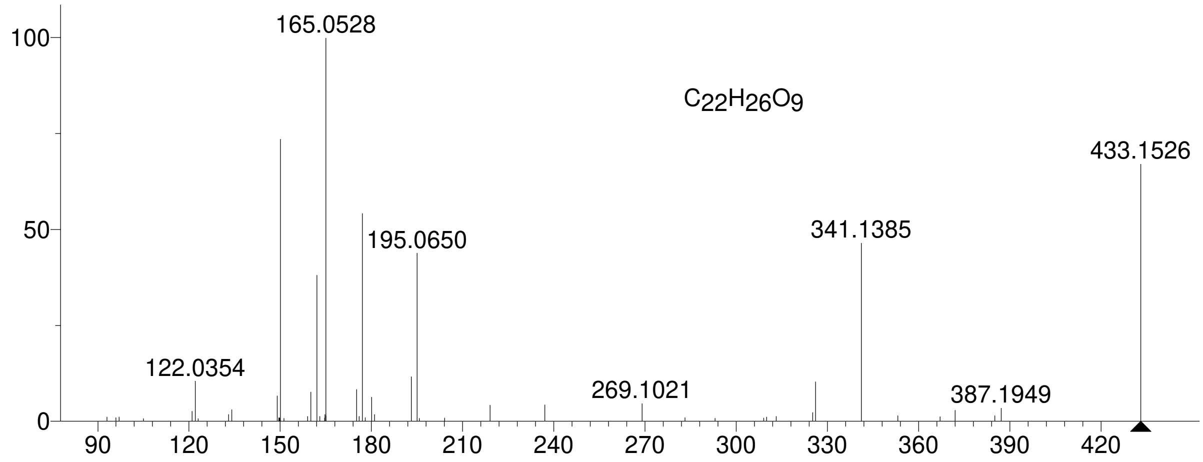

Hit 2 : dihydroferuloyl-beta-keto acid(4-O-8)G 1 (curcumin) [M-H]- P=433.2
C22H26O9; MF: 0; RMF: 10; Prob 50.0%; Lib: phytofrag2018_final_060219; ID: 1023.

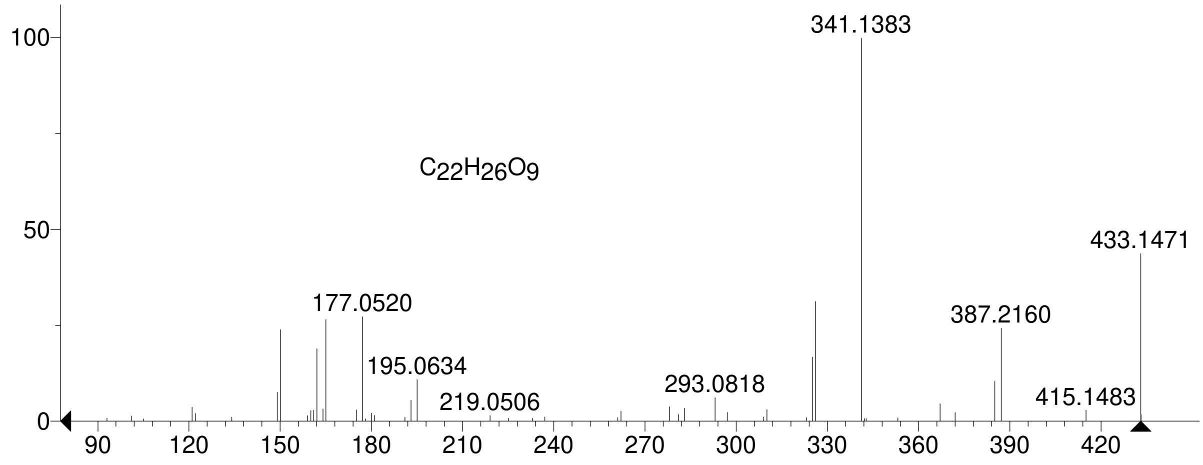

Unknown: Unknown (2.45_358.1225m/z)
Compound in Library Factor = 0

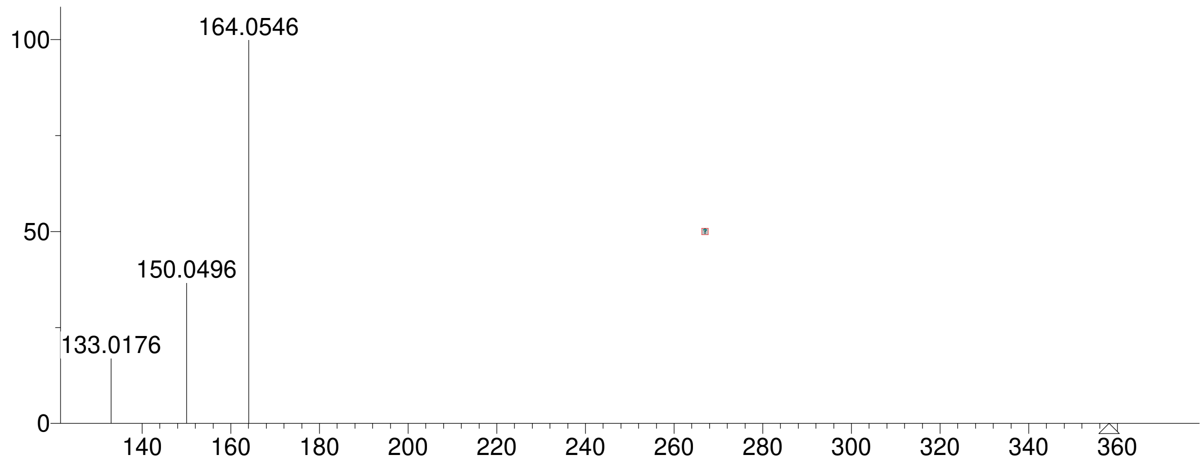

Hit 1 : Formylciprofloxacin [M-H]- HCD 57V P=358.1
C18H18FN3O4; MF: 0; RMF: 33; Prob 100.0%; CAS: 93594-39-9; Lib: nist_msms; ID: 439514.

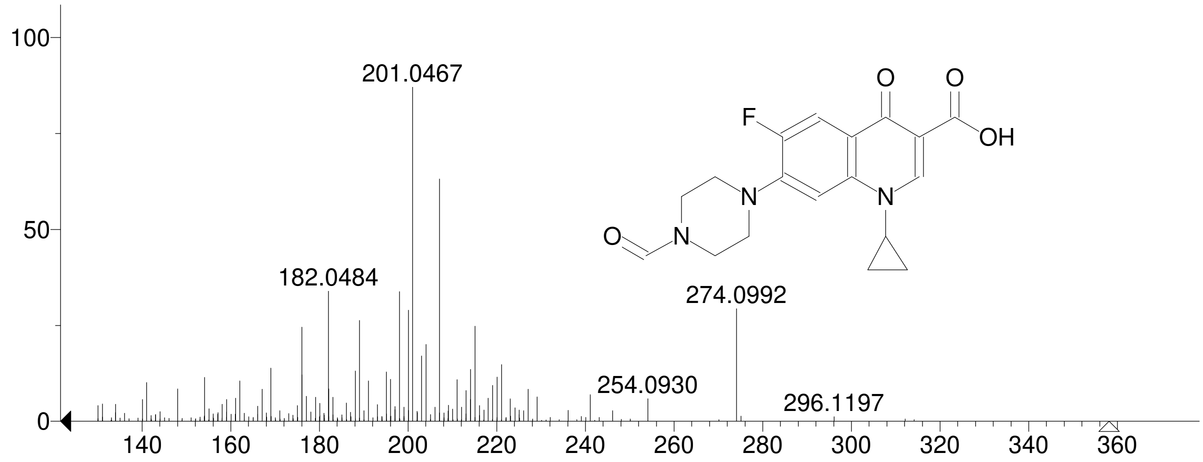

Unknown: Unknown (2.45_459.0745m/z)
Compound in Library Factor = 0

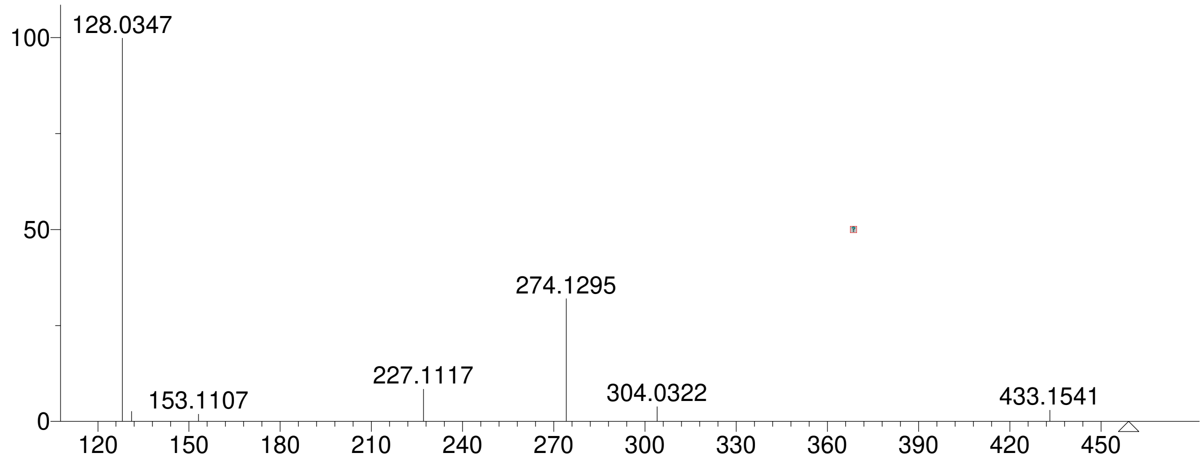

Hit 1 : 3-[(2S,3R,4S,5S,6R)-6-[[(2R,3R,4R)-3,4-dihydroxy-4-(hydroxymethyl)oxolan-2-yl]oxymethyl]-3,4,5-trihydro
C17H24O12; MF: 0; RMF: 67; Prob 100.0%; Lib: mona-export-vf-npl_ltq; ID: 4683.

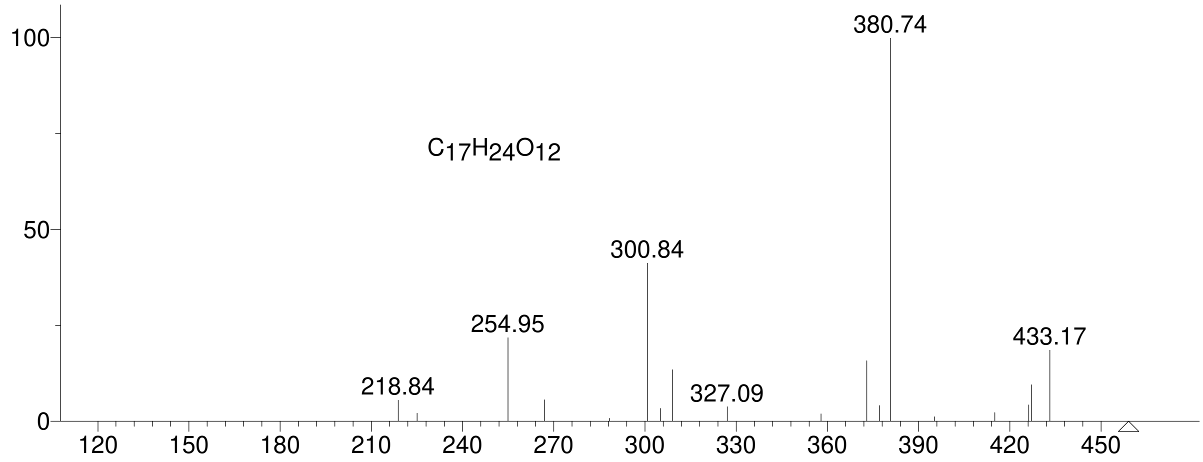

Unknown: Unknown (2.45_485.1873m/z)
Compound in Library Factor = 0

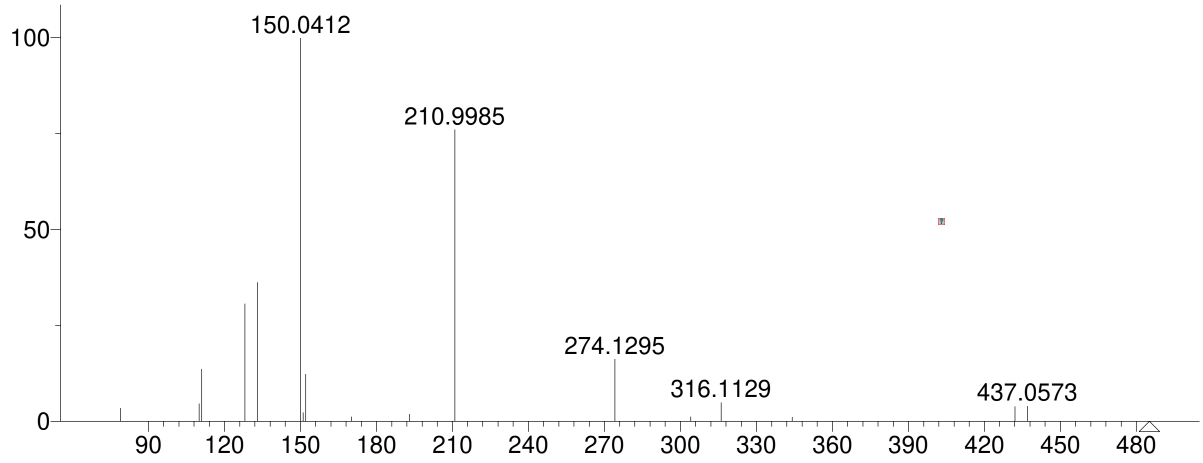

Unknown: Unknown (2.45_549.0886m/z)
Compound in Library Factor = -3273

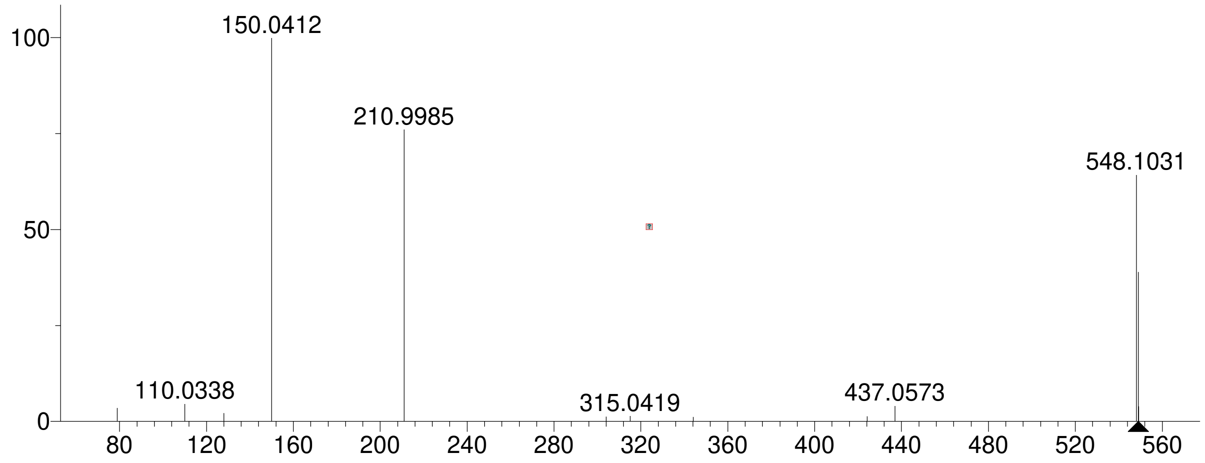

Hit 1 : 5-(3-(2-(7-Chloroquinolin-2-yl)ethenyl)phenyl)-8-dimethylcarbamyl-4,6-dithiaoctanoic acid [M+Cl]- HCD 1
C26H27ClN2O3S2; MF: 6; RMF: 161; Prob 20.3%; CAS: 115104-28-4; Lib: nist_msms; ID: 538815.

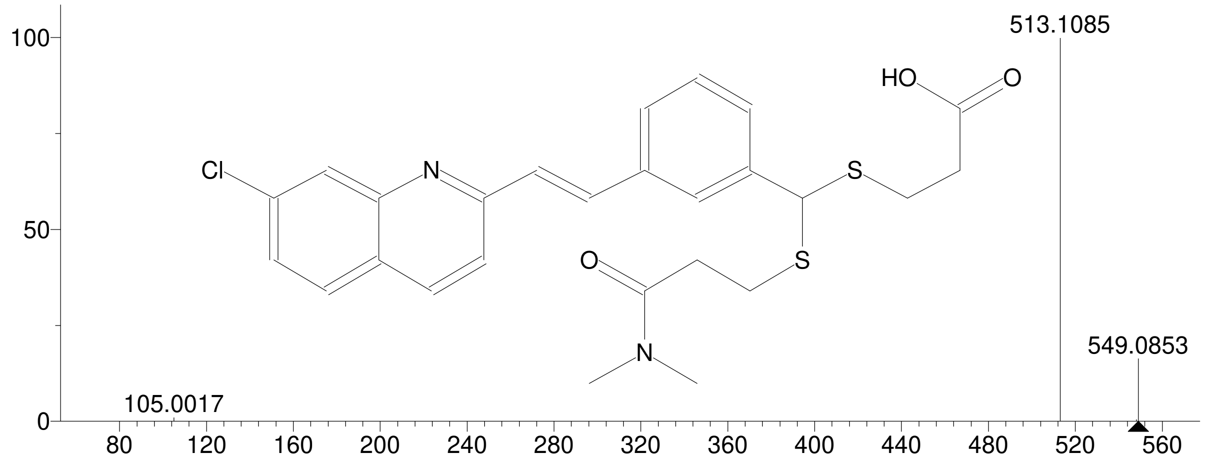

Hit 2 : Quercetin-3-(6''-malonyl)-Glucoside [M-H]- 5-60V P=549.1
C24H22O15; MF: 0; RMF: 80; Prob 15.9%; Lib: mona-export-lc-ms-ms_negative_mode; ID: 8837.

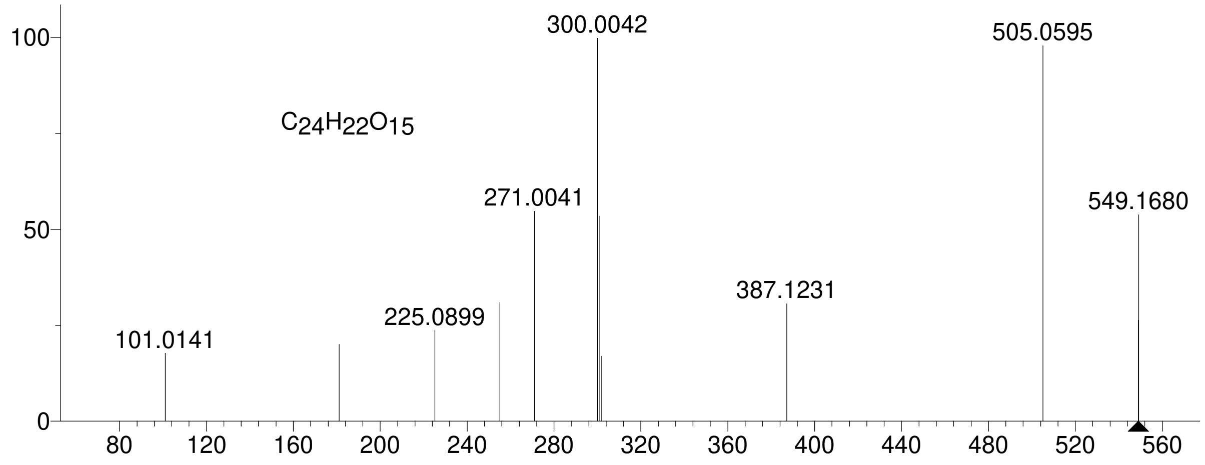

Hit 3 : Quercetin-3-(6"-malonyl)-Glucoside; LC-ESI-QTOF; MS2; CE [M-H]- P=549.1
C24H22O15; MF: 0; RMF: 75; Prob 15.9%; Lib: msms-neg-massbank; ID: 4722.

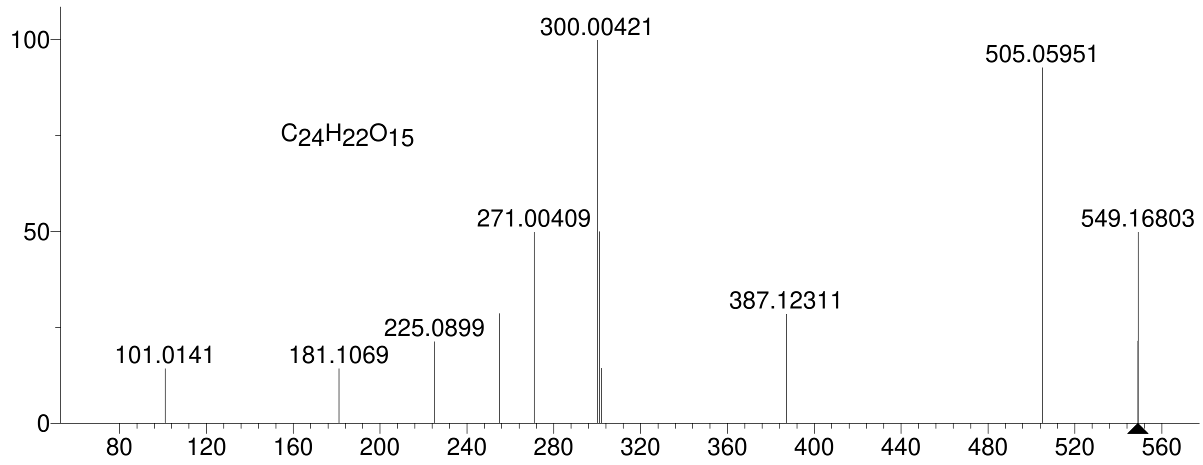

Hit 4 : Quercetin 3-O-malonylglucoside [M-H]- 10 P=549.1
C24H22O15; MF: 0; RMF: 32; Prob 15.9%; Lib: mona-export-lc-ms-ms_negative_mode; ID: 27259.

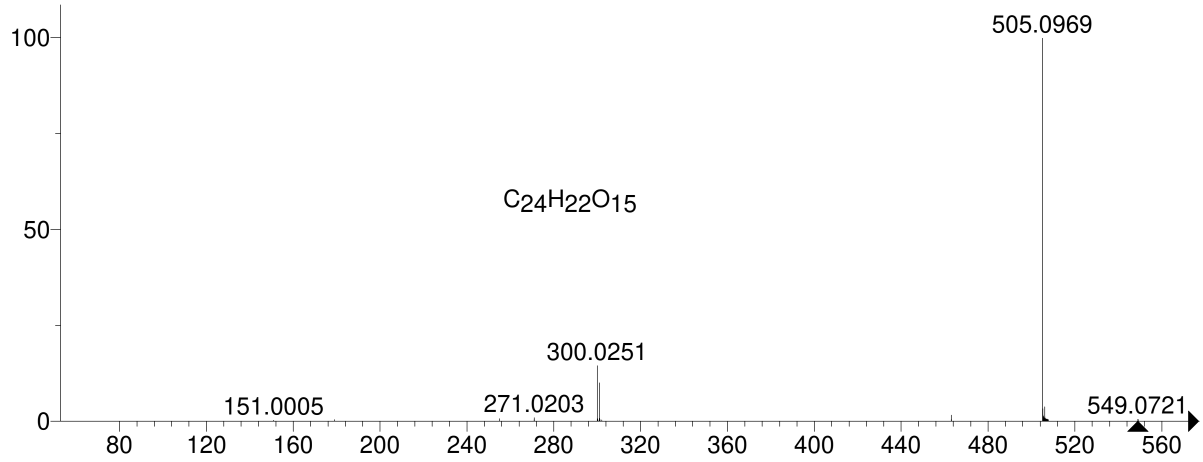

Hit 5 : Flavonol base + 4O, O-MalonylHex [M-H]- 6V P=549.1
C24H22O15; MF: 0; RMF: 21; Prob 15.9%; Lib: anthocyanins mona; ID: 6952.

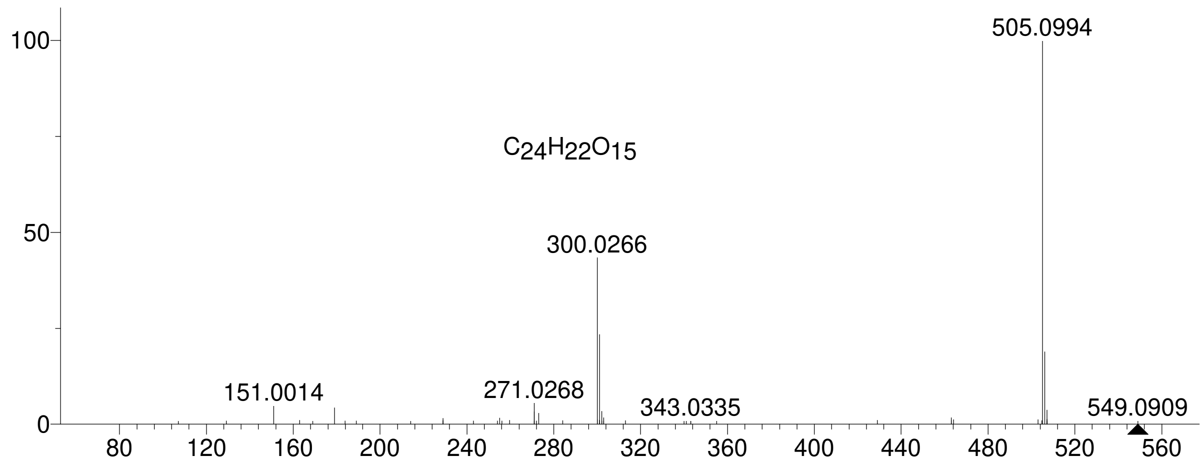

** Search Report Page 1 of 1 **

Unknown: Unknown (2.46_302.1233m/z)
Compound in Library Factor = -2176

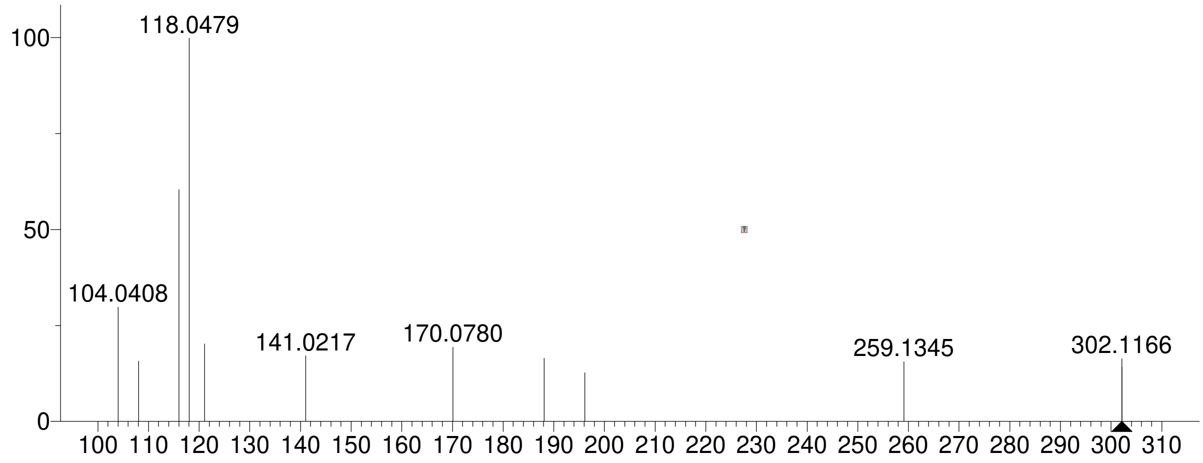

Hit 1 : Pipemidic acid [M-H]- HCD 4V P=302.1
C14H17N5O3; MF: 36; RMF: 518; Prob 68.7%; CAS: 51940-44-4; Lib: nist_msms; ID: 492769.

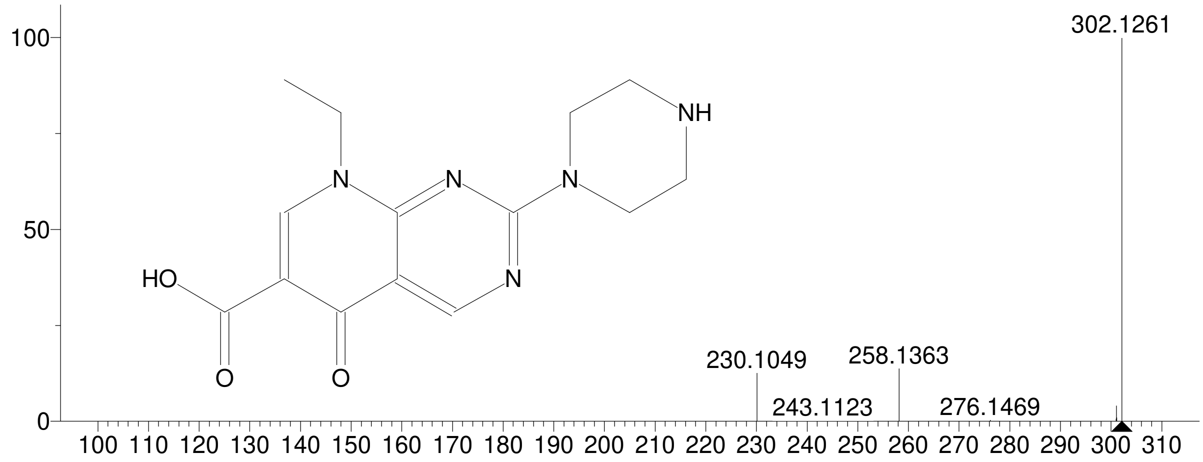

Hit 2 : p-Tolyl-β-D-glucuronide [M+NH4]+ HCD 6V P=302.1
C13H16O7; MF: 16; RMF: 232; Prob 31.3%; CAS: 17680-99-8; Lib: nist_msms; ID: 299054.

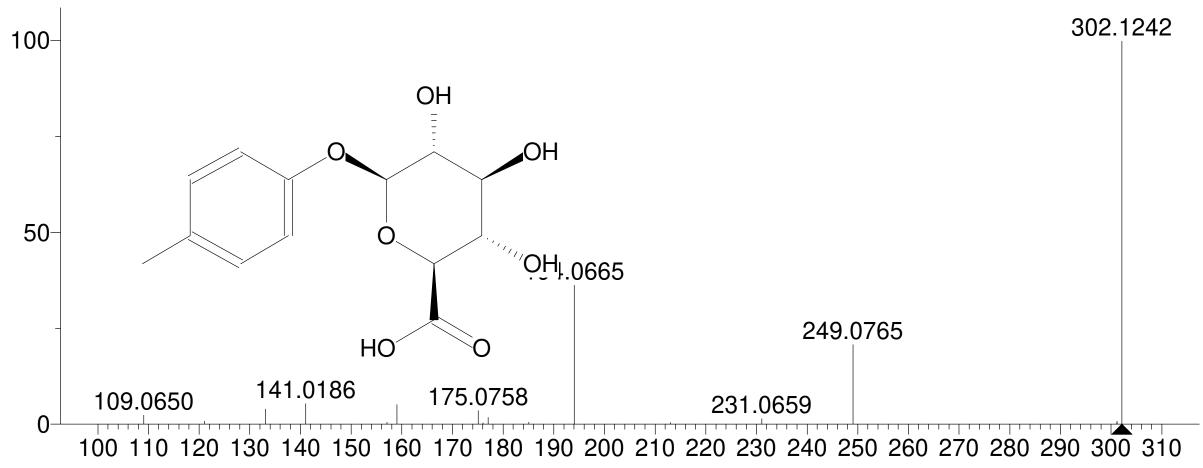

Unknown: Unknown (2.46_389.1183m/z)
Compound in Library Factor = 0

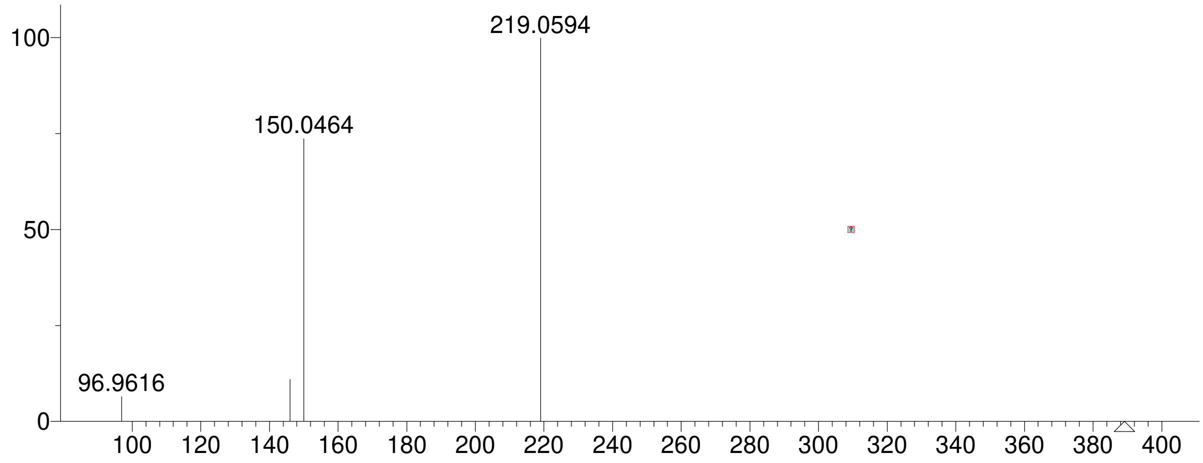

Unknown: Unknown (2.46_587.1247m/z)
Compound in Library Factor = 0

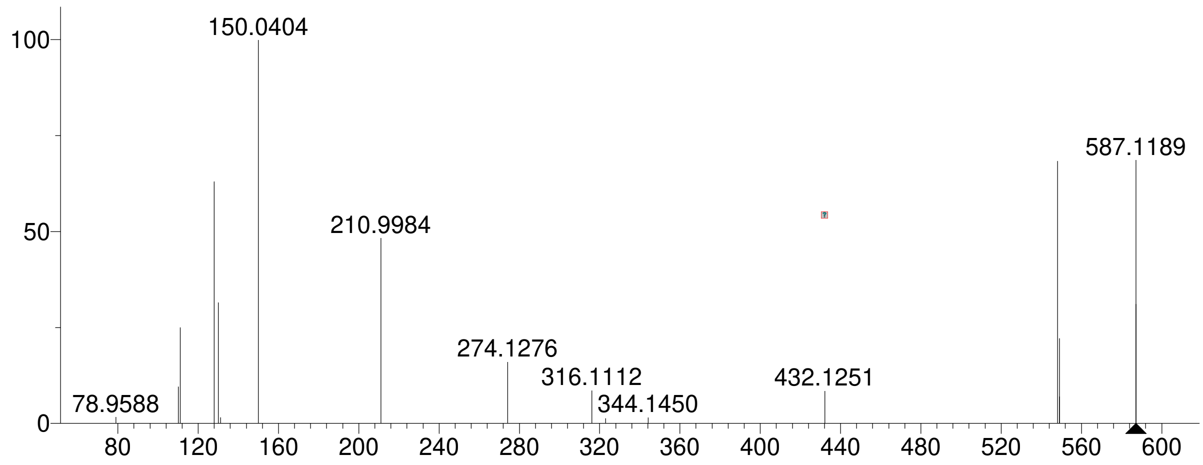

Unknown: Unknown (2.48_316.1028m/z)
Compound in Library Factor = -3273

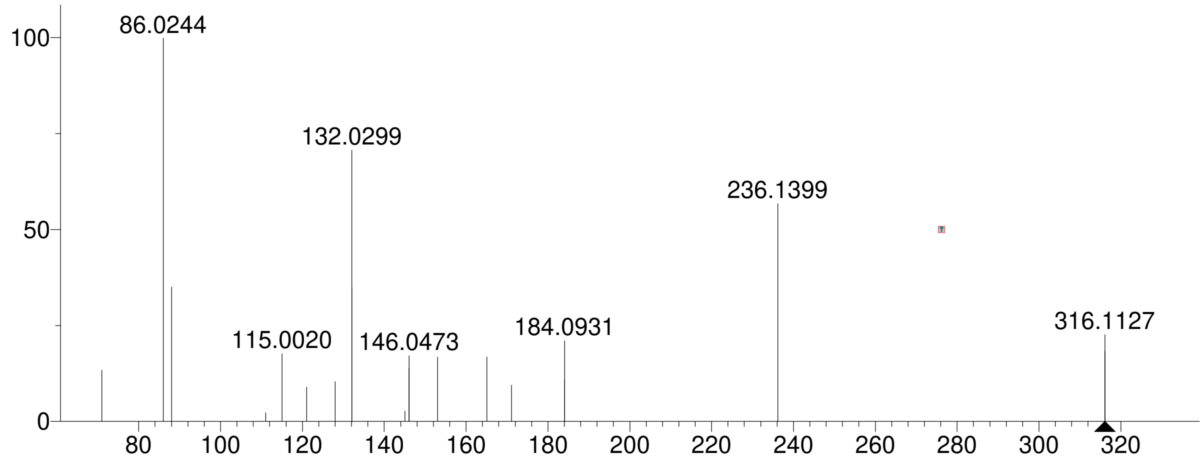

Hit 1 : Topramezone [M+H]+=>334.1=>316.1 IT 35% P=316.1
C16H17N3O5S; MF: 1; RMF: 203; Prob 4.72%; CAS: 210631-68-8; Lib: nist_msms; ID: 541837.

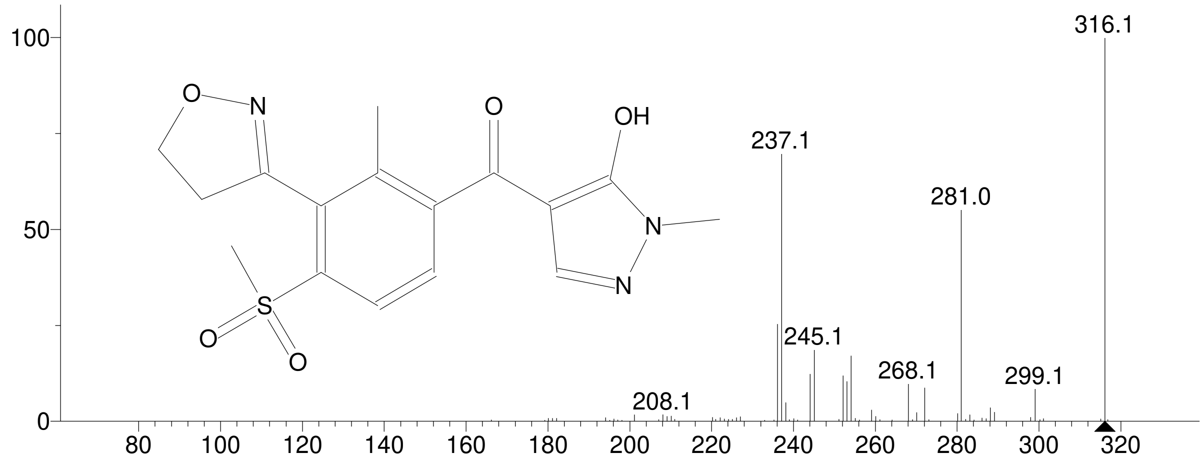

Hit 2 : Dipyridamole [M+H-C3H8O2]+=>399.2=>316.1 IT 35% P=316.1
C24H40N8O4; MF: 0; RMF: 148; Prob 4.53%; CAS: 58-32-2; Lib: nist_msms; ID: 314354.

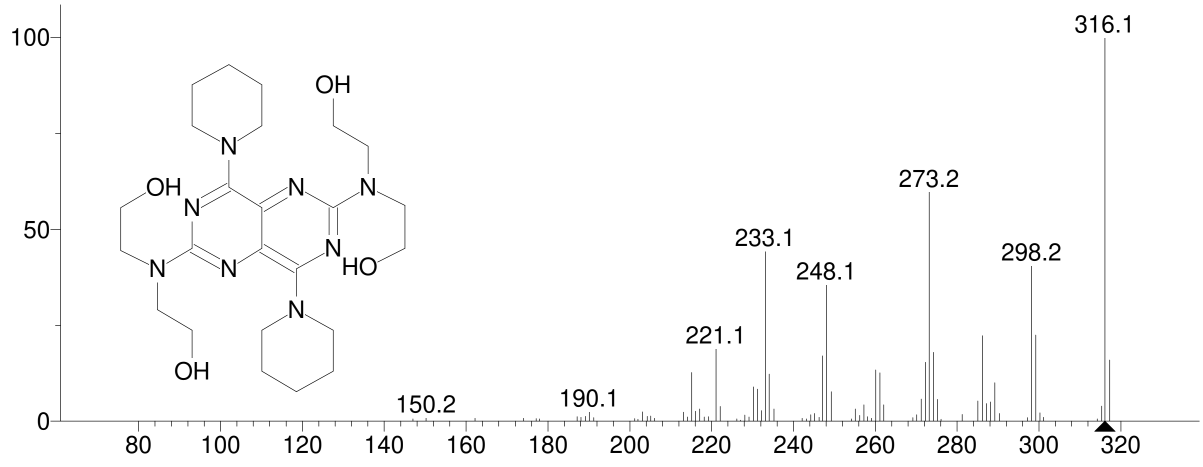

Hit 3 : Caylin-2 [M+H]+=>507.1=>316.1 IT 35% P=316.1
C32H30F6N4O4; MF: 0; RMF: 132; Prob 4.53%; CAS: 1392830-09-9; Lib: nist_msms; ID: 417382.

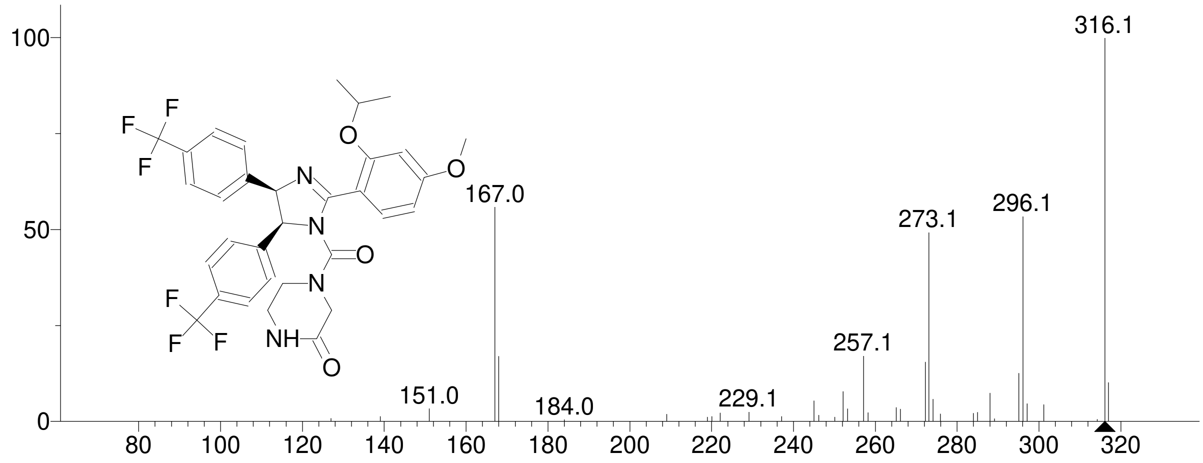

Hit 4 : Celastramycin A [M-H-HCl]-=>316.1 IT 35% P=316.1
C17H18Cl3NO3; MF: 0; RMF: 127; Prob 4.53%; CAS: 491600-94-3; Lib: nist_msms; ID: 376374.

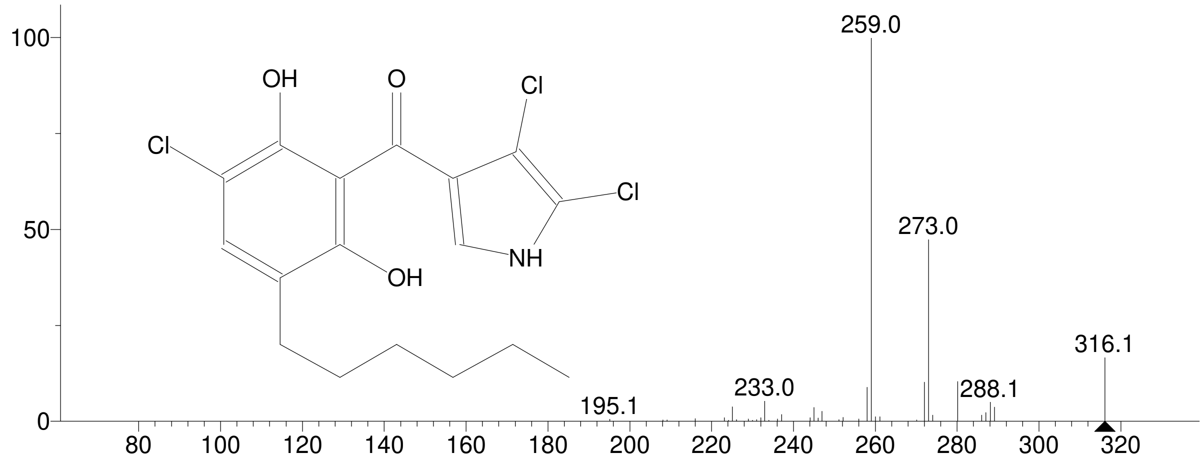

Hit 5 : N-Acetylmuramic Acid [M+Na]+ 35HCD P=316.1
C11H19NO8; MF: 0; RMF: 109; Prob 4.53%; Lib: mona-export-vf-npl_qexactive; ID: 27070.

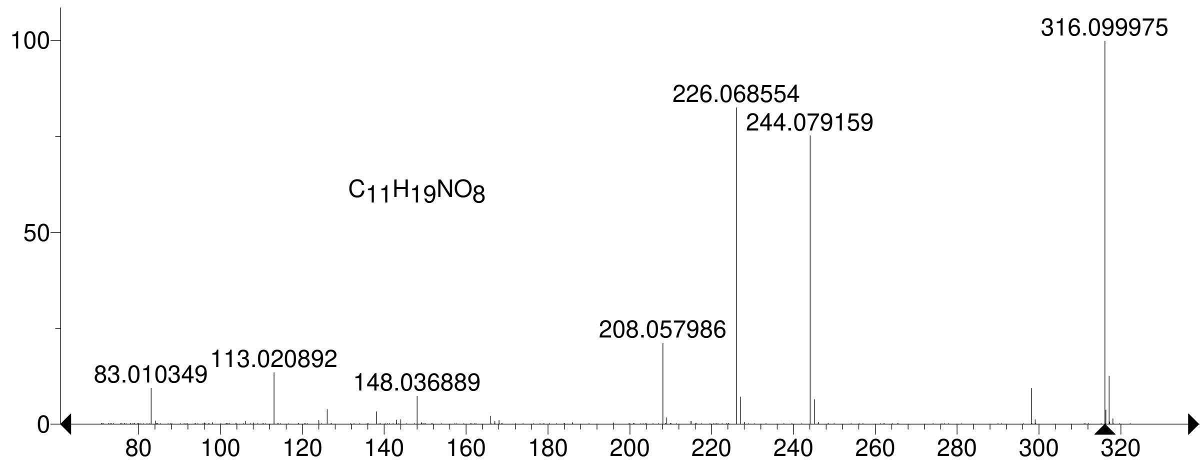

** Search Report Page 1 of 1 **

Unknown: Unknown (2.48_446.1391m/z)
Compound in Library Factor = 0

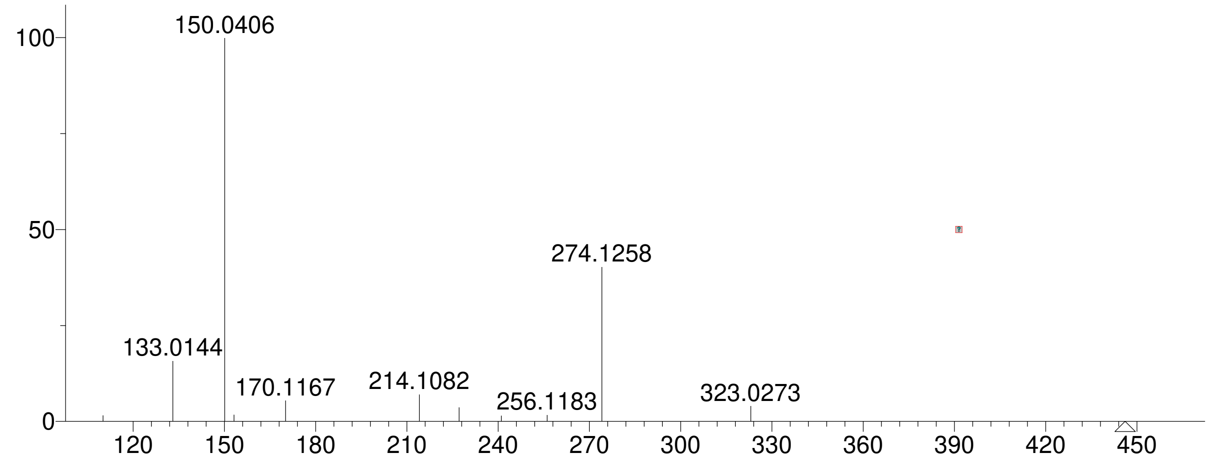

Hit 1 : Benzoic acid, 4-[4-[[5-(4,5-dimethyl-2-nitrophenyl)-2-furanyl]methylene]-4,5-dihydro-3-methyl-5-oxo-1H-pyr
C24H19N3O6; MF: 0; RMF: 100; Prob 50.0%; CAS: 328968-36-1; Lib: nist_msms; ID: 247875.

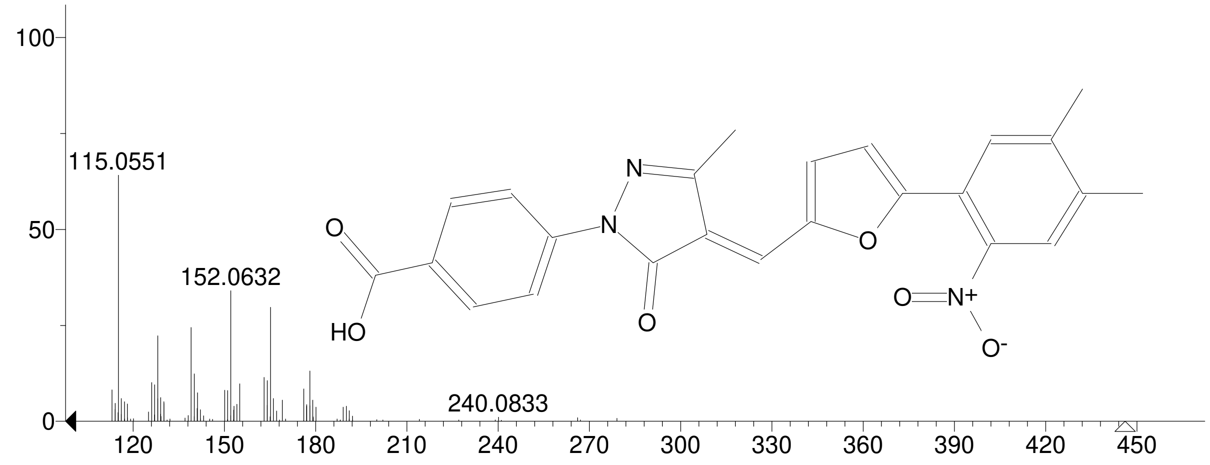

Hit 2 : Dehydroaripiprazole [M+H]+ HCD 66V P=446.1
C23H25Cl2N3O2; MF: 0; RMF: 3; Prob 50.0%; CAS: 129722-25-4; Lib: nist_msms; ID: 323099.

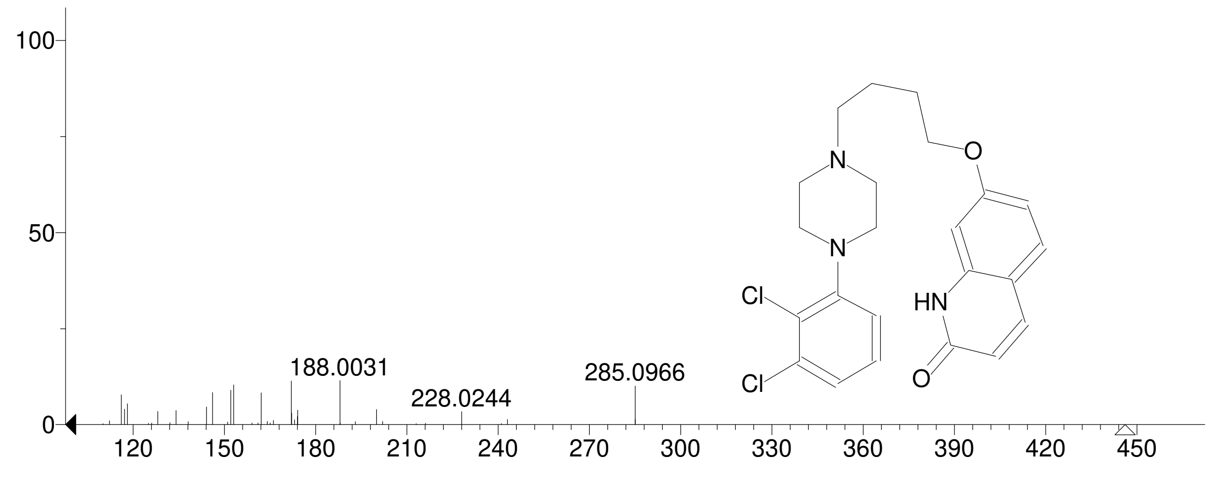

Unknown: Unknown (2.49_346.1605m/z)
Compound in Library Factor = 0

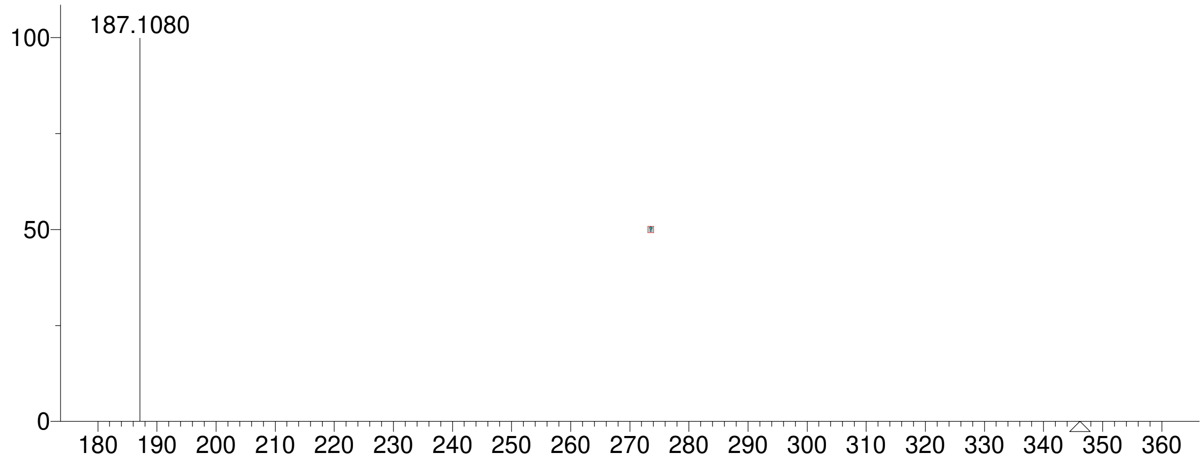

Unknown: Unknown (2.49_359.1554m/z)
Compound in Library Factor = 0

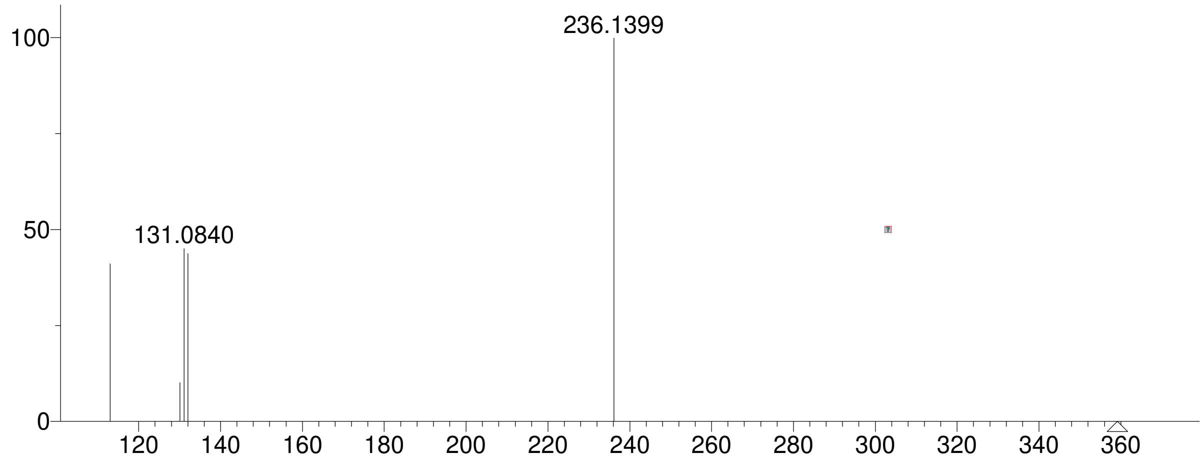

Hit 1 : Ala-Gly-Asp-Val [M-H]- HCD 57eV P=359.2
C14H24N4O7; MF: 0; RMF: 59; Prob 100.0%; Lib: nist_msms2; ID: 72487.

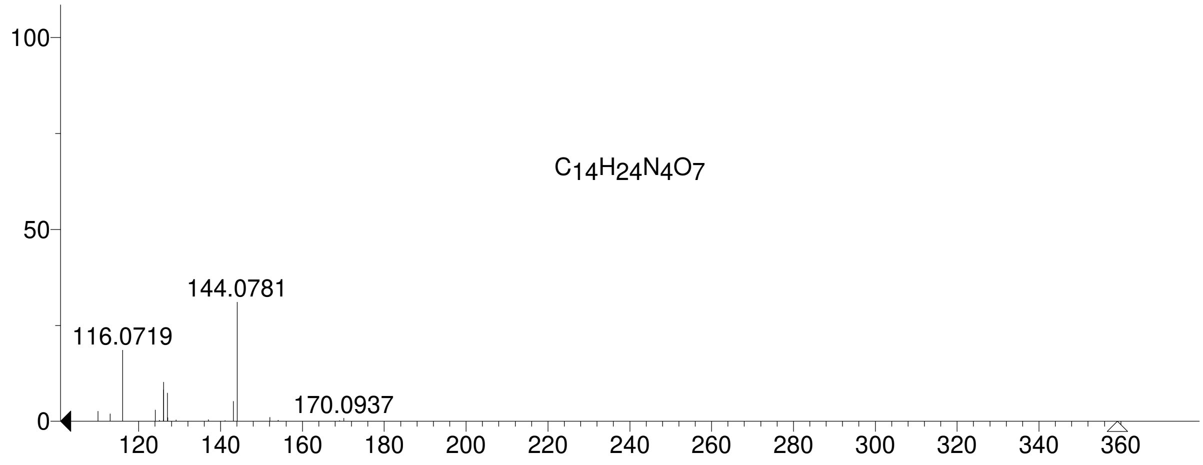

Unknown: Unknown (2.49_508.1087m/z)
Compound in Library Factor = 0

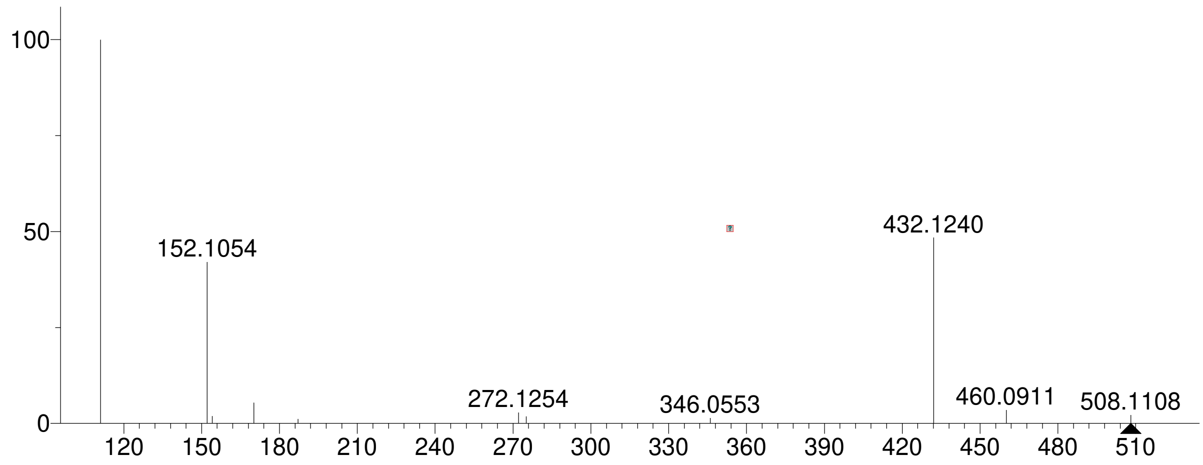

** Search Report Page 1 of 1 **

Unknown: Unknown (2.50_274.1273m/z)
Compound in Library Factor = 0

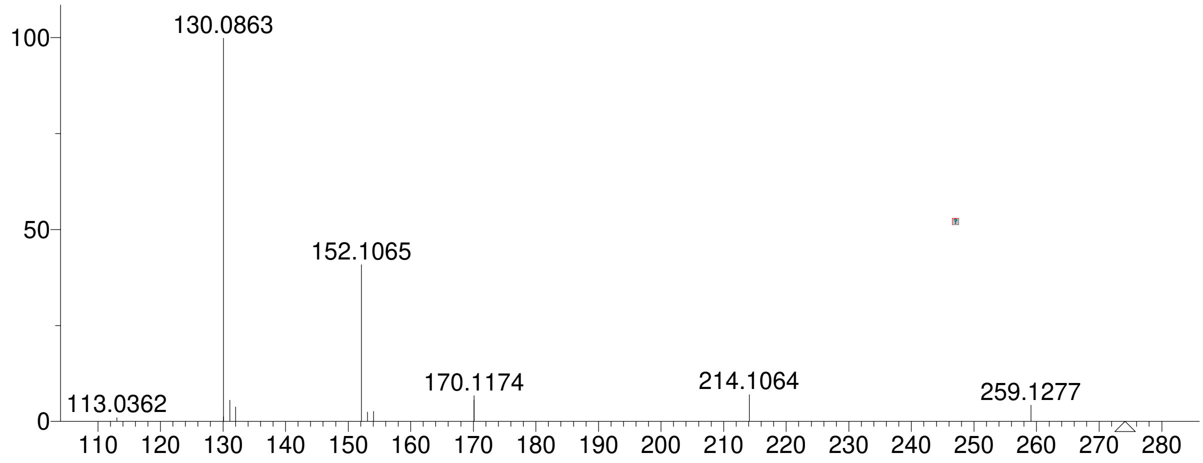

** Search Report Page 1 of 1 **

Unknown: Unknown (2.50_454.1920m/z)
Compound in Library Factor = 0

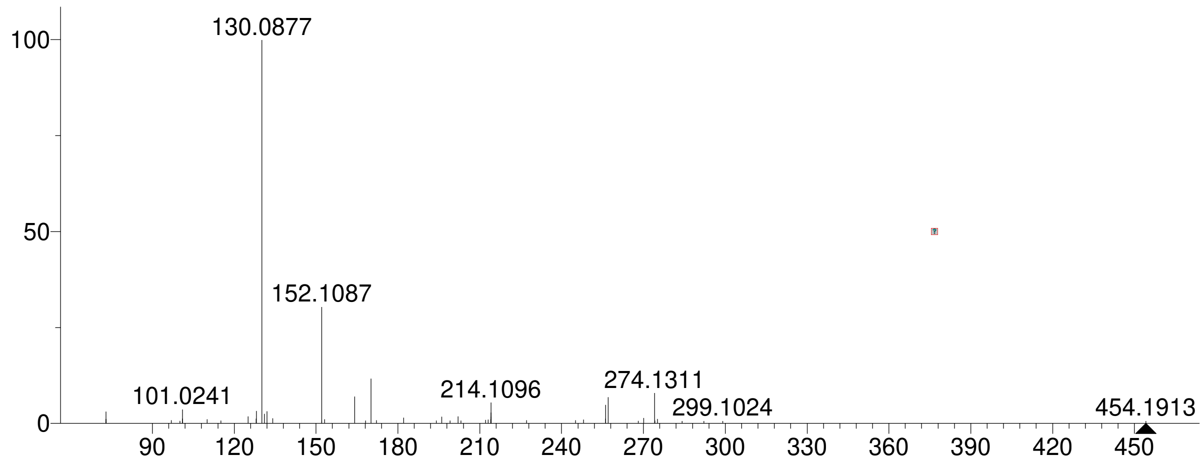

Hit 1 : Acetic acid, 2-[4-[(5,6-diphenyl-2-pyrazinyl)(1-methylethyl)amino]butoxy]- [M+Cl]- HCD 18V P=454.2
C25H29N3O3; MF: 0; RMF: 11; Prob 100.0%; CAS: 475085-57-5; Lib: nist_msms; ID: 551808.

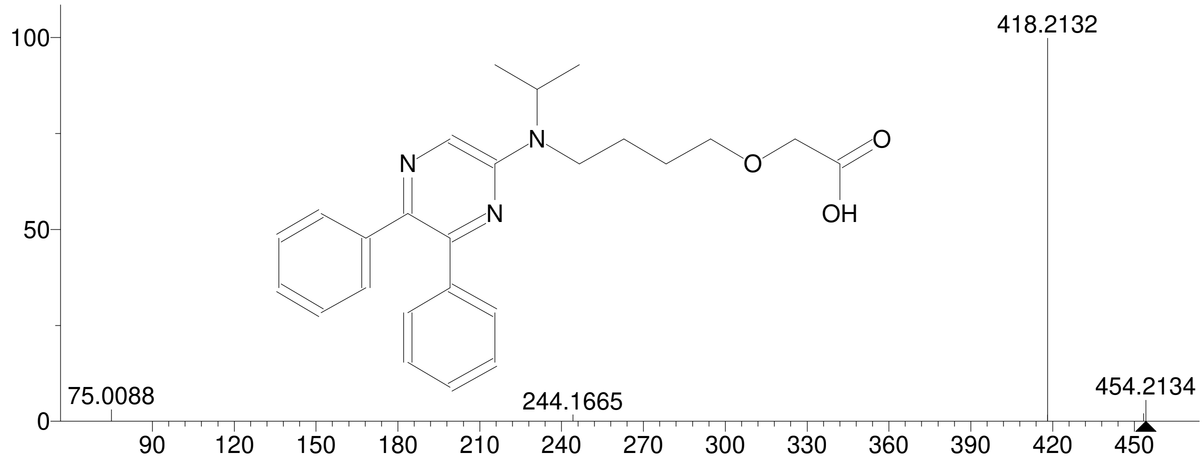

Unknown: Unknown (2.52_237.0860m/z)
Compound in Library Factor = 0

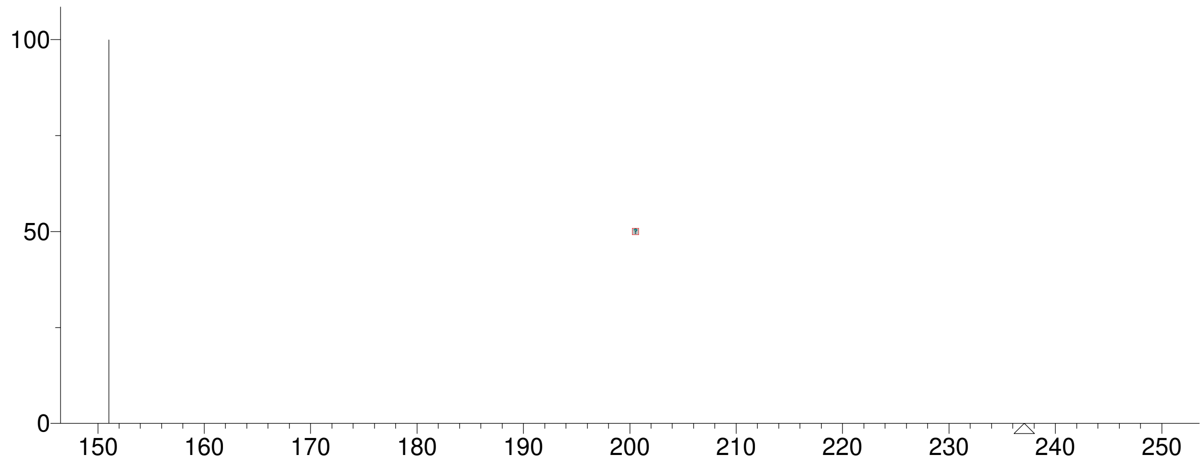

Unknown: Unknown (2.52_330.1186m/z)
Compound in Library Factor = -472

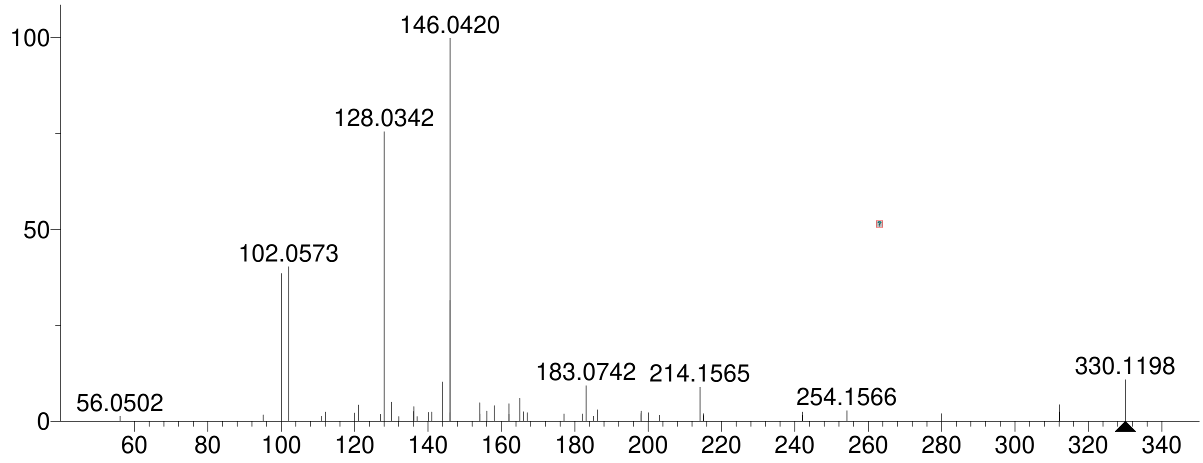

Hit 1 : Dopamine 4-β-D-glucuronide [M+H]+ HCD 9V P=330.1
C14H19NO8; MF: 136; RMF: 240; Prob 96.3%; CAS: 38632-24-5; Lib: nist_msms; ID: 210217.

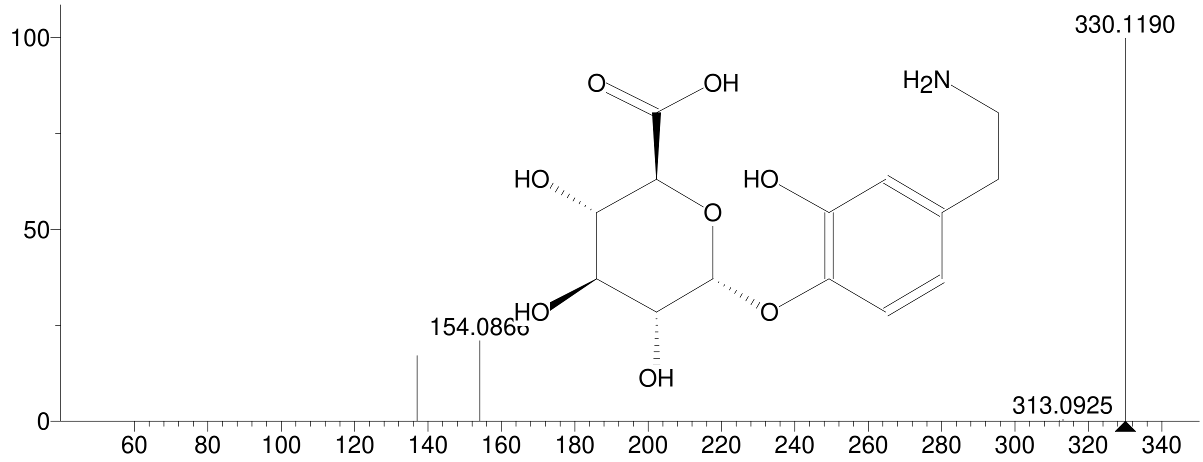

Hit 2 : Nelfinavir [M+H-C14H26N2O]+ HCD 16V P=330.1
C32H45N3O4S; MF: 2; RMF: 172; Prob 3.72%; CAS: 159989-64-7; Lib: nist_msms; ID: 367188.

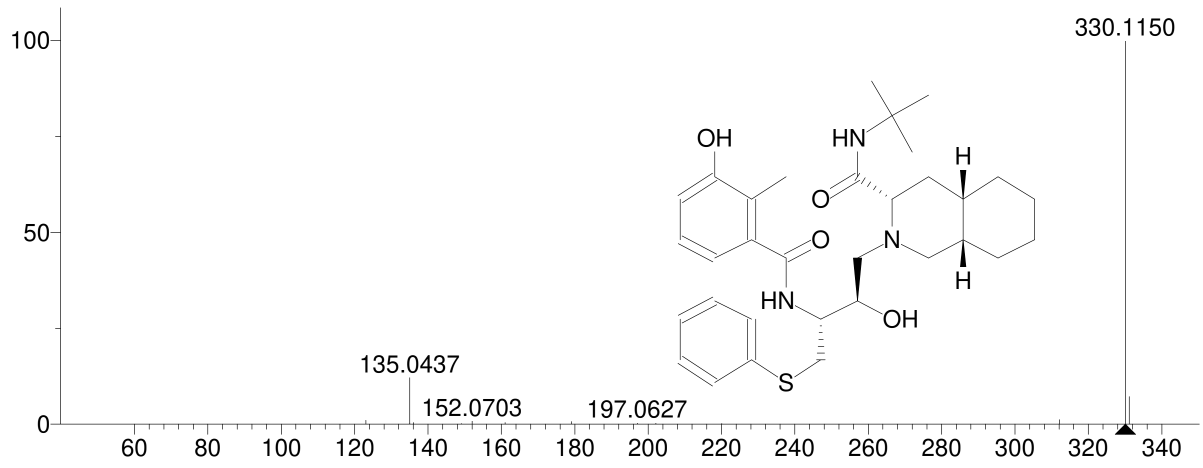

** Search Report Page 1 of 1 **

Unknown: Unknown (2.52_393.1852m/z)
Compound in Library Factor = -3273

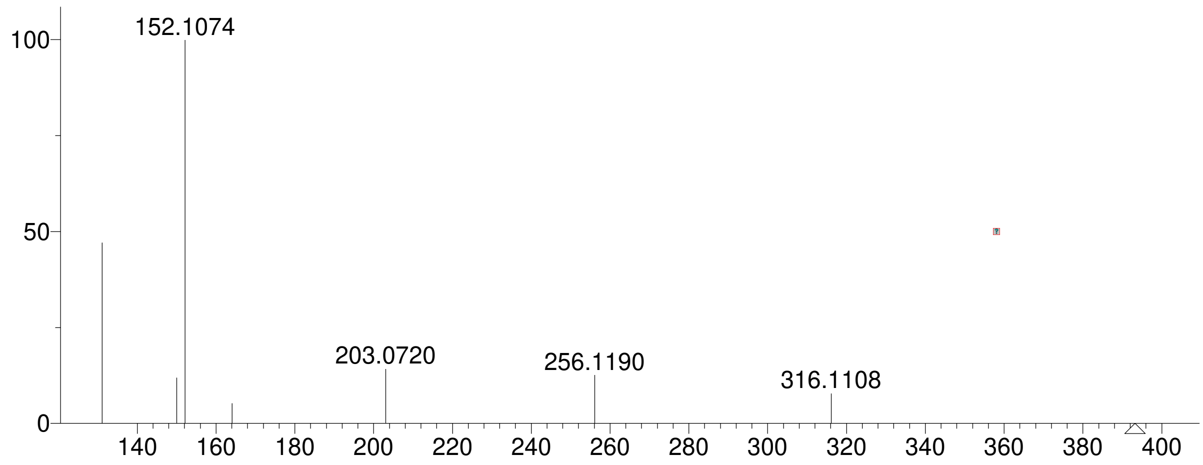

Hit 1 : Simmondsin [M+NH4]+ HCD 27V P=393.2
C16H25NO9; MF: 1; RMF: 63; Prob 51.0%; CAS: 51771-52-9; Lib: nist_msms; ID: 460444.

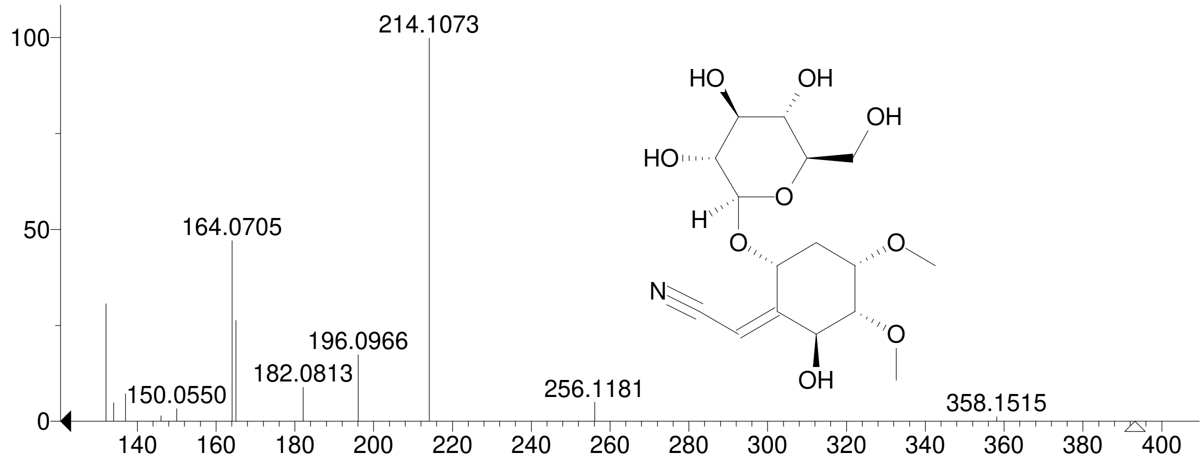

Hit 2 : Dovitinib [M+H]+ HCD 47V P=393.2
C21H21FN6O; MF: 0; RMF: 17; Prob 49.0%; CAS: 405169-16-6; Lib: nist_msms; ID: 381641.

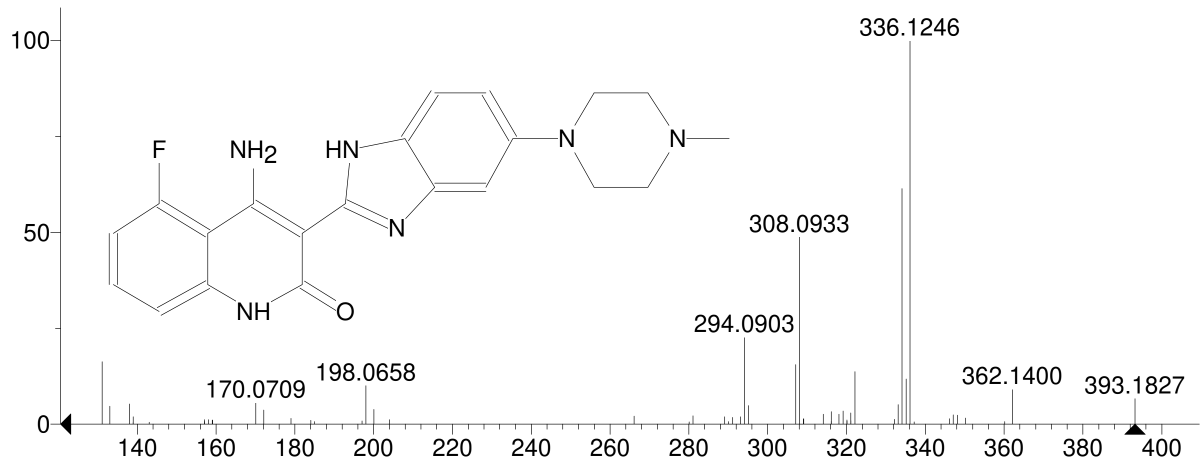

** Search Report Page 1 of 1 **

Unknown: Unknown (2.52_432.1250m/z)
Compound in Library Factor = 0

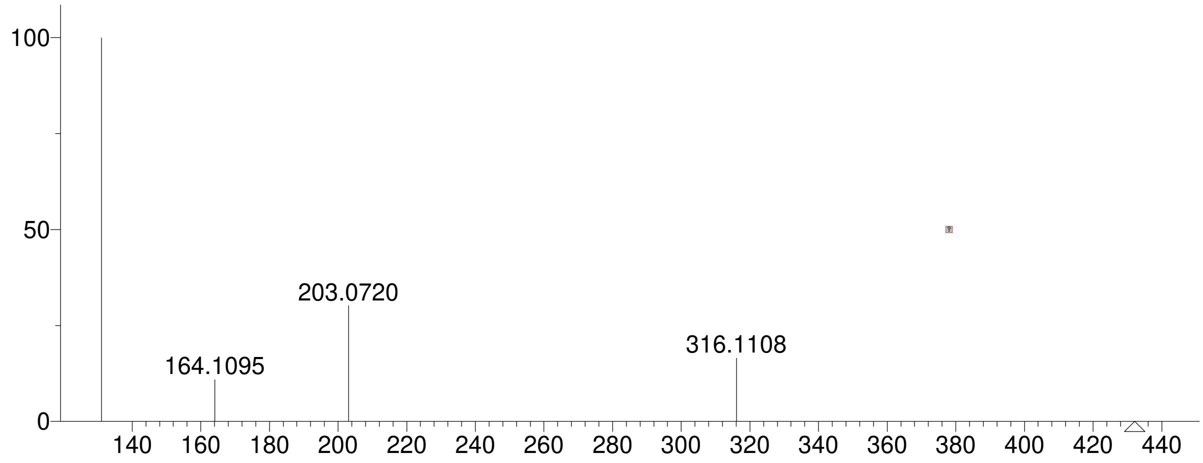

** Search Report Page 1 of 1 **

Unknown: Unknown (2.52_572.1141m/z)
Compound in Library Factor = -3273

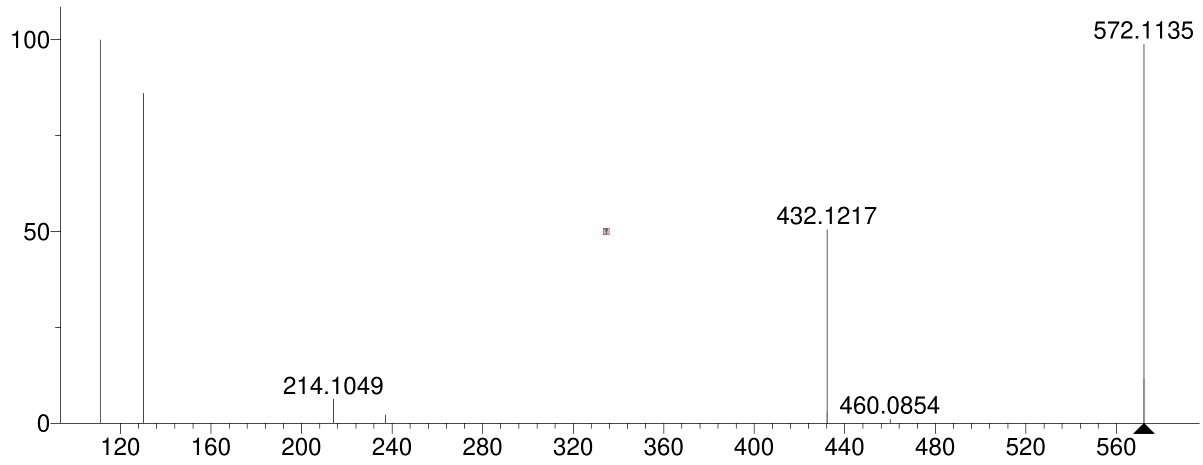

Hit 1 : (Ala11,D-Leu15)-Orexin B, human [M+5H]5+ HCD 8eV P=572.1
C120H206N44O35S; MF: 1; RMF: 168; Prob 100.0%; CAS: 532932-99-3; Lib: nist_msms2; ID: 45808.

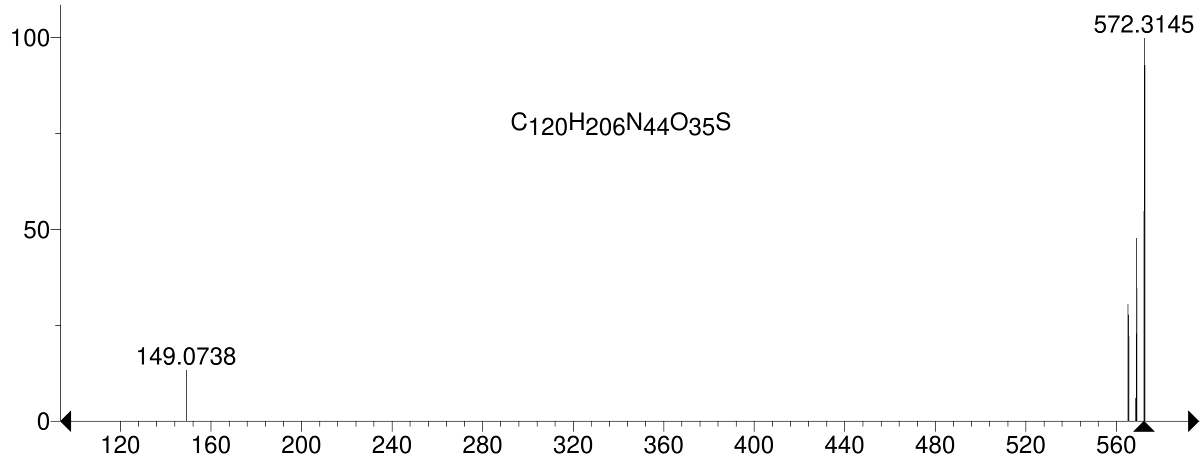

** Search Report Page 1 of 1 **

Unknown: Unknown (2.52_616.2450m/z)
Compound in Library Factor = 0

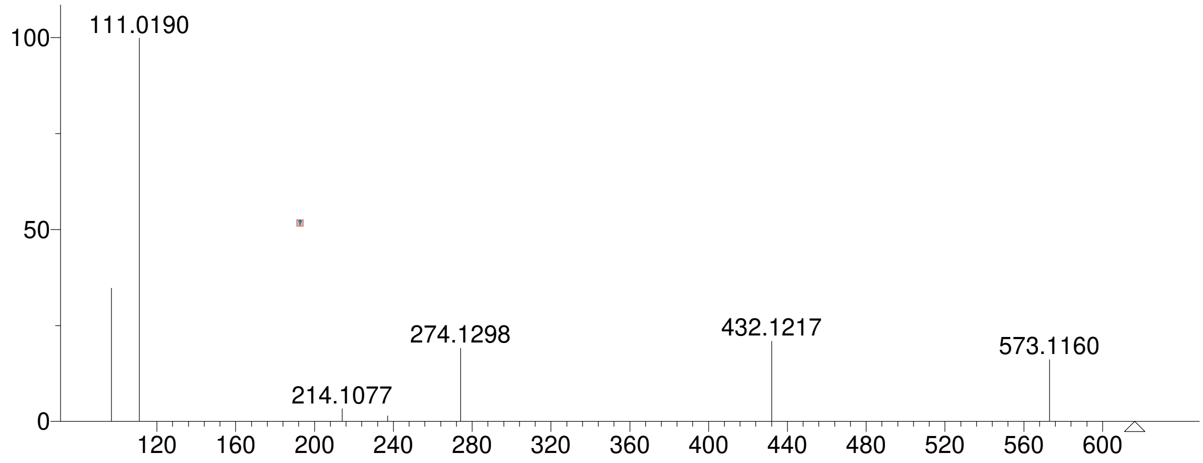

Unknown: Unknown (2.53_373.1231m/z)
Compound in Library Factor = -3273

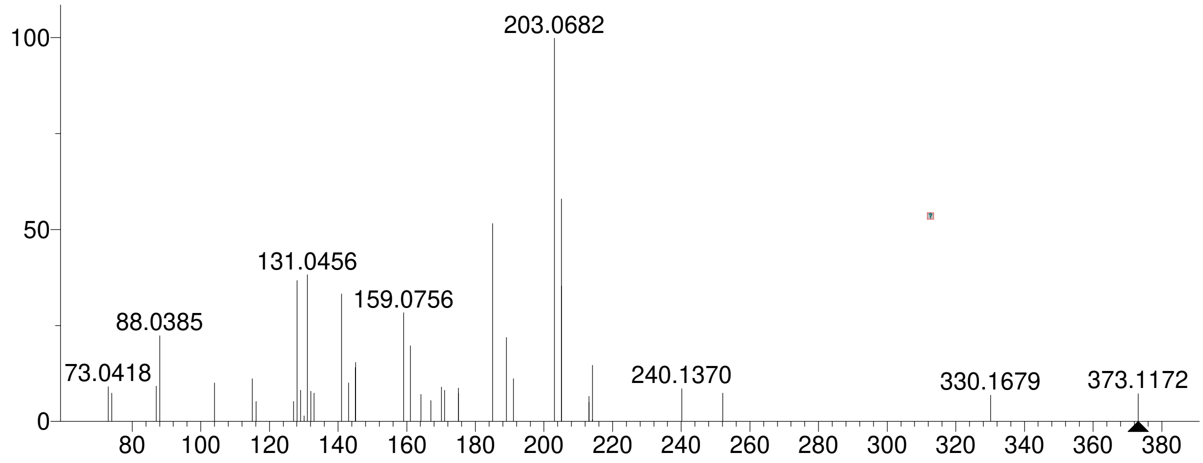

Hit 1 : 2,4-Thiazolidinedione, 5-[[4-[2-(5-ethyl-2-pyridinyl)-2-hydroxyethoxy]phenyl]methyl]- [M+H]+ HCD 14V P:
C19H20N2O4S; MF: 6; RMF: 119; Prob 29.1%; CAS: 101931-00-4; Lib: nist_msms; ID: 443088.

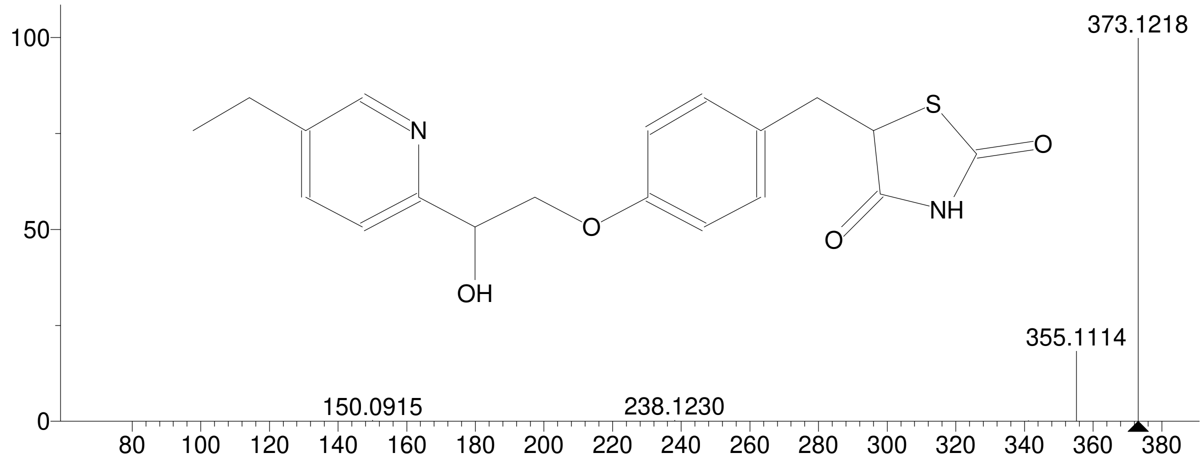

Hit 2 : 2,4-Thiazolidinedione, 5-[[4-[2-[5-(1-hydroxyethyl)-2-pyridinyl]ethoxy]phenyl]methyl]- [M+H]+ HCD 18V P
C19H20N2O4S; MF: 2; RMF: 105; Prob 24.6%; CAS: 146062-44-4; Lib: nist_msms; ID: 443052.

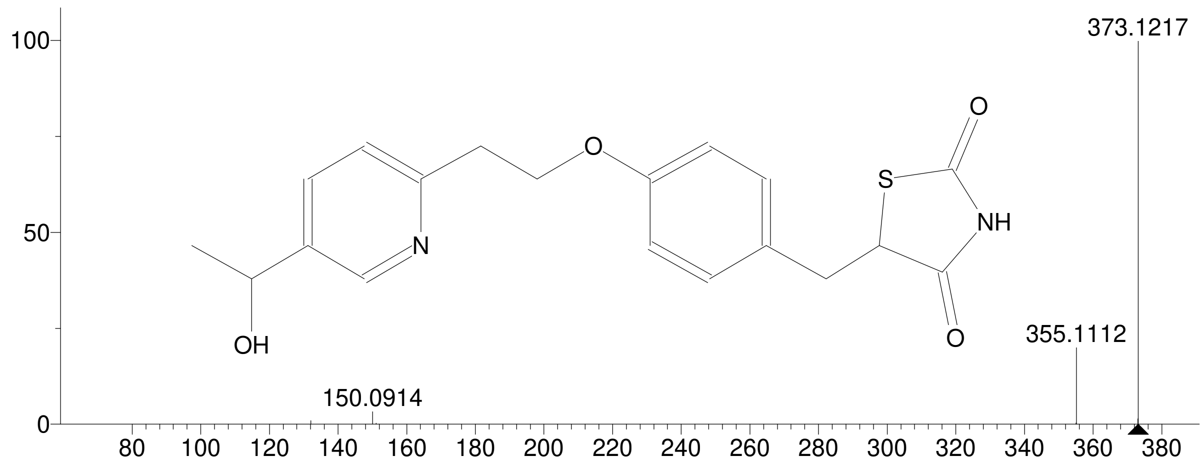

Hit 3 : Dapagliflozin [M+H-2H2O]+ HCD 18V P=373.1
C21H25ClO6; MF: 1; RMF: 43; Prob 23.6%; CAS: 461432-26-8; Lib: nist_msms; ID: 548869.

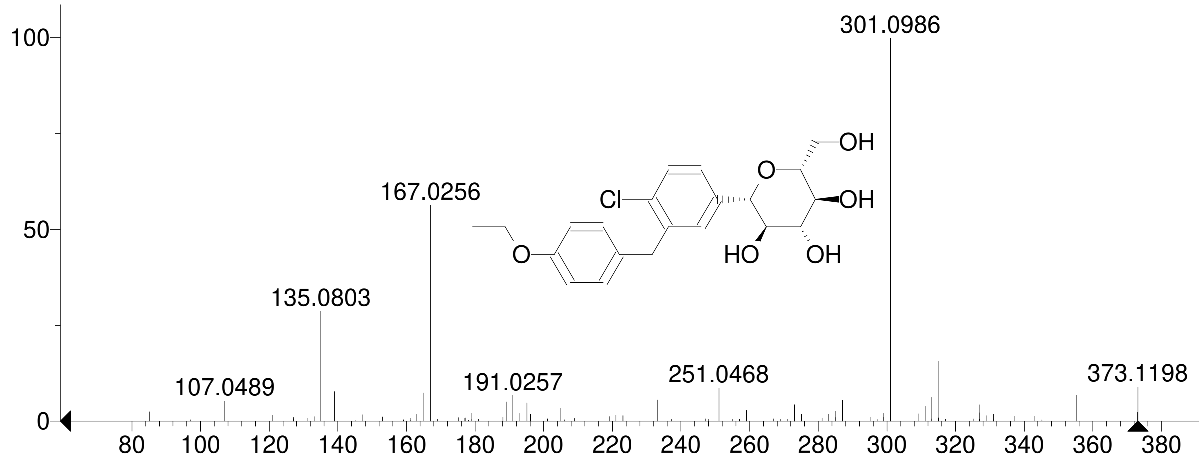

Hit 4 : Gox(8-O-4)G 2 (lignin) [M-H]- P=373.1
C20H22O7; MF: 0; RMF: 38; Prob 22.7%; Lib: phytofrag2018_final_060219; ID: 555.

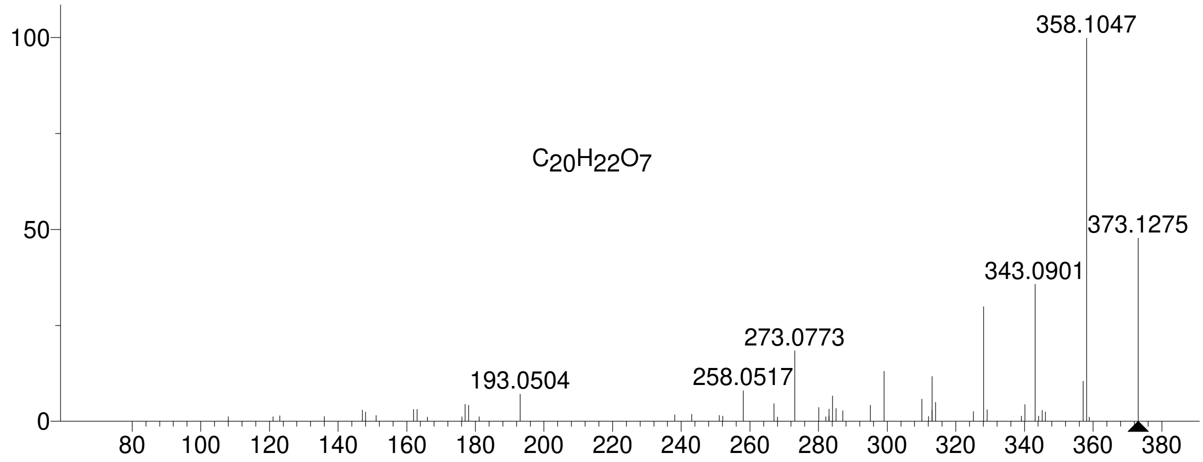

Unknown: Unknown (2.53_415.1819m/z)
Compound in Library Factor = 0

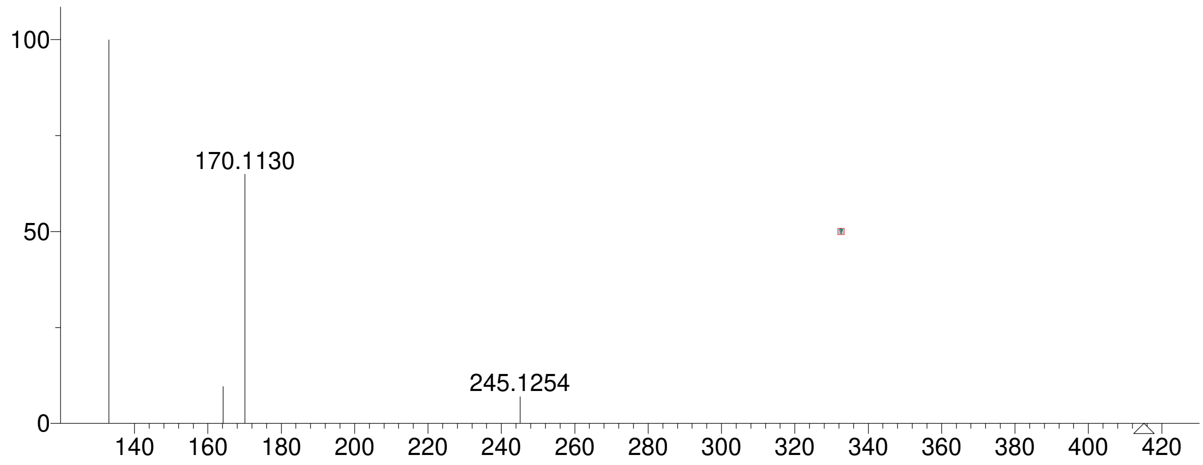

** Search Report Page 1 of 1 **

Unknown: Unknown (2.53_627.1317m/z)
Compound in Library Factor = 0

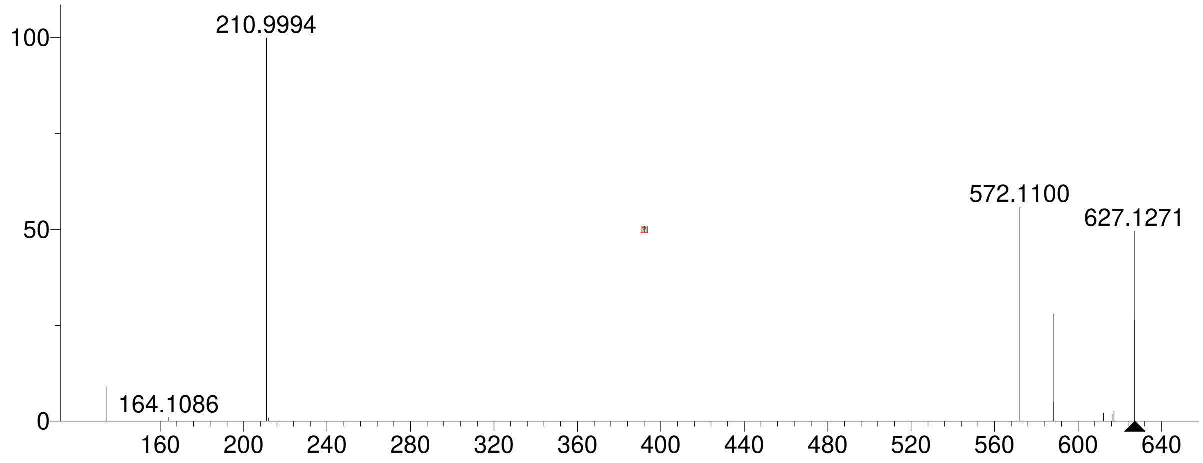

Unknown: Unknown (2.54_348.1113m/z)
Compound in Library Factor = 0

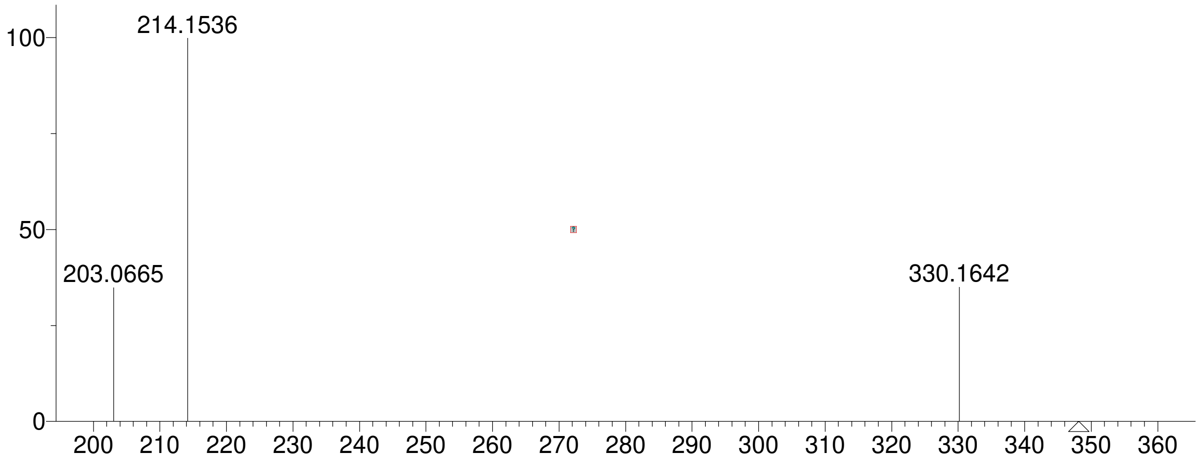

Unknown: Unknown (2.54_430.1438m/z)
Compound in Library Factor = 0

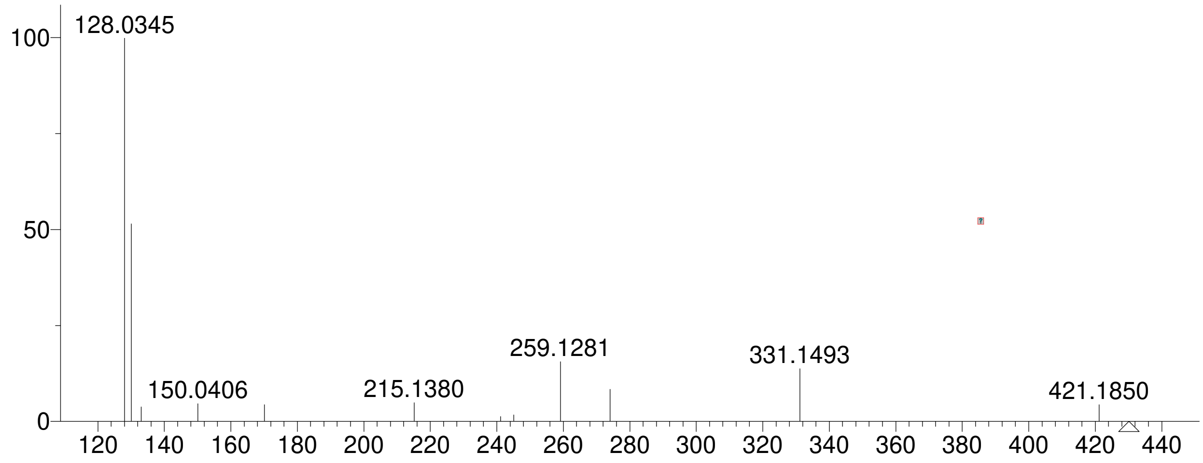

Hit 1 : Tubacin [M+H-C15H20N2O4]+ HCD 137V P=430.1
C41H43N3O7S; MF: 0; RMF: 16; Prob 100.0%; CAS: 537049-40-4; Lib: nist_msms; ID: 262955.

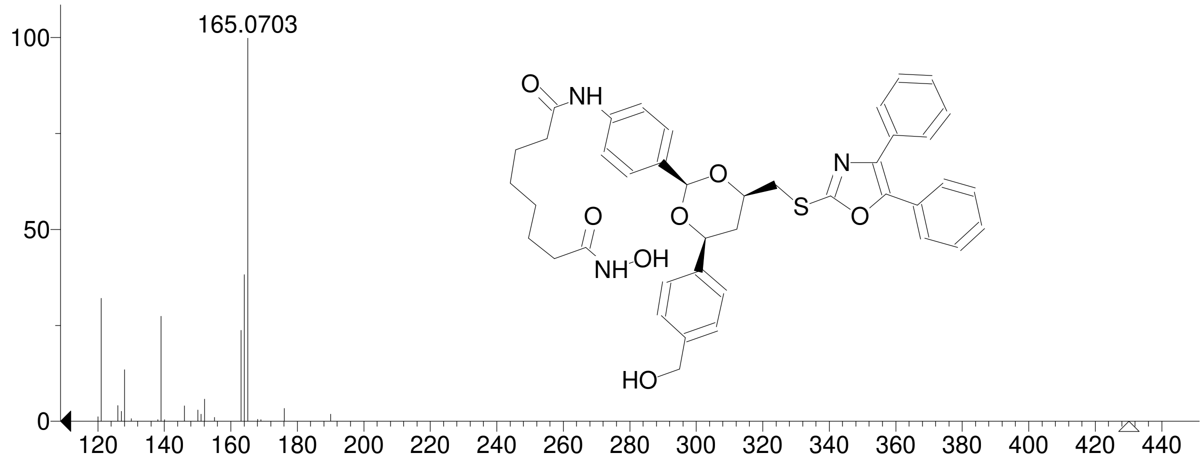

Unknown: Unknown (2.55_389.1211m/z)
Compound in Library Factor = 0

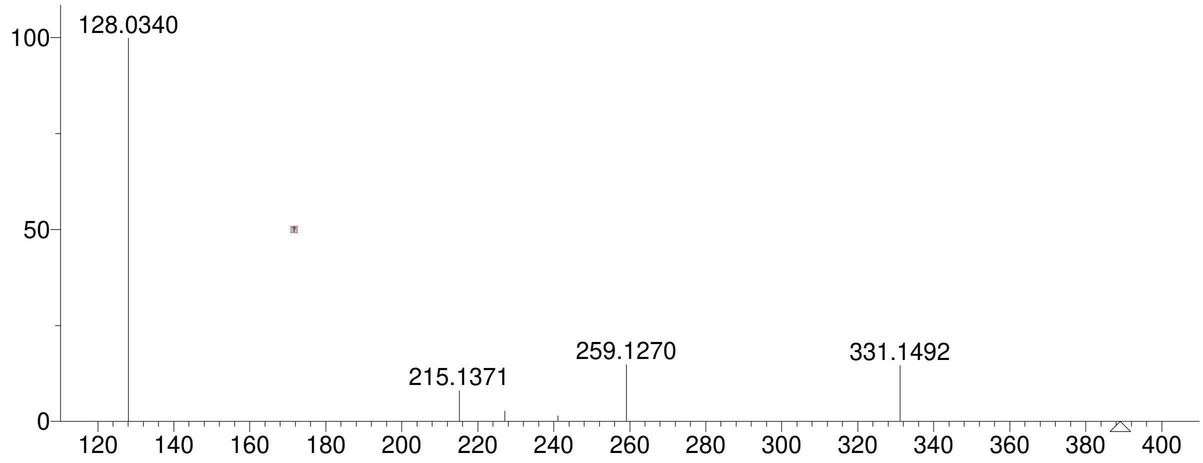

Hit 1 : Ginkgolide B [M+H-2H2O]+ IT-FT 35% P=389.1
C20H24O10; MF: 0; RMF: 52; Prob 20.0%; CAS: 15291-77-7; Lib: nist_msms; ID: 352924.

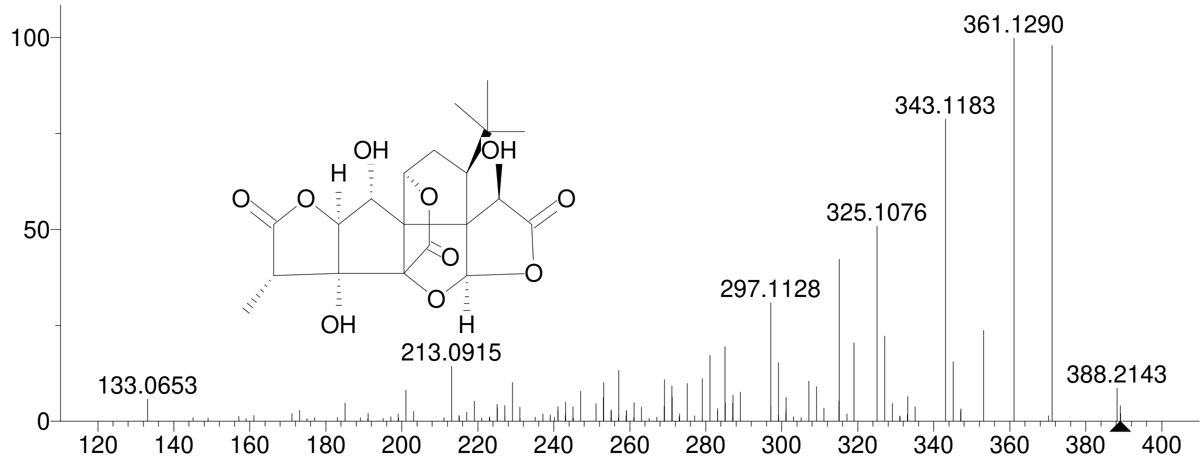

Hit 2 : NCGC00385097-01_C20H22O9_beta-D-Glucopyranoside, 4-hydroxy-2-(hydroxymethyl)phenyl, 6-benzoat
C20H22O9; MF: 0; RMF: 42; Prob 20.0%; Lib: mona-export-gnps; ID: 12625.

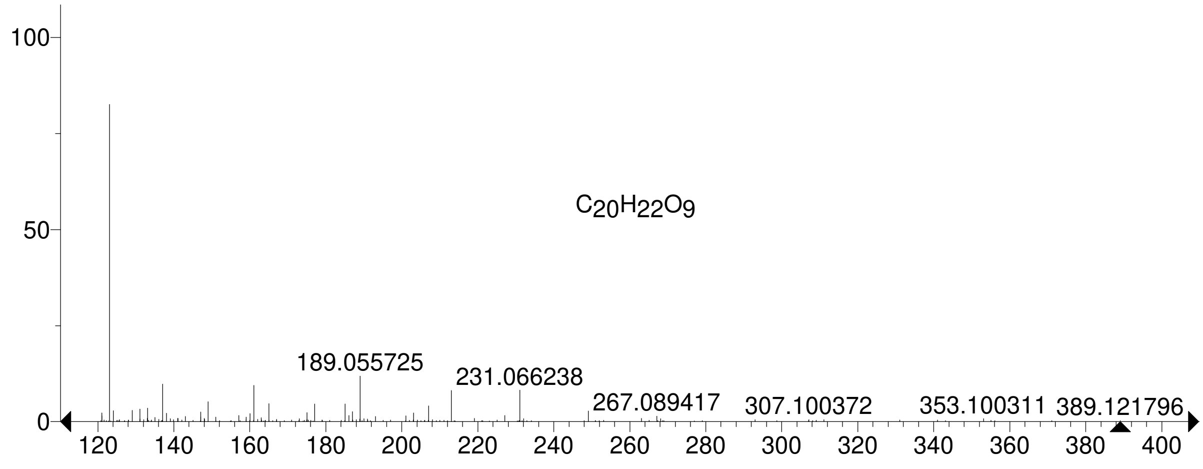

Hit 3 : G(8-O-4)ferulic acid (/Matsuda et al., 2010) [M-H]- P=389.1
C20H22O8; MF: 0; RMF: 11; Prob 20.0%; Lib: phytofrag2018_final_060219; ID: 371.

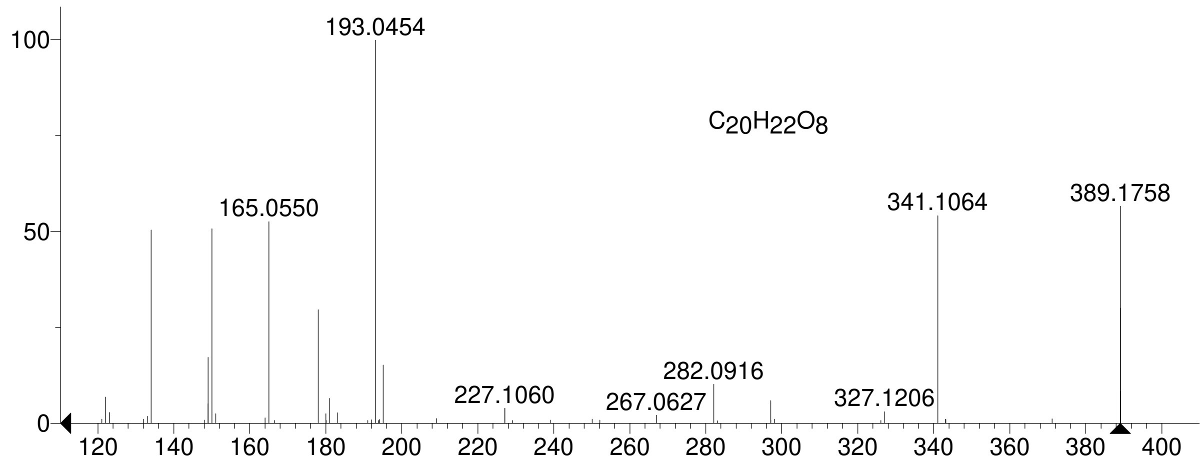

Hit 4 : G(8-O-4)ferulic acid + hexose 4 (fragment) (COc1cc(ccc1O)C(O)C(CO)Oc1ccc(\C=C\C(O)=O)cc1OC) [M-
C20H22O8; MF: 0; RMF: 5; Prob 20.0%; Lib: phytofrag2018_final_060219; ID: 383.

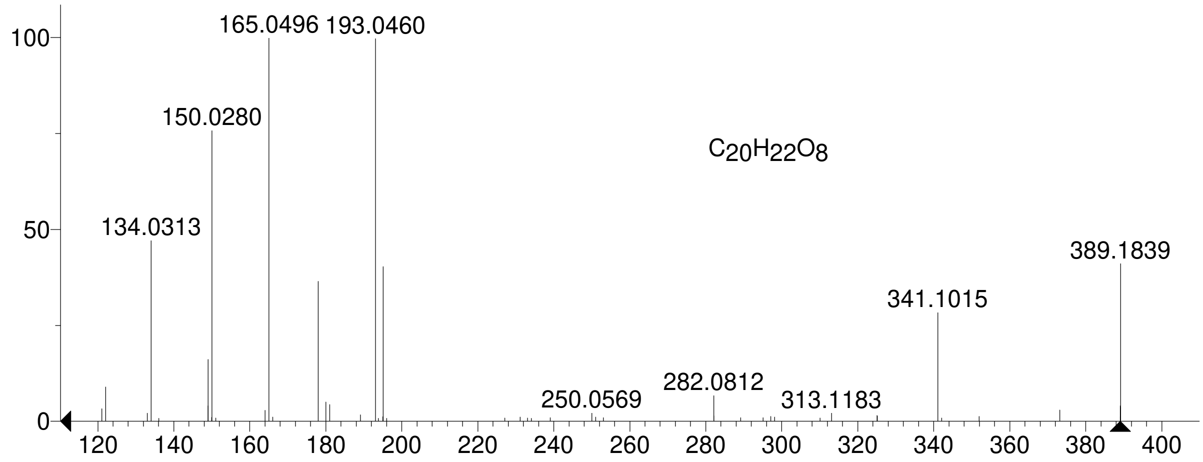

Hit 5 : G(8-O-4)ferulic acid 9 () [M-H]- P=389.1
C20H22O8; MF: 0; RMF: 5; Prob 20.0%; Lib: phytofrag2018_final_060219; ID: 403.

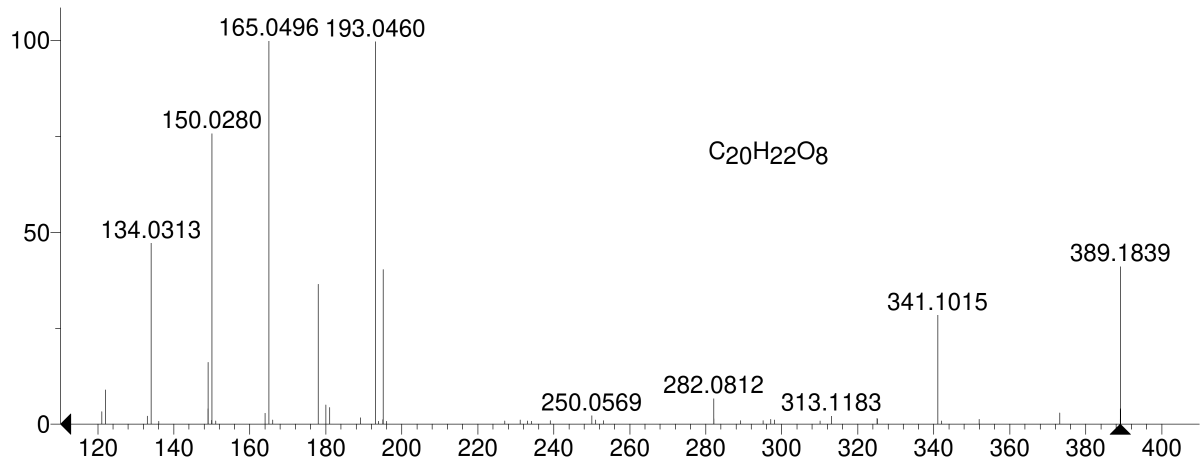

Unknown: Unknown (2.55_611.1352m/z)
Compound in Library Factor = -1309

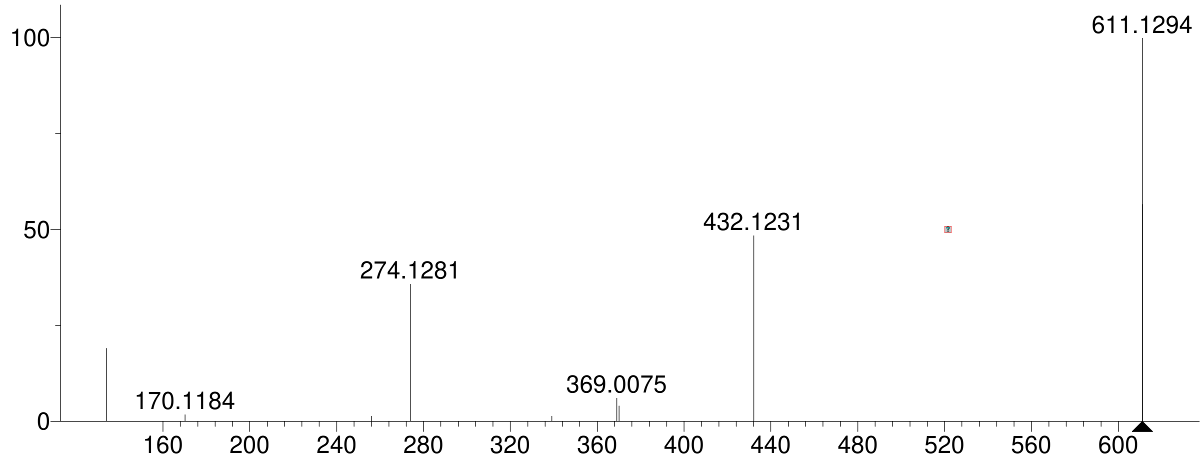

Hit 1 : (-)-Epigallocatechin [2M-H]- HCD 6V P=611.1
C15H14O7; MF: 108; RMF: 622; Prob 58.5%; CAS: 970-74-1; Lib: nist_msms; ID: 235135.

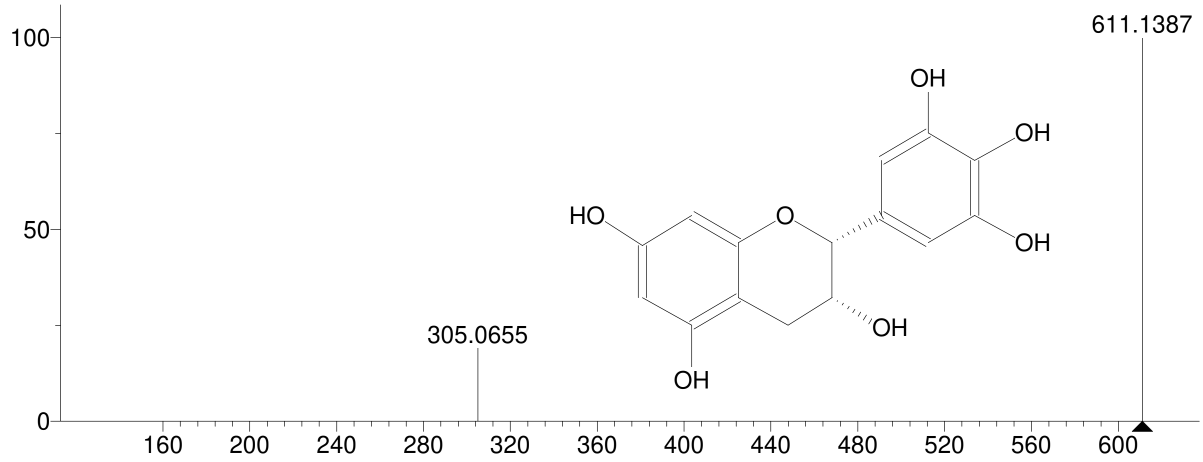

Hit 2 : (-)-Gallocatechin [2M-H]- HCD 6V P=611.1
C15H14O7; MF: 92; RMF: 621; Prob 33.7%; CAS: 3371-27-5; Lib: nist_msms; ID: 235923.

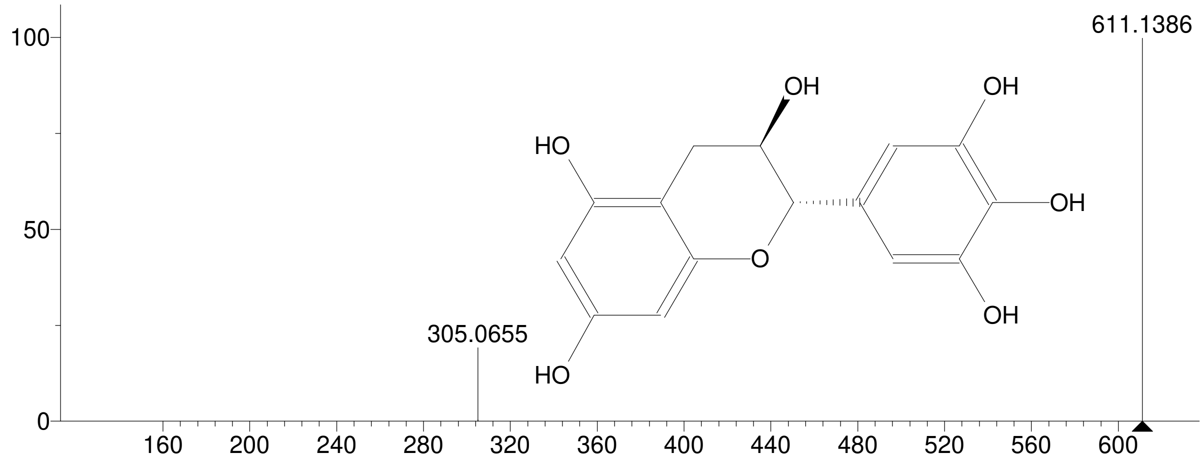

Hit 3 : Melphalan [2M-H+4i]- HCD 5V P=611.1
C13H18Cl2N2O2; MF: 52; RMF: 579; Prob 7.81%; CAS: 148-82-3; Lib: nist_msms; ID: 522785.

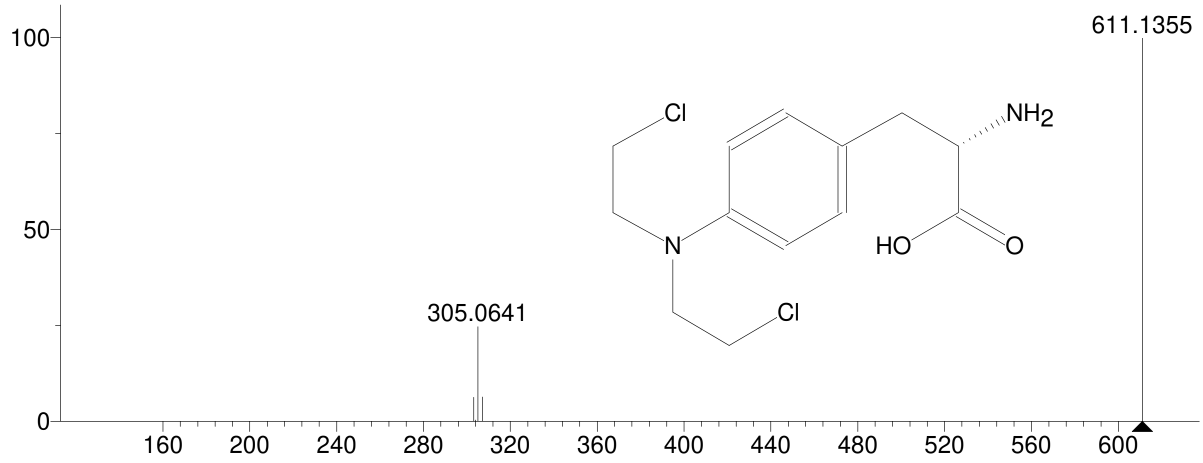

Unknown: Unknown (2.57_146.0451m/z)
Compound in Library Factor = -712

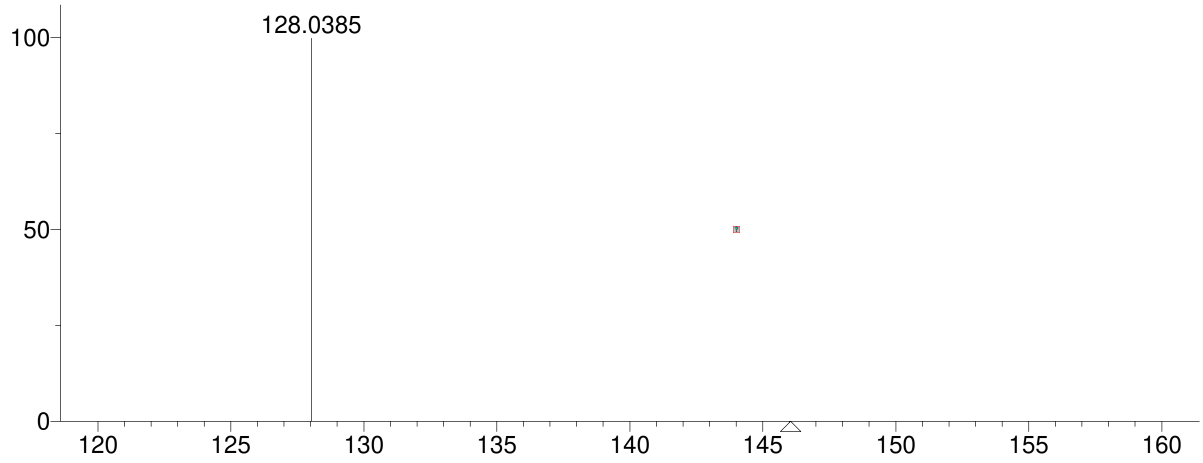

Hit 1 : L-GLUTAMIC ACID
C5H9NO4; MF: 364; RMF: 999; Prob 15.0%; Lib: embl neg mcg; ID: 601.

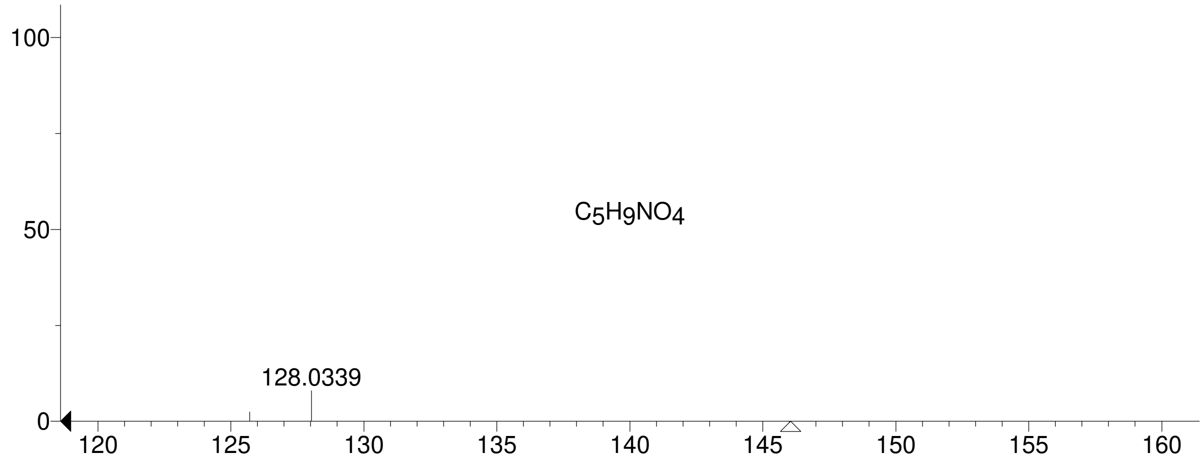

Hit 2 : Glutamic acid [M-H]- 35eV P=146
C5H9NO4; MF: 364; RMF: 999; Prob 15.0%; Lib: mona-export-fiehn_hilic; ID: 2731.

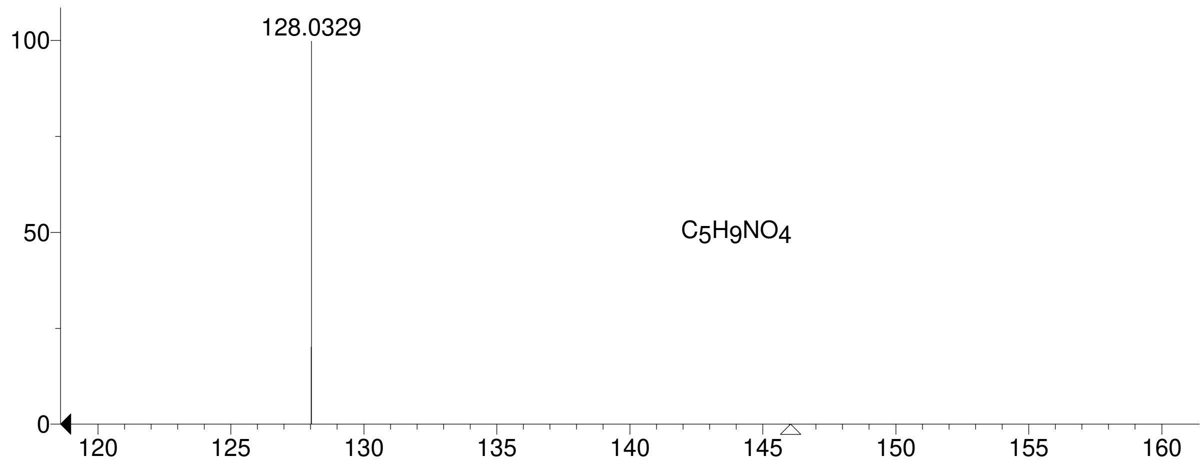

Hit 3 : L-Glutamic acid [M-H]- IT-FT 35% P=146
C5H9NO4; MF: 364; RMF: 999; Prob 15.0%; CAS: 56-86-0; Lib: nist_msms; ID: 140307.

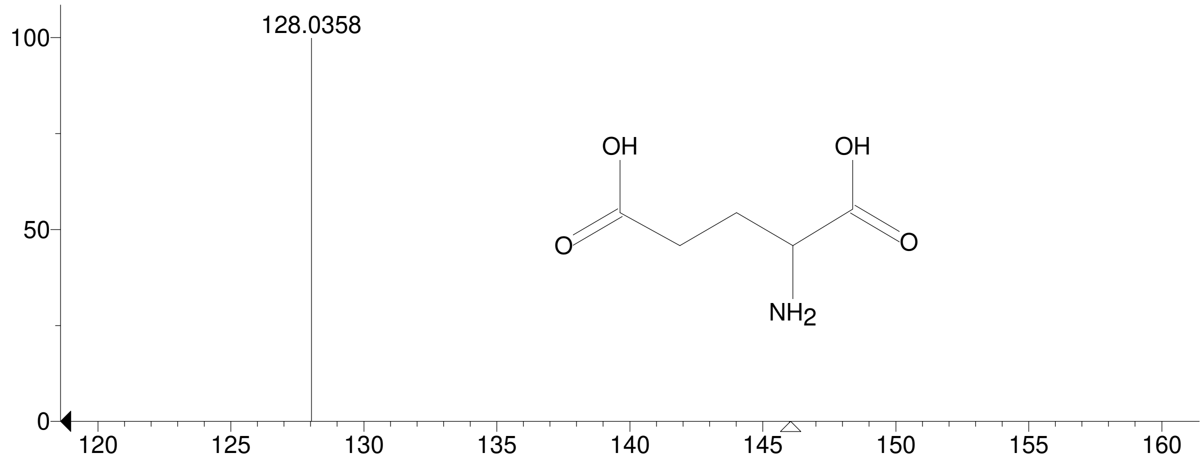

Hit 4 : N-Methyl-D-aspartic acid [M-H]- IT-FT 35% P=146
C5H9NO4; MF: 364; RMF: 999; Prob 15.0%; CAS: 6384-92-5; Lib: nist_msms; ID: 203419.

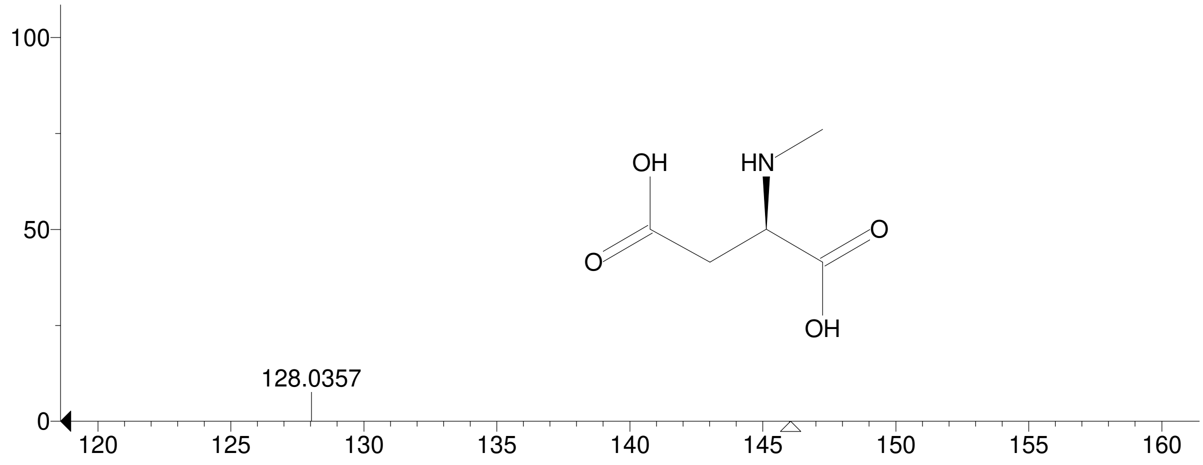

Hit 5 : α-Guanidinoglutaric acid [M-H-CH2N2]- IT-FT 35% P=146
C6H11N3O4; MF: 364; RMF: 999; Prob 15.0%; CAS: 73477-53-9; Lib: nist_msms; ID: 337247.

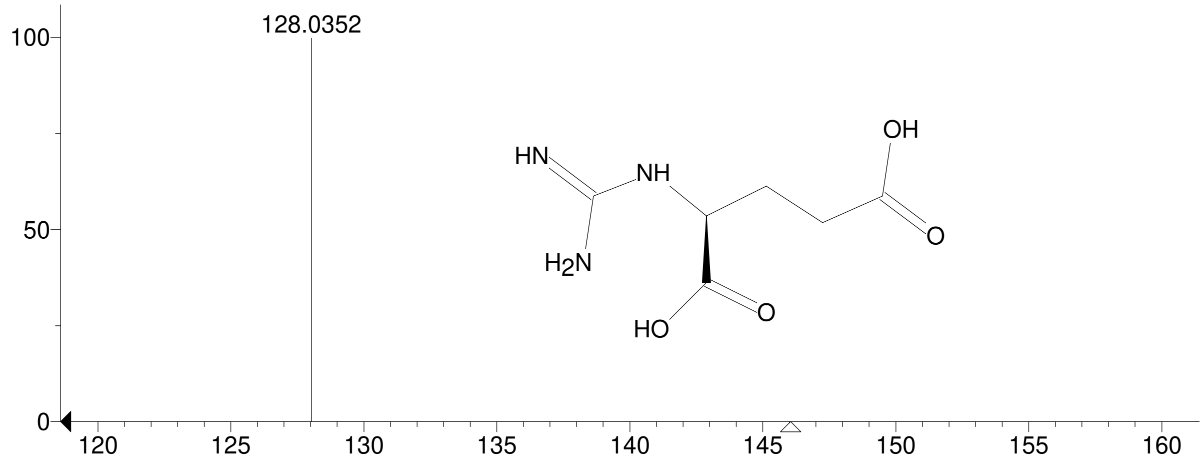

Unknown: Unknown (2.57_389.1166m/z)
Compound in Library Factor = 0

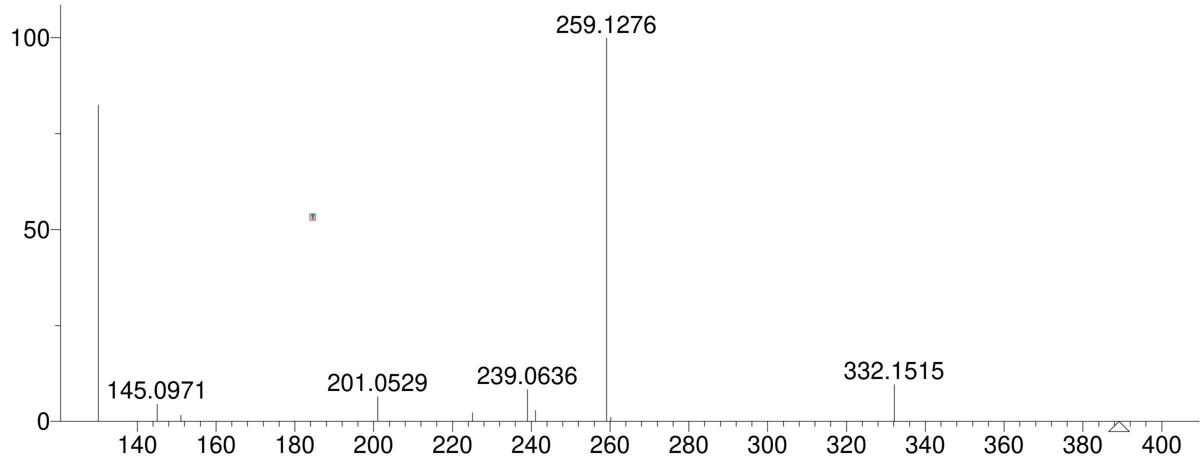

Unknown: Unknown (2.57_602.1827m/z)
Compound in Library Factor = 0

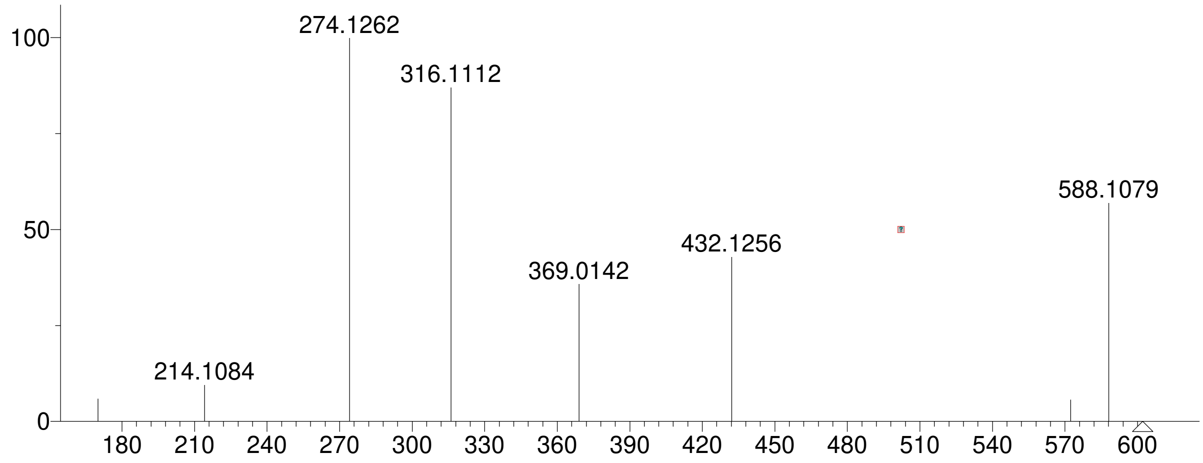

Unknown: Unknown (2.58_254.0658m/z)
Compound in Library Factor = -2176

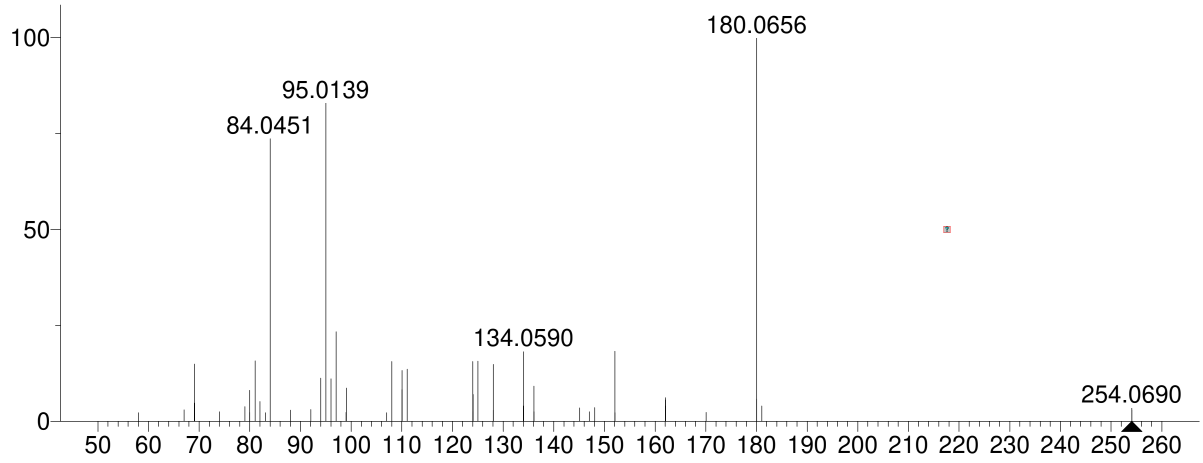

Hit 1 : Nitrothal isopropyl [M+H-C3H6]+ HCD 1V P=254.1
C14H17NO6; MF: 30; RMF: 464; Prob 53.6%; CAS: 10552-74-6; Lib: nist_msms; ID: 398573.

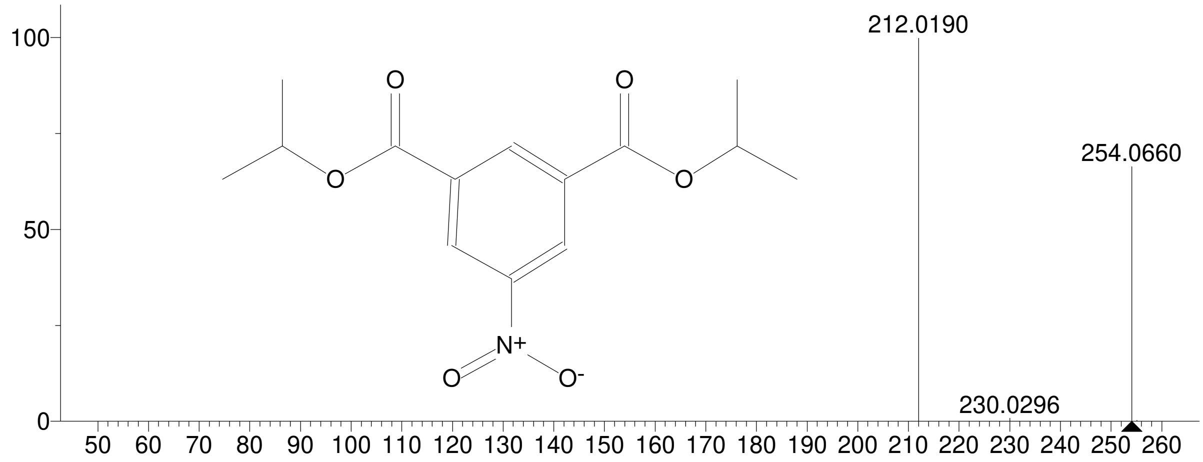

Hit 2 : Unknown (carbon number 11); PlaSMA ID-419 [M-H]- P=254.1
C11H13NO6; MF: 6; RMF: 92; Prob 18.0%; Lib: biomsms-neg-plasma; ID: 1119.

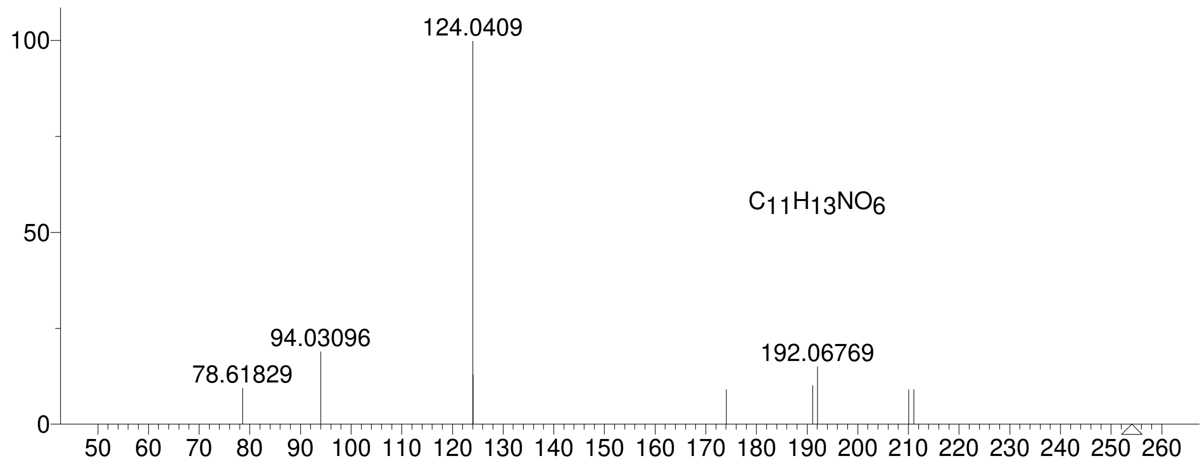

Hit 3 : methyl 2-[(2,3-dihydroxybenzoyl)amino]-3-hydroxypropanoate [M-H]- 65HCD P=254.1
C11H13NO6; MF: 1; RMF: 43; Prob 14.5%; Lib: mona-export-lc-ms-ms_negative_mode; ID: 22579.

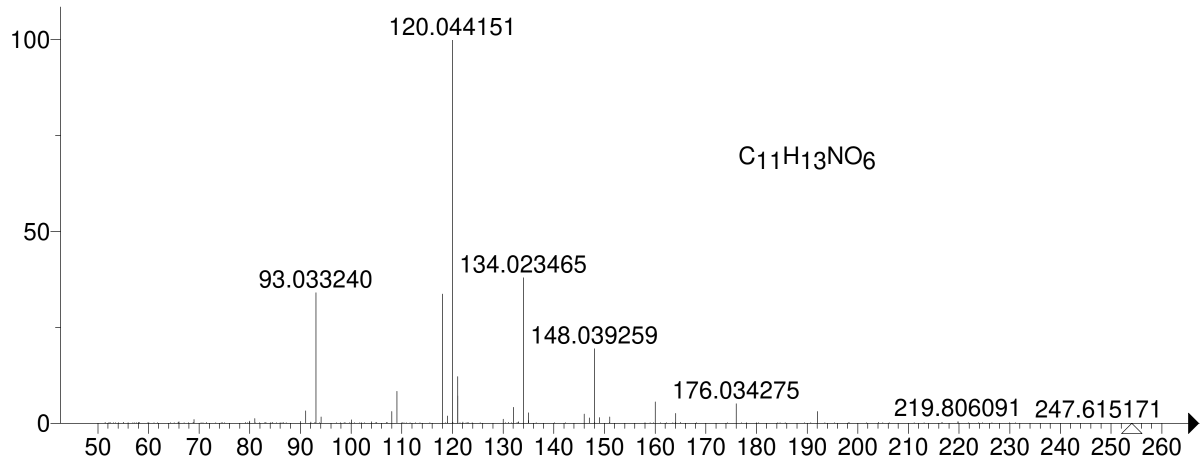

Hit 4 : Caramboxin [M-H]- P=254.1
C11H13NO6; MF: 0; RMF: 9; Prob 13.9%; Lib: msms-neg-gnps; ID: 65.

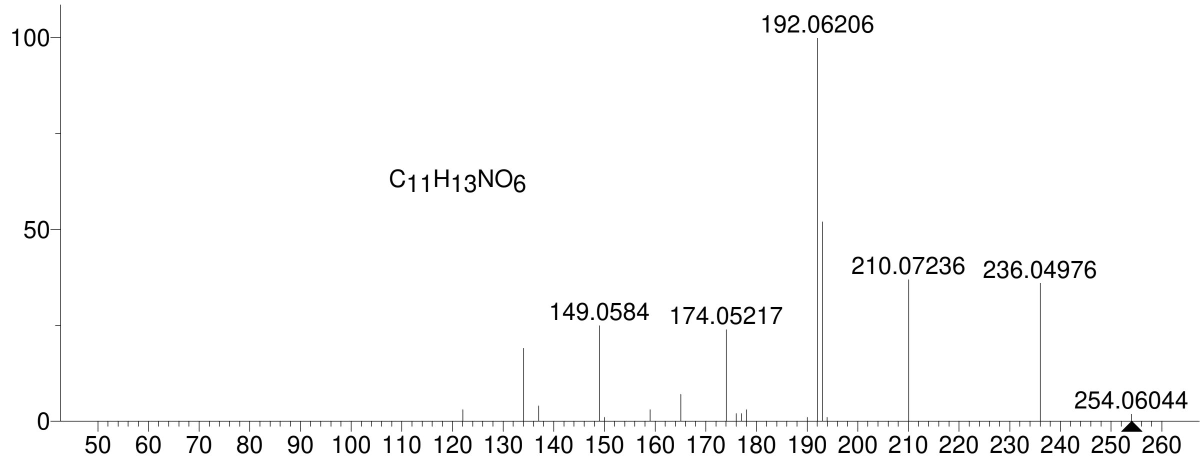

Unknown: Unknown (2.58_421.1816m/z)
Compound in Library Factor = -3273

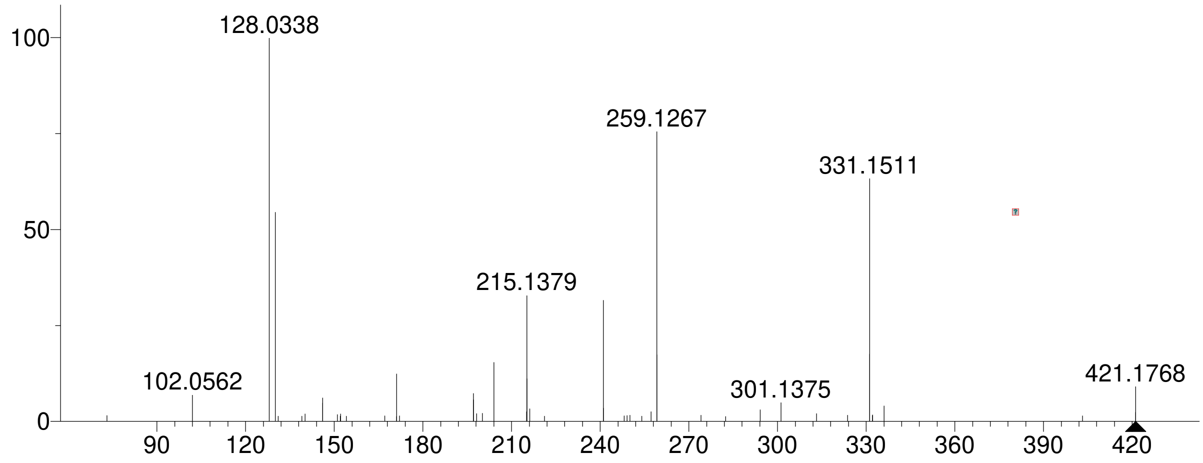

Hit 1 : Glp-His-Pro-Gly [M+H]+ QTOF 3V P=421.2
C18H24N6O6; MF: 7; RMF: 157; Prob 12.5%; Lib: nist_msms2; ID: 32614.

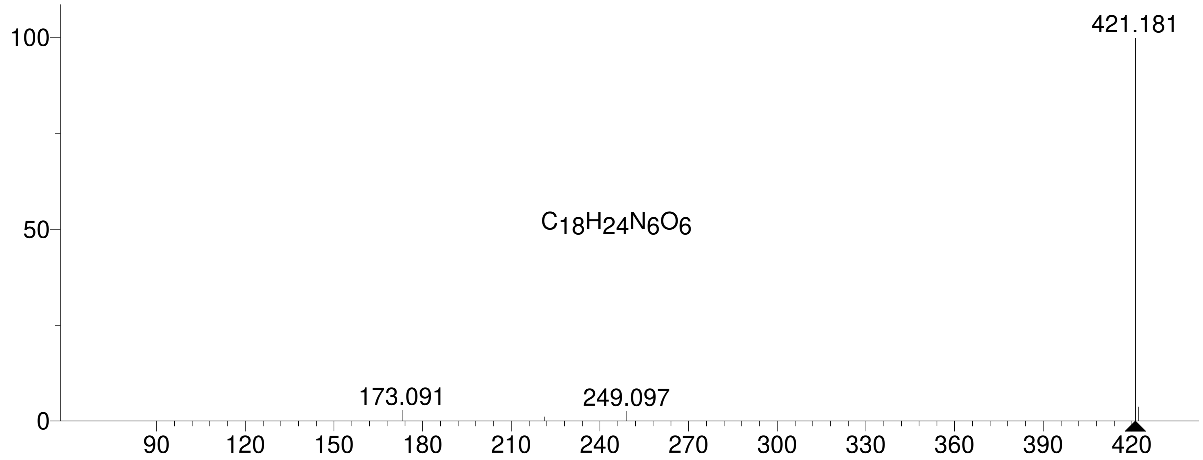

Hit 2 : NCGC00347584-02!5-chloro-2,4-dihydroxy-3-[(E)-4-hydroxy-3-methyl-5-(1,2,6-trimethyl-3-oxocyclohexyl)p
C23H31ClO5; MF: 3; RMF: 134; Prob 10.6%; Lib: mona-export-gnps; ID: 8243.

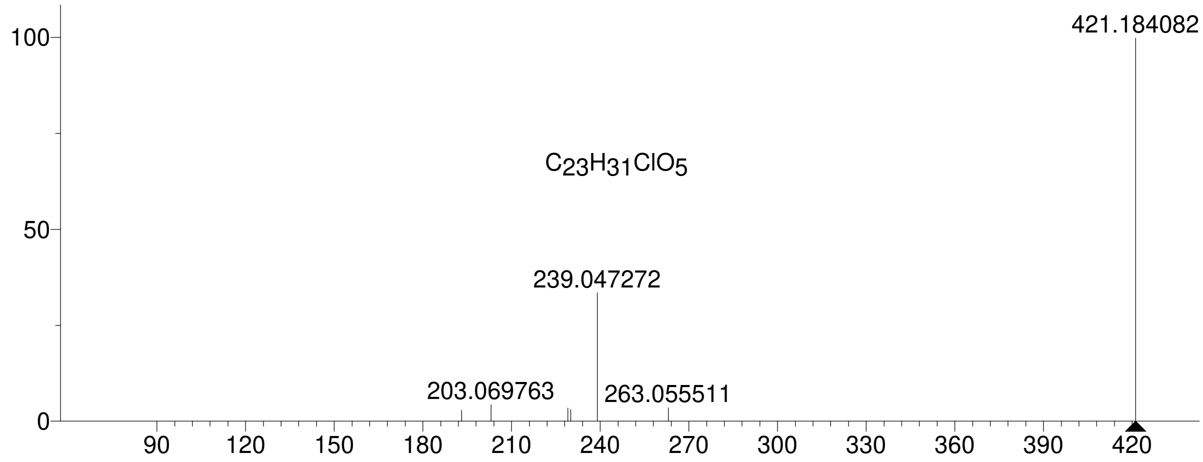

Hit 3 : 3-Cyclohexyl-6-{4-[3-(trifluoromethyl)phenyl]-1-piperazinyl}-2,4(1H,3H)-pyrimidinedione [M-H]- HCD 29V
C21H25F3N4O2; MF: 2; RMF: 134; Prob 10.2%; Lib: nist_msms; ID: 348660.

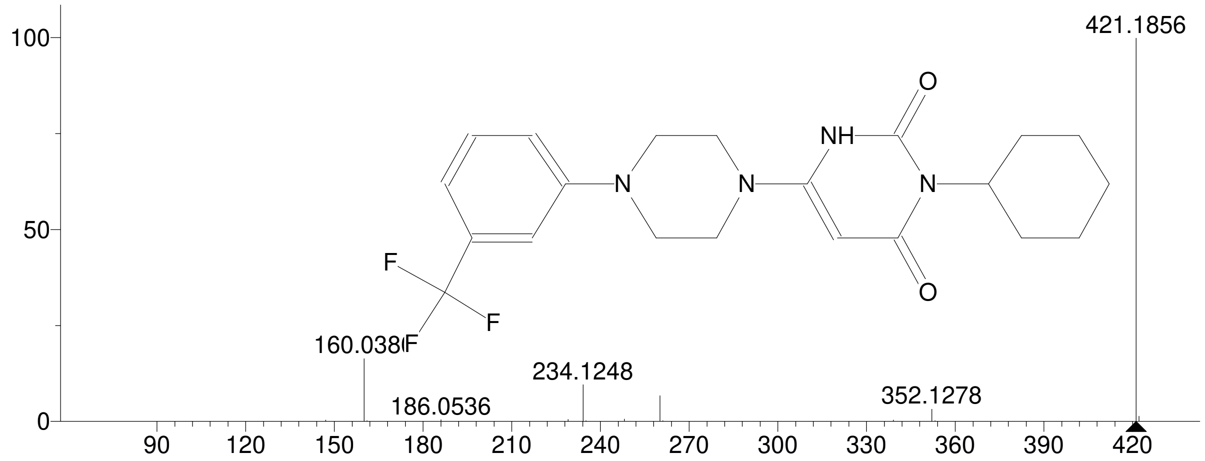

Hit 4 : (2R,3S,4S,9R,12R,13R,14S,15R,16R,17S)-3,4,12,13,15,16-hexahydroxy-2,6,14,17-tetramethyl-10-oxatetr
C20H30O8; MF: 1; RMF: 89; Prob 9.79%; Lib: mona-export-vf-npl_qexactive; ID: 16420.

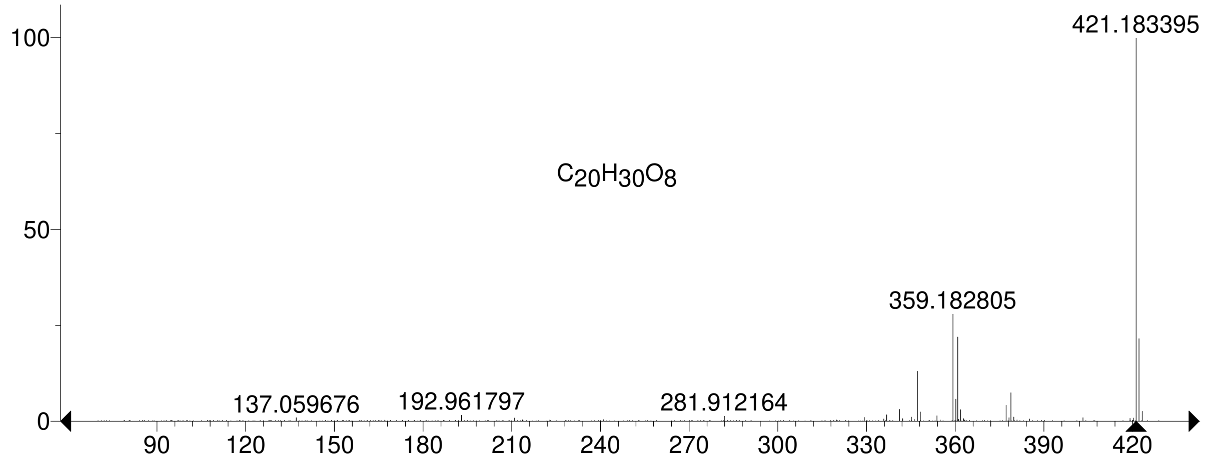

Hit 5 : (+)-Cloprostenol methyl ester [M+H-H2O]+ IT-FT 35% P=421.2
C23H31ClO6; MF: 1; RMF: 46; Prob 9.79%; CAS: 56687-85-5; Lib: nist_msms; ID: 544266.

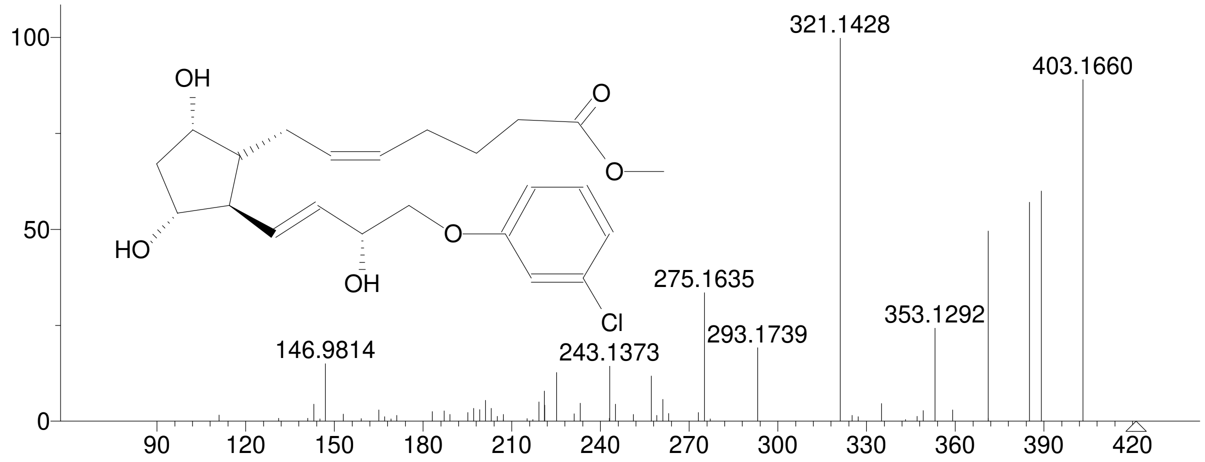

** Search Report Page 1 of 1 **

Unknown: Unknown (2.58_603.1852m/z)
Compound in Library Factor = 0

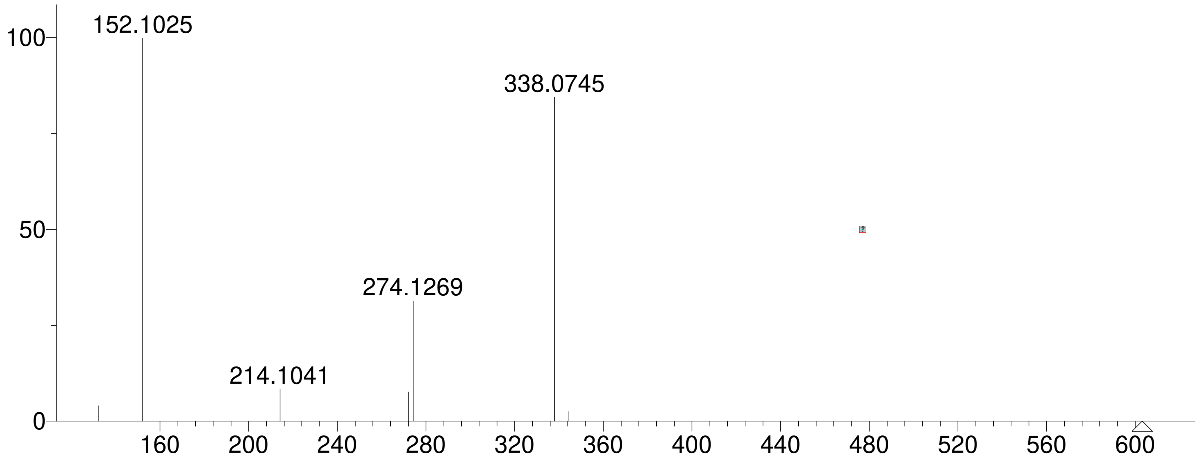

** Search Report Page 1 of 1 **

Unknown: Unknown (2.59_284.0759m/z)
Compound in Library Factor = 0

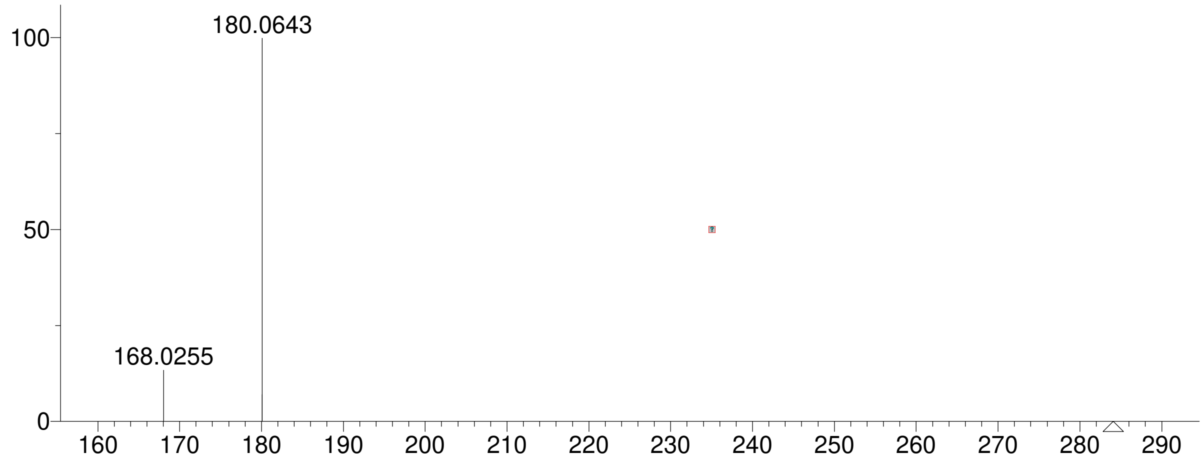

Unknown: Unknown (2.59_386.1190m/z)
Compound in Library Factor = 0

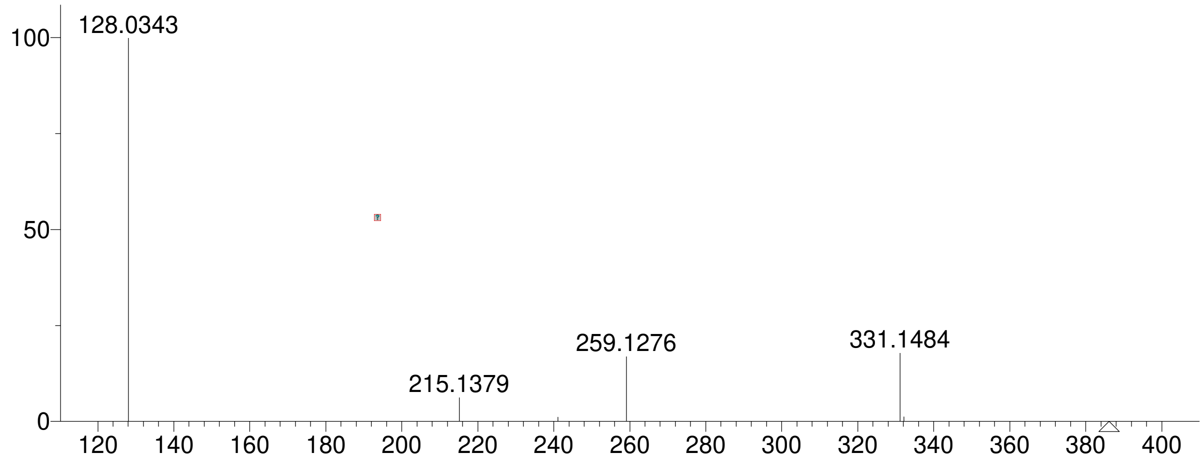

** Search Report Page 1 of 1 **

Unknown: Unknown (2.59_444.1613m/z)
Compound in Library Factor = -3273

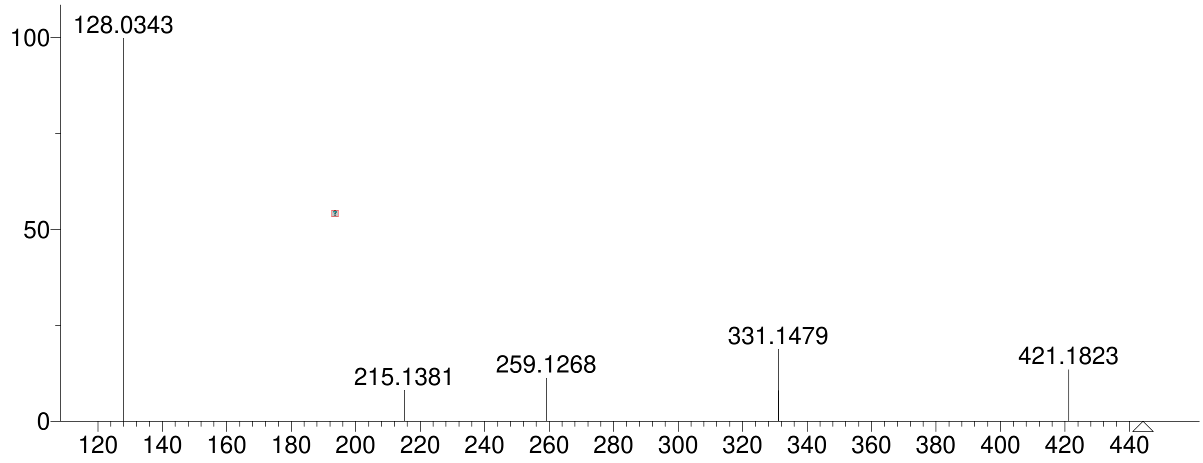

Hit 1 : Tetrahydrofolic acid [M-H]- HCD 71V P=444.2
C19H23N7O6; MF: 3; RMF: 320; Prob 53.1%; CAS: 135-16-0; Lib: nist_msms; ID: 185413.

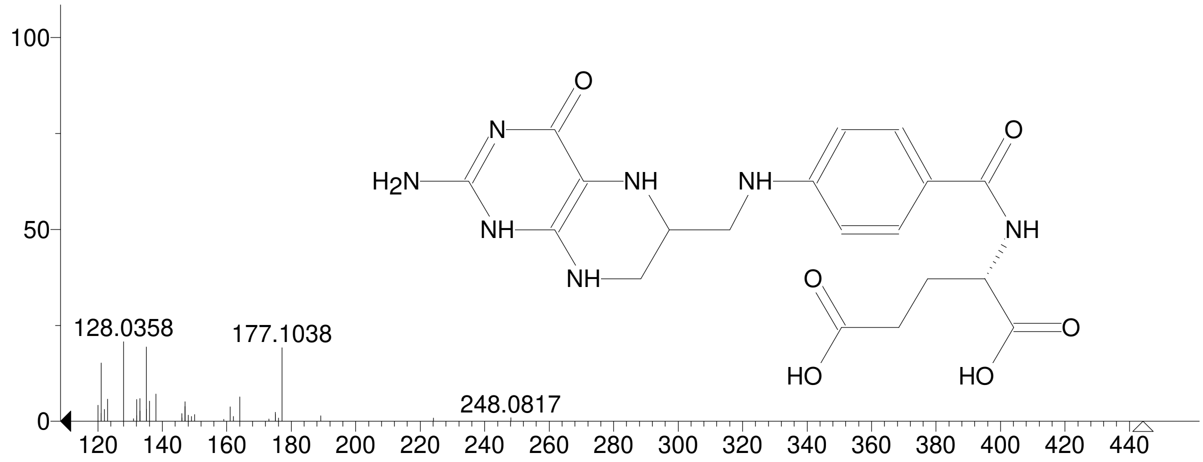

Hit 2 : Pitavastatin [M+Na]+ IT 35% P=444.2
C25H24FNO4; MF: 0; RMF: 23; Prob 46.9%; CAS: 147511-69-1; Lib: nist_msms; ID: 369448.

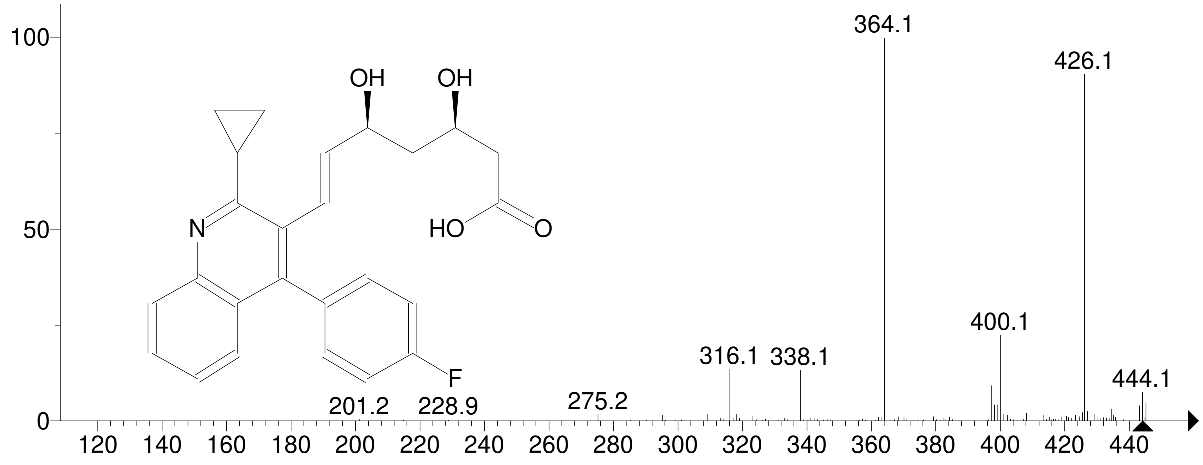

Unknown: Unknown (2.61_345.1296m/z)
Compound in Library Factor = 0

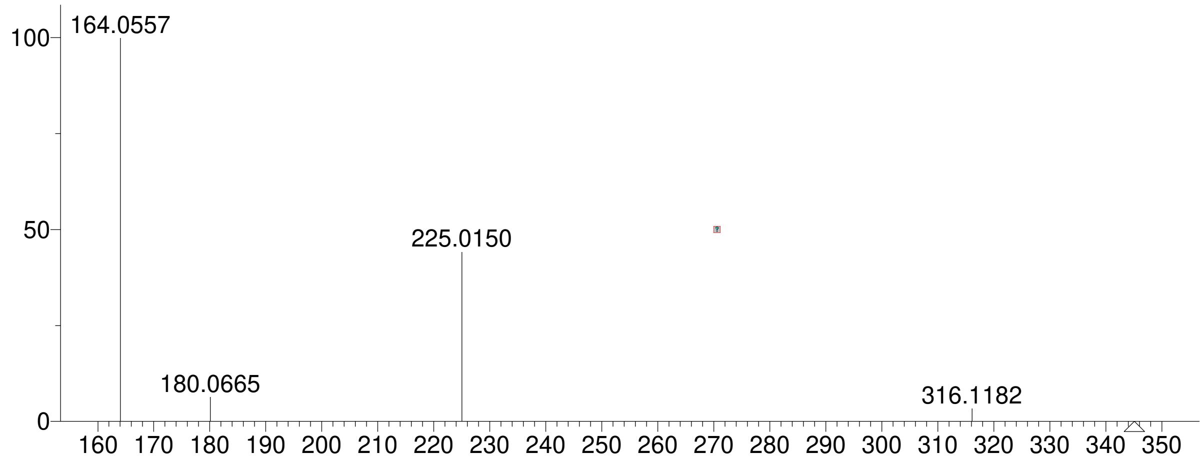

Hit 1 : NCGC00380950-01_C19H20O6_6a,7,8,12a-Tetrahydroxy-3-methyl-2,3,4,5,6,6a,7,12a-octahydro-1,12-tetr
C19H20O6; MF: 0; RMF: 25; Prob 25.0%; Lib: mona-export-gnps; ID: 14170.

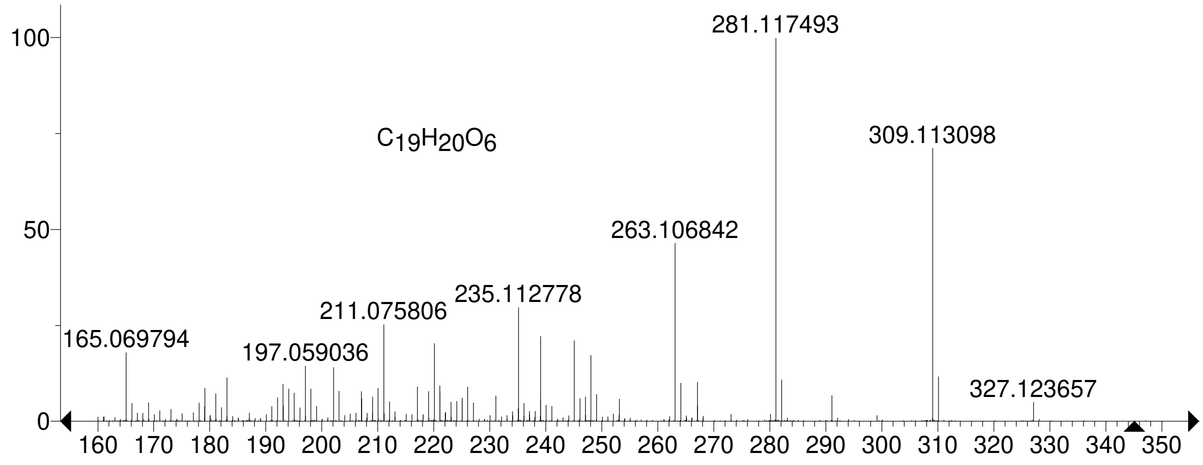

Hit 2 : NCGC00180657-03!(3S,6R,6aS,7S)-6,6a,7,8-tetrahydroxy-3-methyl-3,4,5,6,7,12a-hexahydro-2H-benzo[a]
C19H20O6; MF: 0; RMF: 7; Prob 25.0%; Lib: mona-export-gnps; ID: 9740.

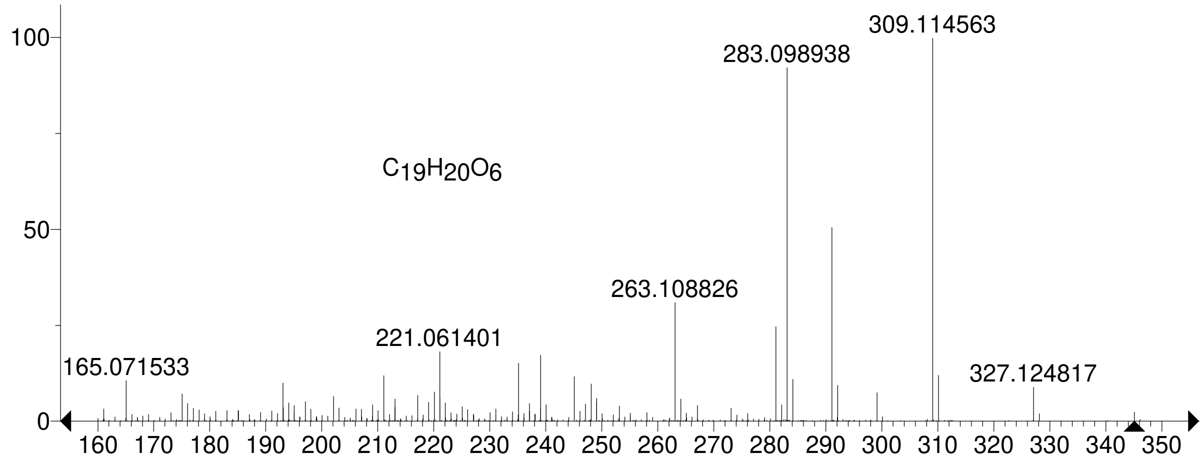

Hit 3 : NCGC00384644-01!(E)-1-(2-hydroxy-3,4,5,6-tetramethoxyphenyl)-3-phenylprop-2-en-1-one [M+H]+ P=3[illegible]
C19H20O6; MF: 0; RMF: 6; Prob 25.0%; Lib: mona-export-gnps; ID: 9870.

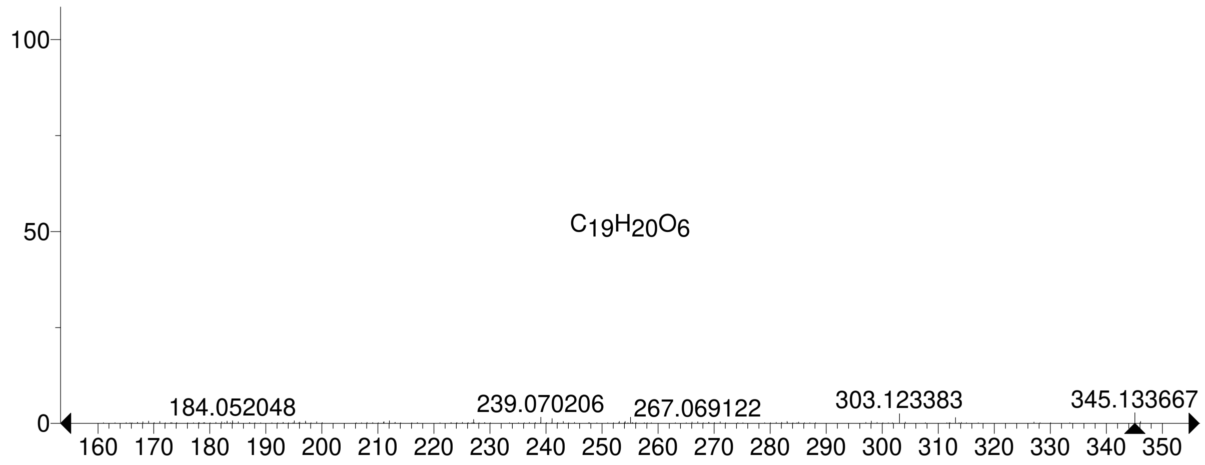

Hit 4 : NCGC00385790-01_C19H20O6_(3S,5S,5a'S,7'S,8'S,8a'R)-5-(3-Furyl)-8'-hydroxy-7'-methyl-3',4,5,5',5a',7',
C19H20O6; MF: 0; RMF: 2; Prob 25.0%; Lib: mona-export-gnps; ID: 13450.

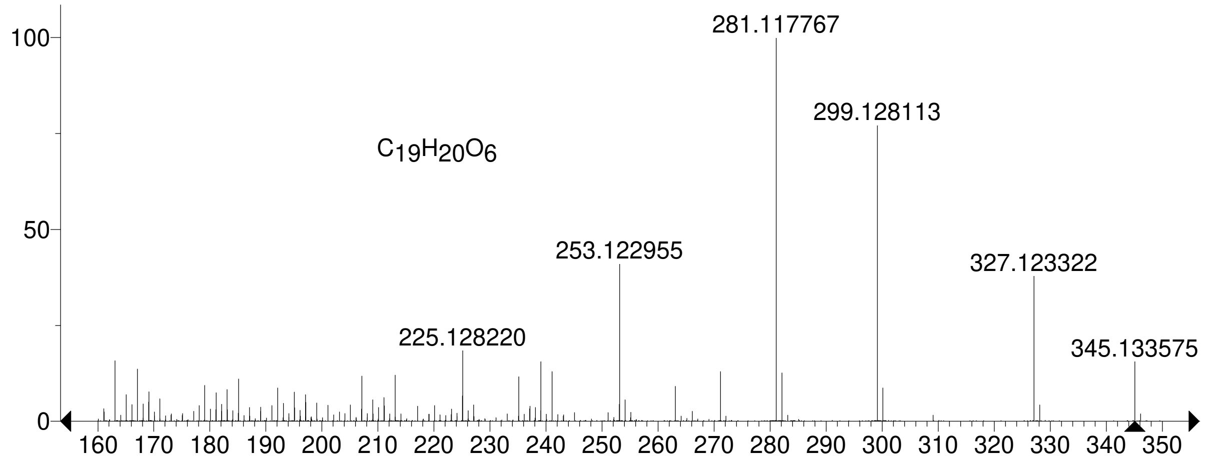

** Search Report Page 1 of 1 **

Unknown: Unknown (2.62_358.0532m/z)
Compound in Library Factor = 0

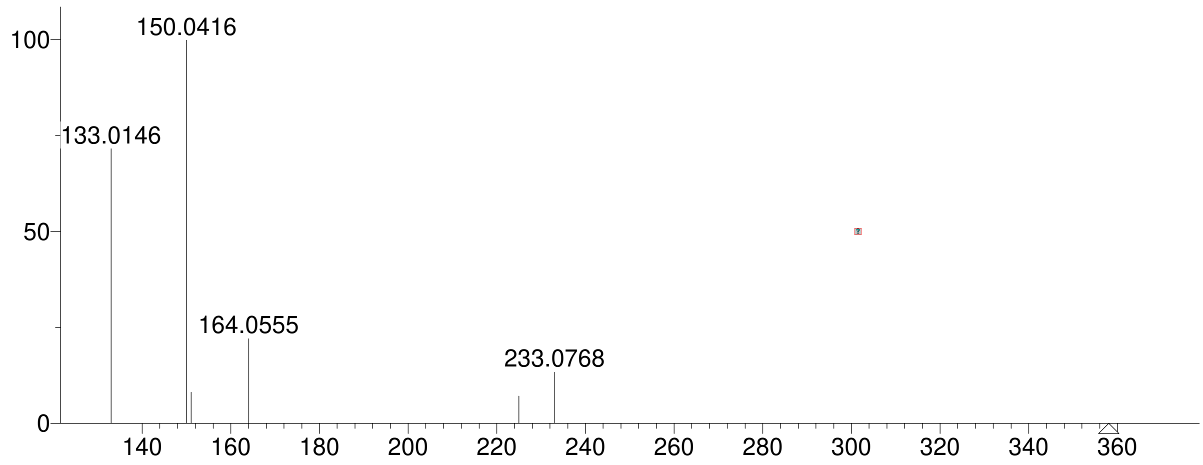

Unknown: Unknown (2.63_281.0326m/z)
Compound in Library Factor = -1486

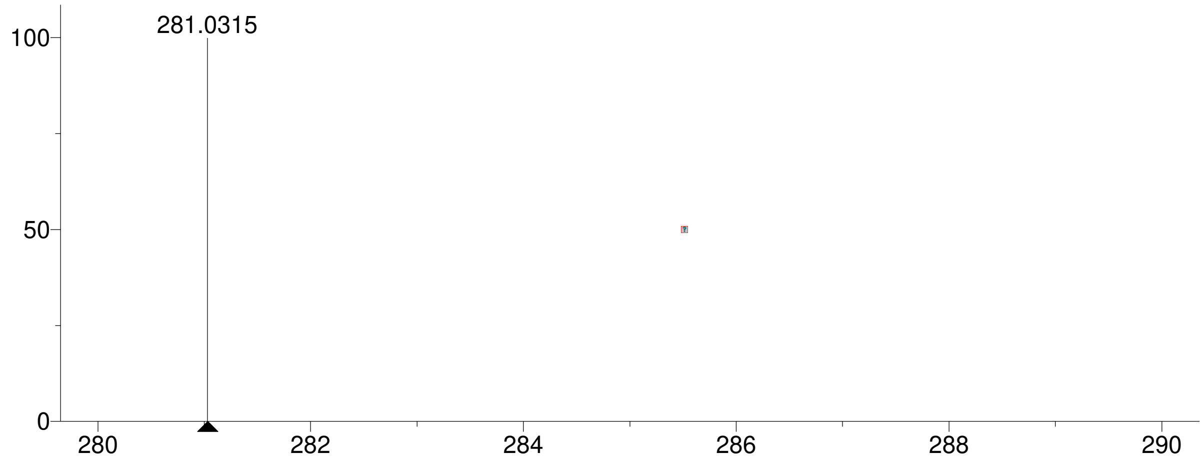

Hit 1 : 2-Aminoethyl dihydrogen phosphate [2M-H]- HCD 7V P=281
C2H8NO4P; MF: 364; RMF: 999; Prob 44.3%; CAS: 1071-23-4; Lib: nist_msms; ID: 299770.

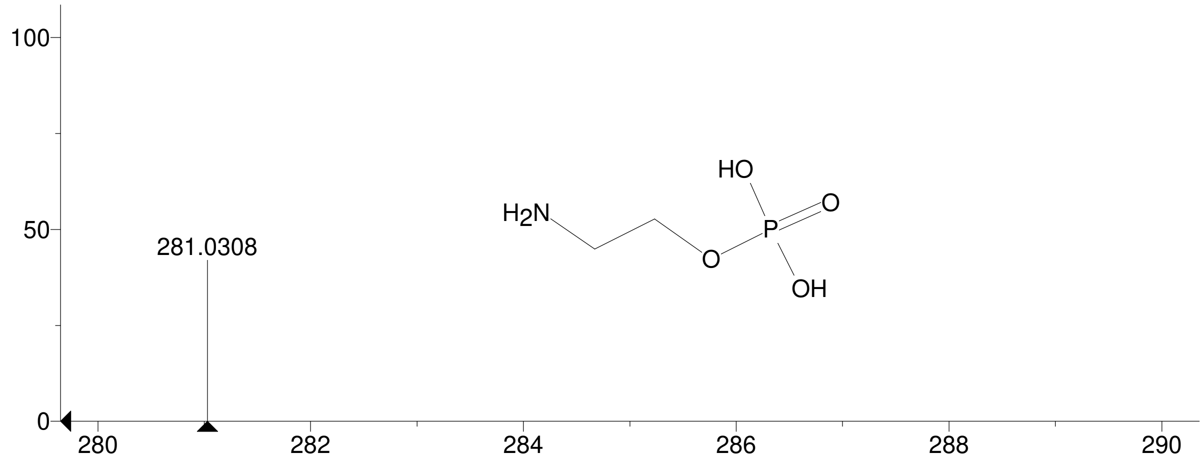

Hit 2 : Diclofensine [M+H-C2H5N+2i]+ HCD 37V P=281
C17H17Cl2NO; MF: 364; RMF: 999; Prob 44.3%; CAS: 67165-56-4; Lib: nist_msms; ID: 510749.

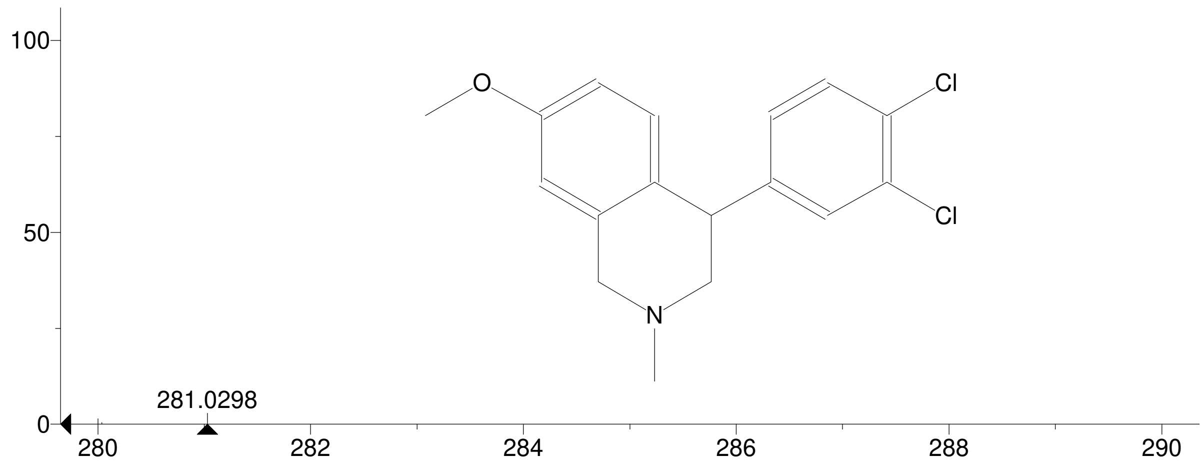

Hit 3 : (E)-1-[4-4-Methoxyphenyl]-2-(3,5-dichlorophenyl)ethene [M+H+2i]+ HCD 14V P=281
C15H12Cl2O; MF: 330; RMF: 954; Prob 11.4%; CAS: 688348-37-0; Lib: nist_msms; ID: 511264.

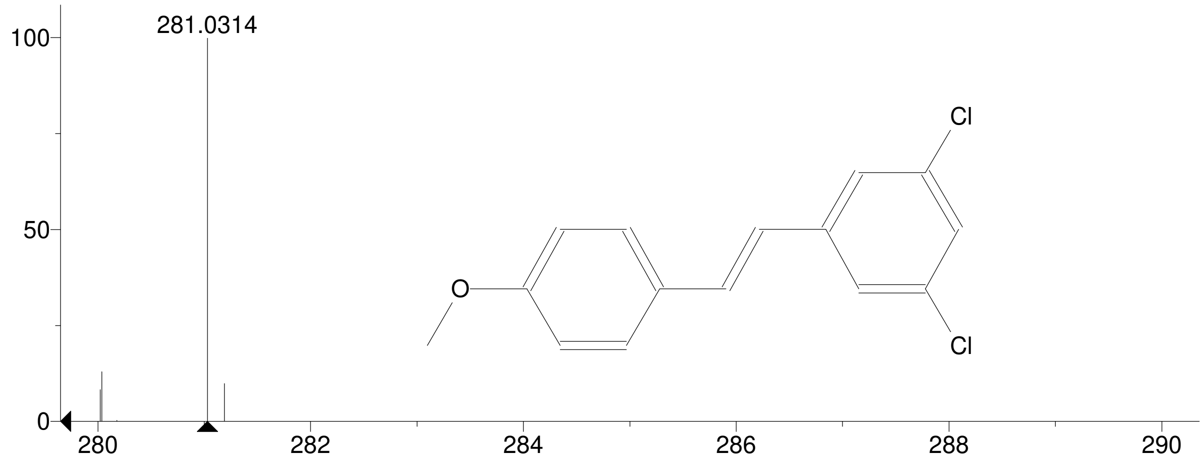

Unknown: Unknown (2.63_376.0657m/z)
Compound in Library Factor = 0

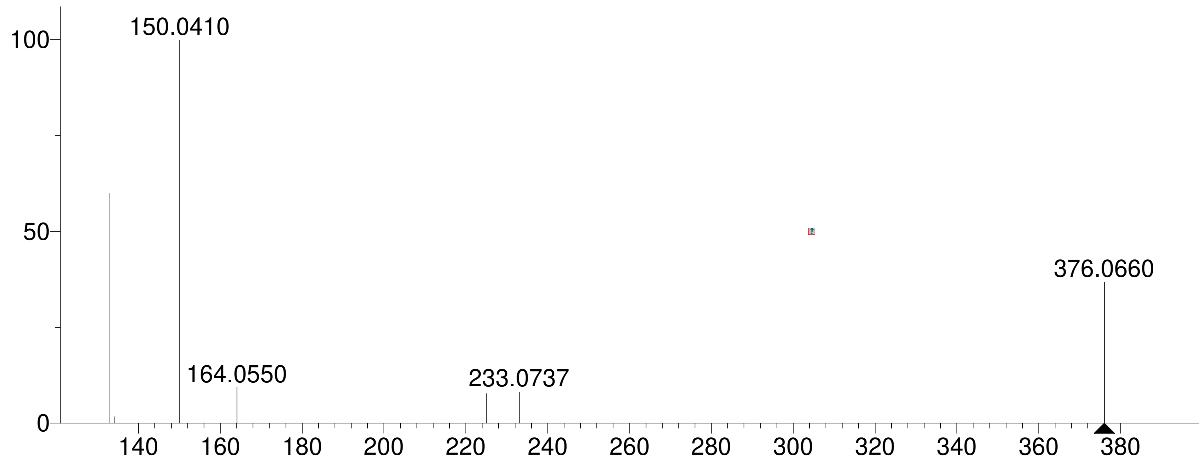

Unknown: Unknown (2.63_403.1348m/z)
Compound in Library Factor = -3273

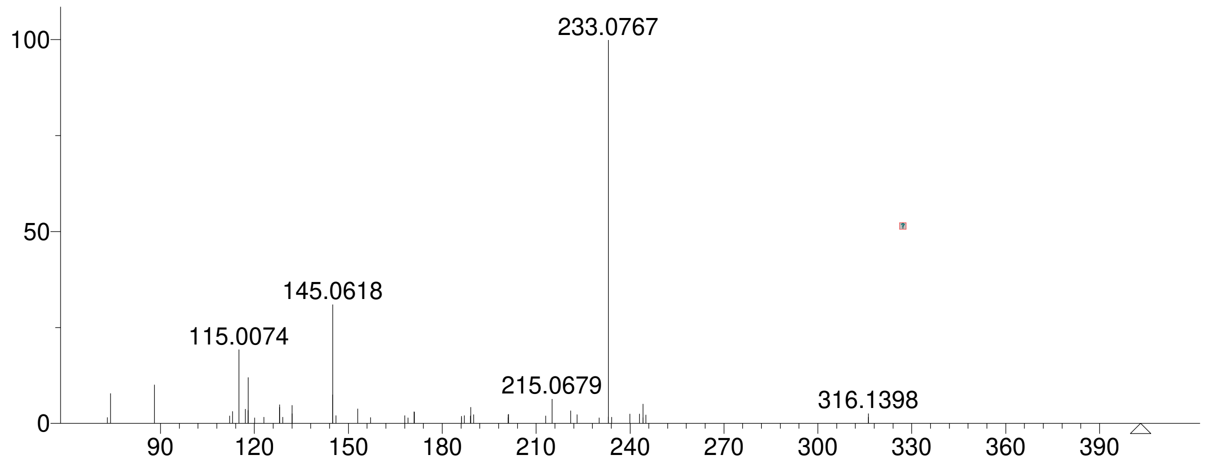

Hit 1 : Naringin dihydrochalcone [M+H-C6H12O6]+ HCD 40V P=403.1
C27H34O14; MF: 6; RMF: 72; Prob 10.9%; CAS: 18916-17-1; Lib: nist_msms; ID: 237396.

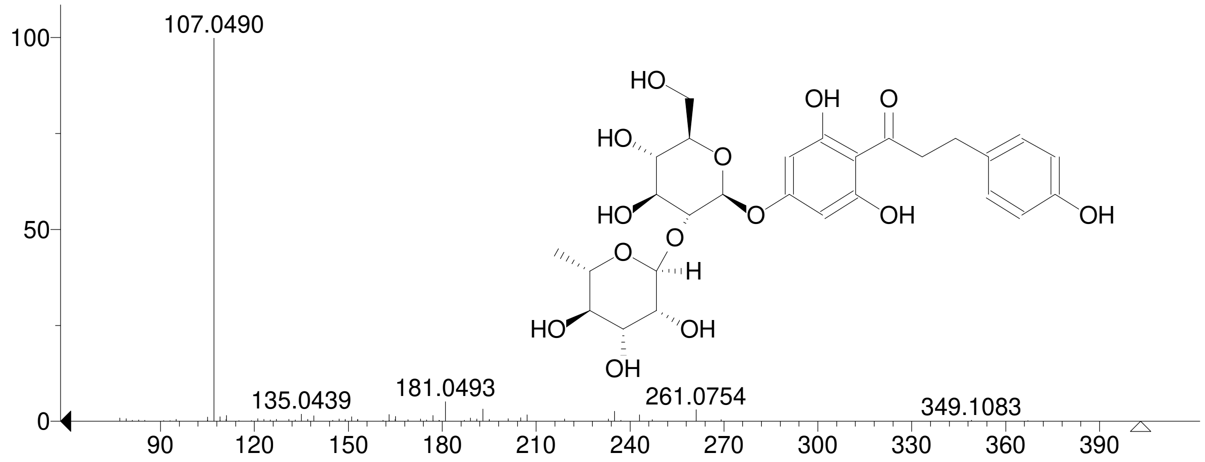

Hit 2 : NCGC00380227-01!(E)-3-[4-[1,3-dihydroxy-1-(4-hydroxy-3-methoxyphenyl)propan-2-yl]oxy-3,5-dimethoxyp
C21H24O9; MF: 4; RMF: 121; Prob 10.1%; Lib: mona-export-gnps; ID: 8693.

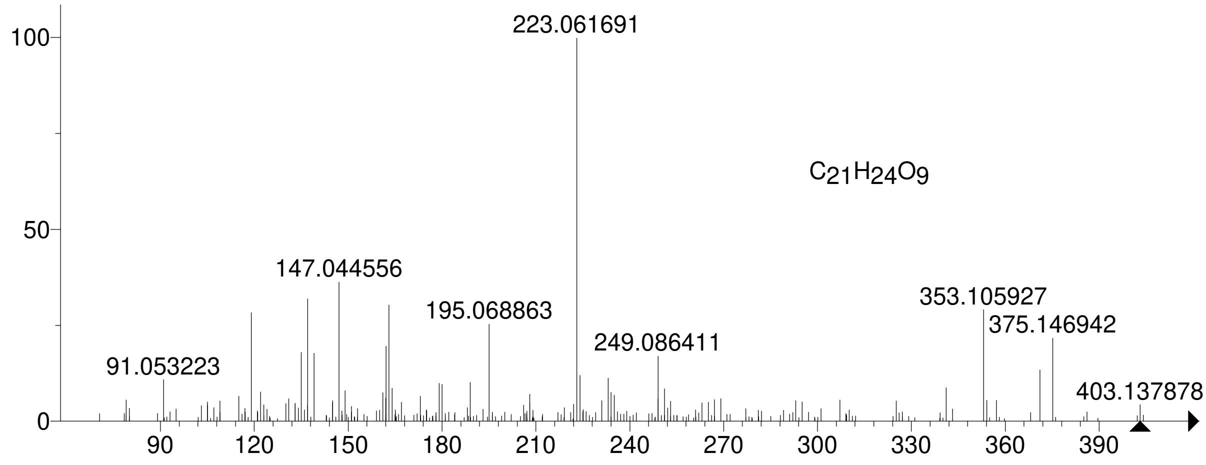

Hit 3 : Nobiletin [M+H]+ HCD 92V P=403.1
C21H22O8; MF: 2; RMF: 67; Prob 9.29%; CAS: 478-01-3; Lib: nist_msms; ID: 178340.

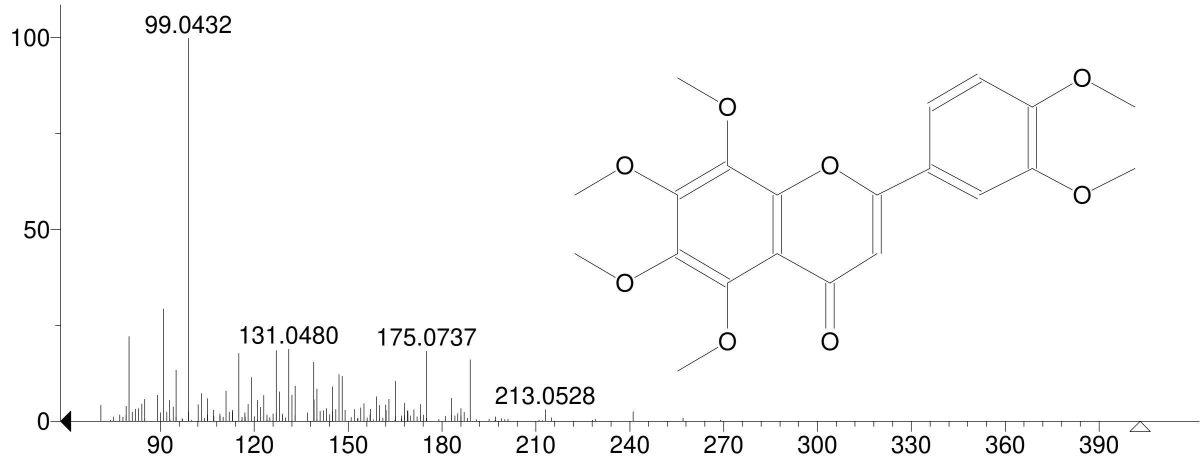

Hit 4 : 4-Deoxyphloridzin [M+H-H2O]+ HCD 24V P=403.1
C21H24O9; MF: 2; RMF: 37; Prob 9.29%; CAS: 31018-48-1; Lib: nist_msms; ID: 494859.

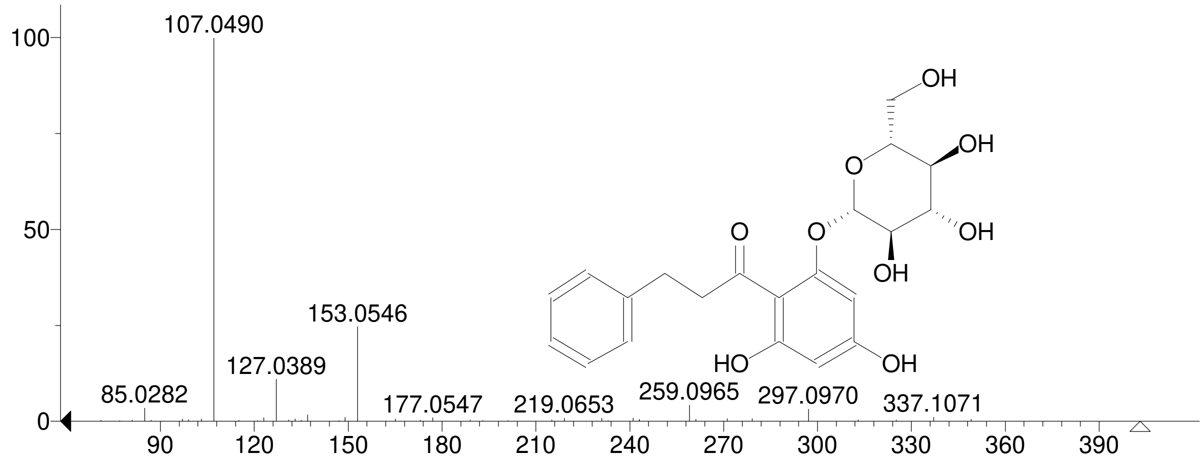

Hit 5 : 3',4',5',5,6,7-Hexamethoxyflavone [M+H]+ HCD 104V P=403.1
C21H22O8; MF: 1; RMF: 56; Prob 8.92%; CAS: 29043-07-0; Lib: nist_msms; ID: 462291.

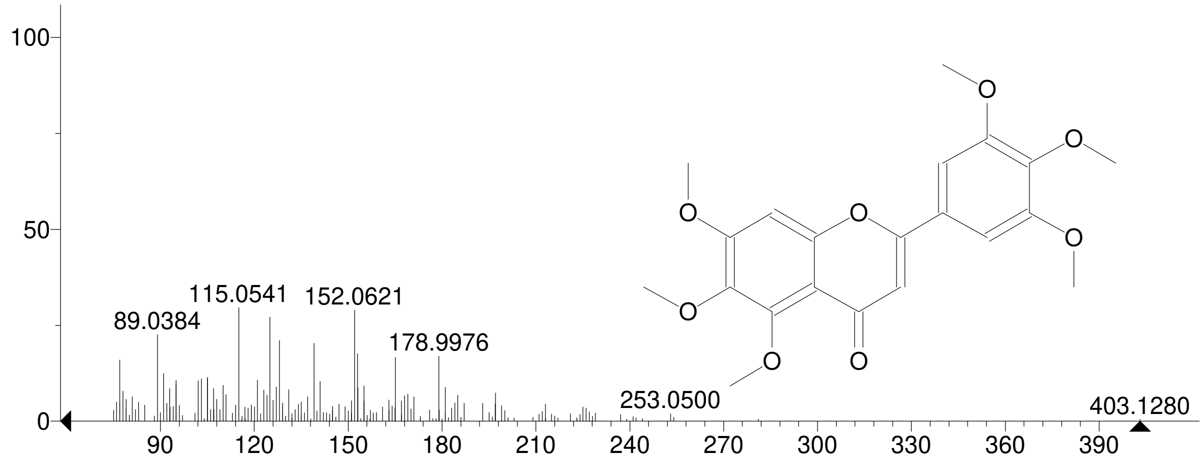

Unknown: Unknown (2.63_549.0872m/z)
Compound in Library Factor = -3273

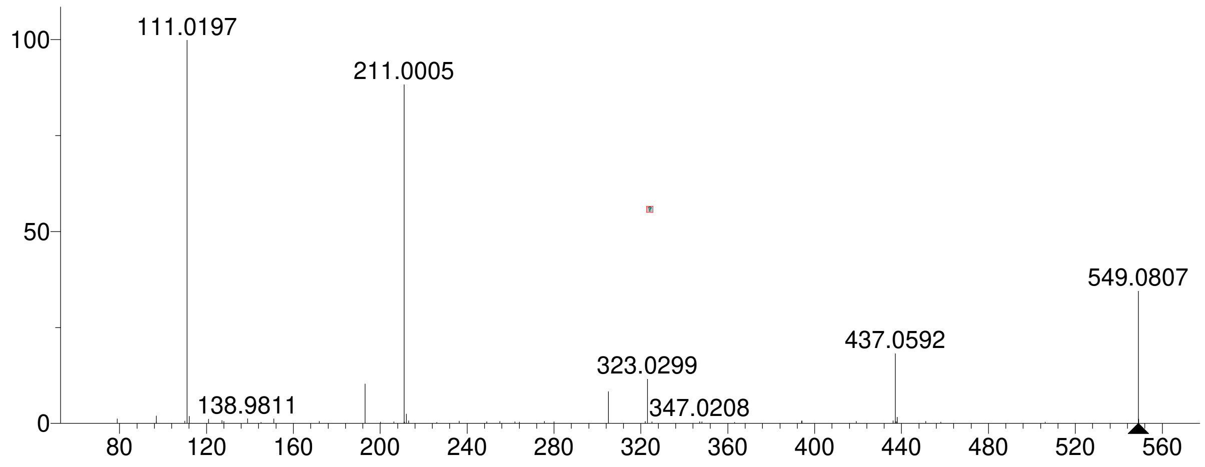

Hit 1 : 5-(3-(2-(7-Chloroquinolin-2-yl)ethenyl)phenyl)-8-dimethylcarbamyl-4,6-dithiaoctanoic acid [M+Cl]- HCD 1
C26H27ClN2O3S2; MF: 3; RMF: 127; Prob 18.4%; CAS: 115104-28-4; Lib: nist_msms; ID: 538815.

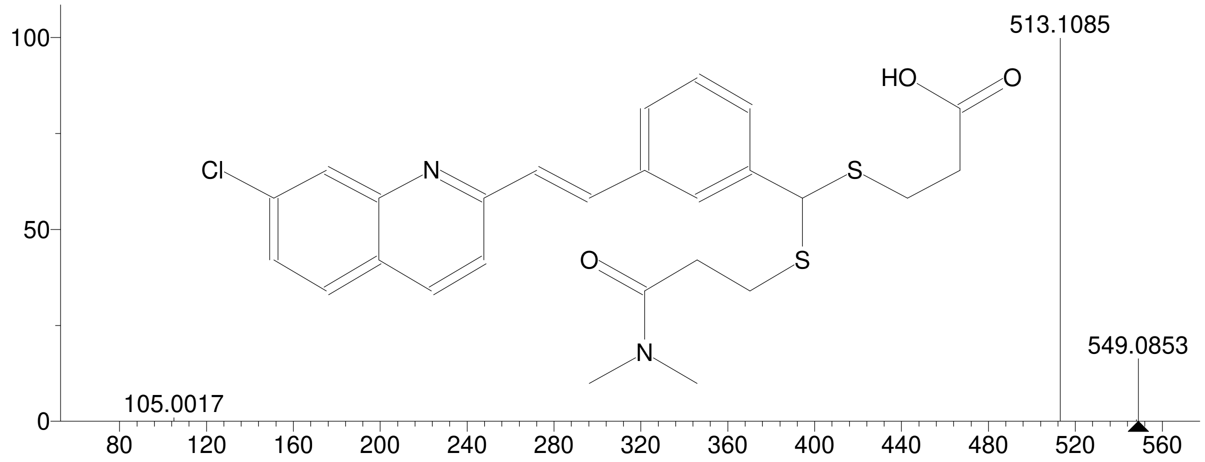

Hit 2 : Quercetin-3-(6''-malonyl)-Glucoside [M-H]- 5-60V P=549.1
C24H22O15; MF: 0; RMF: 76; Prob 16.3%; Lib: mona-export-lc-ms-ms_negative_mode; ID: 8837.

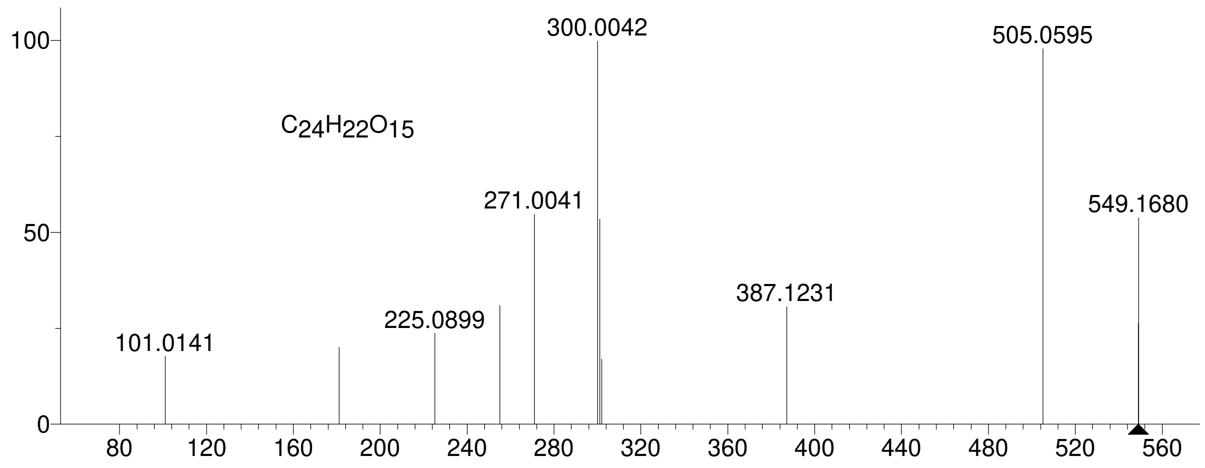

Hit 3 : Quercetin-3-(6''-malonyl)-Glucoside; LC-ESI-QTOF; MS2; CE [M-H]- P=549.1
C24H22O15; MF: 0; RMF: 71; Prob 16.3%; Lib: msms-neg-massbank; ID: 4722.

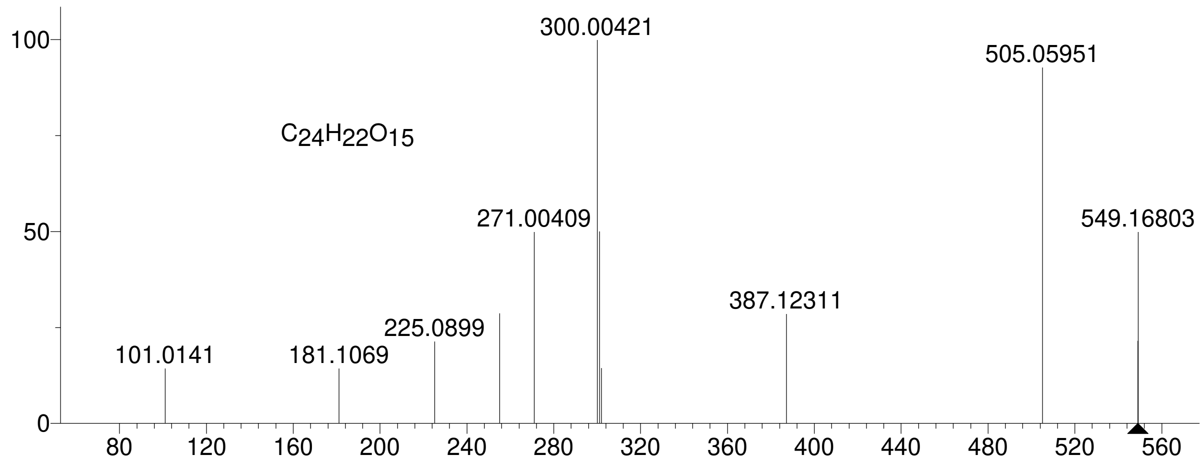

Hit 4 : Quercetin 3-O-malonylglucoside [M-H]- 10 P=549.1
C24H22O15; MF: 0; RMF: 33; Prob 16.3%; Lib: mona-export-lc-ms-ms_negative_mode; ID: 27259.

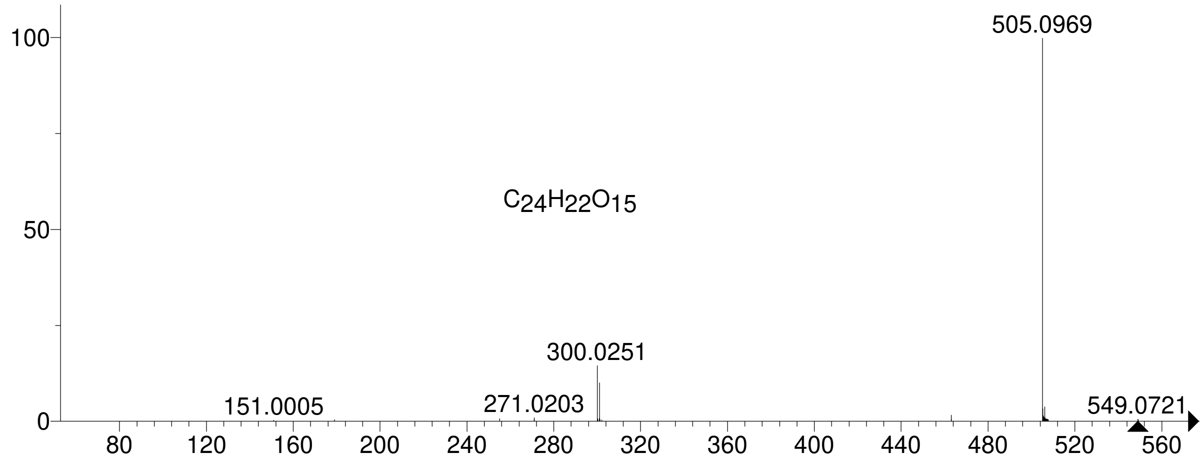

Hit 5 : Flavonol base + 4O, O-MalonylHex [M-H]- 6V P=549.1
C24H22O15; MF: 0; RMF: 20; Prob 16.3%; Lib: anthocyanins mona; ID: 6952.

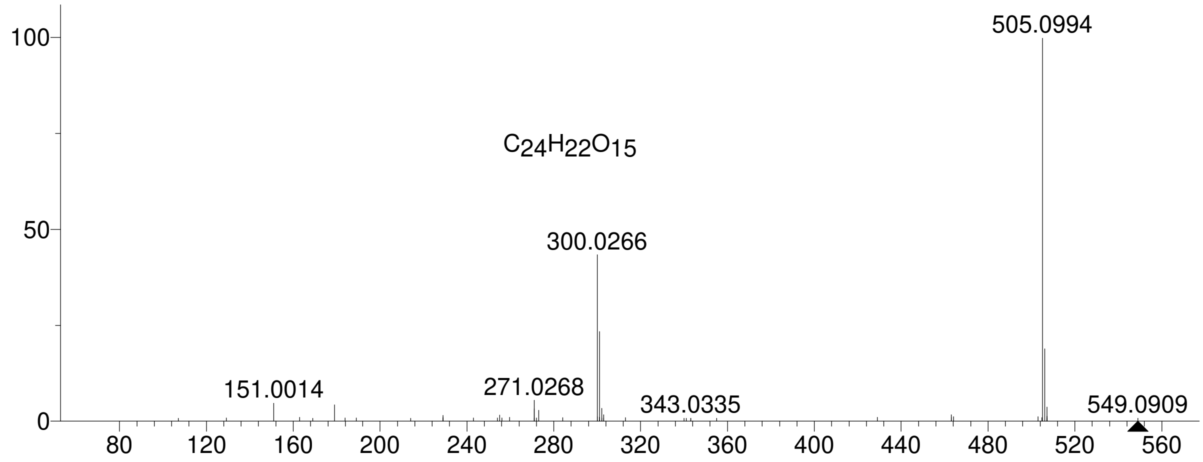

Unknown: Unknown (2.64_265.0374m/z)
Compound in Library Factor = -3273

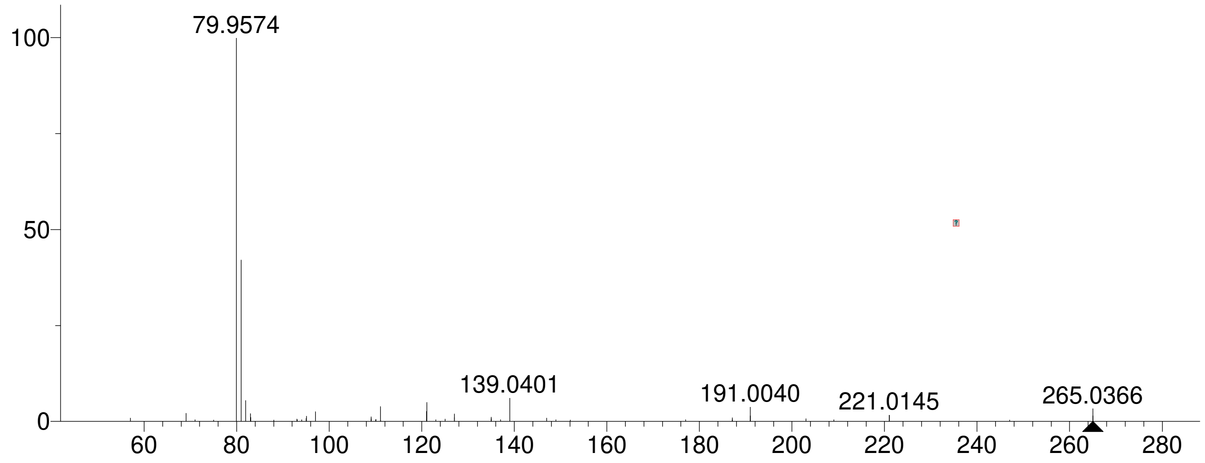

Hit 1 : Aclonifen [M+H]+ HCD 10V P=265
C12H9ClN2O3; MF: 33; RMF: 576; Prob 100.0%; CAS: 74070-46-5; Lib: nist_msms; ID: 473154.

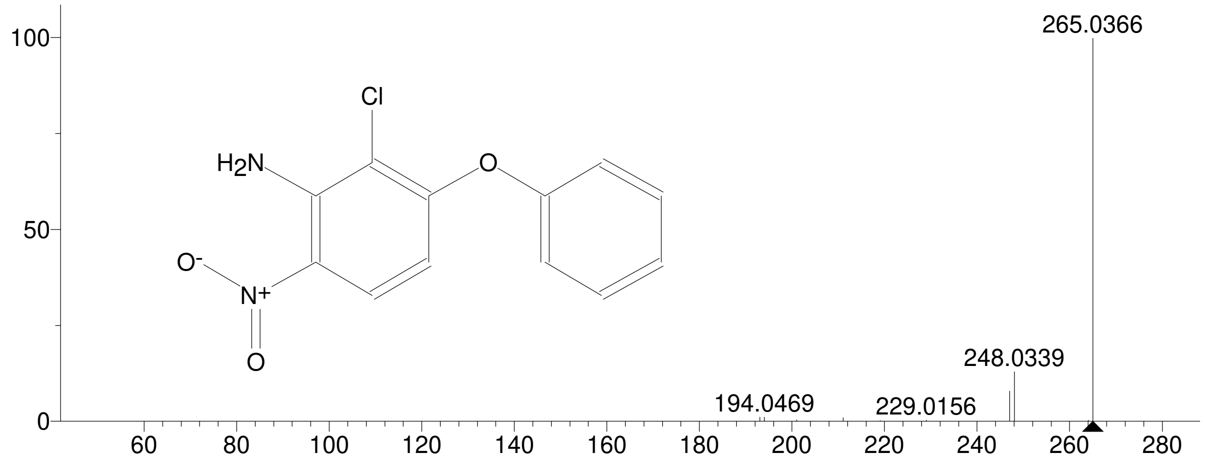

** Search Report Page 1 of 1 **

Unknown: Unknown (2.66_454.2136m/z)
Compound in Library Factor = 0

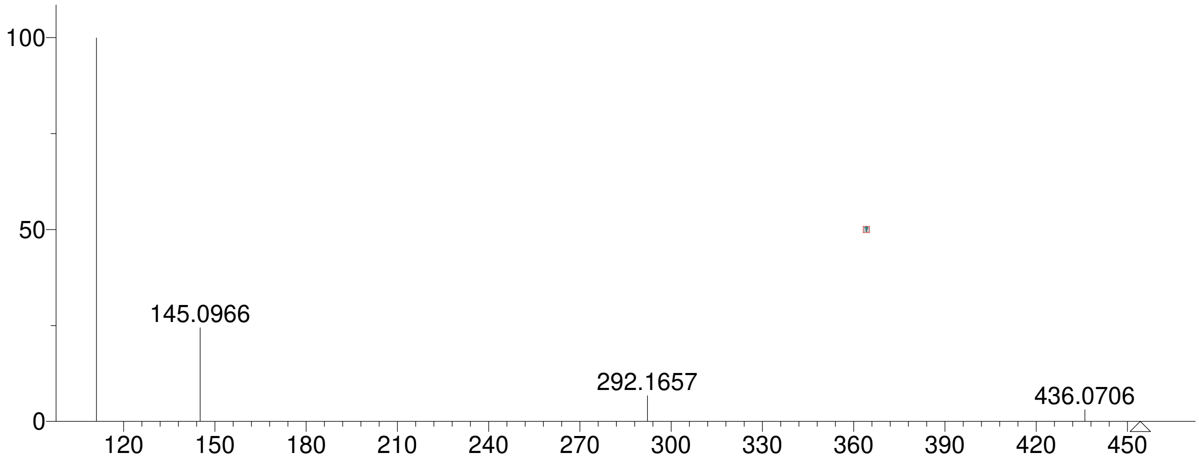

Unknown: Unknown (2.66_477.2077m/z)
Compound in Library Factor = -1848

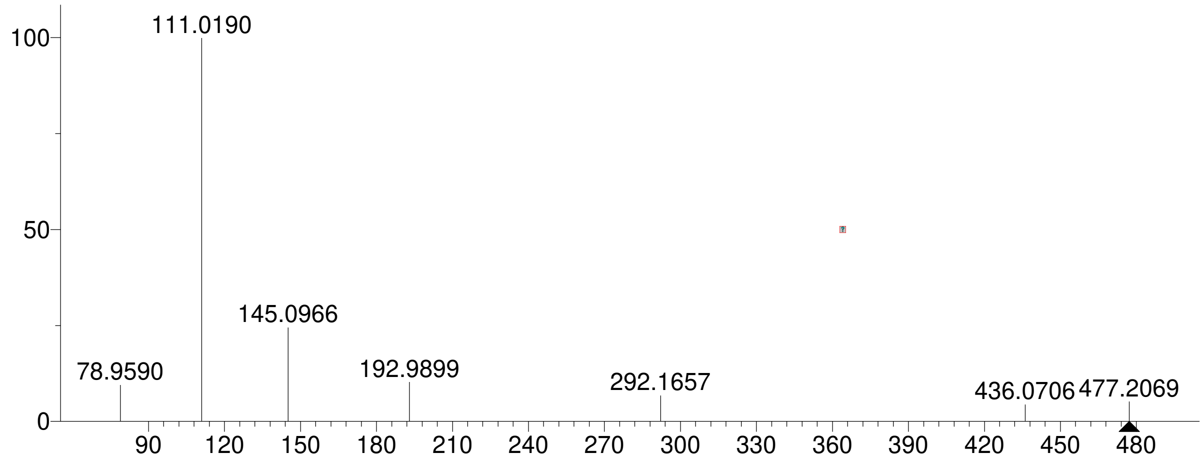

Hit 1 : 4-(2-Hydroxyethyl)piperazine-1-ethanesulfonic acid [2M+H]+ HCD 4V P=477.2
C8H18N2O4S; MF: 34; RMF: 470; Prob 66.1%; CAS: 7365-45-9; Lib: nist_msms; ID: 58512.

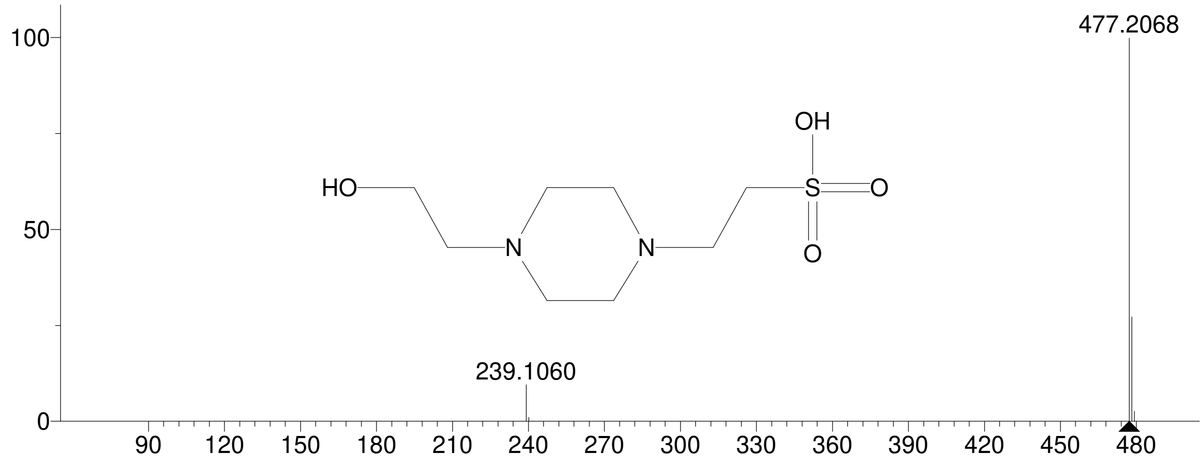

Hit 2 : 5-O-methyllicoricidin [M+K]+ 35HCD P=477.2
C27H34O5; MF: 0; RMF: 24; Prob 16.9%; Lib: mona-export-vf-npl_qexactive; ID: 964.

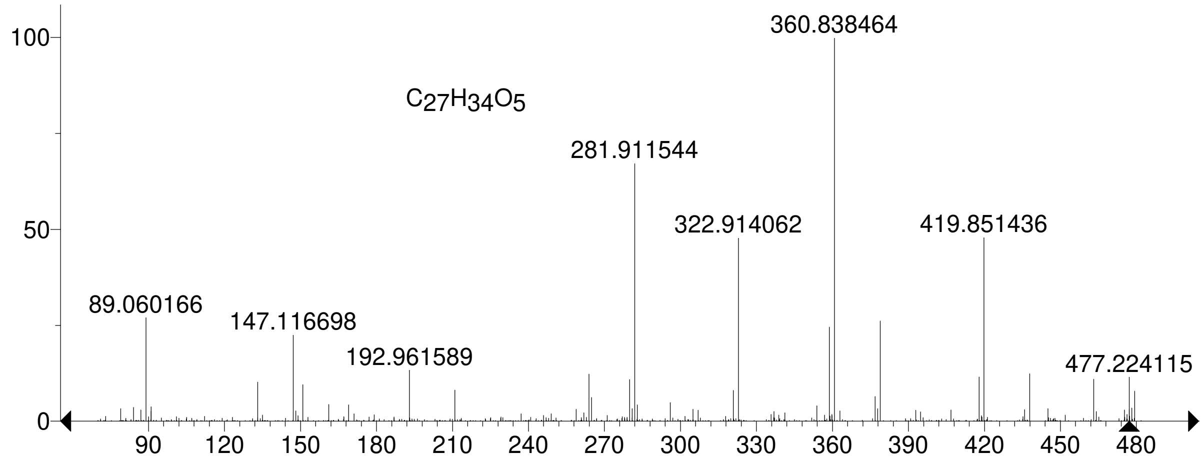

Hit 3 : 1,7-bis(4-hydroxyphenyl)-5-[(2R,3R,4S,5S,6R)-3,4,5-trihydroxy-6-(hydroxymethyl)oxan-2-yl]oxyheptan-3-or
C25H32O9; MF: 0; RMF: 2; Prob 16.9%; Lib: mona-export-vf-npl_qexactive; ID: 7567.

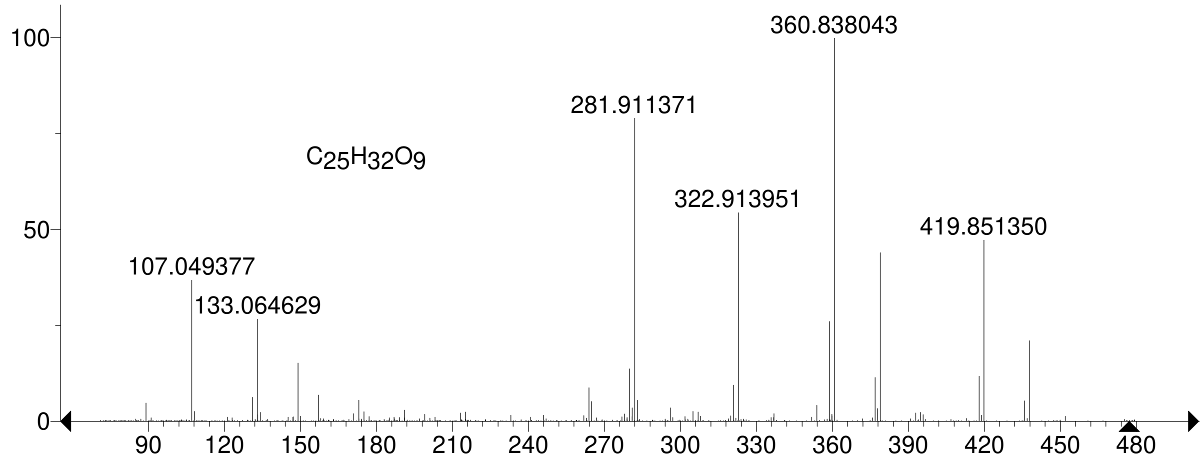

Unknown: Unknown (2.66_521.2086m/z)
Compound in Library Factor = 0

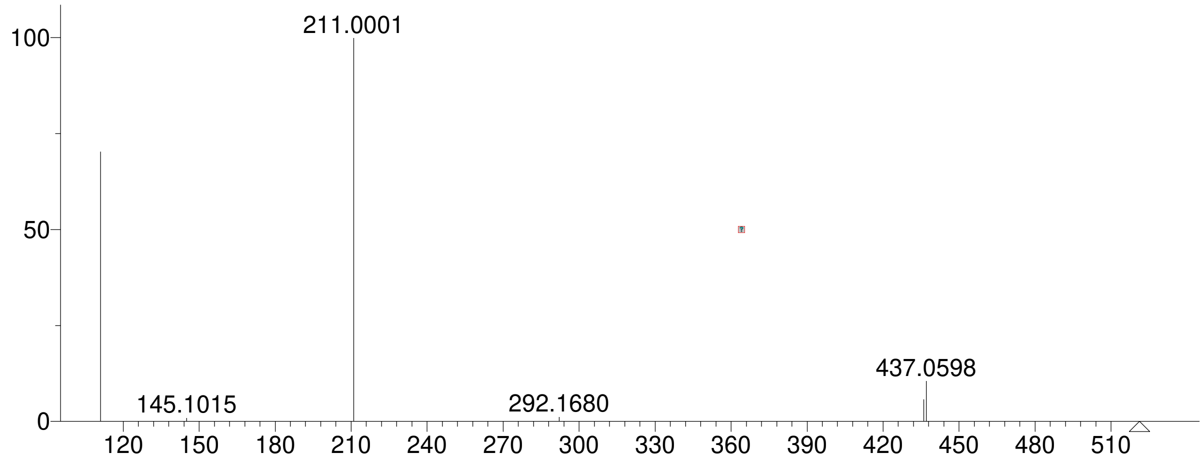

Unknown: Unknown (2.68_283.0681m/z)
Compound in Library Factor = -691

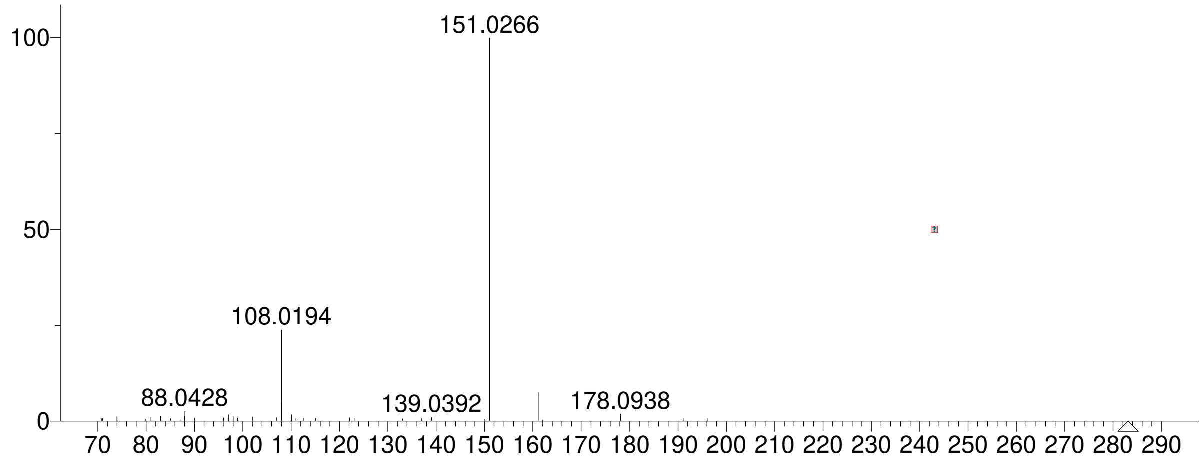

Hit 1 : Xanthosine [M-H]- 5-60V P=283.1
C10H12N4O6; MF: 304; RMF: 838; Prob 49.3%; Lib: mona-export-lc-ms-ms_negative_mode; ID: 8601.

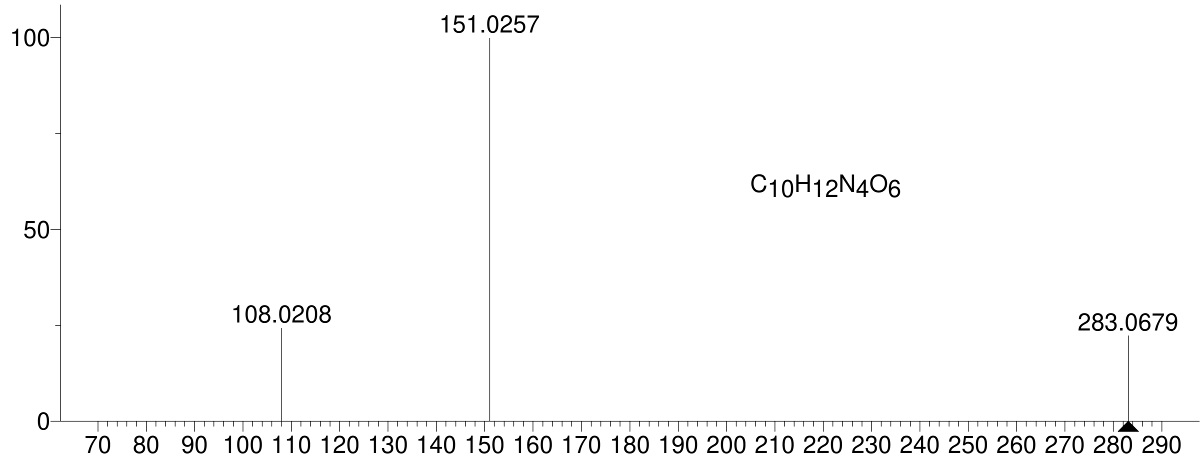

Hit 2 : Xanthosine; LC-ESI-QTOF; MS2; CE [M-H]- P=283.1
C10H12N4O6; MF: 304; RMF: 838; Prob 49.3%; Lib: msms-neg-massbank; ID: 4496.

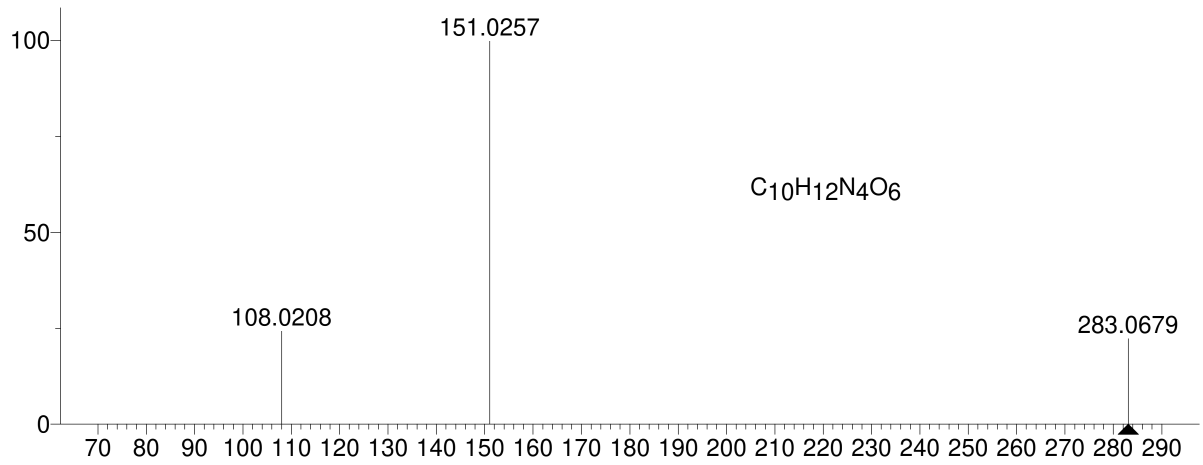

Hit 3 : Xanthosine (Not validated); PlaSMA ID-511 [M-H]- P=283.1
C10H12N4O6; MF: 137; RMF: 796; Prob 1.14%; Lib: biomsms-neg-plasma; ID: 469.

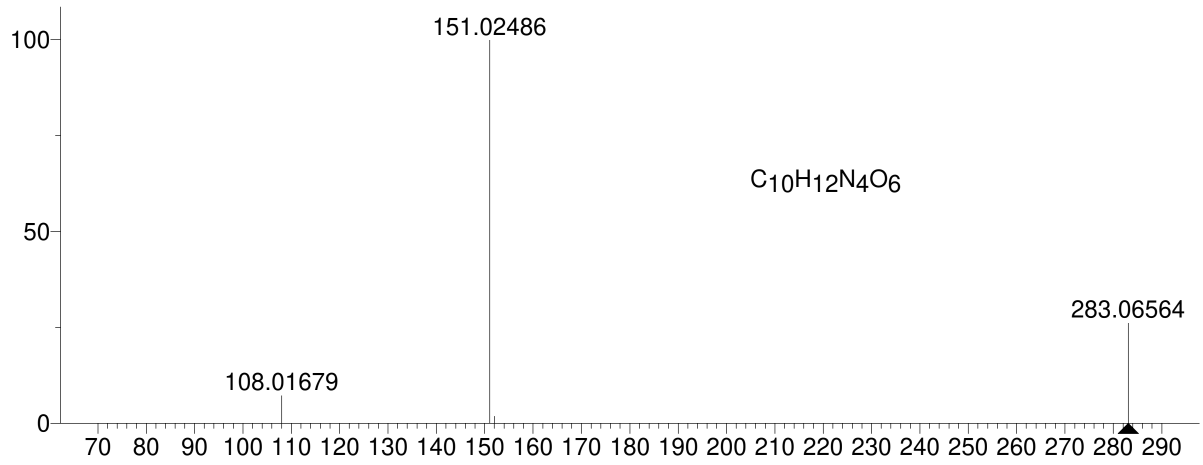

Hit 4 : XANTHOSINE
C10H12N4O6; MF: 46; RMF: 758; Prob 0.09%; Lib: embl neg mcg; ID: 237.

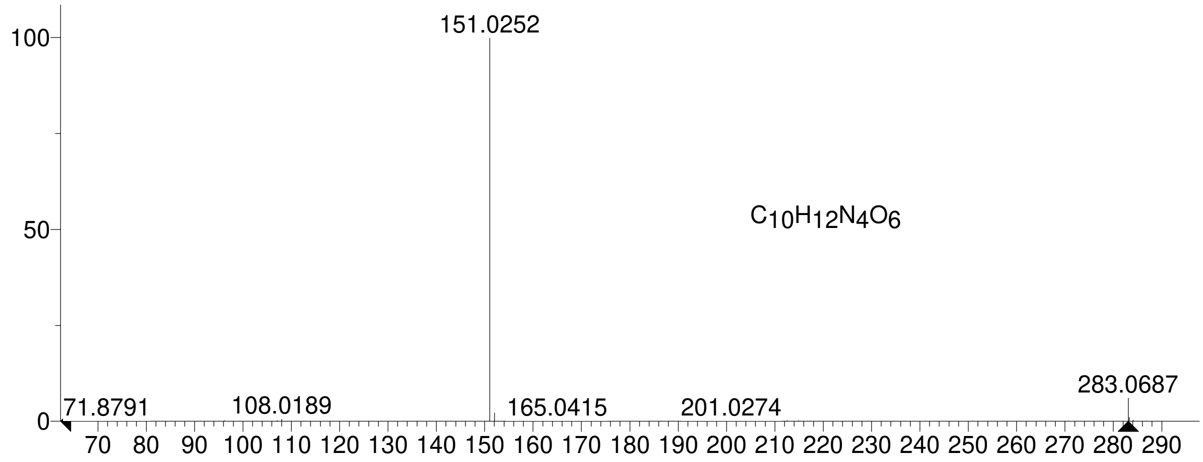

Hit 5 : XANTHOSINE [M-H]- P=283.1
C10H12N4O6; MF: 46; RMF: 758; Prob 0.09%; Lib: msms neg mona_ipb_gnps_plasma; ID: 22900.

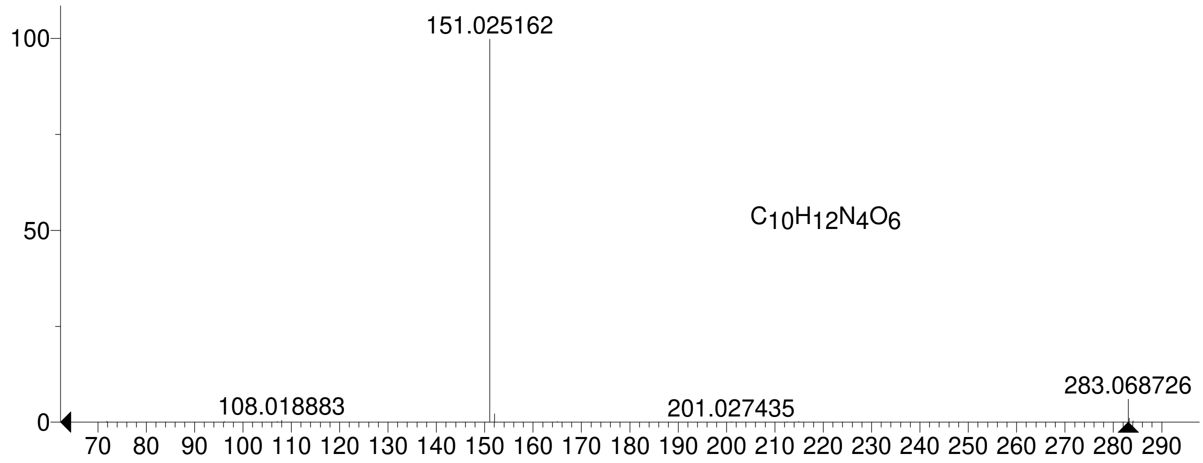

Unknown: Unknown (2.68_426.1259m/z)
Compound in Library Factor = 0

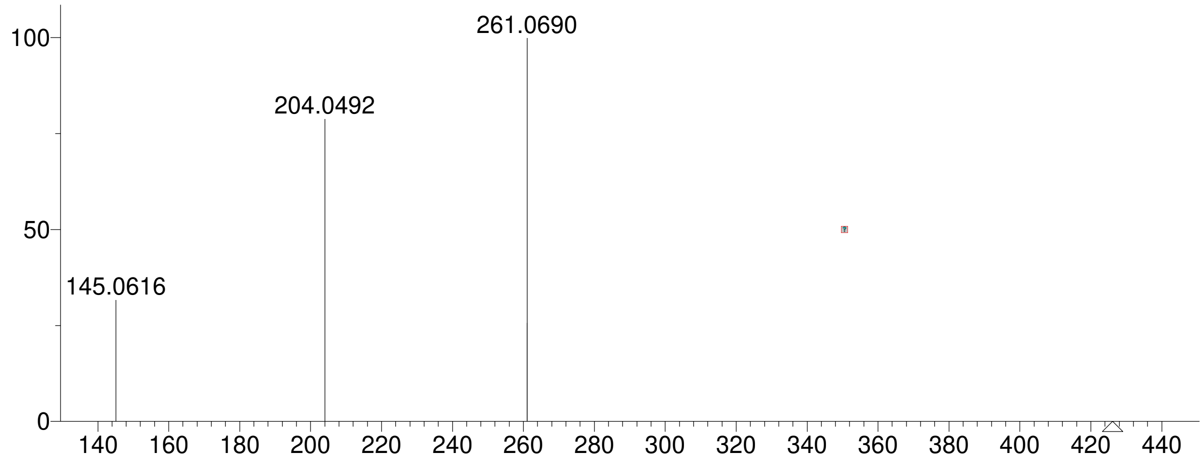

Unknown: Unknown (2.68_431.1296m/z)
Compound in Library Factor = 0

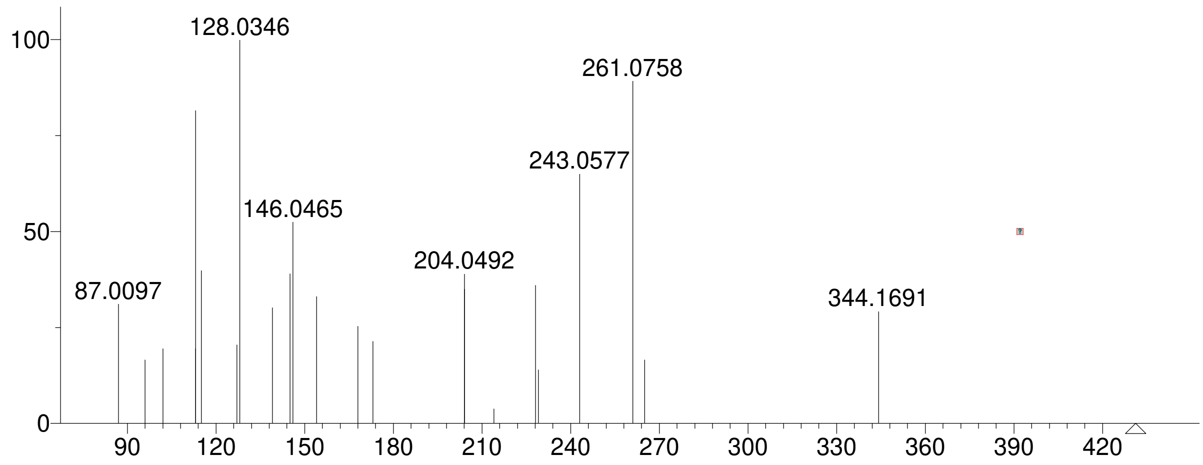

Hit 1 : Agnuside [M+H-2H2O]+ HCD 94V P=431.1
C22H26O11; MF: 0; RMF: 34; Prob 20.0%; CAS: 11027-63-7; Lib: nist_msms; ID: 389587.

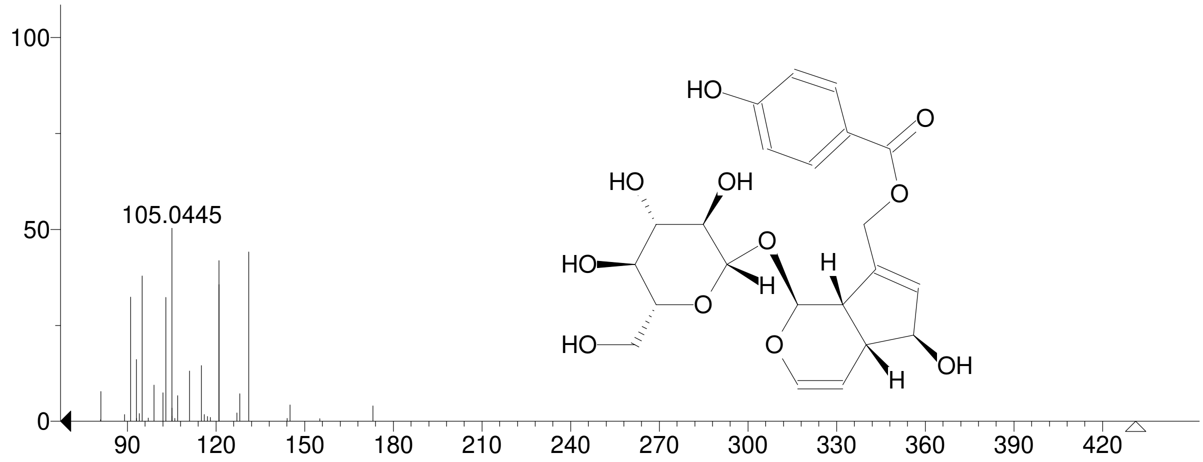

Hit 2 : Formononetin-7-O-glucoside [M+H]+ 50eV P=431.1
C22H22O9; MF: 0; RMF: 18; Prob 20.0%; Lib: anthocyanins mona; ID: 1127.

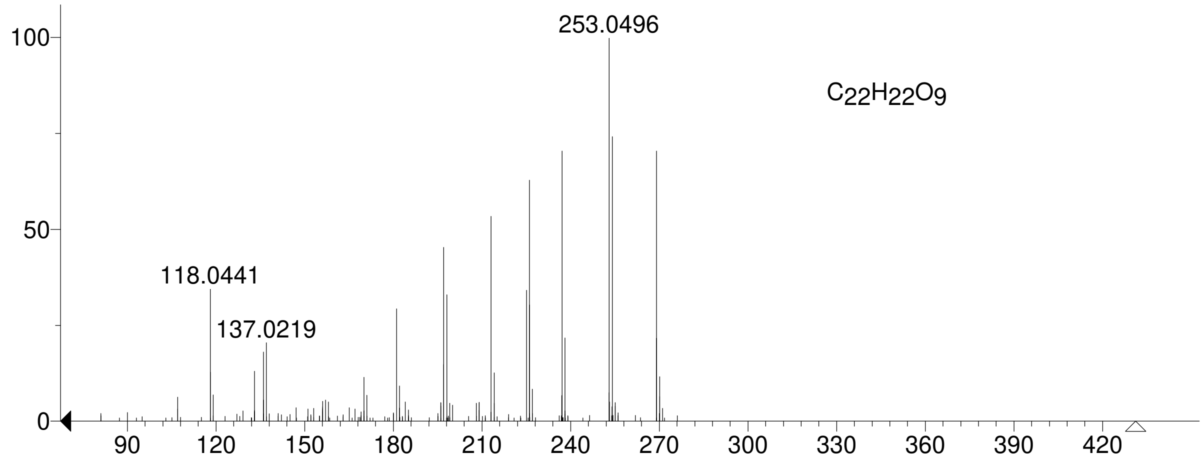

Hit 3 : NCGC00169054-02!3-(4-methoxyphenyl)-7-[(2S,3R,4S,5S,6R)-3,4,5-trihydroxy-6-(hydroxymethyl)oxan-2-y
C22H22O9; MF: 0; RMF: 5; Prob 20.0%; Lib: mona-export-gnps; ID: 9246.

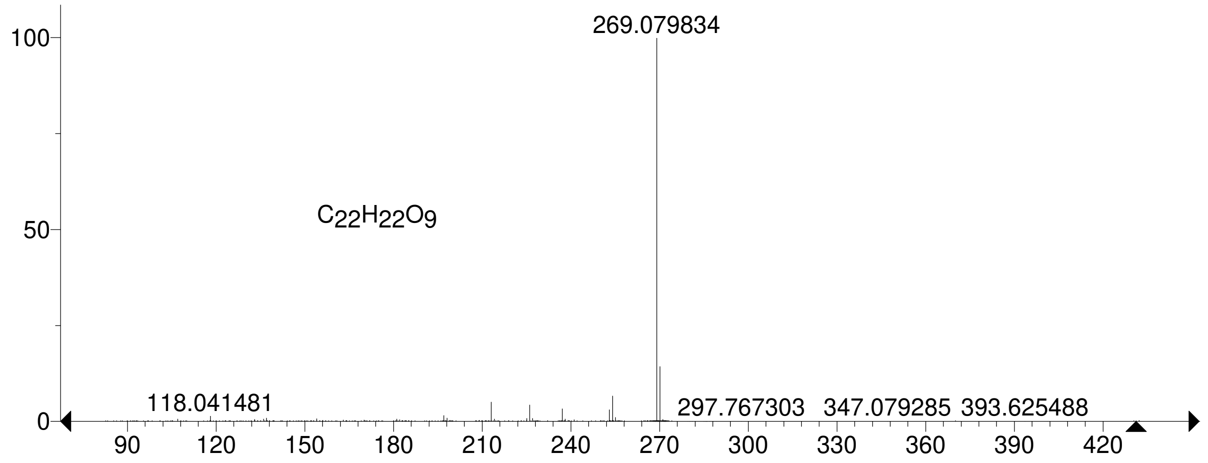

Hit 4 : calycosin-7-O-beta-D-glucoside [M+H]+ 40 P=431.1
C22H22O9; MF: 0; RMF: 5; Prob 20.0%; Lib: mona-export-vf-npl_qtof; ID: 916.

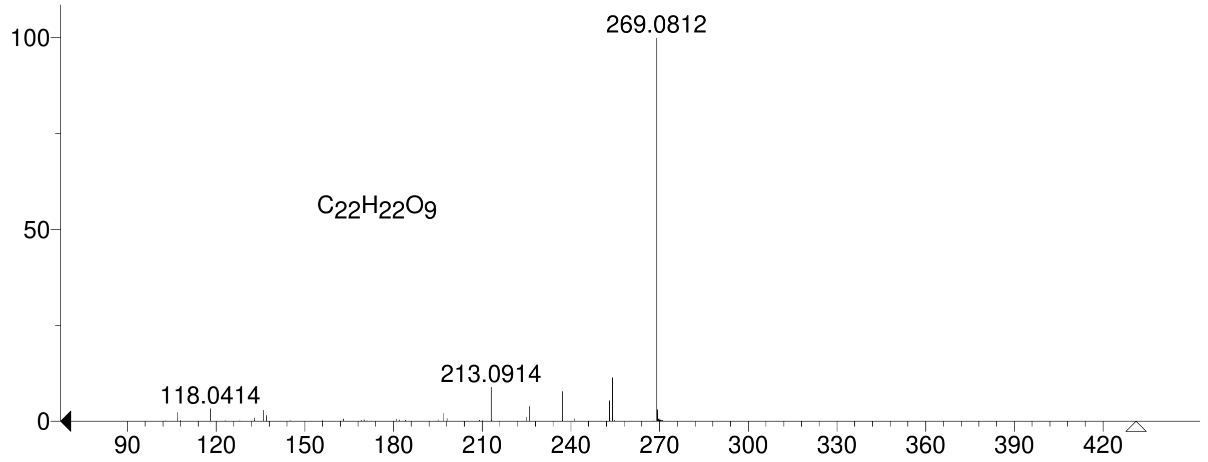

Hit 5 : (1S,3R,6R,7S,8S,10R,13S,16S,17R)-8-tert-butyl-6,17-dihydroxy-16-methyl-2,4,14,19-tetraoxahexacyclo[8.
C20H24O9; MF: 0; RMF: 2; Prob 20.0%; Lib: mona-export-vf-npl_qexactive; ID: 10727.

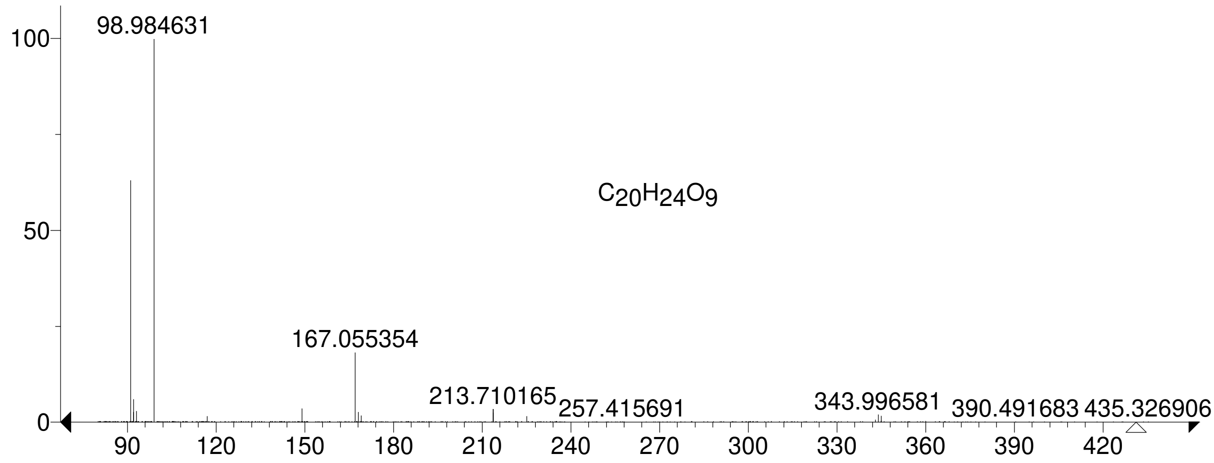

Unknown: Unknown (2.68_616.2528m/z)
Compound in Library Factor = 0

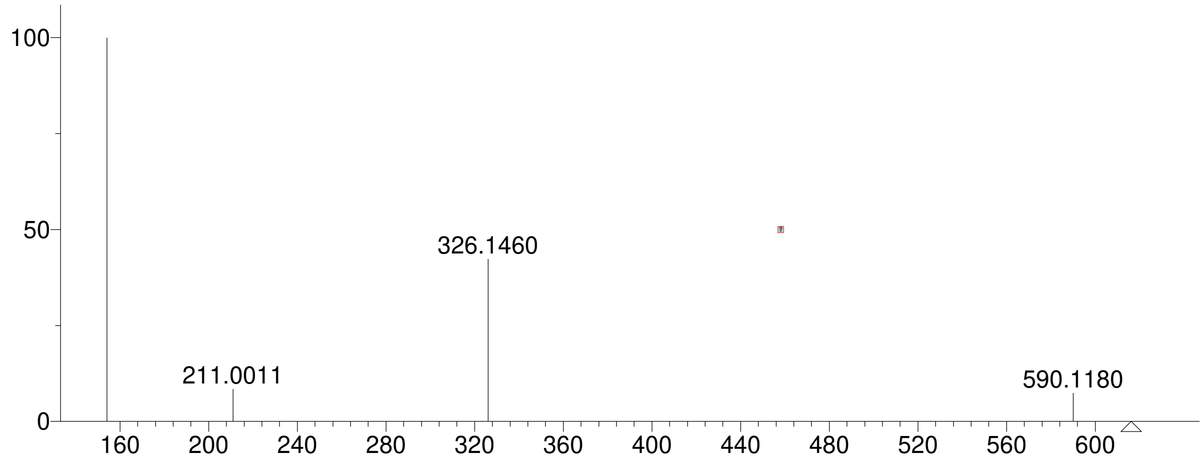

Unknown: Unknown (2.68_732.2837m/z)
Compound in Library Factor = 0

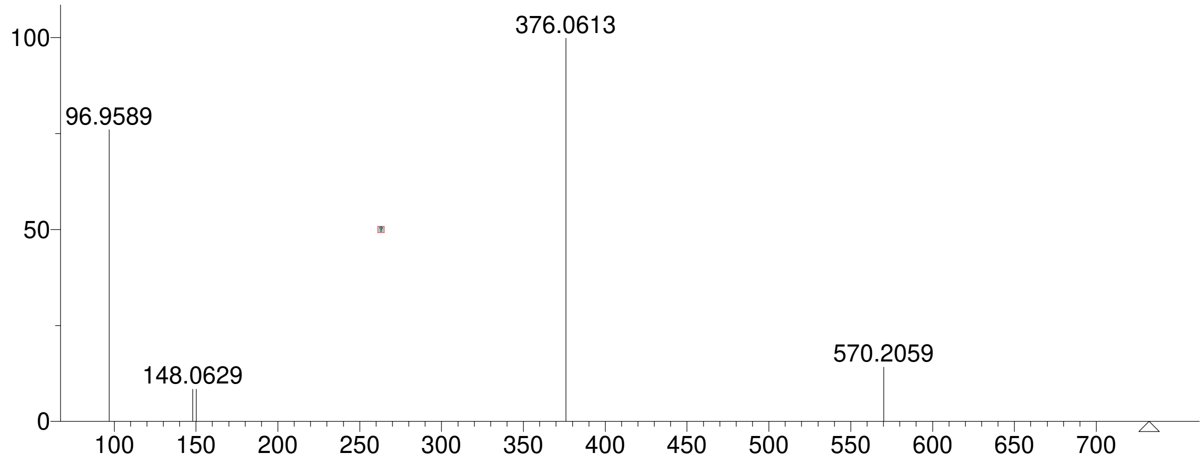

Unknown: Unknown (2.70_588.1090m/z)
Compound in Library Factor = 0

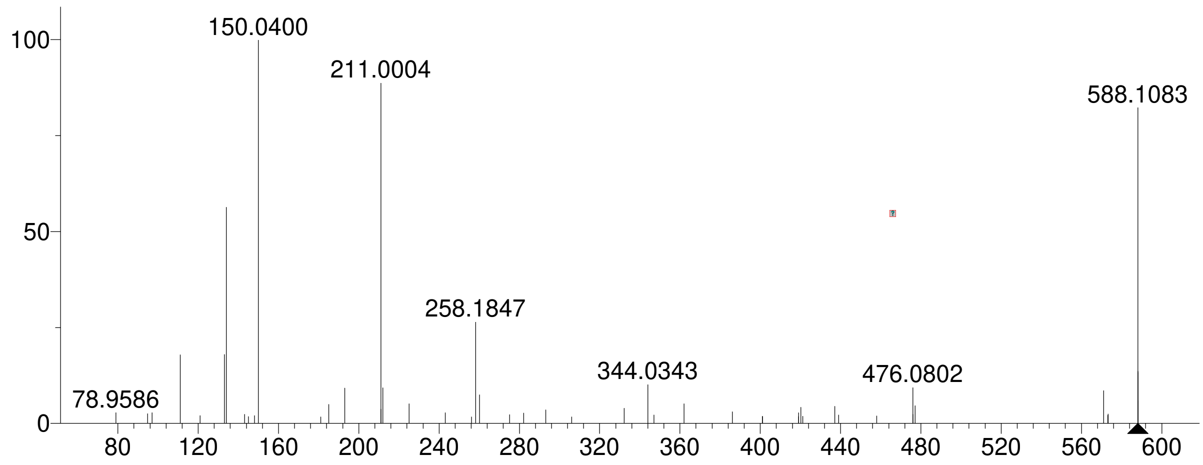

Unknown: Unknown (2.70_595.1416m/z)
Compound in Library Factor = -2176

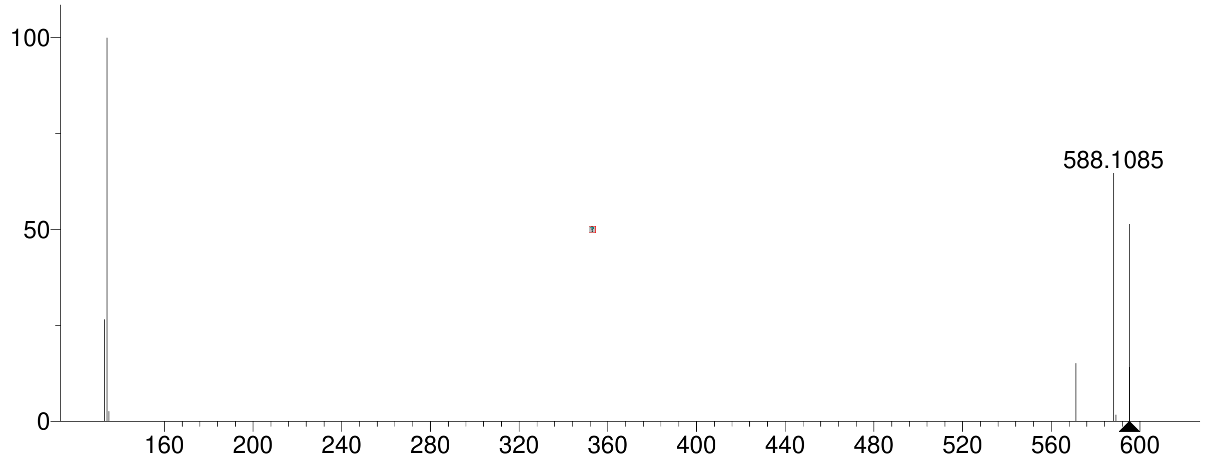

Hit 1 : Tiliroside [M+H]+ HCD 2V P=595.1
C30H26O13; MF: 30; RMF: 401; Prob 27.3%; CAS: 20316-62-5; Lib: nist_msms; ID: 390877.

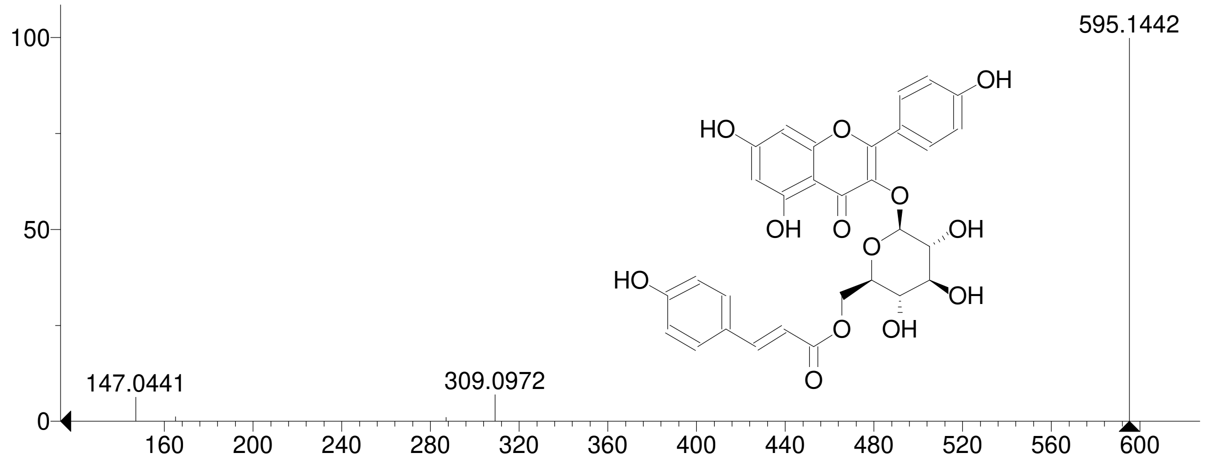

Hit 2 : Flavonol base + 3O, O-Hex, O-coumaroyl [M+H]+ 6V P=595.1
C30H26O13; MF: 5; RMF: 289; Prob 8.32%; Lib: anthocyanins mona; ID: 8311.

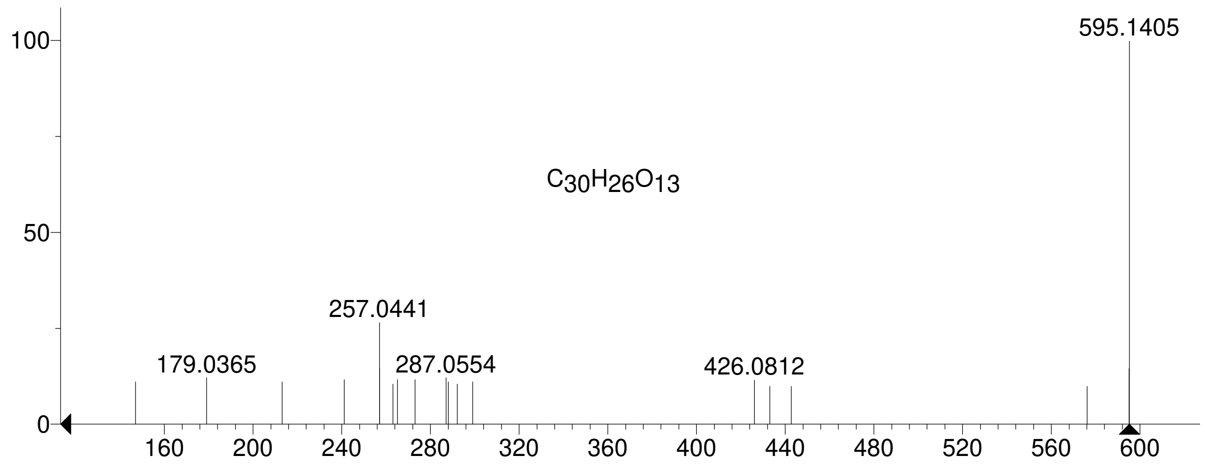

Hit 3 : 7-O-??-(6???-trans-p-coumaroyl)-glucopyranosyl-8,3???,4???-trihydroxyflavanone [M-H]- P=595.1
C30H28O13; MF: 4; RMF: 295; Prob 8.00%; Lib: msms-neg-gnps; ID: 78.

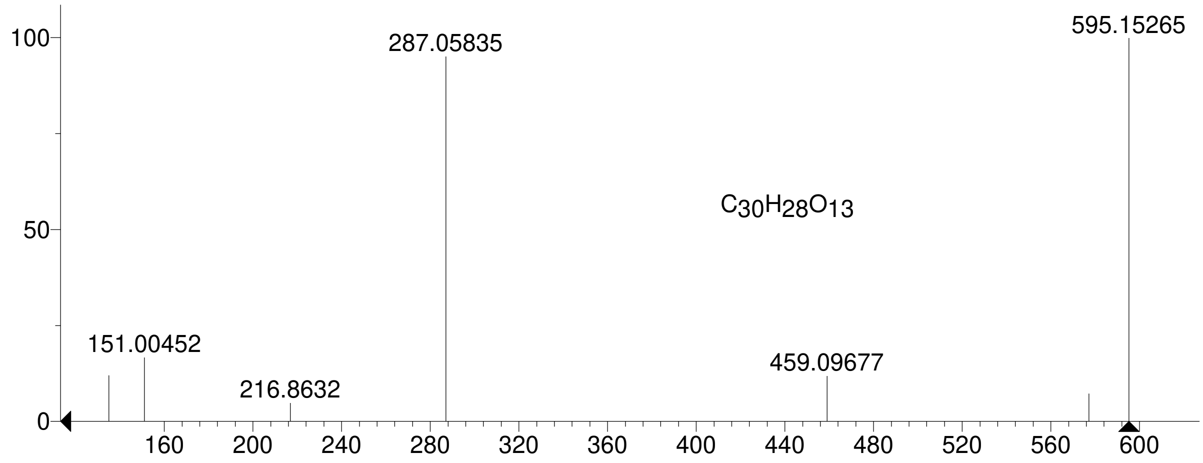

Hit 4 : Kaempferol-3-O-glucoside-6"-p-coumaroyl [M+H]+ 10eV P=595.1
C30H26O13; MF: 3; RMF: 332; Prob 7.69%; Lib: anthocyanins mona; ID: 1220.

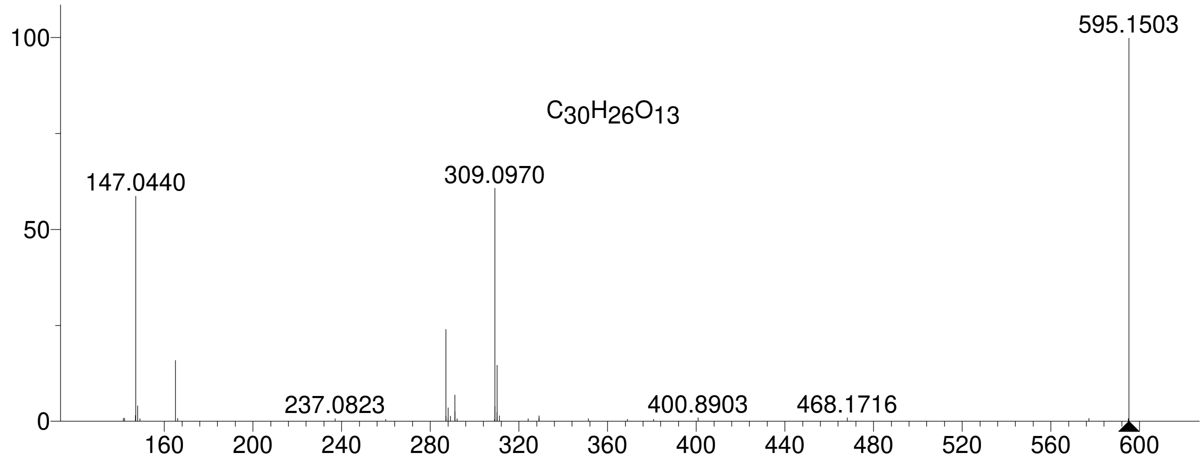

Hit 5 : 4???-O-??-(6???-trans-p-coumaroyl)-glucopyranosyl okanin [M-H]- P=595.1
C30H28O13; MF: 2; RMF: 129; Prob 7.39%; Lib: msms-neg-gnps; ID: 62.

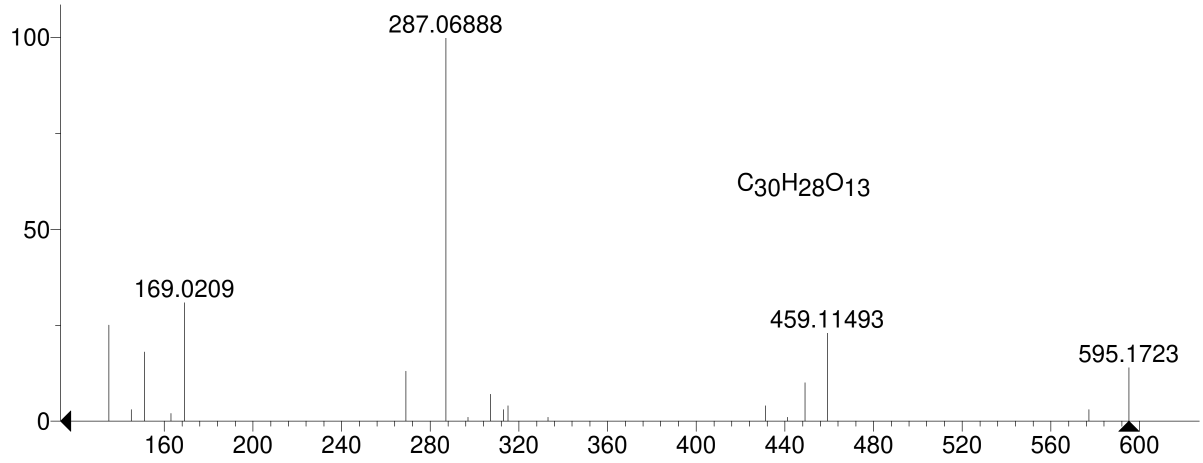

Unknown: Unknown (2.71_355.1970m/z)
Compound in Library Factor = -3273

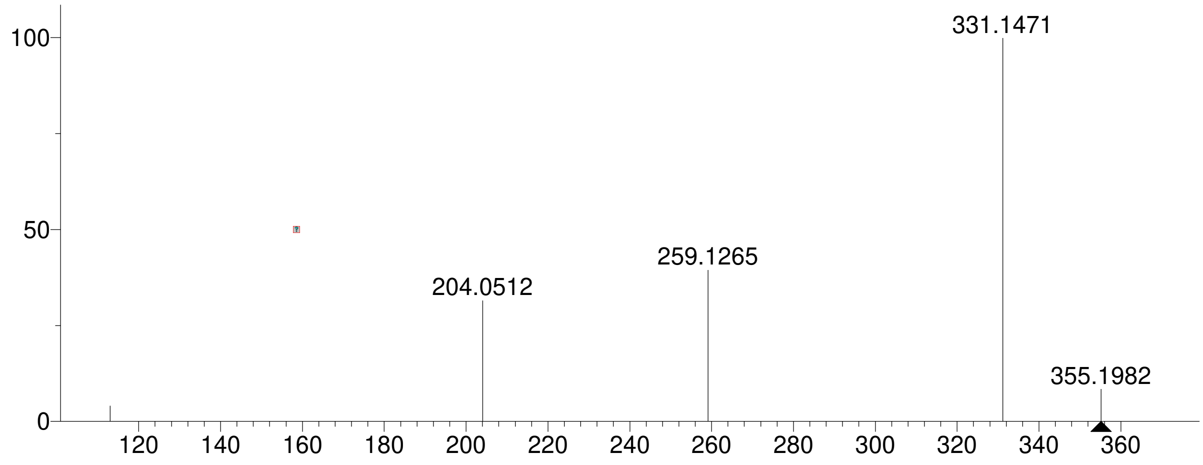

Hit 1 : PyroGlu-Pro-Lys [M+H]+ QQQ 12V P=355.2
C16H26N4O5; MF: 13; RMF: 211; Prob 9.78%; Lib: nist_msms; ID: 44842.

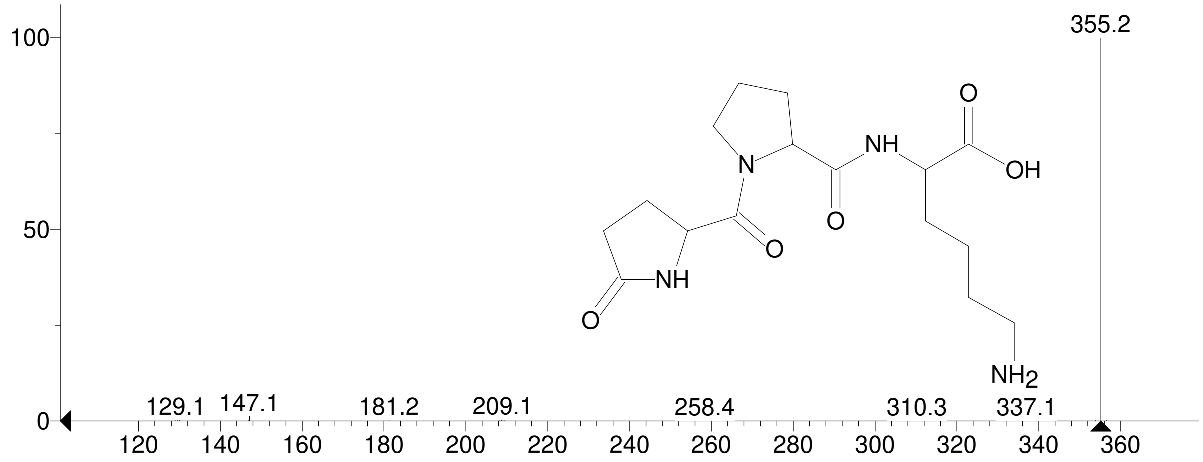

Hit 2 : Quinoline, 4-[5,6-dihydro-2-(2-pyridinyl)-4H-pyrrolo[1,2-b]pyrazol-3-yl]-7-[2-(4-morpholinyl)ethoxy]- [M+H]+
C26H27N5O2; MF: 8; RMF: 188; Prob 7.88%; CAS: 700874-71-1; Lib: nist_msms; ID: 369275.

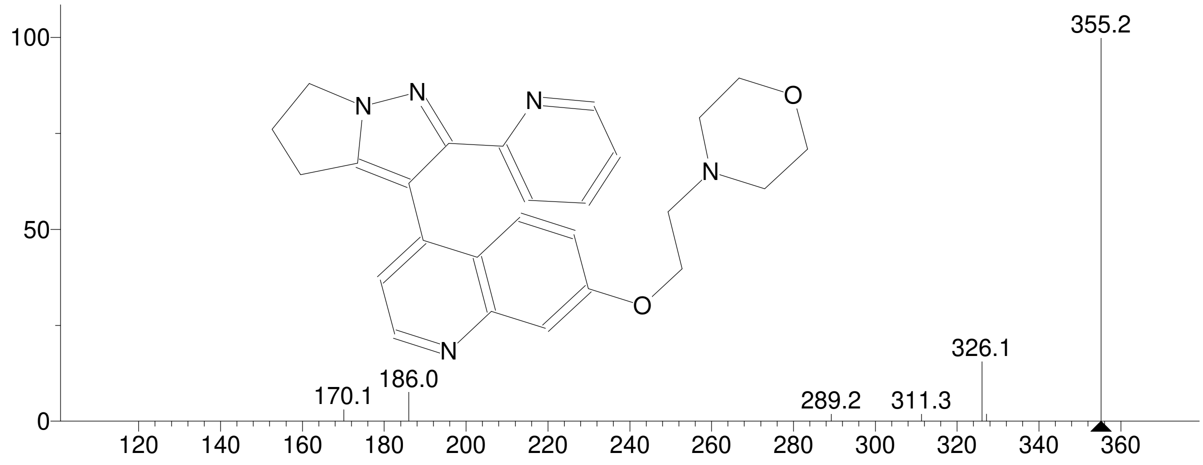

Hit 3 : Carbenoxolone [M-H]-=>469.3=>355.2 IT 35% P=355.2
C34H50O7; MF: 0; RMF: 62; Prob 5.88%; CAS: 5697-56-3; Lib: nist_msms; ID: 153421.

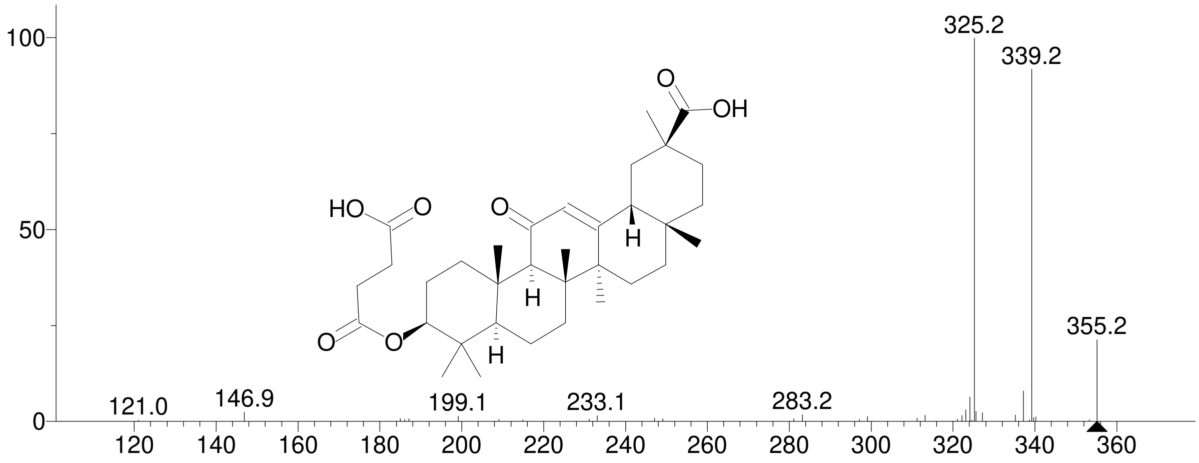

Hit 4 : Nobiletin [M+H]+=>388.4=>355.2 IT 35% P=355.2
C21H22O8; MF: 0; RMF: 60; Prob 5.88%; CAS: 478-01-3; Lib: nist_msms; ID: 178356.

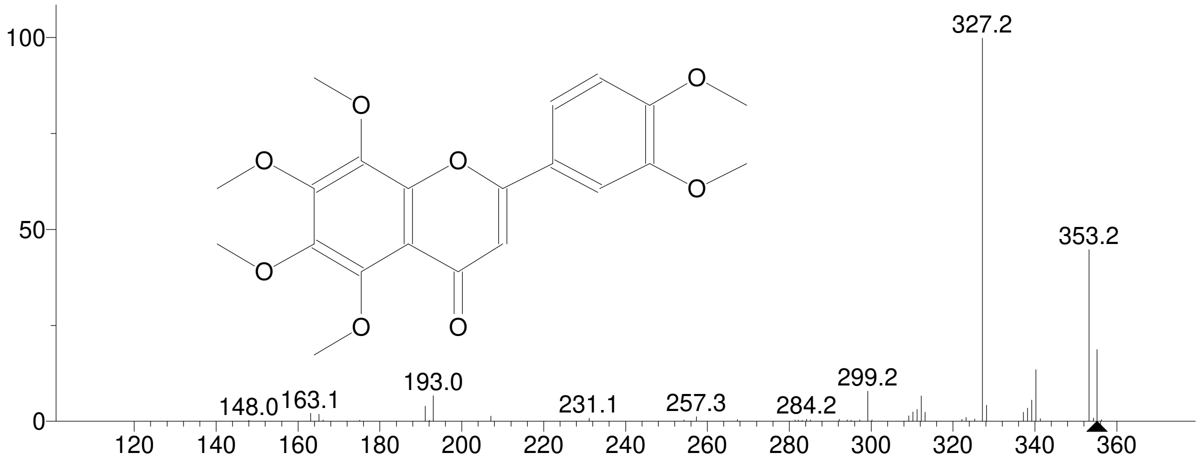

Hit 5 : 15-Ketoprostaglandin E2 [M+Na]+=>355.2 IT 35% P=355.2
C20H30O5; MF: 0; RMF: 48; Prob 5.88%; CAS: 26441-05-4; Lib: nist_msms; ID: 169440.

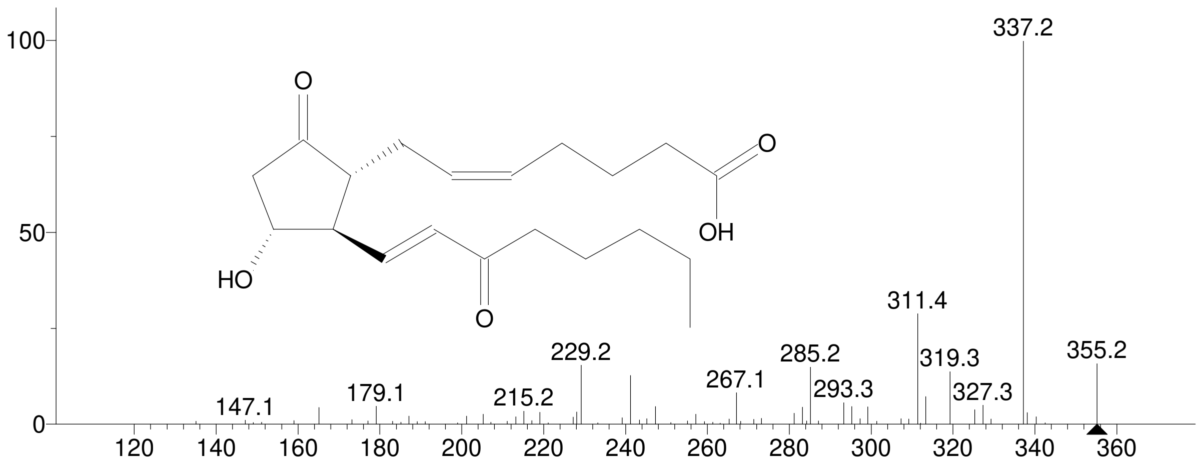

Unknown: Unknown (2.71_432.1260m/z)
Compound in Library Factor = 0

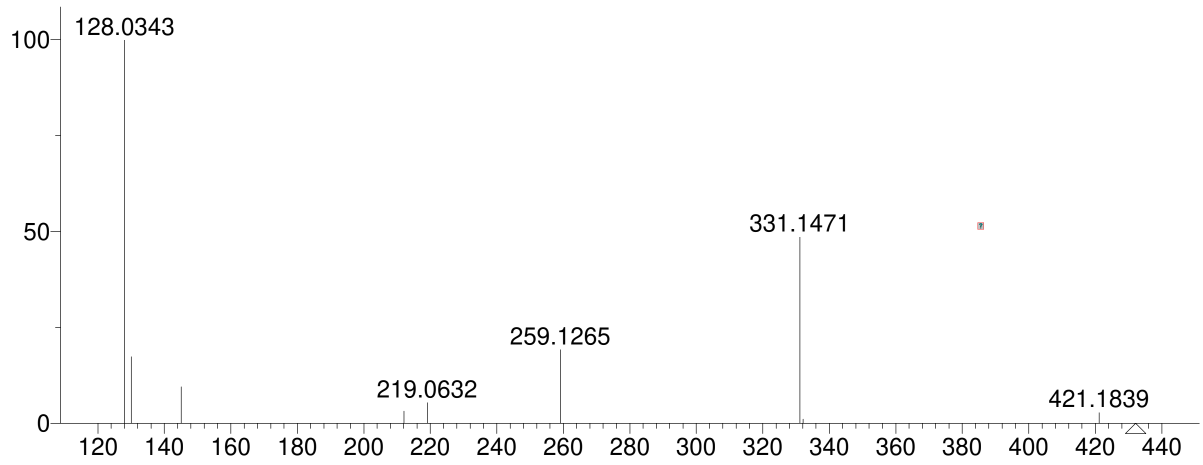

Unknown: Unknown (2.72_355.0239m/z)
Compound in Library Factor = 0

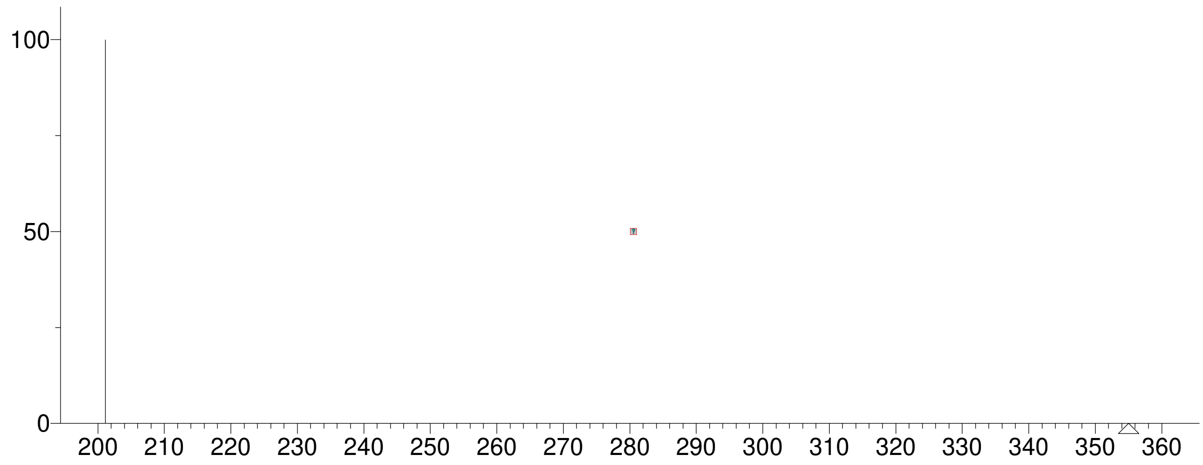

** Search Report Page 1 of 2 **

Unknown: Unknown (2.72_573.0992m/z)
Compound in Library Factor = 0

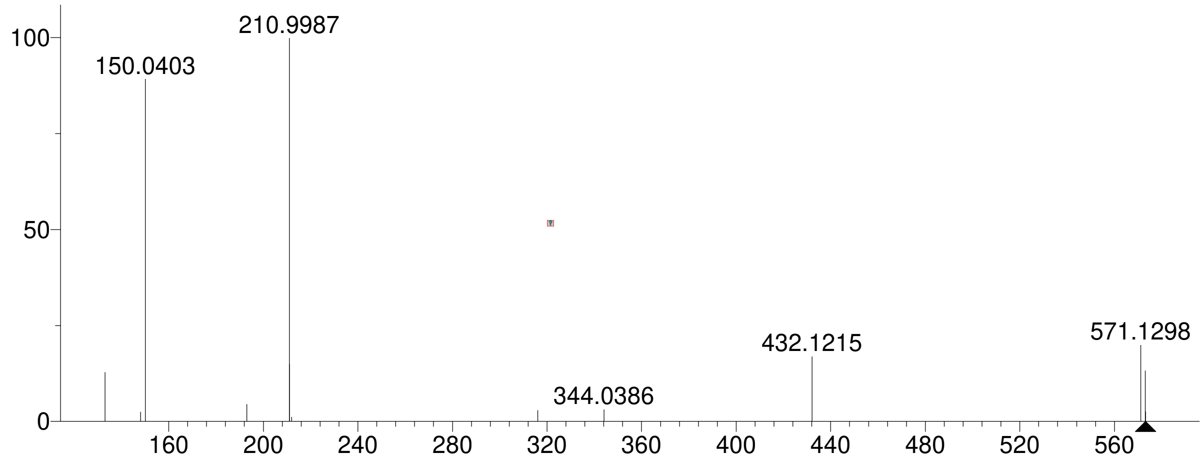

Hit 1 : NCGC00385638-01!2,2'-bis(3,4-dihydroxyphenyl)-3',4,5',6-tetrahydroxyspiro[2H-1-benzofuran-3,9'-3,4-dih
C30H22O12; MF: 0; RMF: 51; Prob 25.0%; Lib: mona-export-gnps; ID: 8139.

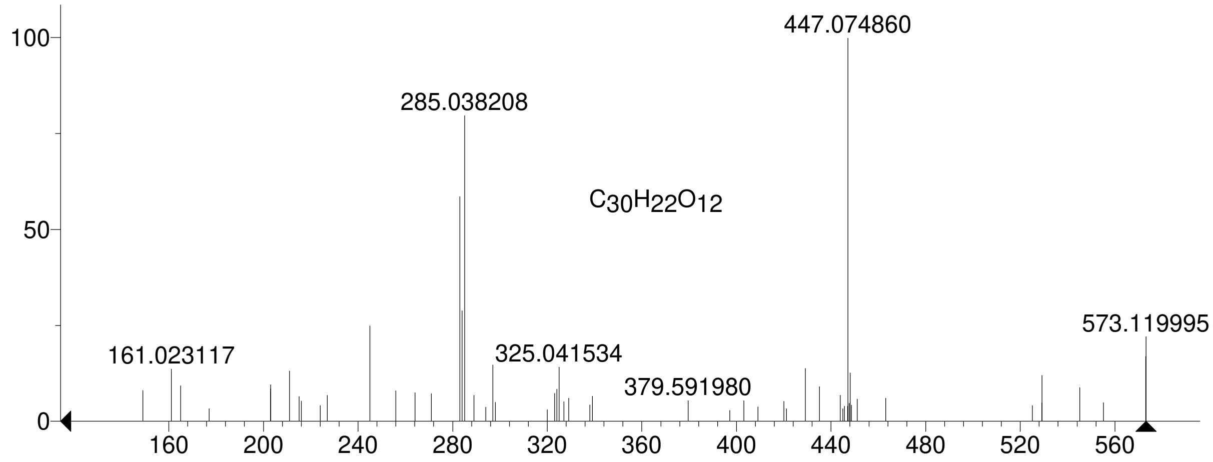

Hit 2 : Theaflavin 3-gallate [M+H-H2O]+=>573.1 IT 35% P=573.1
C36H28O16; MF: 0; RMF: 10; Prob 25.0%; CAS: 30462-34-1; Lib: nist_msms; ID: 394282.

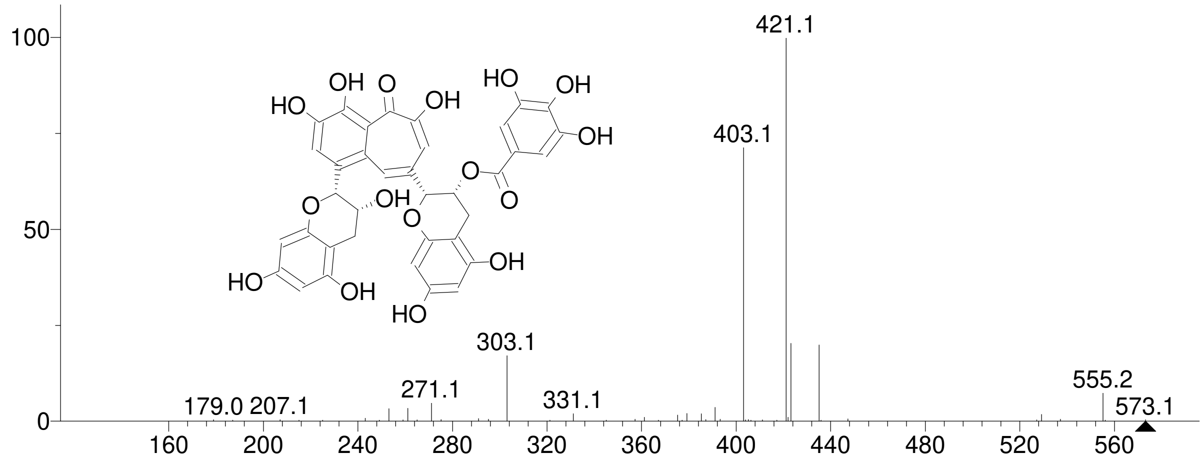

Hit 3 : β-Nicotinamide adenine dinucleotide, reduced [M+Na]+=>671.1=>573.1 IT 35% P=573.1
C21H29N7O14P2; MF: 0; RMF: 5; Prob 25.0%; CAS: 58-68-4; Lib: nist_msms; ID: 114554.

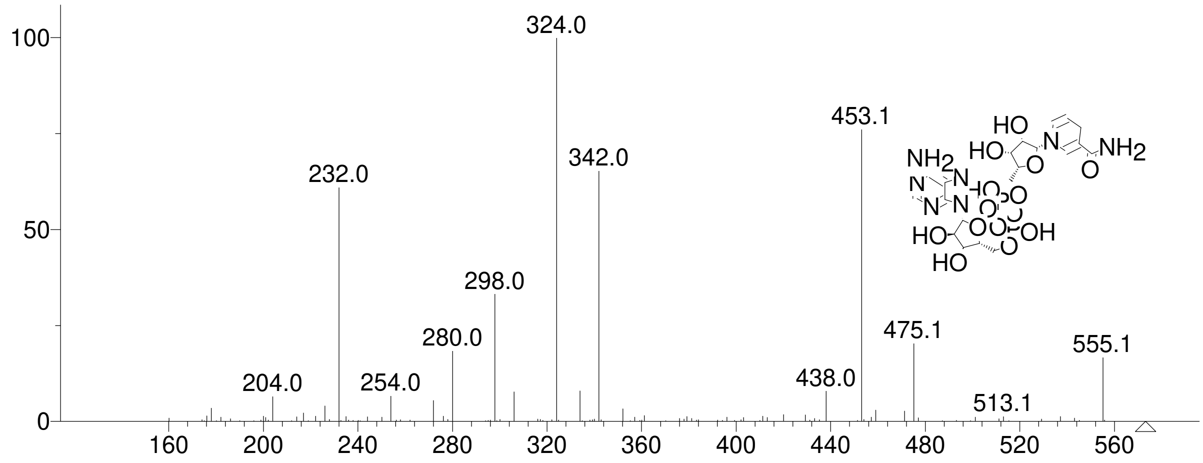

Hit 4 : 3-[5,7-dihydroxy-4-(4-hydroxyphenyl)-2-oxo-3,4-dihydrochromen-3-yl]-5,6,7,8-tetrahydroxy-4-(4-hydroxyphe
C30H22O12; MF: 0; RMF: 4; Prob 25.0%; Lib: mona-export-lc-ms-ms_negative_mode; ID: 23407.

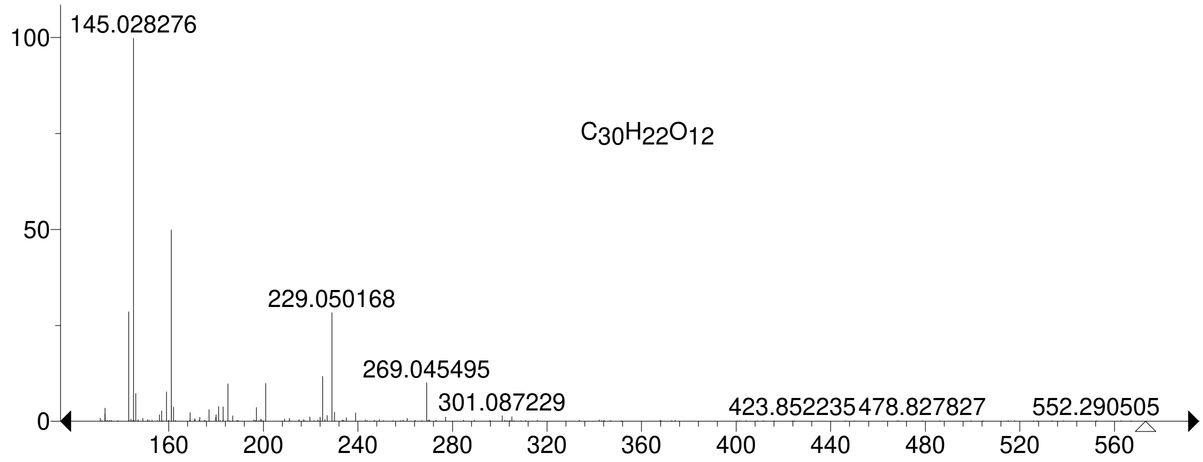

Unknown: Unknown (2.72_596.1269m/z)
Compound in Library Factor = 0

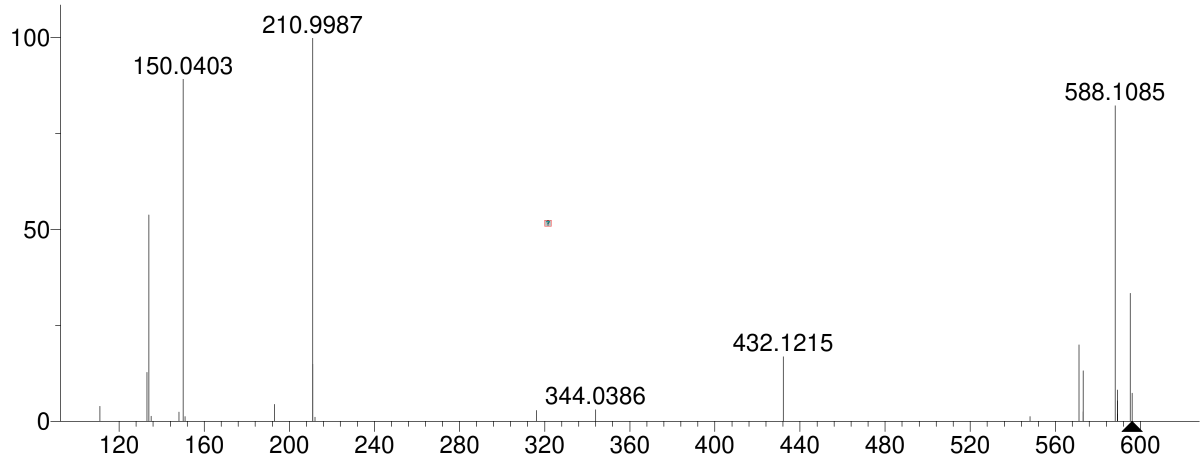

** Search Report Page 1 of 1 **

Unknown: Unknown (2.72_603.1817m/z)
Compound in Library Factor = 0

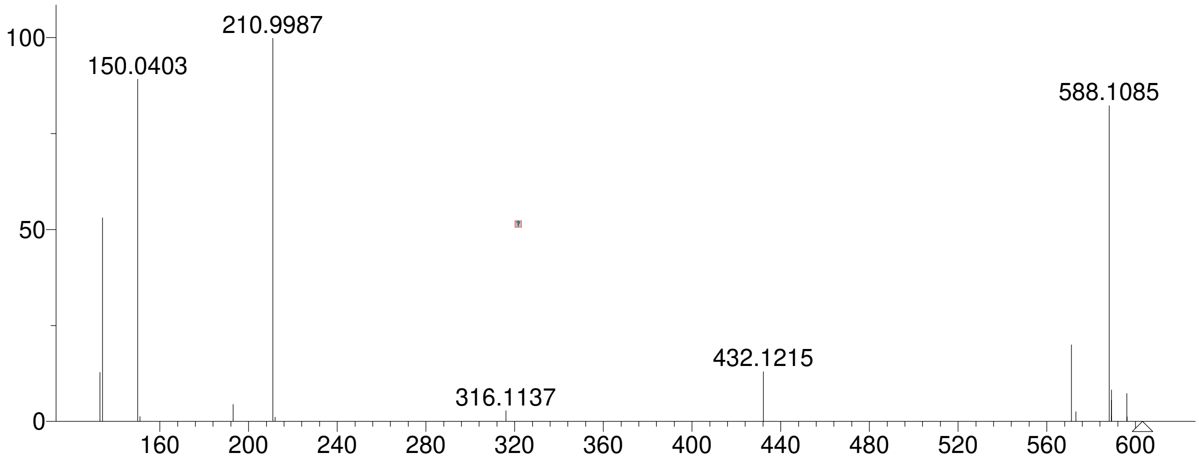

** Search Report Page 1 of 2 **

Unknown: Unknown (2.73_403.1335m/z)
Compound in Library Factor = 0

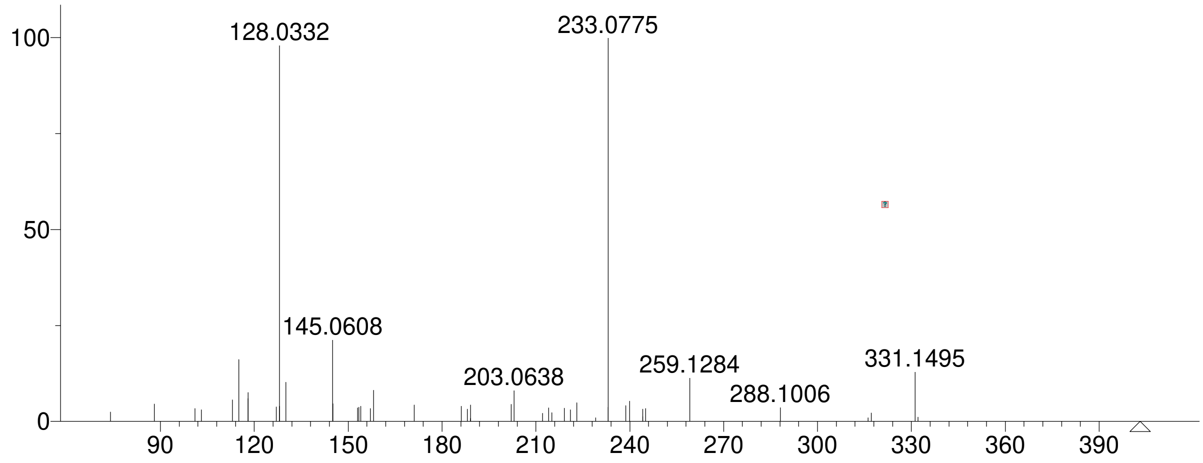

Hit 1 : DEHYDROCHOLIC ACID [M+H]+ 35% P=403.1
C24H34O5; MF: 0; RMF: 38; Prob 25.0%; Lib: mona-export-itree; ID: 2247.

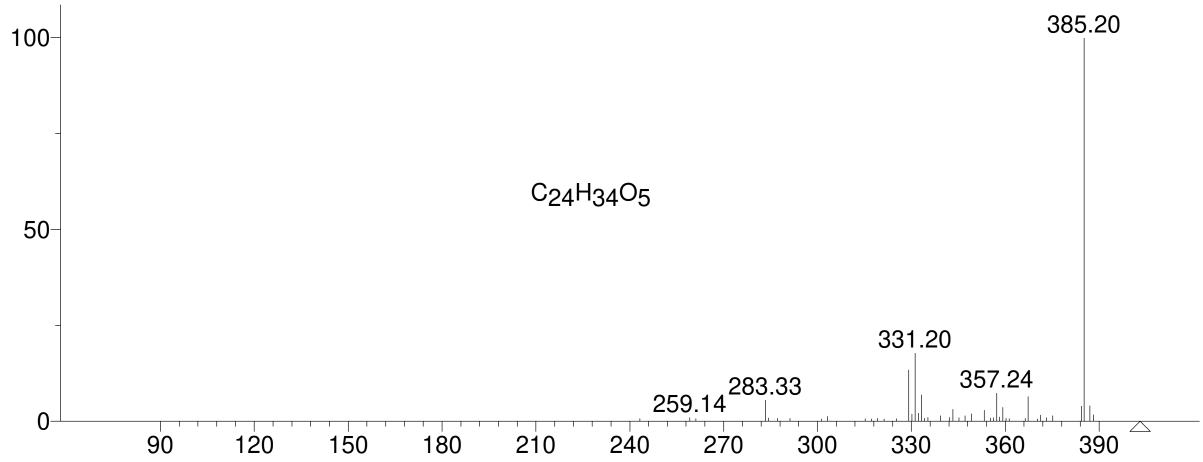

Hit 2 : methyl 2-(4,5-dihydroxy-6-methoxy-3,6-dimethyl-2-oxocyclohex-3-en-1-yl)oxy-4-hydroxy-3,6-dimethylbenzc
C19H24O8; MF: 0; RMF: 29; Prob 25.0%; Lib: mona-export-vf-npl_qexactive; ID: 23486.

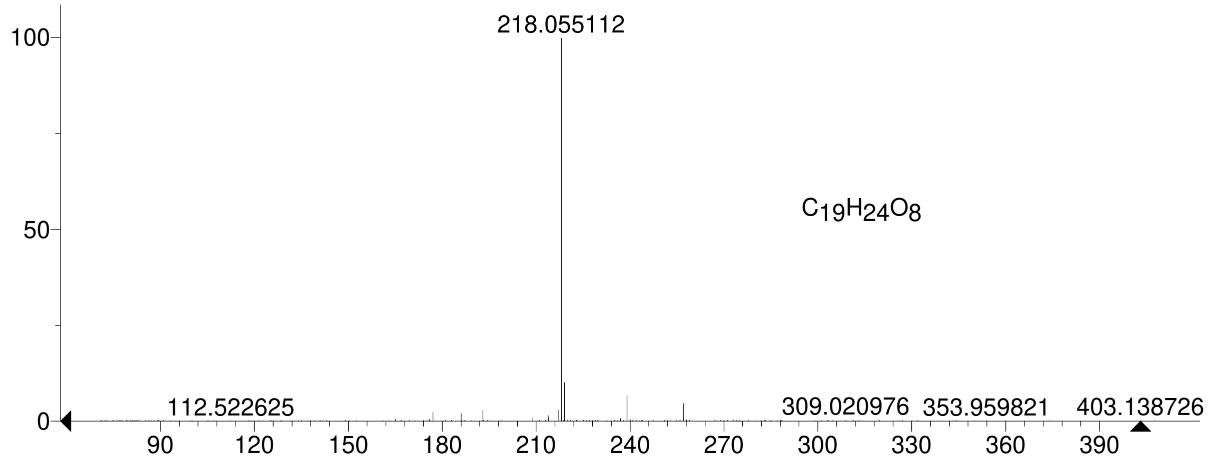

Hit 3 : NCGC00347547-02!methyl 2-(4,5-dihydroxy-6-methoxy-3,6-dimethyl-2-oxocyclohex-3-en-1-yl)oxy-4-hydro:
C19H24O8; MF: 0; RMF: 20; Prob 25.0%; Lib: mona-export-gnps; ID: 8950.

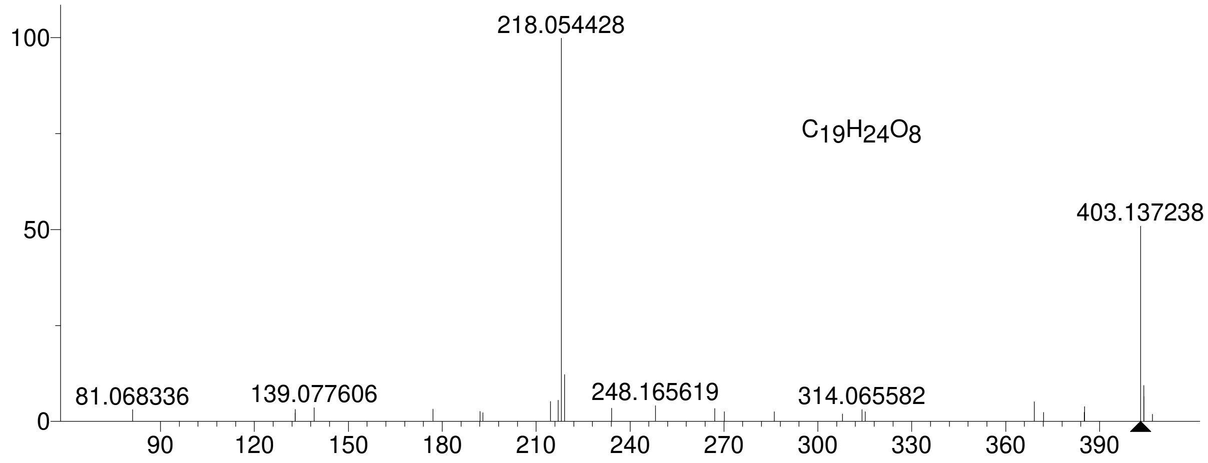

Hit 4 : Egtazic acid [M+Na]+ HCD 52V P=403.1
C14H24N2O10; MF: 0; RMF: 20; Prob 25.0%; CAS: 67-42-5; Lib: nist_msms; ID: 81815.

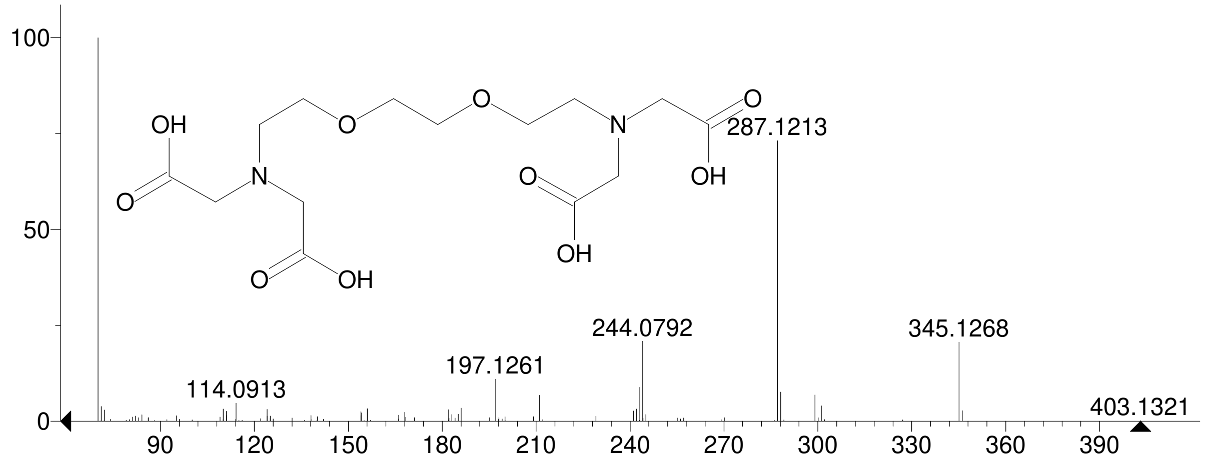

** Search Report Page 1 of 1 **

Unknown: Unknown (2.73_508.2128m/z)
Compound in Library Factor = 0

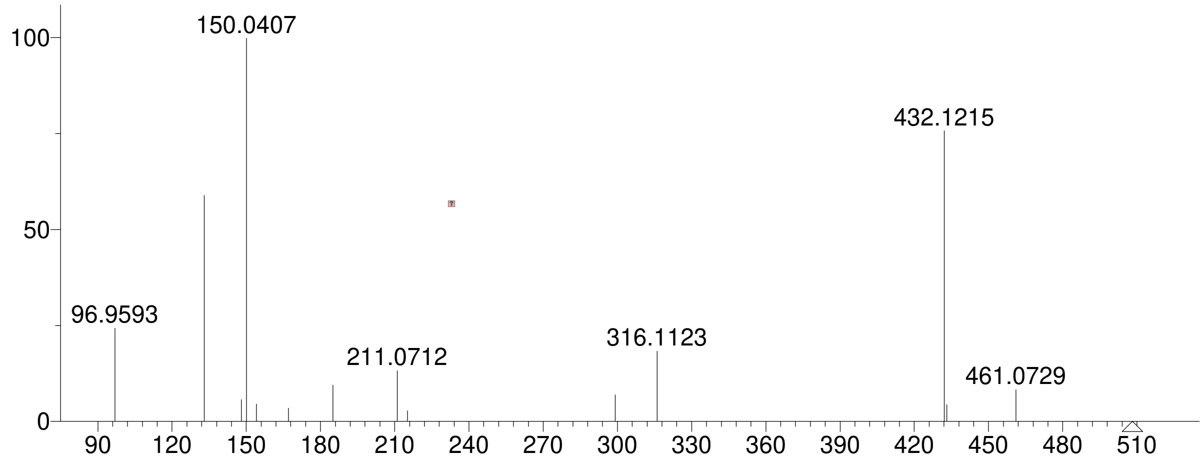

Hit 1 : β-Lipotropin (61-69) [M-2H]2- HCD 41eV P=508.2
C45H66N10O15S; MF: 0; RMF: 27; Prob 100.0%; Lib: nist_msms2; ID: 77123.

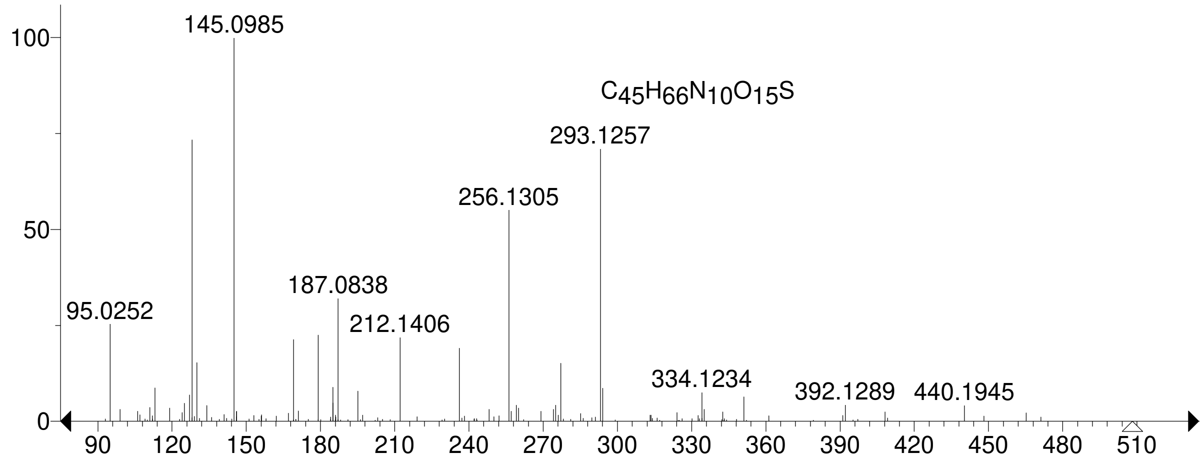

Unknown: Unknown (2.75_309.1075m/z)
Compound in Library Factor = -236

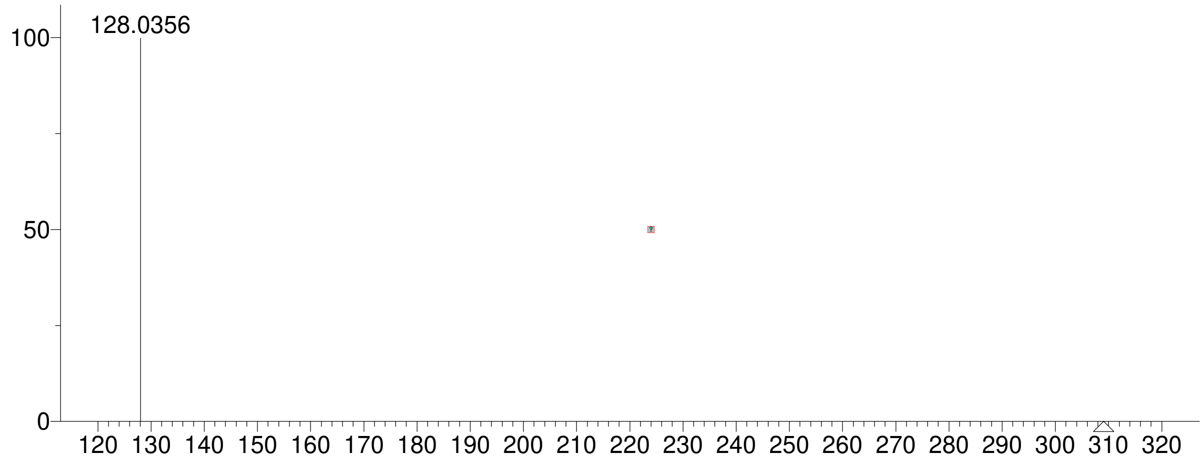

Hit 1 : gamma-Glu-Tyr [M-H]- 35eV P=309.1
C14H18N2O6; MF: 299; RMF: 944; Prob 97.4%; Lib: msms-neg-fiehnhilic; ID: 990.

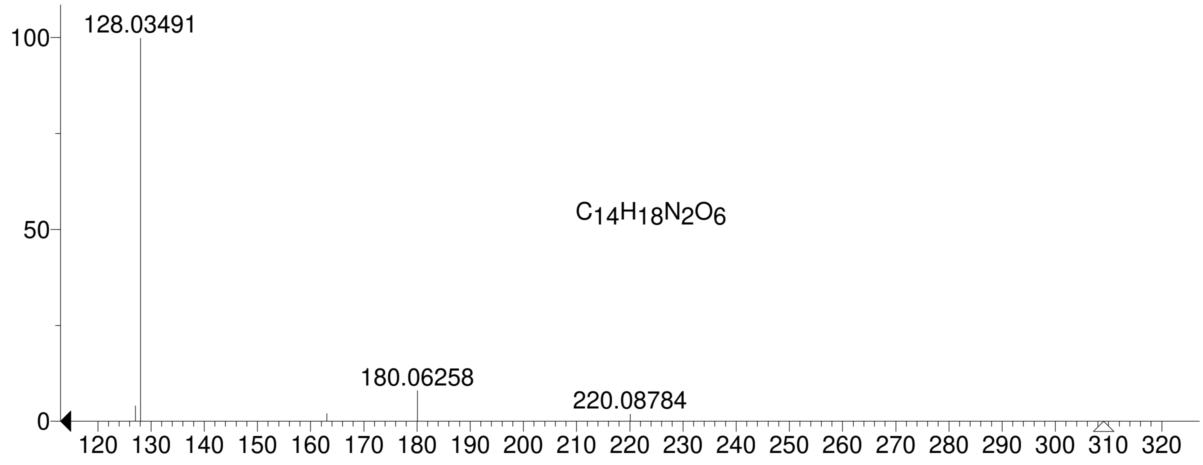

Hit 2 : gamma-Glutamyltyrosine [M-H]- 20-30-40%) P=309.1
C14H18N2O6; MF: 44; RMF: 681; Prob 0.97%; Lib: msms-neg-fiehnhilic; ID: 331.

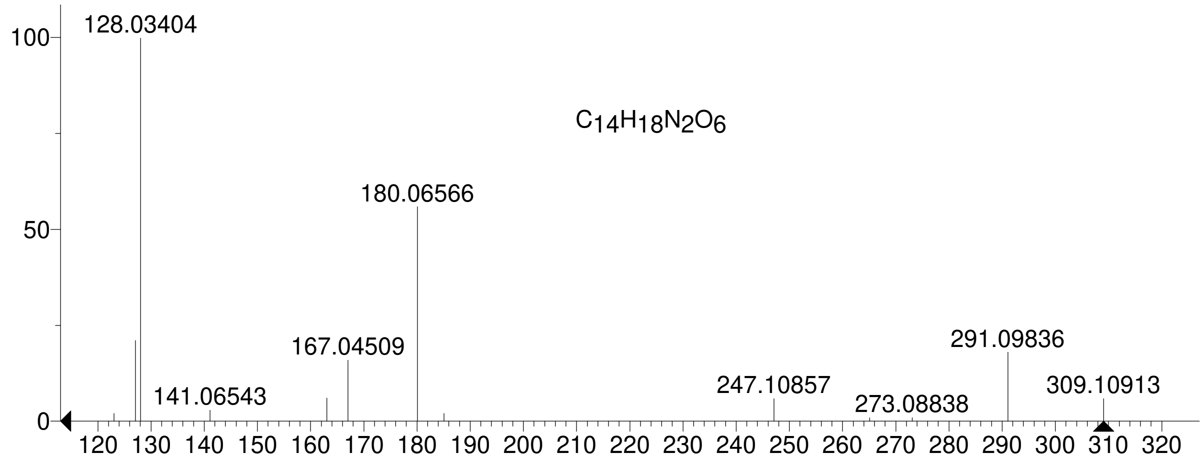

Hit 3 : Glutamyltyrosine; PlaSMA ID-626 [M-H]- P=309.1
C14H18N2O6; MF: 32; RMF: 726; Prob 0.64%; Lib: biomsms-neg-plasma; ID: 743.

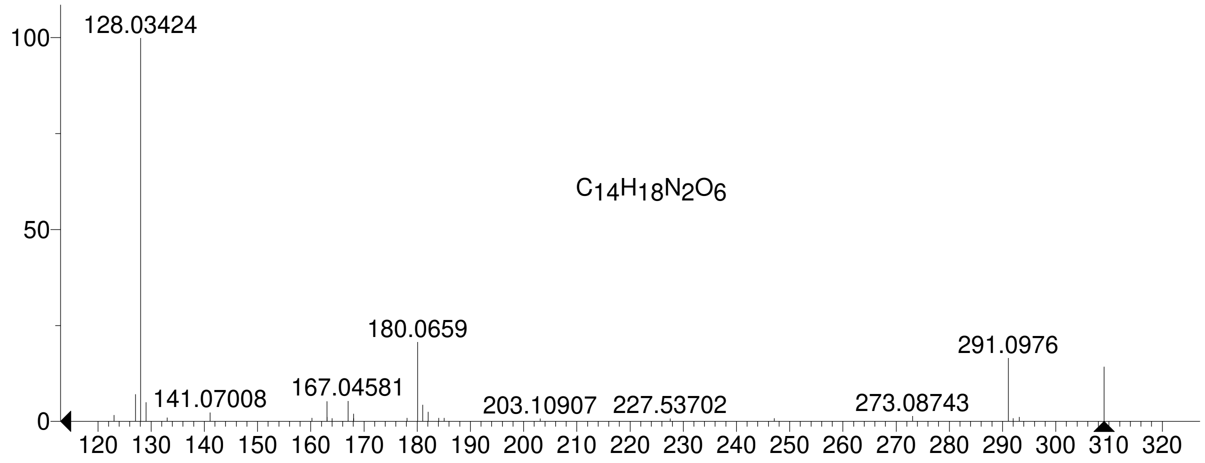

Hit 4 : Tyr-Glu [M-H]- HCD 49eV P=309.1
C14H18N2O6; MF: 15; RMF: 569; Prob 0.35%; Lib: nist_msms; ID: 571144.

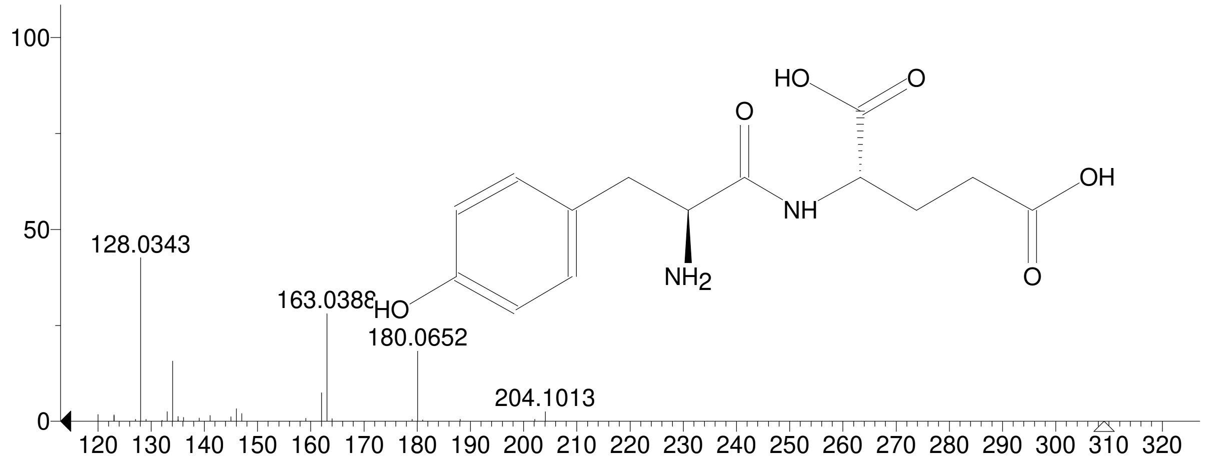

Hit 5 : Tyr-Glu [M-H]- HCD 49eV P=309.1
C14H18N2O6; MF: 15; RMF: 569; Prob 0.35%; CAS: 2545-89-3; Lib: nist_msms2; ID: 84877.

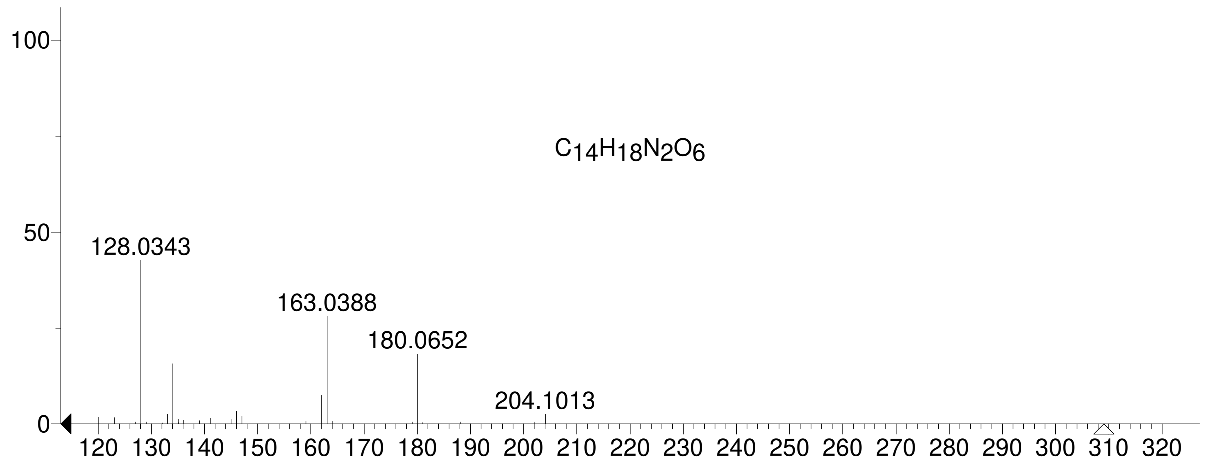

Unknown: Unknown (2.75_346.1610m/z)
Compound in Library Factor = 0

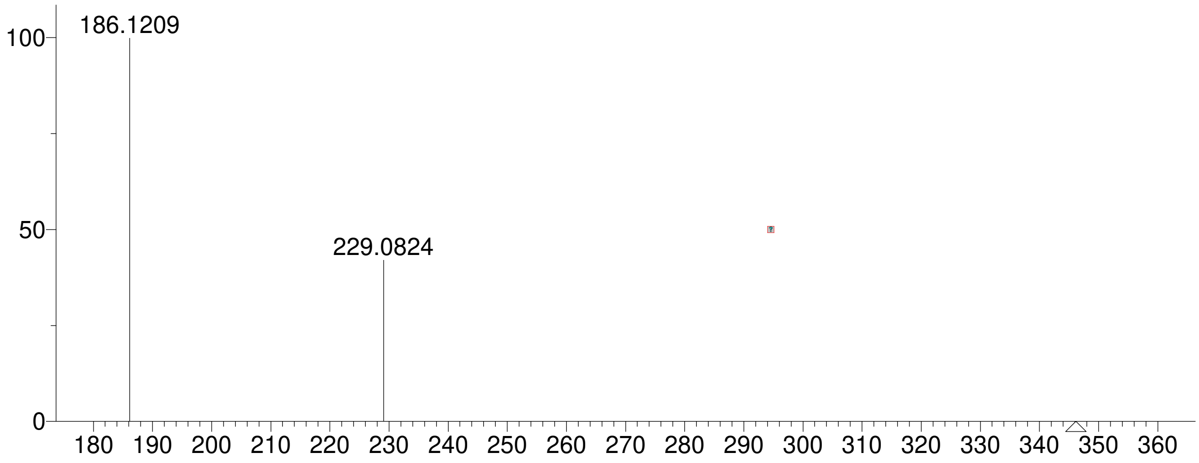

Unknown: Unknown (2.75_400.1699m/z)
Compound in Library Factor = 0

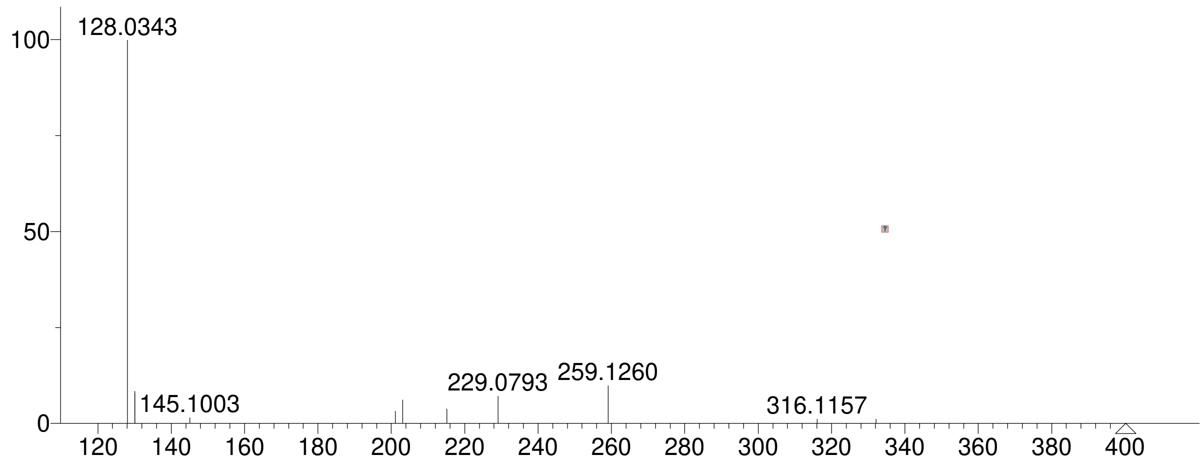

Hit 1 : Quetiapine sulfoxide [M+H]+ HCD 104V P=400.2
C21H25N3O3S; MF: 0; RMF: 19; Prob 100.0%; CAS: 329216-63-9; Lib: nist_msms; ID: 452544.

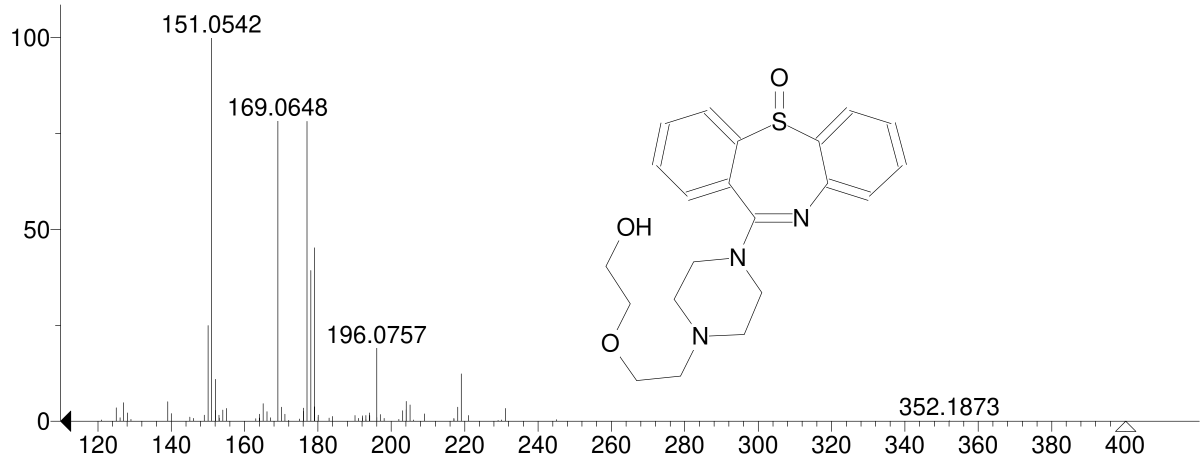

Unknown: Unknown (2.75_417.1144m/z)
Compound in Library Factor = 0

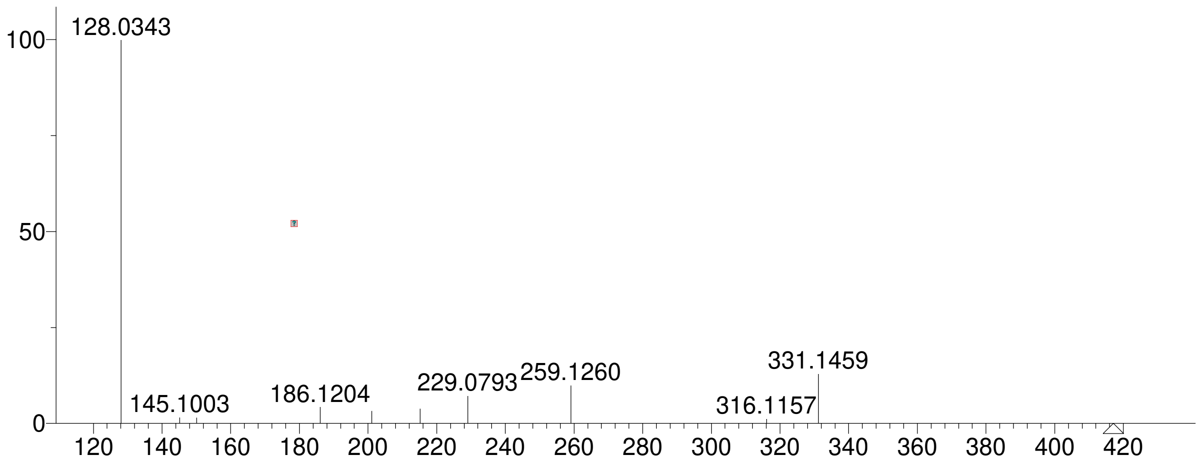

Hit 1 : Daidzin [M+H]+ HCD 133V P=417.1
C21H20O9; MF: 0; RMF: 22; Prob 25.0%; CAS: 552-66-9; Lib: nist_msms; ID: 299121.

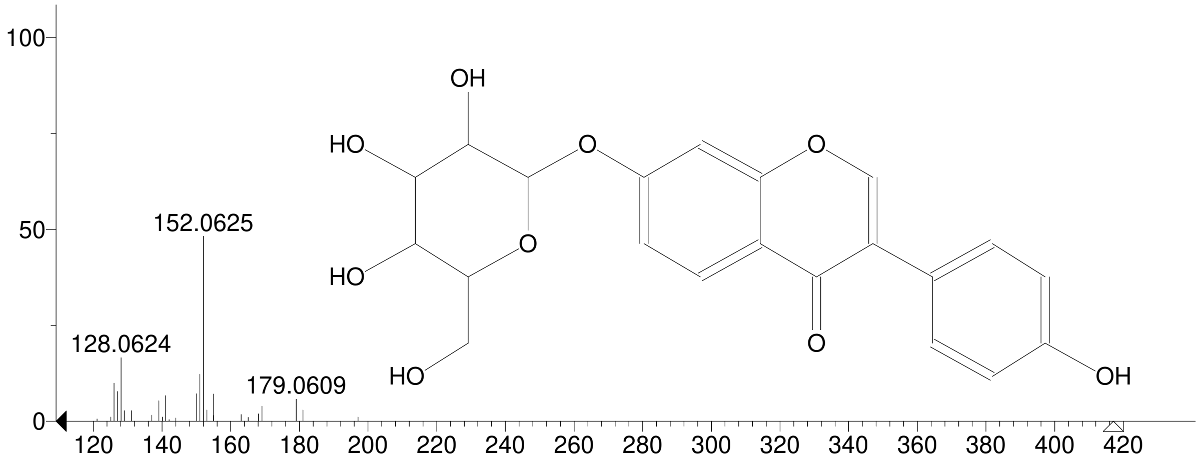

Hit 2 : Engeletin [M+H-H2O]+ HCD 133V P=417.1
C21H22O10; MF: 0; RMF: 15; Prob 25.0%; CAS: 572-31-6; Lib: nist_msms; ID: 236880.

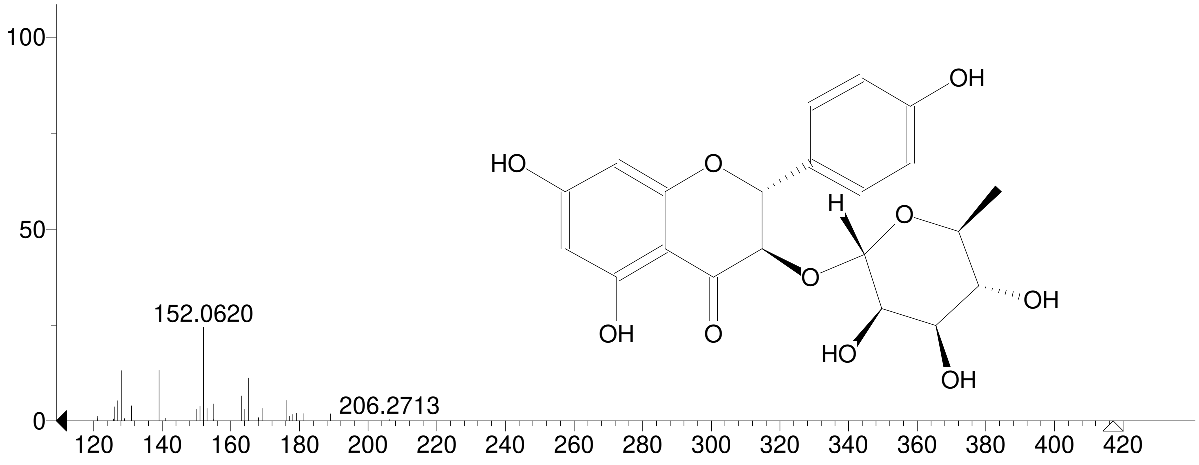

Hit 3 : Frangulin A [M+H]+ HCD 108V P=417.1
C21H20O9; MF: 0; RMF: 13; Prob 25.0%; CAS: 521-62-0; Lib: nist_msms; ID: 258728.

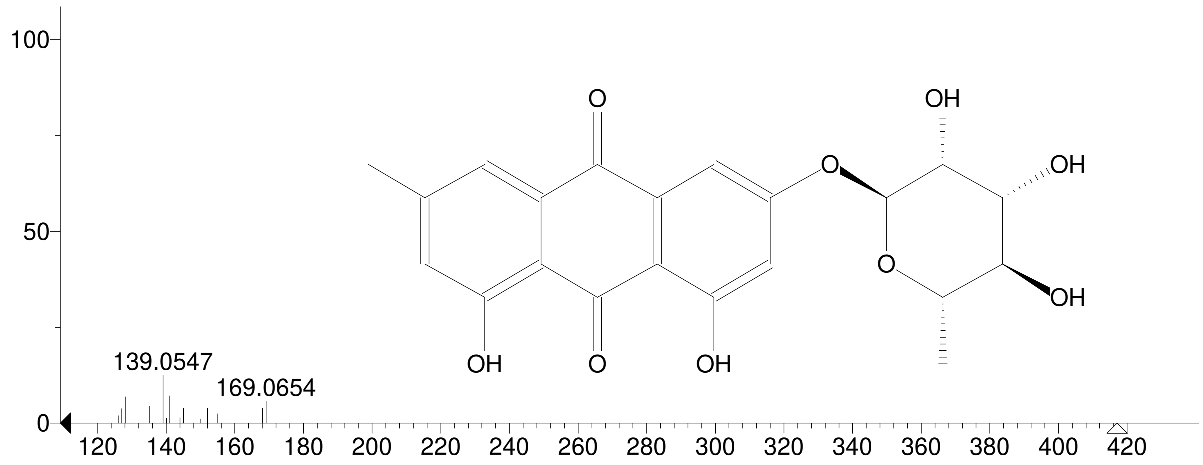

Hit 4 : aloesin [M+Na]+ 35HCD P=417.1
C19H22O9; MF: 0; RMF: 2; Prob 25.0%; Lib: mona-export-vf-npl_qexactive; ID: 4465.

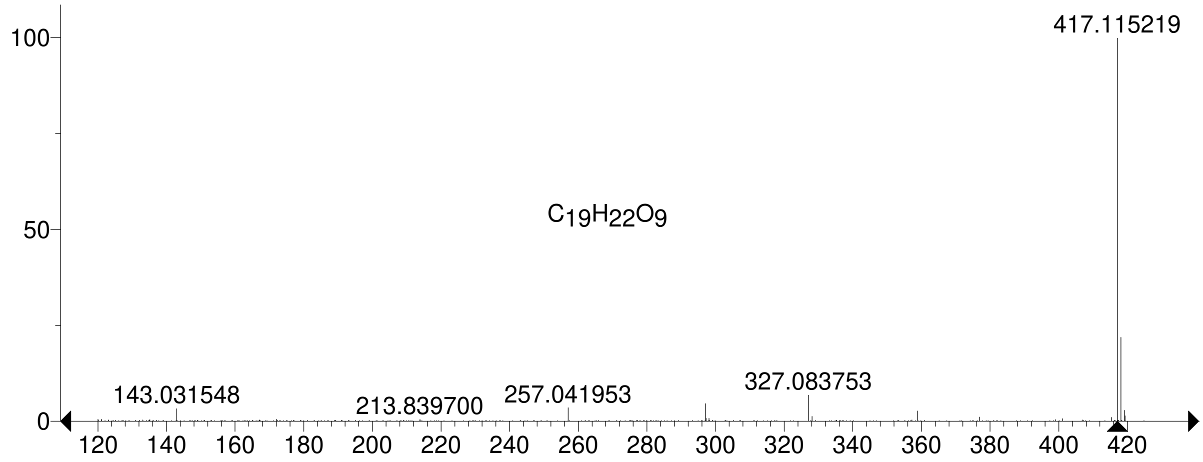

Unknown: Unknown (2.75_698.2882m/z)
Compound in Library Factor = 0

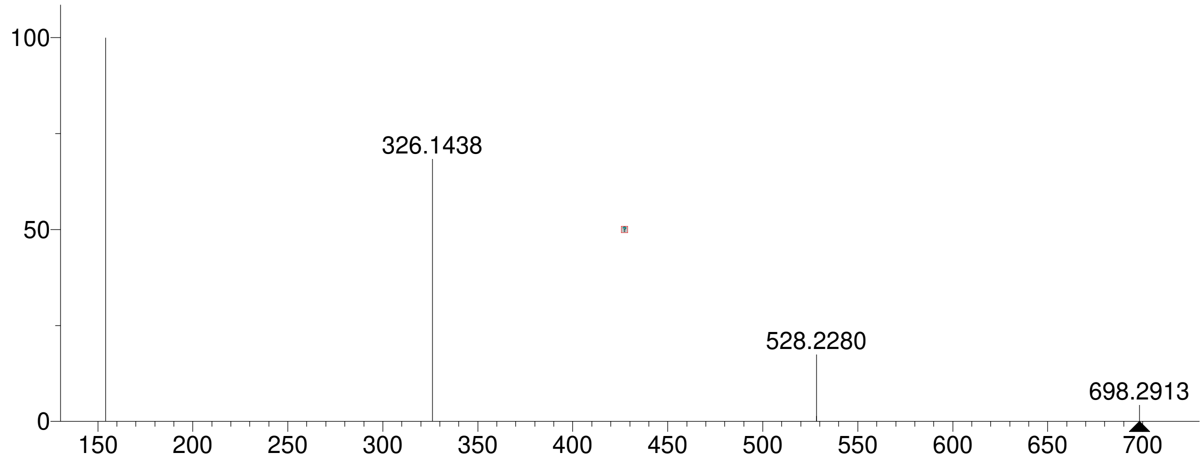

Unknown: Unknown (2.76_422.1836m/z)
Compound in Library Factor = 0

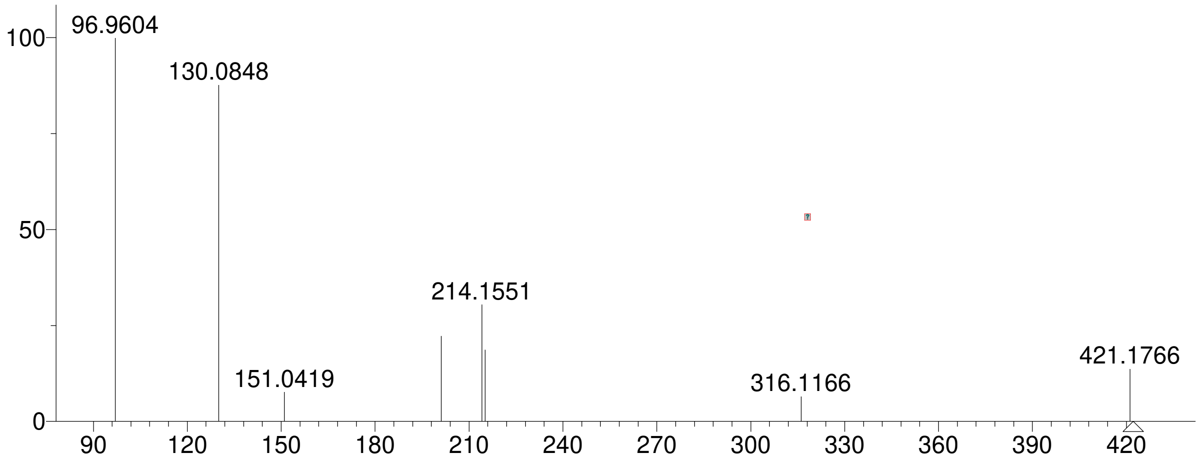

Hit 1 : [Des-Tyr1, DAla] Met-Enkephalins, amid [M-H]- IT 35% P=422.2
C19H29N5O4S; MF: 0; RMF: 25; Prob 100.0%; Lib: nist_msms2; ID: 74806.

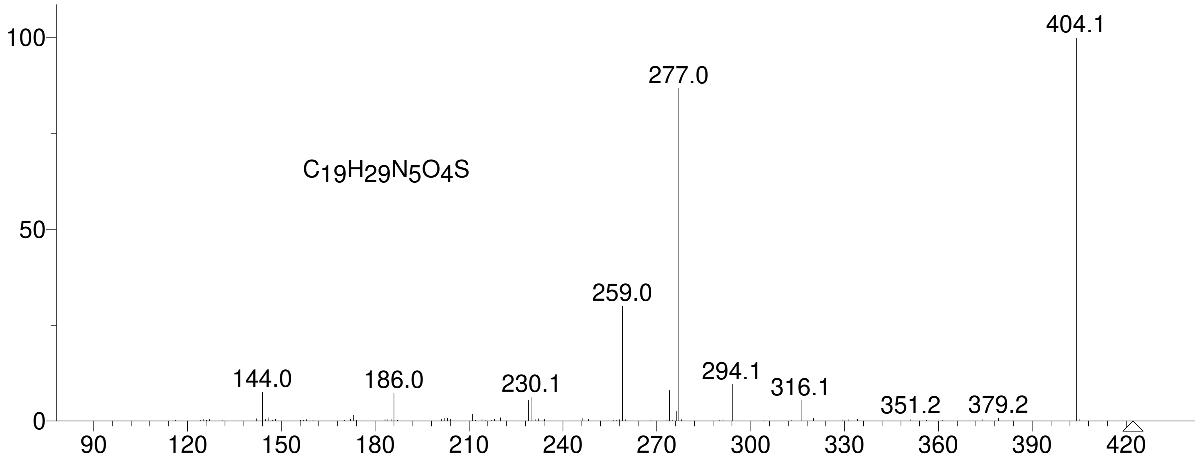

Unknown: Unknown (2.76_639.2605m/z)
Compound in Library Factor = 0

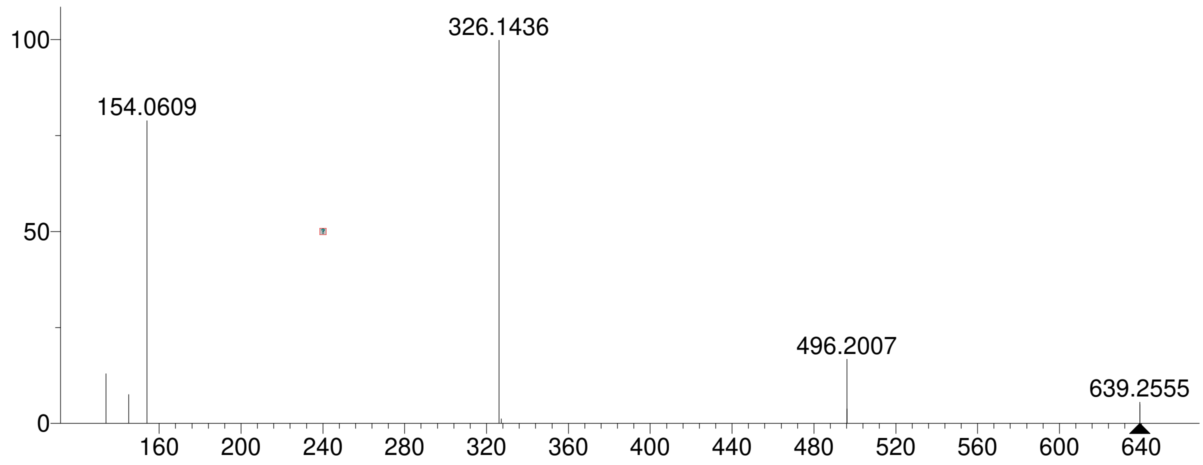

Hit 1 : Caspase 8 Substrate 1, chromogenic [M+H]+ QTOF 5V P=639.3
C27H38N6O12; MF: 0; RMF: 66; Prob 100.0%; Lib: nist_msms2; ID: 39457.

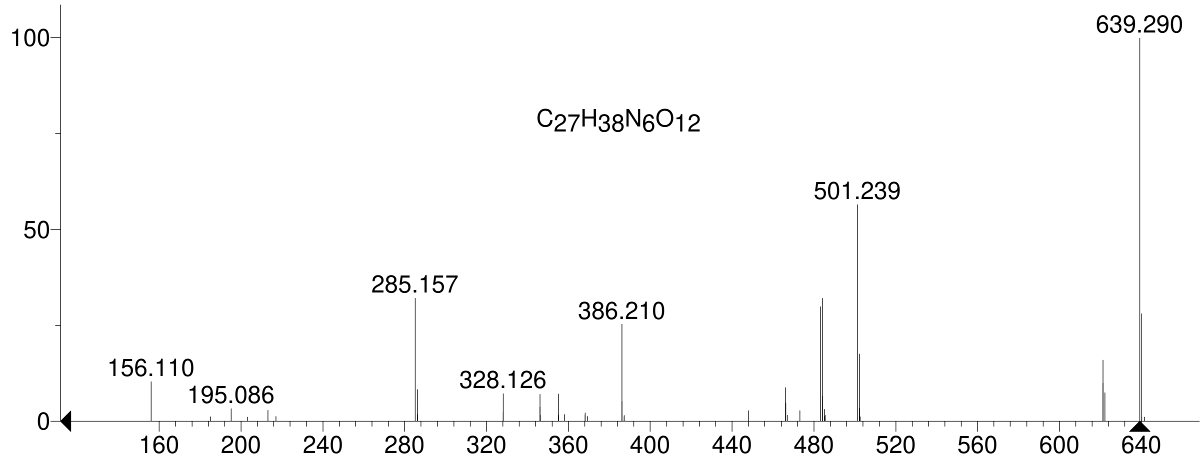

Unknown: Unknown (2.78_255.1333m/z)
Compound in Library Factor = -3273

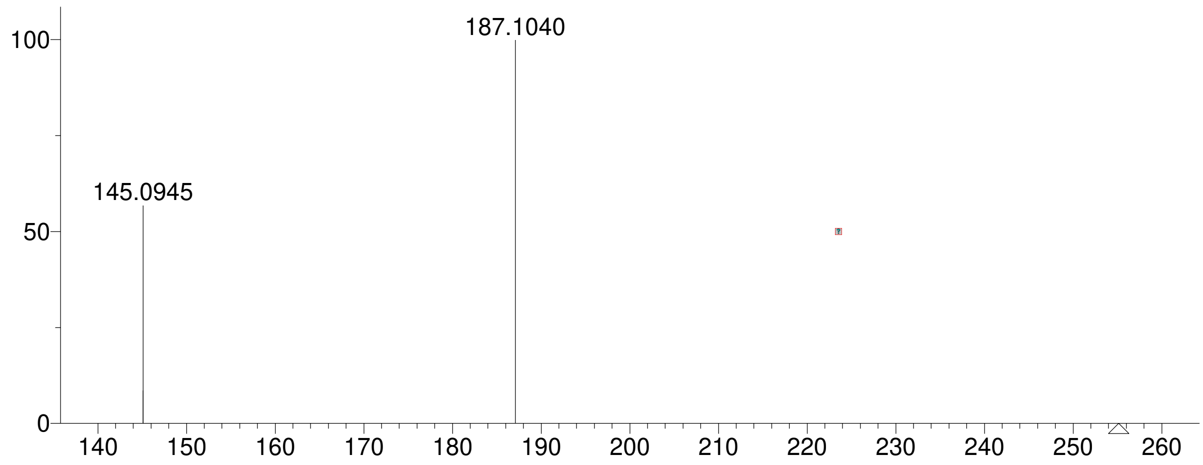

Hit 1 : Secobarbital [M+OH]- IT 35% P=255.1
C12H18N2O3; MF: 4; RMF: 294; Prob 54.2%; CAS: 76-73-3; Lib: nist_msms; ID: 14139.

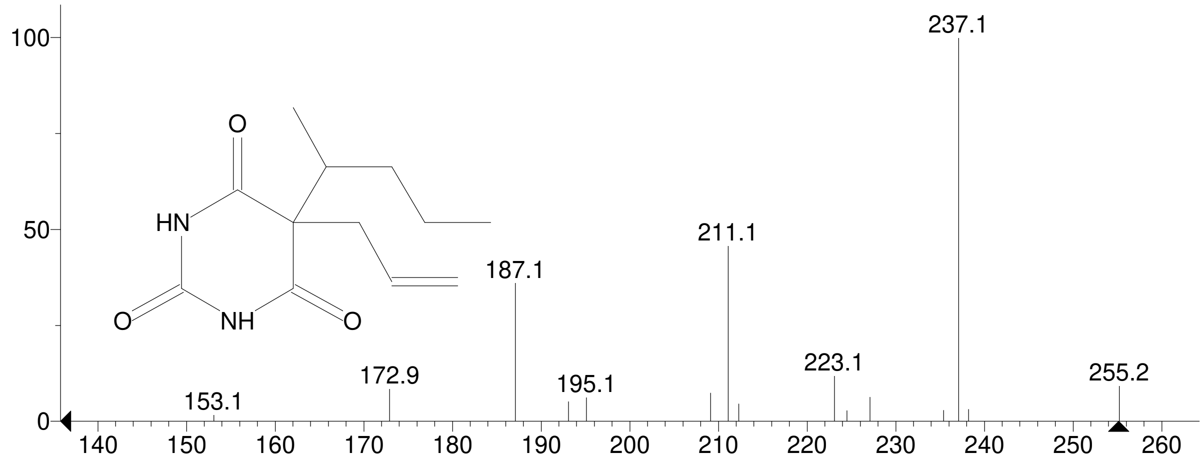

Hit 2 : Helenine [M+Na]+ 65HCD P=255.1
C15H20O2; MF: 0; RMF: 28; Prob 45.8%; Lib: mona-export-vf-npl_qexactive; ID: 2649.

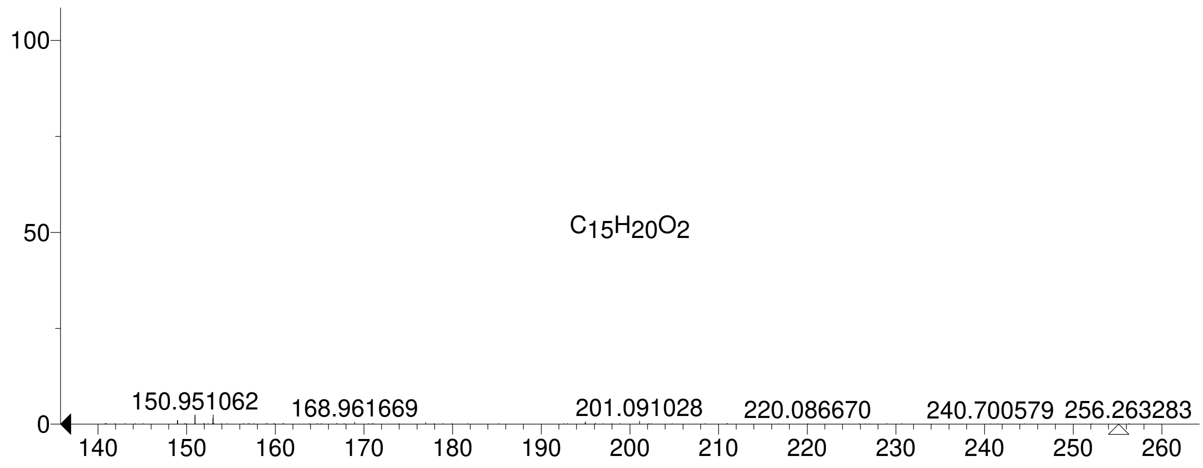

Unknown: Unknown (2.78_395.0754m/z)
Compound in Library Factor = 0

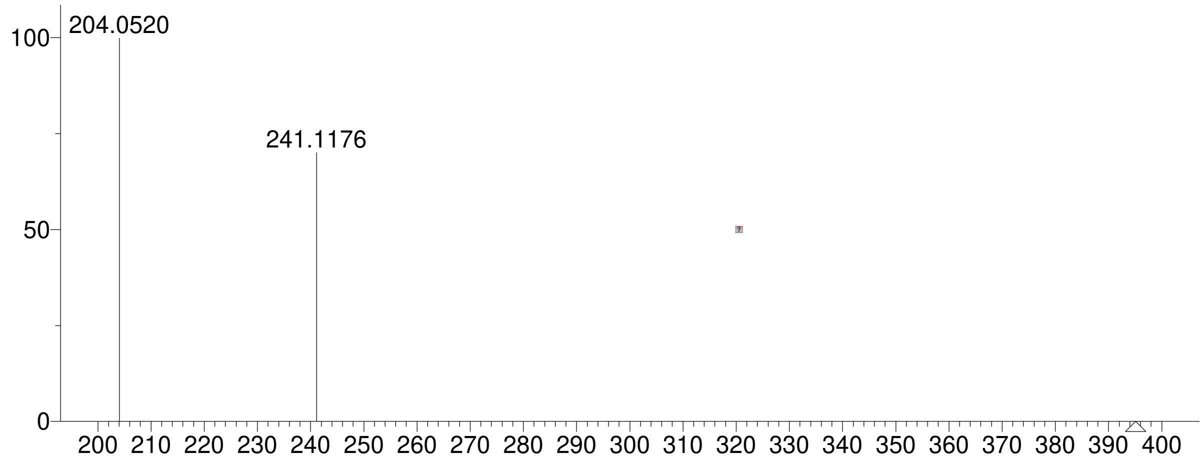

Hit 1 : Doxorubicin [M-H-C6H13NO3]- HCD 126V P=395.1
C27H29NO11; MF: 0; RMF: 120; Prob 25.0%; CAS: 23214-92-8; Lib: nist_msms; ID: 345538.

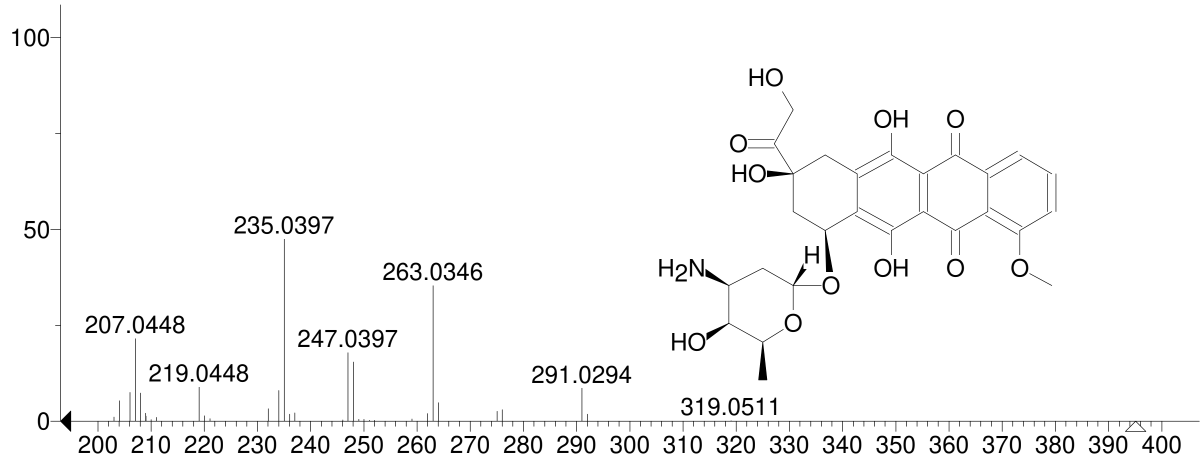

Hit 2 : Doxorubicinone [M-H-H2O]- HCD 63V P=395.1
C21H18O9; MF: 0; RMF: 43; Prob 25.0%; CAS: 24385-10-2; Lib: nist_msms; ID: 437424.

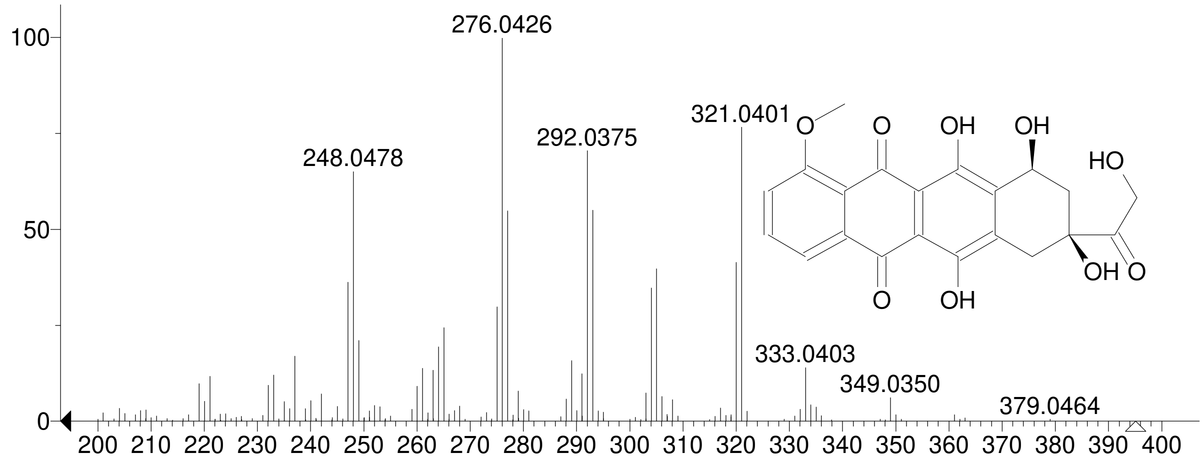

Hit 3 : Isoorientin [M+H-3H2O]+ HCD 71V P=395.1
C21H20O11; MF: 0; RMF: 35; Prob 25.0%; CAS: 4261-42-1; Lib: nist_msms; ID: 390618.

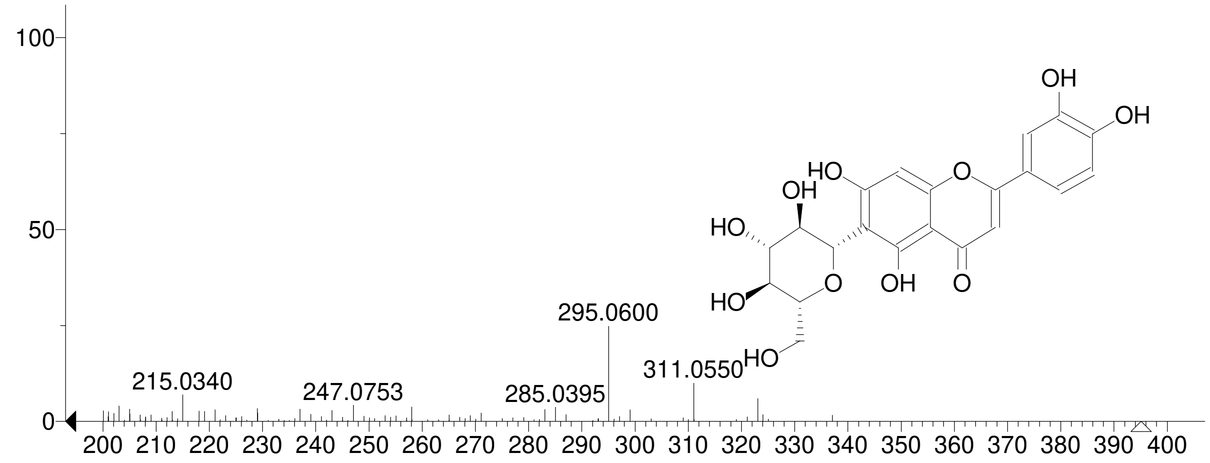

Hit 4 : (Z)-3-[4-methoxy-2-[(2S,3R,4S,5S,6R)-3,4,5-trihydroxy-6-(hydroxymethyl)oxan-2-yl]oxyphenyl]prop-2-enoic
C16H20O9; MF: 0; RMF: 21; Prob 25.0%; Lib: mona-export-vf-npl_qexactive; ID: 7762.

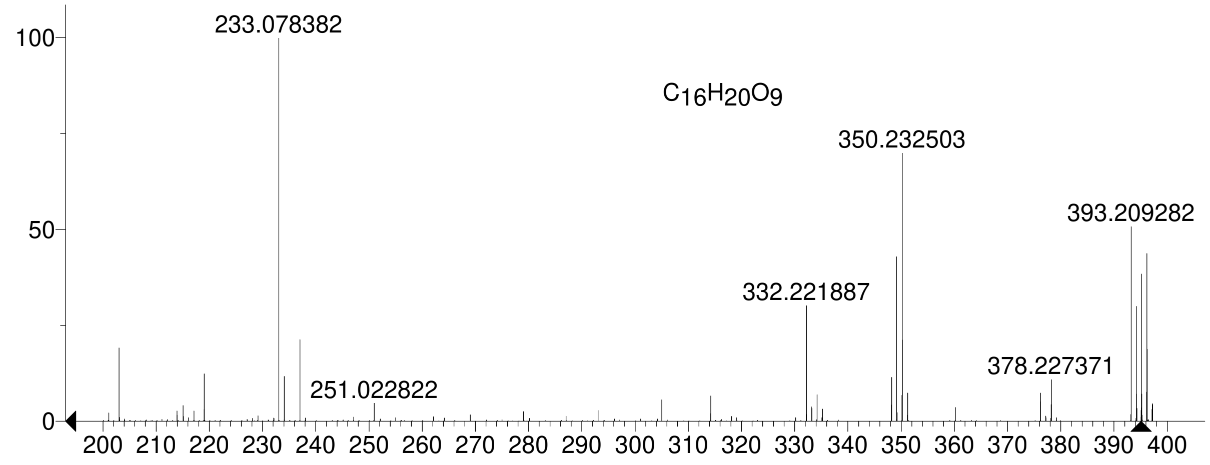

Unknown: Unknown (2.78_666.2620m/z)
Compound in Library Factor = 0

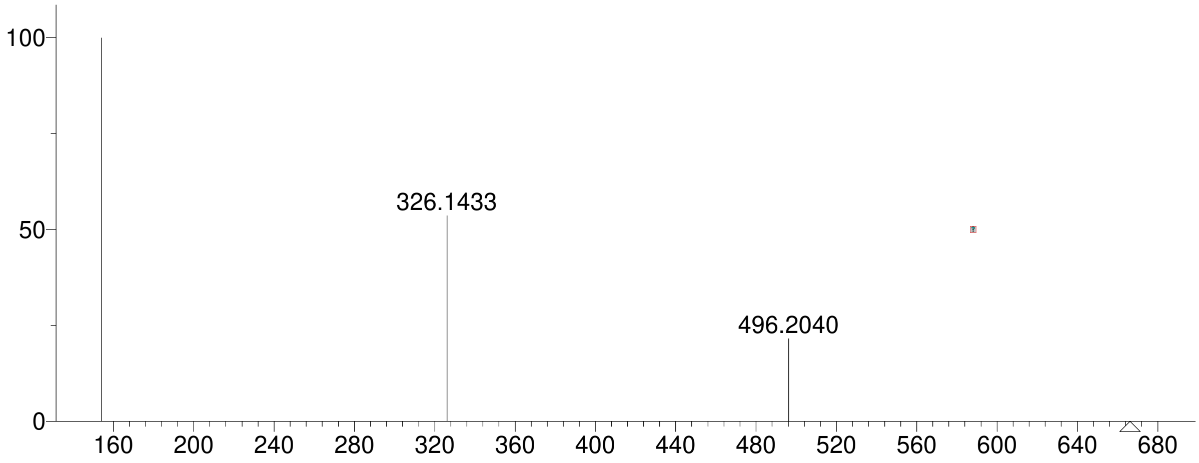

** Search Report Page 1 of 1 **

Unknown: Unknown (2.79_325.0165m/z)
Compound in Library Factor = 0

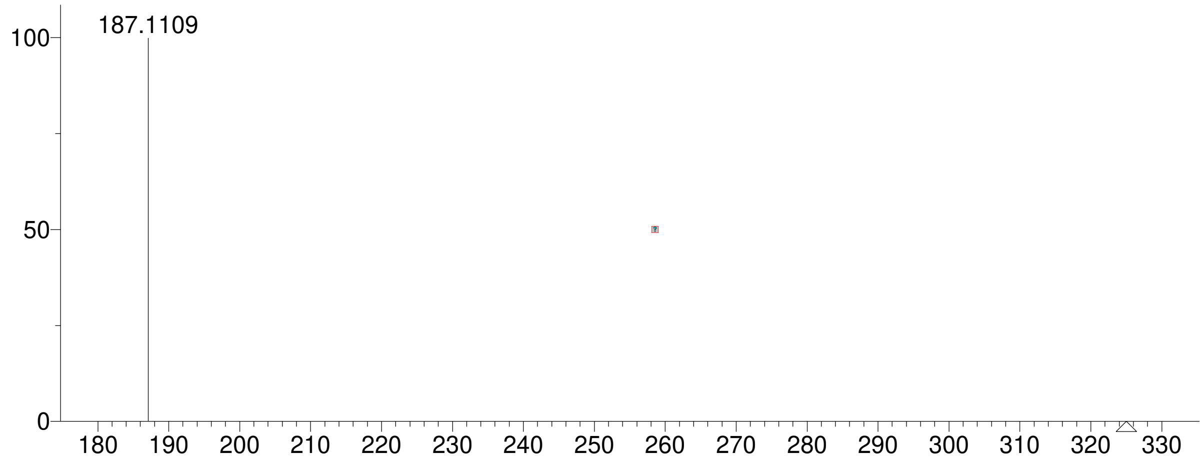

Unknown: Unknown (2.79_373.1220m/z)
Compound in Library Factor = 0

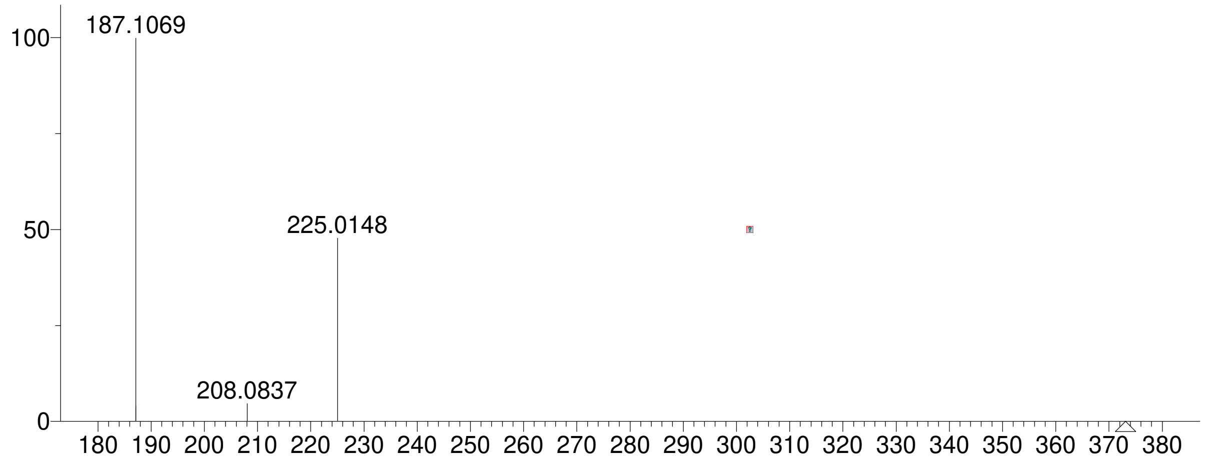

Hit 1 : 2,4-Thiazolidinedione, 5-[[4-[2-(5-ethyl-2-pyridinyl)-2-hydroxyethoxy]phenyl]methyl]- [M+H]+ HCD 82V P:
C19H20N2O4S; MF: 0; RMF: 25; Prob 50.0%; CAS: 101931-00-4; Lib: nist_msms; ID: 443098.

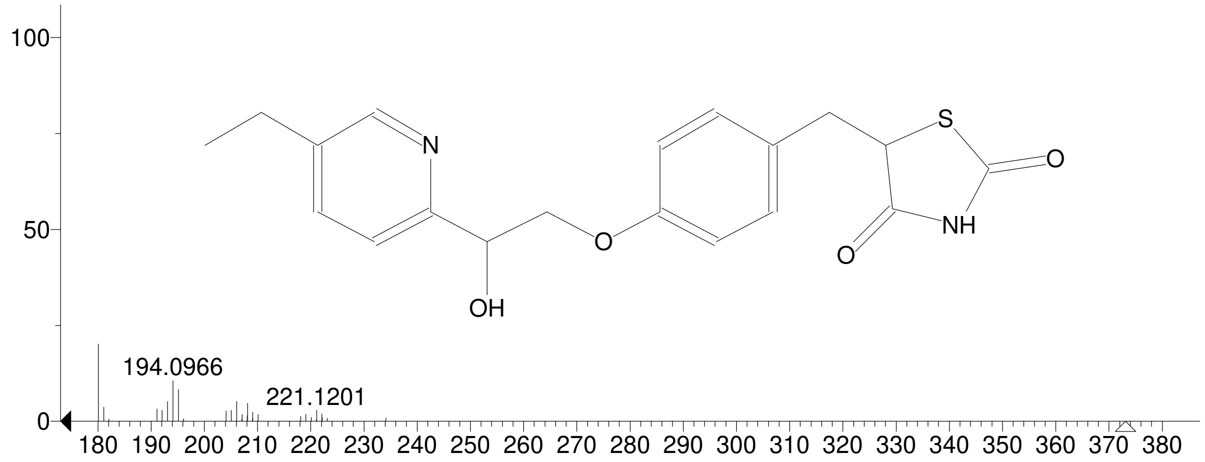

Hit 2 : Dapagliflozin [M+H-2H2O]+ IT 35% P=373.1
C21H25ClO6; MF: 0; RMF: 16; Prob 50.0%; CAS: 461432-26-8; Lib: nist_msms; ID: 548881.

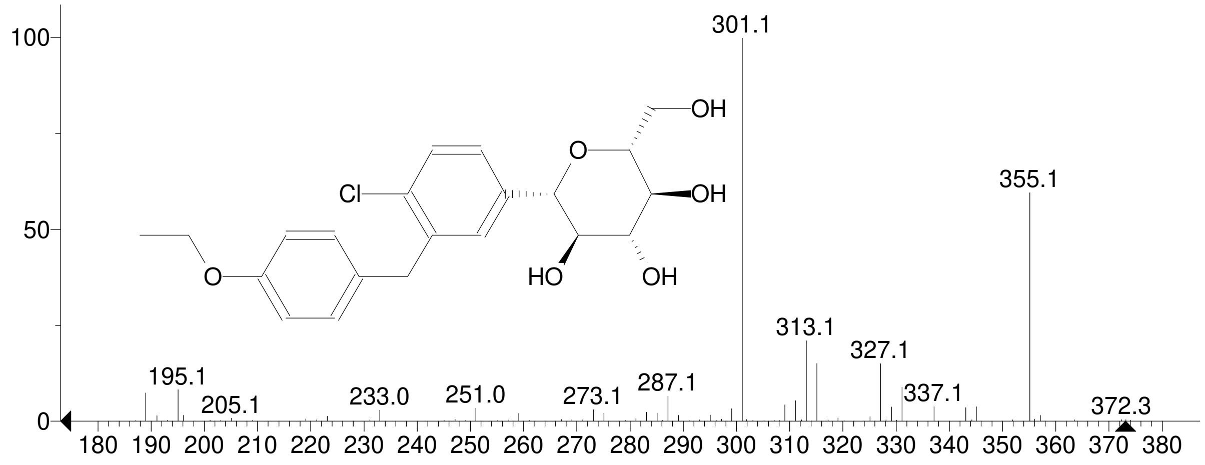

Unknown: Unknown (2.81_215.1384m/z)
Compound in Library Factor = -344

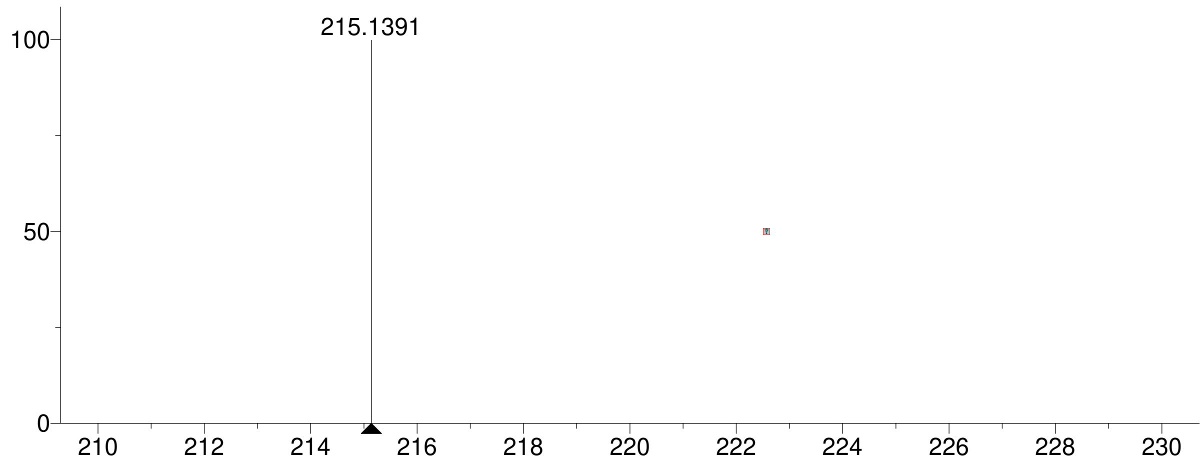

Hit 1 : Val-Pro [M+H]+ QTOF 30V P=215.1
C10H18N2O3; MF: 364; RMF: 999; Prob 14.4%; Lib: nist_msms; ID: 49019.

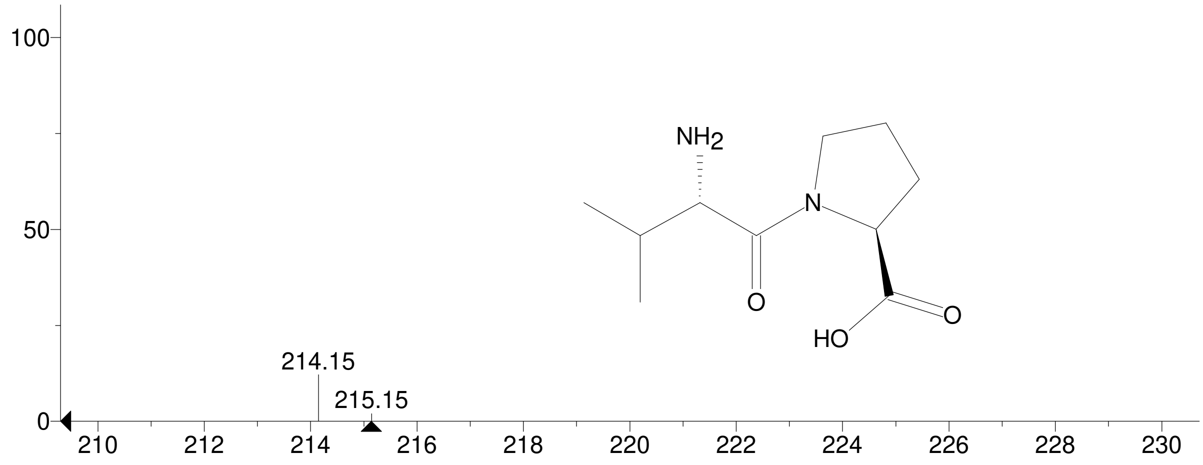

Hit 2 : D-Desthiobiotin [M+H]+ HCD 19V P=215.1
C10H18N2O3; MF: 364; RMF: 999; Prob 14.4%; CAS: 533-48-2; Lib: nist_msms; ID: 489068.

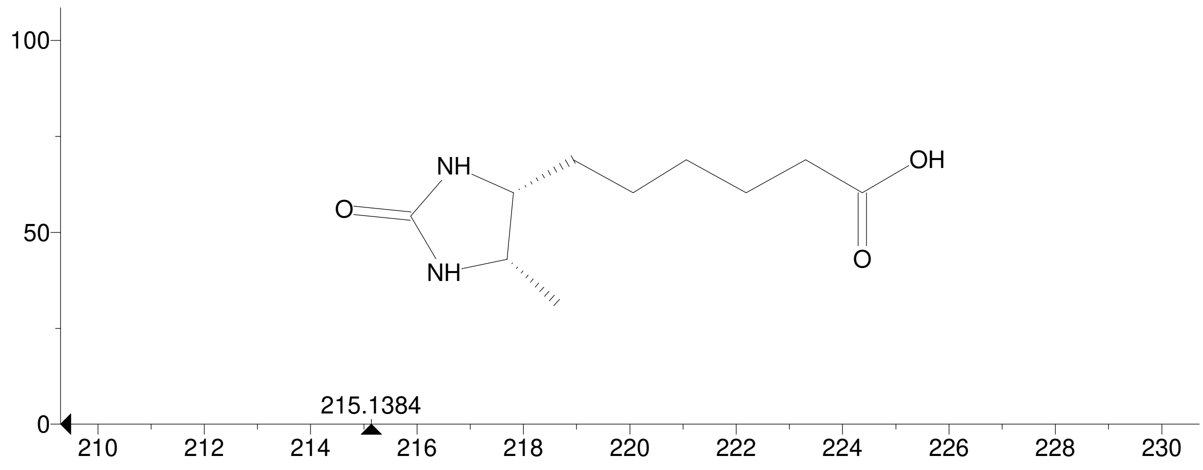

Hit 3 : Pro-Val [M+H]+ HCD 12eV P=215.1
C10H18N2O3; MF: 364; RMF: 999; Prob 14.4%; Lib: nist_msms; ID: 563234.

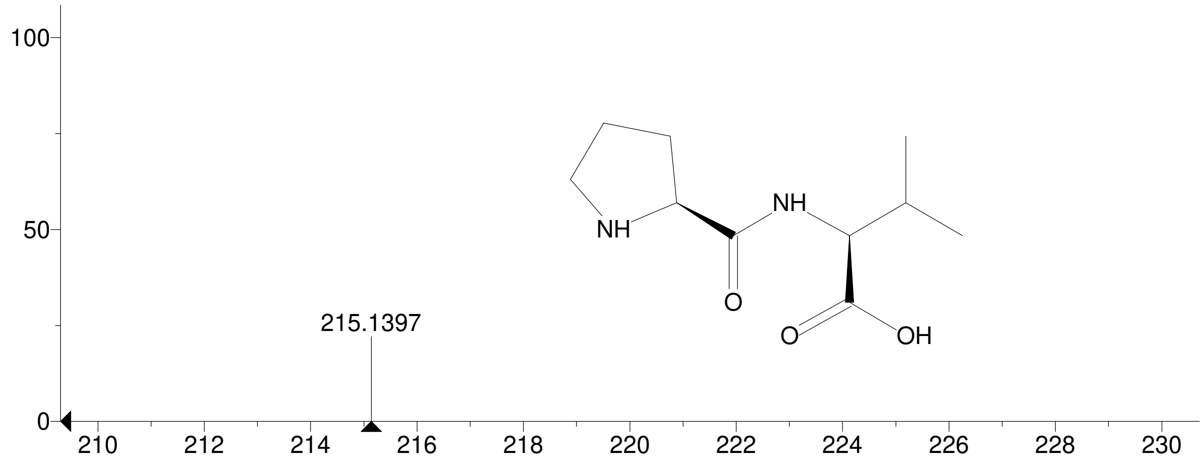

Hit 4 : Val-Val [M-H]- HCD 34eV P=215.1
C10H20N2O3; MF: 364; RMF: 999; Prob 14.4%; CAS: 3918-94-3; Lib: nist_msms; ID: 568163.

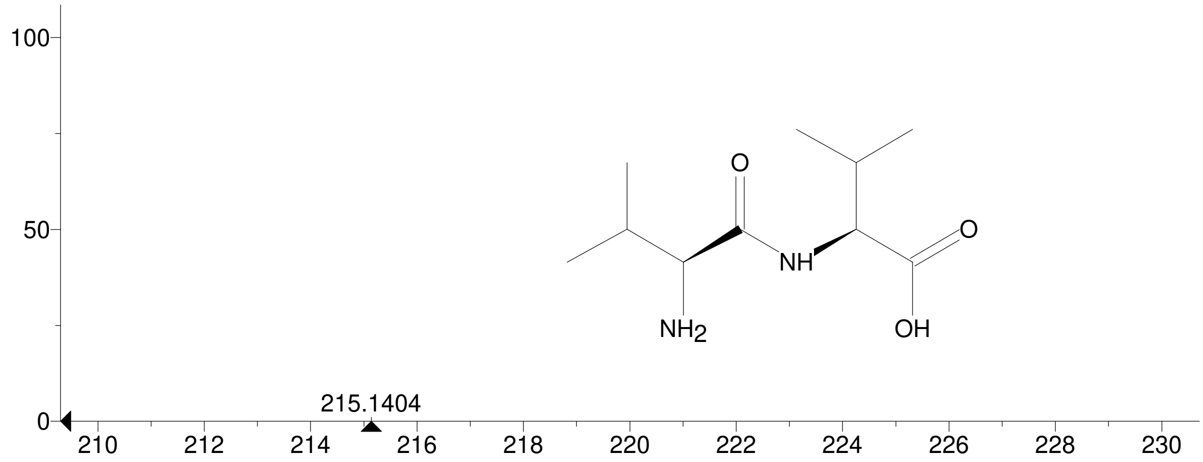

Hit 5 : Pro-Val [M+H]+ HCD 12eV P=215.1
C10H18N2O3; MF: 364; RMF: 999; Prob 14.4%; CAS: 52899-09-9; Lib: nist_msms2; ID: 43035.

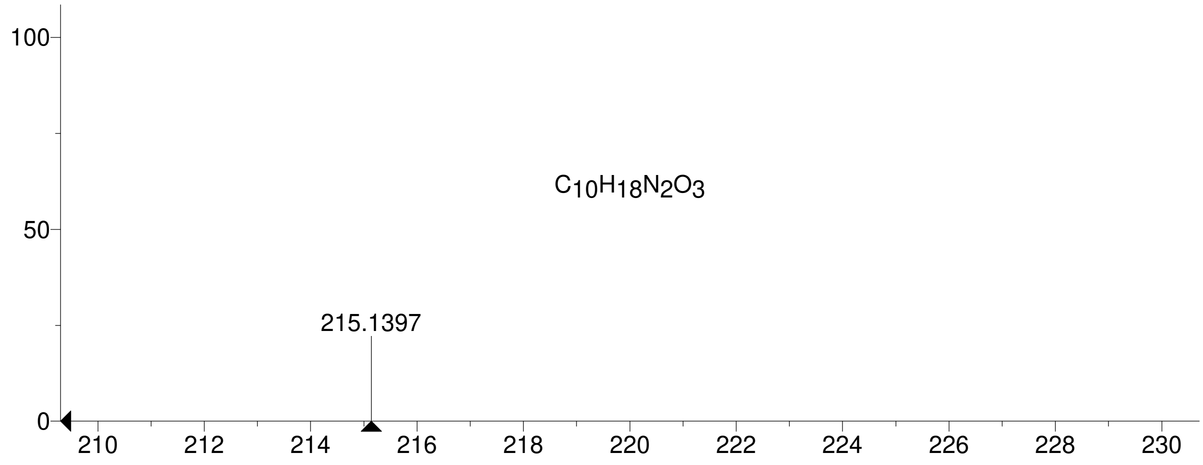

Unknown: Unknown (2.82_409.0901m/z)
Compound in Library Factor = -359

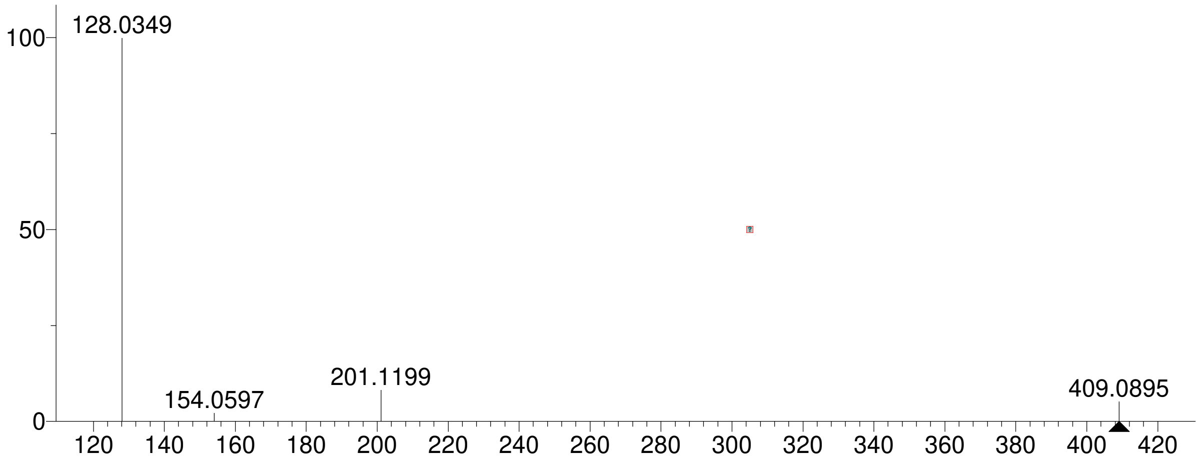

Hit 1 : Irisflorentin [M+Na]+ HCD 24V P=409.1
C20H18O8; MF: 328; RMF: 951; Prob 95.3%; CAS: 41743-73-1; Lib: nist_msms; ID: 240385.

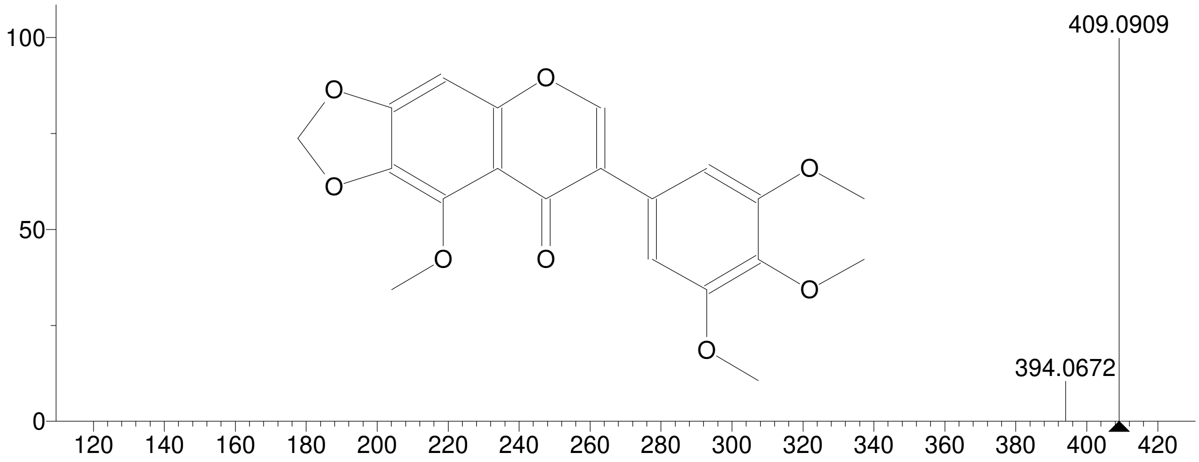

Hit 2 : Nicosulfuron [M-H]- 15% (P=409.1
C15H18N6O6S; MF: 166; RMF: 584; Prob 2.34%; Lib: mona-export-lc-ms-ms_negative_mode; ID: 2153.

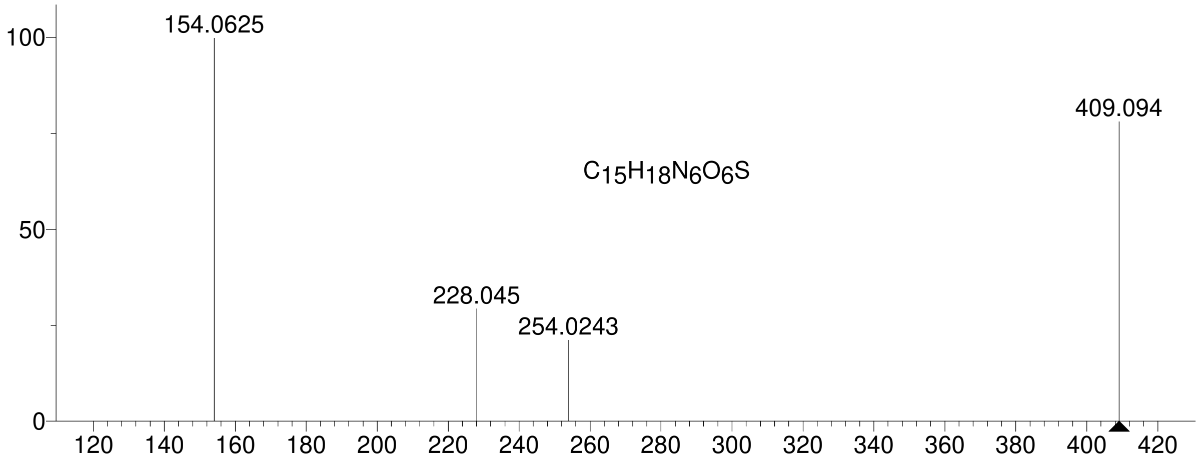

Hit 3 : Nicosulfuron; LC-ESI-ITFT; MS2; CE [M-H]- 15 P=409.1
C15H18N6O6S; MF: 166; RMF: 584; Prob 2.34%; Lib: msms-neg-massbank; ID: 2011.

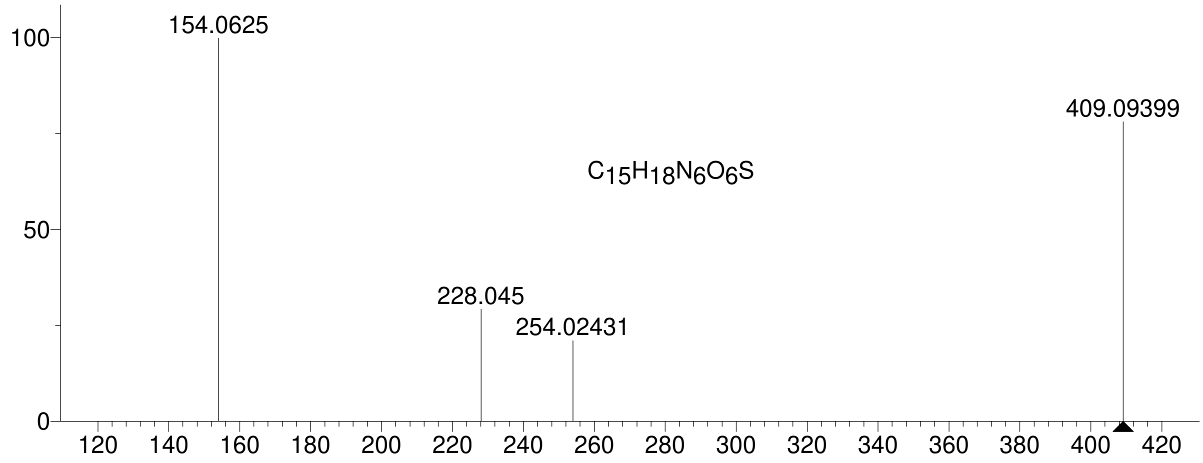

** Search Report Page 1 of 1 **

Unknown: Unknown (2.82_435.1505m/z)
Compound in Library Factor = 0

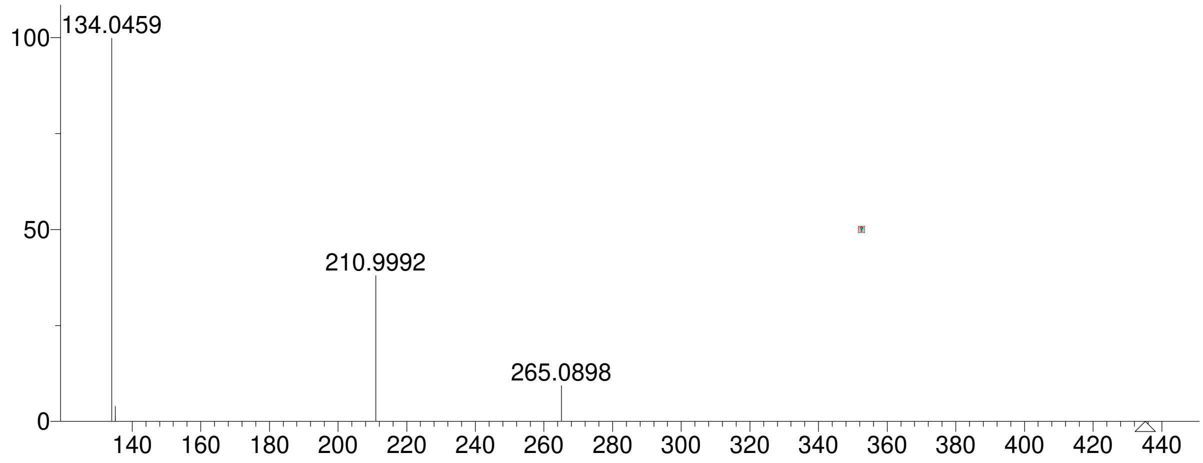

Unknown: Unknown (2.83_344.1819m/z)
Compound in Library Factor = 0

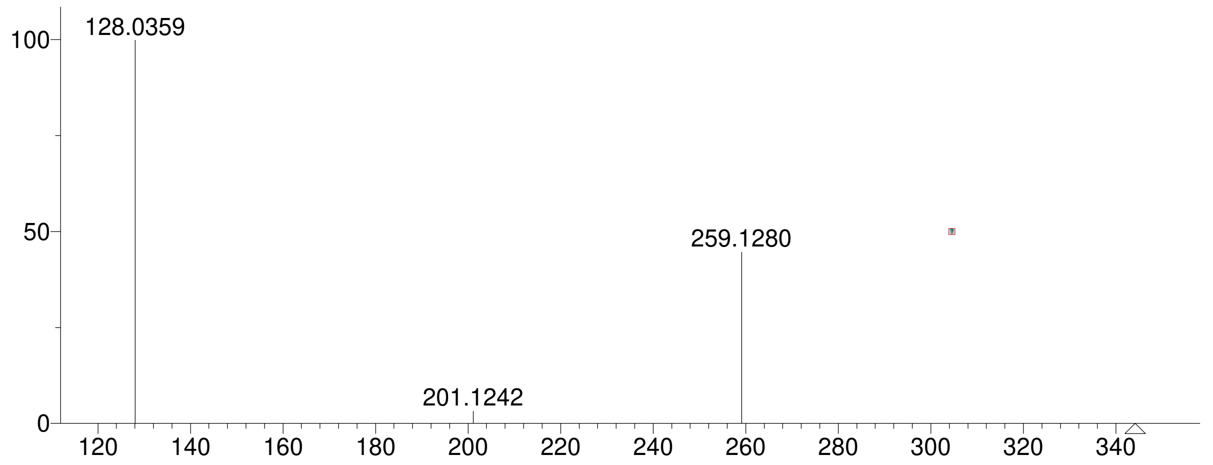

Hit 1 : 4-amino-2-hydroxy-5-[[1-hydroxy-1-(5-oxo-6-bicyclo[4.1.0]hept-3-enyl)propan-2-yl]amino]-5-oxopentanoic a
C15H22N2O6; MF: 0; RMF: 27; Prob 100.0%; Lib: mona-export-vf-npl_qexactive; ID: 16561.

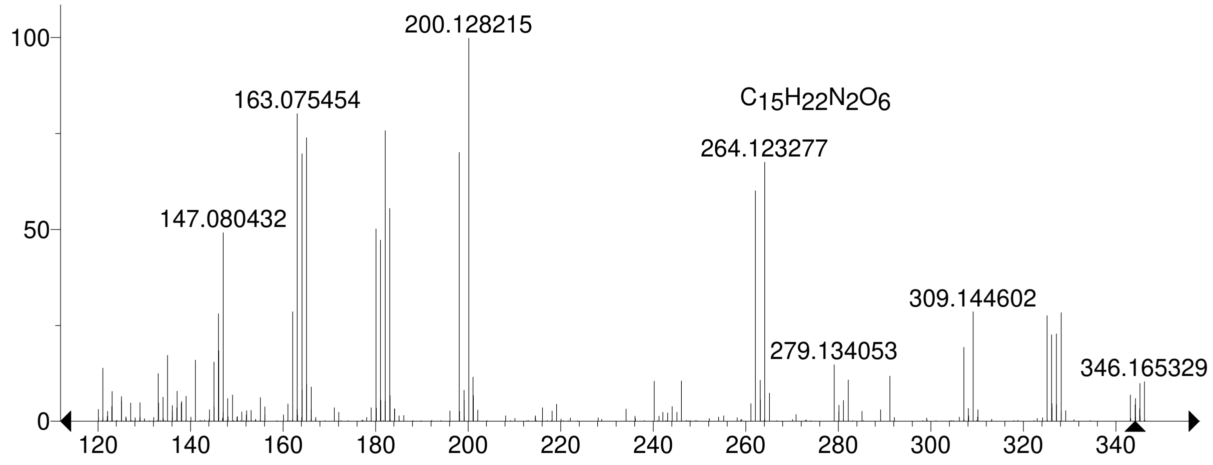

Unknown: Unknown (2.83_373.1250m/z)
Compound in Library Factor = 0

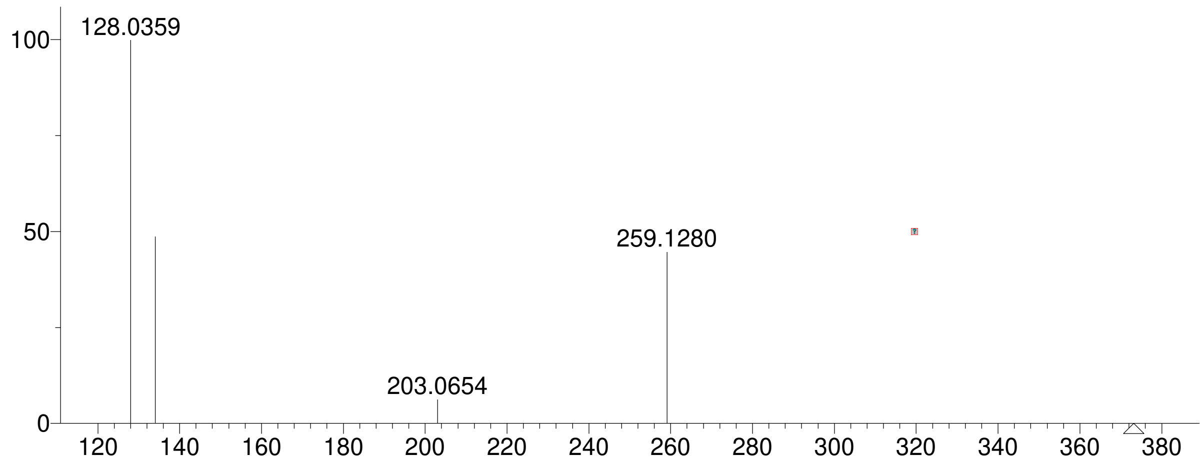

Hit 1 : Gox(8-O-4)G 1 (lignin) [M-H]- P=373.1
C20H22O7; MF: 0; RMF: 58; Prob 7.14%; Lib: phytofrag2018_final_060219; ID: 554.

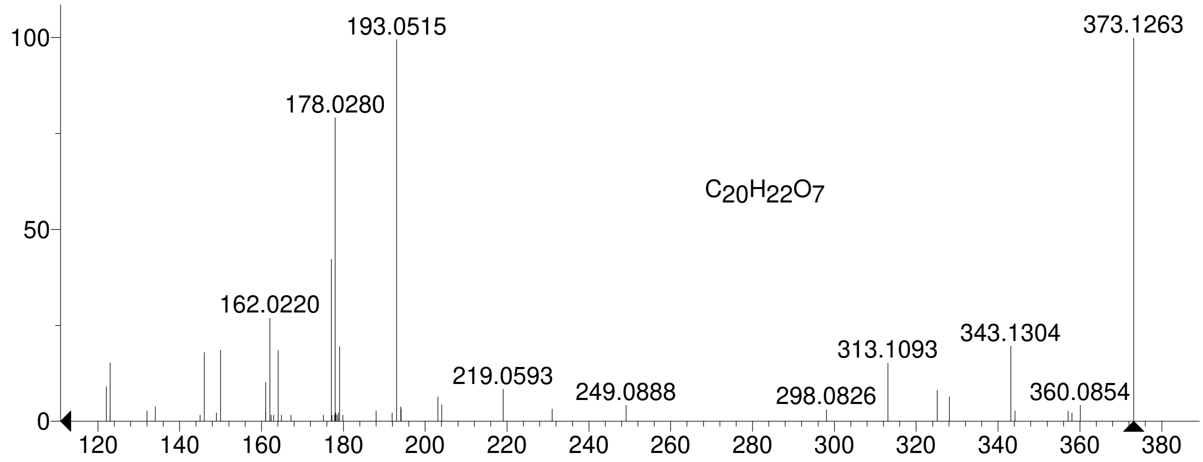

Hit 2 : NCGC00385104-01_C20H22O8_beta-D-Glucopyranoside, 2-(hydroxymethyl)phenyl, 6-benzoate [M-H2O-
C20H22O8; MF: 0; RMF: 38; Prob 7.14%; Lib: mona-export-gnps; ID: 14283.

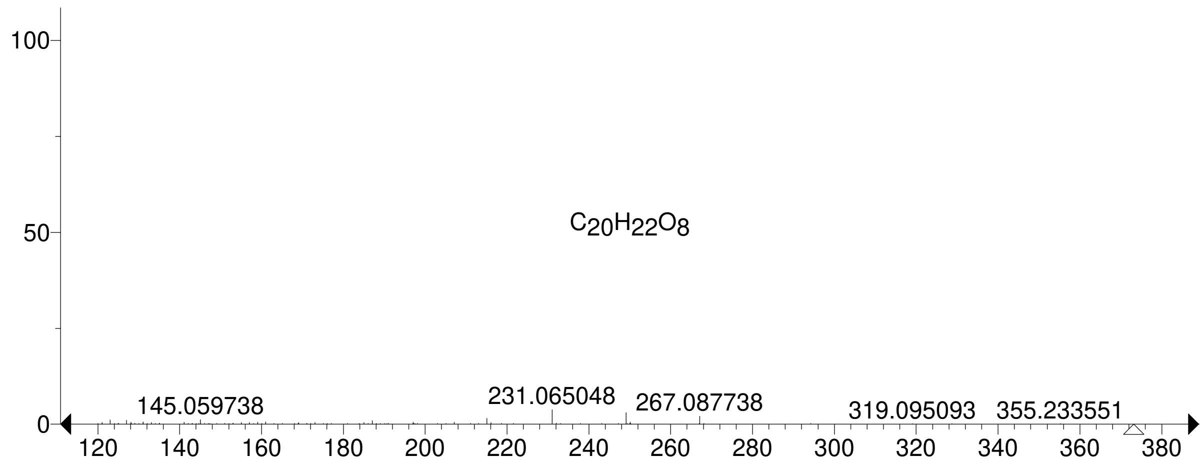

Hit 3 : NCGC00095850-04!5,6,7,8-tetramethoxy-2-(4-methoxyphenyl)chromen-4-one [M+H]+ P=373.1
C20H20O7; MF: 0; RMF: 28; Prob 7.14%; Lib: mona-export-gnps; ID: 9185.

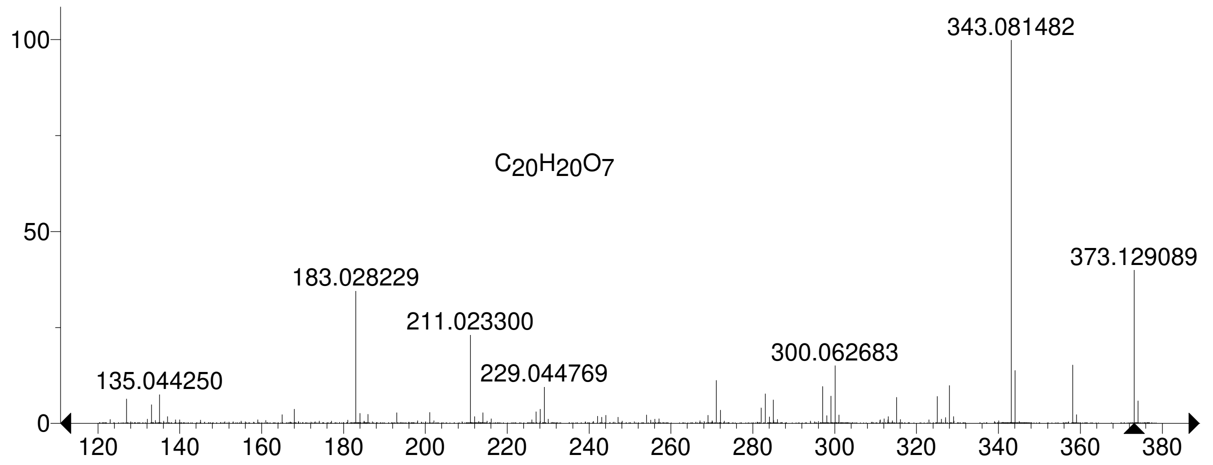

Hit 4 : [3-(4-hydroxy-3-methoxybenzoyl)-2,3-dimethyloxiran-2-yl]-(4-hydroxy-3-methoxyphenyl)methanone [M+H]·
C20H20O7; MF: 0; RMF: 18; Prob 7.14%; Lib: mona-export-vf-npl_qexactive; ID: 19627.

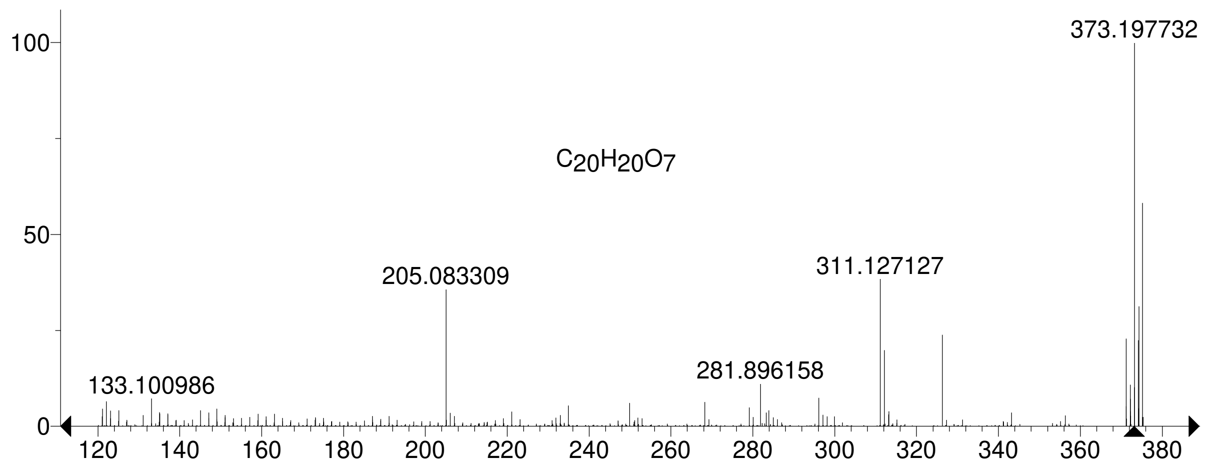

Hit 5 : Sinensetin [M+H]+ 65HCD P=373.1
C20H20O7; MF: 0; RMF: 16; Prob 7.14%; Lib: mona-export-vf-npl_qexactive; ID: 14397.

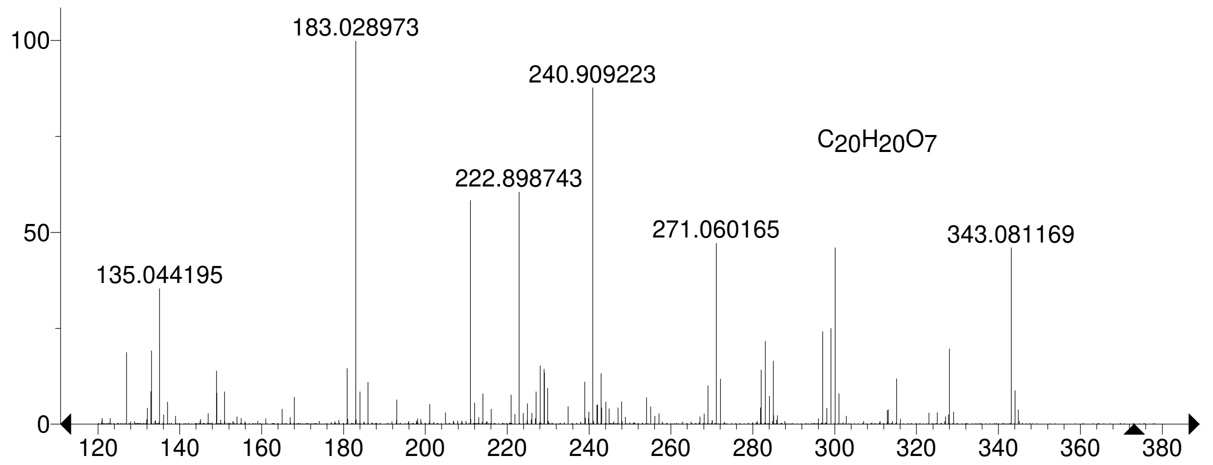

** Search Report Page 1 of 1 **

Unknown: Unknown (2.83_492.2181m/z)
Compound in Library Factor = 0

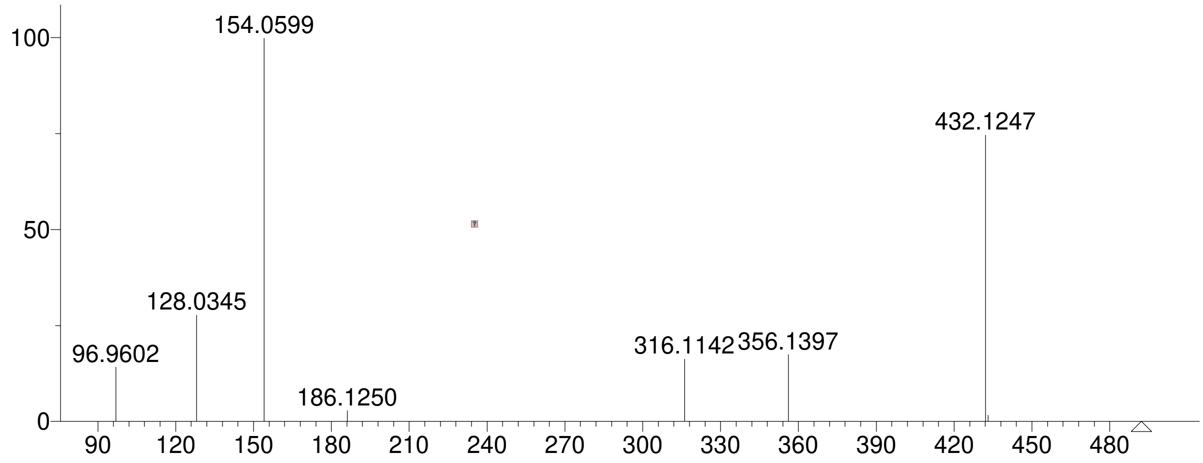

Unknown: Unknown (2.83_526.2034m/z)
Compound in Library Factor = 0

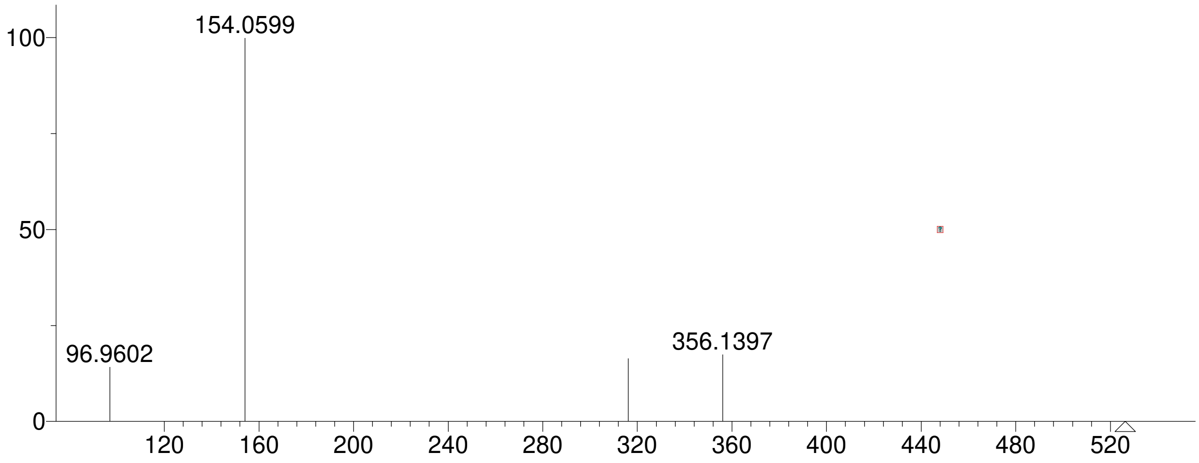

Unknown: Unknown (2.83_603.1866m/z)
Compound in Library Factor = 0

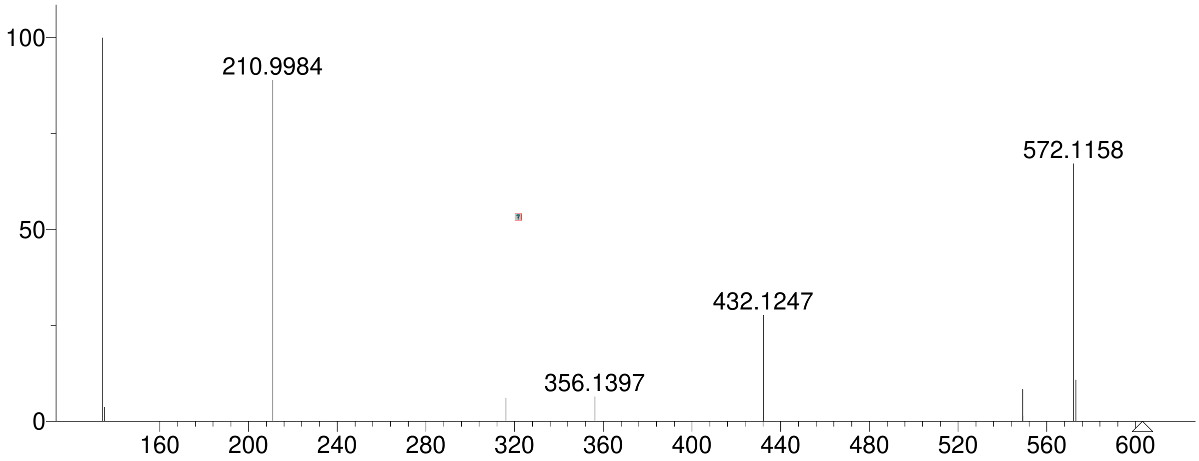

** Search Report Page 1 of 1 **

Unknown: Unknown (2.84_349.1602m/z)
Compound in Library Factor = 0

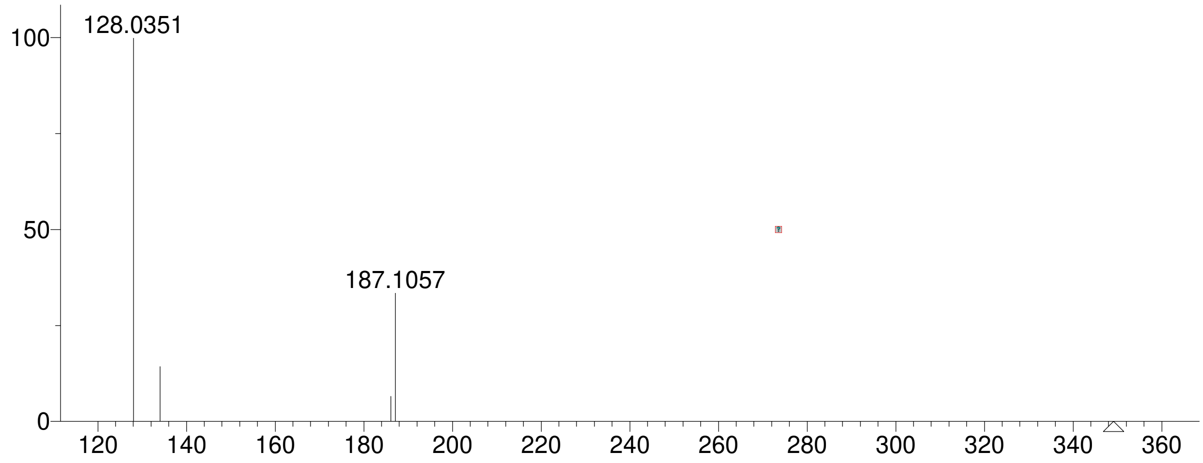

** Search Report Page 1 of 1 **

Unknown: Unknown (2.84_432.1264m/z)
Compound in Library Factor = 0

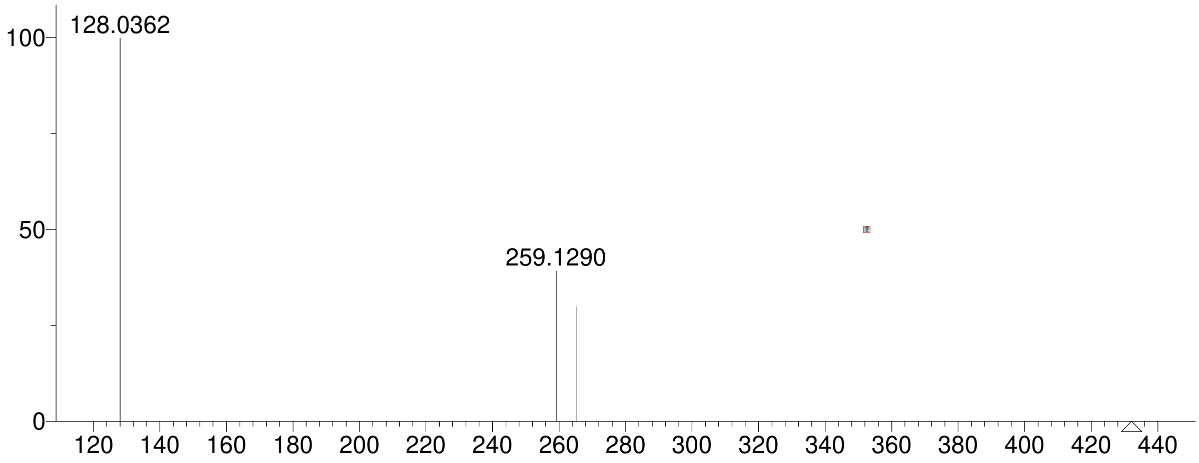

Unknown: Unknown (2.85_572.1147m/z)
Compound in Library Factor = -3273

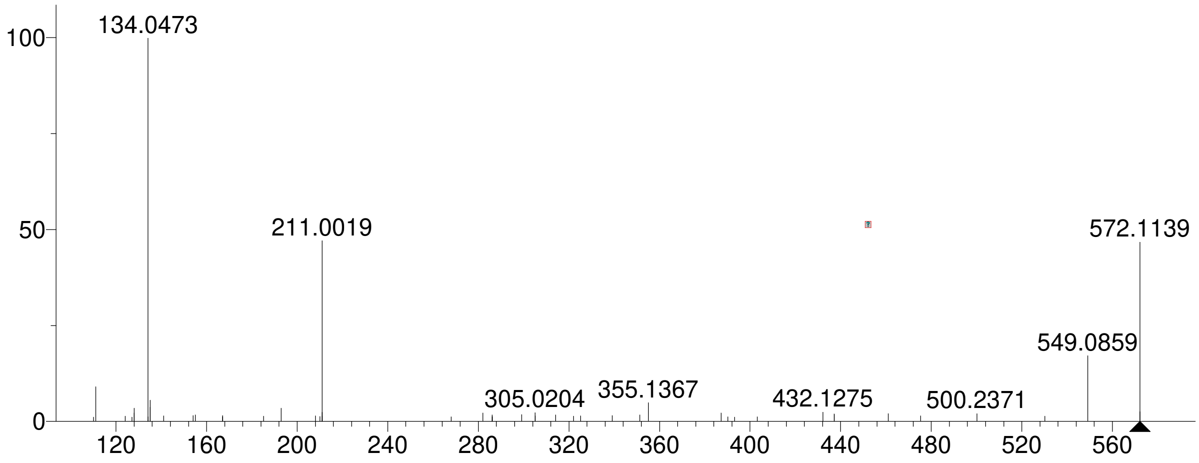

Hit 1 : (Ala11,D-Leu15)-Orexin B, human [M+5H]5+ HCD 8eV P=572.1
C120H206N44O35S; MF: 1; RMF: 126; Prob 100.0%; CAS: 532932-99-3; Lib: nist_msms2; ID: 45808.

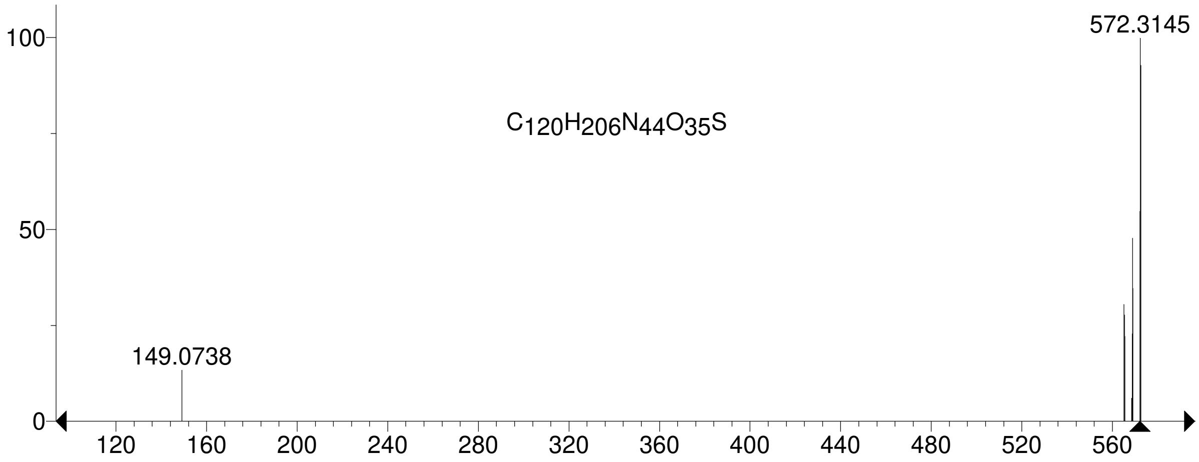

Unknown: Unknown (2.85_695.2510m/z)
Compound in Library Factor = 0

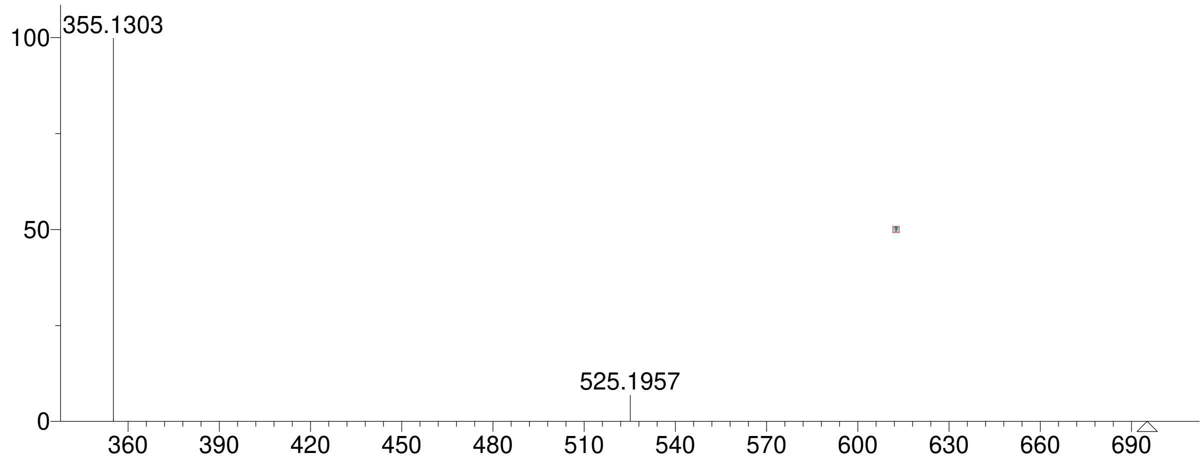

Hit 1 : Calcitonin (1-7), human [M+H]+ HCD 75eV P=695.2
C25H42N8O11S2; MF: 0; RMF: 27; Prob 100.0%; Lib: nist_msms2; ID: 62820.

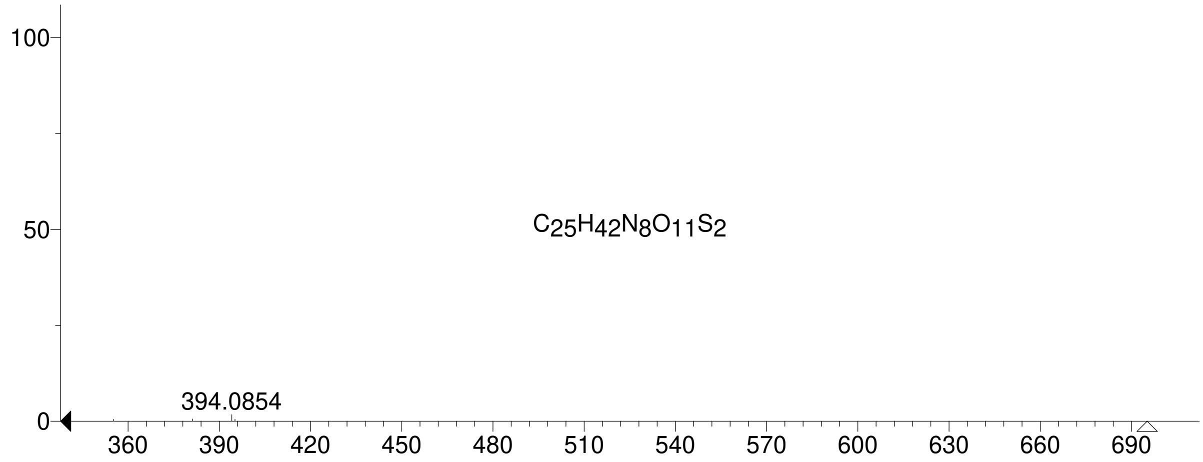

Unknown: Unknown (2.88_399.1391m/z)
Compound in Library Factor = -3273

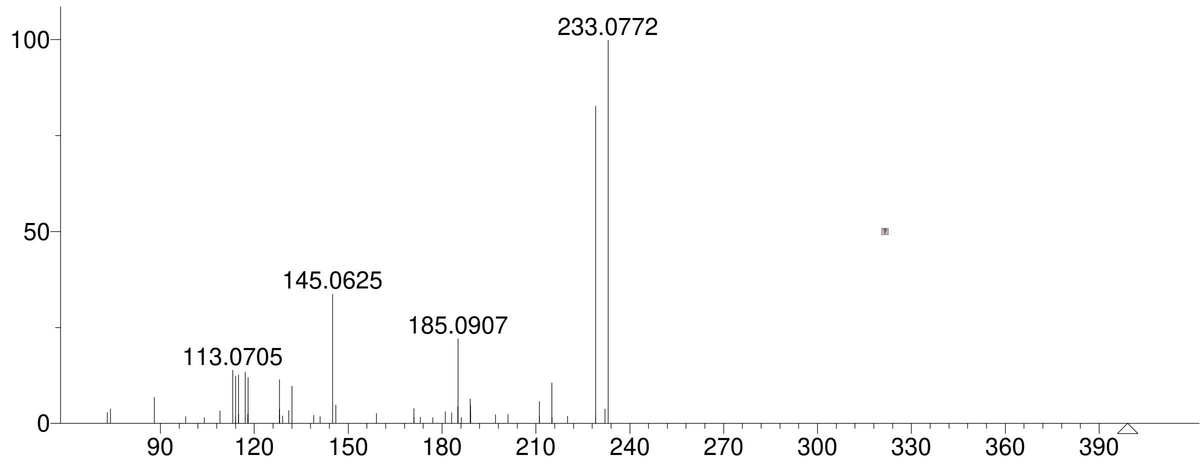

Hit 1 : 4-[4-[hydroxy-(4-hydroxy-3-methoxyphenyl)methyl]-3-(hydroxymethyl)oxolan-2-yl]-2-methoxyphenol [M+Na
C20H24O7; MF: 7; RMF: 109; Prob 15.3%; Lib: mona-export-vf-npl_qexactive; ID: 7275.

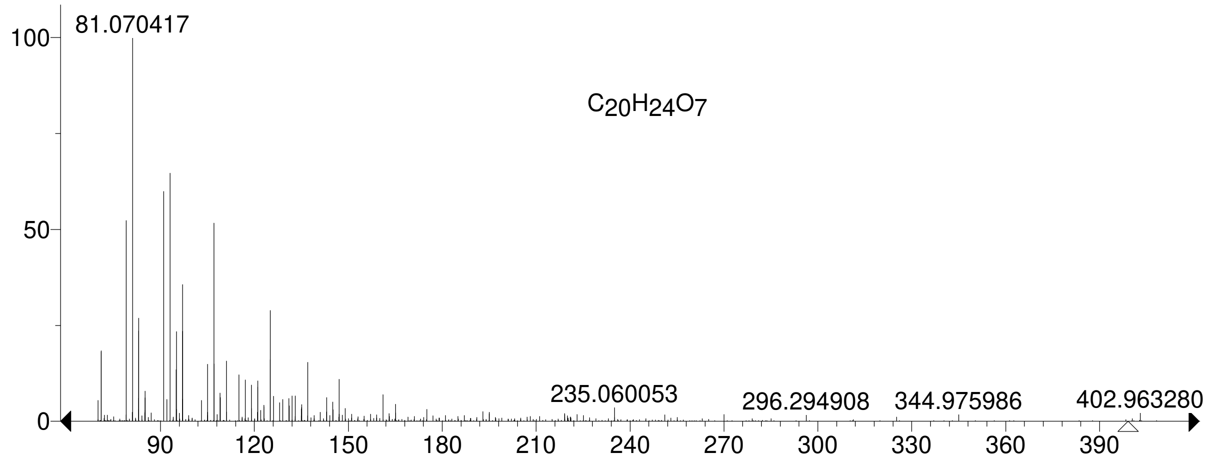

Hit 2 : [1,3-dihydroxy-1-(7-methoxy-2-oxochromen-6-yl)-3-methylbutan-2-yl] (E)-2-methylbut-2-enoate [M+Na]+ 6
C20H24O7; MF: 4; RMF: 129; Prob 13.5%; Lib: mona-export-vf-npl_qexactive; ID: 5457.

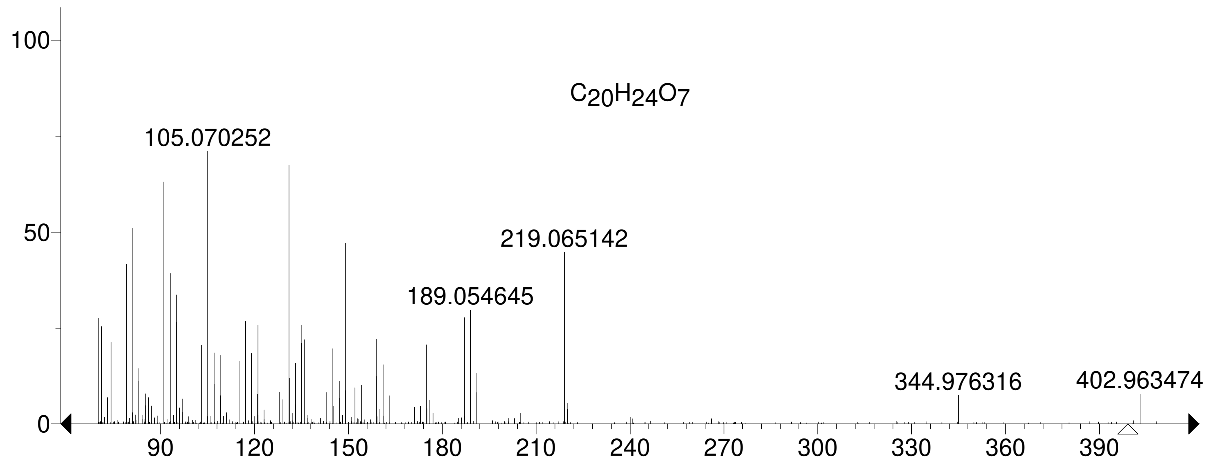

Hit 3 : [2,3-dihydroxy-1-(7-methoxy-2-oxochromen-6-yl)-3-methylbutyl] (Z)-2-methylbut-2-enoate [M+Na]+ 65HCI
C20H24O7; MF: 4; RMF: 110; Prob 13.5%; Lib: mona-export-vf-npl_qexactive; ID: 5712.

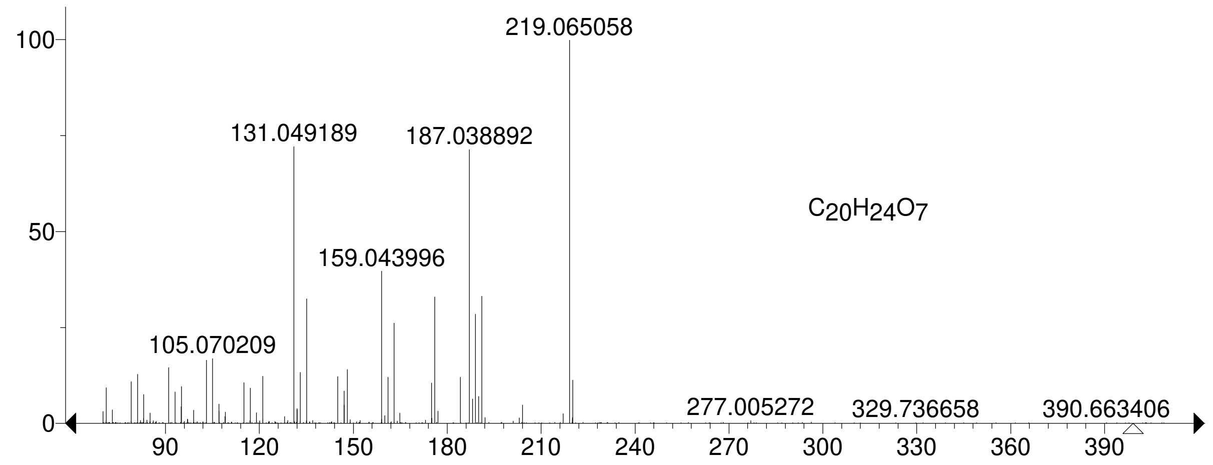

Hit 4 : [1,3-dihydroxy-1-(7-methoxy-2-oxochromen-6-yl)-3-methylbutan-2-yl] 3-methylbut-2-enoate [M+Na]+ 65H
C20H24O7; MF: 1; RMF: 58; Prob 11.9%; Lib: mona-export-vf-npl_qexactive; ID: 24777.

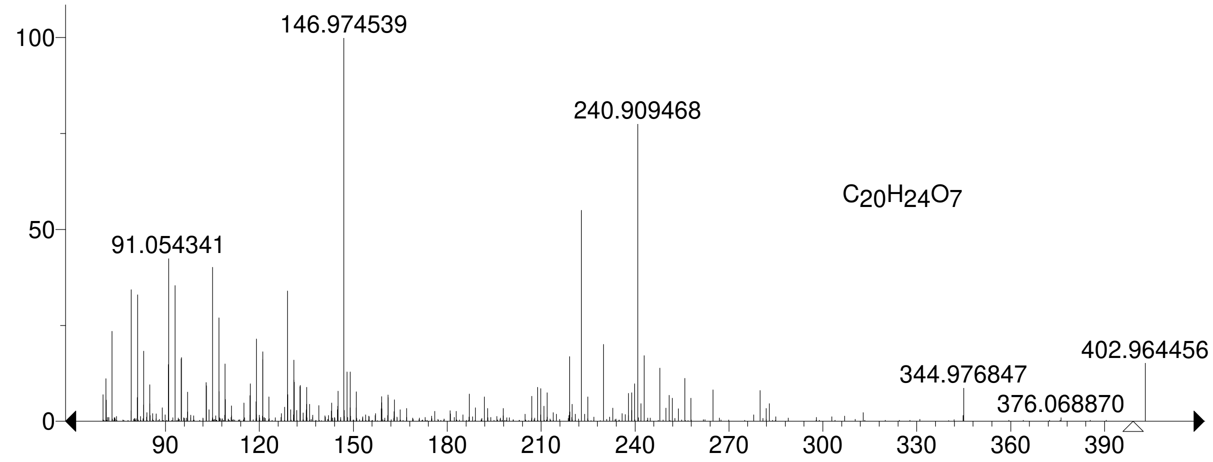

Hit 5 : (1R,2S,4R,6R,7S,9S,11R,12S)-11-hydroxy-4-methyl-10,15-dimethylidene-14-oxo-5,8,13-trioxatetracyclo[1(
C20H24O7; MF: 0; RMF: 42; Prob 11.4%; Lib: mona-export-vf-npl_qexactive; ID: 10422.

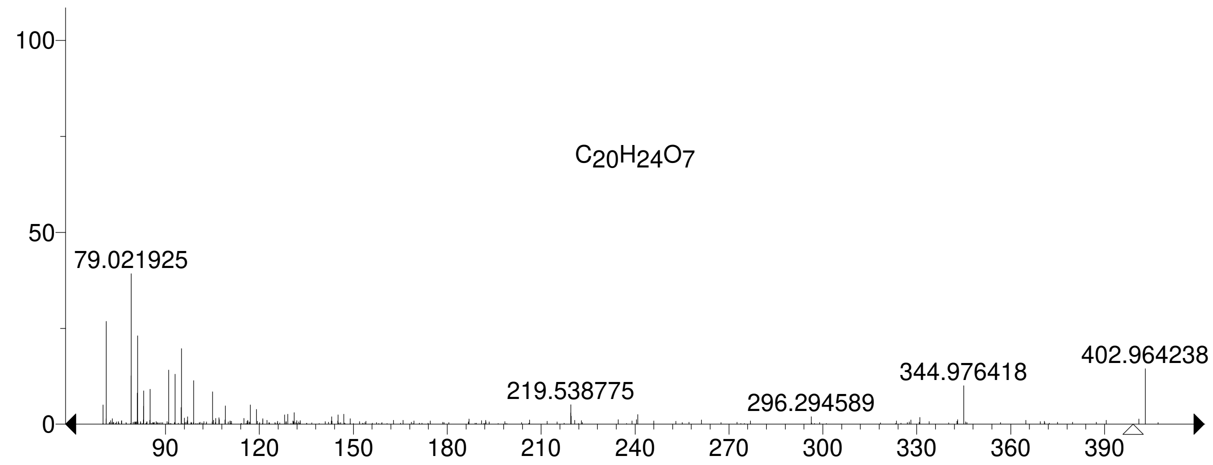

Unknown: Unknown (2.88_588.1091m/z)
Compound in Library Factor = 0

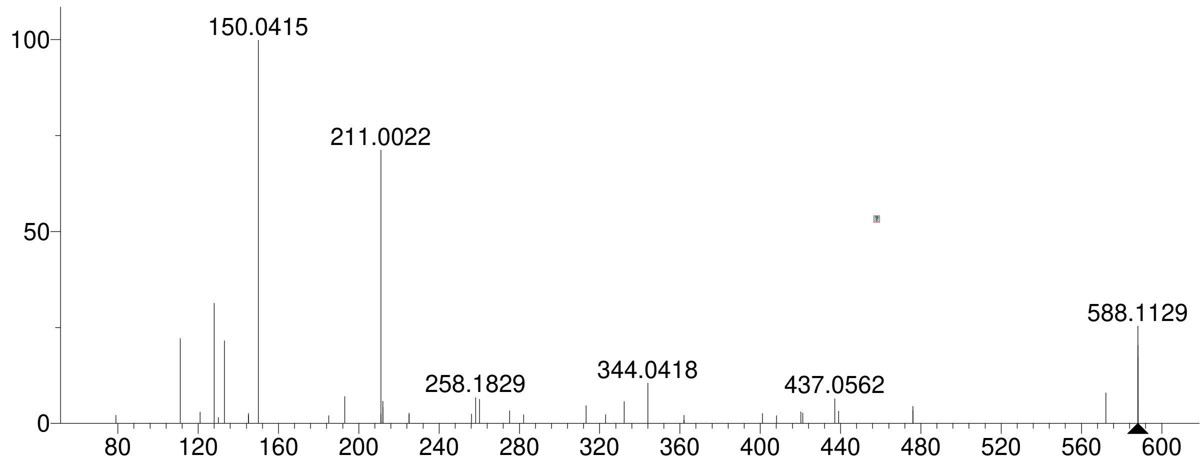

** Search Report Page 1 of 1 **

Unknown: Unknown (2.90_445.1808m/z)
Compound in Library Factor = 0

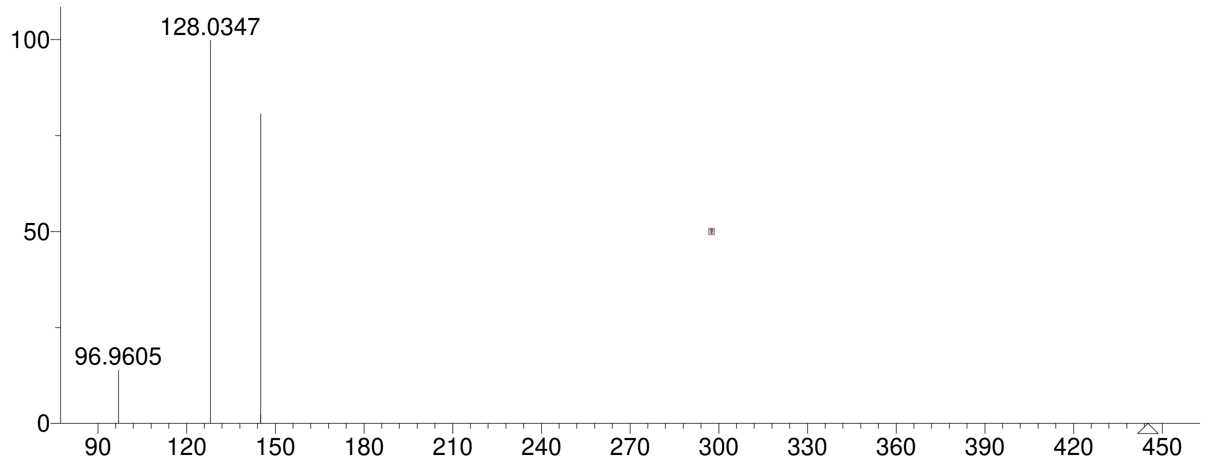

Hit 1 : [(6Z,10Z)-6-(acetyloxymethyl)-10-(hydroperoxymethyl)-3-methylidene-2-oxo-3a,4,5,8,9,11a-hexahydrocyclc
C22H30O8; MF: 0; RMF: 58; Prob 50.0%; Lib: mona-export-vf-npl_qexactive; ID: 19874.

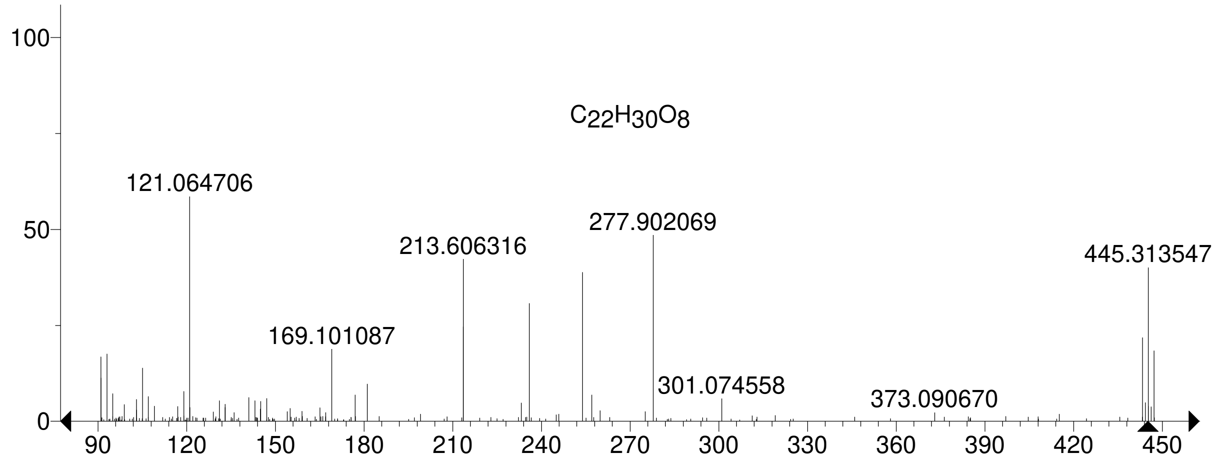

Hit 2 : NCGC00381245-01_C22H30O8_2-[6-{[(2Z)-4-Acetoxy-2-methyl-2-butenoyl]oxy}-2-hydroxy-3-(3-hydroxy-1
C22H30O8; MF: 0; RMF: 20; Prob 50.0%; Lib: mona-export-gnps; ID: 10571.

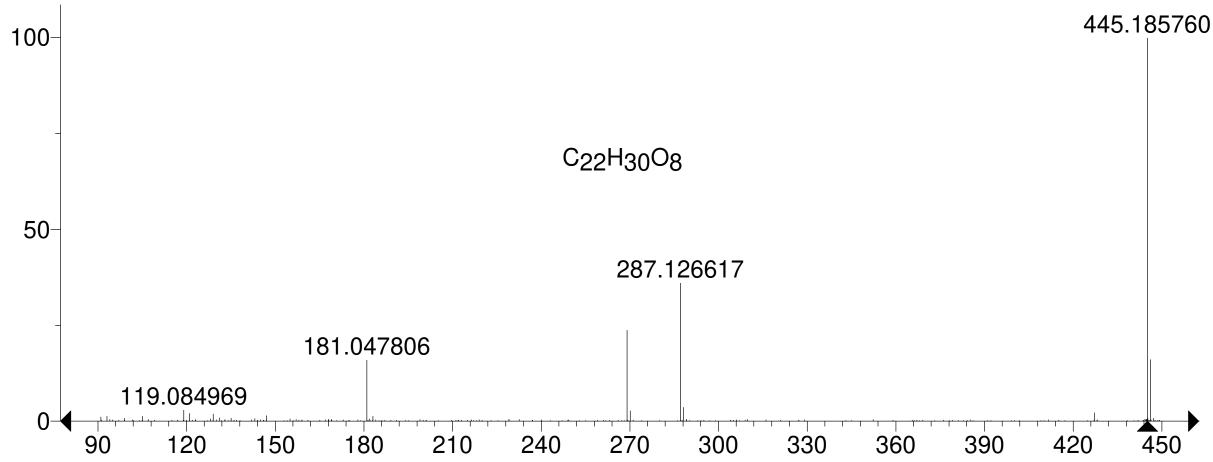

Unknown: Unknown (2.90_445.1883m/z)
Compound in Library Factor = 0

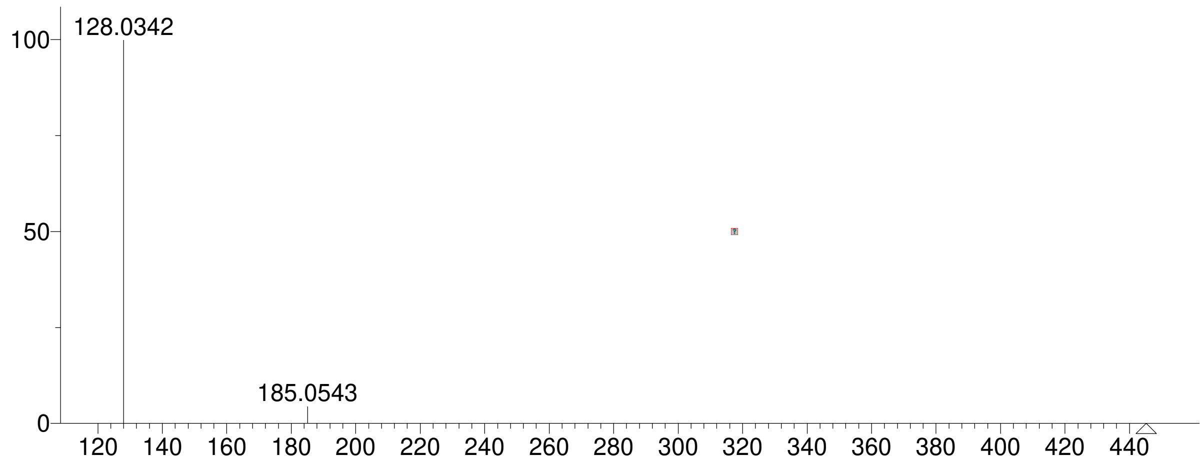

Hit 1 : epiyangambin [M-H]- 45HCD P=445.2
C24H30O8; MF: 0; RMF: 51; Prob 100.0%; Lib: mona-export-lc-ms-ms_negative_mode; ID: 18558.

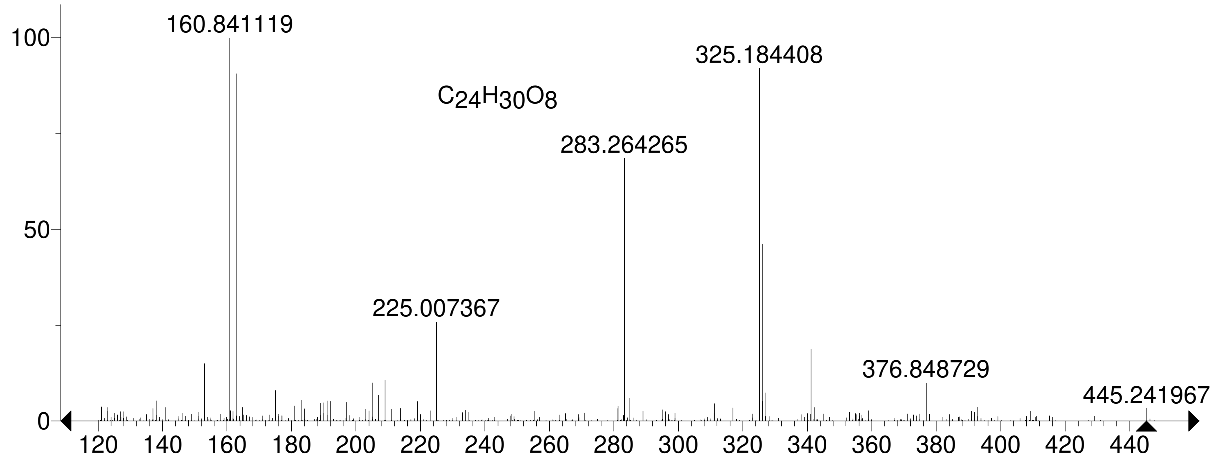

Unknown: Unknown (2.92_666.2620m/z)
Compound in Library Factor = 0

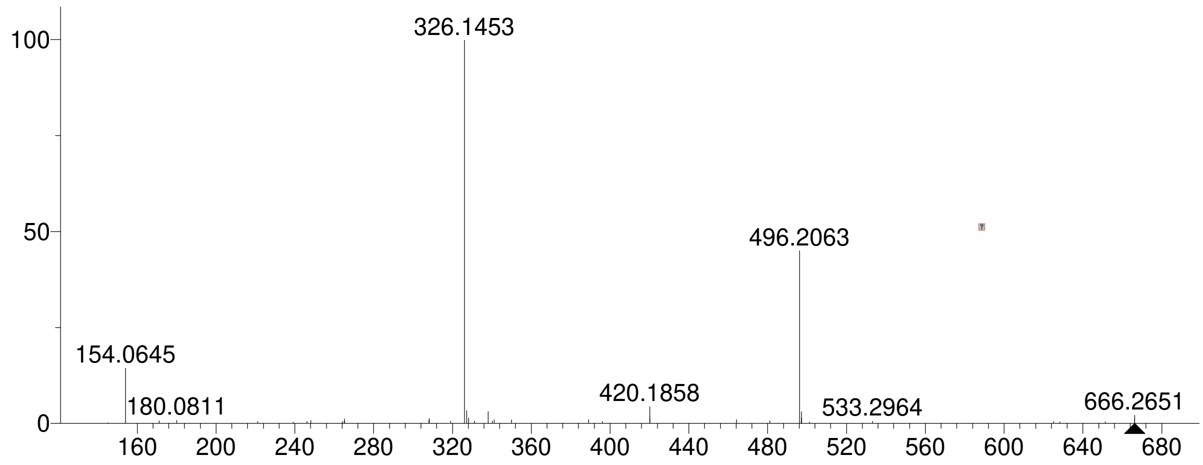

Unknown: Unknown (2.93_325.1052m/z)
Compound in Library Factor = -327

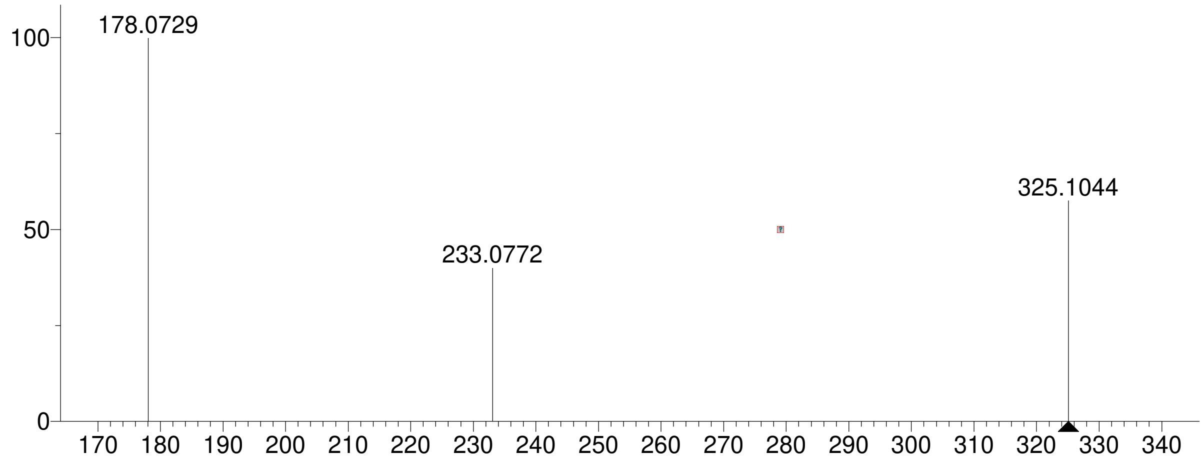

Hit 1 : 8-Desoxygartanin [M+H-C4H8]+ HCD 9V P=325.1
C23H24O5; MF: 287; RMF: 930; Prob 96.8%; CAS: 33390-41-9; Lib: nist_msms; ID: 227545.

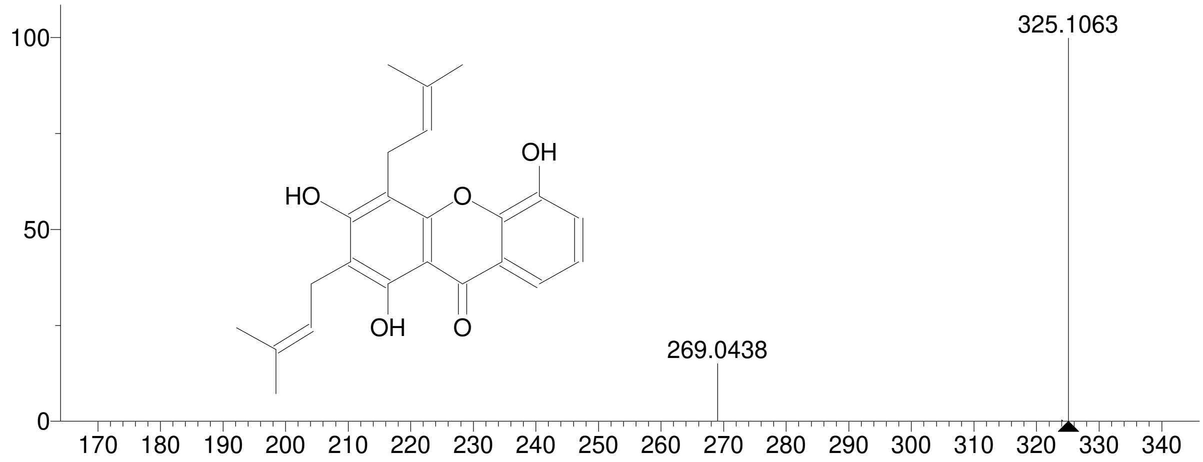

Hit 2 : 6-Hydroxywarfarin [M+H]+ HCD 5V P=325.1
C19H16O5; MF: 92; RMF: 533; Prob 1.35%; CAS: 17834-02-5; Lib: nist_msms; ID: 122405.

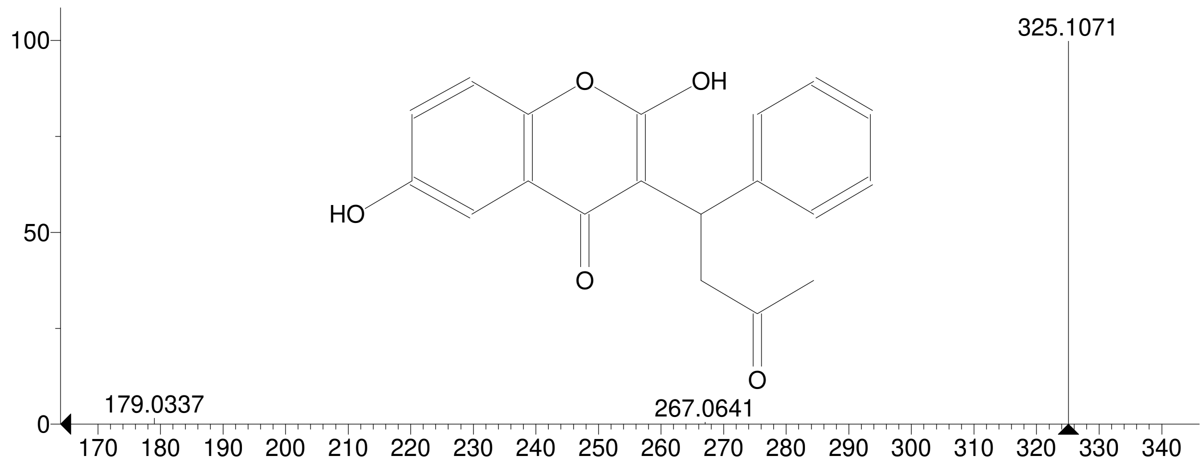

Hit 3 : 7-Hydroxywarfarin [M+H]+ HCD 5V P=325.1
C19H16O5; MF: 87; RMF: 527; Prob 1.09%; CAS: 17834-03-6; Lib: nist_msms; ID: 122448.

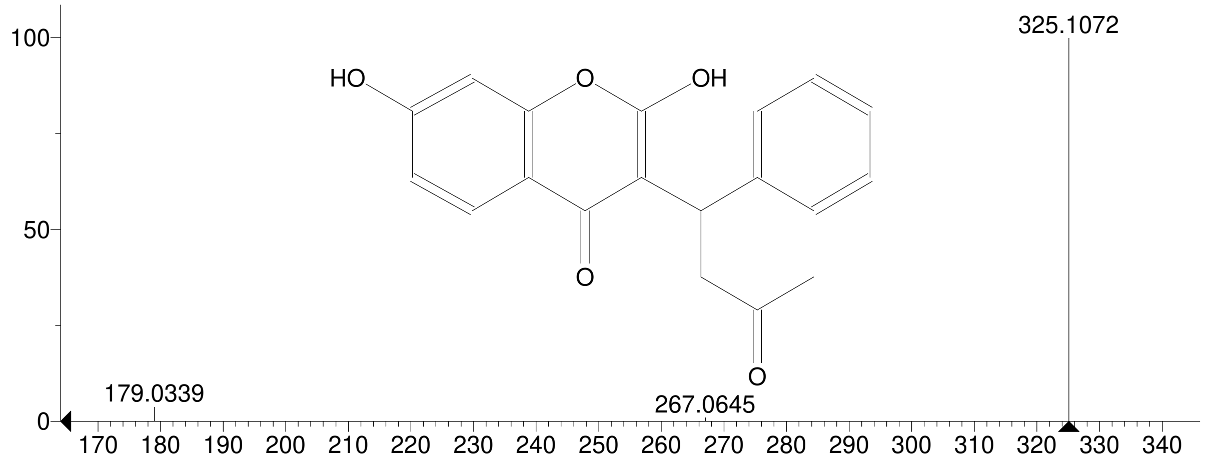

Hit 4 : Methopterine [M-H-C5H7O3N]- HCD 16V P=325.1
C20H21N7O6; MF: 64; RMF: 532; Prob 0.39%; CAS: 2410-93-7; Lib: nist_msms; ID: 206259.

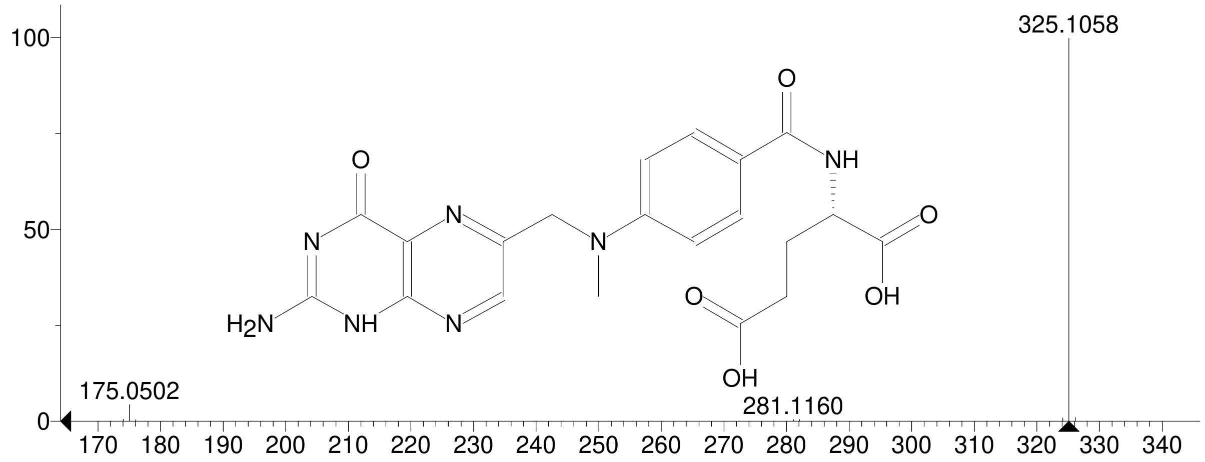

Hit 5 : PA 9:0; PA 2:0-7:0; [M-H]- [M-H]- P=325.1
C12H23O8P; MF: 16; RMF: 381; Prob 0.08%; Lib: msdial-insilicomsms-lipids-neg; ID: 315681.

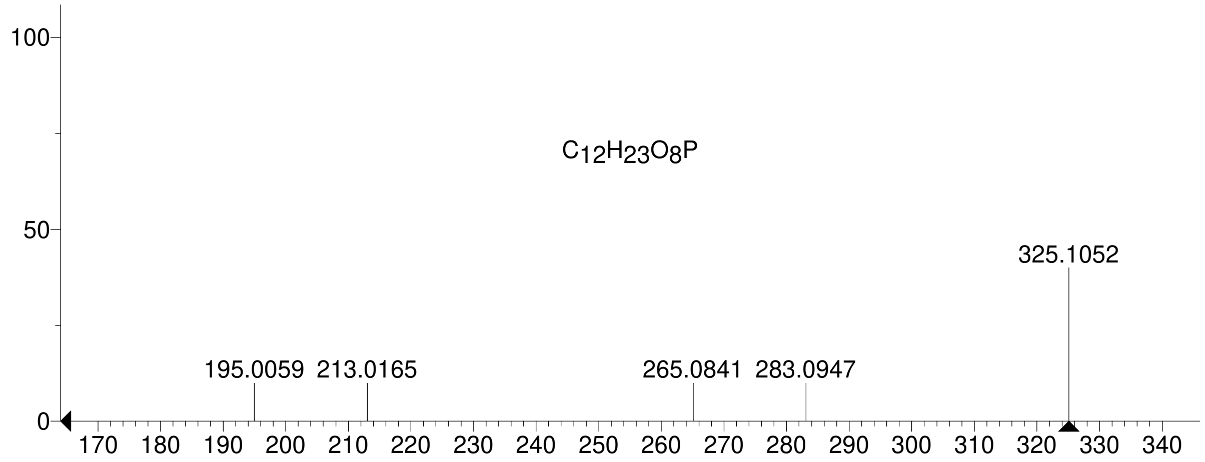

Unknown: Unknown (2.93_417.1150m/z)
Compound in Library Factor = -3273

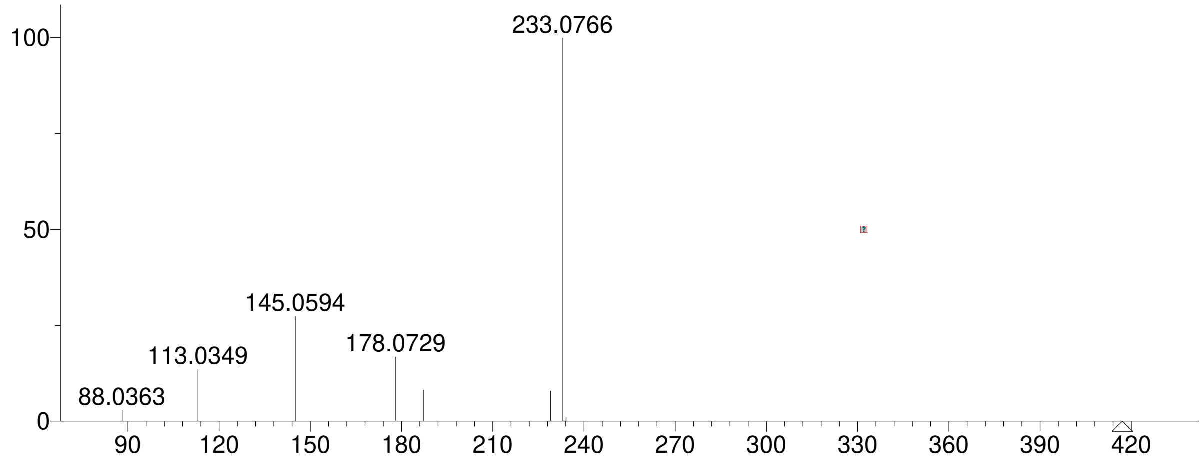

Hit 1 : Frangulin A [M+H]+ HCD 50V P=417.1
C21H20O9; MF: 3; RMF: 118; Prob 12.4%; CAS: 521-62-0; Lib: nist_msms; ID: 258725.

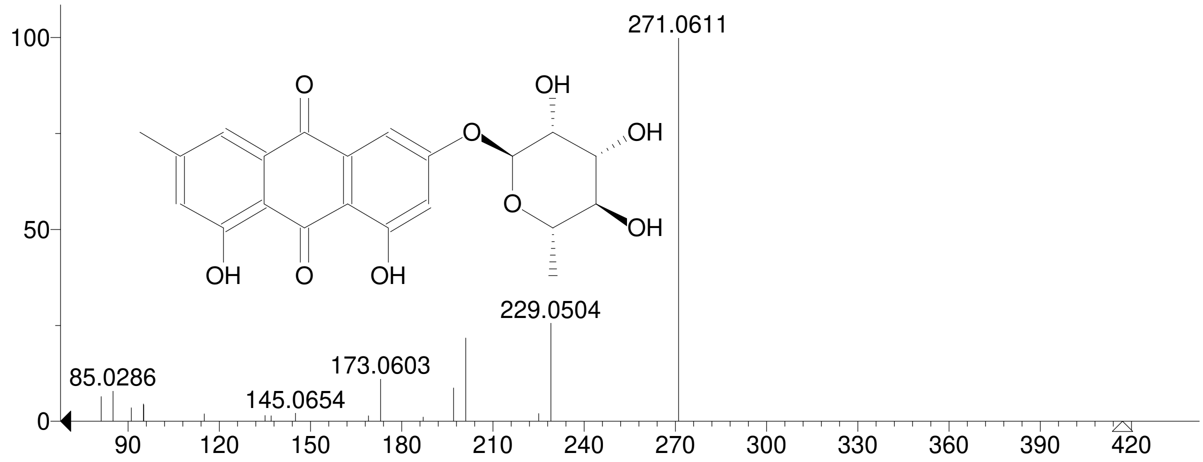

Hit 2 : Aloin [M-H]- 35HCD P=417.1
C21H22O9; MF: 0; RMF: 81; Prob 10.9%; Lib: mona-export-lc-ms-ms_negative_mode; ID: 23720.

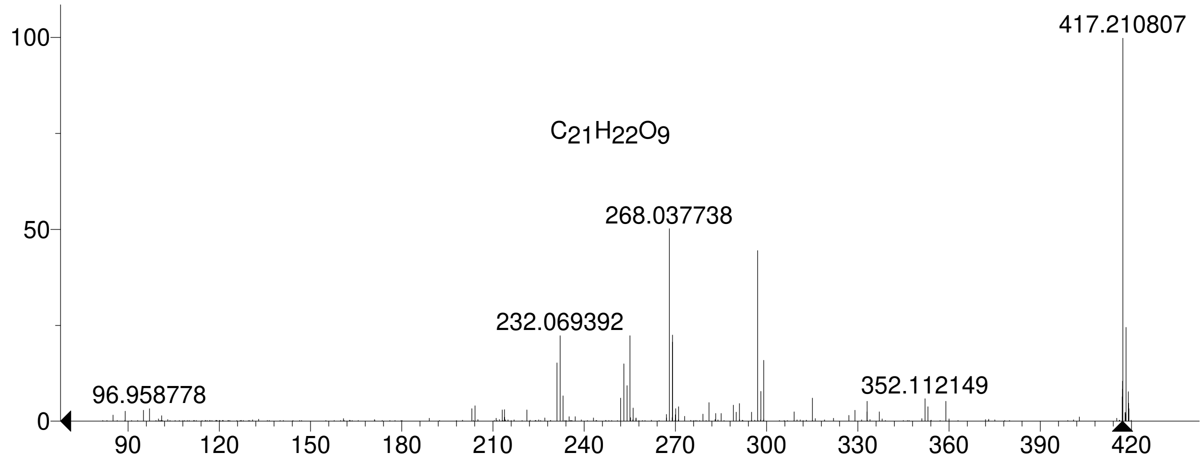

Hit 3 : aloesin [M+Na]+ 65HCD P=417.1
C19H22O9; MF: 0; RMF: 63; Prob 10.9%; Lib: mona-export-vf-npl_qexactive; ID: 4467.

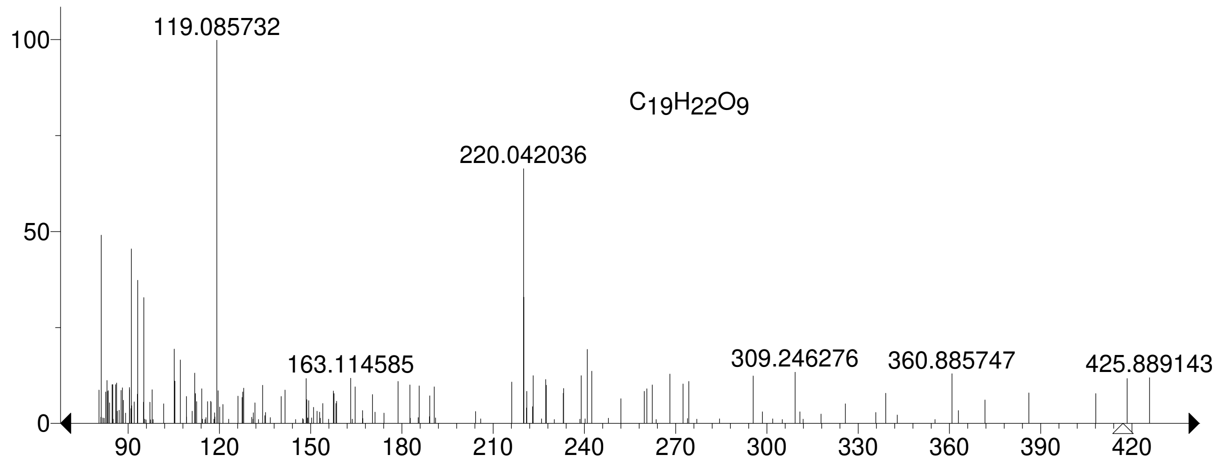

Hit 4 : Daidzein-8-C-glucoside [M+H]+ 50eV P=417.1
C21H20O9; MF: 0; RMF: 56; Prob 10.9%; Lib: anthocyanins mona; ID: 3706.

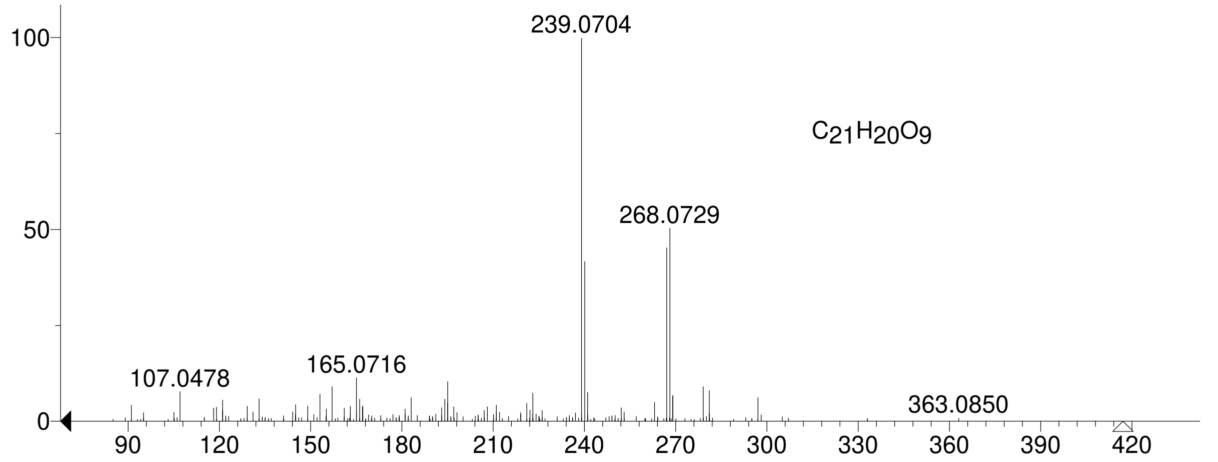

Hit 5 : Engeletin [M+H-H2O]+ HCD 75V P=417.1
C21H22O10; MF: 0; RMF: 51; Prob 10.9%; CAS: 572-31-6; Lib: nist_msms; ID: 236877.

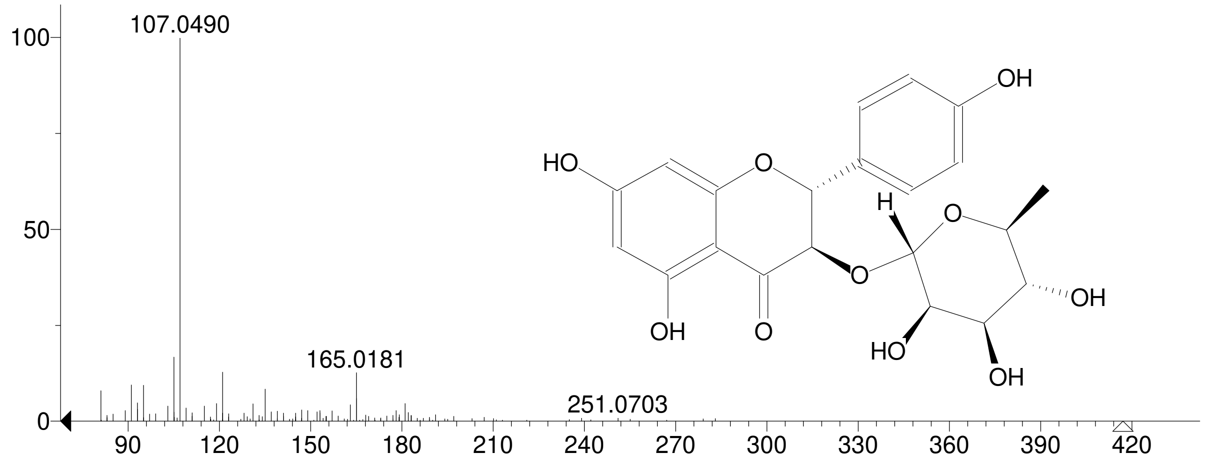

Unknown: Unknown (2.94_474.1730m/z)
Compound in Library Factor = 0

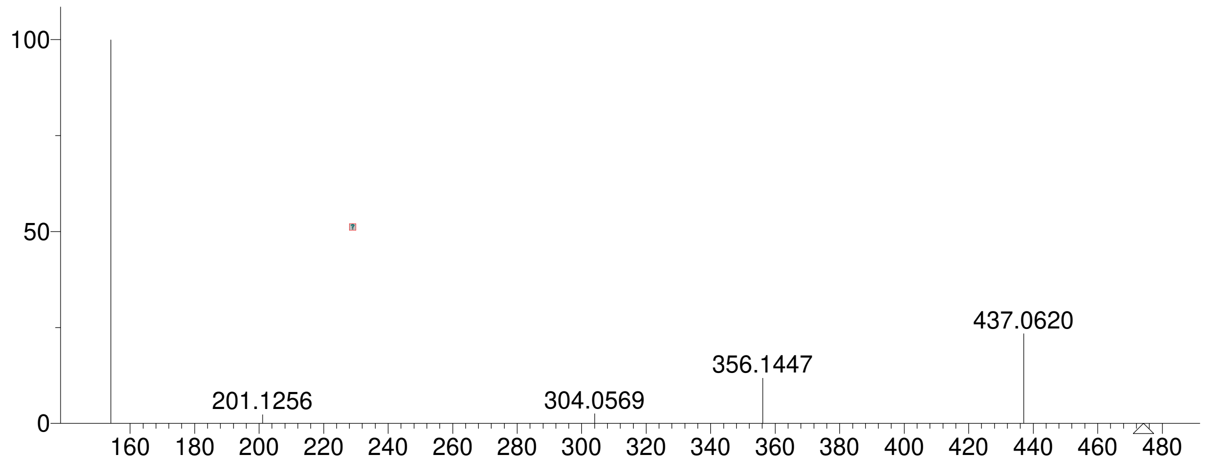

Hit 1 : Podophyllin Acetate [M+NH4]+ 45HCD P=474.2
C24H24O9; MF: 0; RMF: 53; Prob 33.3%; Lib: mona-export-vf-npl_qexactive; ID: 11162.

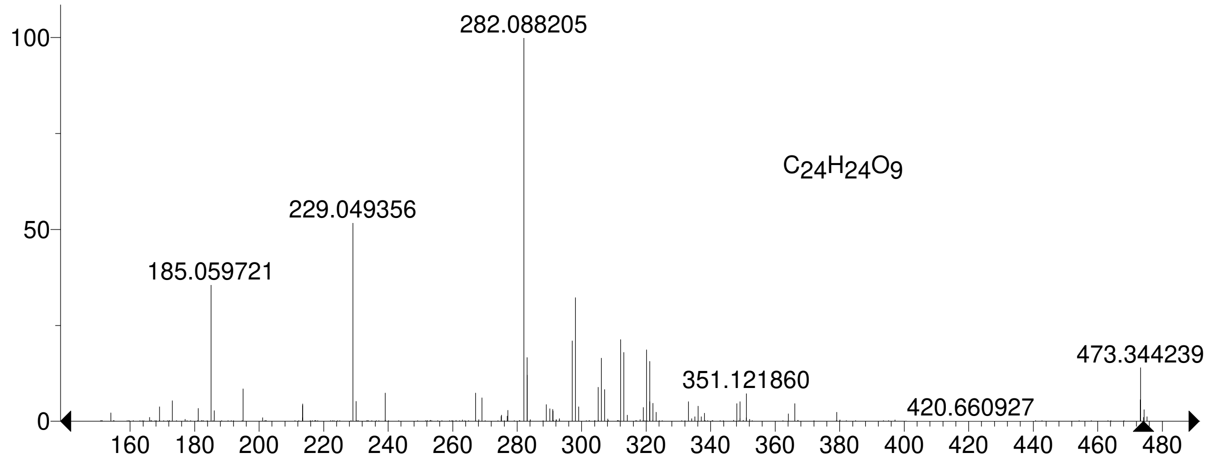

Hit 2 : steganacin [M+NH4]+ 65HCD P=474.2
C24H24O9; MF: 0; RMF: 5; Prob 33.3%; Lib: mona-export-vf-npl_qexactive; ID: 19395.

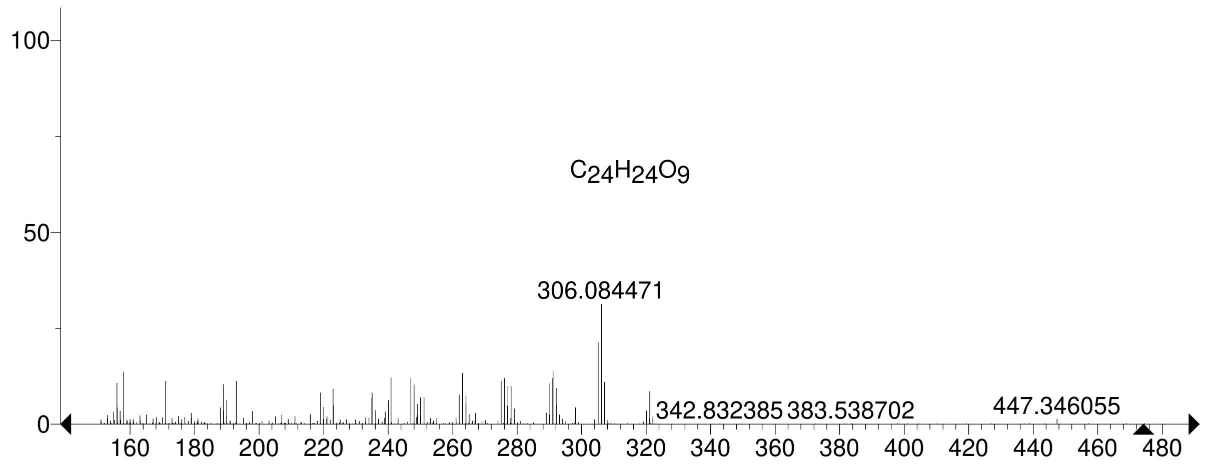

Hit 3 : NCGC00180175-03! [M+NH4]+ P=474.2
C24H24O9; MF: 0; RMF: 3; Prob 33.3%; Lib: mona-export-gnps; ID: 7998.

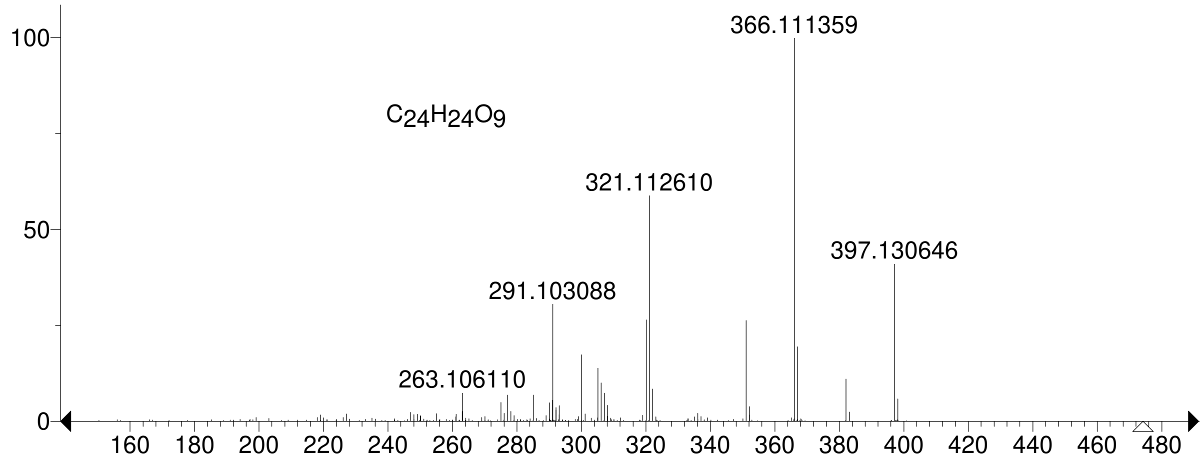

** Search Report Page 1 of 1 **

Unknown: Unknown (2.95_681.2962m/z)
Compound in Library Factor = -1848

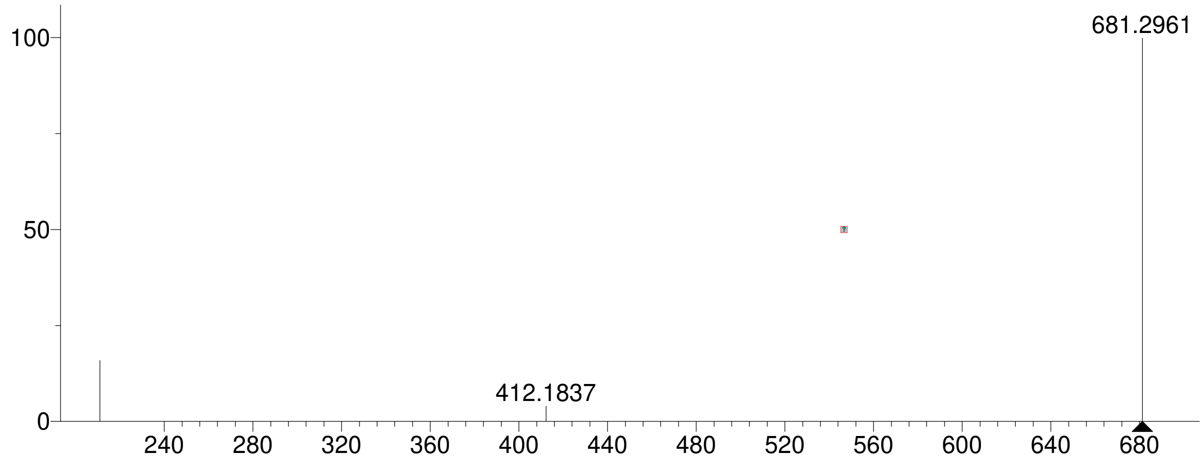

Hit 1 : S4:17(P3:15/F1:2); PlaSMA ID-1857 [M+FA-H]- P=681.3
C29H48O15; MF: 37; RMF: 710; Prob 100.0%; Lib: biomsms-neg-plasma; ID: 344.

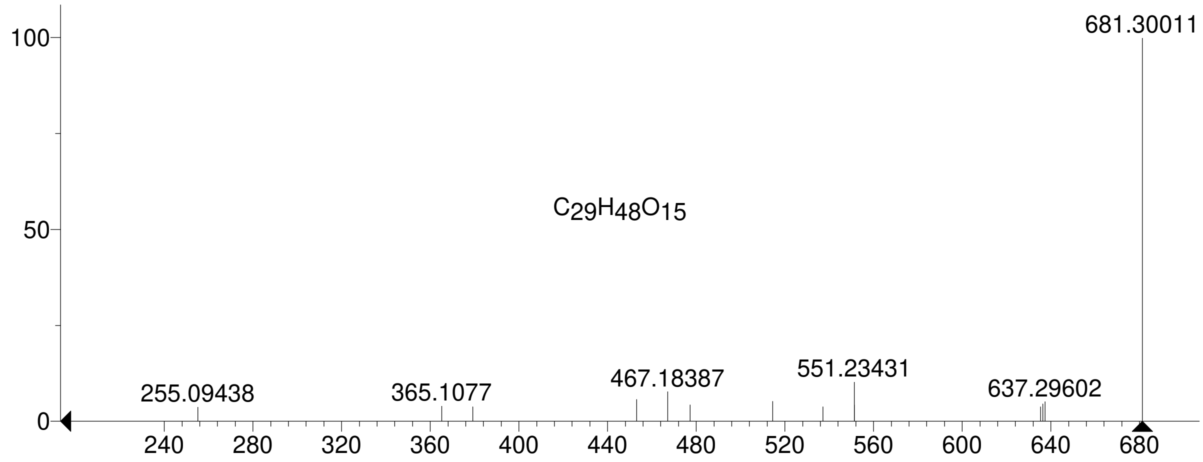

Unknown: Unknown (2.96_279.0972m/z)
Compound in Library Factor = -516

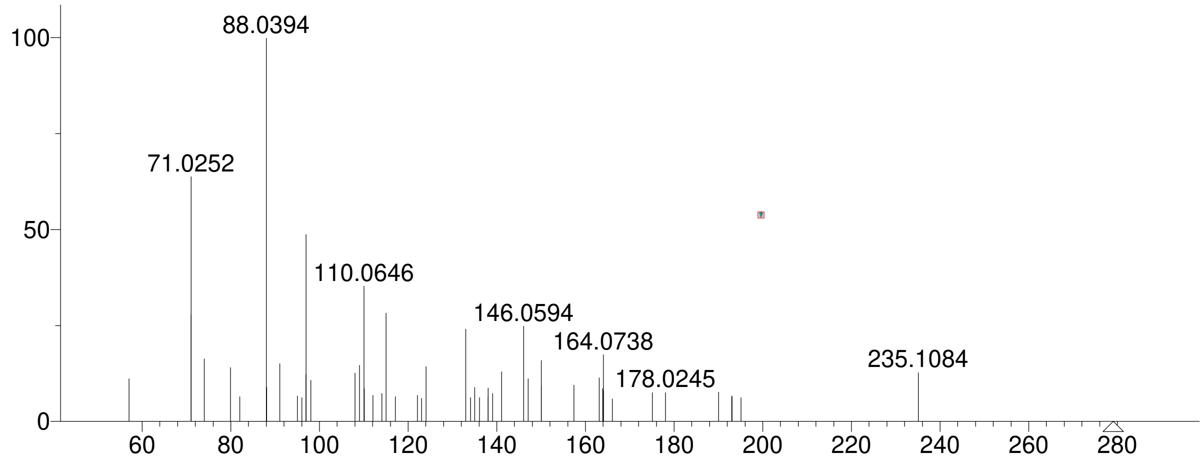

Hit 1 : Phe-Asp [M-H]- HCD 25eV P=279.1
C13H16N2O5; MF: 356; RMF: 579; Prob 49.6%; Lib: nist_msms; ID: 570583.

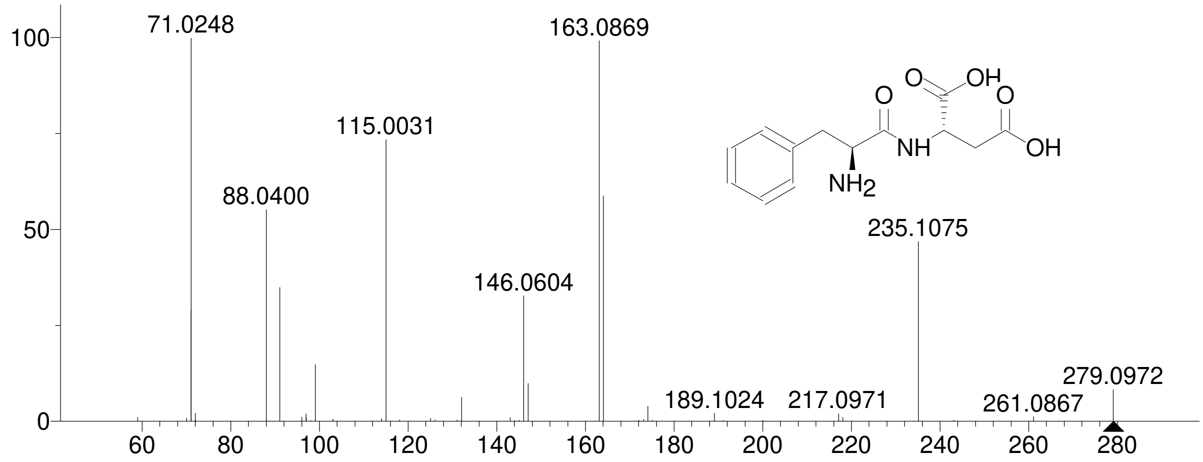

Hit 2 : Phe-Asp [M-H]- HCD 25eV P=279.1
C13H16N2O5; MF: 356; RMF: 579; Prob 49.6%; CAS: 22828-05-3; Lib: nist_msms2; ID: 84316.

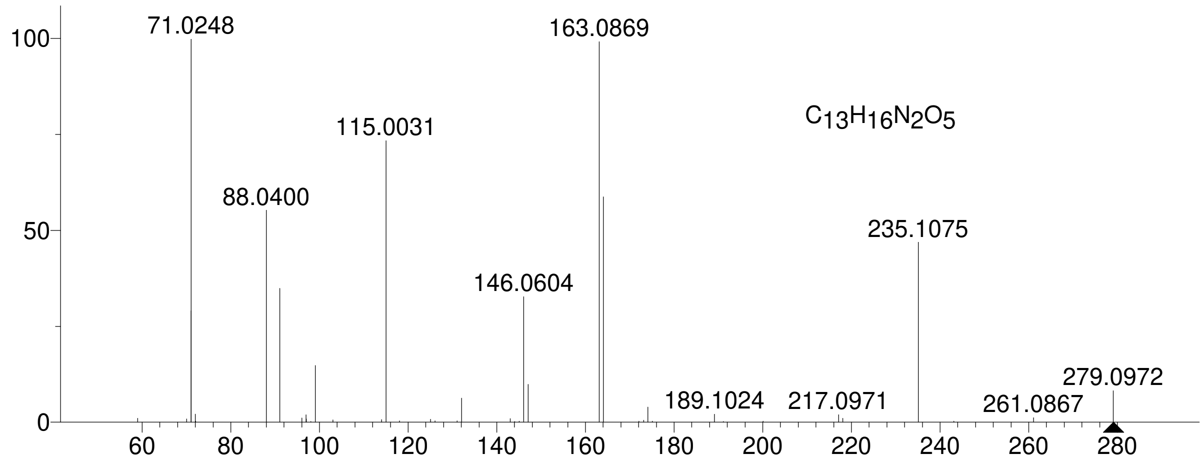

Hit 3 : Asp-Phe [M-H]- 20-30-40%) P=279.1
C13H16N2O5; MF: 14; RMF: 145; Prob 0.49%; Lib: msms-neg-fiehnhilic; ID: 208.

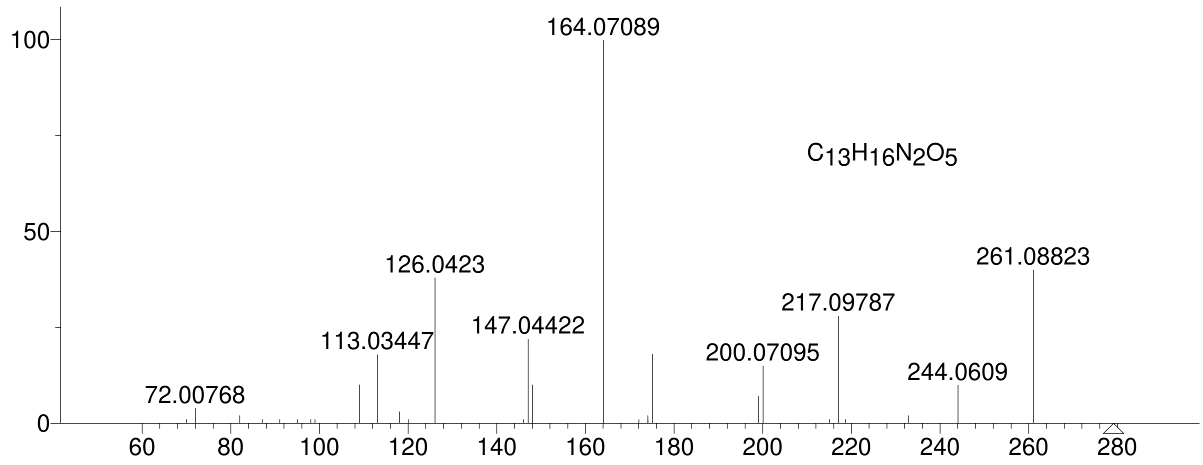

Hit 4 : gamma-Glutamylmethionine [M+H]+ 35eV P=279.1
C10H18N2O5S; MF: 0; RMF: 27; Prob 0.31%; Lib: mona-export-fiehn_hilic; ID: 1953.

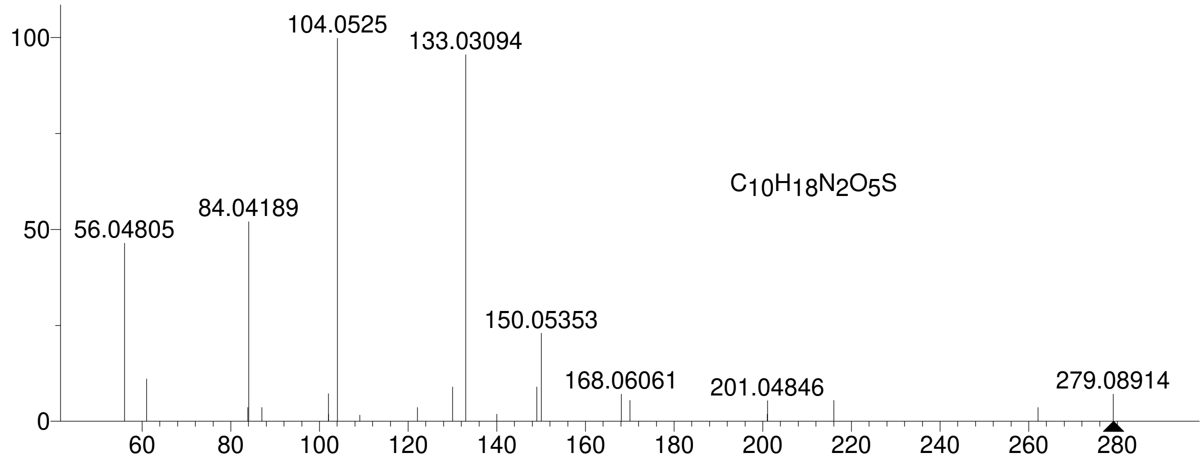

Unknown: Unknown (2.96_399.1027m/z)
Compound in Library Factor = 0

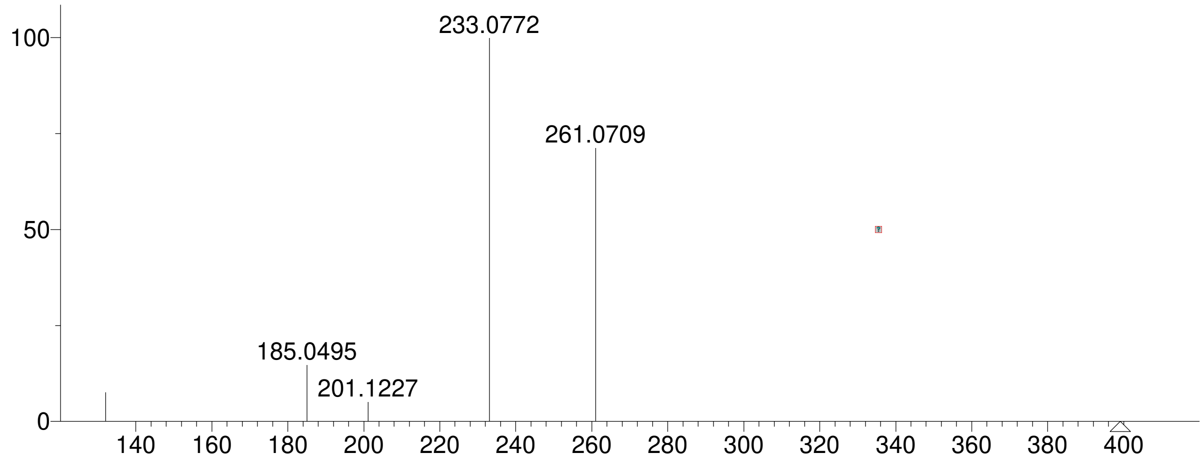

Hit 1 : (2R,3R,4S,5S,6R)-2-[3-hydroxy-2-(4-hydroxy-3-methoxyphenyl)propoxy]-6-(hydroxymethyl)oxane-3,4,5-tric
C16H24O9; MF: 0; RMF: 16; Prob 50.0%; Lib: mona-export-vf-npl_qexactive; ID: 10759.

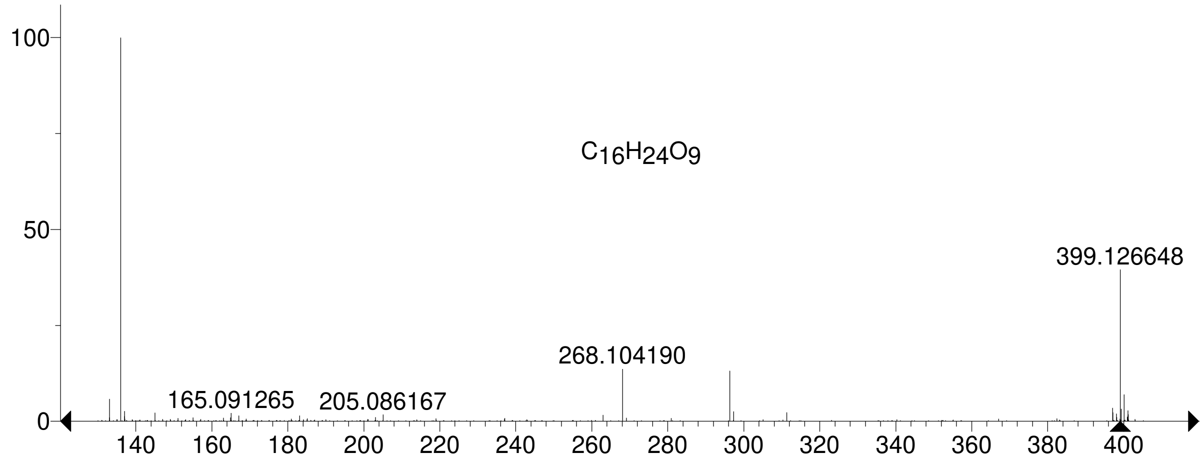

Hit 2 : 2,3,9,10-tetramethoxy-6a,11a-dihydro-6H-[1]benzofuro[3,2-c]chromene-4,8-diol [M+Na]+ 65HCD P=399.
C19H20O8; MF: 0; RMF: 11; Prob 50.0%; Lib: mona-export-vf-npl_qexactive; ID: 18612.

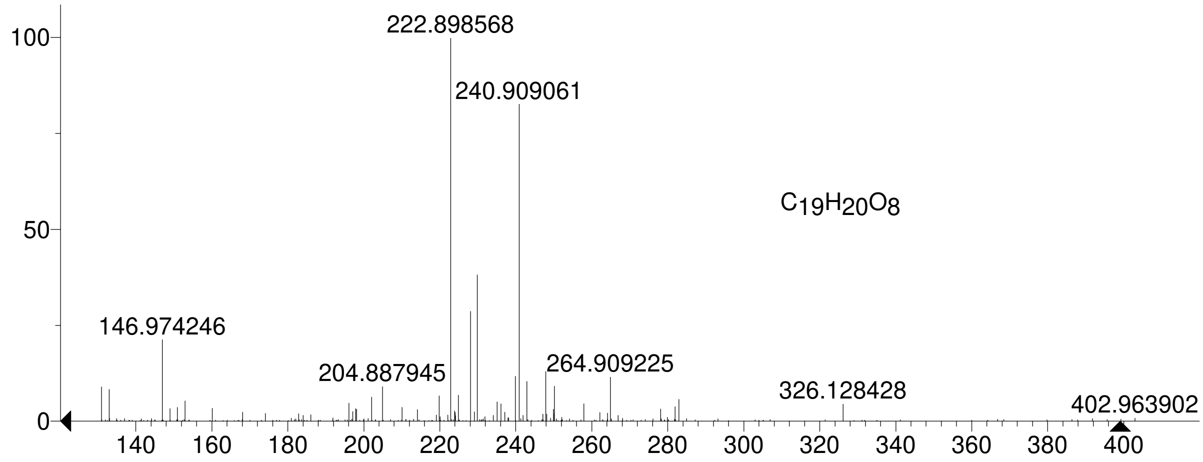

Unknown: Unknown (2.96_403.1344m/z)
Compound in Library Factor = 0

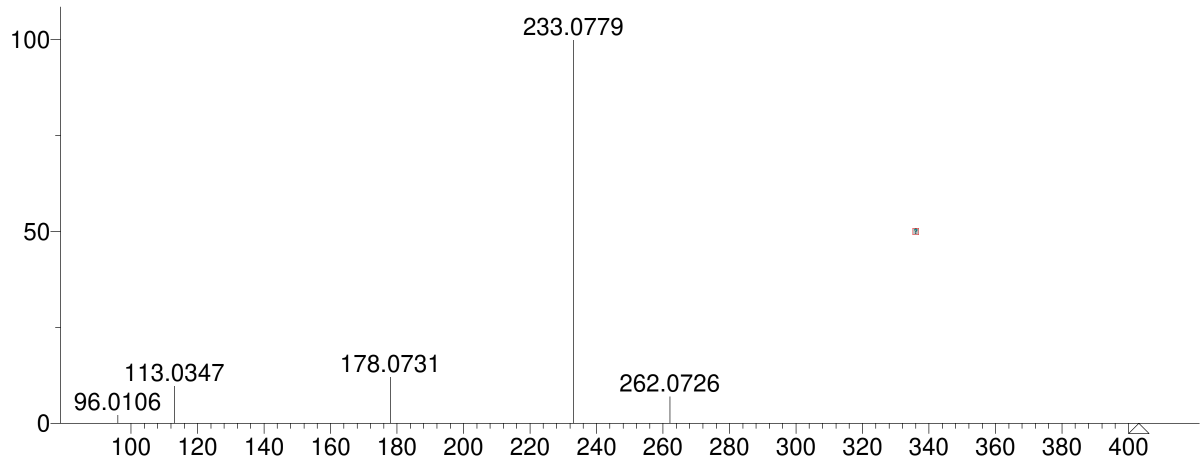

Hit 1 : NCGC00380227-01!(E)-3-[4-[1,3-dihydroxy-1-(4-hydroxy-3-methoxyphenyl)propan-2-yl]oxy-3,5-dimethoxyp
C21H24O9; MF: 0; RMF: 118; Prob 50.0%; Lib: mona-export-gnps; ID: 8693.

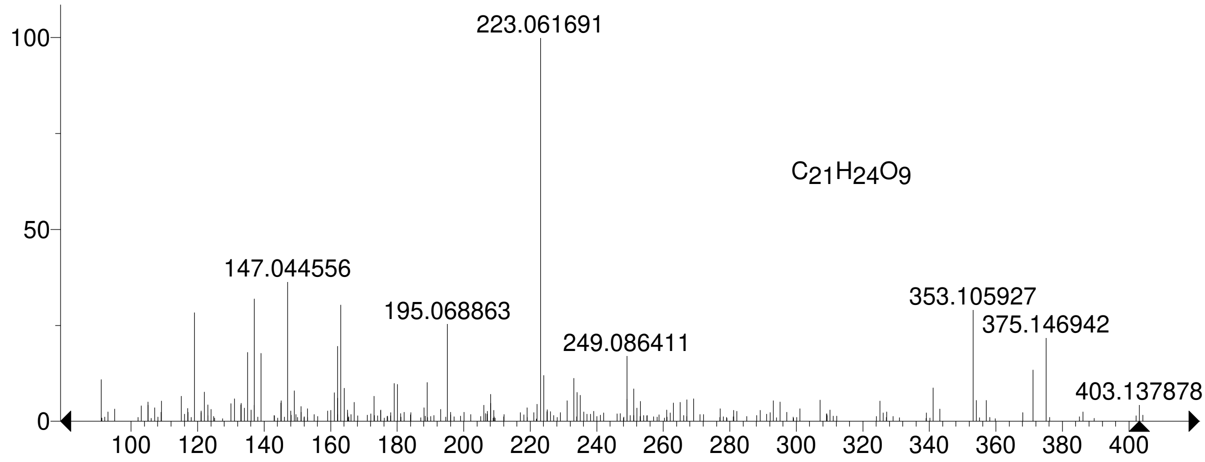

Hit 2 : NCGC00384517-01!(2S)-2-phenyl-7-[(2S,3R,4S,5S,6R)-3,4,5-trihydroxy-6-(hydroxymethyl)oxan-2-yl]oxy-2
C21H22O8; MF: 0; RMF: 12; Prob 50.0%; Lib: mona-export-gnps; ID: 7455.

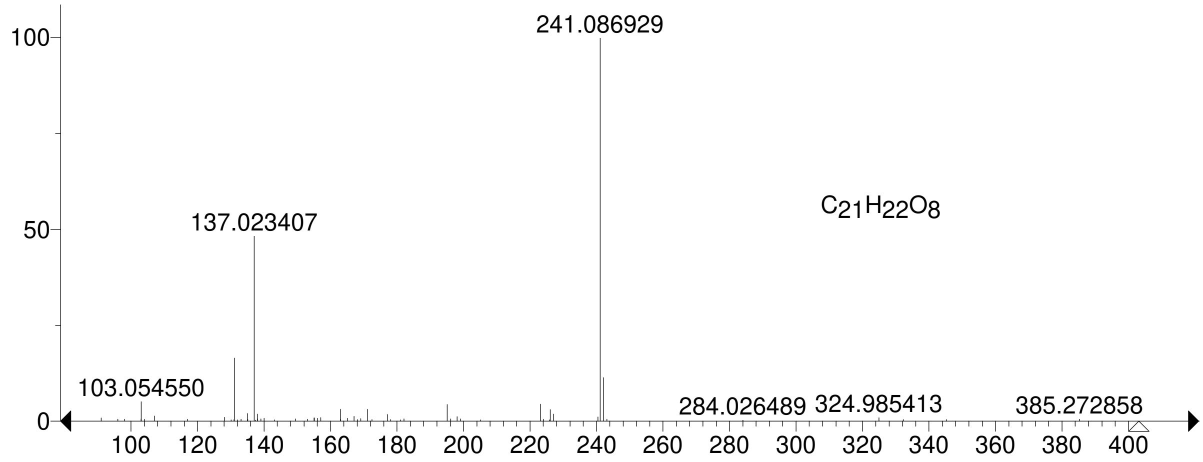

Unknown: Unknown (2.96_431.1298m/z)
Compound in Library Factor = 0

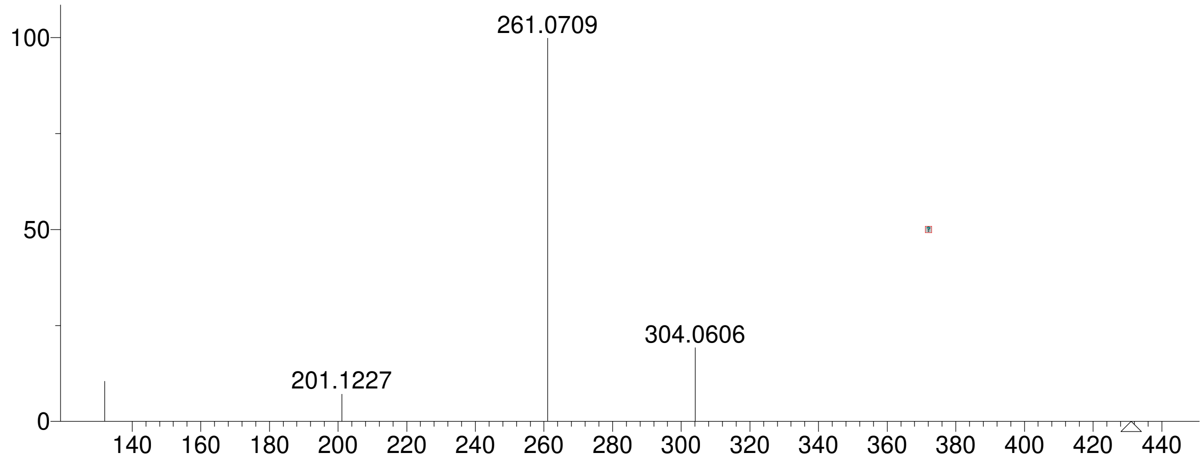

Hit 1 : O-Desmethylgefitinib [M-H]- HCD 68V P=431.1
C21H22ClFN4O3; MF: 0; RMF: 13; Prob 33.3%; CAS: 847949-49-9; Lib: nist_msms; ID: 451781.

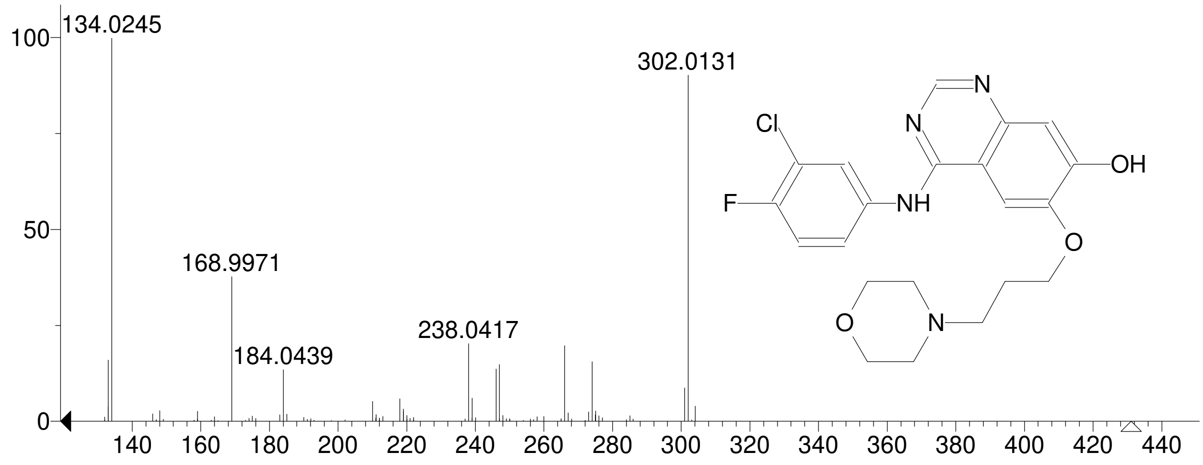

Hit 2 : Formononetin-7-O-glucoside [M+H]+ 50eV P=431.1
C22H22O9; MF: 0; RMF: 7; Prob 33.3%; Lib: anthocyanins mona; ID: 1127.

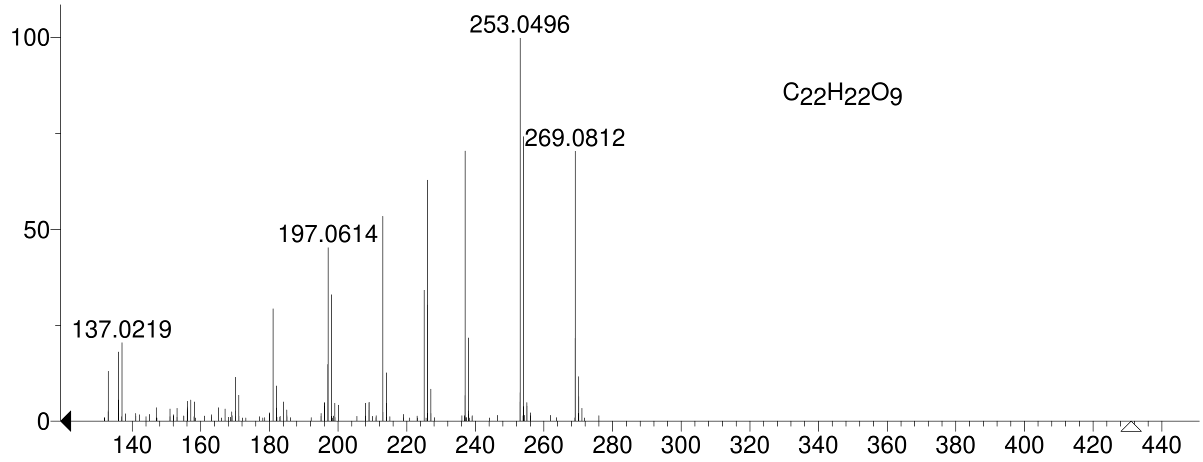

Hit 3 : calycosin-7-O-beta-D-glucoside [M+H]+ 35HCD P=431.1
C22H22O9; MF: 0; RMF: 2; Prob 33.3%; Lib: mona-export-vf-npl_qexactive; ID: 13249.

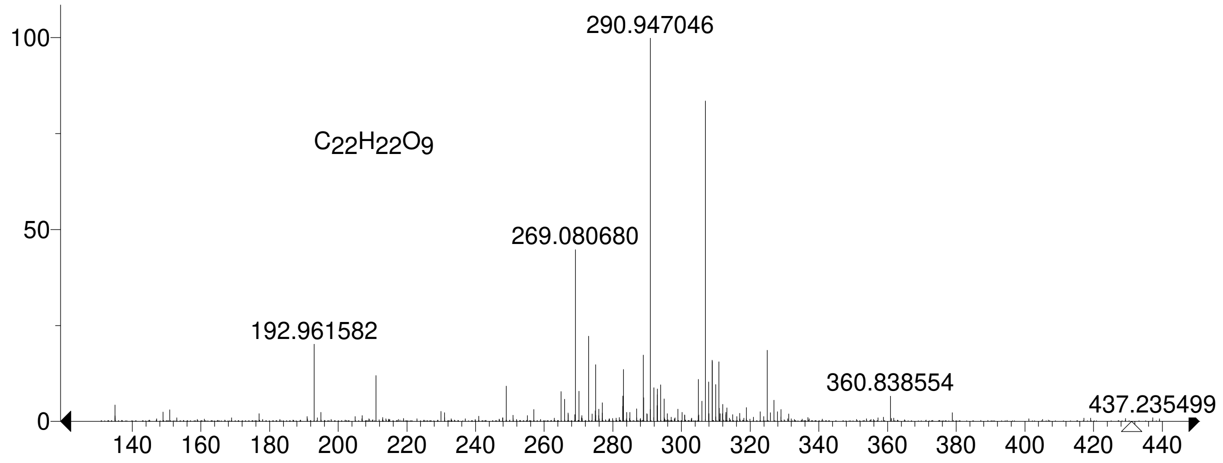

Unknown: Unknown (2.99_416.1298m/z)
Compound in Library Factor = 0

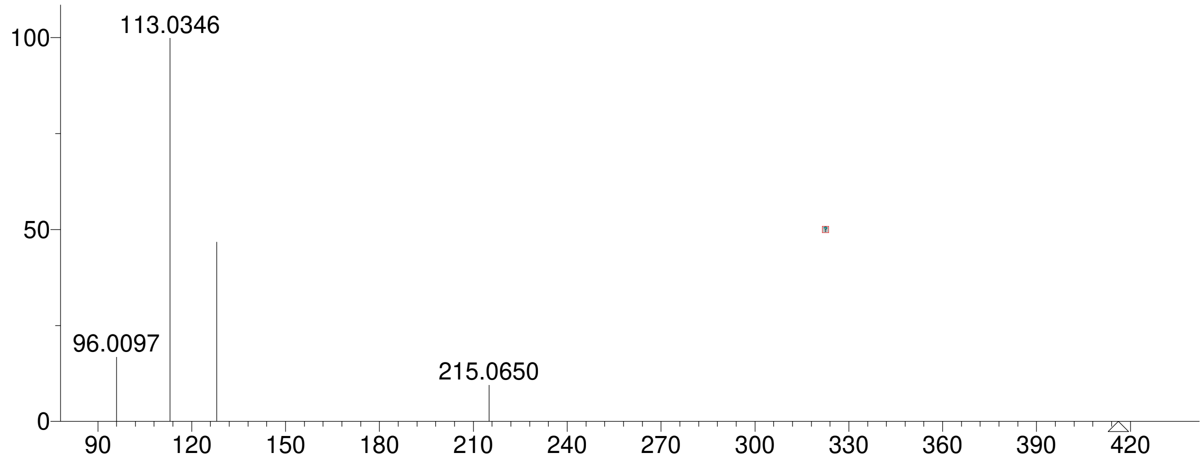

Unknown: Unknown (2.99_464.1857m/z)
Compound in Library Factor = 0

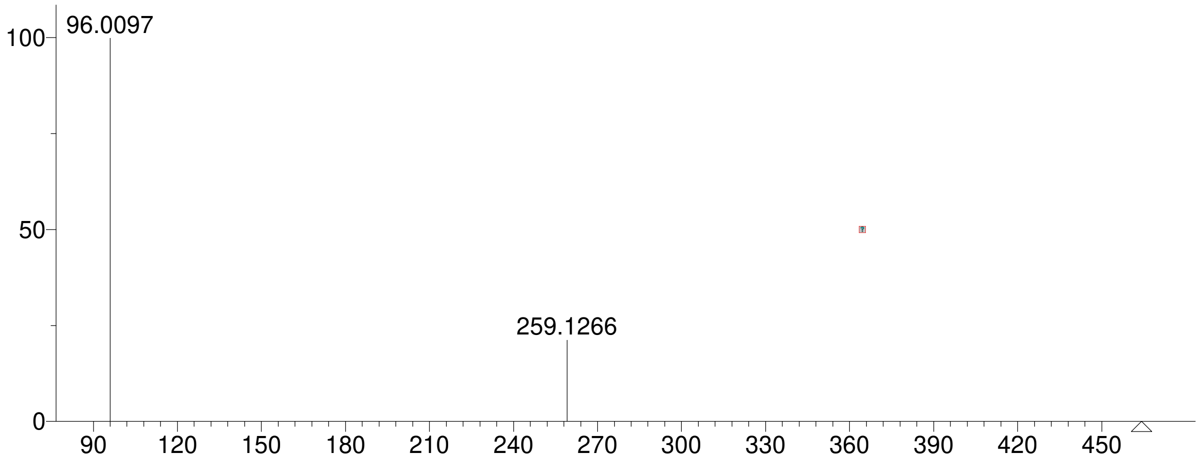

Unknown: Unknown (20.00_341.9273m/z)
Compound in Library Factor = 0

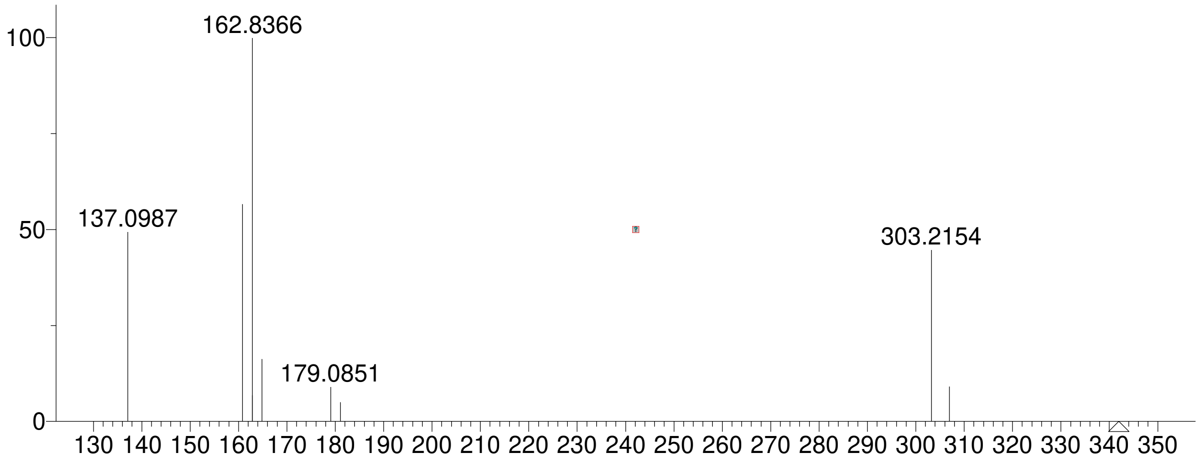

Unknown: Unknown (20.03_277.1435m/z)
Compound in Library Factor = -1072

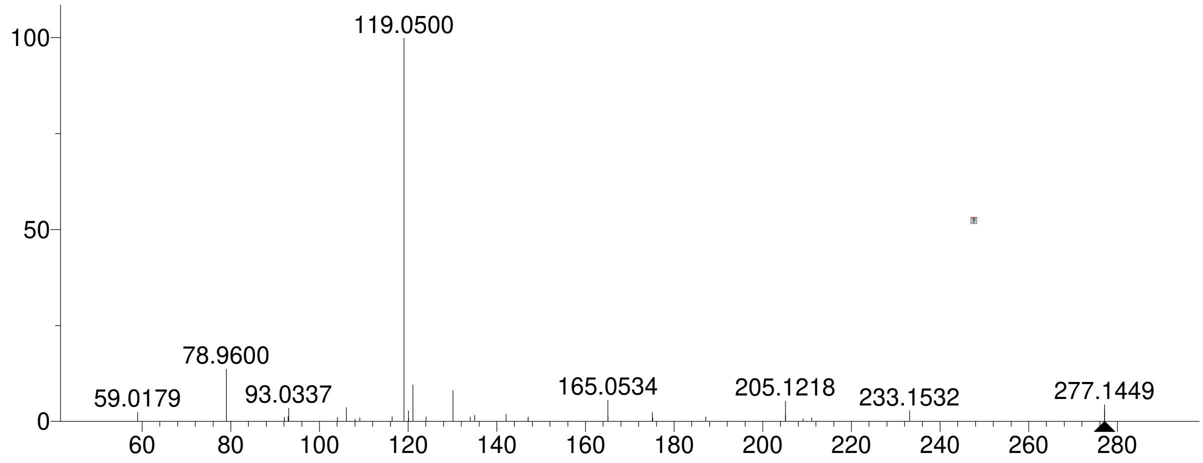

Hit 1 : 2,5,7,8-Tetramethyl-2-(β-carboxyethyl)-6-hydroxychroman [M-H]- HCD 9V P=277.1
C16H22O4; MF: 112; RMF: 462; Prob 69.1%; CAS: 4072-32-6; Lib: nist_msms; ID: 340391.

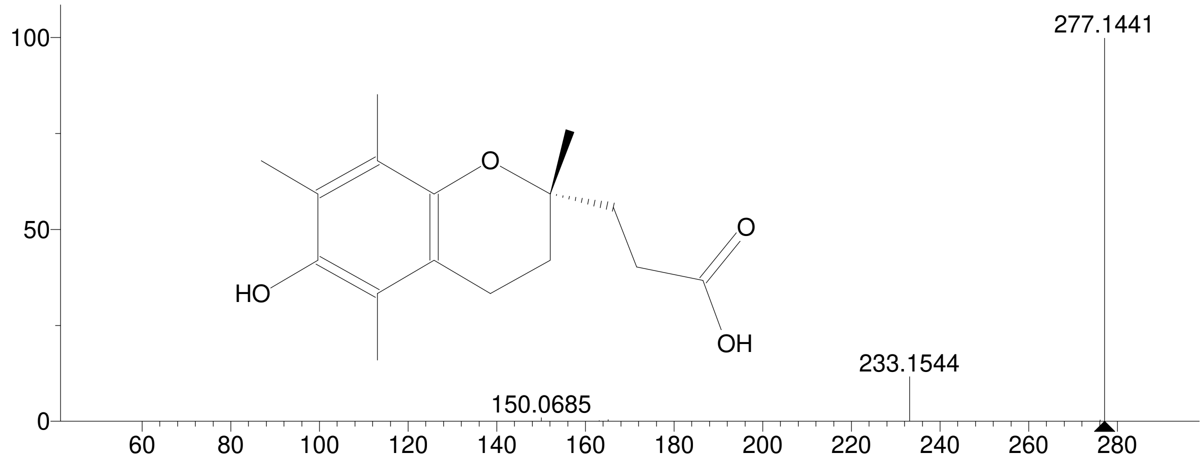

Hit 2 : Ferimzone [M+Na]+ HCD 13V P=277.1
C15H18N4; MF: 39; RMF: 361; Prob 9.41%; CAS: 89269-64-7; Lib: nist_msms; ID: 293640.

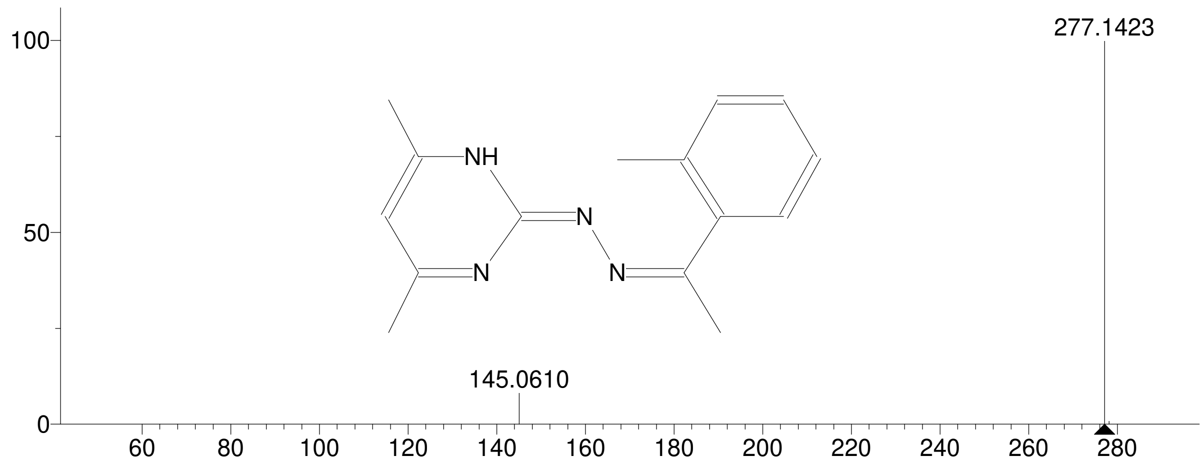

Hit 3 : Mono-2-ethylhexyl phthalate [M-H]- HCD 9V P=277.1
C16H22O4; MF: 26; RMF: 171; Prob 6.07%; CAS: 4376-20-9; Lib: nist_msms; ID: 206479.

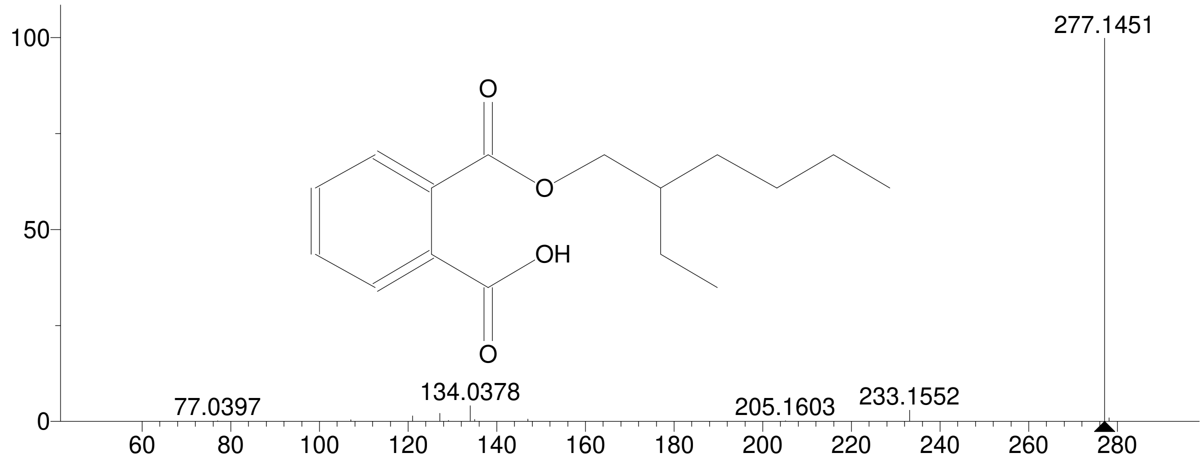

Hit 4 : Di-n-butyl phthalate [M-H]- 15(no P=277.1
C16H22O4; MF: 9; RMF: 151; Prob 3.31%; Lib: mona-export-lc-ms-ms_negative_mode; ID: 4903.

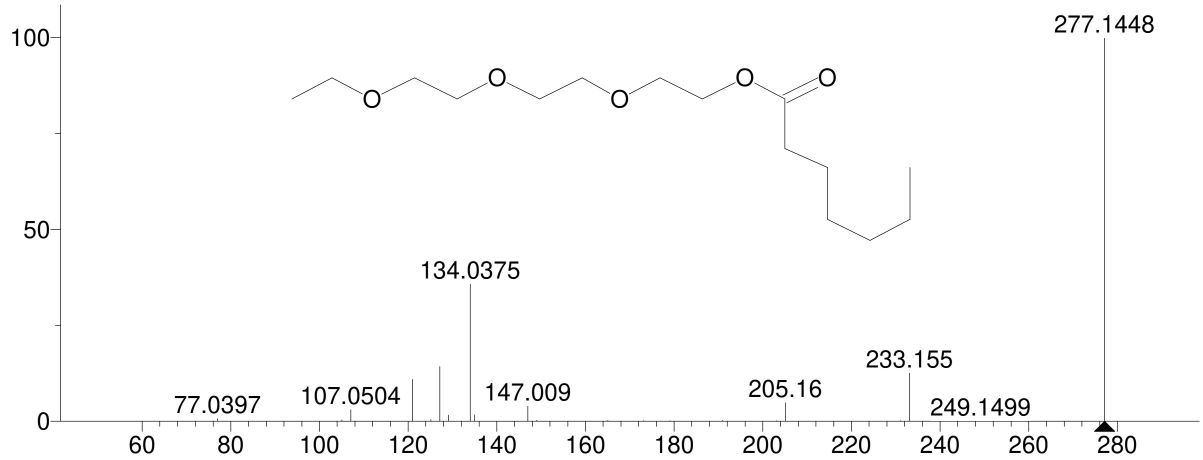

Hit 5 : Gliocladic acid_130132 [M+Na]+ P=277.1
C14H22O4; MF: 1; RMF: 121; Prob 2.47%; Lib: mona-export-gnps; ID: 5077.

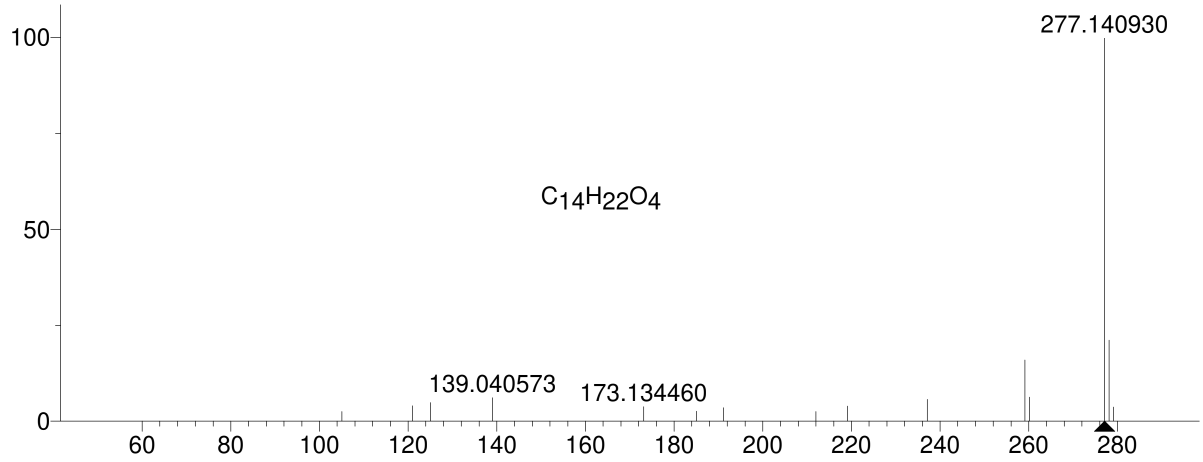

Unknown: Unknown (20.04_567.2180m/z)
Compound in Library Factor = 0

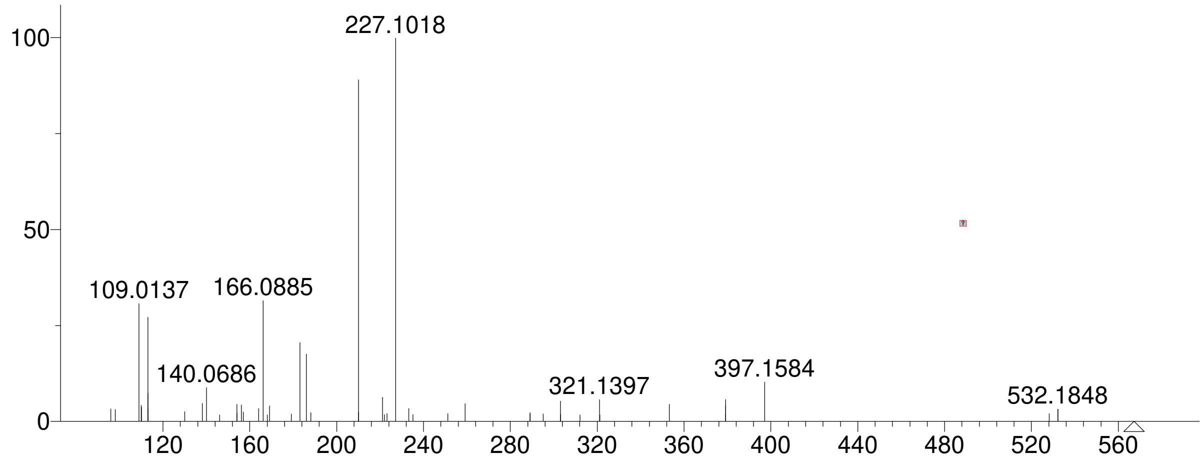

Hit 1 : 10-Deacetylbaccatin III [M+Na]+ HCD 51V P=567.2
C29H36O10; MF: 0; RMF: 48; Prob 20.0%; CAS: 32981-86-5; Lib: nist_msms; ID: 502979.

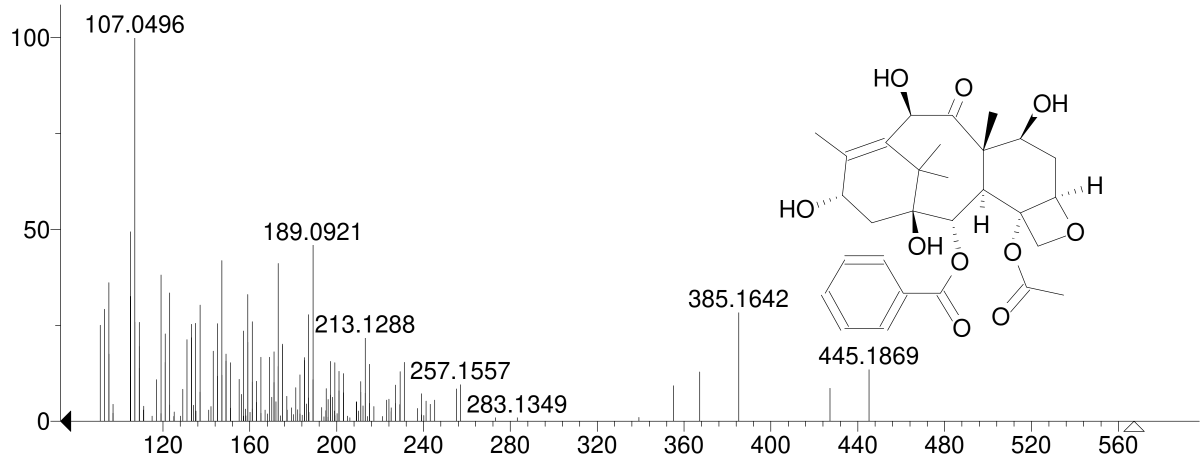

Hit 2 : methyl (2E)-3-[18-(acetyloxy)-7-(furan-3-yl)-17-hydroxy-1,8,15,15-tetramethyl-5-oxo-3,6,14-trioxapentacycl
C29H36O10; MF: 0; RMF: 35; Prob 20.0%; Lib: mona-export-vf-npl_qexactive; ID: 22573.

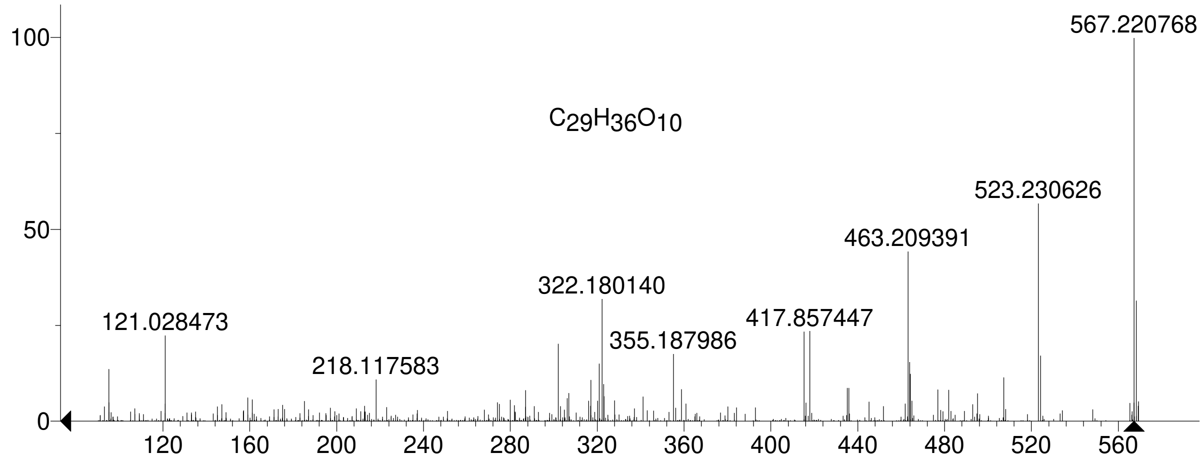

Hit 3 : NCGC00347452-02!4-[4-(2,4-dihydroxy-3,6-dimethylbenzoyl)oxy-2-methoxy-3,5,6-trimethylbenzoyl]oxy-2-r
C31H34O10; MF: 0; RMF: 30; Prob 20.0%; Lib: mona-export-gnps; ID: 7149.

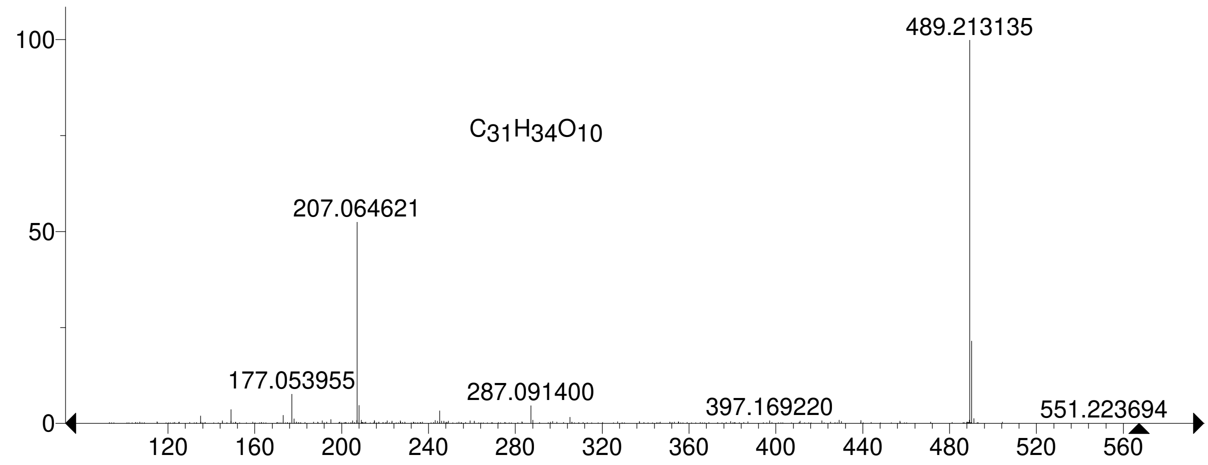

Hit 4 : NCGC00380342-01!methyl 3-[3,4-dihydroxy-2-(3-methylbut-2-enyl)phenyl]-2-[[4-hydroxy-3-(3-methylbut-2-
C30H34O8; MF: 0; RMF: 14; Prob 20.0%; Lib: mona-export-gnps; ID: 6760.

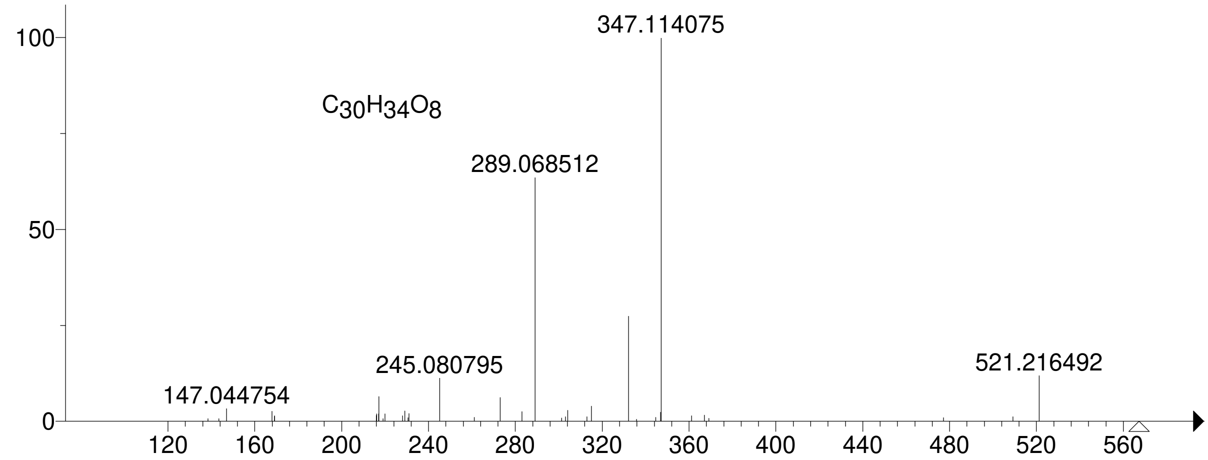

Hit 5 : NCGC00384820-01_C29H36O10_2-Propenoic acid, 3-[5-(acetyloxy)-9-(3-furanyl)dodecahydro-4-hydroxy-
C29H36O10; MF: 0; RMF: 4; Prob 20.0%; Lib: mona-export-gnps; ID: 13032.

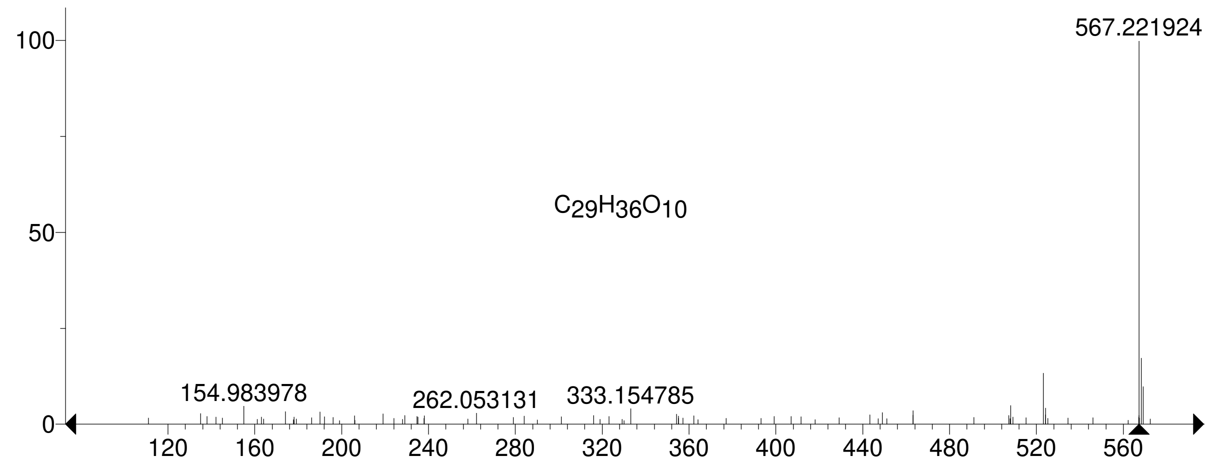

Unknown: Unknown (20.10_325.1649m/z)
Compound in Library Factor = -2387

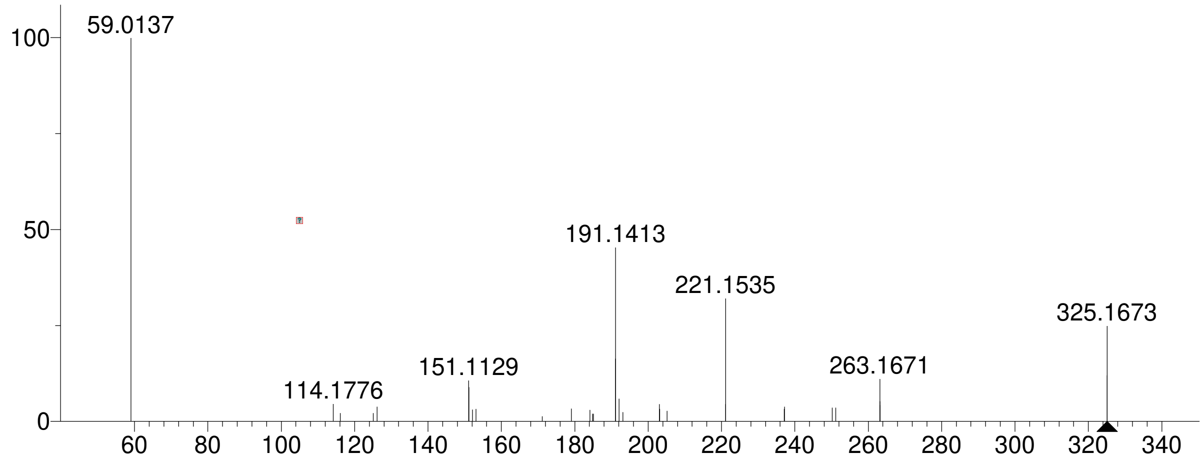

Hit 1 : [5-acetyloxy-3-(hydroxymethyl)-2-oxo-6-propan-2-ylcyclohex-3-en-1-yl] 3-methylbutanoate [M-H]- 35HCD
C17H26O6; MF: 26; RMF: 286; Prob 23.1%; Lib: mona-export-lc-ms-ms_negative_mode; ID: 19385.

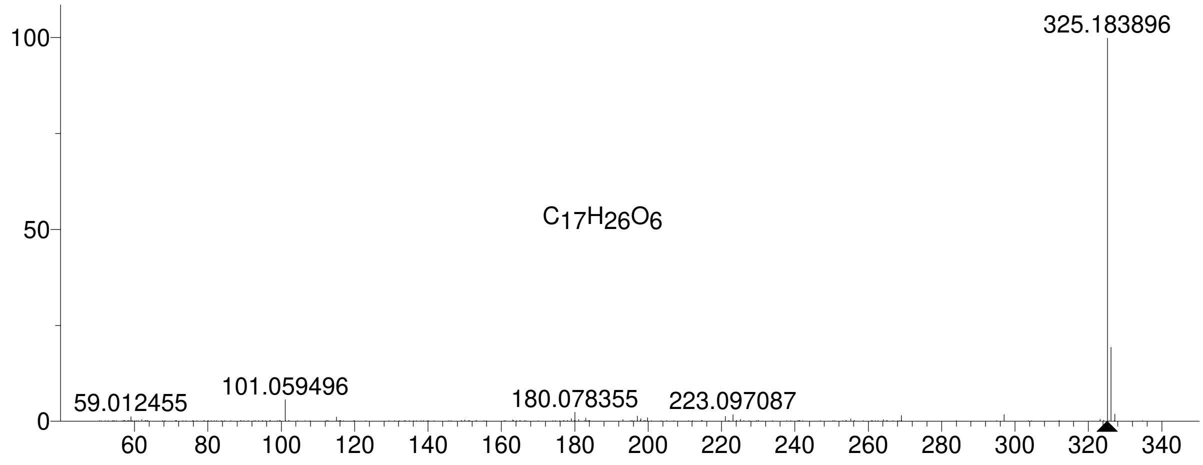

Hit 2 : Tributyrin [M+Na]+ HCD 13V P=325.2
C15H26O6; MF: 22; RMF: 306; Prob 19.5%; CAS: 60-01-5; Lib: nist_msms; ID: 144821.

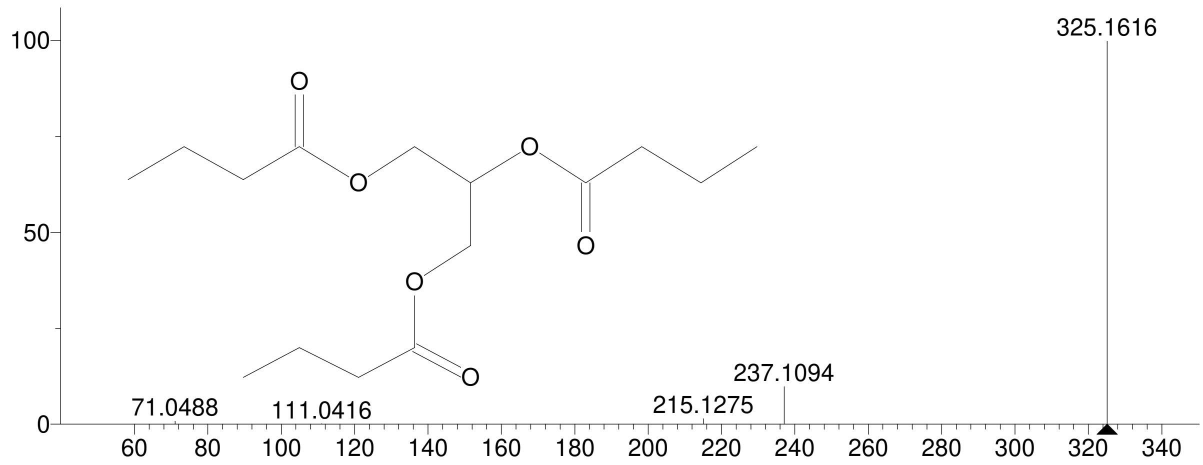

Hit 3 : Chamissonolide [M+H]+ HCD 1V P=325.2
C17H24O6; MF: 20; RMF: 315; Prob 18.0%; CAS: 78853-98-2; Lib: nist_msms; ID: 500759.

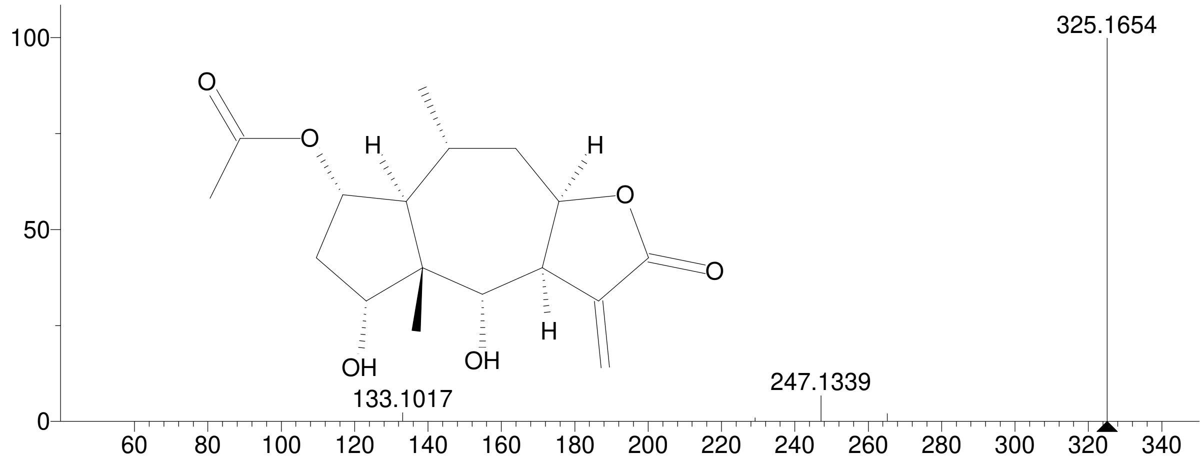

Hit 4 : MMV023985 [M+H]+ 20-30-40%) P=325.2
C18H20N4O2; MF: 8; RMF: 286; Prob 12.0%; Lib: mona-export-pathogen_box; ID: 292.

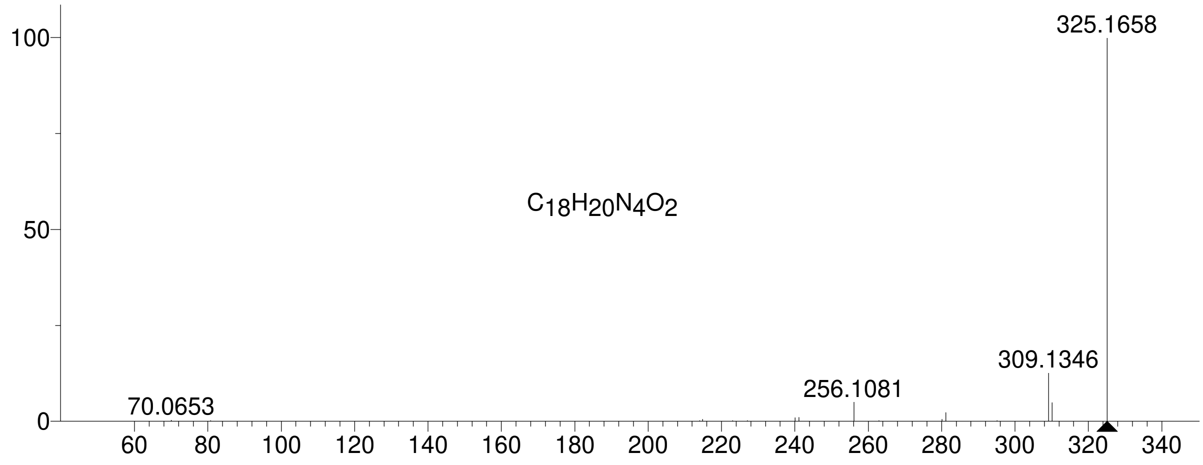

Hit 5 : 2H-Benzimidazol-2-one, 1-ethyl-1,3-dihydro- [2M+H]+ HCD 3V P=325.2
C9H10N2O; MF: 4; RMF: 182; Prob 10.1%; CAS: 10045-45-1; Lib: nist_msms; ID: 364753.

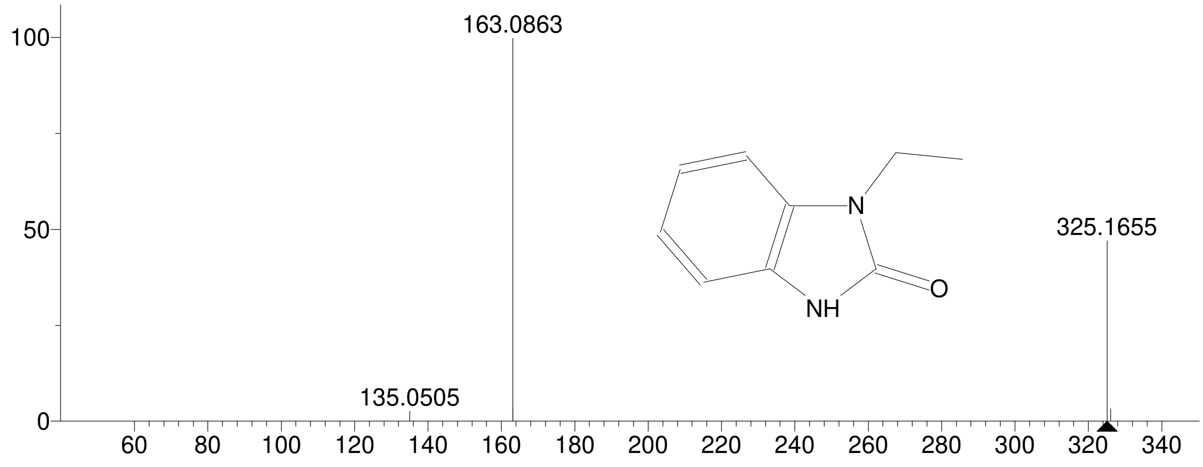

Unknown: Unknown (20.10_450.0290m/z)
Compound in Library Factor = 0

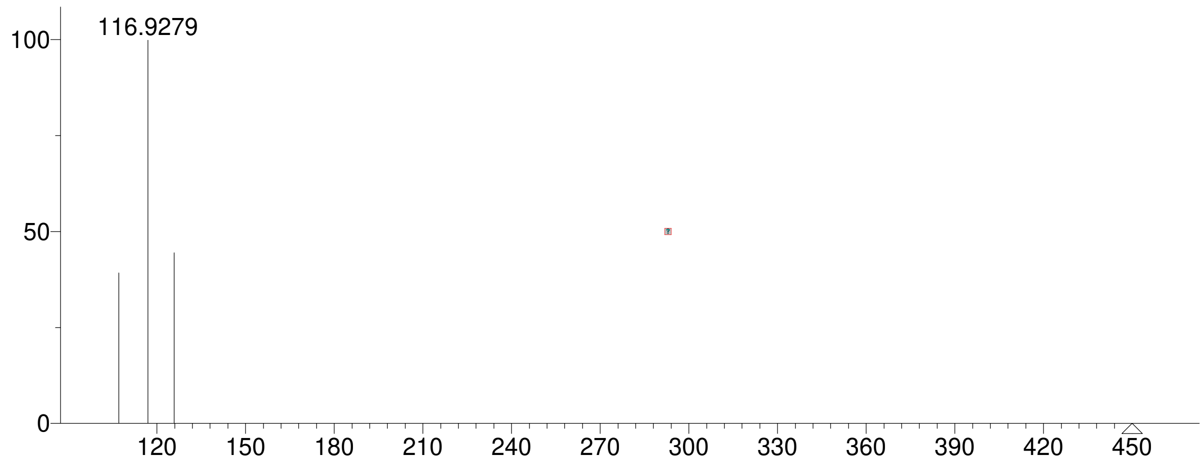

** Search Report Page 1 of 1 **

Unknown: Unknown (20.11_687.3608m/z)
Compound in Library Factor = 0

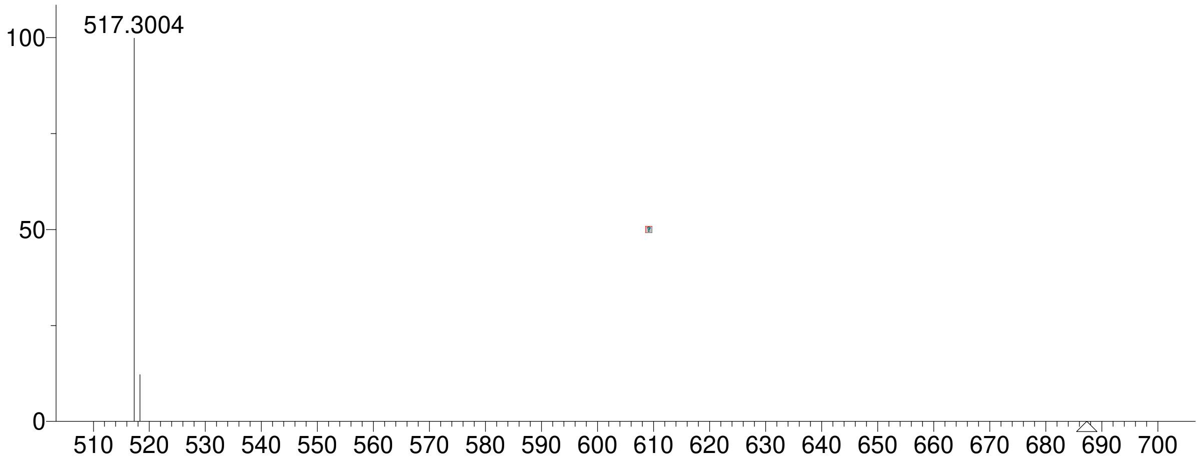

Unknown: Unknown (20.15_425.1919m/z)
Compound in Library Factor = -3273

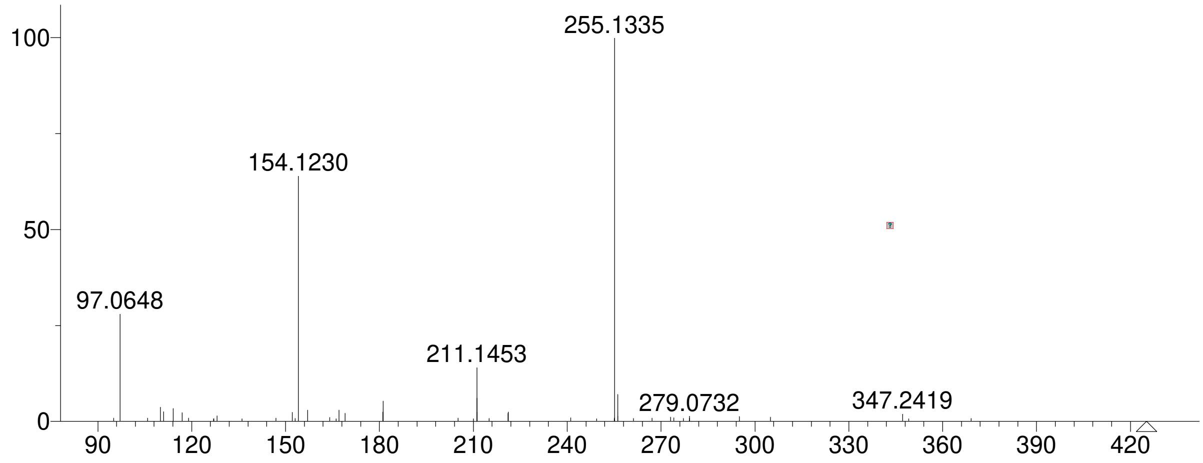

Hit 1 : Evodin [M+H-CH2O2]+ IT-FT 35% P=425.2
C26H30O8; MF: 1; RMF: 53; Prob 9.42%; CAS: 1180-71-8; Lib: nist_msms; ID: 223331.

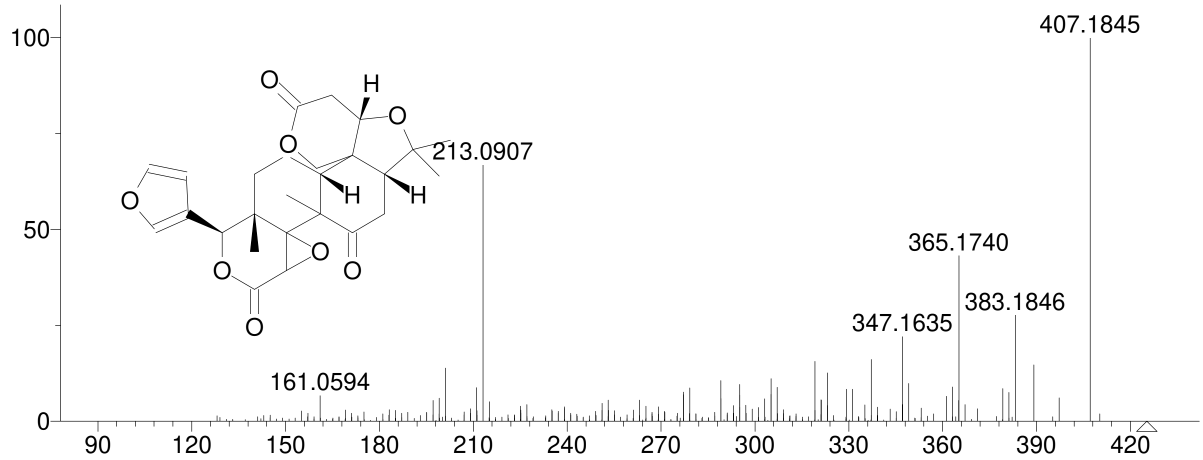

Hit 2 : NCGC00385788-01!3-(2,4-dihydroxyphenyl)-5,7-dihydroxy-6,8-bis(3-methylbut-2-enyl)-2,3-dihydrochromen
C25H28O6; MF: 0; RMF: 39; Prob 9.05%; Lib: mona-export-gnps; ID: 7334.

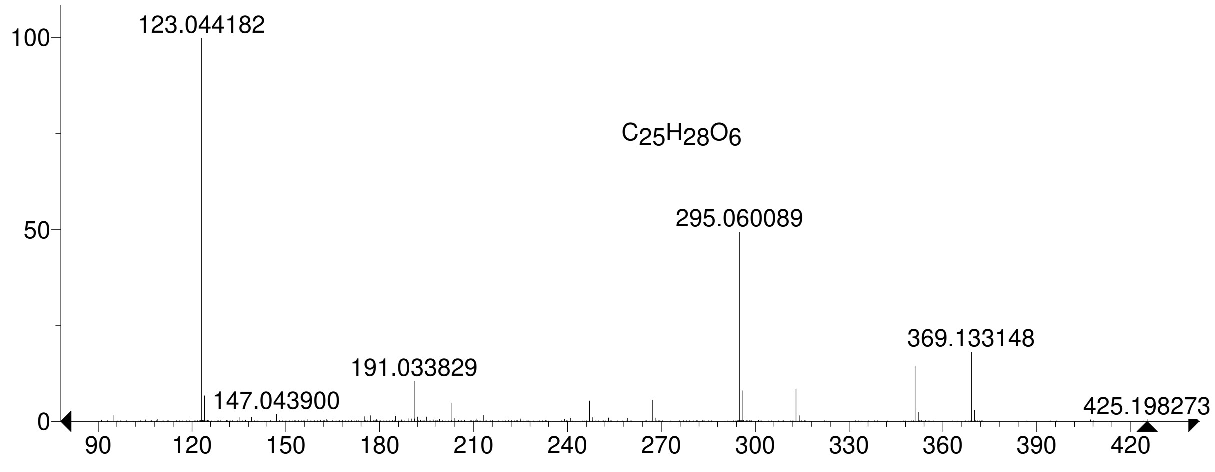

Hit 3 : 2-[5-[2-[2-[5-(2-hydroxypropyl)oxolan-2-yl]propanoyloxy]propyl]oxolan-2-yl]propanoic acid [M+K]+ 45HCD
C20H34O7; MF: 0; RMF: 27; Prob 9.05%; Lib: mona-export-vf-npl_qexactive; ID: 9131.

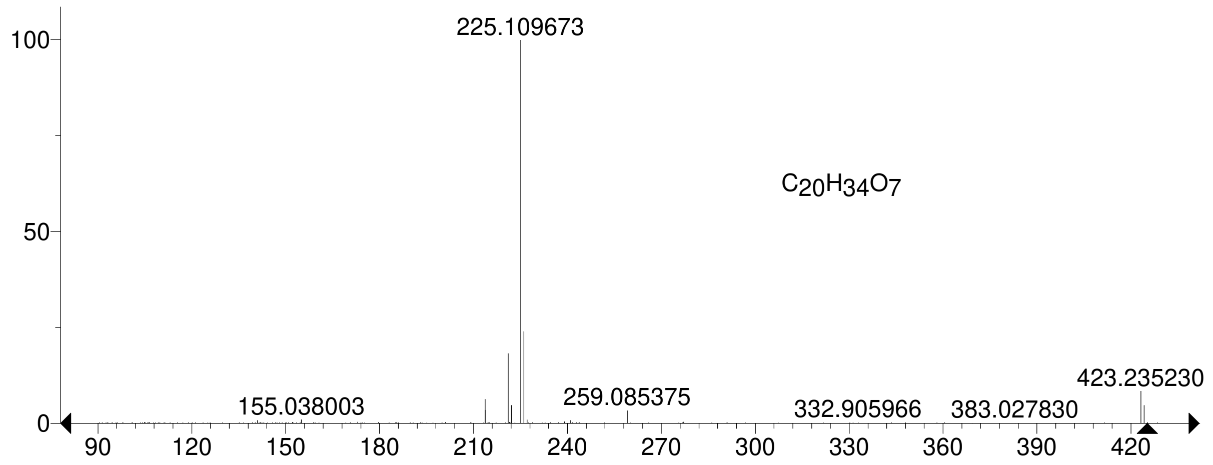

Hit 4 : NCGC00385984-01!2-(3,5-dihydroxyphenyl)-5,7-dihydroxy-6,8-bis(3-methylbut-2-enyl)-2,3-dihydrochromei
C25H28O6; MF: 0; RMF: 12; Prob 9.05%; Lib: mona-export-gnps; ID: 6690.

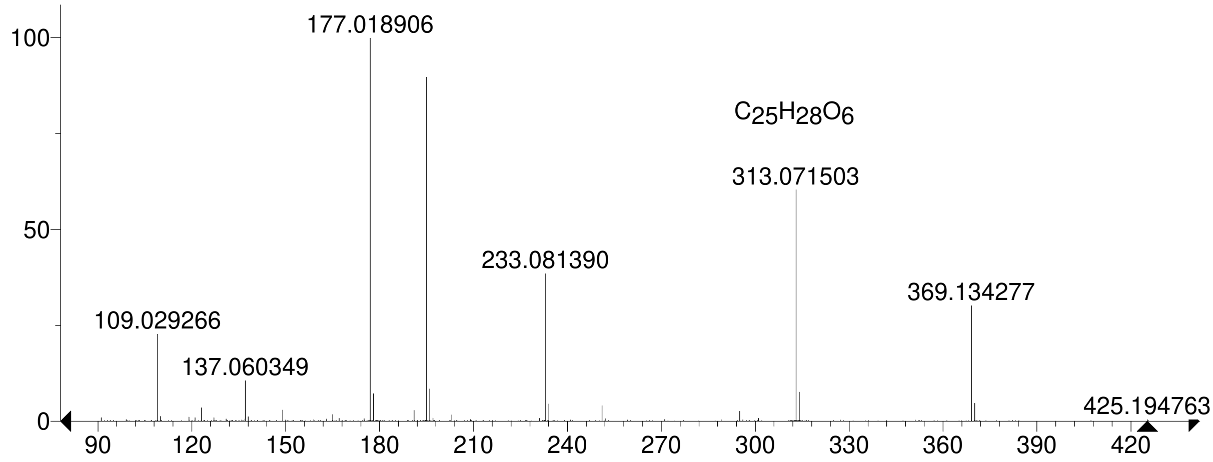

Hit 5 : Pantethine [M+H-C6H10O3]+ HCD 63V P=425.2
C22H42N4O8S2; MF: 0; RMF: 10; Prob 9.05%; CAS: 16816-67-4; Lib: nist_msms; ID: 218929.

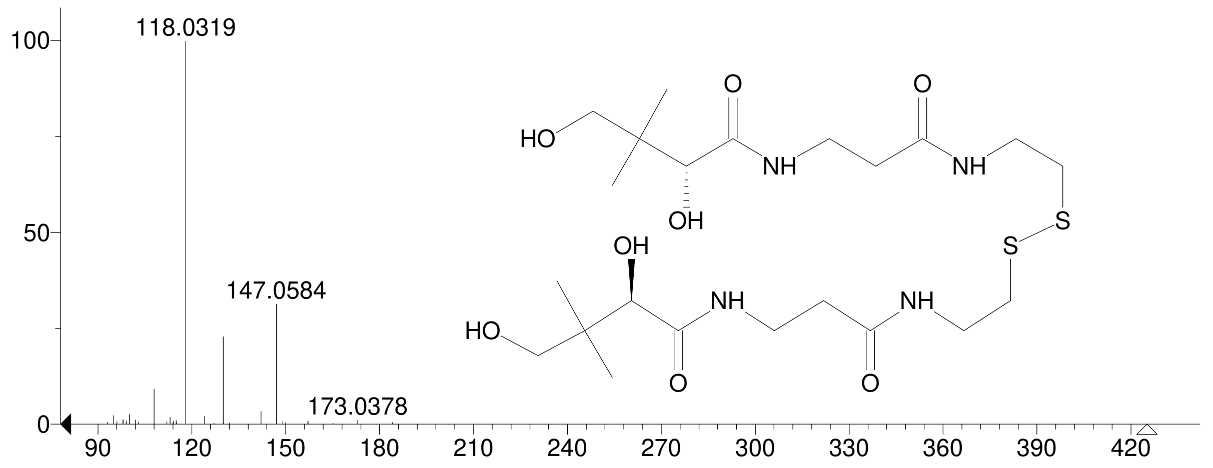

Unknown: Unknown (20.29_381.0999m/z)
Compound in Library Factor = -275

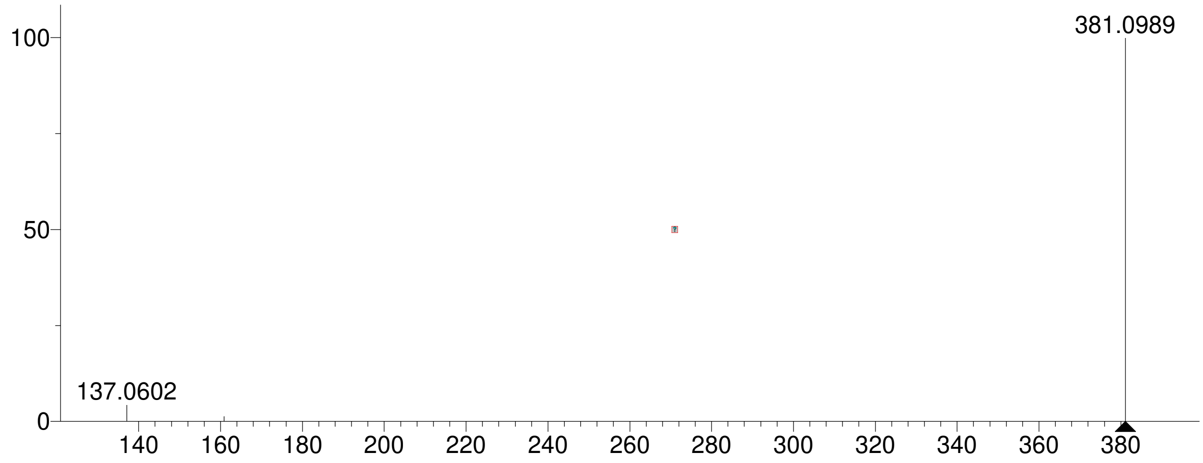

Hit 1 : Lactobionic acid [M+Na]+ HCD 19V P=381.1
C12H22O12; MF: 313; RMF: 966; Prob 97.7%; CAS: 96-82-2; Lib: nist_msms; ID: 407480.

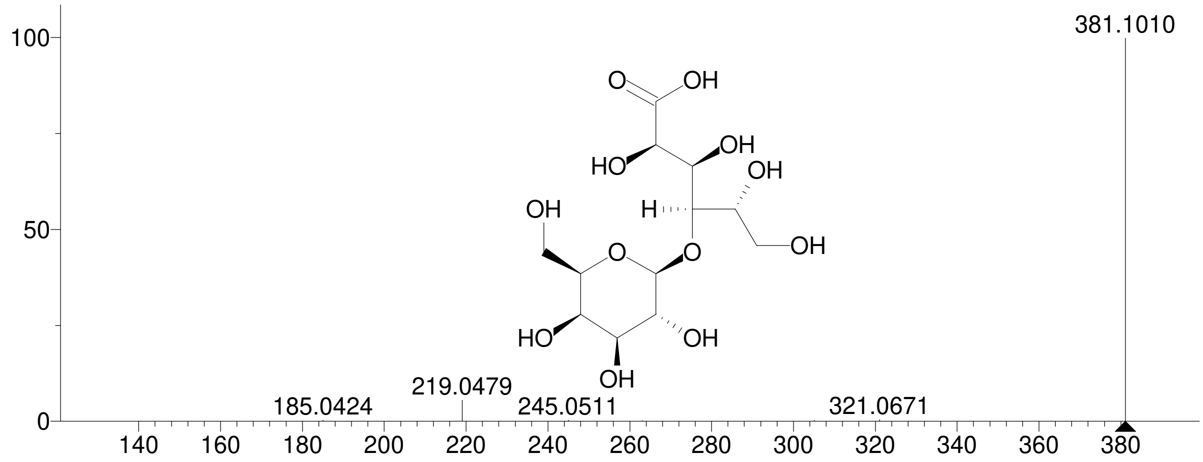

Hit 2 : Swertisin [M+H-CH6O3]+ HCD 11V P=381.1
C22H22O10; MF: 93; RMF: 744; Prob 0.97%; CAS: 6991-10-2; Lib: nist_msms; ID: 411740.

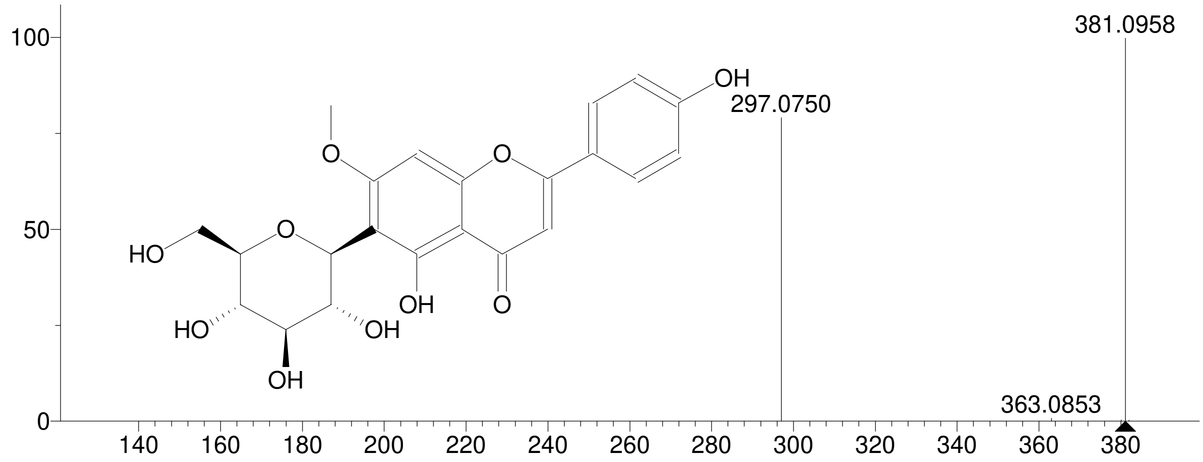

Hit 3 : Teniposide [M-H-C11H14O6S]- HCD 19V P=381.1
C32H32O13S; MF: 85; RMF: 827; Prob 0.72%; CAS: 29767-20-2; Lib: nist_msms; ID: 419605.

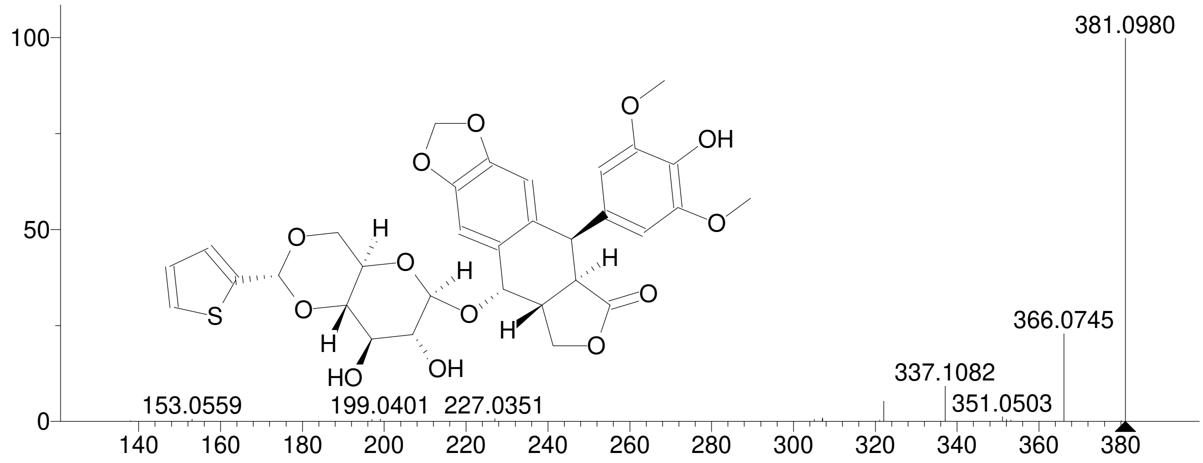

Hit 4 : 3-[1-[3-(Dimethylamino)propyl]-5-methoxy-1H-indol-3-yl]-4-(1H-indol-3-yl)-1H-pyrrole-2,5-dione [M+H]+=>
C26H26N4O3; MF: 20; RMF: 619; Prob 0.11%; CAS: 133053-19-7; Lib: nist_msms; ID: 321304.

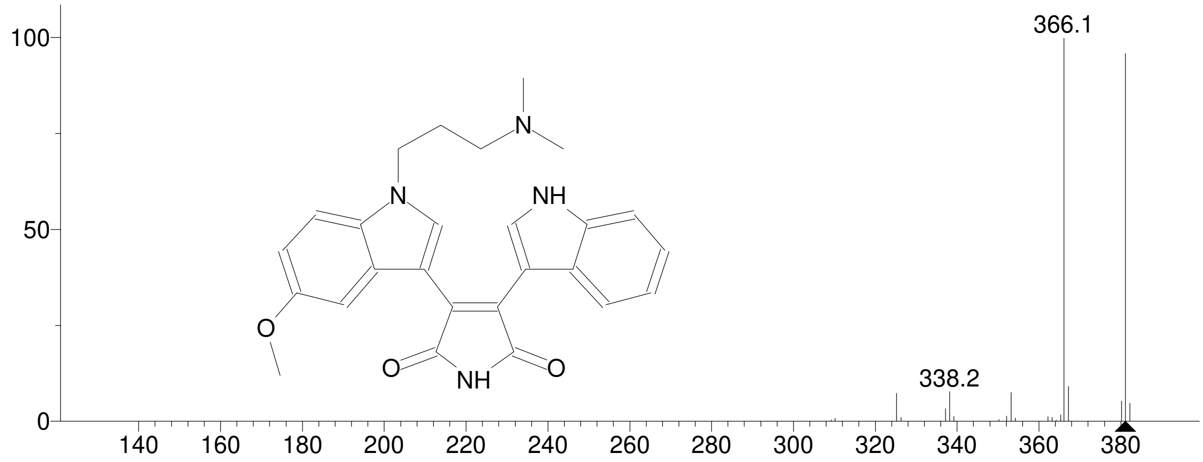

Hit 5 : 1-[[4,5-Bis(4-methoxyphenyl)-2-thiazolyl]carbonyl]-4-methylpiperazine [M+H]+=>381.1 IT 35% P=381.1
C23H25N3O3S; MF: 18; RMF: 646; Prob 0.10%; CAS: 130717-22-5; Lib: nist_msms; ID: 538941.

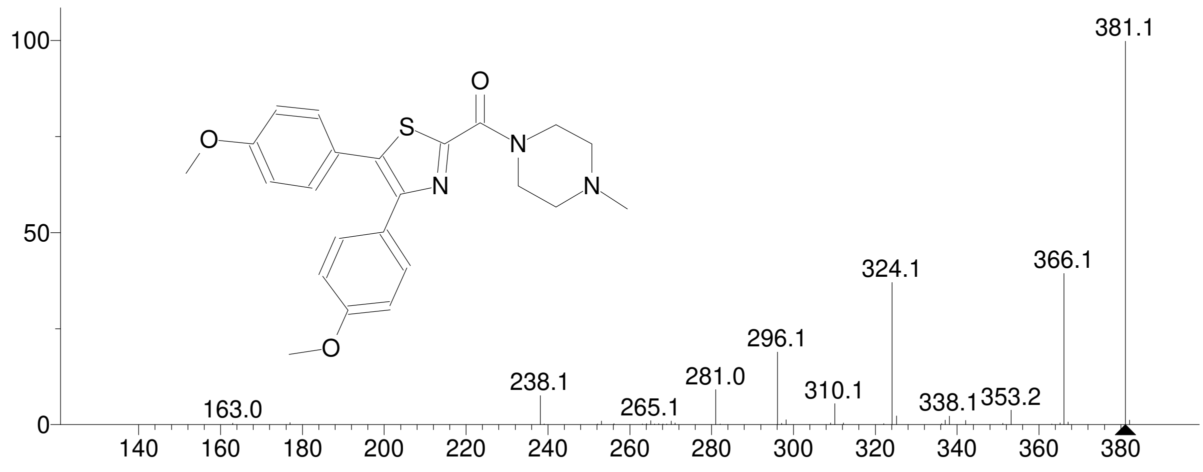

Unknown: Unknown (20.41_303.2169m/z)
Compound in Library Factor = -2176

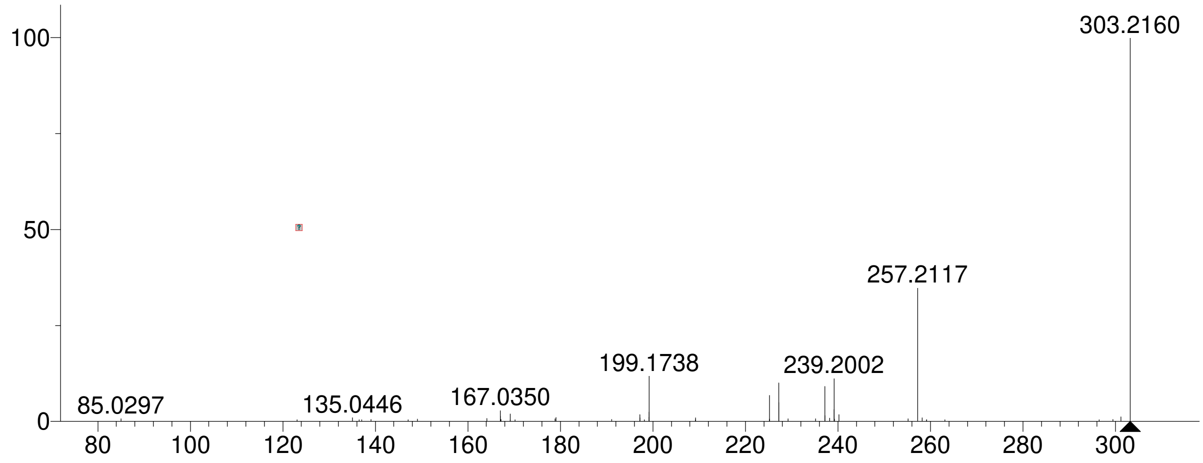

Hit 1 : Lys-Arg [M+H]+ QTOF 8V P=303.2
C12H26N6O3; MF: 72; RMF: 628; Prob 43.8%; Lib: nist_msms; ID: 48416.

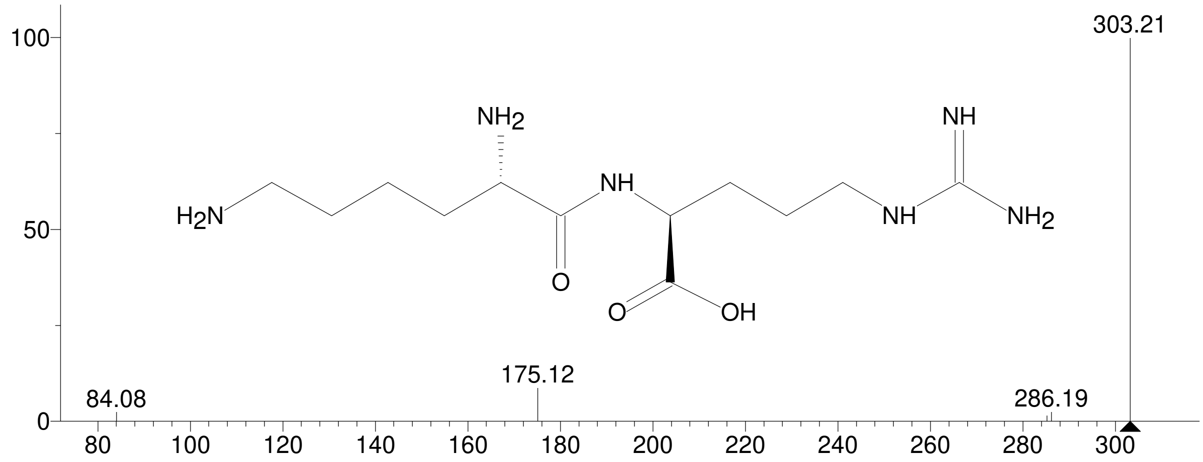

Hit 2 : Arg-Lys [M+H]+ HCD 13eV P=303.2
C12H26N6O3; MF: 67; RMF: 648; Prob 35.3%; CAS: 40968-46-5; Lib: nist_msms; ID: 565904.

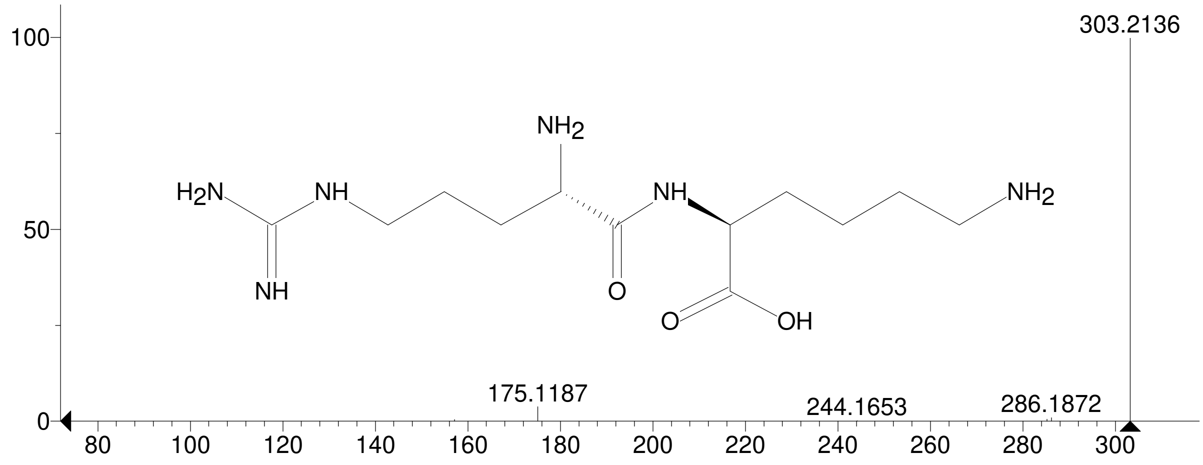

Hit 3 : Aleuritic acid [M-H]- IT-FT 35% P=303.2
C16H32O5; MF: 26; RMF: 190; Prob 8.05%; CAS: 533-87-9; Lib: nist_msms; ID: 411121.

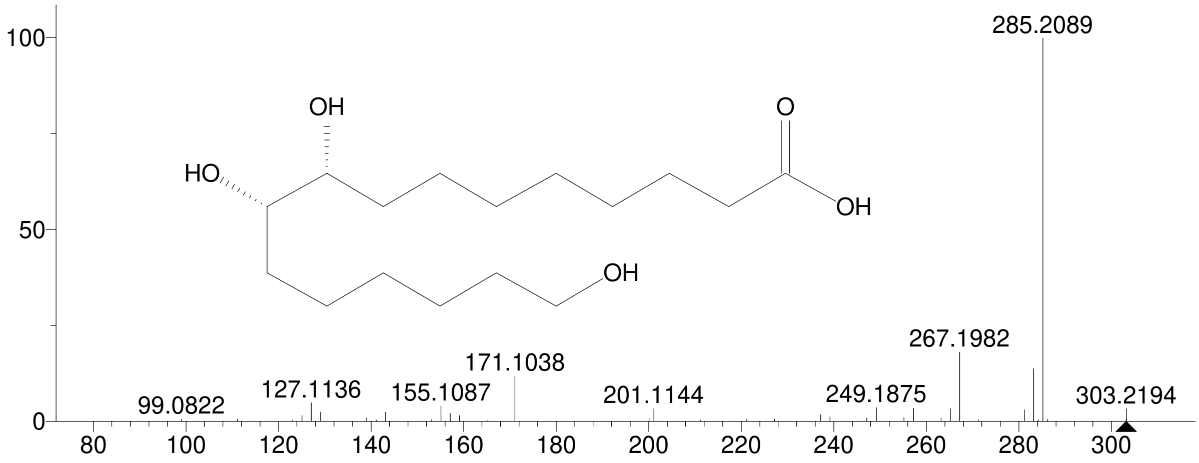

Hit 4 : Lys-Arg [M+H]+ HCD 15eV P=303.2
C12H26N6O3; MF: 25; RMF: 606; Prob 7.73%; CAS: 29586-66-1; Lib: nist_msms2; ID: 41398.

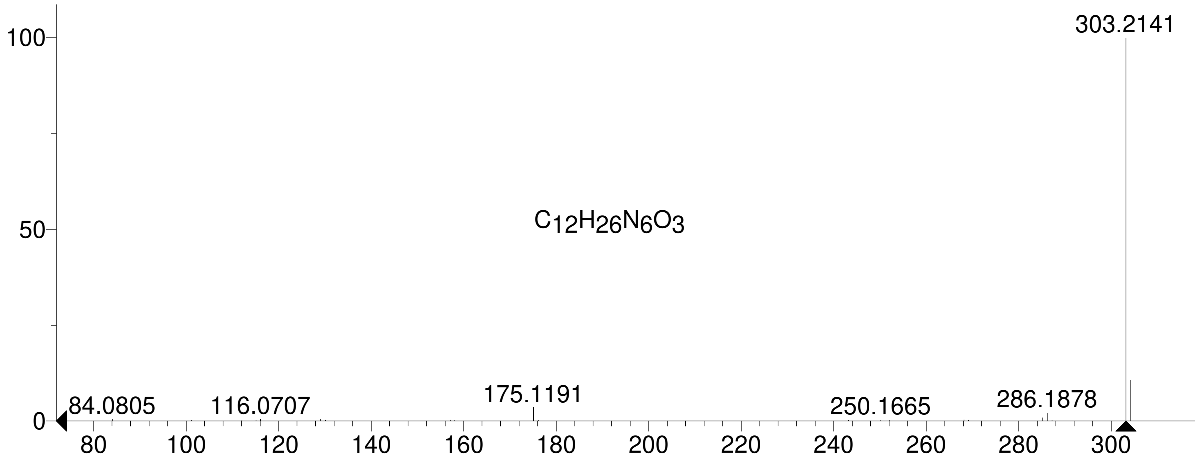

Hit 5 : Aleuretic Acid [M-H]- 35HCD P=303.2
C16H32O5; MF: 13; RMF: 569; Prob 5.15%; Lib: mona-export-lc-ms-ms_negative_mode; ID: 20798.

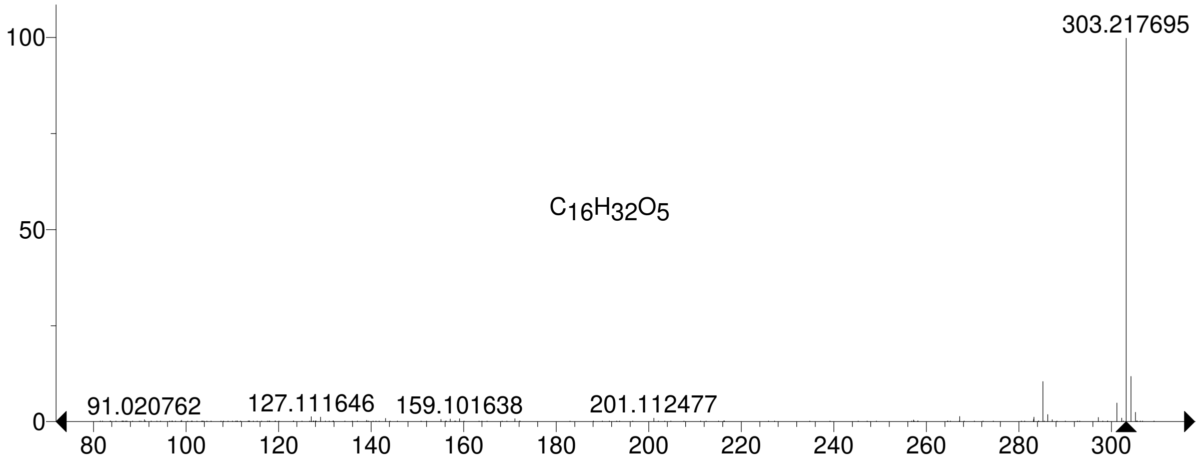

Unknown: Unknown (20.47_239.1385m/z)
Compound in Library Factor = 0

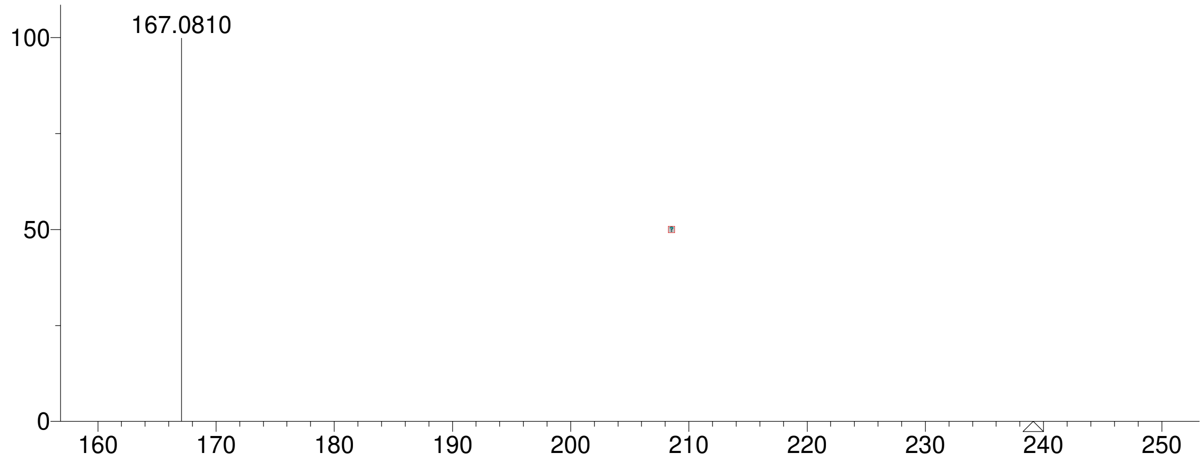

Unknown: Unknown (20.52_305.0656m/z)
Compound in Library Factor = -3273

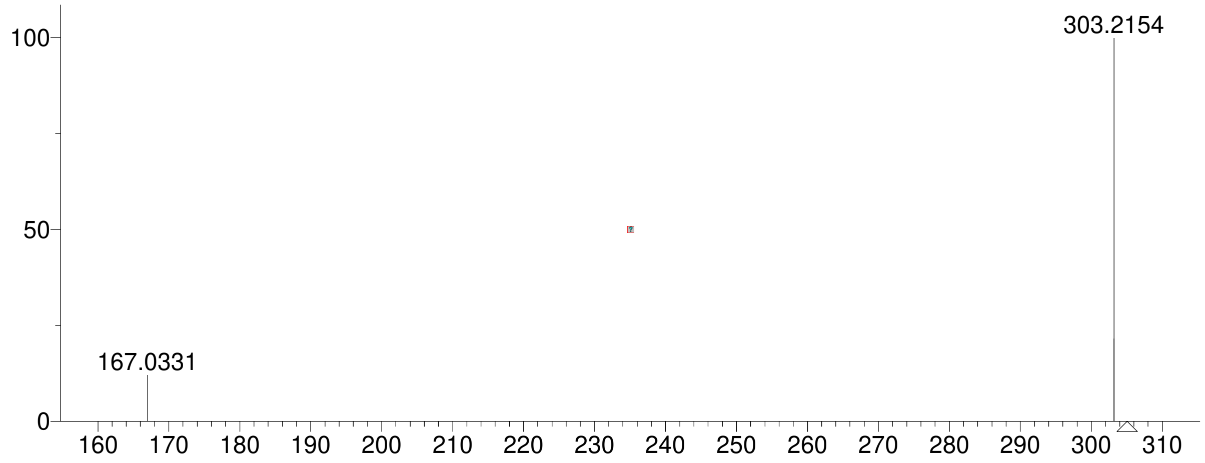

Hit 1 : (2S,3S)-2-(3,4,5-trihydroxyphenyl)-3,4-dihydro-2H-chromene-3,5,7-triol [M-H]- 45HCD P=305.1
C15H14O7; MF: 3; RMF: 173; Prob 6.89%; Lib: mona-export-lc-ms-ms_negative_mode; ID: 20160.

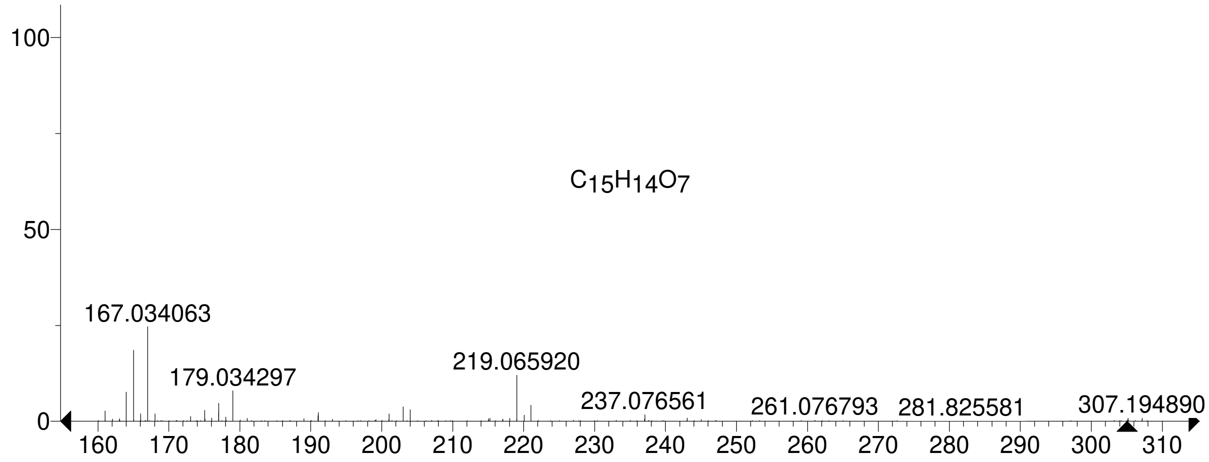

Hit 2 : Epigallocatechin [M-H]- P=305.1
C15H14O7; MF: 2; RMF: 109; Prob 6.62%; Lib: msms-neg-gnps; ID: 39.

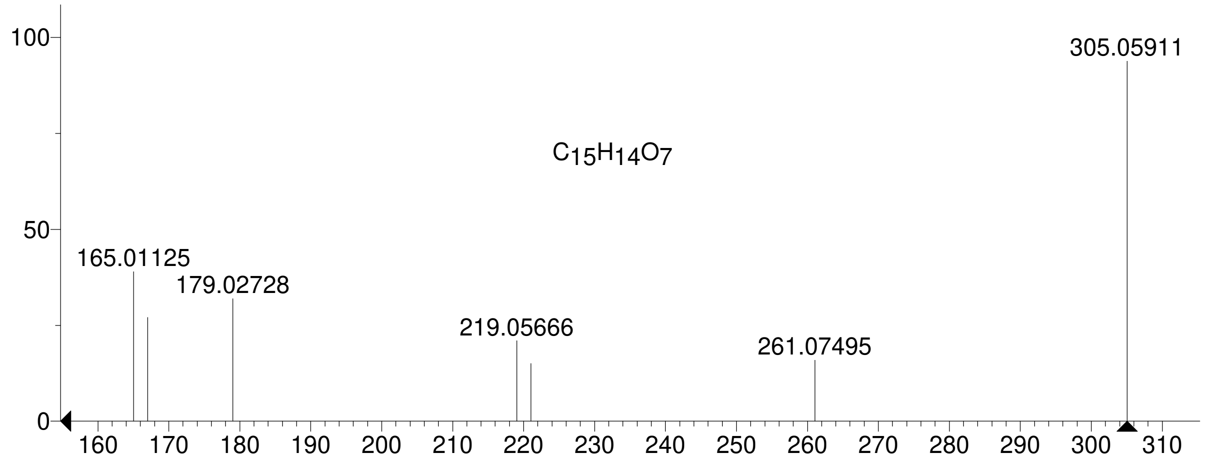

Hit 3 : (-)-Epigallocatechin [M-H]- HCD 33V P=305.1
C15H14O7; MF: 1; RMF: 174; Prob 6.37%; CAS: 970-74-1; Lib: nist_msms; ID: 235103.

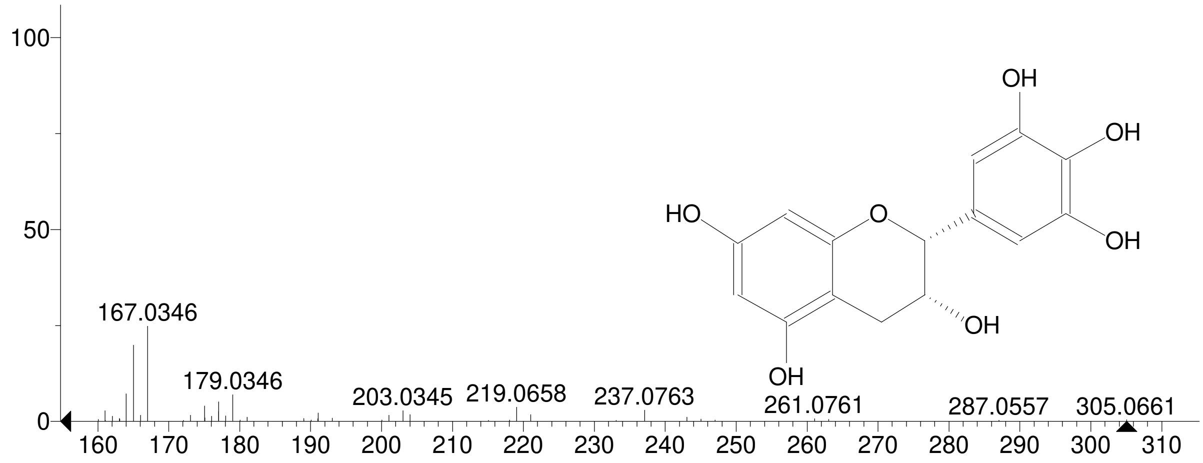

Hit 4 : (-)-Gallocatechin [M-H]- HCD 33V P=305.1
C15H14O7; MF: 1; RMF: 171; Prob 6.37%; CAS: 3371-27-5; Lib: nist_msms; ID: 235885.

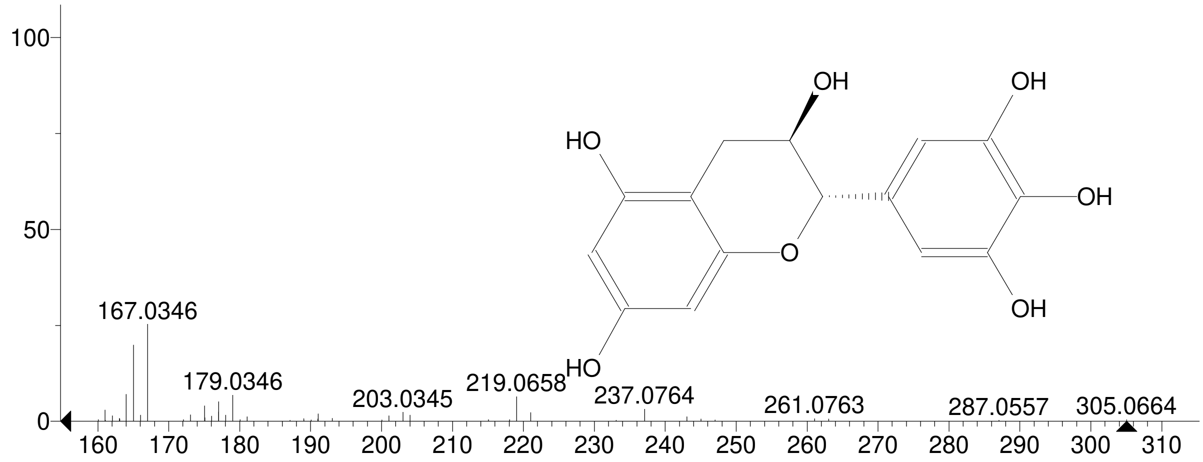

Hit 5 : 3,5,7,3',4'-Pentahydroxyflavanone [M+H]+ HCD 24V P=305.1
C15H12O7; MF: 1; RMF: 118; Prob 6.37%; CAS: 480-18-2; Lib: nist_msms; ID: 136818.

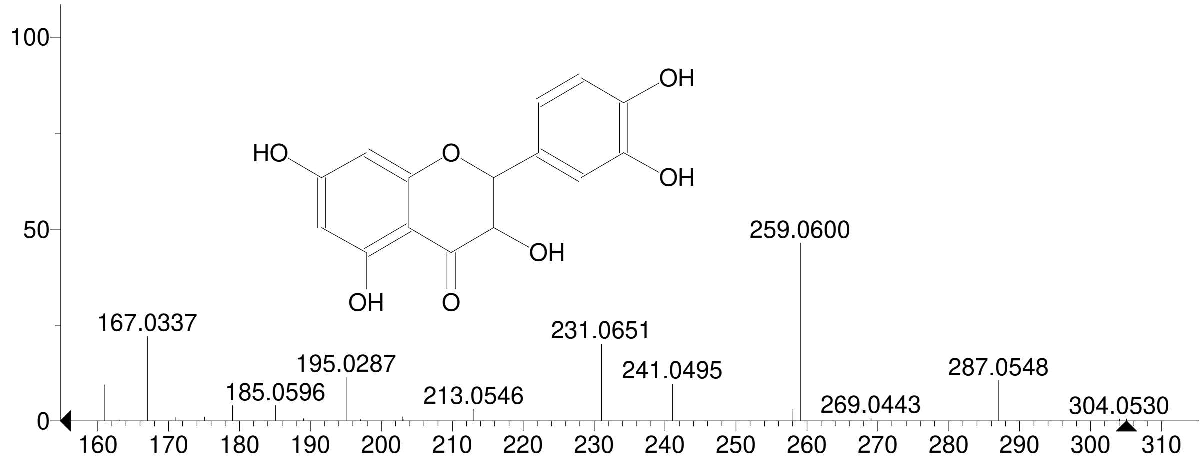

** Search Report Page 1 of 1 **

Unknown: Unknown (20.52_568.2849m/z)
Compound in Library Factor = -1309

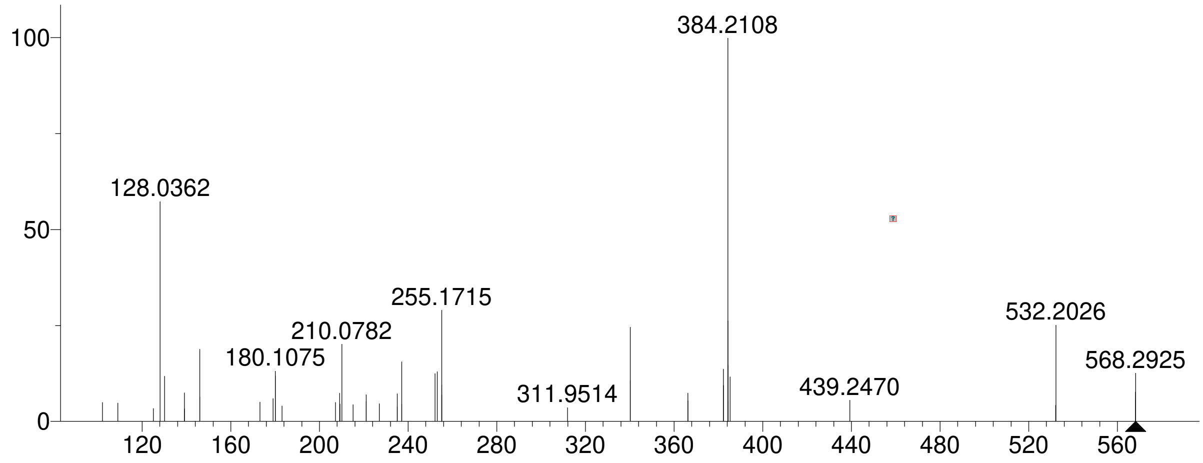

Hit 1 : Amikacin [M+H-H2O]+ HCD 22V P=568.3
C22H43N5O13; MF: 54; RMF: 417; Prob 83.6%; CAS: 37517-28-5; Lib: nist_msms; ID: 153483.

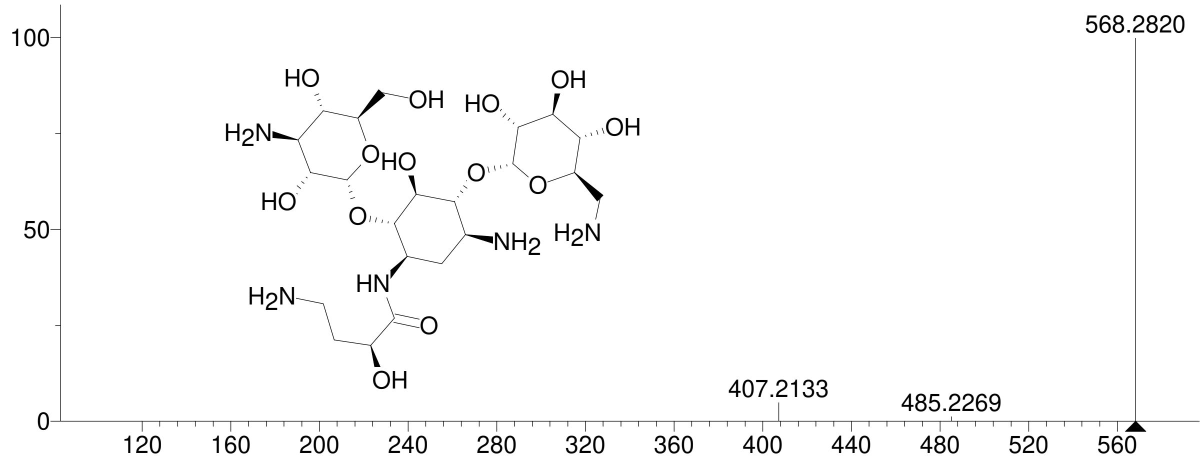

Hit 2 : ACTH (1-39), human [M+8H]8+ HCD 21eV P=568.3
C207H308N56O58S; MF: 0; RMF: 42; Prob 16.4%; CAS: 12279-41-3; Lib: nist_msms2; ID: 51598.

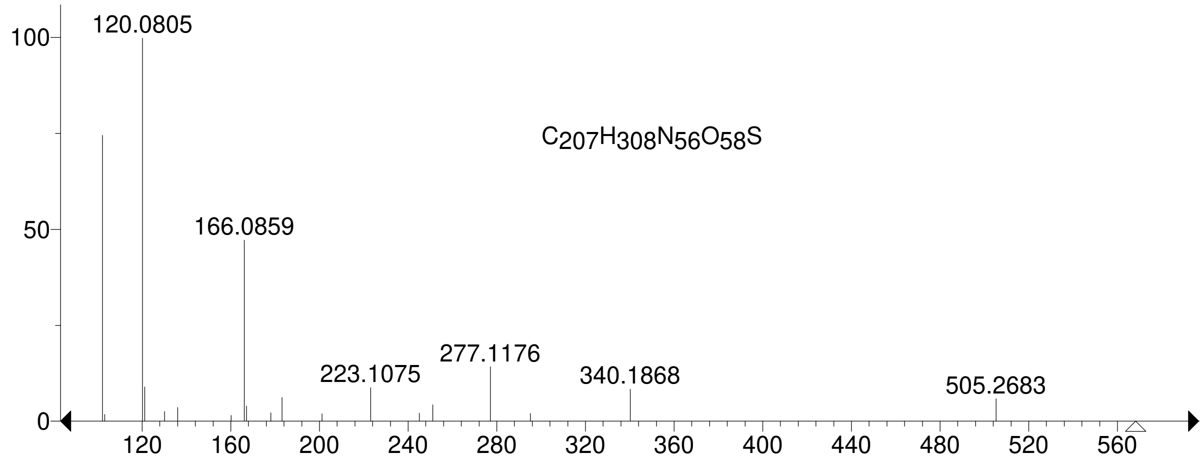

Unknown: Unknown (20.56_303.2164m/z)
Compound in Library Factor = -2387

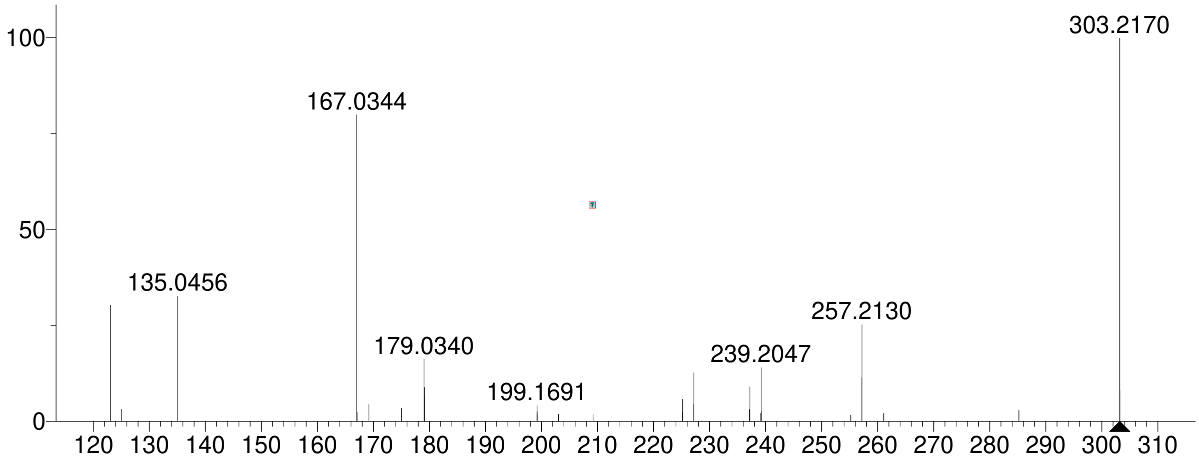

Hit 1 : Aleuritic acid [M-H]- IT-FT 35% P=303.2
C16H32O5; MF: 32; RMF: 204; Prob 35.2%; CAS: 533-87-9; Lib: nist_msms; ID: 411121.

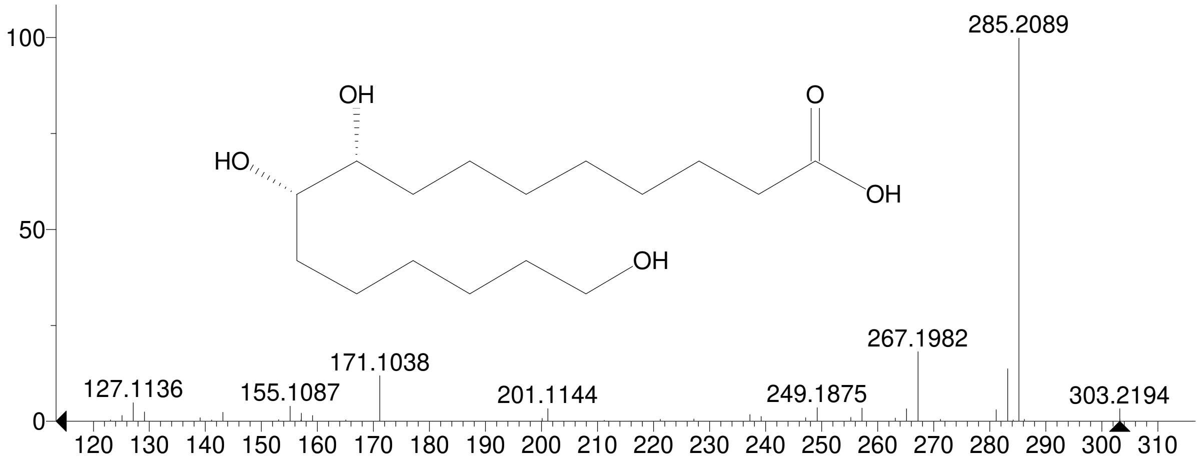

Hit 2 : Arg-Lys [M+H]+ QTOF 5V P=303.2
C12H26N6O3; MF: 15; RMF: 412; Prob 19.2%; CAS: 40968-46-5; Lib: nist_msms; ID: 55168.

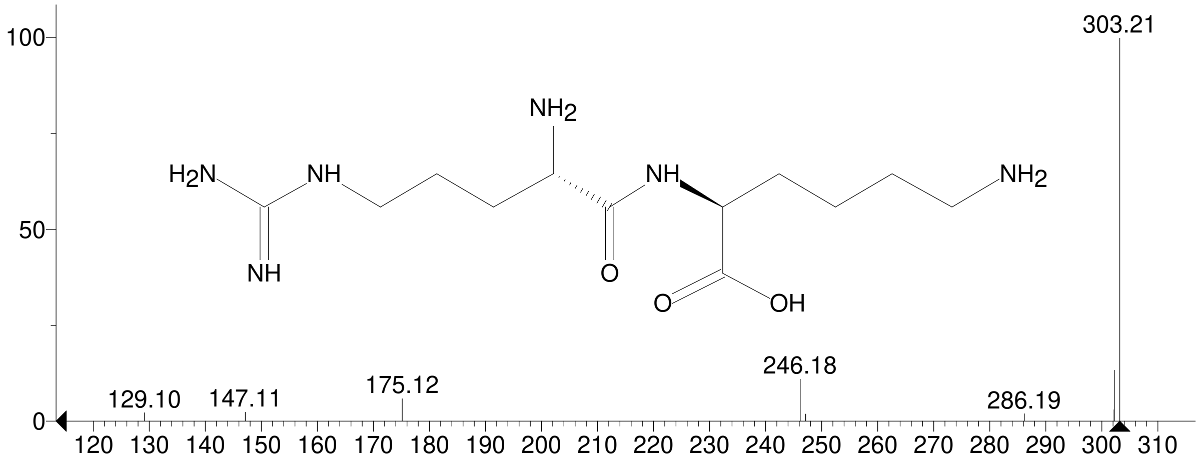

Hit 3 : Lys-Arg [M+H]+ QTOF 8V P=303.2
C12H26N6O3; MF: 12; RMF: 471; Prob 17.0%; Lib: nist_msms; ID: 48416.

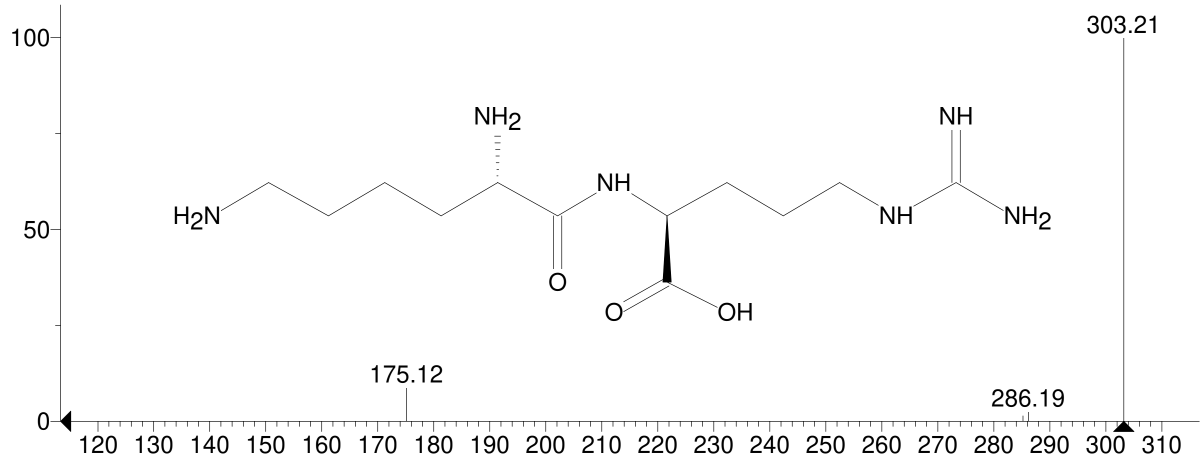

Hit 4 : Aleuretic Acid [M-H]- 35HCD P=303.2
C16H32O5; MF: 11; RMF: 430; Prob 16.3%; Lib: mona-export-lc-ms-ms_negative_mode; ID: 20798.

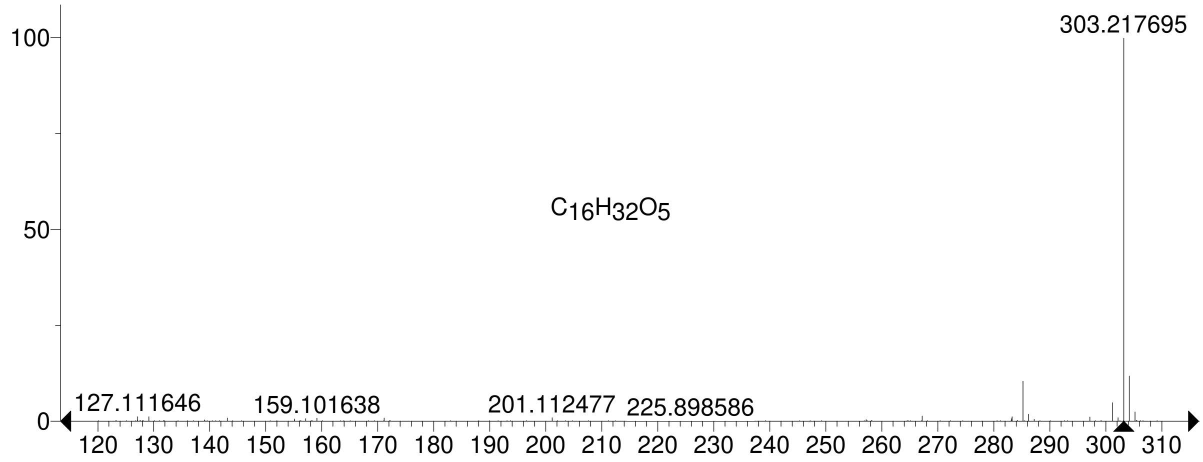

Hit 5 : Lys-Arg [M+H]+ HCD 15eV P=303.2
C12H26N6O3; MF: 3; RMF: 454; Prob 12.2%; CAS: 29586-66-1; Lib: nist_msms2; ID: 41398.

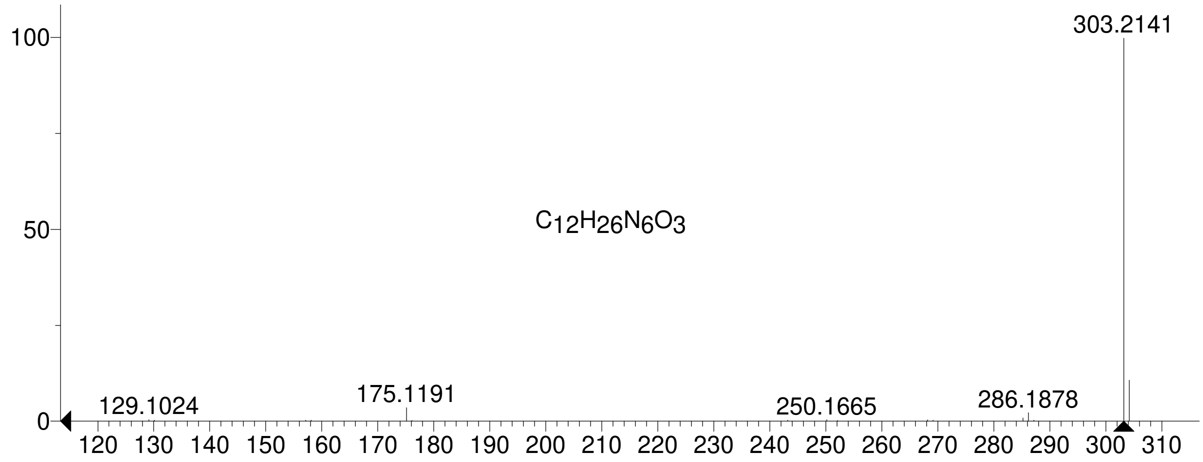

Unknown: Unknown (20.66_440.1122m/z)
Compound in Library Factor = 0

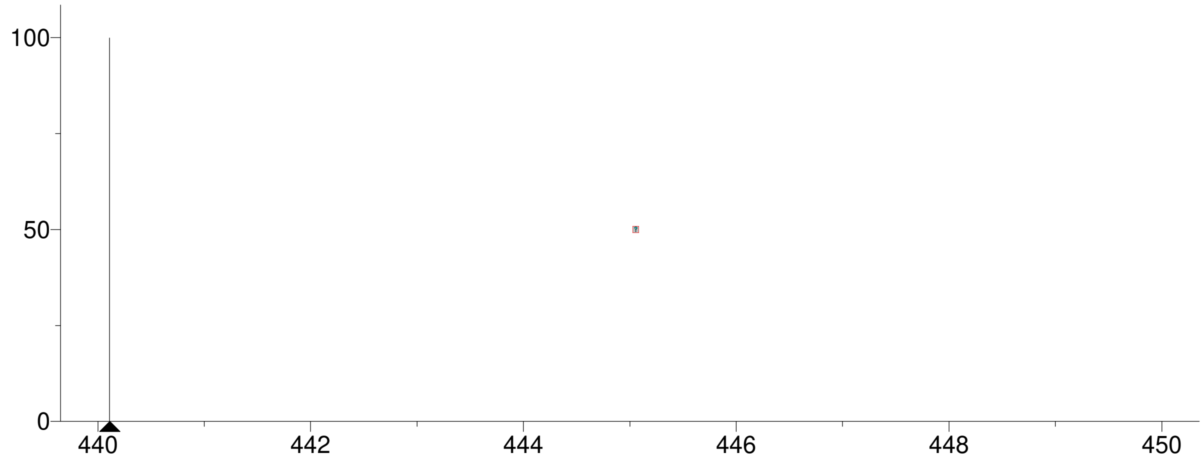

Unknown: Unknown (20.72_403.1753m/z)
Compound in Library Factor = -3273

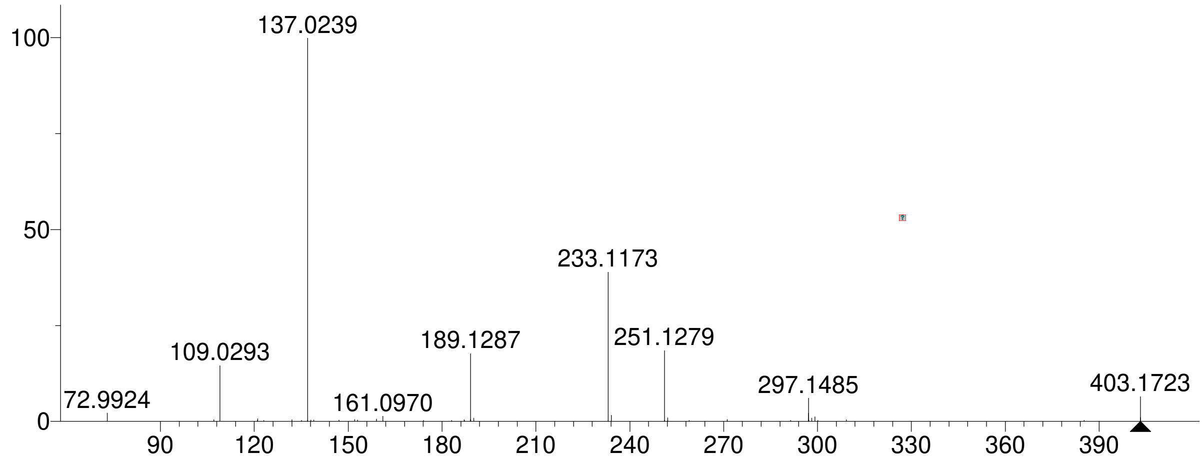

Hit 1 : Acetic acid, 2-[4-[3-(4-acetyl-3-hydroxy-2-propylphenoxy)propoxy]phenoxy]- [M+H]+ HCD 20V P=403.2
C22H26O7; MF: 12; RMF: 133; Prob 10.9%; CAS: 79558-09-1; Lib: nist_msms; ID: 245666.

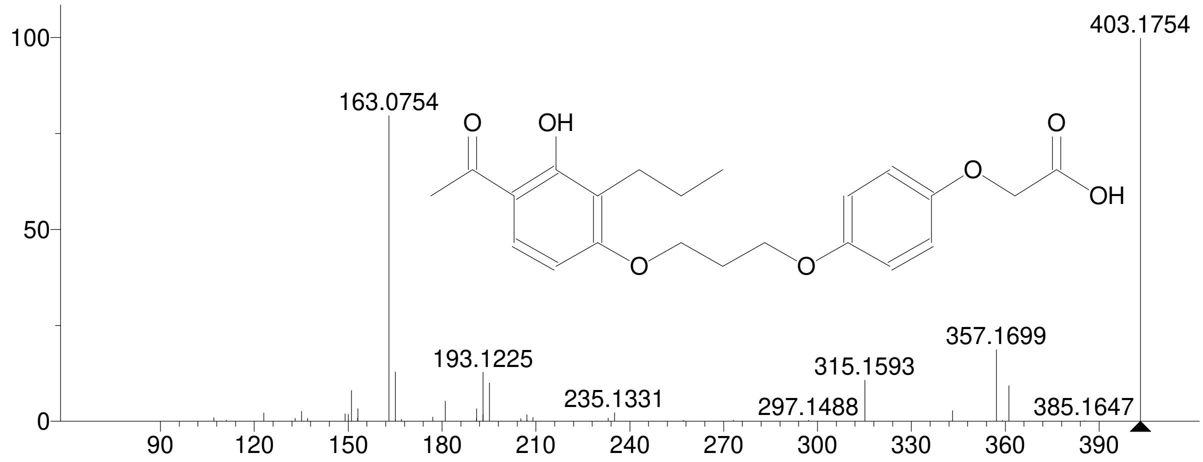

Hit 2 : NCGC00380826-01![1-(3,4-dihydroxy-5-methoxyphenyl)-7-(3,4-dihydroxyphenyl)heptan-3-yl] acetate [M-H
C22H28O7; MF: 11; RMF: 181; Prob 10.5%; Lib: msms-neg-gnps; ID: 1036.

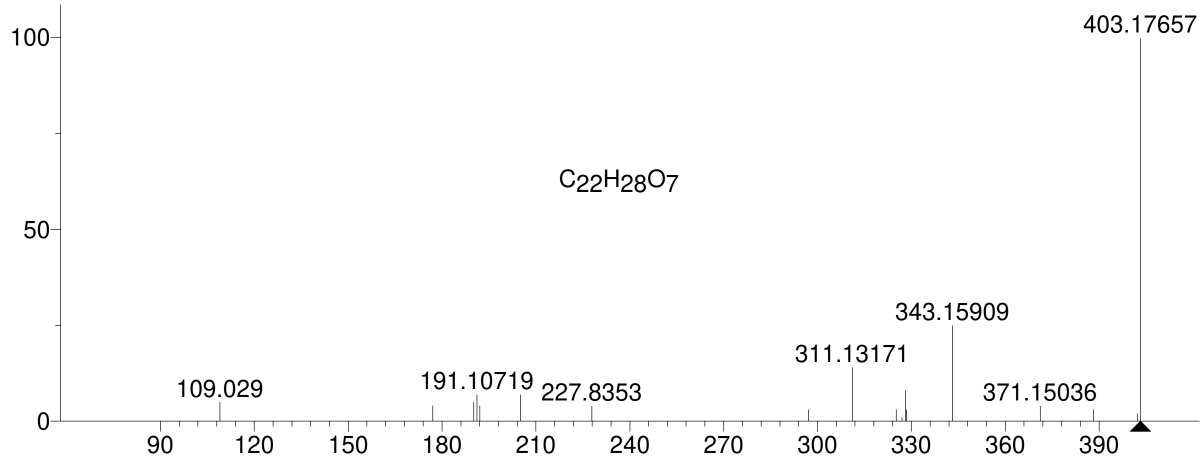

Hit 3 : NCGC00347636-02![2-[2-(2-methylpropanoyloxy)propan-2-yl]-7-oxo-2,3-dihydrofuro[3,2-g]chromen-3-yl] 2-
C22H26O7; MF: 3; RMF: 88; Prob 7.81%; Lib: mona-export-gnps; ID: 9715.

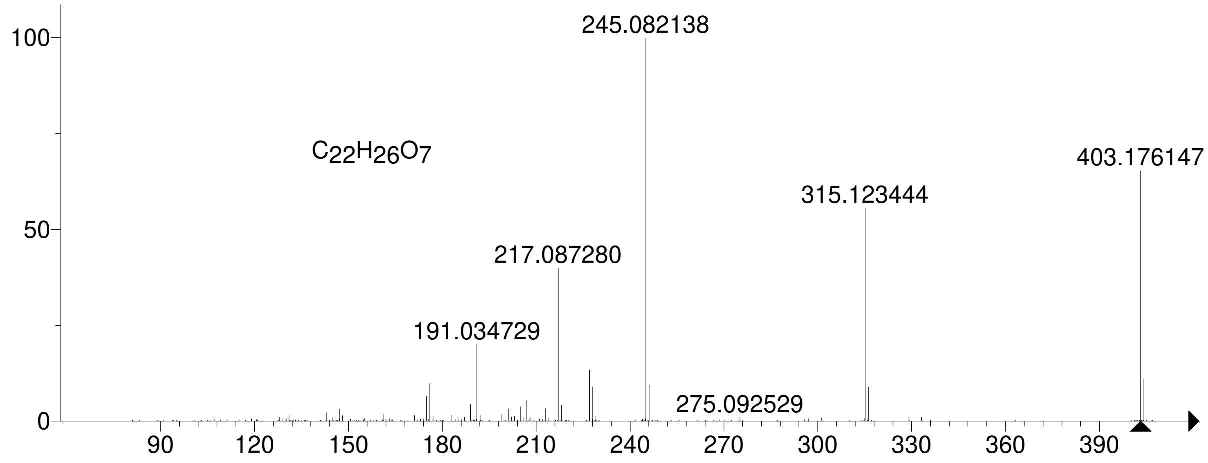

Hit 4 : NCGC00384925-01_C22H28O8_[(2'R,3R,4'R,4a'R,5S,5'R,7'S,8a'S)-5-(3-Furyl)-4',7'-dihydroxy-2'-methyl-2
C22H28O8; MF: 2; RMF: 75; Prob 7.51%; Lib: mona-export-gnps; ID: 11188.

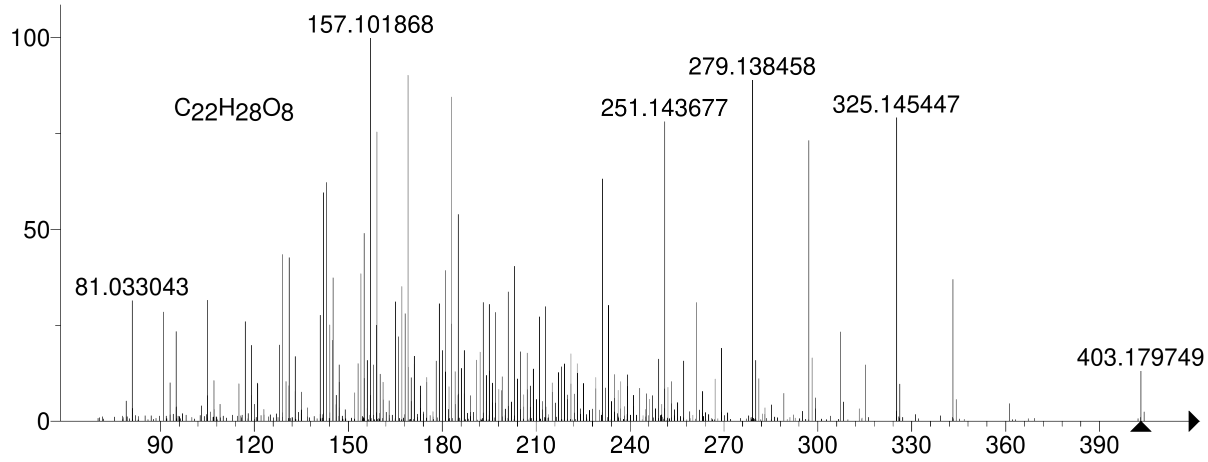

Hit 5 : NCGC00169274-03_C22H26O7_6(2H)-Benzofuranone, 3,3a,4,5-tetrahydro-5,7-dimethoxy-2-(7-methoxy-1
C22H26O7; MF: 1; RMF: 71; Prob 7.22%; Lib: mona-export-gnps; ID: 12133.

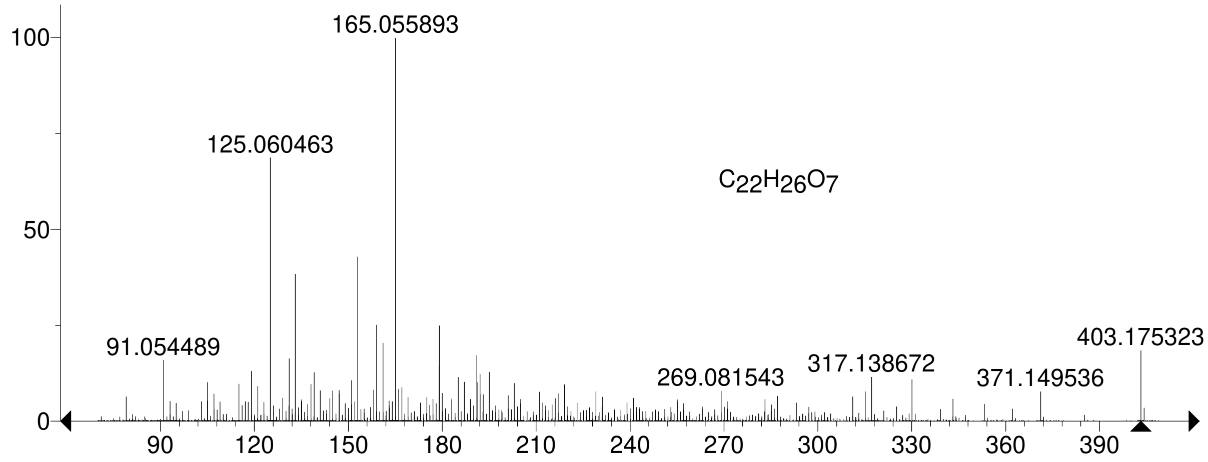

Unknown: Unknown (20.79_650.2270m/z)
Compound in Library Factor = 0

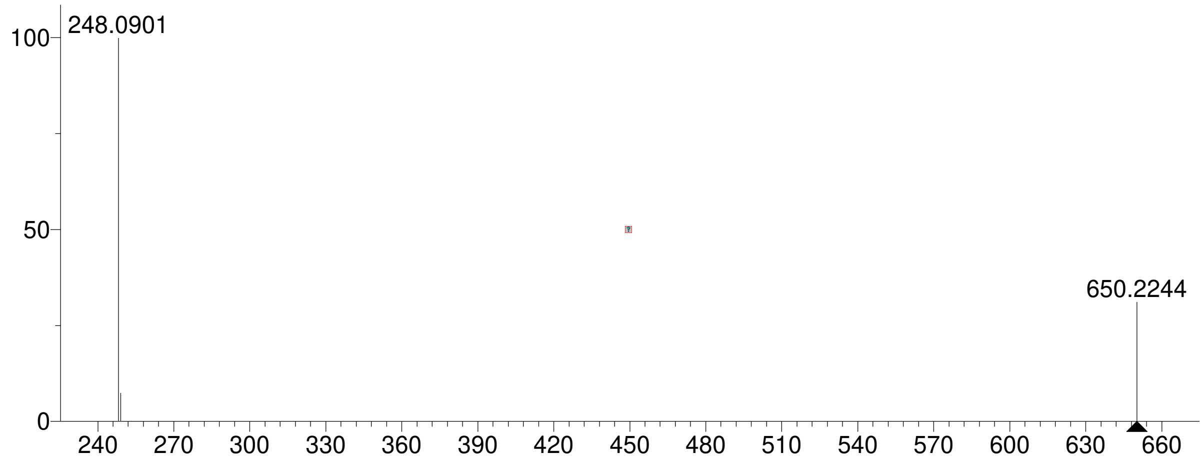

Unknown: Unknown (20.86_263.1646m/z)
Compound in Library Factor = -791

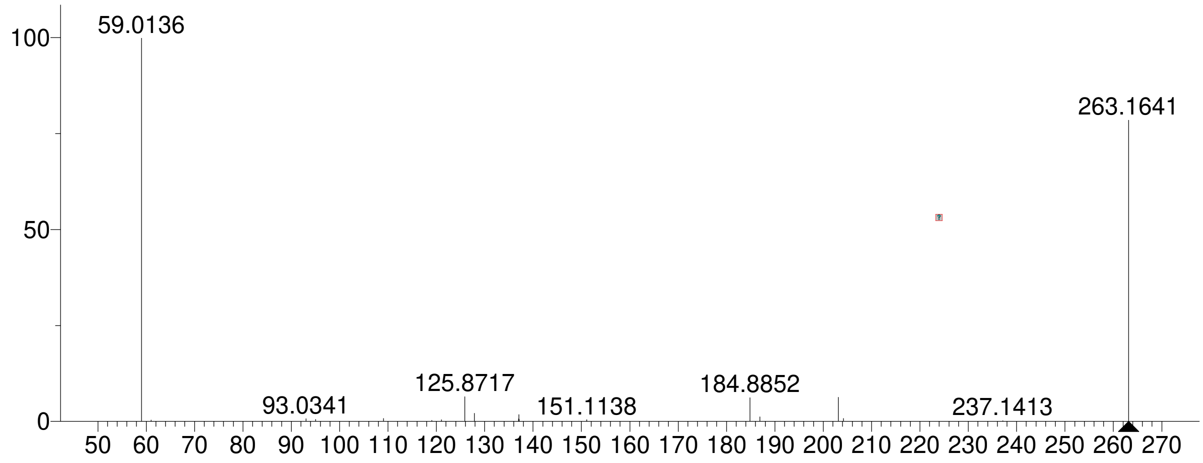

Hit 1 : Dinor-12-oxophytodienoic acid [M-H]- HCD 21V P=263.2
C16H24O3; MF: 113; RMF: 634; Prob 79.1%; CAS: 197247-23-7; Lib: nist_msms; ID: 87586.

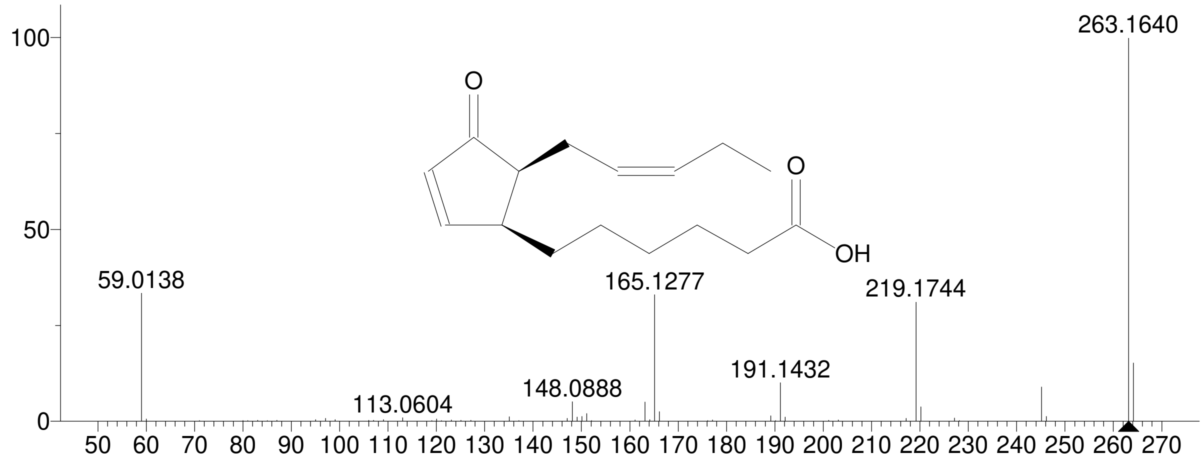

Hit 2 : Brefeldin A [M+H-H2O]+ HCD 13V P=263.2
C16H24O4; MF: 18; RMF: 403; Prob 6.40%; CAS: 20350-15-6; Lib: nist_msms; ID: 271402.

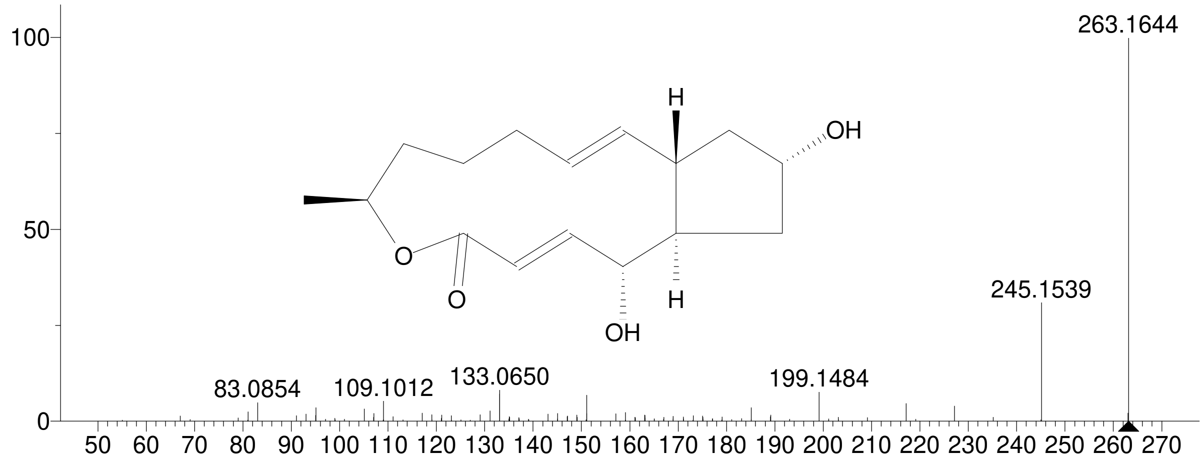

Hit 3 : NCGC00381408-02_C16H24O4_4-Hydroxy-1-methoxy-6,6,9a-trimethyl-4,5,5a,6,7,8,9,9a-octahydronaphtl
C16H24O4; MF: 6; RMF: 430; Prob 4.26%; Lib: mona-export-gnps; ID: 10408.

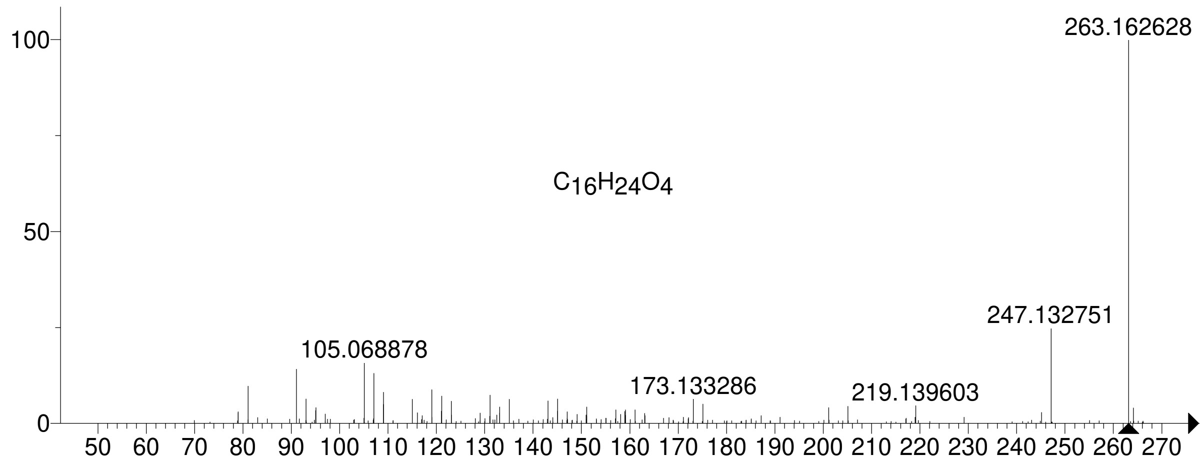

Hit 4 : NCGC00381353-01_C16H24O4_[4a-Formyl-5-(hydroxymethyl)-1,2-dimethyl-1,2,3,4,4a,7,8,8a-octahydro-1
C16H24O4; MF: 2; RMF: 155; Prob 3.60%; Lib: mona-export-gnps; ID: 12572.

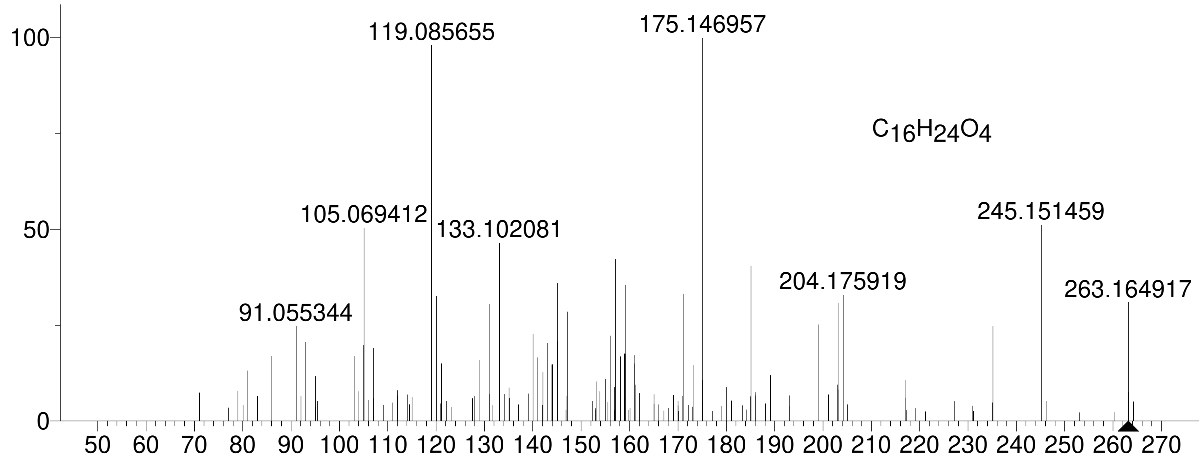

Hit 5 : 13,14-Dihydro-15-ketotetranorprostaglandin D2 [M+H-2H2O]+ IT-FT 35% P=263.2
C16H26O5; MF: 0; RMF: 50; Prob 3.32%; CAS: 1204116-69-7; Lib: nist_msms; ID: 546205.

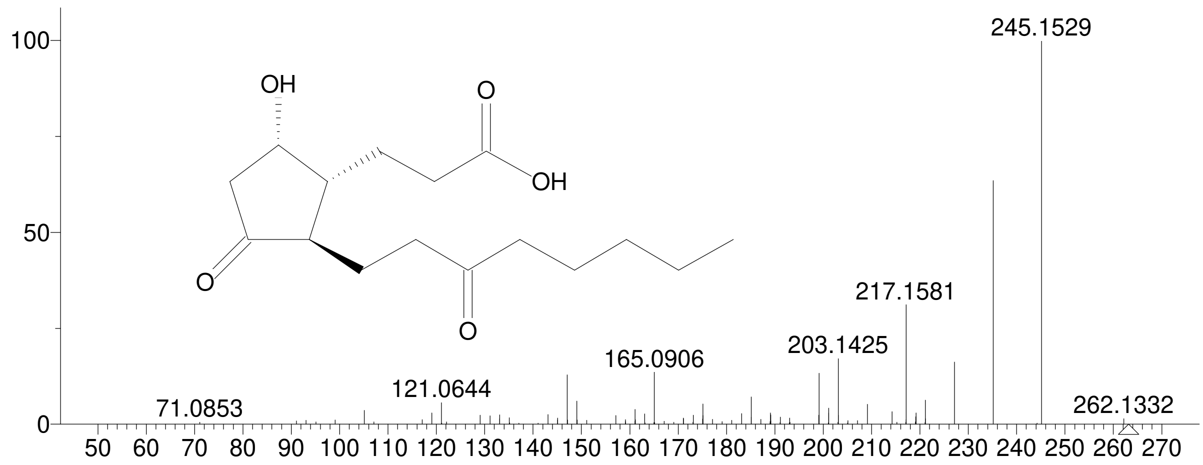

Unknown: Unknown (20.86_423.2131m/z)
Compound in Library Factor = 0

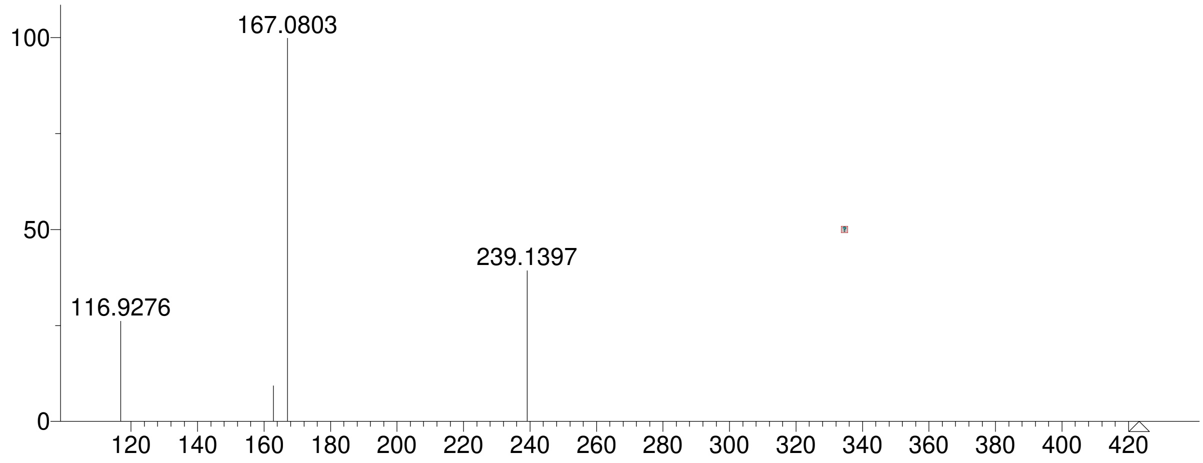

Hit 1 : Cinobufotalin [M+H-2H2O]+ HCD 63V P=423.2
C26H34O7; MF: 0; RMF: 115; Prob 25.0%; CAS: 1108-68-5; Lib: nist_msms; ID: 391347.

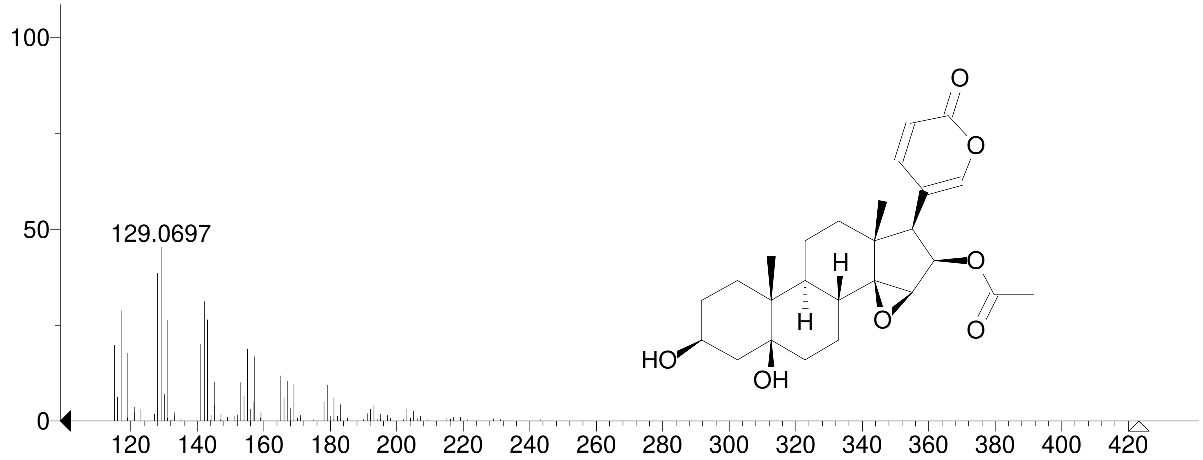

Hit 2 : [4,5-dihydroxy-3,4-bis(hydroxymethyl)-4a,8,8-trimethyl-5,6,7,8a-tetrahydro-1H-naphthalen-1-yl] hexanoate
C21H36O6; MF: 0; RMF: 39; Prob 25.0%; Lib: mona-export-vf-npl_qexactive; ID: 8154.

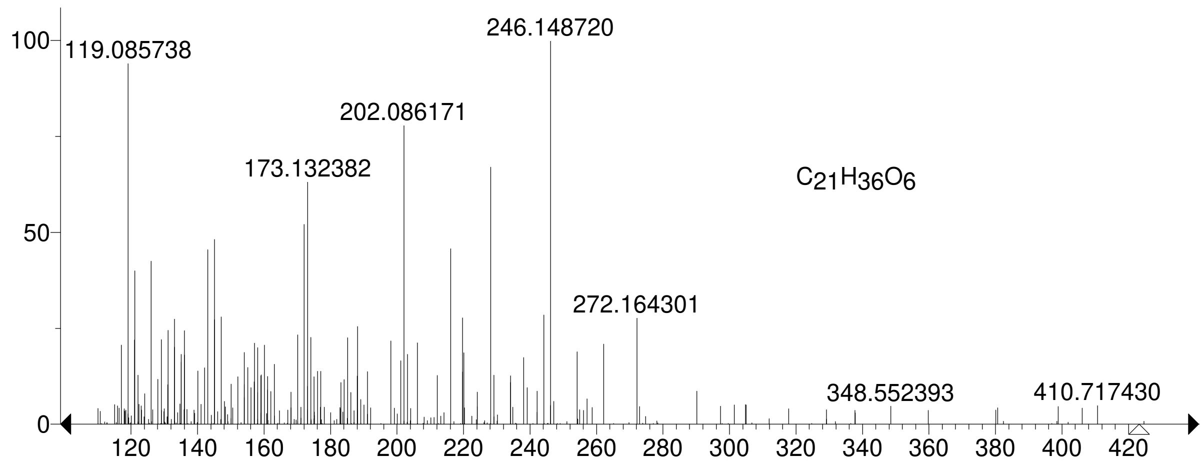

Hit 3 : NCGC00384666-01!(2S)-5,7-dihydroxy-2-[4-methoxy-3,5-bis(3-methylbut-2-enyl)phenyl]-2,3-dihydrochrom
C26H30O5; MF: 0; RMF: 23; Prob 25.0%; Lib: mona-export-gnps; ID: 7800.

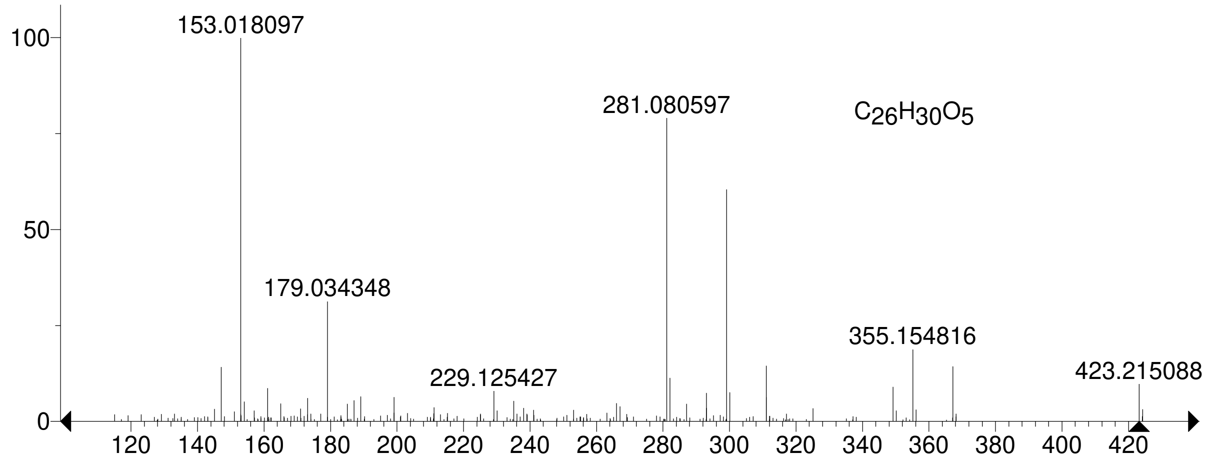

Hit 4 : 4-Methylabyssinone V [M+H]+ 65HCD P=423.2
C26H30O5; MF: 0; RMF: 17; Prob 25.0%; Lib: mona-export-vf-npl_qexactive; ID: 6033.

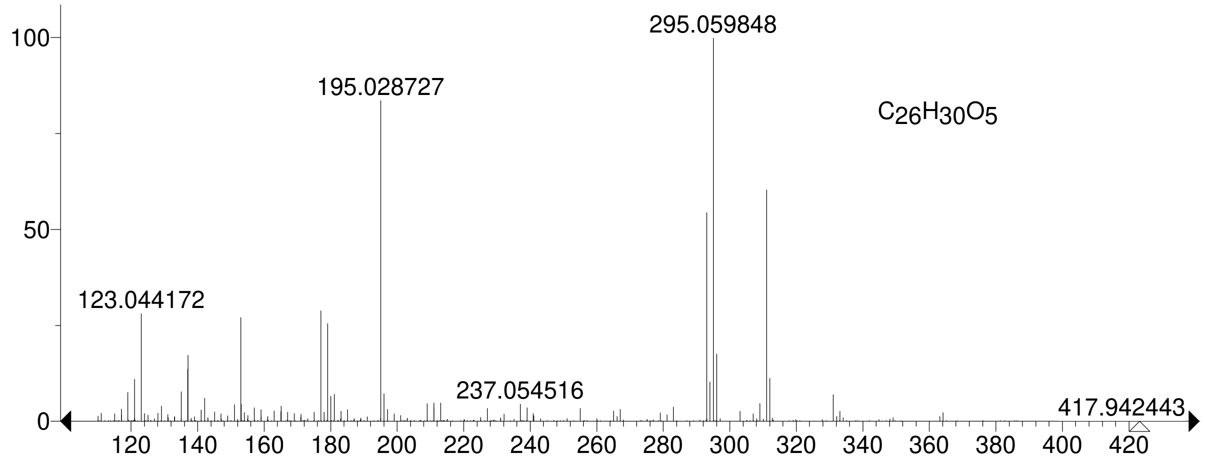

Unknown: Unknown (20.86_450.0296m/z)
Compound in Library Factor = 0

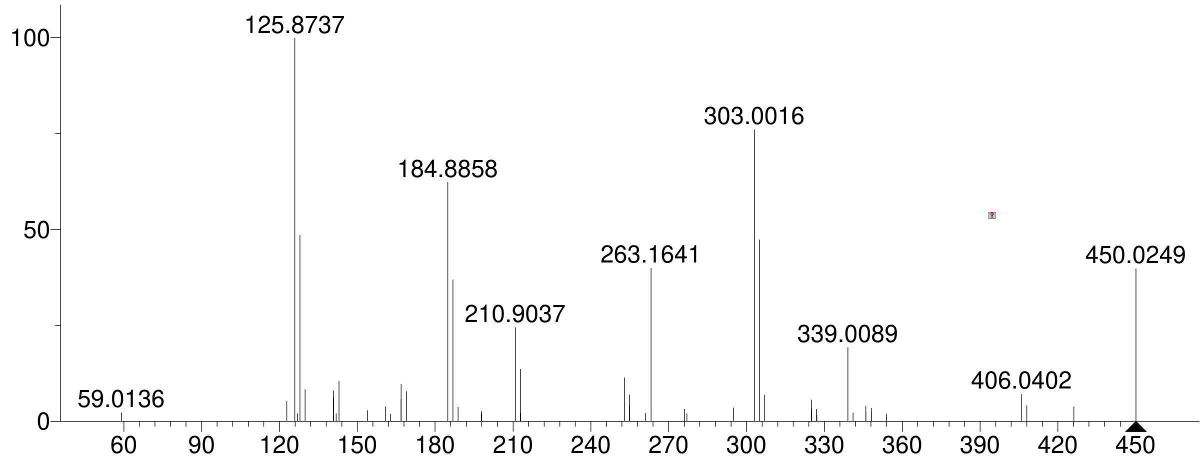

** Search Report Page 1 of 1 **

Unknown: Unknown (20.86_486.0058m/z)
Compound in Library Factor = 0

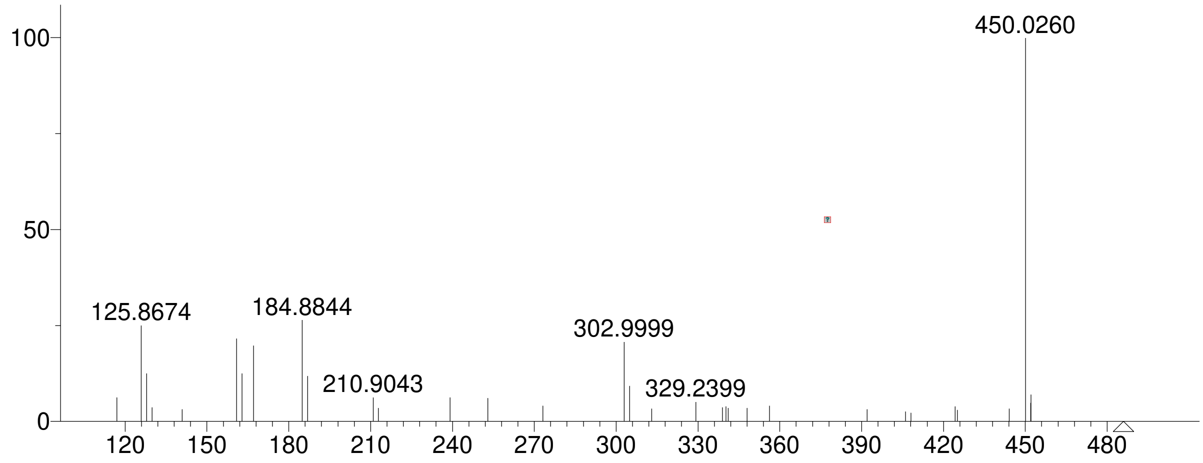

** Search Report Page 1 of 1 **

Unknown: Unknown (20.87_452.0266m/z)
Compound in Library Factor = -3273

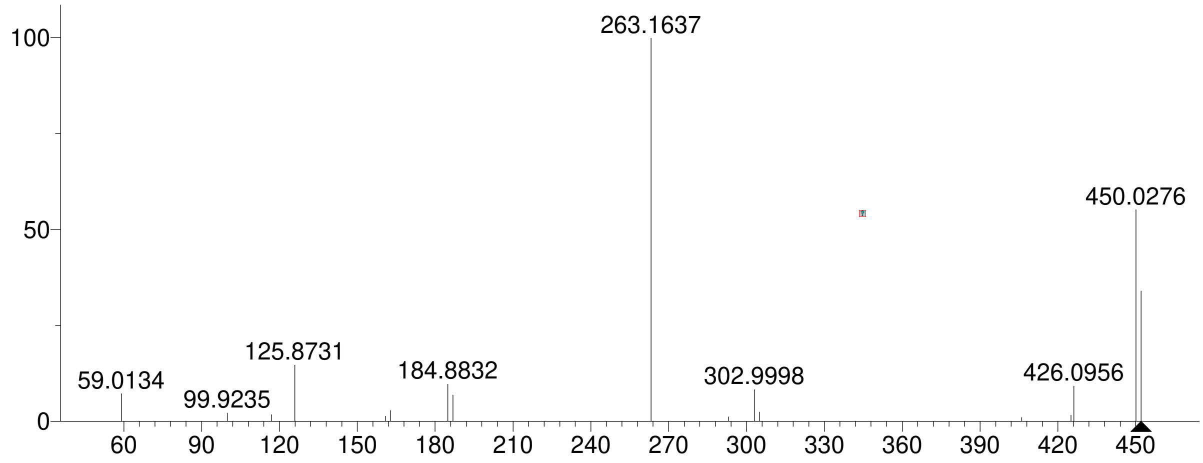

Hit 1 : Copper gluconate [M-H]- HCD 6V P=452
C12H22CuO14; MF: 20; RMF: 365; Prob 100.0%; CAS: 527-09-3; Lib: nist_msms; ID: 152771.

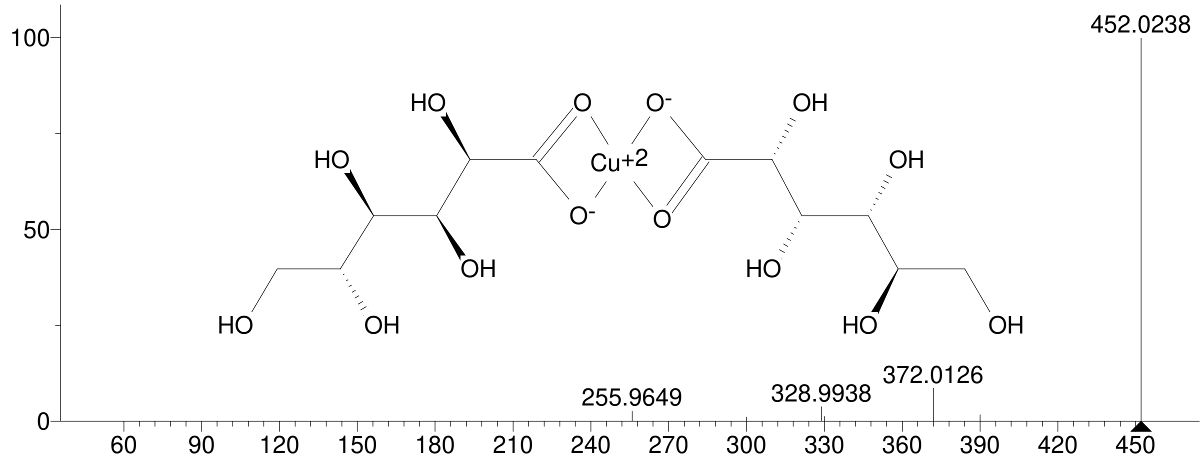

** Search Report Page 1 of 1 **

Unknown: Unknown (20.87_470.0872m/z)
Compound in Library Factor = 0

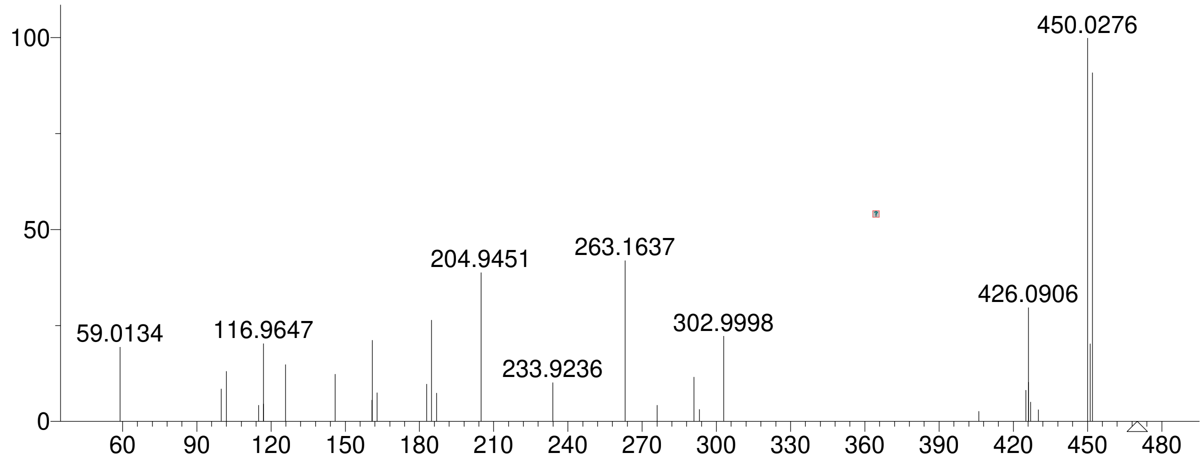

Hit 1 : 2-Thiophenecarbonitrile, 5-(5,6-dimethoxy-1H-benzimidazol-1-yl)-3-[[2-(methylsulfonyl)phenyl]methoxy]- [M
C22H19N3O5S2; MF: 0; RMF: 36; Prob 100.0%; CAS: 862812-98-4; Lib: nist_msms; ID: 372407.

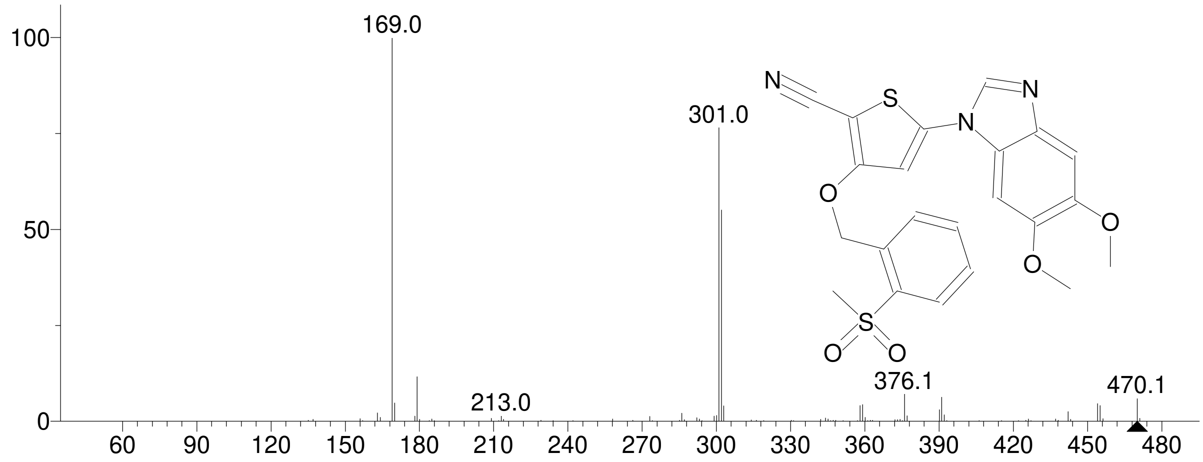

Unknown: Unknown (20.87_740.2260m/z)
Compound in Library Factor = 0

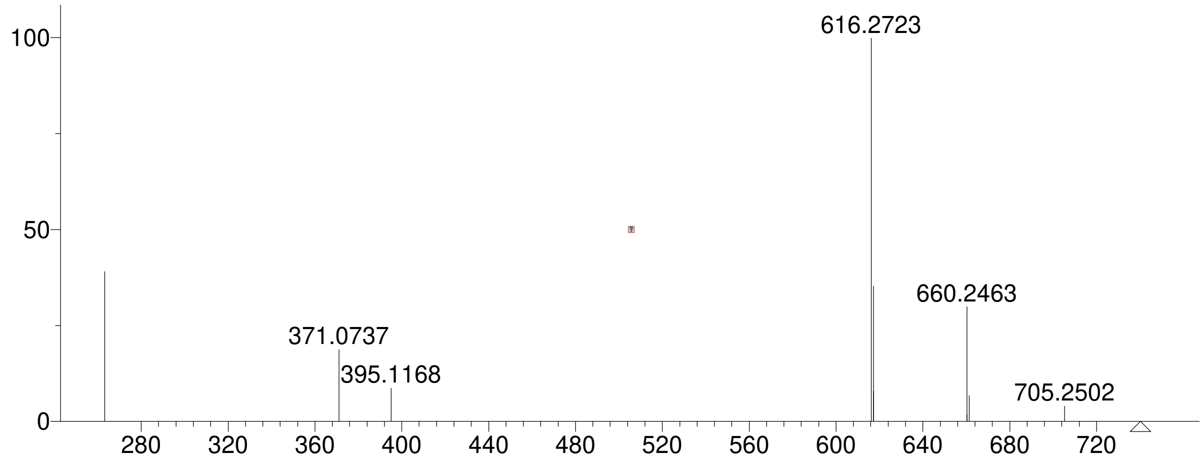

Unknown: Unknown (20.88_325.1649m/z)
Compound in Library Factor = -3273

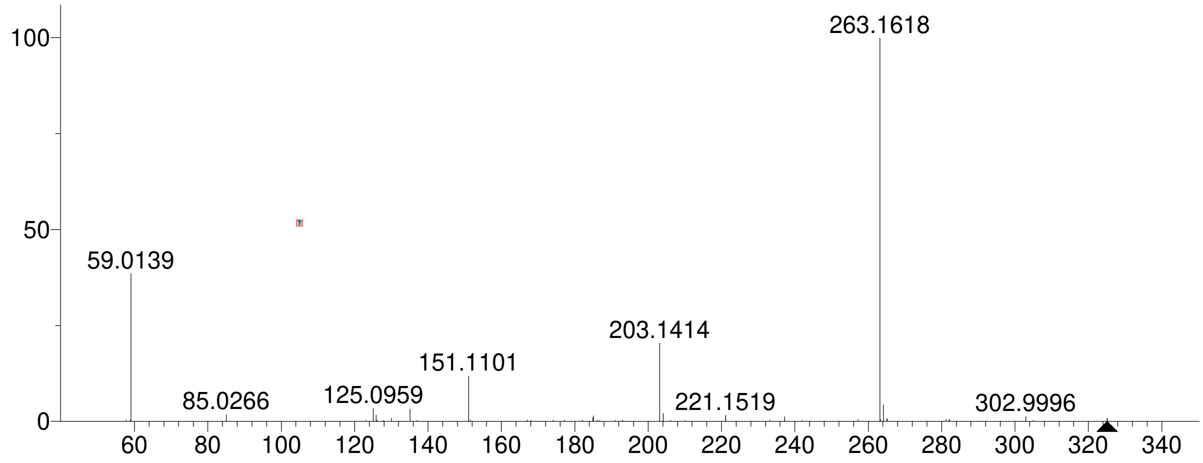

Hit 1 : Chamissonolide [M+H]+ HCD 1V P=325.2
C17H24O6; MF: 5; RMF: 75; Prob 16.1%; CAS: 78853-98-2; Lib: nist_msms; ID: 500759.

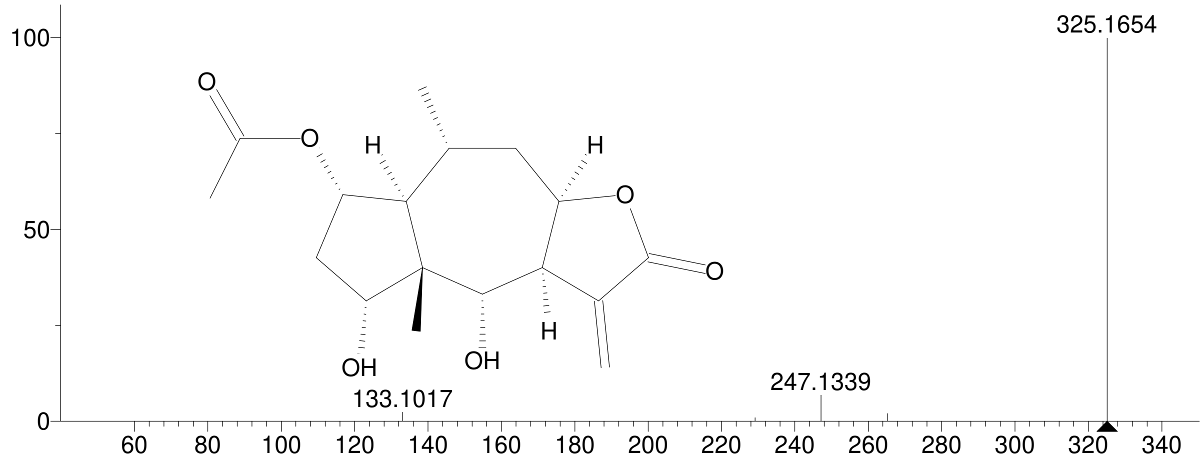

Hit 2 : [5-acetyloxy-3-(hydroxymethyl)-2-oxo-6-propan-2-ylcyclohex-3-en-1-yl] 3-methylbutanoate [M-H]- 35HCD
C17H26O6; MF: 4; RMF: 86; Prob 15.5%; Lib: mona-export-lc-ms-ms_negative_mode; ID: 19385.

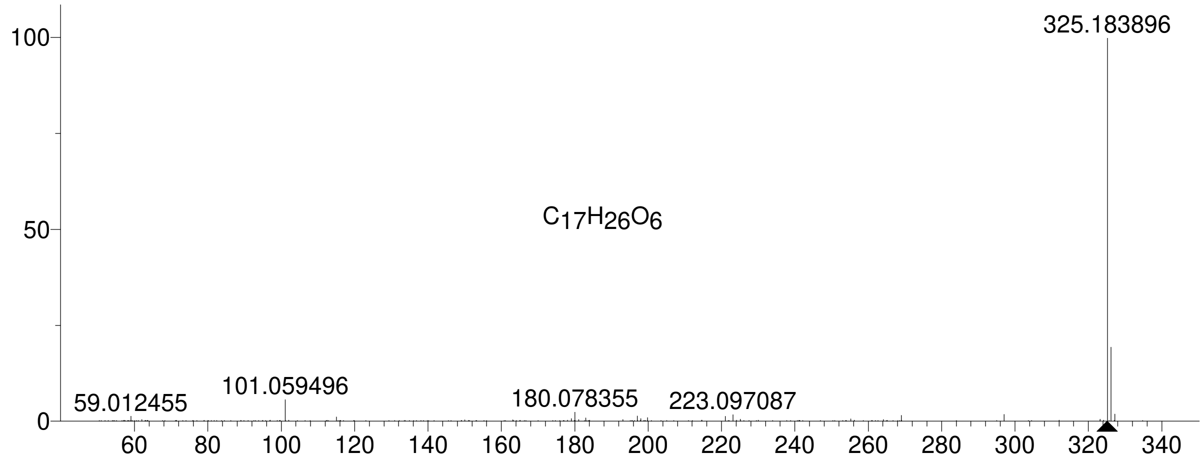

Hit 3 : Tributyrin [M+Na]+ HCD 13V P=325.2
C15H26O6; MF: 3; RMF: 66; Prob 14.9%; CAS: 60-01-5; Lib: nist_msms; ID: 144821.

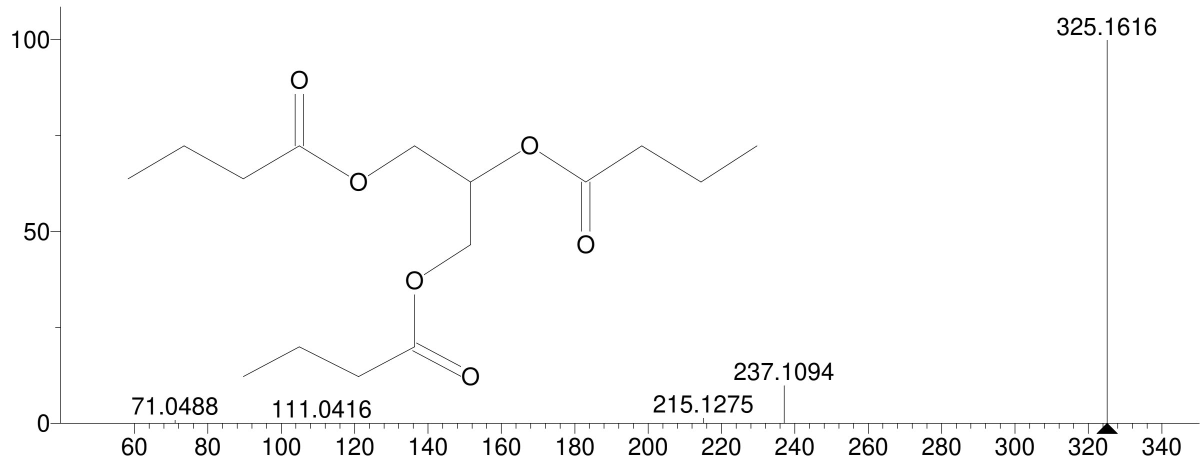

Hit 4 : MMV023985 [M+H]+ 20-30-40%) P=325.2
C18H20N4O2; MF: 1; RMF: 61; Prob 13.8%; Lib: mona-export-pathogen_box; ID: 292.

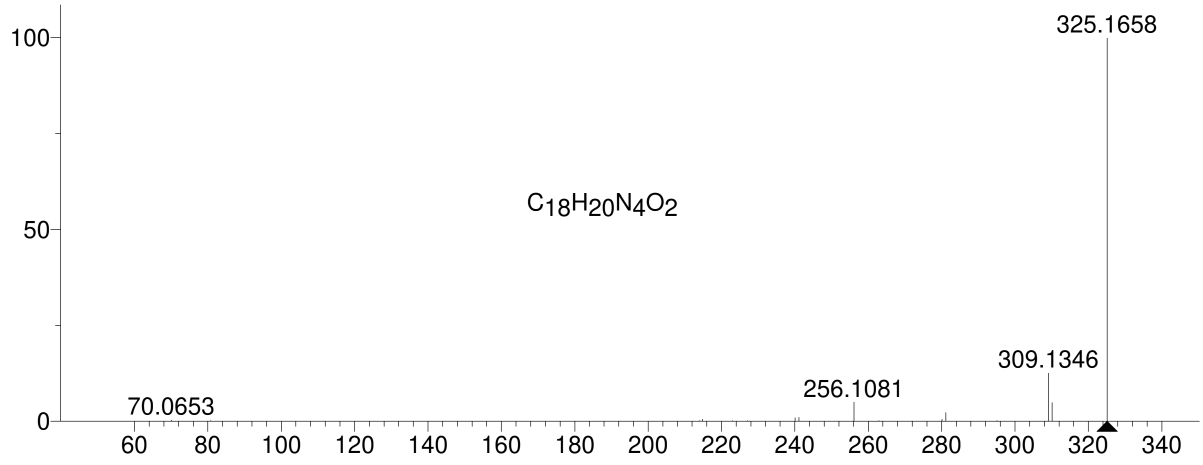

Hit 5 : 2H-Benzimidazol-2-one, 1-ethyl-1,3-dihydro- [2M+H]+ HCD 3V P=325.2
C9H10N2O; MF: 0; RMF: 39; Prob 13.2%; CAS: 10045-45-1; Lib: nist_msms; ID: 364753.

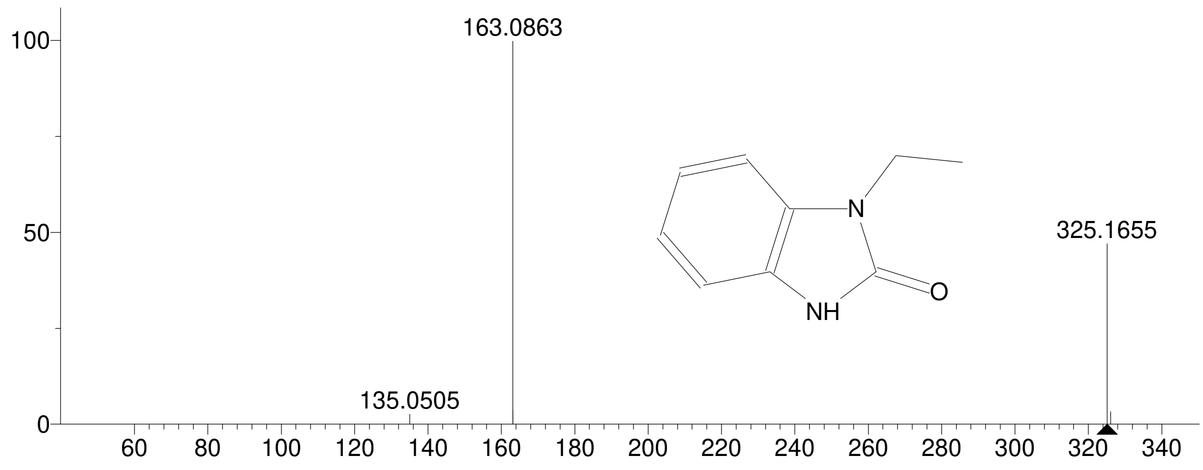

** Search Report Page 1 of 1 **

Unknown: Unknown (20.88_425.0896m/z)
Compound in Library Factor = 0

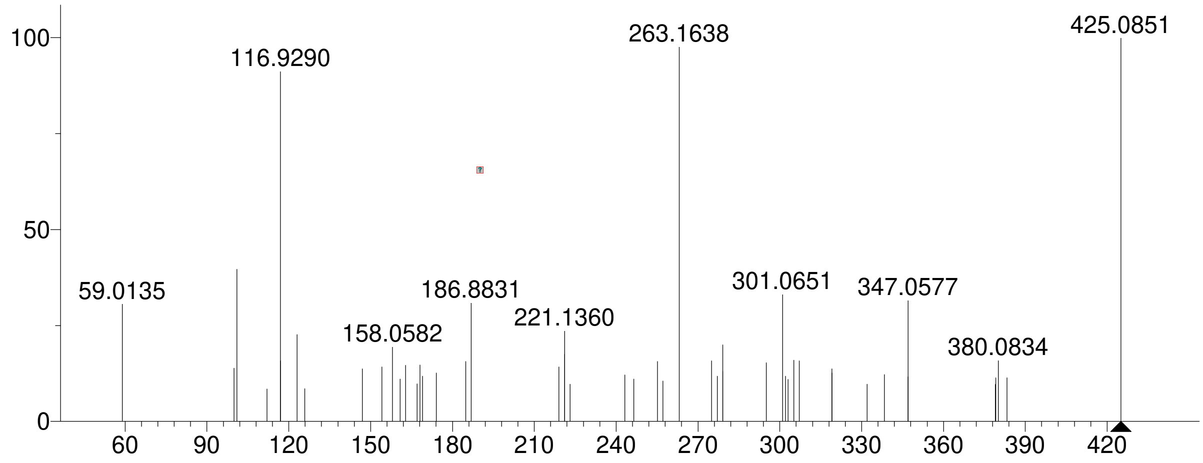

Unknown: Unknown (20.89_393.1518m/z)
Compound in Library Factor = 0

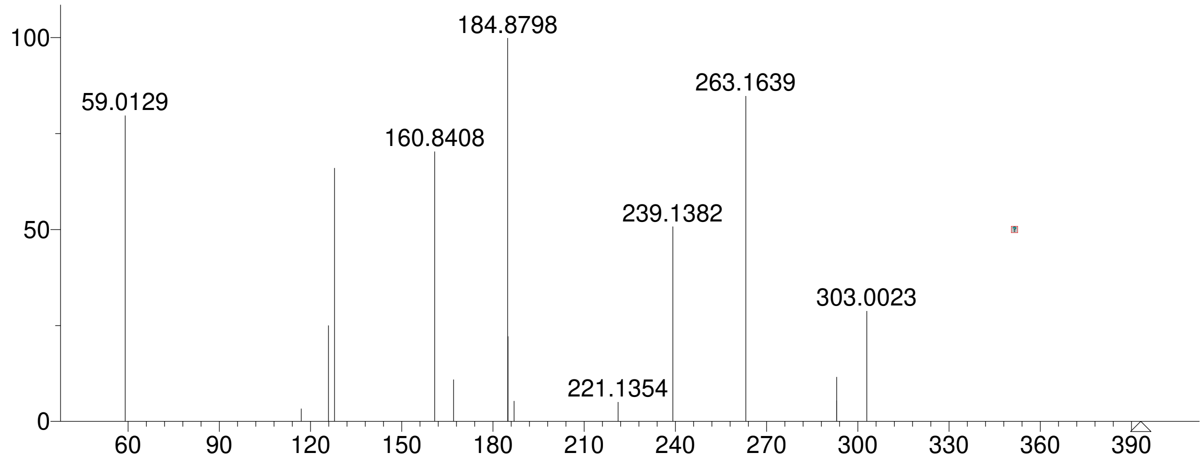

Hit 1 : NCGC00168813-03_C20H24O8_1H-2-Benzopyran-6-acetic acid, 8a-ethenyloctahydro-5-hydroxy-7-[[2-(hy
C20H24O8; MF: 0; RMF: 44; Prob 50.0%; Lib: mona-export-gnps; ID: 13917.

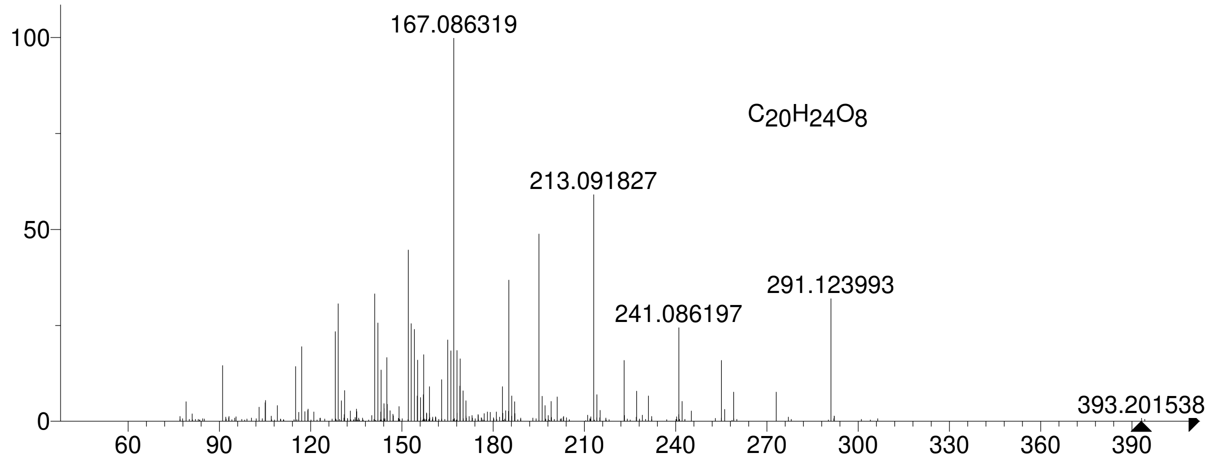

Hit 2 : [(5R,6S,7S,8aR)-8a-ethenyl-5-hydroxy-6-(3-methoxy-3-oxoprop-1-en-2-yl)-4-methylidene-3-oxo-1,4a,5,6,7,
C20H24O8; MF: 0; RMF: 17; Prob 50.0%; Lib: mona-export-vf-npl_qexactive; ID: 9920.

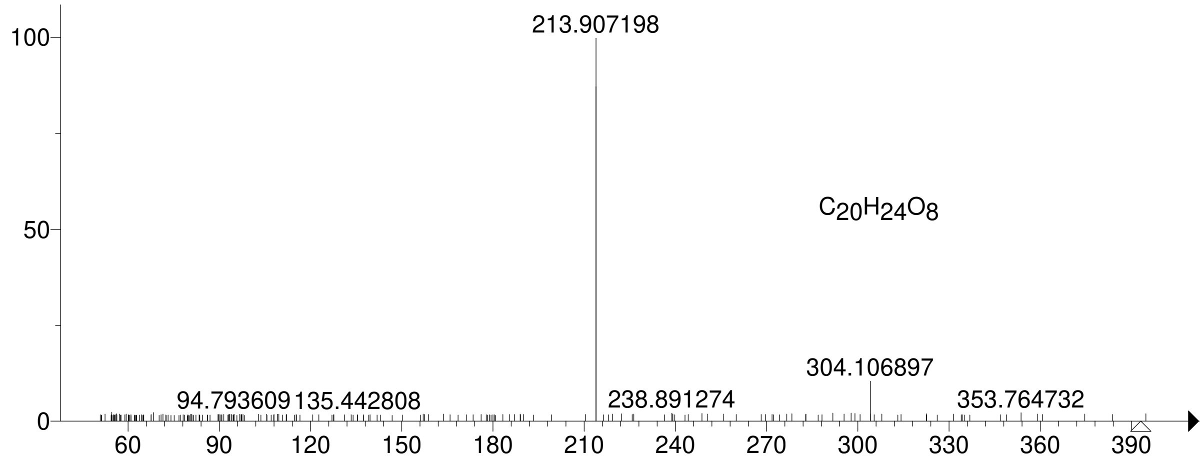

Unknown: Unknown (20.89_704.2504m/z)
Compound in Library Factor = 0

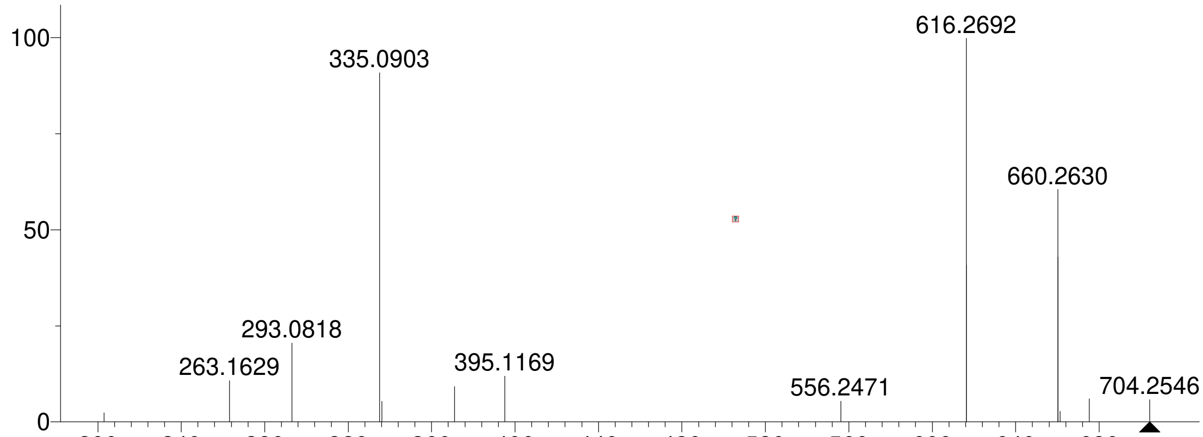

Hit 1 : DFLNHc [M-H+HCO2]2- IT 35% P=704.2
C52H88N2O39; MF: 0; RMF: 12; Prob 100.0%; Lib: nist_msms; ID: 532655.

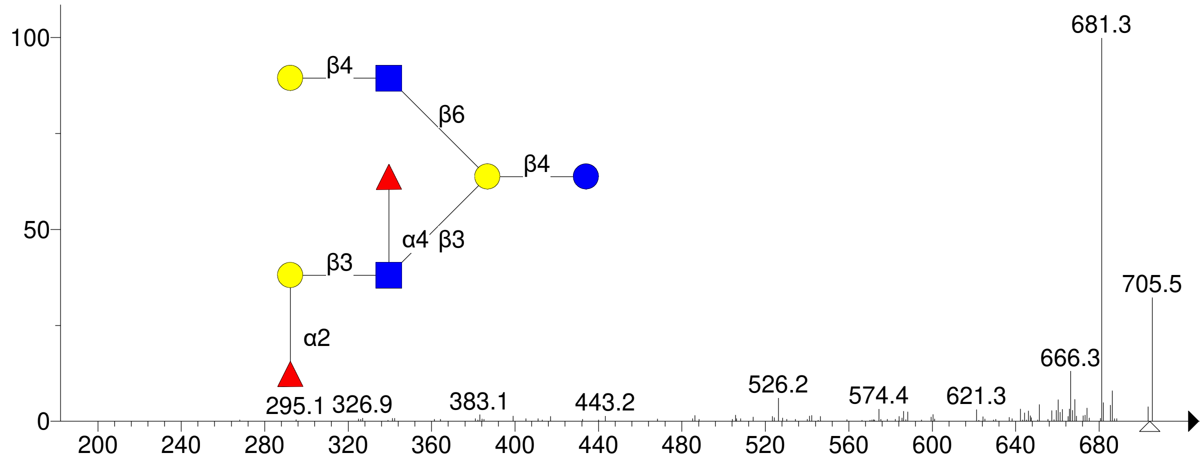

Unknown: Unknown (20.97_682.2865m/z)
Compound in Library Factor = 0

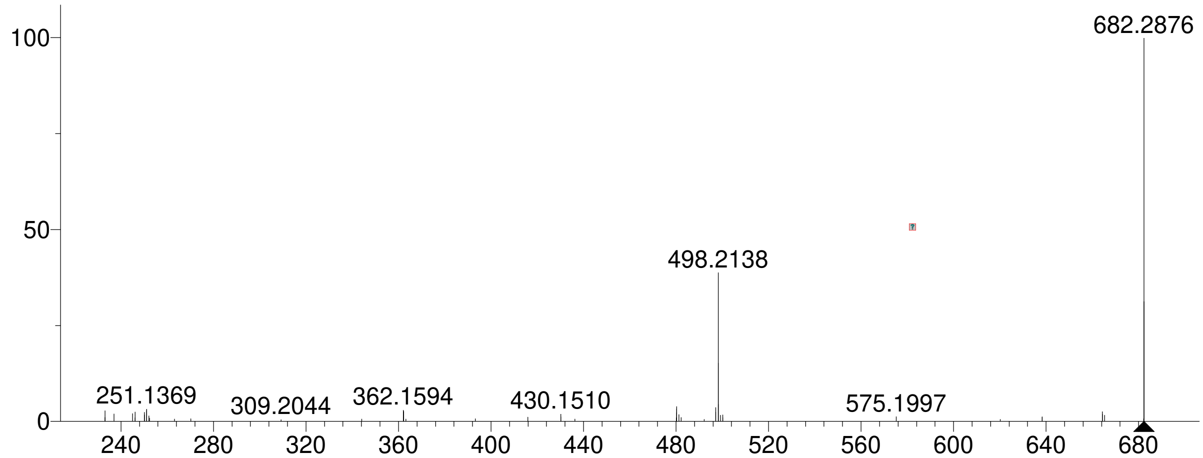

Unknown: Unknown (21.20_425.1915m/z)
Compound in Library Factor = 0

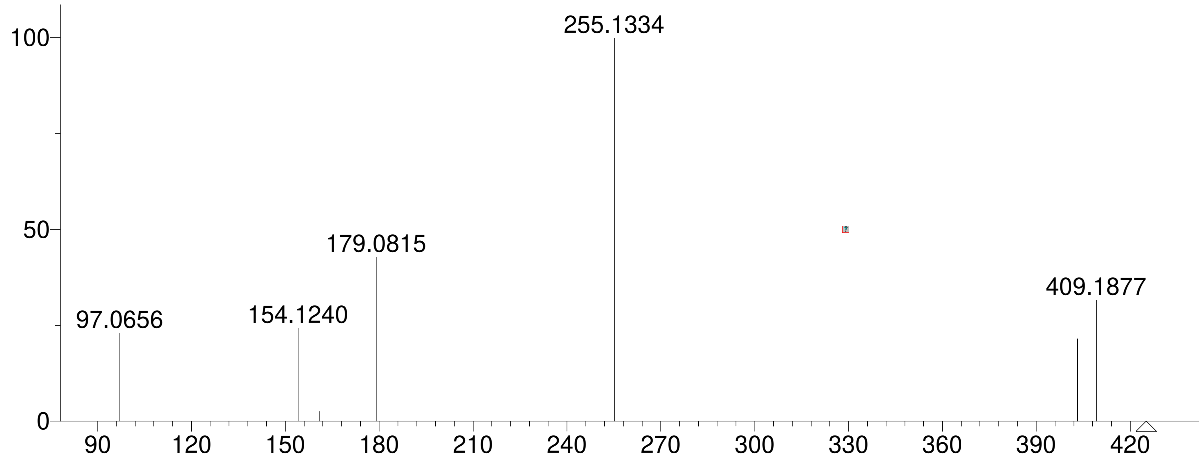

Hit 1 : 2-[5-[2-[2-[5-(2-hydroxypropyl)oxolan-2-yl]propanoyloxy]propyl]oxolan-2-yl]propanoic acid [M+K]+ 65HCD
C20H34O7; MF: 0; RMF: 16; Prob 33.3%; Lib: mona-export-vf-npl_qexactive; ID: 9132.

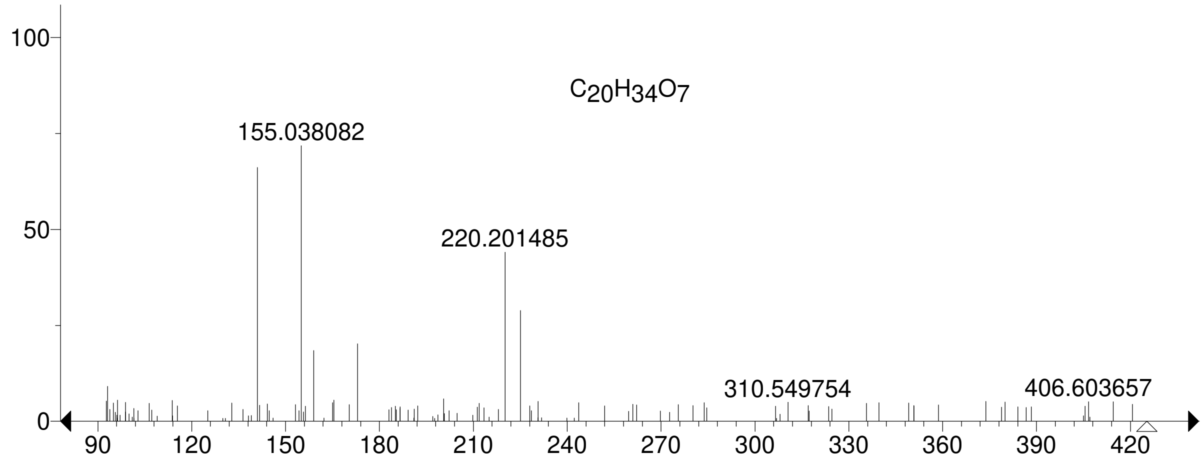

Hit 2 : MLS001183714-01!Clindamycin [M+H]+ P=425.2
C18H33ClN2O5S; MF: 0; RMF: 7; Prob 33.3%; Lib: mona-export-gnps; ID: 3814.

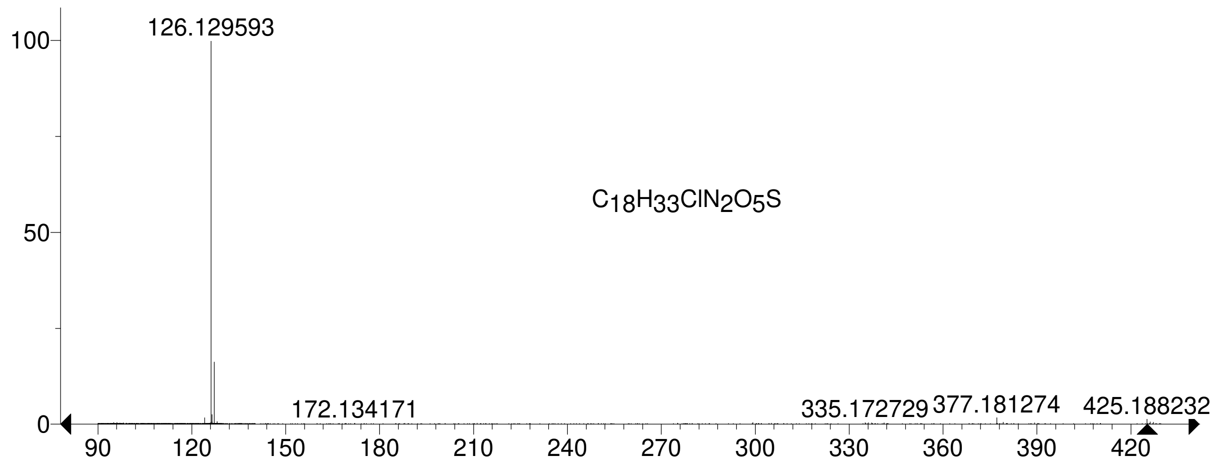

Hit 3 : MLS001304044-01! [M+H]+ P=425.2
C18H36Cl2N2O6S; MF: 0; RMF: 5; Prob 33.3%; Lib: mona-export-gnps; ID: 3214.

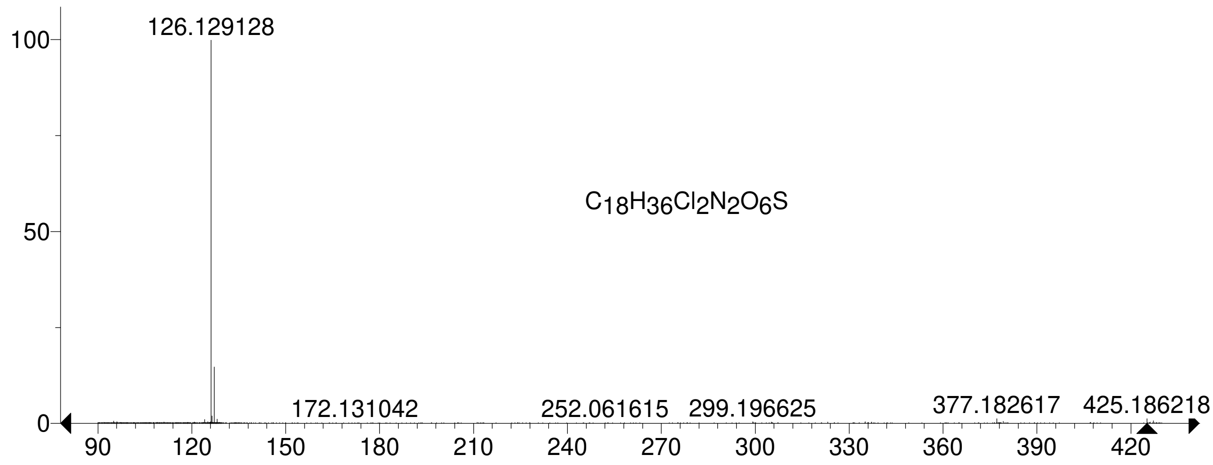

Unknown: Unknown (21.21_403.1734m/z)
Compound in Library Factor = -3273

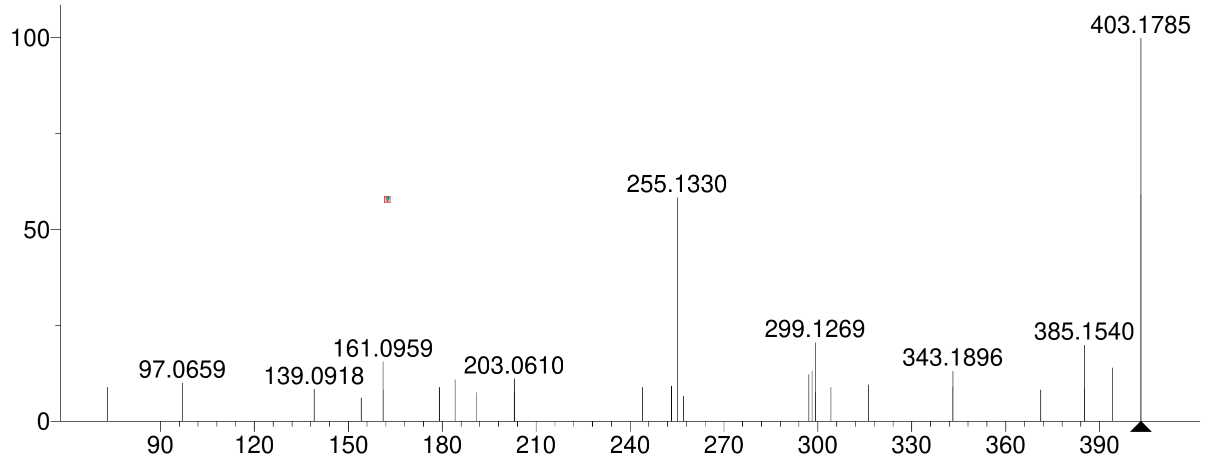

Hit 1 : NCGC00380826-01![1-(3,4-dihydroxy-5-methoxyphenyl)-7-(3,4-dihydroxyphenyl)heptan-3-yl] acetate [M-H
$C_{22}H_{28}O_7$; MF: 5; RMF: 362; Prob 8.75%; Lib: msms-neg-gnps; ID: 1036.

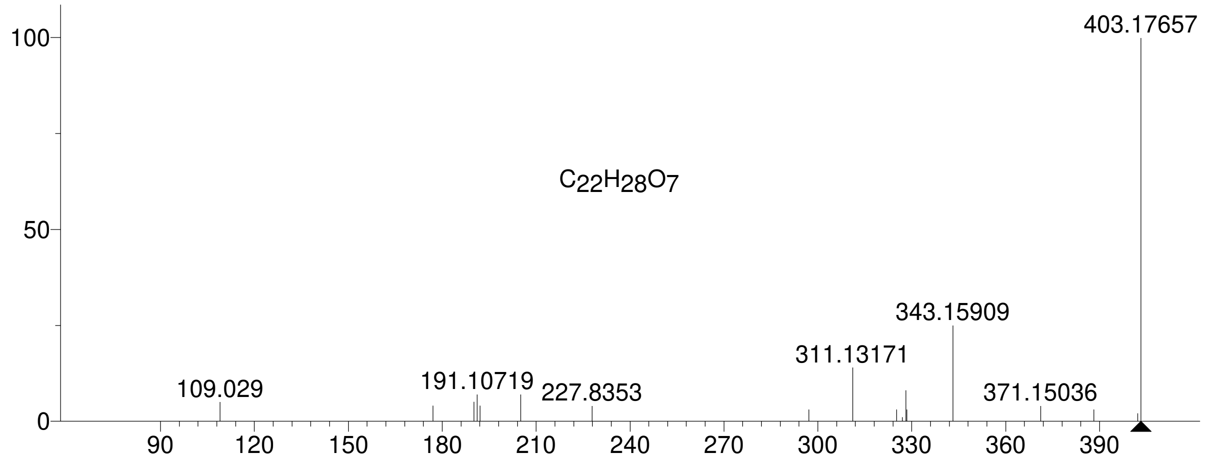

Hit 2 : Acetic acid, 2-[4-[3-(4-acetyl-3-hydroxy-2-propylphenoxy)propoxy]phenoxy]- [M+H]+ HCD 24V P=403.2
$C_{22}H_{26}O_7$; MF: 5; RMF: 167; Prob 8.75%; CAS: 79558-09-1; Lib: nist_msms; ID: 245667.

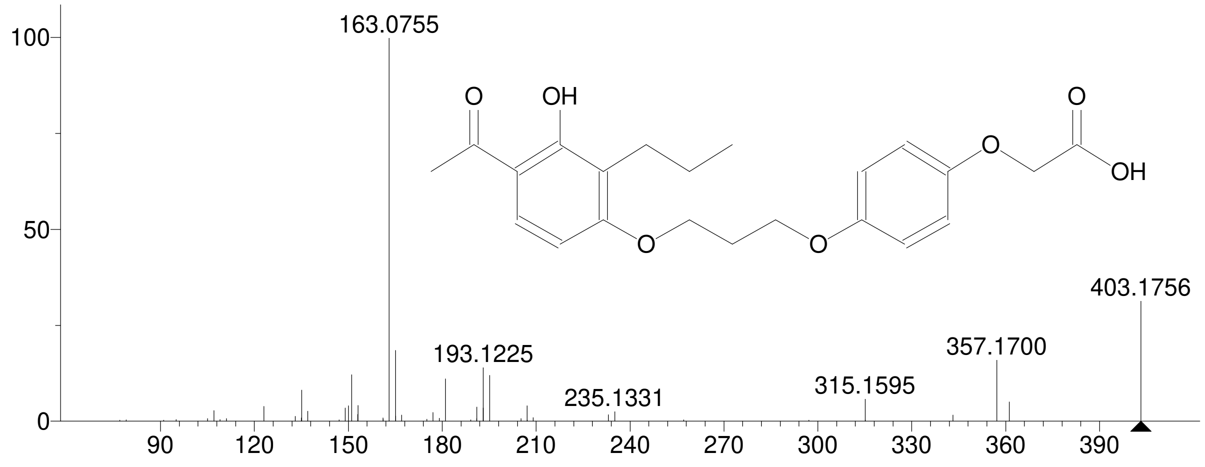

Hit 3 : (2R,3R,3aR,5R)-5,7-dimethoxy-2-(7-methoxy-1,3-benzodioxol-5-yl)-3-methyl-3a-prop-2-enyl-2,3,4,5-tetrahy
C22H26O7; MF: 4; RMF: 150; Prob 8.41%; Lib: mona-export-vf-npl_qexactive; ID: 23182.

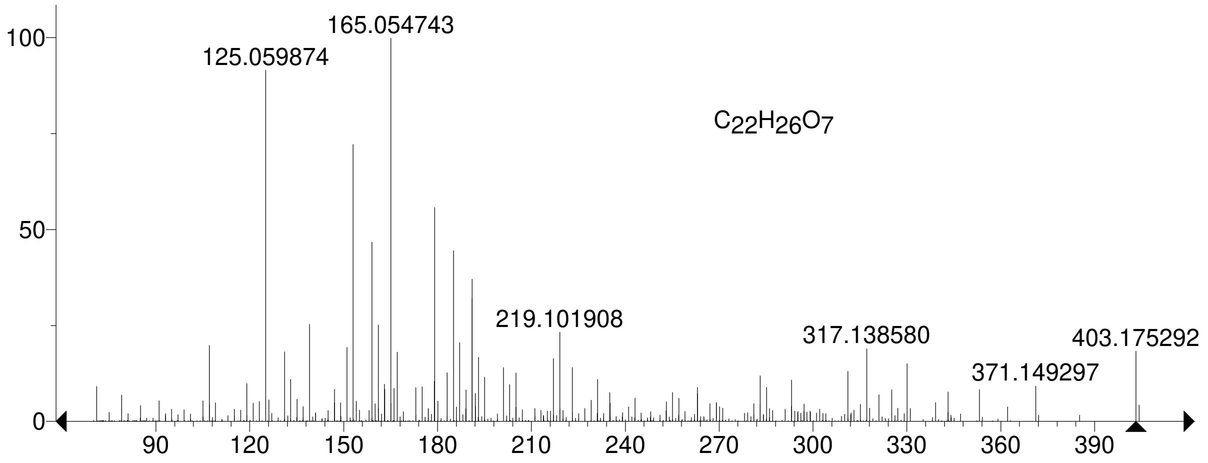

Hit 4 : gmelinol [M+H]+ 10 P=403.2
; MF: 2; RMF: 220; Prob 7.76%; Lib: mona-export-vf-npl_qtof; ID: 1874.

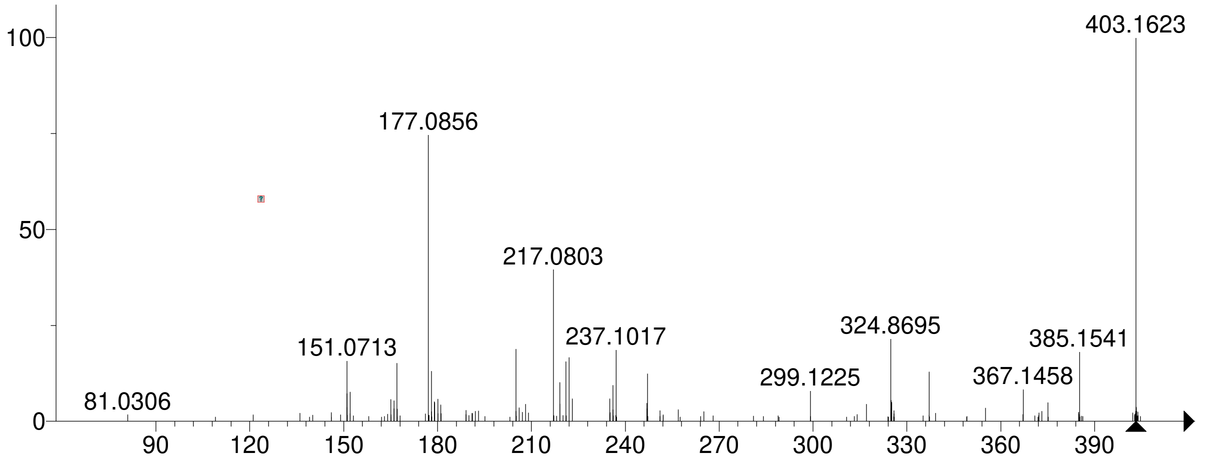

Hit 5 : NCGC00347636-02![2-[2-(2-methylpropanoyloxy)propan-2-yl]-7-oxo-2,3-dihydrofuro[3,2-g]chromen-3-yl] 2-
C22H26O7; MF: 2; RMF: 197; Prob 7.76%; Lib: mona-export-gnps; ID: 9715.

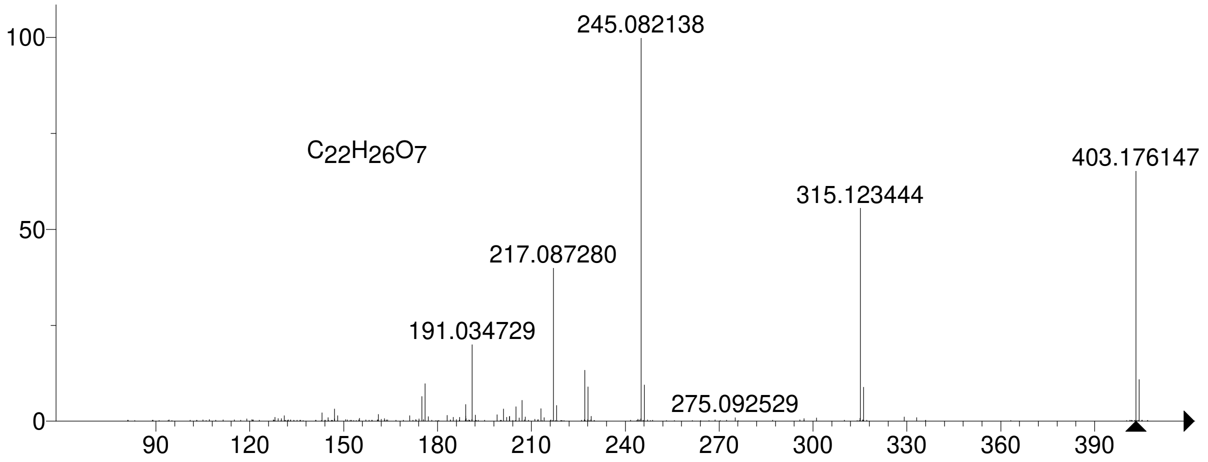

Unknown: Unknown (21.29_327.2164m/z)
Compound in Library Factor = -755

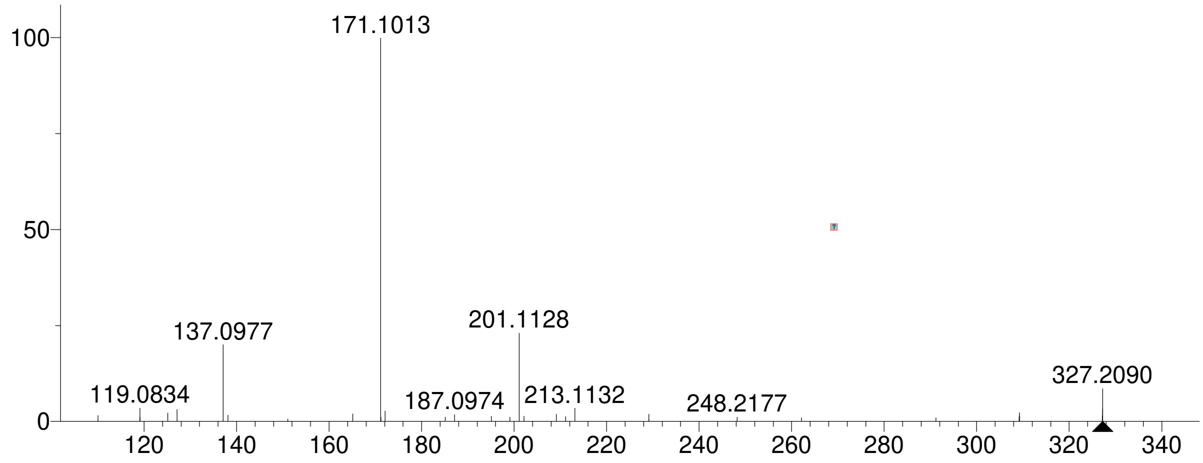

Hit 1 : FA 18:2+3O; PlaSMA ID-711 [M-H]- P=327.2
C18H32O5; MF: 214; RMF: 428; Prob 31.1%; Lib: biomsms-neg-plasma; ID: 437.

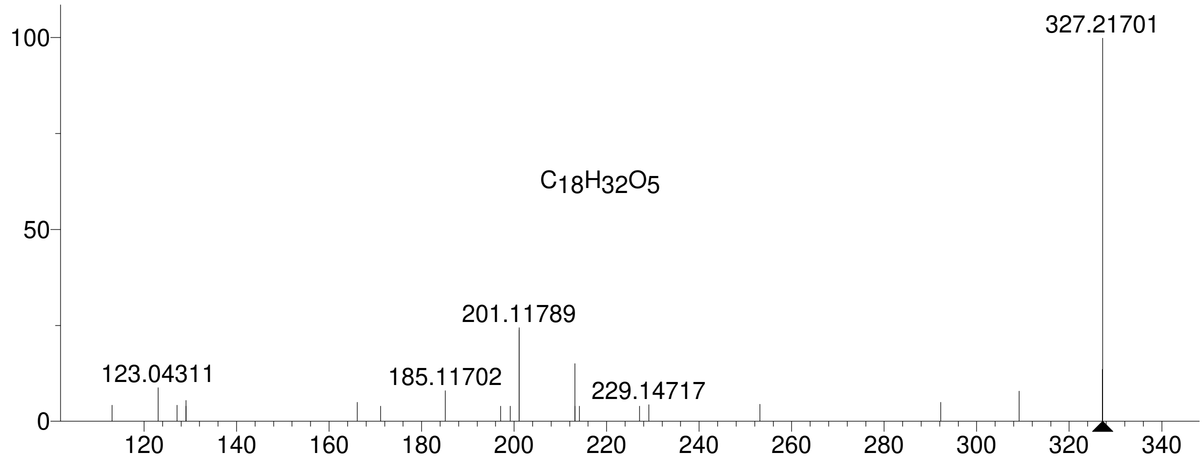

Hit 2 : Unknown (carbon number 18); PlaSMA ID-715 [M-H]- P=327.2
C18H32O5; MF: 214; RMF: 368; Prob 31.1%; Lib: biomsms-neg-plasma; ID: 1234.

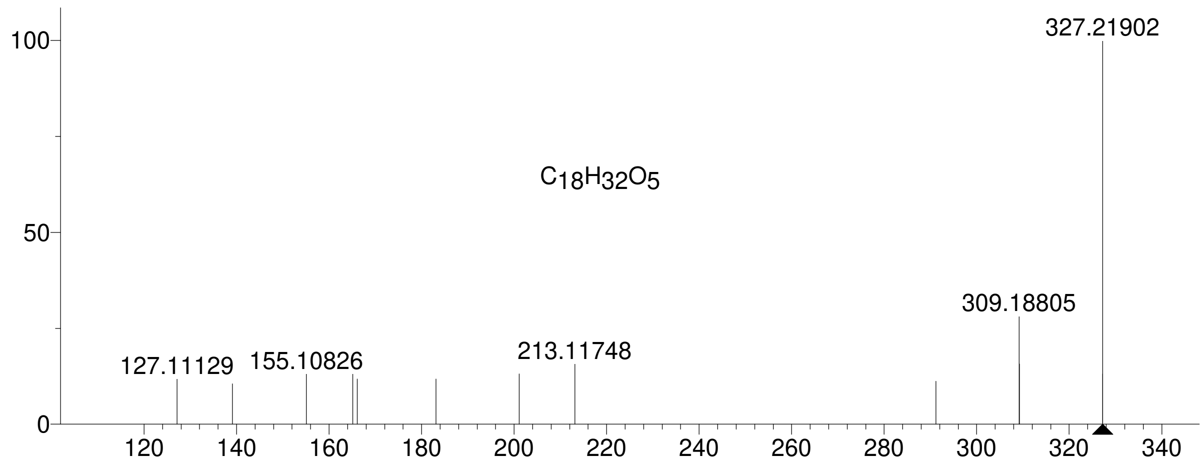

Hit 3 : FA 18:2+3O; PlaSMA ID-717 [M-H]- P=327.2
C18H32O5; MF: 201; RMF: 549; Prob 20.1%; Lib: biomsms-neg-plasma; ID: 436.

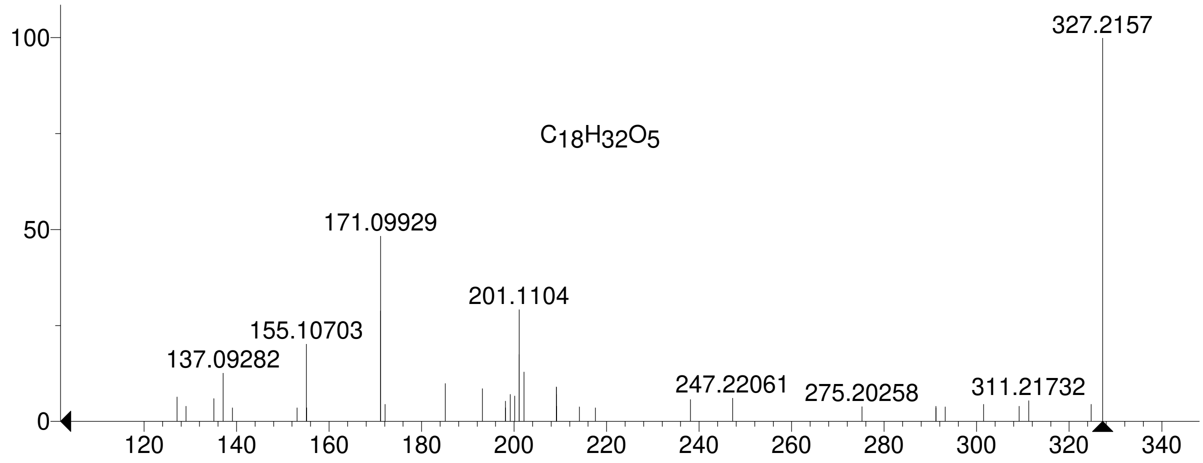

Hit 4 : 9,12,13-Trihydroxy-10(E),15(Z)-octadecadienoic acid () [M-H]- P=327.2
C18H32O5; MF: 188; RMF: 516; Prob 13.0%; Lib: phytofrag2018_final_060219; ID: 128.

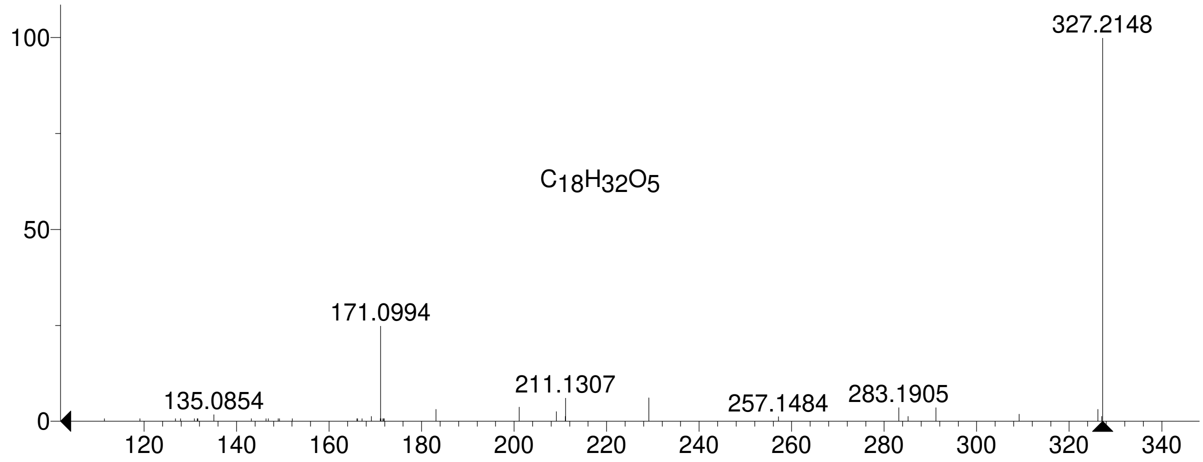

Hit 5 : FA 18:2+3O; PlaSMA ID-720 [M-H]- P=327.2
C18H32O5; MF: 156; RMF: 381; Prob 3.46%; Lib: biomsms-neg-plasma; ID: 434.

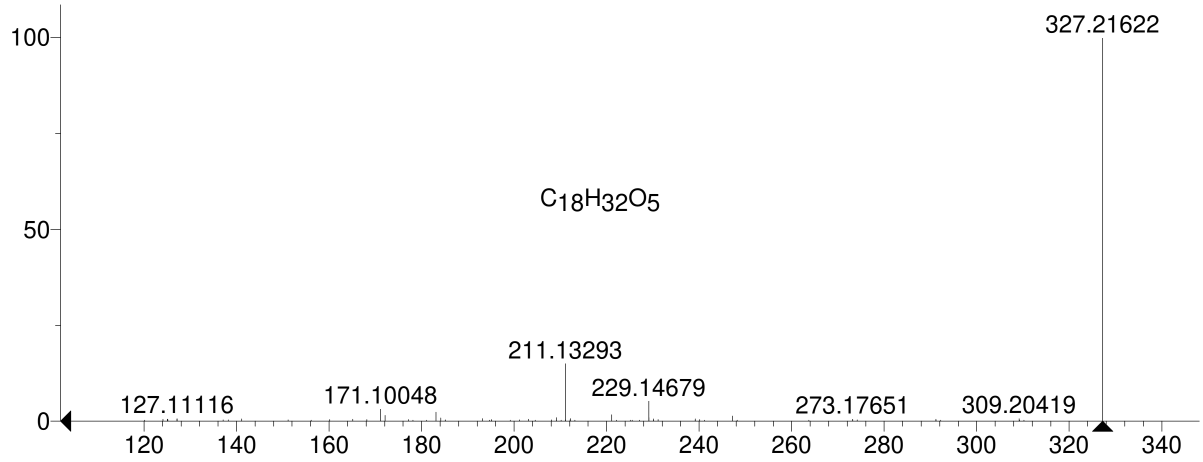

Unknown: Unknown (21.37_290.1390m/z)
Compound in Library Factor = -2387

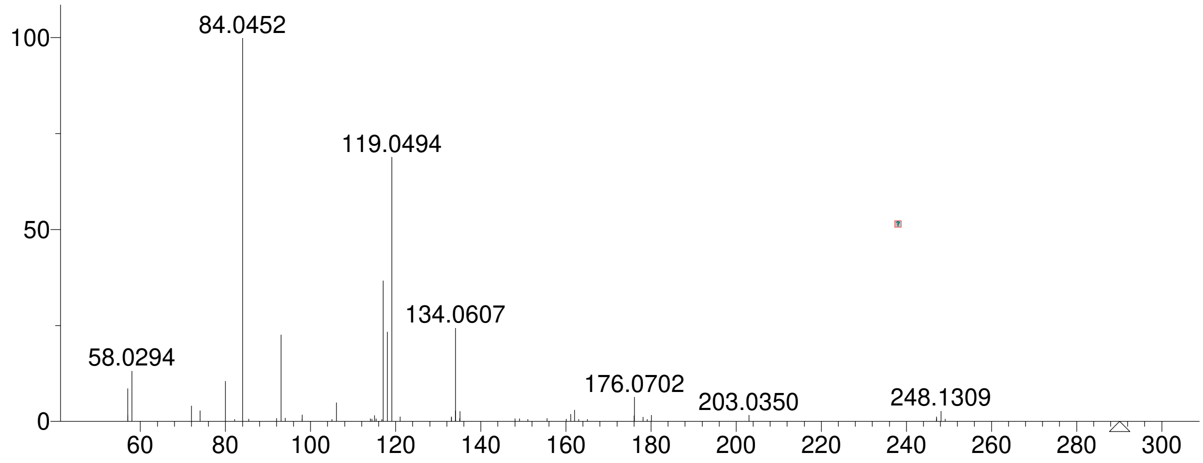

Hit 1 : Benzoylecgonine [M+H]+ 50eV P=290.1
C16H19NO4; MF: 31; RMF: 361; Prob 49.1%; Lib: mona-export-massbank; ID: 526.

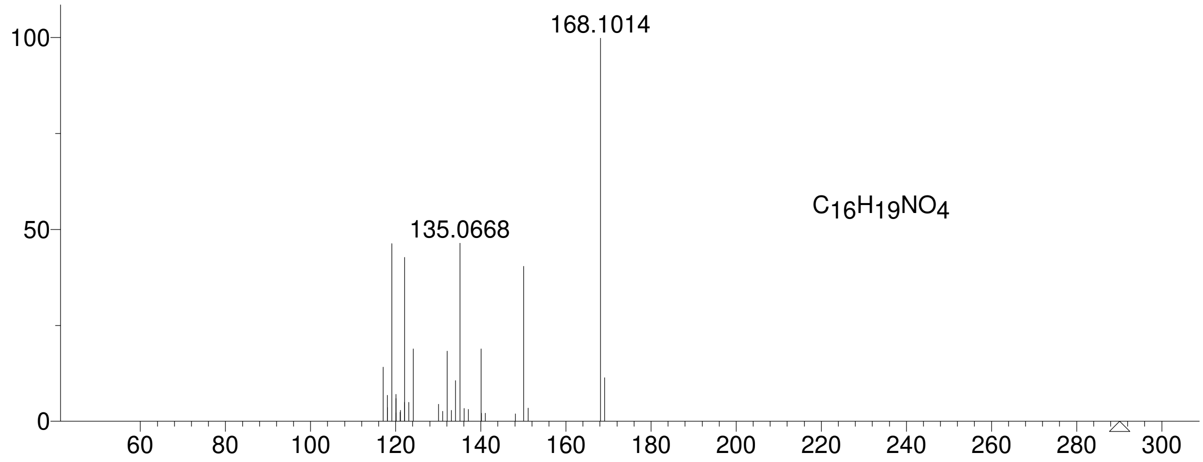

Hit 2 : Norcocaine [M+H]+ HCD 34V P=290.1
C16H19NO4; MF: 10; RMF: 137; Prob 20.9%; CAS: 18717-72-1; Lib: nist_msms; ID: 325503.

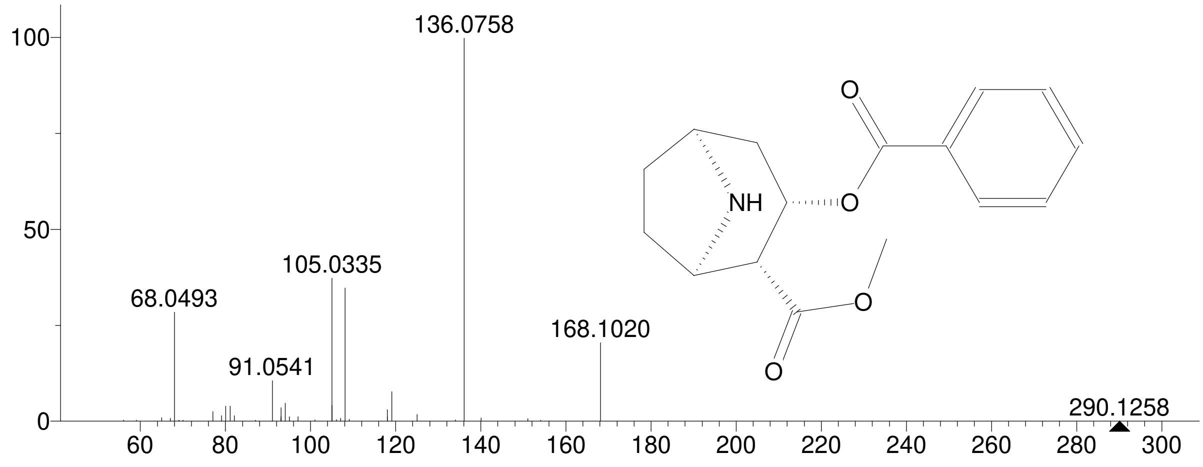

Hit 3 : OPHTHALMIC ACID [M+H]+ P=290.1
C11H19N3O6; MF: 2; RMF: 123; Prob 15.6%; Lib: mona-export-gnps; ID: 5471.

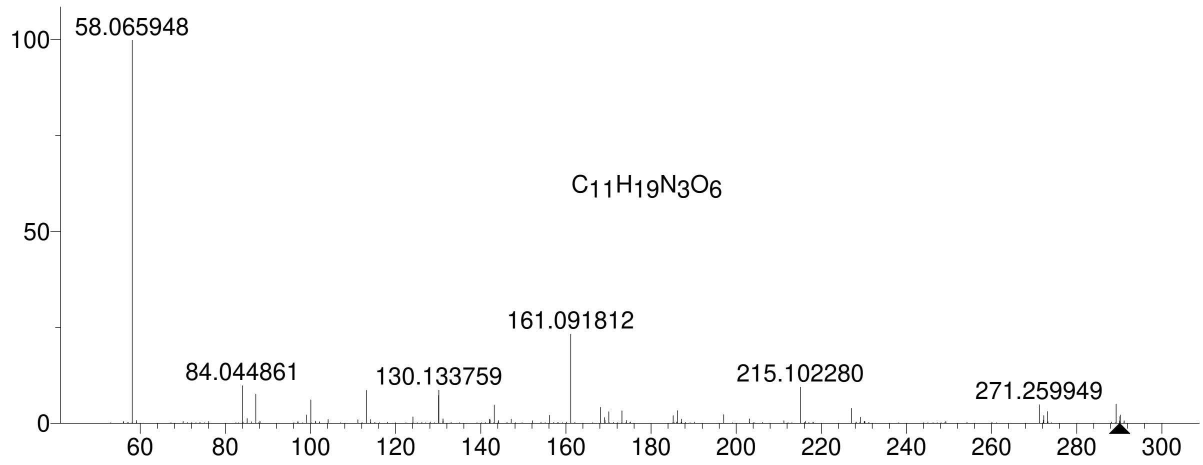

Hit 4 : Anabasamine Hydrochloride [M+H]+ 65HCD P=290.1
C16H20ClN3; MF: 0; RMF: 72; Prob 14.4%; Lib: mona-export-vf-npl_qexactive; ID: 26781.

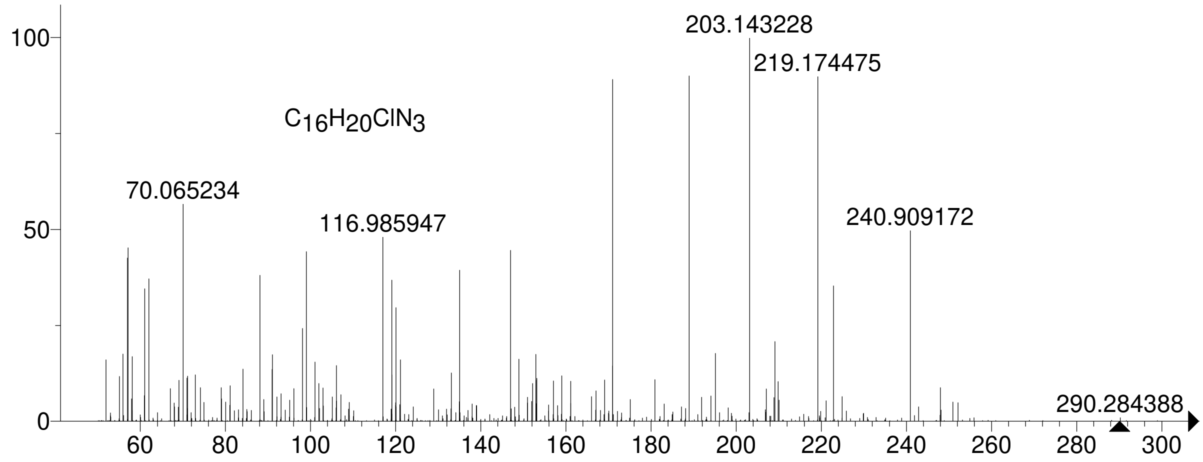

Unknown: Unknown (21.39_349.1281m/z)
Compound in Library Factor = -3273

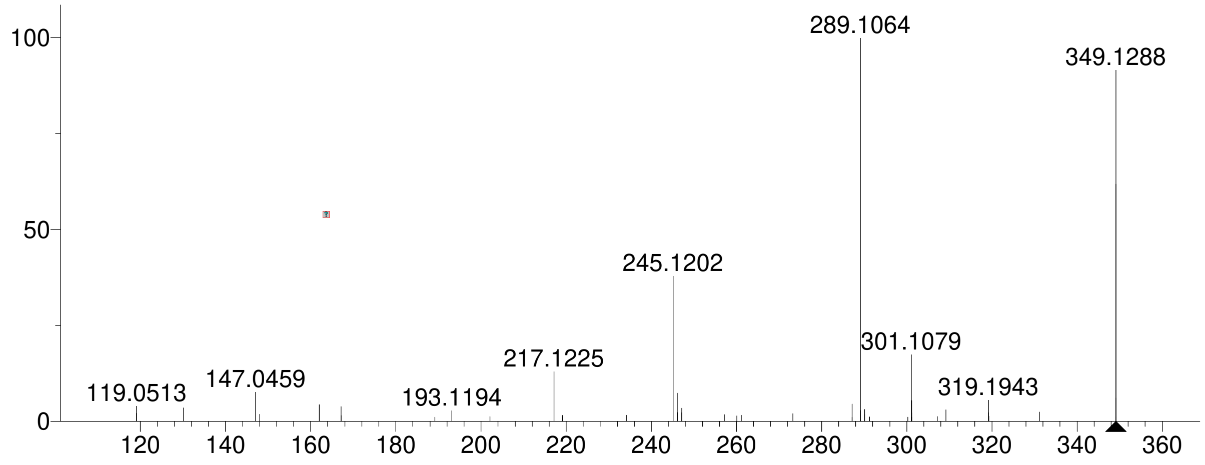

Hit 1 : Phenol, 3-[4-(4-morpholinyl)pyrido[3',2':4,5]furo[3,2-d]pyrimidin-2-yl]- [M+H]+ HCD 31V P=349.1
C19H16N4O3; MF: 12; RMF: 388; Prob 38.1%; CAS: 371935-74-9; Lib: nist_msms; ID: 375610.

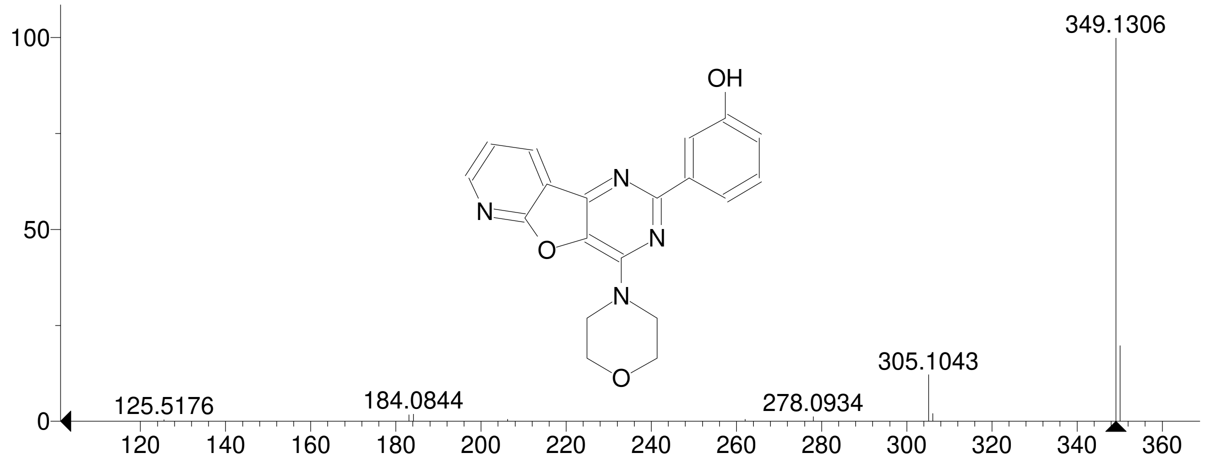

Hit 2 : NCGC00380409-01_C16H22O7_(4E)-7-Acetoxy-6-hydroxy-2-methyl-10-oxo-3,6,7,8,9,10-hexahydro-2H-o
C16H22O7; MF: 8; RMF: 392; Prob 32.2%; Lib: mona-export-gnps; ID: 12852.

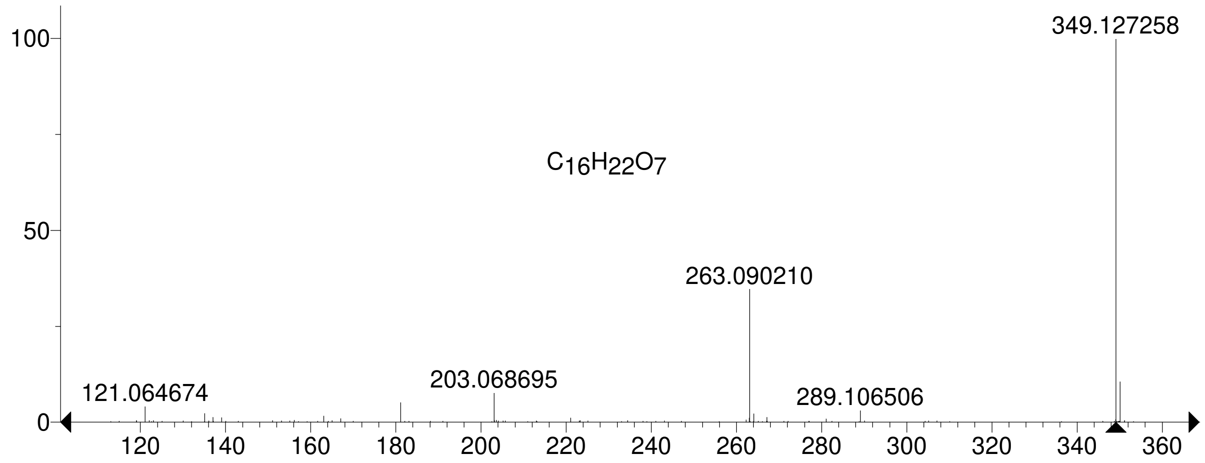

Hit 3 : [(4E)-7-acetyloxy-6-hydroxy-2-methyl-10-oxo-2,3,6,7,8,9-hexahydrooxecin-3-yl] (E)-but-2-enoate [M+Na]+
C16H22O7; MF: 6; RMF: 172; Prob 29.7%; Lib: mona-export-vf-npl_qexactive; ID: 1693.

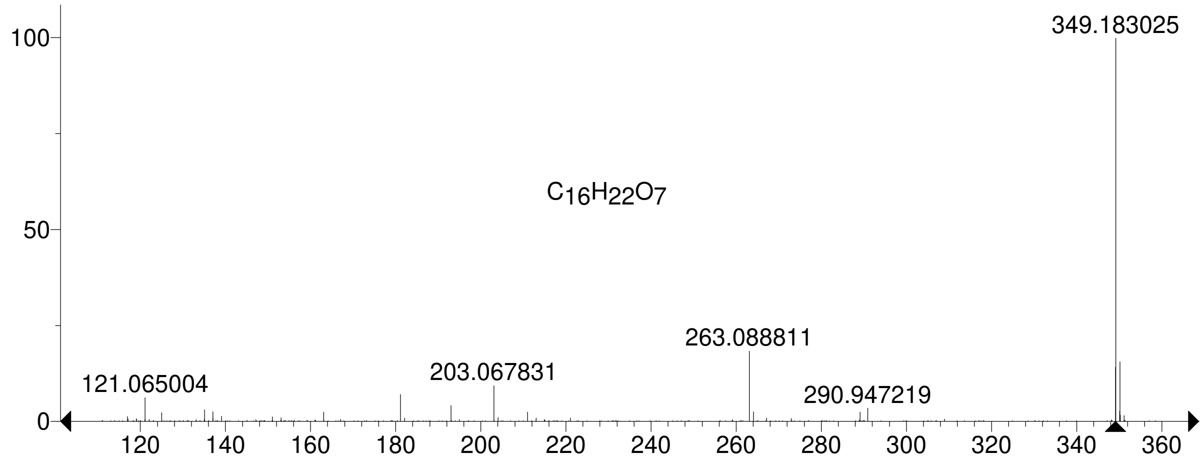

Unknown: Unknown (21.51_329.2325m/z)
Compound in Library Factor = -246

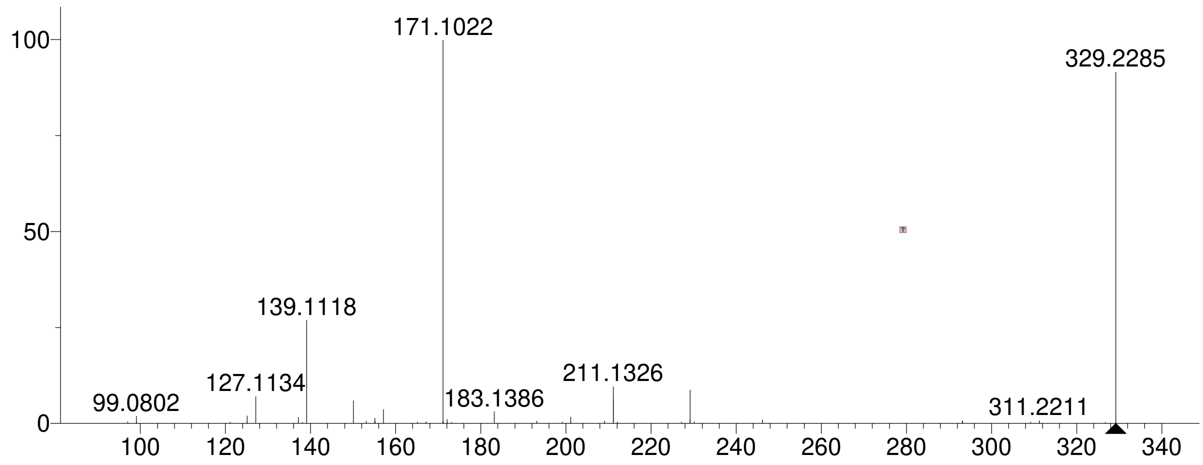

Hit 1 : FA 18:1+3O; PlaSMA ID-738 [M-H]- P=329.2
C18H34O5; MF: 588; RMF: 762; Prob 98.2%; Lib: biomsms-neg-plasma; ID: 444.

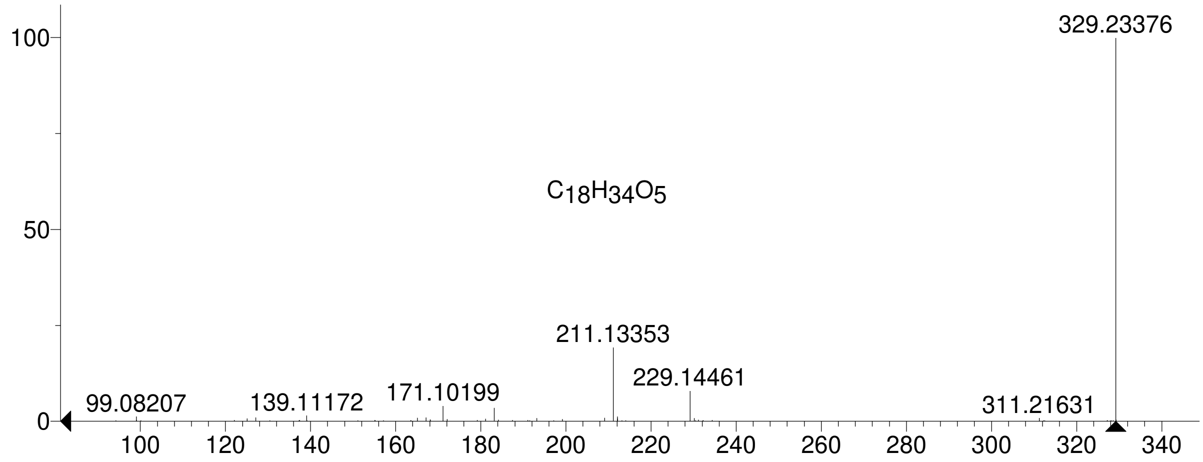

Hit 2 : FA 18:1+3O; PlaSMA ID-737 [M-H]- P=329.2
C18H34O5; MF: 402; RMF: 728; Prob 1.55%; Lib: biomsms-neg-plasma; ID: 448.

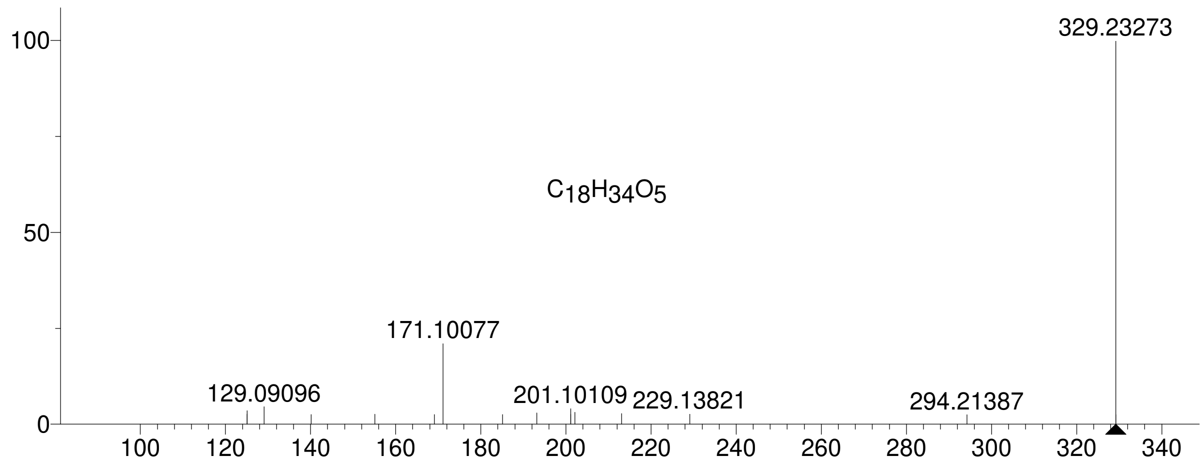

Hit 3 : FA 18:1+3O; PlaSMA ID-735 [M-H]- P=329.2
C18H34O5; MF: 323; RMF: 758; Prob 0.17%; Lib: biomsms-neg-plasma; ID: 443.

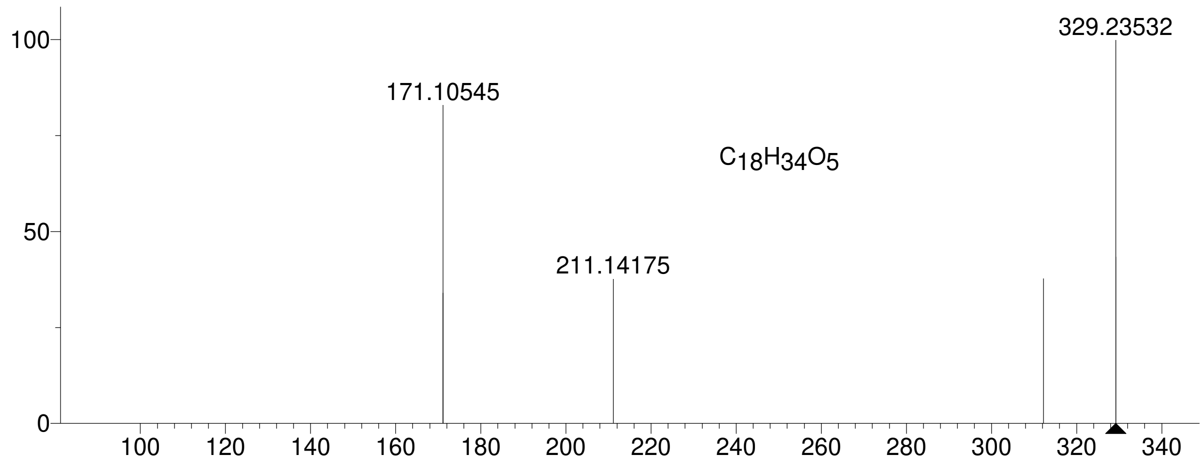

Hit 4 : 9,12,13-Trihydroxy-10(E)-octadecadienoic acid () [M-H]- P=329.2
C18H34O5; MF: 273; RMF: 620; Prob 0.03%; Lib: phytofrag2018_final_060219; ID: 129.

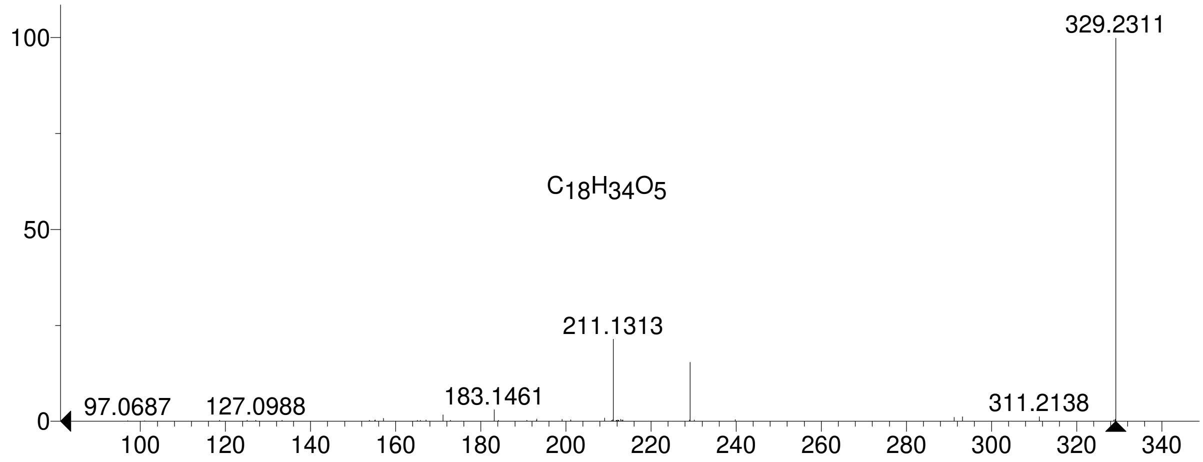

Hit 5 : FA 18:1+3O; PlaSMA ID-739 [M-H]- P=329.2
C18H34O5; MF: 111; RMF: 533; Prob 0.00%; Lib: biomsms-neg-plasma; ID: 446.

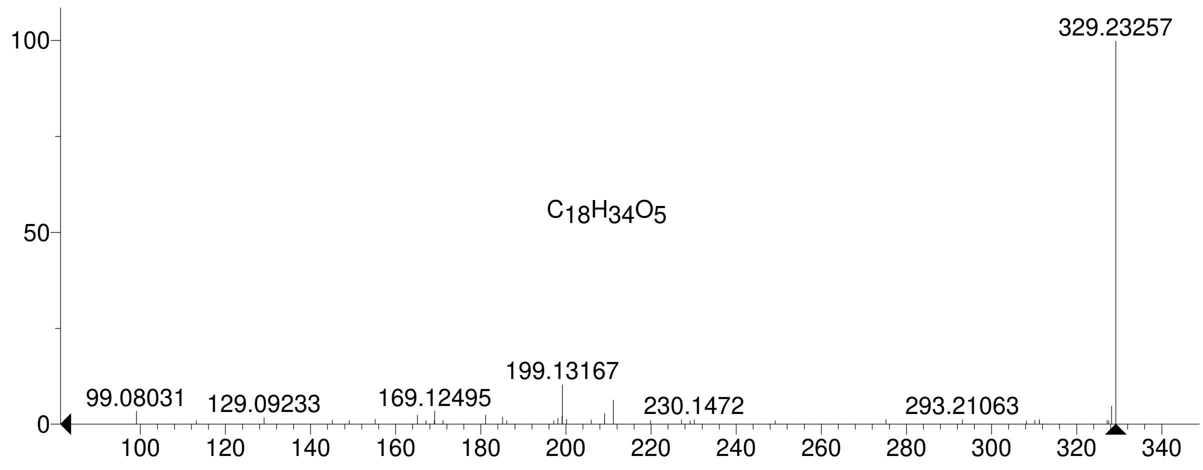

Unknown: Unknown (21.52_522.2801m/z)
Compound in Library Factor = 0

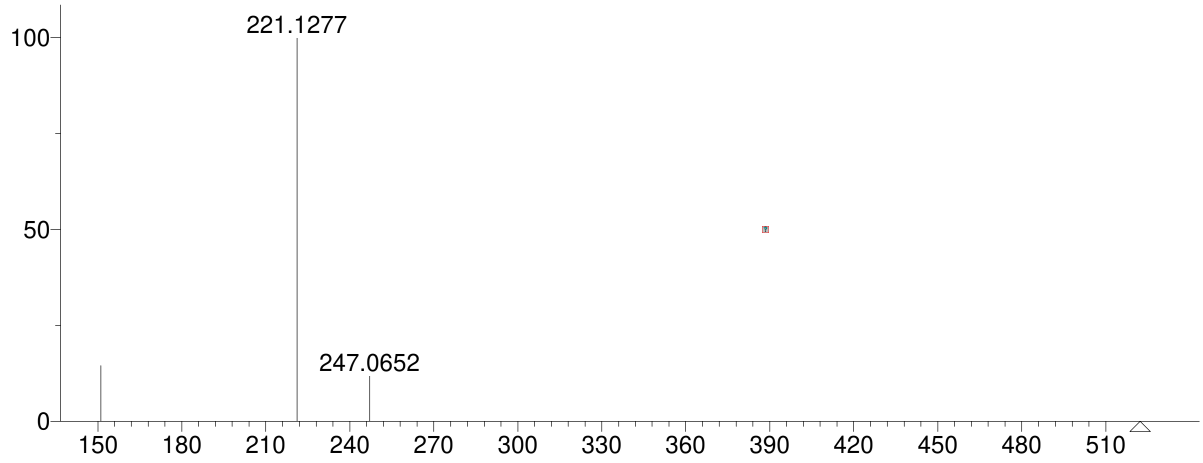

Unknown: Unknown (21.54_257.1494m/z)
Compound in Library Factor = 0

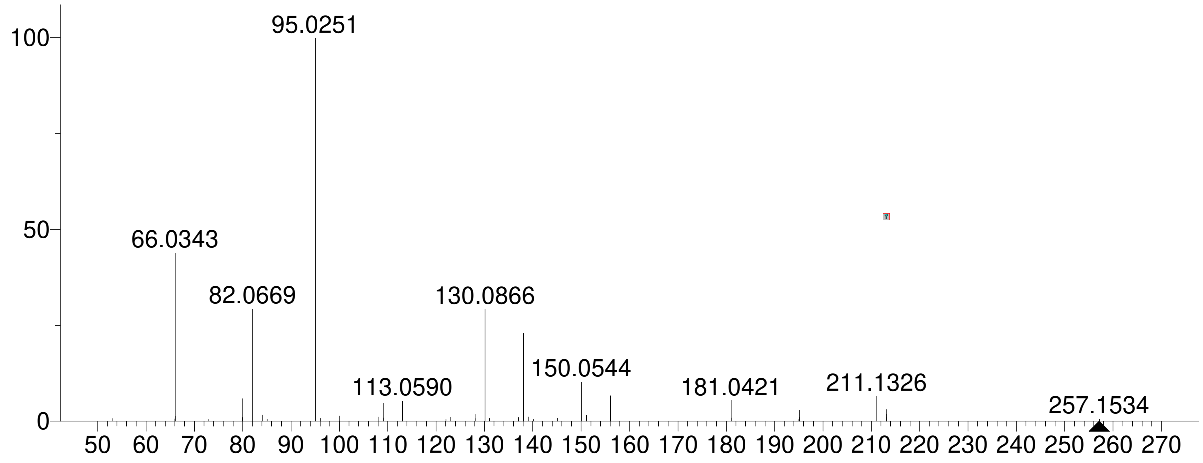

Hit 1 : NCGC00380601-01!N-[1-(4-methoxy-6-oxopyran-2-yl)-2-methylpropyl]acetamide [M+NH4]+ P=257.2
C12H17NO4; MF: 0; RMF: 44; Prob 20.0%; Lib: mona-export-gnps; ID: 9337.

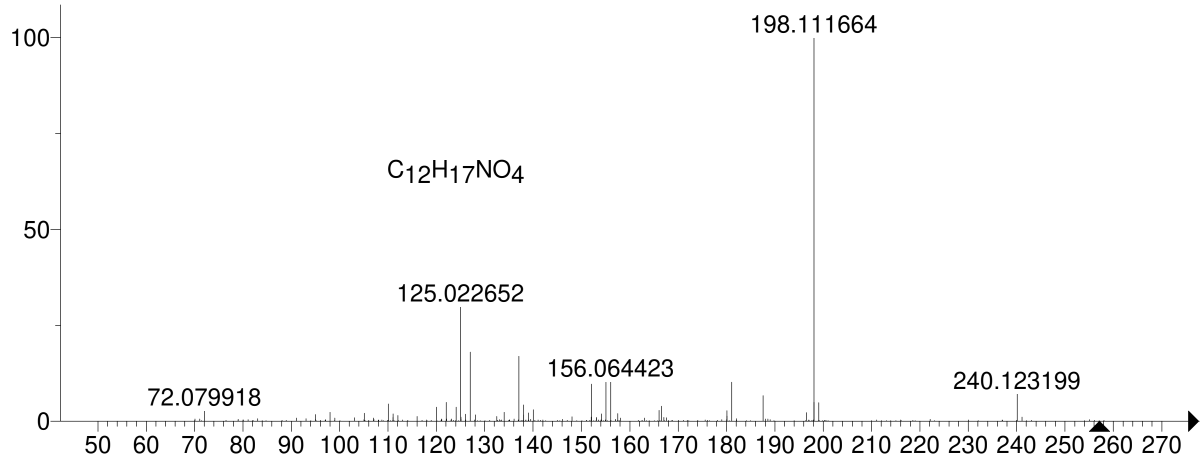

Hit 2 : NCGC00385875-01_C15H22O2_(1R,4Z,9S)-4-(Hydroxymethyl)-11,11-dimethyl-8-methylenebicyclo[7.2.0]u
C15H22O2; MF: 0; RMF: 17; Prob 20.0%; Lib: mona-export-gnps; ID: 10431.

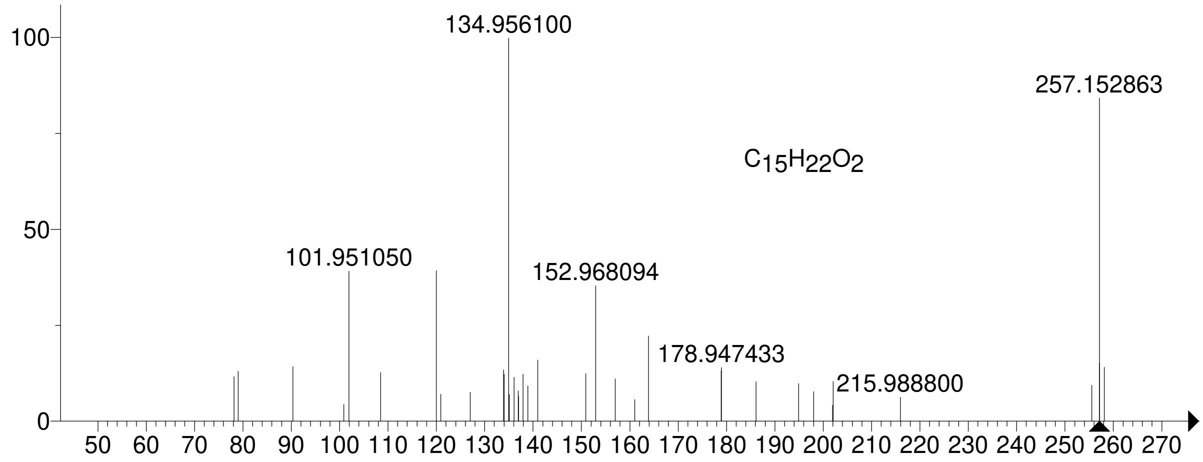

Hit 3 : (4S,5Z,6S)-4-(2-methoxy-2-oxoethyl)-5-[2-[(E)-3-phenylprop-2-enoyl]oxyethylidene]-6-[(2S,3R,4S,5S,6R)-3
C15H22O2; MF: 0; RMF: 10; Prob 20.0%; Lib: mona-export-vf-npl_qexactive; ID: 9355.

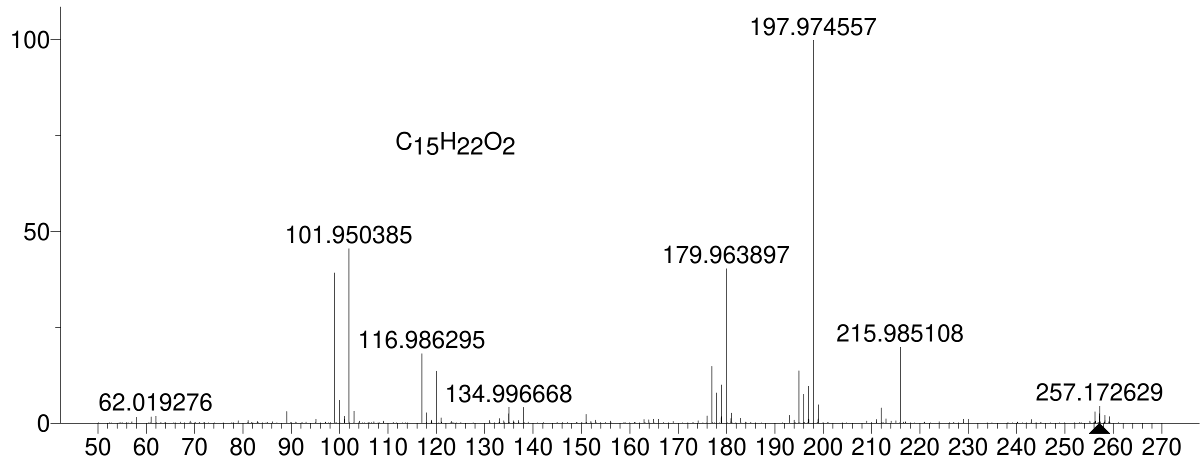

Hit 4 : (1R,4Z,9S)-4-(hydroxymethyl)-11,11-dimethyl-8-methylidenebicyclo[7.2.0]undec-4-en-3-one [M+Na]+ 35H
C15H22O2; MF: 0; RMF: 7; Prob 20.0%; Lib: mona-export-vf-npl_qexactive; ID: 21136.

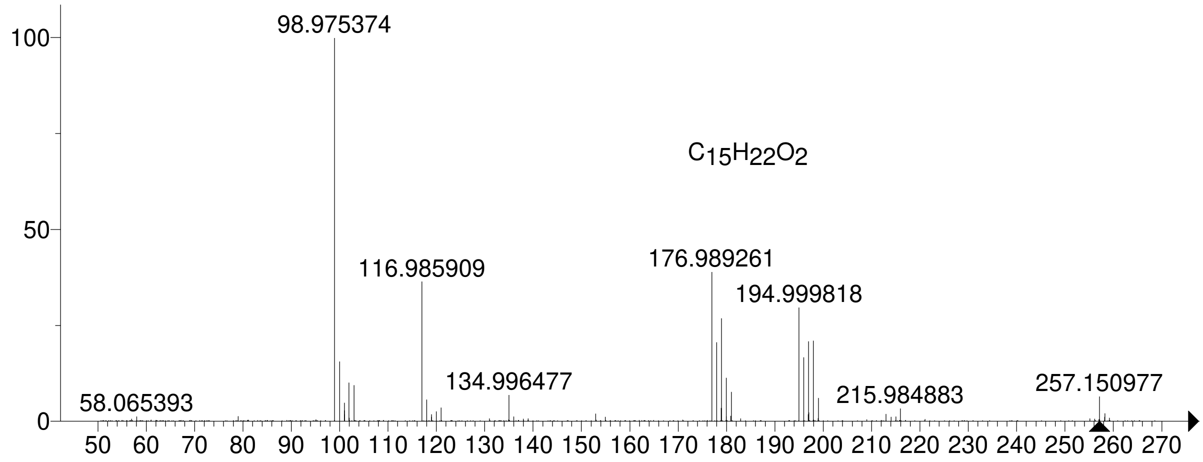

Hit 5 : Curcumenol [M+Na]+ 35HCD P=257.2
C15H22O2; MF: 0; RMF: 6; Prob 20.0%; Lib: mona-export-vf-npl_qexactive; ID: 18640.

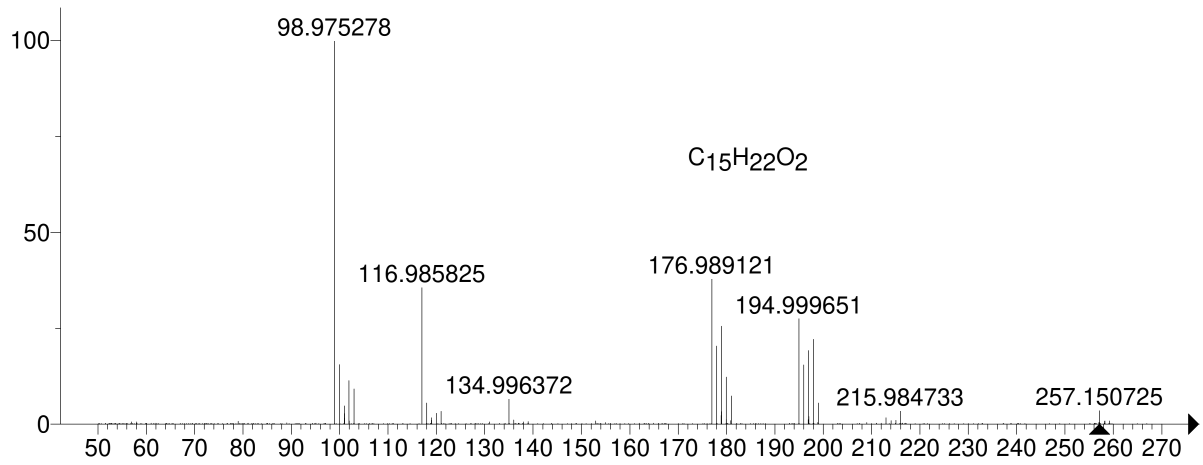

Unknown: Unknown (21.55_532.2172m/z)
Compound in Library Factor = 0

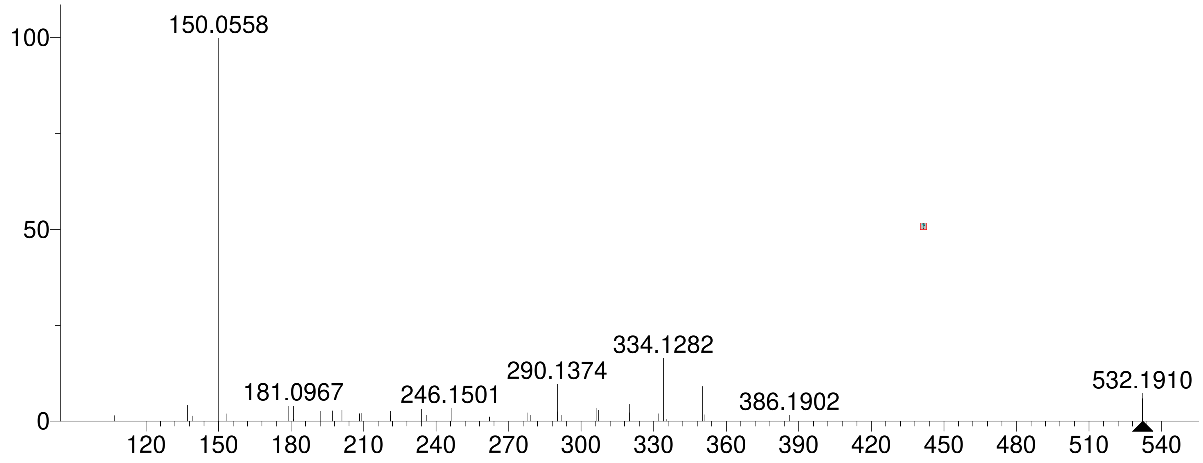

Unknown: Unknown (21.57_647.3769m/z)
Compound in Library Factor = -165

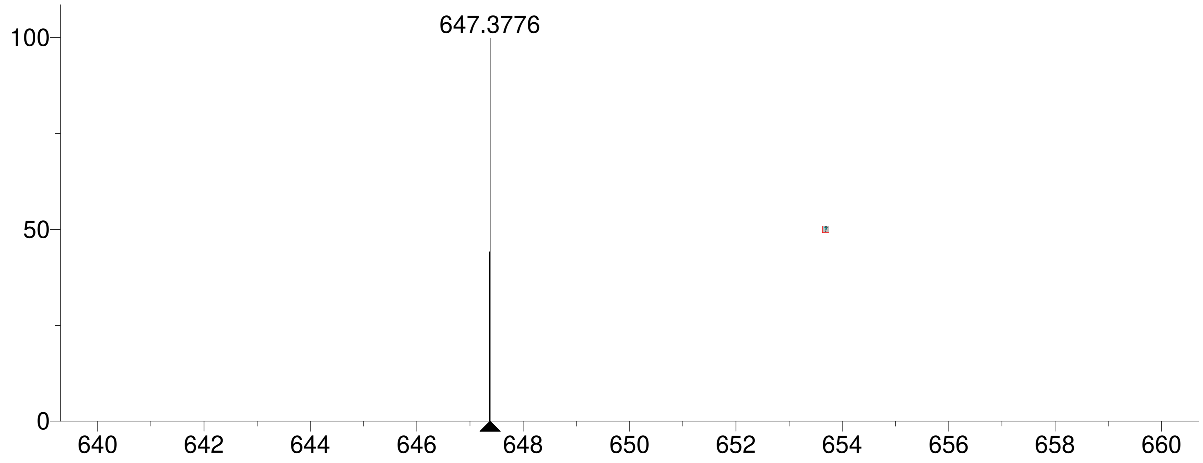

Hit 1 : Hymeglusin [2M-H]- HCD 25V P=647.4
C18H28O5; MF: 569; RMF: 946; Prob 96.8%; CAS: 29066-42-0; Lib: nist_msms; ID: 376632.

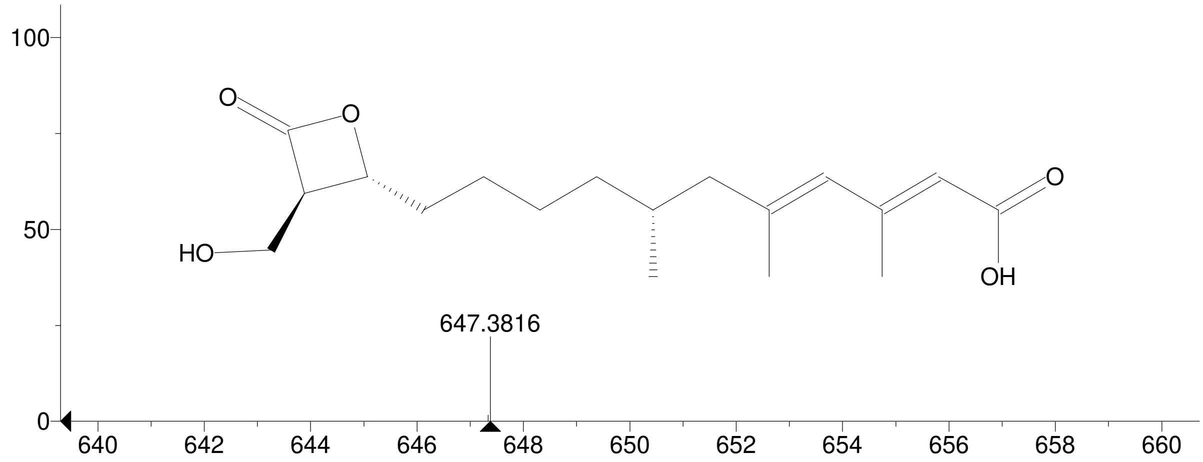

Hit 2 : Unknown (carbon number 36); PlaSMA ID-1785 [M-H]- P=647.4
C36H56O10; MF: 290; RMF: 833; Prob 0.96%; Lib: biomsms-neg-plasma; ID: 1567.

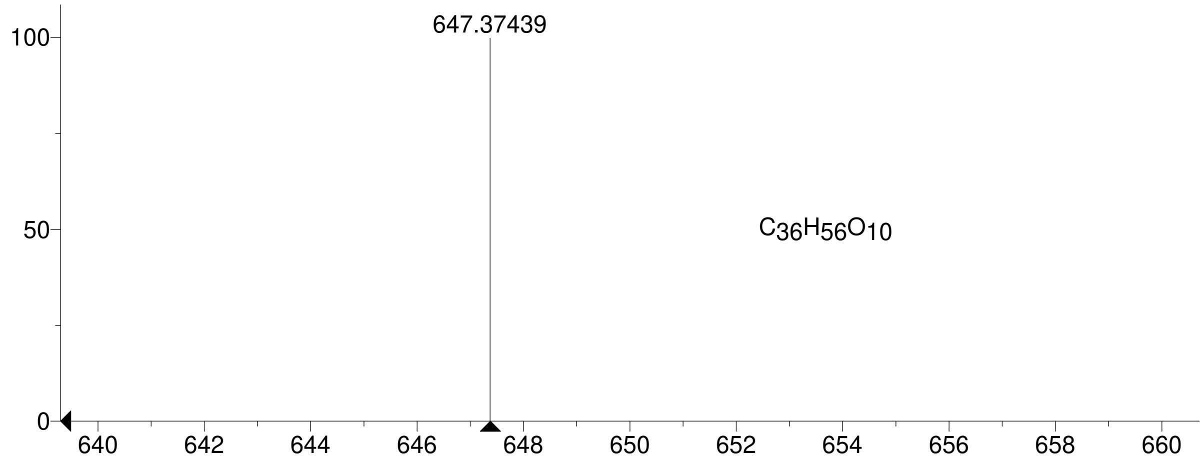

Hit 3 : PA 33:7 [M-H]- 40eV P=647.4
C36H57O8P; MF: 290; RMF: 833; Prob 0.96%; Lib: mona-export-in-silico_spectra; ID: 59358.

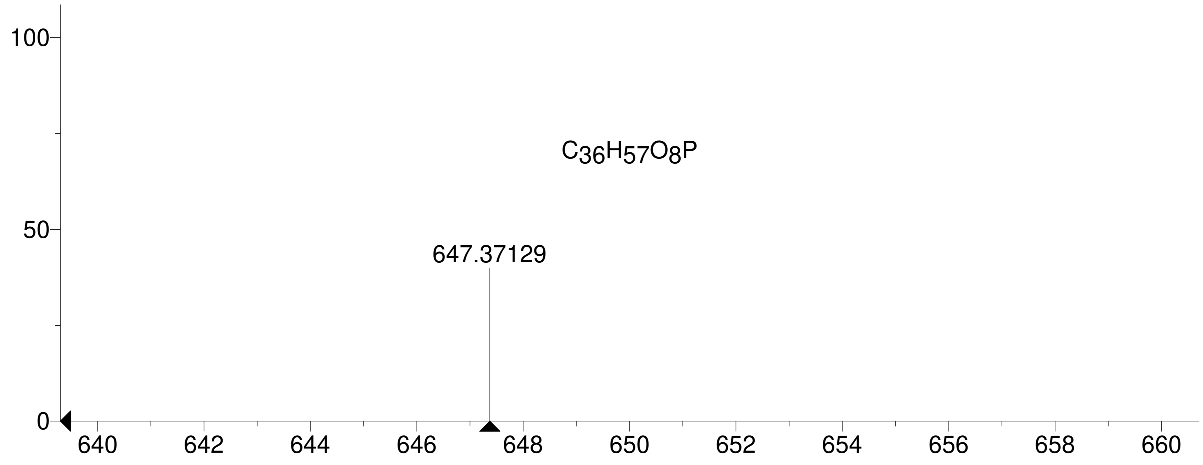

Hit 4 : PMeOH 32:7; PMeOH 16:3-16:4; [M-H]- [M-H]- P=647.4
C36H57O8P; MF: 290; RMF: 833; Prob 0.96%; Lib: msdial-insilicomsms-lipids-neg; ID: 328815.

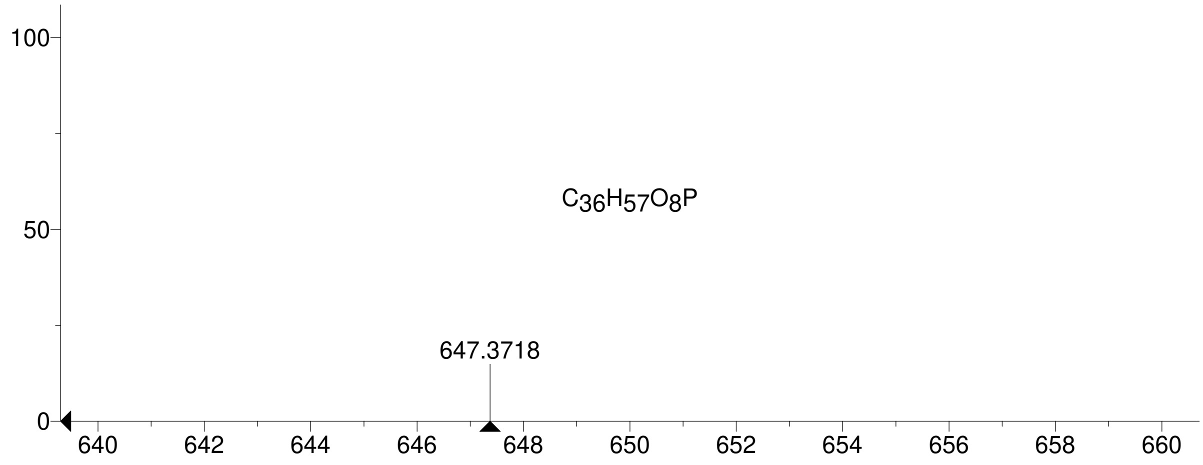

Hit 5 : NCGC00381031-01_C36H56O10_Olean-12-en-28-oic acid, 3-(beta-D-glucopyranuronosyloxy)-23-hydroxy
C36H56O10; MF: 251; RMF: 828; Prob 0.22%; Lib: msms-neg-gnps; ID: 1840.

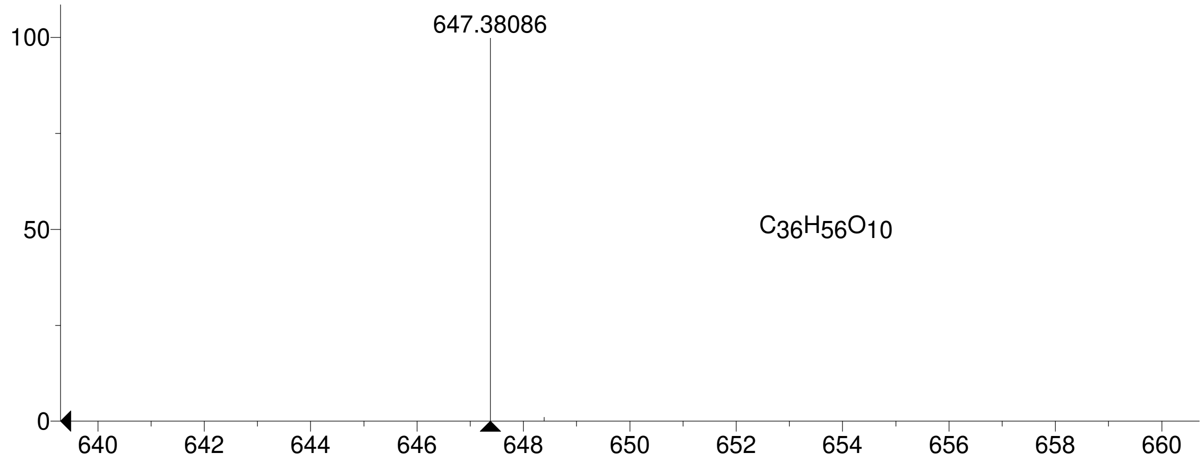

Unknown: Unknown (21.62_411.9511m/z)
Compound in Library Factor = 0

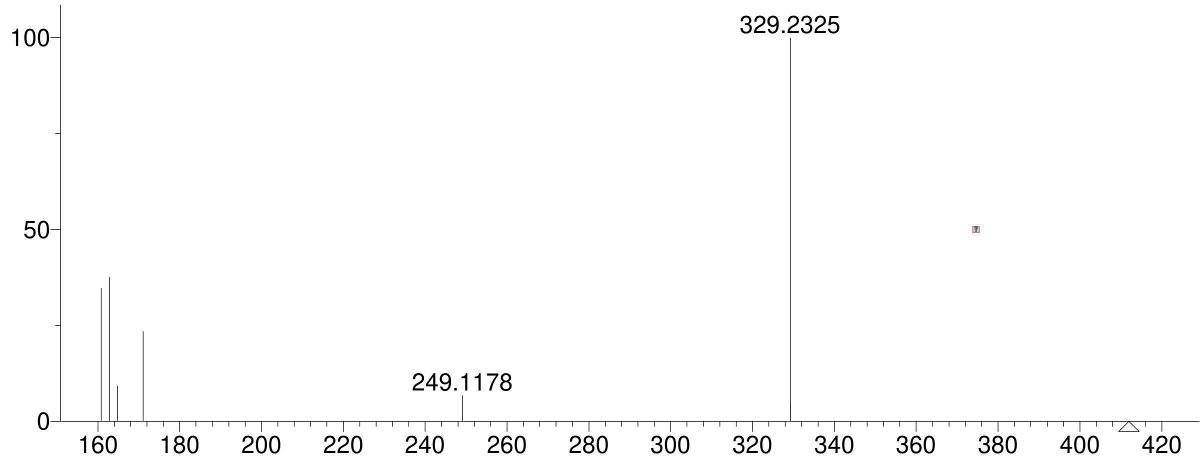

Unknown: Unknown (21.62_624.0987m/z)
Compound in Library Factor = 0

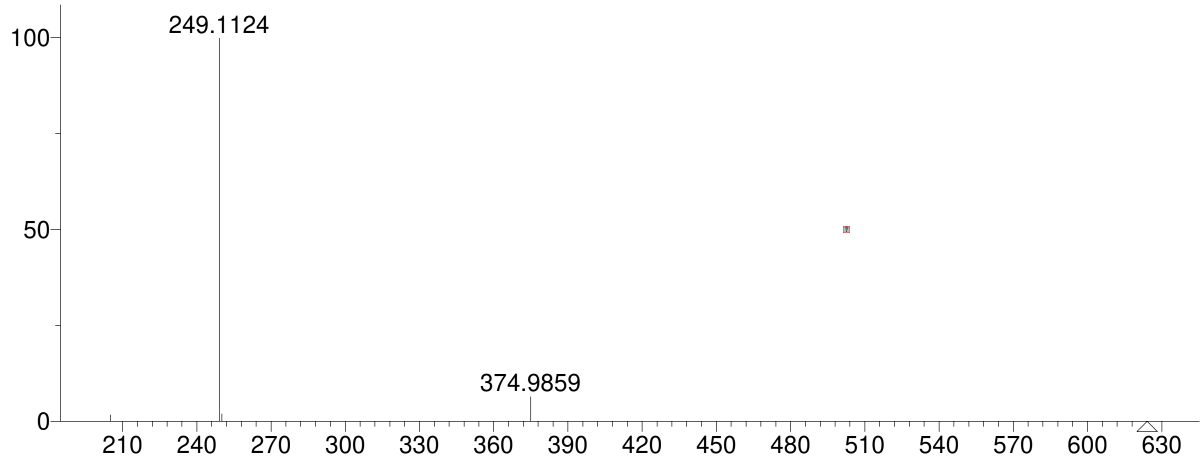

Unknown: Unknown (21.63_329.2325m/z)
Compound in Library Factor = -517

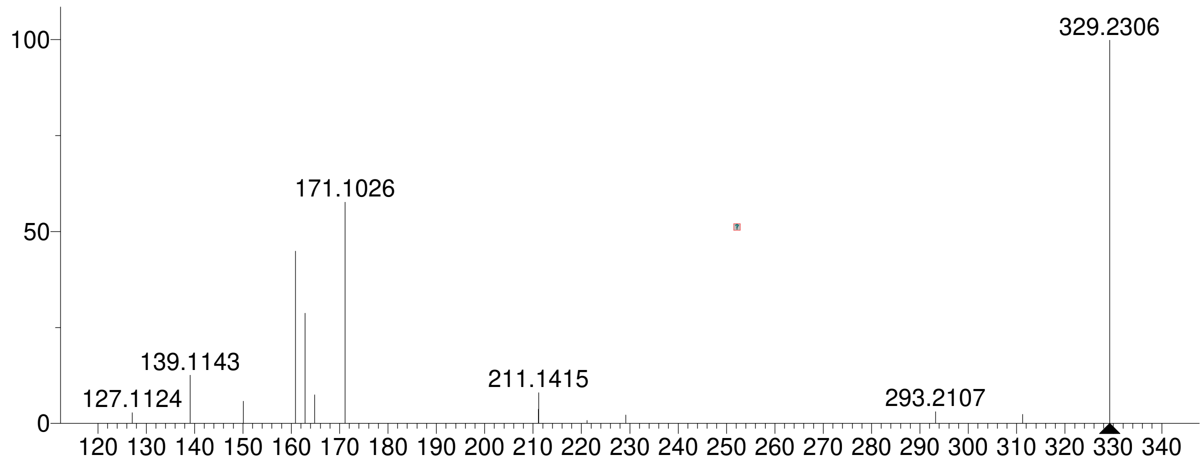

Hit 1 : FA 18:1+3O; PlaSMA ID-735 [M-H]- P=329.2
C18H34O5; MF: 234; RMF: 764; Prob 77.8%; Lib: biomsms-neg-plasma; ID: 443.

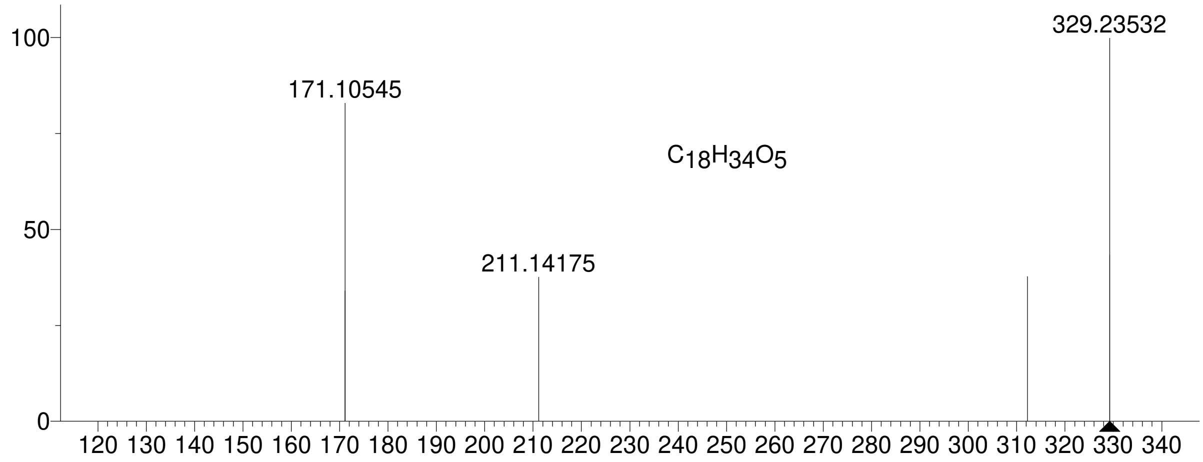

Hit 2 : FA 18:1+3O; PlaSMA ID-738 [M-H]- P=329.2
C18H34O5; MF: 201; RMF: 658; Prob 20.4%; Lib: biomsms-neg-plasma; ID: 444.

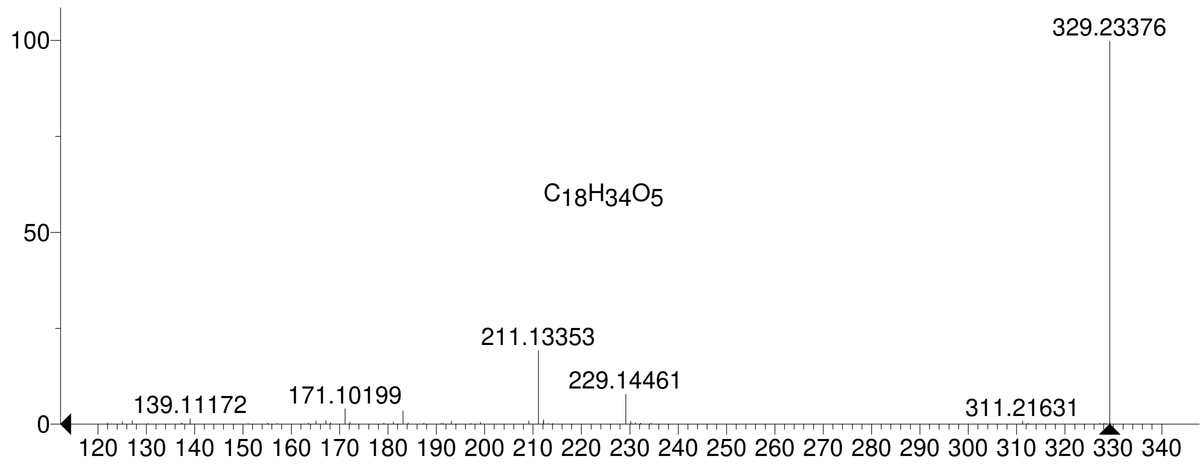

Hit 3 : FA 18:1+3O; PlaSMA ID-737 [M-H]- P=329.2
C18H34O5; MF: 74; RMF: 641; Prob 0.88%; Lib: biomsms-neg-plasma; ID: 448.

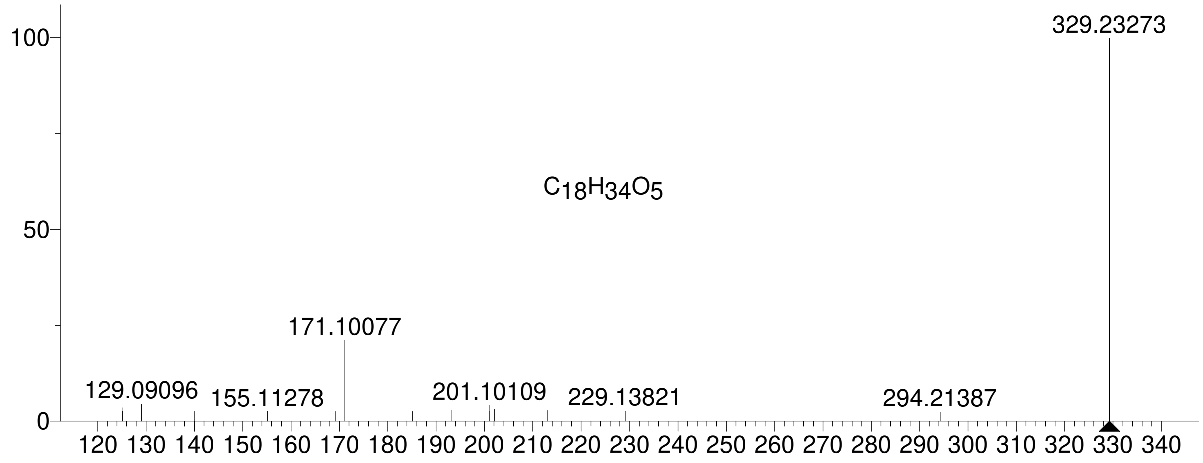

Hit 4 : FA 18:1+3O; PlaSMA ID-741 [M-H]- P=329.2
C18H34O5; MF: 56; RMF: 577; Prob 0.45%; Lib: biomsms-neg-plasma; ID: 449.

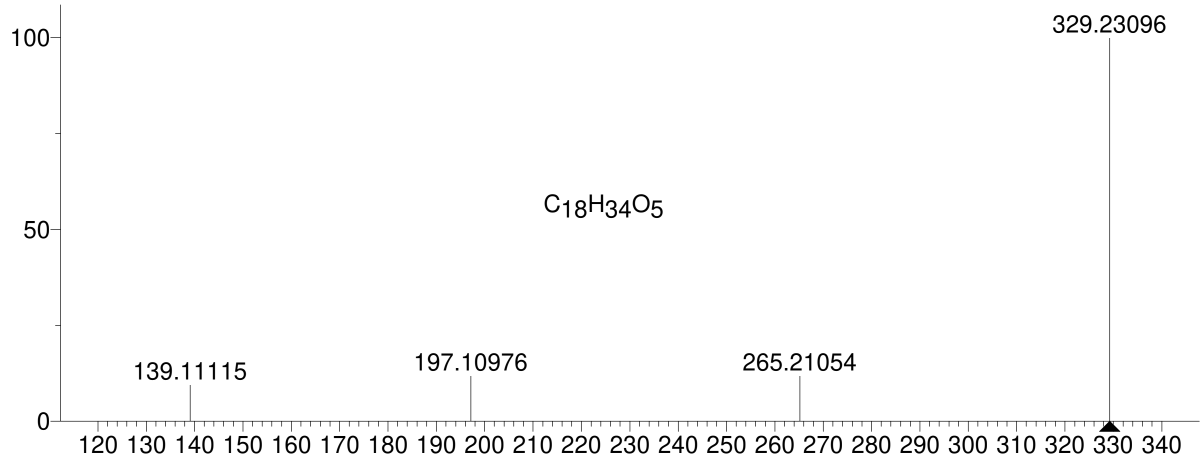

Hit 5 : Unknown (carbon number 18); PlaSMA ID-736 [M-H]- P=329.2
C18H34O5; MF: 30; RMF: 501; Prob 0.13%; Lib: biomsms-neg-plasma; ID: 1245.

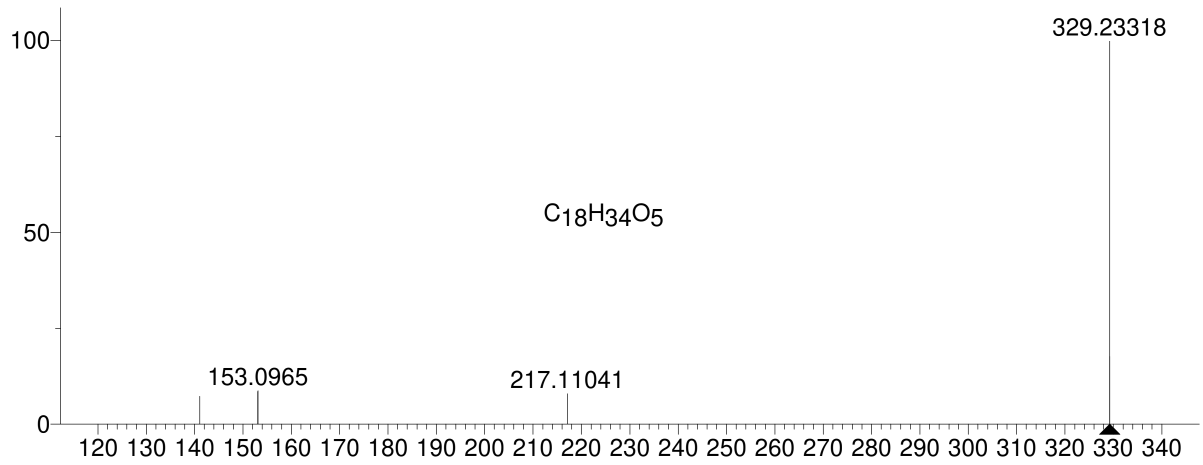

** Search Report Page 1 of 1 **

Unknown: Unknown (21.63_409.9537m/z)
Compound in Library Factor = 0

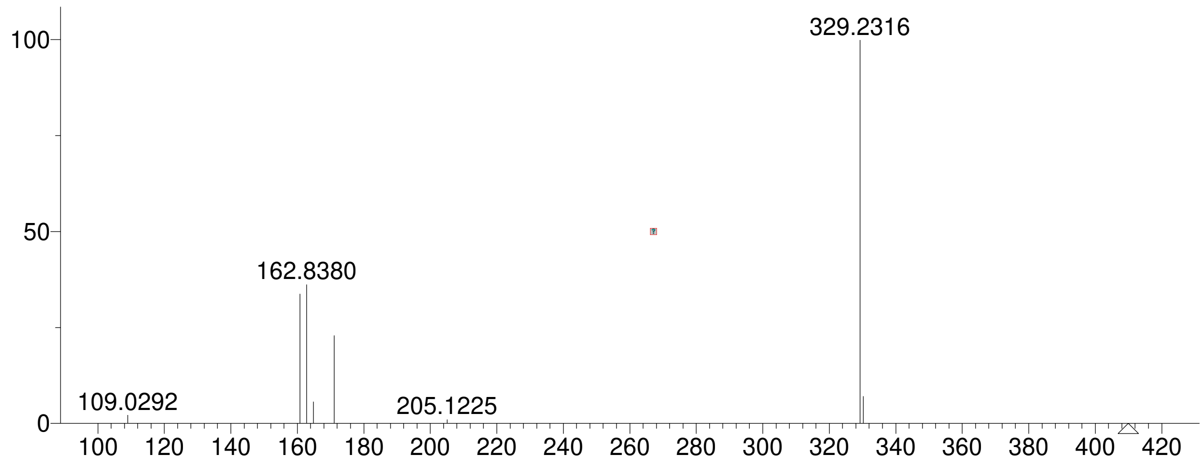

Unknown: Unknown (21.64_249.1126m/z)
Compound in Library Factor = -1072

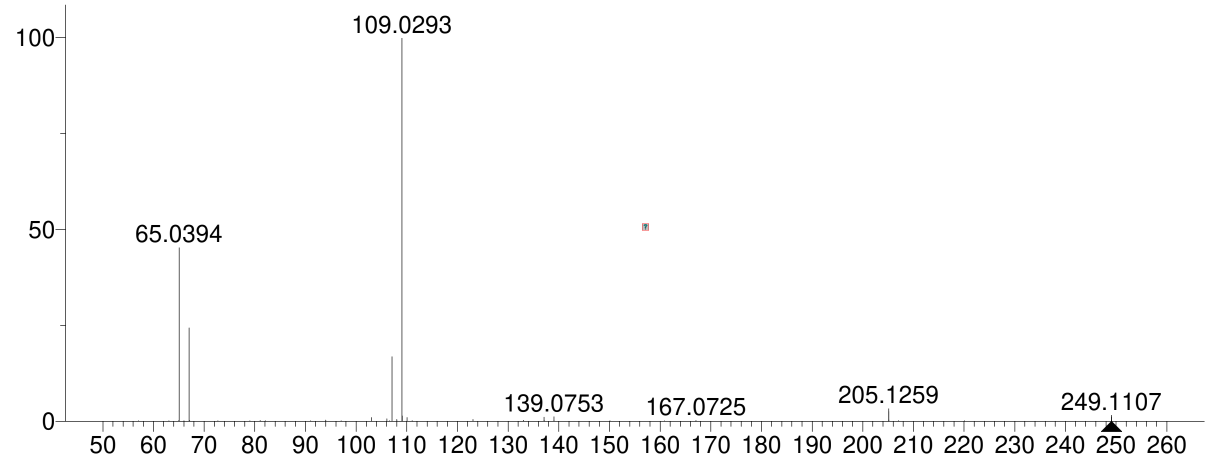

Hit 1 : 2,8-Dimethyl-2-(β-carboxyethyl)-6-hydroxychroman [M-H]- QTOF 4V P=249.1
$C_{14}H_{18}O_4$; MF: 150; RMF: 547; Prob 78.4%; CAS: 1221504-67-1; Lib: nist_msms; ID: 63168.

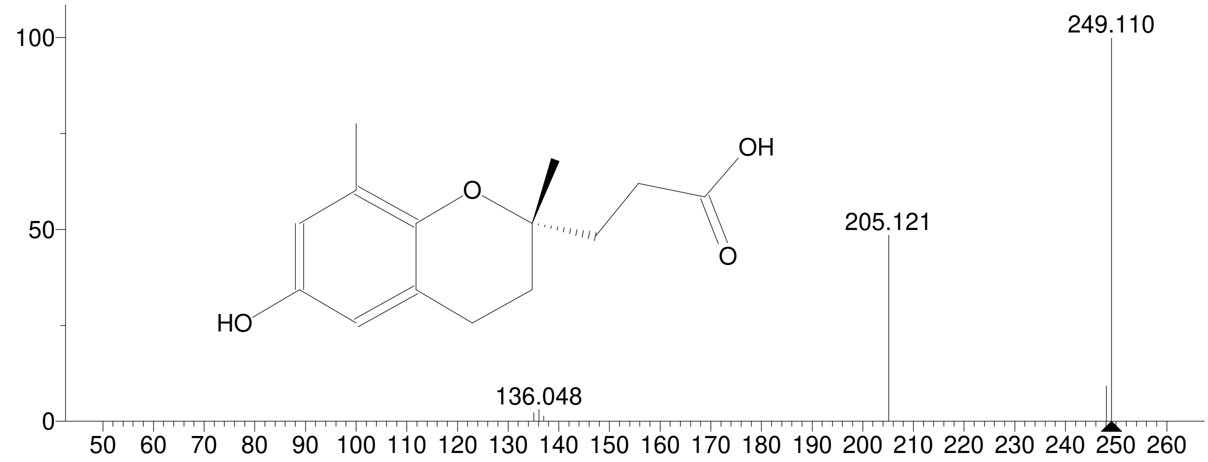

Hit 2 : Trolox [M-H]- HCD 9V P=249.1
$C_{14}H_{18}O_4$; MF: 77; RMF: 454; Prob 10.7%; CAS: 53188-07-1; Lib: nist_msms; ID: 159177.

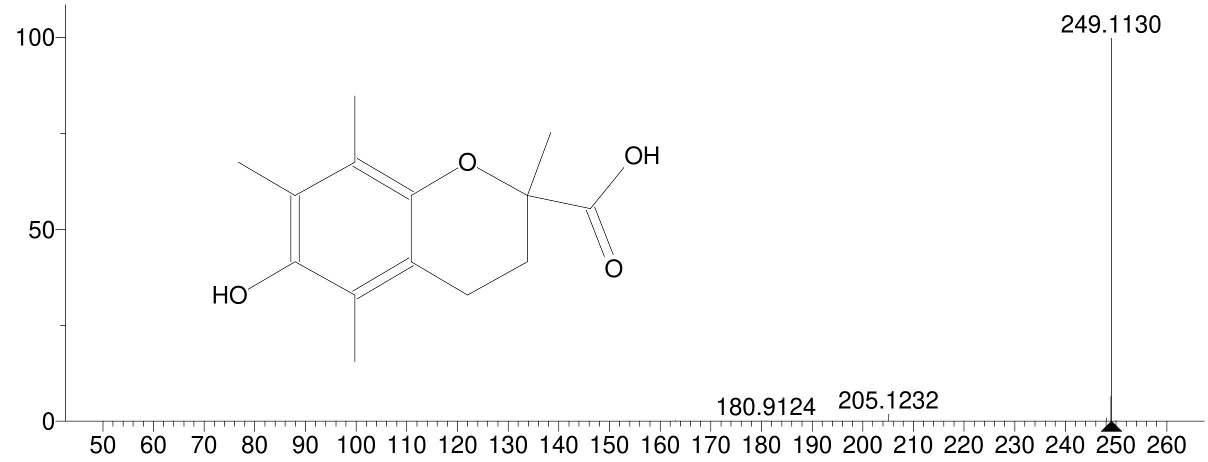

Hit 3 : 6,7-Diethoxy-4-methylcoumarin [M+H]+ HCD 79V P=249.1
C14H16O4; MF: 58; RMF: 313; Prob 5.18%; CAS: 314744-06-4; Lib: nist_msms; ID: 109458.

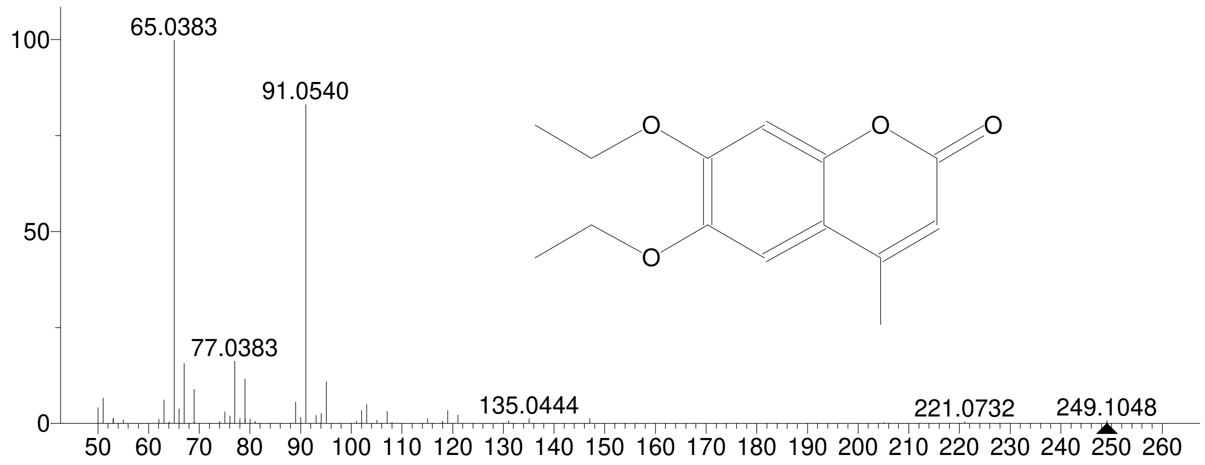

Hit 4 : Nevirapine [M+H-H2O]+ HCD 71V P=249.1
C15H14N4O; MF: 3; RMF: 68; Prob 1.00%; CAS: 129618-40-2; Lib: nist_msms; ID: 366892.

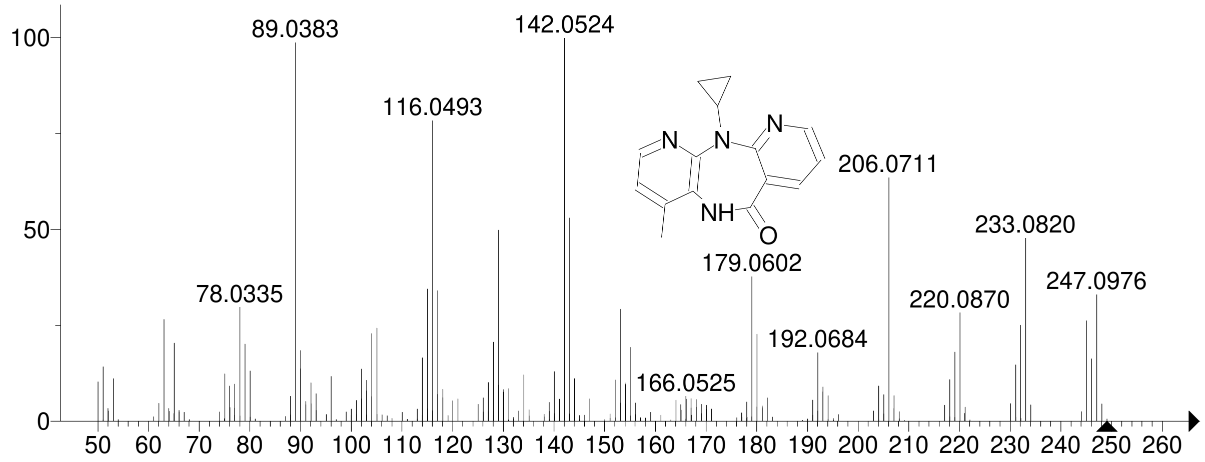

Hit 5 : (+)-2,5-epi-Goniothalesdiol [M+H-H2O]+ HCD 39V P=249.1
C14H18O5; MF: 2; RMF: 67; Prob 0.96%; Lib: nist_msms; ID: 94184.

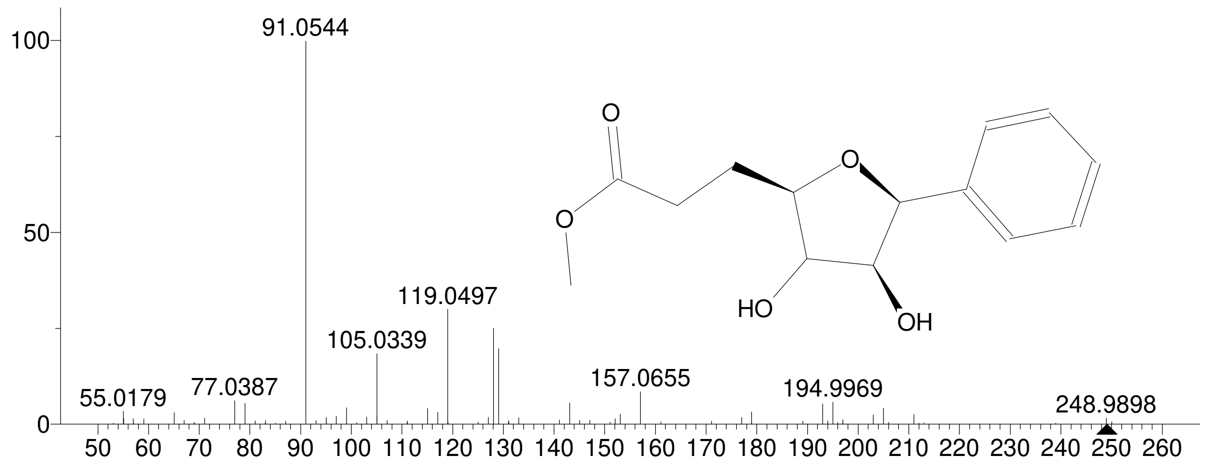

Unknown: Unknown (21.65_374.1960m/z)
Compound in Library Factor = -344

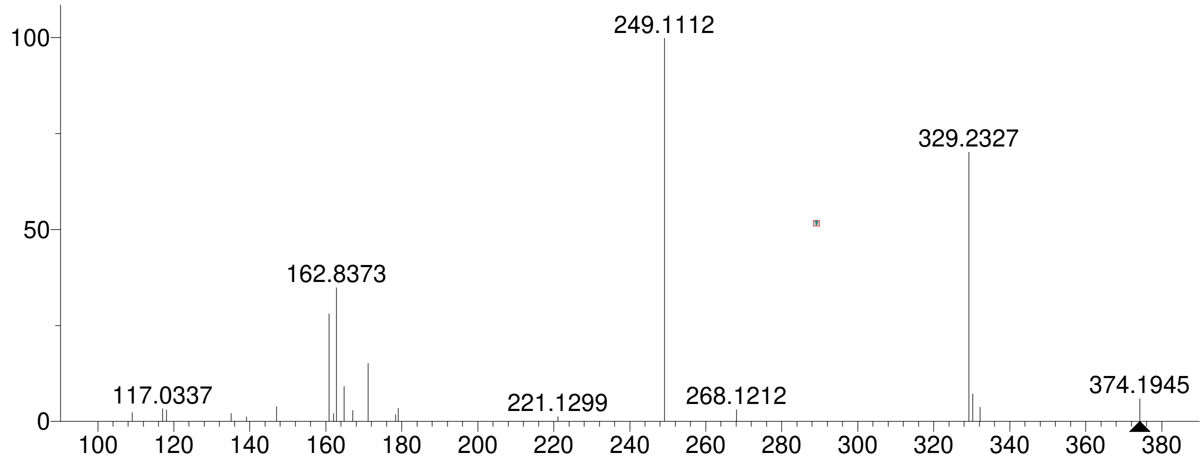

Hit 1 : Hetisine; PlaSMA ID-919 [M+FA-H]- P=374.2
C20H27NO3; MF: 364; RMF: 999; Prob 97.6%; Lib: biomsms-neg-plasma; ID: 214.

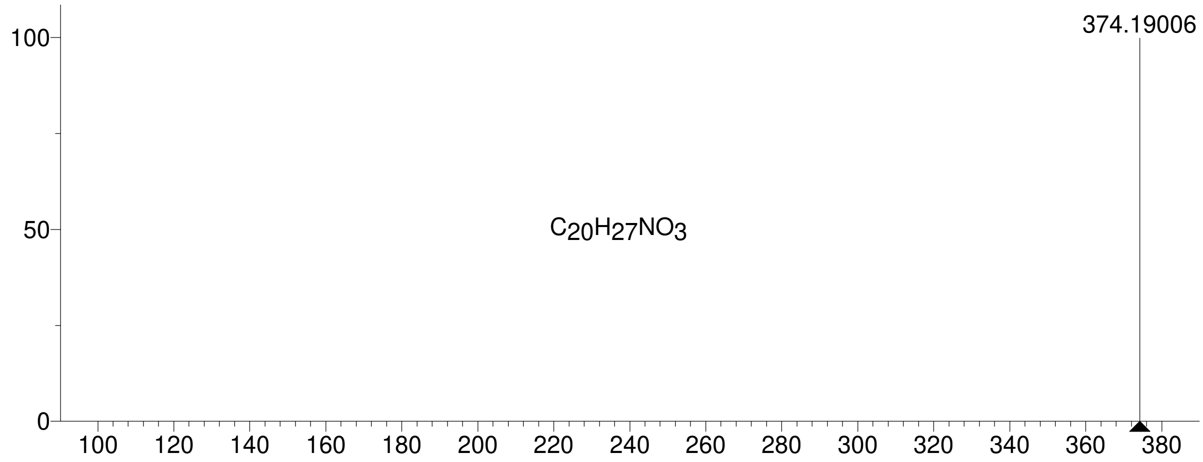

Hit 2 : Hetisine [M+FA-H]- 10 P=374.2
C20H27NO3; MF: 197; RMF: 840; Prob 2.26%; Lib: msms neg mona_ipb_gnps_plasma; ID: 18798.

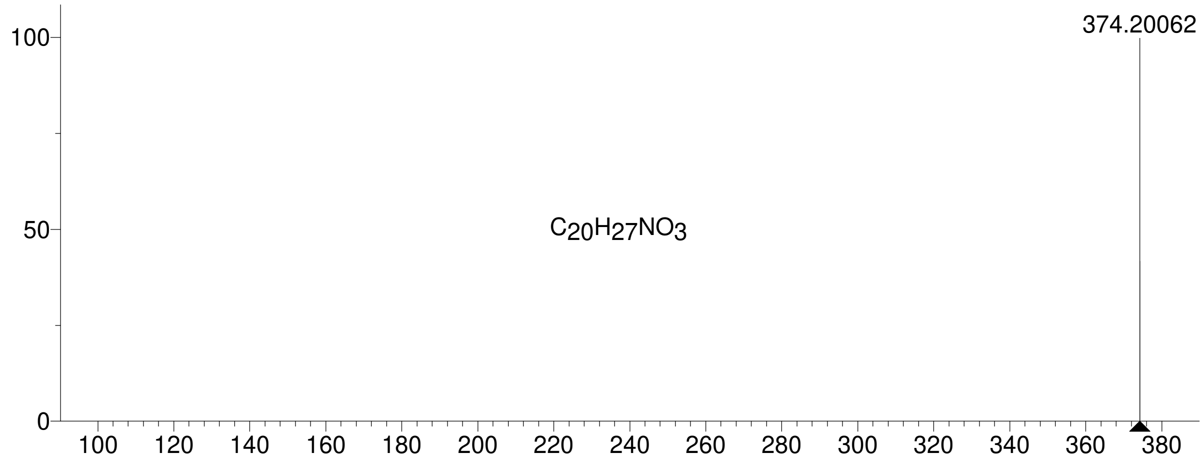

Hit 3 : Spirotetramat [M+H]+ HCD 3V P=374.2
C21H27NO5; MF: 54; RMF: 262; Prob 0.07%; CAS: 203313-25-1; Lib: nist_msms; ID: 161965.

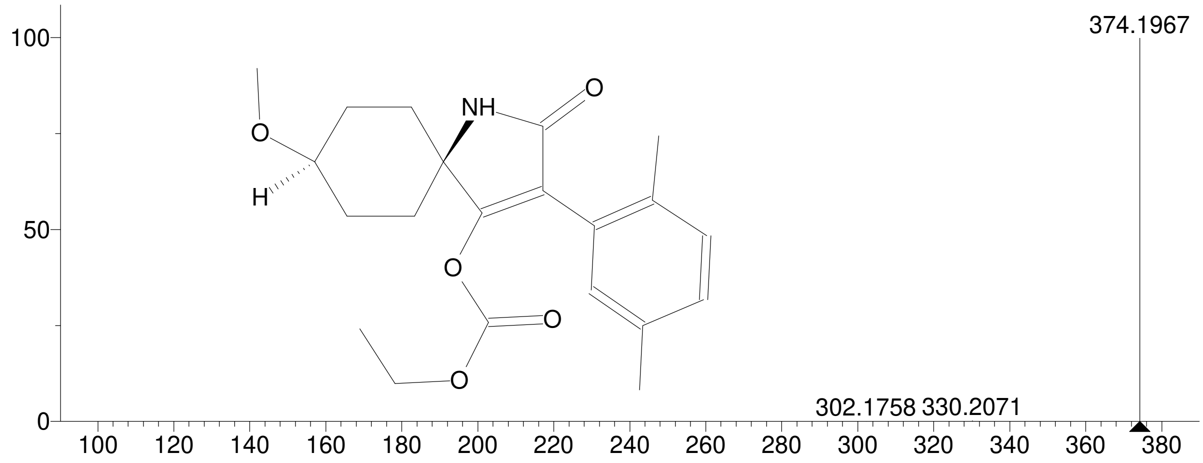

Hit 4 : N-Desmethyltrimebutine [M+H]+ HCD 1V P=374.2
C21H27NO5; MF: 19; RMF: 176; Prob 0.01%; CAS: 294882-33-0; Lib: nist_msms; ID: 168311.

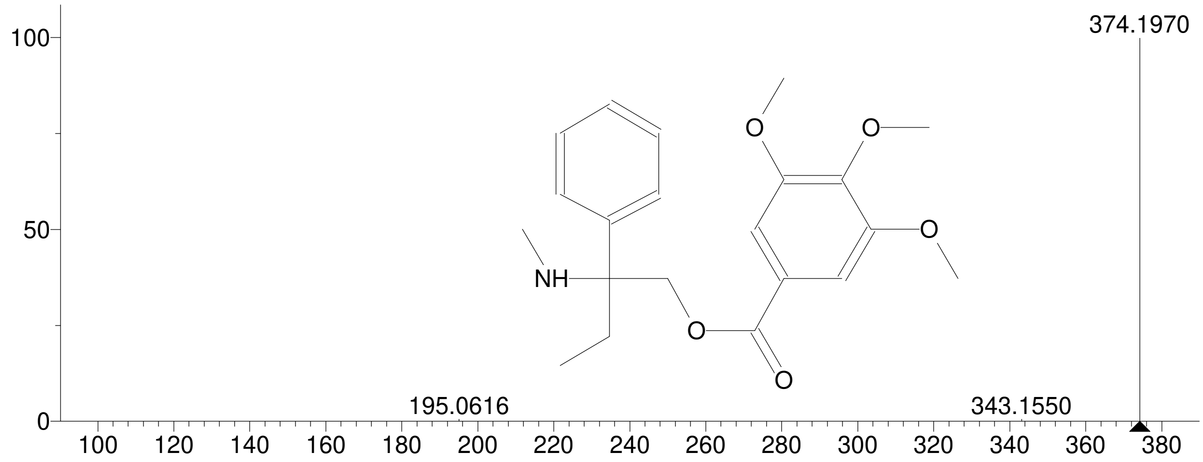

Hit 5 : Dipivefrine [M+Na]+ HCD 37V P=374.2
C19H29NO5; MF: 15; RMF: 136; Prob 0.01%; CAS: 52365-63-6; Lib: nist_msms; ID: 127584.

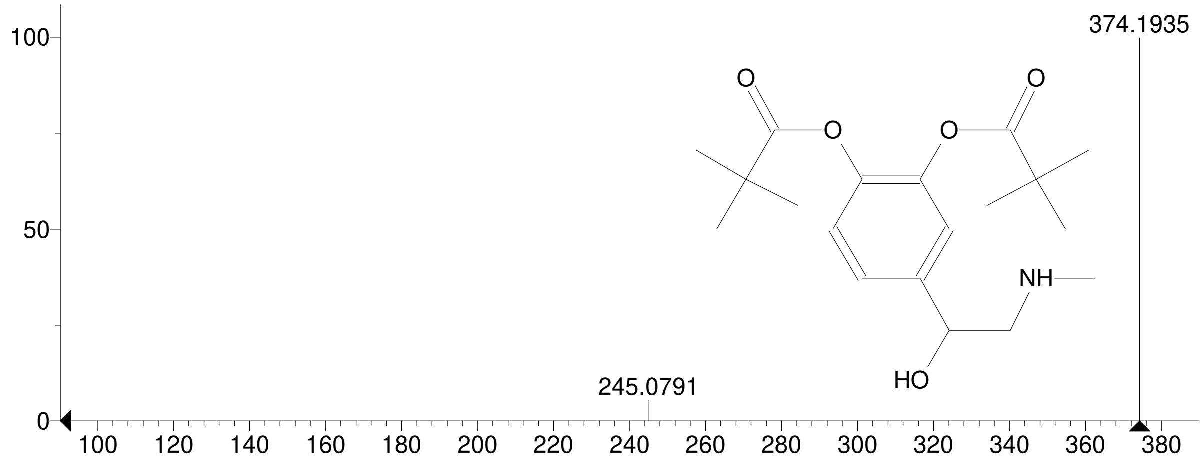

** Search Report Page 1 of 1 **

Unknown: Unknown (21.66_419.9823m/z)
Compound in Library Factor = 0

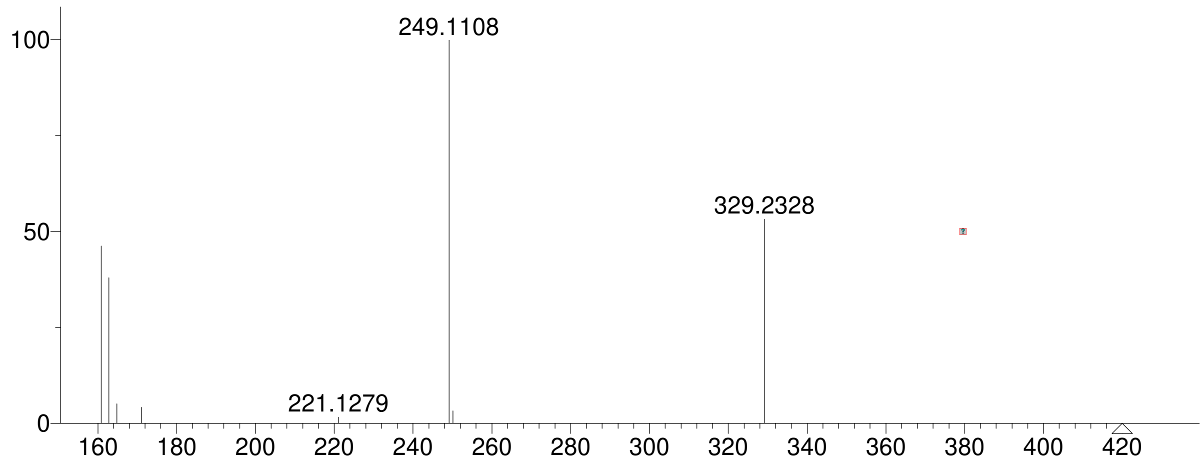

Unknown: Unknown (21.68_267.1588m/z)
Compound in Library Factor = -462

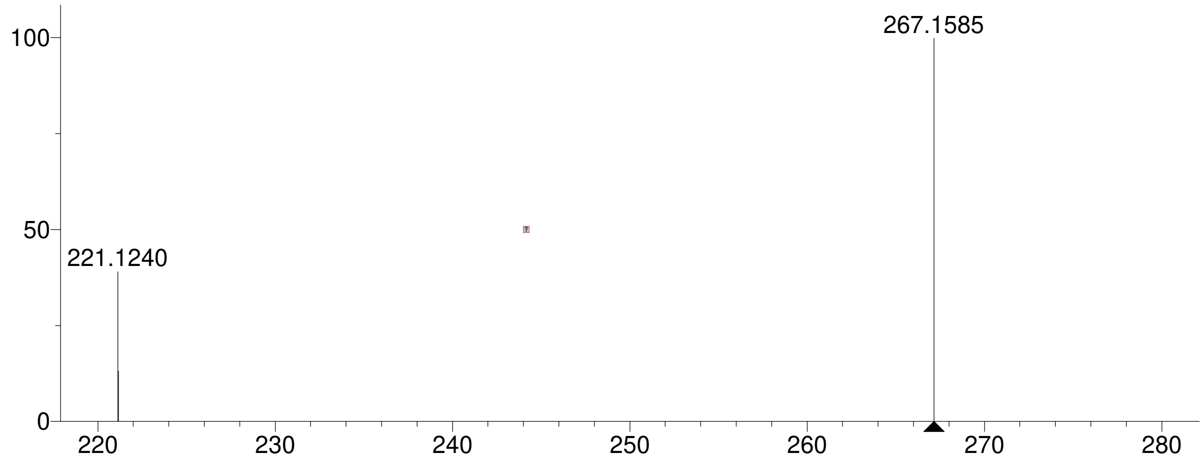

Hit 1 : Dihydroartemisinin [M+H-H2O]+ HCD 7V P=267.2
C15H24O5; MF: 167; RMF: 787; Prob 77.5%; CAS: 71939-50-9; Lib: nist_msms; ID: 121293.

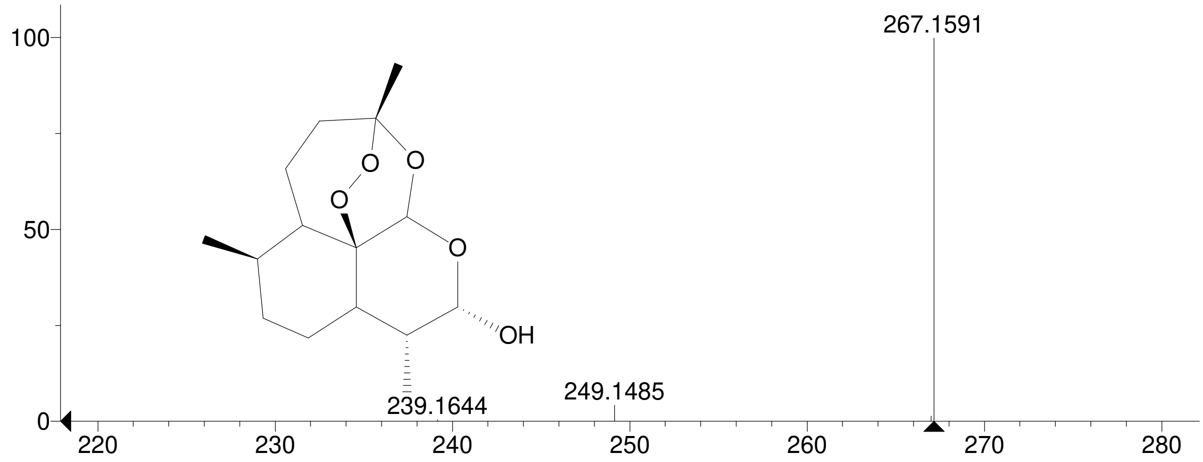

Hit 2 : NCGC00385871-01_C15H22O4_2,8-Dihydroxy-5,5,8-trimethyl-11-oxatetracyclo[7.3.1.0~1,9~.0~3,7~]tride
C15H22O4; MF: 22; RMF: 240; Prob 2.32%; Lib: mona-export-gnps; ID: 10011.

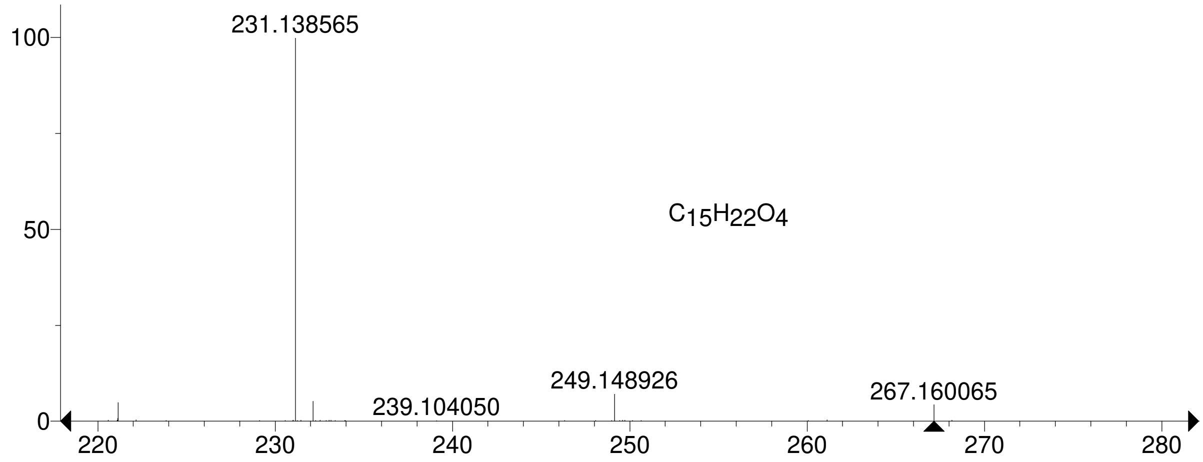

Hit 3 : 3-[4-methyl-1-(2-methylpropanoyl)-3-oxocyclohexyl]butanoic acid [M-H]- 35HCD P=267.2
C15H24O4; MF: 19; RMF: 596; Prob 2.05%; Lib: mona-export-lc-ms-ms_negative_mode; ID: 19214.

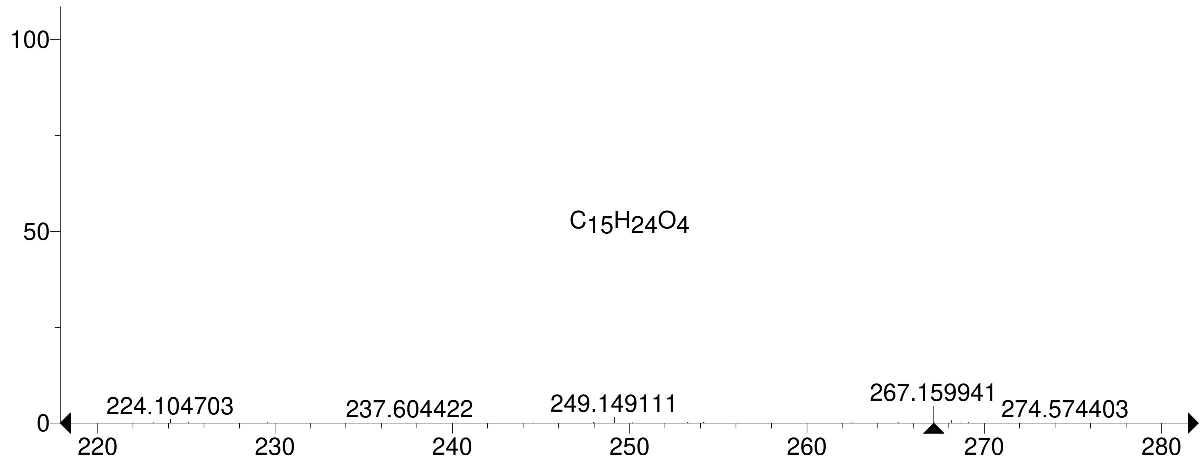

Hit 4 : NCGC00385633-01_C15H22O4_Naphtho[1,2-c]furan-3(1H)-one, 5,5a,6,7,8,9,9a,9b-octahydro-5-hydroxy-
C15H22O4; MF: 18; RMF: 481; Prob 1.97%; Lib: mona-export-gnps; ID: 13883.

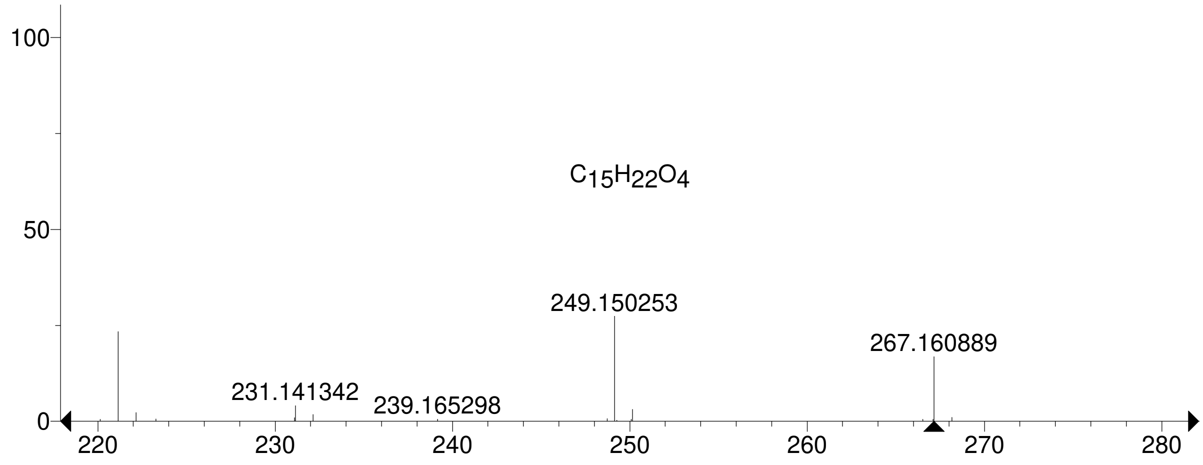

Hit 5 : NCGC00380321-01_C15H22O4_11-(Hydroxymethyl)-2,11-dimethyl-3-oxotricyclo[4.3.2.0~1,5~]undecane-9
C15H22O4; MF: 12; RMF: 313; Prob 1.55%; Lib: mona-export-gnps; ID: 10316.

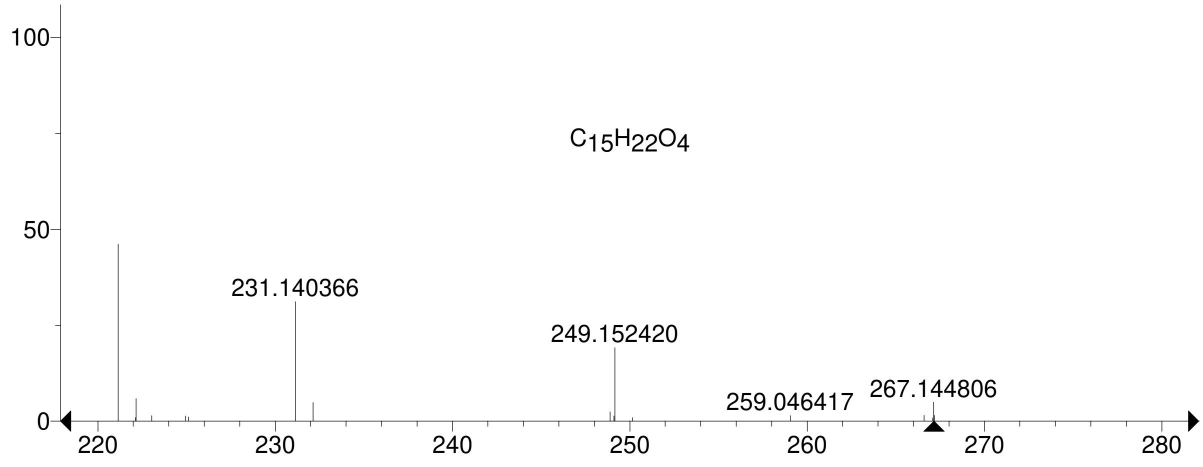

Unknown: Unknown (21.69_351.2153m/z)
Compound in Library Factor = -1848

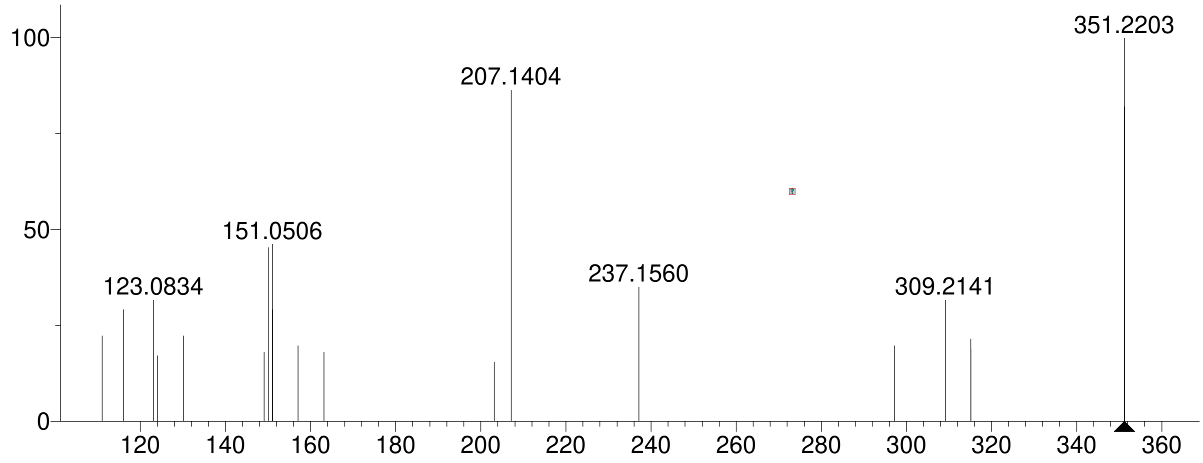

Hit 1 : 15-Ketoprostaglandin E2 [M+H]+ HCD 1V P=351.2
C20H30O5; MF: 162; RMF: 725; Prob 36.8%; CAS: 26441-05-4; Lib: nist_msms; ID: 169378.

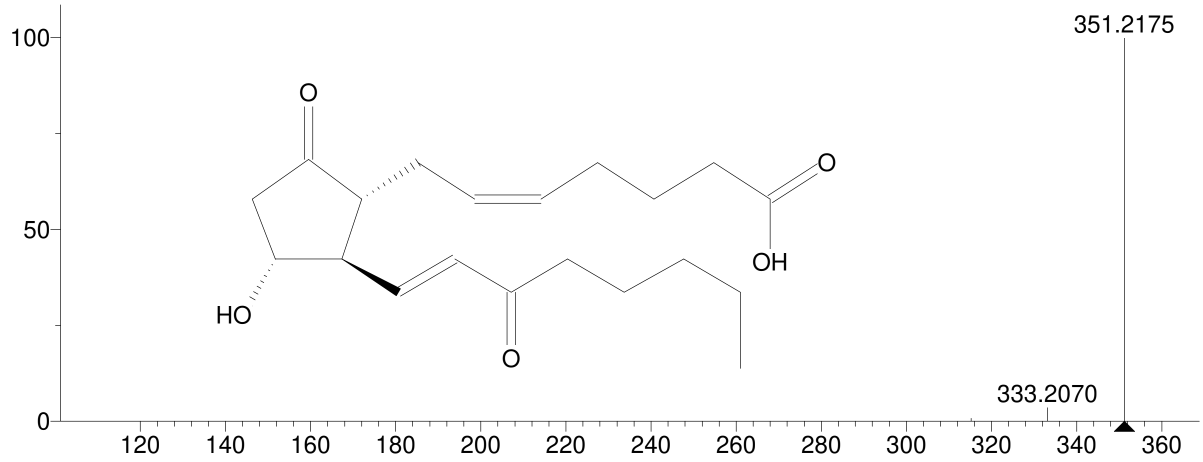

Hit 2 : 15-Ketoprostaglandin F2α [M-H]- HCD 3V P=351.2
C20H32O5; MF: 145; RMF: 730; Prob 20.1%; CAS: 35850-13-6; Lib: nist_msms; ID: 155319.

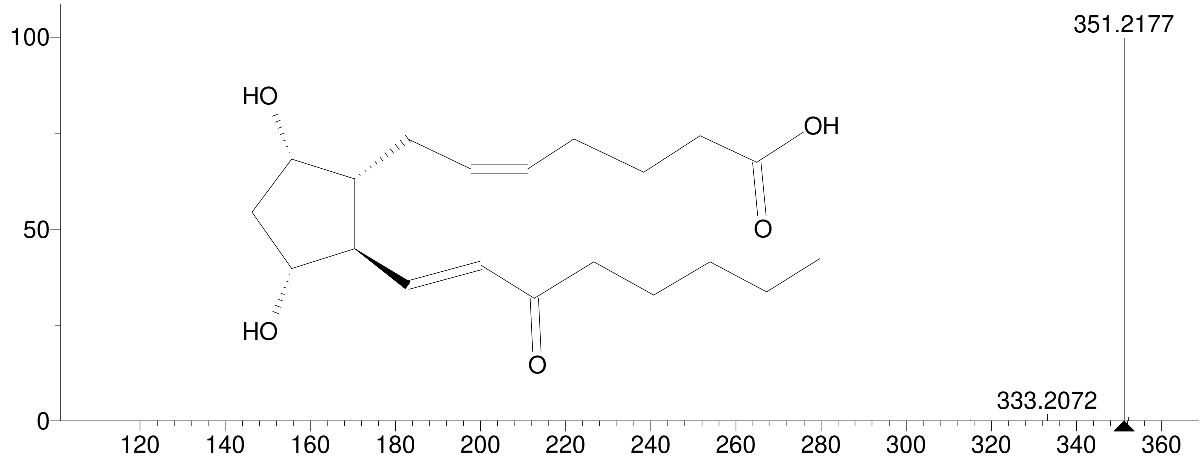

Hit 3 : 11,15-Dioxo-9S-hydroxy-5Z-prostenoic acid [M-H]- HCD 1V P=351.2
C20H32O5; MF: 133; RMF: 722; Prob 13.4%; CAS: 59894-07-4; Lib: nist_msms; ID: 87215.

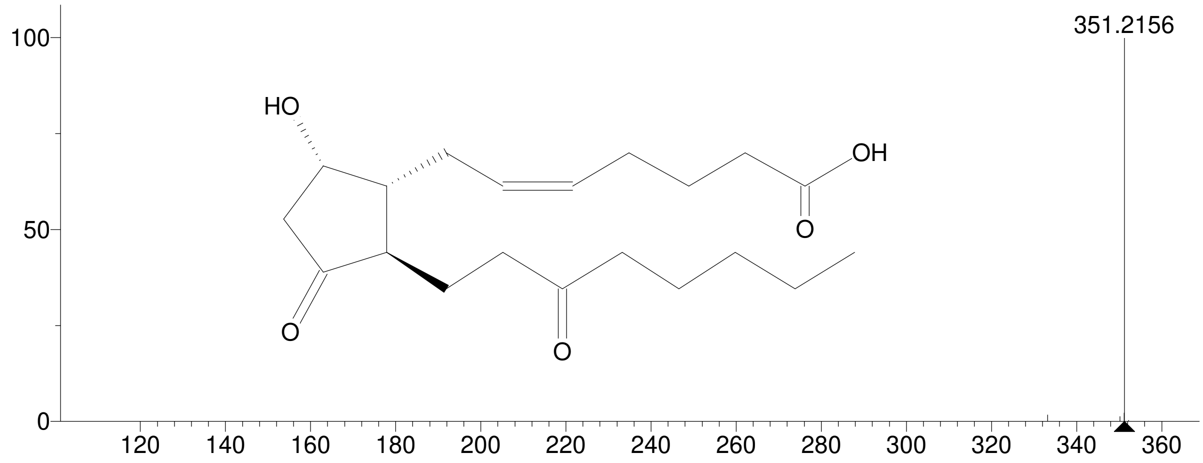

Hit 4 : 19(R)-Hydroxyprostaglandin E2 [M+H-H2O]+ HCD 1V P=351.2
C20H32O6; MF: 128; RMF: 723; Prob 10.8%; CAS: 64625-54-3; Lib: nist_msms; ID: 169989.

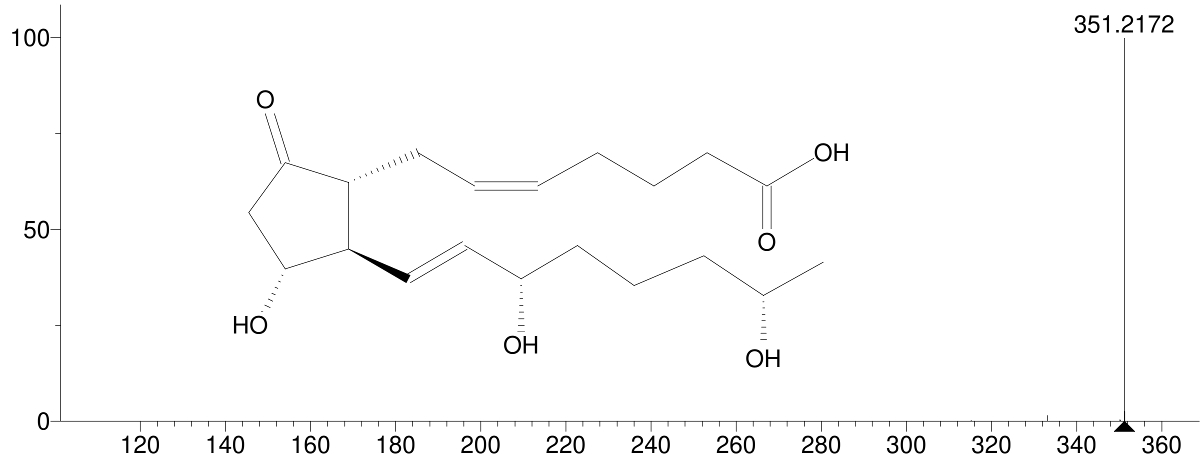

Hit 5 : 6-Ketoprostaglandin E1 [M+H-H2O]+ HCD 1V P=351.2
C20H32O6; MF: 112; RMF: 620; Prob 6.21%; CAS: 67786-53-2; Lib: nist_msms; ID: 161827.

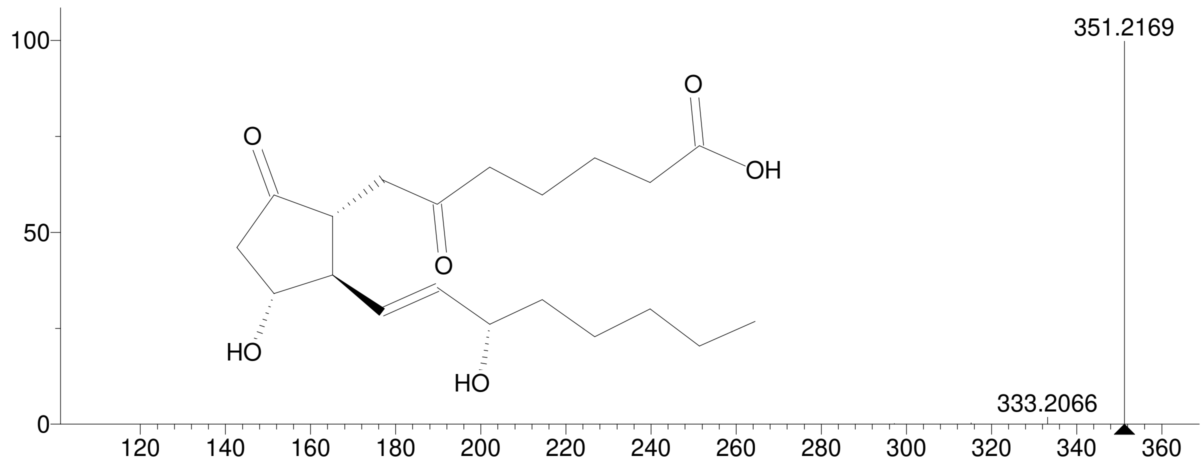

Unknown: Unknown (21.75_358.1645m/z)
Compound in Library Factor = -2176

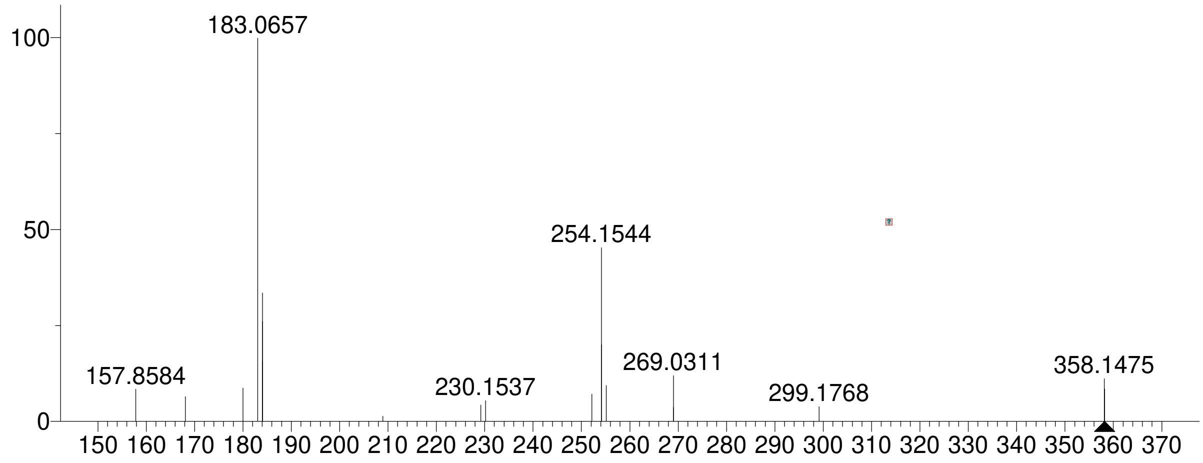

Hit 1 : Pitavastatin [M-H-CH2O3]- HCD 10V P=358.2
C25H24FNO4; MF: 51; RMF: 508; Prob 57.1%; CAS: 147511-69-1; Lib: nist_msms; ID: 369473.

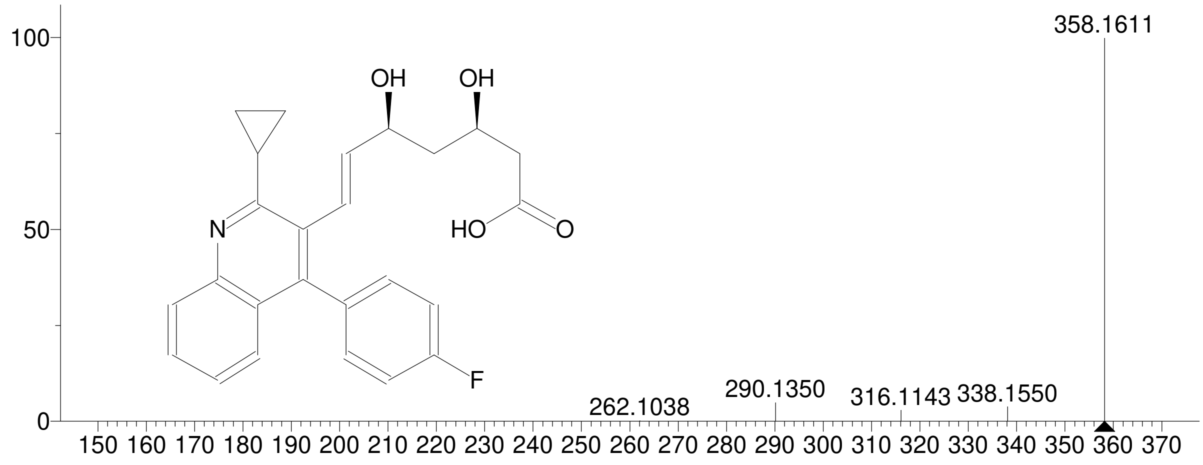

Hit 2 : 3',4',5'-Trimethoxytropacocaine [M+Na]+ HCD 9V P=358.2
C18H25NO5; MF: 22; RMF: 182; Prob 16.2%; CAS: 60820-69-1; Lib: nist_msms; ID: 383514.

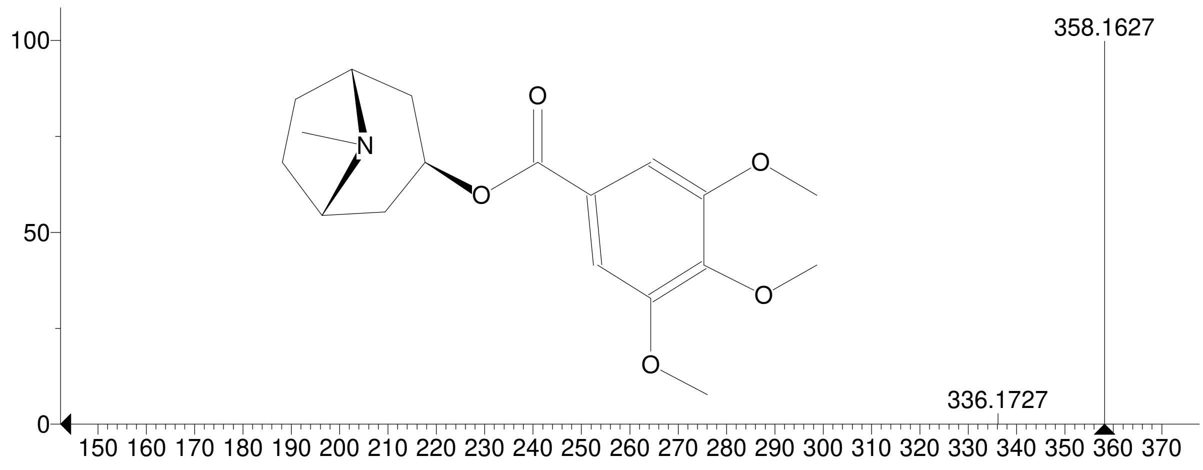

Hit 3 : MMV688361 [M+H]+ 20-30-40%) P=358.2
C21H19N5O; MF: 7; RMF: 153; Prob 9.81%; Lib: mona-export-pathogen_box; ID: 186.

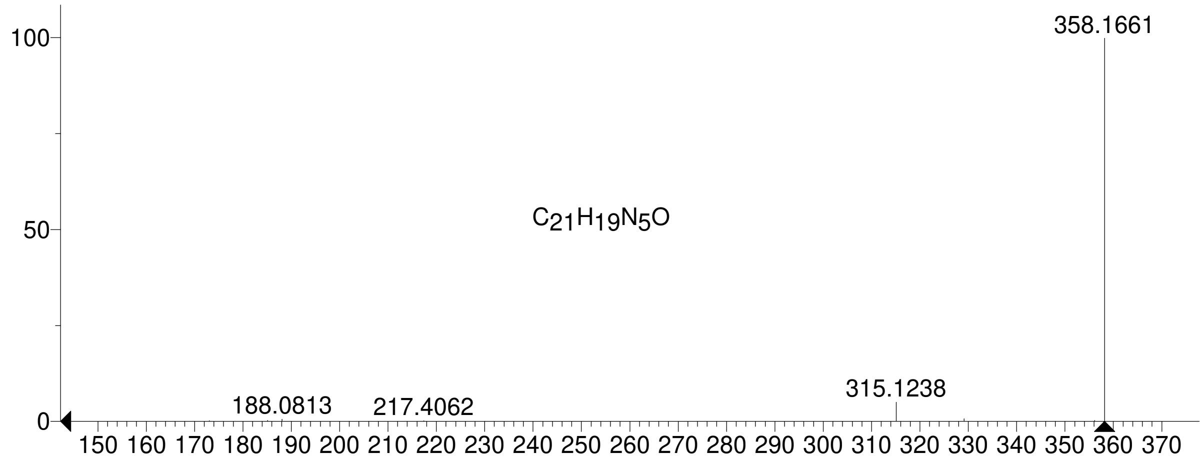

Hit 4 : Senecionine [M+Na]+ HCD 19V P=358.2
C18H25NO5; MF: 6; RMF: 173; Prob 9.43%; CAS: 130-01-8; Lib: nist_msms; ID: 219389.

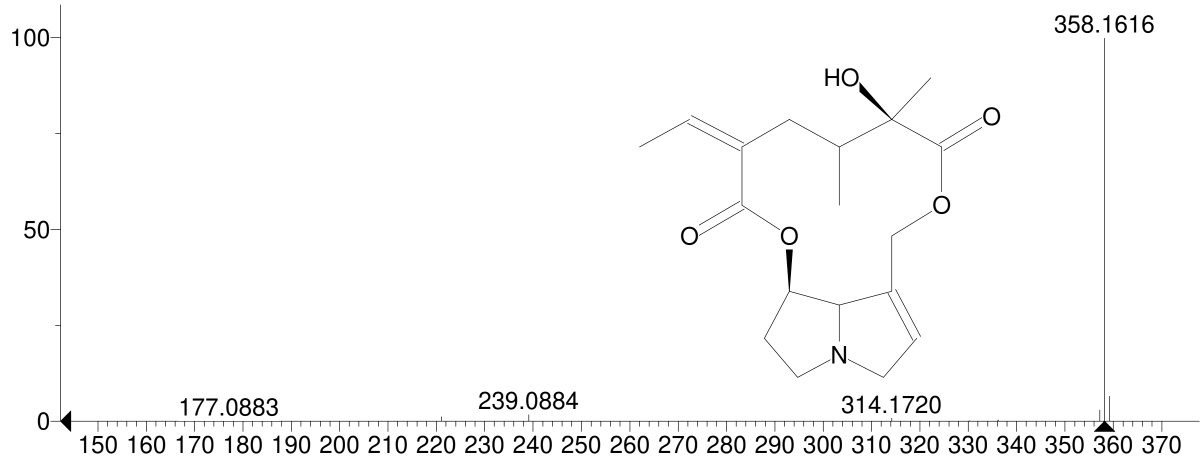

Hit 5 : (6aS)-1,2,10-trimethoxy-6-methyl-6-oxido-5,6,6a,7-tetrahydro-4H-dibenzo[de,g]quinoline-6-ium-9-ol [M+H]
C20H23NO5; MF: 0; RMF: 10; Prob 7.41%; Lib: mona-export-vf-npl_qexactive; ID: 1684.

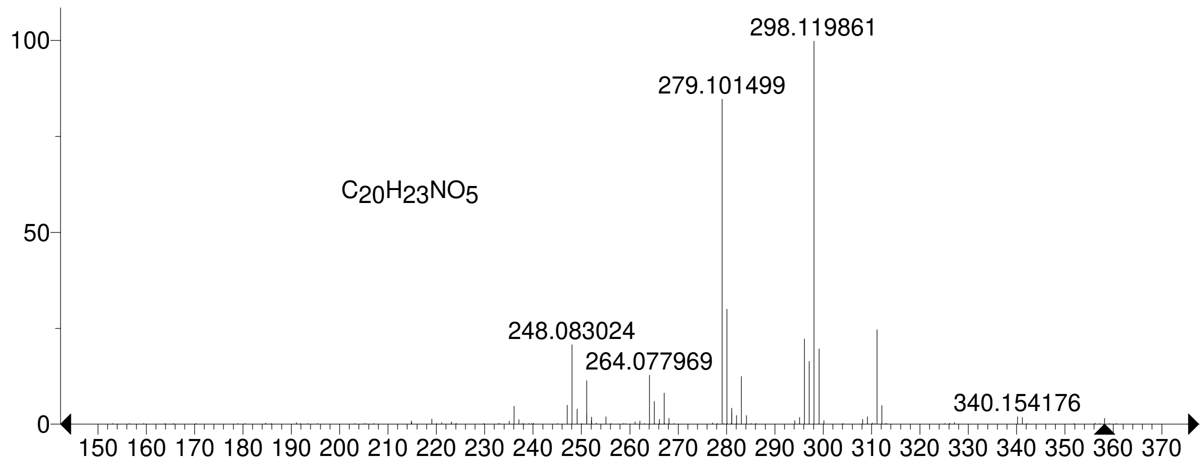

** Search Report Page 1 of 1 **

Unknown: Unknown (21.75_532.2178m/z)
Compound in Library Factor = 0

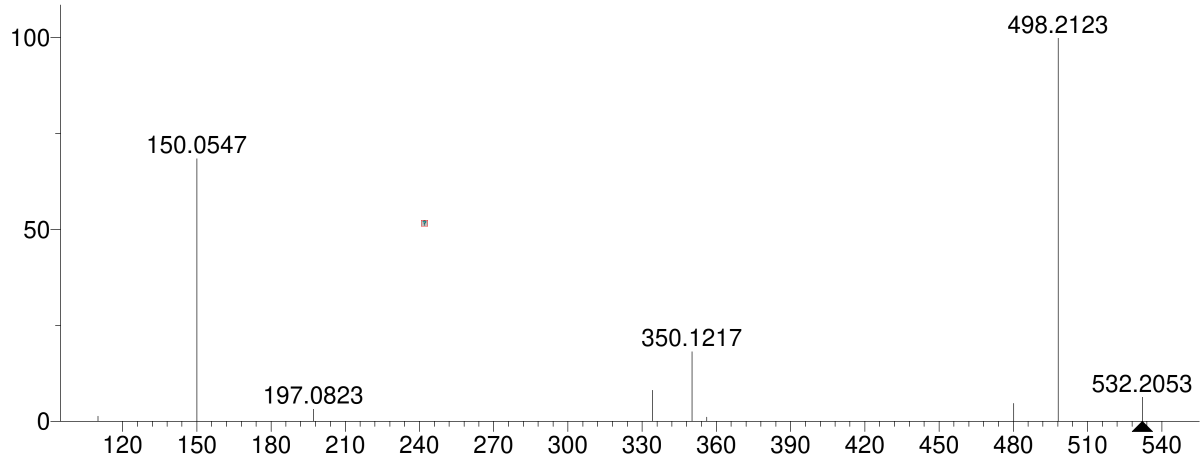

Unknown: Unknown (21.77_329.2323m/z)
Compound in Library Factor = -396

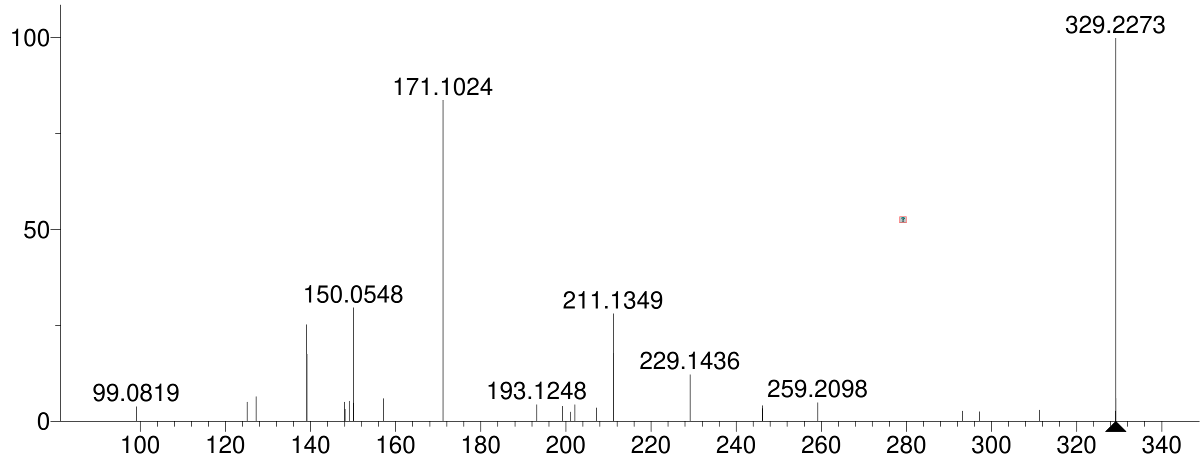

Hit 1 : FA 18:1+3O; PlaSMA ID-738 [M-H]- P=329.2
C18H34O5; MF: 348; RMF: 669; Prob 95.1%; Lib: biomsms-neg-plasma; ID: 444.

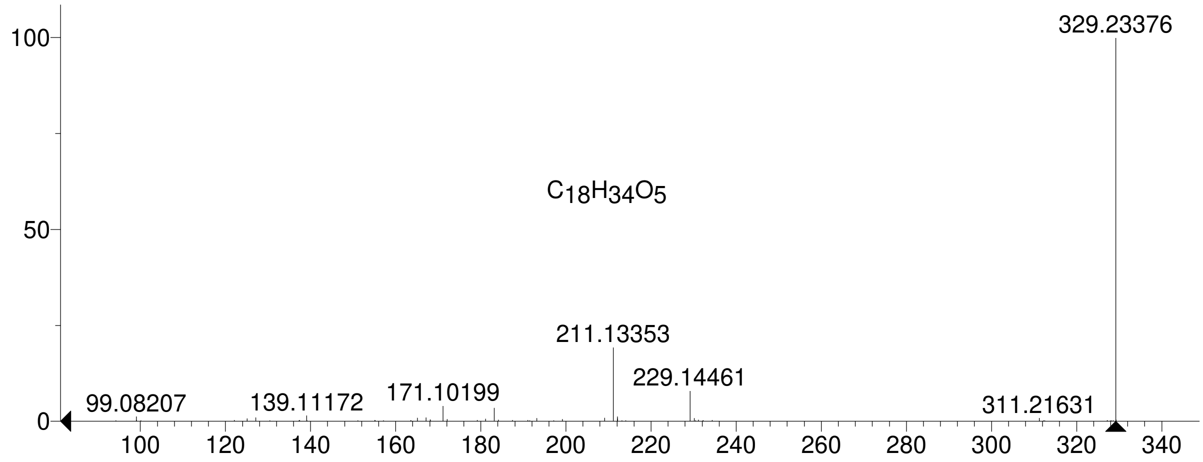

Hit 2 : FA 18:1+3O; PlaSMA ID-737 [M-H]- P=329.2
C18H34O5; MF: 216; RMF: 632; Prob 3.81%; Lib: biomsms-neg-plasma; ID: 448.

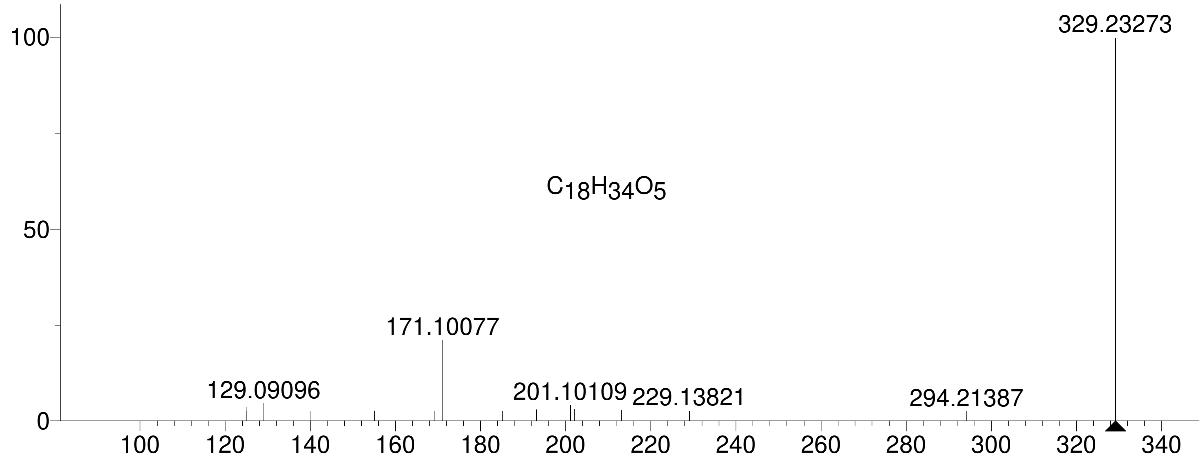

Hit 3 : FA 18:1+3O; PlaSMA ID-735 [M-H]- P=329.2
C18H34O5; MF: 180; RMF: 692; Prob 0.94%; Lib: biomsms-neg-plasma; ID: 443.

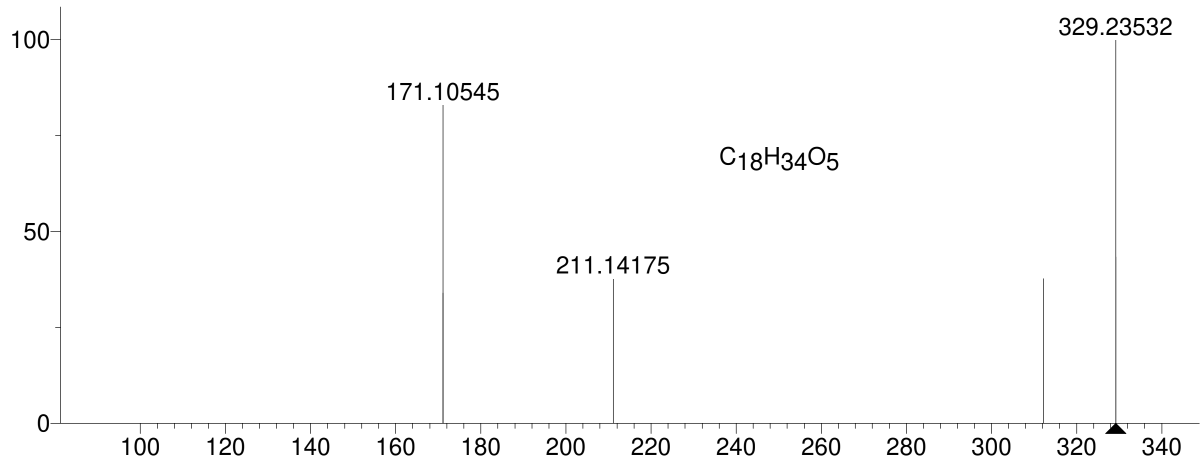

Hit 4 : 9,12,13-Trihydroxy-10(E)-octadecadienoic acid () [M-H]- P=329.2
C18H34O5; MF: 107; RMF: 566; Prob 0.12%; Lib: phytofrag2018_final_060219; ID: 129.

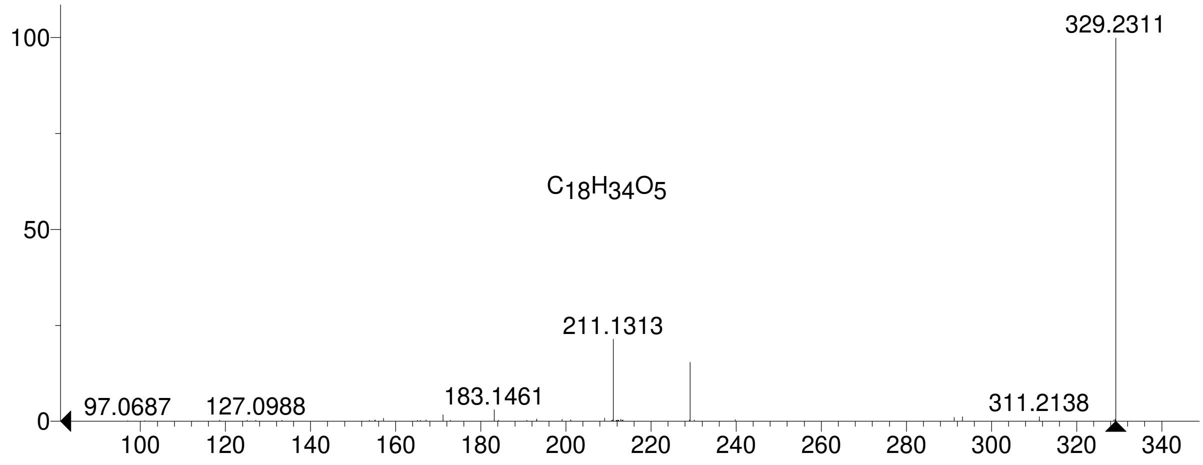

Hit 5 : FA 18:1+3O; PlaSMA ID-741 [M-H]- P=329.2
C18H34O5; MF: 55; RMF: 509; Prob 0.02%; Lib: biomsms-neg-plasma; ID: 449.

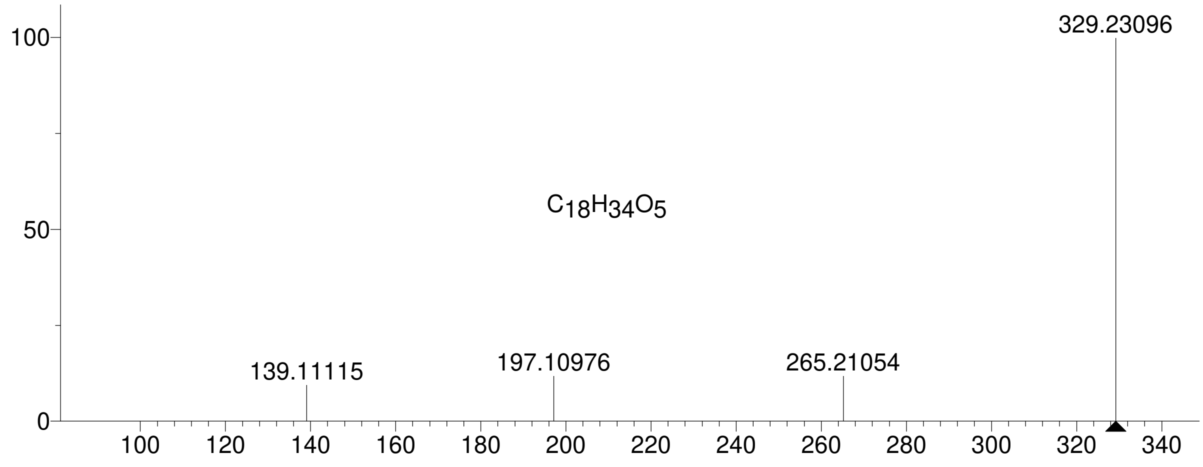

Unknown: Unknown (21.83_392.0549m/z)
Compound in Library Factor = 0

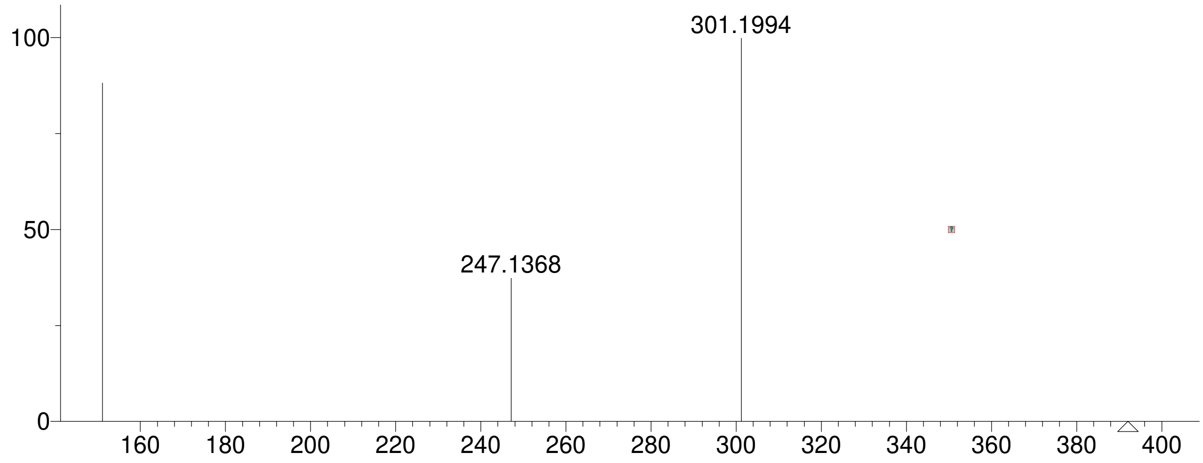

Unknown: Unknown (21.85_247.1332m/z)
Compound in Library Factor = -2176

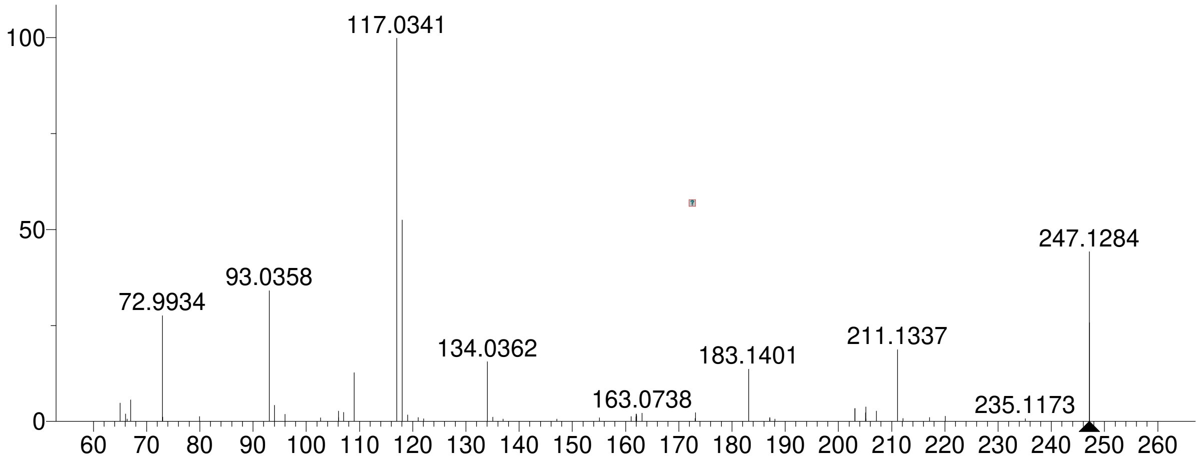

Hit 1 : 2-cis-4-trans-Abscisic acid [M+H-H2O]+ HCD 9V P=247.1
C15H20O4; MF: 81; RMF: 328; Prob 21.7%; CAS: 14375-45-2; Lib: nist_msms; ID: 127783.

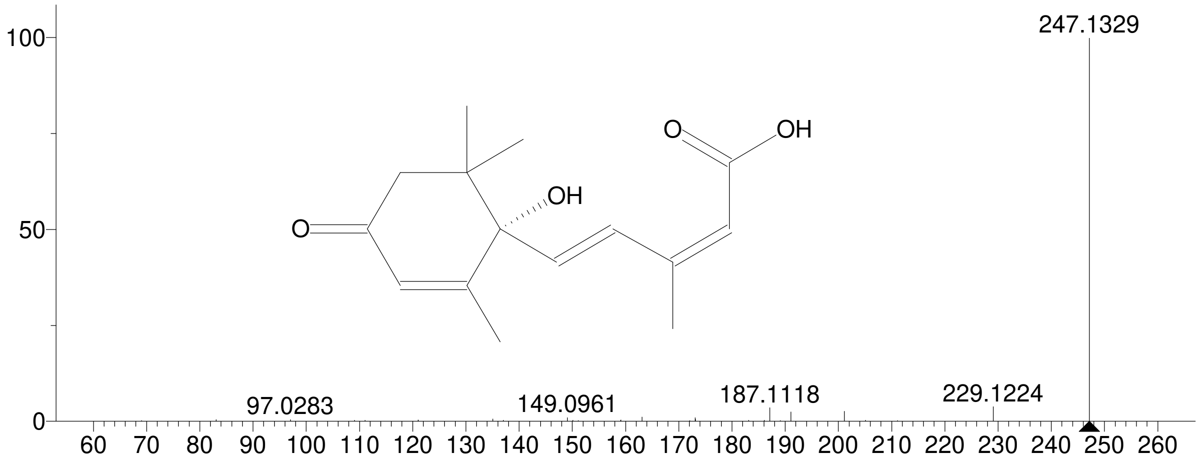

Hit 2 : α-Santonin [M+H]+ HCD 4V P=247.1
C15H18O3; MF: 74; RMF: 566; Prob 16.6%; CAS: 481-06-1; Lib: nist_msms; ID: 456743.

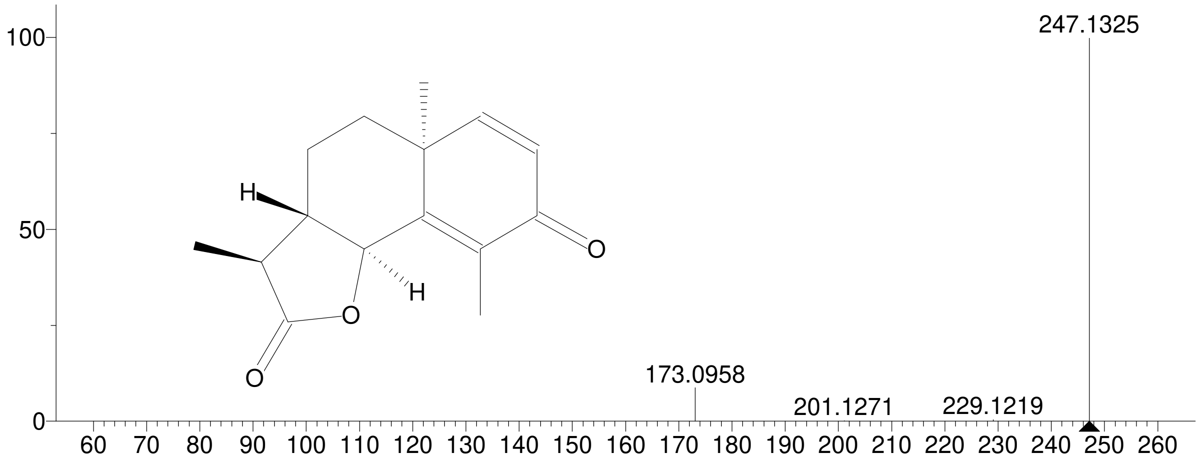

Hit 3 : (+)-Abscisic acid [M+H-H2O]+ HCD 9V P=247.1
C15H20O4; MF: 71; RMF: 316; Prob 14.7%; CAS: 21293-29-8; Lib: nist_msms; ID: 539021.

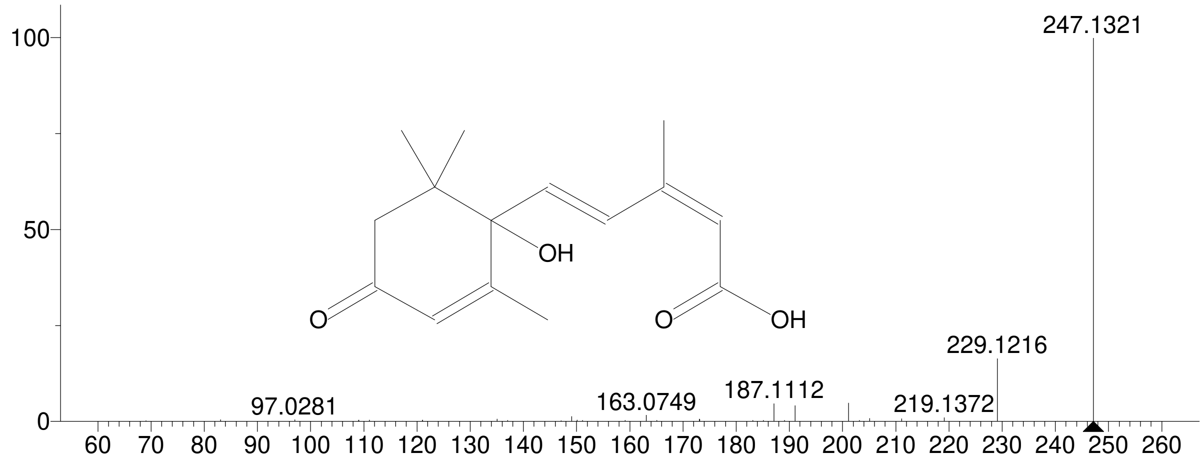

Hit 4 : 2,7,8-Trimethyl-2-(β-carboxyethyl)-6-hydroxychroman [M+H-H2O]+ HCD 12V P=247.1
C15H20O4; MF: 46; RMF: 311; Prob 4.47%; CAS: 178167-75-4; Lib: nist_msms; ID: 340433.

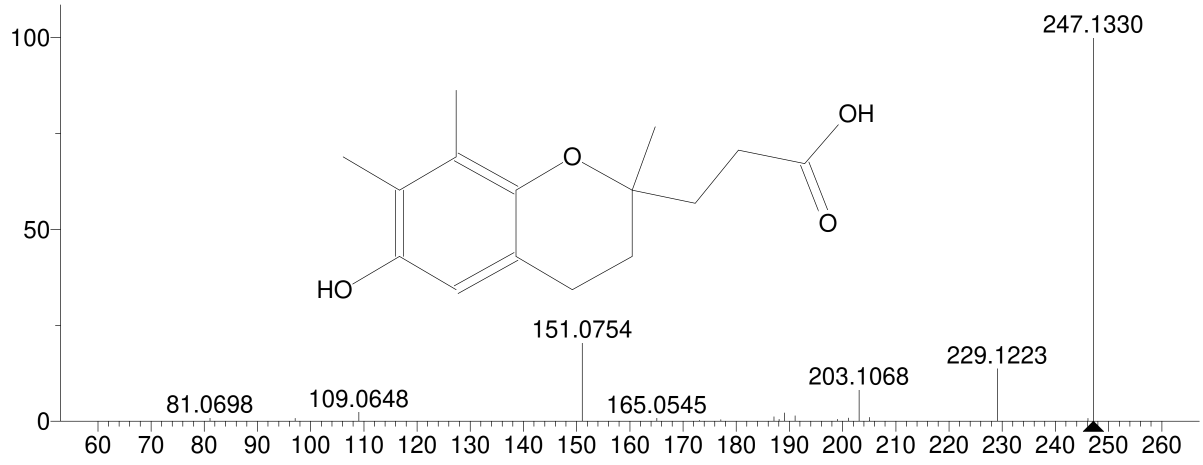

Hit 5 : Chamissonolide [M+H-C2H6O3]+ HCD 9V P=247.1
C17H24O6; MF: 40; RMF: 296; Prob 3.51%; CAS: 78853-98-2; Lib: nist_msms; ID: 500793.

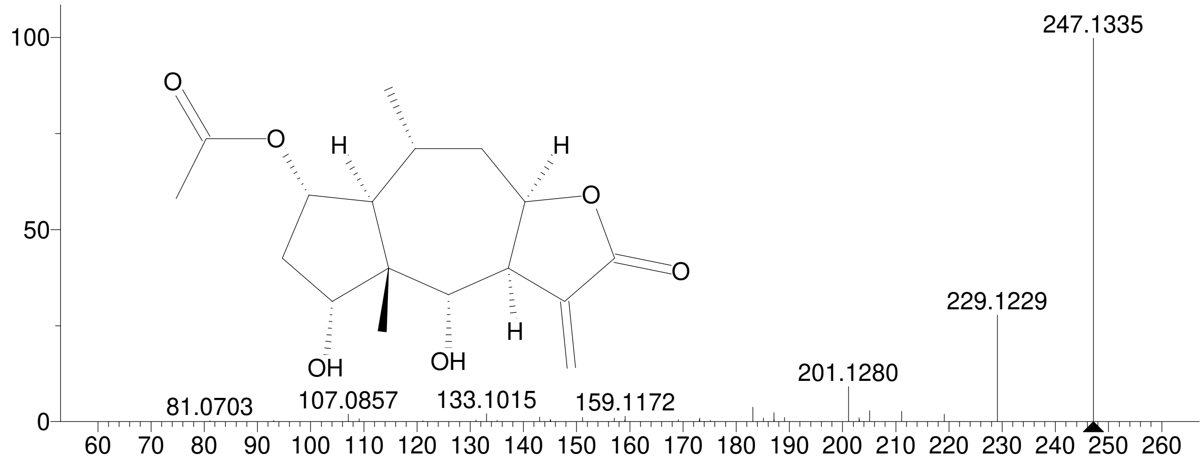

Unknown: Unknown (21.88_229.1433m/z)
Compound in Library Factor = 588

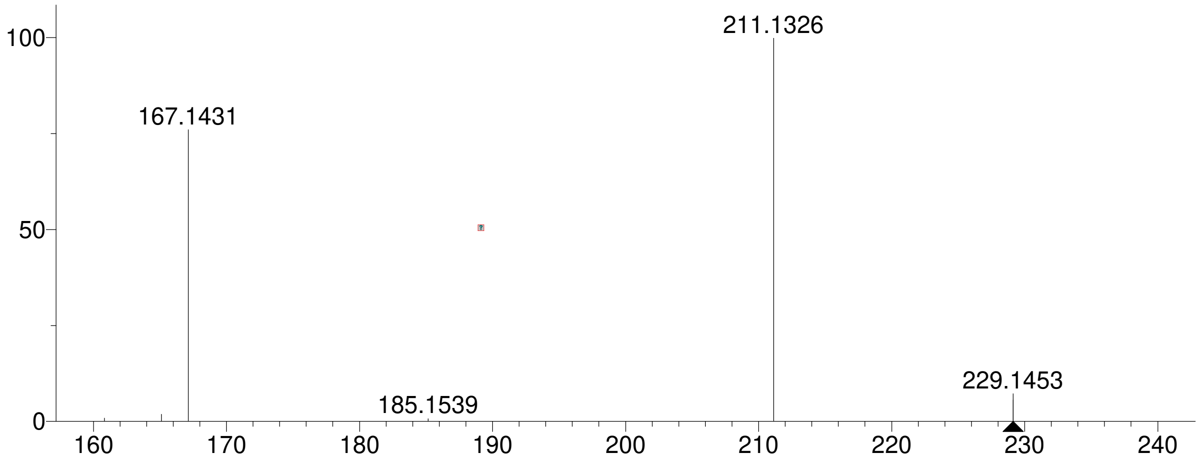

Hit 1 : Dodecanedioic acid [M-H]- HCD 29V P=229.1
C12H22O4; MF: 890; RMF: 970; Prob 99.0%; CAS: 693-23-2; Lib: nist_msms; ID: 289814.

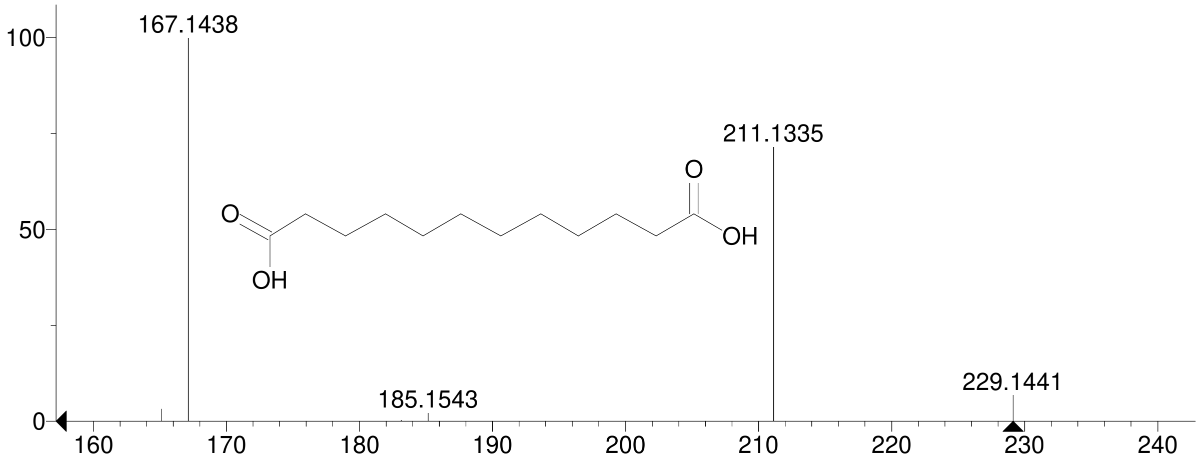

Hit 2 : Unknown (carbon number 12); PlaSMA ID-339 [M-H]- P=229.1
C12H22O4; MF: 574; RMF: 868; Prob 0.98%; Lib: biomsms-neg-plasma; ID: 1074.

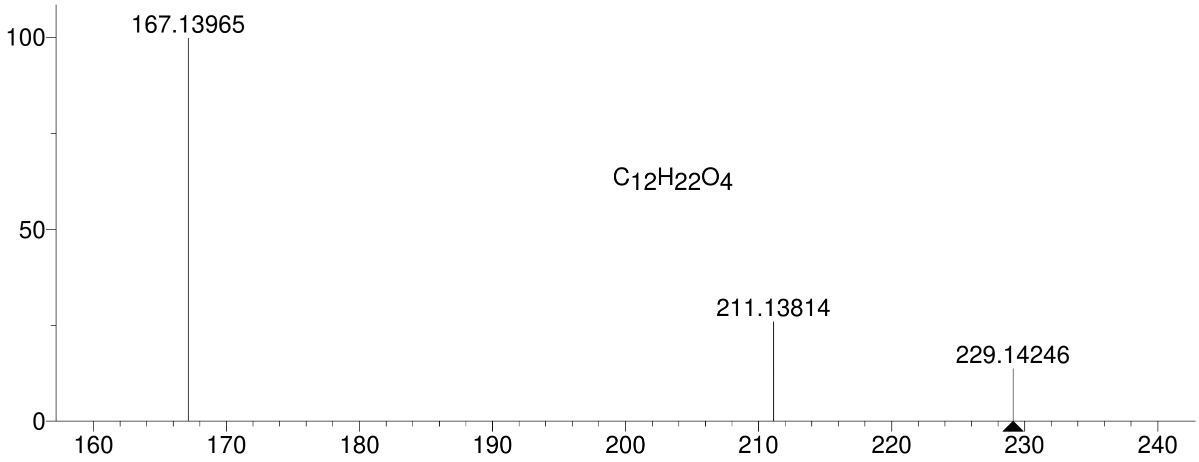

Hit 3 : Unknown (carbon number 12); PlaSMA ID-340 [M-H]- P=229.1
C12H22O4; MF: 255; RMF: 750; Prob 0.00%; Lib: biomsms-neg-plasma; ID: 1075.

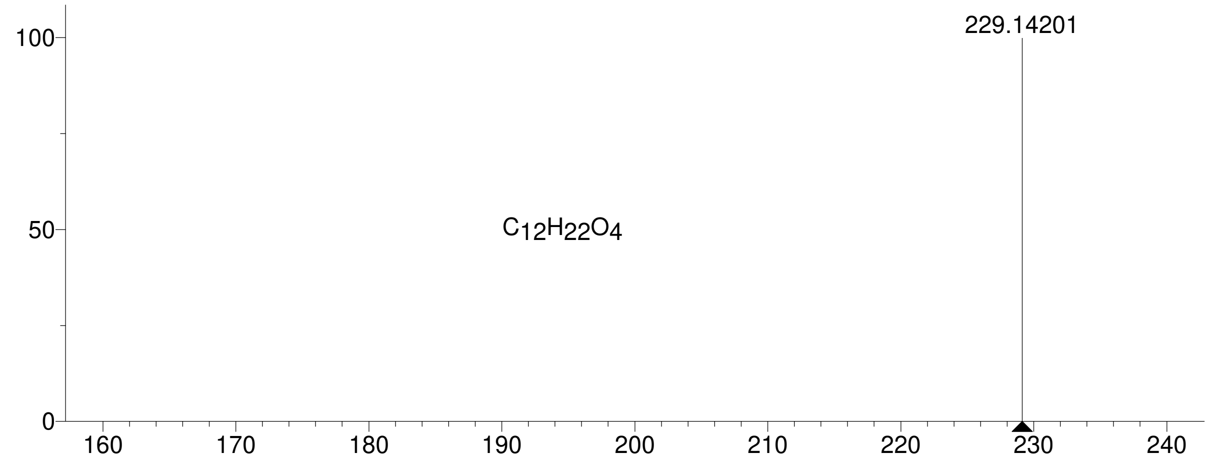

Hit 4 : Unknown (carbon number 12); PlaSMA ID-341 [M-H]- P=229.1
C12H22O4; MF: 255; RMF: 750; Prob 0.00%; Lib: biomsms-neg-plasma; ID: 1076.

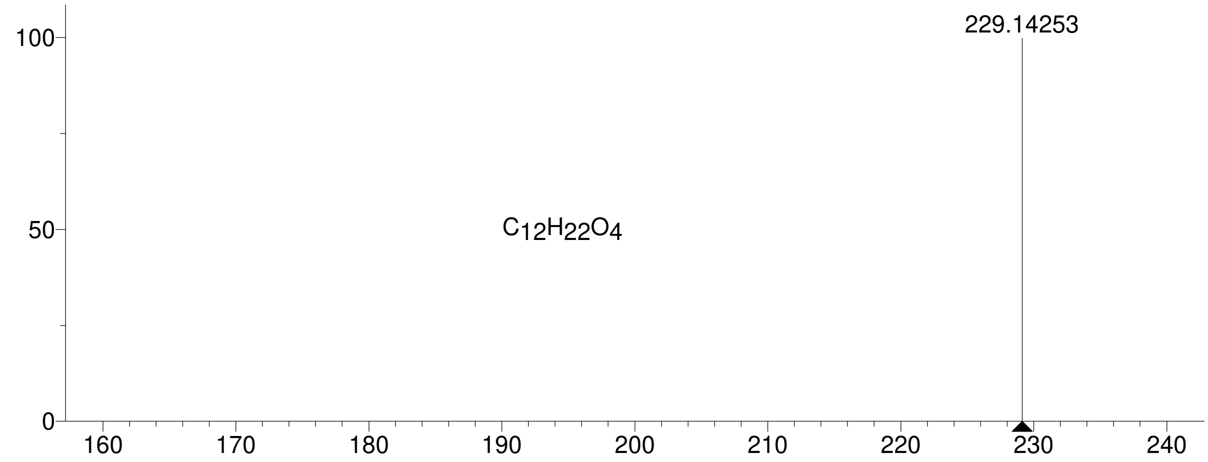

Hit 5 : Unknown (carbon number 12); PlaSMA ID-342 [M-H]- P=229.1
C12H22O4; MF: 132; RMF: 450; Prob 0.00%; Lib: biomsms-neg-plasma; ID: 1077.

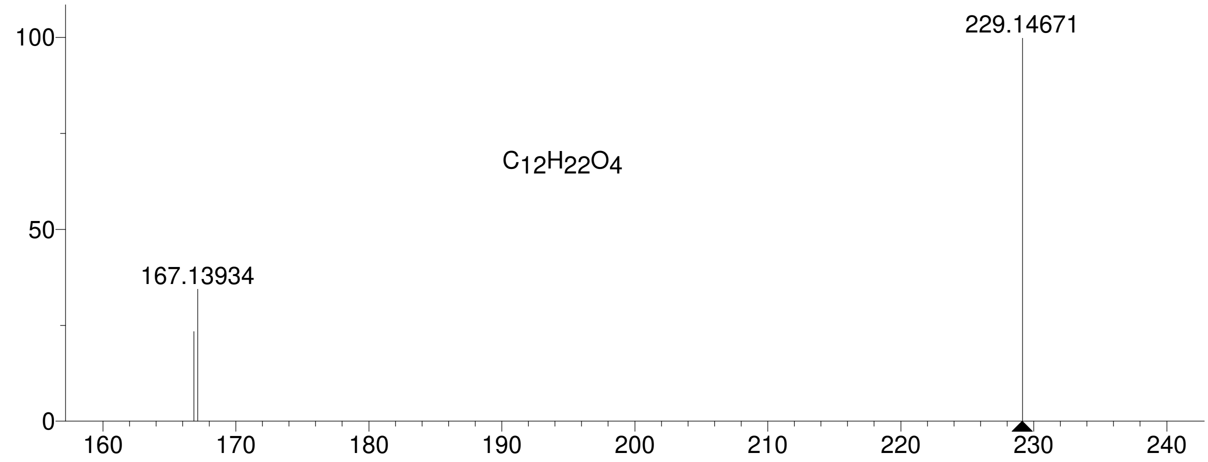

Unknown: Unknown (21.97_438.0470m/z)
Compound in Library Factor = 0

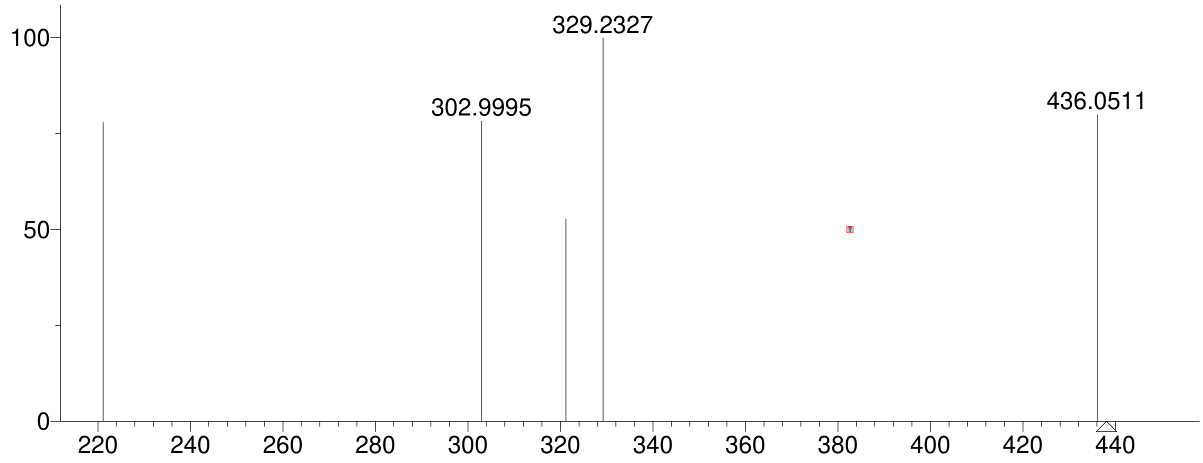

Unknown: Unknown (21.98_329.2317m/z)
Compound in Library Factor = -357

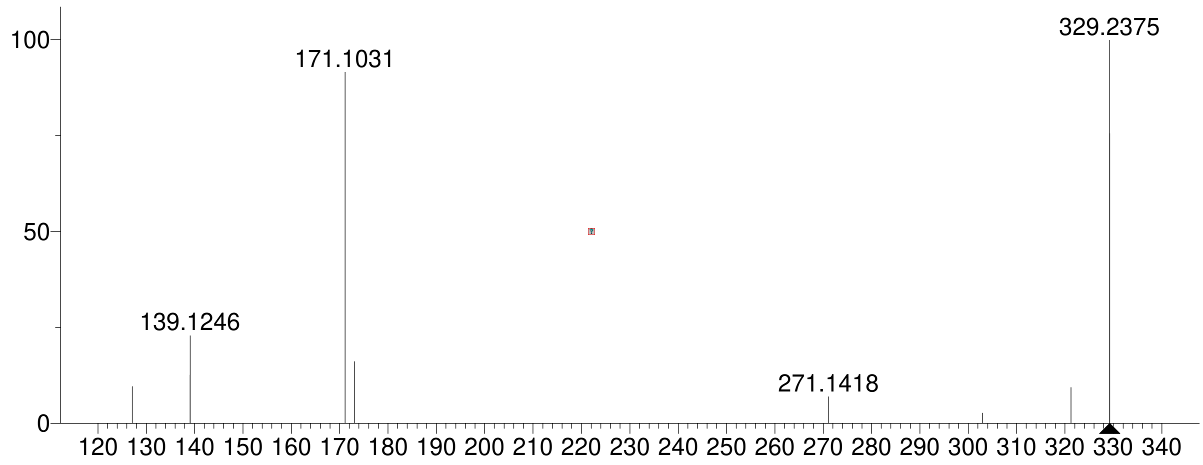

Hit 1 : FA 18:1+3O; PlaSMA ID-735 [M-H]- P=329.2
C18H34O5; MF: 320; RMF: 767; Prob 97.6%; Lib: biomsms-neg-plasma; ID: 443.

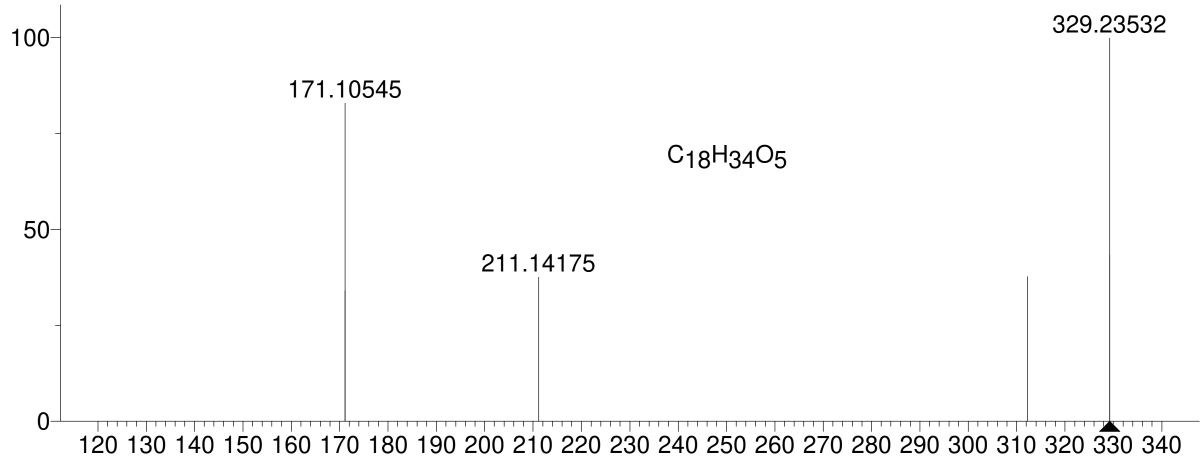

Hit 2 : Unknown (carbon number 18); PlaSMA ID-736 [M-H]- P=329.2
C18H34O5; MF: 143; RMF: 605; Prob 1.87%; Lib: biomsms-neg-plasma; ID: 1245.

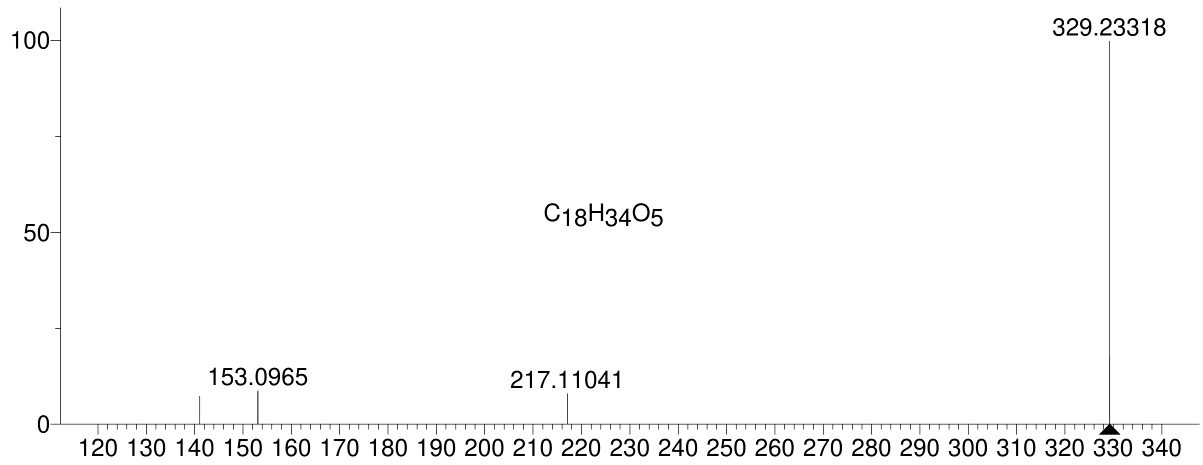

Hit 3 : FA 18:1+3O; PlaSMA ID-737 [M-H]- P=329.2
C18H34O5; MF: 77; RMF: 613; Prob 0.29%; Lib: biomsms-neg-plasma; ID: 448.

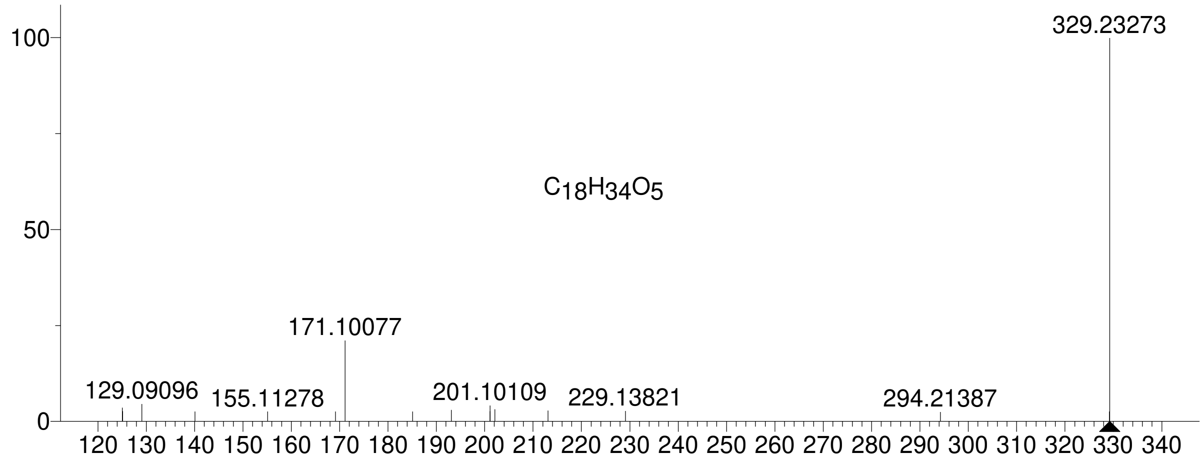

Hit 4 : FA 18:1+3O; PlaSMA ID-741 [M-H]- P=329.2
C18H34O5; MF: 48; RMF: 523; Prob 0.08%; Lib: biomsms-neg-plasma; ID: 449.

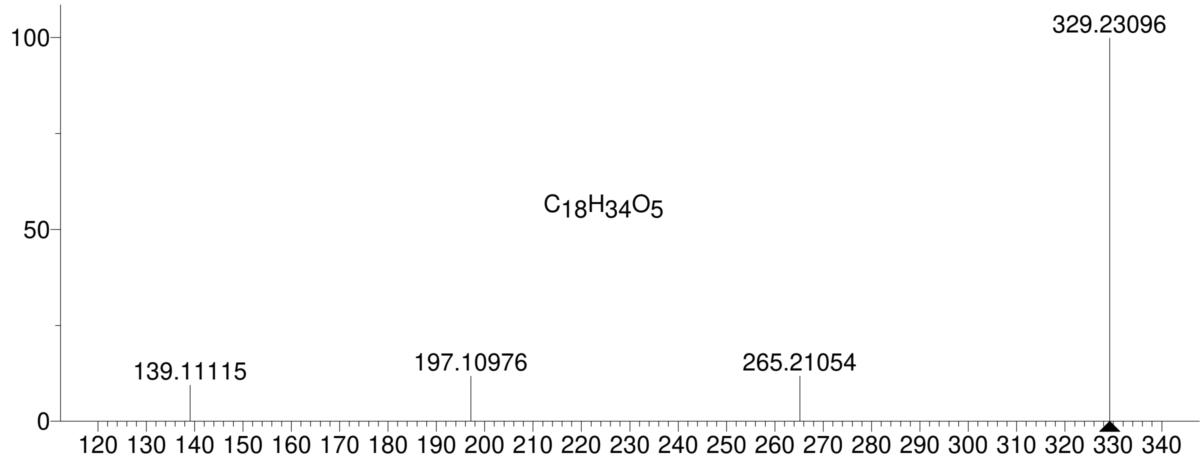

Hit 5 : FA 18:1+3O; PlaSMA ID-738 [M-H]- P=329.2
C18H34O5; MF: 43; RMF: 561; Prob 0.06%; Lib: biomsms-neg-plasma; ID: 444.

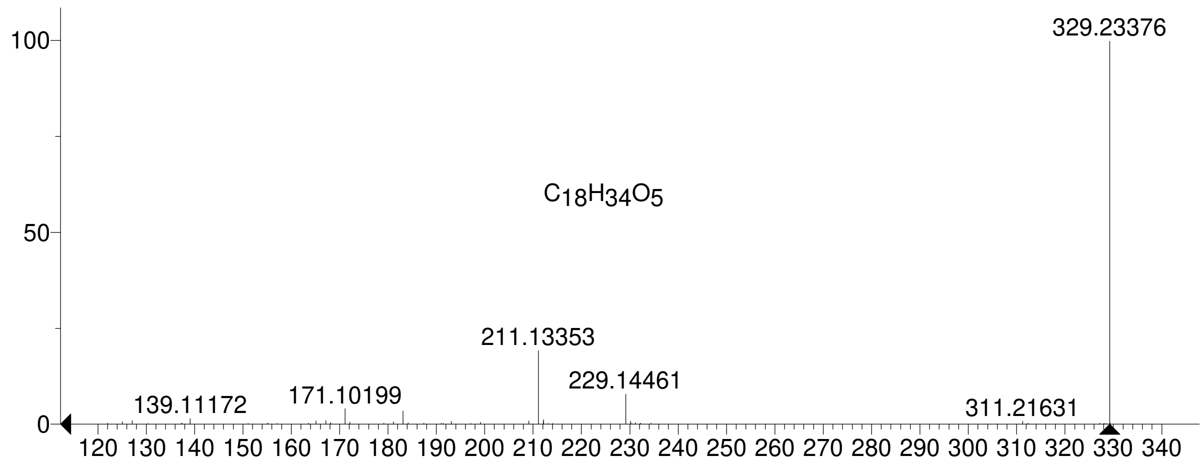

Unknown: Unknown (21.98_351.2156m/z)
Compound in Library Factor = -2176

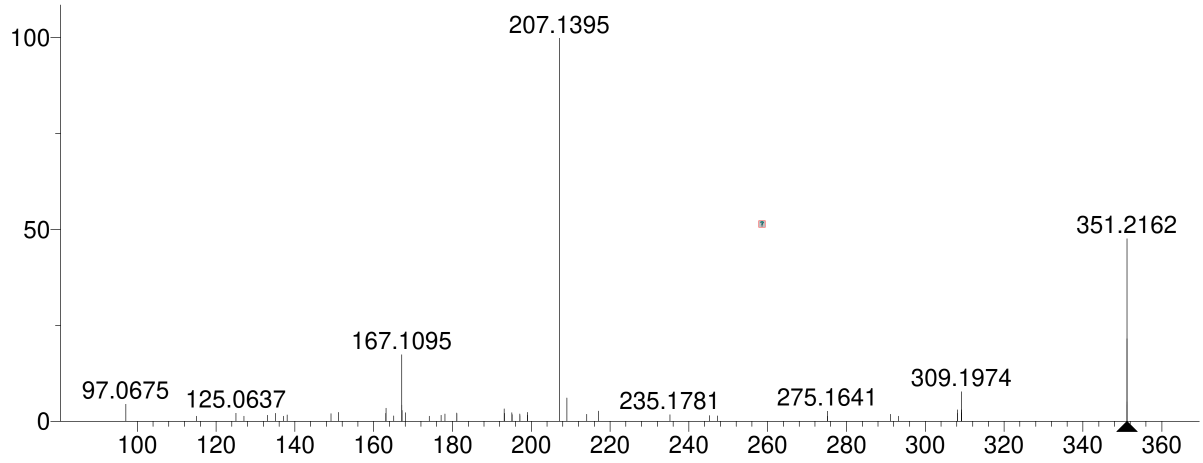

Hit 1 : 15-Ketoprostaglandin E2 [M+H]+ HCD 1V P=351.2
C20H30O5; MF: 175; RMF: 762; Prob 36.9%; CAS: 26441-05-4; Lib: nist_msms; ID: 169378.

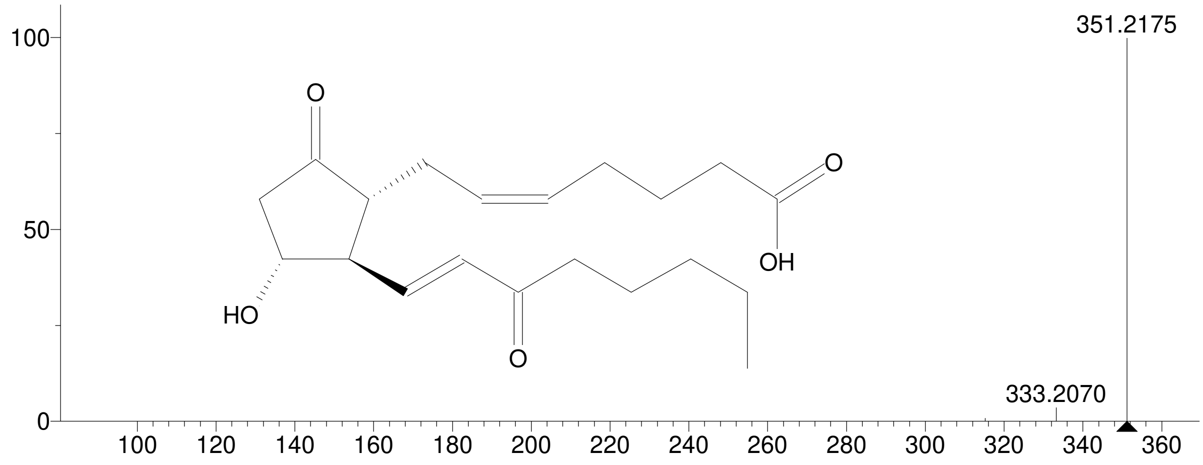

Hit 2 : 15-Ketoprostaglandin F2α [M-H]- HCD 3V P=351.2
C20H32O5; MF: 158; RMF: 767; Prob 20.1%; CAS: 35850-13-6; Lib: nist_msms; ID: 155319.

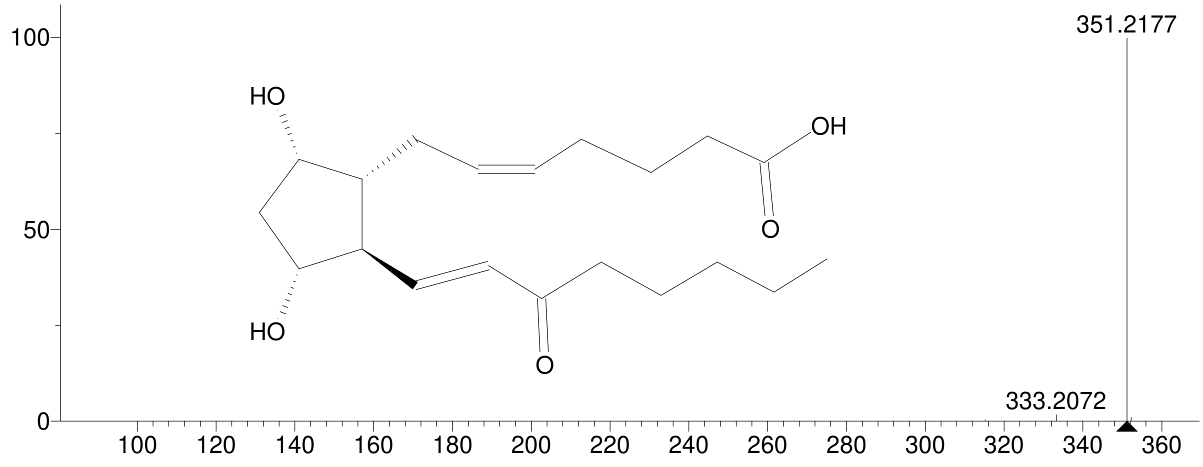

Hit 3 : 11,15-Dioxo-9S-hydroxy-5Z-prostenoic acid [M-H]- HCD 1V P=351.2
C20H32O5; MF: 145; RMF: 759; Prob 13.0%; CAS: 59894-07-4; Lib: nist_msms; ID: 87215.

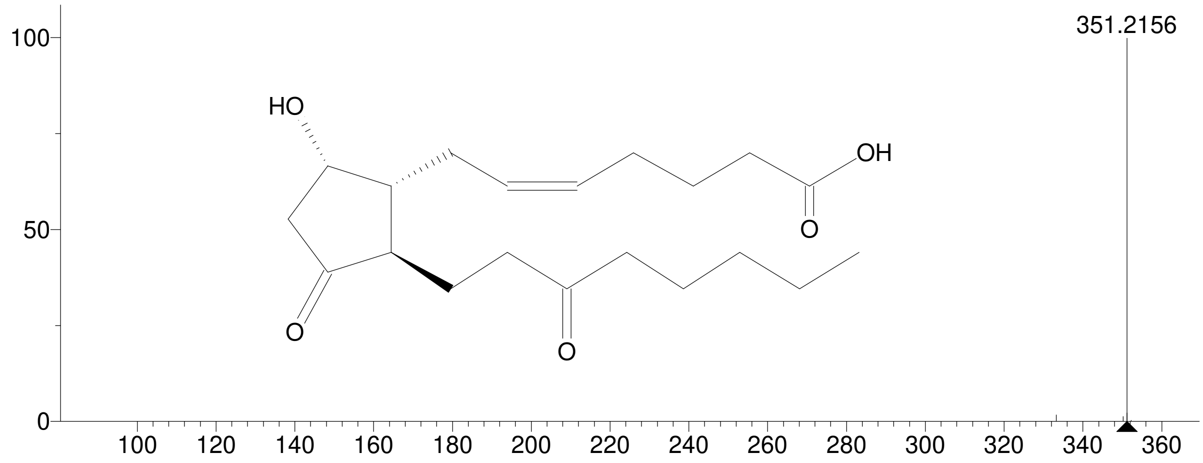

Hit 4 : 19(R)-Hydroxyprostaglandin E2 [M+H-H2O]+ HCD 1V P=351.2
C20H32O6; MF: 140; RMF: 760; Prob 10.5%; CAS: 64625-54-3; Lib: nist_msms; ID: 169989.

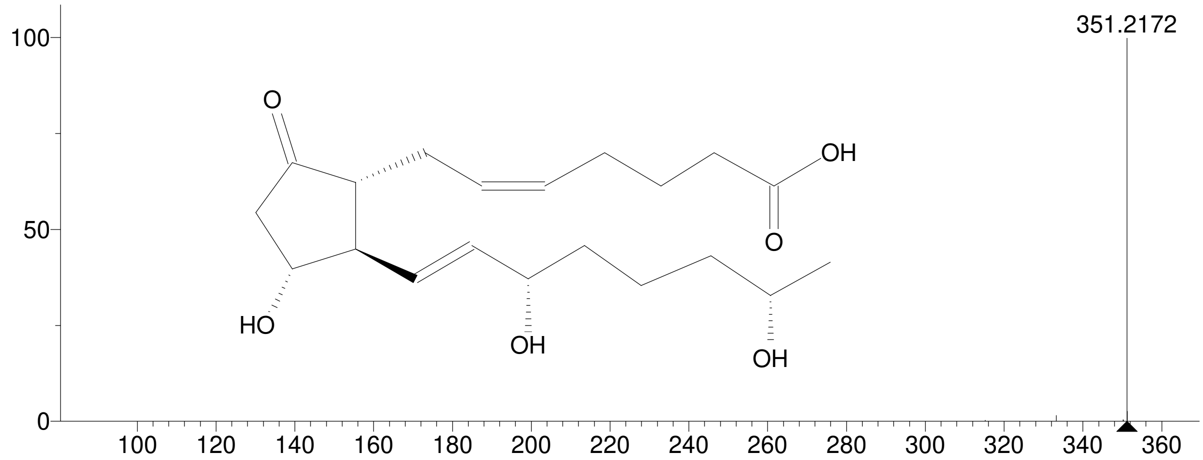

Hit 5 : 6-Ketoprostaglandin E1 [M+H-H2O]+ HCD 1V P=351.2
C20H32O6; MF: 137; RMF: 694; Prob 9.26%; CAS: 67786-53-2; Lib: nist_msms; ID: 161827.

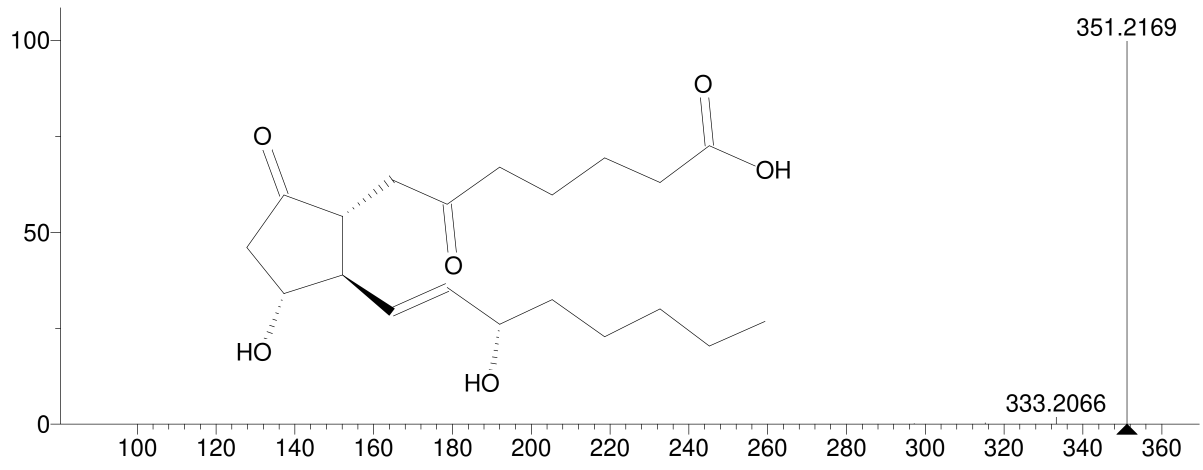

Unknown: Unknown (21.99_436.0494m/z)
Compound in Library Factor = 0

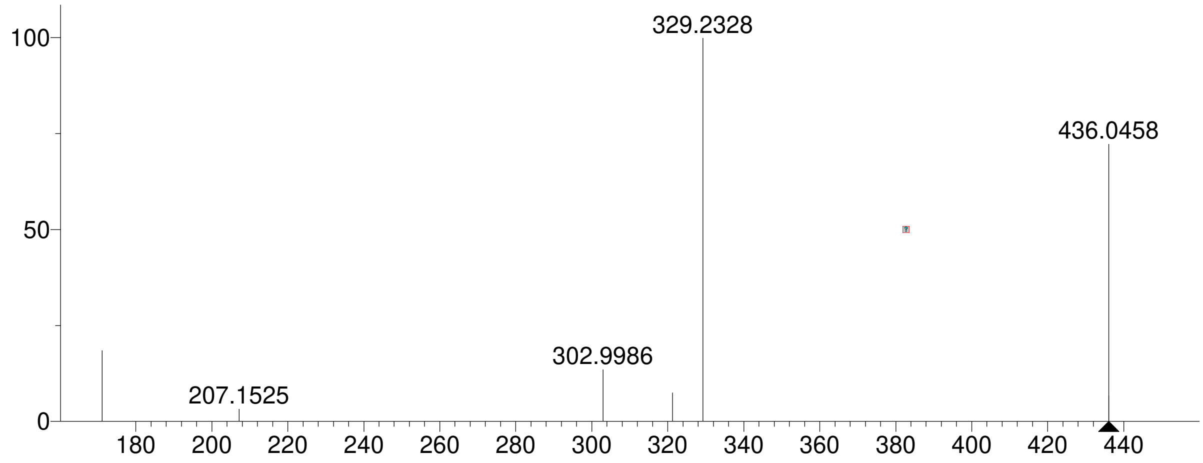

Unknown: Unknown (21.99_456.1076m/z)
Compound in Library Factor = 0

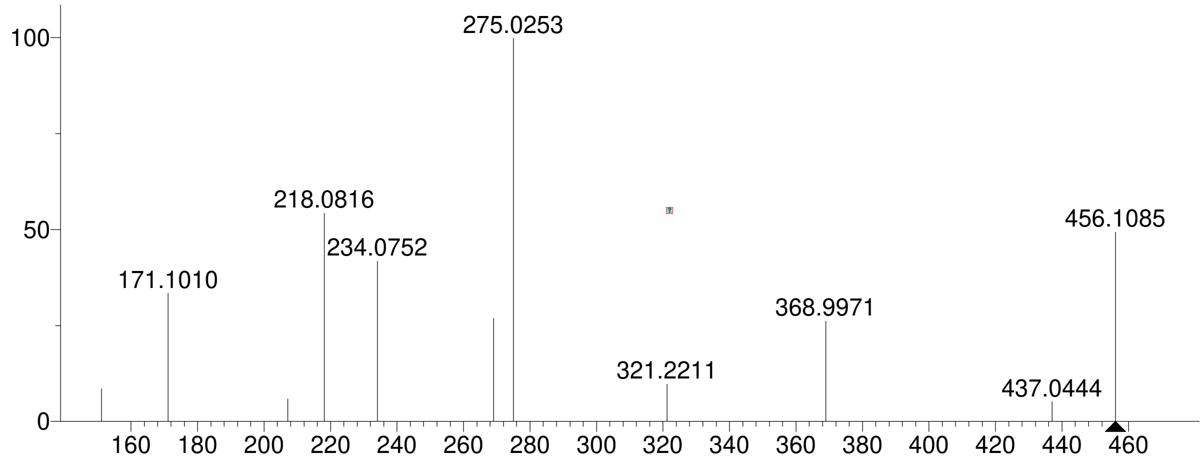

Unknown: Unknown (22.03_220.0968m/z)
Compound in Library Factor = -1523

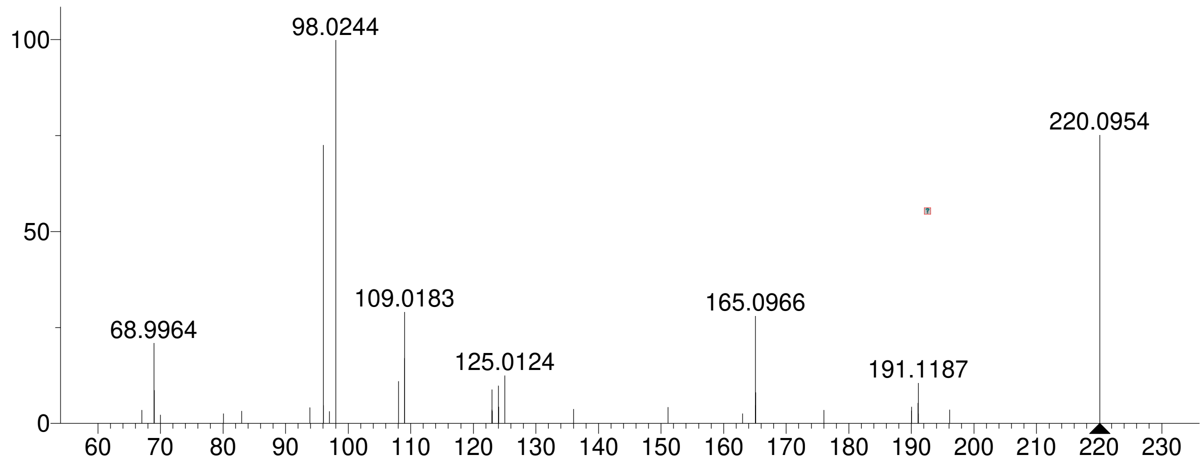

Hit 1 : 6-Methoxymethylone [M+H-H2O]+ HCD 9V P=220.1
C12H15NO4; MF: 144; RMF: 759; Prob 37.3%; Lib: nist_msms; ID: 551027.

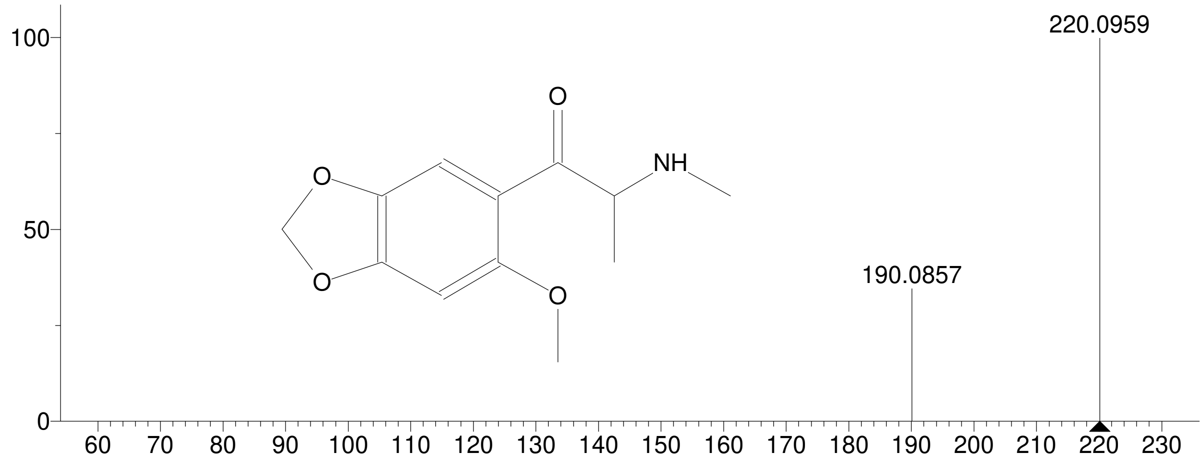

Hit 2 : 5-Methoxymethylone [M+H-H2O]+ HCD 8V P=220.1
C12H15NO4; MF: 137; RMF: 679; Prob 28.6%; Lib: nist_msms; ID: 547148.

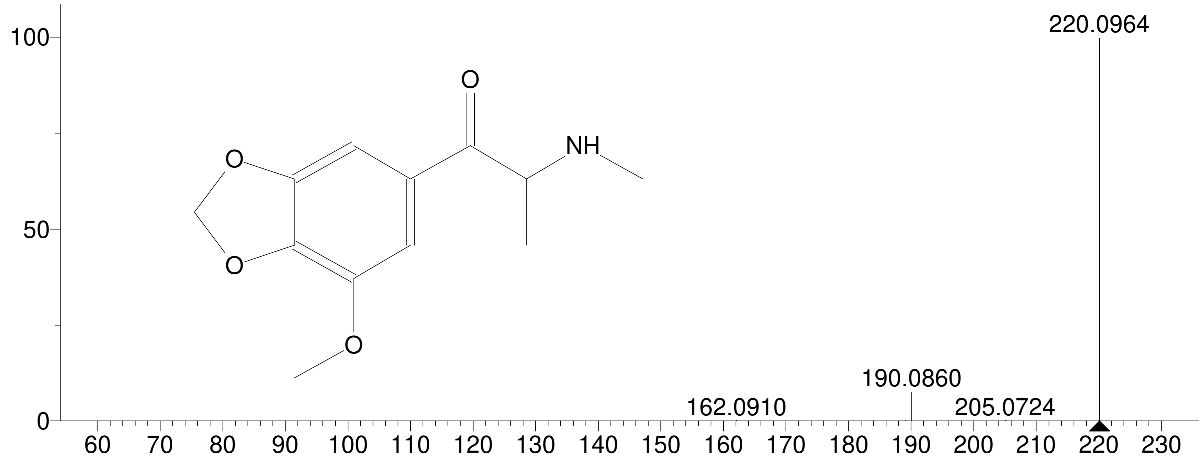

Hit 3 : 3-Hydroxycarbofuran [M+H-H2O]+ HCD 2V P=220.1
C12H15NO4; MF: 136; RMF: 669; Prob 27.5%; CAS: 16655-82-6; Lib: nist_msms; ID: 471676.

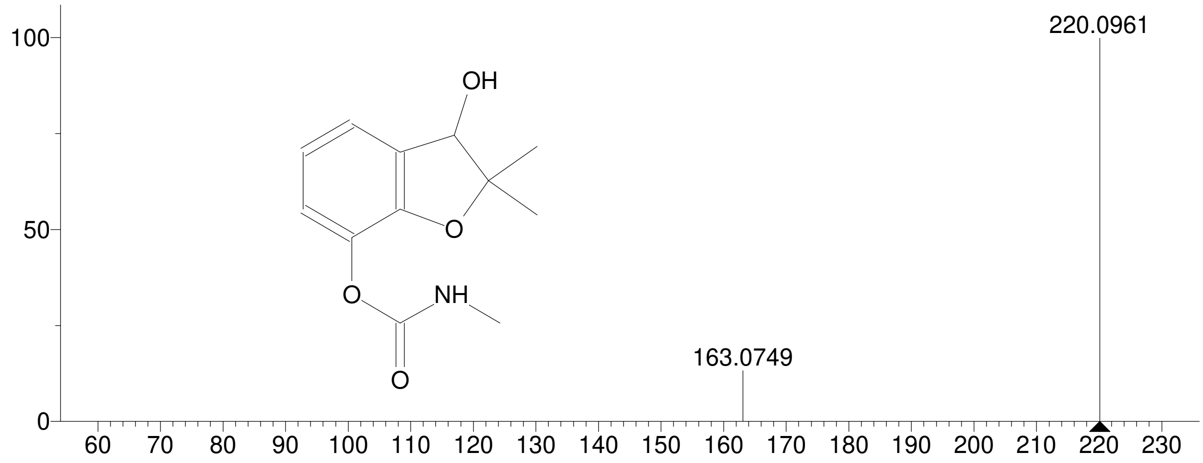

Hit 4 : N-Phenylacetyl-L-homoserine lactone [M+H]+ HCD 4V P=220.1
C12H13NO3; MF: 17; RMF: 362; Prob 1.48%; CAS: 55154-48-8; Lib: nist_msms; ID: 423889.

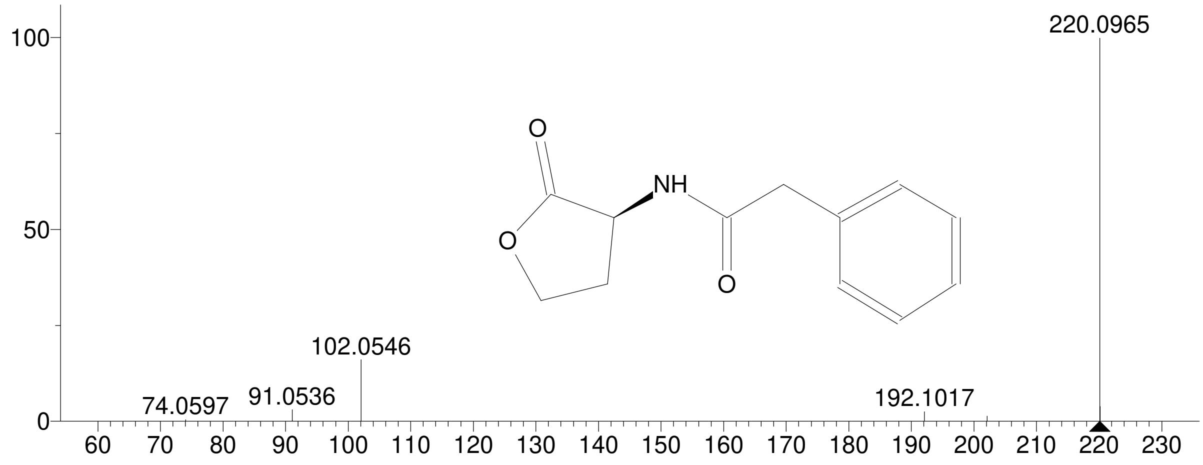

Hit 5 : Mepirizole [M+H-CH3]+ HCD 13V P=220.1
C11H14N4O2; MF: 10; RMF: 296; Prob 1.13%; CAS: 18694-40-1; Lib: nist_msms; ID: 188207.

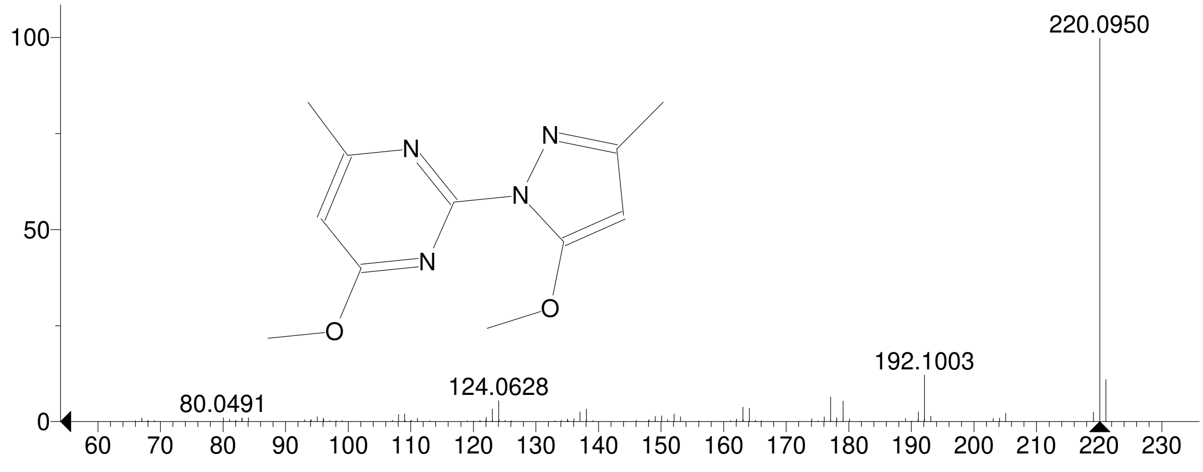

Unknown: Unknown (22.03_395.0298m/z)
Compound in Library Factor = 0

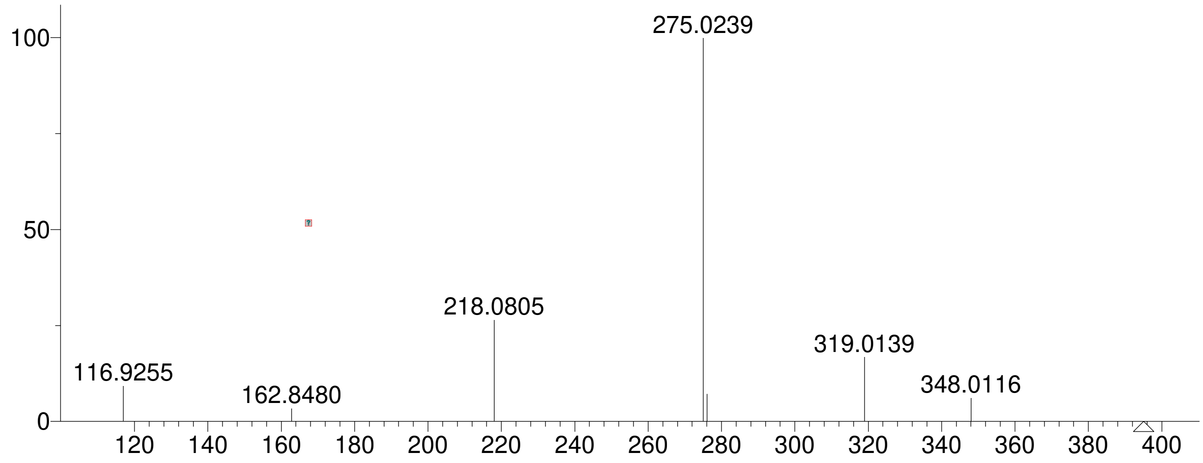

Hit 1 : (4R,8S)-17-chloro-2,18-dimethoxy-7,9,13-trioxapentacyclo[10.8.0.0A^'3,A^'1a^'o.0a^'',a^,,.0A^'1a^'',A^'1a^'
C19H13ClO6; MF: 0; RMF: 51; Prob 100.0%; Lib: mona-export-vf-npl_qexactive; ID: 22920.

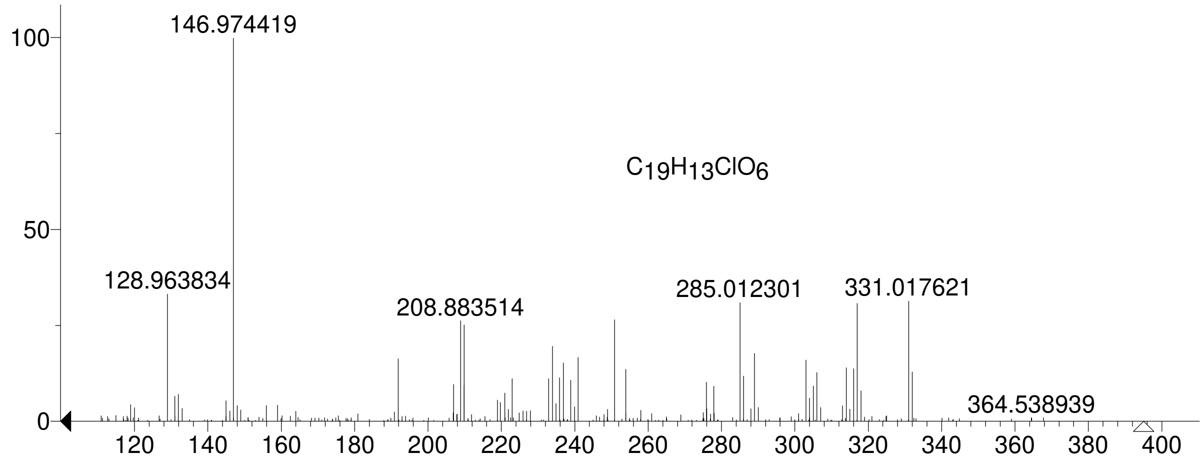

Unknown: Unknown (22.04_250.1077m/z)
Compound in Library Factor = -2387

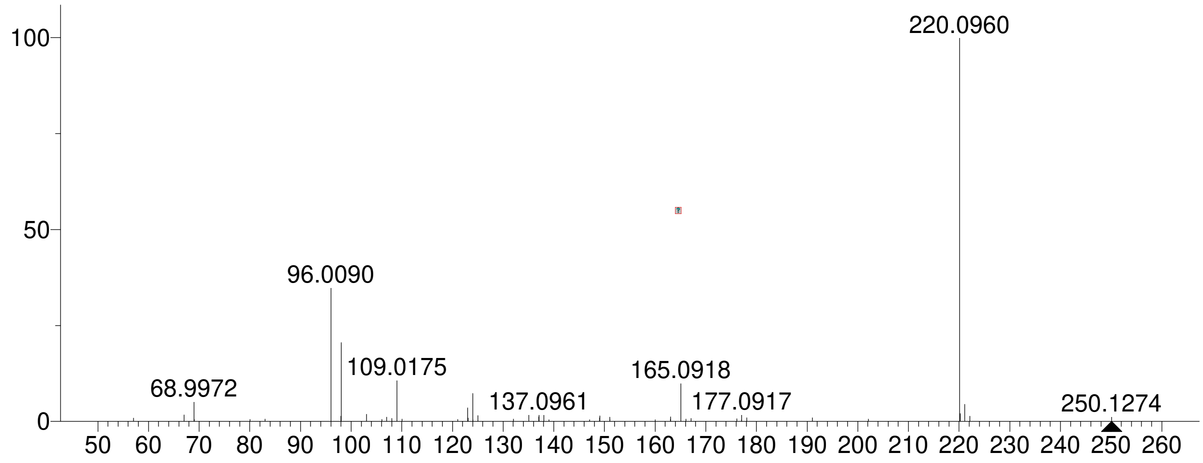

Hit 1 : N-Acetyl-L-tyrosine ethyl ester [M-H]- HCD 22V P=250.1
C13H17NO4; MF: 16; RMF: 283; Prob 46.5%; CAS: 36546-50-6; Lib: nist_msms; ID: 220864.

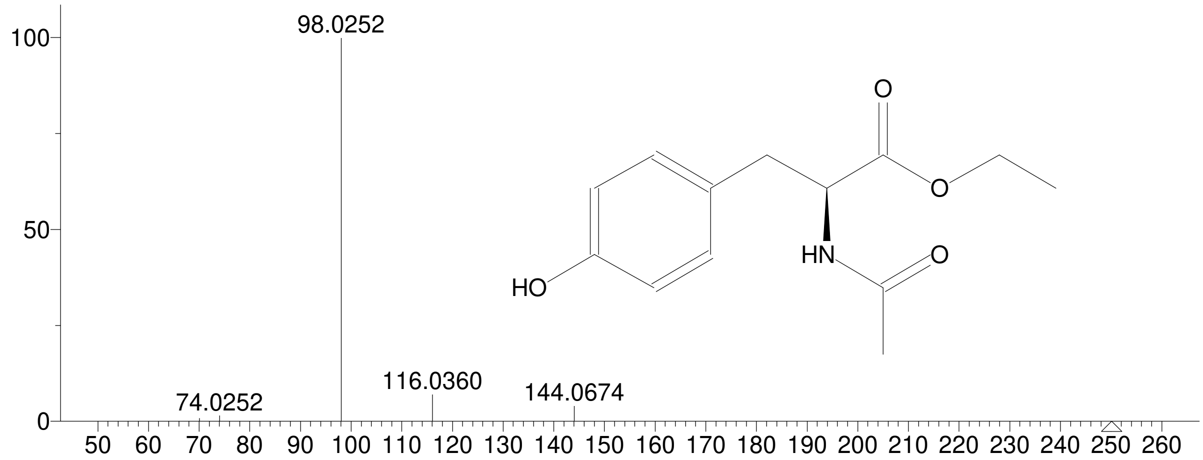

Hit 2 : Goniothalenol [M+NH4]+ 35HCD P=250.1
C13H12O4; MF: 0; RMF: 9; Prob 26.8%; Lib: mona-export-vf-npl_qexactive; ID: 18316.

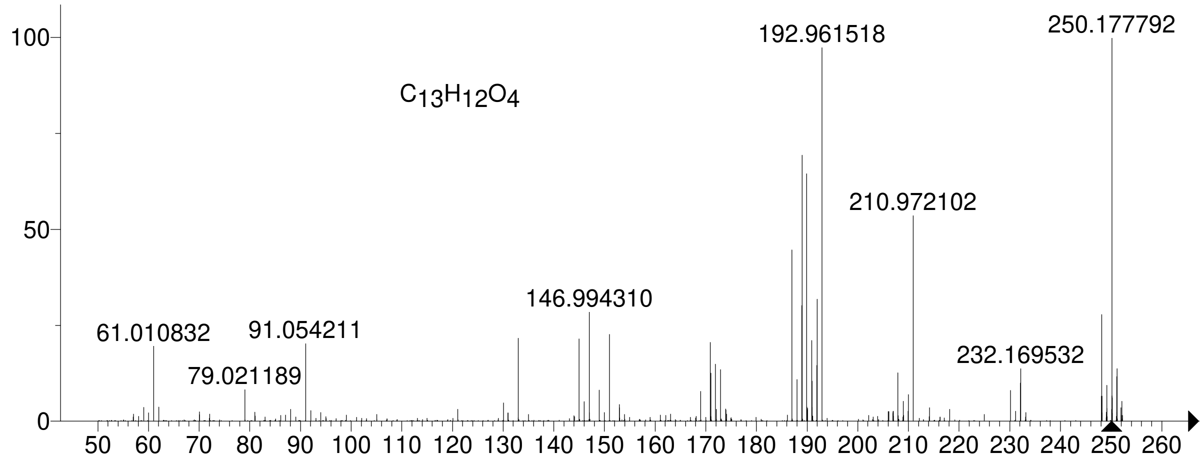

Hit 3 : N-Acetyl-3,4-methylenedioxymethcathinone [M+H]+ HCD 45V P=250.1
C13H15NO4; MF: 0; RMF: 2; Prob 26.8%; CAS: 1227293-15-3; Lib: nist_msms; ID: 423651.

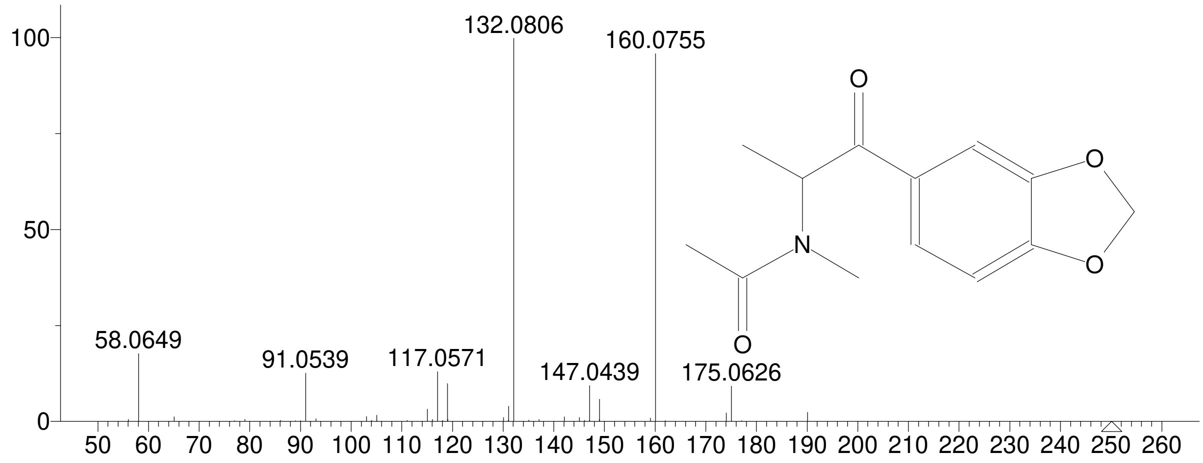

** Search Report Page 1 of 1 **

Unknown: Unknown (22.08_396.2271m/z)
Compound in Library Factor = 0

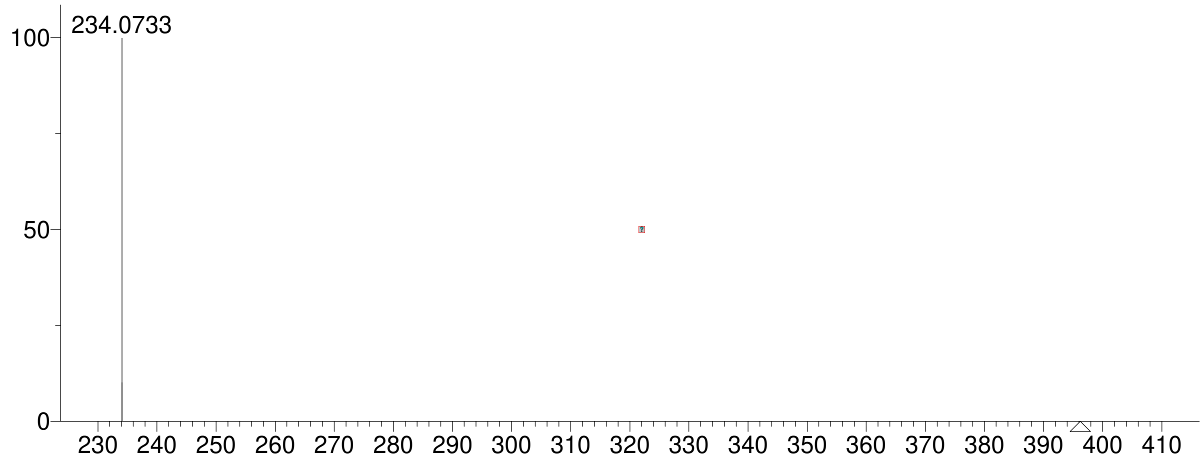

Unknown: Unknown (22.20_252.1226m/z)
Compound in Library Factor = -2176

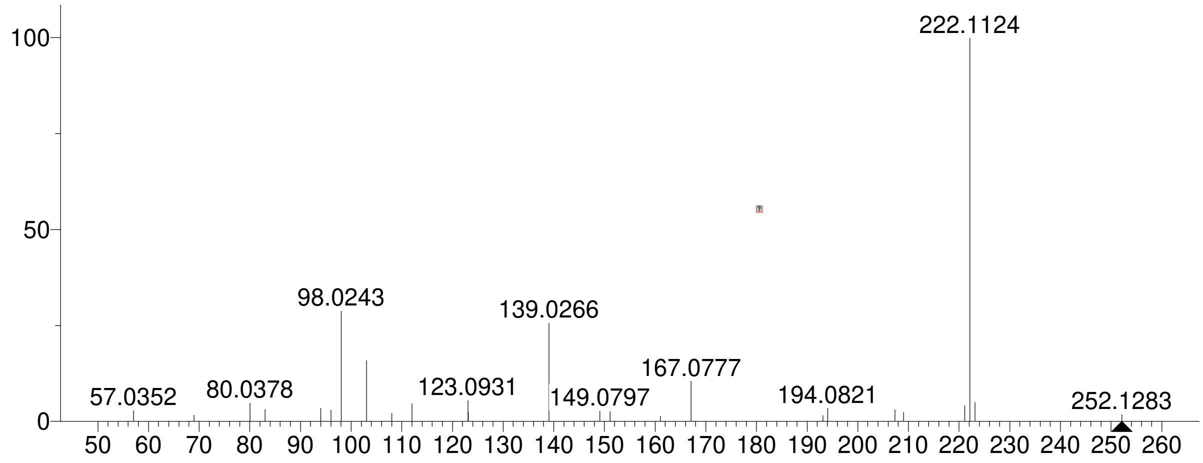

Hit 1 : Dimethachlor-OXA [M+H]+ 10eV P=252.1
C13H17NO4; MF: 27; RMF: 197; Prob 37.5%; Lib: mona-export-massbank; ID: 785.

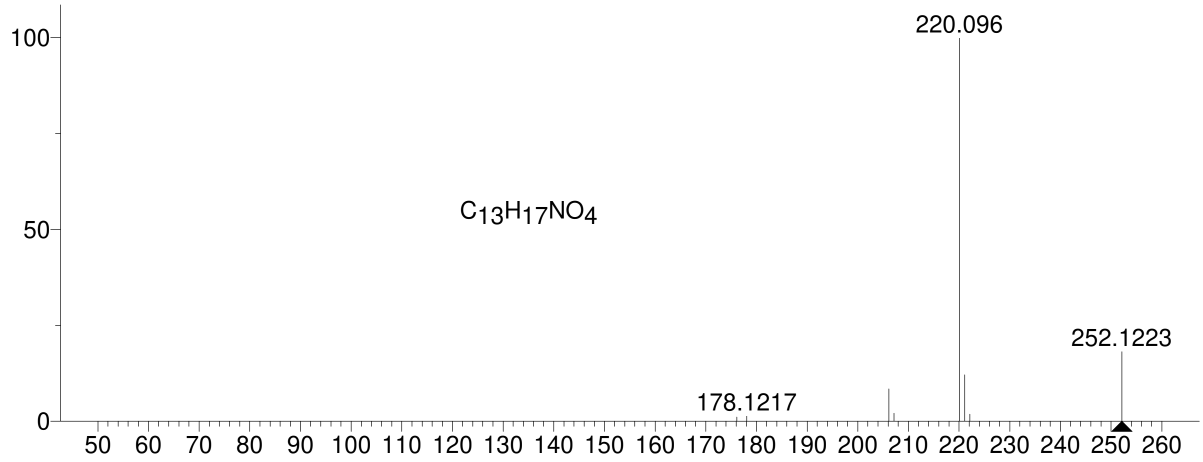

Hit 2 : Dipropyl pyridine-2,5-dicarboxylate [M+H]+ HCD 7V P=252.1
C13H17NO4; MF: 4; RMF: 97; Prob 13.7%; CAS: 136-45-8; Lib: nist_msms; ID: 282969.

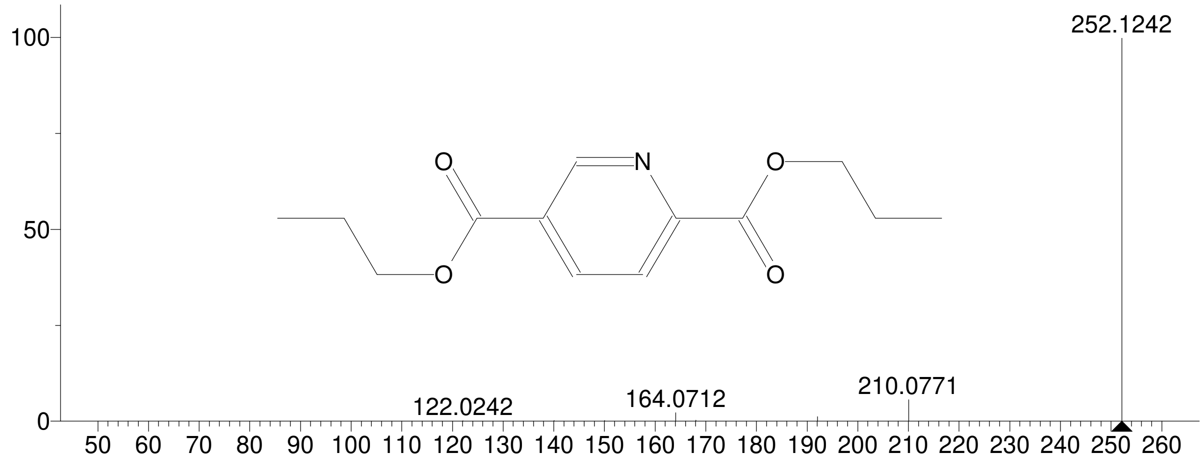

Hit 3 : N-Acetyl-L-tyrosine ethyl ester [M+H]+ HCD 1V P=252.1
C13H17NO4; MF: 3; RMF: 84; Prob 13.2%; CAS: 36546-50-6; Lib: nist_msms; ID: 220796.

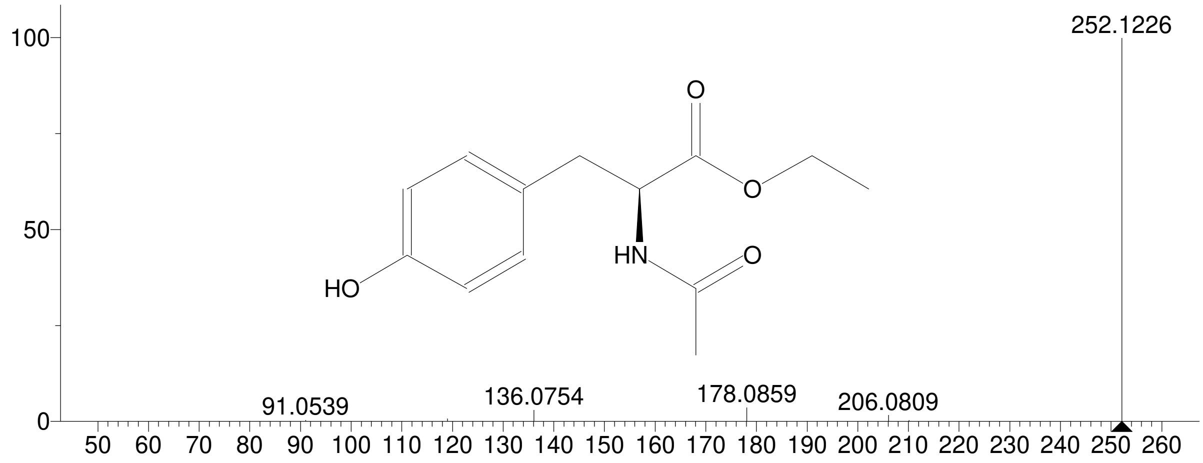

Hit 4 : Dimethachlor OXA [M+H]+ 15% (P=252.1
C13H17NO4; MF: 1; RMF: 66; Prob 12.2%; Lib: mona-export-massbank; ID: 7734.

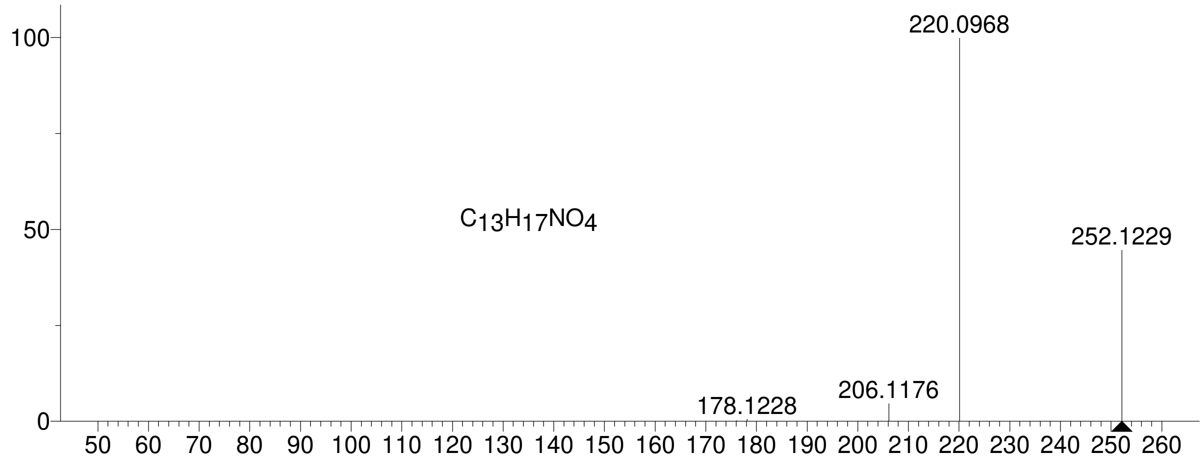

Hit 5 : N-[1-(4-methoxy-6-oxopyran-2-yl)-2-methylbutyl]acetamide [M-H]- 65HCD P=252.1
C13H19NO4; MF: 0; RMF: 29; Prob 11.7%; Lib: mona-export-lc-ms-ms_negative_mode; ID: 22408.

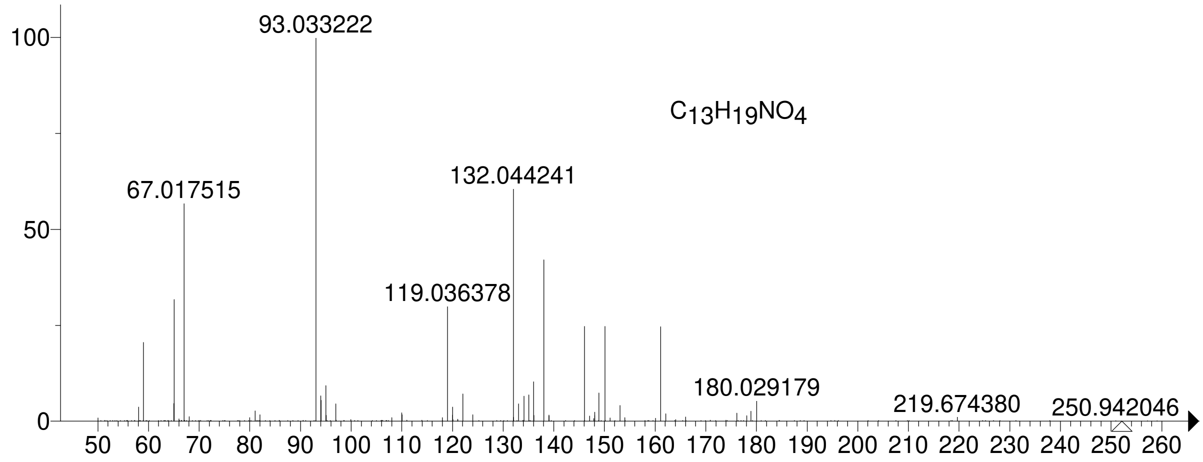

** Search Report Page 1 of 2 **

Unknown: Unknown (22.23_391.1752m/z)
Compound in Library Factor = -3273

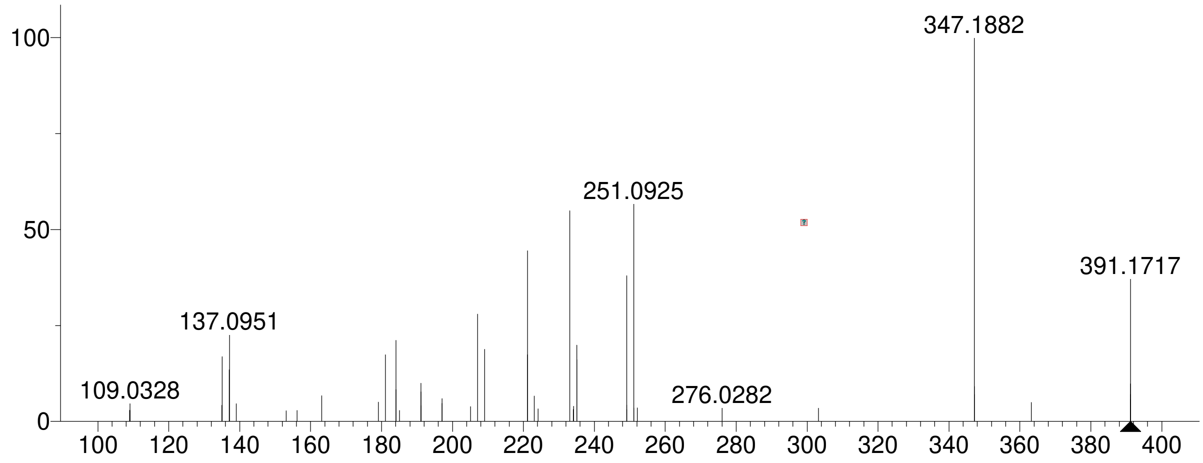

Hit 1 : Tafluprost (free acid) [M-H-H2O]- HCD 15V P=391.2
C22H28F2O5; MF: 17; RMF: 213; Prob 27.1%; CAS: 209860-88-8; Lib: nist_msms; ID: 156343.

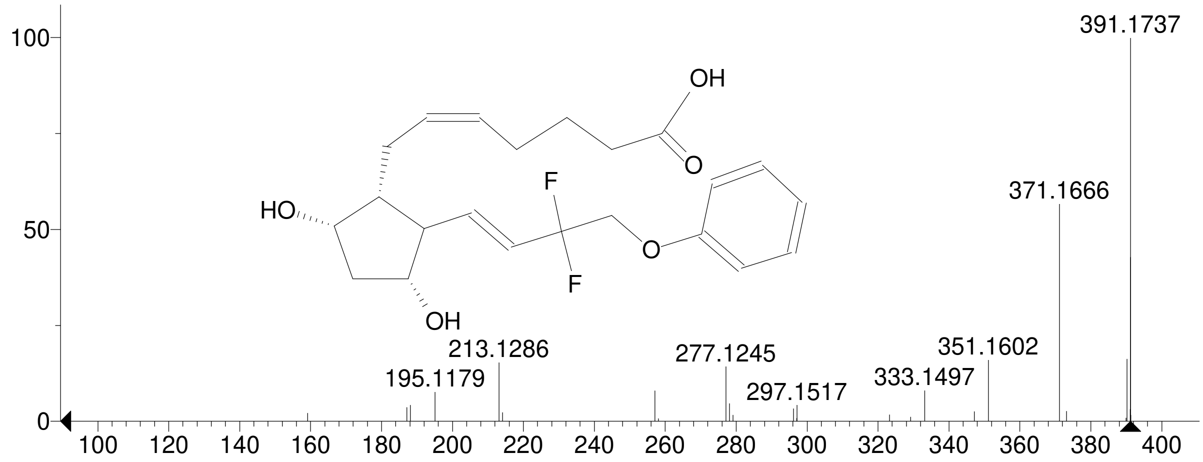

Hit 2 : Salvinorin B [M+H]+ HCD 15V P=391.2
C21H26O7; MF: 14; RMF: 187; Prob 24.0%; CAS: 92545-30-7; Lib: nist_msms; ID: 327112.

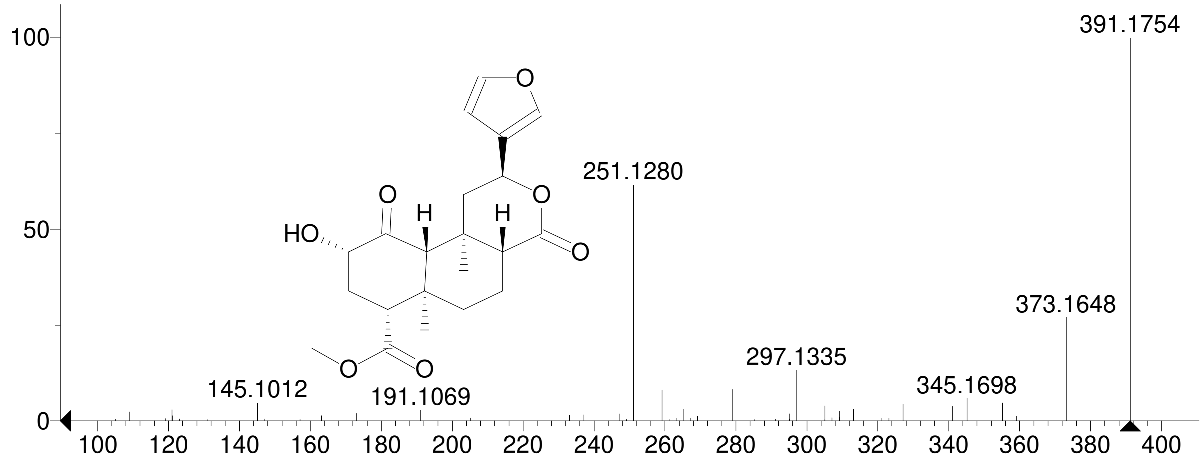

Hit 3 : (2S,3S,4S,8R,9S,13R,14R,15S,16R)-3,4,8,14,15-pentahydroxy-2,13,16-trimethyl-6-methylidene-10-oxatet[illegible]
C19H28O7; MF: 9; RMF: 201; Prob 19.3%; Lib: mona-export-vf-npl_qexactive; ID: 21643.

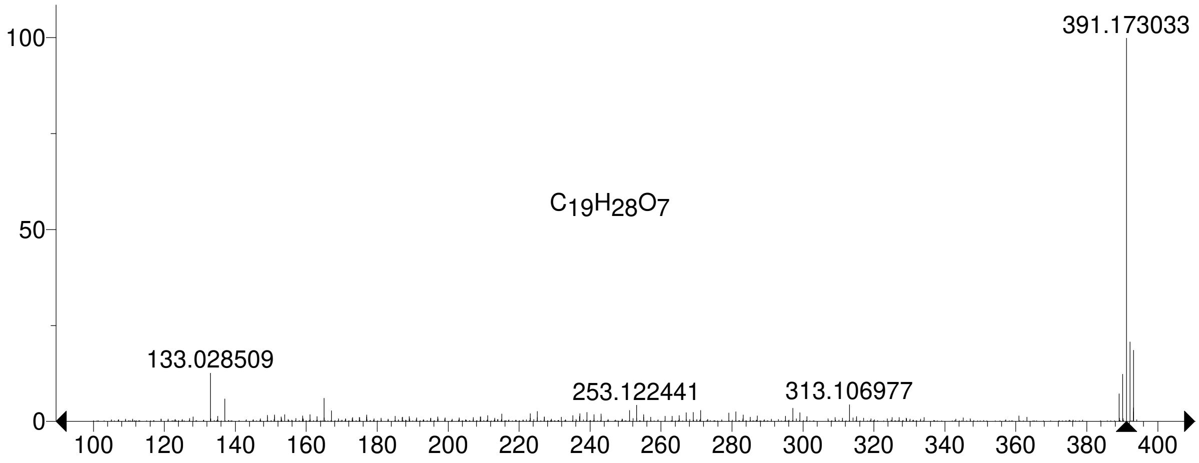

Hit 4 : Trp-Trp [M+H]+ QTOF 2V P=391.2
C22H22N4O3; MF: 5; RMF: 194; Prob 16.3%; CAS: 20696-60-0; Lib: nist_msms; ID: 31562.

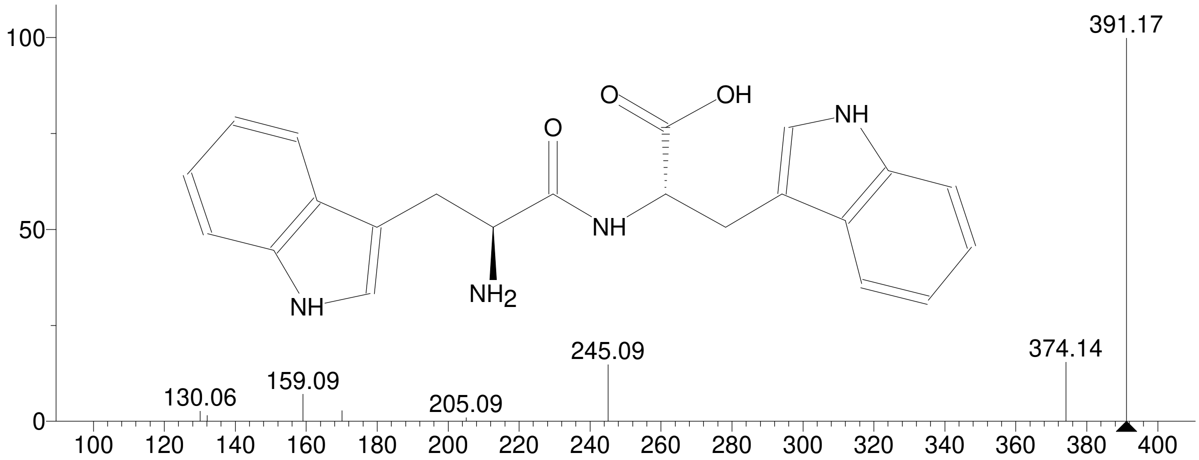

Hit 5 : 6-formyl-10-(hydroxymethyl)-5-methoxy-3-methylidene-2-oxo-2H,3H,3aH,4H,5H,8H,9H,11aH-cyclodeca[b][illegible]
C21H28O7; MF: 0; RMF: 37; Prob 13.2%; Lib: mona-export-lc-ms-ms_negative_mode; ID: 21660.

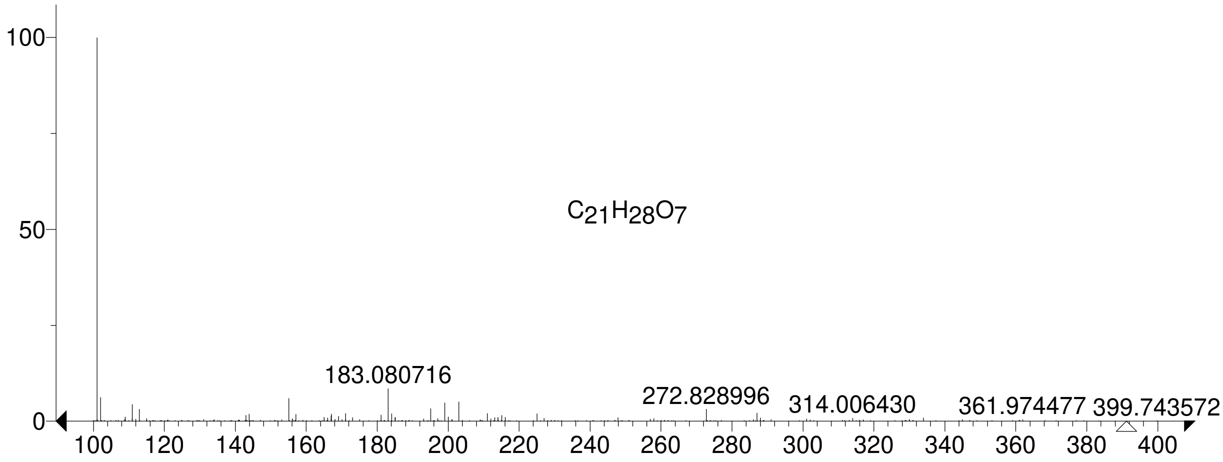

** Search Report Page 1 of 2 **

Unknown: Unknown (22.33_351.2152m/z)
Compound in Library Factor = -284

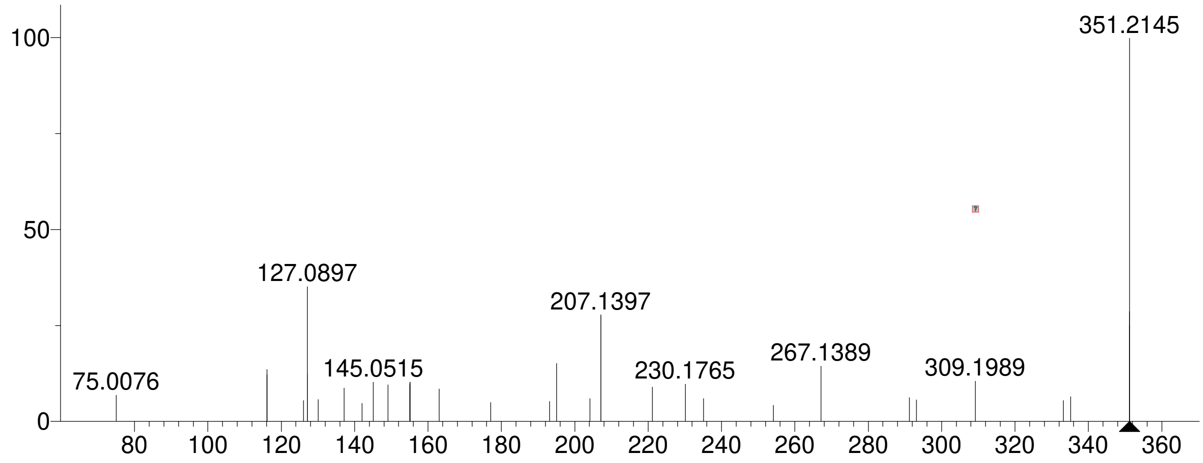

Hit 1 : 11,15-Dioxo-9S-hydroxy-5Z-prostenoic acid [M-H]- HCD 4V P=351.2
C20H32O5; MF: 382; RMF: 839; Prob 98.3%; CAS: 59894-07-4; Lib: nist_msms; ID: 87217.

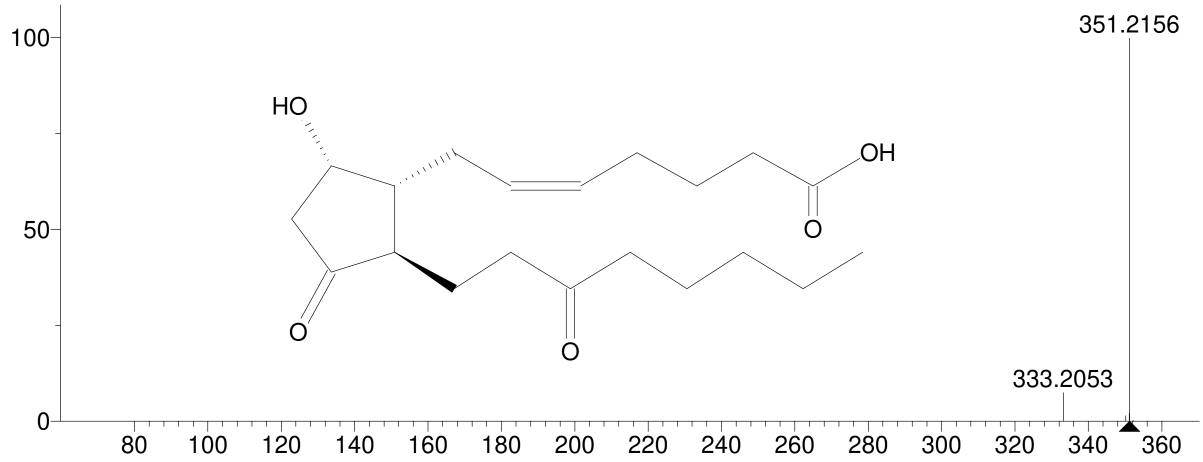

Hit 2 : 15-Ketoprostaglandin E2 [M+H]+ HCD 1V P=351.2
C20H30O5; MF: 179; RMF: 794; Prob 0.98%; CAS: 26441-05-4; Lib: nist_msms; ID: 169378.

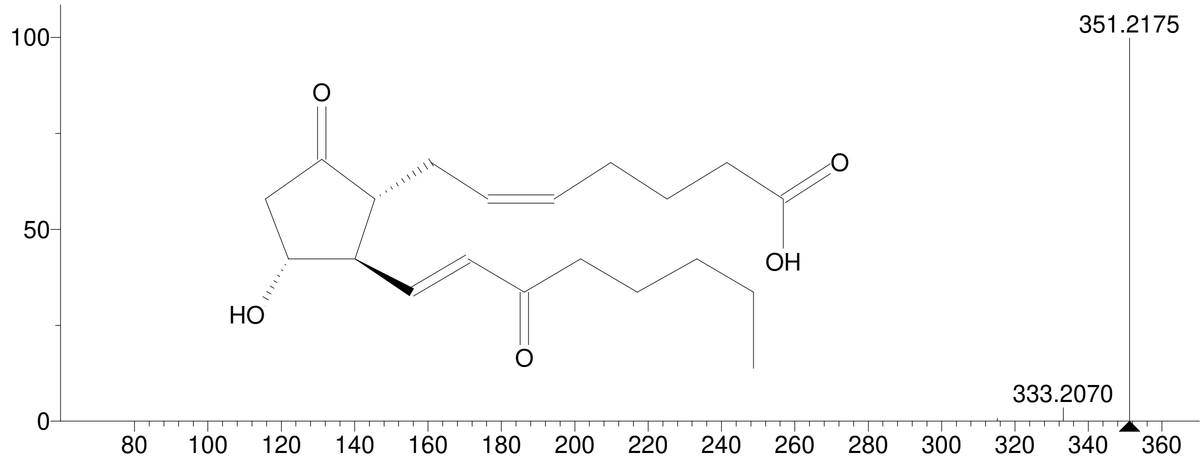

Hit 3 : 6-Ketoprostaglandin E1 [M+H-H2O]+ HCD 1V P=351.2
C20H32O6; MF: 141; RMF: 768; Prob 0.23%; CAS: 67786-53-2; Lib: nist_msms; ID: 161827.

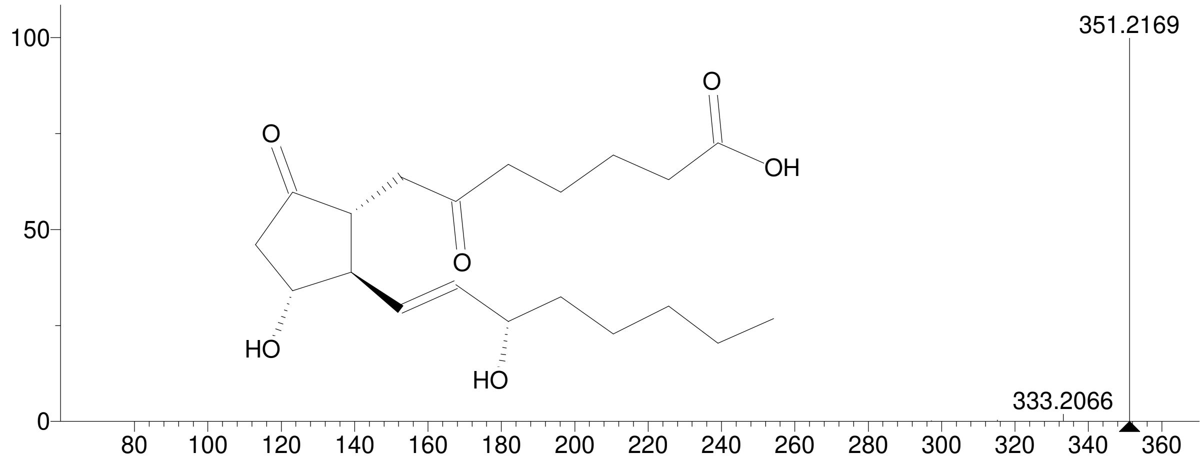

Hit 4 : 15-Ketoprostaglandin F2α [M-H]- HCD 4V P=351.2
C20H32O5; MF: 125; RMF: 766; Prob 0.13%; CAS: 35850-13-6; Lib: nist_msms; ID: 155320.

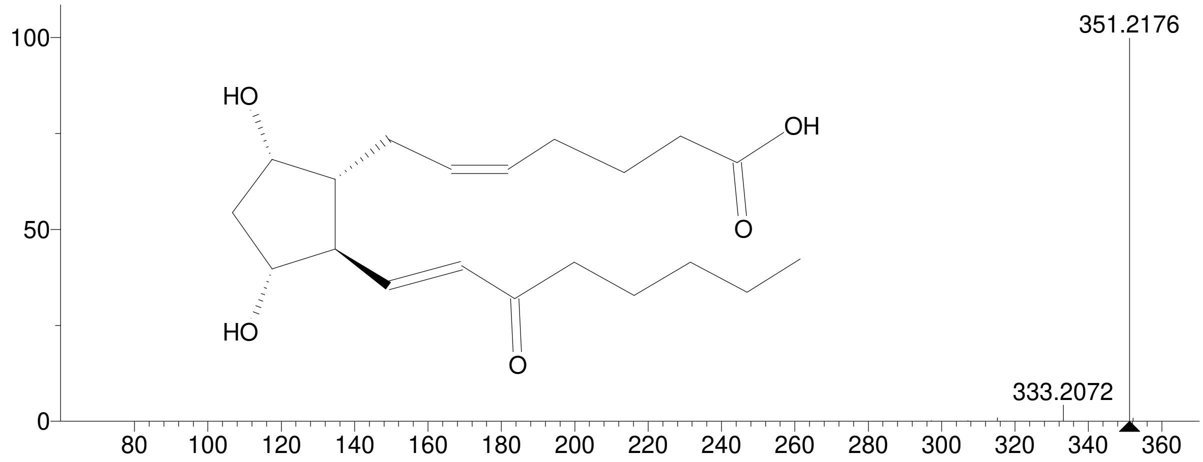

Hit 5 : 8-iso-15-Ketoprostaglandin F2α [M-H]- HCD 7V P=351.2
C20H32O5; MF: 118; RMF: 699; Prob 0.10%; CAS: 191919-01-4; Lib: nist_msms; ID: 158459.

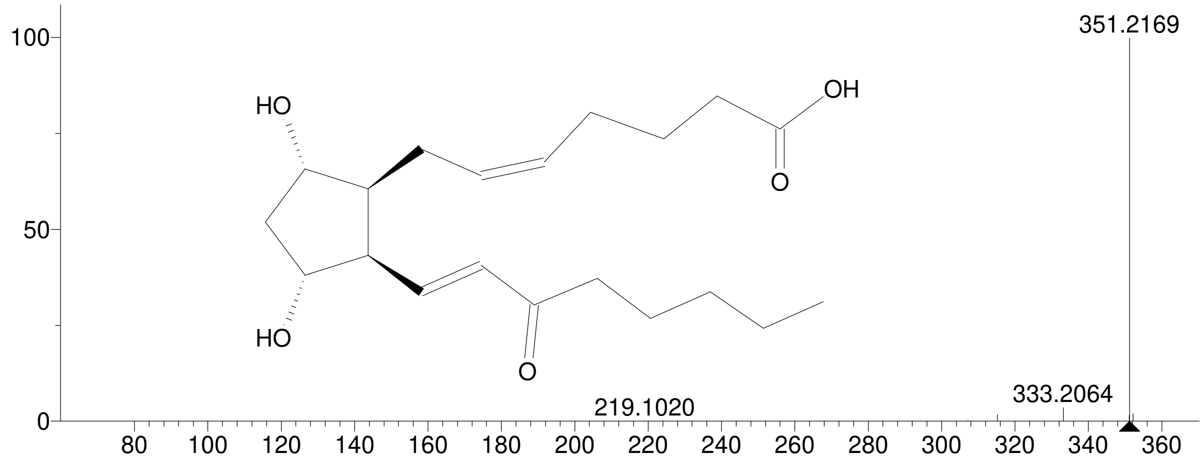

** Search Report Page 1 of 2 **

Unknown: Unknown (22.52_251.1635m/z)
Compound in Library Factor = -302

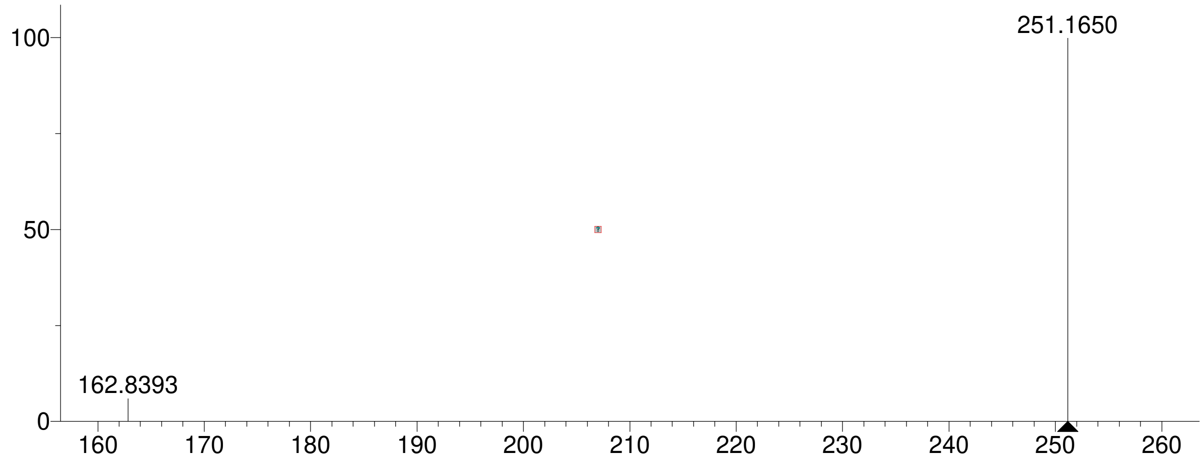

Hit 1 : (6aR)-5S-Hydroxy-4S-((R,E)-3aS-hydroxyoct-1-en-1-yl)hexahydro-2H-cyclopenta[b]furan-2-one [M+H-H2(
C15H24O4; MF: 352; RMF: 971; Prob 49.4%; CAS: 53110-06-8; Lib: nist_msms; ID: 158153.

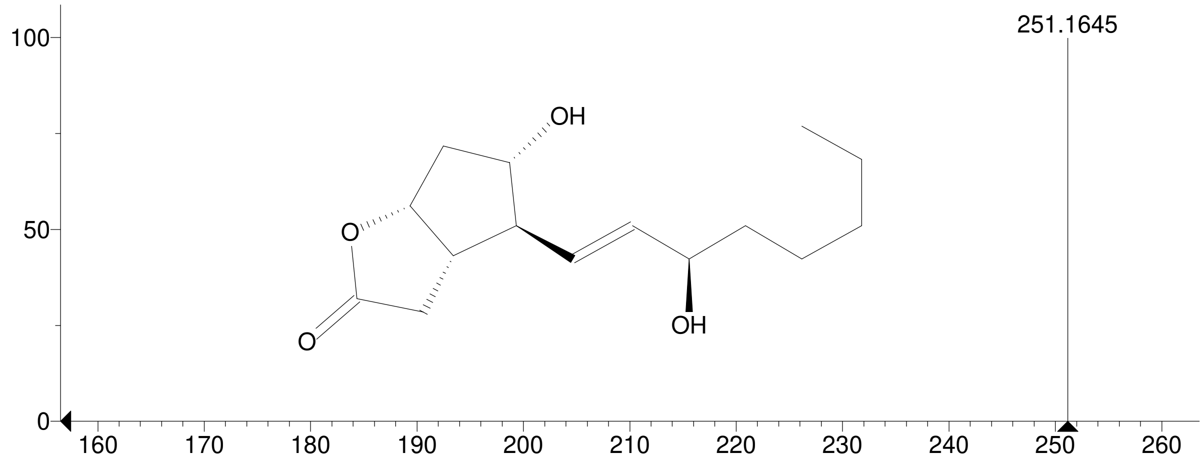

Hit 2 : 3,5-Di-tert-butyl-4-hydroxybenzoic acid [M+H]+ HCD 80V P=251.2
C15H22O3; MF: 352; RMF: 971; Prob 49.4%; CAS: 1421-49-4; Lib: nist_msms; ID: 299242.

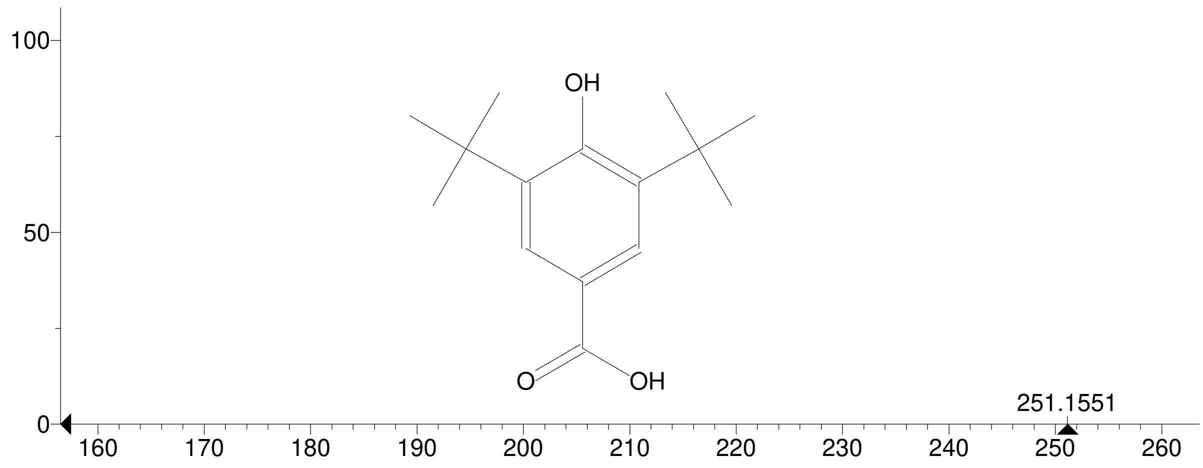

Hit 3 : Corey PG-lactone diol [M+H-H2O]+ HCD 10V P=251.2
C15H24O4; MF: 171; RMF: 904; Prob 0.86%; CAS: 26054-67-1; Lib: nist_msms; ID: 374216.

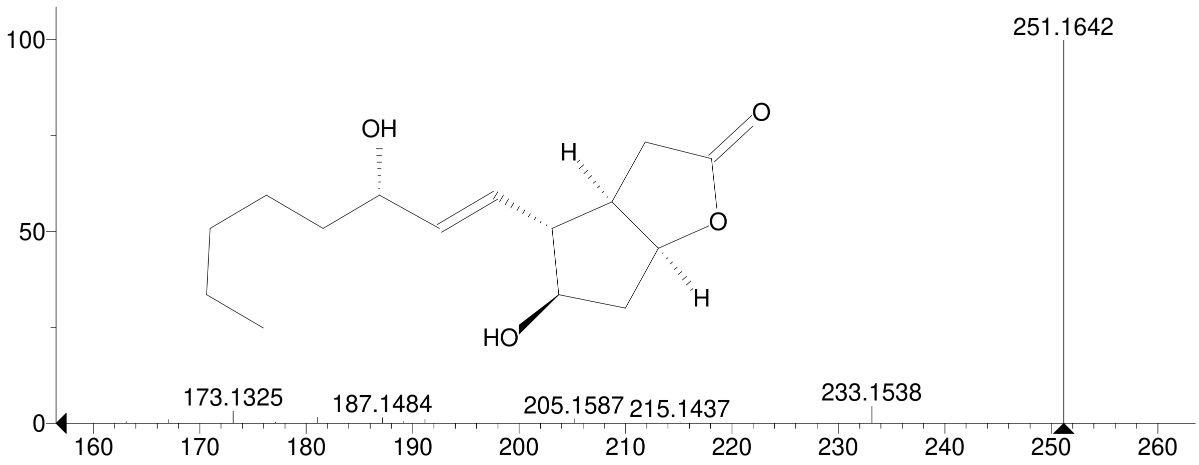

Hit 4 : NCGC00380529-01_C15H22O3_4,7-Methanoazulen-6(1H)-one, 2,4,5,7,8,8a-hexahydro-7-hydroxy-9-(hyd
C15H22O3; MF: 1; RMF: 262; Prob 0.01%; Lib: mona-export-gnps; ID: 10529.

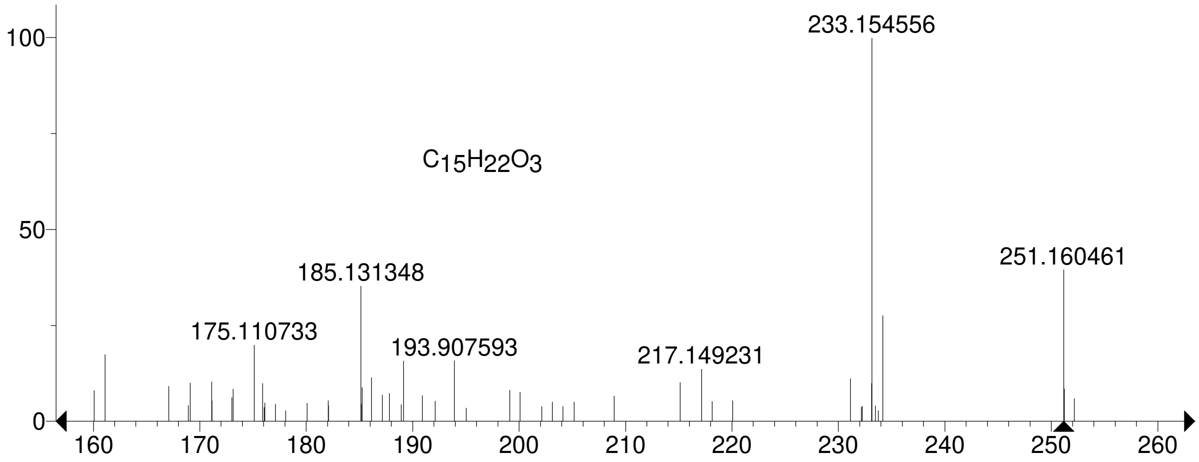

Hit 5 : 8-hydroxy-11-(hydroxymethyl)-1,5,11-trimethyltricyclo[6.2.1.0A^'2,a^!P]undec-2-en-9-one [M+H]+ 35HCD
C15H22O3; MF: 0; RMF: 188; Prob 0.01%; Lib: mona-export-vf-npl_qexactive; ID: 22627.

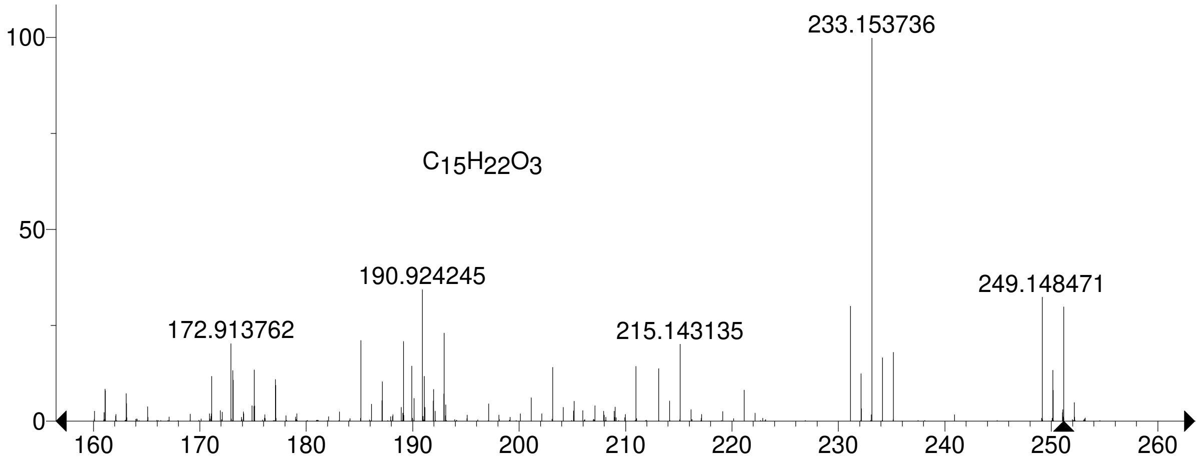

Unknown: Unknown (22.52_421.1852m/z)
Compound in Library Factor = -3273

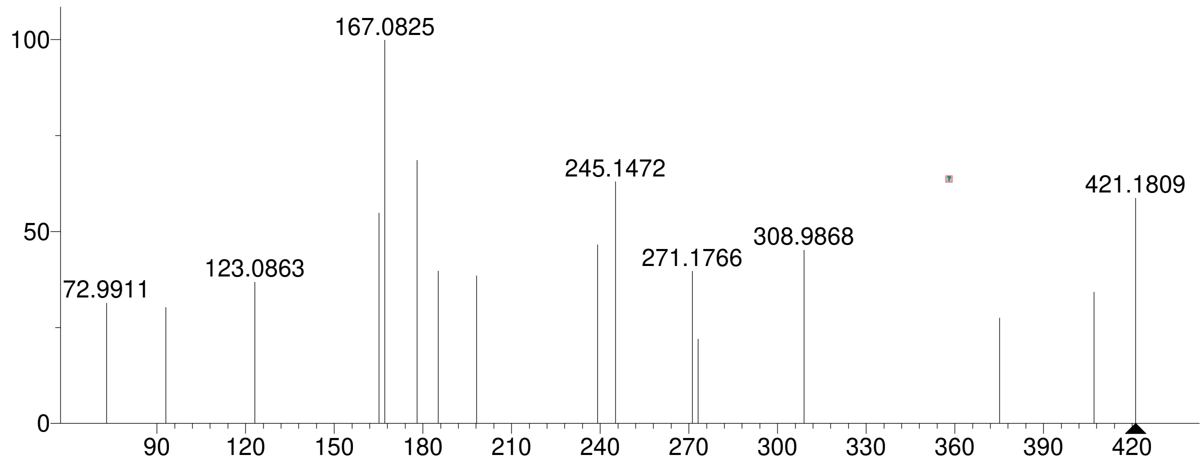

Hit 1 : Met-Asp-Arg [M+H]+ QTOF 5V P=421.2
C15H28N6O6S; MF: 28; RMF: 332; Prob 21.0%; Lib: nist_msms; ID: 52198.

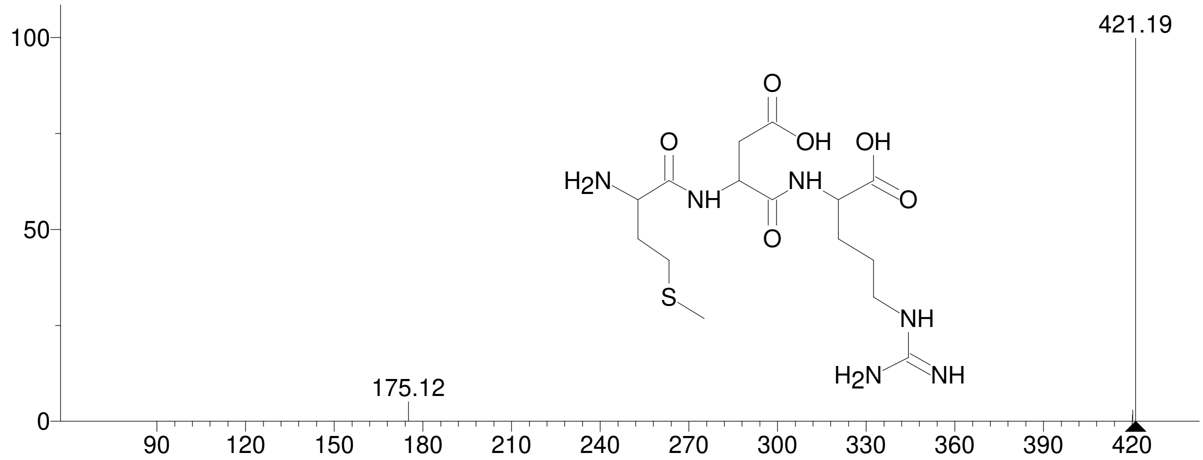

Hit 2 : Glp-His-Pro-Gly [M+H]+ QTOF 3V P=421.2
C18H24N6O6; MF: 23; RMF: 332; Prob 16.9%; Lib: nist_msms2; ID: 32614.

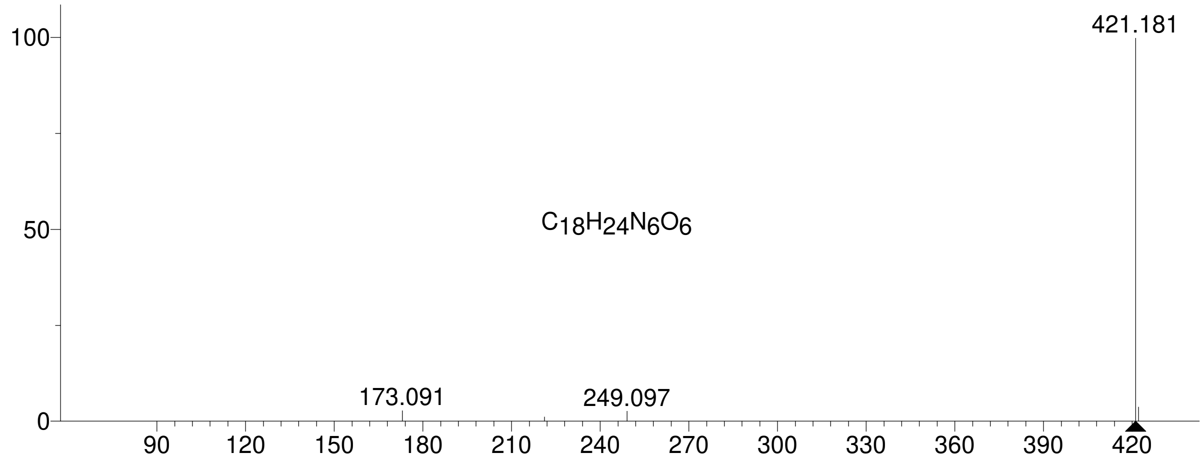

Hit 3 : Asp-Met-Arg [M+H]+ QQQ 8V P=421.2
C15H28N6O6S; MF: 21; RMF: 292; Prob 15.6%; Lib: nist_msms; ID: 41649.

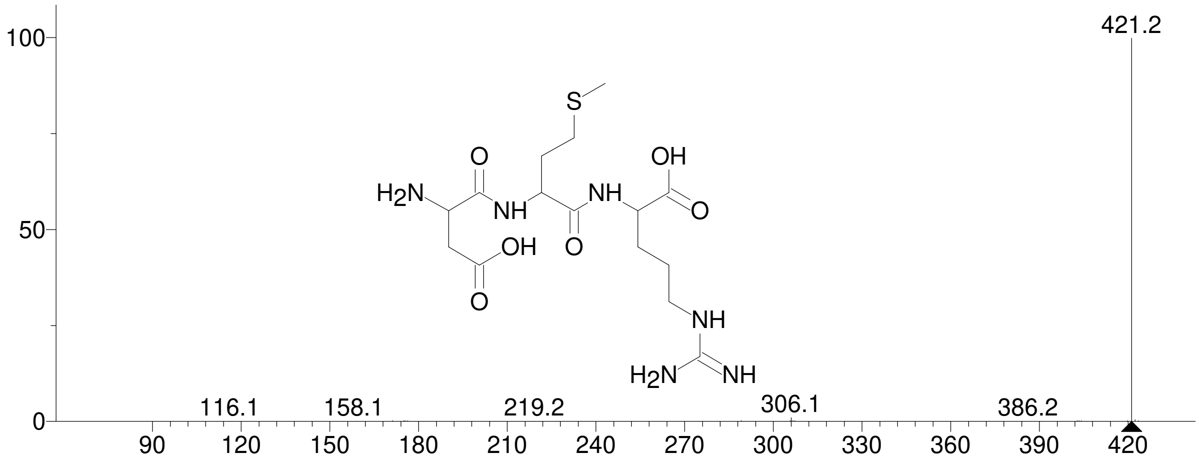

Hit 4 : 3-Cyclohexyl-6-{4-[3-(trifluoromethyl)phenyl]-1-piperazinyl}-2,4(1H,3H)-pyrimidinedione [M-H]- HCD 29V
C21H25F3N4O2; MF: 6; RMF: 256; Prob 9.46%; Lib: nist_msms; ID: 348660.

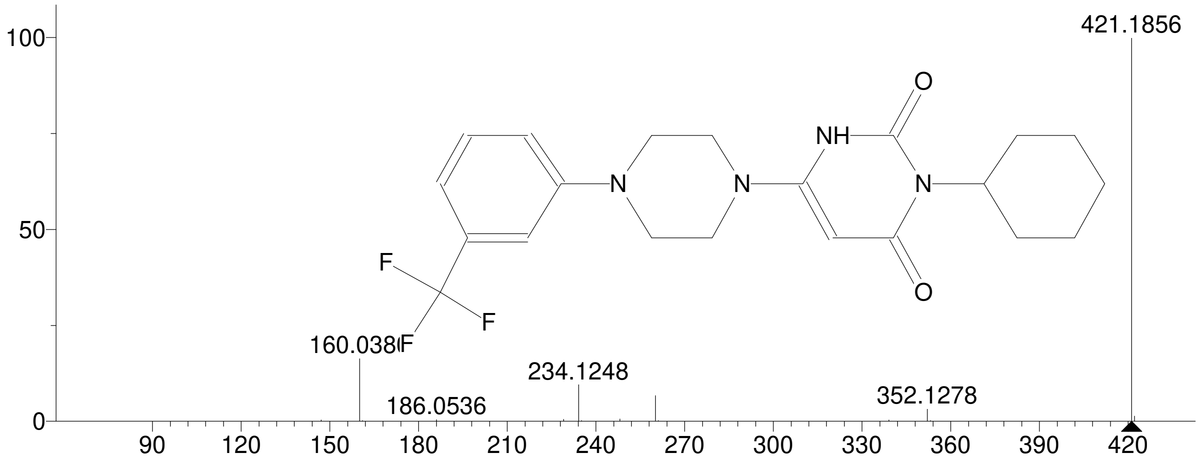

Hit 5 : (2R,3S,4S,9R,12R,13R,14S,15R,16R,17S)-3,4,12,13,15,16-hexahydroxy-2,6,14,17-tetramethyl-10-oxatetr
C20H30O8; MF: 1; RMF: 186; Prob 7.62%; Lib: mona-export-vf-npl_qexactive; ID: 16420.

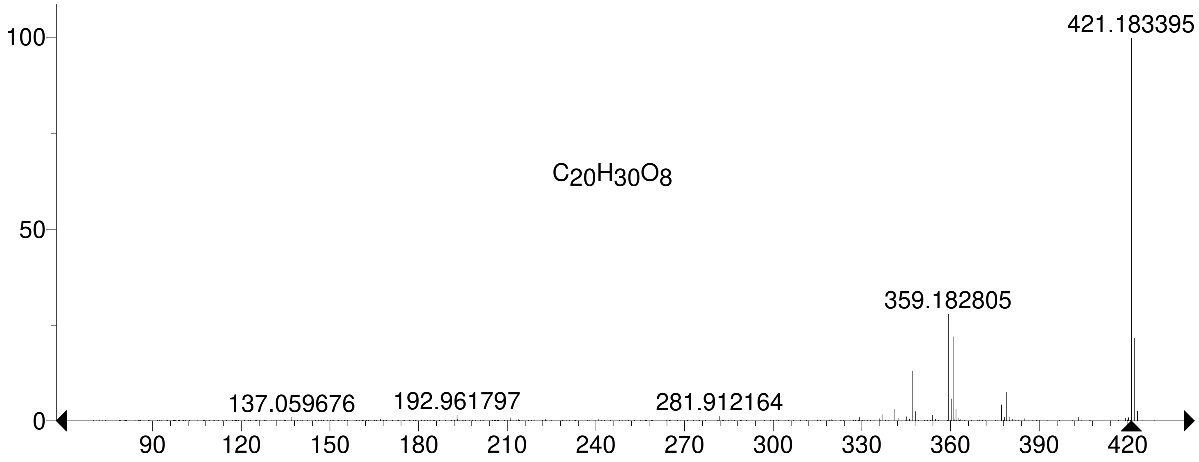

Unknown: Unknown (22.59_250.1079m/z)
Compound in Library Factor = -2176

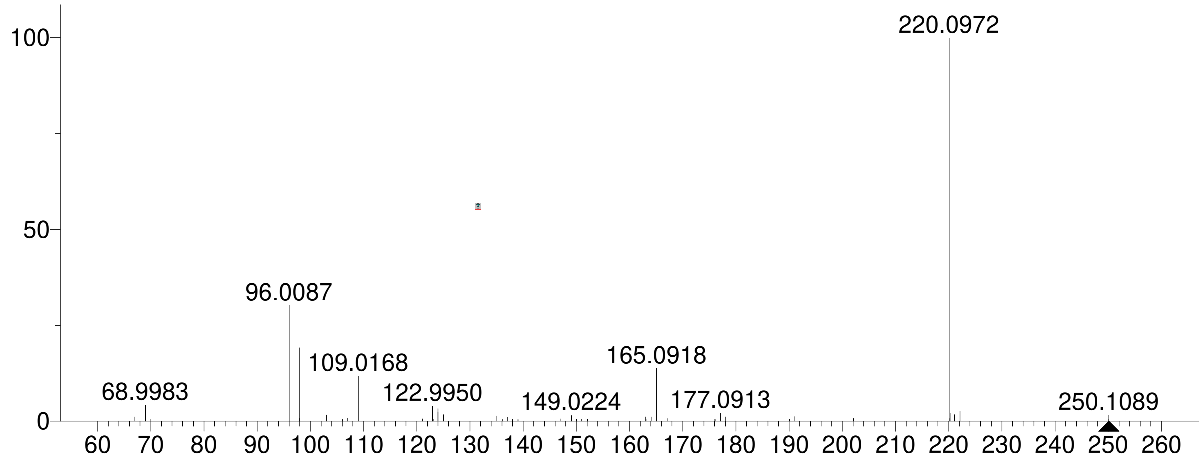

Hit 1 : N-Acetyl-L-tyrosine ethyl ester [M-H]- HCD 5V P=250.1
C13H17NO4; MF: 33; RMF: 165; Prob 50.0%; CAS: 36546-50-6; Lib: nist_msms; ID: 220855.

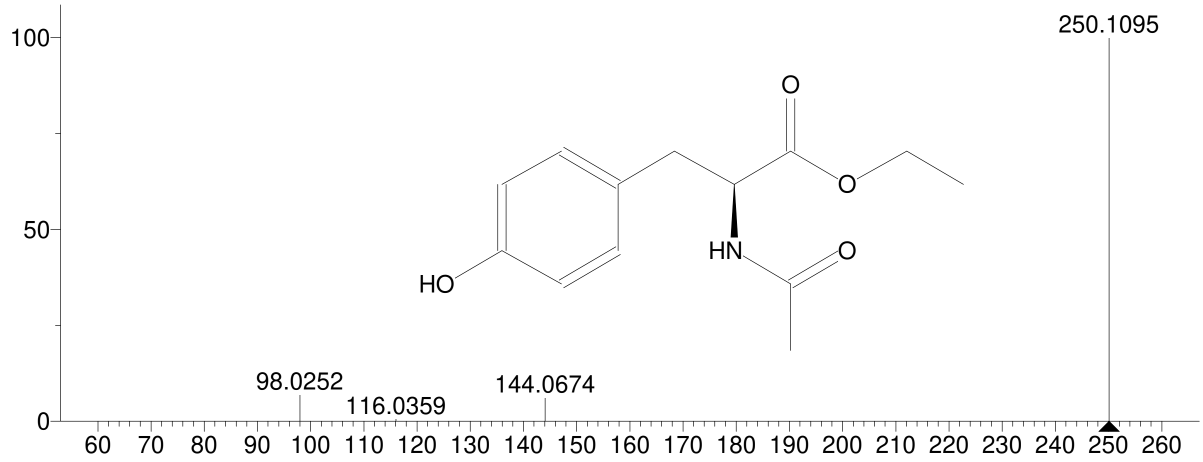

Hit 2 : N-Acetyl-3,4-methylenedioxymethcathinone [M+H]+ HCD 7V P=250.1
C13H15NO4; MF: 4; RMF: 80; Prob 14.2%; CAS: 1227293-15-3; Lib: nist_msms; ID: 423641.

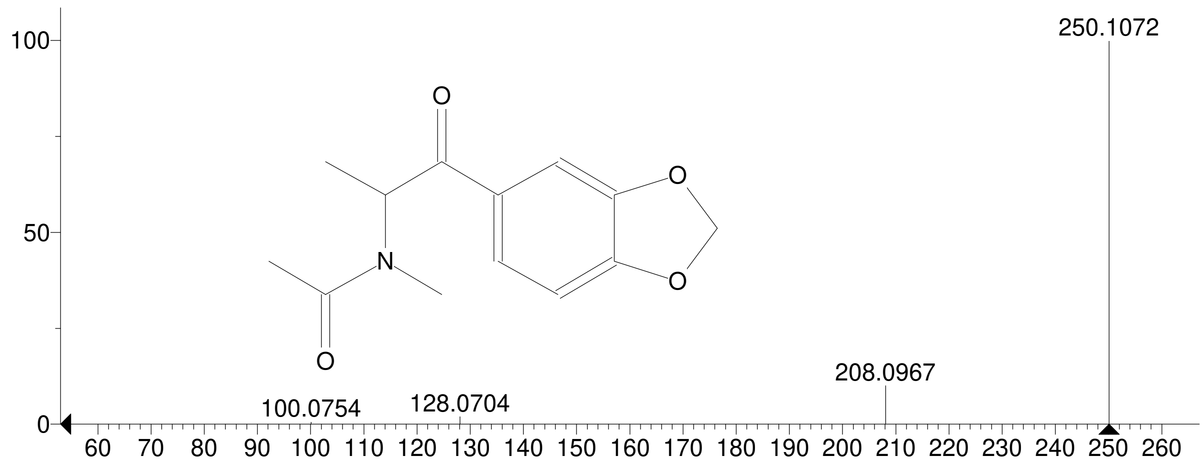

Hit 3 : Dimethachlor OXA; LC-ESI-ITFT; MS2; CE [M-H]- 15 P=250.1
C13H17NO4; MF: 0; RMF: 28; Prob 12.0%; Lib: msms-neg-massbank; ID: 2796.

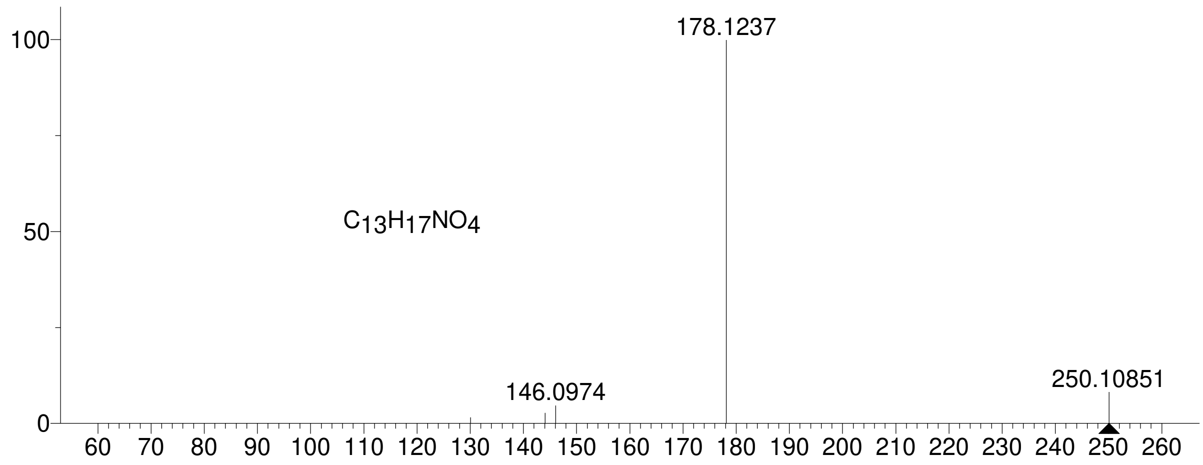

Hit 4 : Dimethachlor OXA [M-H]- 15% (P=250.1
C13H17NO4; MF: 0; RMF: 26; Prob 12.0%; Lib: mona-export-lc-ms-ms_negative_mode; ID: 3042.

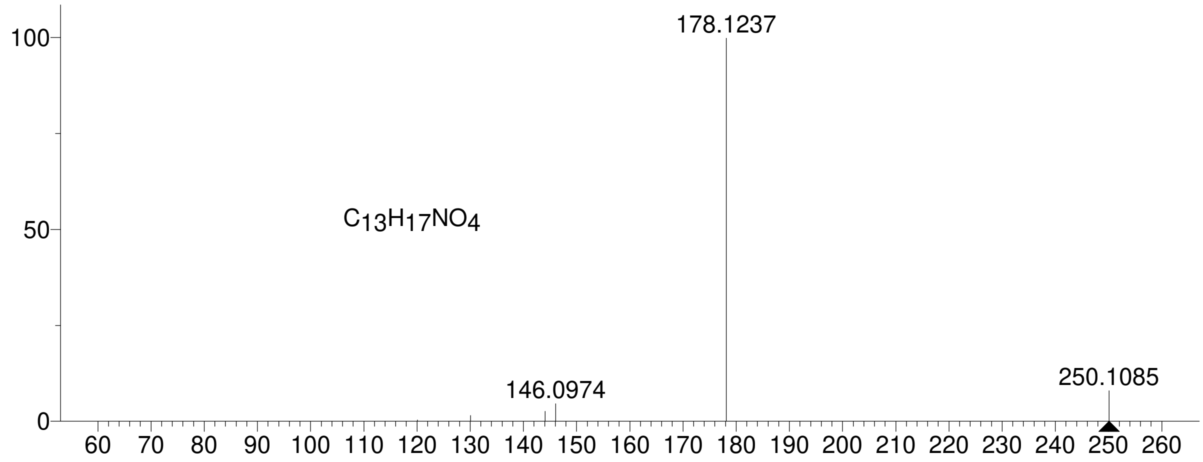

Hit 5 : Goniothalenol [M+NH4]+ 35HCD P=250.1
C13H12O4; MF: 0; RMF: 12; Prob 12.0%; Lib: mona-export-vf-npl_qexactive; ID: 18316.

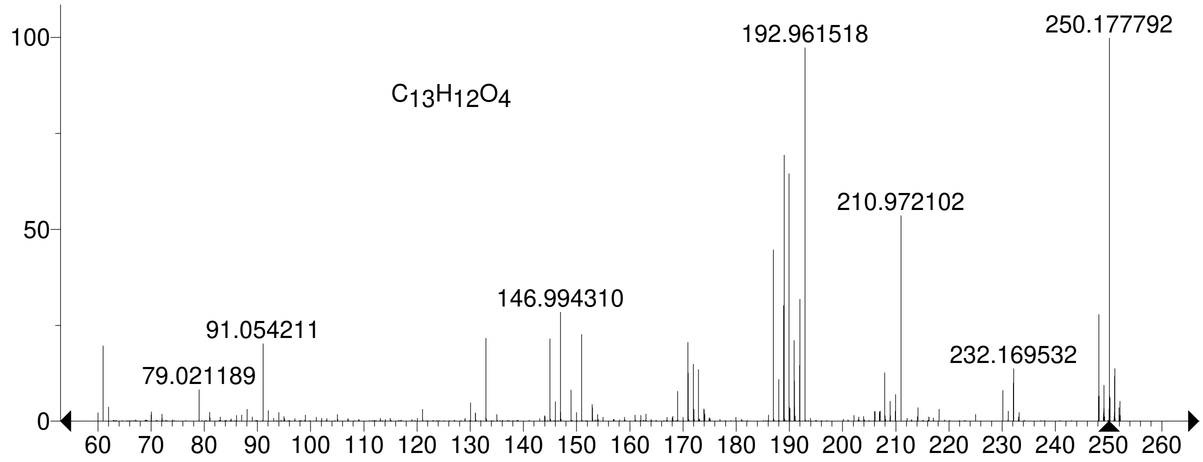

Unknown: Unknown (22.59_250.2564m/z)
Compound in Library Factor = 0

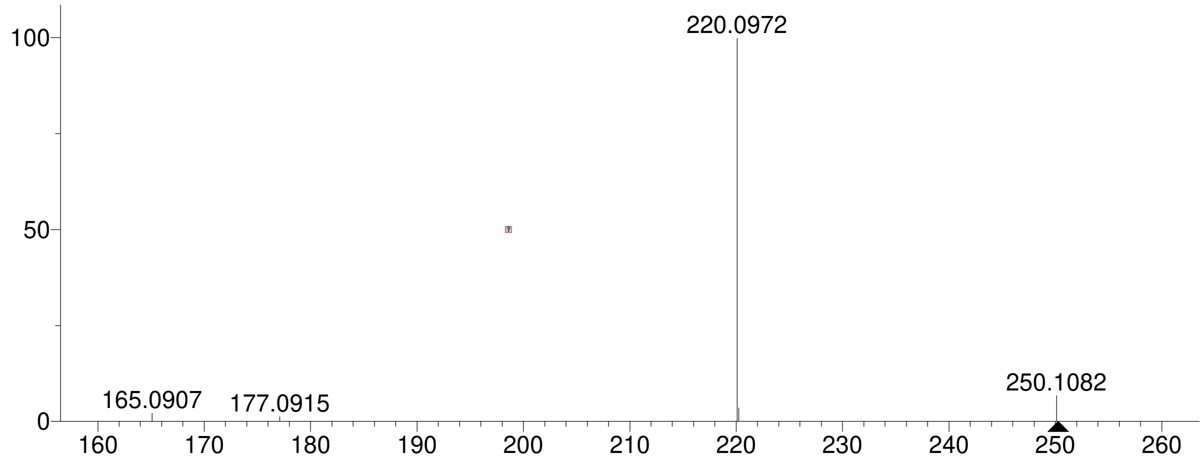

Unknown: Unknown (22.60_220.0971m/z)
Compound in Library Factor = -1523

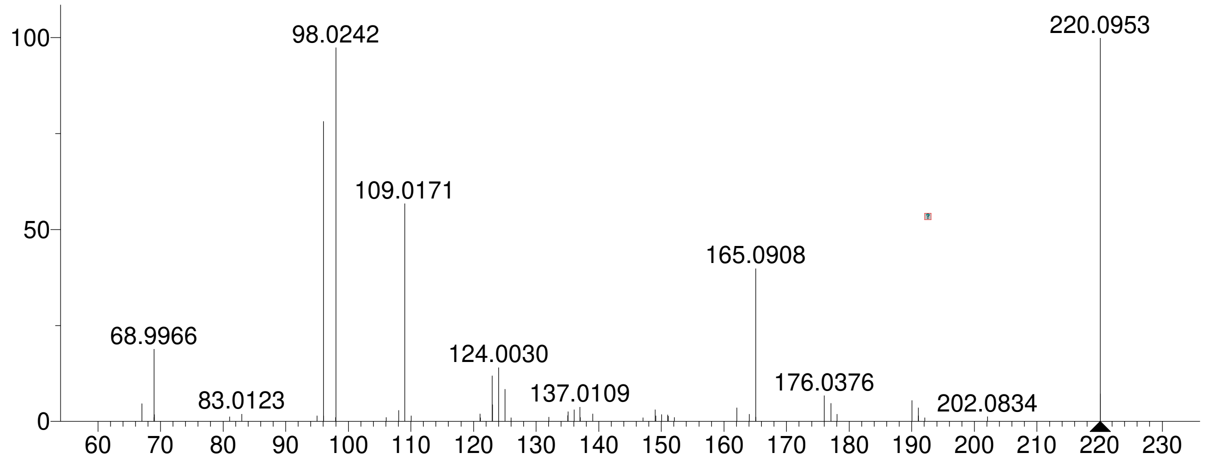

Hit 1 : 6-Methoxymethylone [M+H-H2O]+ HCD 9V P=220.1
C12H15NO4; MF: 168; RMF: 807; Prob 43.5%; Lib: nist_msms; ID: 551027.

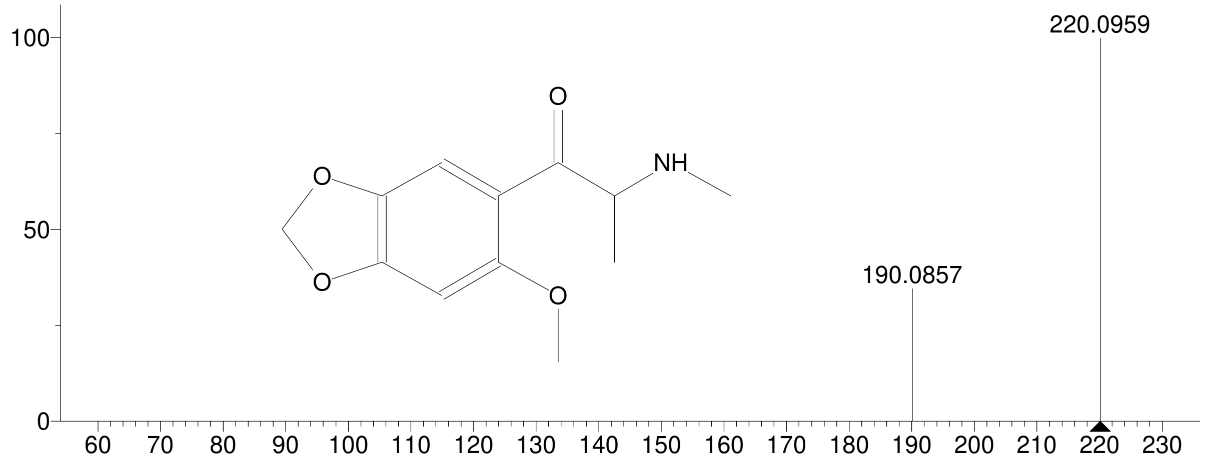

Hit 2 : 5-Methoxymethylone [M+H-H2O]+ HCD 8V P=220.1
C12H15NO4; MF: 156; RMF: 726; Prob 29.0%; Lib: nist_msms; ID: 547148.

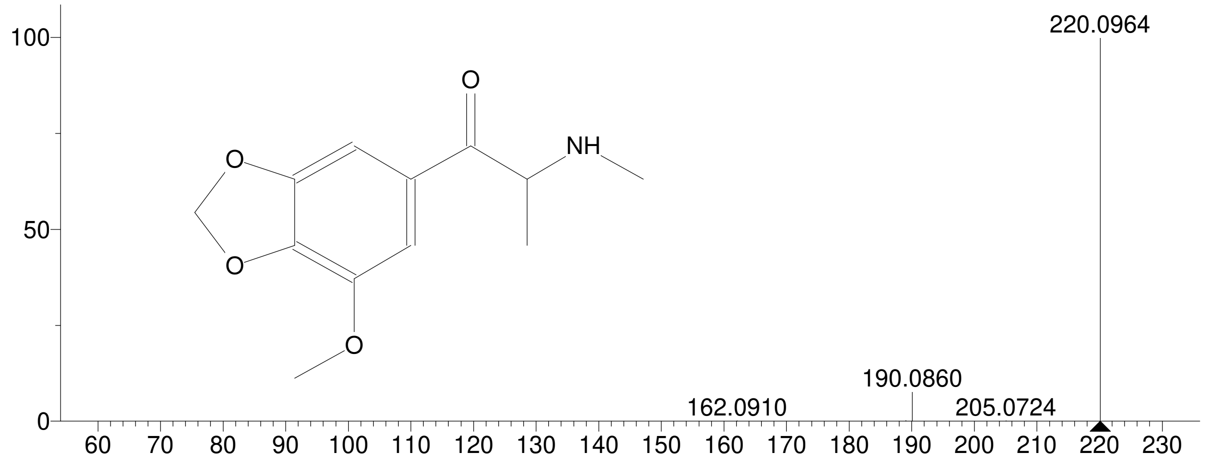

Hit 3 : 3-Hydroxycarbofuran [M+H-H2O]+ HCD 2V P=220.1
C12H15NO4; MF: 151; RMF: 709; Prob 23.3%; CAS: 16655-82-6; Lib: nist_msms; ID: 471676.

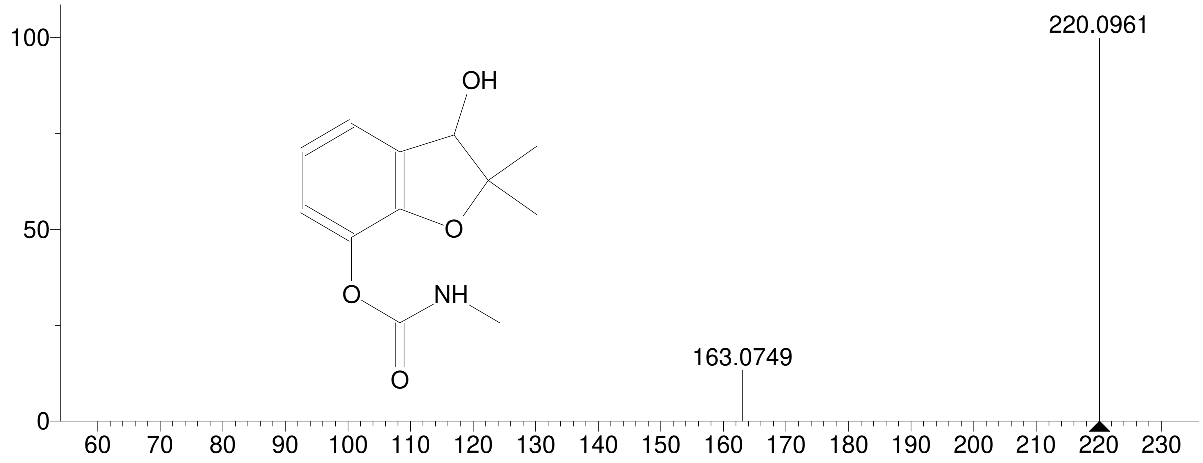

Hit 4 : Mepirizole [M+H-CH3]+ IT-FT 35% P=220.1
C11H14N4O2; MF: 11; RMF: 109; Prob 0.79%; CAS: 18694-40-1; Lib: nist_msms; ID: 188218.

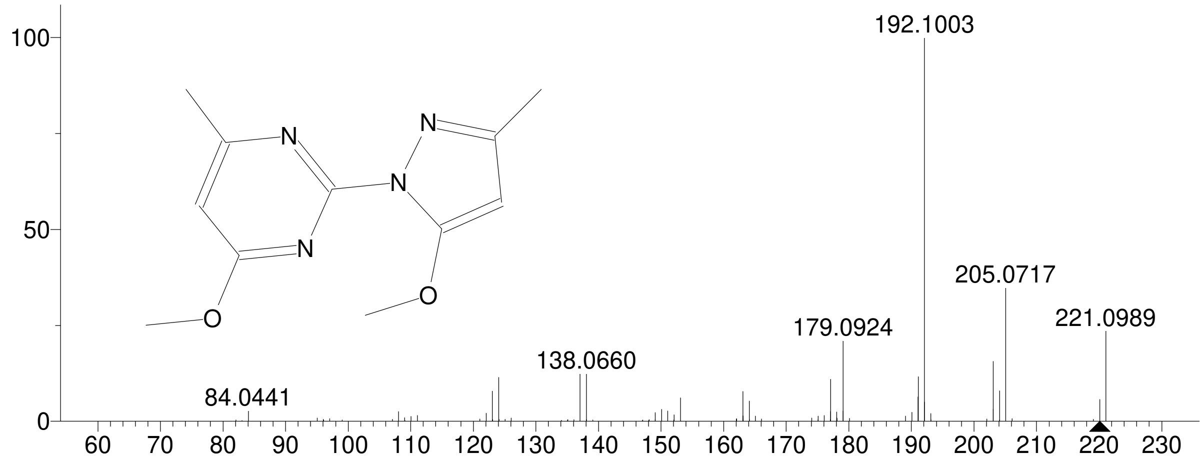

Hit 5 : NCGC00385964-01!3-ethyl-6-hydroxy-3H-2-benzofuran-1-one [M+ACN+H]+ P=220.1
C10H10O3; MF: 6; RMF: 187; Prob 0.63%; Lib: mona-export-gnps; ID: 9039.

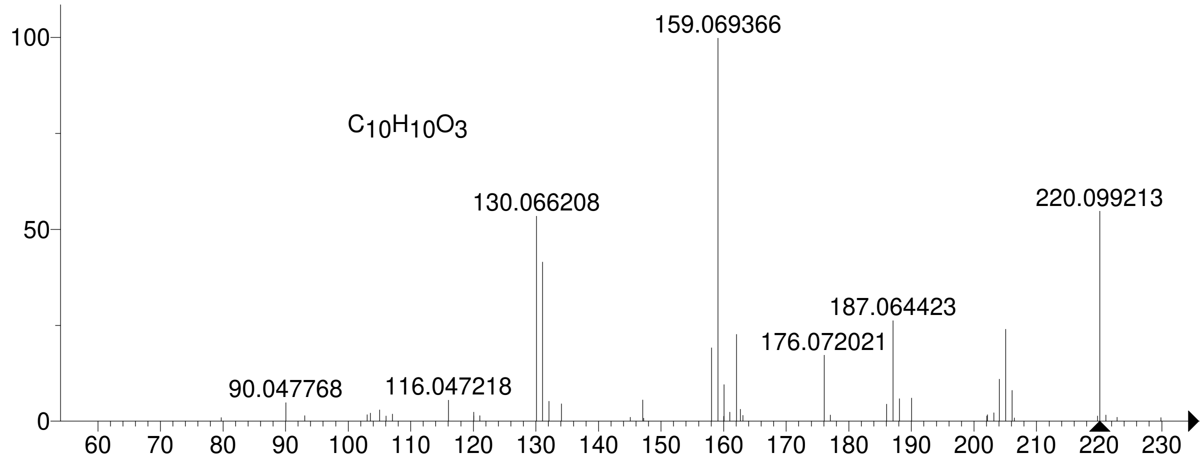

Unknown: Unknown (22.60_318.0942m/z)
Compound in Library Factor = 0

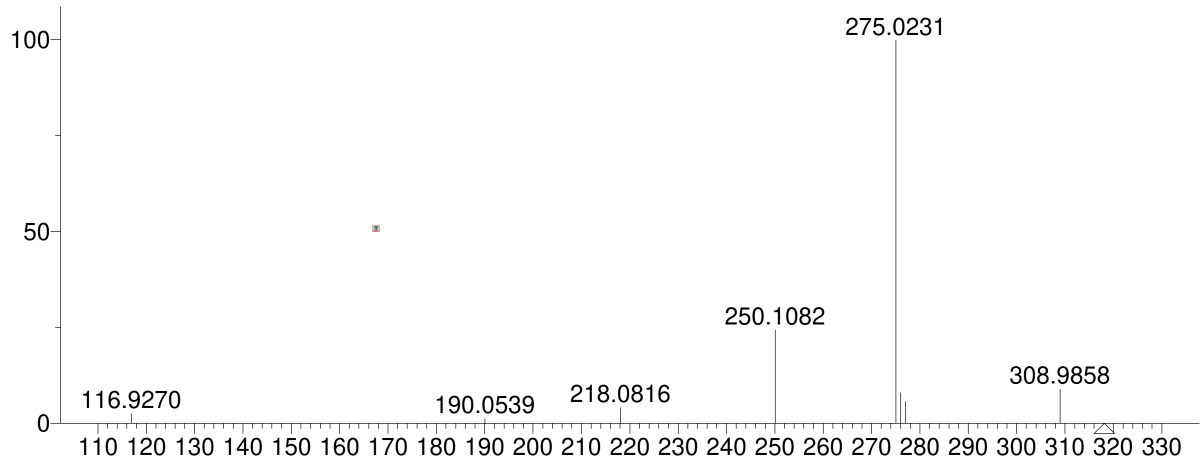

Hit 1 : Hematein [M+NH4]+ 35HCD P=318.1
C16H12O6; MF: 0; RMF: 35; Prob 20.0%; Lib: mona-export-vf-npl_qexactive; ID: 26926.

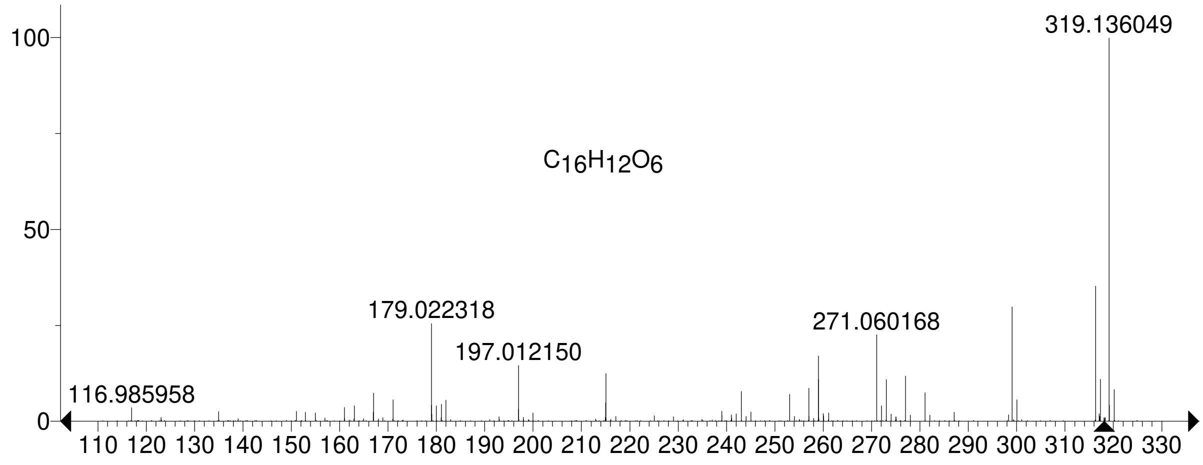

Hit 2 : Prulaurasin [M+Na]+ 35HCD P=318.1
C14H17NO6; MF: 0; RMF: 22; Prob 20.0%; Lib: mona-export-vf-npl_qexactive; ID: 7528.

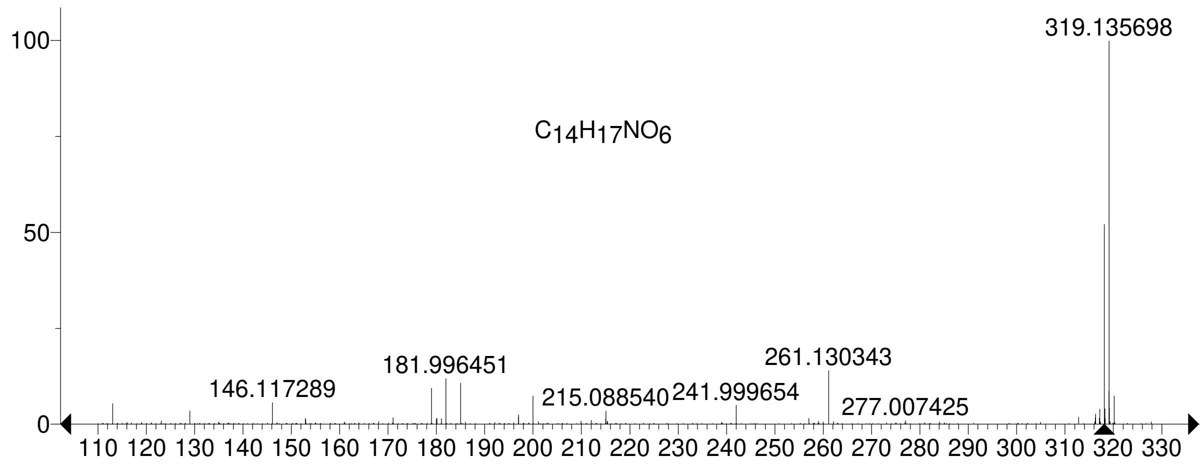

Hit 3 : Fallacinol [M+NH4]+ 35HCD P=318.1
C16H12O6; MF: 0; RMF: 8; Prob 20.0%; Lib: mona-export-vf-npl_qexactive; ID: 4921.

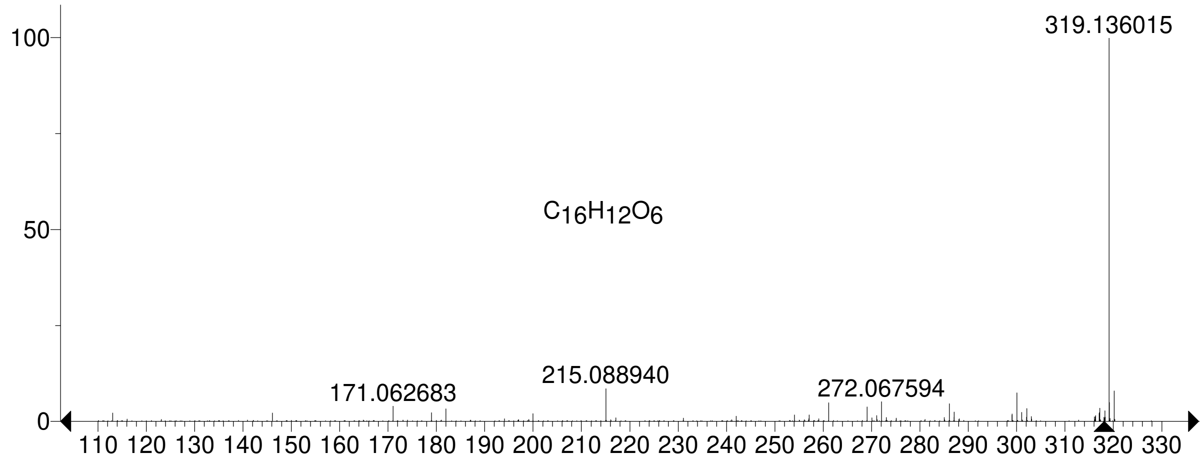

Hit 4 : NCGC00385951-01!1,3,6-trihydroxy-4,5-dimethoxy-10-methylacridin-9-one [M+H]+ P=318.1
C16H15NO6; MF: 0; RMF: 5; Prob 20.0%; Lib: mona-export-gnps; ID: 9043.

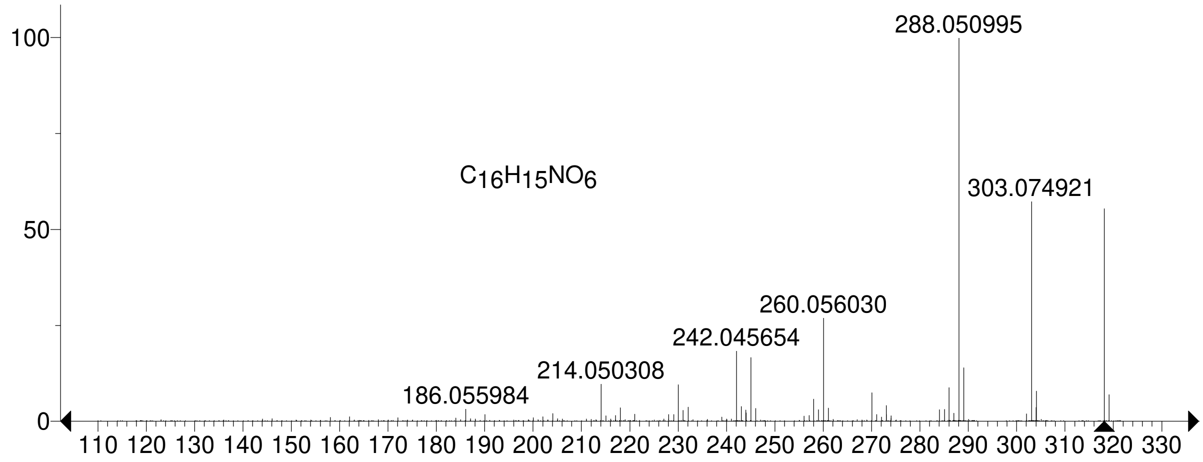

Hit 5 : carviolin [M+NH4]+ 65HCD P=318.1
C16H12O6; MF: 0; RMF: 4; Prob 20.0%; Lib: mona-export-vf-npl_qexactive; ID: 6510.

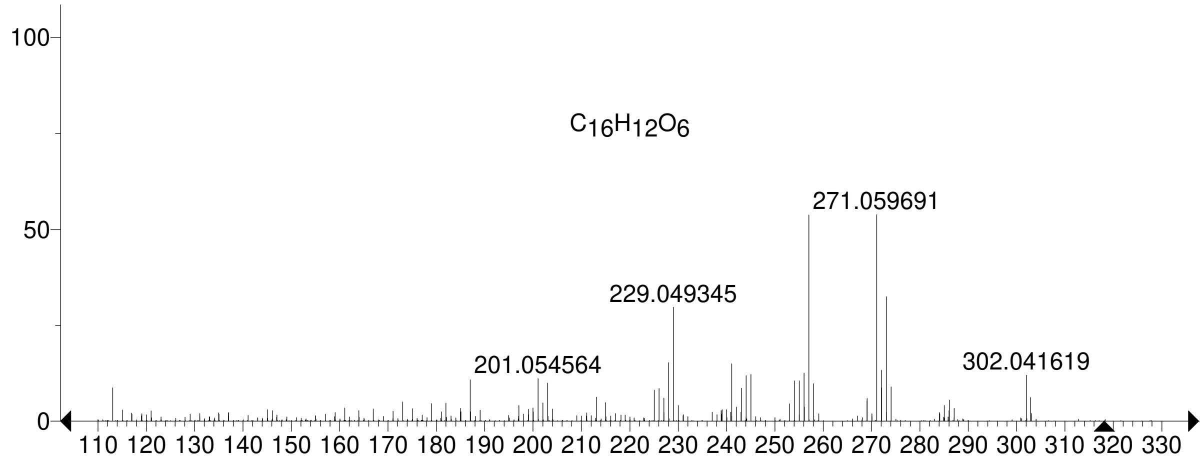

Unknown: Unknown (22.60_395.0300m/z)
Compound in Library Factor = -3273

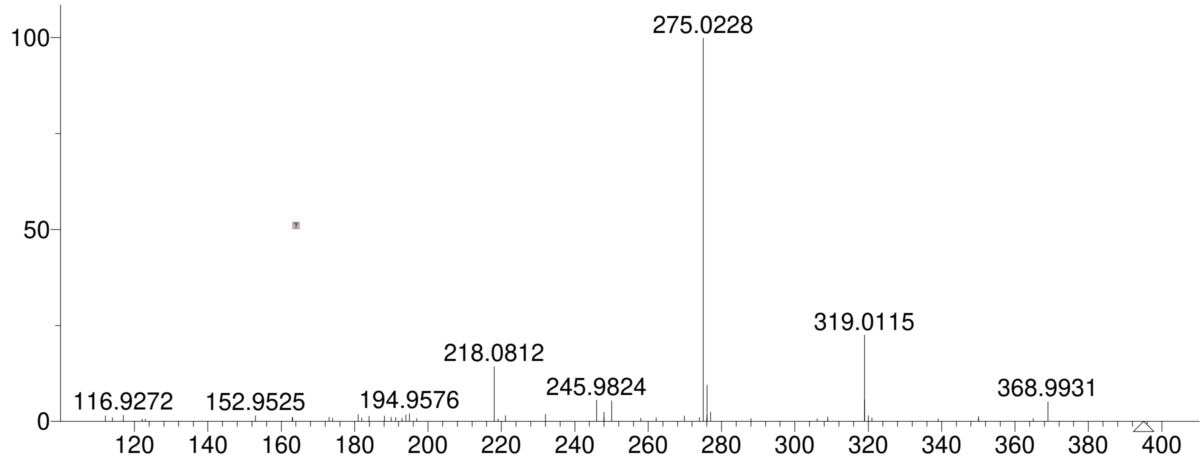

Hit 1 : (4R,8S)-17-chloro-2,18-dimethoxy-7,9,13-trioxapentacyclo[10.8.0.0A^'3,A^'1a^'o.0a^",a^,,.0A^'1a^",A^'1a^'
C19H13ClO6; MF: 1; RMF: 64; Prob 100.0%; Lib: mona-export-vf-npl_qexactive; ID: 22920.

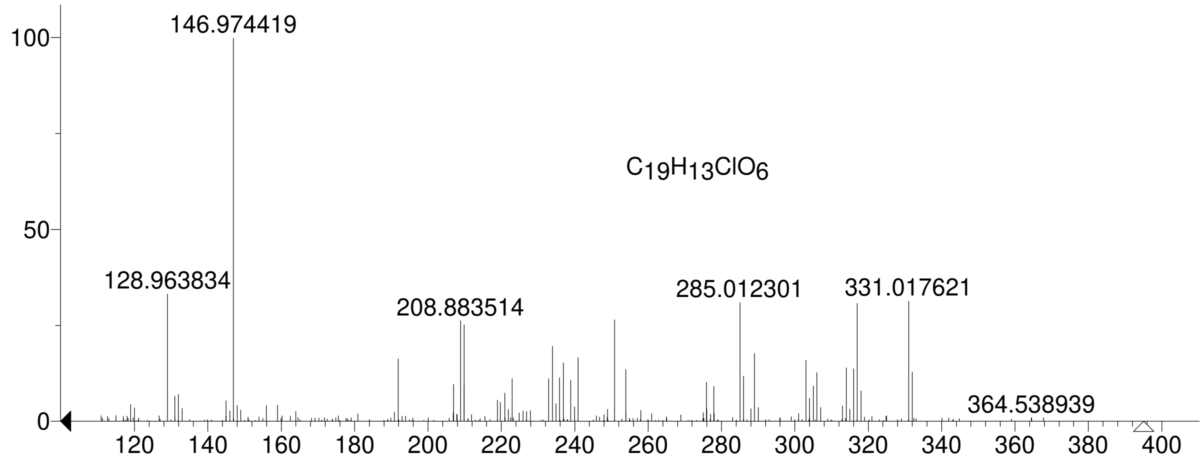

** Search Report Page 1 of 1 **

Unknown: Unknown (22.60_445.0120m/z)
Compound in Library Factor = 0

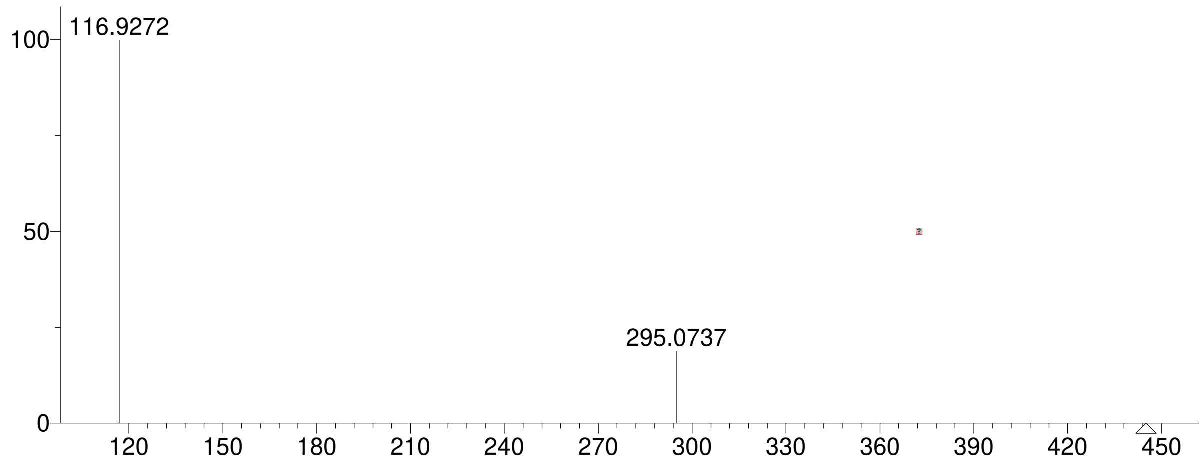

Unknown: Unknown (22.61_351.2159m/z)
Compound in Library Factor = -409

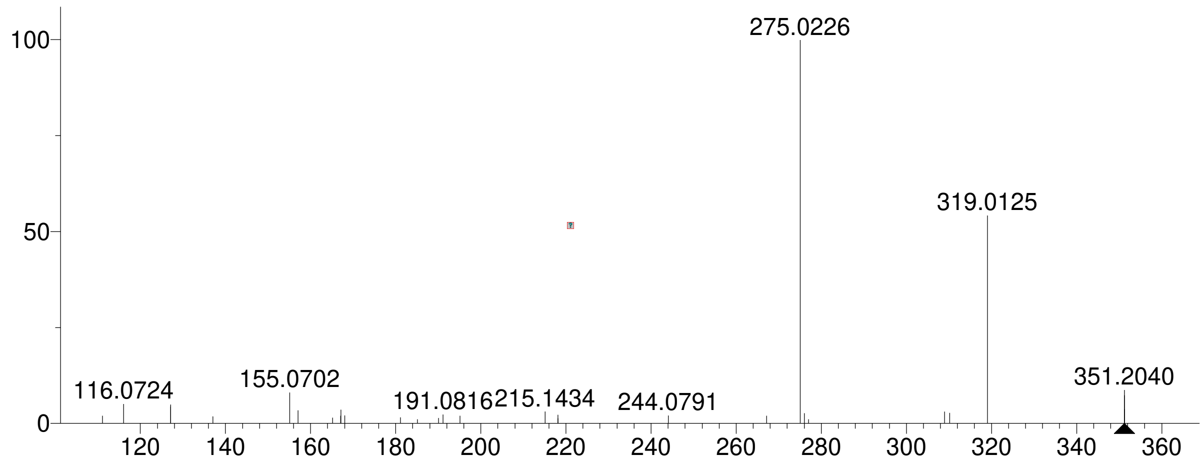

Hit 1 : 11,15-Dioxo-9S-hydroxy-5Z-prostenoic acid [M-H]- HCD 1V P=351.2
C20H32O5; MF: 211; RMF: 687; Prob 77.2%; CAS: 59894-07-4; Lib: nist_msms; ID: 87215.

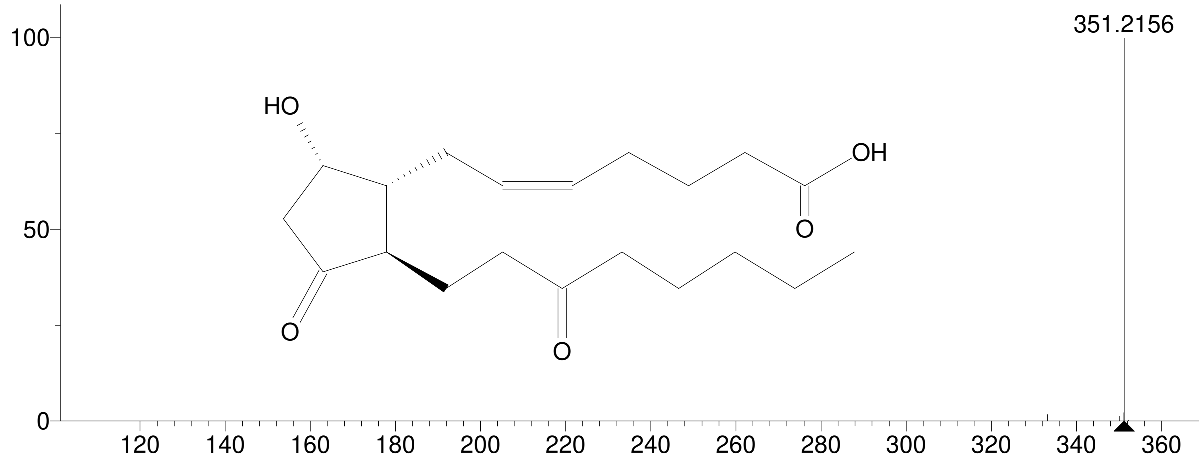

Hit 2 : 15-Ketoprostaglandin E2 [M+H]+ HCD 1V P=351.2
C20H30O5; MF: 43; RMF: 336; Prob 1.76%; CAS: 26441-05-4; Lib: nist_msms; ID: 169378.

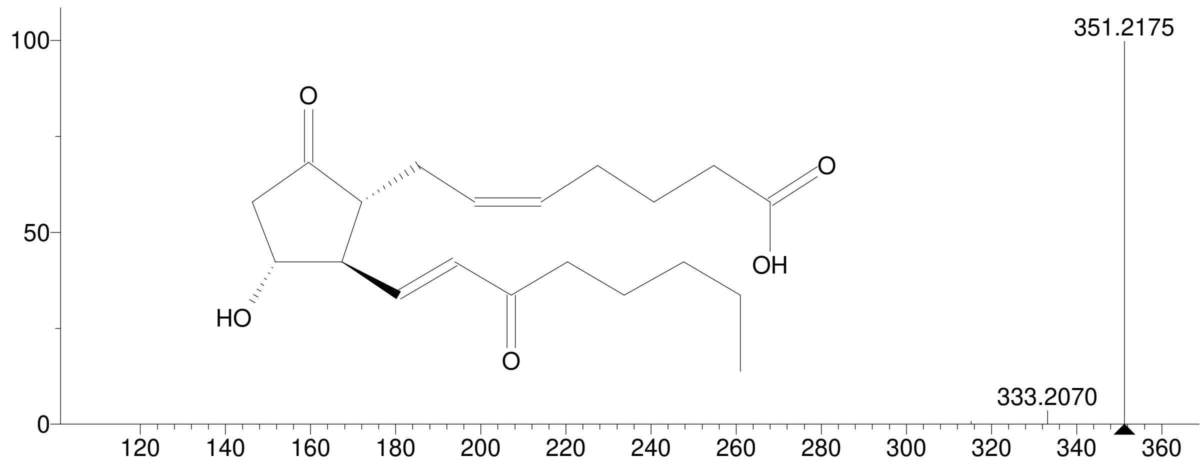

Hit 3 : 6-Ketoprostaglandin E1 [M+H-H2O]+ HCD 1V P=351.2
C20H32O6; MF: 40; RMF: 327; Prob 1.55%; CAS: 67786-53-2; Lib: nist_msms; ID: 161827.

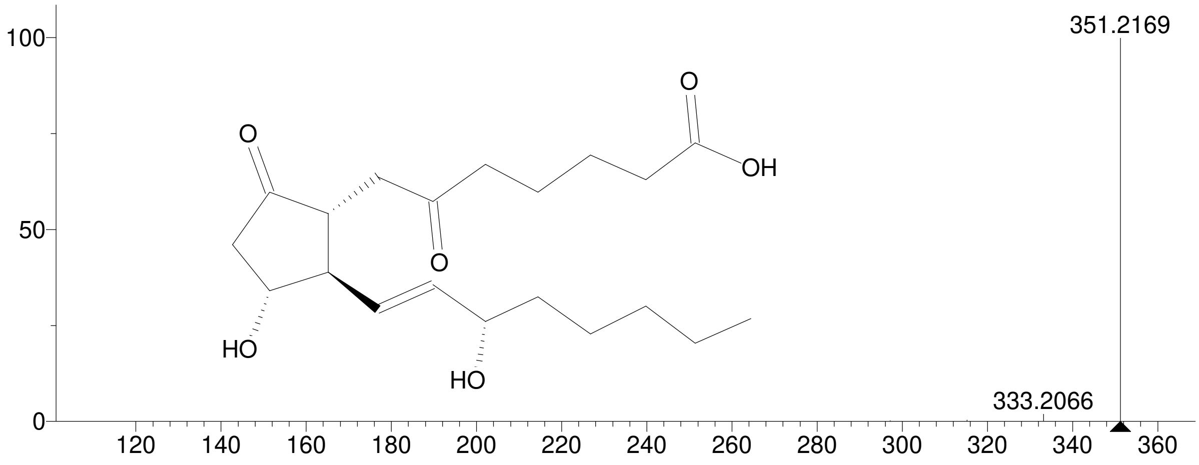

Hit 4 : 15-Ketoprostaglandin F2α [M-H]- HCD 3V P=351.2
C20H32O5; MF: 36; RMF: 338; Prob 1.31%; CAS: 35850-13-6; Lib: nist_msms; ID: 155319.

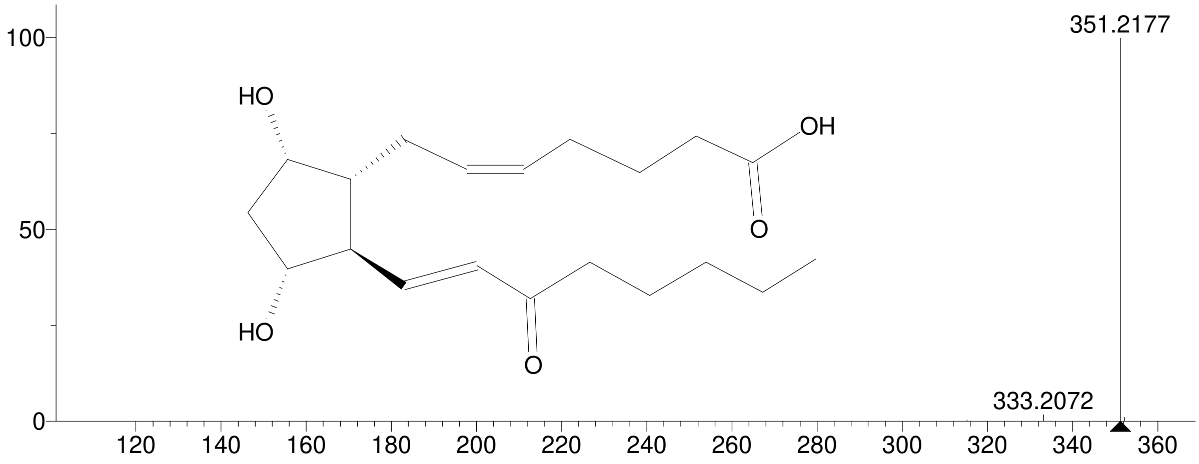

Hit 5 : 19(R)-Hydroxyprostaglandin E2 [M+H-H2O]+ HCD 1V P=351.2
C20H32O6; MF: 31; RMF: 335; Prob 1.05%; CAS: 64625-54-3; Lib: nist_msms; ID: 169989.

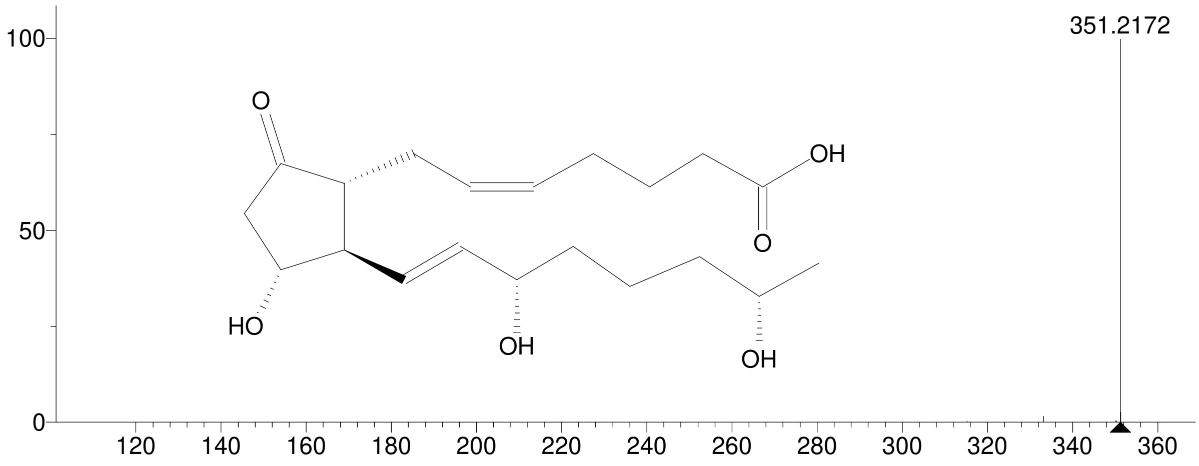

Unknown: Unknown (22.61_523.2050m/z)
Compound in Library Factor = 0

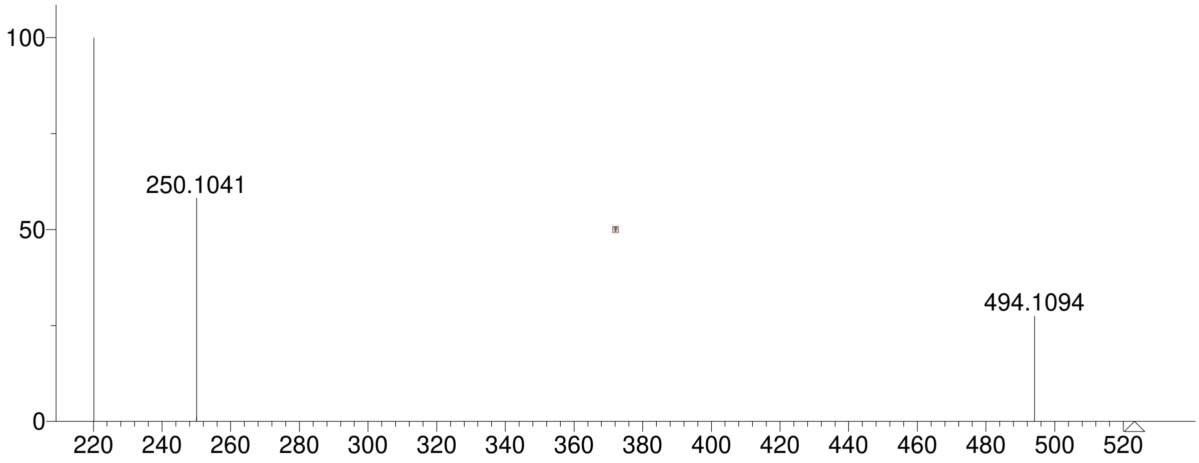

Hit 1 : Oxyacanthine [M+H-C2H5N]+=>523.2 IT 35% P=523.2
C37H40N2O6; MF: 0; RMF: 18; Prob 50.0%; CAS: 548-40-3; Lib: nist_msms; ID: 455875.

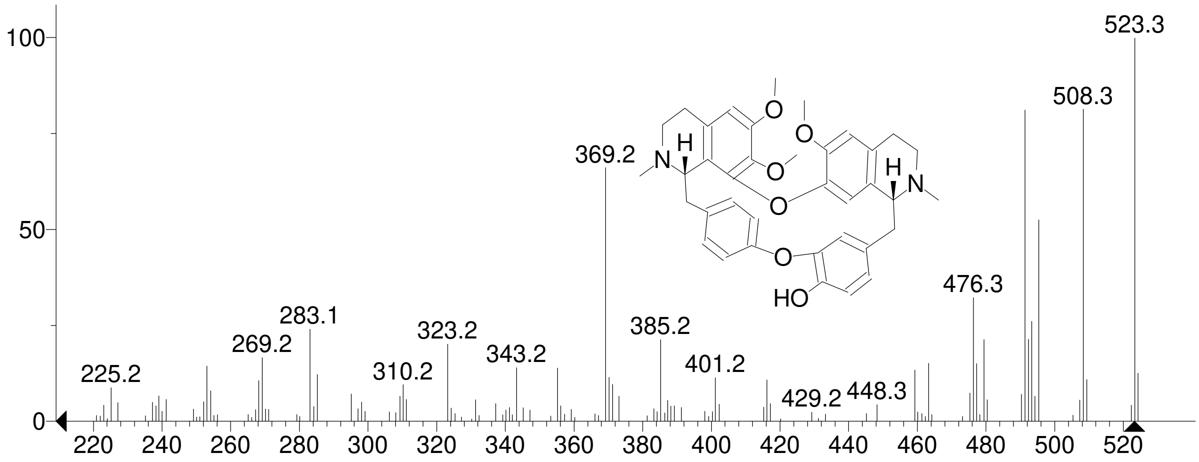

Hit 2 : 3-Deoxo-3Beta-Hydroxymexicanolide 16-Enol Ether [M+K]+ 35HCD P=523.2
C28H36O7; MF: 0; RMF: 3; Prob 50.0%; Lib: mona-export-vf-npl_qexactive; ID: 25726.

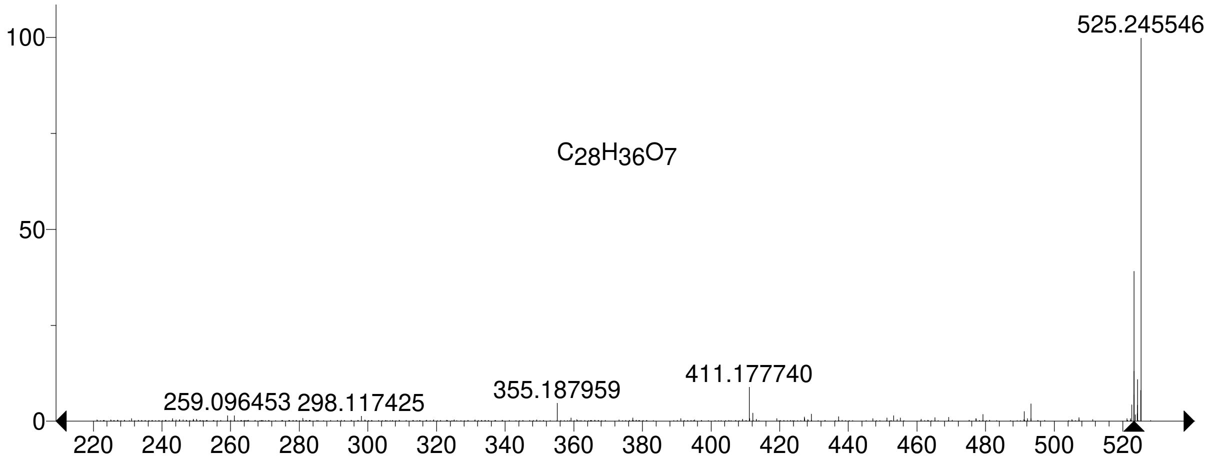

Unknown: Unknown (22.62_252.1142m/z)
Compound in Library Factor = 0

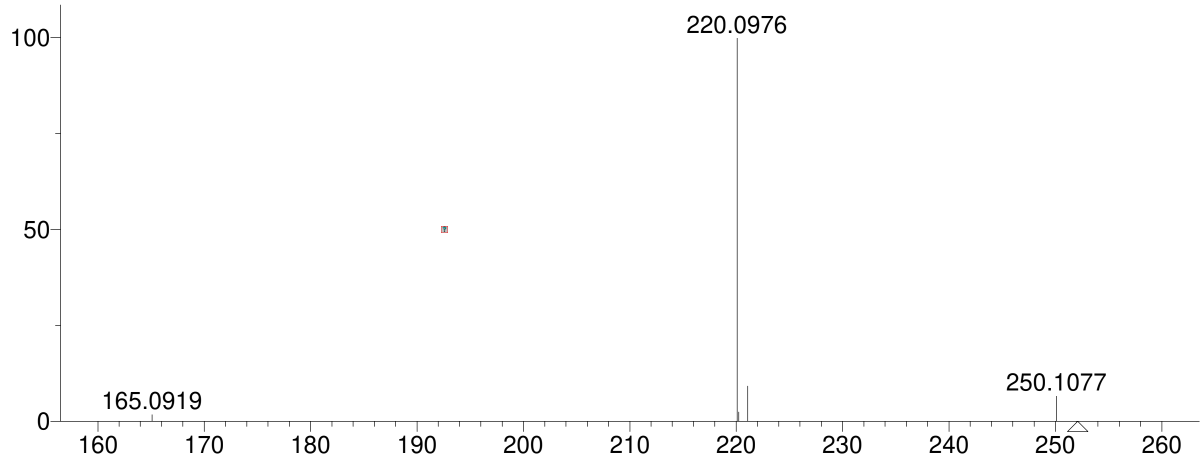

Hit 1 : 7-Aminonitrazepam [M+H]+ HCD 65V P=252.1
C15H13N3O; MF: 0; RMF: 13; Prob 100.0%; CAS: 4928-02-3; Lib: nist_msms; ID: 193640.

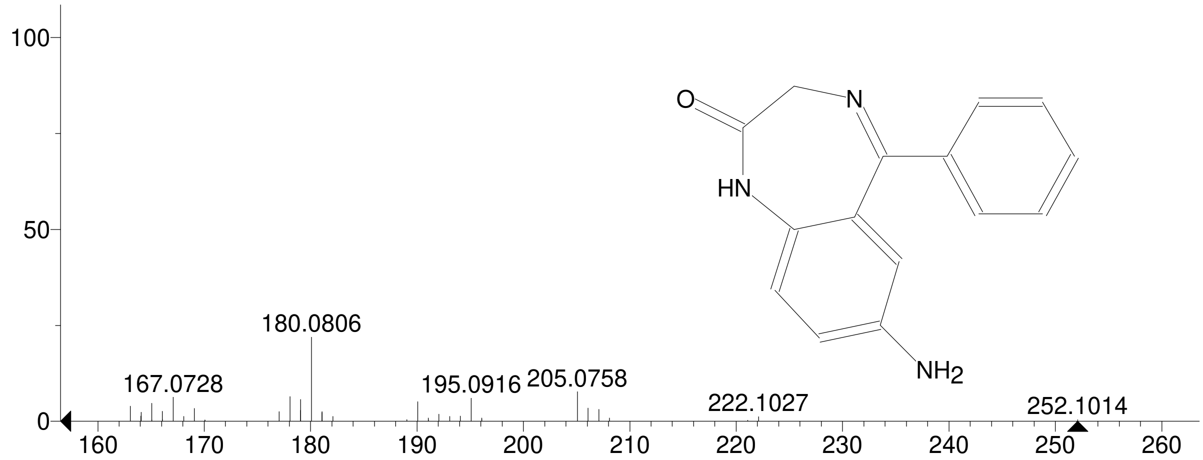

Unknown: Unknown (22.64_291.1324m/z)
Compound in Library Factor = 0

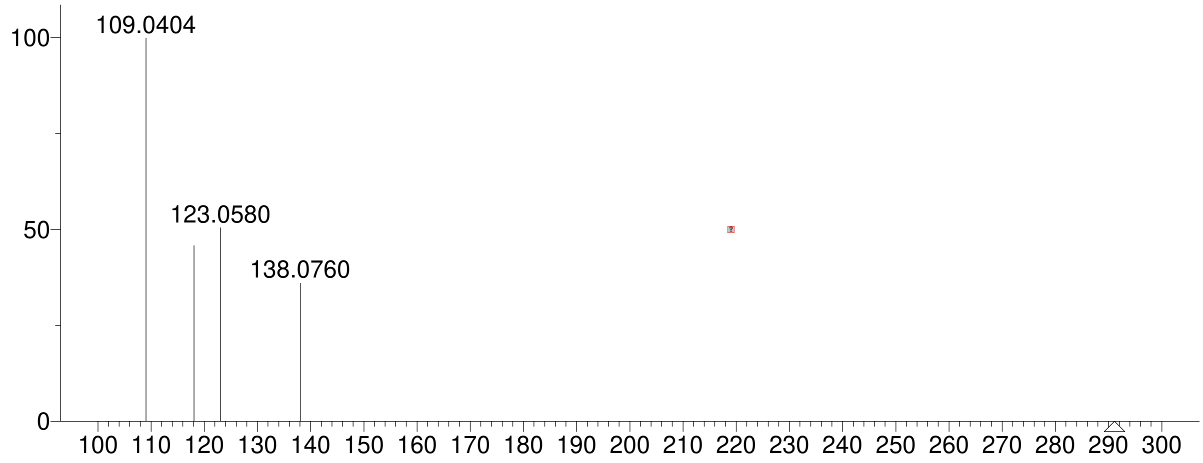

** Search Report Page 1 of 1 **

Unknown: Unknown (22.89_423.0631m/z)
Compound in Library Factor = -1309

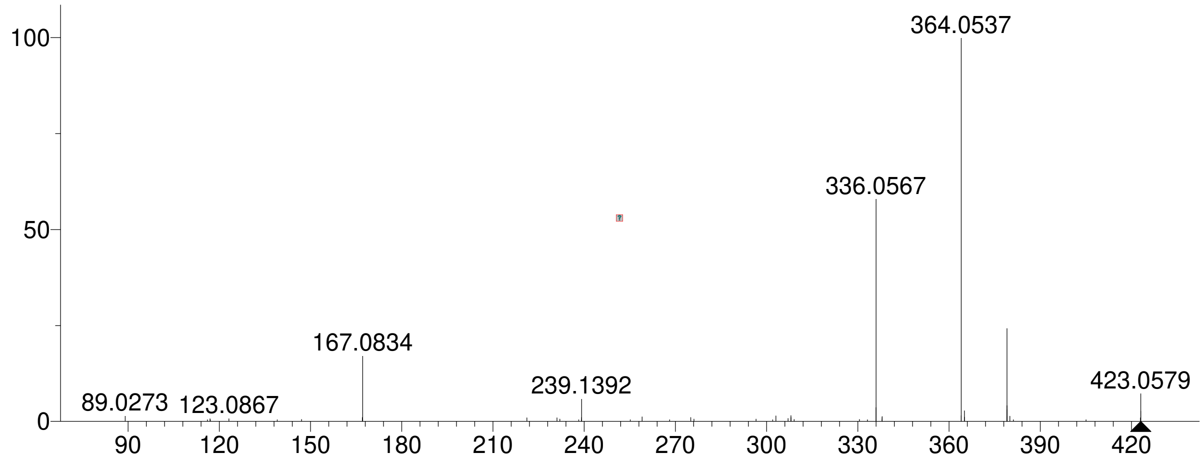

Hit 1 : α-Carboxybenzylpenicillin [M-H+2Na]+ HCD 4V P=423.1
C17H18N2O6S; MF: 64; RMF: 254; Prob 83.2%; CAS: 4697-36-3; Lib: nist_msms; ID: 488820.

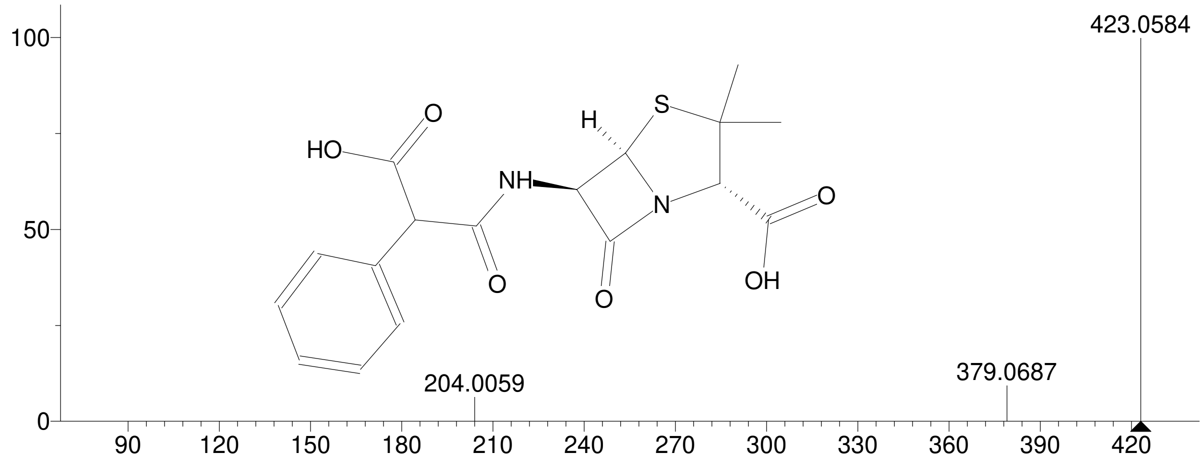

Hit 2 : Cefuroxime [M-H]- HCD 4V P=423.1
C16H16N4O8S; MF: 13; RMF: 209; Prob 16.8%; CAS: 55268-75-2; Lib: nist_msms; ID: 152660.

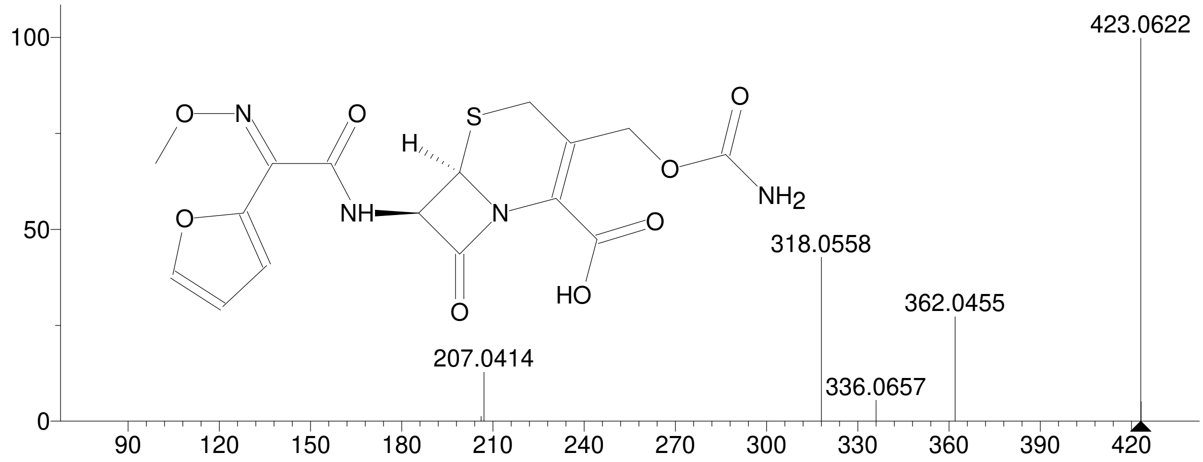

Unknown: Unknown (23.15_477.3206m/z)
Compound in Library Factor = 0

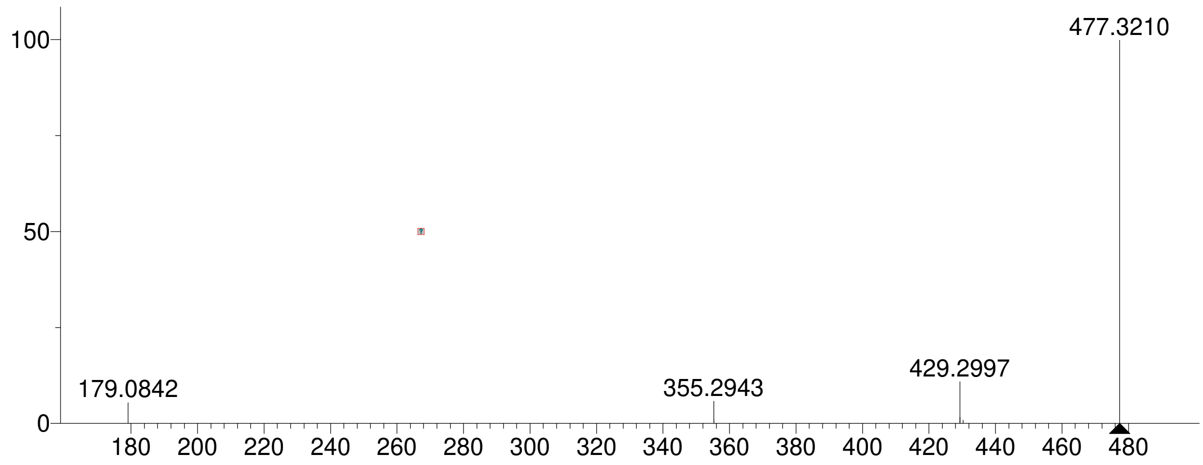

Hit 1 : NCGC00380884-01!(2S,3R,10R,13R,14S)-2,3,14-trihydroxy-10,13-dimethyl-17-[(2R,3R,5R)-2,3,5-trihydro
C28H46O7; MF: 0; RMF: 172; Prob 100.0%; Lib: mona-export-gnps; ID: 8681.

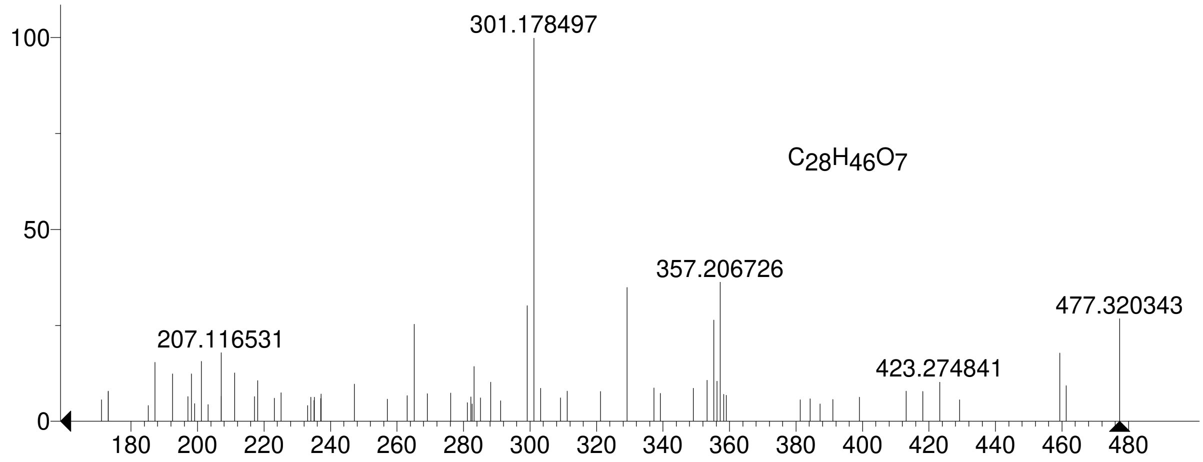

Unknown: Unknown (23.19_368.2933m/z)
Compound in Library Factor = 0

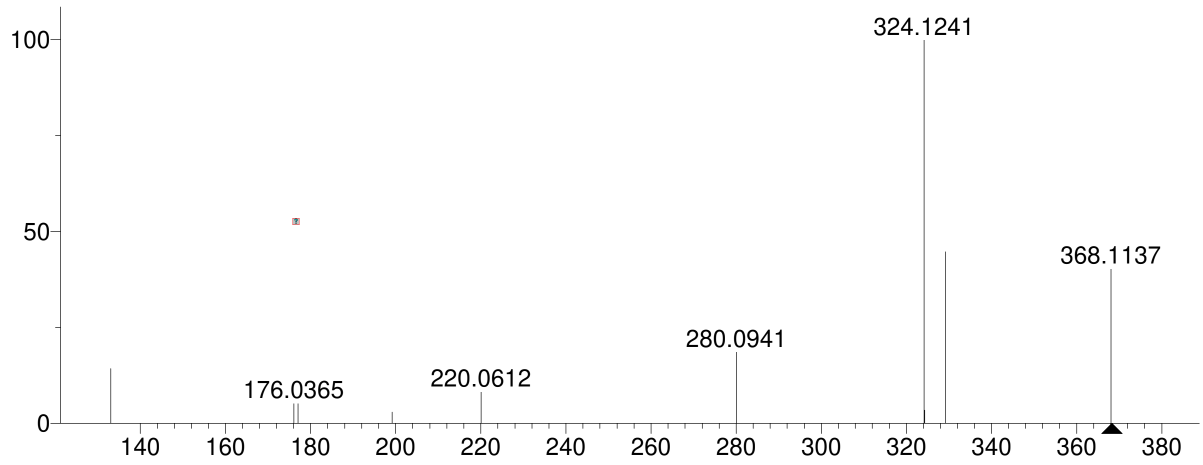

Unknown: Unknown (23.19_390.0951m/z)
Compound in Library Factor = -3273

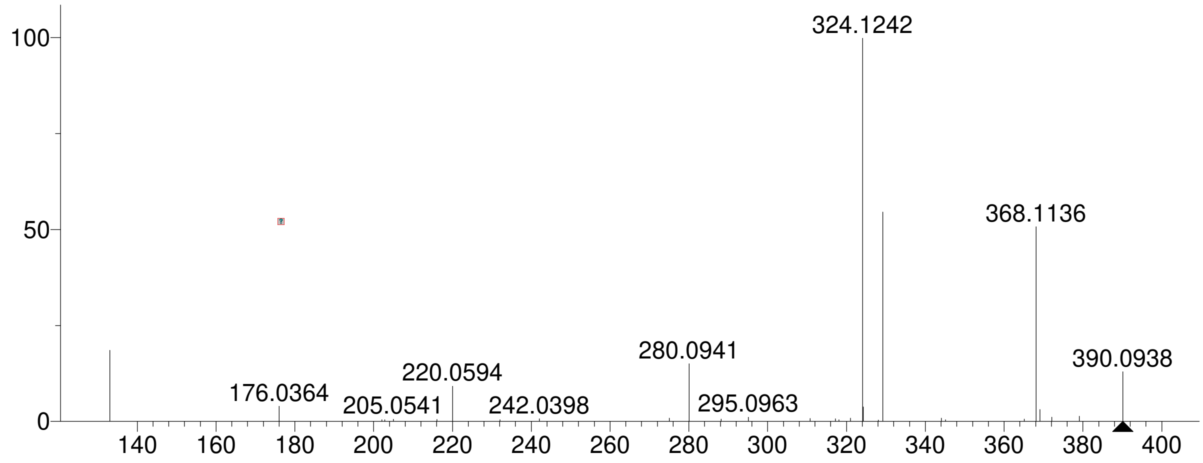

Hit 1 : Picoxystrobin [M+Na]+ HCD 19V P=390.1
C18H16F3NO4; MF: 8; RMF: 190; Prob 40.1%; CAS: 117428-22-5; Lib: nist_msms; ID: 476279.

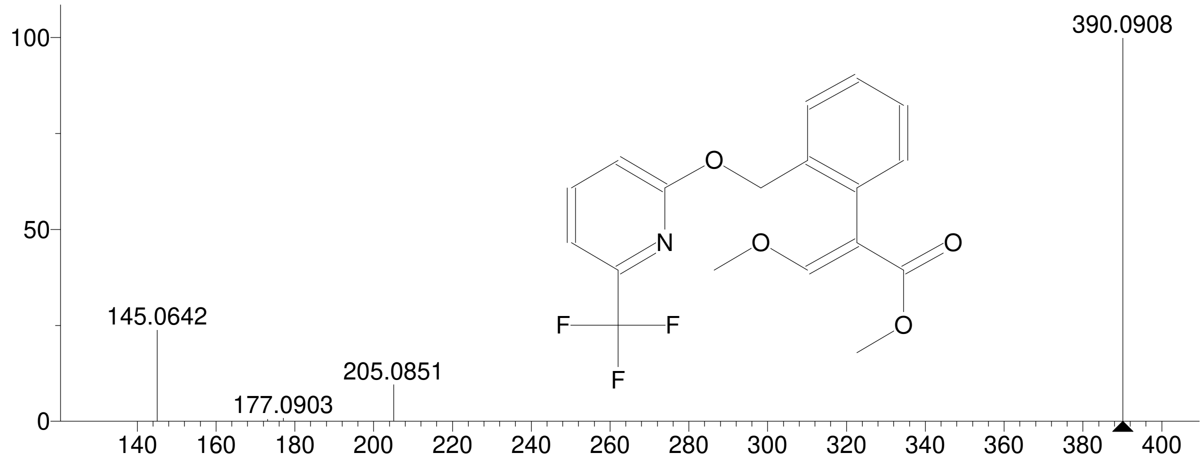

Hit 2 : Pesticide4_Picoxystrobin_C18H16F3NO4_Acanto [M+Na]+ P=390.1
C18H16F3NO4; MF: 0; RMF: 124; Prob 29.9%; Lib: mona-export-gnps; ID: 14037.

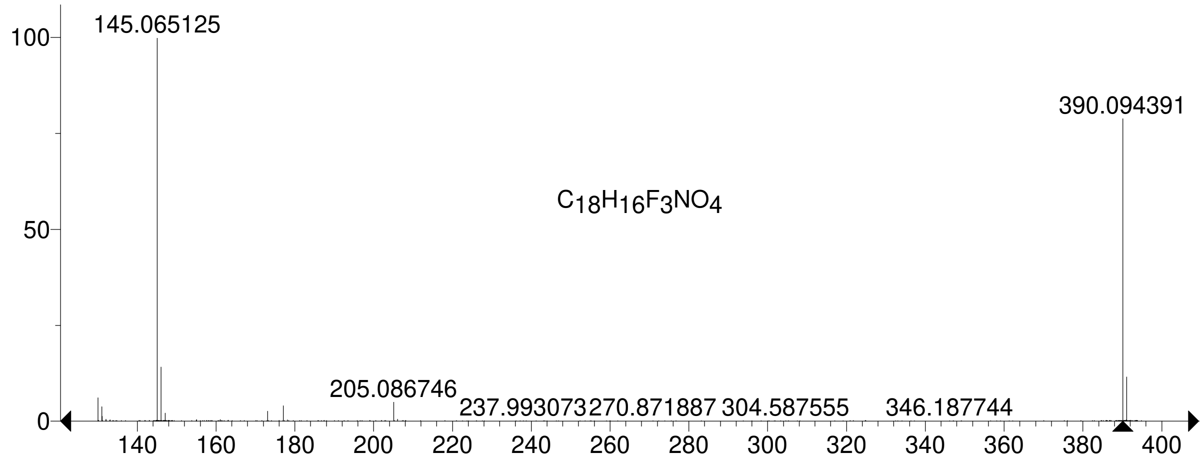

Hit 3 : Bicuculline (+) [M+Na]+ 35HCD P=390.1
C20H17NO6; MF: 0; RMF: 13; Prob 29.9%; Lib: mona-export-vf-npl_qexactive; ID: 3340.

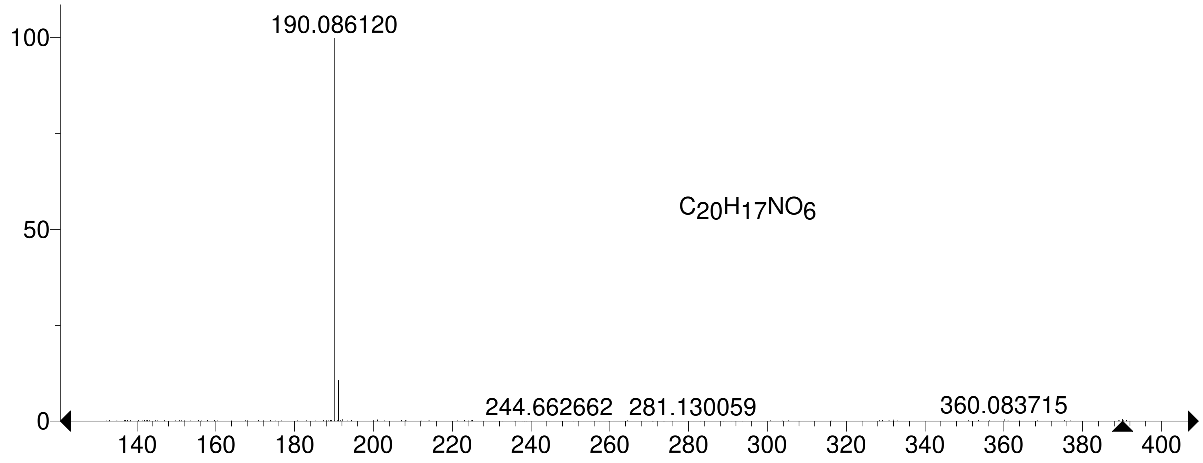

Unknown: Unknown (23.19_467.0295m/z)
Compound in Library Factor = 0

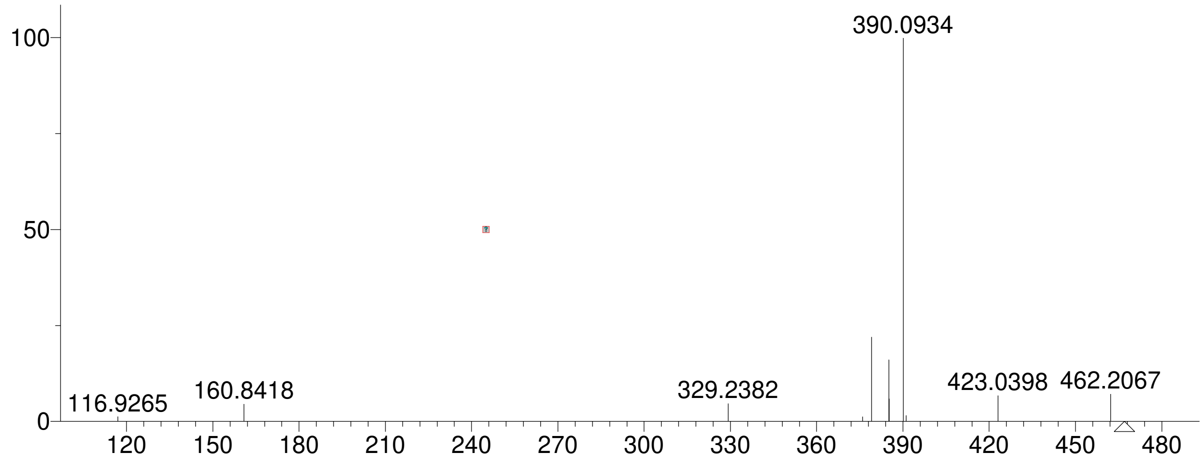

** Search Report Page 1 of 2 **

Unknown: Unknown (23.20_324.1232m/z)
Compound in Library Factor = -389

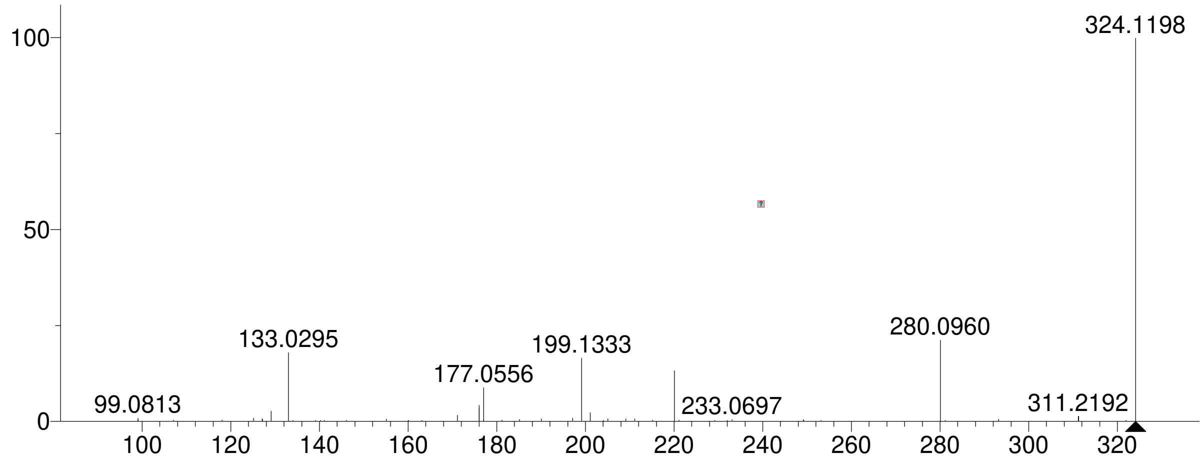

Hit 1 : Tapentadol O-sulfate [M+Na]+ HCD 12V P=324.1
C14H23NO4S; MF: 256; RMF: 866; Prob 94.5%; CAS: 1300037-87-9; Lib: nist_msms; ID: 328508.

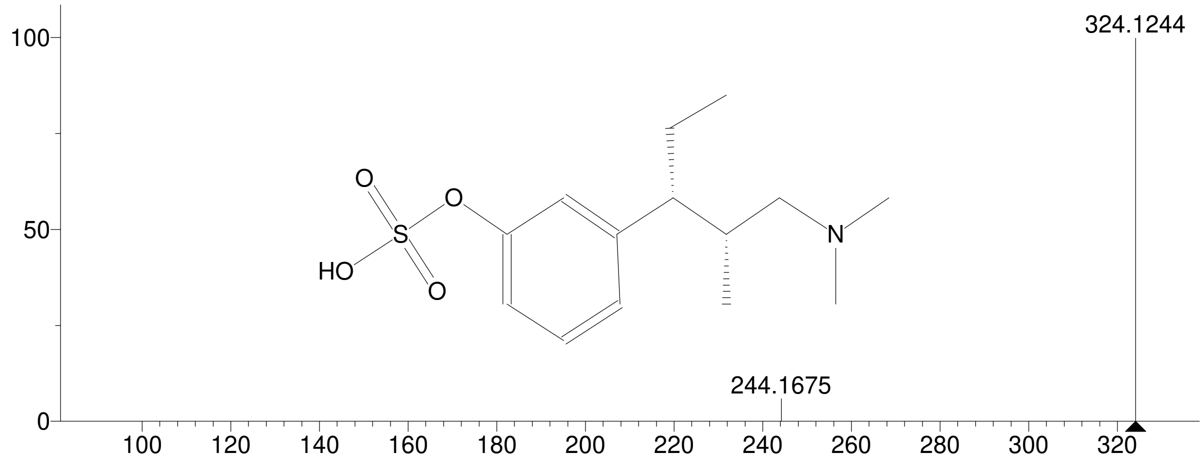

Hit 2 : Stylopine [M+H]+ HCD 19V P=324.1
C19H17NO4; MF: 82; RMF: 695; Prob 1.92%; CAS: 84-39-9; Lib: nist_msms; ID: 219734.

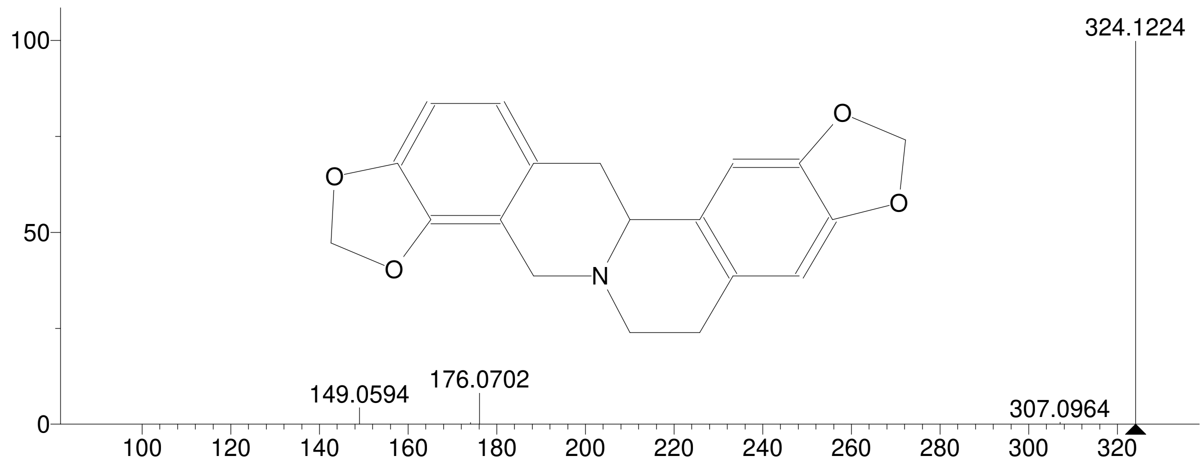

Hit 3 : Flutolanil [M+H]+ HCD 9V P=324.1
C17H16F3NO2; MF: 72; RMF: 658; Prob 1.36%; CAS: 66332-96-5; Lib: nist_msms; ID: 486552.

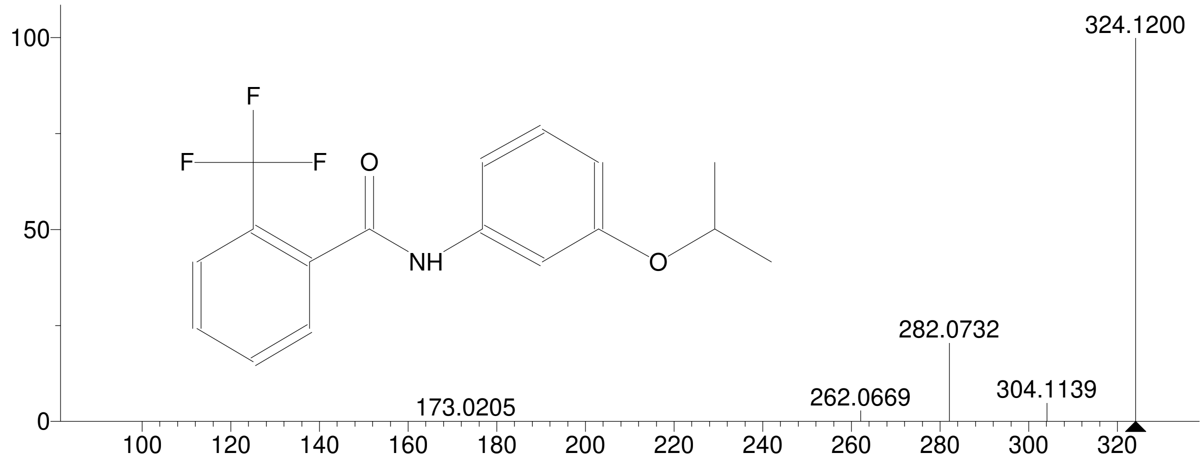

Hit 4 : Brucine [M+H-C4H9N]+ HCD 16V P=324.1
C23H26N2O4; MF: 65; RMF: 780; Prob 1.04%; CAS: 357-57-3; Lib: nist_msms; ID: 216817.

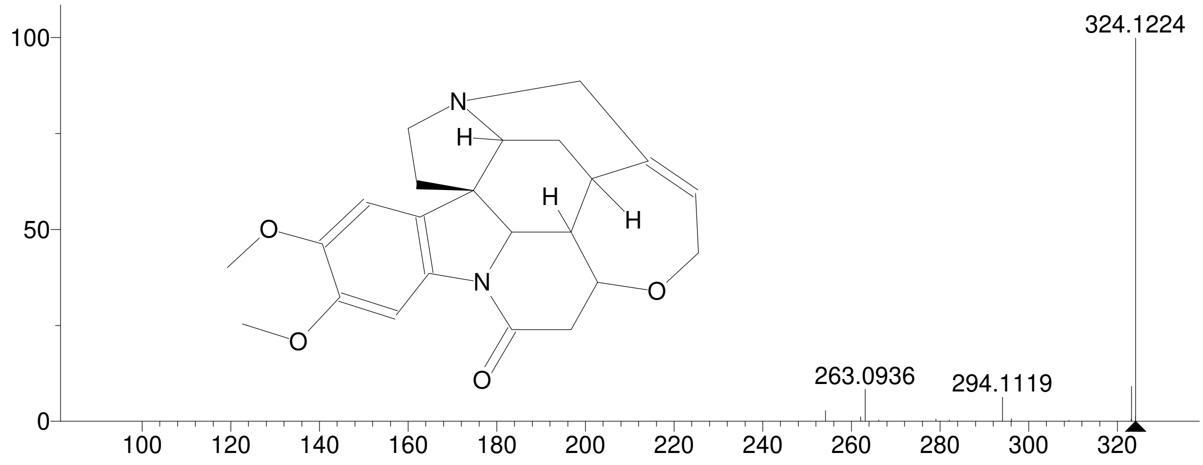

Hit 5 : Furalaxyl [M+Na]+ HCD 29V P=324.1
C17H19NO4; MF: 53; RMF: 630; Prob 0.69%; CAS: 57646-30-7; Lib: nist_msms; ID: 474805.

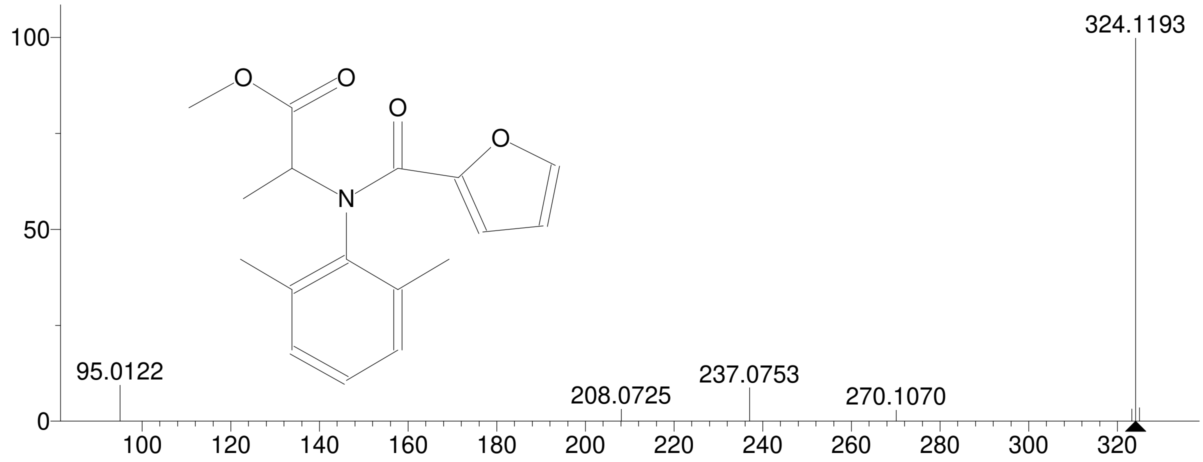

Unknown: Unknown (23.20_368.1134m/z)
Compound in Library Factor = -3273

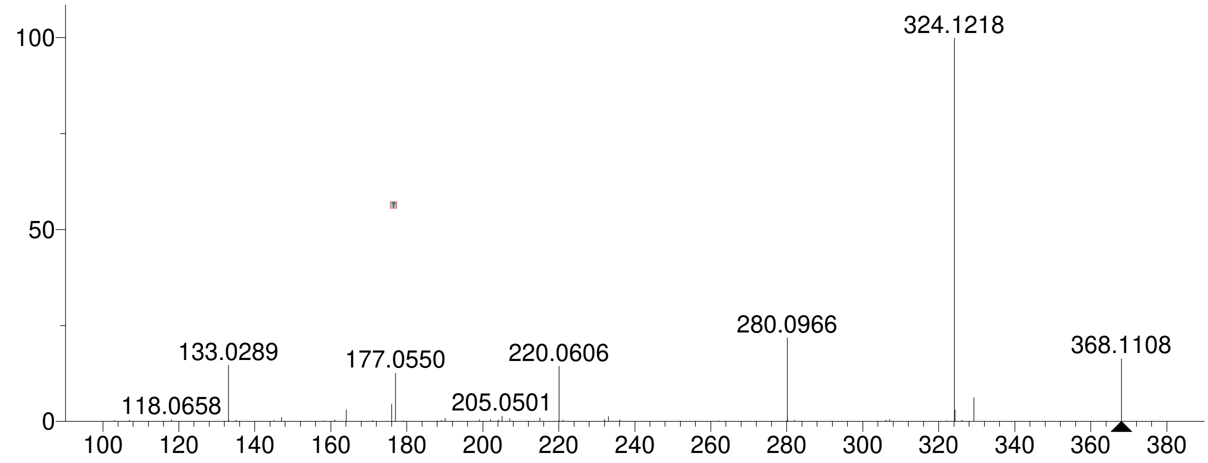

Hit 1 : Bicuculline [M+H]+ HCD 14V P=368.1
C20H17NO6; MF: 13; RMF: 280; Prob 16.8%; CAS: 485-49-4; Lib: nist_msms; ID: 216706.

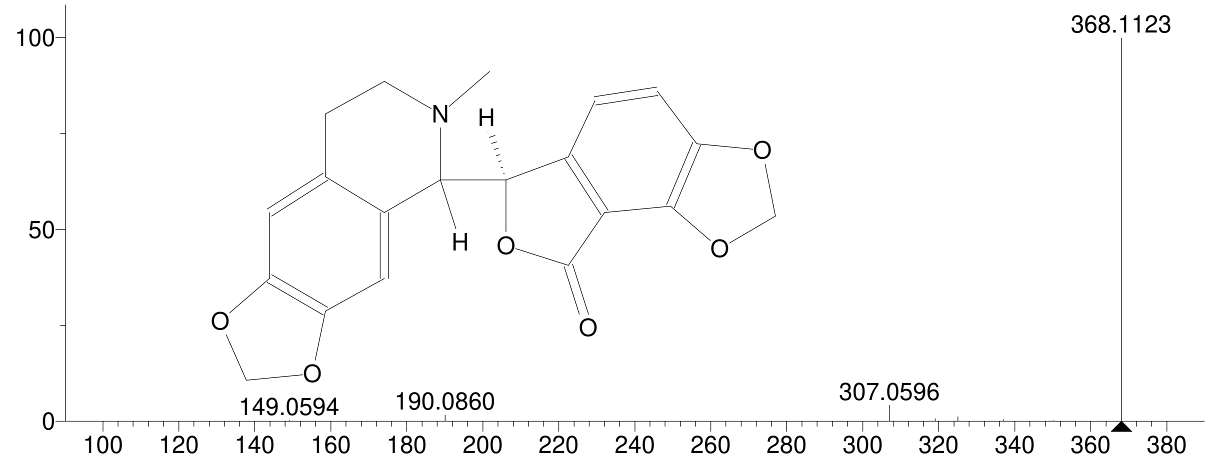

Hit 2 : Picoxystrobin [M+H]+ HCD 1V P=368.1
C18H16F3NO4; MF: 13; RMF: 238; Prob 16.8%; CAS: 117428-22-5; Lib: nist_msms; ID: 476221.

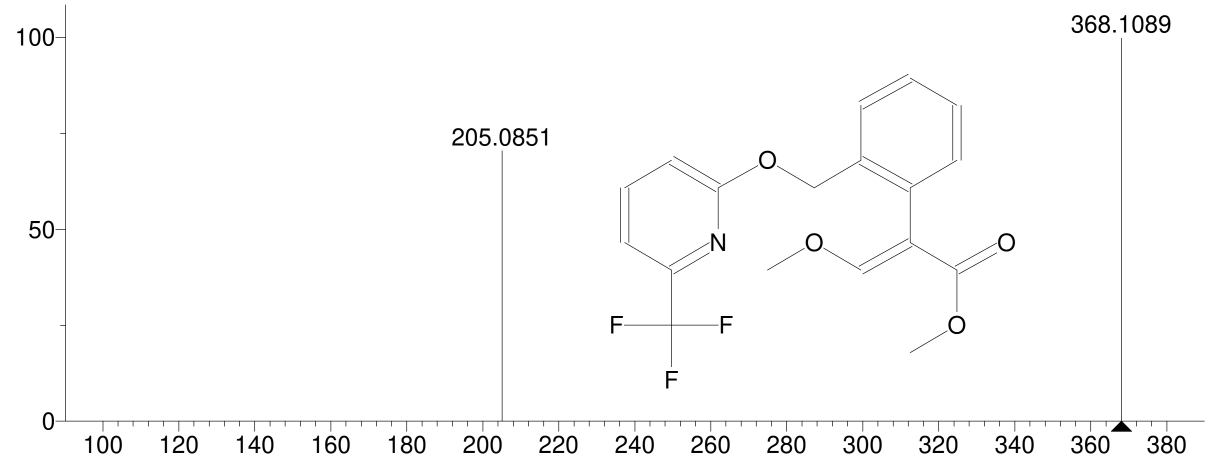

Hit 3 : 2-Chloro-N6-cyclopentyladenosine [M-H]- 20-30-40%) P=368.1
C15H20ClN5O4; MF: 4; RMF: 196; Prob 12.2%; Lib: msms-neg-fiehnhilic; ID: 44.

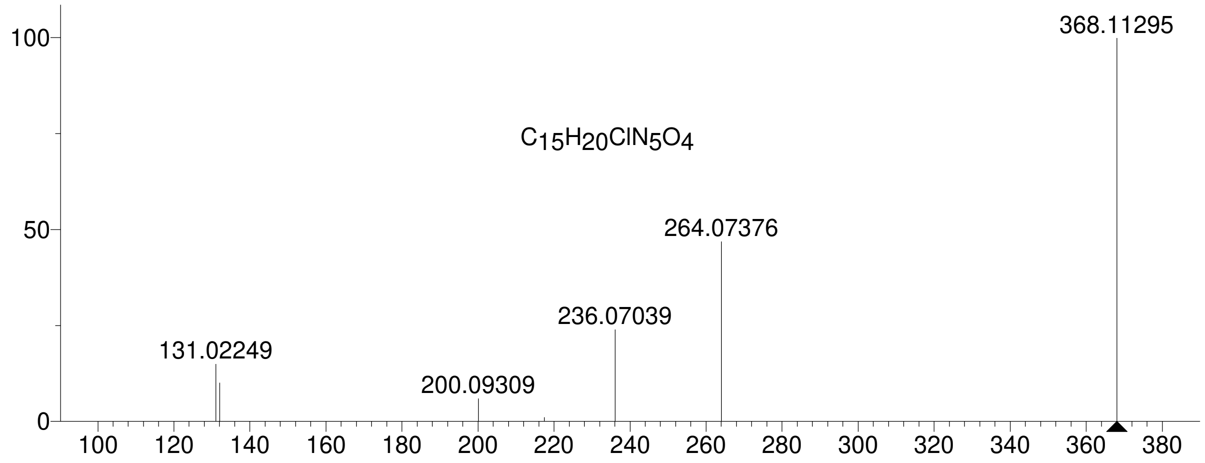

Hit 4 : NCGC00015169-03_C20H17NO6_6-(6-Methyl-5,6,7,8-tetrahydro[1,3]dioxolo[4,5-g]isoquinolin-5-yl)furo[3,4
C20H17NO6; MF: 4; RMF: 126; Prob 12.2%; Lib: mona-export-gnps; ID: 13752.

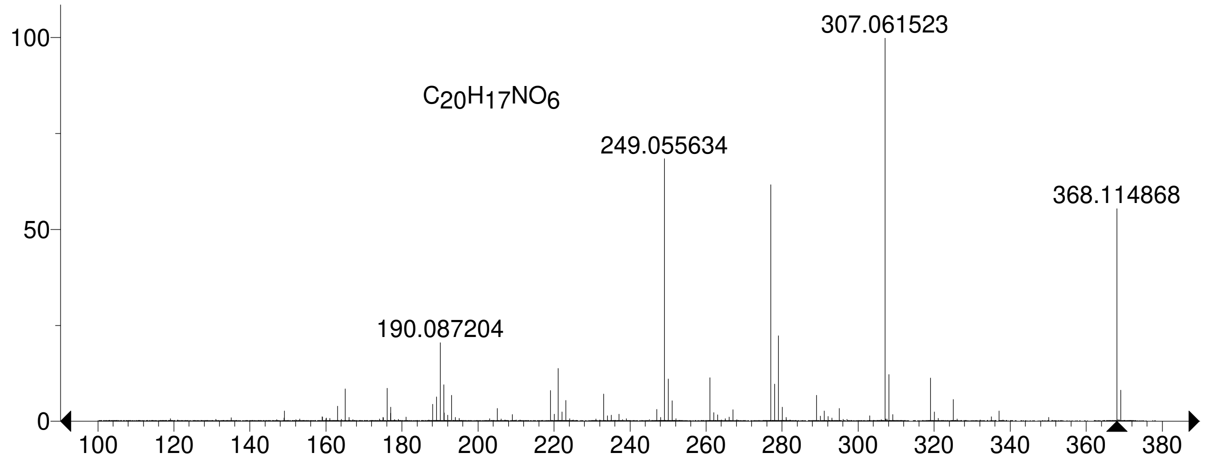

Hit 5 : CCPA [M-H]- 35eV P=368.1
C15H20ClN5O4; MF: 1; RMF: 226; Prob 10.7%; Lib: msms-neg-fiehnhilic; ID: 913.

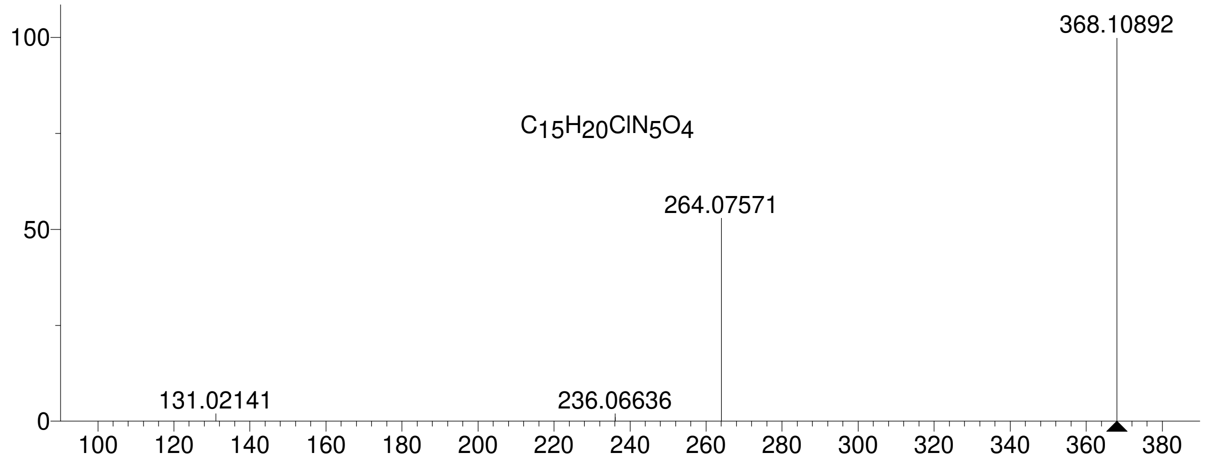

** Search Report Page 1 of 1 **

Unknown: Unknown (23.20_424.0300m/z)
Compound in Library Factor = 0

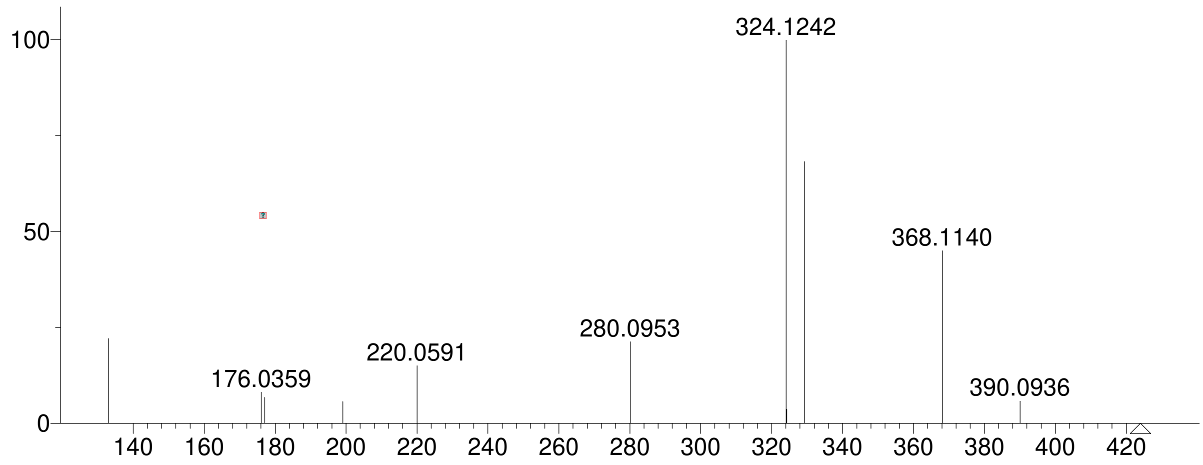

Unknown: Unknown (23.21_365.1585m/z)
Compound in Library Factor = 0

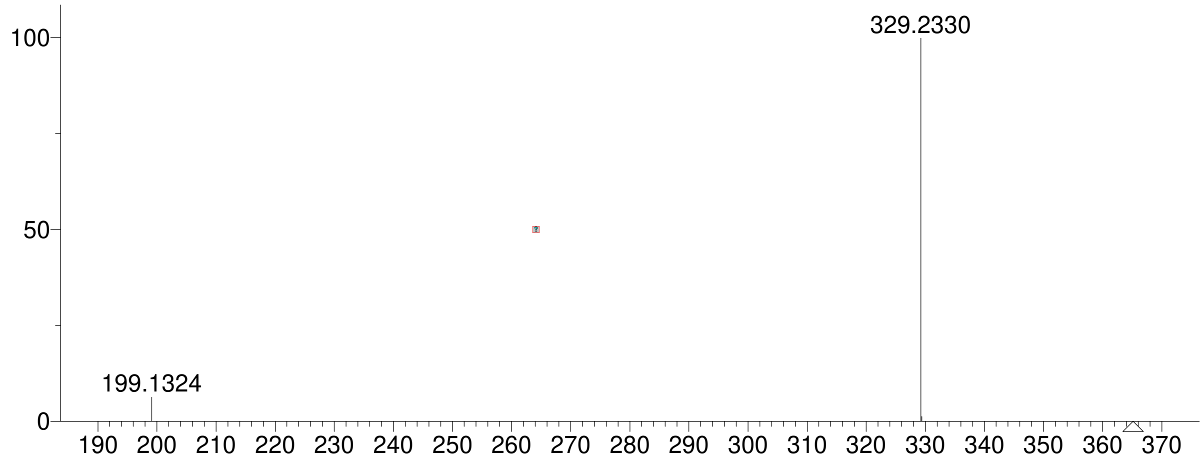

** Search Report Page 1 of 1 **

Unknown: Unknown (23.22_474.1541m/z)
Compound in Library Factor = 0

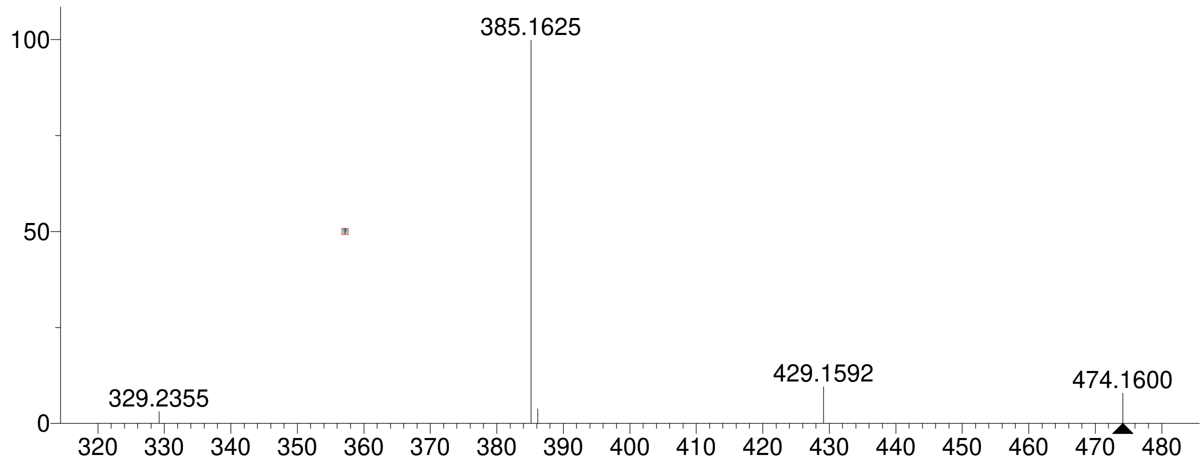

Unknown: Unknown (23.29_396.2273m/z)
Compound in Library Factor = -2176

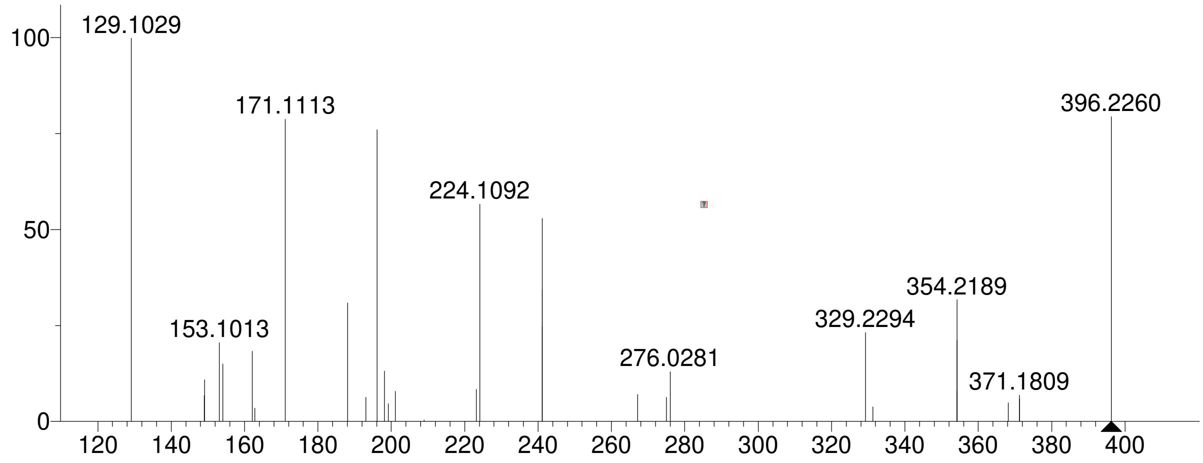

Hit 1 : 1H-Indazole-1-pentanoic acid, 3-[(tricyclo[3.3.1.1(3,7)]dec-1-ylamino)carbonyl]- [M+H]+ HCD 11V P=396
C23H29N3O3; MF: 30; RMF: 324; Prob 47.1%; CAS: 1630022-94-4; Lib: nist_msms; ID: 361142.

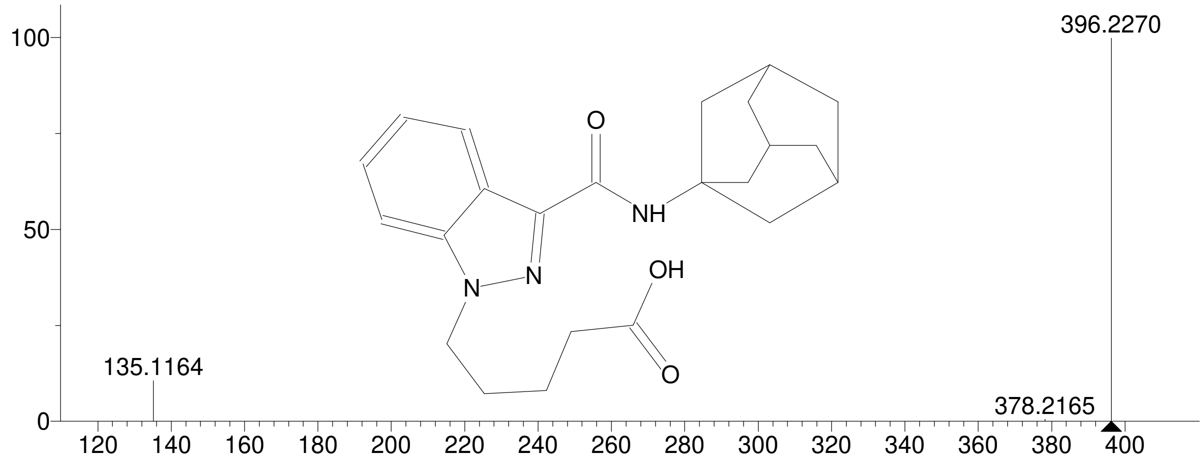

Hit 2 : Phosphonic acid, P-[[4-[(1-oxotetradecyl)amino]phenyl]methyl]- [M-H]- HCD 27V P=396.2
C21H36NO4P; MF: 25; RMF: 313; Prob 37.9%; CAS: 1096770-84-1; Lib: nist_msms; ID: 262687.

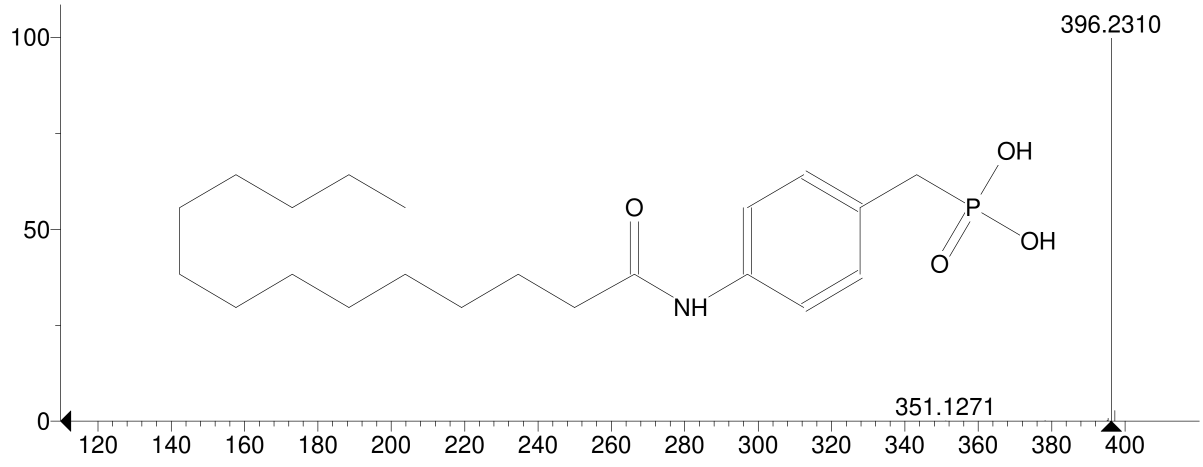

Hit 3 : NCGC00015878-08_C21H26N2O3_Methyl (16alpha,17alpha)-17-hydroxyyohimban-16-carboxylate [M+A(
C21H26N2O3; MF: 3; RMF: 207; Prob 15.0%; Lib: mona-export-gnps; ID: 13933.

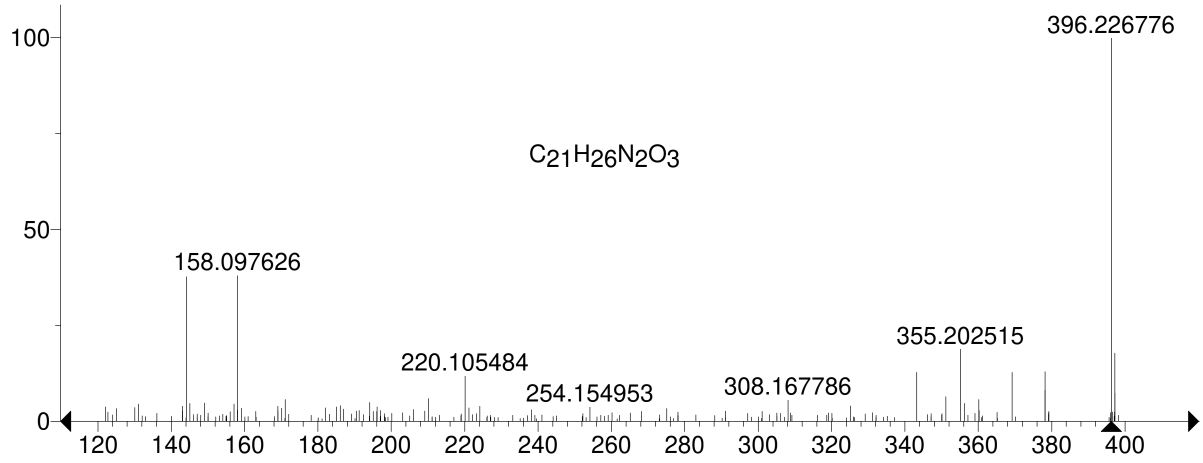

Unknown: Unknown (23.40_329.2324m/z)
Compound in Library Factor = -182

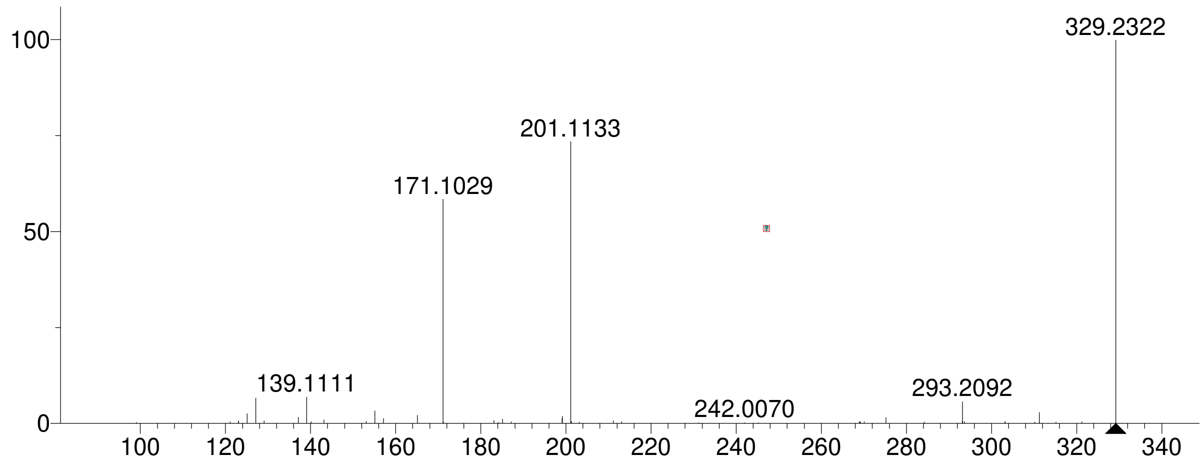

Hit 1 : FA 18:1+3O; PlaSMA ID-737 [M-H]- P=329.2
C18H34O5; MF: 420; RMF: 740; Prob 98.7%; Lib: biomsms-neg-plasma; ID: 448.

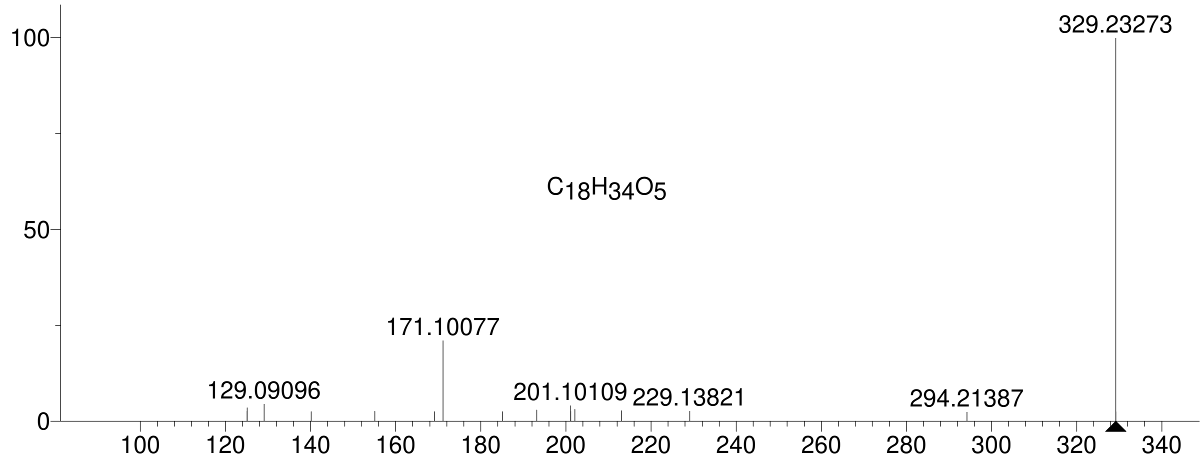

Hit 2 : FA 18:1+3O; PlaSMA ID-738 [M-H]- P=329.2
C18H34O5; MF: 136; RMF: 626; Prob 0.98%; Lib: biomsms-neg-plasma; ID: 444.

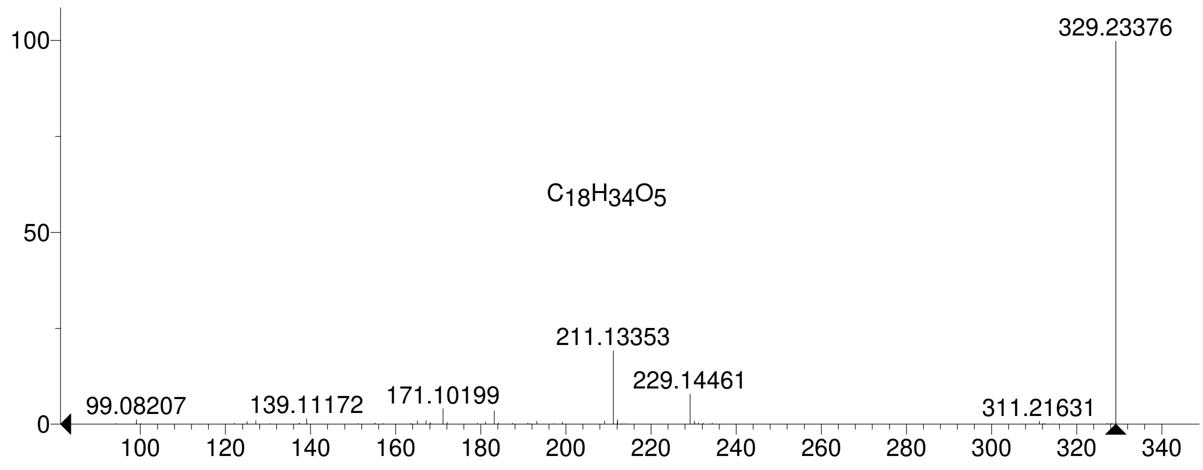

Hit 3 : FA 18:1+3O; PlaSMA ID-735 [M-H]- P=329.2
C18H34O5; MF: 56; RMF: 596; Prob 0.10%; Lib: biomsms-neg-plasma; ID: 443.

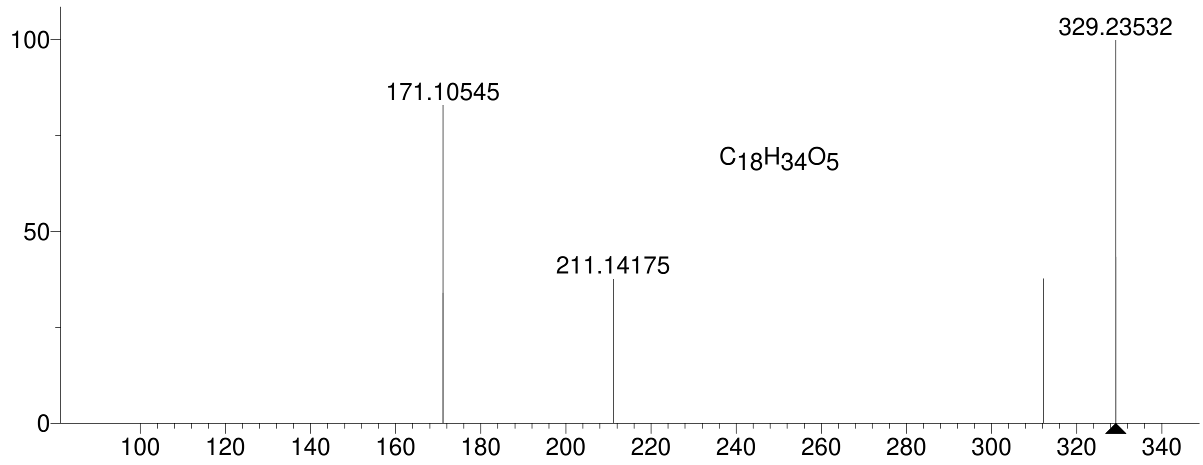

Hit 4 : FA 18:1+3O; PlaSMA ID-739 [M-H]- P=329.2
C18H34O5; MF: 56; RMF: 528; Prob 0.10%; Lib: biomsms-neg-plasma; ID: 446.

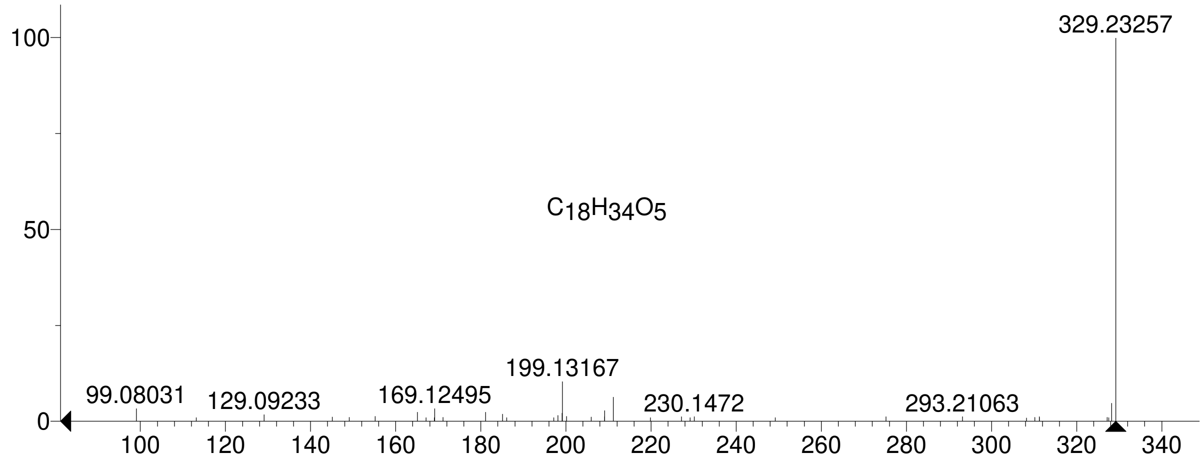

Hit 5 : FA 18:1+3O; PlaSMA ID-741 [M-H]- P=329.2
C18H34O5; MF: 29; RMF: 569; Prob 0.03%; Lib: biomsms-neg-plasma; ID: 449.

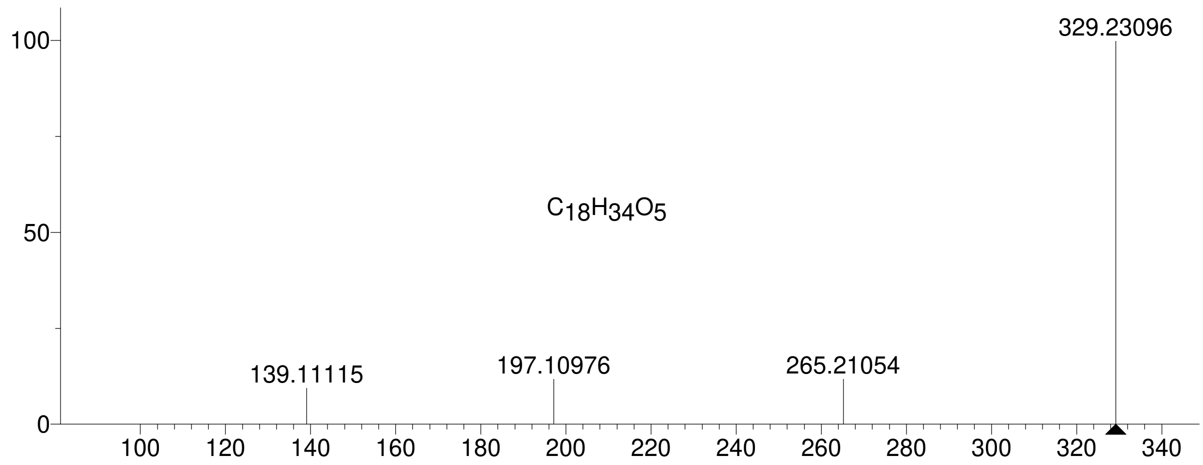

Unknown: Unknown (23.45_372.1799m/z)
Compound in Library Factor = 0

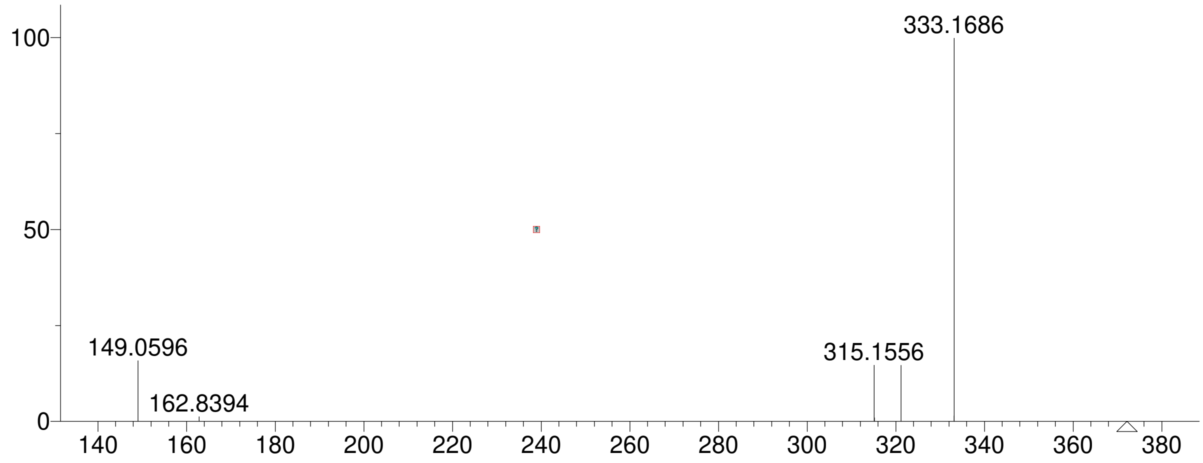

Hit 1 : 5,7-dihydroxy-2-(4-methoxyphenyl)-6-(3-methylbut-2-enyl)-2,3-dihydrochromen-4-one [M+NH4]+ 65HCD
C21H22O5; MF: 0; RMF: 29; Prob 50.0%; Lib: mona-export-vf-npl_qexactive; ID: 9528.

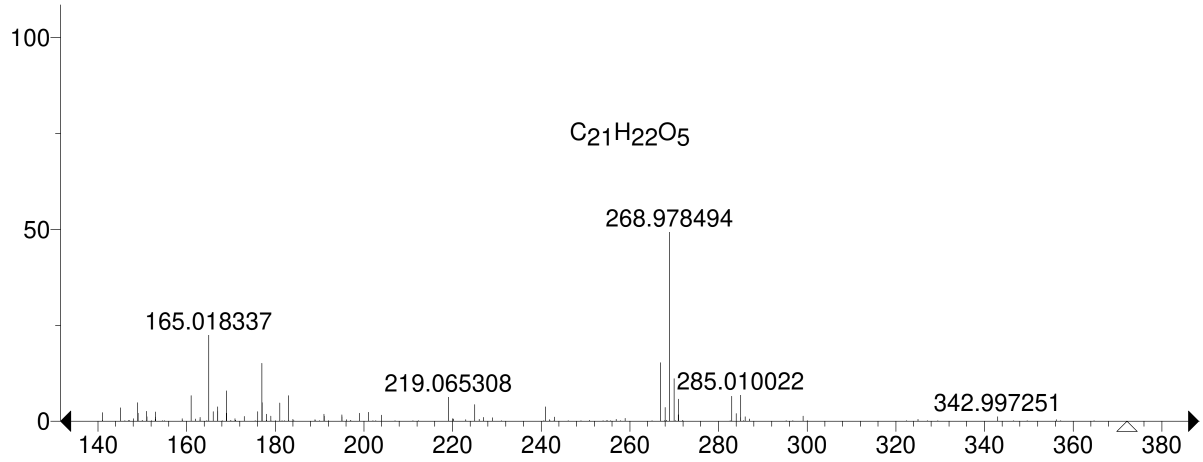

Hit 2 : xanthohumol [M+NH4]+ 65HCD P=372.2
C21H22O5; MF: 0; RMF: 7; Prob 50.0%; Lib: mona-export-vf-npl_qexactive; ID: 10932.

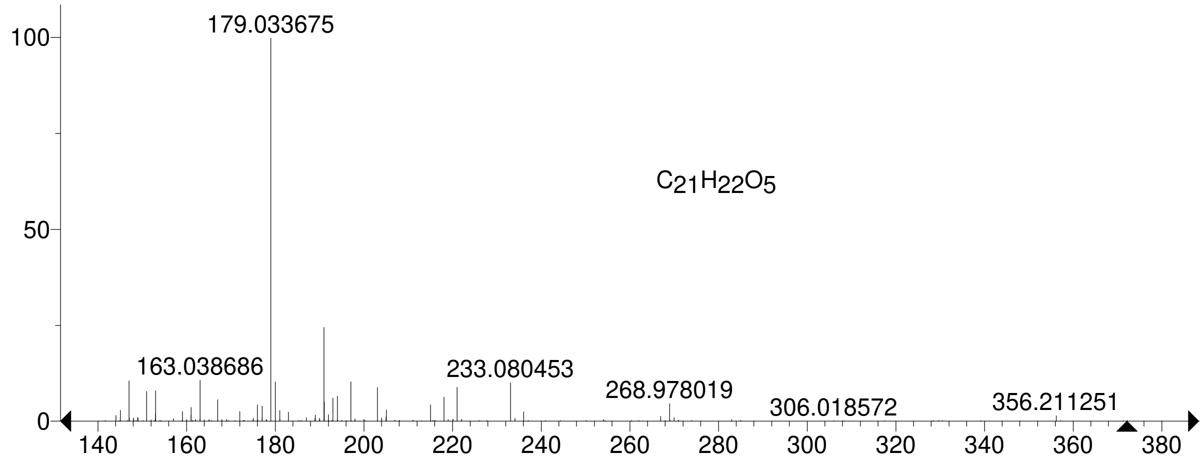

** Search Report Page 1 of 1 **

Unknown: Unknown (23.48_323.1839m/z)
Compound in Library Factor = 0

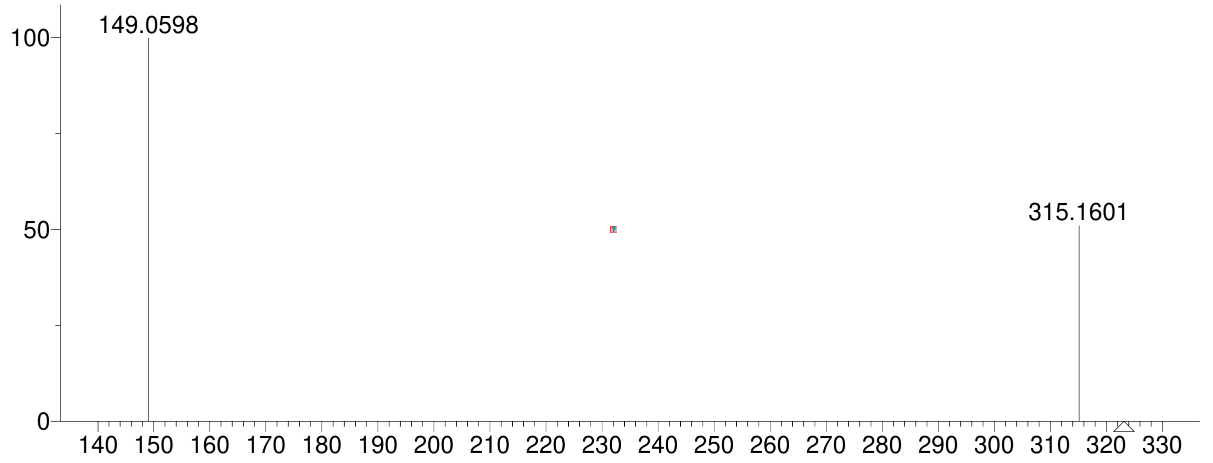

Hit 1 : Zeranol [M+H]+ 20-30-40%) P=323.2
C18H26O5; MF: 0; RMF: 164; Prob 50.0%; Lib: mona-export-fiehn_hilic; ID: 867.

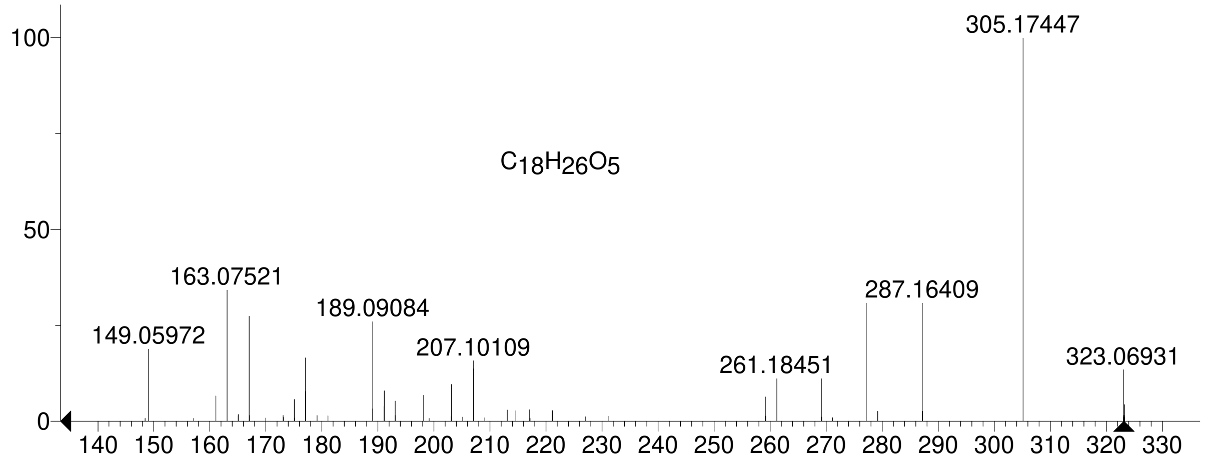

Hit 2 : (5E)-2-heptyl-3,4,7-trihydroxy-2,3,4,7,8,9-hexahydrooxecin-10-one [M+Na]+ 65HCD P=323.2
C16H28O5; MF: 0; RMF: 35; Prob 50.0%; Lib: mona-export-vf-npl_qexactive; ID: 2283.

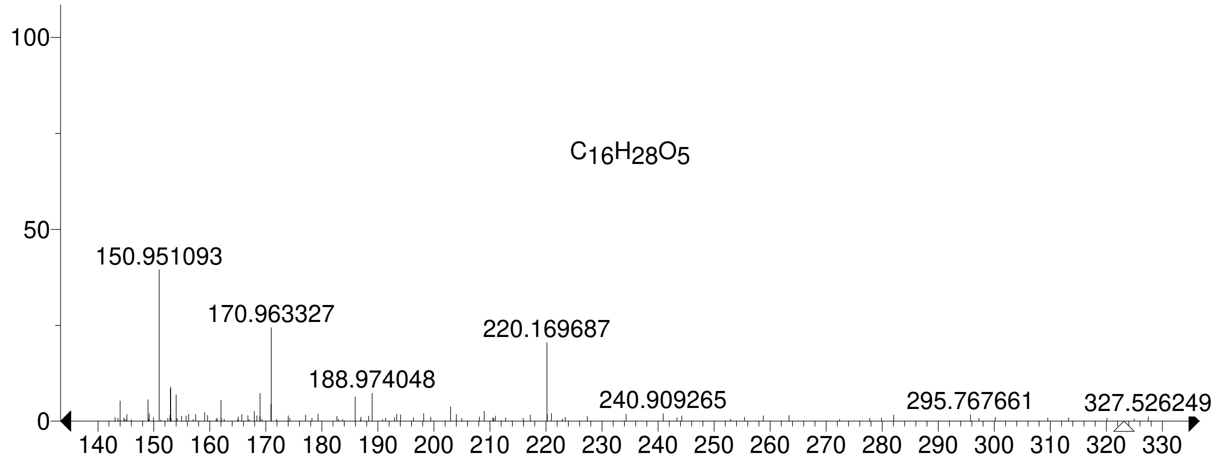

Unknown: Unknown (23.49_325.2009m/z)
Compound in Library Factor = -2176

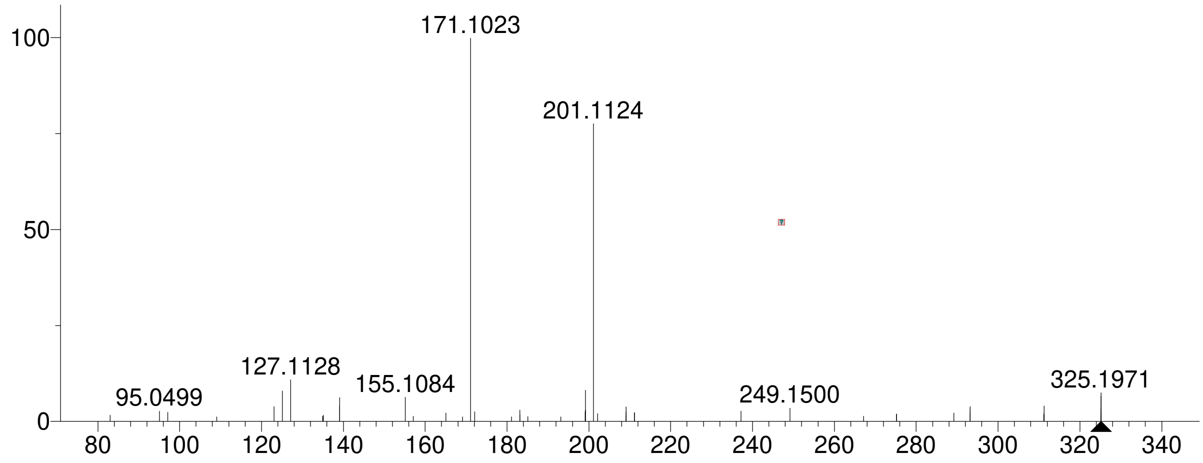

Hit 1 : Hymeglusin [M+H]+ HCD 3V P=325.2
C18H28O5; MF: 102; RMF: 530; Prob 38.3%; CAS: 29066-42-0; Lib: nist_msms; ID: 376516.

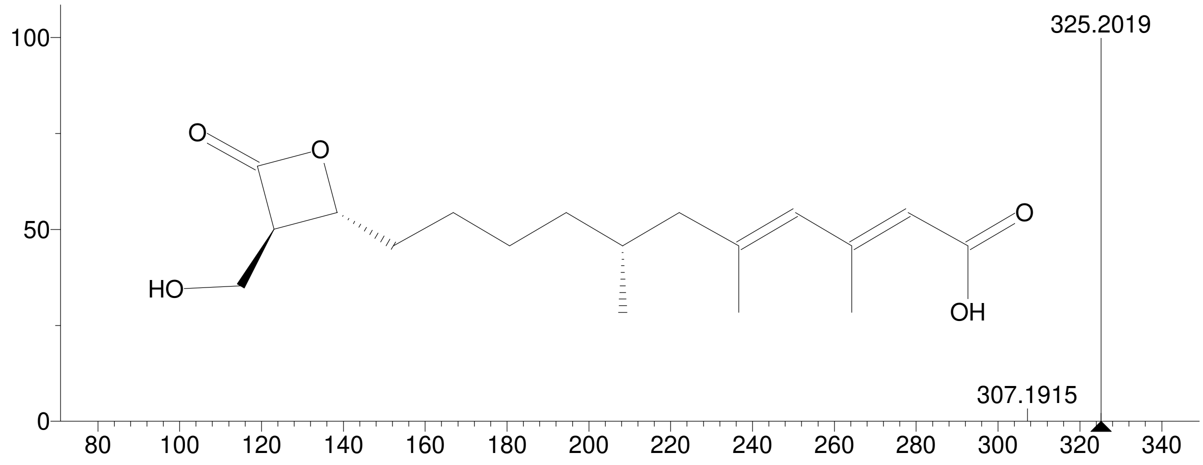

Hit 2 : Unknown (carbon number 18); PlaSMA ID-697 [M-H]- P=325.2
C18H30O5; MF: 80; RMF: 407; Prob 15.2%; Lib: biomsms-neg-plasma; ID: 1224.

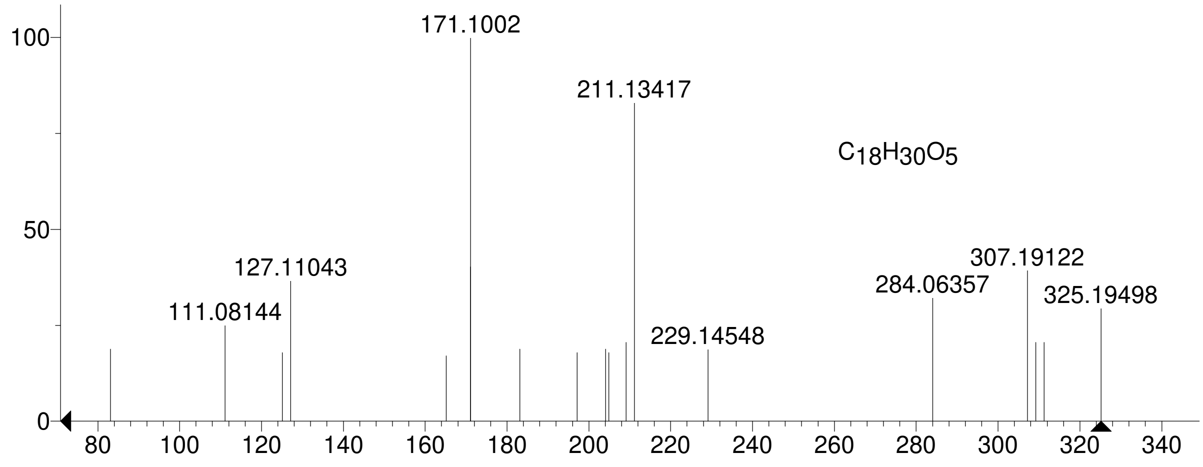

Hit 3 : FA 18:3+3O; PlaSMA ID-694 [M-H]- P=325.2
C18H30O5; MF: 58; RMF: 289; Prob 5.99%; Lib: biomsms-neg-plasma; ID: 424.

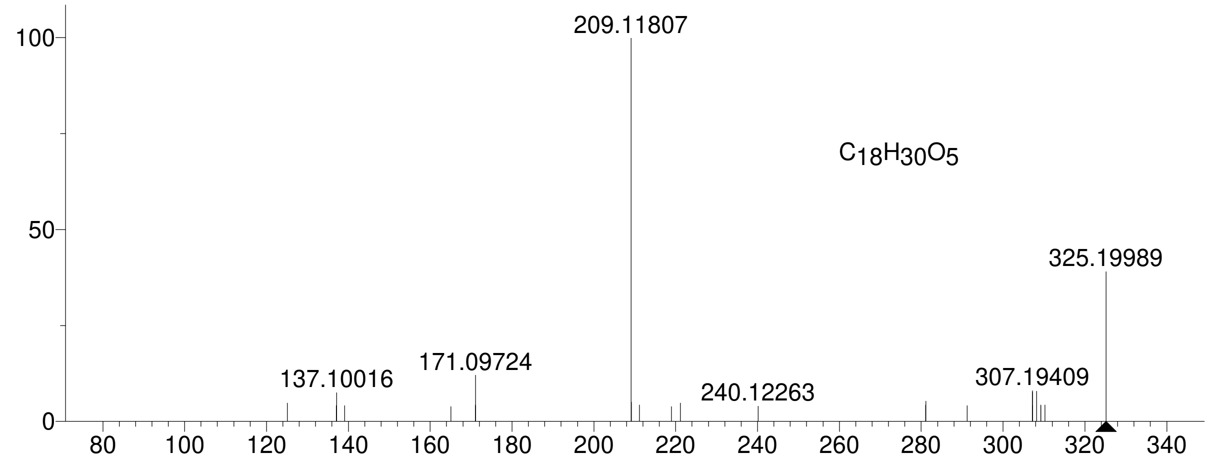

Hit 4 : Dinorprostaglandin E1 [M-H]- HCD 4V P=325.2
C18H30O5; MF: 48; RMF: 373; Prob 4.22%; CAS: 7046-40-4; Lib: nist_msms; ID: 88066.

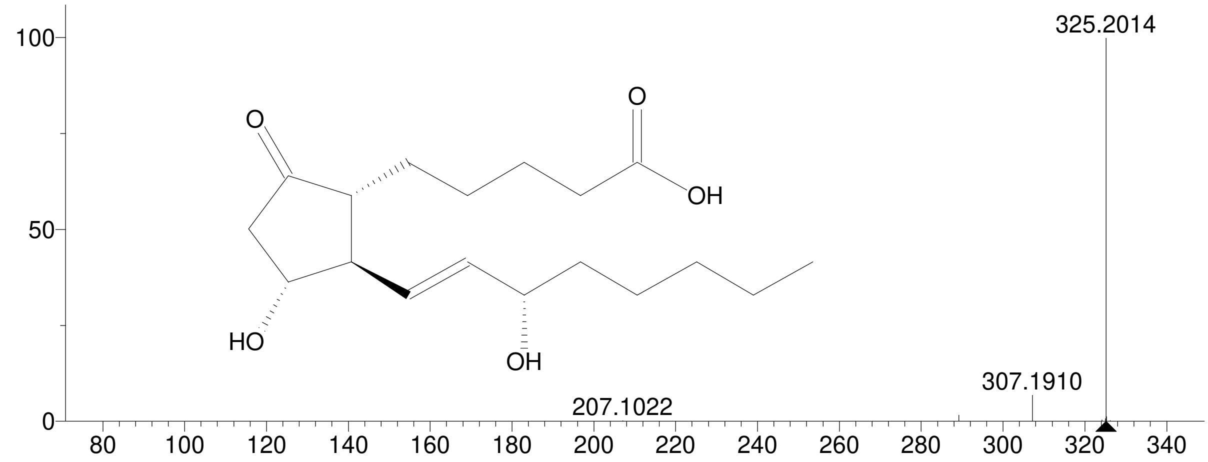

Hit 5 : Unknown (carbon number 19); PlaSMA ID-693 [M-H]- P=325.2
C19H26N4O; MF: 27; RMF: 252; Prob 1.79%; Lib: biomsms-neg-plasma; ID: 1223.

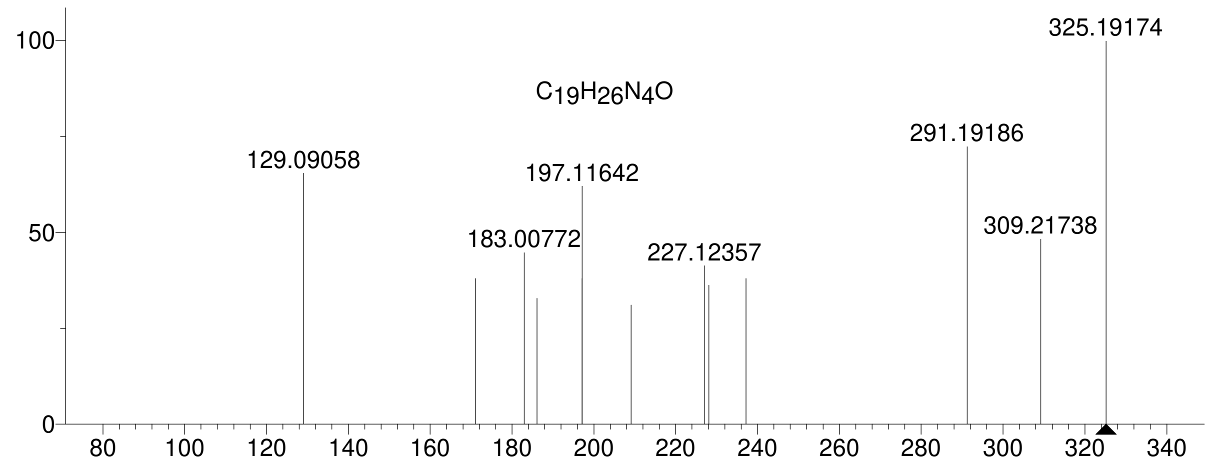

Unknown: Unknown (23.49_413.1259m/z)
Compound in Library Factor = -2387

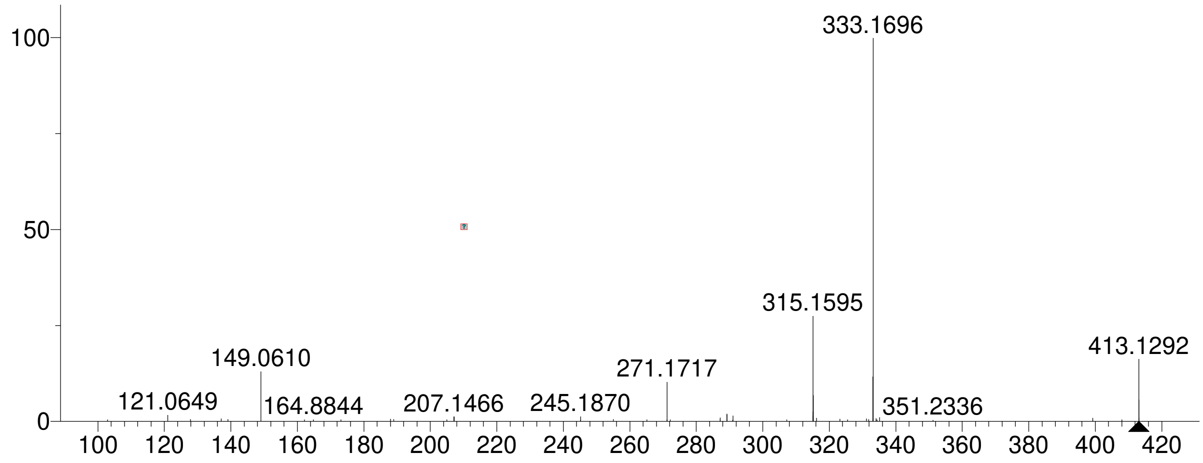

Hit 1 : 7H-1,2,4-Triazolo[3,4-b][1,3,4]thiadiazine, 3-(2,5-dimethoxyphenyl)-6-(3,4-dimethoxyphenyl)- [M+H]+ HCD
C20H20N4O4S; MF: 13; RMF: 266; Prob 23.7%; CAS: 1013750-77-0; Lib: nist_msms; ID: 367682.

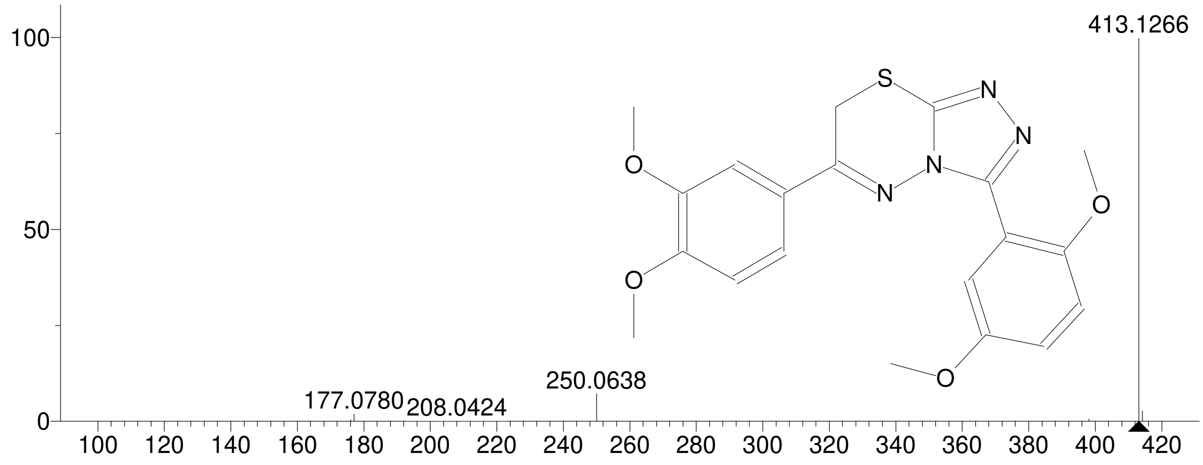

Hit 2 : Cyflufenamide [M+H]+ HCD 8V P=413.1
C20H17F5N2O2; MF: 2; RMF: 250; Prob 16.3%; CAS: 180409-60-3; Lib: nist_msms; ID: 477205.

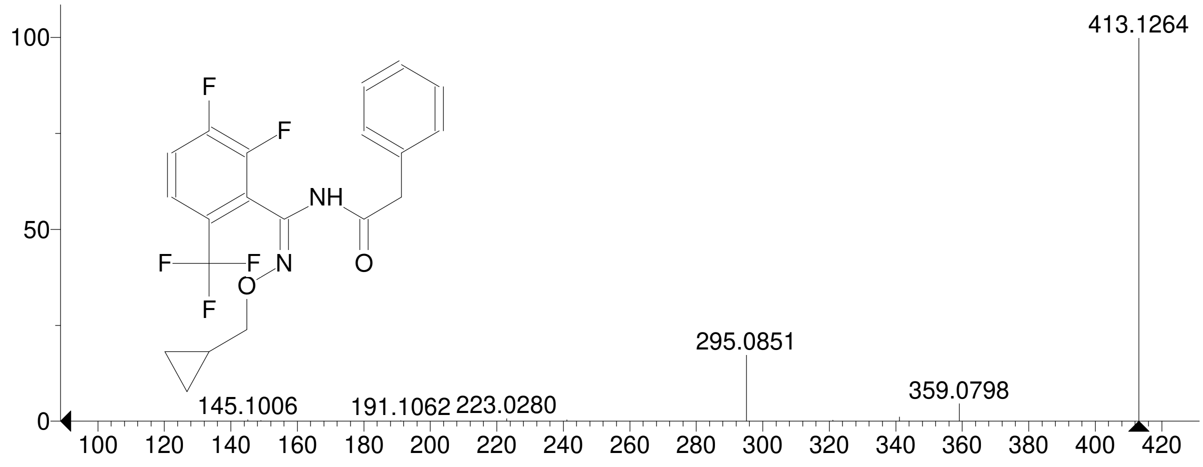

Hit 3 : NCGC00380419-01_C19H24O8S_3-[(11,13-Dihydroxy-4-methyl-2,10-dioxo-1,4,5,6,7,8,9,10-octahydro-2H
C19H24O8S; MF: 0; RMF: 56; Prob 15.0%; Lib: mona-export-gnps; ID: 14573.

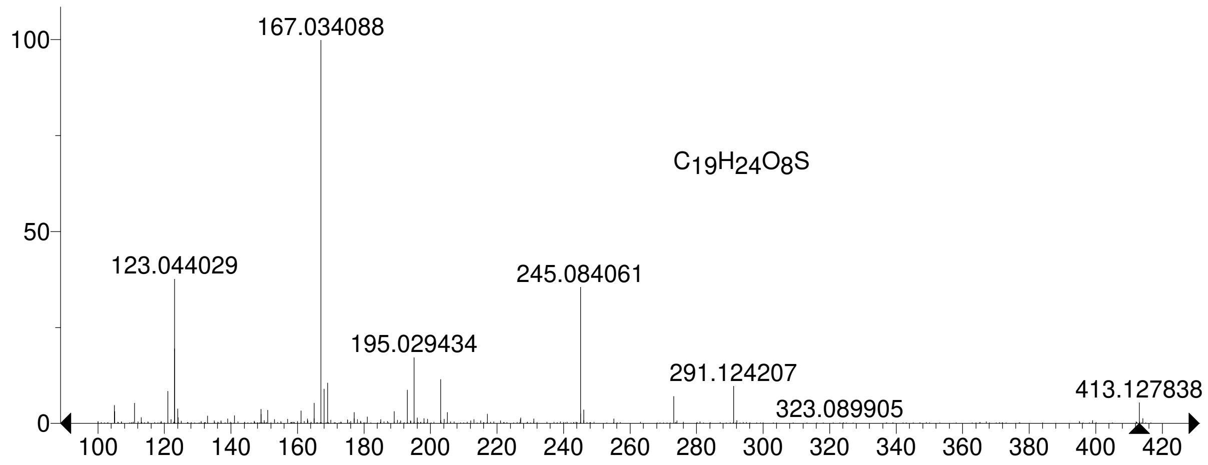

Hit 4 : Beta-Peltatin [M-H]- 35HCD P=413.1
C22H22O8; MF: 0; RMF: 33; Prob 15.0%; Lib: mona-export-lc-ms-ms_negative_mode; ID: 19961.

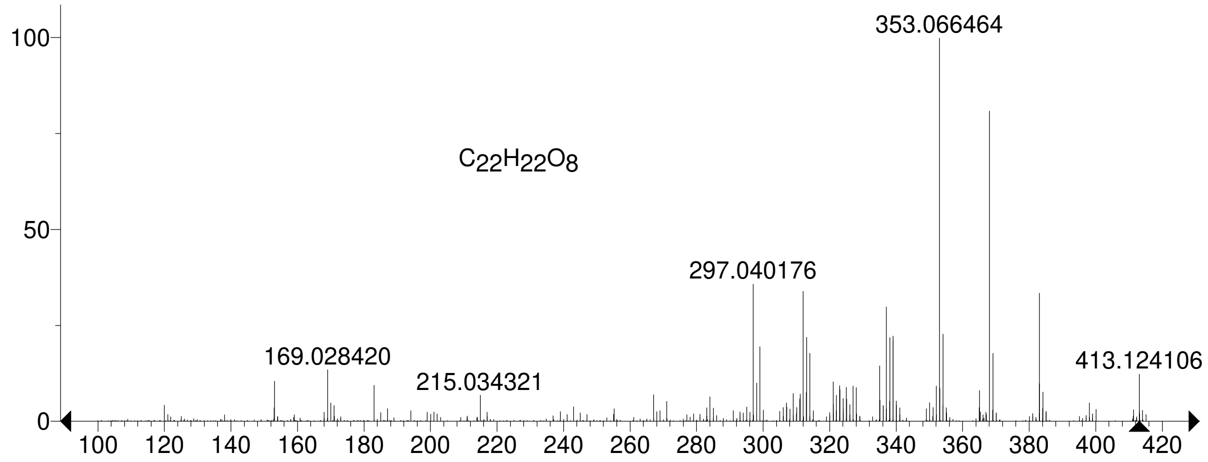

Hit 5 : 2,4,18-trihydroxy-15-(3-hydroxy-3-methylbutyl)-7,9,13-trioxapentacyclo[10.8.0.0A^'3,A^'1a^'o.0a^'',a^,,.0A^'
C22H20O8; MF: 0; RMF: 24; Prob 15.0%; Lib: mona-export-vf-npl_qexactive; ID: 1267.

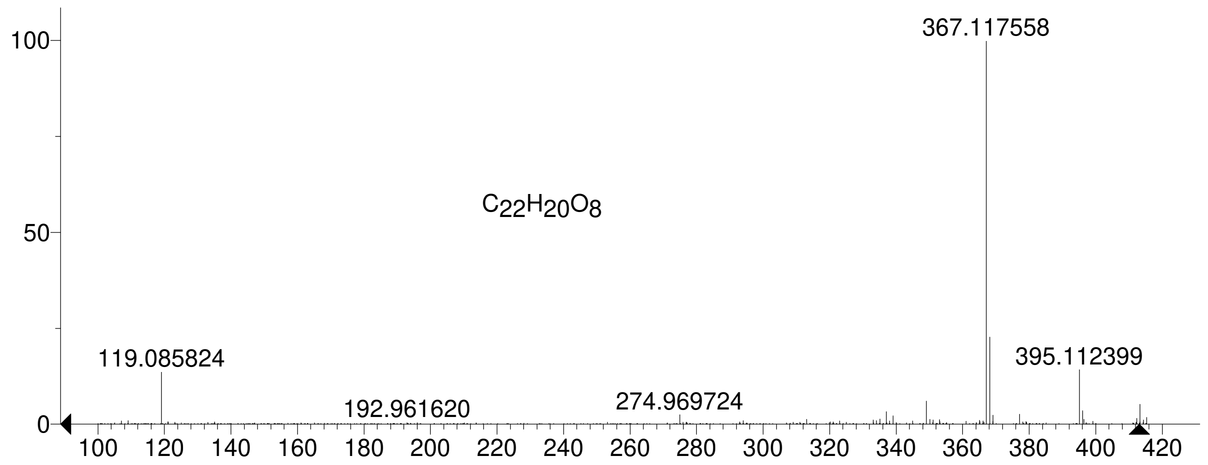

Unknown: Unknown (23.59_325.2009m/z)
Compound in Library Factor = -1848

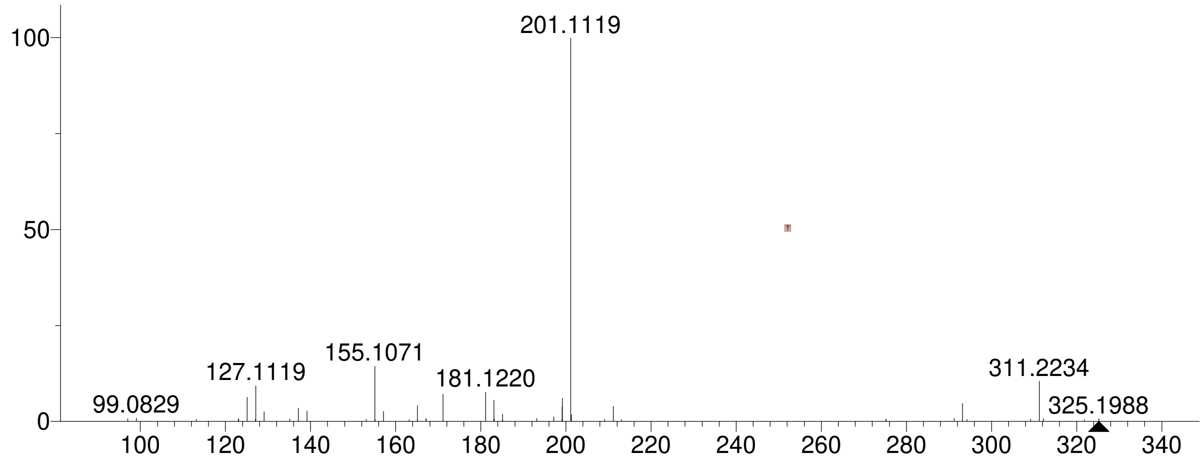

Hit 1 : Unknown (carbon number 18); PlaSMA ID-697 [M-H]- P=325.2
C18H30O5; MF: 111; RMF: 274; Prob 43.5%; Lib: biomsms-neg-plasma; ID: 1224.

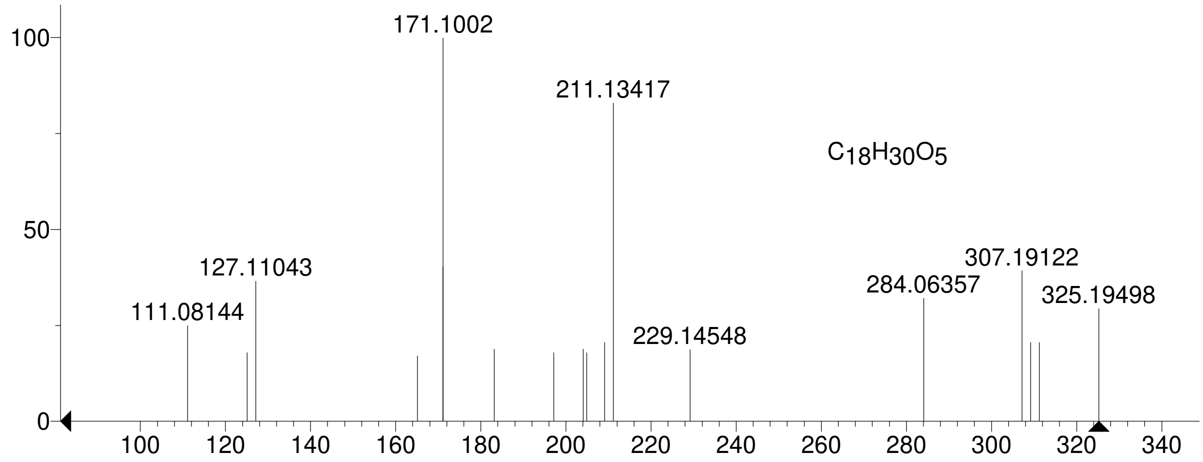

Hit 2 : Unknown (carbon number 19); PlaSMA ID-693 [M-H]- P=325.2
C19H26N4O; MF: 72; RMF: 183; Prob 10.3%; Lib: biomsms-neg-plasma; ID: 1223.

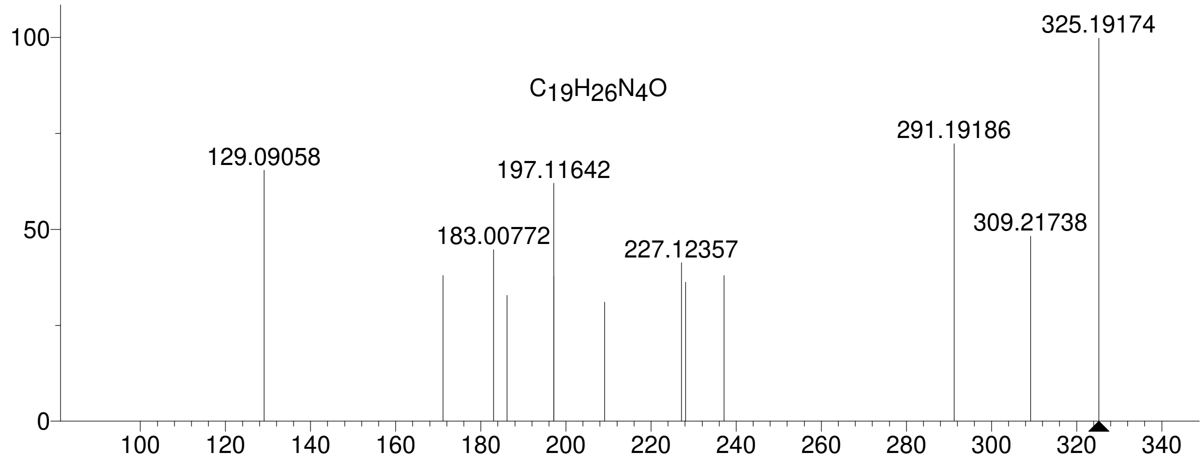

Hit 3 : 2,3-Dinor-8-isoprostaglandin-F2α [M-H]- HCD 26V P=325.2
C18H30O5; MF: 51; RMF: 182; Prob 4.36%; CAS: 221664-05-7; Lib: nist_msms; ID: 88183.

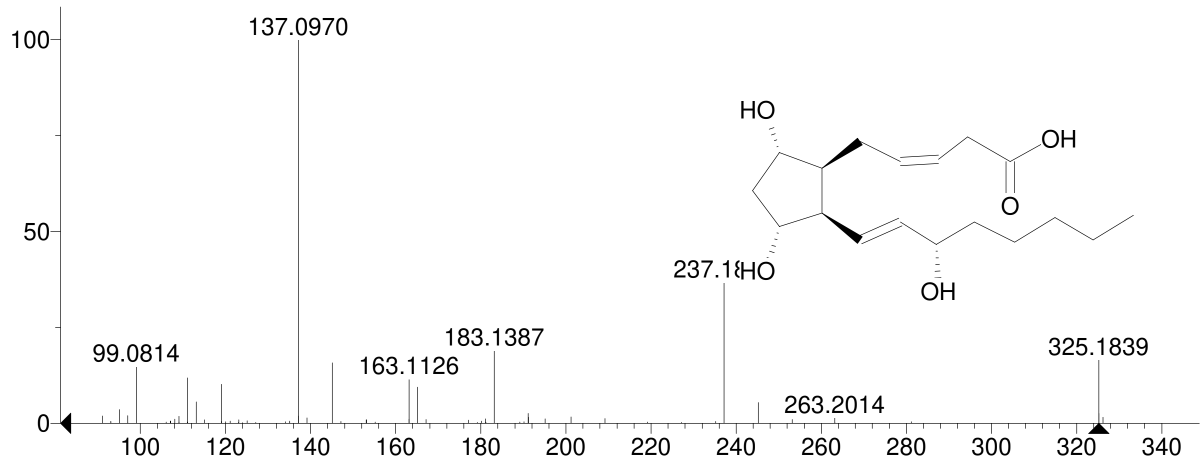

Hit 4 : FA 18:3+3O; PlaSMA ID-694 [M-H]- P=325.2
C18H30O5; MF: 41; RMF: 161; Prob 3.08%; Lib: biomsms-neg-plasma; ID: 424.

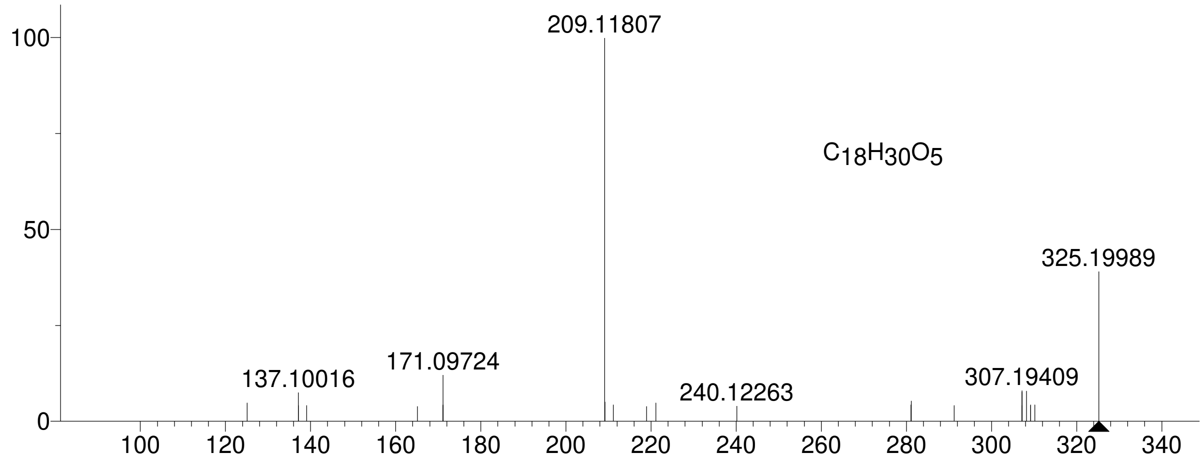

Hit 5 : Unknown (carbon number 18); PlaSMA ID-698 [M-H]- P=325.2
C18H30O5; MF: 27; RMF: 104; Prob 1.93%; Lib: biomsms-neg-plasma; ID: 1225.

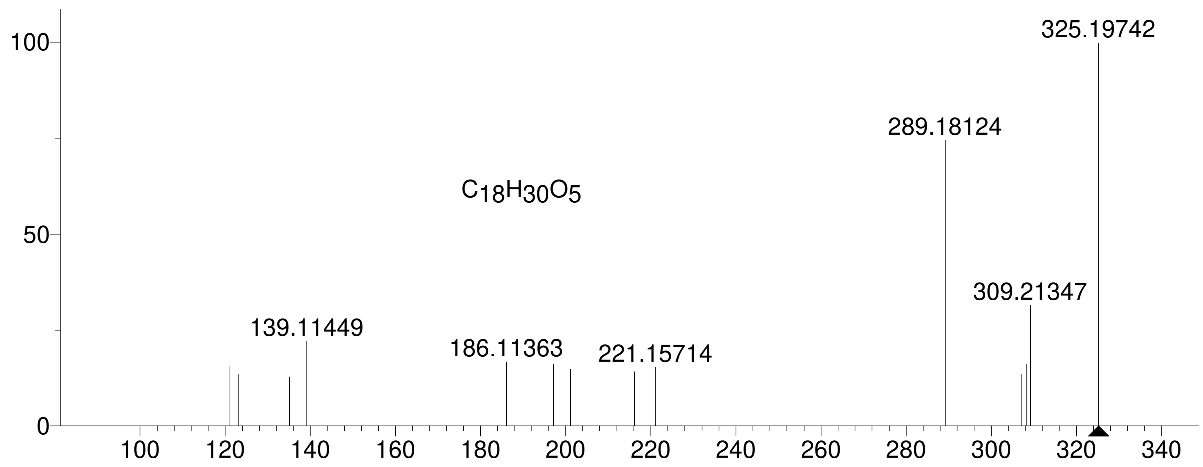

Unknown: Unknown (23.71_329.2328m/z)
Compound in Library Factor = -773

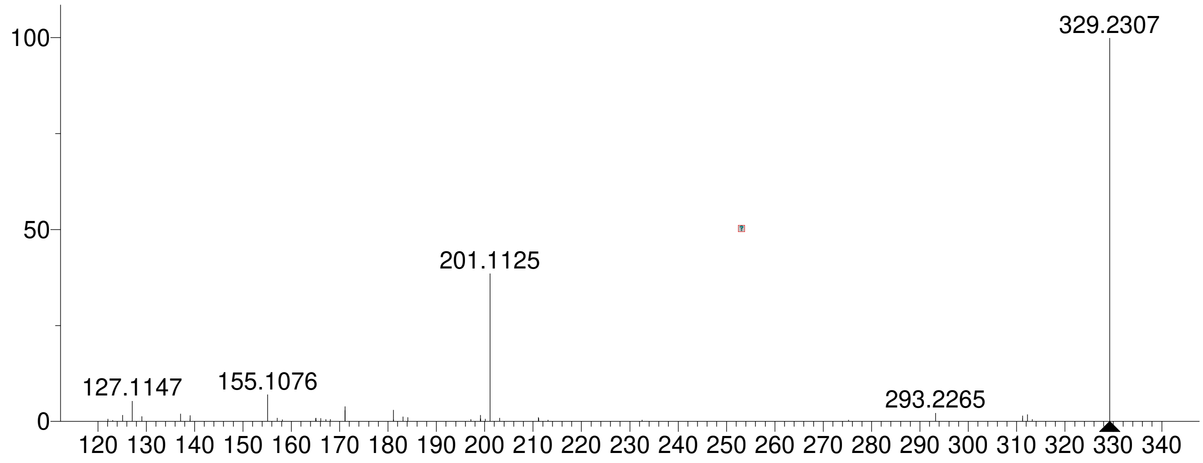

Hit 1 : FA 18:1+3O; PlaSMA ID-735 [M-H]- P=329.2
C18H34O5; MF: 282; RMF: 599; Prob 82.4%; Lib: biomsms-neg-plasma; ID: 443.

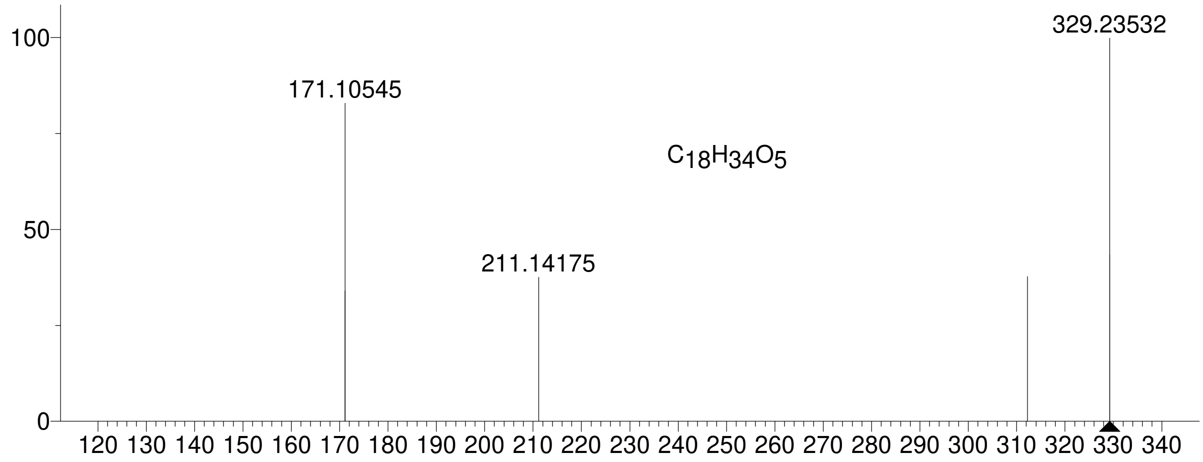

Hit 2 : FA 18:1+3O; PlaSMA ID-737 [M-H]- P=329.2
C18H34O5; MF: 219; RMF: 719; Prob 13.8%; Lib: biomsms-neg-plasma; ID: 448.

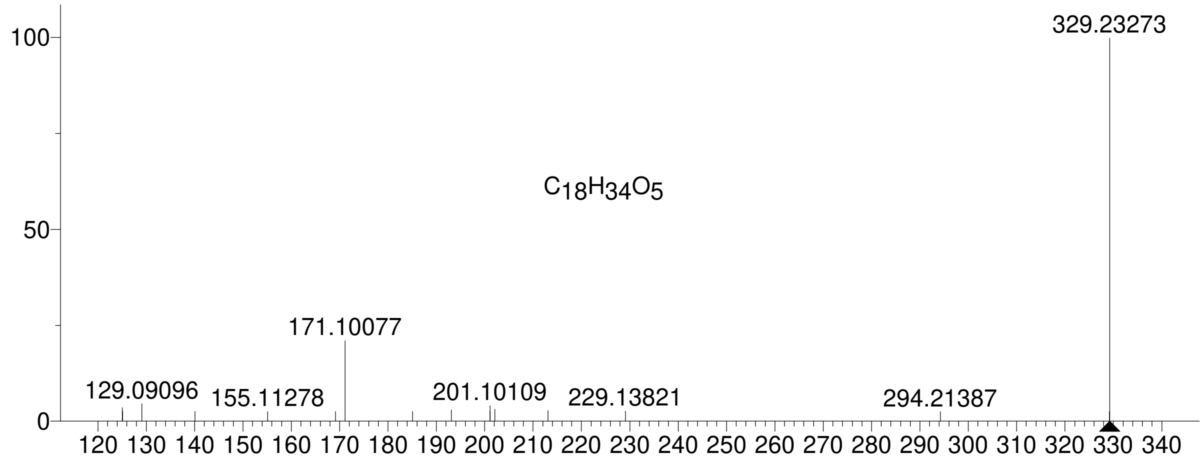

Hit 3 : FA 18:1+3O; PlaSMA ID-738 [M-H]- P=329.2
C18H34O5; MF: 172; RMF: 701; Prob 2.89%; Lib: biomsms-neg-plasma; ID: 444.

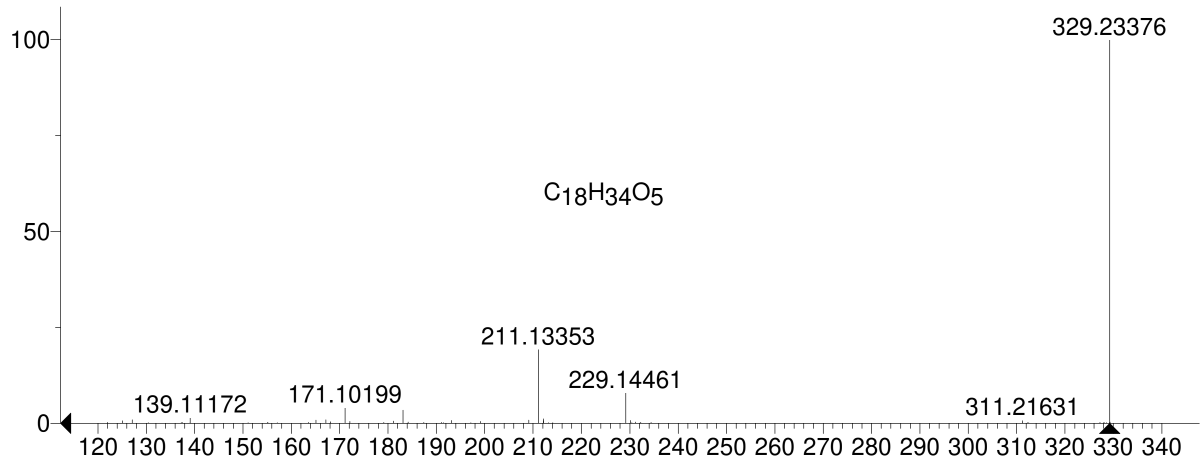

Hit 4 : FA 18:1+3O; PlaSMA ID-742 [M-H]- P=329.2
C18H34O5; MF: 91; RMF: 671; Prob 0.30%; Lib: biomsms-neg-plasma; ID: 445.

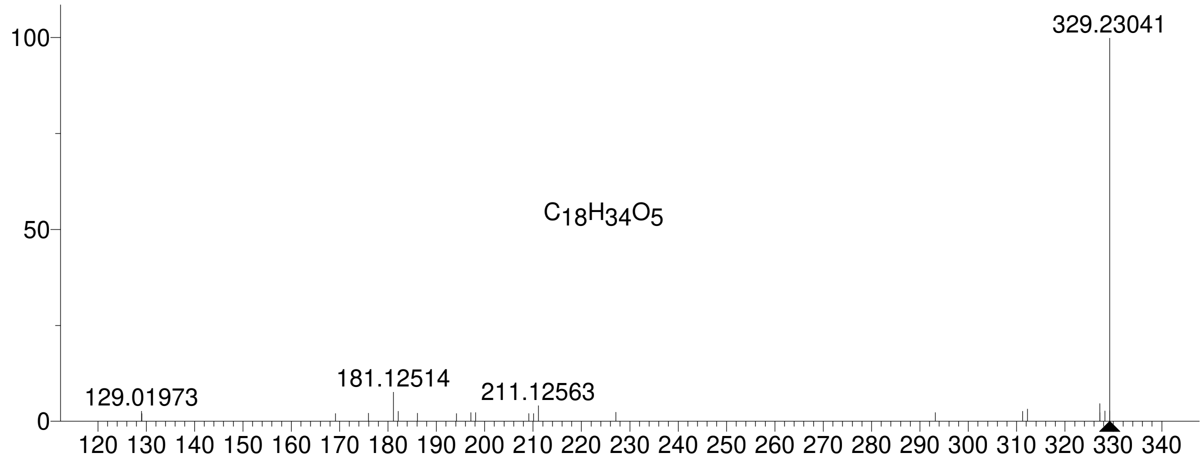

Hit 5 : FA 18:1+3O; PlaSMA ID-739 [M-H]- P=329.2
C18H34O5; MF: 83; RMF: 645; Prob 0.22%; Lib: biomsms-neg-plasma; ID: 446.

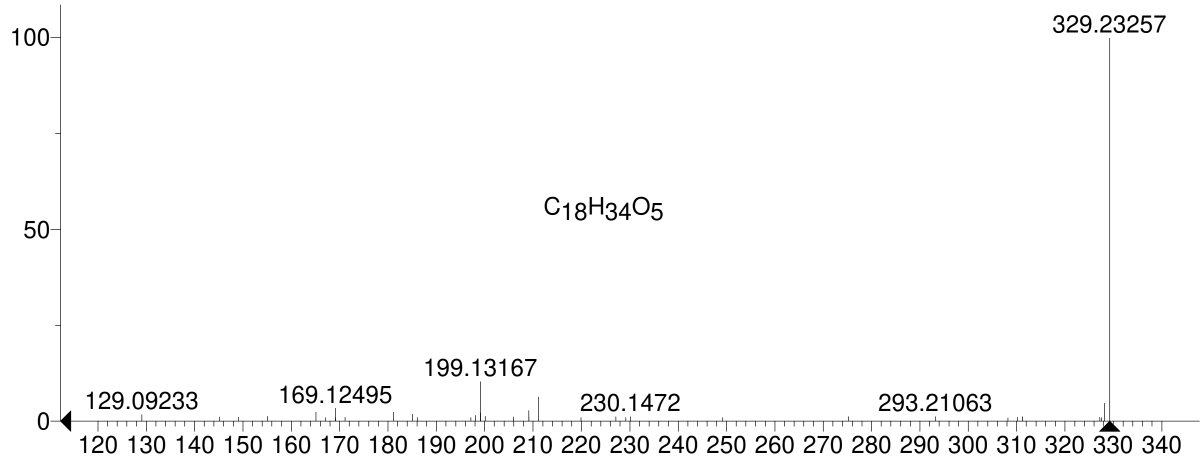

** Search Report Page 1 of 1 **

Unknown: Unknown (23.72_329.4029m/z)
Compound in Library Factor = 0

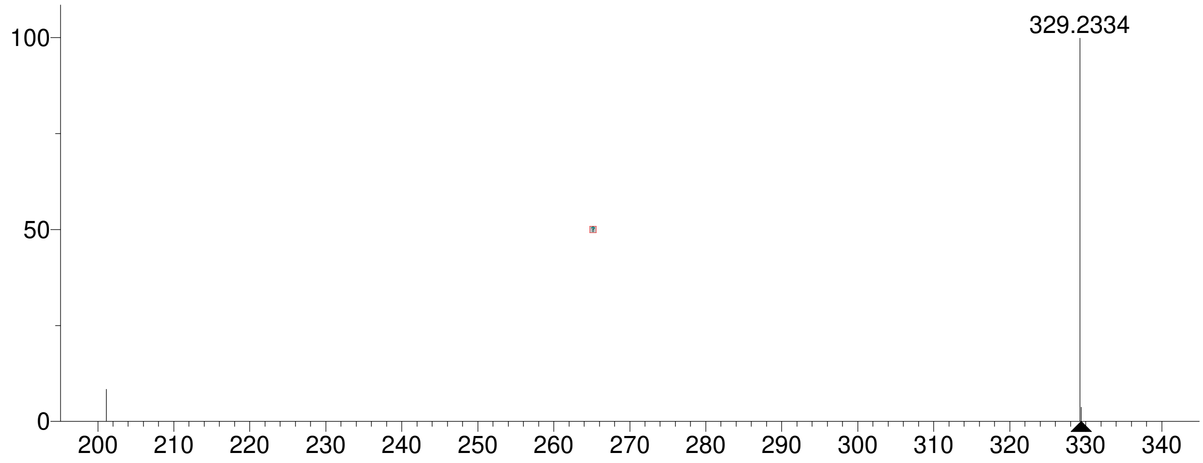

Hit 1 : Unoprostone isopropyl ester [M+H-H2O]+=>329.4 IT 35% P=329.4
C25H44O5; MF: 0; RMF: 10; Prob 100.0%; CAS: 120373-24-2; Lib: nist_msms; ID: 172883.

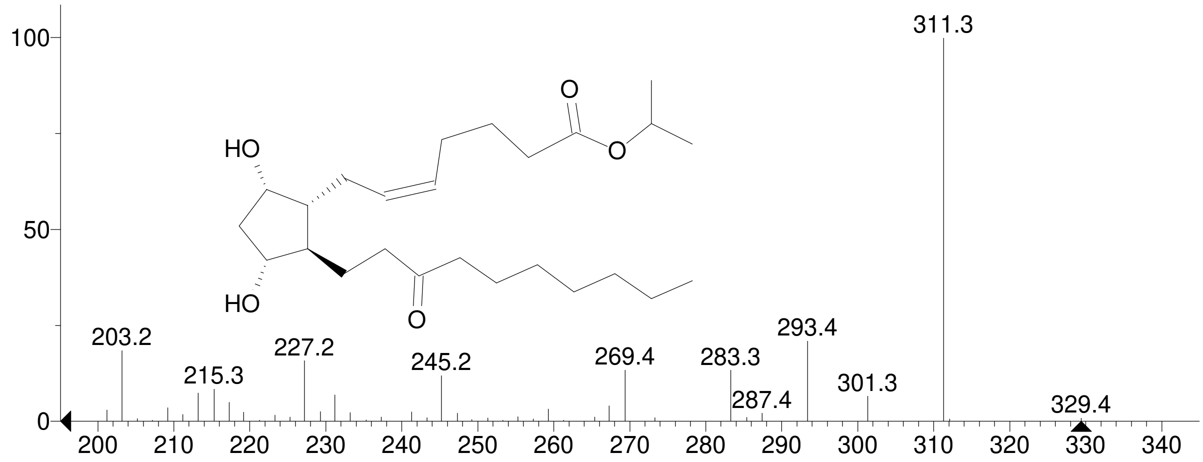

Unknown: Unknown (23.72_397.2191m/z)
Compound in Library Factor = 0

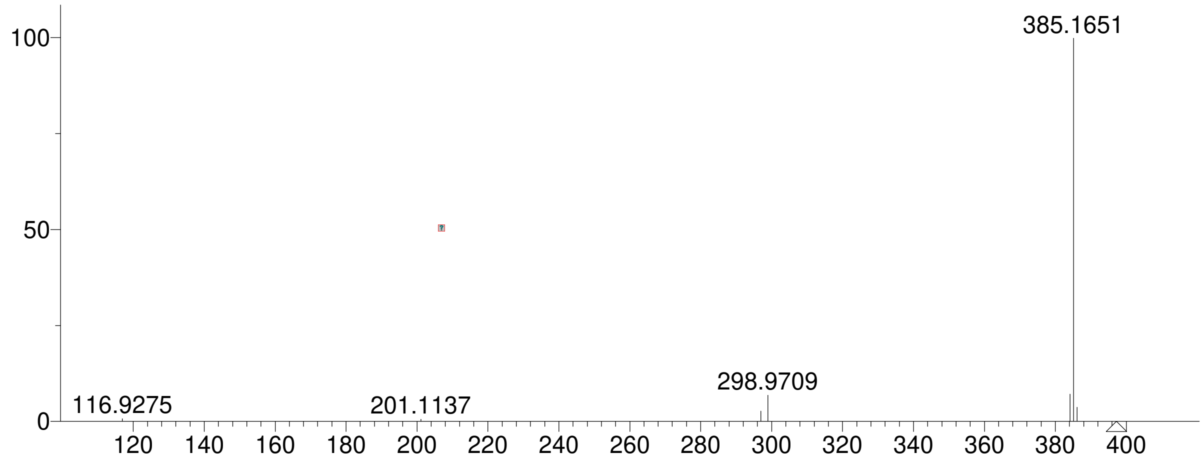

** Search Report Page 1 of 1 **

Unknown: Unknown (23.73_474.1550m/z)
Compound in Library Factor = 0

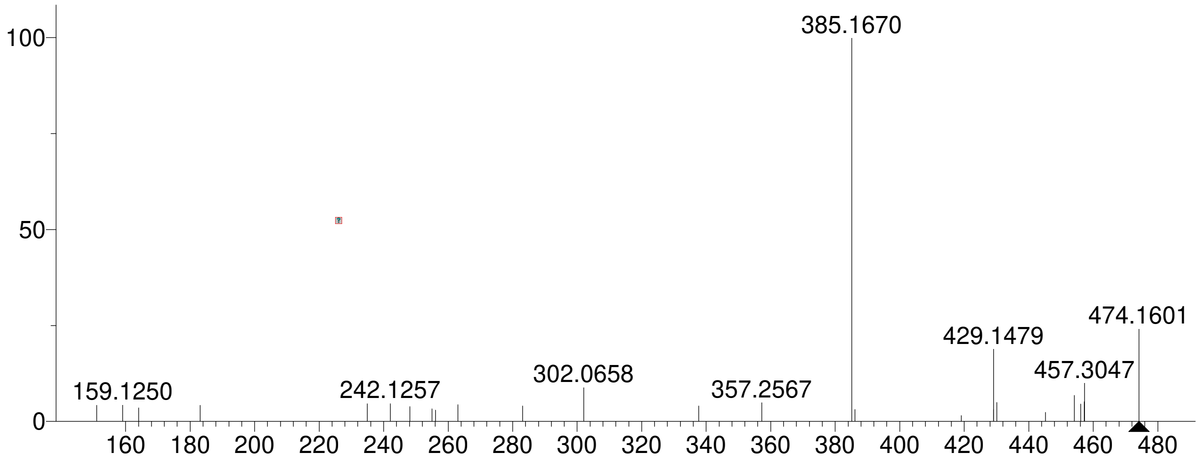

Unknown: Unknown (23.77_353.2309m/z)
Compound in Library Factor = -1523

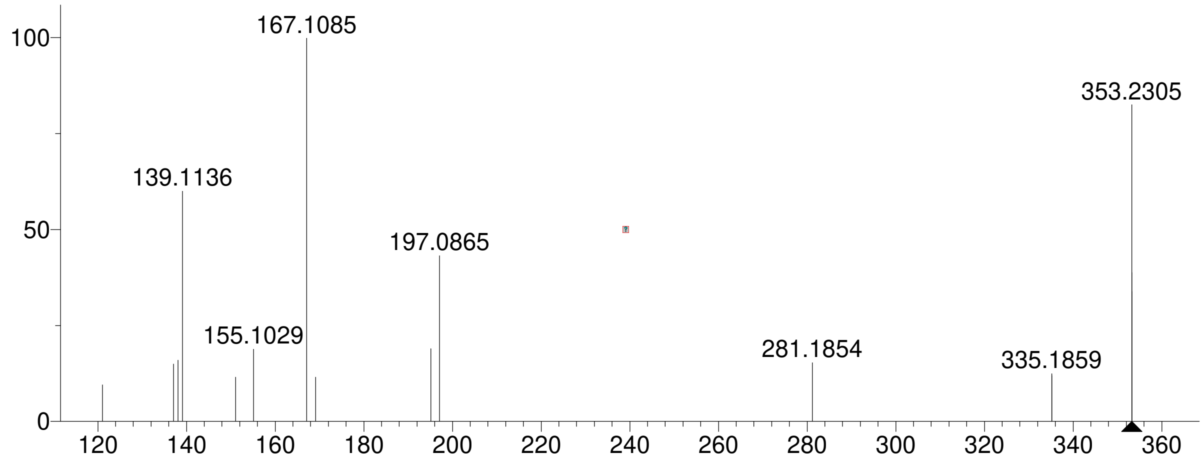

Hit 1 : 15-Ketoprostaglandin E1 [M+H]+ HCD 3V P=353.2
C20H32O5; MF: 187; RMF: 675; Prob 47.7%; CAS: 22973-19-9; Lib: nist_msms; ID: 169227.

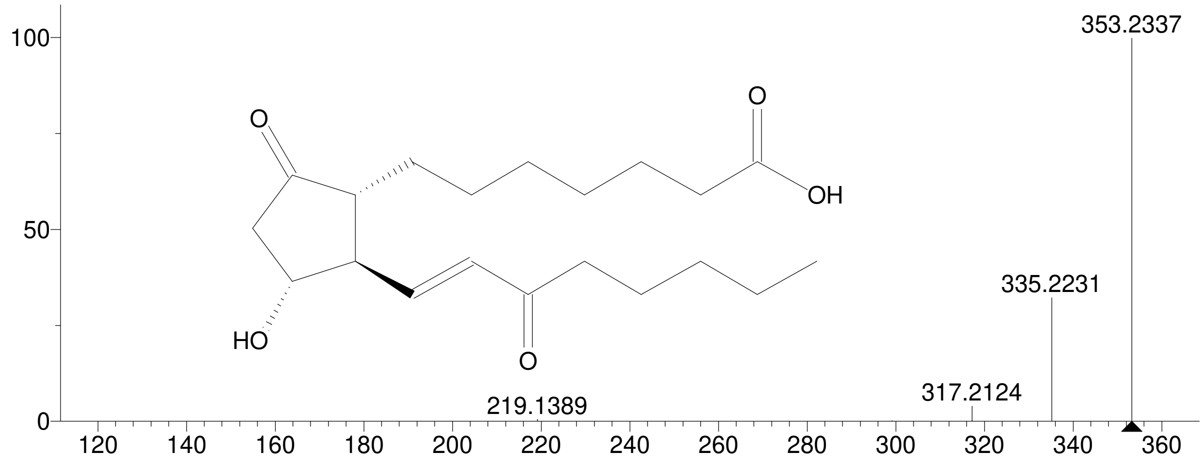

Hit 2 : Prostaglandin D1 [M-H]- HCD 5V P=353.2
C20H34O5; MF: 145; RMF: 680; Prob 10.7%; CAS: 17968-82-0; Lib: nist_msms; ID: 320117.

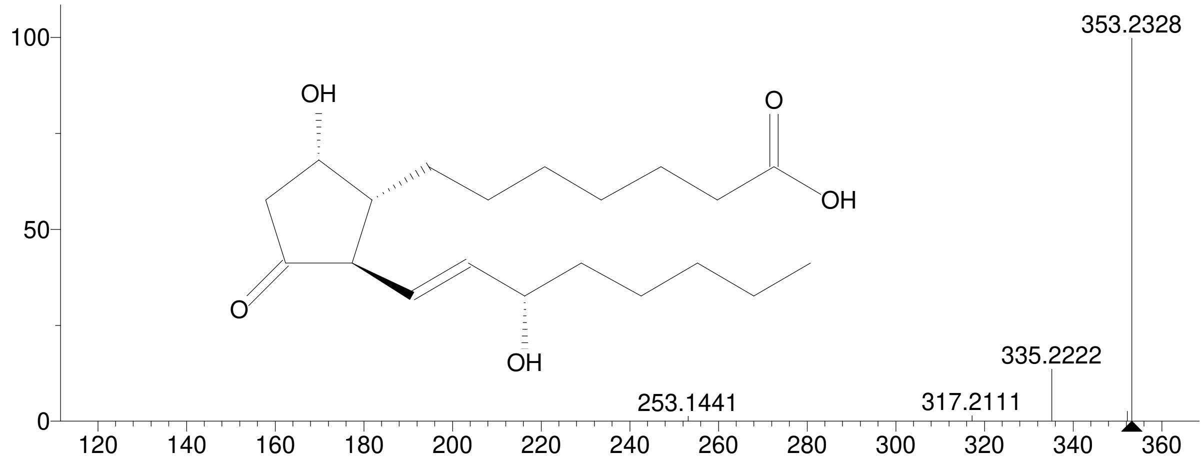

Hit 3 : Prostaglandin E1 [M-H]- HCD 7V P=353.2
C20H34O5; MF: 138; RMF: 674; Prob 8.19%; CAS: 745-65-3; Lib: nist_msms; ID: 320381.

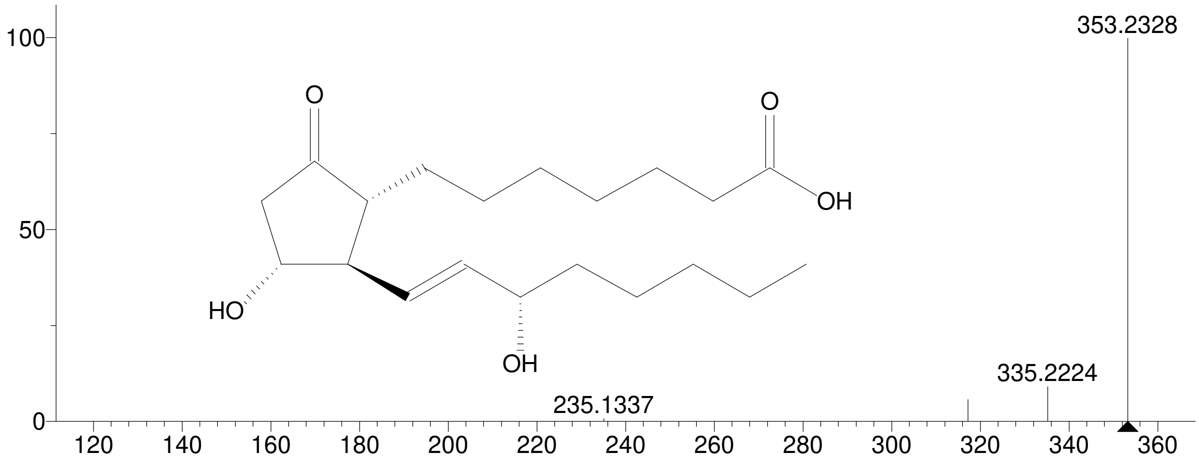

Hit 4 : 9S,11R-Dihydroxy-15-oxo-13E-prostenoic acid [M-H]- HCD 7V P=353.2
C20H34O5; MF: 135; RMF: 706; Prob 7.24%; CAS: 21562-58-3; Lib: nist_msms; ID: 155079.

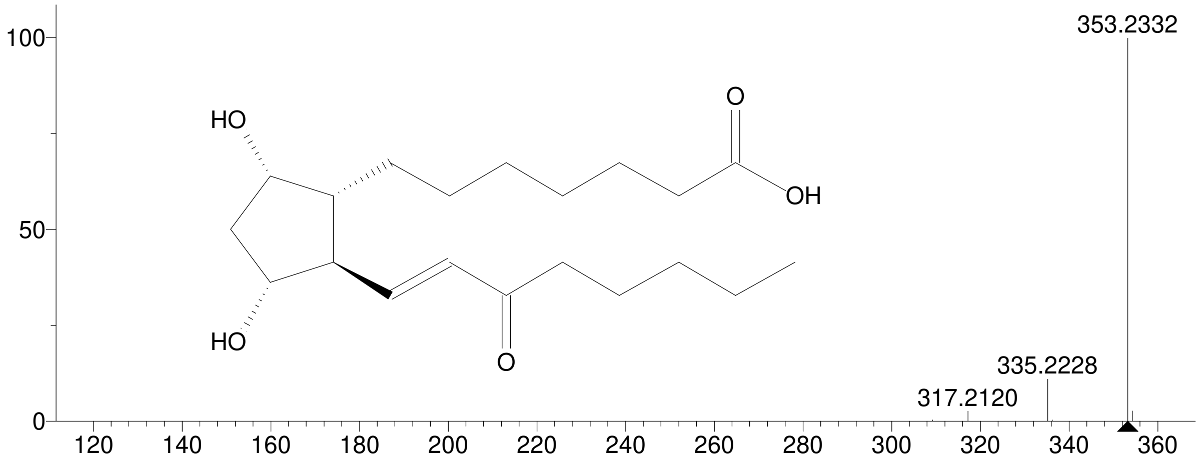

Hit 5 : 6-Ketoprostaglandin F1α [M+H-H2O]+ HCD 1V P=353.2
C20H34O6; MF: 133; RMF: 700; Prob 6.68%; CAS: 58962-34-8; Lib: nist_msms; ID: 166451.

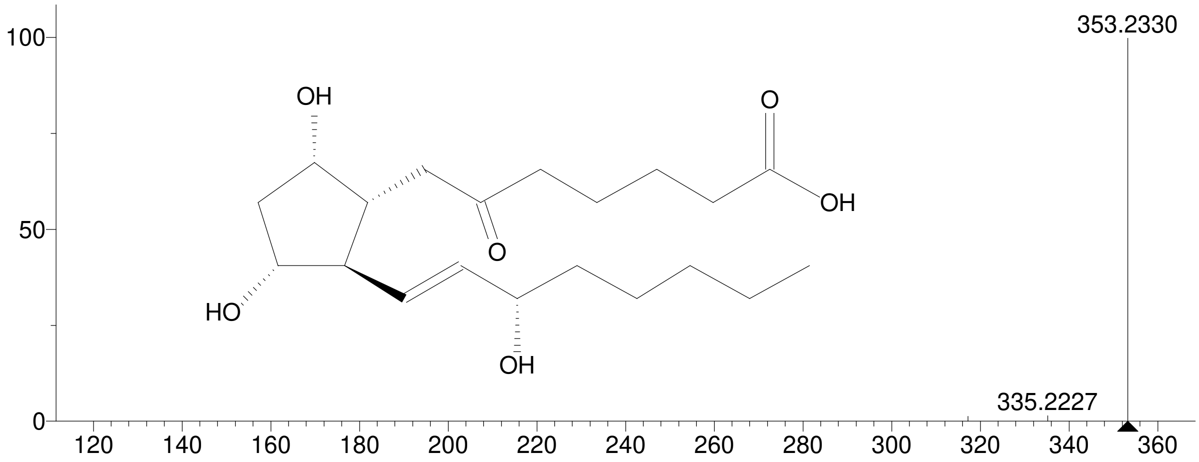

** Search Report Page 1 of 1 **

Unknown: Unknown (23.82_351.1802m/z)
Compound in Library Factor = -3273

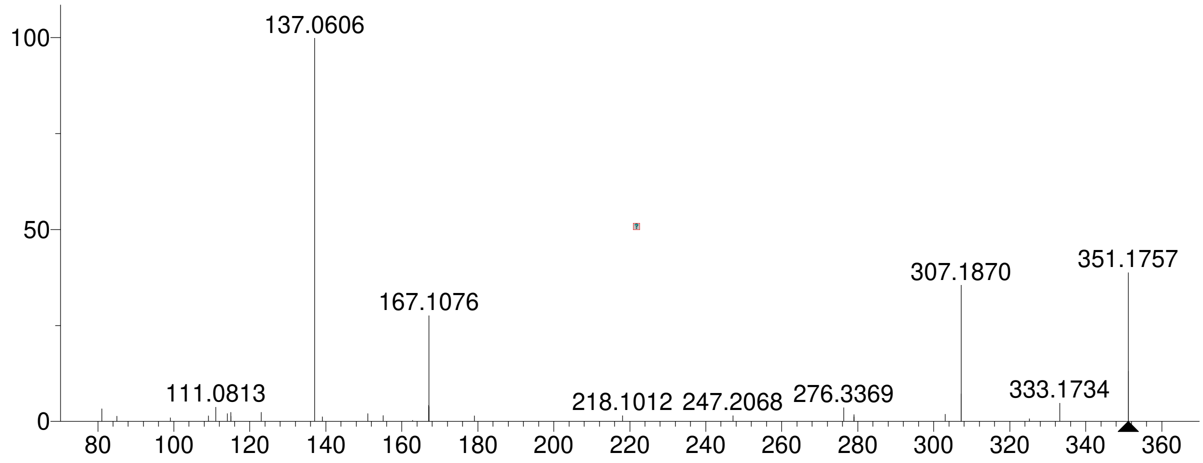

Hit 1 : 3-[(E)-3,4-dihydroxy-3,5-dimethylhept-1-enyl]-7,8-dihydroxy-7-methyl-8,8a-dihydro-1H-isochromen-6-one [
C19H28O6; MF: 5; RMF: 137; Prob 100.0%; Lib: mona-export-lc-ms-ms_negative_mode; ID: 22067.

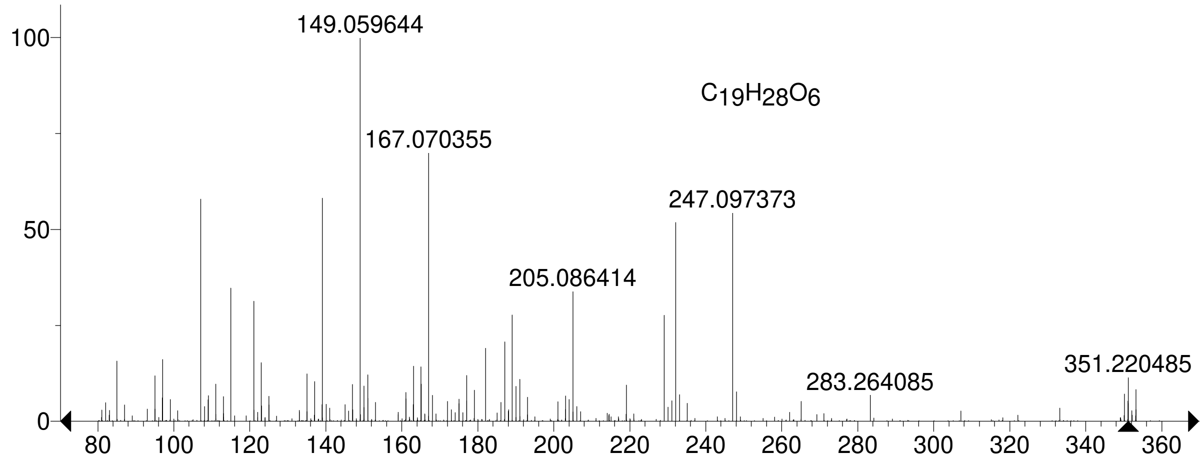

Unknown: Unknown (23.84_325.2006m/z)
Compound in Library Factor = -755

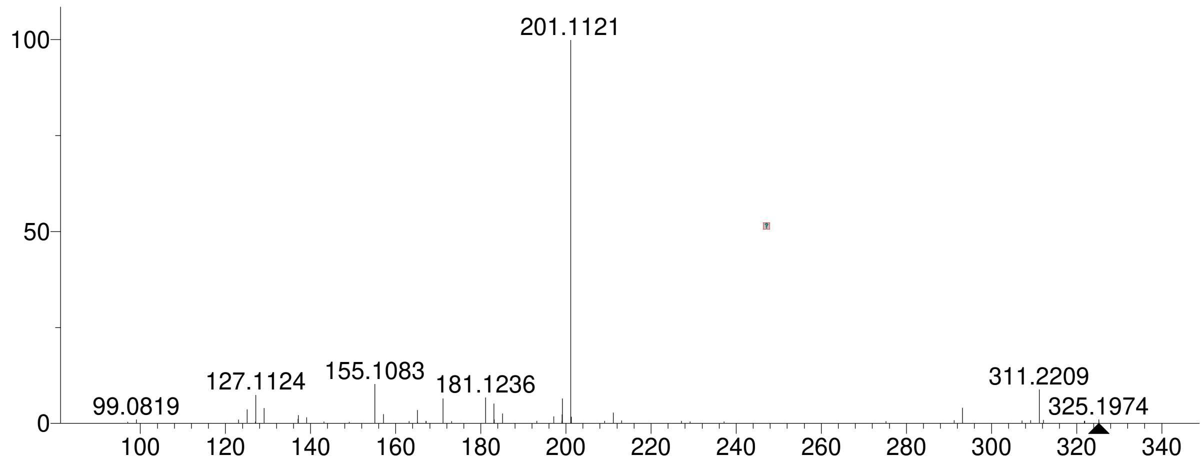

Hit 1 : Unknown (carbon number 18); PlaSMA ID-697 [M-H]- P=325.2
C18H30O5; MF: 205; RMF: 325; Prob 81.6%; Lib: biomsms-neg-plasma; ID: 1224.

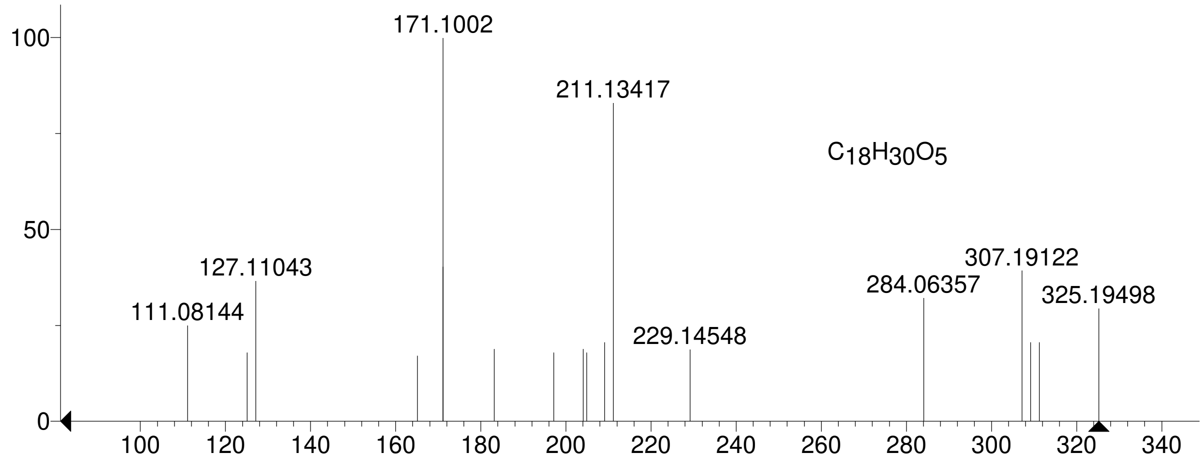

Hit 2 : Unknown (carbon number 19); PlaSMA ID-693 [M-H]- P=325.2
C19H26N4O; MF: 110; RMF: 221; Prob 6.61%; Lib: biomsms-neg-plasma; ID: 1223.

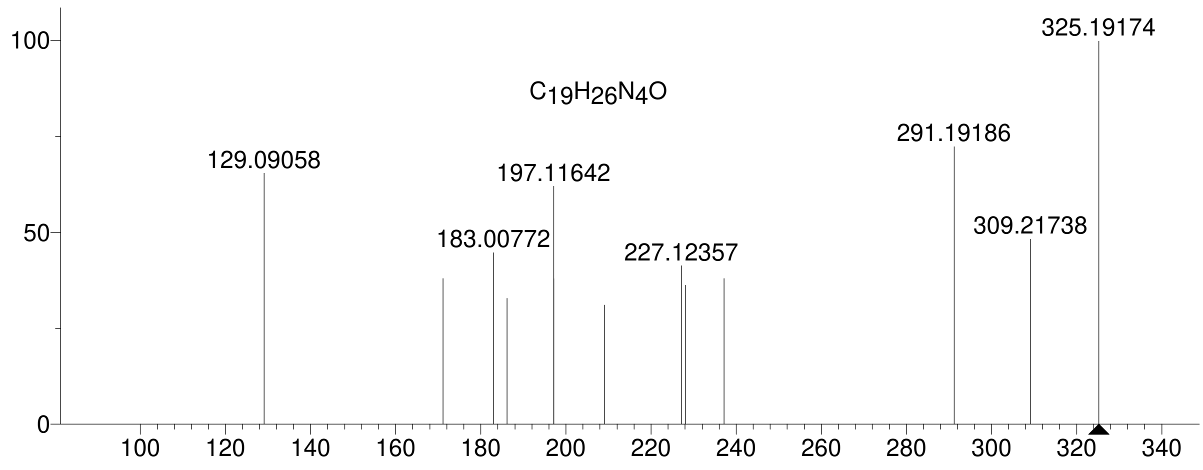

Hit 3 : Hymeglusin [M+H]+ HCD 3V P=325.2
C18H28O5; MF: 63; RMF: 297; Prob 1.38%; CAS: 29066-42-0; Lib: nist_msms; ID: 376516.

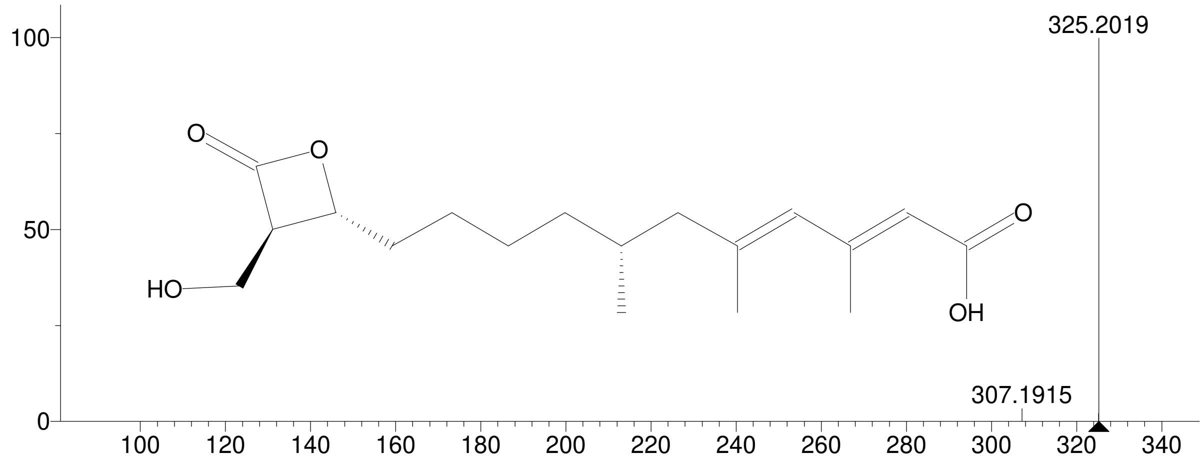

Hit 4 : FA 18:3+3O; PlaSMA ID-694 [M-H]- P=325.2
C18H30O5; MF: 56; RMF: 181; Prob 1.05%; Lib: biomsms-neg-plasma; ID: 424.

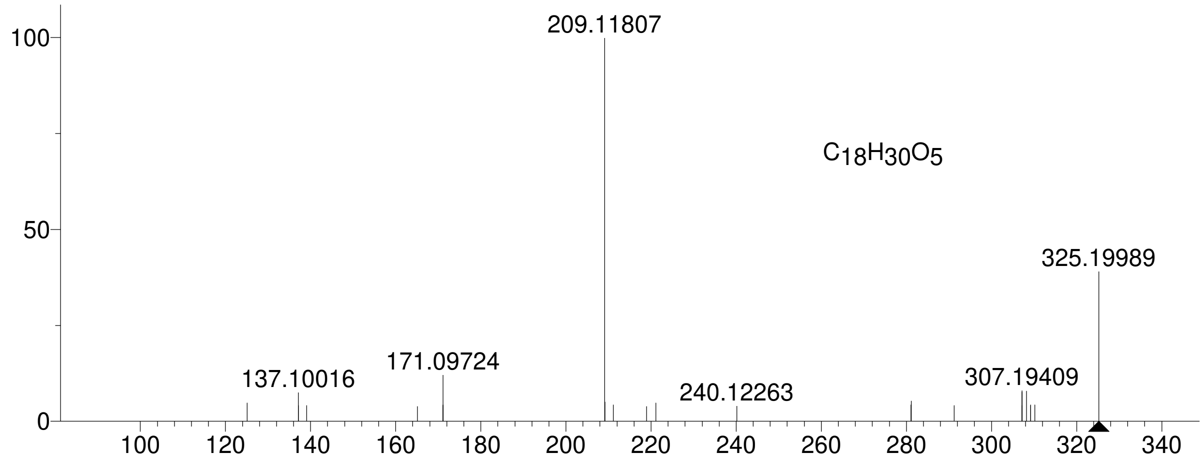

Hit 5 : 3-Oxocholic acid [M-H]-=>361.3=>325.2 IT 35% P=325.2
C24H38O5; MF: 40; RMF: 258; Prob 0.61%; CAS: 2304-89-4; Lib: nist_msms; ID: 117921.

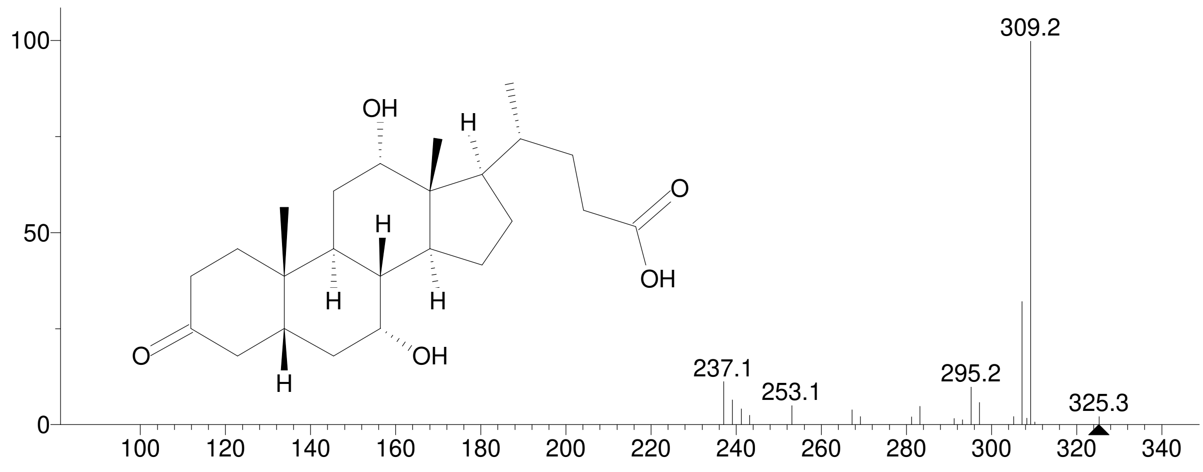

** Search Report Page 1 of 1 **

Unknown: Unknown (24.03_384.0615m/z)
Compound in Library Factor = 0

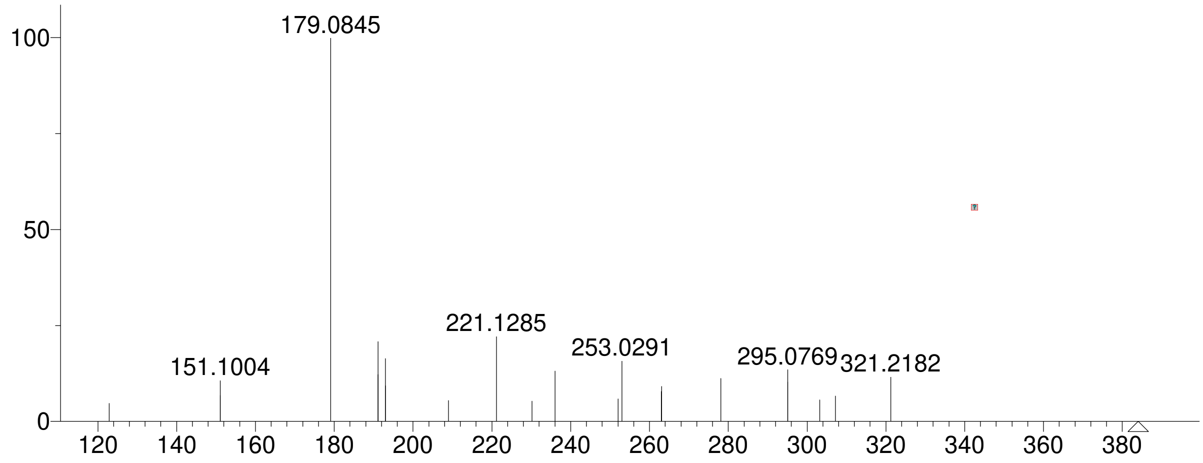

Hit 1 : Dehydrofelodipine [M+H+2i]+ HCD 46V P=384.1
C18H17Cl2NO4; MF: 0; RMF: 3; Prob 100.0%; CAS: 96382-71-7; Lib: nist_msms; ID: 517307.

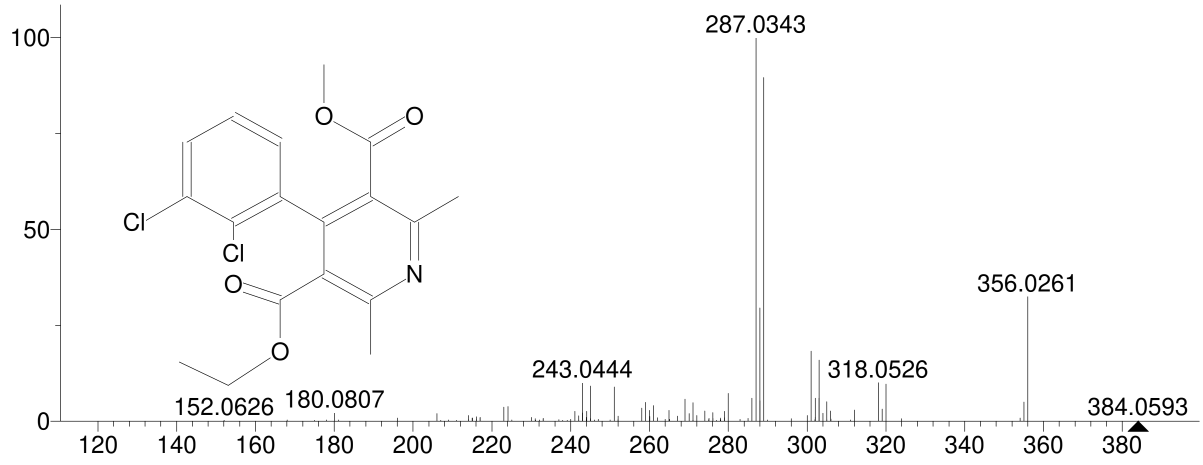

Unknown: Unknown (24.06_793.4383m/z)
Compound in Library Factor = -1072

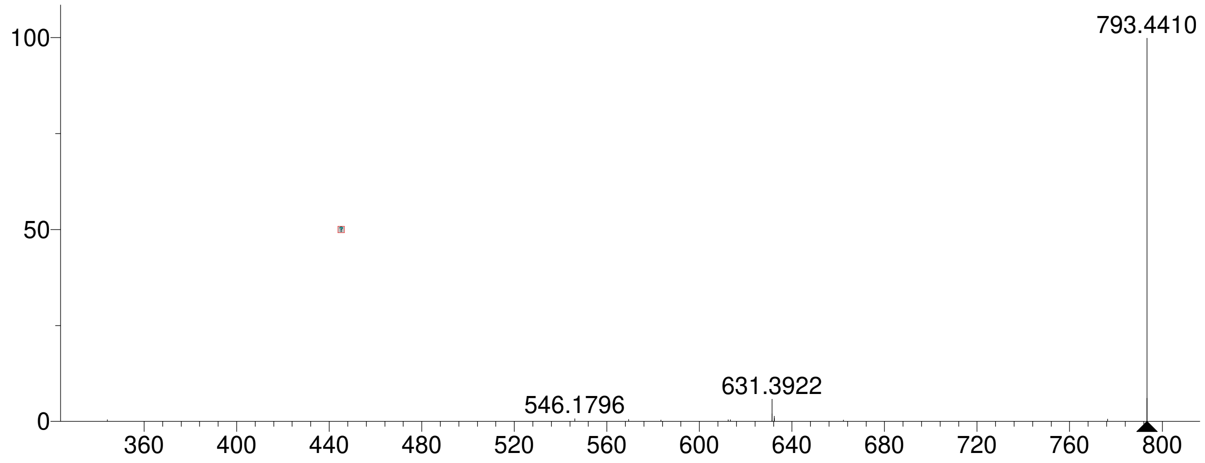

Hit 1 : Unknown (carbon number 42); PlaSMA ID-2040 [M-H]- P=793.4
C42H66O14; MF: 190; RMF: 875; Prob 74.7%; Lib: biomsms-neg-plasma; ID: 1628.

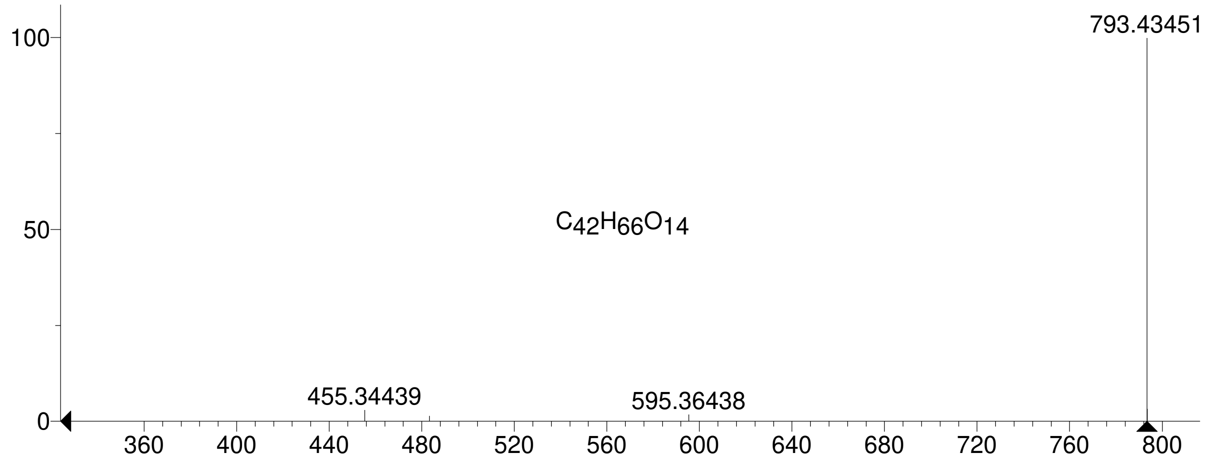

Hit 2 : 3-GlcA-28-Glc oleanolic acid (NMR) [M-H]- 45eV P=793.4
C42H66O14; MF: 118; RMF: 750; Prob 10.4%; Lib: msms-neg-metabobase; ID: 1118.

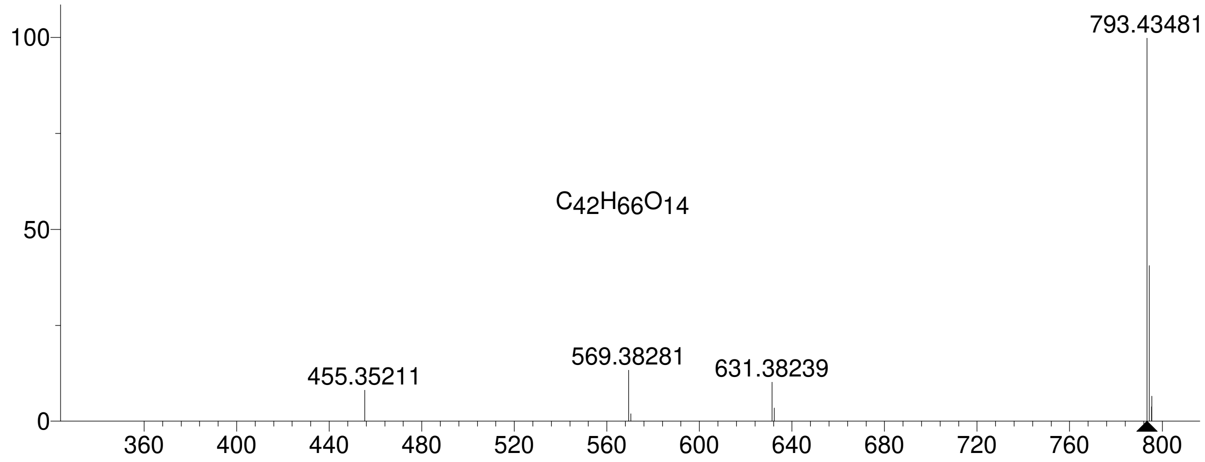

Hit 3 : (2S,3S,4S,5R,6R)-6-[[(3S,6aR,6bS,8aS,14bR)-4,4,6a,6b,11,11,14b-heptamethyl-8a-[(2S,3R,4S,5S,6R)-3,4
C42H66O14; MF: 111; RMF: 768; Prob 7.97%; Lib: mona-export-lc-ms-ms_negative_mode; ID: 21494.

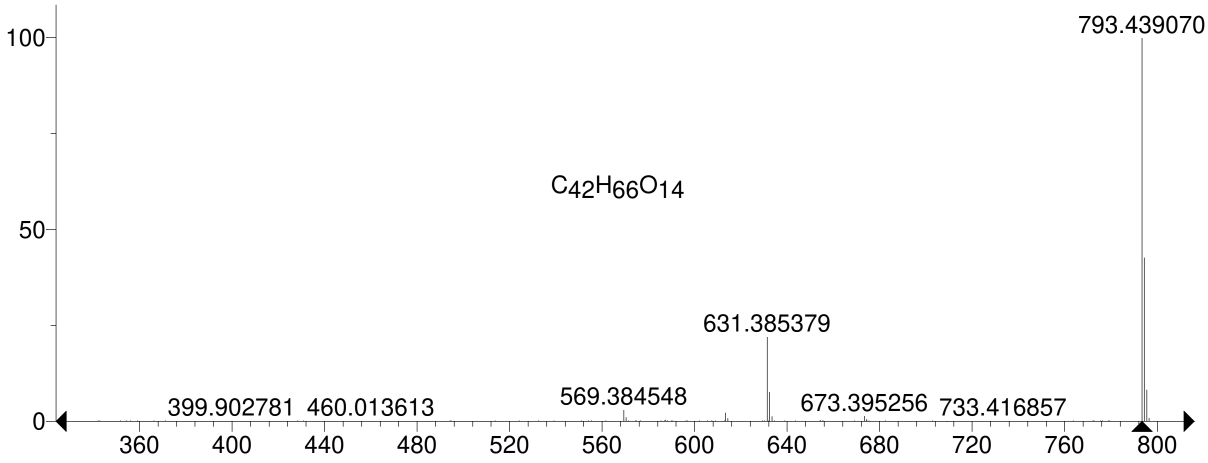

Hit 4 : Unknown (carbon number 42); PlaSMA ID-2041 [M-H]- P=793.4
C42H66O14; MF: 95; RMF: 780; Prob 4.59%; Lib: biomsms-neg-plasma; ID: 1629.

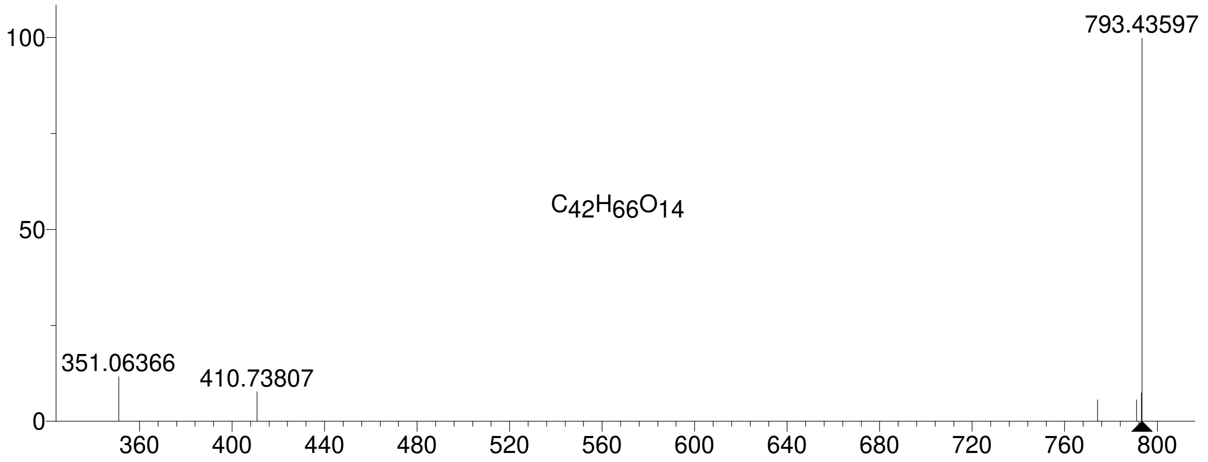

Hit 5 : Oleanolic acid base + O-HexA-Hex; PlaSMA ID-2042 [M-H]- P=793.4
C42H66O14; MF: 25; RMF: 871; Prob 0.66%; Lib: biomsms-neg-plasma; ID: 248.

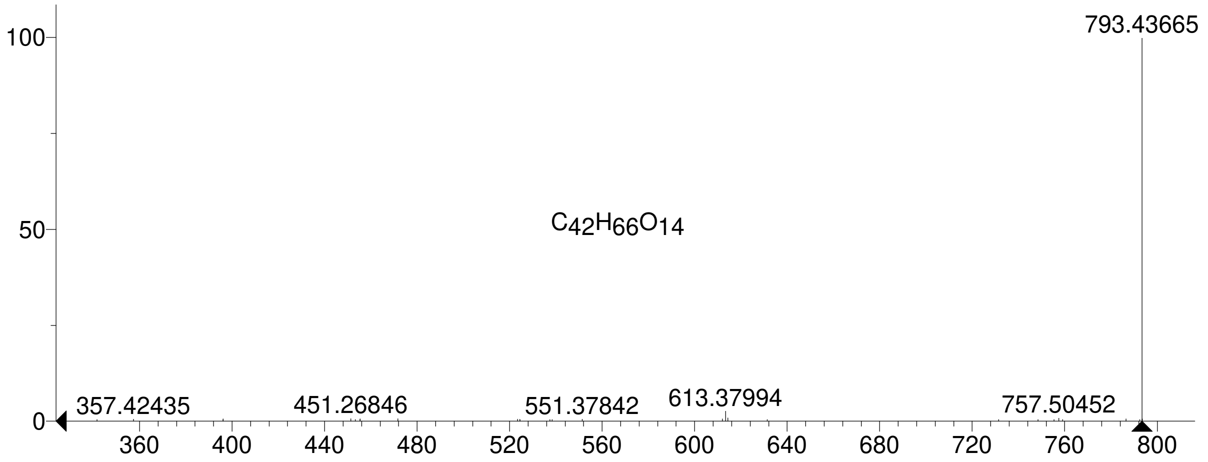

Unknown: Unknown (24.15_329.2313m/z)
Compound in Library Factor = 0

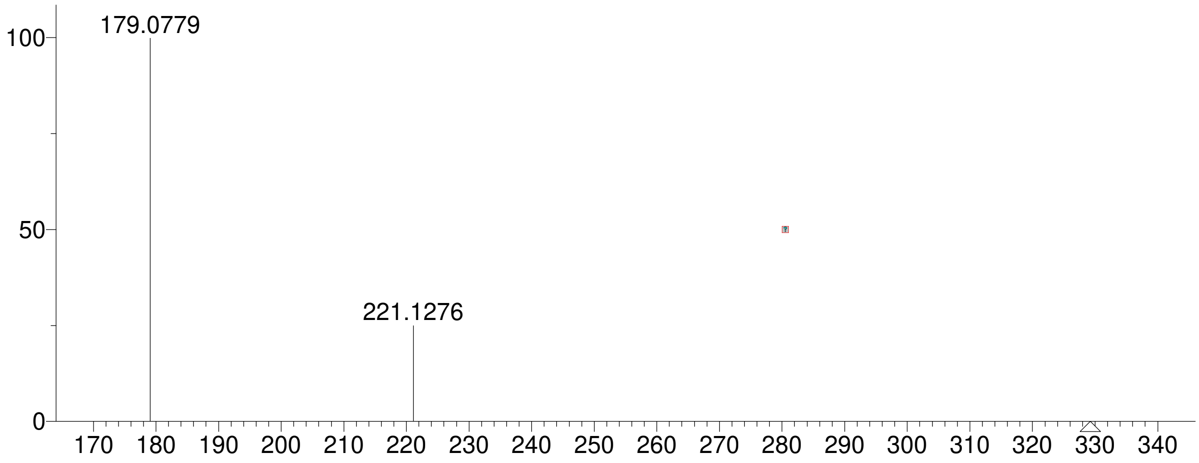

Unknown: Unknown (24.27_309.2058m/z)
Compound in Library Factor = -2387

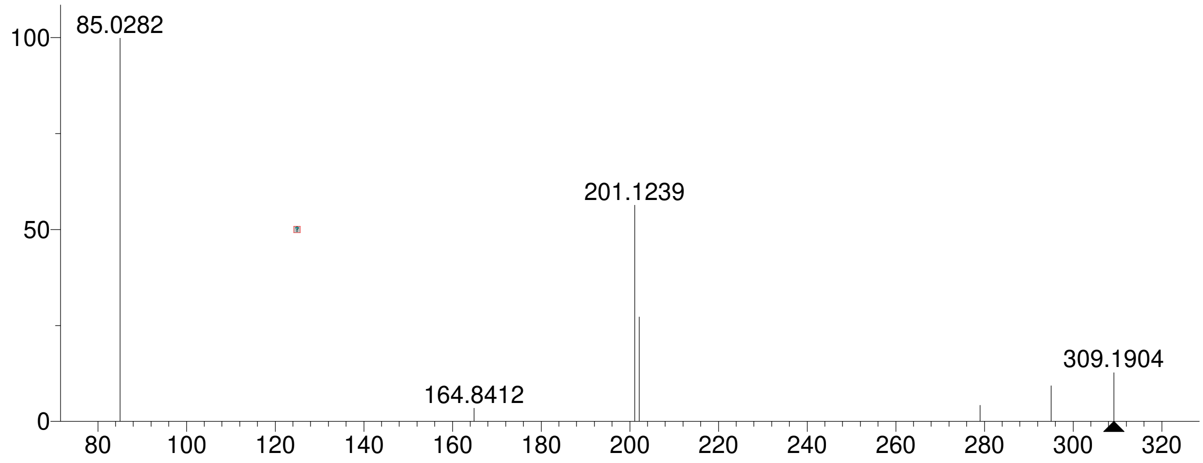

Hit 1 : 3-Cyclopentene-1-octanoic acid, 2-(3-hydroxy-1-penten-1-yl)-5-oxo- [M+H]+ HCD 6V P=309.2
C18H28O4; MF: 32; RMF: 615; Prob 12.7%; CAS: 1035557-09-5; Lib: nist_msms; ID: 245895.

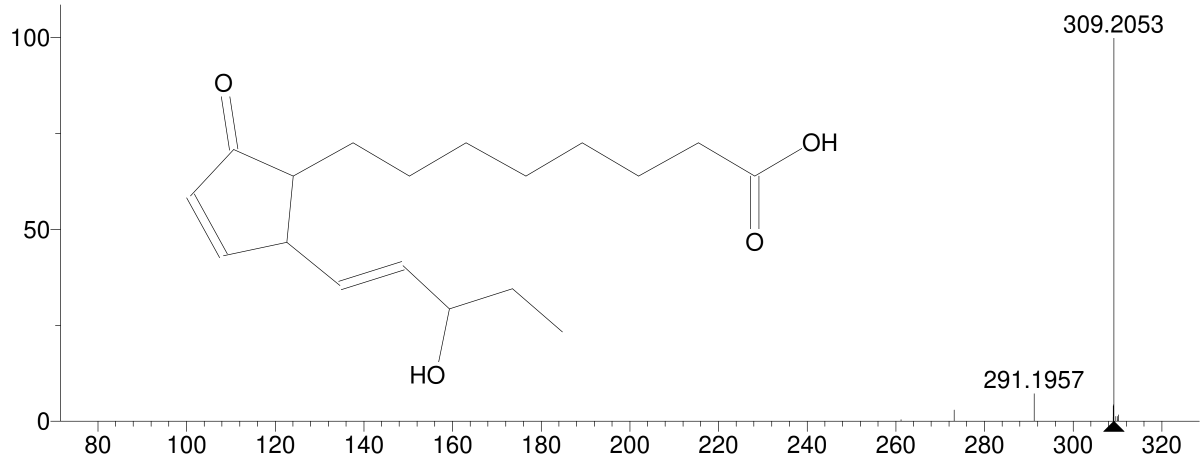

Hit 2 : 2,2,4-Trimethyl-1,3-pentanediol diisobutyrate [M+Na]+ HCD 21V P=309.2
C16H30O4; MF: 13; RMF: 232; Prob 6.15%; CAS: 6846-50-0; Lib: nist_msms; ID: 241979.

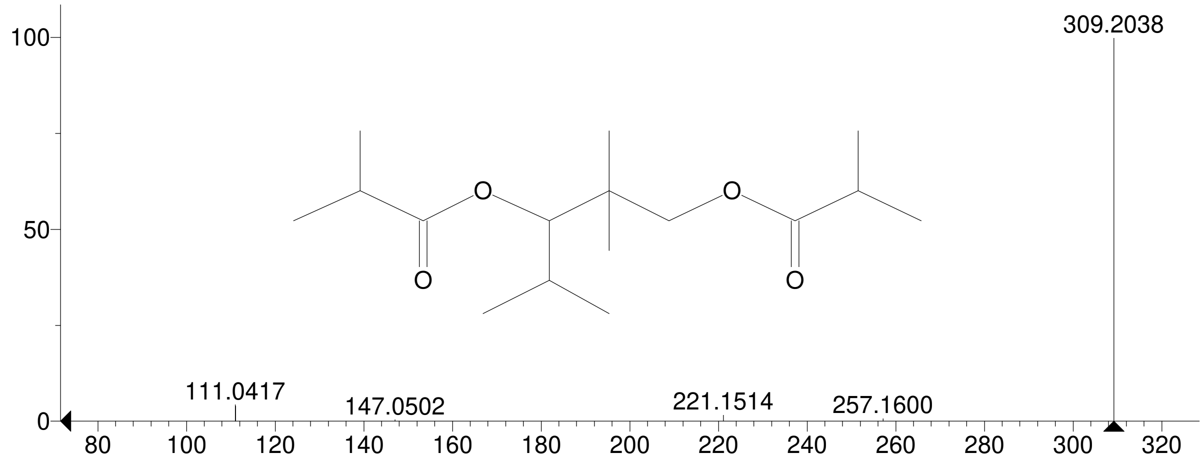

Hit 3 : Unknown (carbon number 18); PlaSMA ID-632 [M-H]- P=309.2
C18H30O4; MF: 7; RMF: 240; Prob 4.83%; Lib: biomsms-neg-plasma; ID: 1197.

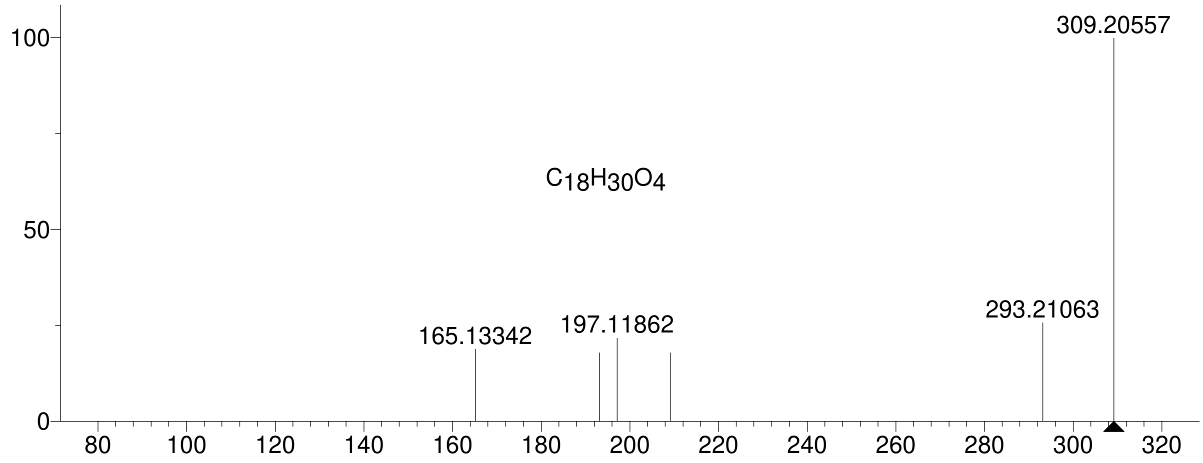

Hit 4 : Unknown (carbon number 17); PlaSMA ID-635 [M-H]- P=309.2
C17H30N2OS; MF: 6; RMF: 208; Prob 4.65%; Lib: biomsms-neg-plasma; ID: 1198.

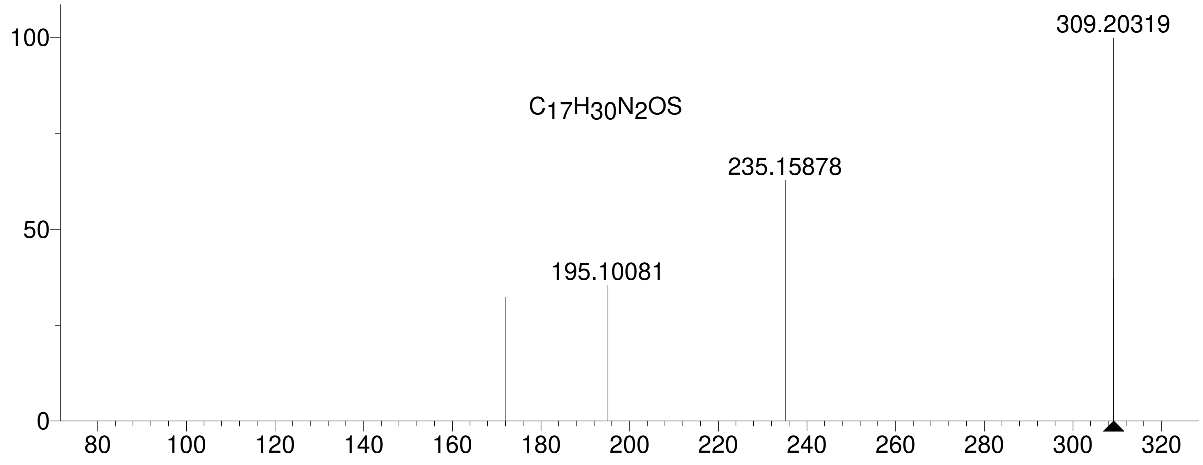

Hit 5 : (E)-4-[(1R,2S,3S,4R,8aS)-2,3,4-trihydroxy-2,5,5,8a-tetramethyl-3,4,4a,6,7,8-hexahydro-1H-naphthalen-1-y
C18H30O4; MF: 5; RMF: 150; Prob 4.47%; Lib: mona-export-lc-ms-ms_negative_mode; ID: 23195.

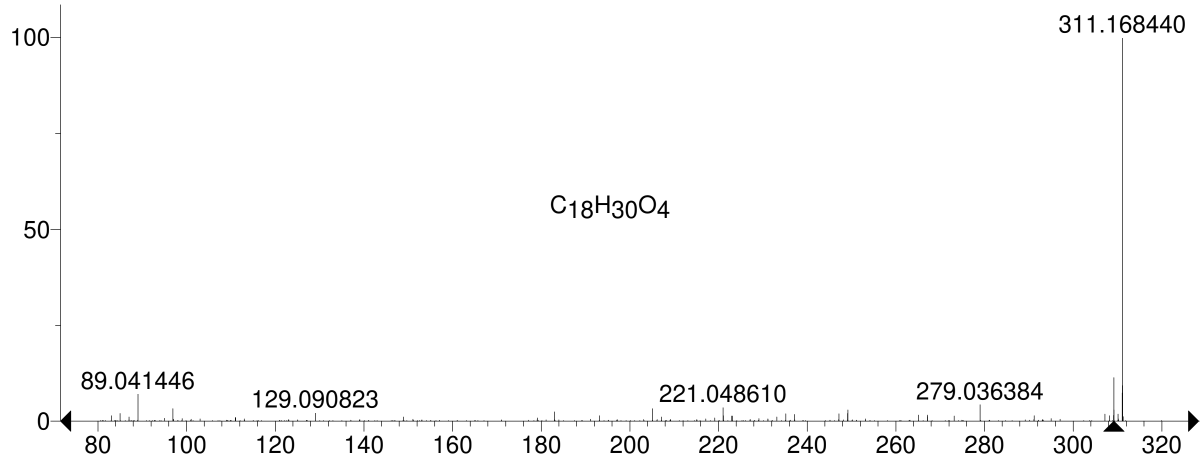

Unknown: Unknown (24.27_329.2310m/z)
Compound in Library Factor = -2176

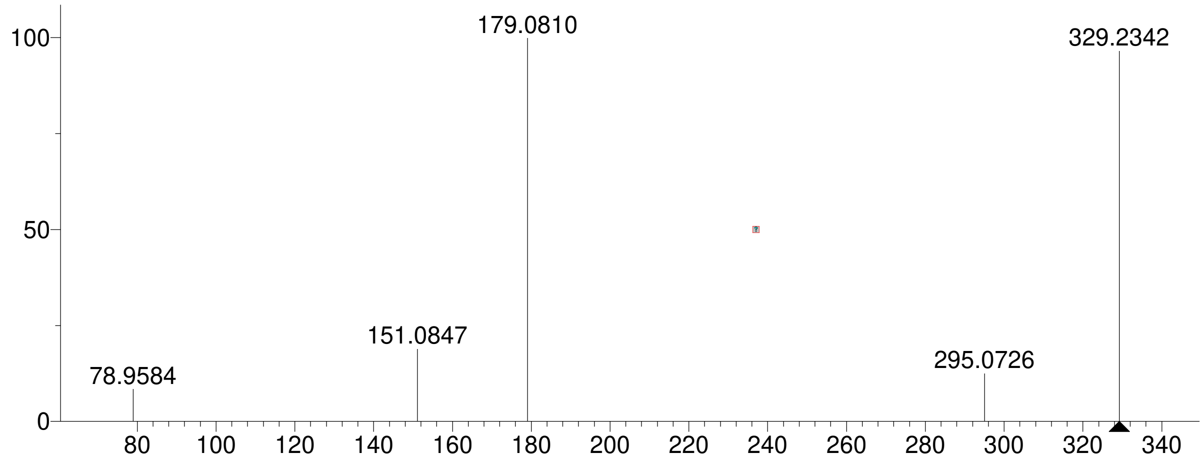

Hit 1 : FA 18:1+3O; PlaSMA ID-741 [M-H]- P=329.2
C18H34O5; MF: 58; RMF: 564; Prob 37.1%; Lib: biomsms-neg-plasma; ID: 449.

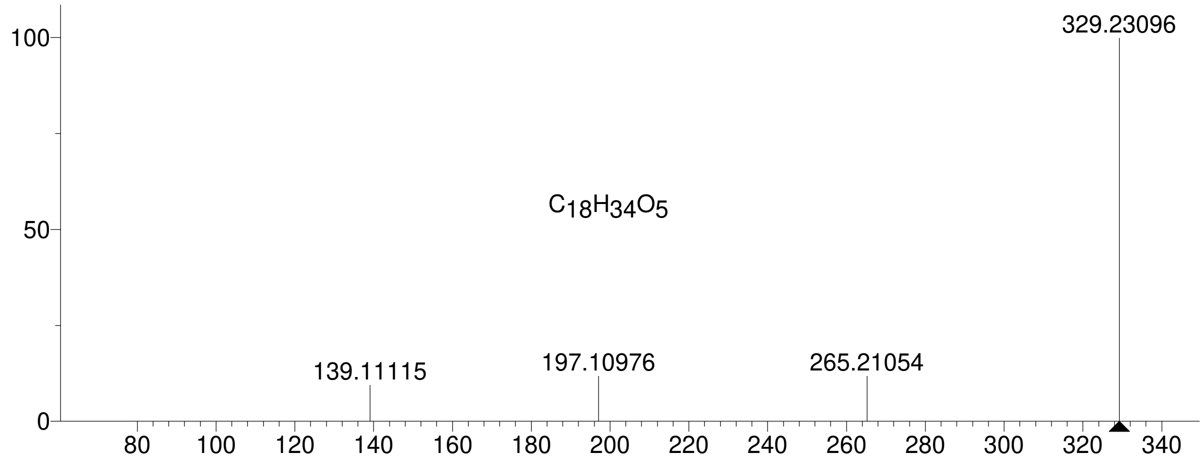

Hit 2 : Unknown (carbon number 18); PlaSMA ID-736 [M-H]- P=329.2
C18H34O5; MF: 37; RMF: 531; Prob 15.8%; Lib: biomsms-neg-plasma; ID: 1245.

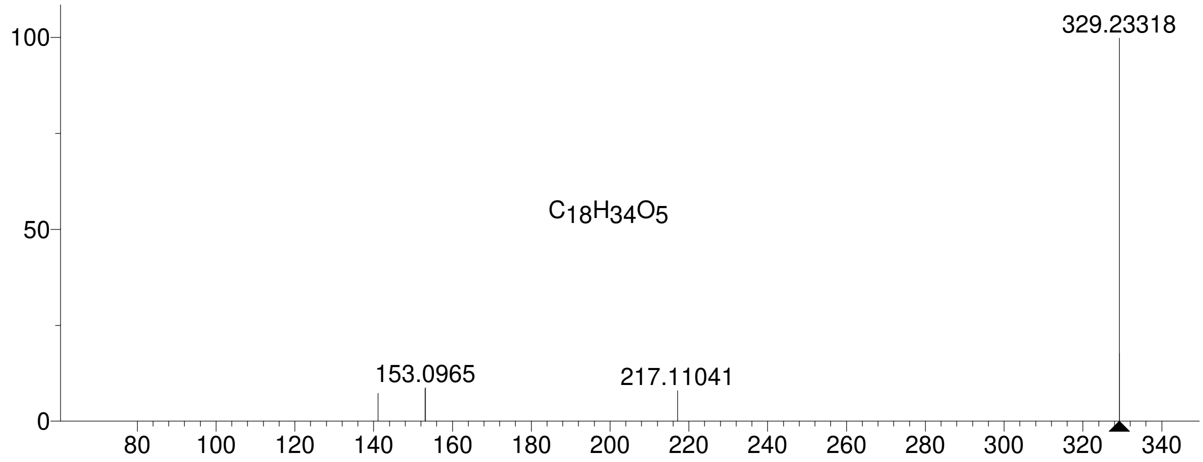

Hit 3 : FA 18:1+3O; PlaSMA ID-740 [M-H]- P=329.2
C18H34O5; MF: 24; RMF: 522; Prob 10.2%; Lib: biomsms-neg-plasma; ID: 447.

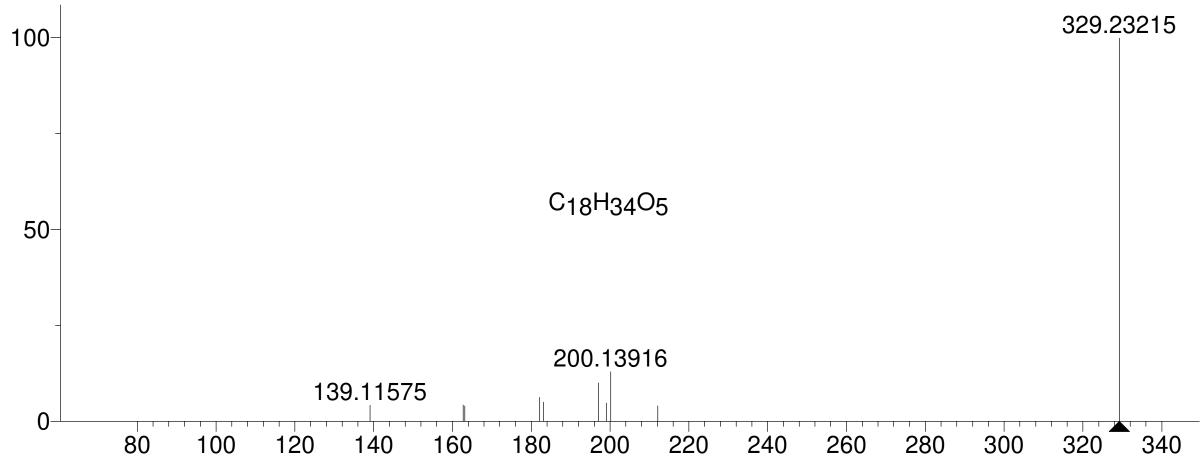

Hit 4 : FA 18:1+3O; PlaSMA ID-737 [M-H]- P=329.2
C18H34O5; MF: 17; RMF: 507; Prob 7.80%; Lib: biomsms-neg-plasma; ID: 448.

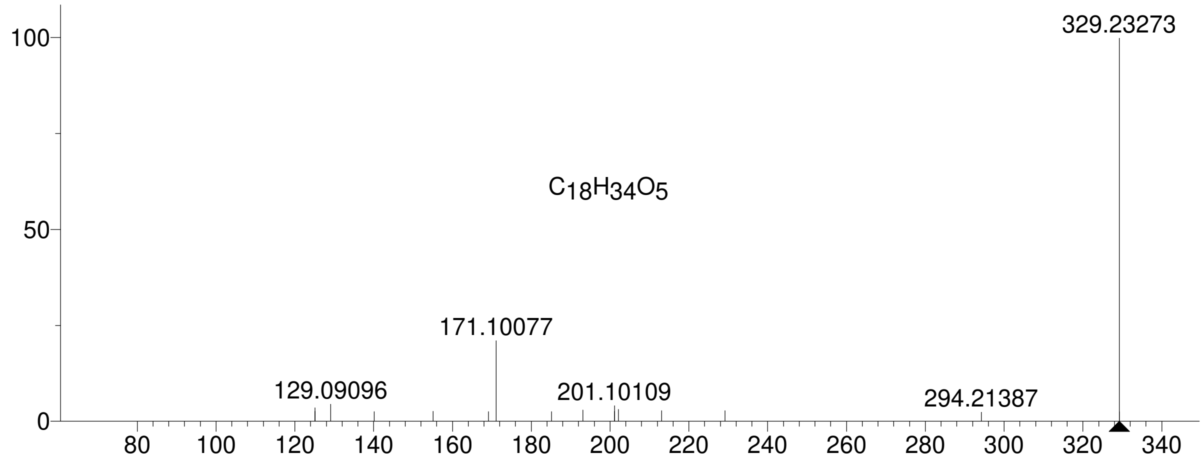

Hit 5 : FA 18:1+3O; PlaSMA ID-742 [M-H]- P=329.2
C18H34O5; MF: 12; RMF: 518; Prob 6.29%; Lib: biomsms-neg-plasma; ID: 445.

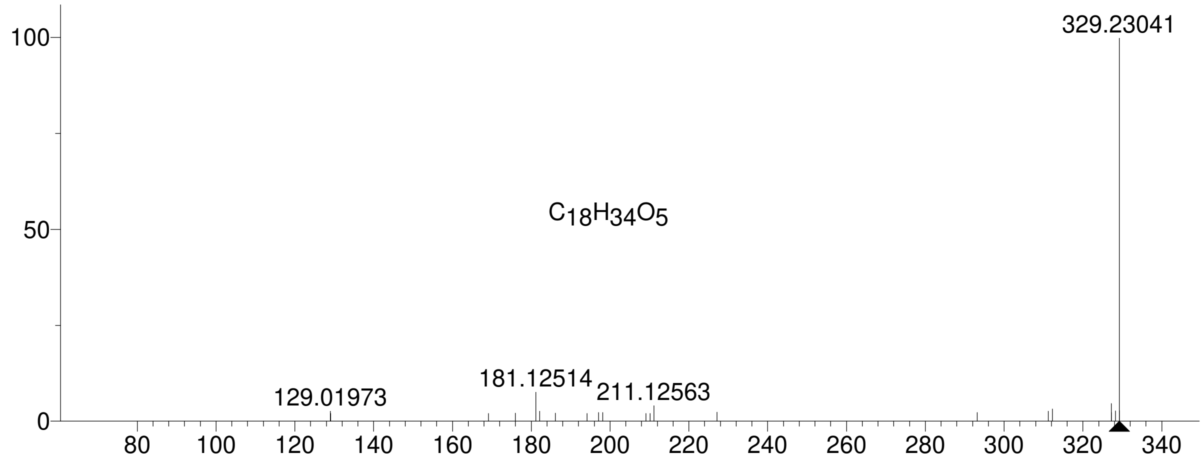

** Search Report Page 1 of 2 **

Unknown: Unknown (24.35_402.2011m/z)
Compound in Library Factor = -3273

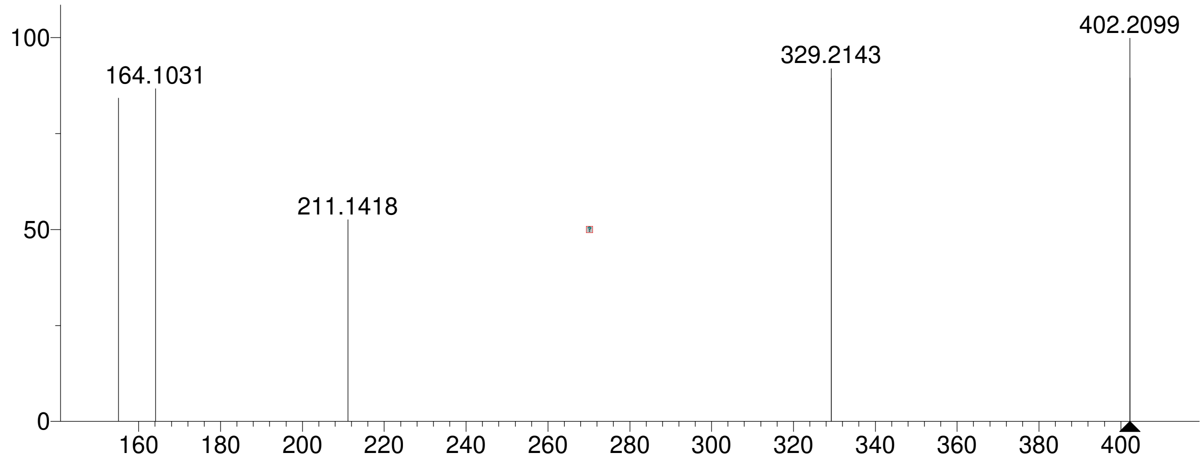

Hit 1 : 3-(1-(Cyclohexylmethyl)-1H-indazole-3-carboxamido)-2,2-dimethylsuccinic acid [M+H]+ HCD 12V P=402
C21H27N3O5; MF: 12; RMF: 321; Prob 17.7%; Lib: nist_msms; ID: 549930.

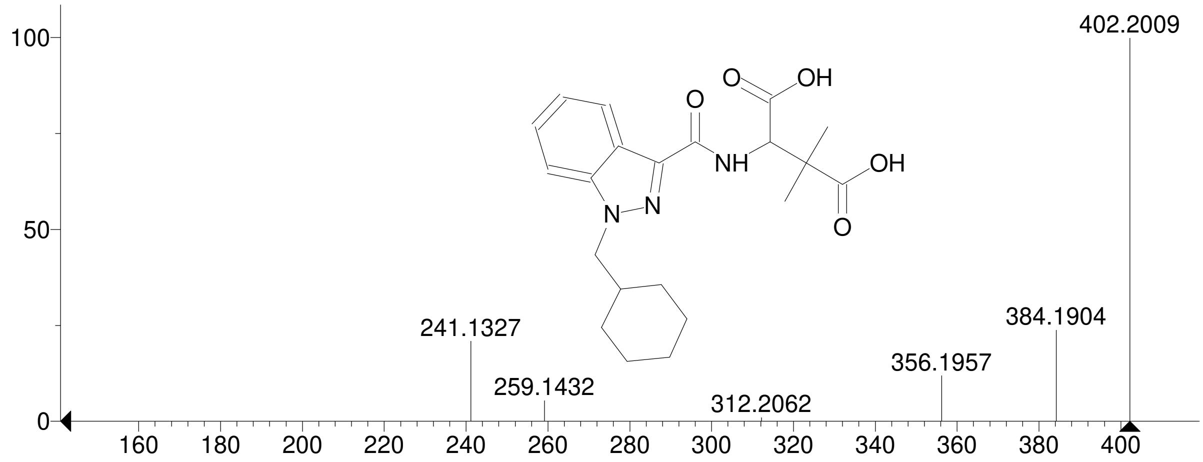

Hit 2 : Schizandrin A [M+H-CH3]+ HCD 20V P=402.2
C24H32O6; MF: 2; RMF: 308; Prob 12.5%; CAS: 61281-38-7; Lib: nist_msms; ID: 388069.

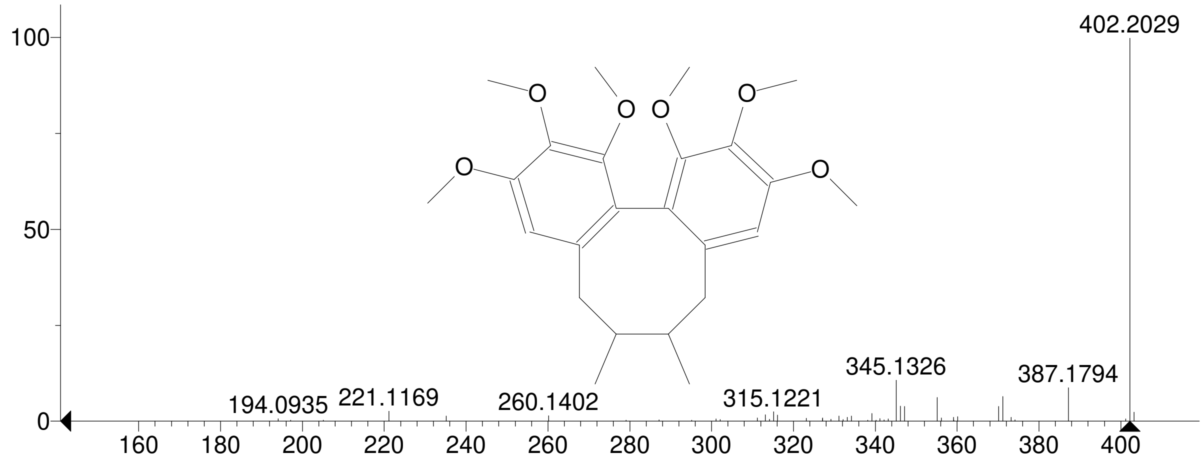

Hit 3 : cis-Moxifloxacin acyl-β-D-glucuronide [M+H]+=>402.2 IT 35% P=402.2
C27H32FN3O10; MF: 1; RMF: 63; Prob 12.0%; CAS: 733002-61-4; Lib: nist_msms; ID: 463695.

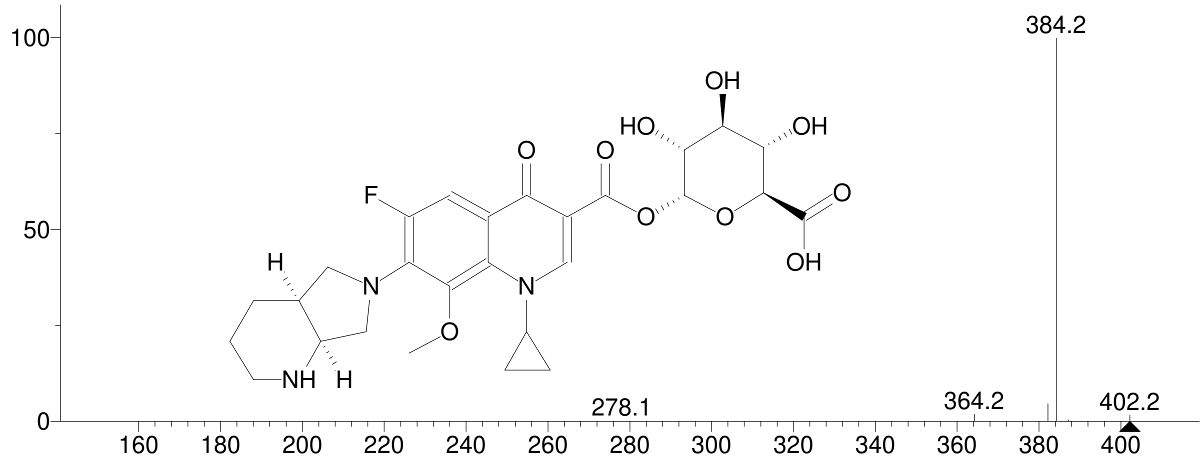

Hit 4 : Epothilone B [M+H]+=>420.2=>402.2 IT 35% P=402.2
C27H41NO6S; MF: 0; RMF: 54; Prob 11.6%; CAS: 152044-54-7; Lib: nist_msms; ID: 252223.

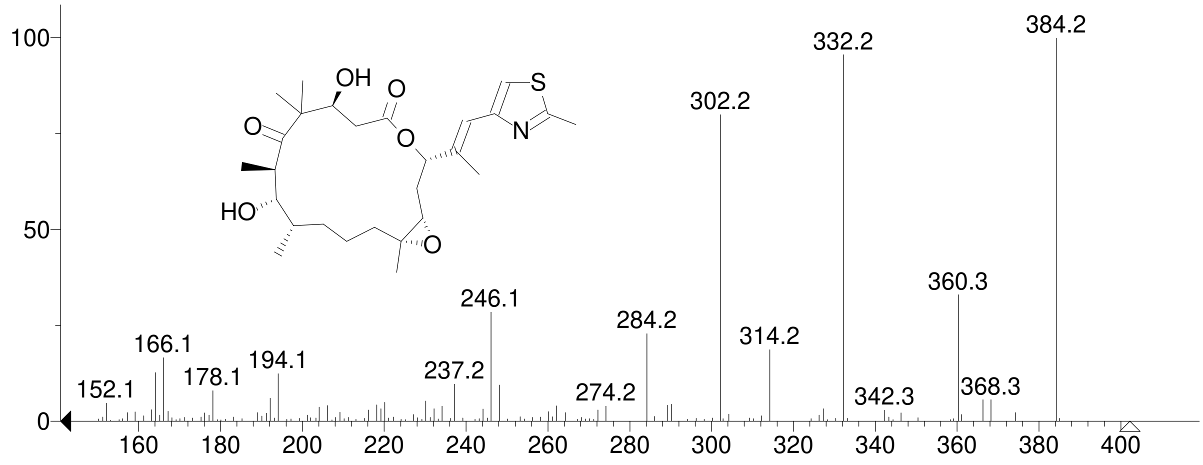

Hit 5 : Cytochalasin C [M+H-H2O]+=>430.2=>402.2 IT 35% P=402.2
C30H37NO6; MF: 0; RMF: 30; Prob 11.6%; CAS: 22144-76-9; Lib: nist_msms; ID: 363302.

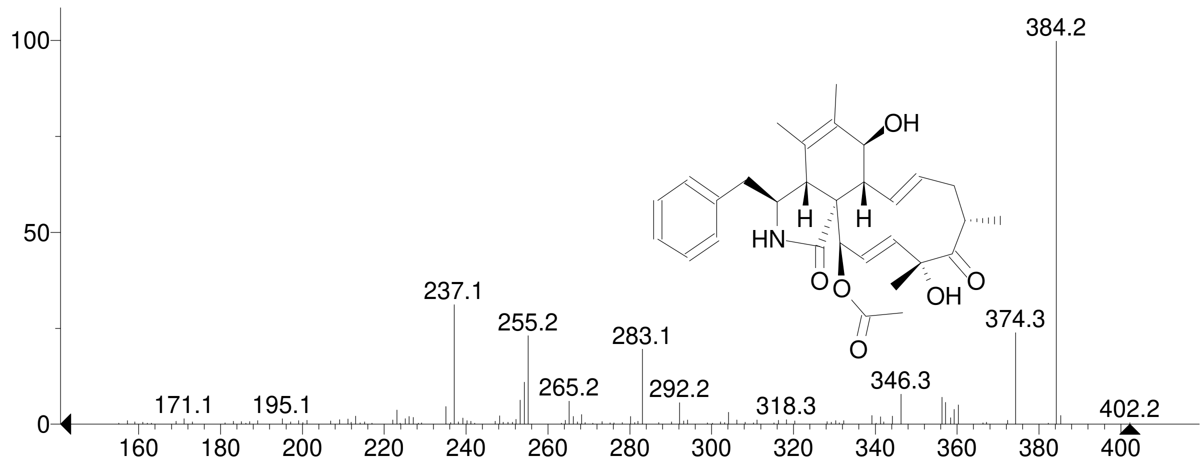

Unknown: Unknown (24.35_422.1384m/z)
Compound in Library Factor = 0

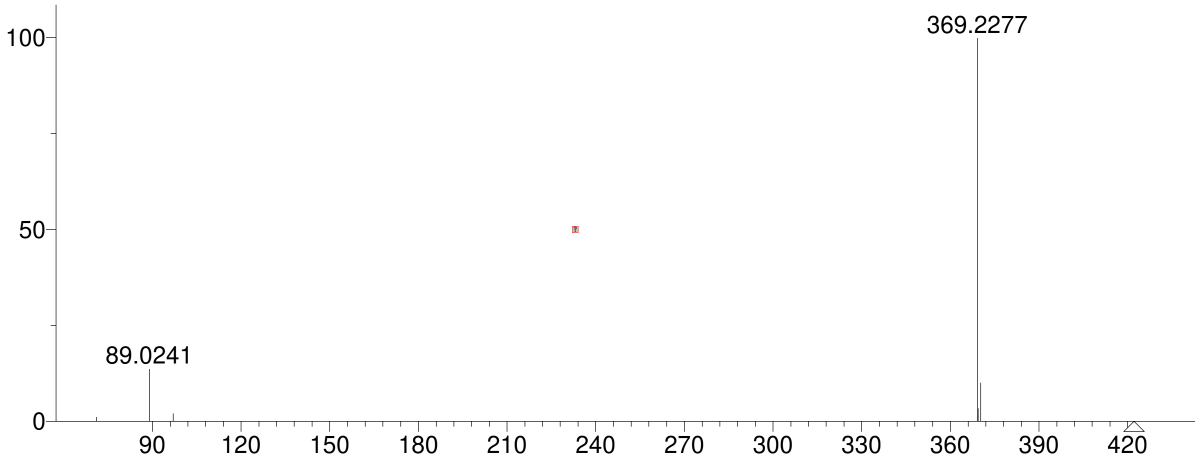

Hit 1 : peniprequinolone [M+K]+ 35HCD P=422.1
C22H25NO5; MF: 0; RMF: 1; Prob 100.0%; Lib: mona-export-vf-npl_qexactive; ID: 22066.

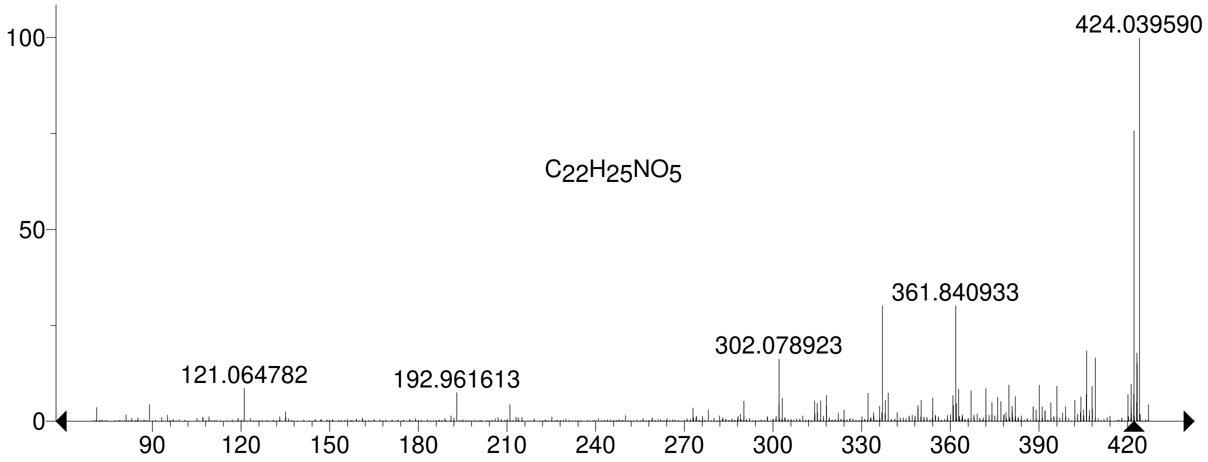

Unknown: Unknown (24.36_468.1444m/z)
Compound in Library Factor = 0

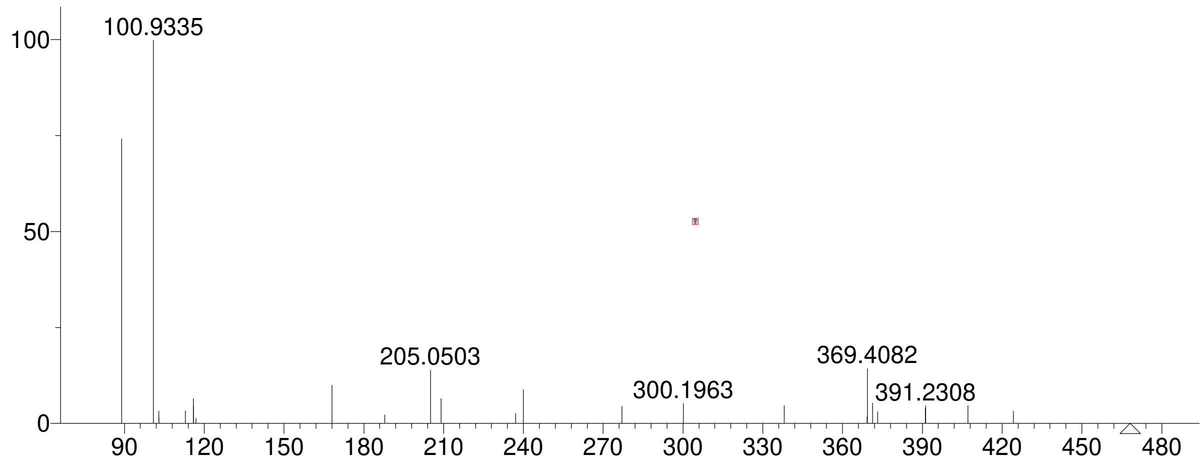

Unknown: Unknown (24.37_369.2277m/z)
Compound in Library Factor = -517

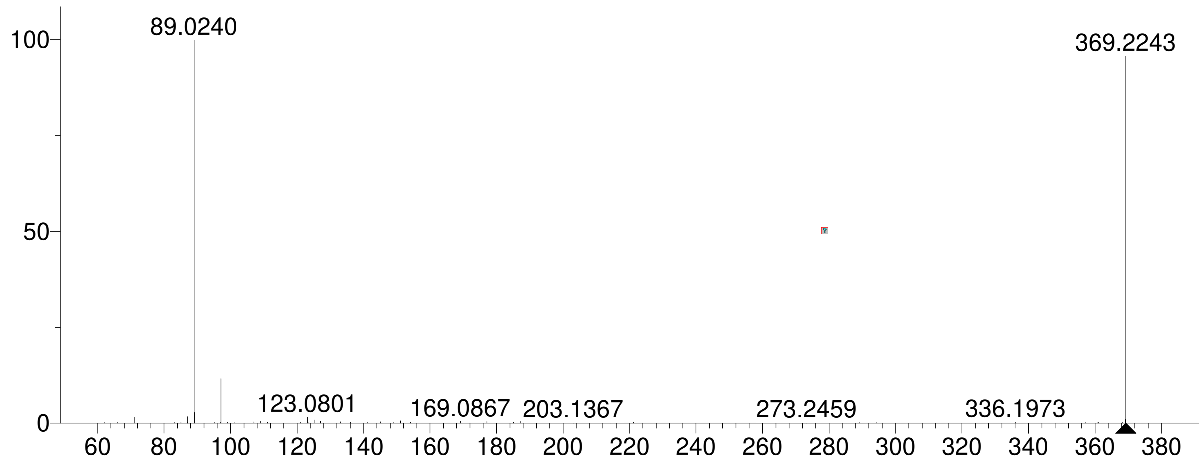

Hit 1 : 15(R),19(R)-Hydroxyprostaglandin E1 [M-H]- HCD 7V P=369.2
C20H34O6; MF: 232; RMF: 911; Prob 93.5%; Lib: nist_msms; ID: 85452.

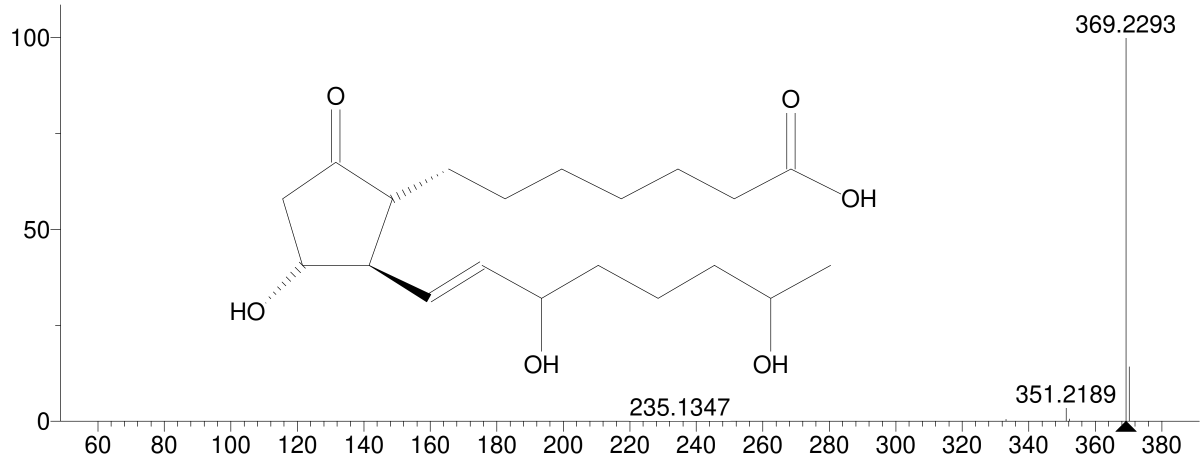

Hit 2 : 6,15-Diketo-13,14-dihydroprostaglandin F1α [M-H]- HCD 23V P=369.2
C20H34O6; MF: 105; RMF: 598; Prob 4.07%; CAS: 63983-53-9; Lib: nist_msms; ID: 157291.

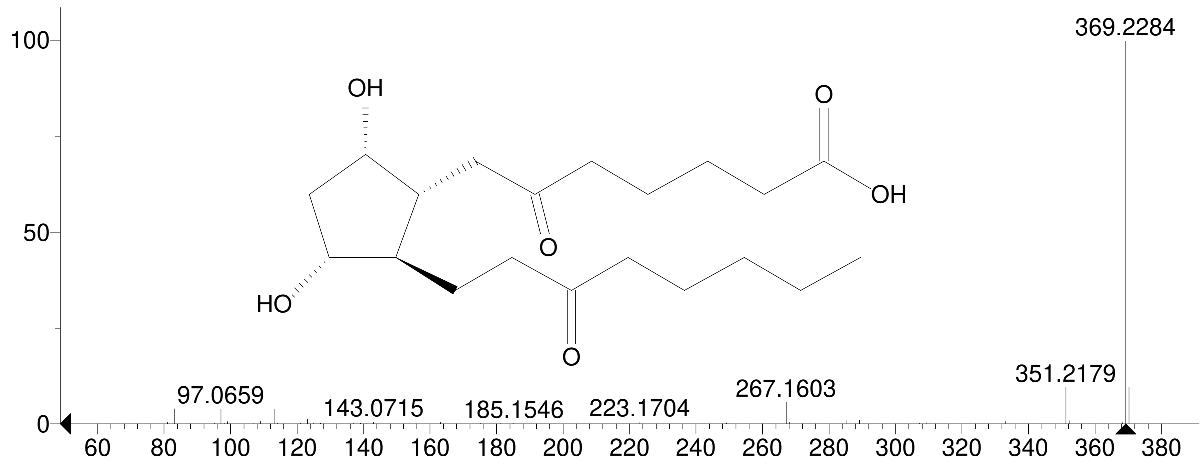

Hit 3 : Pro-Pro-Arg [M+H]+ QTOF 5V P=369.2
C16H28N6O4; MF: 78; RMF: 629; Prob 1.19%; Lib: nist_msms; ID: 52893.

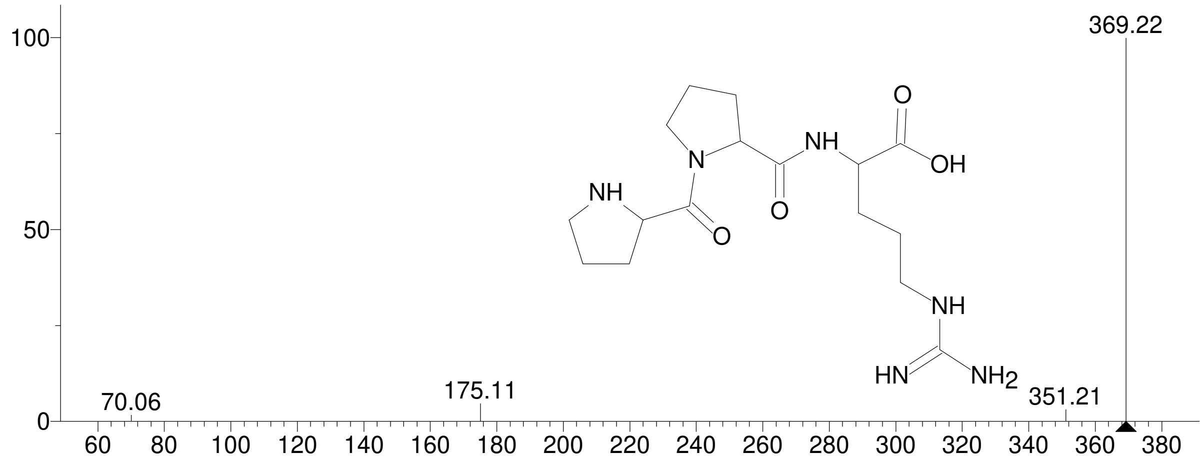

Hit 4 : Thromboxane B2 [M-H]- QTOF 2V P=369.2
C20H34O6; MF: 57; RMF: 799; Prob 0.51%; CAS: 54397-85-2; Lib: nist_msms; ID: 30001.

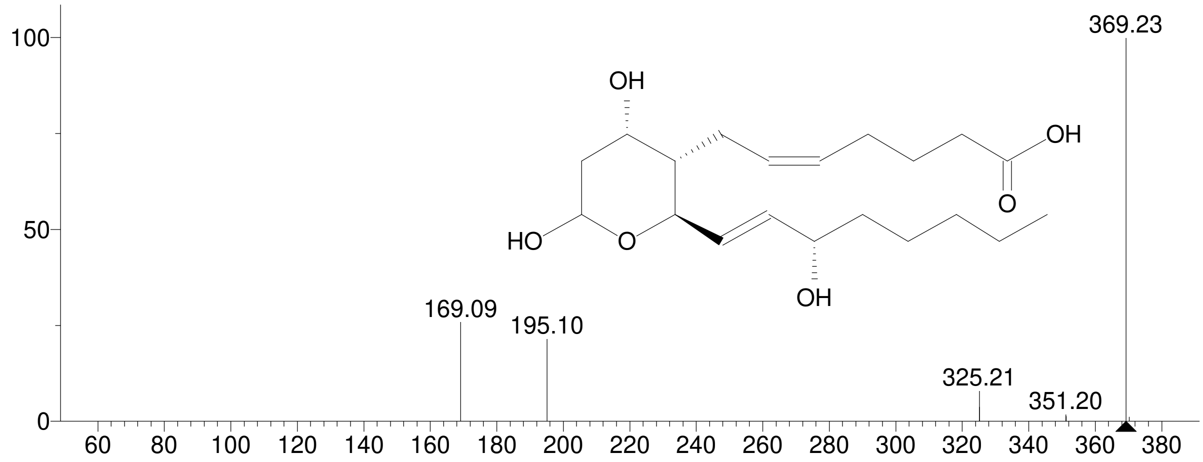

Hit 5 : 19(R)-Hydroxyprostaglandin F2α [M-H]- HCD 29V P=369.2
C20H34O6; MF: 37; RMF: 303; Prob 0.23%; CAS: 64625-53-2; Lib: nist_msms; ID: 85679.

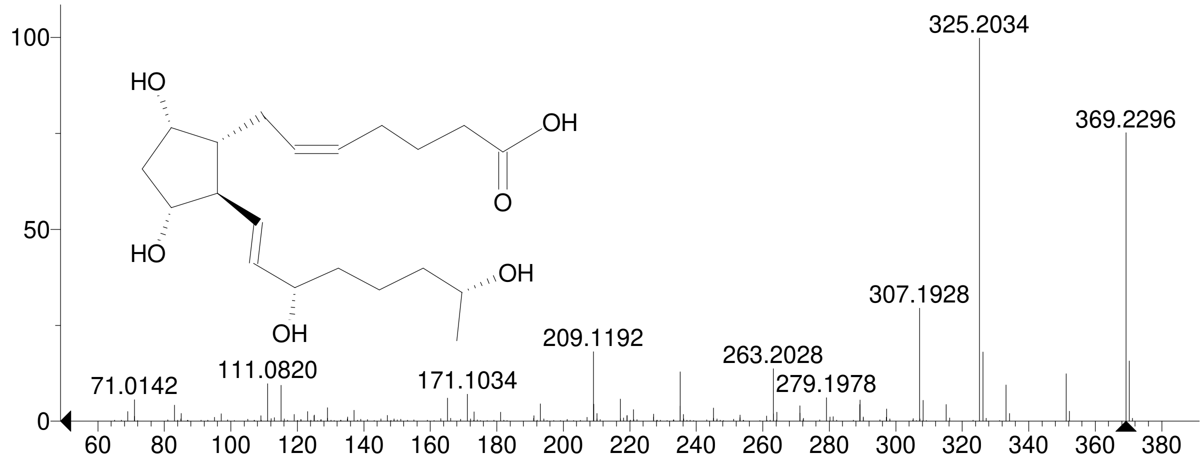

** Search Report Page 1 of 1 **

Unknown: Unknown (24.37_369.4076m/z)
Compound in Library Factor = 0

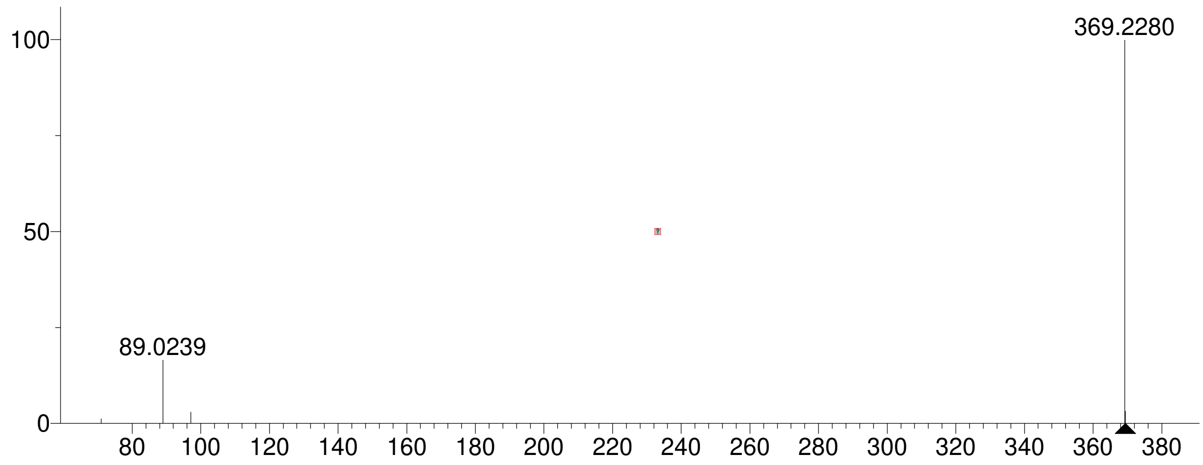

Unknown: Unknown (24.38_437.2143m/z)
Compound in Library Factor = -2176

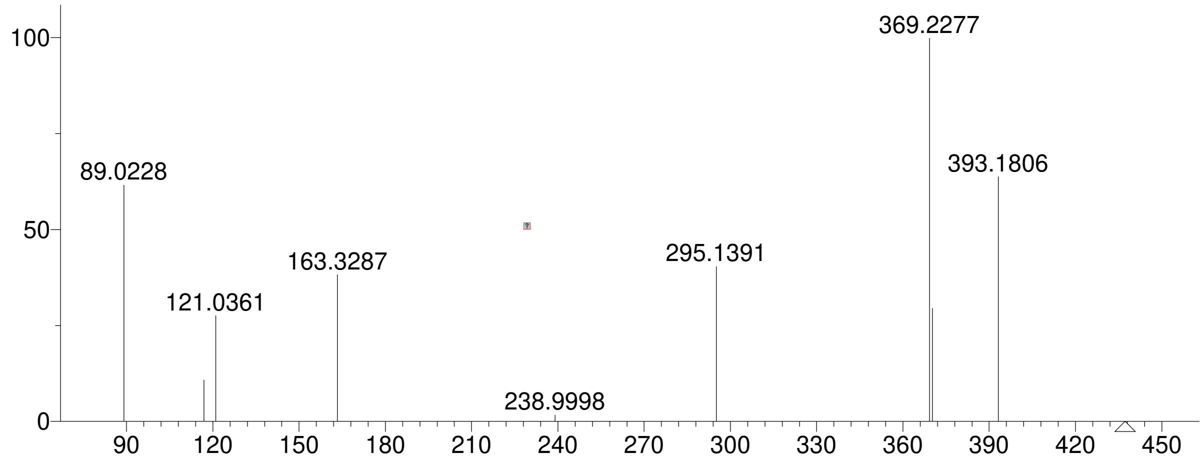

Hit 1 : 1-Hydroxy-3,6,7-Trimethoxy-2,8-Diprenylxanthone M-H 35 P=437.2
C26H30O6; MF: 20; RMF: 315; Prob 52.3%; Lib: mona-export-lc-ms-ms_negative_mode; ID: 26360.

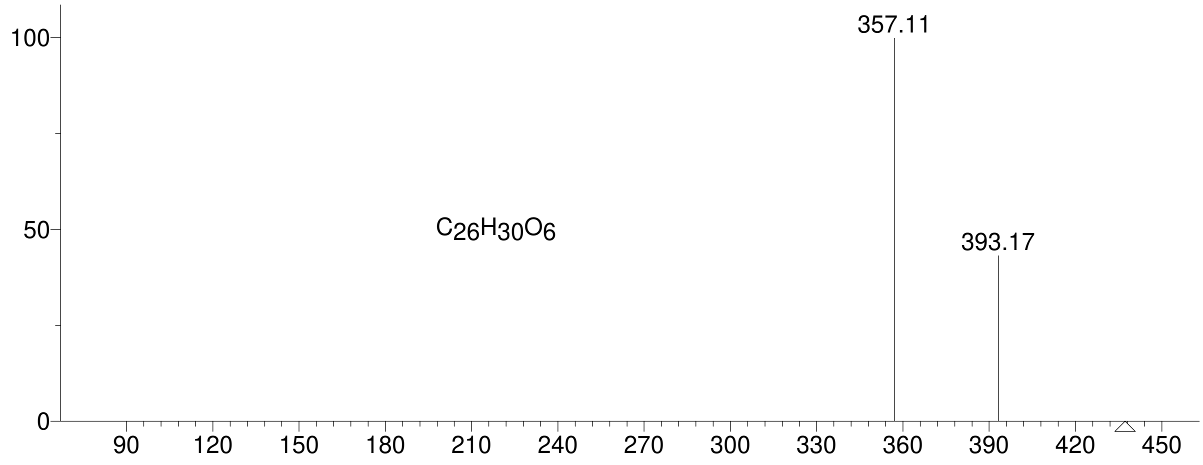

Hit 2 : Ouabagenin [M-H]- IT 35% P=437.2
C23H34O8; MF: 0; RMF: 168; Prob 23.8%; CAS: 508-52-1; Lib: nist_msms; ID: 118282.

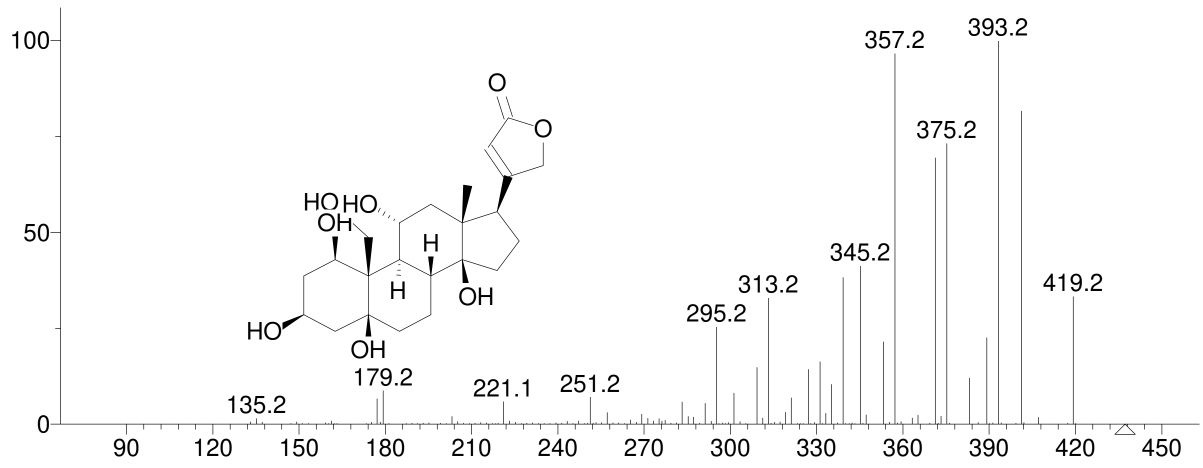

Hit 3 : NCGC00385056-01_C23H32O8_Propanoic acid, 2-methyl-, [(4S)-4-(acetyloxy)-5-[4-(acetyloxy)-1-methylb
C23H32O8; MF: 0; RMF: 1; Prob 23.8%; Lib: mona-export-gnps; ID: 10830.

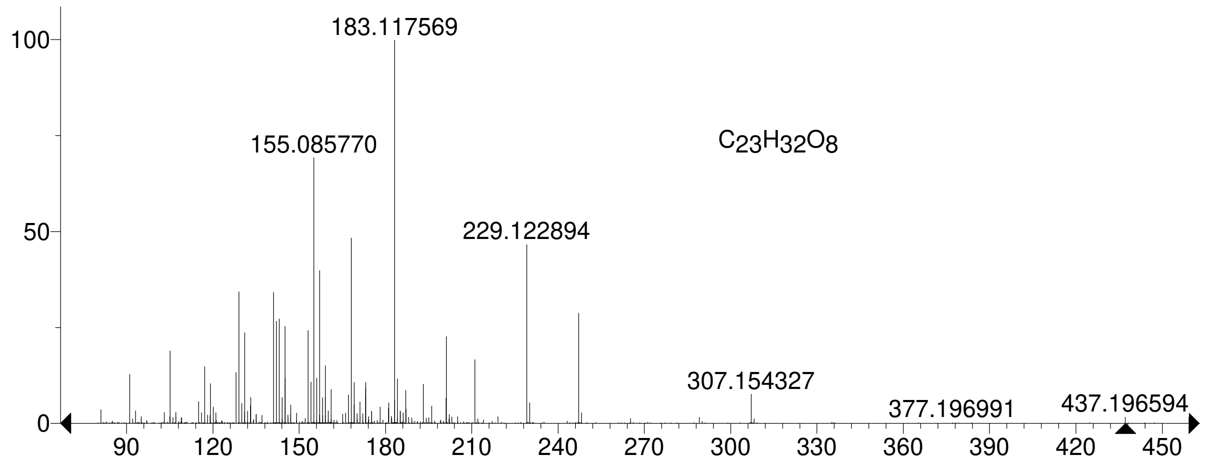

Unknown: Unknown (24.50_367.2141m/z)
Compound in Library Factor = -1309

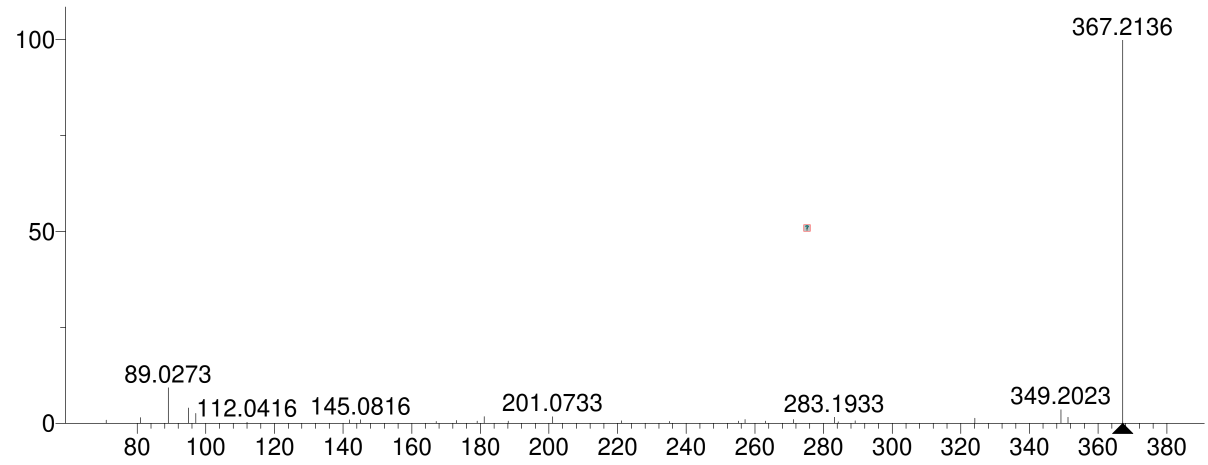

Hit 1 : 15(R),19(R)-Hydroxyprostaglandin E2 [M-H]- HCD 7V P=367.2
C20H32O6; MF: 174; RMF: 914; Prob 75.0%; CAS: 54142-29-9; Lib: nist_msms; ID: 95386.

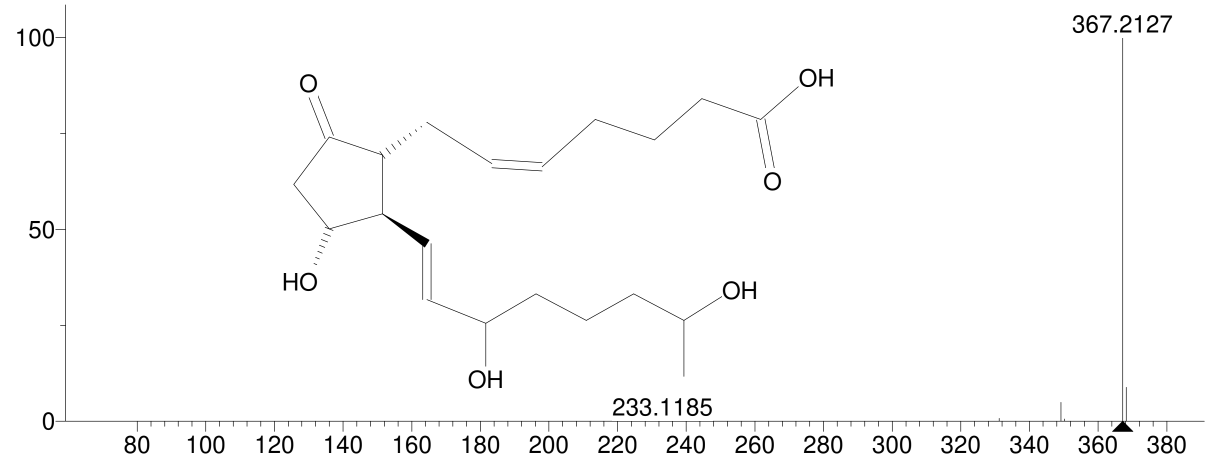

Hit 2 : 20-Hydroxyprostaglandin E2 [M-H]- HCD 7V P=367.2
C20H32O6; MF: 125; RMF: 884; Prob 15.4%; CAS: 57930-95-7; Lib: nist_msms; ID: 85513.

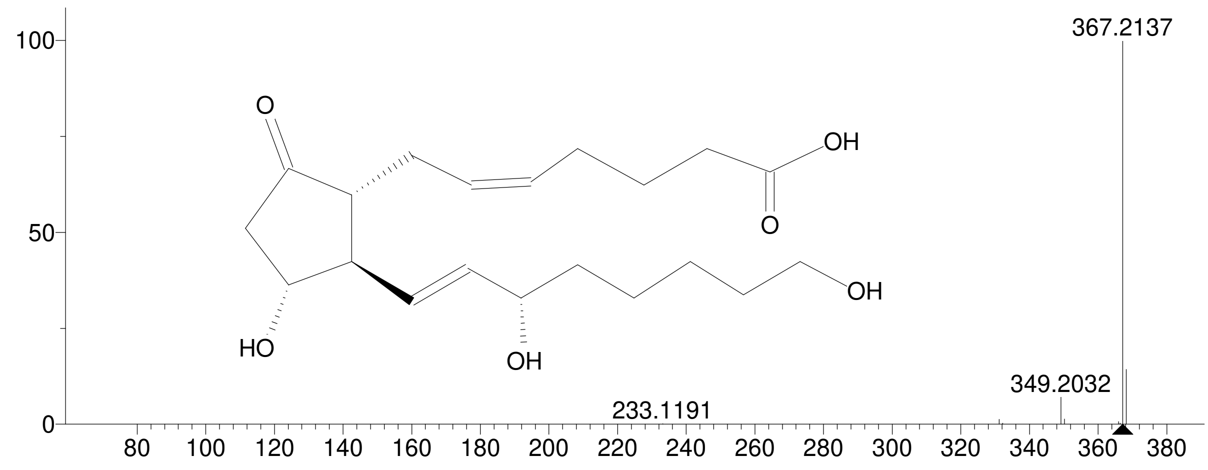

Hit 3 : Prostaglandin G2 [M-H]- HCD 14V P=367.2
C20H32O6; MF: 69; RMF: 825; Prob 2.93%; CAS: 51982-36-6; Lib: nist_msms; ID: 88334.

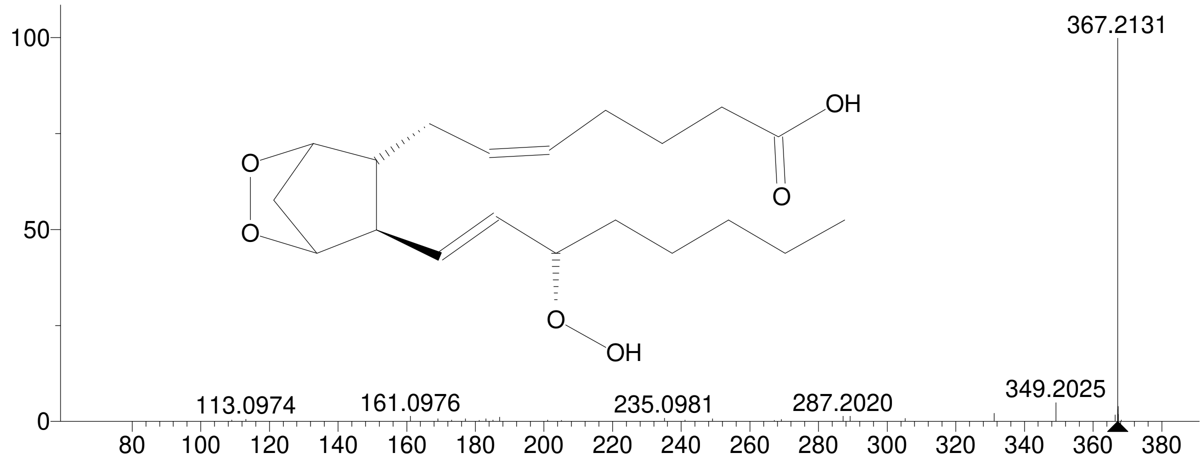

Hit 4 : 6-Ketoprostaglandin E1 [M-H]- QTOF 7V P=367.2
C20H32O6; MF: 66; RMF: 463; Prob 2.59%; CAS: 67786-53-2; Lib: nist_msms; ID: 22083.

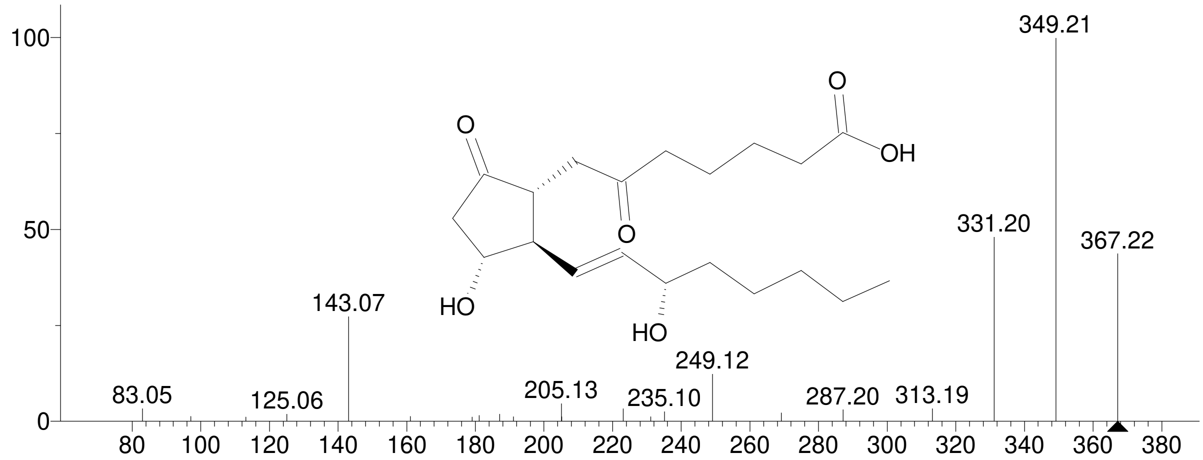

Hit 5 : .DELTA.17-6-Ketoprostaglandin F1α [M-H]- HCD 14V P=367.2
C20H32O6; MF: 62; RMF: 917; Prob 2.19%; CAS: 68324-95-8; Lib: nist_msms; ID: 158396.

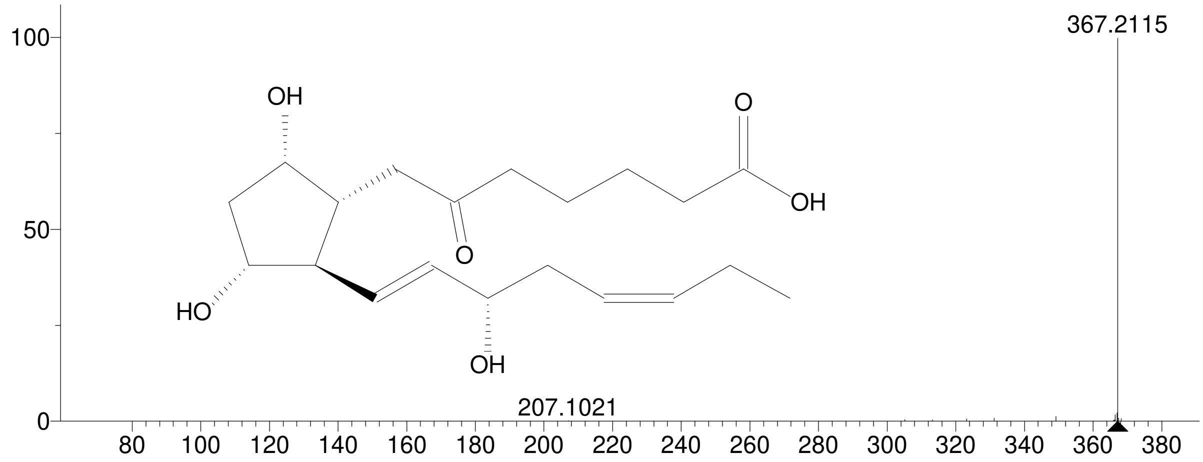

Unknown: Unknown (24.63_329.2314m/z)
Compound in Library Factor = -517

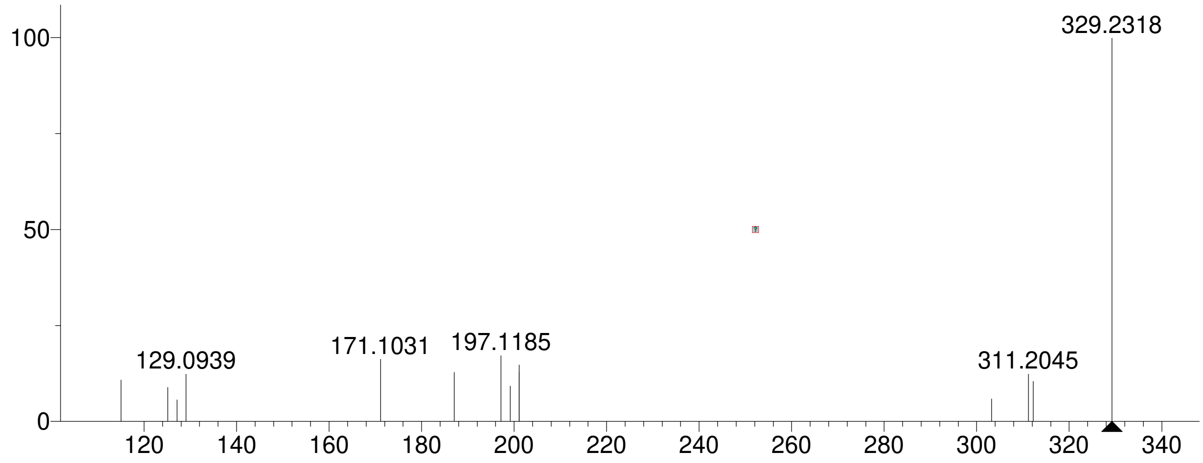

Hit 1 : FA 18:1+3O; PlaSMA ID-737 [M-H]- P=329.2
C18H34O5; MF: 240; RMF: 732; Prob 89.3%; Lib: biomsms-neg-plasma; ID: 448.

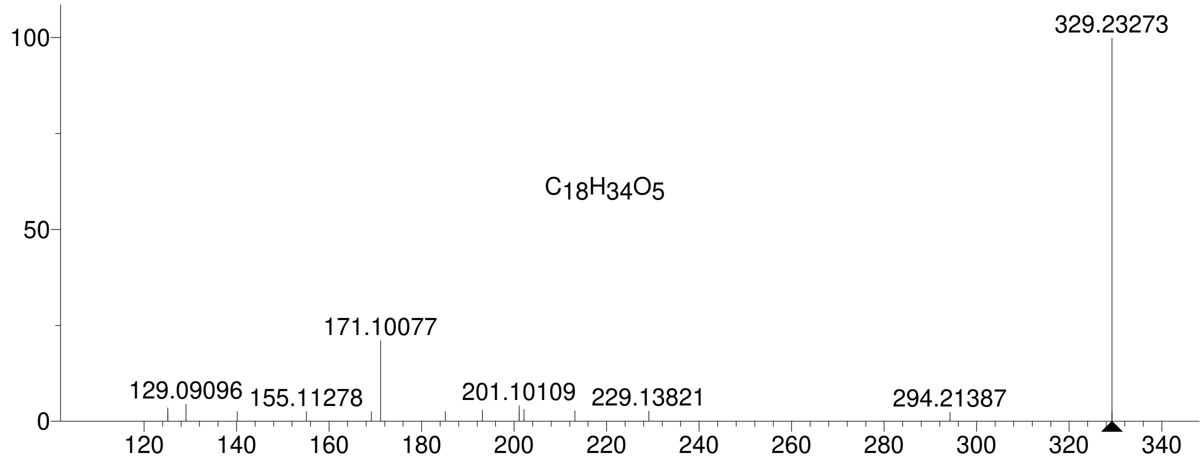

Hit 2 : FA 18:1+3O; PlaSMA ID-735 [M-H]- P=329.2
C18H34O5; MF: 118; RMF: 587; Prob 4.42%; Lib: biomsms-neg-plasma; ID: 443.

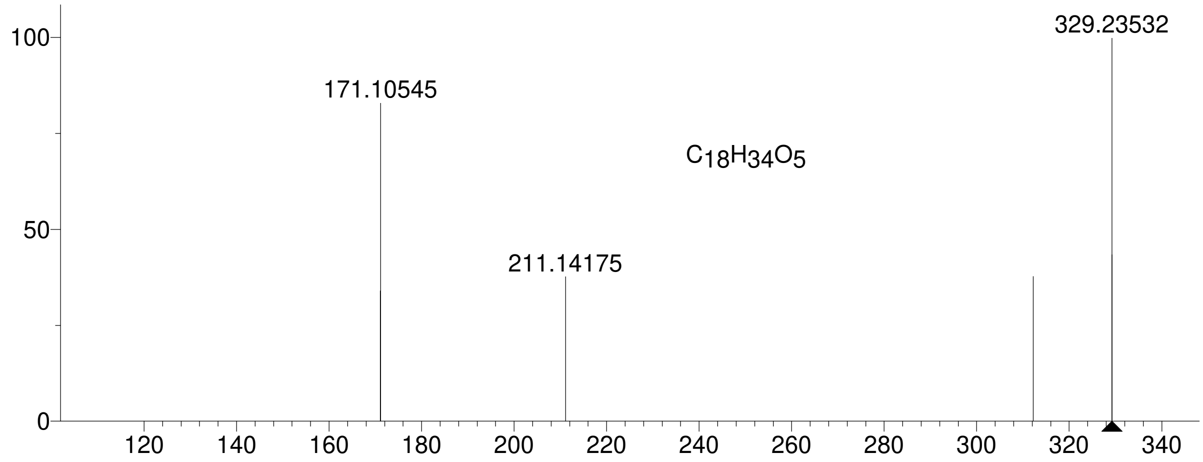

Hit 3 : FA 18:1+3O; PlaSMA ID-741 [M-H]- P=329.2
C18H34O5; MF: 116; RMF: 681; Prob 4.07%; Lib: biomsms-neg-plasma; ID: 449.

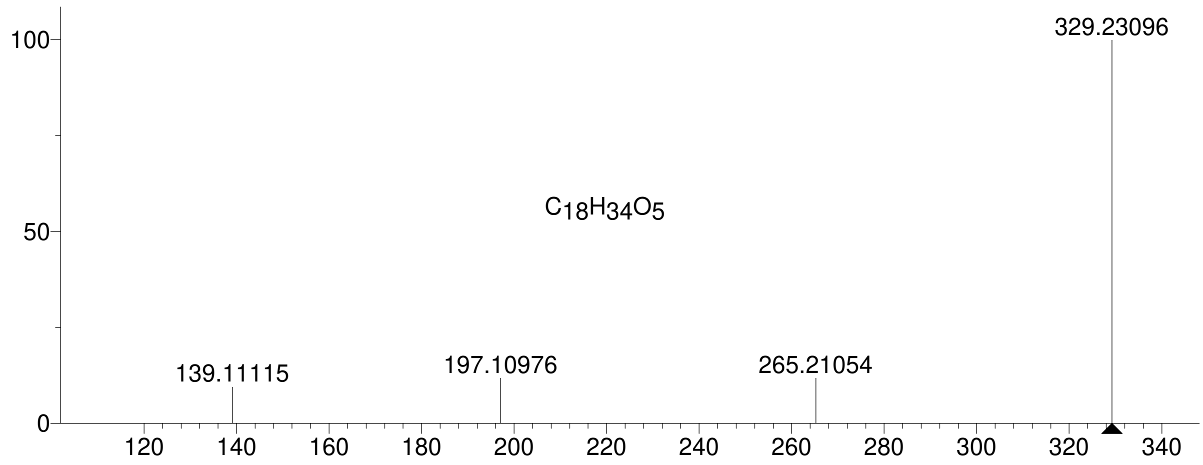

Hit 4 : FA 18:1+3O; PlaSMA ID-738 [M-H]- P=329.2
C18H34O5; MF: 86; RMF: 620; Prob 1.13%; Lib: biomsms-neg-plasma; ID: 444.

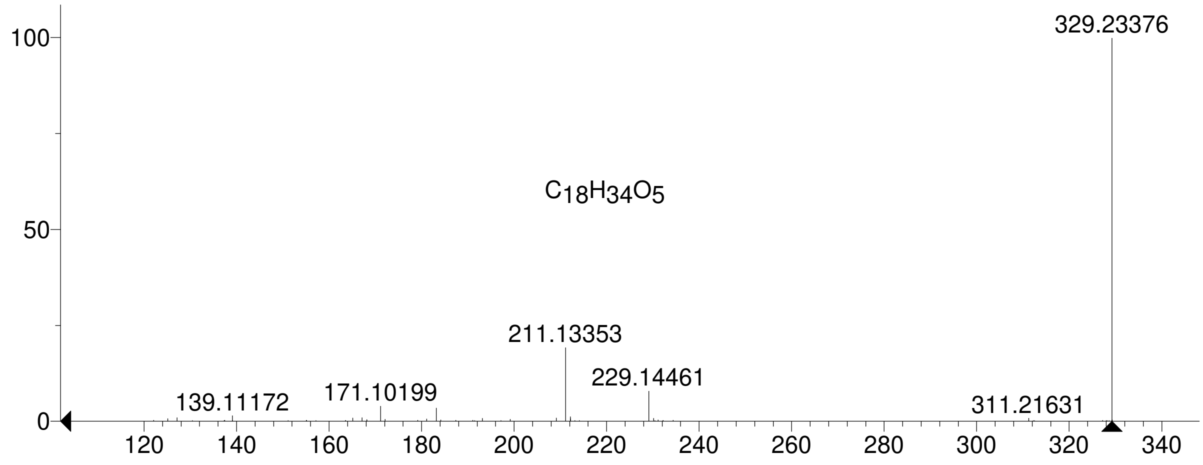

Hit 5 : FA 18:1+3O; PlaSMA ID-742 [M-H]- P=329.2
C18H34O5; MF: 59; RMF: 651; Prob 0.33%; Lib: biomsms-neg-plasma; ID: 445.

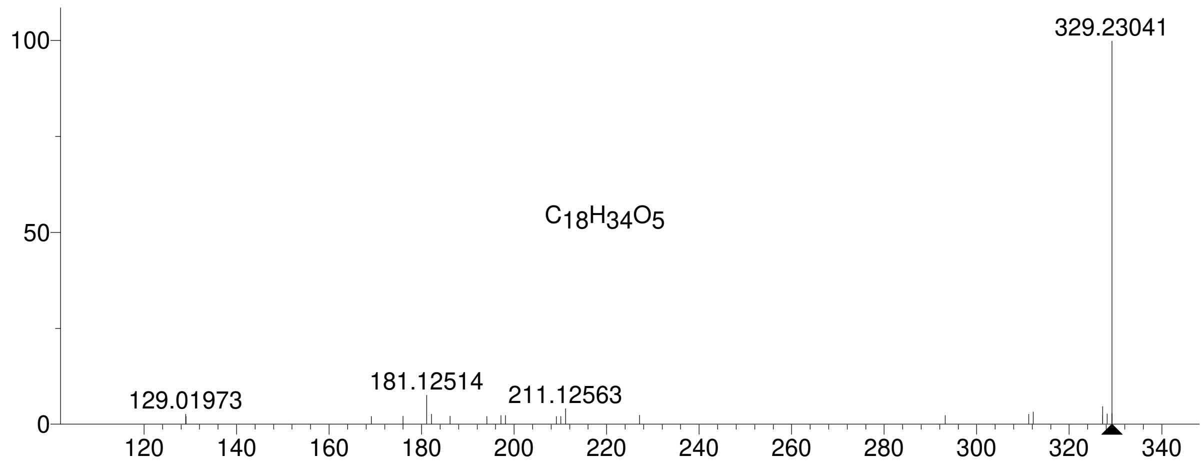

Unknown: Unknown (24.75_329.2316m/z)
Compound in Library Factor = -691

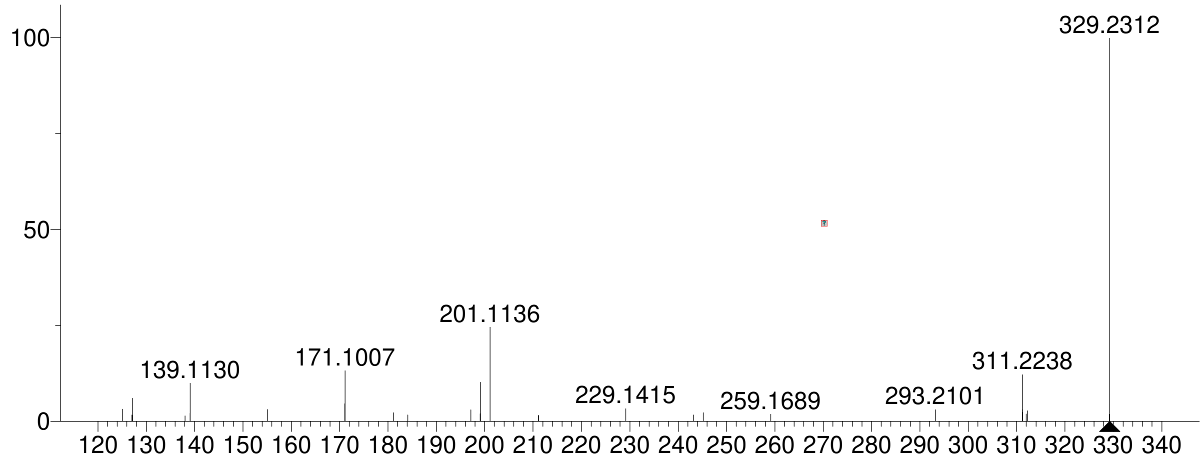

Hit 1 : FA 18:1+3O; PlaSMA ID-738 [M-H]- P=329.2
C18H34O5; MF: 283; RMF: 712; Prob 82.6%; Lib: biomsms-neg-plasma; ID: 444.

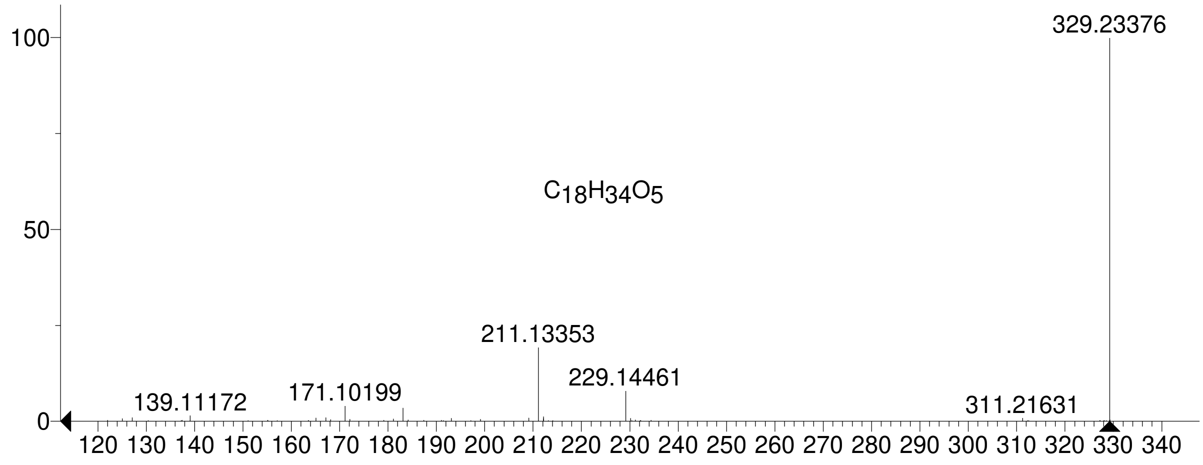

Hit 2 : FA 18:1+3O; PlaSMA ID-737 [M-H]- P=329.2
C18H34O5; MF: 189; RMF: 686; Prob 6.76%; Lib: biomsms-neg-plasma; ID: 448.

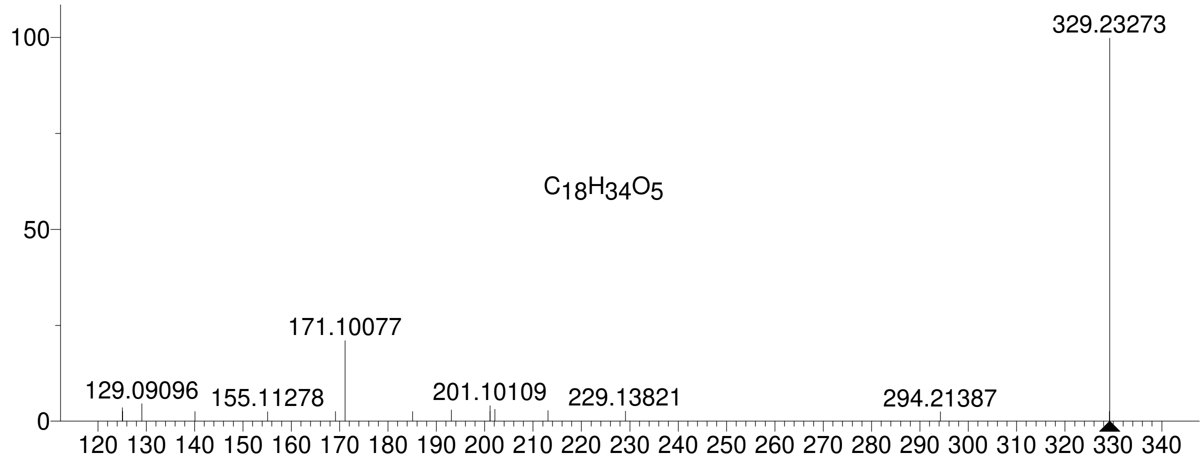

Hit 3 : FA 18:1+3O; PlaSMA ID-735 [M-H]- P=329.2
C18H34O5; MF: 180; RMF: 584; Prob 4.91%; Lib: biomsms-neg-plasma; ID: 443.

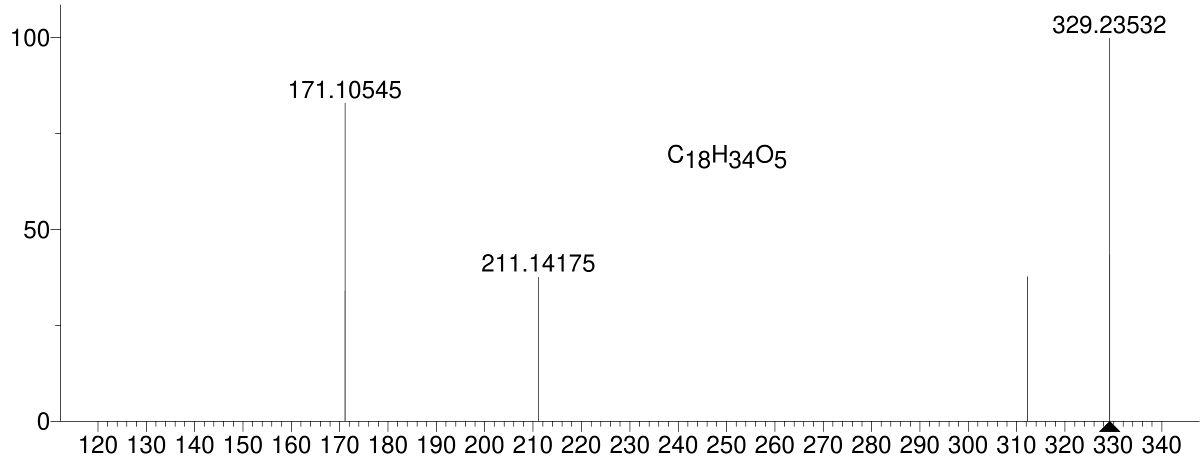

Hit 4 : 9,12,13-Trihydroxy-10(E)-octadecadienoic acid () [M-H]- P=329.2
C18H34O5; MF: 174; RMF: 667; Prob 3.86%; Lib: phytofrag2018_final_060219; ID: 129.

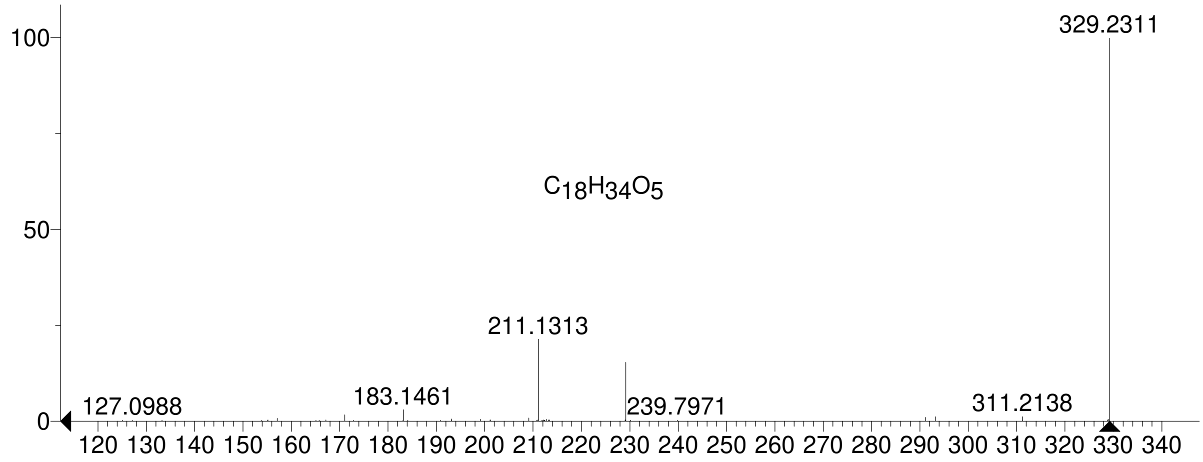

Hit 5 : FA 18:1+3O; PlaSMA ID-741 [M-H]- P=329.2
C18H34O5; MF: 129; RMF: 680; Prob 0.82%; Lib: biomsms-neg-plasma; ID: 449.

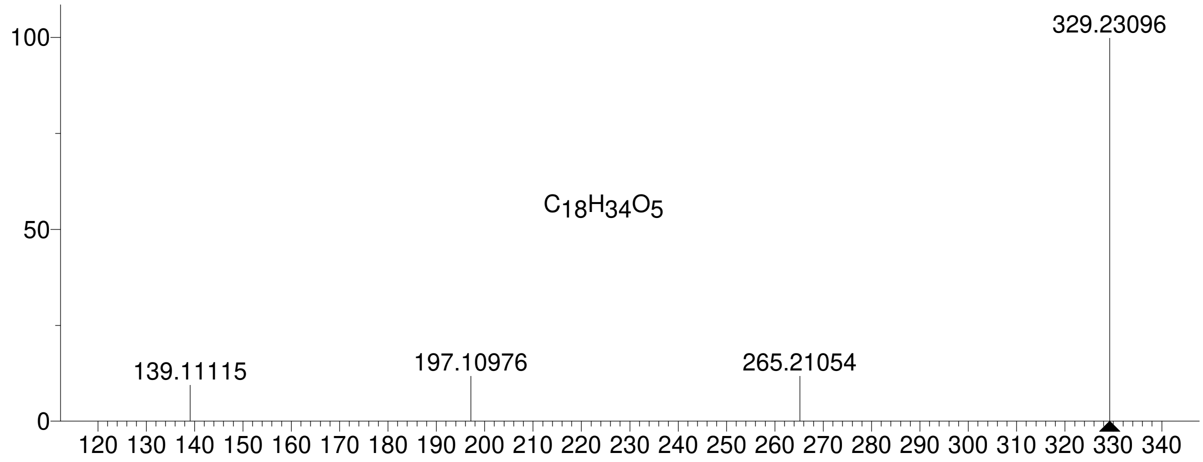

Unknown: Unknown (24.81_353.2314m/z)
Compound in Library Factor = -1453

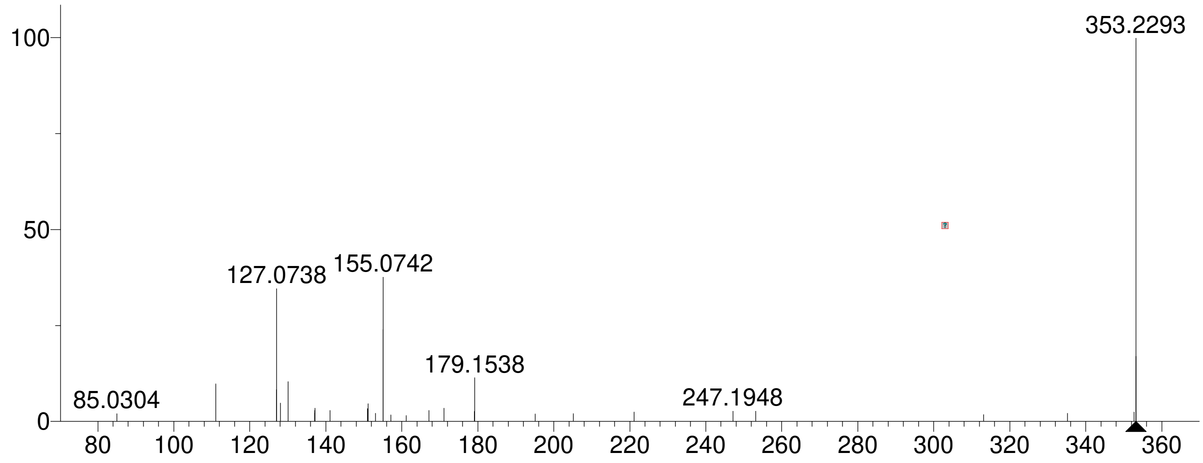

Hit 1 : Isoprostaglandin-F2α-IV [M-H]- HCD 22V P=353.2
C20H34O5; MF: 239; RMF: 643; Prob 41.9%; CAS: 331962-00-6; Lib: nist_msms; ID: 88135.

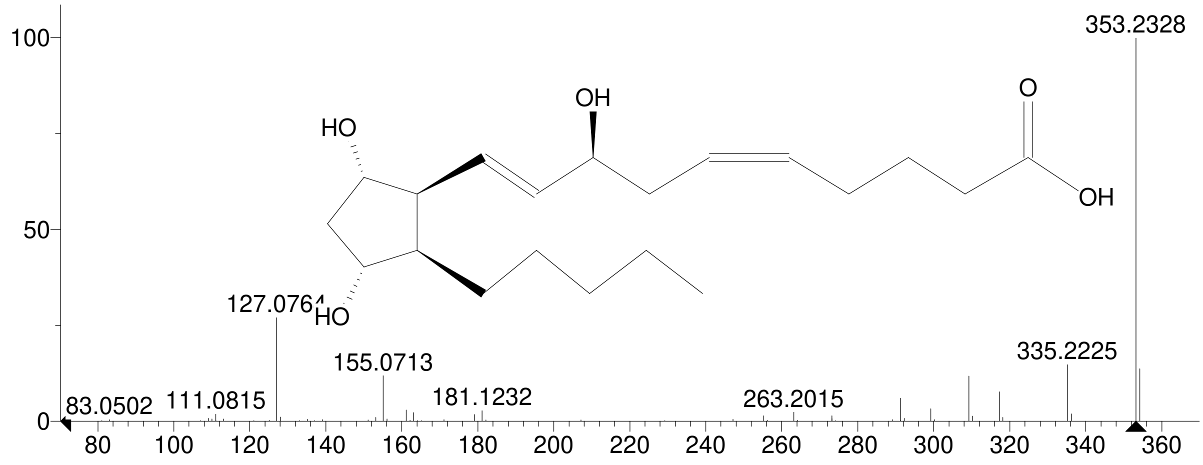

Hit 2 : Prostaglandin D1 [M-H]- HCD 5V P=353.2
C20H34O5; MF: 231; RMF: 875; Prob 31.3%; CAS: 17968-82-0; Lib: nist_msms; ID: 320117.

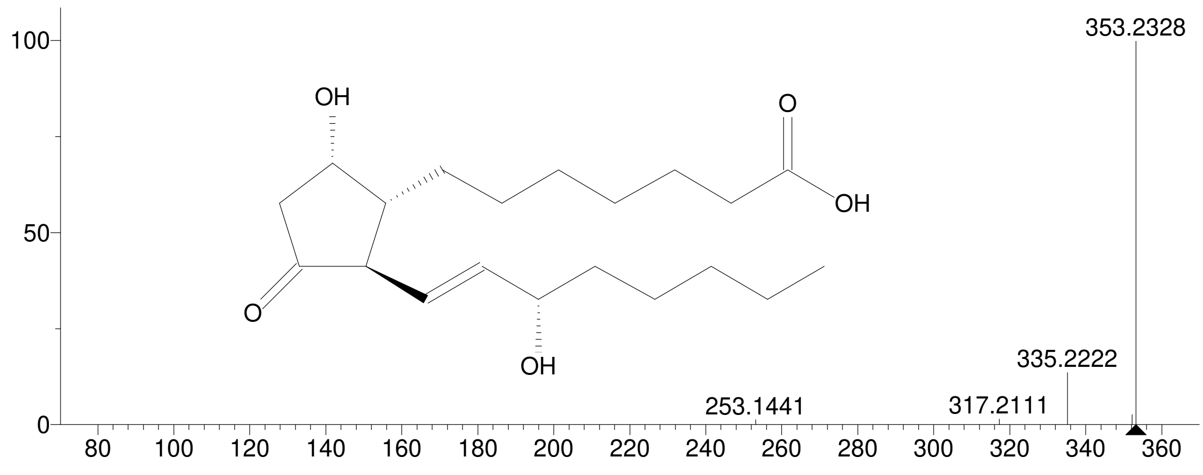

Hit 3 : 15-Ketoprostaglandin E1 [M+H]+ HCD 3V P=353.2
C20H32O5; MF: 188; RMF: 807; Prob 6.89%; CAS: 22973-19-9; Lib: nist_msms; ID: 169227.

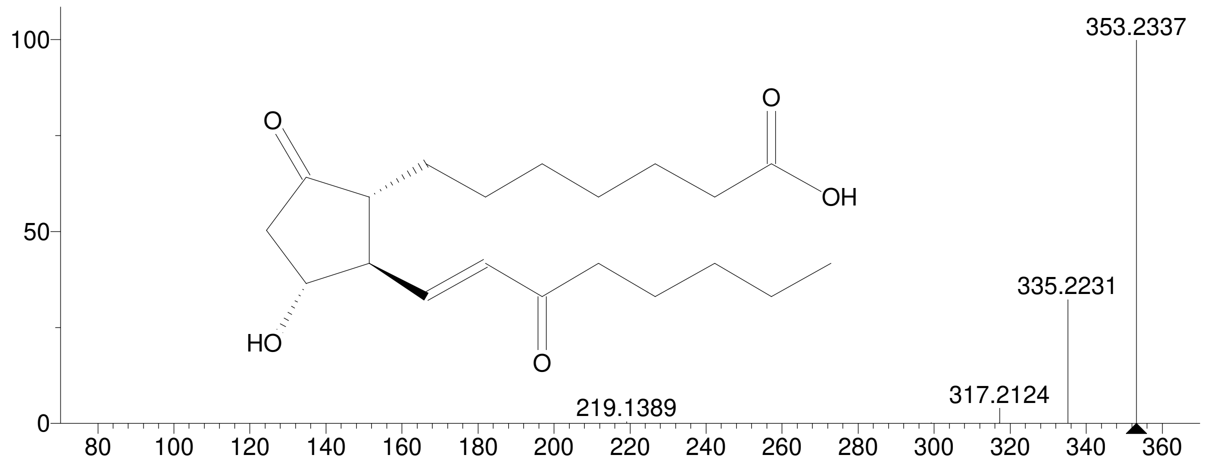

Hit 4 : 6-Ketoprostaglandin F1α [M+H-H2O]+ HCD 1V P=353.2
C20H34O6; MF: 187; RMF: 911; Prob 6.63%; CAS: 58962-34-8; Lib: nist_msms; ID: 166451.

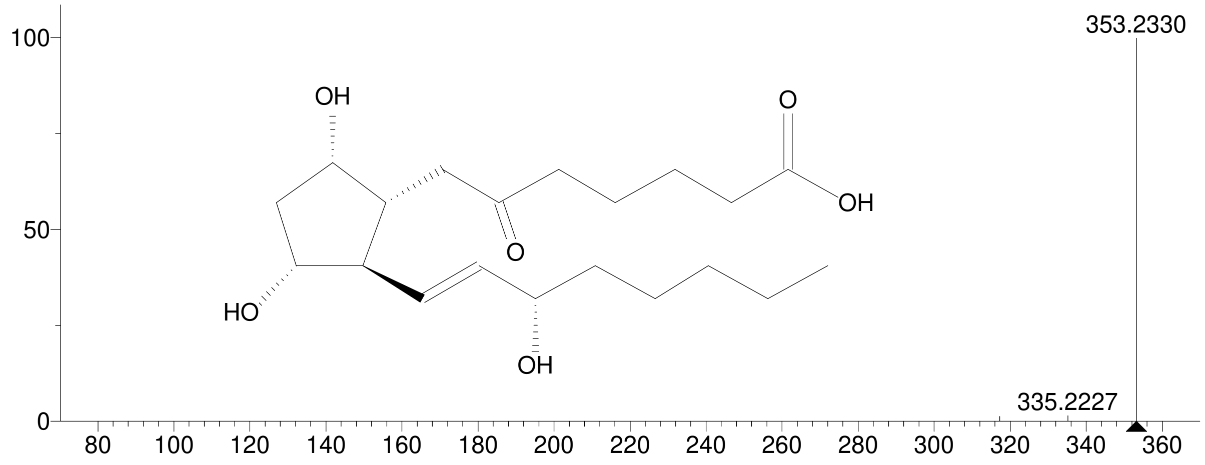

Hit 5 : Prostaglandin I2 [M+H]+ HCD 1V P=353.2
C20H32O5; MF: 180; RMF: 911; Prob 5.07%; CAS: 35121-78-9; Lib: nist_msms; ID: 166889.

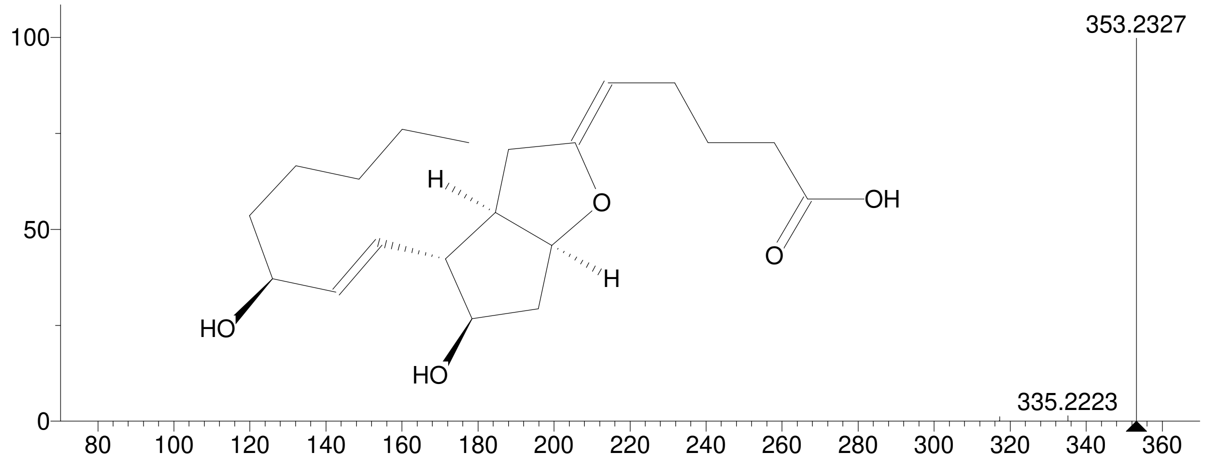

Unknown: Unknown (24.92_325.1647m/z)
Compound in Library Factor = -3273

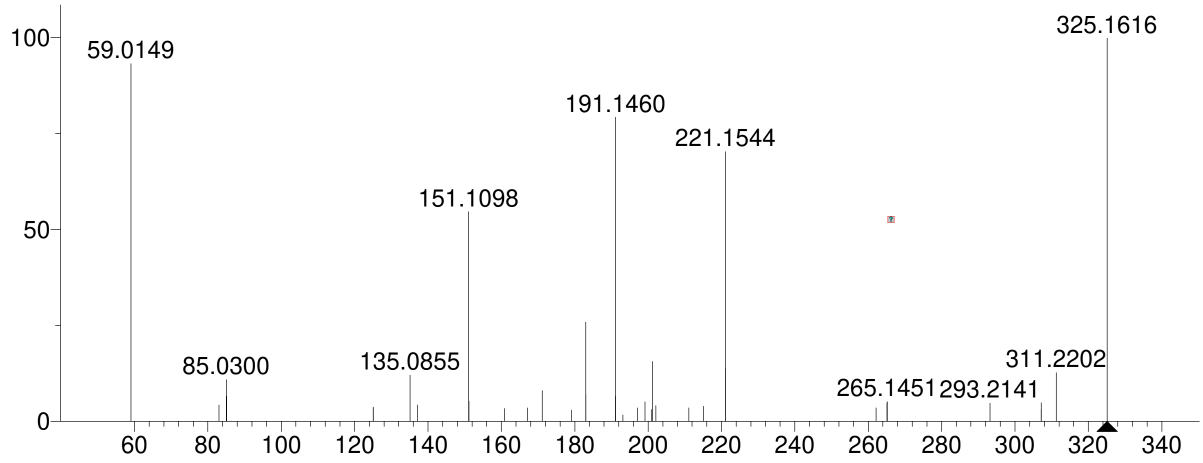

Hit 1 : Chamissonolide [M+H]+ HCD 1V P=325.2
C17H24O6; MF: 16; RMF: 447; Prob 17.2%; CAS: 78853-98-2; Lib: nist_msms; ID: 500759.

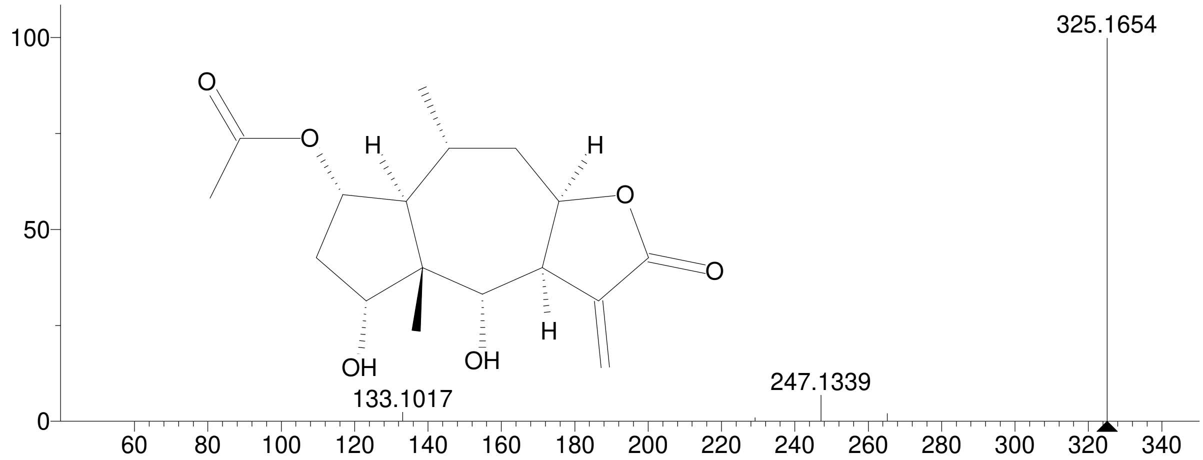

Hit 2 : Tributyrin [M+Na]+ HCD 16V P=325.2
C15H26O6; MF: 15; RMF: 416; Prob 16.5%; CAS: 60-01-5; Lib: nist_msms; ID: 144822.

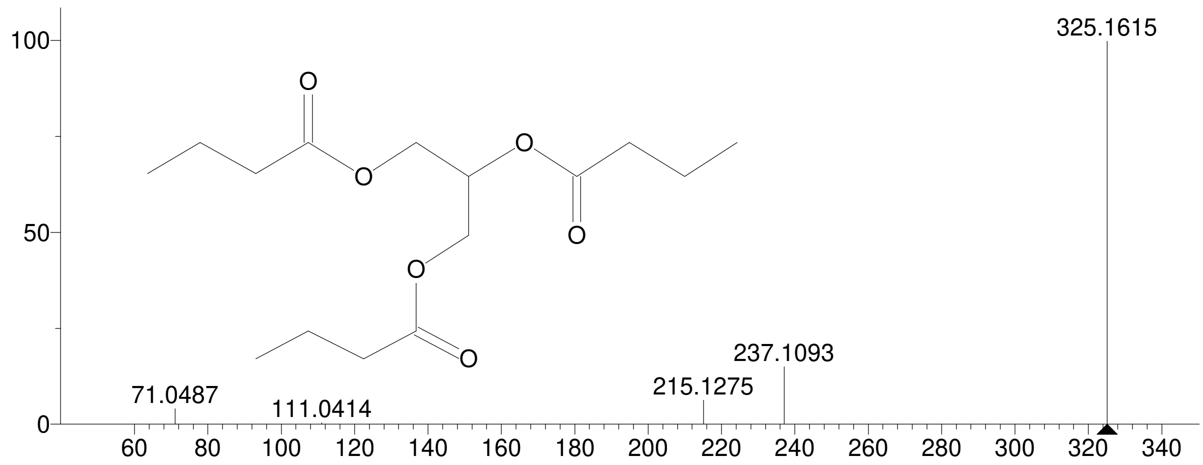

Hit 3 : MMV023985 [M+H]+ 20-30-40%) P=325.2
C18H20N4O2; MF: 14; RMF: 393; Prob 15.9%; Lib: mona-export-pathogen_box; ID: 292.

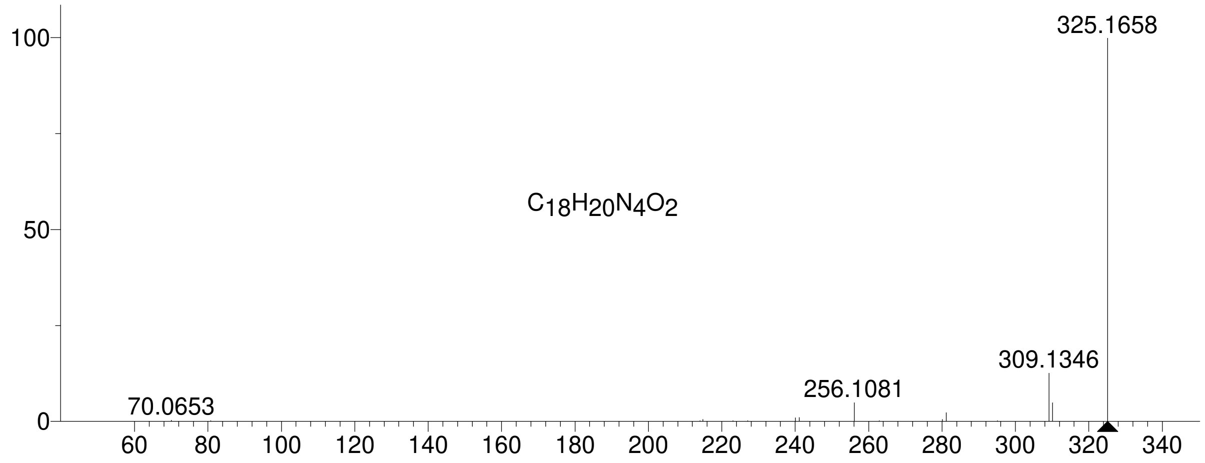

Hit 4 : 2H-Benzimidazol-2-one, 1-ethyl-1,3-dihydro- [2M+H]+ HCD 3V P=325.2
C9H10N2O; MF: 7; RMF: 251; Prob 12.2%; CAS: 10045-45-1; Lib: nist_msms; ID: 364753.

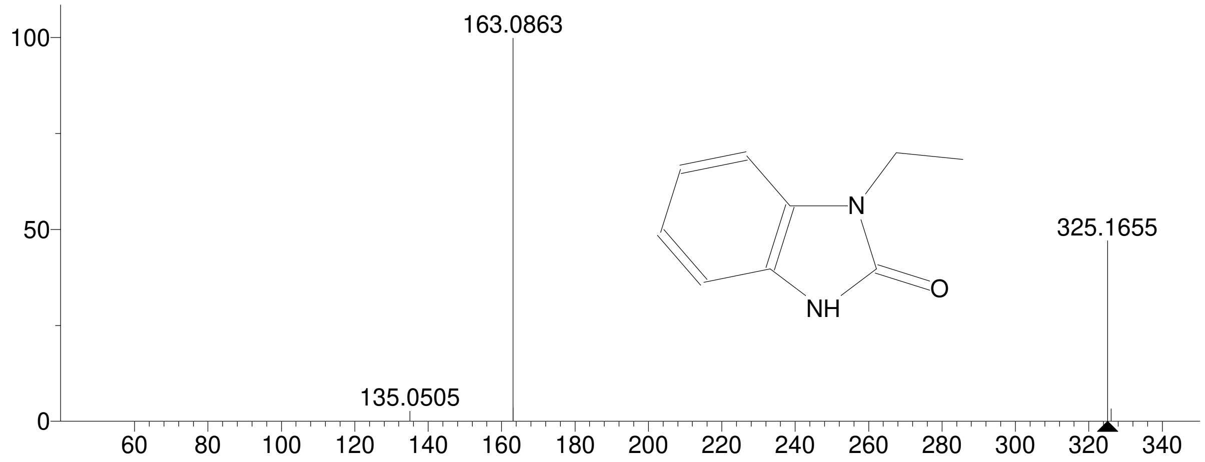

Hit 5 : [5-acetyloxy-3-(hydroxymethyl)-2-oxo-6-propan-2-ylcyclohex-3-en-1-yl] 3-methylbutanoate [M-H]- 65HCD
C17H26O6; MF: 2; RMF: 142; Prob 9.81%; Lib: mona-export-lc-ms-ms_negative_mode; ID: 19387.

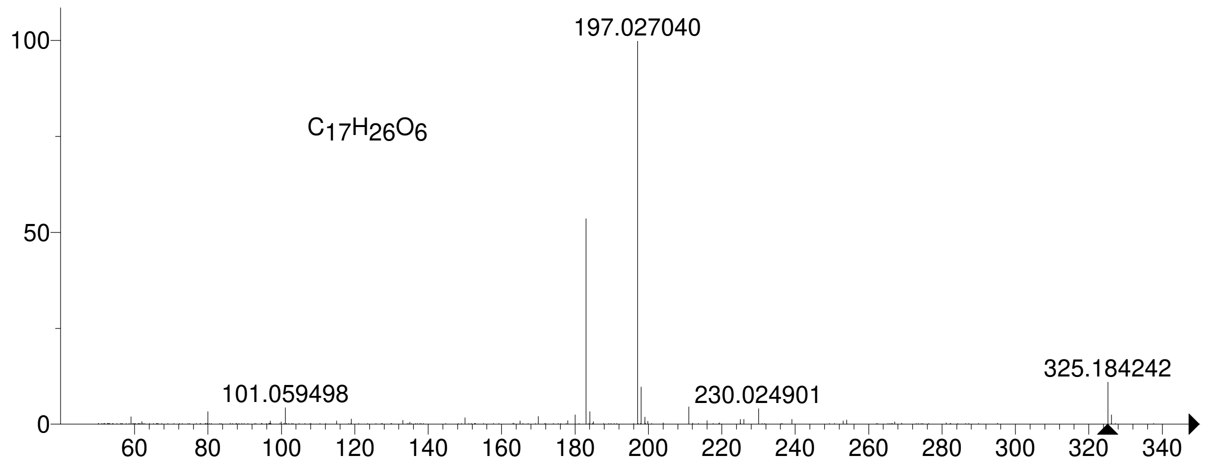

Unknown: Unknown (24.99_631.3474m/z)
Compound in Library Factor = 0

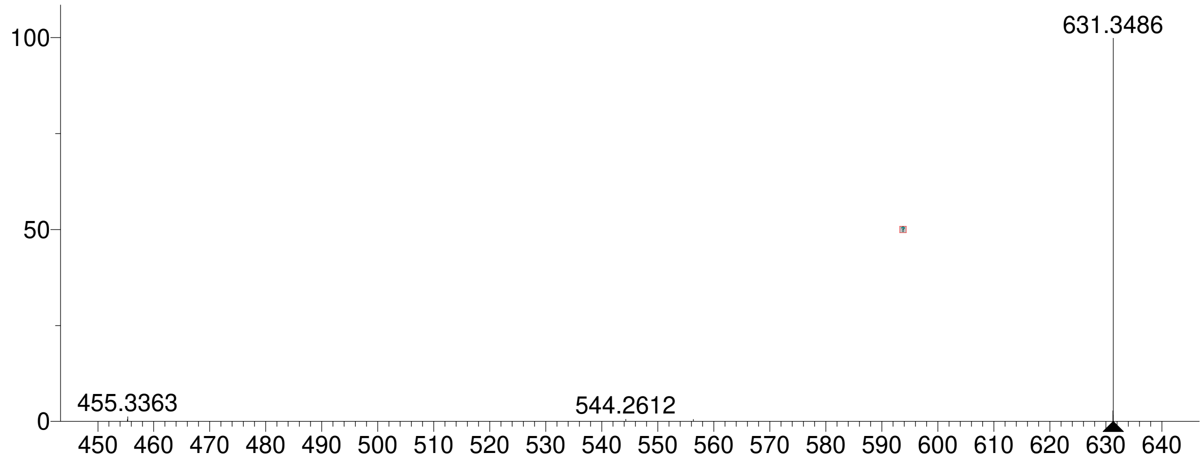

Unknown: Unknown (25.02_311.2217m/z)
Compound in Library Factor = -177

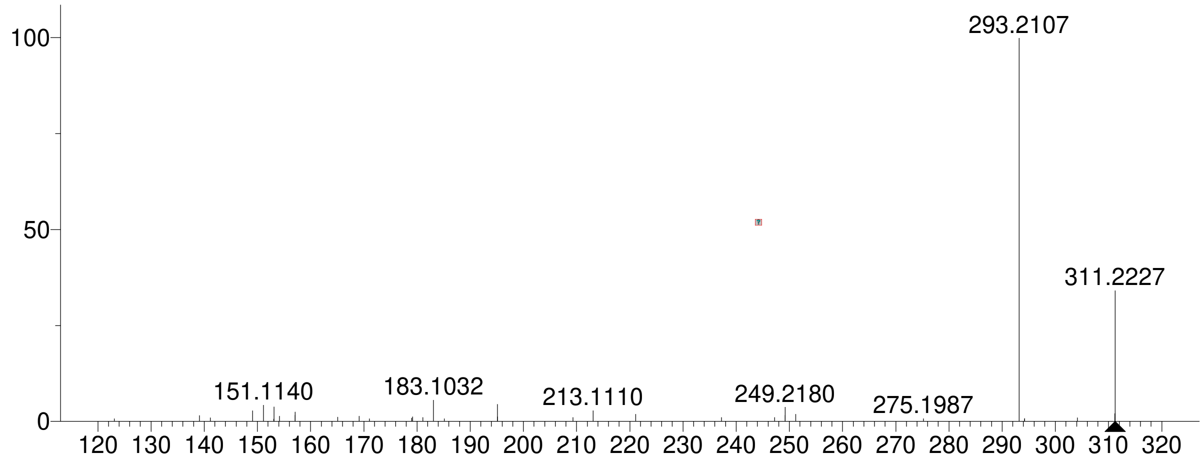

Hit 1 : 9-Oxo-11-(3-pentyl-2-oxiranyl)-10E-undecenoic acid [M+H]+ HCD 3V P=311.2
C18H30O4; MF: 581; RMF: 738; Prob 98.0%; CAS: 478931-82-7; Lib: nist_msms; ID: 91854.

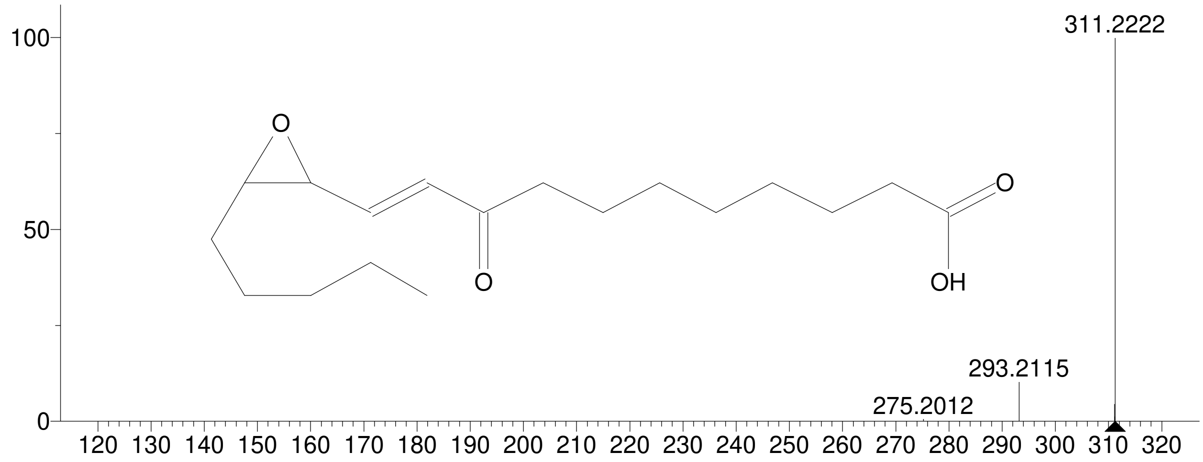

Hit 2 : Unknown (carbon number 18); PlaSMA ID-652 [M-H]- P=311.2
C18H32O4; MF: 329; RMF: 577; Prob 0.98%; Lib: biomsms-neg-plasma; ID: 1205.

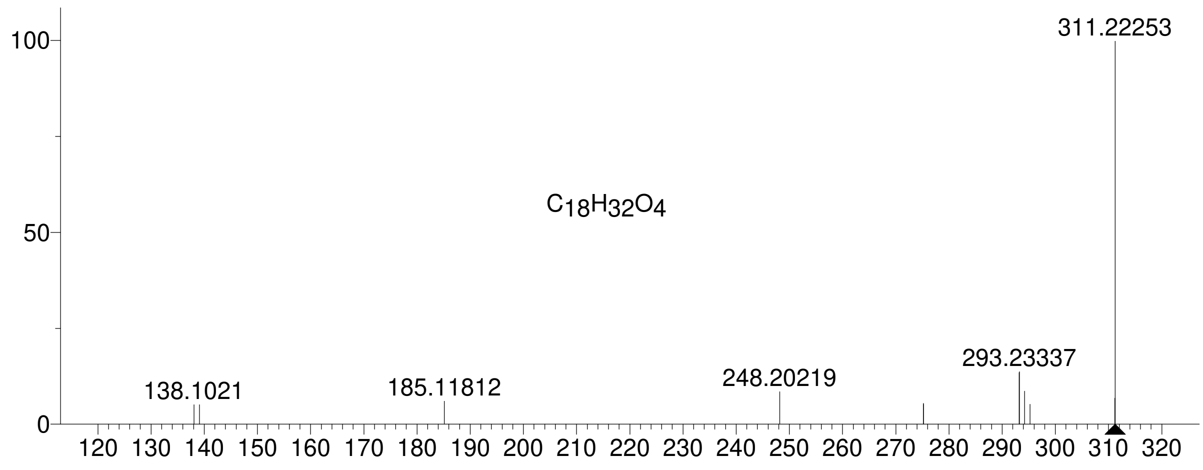

Hit 3 : Unknown (carbon number 17); PlaSMA ID-642 [M-H]- P=311.2
C17H32N2OS; MF: 327; RMF: 616; Prob 0.90%; Lib: biomsms-neg-plasma; ID: 1201.

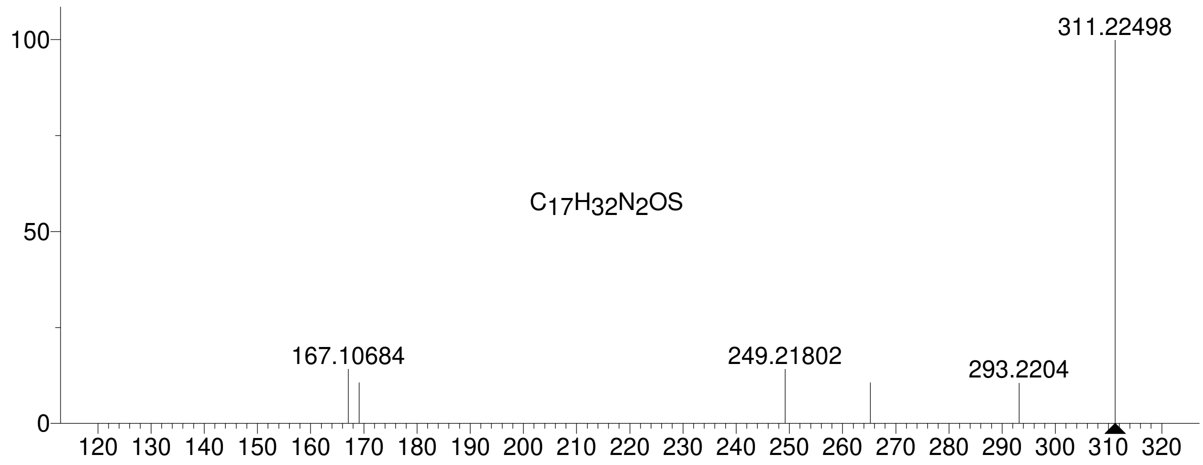

Hit 4 : FA 18:2+2O; PlaSMA ID-643 [M-H]- P=311.2
C18H32O4; MF: 228; RMF: 648; Prob 0.06%; Lib: biomsms-neg-plasma; ID: 431.

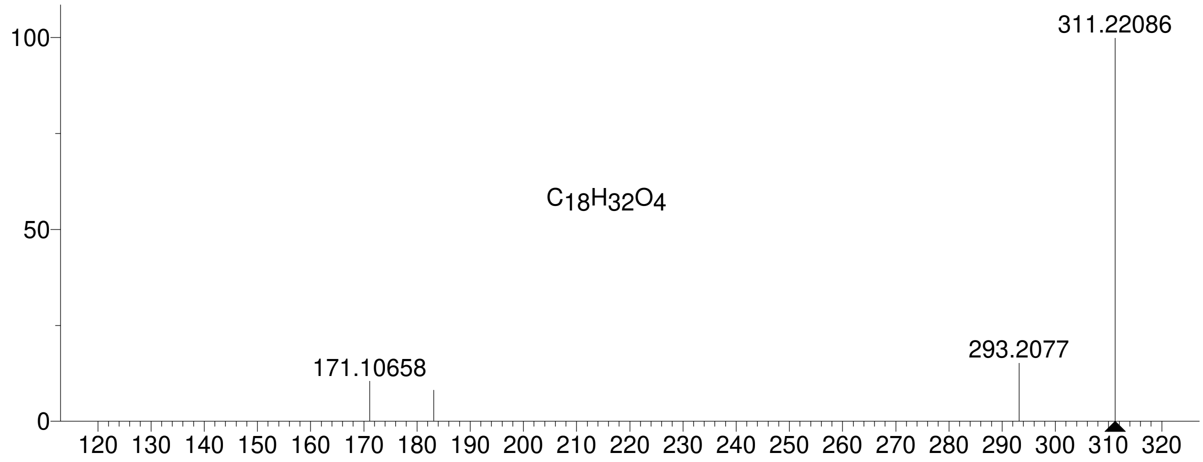

Hit 5 : Unknown (carbon number 19); PlaSMA ID-644 [M-CH3]- P=311.2
C19H34O4; MF: 139; RMF: 511; Prob 0.00%; Lib: biomsms-neg-plasma; ID: 1202.

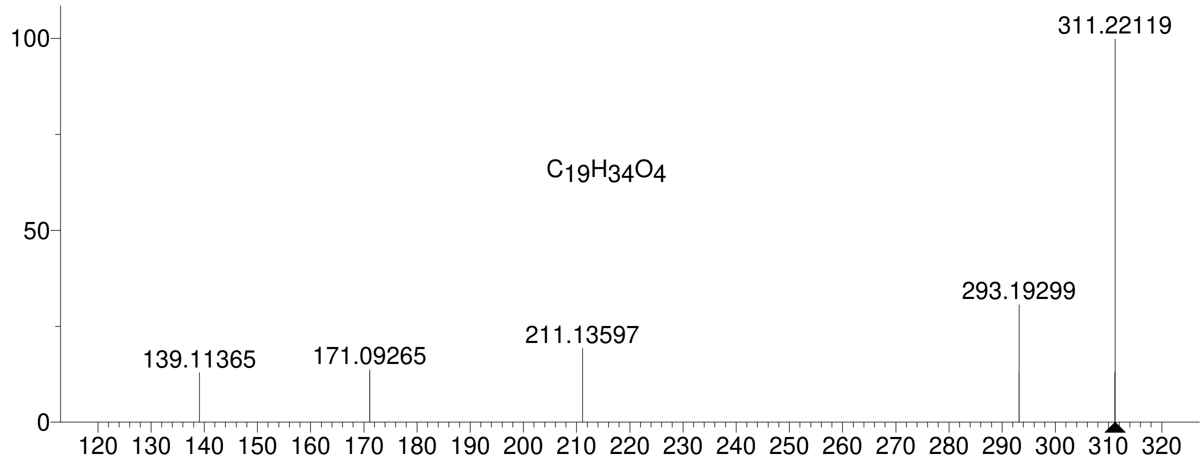

Unknown: Unknown (25.05_329.2324m/z)
Compound in Library Factor = -542

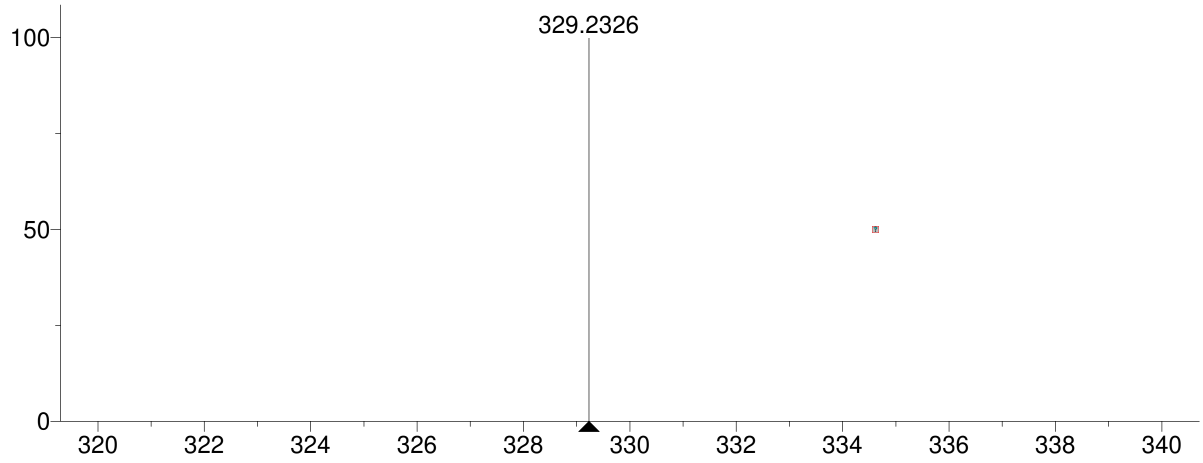

Hit 1 : FA 18:1+3O; PlaSMA ID-739 [M-H]- P=329.2
C18H34O5; MF: 364; RMF: 999; Prob 14.7%; Lib: biomsms-neg-plasma; ID: 446.

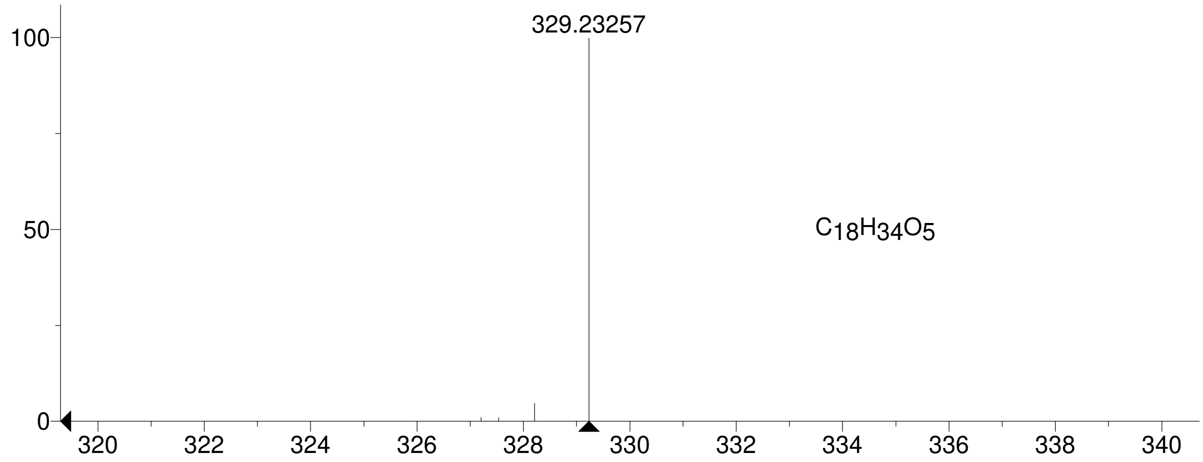

Hit 2 : FA 18:1+3O; PlaSMA ID-740 [M-H]- P=329.2
C18H34O5; MF: 364; RMF: 999; Prob 14.7%; Lib: biomsms-neg-plasma; ID: 447.

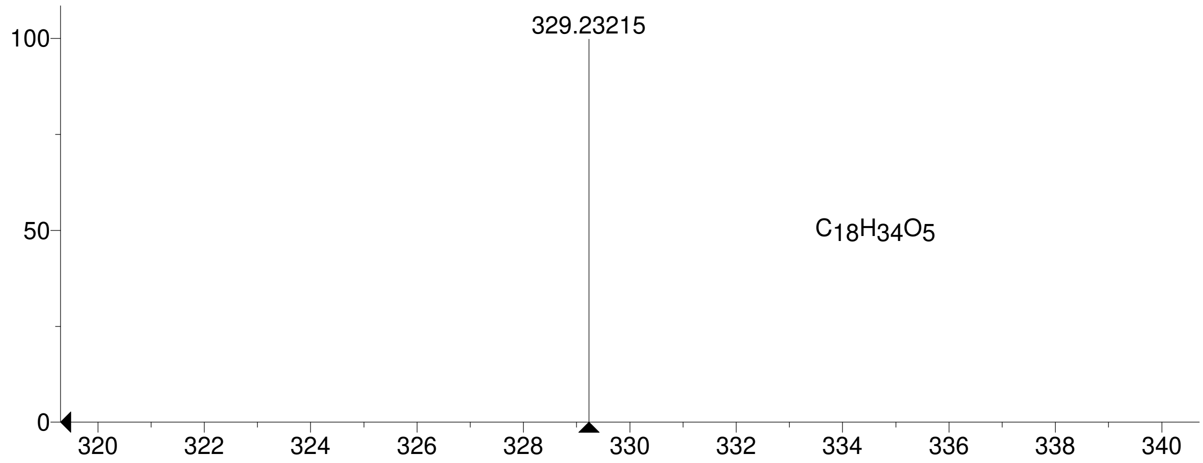

Hit 3 : FA 18:1+3O; PlaSMA ID-737 [M-H]- P=329.2
C18H34O5; MF: 364; RMF: 999; Prob 14.7%; Lib: biomsms-neg-plasma; ID: 448.

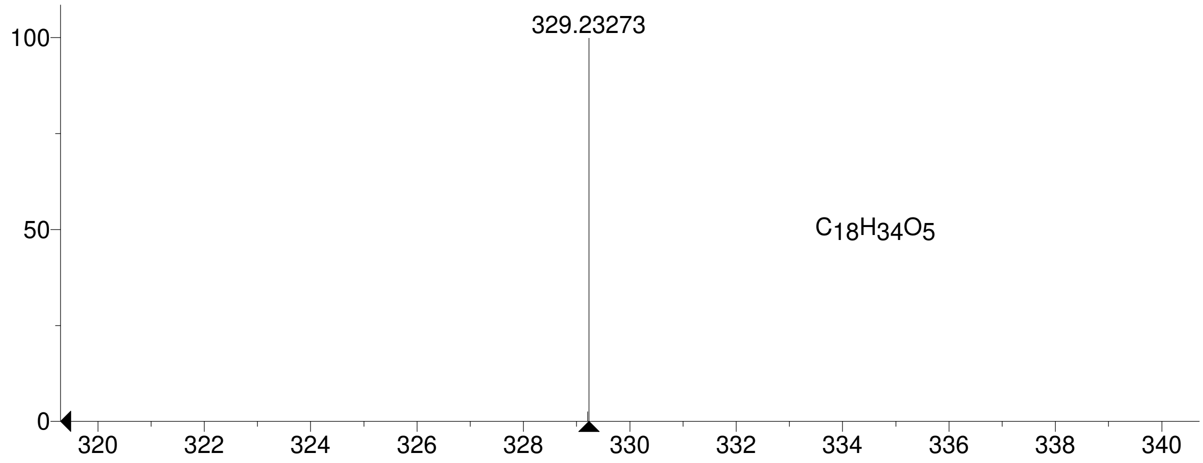

Hit 4 : FA 18:1+3O; PlaSMA ID-741 [M-H]- P=329.2
C18H34O5; MF: 364; RMF: 999; Prob 14.7%; Lib: biomsms-neg-plasma; ID: 449.

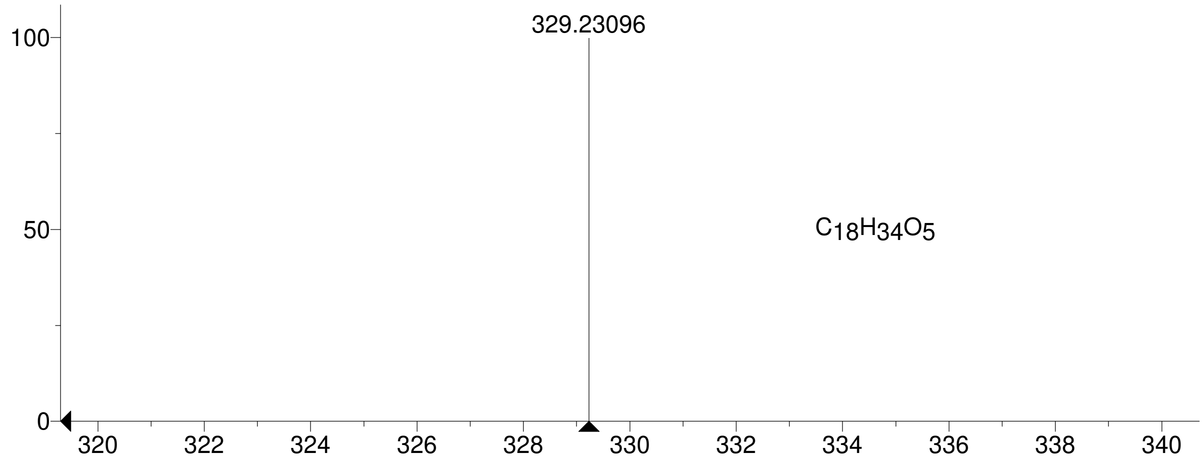

Hit 5 : 9,12,13-Trihydroxy-10(E)-octadecadienoic acid () [M-H]- P=329.2
C18H34O5; MF: 364; RMF: 999; Prob 14.7%; Lib: phytofrag2018_final_060219; ID: 129.

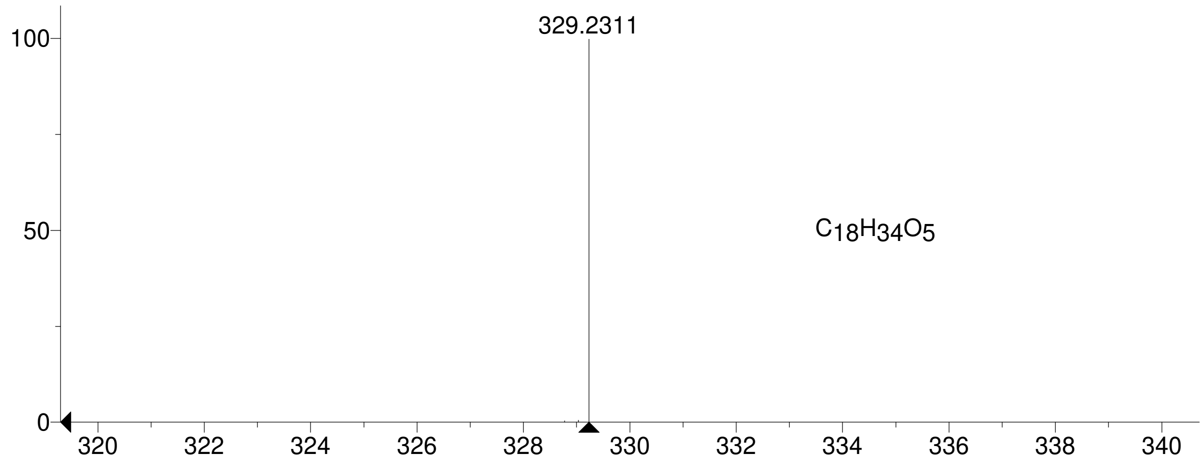

** Search Report Page 1 of 1 **

Unknown: Unknown (25.10_398.0393m/z)
Compound in Library Factor = 0

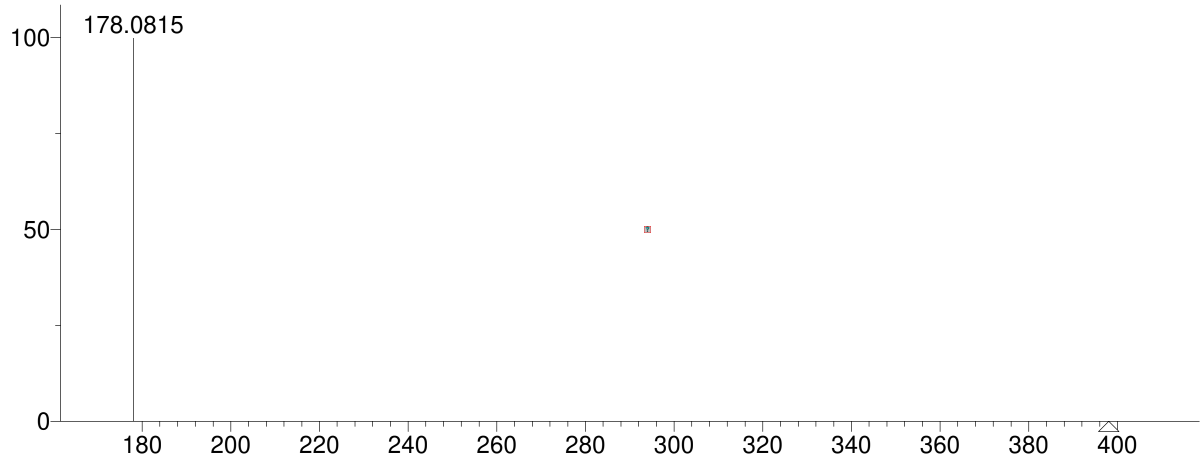

Unknown: Unknown (25.16_219.1379m/z)
Compound in Library Factor = -396

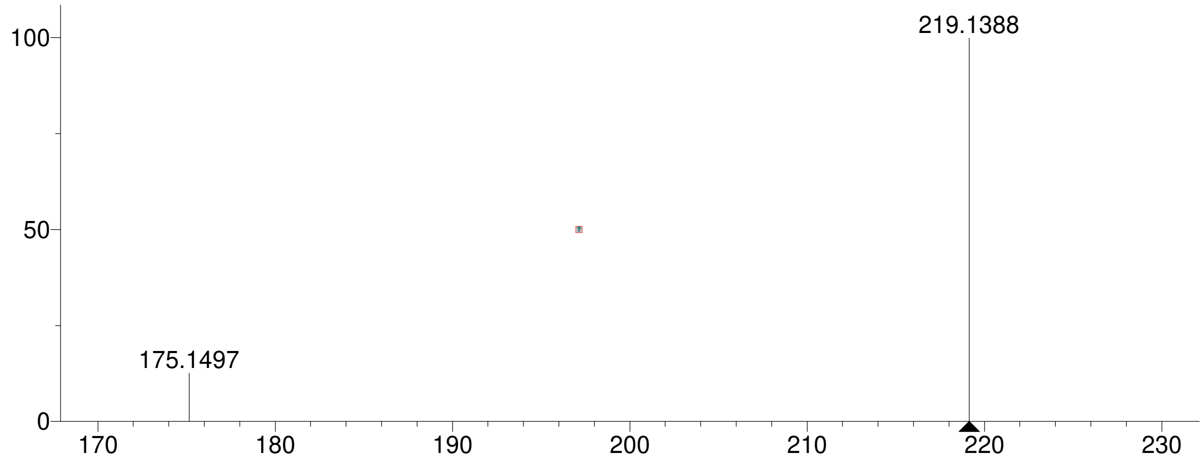

Hit 1 : 1-Heptanone, 1-(4-methoxyphenyl)-2-(1-pyrrolidinyl)- [M+H-C4H9N]+ HCD 6V P=219.1
C18H27NO2; MF: 321; RMF: 938; Prob 58.1%; CAS: 1801552-04-4; Lib: nist_msms; ID: 370217.

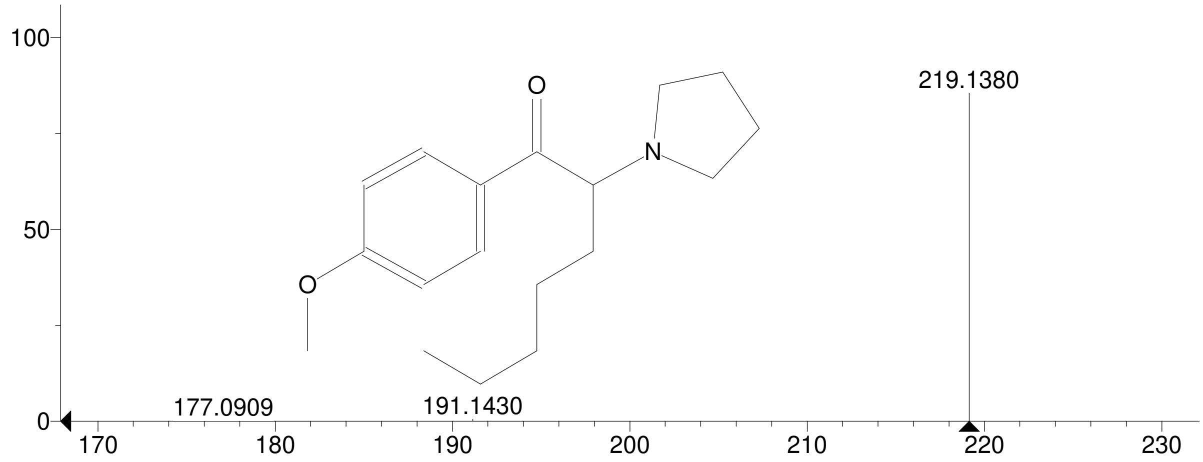

Hit 2 : Empenthrin [M+H-C4H8]+ HCD 10V P=219.1
C18H26O2; MF: 310; RMF: 944; Prob 39.9%; CAS: 54406-48-3; Lib: nist_msms; ID: 475486.

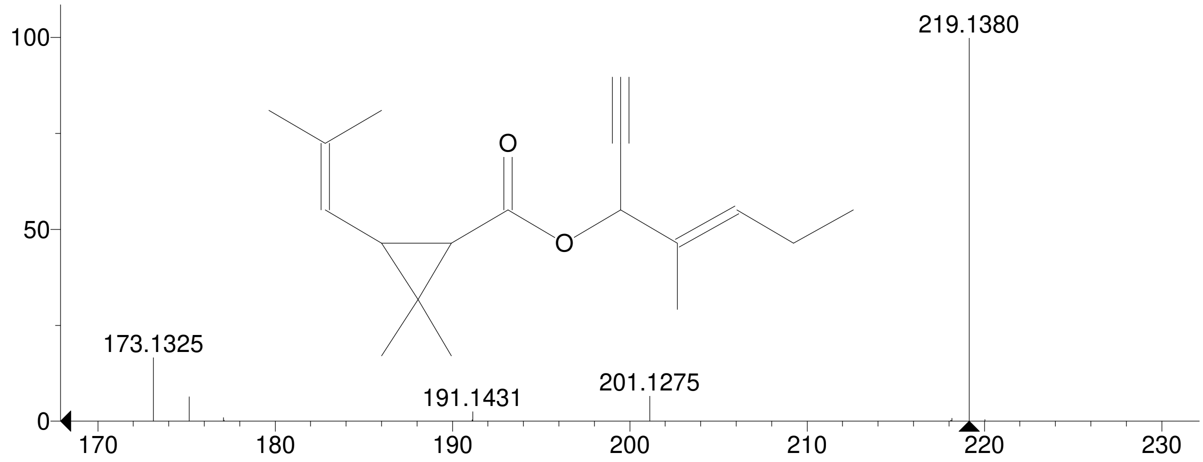

Hit 3 : Hydroxyebastine [M+H-C18H21NO]+ HCD 17V P=219.1
C32H39NO3; MF: 179; RMF: 834; Prob 1.62%; CAS: 210686-41-2; Lib: nist_msms; ID: 441211.

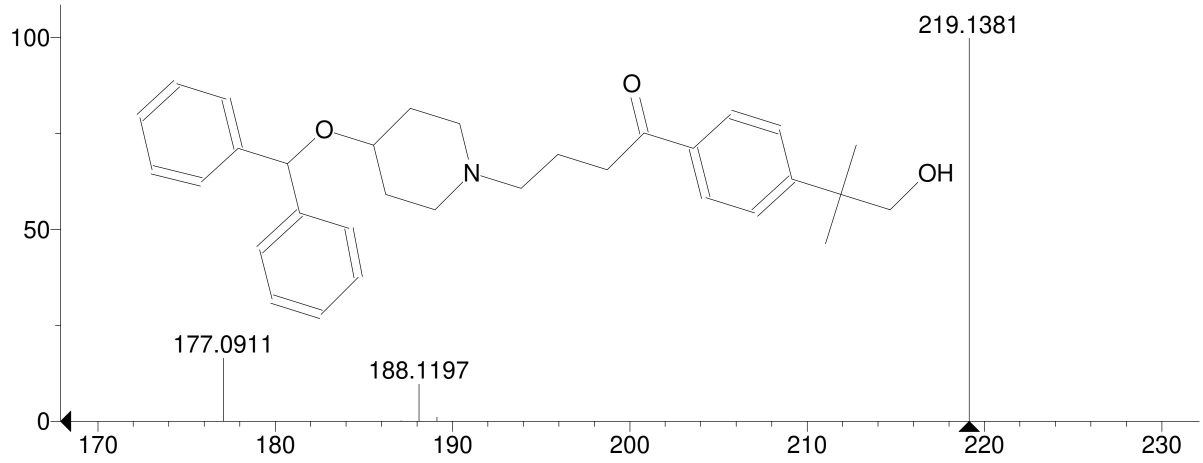

Hit 4 : 3-Furancarboxylic acid, tetrahydro-4-methylene-2-octyl-5-oxo-, (2R,3S)-rel- [M+H-2H2O]+ HCD 8V P=2
C14H22O4; MF: 125; RMF: 917; Prob 0.31%; CAS: 191282-48-1; Lib: nist_msms; ID: 427596.

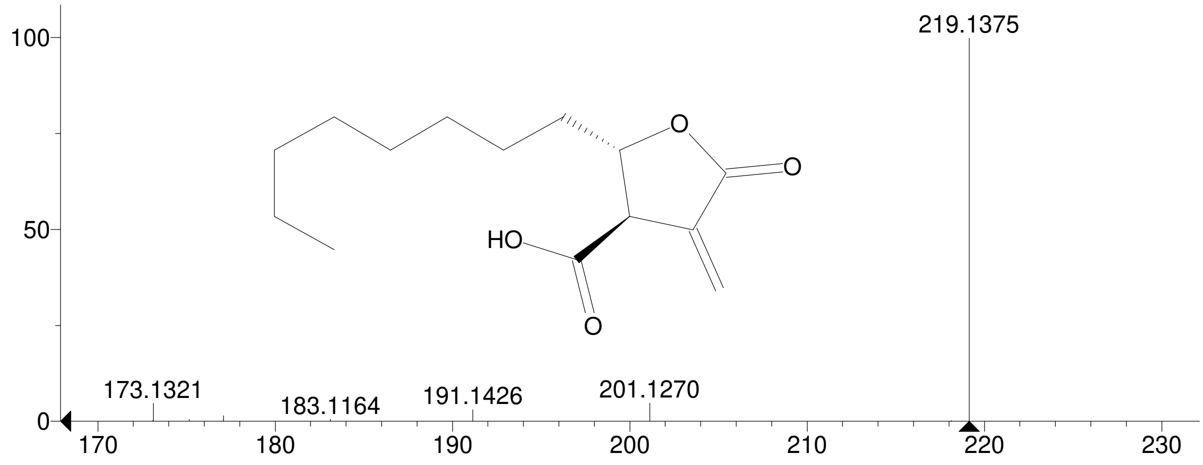

Hit 5 : (+)-Abscisic acid [M-H-CO2]- HCD 19V P=219.1
C15H20O4; MF: 100; RMF: 807; Prob 0.09%; CAS: 21293-29-8; Lib: nist_msms; ID: 539122.

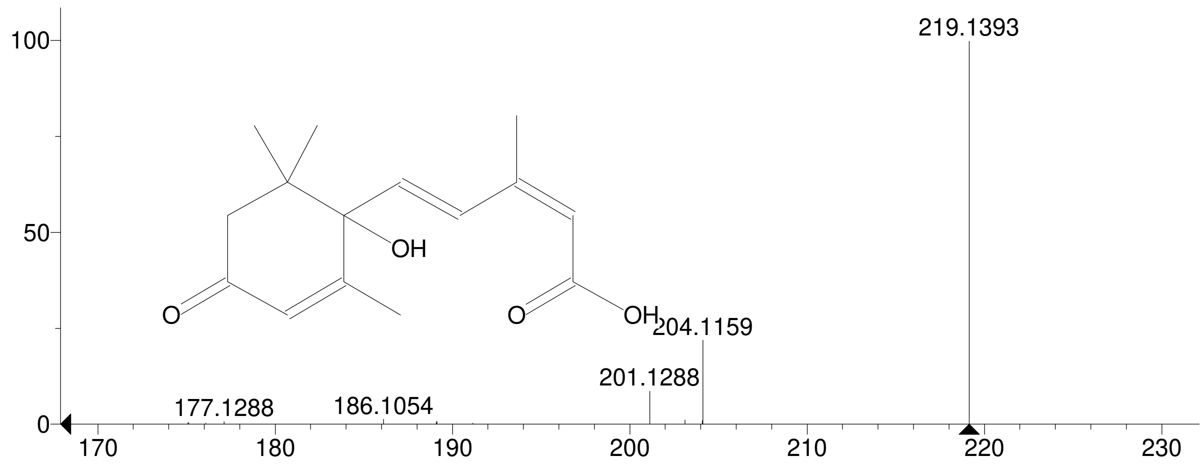

Unknown: Unknown (25.16_376.0089m/z)
Compound in Library Factor = 0

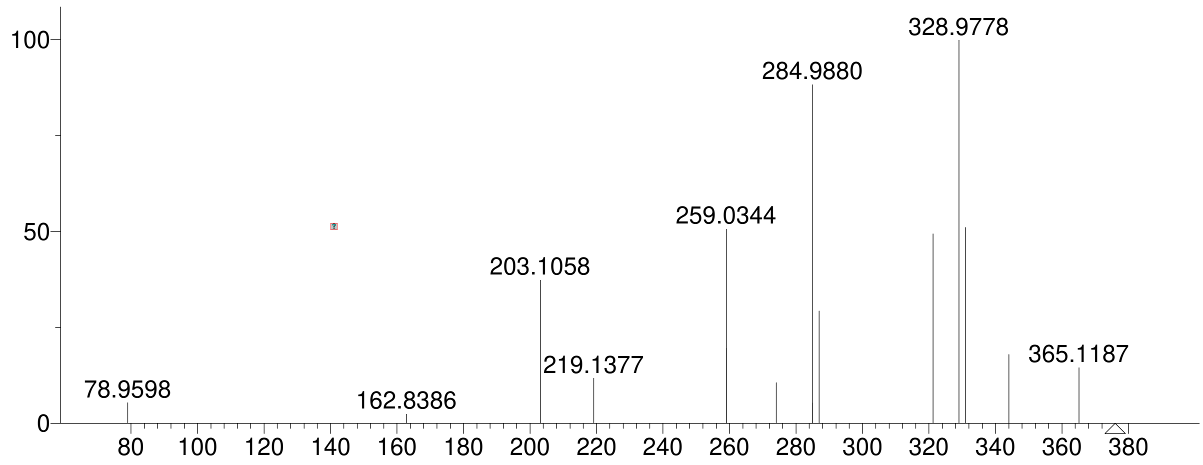

** Search Report Page 1 of 1 **

Unknown: Unknown (25.16_394.0712m/z)
Compound in Library Factor = -3273

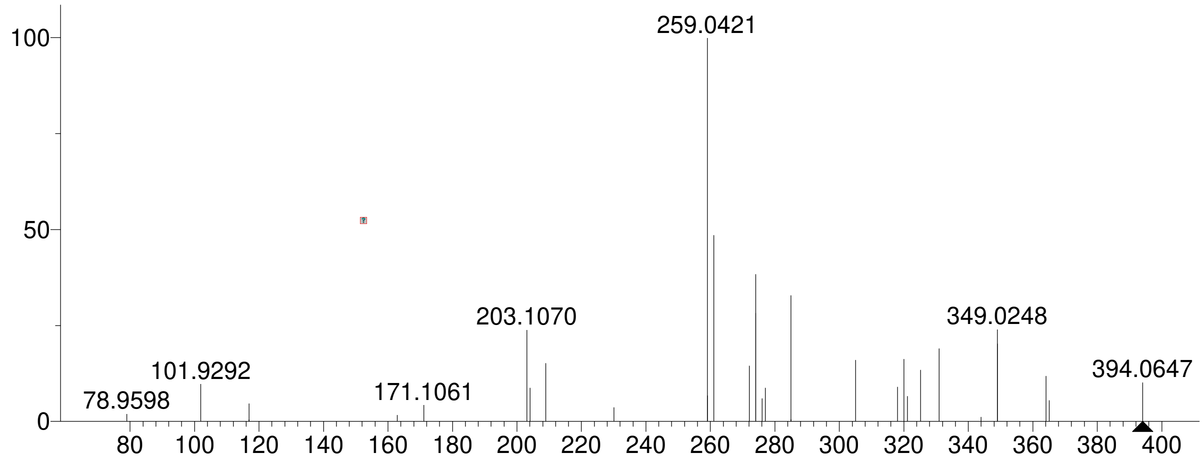

Hit 1 : rac-4-Sulfoxypropranolol [M+K]+ HCD 11V P=394.1
C16H21NO6S; MF: 13; RMF: 148; Prob 100.0%; CAS: 87075-33-0; Lib: nist_msms; ID: 443558.

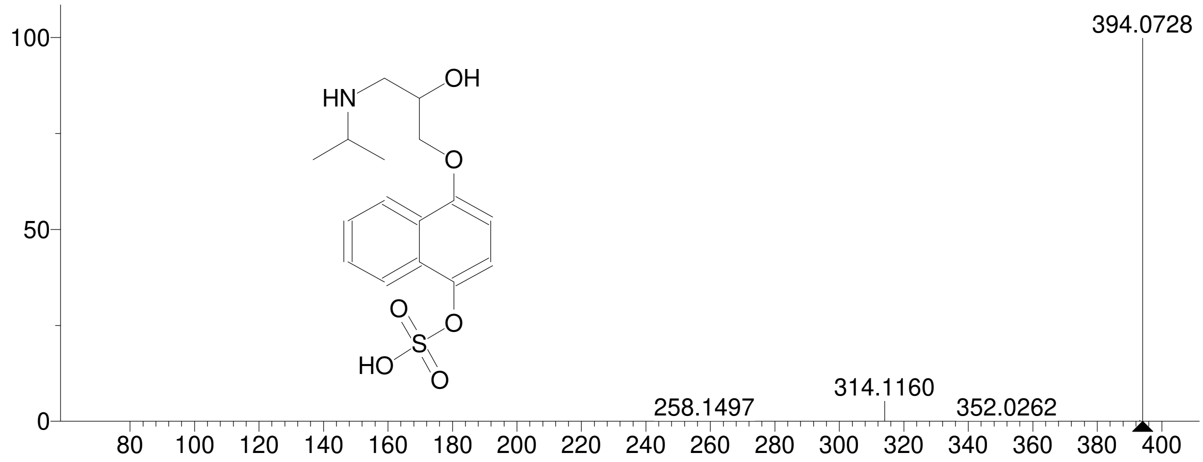

Unknown: Unknown (25.17_249.1489m/z)
Compound in Library Factor = -226

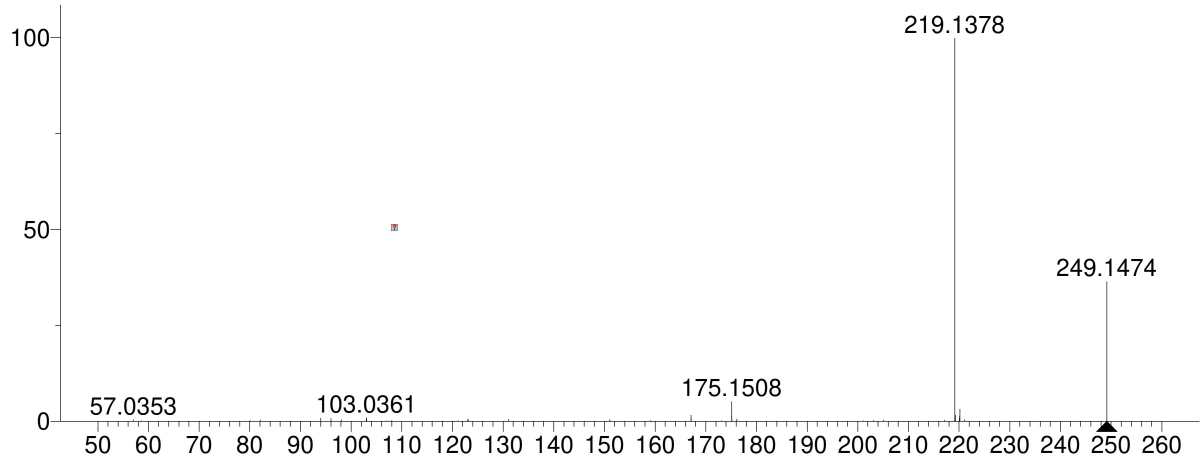

Hit 1 : Atractylenolide III [M+H]+ HCD 0V P=249.1
C15H20O3; MF: 328; RMF: 954; Prob 93.2%; CAS: 73030-71-4; Lib: nist_msms; ID: 459539.

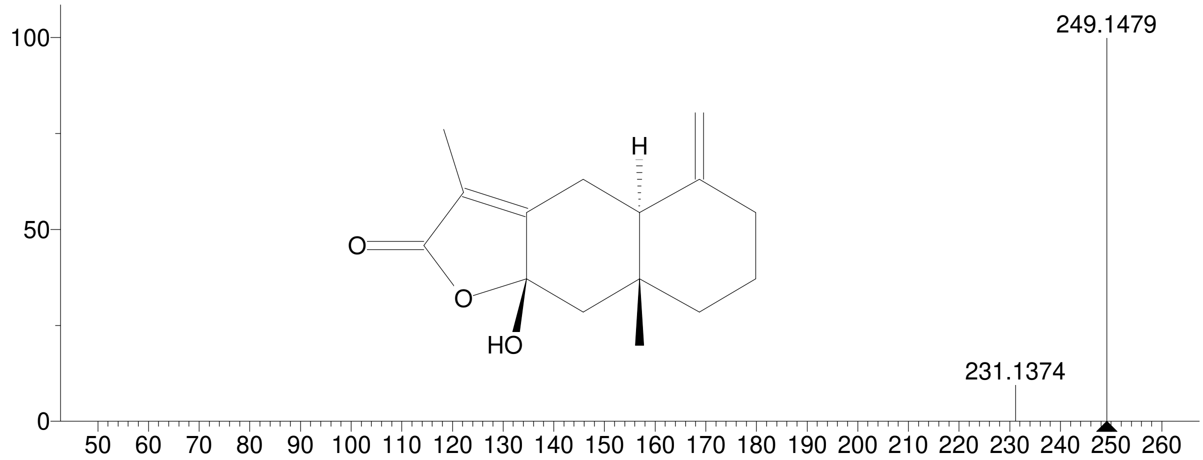

Hit 2 : Parthenolide [M+H]+ HCD 9V P=249.1
C15H20O3; MF: 66; RMF: 395; Prob 0.93%; CAS: 20554-84-1; Lib: nist_msms; ID: 225068.

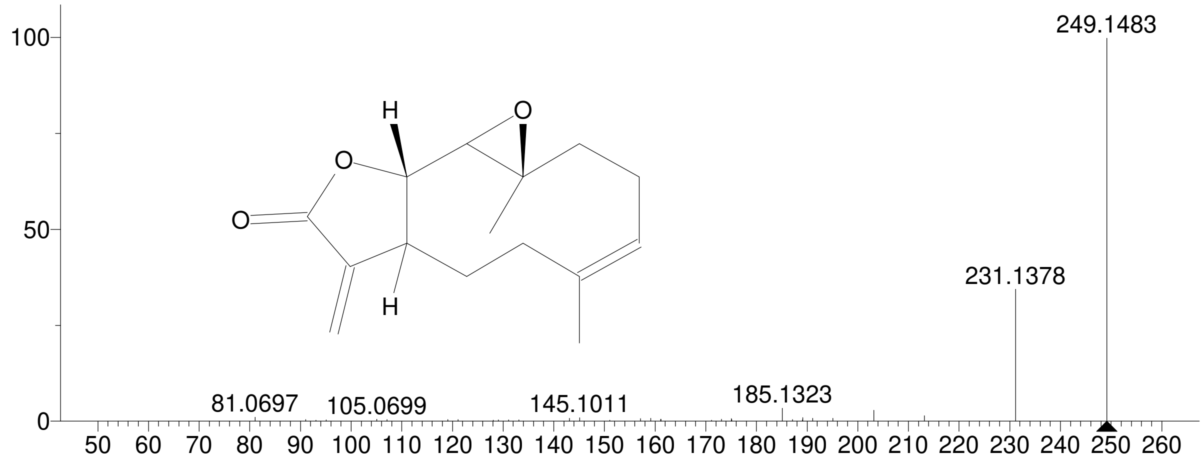

Hit 3 : 3,5-Di-tert-butyl-4-hydroxybenzoic acid [M-H]- HCD 22V P=249.1
C15H22O3; MF: 55; RMF: 481; Prob 0.63%; CAS: 1421-49-4; Lib: nist_msms; ID: 299282.

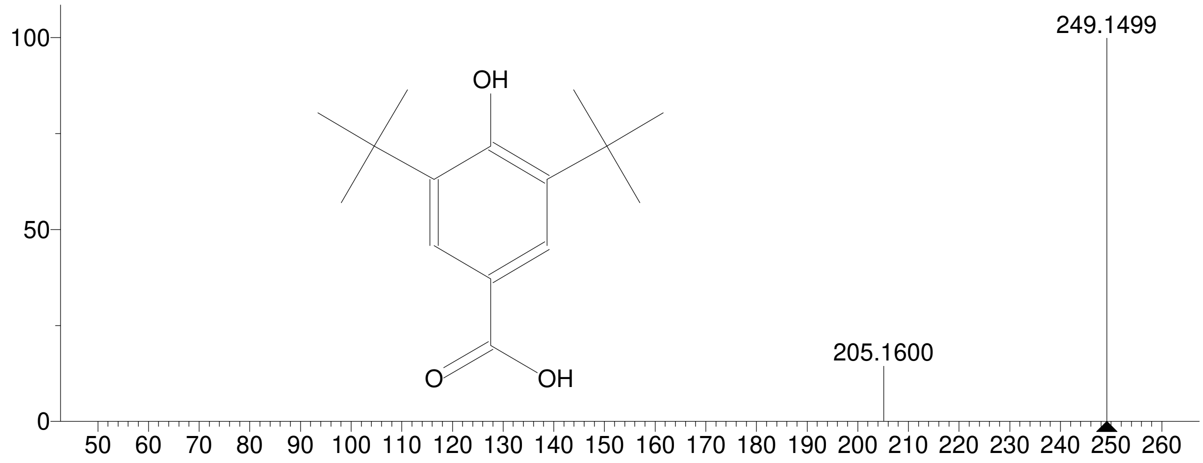

Hit 4 : Gemfibrozil [M-H]- HCD 3V P=249.1
C15H22O3; MF: 49; RMF: 450; Prob 0.50%; CAS: 25812-30-0; Lib: nist_msms; ID: 376181.

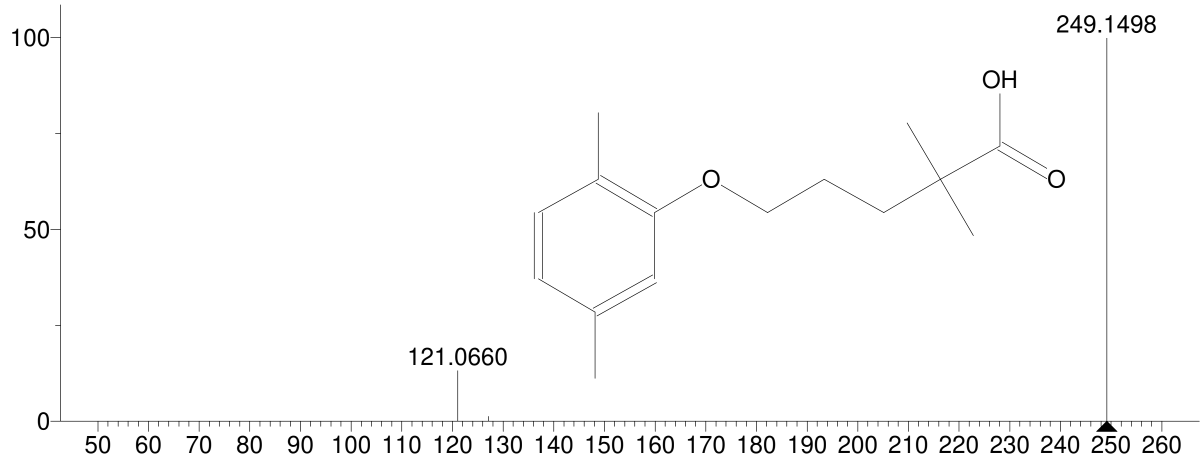

Hit 5 : Hydroxyvalerenic Acid [M-H]- 35HCD P=249.1
C15H22O3; MF: 36; RMF: 361; Prob 0.32%; Lib: mona-export-lc-ms-ms_negative_mode; ID: 17834.

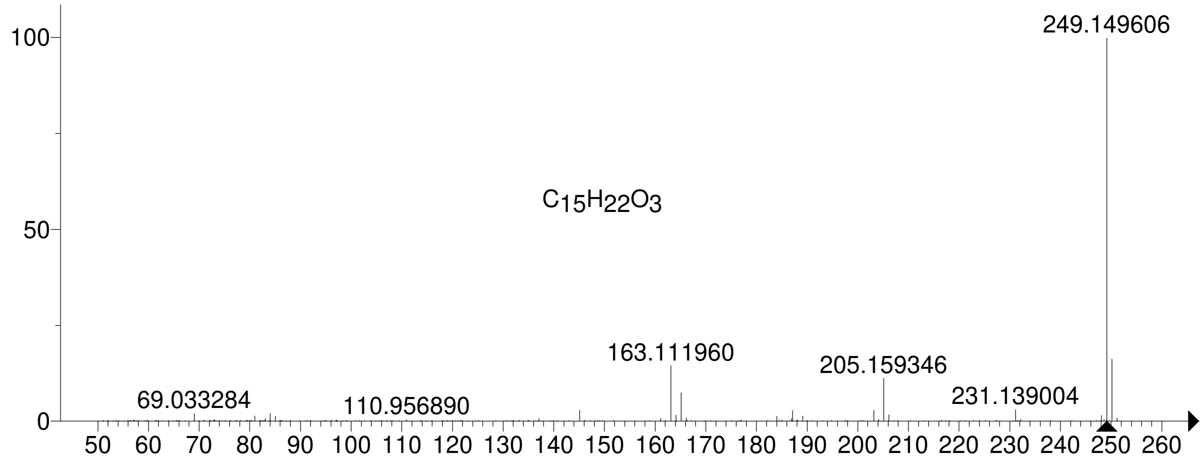

Unknown: Unknown (25.17_317.1350m/z)
Compound in Library Factor = 0

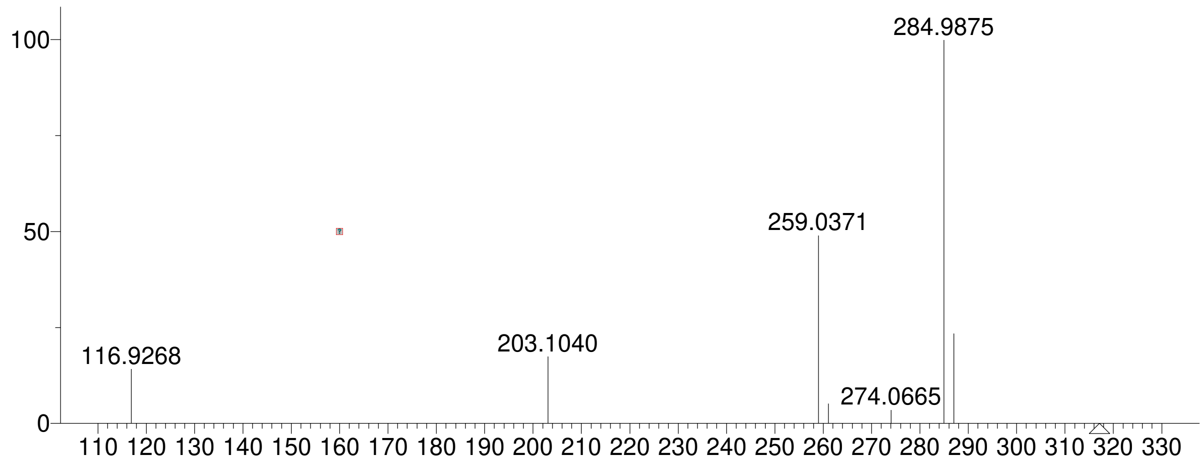

Hit 1 : NCGC00385420-01!(E)-1-(1,3,6,8-tetramethoxynaphthalen-2-yl)but-2-en-1-one [M+H]+ P=317.1
C18H20O5; MF: 0; RMF: 7; Prob 100.0%; Lib: mona-export-gnps; ID: 9360.

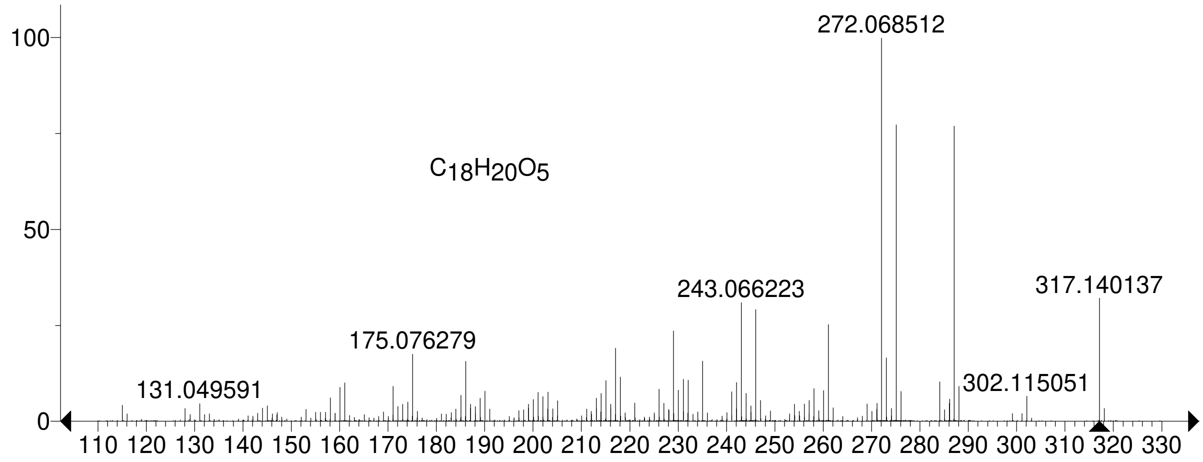

Unknown: Unknown (25.17_444.0520m/z)
Compound in Library Factor = 0

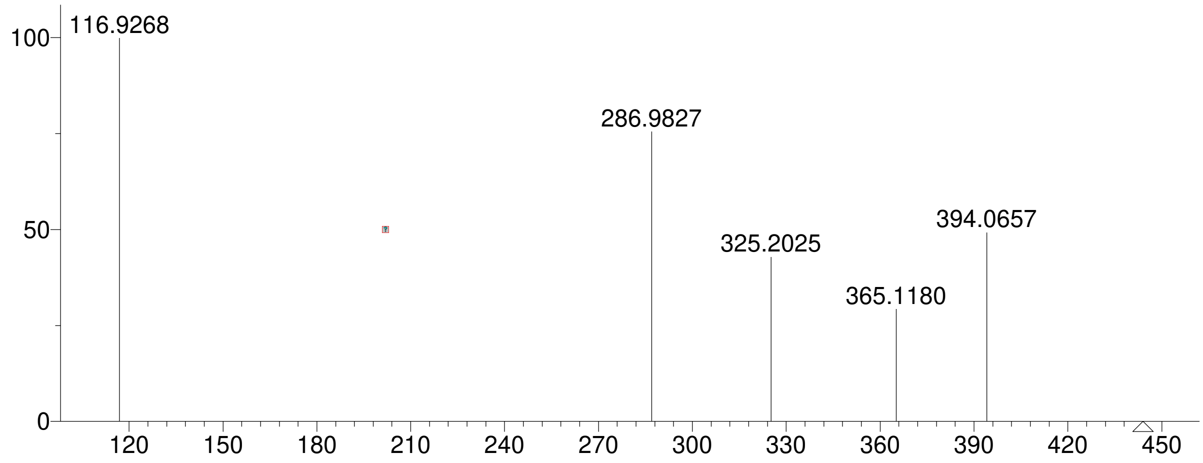

Unknown: Unknown (25.19_374.0136m/z)
Compound in Library Factor = 0

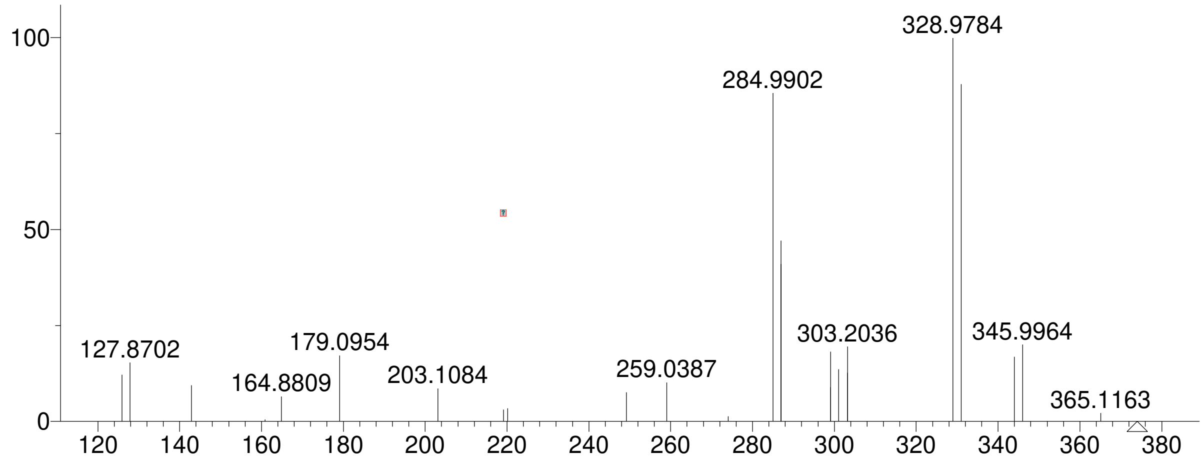

Hit 1 : Mast Cell Degranulating Peptide HR-1 [M+4H]4+ QQQ 15V P=374
C71H133N19O15; MF: 0; RMF: 14; Prob 100.0%; Lib: nist_msms2; ID: 10928.

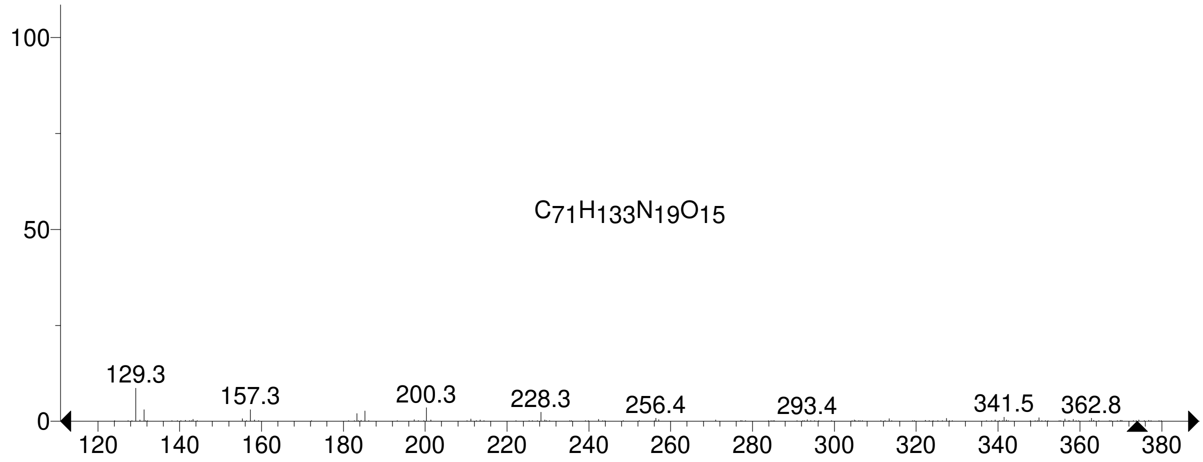

Unknown: Unknown (25.20_329.2324m/z)
Compound in Library Factor = -611

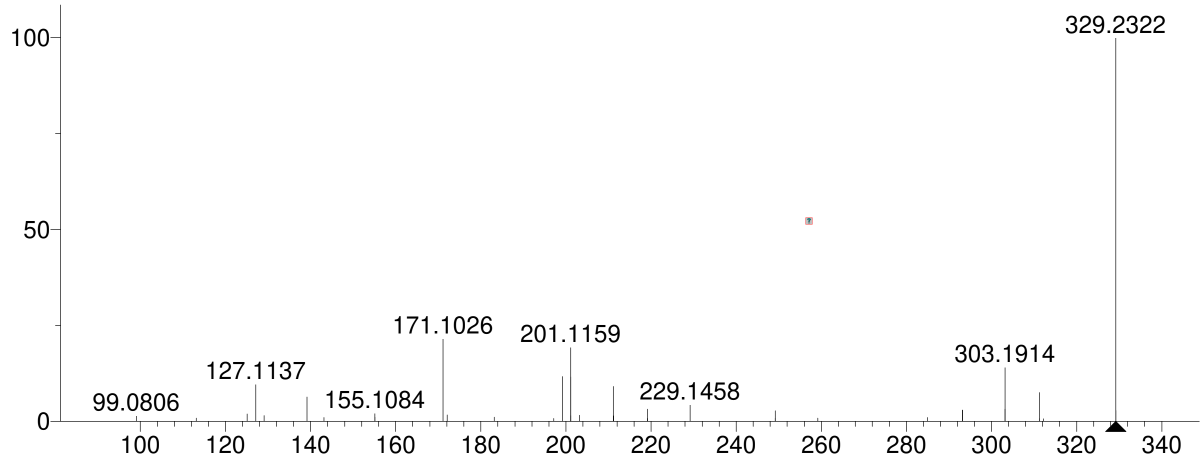

Hit 1 : FA 18:1+3O; PlaSMA ID-738 [M-H]- P=329.2
C18H34O5; MF: 395; RMF: 737; Prob 61.4%; Lib: biomsms-neg-plasma; ID: 444.

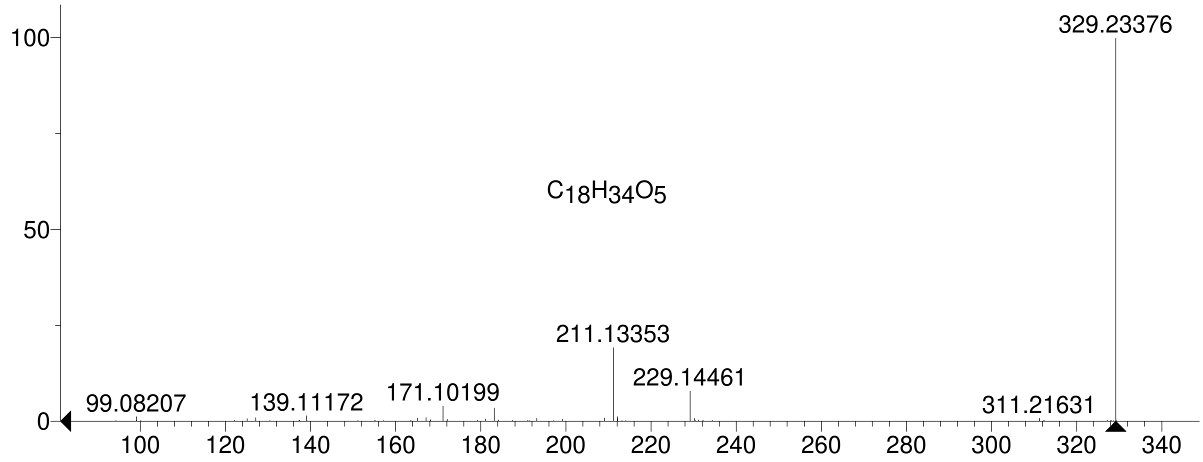

Hit 2 : FA 18:1+3O; PlaSMA ID-735 [M-H]- P=329.2
C18H34O5; MF: 379; RMF: 637; Prob 35.3%; Lib: biomsms-neg-plasma; ID: 443.

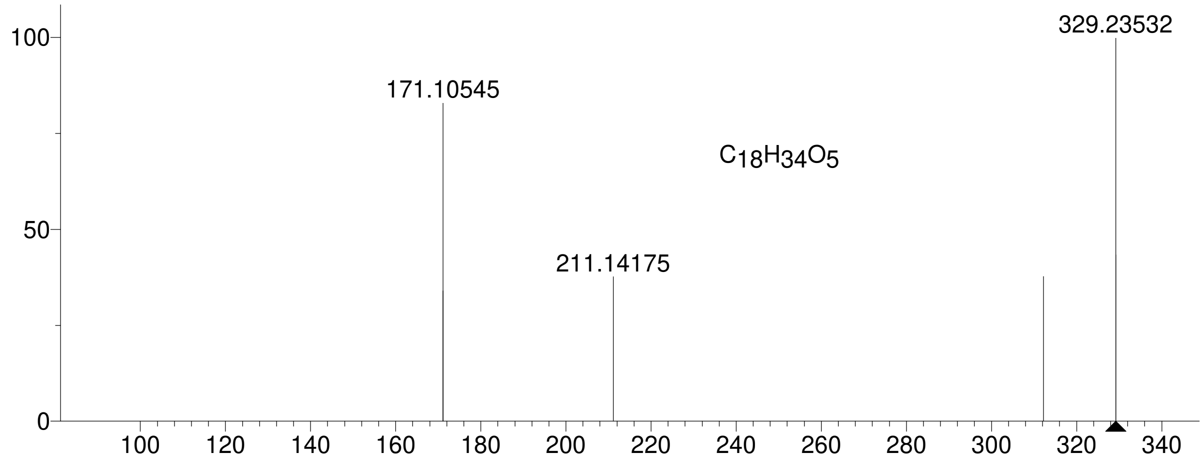

Hit 3 : FA 18:1+3O; PlaSMA ID-737 [M-H]- P=329.2
C18H34O5; MF: 288; RMF: 730; Prob 2.98%; Lib: biomsms-neg-plasma; ID: 448.

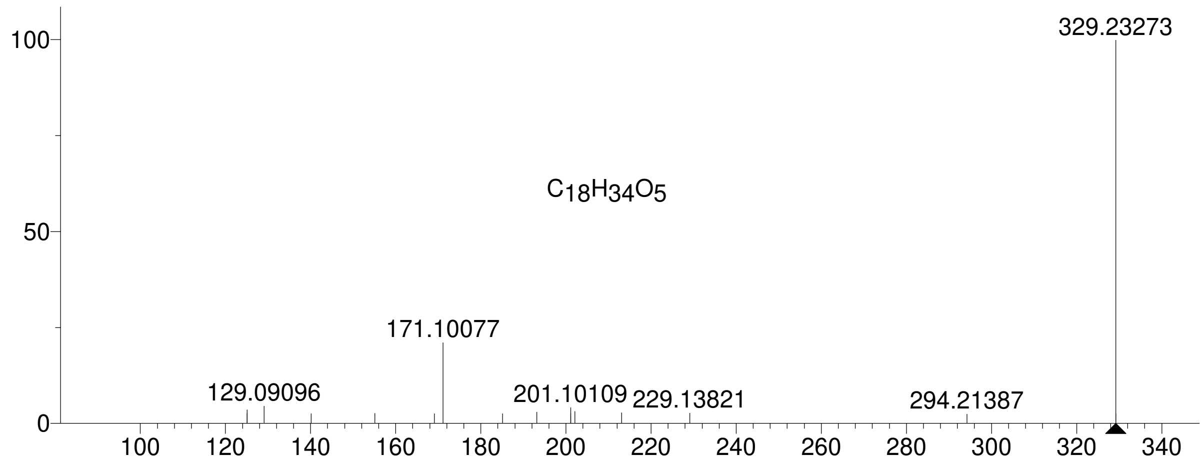

Hit 4 : 9,12,13-Trihydroxy-10(E)-octadecadienoic acid () [M-H]- P=329.2
C18H34O5; MF: 193; RMF: 666; Prob 0.24%; Lib: phytofrag2018_final_060219; ID: 129.

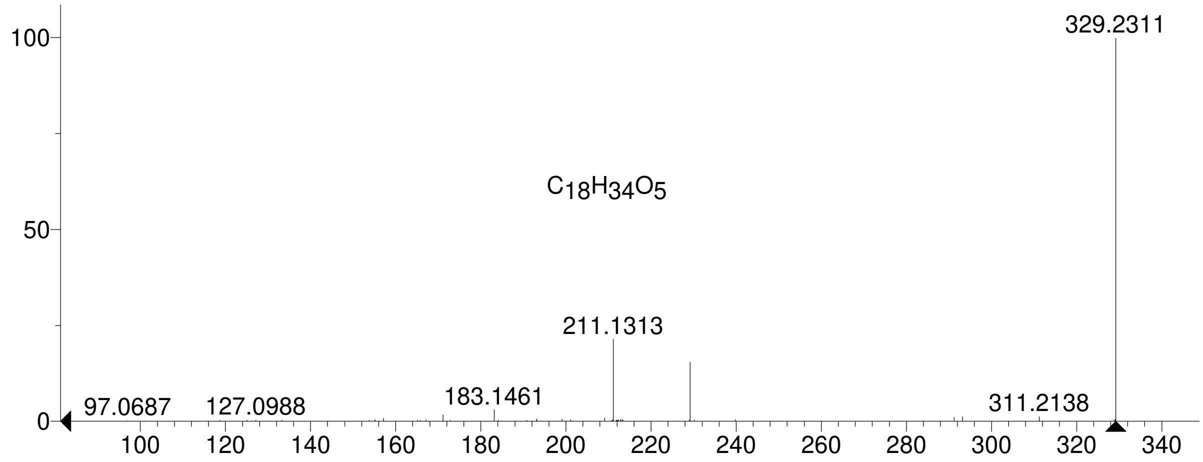

Hit 5 : FA 18:1+3O; PlaSMA ID-739 [M-H]- P=329.2
C18H34O5; MF: 128; RMF: 636; Prob 0.03%; Lib: biomsms-neg-plasma; ID: 446.

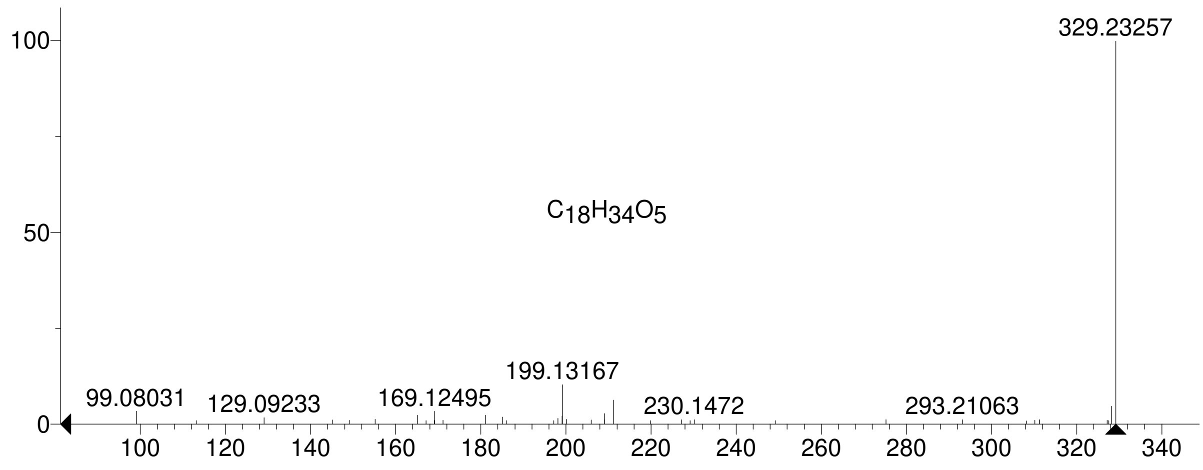

** Search Report Page 1 of 1 **

Unknown: Unknown (25.44_397.1540m/z)
Compound in Library Factor = -3273

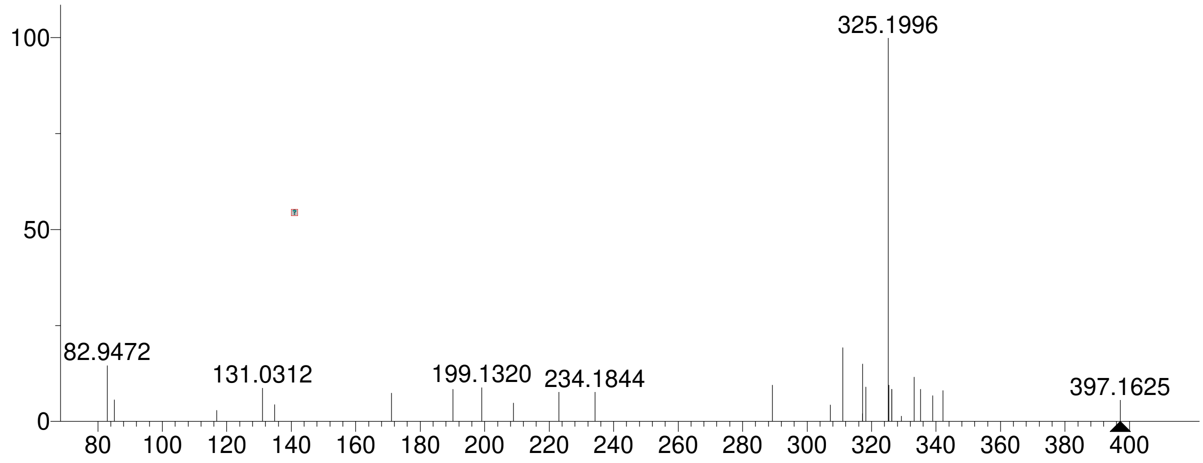

Hit 1 : 1H-Indole-3-carboxylic acid, 1-(5-hydroxypentyl)-, 8-quinolinyl ester [M+Na]+ HCD 19V P=397.2
C23H22N2O3; MF: 7; RMF: 128; Prob 51.0%; Lib: nist_msms; ID: 270095.

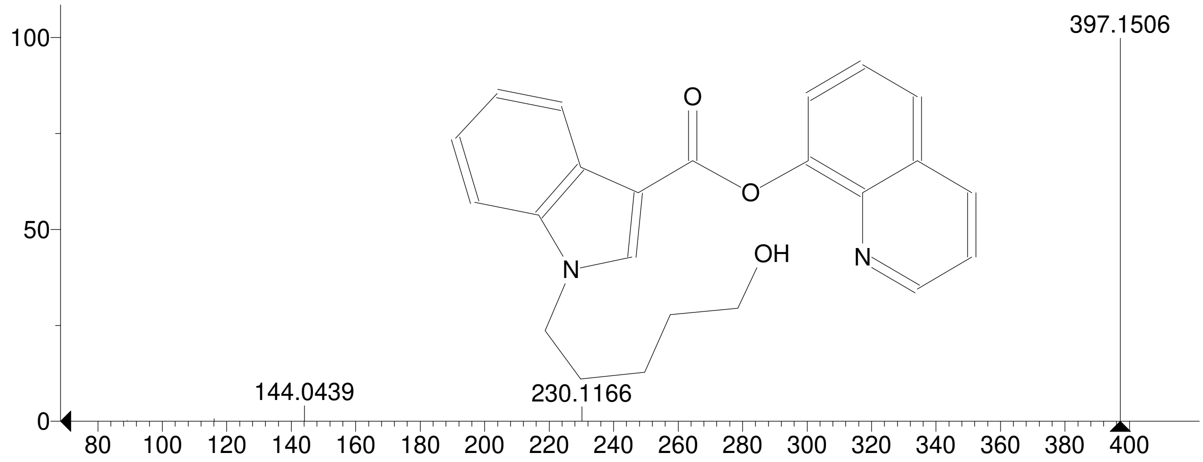

Hit 2 : 1H-Indole-3-carboxylic acid, 1-(4-hydroxypentyl)-, 8-quinolinyl ester [M+Na]+ HCD 19V P=397.2
C23H22N2O3; MF: 6; RMF: 125; Prob 49.0%; Lib: nist_msms; ID: 270048.

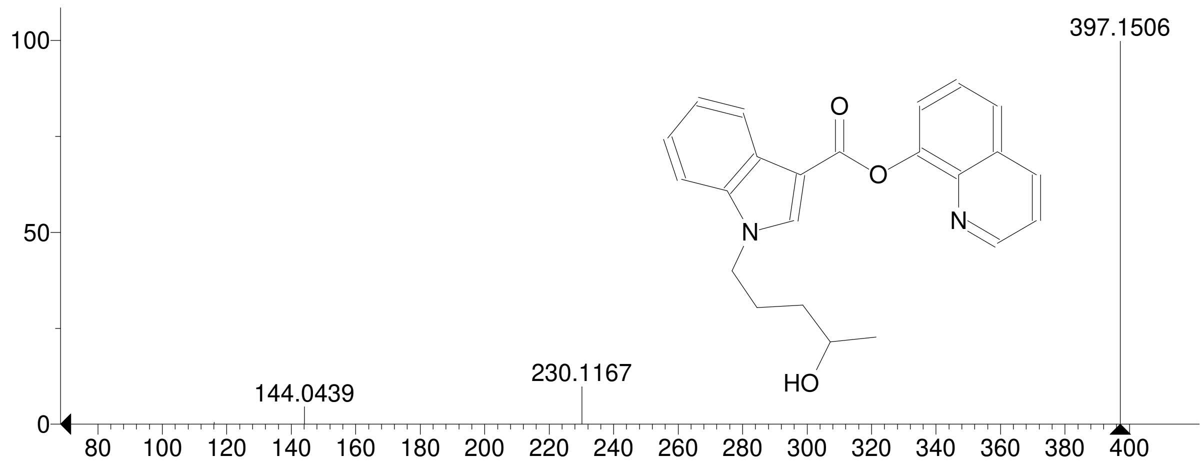

** Search Report Page 1 of 1 **

Unknown: Unknown (25.47_579.1037m/z)
Compound in Library Factor = 0

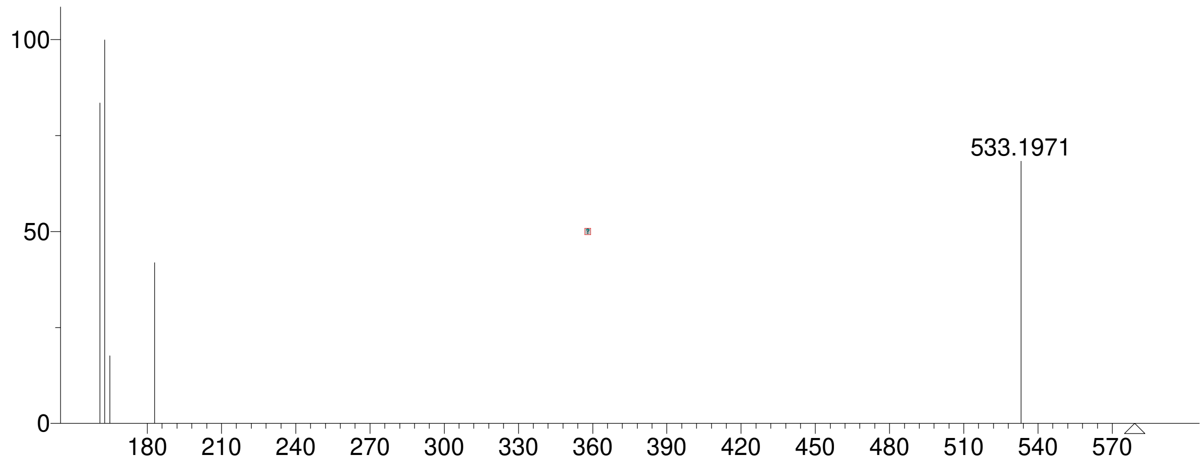

Unknown: Unknown (25.48_534.1043m/z)
Compound in Library Factor = 0

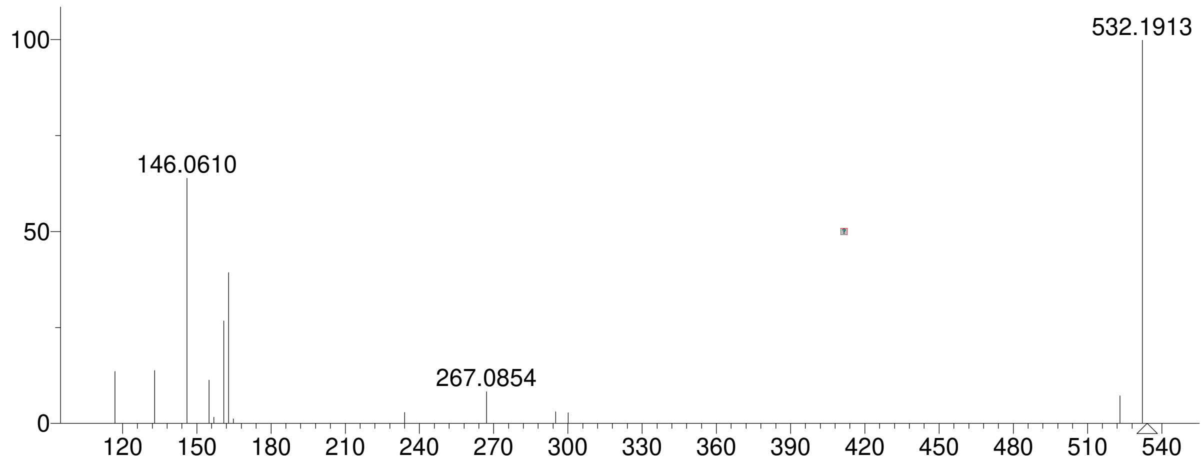

** Search Report Page 1 of 1 **

Unknown: Unknown (25.60_426.1896m/z)
Compound in Library Factor = 0

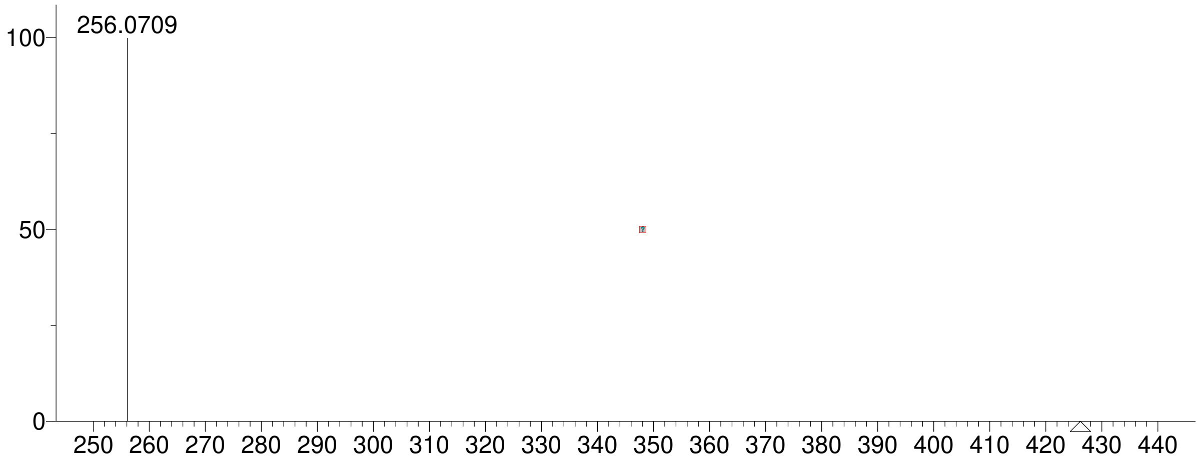

Unknown: Unknown (25.97_456.1623m/z)
Compound in Library Factor = 0

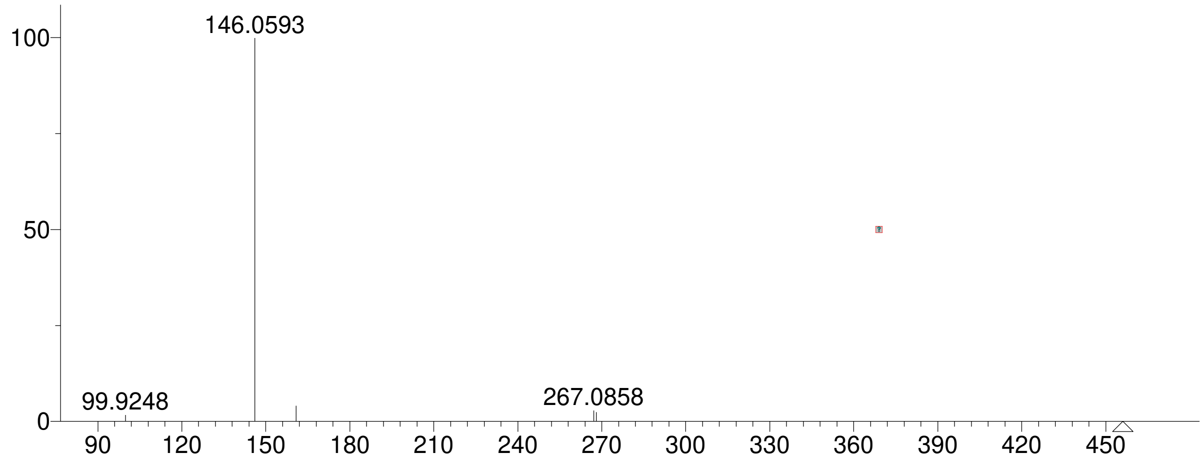

Hit 1 : Folinic acid [M+H-H2O]+ HCD 86V P=456.2
C20H23N7O7; MF: 0; RMF: 67; Prob 50.0%; CAS: 58-05-9; Lib: nist_msms; ID: 138161.

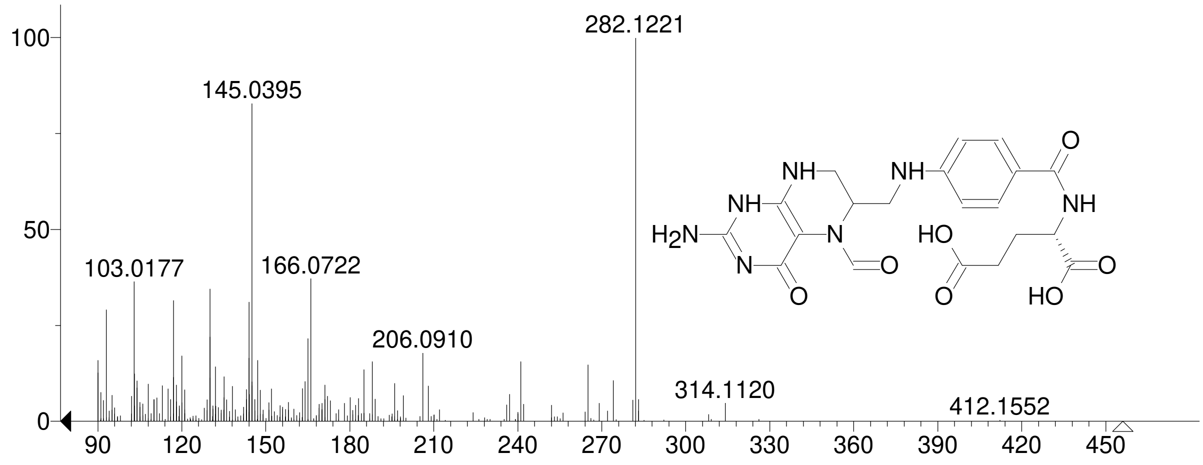

Hit 2 : Codeine-6-β-D-glucuronide [M-H-H2O]- HCD 72V P=456.2
C24H29NO9; MF: 0; RMF: 52; Prob 50.0%; CAS: 20736-11-2; Lib: nist_msms; ID: 322871.

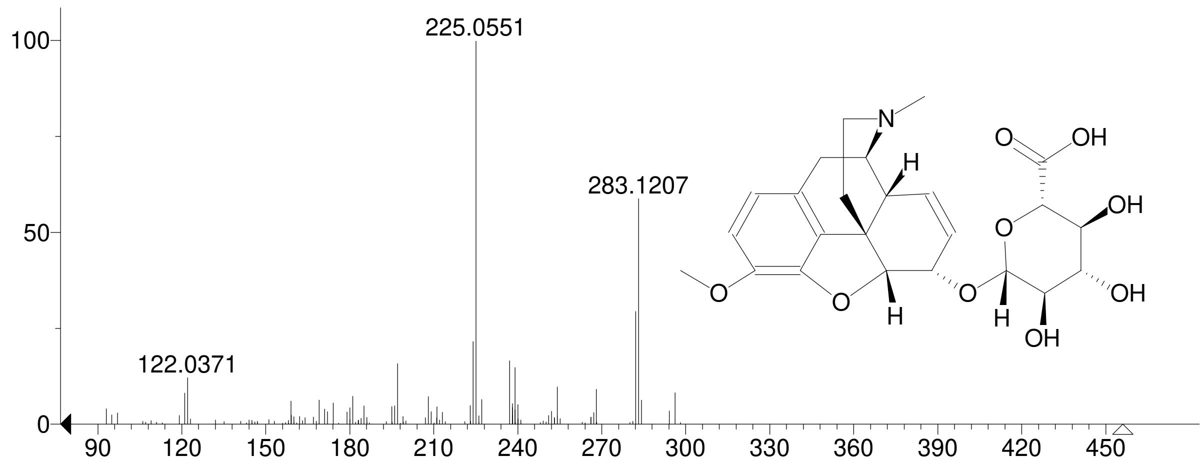

Unknown: Unknown (26.20_456.1626m/z)
Compound in Library Factor = -3273

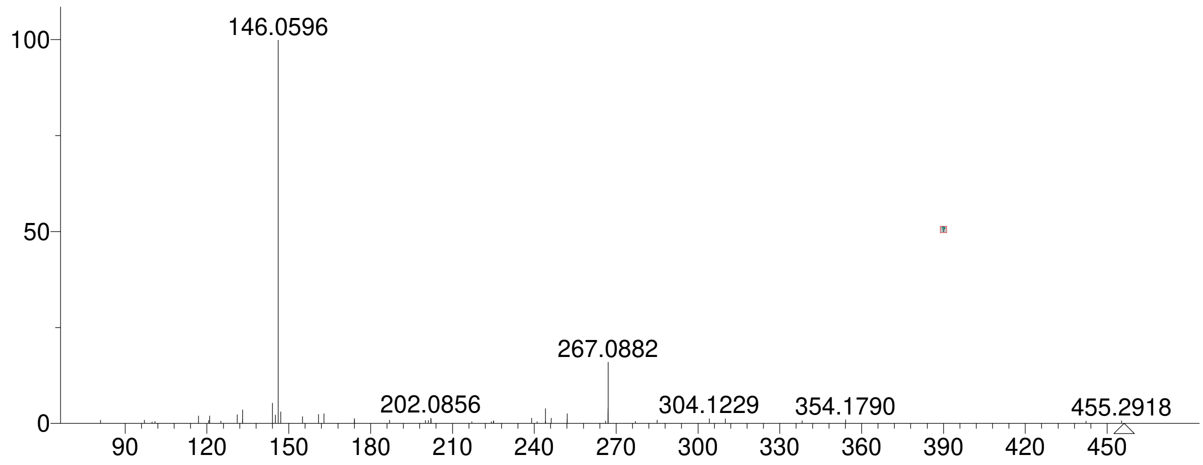

Hit 1 : Codeine-6-β-D-glucuronide [M-H-H2O]- HCD 72V P=456.2
C24H29NO9; MF: 6; RMF: 88; Prob 37.0%; CAS: 20736-11-2; Lib: nist_msms; ID: 322871.

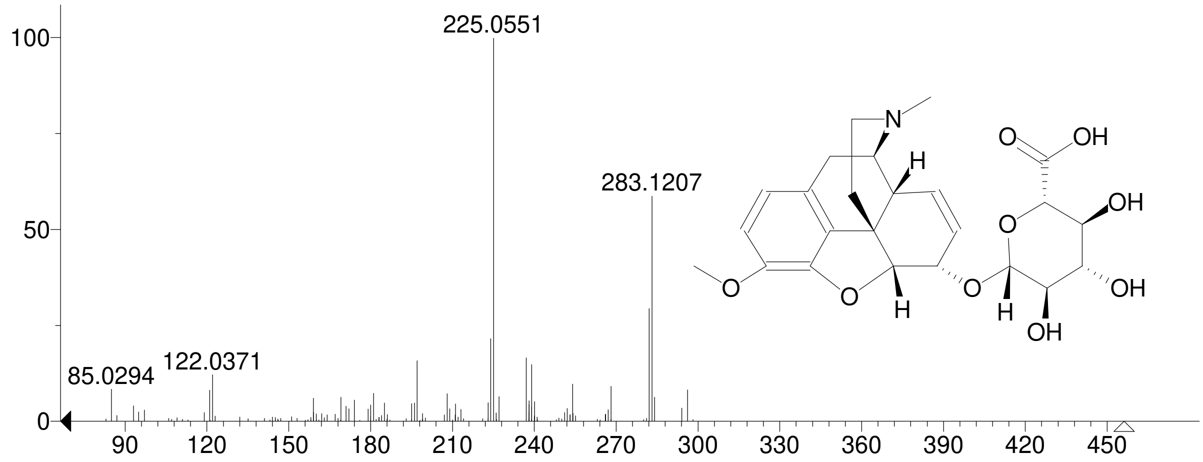

Hit 2 : Folinic acid [M+H-H2O]+ HCD 72V P=456.2
C20H23N7O7; MF: 4; RMF: 89; Prob 34.1%; CAS: 58-05-9; Lib: nist_msms; ID: 138160.

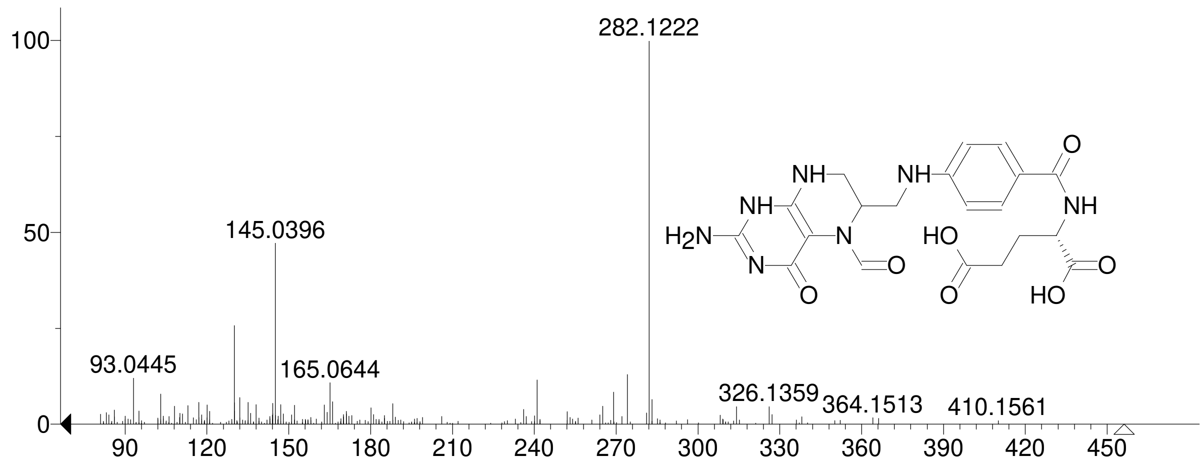

Hit 3 : Methopterine [M+H]+ HCD 82V P=456.2
C20H21N7O6; MF: 0; RMF: 16; Prob 28.8%; CAS: 2410-93-7; Lib: nist_msms; ID: 206196.

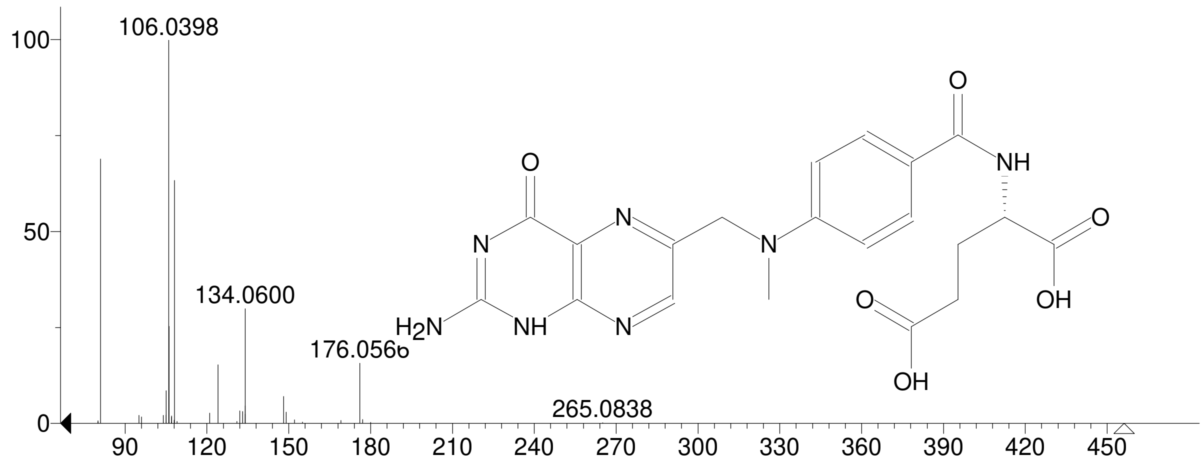

** Search Report Page 1 of 1 **

Unknown: Unknown (26.20_579.1033m/z)
Compound in Library Factor = 0

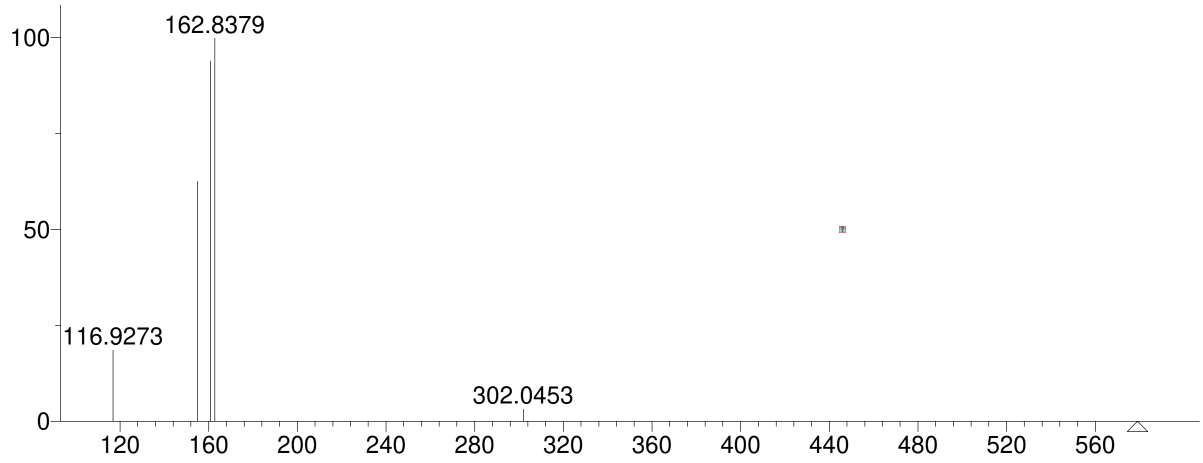

** Search Report Page 1 of 1 **

Unknown: Unknown (26.21_534.1052m/z)
Compound in Library Factor = 0

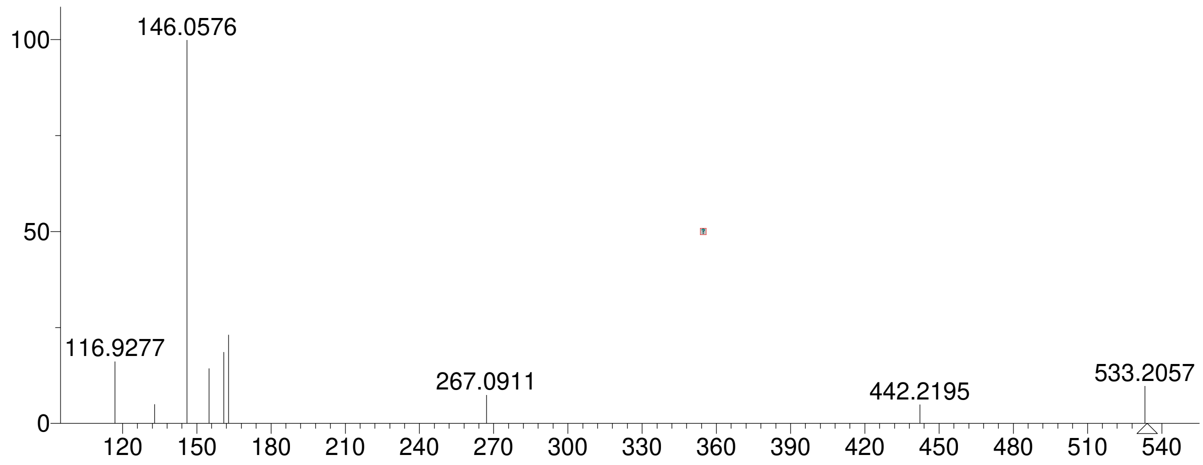

Hit 1 : 3'-α-Sialyl-N-acetyllactosamine [M+K]+=>552.1=>534.1 IT 35% P=534.1
C25H42N2O19; MF: 0; RMF: 21; Prob 50.0%; CAS: 81693-22-3; Lib: nist_msms; ID: 533666.

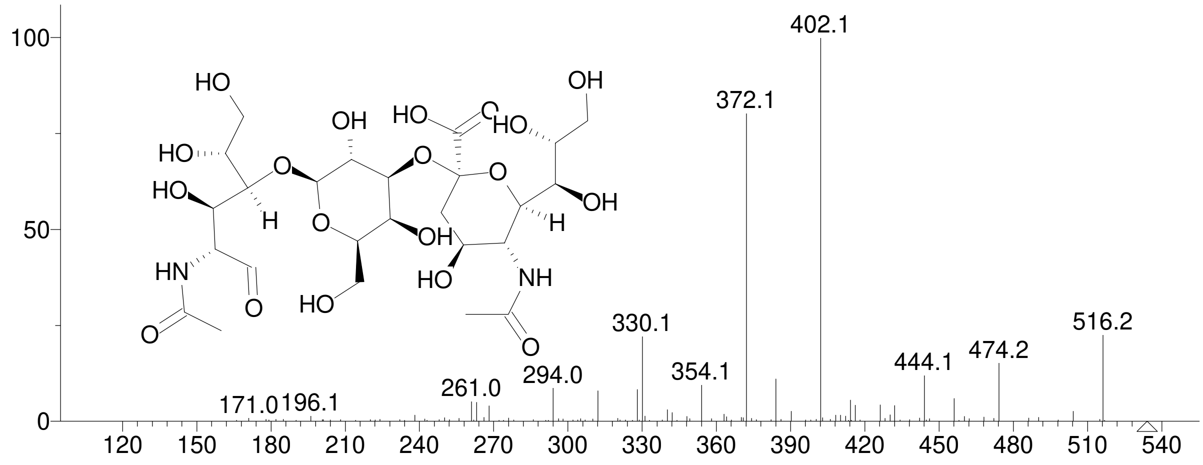

Hit 2 : 3'-Sialyllactose [M+K]+=>552.1=>534.1 IT 35% P=534.1
C23H39NO19; MF: 0; RMF: 10; Prob 50.0%; CAS: 35890-38-1; Lib: nist_msms; ID: 530573.

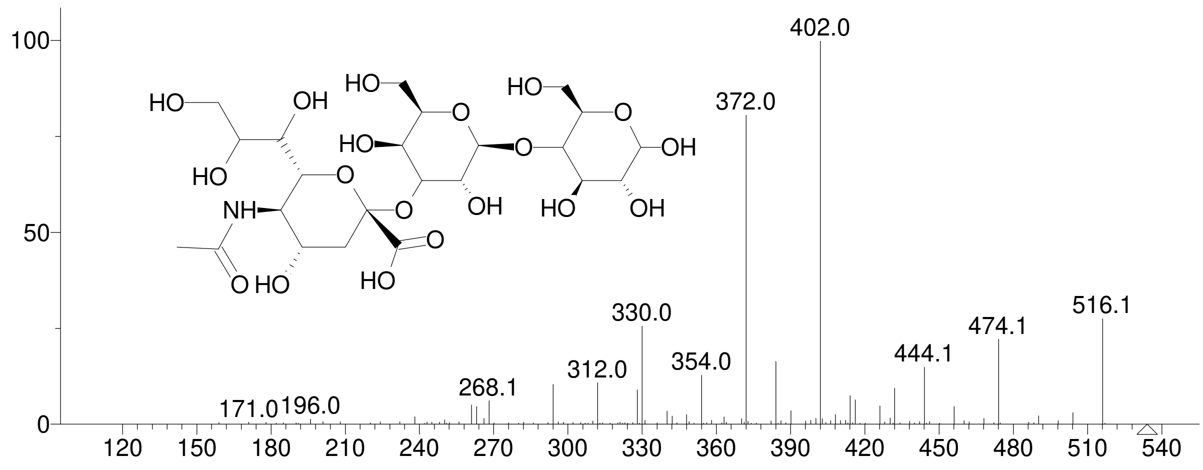

Unknown: Unknown (26.22_534.1060m/z)
Compound in Library Factor = 0

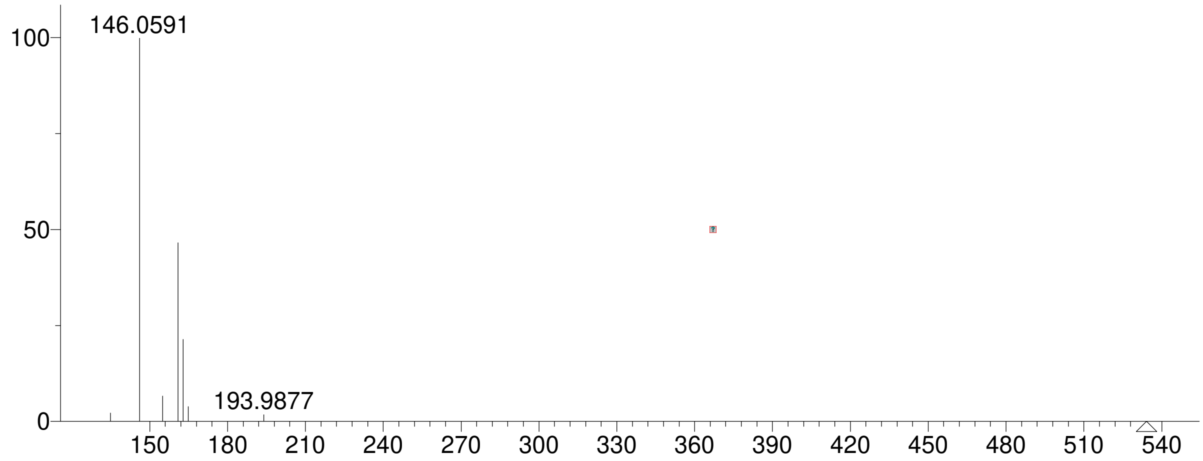

Unknown: Unknown (26.29_329.2326m/z)
Compound in Library Factor = -898

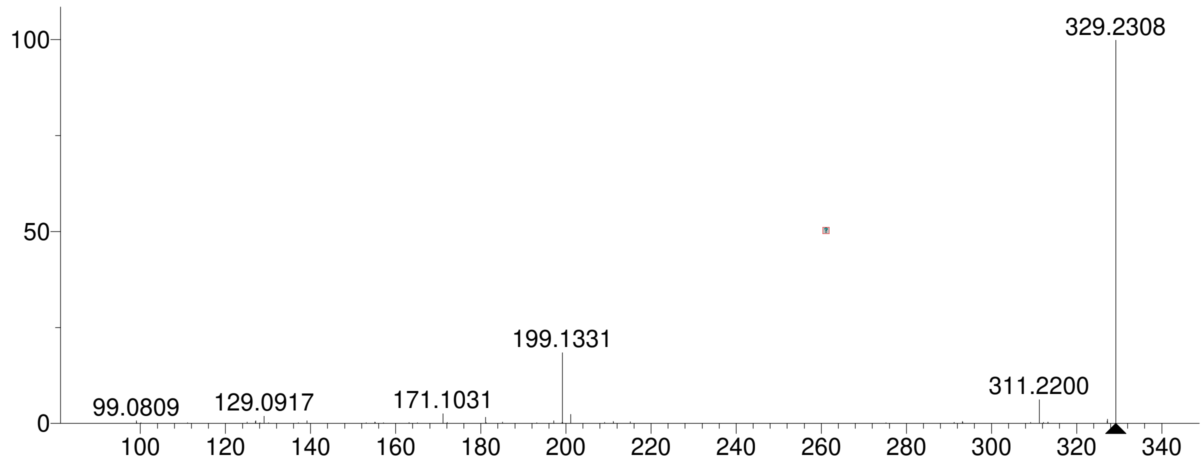

Hit 1 : FA 18:1+3O; PlaSMA ID-738 [M-H]- P=329.2
C18H34O5; MF: 342; RMF: 799; Prob 47.0%; Lib: biomsms-neg-plasma; ID: 444.

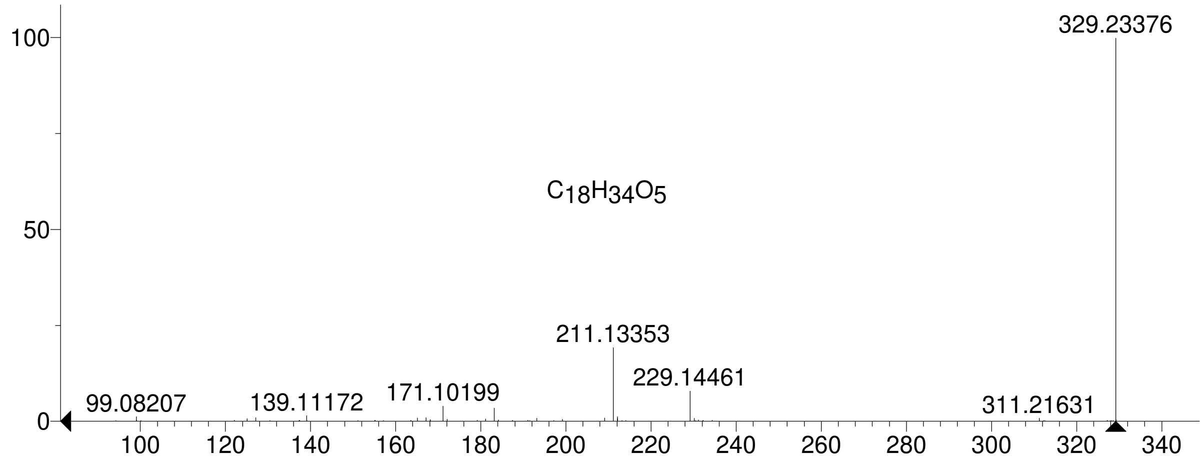

Hit 2 : FA 18:1+3O; PlaSMA ID-739 [M-H]- P=329.2
C18H34O5; MF: 338; RMF: 832; Prob 39.7%; Lib: biomsms-neg-plasma; ID: 446.

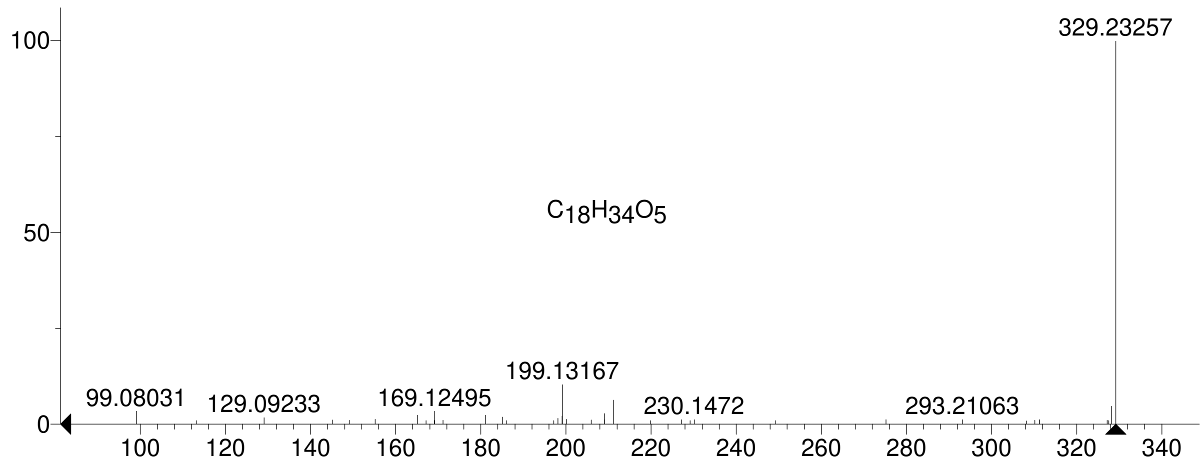

Hit 3 : FA 18:1+3O; PlaSMA ID-742 [M-H]- P=329.2
C18H34O5; MF: 309; RMF: 790; Prob 11.3%; Lib: biomsms-neg-plasma; ID: 445.

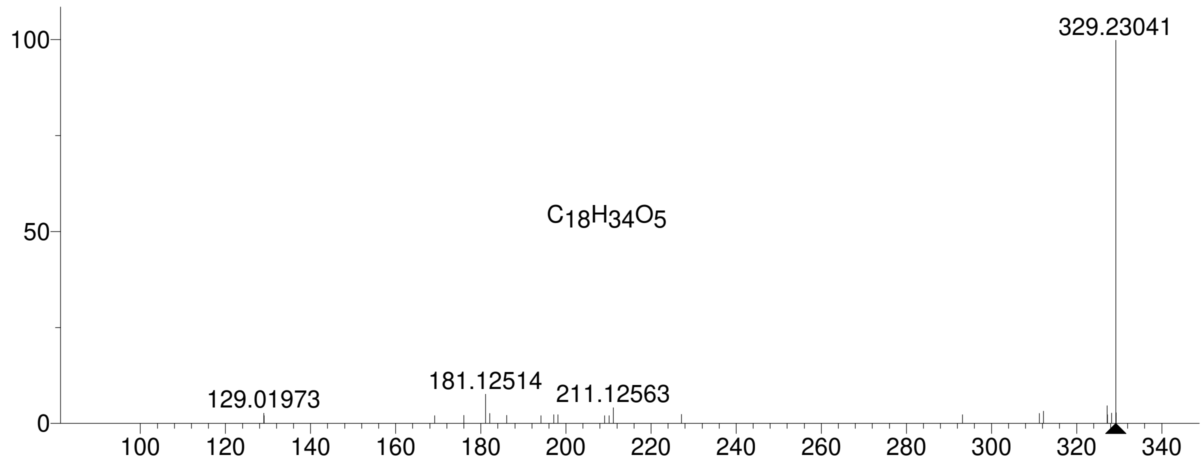

Hit 4 : FA 18:1+3O; PlaSMA ID-737 [M-H]- P=329.2
C18H34O5; MF: 236; RMF: 761; Prob 1.53%; Lib: biomsms-neg-plasma; ID: 448.

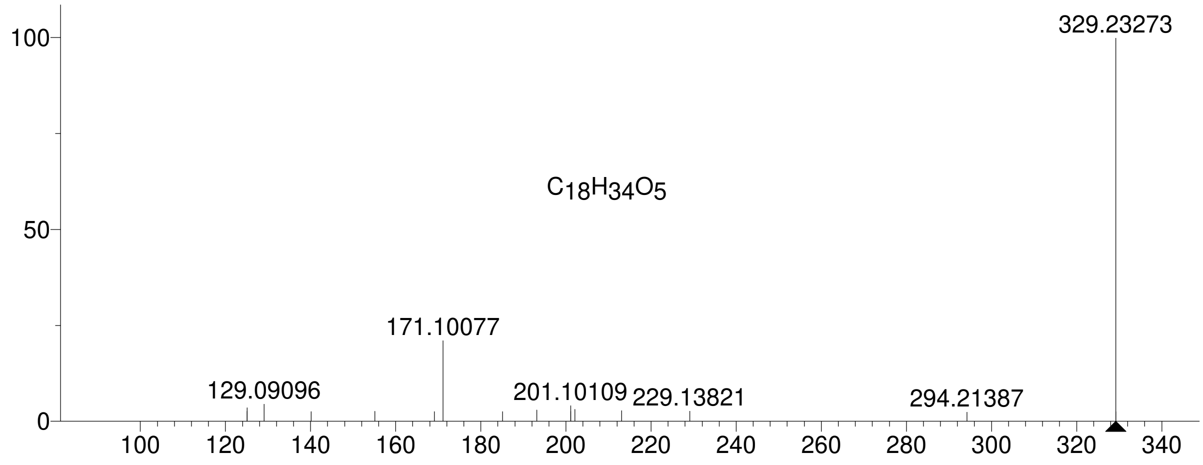

Hit 5 : FA 18:1+3O; PlaSMA ID-740 [M-H]- P=329.2
C18H34O5; MF: 187; RMF: 779; Prob 0.31%; Lib: biomsms-neg-plasma; ID: 447.

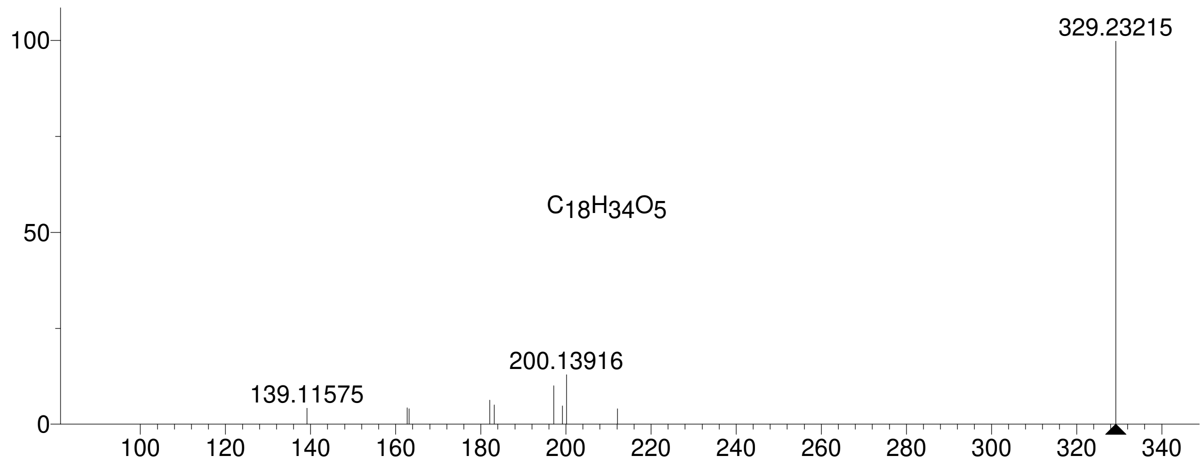

Unknown: Unknown (26.29_428.1493m/z)
Compound in Library Factor = 0

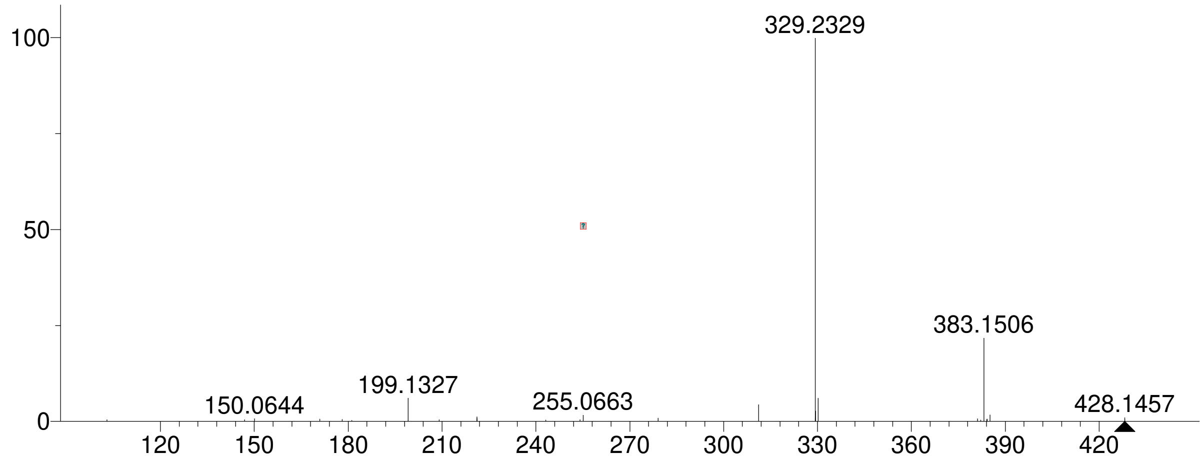

Hit 1 : Roquefortine C [M+K]+ 35HCD P=428.1
C22H23N5O2; MF: 0; RMF: 23; Prob 100.0%; Lib: mona-export-vf-npl_qexactive; ID: 16408.

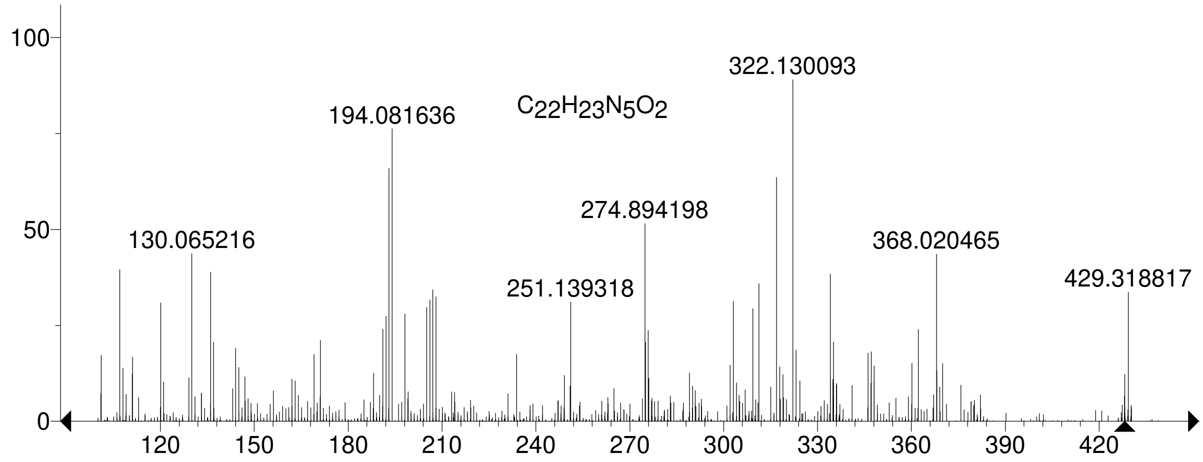

Unknown: Unknown (26.38_353.2312m/z)
Compound in Library Factor = -1249

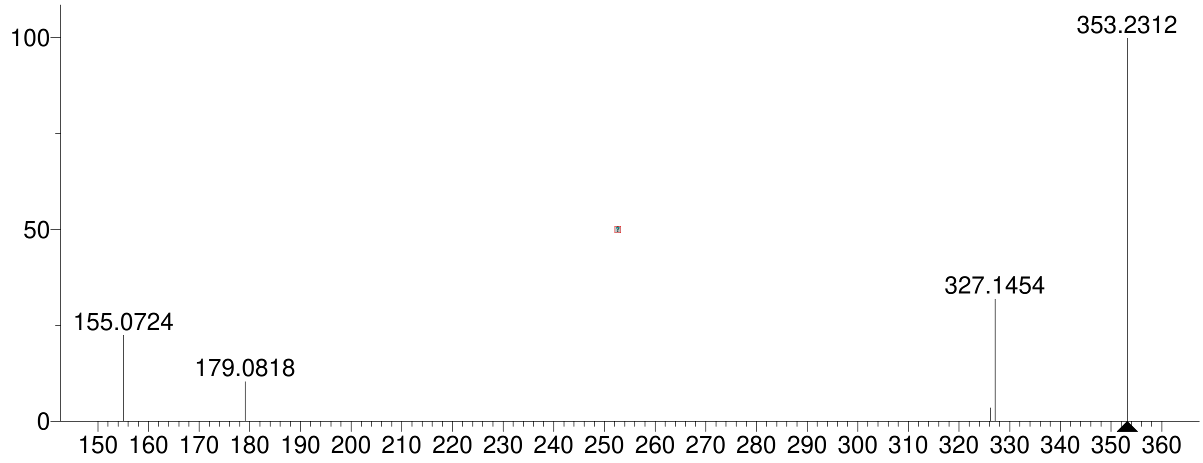

Hit 1 : 6-Ketoprostaglandin F1α [M+H-H2O]+ HCD 1V P=353.2
C20H34O6; MF: 232; RMF: 848; Prob 37.8%; CAS: 58962-34-8; Lib: nist_msms; ID: 166451.

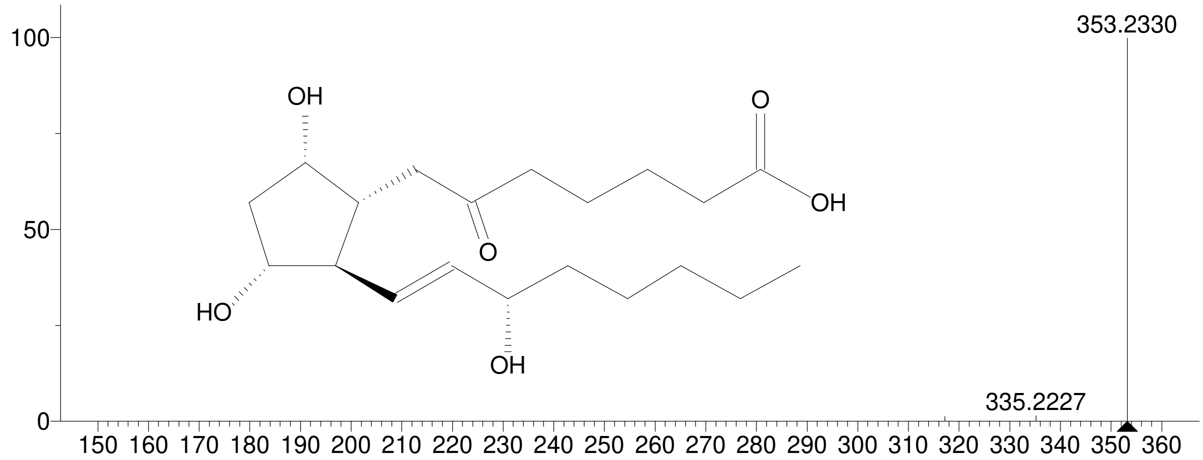

Hit 2 : Prostaglandin I2 [M+H]+ HCD 1V P=353.2
C20H32O5; MF: 230; RMF: 848; Prob 34.9%; CAS: 35121-78-9; Lib: nist_msms; ID: 166889.

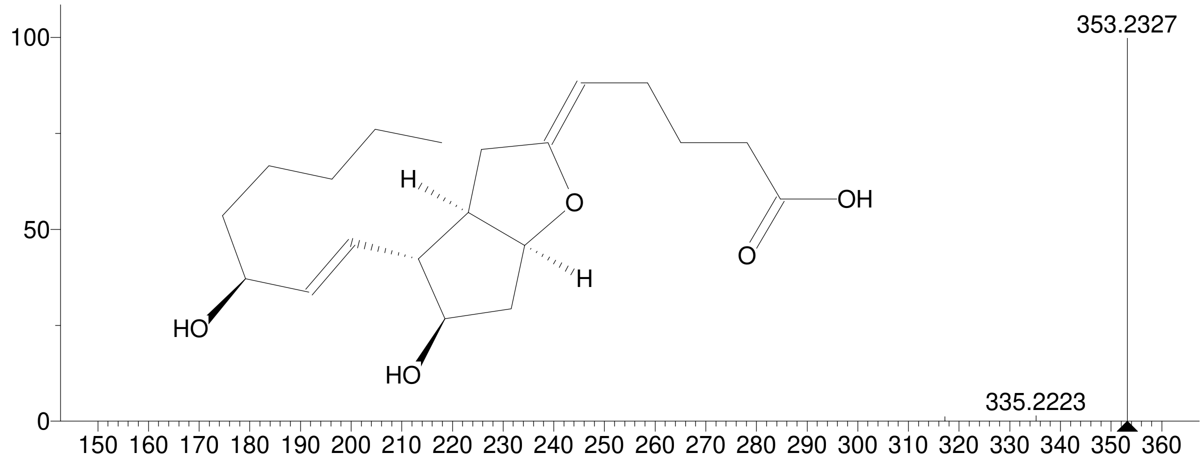

Hit 3 : 9S,11R-Dihydroxy-15-oxo-13E-prostenoic acid [M-H]- HCD 3V P=353.2
C20H34O5; MF: 208; RMF: 841; Prob 13.8%; CAS: 21562-58-3; Lib: nist_msms; ID: 155077.

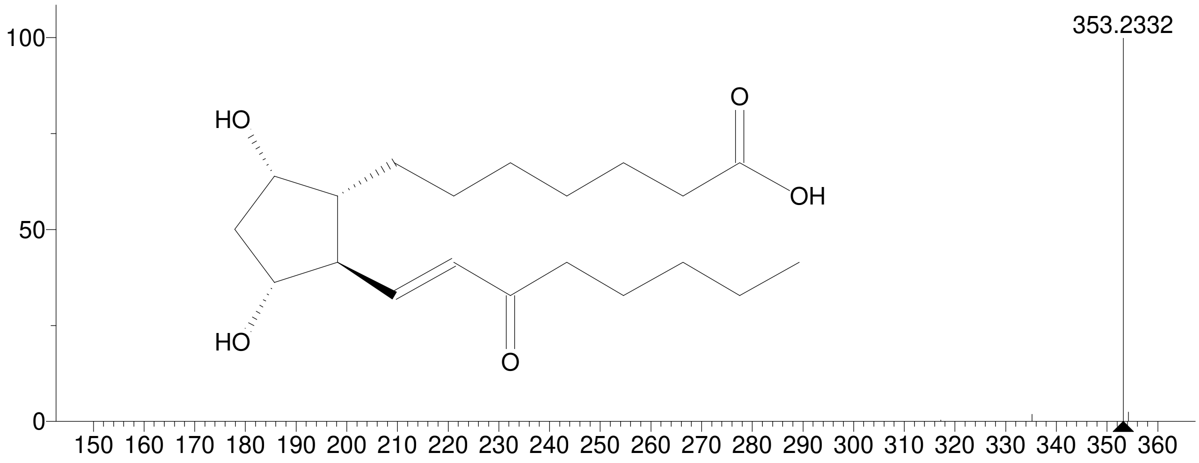

Hit 4 : 15-Ketoprostaglandin E1 [M+H]+ HCD 1V P=353.2
C20H32O5; MF: 175; RMF: 814; Prob 3.61%; CAS: 22973-19-9; Lib: nist_msms; ID: 169226.

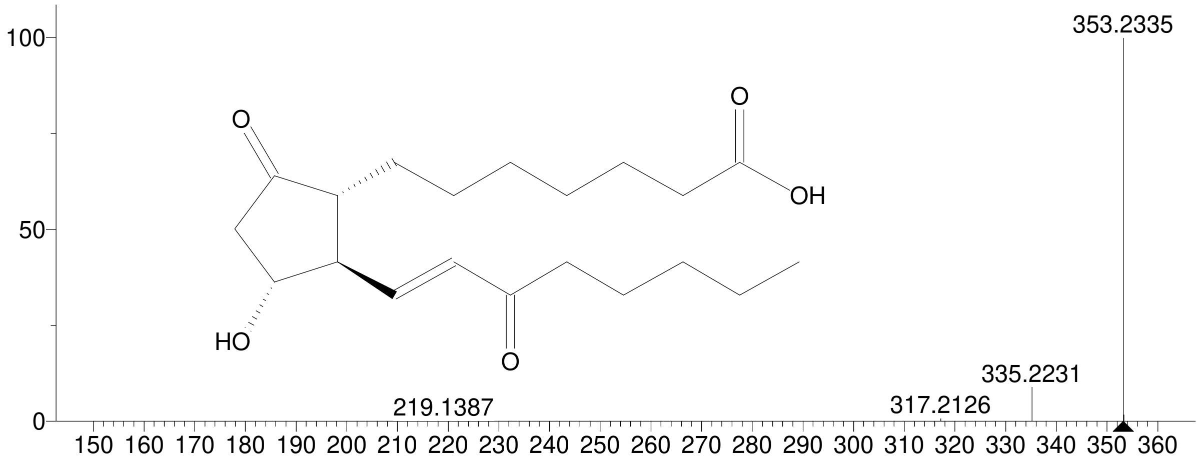

Hit 5 : Prostaglandin E1 [M-H]- HCD 7V P=353.2
C20H34O5; MF: 162; RMF: 799; Prob 2.33%; CAS: 745-65-3; Lib: nist_msms; ID: 320381.

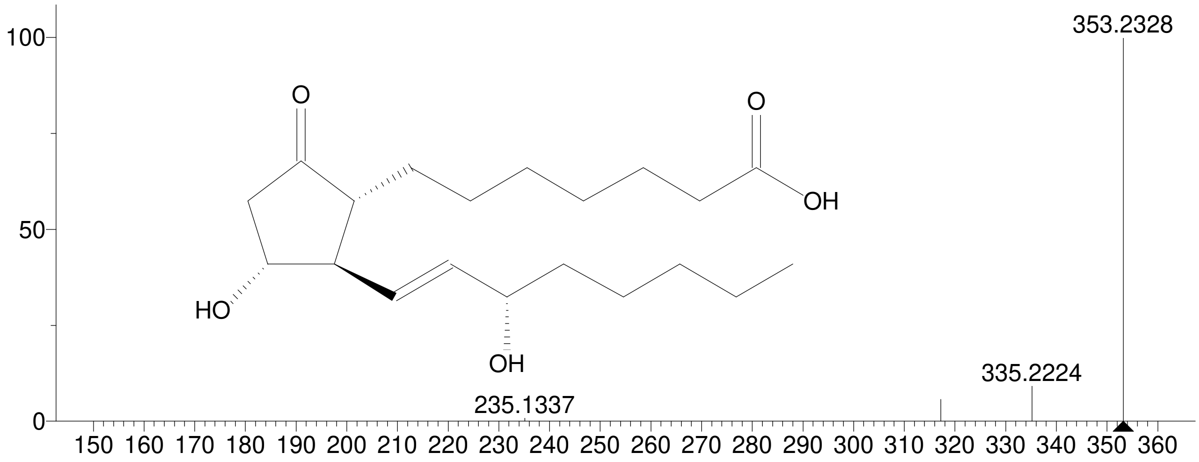

Unknown: Unknown (26.44_417.2629m/z)
Compound in Library Factor = -374

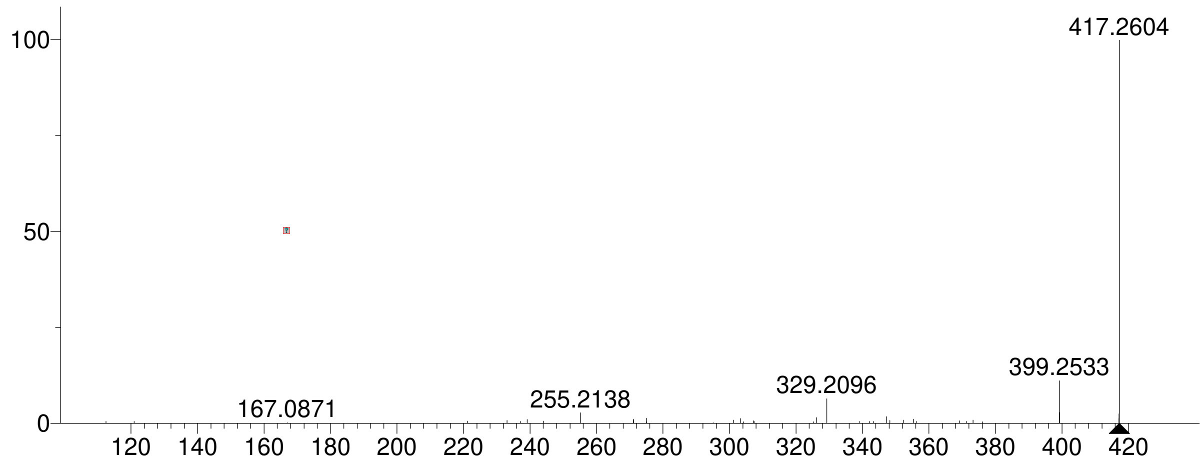

Hit 1 : Prostaglandin E2 isopropyl ester [M+Na]+ QTOF 17V P=417.3
C23H38O5; MF: 286; RMF: 899; Prob 90.2%; CAS: 71845-66-4; Lib: nist_msms; ID: 62065.

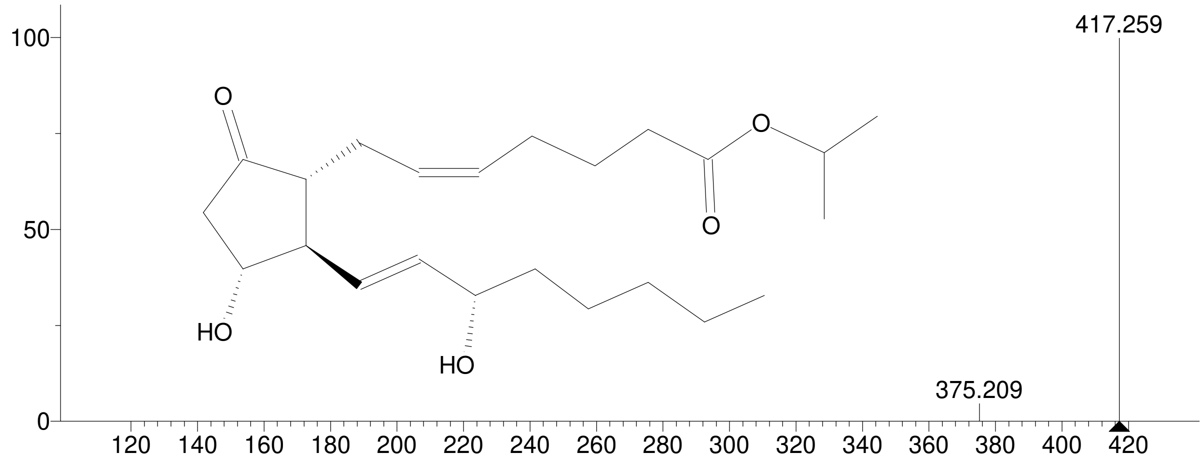

Hit 2 : Manoalide [M+H]+ HCD 12V P=417.3
C25H36O5; MF: 176; RMF: 852; Prob 5.77%; CAS: 75088-80-1; Lib: nist_msms; ID: 362740.

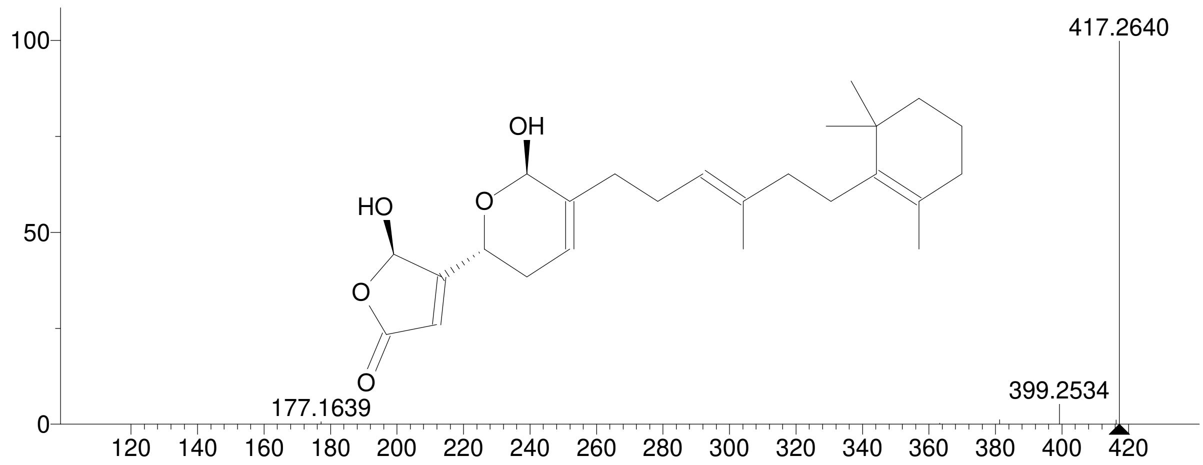

Hit 3 : Alfentanyl [M+H]+ HCD 12V P=417.3
C21H32N6O3; MF: 165; RMF: 798; Prob 3.95%; CAS: 71195-58-9; Lib: nist_msms; ID: 322110.

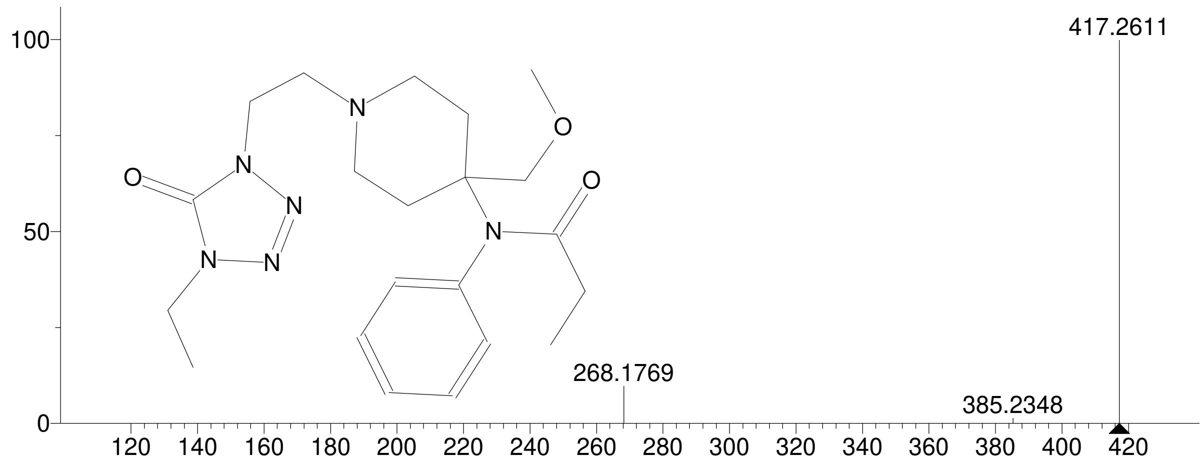

Hit 4 : NCGC00384505-01_C25H36O5_2-Butenoic acid, 2-methyl-, (4R,6R)-3,3,7,9-tetramethyl-6-(3-methyl-1-ox
C25H36O5; MF: 0; RMF: 3; Prob 0.09%; Lib: mona-export-gnps; ID: 11433.

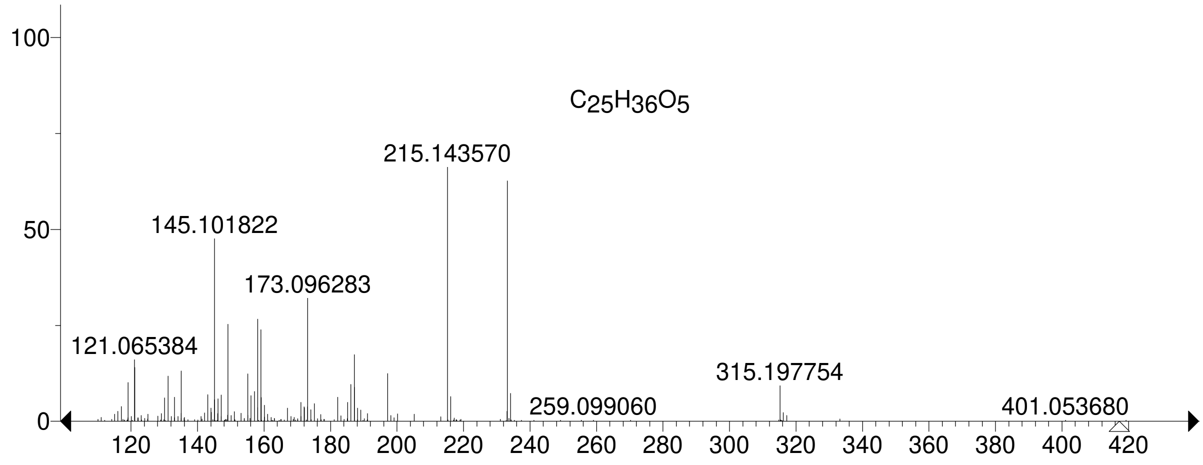

Unknown: Unknown (26.47_329.2326m/z)
Compound in Library Factor = -1249

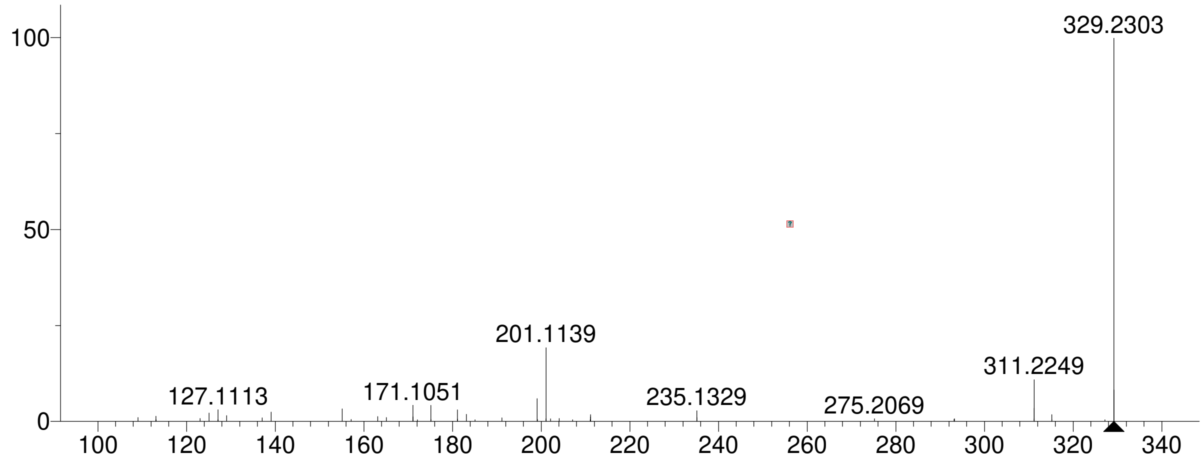

Hit 1 : FA 18:1+3O; PlaSMA ID-737 [M-H]- P=329.2
C18H34O5; MF: 233; RMF: 706; Prob 73.8%; Lib: biomsms-neg-plasma; ID: 448.

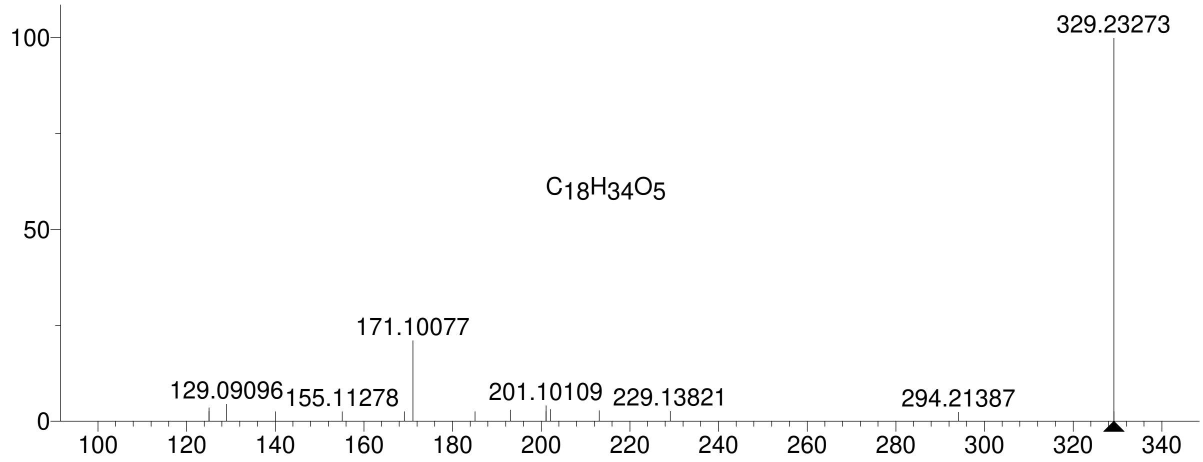

Hit 2 : FA 18:1+3O; PlaSMA ID-738 [M-H]- P=329.2
C18H34O5; MF: 183; RMF: 703; Prob 15.0%; Lib: biomsms-neg-plasma; ID: 444.

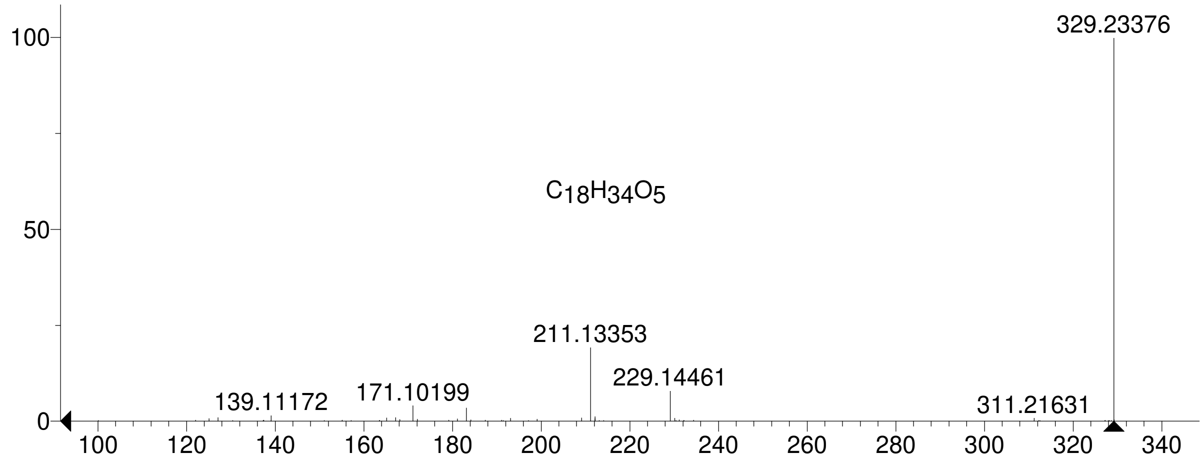

Hit 3 : FA 18:1+3O; PlaSMA ID-739 [M-H]- P=329.2
C18H34O5; MF: 164; RMF: 689; Prob 7.29%; Lib: biomsms-neg-plasma; ID: 446.

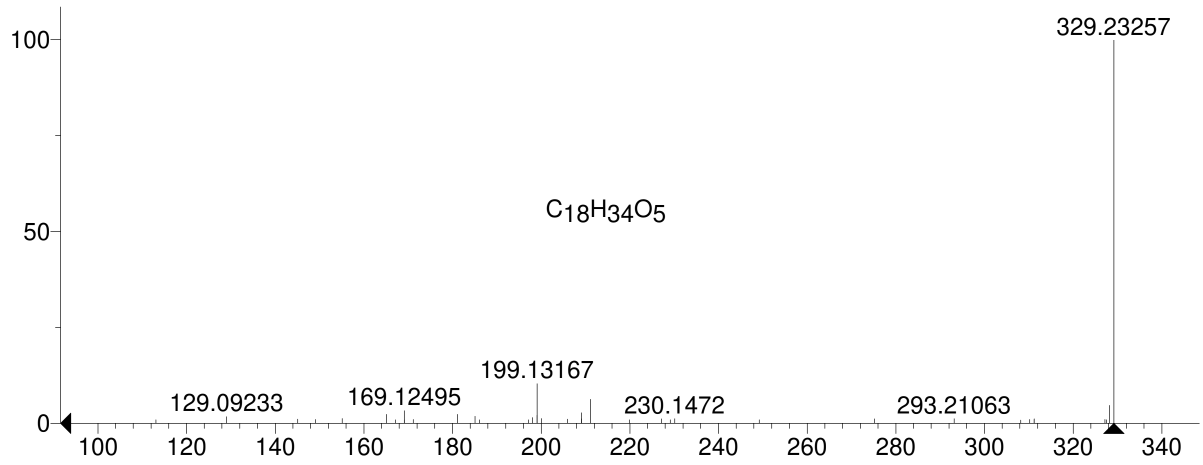

Hit 4 : FA 18:1+3O; PlaSMA ID-735 [M-H]- P=329.2
C18H34O5; MF: 123; RMF: 540; Prob 1.66%; Lib: biomsms-neg-plasma; ID: 443.

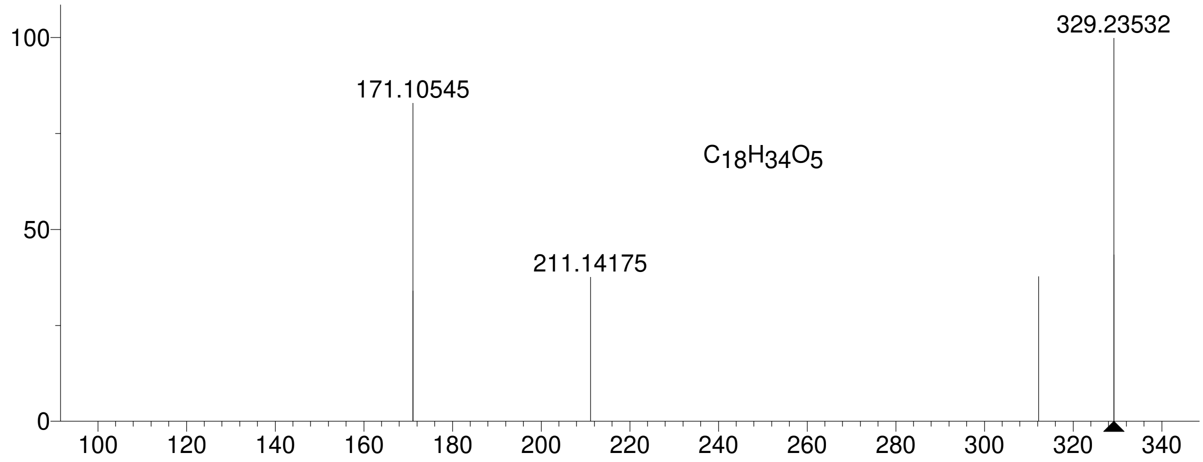

Hit 5 : FA 18:1+3O; PlaSMA ID-742 [M-H]- P=329.2
C18H34O5; MF: 120; RMF: 673; Prob 1.47%; Lib: biomsms-neg-plasma; ID: 445.

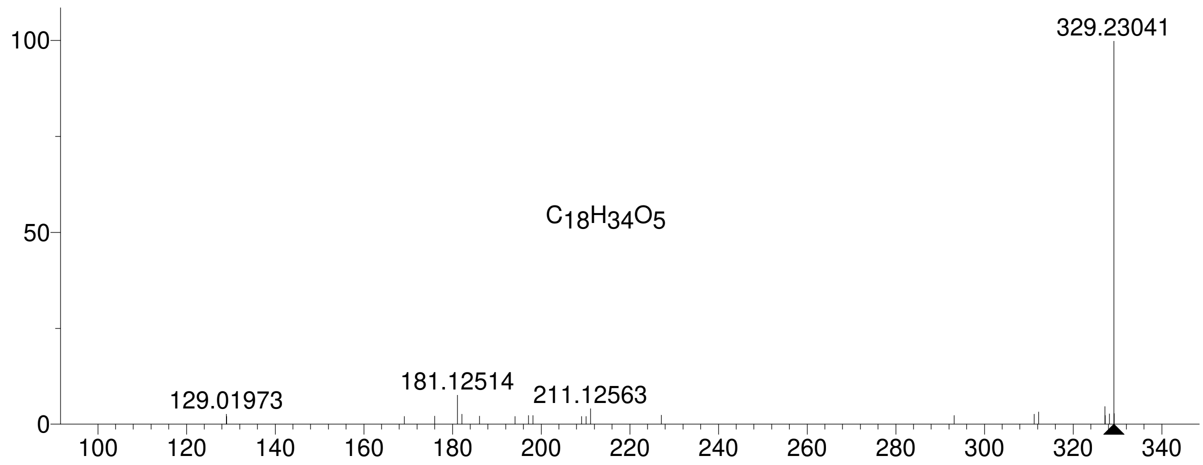

Unknown: Unknown (26.47_428.1491m/z)
Compound in Library Factor = 0

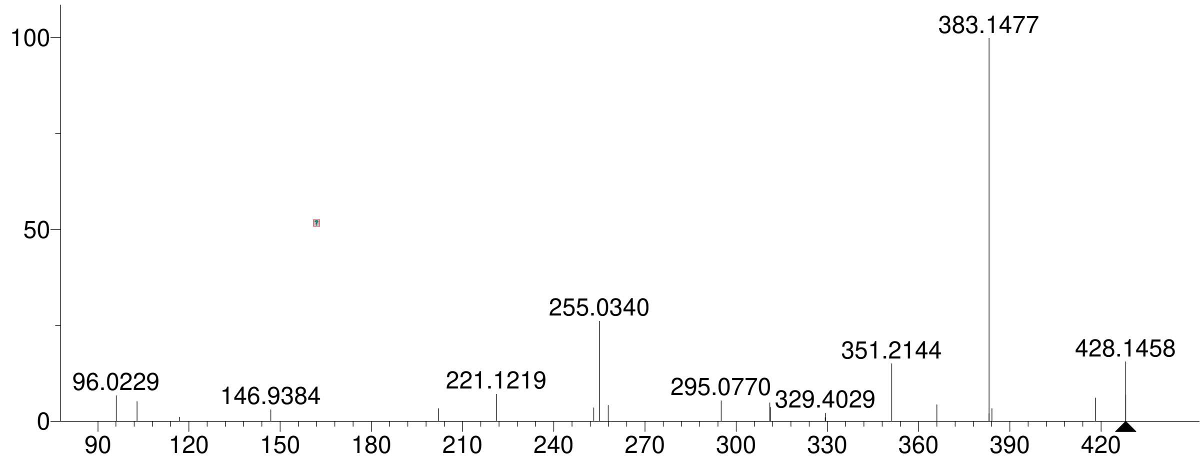

Hit 1 : Roquefortine C [M+K]+ 35HCD P=428.1
C22H23N5O2; MF: 0; RMF: 21; Prob 100.0%; Lib: mona-export-vf-npl_qexactive; ID: 16408.

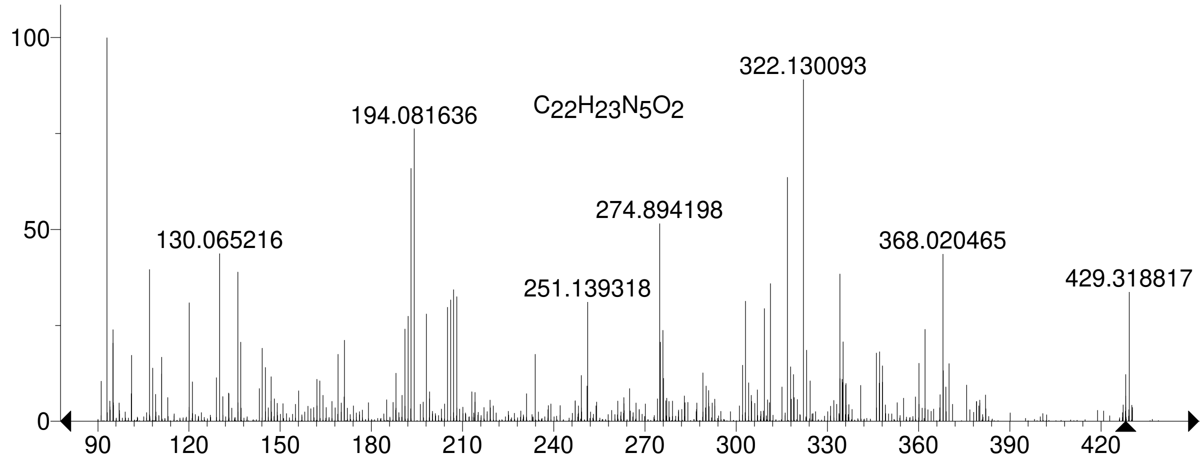

Unknown: Unknown (26.68_353.2316m/z)
Compound in Library Factor = -516

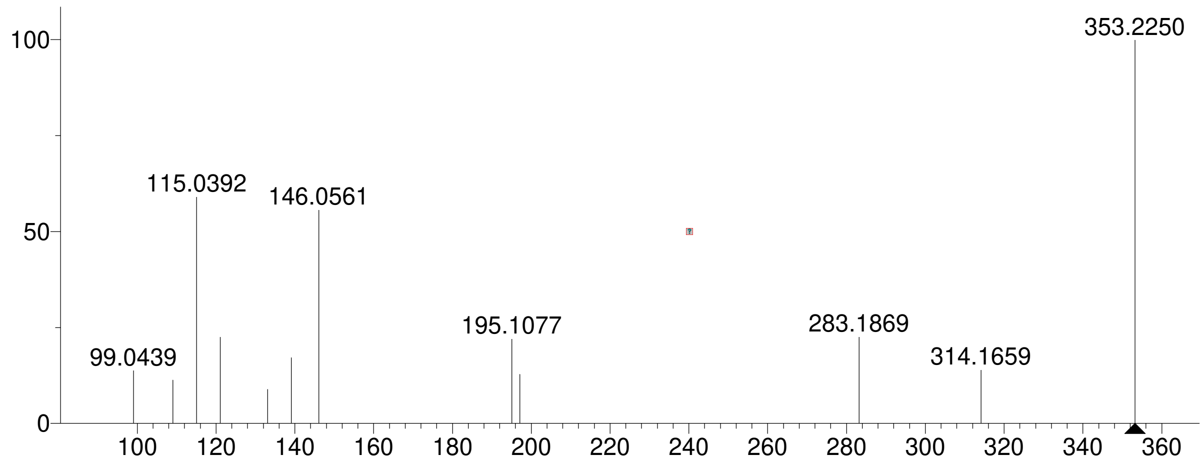

Hit 1 : 6-Ketoprostaglandin F1α [M+H-H2O]+ HCD 1V P=353.2
C20H34O6; MF: 352; RMF: 986; Prob 36.0%; CAS: 58962-34-8; Lib: nist_msms; ID: 166451.

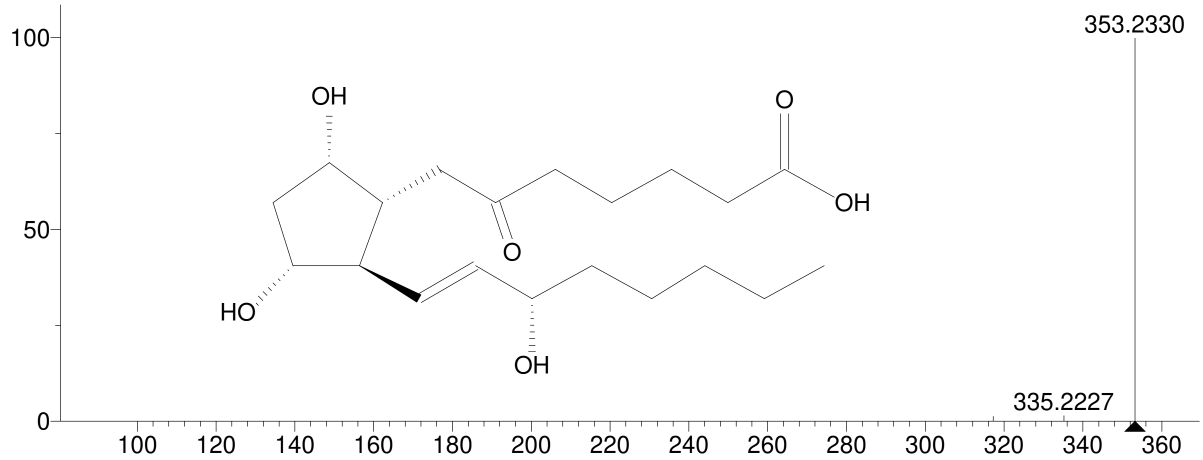

Hit 2 : Prostaglandin I2 [M+H]+ HCD 1V P=353.2
C20H32O5; MF: 351; RMF: 986; Prob 34.6%; CAS: 35121-78-9; Lib: nist_msms; ID: 166889.

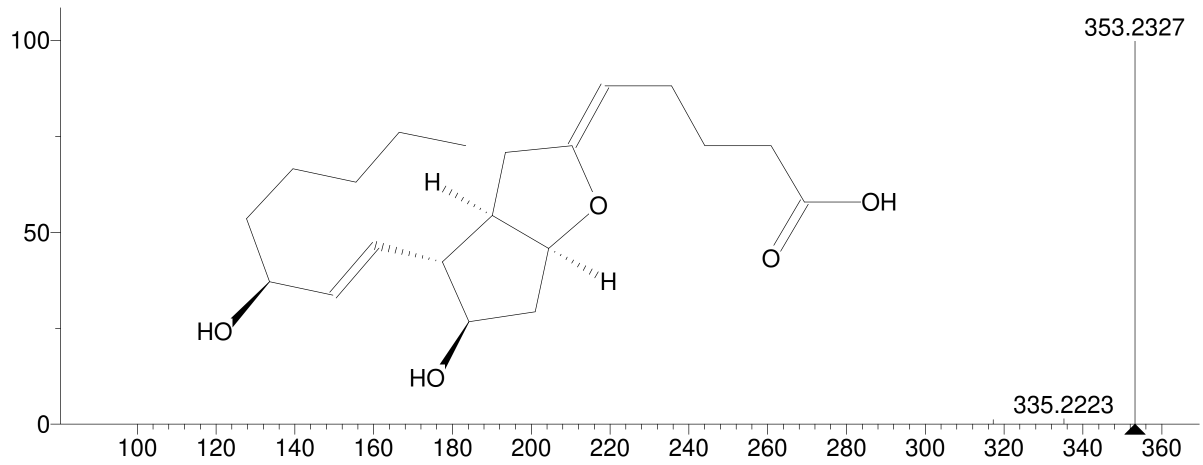

Hit 3 : 9S,11R-Dihydroxy-15-oxo-13E-prostenoic acid [M-H]- HCD 3V P=353.2
C20H34O5; MF: 340; RMF: 978; Prob 23.7%; CAS: 21562-58-3; Lib: nist_msms; ID: 155077.

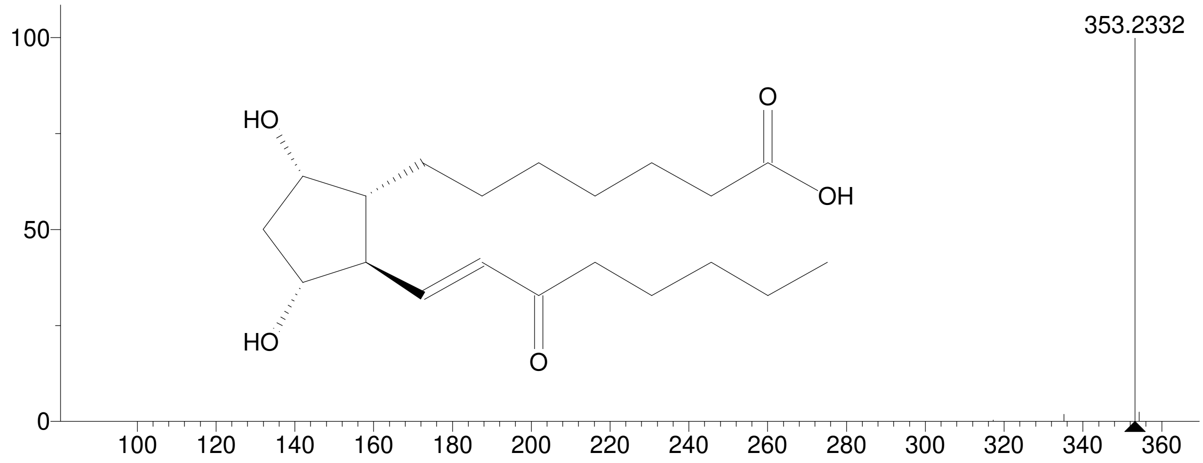

Hit 4 : 11,15-Dioxo-9S-hydroxy-5Z-prostenoic acid [M+H]+ HCD 1V P=353.2
C20H32O5; MF: 289; RMF: 952; Prob 4.78%; CAS: 59894-07-4; Lib: nist_msms; ID: 87155.

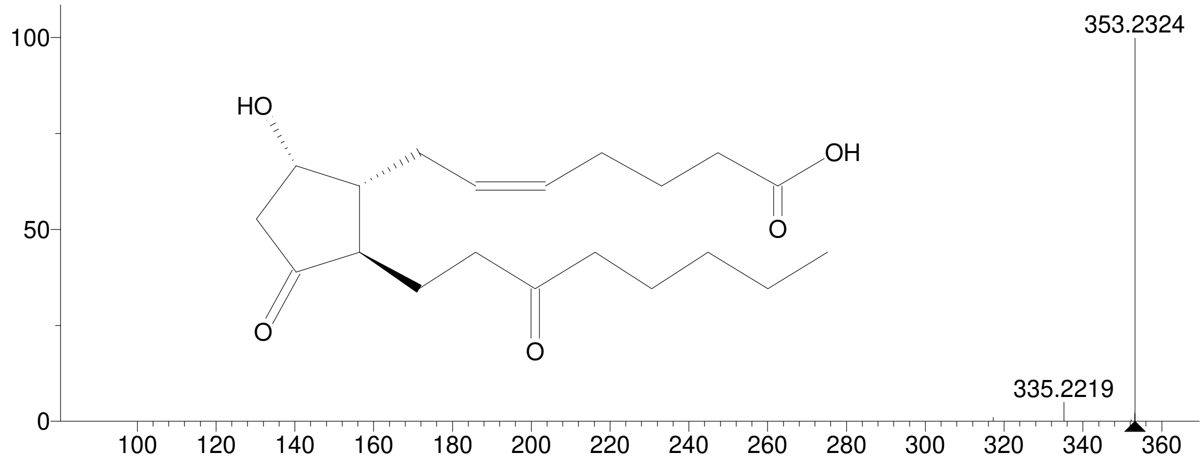

Hit 5 : 15-Ketoprostaglandin E1 [M+H]+ HCD 1V P=353.2
C20H32O5; MF: 172; RMF: 810; Prob 0.27%; CAS: 22973-19-9; Lib: nist_msms; ID: 169226.

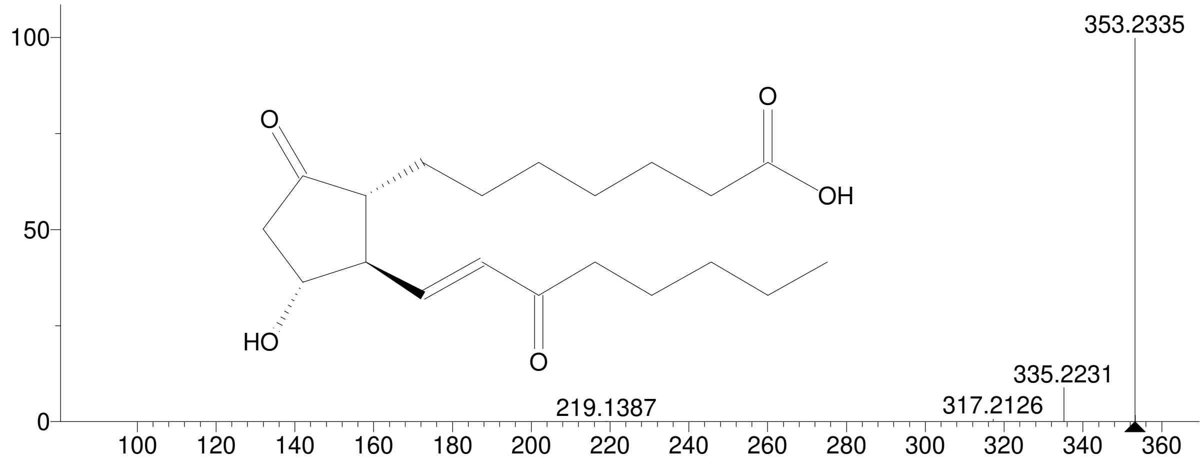

Unknown: Unknown (26.84_413.3049m/z)
Compound in Library Factor = -1750

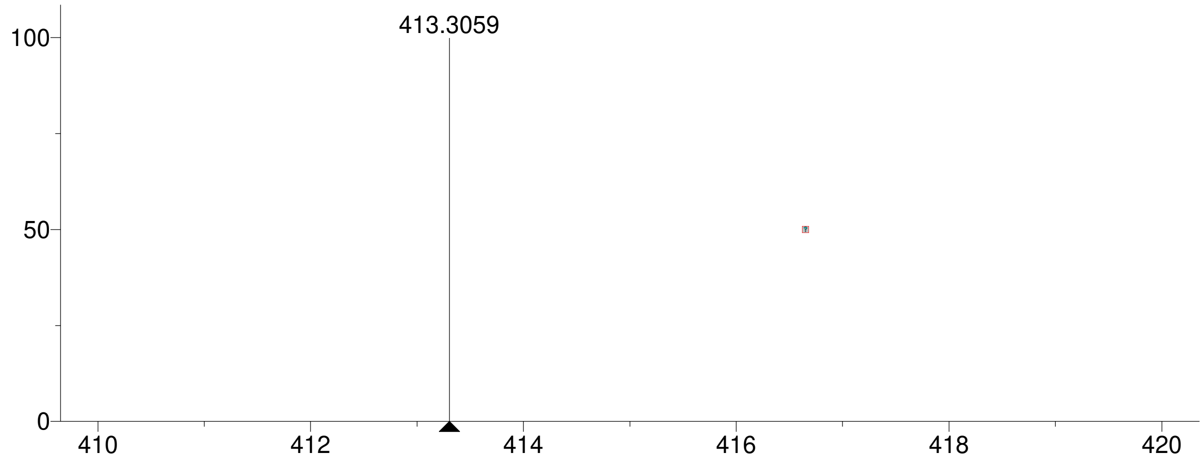

Hit 1 : 7α-Hydroxy-3-oxo-4-cholestenoic acid [M+H-H2O]+ HCD 41V P=413.3
C27H42O4; MF: 364; RMF: 999; Prob 22.5%; CAS: 1246298-65-6; Lib: nist_msms; ID: 101916.

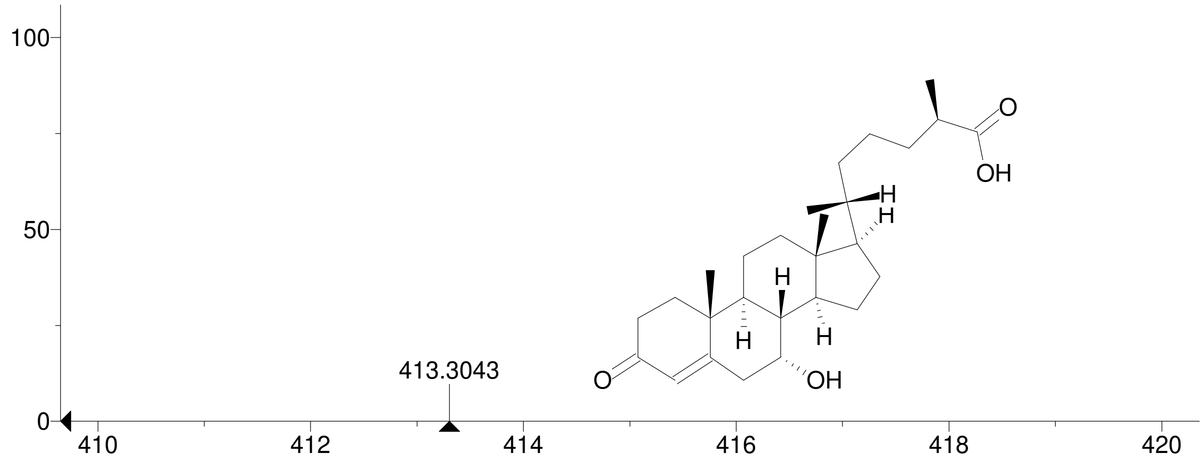

Hit 2 : Hecogenin [M+H-H2O]+ HCD 55V P=413.3
C27H42O4; MF: 364; RMF: 999; Prob 22.5%; CAS: 467-55-0; Lib: nist_msms; ID: 404024.

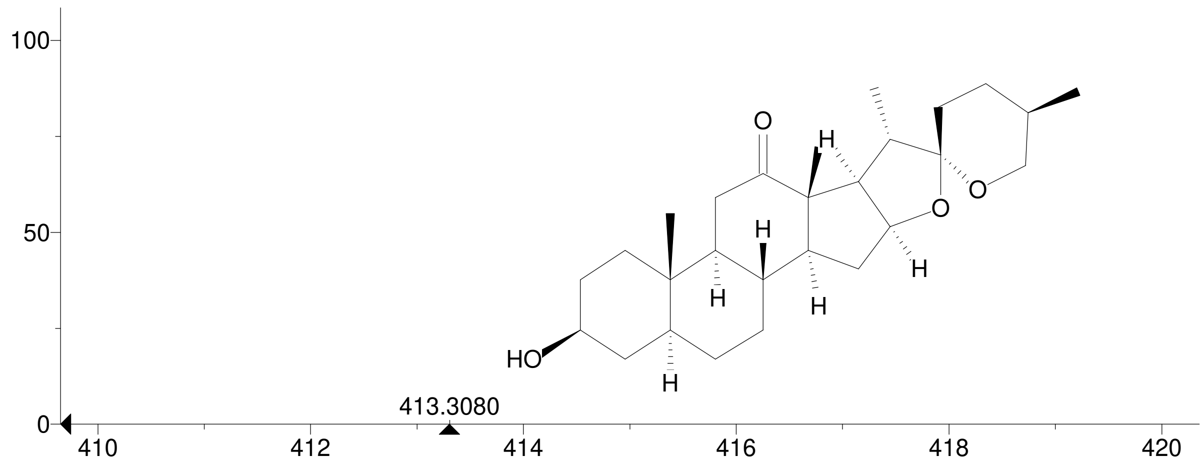

Hit 3 : Testosterone cypionate [M+H]+ HCD 16V P=413.3
C27H40O3; MF: 364; RMF: 999; Prob 22.5%; CAS: 58-20-8; Lib: nist_msms; ID: 383858.

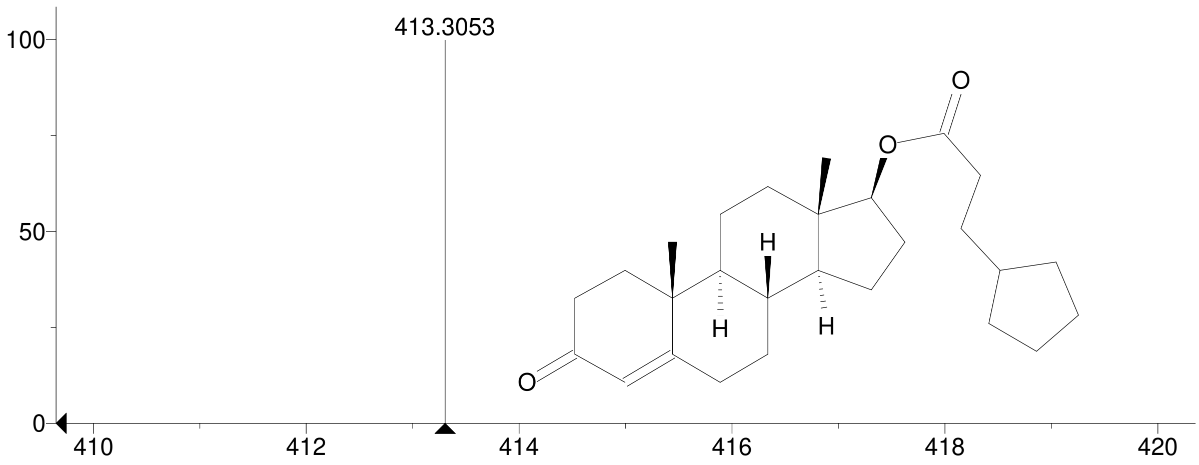

Hit 4 : 3-Oxo-4-cholestenoic acid [M-H]- HCD 61V P=413.3
C27H42O3; MF: 362; RMF: 996; Prob 20.8%; CAS: 309762-85-4; Lib: nist_msms; ID: 265773.

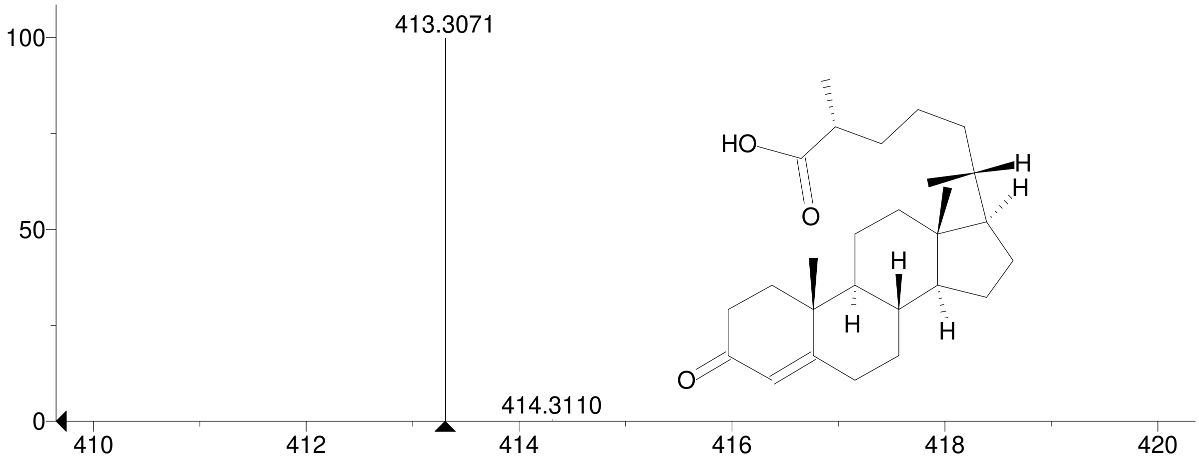

Hit 5 : 2-methyl-2-(3-pentadecylphenoxy)propanoic acid [M+Na]+ P=413.3
C25H42O3; MF: 335; RMF: 959; Prob 6.10%; Lib: mona-export-gnps; ID: 4860.

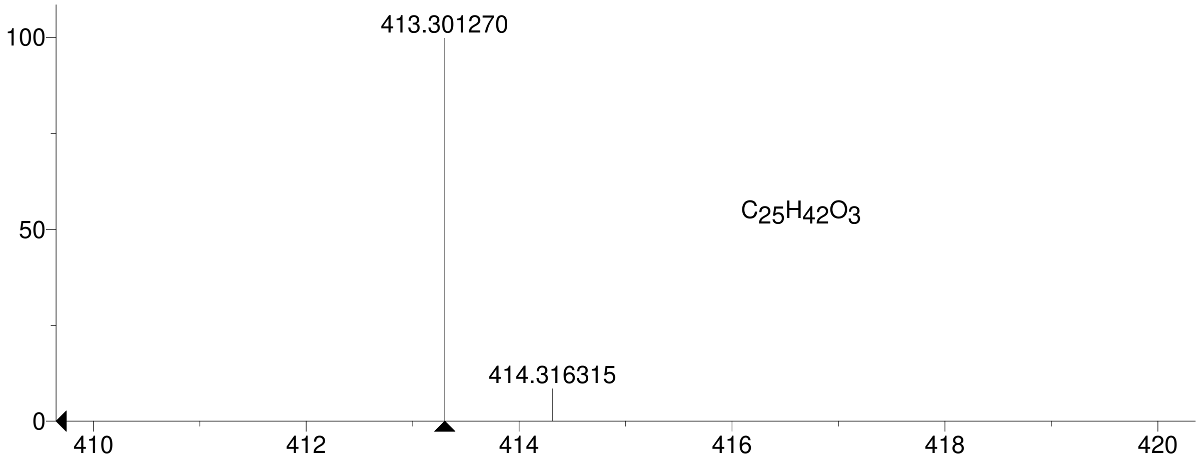

Unknown: Unknown (26.86_475.3054m/z)
Compound in Library Factor = 0

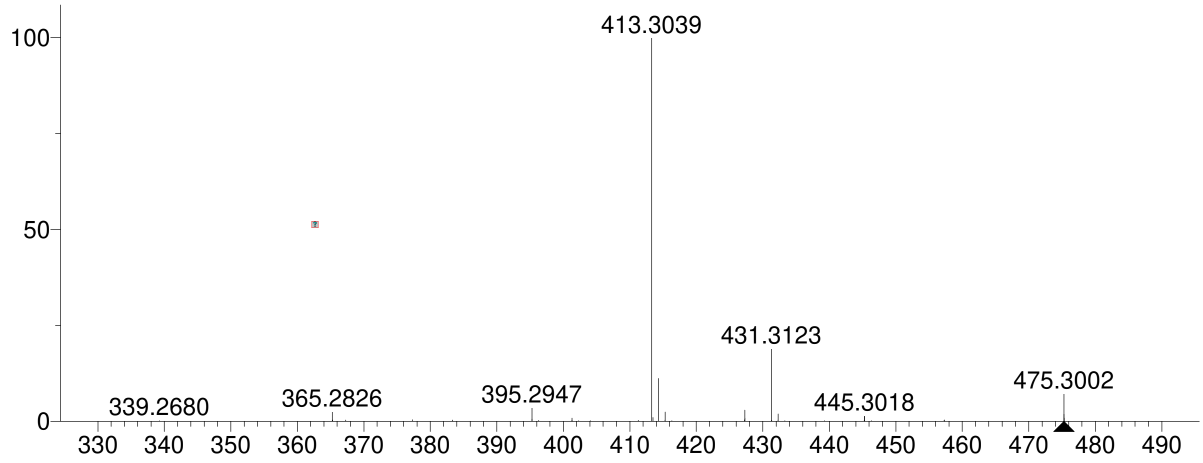

** Search Report Page 1 of 1 **

Unknown: Unknown (26.98_426.0525m/z)
Compound in Library Factor = 0

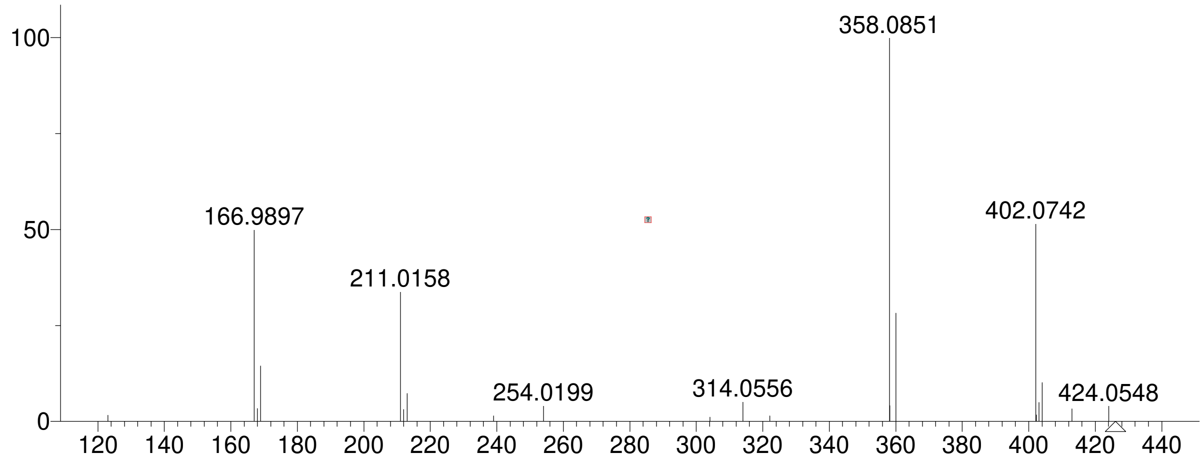

Unknown: Unknown (26.99_500.9910m/z)
Compound in Library Factor = 0

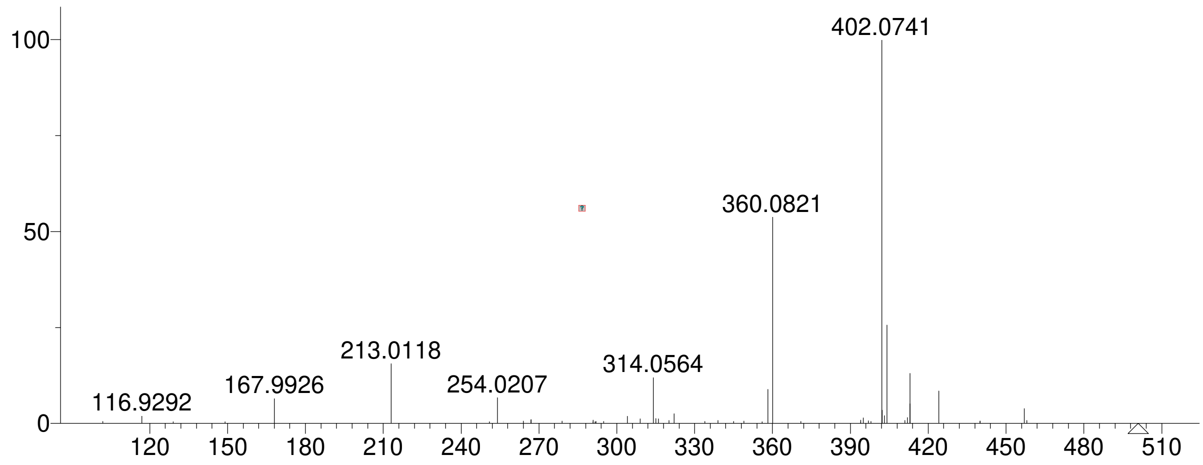

Unknown: Unknown (27.00_402.2621m/z)
Compound in Library Factor = -3273

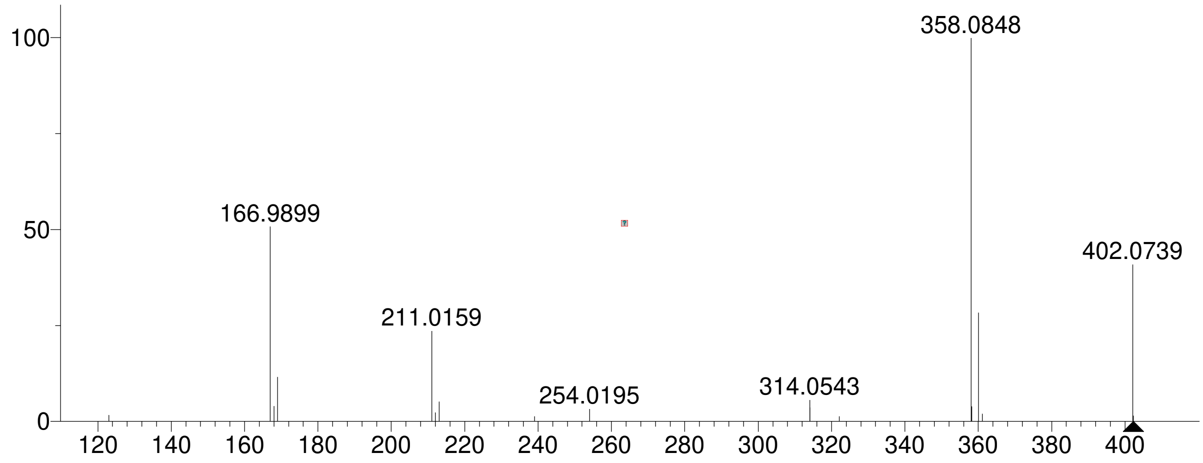

Hit 1 : β-Estradiol 3,17-dipropionate [M+NH4]+ HCD 4V P=402.3
C24H32O4; MF: 3; RMF: 75; Prob 11.1%; CAS: 113-38-2; Lib: nist_msms; ID: 302458.

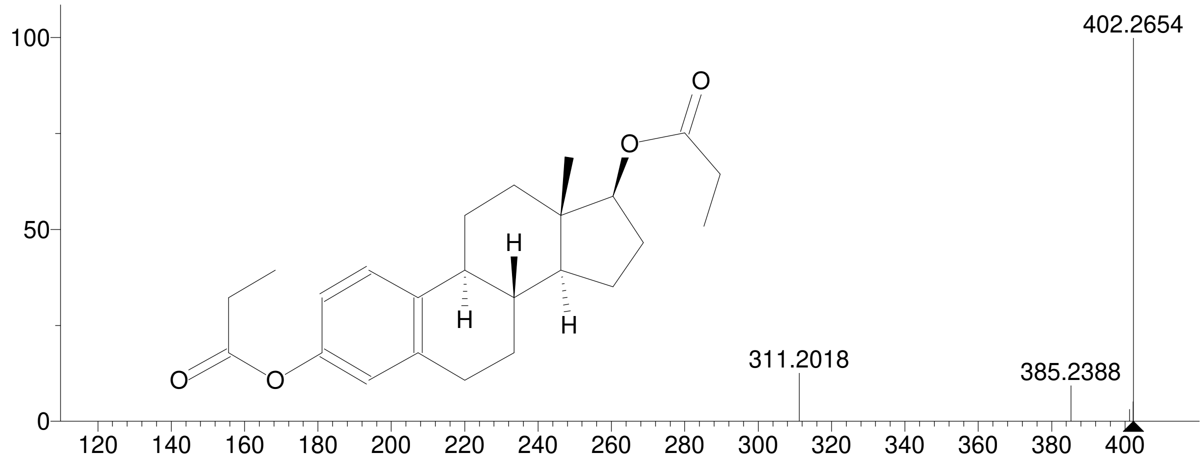

Hit 2 : Leukotriene B4 ethanolamide [M+Na]+ HCD 24V P=402.3
C22H37NO4; MF: 1; RMF: 65; Prob 10.2%; CAS: 877459-63-7; Lib: nist_msms; ID: 88441.

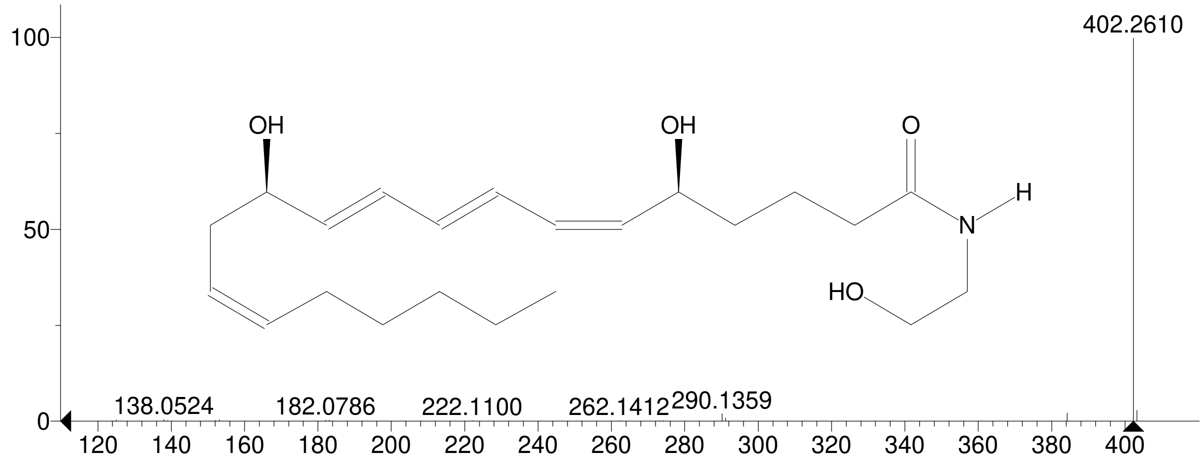

Hit 3 : NCGC00386058-01_C24H35NO4_(1S,2R,3R,6S,7R,8S,11S,14S,15S,18R)-18-Hydroxy-8-isobutyl-5,6,15-t
C24H35NO4; MF: 0; RMF: 23; Prob 9.83%; Lib: mona-export-gnps; ID: 12370.

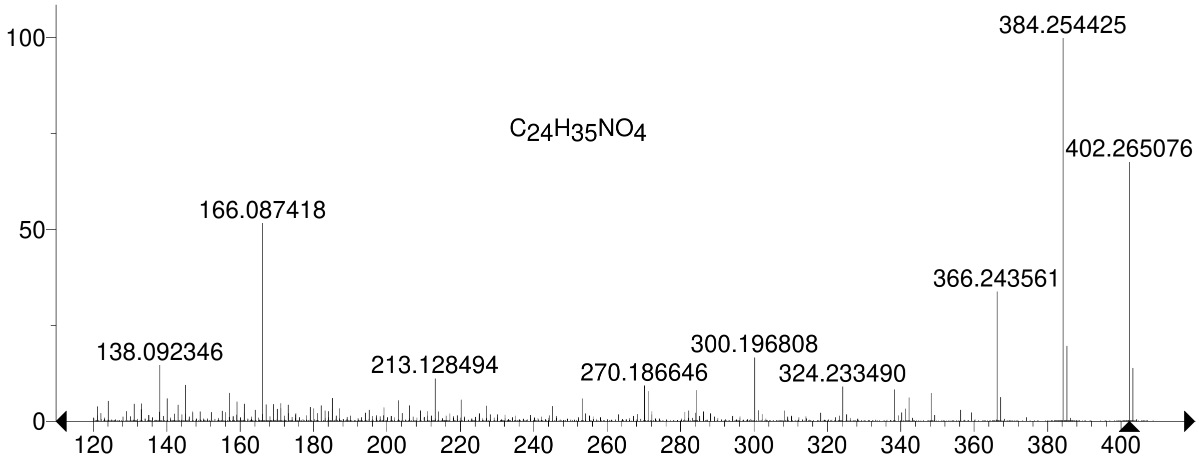

Hit 4 : NCGC00380732-01_C24H35NO4_1H-Cycloundec[d]isoindole-1,15(2H)-dione, 3,3a,4,6a,9,10,11,12-octah
C24H35NO4; MF: 0; RMF: 16; Prob 9.83%; Lib: mona-export-gnps; ID: 11653.

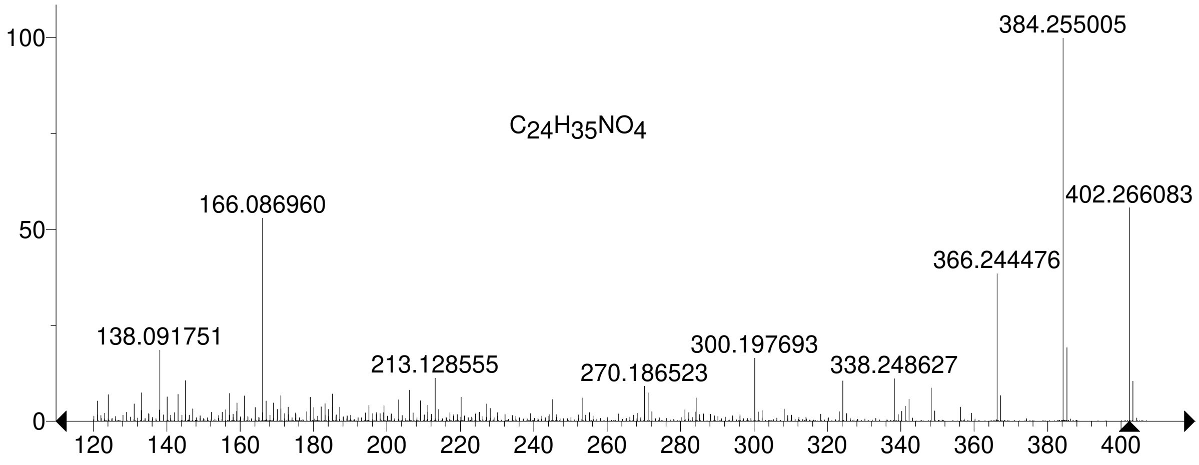

Hit 5 : NCGC00380312-01_C24H35NO4_ [M+H]+ P=402.3
C24H35NO4; MF: 0; RMF: 13; Prob 9.83%; Lib: mona-export-gnps; ID: 10374.

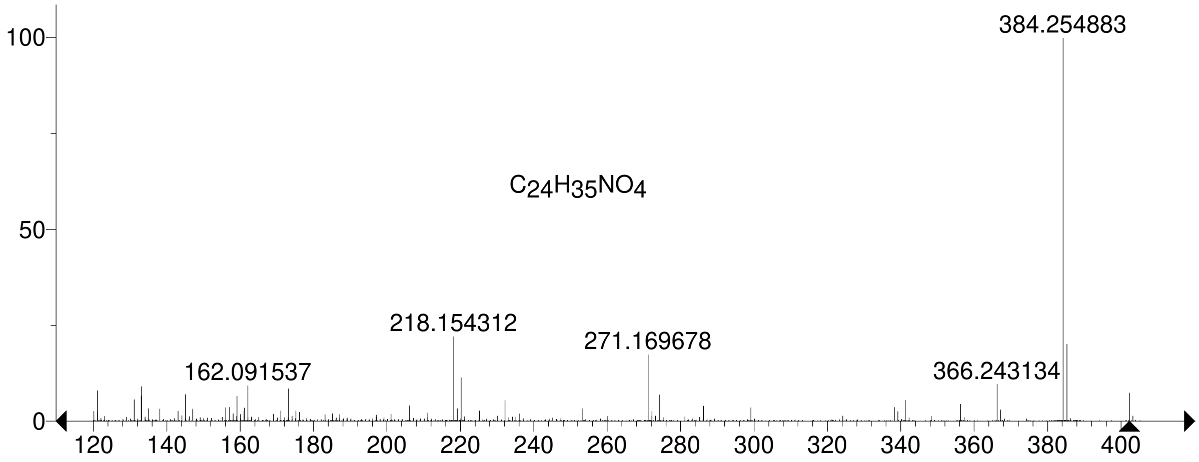

Unknown: Unknown (27.00_404.0719m/z)
Compound in Library Factor = -3273

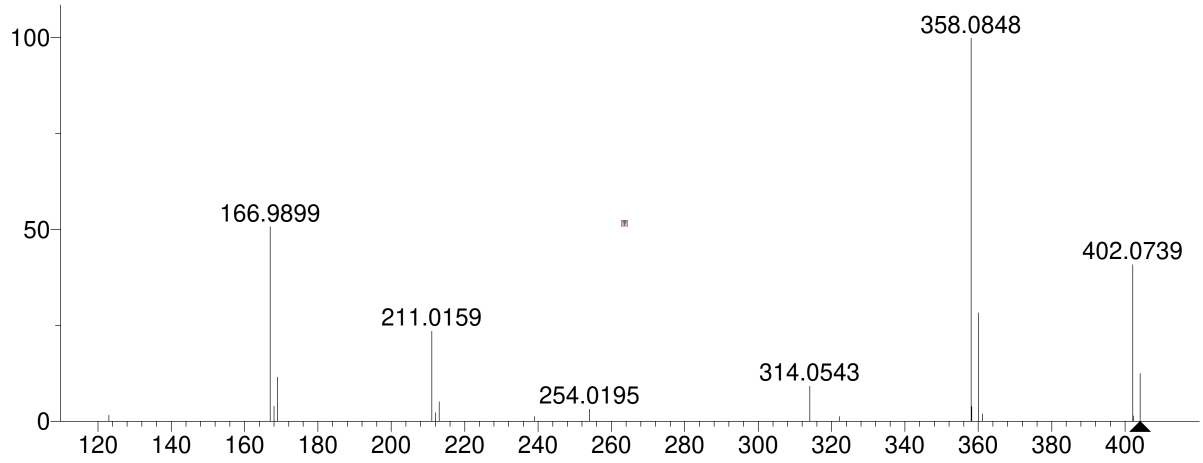

Hit 1 : 2-hydroxypentyl glucosinolate (CCR vessel) [M-H]- P=404.1
C12H23NO10S2; MF: 9; RMF: 202; Prob 56.6%; Lib: phytofrag2018_final_060219; ID: 921.

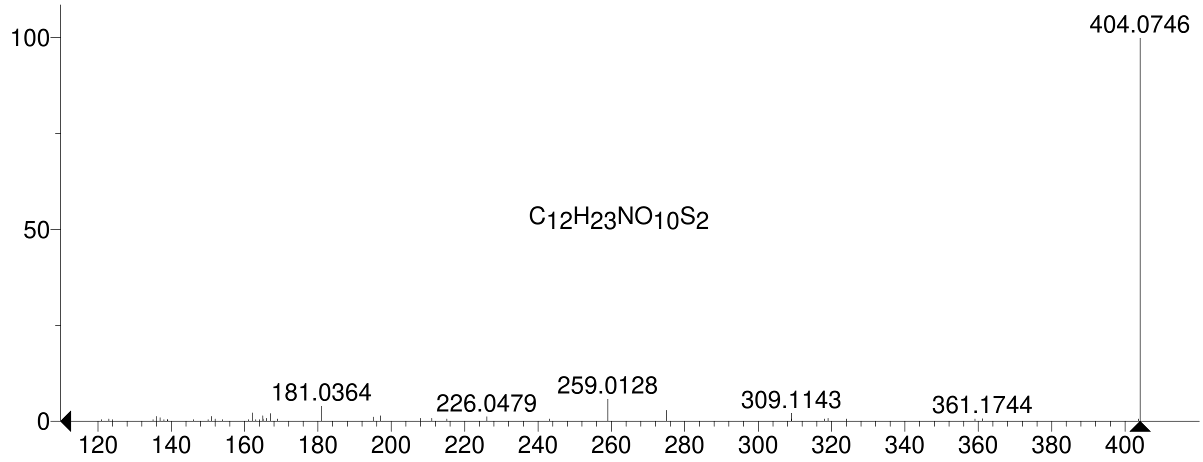

Hit 2 : 5-hydroxyalkyl glucosinolate () [M-H]- P=404.1
C12H23NO10S2; MF: 2; RMF: 196; Prob 43.4%; Lib: phytofrag2018_final_060219; ID: 87.

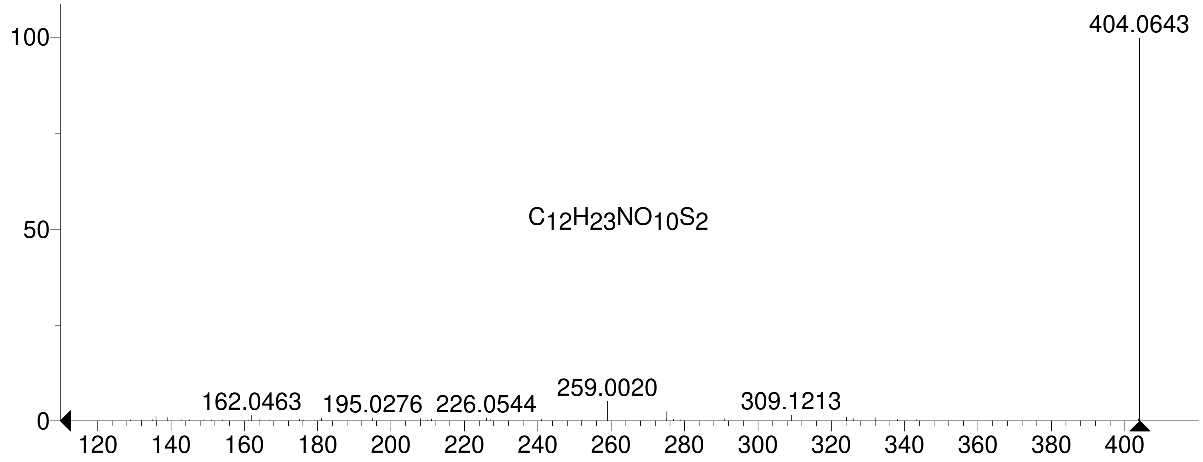

** Search Report Page 1 of 1 **

Unknown: Unknown (27.00_404.2600m/z)
Compound in Library Factor = 0

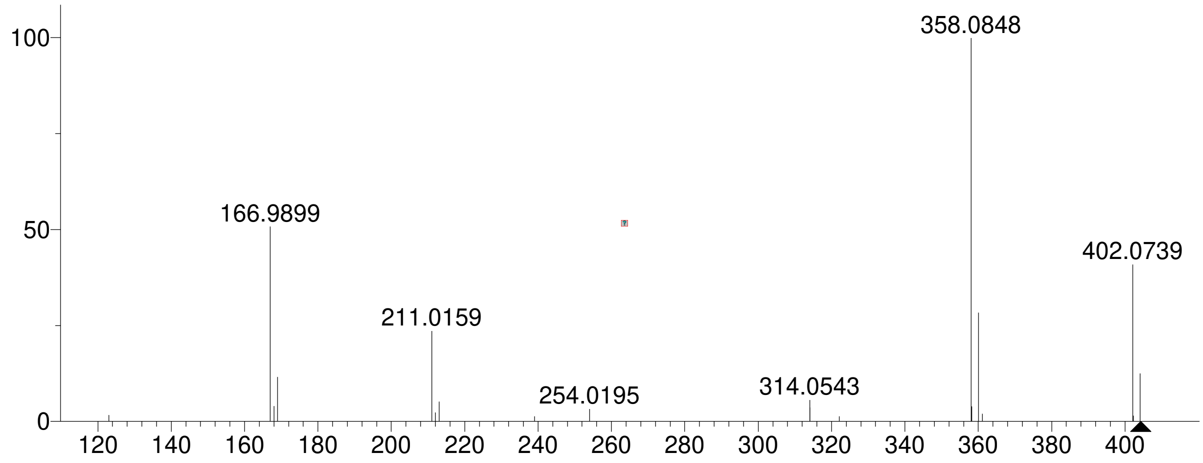

Hit 1 : Lys-Thr-Arg [M+H]+ IT 35% P=404.3
C16H33N7O5; MF: 0; RMF: 27; Prob 50.0%; Lib: nist_msms; ID: 47215.

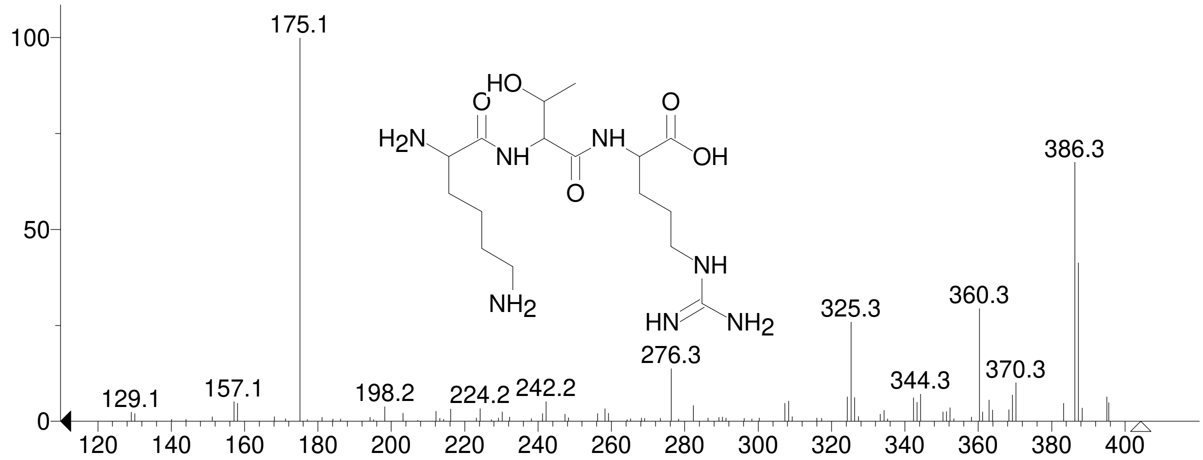

Hit 2 : Arg-Thr-Lys [M+H]+ QQQ 14V P=404.3
C16H33N7O5; MF: 0; RMF: 9; Prob 50.0%; Lib: nist_msms; ID: 41554.

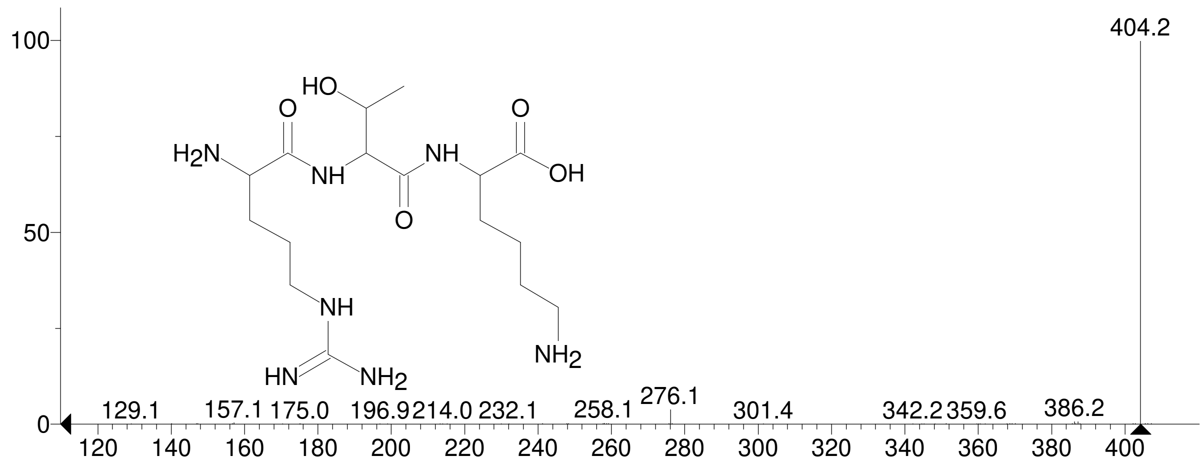

** Search Report Page 1 of 1 **

Unknown: Unknown (27.00_490.9614m/z)
Compound in Library Factor = 0

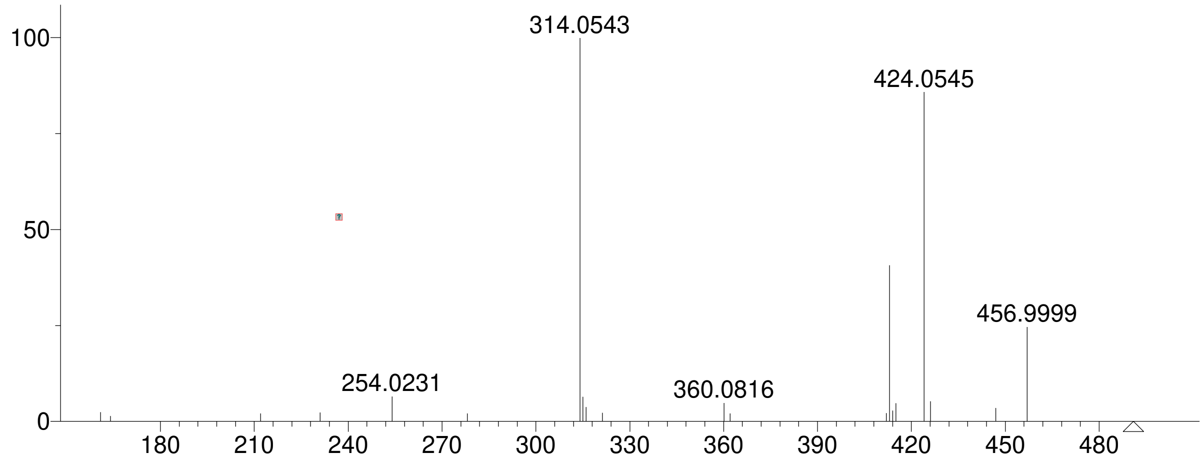

Unknown: Unknown (27.01_358.0842m/z)
Compound in Library Factor = -1848

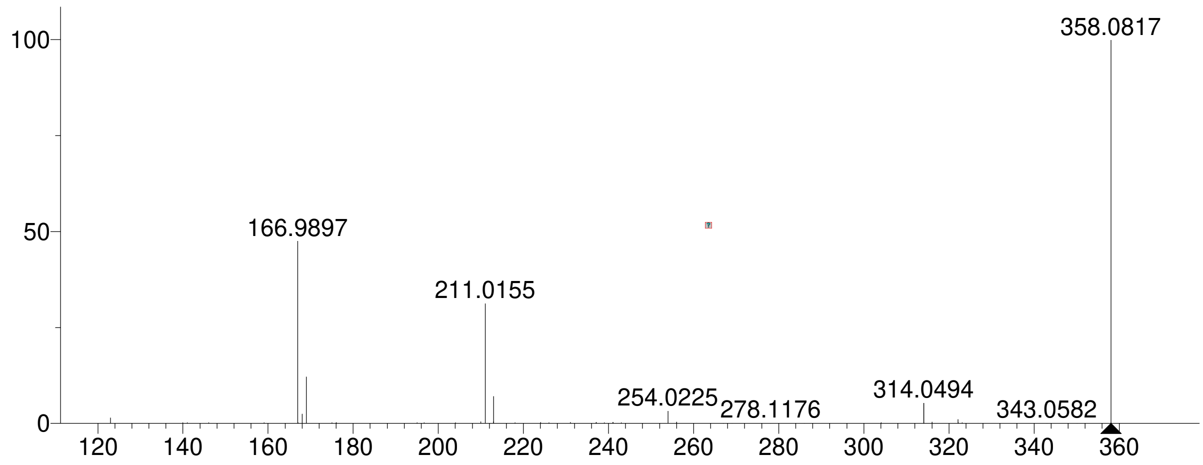

Hit 1 : Indomethacin [M+H]+ HCD 7V P=358.1
C19H16ClNO4; MF: 82; RMF: 651; Prob 73.0%; CAS: 53-86-1; Lib: nist_msms; ID: 332462.

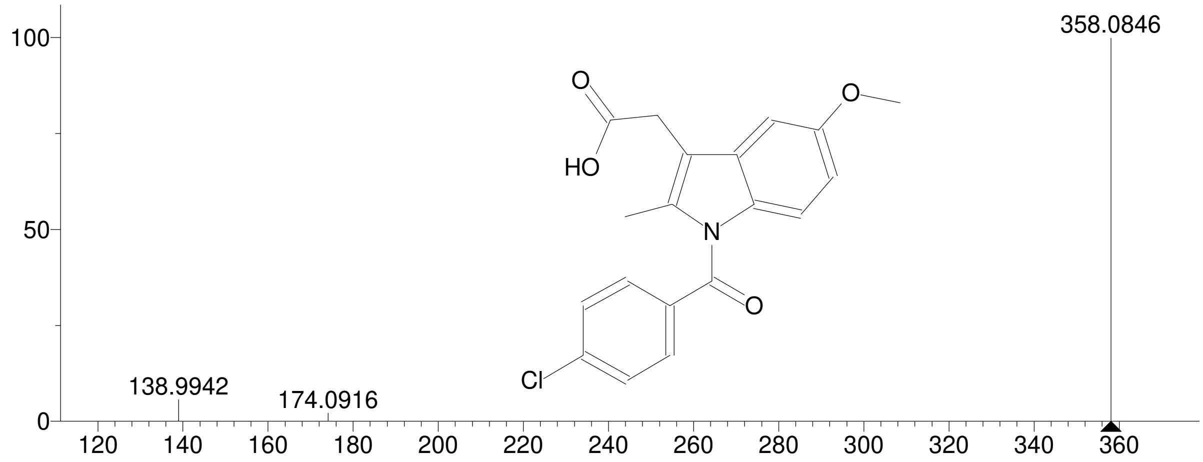

Hit 2 : 2-Furancarboxamide, 5-(4-chlorophenyl)-N-(3,5-dimethoxyphenyl)- [M+H]+ HCD 14V P=358.1
C19H16ClNO4; MF: 49; RMF: 610; Prob 19.1%; CAS: 944261-79-4; Lib: nist_msms; ID: 373202.

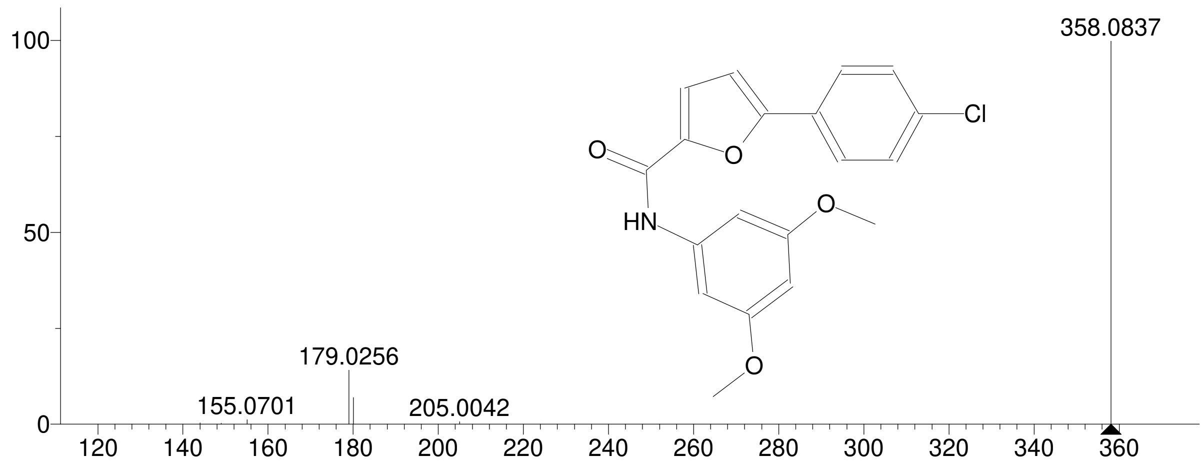

Hit 3 : Indomethacin heptyl ester [M+H-C7H14]+ HCD 21V P=358.1
C26H30ClNO4; MF: 4; RMF: 47; Prob 4.07%; CAS: 282728-47-6; Lib: nist_msms; ID: 359332.

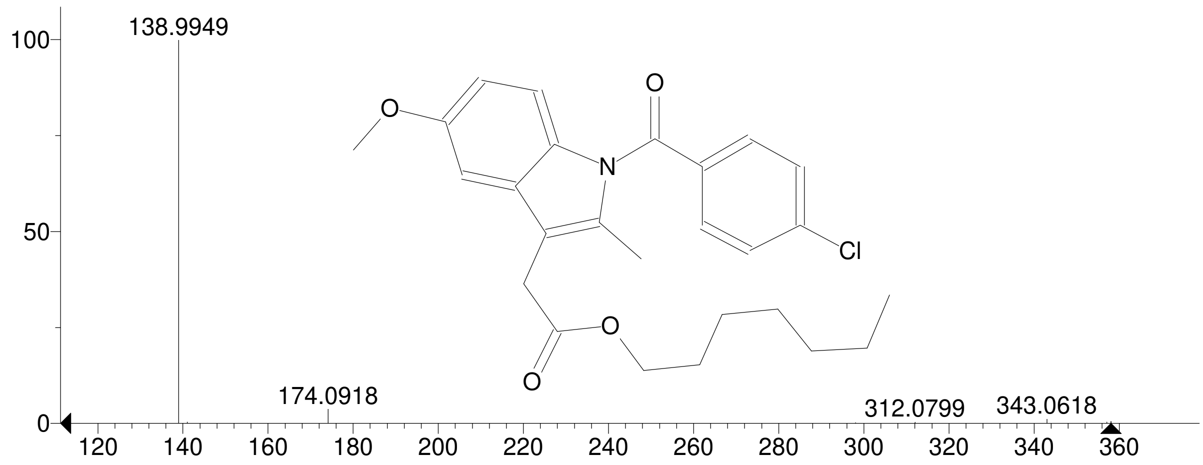

Hit 4 : "Indomethacin (Indocid, Indocin)" [M+H]+ P=358.1
C19H16ClNO4; MF: 2; RMF: 193; Prob 3.75%; Lib: mona-export-gnps; ID: 583.

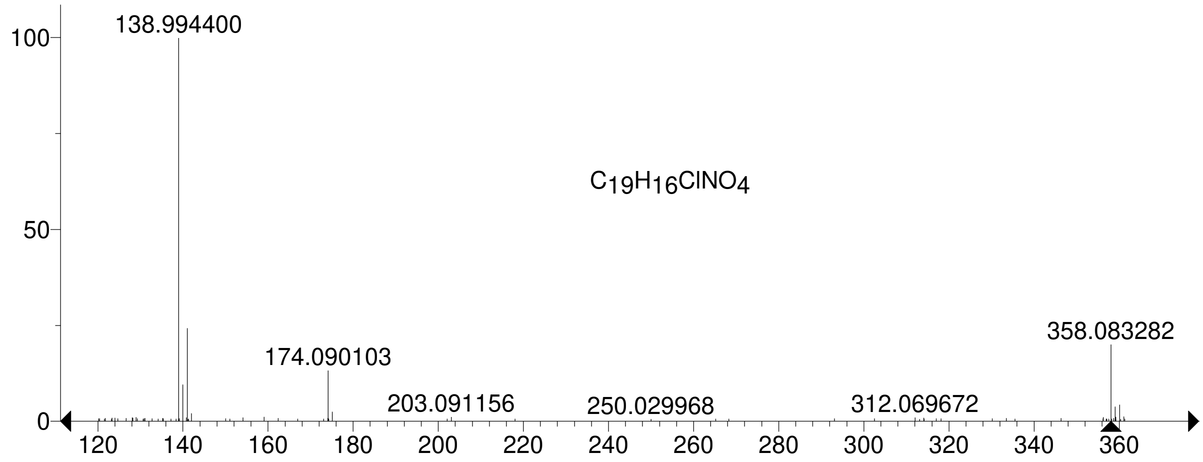

Unknown: Unknown (27.01_360.0815m/z)
Compound in Library Factor = -3273

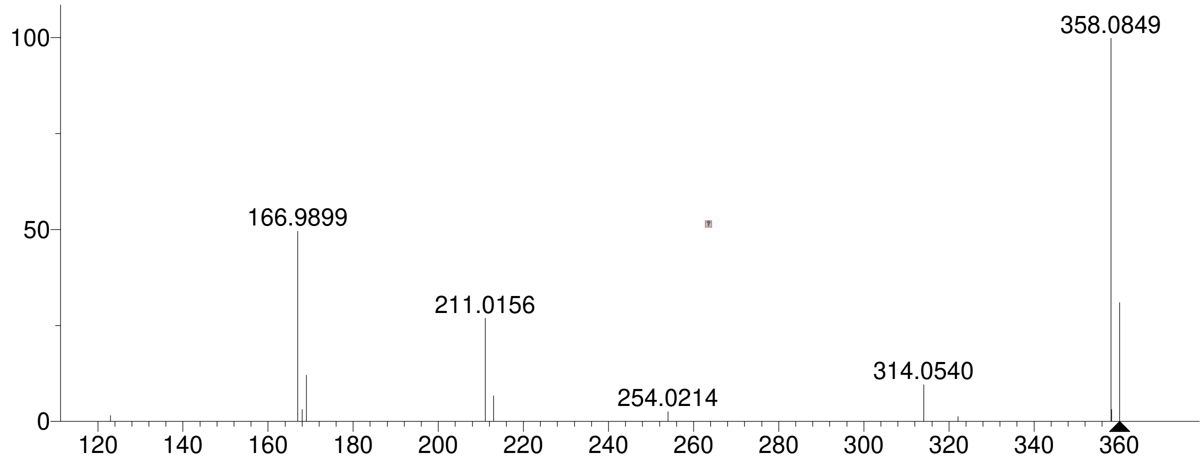

Hit 1 : MMV688755 [M+H]+ 20-30-40%) P=360.1
C14H12F3N3O5; MF: 1; RMF: 151; Prob 51.0%; Lib: mona-export-pathogen_box; ID: 371.

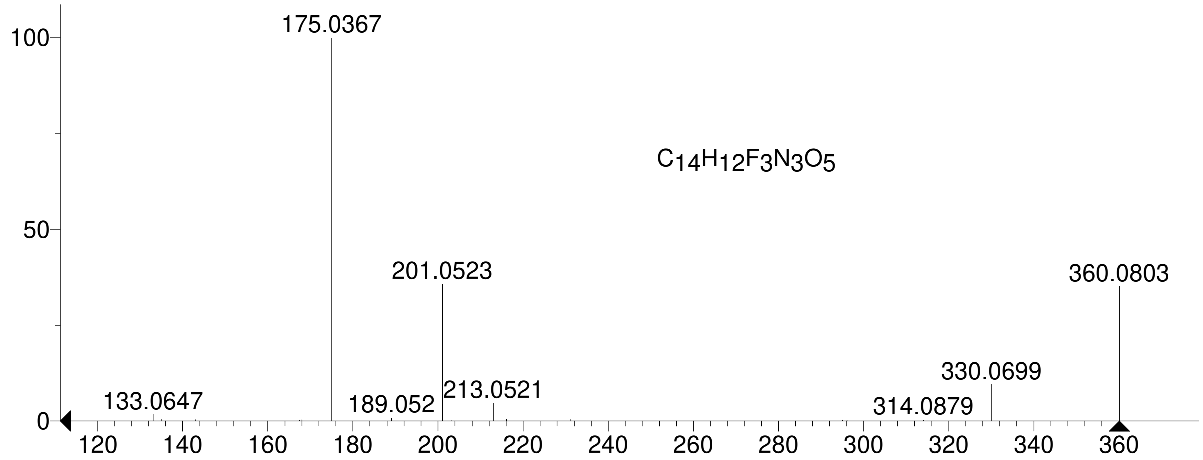

Hit 2 : Casticin [M+H-CH3]+ HCD 25V P=360.1
C19H18O8; MF: 0; RMF: 166; Prob 49.0%; CAS: 479-91-4; Lib: nist_msms; ID: 391649.

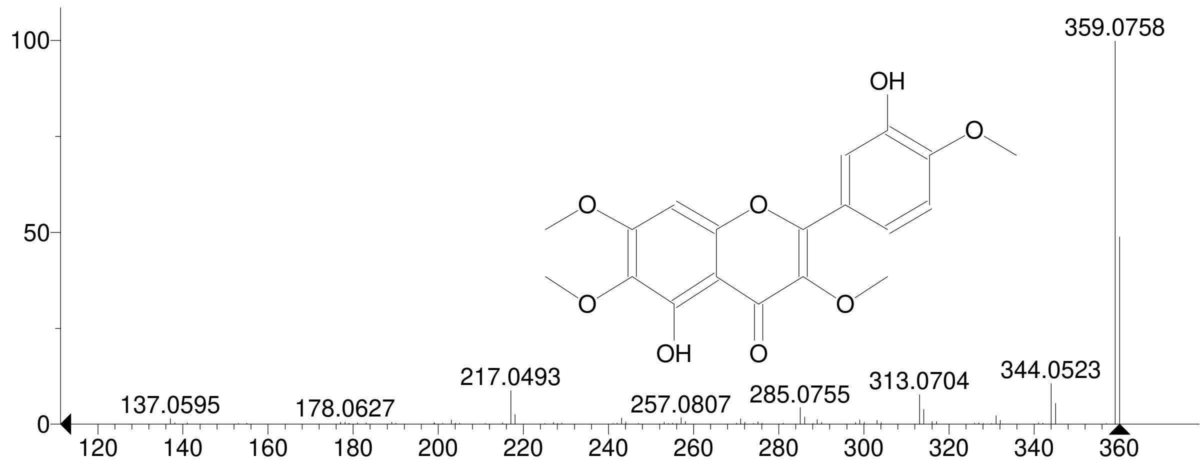

** Search Report Page 1 of 1 **

Unknown: Unknown (27.01_402.0746m/z)
Compound in Library Factor = -3273

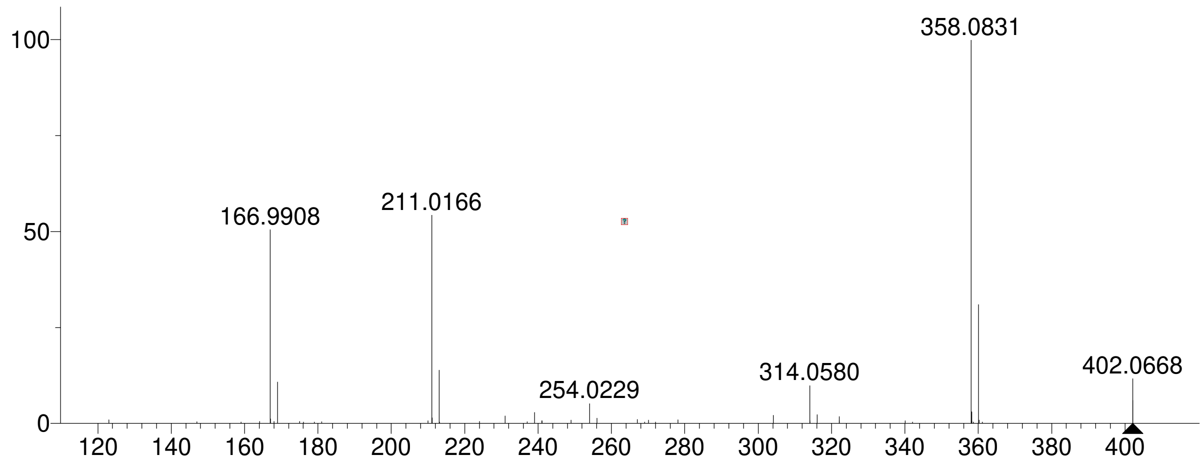

Hit 1 : NCGC00162403-05_C20H18ClNO6_L-Phenylalanine, N-[[(3R)-5-chloro-3,4-dihydro-8-hydroxy-3-methyl-1-
C20H18ClNO6; MF: 74; RMF: 401; Prob 100.0%; Lib: mona-export-gnps; ID: 13740.

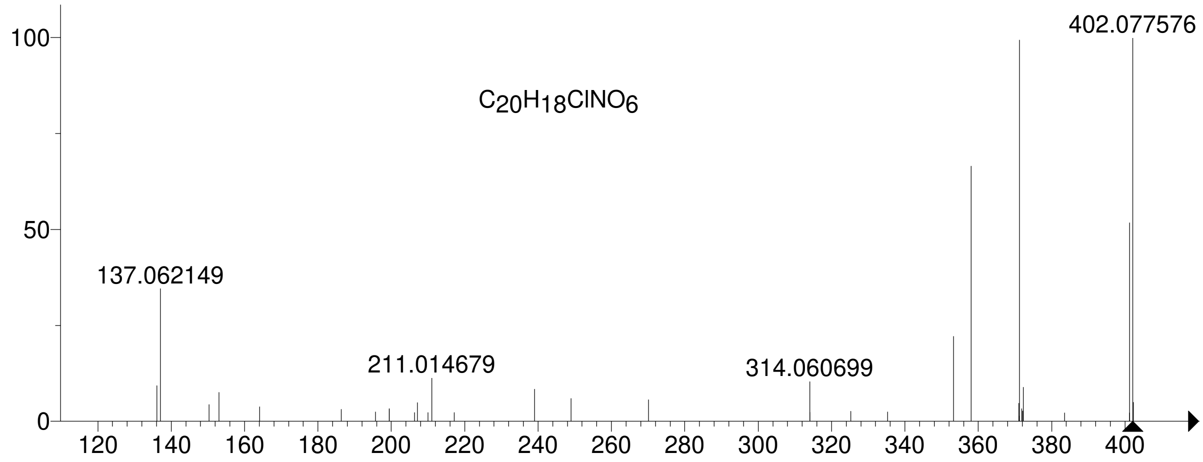

Unknown: Unknown (27.02_424.0560m/z)
Compound in Library Factor = 0

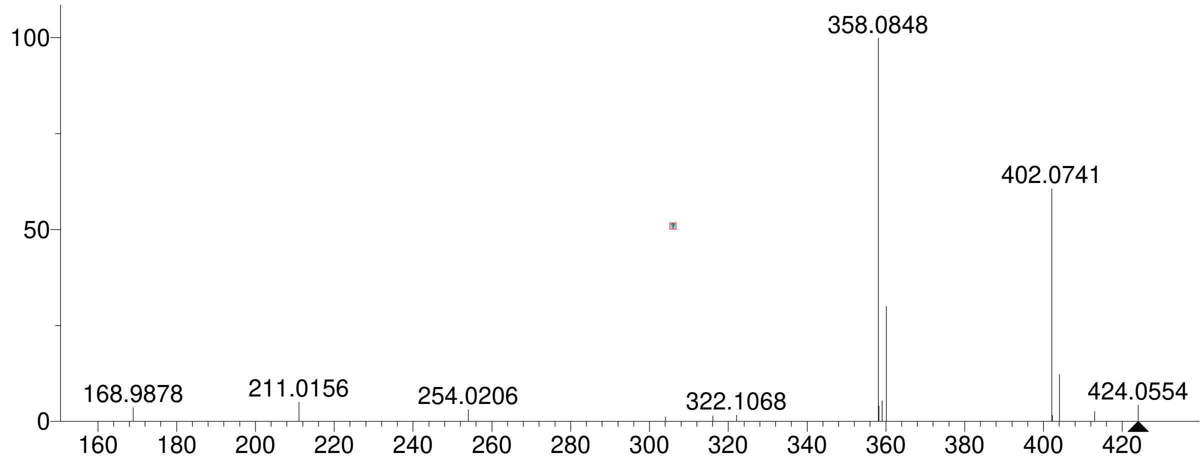

Unknown: Unknown (27.02_457.9918m/z)
Compound in Library Factor = 0

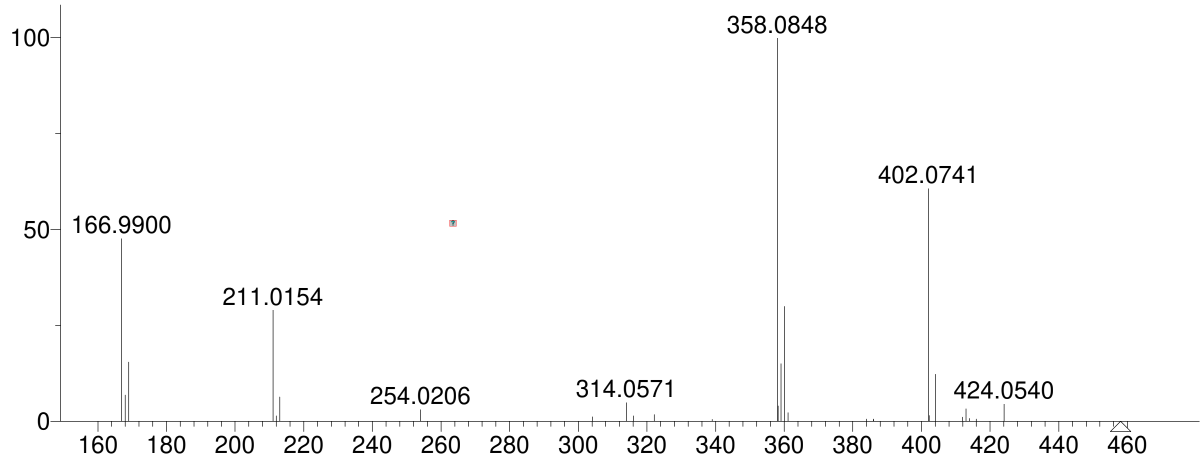

Unknown: Unknown (27.48_353.2326m/z)
Compound in Library Factor = -997

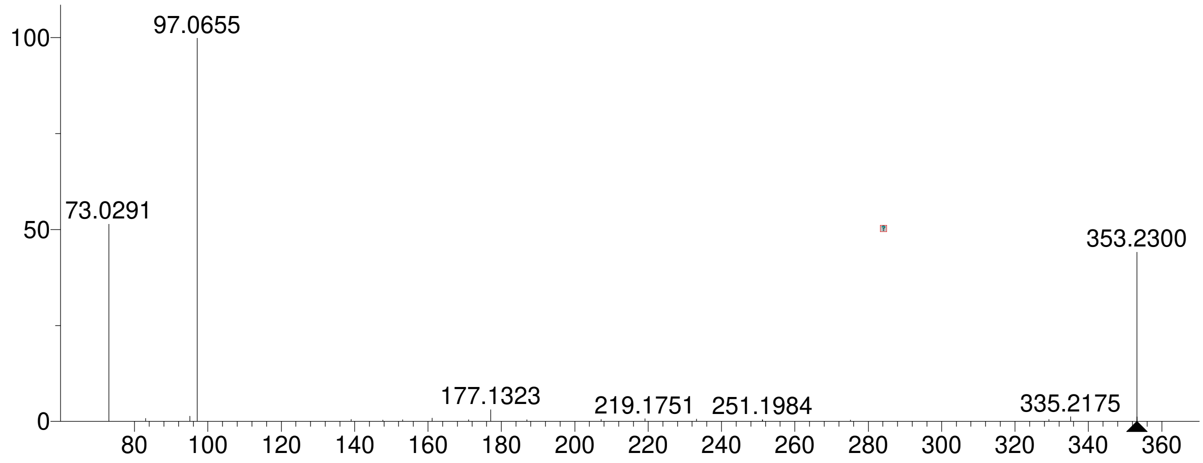

Hit 1 : 6-Ketoprostaglandin F1α [M+H-H2O]+ HCD 1V P=353.2
C20H34O6; MF: 310; RMF: 961; Prob 34.2%; CAS: 58962-34-8; Lib: nist_msms; ID: 166451.

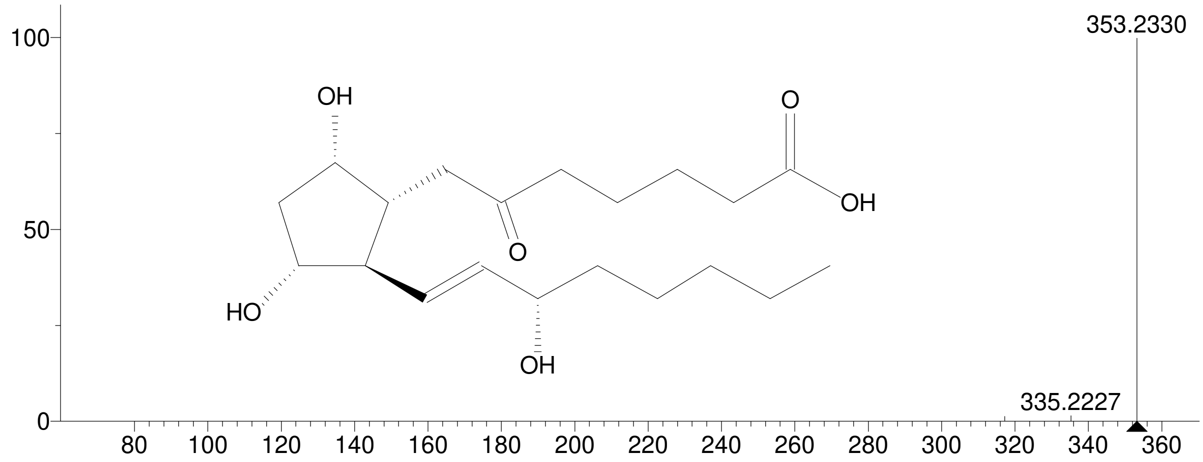

Hit 2 : Prostaglandin I2 [M+H]+ HCD 1V P=353.2
C20H32O5; MF: 301; RMF: 961; Prob 24.9%; CAS: 35121-78-9; Lib: nist_msms; ID: 166889.

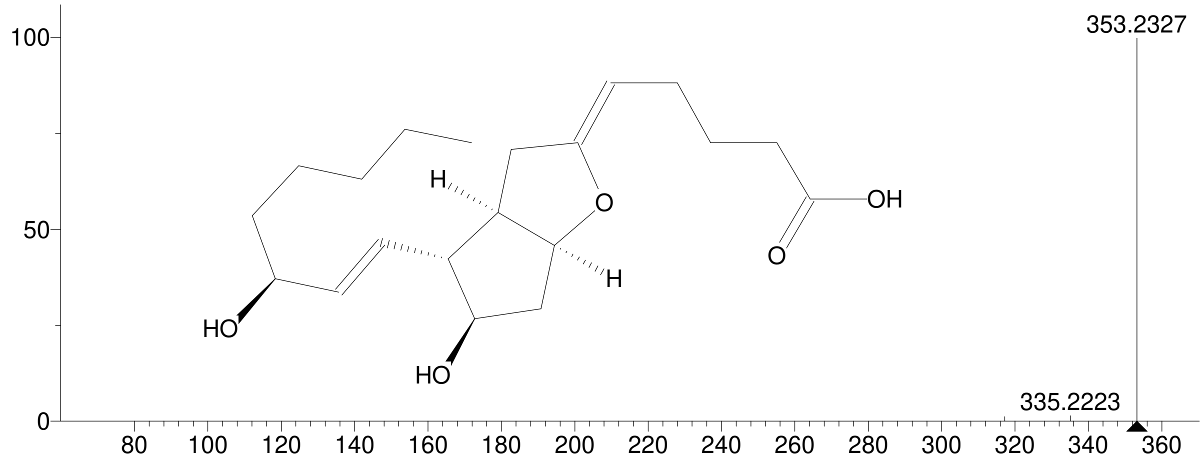

Hit 3 : 9S,11R-Dihydroxy-15-oxo-13E-prostenoic acid [M-H]- HCD 4V P=353.2
C20H34O5; MF: 287; RMF: 953; Prob 15.6%; CAS: 21562-58-3; Lib: nist_msms; ID: 155078.

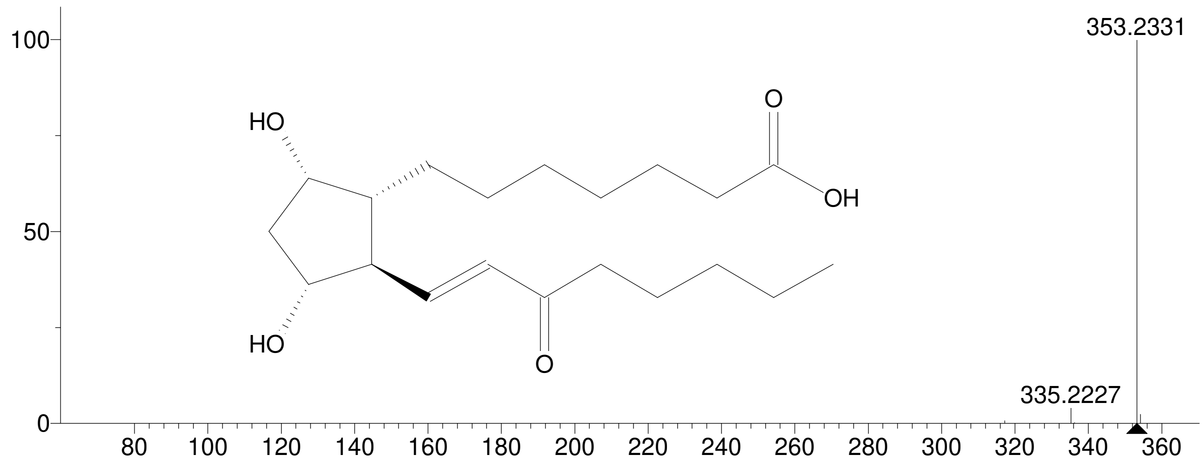

Hit 4 : 15-Ketoprostaglandin E1 [M+H]+ HCD 3V P=353.2
C20H32O5; MF: 287; RMF: 869; Prob 15.6%; CAS: 22973-19-9; Lib: nist_msms; ID: 169227.

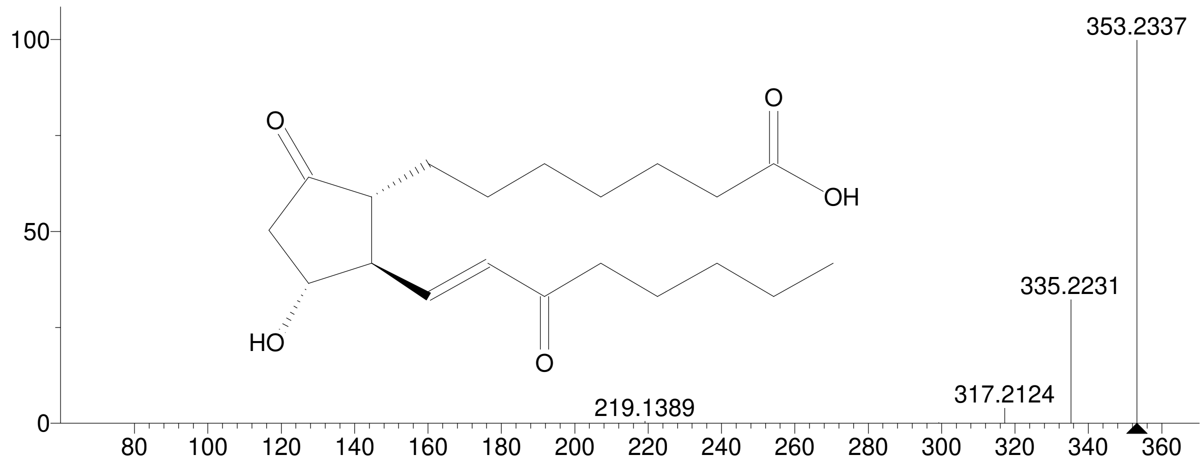

Hit 5 : Prostaglandin D1 [M-H]- HCD 5V P=353.2
C20H34O5; MF: 260; RMF: 925; Prob 4.57%; CAS: 17968-82-0; Lib: nist_msms; ID: 320117.

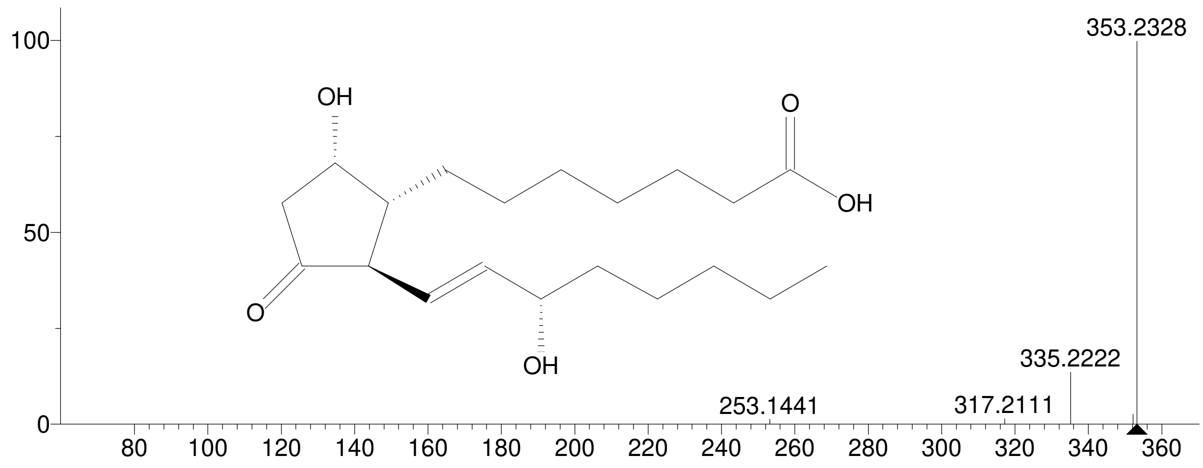

Unknown: Unknown (27.48_452.1495m/z)
Compound in Library Factor = 0

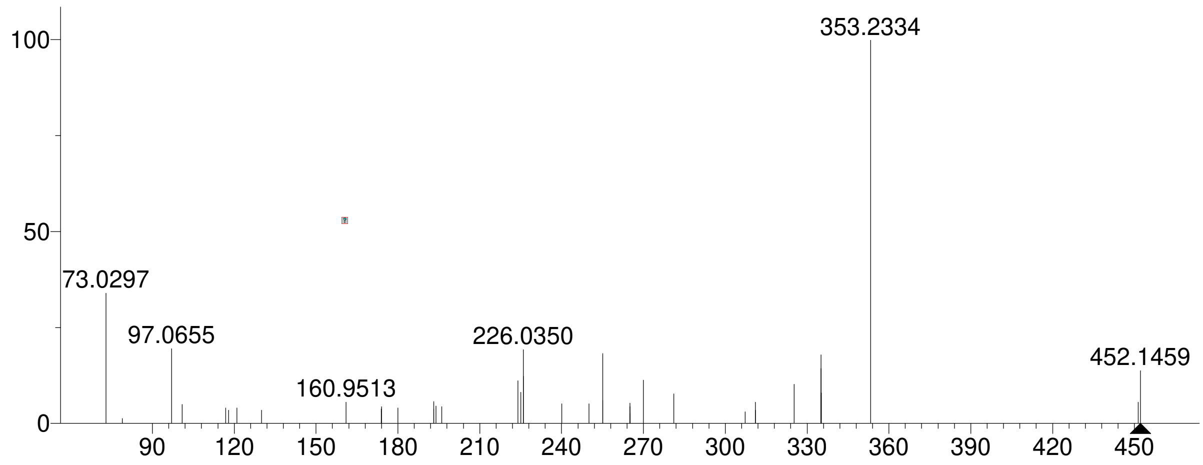

Hit 1 : NOSCAPINE [M+K]+ 35% P=452.2
C22H23NO7; MF: 0; RMF: 57; Prob 100.0%; Lib: mona-export-itree; ID: 697.

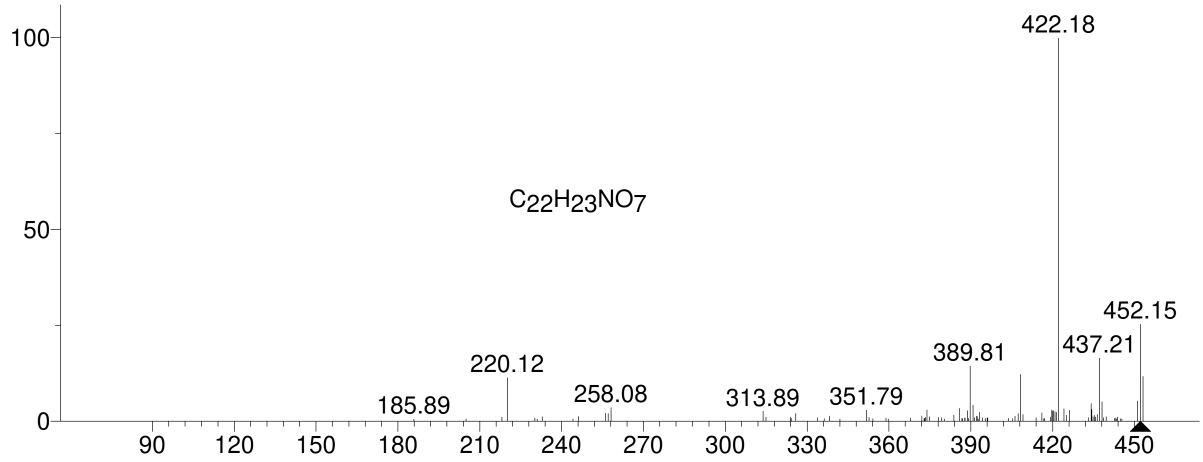

Unknown: Unknown (27.72_417.2637m/z)
Compound in Library Factor = -289

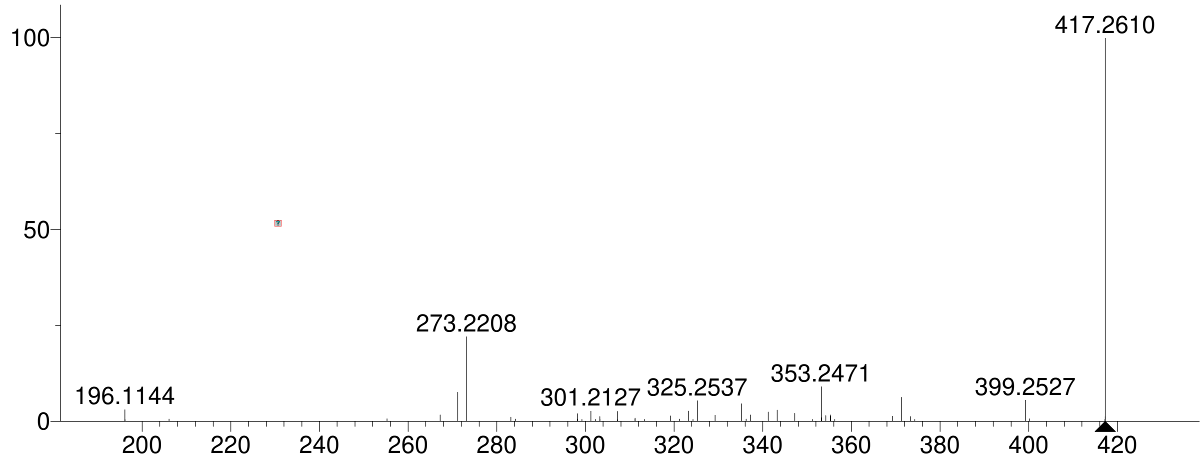

Hit 1 : Prostaglandin E2 isopropyl ester [M+Na]+ QTOF 17V P=417.3
C23H38O5; MF: 317; RMF: 941; Prob 98.8%; CAS: 71845-66-4; Lib: nist_msms; ID: 62065.

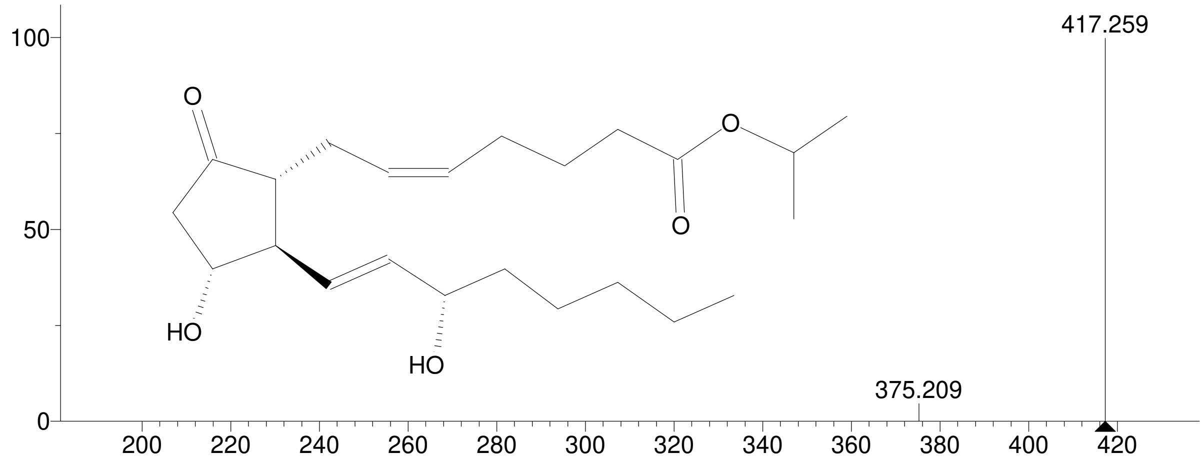

Hit 2 : Alfentanyl [M+H]+ HCD 12V P=417.3
C21H32N6O3; MF: 102; RMF: 667; Prob 0.98%; CAS: 71195-58-9; Lib: nist_msms; ID: 322110.

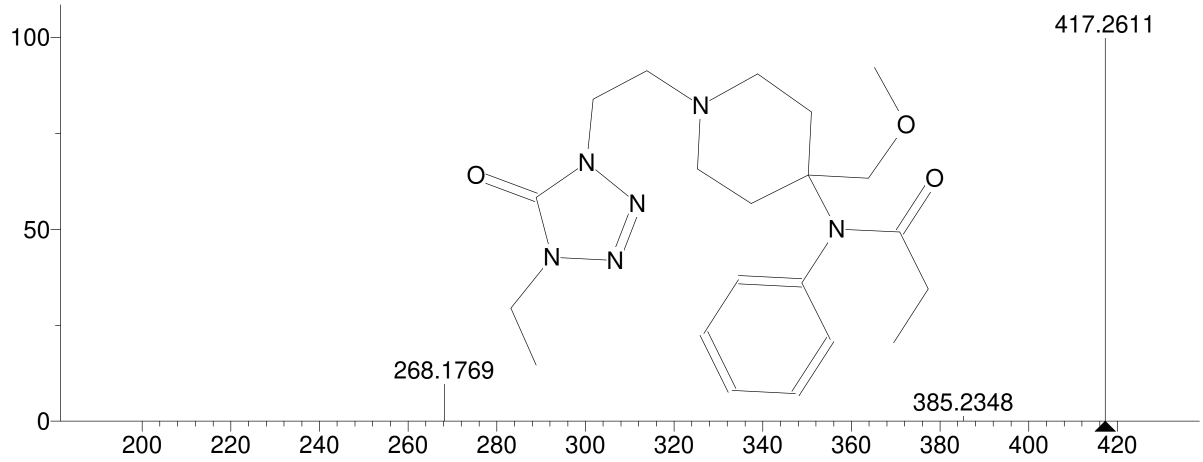

Hit 3 : Manoalide [M+H]+ HCD 12V P=417.3
C25H36O5; MF: 63; RMF: 703; Prob 0.23%; CAS: 75088-80-1; Lib: nist_msms; ID: 362740.

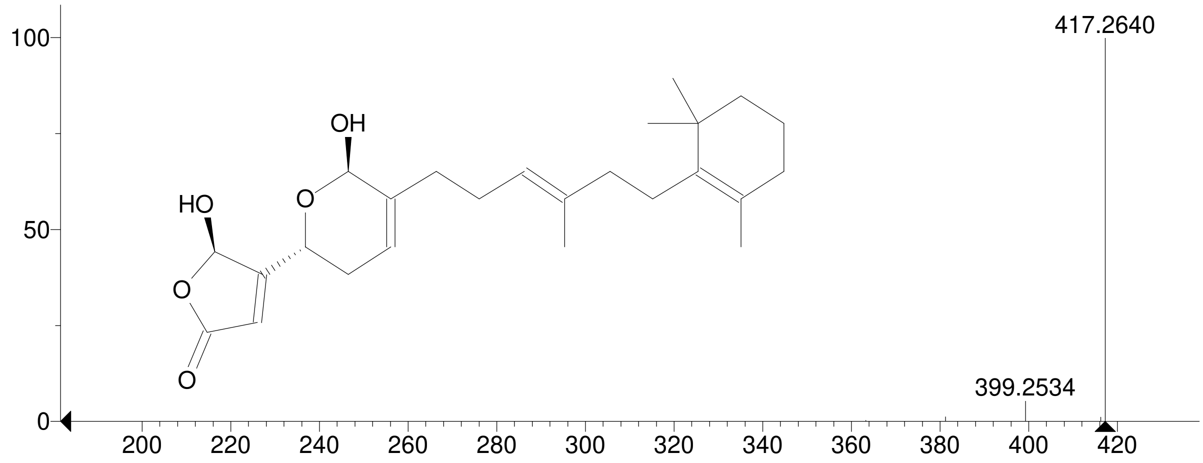

** Search Report Page 1 of 1 **

Unknown: Unknown (27.73_562.1866m/z)
Compound in Library Factor = 0

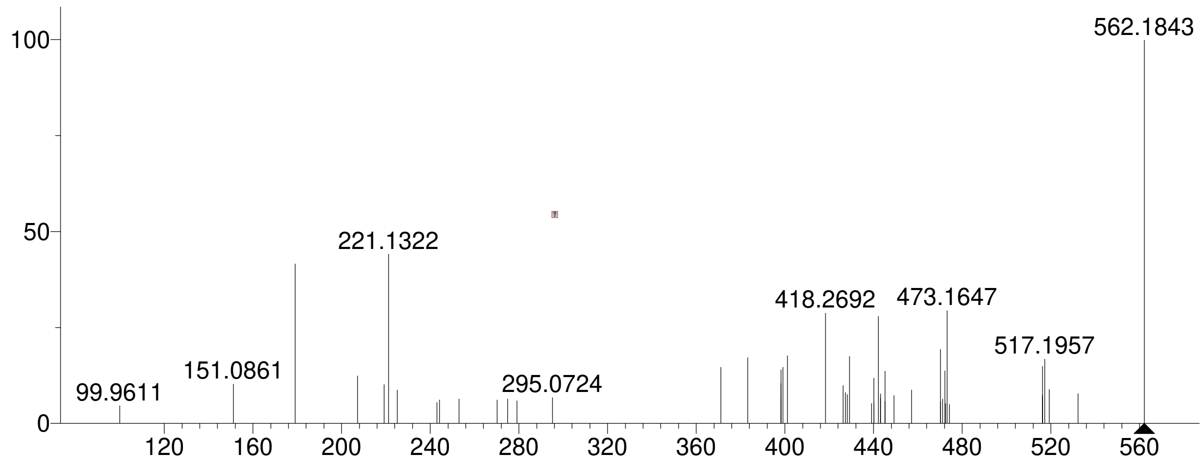

Unknown: Unknown (28.04_299.1632m/z)
Compound in Library Factor = -1090

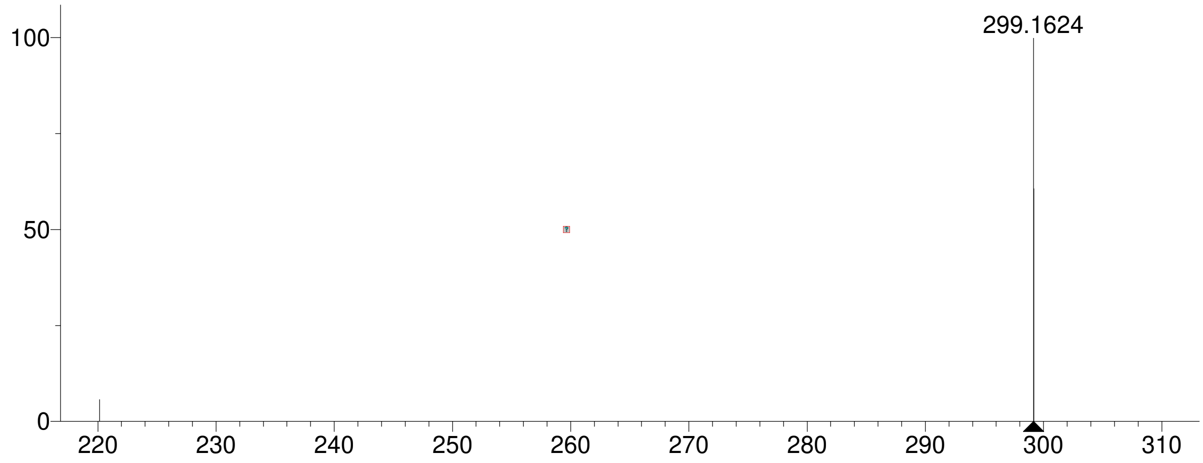

Hit 1 : Olomoucine [M+H]+ HCD 14V P=299.2
C15H18N6O; MF: 220; RMF: 773; Prob 50.6%; CAS: 101622-51-9; Lib: nist_msms; ID: 167756.

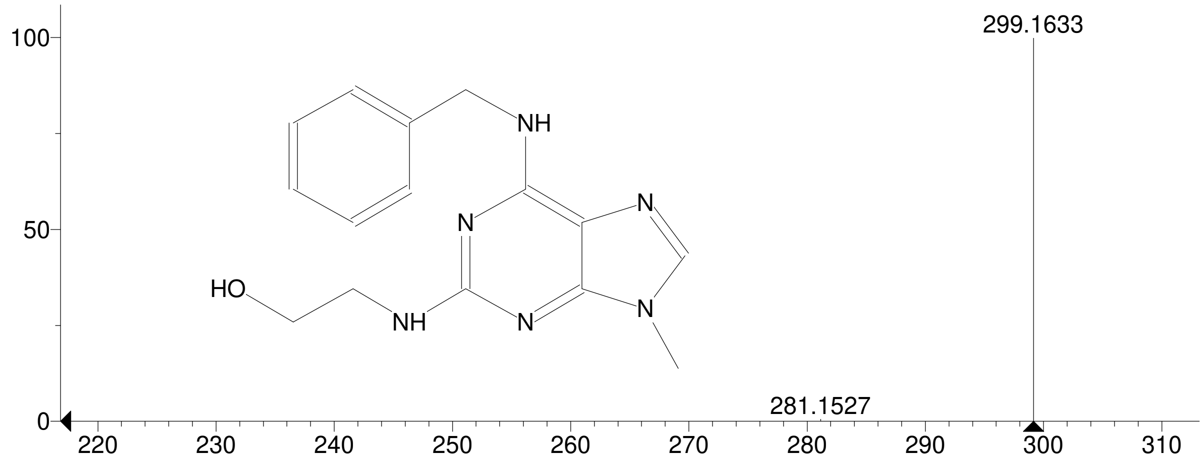

Hit 2 : Urea, N-cyclopropyl-N'-[3-[6-(4-morpholinylmethyl)-1H-benzimidazol-2-yl]-1H-pyrazol-4-yl]- [M+H-C4H5NC
C19H23N7O2; MF: 214; RMF: 772; Prob 39.8%; CAS: 896466-04-9; Lib: nist_msms; ID: 362571.

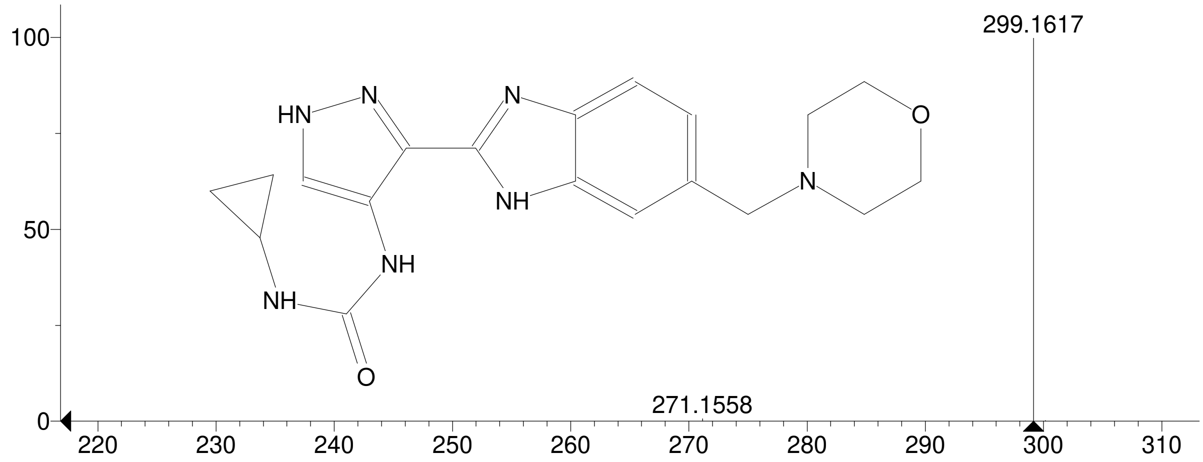

Hit 3 : Aurapten [M+H]+ HCD 8V P=299.2
C19H22O3; MF: 148; RMF: 760; Prob 6.28%; CAS: 495-02-3; Lib: nist_msms; ID: 425033.

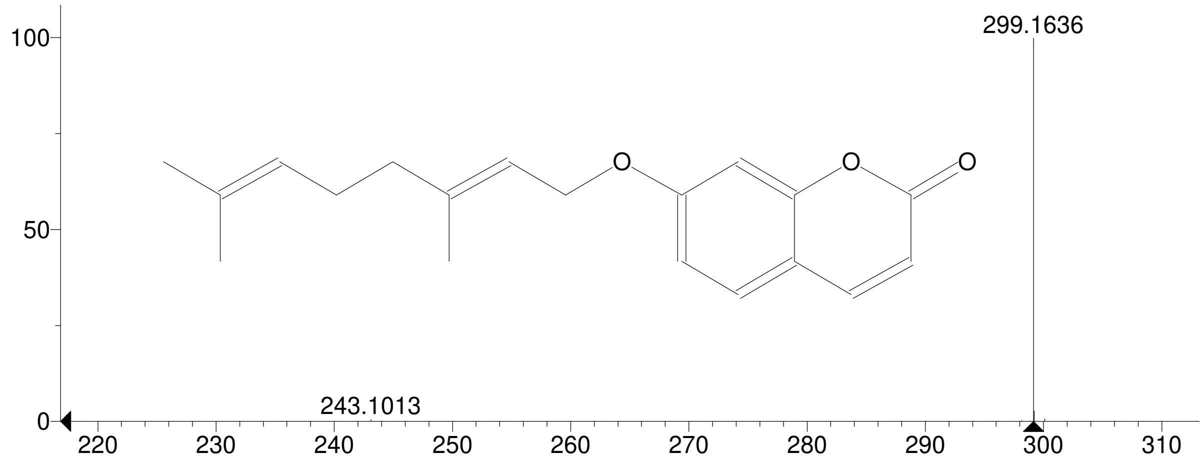

Hit 4 : Iso-olomoucine [M+H]+ HCD 23V P=299.2
C15H18N6O; MF: 105; RMF: 746; Prob 1.38%; CAS: 101622-50-8; Lib: nist_msms; ID: 415534.

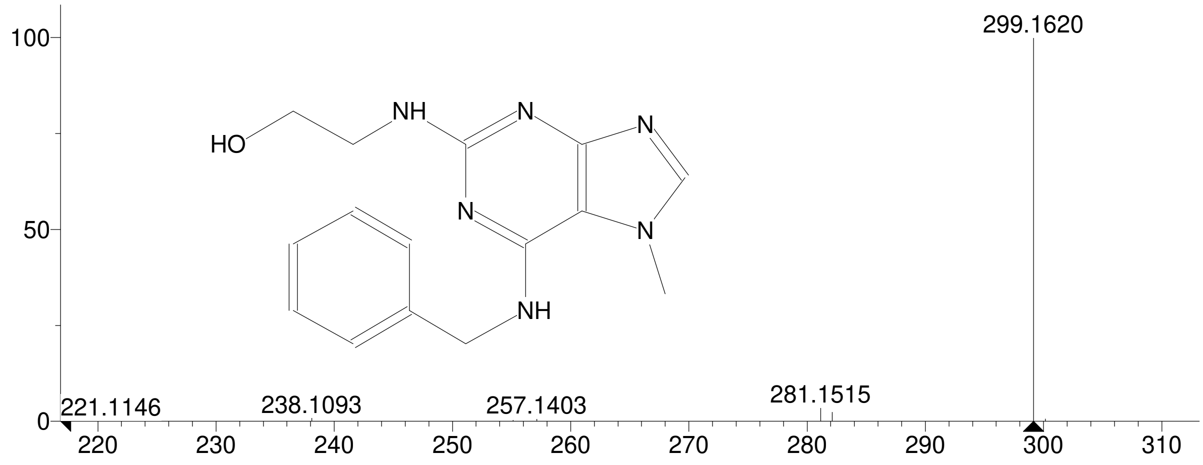

Hit 5 : Oridonin [M+H-CH6O3]+ HCD 13V P=299.2
C20H28O6; MF: 100; RMF: 721; Prob 1.11%; CAS: 28957-04-2; Lib: nist_msms; ID: 238338.

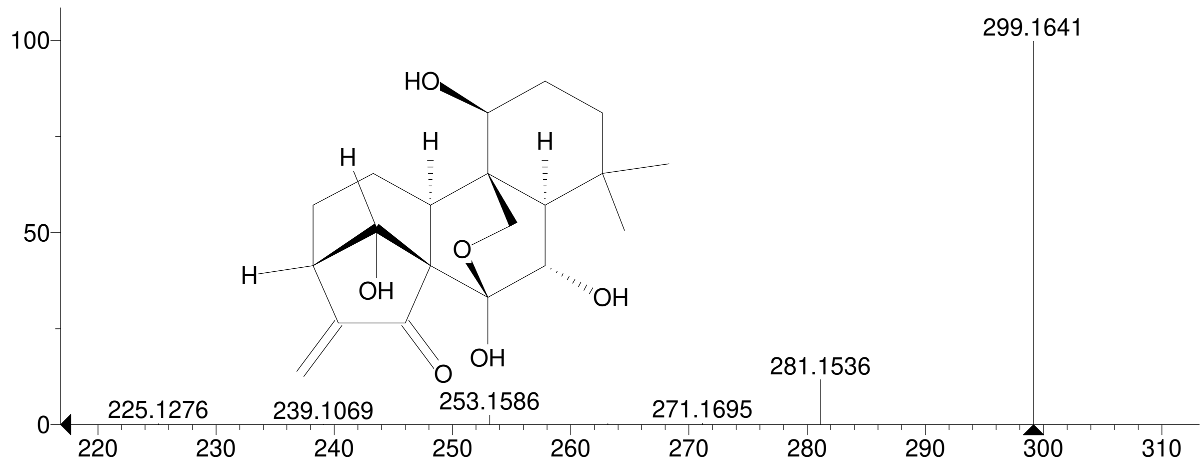

Unknown: Unknown (28.15_417.2630m/z)
Compound in Library Factor = -1142

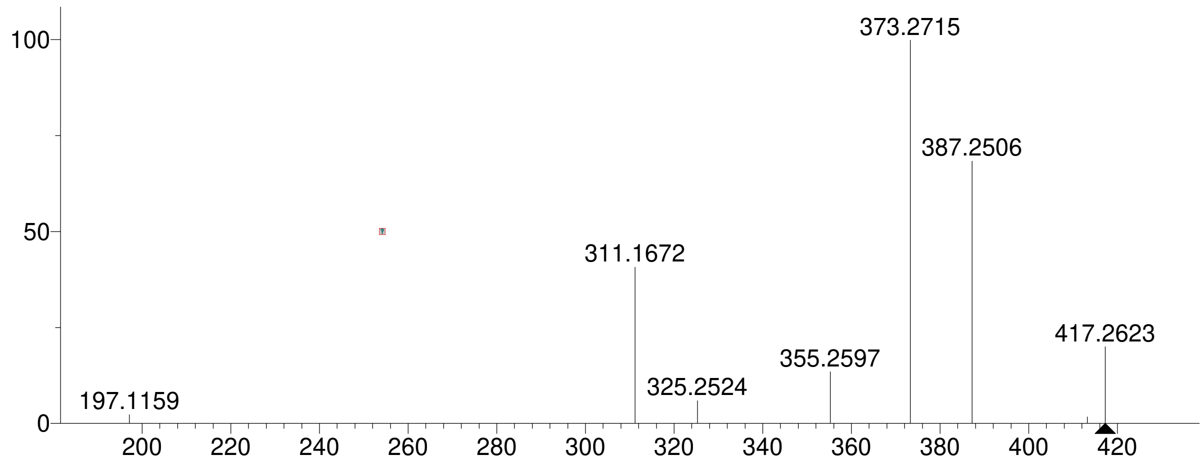

Hit 1 : Prostaglandin E2 isopropyl ester [M+Na]+ QTOF 17V P=417.3
C23H38O5; MF: 91; RMF: 460; Prob 77.5%; CAS: 71845-66-4; Lib: nist_msms; ID: 62065.

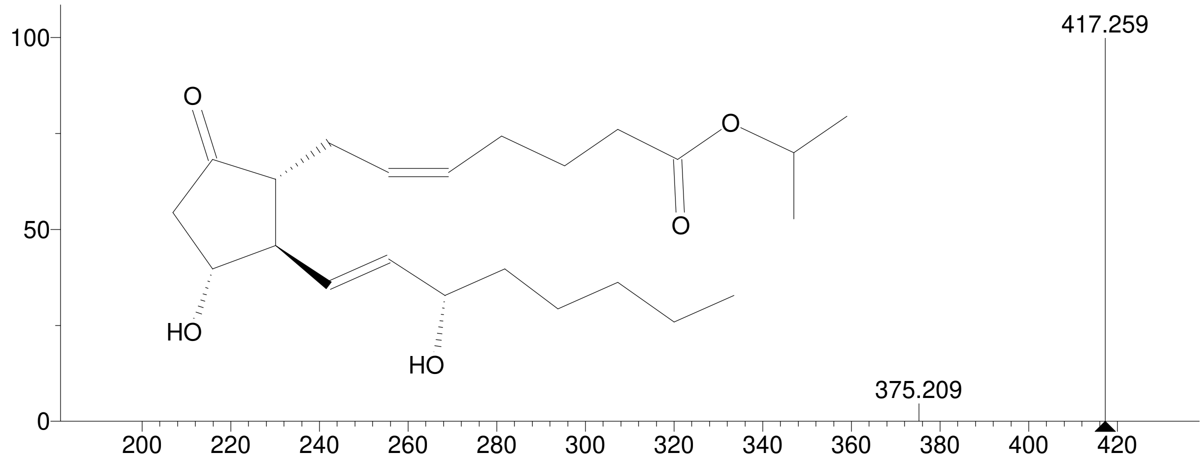

Hit 2 : Alfentanyl [M+H]+ HCD 12V P=417.3
C21H32N6O3; MF: 27; RMF: 268; Prob 12.7%; CAS: 71195-58-9; Lib: nist_msms; ID: 322110.

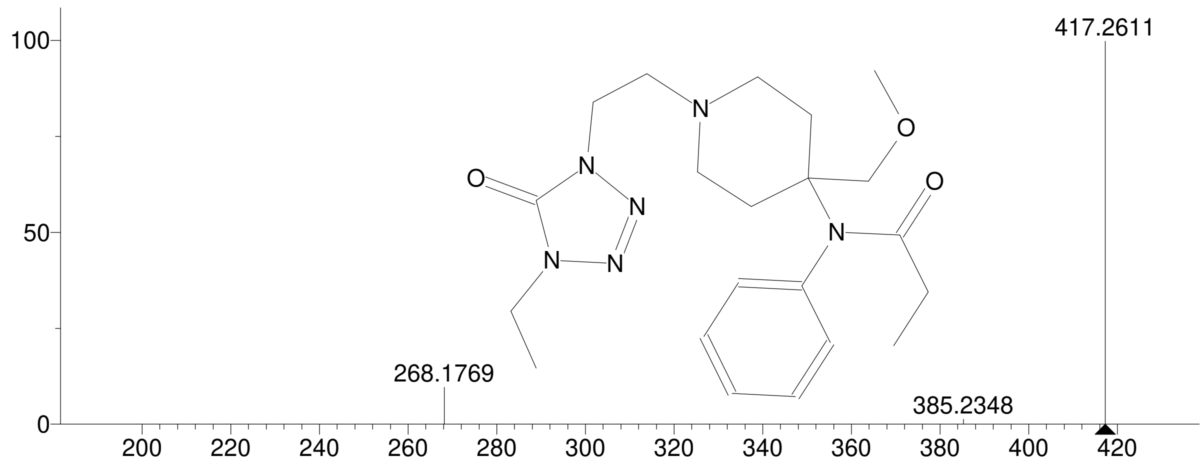

Hit 3 : Manoalide [M+H]+ HCD 12V P=417.3
C25H36O5; MF: 20; RMF: 271; Prob 9.75%; CAS: 75088-80-1; Lib: nist_msms; ID: 362740.

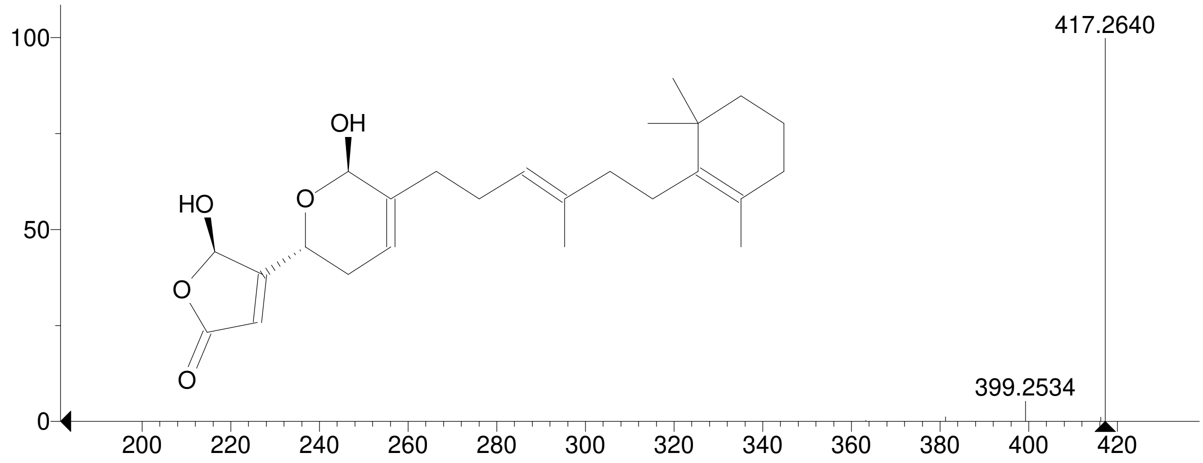

Unknown: Unknown (28.17_431.2428m/z)
Compound in Library Factor = -218

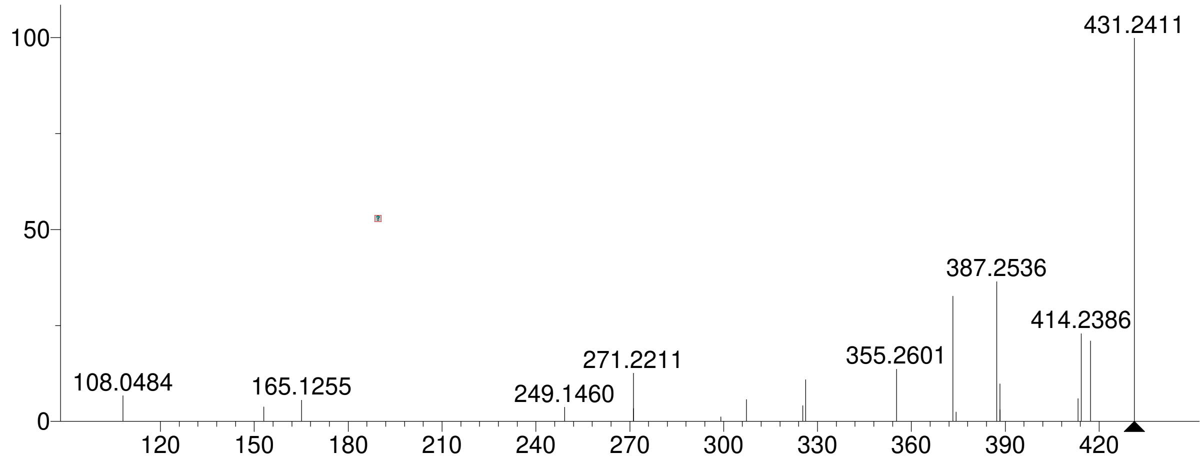

Hit 1 : Bufalin; PlaSMA ID-1130 [M+FA-H]- P=431.2
C24H34O4; MF: 364; RMF: 999; Prob 79.0%; Lib: biomsms-neg-plasma; ID: 160.

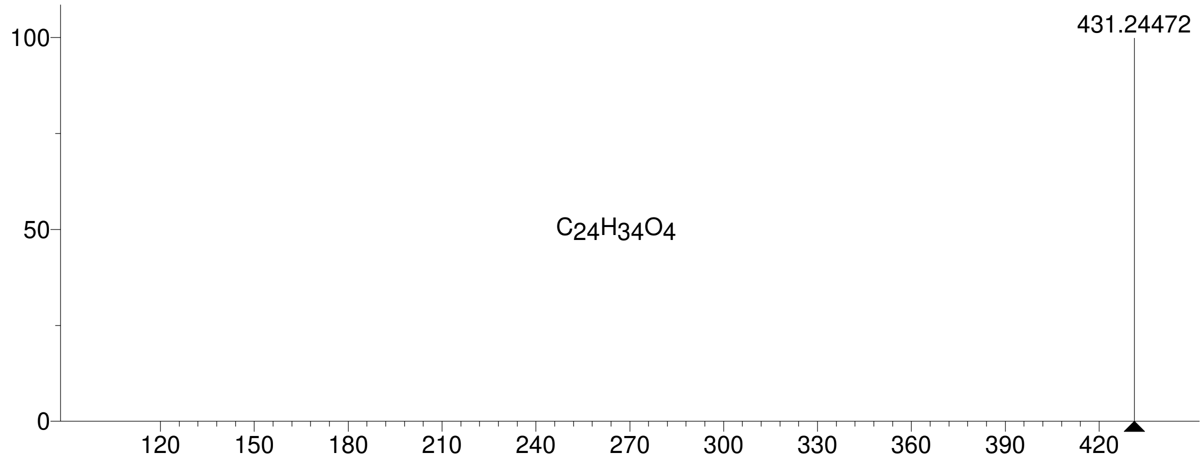

Hit 2 : Budesonide [M+H]+ HCD 4V P=431.2
C25H34O6; MF: 316; RMF: 830; Prob 16.4%; CAS: 51333-22-3; Lib: nist_msms; ID: 152670.

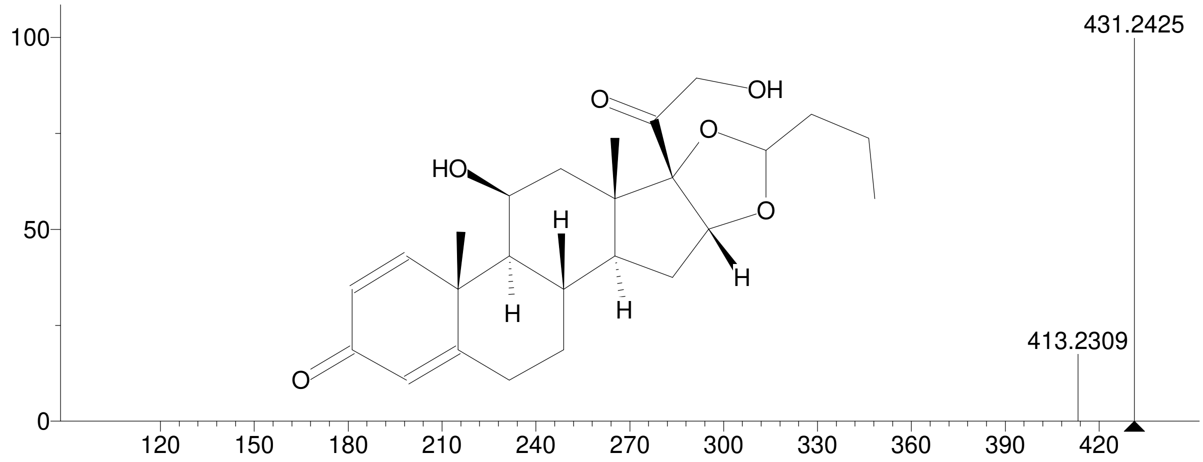

Hit 3 : Bufalin [M+FA-H]- 10 P=431.2
C24H34O4; MF: 286; RMF: 913; Prob 4.54%; Lib: msms neg mona_ipb_gnps_plasma; ID: 21271.

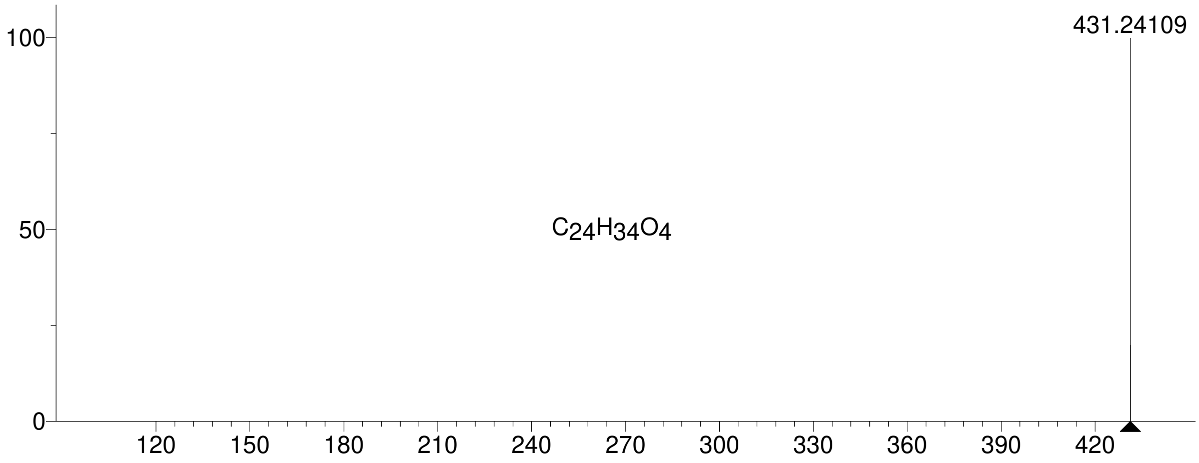

Hit 4 : 3β,5β-Tetrahydrocortisone 3,21-diacetate [M+H-H2O]+ HCD 8V P=431.2
C25H36O7; MF: 35; RMF: 539; Prob 0.04%; CAS: 4003-93-4; Lib: nist_msms; ID: 551376.

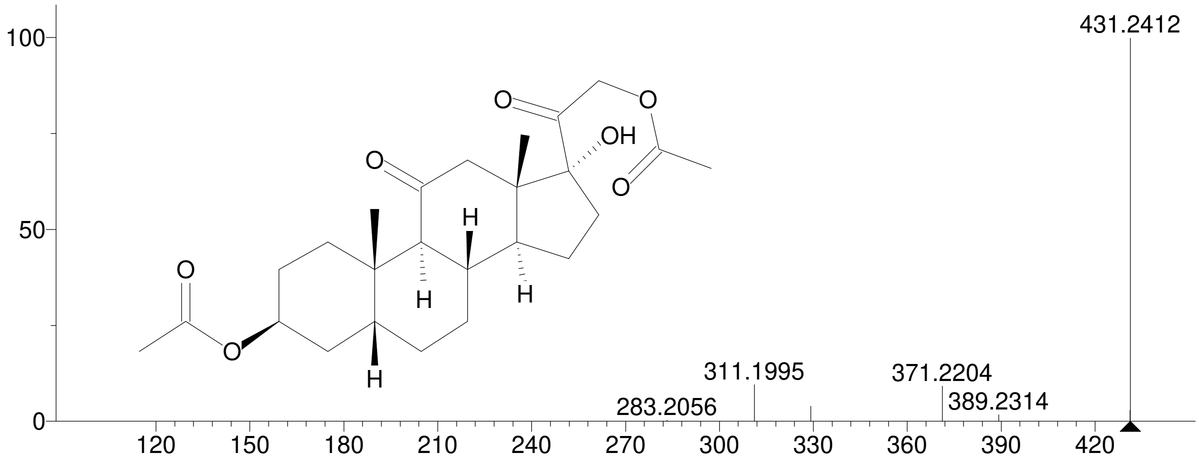

Hit 5 : Phe-His-Lys [M+H]+ QTOF 5V P=431.2
C21H30N6O4; MF: 8; RMF: 537; Prob 0.01%; Lib: nist_msms; ID: 50393.

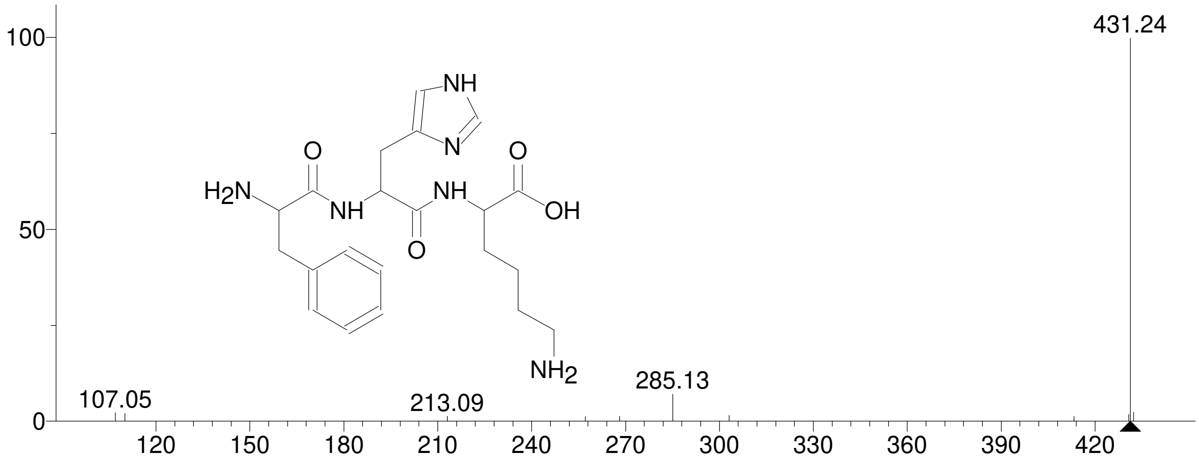

Unknown: Unknown (28.41_417.2631m/z)
Compound in Library Factor = -2176

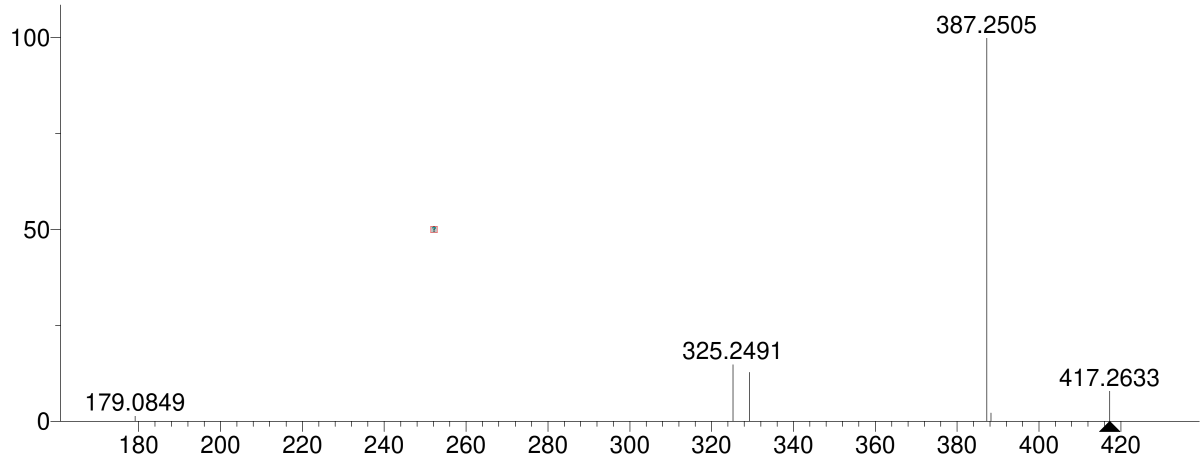

Hit 1 : Prostaglandin E2 isopropyl ester [M+Na]+ QTOF 17V P=417.3
C23H38O5; MF: 43; RMF: 262; Prob 56.5%; CAS: 71845-66-4; Lib: nist_msms; ID: 62065.

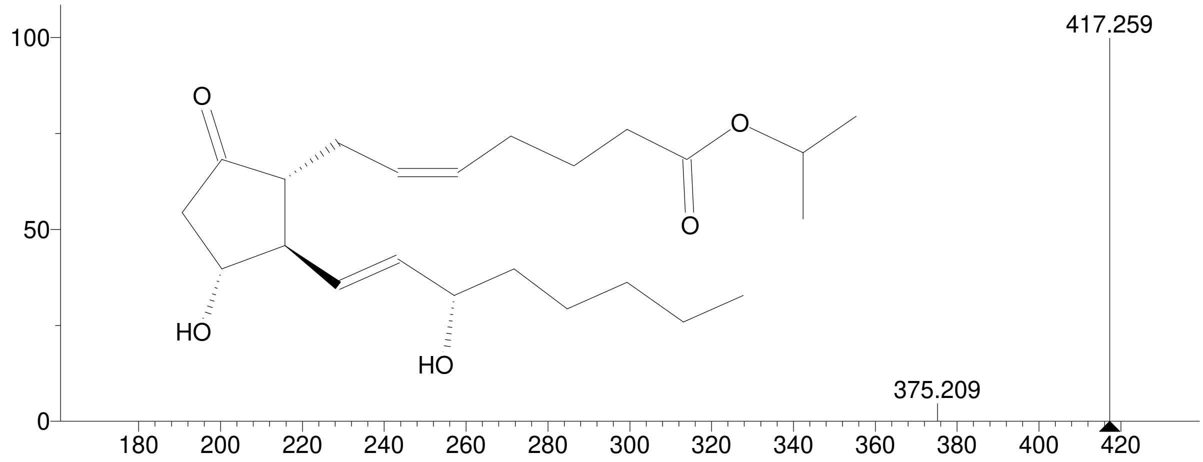

Hit 2 : Alfentanyl [M+H]+ HCD 12V P=417.3
C21H32N6O3; MF: 22; RMF: 227; Prob 24.1%; CAS: 71195-58-9; Lib: nist_msms; ID: 322110.

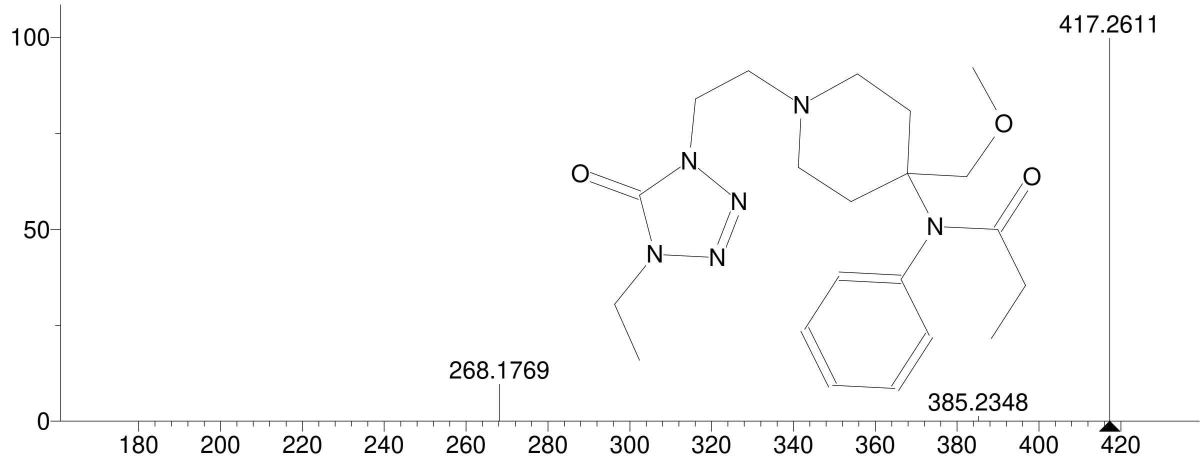

Hit 3 : Manoalide [M+H]+ HCD 12V P=417.3
C25H36O5; MF: 17; RMF: 229; Prob 19.4%; CAS: 75088-80-1; Lib: nist_msms; ID: 362740.

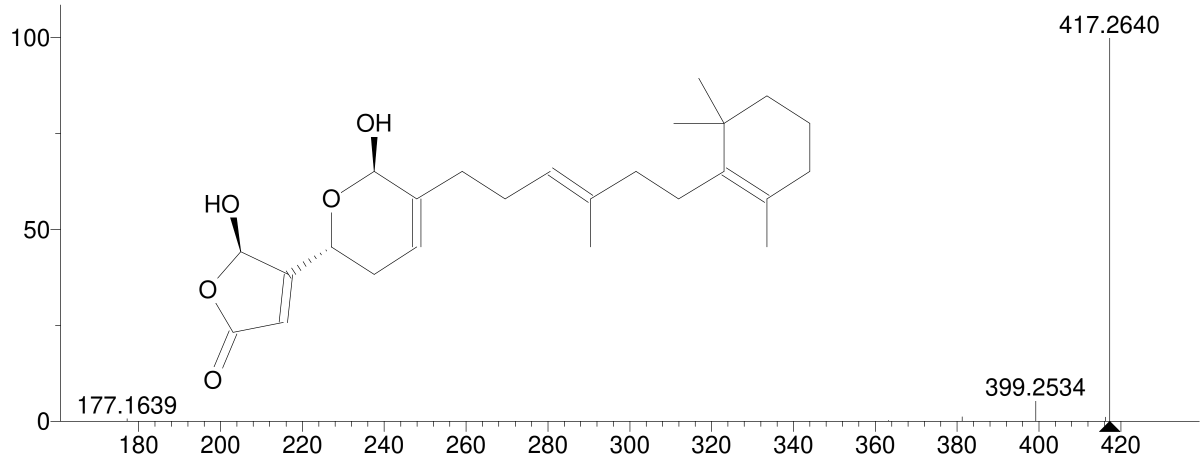

Unknown: Unknown (28.87_433.2581m/z)
Compound in Library Factor = -2387

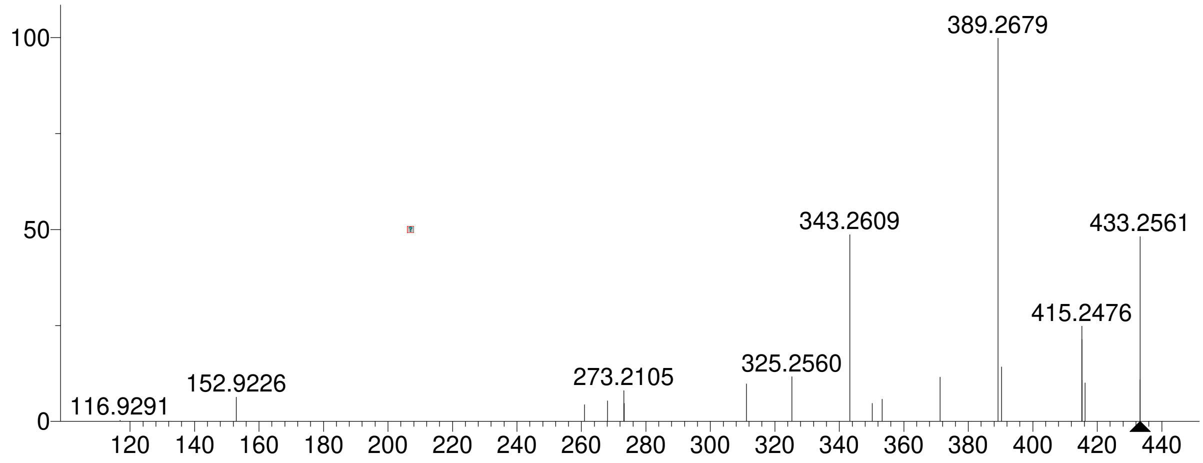

Hit 1 : 3β-Tetrahydrocortisol, 3,21-diacetate [M+H-H2O]+ HCD 8V P=433.3
C25H38O7; MF: 25; RMF: 357; Prob 39.6%; CAS: 6820-57-1; Lib: nist_msms; ID: 454119.

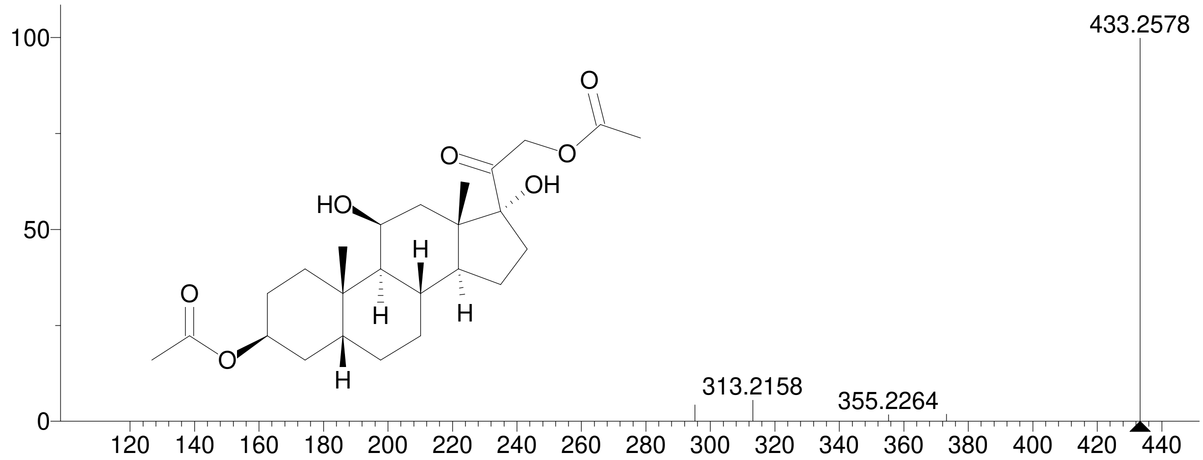

Hit 2 : Arg-Phe-Lys [M+H-NH3]+ QTOF 15V P=433.3
C21H35N7O4; MF: 11; RMF: 285; Prob 24.8%; Lib: nist_msms; ID: 53463.

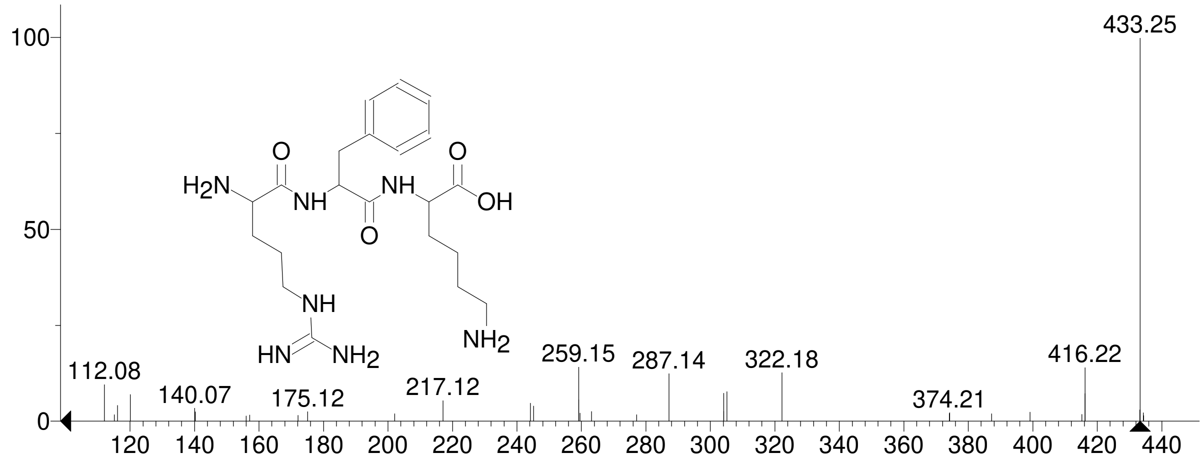

Hit 3 : [(2R)-2-[(E,2S,4R)-4,6-dimethyloct-6-en-2-yl]-6-oxo-2,3-dihydropyran-3-yl] (2E,4E,6S)-8-hydroxy-6-(hydrox
C25H38O6; MF: 6; RMF: 164; Prob 20.0%; Lib: mona-export-lc-ms-ms_negative_mode; ID: 22226.

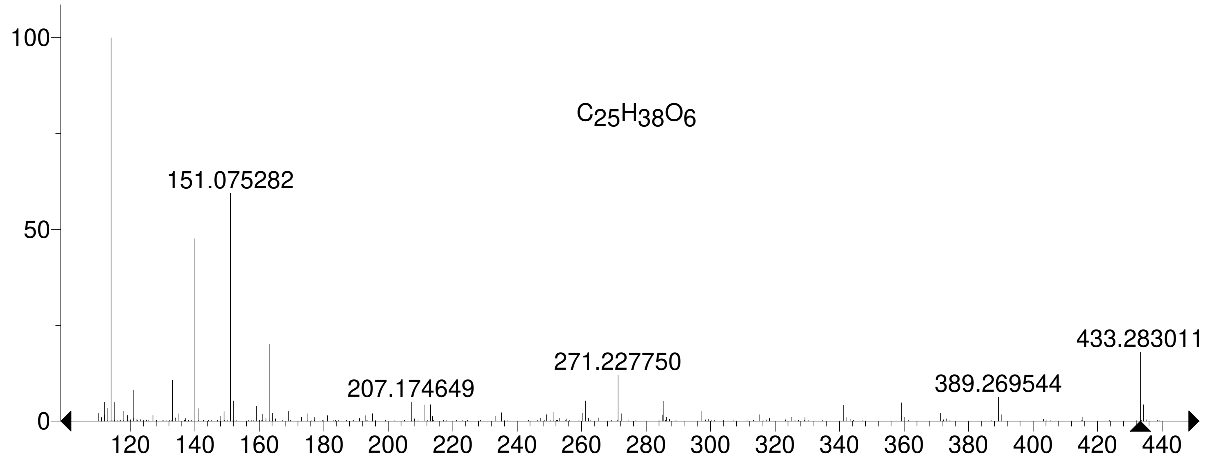

Hit 4 : "MLS001076086-01!11BETA,17,21-TRIHYDROXYPREGN-4-ENE-3,20-DIONE 17-BUTYRATE" [M+H]+ F
C25H36O6; MF: 0; RMF: 7; Prob 15.7%; Lib: mona-export-gnps; ID: 3390.

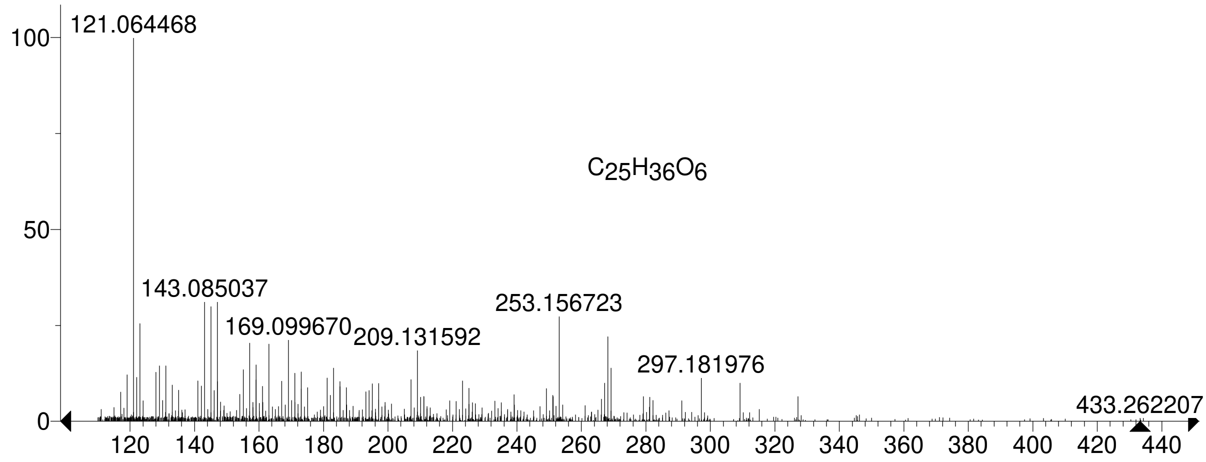

Unknown: Unknown (29.19_309.1732m/z)
Compound in Library Factor = -1309

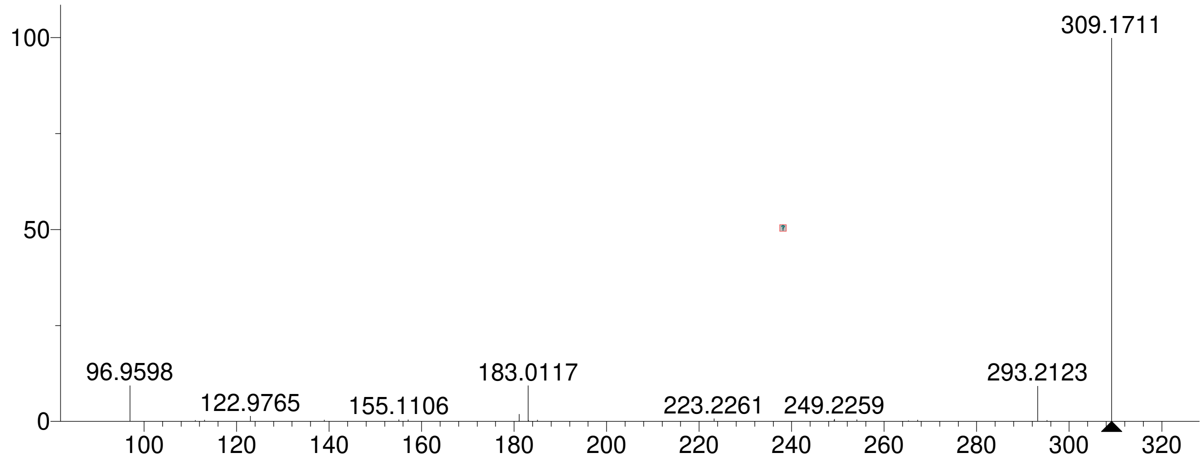

Hit 1 : MMV676401 [M+H]+ 20-30-40%) P=309.2
C18H20N4O; MF: 105; RMF: 808; Prob 72.3%; Lib: mona-export-pathogen_box; ID: 17.

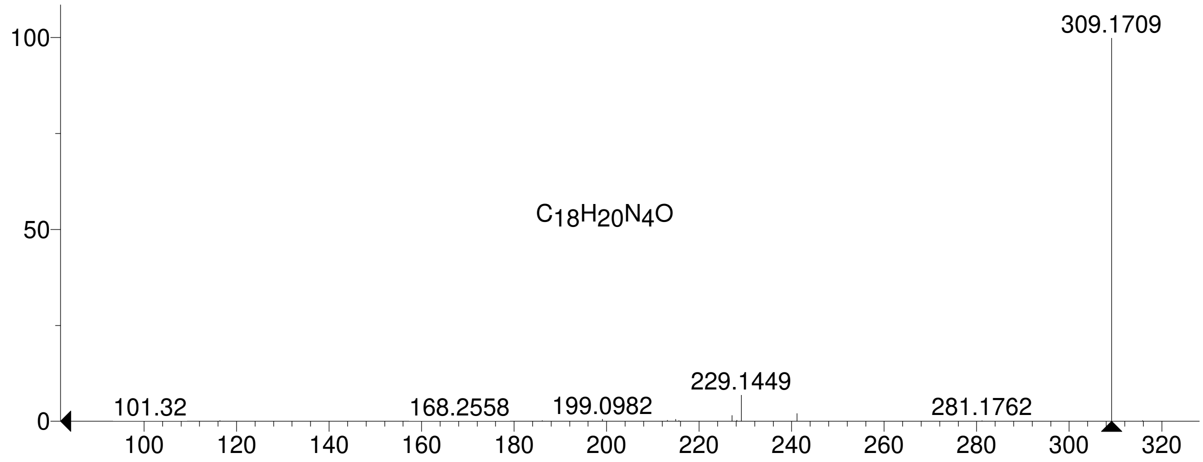

Hit 2 : Mestranol [M-H]- 20-30-40%) P=309.2
C21H26O2; MF: 80; RMF: 796; Prob 22.1%; Lib: mona-export-fiehn_hilic; ID: 1322.

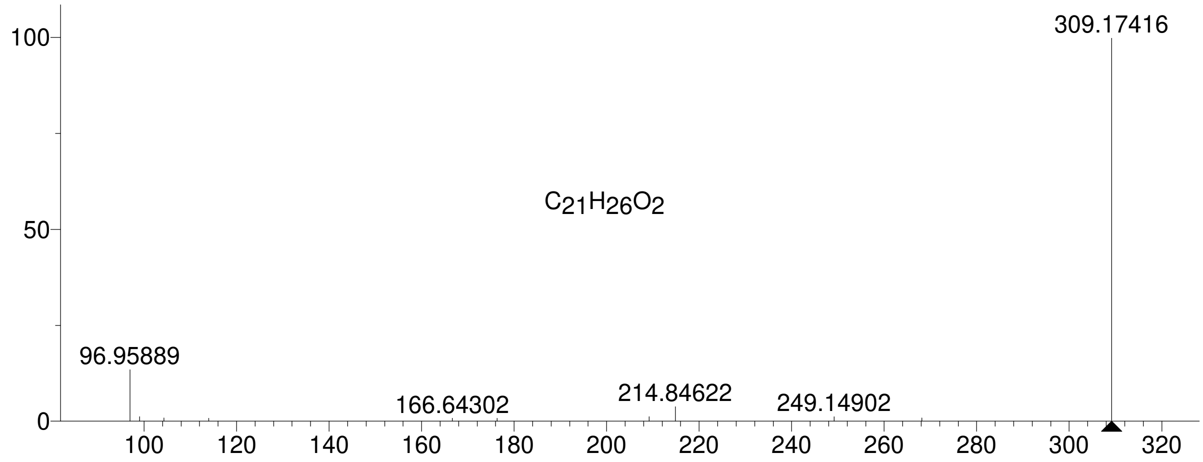

Hit 3 : C12-AE1S (TENTATIVE) [M-H]- 60NCE P=309.2
C14H30O5S; MF: 27; RMF: 835; Prob 4.36%; Lib: mona-export-lc-ms-ms_negative_mode; ID: 14009.

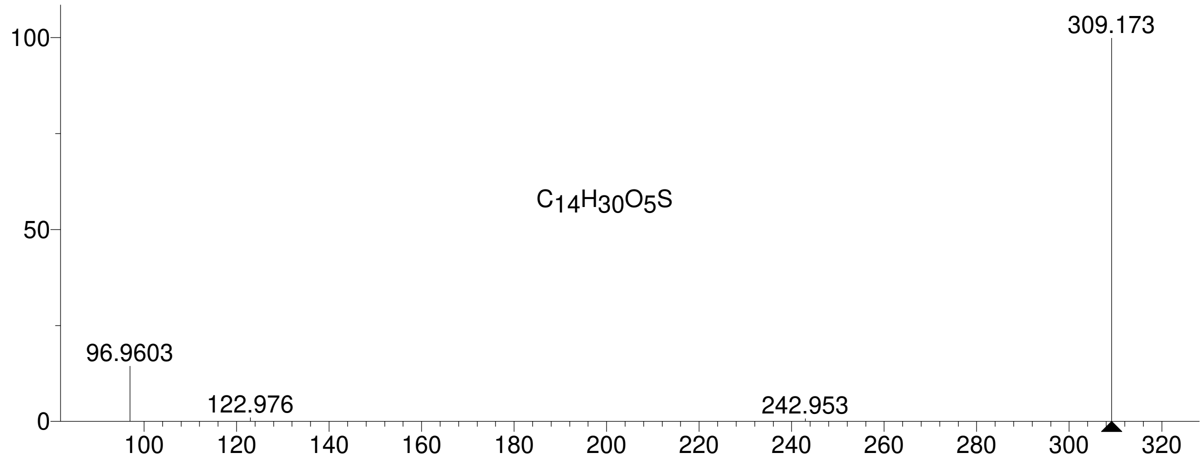

Hit 4 : Ethylene glycol dodecyl ether sulfate [M-H]- QQQ 28V P=309.2
C14H30O5S; MF: 0; RMF: 7; Prob 1.28%; CAS: 48073-44-5; Lib: nist_msms; ID: 6887.

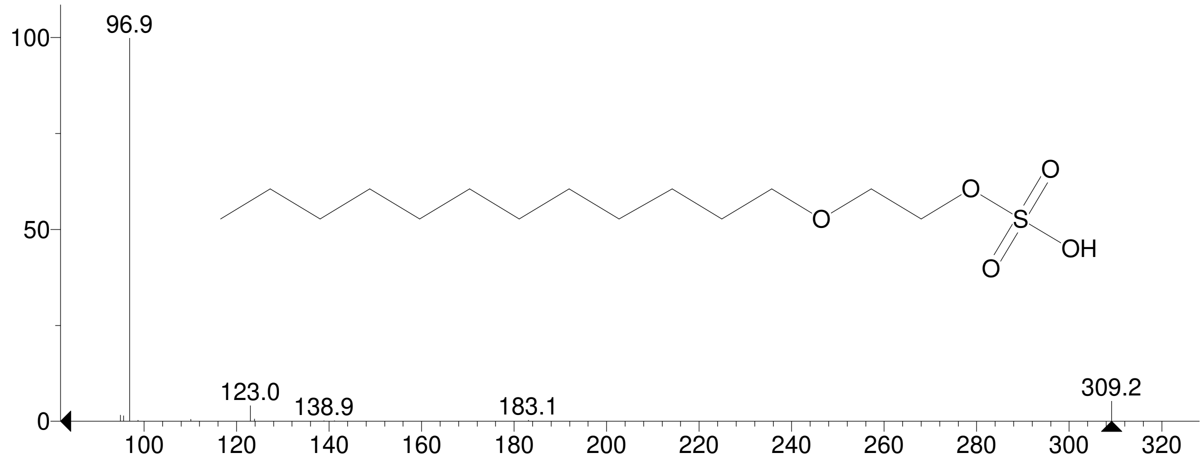

Unknown: Unknown (29.81_437.2899m/z)
Compound in Library Factor = 0

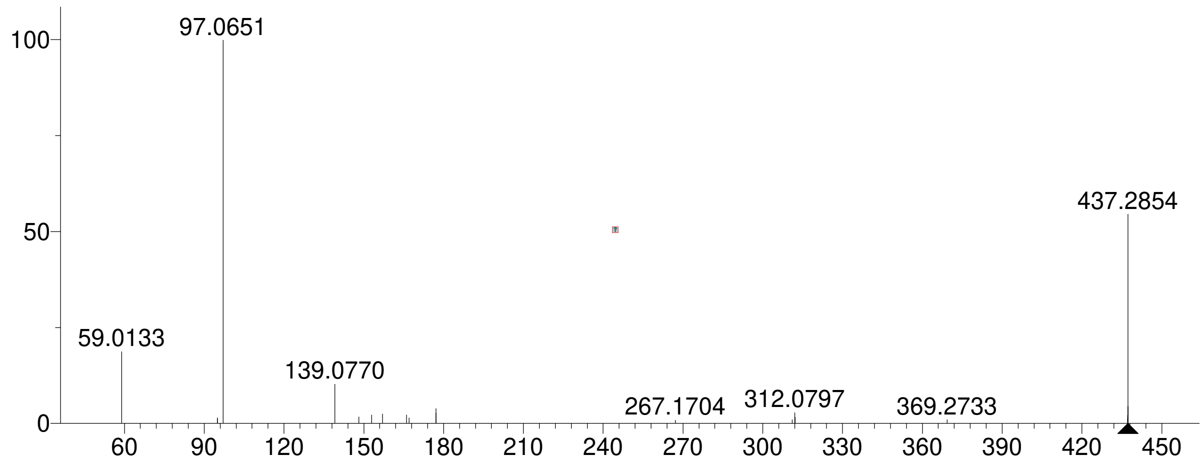

Unknown: Unknown (3.00_695.2537m/z)
Compound in Library Factor = 0

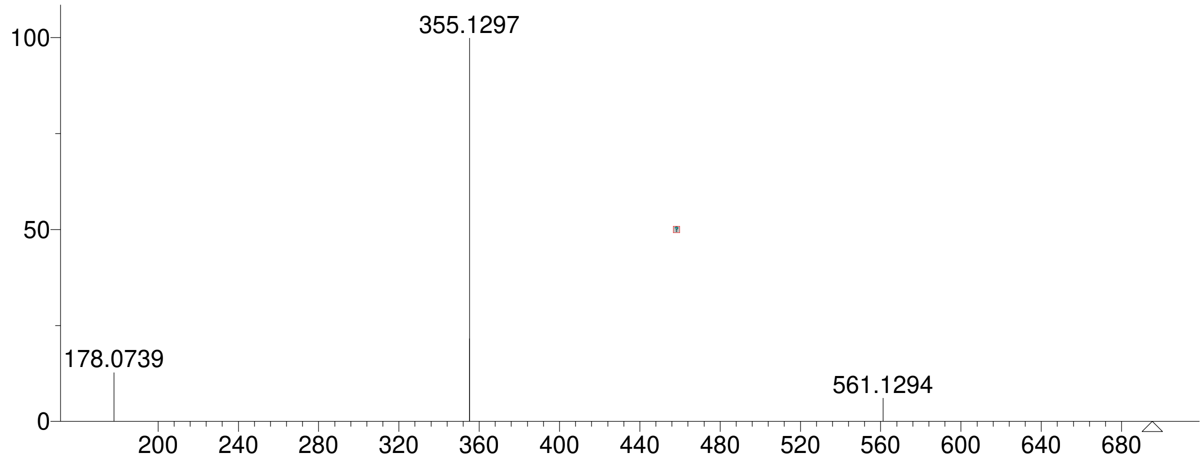

Hit 1 : Calcitonin (1-7), human [M+H]+ HCD 43eV P=695.2
C25H42N8O11S2; MF: 0; RMF: 4; Prob 100.0%; Lib: nist_msms2; ID: 62813.

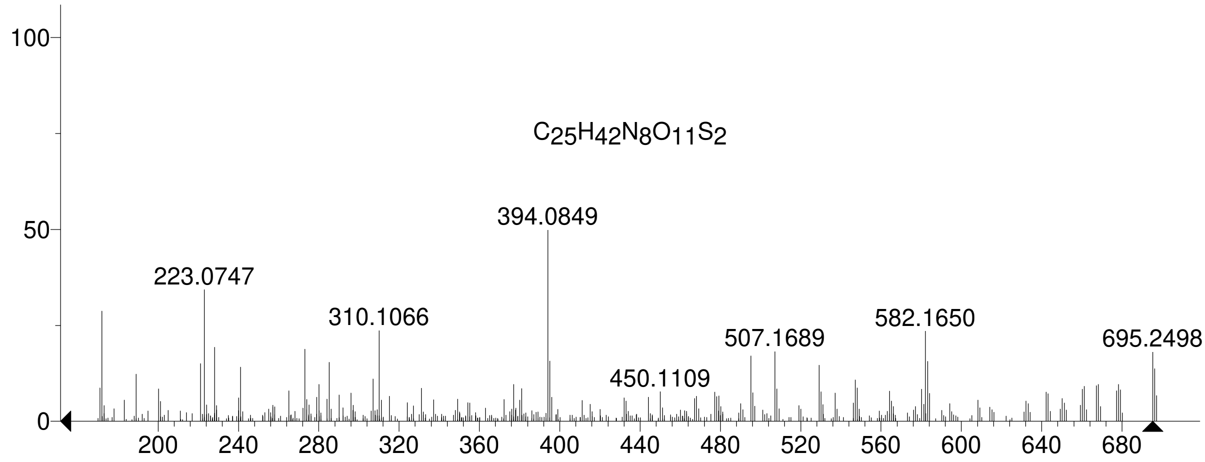

Unknown: Unknown (3.01_387.1394m/z)
Compound in Library Factor = 0

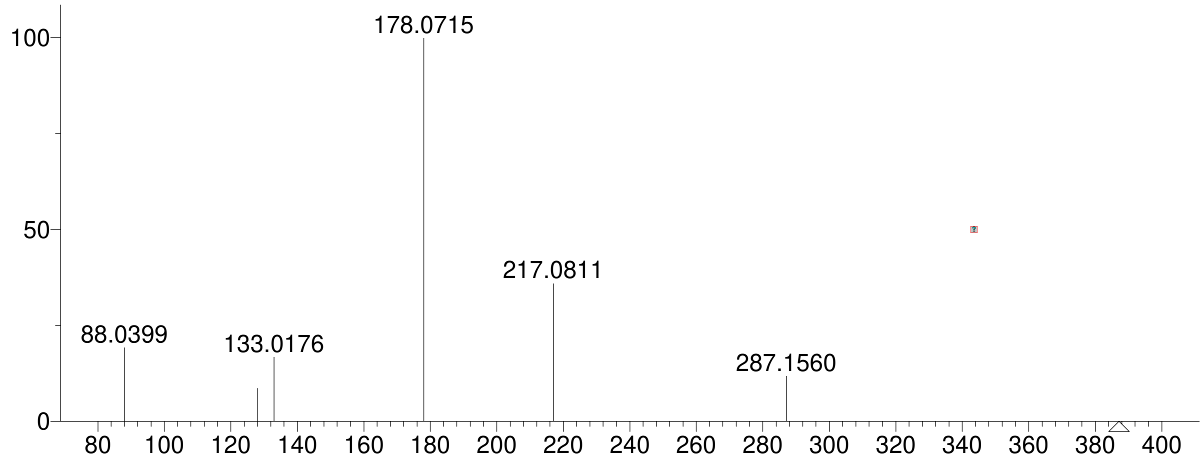

Hit 1 : (5R,6S,8Z,11S)-5,6,15-trihydroxy-17-methoxy-11-methyl-12-oxabicyclo[12.4.0]octadeca-1(14),8,15,17-tetra
C19H24O7; MF: 0; RMF: 36; Prob 33.3%; Lib: mona-export-vf-npl_qexactive; ID: 18340.

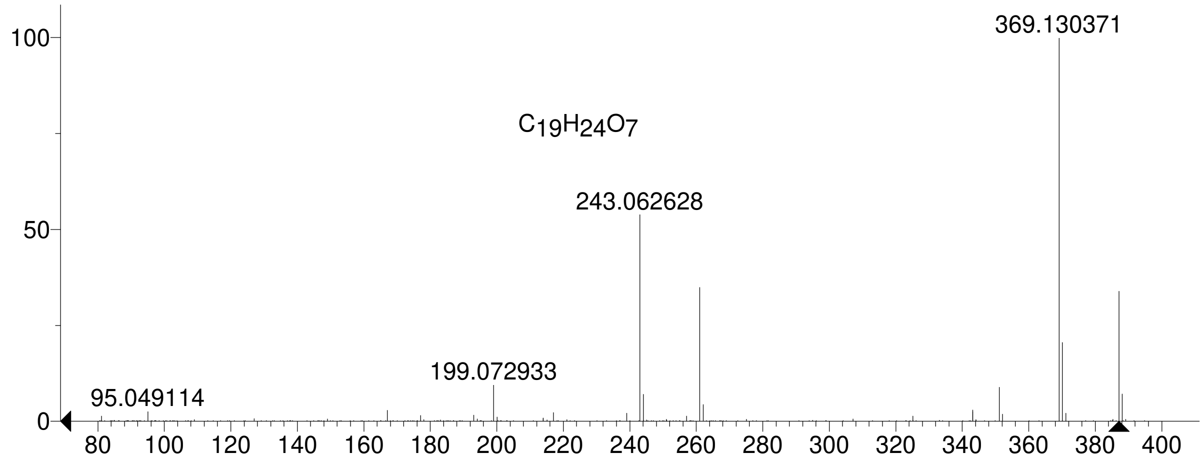

Hit 2 : [(3aR,4S,6R,11aR)-6,9-dihydroxy-6,10-dimethyl-3-methylidene-2,7-dioxo-3a,4,5,8,9,11a-hexahydrocyclode
C19H24O7; MF: 0; RMF: 25; Prob 33.3%; Lib: mona-export-vf-npl_qexactive; ID: 10212.

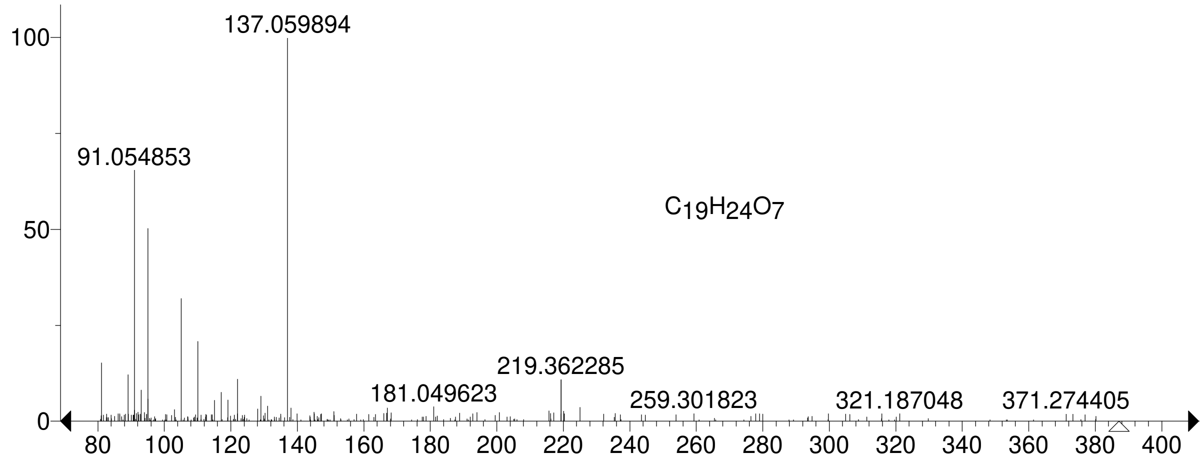

Hit 3 : NCGC00347789-02_C19H24O7_(3S,5Z,8S,9R)-8,9,16-Trihydroxy-14-methoxy-3-methyl-3,4,9,10,11,12-he
C19H24O7; MF: 0; RMF: 21; Prob 33.3%; Lib: mona-export-gnps; ID: 11355.

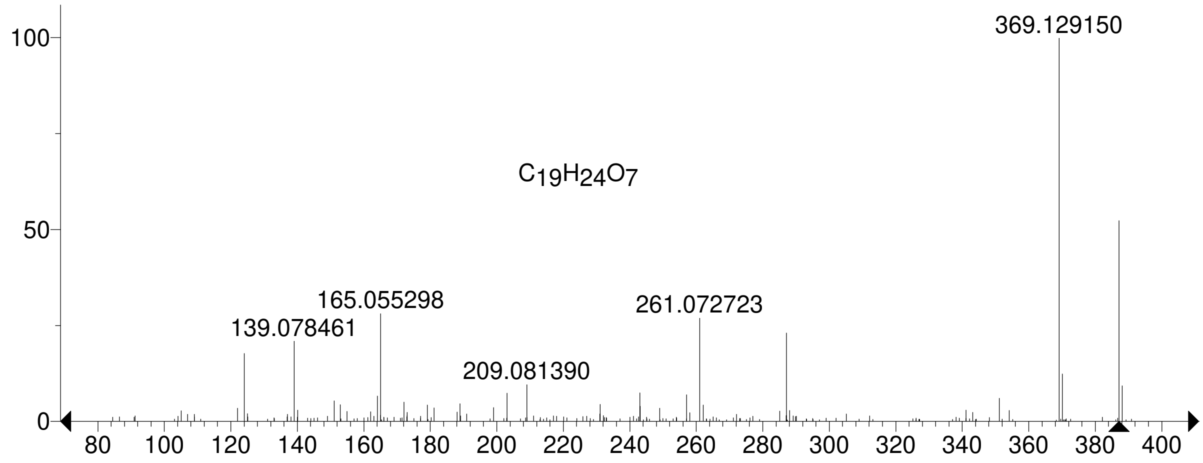

Unknown: Unknown (3.01_508.2138m/z)
Compound in Library Factor = -3273

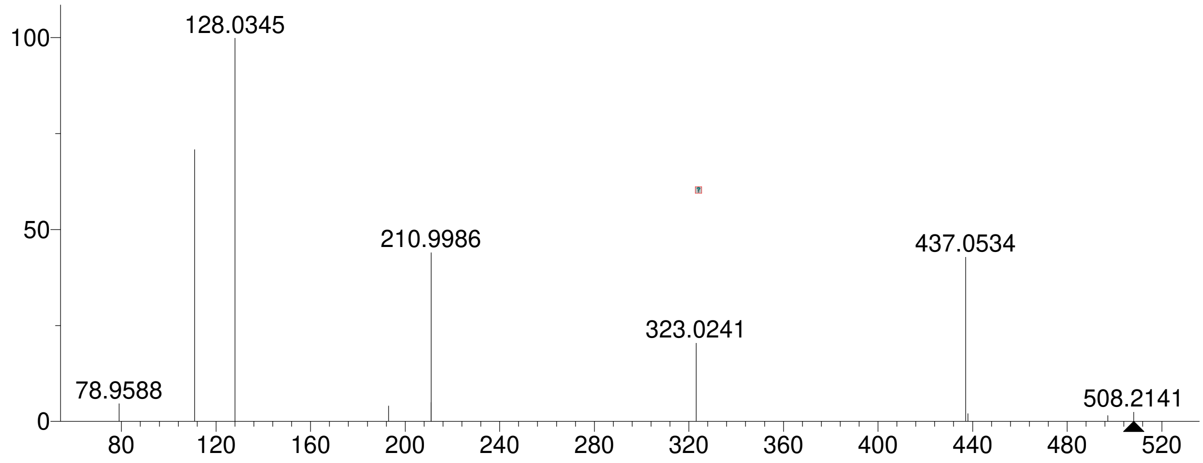

Hit 1 : β-Lipotropin (61-69) [M-2H]2- HCD 59eV P=508.2
C45H66N10O15S; MF: 1; RMF: 232; Prob 100.0%; Lib: nist_msms2; ID: 77125.

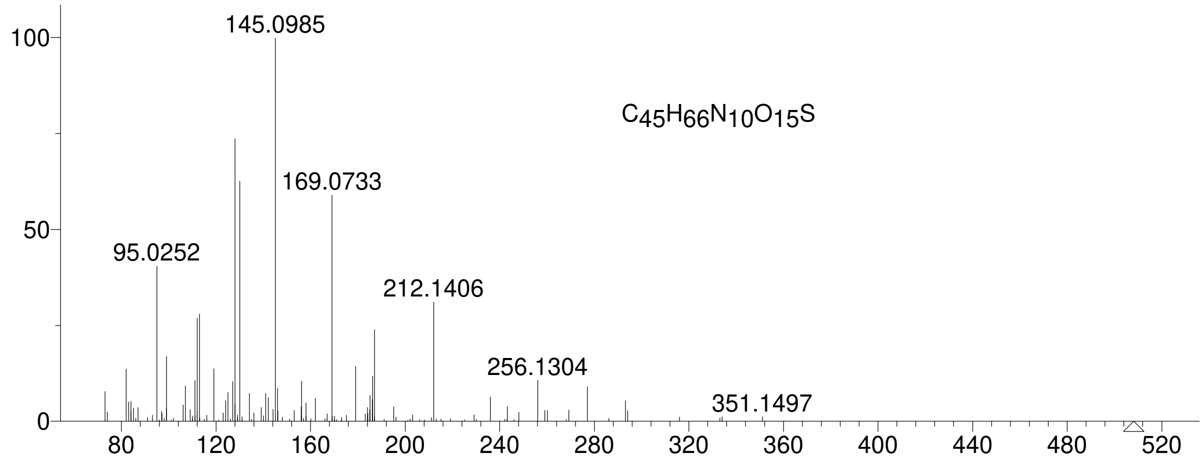

** Search Report Page 1 of 2 **

Unknown: Unknown (3.02_549.0867m/z)
Compound in Library Factor = -3273

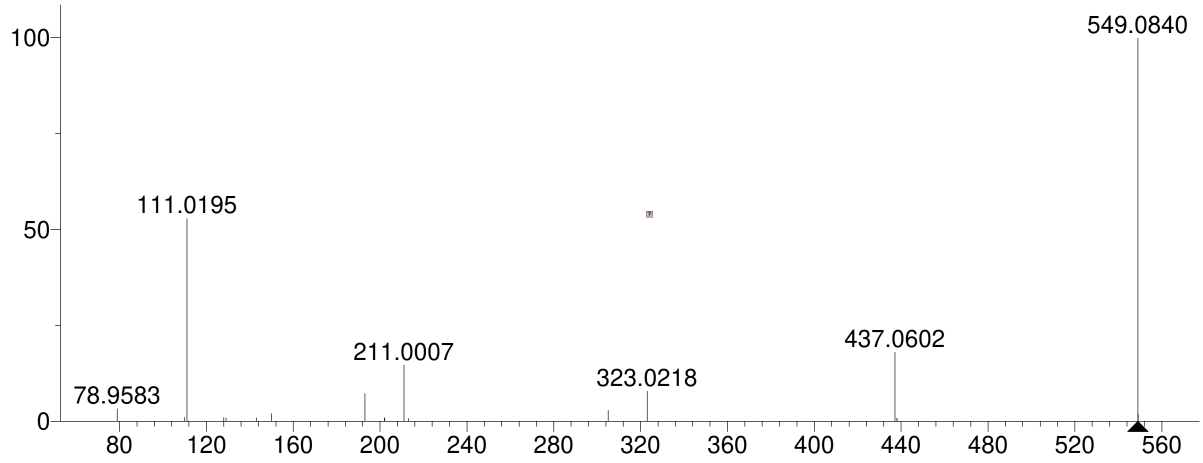

Hit 1 : 5-(3-(2-(7-Chloroquinolin-2-yl)ethenyl)phenyl)-8-dimethylcarbamyl-4,6-dithiaoctanoic acid [M+Cl]- HCD 1
C26H27ClN2O3S2; MF: 8; RMF: 254; Prob 21.1%; CAS: 115104-28-4; Lib: nist_msms; ID: 538815.

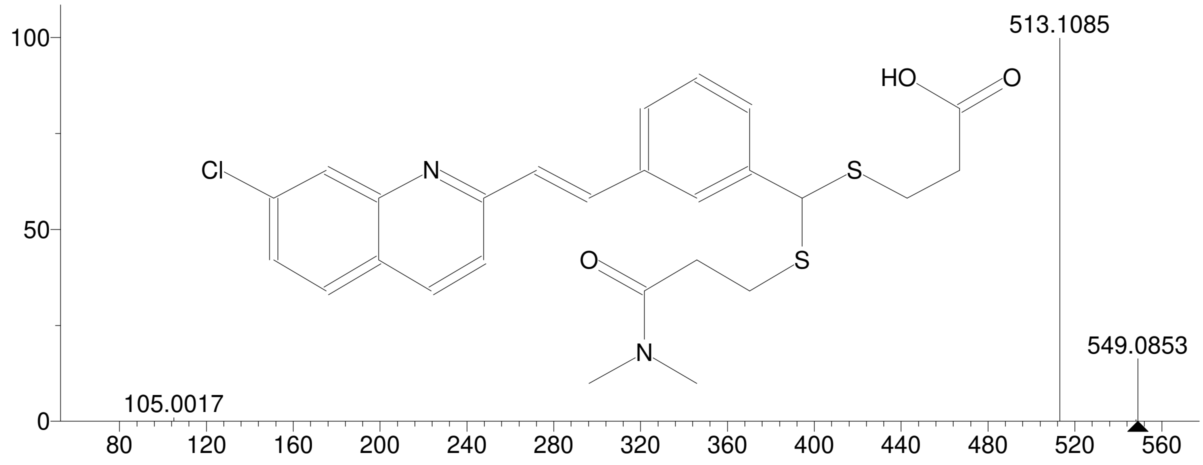

Hit 2 : Quercetin-3-(6''-malonyl)-Glucoside [M-H]- 5-60V P=549.1
C24H22O15; MF: 1; RMF: 152; Prob 16.1%; Lib: mona-export-lc-ms-ms_negative_mode; ID: 8837.

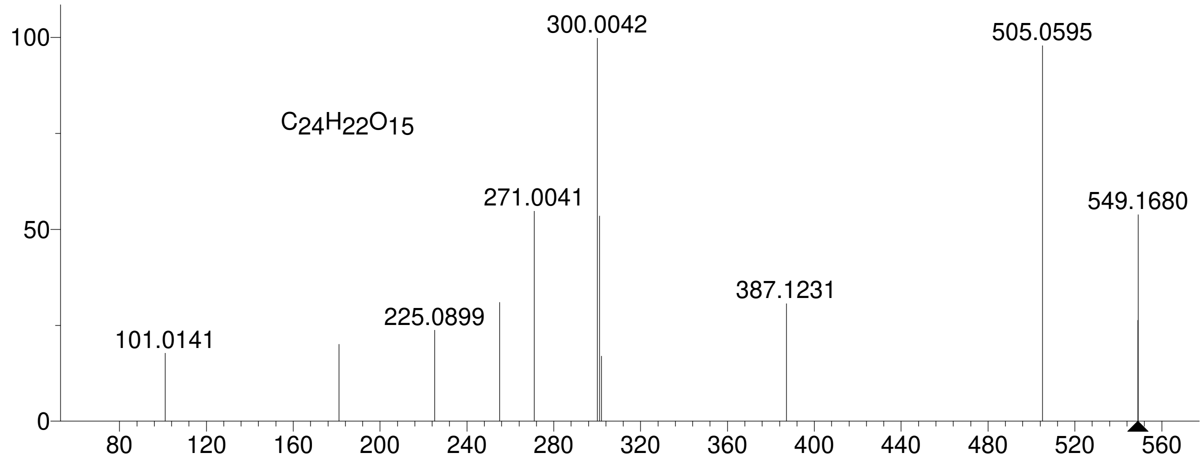

Hit 3 : Quercetin-3-(6"-malonyl)-Glucoside; LC-ESI-QTOF; MS2; CE [M-H]- P=549.1
C24H22O15; MF: 1; RMF: 143; Prob 16.1%; Lib: msms-neg-massbank; ID: 4722.

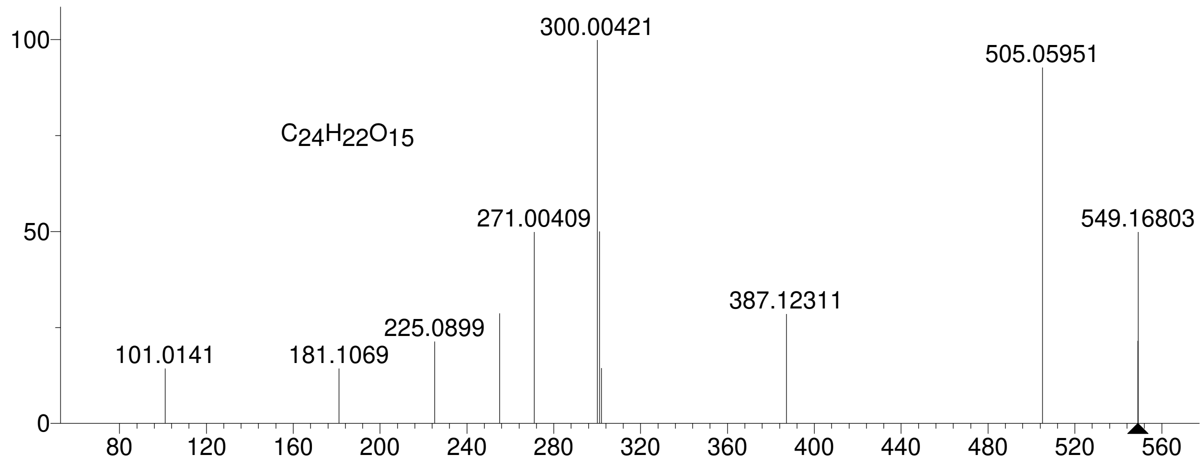

Hit 4 : Quercetin 3-O-malonylglucoside [M-H]- 10 P=549.1
C24H22O15; MF: 0; RMF: 51; Prob 15.5%; Lib: mona-export-lc-ms-ms_negative_mode; ID: 27259.

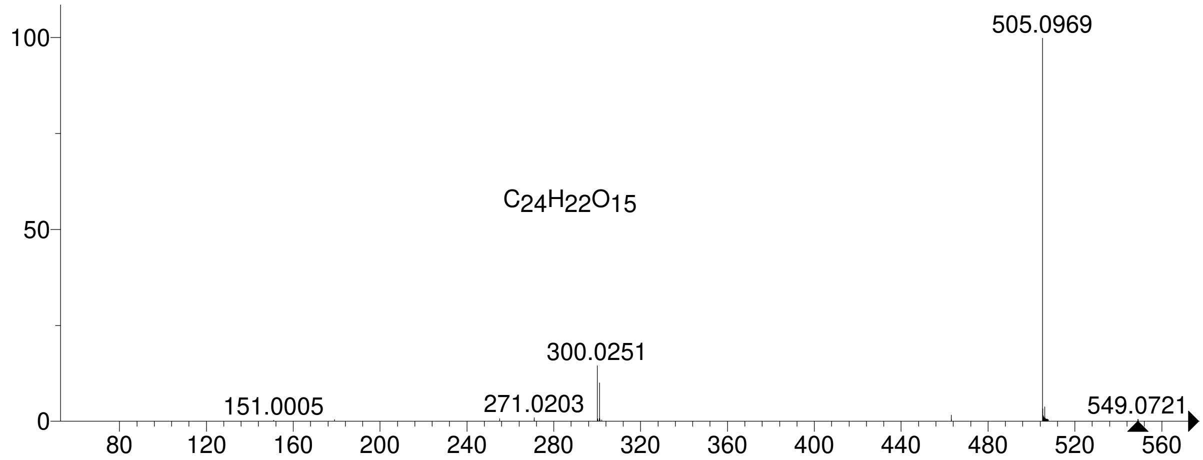

Hit 5 : Flavonol base + 4O, O-MalonylHex [M-H]- 6V P=549.1
C24H22O15; MF: 0; RMF: 40; Prob 15.5%; Lib: anthocyanins mona; ID: 6952.

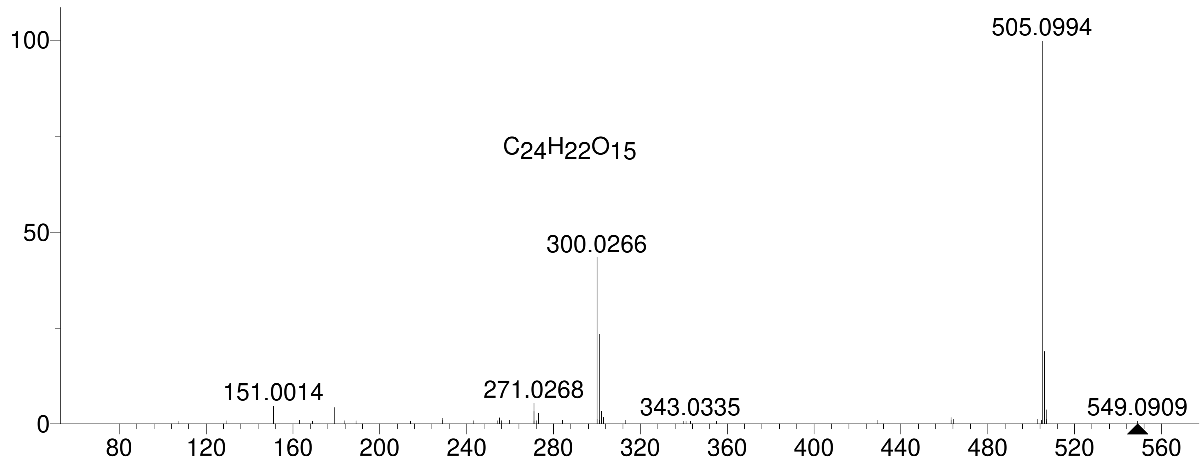

Unknown: Unknown (3.03_515.1643m/z)
Compound in Library Factor = 0

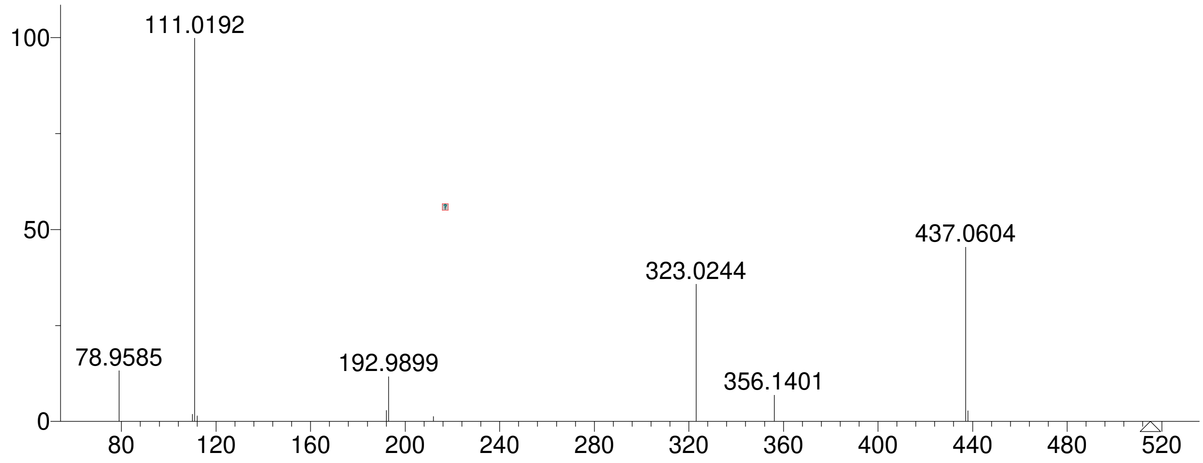

Hit 1 : 1,7-bis(4-hydroxyphenyl)-5-[(2R,3R,4S,5S,6R)-3,4,5-trihydroxy-6-(hydroxymethyl)oxan-2-yl]oxyheptan-3-or
C25H32O9; MF: 0; RMF: 6; Prob 100.0%; Lib: mona-export-vf-npl_qexactive; ID: 7561.

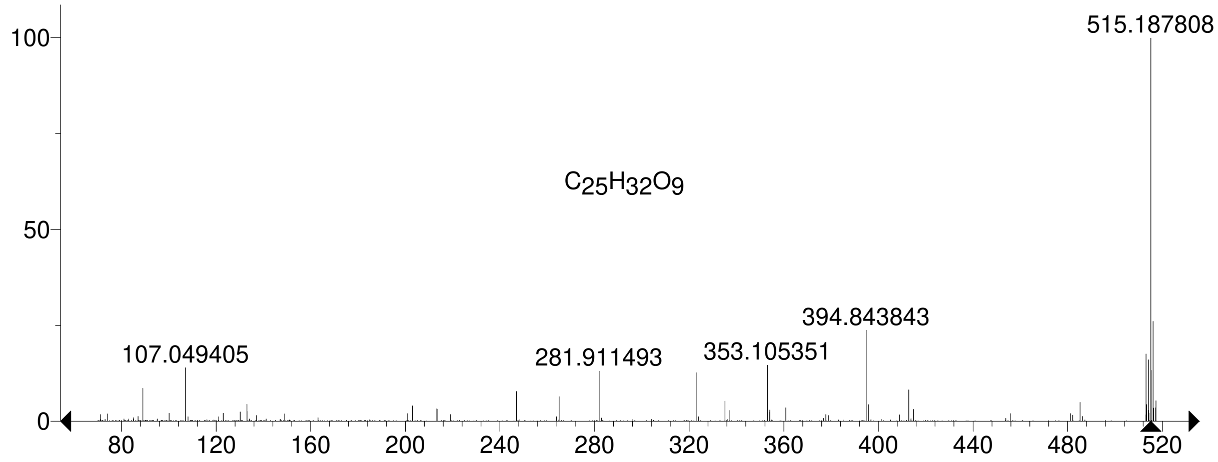

Unknown: Unknown (3.03_561.1346m/z)
Compound in Library Factor = 0

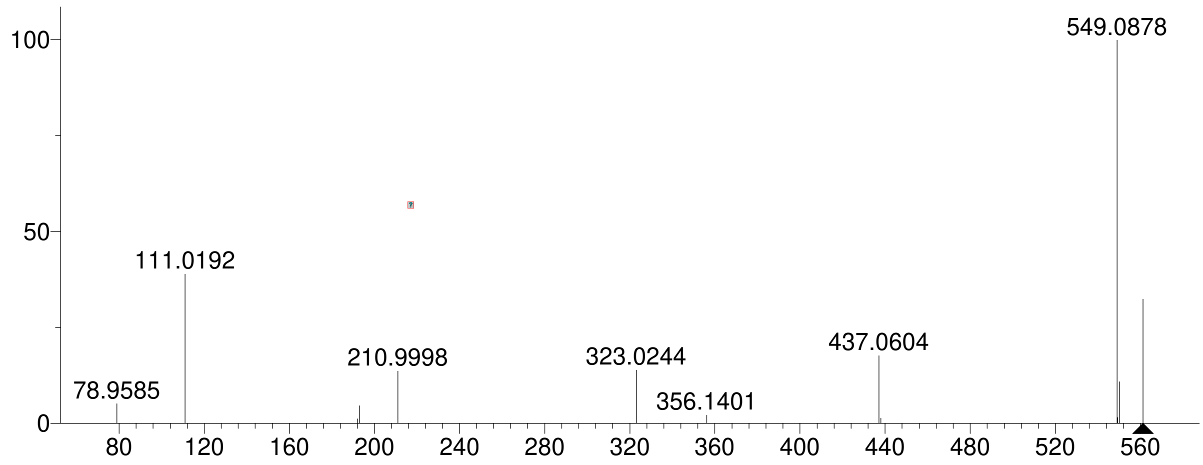

Unknown: Unknown (3.03_719.3480m/z)
Compound in Library Factor = -3273

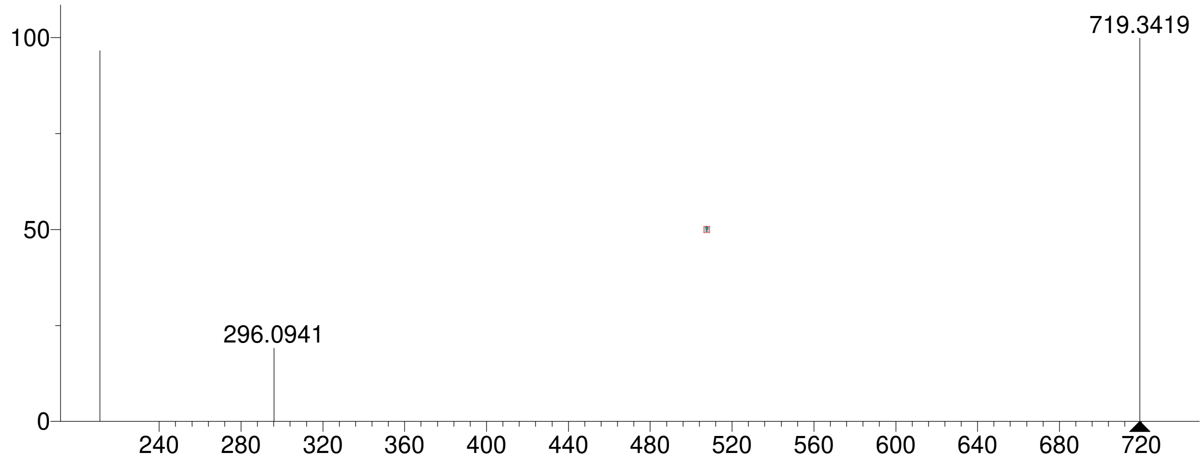

Hit 1 : Etoxazole [2M+H]+ HCD 14V P=719.3
C21H23F2NO2; MF: 31; RMF: 396; Prob 100.0%; CAS: 153233-91-1; Lib: nist_msms; ID: 482644.

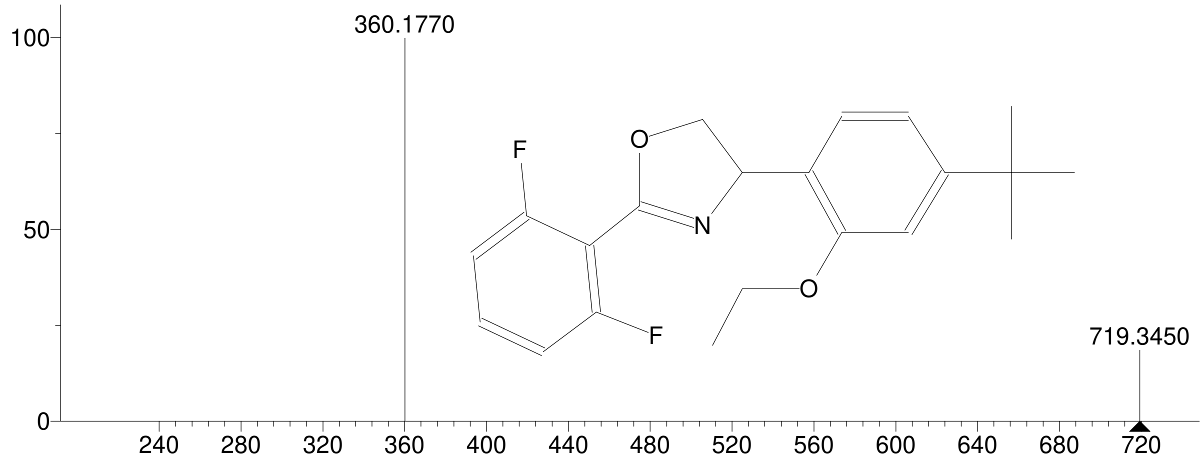

Unknown: Unknown (3.04_296.0986m/z)
Compound in Library Factor = -662

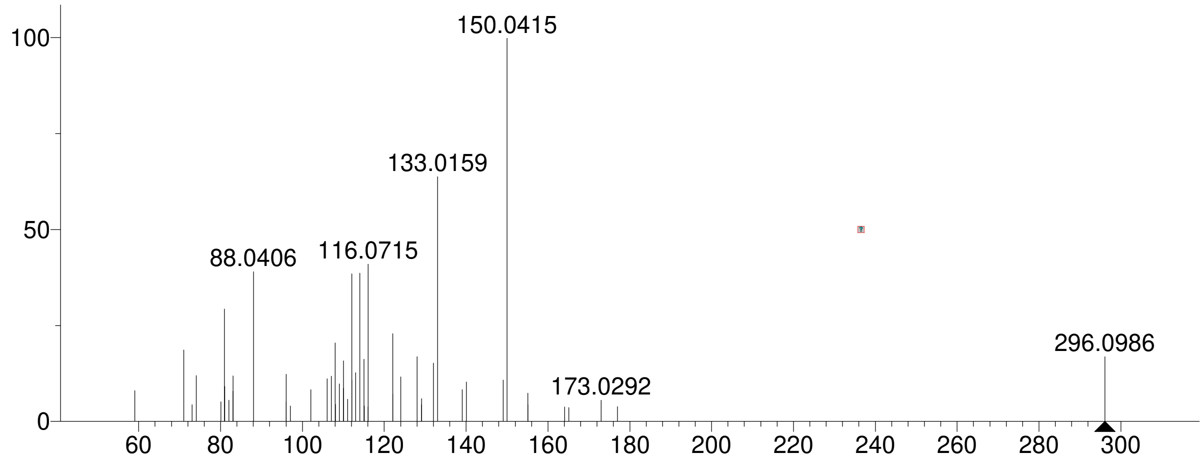

Hit 1 : 3'-O-Methylguanosine [M-H]- 20-30-40%) P=296.1
C11H15N5O5; MF: 336; RMF: 535; Prob 92.3%; Lib: mona-export-fiehn_hilic; ID: 937.

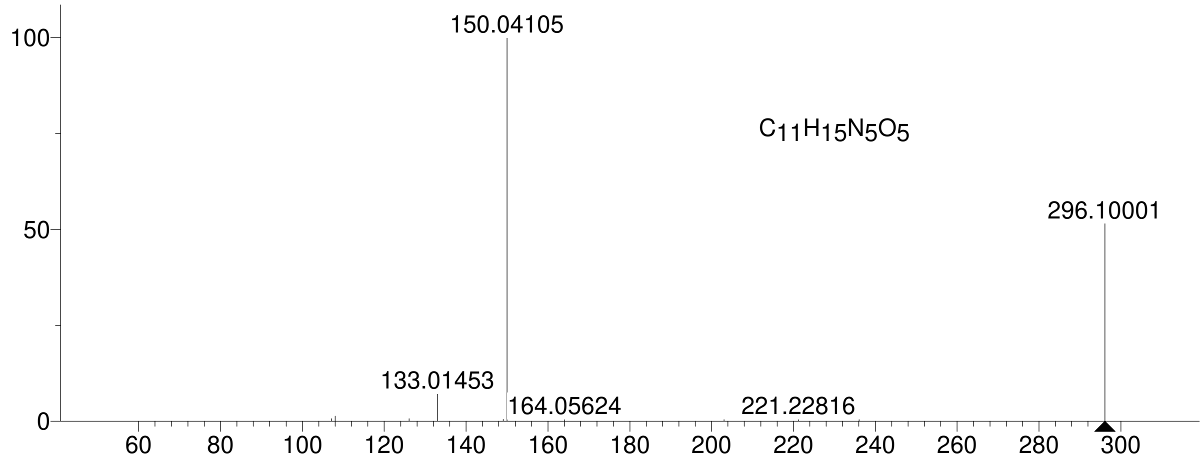

Hit 2 : Fluotrimazole [M+H-C2H3N3]+=>296.1 IT 35% P=296.1
C22H16F3N3; MF: 228; RMF: 917; Prob 6.05%; CAS: 31251-03-3; Lib: nist_msms; ID: 284480.

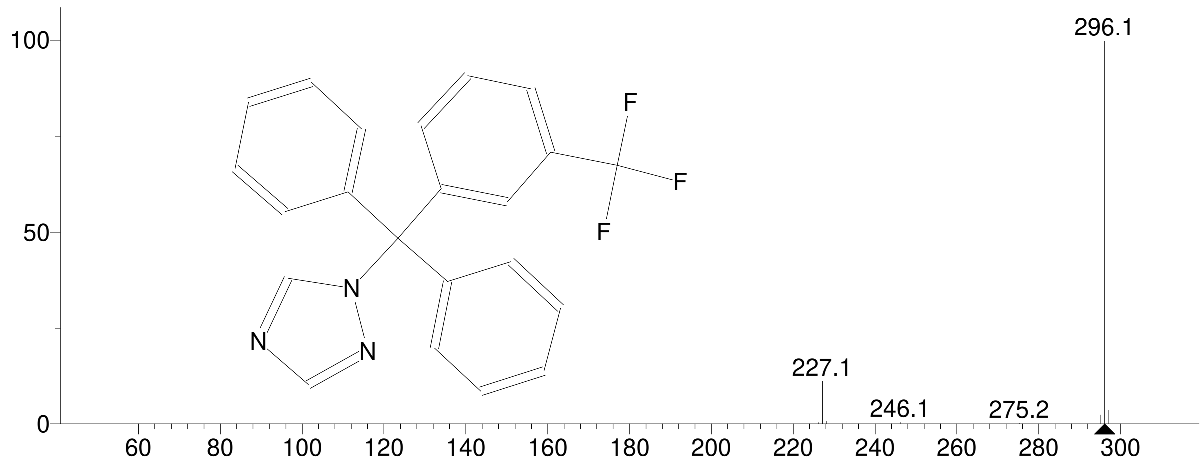

Hit 3 : 2'-O-Methylguanosine [M-H]- 20-30-40%) P=296.1
C11H15N5O5; MF: 181; RMF: 427; Prob 1.26%; Lib: msms-neg-fiehnhilic; ID: 30.

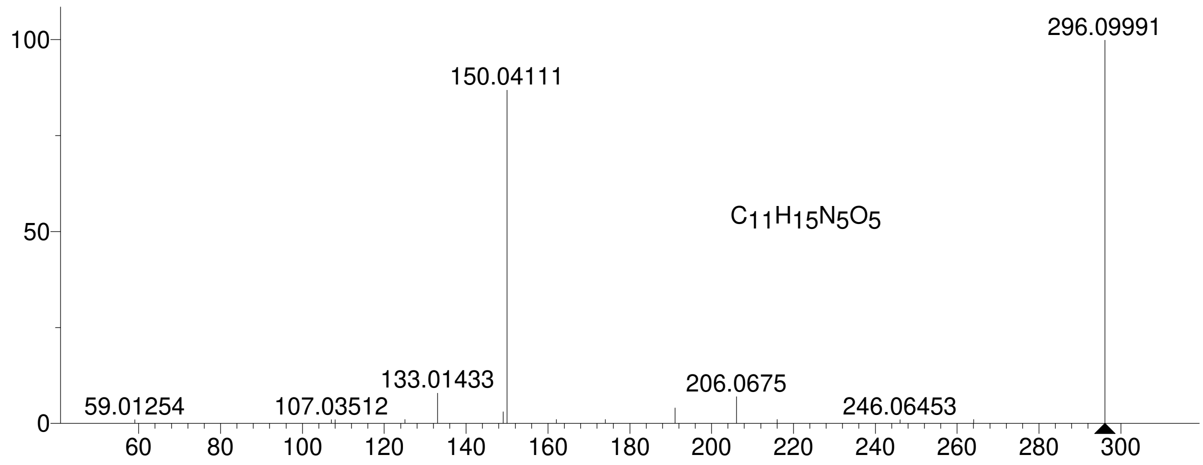

Hit 4 : N2-Methylguanosine [M-H]- 20-30-40%) P=296.1
C11H15N5O5; MF: 125; RMF: 210; Prob 0.24%; Lib: msms-neg-fiehnhilic; ID: 512.

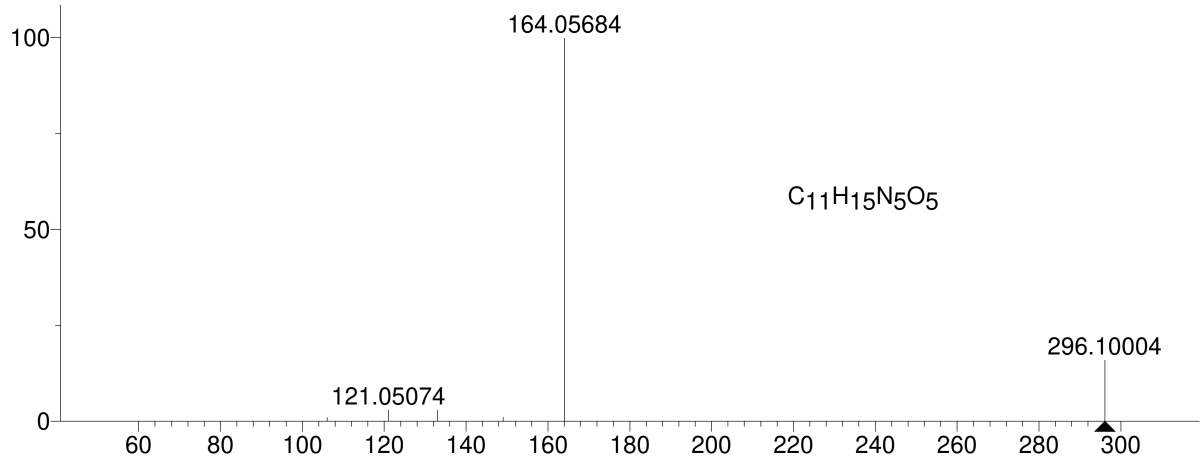

Hit 5 : 1-Methylguanosine [M-H]- 20-30-40%) P=296.1
C11H15N5O5; MF: 100; RMF: 217; Prob 0.07%; Lib: msms-neg-fiehnhilic; ID: 16.

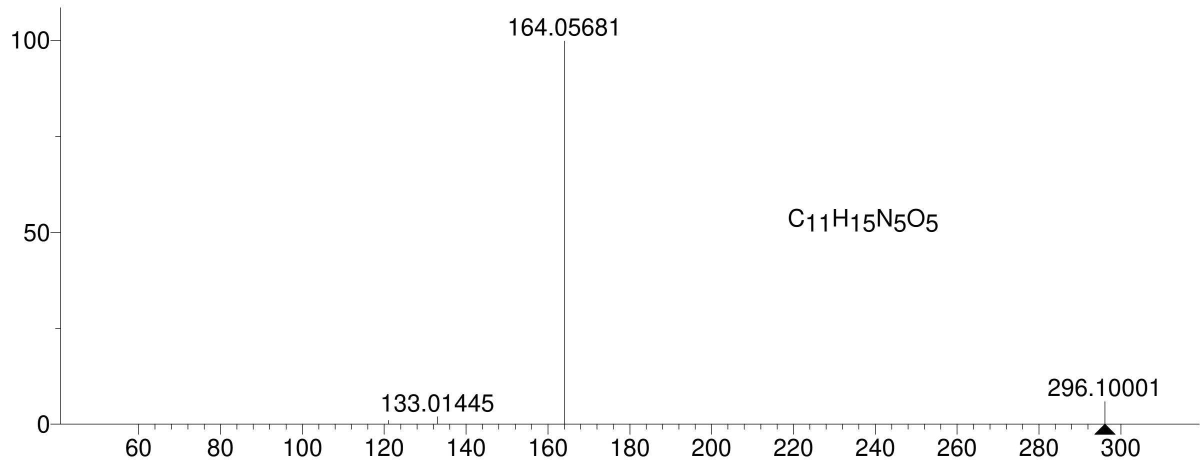

Unknown: Unknown (3.05_372.0697m/z)
Compound in Library Factor = 0

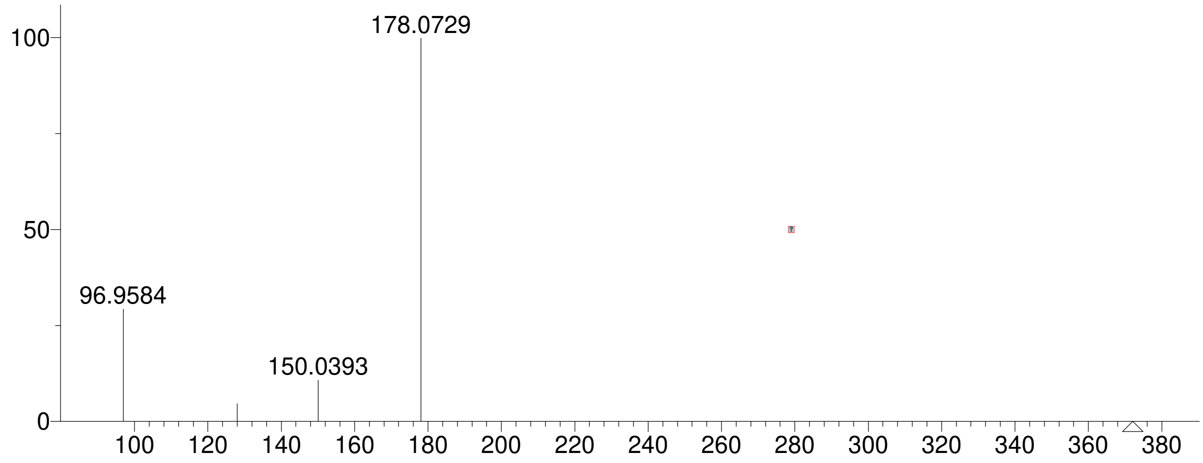

Unknown: Unknown (3.05_526.2030m/z)
Compound in Library Factor = 0

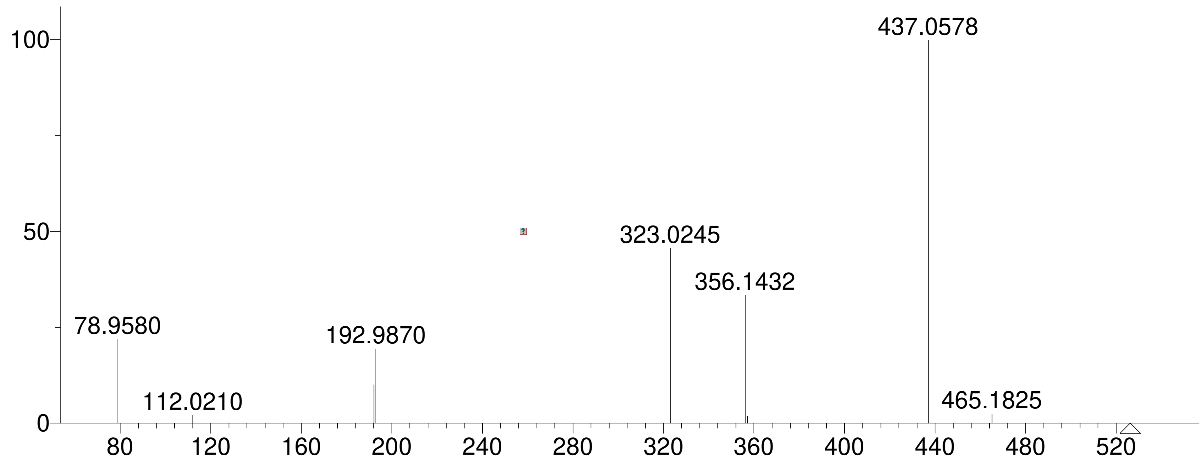

** Search Report Page 1 of 1 **

Unknown: Unknown (3.05_680.2788m/z)
Compound in Library Factor = 0

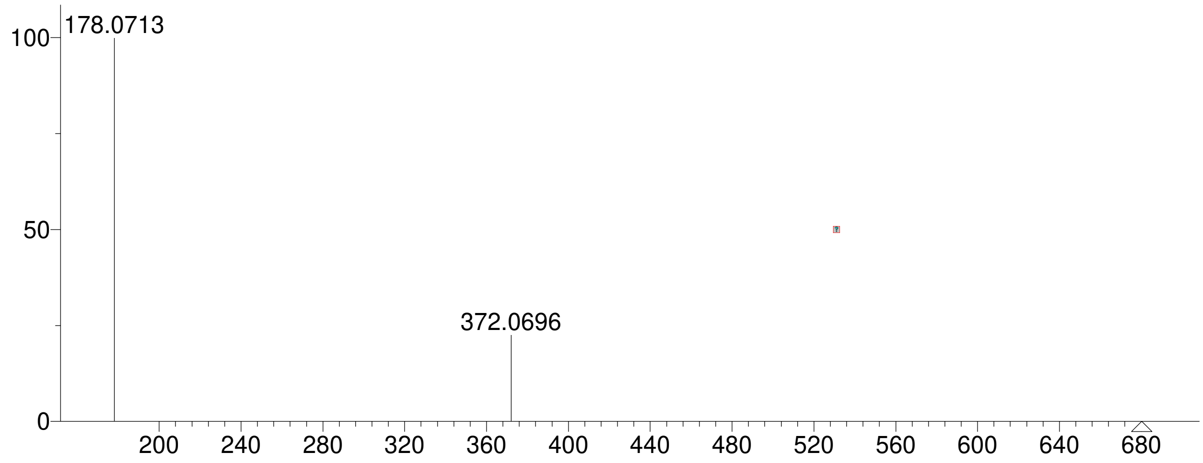

Unknown: Unknown (3.06_316.1510m/z)
Compound in Library Factor = 0

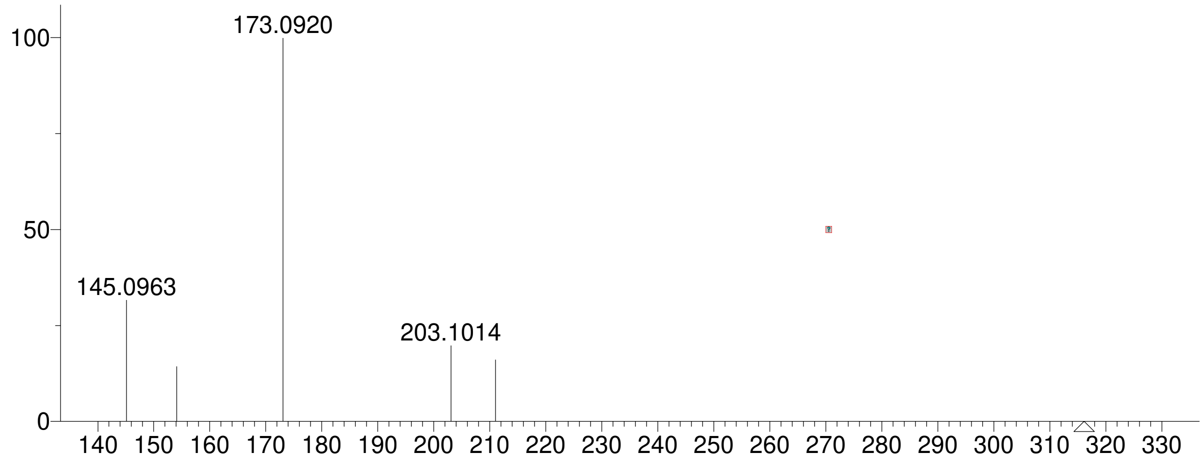

Unknown: Unknown (3.07_377.1911m/z)
Compound in Library Factor = -3273

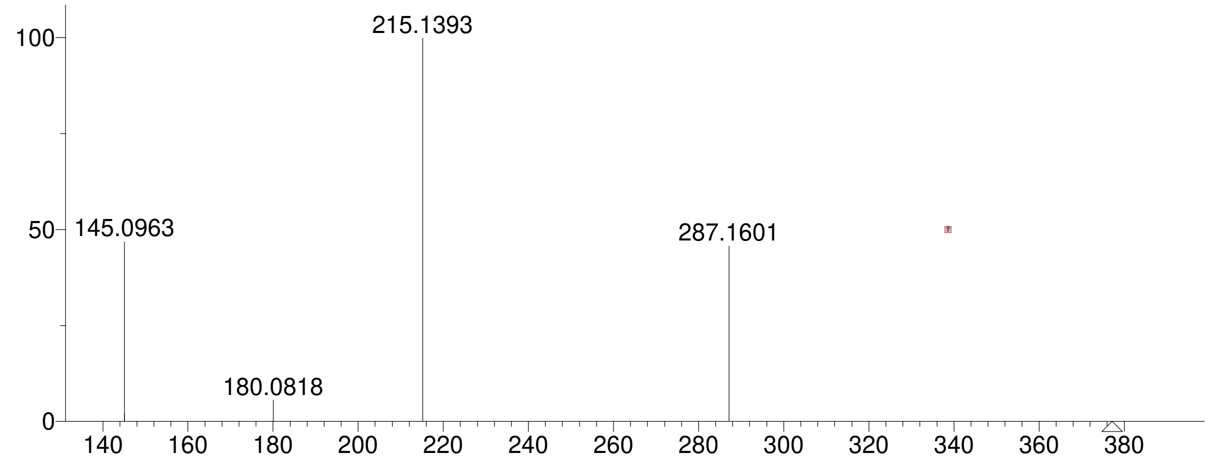

Hit 1 : (E)-3-(4-acetyloxy-2,3-dihydroxy-2,5,5,8a-tetramethyl-3,4,4a,6,7,8-hexahydro-1H-naphthalen-1-yl)prop-2-e
C19H30O6; MF: 1; RMF: 90; Prob 20.6%; Lib: mona-export-vf-npl_qexactive; ID: 20128.

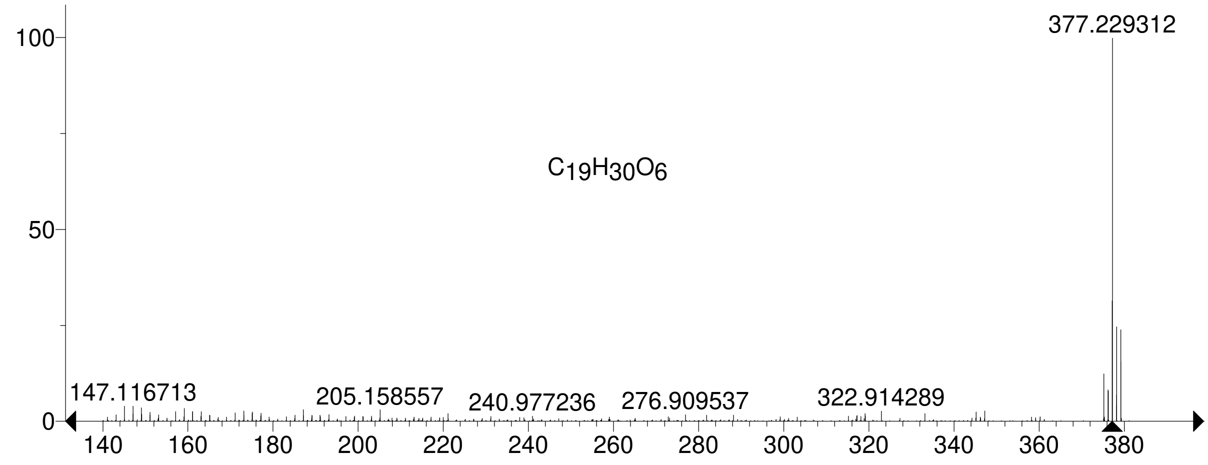

Hit 2 : Pentanoic acid, 5-[(3aR,4R,5R,6aS)-3,3-difluorohexahydro-5-hydroxy-4-[(1E,3S,4S)-3-hydroxy-4-methyl-1-
C22H30F2O5; MF: 0; RMF: 55; Prob 19.8%; CAS: 788799-13-3; Lib: nist_msms; ID: 347296.

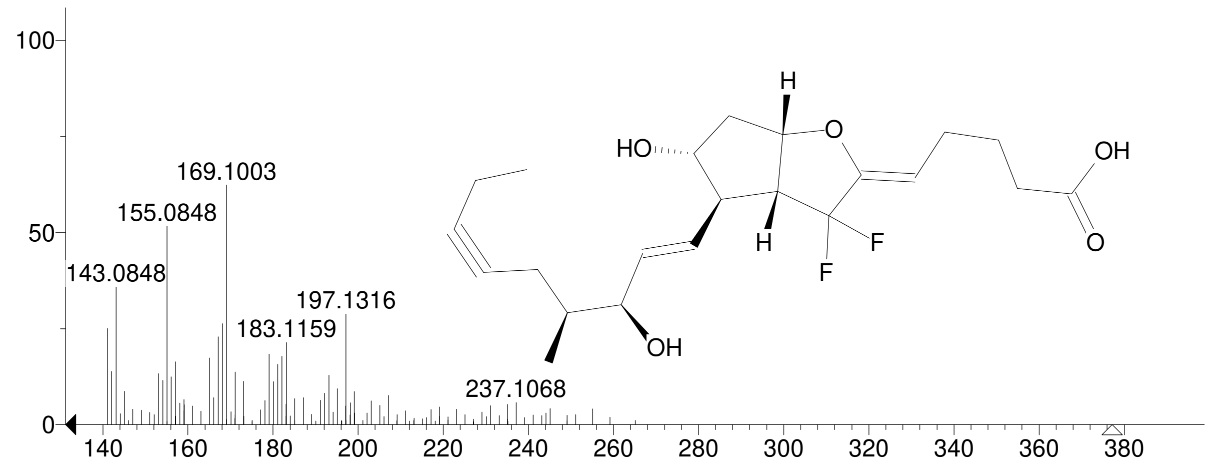

Hit 3 : Pentanoic acid, 5-[(3aR,4R,5R,6aS)-3,3-difluorohexahydro-5-hydroxy-4-[(1E,3R,4S)-3-hydroxy-4-methyl-1-
C22H30F2O5; MF: 0; RMF: 46; Prob 19.8%; CAS: 773825-80-2; Lib: nist_msms; ID: 357840.

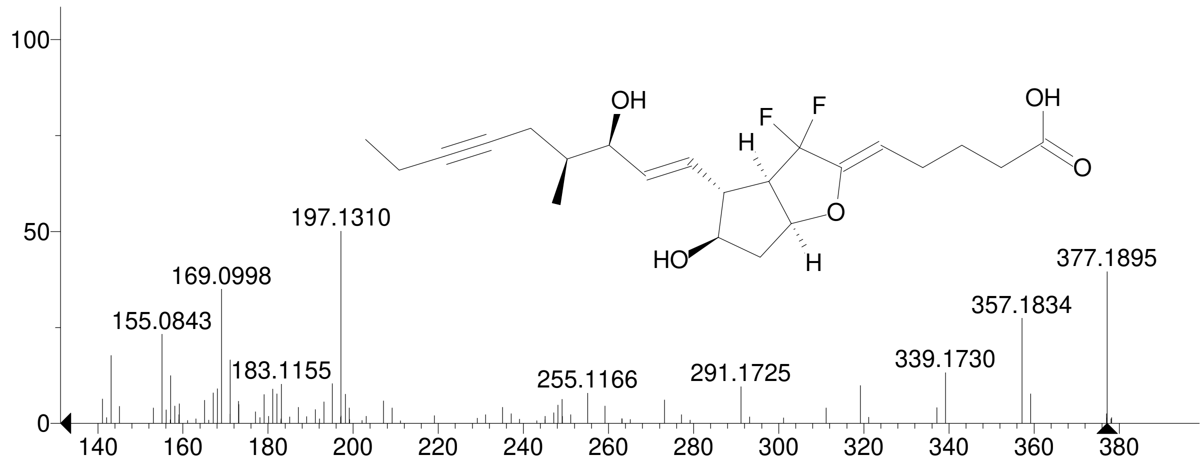

Hit 4 : [3-Me-His2] TRH [M+H]+ HCD 45eV P=377.2
C17H24N6O4; MF: 0; RMF: 14; Prob 19.8%; CAS: 34367-54-9; Lib: nist_msms; ID: 564165.

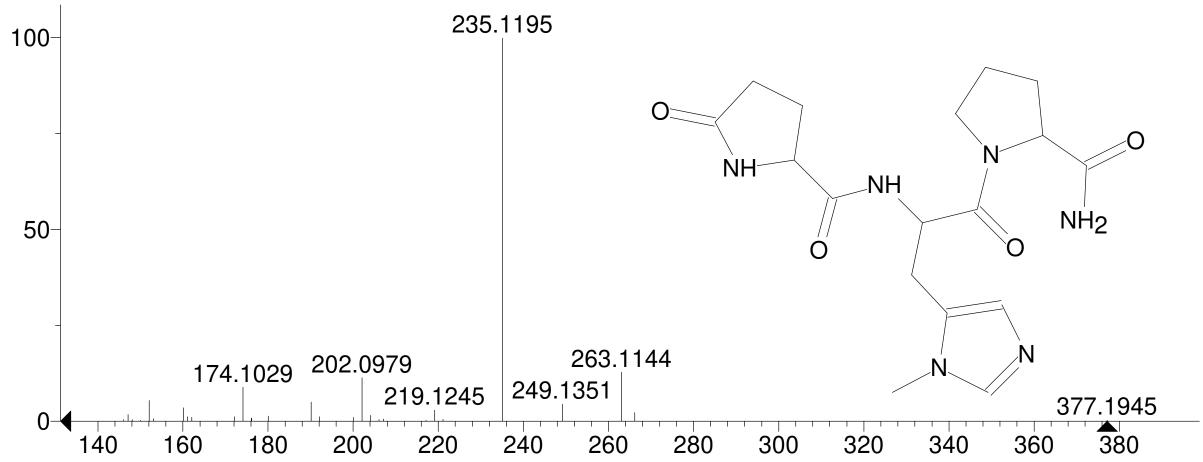

Hit 5 : [3-Me-His2] TRH [M+H]+ HCD 45eV P=377.2
C17H24N6O4; MF: 0; RMF: 14; Prob 19.8%; Lib: nist_msms2; ID: 46584.

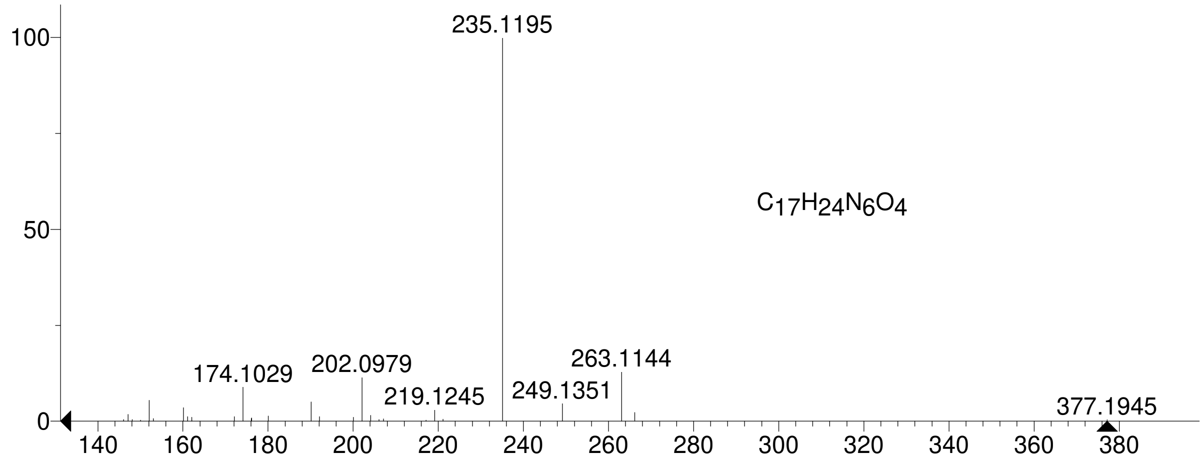

Unknown: Unknown (3.08_282.1075m/z)
Compound in Library Factor = 0

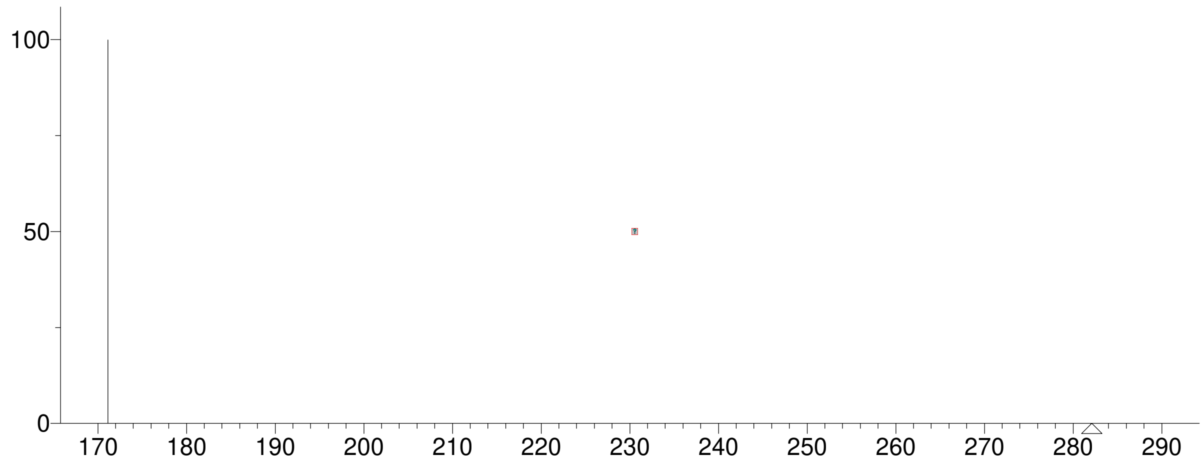

Unknown: Unknown (3.09_477.2075m/z)
Compound in Library Factor = -1072

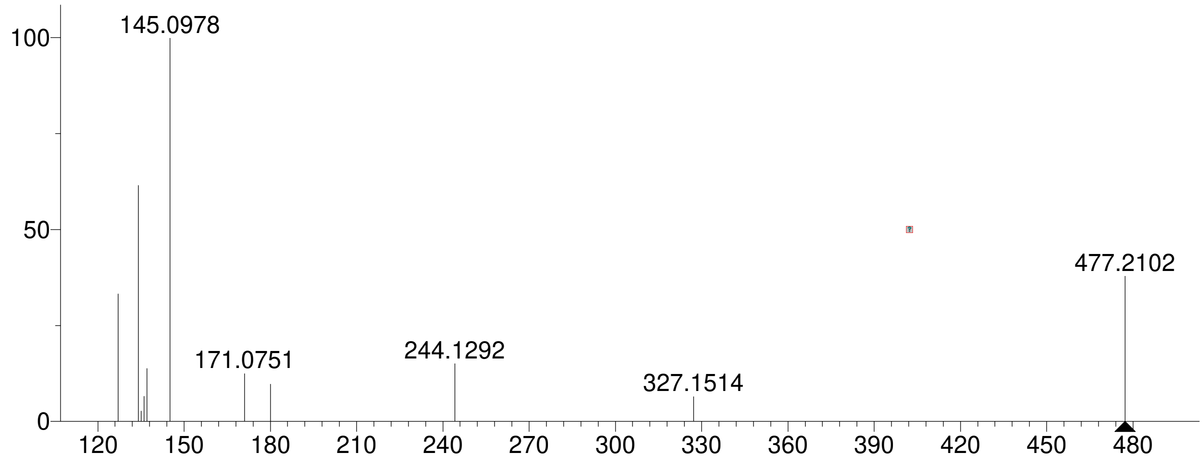

Hit 1 : 4-(2-Hydroxyethyl)piperazine-1-ethanesulfonic acid [2M+H]+ HCD 4V P=477.2
C8H18N2O4S; MF: 74; RMF: 673; Prob 79.0%; CAS: 7365-45-9; Lib: nist_msms; ID: 58512.

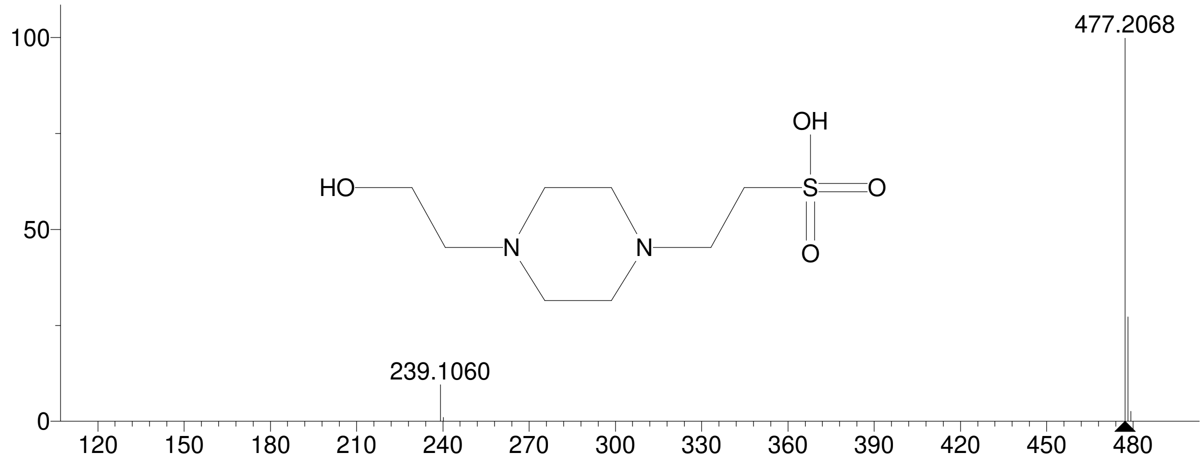

Hit 2 : 5-O-methyllicoricidin [M+K]+ 35HCD P=477.2
C27H34O5; MF: 0; RMF: 54; Prob 10.5%; Lib: mona-export-vf-npl_qexactive; ID: 964.

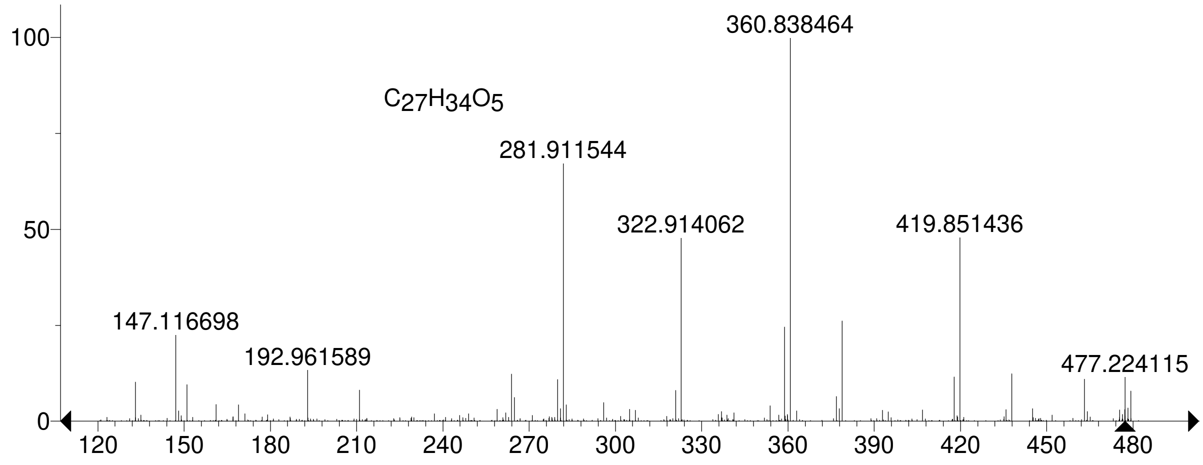

Hit 3 : 1,7-bis(4-hydroxyphenyl)-5-[(2R,3R,4S,5S,6R)-3,4,5-trihydroxy-6-(hydroxymethyl)oxan-2-yl]oxyheptan-3-or
C25H32O9; MF: 0; RMF: 17; Prob 10.5%; Lib: mona-export-vf-npl_qexactive; ID: 7567.

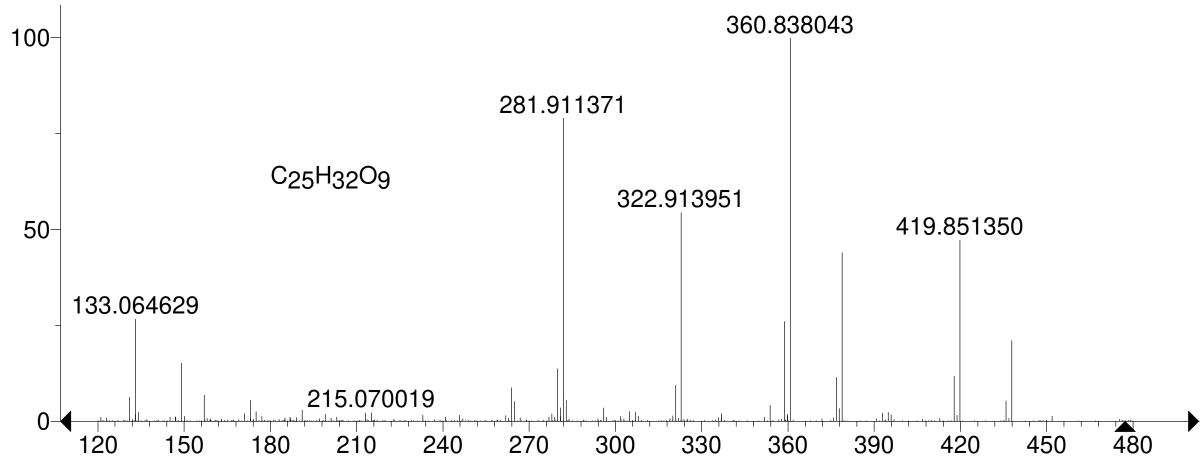

Unknown: Unknown (3.09_496.2027m/z)
Compound in Library Factor = -3273

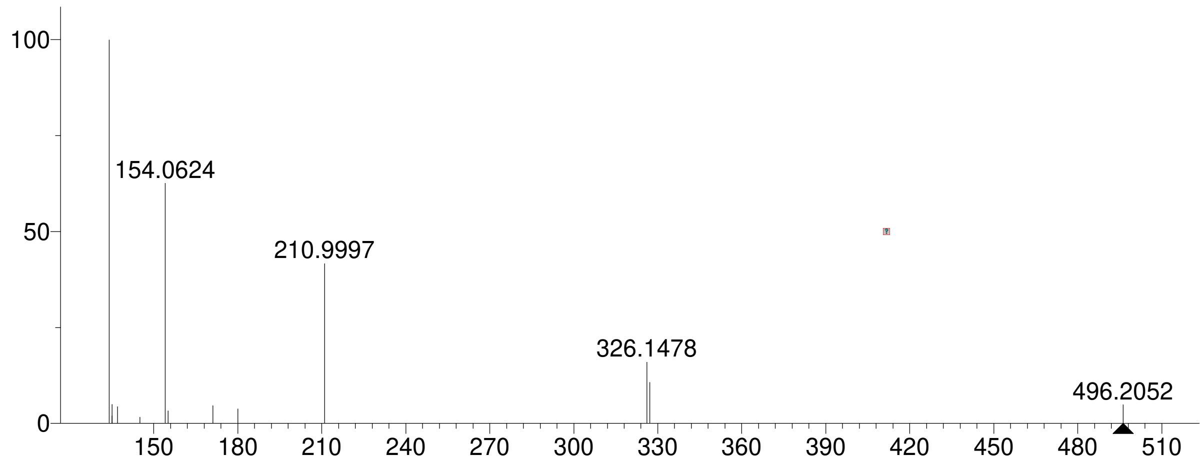

Hit 1 : (2R,3S,4S,5R,6S)-2-[(3,4,5-trihydroxyoxan-2-yl)oxymethyl]-6-(3,4,5-trimethoxyphenoxy)oxane-3,4,5-triol [M
C20H30O13; MF: 6; RMF: 238; Prob 22.8%; Lib: mona-export-vf-npl_qexactive; ID: 9258.

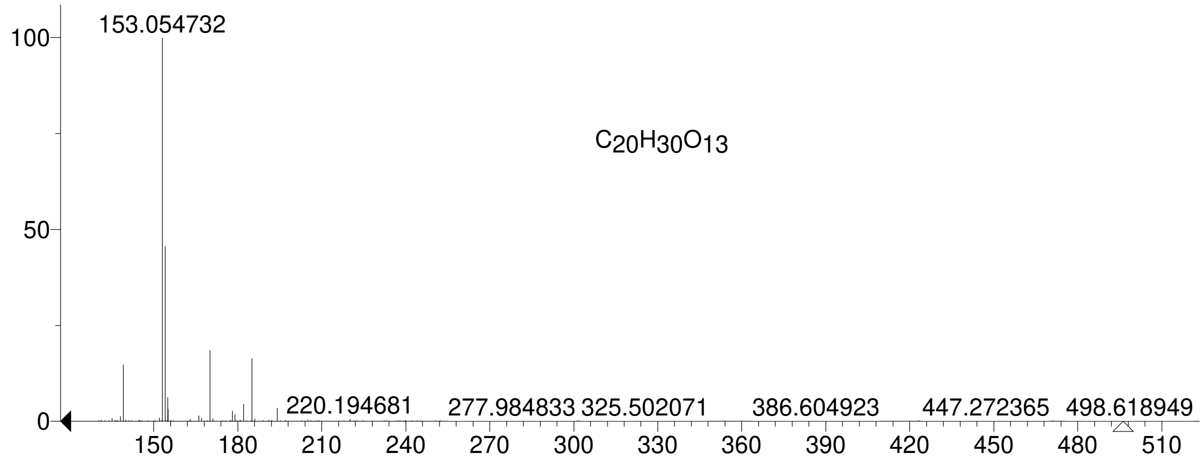

Hit 2 : (2R,3S,4S,5R,6S)-2-[[(2S,3R,4R)-3,4-dihydroxy-4-(hydroxymethyl)oxolan-2-yl]oxymethyl]-6-(3,4,5-trimetho
C20H30O13; MF: 4; RMF: 217; Prob 21.1%; Lib: mona-export-vf-npl_qexactive; ID: 9783.

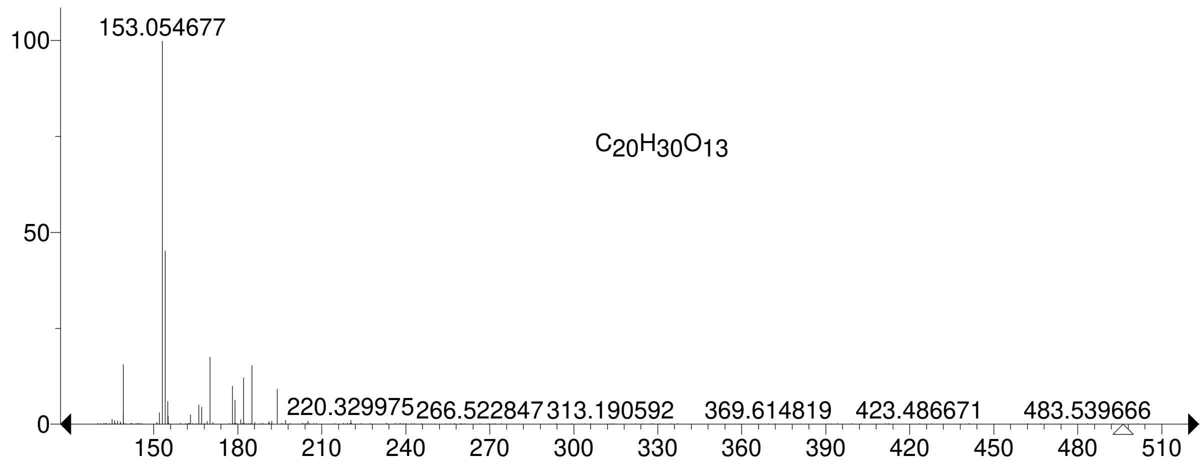

Hit 3 : NCGC00180666-02!(2R,3S,4S,5R,6S)-2-[(3,4,5-trihydroxyoxan-2-yl)oxymethyl]-6-(3,4,5-trimethoxyphenox
C20H30O13; MF: 2; RMF: 146; Prob 19.4%; Lib: mona-export-gnps; ID: 8997.

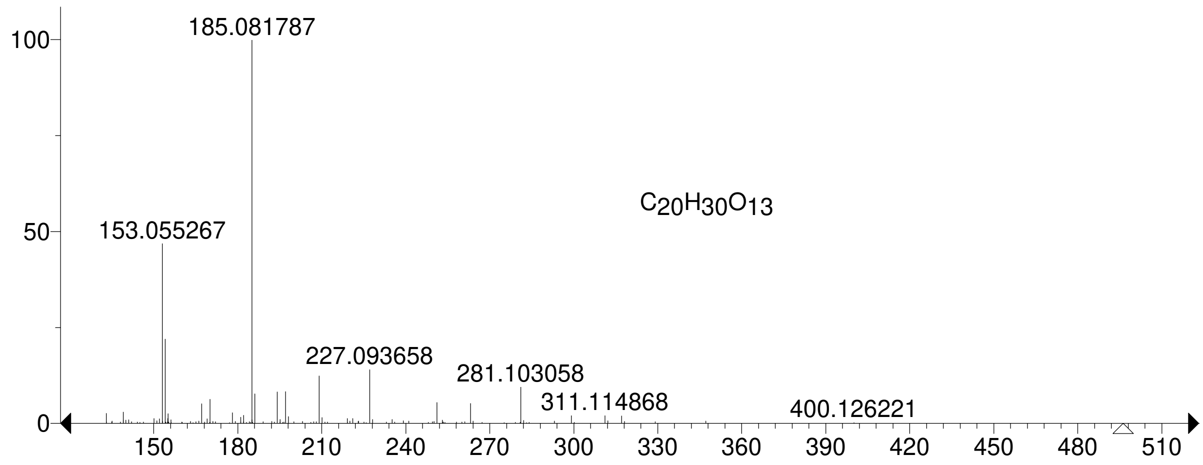

Hit 4 : NCGC00180025-03!(2R,3S,4S,5R,6S)-2-[[(2S,3R,4R)-3,4-dihydroxy-4-(hydroxymethyl)oxolan-2-yl]oxymetl
C20H30O13; MF: 1; RMF: 146; Prob 18.7%; Lib: mona-export-gnps; ID: 8565.

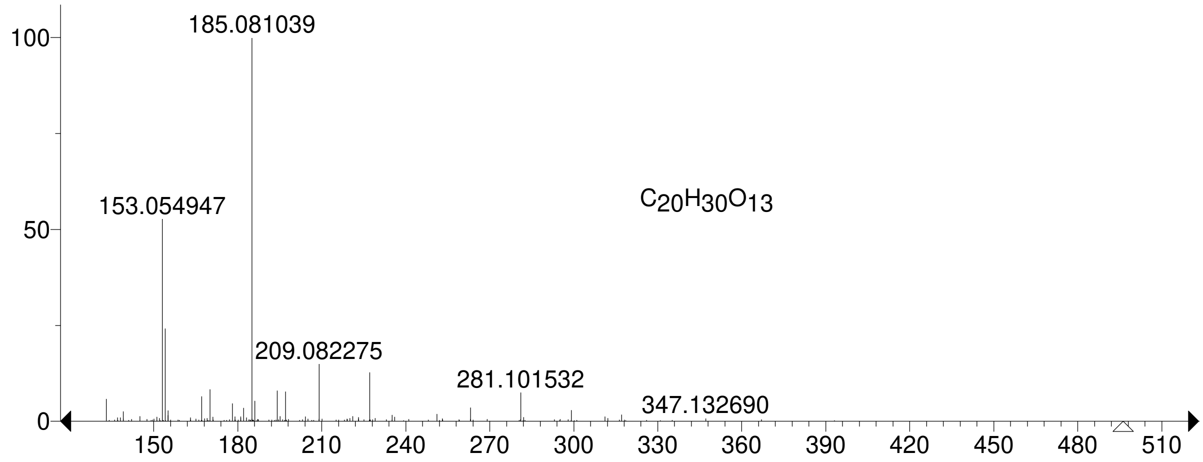

Hit 5 : Benzoic acid, 3-[[(3-carboxycyclohexyl)amino]carbonyl]-4-[3-[4-[4-(cyclohexyloxy)butoxy]phenyl]propoxy]-
C34H45NO8; MF: 0; RMF: 4; Prob 17.9%; CAS: 712313-35-4; Lib: nist_msms; ID: 190533.

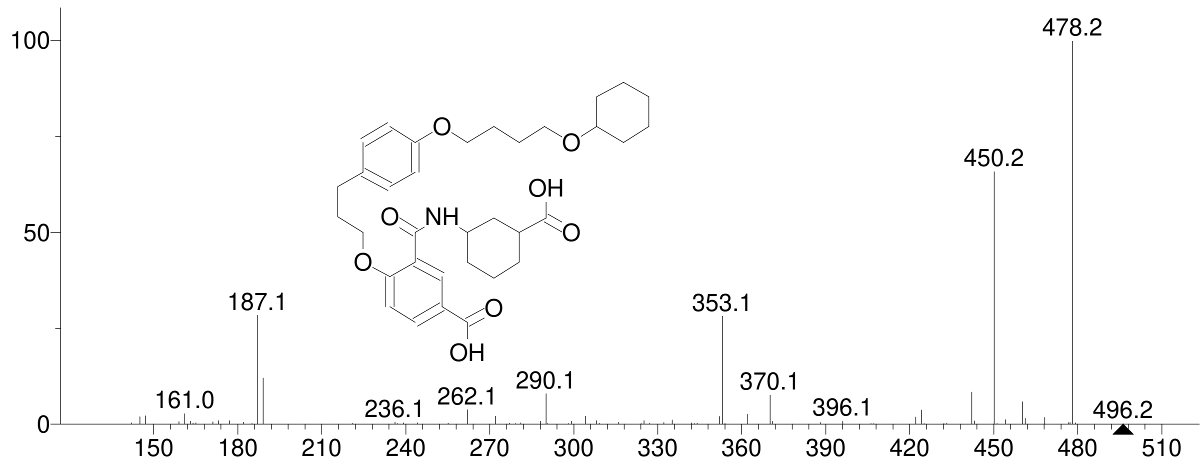

Unknown: Unknown (3.10_244.1292m/z)
Compound in Library Factor = -674

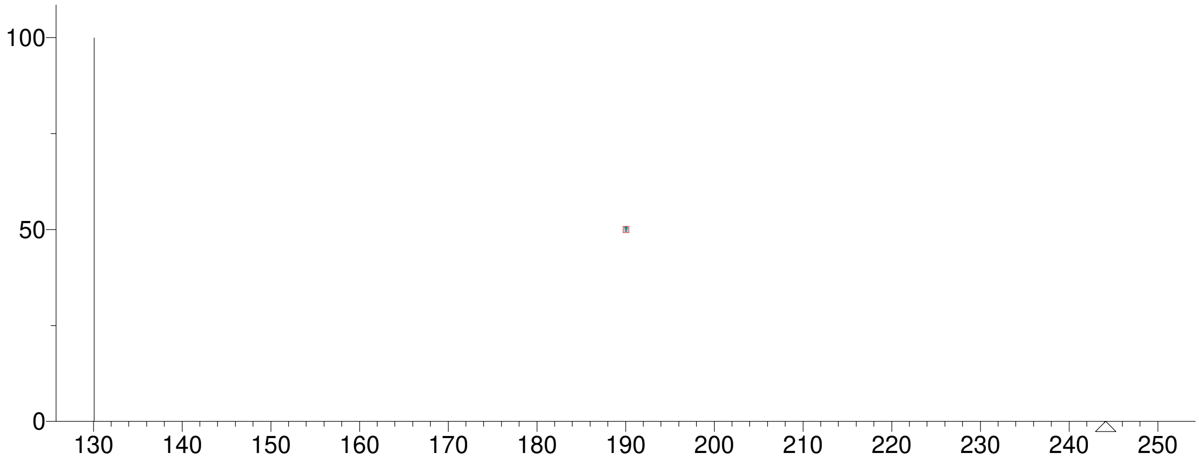

Hit 1 : Asn-Leu [M-H]- HCD 39eV P=244.1
C10H19N3O4; MF: 187; RMF: 909; Prob 26.1%; Lib: nist_msms; ID: 570276.

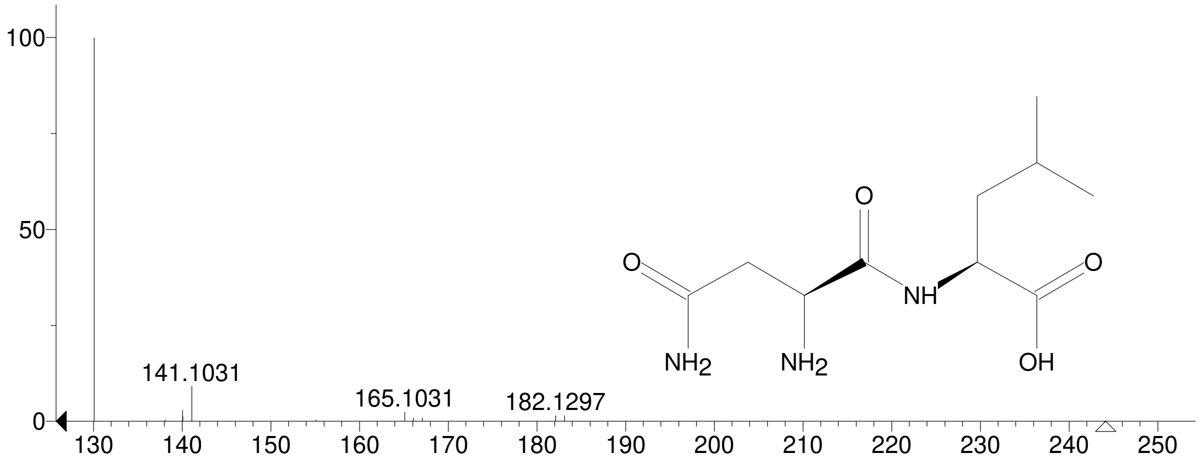

Hit 2 : Asn-Leu [M-H]- HCD 39eV P=244.1
C10H19N3O4; MF: 187; RMF: 909; Prob 26.1%; CAS: 22601-71-4; Lib: nist_msms2; ID: 84002.

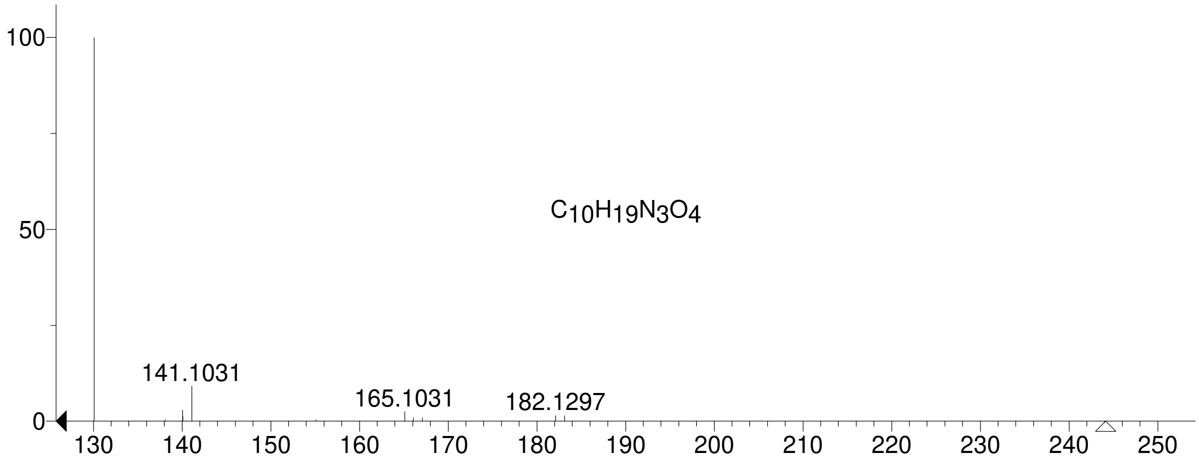

Hit 3 : Asn-Ile [M-H]- HCD 39eV P=244.1
C10H19N3O4; MF: 184; RMF: 914; Prob 23.1%; Lib: nist_msms; ID: 570863.

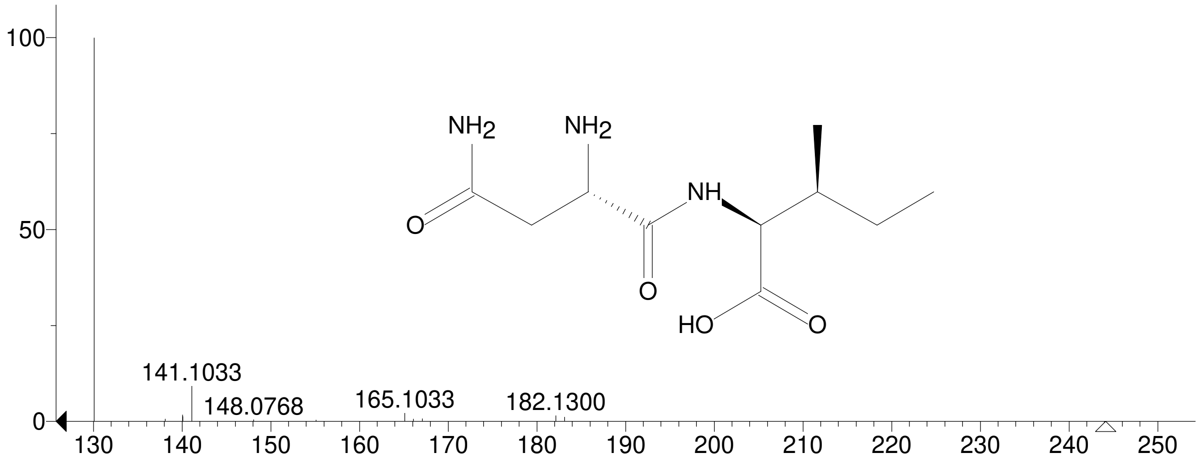

Hit 4 : Asn-Ile [M-H]- HCD 39eV P=244.1
C10H19N3O4; MF: 184; RMF: 914; Prob 23.1%; CAS: 438533-59-6; Lib: nist_msms2; ID: 84596.

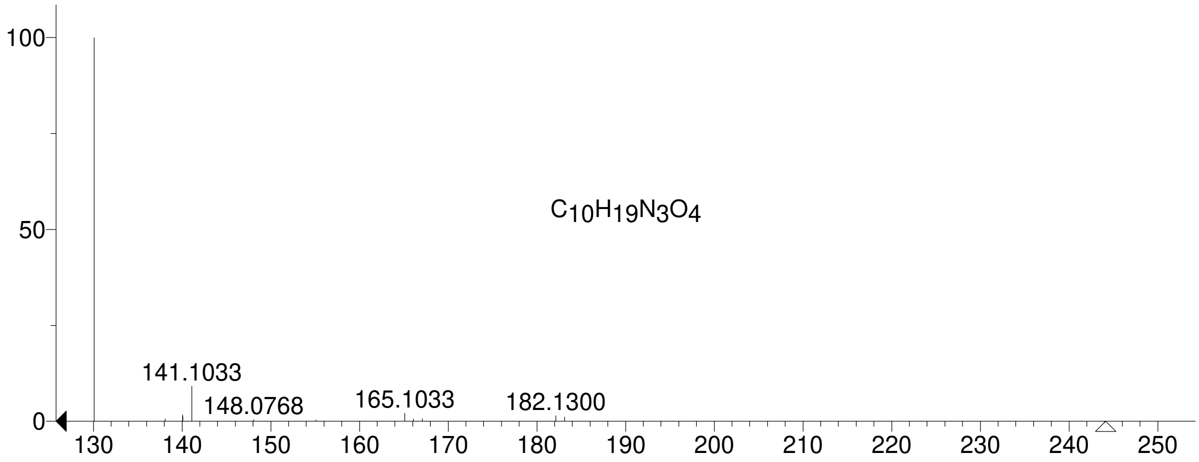

Hit 5 : Unknown (carbon number 10); PlaSMA ID-380 [M-H]- P=244.1
C10H19N3O4; MF: 24; RMF: 440; Prob 0.57%; Lib: biomsms-neg-plasma; ID: 1104.

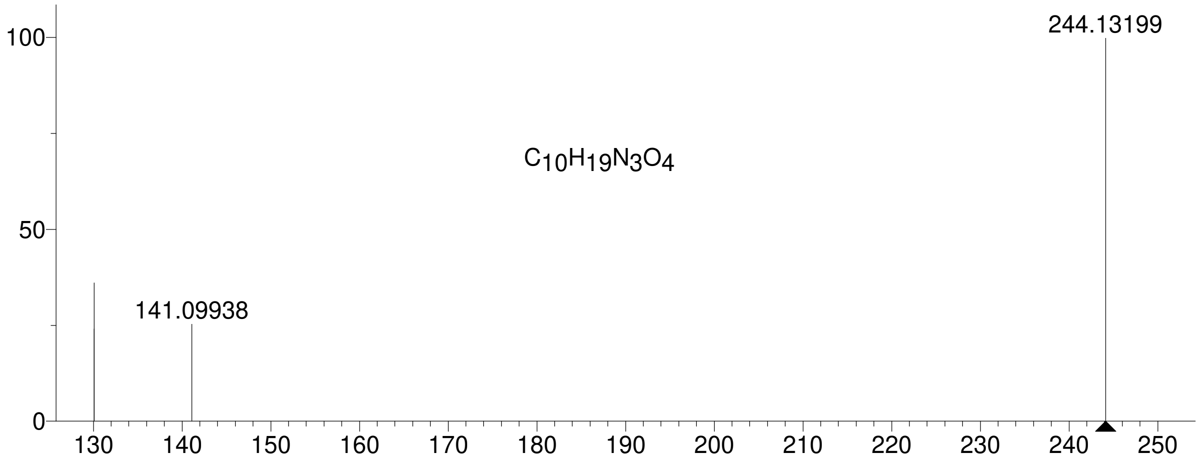

** Search Report Page 1 of 1 **

Unknown: Unknown (3.10_481.2037m/z)
Compound in Library Factor = 0

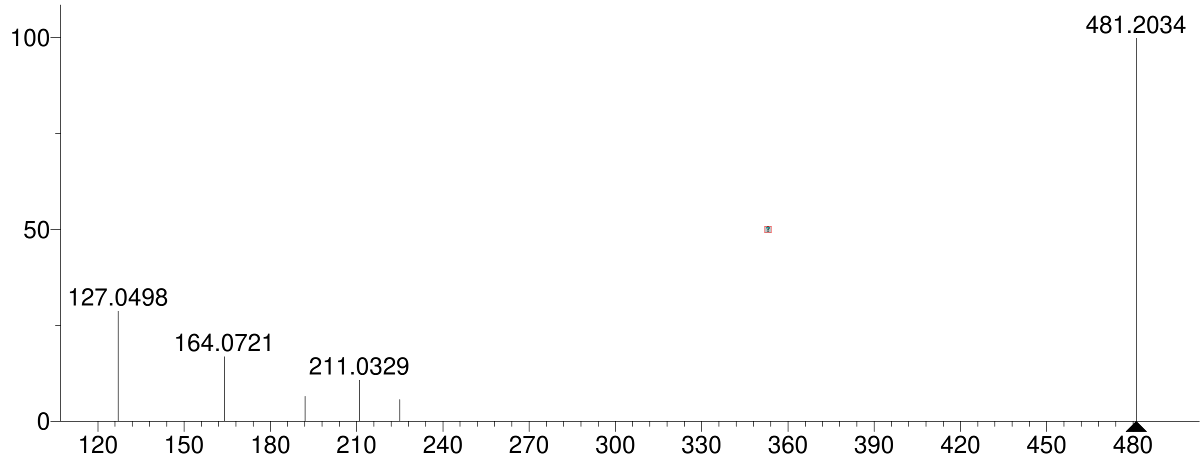

Unknown: Unknown (3.10_488.1861m/z)
Compound in Library Factor = 0

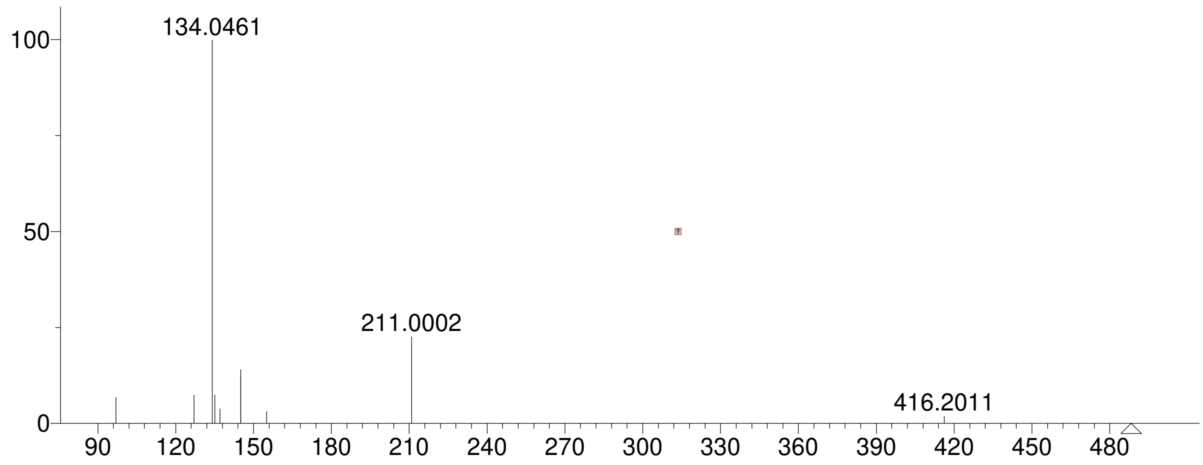

Unknown: Unknown (3.10_536.2114m/z)
Compound in Library Factor = 0

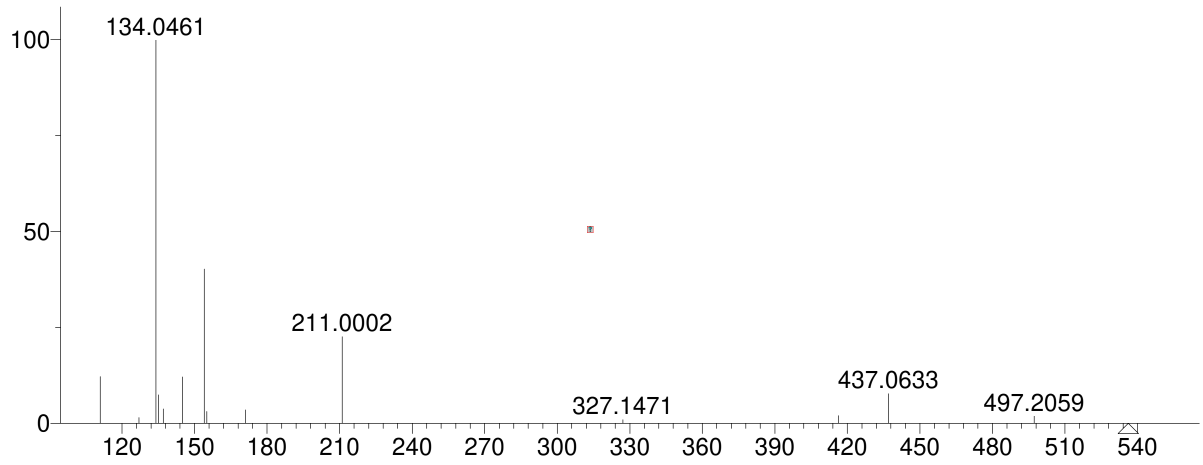

Hit 1 : Lewis A trisaccharide [M+Li]+ HCD 96V P=536.2
C20H35NO15; MF: 0; RMF: 41; Prob 100.0%; CAS: 56570-03-7; Lib: nist_msms; ID: 211089.

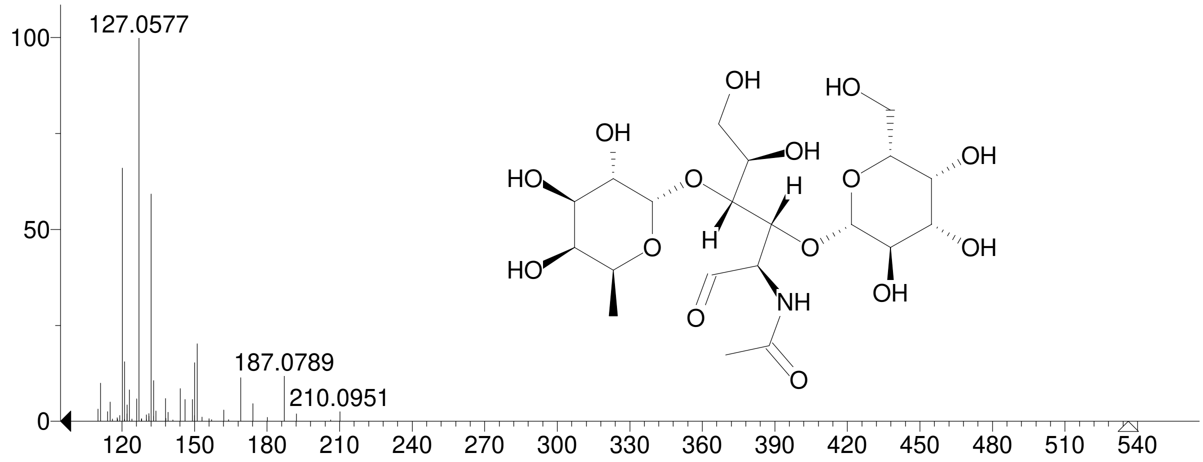

Unknown: Unknown (3.10_572.1142m/z)
Compound in Library Factor = 0

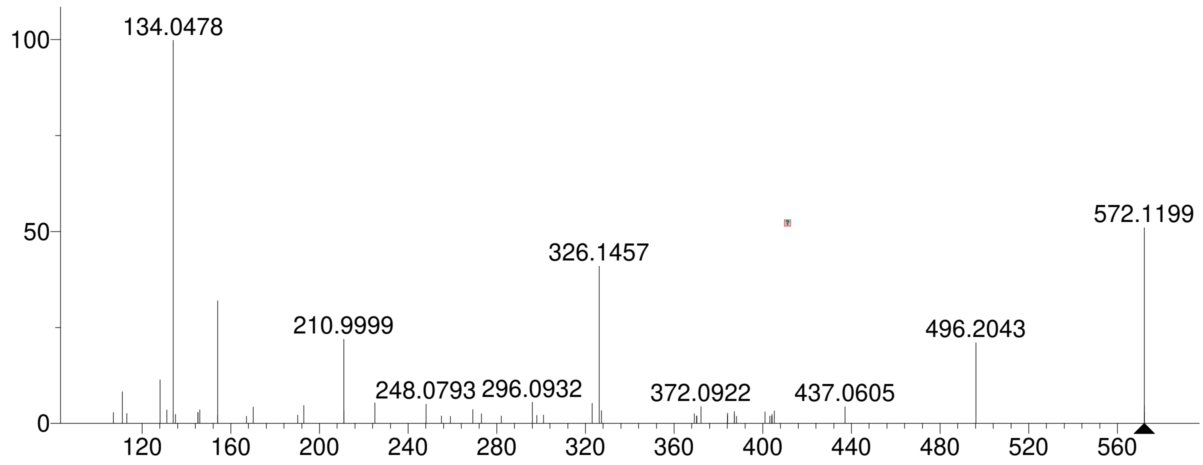

Hit 1 : (Ala11,D-Leu15)-Orexin B, human [M+5H]5+ HCD 8eV P=572.1
C120H206N44O35S; MF: 0; RMF: 113; Prob 100.0%; CAS: 532932-99-3; Lib: nist_msms2; ID: 45808.

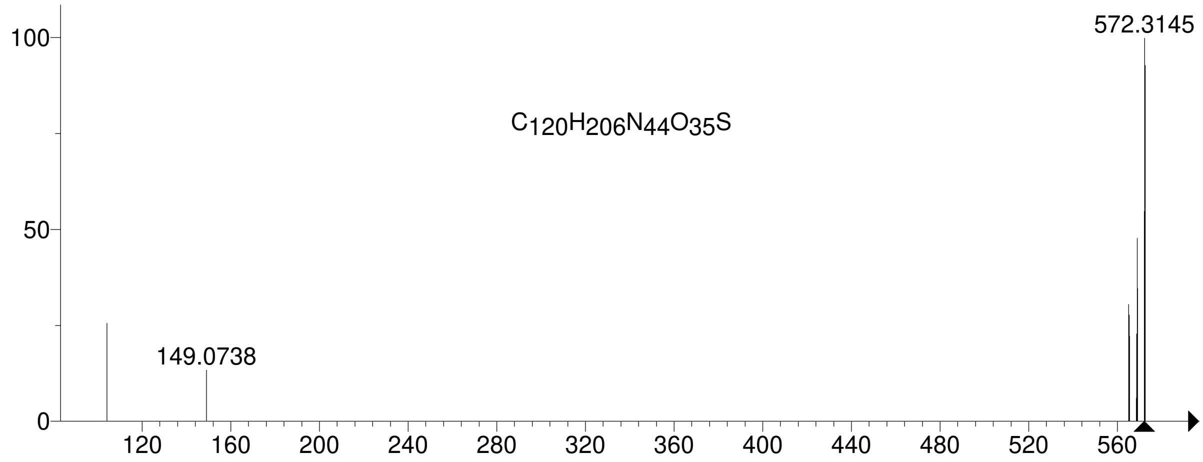

Unknown: Unknown (3.11_444.1596m/z)
Compound in Library Factor = 0

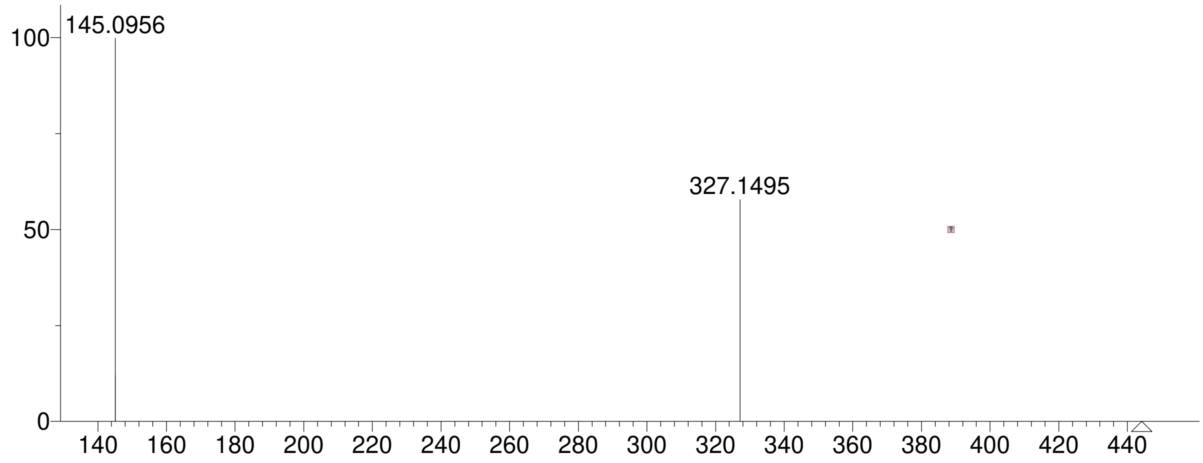

Unknown: Unknown (3.11_666.2614m/z)
Compound in Library Factor = 0

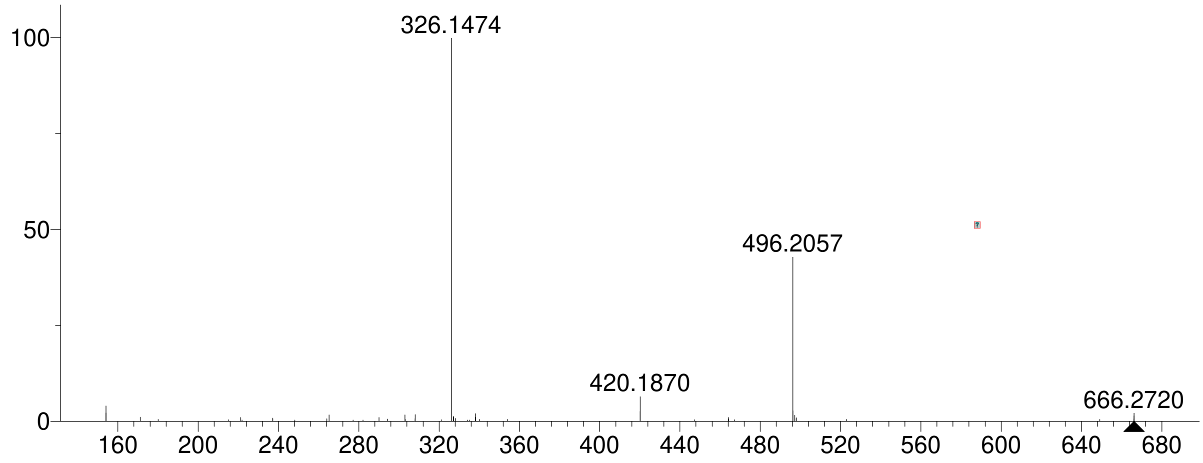

Hit 1 : NCGC00385630-01![6-[(5,7-dihydroxy-4-methyl-1,2,3,4-tetrahydronaphthalen-2-yl)oxy]-4,5-dihydroxy-3-[(E
C35H36O12; MF: 0; RMF: 3; Prob 100.0%; Lib: mona-export-gnps; ID: 7956.

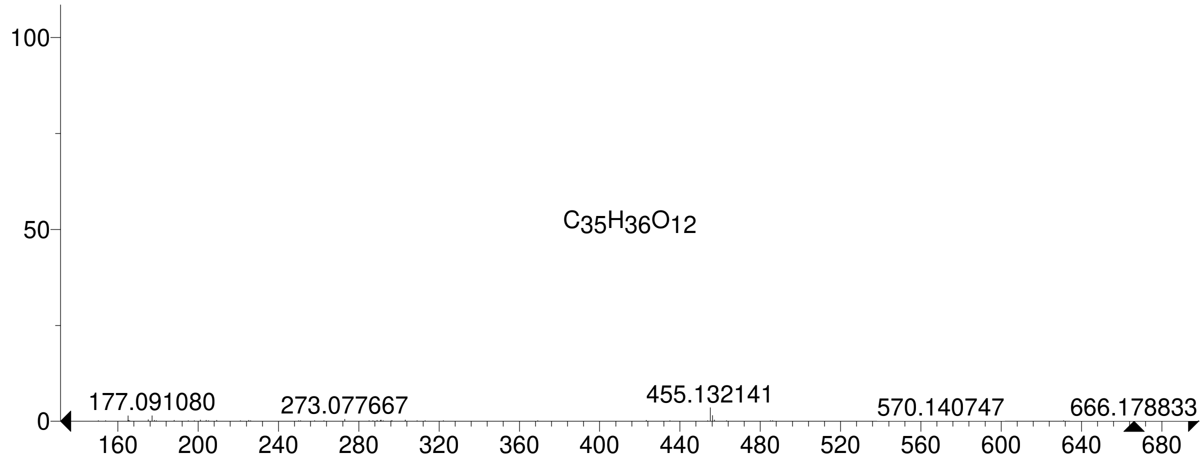

** Search Report Page 1 of 1 **

Unknown: Unknown (3.12_297.0096m/z)
Compound in Library Factor = 0

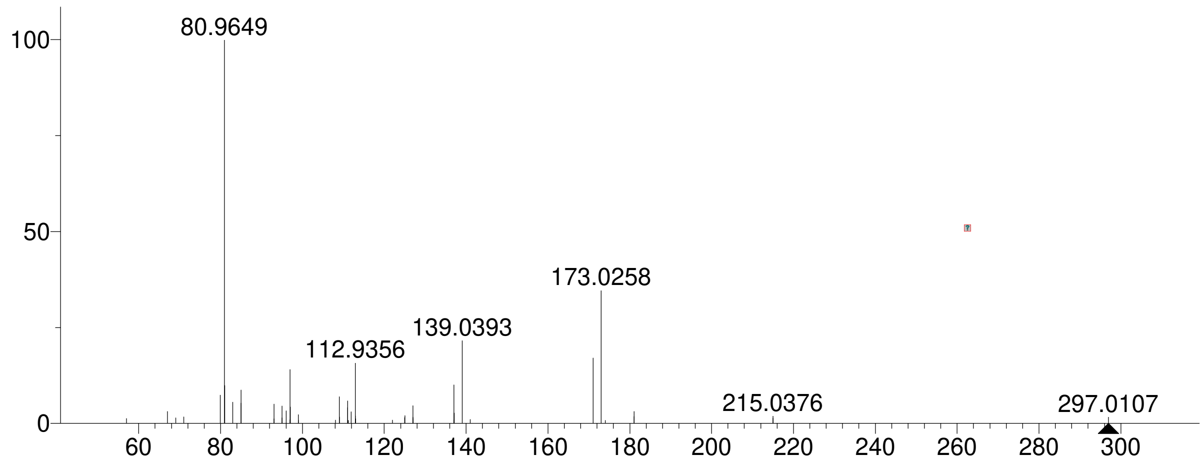

Unknown: Unknown (3.12_354.1044m/z)
Compound in Library Factor = -674

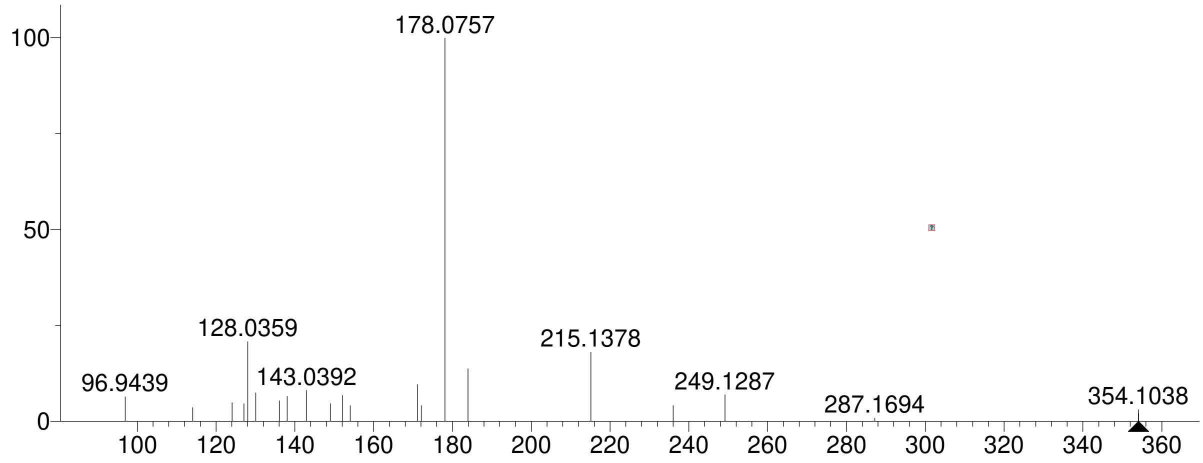

Hit 1 : Nizatidine [M+Na]+ HCD 10V P=354.1
C12H21N5O2S2; MF: 109; RMF: 695; Prob 83.7%; CAS: 76963-41-2; Lib: nist_msms; ID: 152083.

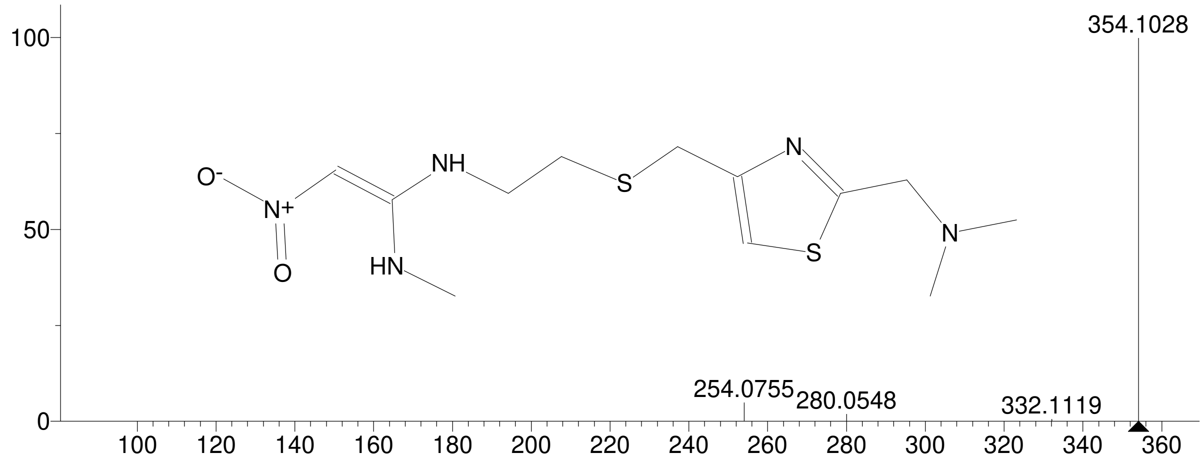

Hit 2 : 5'-O-Acetyladenosine [M+HCO2]- HCD 5V P=354.1
C12H15N5O5; MF: 7; RMF: 113; Prob 6.02%; CAS: 2140-25-2; Lib: nist_msms; ID: 228060.

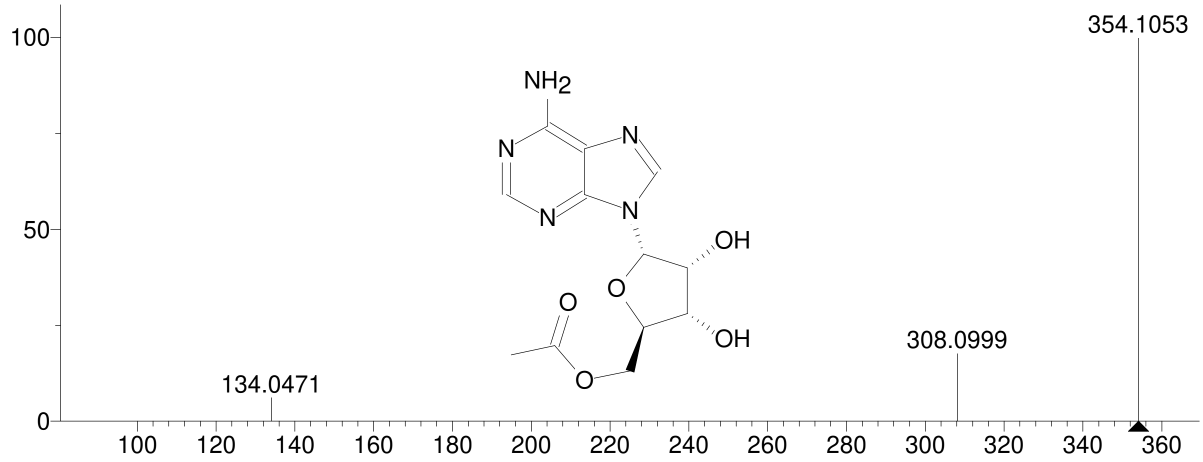

Hit 3 : rac-4-Sulfoxypropranolol [M-H]- HCD 17V P=354.1
C16H21NO6S; MF: 4; RMF: 106; Prob 5.32%; CAS: 87075-33-0; Lib: nist_msms; ID: 443588.

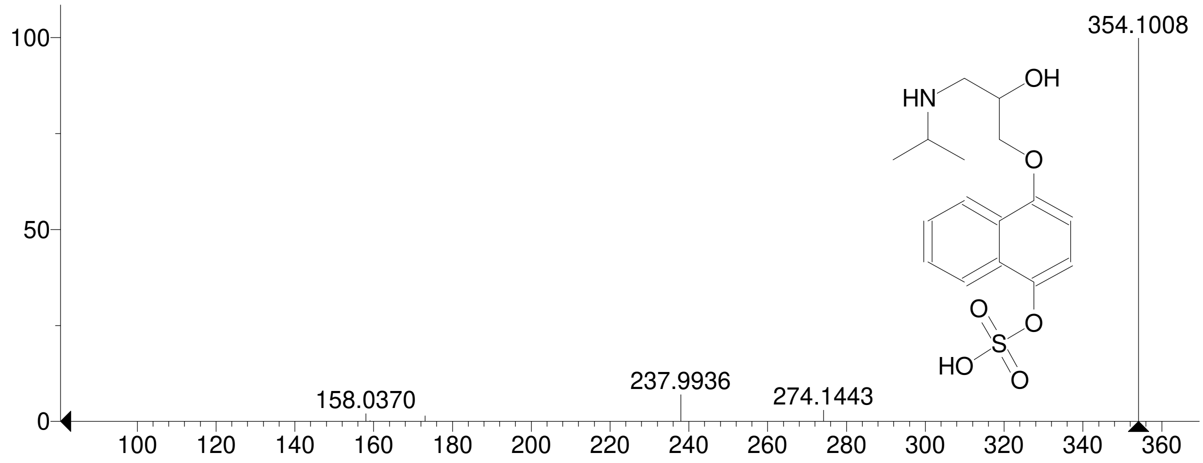

Hit 4 : 1H-Indole-2-propanoic acid, 1-[(4-chlorophenyl)methyl]-5-fluoro-α,α,3-trimethyl- [M-H-H2O]- HCD 24V P
C21H21ClFNO2; MF: 2; RMF: 105; Prob 4.90%; CAS: 103253-15-2; Lib: nist_msms; ID: 430035.

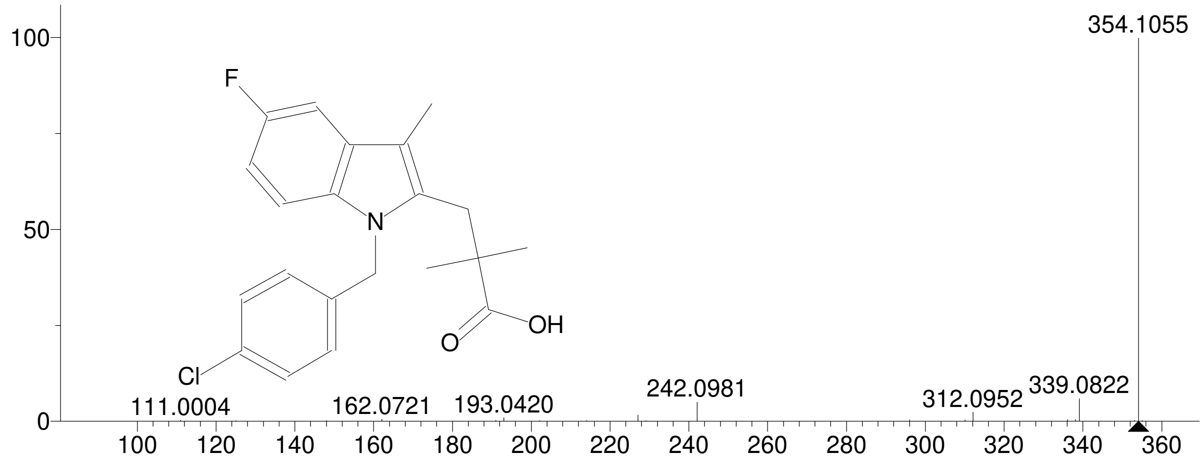

Unknown: Unknown (3.14_330.1175m/z)
Compound in Library Factor = 0

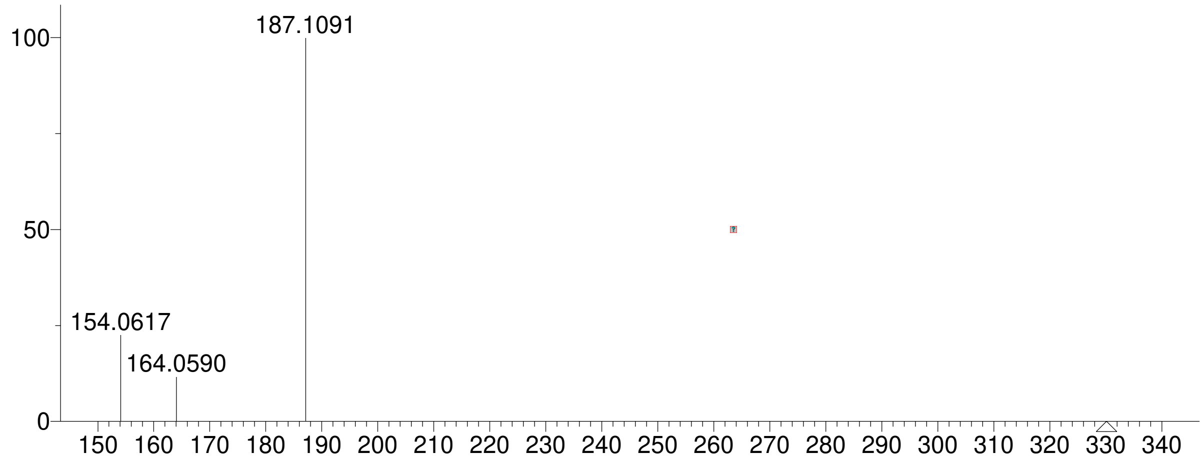

Unknown: Unknown (3.14_360.1762m/z)
Compound in Library Factor = 0

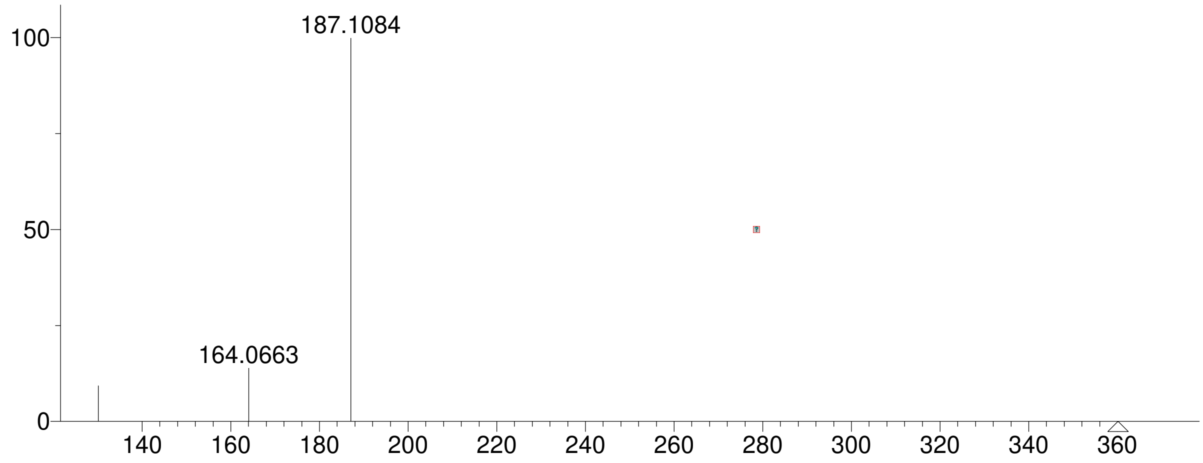

Hit 1 : Pesticide6_Etoxazole_C21H23F2NO2_4-(4-tert-Butyl-2-ethoxyphenyl)-2-(2,6-difluorophenyl)-4,5-dihydro-1
C21H23F2NO2; MF: 0; RMF: 28; Prob 100.0%; Lib: mona-export-gnps; ID: 12999.

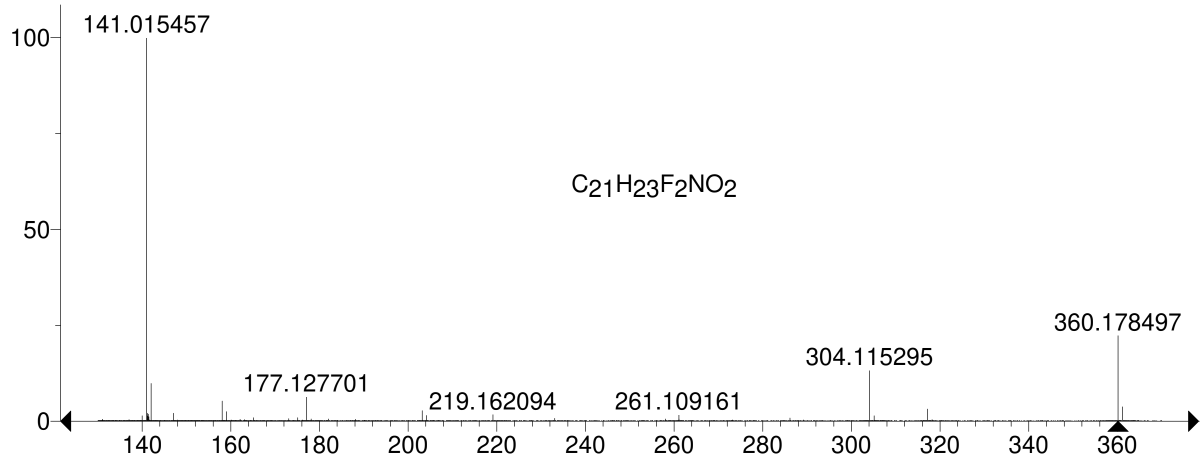

Unknown: Unknown (3.14_574.2016m/z)
Compound in Library Factor = 0

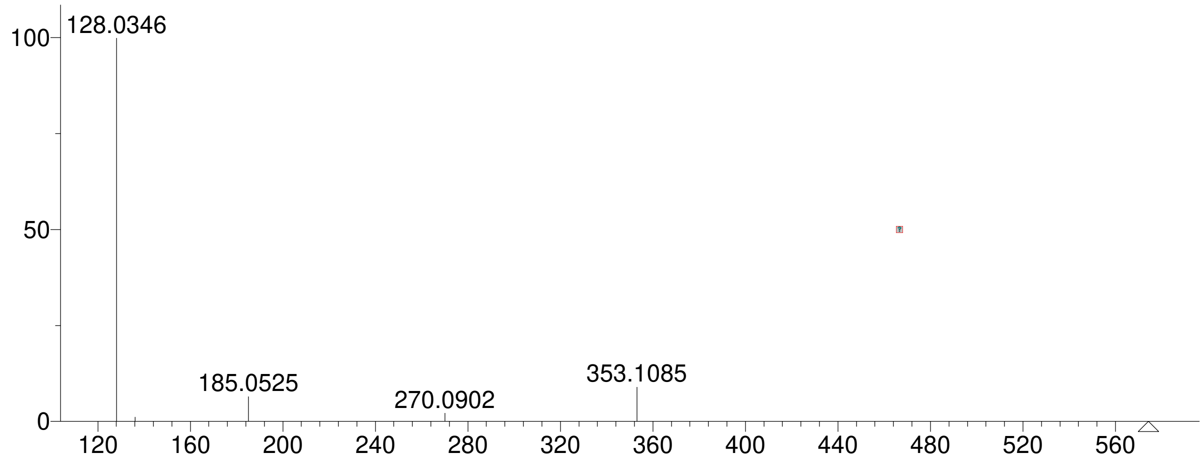

Hit 1 : Lewis A trisaccharide [M+HCO2]- IT-FT 35% P=574.2
C20H35NO15; MF: 0; RMF: 21; Prob 100.0%; CAS: 56570-03-7; Lib: nist_msms; ID: 532836.

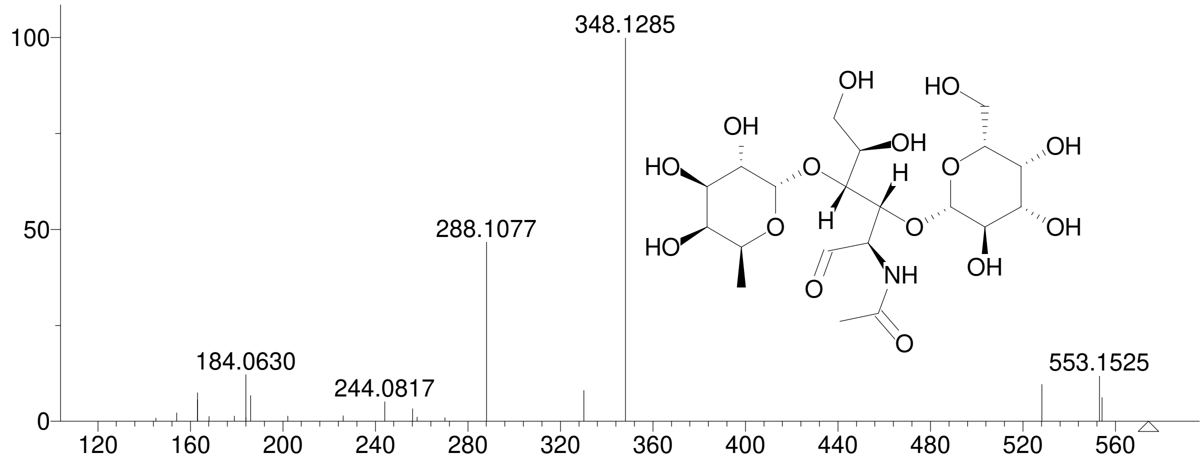

Unknown: Unknown (3.14_966.3584m/z)
Compound in Library Factor = 0

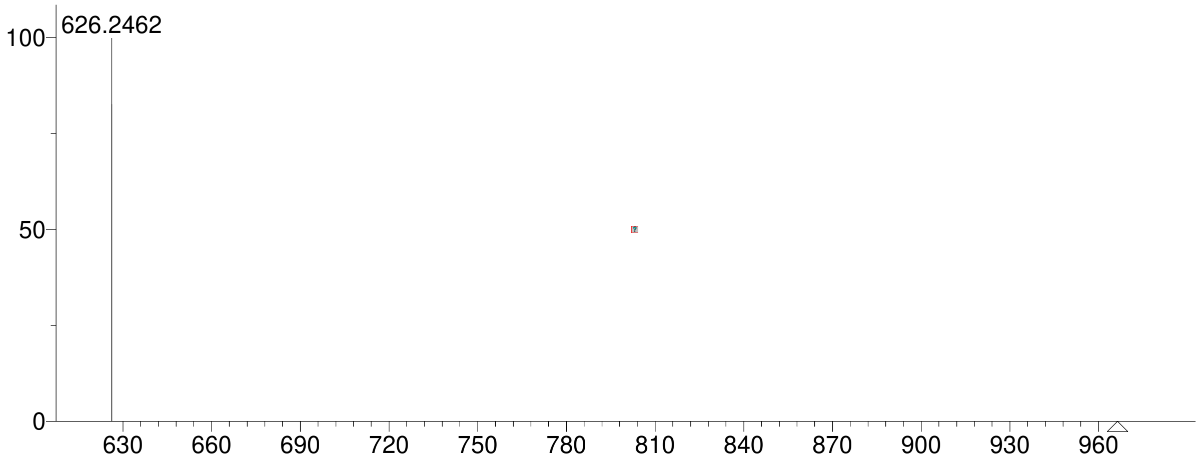

Unknown: Unknown (3.15_164.0708m/z)
Compound in Library Factor = -349

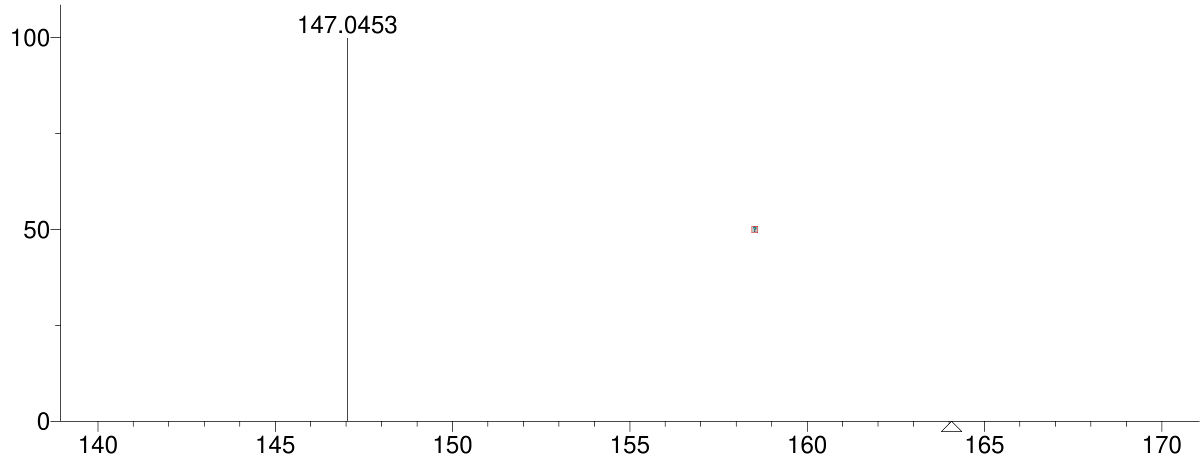

Hit 1 : L-Phenylalanine [M-H]- 20 P=164.1
C9H11NO2; MF: 364; RMF: 999; Prob 17.0%; Lib: msms neg mona_ipb_gnps_plasma; ID: 569.

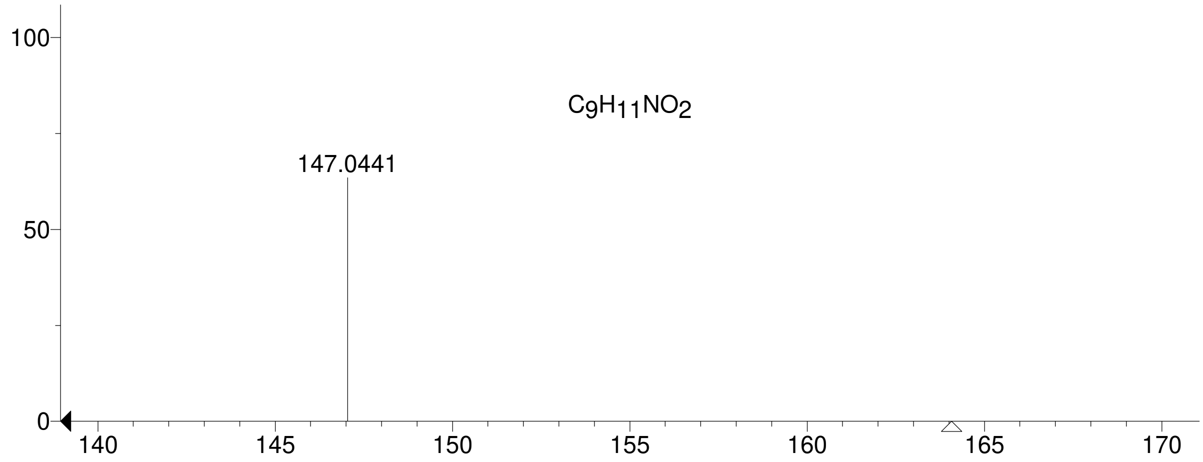

Hit 2 : Phenylalanine [M-H]- 35eV P=164.1
C9H11NO2; MF: 364; RMF: 999; Prob 17.0%; Lib: mona-export-fiehn_hilic; ID: 2924.

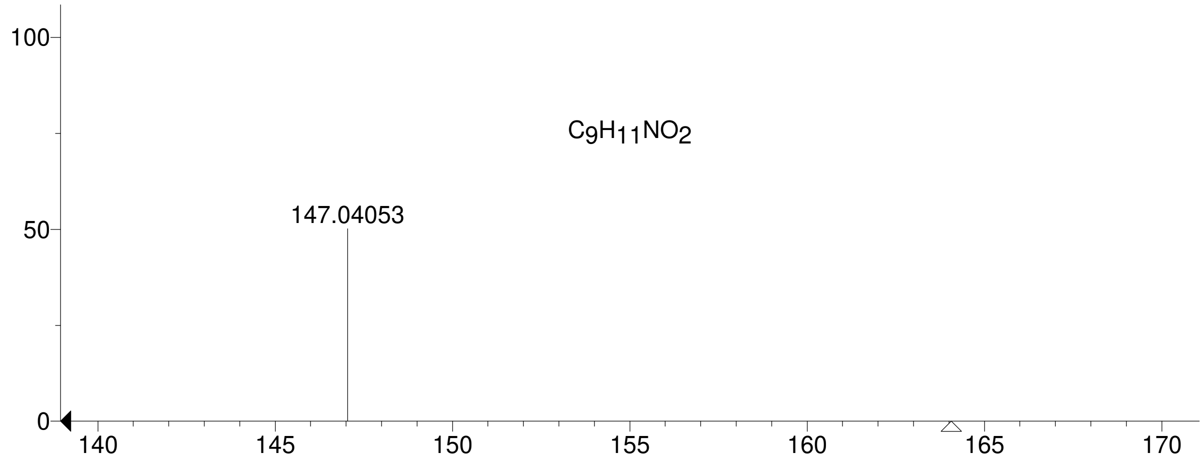

Hit 3 : DL-Phenylalanine [M-H]- IT-FT 35% P=164.1
C9H11NO2; MF: 364; RMF: 999; Prob 17.0%; CAS: 150-30-1; Lib: nist_msms; ID: 141494.

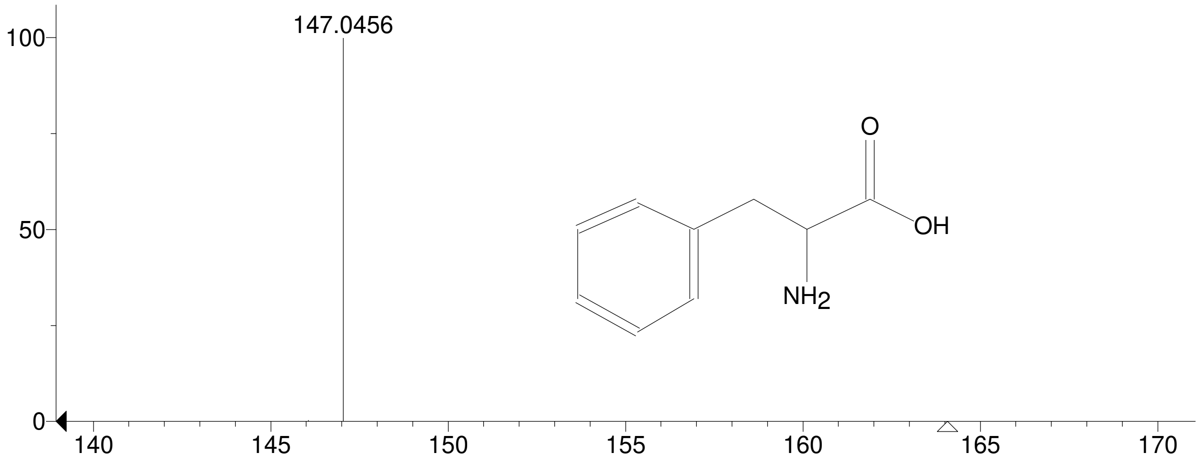

Hit 4 : N-Acetyl-L-phenylalanine [M-H-C2H2O]- IT-FT 35% P=164.1
C11H13NO3; MF: 364; RMF: 999; Prob 17.0%; CAS: 2018-61-3; Lib: nist_msms; ID: 290399.

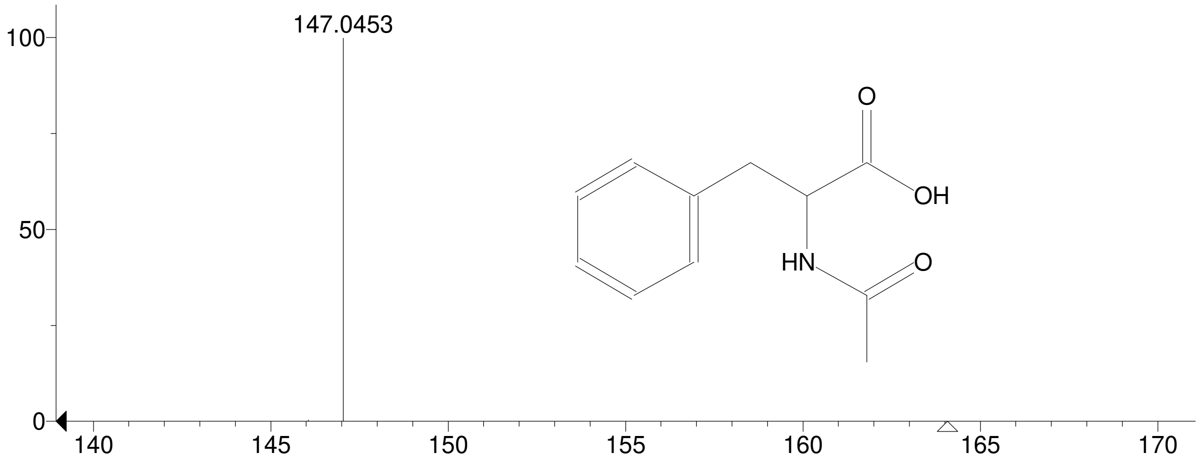

Hit 5 : N-(Phenylacetyl)-L-phenylalanine [M-H-C8H6O]- HCD 52V P=164.1
C17H17NO3; MF: 364; RMF: 999; Prob 17.0%; CAS: 738-75-0; Lib: nist_msms; ID: 485931.

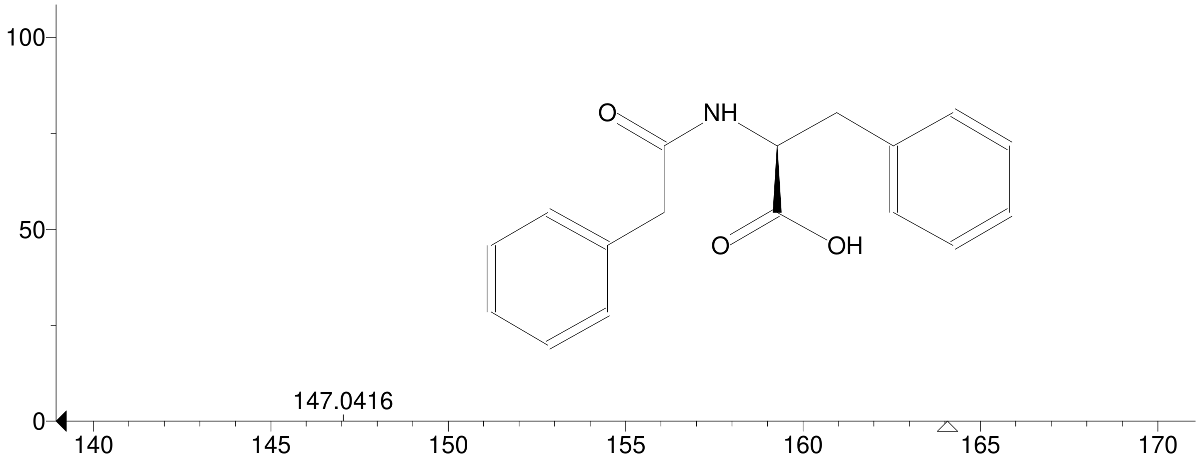

Unknown: Unknown (3.15_413.1547m/z)
Compound in Library Factor = -3273

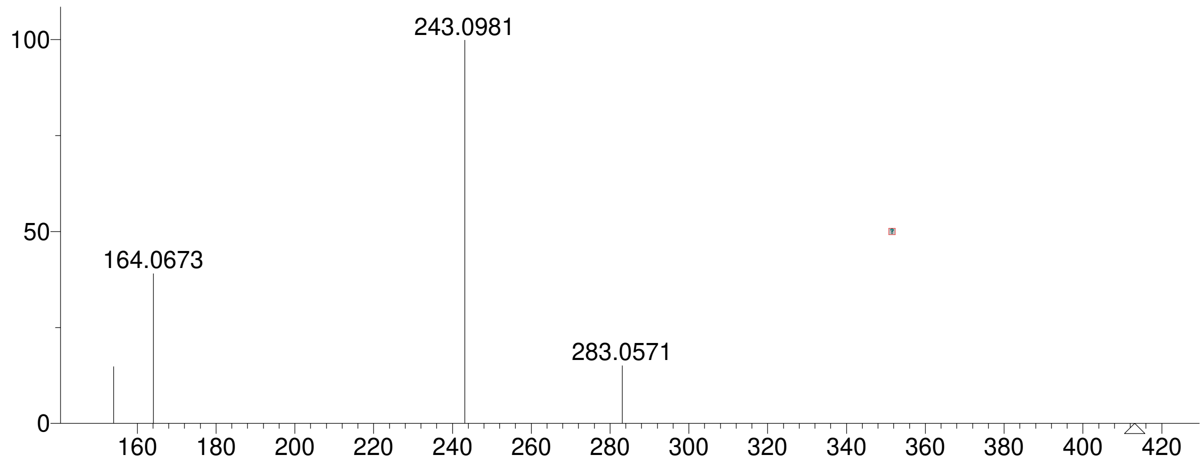

Hit 1 : 2-amino-5-[2-[[2,3-dihydroxy-2-(1-hydroxyethyl)butanoyl]oxymethyl]-4-hydroxyanilino]-5-oxopentanoic acid
C18H26N2O9; MF: 1; RMF: 112; Prob 33.8%; Lib: mona-export-lc-ms-ms_negative_mode; ID: 22977.

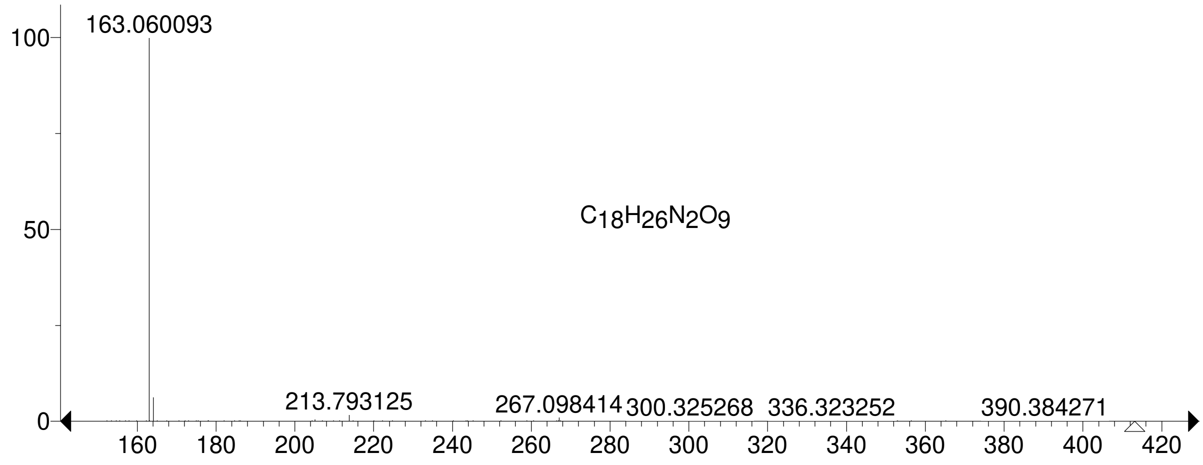

Hit 2 : PG 10:0; PG 3:0-7:0; [M-H]- [M-H]- P=413.2
C16H31O10P; MF: 1; RMF: 99; Prob 33.8%; Lib: msdial-insilicomsms-lipids-neg; ID: 324631.

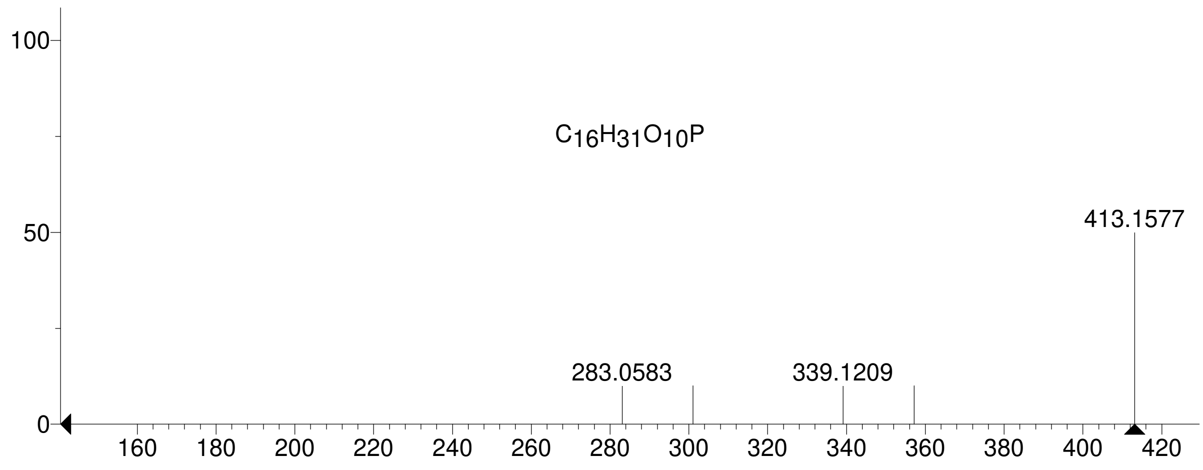

Hit 3 : 2-[2-[(Z)-pent-2-enyl]-3-[3,4,5-trihydroxy-6-(hydroxymethyl)oxan-2-yl]oxycyclopentyl]acetic acid [M+K]+ 65
C18H30O8; MF: 0; RMF: 32; Prob 32.5%; Lib: anthocyanins mona; ID: 9976.

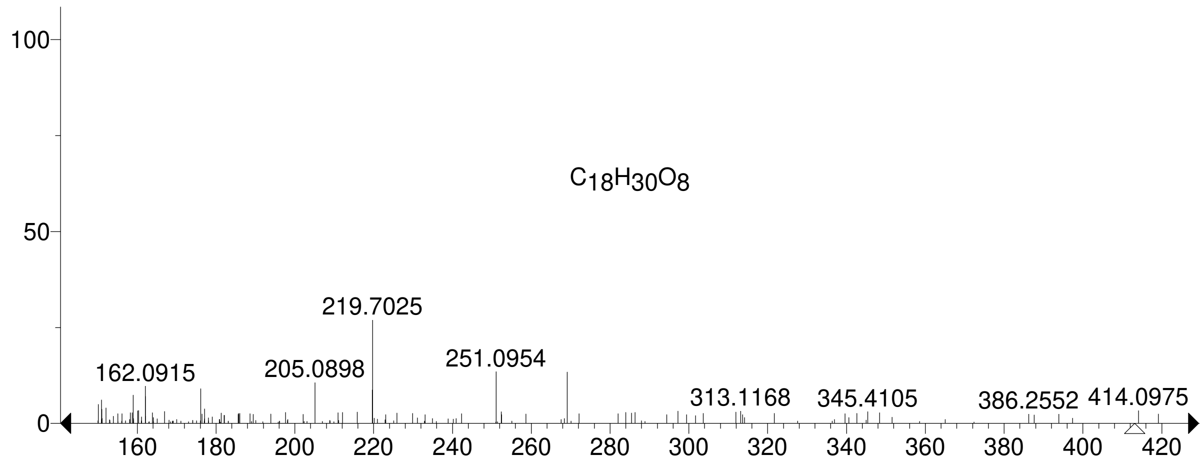

Unknown: Unknown (3.15_488.1526m/z)
Compound in Library Factor = 0

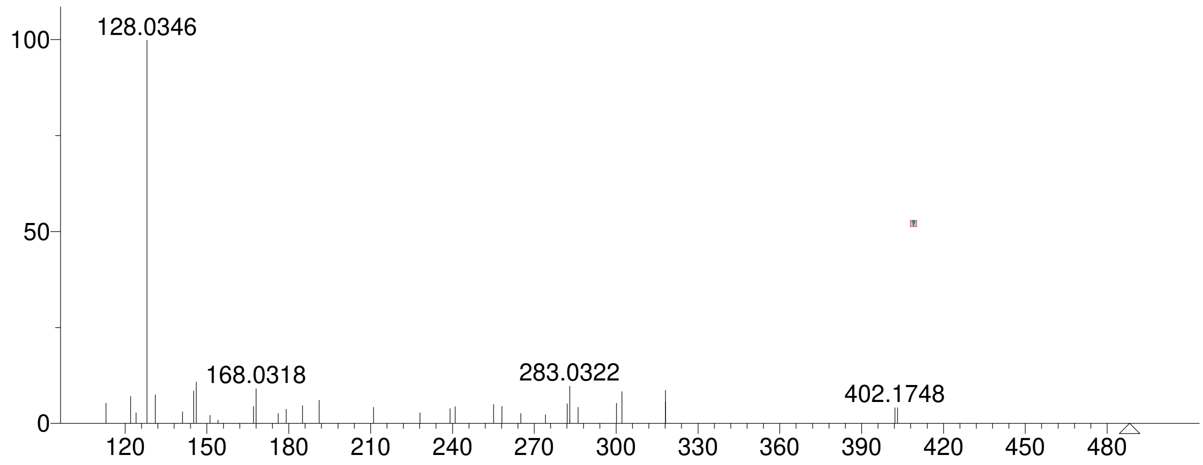

** Search Report Page 1 of 1 **

Unknown: Unknown (3.16_281.0867m/z)
Compound in Library Factor = 0

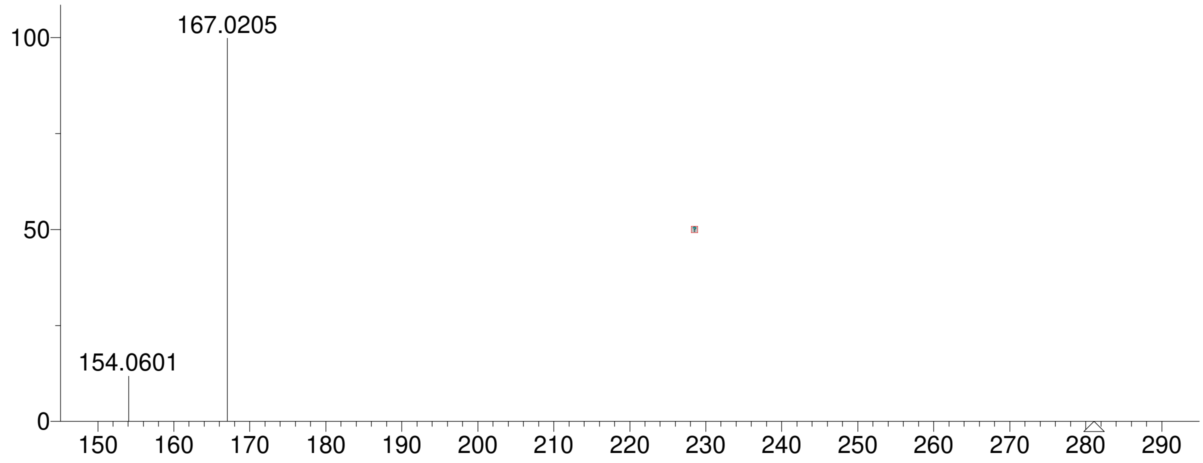

Hit 1 : 1H- Pyrazole-4-carboxylic acid, 1-(2,4-difluorophenyl)-5-[[2-[[(1,1-dimethylethyl)amino] thioxomethyl]hydraz
C17H19F2N5O2S; MF: 0; RMF: 61; Prob 100.0%; CAS: 264233-05-8; Lib: nist_msms; ID: 273556.

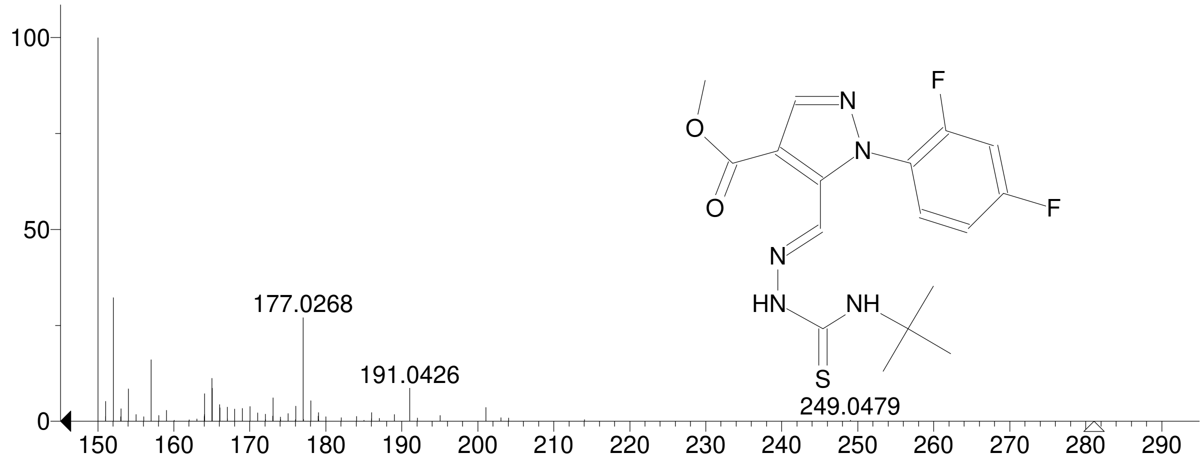

Unknown: Unknown (3.16_303.0530m/z)
Compound in Library Factor = -3273

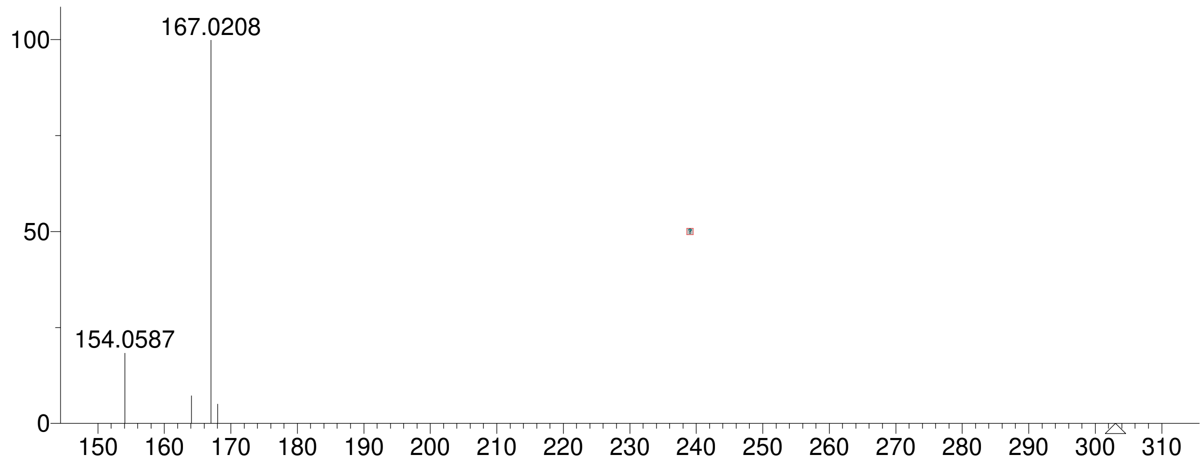

Hit 1 : Sulfameter [M+Na]+ HCD 27V P=303.1
C11H12N4O3S; MF: 1; RMF: 63; Prob 17.2%; CAS: 651-06-9; Lib: nist_msms; ID: 310116.

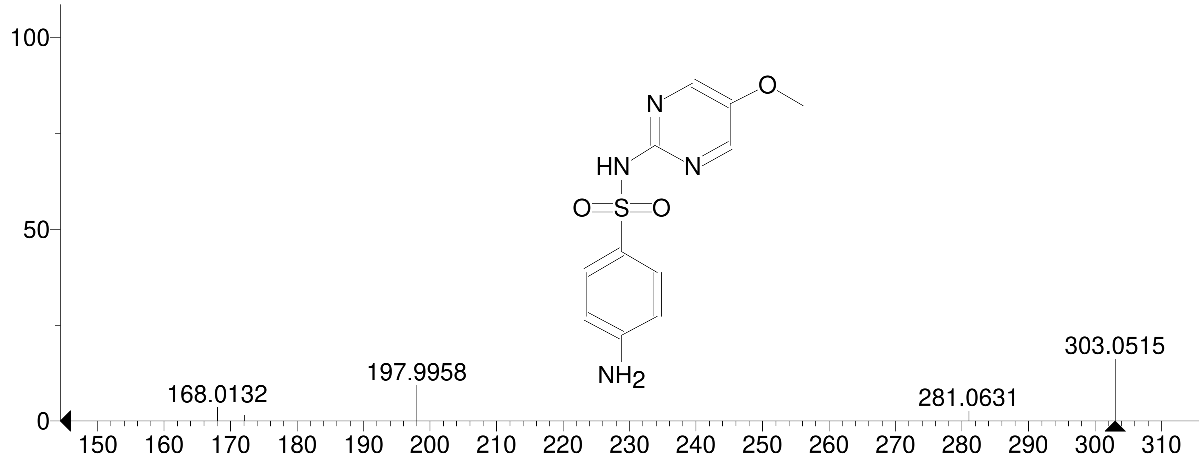

Hit 2 : Dihydroquercetin [M-H]- 40ev P=303.1
C15H12O7; MF: 0; RMF: 50; Prob 16.6%; Lib: mona-export-lc-ms-ms_negative_mode; ID: 9365.

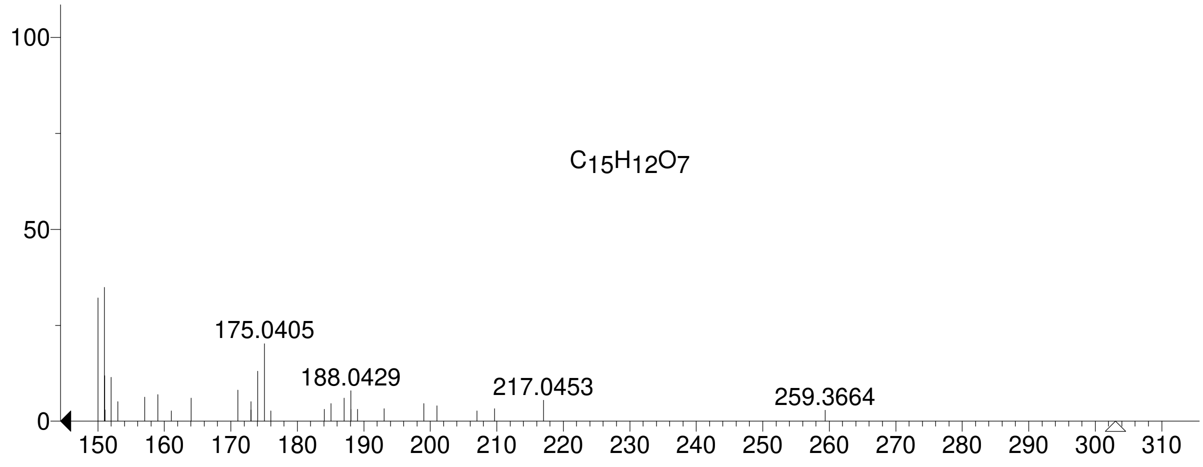

Hit 3 : 3,5,7,3',4'-Pentahydroxyflavanone [M-H]- HCD 48V P=303.1
C15H12O7; MF: 0; RMF: 48; Prob 16.6%; CAS: 480-18-2; Lib: nist_msms; ID: 148075.

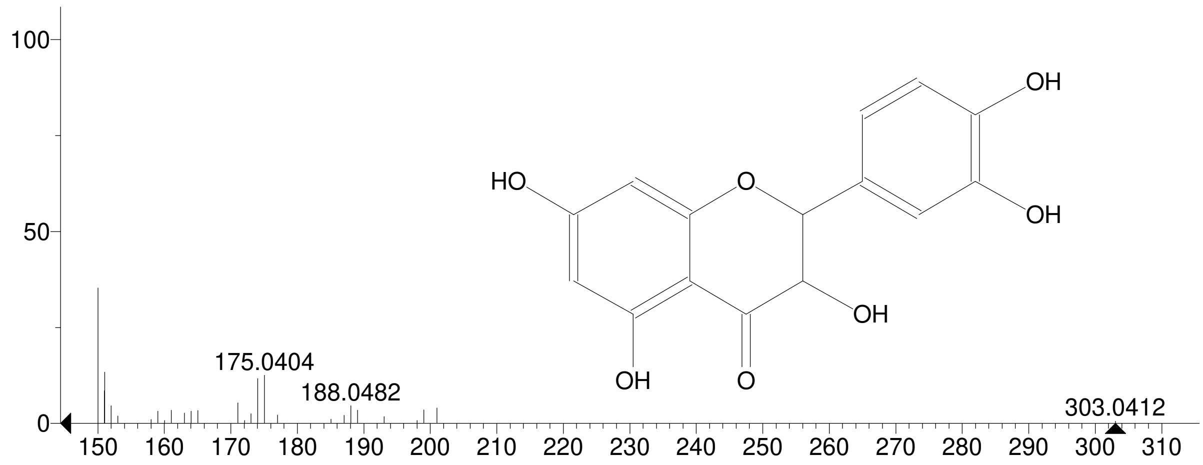

Hit 4 : taxifolin [M-H]- 40 P=303.1
C15H12O7; MF: 0; RMF: 36; Prob 16.6%; Lib: mona-export-lc-ms-ms_negative_mode; ID: 27780.

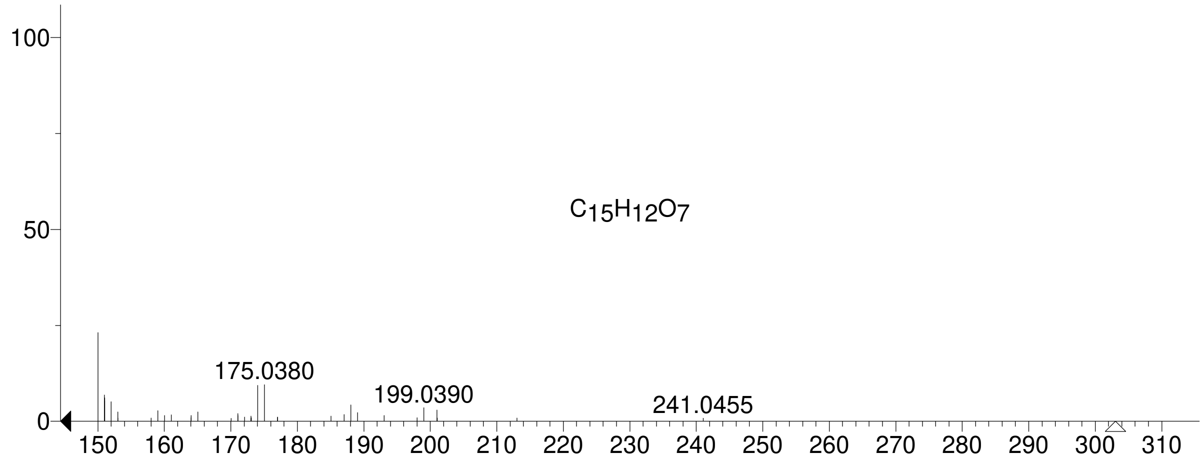

Hit 5 : NCGC00381232-01!5,7-dihydroxy-2-(2,3,4-trihydroxyphenyl)chromen-4-one [M+H]+ P=303.1
C15H10O7; MF: 0; RMF: 25; Prob 16.6%; Lib: mona-export-gnps; ID: 9293.

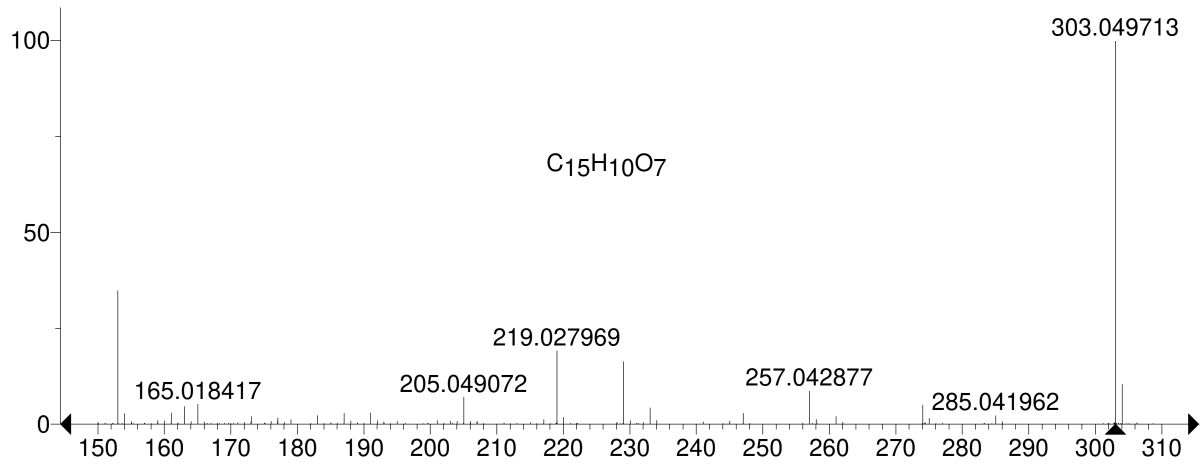

Unknown: Unknown (3.17_351.0928m/z)
Compound in Library Factor = 0

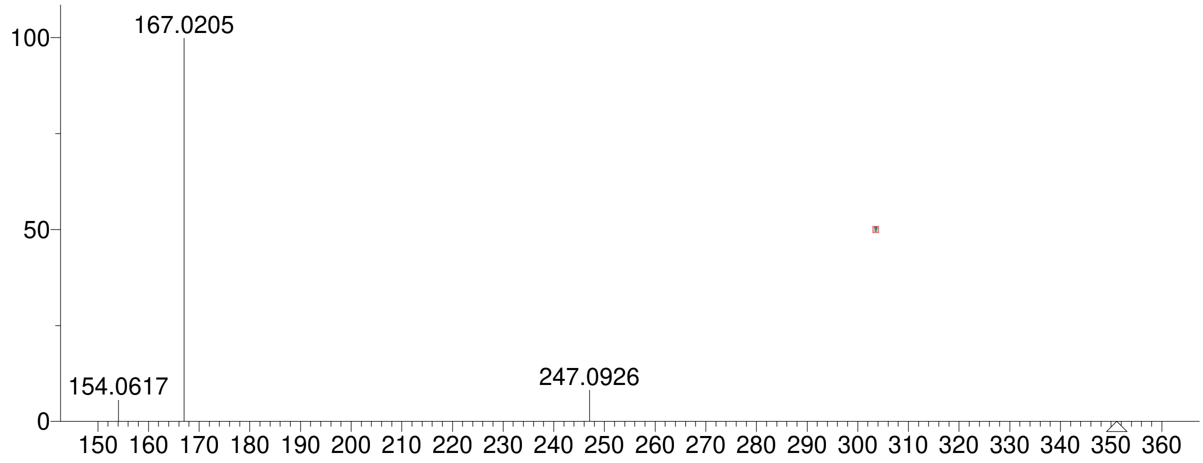

** Search Report Page 1 of 1 **

Unknown: Unknown (3.17_417.1498m/z)
Compound in Library Factor = 0

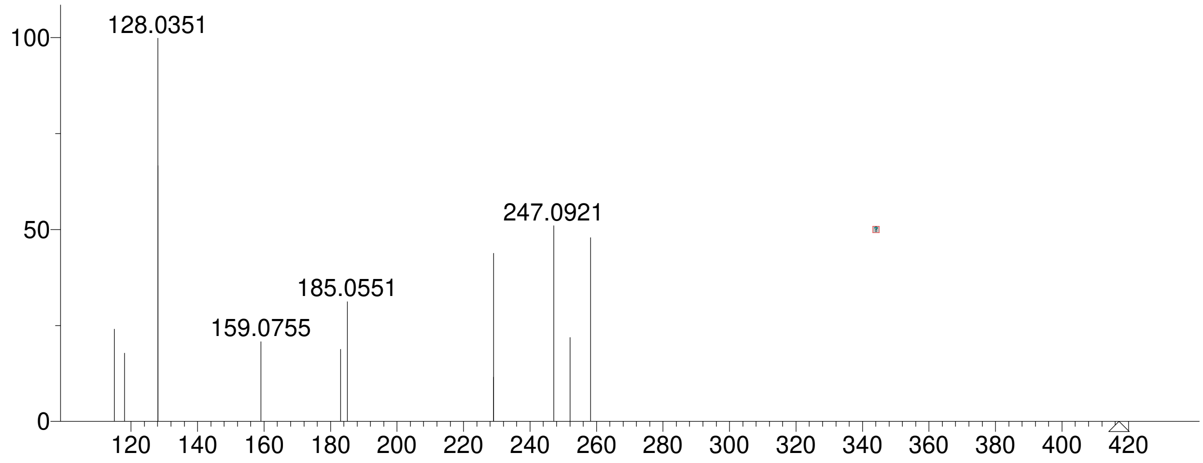

** Search Report Page 1 of 1 **

Unknown: Unknown (3.18_719.3497m/z)
Compound in Library Factor = -3273

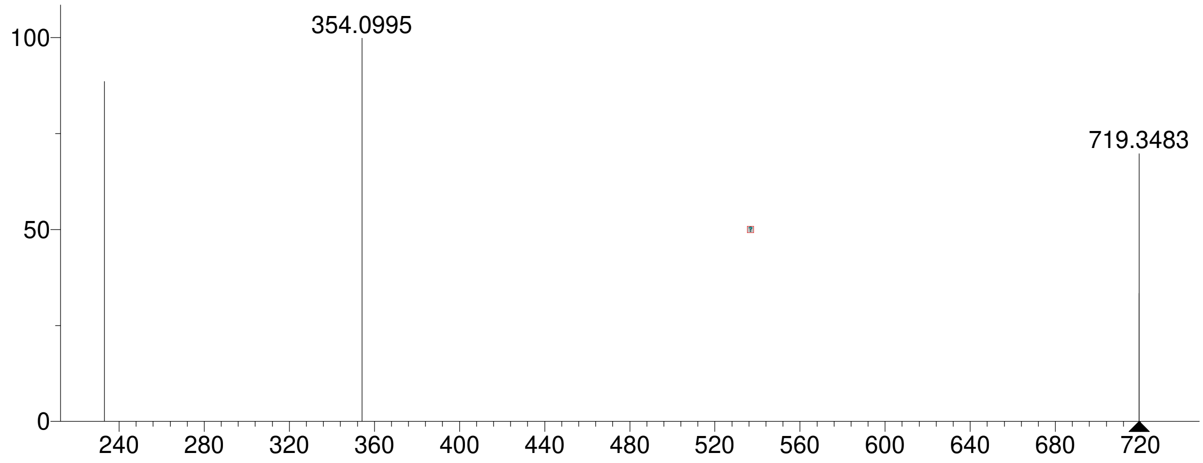

Hit 1 : Etoxazole [2M+H]+ HCD 14V P=719.3
C21H23F2NO2; MF: 20; RMF: 326; Prob 100.0%; CAS: 153233-91-1; Lib: nist_msms; ID: 482644.

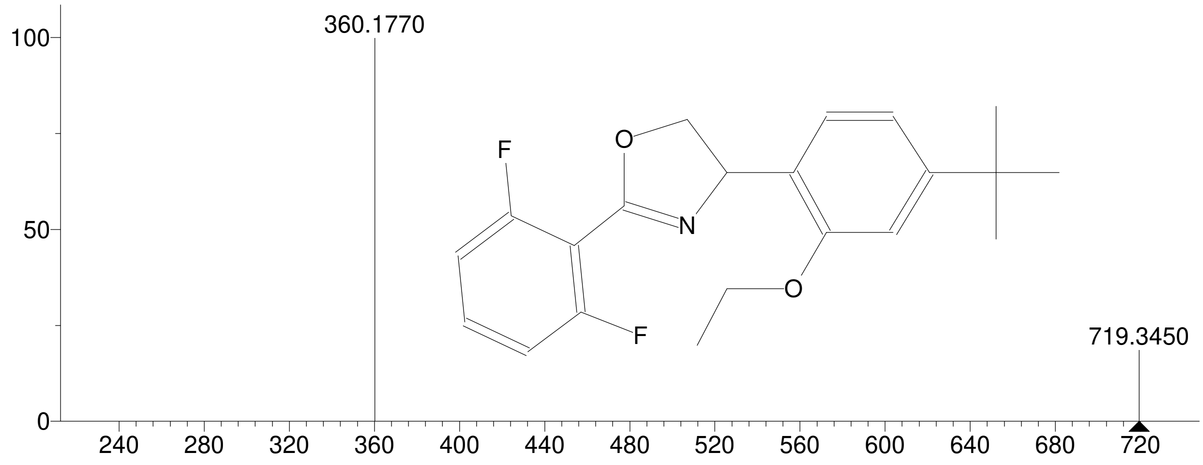

Unknown: Unknown (3.19_445.1446m/z)
Compound in Library Factor = -3273

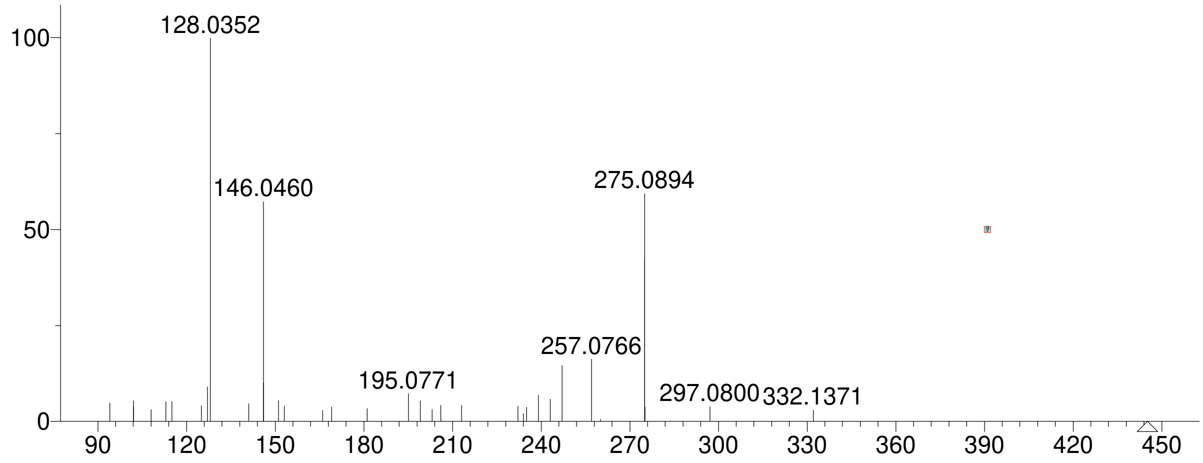

Hit 1 : (1R,3R,4S,6S,8R,9R,10S,11S)-9-(acetyloxy)-8-hydroxy-3,8-dimethyl-12-methylidene-7,13-dioxo-5,14-dioxa
C21H26O9; MF: 1; RMF: 49; Prob 34.2%; Lib: mona-export-vf-npl_qexactive; ID: 6949.

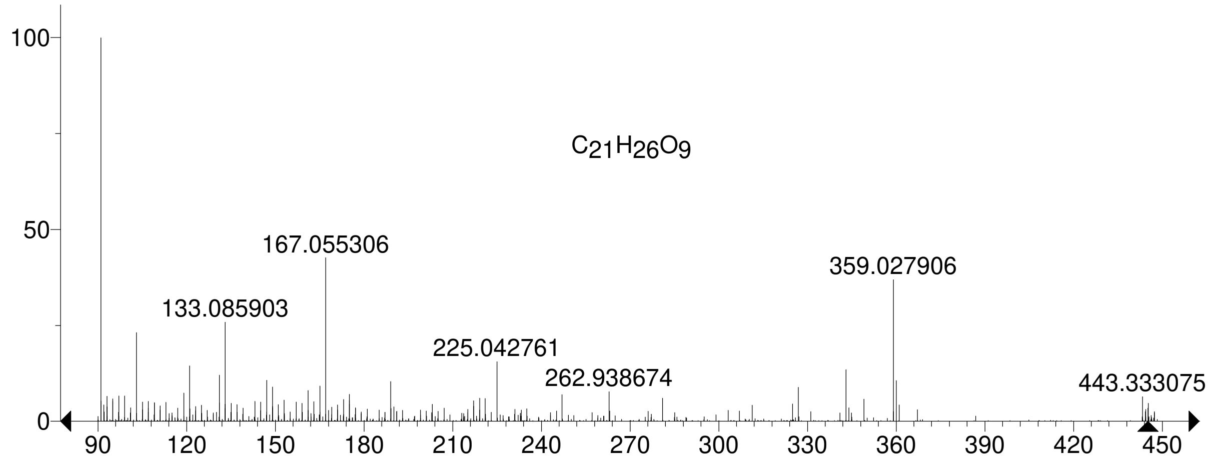

Hit 2 : 1,3-Cyclobutanedicarboxylic acid, 2,4-diphenyl-, 1-(1-naphthalenyl) ester, (1β,2R,3α,4R)- [M+Na]+ HCD
C28H22O4; MF: 0; RMF: 6; Prob 32.9%; CAS: 1541209-75-9; Lib: nist_msms; ID: 349631.

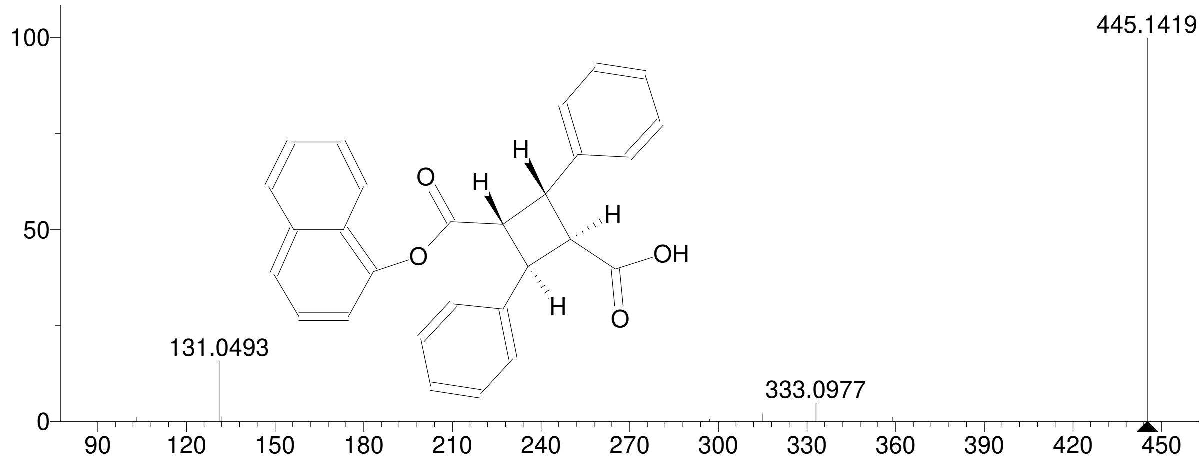

Hit 3 : Acetamiprid [2M+H]+ IT 35% P=445.1
C10H11ClN4; MF: 0; RMF: 5; Prob 32.9%; CAS: 135410-20-7; Lib: nist_msms; ID: 306864.

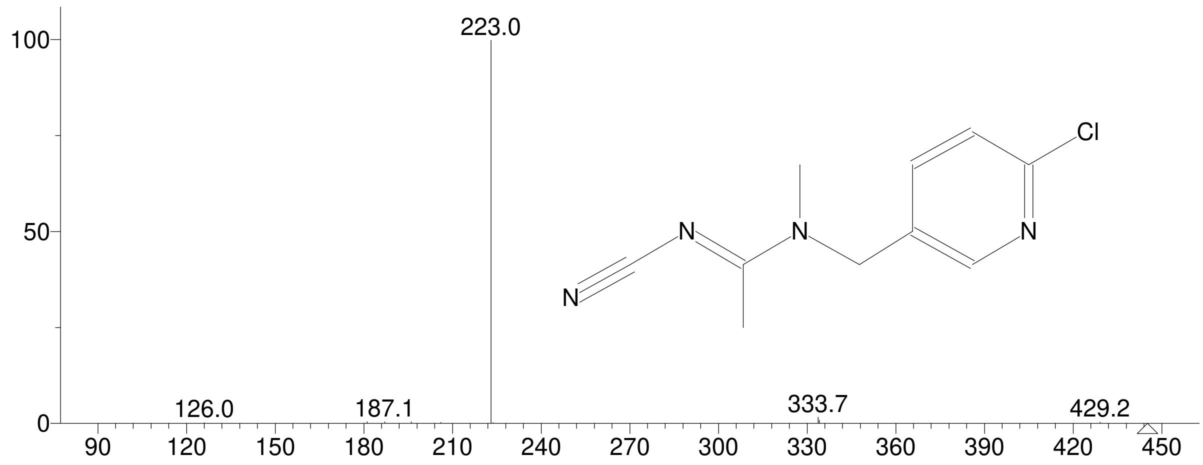

Unknown: Unknown (3.20_387.1382m/z)
Compound in Library Factor = -3273

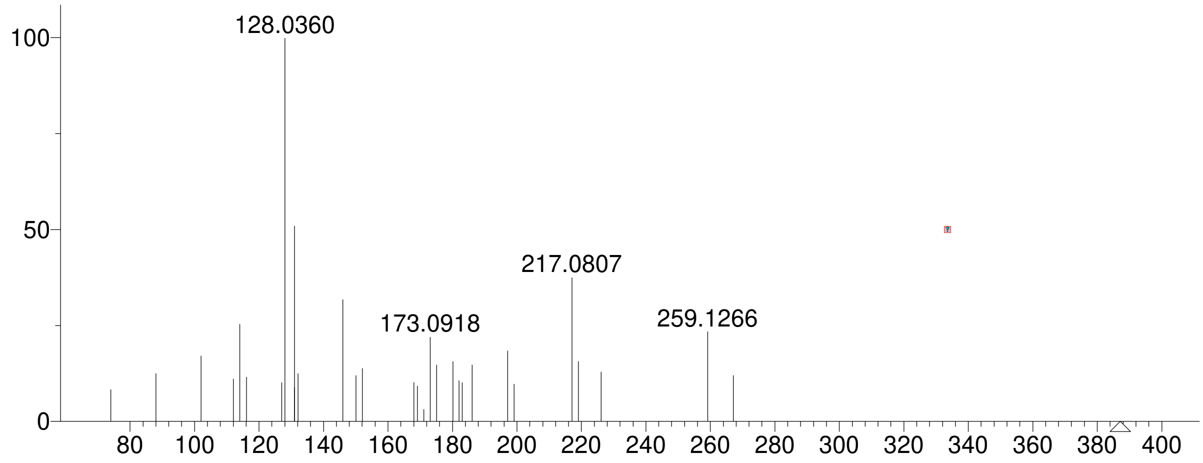

Hit 1 : [(3aR,4S,6R,11aR)-6,9-dihydroxy-6,10-dimethyl-3-methylidene-2,7-dioxo-3a,4,5,8,9,11a-hexahydrocyclode
$C_{19}H_{24}O_7$; MF: 1; RMF: 51; Prob 34.2%; Lib: mona-export-vf-npl_qexactive; ID: 10210.

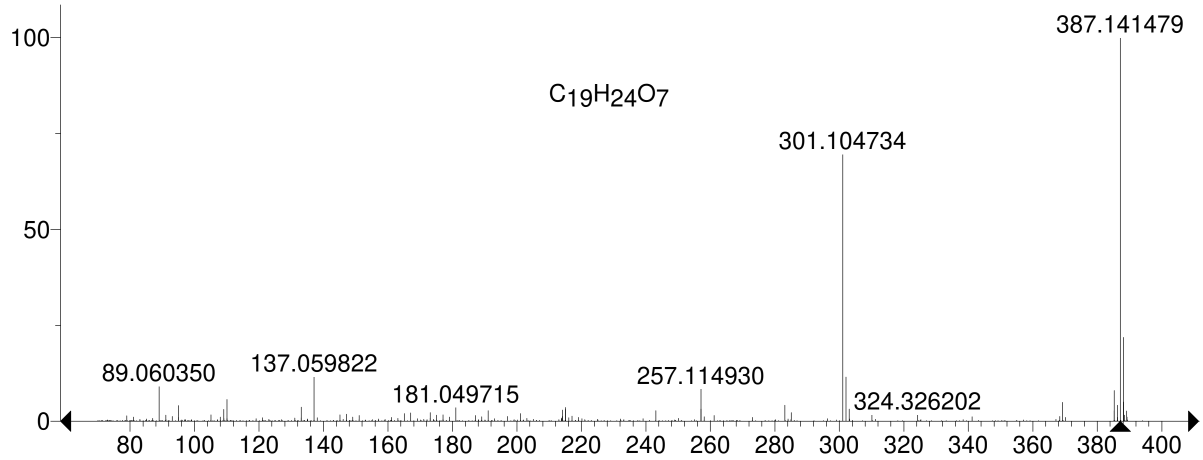

Hit 2 : (5R,6S,8Z,11S)-5,6,15-trihydroxy-17-methoxy-11-methyl-12-oxabicyclo[12.4.0]octadeca-1(14),8,15,17-tetra
$C_{19}H_{24}O_7$; MF: 0; RMF: 46; Prob 32.9%; Lib: mona-export-vf-npl_qexactive; ID: 18340.

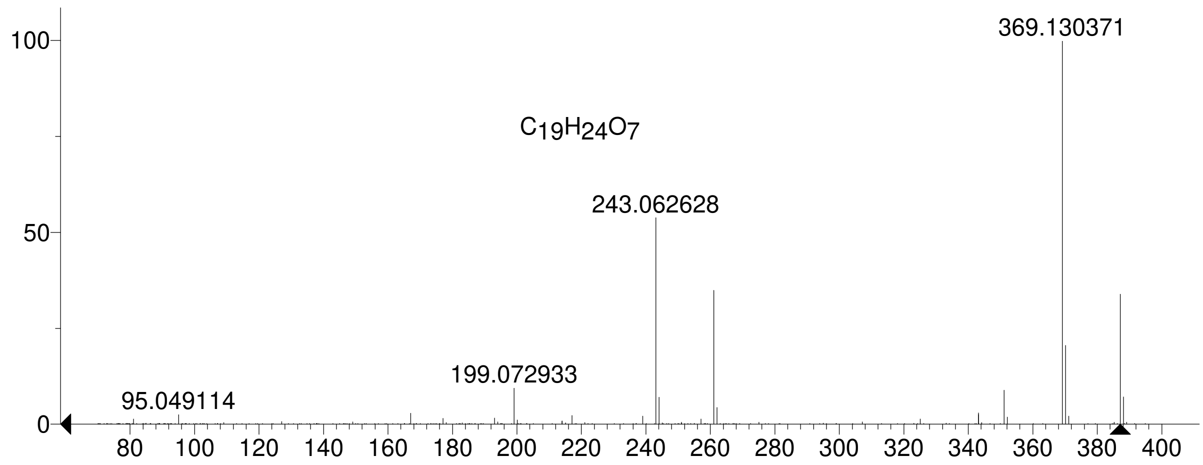

Hit 3 : NCGC00347789-02_C19H24O7_(3S,5Z,8S,9R)-8,9,16-Trihydroxy-14-methoxy-3-methyl-3,4,9,10,11,12-he
C19H24O7; MF: 0; RMF: 23; Prob 32.9%; Lib: mona-export-gnps; ID: 11355.

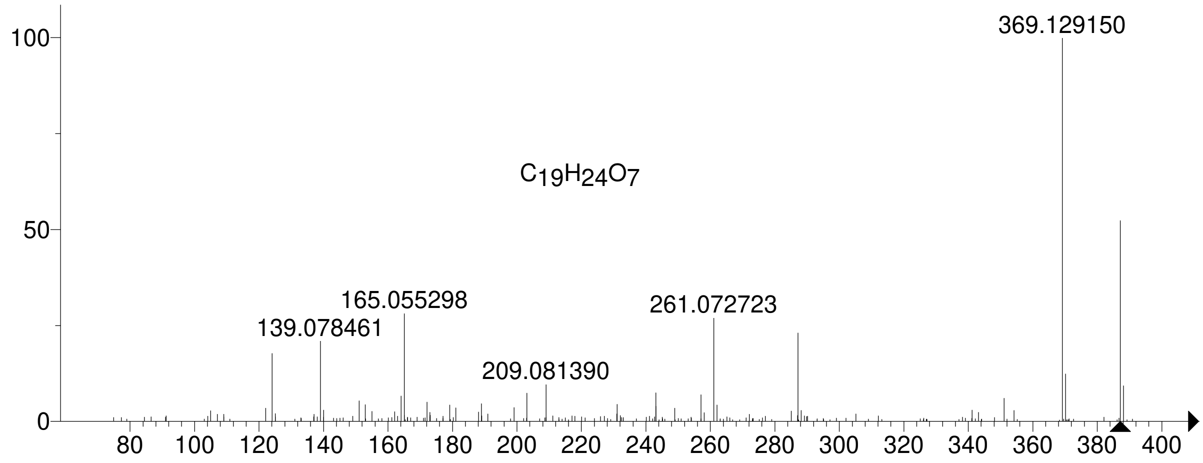

** Search Report Page 1 of 1 **

Unknown: Unknown (3.20_545.2095m/z)
Compound in Library Factor = 0

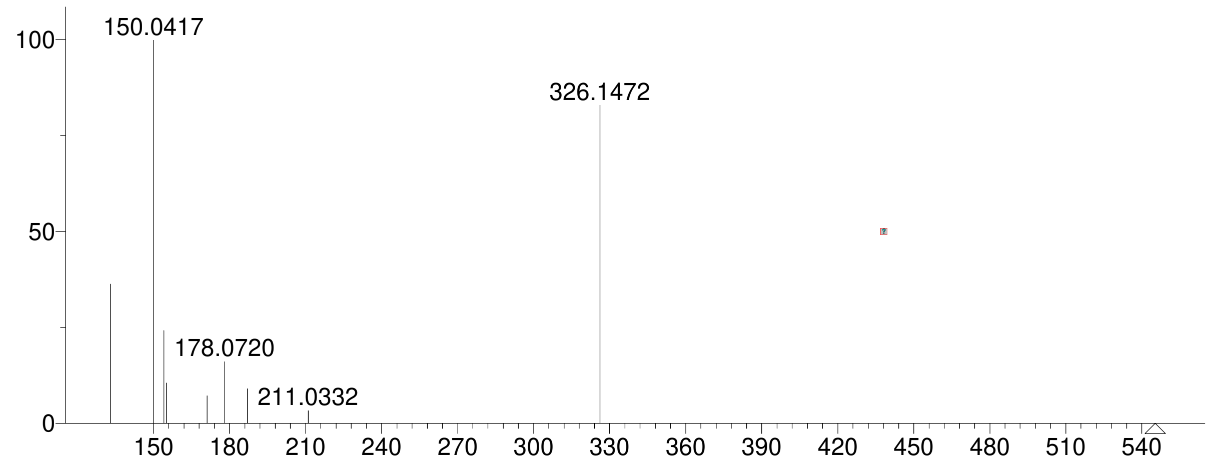

Hit 1 : methyl 3-[3,4-dihydroxy-5-(3-methylbut-2-enyl)phenyl]-2-[[4-hydroxy-3-(3-methylbut-2-enyl)phenyl]methyl]-4
C30H34O8; MF: 0; RMF: 34; Prob 50.0%; Lib: mona-export-vf-npl_qexactive; ID: 8106.

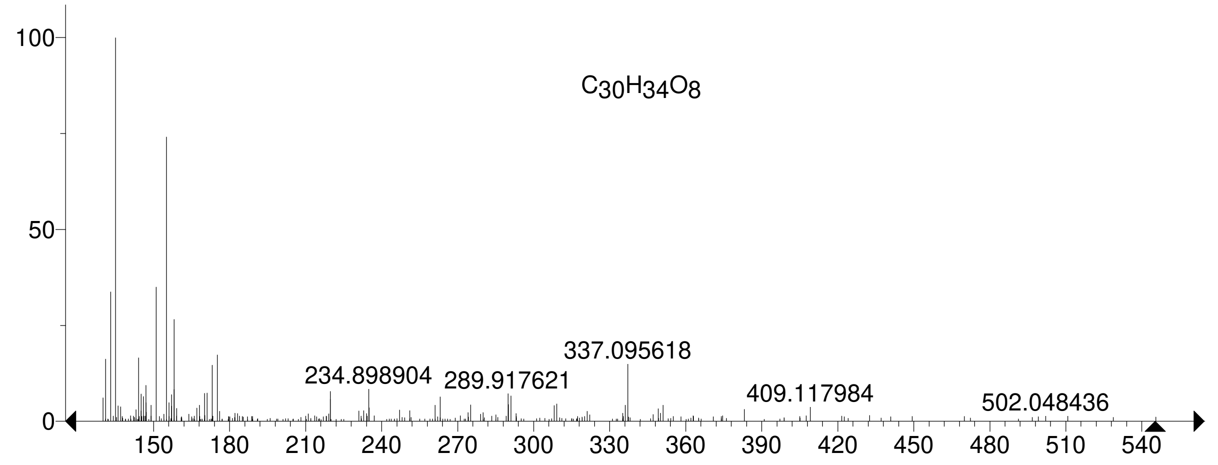

Hit 2 : cadambine [M+H]+ P=545.2
C27H32N2O10; MF: 0; RMF: 30; Prob 50.0%; Lib: mona-export-gnps; ID: 14914.

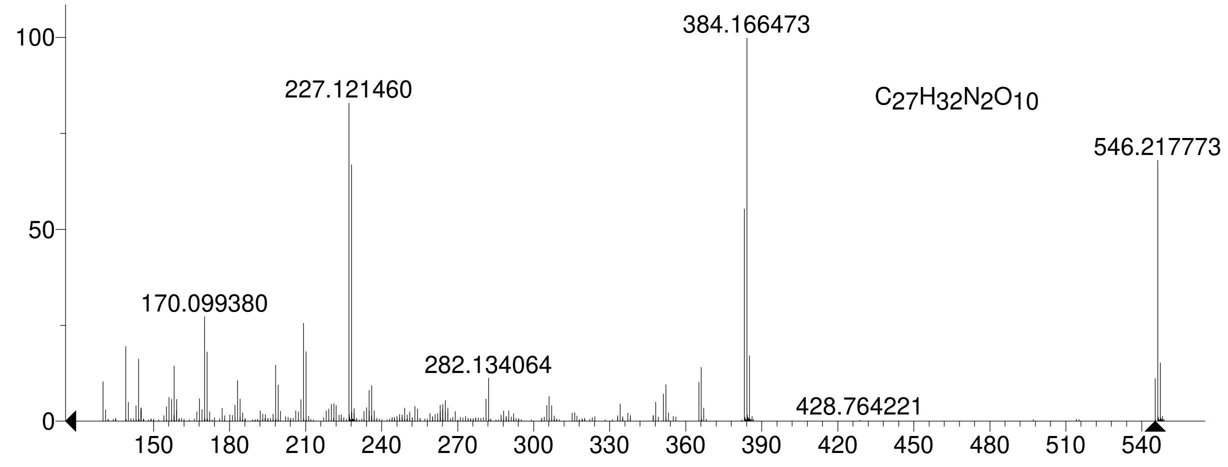

** Search Report Page 1 of 1 **

Unknown: Unknown (3.20_680.2779m/z)
Compound in Library Factor = 0

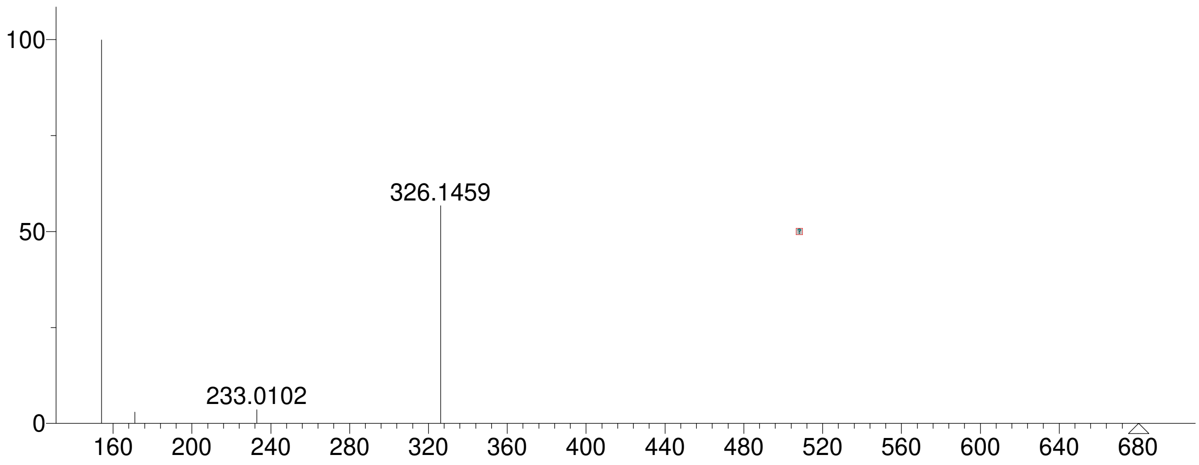

Unknown: Unknown (3.21_494.1776m/z)
Compound in Library Factor = 0

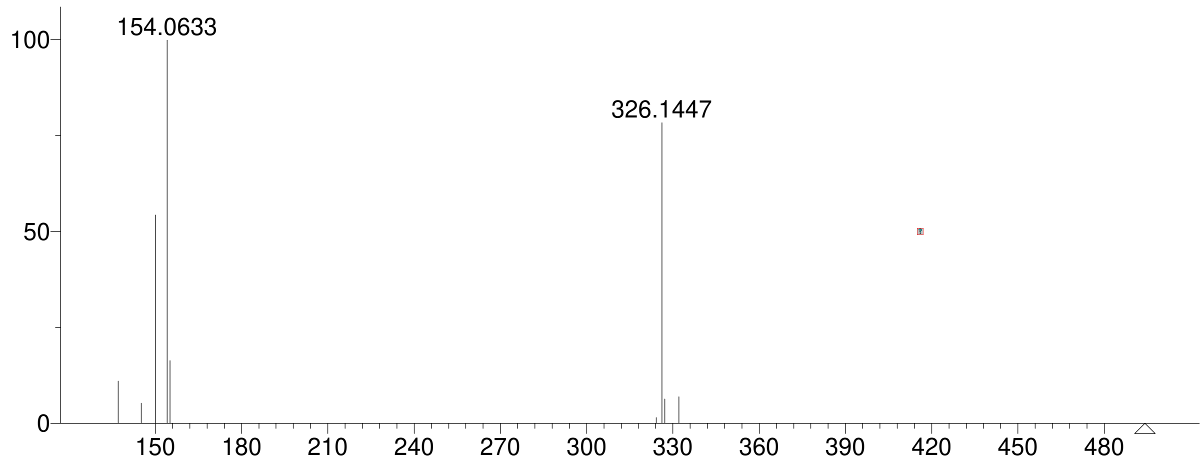

Unknown: Unknown (3.21_555.1331m/z)
Compound in Library Factor = 0

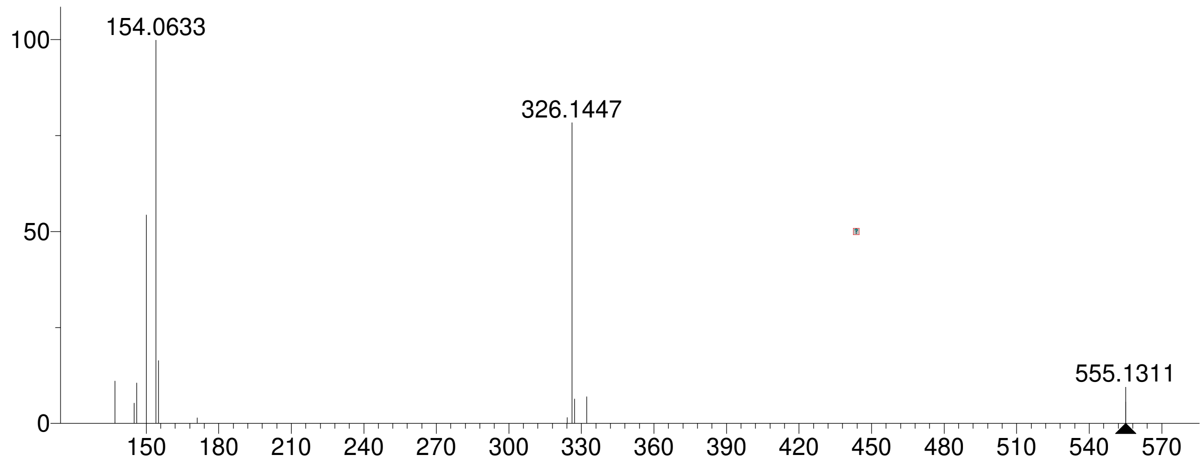

Hit 1 : NCGC00385087-01!3-[5,7-dihydroxy-2-(4-methoxyphenyl)-4-oxo-2,3-dihydrochromen-3-yl]-5,7-dihydroxy-2
C31H24O10; MF: 0; RMF: 51; Prob 100.0%; Lib: mona-export-gnps; ID: 7535.

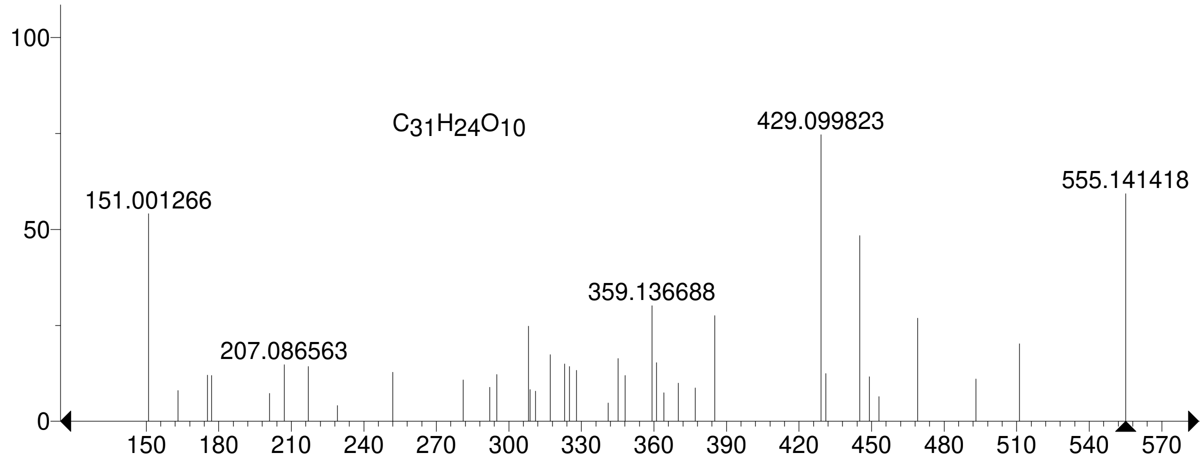

Unknown: Unknown (3.22_496.2038m/z)
Compound in Library Factor = -3273

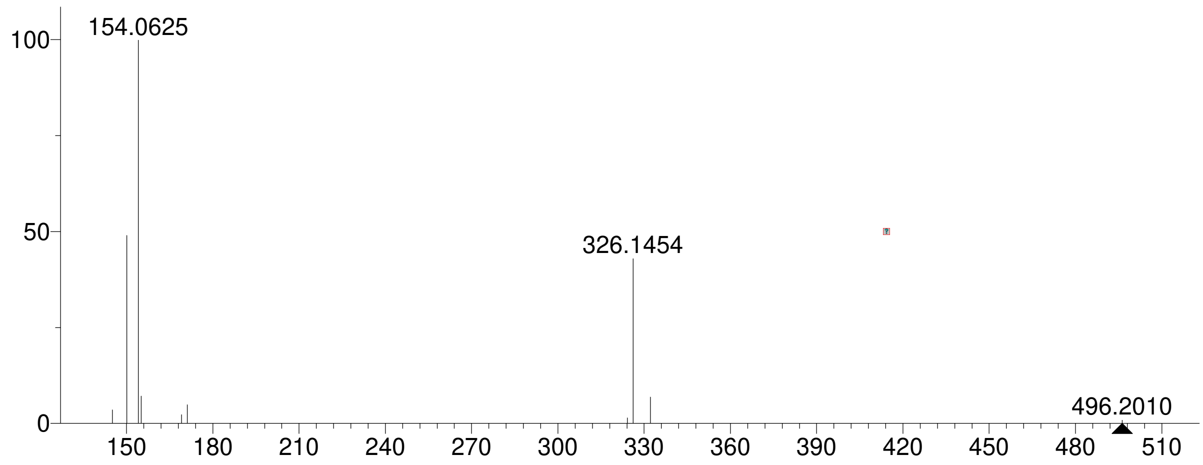

Hit 1 : NCGC00180666-02!(2R,3S,4S,5R,6S)-2-[(3,4,5-trihydroxyoxan-2-yl)oxymethyl]-6-(3,4,5-trimethoxyphenox
C20H30O13; MF: 2; RMF: 199; Prob 21.1%; Lib: mona-export-gnps; ID: 8997.

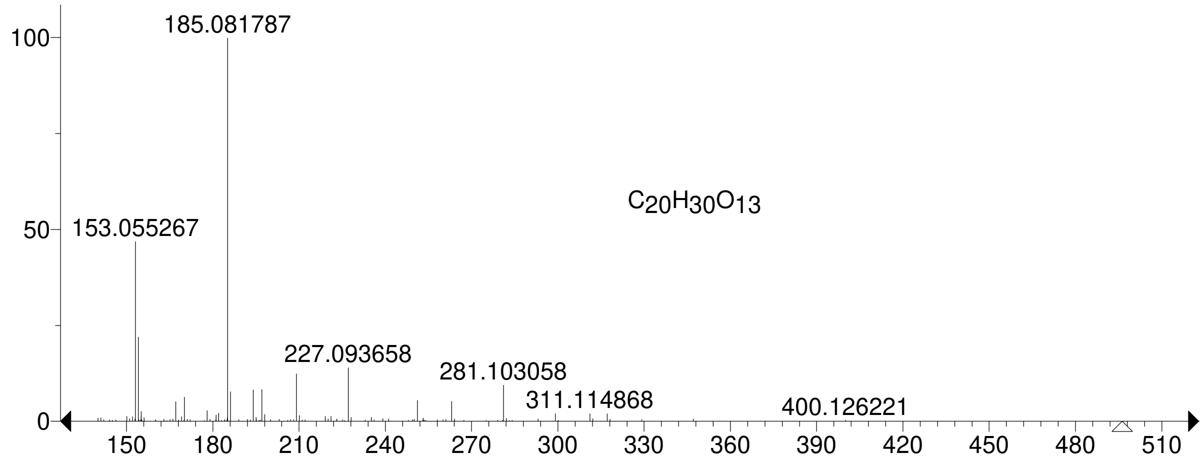

Hit 2 : (2R,3S,4S,5R,6S)-2-[(3,4,5-trihydroxyoxan-2-yl)oxymethyl]-6-(3,4,5-trimethoxyphenoxy)oxane-3,4,5-triol [M
C20H30O13; MF: 1; RMF: 334; Prob 20.3%; Lib: mona-export-vf-npl_qexactive; ID: 9258.

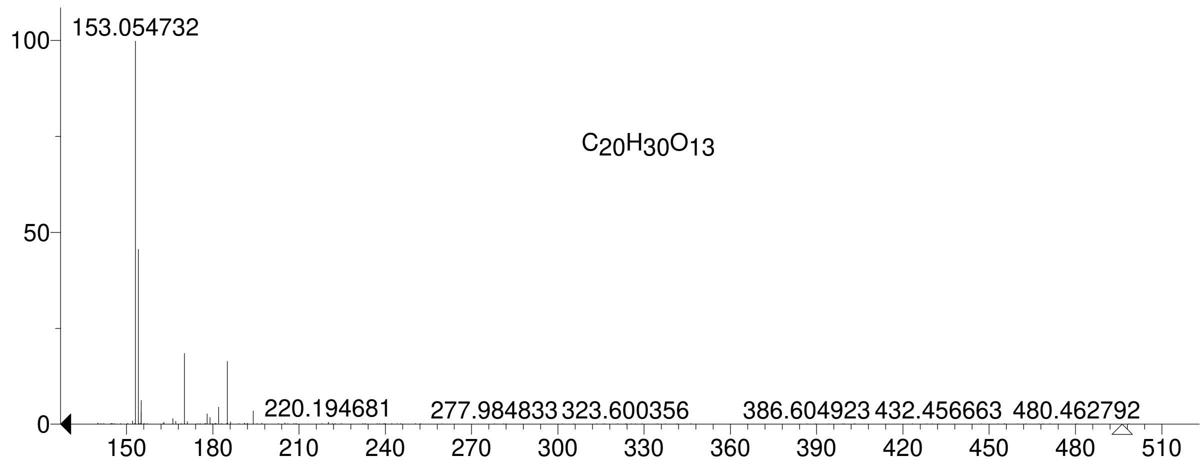

Hit 3 : (2R,3S,4S,5R,6S)-2-[[(2S,3R,4R)-3,4-dihydroxy-4-(hydroxymethyl)oxolan-2-yl]oxymethyl]-6-(3,4,5-trimetho
C20H30O13; MF: 0; RMF: 302; Prob 19.5%; Lib: mona-export-vf-npl_qexactive; ID: 9783.

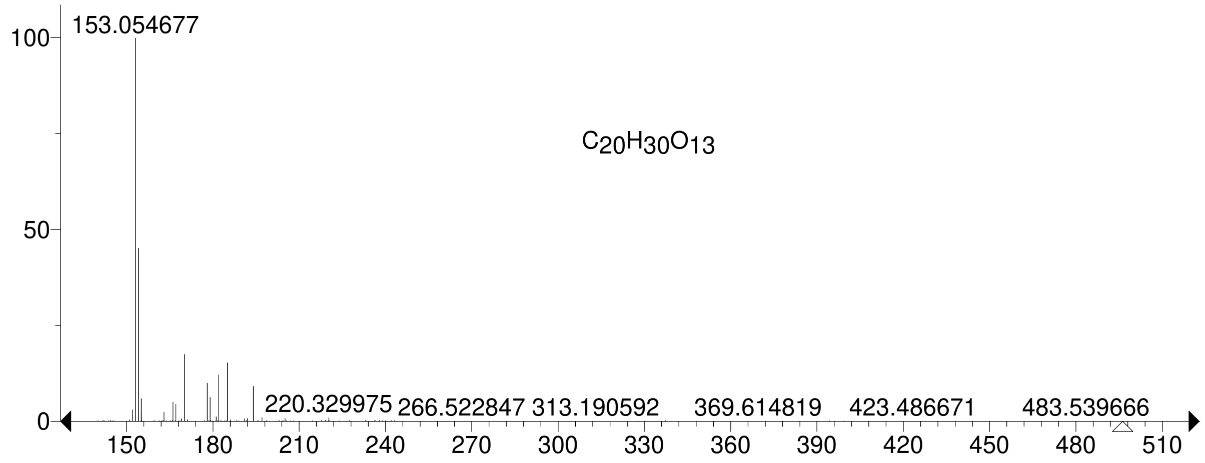

Hit 4 : NCGC00180025-03!(2R,3S,4S,5R,6S)-2-[[(2S,3R,4R)-3,4-dihydroxy-4-(hydroxymethyl)oxolan-2-yl]oxymetl
C20H30O13; MF: 0; RMF: 201; Prob 19.5%; Lib: mona-export-gnps; ID: 8565.

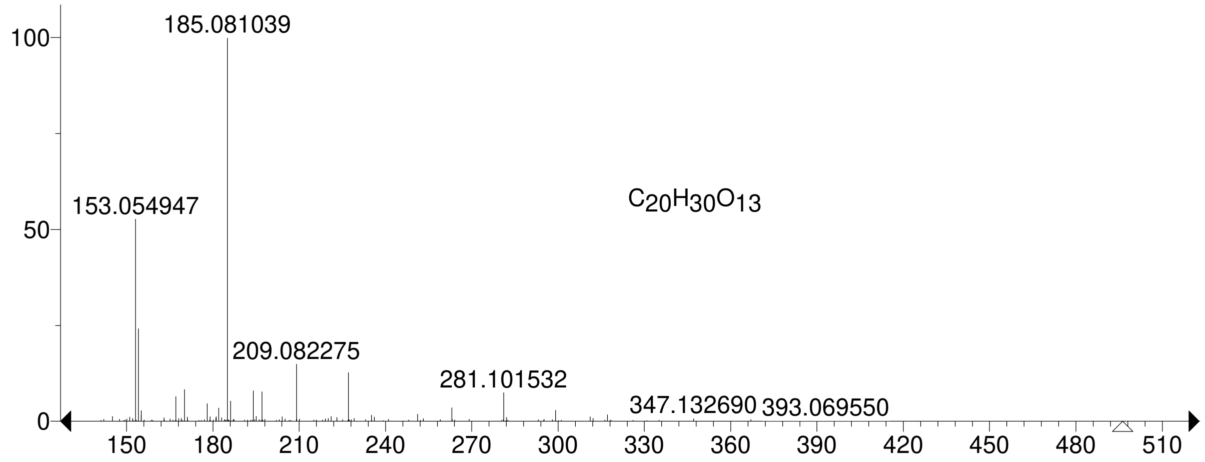

Hit 5 : Benzoic acid, 3-[[(3-carboxycyclohexyl)amino]carbonyl]-4-[3-[4-[4-(cyclohexyloxy)butoxy]phenyl]propoxy]-
C34H45NO8; MF: 0; RMF: 1; Prob 19.5%; CAS: 712313-35-4; Lib: nist_msms; ID: 190533.

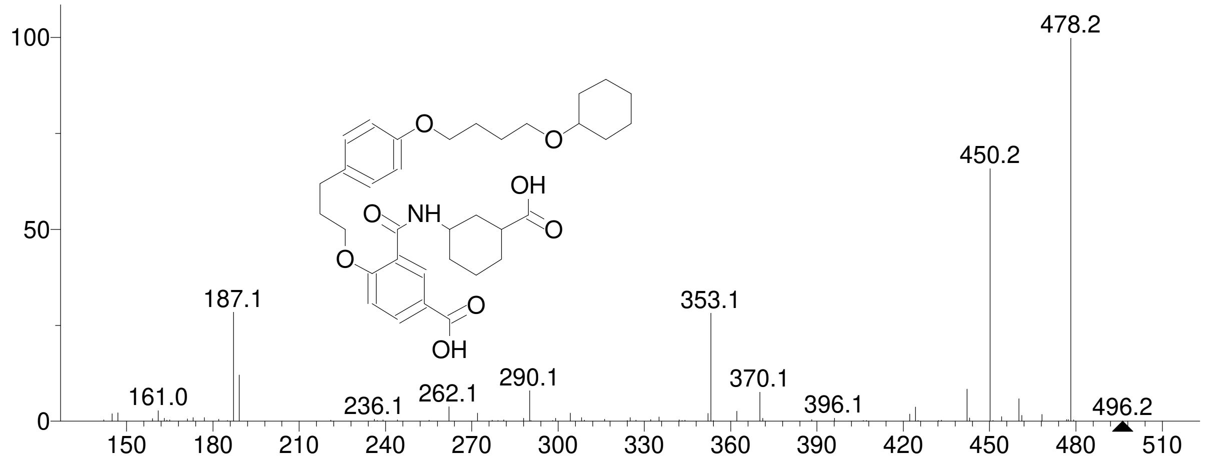

Unknown: Unknown (3.22_666.2619m/z)
Compound in Library Factor = 0

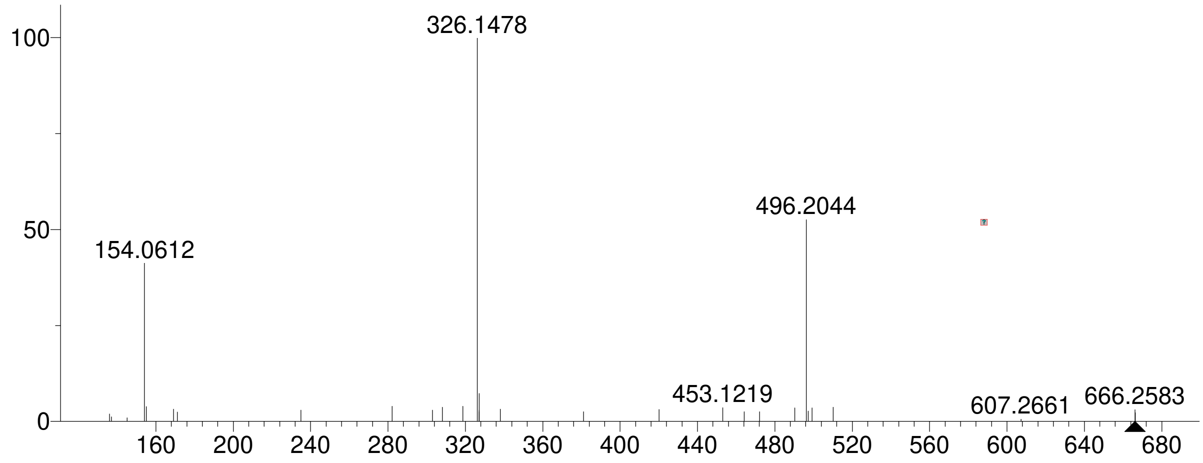

Unknown: Unknown (3.23_413.1558m/z)
Compound in Library Factor = -3273

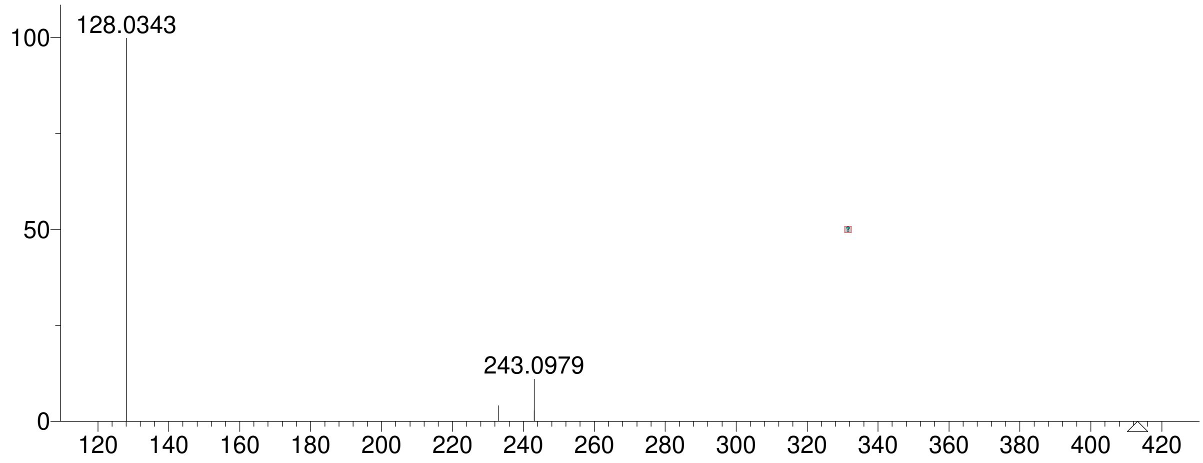

Hit 1 : 2-amino-5-[2-[[2,3-dihydroxy-2-(1-hydroxyethyl)butanoyl]oxymethyl]-4-hydroxyanilino]-5-oxopentanoic acid
C18H26N2O9; MF: 2; RMF: 198; Prob 26.5%; Lib: mona-export-lc-ms-ms_negative_mode; ID: 22977.

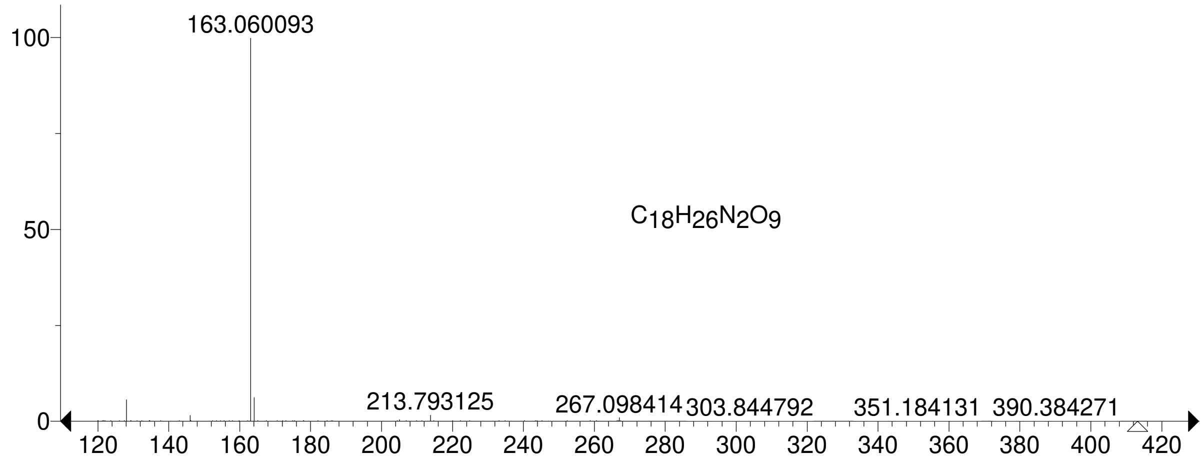

Hit 2 : NCGC00385396-01_C23H24O7_Benzeneacetic acid, 4-hydroxy-, (3S,3aR,4S,9aS,9bR)-2,3,3a,4,5,7,9a,9
C23H24O7; MF: 0; RMF: 40; Prob 24.5%; Lib: mona-export-gnps; ID: 14019.

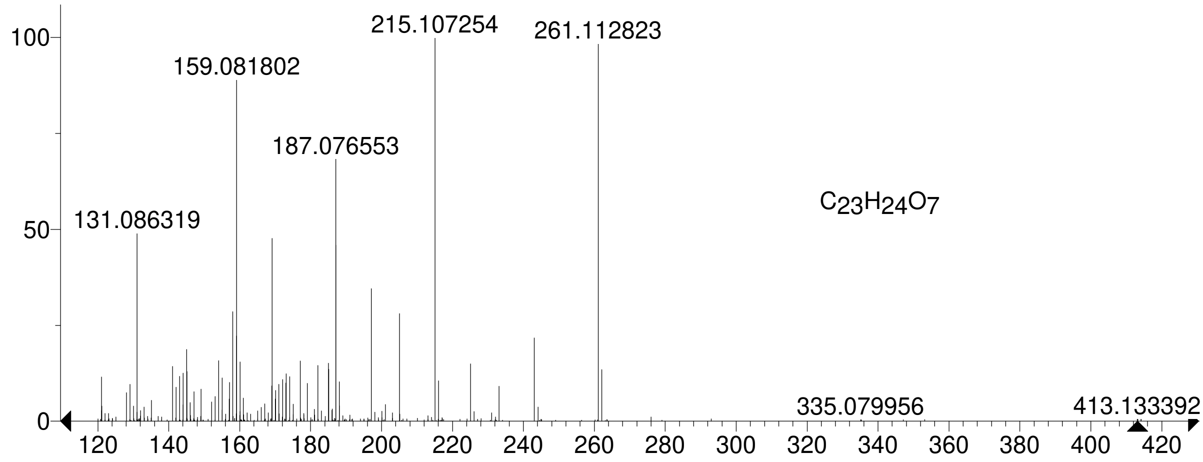

Hit 3 : NCGC00380168-01!3-[2-(2-hydroxy-4-methoxy-6-methylphenoxy)-4-methoxy-6-methylphenoxy]-5-methylb
C23H24O7; MF: 0; RMF: 19; Prob 24.5%; Lib: mona-export-gnps; ID: 6039.

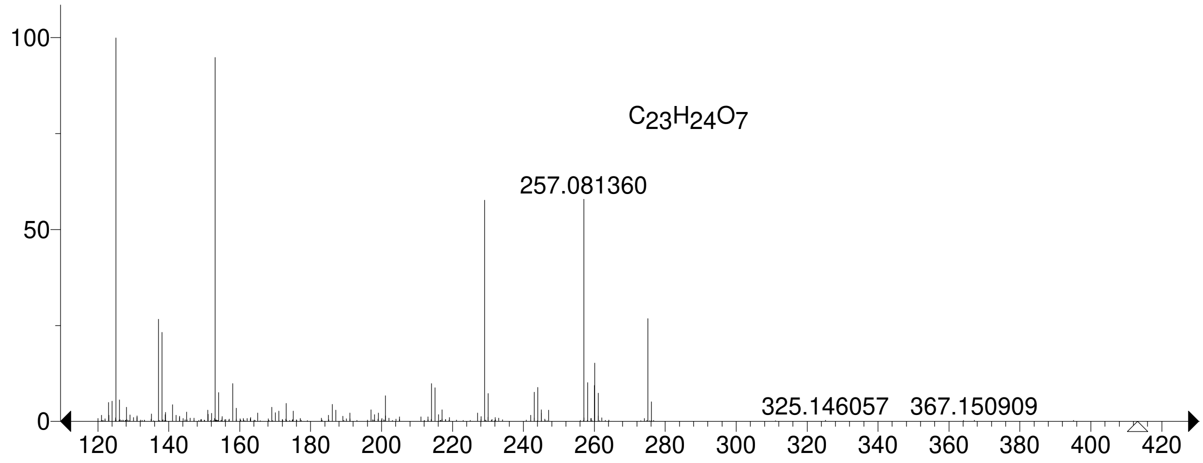

Hit 4 : 2-[2-[(Z)-pent-2-enyl]-3-[3,4,5-trihydroxy-6-(hydroxymethyl)oxan-2-yl]oxycyclopentyl]acetic acid [M+K]+ 45
C18H30O8; MF: 0; RMF: 5; Prob 24.5%; Lib: anthocyanins mona; ID: 9972.

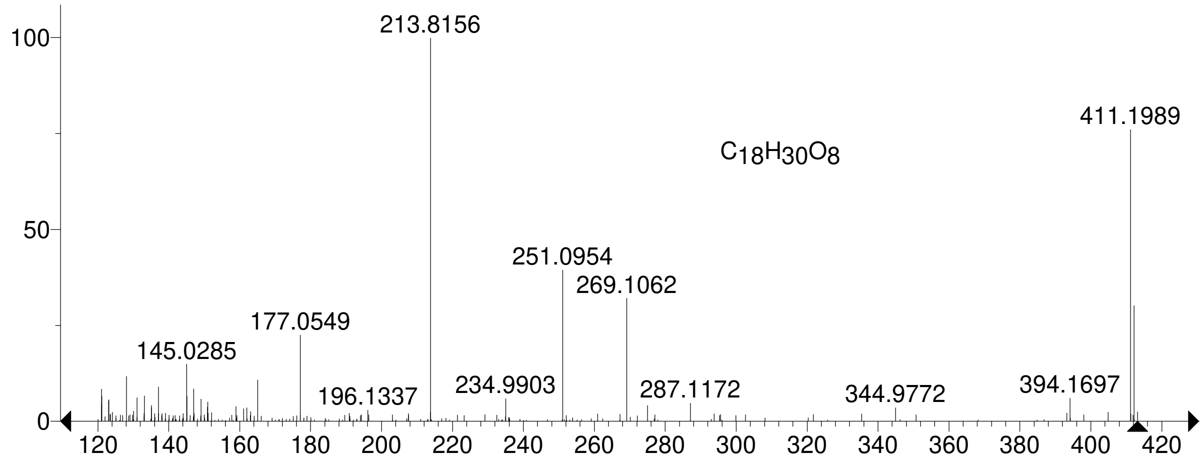

Unknown: Unknown (3.24_403.1341m/z)
Compound in Library Factor = 0

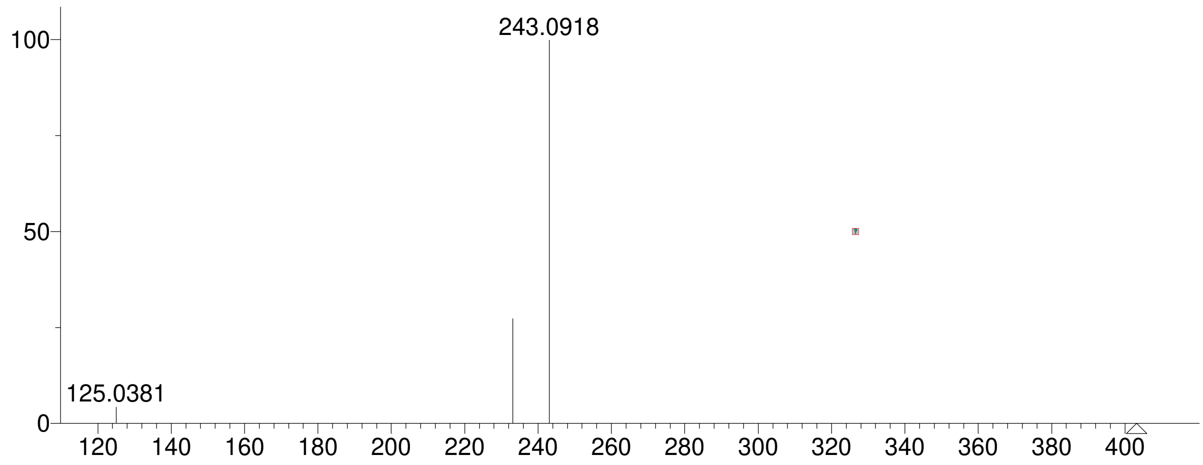

Hit 1 : NCGC00380227-01!(E)-3-[4-[1,3-dihydroxy-1-(4-hydroxy-3-methoxyphenyl)propan-2-yl]oxy-3,5-dimethoxyp
C21H24O9; MF: 0; RMF: 51; Prob 33.3%; Lib: mona-export-gnps; ID: 8693.

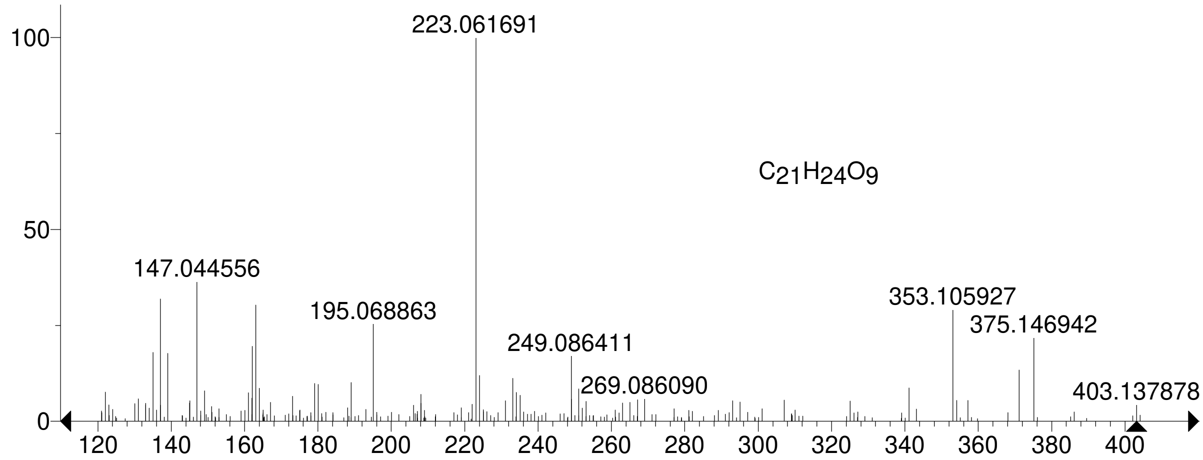

Hit 2 : Egtazic acid [M+Na]+ HCD 52V P=403.1
C14H24N2O10; MF: 0; RMF: 10; Prob 33.3%; CAS: 67-42-5; Lib: nist_msms; ID: 81815.

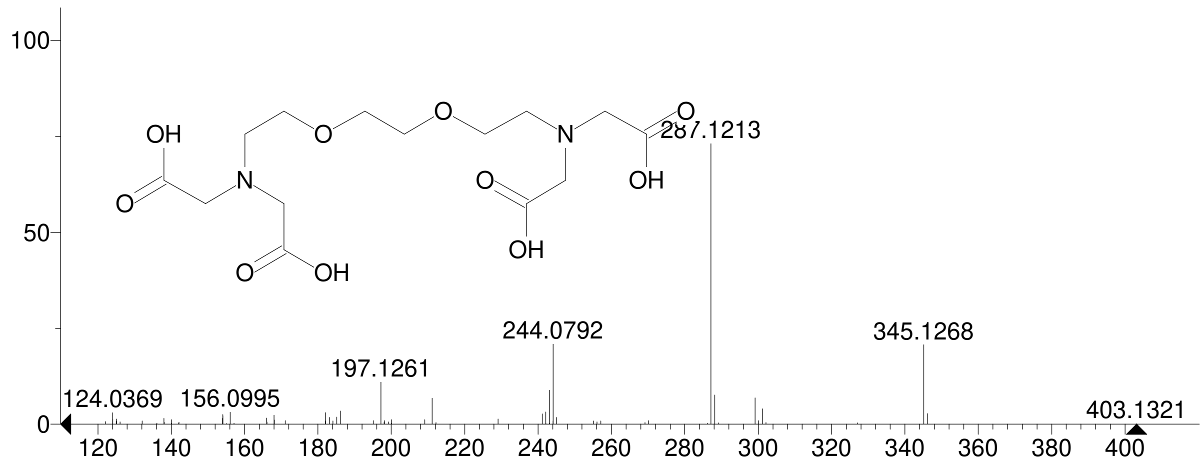

Hit 3 : methyl 2-(4,5-dihydroxy-6-methoxy-3,6-dimethyl-2-oxocyclohex-3-en-1-yl)oxy-4-hydroxy-3,6-dimethylbenzc
C19H24O8; MF: 0; RMF: 3; Prob 33.3%; Lib: mona-export-vf-npl_qexactive; ID: 23485.

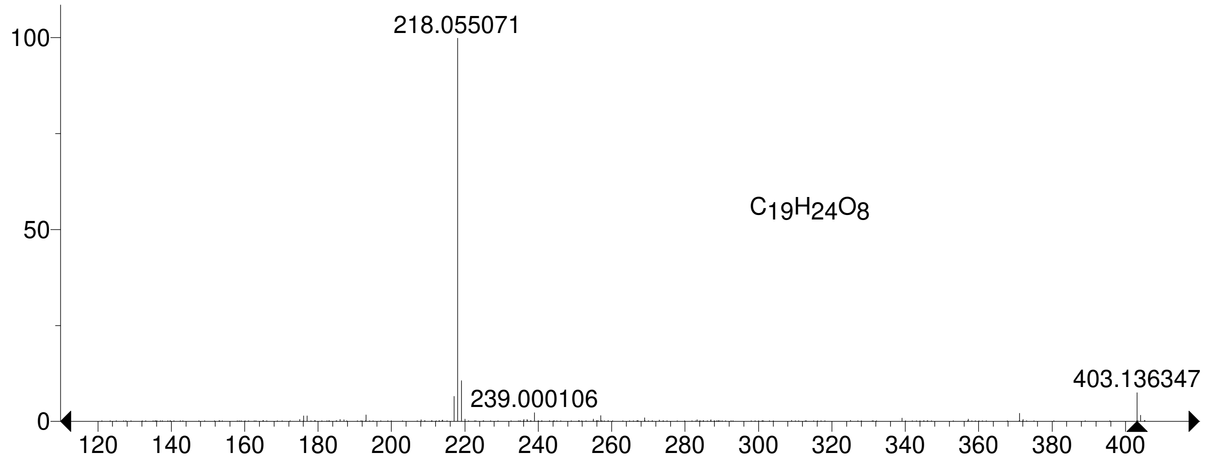

Unknown: Unknown (3.24_659.1476m/z)
Compound in Library Factor = 0

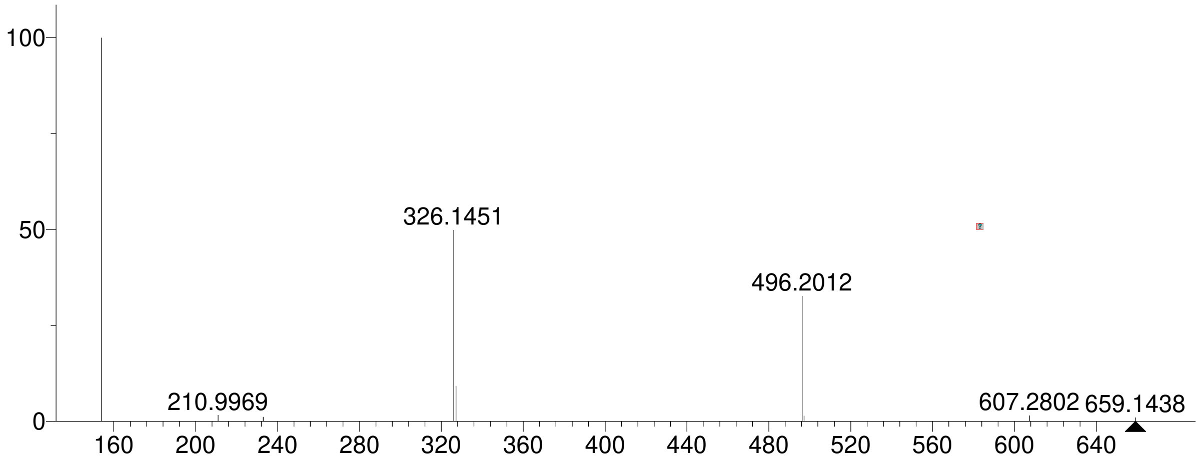

Unknown: Unknown (3.25_406.1342m/z)
Compound in Library Factor = 0

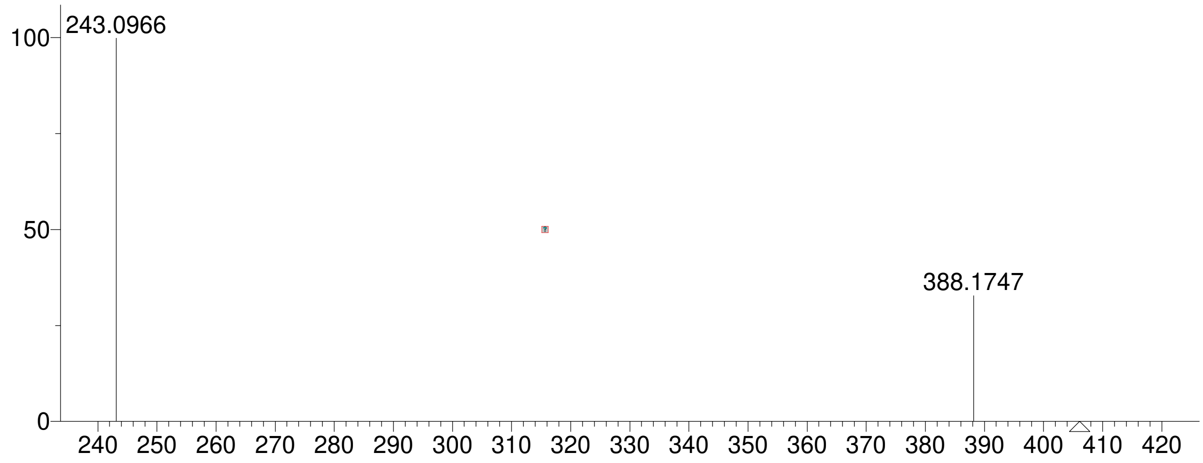

Hit 1 : N-Acetyl-D-lactosamine [M+Na]+ QTOF 15V P=406.1
C14H25NO11; MF: 0; RMF: 48; Prob 100.0%; CAS: 32181-59-2; Lib: nist_msms; ID: 27969.

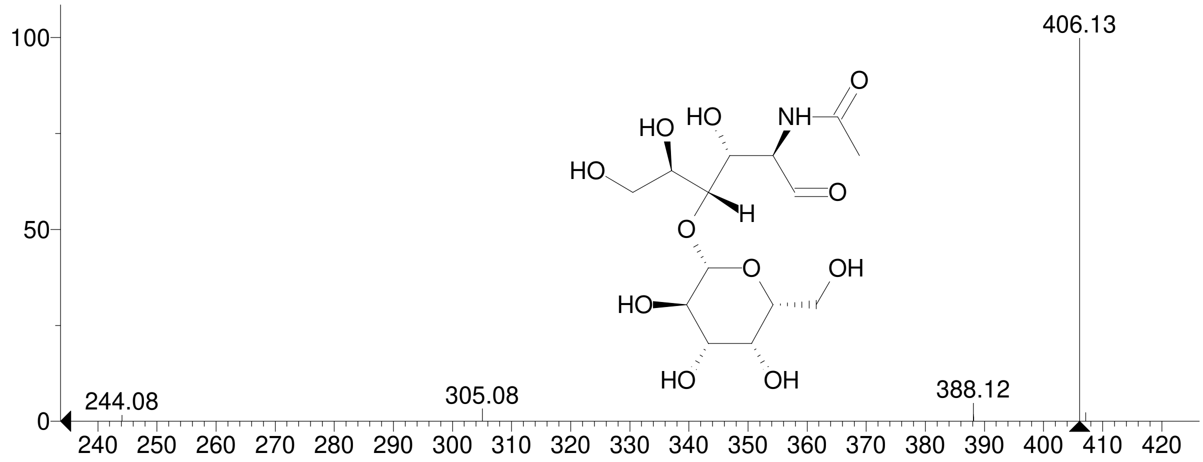

Unknown: Unknown (3.27_571.1256m/z)
Compound in Library Factor = -2387

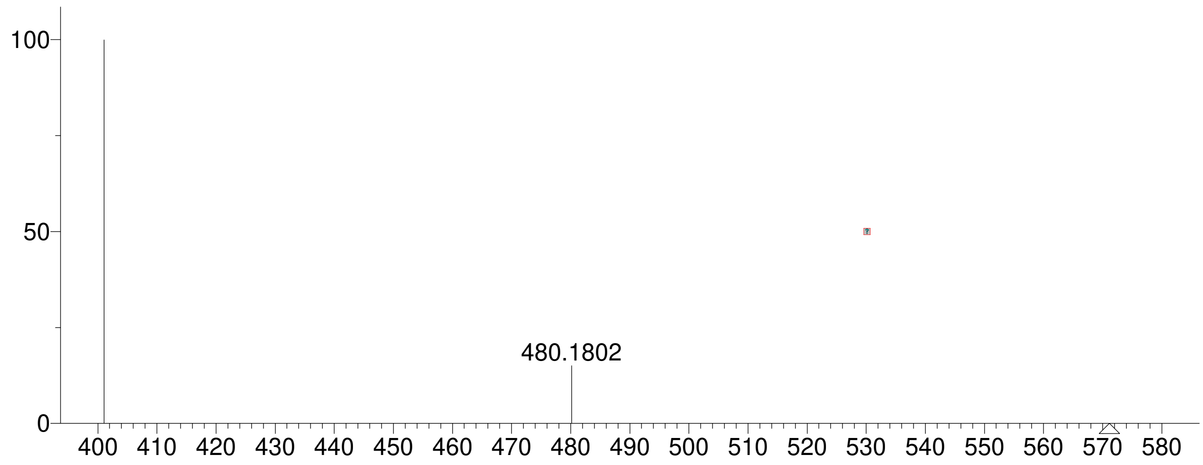

Hit 1 : NCGC00380623-01! [M-H]- P=571.1
C31H24O11; MF: 14; RMF: 490; Prob 59.3%; Lib: msms-neg-gnps; ID: 661.

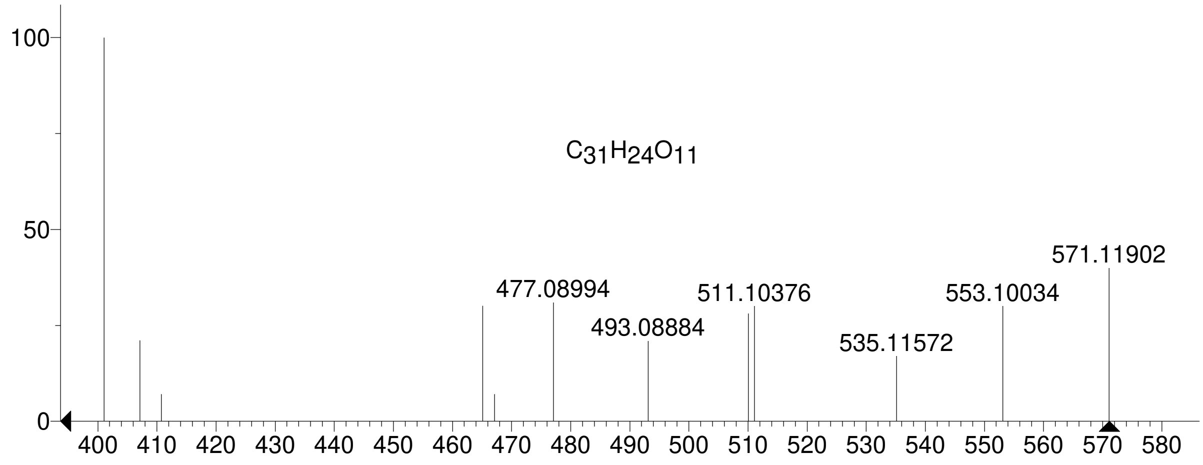

Hit 2 : methyl 8,11,15,20,22-pentahydroxy-24-methyl-13,18,27-trioxo-6-oxaheptacyclo[15.10.2.0A^'1,A^'1a^'1.0A^
C31H24O11; MF: 3; RMF: 401; Prob 40.7%; Lib: mona-export-lc-ms-ms_negative_mode; ID: 23337.

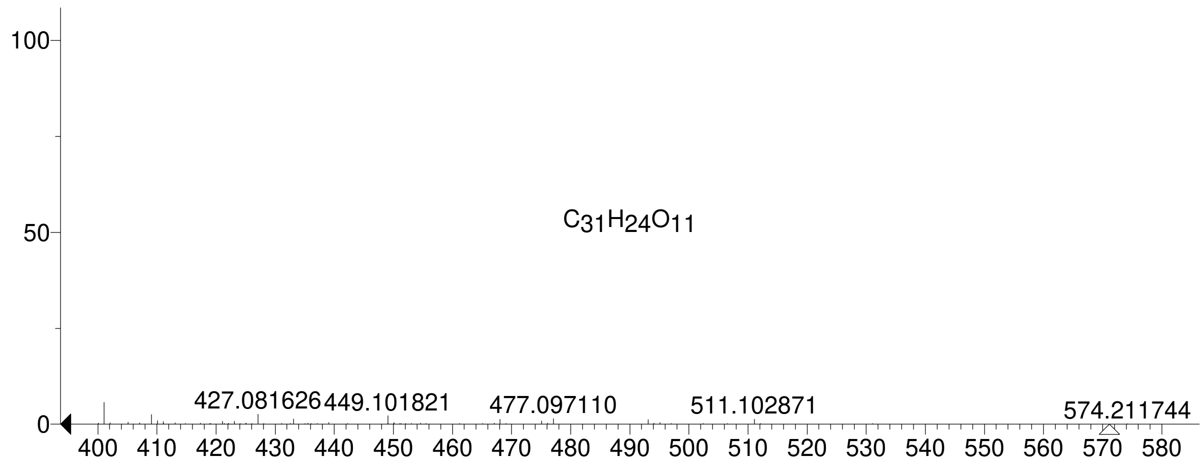

Unknown: Unknown (3.28_395.0753m/z)
Compound in Library Factor = 0

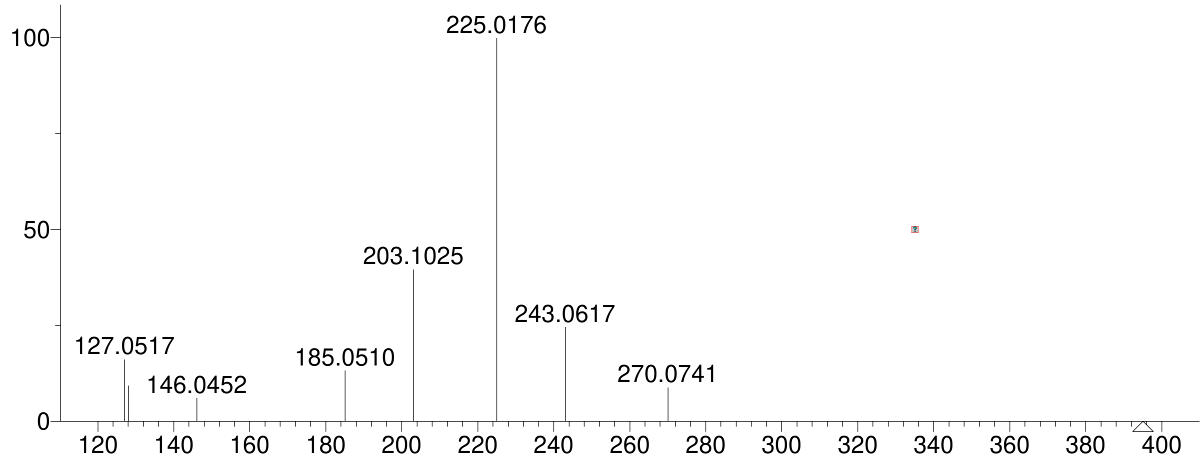

Hit 1 : (Z)-3-[4-methoxy-2-[(2S,3R,4S,5S,6R)-3,4,5-trihydroxy-6-(hydroxymethyl)oxan-2-yl]oxyphenyl]prop-2-enoic
C16H20O9; MF: 0; RMF: 55; Prob 33.3%; Lib: mona-export-vf-npl_qexactive; ID: 7762.

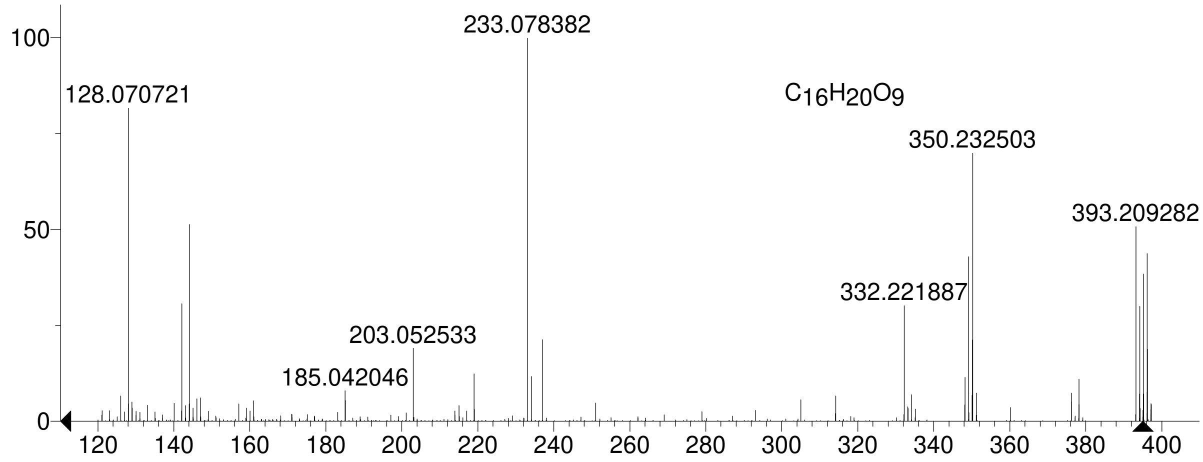

Hit 2 : Isoorientin [M+H-3H2O]+ HCD 59V P=395.1
C21H20O11; MF: 0; RMF: 52; Prob 33.3%; CAS: 4261-42-1; Lib: nist_msms; ID: 390617.

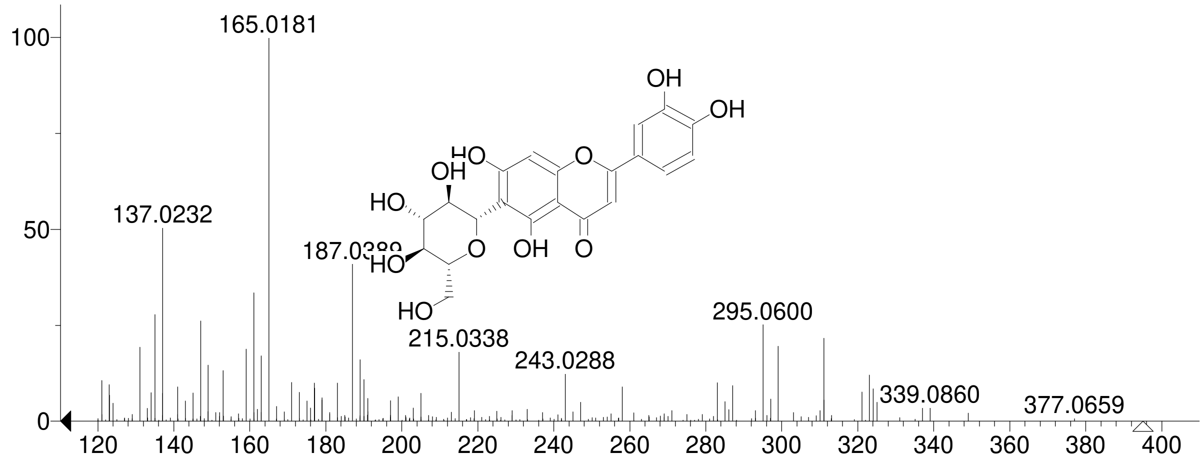

Hit 3 : (E)-3-[4-methoxy-2-[(2S,3R,4S,5S,6R)-3,4,5-trihydroxy-6-(hydroxymethyl)oxan-2-yl]oxyphenyl]prop-2-enoic
C16H20O9; MF: 0; RMF: 15; Prob 33.3%; Lib: mona-export-vf-npl_qexactive; ID: 10003.

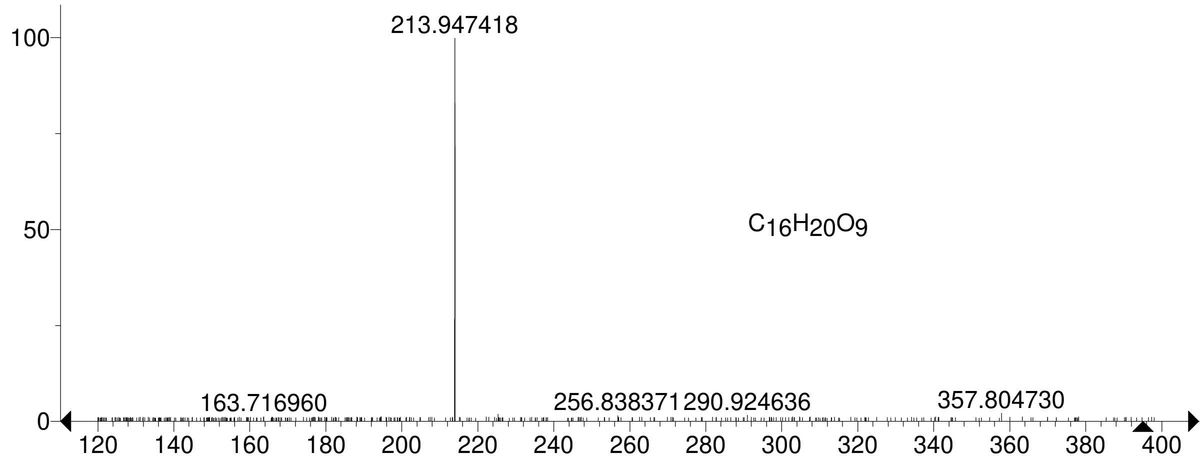

Unknown: Unknown (3.28_431.1301m/z)
Compound in Library Factor = 0

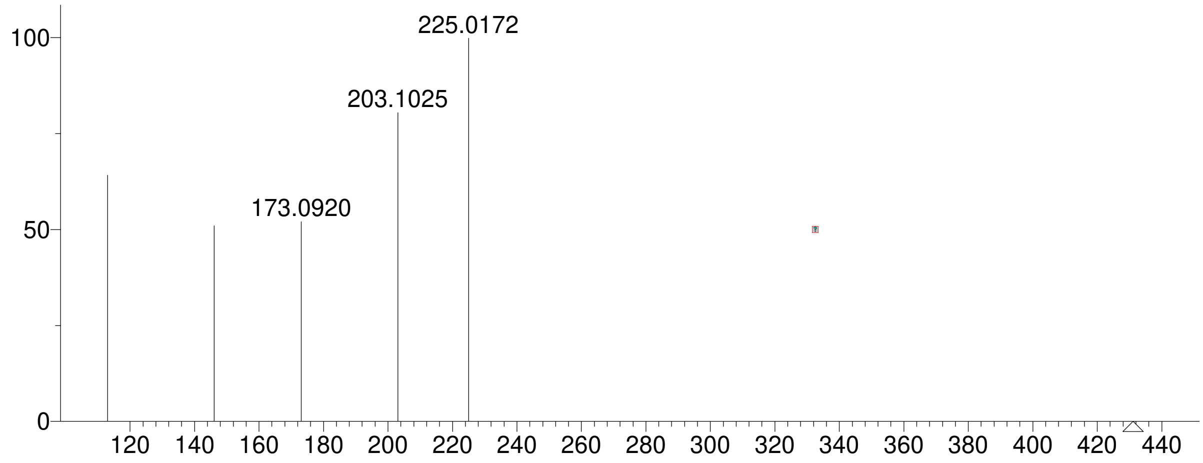

Hit 1 : O-Desmethylgefitinib [M-H]- HCD 68V P=431.1
C21H22ClFN4O3; MF: 0; RMF: 24; Prob 25.0%; CAS: 847949-49-9; Lib: nist_msms; ID: 451781.

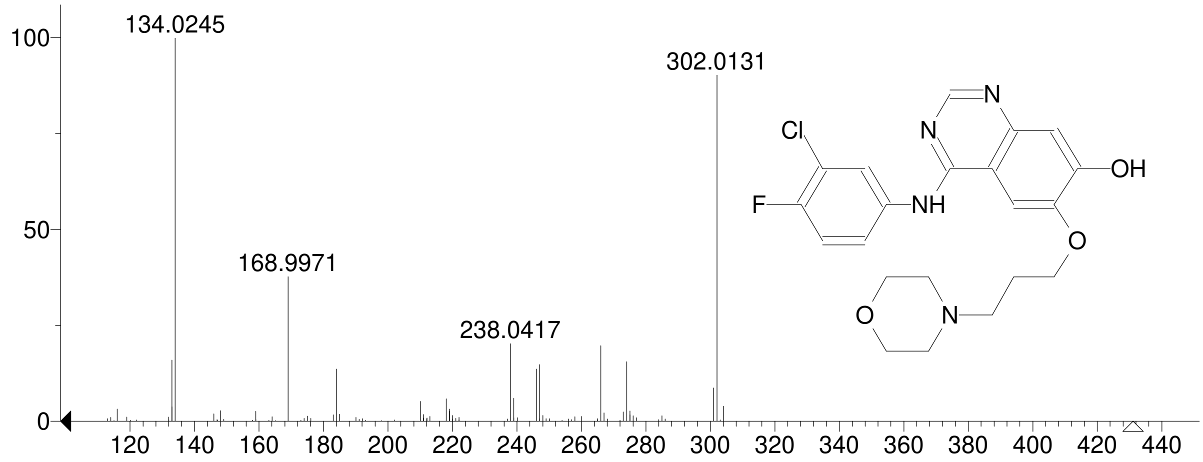

Hit 2 : Agnuside [M+H-2H2O]+ IT 35% P=431.1
C22H26O11; MF: 0; RMF: 22; Prob 25.0%; CAS: 11027-63-7; Lib: nist_msms; ID: 389591.

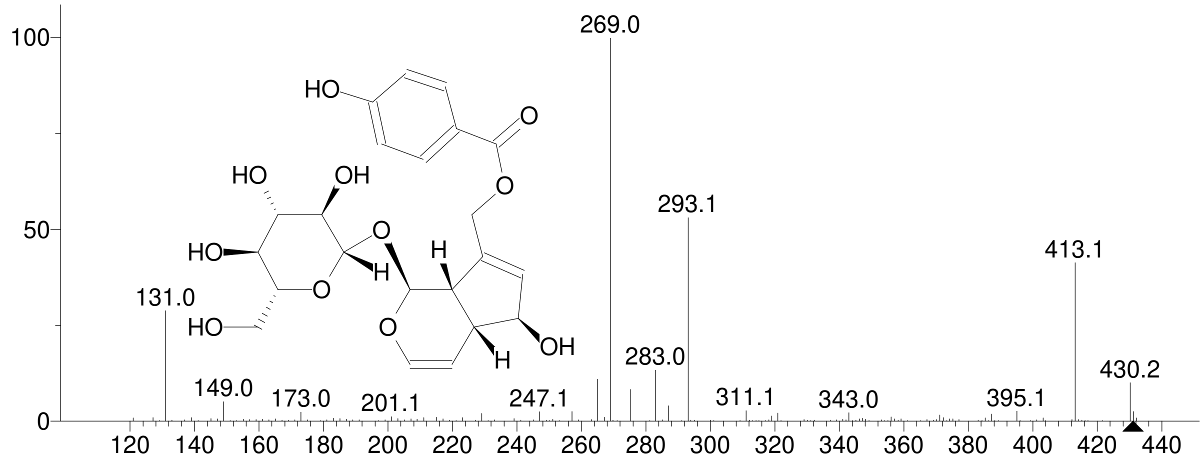

Hit 3 : Formononetin-7-O-glucoside [M+H]+ 50eV P=431.1
C22H22O9; MF: 0; RMF: 10; Prob 25.0%; Lib: anthocyanins mona; ID: 1127.

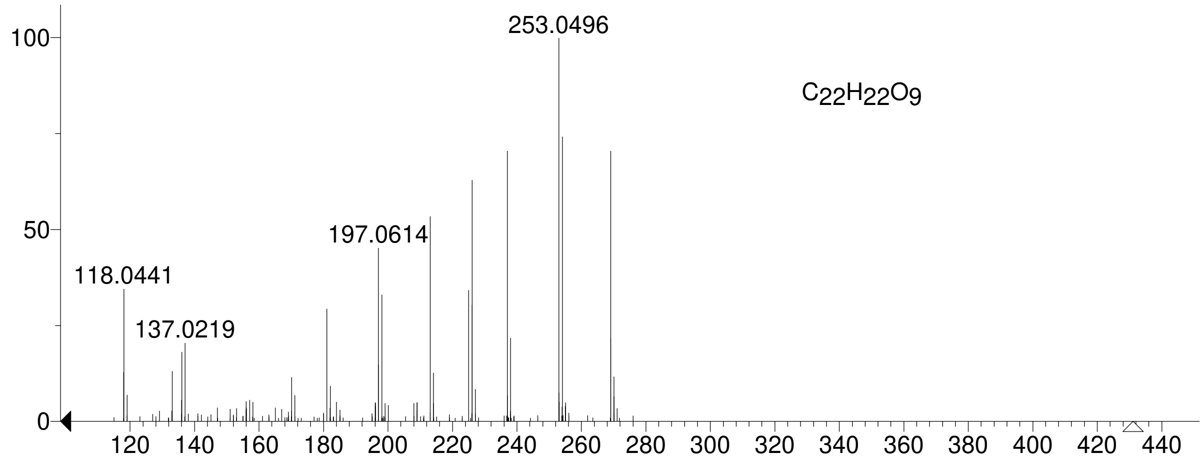

Hit 4 : calycosin-7-O-beta-D-glucoside [M+H]+ 35HCD P=431.1
C22H22O9; MF: 0; RMF: 5; Prob 25.0%; Lib: mona-export-vf-npl_qexactive; ID: 13249.

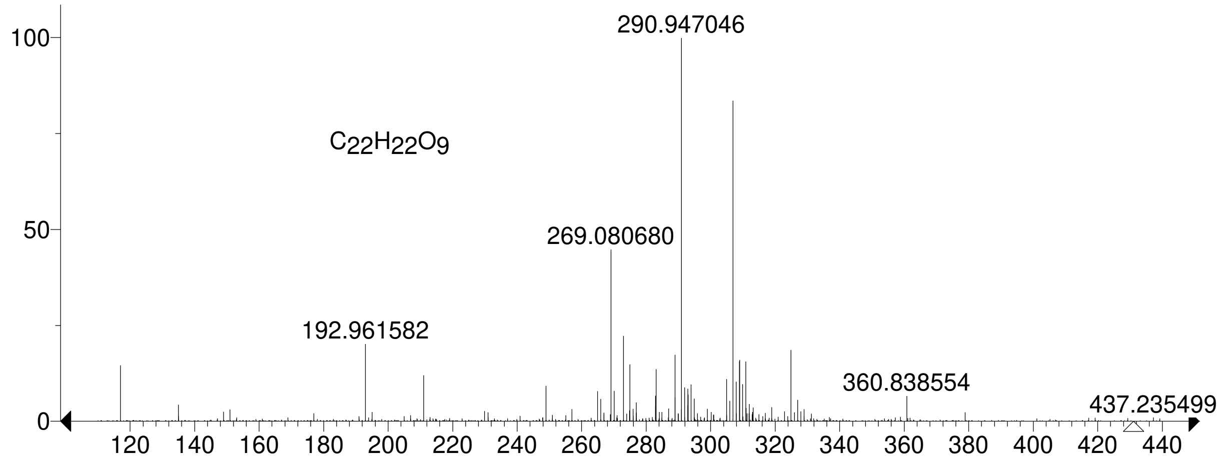

** Search Report Page 1 of 1 **

Unknown: Unknown (3.30_498.1345m/z)
Compound in Library Factor = 0

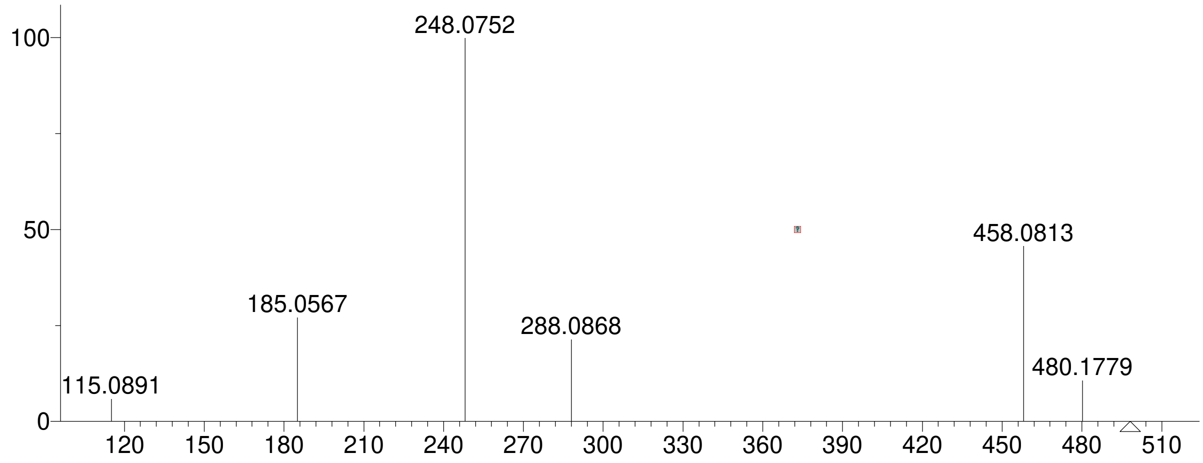

Unknown: Unknown (3.31_401.1551m/z)
Compound in Library Factor = -3273

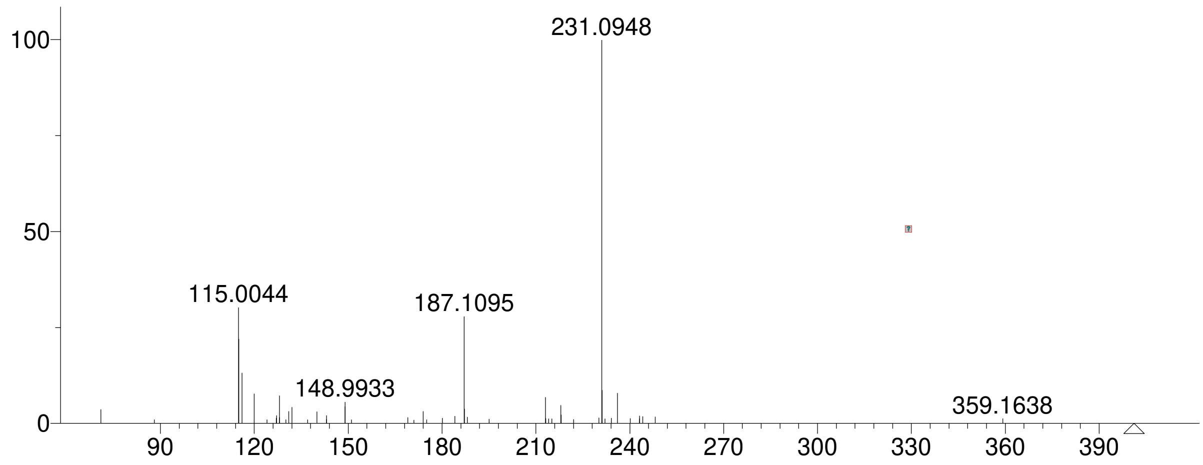

Hit 1 : NCGC00168917-02!5-[(3S,3aR,6S,6aR)-6-(3,4,5-trimethoxyphenyl)-1,3,3a,4,6,6a-hexahydrofuro[3,4-c]fura
C22H24O7; MF: 2; RMF: 117; Prob 9.69%; Lib: mona-export-gnps; ID: 8887.

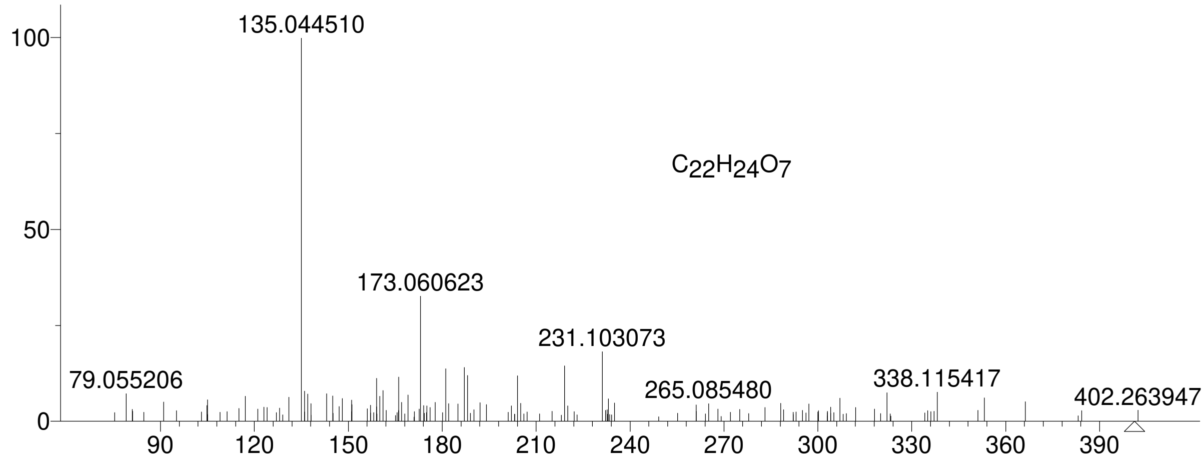

Hit 2 : 6-formyl-5-hydroxy-10-(hydroxymethyl)-3-methylidene-2-oxo-2H,3H,3aH,4H,5H,8H,9H,11aH-cyclodeca[b]fu
C20H26O7; MF: 1; RMF: 60; Prob 9.31%; Lib: mona-export-vf-npl_qexactive; ID: 22438.

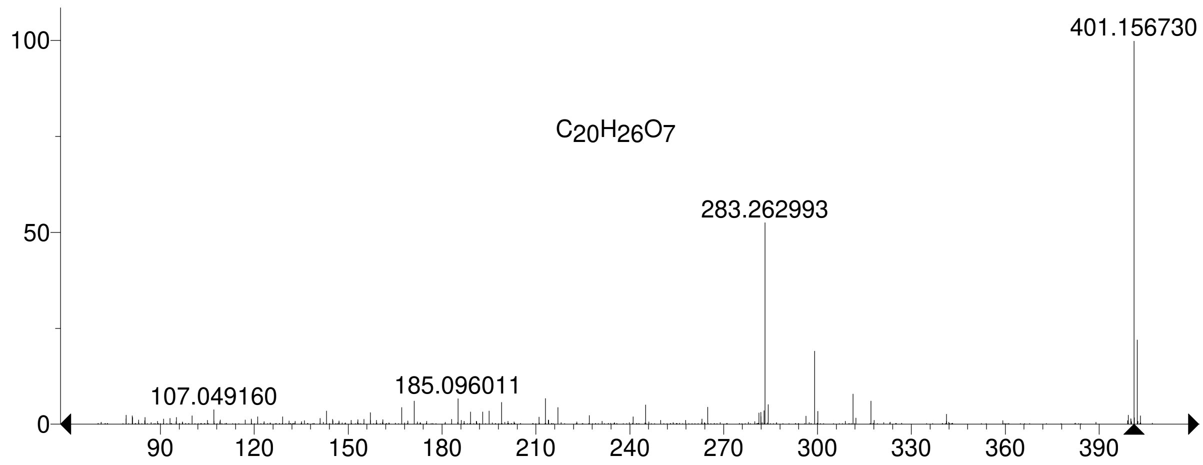

Hit 3 : (2aR,4S,6R,7R,9R,9aR)-5'-(furan-3-yl)-4,9-dihydroxy-2a-(hydroxymethyl)-7-methylspiro[1,3,4,5,5a,7,8,9-oc
C20H26O7; MF: 1; RMF: 56; Prob 9.31%; Lib: mona-export-vf-npl_qexactive; ID: 23516.

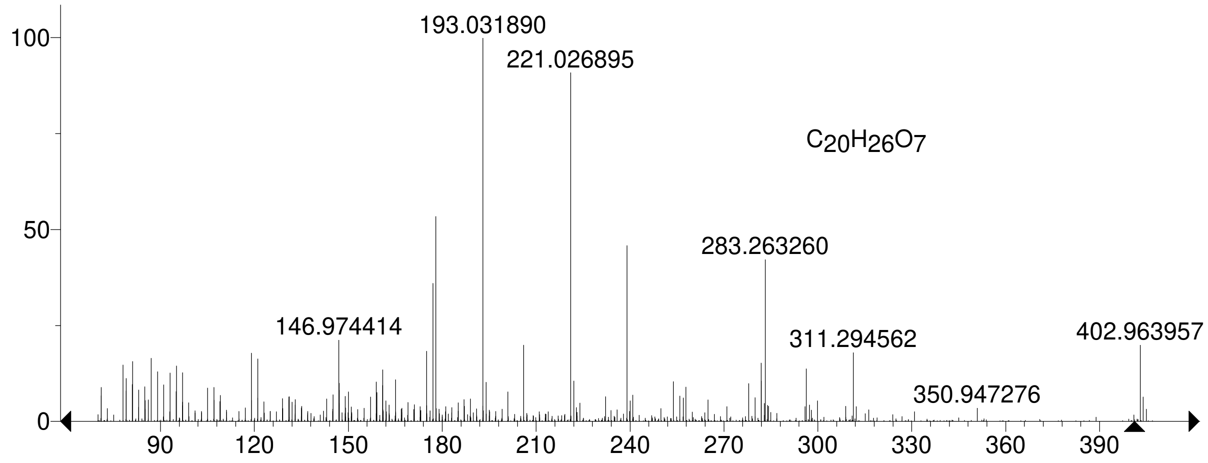

Hit 4 : rel-Dimethylenedioxy-dimethoxy-epoxylignan [M+H]+ P=401.2
C22H24O7; MF: 0; RMF: 315; Prob 8.95%; Lib: mona-export-gnps; ID: 2894.

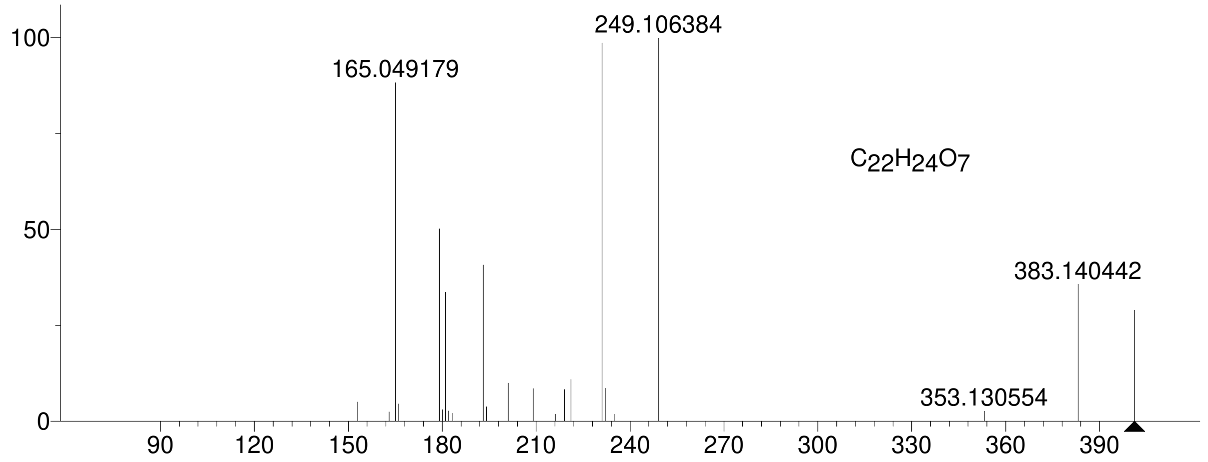

Hit 5 : NCGC00347752-02![2-(3'-ethenyl-3',5-dimethyl-2,4,5'-trioxospiro[chromene-3,2'-cyclopentane]-1'-yl)-3-hyd
C22H24O7; MF: 0; RMF: 41; Prob 8.95%; Lib: mona-export-gnps; ID: 7415.

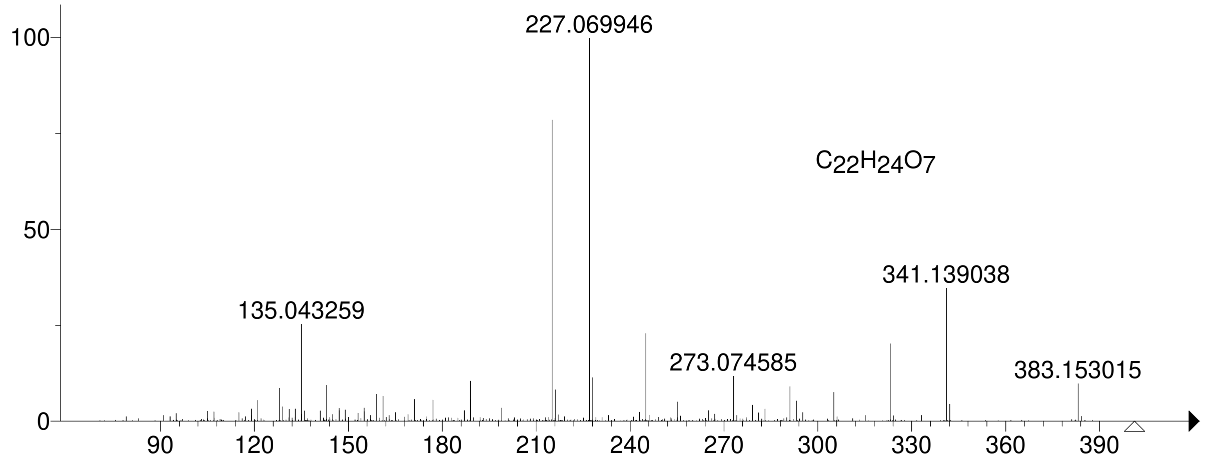

Unknown: Unknown (3.31_441.1486m/z)
Compound in Library Factor = 0

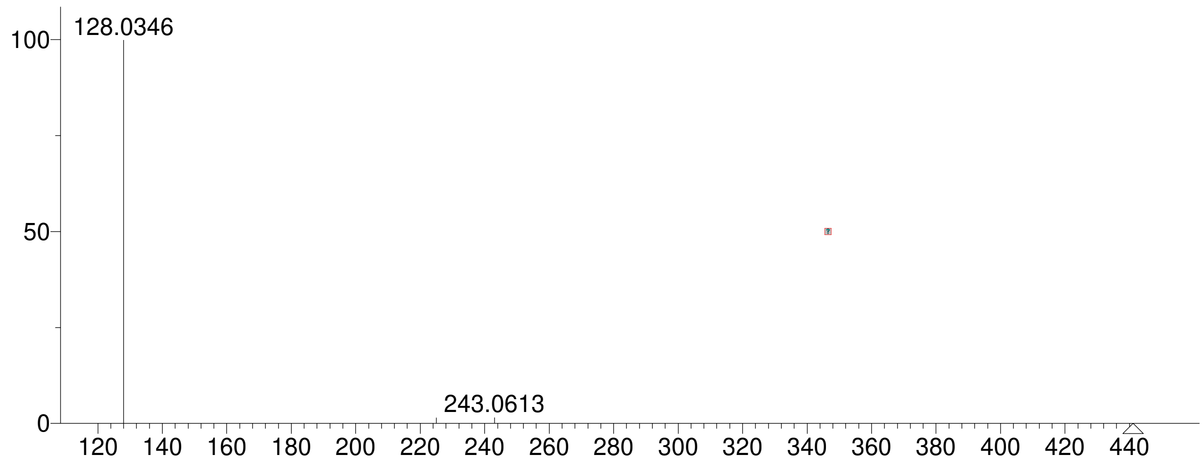

Hit 1 : MLS002695932-01! [M+H]+ P=441.1
C20H24O11; MF: 0; RMF: 5; Prob 50.0%; Lib: mona-export-gnps; ID: 3878.

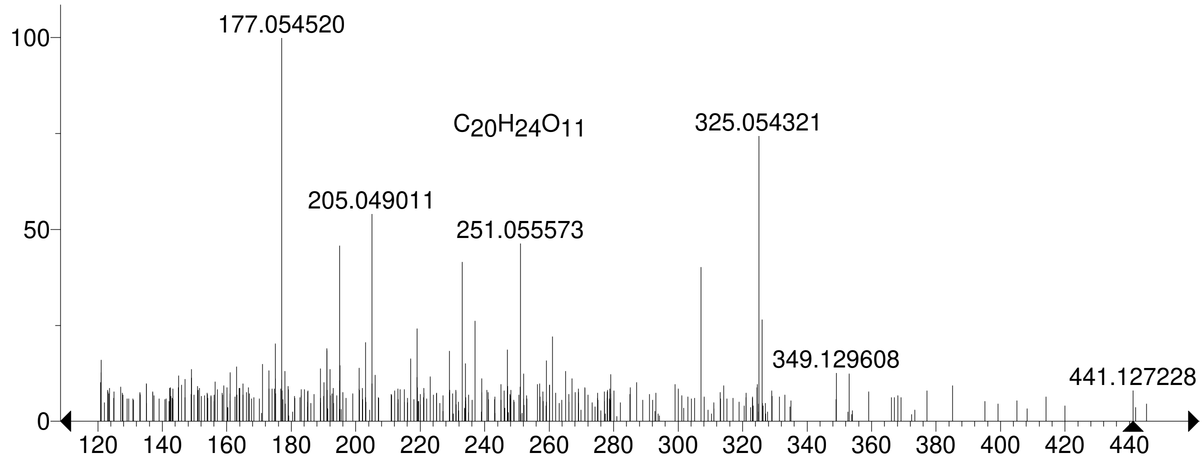

Hit 2 : syringaresinol [M+Na]+ 45HCD P=441.2
C22H26O8; MF: 0; RMF: 1; Prob 50.0%; Lib: mona-export-vf-npl_qexactive; ID: 4436.

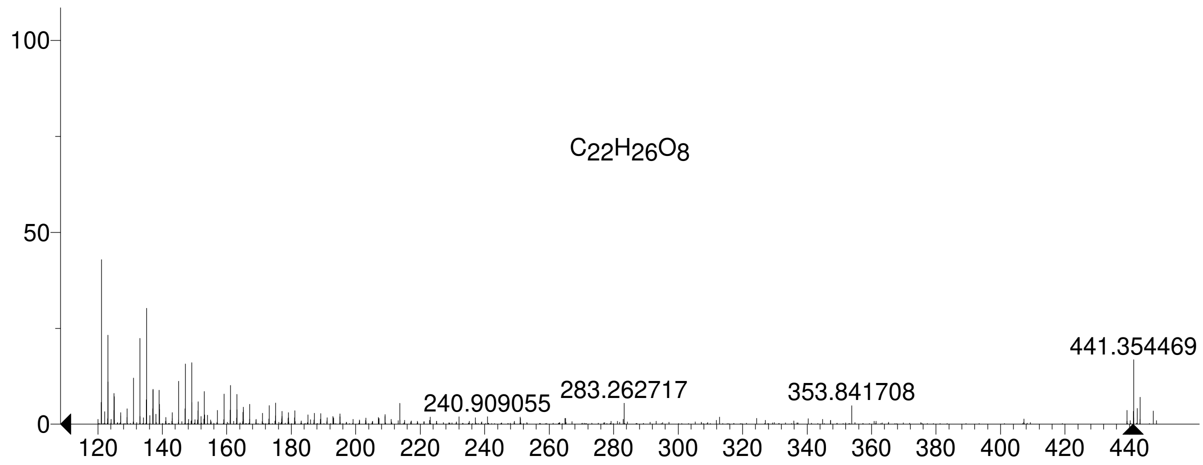

** Search Report Page 1 of 1 **

Unknown: Unknown (3.31_458.1417m/z)
Compound in Library Factor = 0

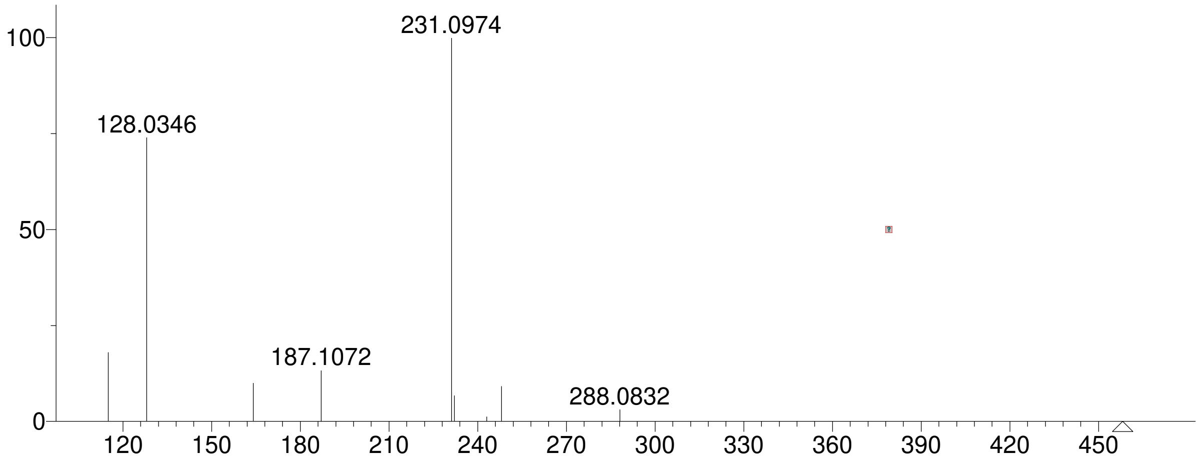

Unknown: Unknown (3.31_473.0879m/z)
Compound in Library Factor = 0

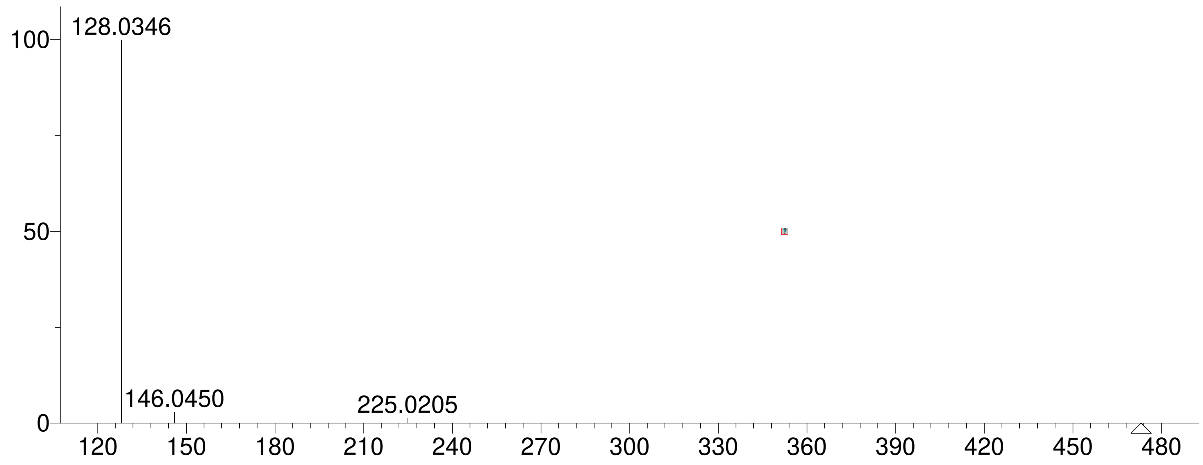

** Search Report Page 1 of 1 **

Unknown: Unknown (3.31_478.2042m/z)
Compound in Library Factor = 0

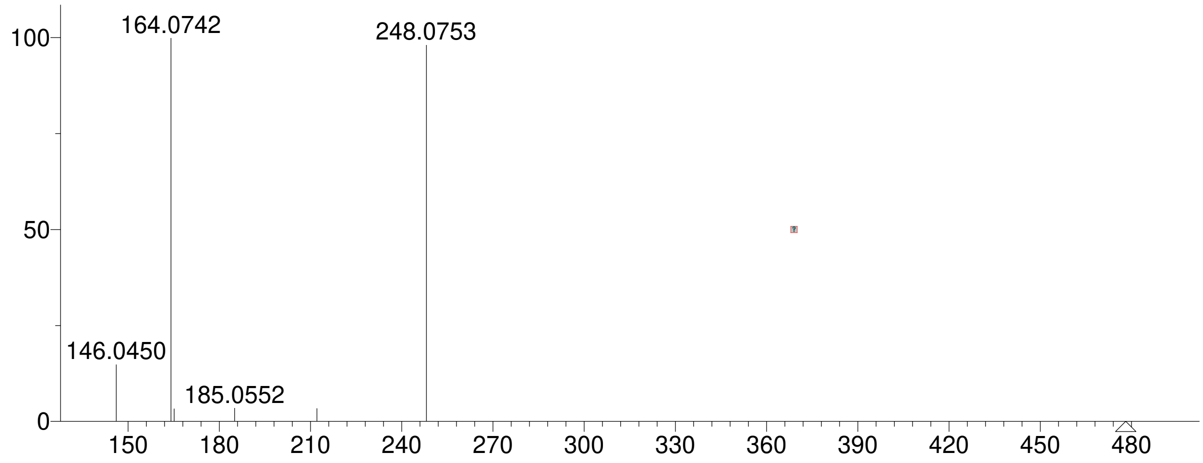

Hit 1 : NCGC00169156-03_C24H28O9_[(2'R,3R,4a'S,5S,5'R,6'S,8a'S)-6'-Acetoxy-5-(3-furyl)-2'-methyl-2,4'-dioxo
C24H28O9; MF: 0; RMF: 37; Prob 50.0%; Lib: mona-export-gnps; ID: 13458.

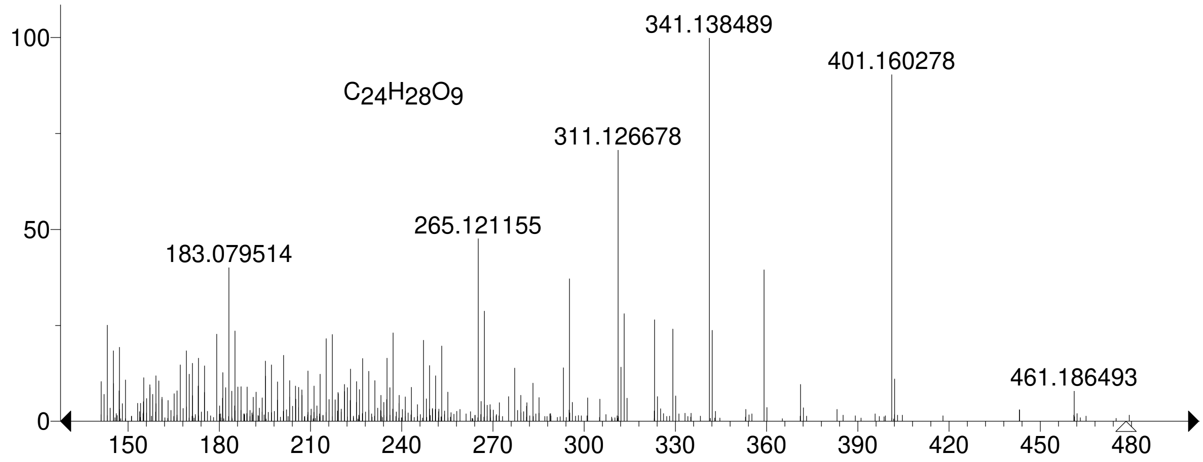

Hit 2 : (2R,2'S,4'aS,5'R,5''S,6'R,8'aS)-2'-(acetyloxy)-5''-(furan-3-yl)-6'-methyl-2'',8'-dioxo-octahydrodispiro[oxirane-
C24H28O9; MF: 0; RMF: 23; Prob 50.0%; Lib: mona-export-vf-npl_qexactive; ID: 9886.

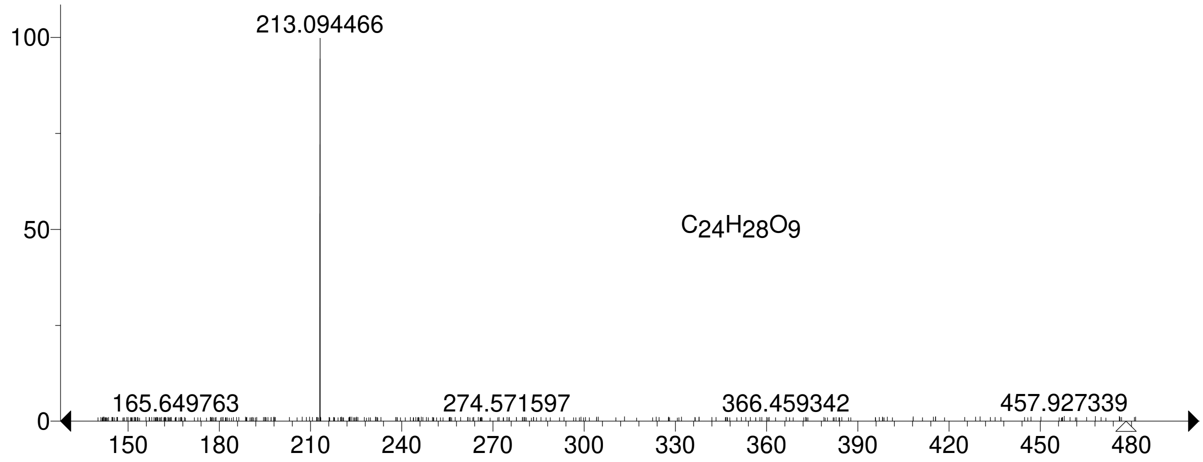

** Search Report Page 1 of 1 **

Unknown: Unknown (3.32_360.1396m/z)
Compound in Library Factor = 0

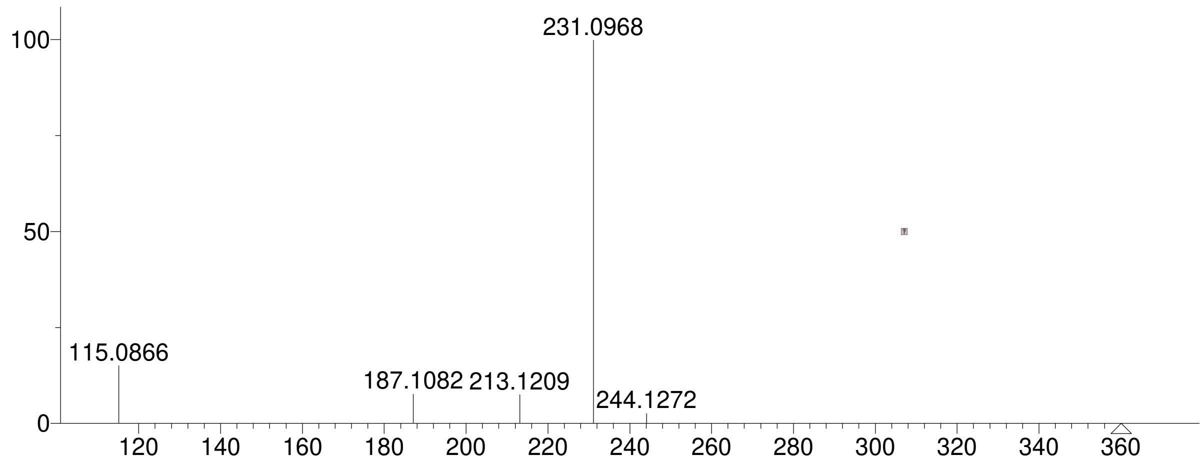

Hit 1 : Clethodim [M+H]+ IT-FT 35% P=360.1
C17H26ClNO3S; MF: 0; RMF: 14; Prob 100.0%; CAS: 99129-21-2; Lib: nist_msms; ID: 491497.

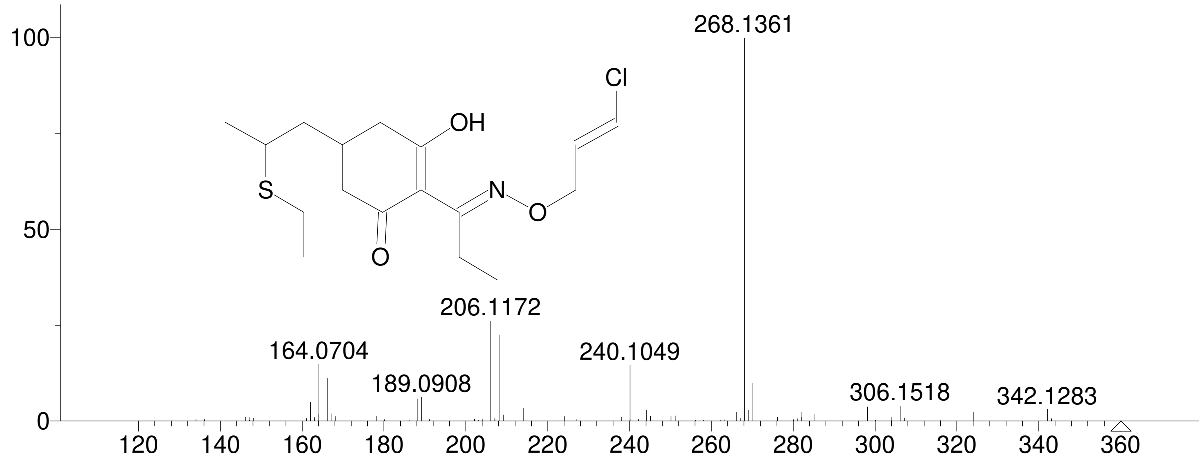

Unknown: Unknown (3.32_381.1868m/z)
Compound in Library Factor = -3273

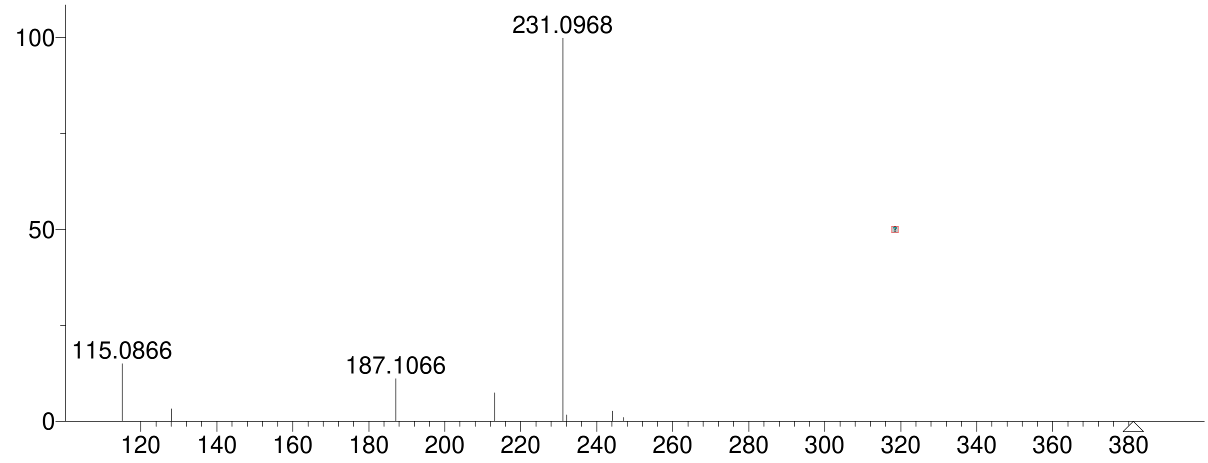

Hit 1 : [Glu1] TRH [M+H]+ QTOF 33V P=381.2
C16H24N6O5; MF: 3; RMF: 170; Prob 51.0%; Lib: nist_msms2; ID: 32559.

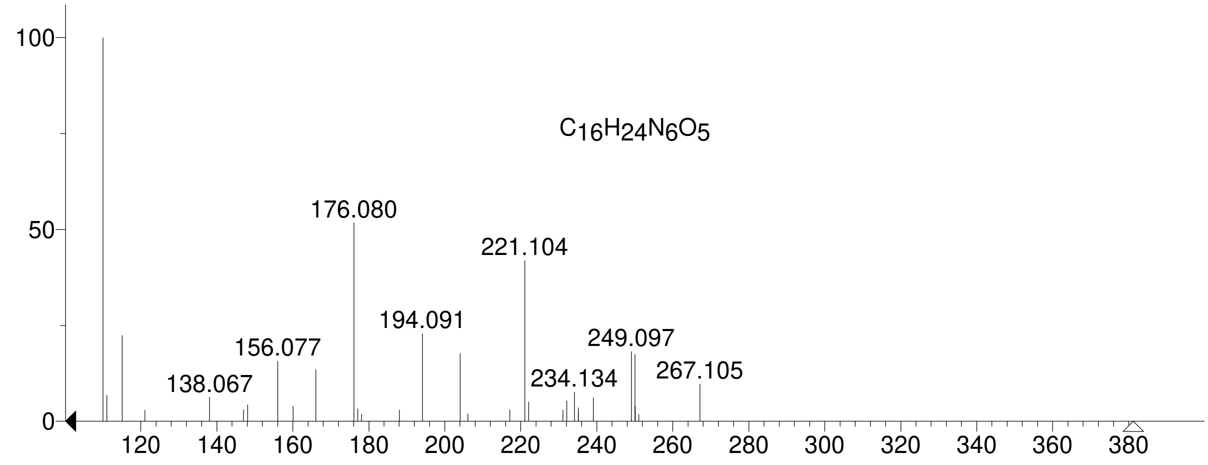

Hit 2 : [Glu1] TRH [M+H]+ HCD 34eV P=381.2
C16H24N6O5; MF: 2; RMF: 173; Prob 49.0%; CAS: 51826-17-6; Lib: nist_msms; ID: 564476.

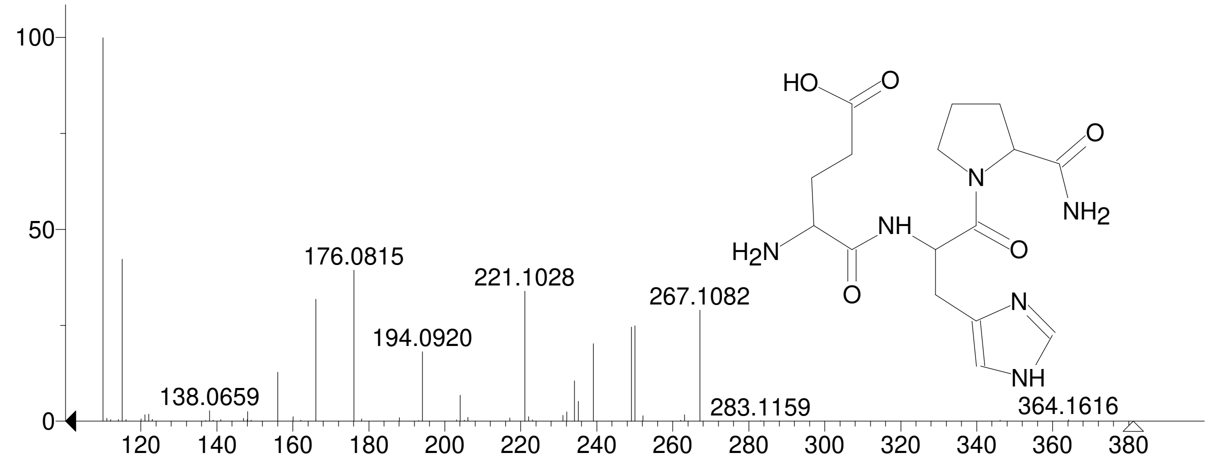

Unknown: Unknown (3.32_427.0456m/z)
Compound in Library Factor = 0

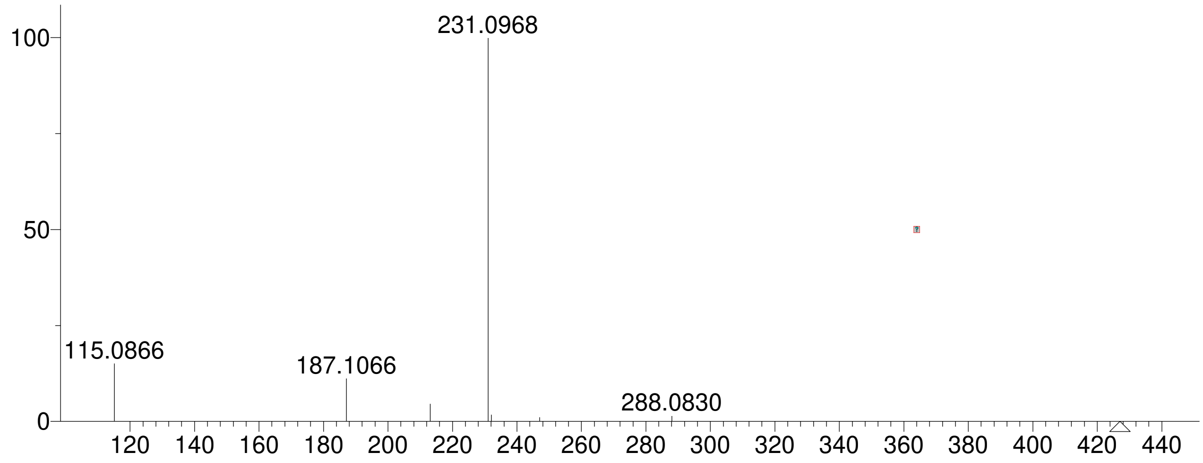

Unknown: Unknown (3.32_568.1889m/z)
Compound in Library Factor = -2176

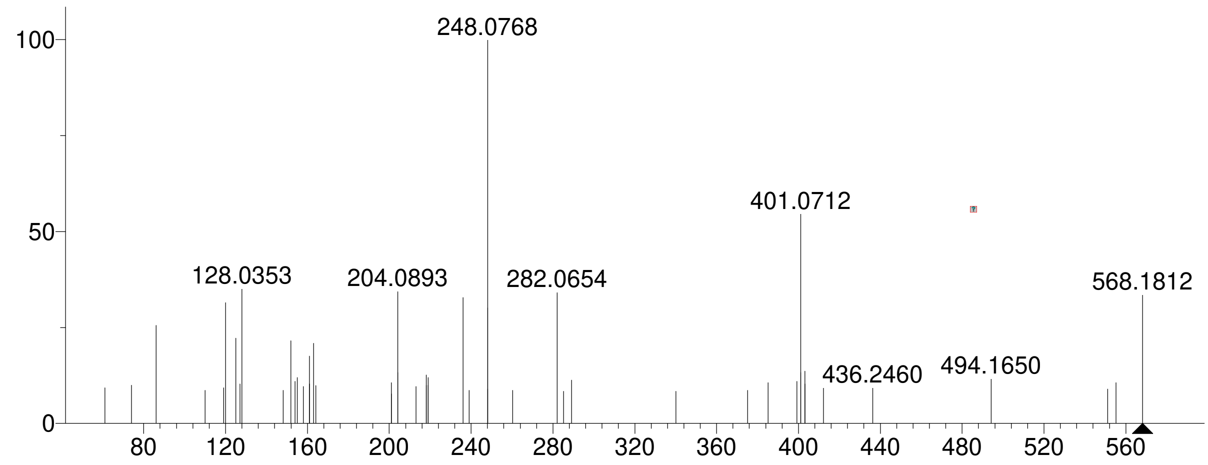

Hit 1 : Lacto-N-triaose [M+Na]+ HCD 28V P=568.2
C20H35NO16; MF: 20; RMF: 374; Prob 68.7%; CAS: 75645-27-1; Lib: nist_msms; ID: 533058.

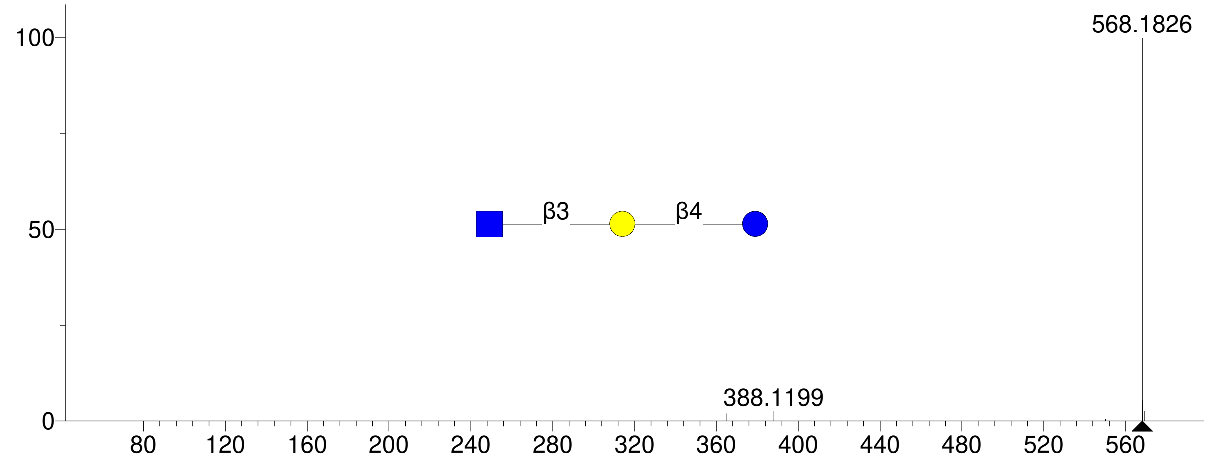

Hit 2 : ophiocordin [M+NH4]+ 35HCD P=568.2
C28H26N2O10; MF: 0; RMF: 2; Prob 31.3%; Lib: mona-export-vf-npl_qexactive; ID: 22744.

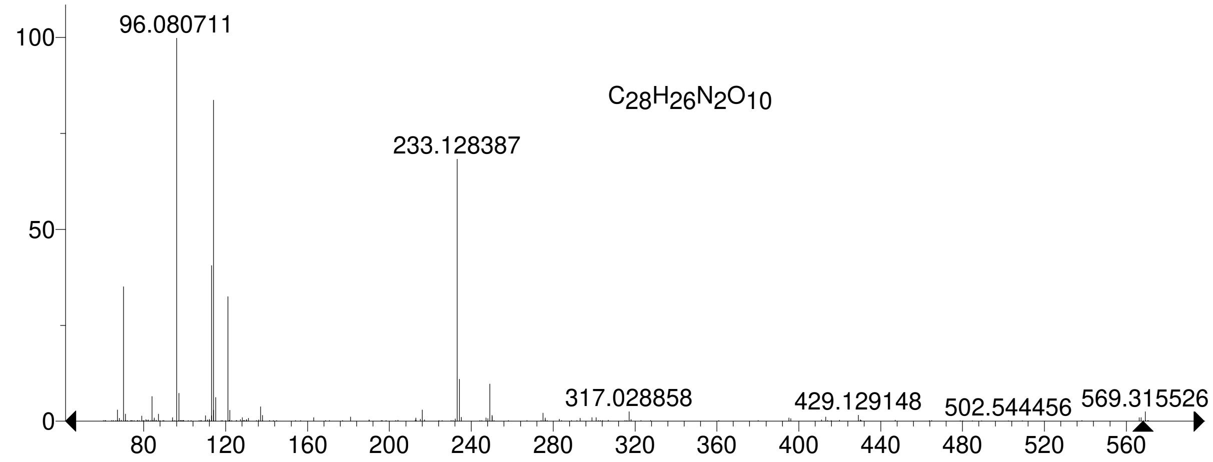

** Search Report Page 1 of 1 **

Unknown: Unknown (3.32_628.1471m/z)
Compound in Library Factor = 0

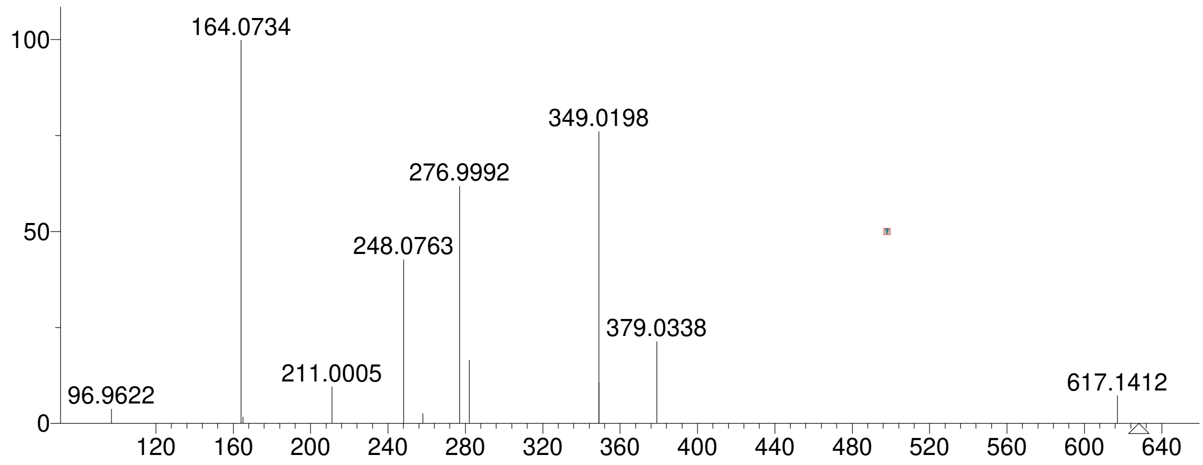

Unknown: Unknown (3.33_706.1670m/z)
Compound in Library Factor = 0

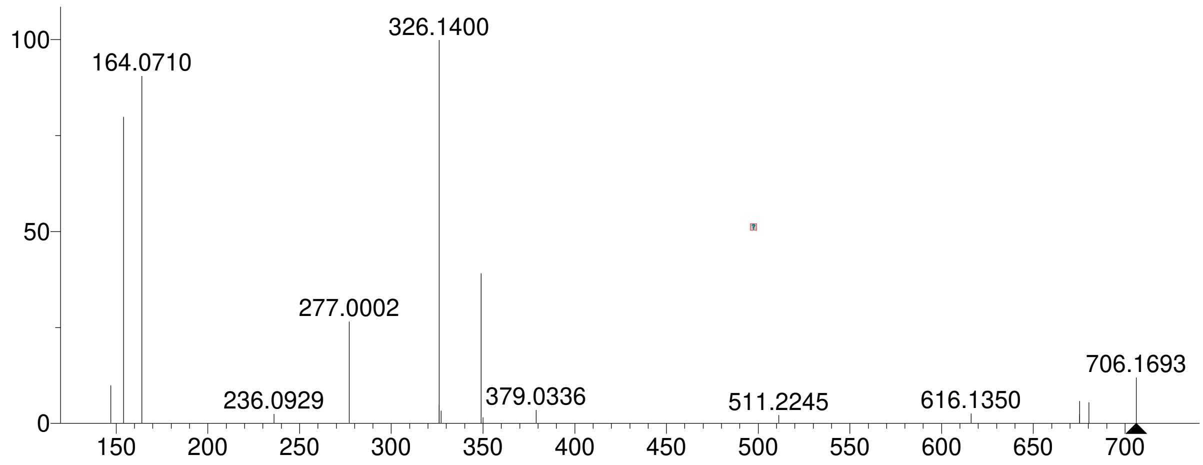

Unknown: Unknown (3.34_326.1231m/z)
Compound in Library Factor = 0

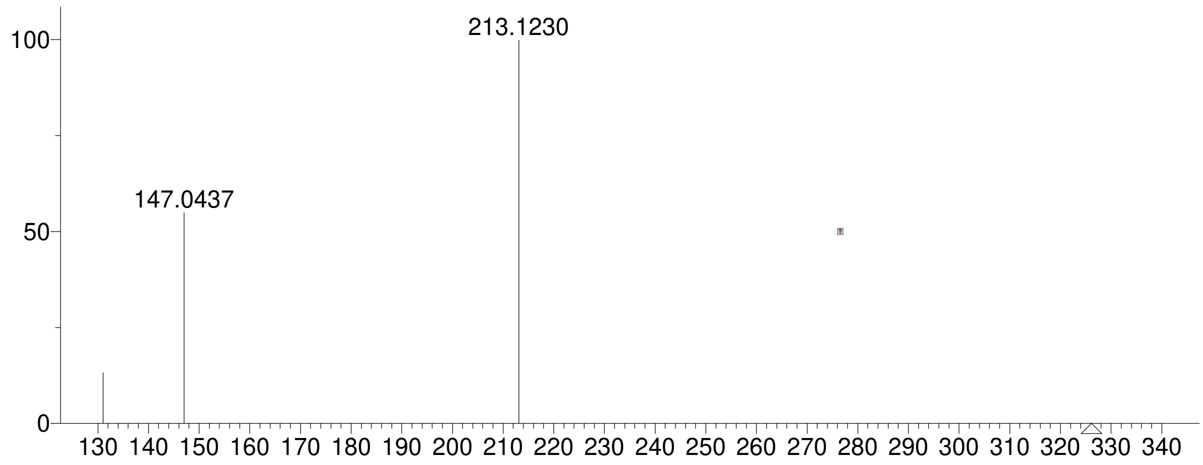

Unknown: Unknown (3.34_406.0795m/z)
Compound in Library Factor = 0

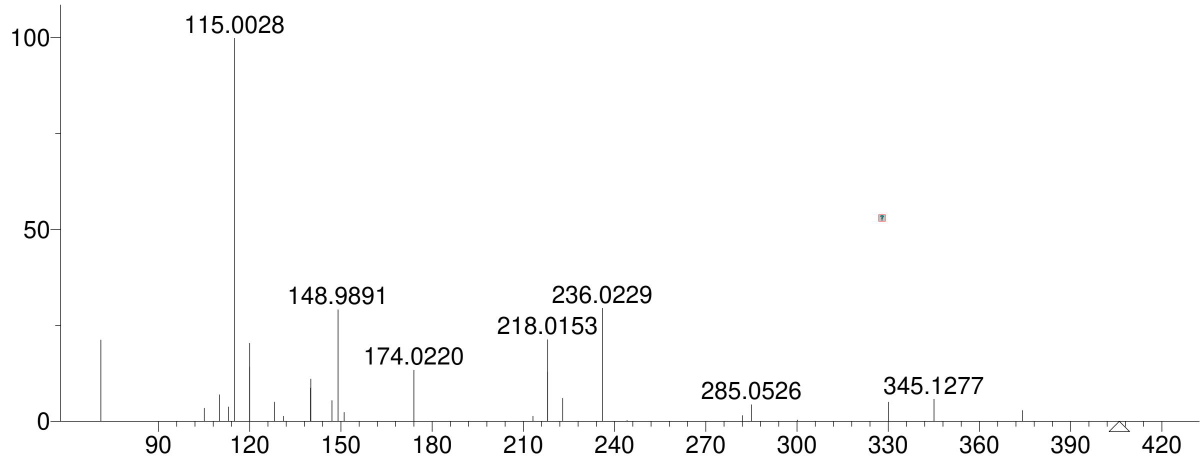

Hit 1 : Bicuculline (+) [M+K]+ 35% P=406.1
C20H17NO6; MF: 0; RMF: 12; Prob 100.0%; Lib: mona-export-itree; ID: 1031.

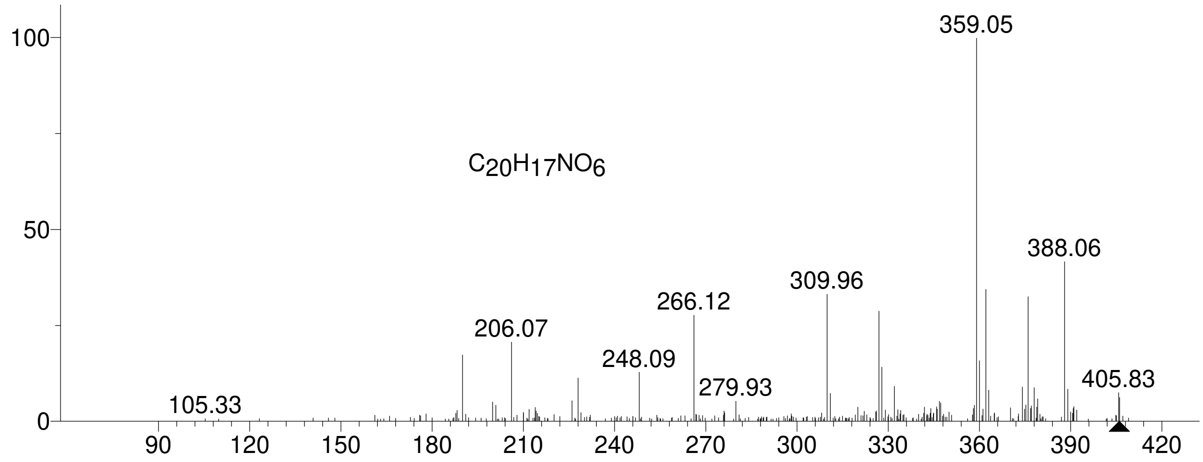

Unknown: Unknown (3.34_675.2356m/z)
Compound in Library Factor = -1072

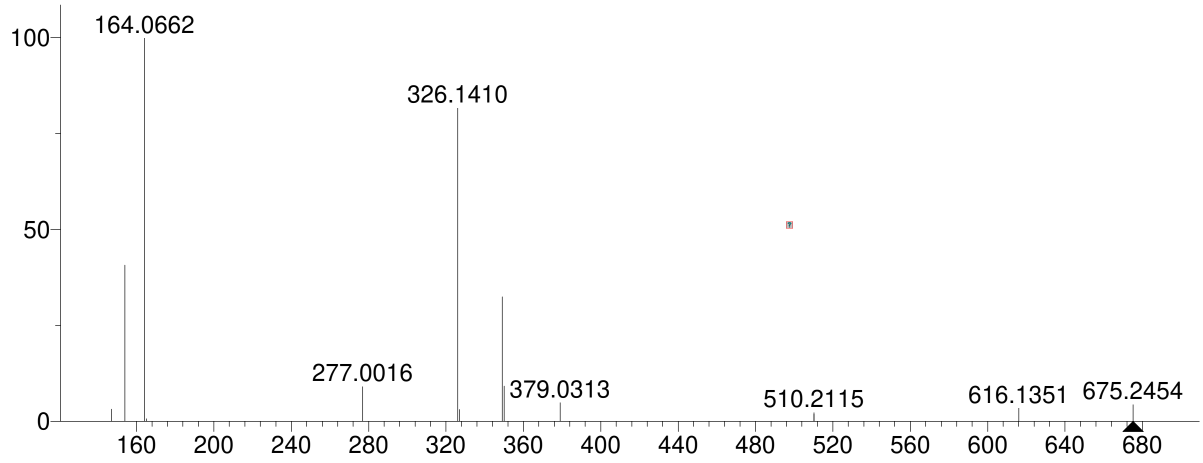

Hit 1 : Blood Group H Disaccharide [2M+Na]+ HCD 2V P=675.2
C12H22O10; MF: 70; RMF: 404; Prob 87.3%; CAS: 24656-24-4; Lib: nist_msms; ID: 530868.

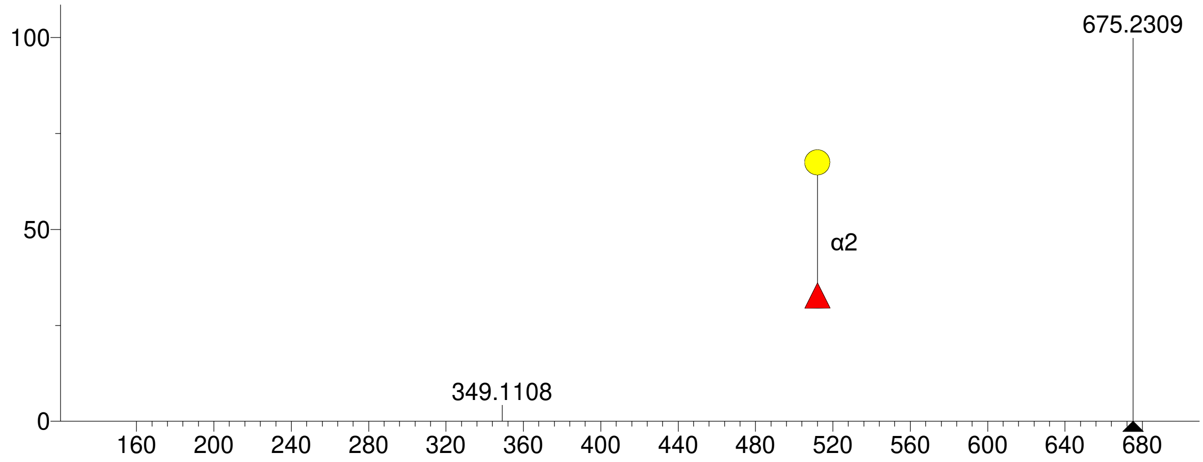

Hit 2 : Icariin [M-H]- 45eV P=675.2
C33H40O15; MF: 0; RMF: 56; Prob 12.7%; Lib: msms-neg-metabobase; ID: 693.

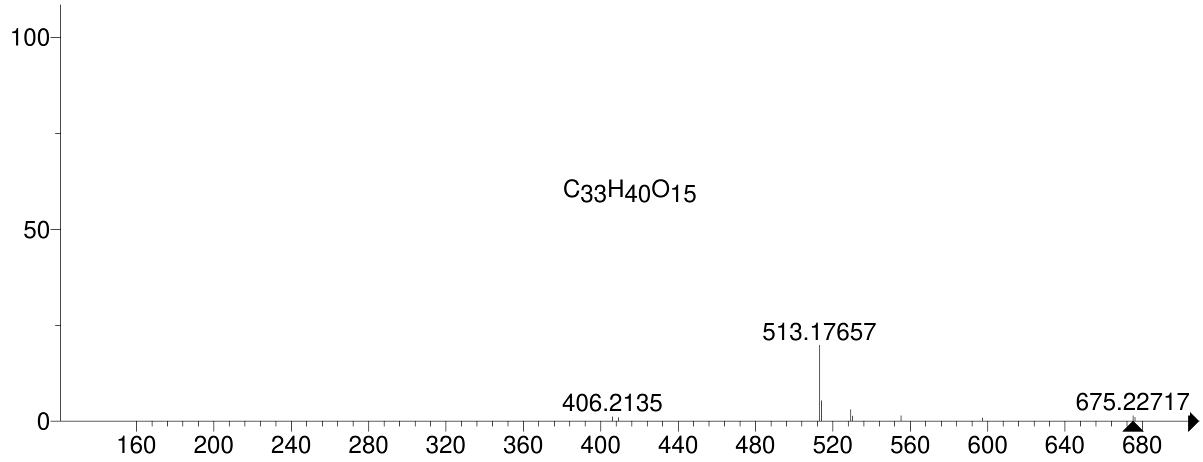

Unknown: Unknown (3.35_563.1505m/z)
Compound in Library Factor = 0

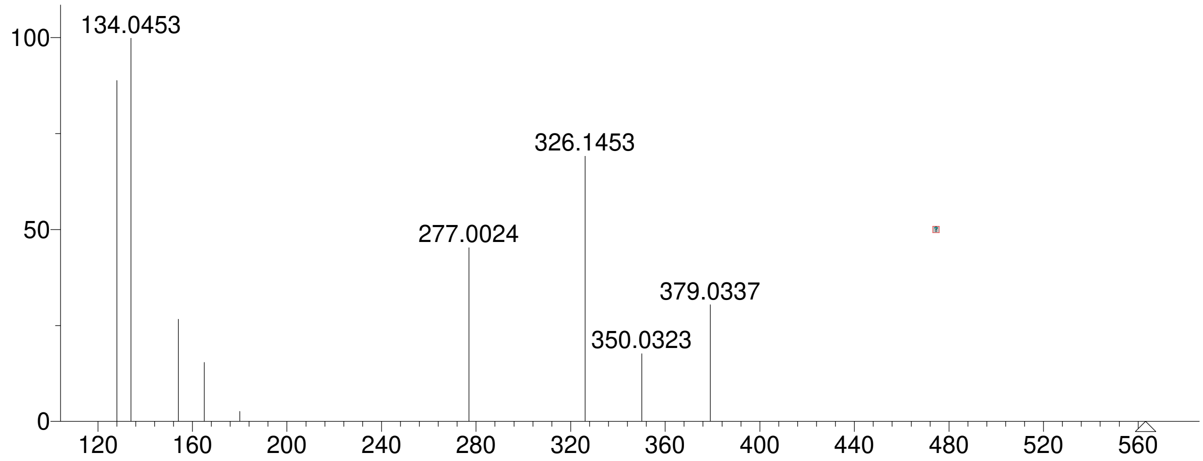

Unknown: Unknown (3.36_573.2029m/z)
Compound in Library Factor = 0

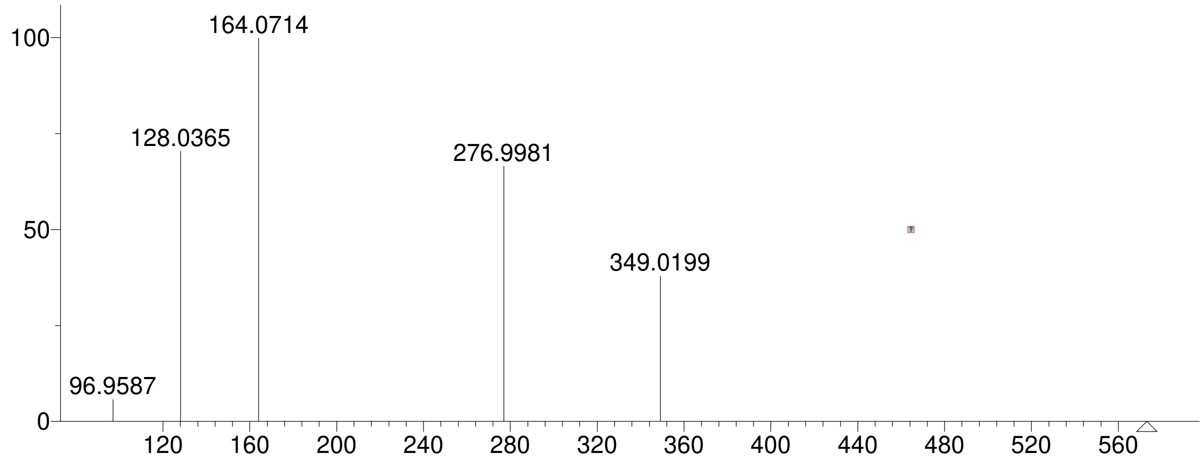

** Search Report Page 1 of 1 **

Unknown: Unknown (3.36_680.2761m/z)
Compound in Library Factor = 0

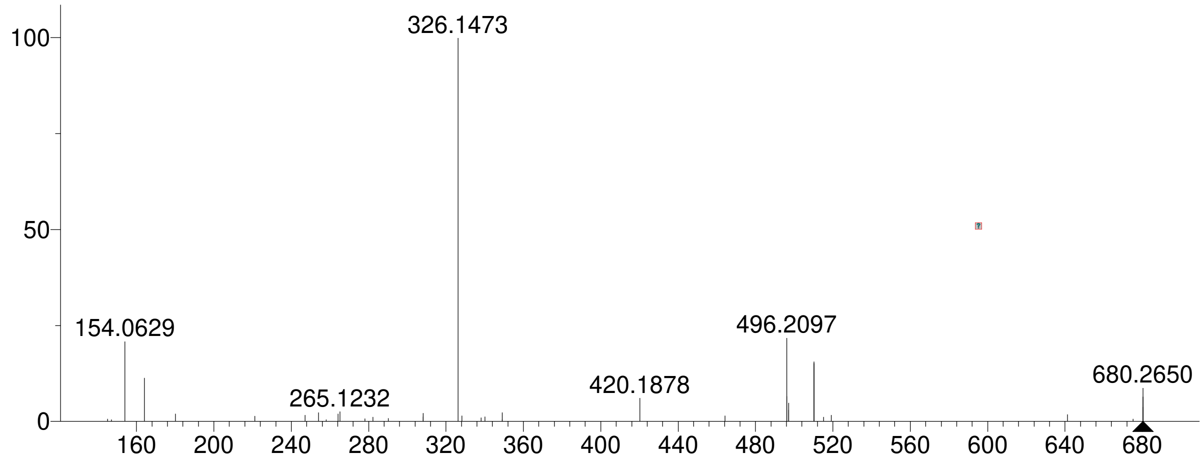

Unknown: Unknown (3.37_164.0707m/z)
Compound in Library Factor = -349

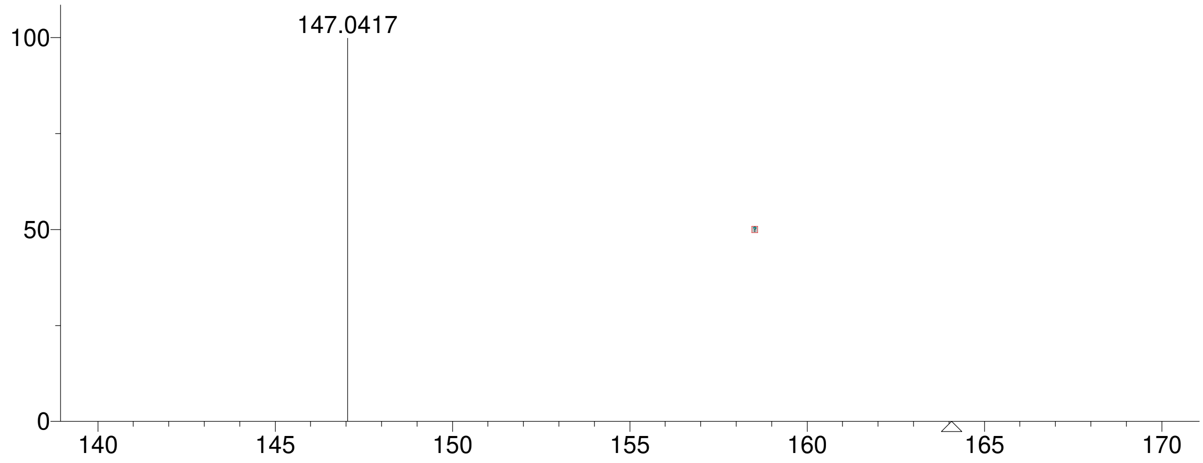

Hit 1 : L-Phenylalanine [M-H]- 20 P=164.1
C9H11NO2; MF: 364; RMF: 999; Prob 17.0%; Lib: msms neg mona_ipb_gnps_plasma; ID: 569.

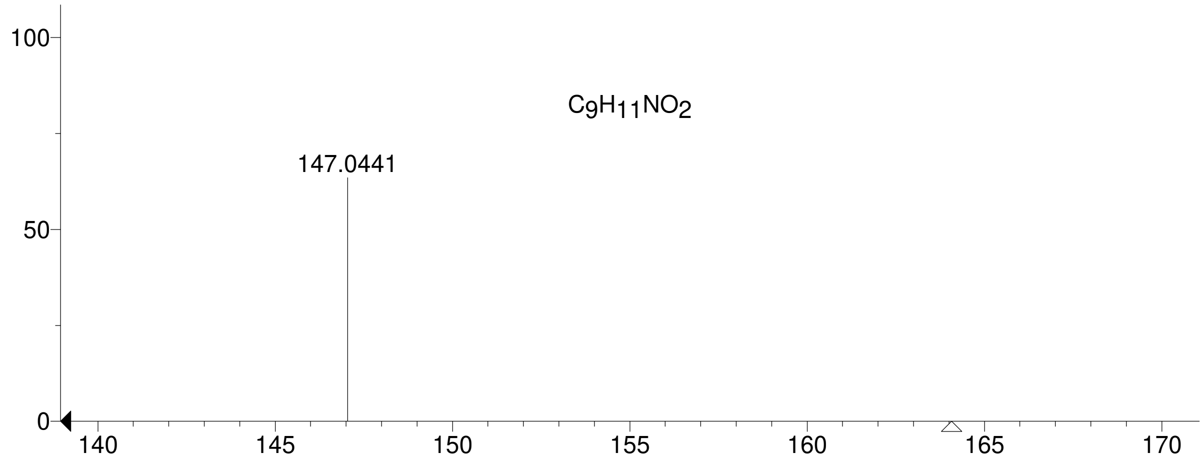

Hit 2 : Phenylalanine [M-H]- 35eV P=164.1
C9H11NO2; MF: 364; RMF: 999; Prob 17.0%; Lib: mona-export-fiehn_hilic; ID: 2924.

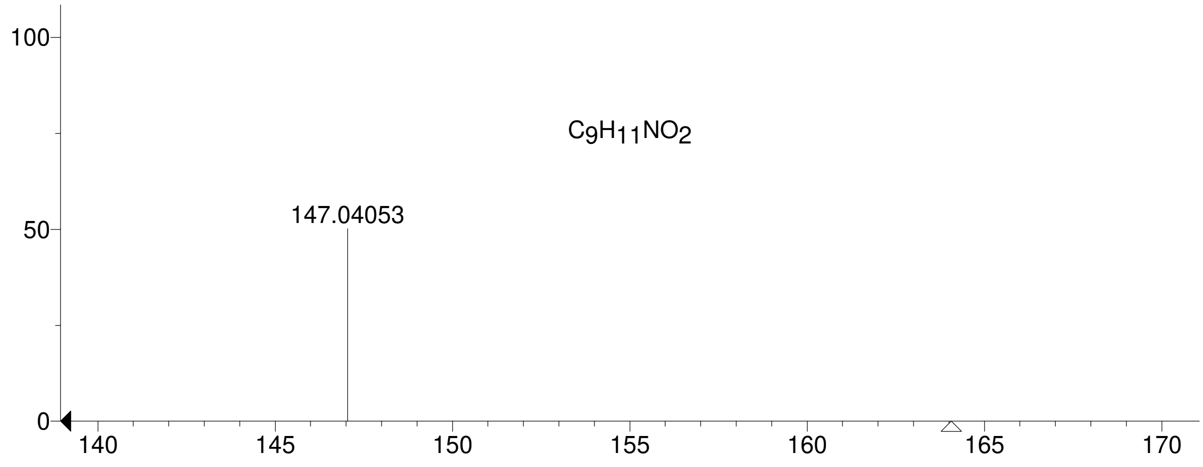

Hit 3 : DL-Phenylalanine [M-H]- IT-FT 35% P=164.1
C9H11NO2; MF: 364; RMF: 999; Prob 17.0%; CAS: 150-30-1; Lib: nist_msms; ID: 141494.

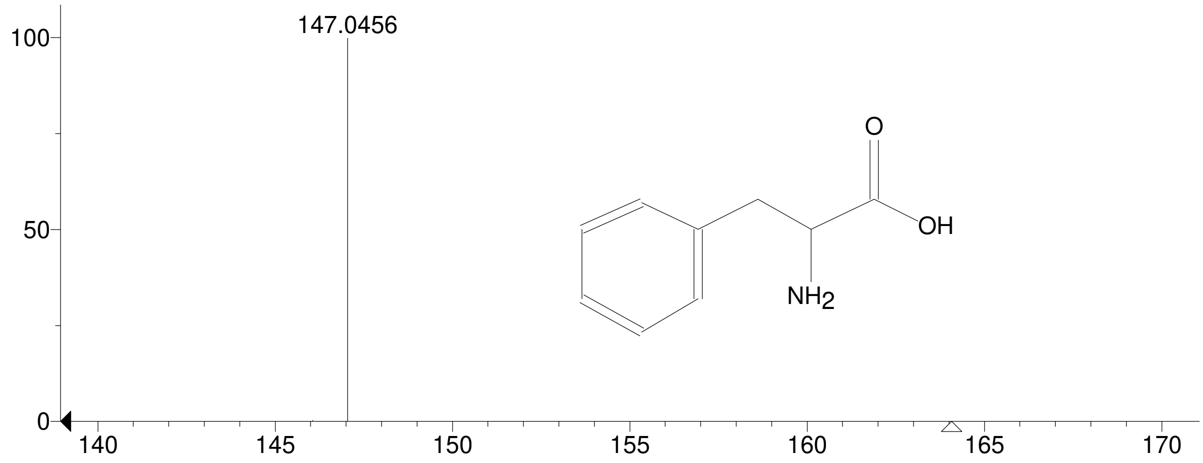

Hit 4 : N-Acetyl-L-phenylalanine [M-H-C2H2O]- IT-FT 35% P=164.1
C11H13NO3; MF: 364; RMF: 999; Prob 17.0%; CAS: 2018-61-3; Lib: nist_msms; ID: 290399.

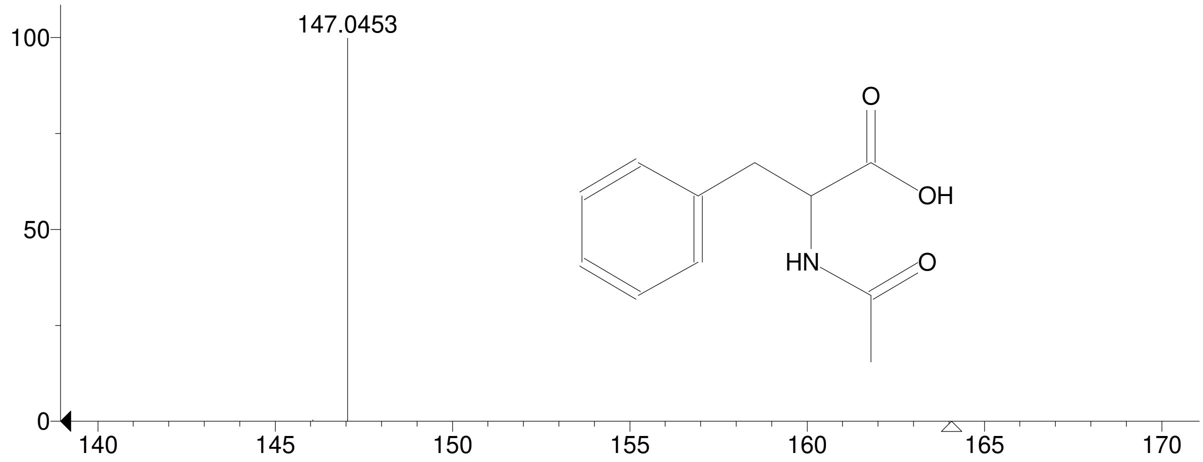

Hit 5 : N-(Phenylacetyl)-L-phenylalanine [M-H-C8H6O]- HCD 52V P=164.1
C17H17NO3; MF: 364; RMF: 999; Prob 17.0%; CAS: 738-75-0; Lib: nist_msms; ID: 485931.

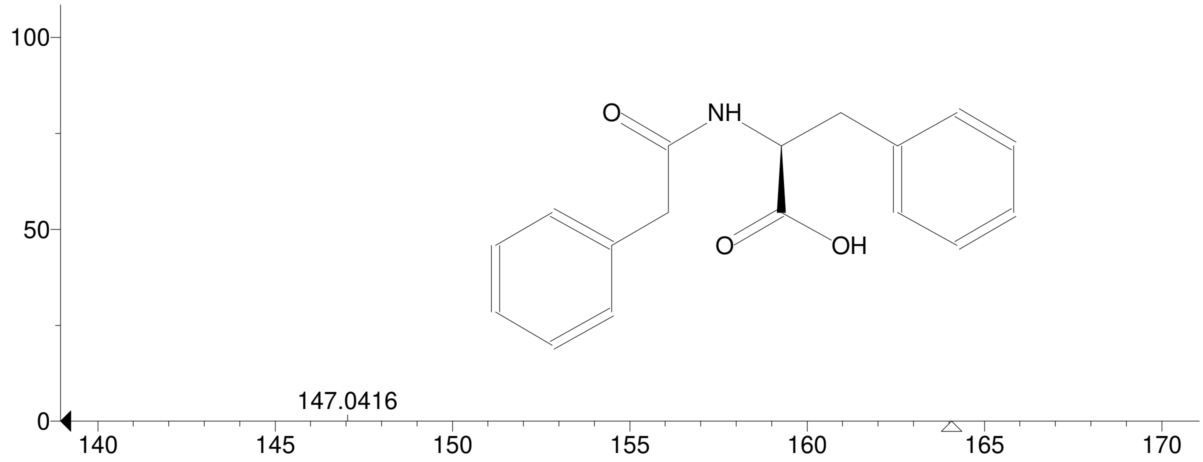

Unknown: Unknown (3.37_413.1530m/z)
Compound in Library Factor = -3273

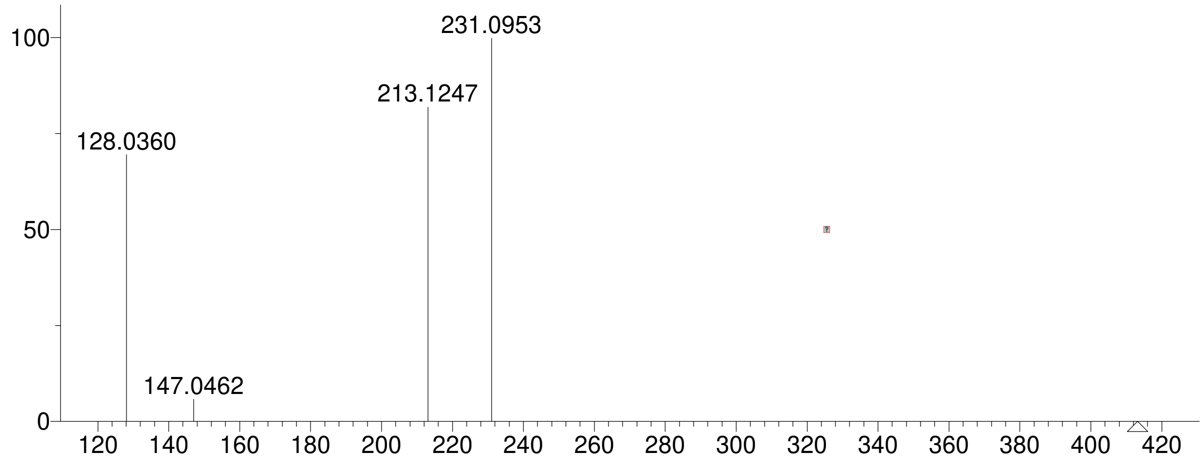

Hit 1 : 2-amino-5-[2-[[2,3-dihydroxy-2-(1-hydroxyethyl)butanoyl]oxymethyl]-4-hydroxyanilino]-5-oxopentanoic acid
C18H26N2O9; MF: 1; RMF: 112; Prob 100.0%; Lib: mona-export-lc-ms-ms_negative_mode; ID: 22977.

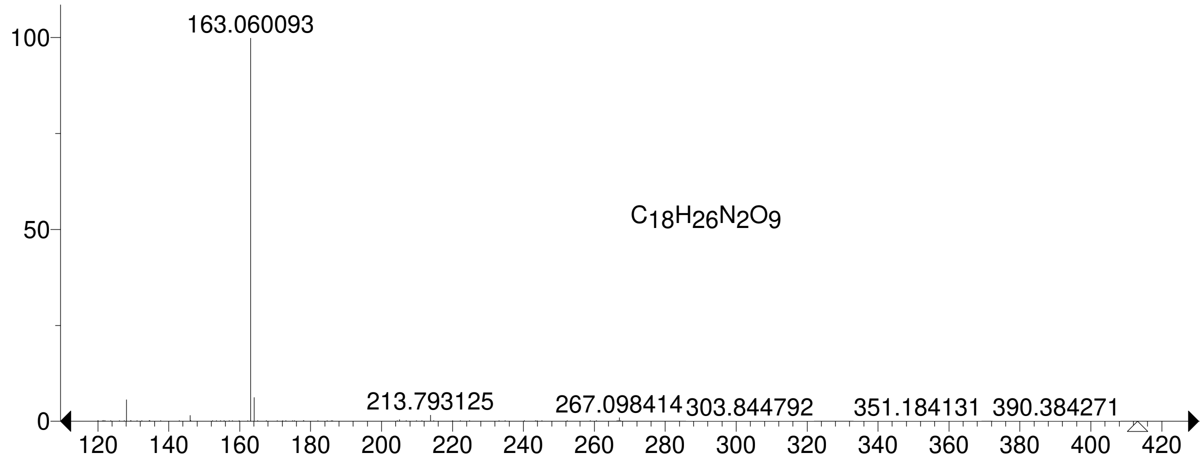

Unknown: Unknown (3.38_293.1133m/z)
Compound in Library Factor = -589

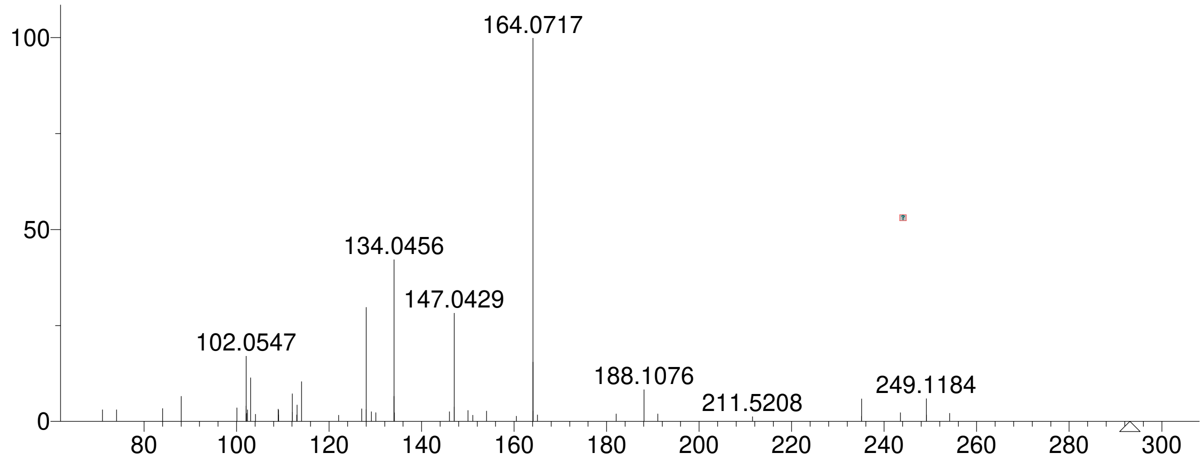

Hit 1 : Phe-Glu [M-H]- HCD 26eV P=293.1
C14H18N2O5; MF: 305; RMF: 628; Prob 49.0%; Lib: nist_msms; ID: 570595.

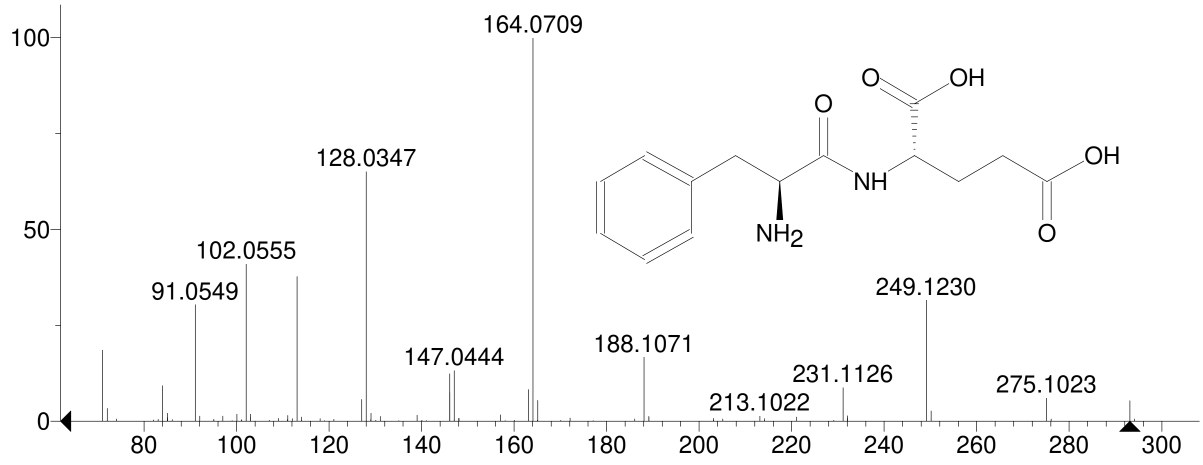

Hit 2 : Phe-Glu [M-H]- HCD 26eV P=293.1
C14H18N2O5; MF: 305; RMF: 628; Prob 49.0%; CAS: 3617-45-6; Lib: nist_msms2; ID: 84328.

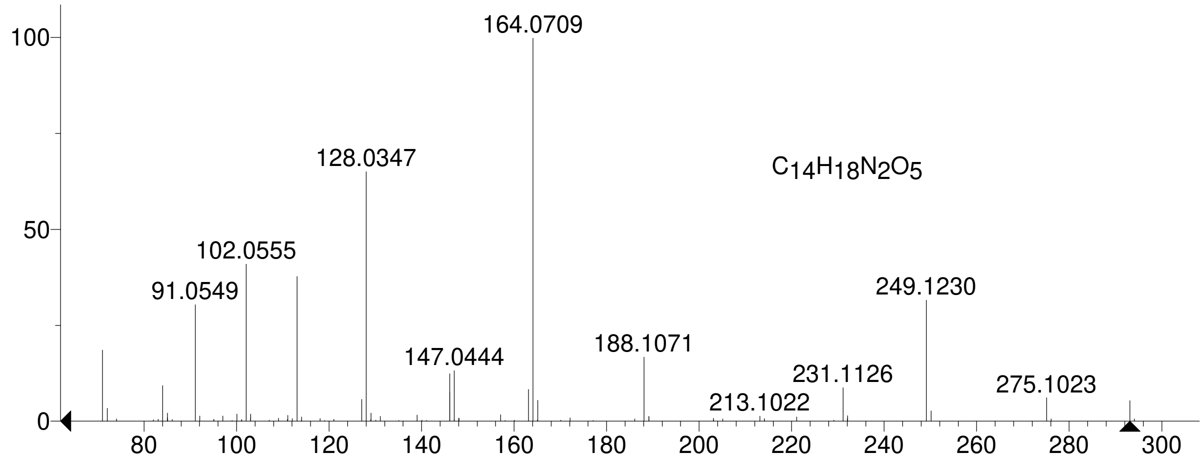

Hit 3 : Glu-Phe [M-H]- HCD 26eV P=293.1
C14H18N2O5; MF: 127; RMF: 491; Prob 0.92%; Lib: nist_msms; ID: 570031.

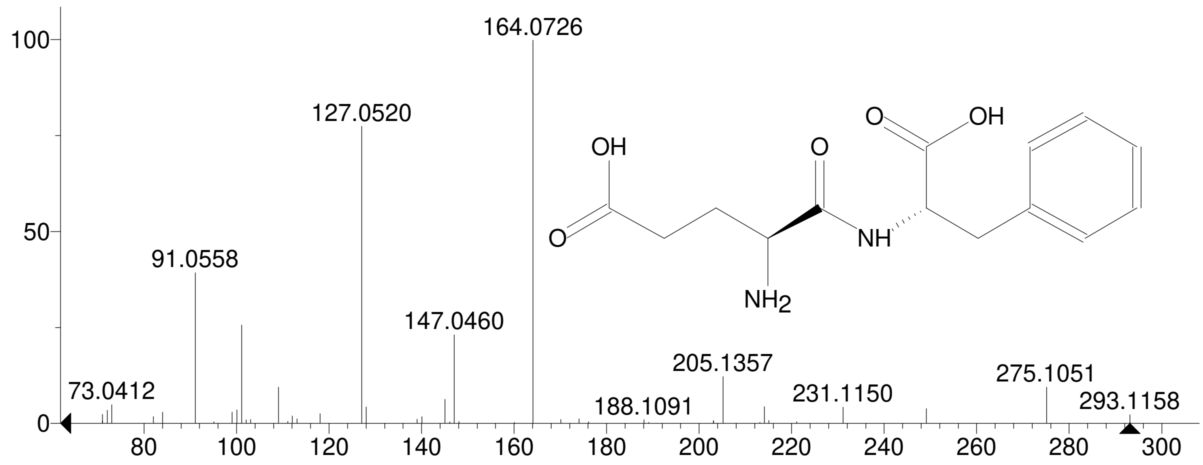

Hit 4 : Glu-Phe [M-H]- HCD 26eV P=293.1
C14H18N2O5; MF: 127; RMF: 491; Prob 0.92%; CAS: 20556-22-3; Lib: nist_msms2; ID: 83757.

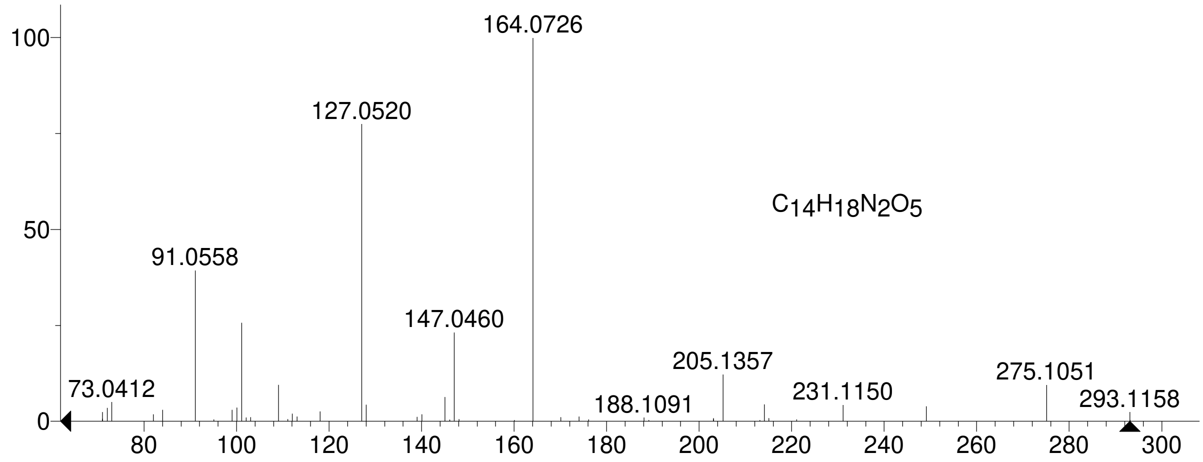

Hit 5 : Unknown (carbon number 14); PlaSMA ID-549 [M-H]- P=293.1
C14H18N2O5; MF: 29; RMF: 210; Prob 0.07%; Lib: biomsms-neg-plasma; ID: 1167.

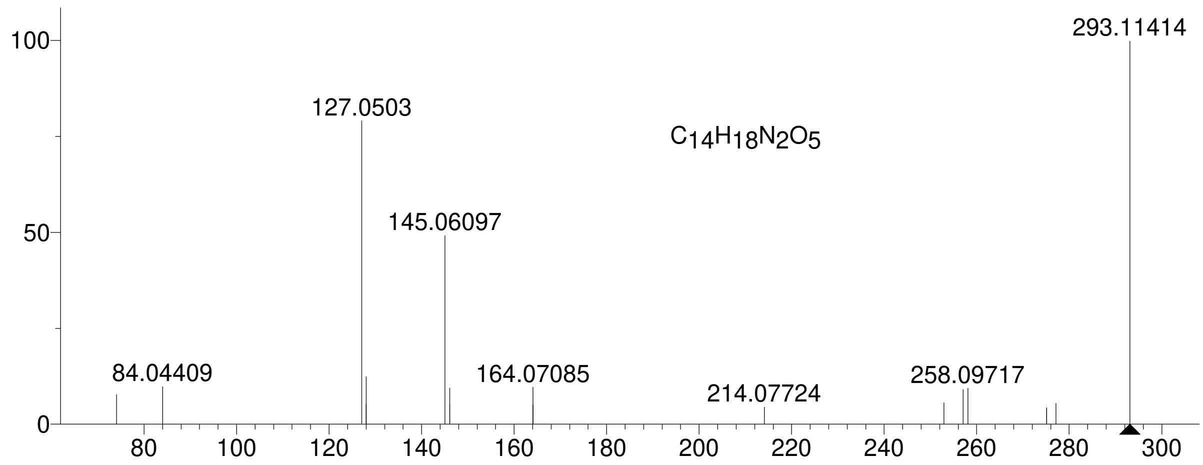

** Search Report Page 1 of 1 **

Unknown: Unknown (3.38_426.1871m/z)
Compound in Library Factor = 0

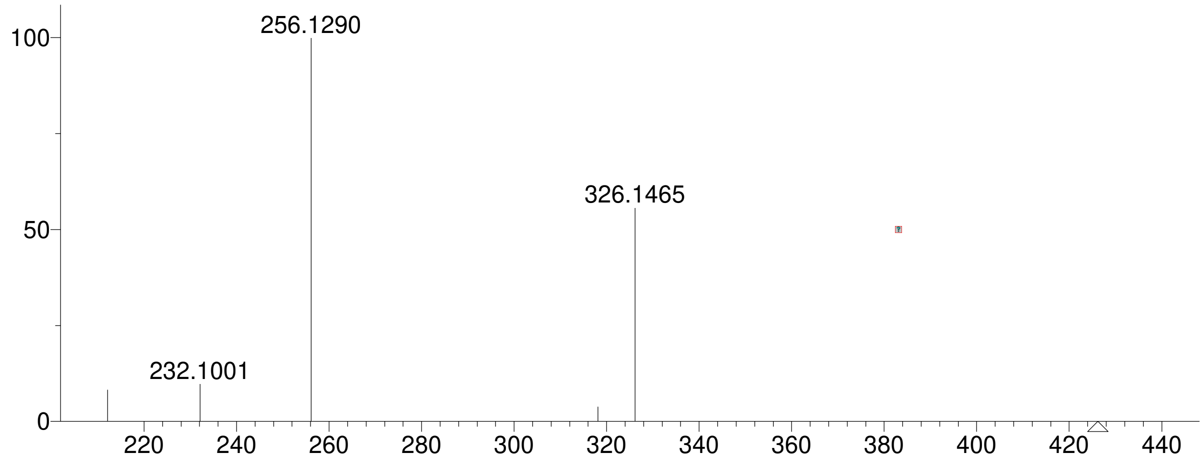

Hit 1 : Leucokinin II [M+2H]2+ HCD 30eV P=426.2
C39H50N10O12; MF: 0; RMF: 60; Prob 50.0%; Lib: nist_msms2; ID: 59914.

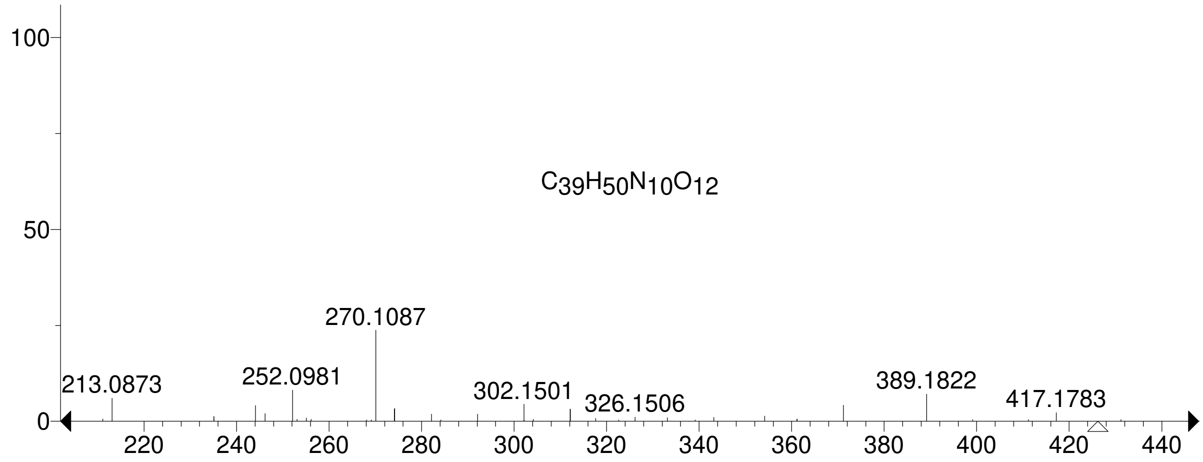

Hit 2 : Ritonavir [M+H-C14H21N3O2S]+ IT 35% P=426.2
C37H48N6O5S2; MF: 0; RMF: 7; Prob 50.0%; CAS: 155213-67-5; Lib: nist_msms; ID: 366790.

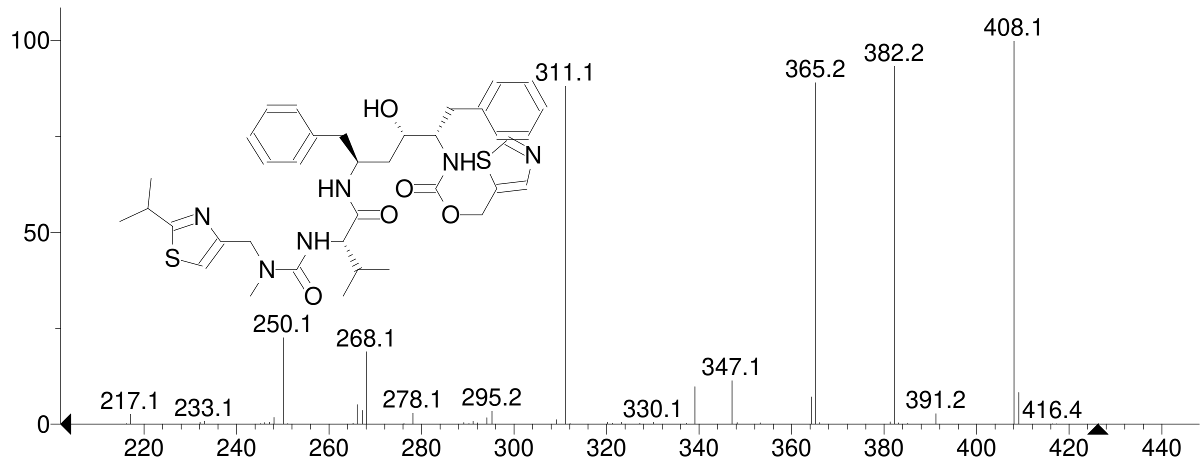

** Search Report Page 1 of 1 **

Unknown: Unknown (3.39_369.0410m/z)
Compound in Library Factor = 0

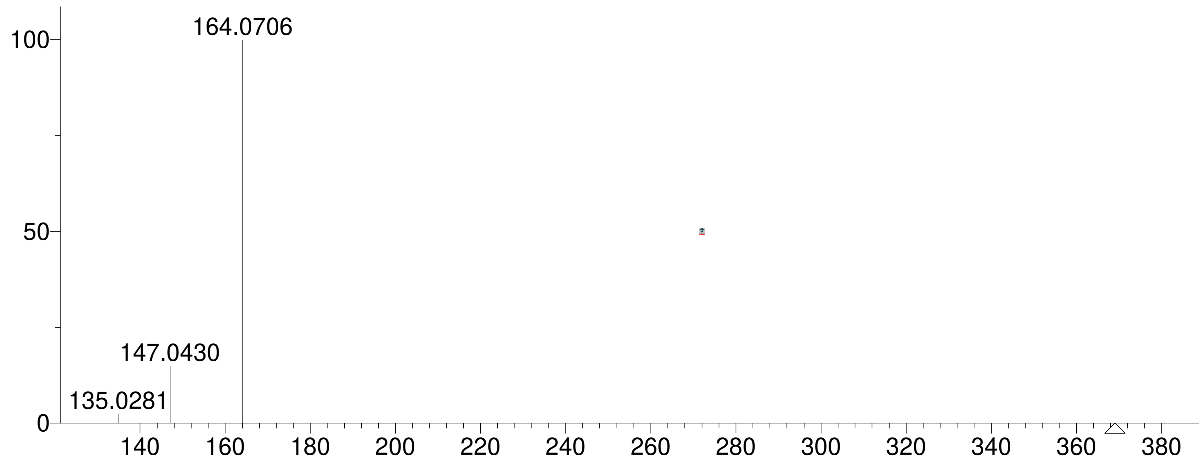

Unknown: Unknown (3.39_417.1159m/z)
Compound in Library Factor = 0

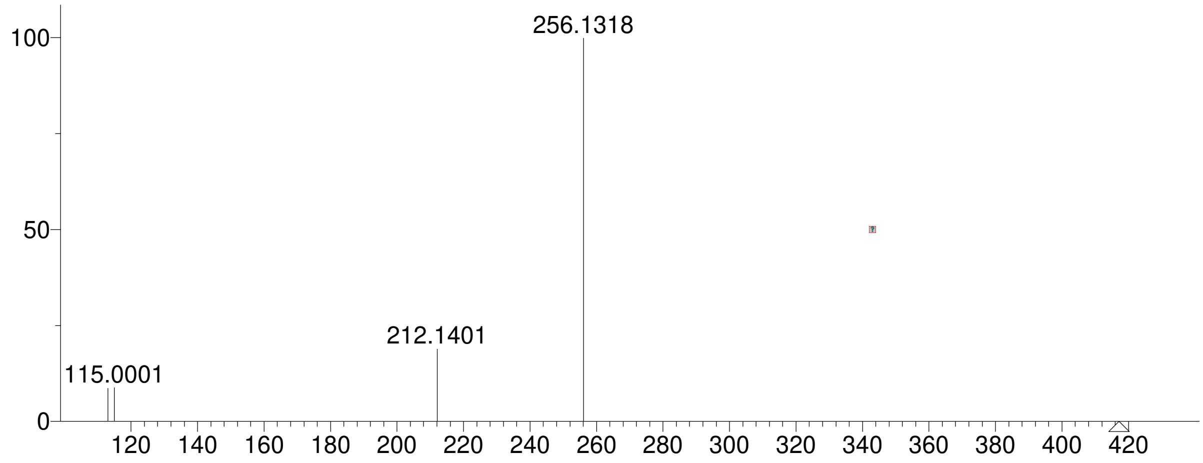

Hit 1 : 7-hydroxy-2-[4-[(2S,3R,4S,5S,6R)-3,4,5-trihydroxy-6-(hydroxymethyl)oxan-2-yl]oxyphenyl]-2,3-dihydrochro
C21H22O9; MF: 0; RMF: 33; Prob 16.7%; Lib: mona-export-lc-ms-ms_negative_mode; ID: 28010.

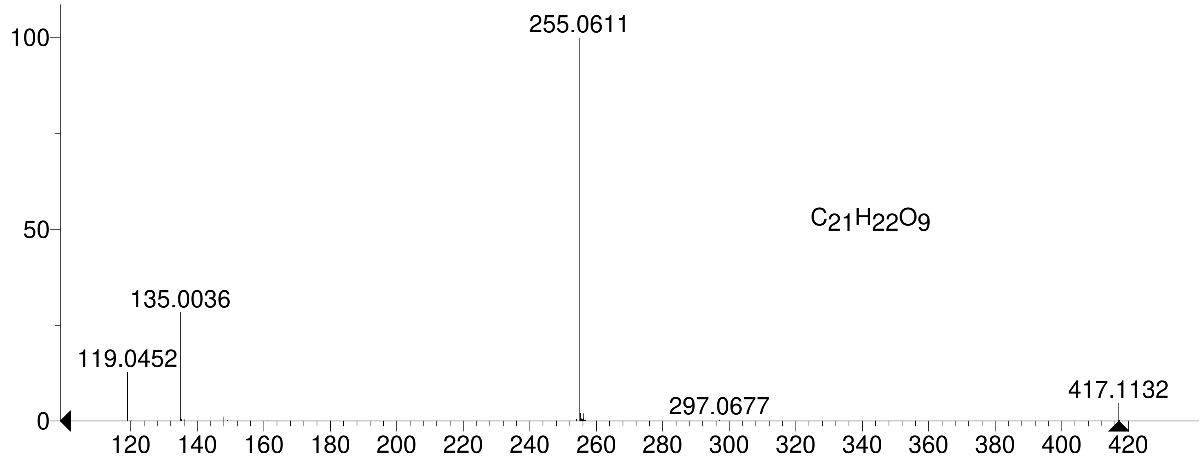

Hit 2 : Engeletin [M+H-H2O]+ HCD 133V P=417.1
C21H22O10; MF: 0; RMF: 21; Prob 16.7%; CAS: 572-31-6; Lib: nist_msms; ID: 236880.

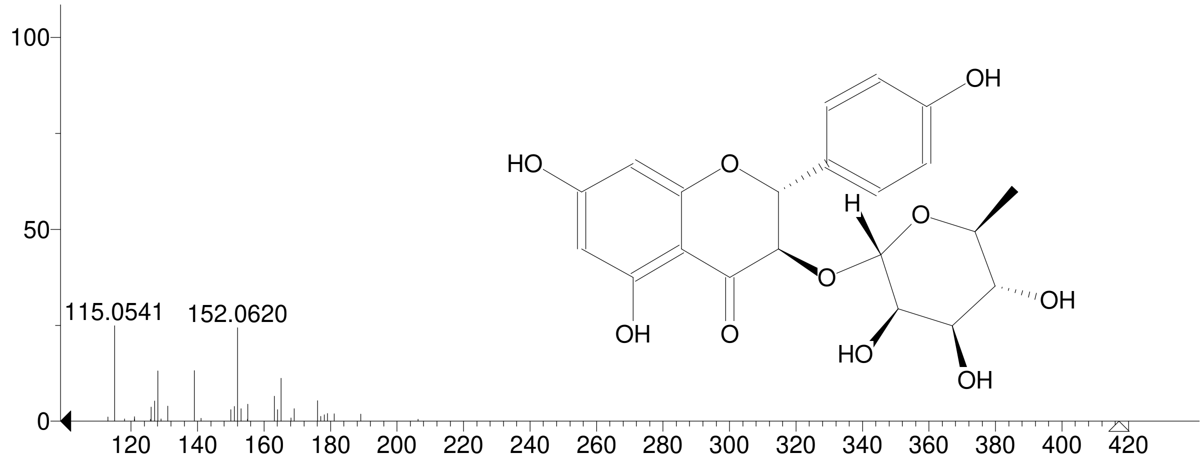

Hit 3 : Daidzin [M+H]+ HCD 133V P=417.1
C21H20O9; MF: 0; RMF: 20; Prob 16.7%; CAS: 552-66-9; Lib: nist_msms; ID: 299121.

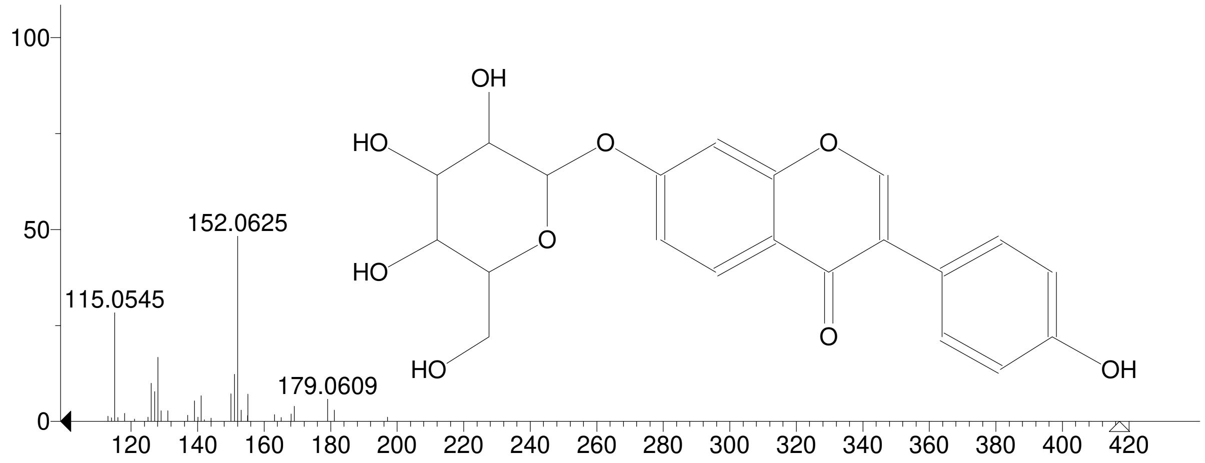

Hit 4 : Isoflavone base + 2O, O-Hex [M+H]+ 6V P=417.1
C21H20O9; MF: 0; RMF: 16; Prob 16.7%; Lib: mona anthocyanidin-o-glycosides; ID: 6910.

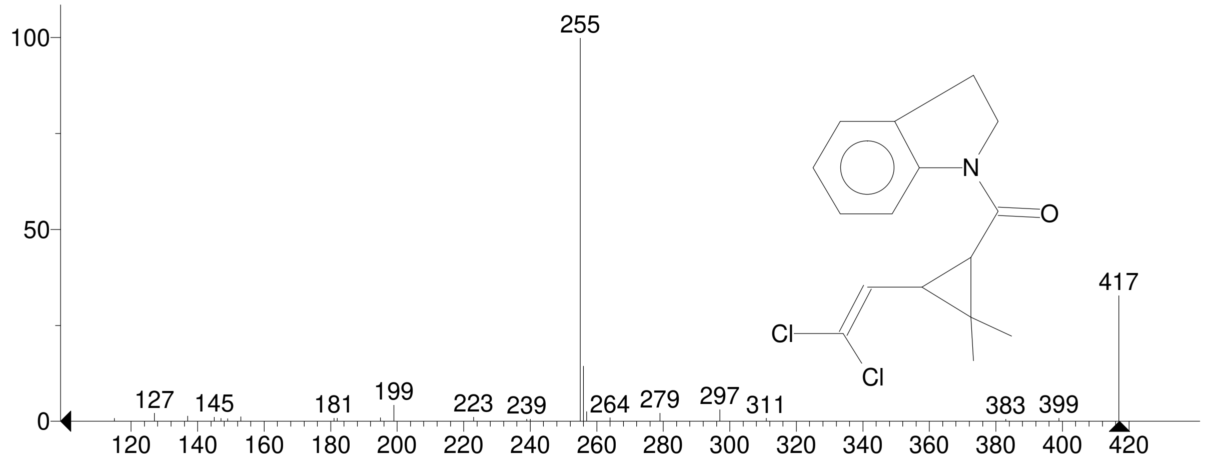

Hit 5 : Daidzein-8-C-glucoside [M+H]+ 50eV P=417.1
C21H20O9; MF: 0; RMF: 14; Prob 16.7%; Lib: mona anthocyanidin-o-glycosides; ID: 3705.

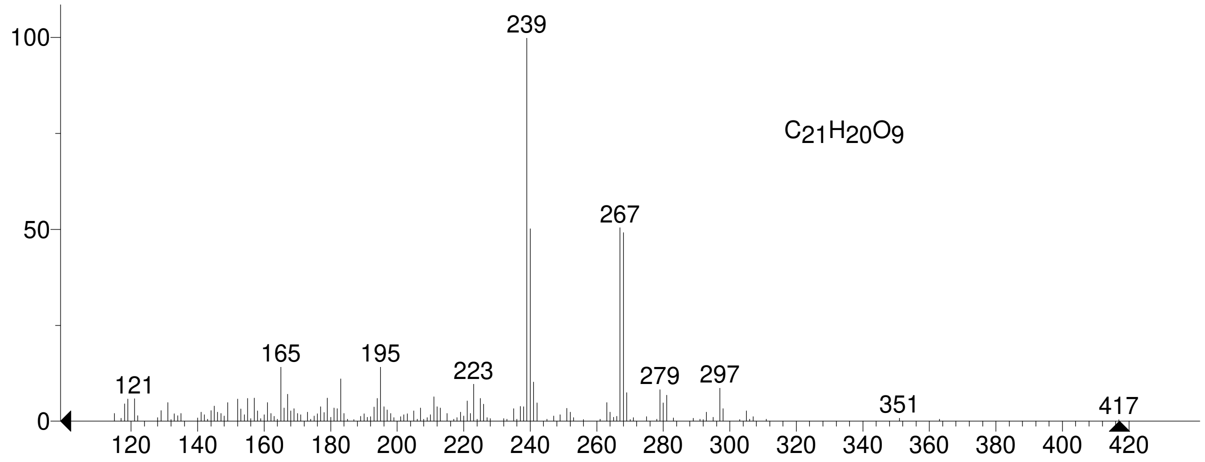

Unknown: Unknown (3.39_460.1571m/z)
Compound in Library Factor = -3273

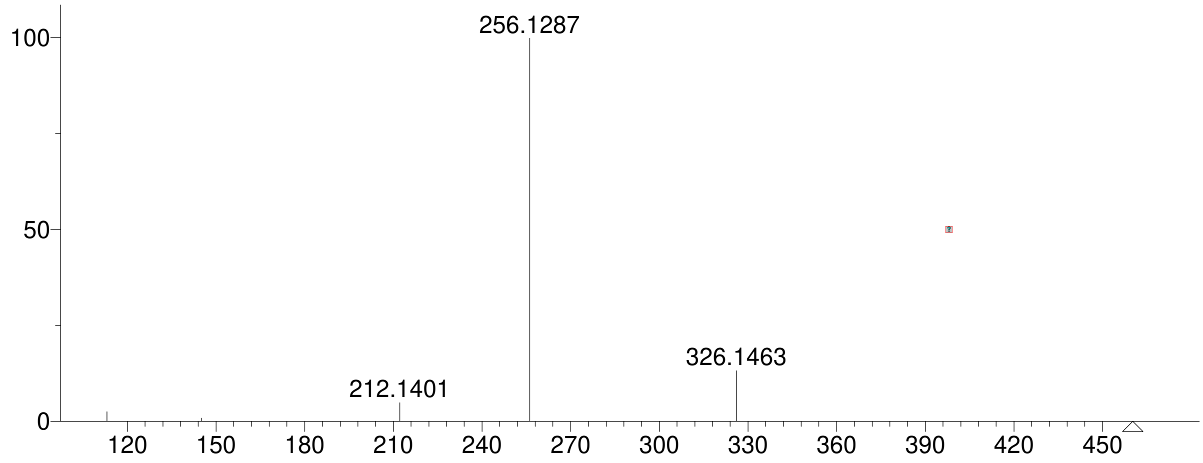

Hit 1 : Hydromorphone-3-β-D-glucuronide [M-H]- HCD 59V P=460.2
C23H27NO9; MF: 3; RMF: 345; Prob 22.1%; CAS: 40505-76-8; Lib: nist_msms; ID: 332372.

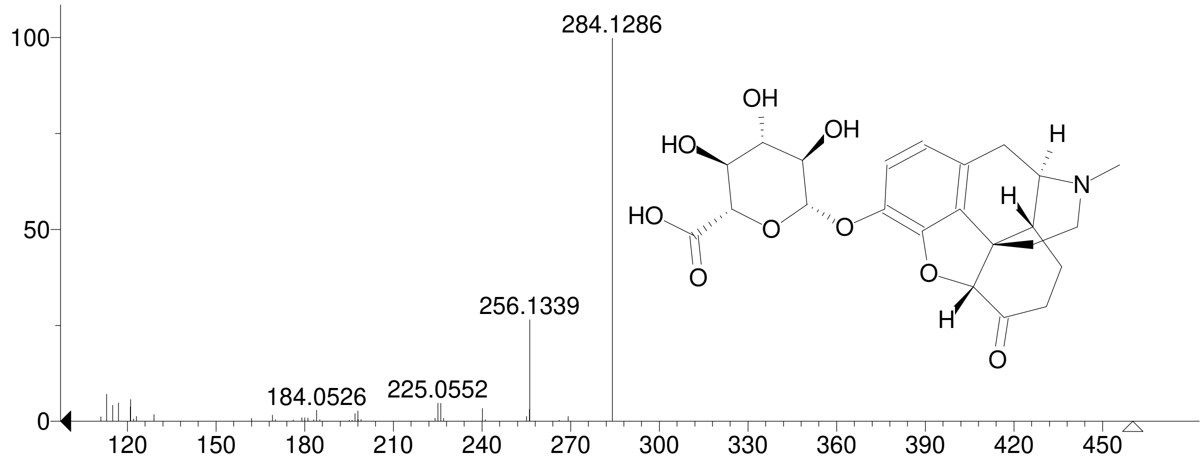

Hit 2 : Oxymorphone 3-β-D-glucuronide [M+H-H2O]+ HCD 46V P=460.2
C23H27NO10; MF: 0; RMF: 69; Prob 19.5%; CAS: 770735-01-8; Lib: nist_msms; ID: 326438.

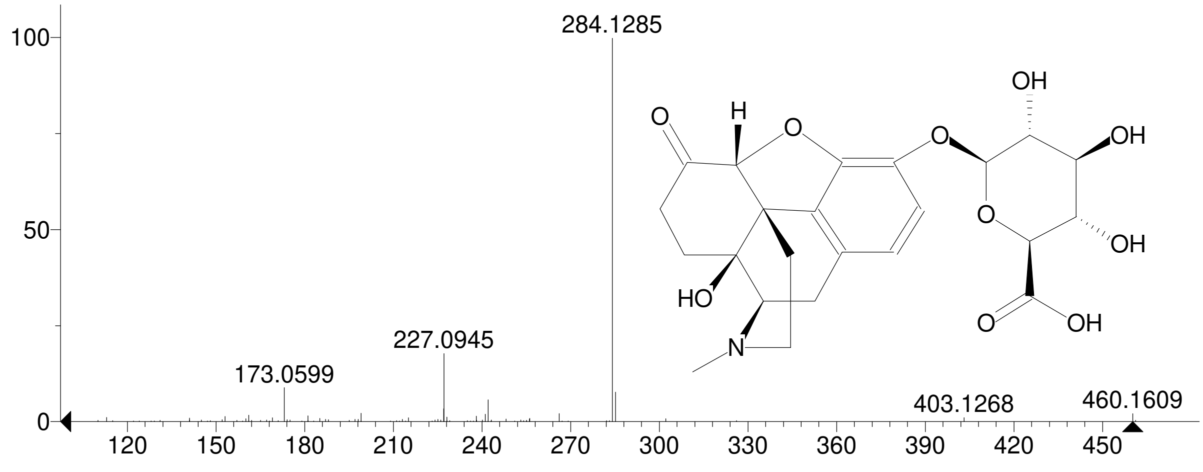

Hit 3 : Morphine-3-glucuronide [M-H]- HCD 73V P=460.2
C23H27NO9; MF: 0; RMF: 47; Prob 19.5%; CAS: 20290-09-9; Lib: nist_msms; ID: 324944.

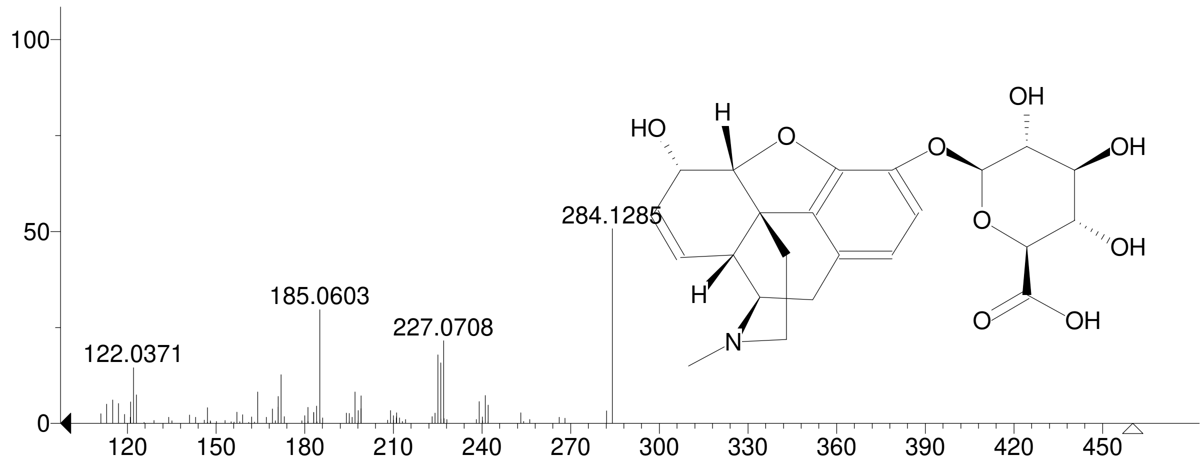

Hit 4 : Morphine-6-glucuronide [M-H]- HCD 73V P=460.2
C23H27NO9; MF: 0; RMF: 32; Prob 19.5%; CAS: 20290-10-2; Lib: nist_msms; ID: 325026.

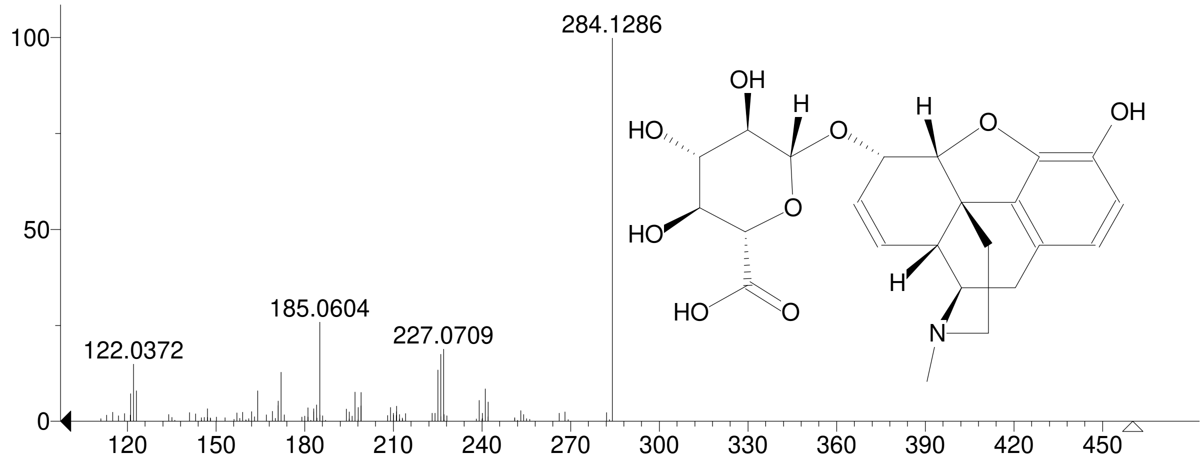

Hit 5 : Villol [M+NH4]+ 45HCD P=460.2
C23H22O9; MF: 0; RMF: 3; Prob 19.5%; Lib: mona-export-vf-npl_qexactive; ID: 8051.

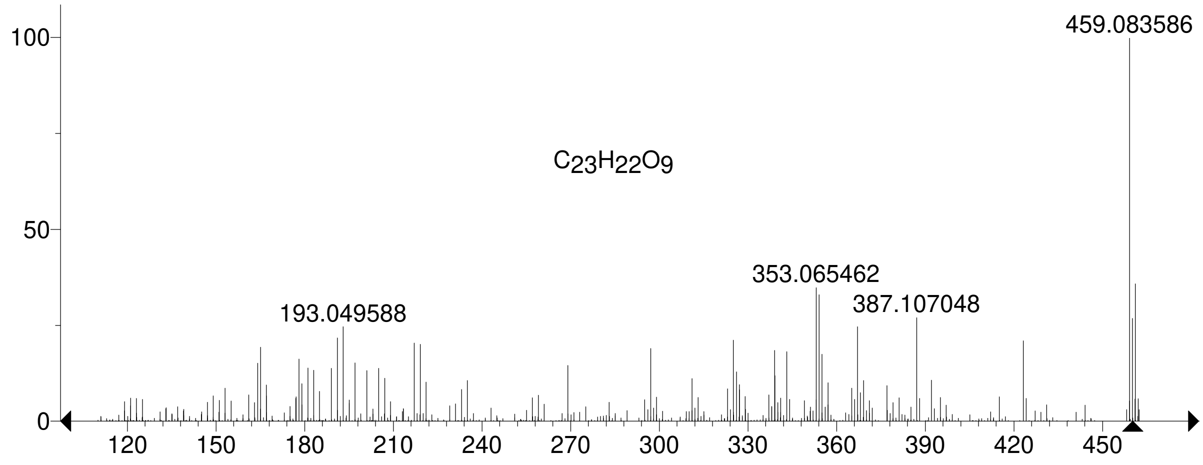

** Search Report Page 1 of 1 **

Unknown: Unknown (3.40_297.0095m/z)
Compound in Library Factor = 0

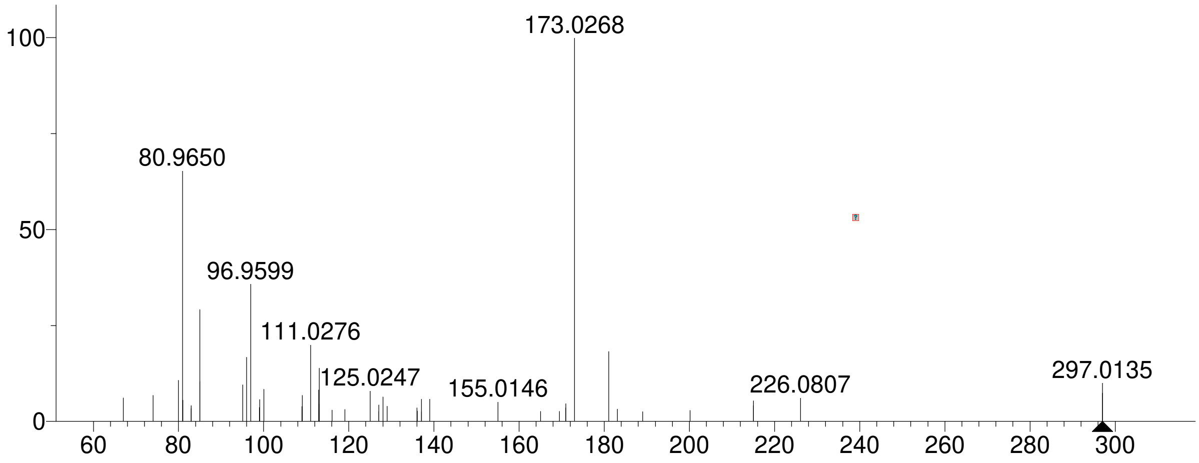

Unknown: Unknown (3.40_343.1484m/z)
Compound in Library Factor = 0

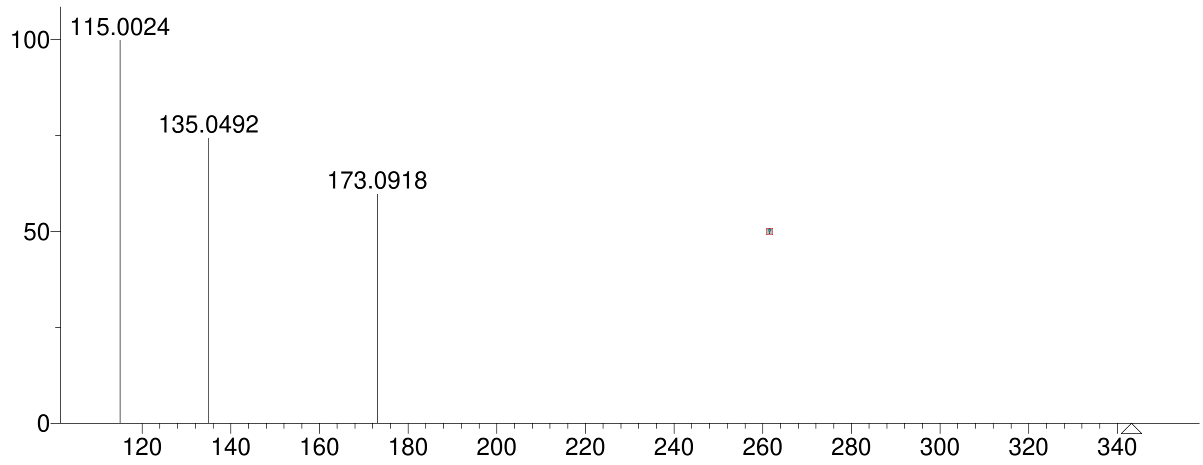

** Search Report Page 1 of 1 **

Unknown: Unknown (3.40_375.1737m/z)
Compound in Library Factor = 0

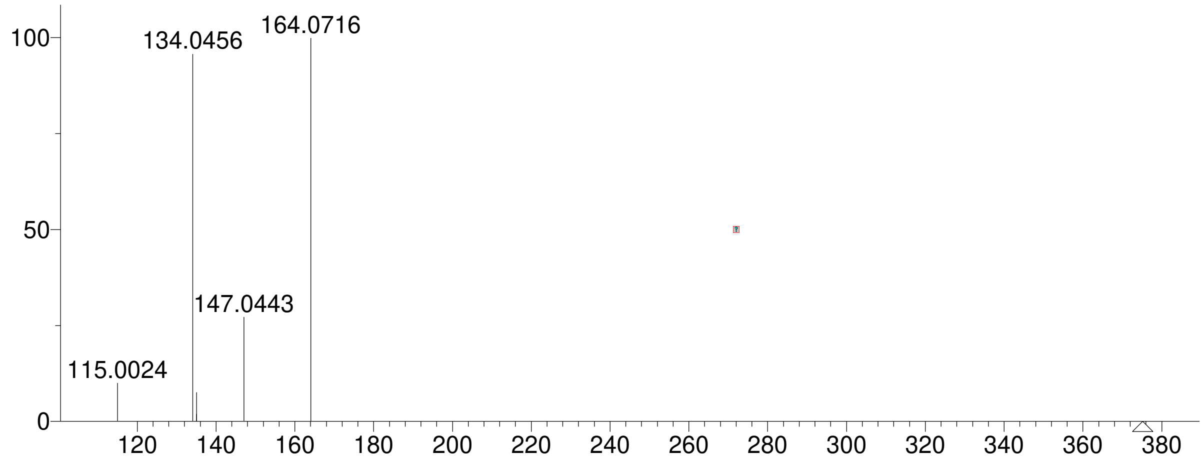

Hit 1 : Cyhalofop-butyl [M+NH4]+ HCD 82V P=375.2
C20H20FNO4; MF: 0; RMF: 96; Prob 100.0%; CAS: 122008-85-9; Lib: nist_msms; ID: 399034.

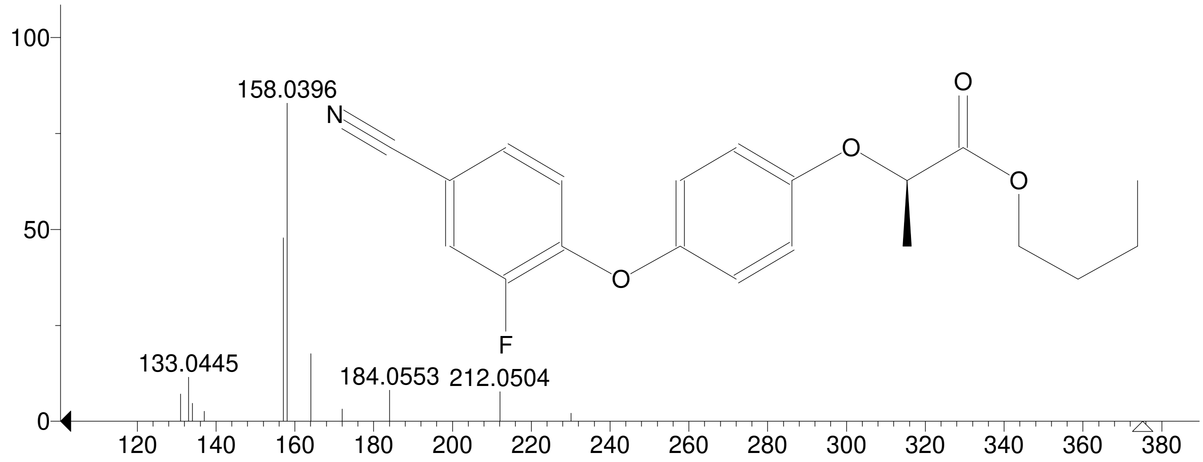

Unknown: Unknown (3.40_406.0800m/z)
Compound in Library Factor = 0

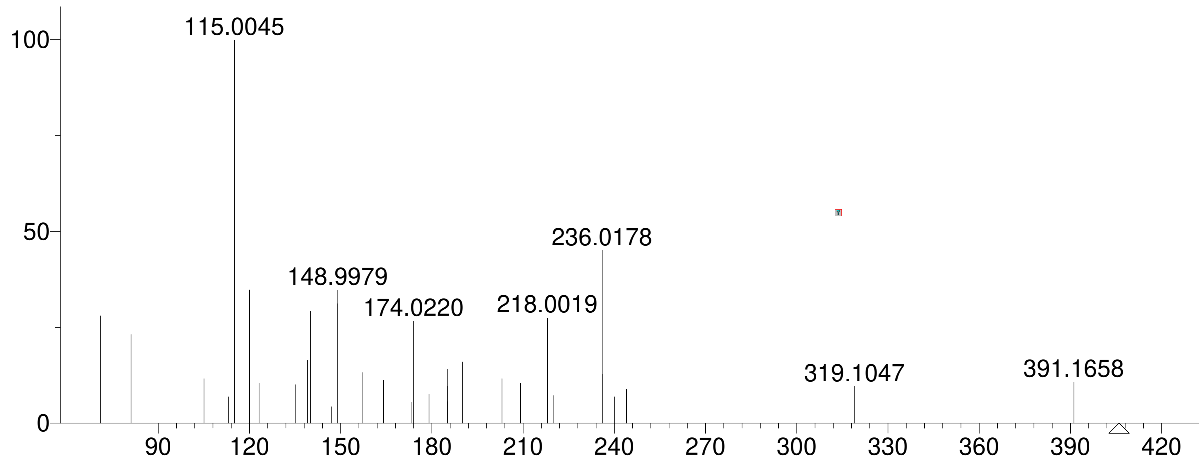

Hit 1 : Bicuculline (+) [M+K]+ 35% P=406.1
C20H17NO6; MF: 0; RMF: 8; Prob 100.0%; Lib: mona-export-itree; ID: 1031.

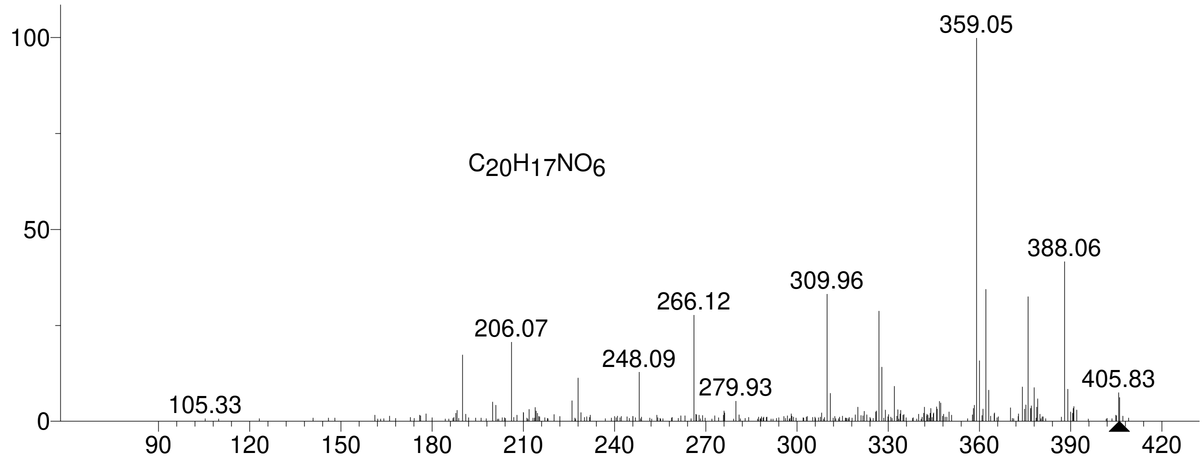

Unknown: Unknown (3.41_516.1806m/z)
Compound in Library Factor = 0

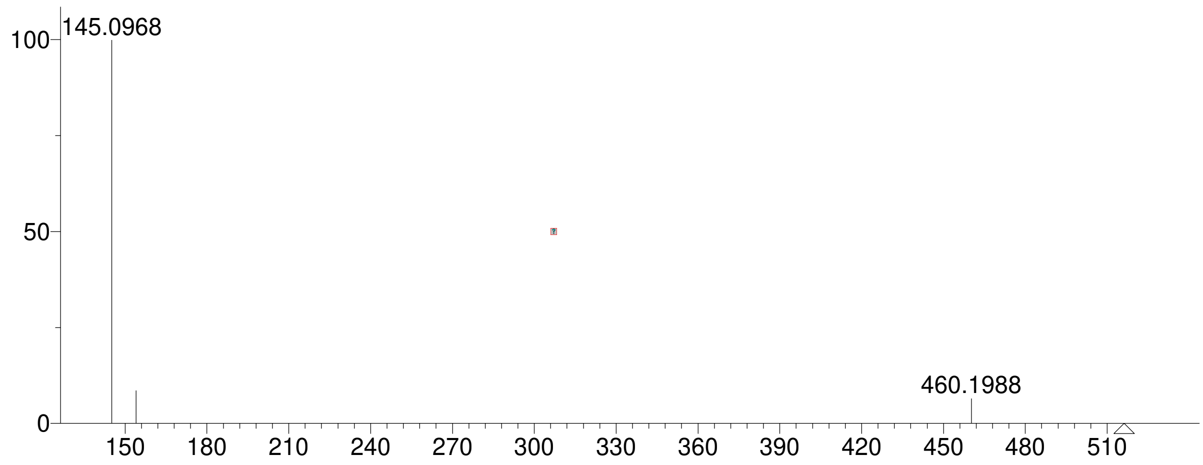

Unknown: Unknown (3.42_364.1145m/z)
Compound in Library Factor = 0

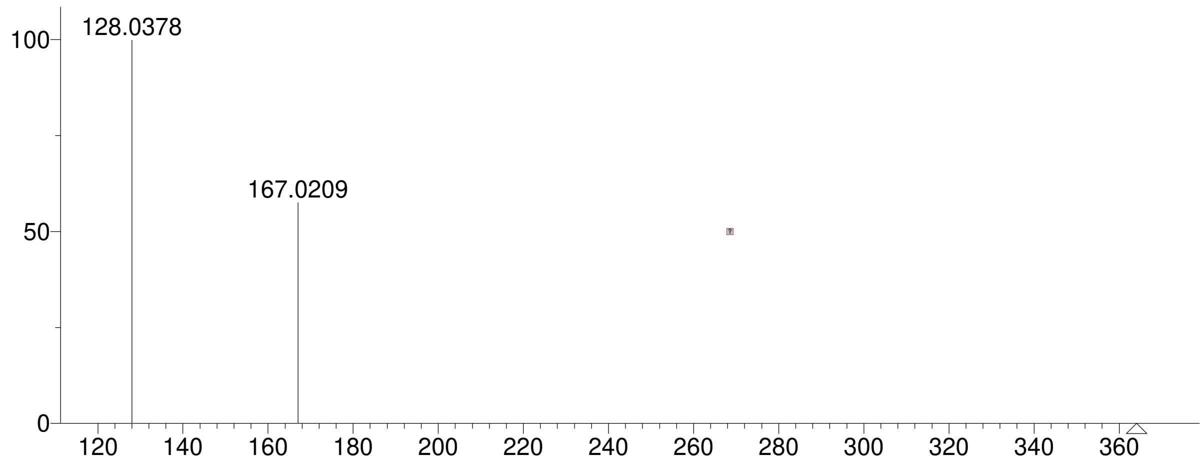

** Search Report Page 1 of 1 **

Unknown: Unknown (3.43_625.2575m/z)
Compound in Library Factor = 0

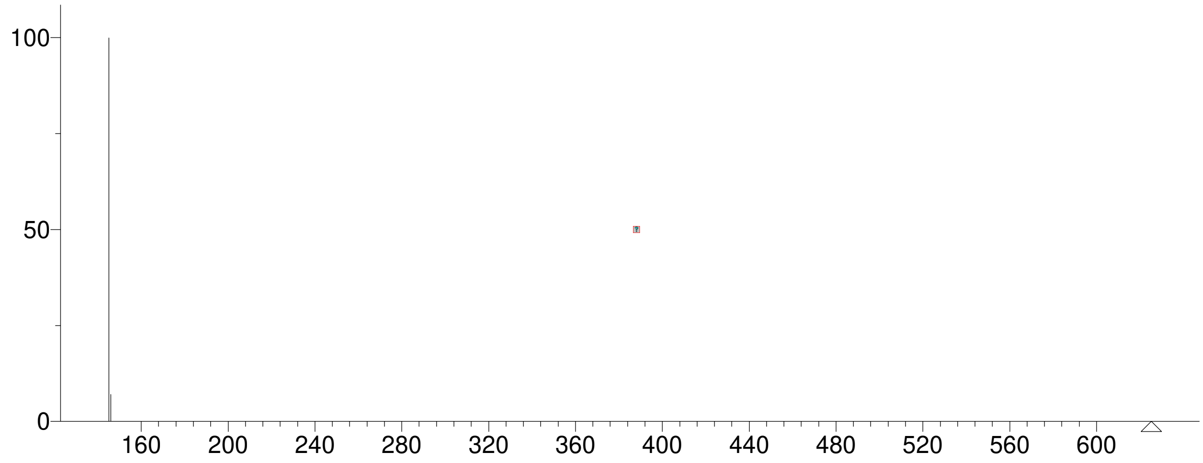

Hit 1 : N-Caffeoyl-O-methyltyramine [2M-H]- HCD 100V P=625.3
C18H19NO4; MF: 0; RMF: 14; Prob 100.0%; CAS: 189307-47-9; Lib: nist_msms; ID: 221342.

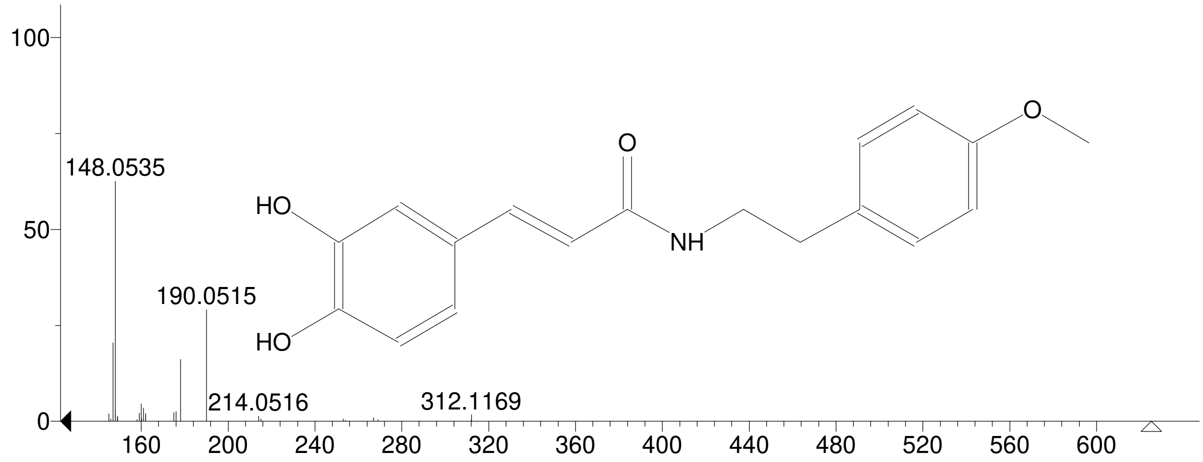

** Search Report Page 1 of 1 **

Unknown: Unknown (3.44_339.0301m/z)
Compound in Library Factor = 0

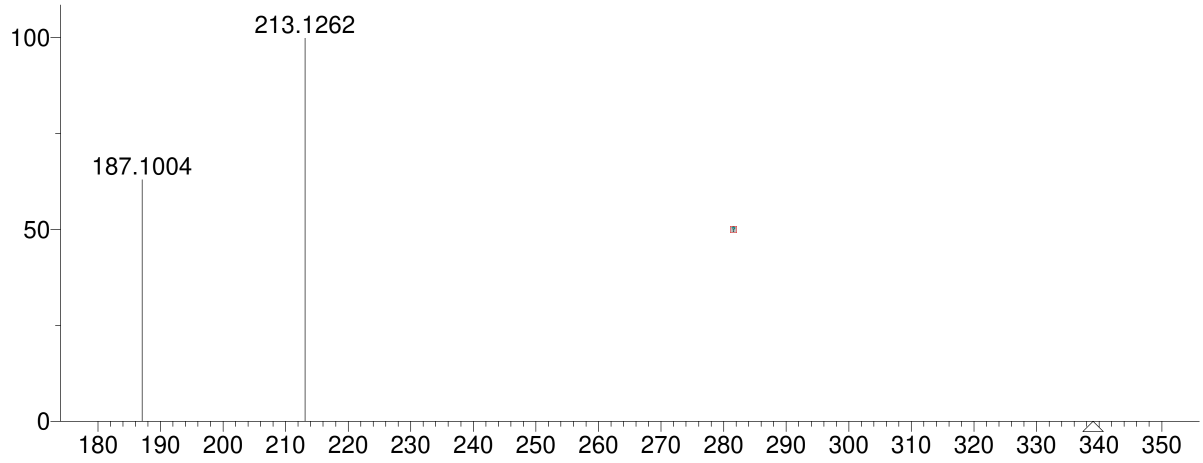

Unknown: Unknown (3.44_341.1440m/z)
Compound in Library Factor = 0

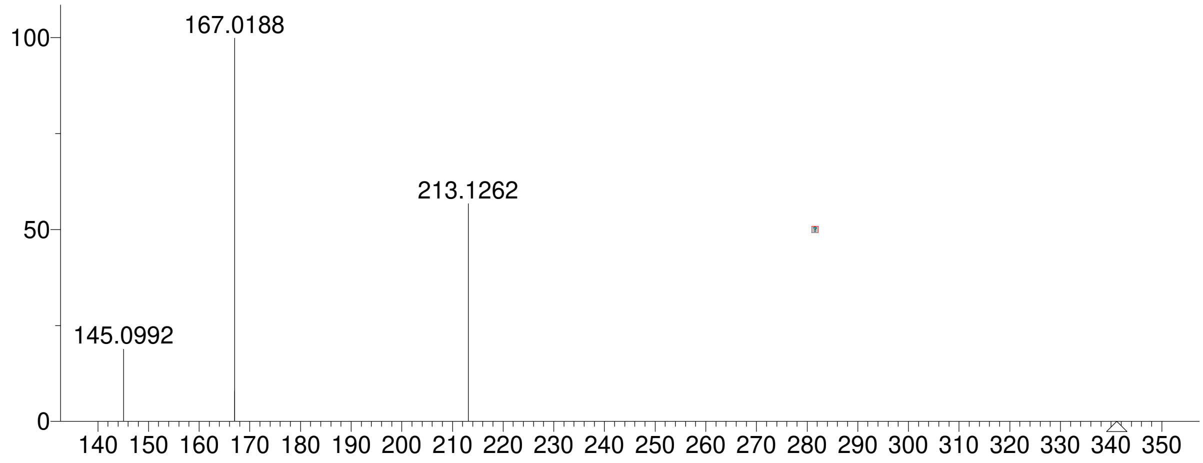

Unknown: Unknown (3.44_388.1752m/z)
Compound in Library Factor = 0

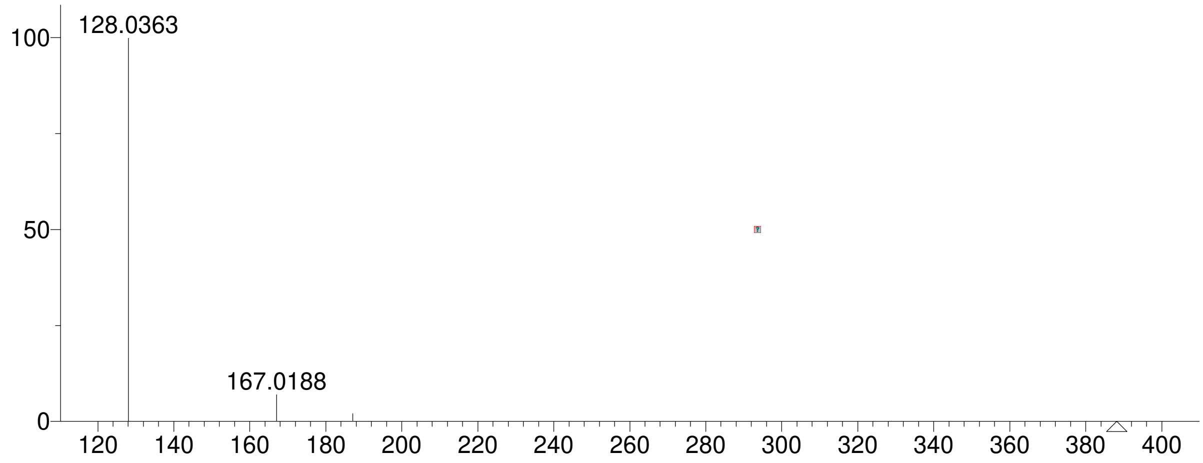

Hit 1 : 5,7-dihydroxy-2-(4-hydroxy-3-methoxyphenyl)-6-(3-methylbut-2-enyl)-2,3-dihydrochromen-4-one [M+NH4]
C21H22O6; MF: 0; RMF: 17; Prob 100.0%; Lib: mona-export-vf-npl_qexactive; ID: 9003.

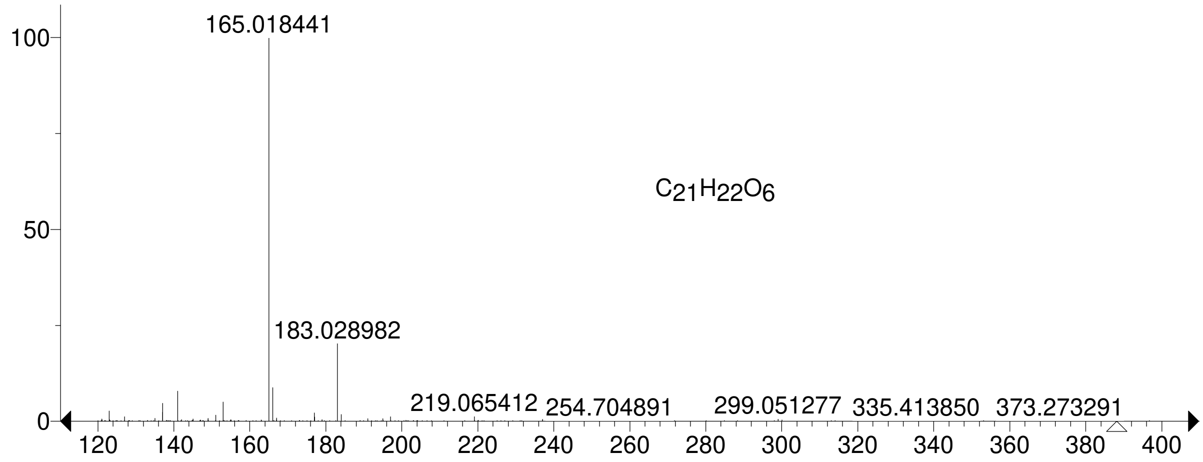

Unknown: Unknown (3.44_445.1454m/z)
Compound in Library Factor = -3273

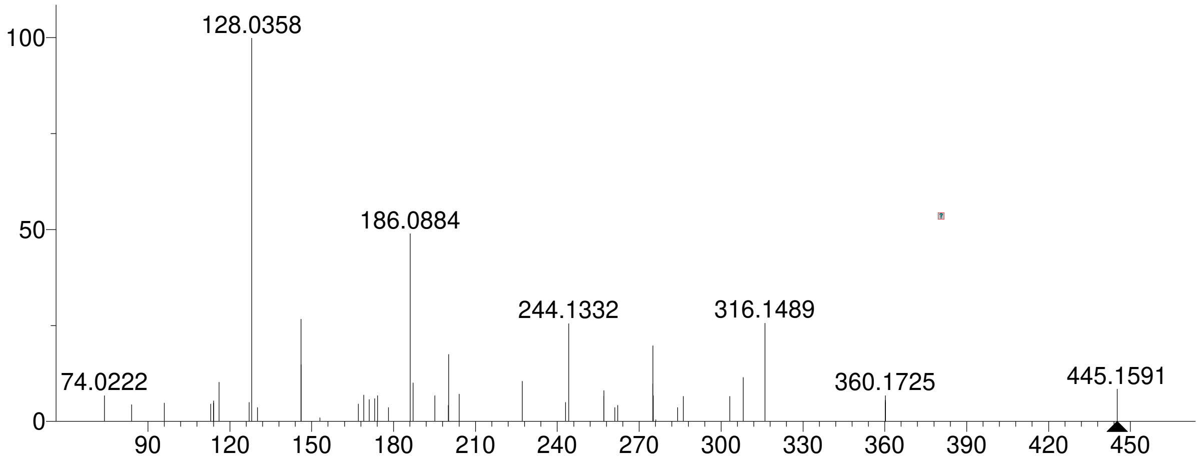

Hit 1 : Acetamiprid [2M+H]+ HCD 1V P=445.1
C10H11ClN4; MF: 9; RMF: 179; Prob 30.6%; CAS: 135410-20-7; Lib: nist_msms; ID: 306855.

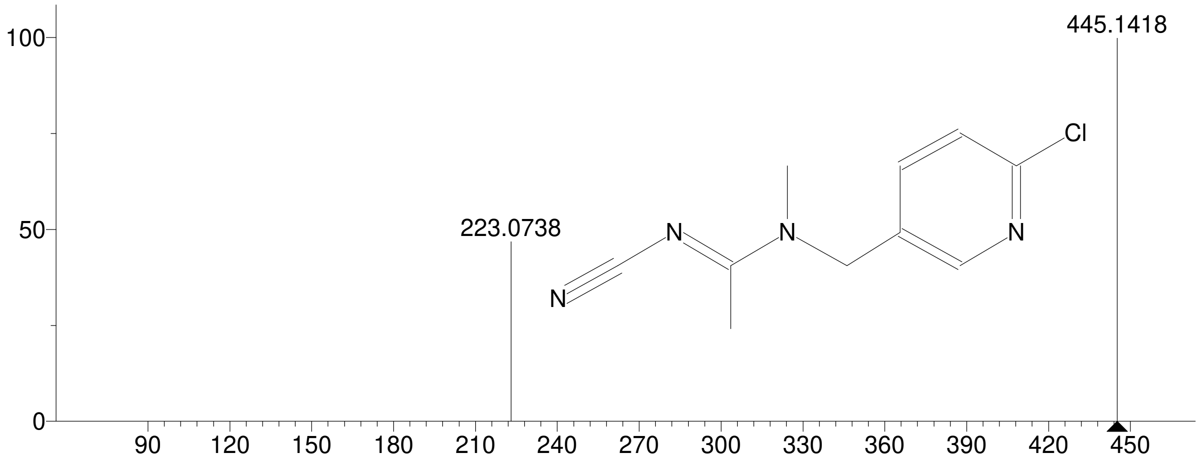

Hit 2 : 1,3-Cyclobutanedicarboxylic acid, 2,4-diphenyl-, 1-(1-naphthalenyl) ester, (1β,2R,3α,4R)- [M+Na]+ HCD
C28H22O4; MF: 3; RMF: 117; Prob 24.1%; CAS: 1541209-75-9; Lib: nist_msms; ID: 349631.

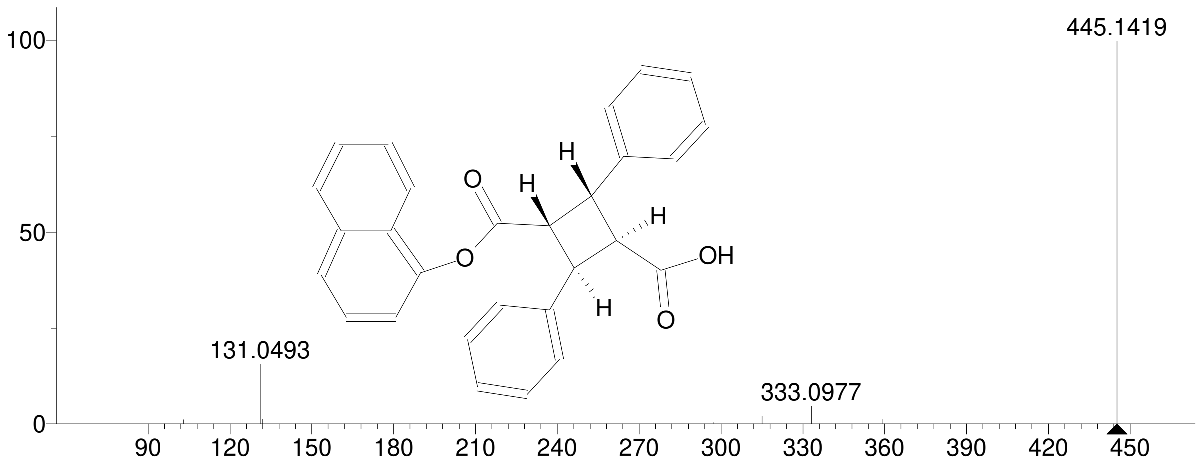

Hit 3 : LPI 7:0; [M-H]- [M-H]- P=445.1
C16H31O12P; MF: 2; RMF: 93; Prob 23.1%; Lib: msdial-insilicomsms-lipids-neg; ID: 311147.

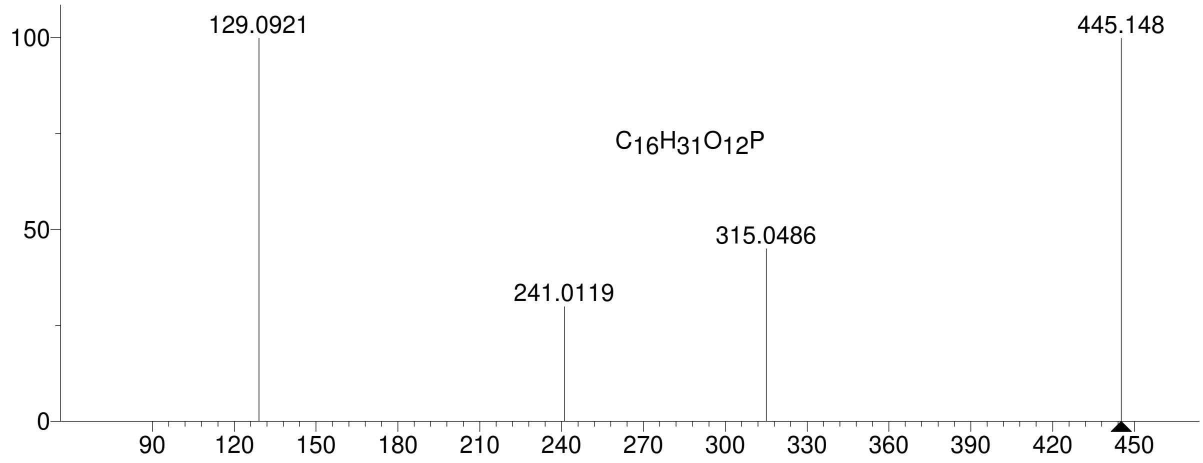

Hit 4 : (1R,3R,4S,6S,8R,9R,10S,11S)-9-(acetyloxy)-8-hydroxy-3,8-dimethyl-12-methylidene-7,13-dioxo-5,14-dioxa
C21H26O9; MF: 1; RMF: 57; Prob 22.2%; Lib: mona-export-vf-npl_qexactive; ID: 6949.

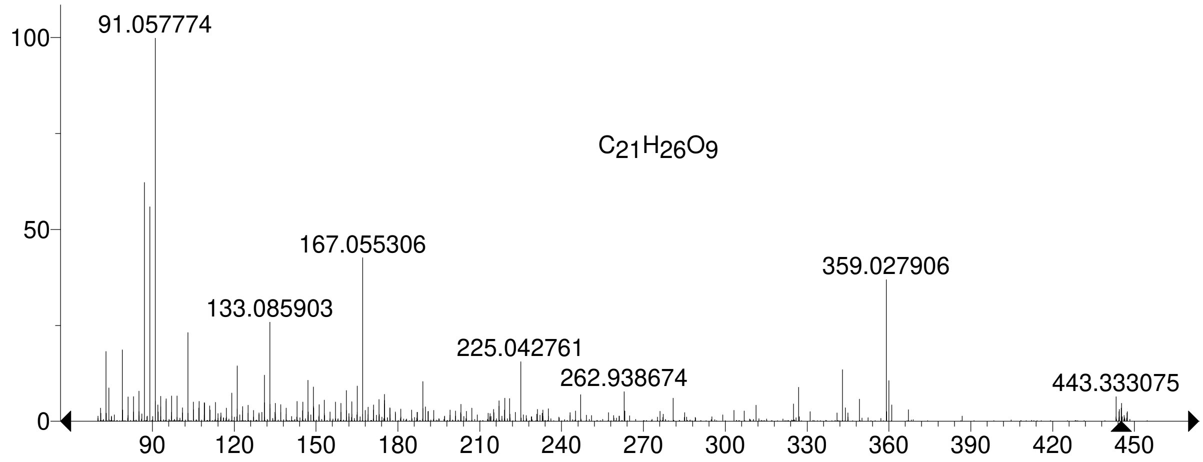

** Search Report Page 1 of 1 **

Unknown: Unknown (3.45_300.1440m/z)
Compound in Library Factor = -1523

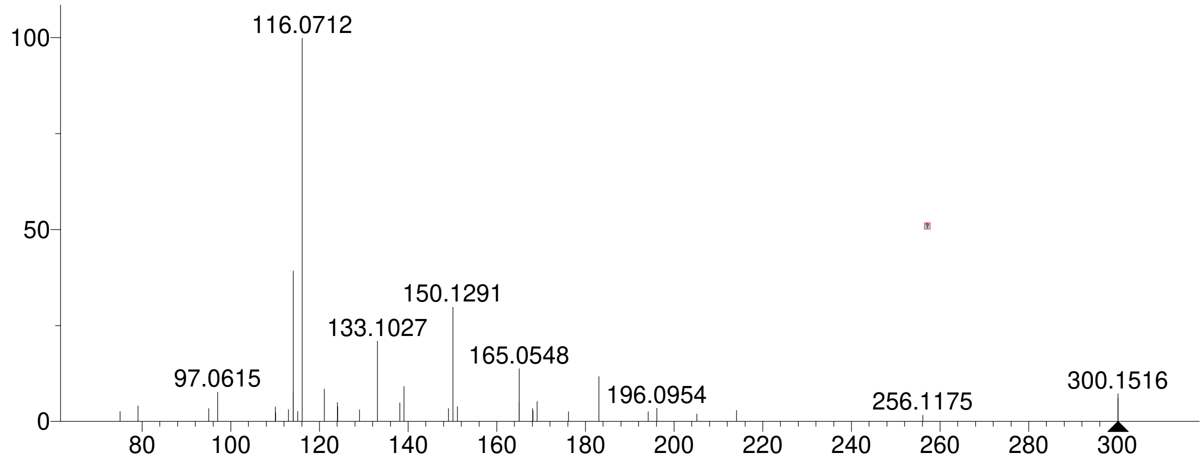

Hit 1 : Hematoporphyrin [M+2H]2+ HCD 8V P=300.1
C34H38N4O6; MF: 42; RMF: 622; Prob 81.4%; CAS: 14459-29-1; Lib: nist_msms; ID: 288998.

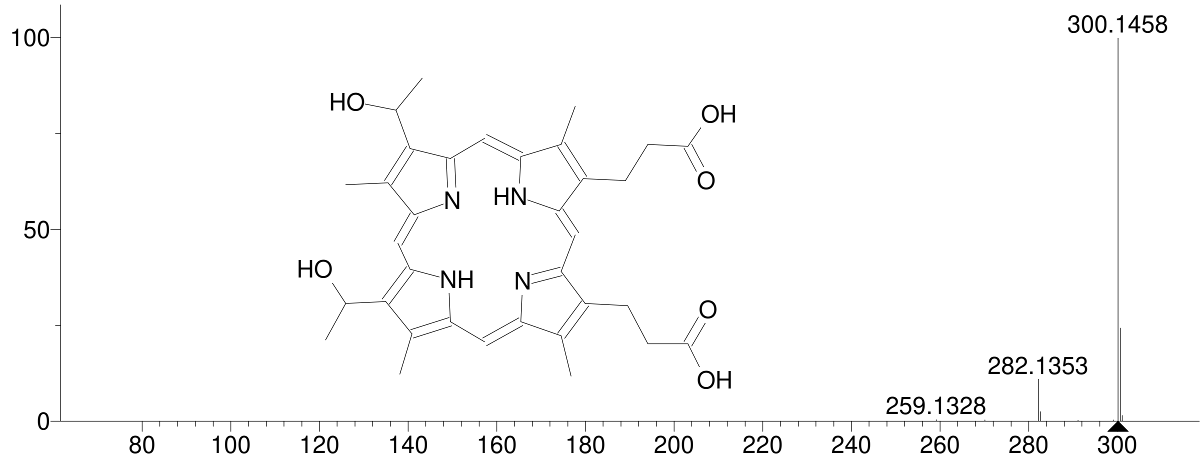

Hit 2 : methyl 3-(3,4-dihydroxy-5-phenyloxolan-2-yl)-3-hydroxypropanoate [M+NH4]+ 65HCD P=300.1
C14H18O6; MF: 1; RMF: 66; Prob 18.6%; Lib: mona-export-vf-npl_qexactive; ID: 24723.

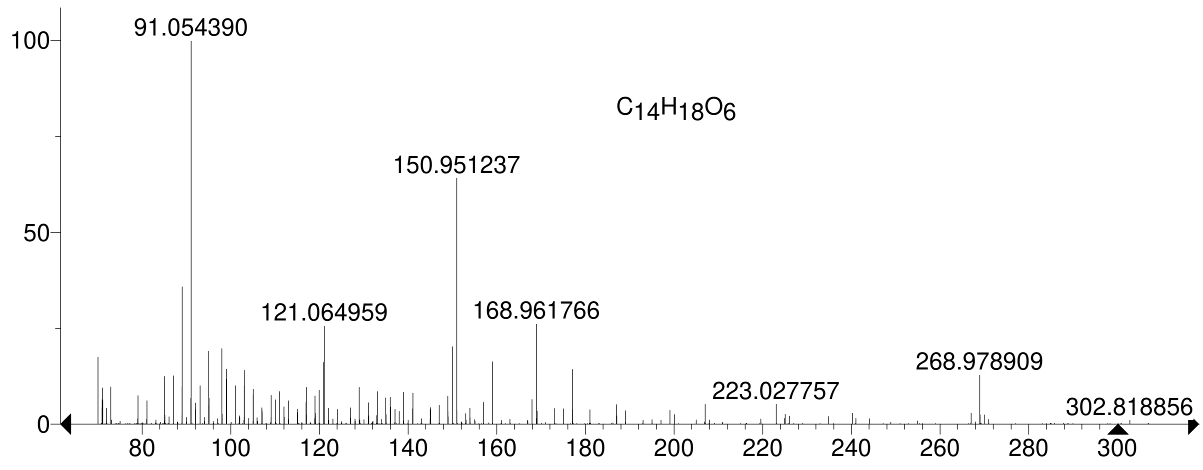

Unknown: Unknown (3.45_308.1107m/z)
Compound in Library Factor = 0

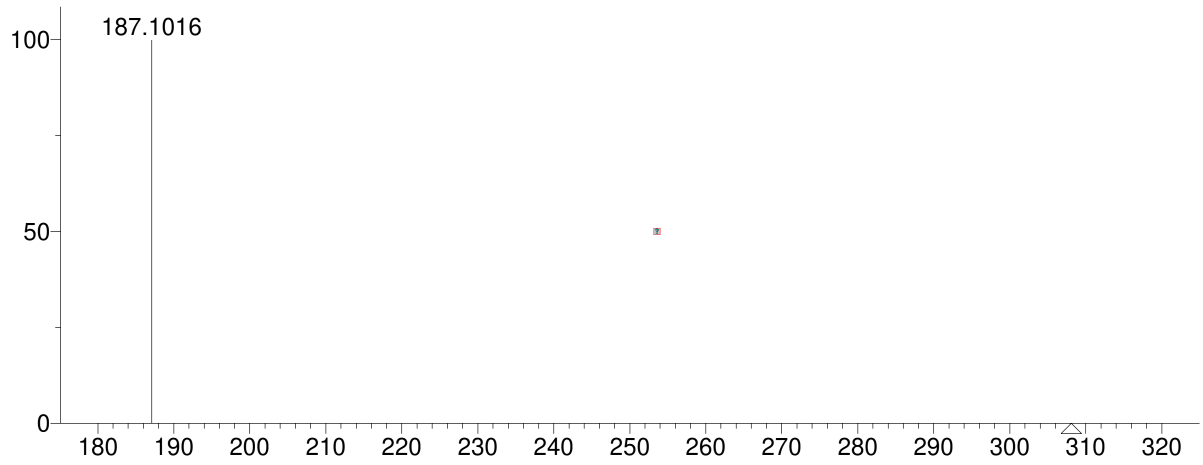

Hit 1 : altenusin [M+NH4]+ 35HCD P=308.1
C15H14O6; MF: 0; RMF: 30; Prob 100.0%; Lib: mona-export-vf-npl_qexactive; ID: 24511.

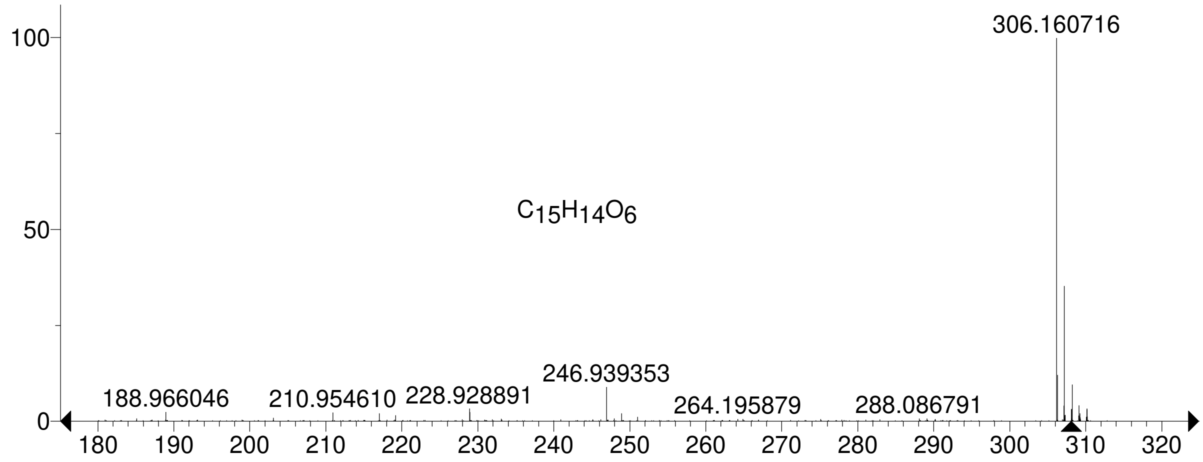

Unknown: Unknown (3.45_450.2091m/z)
Compound in Library Factor = 0

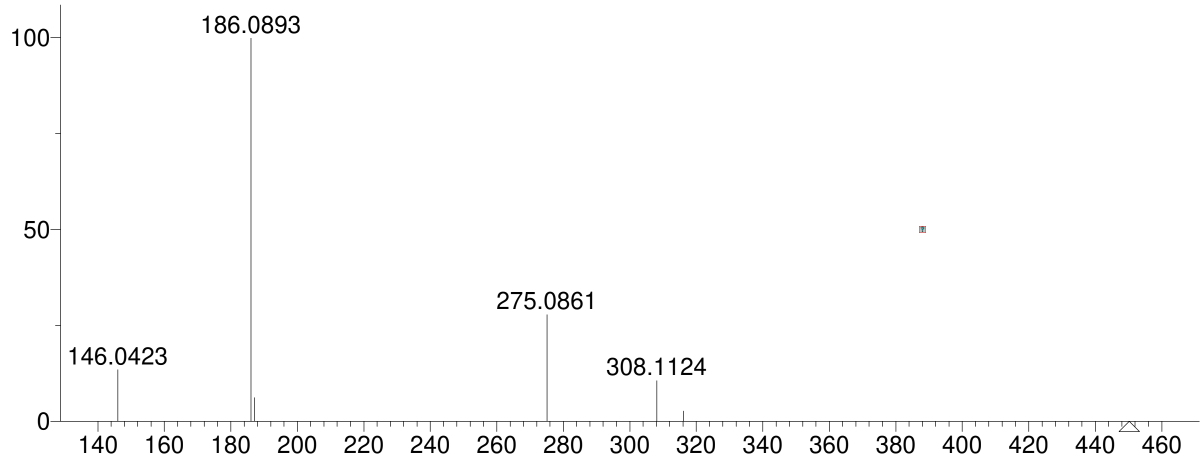

Hit 1 : NCGC00385574-01![5-acetyloxy-1,7-bis(3,4-dihydroxyphenyl)heptan-3-yl] acetate [M+NH4]+ P=450.2
C23H28O8; MF: 0; RMF: 38; Prob 100.0%; Lib: mona-export-gnps; ID: 9703.

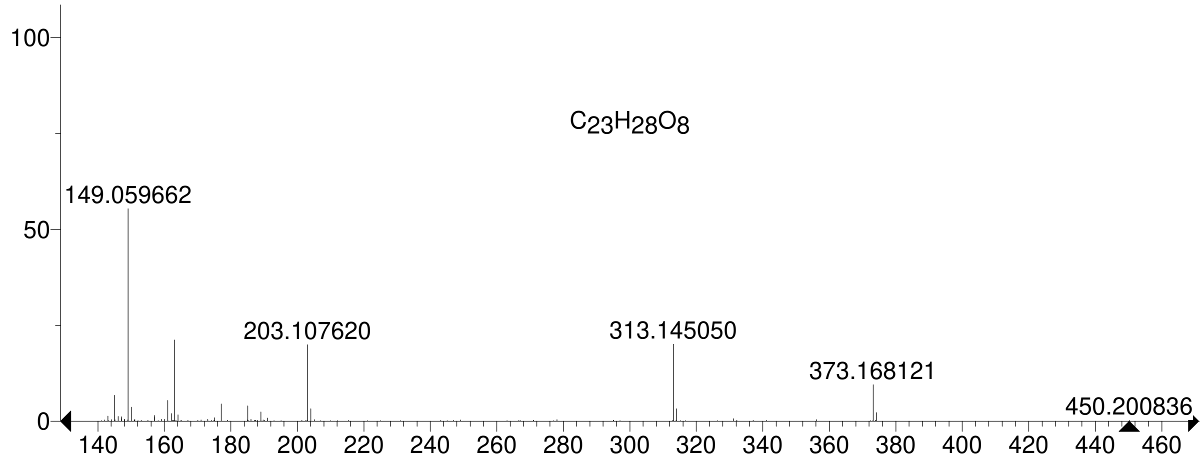

Unknown: Unknown (3.47_434.1644m/z)
Compound in Library Factor = 0

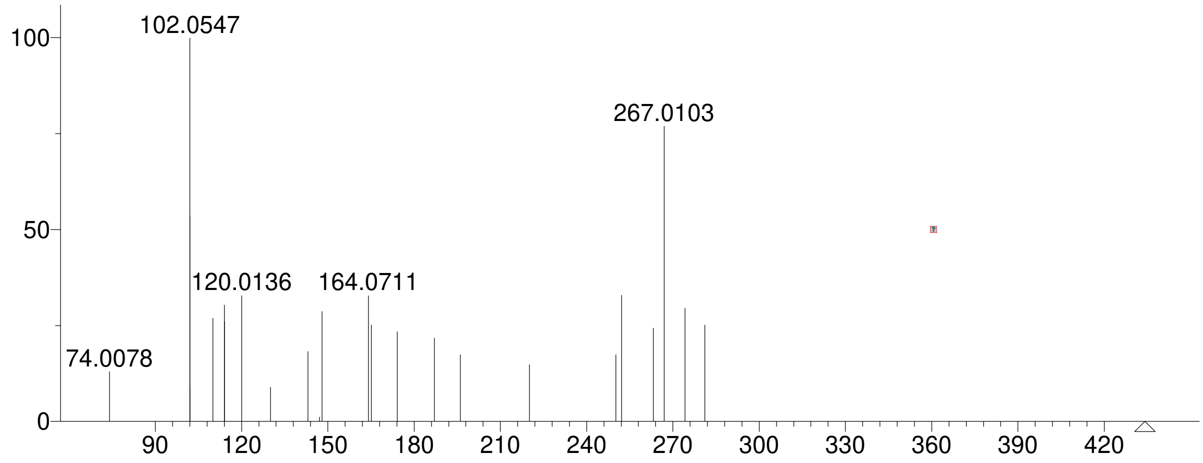

Unknown: Unknown (3.47_450.2062m/z)
Compound in Library Factor = 0

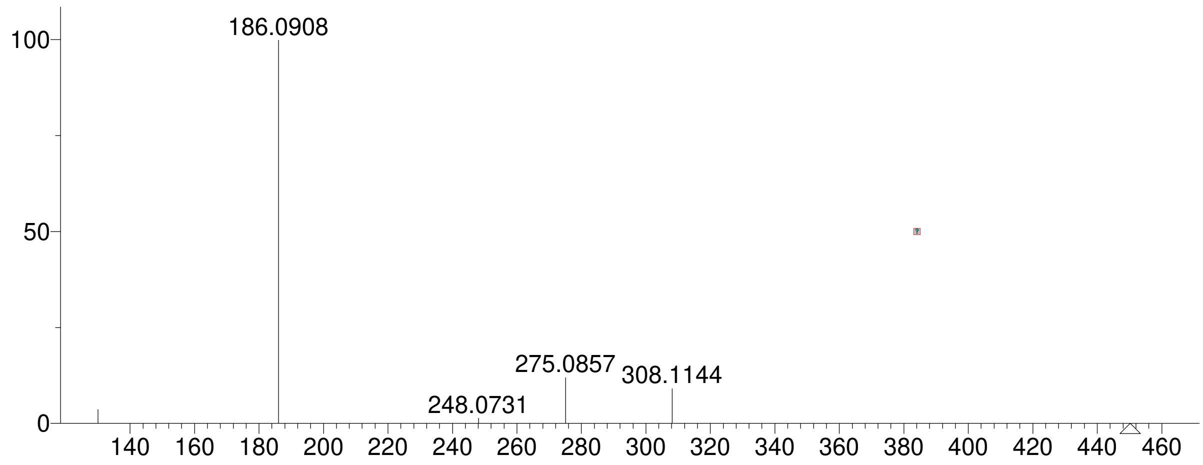

Unknown: Unknown (3.48_279.1333m/z)
Compound in Library Factor = -3273

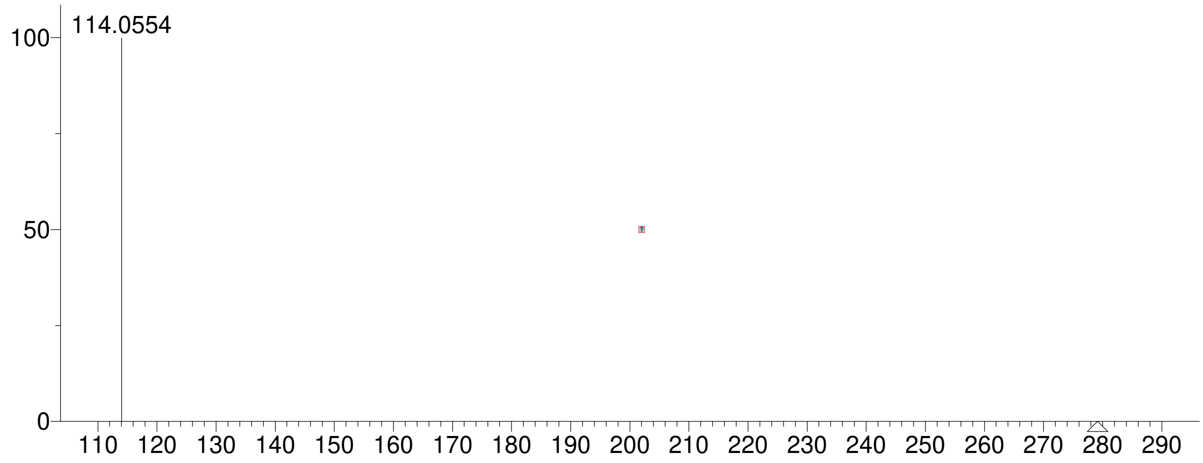

Hit 1 : Tyr-Pro [M+H]+ HCD 22eV P=279.1
C14H18N2O4; MF: 3; RMF: 154; Prob 50.0%; Lib: nist_msms; ID: 561803.

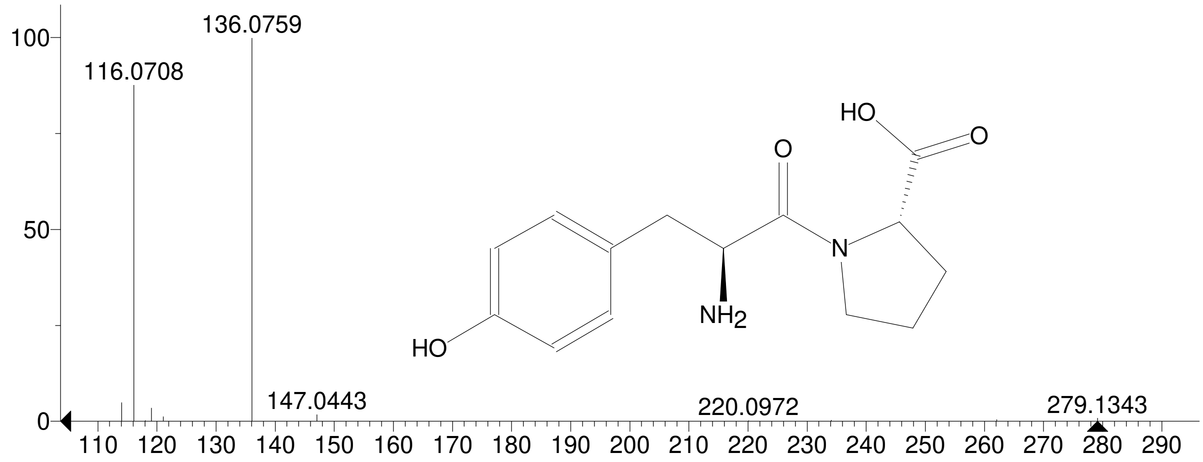

Hit 2 : Tyr-Pro [M+H]+ HCD 22eV P=279.1
C14H18N2O4; MF: 3; RMF: 154; Prob 50.0%; CAS: 51871-47-7; Lib: nist_msms2; ID: 41603.

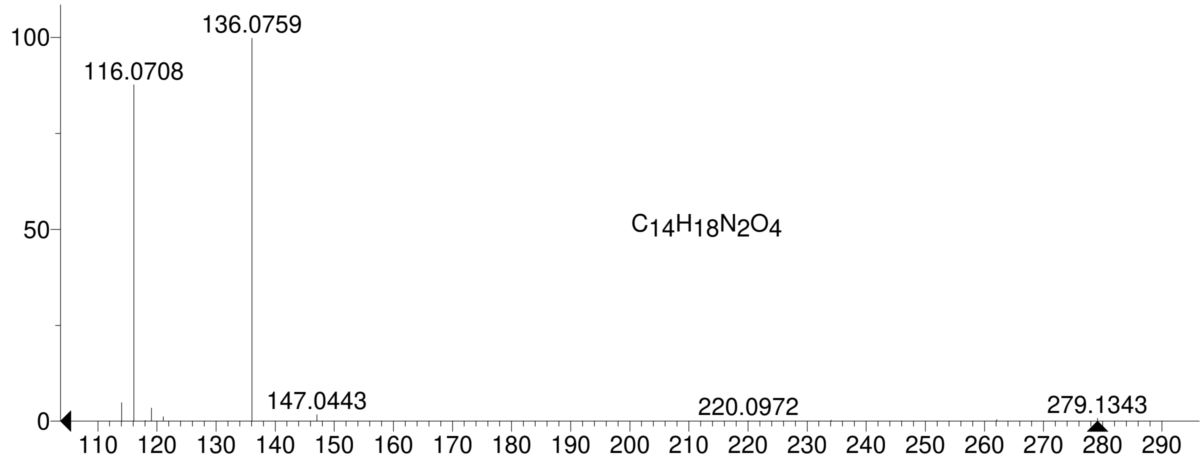

Unknown: Unknown (3.48_375.1382m/z)
Compound in Library Factor = 0

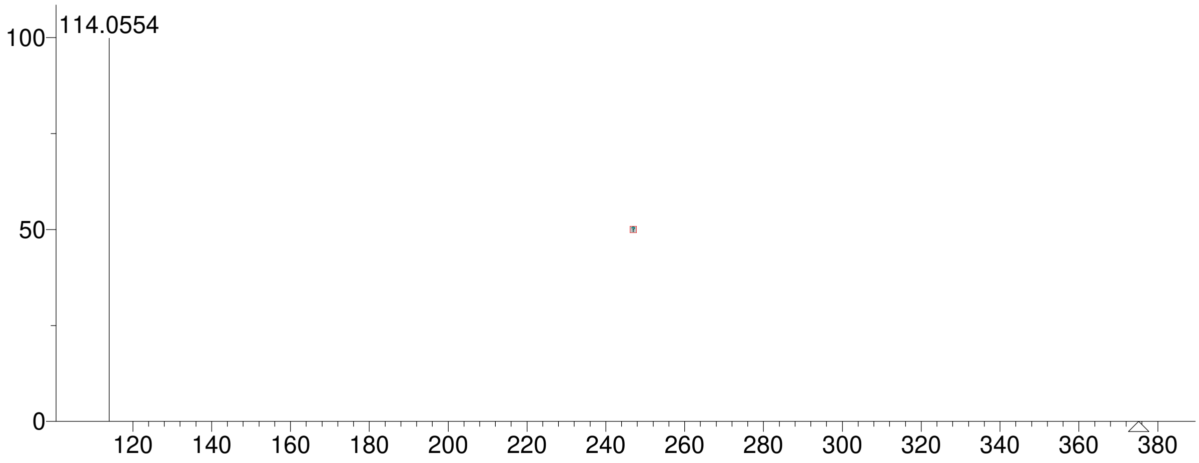

** Search Report Page 1 of 1 **

Unknown: Unknown (3.48_488.1762m/z)
Compound in Library Factor = -3273

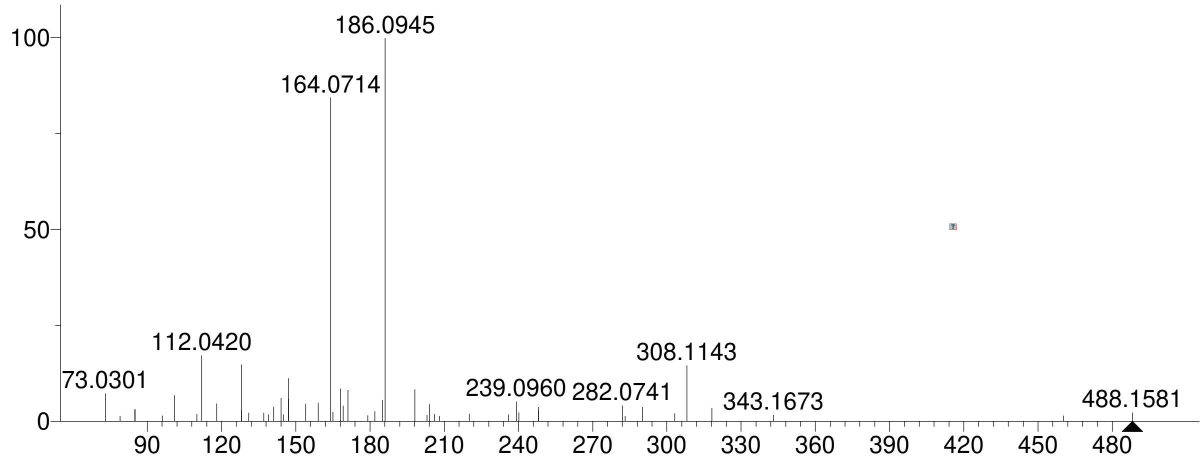

Hit 1 : plumieride [M+NH4]+ 65HCD P=488.2
C21H26O12; MF: 2; RMF: 88; Prob 52.0%; Lib: anthocyanins mona; ID: 8440.

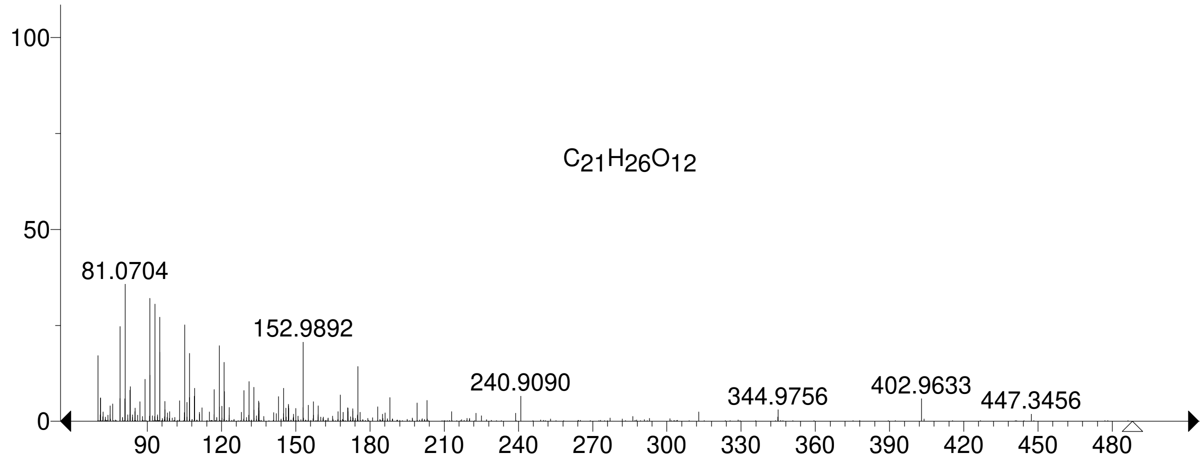

Hit 2 : NCGC00380091-01![2,6-dihydroxy-5-[3,4,5-trihydroxy-6-(hydroxymethyl)oxan-2-yl]oxycyclohex-3-en-1-yl] (
C21H26O12; MF: 0; RMF: 19; Prob 48.0%; Lib: mona-export-gnps; ID: 8124.

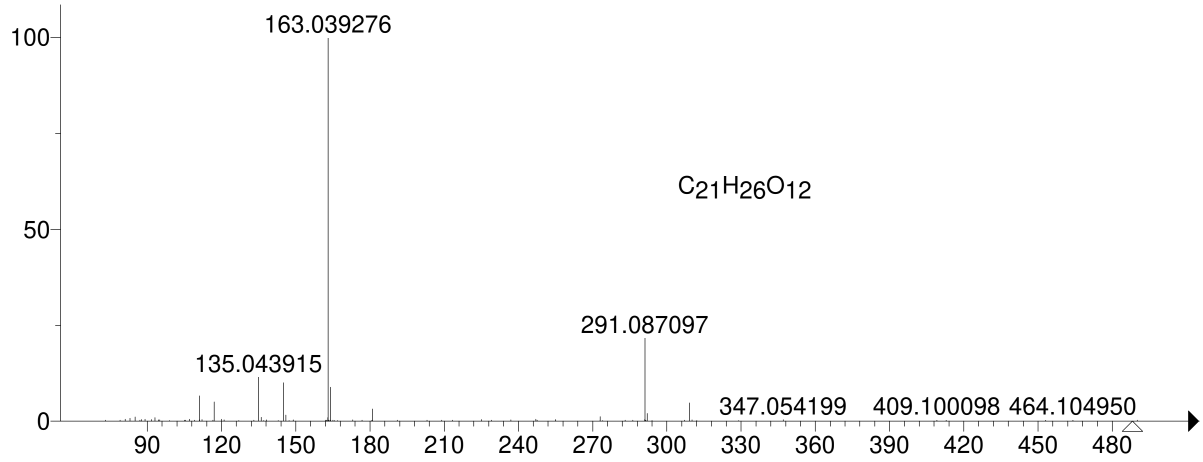

Unknown: Unknown (3.49_571.1260m/z)
Compound in Library Factor = 0

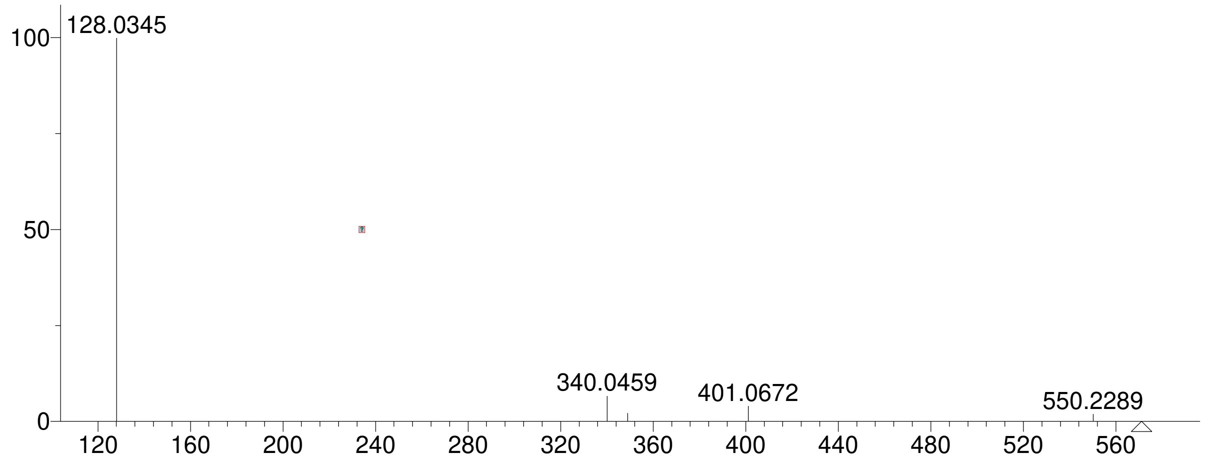

Hit 1 : NCGC00380623-01! [M-H]- P=571.1
C31H24O11; MF: 0; RMF: 79; Prob 50.0%; Lib: mona-export-gnps; ID: 6637.

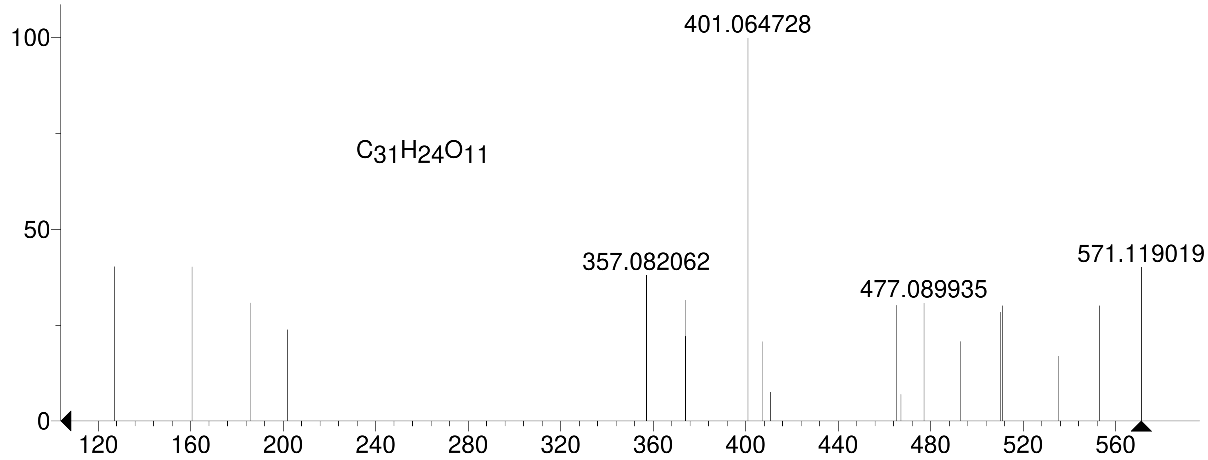

Hit 2 : methyl 8,11,15,20,22-pentahydroxy-24-methyl-13,18,27-trioxo-6-oxaheptacyclo[15.10.2.0A^'1,A^'1a^'1.0A^
C31H24O11; MF: 0; RMF: 32; Prob 50.0%; Lib: mona-export-lc-ms-ms_negative_mode; ID: 23336.

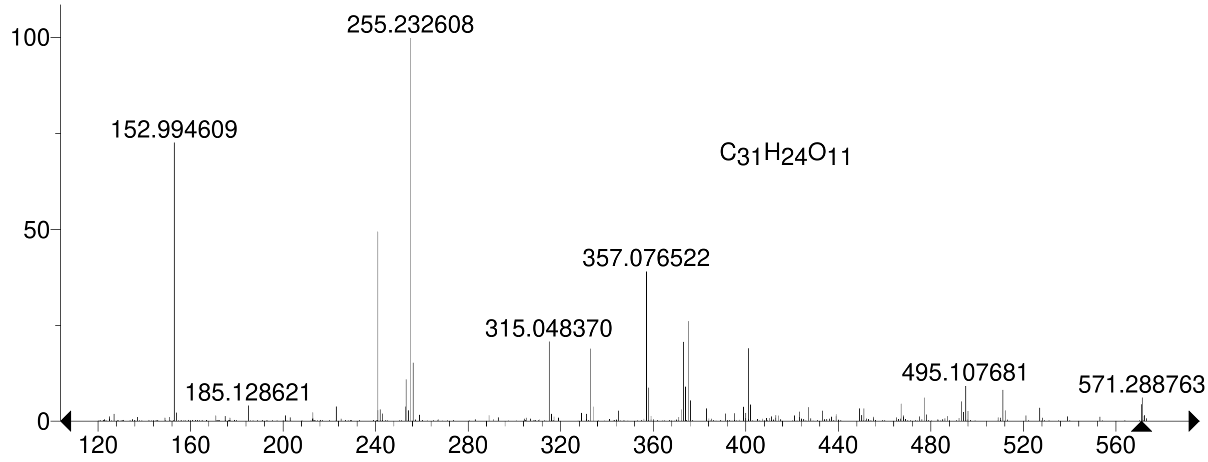

** Search Report Page 1 of 1 **

Unknown: Unknown (3.49_626.1751m/z)
Compound in Library Factor = 0

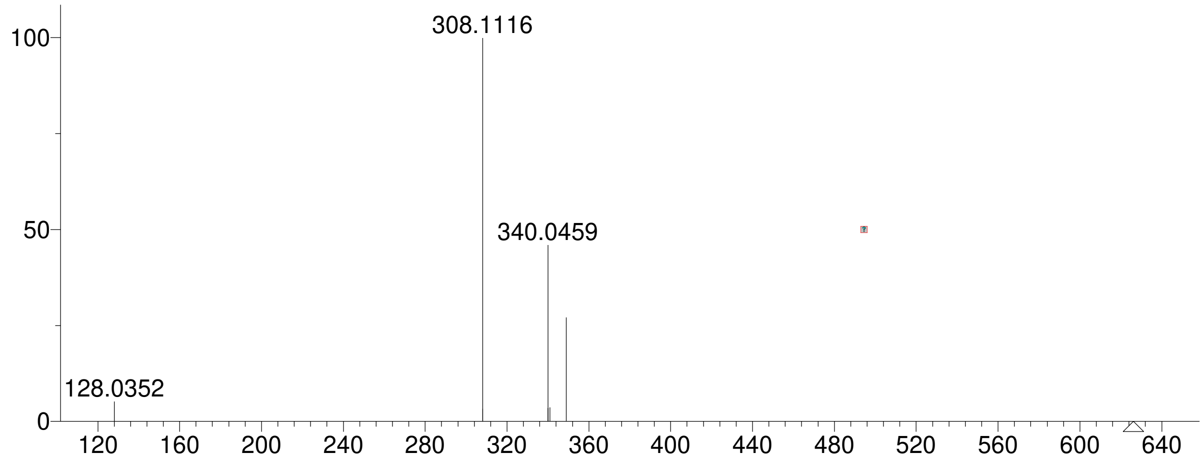

Unknown: Unknown (3.49_650.2290m/z)
Compound in Library Factor = 0

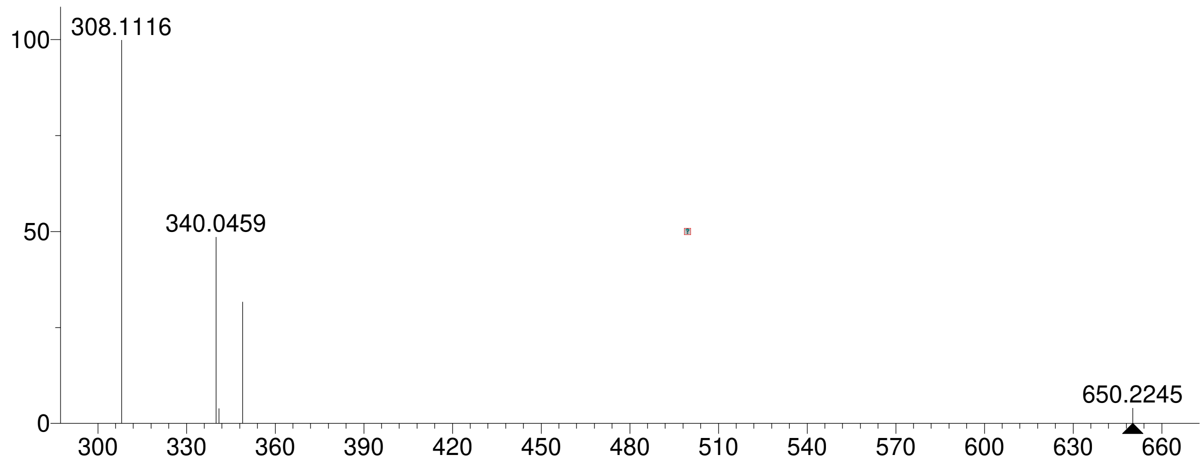

Unknown: Unknown (3.50_333.0811m/z)
Compound in Library Factor = 0

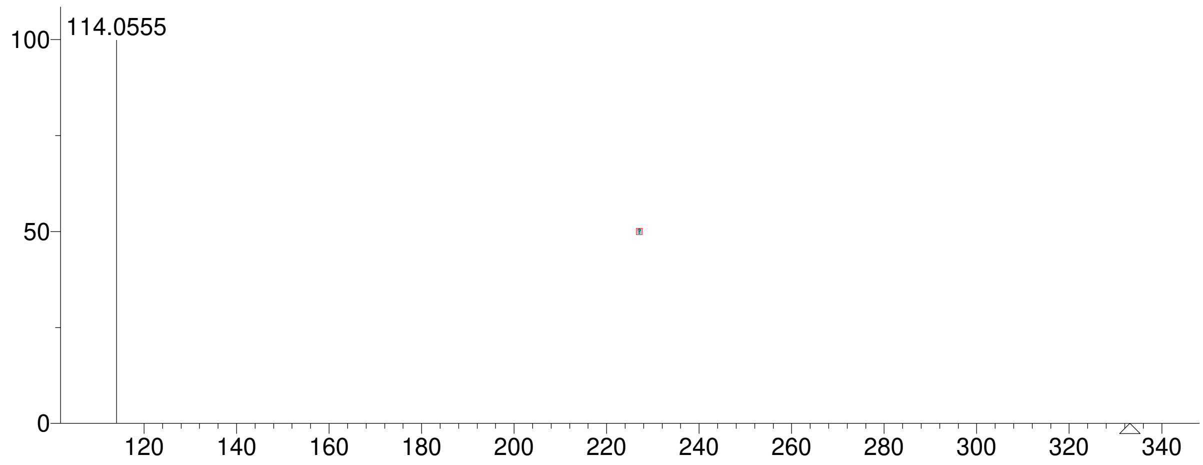

Unknown: Unknown (3.50_550.2247m/z)
Compound in Library Factor = -3273

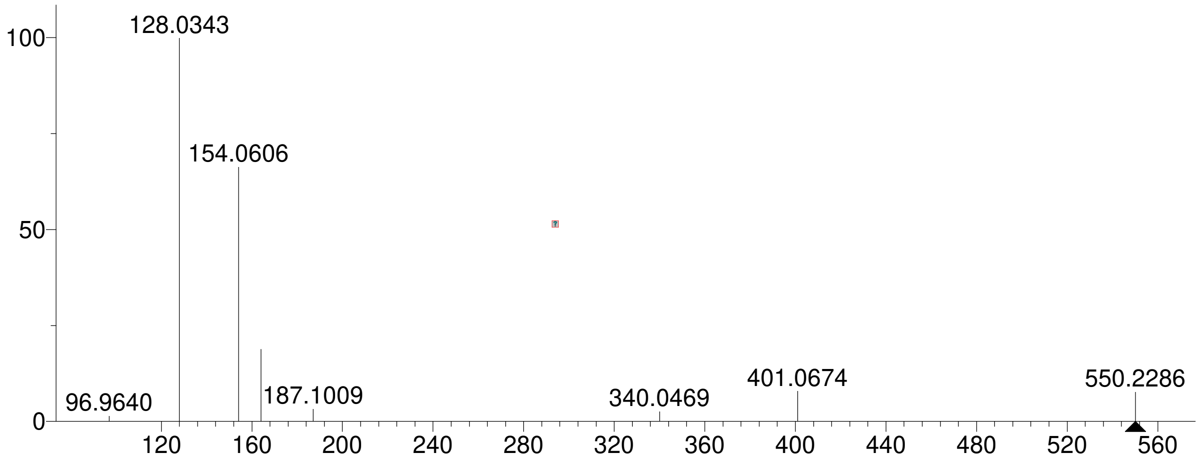

Hit 1 : 1,2-Dioleoyl-sn-glycero-3-phospho-(1'-myo-inositol-3',4',5'-trisphosphate) [M-2H]2- HCD 12V P=550.2
C45H86O22P4; MF: 4; RMF: 152; Prob 51.0%; CAS: 207603-22-3; Lib: nist_msms; ID: 102908.

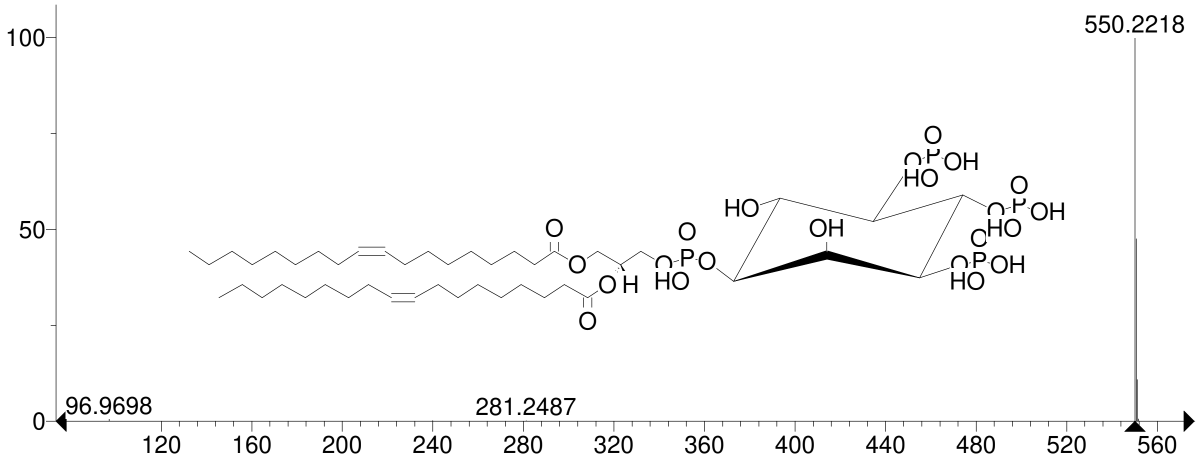

Hit 2 : Hygromycin B [M+Na]+ HCD 44V P=550.2
C20H37N3O13; MF: 3; RMF: 226; Prob 49.0%; CAS: 31282-04-9; Lib: nist_msms; ID: 373748.

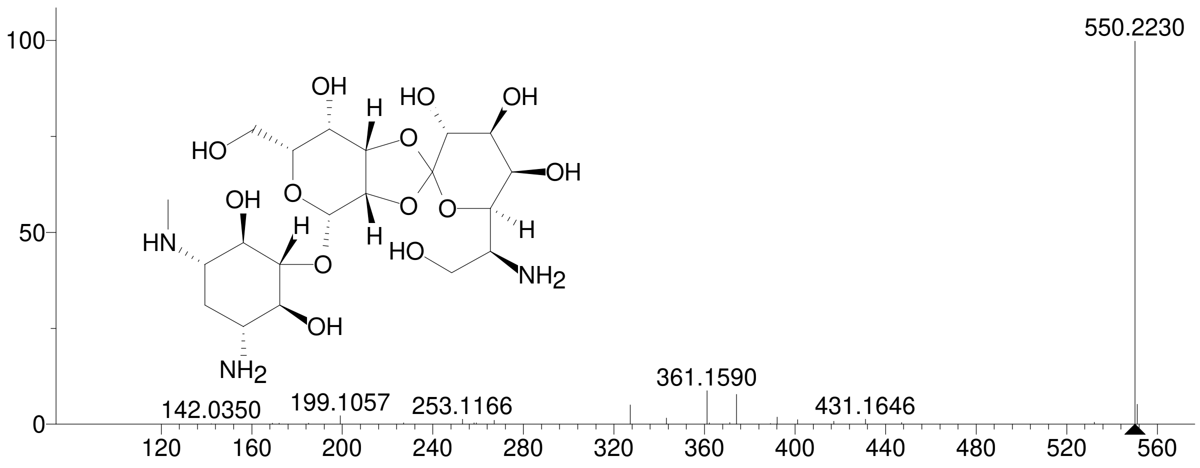

Unknown: Unknown (3.51_427.0490m/z)
Compound in Library Factor = 0

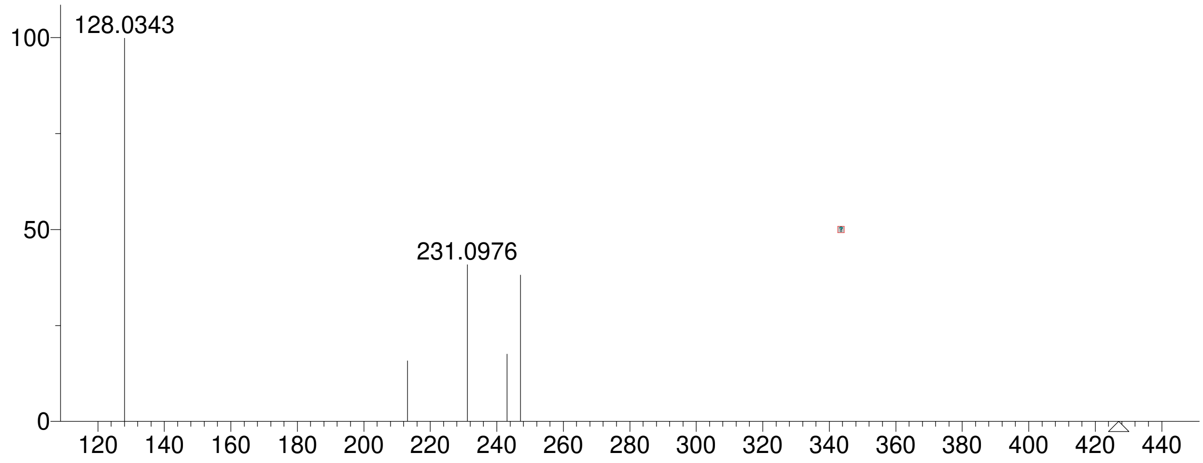

Unknown: Unknown (3.52_463.1011m/z)
Compound in Library Factor = -3273

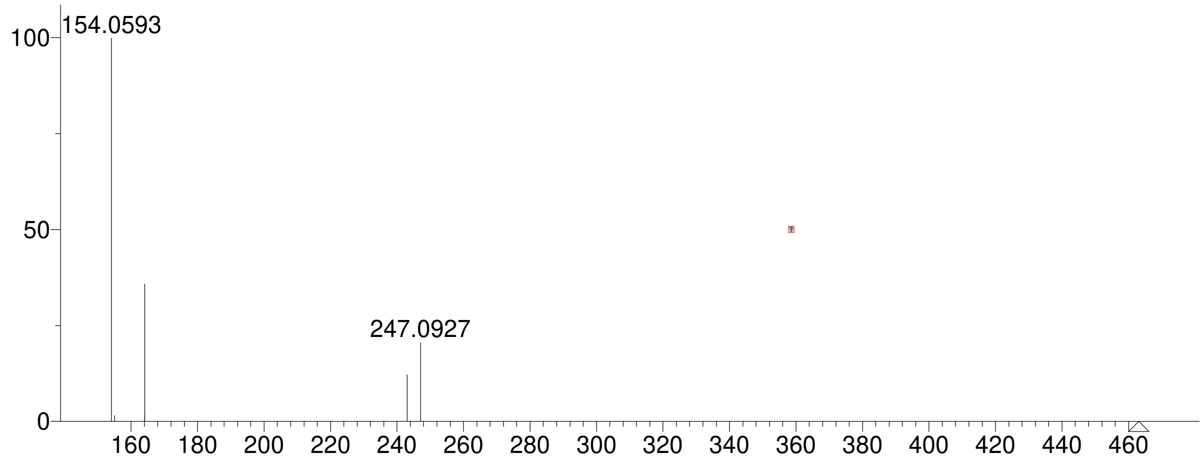

Hit 1 : 2,3-Dehydrosilybin A [M+H-H2O]+ HCD 148V P=463.1
C25H20O10; MF: 1; RMF: 126; Prob 6.91%; CAS: 25166-14-7; Lib: nist_msms; ID: 386721.

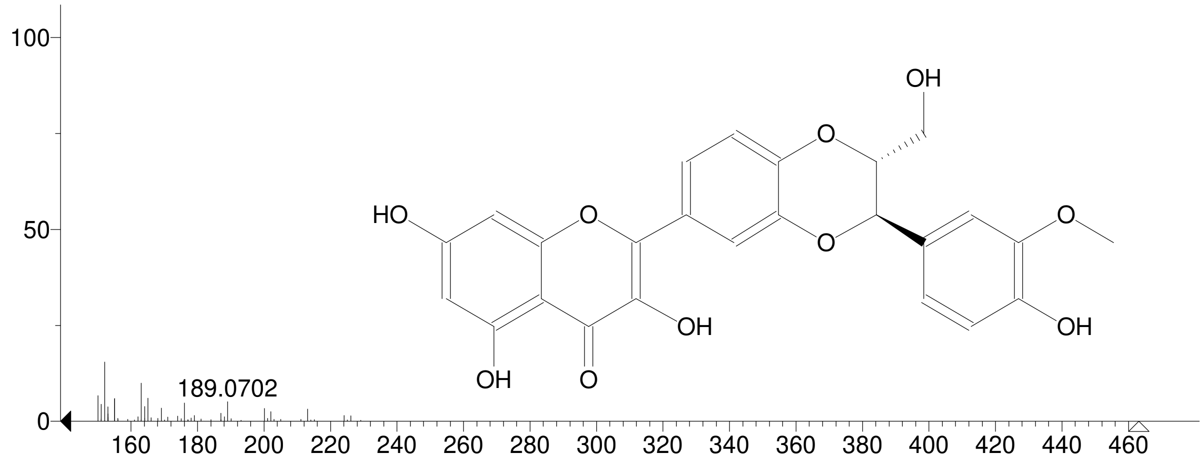

Hit 2 : Rutarin [M+K]+ 65HCD P=463.1
C20H24O10; MF: 0; RMF: 27; Prob 6.64%; Lib: anthocyanins mona; ID: 6621.

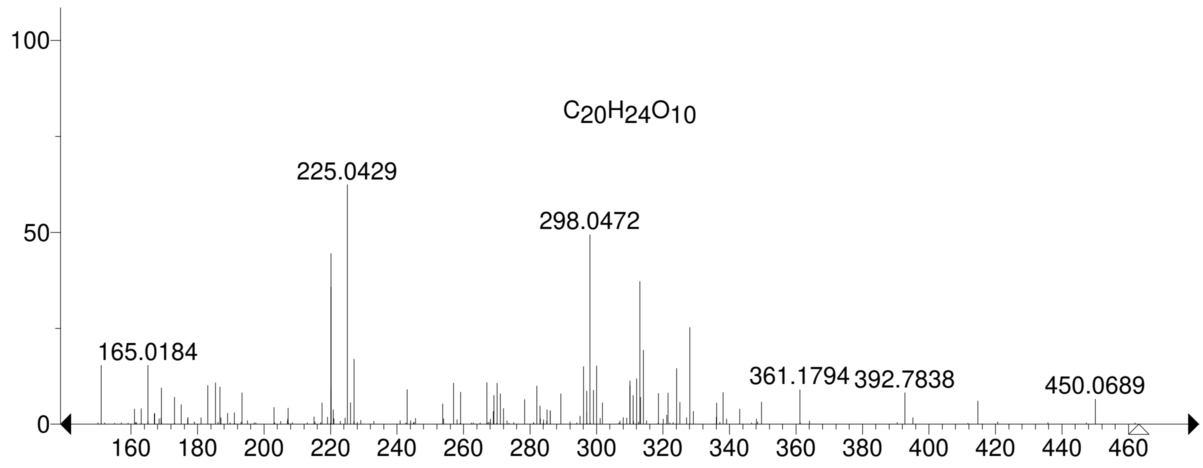

Hit 3 : Delphinidin-3,5-diglucoside cation [Cat-2H]-=>463.1 IT 35% P=463.1
C27H31O17; MF: 0; RMF: 18; Prob 6.64%; CAS: 47851-70-7; Lib: nist_msms; ID: 501126.

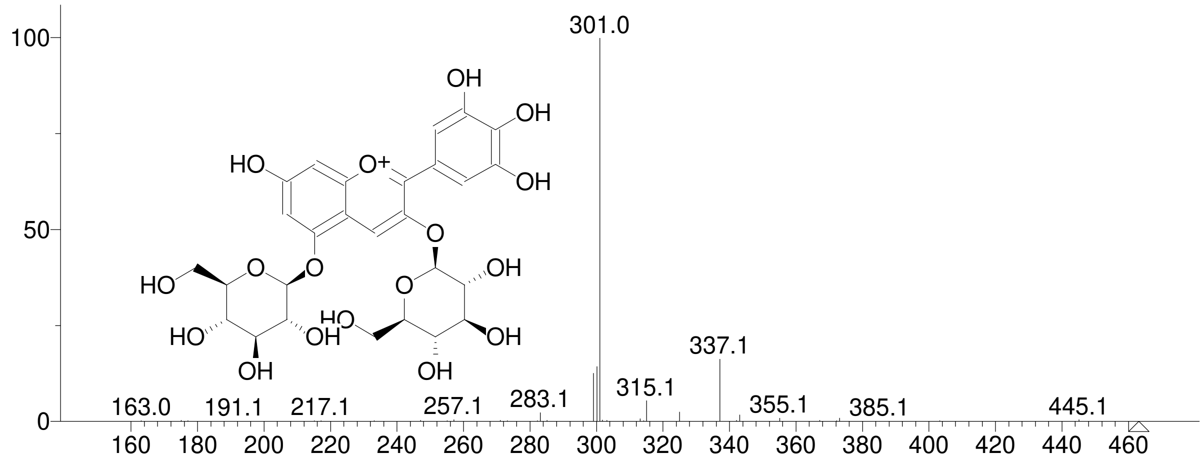

Hit 4 : Silydianin [M-H-H2O]- IT-FT 35% P=463.1
C25H22O10; MF: 0; RMF: 13; Prob 6.64%; CAS: 29782-68-1; Lib: nist_msms; ID: 233871.

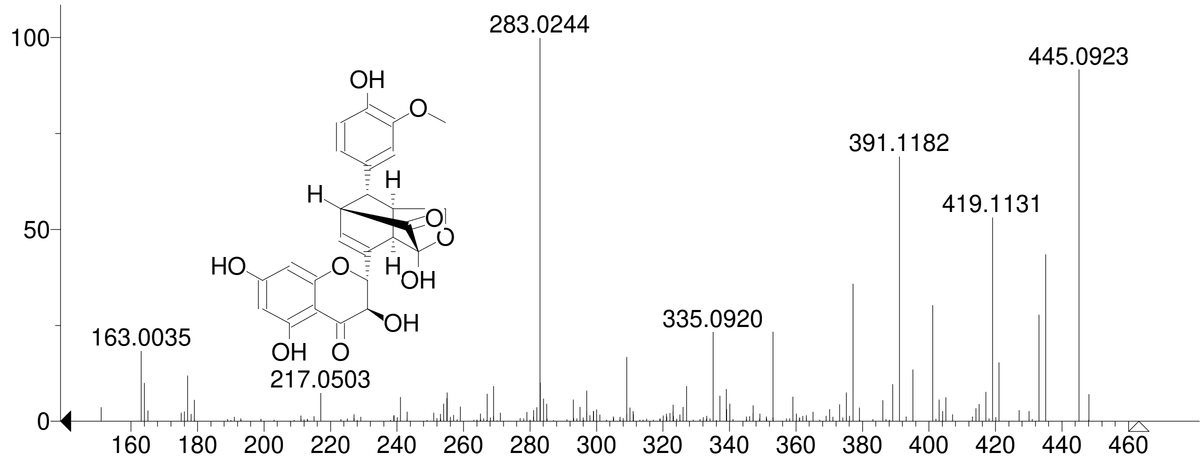

Hit 5 : 2-(2-hydroxypropan-2-yl)-3-[(2R,3R,4S,5S,6R)-3,4,5-trihydroxy-6-(hydroxymethyl)oxan-2-yl]oxy-2,3-dihydrc
C20H24O10; MF: 0; RMF: 11; Prob 6.64%; Lib: mona-export-vf-npl_qexactive; ID: 1788.

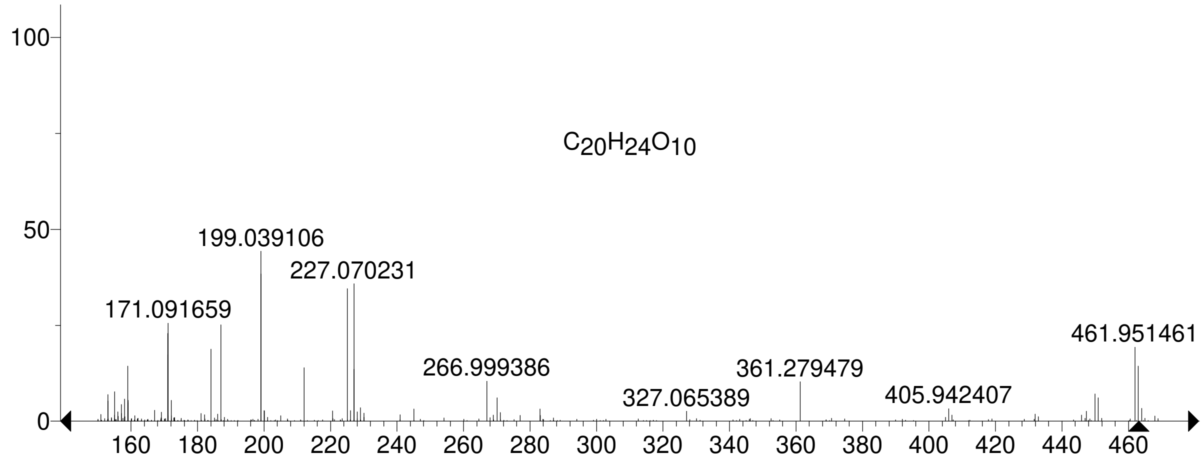

Unknown: Unknown (3.53_417.1497m/z)
Compound in Library Factor = 0

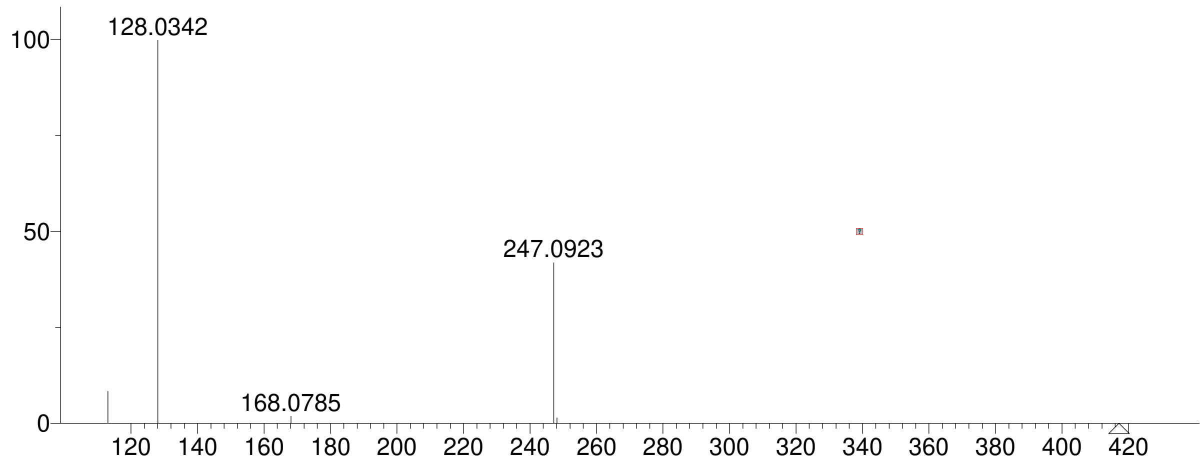

Hit 1 : Halopemide [M+H]+ HCD 133V P=417.1
C21H22ClFN4O2; MF: 0; RMF: 58; Prob 100.0%; CAS: 59831-65-1; Lib: nist_msms; ID: 321102.

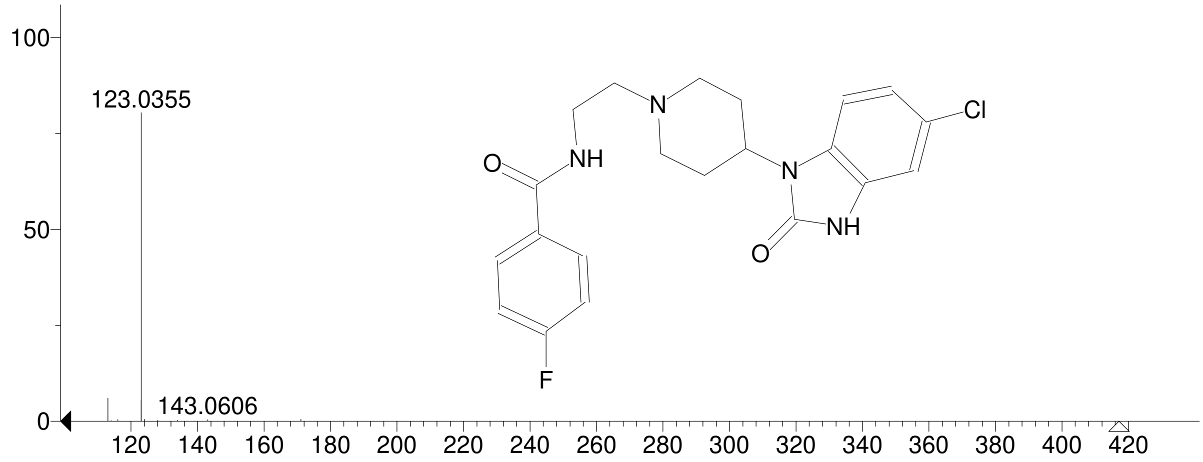

Unknown: Unknown (3.54_222.0757m/z)
Compound in Library Factor = 0

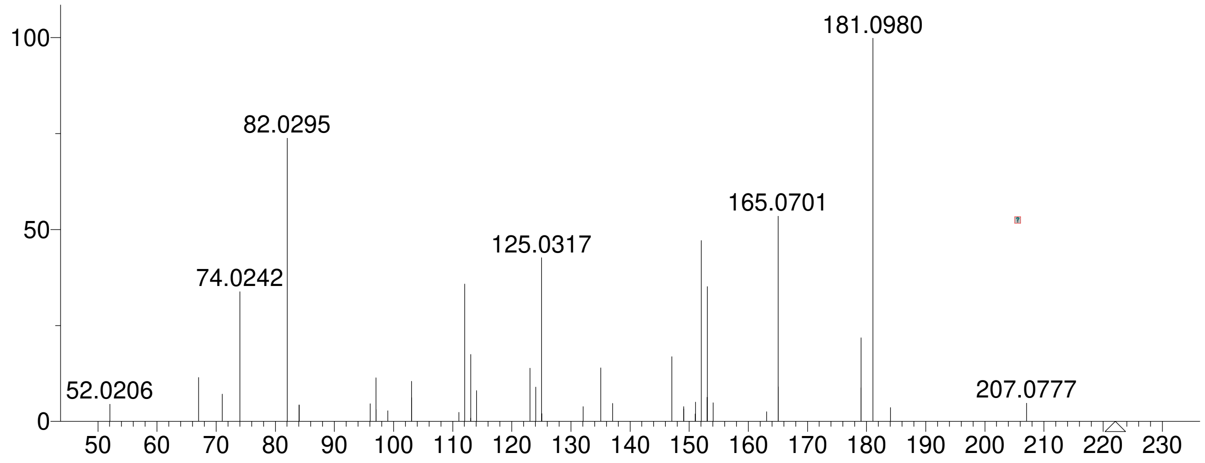

Hit 1 : N-Acetyl-L-tyrosine [M-H]- HCD 35V P=222.1
C11H13NO4; MF: 0; RMF: 32; Prob 33.3%; CAS: 537-55-3; Lib: nist_msms; ID: 280196.

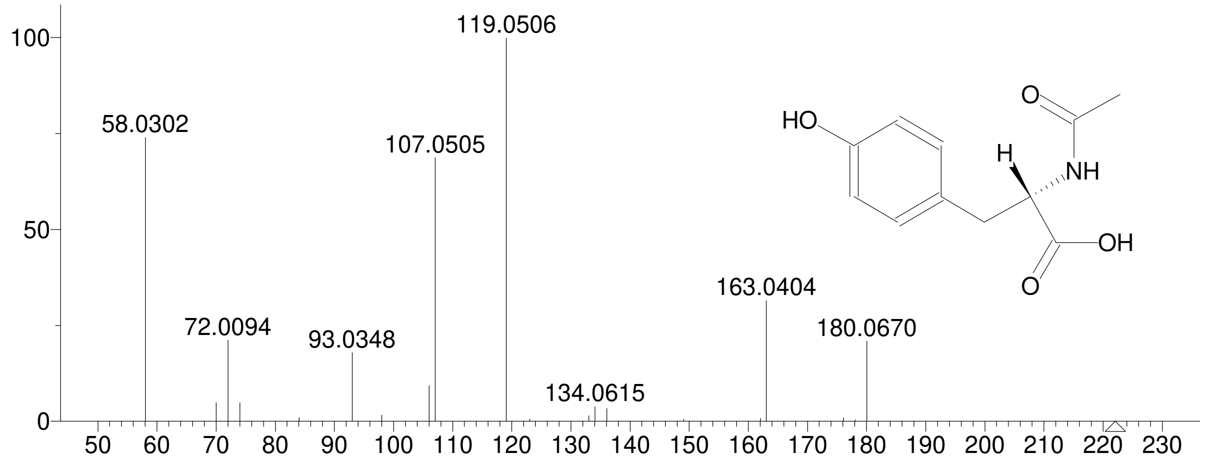

Hit 2 : NCGC00381349-01_C11H13NO5_1H-Pyrrole-3-propanoic acid, 5-acetyl-4-(carboxymethyl)- [M-H2O+H]+
C11H13NO5; MF: 0; RMF: 26; Prob 33.3%; Lib: mona-export-gnps; ID: 11085.

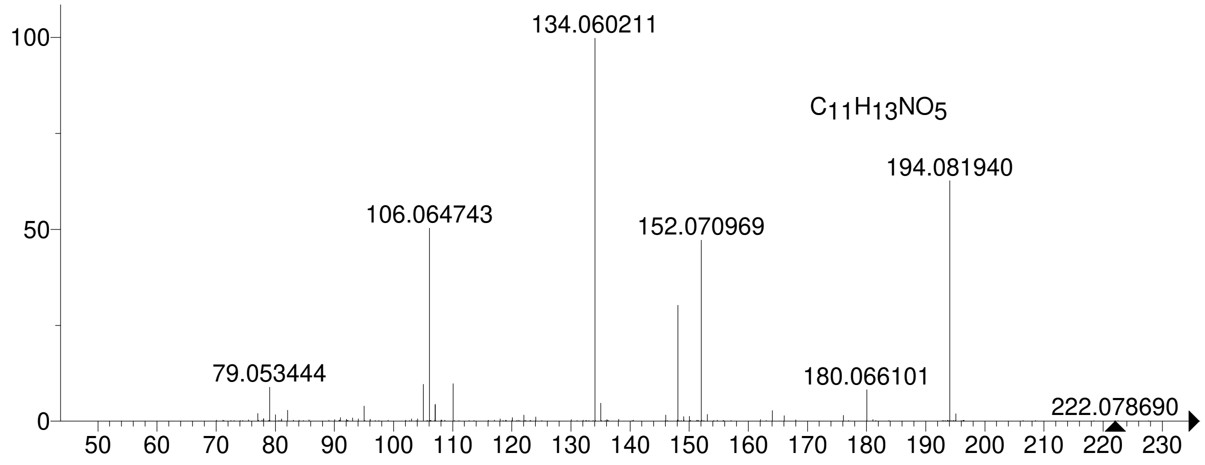

Hit 3 : N-Benzoylaminopurine [M+H-H2O]+ HCD 15V P=222.1
C12H9N5O; MF: 0; RMF: 23; Prob 33.3%; CAS: 4005-49-6; Lib: nist_msms; ID: 144559.

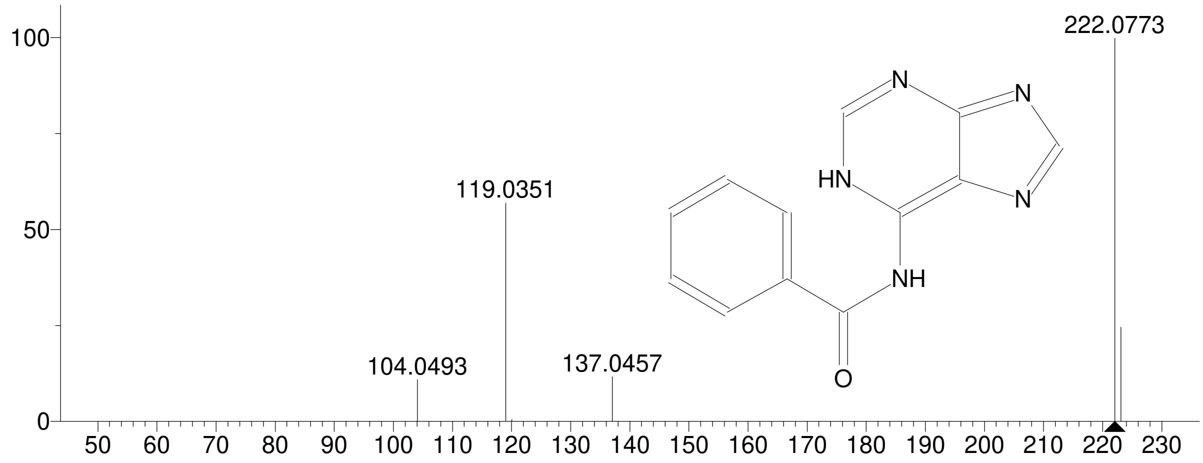

Unknown: Unknown (3.54_232.9748m/z)
Compound in Library Factor = 0

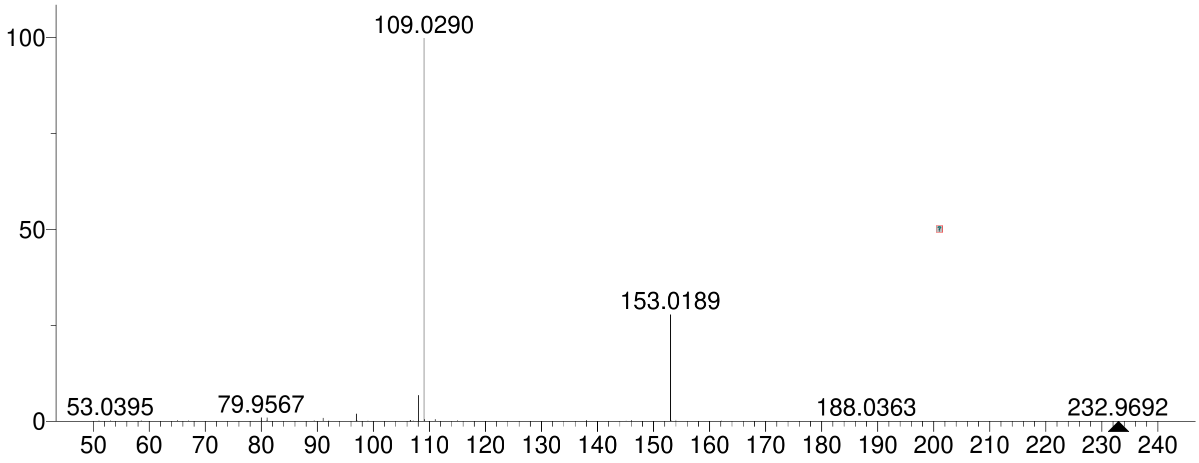

Unknown: Unknown (3.54_346.1590m/z)
Compound in Library Factor = 0

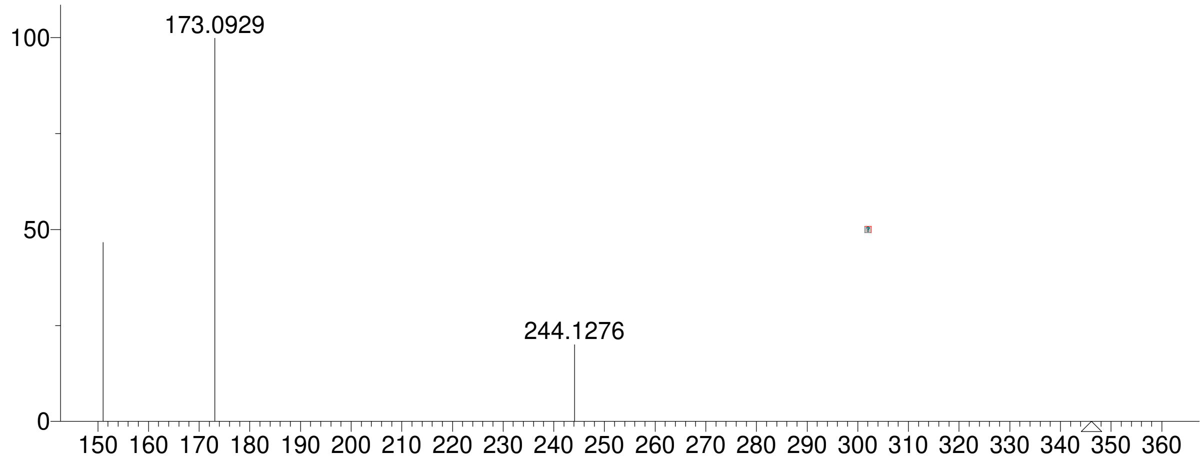

** Search Report Page 1 of 1 **

Unknown: Unknown (3.54_478.2388m/z)
Compound in Library Factor = 0

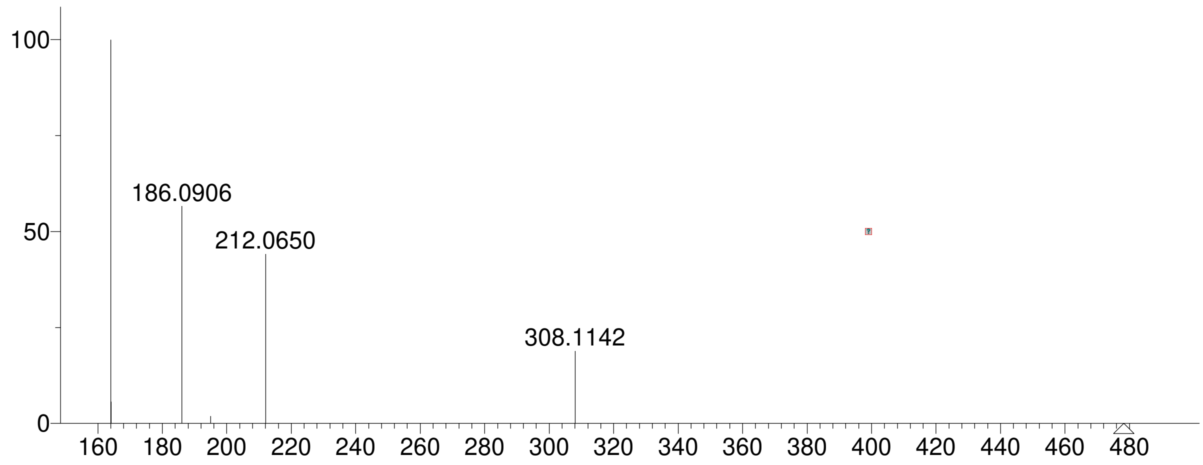

Unknown: Unknown (3.54_502.1686m/z)
Compound in Library Factor = 0

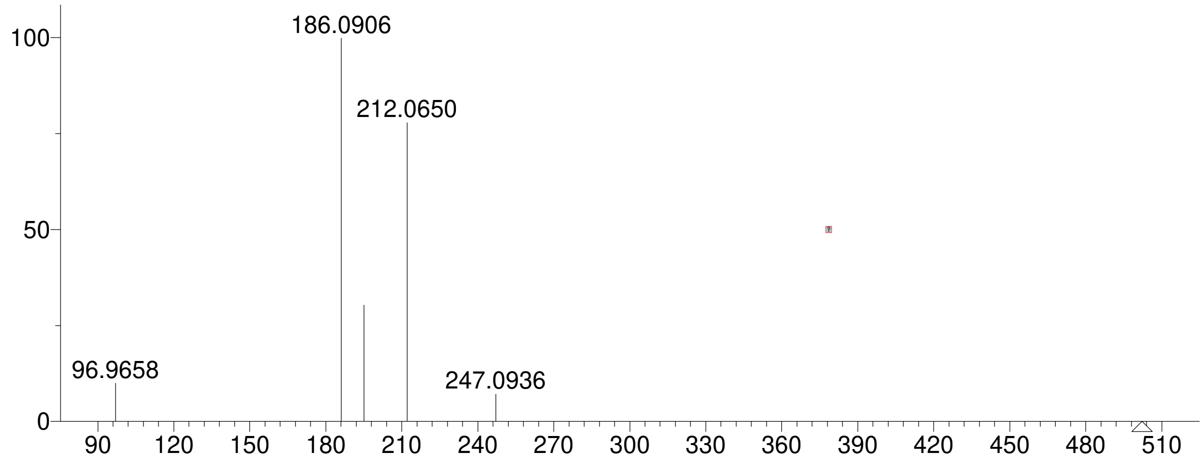

Hit 1 : NCGC00380440-01!2-[[(E)-3-[2-(4-hydroxy-3-methoxyphenyl)-3-(hydroxymethyl)-7-methoxy-2,3-dihydro-1-
C25H27NO10; MF: 0; RMF: 14; Prob 100.0%; Lib: mona-export-gnps; ID: 8737.

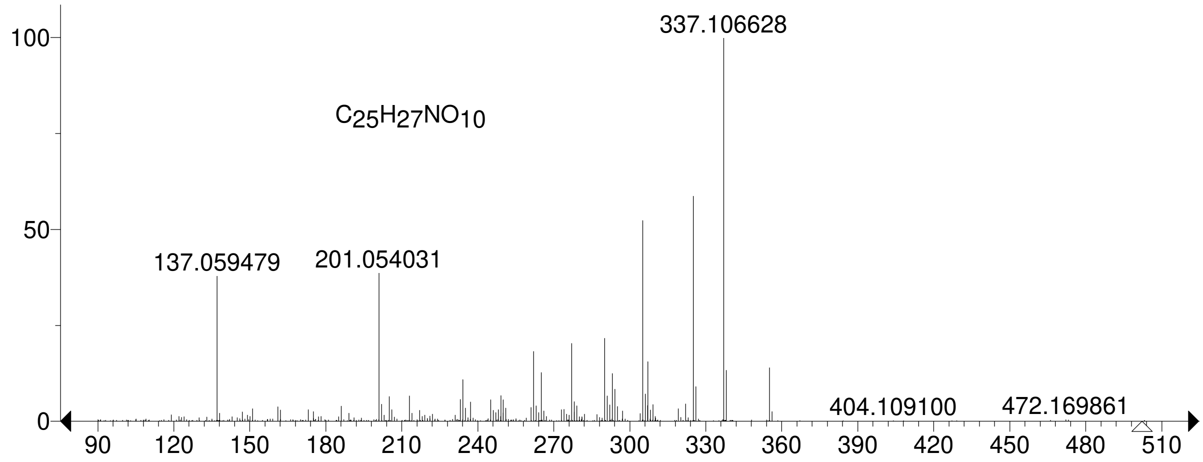

Unknown: Unknown (3.55_647.2653m/z)
Compound in Library Factor = 0

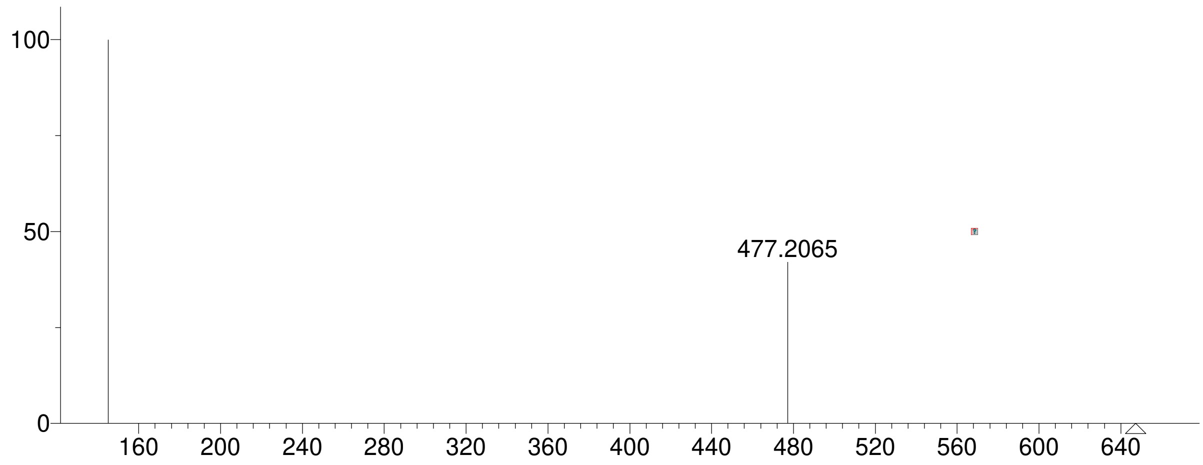

Unknown: Unknown (3.58_508.1924m/z)
Compound in Library Factor = 0

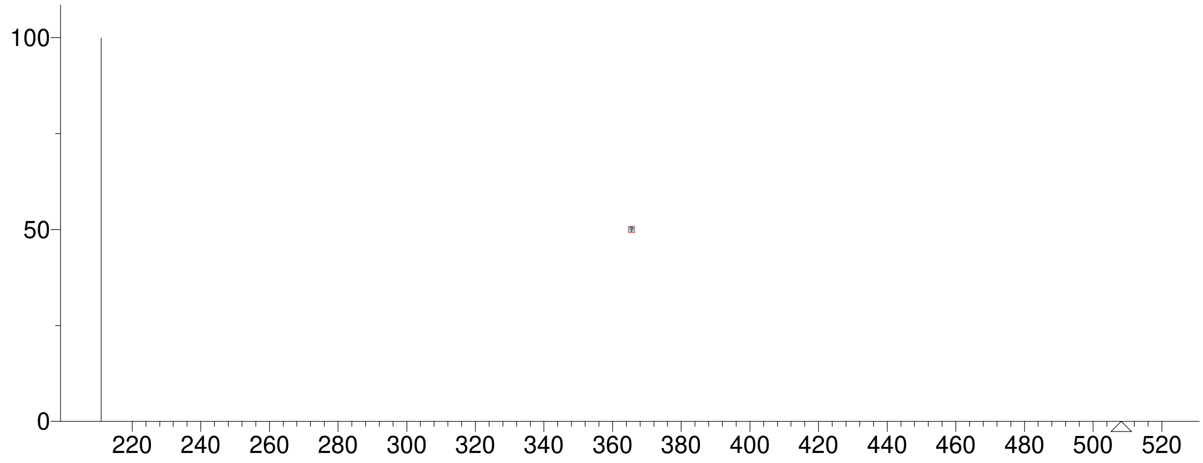

Unknown: Unknown (3.59_401.1552m/z)
Compound in Library Factor = -3273

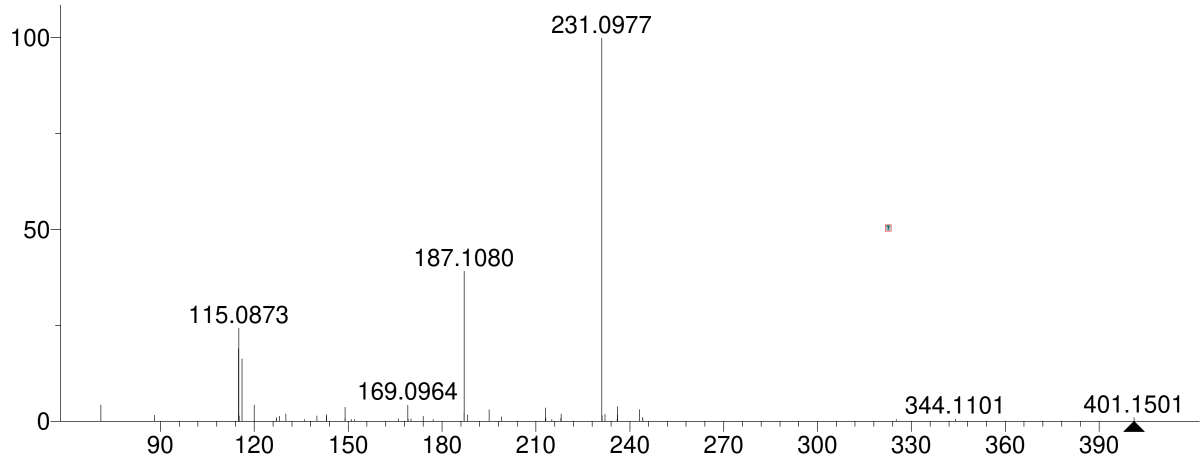

Hit 1 : NCGC00384788-01_C20H26O7_Butanoic acid, 2-methyl-, (10Z)-6-formyl-2,3,3a,4,5,8,9,11a-octahydro-5-l
C20H26O7; MF: 6; RMF: 101; Prob 9.98%; Lib: mona-export-gnps; ID: 12632.

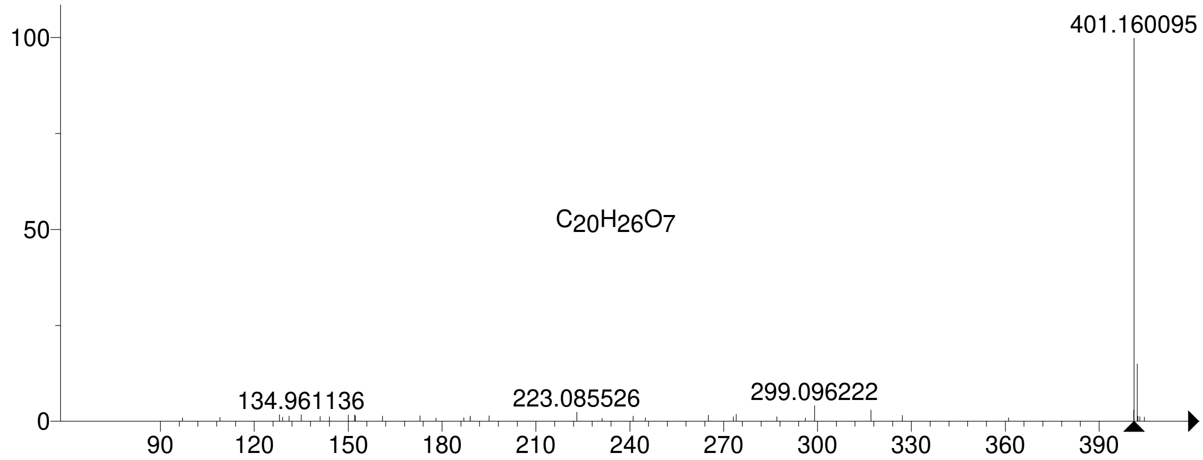

Hit 2 : rel-Dimethylenedioxy-dimethoxy-epoxylignan [M+H]+ P=401.2
C22H24O7; MF: 3; RMF: 343; Prob 8.82%; Lib: mona-export-gnps; ID: 2894.

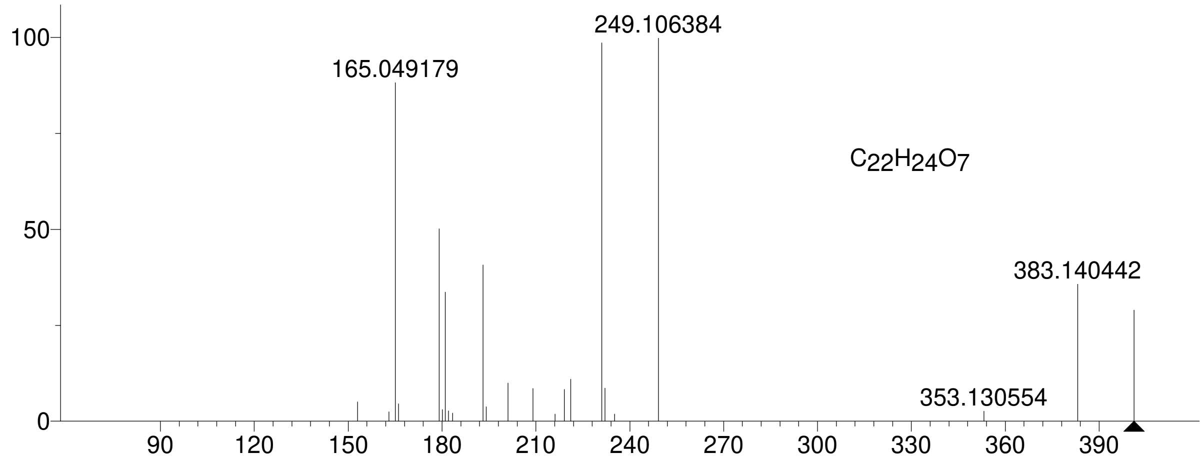

Hit 3 : 6-formyl-5-hydroxy-10-(hydroxymethyl)-3-methylidene-2-oxo-2H,3H,3aH,4H,5H,8H,9H,11aH-cyclodeca[b]fu
C20H26O7; MF: 3; RMF: 95; Prob 8.82%; Lib: mona-export-vf-npl_qexactive; ID: 22438.

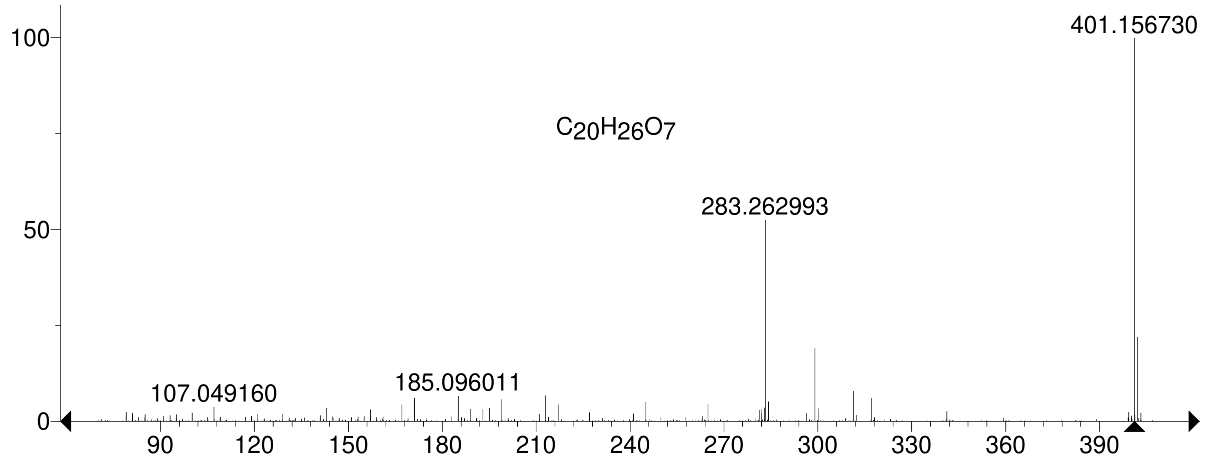

Hit 4 : Cnicin [M+Na]+ HCD 20V P=401.2
C20H26O7; MF: 3; RMF: 67; Prob 8.82%; CAS: 24394-09-0; Lib: nist_msms; ID: 236002.

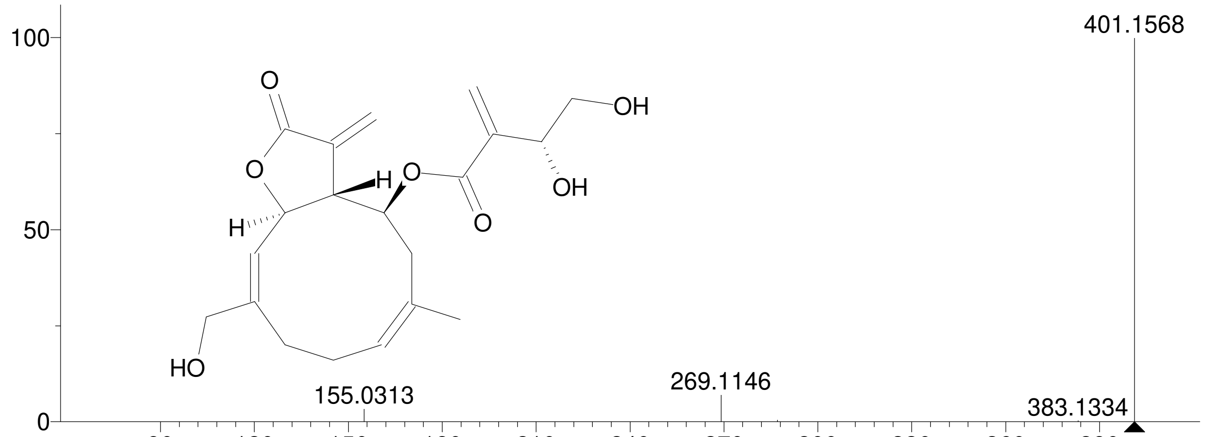

Hit 5 : NCGC00168917-02!5-[(3S,3aR,6S,6aR)-6-(3,4,5-trimethoxyphenyl)-1,3,3a,4,6,6a-hexahydrofuro[3,4-c]fura
C22H24O7; MF: 1; RMF: 109; Prob 8.13%; Lib: mona-export-gnps; ID: 8887.

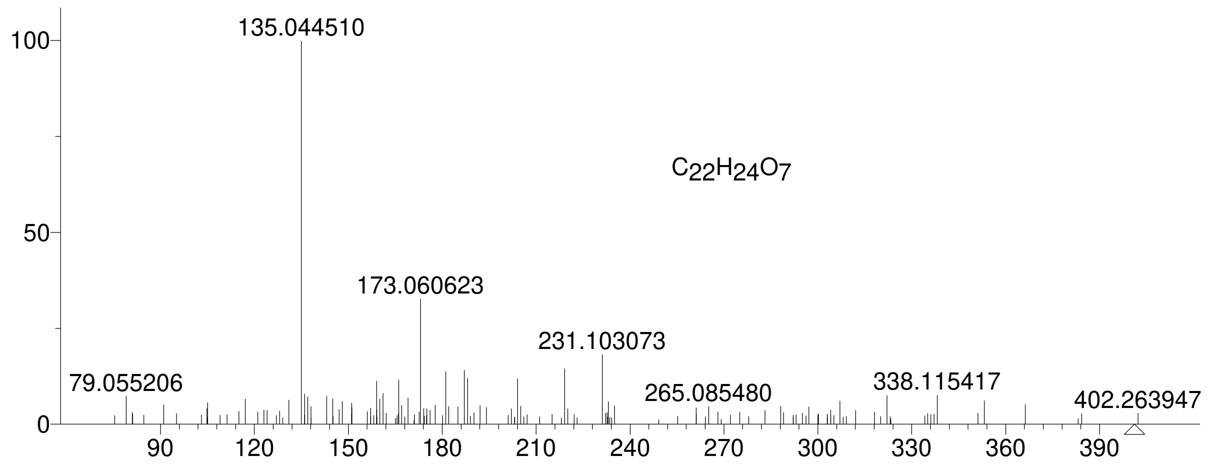

** Search Report Page 1 of 1 **

Unknown: Unknown (3.59_416.0597m/z)
Compound in Library Factor = 0

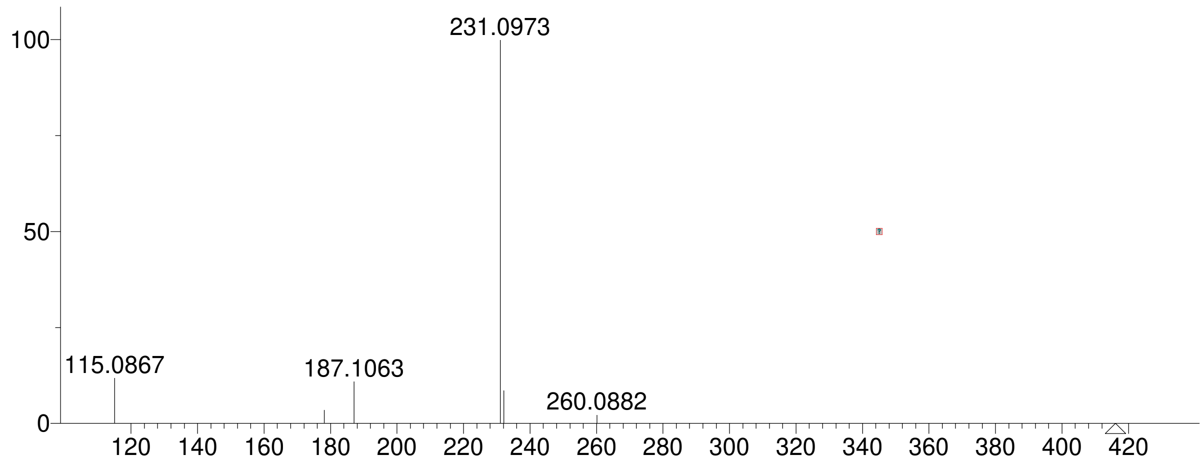

Unknown: Unknown (3.59_430.1452m/z)
Compound in Library Factor = 0

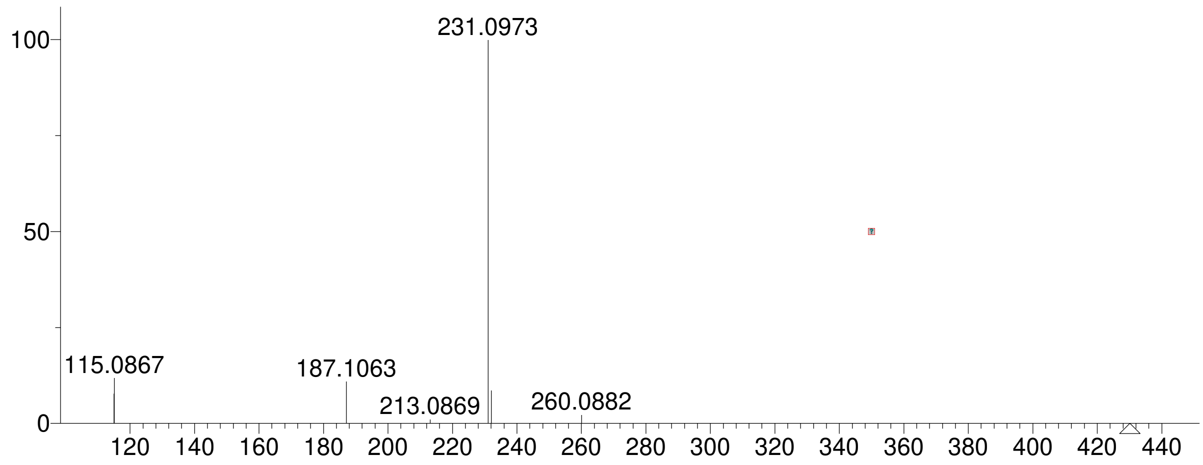

Unknown: Unknown (3.61_365.1336m/z)
Compound in Library Factor = 0

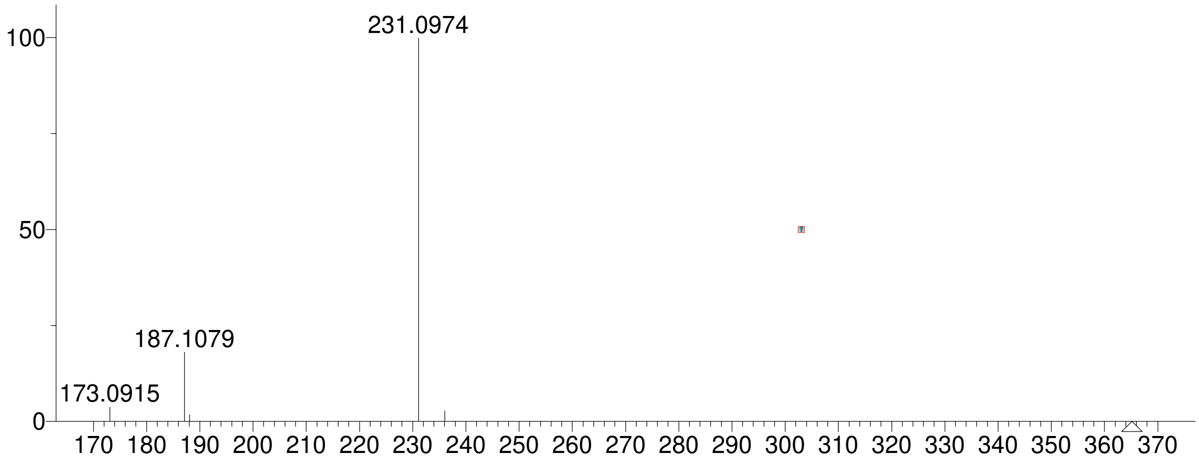

Hit 1 : [5-acetyloxy-3-(hydroxymethyl)-2-oxo-6-propan-2-ylcyclohex-3-en-1-yl] 3-methylbutanoate [M+K]+ 35HCD
C17H26O6; MF: 0; RMF: 15; Prob 100.0%; Lib: mona-export-vf-npl_qexactive; ID: 8245.

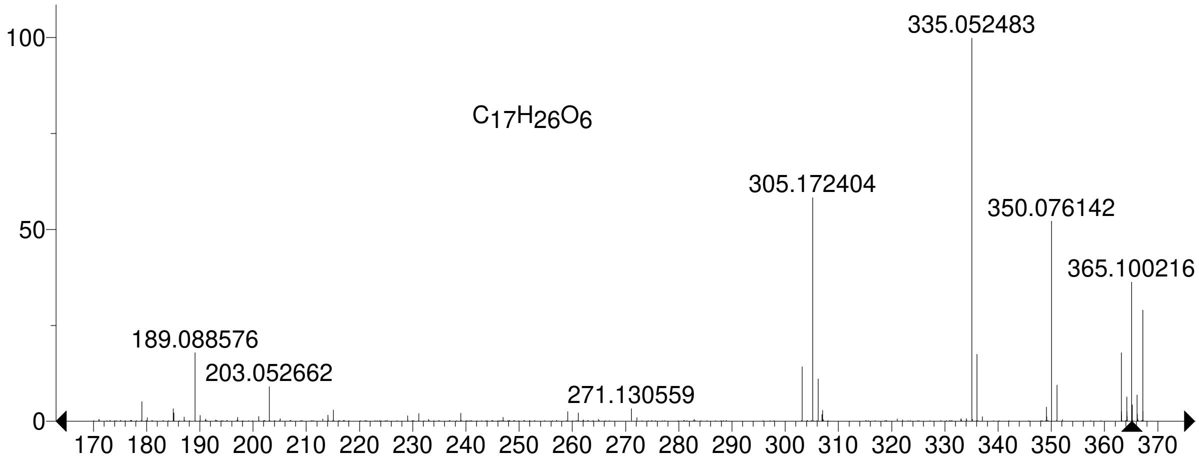

Unknown: Unknown (3.61_406.0798m/z)
Compound in Library Factor = 0

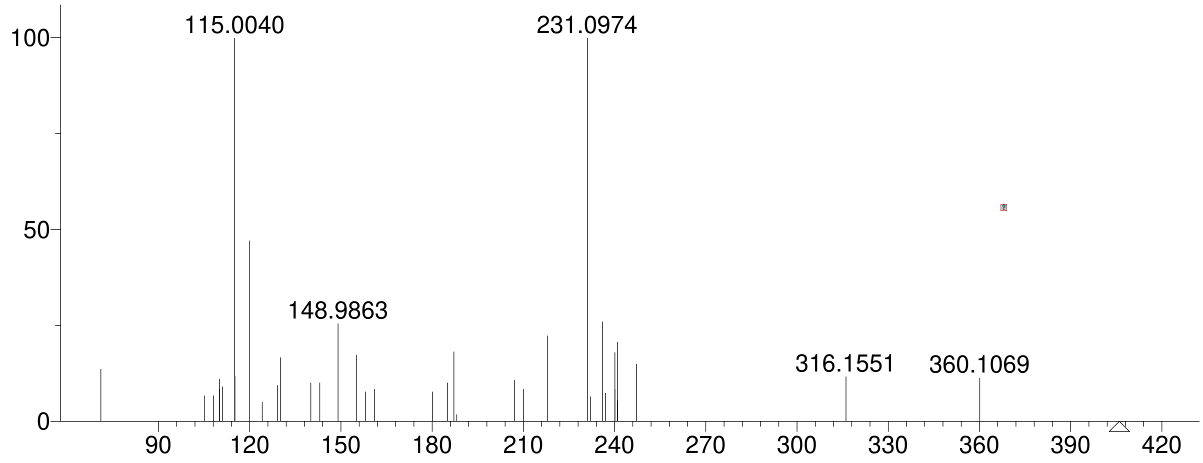

Hit 1 : Bicuculline (+) [M+K]+ 35% P=406.1
C20H17NO6; MF: 0; RMF: 7; Prob 100.0%; Lib: mona-export-itree; ID: 1031.

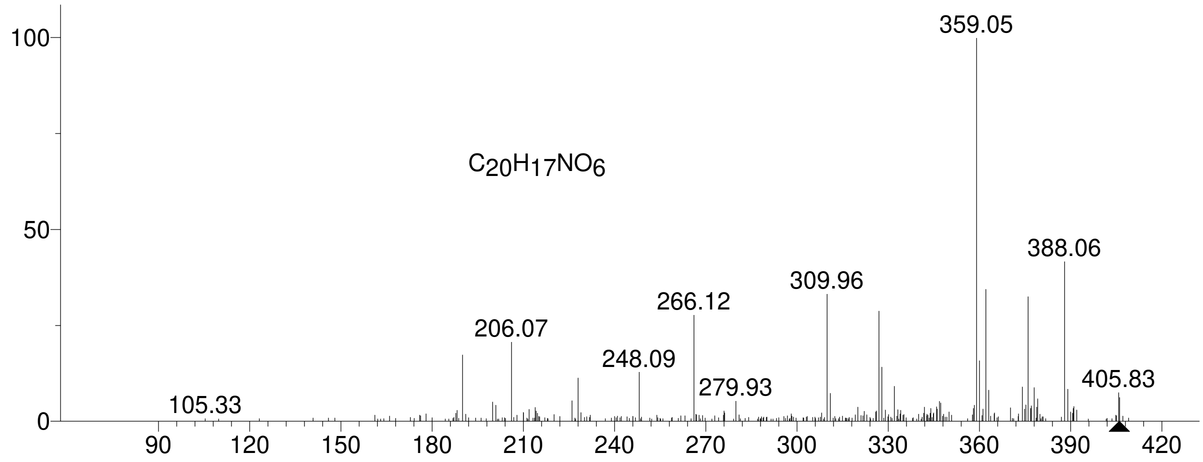

Unknown: Unknown (3.62_441.1861m/z)
Compound in Library Factor = 0

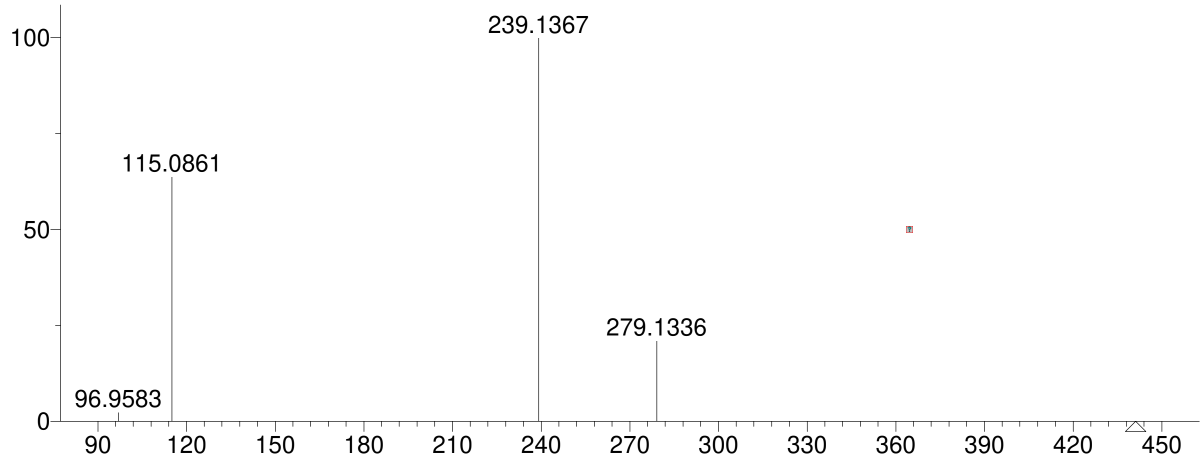

Hit 1 : NCGC00380134-01_C25H30O8_3-Furancarboxylic acid, (3R,5S,5aS,6S,9aR)-octahydro-9-hydroxy-2,2,5a
C25H30O8; MF: 0; RMF: 12; Prob 100.0%; Lib: mona-export-gnps; ID: 11250.

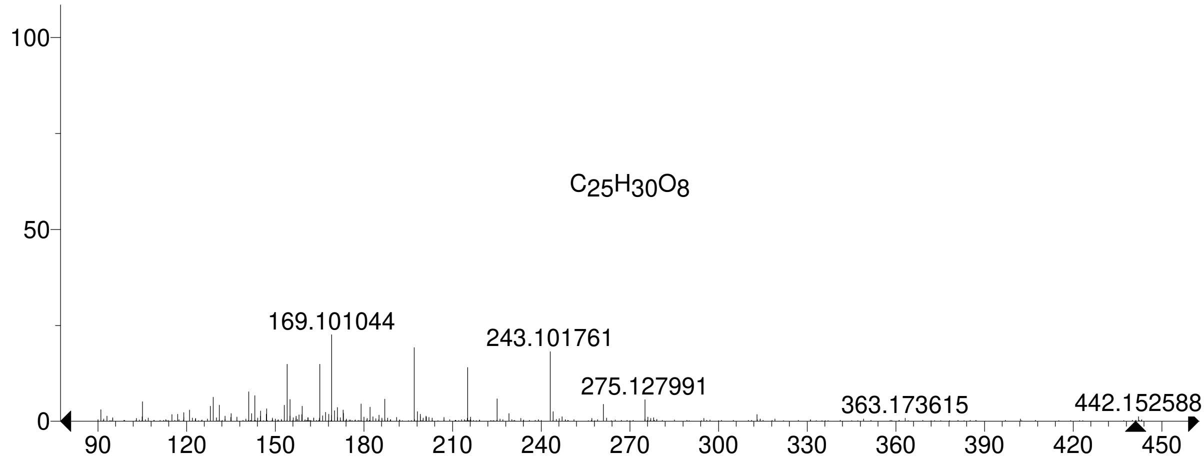

Unknown: Unknown (3.62_609.2047m/z)
Compound in Library Factor = 0

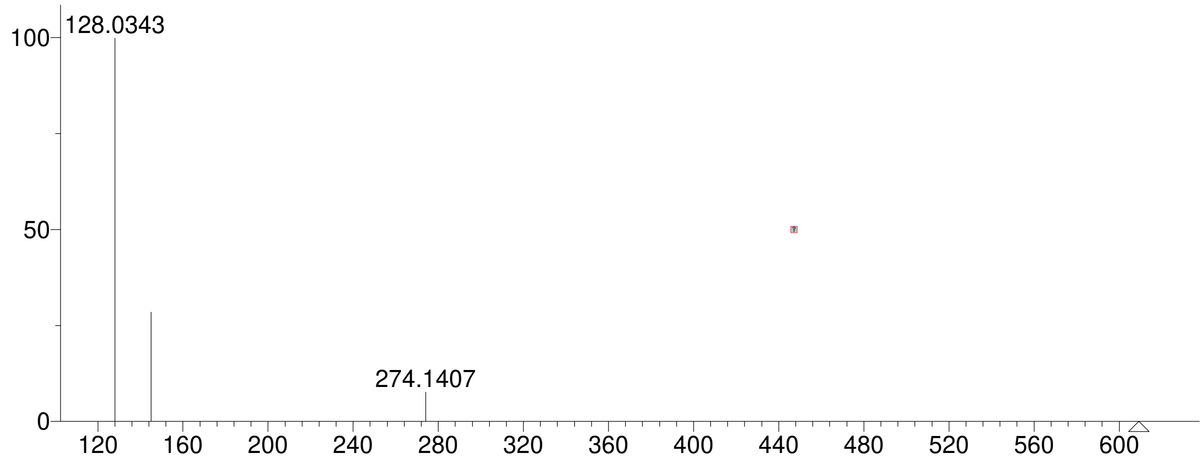

Unknown: Unknown (3.63_515.1976m/z)
Compound in Library Factor = 0

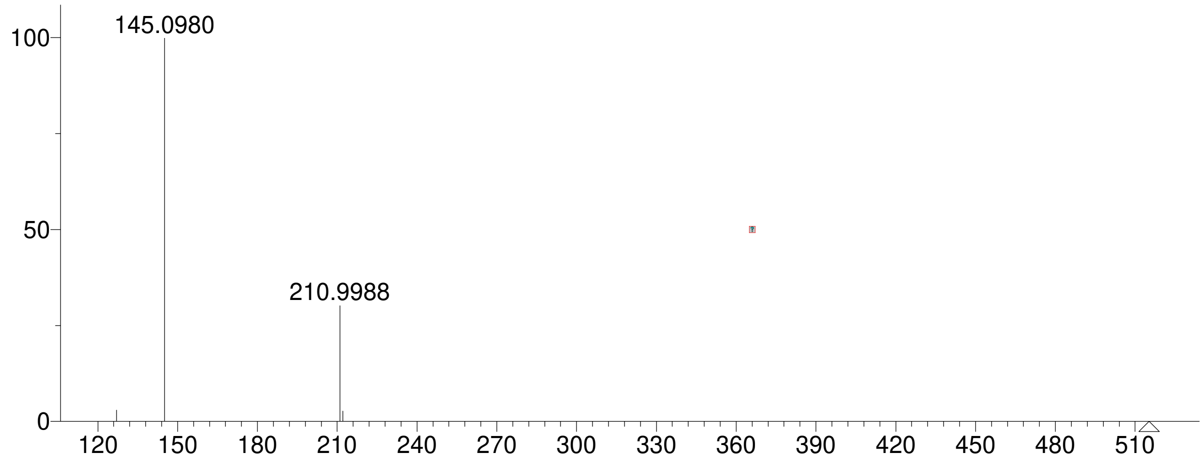

Unknown: Unknown (3.64_344.1815m/z)
Compound in Library Factor = -3273

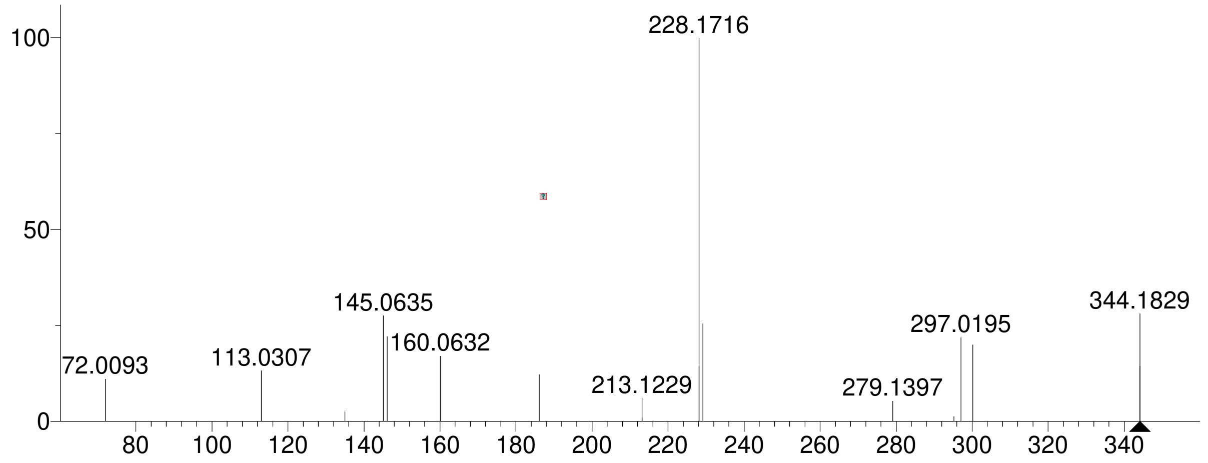

Hit 1 : NCGC00347618-02!3-hydroxy-N-(1-hydroxy-4-methylpentan-2-yl)-5-oxo-6-phenylhexanamide [M+Na]+ P
C18H27NO4; MF: 4; RMF: 232; Prob 54.2%; Lib: mona-export-gnps; ID: 9879.

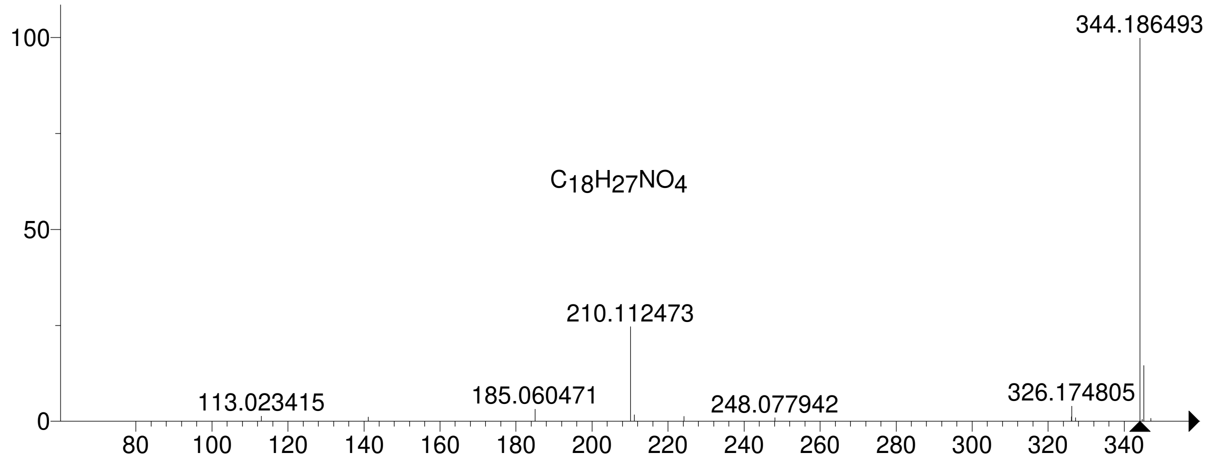

Hit 2 : 4-amino-2-hydroxy-5-[[1-hydroxy-1-(5-oxo-6-bicyclo[4.1.0]hept-3-enyl)propan-2-yl]amino]-5-oxopentanoic a
C15H22N2O6; MF: 0; RMF: 52; Prob 45.8%; Lib: mona-export-vf-npl_qexactive; ID: 16561.

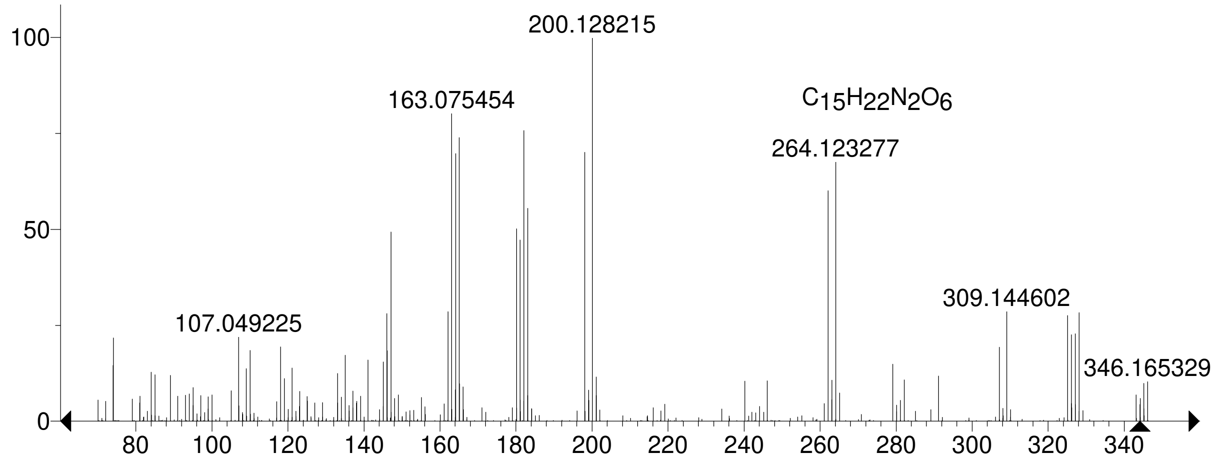

Unknown: Unknown (3.64_467.0791m/z)
Compound in Library Factor = -3273

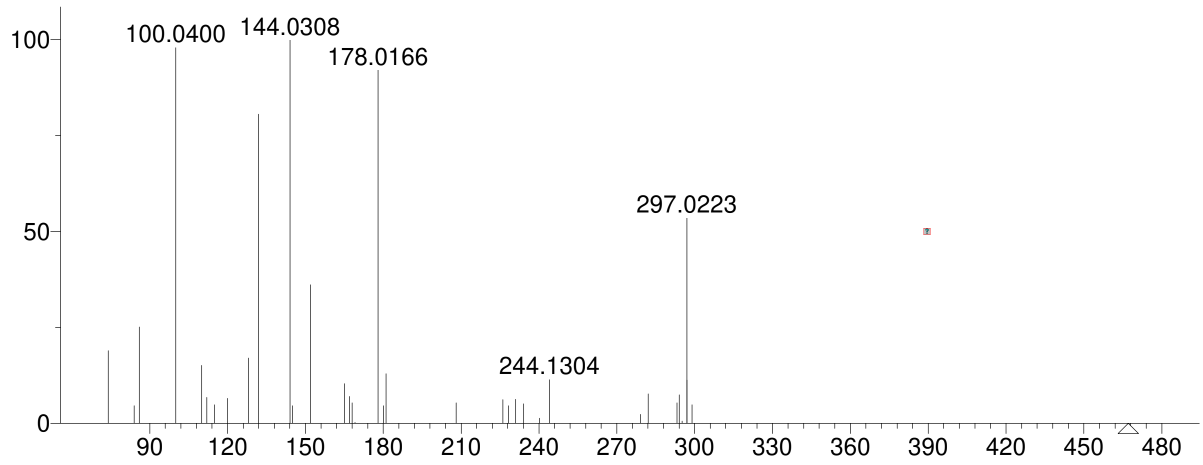

Hit 1 : Hamamelitannin [M+H-H2O]+ IT-FT 35% P=467.1
C20H20O14; MF: 2; RMF: 115; Prob 35.1%; CAS: 469-32-9; Lib: nist_msms; ID: 466278.

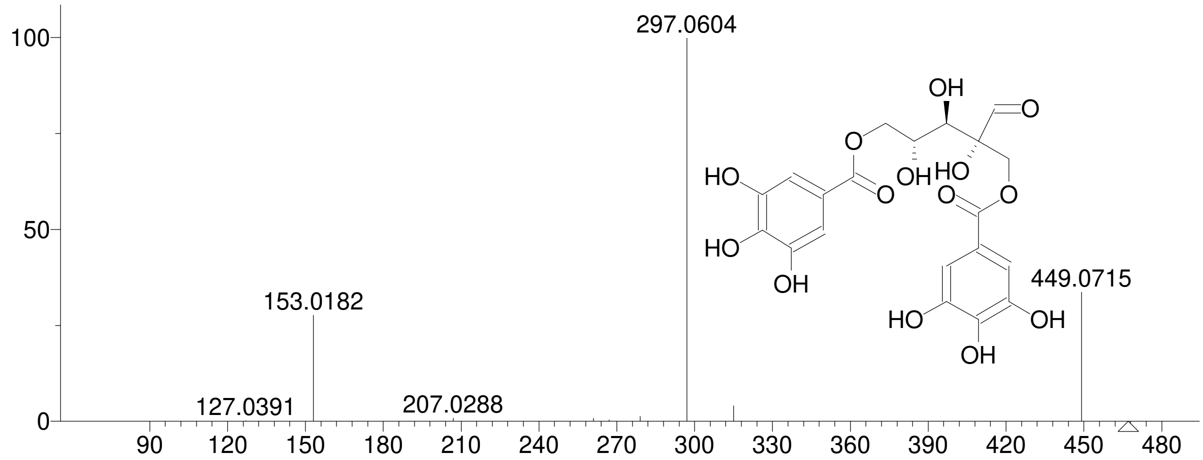

Hit 2 : ginnalin A [M-H]- 35HCD P=467.1
C20H20O13; MF: 0; RMF: 43; Prob 32.4%; Lib: mona-export-lc-ms-ms_negative_mode; ID: 18866.

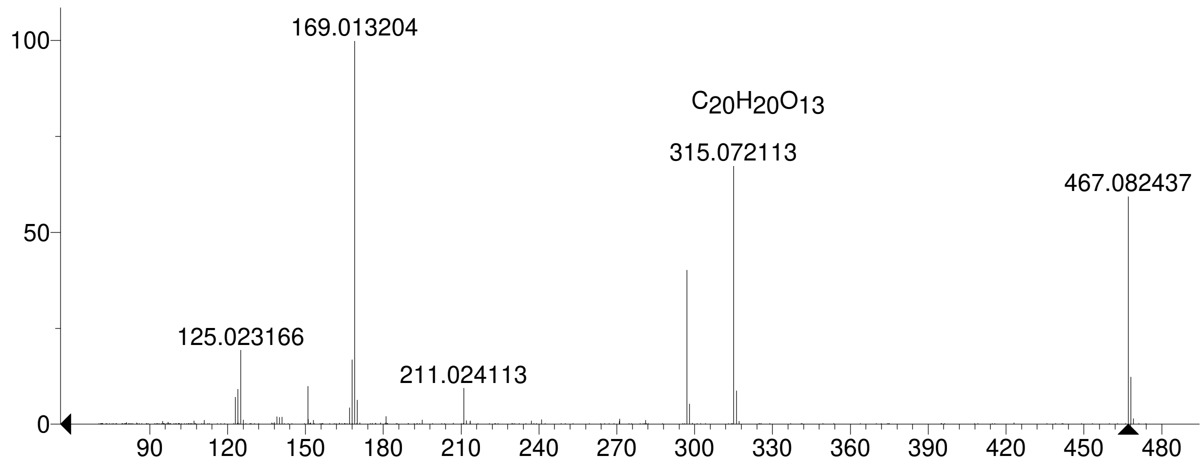

Hit 3 : NCGC00347845-02![(2R,3S,4S,5S)-3,4-dihydroxy-5-(3,4,5-trihydroxybenzoyl)oxyoxan-2-yl]methyl 3,4,5-tril
C20H20O13; MF: 0; RMF: 20; Prob 32.4%; Lib: msms-neg-gnps; ID: 742.

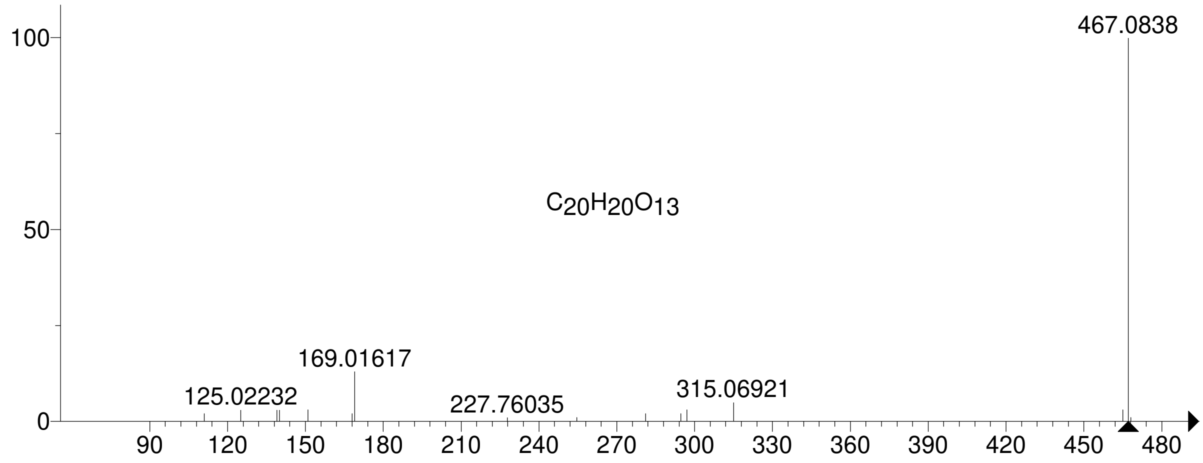

** Search Report Page 1 of 1 **

Unknown: Unknown (3.64_474.1938m/z)
Compound in Library Factor = 0

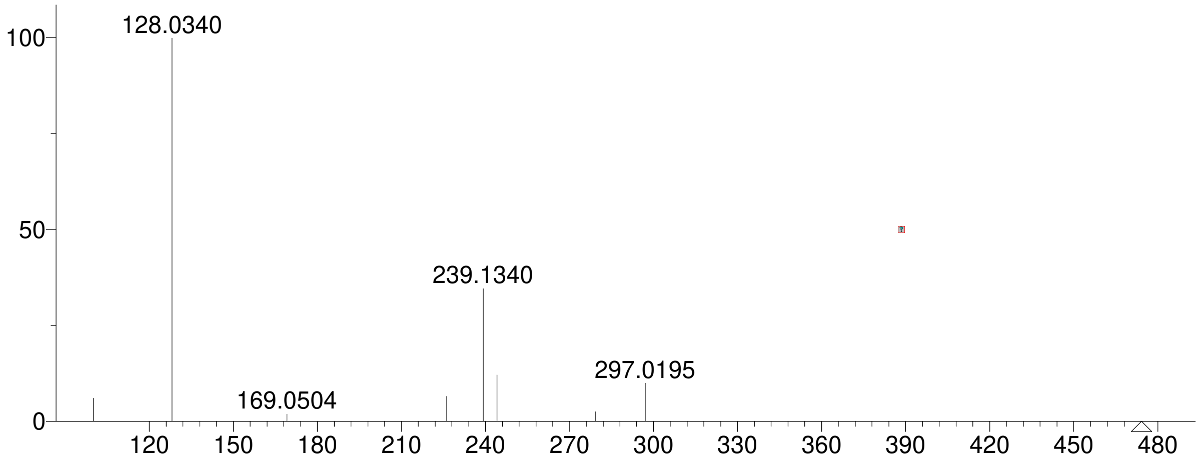

** Search Report Page 1 of 1 **

Unknown: Unknown (3.64_524.1018m/z)
Compound in Library Factor = 0

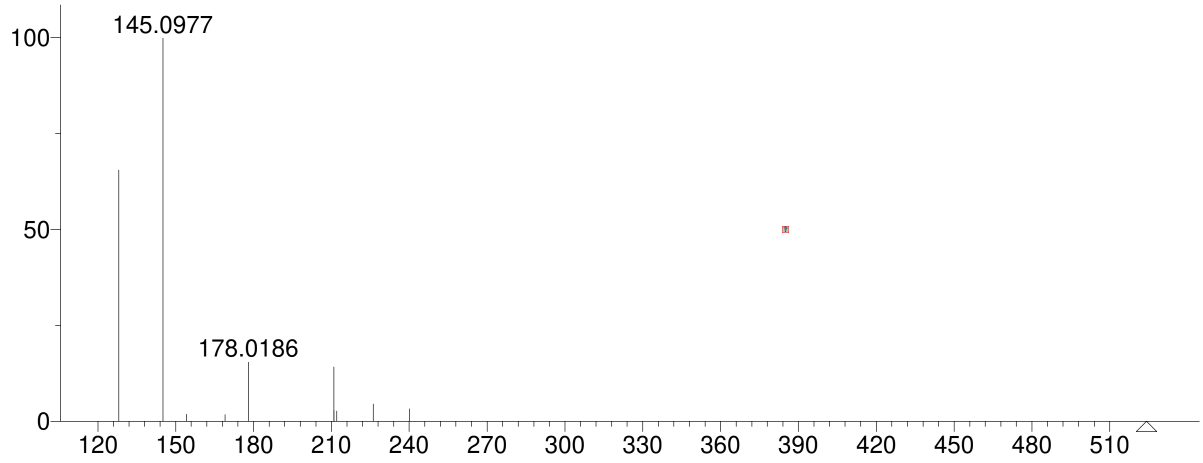

Unknown: Unknown (3.66_210.1117m/z)
Compound in Library Factor = 0

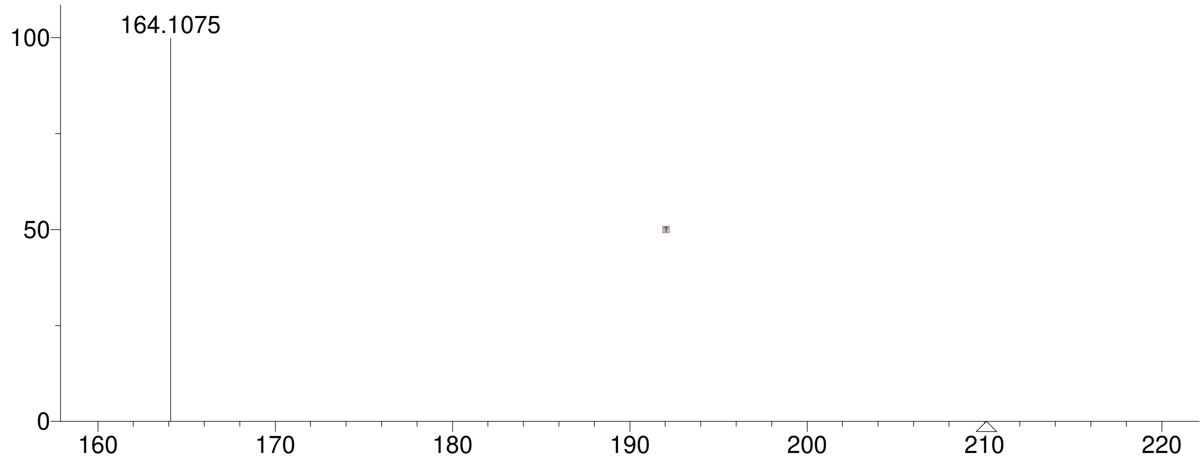

Hit 1 : 4-oxo-5-phenylpentanoic acid [M+NH4]+ 65HCD P=210.1
C11H12O3; MF: 0; RMF: 68; Prob 100.0%; Lib: mona-export-vf-npl_qexactive; ID: 21114.

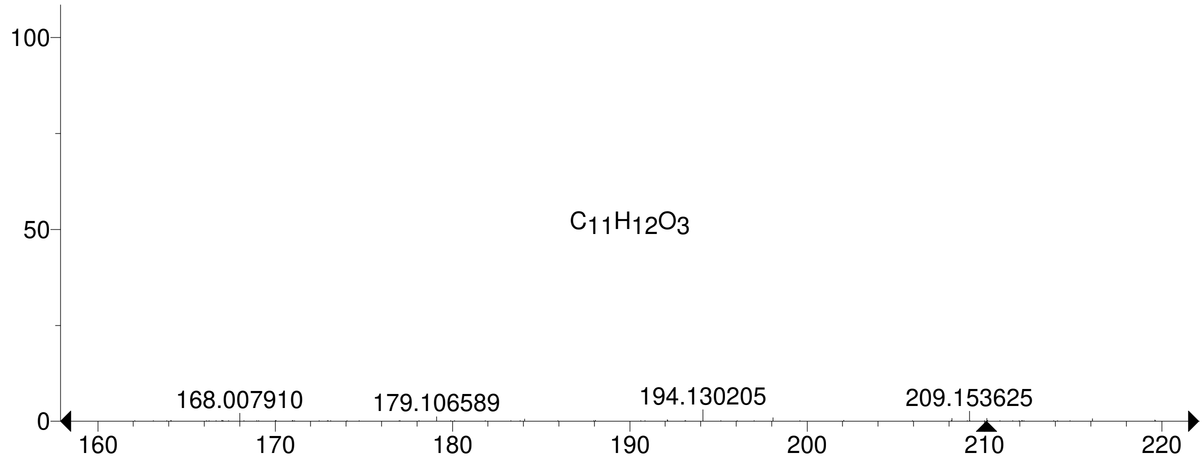

Unknown: Unknown (3.66_485.2124m/z)
Compound in Library Factor = 0

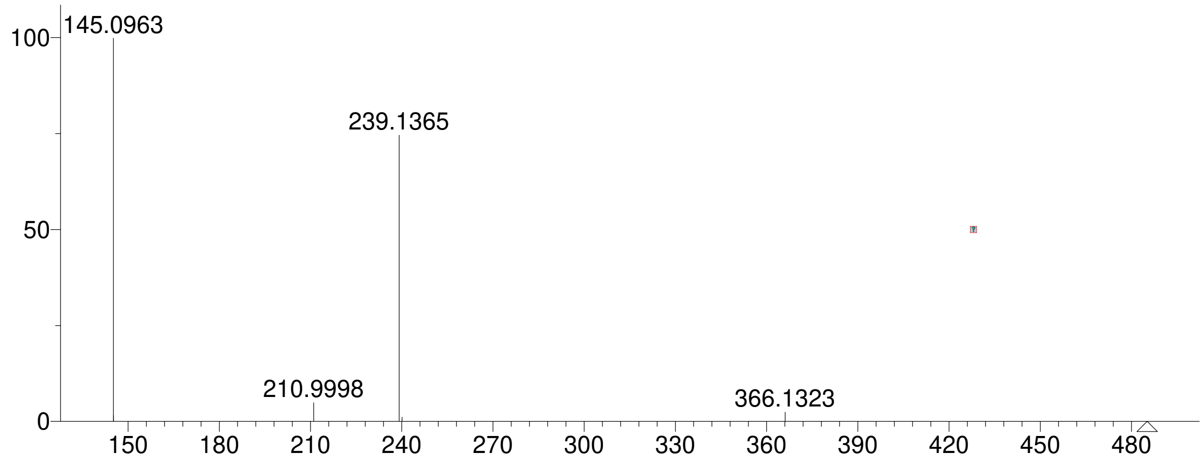

Hit 1 : NCGC00380618-01_C27H32O8_(1'R,3'R,8'R,12'E,18'E,20'Z,24'R,25'S)-5',13',25'-Trimethyl-11'H,17'H,22'H
C27H32O8; MF: 0; RMF: 119; Prob 25.0%; Lib: mona-export-gnps; ID: 10939.

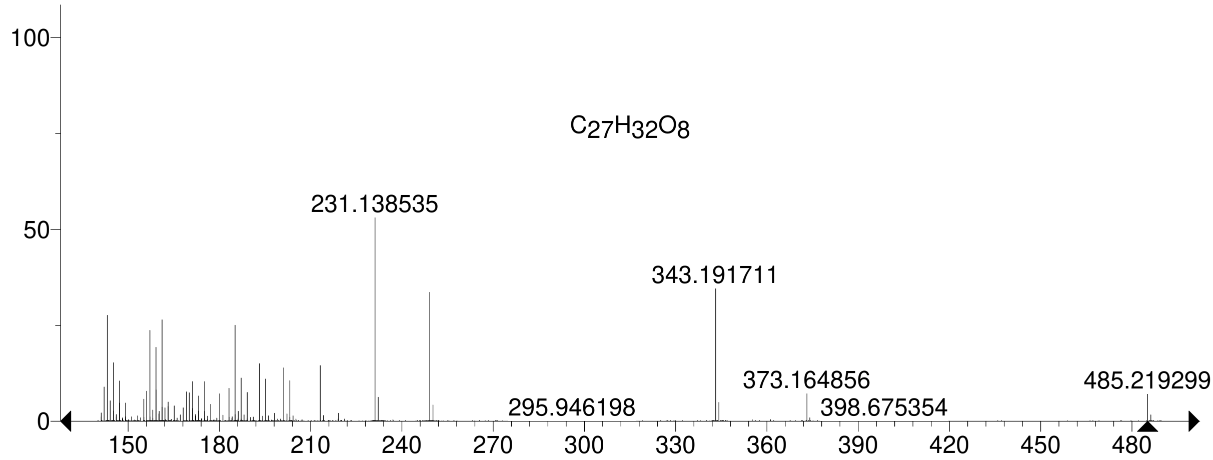

Hit 2 : 3-[7-hydroxy-8-(2-hydroxypropan-2-yl)-11-methoxy-4,5a,9-trimethyl-1-oxo-3,6,7,8,9a,10-hexahydro-[2]benz
C25H34O8; MF: 0; RMF: 87; Prob 25.0%; Lib: mona-export-vf-npl_qexactive; ID: 2220.

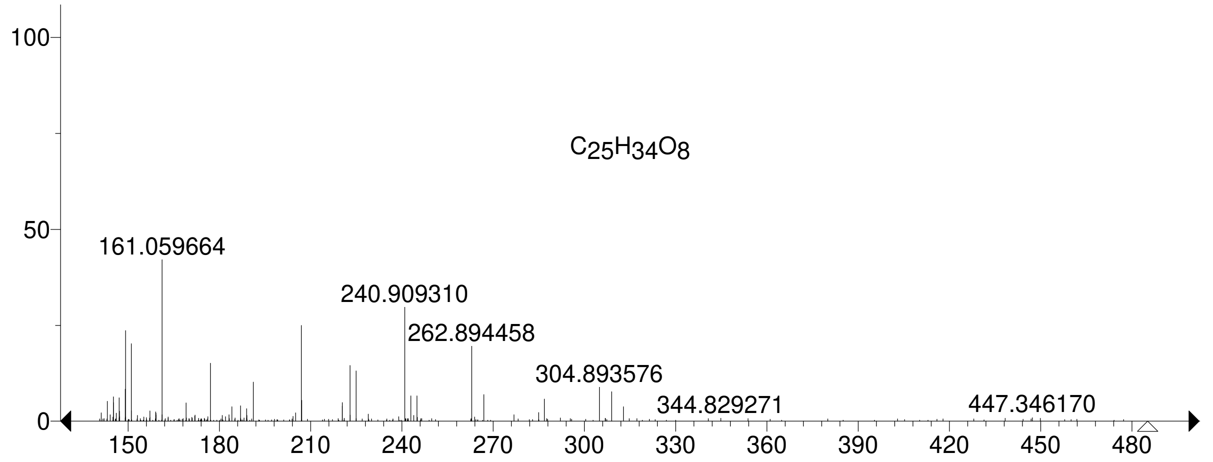

Hit 3 : (2R,3R,4S,5S,6R)-2-[1,7-bis(4-hydroxyphenyl)heptan-3-yloxy]-6-(hydroxymethyl)oxane-3,4,5-triol [M+Na]+
C25H34O8; MF: 0; RMF: 30; Prob 25.0%; Lib: mona-export-vf-npl_qexactive; ID: 7730.

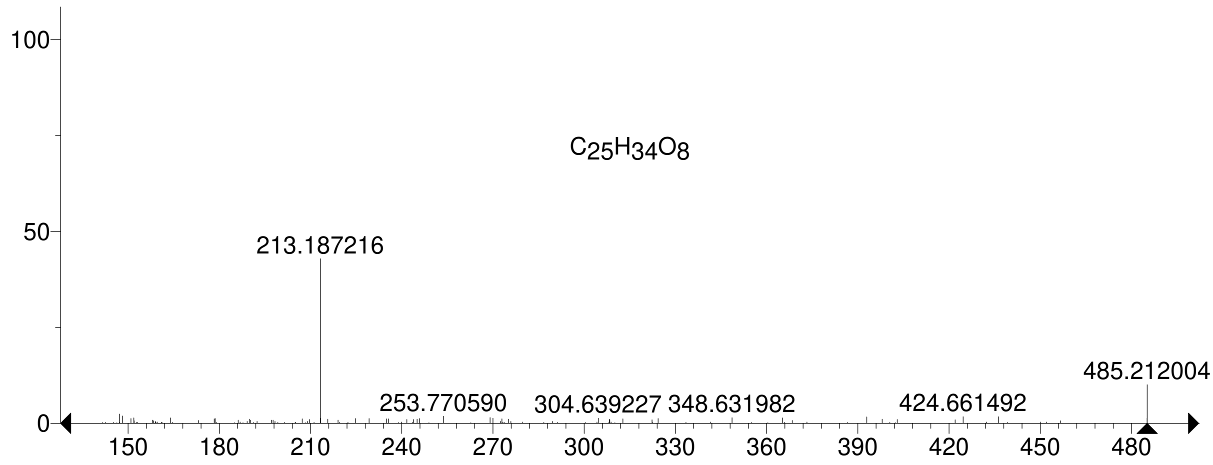

Hit 4 : methyl 4,10-diacetyloxy-5,9-dihydroxy-1,4a-dimethyl-7-propan-2-yl-2,3,4,9,10,10a-hexahydrophenanthrene
C25H34O8; MF: 0; RMF: 15; Prob 25.0%; Lib: mona-export-vf-npl_qexactive; ID: 22514.

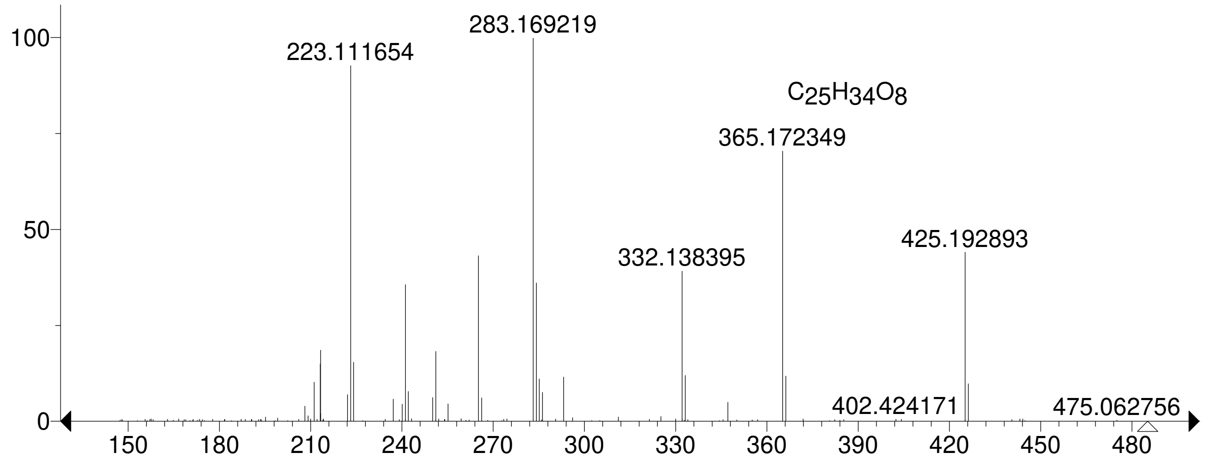

Unknown: Unknown (3.67_227.0677m/z)
Compound in Library Factor = 0

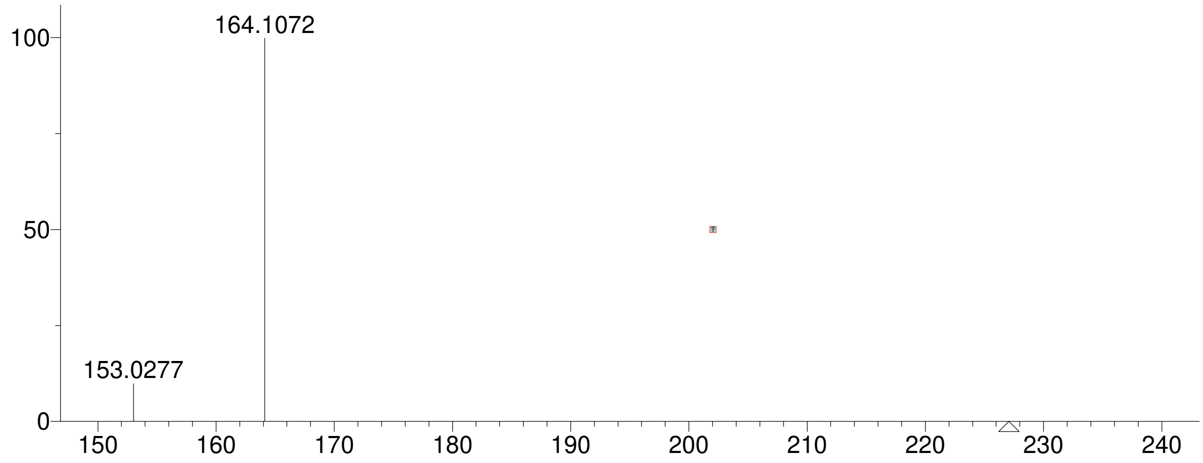

Hit 1 : AZELAIC ACID [M+K]+ 65HCD P=227.1
C9H16O4; MF: 0; RMF: 13; Prob 100.0%; Lib: mona-export-vf-npl_qexactive; ID: 11751.

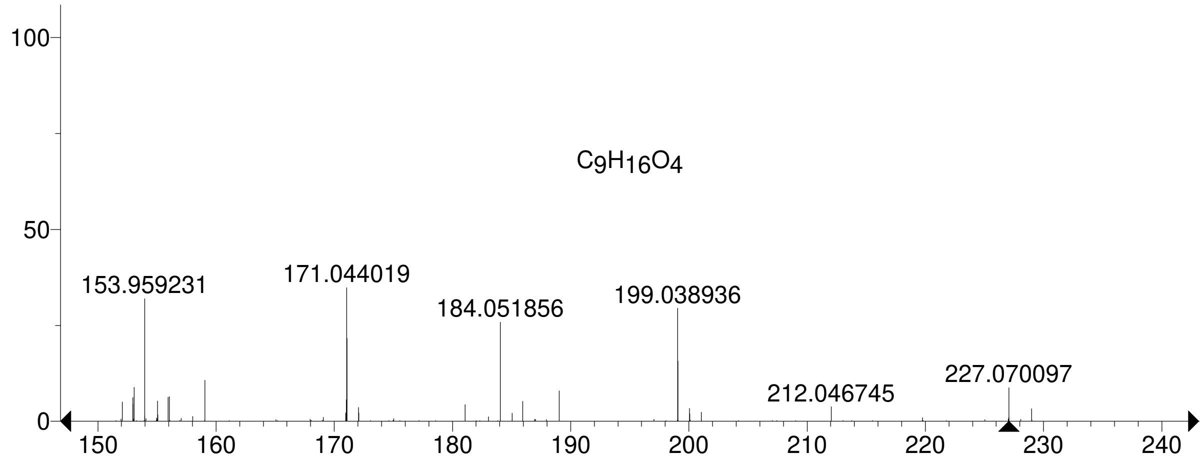

Unknown: Unknown (3.67_414.1854m/z)
Compound in Library Factor = 0

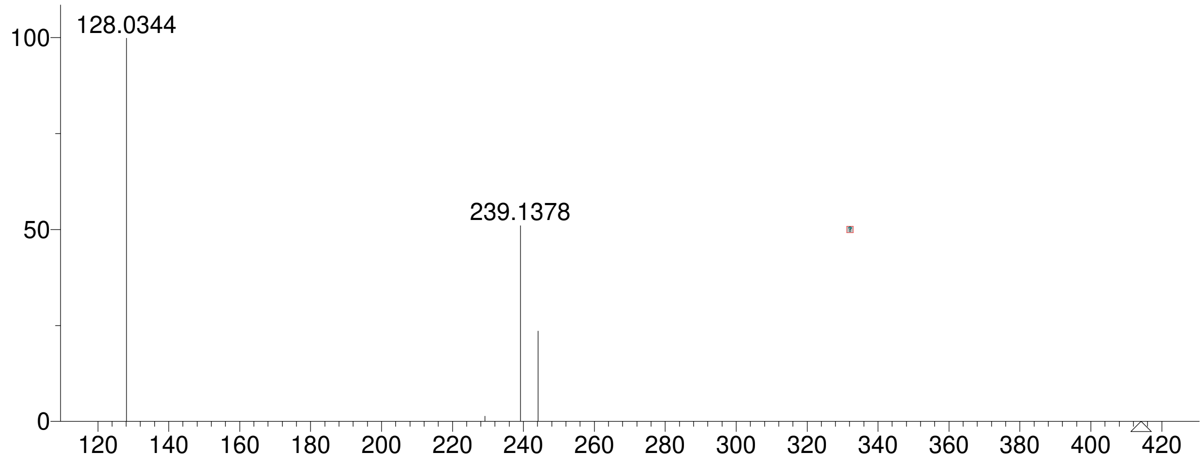

Hit 1 : ACTH (5-10) [M-2H]2- HCD 41eV P=414.2
C39H50N12O9; MF: 0; RMF: 63; Prob 100.0%; Lib: nist_msms2; ID: 71787.

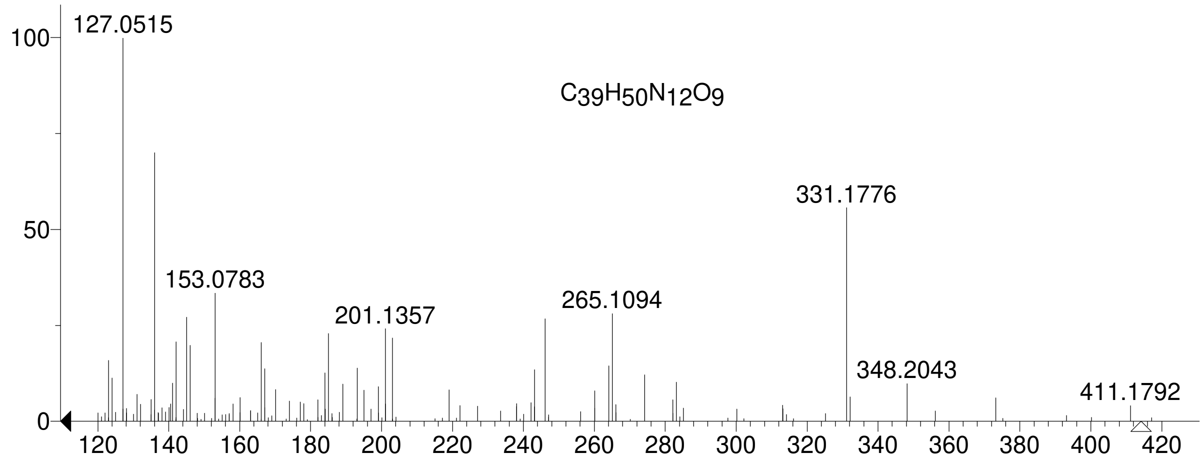

Unknown: Unknown (3.67_453.1852m/z)
Compound in Library Factor = 0

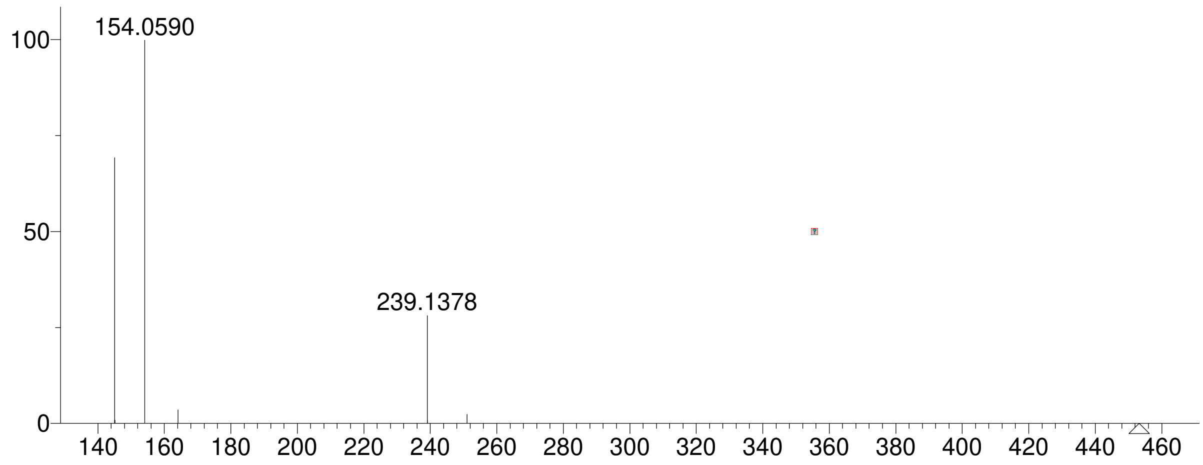

Hit 1 : (1-acetyloxy-3-hydroxy-6,8a-dimethyl-7-oxo-3-propan-2-yl-2,3a,4,8-tetrahydro-1H-azulen-4-yl) 4-hydroxybe
C24H30O7; MF: 0; RMF: 83; Prob 50.0%; Lib: mona-export-vf-npl_qexactive; ID: 17757.

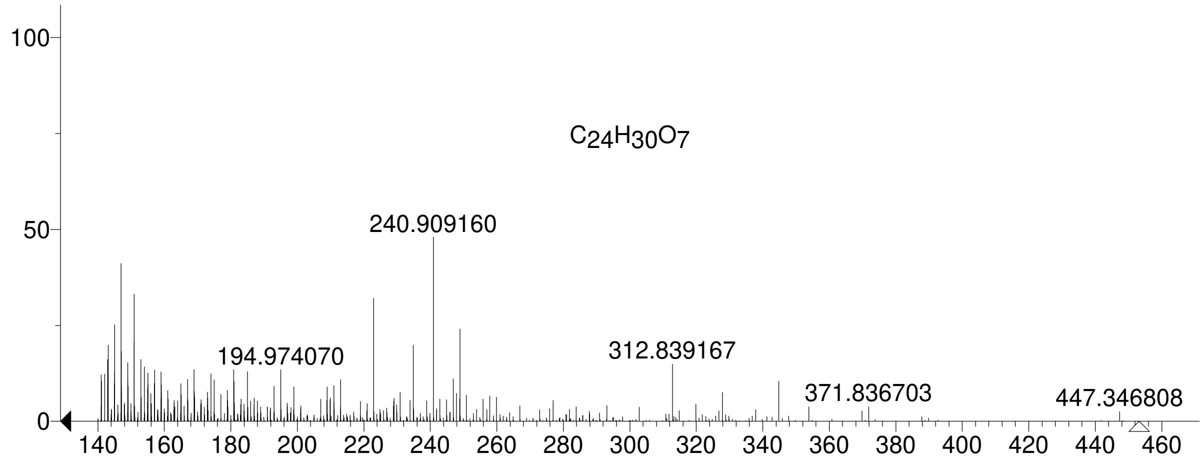

Hit 2 : [8-[2-(3-methylbutanoyloxy)propan-2-yl]-2-oxo-8,9-dihydrofuro[2,3-h]chromen-9-yl] 3-methylbutanoate [M+
C24H30O7; MF: 0; RMF: 9; Prob 50.0%; Lib: mona-export-vf-npl_qexactive; ID: 5397.

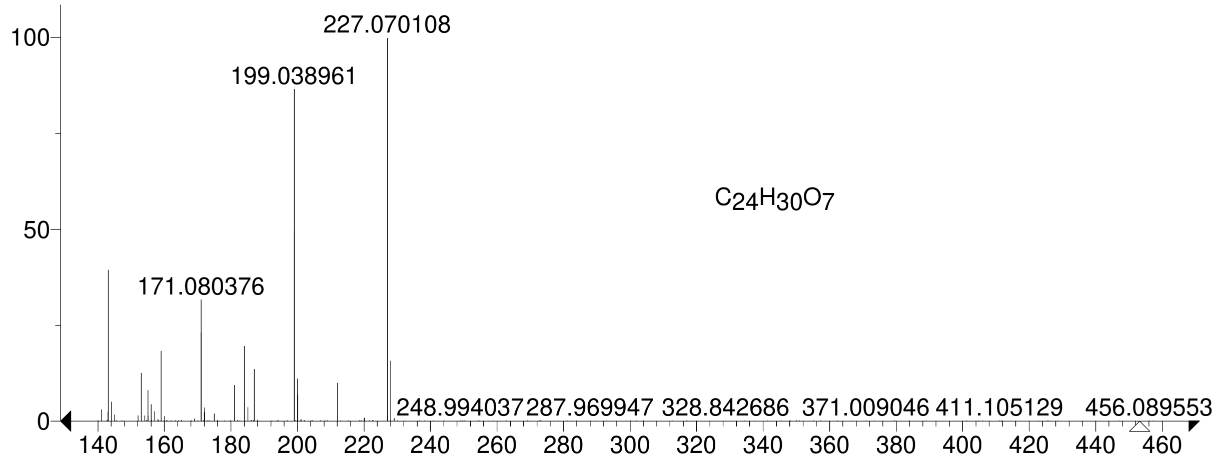

** Search Report Page 1 of 1 **

Unknown: Unknown (3.67_626.1783m/z)
Compound in Library Factor = 0

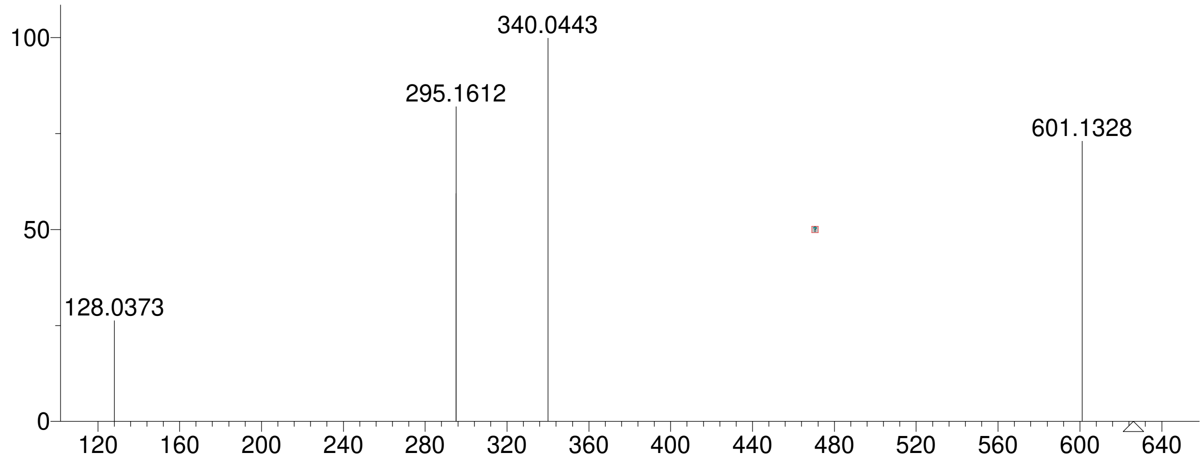

Unknown: Unknown (3.69_389.2056m/z)
Compound in Library Factor = 0

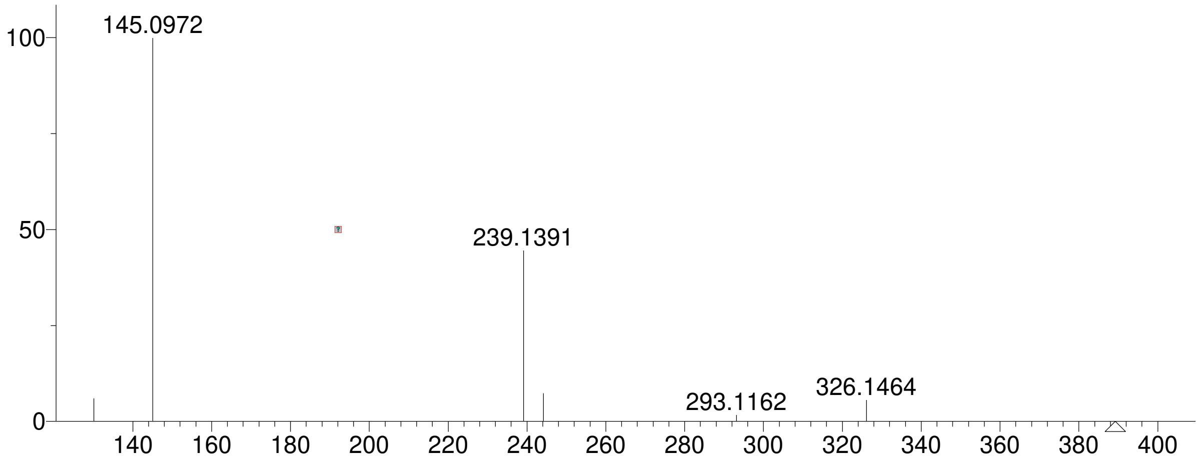

Unknown: Unknown (3.69_626.2405m/z)
Compound in Library Factor = 0

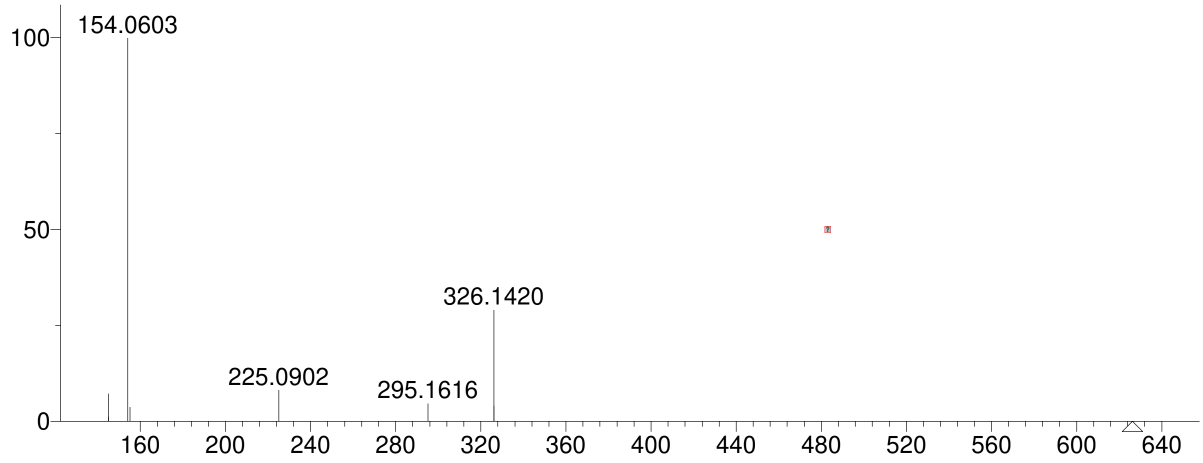

Unknown: Unknown (3.70_313.1013m/z)
Compound in Library Factor = 0

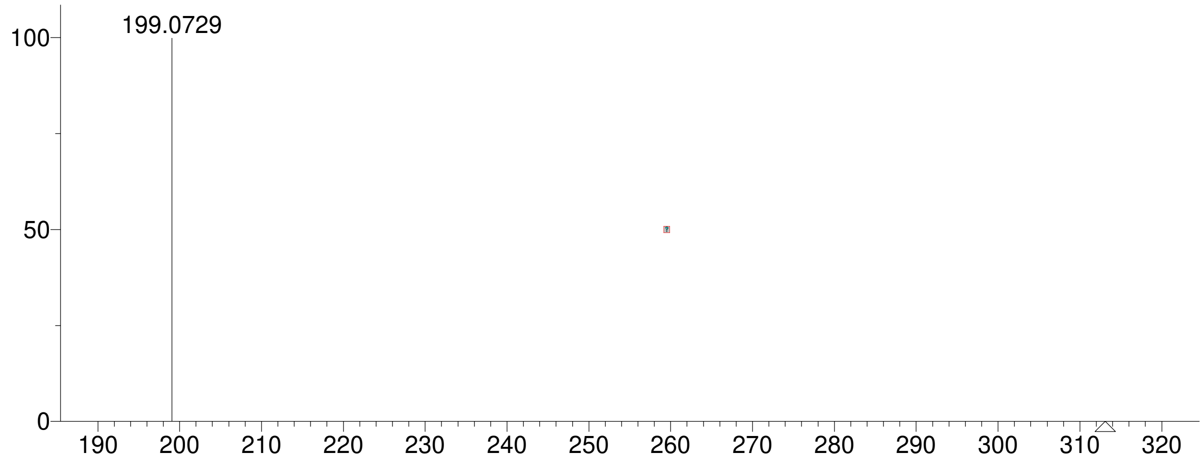

Hit 1 : Flumioxazin [M+H-C2H2O]+ HCD 81V P=313.1
C19H15FN2O4; MF: 0; RMF: 154; Prob 100.0%; CAS: 103361-09-7; Lib: nist_msms; ID: 486496.

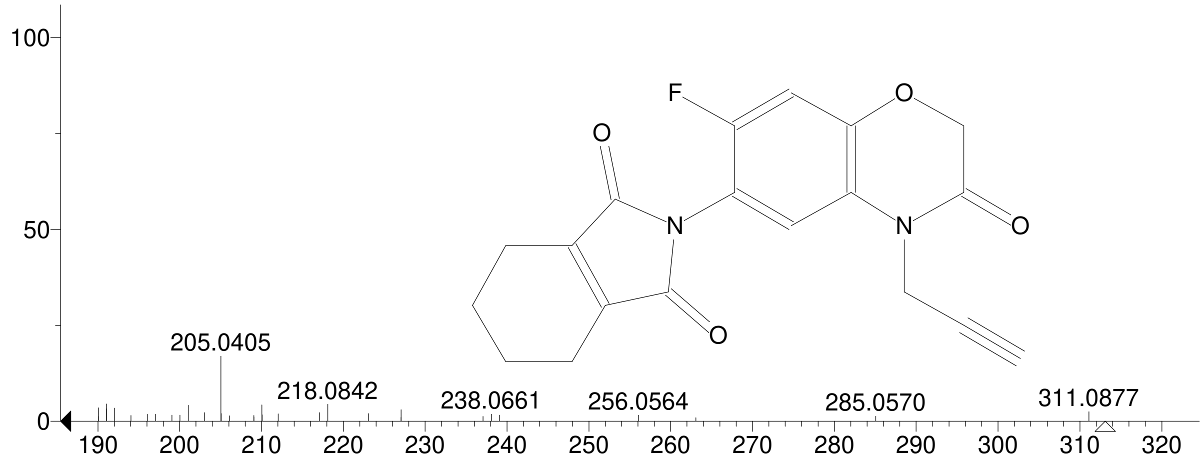

Unknown: Unknown (3.70_344.1329m/z)
Compound in Library Factor = -3273

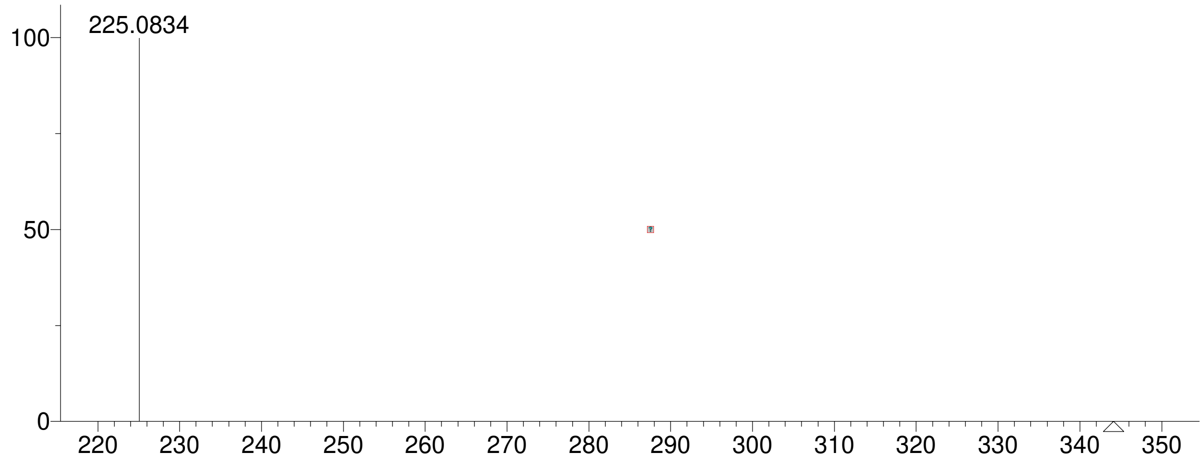

Hit 1 : (1S,4R,7S,8S,11S)-9-tert-butyl-7,9-dihydroxy-3,5,12-trioxatetracyclo[6.6.0.0A^'1,A^'1A^'1.0a^",a^,,]tetradec
C15H18O8; MF: 1; RMF: 236; Prob 100.0%; Lib: mona-export-vf-npl_qexactive; ID: 8442.

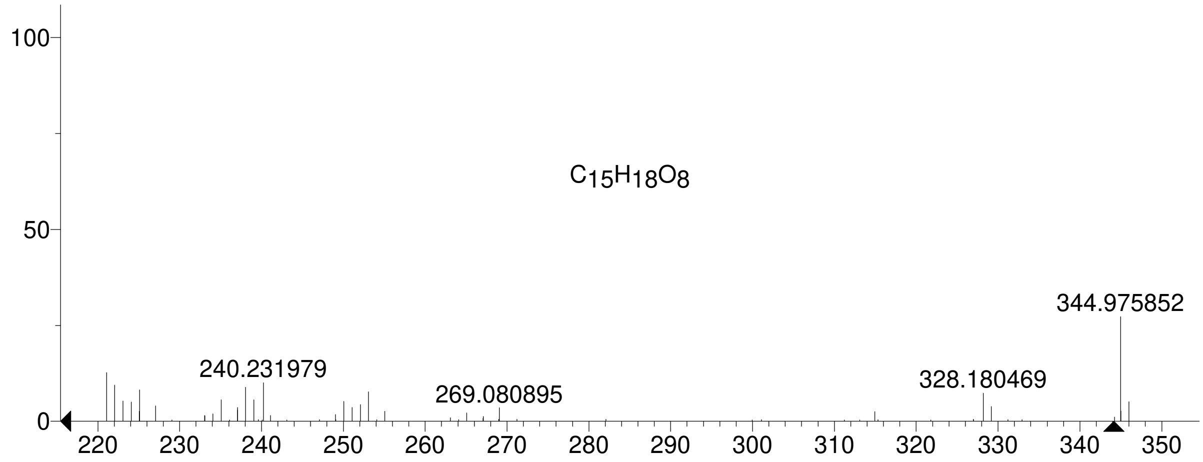

Unknown: Unknown (3.70_360.1763m/z)
Compound in Library Factor = 0

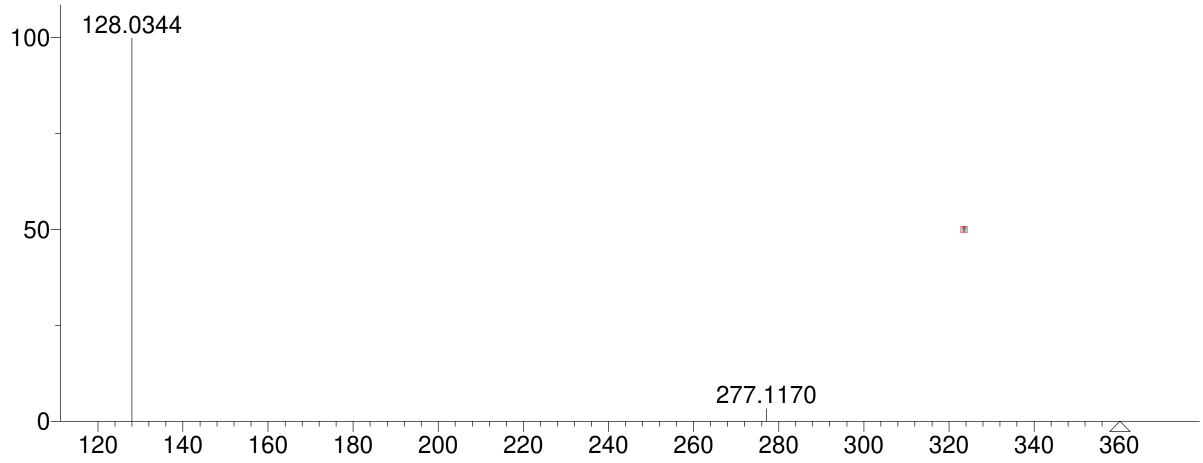

Hit 1 : Pesticide6_Etoxazole_C21H23F2NO2_4-(4-tert-Butyl-2-ethoxyphenyl)-2-(2,6-difluorophenyl)-4,5-dihydro-1
C21H23F2NO2; MF: 0; RMF: 7; Prob 100.0%; Lib: mona-export-gnps; ID: 12999.

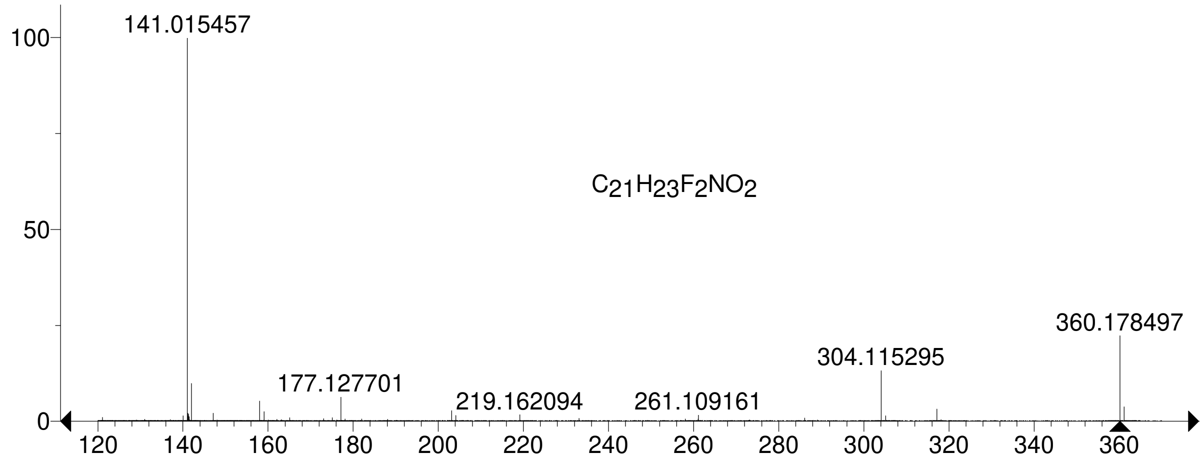

Unknown: Unknown (3.70_455.1661m/z)
Compound in Library Factor = -1848

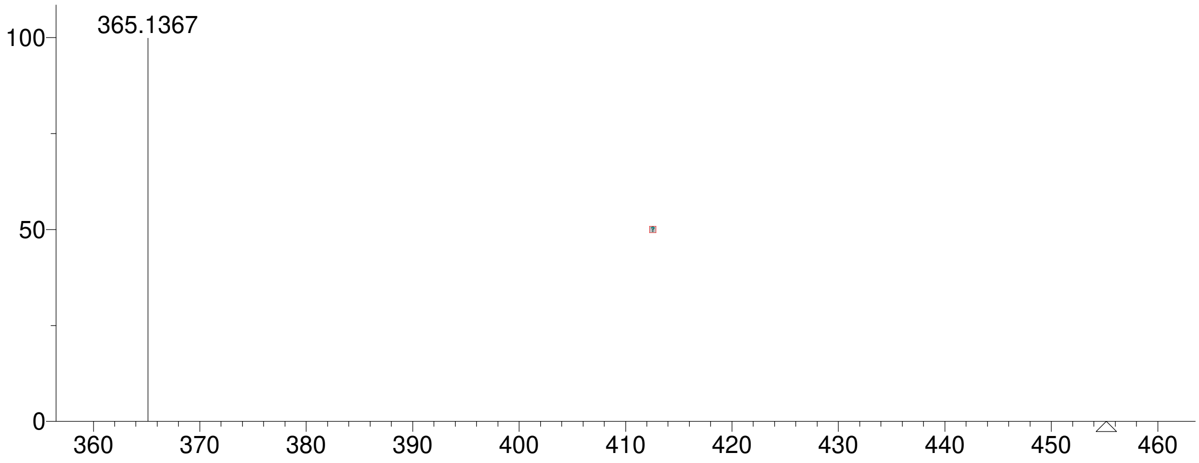

Hit 1 : C20H28N2O10; PlaSMA ID-1228 [M-H]- P=455.2
C20H28N2O10; MF: 39; RMF: 507; Prob 80.9%; Lib: biomsms-neg-plasma; ID: 855.

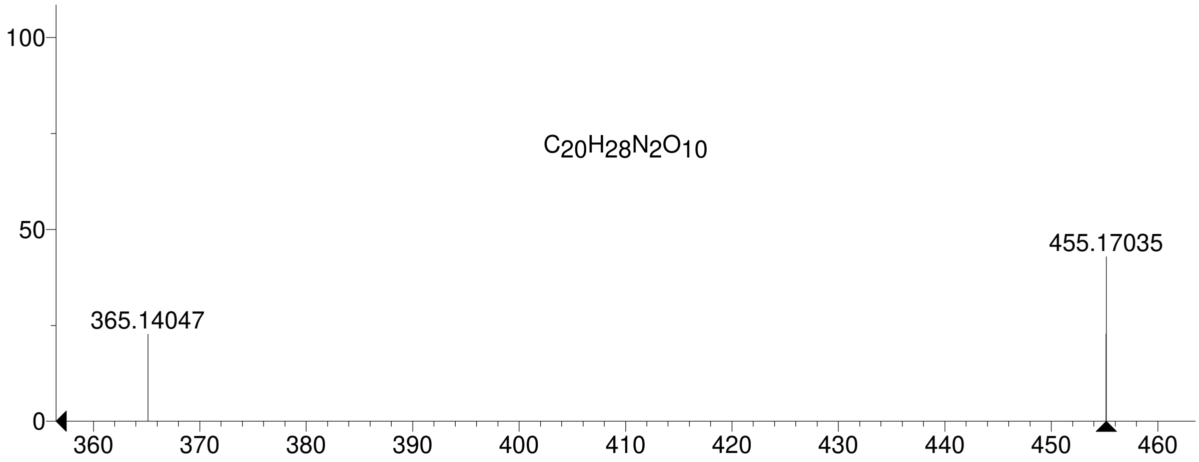

Hit 2 : methyl 3-(3,4-dihydroxyphenyl)-2-[[4-hydroxy-3-(3-methylbut-2-enyl)phenyl]methyl]-4-methoxy-5-oxofuran-2
C25H26O8; MF: 0; RMF: 134; Prob 19.1%; Lib: mona-export-vf-npl_qexactive; ID: 9394.

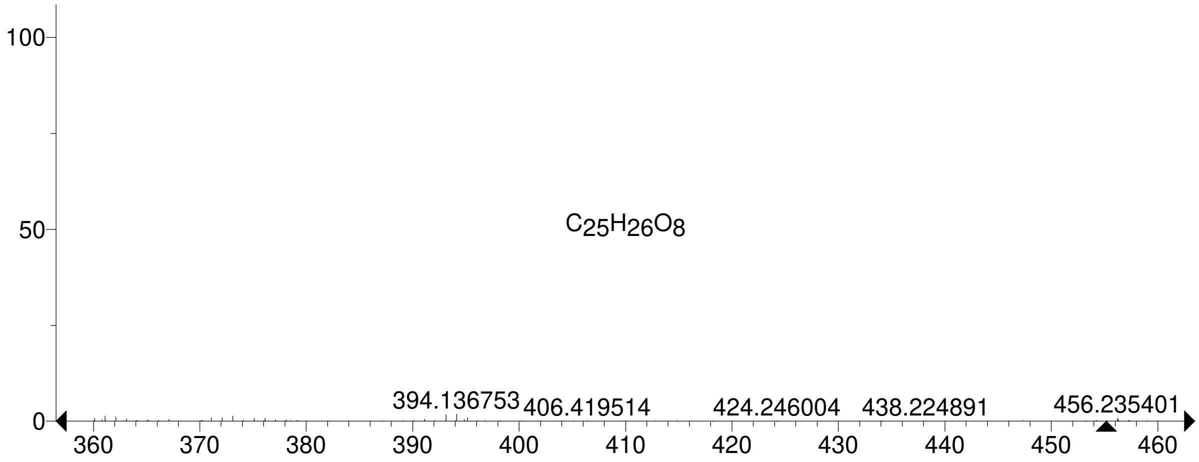

** Search Report Page 1 of 1 **

Unknown: Unknown (3.70_587.2200m/z)
Compound in Library Factor = 0

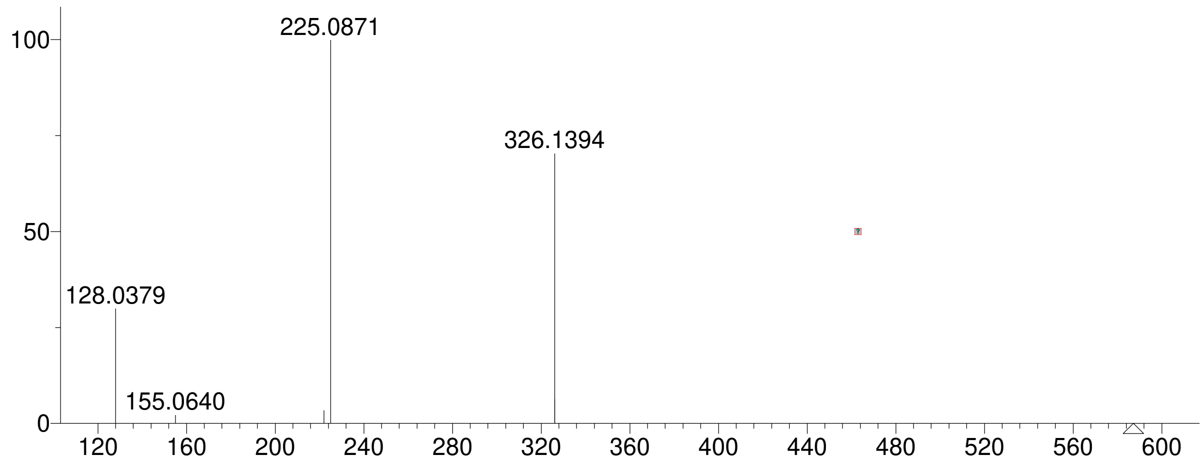

Unknown: Unknown (3.70_966.3585m/z)
Compound in Library Factor = 0

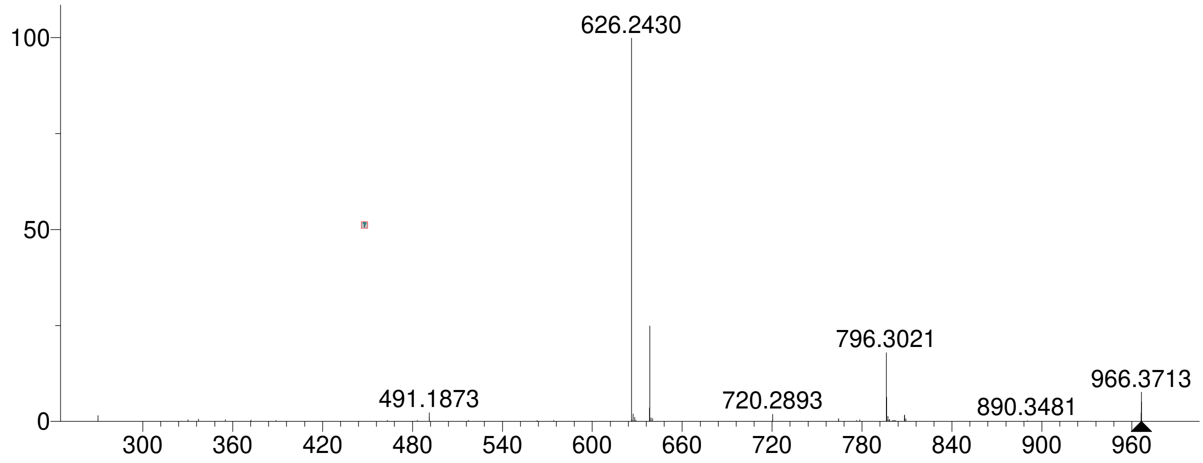

** Search Report Page 1 of 1 **

Unknown: Unknown (3.71_314.1338m/z)
Compound in Library Factor = 0

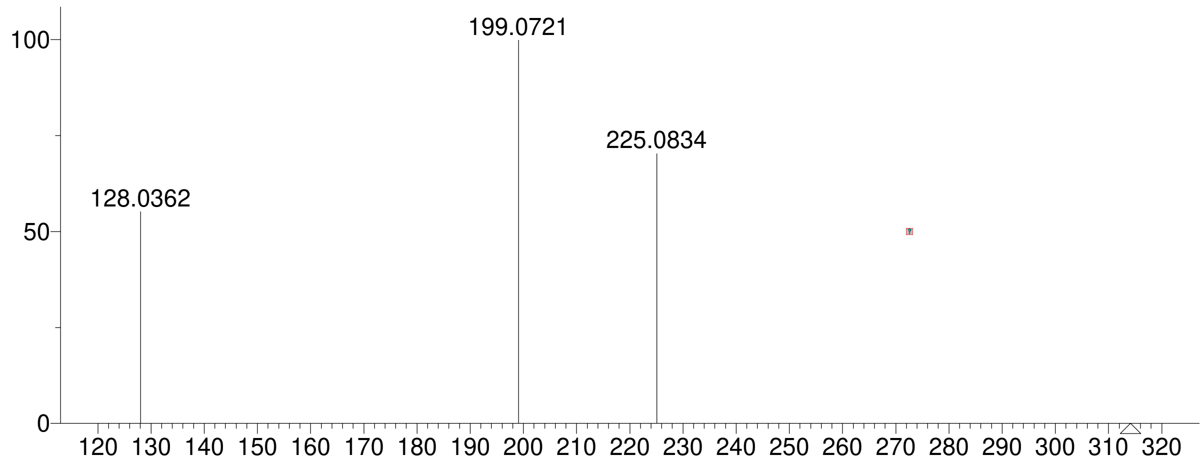

Hit 1 : Formylciprofloxacin [M-H-CO2]- HCD 40V P=314.1
C18H18FN3O4; MF: 0; RMF: 78; Prob 100.0%; CAS: 93594-39-9; Lib: nist_msms; ID: 439525.

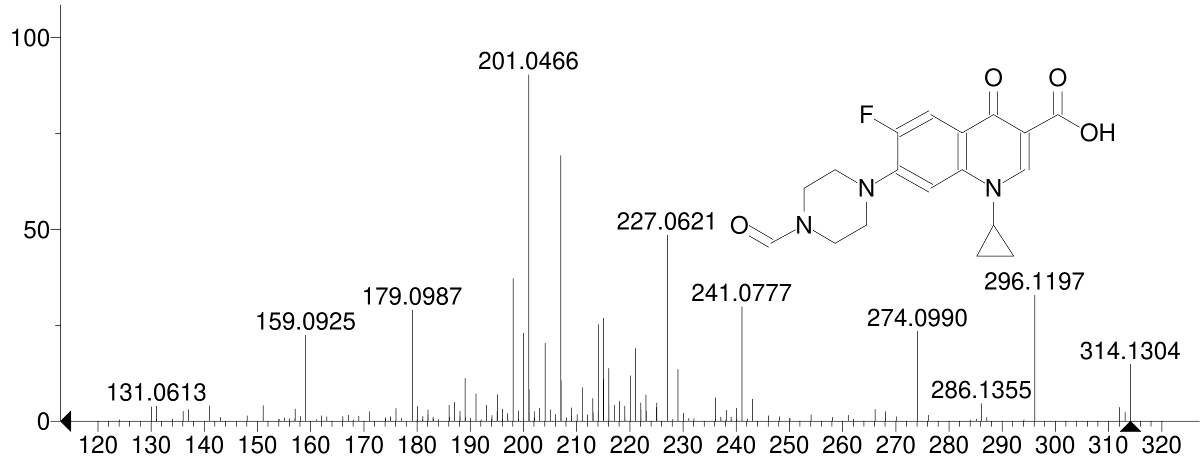

Unknown: Unknown (3.71_524.1011m/z)
Compound in Library Factor = 0

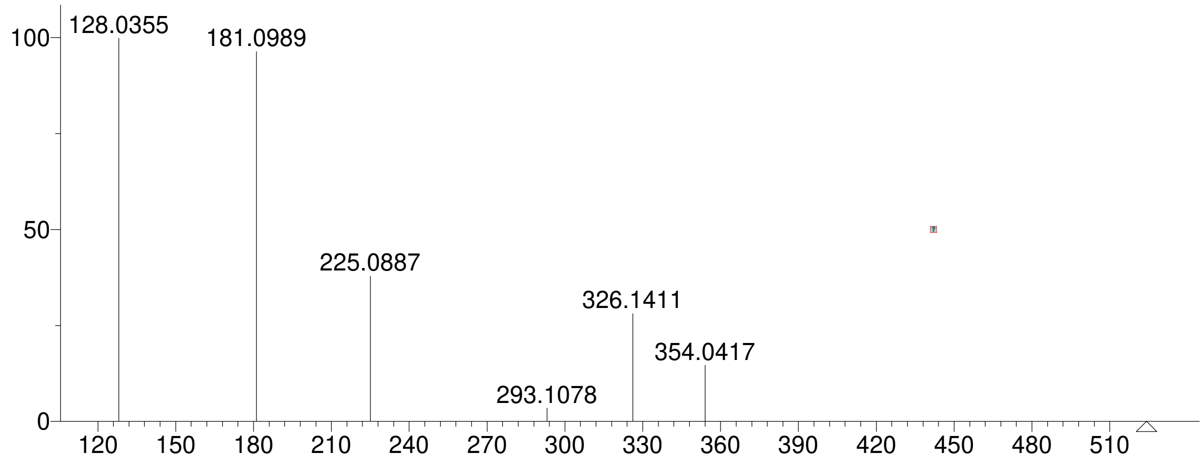

Hit 1 : Acrinathrin [M+H]+=>524.1 IT 35% P=524.1
C26H21F6NO5; MF: 0; RMF: 36; Prob 100.0%; CAS: 101007-06-1; Lib: nist_msms; ID: 476079.

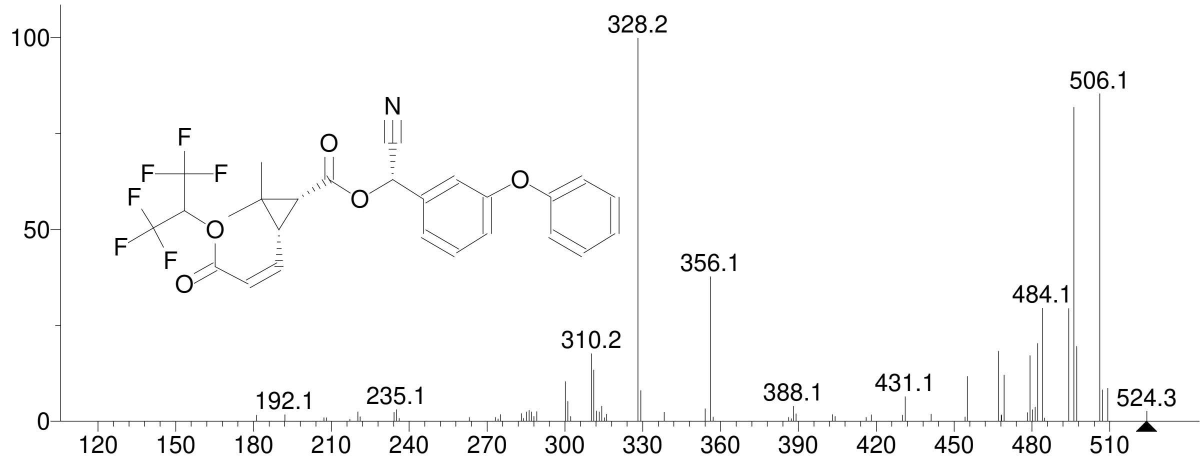

Unknown: Unknown (3.71_680.2771m/z)
Compound in Library Factor = 0

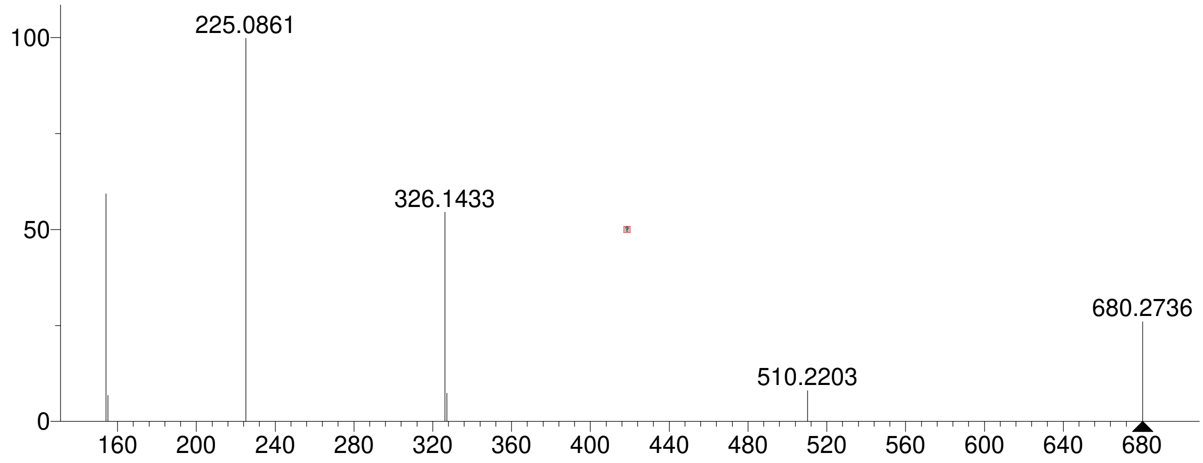

Unknown: Unknown (3.72_225.0870m/z)
Compound in Library Factor = -219

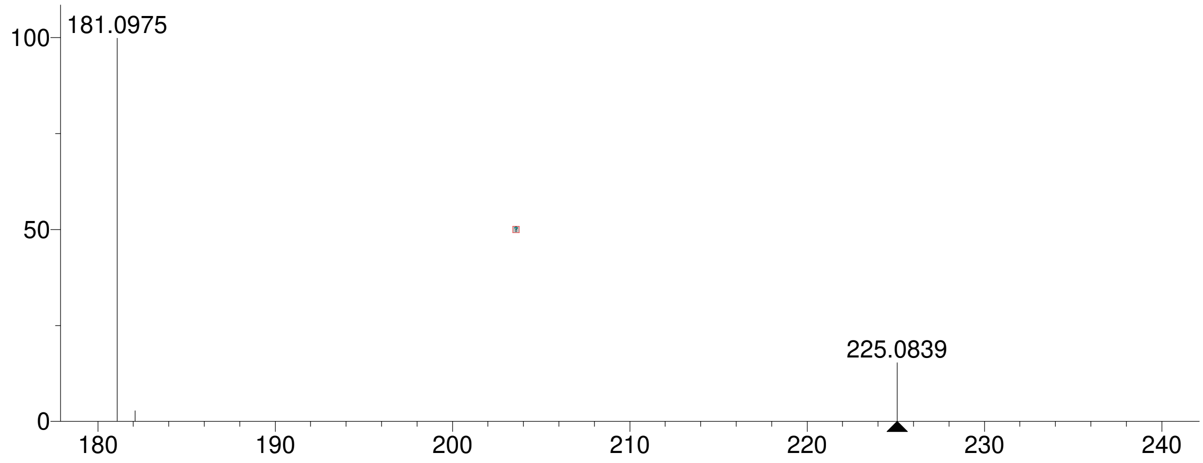

Hit 1 : Porphobilinogen [M-H]- HCD 24V P=225.1
C10H14N2O4; MF: 560; RMF: 961; Prob 99.0%; CAS: 487-90-1; Lib: nist_msms; ID: 125588.

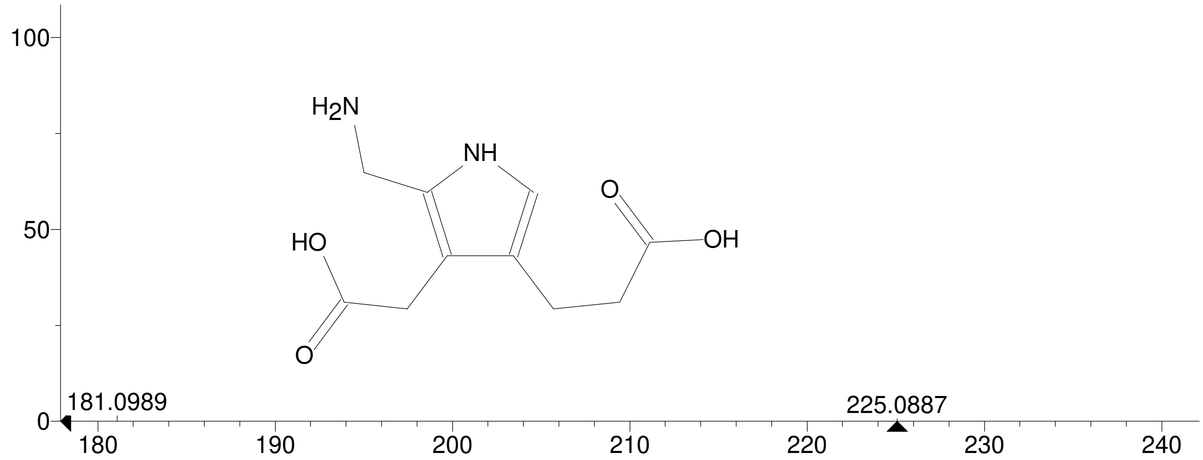

Hit 2 : 3-Hydroxy-DL-kynurenine [M+H]+ QTOF 5-45V P=225.1
C10H12N2O4; MF: 254; RMF: 894; Prob 0.98%; Lib: mona-export-respect; ID: 3984.

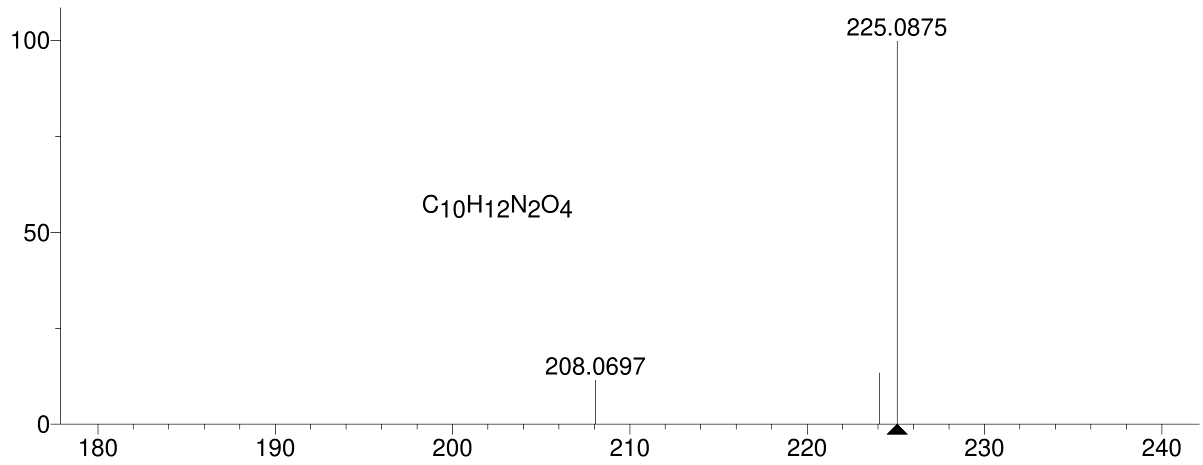

Hit 3 : Thymidine [M+H-H2O]+ HCD 4V P=225.1
C10H14N2O5; MF: 103; RMF: 360; Prob 0.02%; CAS: 50-89-5; Lib: nist_msms; ID: 296349.

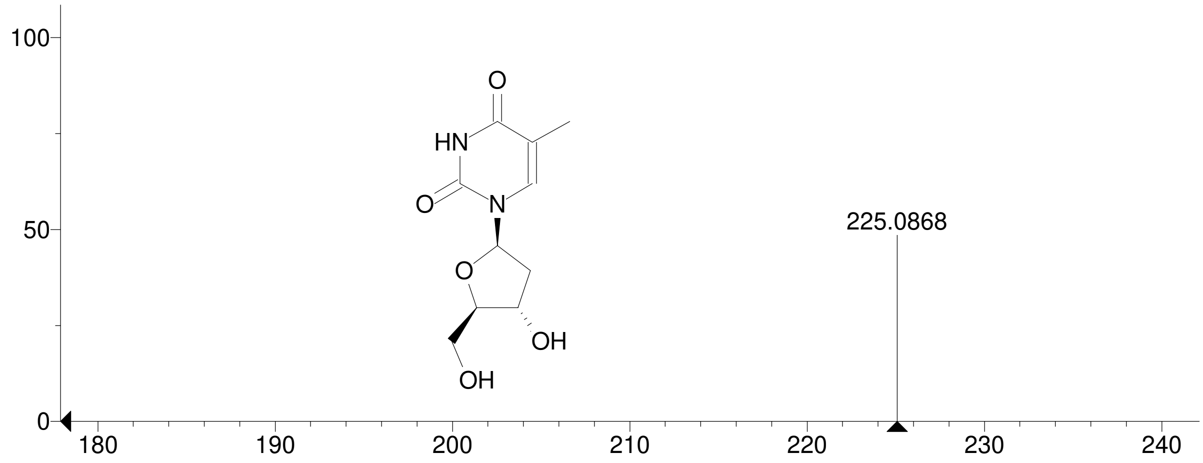

Hit 4 : Carbidopa [M-H]- HCD 9V P=225.1
C10H14N2O4; MF: 85; RMF: 388; Prob 0.01%; CAS: 28860-95-9; Lib: nist_msms; ID: 313764.

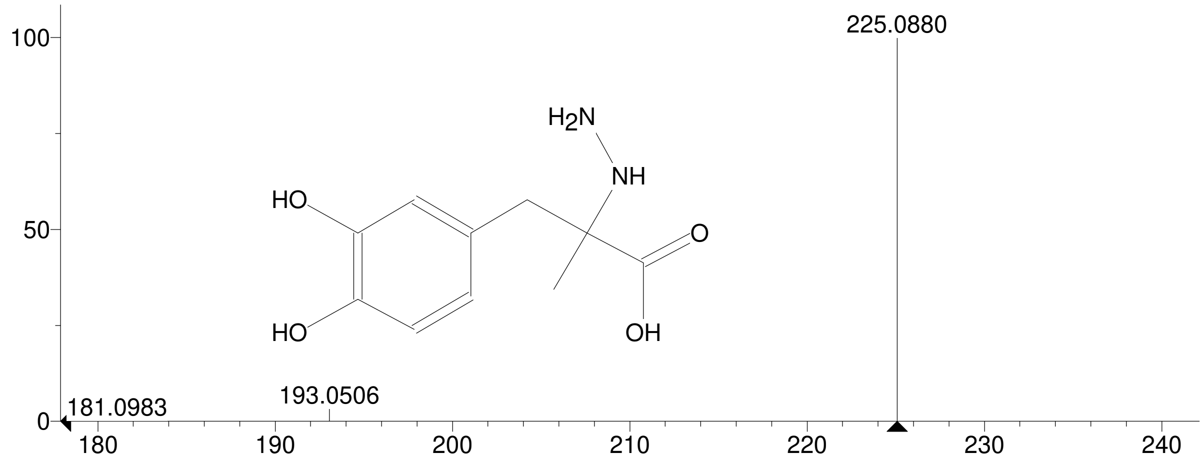

Hit 5 : 3-Hydroxykynurenine [M+H]+ HCD 0V P=225.1
C10H12N2O4; MF: 48; RMF: 337; Prob 0.00%; CAS: 2147-61-7; Lib: nist_msms; ID: 162769.

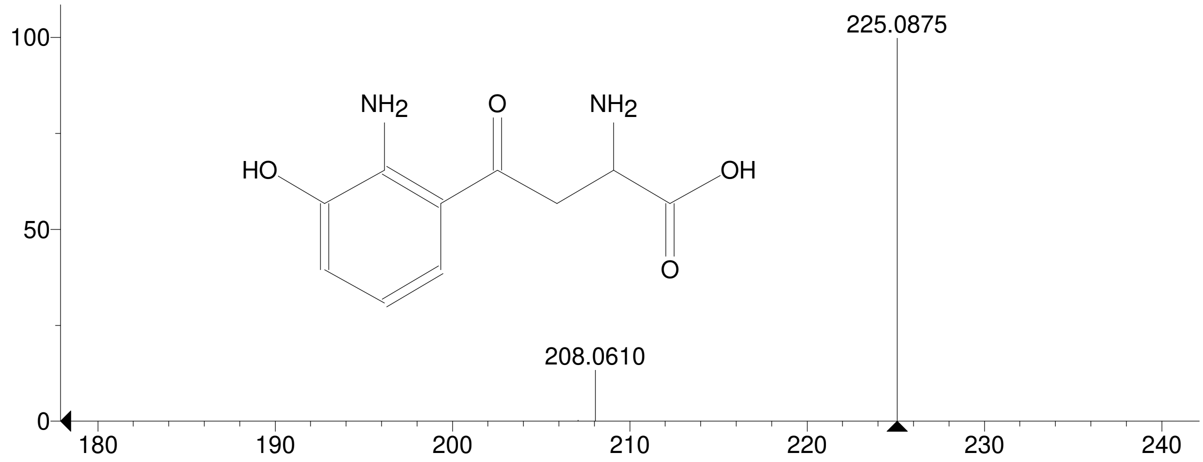

Unknown: Unknown (3.74_562.1180m/z)
Compound in Library Factor = 0

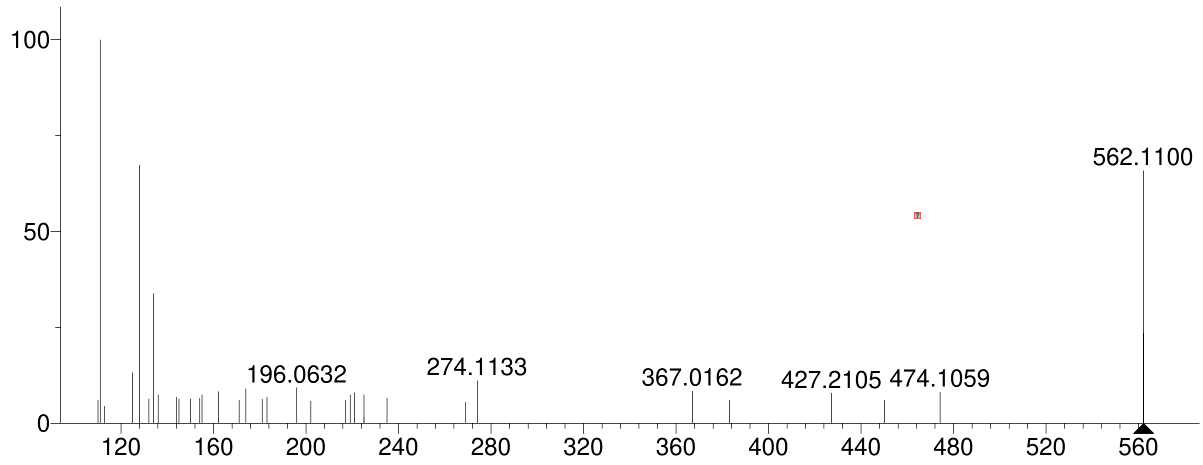

** Search Report Page 1 of 1 **

Unknown: Unknown (3.76_582.2041m/z)
Compound in Library Factor = -3273

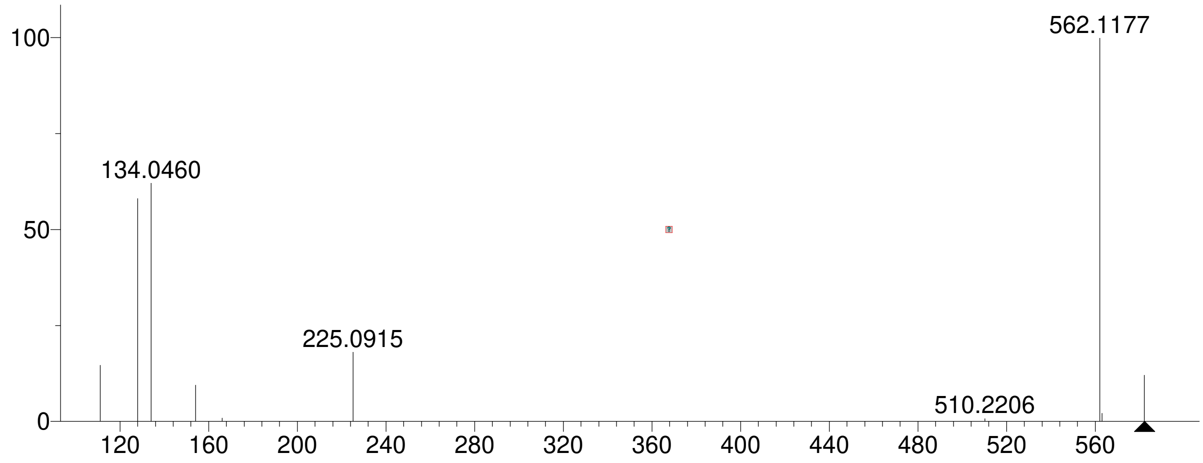

Hit 1 : Benzeneacetic acid, 3-[3-[[[2-chloro-3-(trifluoromethyl)phenyl]methyl](2,2-diphenylethyl)amino]propoxy]- [M
C33H31ClF3NO3; MF: 19; RMF: 277; Prob 57.3%; CAS: 405911-09-3; Lib: nist_msms; ID: 247416.

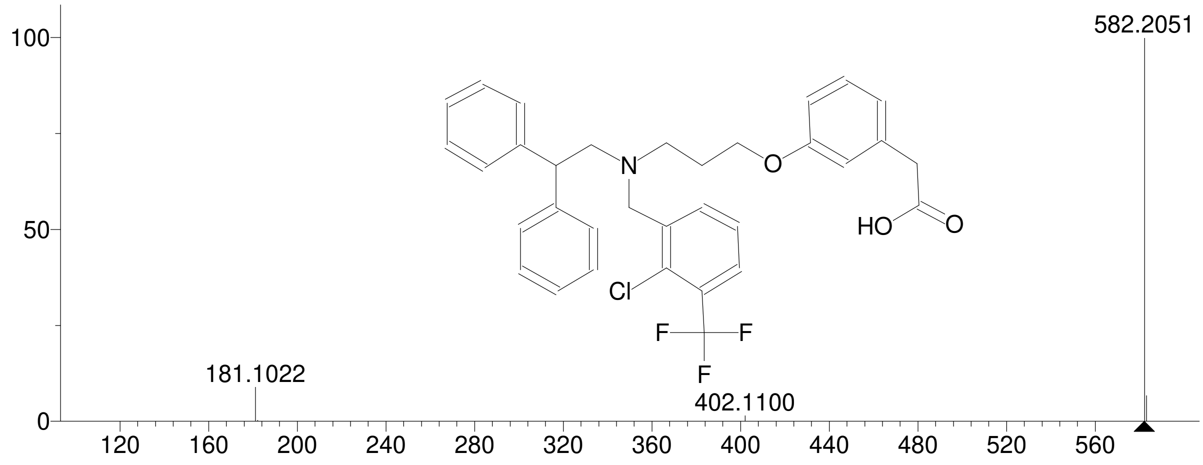

Hit 2 : Isoglobotriaose-β-N(Acetyl)-Propargyl [M-H]- HCD 17V P=582.2
C23H37NO16; MF: 11; RMF: 210; Prob 42.7%; Lib: nist_msms; ID: 185069.

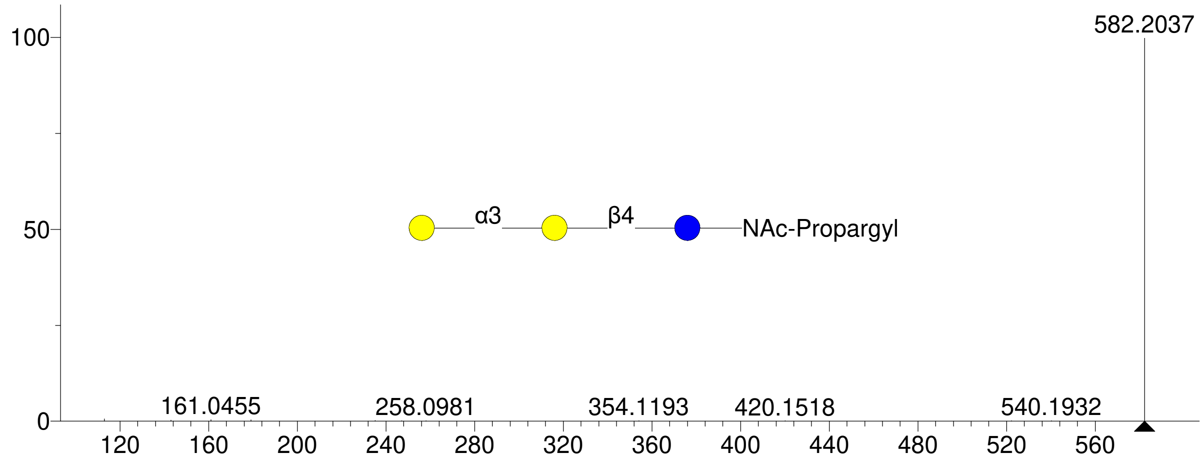

** Search Report Page 1 of 1 **

Unknown: Unknown (3.76_585.1452m/z)
Compound in Library Factor = -3273

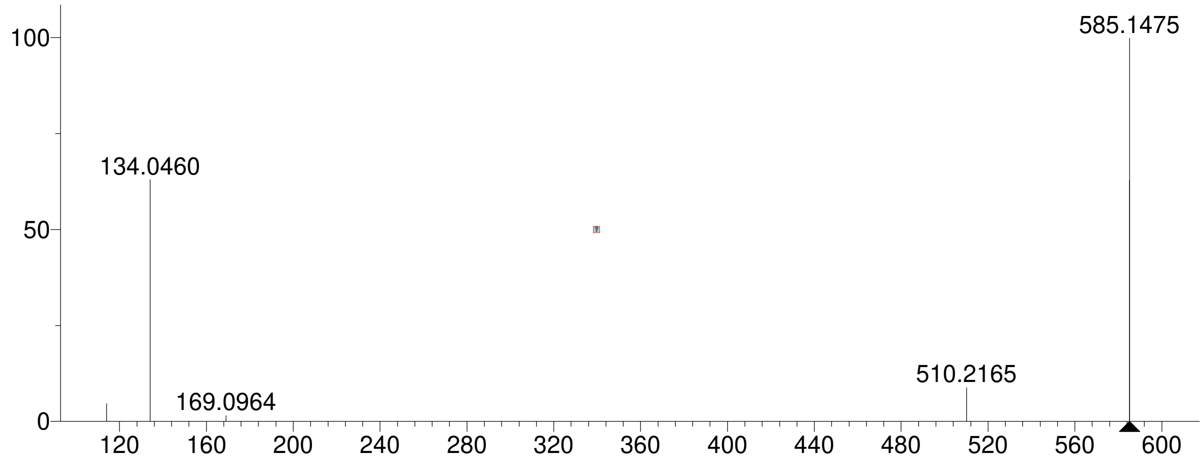

Hit 1 : Neomangiferin [M+H]+ HCD 11V P=585.1
C25H28O16; MF: 126; RMF: 733; Prob 100.0%; CAS: 64809-67-2; Lib: nist_msms; ID: 239539.

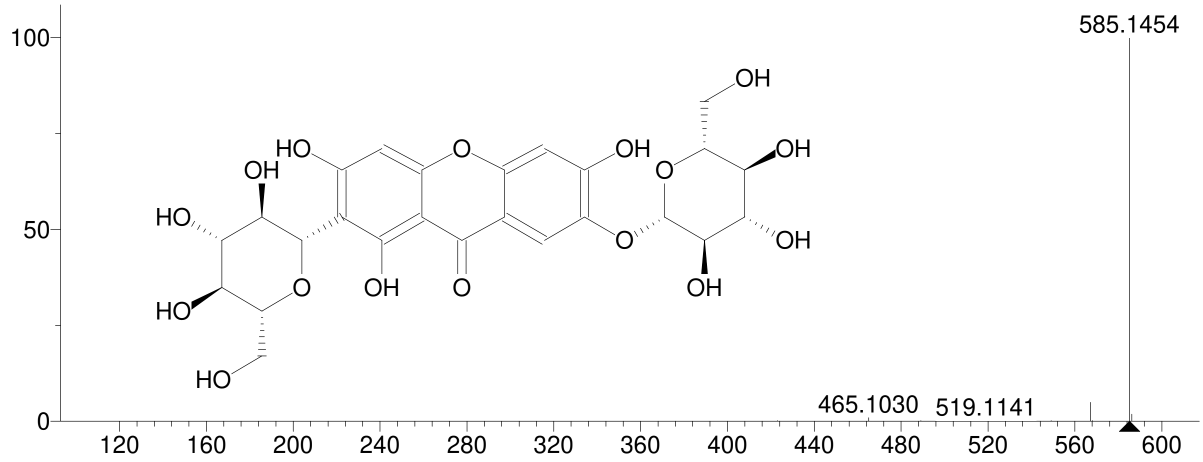

Unknown: Unknown (3.77_213.0868m/z)
Compound in Library Factor = 0

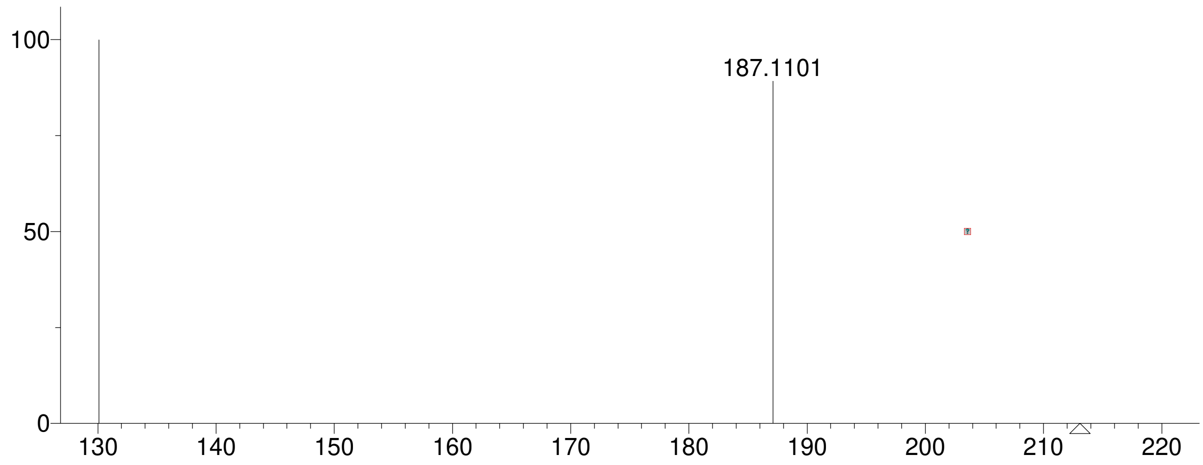

Unknown: Unknown (3.77_417.1521m/z)
Compound in Library Factor = -3273

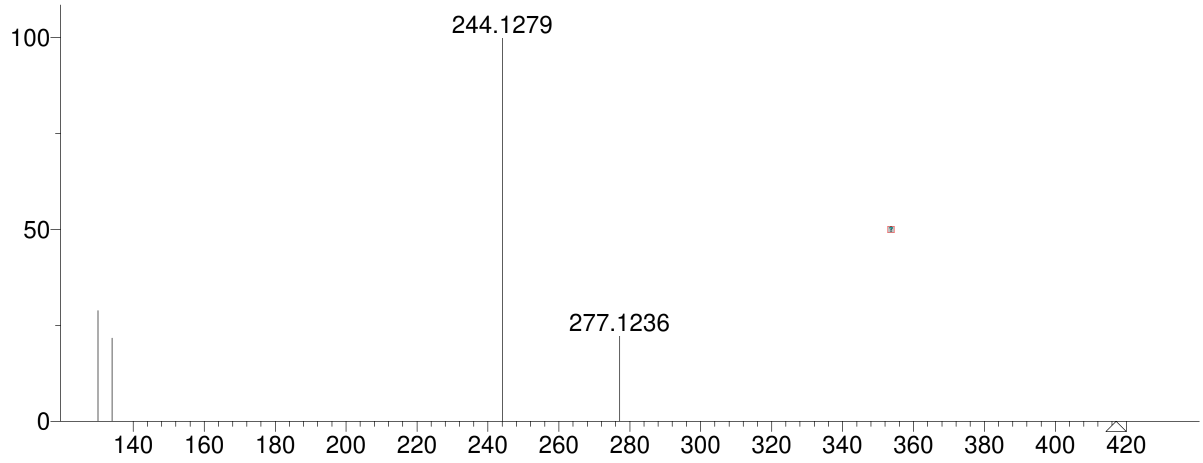

Hit 1 : Halopemide [M+H]+ HCD 108V P=417.1
C21H22ClFN4O2; MF: 1; RMF: 88; Prob 25.7%; CAS: 59831-65-1; Lib: nist_msms; ID: 321101.

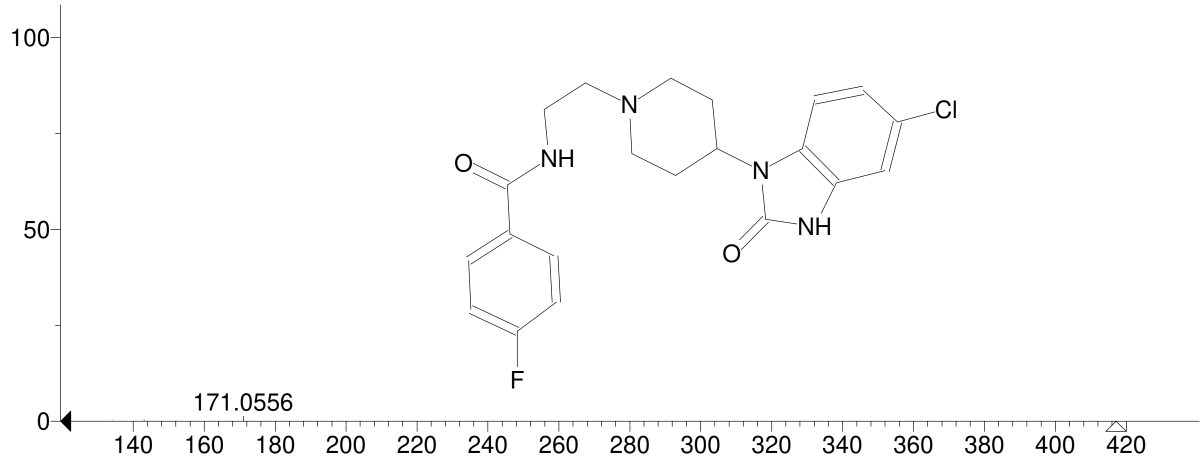

Hit 2 : NCGC00380812-02_C23H22N4O5_1H-Imidazo[1,2-a]indole-9-propanoic acid, 2,3,9,9a-tetrahydro-9-hydro
C23H22N4O5; MF: 0; RMF: 113; Prob 24.8%; Lib: mona-export-gnps; ID: 13176.

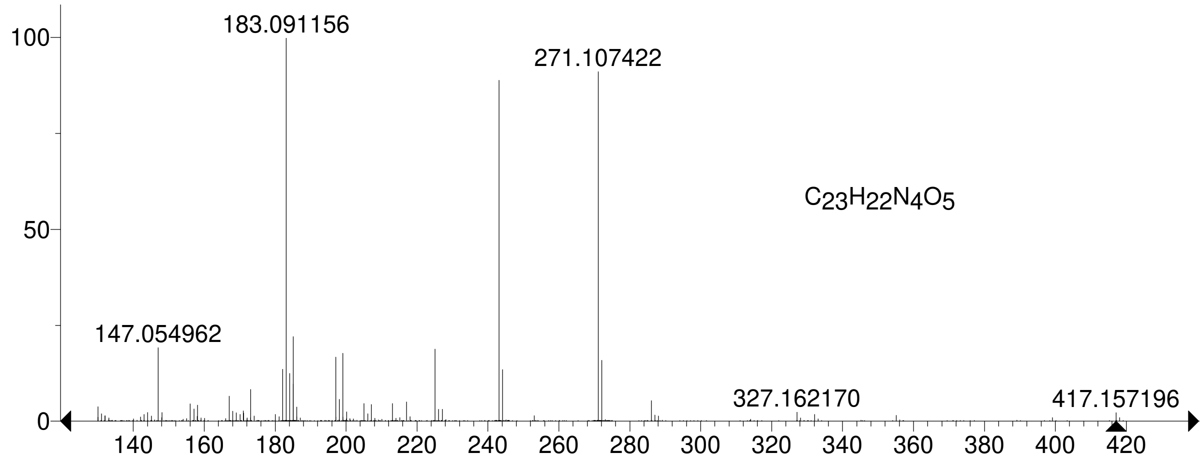

Hit 3 : NCGC00380812-01!3-(4-hydroxy-2,2-dimethyl-1-oxo-3,3a-dihydroimidazo[1,2-a]indol-4-yl)-2-(4-oxoquinaz
C23H22N4O5; MF: 0; RMF: 99; Prob 24.8%; Lib: mona-export-gnps; ID: 7434.

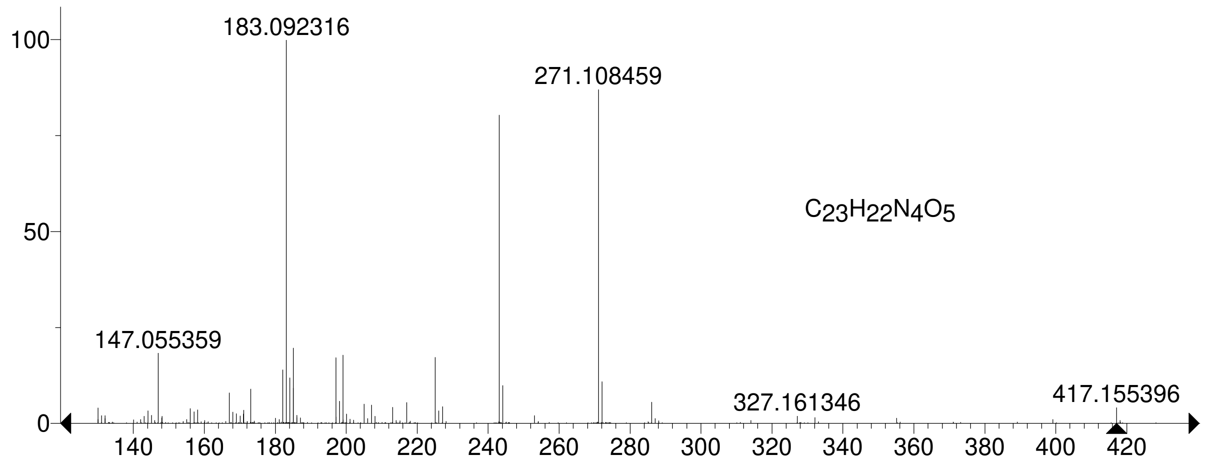

Hit 4 : NCGC00384503-01_C22H24O8_(2E)-2,11-Dimethyl-7-methylene-6,12-dioxo-5,14-dioxatricyclo[9.2.1.0~4,
C22H24O8; MF: 0; RMF: 8; Prob 24.8%; Lib: mona-export-gnps; ID: 10322.

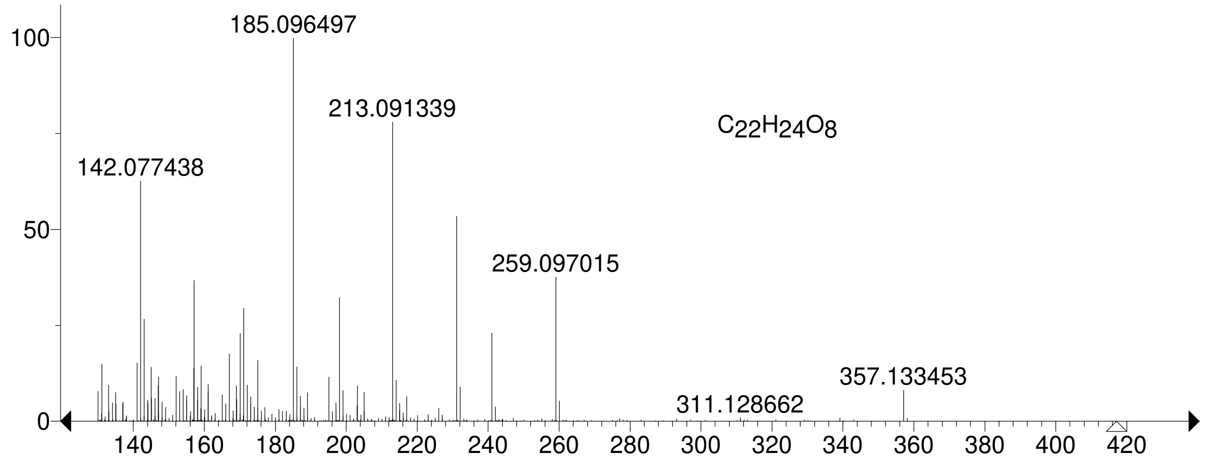

** Search Report Page 1 of 1 **

Unknown: Unknown (3.77_437.0672m/z)
Compound in Library Factor = 0

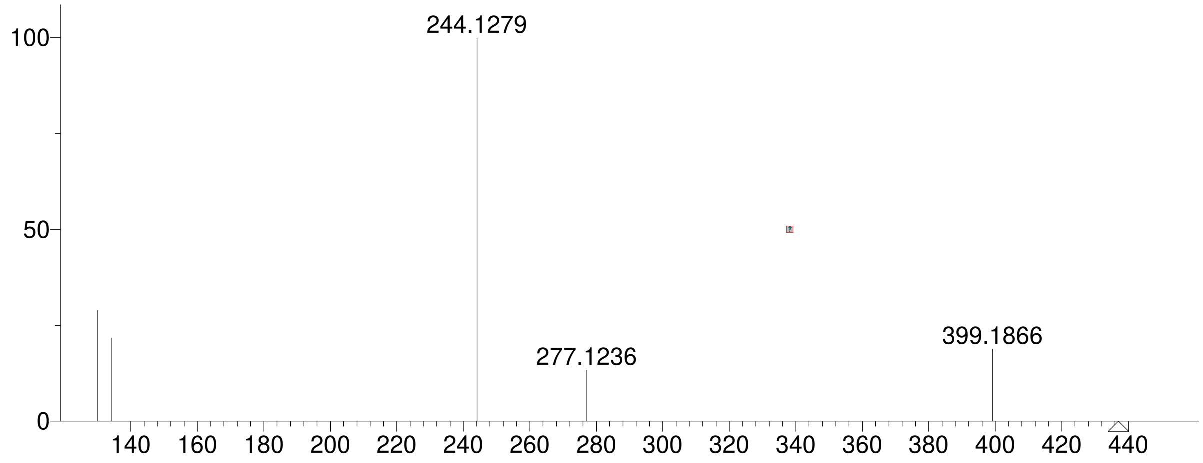

Unknown: Unknown (3.77_626.2391m/z)
Compound in Library Factor = 0

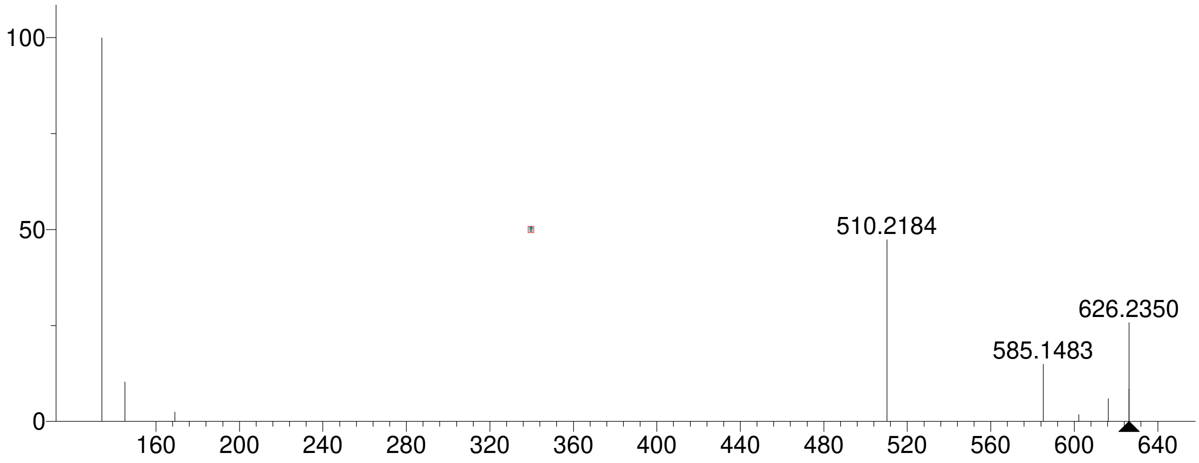

Unknown: Unknown (3.77_966.3249m/z)
Compound in Library Factor = 0

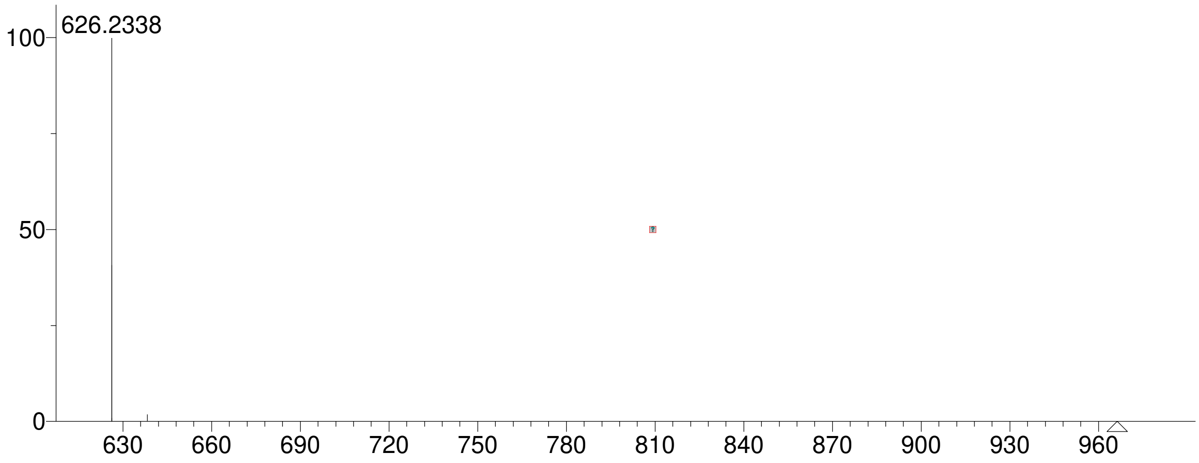

Unknown: Unknown (3.78_417.1968m/z)
Compound in Library Factor = -3273

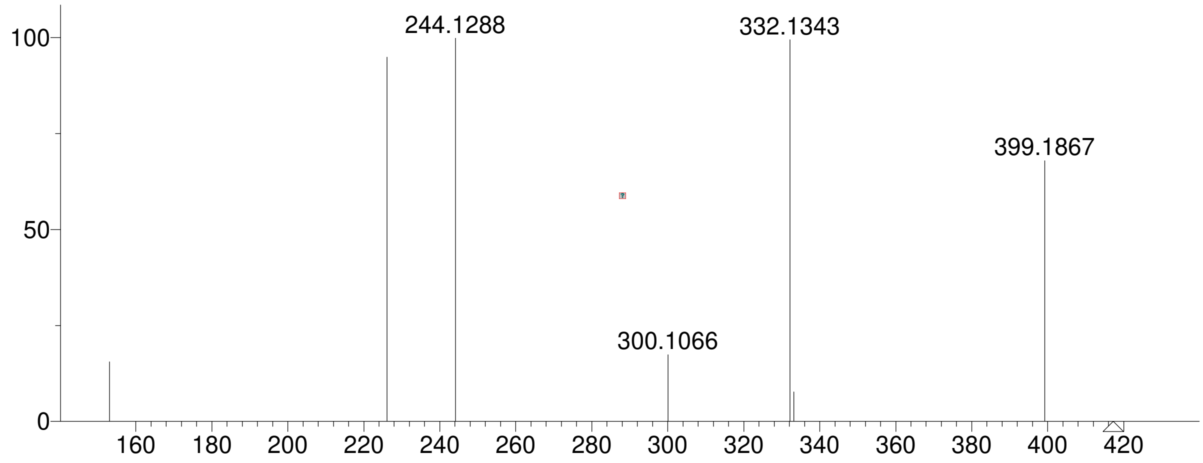

Hit 1 : Glycine, N,N'-[1,2-ethanediylbis(oxy-2,1-phenylene)]bis[N-(carboxymethyl)- [M-H]-=>417.2 IT 35% P=41
C22H24N2O10; MF: 1; RMF: 203; Prob 9.38%; CAS: 85233-19-8; Lib: nist_msms; ID: 414018.

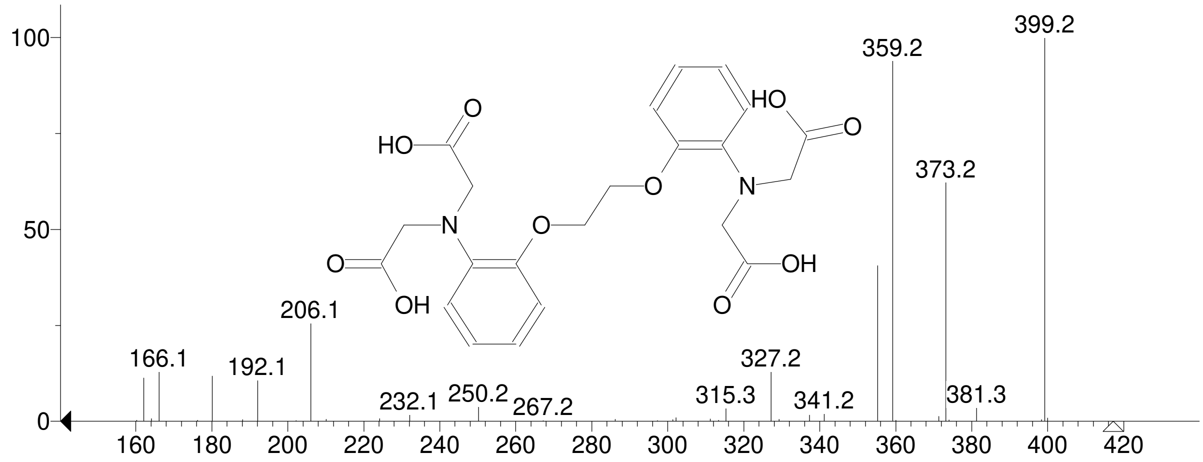

Hit 2 : 19-Norandrosterone glucuronide [M+H-H2O]+=>417.2 IT 35% P=417.2
C24H36O8; MF: 1; RMF: 96; Prob 9.38%; CAS: 294213-86-8; Lib: nist_msms; ID: 330392.

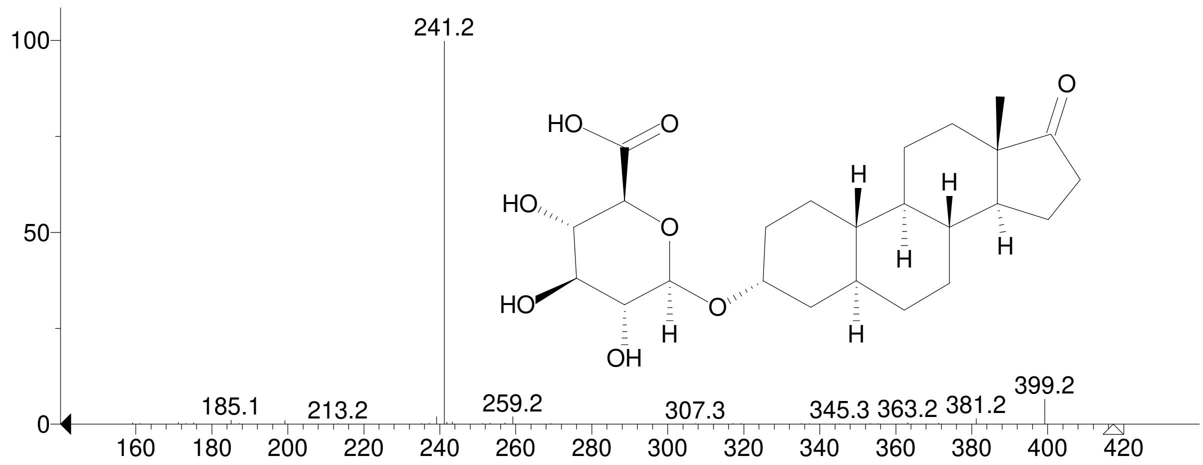

Hit 3 : Withanolide A [M+H]+=>417.2 IT 35% P=417.2
C28H38O6; MF: 0; RMF: 183; Prob 9.02%; CAS: 32911-62-9; Lib: nist_msms; ID: 227142.

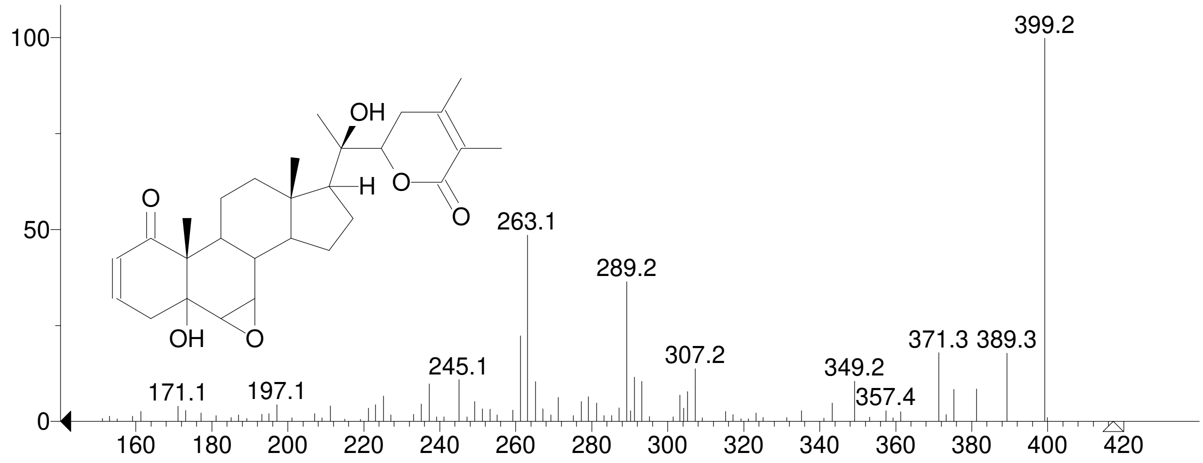

Hit 4 : 12-Deoxywithastramonolide [M+H-H2O]+=>417.2 IT 35% P=417.2
C28H38O6; MF: 0; RMF: 183; Prob 9.02%; CAS: 60124-17-6; Lib: nist_msms; ID: 502681.

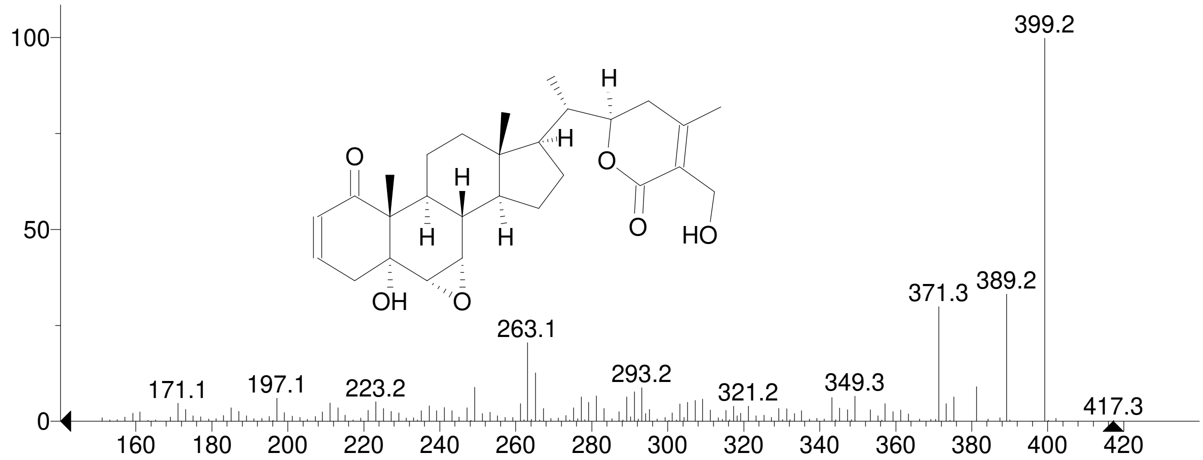

Hit 5 : Dipyridamole [M+H]+=>460.3=>417.2 IT 35% P=417.2
C24H40N8O4; MF: 0; RMF: 166; Prob 9.02%; CAS: 58-32-2; Lib: nist_msms; ID: 314333.

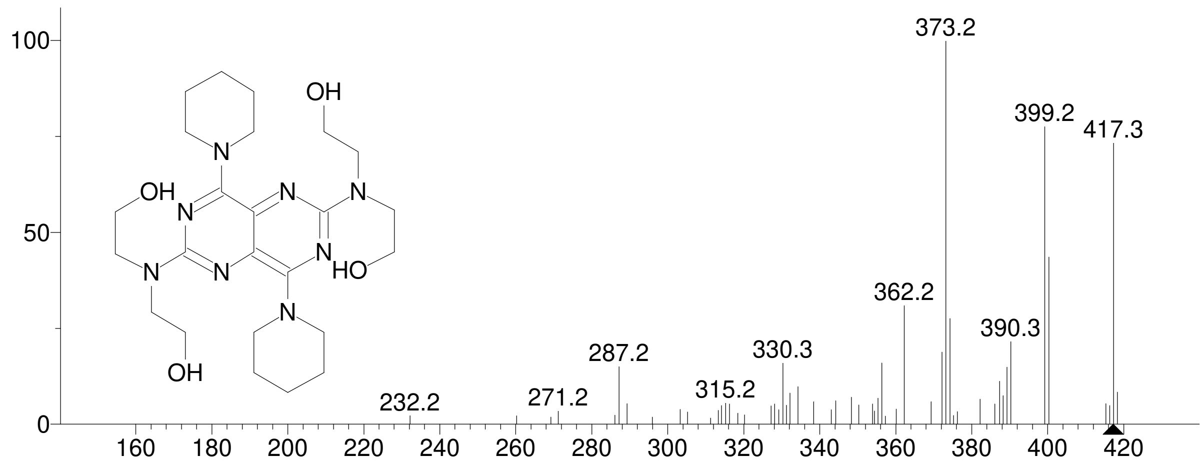

** Search Report Page 1 of 1 **

Unknown: Unknown (3.78_502.1928m/z)
Compound in Library Factor = 0

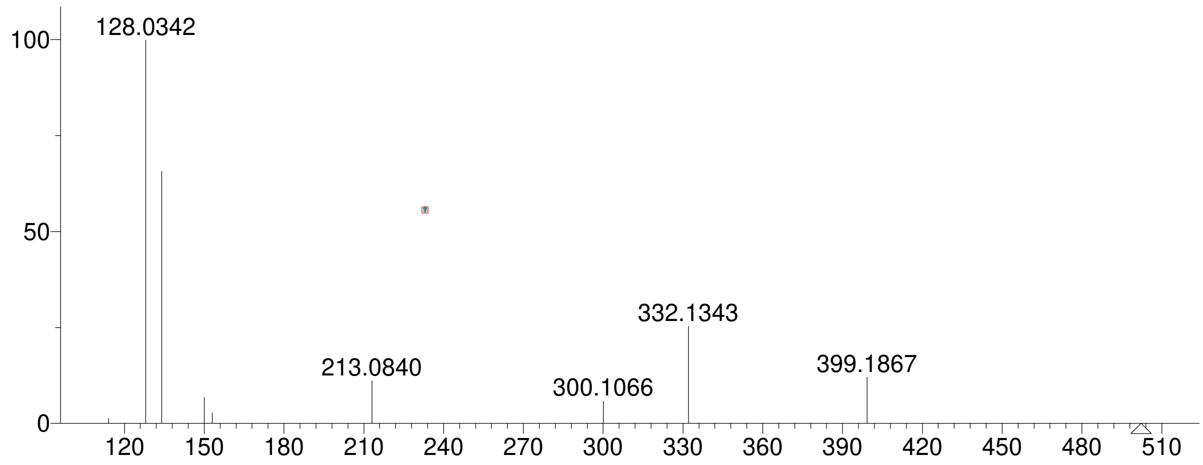

Hit 1 : Irinotecan [M+H-C5H11N]+ IT 35% P=502.2
C33H38N4O6; MF: 0; RMF: 35; Prob 50.0%; CAS: 97682-44-5; Lib: nist_msms; ID: 218340.

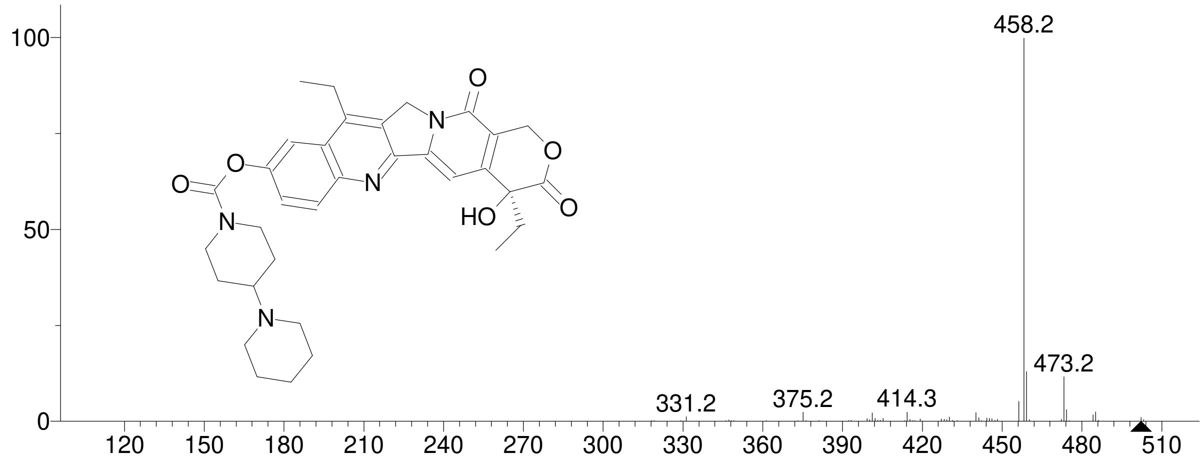

Hit 2 : [(2R,3S,4S,5R,6R)-3,4,5-trihydroxy-6-[2-(3-hydroxy-5-oxooxolan-3-yl)propoxy]oxan-2-yl]methyl (E)-3-(3,4-c
C22H28O12; MF: 0; RMF: 2; Prob 50.0%; Lib: mona-export-vf-npl_qexactive; ID: 12996.

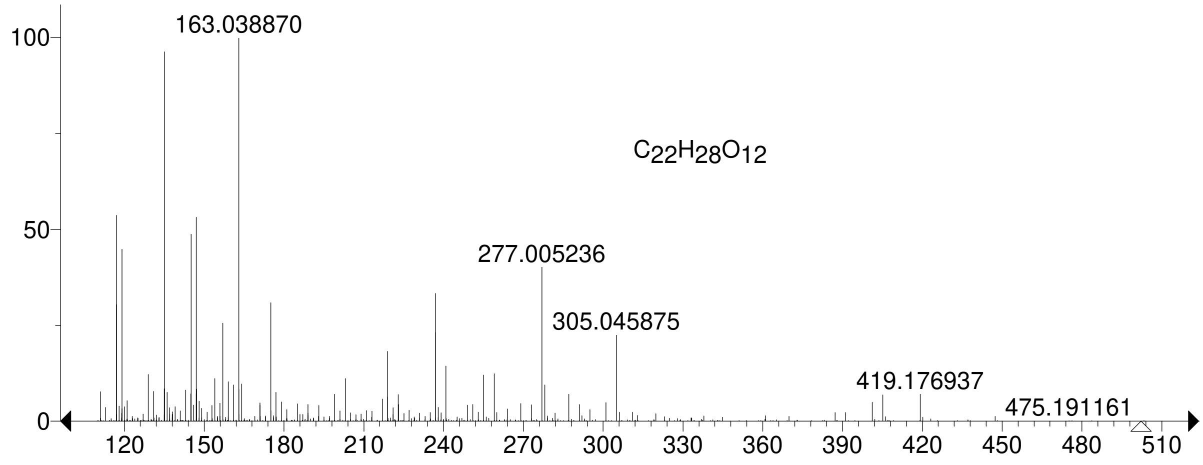

Unknown: Unknown (3.78_796.2983m/z)
Compound in Library Factor = 0

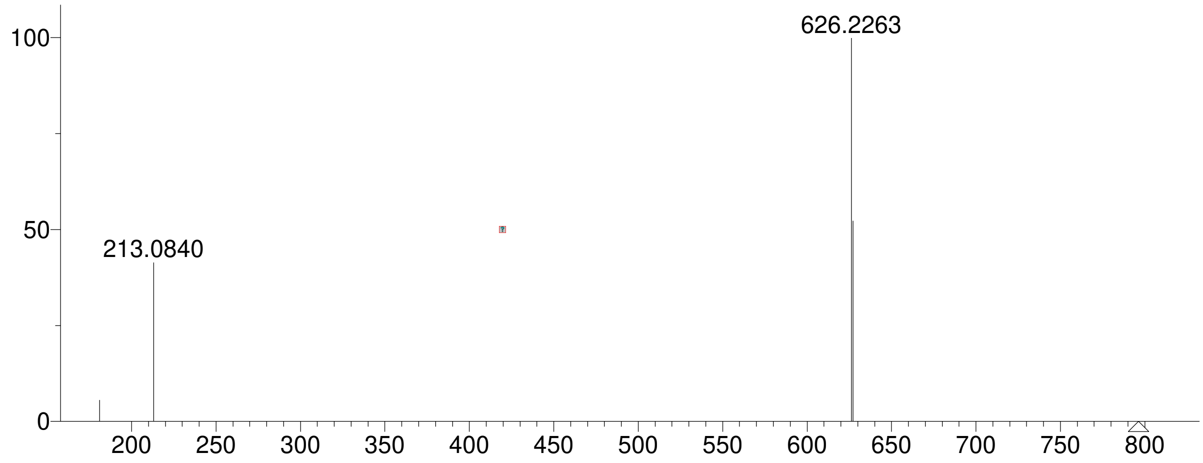

Unknown: Unknown (3.79_187.1070m/z)
Compound in Library Factor = -218

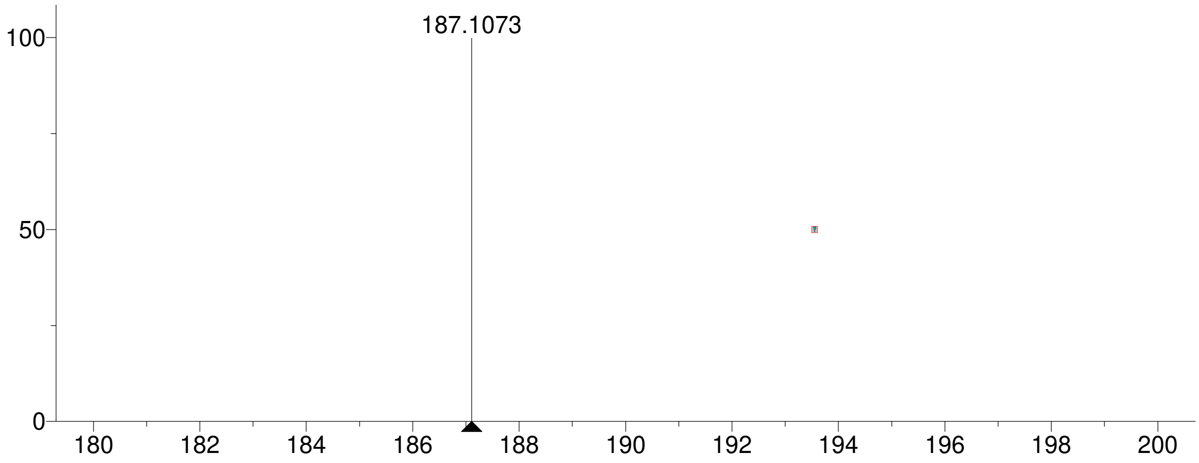

Hit 1 : Unknown (carbon number 8); PlaSMA ID-222 [M-H]- P=187.1
C8H16N2O3; MF: 364; RMF: 999; Prob 5.19%; Lib: biomsms-neg-plasma; ID: 1025.

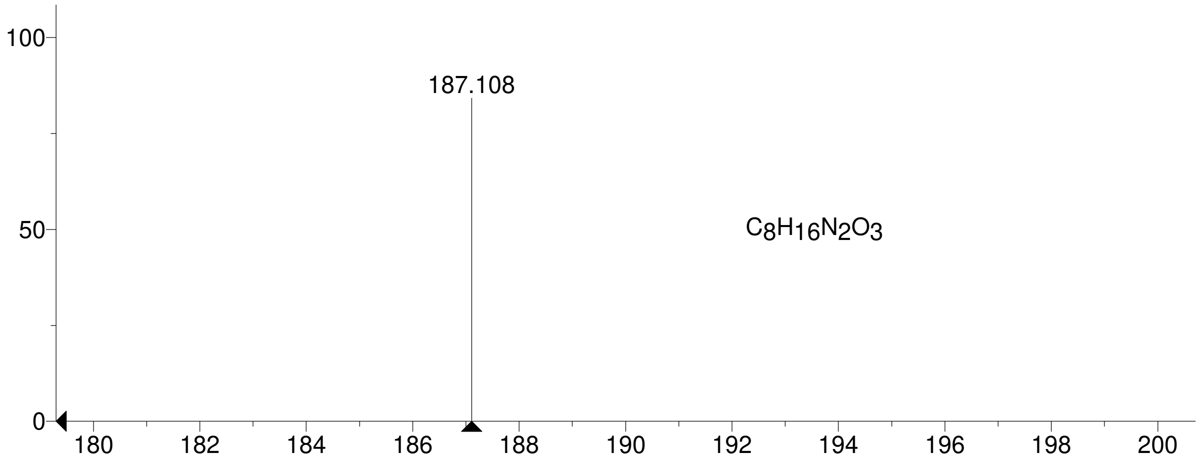

Hit 2 : Gly-Leu [M-H]- 20-30-40%) P=187.1
C8H16N2O3; MF: 364; RMF: 999; Prob 5.19%; Lib: mona-export-fiehn_hilic; ID: 1235.

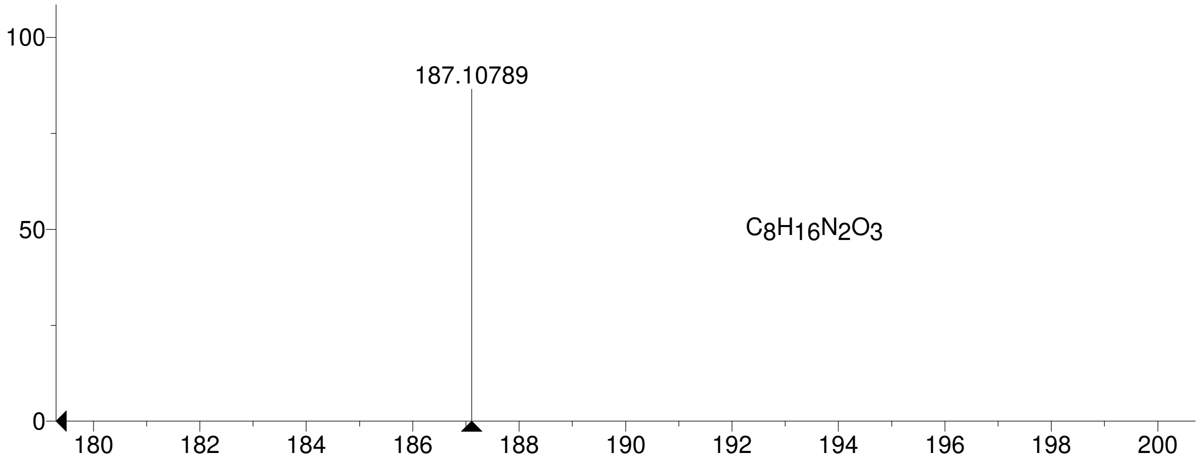

Hit 3 : Ala-Pro [M+H]+ QTOF 7V P=187.1
C8H14N2O3; MF: 364; RMF: 999; Prob 5.19%; CAS: 13485-59-1; Lib: nist_msms; ID: 17958.

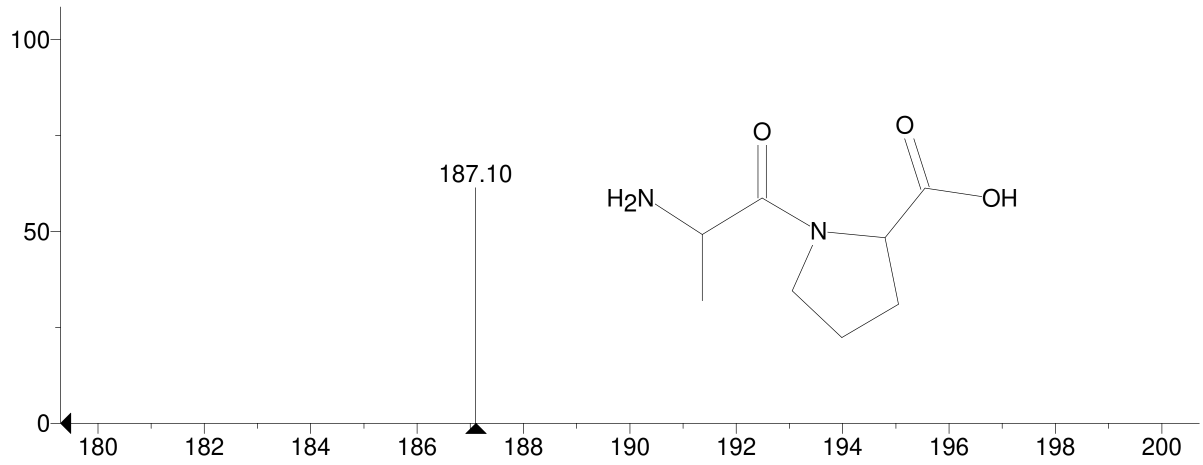

Hit 4 : Glycyl-L-norleucine [M-H]- HCD 46V P=187.1
C8H16N2O3; MF: 364; RMF: 999; Prob 5.19%; CAS: 19257-04-6; Lib: nist_msms; ID: 558394.

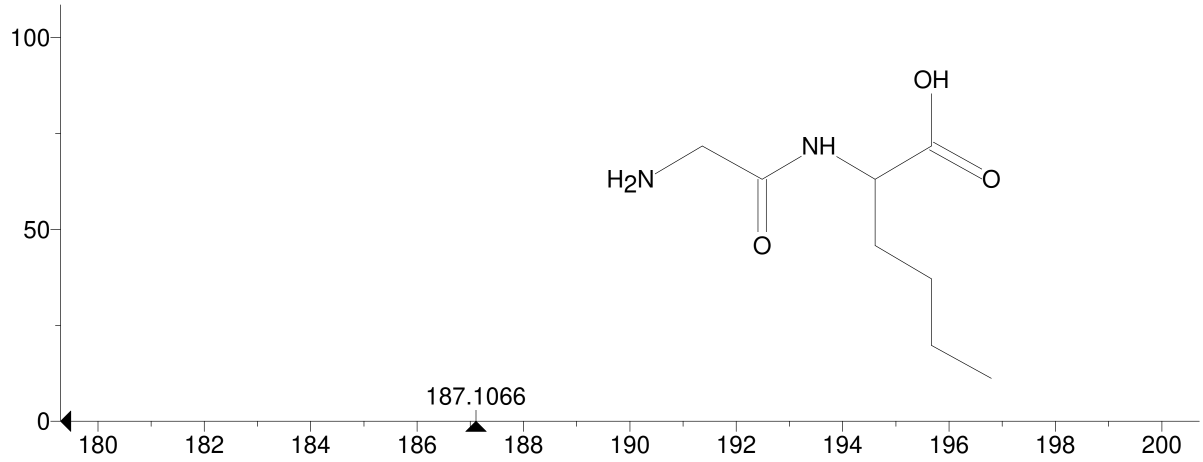

Hit 5 : Ala-Val [M-H]- HCD 29eV P=187.1
C8H16N2O3; MF: 364; RMF: 999; Prob 5.19%; Lib: nist_msms; ID: 567290.

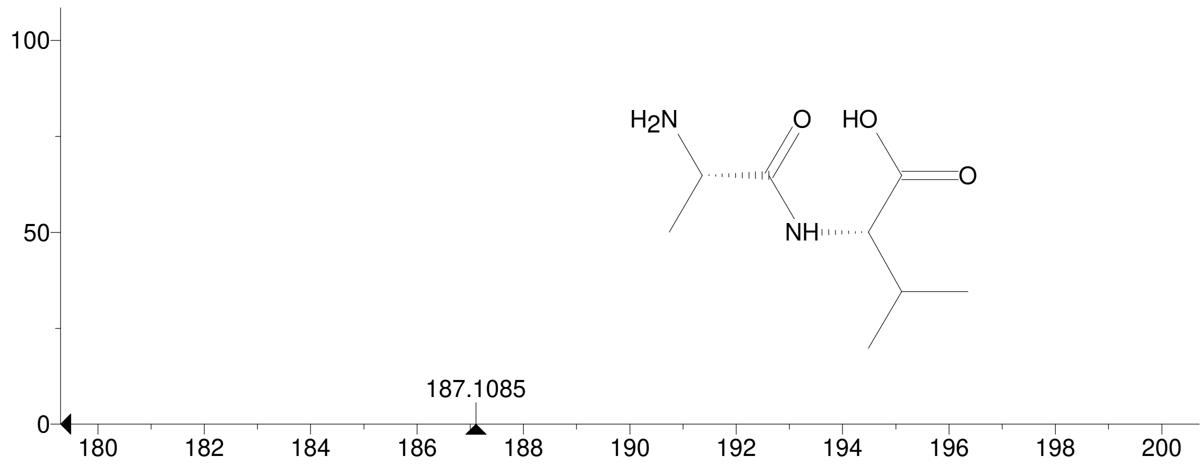

** Search Report Page 1 of 1 **

Unknown: Unknown (3.79_353.1447m/z)
Compound in Library Factor = 0

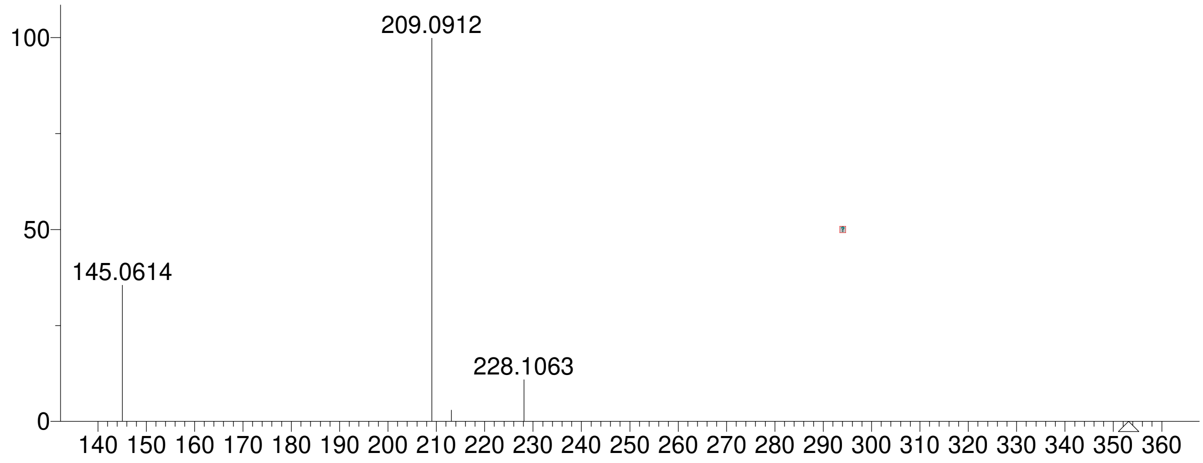

Unknown: Unknown (3.79_427.1698m/z)
Compound in Library Factor = -3273

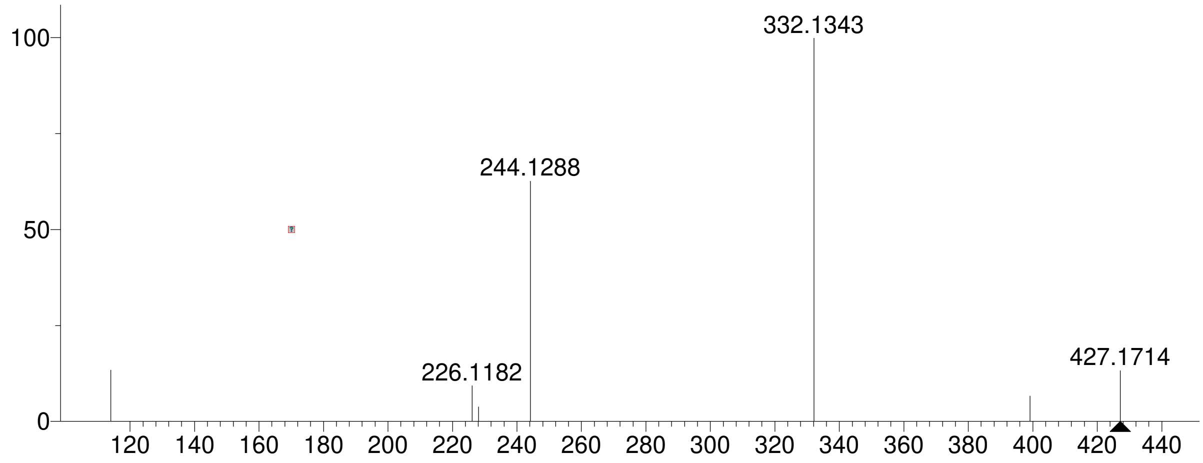

Hit 1 : PG 11:0; PG 2:0-9:0; [M-H]- [M-H]- P=427.2
C17H33O10P; MF: 3; RMF: 129; Prob 20.5%; Lib: msdial-insilicomsms-lipids-neg; ID: 324573.

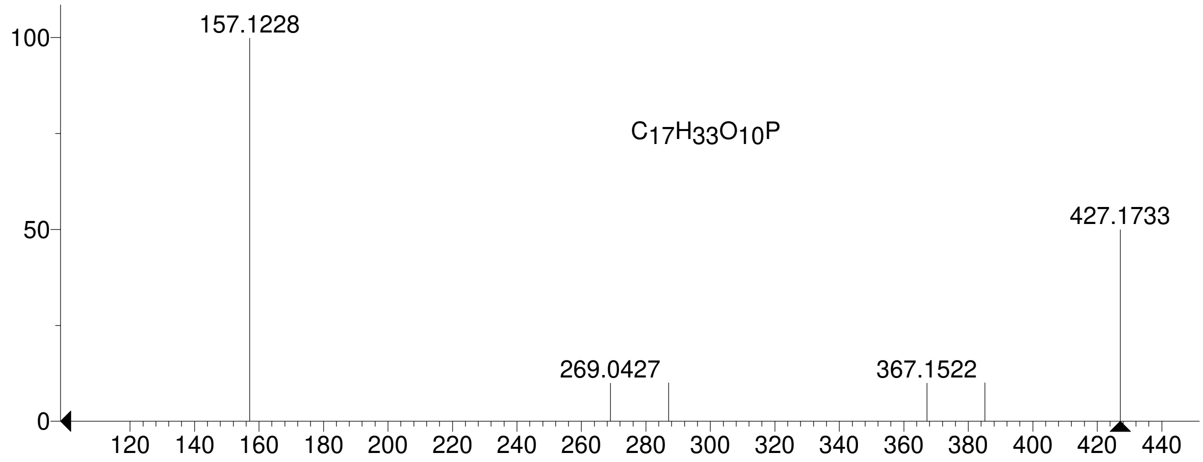

Hit 2 : PG 11:0; PG 3:0-8:0; [M-H]- [M-H]- P=427.2
C17H33O10P; MF: 3; RMF: 129; Prob 20.5%; Lib: msdial-insilicomsms-lipids-neg; ID: 324632.

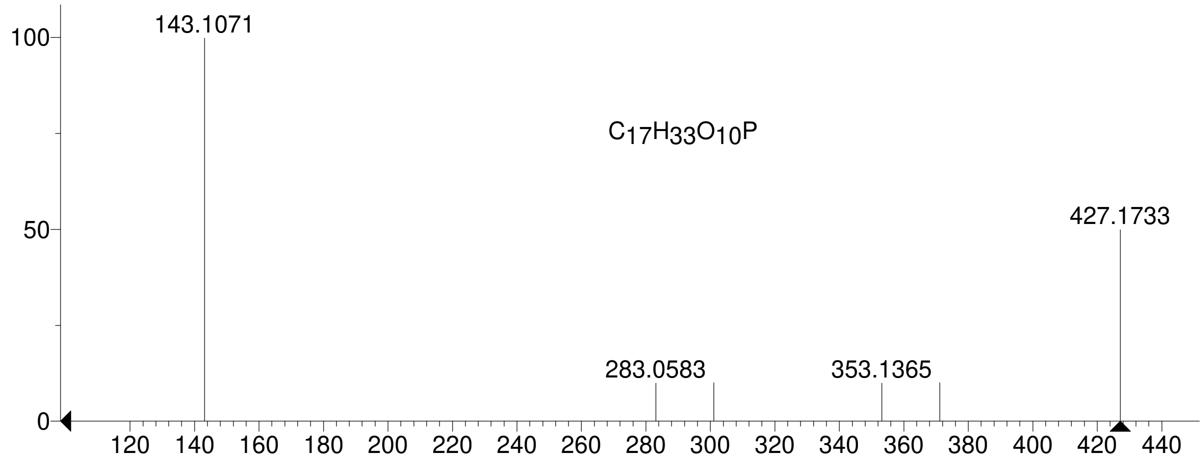

Hit 3 : PG 11:0; PG 4:0-7:0; [M-H]- [M-H]- P=427.2
C17H33O10P; MF: 3; RMF: 129; Prob 20.5%; Lib: msdial-insilicomsms-lipids-neg; ID: 324690.

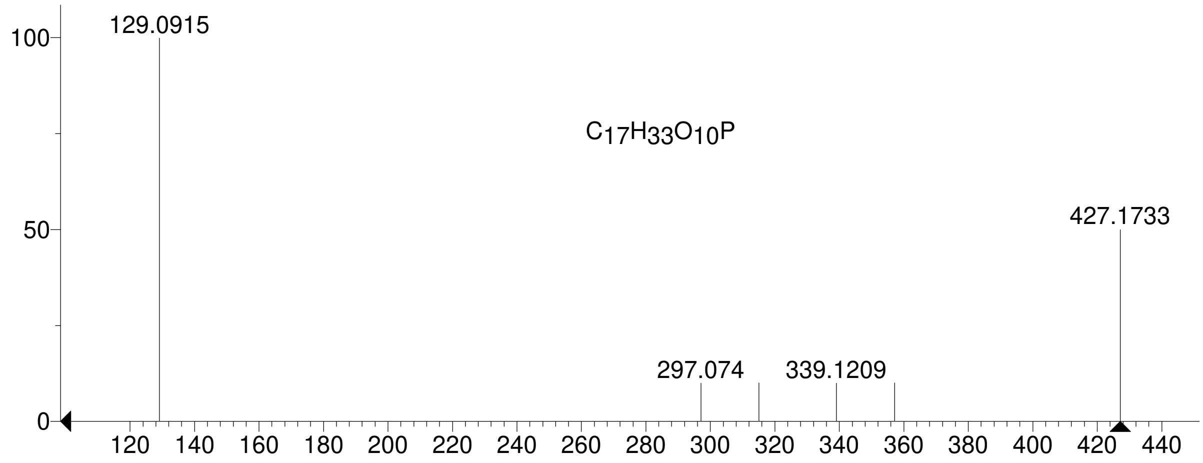

Hit 4 : PG 11:0; PG 5:0-6:0; [M-H]- [M-H]- P=427.2
C17H33O10P; MF: 3; RMF: 129; Prob 20.5%; Lib: msdial-insilicomsms-lipids-neg; ID: 324747.

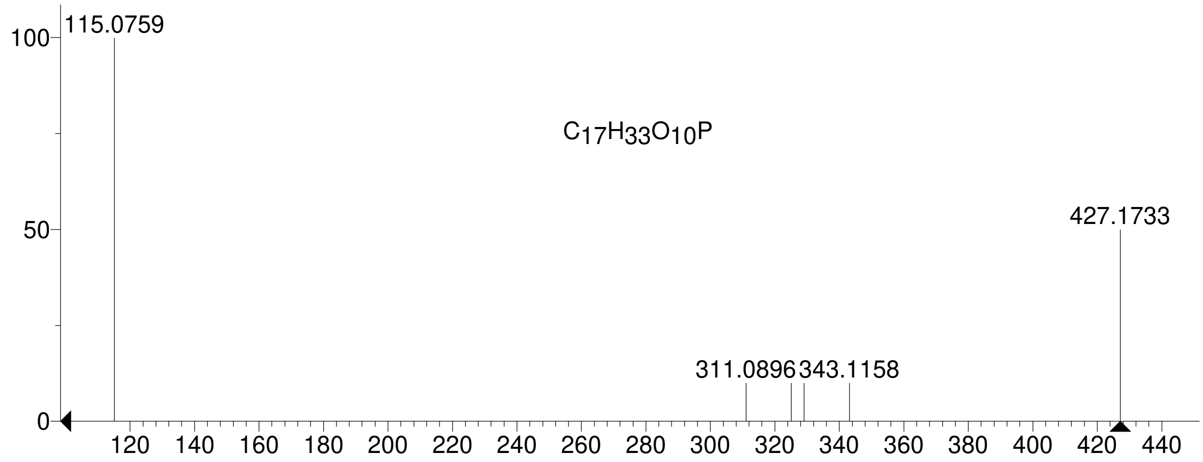

Hit 5 : [(6E,10Z)-10-(acetyloxymethyl)-6-formyl-3-methylidene-2-oxo-3a,4,5,8,9,11a-hexahydrocyclodeca[b]furan-
C22H28O7; MF: 0; RMF: 29; Prob 18.1%; Lib: mona-export-vf-npl_qexactive; ID: 23323.

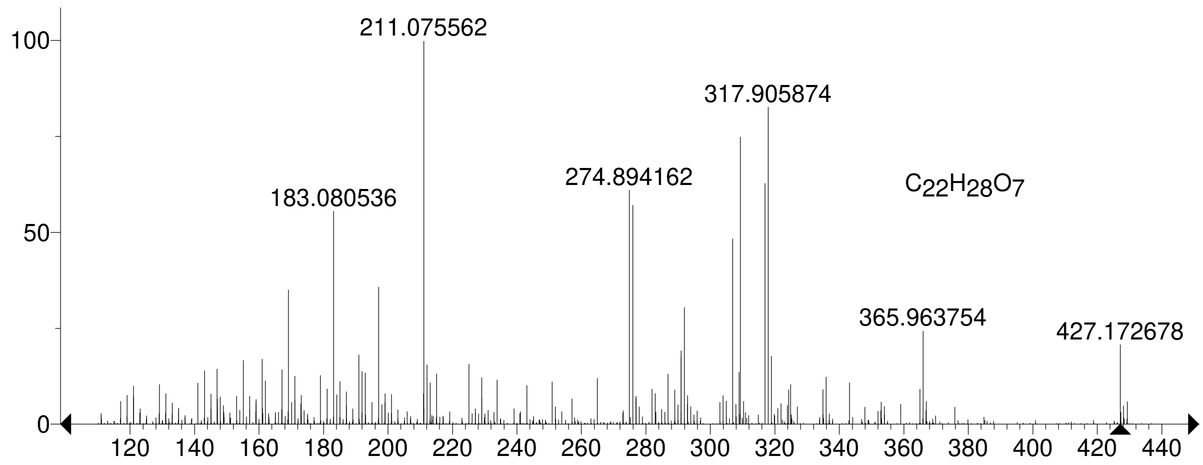

Unknown: Unknown (3.80_499.2274m/z)
Compound in Library Factor = 0

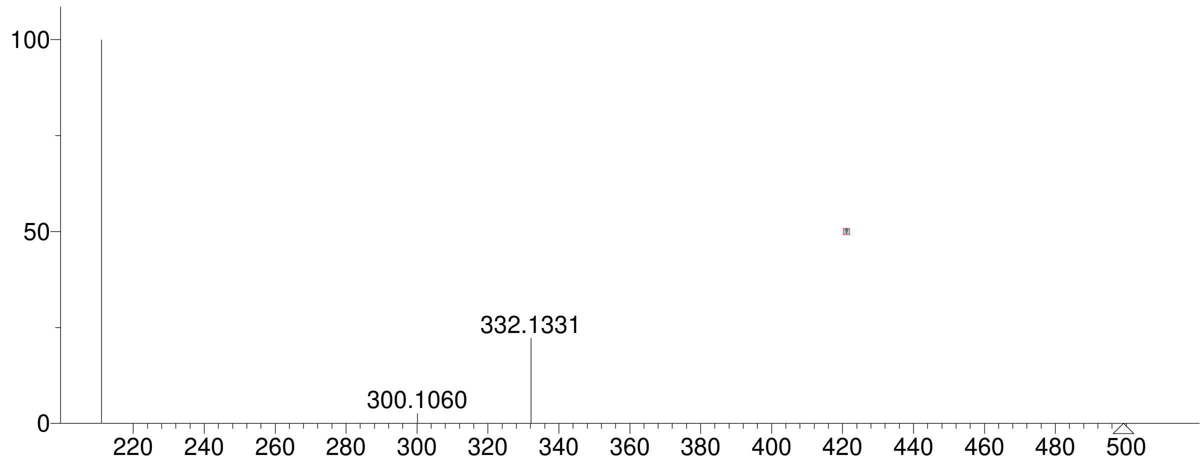

Unknown: Unknown (3.81_231.1334m/z)
Compound in Library Factor = -636

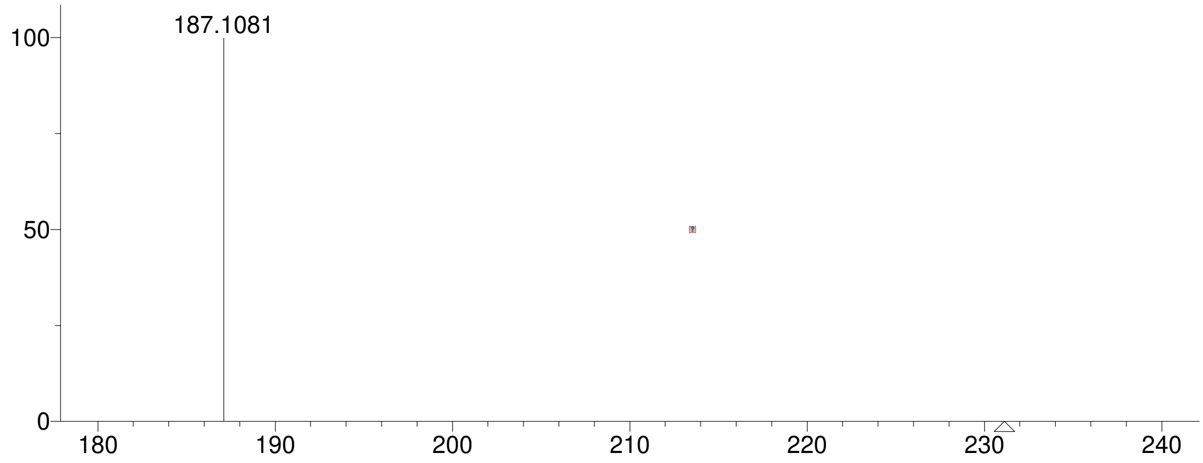

Hit 1 : Leu-Thr [M-H]- HCD 30eV P=231.1
C10H20N2O4; MF: 364; RMF: 999; Prob 19.9%; Lib: nist_msms; ID: 566949.

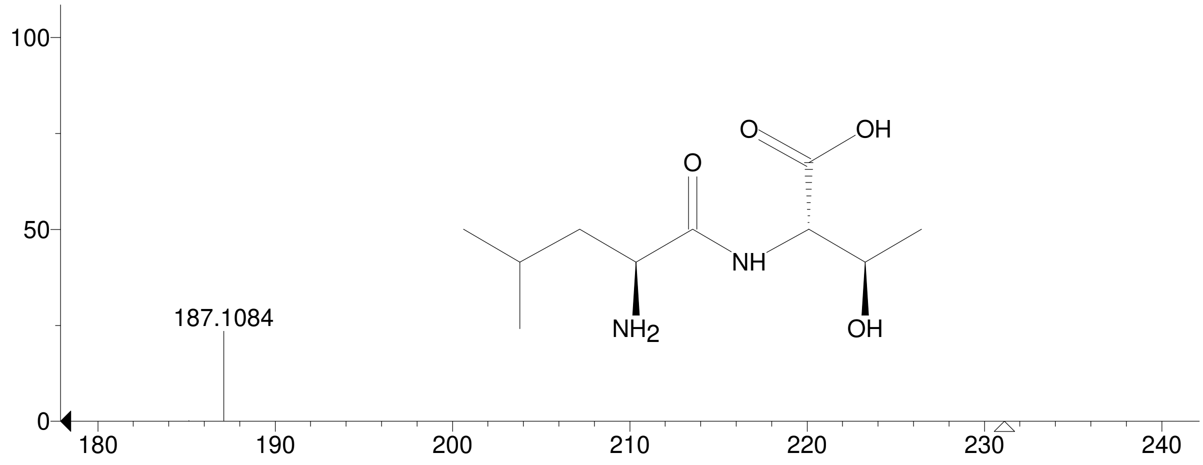

Hit 2 : Thr-Leu [M-H]- HCD 30eV P=231.1
C10H20N2O4; MF: 364; RMF: 999; Prob 19.9%; CAS: 50299-12-2; Lib: nist_msms; ID: 568153.

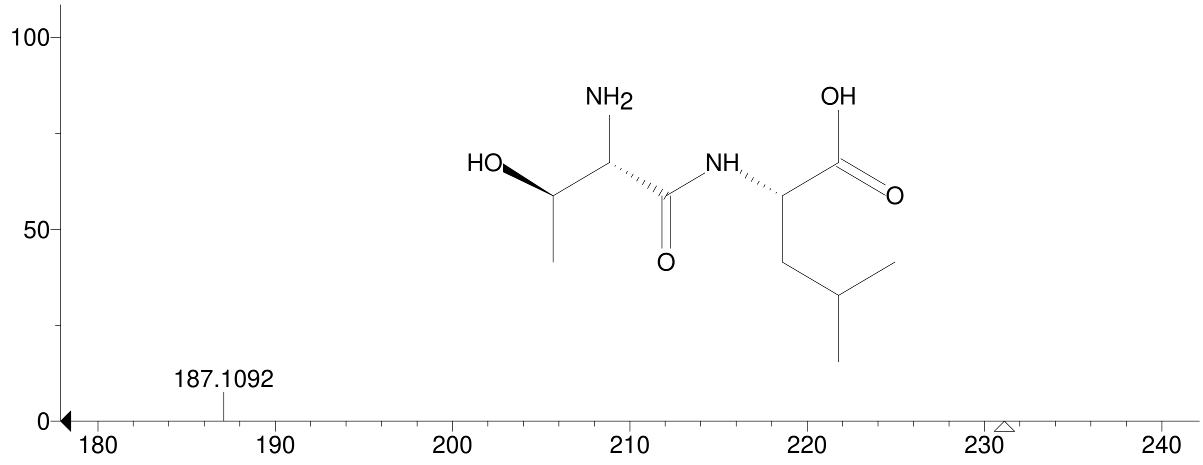

Hit 3 : Leu-Thr [M-H]- HCD 30eV P=231.1
C10H20N2O4; MF: 364; RMF: 999; Prob 19.9%; CAS: 38062-82-7; Lib: nist_msms2; ID: 69806.

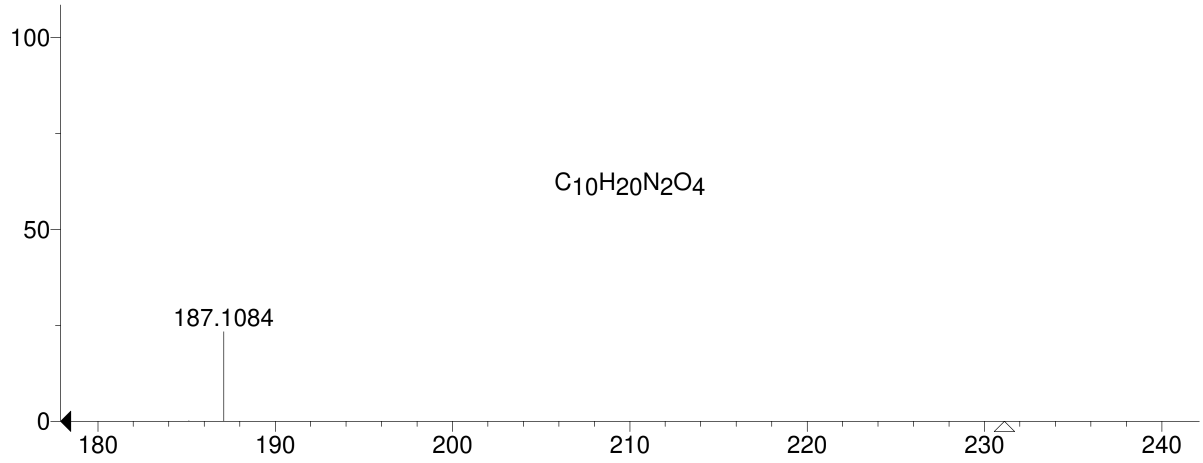

Hit 4 : Ile-Thr [M-H]- HCD 30eV P=231.1
C10H20N2O4; MF: 364; RMF: 999; Prob 19.9%; Lib: nist_msms; ID: 566888.

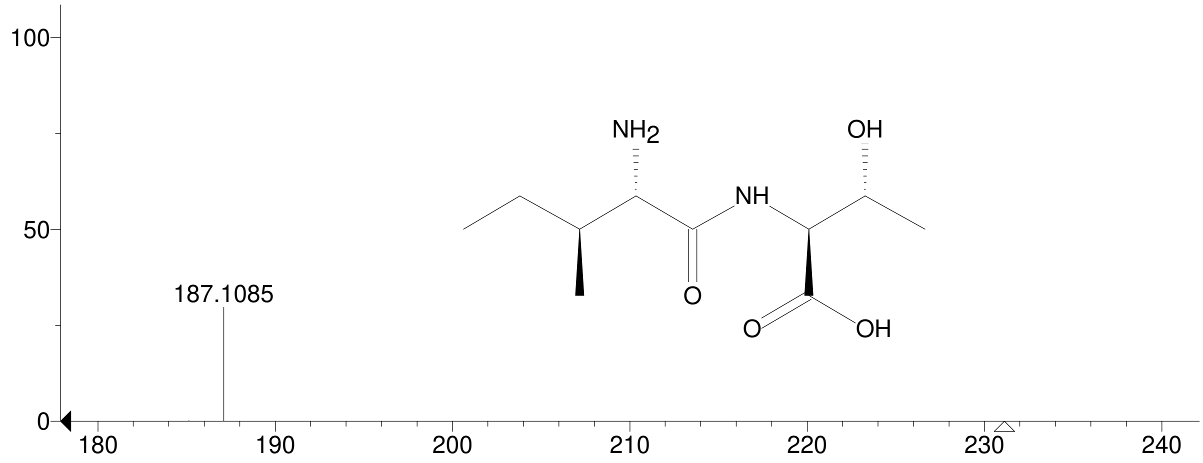

Hit 5 : Ile-Thr [M-H]- HCD 30eV P=231.1
C10H20N2O4; MF: 364; RMF: 999; Prob 19.9%; CAS: 59652-61-8; Lib: nist_msms2; ID: 69745.

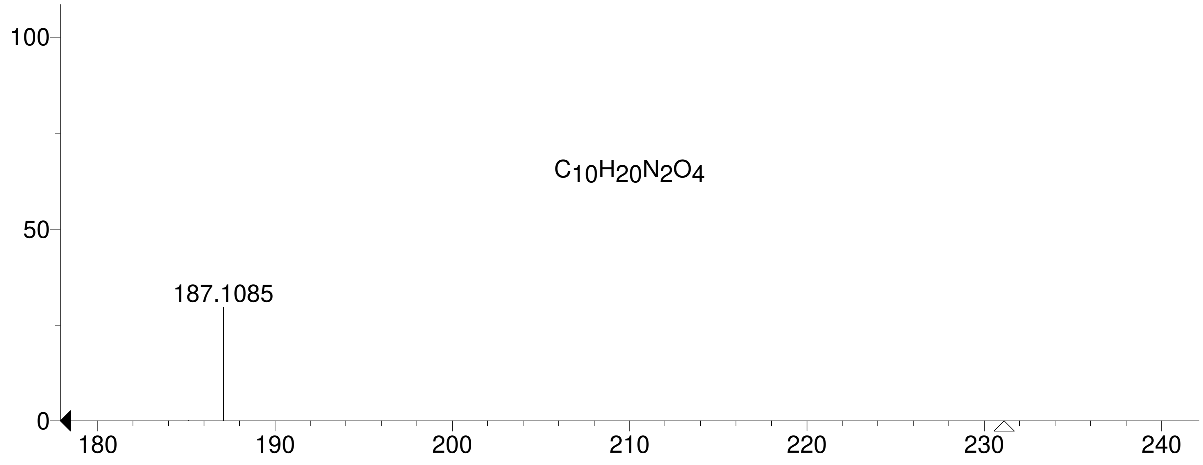

** Search Report Page 1 of 1 **

Unknown: Unknown (3.81_355.1607m/z)
Compound in Library Factor = -2176

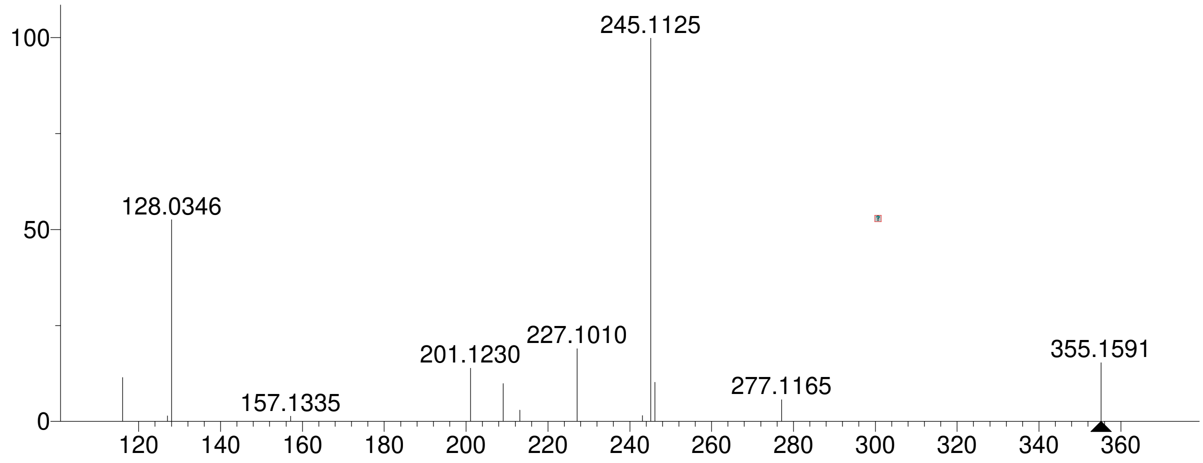

Hit 1 : pGlu-Glu-Pro-amide [M+H]+ HCD 3eV P=355.2
C15H22N4O6; MF: 37; RMF: 249; Prob 77.9%; CAS: 85541-78-2; Lib: nist_msms; ID: 564354.

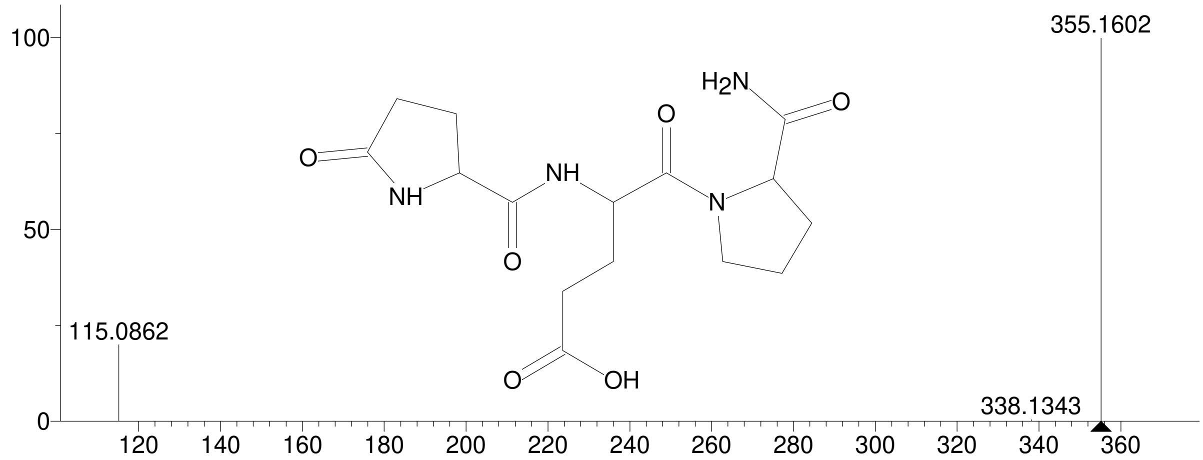

Hit 2 : Isoxaben [M+Na]+ HCD 10V P=355.2
C18H24N2O4; MF: 8; RMF: 238; Prob 22.1%; CAS: 82558-50-7; Lib: nist_msms; ID: 397188.

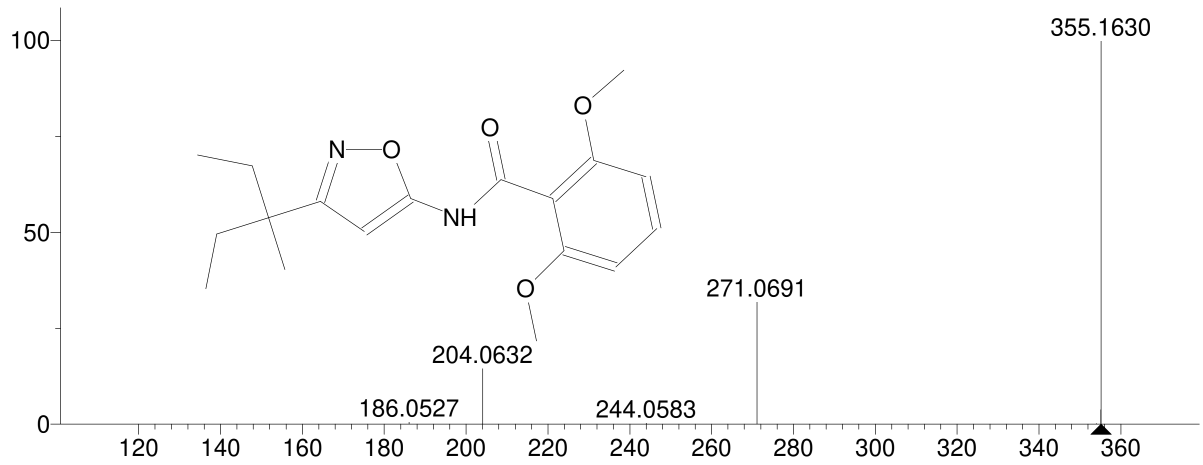

** Search Report Page 1 of 1 **

Unknown: Unknown (3.81_389.1545m/z)
Compound in Library Factor = 0

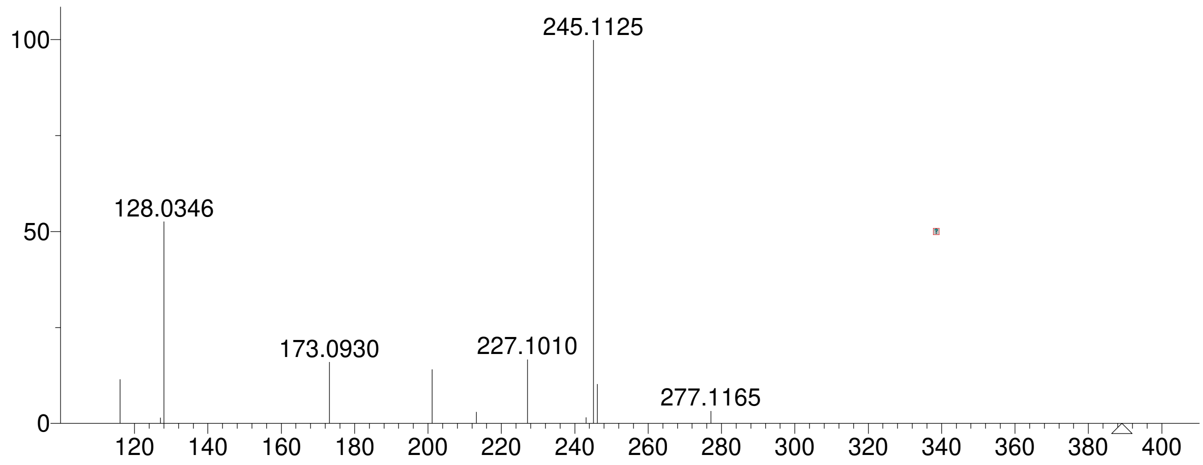

Hit 1 : Diacetoxyscirpenol [M+Na]+ IT 35% P=389.2
C19H26O7; MF: 0; RMF: 49; Prob 100.0%; CAS: 2270-40-8; Lib: nist_msms; ID: 379600.

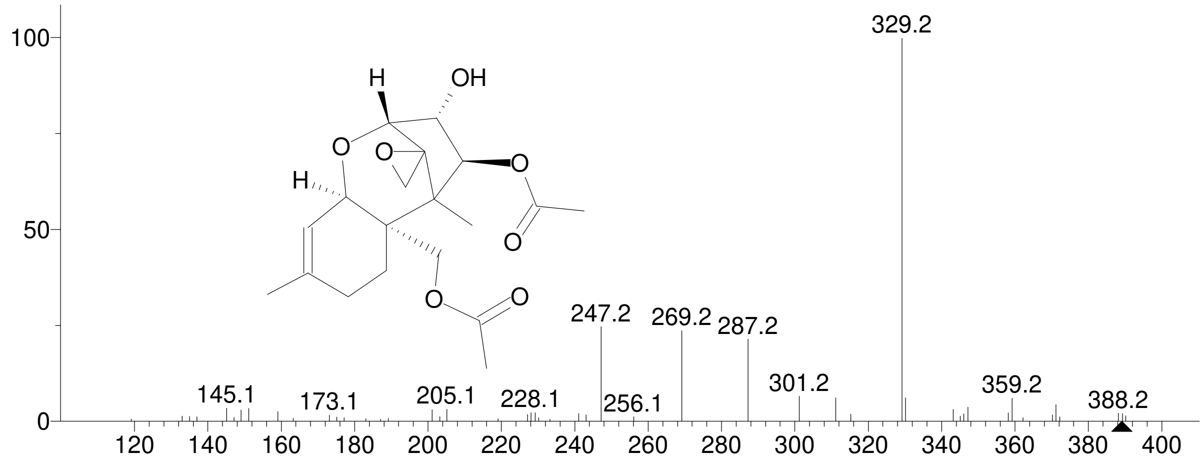

Unknown: Unknown (3.81_530.1991m/z)
Compound in Library Factor = 0

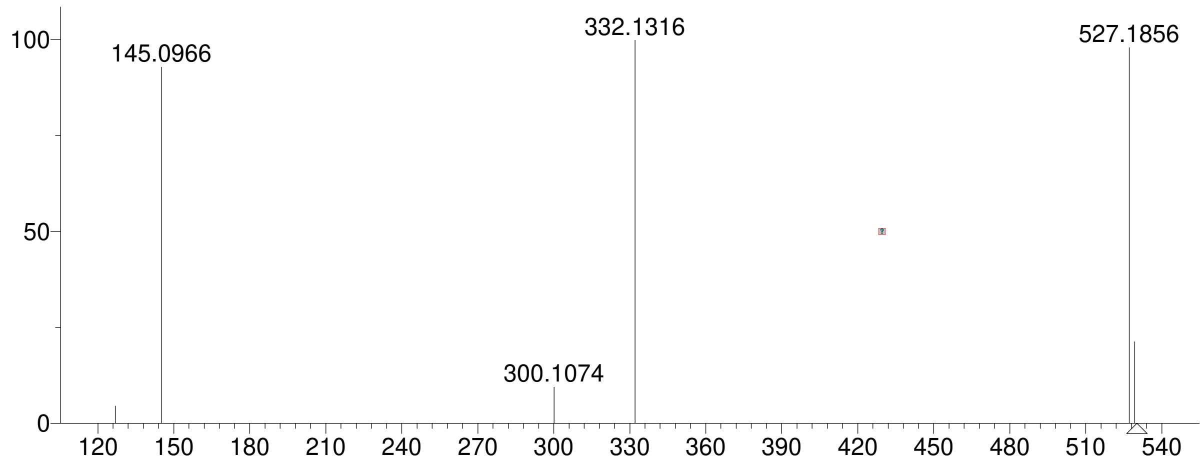

Unknown: Unknown (3.82_387.1751m/z)
Compound in Library Factor = -3273

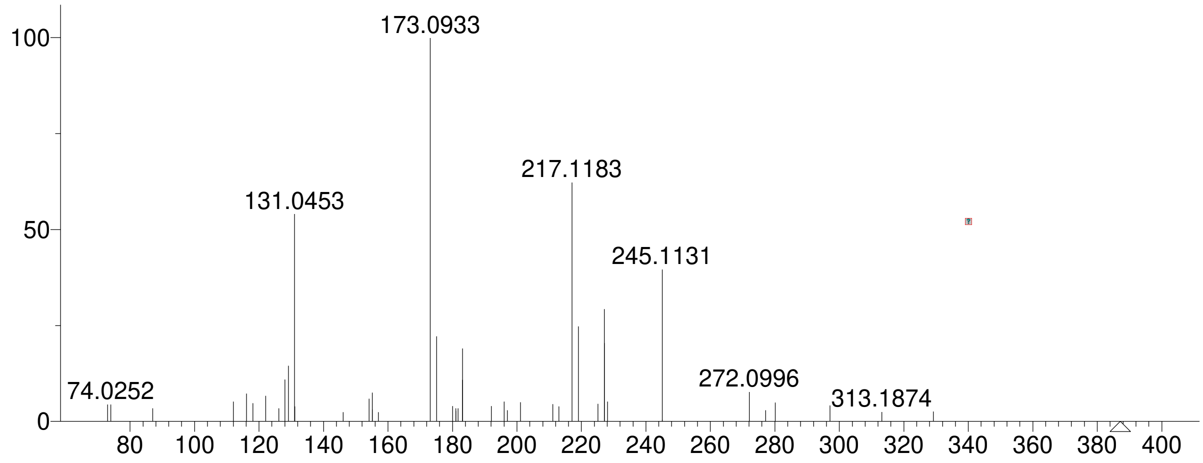

Hit 1 : Neurotensin (1-6) [M-2H]2- HCD 31eV P=387.2
C35H52N8O12; MF: 2; RMF: 49; Prob 21.3%; Lib: nist_msms2; ID: 74177.

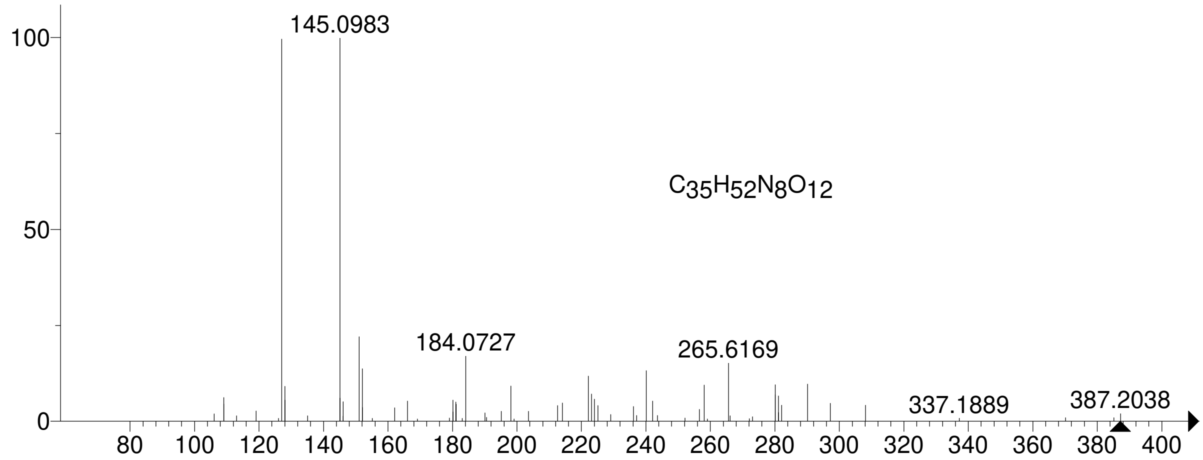

Hit 2 : NCGC00347508-02!4-[2-[[2,3-dihydroxy-2-(1-hydroxyethyl)butanoyl]oxymethyl]anilino]-4-oxobutanoic acid
C17H23NO8; MF: 0; RMF: 49; Prob 19.7%; Lib: mona-export-gnps; ID: 6723.

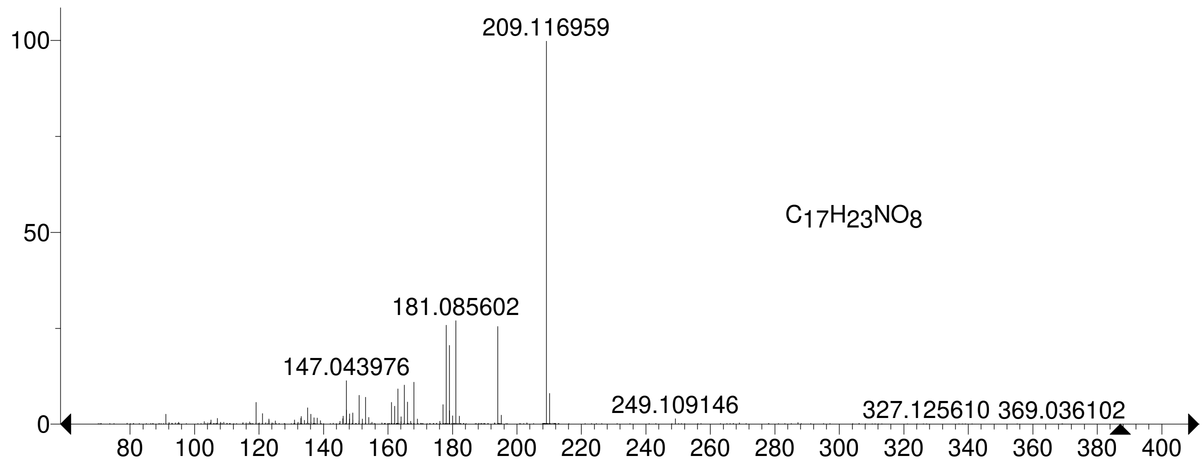

Hit 3 : rac-N'-Nitrosonornicotine 1-N-oxide [2M+H]+ HCD 100V P=387.2
C9H11N3O2; MF: 0; RMF: 21; Prob 19.7%; CAS: 1314916-69-2; Lib: nist_msms; ID: 464580.

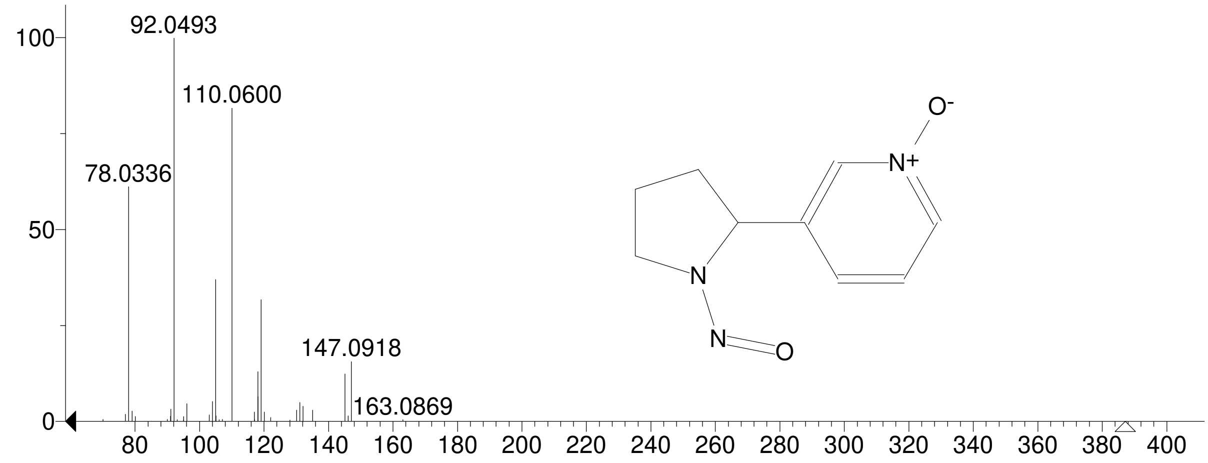

Hit 4 : N6-2-(4-Aminophenyl)ethyladenosine [M+H]+ QTOF 60V P=387.2
C18H22N6O4; MF: 0; RMF: 17; Prob 19.7%; CAS: 89705-21-5; Lib: nist_msms; ID: 17232.

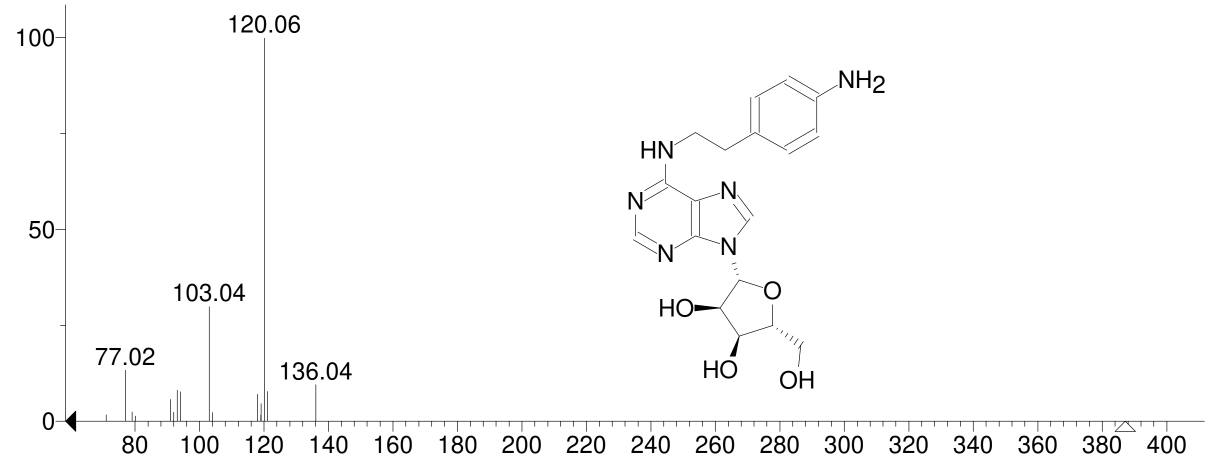

Hit 5 : Rubescensin A [M+Na]+ IT 35% P=387.2
C20H28O6; MF: 0; RMF: 9; Prob 19.7%; CAS: 28957-10-0; Lib: nist_msms; ID: 401361.

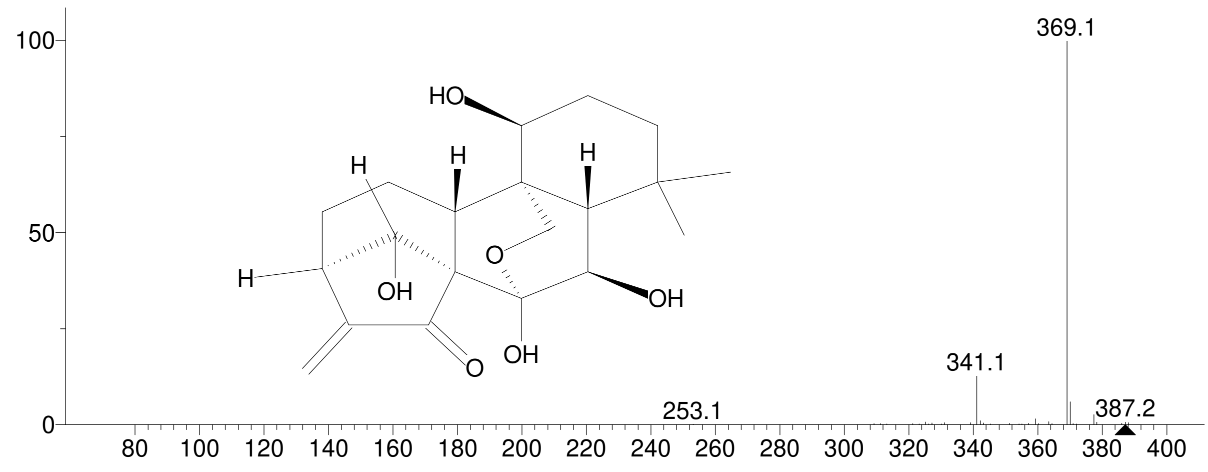

Unknown: Unknown (3.82_415.1715m/z)
Compound in Library Factor = -3273

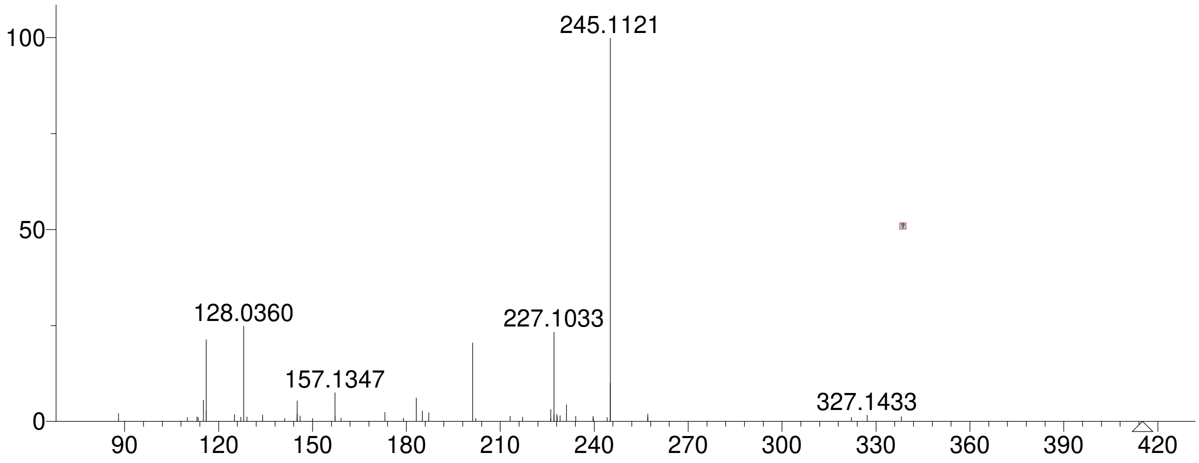

Hit 1 : 6-formyl-10-(hydroxymethyl)-5-methoxy-3-methylidene-2-oxo-2H,3H,3aH,4H,5H,8H,9H,11aH-cyclodeca[b]f
C21H28O7; MF: 4; RMF: 76; Prob 18.9%; Lib: mona-export-vf-npl_qexactive; ID: 16435.

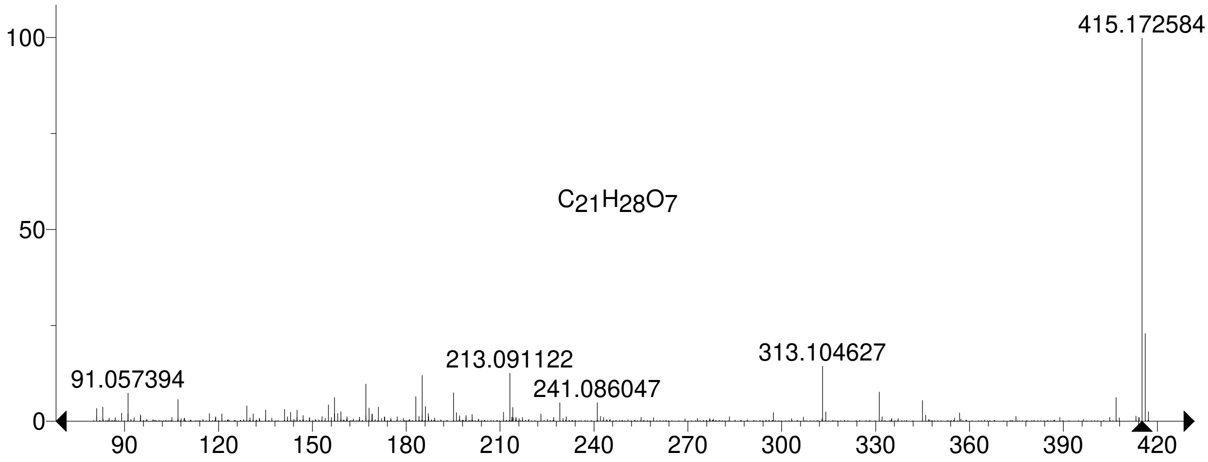

Hit 2 : Garcinone C [M+H]+ HCD 132V P=415.2
C23H26O7; MF: 1; RMF: 38; Prob 16.7%; CAS: 76996-27-5; Lib: nist_msms; ID: 389293.

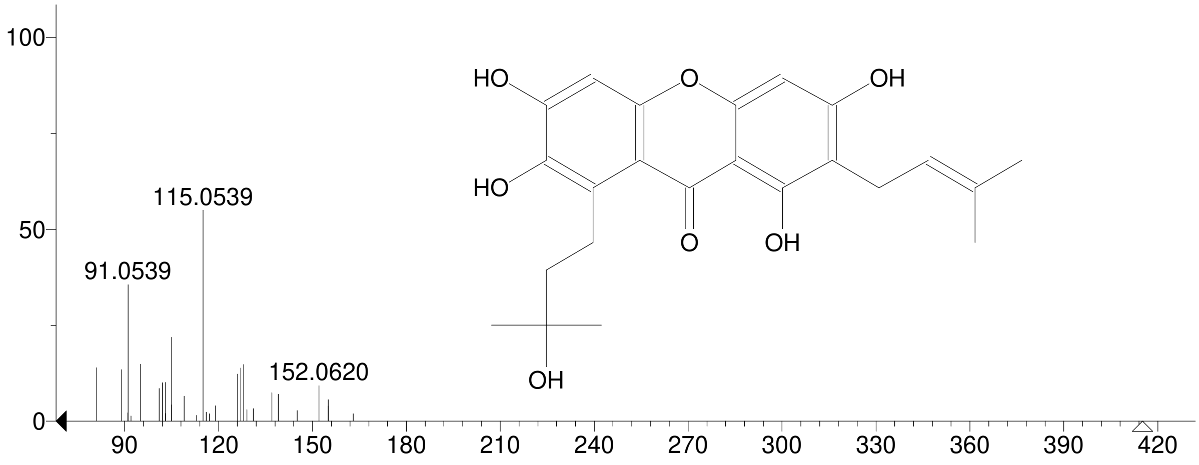

Hit 3 : NCGC00347525-03!2-amino-5-[2-[[2,3-dihydroxy-2-(1-hydroxyethyl)butanoyl]oxymethyl]-4-hydroxyanilino]-C18H26N2O9; MF: 0; RMF: 9; Prob 16.1%; Lib: mona-export-gnps; ID: 9213.

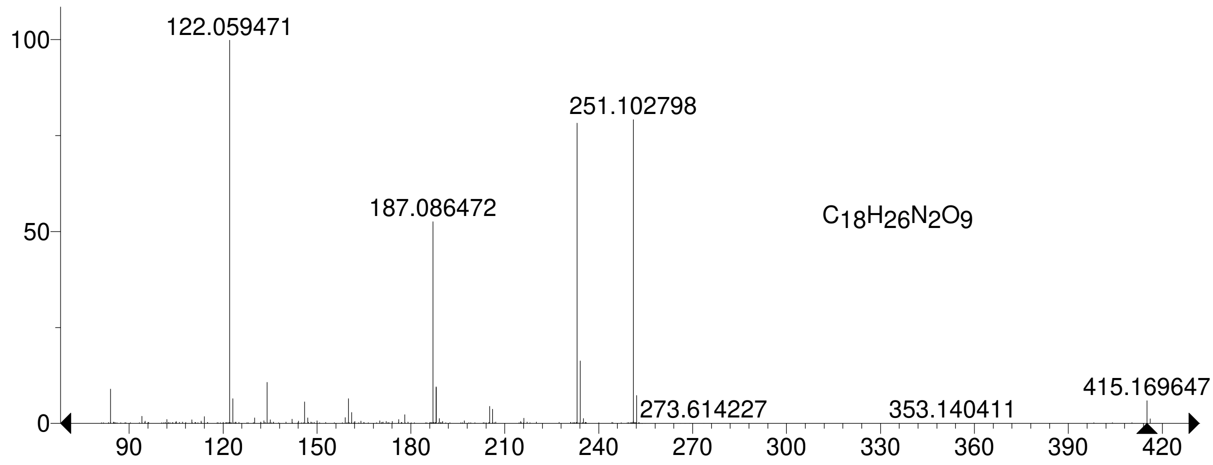

Hit 4 : NCGC00347525-02!2-amino-5-[2-[[2,3-dihydroxy-2-(1-hydroxyethyl)butanoyl]oxymethyl]-4-hydroxyanilino]-C18H26N2O9; MF: 0; RMF: 5; Prob 16.1%; Lib: mona-export-gnps; ID: 6270.

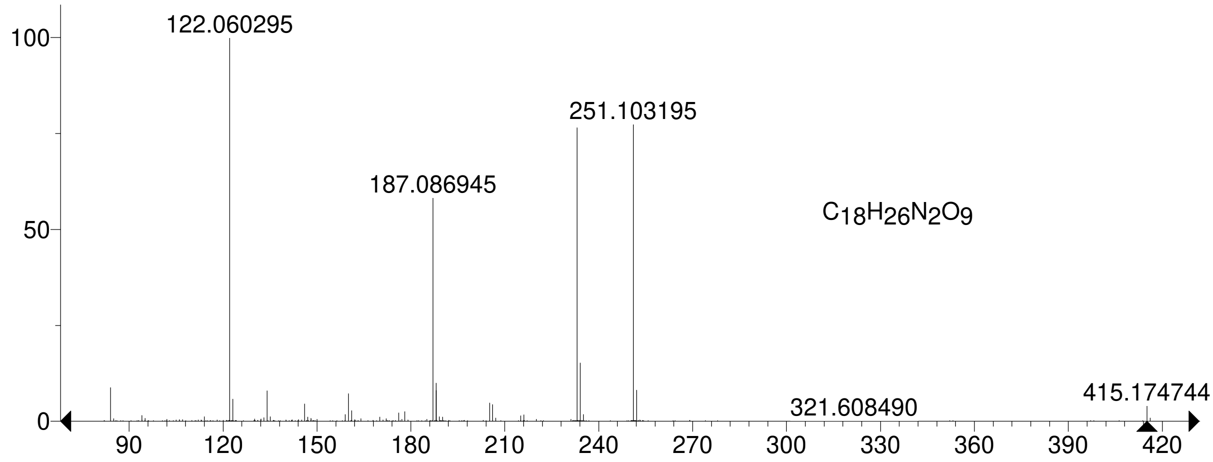

Hit 5 : 2-amino-5-[2-[[2,3-dihydroxy-2-(1-hydroxyethyl)butanoyl]oxymethyl]-4-hydroxyanilino]-5-oxopentanoic acid C18H26N2O9; MF: 0; RMF: 4; Prob 16.1%; Lib: mona-export-vf-npl_qexactive; ID: 23034.

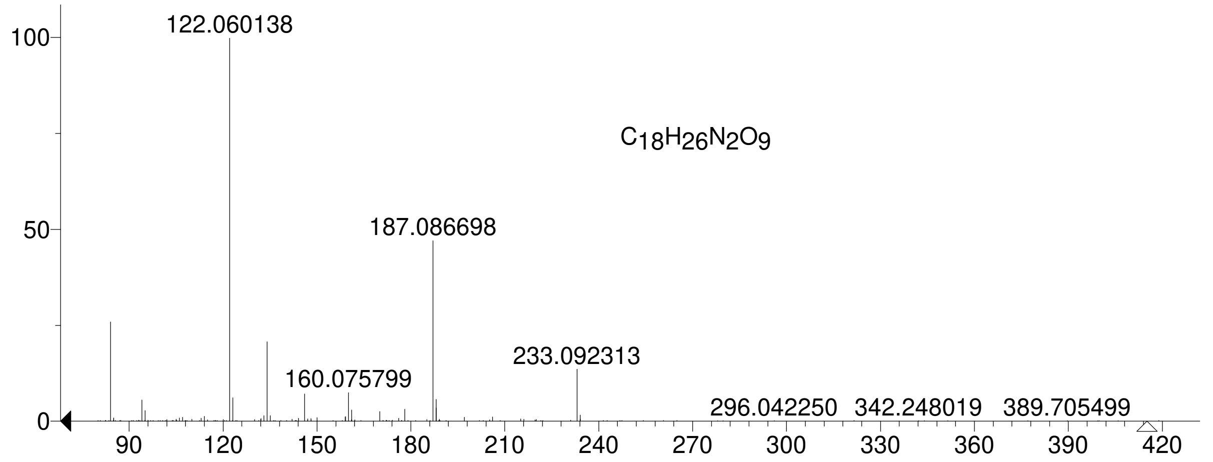

Unknown: Unknown (3.83_529.2611m/z)
Compound in Library Factor = -3273

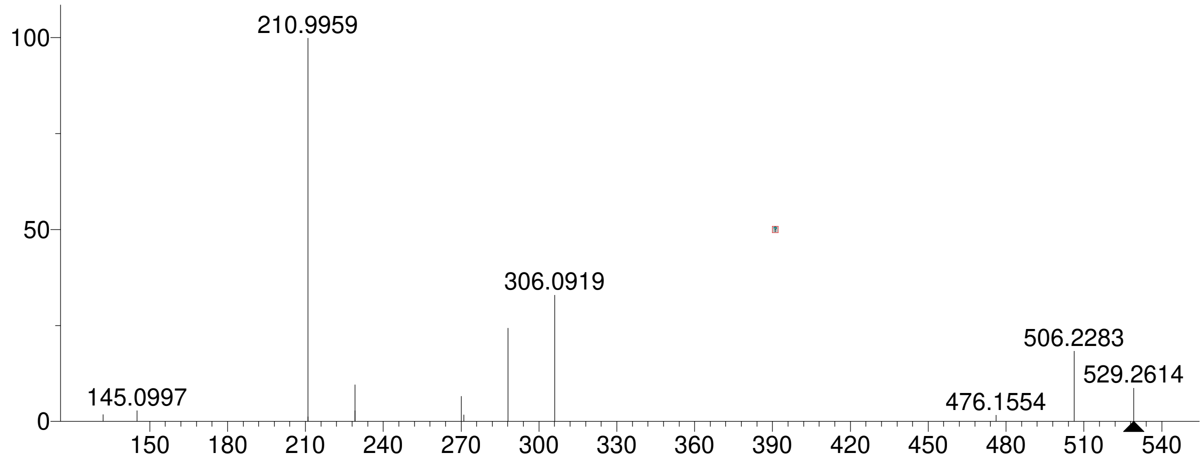

Hit 1 : NCGC00380707-01_C26H42O11_(5xi,6alpha,7alpha,9xi,16xi)-16-(beta-D-Glucopyranosyloxy)-6,7,17-trihy
C26H42O11; MF: 5; RMF: 244; Prob 22.9%; Lib: msms-neg-gnps; ID: 1893.

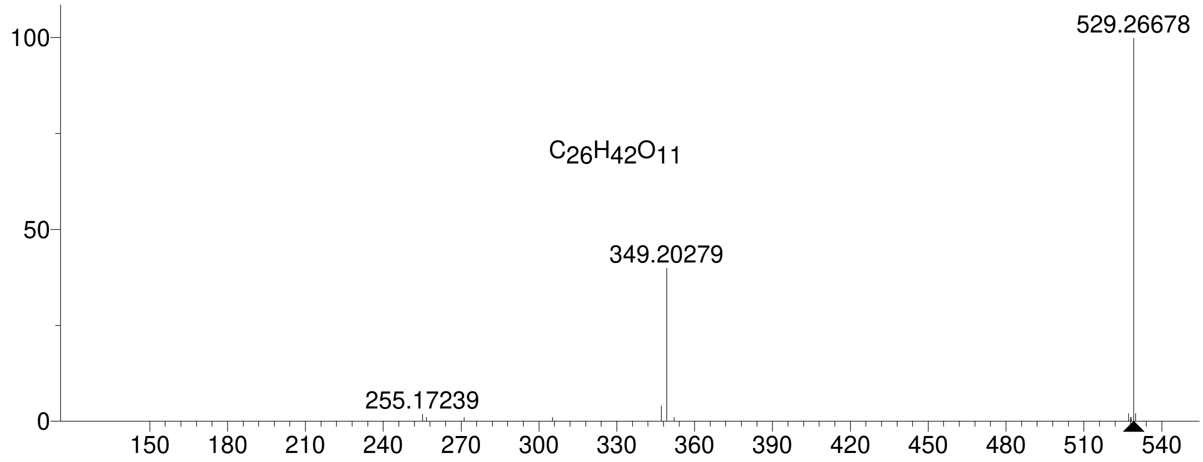

Hit 2 : LPG 20:5; [M-H]- [M-H]- P=529.3
C26H43O9P; MF: 2; RMF: 87; Prob 20.2%; Lib: msdial-insilicomsms-lipids-neg; ID: 311120.

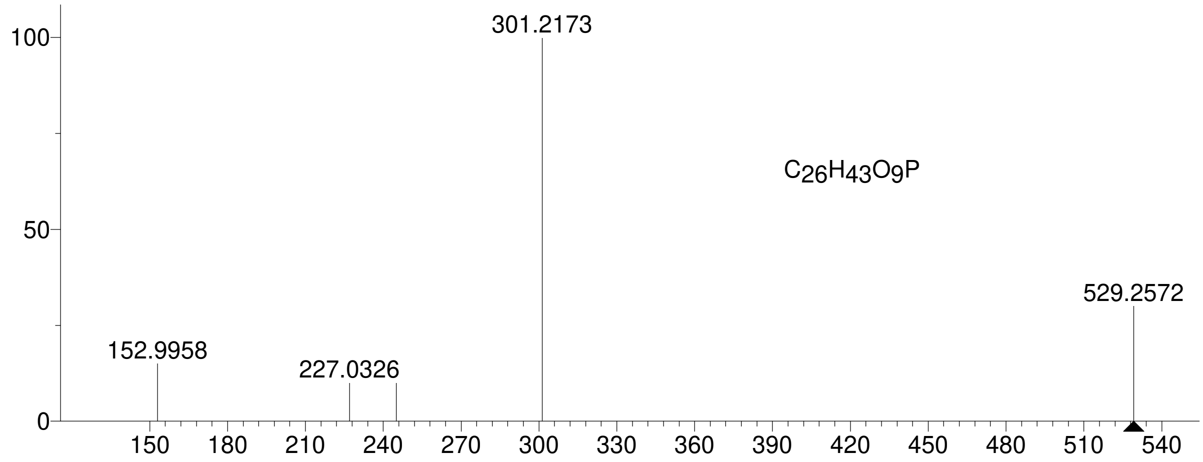

Hit 3 : NCGC00386012-01_C24H42O11_beta-D-Glucopyranoside, (1S,4R,5R)-4-[(1E,3R)-3-hydroxy-1-buten-1-yl
C24H42O11; MF: 1; RMF: 171; Prob 19.4%; Lib: mona-export-gnps; ID: 13699.

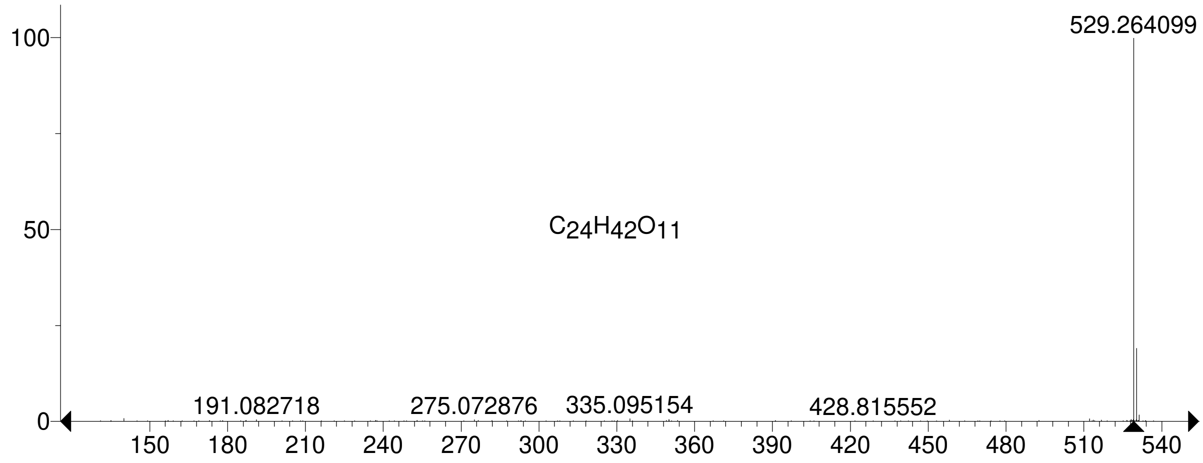

Hit 4 : (2S,3R,5S,9R)-2,3-dihydroxy-14-(hydroxymethyl)-5,9-dimethyl-14-{[(2S,3R,4S,5S,6R)-3,4,5-trihydroxy-6-(h
C26H42O11; MF: 0; RMF: 94; Prob 18.7%; Lib: anthocyanins mona; ID: 5864.

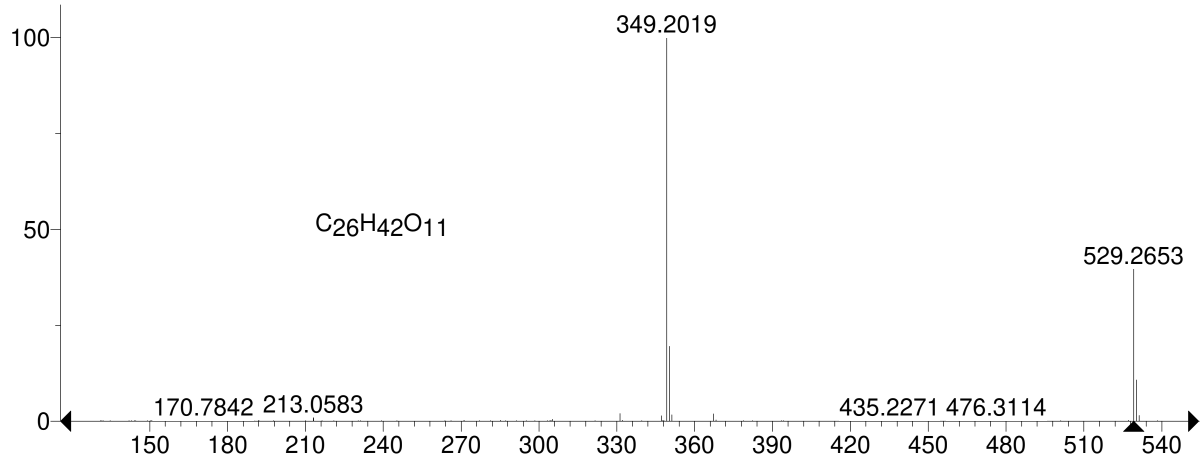

Hit 5 : (2S,3R,5S,9R)-2,3-dihydroxy-14-(hydroxymethyl)-5,9-dimethyl-14-{[(2S,3R,4S,5S,6R)-3,4,5-trihydroxy-6-(h
C26H42O11; MF: 0; RMF: 94; Prob 18.7%; Lib: mona-export-lc-ms-ms_negative_mode; ID: 21950.

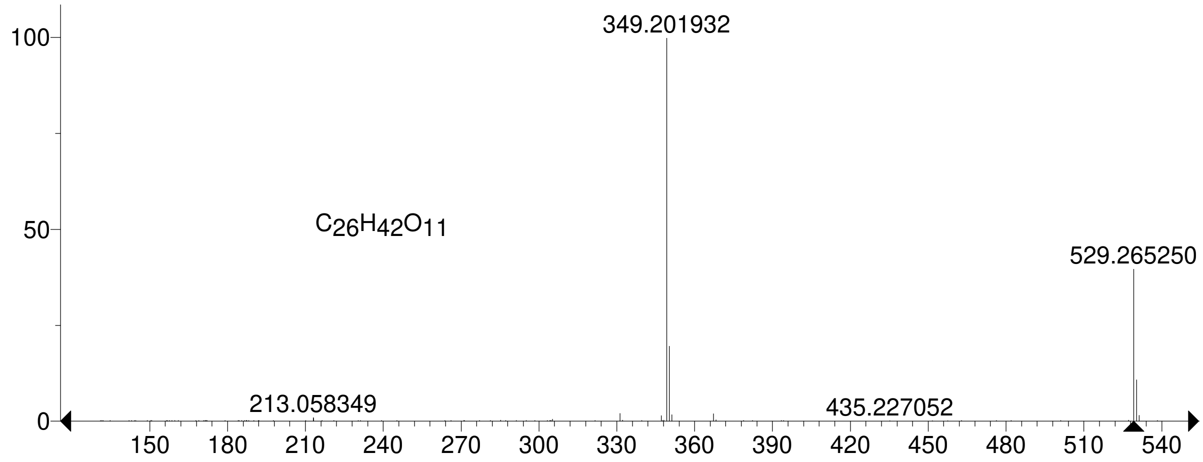

** Search Report Page 1 of 1 **

Unknown: Unknown (3.83_646.2091m/z)
Compound in Library Factor = 0

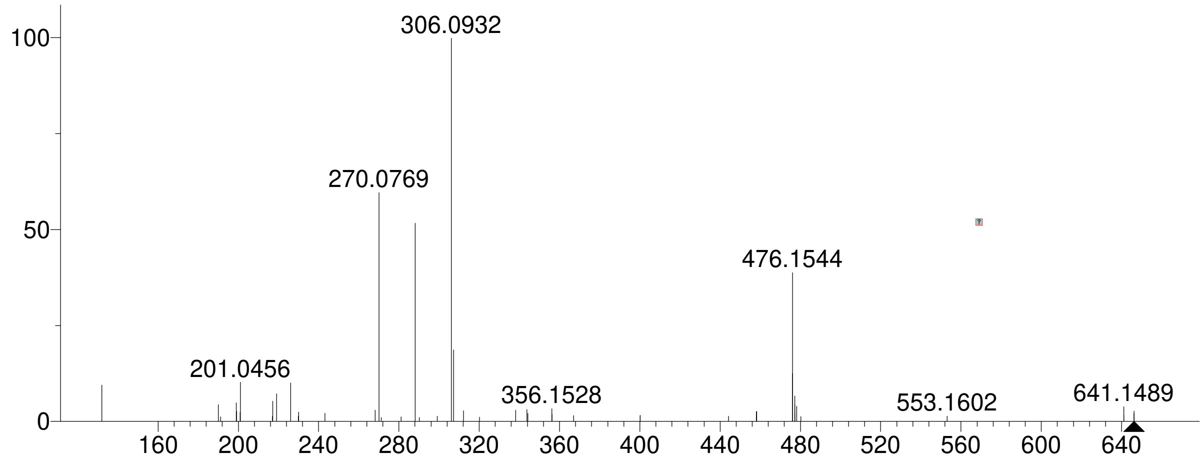

** Search Report Page 1 of 1 **

Unknown: Unknown (3.83_647.2658m/z)
Compound in Library Factor = 0

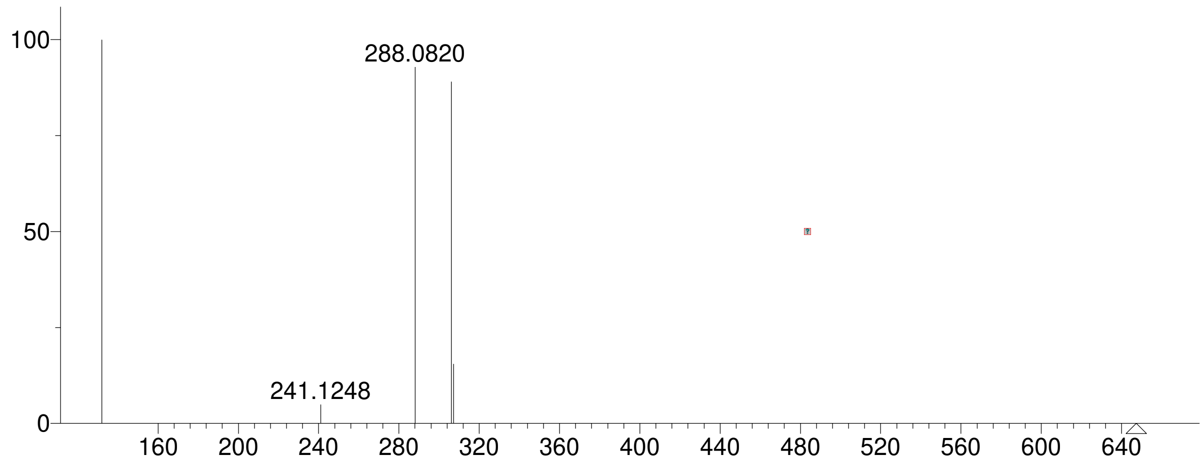

Unknown: Unknown (3.84_218.1024m/z)
Compound in Library Factor = 1080

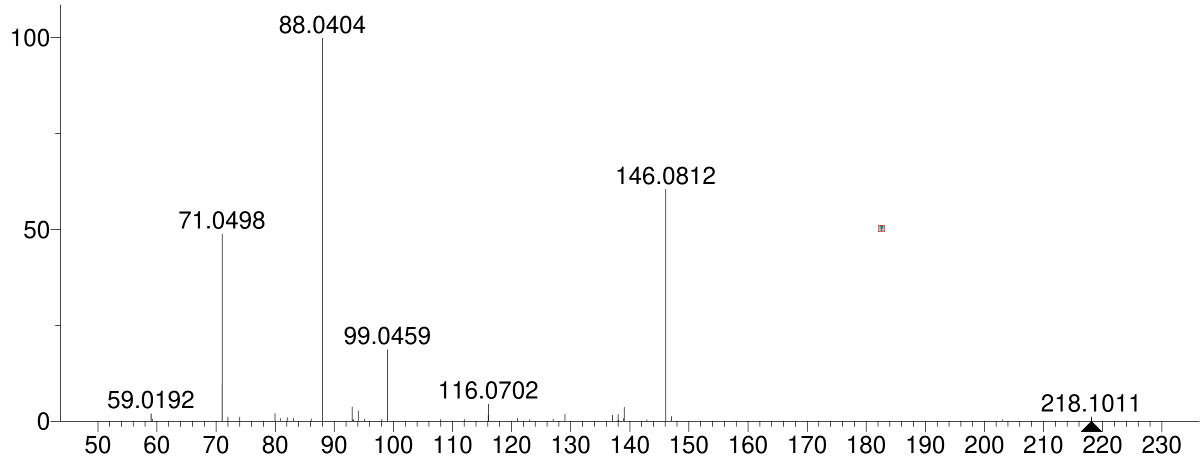

Hit 1 : Pantothenic acid [M-H]- HCD 28V P=218.1
C9H17NO5; MF: 912; RMF: 933; Prob 47.3%; CAS: 599-54-2; Lib: nist_msms; ID: 295331.

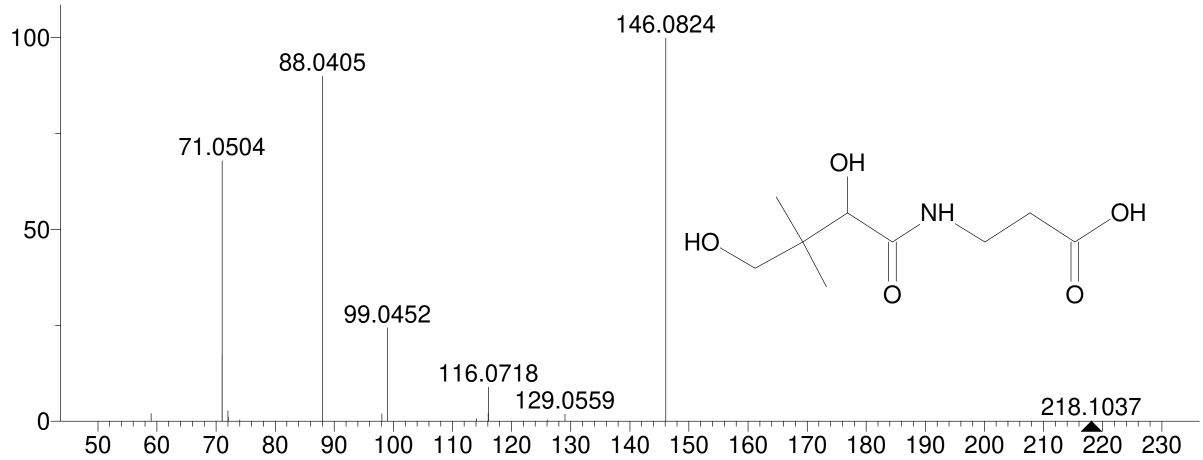

Hit 2 : (+)-Pantothenic acid [M-H]- 20 P=218.1
C9H17NO5; MF: 906; RMF: 931; Prob 37.2%; Lib: msms neg mona_ipb_gnps_plasma; ID: 844.

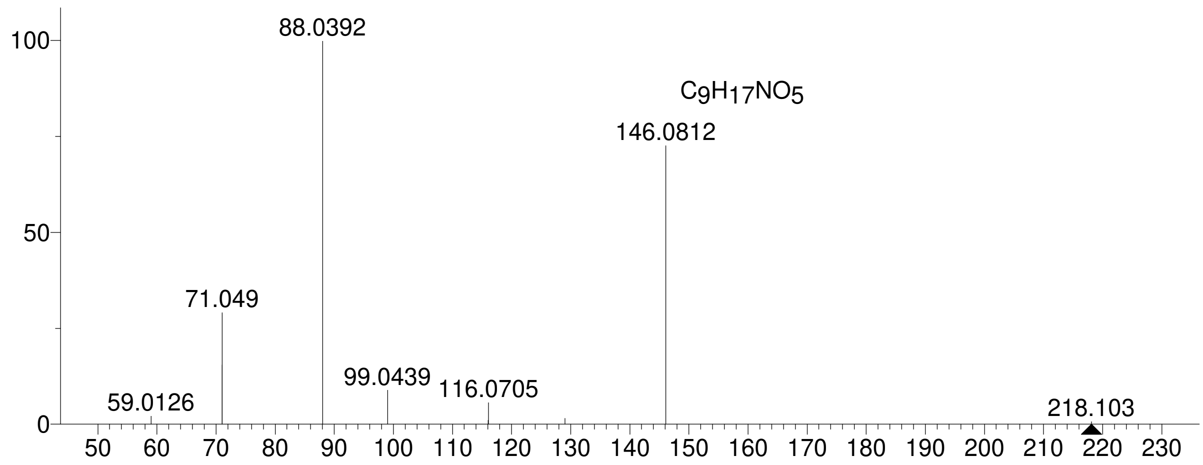

Hit 3 : D-Pantothenic Acid [M-H]- 5-60V P=218.1
C9H17NO5; MF: 799; RMF: 853; Prob 2.46%; Lib: mona-export-lc-ms-ms_negative_mode; ID: 8598.

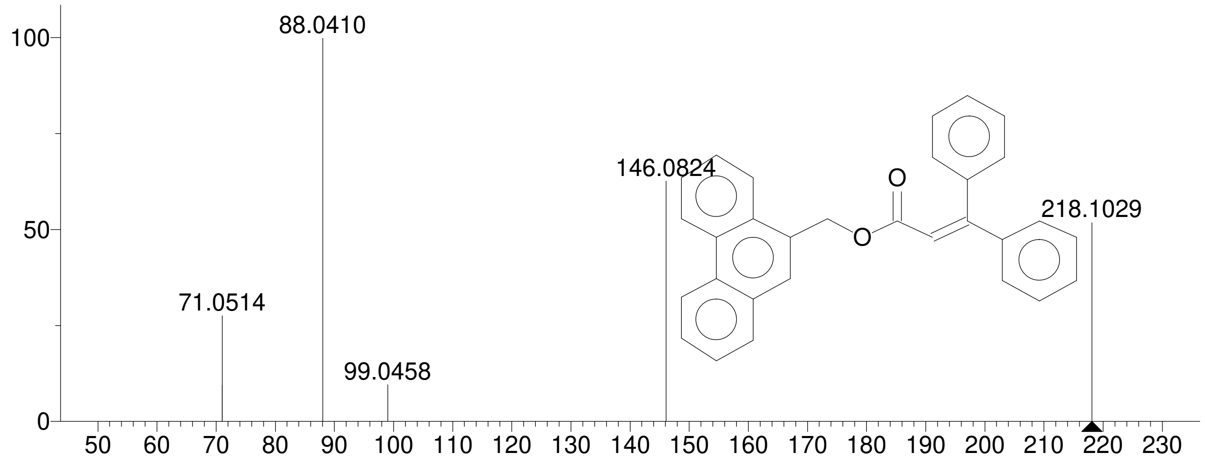

Hit 4 : Sodium pantothenate [M-H]- QTOF 5-45V P=218.1
C9H17NO5; MF: 799; RMF: 853; Prob 2.46%; Lib: mona-export-lc-ms-ms_negative_mode; ID: 11485.

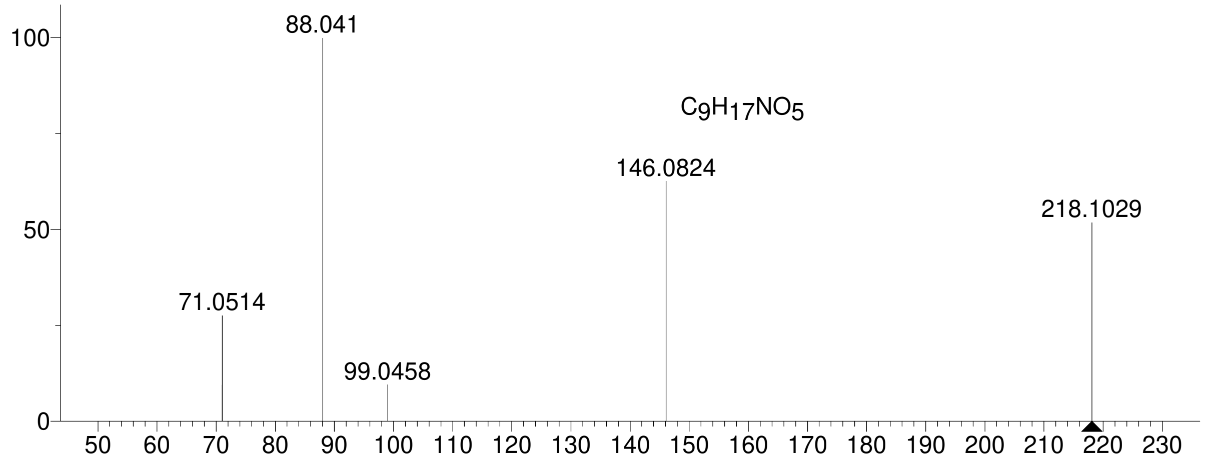

Hit 5 : D-(+)-Pantothenic acid; LC-ESI-QTOF; MS2; CE [M-H]- P=218.1
C9H17NO5; MF: 799; RMF: 853; Prob 2.46%; Lib: msms-neg-massbank; ID: 4493.

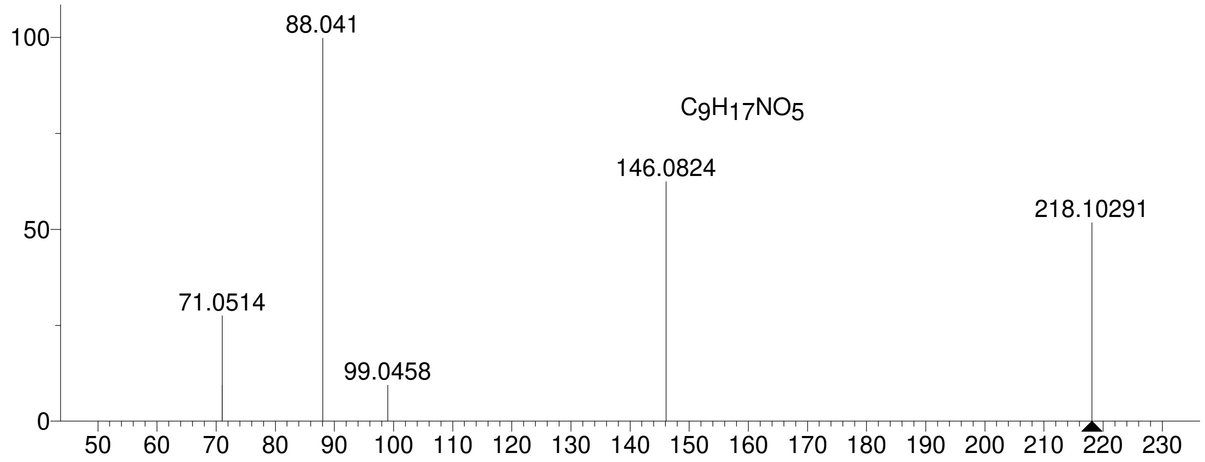

** Search Report Page 1 of 1 **

Unknown: Unknown (3.84_476.1503m/z)
Compound in Library Factor = 0

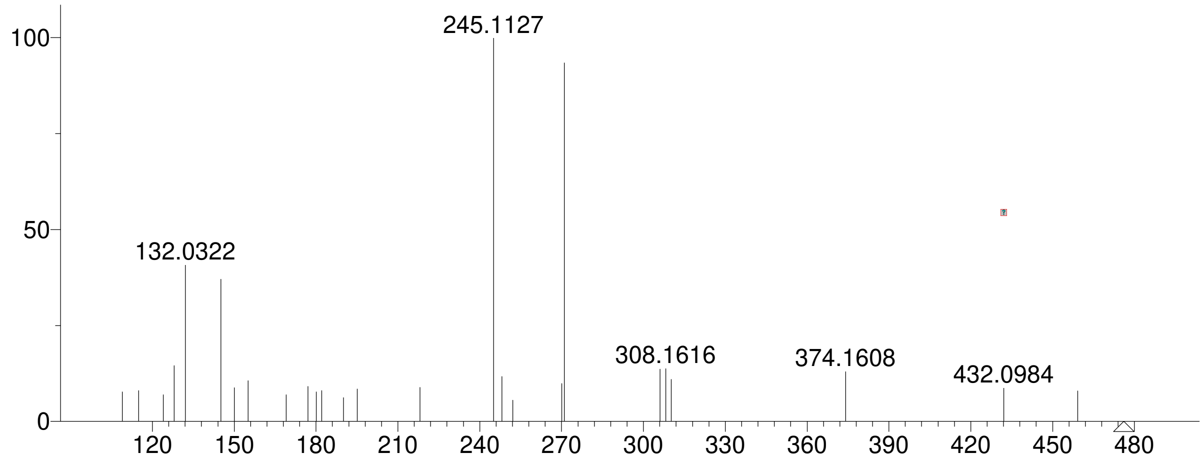

** Search Report Page 1 of 1 **

Unknown: Unknown (3.85_587.2206m/z)
Compound in Library Factor = 0

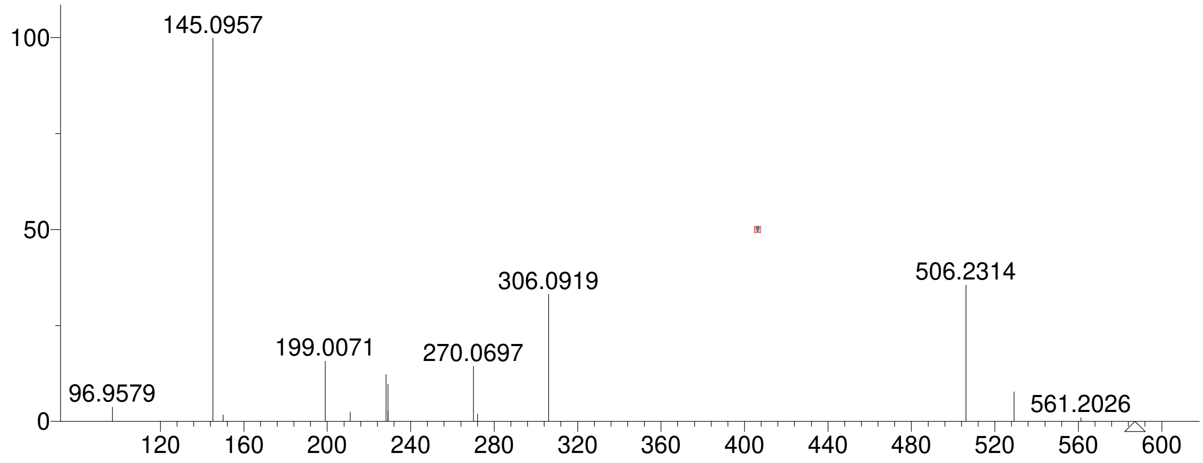

Unknown: Unknown (3.85_641.1467m/z)
Compound in Library Factor = 0

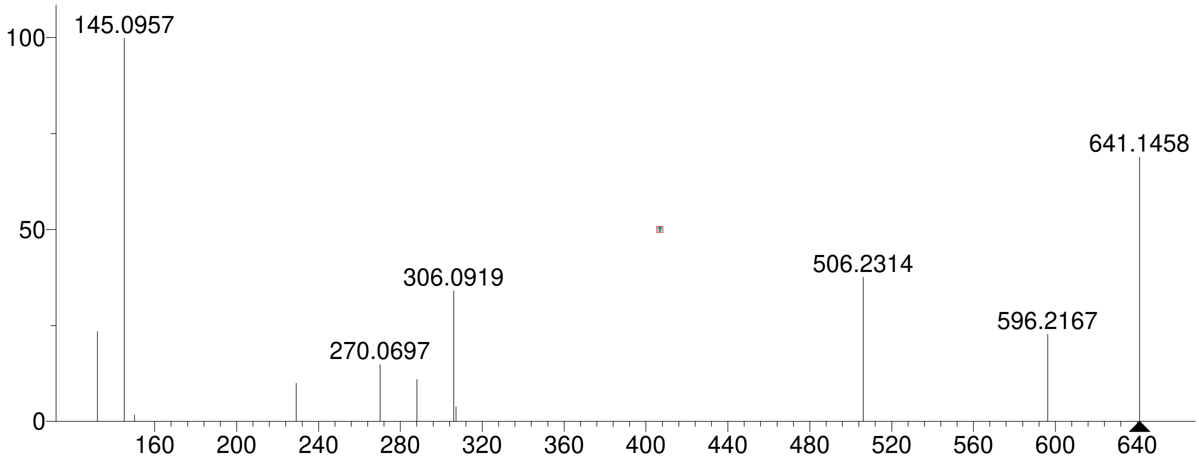

Unknown: Unknown (3.86_384.0969m/z)
Compound in Library Factor = -300

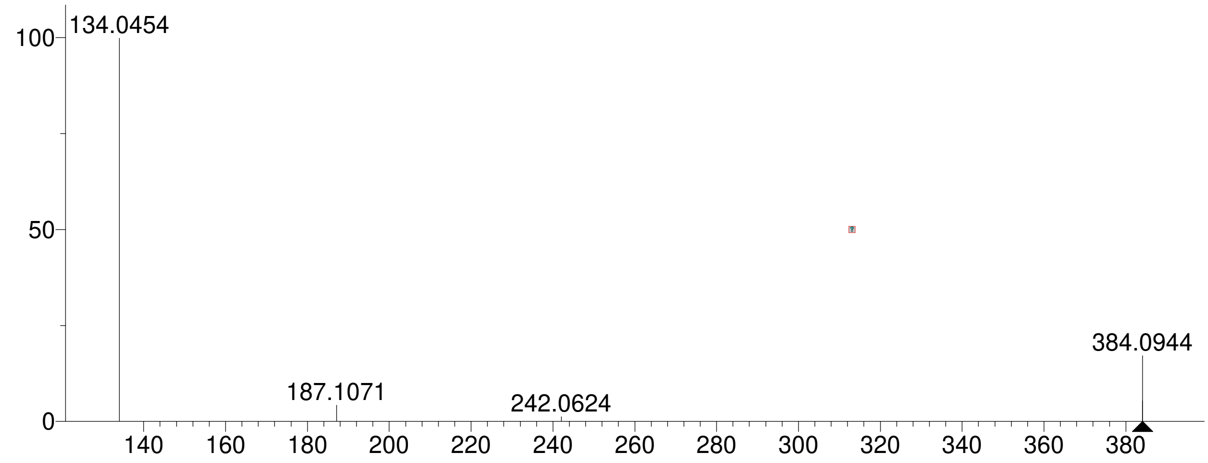

Hit 1 : Bezafibrate [M+Na]+ HCD 11V P=384.1
C19H20ClNO4; MF: 247; RMF: 838; Prob 96.4%; CAS: 41859-67-0; Lib: nist_msms; ID: 163281.

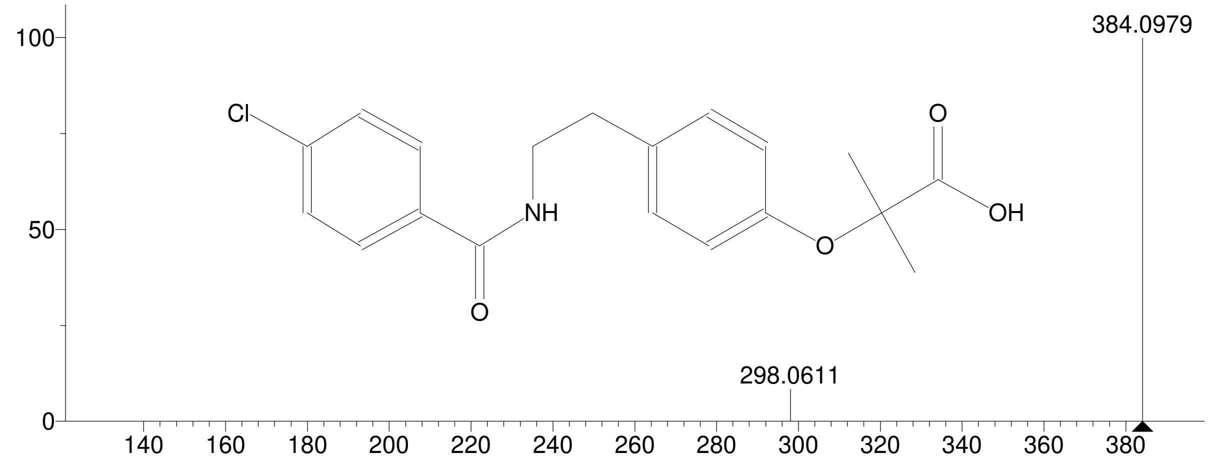

Hit 2 : Ethanone, 2-(2-bromophenyl)-1-(1-pentyl-1H-indol-3-yl)- [M+H]+ HCD 19V P=384.1
C21H22BrNO; MF: 23; RMF: 343; Prob 0.96%; CAS: 864445-60-3; Lib: nist_msms; ID: 253885.

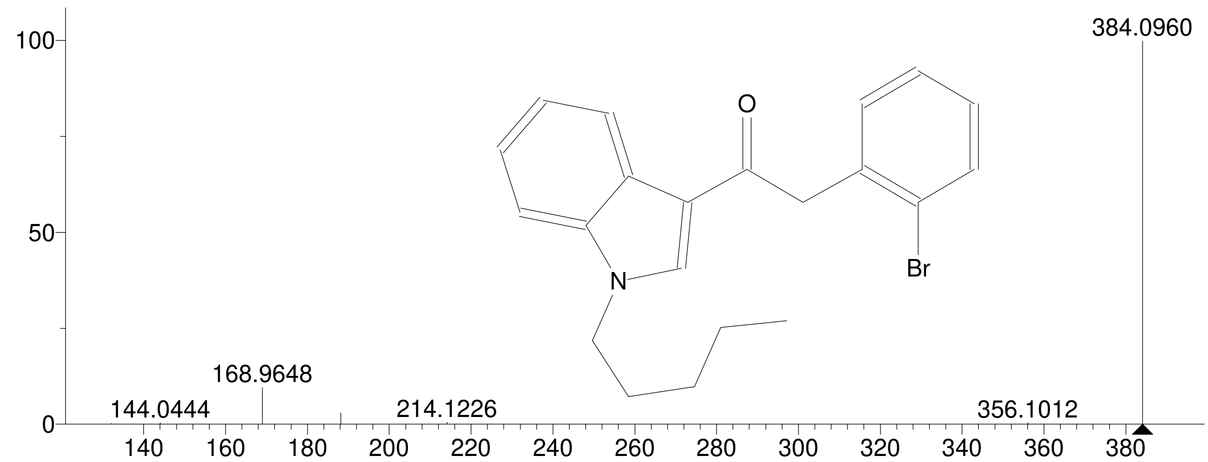

Hit 3 : Flavopiridol [M+H-H2O]+ HCD 19V P=384.1
C21H20ClNO5; MF: 23; RMF: 343; Prob 0.96%; CAS: 146426-40-6; Lib: nist_msms; ID: 375544.

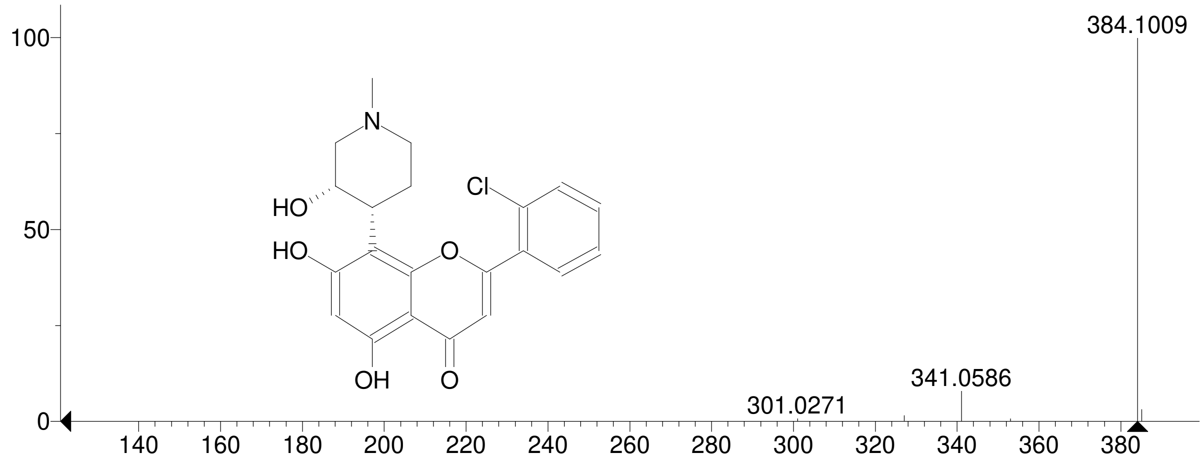

Hit 4 : Rhodamine B cation [Cat-CH3]+=>384.1 IT 35% P=384.1
C28H31N2O3; MF: 17; RMF: 518; Prob 0.75%; CAS: 64381-98-2; Lib: nist_msms; ID: 493948.

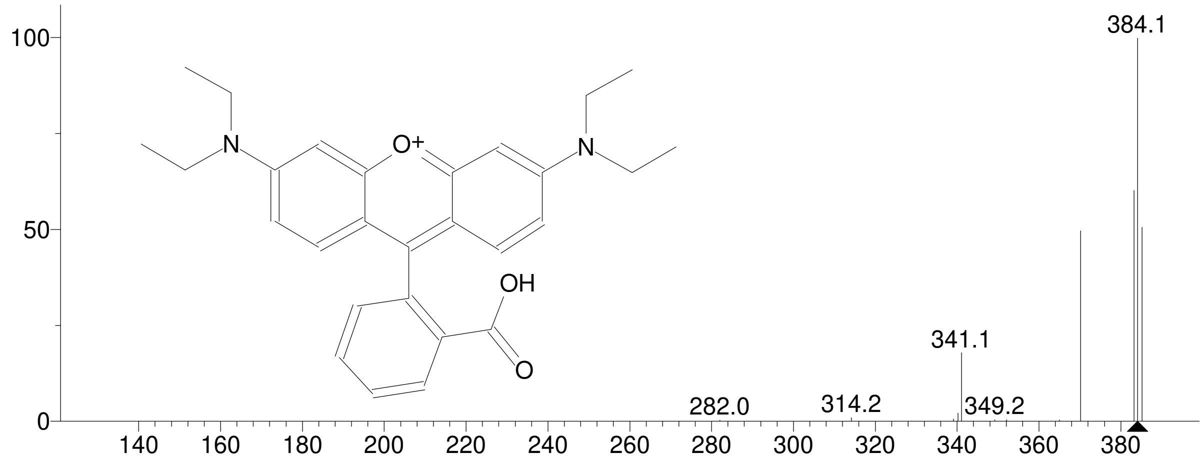

Hit 5 : Torin 2 [M+H-HF]+=>412.2=>384.1 IT 35% P=384.1
C24H15F3N4O; MF: 2; RMF: 237; Prob 0.45%; CAS: 1223001-51-1; Lib: nist_msms; ID: 269358.

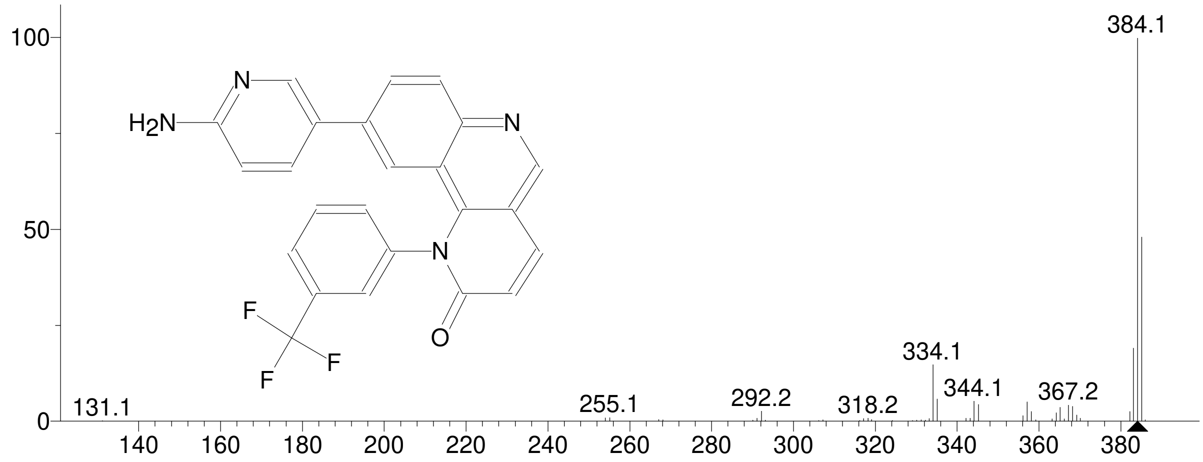

Unknown: Unknown (3.86_506.2338m/z)
Compound in Library Factor = -2176

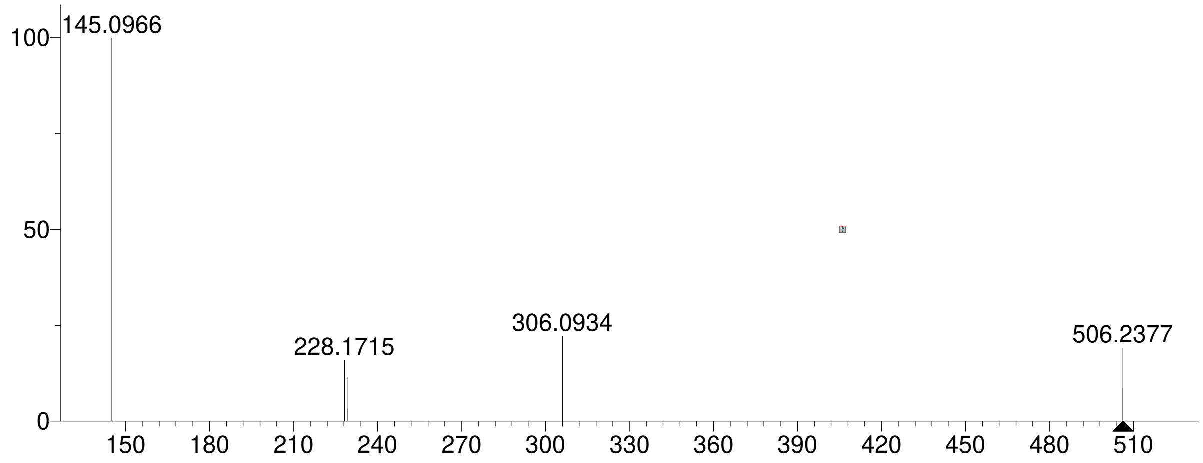

Hit 1 : Amprenavir [M+H]+ HCD 10V P=506.2
C25H35N3O6S; MF: 21; RMF: 415; Prob 42.9%; CAS: 161814-49-9; Lib: nist_msms; ID: 368619.

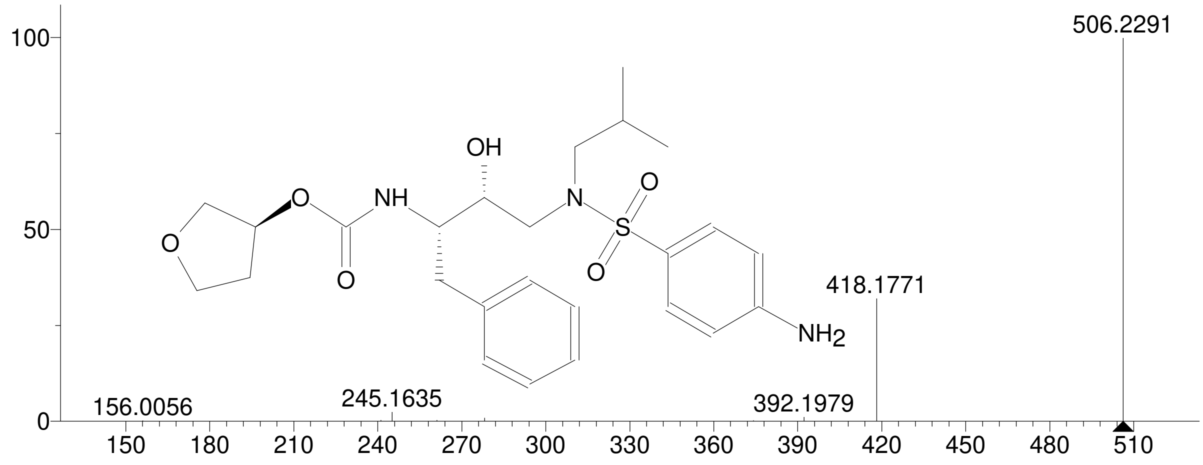

Hit 2 : Virginiamycin component b [M-H-H2O]- HCD 25V P=506.2
C28H35N3O7; MF: 1; RMF: 235; Prob 19.5%; Lib: nist_msms; ID: 353199.

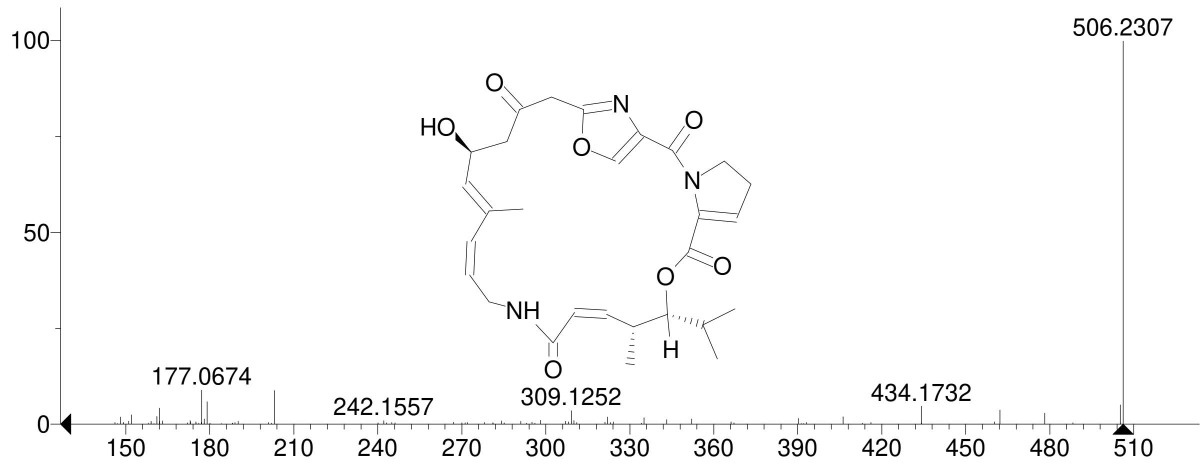

Hit 3 : Benidipine [M+H]+ 35eV P=506.2
C28H31N3O6; MF: 0; RMF: 151; Prob 18.8%; Lib: mona-export-fiehn_hilic; ID: 1784.

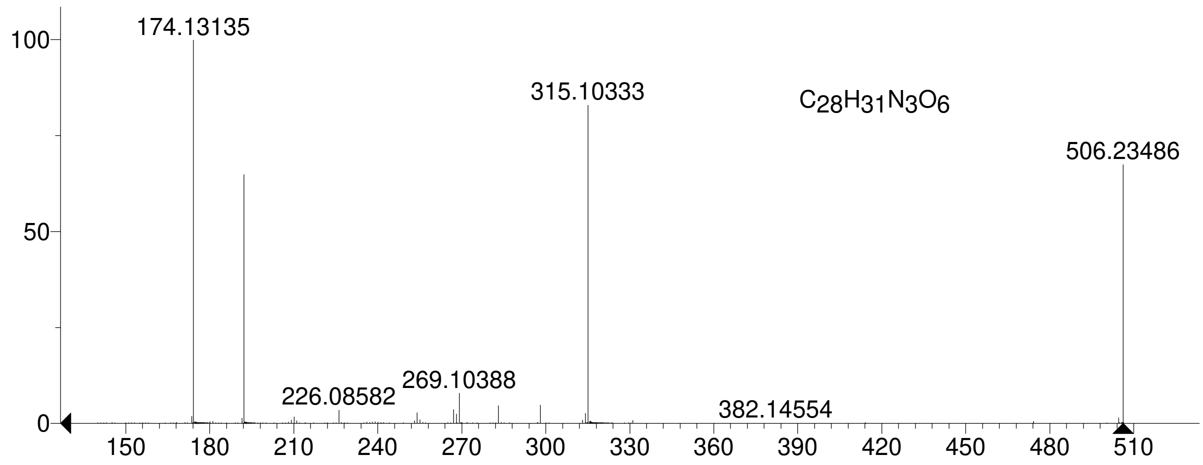

Hit 4 : Amprenavir (Agenerase) [M+H]+ P=506.2
C25H35N3O6S; MF: 0; RMF: 65; Prob 18.8%; Lib: mona-export-gnps; ID: 547.

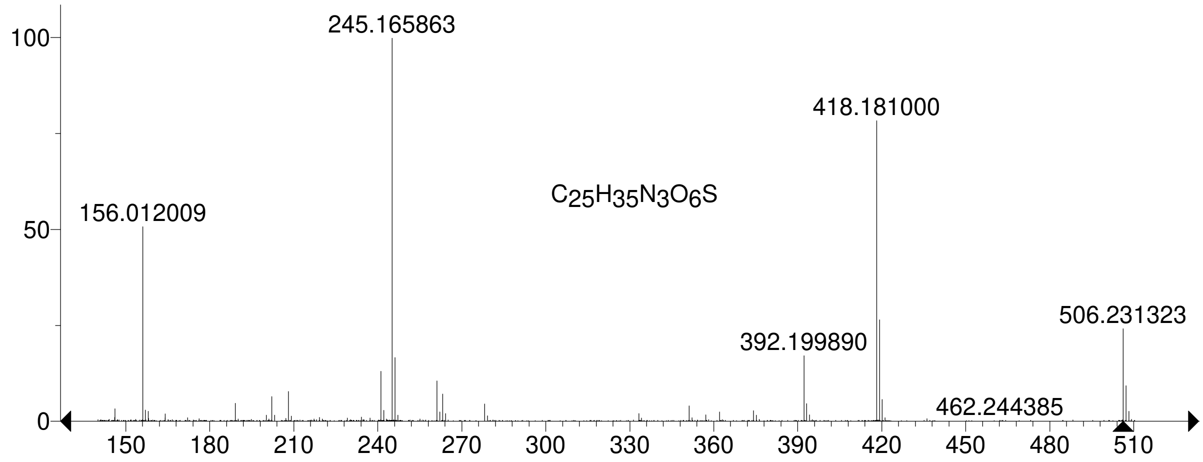

** Search Report Page 1 of 1 **

Unknown: Unknown (3.87_596.2166m/z)
Compound in Library Factor = 0

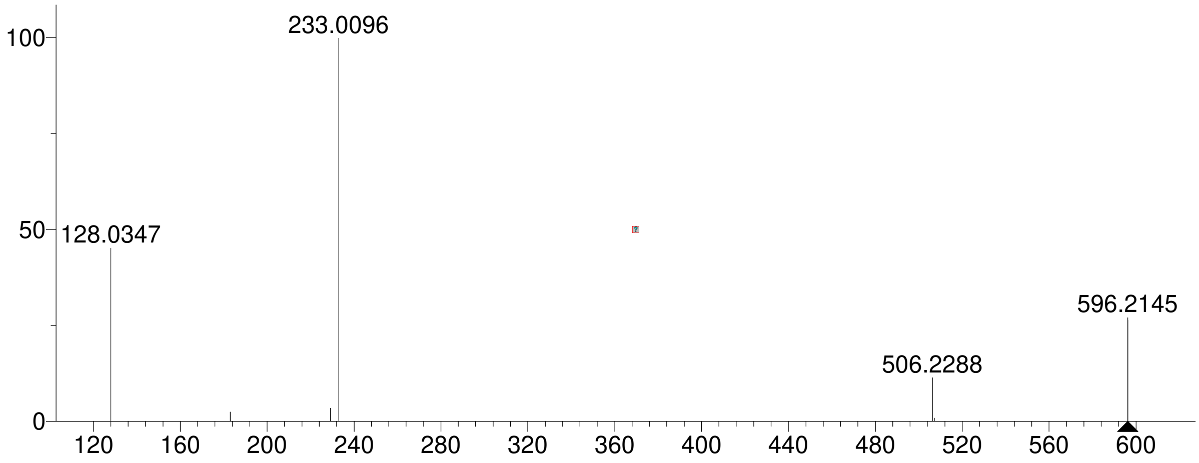

** Search Report Page 1 of 1 **

Unknown: Unknown (3.88_609.2043m/z)
Compound in Library Factor = -3273

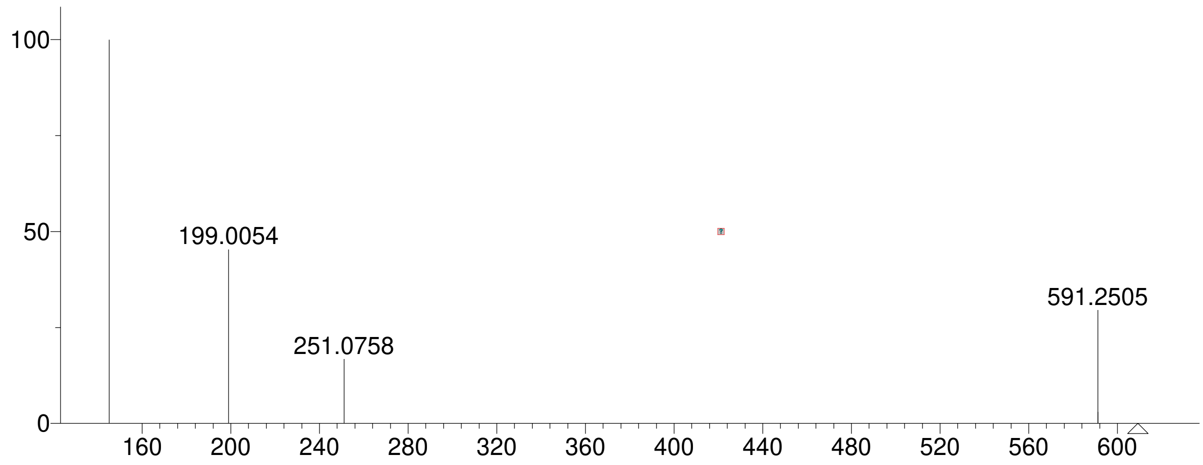

Hit 1 : Hesperidin M-H 35 P=609.2
C28H34O15; MF: 4; RMF: 199; Prob 100.0%; Lib: mona anthocyanidin-o-glycosides; ID: 2976.

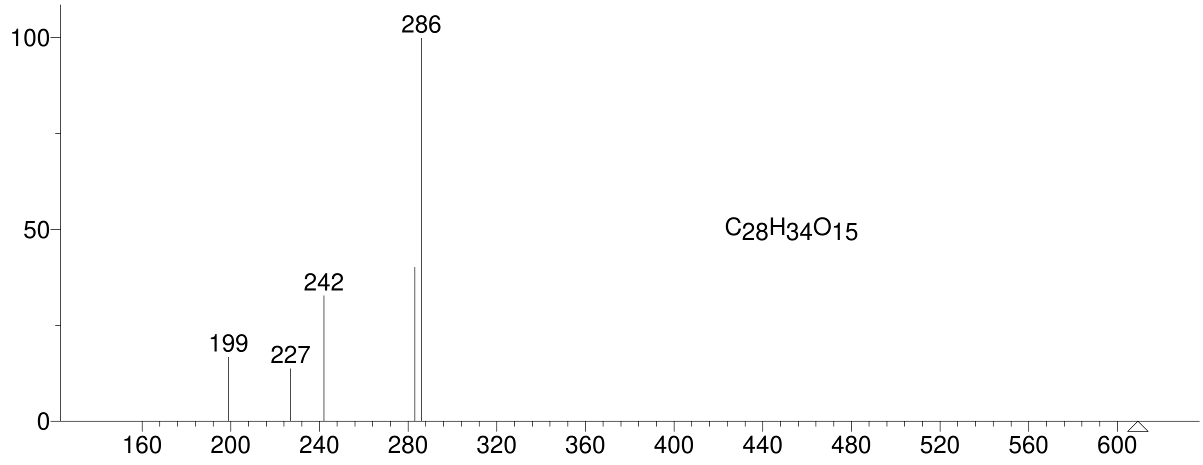

** Search Report Page 1 of 1 **

Unknown: Unknown (3.91_530.1959m/z)
Compound in Library Factor = 0

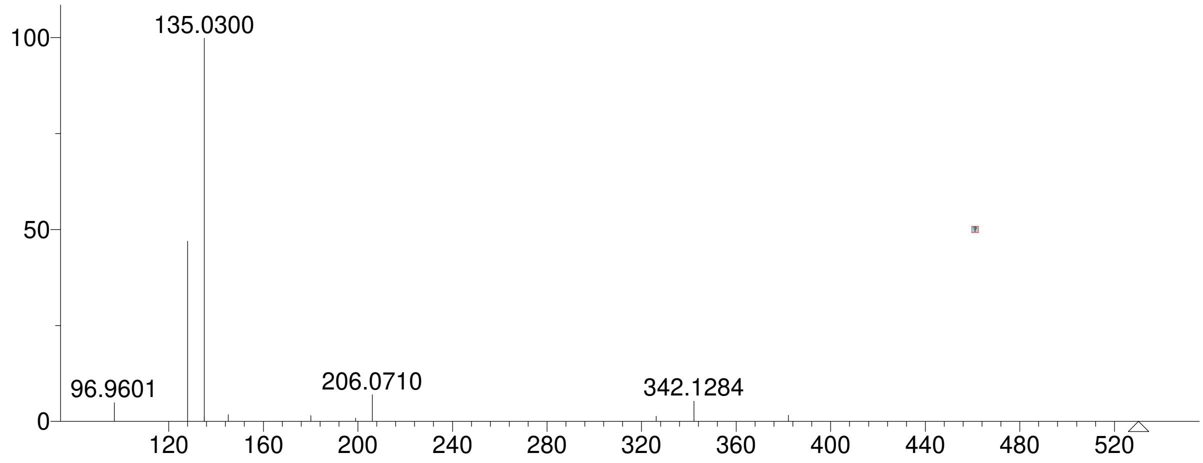

Hit 1 : Nilotinib [M+H]+ HCD 174V P=530.2
C28H22F3N7O; MF: 0; RMF: 50; Prob 50.0%; CAS: 641571-10-0; Lib: nist_msms; ID: 165145.

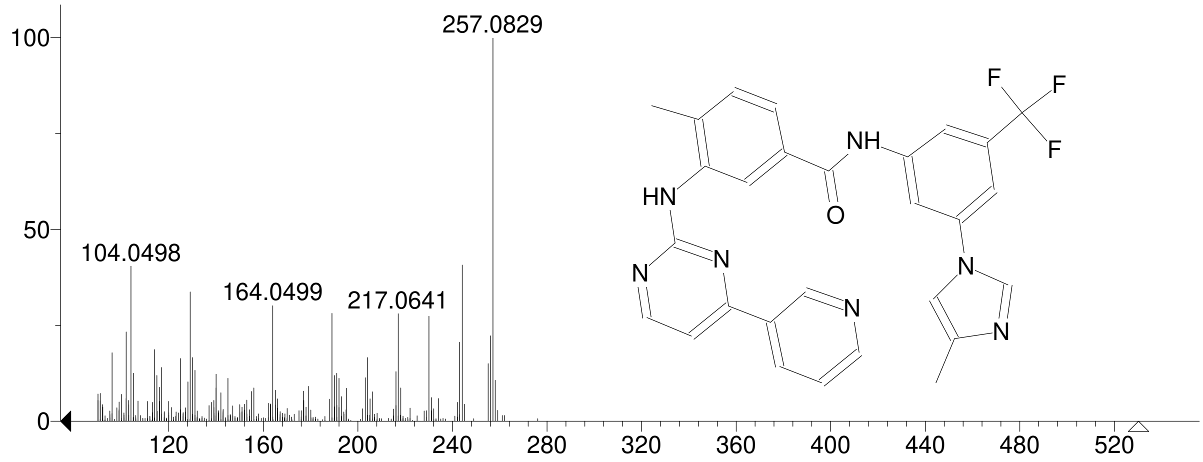

Hit 2 : Cepharanthine [M+H]+=>545.2=>530.2 IT 35% P=530.2
C37H38N2O6; MF: 0; RMF: 19; Prob 50.0%; CAS: 481-49-2; Lib: nist_msms; ID: 217075.

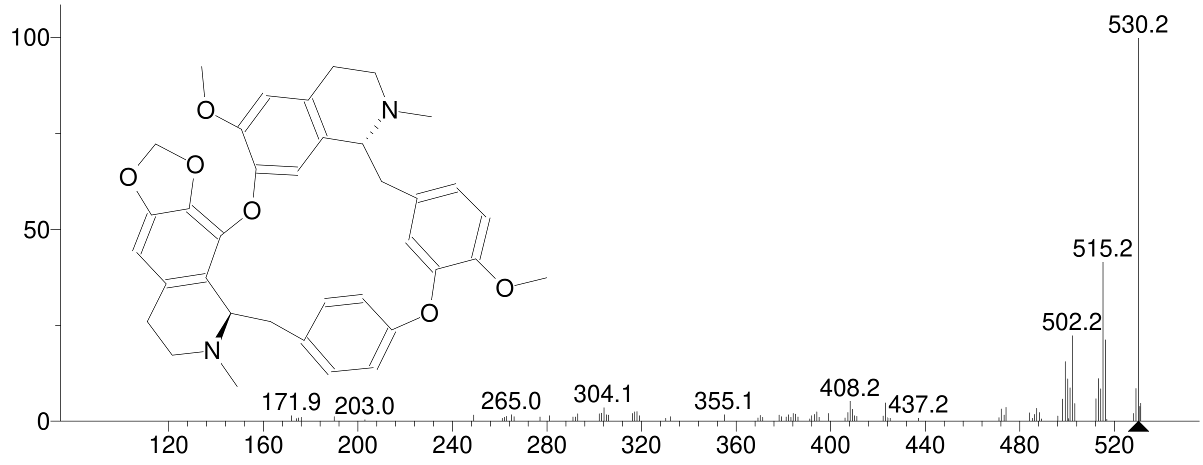

Unknown: Unknown (3.91_591.2530m/z)
Compound in Library Factor = -3273

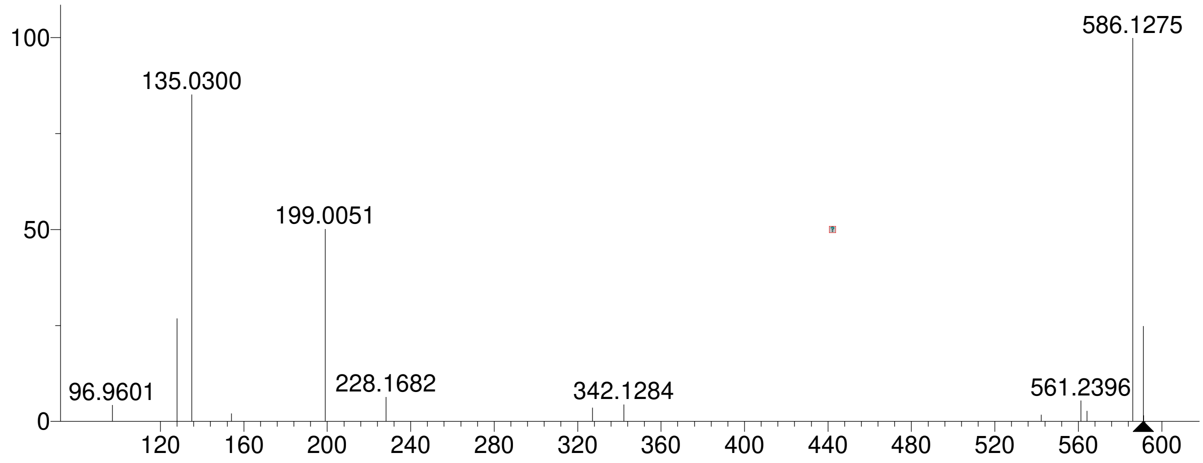

Hit 1 : LPI 18:4; [M-H]- [M-H]- P=591.3
C27H45O12P; MF: 8; RMF: 250; Prob 57.3%; Lib: msdial-insilicomsms-lipids-neg; ID: 311171.

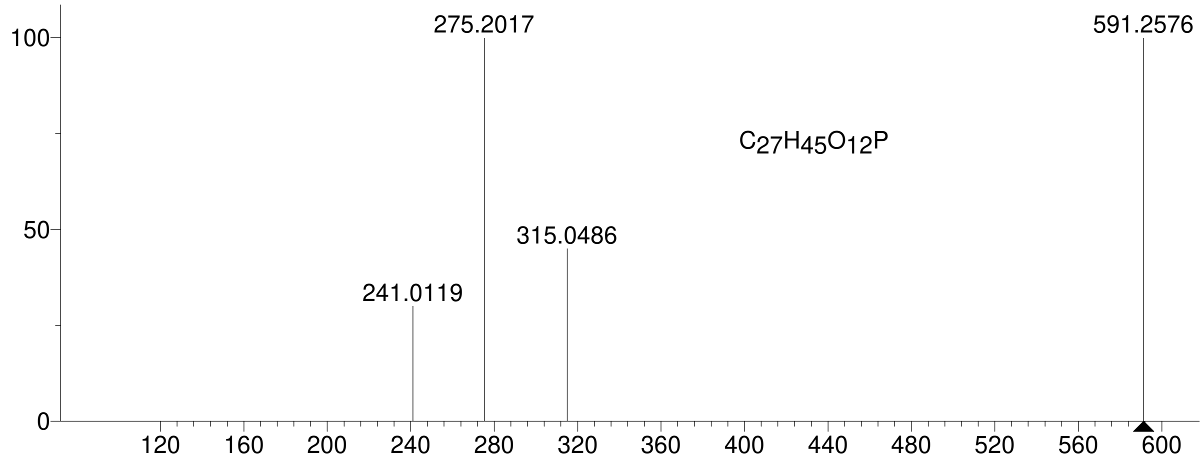

Hit 2 : 3-[(3R,5S,10R,13R,14S,17R)-5,14-dihydroxy-10,13-dimethyl-3-[(2R,3R,4S,5S,6R)-3,4,5-trihydroxy-6-(hydr
C29H44O10; MF: 0; RMF: 33; Prob 42.7%; Lib: mona-export-vf-npl_qexactive; ID: 14698.

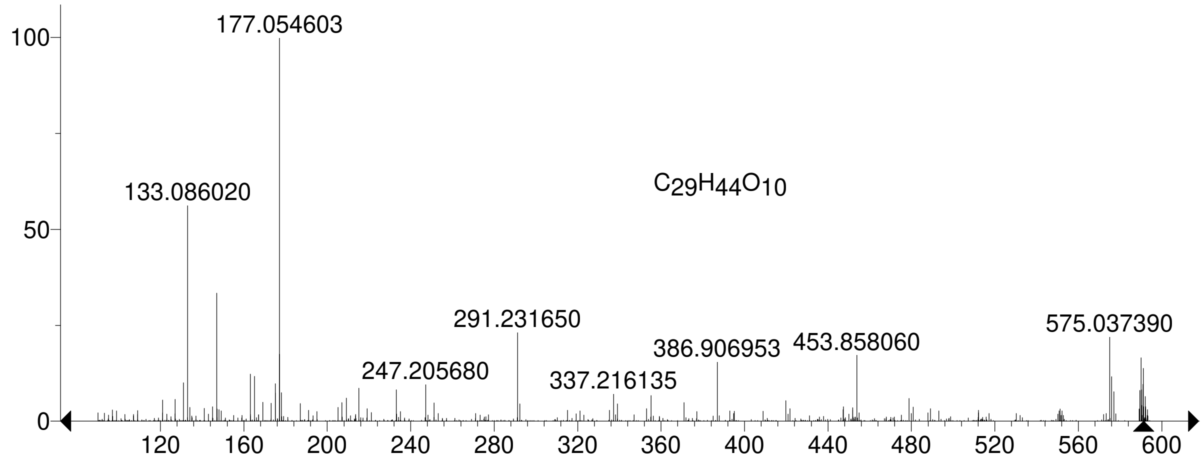

Unknown: Unknown (3.91_680.2762m/z)
Compound in Library Factor = 0

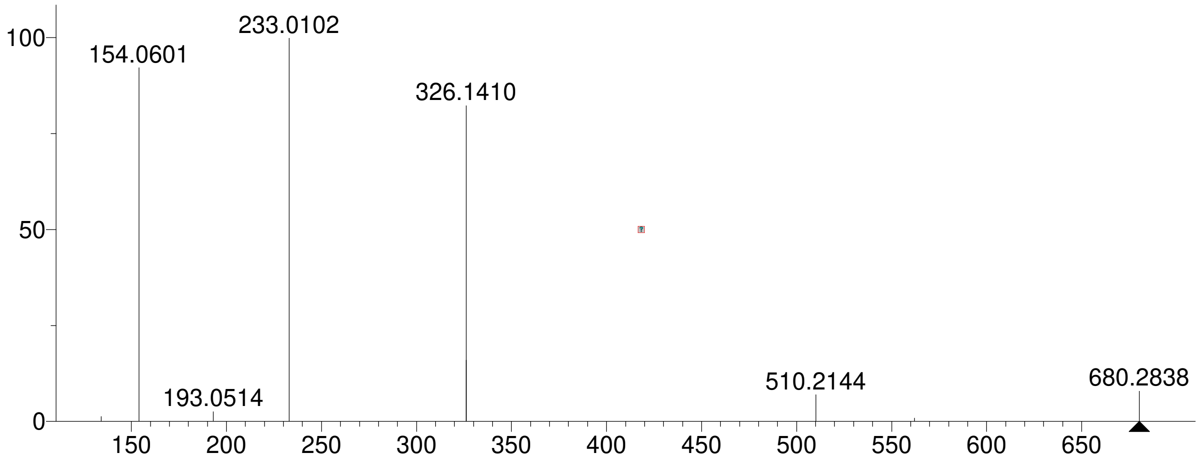

Unknown: Unknown (3.92_277.1179m/z)
Compound in Library Factor = 0

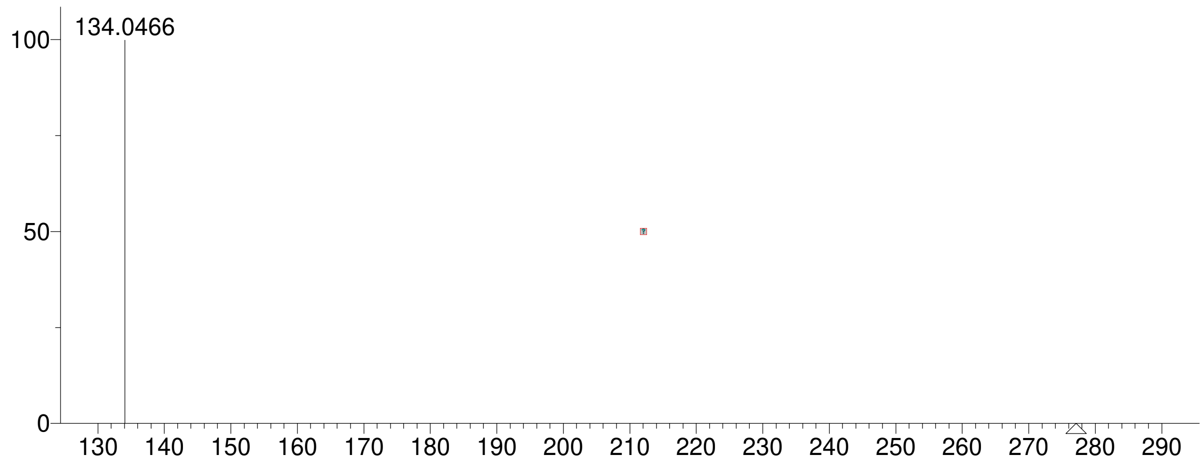

Unknown: Unknown (3.92_382.0995m/z)
Compound in Library Factor = -462

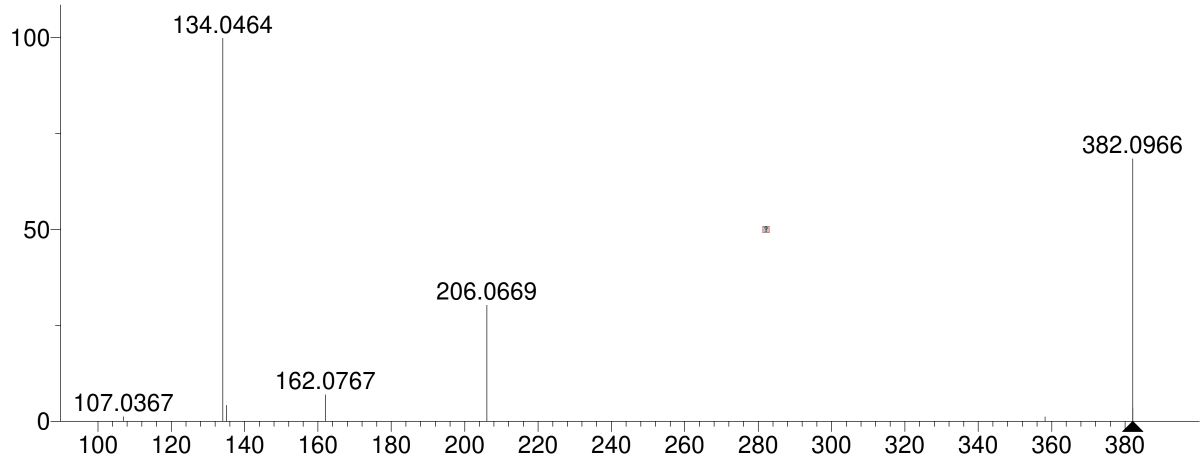

Hit 1 : Fluxapyroxad [M+H]+ HCD 7V P=382.1
C18H12F5N3O; MF: 149; RMF: 738; Prob 94.6%; CAS: 907204-31-3; Lib: nist_msms; ID: 469925.

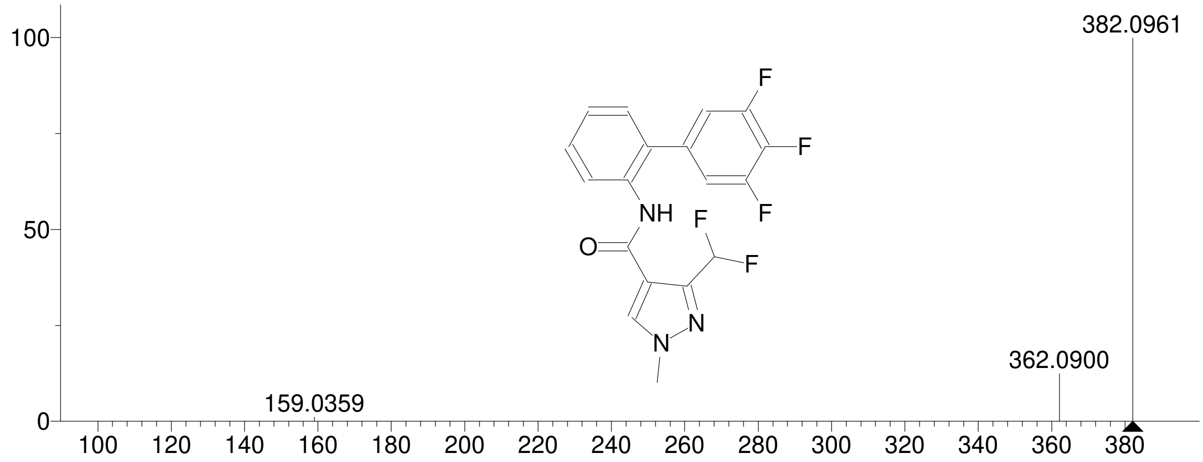

Hit 2 : 5-(3-(2-(7-Chloroquinolin-2-yl)ethenyl)phenyl)-8-dimethylcarbamyl-4,6-dithiaoctanoic acid [M+H]+=>382.1
C26H27ClN2O3S2; MF: 0; RMF: 53; Prob 2.68%; CAS: 115104-28-4; Lib: nist_msms; ID: 538755.

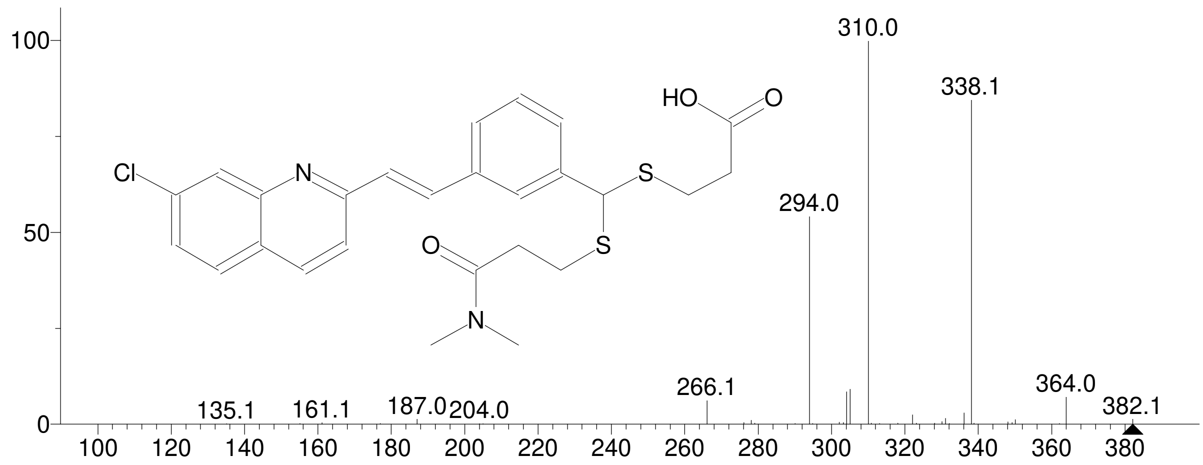

Hit 3 : Flavopiridol [M-H]-=>382.1 IT 35% P=382.1
C21H20ClNO5; MF: 0; RMF: 52; Prob 2.68%; CAS: 146426-40-6; Lib: nist_msms; ID: 375595.

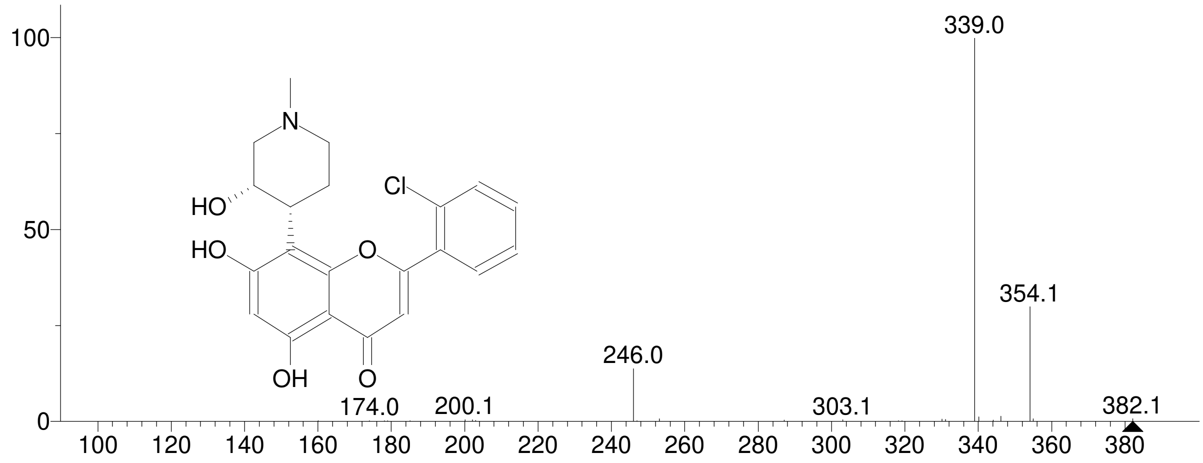

Unknown: Unknown (3.93_332.1695m/z)
Compound in Library Factor = 0

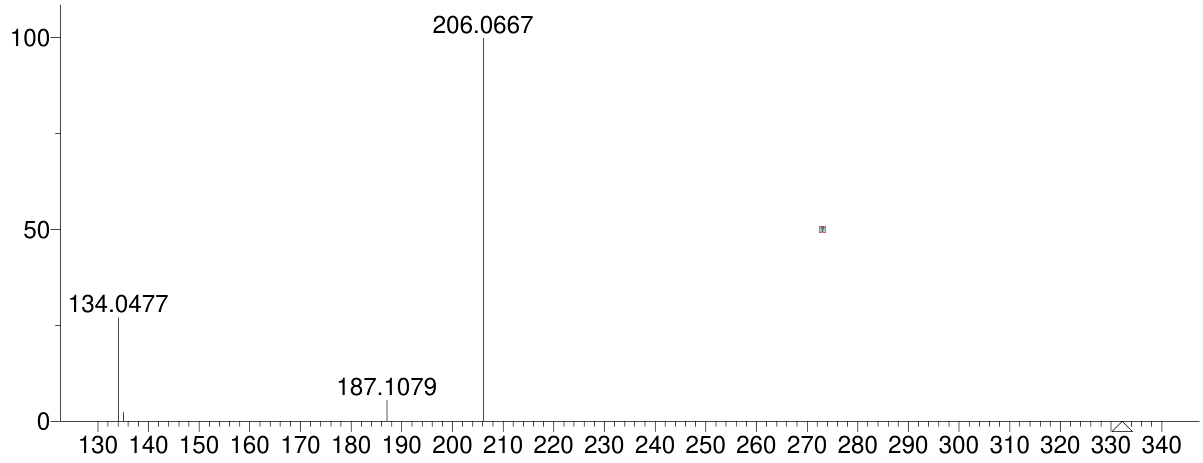

Unknown: Unknown (3.93_458.1416m/z)
Compound in Library Factor = 0

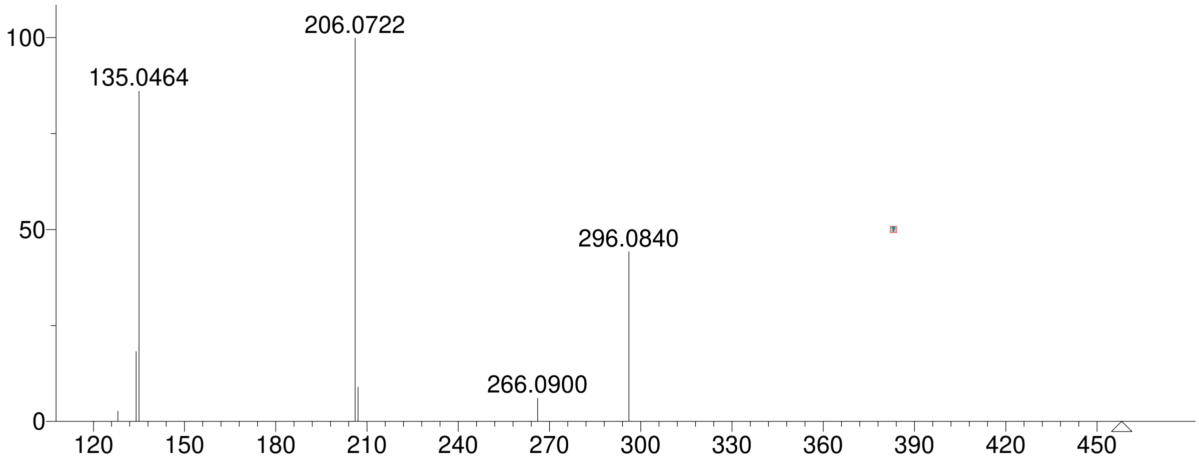

Unknown: Unknown (3.93_562.1182m/z)
Compound in Library Factor = 0

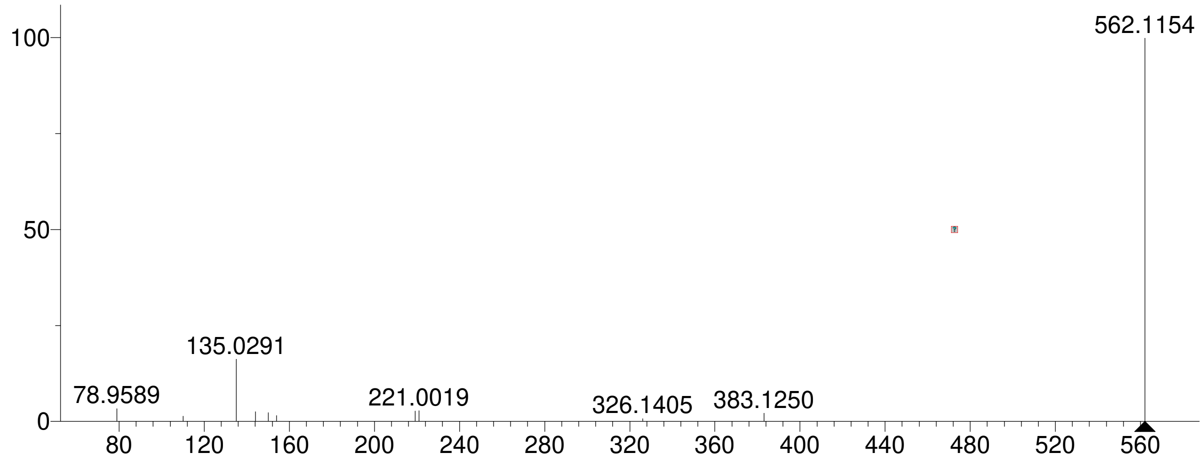

** Search Report Page 1 of 1 **

Unknown: Unknown (3.94_586.1290m/z)
Compound in Library Factor = 0

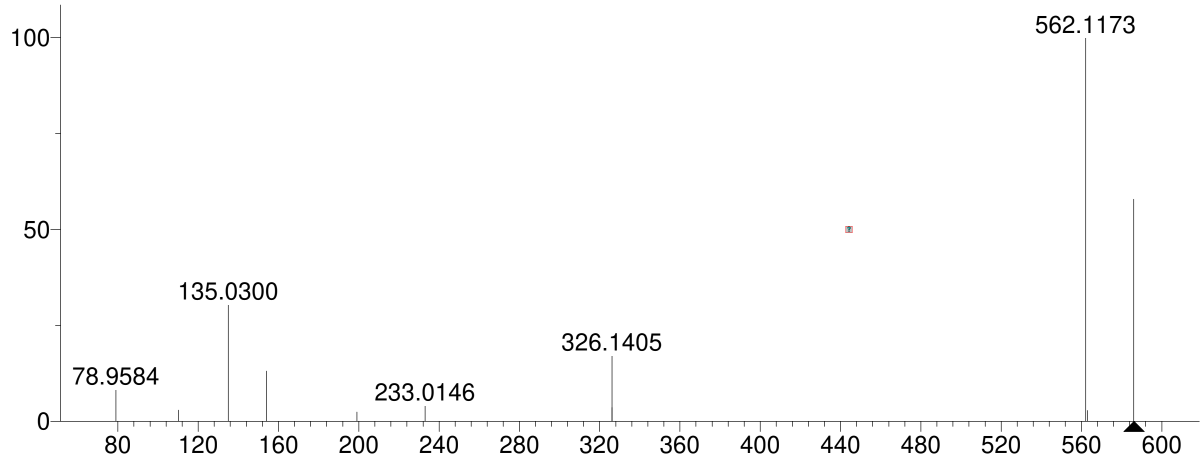

Unknown: Unknown (3.95_358.1973m/z)
Compound in Library Factor = -2176

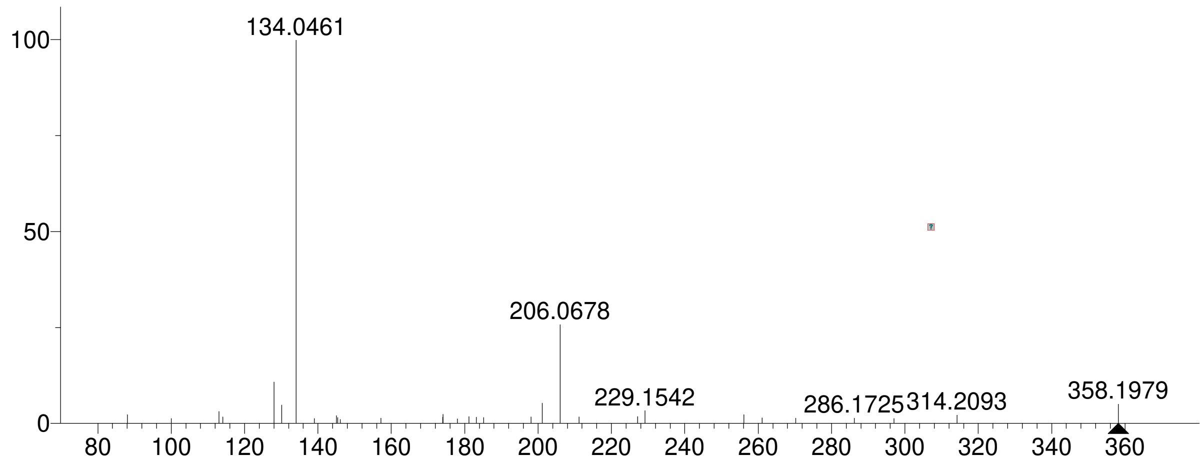

Hit 1 : 4-Hydroxyatorvastatin lactone [M-H-H2O]-=>493.2=>358.2 IT 35% P=358.2
C33H33FN2O5; MF: 29; RMF: 447; Prob 17.4%; CAS: 163217-70-7; Lib: nist_msms; ID: 440854.

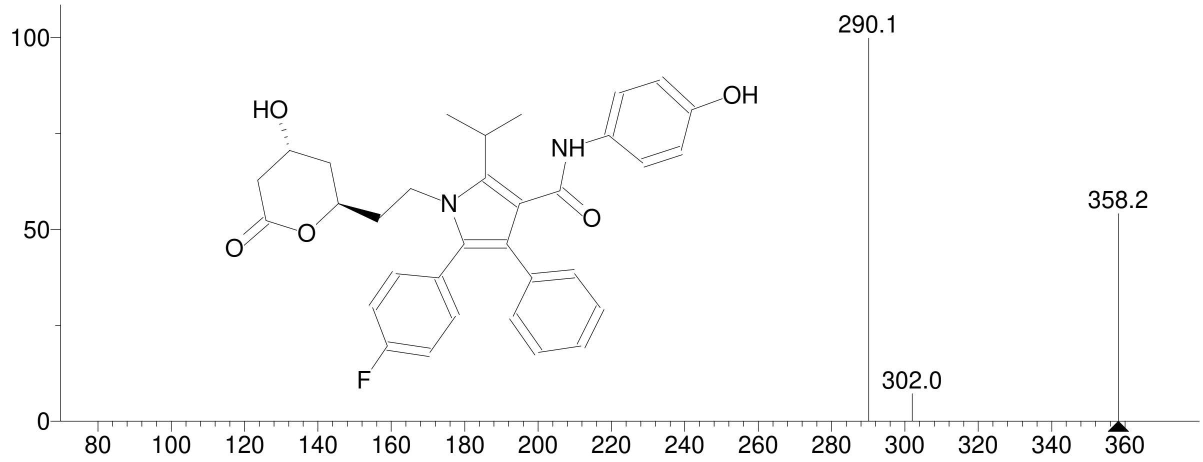

Hit 2 : 1H-Indole-3-carboxamide, 1-(5-fluoropentyl)-N-8-quinolinyl- [M+H]+=>358.2 IT 35% P=358.2
C23H22FN3O; MF: 1; RMF: 141; Prob 5.03%; Lib: nist_msms; ID: 344362.

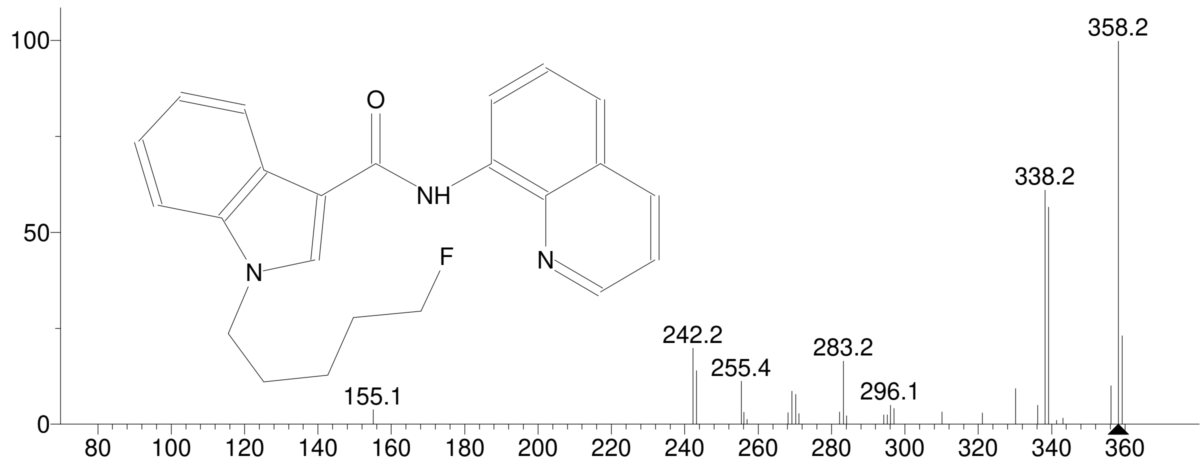

Hit 3 : 1H-Indole-3,5-dicarboxylic acid, 1-[3-[4-(decyloxy)phenoxy]-2-oxopropyl]-, 3-methyl ester [M-H]-=>358.2 I
C30H37NO7; MF: 1; RMF: 111; Prob 5.03%; CAS: 888320-29-4; Lib: nist_msms; ID: 374612.

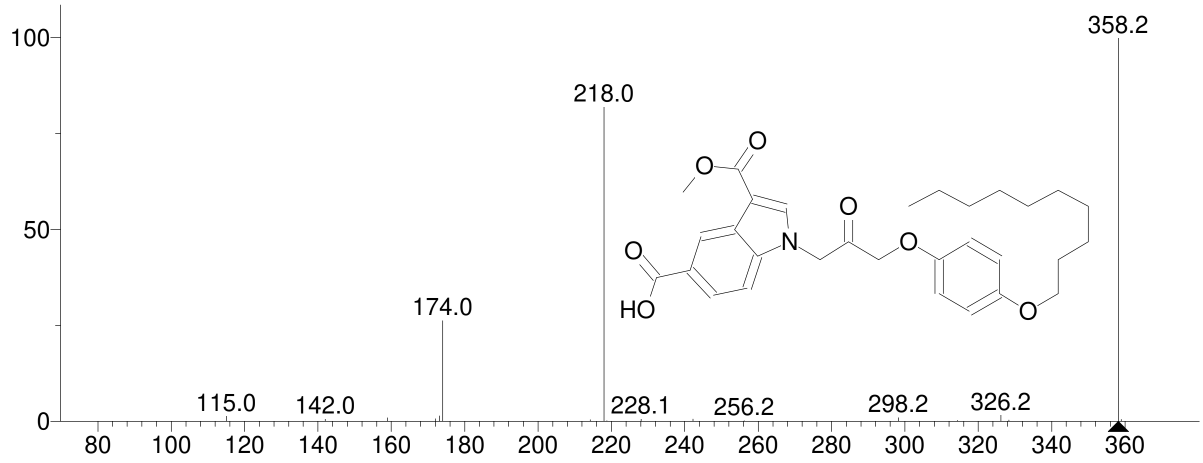

Hit 4 : Nalbuphine [M+H]+ 20-30-40%) P=358.2
C21H27NO4; MF: 0; RMF: 104; Prob 4.83%; Lib: mona-export-fiehn_hilic; ID: 599.

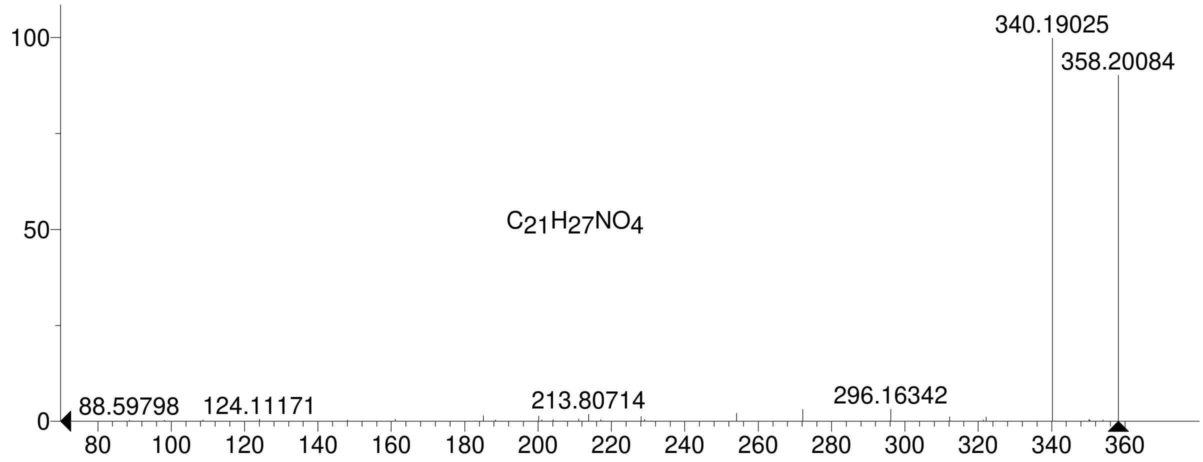

Hit 5 : Leukotriene D4 methyl ester [M+H-H2O]+=>390.2=>358.2 IT 35% P=358.2
C26H42N2O6S; MF: 0; RMF: 83; Prob 4.83%; Lib: nist_msms; ID: 92133.

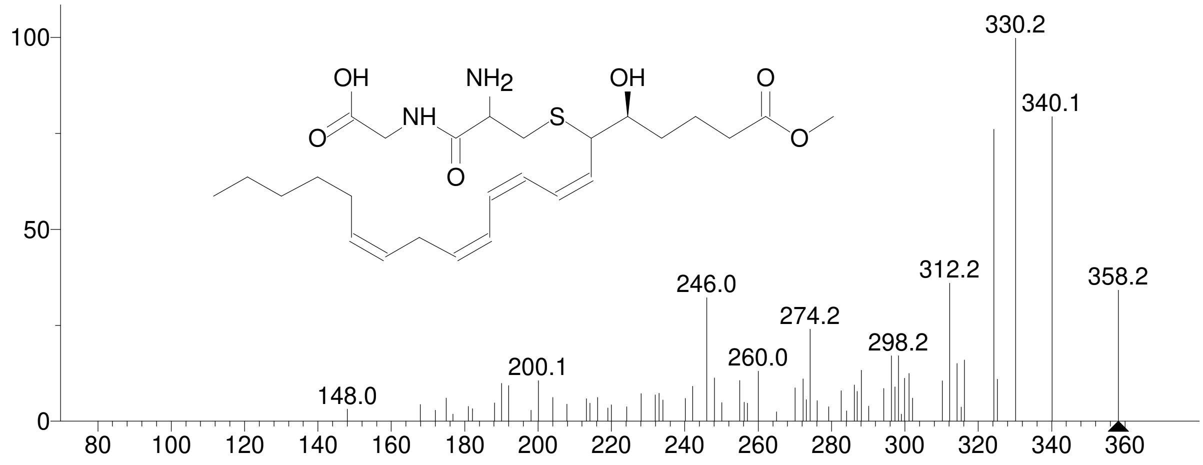

Unknown: Unknown (3.96_259.1287m/z)
Compound in Library Factor = -1142

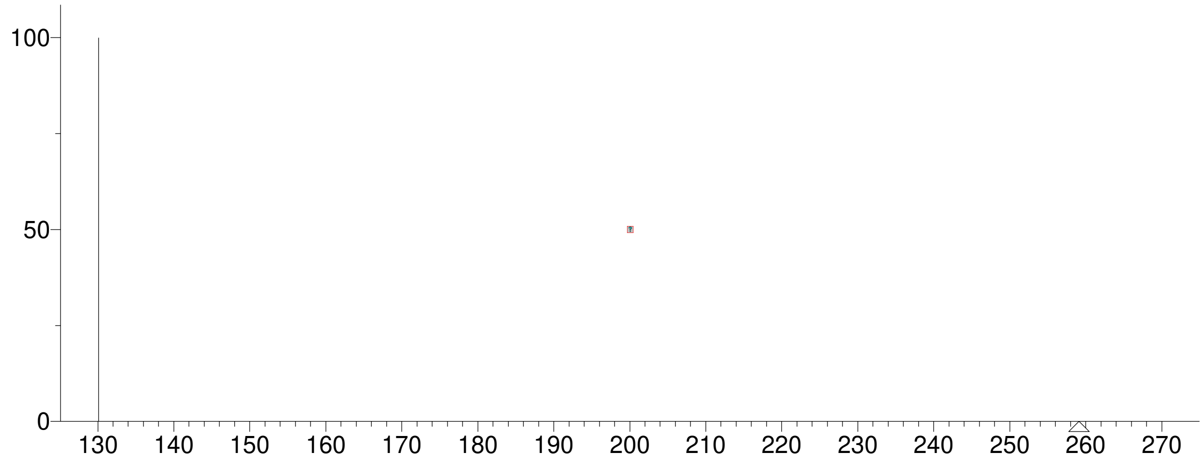

Hit 1 : Glu-Leu [M-H]- HCD 41eV P=259.1
C11H20N2O5; MF: 181; RMF: 918; Prob 40.6%; Lib: nist_msms; ID: 570065.

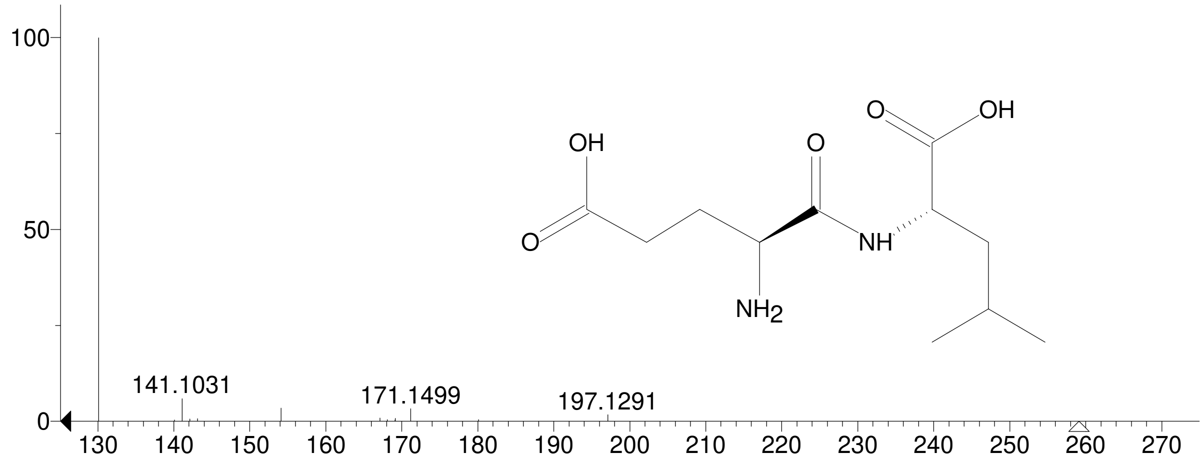

Hit 2 : Glu-Leu [M-H]- HCD 41eV P=259.1
C11H20N2O5; MF: 181; RMF: 918; Prob 40.6%; CAS: 5969-52-8; Lib: nist_msms2; ID: 83791.

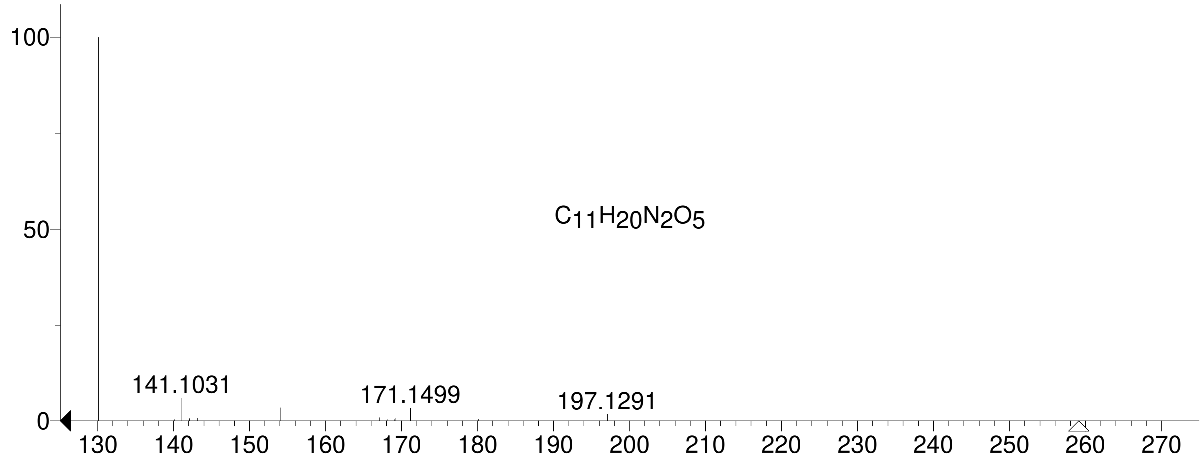

Hit 3 : Ile-Glu [M-H]- HCD 41eV P=259.1
C11H20N2O5; MF: 118; RMF: 874; Prob 6.80%; Lib: nist_msms; ID: 570719.

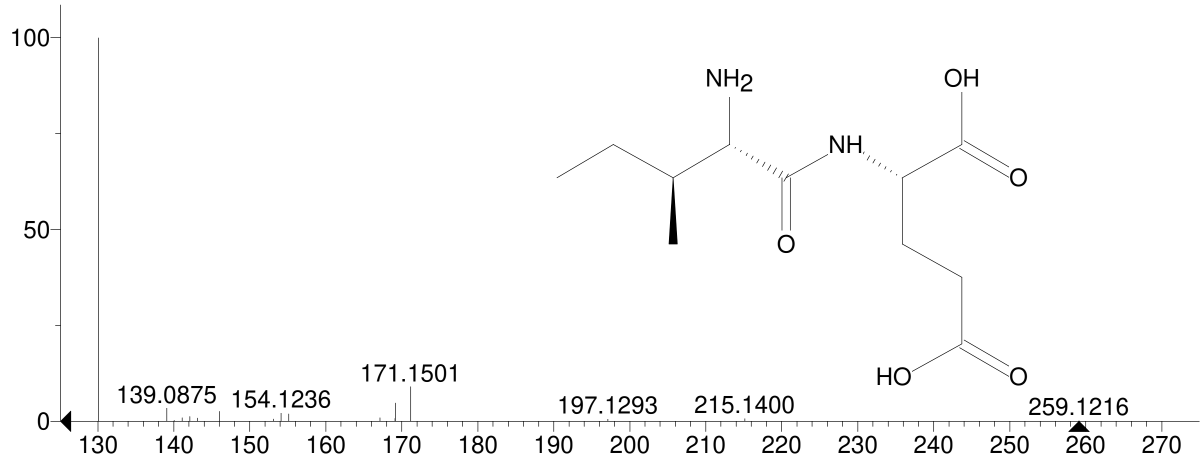

Hit 4 : Ile-Glu [M-H]- HCD 41eV P=259.1
C11H20N2O5; MF: 118; RMF: 874; Prob 6.80%; CAS: 42516-53-0; Lib: nist_msms2; ID: 84452.

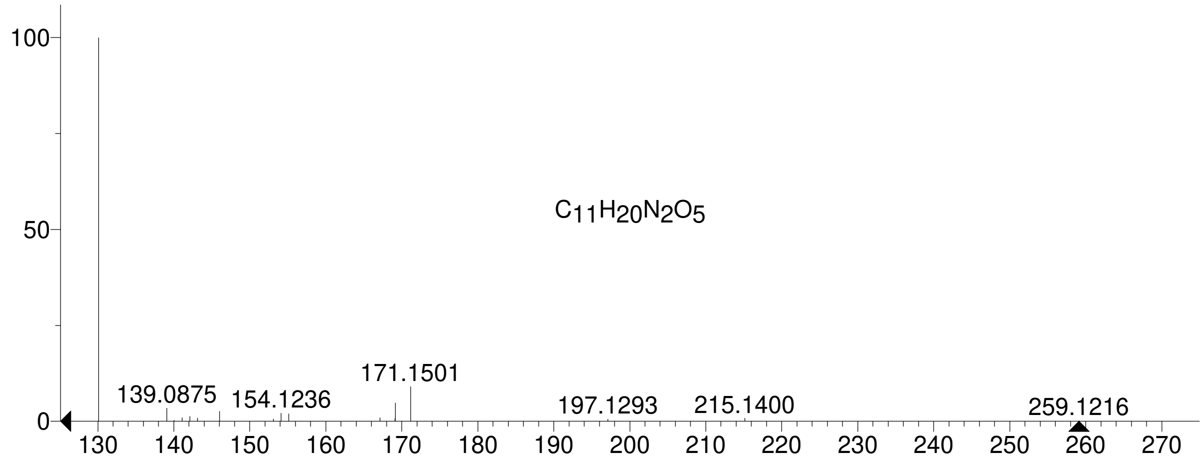

Hit 5 : Leu-Glu [M-H]- HCD 41eV P=259.1
C11H20N2O5; MF: 86; RMF: 842; Prob 1.81%; Lib: nist_msms; ID: 570787.

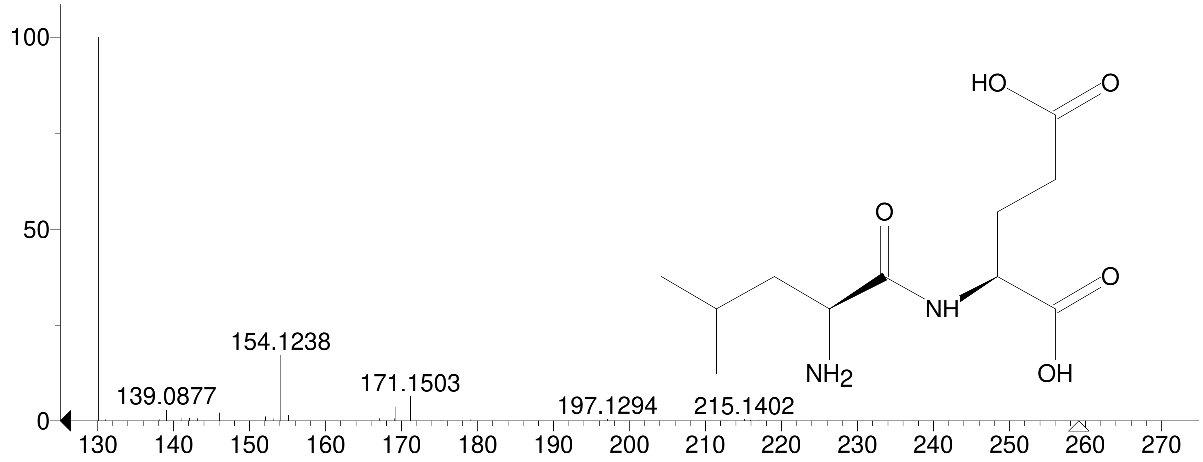

Unknown: Unknown (3.96_415.1709m/z)
Compound in Library Factor = -3273

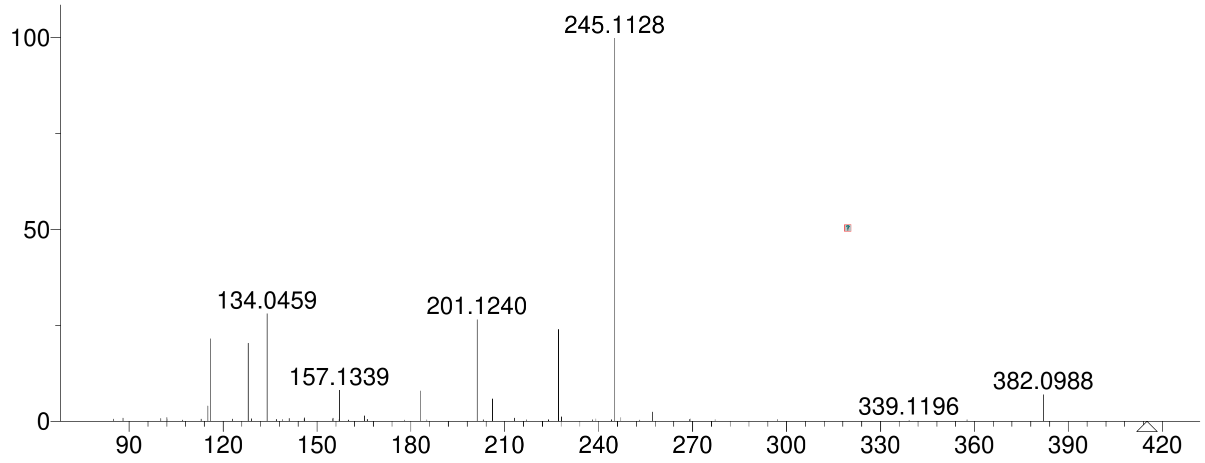

Hit 1 : 6-formyl-10-(hydroxymethyl)-5-methoxy-3-methylidene-2-oxo-2H,3H,3aH,4H,5H,8H,9H,11aH-cyclodeca[b]f
C21H28O7; MF: 1; RMF: 54; Prob 20.6%; Lib: mona-export-vf-npl_qexactive; ID: 16435.

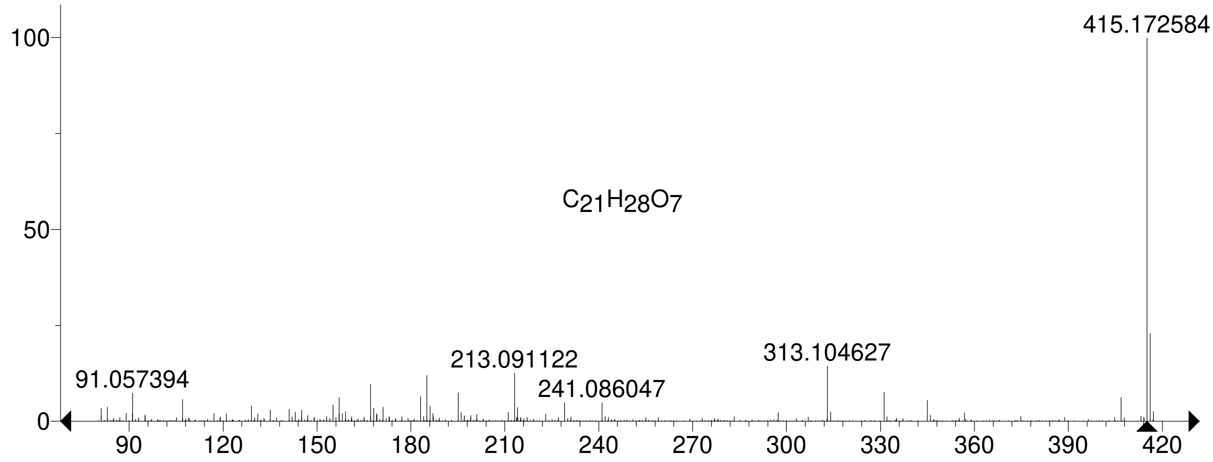

Hit 2 : NCGC00347525-03!2-amino-5-[2-[[2,3-dihydroxy-2-(1-hydroxyethyl)butanoyl]oxymethyl]-4-hydroxyanilino]-
C18H26N2O9; MF: 0; RMF: 8; Prob 19.8%; Lib: mona-export-gnps; ID: 9213.

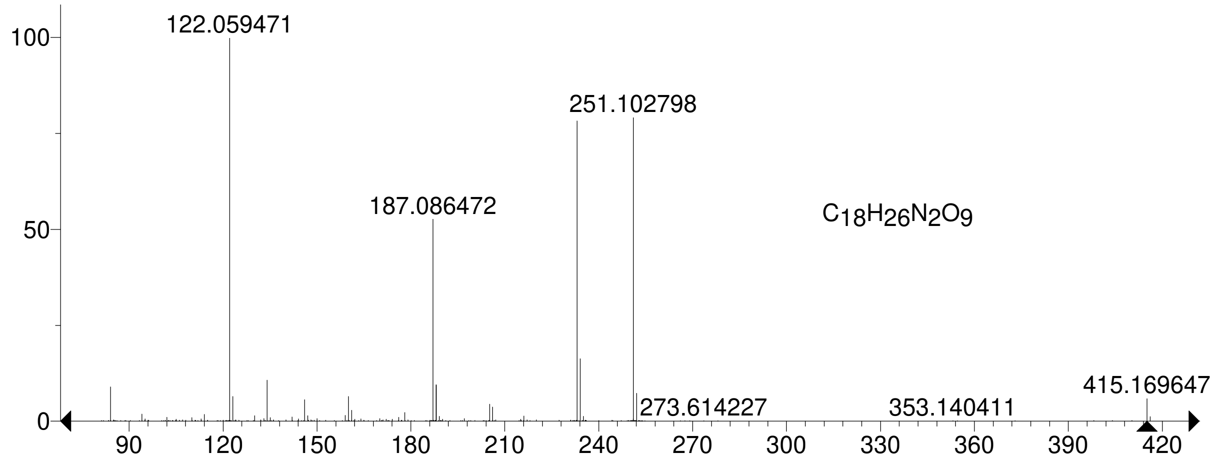

Hit 3 : 2-amino-5-[2-[[2,3-dihydroxy-2-(1-hydroxyethyl)butanoyl]oxymethyl]-4-hydroxyanilino]-5-oxopentanoic acid
C18H26N2O9; MF: 0; RMF: 6; Prob 19.8%; Lib: mona-export-vf-npl_qexactive; ID: 23034.

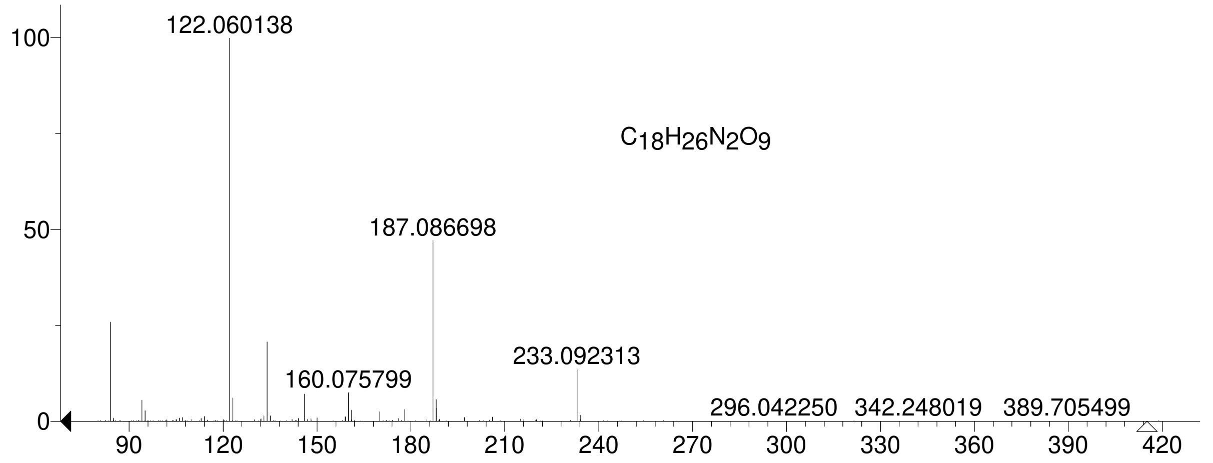

Hit 4 : NCGC00347525-02!2-amino-5-[2-[[2,3-dihydroxy-2-(1-hydroxyethyl)butanoyl]oxymethyl]-4-hydroxyanilino]-
C18H26N2O9; MF: 0; RMF: 5; Prob 19.8%; Lib: mona-export-gnps; ID: 6270.

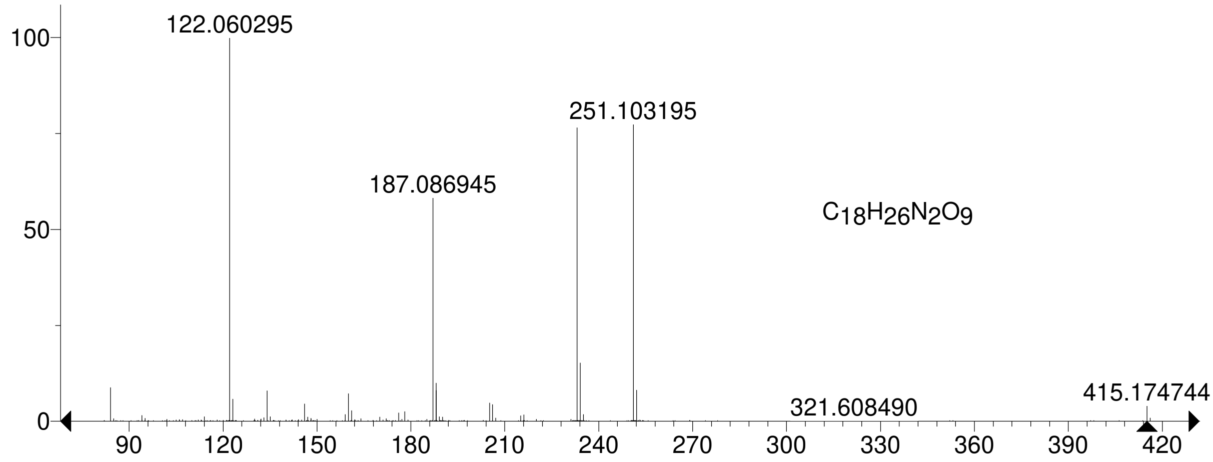

Hit 5 : Diltiazem HCl (Tiazac) [M+H]+ P=415.2
C22H27ClN2O4S; MF: 0; RMF: 4; Prob 19.8%; Lib: mona-export-gnps; ID: 648.

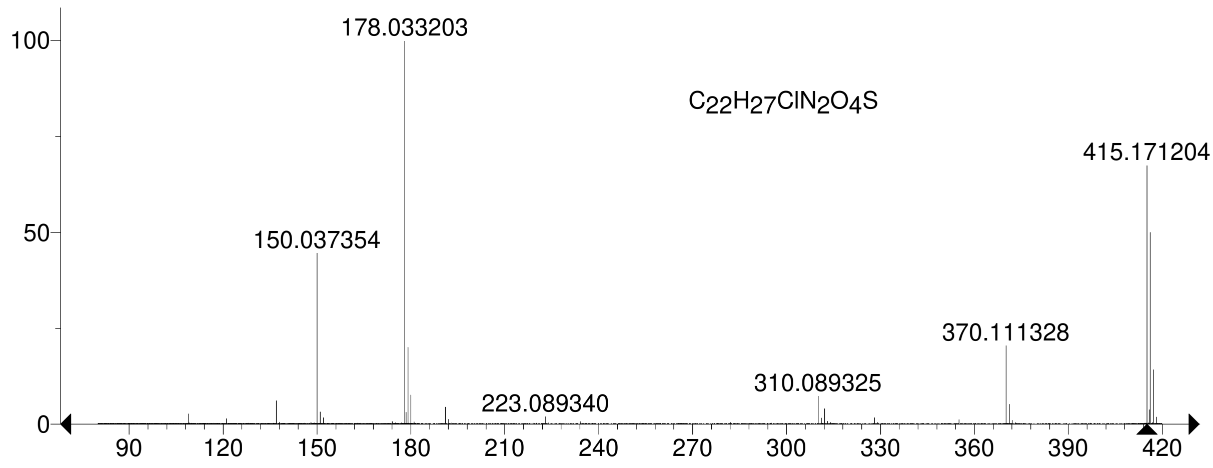

** Search Report Page 1 of 1 **

Unknown: Unknown (3.96_467.0790m/z)
Compound in Library Factor = 0

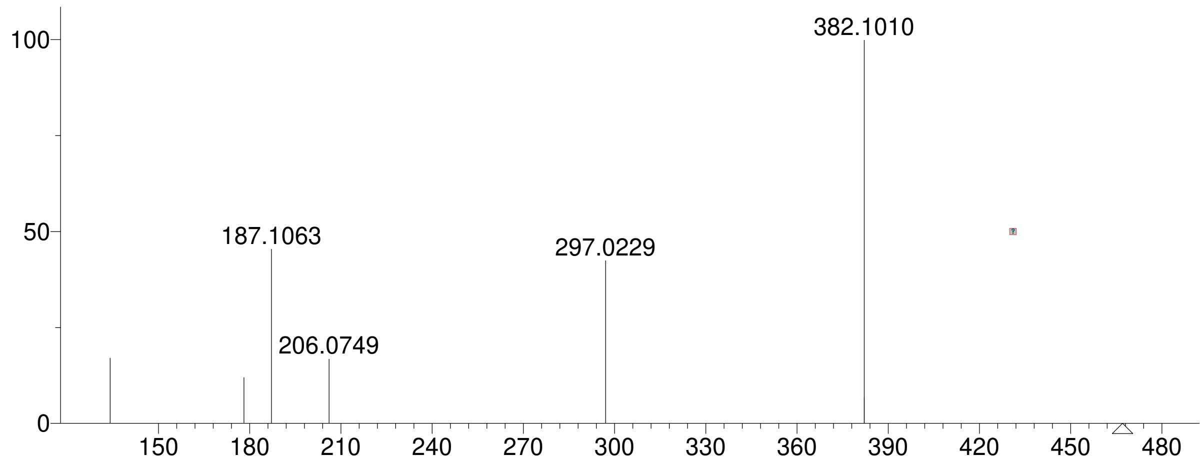

** Search Report Page 1 of 1 **

Unknown: Unknown (3.96_522.2280m/z)
Compound in Library Factor = 0

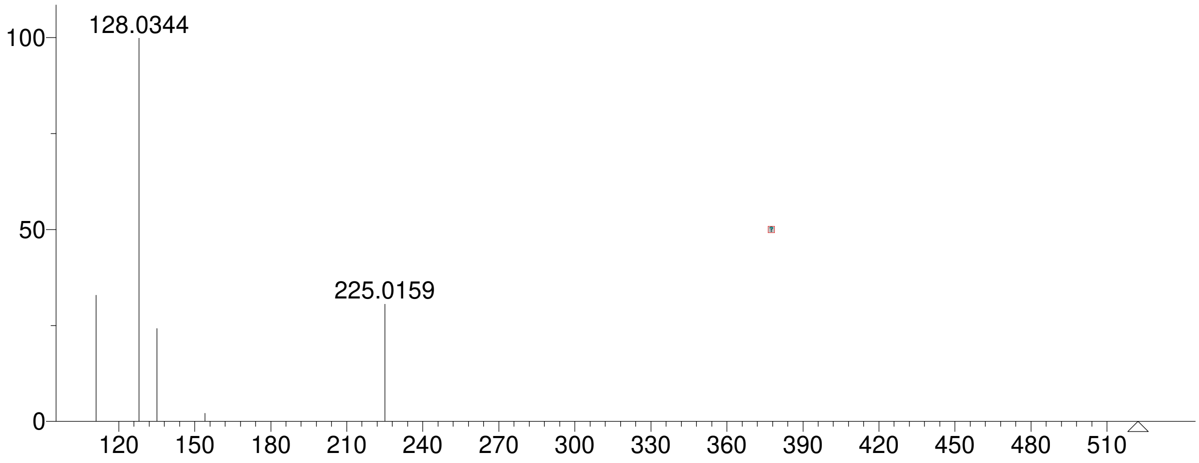

Unknown: Unknown (3.96_532.2012m/z)
Compound in Library Factor = 0

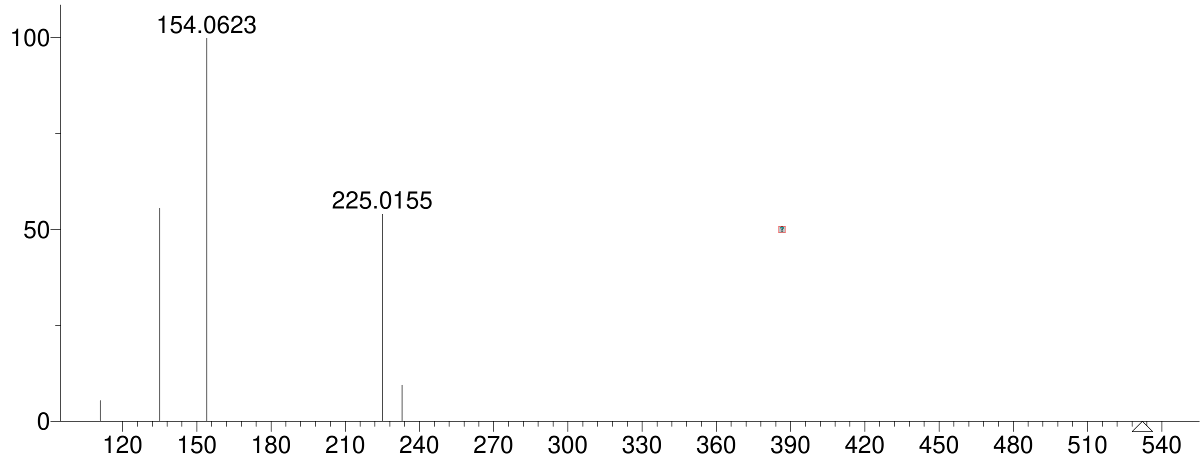

Hit 1 : Cholecystokinin, CCK Octapeptide (26-33), Non-Sulfated [M+2H]2+ HCD 71eV P=532.2
C49H62N10O13S2; MF: 0; RMF: 30; Prob 50.0%; CAS: 25679-24-7; Lib: nist_msms2; ID: 63545.

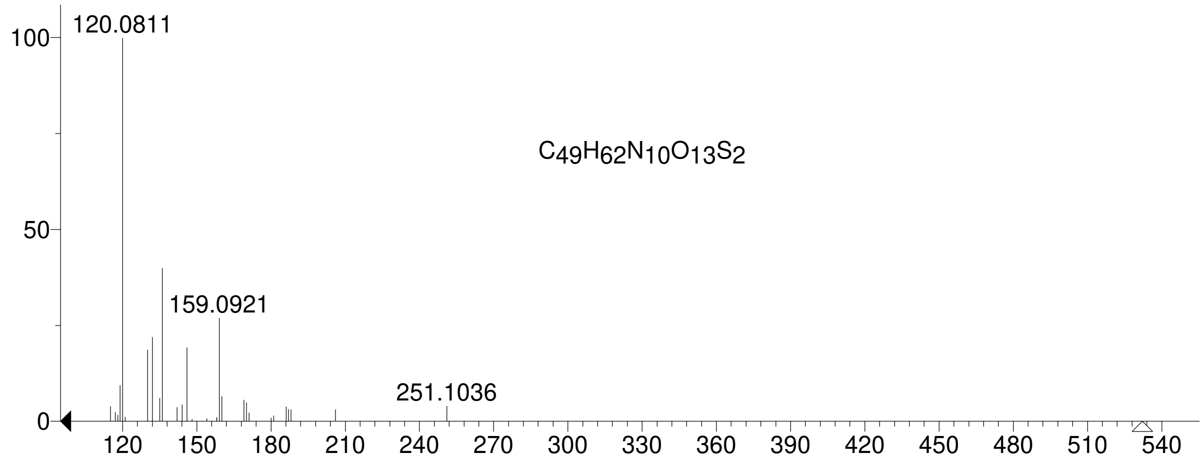

Hit 2 : [(2R,3S,4S,5R,6R)-6-[(2S,3S,4S,5R)-4-acetyloxy-3-hydroxy-2,5-bis(hydroxymethyl)oxolan-2-yl]oxy-3,4,5-tri
C23H30O13; MF: 0; RMF: 12; Prob 50.0%; Lib: mona-export-vf-npl_qexactive; ID: 12469.

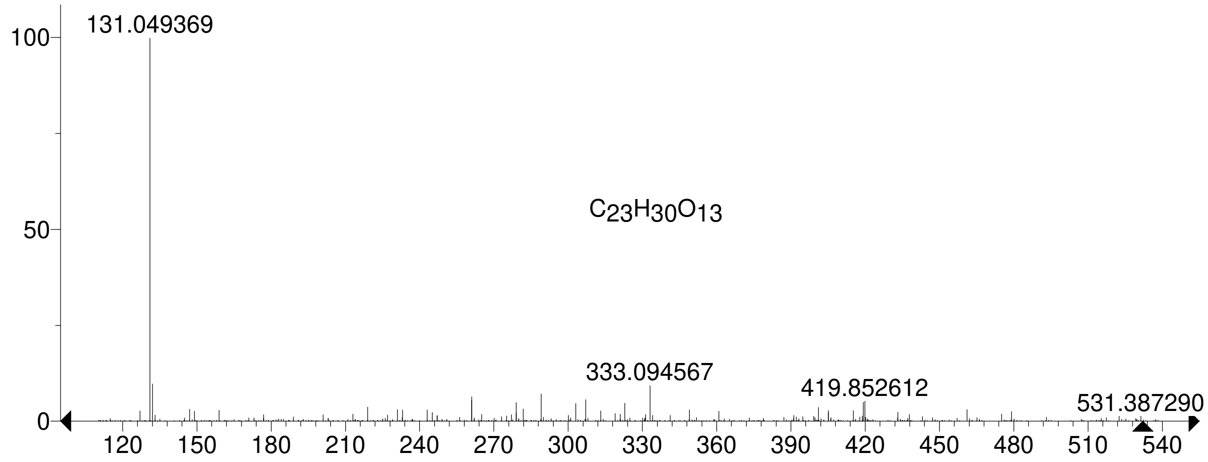

Unknown: Unknown (3.96_602.1248m/z)
Compound in Library Factor = 0

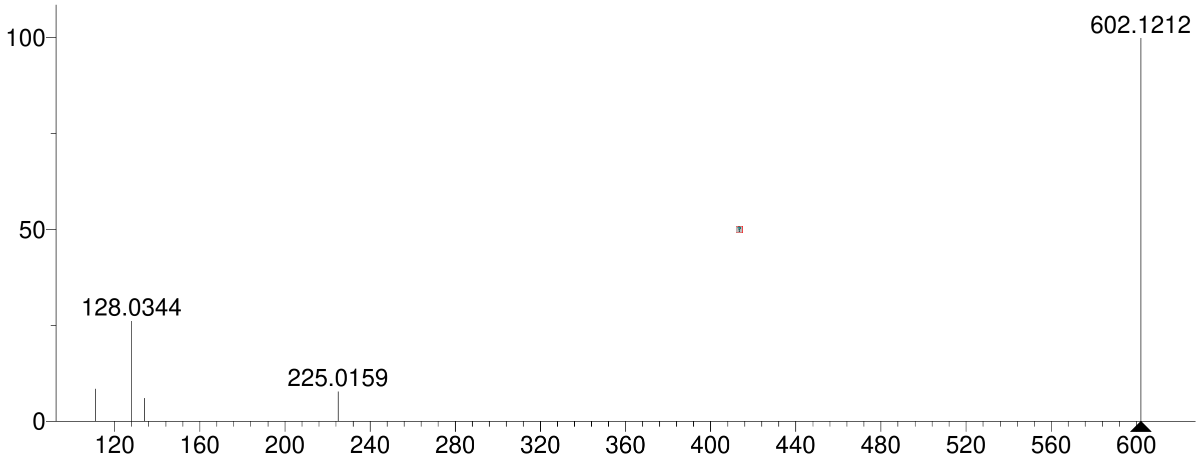

** Search Report Page 1 of 1 **

Unknown: Unknown (3.97_352.0629m/z)
Compound in Library Factor = 0

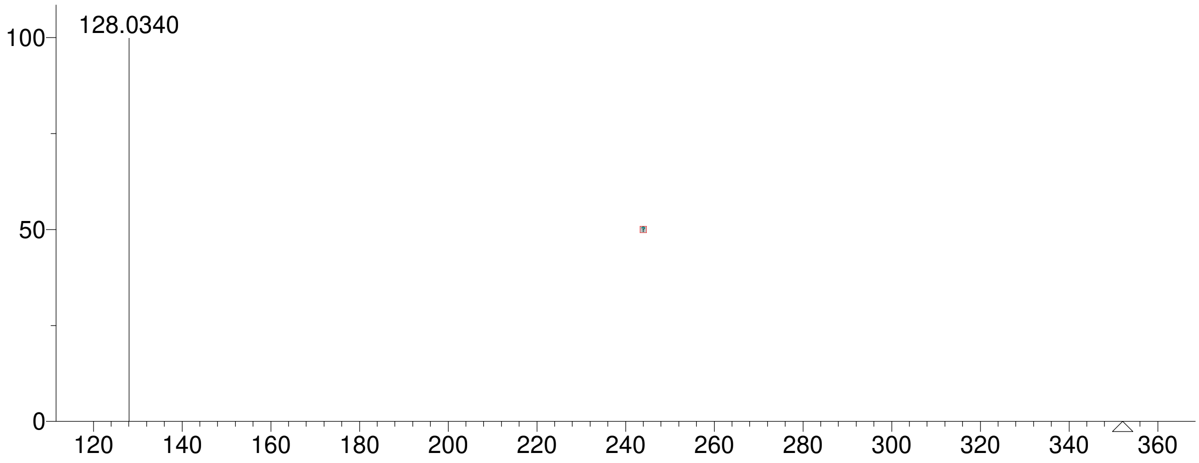

Unknown: Unknown (3.97_458.1999m/z)
Compound in Library Factor = 0

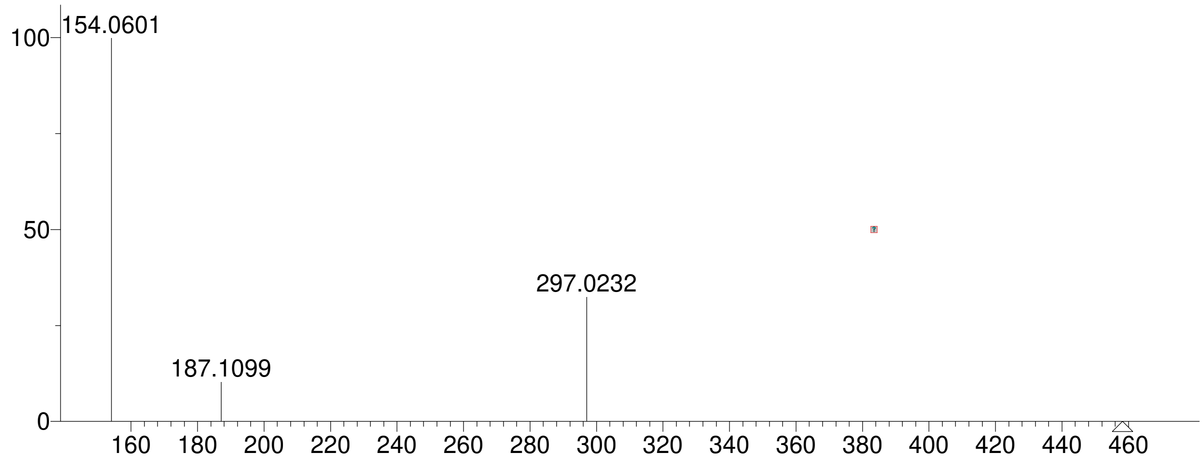

Unknown: Unknown (3.98_298.0917m/z)
Compound in Library Factor = 0

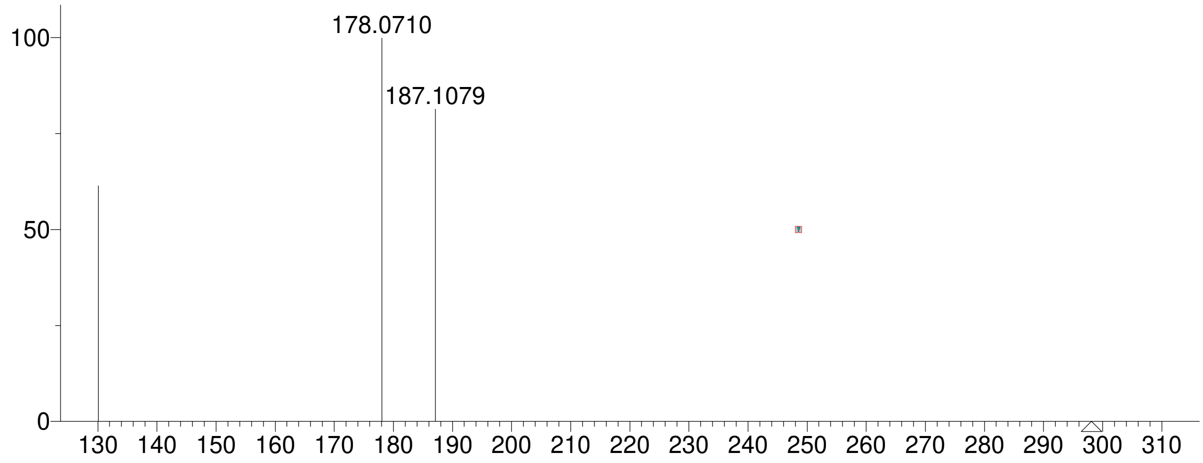

Unknown: Unknown (3.98_330.1636m/z)
Compound in Library Factor = 0

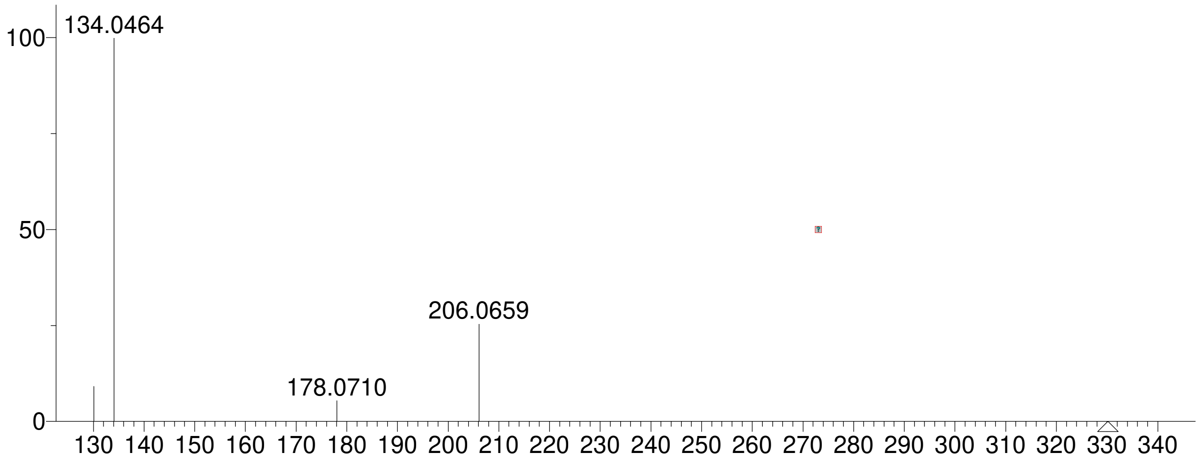

Unknown: Unknown (3.99_719.4187m/z)
Compound in Library Factor = 0

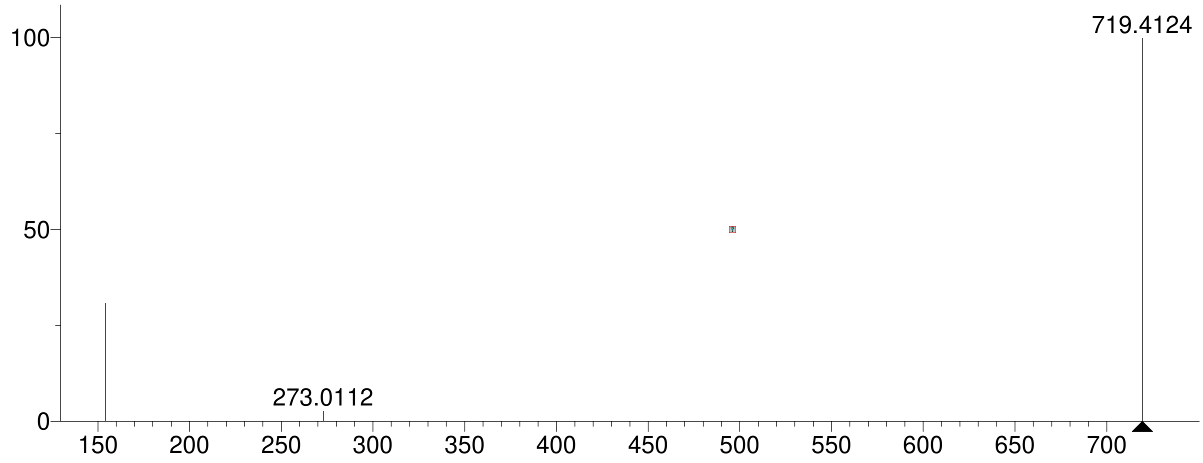

Unknown: Unknown (30.02_467.3007m/z)
Compound in Library Factor = -3273

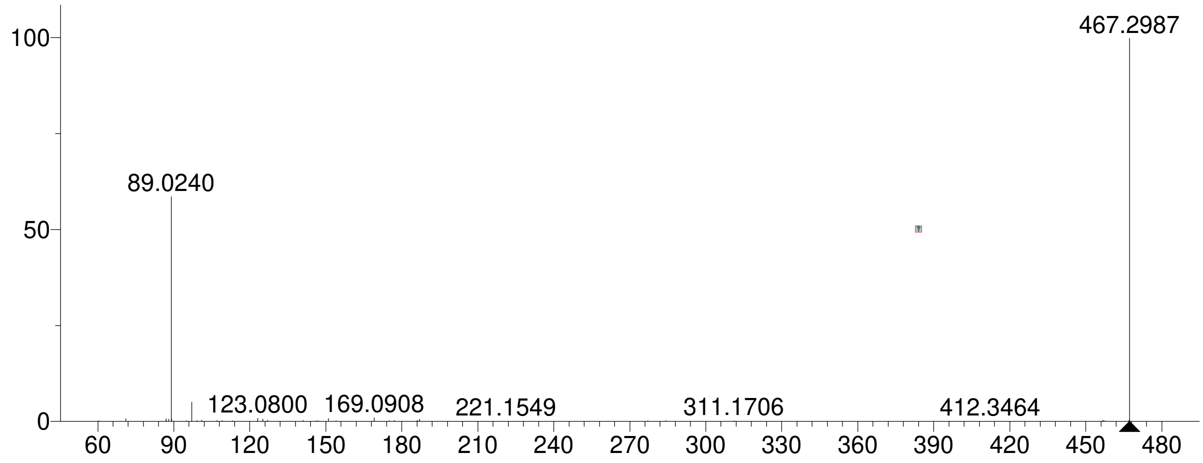

Hit 1 : NCGC00385231-01_C26H44O8_{(1S,2R,4aS,5R,8aS)-2-Hydroxy-5-[(3E)-5-hydroxy-3-methyl-3-penten-1-
C26H44O8; MF: 2; RMF: 46; Prob 26.5%; Lib: mona-export-gnps; ID: 11850.

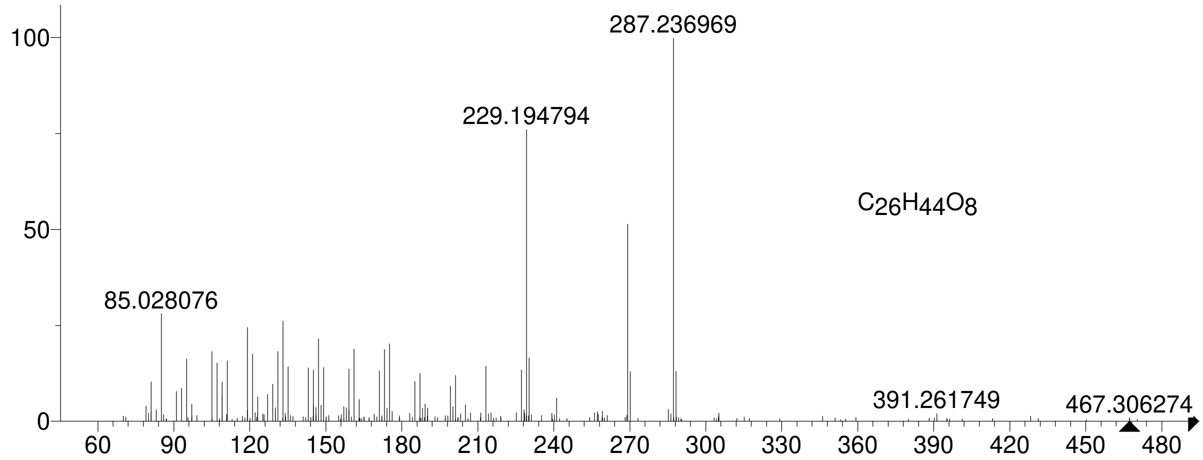

Hit 2 : Kendomycin [M-H]-=>467.3 IT 35% P=467.3
C29H42O6; MF: 0; RMF: 63; Prob 24.5%; CAS: 183202-73-5; Lib: nist_msms; ID: 254473.

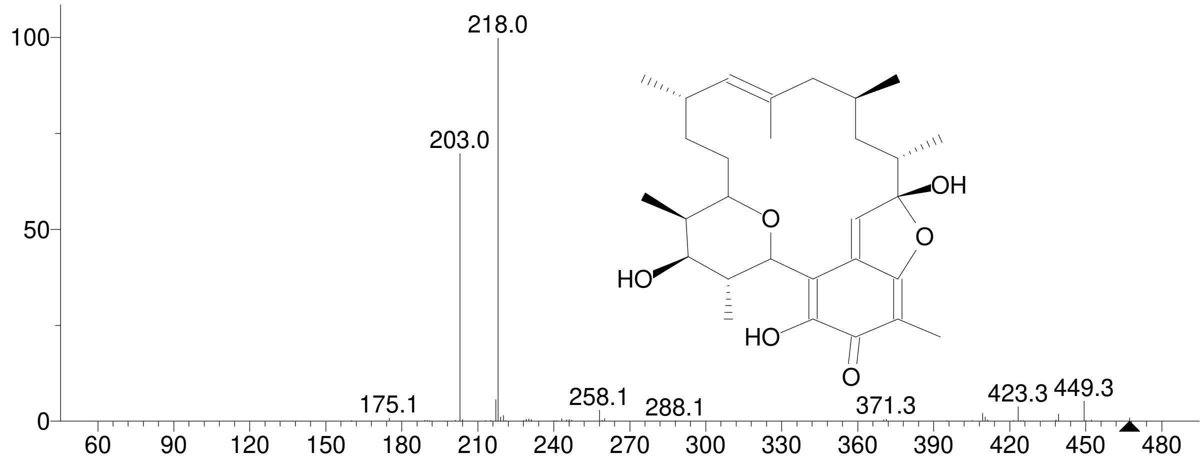

Hit 3 : Ganoderic acid H [M-H-H2O]-=>467.3 IT 35% P=467.3
C32H44O9; MF: 0; RMF: 21; Prob 24.5%; CAS: 98665-19-1; Lib: nist_msms; ID: 226715.

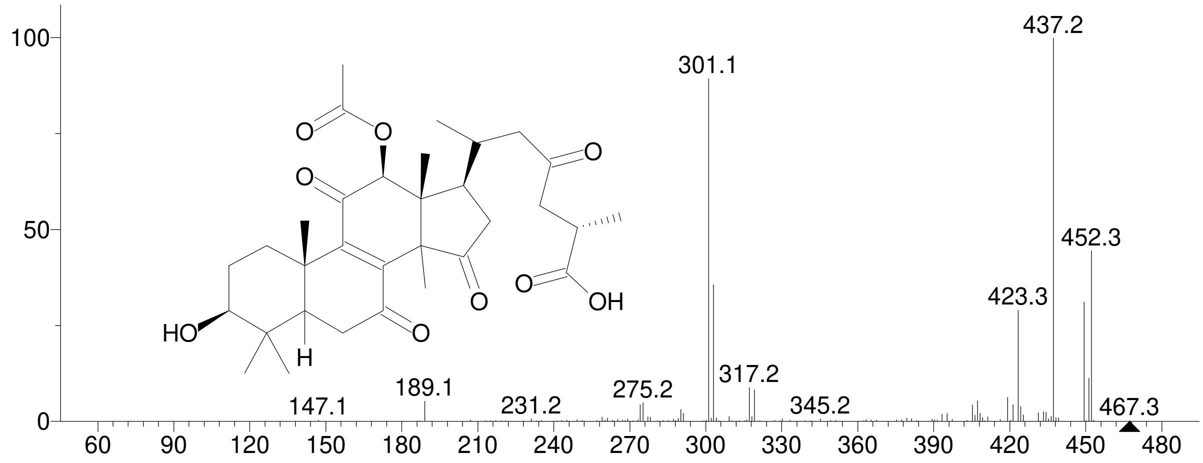

Hit 4 : Cimicifugoside H 2 [M+H-H2O]+=>467.3 IT 35% P=467.3
C35H54O10; MF: 0; RMF: 5; Prob 24.5%; CAS: 161097-77-4; Lib: nist_msms; ID: 226002.

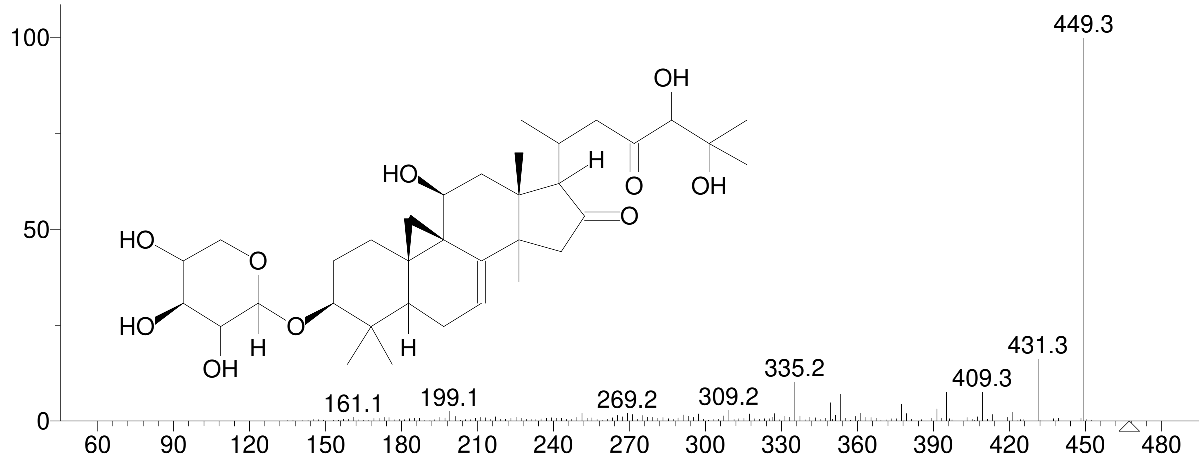

** Search Report Page 1 of 1 **

Unknown: Unknown (31.13_471.3106m/z)
Compound in Library Factor = -2387

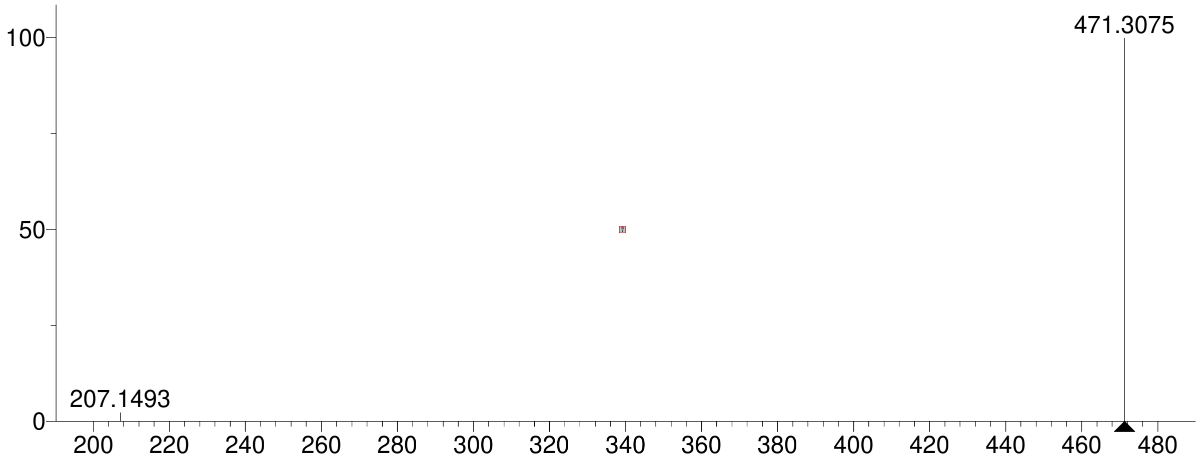

Hit 1 : 3-Ketofusidic acid [M-H-C2H2O]- HCD 28V P=471.3
C31H46O6; MF: 49; RMF: 790; Prob 63.5%; CAS: 4680-37-9; Lib: nist_msms; ID: 445022.

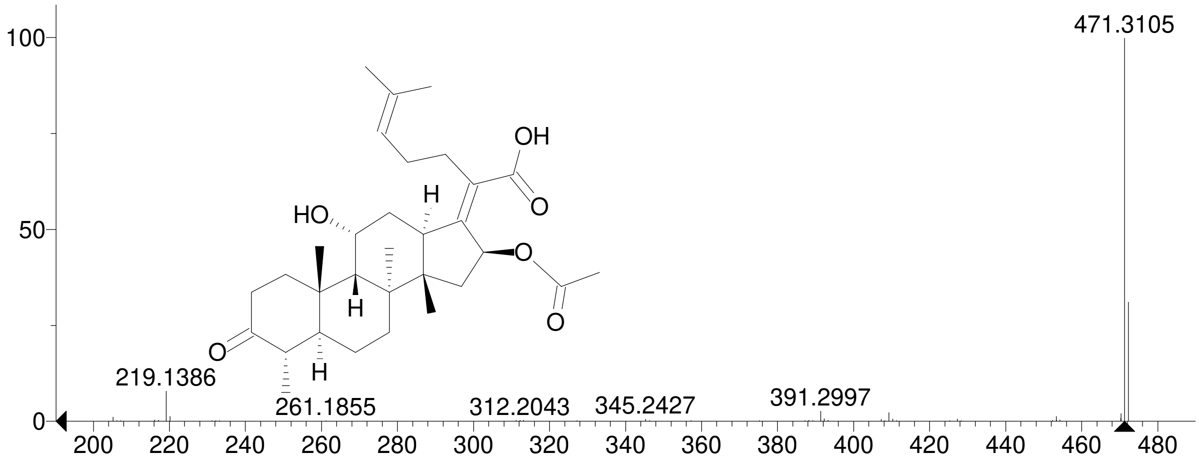

Hit 2 : 11-Ketofusidic acid [M-H-C2H2O]- HCD 28V P=471.3
C31H46O6; MF: 33; RMF: 756; Prob 36.5%; CAS: 16711-91-4; Lib: nist_msms; ID: 445168.

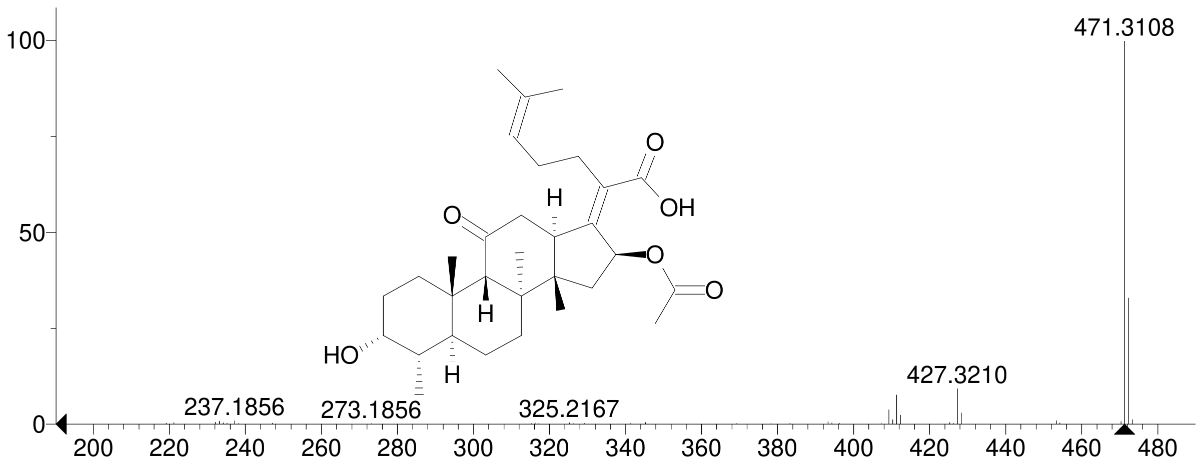

Unknown: Unknown (31.57_401.2691m/z)
Compound in Library Factor = -494

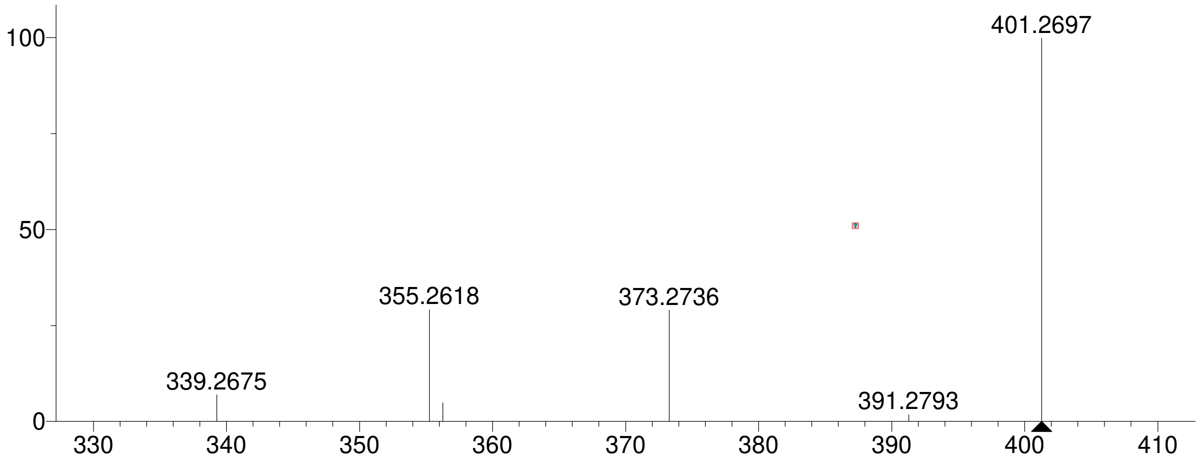

Hit 1 : FAHFA 25:5; FAHFA 18:5/7:0; [M-H]- [M-H]- P=401.3
C25H38O4; MF: 251; RMF: 763; Prob 32.6%; Lib: msdial-insilicomsms-lipids-neg; ID: 272714.

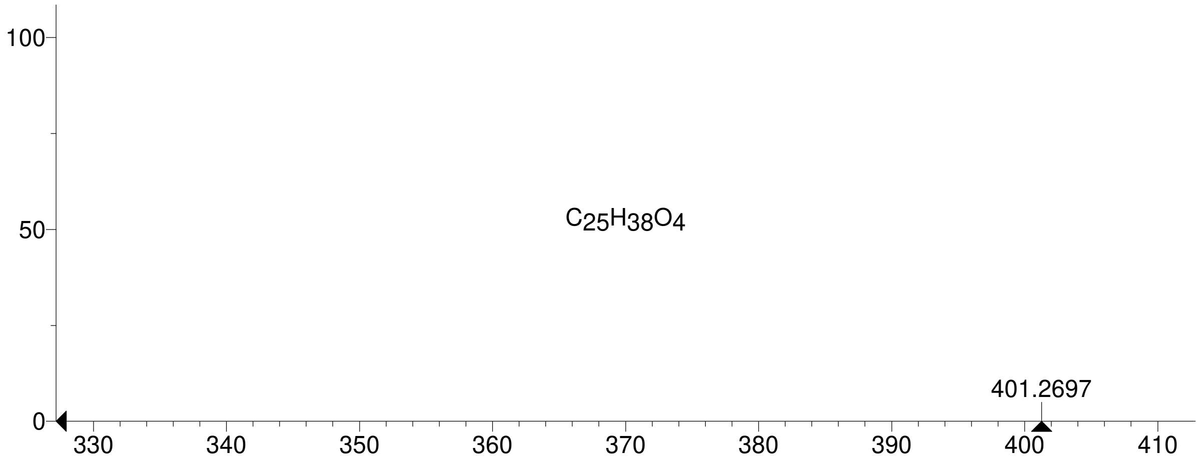

Hit 2 : FAHFA 25:5; FAHFA 20:5/5:0; [M-H]- [M-H]- P=401.3
C25H38O4; MF: 251; RMF: 763; Prob 32.6%; Lib: msdial-insilicomsms-lipids-neg; ID: 273216.

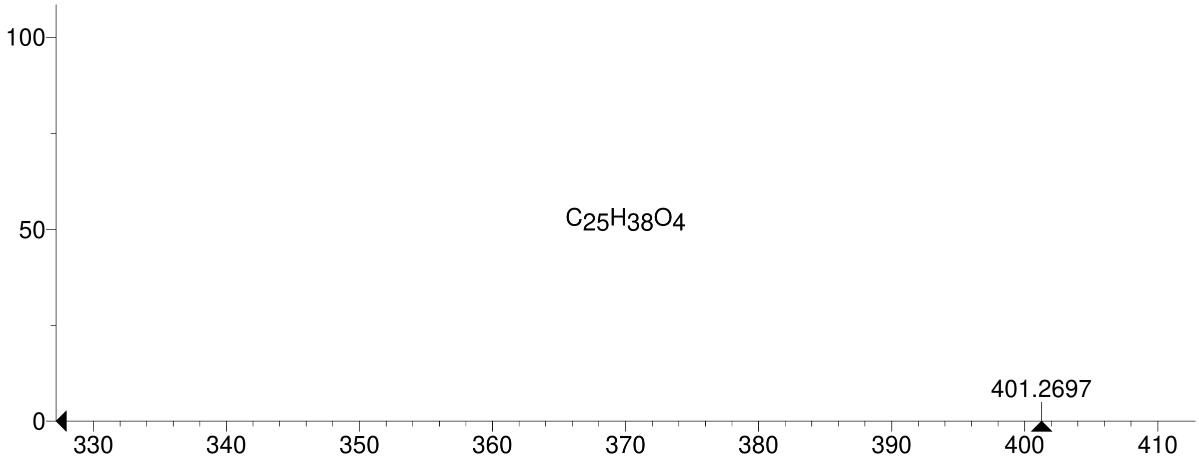

Hit 3 : FAHFA 25:5; FAHFA 22:5/3:0; [M-H]- [M-H]- P=401.3
C25H38O4; MF: 251; RMF: 763; Prob 32.6%; Lib: msdial-insilicomsms-lipids-neg; ID: 273718.

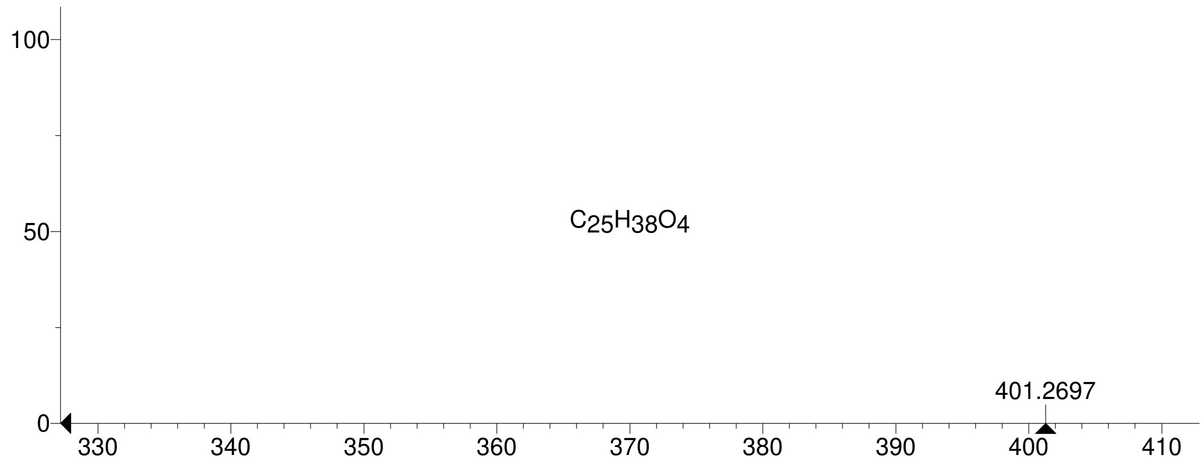

Hit 4 : 1-Arachidonoylglycerol [M+Na]+ QTOF 20V P=401.3
C23H38O4; MF: 127; RMF: 728; Prob 1.51%; CAS: 35474-99-8; Lib: nist_msms; ID: 67120.

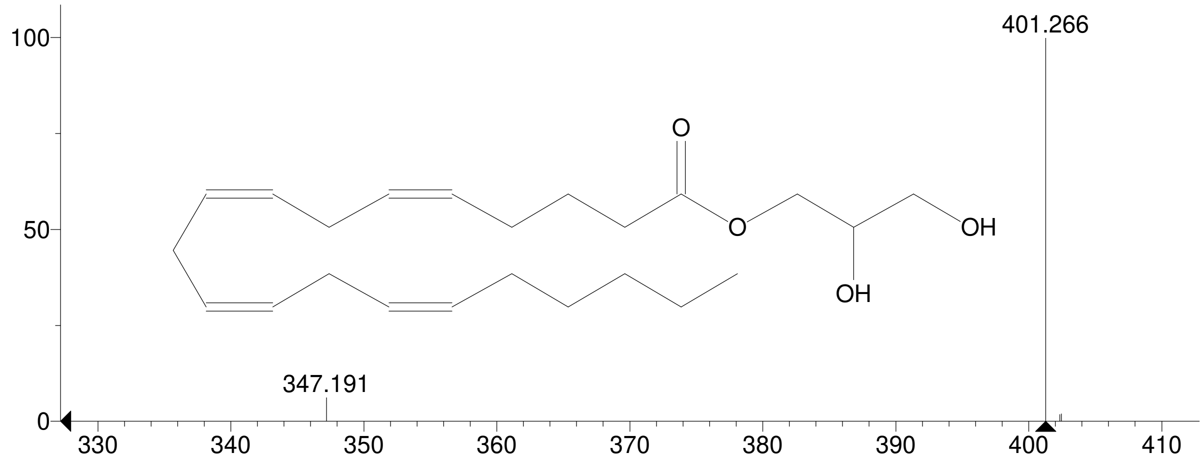

Hit 5 : 9-Deoxy-9-methylene-16,16-dimethylprostaglandin E2 [M+Na]+ HCD 24V P=401.3
C23H38O4; MF: 86; RMF: 722; Prob 0.34%; CAS: 61263-35-2; Lib: nist_msms; ID: 157091.

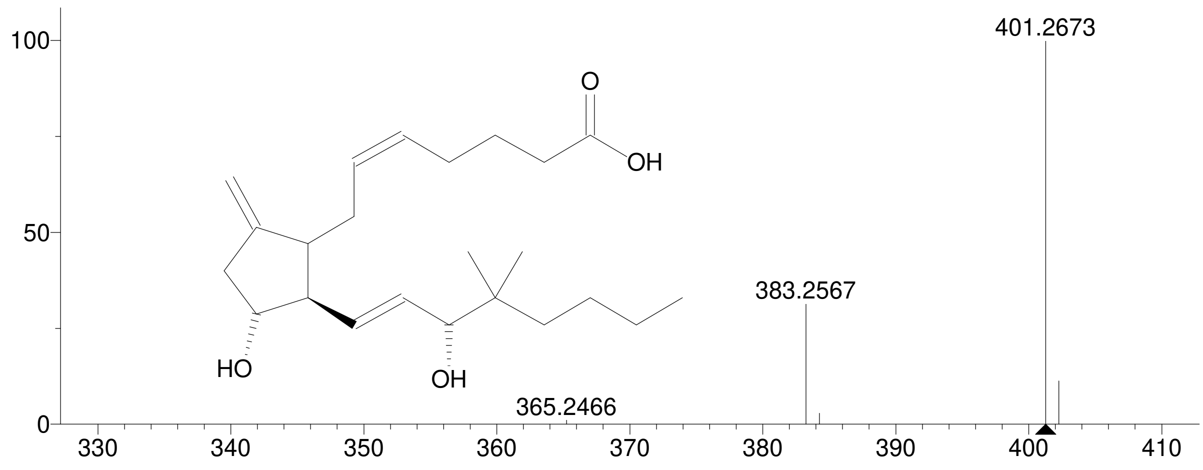

Unknown: Unknown (31.59_417.2638m/z)
Compound in Library Factor = -1848

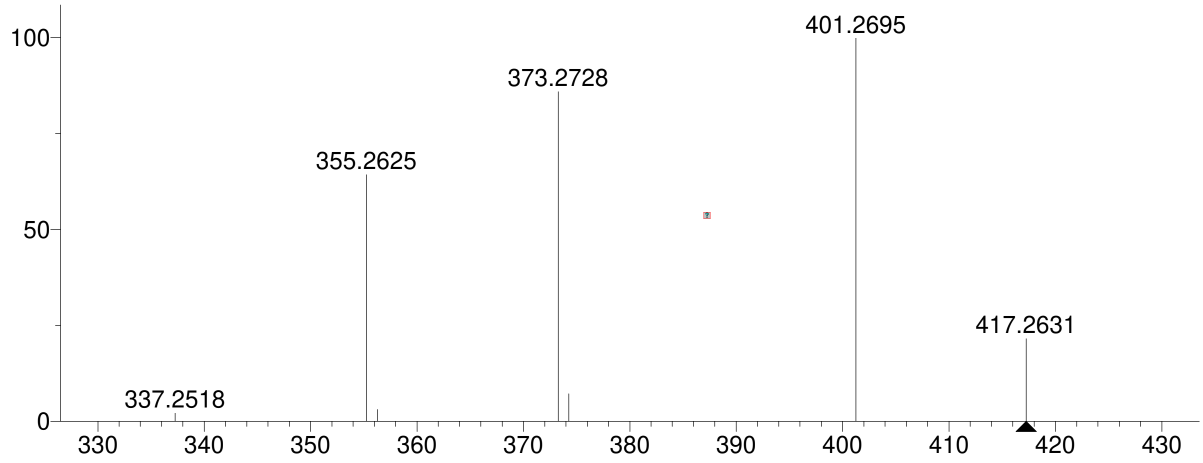

Hit 1 : Prostaglandin E2 isopropyl ester [M+Na]+ QTOF 17V P=417.3
C23H38O5; MF: 80; RMF: 412; Prob 75.2%; CAS: 71845-66-4; Lib: nist_msms; ID: 62065.

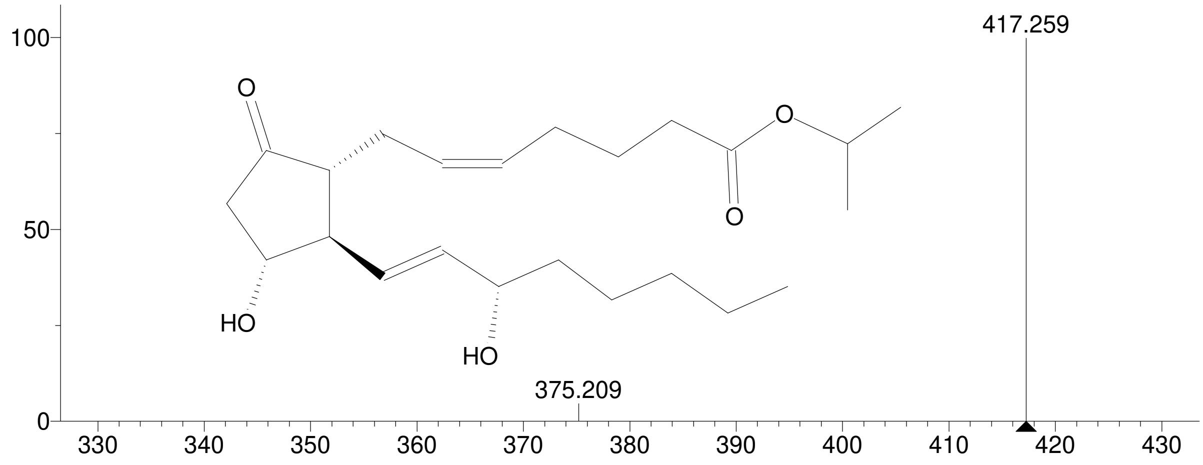

Hit 2 : Alfentanyl [M+H]+ HCD 12V P=417.3
C21H32N6O3; MF: 44; RMF: 274; Prob 18.6%; CAS: 71195-58-9; Lib: nist_msms; ID: 322110.

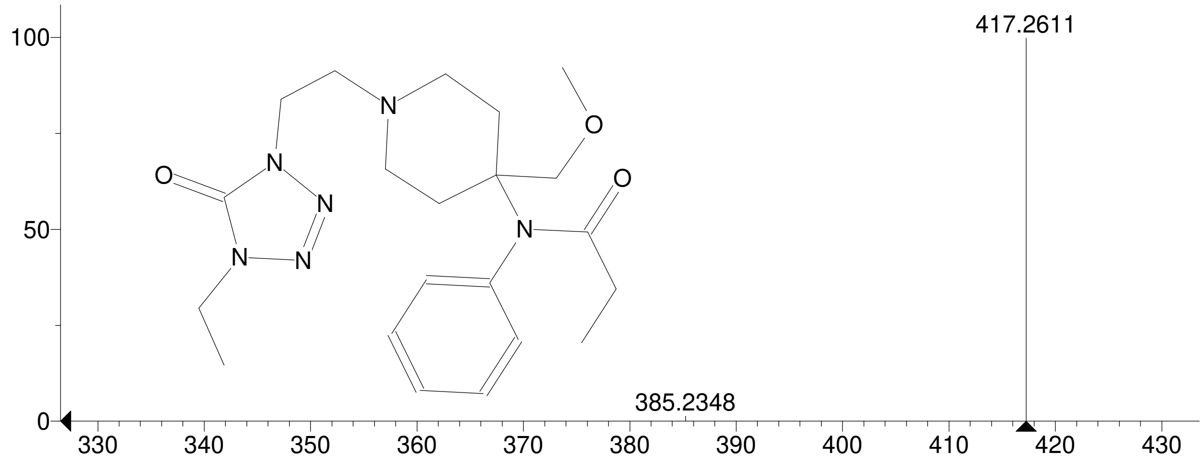

Hit 3 : Manoalide [M+H]+ HCD 12V P=417.3
C25H36O5; MF: 20; RMF: 266; Prob 6.22%; CAS: 75088-80-1; Lib: nist_msms; ID: 362740.

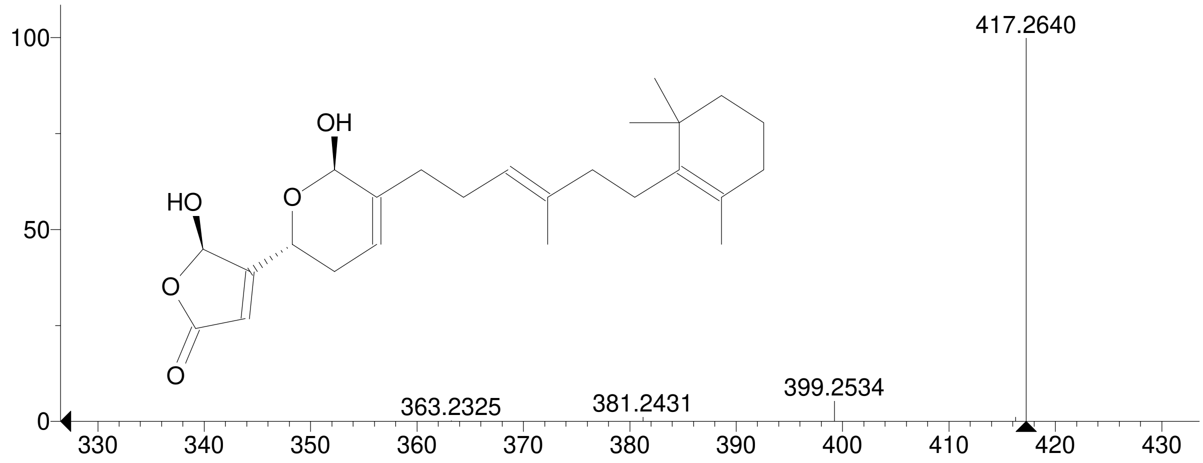

** Search Report Page 1 of 1 **

Unknown: Unknown (31.61_472.0636m/z)
Compound in Library Factor = -3273

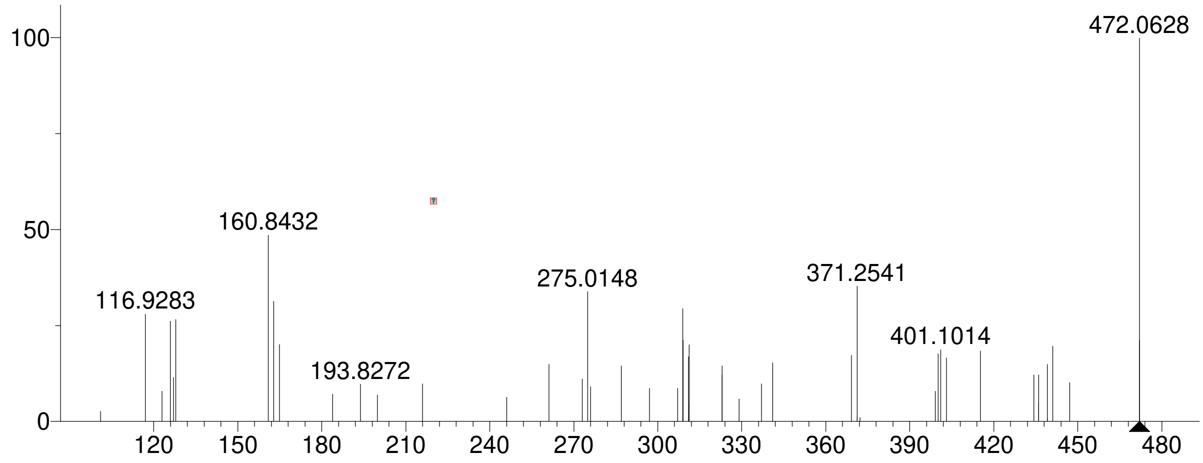

Hit 1 : Acetic acid, 2-[4-[[[2-[3-fluoro-4-(trifluoromethyl)phenyl]-4-methyl-5- thiazolyl]methyl]thio]-2-methylphenoxy]
C21H17F4NO3S2; MF: 17; RMF: 330; Prob 100.0%; CAS: 317318-84-6; Lib: nist_msms; ID: 427976.

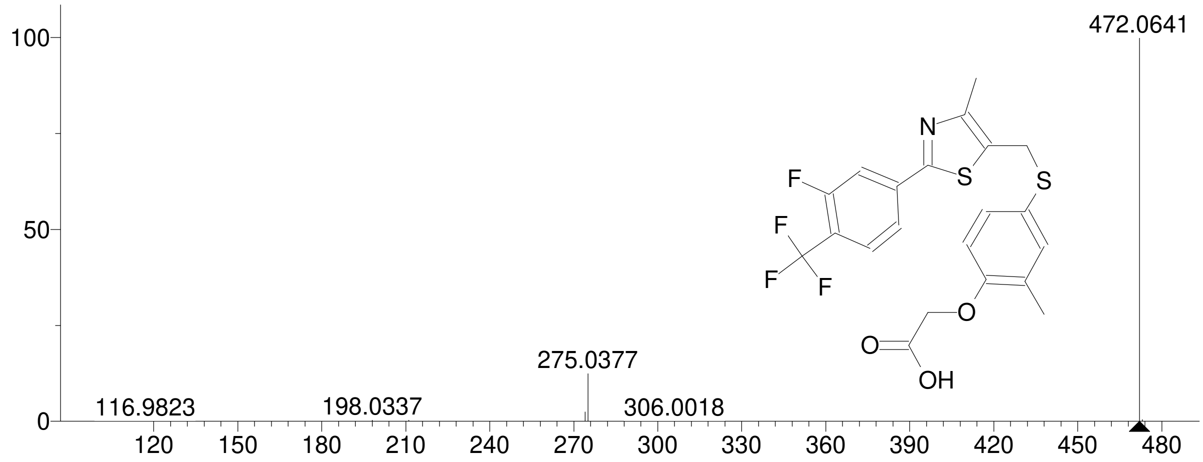

Unknown: Unknown (31.63_492.1212m/z)
Compound in Library Factor = -1309

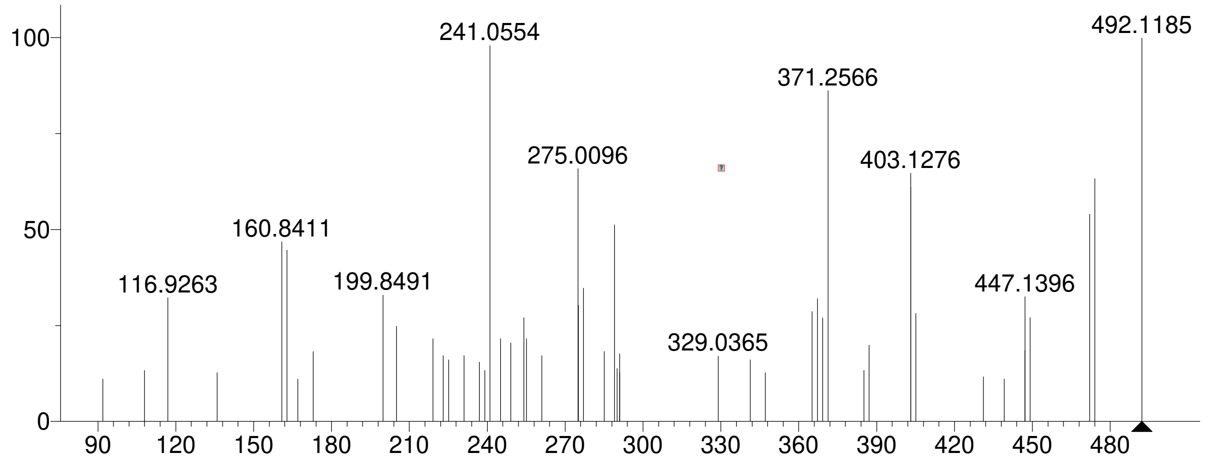

Hit 1 : 6H-Thieno[3,2-f][1,2,4]triazolo[4,3-a][1,4]diazepine-6-acetamide, 4-(4-chlorophenyl)-N-(4-hydroxyphenyl)-2
C25H22ClN5O2S; MF: 60; RMF: 429; Prob 83.5%; CAS: 202590-98-5; Lib: nist_msms; ID: 356211.

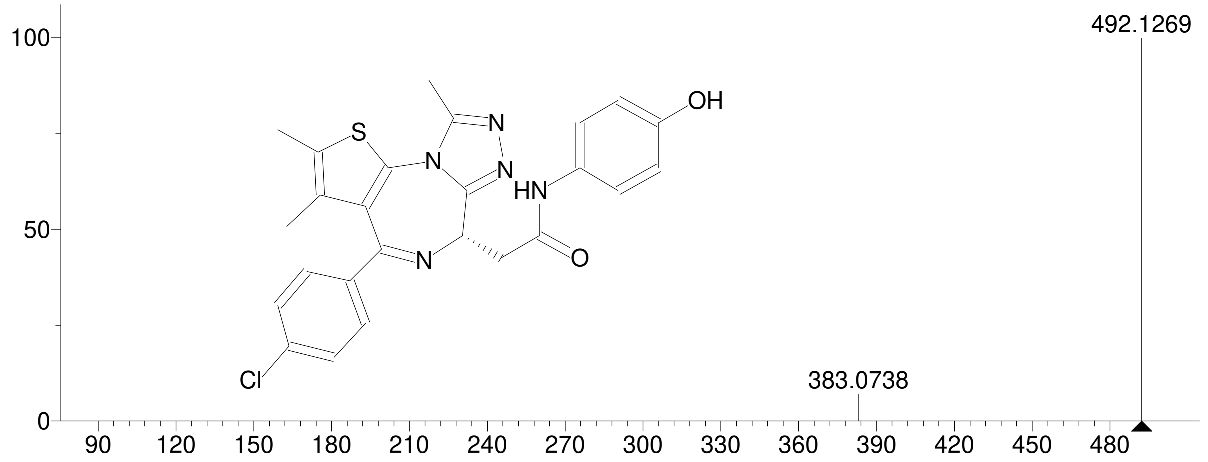

Hit 2 : Bucladesine [M+Na]+ HCD 19V P=492.1
C18H24N5O8P; MF: 7; RMF: 228; Prob 16.5%; CAS: 362-74-3; Lib: nist_msms; ID: 350906.

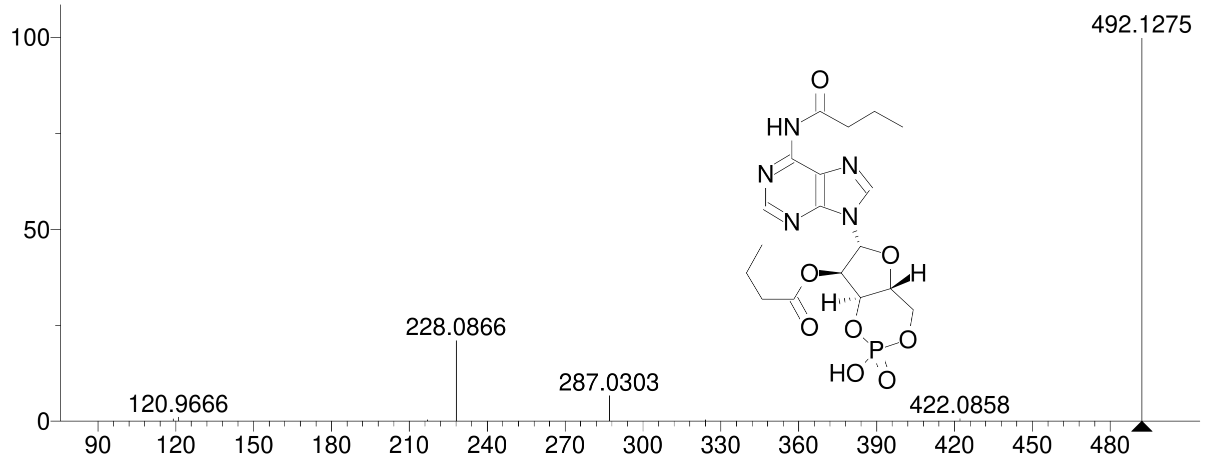

** Search Report Page 1 of 1 **

Unknown: Unknown (31.63_494.1178m/z)
Compound in Library Factor = 0

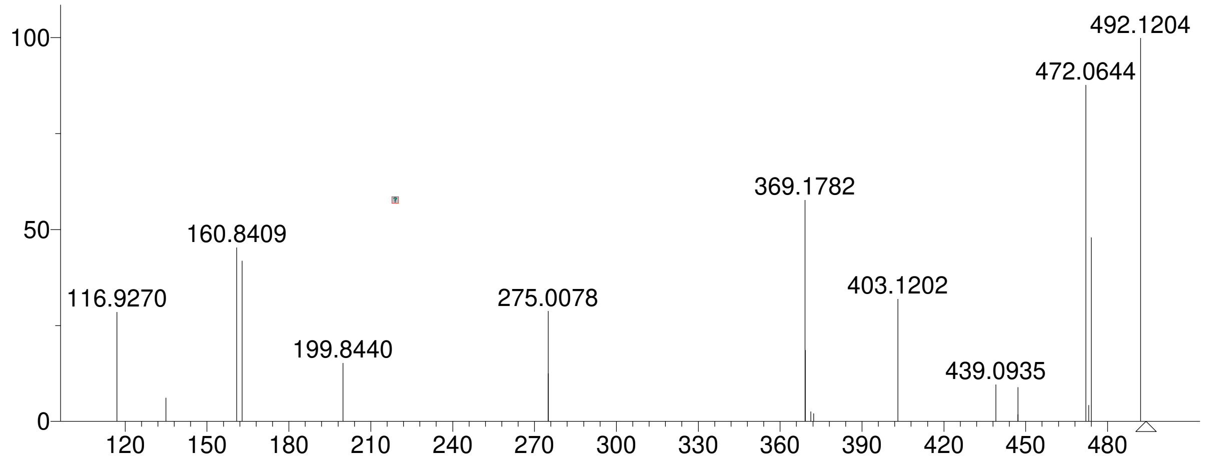

Hit 1 : Halfenprox [M+NH4]+ IT 35% P=494.1
C24H23BrF2O3; MF: 0; RMF: 13; Prob 100.0%; CAS: 111872-58-3; Lib: nist_msms; ID: 306726.

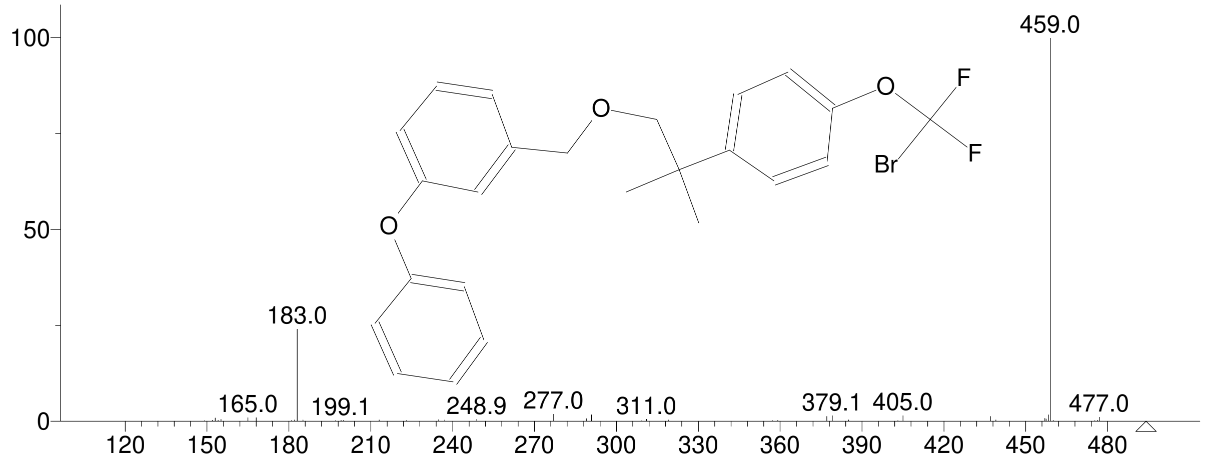

Unknown: Unknown (31.64_415.2490m/z)
Compound in Library Factor = -3273

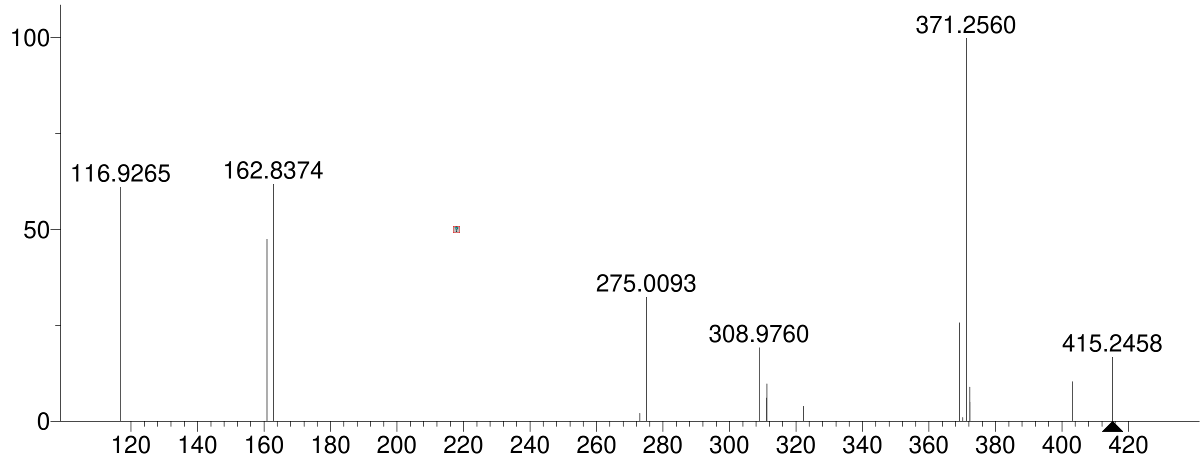

Hit 1 : 9-Oxo-11α,16R-dihydroxy-17-cyclobutyl-5Z,13E-dien-1-oic acid [M+Na]+ HCD 33V P=415.2
C23H36O5; MF: 17; RMF: 278; Prob 35.3%; CAS: 212310-16-2; Lib: nist_msms; ID: 172347.

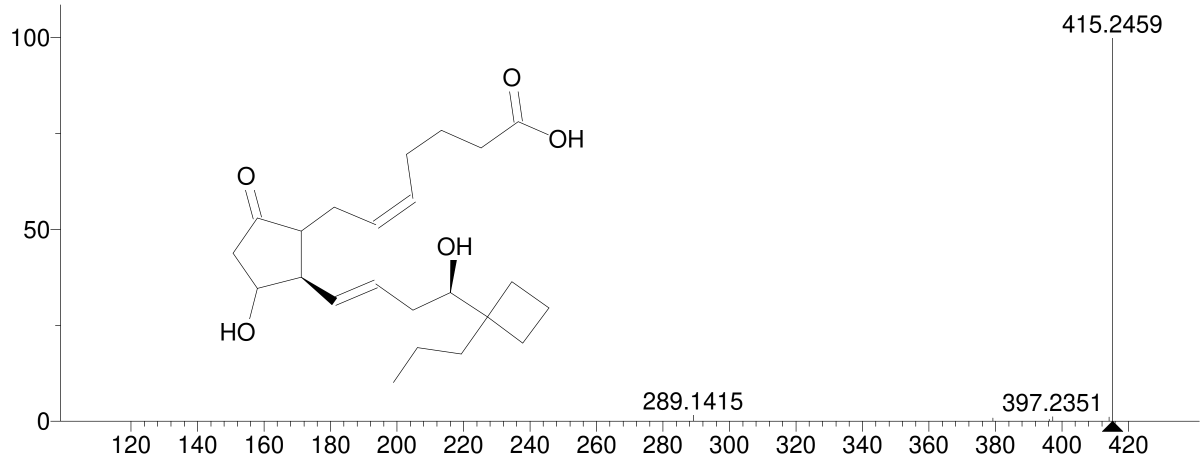

Hit 2 : 3β-Tetrahydrocortisol, 3,21-diacetate [M+H-2H2O]+ HCD 8V P=415.2
C25H38O7; MF: 9; RMF: 206; Prob 26.4%; CAS: 6820-57-1; Lib: nist_msms; ID: 454143.

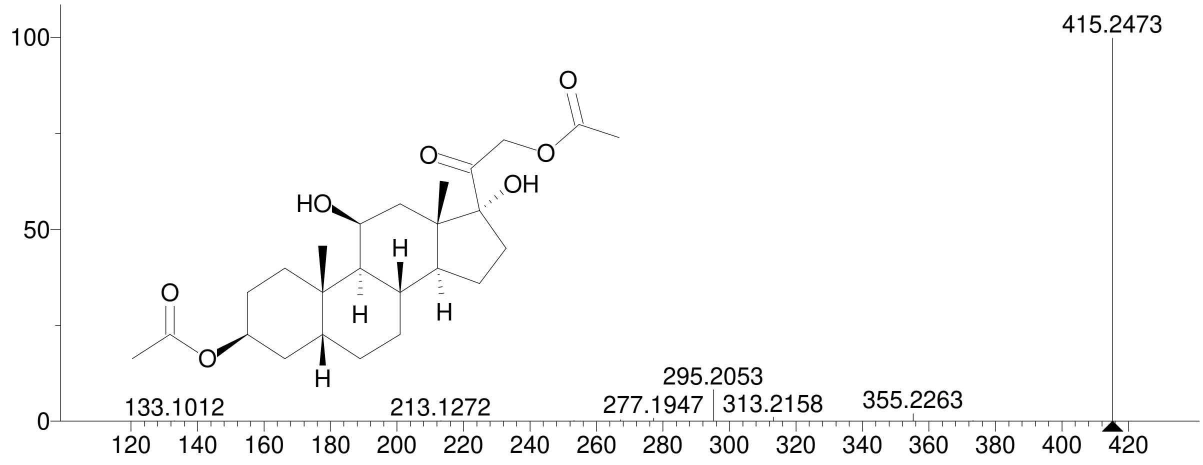

Hit 3 : Alpha-Hydroxydeoxycholic Acid [M+Na]+ 35% P=415.3
C24H40O4; MF: 0; RMF: 71; Prob 19.1%; Lib: mona-export-itree; ID: 2369.

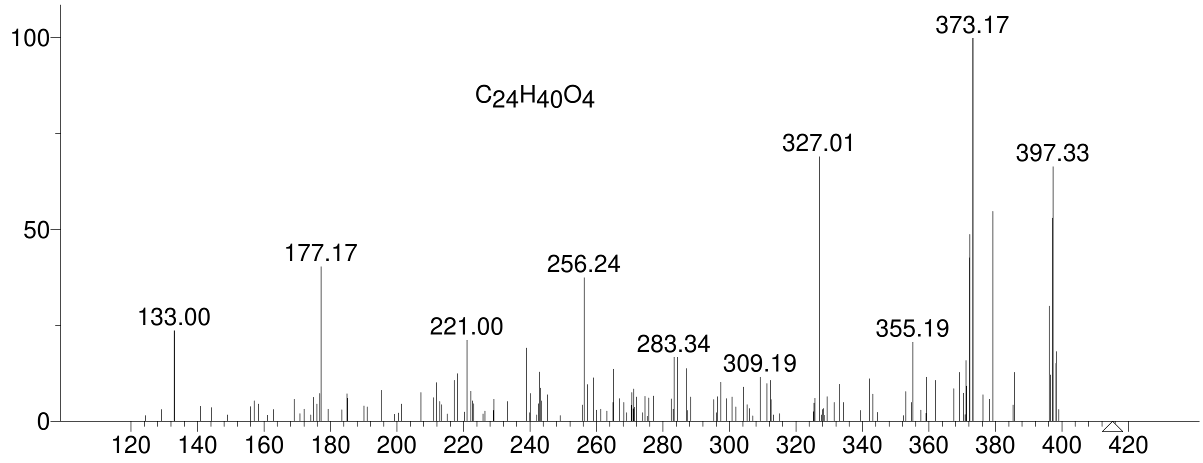

Hit 4 : 16-Phenoxytetranorprostaglandin F2α isopropyl ester [M+H-H2O]+ IT-FT 35% P=415.2
C25H36O6; MF: 0; RMF: 6; Prob 19.1%; CAS: 130209-78-8; Lib: nist_msms; ID: 545962.

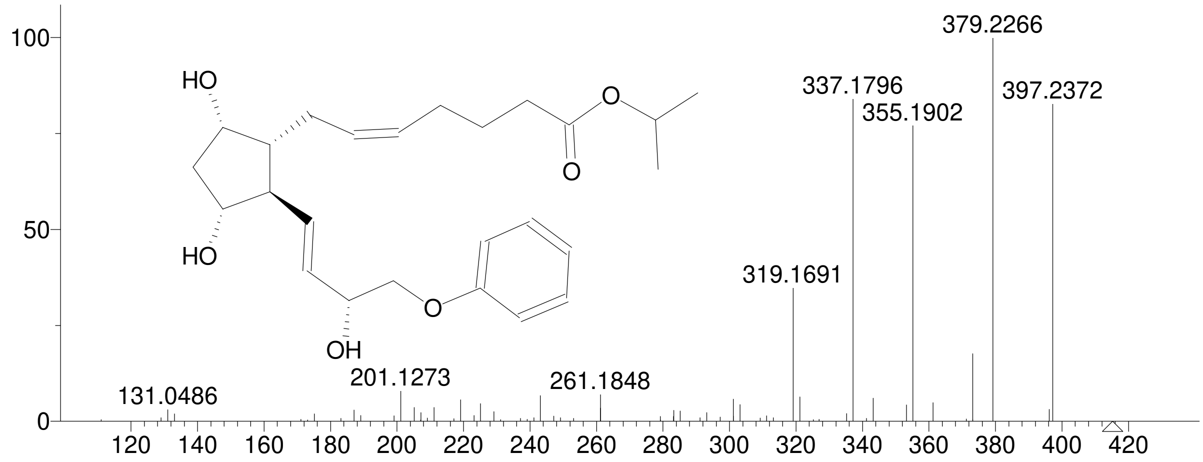

Unknown: Unknown (31.64_449.1237m/z)
Compound in Library Factor = 0

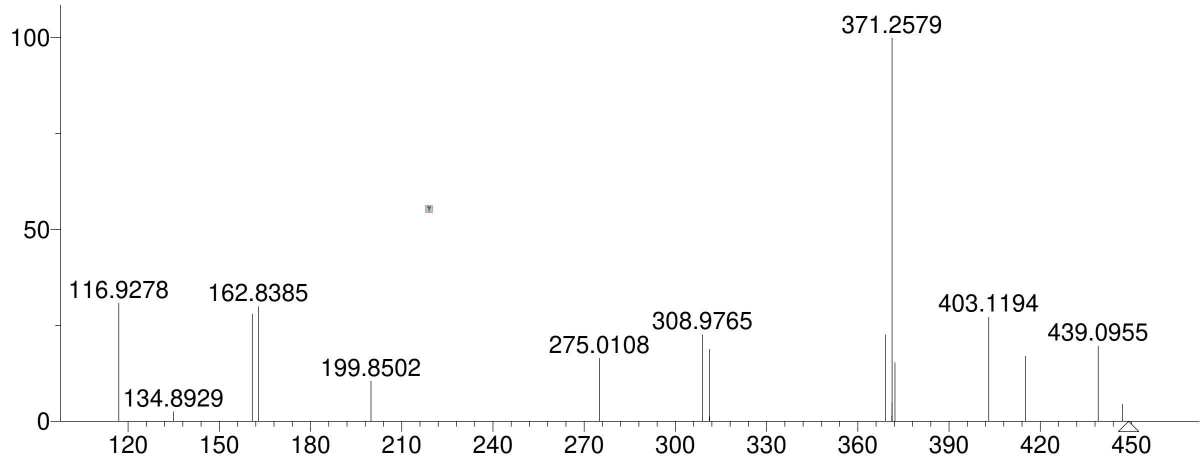

Hit 1 : 6-Hydroxysumatrol [M+Na]+ 35HCD P=449.1
C23H22O8; MF: 0; RMF: 9; Prob 50.0%; Lib: mona-export-vf-npl_qexactive; ID: 7093.

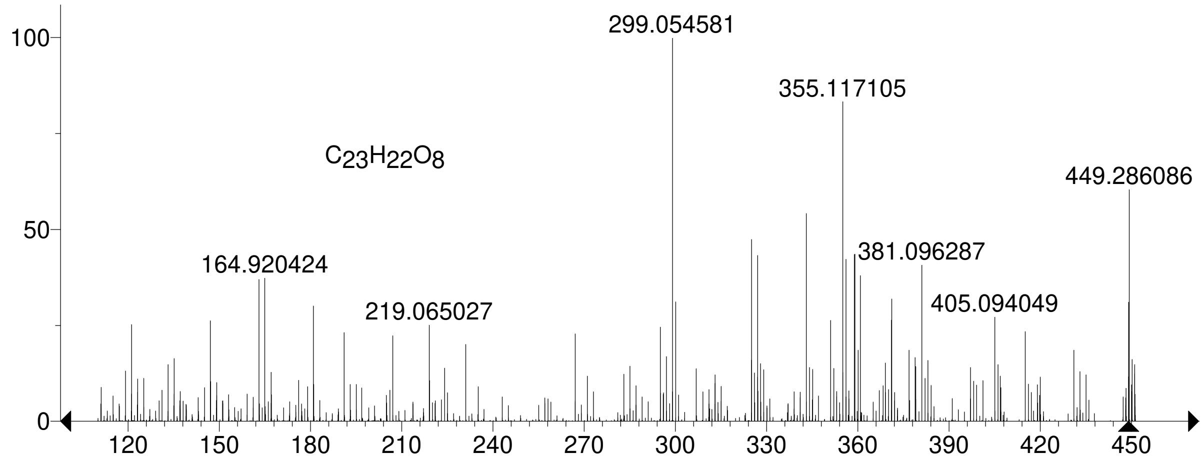

Hit 2 : dabinol [M+Na]+ 35HCD P=449.1
C23H22O8; MF: 0; RMF: 3; Prob 50.0%; Lib: mona-export-vf-npl_qexactive; ID: 10561.

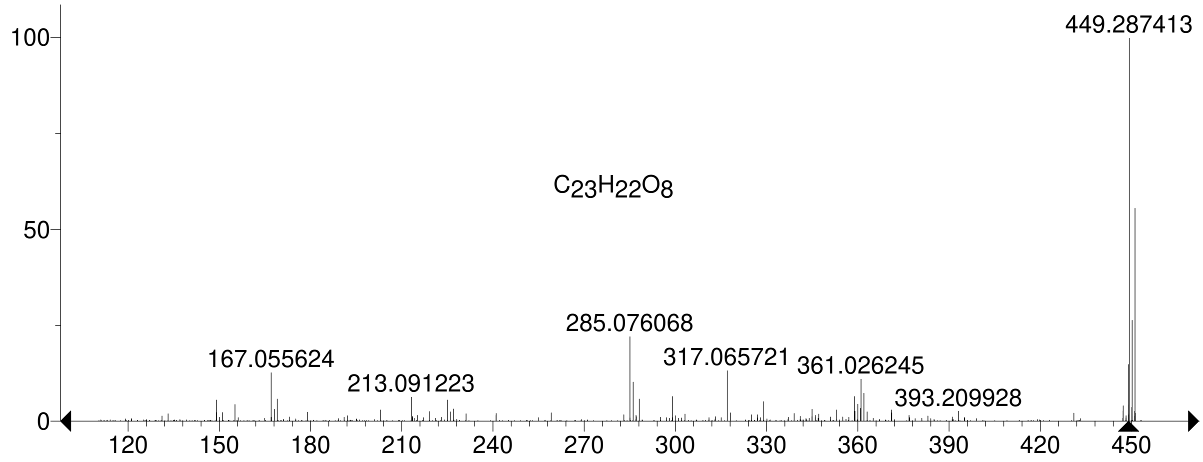

** Search Report Page 1 of 1 **

Unknown: Unknown (31.64_474.0606m/z)
Compound in Library Factor = 0

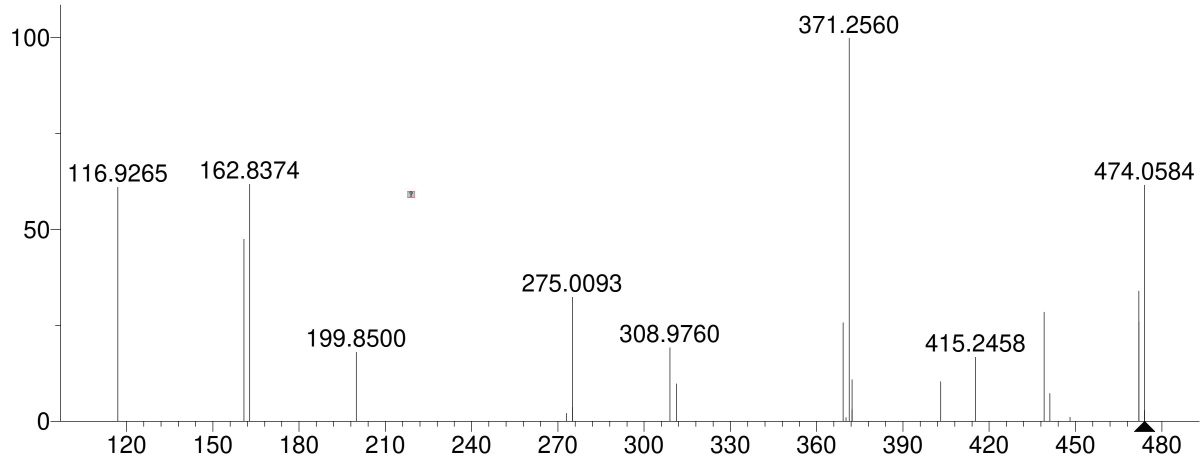

Unknown: Unknown (31.65_347.1988m/z)
Compound in Library Factor = 0

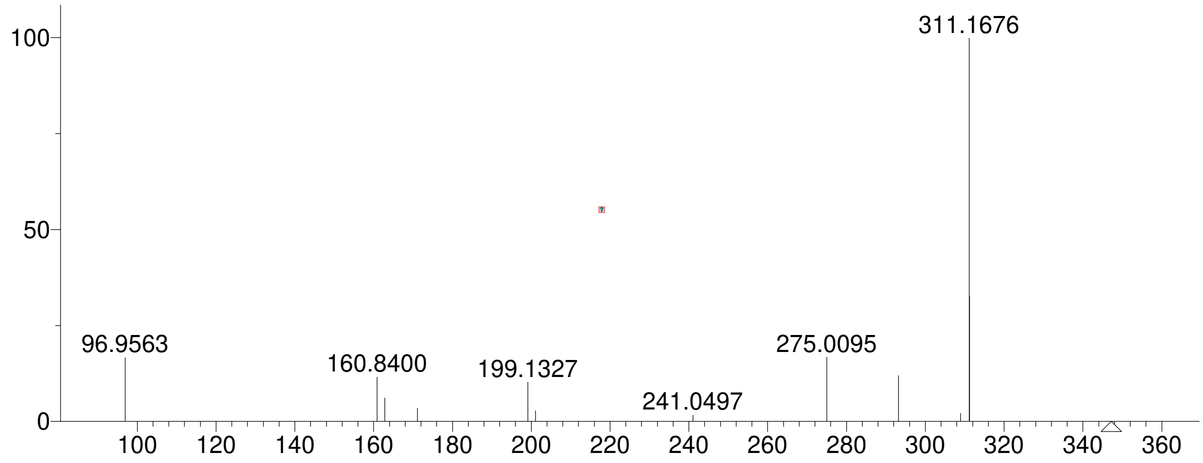

Hit 1 : 5-Heptenoic acid, 7-[(1R,2R,3S,5S)-2-[(1E,3R)-3-(2,3-dihydro-1H-inden-2-yl)-3-hydroxy-1-propen-1-yl]-3-flu
C27H37FO4; MF: 0; RMF: 243; Prob 3.84%; CAS: 208114-93-6; Lib: nist_msms; ID: 545035.

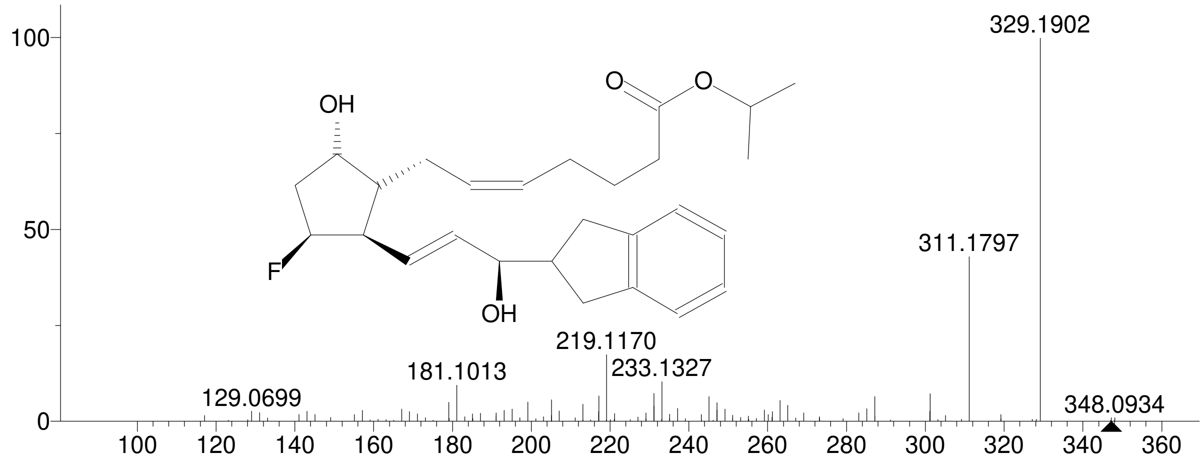

Hit 2 : 5-Heptenoic acid, 7-[(1R,2R,3S,5S)-2-[(1E,3S)-3-(2,3-dihydro-1H-inden-2-yl)-3-hydroxy-1-propen-1-yl]-3-flu
C24H31FO4; MF: 0; RMF: 235; Prob 3.84%; CAS: 1224444-55-6; Lib: nist_msms; ID: 190288.

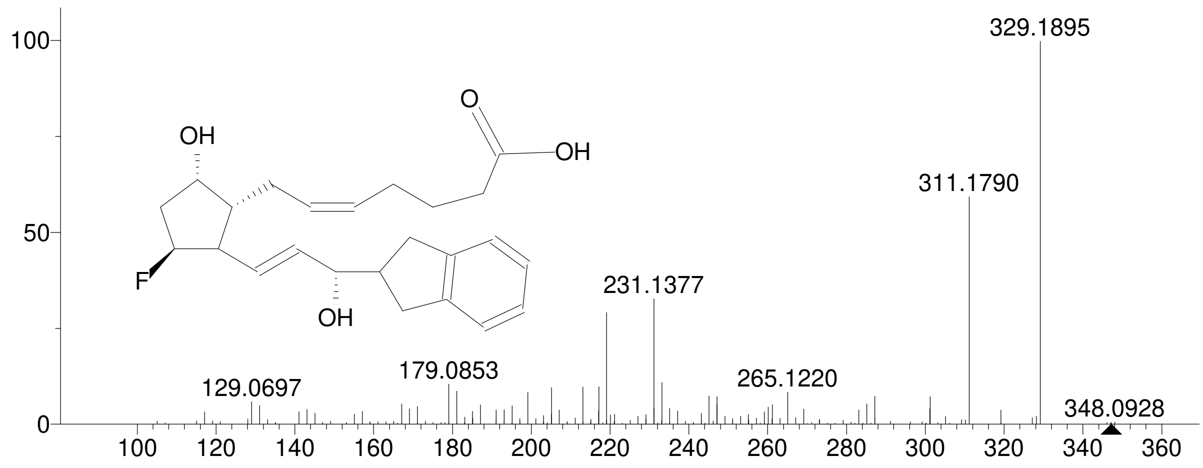

Hit 3 : 5-Heptenoic acid, 7-[(1R,2R,3S,5S)-2-[(1E,3R)-3-(2,3-dihydro-1H-inden-2-yl)-3-hydroxy-1-propen-1-yl]-3-fl
C24H31FO4; MF: 0; RMF: 233; Prob 3.84%; CAS: 246246-19-5; Lib: nist_msms; ID: 366323.

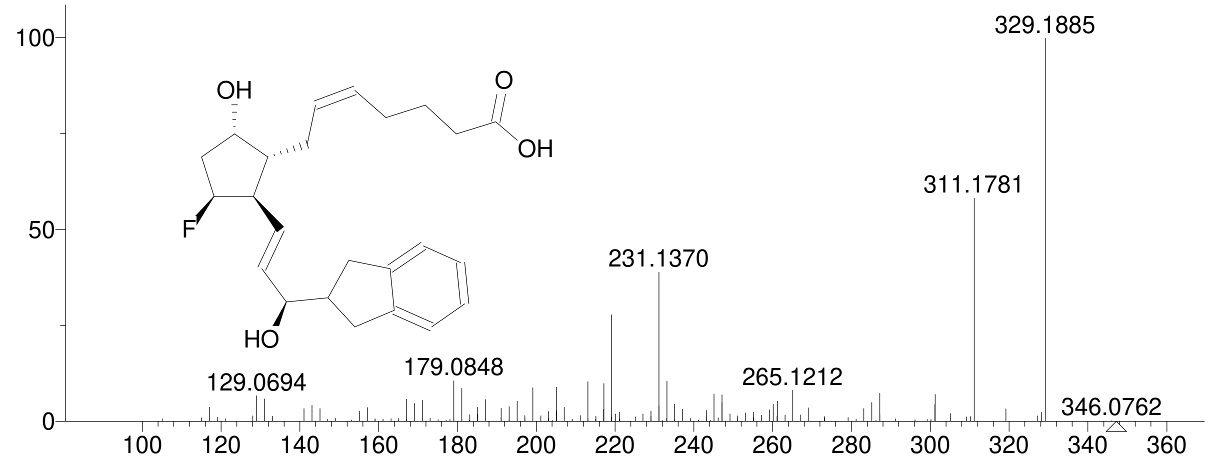

Hit 4 : 5-Heptenoic acid, 7-[(1R,2R,3S,5S)-2-[(1E,3R)-3-(2,3-dihydro-1H-inden-2-yl)-3-hydroxy-1-propen-1-yl]-3-fl
C25H33FO4; MF: 0; RMF: 215; Prob 3.84%; CAS: 1176541-11-9; Lib: nist_msms; ID: 418884.

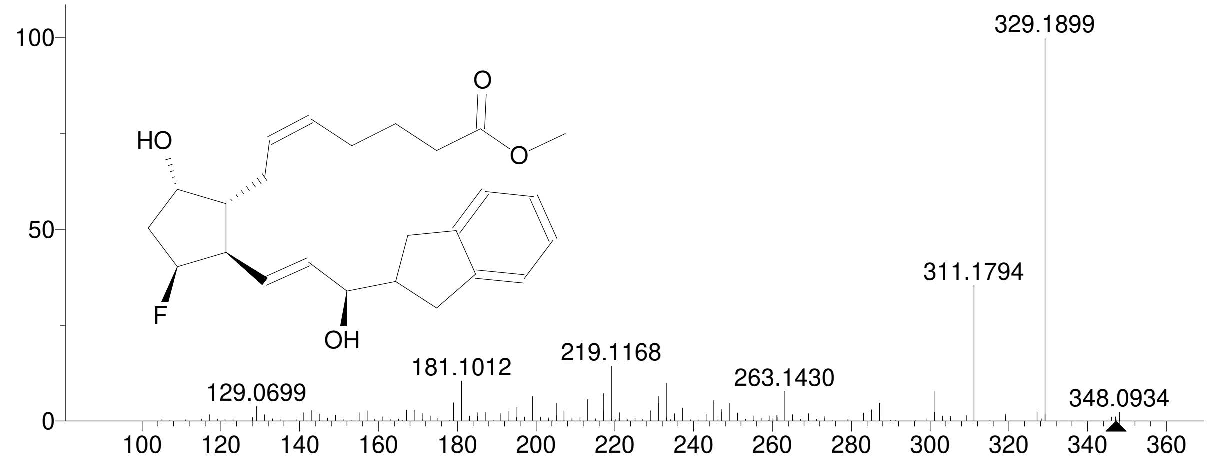

Hit 5 : Ganoderic acid H [M+H-C8H18O7]+ IT-FT 35% P=347.2
C32H44O9; MF: 0; RMF: 67; Prob 3.84%; CAS: 98665-19-1; Lib: nist_msms; ID: 226690.

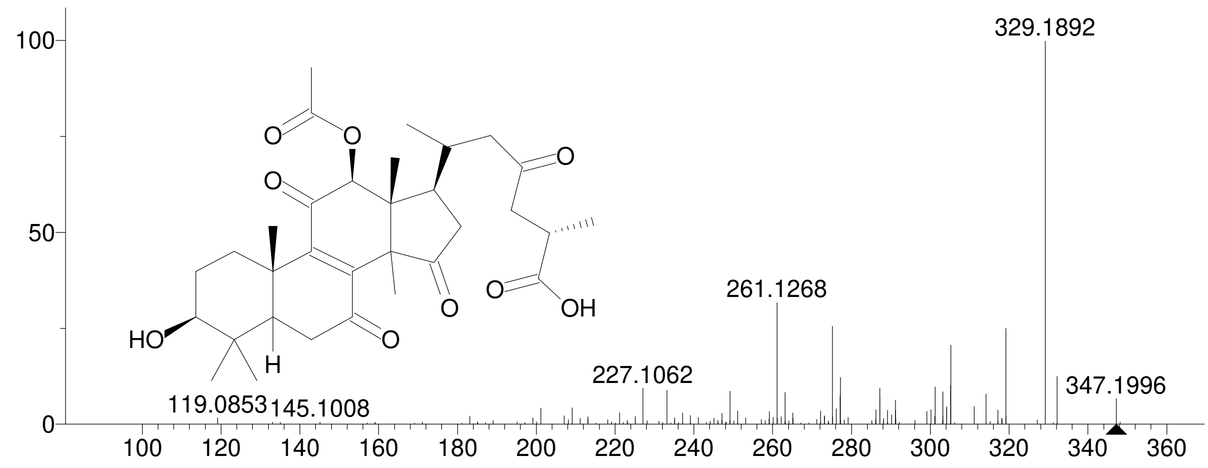

Unknown: Unknown (31.65_415.1858m/z)
Compound in Library Factor = -2176

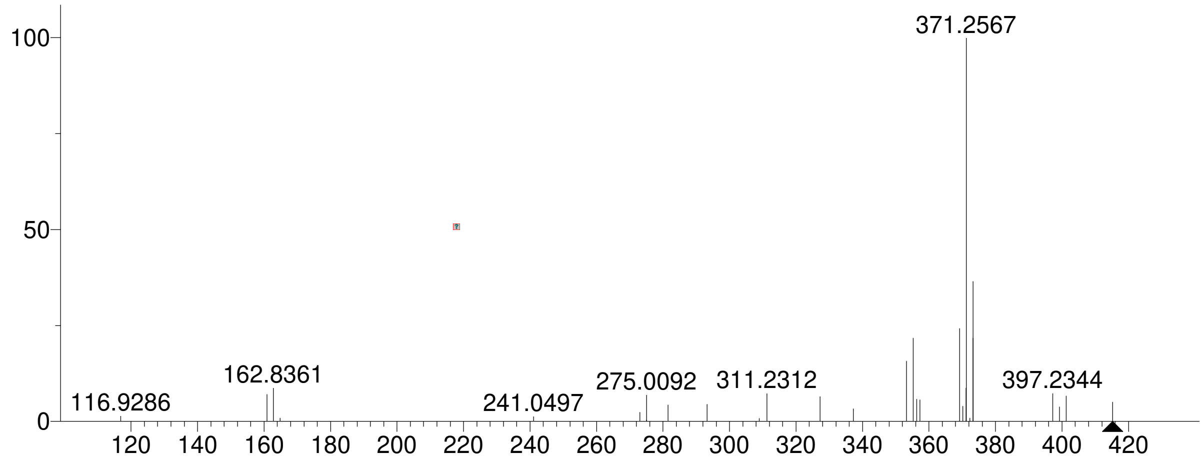

Hit 1 : Dexamethasone [M+Na]+ HCD 24V P=415.2
C22H29FO5; MF: 33; RMF: 434; Prob 38.3%; CAS: 50-02-2; Lib: nist_msms; ID: 310170.

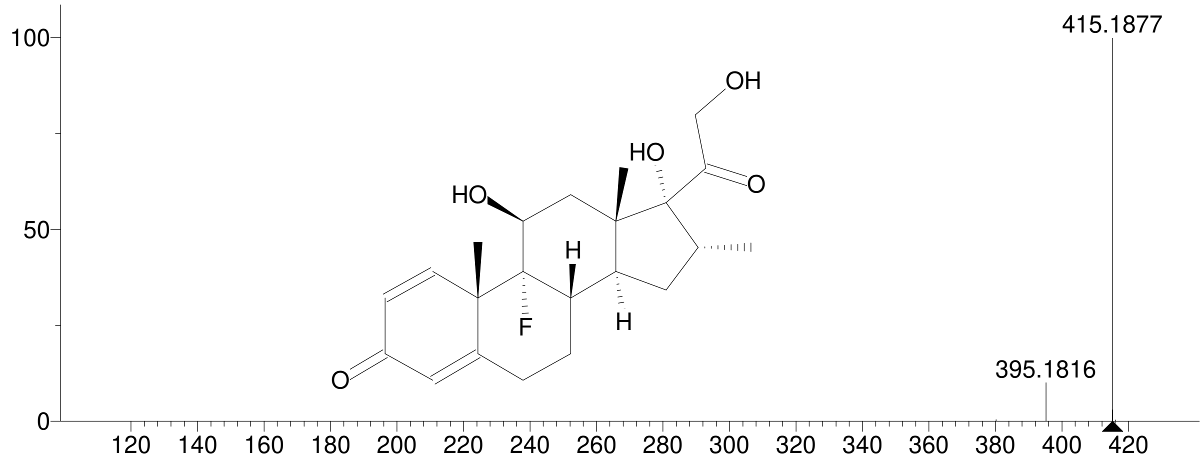

Hit 2 : (±)-N-Formyl-3,4-methylenedioxyamphetamine [2M+H]+ HCD 1V P=415.2
C11H13NO3; MF: 5; RMF: 114; Prob 11.1%; CAS: 67669-00-5; Lib: nist_msms; ID: 243531.

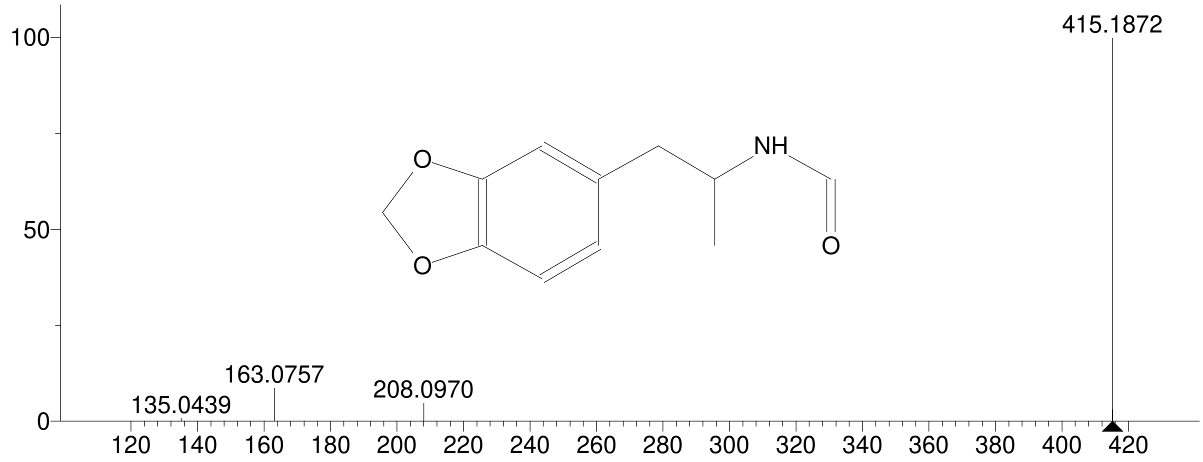

Hit 3 : His-Cys-Arg [M+H]+ QTOF 2V P=415.2
C15H26N8O4S; MF: 5; RMF: 113; Prob 11.1%; Lib: nist_msms; ID: 55680.

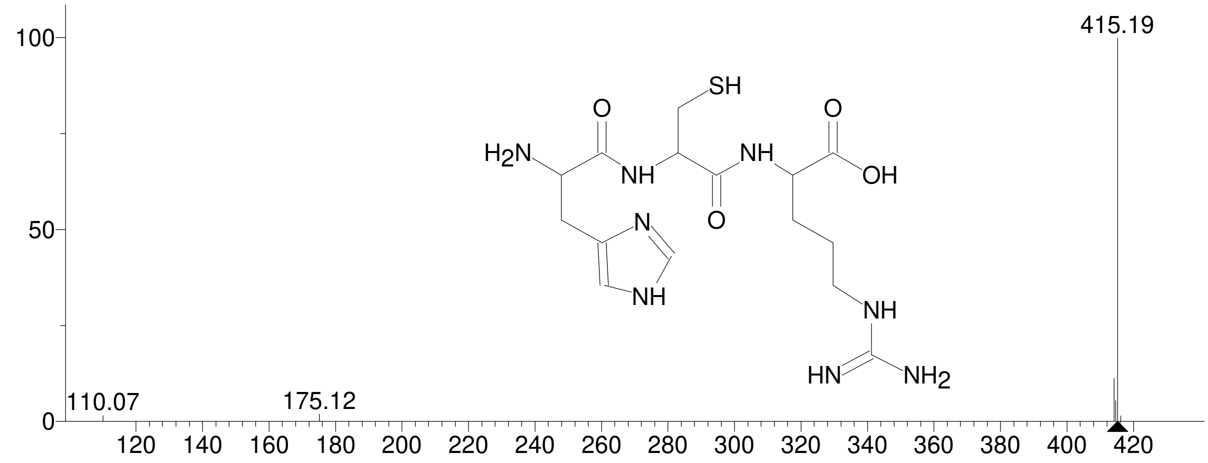

Hit 4 : Betamethasone [M+Na]+ HCD 24V P=415.2
C22H29FO5; MF: 3; RMF: 113; Prob 10.2%; CAS: 378-44-9; Lib: nist_msms; ID: 409063.

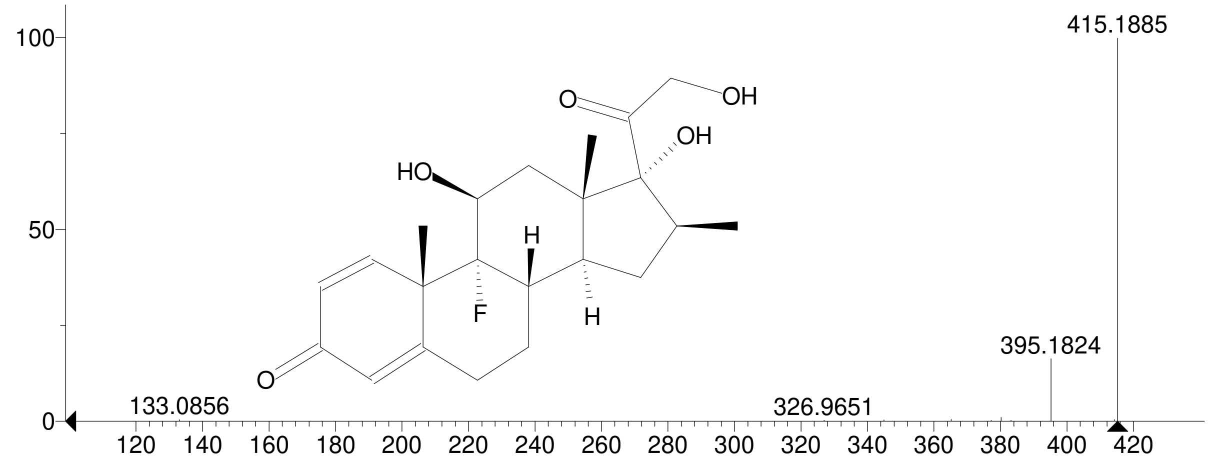

Hit 5 : Cys-His-Arg [M+H]+ QTOF 10V P=415.2
C15H26N8O4S; MF: 3; RMF: 110; Prob 10.2%; Lib: nist_msms; ID: 49594.

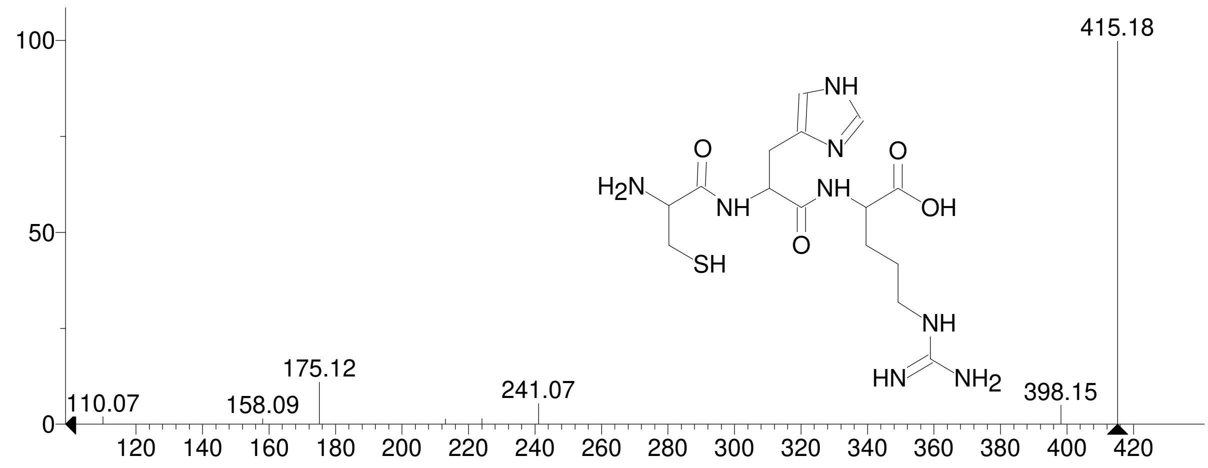

Unknown: Unknown (31.70_315.2532m/z)
Compound in Library Factor = 118

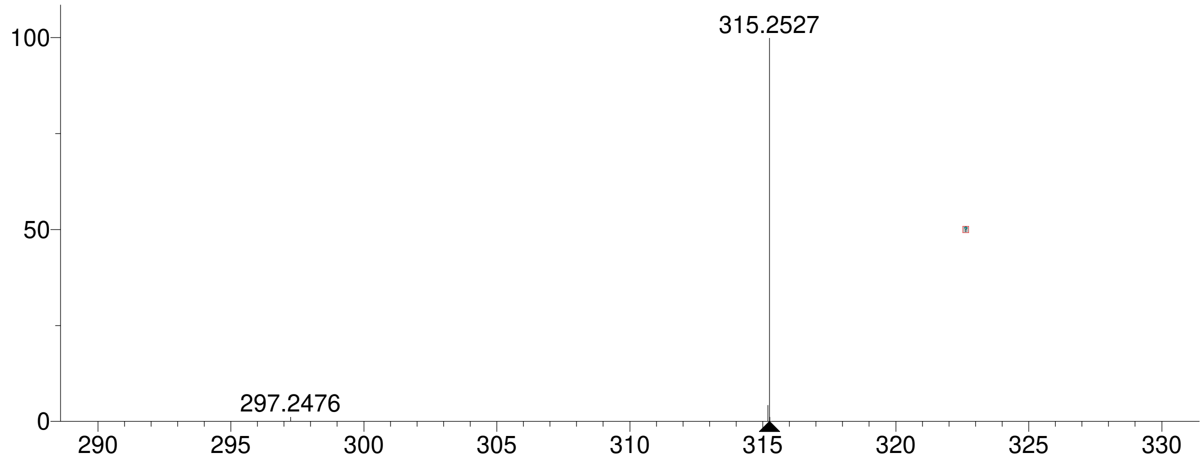

Hit 1 : 12,13-Dihydroxy-9Z-octadecenoic acid [M+H]+ HCD 3V P=315.3
C18H34O4; MF: 687; RMF: 987; Prob 98.3%; CAS: 263399-35-5; Lib: nist_msms; ID: 92801.

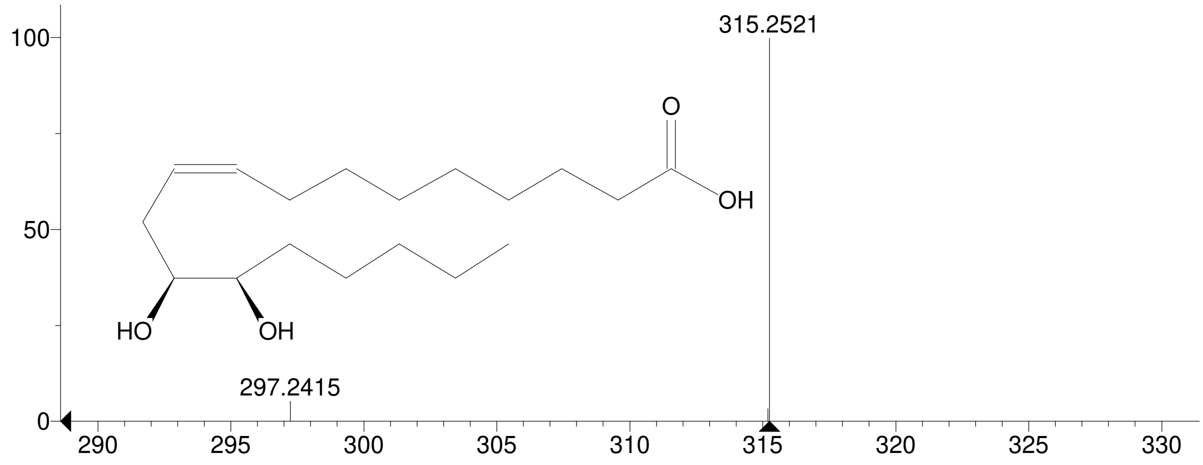

Hit 2 : 9,10-Dihydroxy-12Z-octadecenoic acid [M+H]+ HCD 5V P=315.3
C18H34O4; MF: 360; RMF: 831; Prob 0.98%; CAS: 263399-34-4; Lib: nist_msms; ID: 95803.

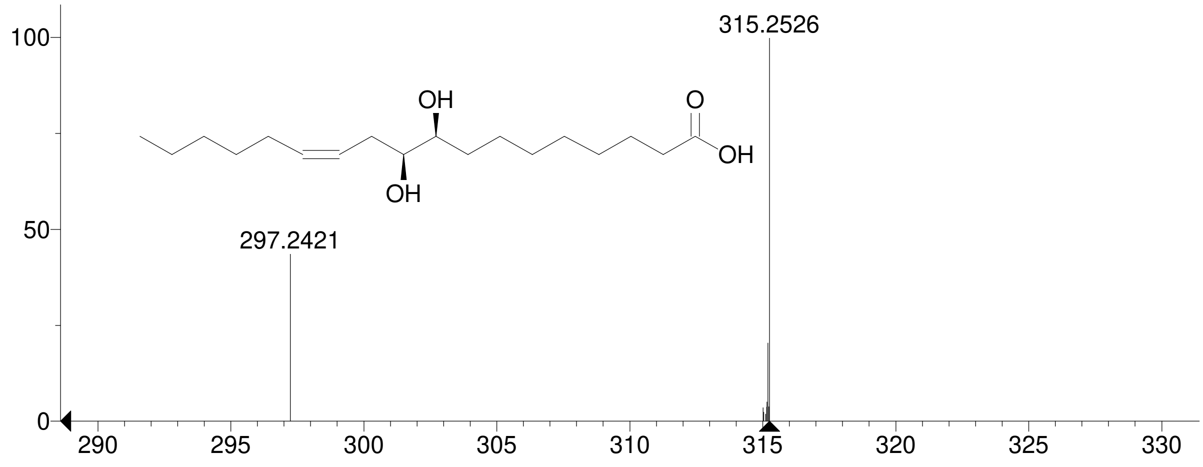

Hit 3 : Dibutyl sebacate [M+H]+ QTOF 5V P=315.3
C18H34O4; MF: 350; RMF: 969; Prob 0.69%; CAS: 109-43-3; Lib: nist_msms; ID: 26918.

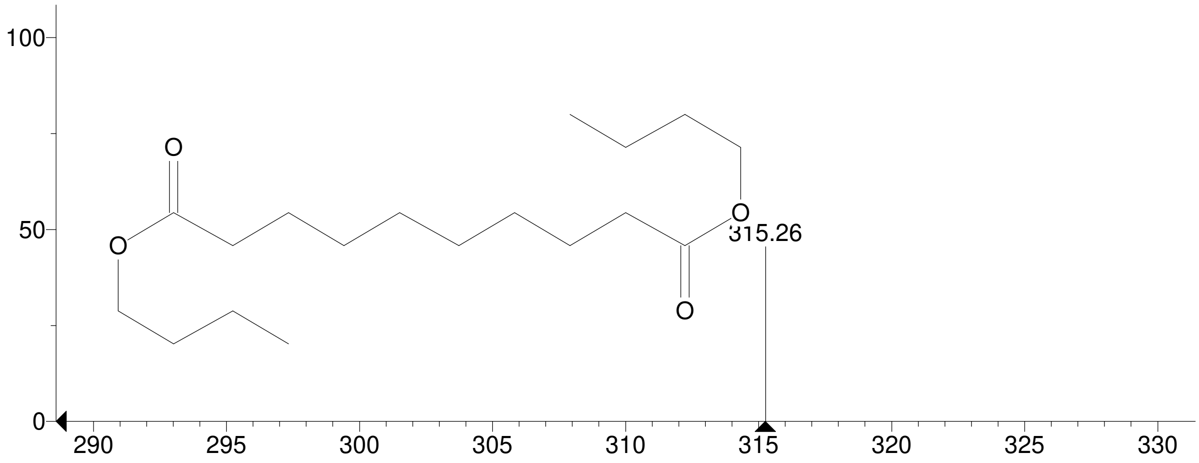

Hit 4 : NCGC00381425-01!8-hydroxy-8-(3-octyloxiran-2-yl)octanoic acid [M+H]+ P=315.3
C18H34O4; MF: 0; RMF: 39; Prob 0.00%; Lib: mona-export-gnps; ID: 7520.

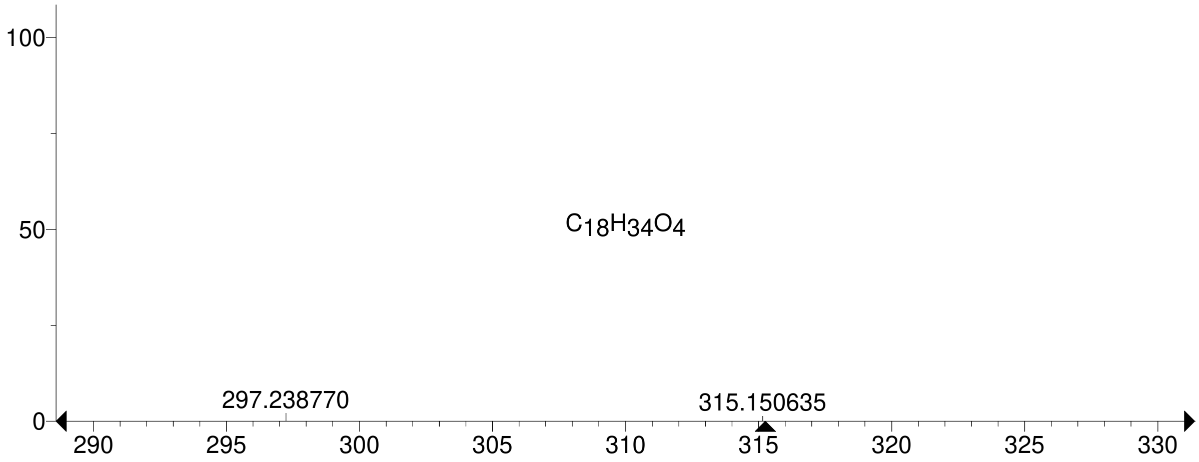

Unknown: Unknown (31.73_492.1208m/z)
Compound in Library Factor = -1848

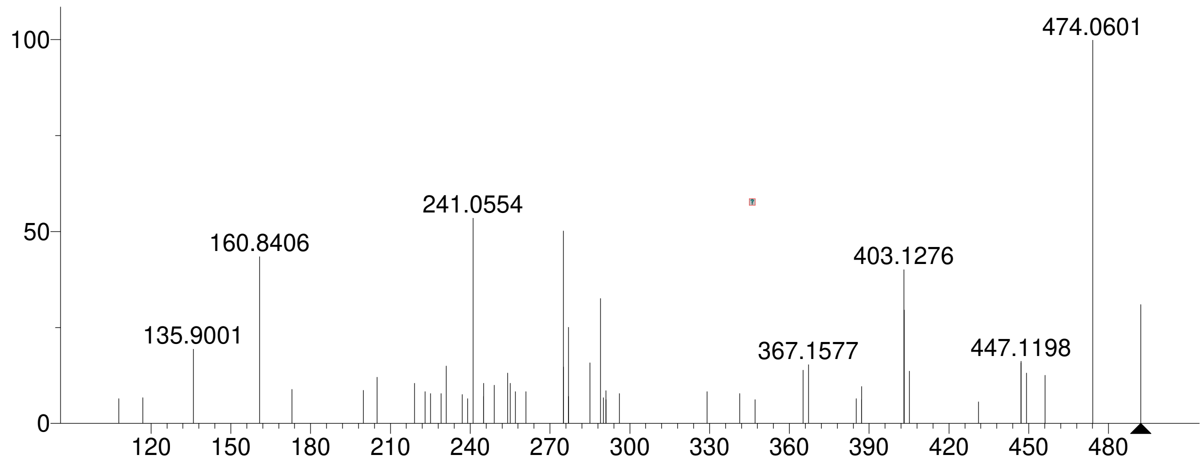

Hit 1 : 6H-Thieno[3,2-f][1,2,4]triazolo[4,3-a][1,4]diazepine-6-acetamide, 4-(4-chlorophenyl)-N-(4-hydroxyphenyl)-2
C25H22ClN5O2S; MF: 38; RMF: 310; Prob 79.3%; CAS: 202590-98-5; Lib: nist_msms; ID: 356211.

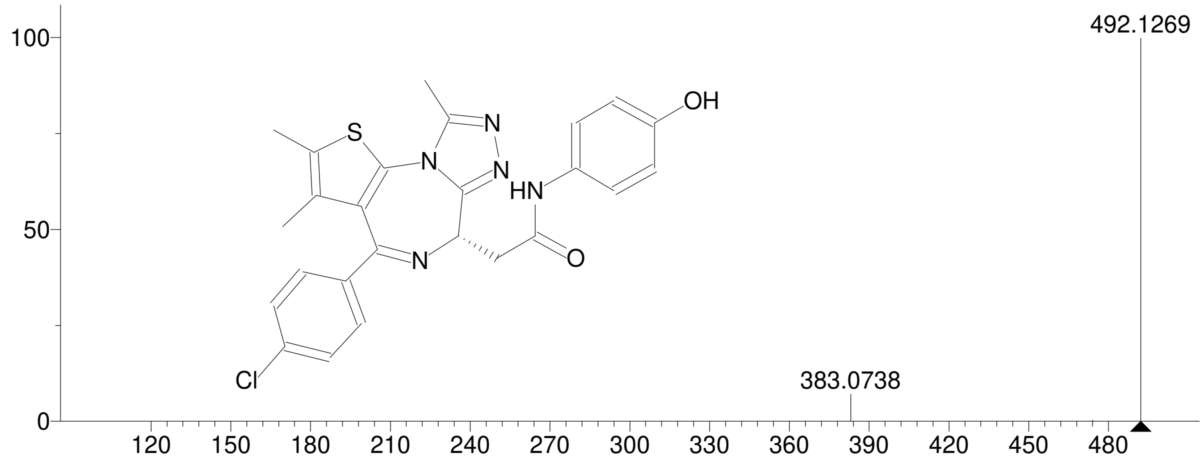

Hit 2 : Bucladesine [M+Na]+ HCD 19V P=492.1
C18H24N5O8P; MF: 5; RMF: 171; Prob 20.7%; CAS: 362-74-3; Lib: nist_msms; ID: 350906.

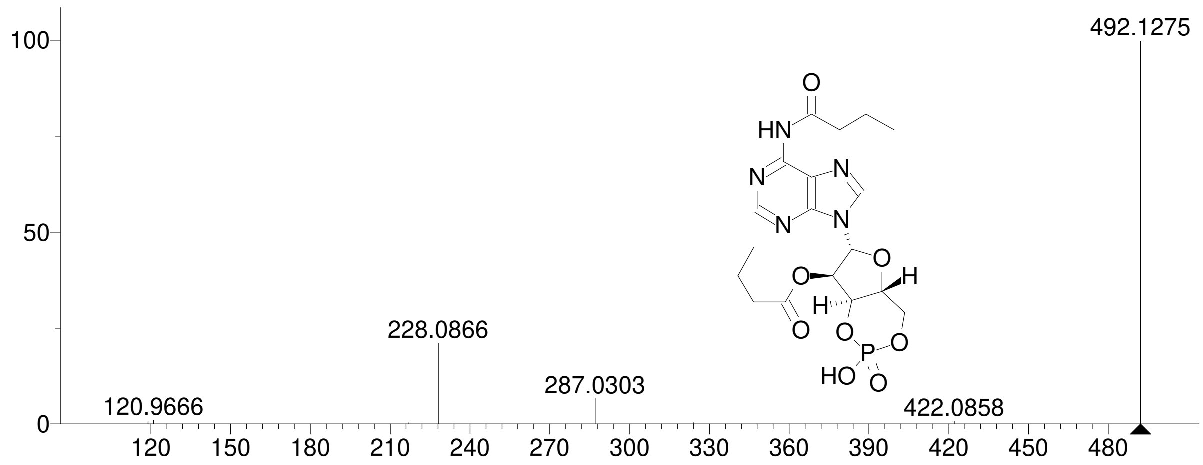

** Search Report Page 1 of 1 **

Unknown: Unknown (31.77_311.3876m/z)
Compound in Library Factor = 0

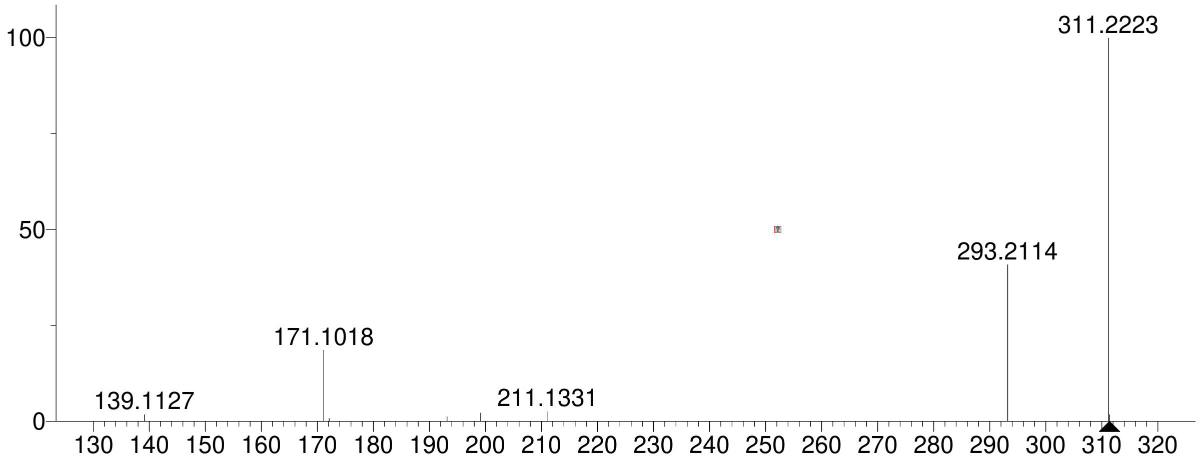

** Search Report Page 1 of 1 **

Unknown: Unknown (31.77_502.1501m/z)
Compound in Library Factor = 0

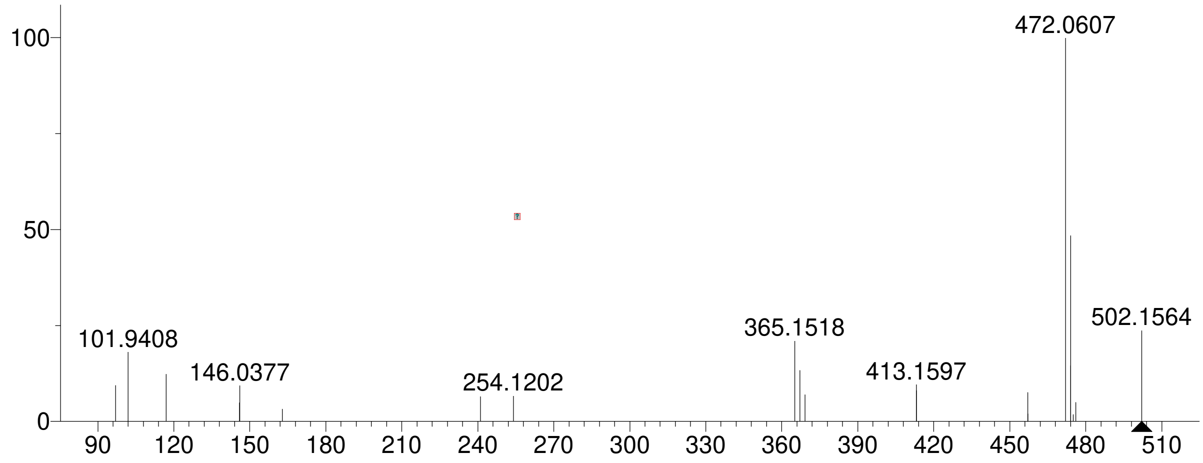

Unknown: Unknown (31.78_293.2108m/z)
Compound in Library Factor = -658

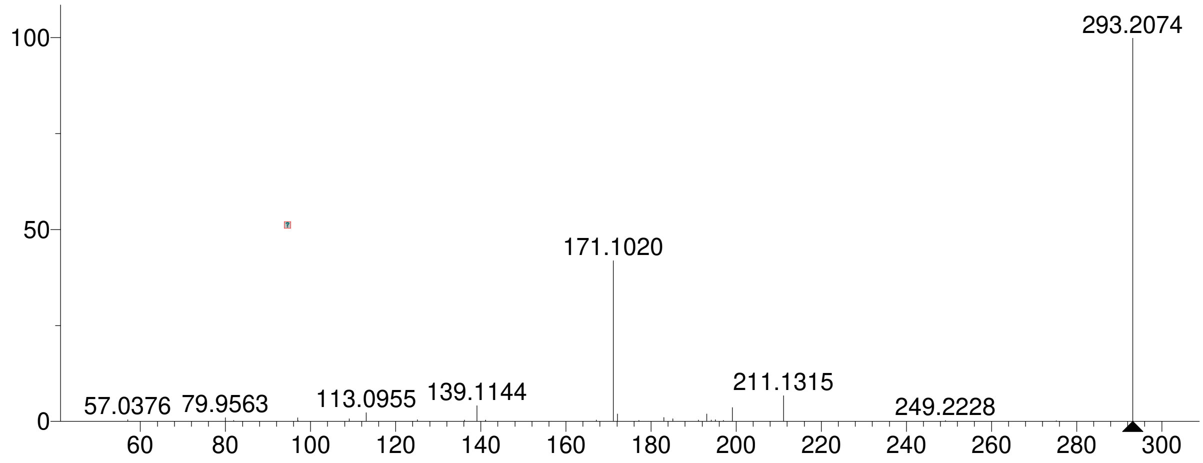

Hit 1 : 9S-Hydroxy-10E,12Z,15Z-octadecatrienoic acid [M-H]- IT-FT 35% P=293.2
C18H30O3; MF: 450; RMF: 650; Prob 88.8%; CAS: 89886-42-0; Lib: nist_msms; ID: 82804.

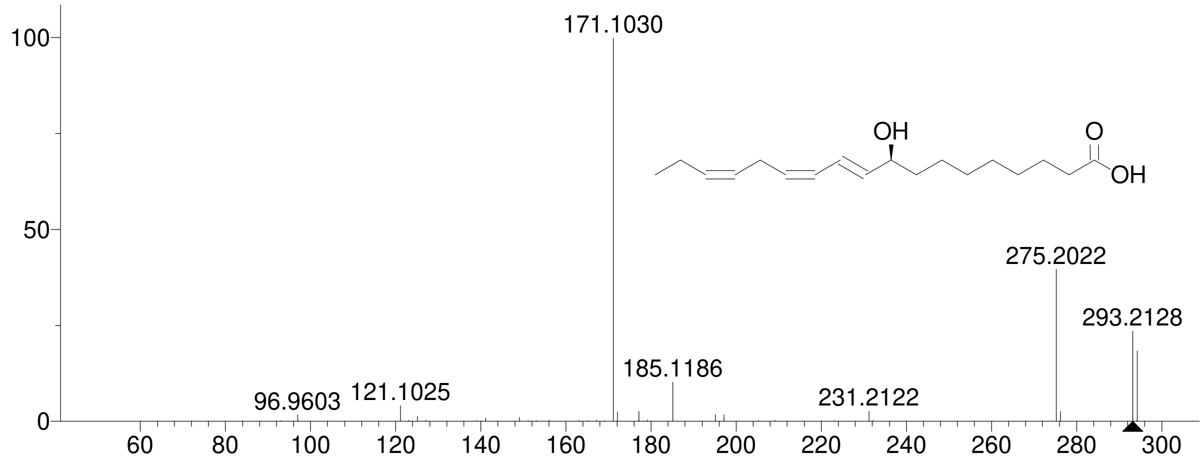

Hit 2 : Unknown (carbon number 18); PlaSMA ID-561 [M-H]- P=293.2
C18H30O3; MF: 364; RMF: 999; Prob 7.91%; Lib: biomsms-neg-plasma; ID: 1171.

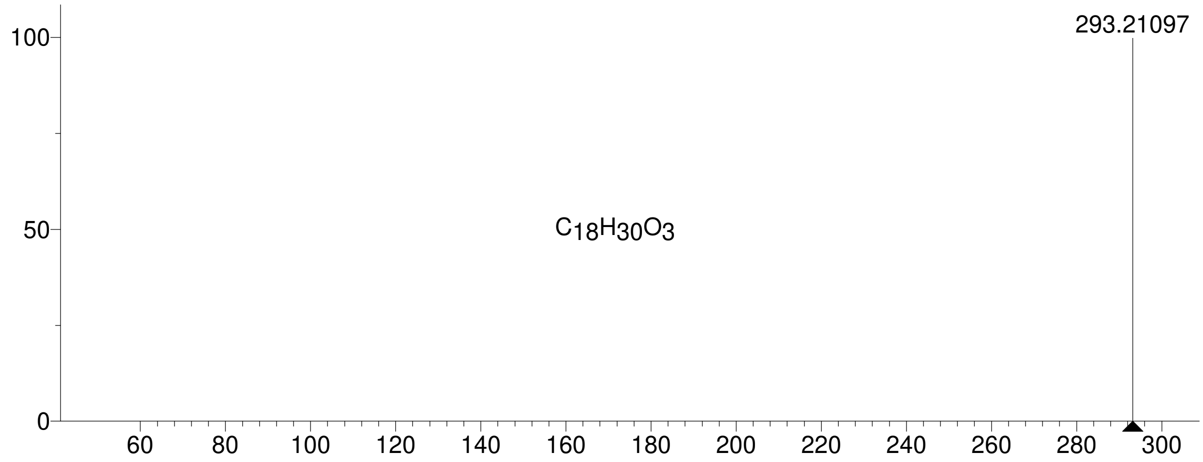

Hit 3 : 9-Hydroperoxy-10E,12Z-octadecadienoic acid [M-H-H2O]- HCD 38V P=293.2
C18H32O4; MF: 316; RMF: 379; Prob 1.64%; CAS: 5502-91-0; Lib: nist_msms; ID: 87782.

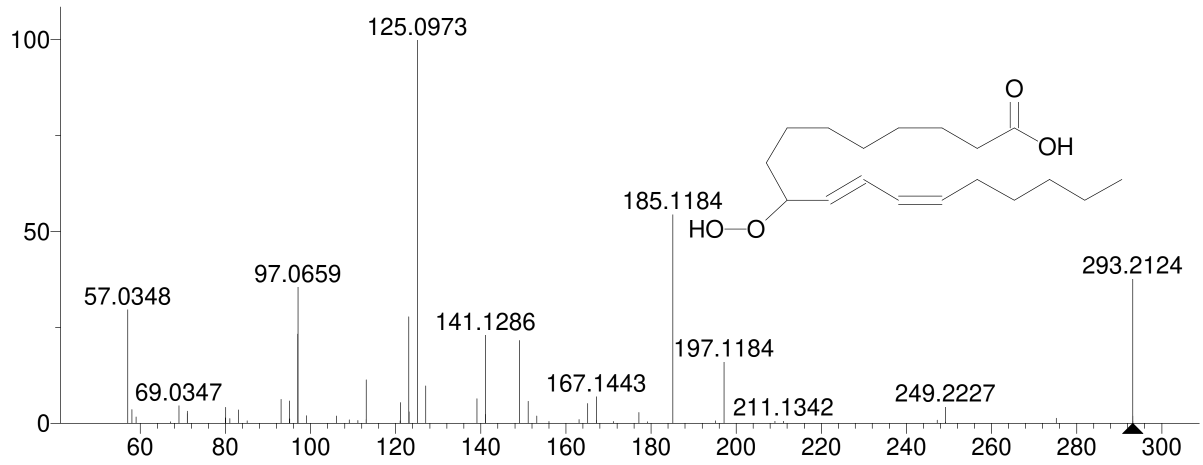

Hit 4 : FA 18:2+O; PlaSMA ID-565 [M-H]- P=293.2
C18H30O3; MF: 274; RMF: 894; Prob 0.36%; Lib: biomsms-neg-plasma; ID: 418.

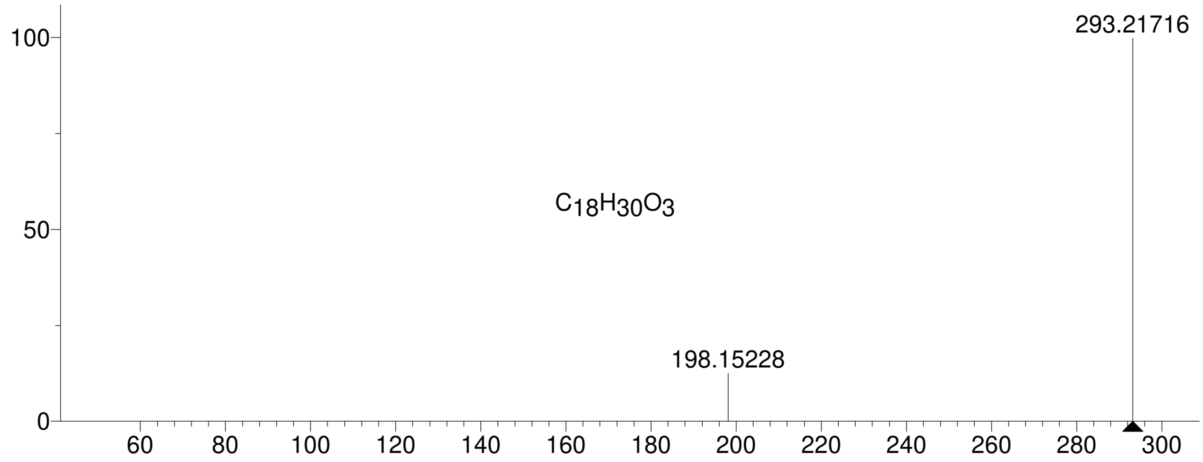

Hit 5 : FA 18:3+1O; PlaSMA ID-558 [M-H]- P=293.2
C18H30O3; MF: 267; RMF: 779; Prob 0.28%; Lib: biomsms-neg-plasma; ID: 414.

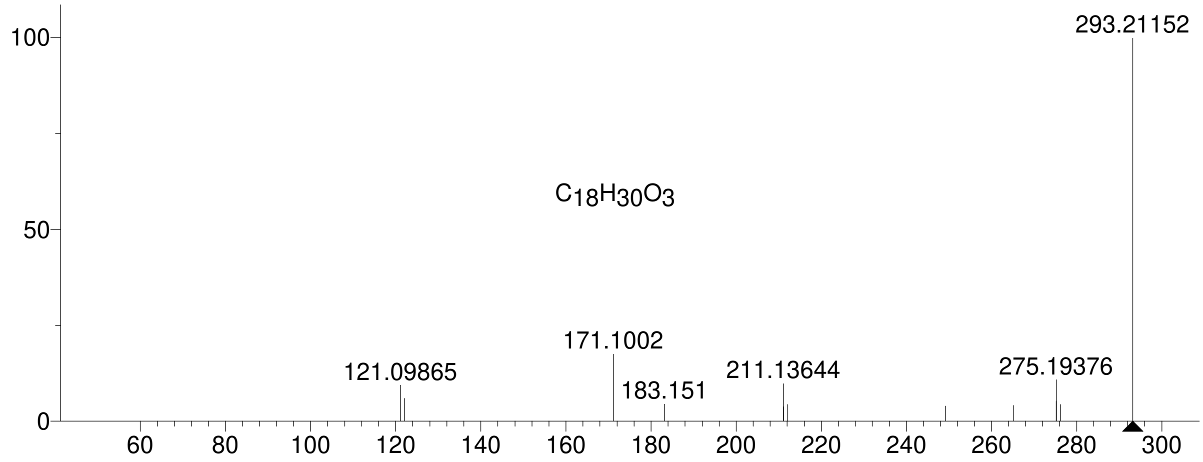

Unknown: Unknown (31.78_311.2225m/z)
Compound in Library Factor = -126

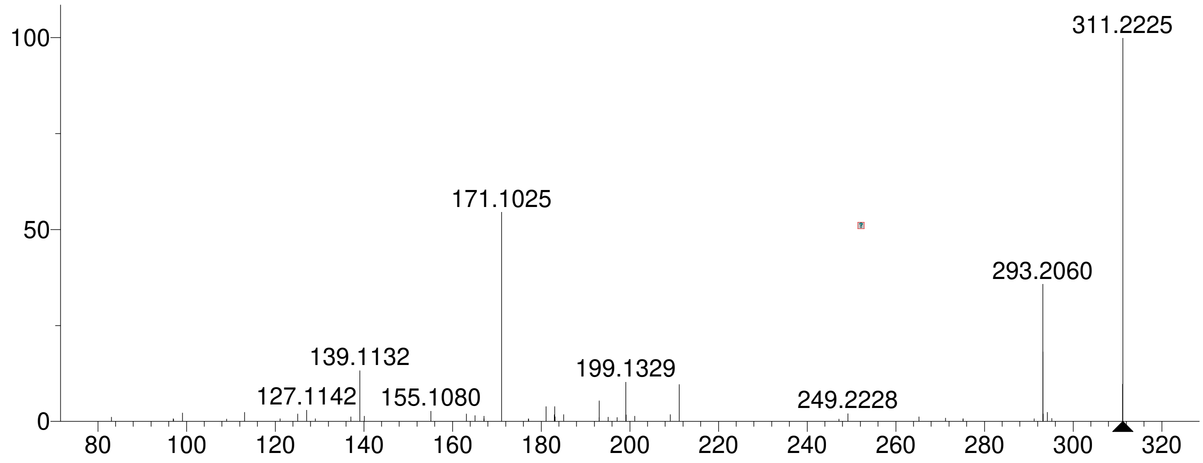

Hit 1 : Unknown (carbon number 19); PlaSMA ID-644 [M-CH3]- P=311.2
C19H34O4; MF: 742; RMF: 837; Prob 97.8%; Lib: biomsms-neg-plasma; ID: 1202.

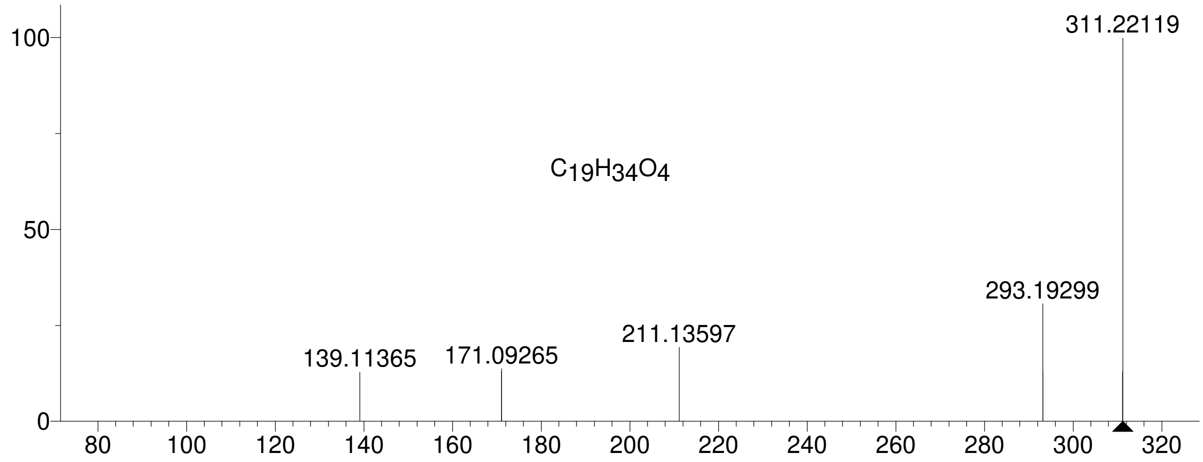

Hit 2 : FA 18:2+2O; PlaSMA ID-643 [M-H]- P=311.2
C18H32O4; MF: 562; RMF: 780; Prob 1.76%; Lib: biomsms-neg-plasma; ID: 431.

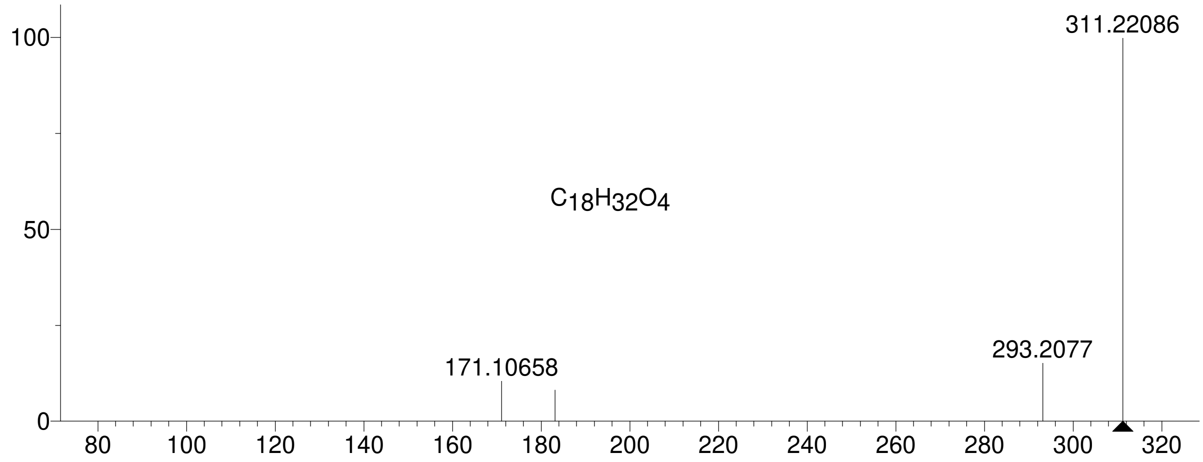

Hit 3 : FA 18:2+2O; PlaSMA ID-641 [M-H]- P=311.2
C18H32O4; MF: 509; RMF: 750; Prob 0.34%; Lib: biomsms-neg-plasma; ID: 432.

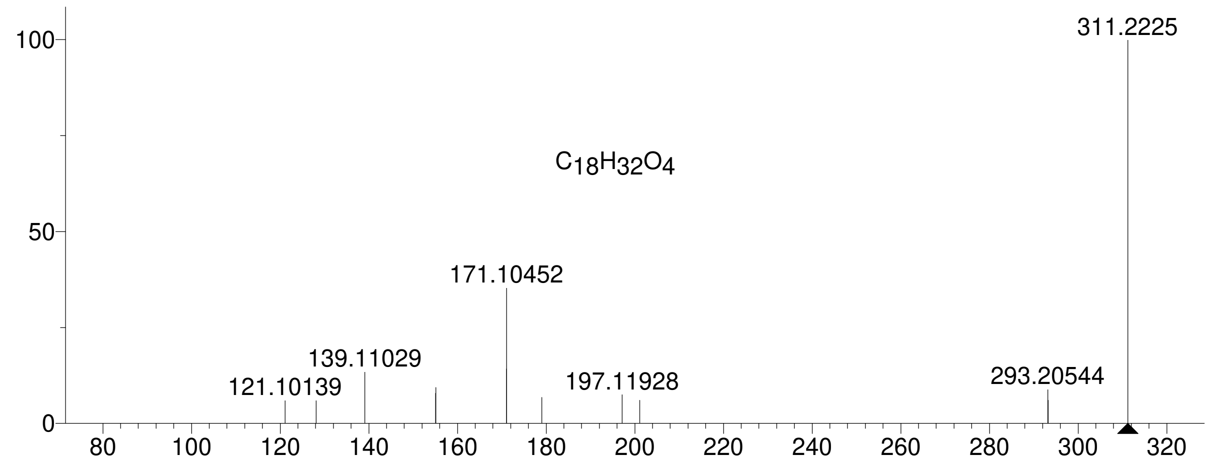

Hit 4 : FA 18:2+2O; PlaSMA ID-645 [M-H]- P=311.2
C18H32O4; MF: 474; RMF: 738; Prob 0.08%; Lib: biomsms-neg-plasma; ID: 429.

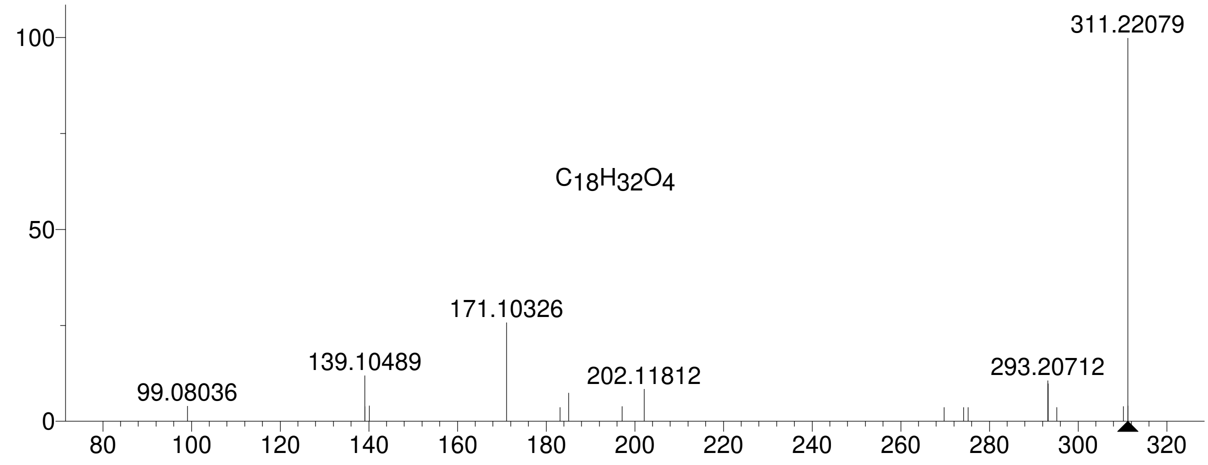

Hit 5 : 9-Oxo-11-(3-pentyl-2-oxiranyl)-10E-undecenoic acid [M+H]+ HCD 12V P=311.2
C18H30O4; MF: 401; RMF: 621; Prob 0.01%; CAS: 478931-82-7; Lib: nist_msms; ID: 91857.

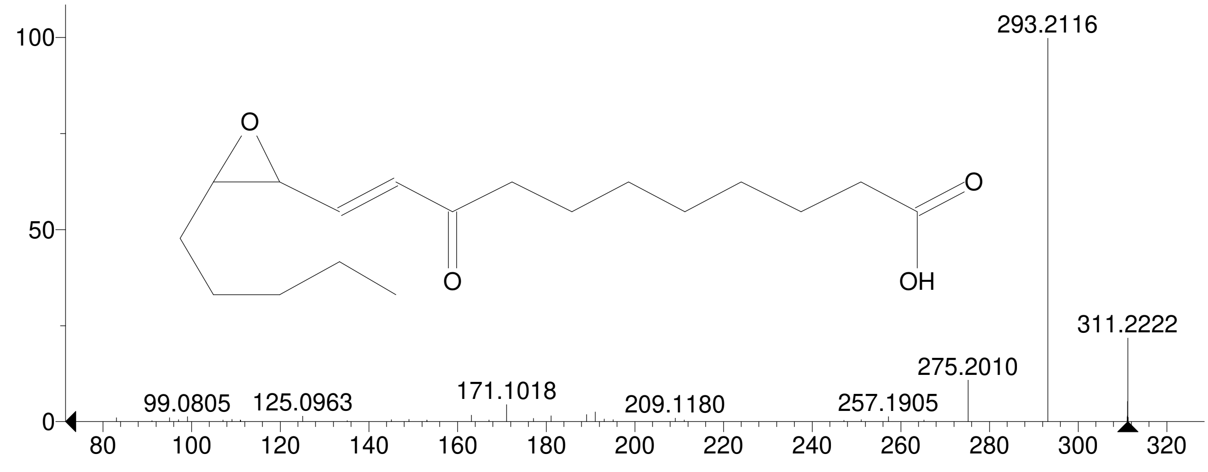

Unknown: Unknown (31.78_472.0633m/z)
Compound in Library Factor = -3273

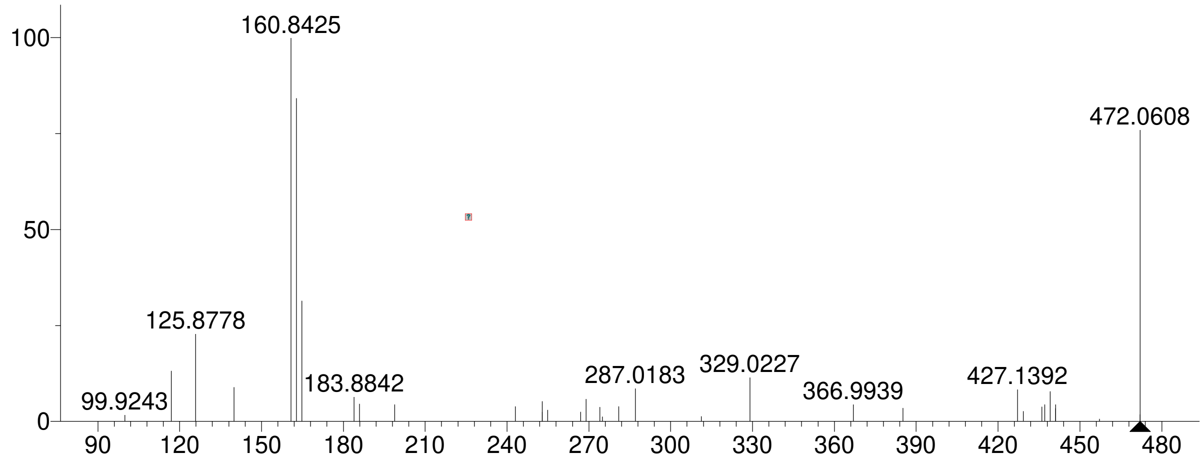

Hit 1 : Acetic acid, 2-[4-[[[2-[3-fluoro-4-(trifluoromethyl)phenyl]-4-methyl-5- thiazolyl]methyl]thio]-2-methylphenoxy]
C21H17F4NO3S2; MF: 11; RMF: 401; Prob 100.0%; CAS: 317318-84-6; Lib: nist_msms; ID: 427976.

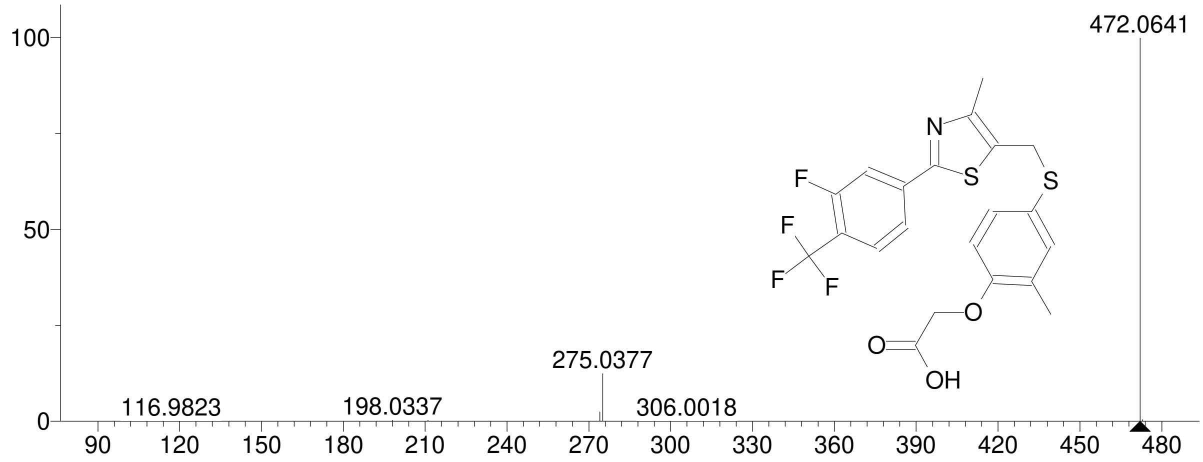

Unknown: Unknown (31.79_459.1517m/z)
Compound in Library Factor = 0

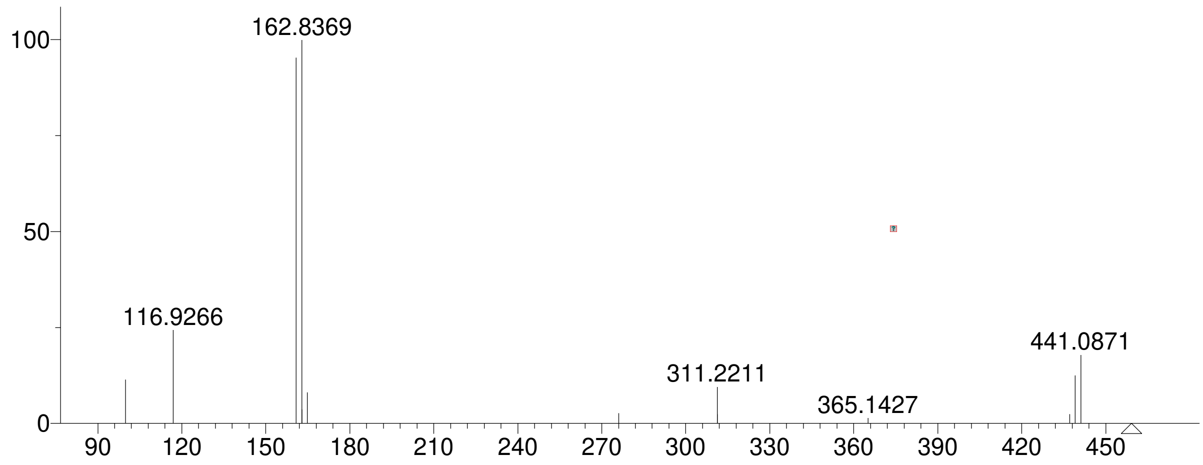

** Search Report Page 1 of 1 **

Unknown: Unknown (31.79_474.0611m/z)
Compound in Library Factor = 0

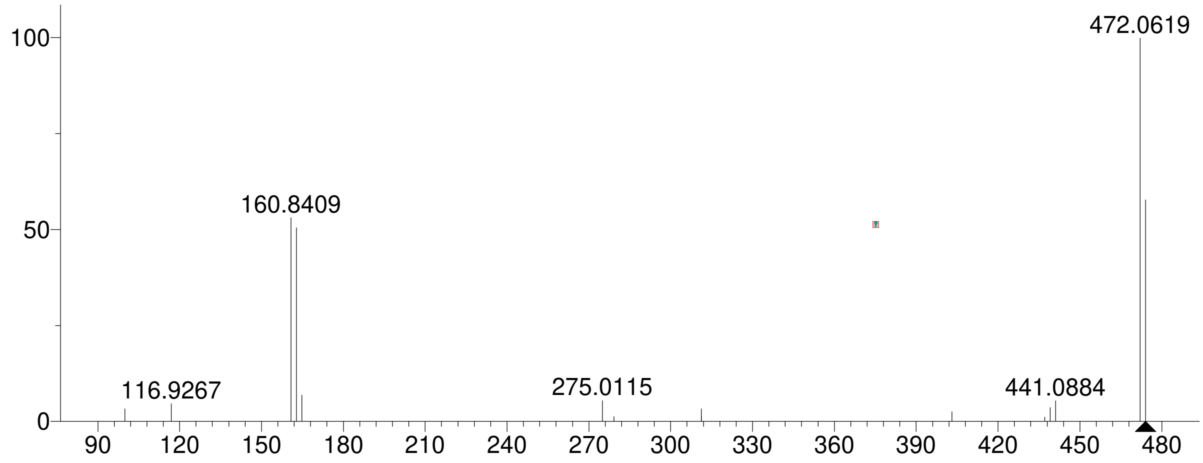

Unknown: Unknown (31.81_379.2088m/z)
Compound in Library Factor = 0

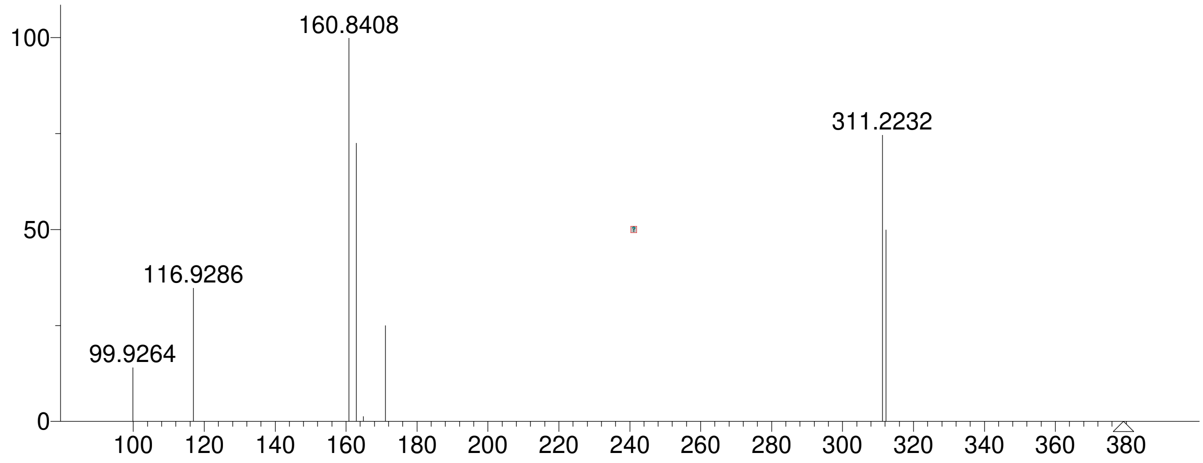

Hit 1 : Gly-Phe-Arg [M+H]+ QQQ 28V P=379.2
C17H26N6O4; MF: 0; RMF: 4; Prob 100.0%; Lib: nist_msms; ID: 38881.

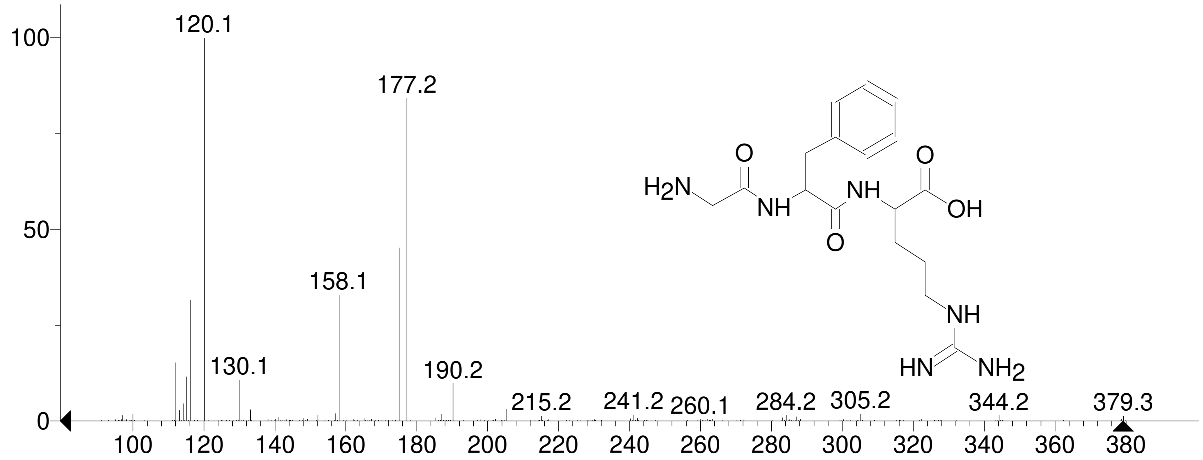

Unknown: Unknown (31.81_415.1855m/z)
Compound in Library Factor = 0

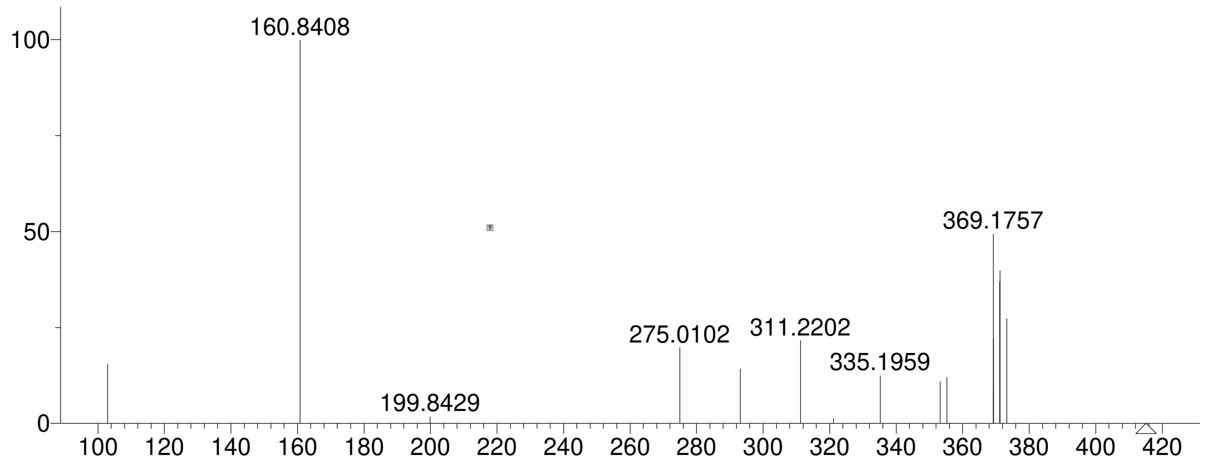

Hit 1 : Dexamethasone [M+Na]+ IT 35% P=415.2
C22H29FO5; MF: 0; RMF: 32; Prob 20.0%; CAS: 50-02-2; Lib: nist_msms; ID: 310175.

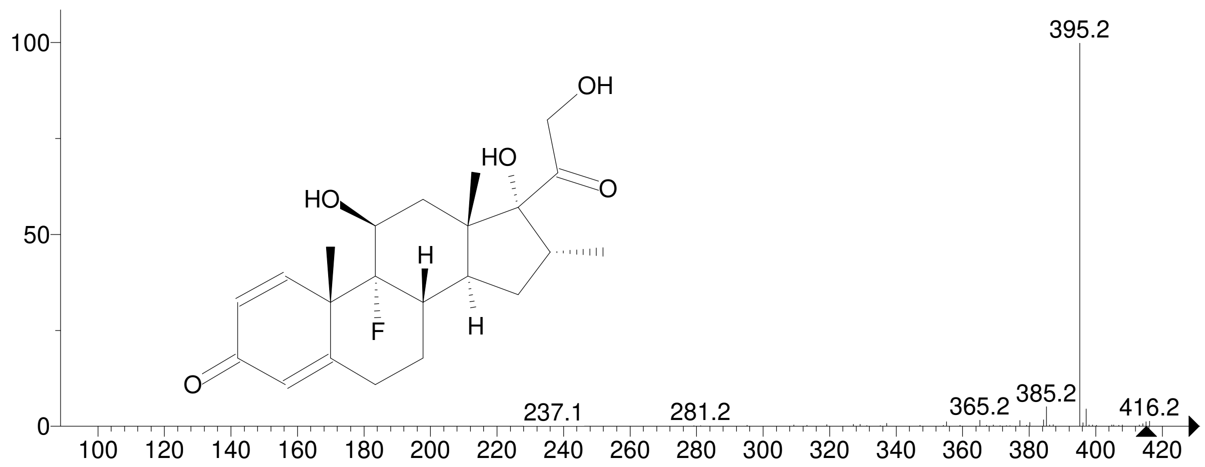

Hit 2 : Betamethasone [M+Na]+ IT 35% P=415.2
C22H29FO5; MF: 0; RMF: 31; Prob 20.0%; CAS: 378-44-9; Lib: nist_msms; ID: 409066.

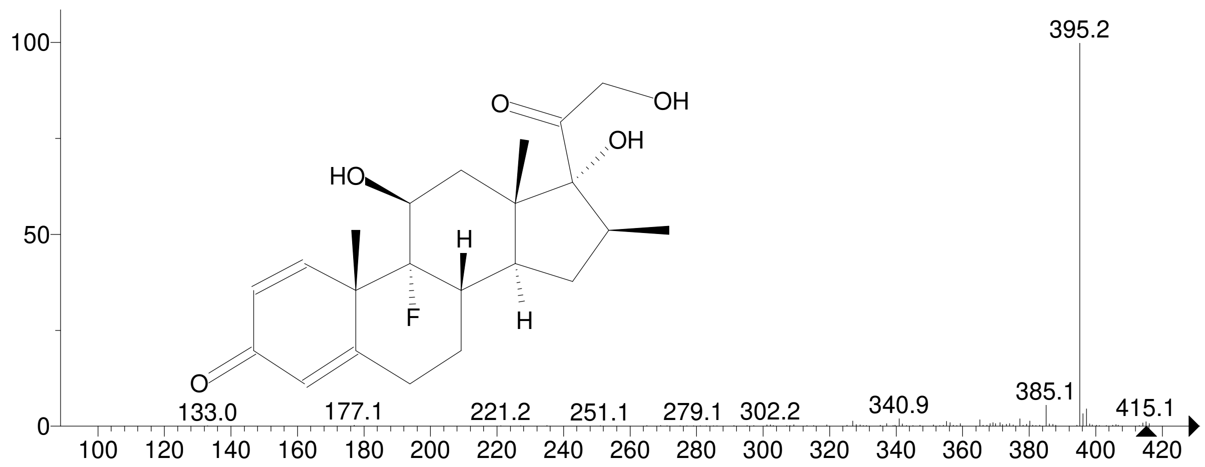

Hit 3 : Acetic acid, 2-[[2'-(5-ethyl-3,4-diphenyl-1H-pyrazol-1-yl)[1,1'-biphenyl]-3-yl]oxy]- [M-H-C2H2O2]- IT 35%
C31H26N2O3; MF: 0; RMF: 28; Prob 20.0%; CAS: 300657-03-8; Lib: nist_msms; ID: 365533.

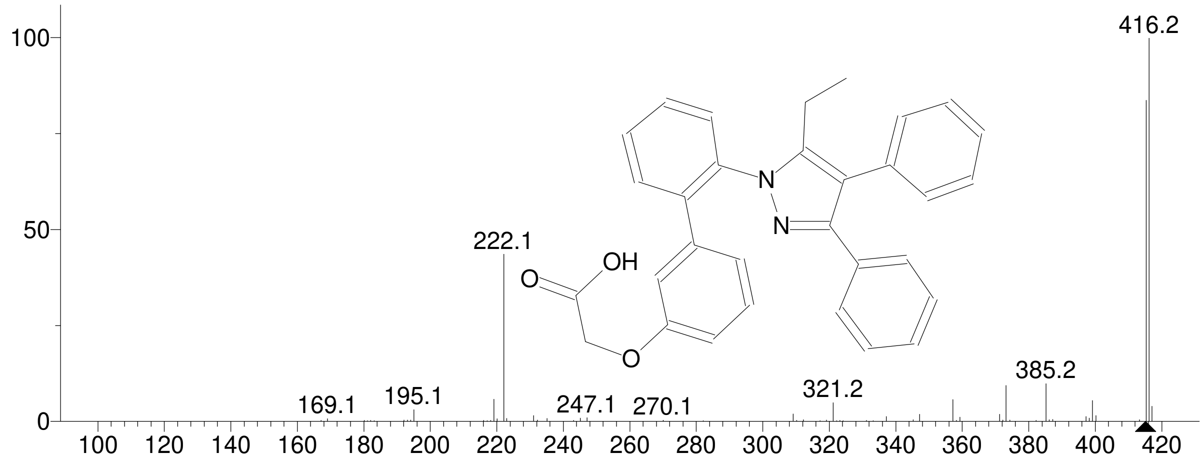

Hit 4 : Cys-His-Arg [M+H]+ IT 35% P=415.2
C15H26N8O4S; MF: 0; RMF: 23; Prob 20.0%; Lib: nist_msms; ID: 47287.

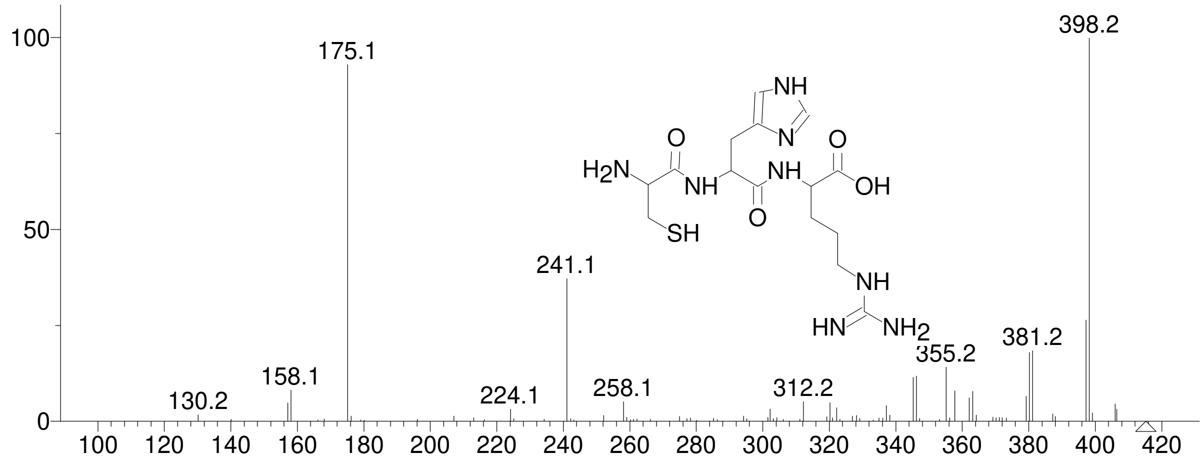

Hit 5 : (±)-N-Formyl-3,4-methylenedioxyamphetamine [2M+H]+ IT 35% P=415.2
C11H13NO3; MF: 0; RMF: 7; Prob 20.0%; CAS: 67669-00-5; Lib: nist_msms; ID: 243548.

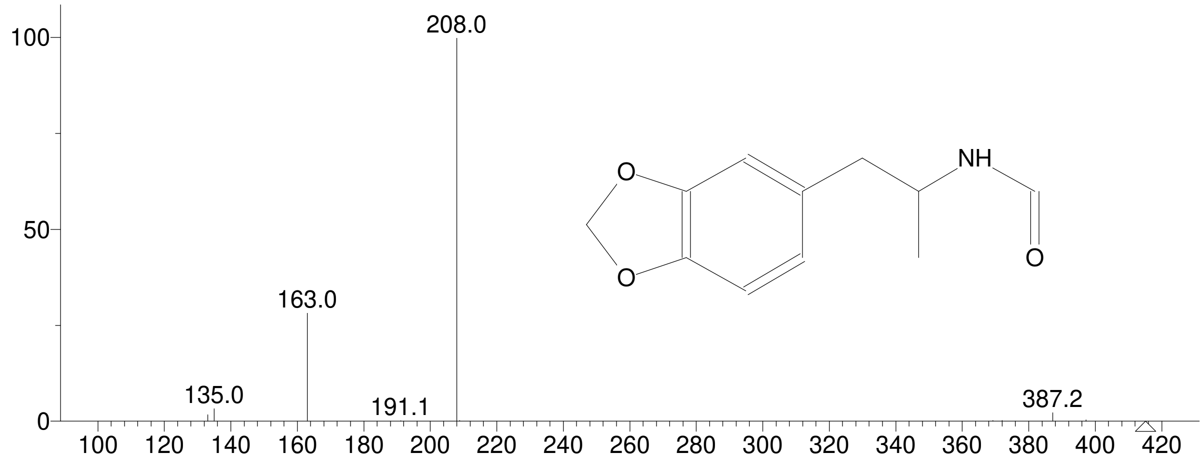

Unknown: Unknown (31.89_315.1943m/z)
Compound in Library Factor = -1096

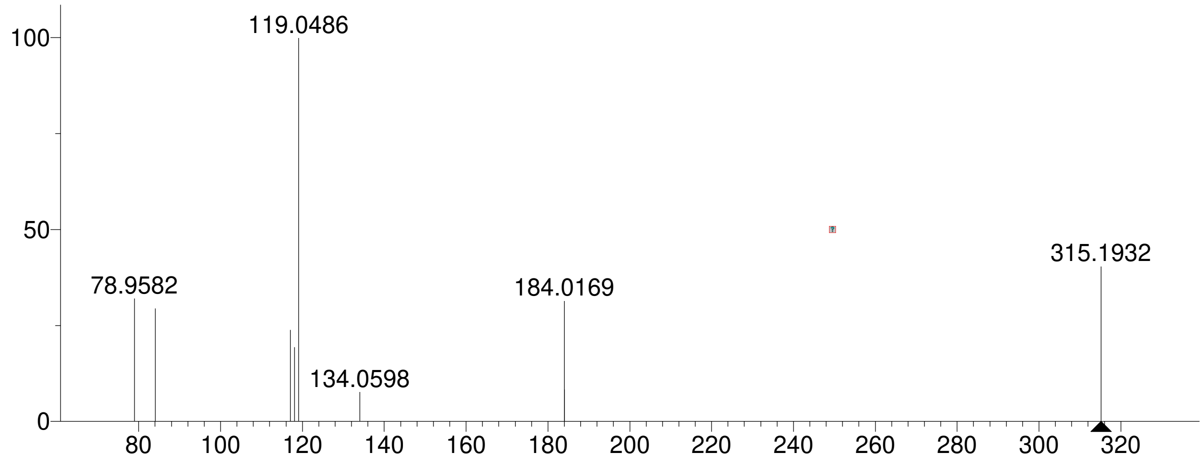

Hit 1 : 20-Carboxyarachidonic acid [M-H-H2O]- HCD 9V P=315.2
C20H30O4; MF: 342; RMF: 983; Prob 70.3%; CAS: 79551-84-1; Lib: nist_msms; ID: 92299.

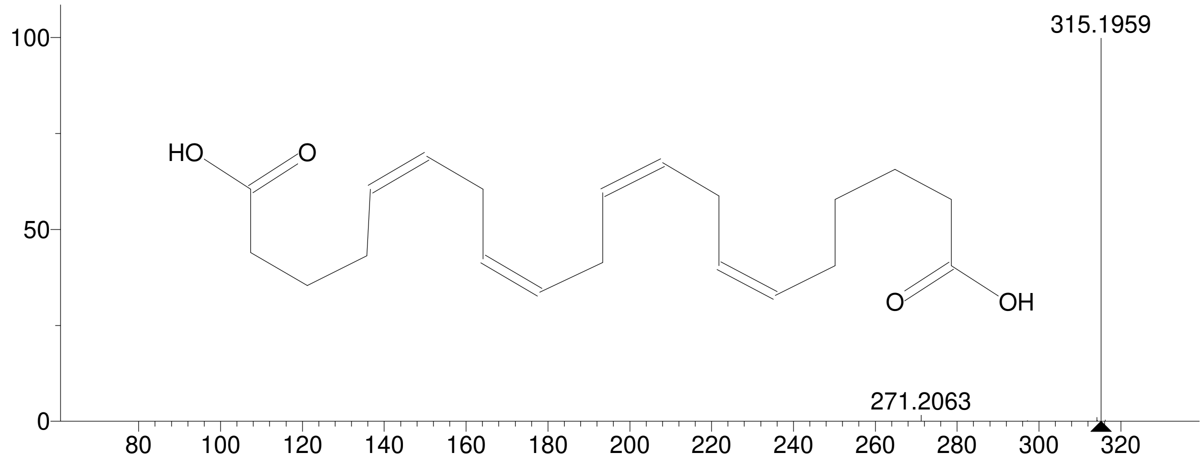

Hit 2 : 5-trans-Prostaglandin D2 [M-H-2H2O]- HCD 12V P=315.2
C20H32O5; MF: 312; RMF: 935; Prob 19.5%; Lib: nist_msms; ID: 341167.

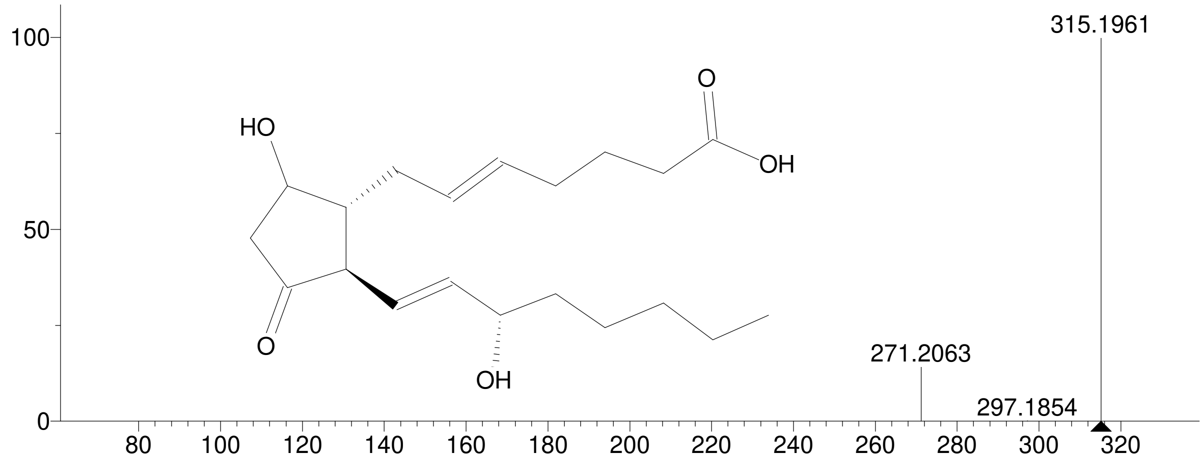

Hit 3 : Prostaglandin B3 [M+H-H2O]+ HCD 8V P=315.2
C20H28O4; MF: 244; RMF: 916; Prob 2.96%; CAS: 36614-32-1; Lib: nist_msms; ID: 85309.

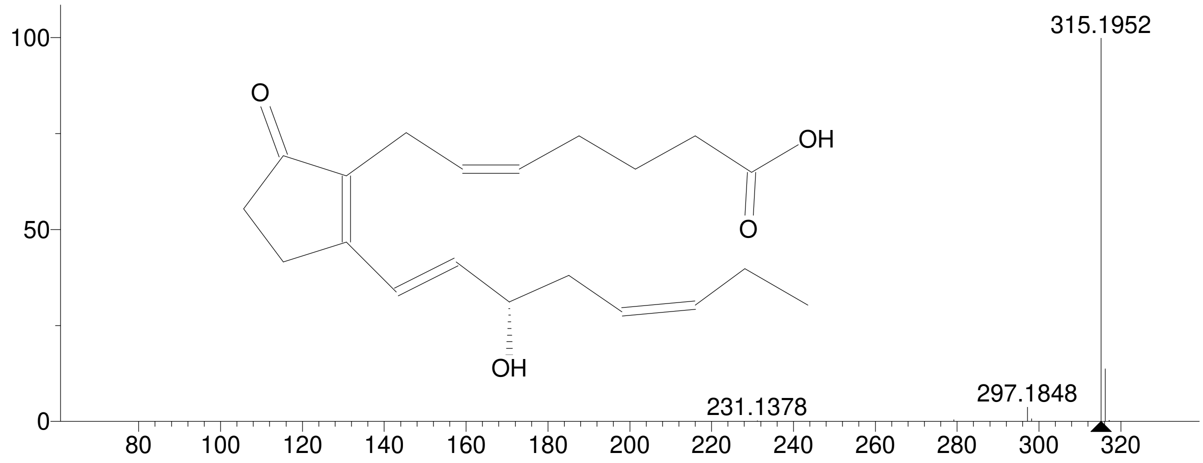

Hit 4 : Prostaglandin A3 [M+H-H2O]+ HCD 5V P=315.2
C20H28O4; MF: 243; RMF: 909; Prob 2.85%; CAS: 36614-31-0; Lib: nist_msms; ID: 85137.

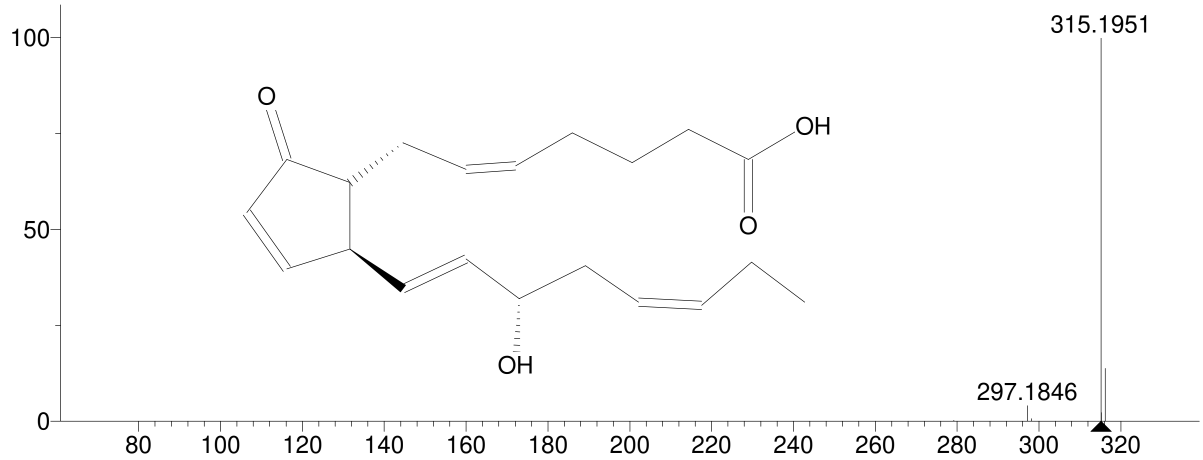

Hit 5 : 9-Oxoprosta-5Z,10,12Z,14E-tetraenoic acid [M-H]- HCD 9V P=315.2
C20H28O3; MF: 240; RMF: 905; Prob 2.51%; CAS: 112839-31-3; Lib: nist_msms; ID: 156892.

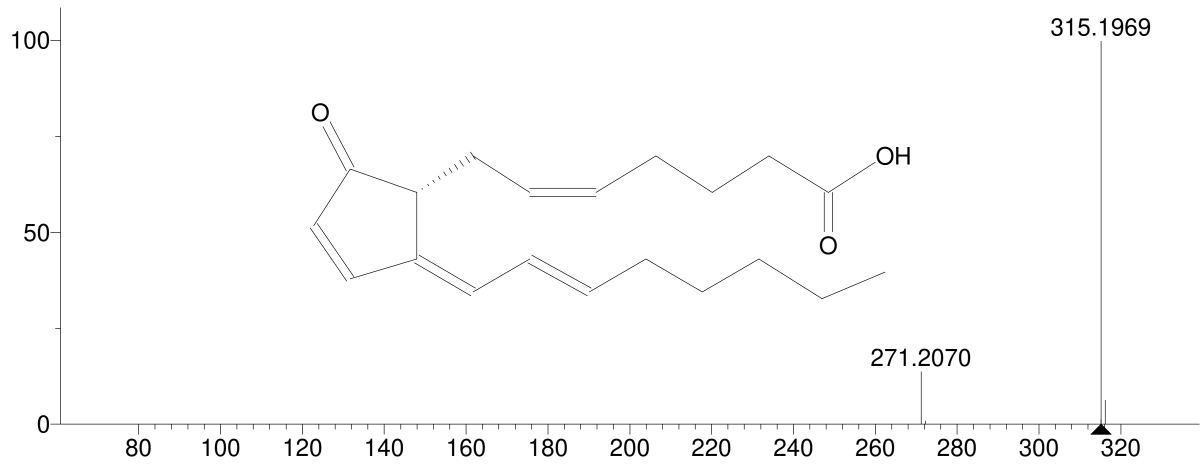

Unknown: Unknown (31.92_383.1756m/z)
Compound in Library Factor = 0

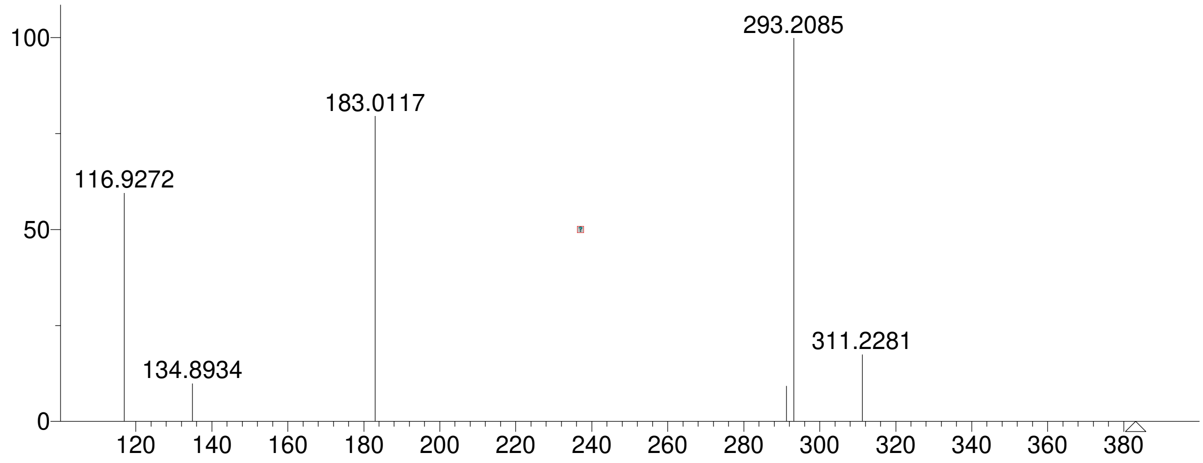

Unknown: Unknown (32.13_315.2537m/z)
Compound in Library Factor = -351

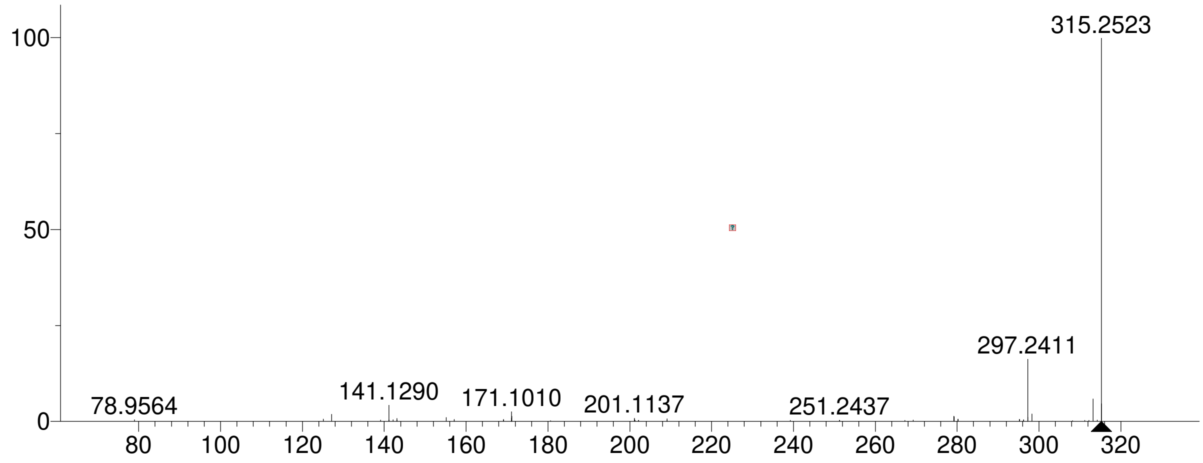

Hit 1 : 12,13-Dihydroxy-9Z-octadecenoic acid [M+H]+ HCD 8V P=315.3
C18H34O4; MF: 232; RMF: 655; Prob 56.3%; CAS: 263399-35-5; Lib: nist_msms; ID: 92803.

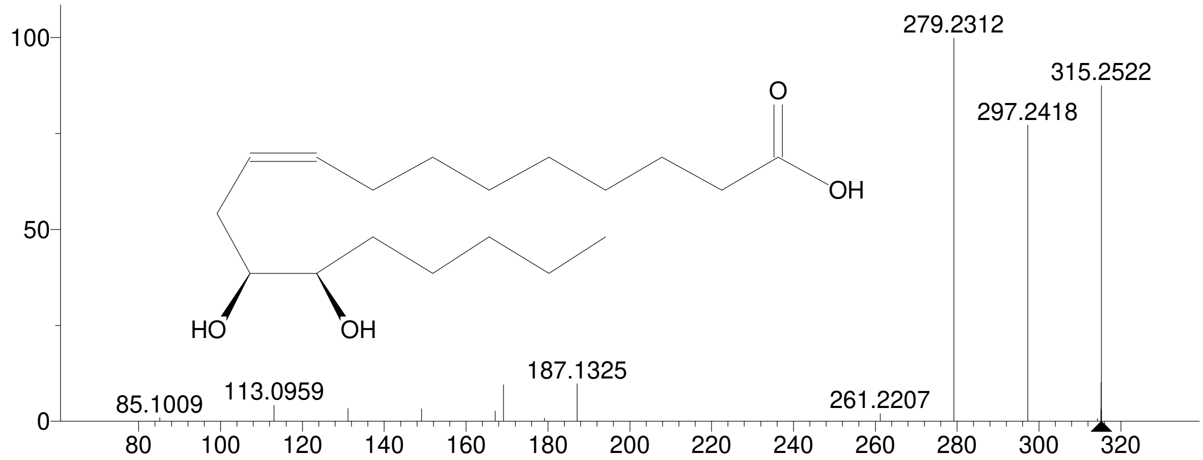

Hit 2 : 9,10-Dihydroxy-12Z-octadecenoic acid [M+H]+ HCD 8V P=315.3
C18H34O4; MF: 225; RMF: 603; Prob 43.1%; CAS: 263399-34-4; Lib: nist_msms; ID: 95804.

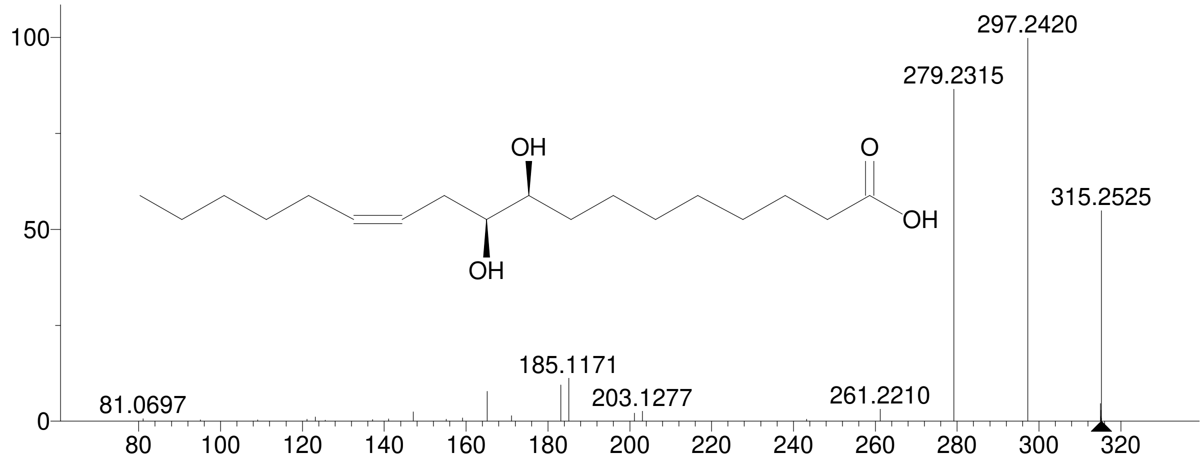

Hit 3 : Dibutyl sebacate [M+H]+ HCD 9V P=315.3
C18H34O4; MF: 24; RMF: 420; Prob 0.43%; CAS: 109-43-3; Lib: nist_msms; ID: 314968.

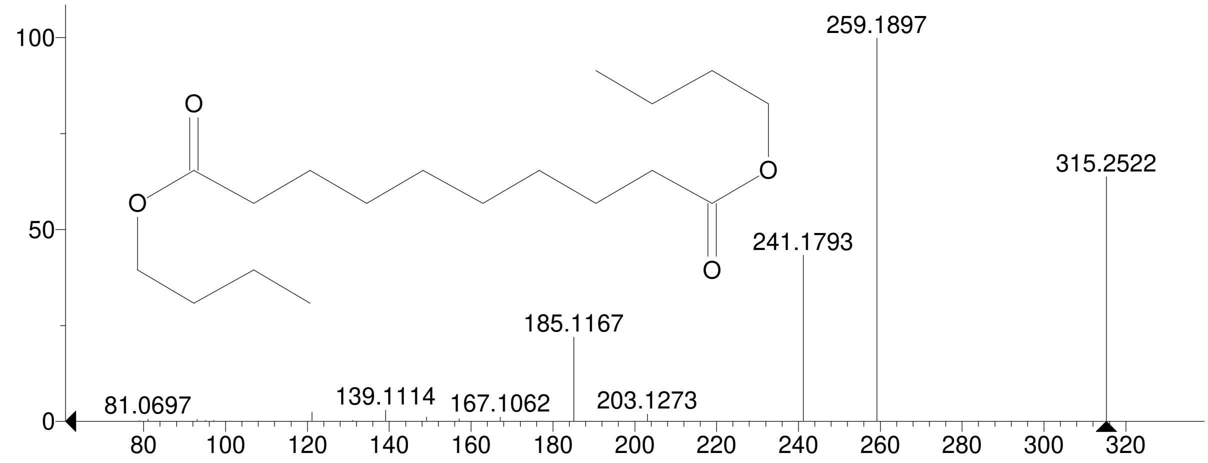

Hit 4 : NCGC00381425-01!8-hydroxy-8-(3-octyloxiran-2-yl)octanoic acid [M+H]+ P=315.3
C18H34O4; MF: 4; RMF: 94; Prob 0.19%; Lib: mona-export-gnps; ID: 7520.

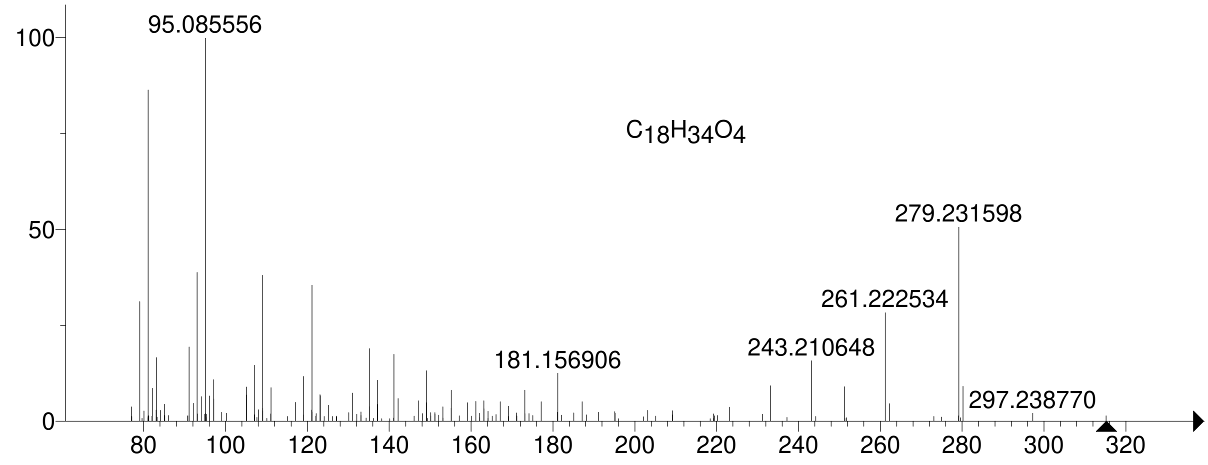

Unknown: Unknown (32.16_347.1989m/z)
Compound in Library Factor = 0

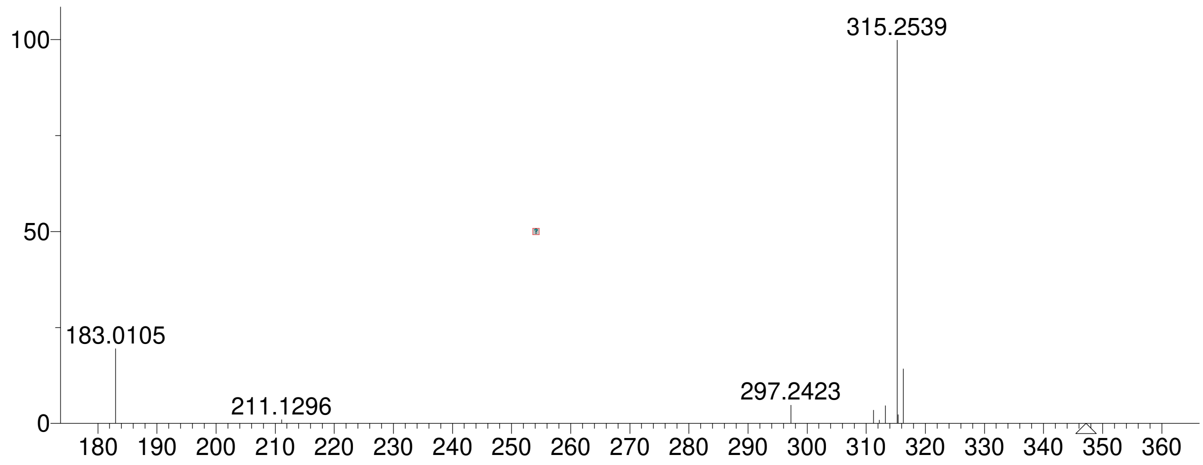

Unknown: Unknown (32.22_313.1798m/z)
Compound in Library Factor = -1249

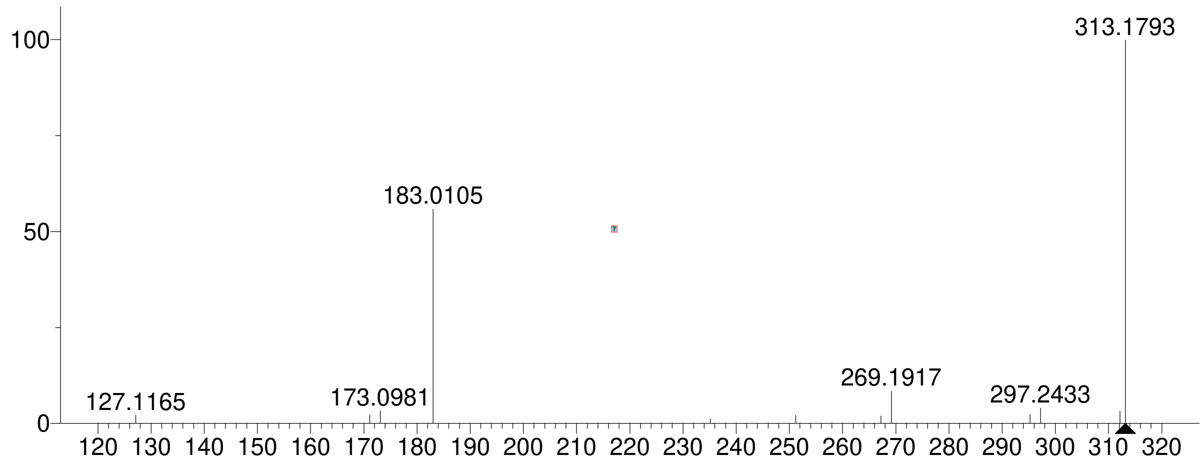

Hit 1 : 1,4a-dimethyl-9-oxo-7-propan-2-yl-3,4,10,10a-tetrahydro-2H-phenanthrene-1-carboxylic acid [M-H]- 45HC
C20H26O3; MF: 234; RMF: 682; Prob 40.3%; Lib: mona-export-lc-ms-ms_negative_mode; ID: 19329.

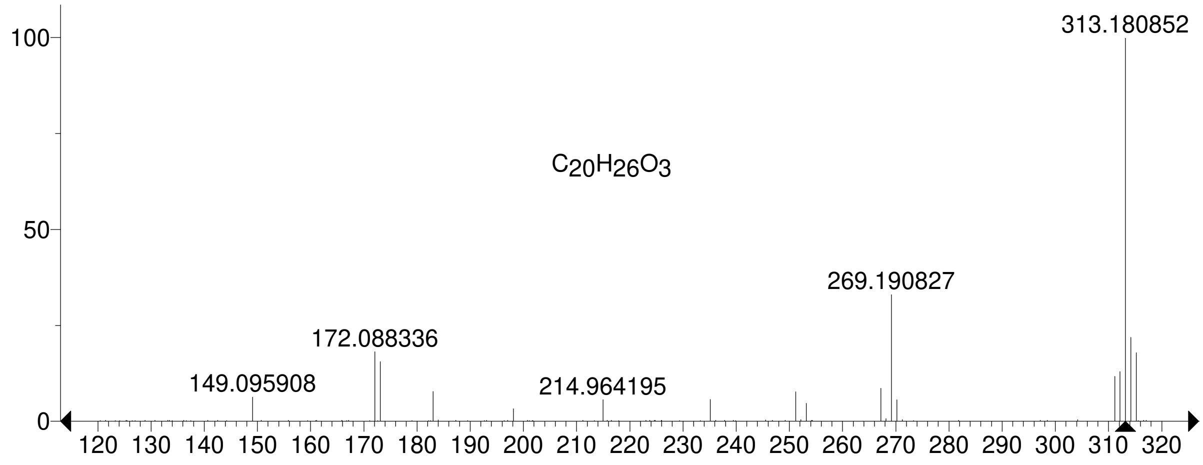

Hit 2 : Trenbolone acetate [M+H]+ HCD 15V P=313.2
C20H24O3; MF: 224; RMF: 859; Prob 28.4%; CAS: 10161-34-9; Lib: nist_msms; ID: 383931.

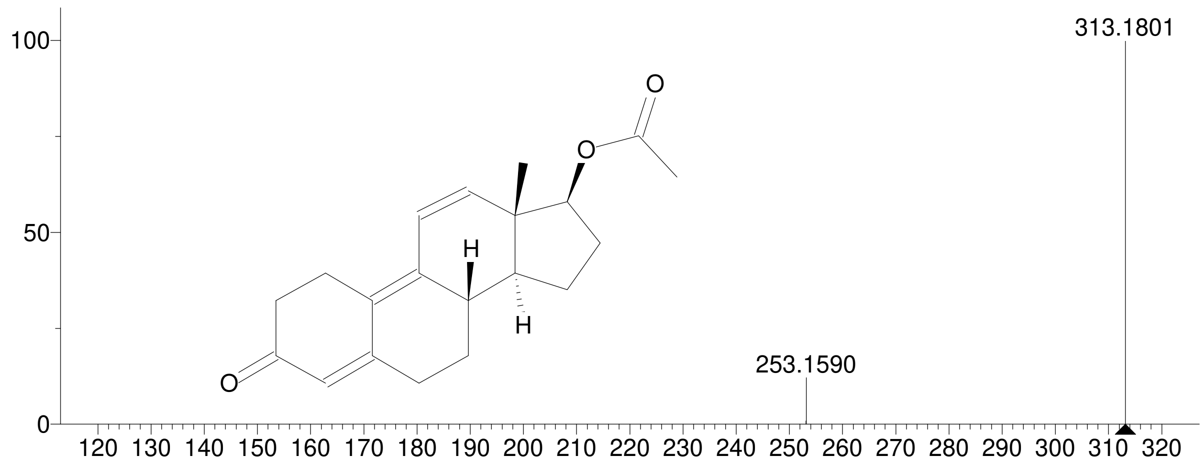

Hit 3 : Prostaglandin A3 [M-H-H2O]- HCD 9V P=313.2
C20H28O4; MF: 219; RMF: 912; Prob 22.9%; CAS: 36614-31-0; Lib: nist_msms; ID: 85161.

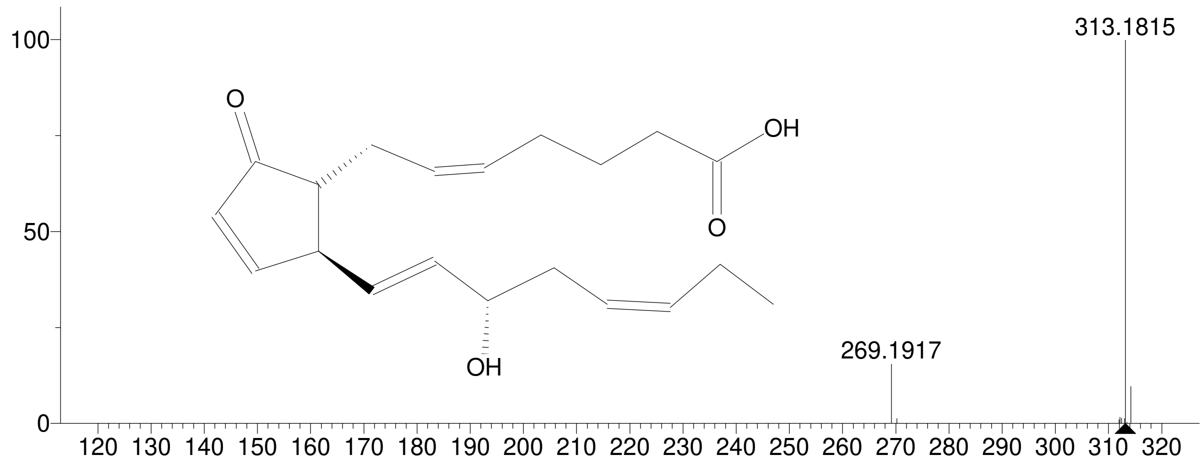

Hit 4 : all-trans-4-Ketoretinoic acid [M-H]- HCD 10V P=313.2
C20H26O3; MF: 191; RMF: 784; Prob 6.61%; CAS: 38030-57-8; Lib: nist_msms; ID: 209978.

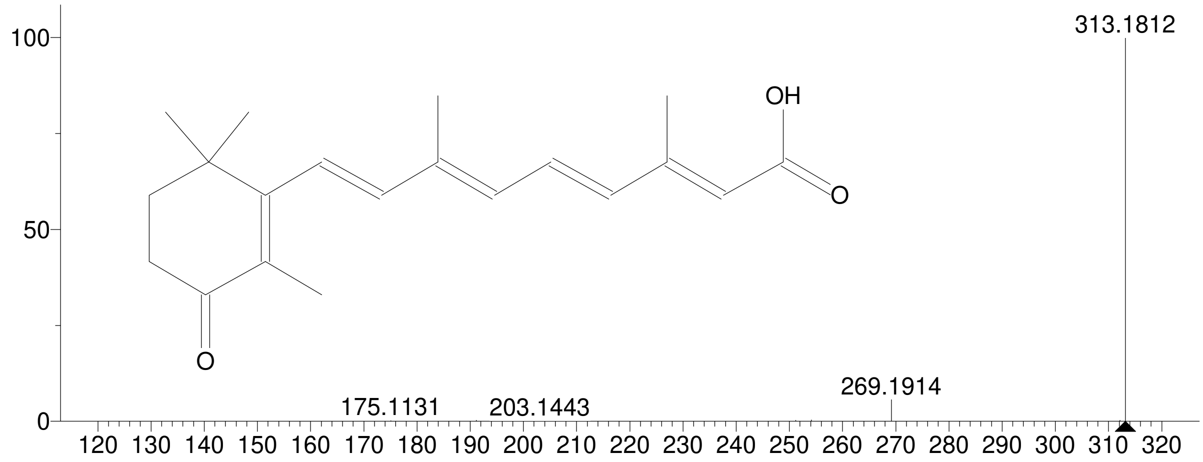

Hit 5 : Prostaglandin B3 [M-H-H2O]- HCD 6V P=313.2
C20H28O4; MF: 127; RMF: 878; Prob 1.08%; CAS: 36614-32-1; Lib: nist_msms; ID: 85344.

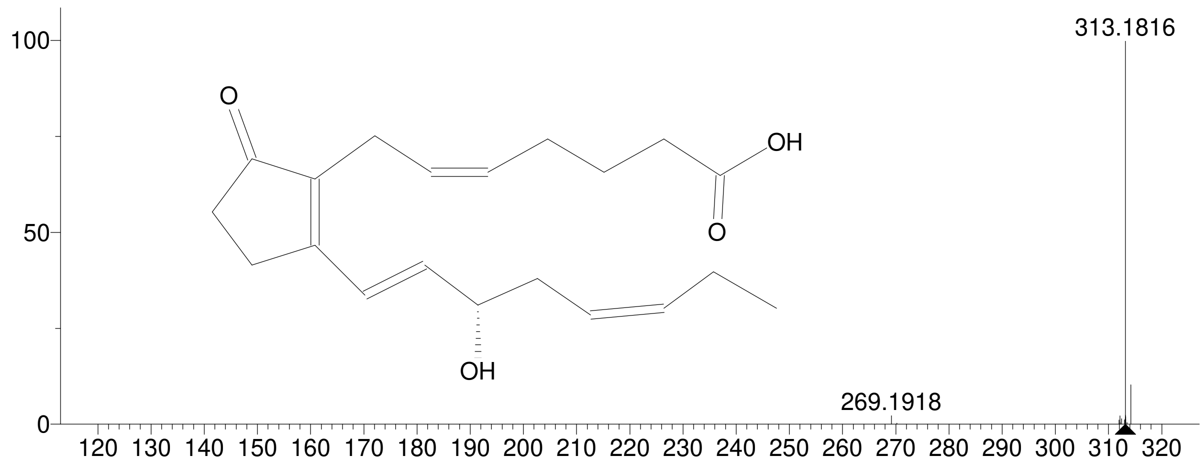

Unknown: Unknown (32.25_347.1991m/z)
Compound in Library Factor = 0

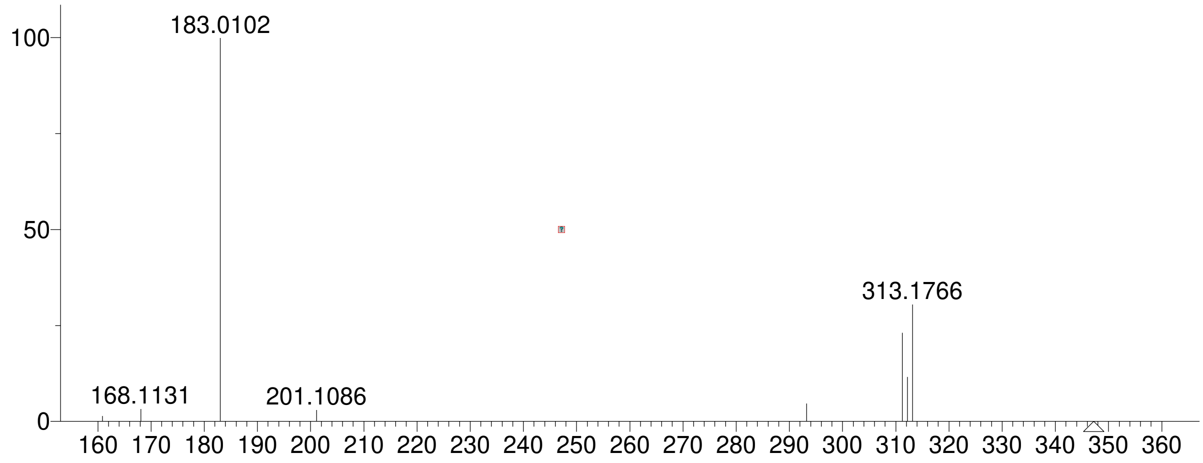

Hit 1 : Ganoderic acid H [M+H-C8H16O6]+=>347.2 IT 35% P=347.2
C32H44O9; MF: 0; RMF: 20; Prob 5.88%; CAS: 98665-19-1; Lib: nist_msms; ID: 226680.

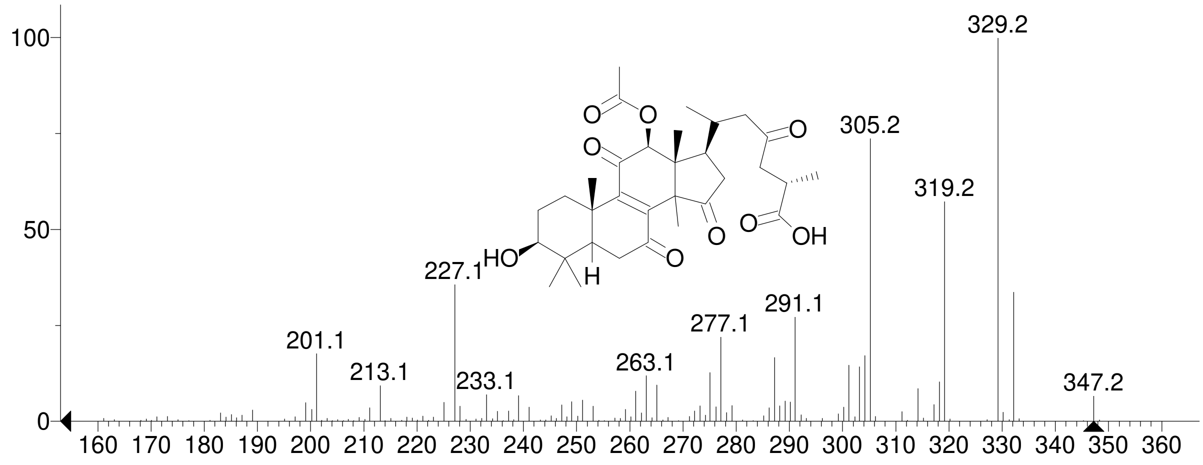

Hit 2 : D-Amygdalin [M+Na]+=>347.2 IT 35% P=347.2
C20H27NO11; MF: 0; RMF: 16; Prob 5.88%; CAS: 29883-15-6; Lib: nist_msms; ID: 162980.

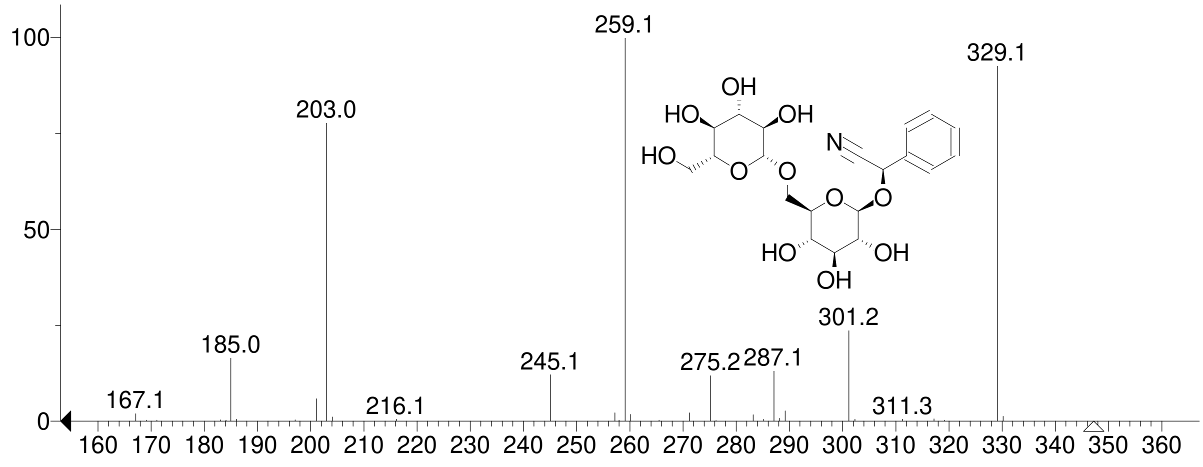

Hit 3 : Cinobufagin [M+H-H2O]+=>347.2 IT 35% P=347.2
C26H34O6; MF: 0; RMF: 13; Prob 5.88%; CAS: 470-37-1; Lib: nist_msms; ID: 234774.

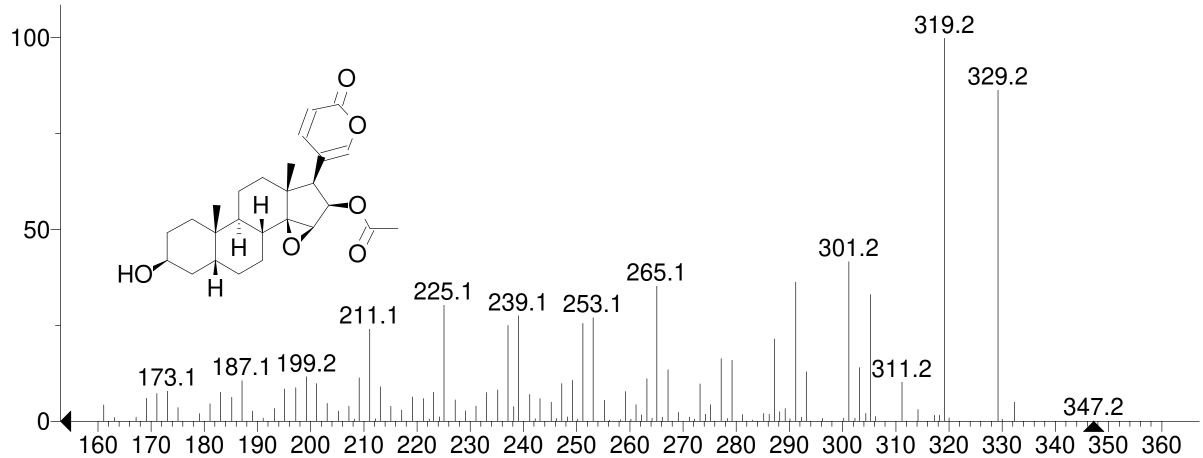

Hit 4 : Dinaciclib [M+H-H2O]+=>362.2=>347.2 IT 35% P=347.2
C21H28N6O2; MF: 0; RMF: 13; Prob 5.88%; CAS: 779353-01-4; Lib: nist_msms; ID: 342309.

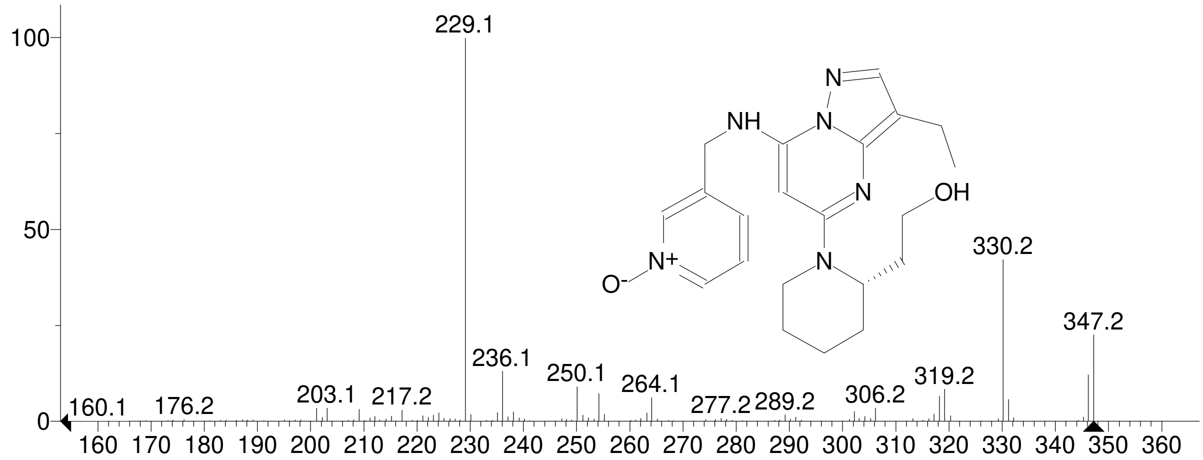

Hit 5 : Alisol B acetate [M+H-C6H14O4]+=>347.2 IT 35% P=347.2
C32H50O5; MF: 0; RMF: 11; Prob 5.88%; CAS: 26575-95-1; Lib: nist_msms; ID: 400486.

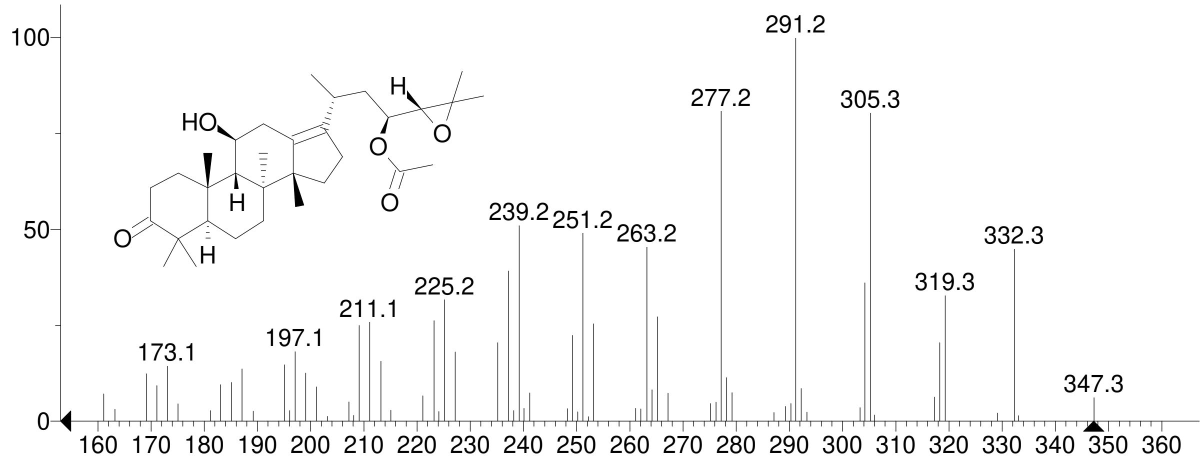

Unknown: Unknown (32.52_415.2478m/z)
Compound in Library Factor = -2387

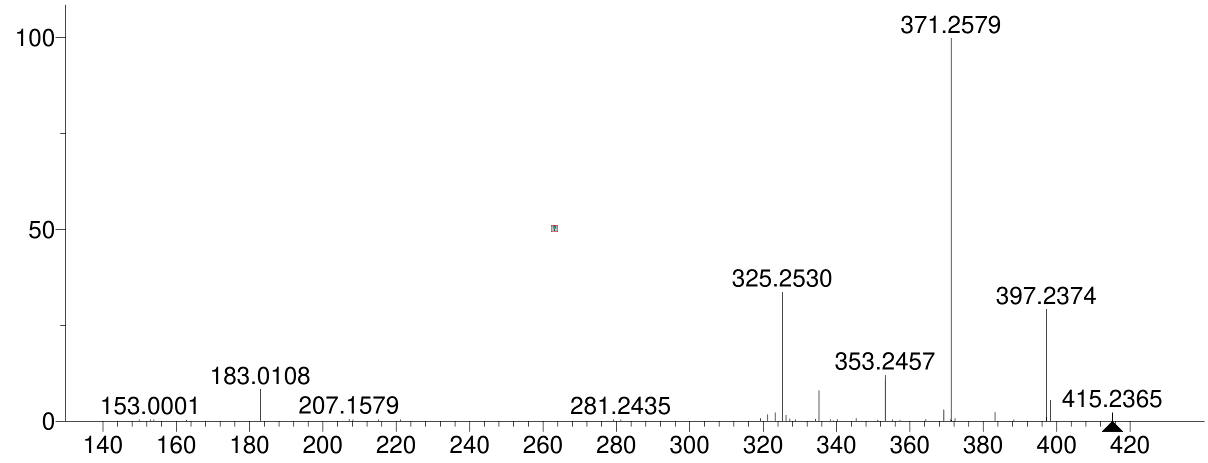

Hit 1 : 9-Oxo-11α,16R-dihydroxy-17-cyclobutyl-5Z,13E-dien-1-oic acid [M+Na]+ HCD 33V P=415.2
C23H36O5; MF: 19; RMF: 139; Prob 32.2%; CAS: 212310-16-2; Lib: nist_msms; ID: 172347.

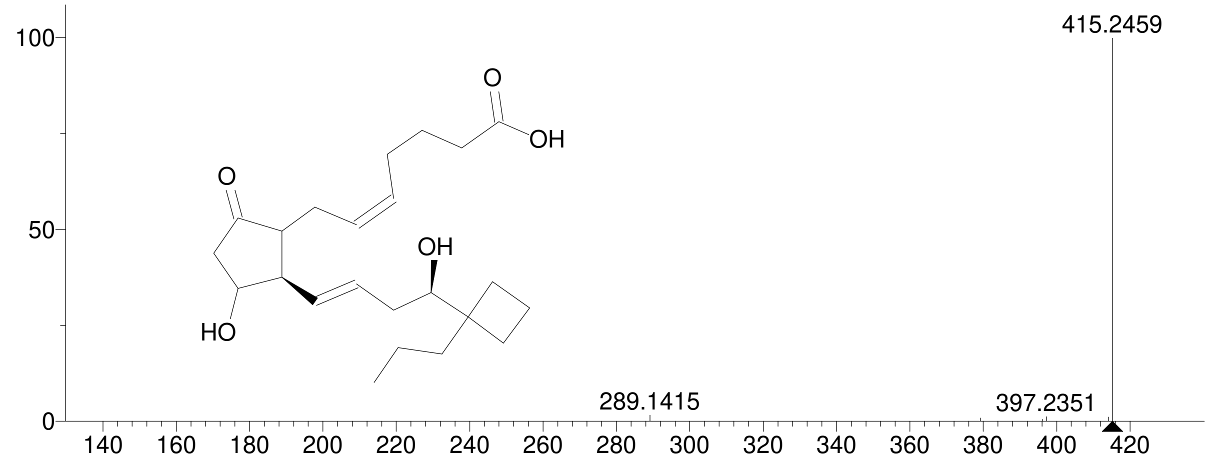

Hit 2 : 3β-Tetrahydrocortisol, 3,21-diacetate [M+H-2H2O]+ HCD 8V P=415.2
C25H38O7; MF: 3; RMF: 94; Prob 18.6%; CAS: 6820-57-1; Lib: nist_msms; ID: 454143.

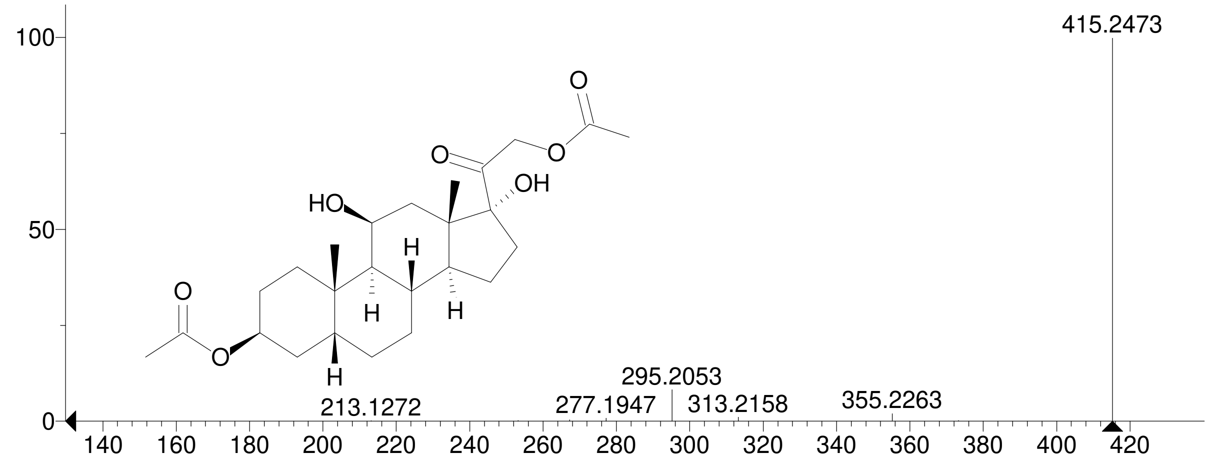

Hit 3 : 16-Phenoxytetranorprostaglandin F2α isopropyl ester [M+H-H2O]+ IT-FT 35% P=415.2
C25H36O6; MF: 0; RMF: 149; Prob 16.4%; CAS: 130209-78-8; Lib: nist_msms; ID: 545962.

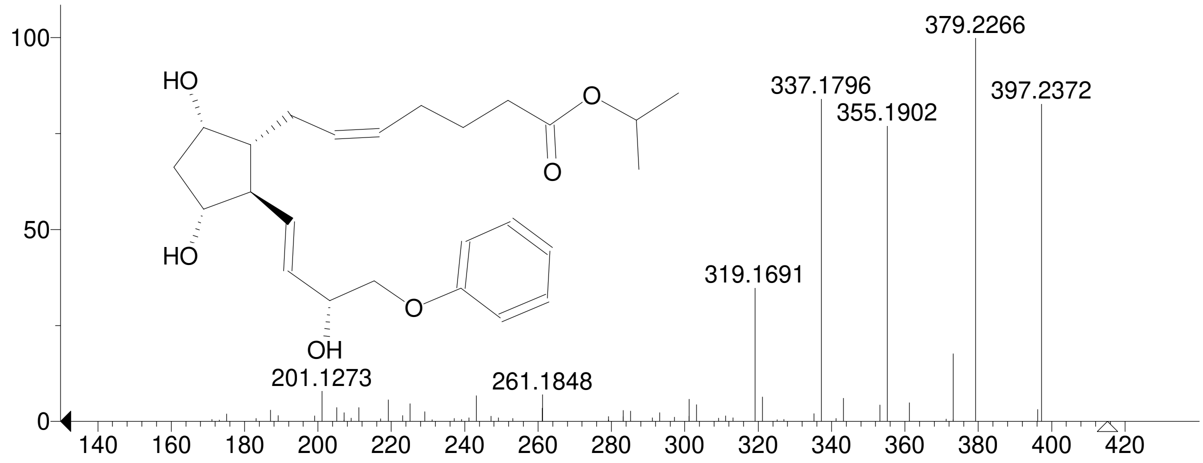

Hit 4 : Alpha-Hydroxydeoxycholic Acid [M+Na]+ 35% P=415.3
C24H40O4; MF: 0; RMF: 26; Prob 16.4%; Lib: mona-export-itree; ID: 2369.

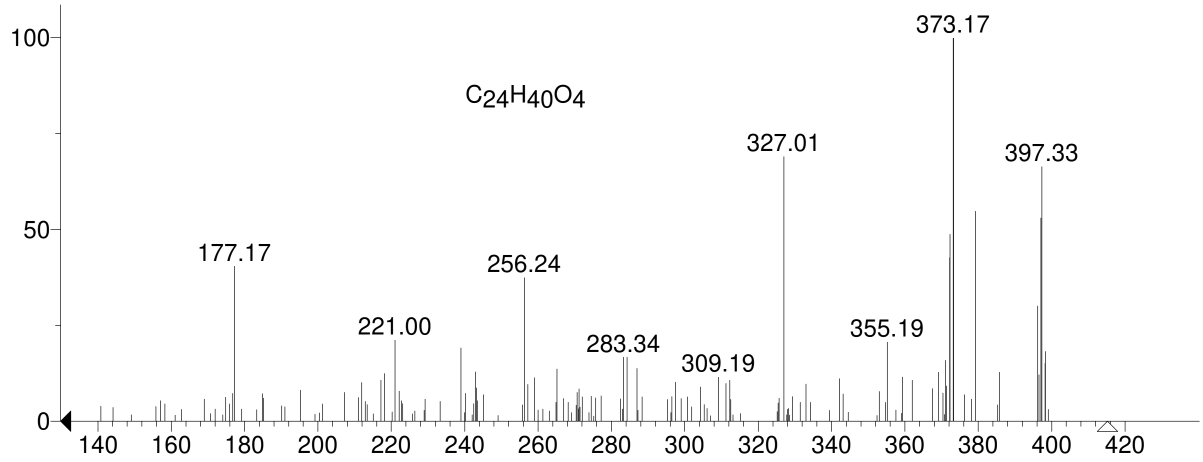

Hit 5 : NCGC00385486-01_C25H34O5_2-Butenoic acid, 2-methyl-, (2R,3R,5R)-2,6,6,9-tetramethyl-11-oxotricyclc
C25H34O5; MF: 0; RMF: 12; Prob 16.4%; Lib: mona-export-gnps; ID: 11887.

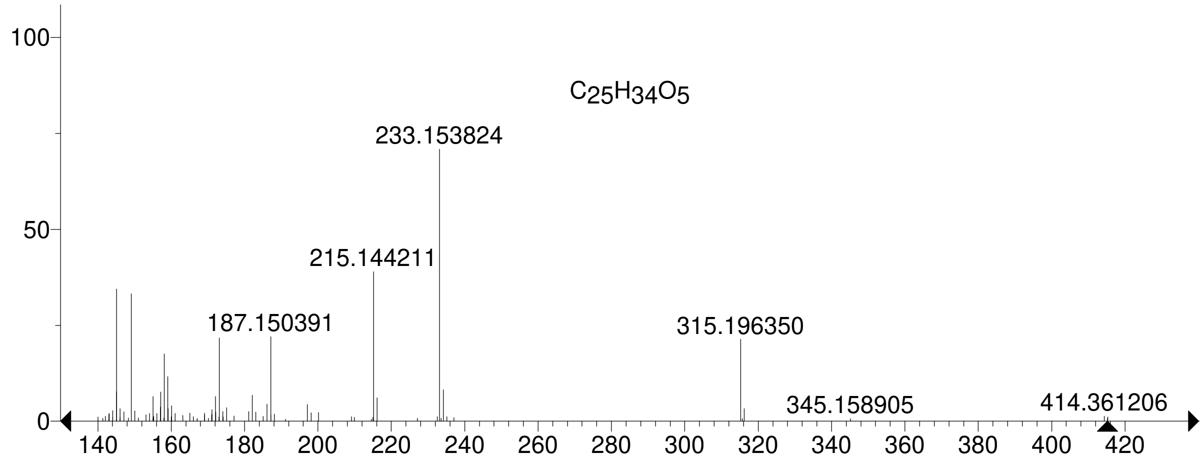

Unknown: Unknown (32.59_399.2534m/z)
Compound in Library Factor = -374

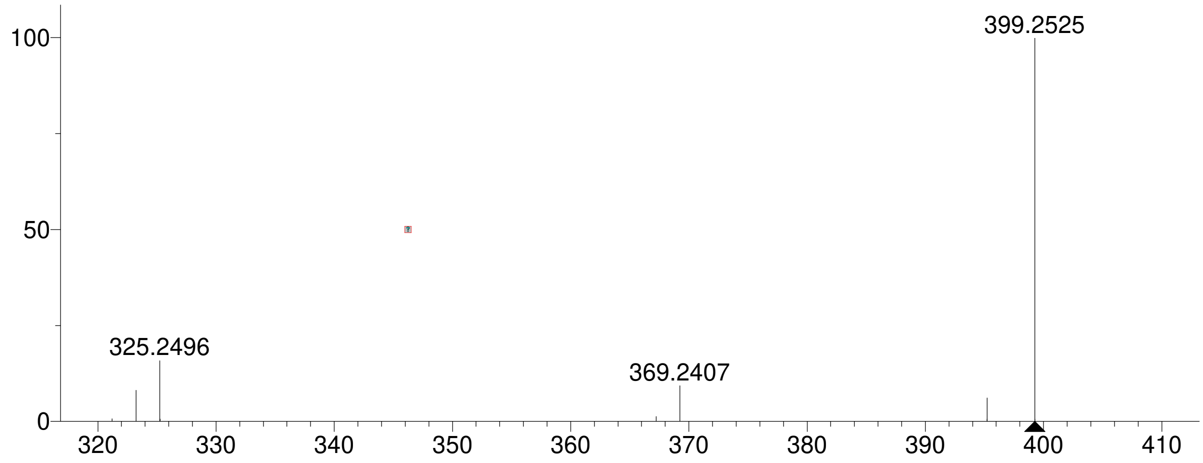

Hit 1 : Tris(2-butoxyethyl) phosphate [M+H]+ HCD 7V P=399.3
C18H39O7P; MF: 283; RMF: 837; Prob 97.2%; CAS: 78-51-3; Lib: nist_msms; ID: 100520.

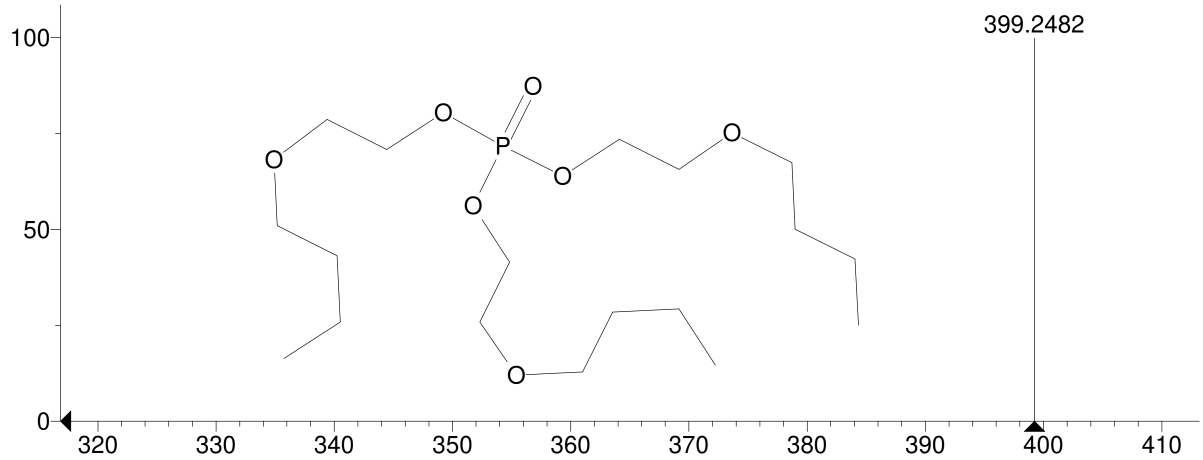

Hit 2 : Manoalide [M+H-H2O]+ HCD 15V P=399.3
C25H36O5; MF: 120; RMF: 778; Prob 2.37%; CAS: 75088-80-1; Lib: nist_msms; ID: 362758.

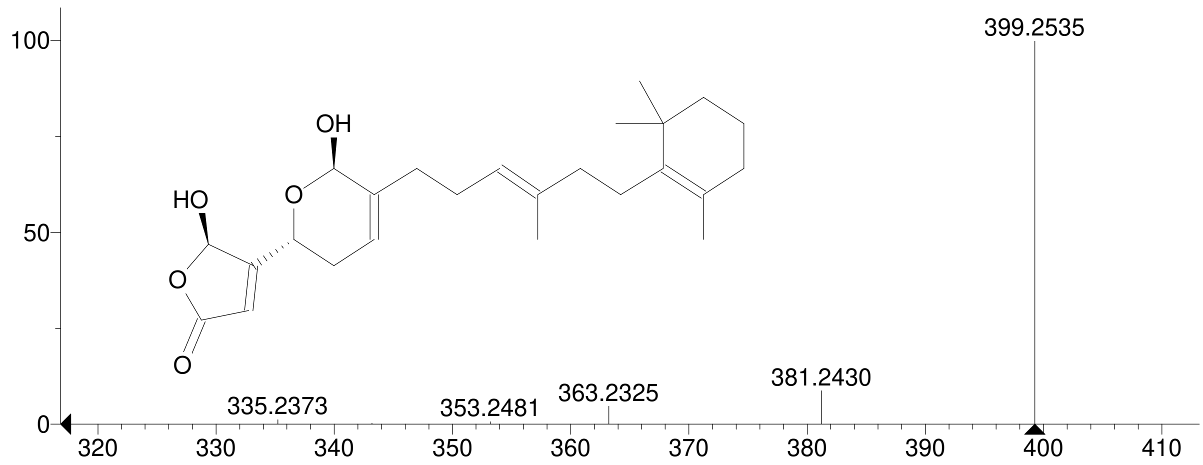

Hit 3 : NCGC00180855-02_C23H36O4_2-Pentenoic acid, 3-[(acetyloxy)methyl]-5-[(1R,4aR,8aR)-decahydro-5,5,8
C23H36O4; MF: 47; RMF: 748; Prob 0.32%; Lib: mona-export-gnps; ID: 10515.

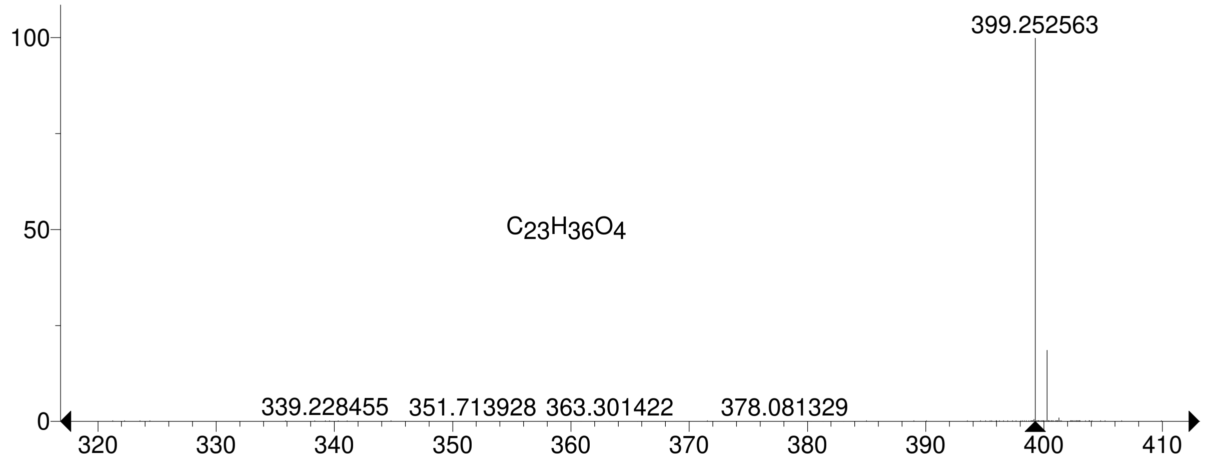

Hit 4 : FAHFA 25:6; FAHFA 22:6/3:0; [M-H]- [M-H]- P=399.3
C25H36O4; MF: 7; RMF: 182; Prob 0.07%; Lib: msdial-insilicomsms-lipids-neg; ID: 273774.

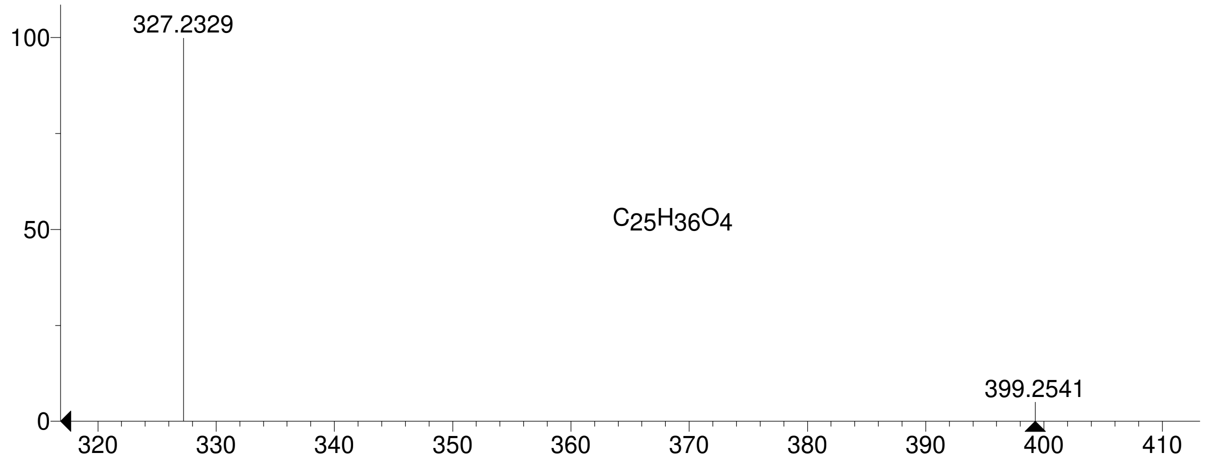

Unknown: Unknown (32.59_413.2327m/z)
Compound in Library Factor = -1072

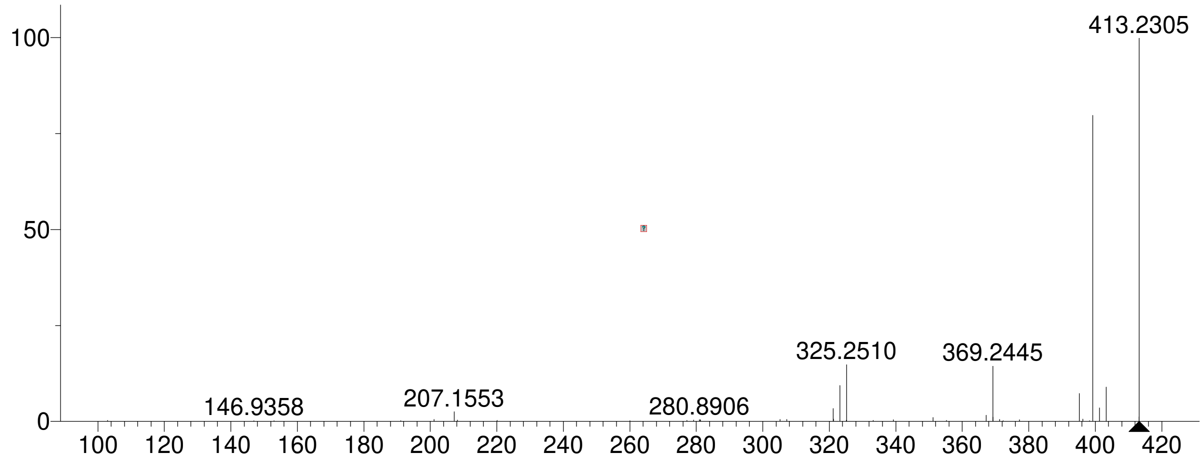

Hit 1 : Mevastatin [M+Na]+ HCD 20V P=413.2
C23H34O5; MF: 93; RMF: 610; Prob 54.8%; CAS: 73573-88-3; Lib: nist_msms; ID: 165105.

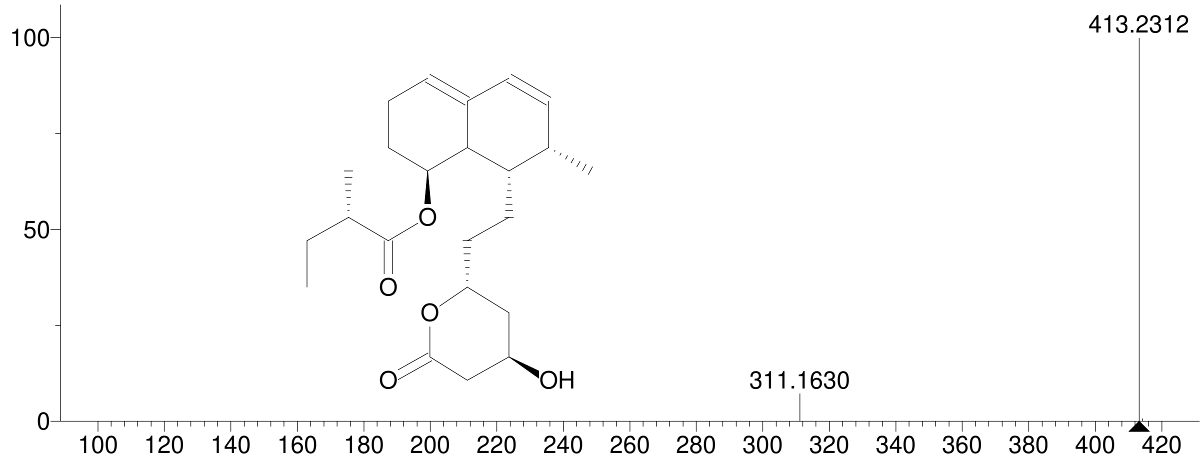

Hit 2 : 17(R)-Resolvin D1 methyl ester [M+Na]+ HCD 37V P=413.2
C23H34O5; MF: 23; RMF: 502; Prob 7.97%; CAS: 937738-64-2; Lib: nist_msms; ID: 378599.

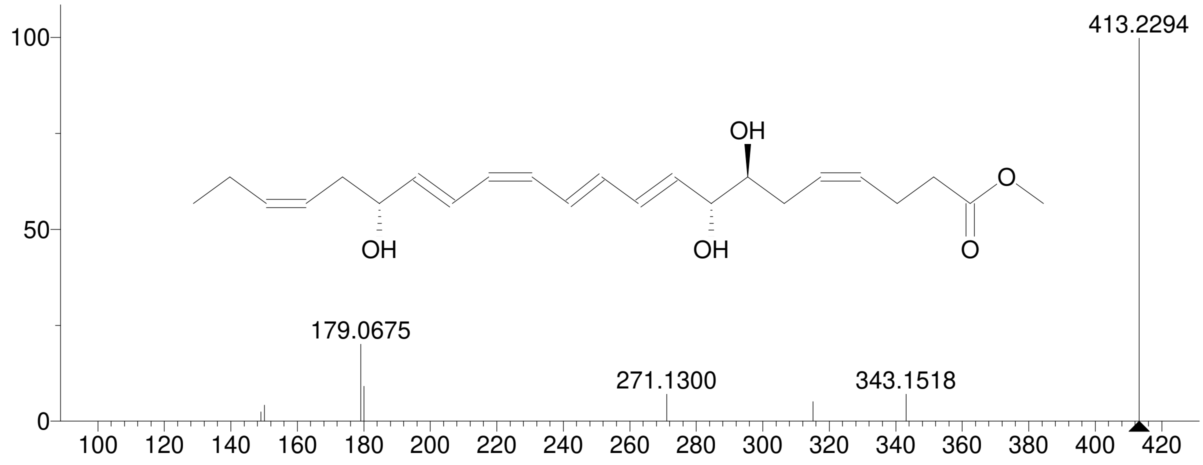

Hit 3 : Digoxigenin [M+Na]+ IT-FT 35% P=413.2
C23H34O5; MF: 19; RMF: 177; Prob 6.73%; CAS: 1672-46-4; Lib: nist_msms; ID: 308679.

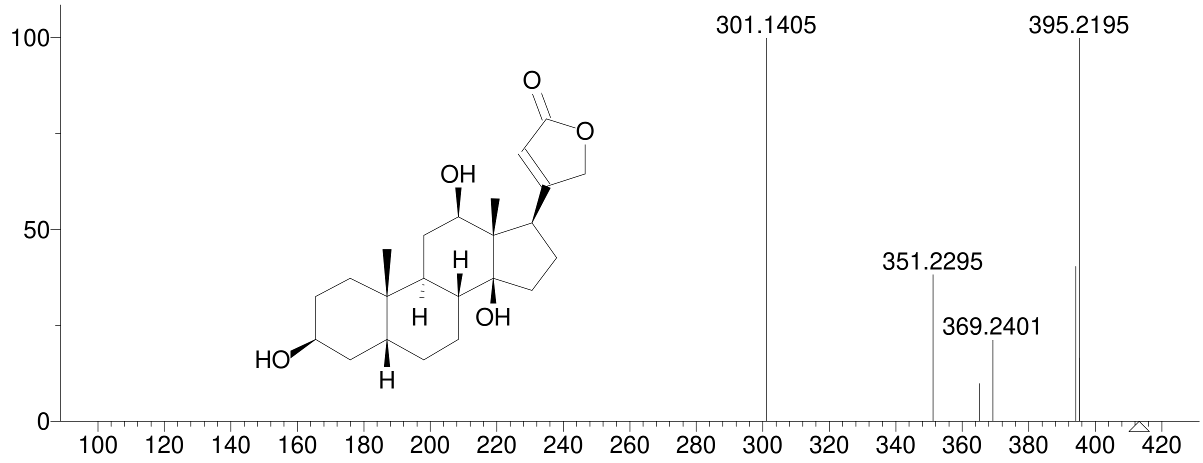

Hit 4 : Resolvin D2 methyl ester [M+Na]+ HCD 28V P=413.2
C23H34O5; MF: 18; RMF: 599; Prob 6.47%; CAS: 810668-53-2; Lib: nist_msms; ID: 378653.

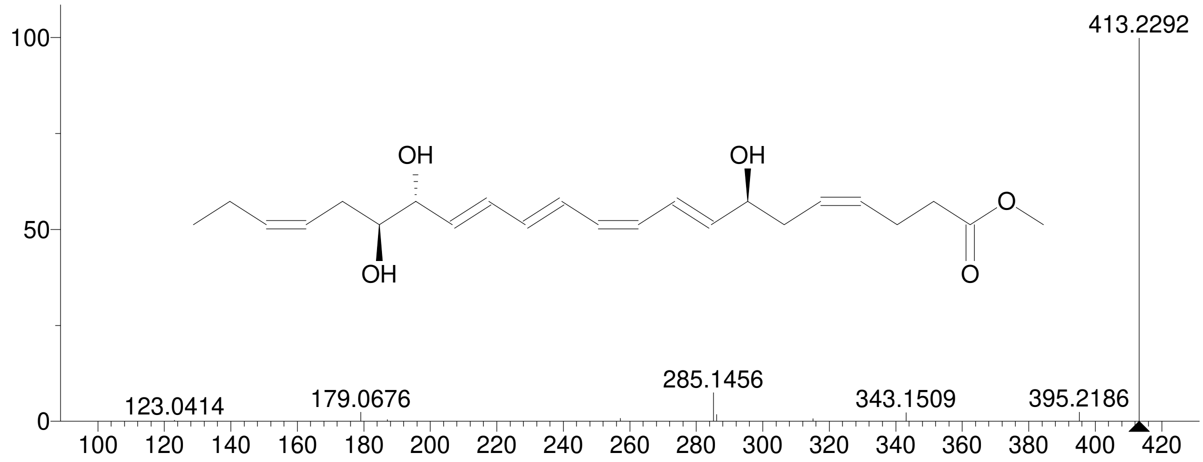

Hit 5 : Treprostinil [M+Na]+ IT-FT 35% P=413.2
C23H34O5; MF: 7; RMF: 151; Prob 4.44%; CAS: 81846-19-7; Lib: nist_msms; ID: 540012.

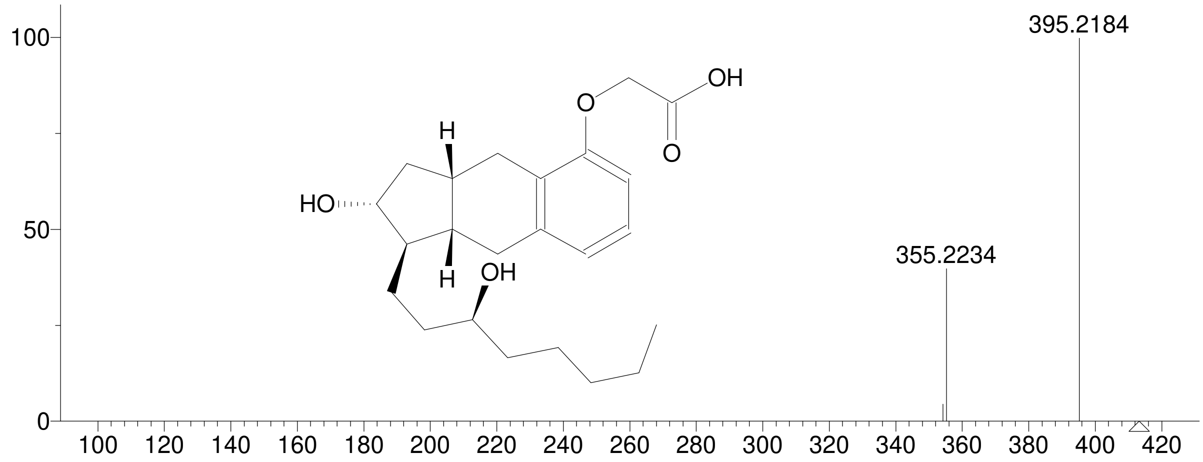

Unknown: Unknown (32.70_399.2531m/z)
Compound in Library Factor = -472

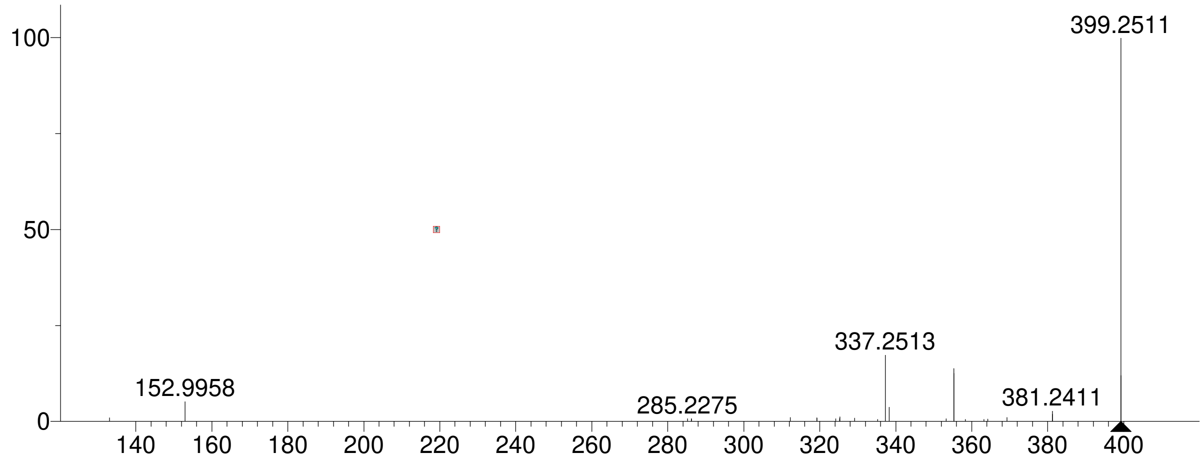

Hit 1 : Tris(2-butoxyethyl) phosphate [M+H]+ HCD 7V P=399.3
C18H39O7P; MF: 160; RMF: 738; Prob 93.7%; CAS: 78-51-3; Lib: nist_msms; ID: 100520.

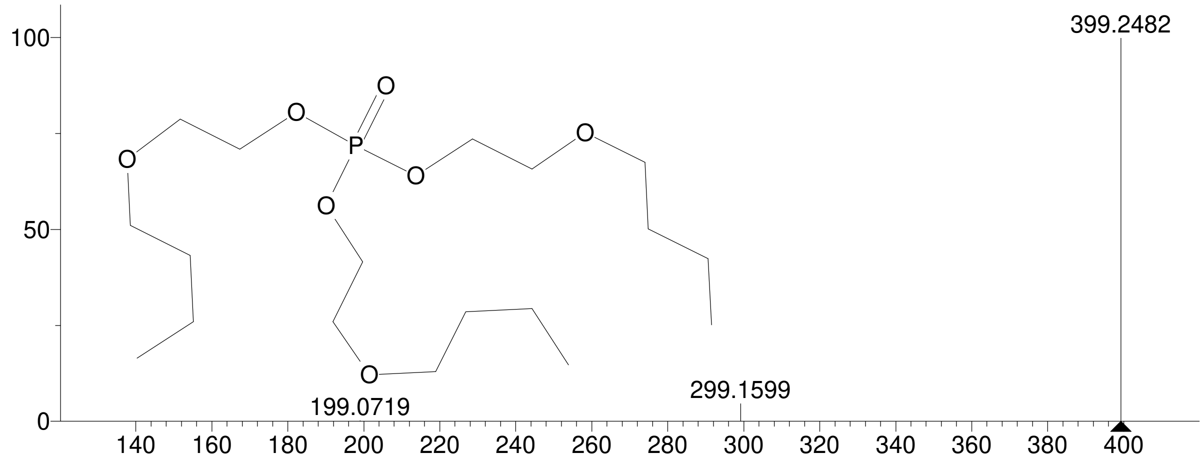

Hit 2 : Manoalide [M+H-H2O]+ HCD 15V P=399.3
C25H36O5; MF: 27; RMF: 670; Prob 3.69%; CAS: 75088-80-1; Lib: nist_msms; ID: 362758.

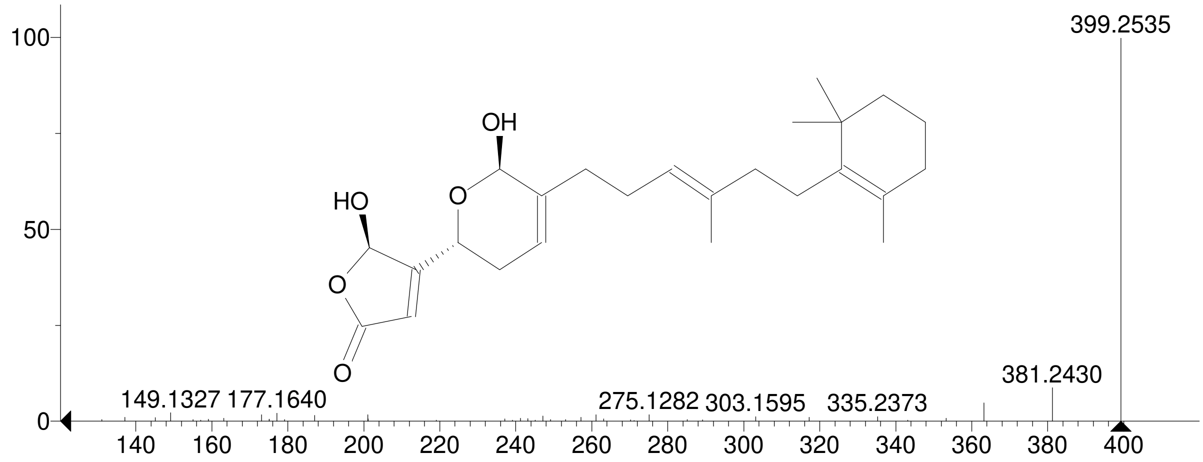

Hit 3 : FAHFA 25:6; FAHFA 22:6/3:0; [M-H]- [M-H]- P=399.3
C25H36O4; MF: 5; RMF: 162; Prob 1.45%; Lib: msdial-insilicomsms-lipids-neg; ID: 273774.

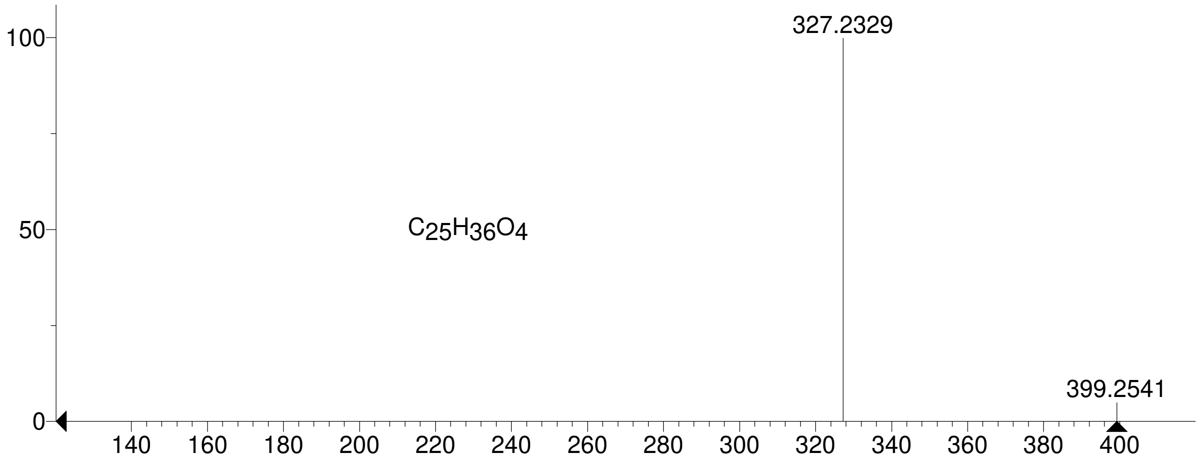

Hit 4 : NCGC00180855-02_C23H36O4_2-Pentenoic acid, 3-[(acetyloxy)methyl]-5-[(1R,4aR,8aR)-decahydro-5,5,8
C23H36O4; MF: 0; RMF: 647; Prob 1.17%; Lib: mona-export-gnps; ID: 10515.

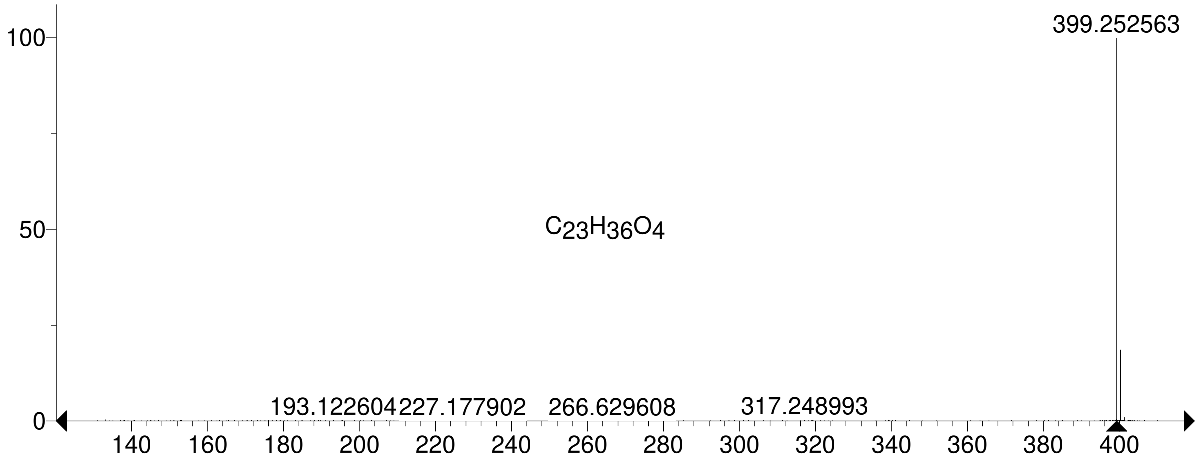

Unknown: Unknown (4.00_609.1559m/z)
Compound in Library Factor = -2176

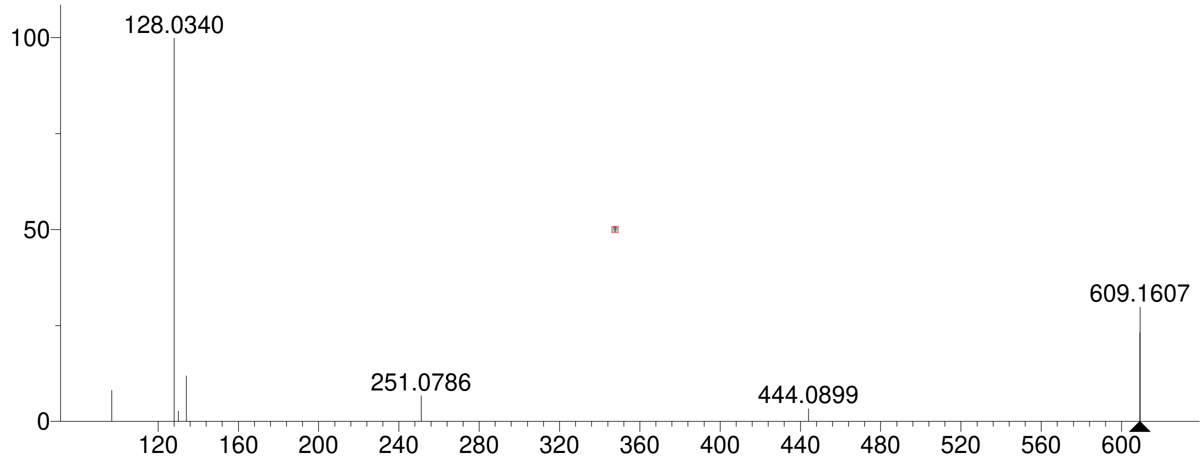

Hit 1 : Amarogentin [M+Na]+ HCD 15V P=609.2
C29H30O13; MF: 47; RMF: 601; Prob 64.7%; CAS: 21018-84-8; Lib: nist_msms; ID: 176811.

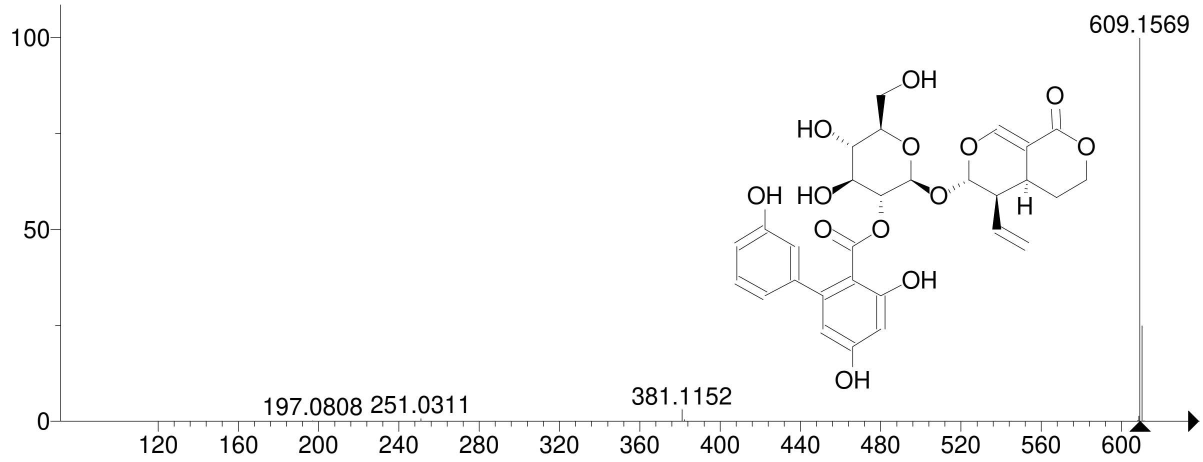

Hit 2 : Luteolin-7,3'-di-O-glucoside [M-H]- 20 P=609.2
C27H30O16; MF: 22; RMF: 485; Prob 19.7%; Lib: mona-export-lc-ms-ms_negative_mode; ID: 11885.

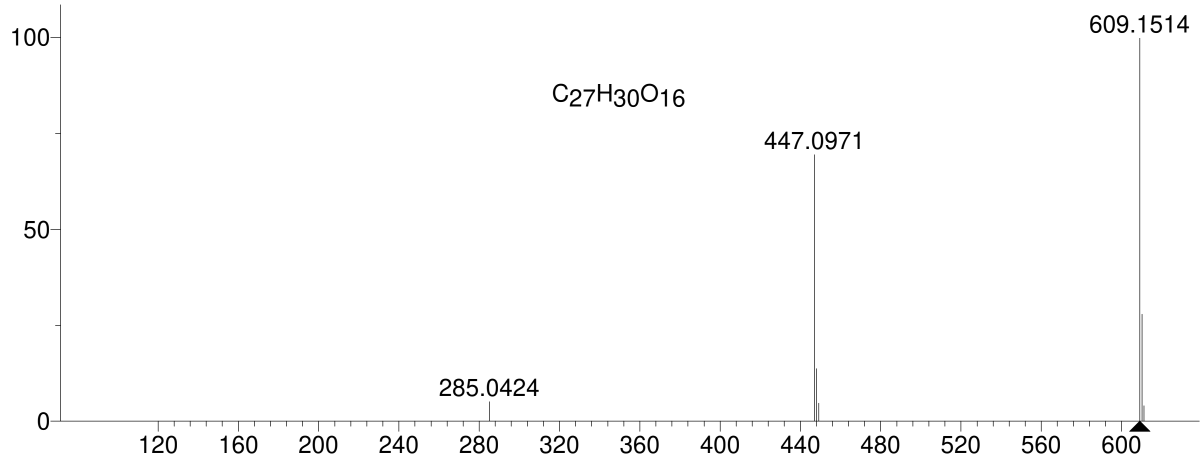

Hit 3 : Quercetin 3-O-neohesperidoside [M-H]- P=609.2
C27H30O16; MF: 0; RMF: 26; Prob 7.79%; Lib: mona-export-gnps; ID: 14858.

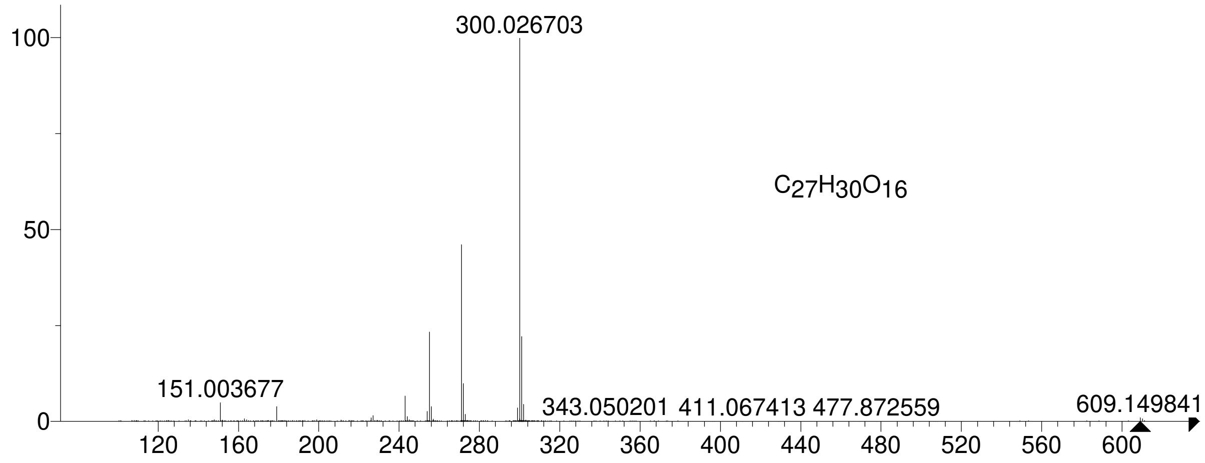

Hit 4 : Kaempferol 3-gentiobioside [M-H]- P=609.2
C27H30O16; MF: 0; RMF: 22; Prob 7.79%; Lib: mona-export-gnps; ID: 15033.

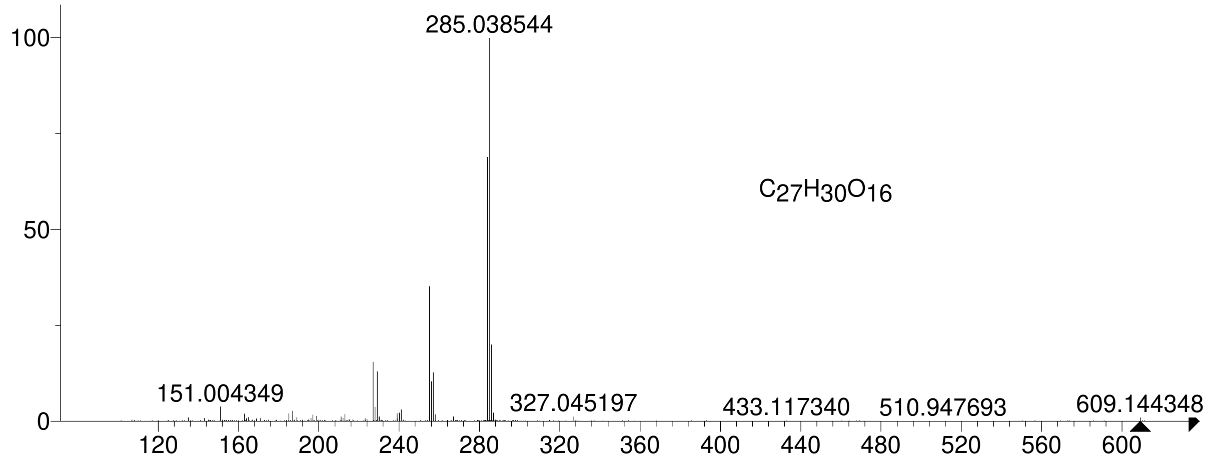

Unknown: Unknown (4.01_488.1964m/z)
Compound in Library Factor = 0

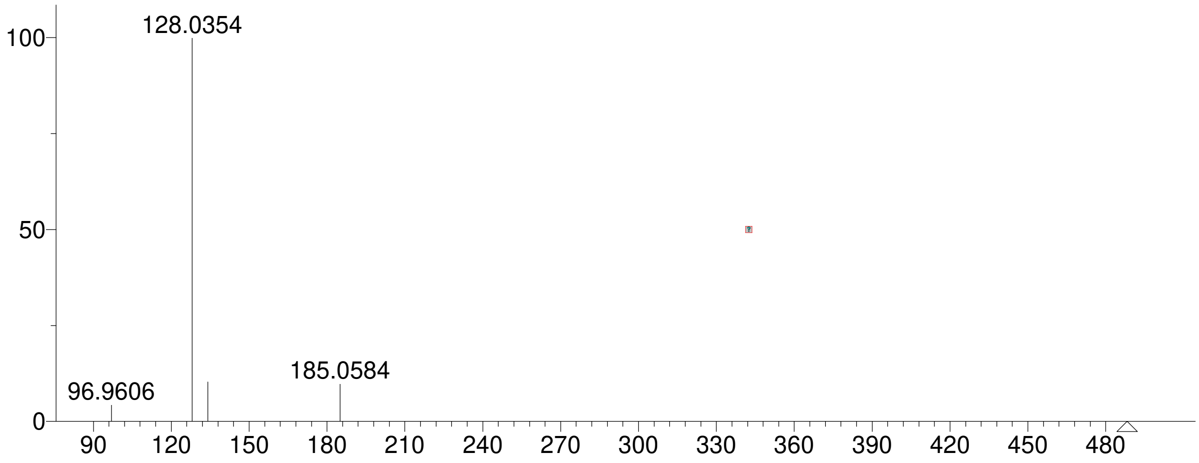

Unknown: Unknown (4.02_570.1346m/z)
Compound in Library Factor = 0

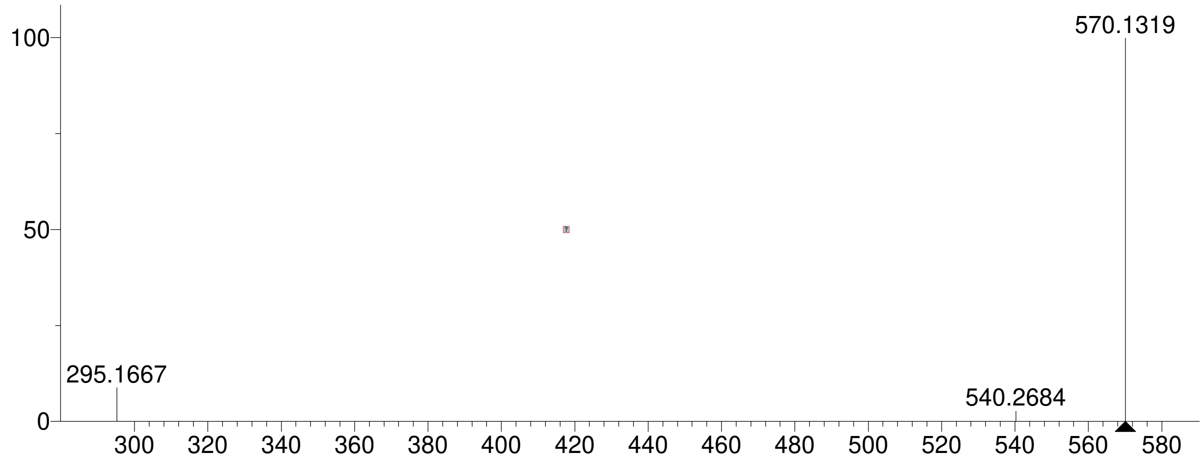

** Search Report Page 1 of 1 **

Unknown: Unknown (4.03_359.1544m/z)
Compound in Library Factor = 0

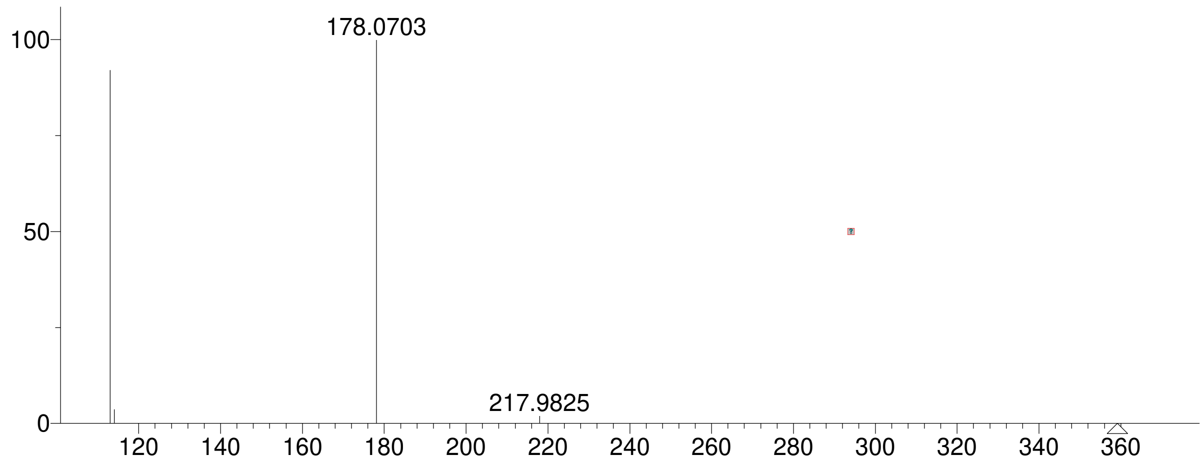

Hit 1 : Ala-Gly-Asp-Val [M-H]- HCD 57eV P=359.2
C14H24N4O7; MF: 0; RMF: 97; Prob 100.0%; Lib: nist_msms2; ID: 72487.

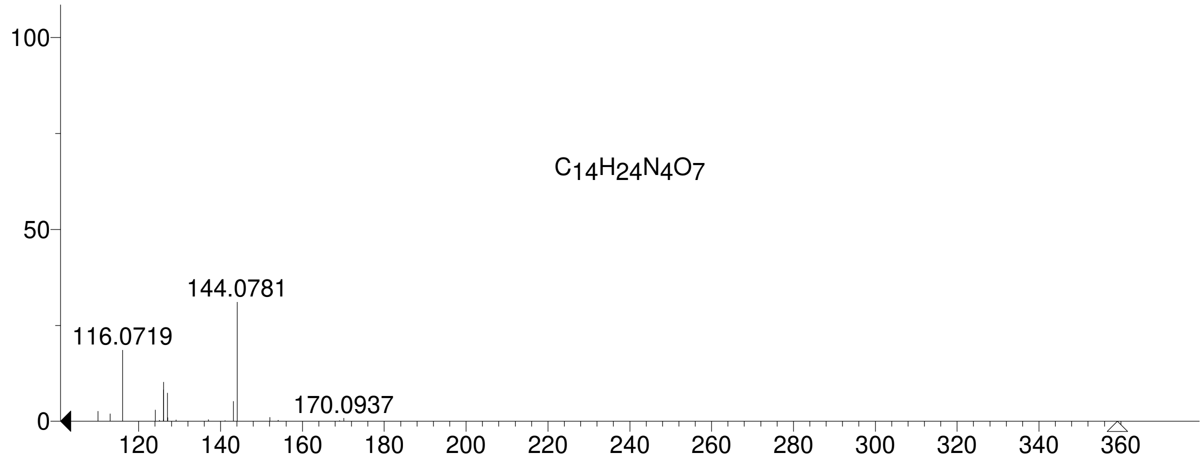

Unknown: Unknown (4.03_674.2536m/z)
Compound in Library Factor = 0

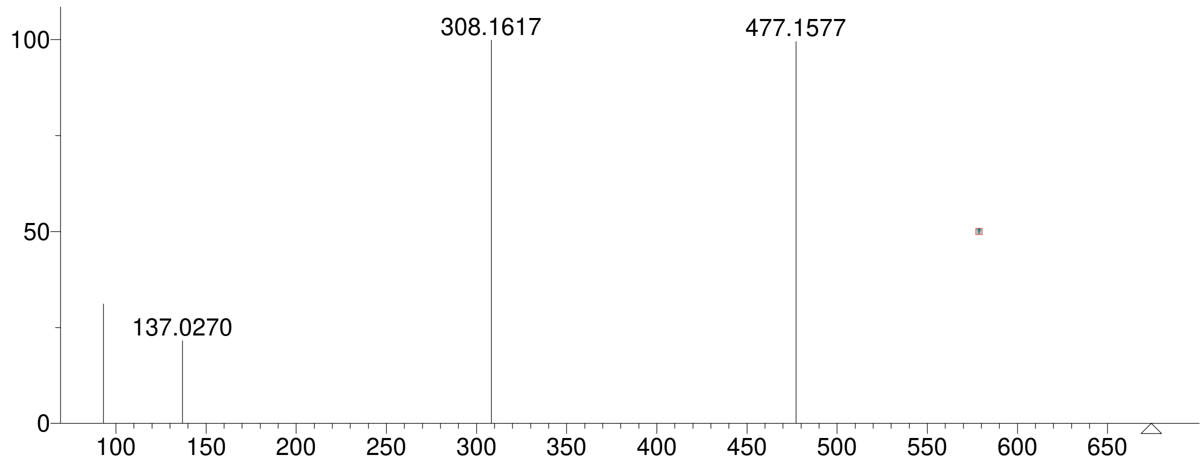

Unknown: Unknown (4.03_693.2946m/z)
Compound in Library Factor = -3273

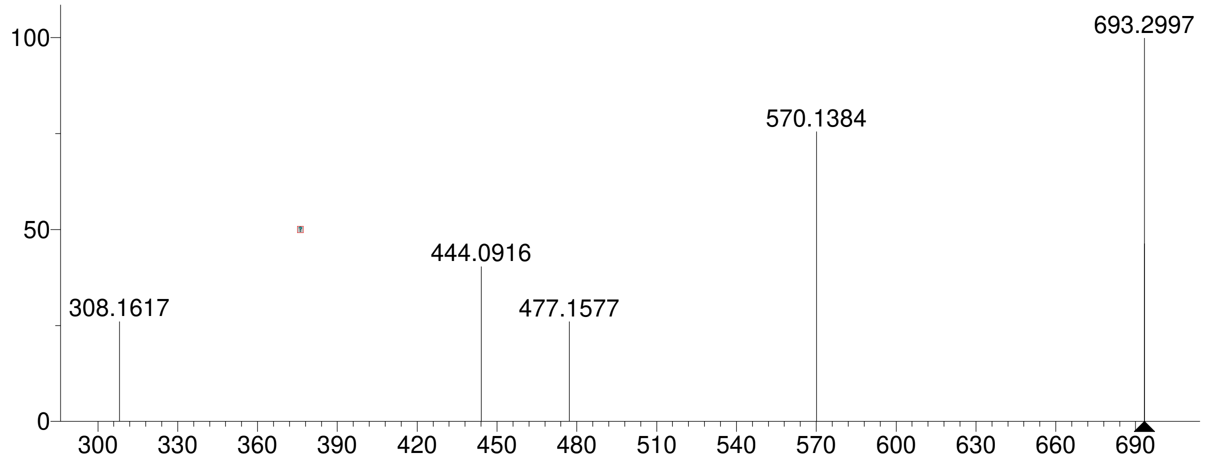

Hit 1 : Strictosidine hexoside ([H][C@@]1(C[C@]2([H])C(=CO[C@@]([H])(O[C@]3([H])O[C@]([H])(CO[C@]4([H])
C33H44N2O14; MF: 8; RMF: 433; Prob 57.3%; Lib: alkaloids; ID: 27.

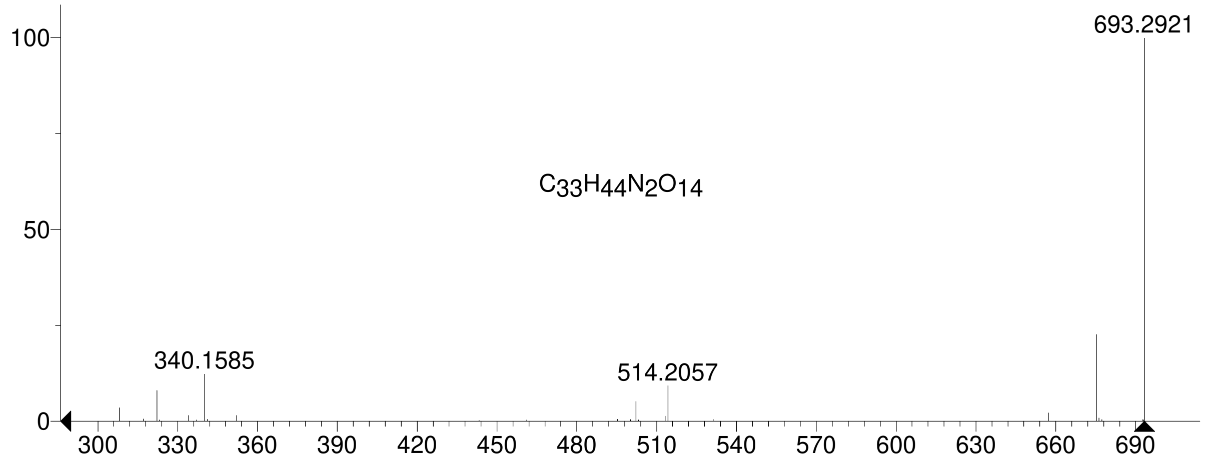

Hit 2 : "7_,13_,17-O-triacetoxy-5_-O-benzoyloxy-7,13,17-O-triacetyl-5-O-benzoyl-3-O-propanoyl-14-oxopremyrsir
C36H46O12; MF: 0; RMF: 183; Prob 42.7%; Lib: mona-export-gnps; ID: 5962.

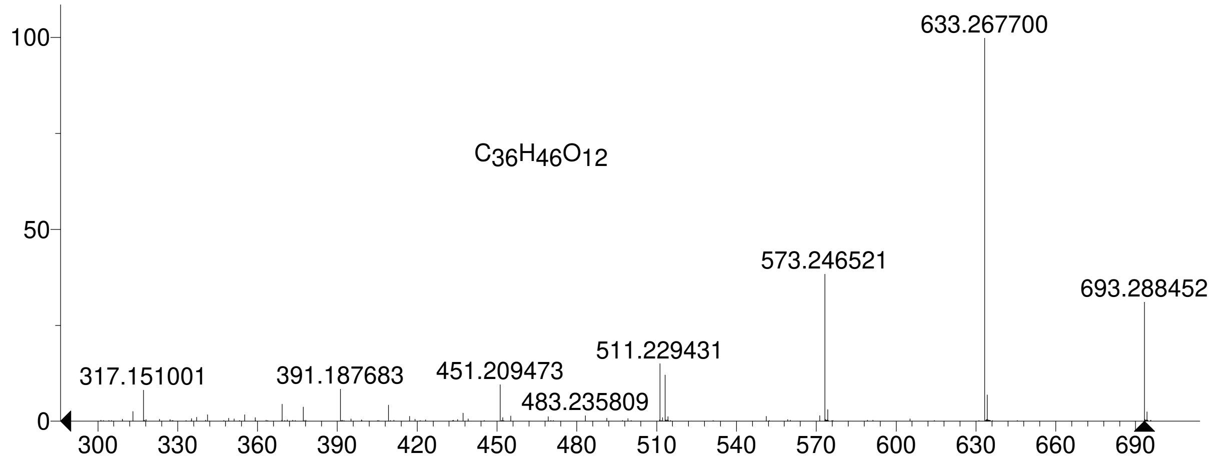

** Search Report Page 1 of 1 **

Unknown: Unknown (4.05_482.1502m/z)
Compound in Library Factor = 0

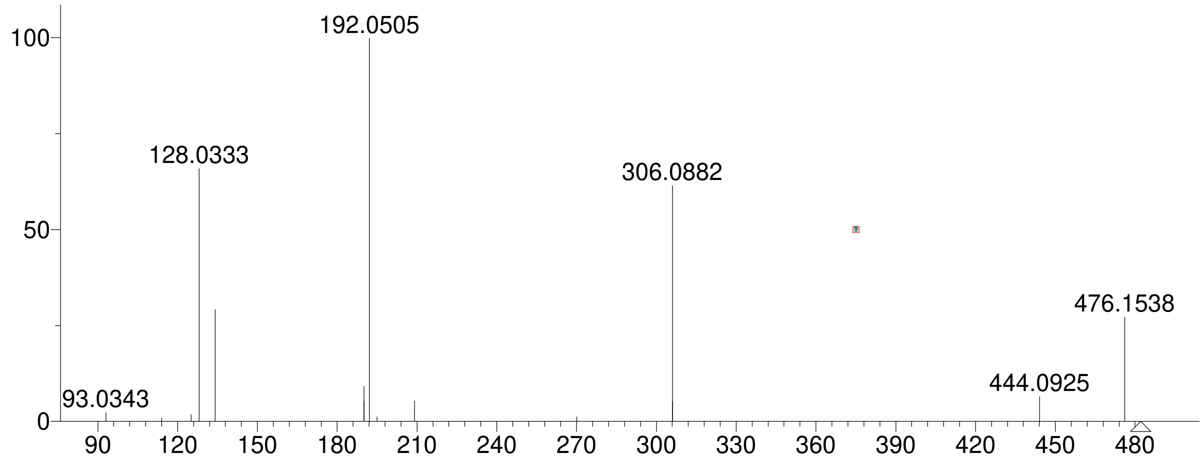

Unknown: Unknown (4.05_502.1993m/z)
Compound in Library Factor = 0

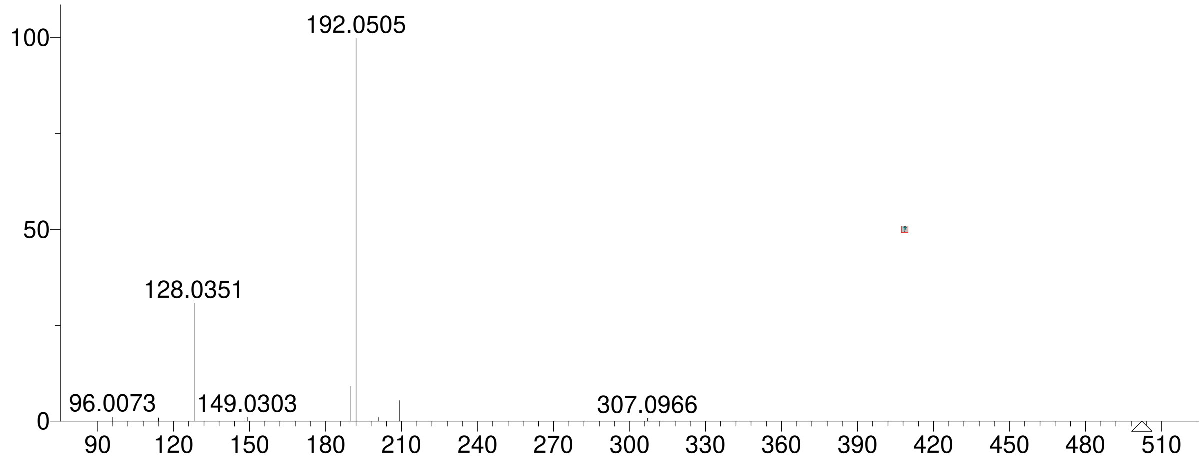

Hit 1 : Irinotecan [M+H-C5H11N]+ HCD 160V P=502.2
C33H38N4O6; MF: 0; RMF: 54; Prob 100.0%; CAS: 97682-44-5; Lib: nist_msms; ID: 218338.

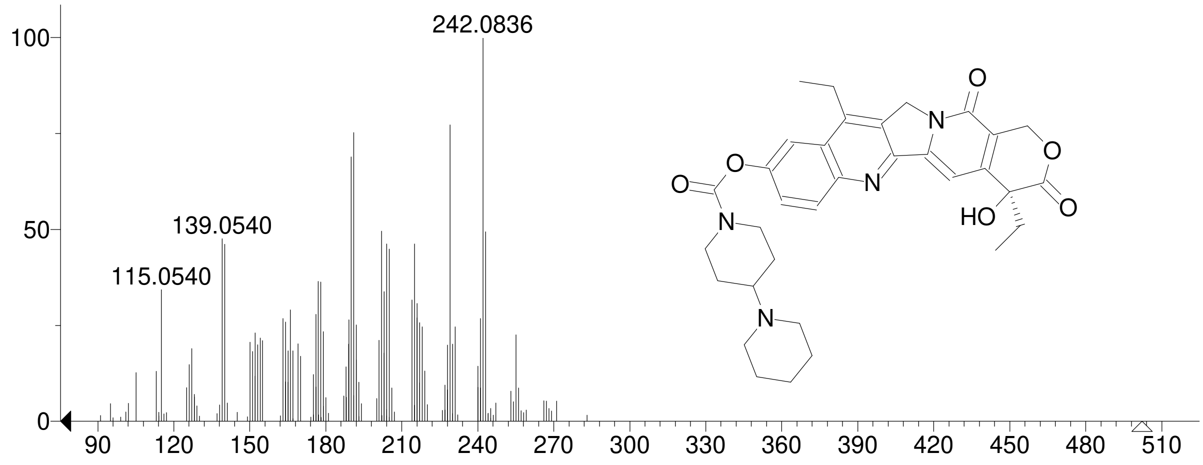

Unknown: Unknown (4.05_625.1502m/z)
Compound in Library Factor = 0

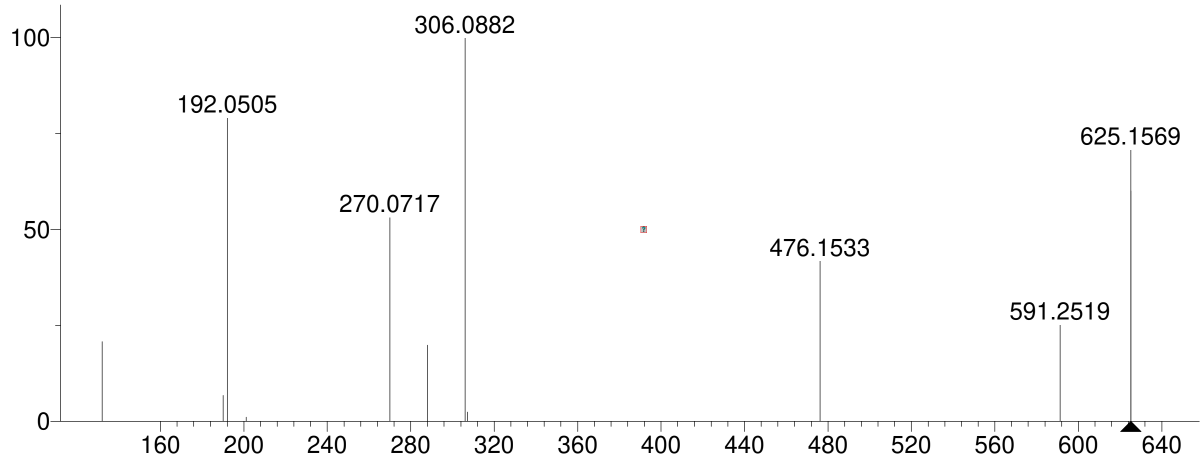

Hit 1 : 3-O-beta-(6''-trans-caffeoyl)-galactopyranosyl quercetin [M-H]- P=625.1
C30H26O15; MF: 0; RMF: 40; Prob 100.0%; Lib: mona-export-gnps; ID: 3005.

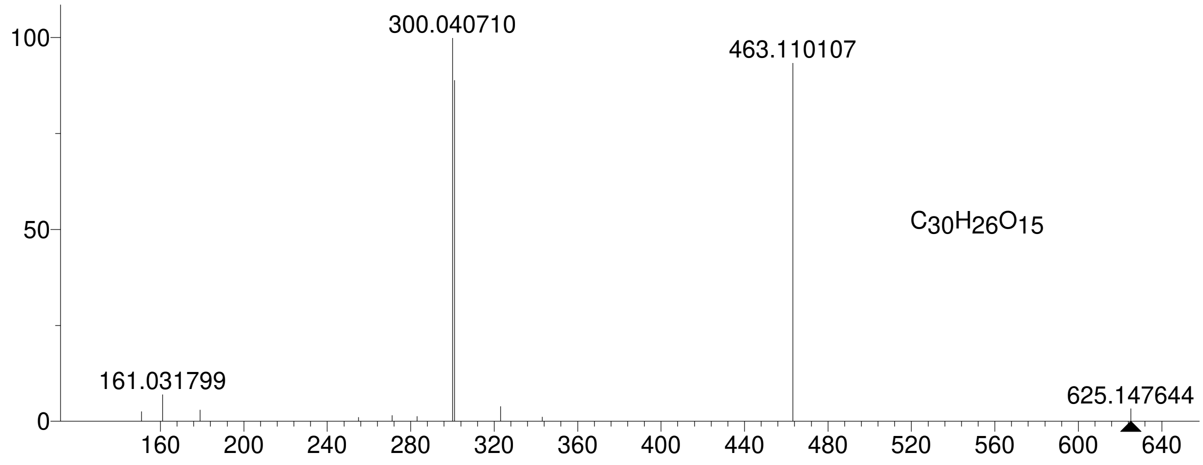

Unknown: Unknown (4.06_431.1293m/z)
Compound in Library Factor = 0

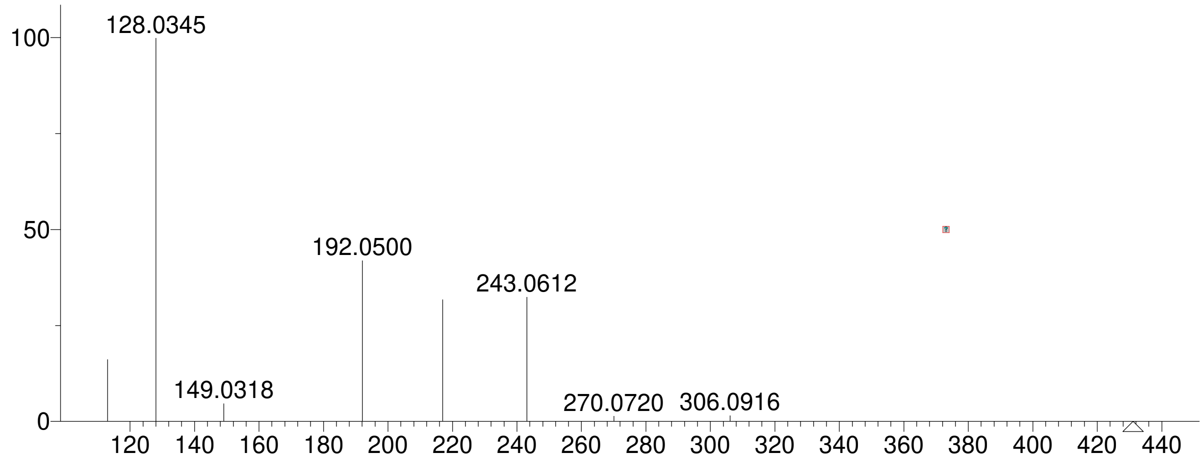

Hit 1 : NCGC00169054-02!3-(4-methoxyphenyl)-7-[(2S,3R,4S,5S,6R)-3,4,5-trihydroxy-6-(hydroxymethyl)oxan-2-y
C22H22O9; MF: 0; RMF: 29; Prob 33.3%; Lib: mona-export-gnps; ID: 9246.

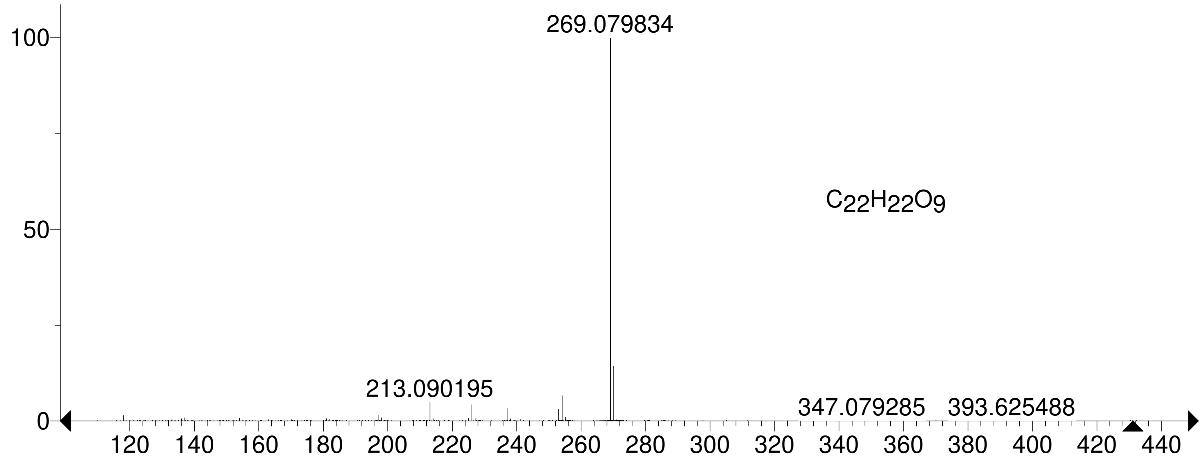

Hit 2 : O-Desmethylgefitinib [M-H]- HCD 68V P=431.1
C21H22ClFN4O3; MF: 0; RMF: 20; Prob 33.3%; CAS: 847949-49-9; Lib: nist_msms; ID: 451781.

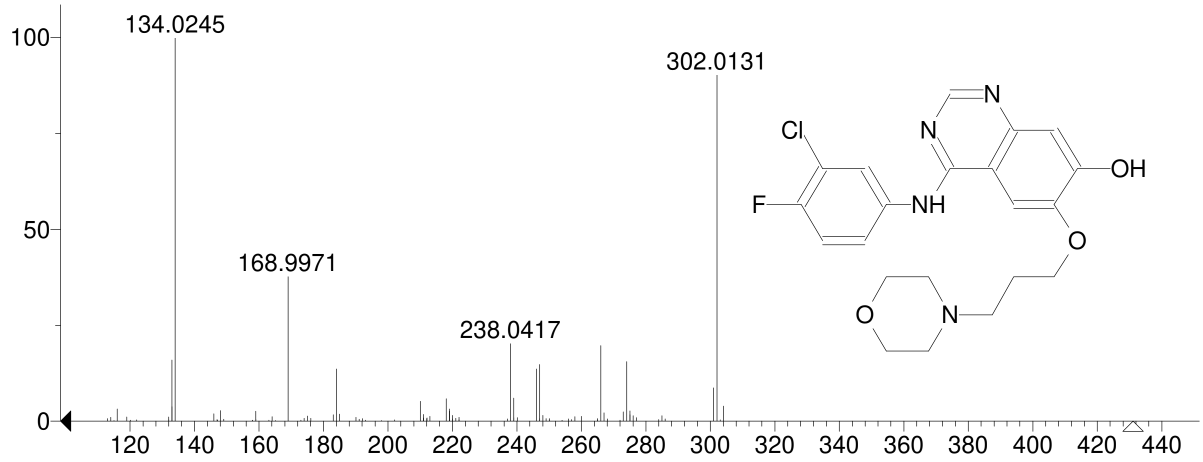

Hit 3 : (1R,3R,6R,8S,10R,13S,16S,17R)-8-tert-butyl-6,17-dihydroxy-16-methyl-2,4,14,19-tetraoxahexacyclo[8.7.2
C20H24O9; MF: 0; RMF: 6; Prob 33.3%; Lib: mona-export-vf-npl_qexactive; ID: 6278.

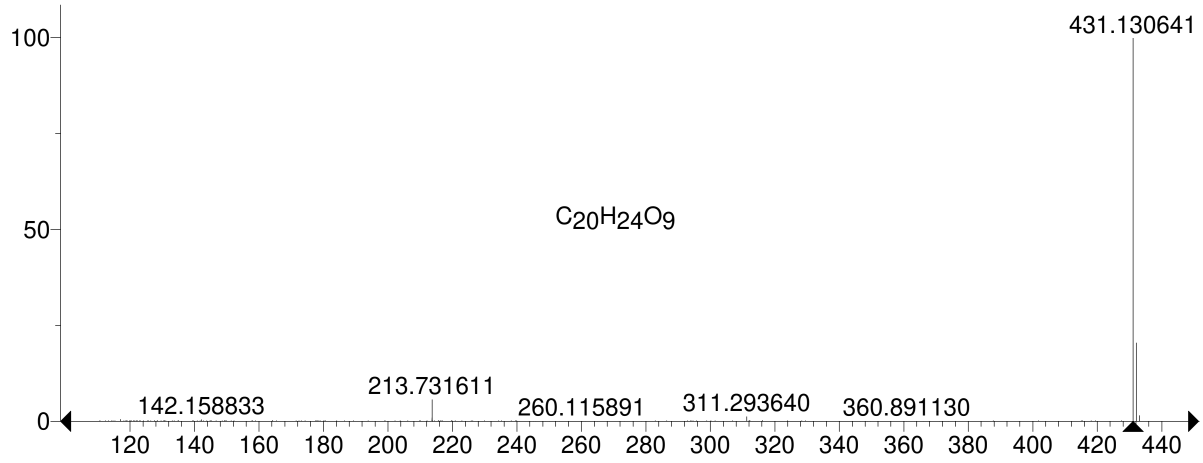

Unknown: Unknown (4.06_476.1504m/z)
Compound in Library Factor = 0

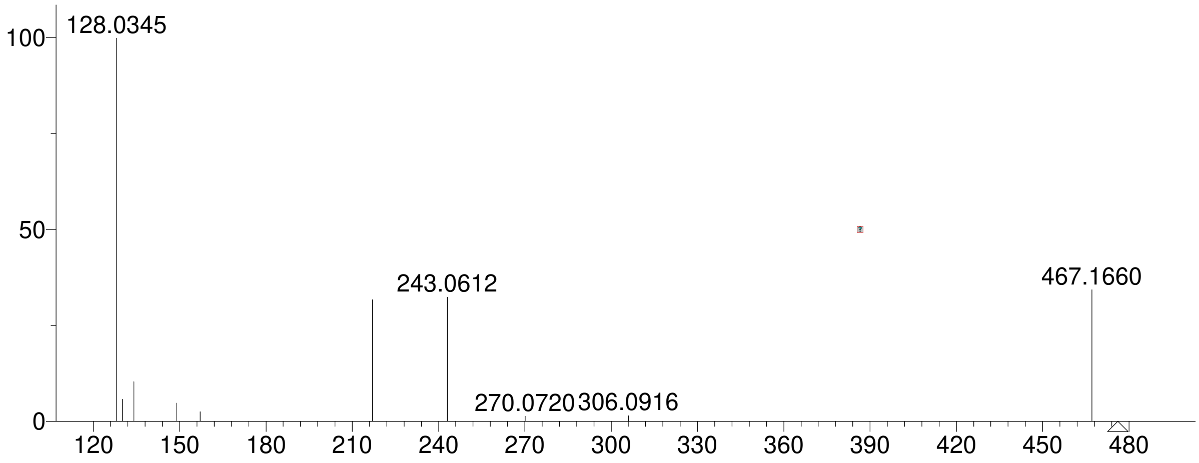

** Search Report Page 1 of 1 **

Unknown: Unknown (4.07_646.2092m/z)
Compound in Library Factor = 0

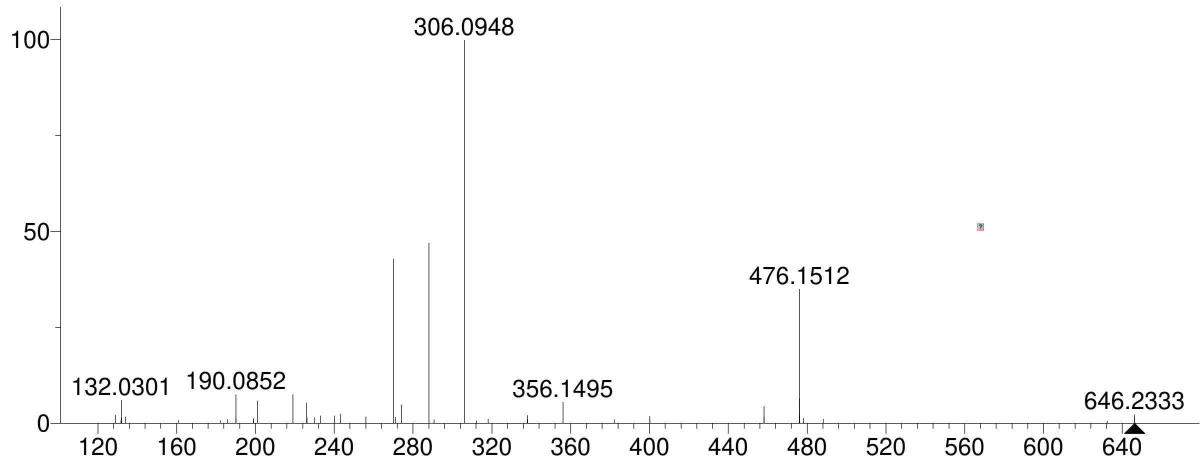

Unknown: Unknown (4.08_342.1293m/z)
Compound in Library Factor = -3273

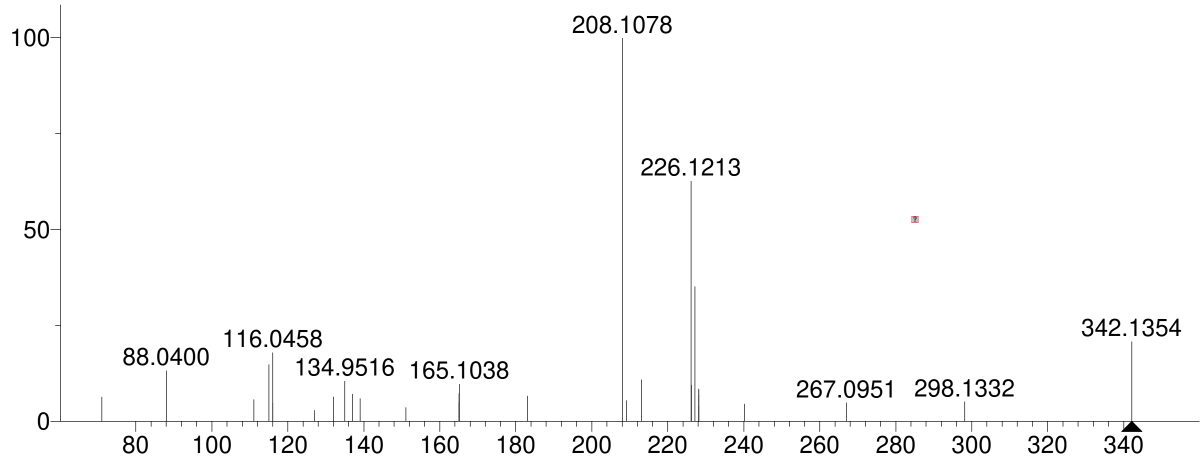

Hit 1 : Rabeprazole [M+H-H2O]+ HCD 18V P=342.1
C18H21N3O3S; MF: 4; RMF: 189; Prob 26.8%; CAS: 117976-89-3; Lib: nist_msms; ID: 208758.

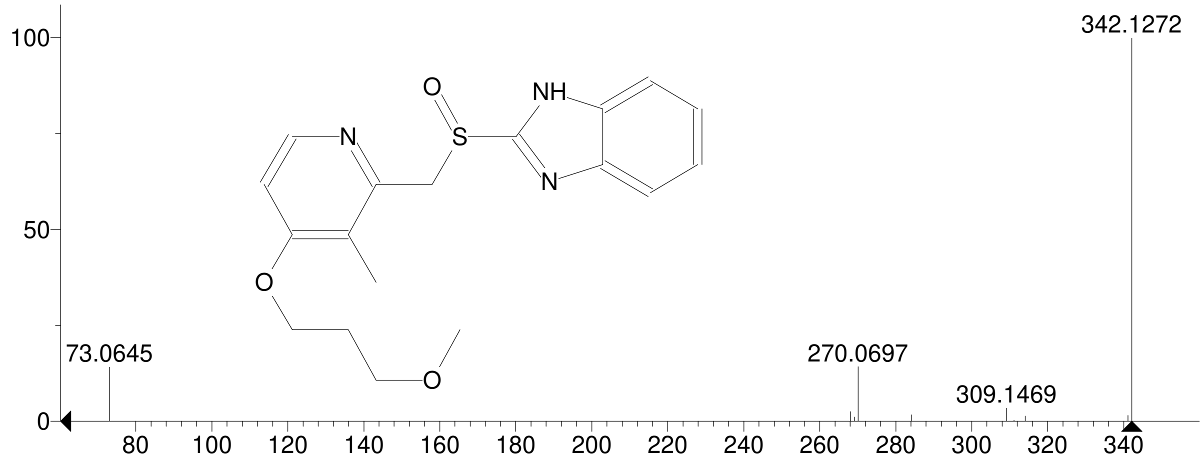

Hit 2 : Clethodim [M+H-H2O]+ HCD 17V P=342.1
C17H26ClNO3S; MF: 3; RMF: 159; Prob 25.7%; CAS: 99129-21-2; Lib: nist_msms; ID: 491505.

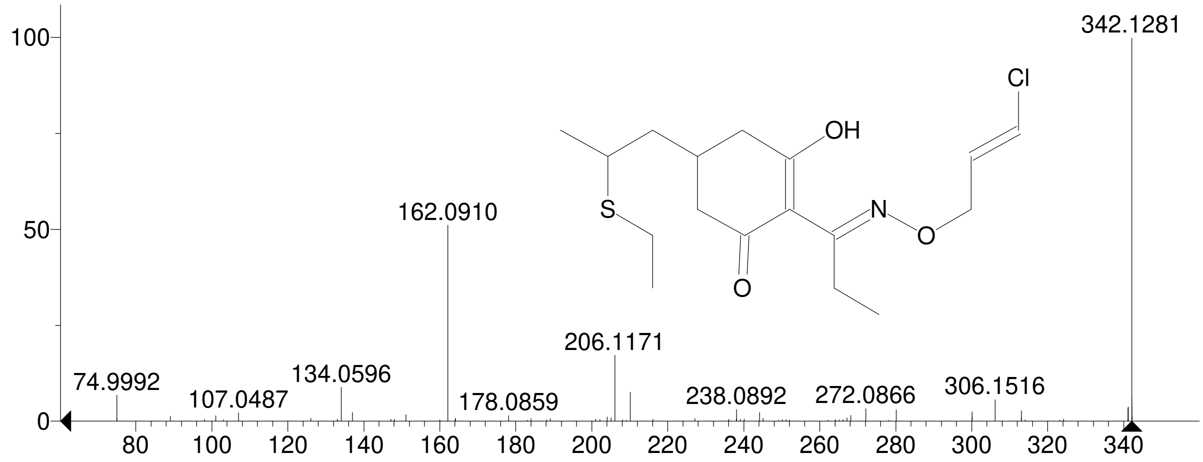

Hit 3 : Unknown (carbon number 19); PlaSMA ID-794 [M-H]- P=342.1
C19H21NO5; MF: 2; RMF: 161; Prob 24.7%; Lib: biomsms-neg-plasma; ID: 1262.

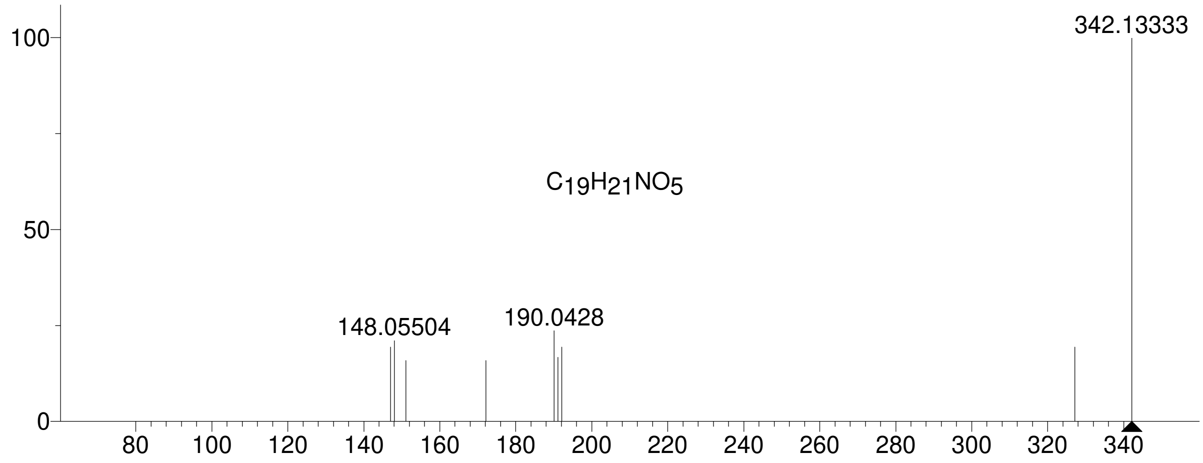

Hit 4 : 8,9-Dihydropiplartine [M+Na]+ P=342.1
C17H21NO5; MF: 0; RMF: 98; Prob 22.8%; Lib: mona-export-gnps; ID: 2931.

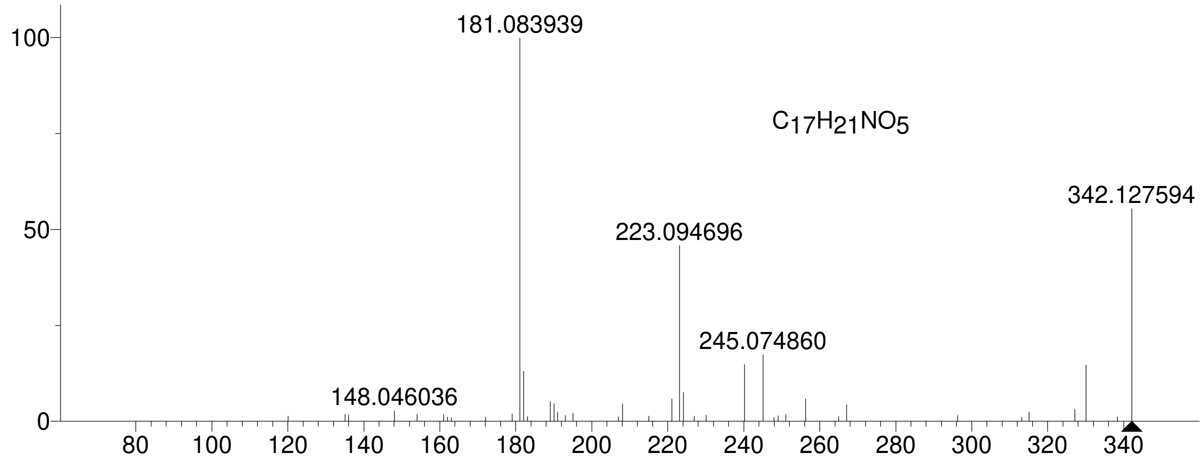

** Search Report Page 1 of 1 **

Unknown: Unknown (4.08_399.1507m/z)
Compound in Library Factor = 0

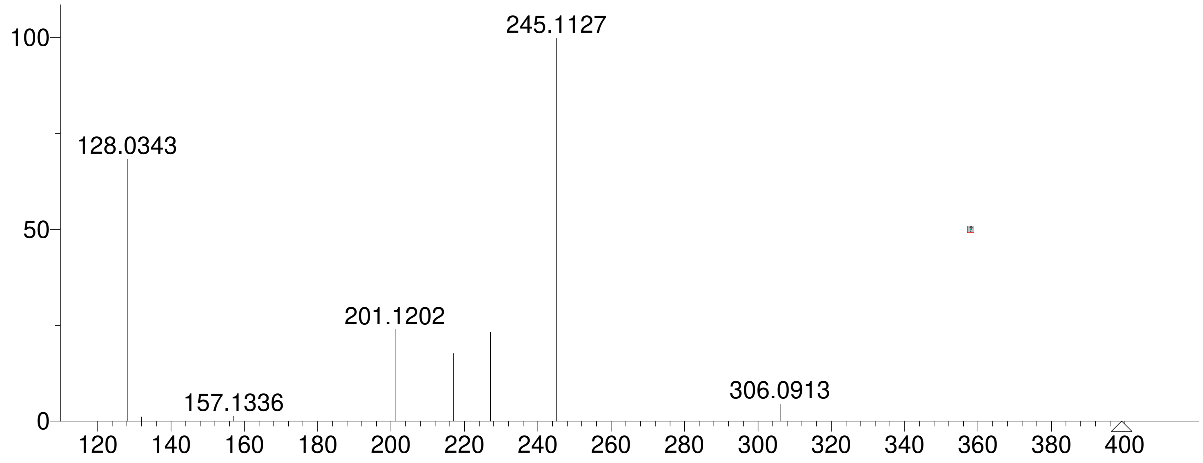

Unknown: Unknown (4.08_699.1225m/z)
Compound in Library Factor = 0

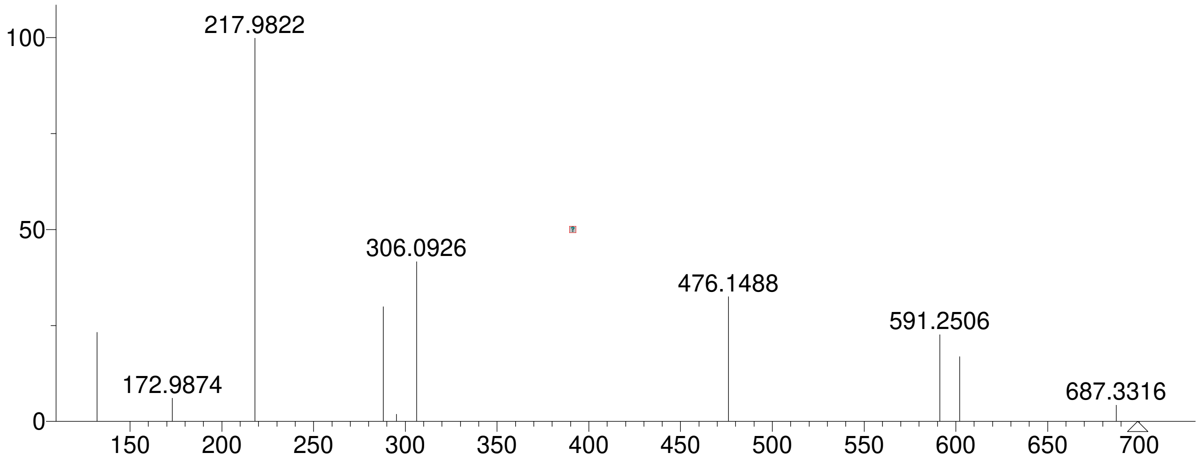

Unknown: Unknown (4.09_415.1708m/z)
Compound in Library Factor = -3273

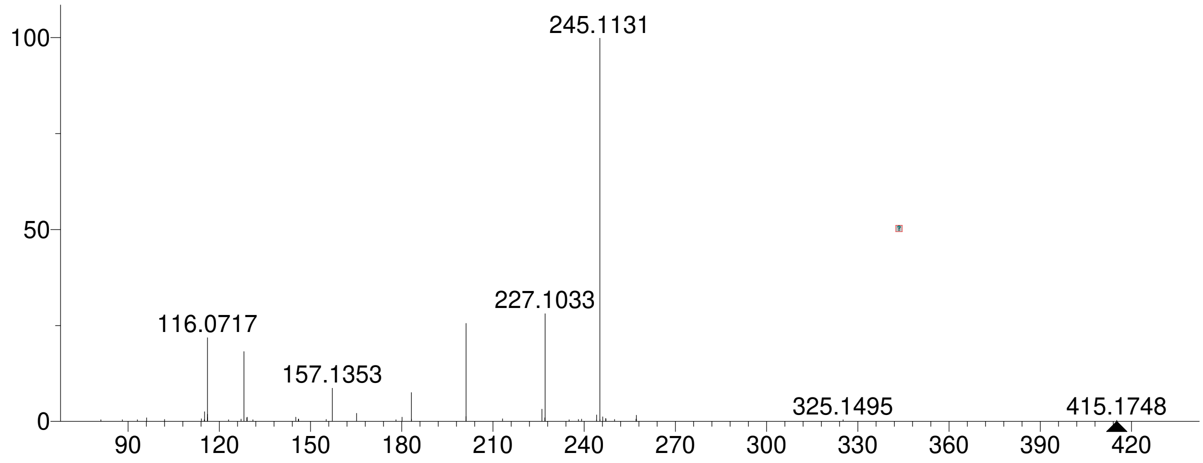

Hit 1 : Diltiazem [M+H]+ QTOF 3V P=415.2
C22H26N2O4S; MF: 4; RMF: 45; Prob 16.3%; CAS: 42399-41-7; Lib: nist_msms; ID: 20519.

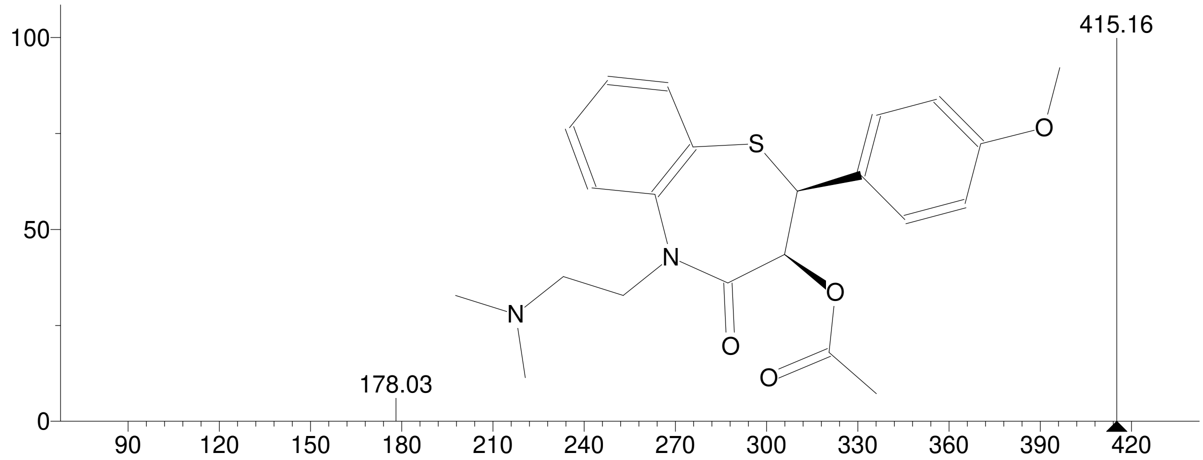

Hit 2 : 6-formyl-10-(hydroxymethyl)-5-methoxy-3-methylidene-2-oxo-2H,3H,3aH,4H,5H,8H,9H,11aH-cyclodeca[b]f
C21H28O7; MF: 1; RMF: 65; Prob 14.4%; Lib: mona-export-vf-npl_qexactive; ID: 16435.

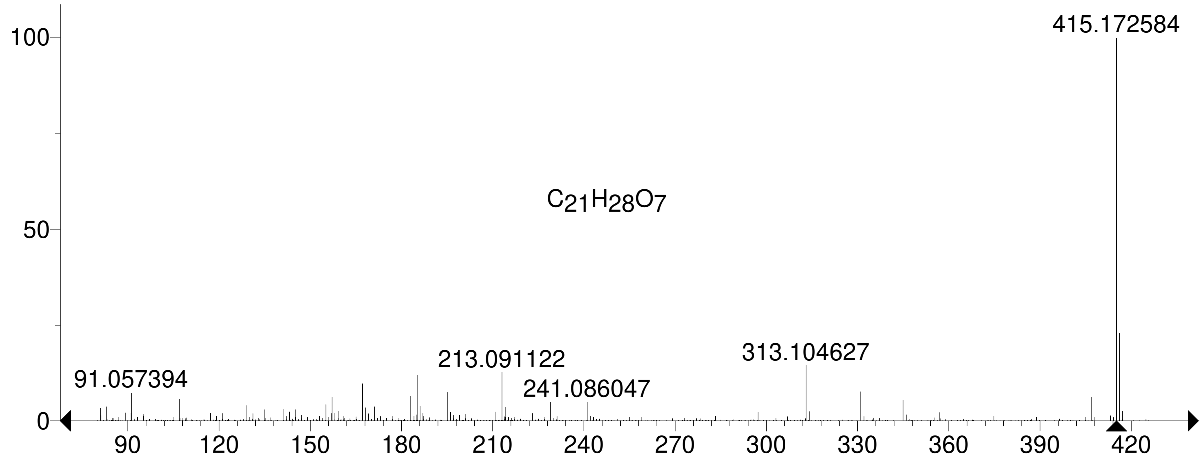

Hit 3 : Diltiazem HCl (Tiazac) [M+H]+ P=415.2
C22H27ClN2O4S; MF: 0; RMF: 16; Prob 13.9%; Lib: mona-export-gnps; ID: 648.

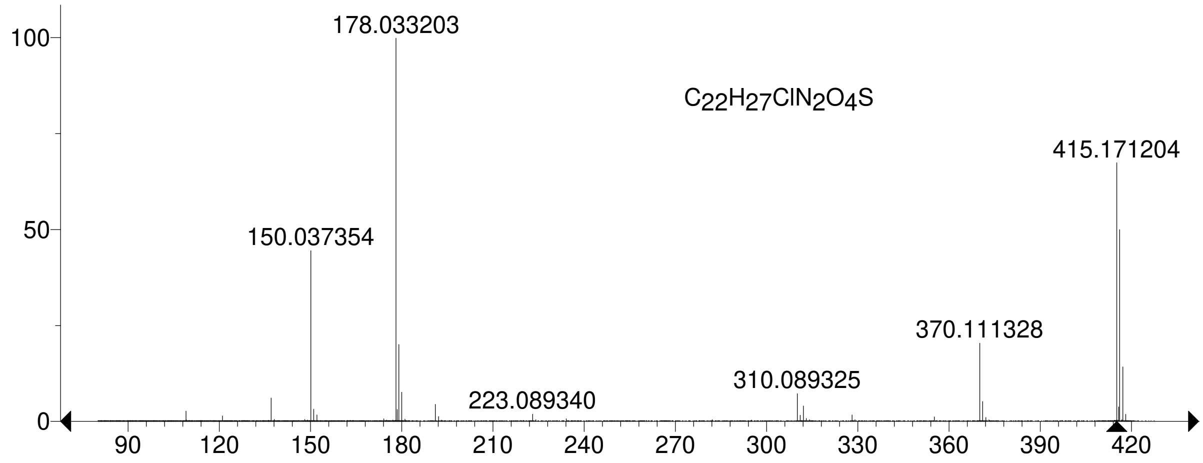

Hit 4 : NCGC00347525-03!2-amino-5-[2-[[2,3-dihydroxy-2-(1-hydroxyethyl)butanoyl]oxymethyl]-4-hydroxyanilino]-
C18H26N2O9; MF: 0; RMF: 9; Prob 13.9%; Lib: mona-export-gnps; ID: 9213.

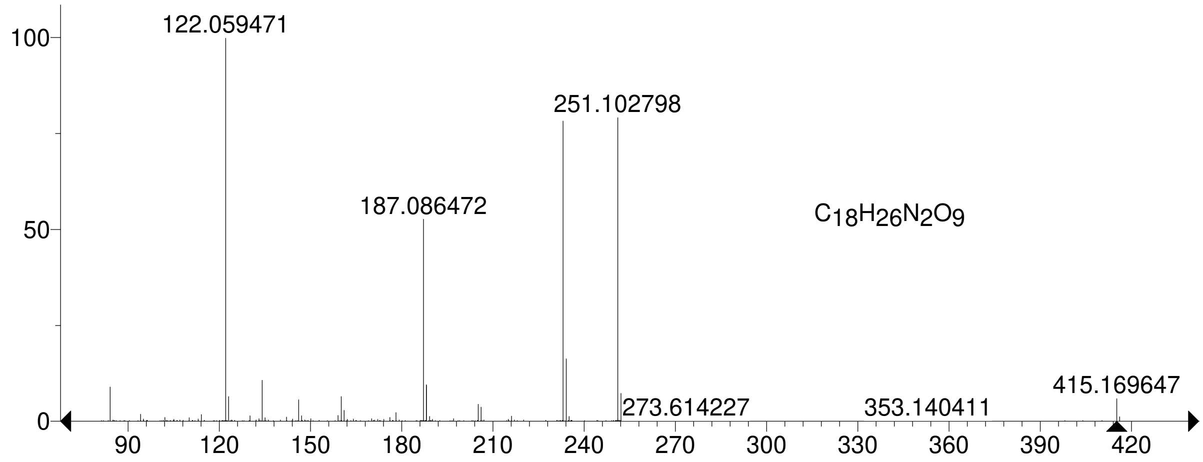

Hit 5 : NCGC00347525-02!2-amino-5-[2-[[2,3-dihydroxy-2-(1-hydroxyethyl)butanoyl]oxymethyl]-4-hydroxyanilino]-
C18H26N2O9; MF: 0; RMF: 5; Prob 13.9%; Lib: mona-export-gnps; ID: 6270.

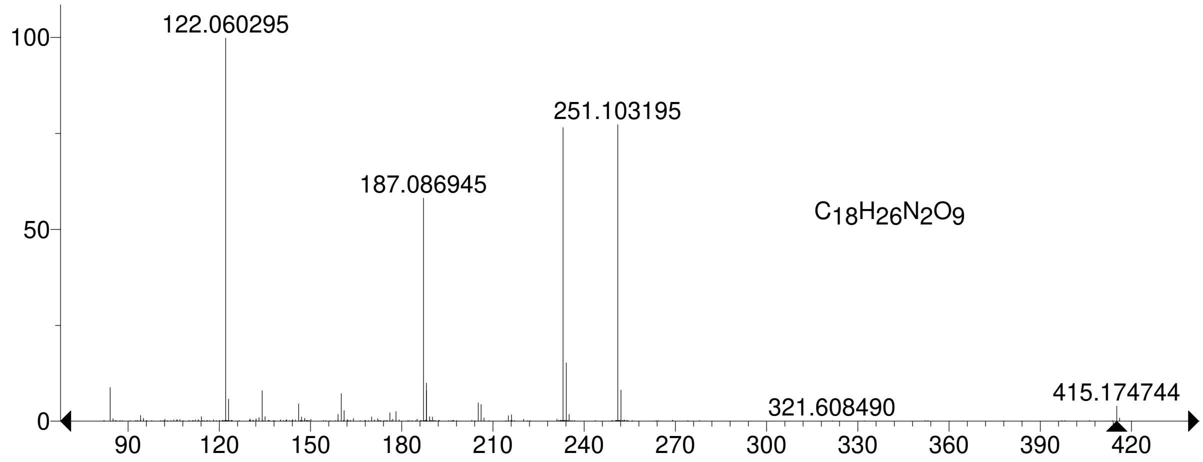

Unknown: Unknown (4.10_298.0913m/z)
Compound in Library Factor = 0

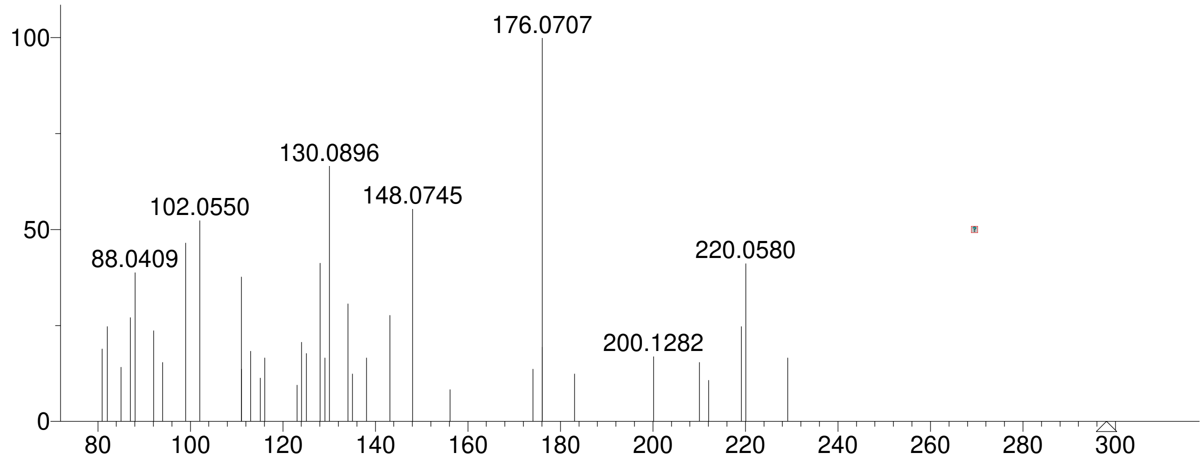

** Search Report Page 1 of 1 **

Unknown: Unknown (4.10_554.1397m/z)
Compound in Library Factor = 0

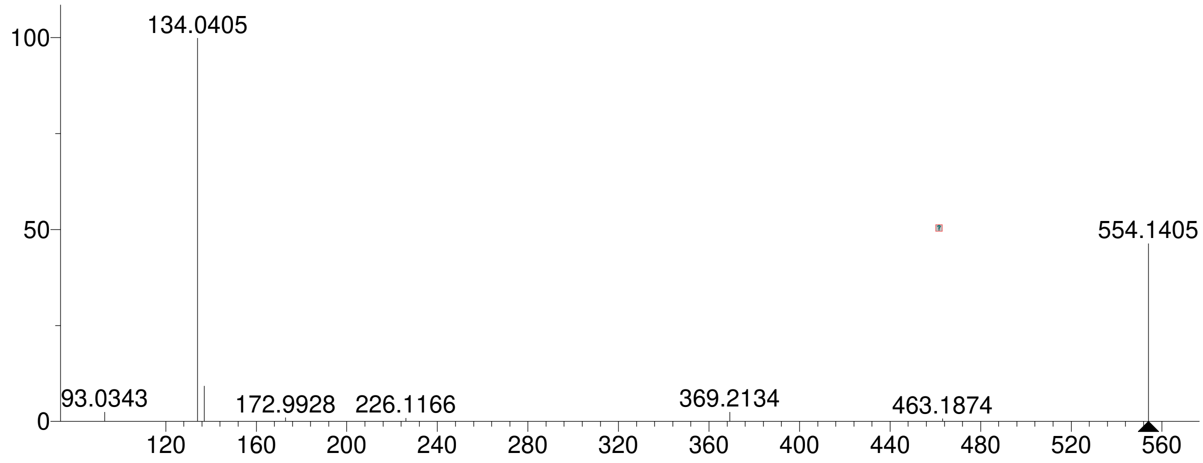

** Search Report Page 1 of 1 **

Unknown: Unknown (4.11_602.1241m/z)
Compound in Library Factor = 0

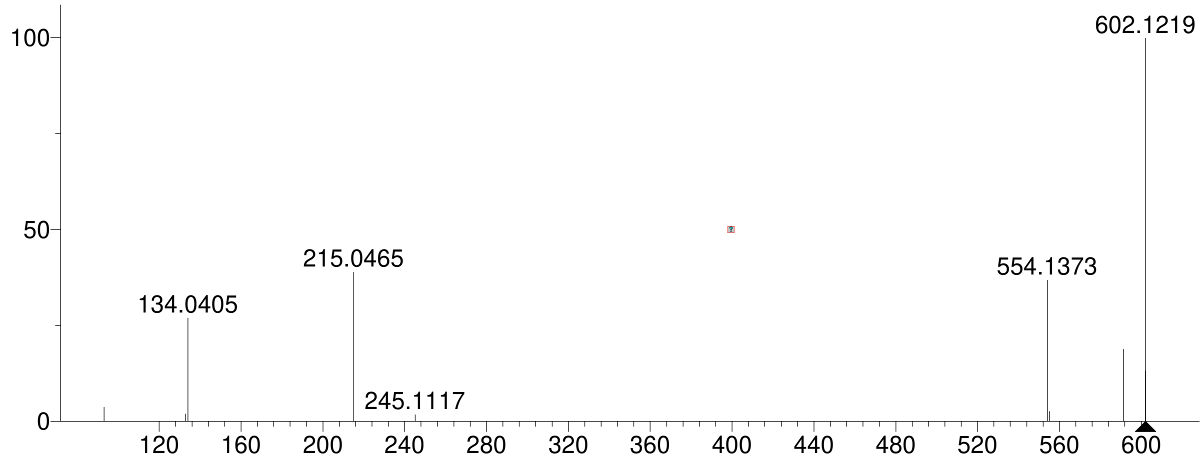

Unknown: Unknown (4.12_137.0237m/z)
Compound in Library Factor = -385

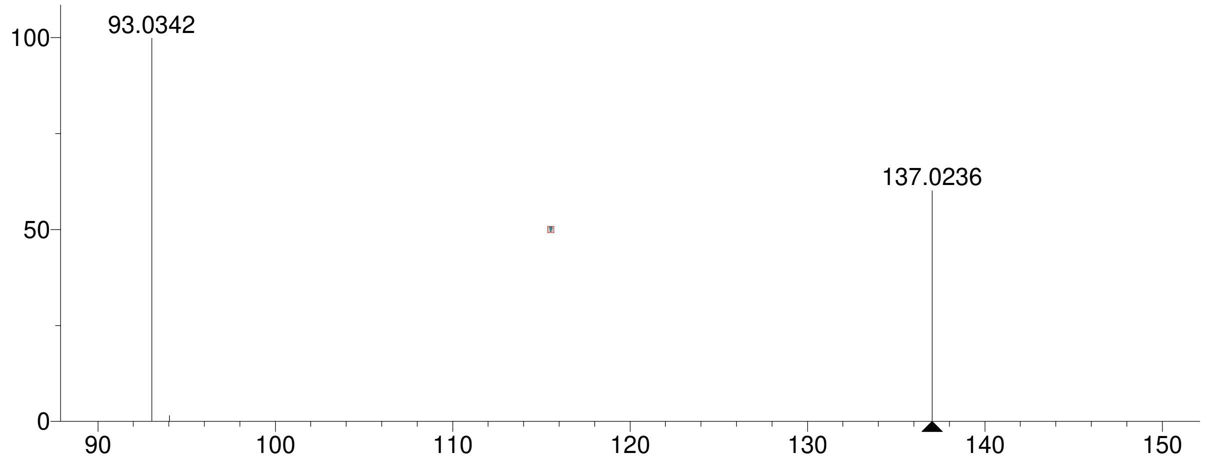

Hit 1 : Salicylic acid [M-H]- HCD 12V P=137
C7H6O3; MF: 747; RMF: 997; Prob 45.6%; CAS: 69-72-7; Lib: nist_msms; ID: 101033.

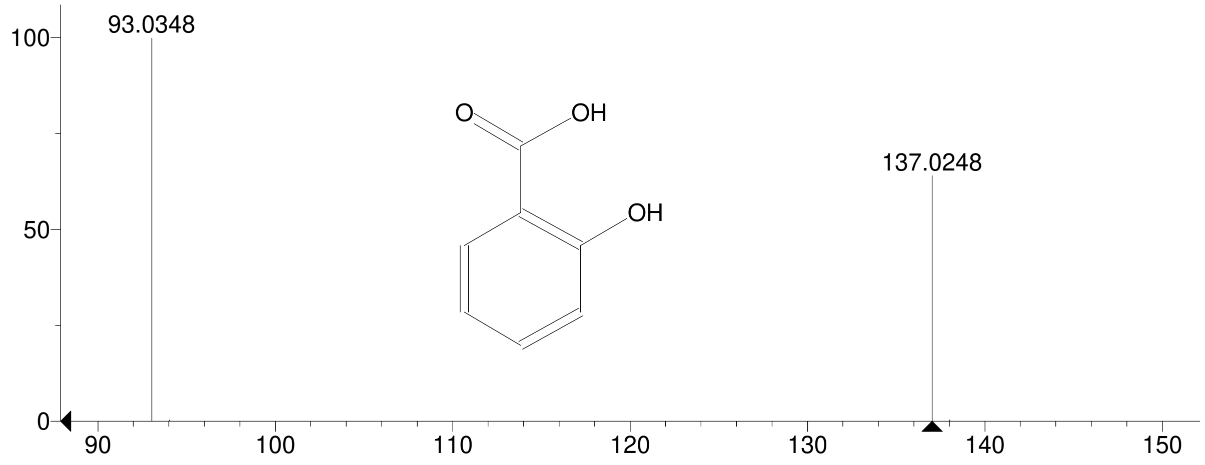

Hit 2 : 3-HYDROXYBENZOATE [M-H]- P=137
C7H6O3; MF: 731; RMF: 990; Prob 26.3%; Lib: mona-export-gnps; ID: 5777.

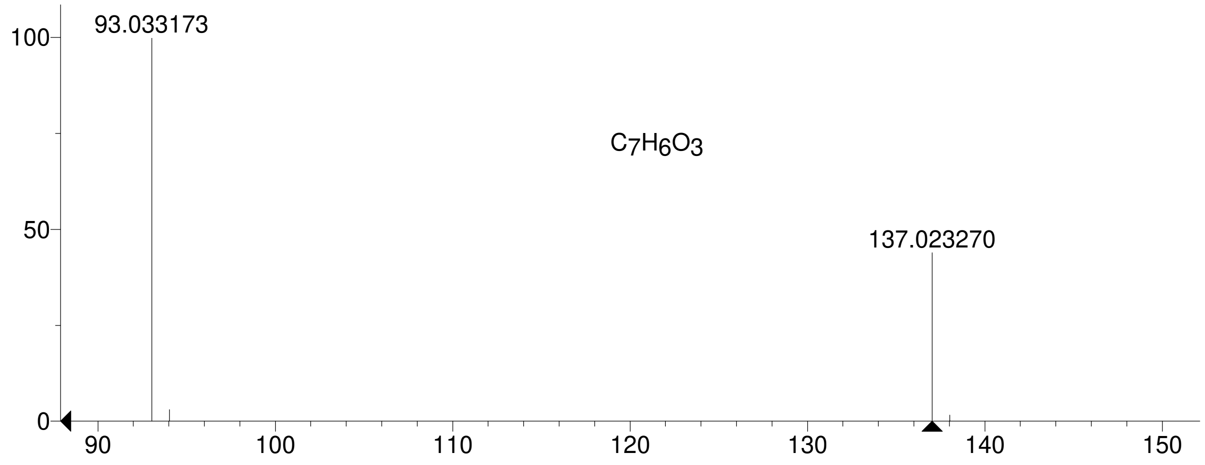

Hit 3 : 4-HYDROXYBENZOATE [M-H]- P=137
C7H6O3; MF: 723; RMF: 965; Prob 19.6%; Lib: msms-neg-gnps; ID: 338.

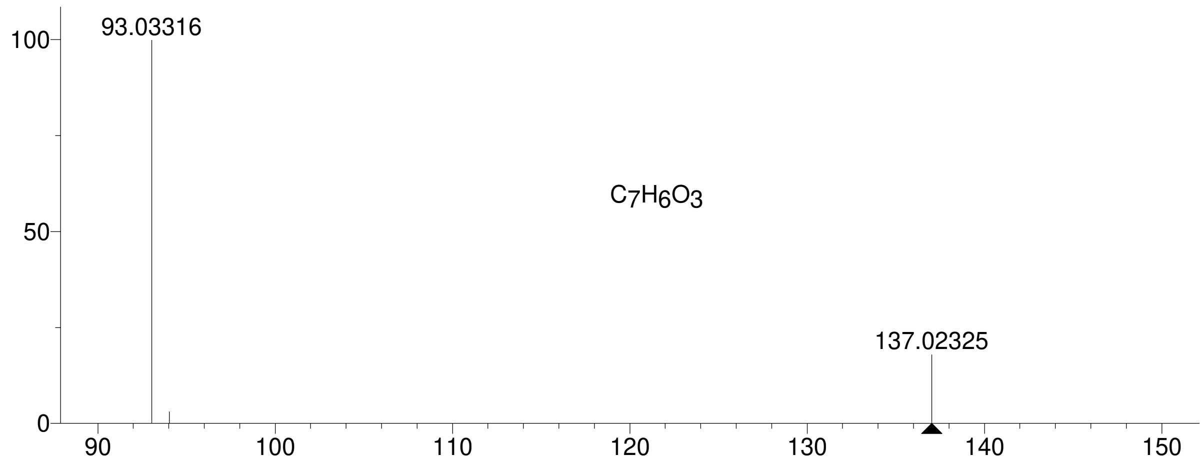

Hit 4 : 4-HYDROXYBENZOATE
C7H6O3; MF: 669; RMF: 958; Prob 3.83%; Lib: embl neg mcg; ID: 406.

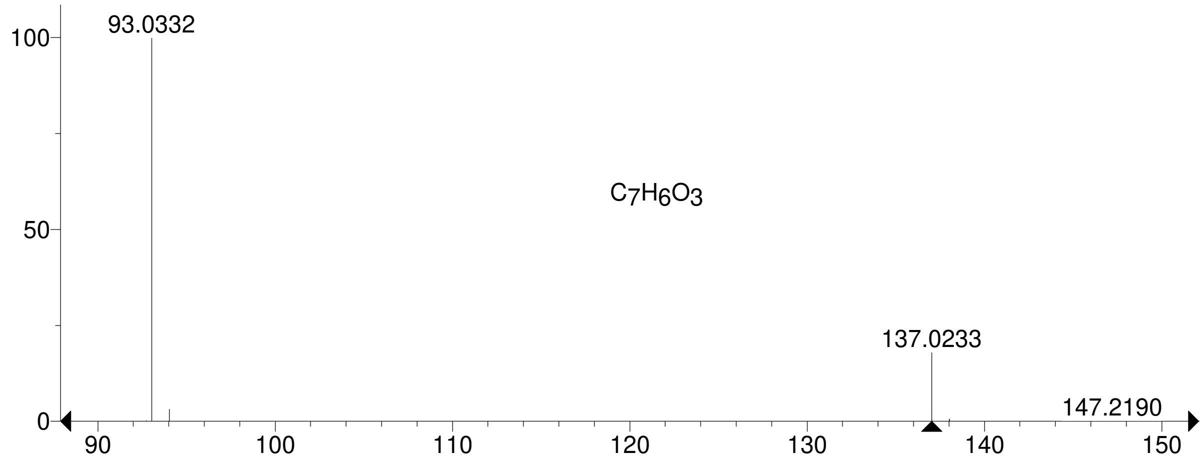

Hit 5 : Unknown (carbon number 7); PlaSMA ID-70 [M-H]- P=137
C7H6O3; MF: 633; RMF: 952; Prob 0.94%; Lib: biomsms-neg-plasma; ID: 973.

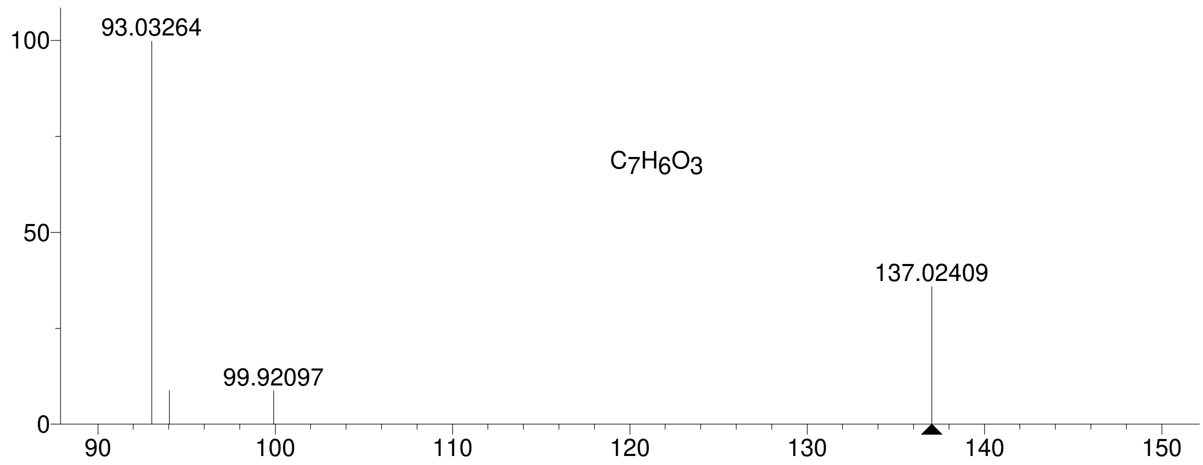

Unknown: Unknown (4.12_216.9805m/z)
Compound in Library Factor = -314

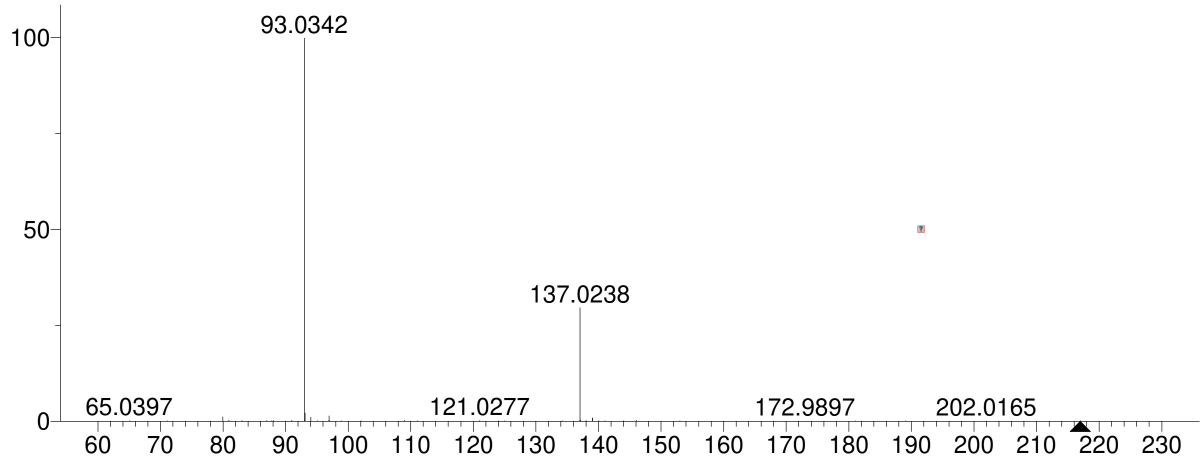

Hit 1 : 5-Sulfosalicylic acid [M-H]- HCD 16V P=217
C7H6O6S; MF: 227; RMF: 393; Prob 100.0%; CAS: 97-05-2; Lib: nist_msms; ID: 143731.

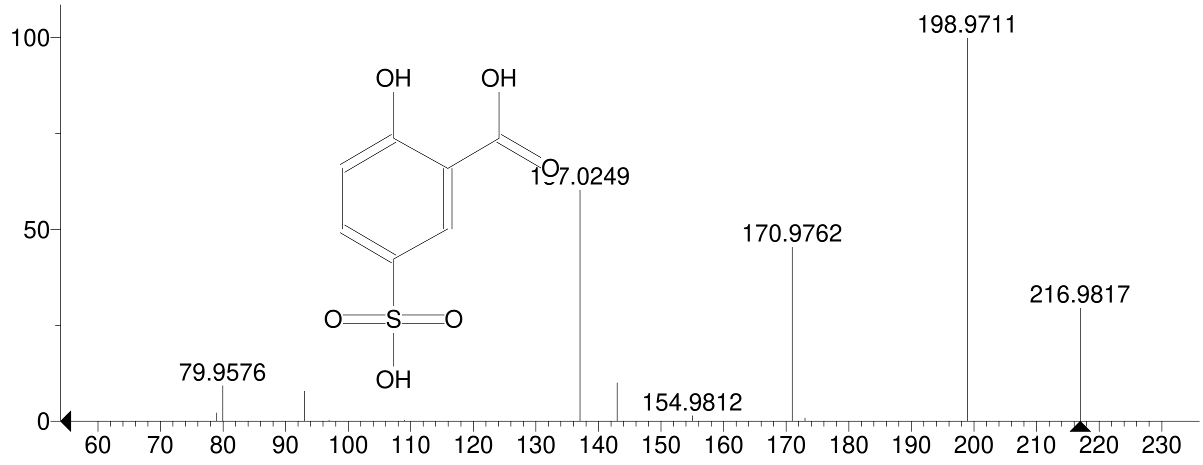

Unknown: Unknown (4.12_369.2131m/z)
Compound in Library Factor = -3273

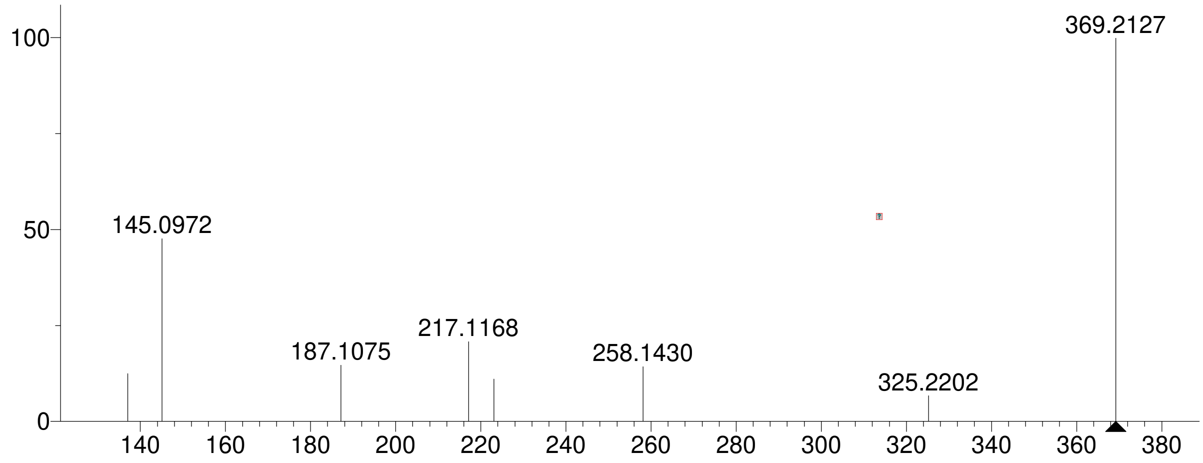

Hit 1 : Neuropeptide S, rat [M+6H]6+ QTOF 2V P=369.2
C95H160N34O27; MF: 4; RMF: 333; Prob 100.0%; Lib: nist_msms2; ID: 35976.

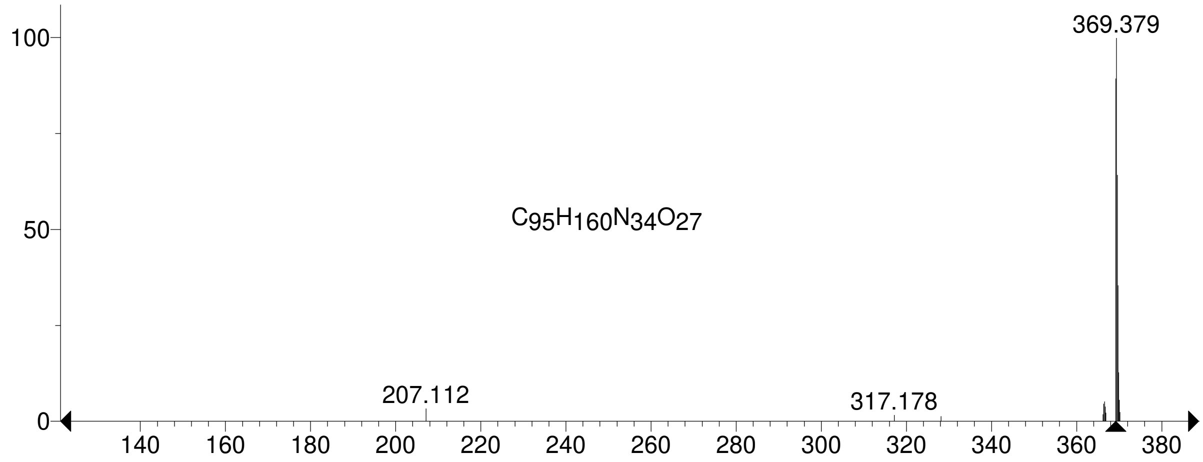

Unknown: Unknown (4.12_428.2033m/z)
Compound in Library Factor = 0

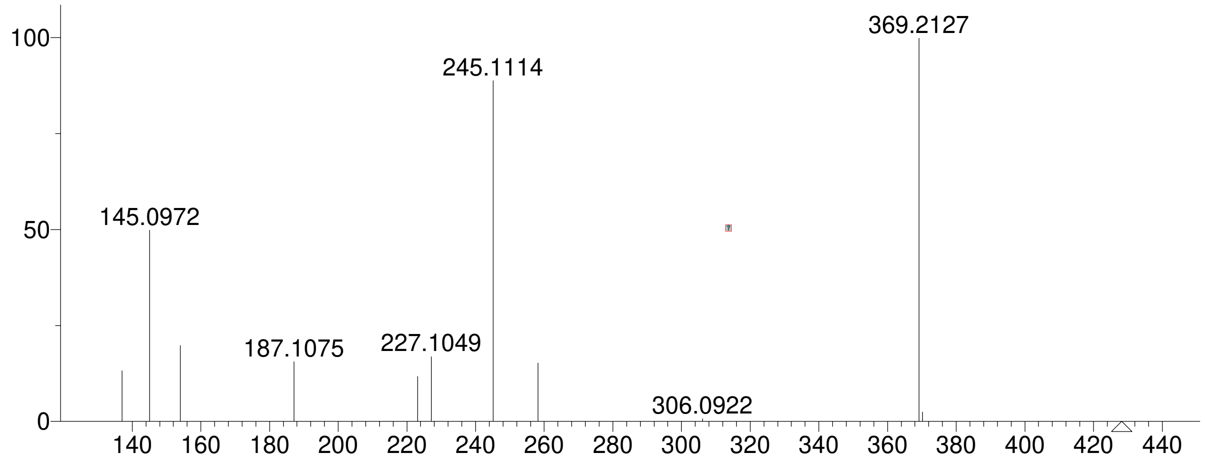

Hit 1 : Desmethylcerivastatin [M+H]+=>428.2 IT 35% P=428.2
C25H32FNO5; MF: 0; RMF: 119; Prob 16.7%; CAS: 690952-00-2; Lib: nist_msms; ID: 451579.

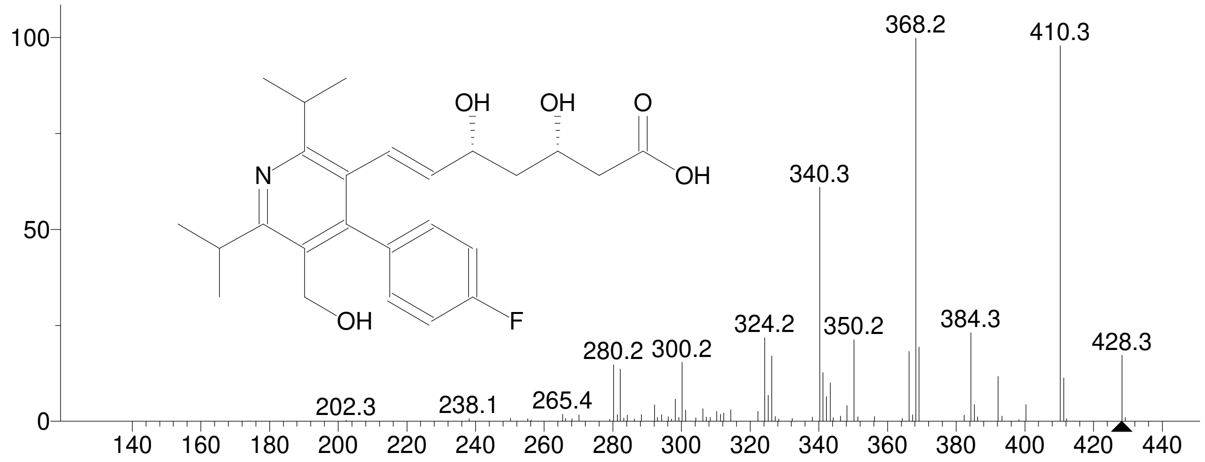

Hit 2 : ROTENONIC ACID, METHYL ETHER [M+NH4]+ 35HCD P=428.2
C24H26O6; MF: 0; RMF: 93; Prob 16.7%; Lib: mona-export-vf-npl_qexactive; ID: 11536.

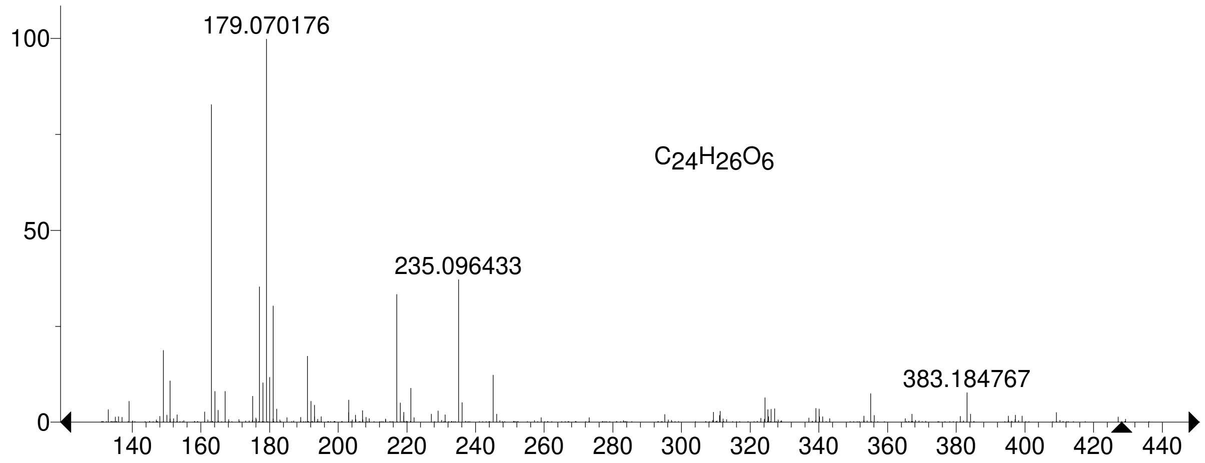

Hit 3 : Epothilone B [M+H-H2O]+=>472.2=>428.2 IT 35% P=428.2
C27H41NO6S; MF: 0; RMF: 37; Prob 16.7%; CAS: 152044-54-7; Lib: nist_msms; ID: 252242.

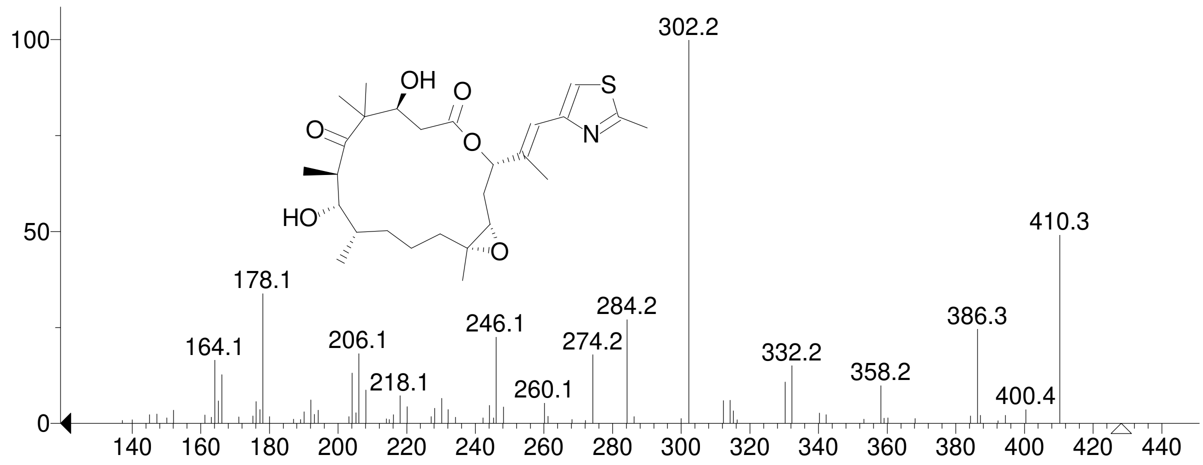

Hit 4 : Ampkinone [M+H]+=>428.2 IT 35% P=428.2
C31H23NO6; MF: 0; RMF: 36; Prob 16.7%; CAS: 1233082-79-5; Lib: nist_msms; ID: 265930.

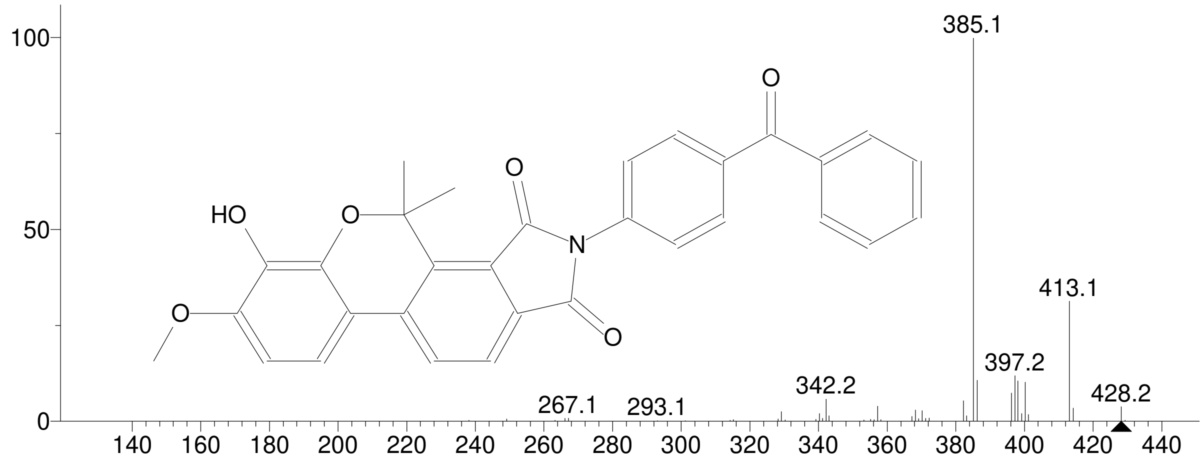

Hit 5 : 4-Hydroxynonenal glutathione [M+H-H2O]+=>428.2 IT 35% P=428.2
C19H33N3O8S; MF: 0; RMF: 33; Prob 16.7%; CAS: 99927-70-5; Lib: nist_msms; ID: 170262.

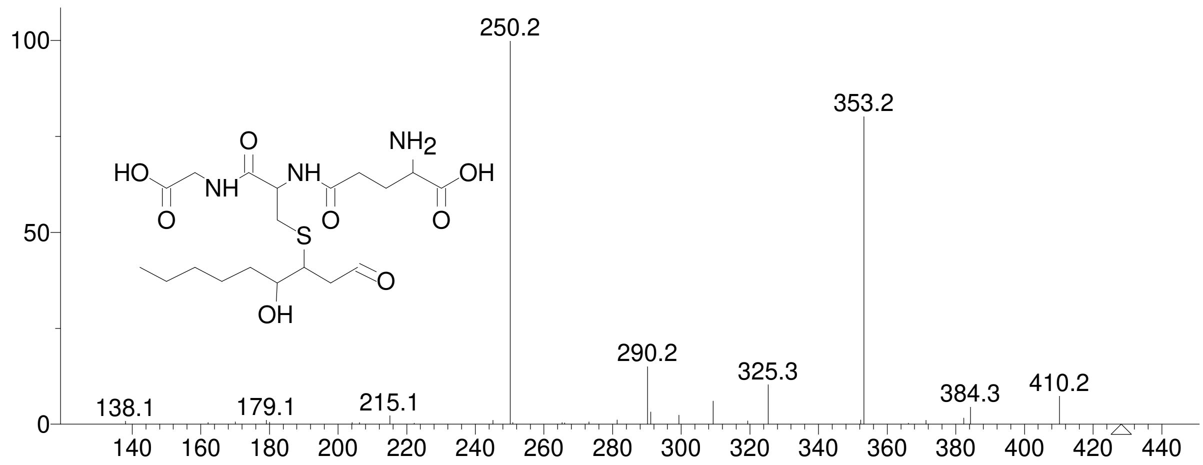

Unknown: Unknown (4.12_446.1679m/z)
Compound in Library Factor = 0

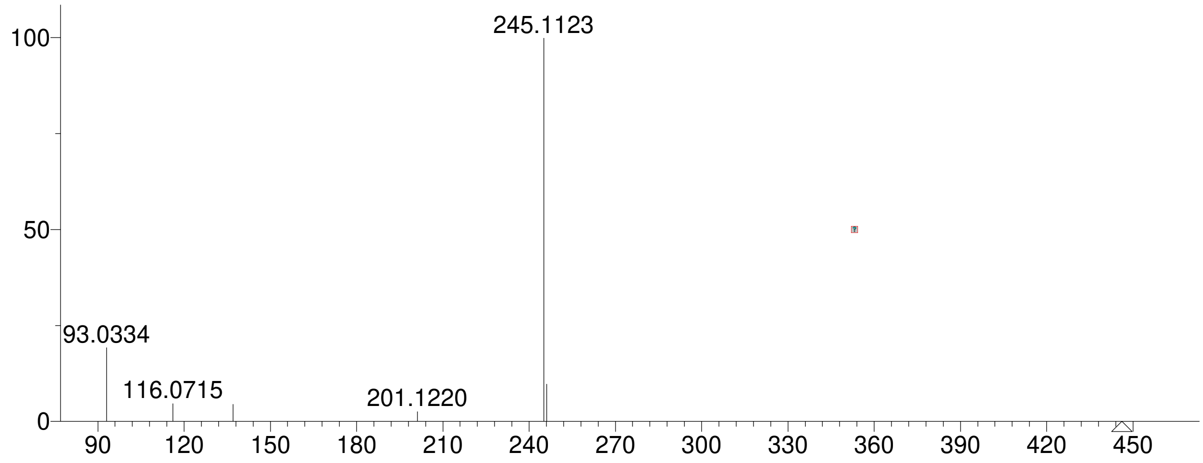

** Search Report Page 1 of 1 **

Unknown: Unknown (4.13_387.1750m/z)
Compound in Library Factor = 0

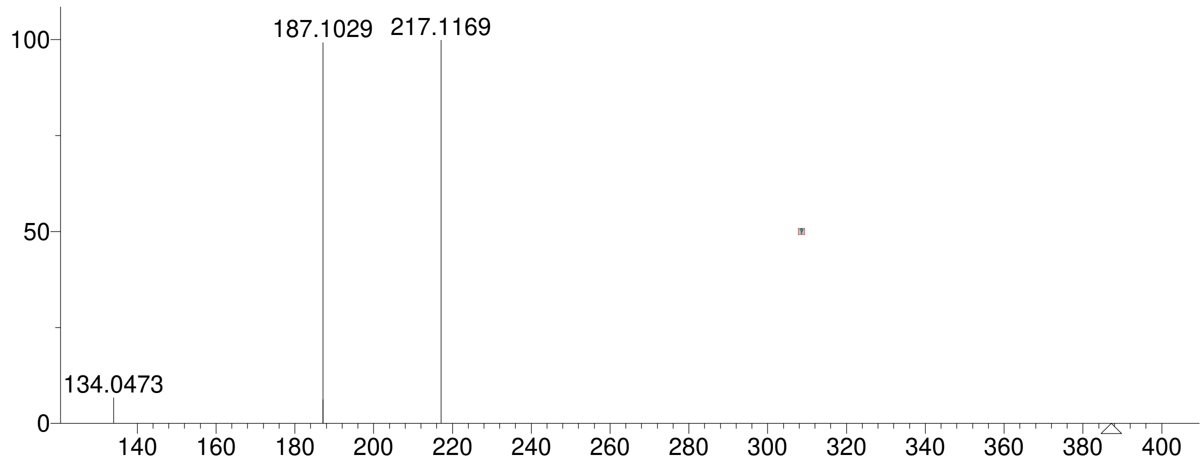

Unknown: Unknown (4.14_472.1561m/z)
Compound in Library Factor = -2176

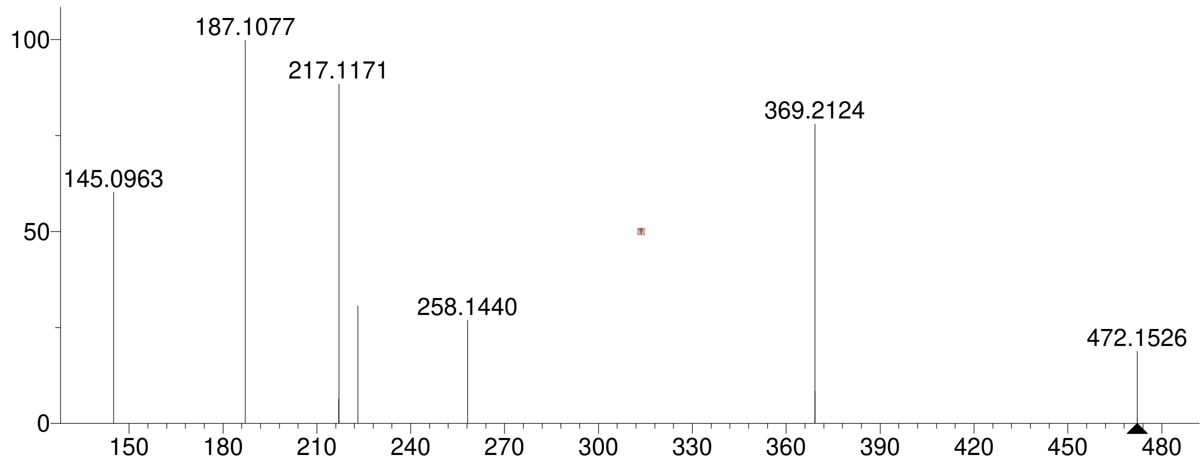

Hit 1 : Folinic acid [M-H]- HCD 18V P=472.2
C20H23N7O7; MF: 23; RMF: 374; Prob 41.2%; CAS: 58-05-9; Lib: nist_msms; ID: 138208.

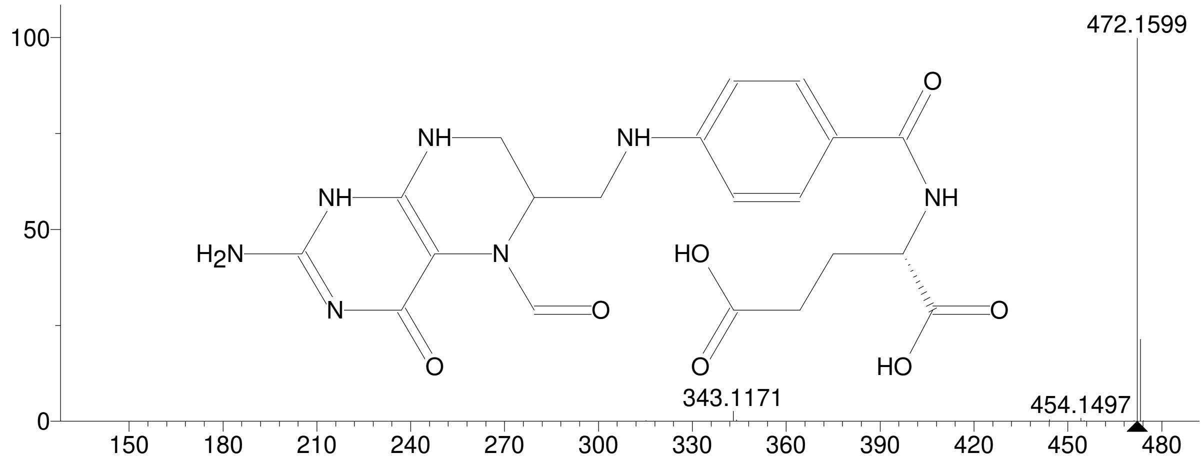

Hit 2 : 10-Formyl-7,8-dihydrofolic acid [M+H]+ HCD 9V P=472.2
C20H21N7O7; MF: 23; RMF: 224; Prob 41.2%; CAS: 28459-40-7; Lib: nist_msms; ID: 213137.

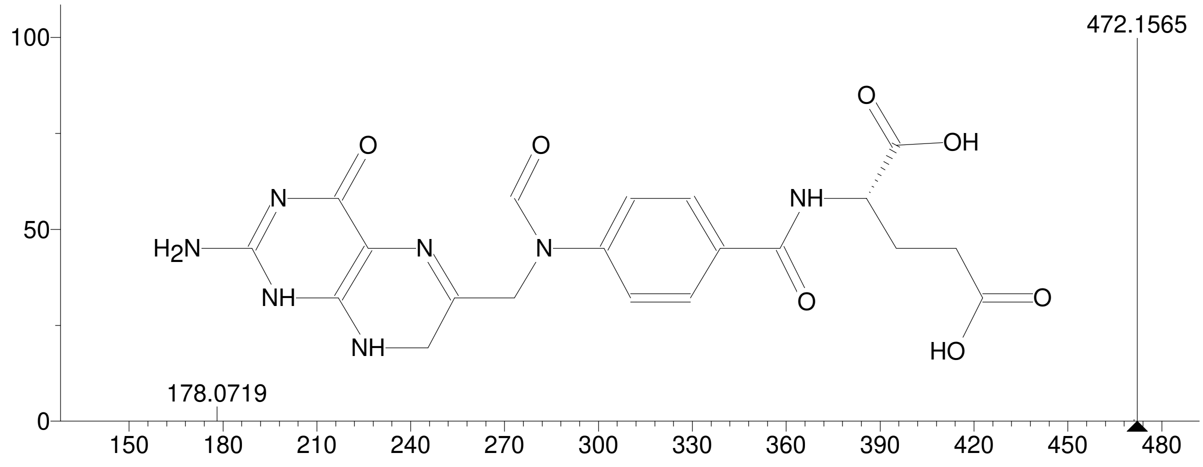

Hit 3 : Raloxifene [M-H]- 35(no P=472.2
C28H27NO4S; MF: 2; RMF: 148; Prob 17.5%; Lib: msms-neg-casmi2016; ID: 107.

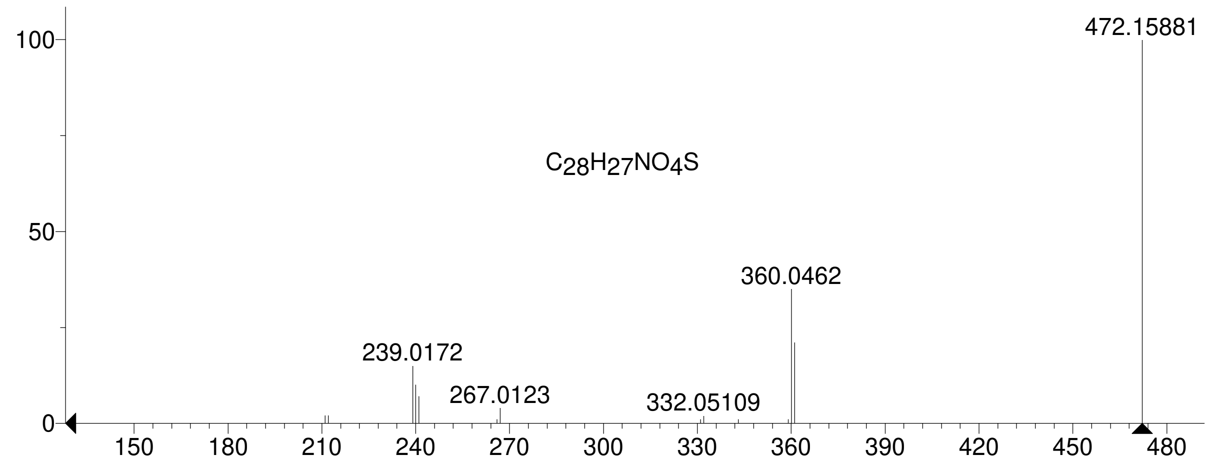

Unknown: Unknown (4.15_530.1287m/z)
Compound in Library Factor = 0

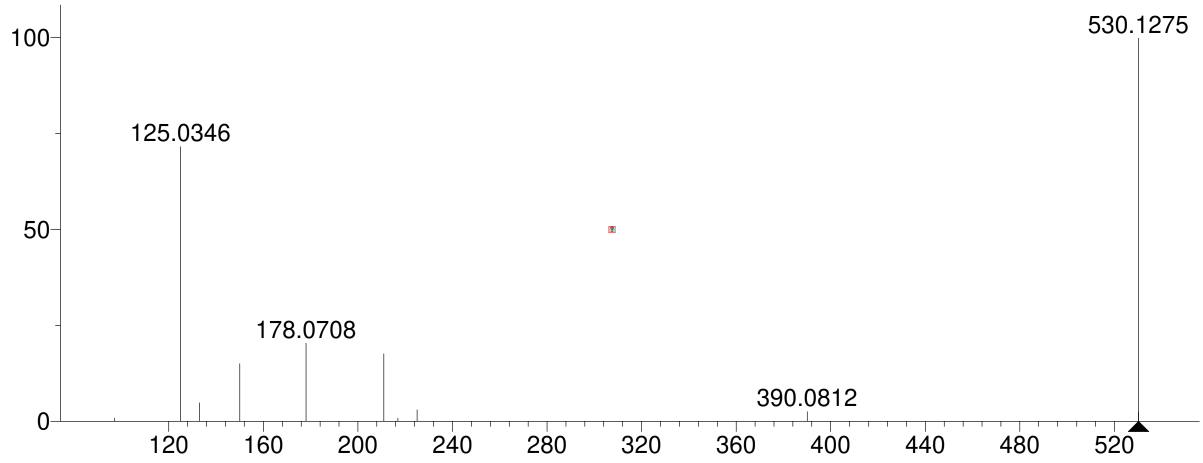

Unknown: Unknown (4.16_285.0533m/z)
Compound in Library Factor = 0

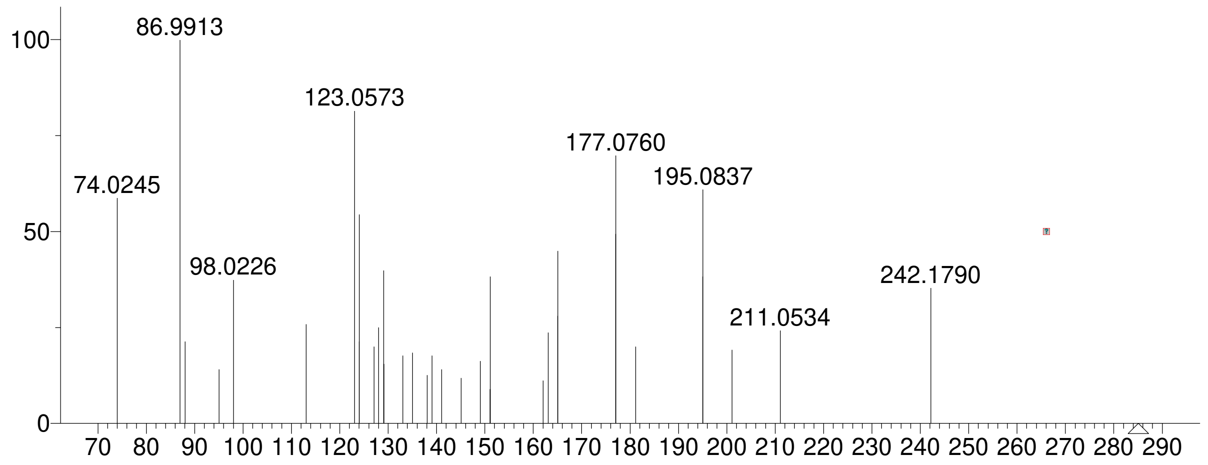

Hit 1 : columbianetin [M+K]+ 65HCD P=285.1
C14H14O4; MF: 0; RMF: 43; Prob 25.0%; Lib: mona-export-vf-npl_qexactive; ID: 4032.

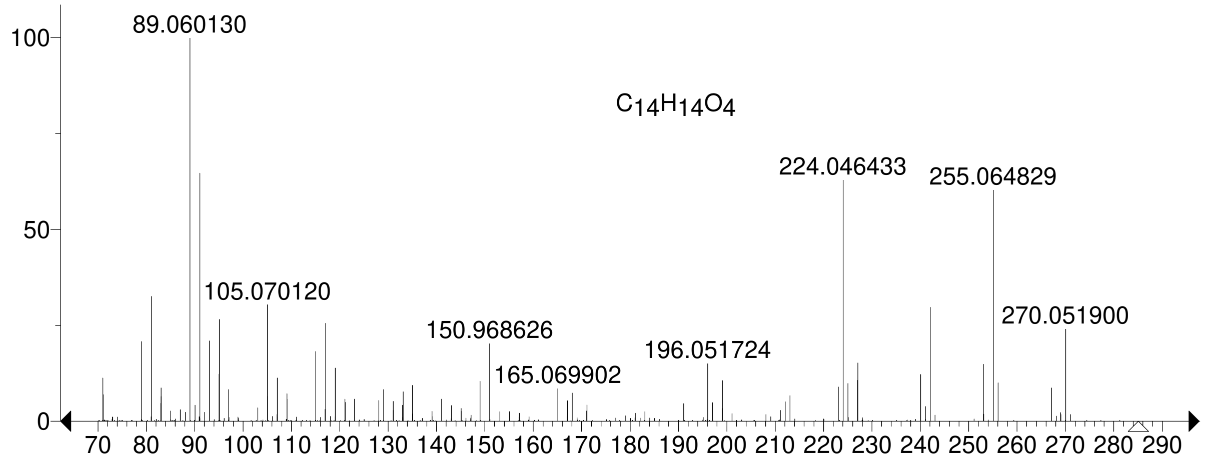

Hit 2 : Prenyletin [M+K]+ 35HCD P=285.1
C14H14O4; MF: 0; RMF: 20; Prob 25.0%; Lib: mona-export-vf-npl_qexactive; ID: 11380.

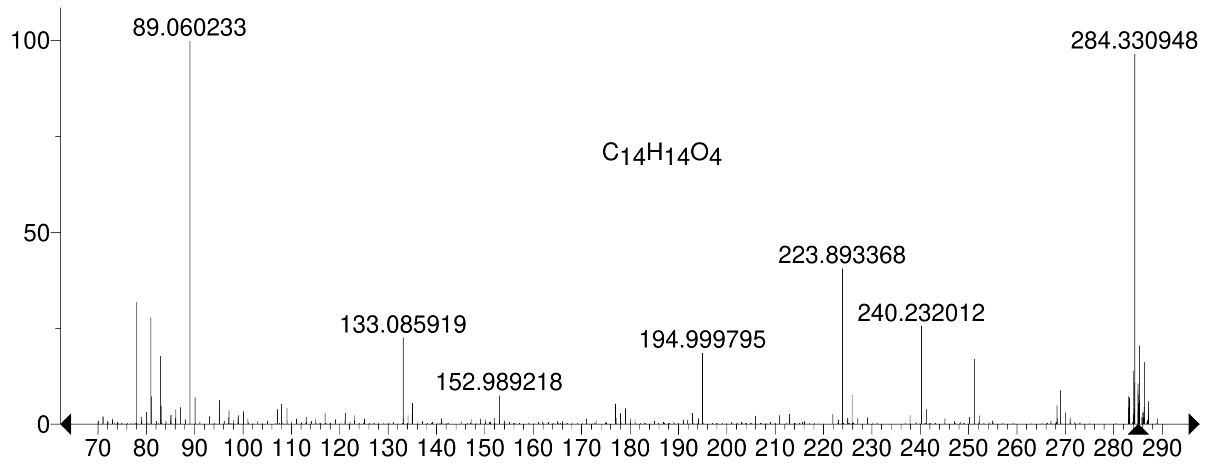

Hit 3 : (2S,3S)-3-hydroxy-4-methoxy-2-[(E)-2-phenylethenyl]-2,3-dihydropyran-6-one [M+K]+ 65HCD P=285.1
C14H14O4; MF: 0; RMF: 19; Prob 25.0%; Lib: mona-export-vf-npl_qexactive; ID: 2397.

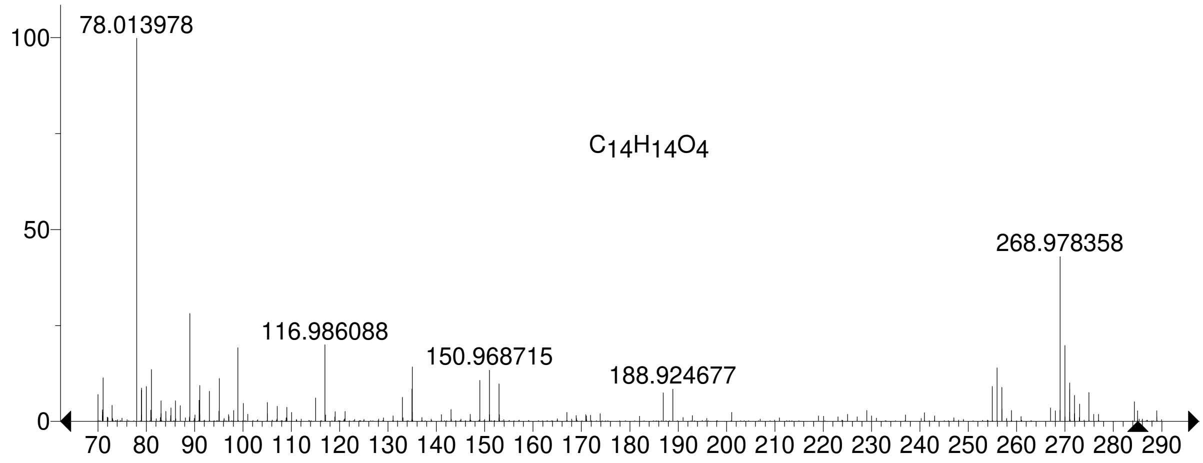

Hit 4 : Griseofulvin [M+H-C4H4O]+ HCD 62V P=285.1
C17H17ClO6; MF: 0; RMF: 9; Prob 25.0%; CAS: 126-07-8; Lib: nist_msms; ID: 310445.

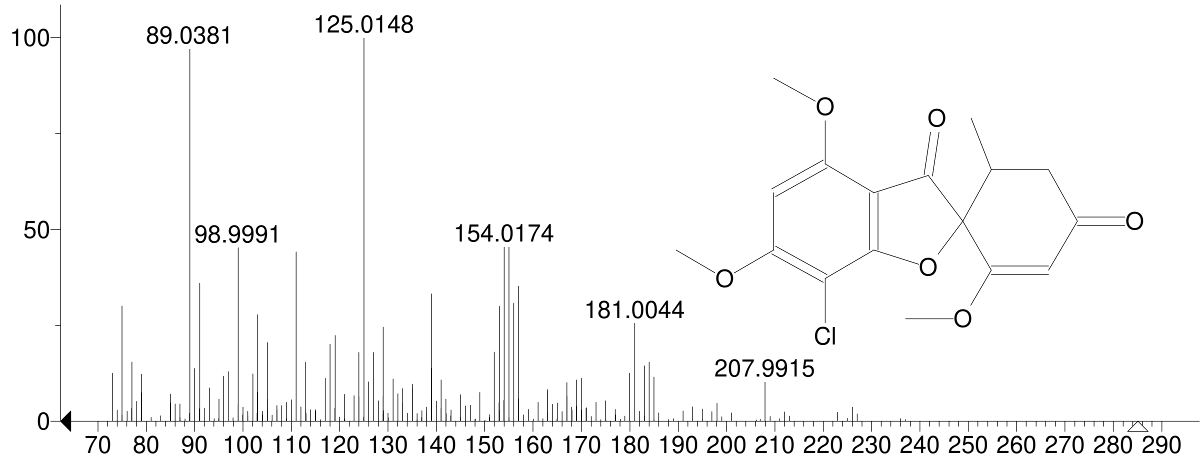

Unknown: Unknown (4.16_529.2142m/z)
Compound in Library Factor = 0

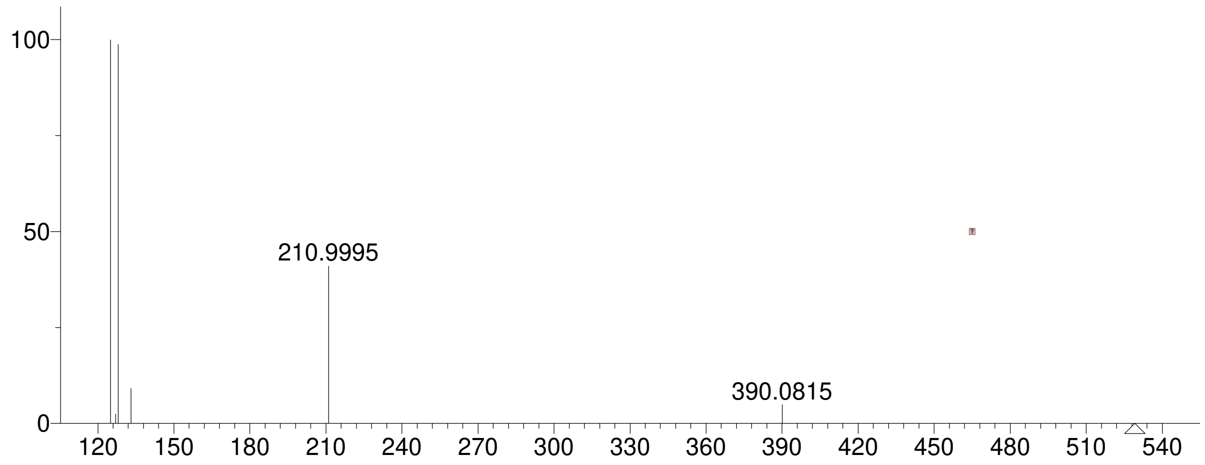

** Search Report Page 1 of 1 **

Unknown: Unknown (4.16_570.1348m/z)
Compound in Library Factor = 0

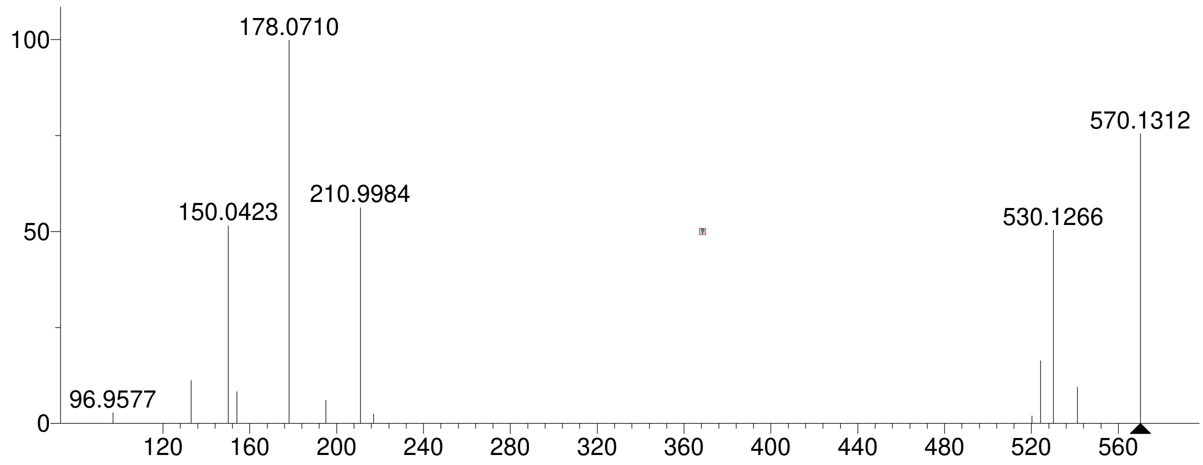

Unknown: Unknown (4.16_616.1406m/z)
Compound in Library Factor = 0

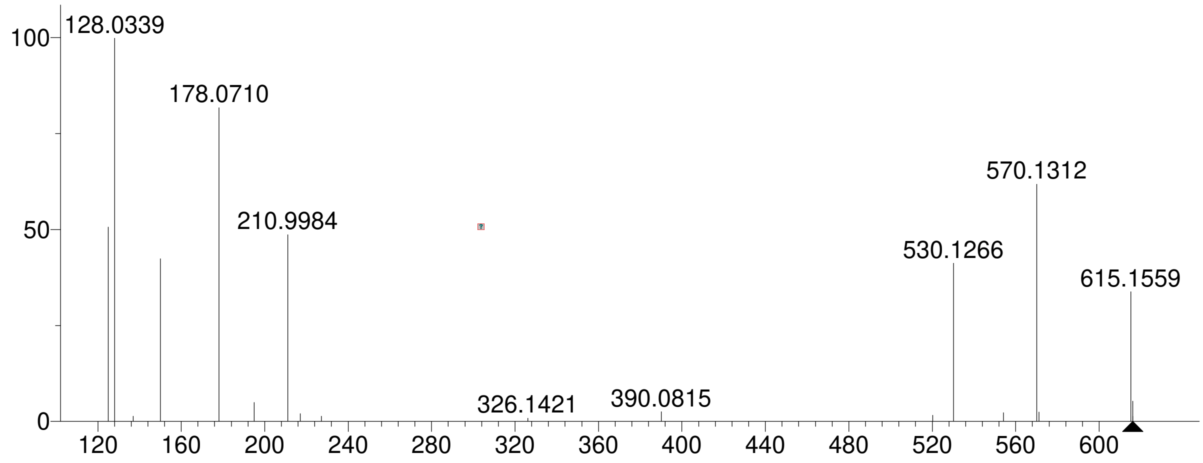

Unknown: Unknown (4.17_559.2353m/z)
Compound in Library Factor = -2387

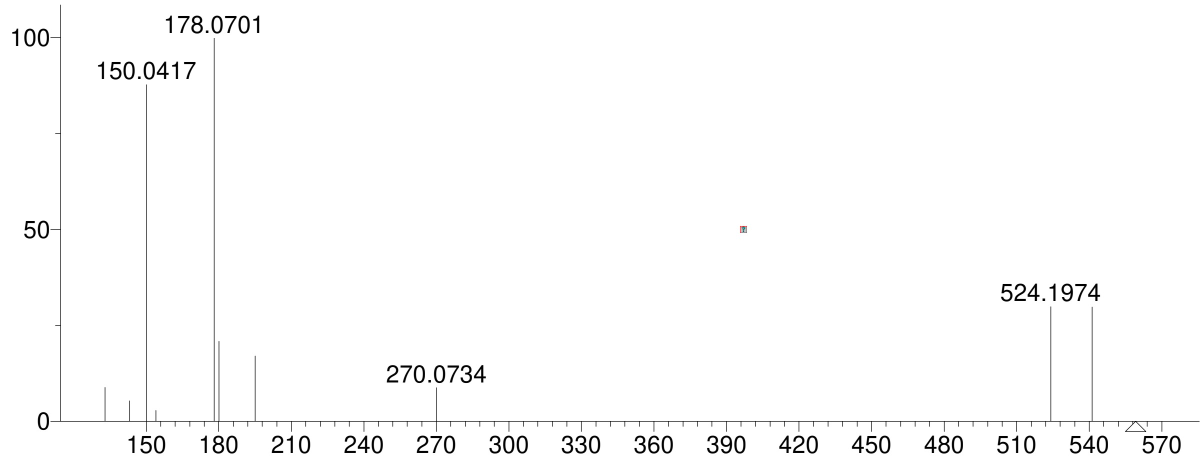

Hit 1 : Olmesartan medoxomil [M+H]+ HCD 5V P=559.2
C29H30N6O6; MF: 18; RMF: 296; Prob 48.3%; CAS: 144689-63-4; Lib: nist_msms; ID: 255791.

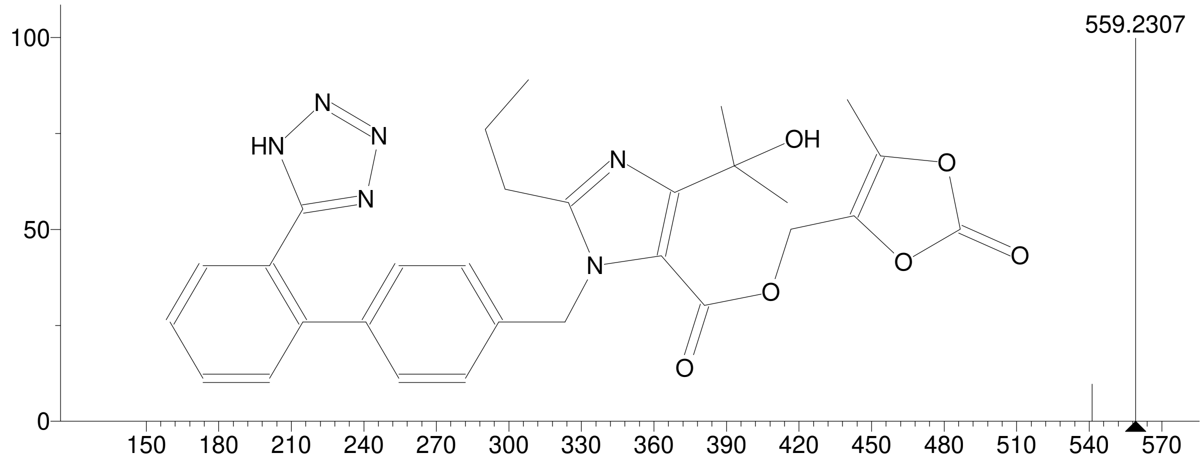

Hit 2 : Olmesartan medoxomil (Benicar) [M+H]+ P=559.2
C29H30N6O6; MF: 1; RMF: 169; Prob 26.4%; Lib: mona-export-gnps; ID: 522.

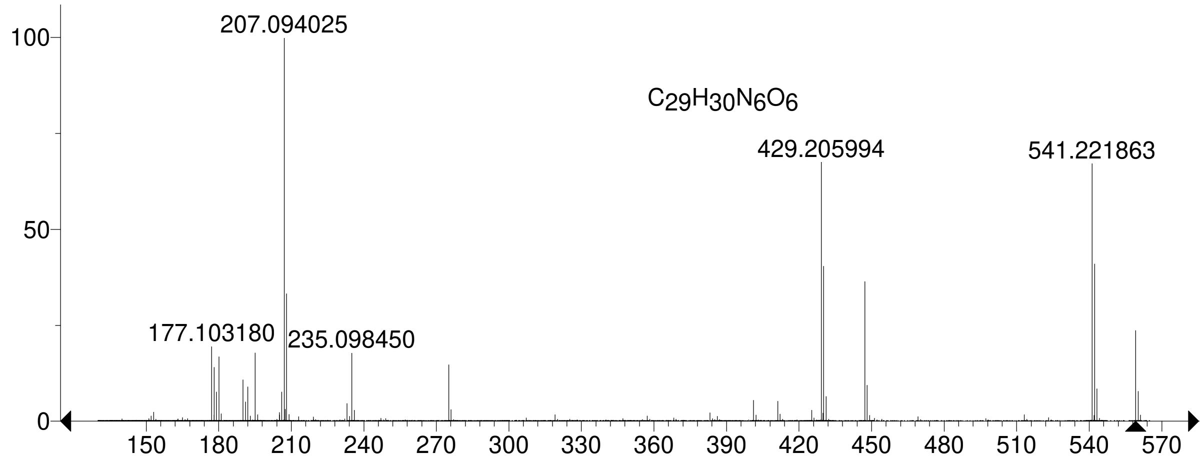

Hit 3 : Okaramine R_120153 [M+Na]+ P=559.2
C32H32N4O4; MF: 0; RMF: 20; Prob 25.3%; Lib: mona-export-gnps; ID: 4971.

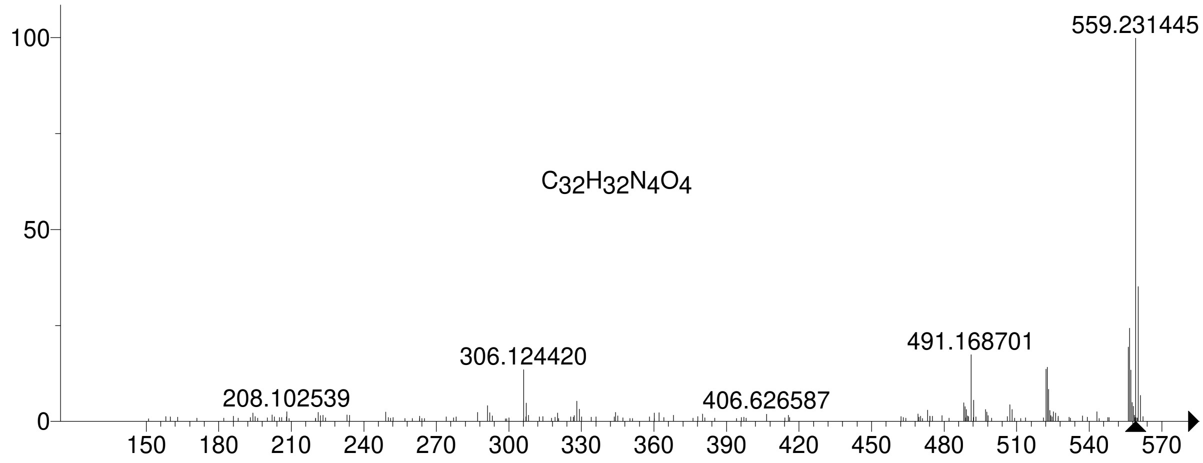

Unknown: Unknown (4.17_698.2881m/z)
Compound in Library Factor = 0

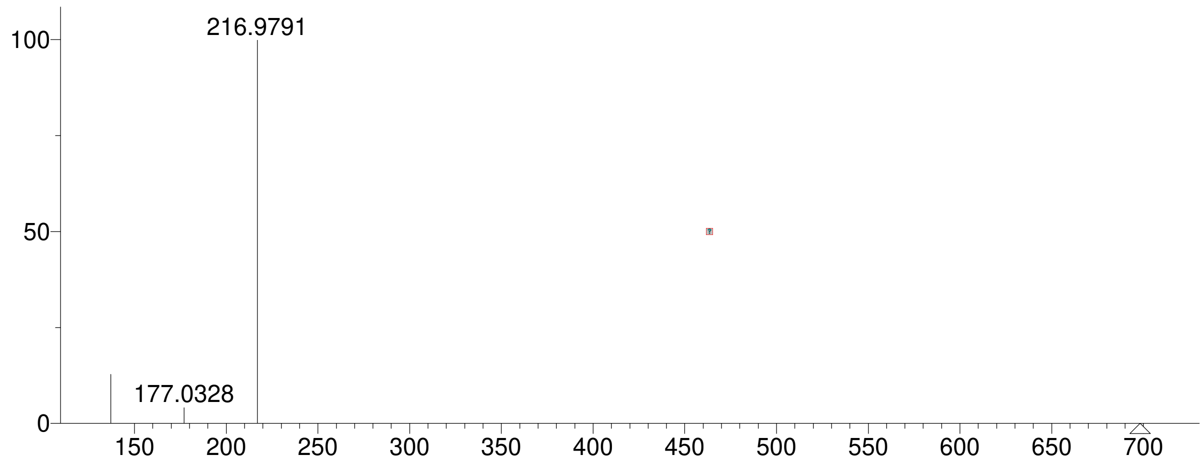

Unknown: Unknown (4.18_693.1751m/z)
Compound in Library Factor = 0

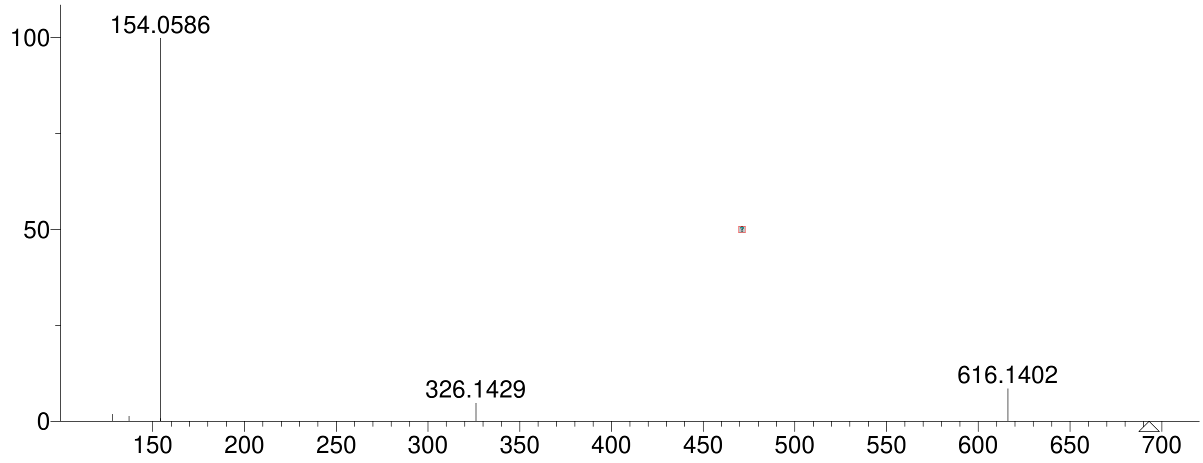

Unknown: Unknown (4.19_347.1229m/z)
Compound in Library Factor = -237

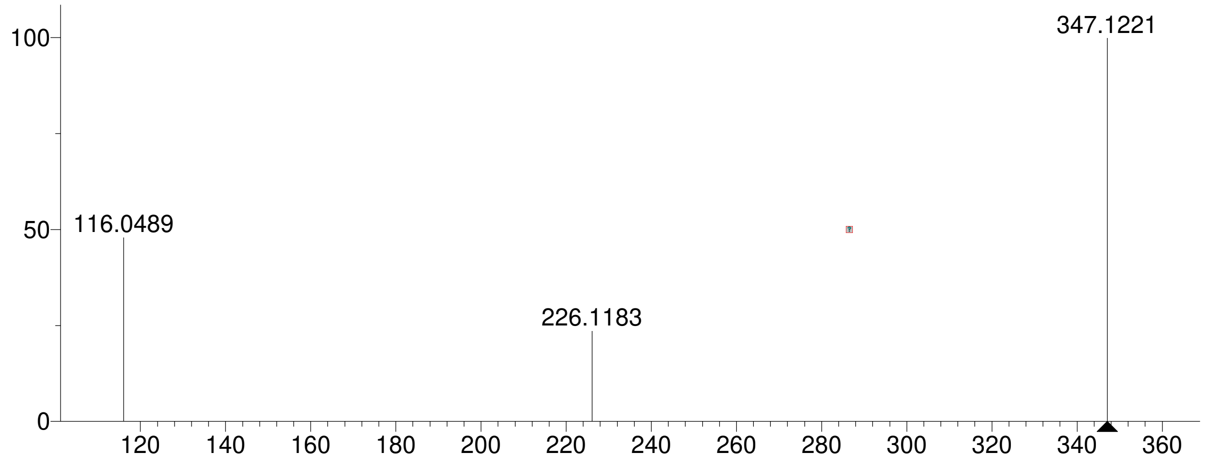

Hit 1 : 1H-Indole-1-acetic acid, 5-fluoro-2-methyl-3-(2-quinolinylmethyl)- [M-H]- HCD 10V P=347.1
C21H17FN2O2; MF: 324; RMF: 948; Prob 98.7%; CAS: 851723-84-7; Lib: nist_msms; ID: 273058.

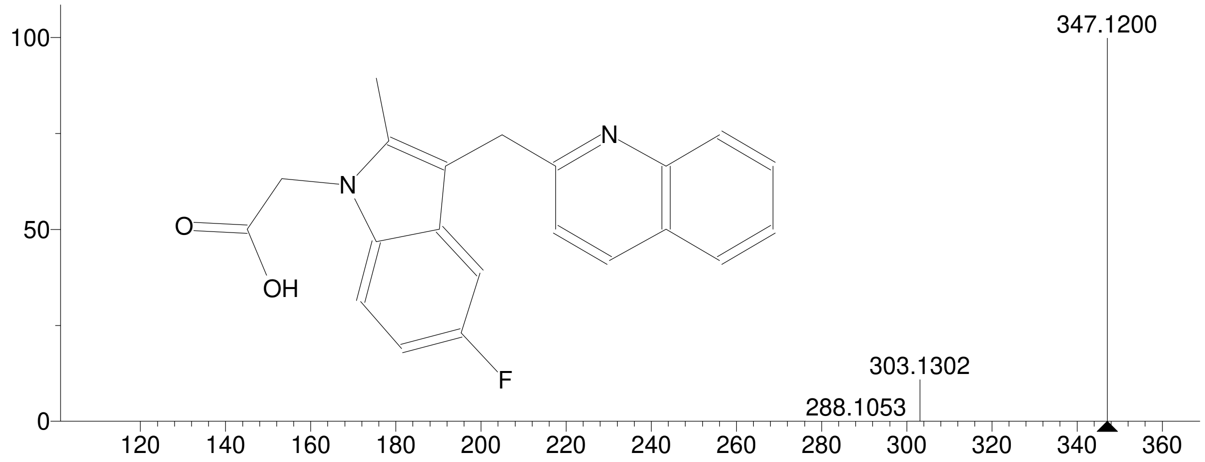

Hit 2 : Nifedipine [M+H]+ HCD 3V P=347.1
C17H18N2O6; MF: 82; RMF: 784; Prob 0.98%; CAS: 21829-25-4; Lib: nist_msms; ID: 105423.

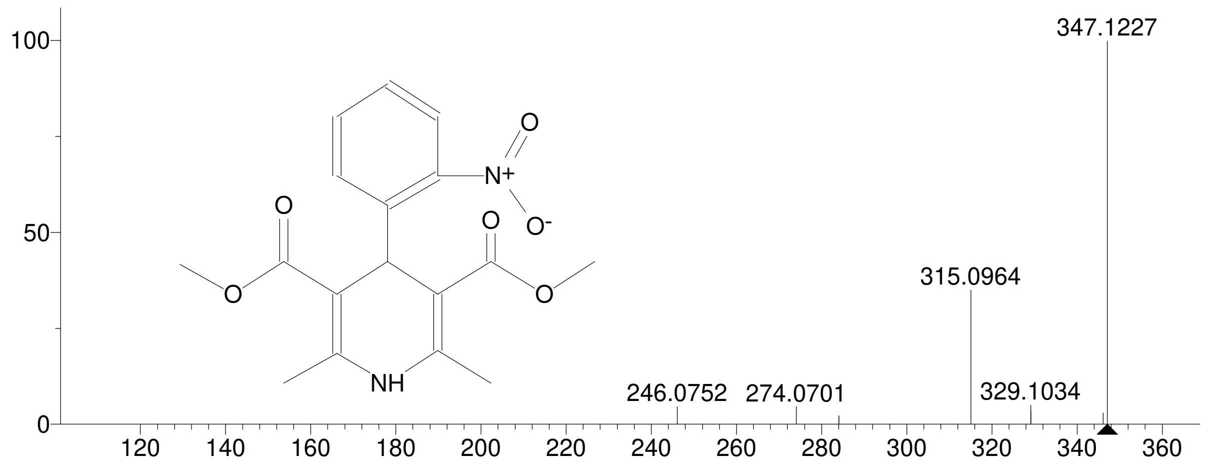

Hit 3 : (E)-1-(2,4-dihydroxyphenyl)-3-[4-(3-methylbut-2-enoxy)phenyl]prop-2-en-1-one [M+Na]+ 35HCD P=347.1
C20H20O4; MF: 0; RMF: 80; Prob 0.10%; Lib: mona-export-vf-npl_qexactive; ID: 10435.

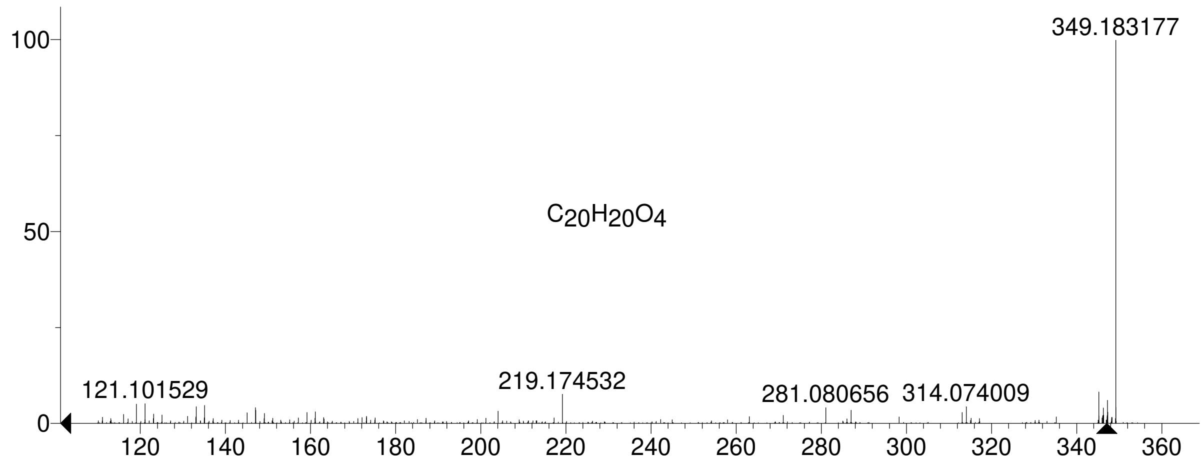

Hit 4 : Nifedipine (Adalat) [M+H]+ P=347.1
C17H18N2O6; MF: 0; RMF: 43; Prob 0.10%; Lib: mona-export-gnps; ID: 626.

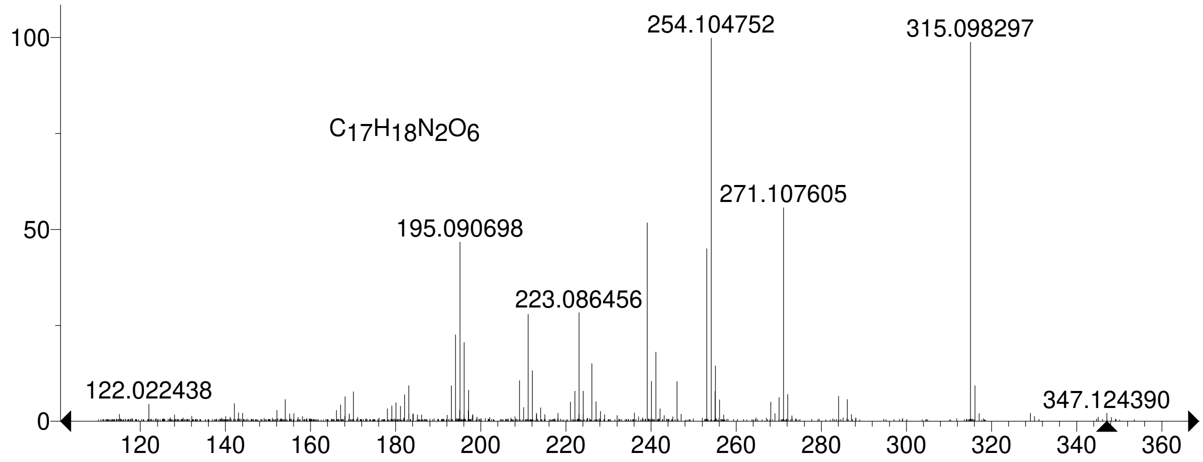

Hit 5 : 4-(8-prop-1-en-2-yl-3,4,8,9-tetrahydro-2H-furo[2,3-h]chromen-3-yl)benzene-1,3-diol [M+Na]+ 35HCD P=3
C20H20O4; MF: 0; RMF: 27; Prob 0.10%; Lib: mona-export-vf-npl_qexactive; ID: 16300.

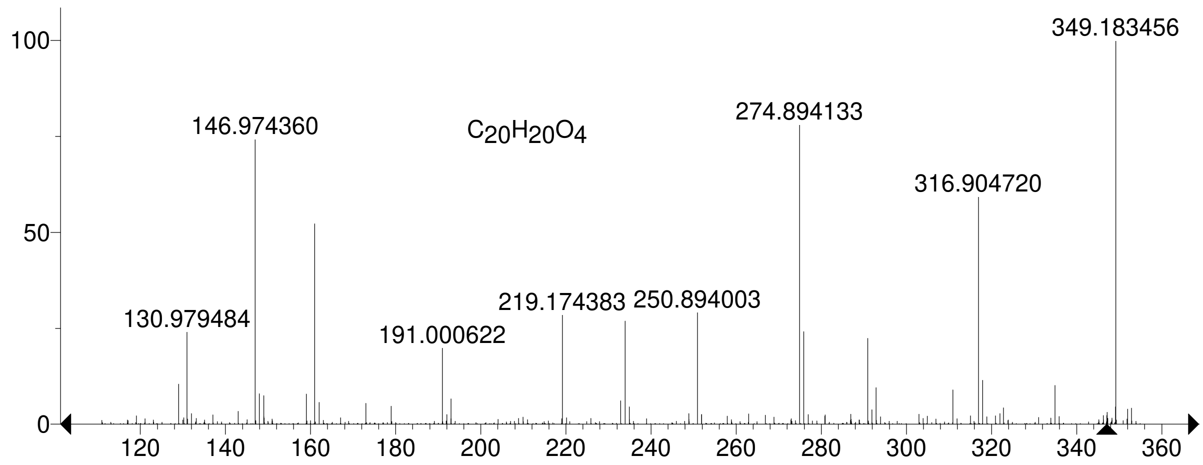

Unknown: Unknown (4.19_359.1451m/z)
Compound in Library Factor = 0

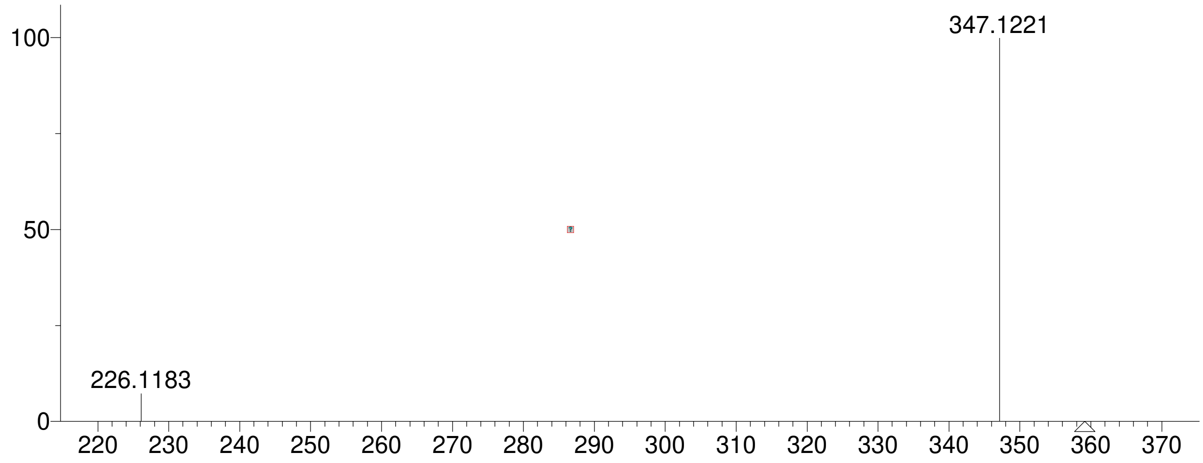

Unknown: Unknown (4.19_461.1884m/z)
Compound in Library Factor = 0

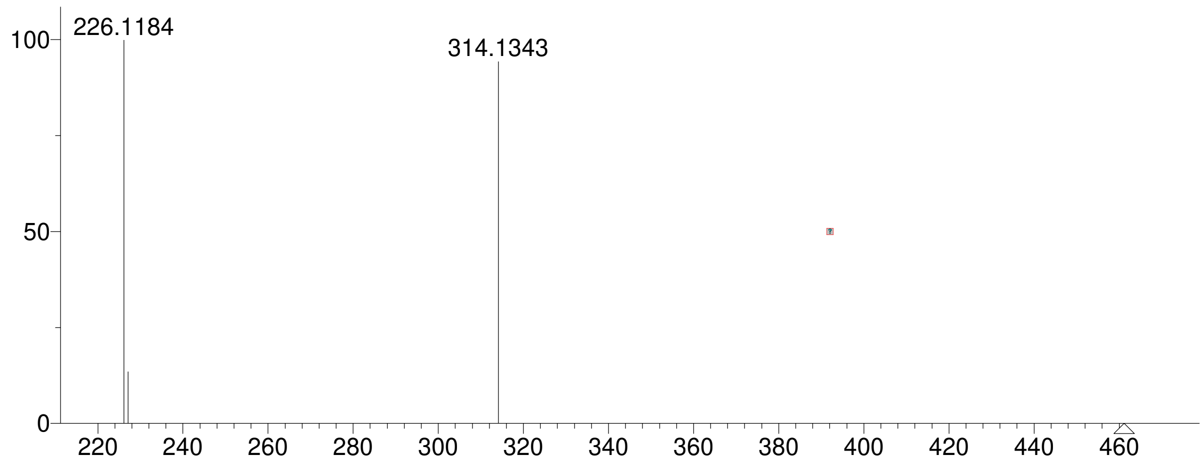

** Search Report Page 1 of 1 **

Unknown: Unknown (4.19_626.1365m/z)
Compound in Library Factor = 0

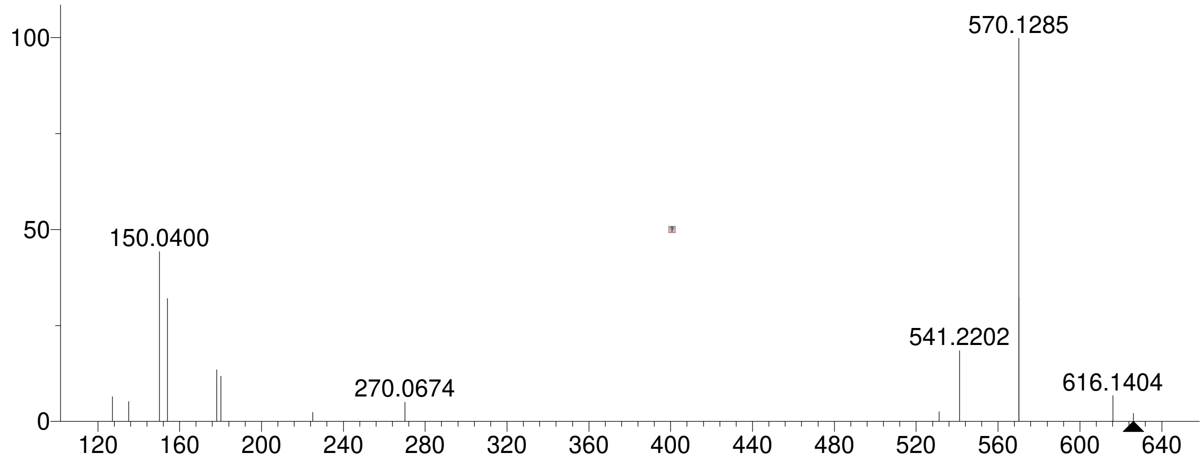

Hit 1 : Calcitonin Gene Related Peptide (8-37), rat [M+5H]5+ QQQ 20V P=626.1
C138H224N42O41; MF: 0; RMF: 22; Prob 100.0%; Lib: nist_msms2; ID: 20967.

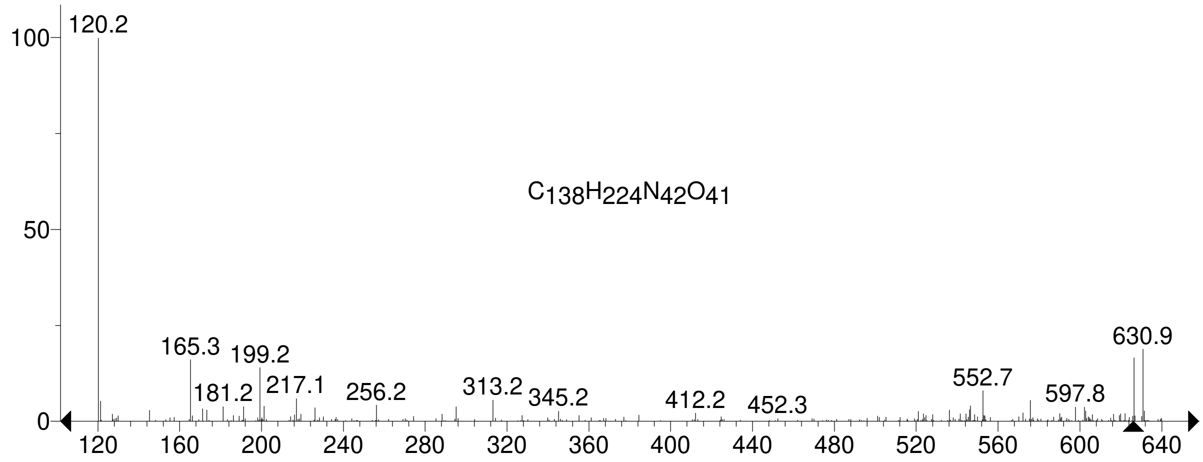

** Search Report Page 1 of 1 **

Unknown: Unknown (4.20_332.1698m/z)
Compound in Library Factor = 0

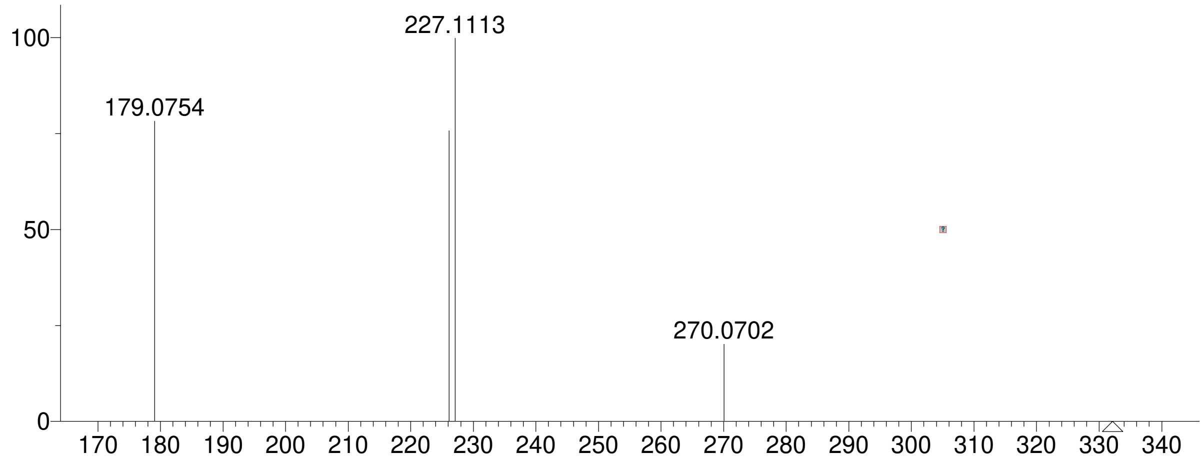

Unknown: Unknown (4.20_587.1140m/z)
Compound in Library Factor = 0

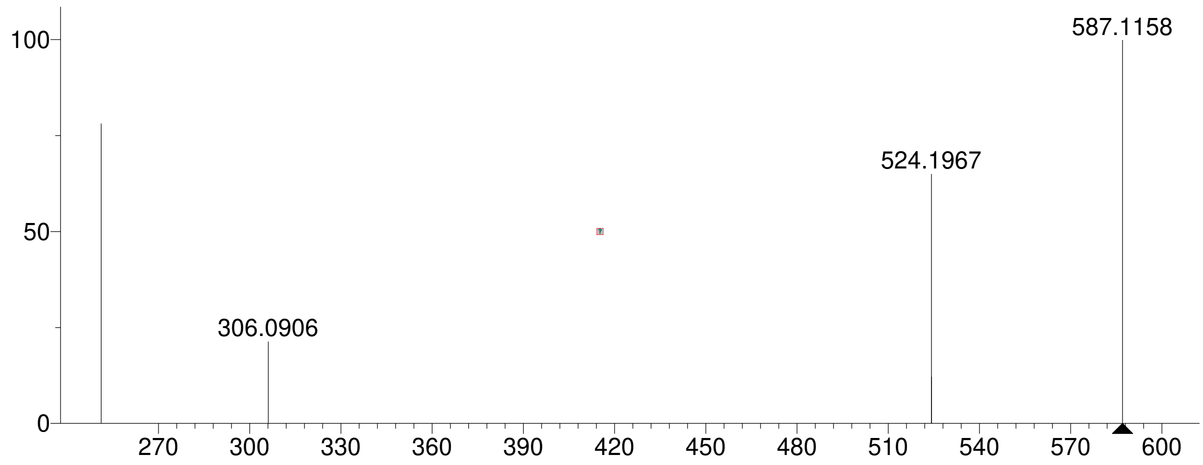

Hit 1 : 5,7-dihydroxy-2-phenyl-6-[3,4,5-trihydroxy-6-(hydroxymethyl)oxan-2-yl]-8-(3,4,5-trihydroxyoxan-2-yl)chrome
C26H28O13; MF: 0; RMF: 28; Prob 50.0%; Lib: anthocyanins mona; ID: 8506.

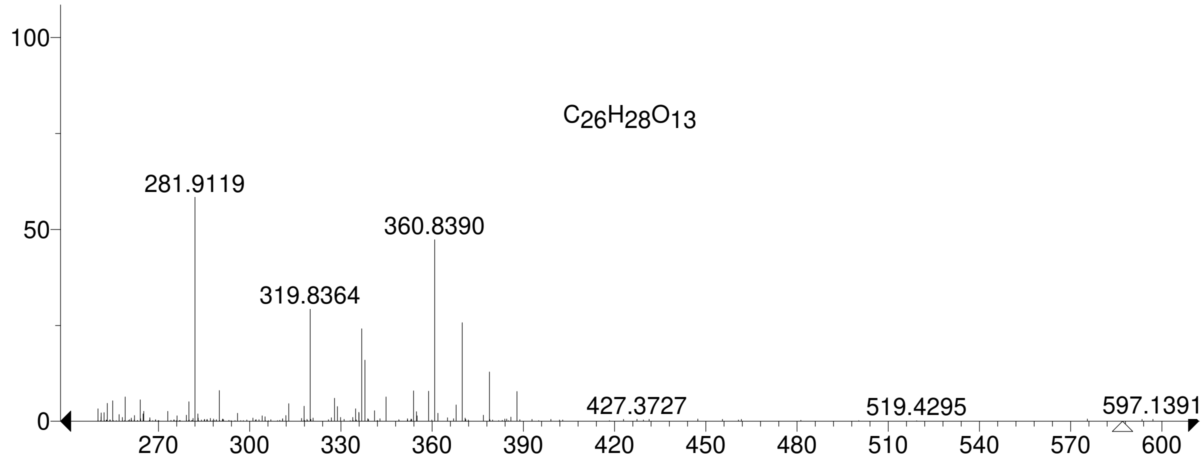

Hit 2 : Theaflavin [M+Na]+ 35HCD P=587.1
C29H24O12; MF: 0; RMF: 15; Prob 50.0%; Lib: mona-export-vf-npl_qexactive; ID: 2698.

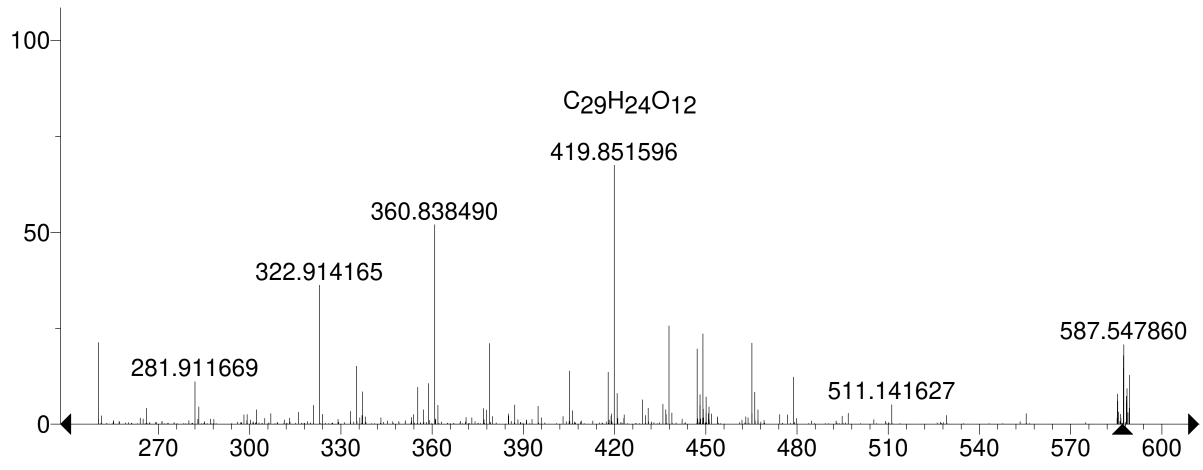

Unknown: Unknown (4.21_458.1986m/z)
Compound in Library Factor = 0

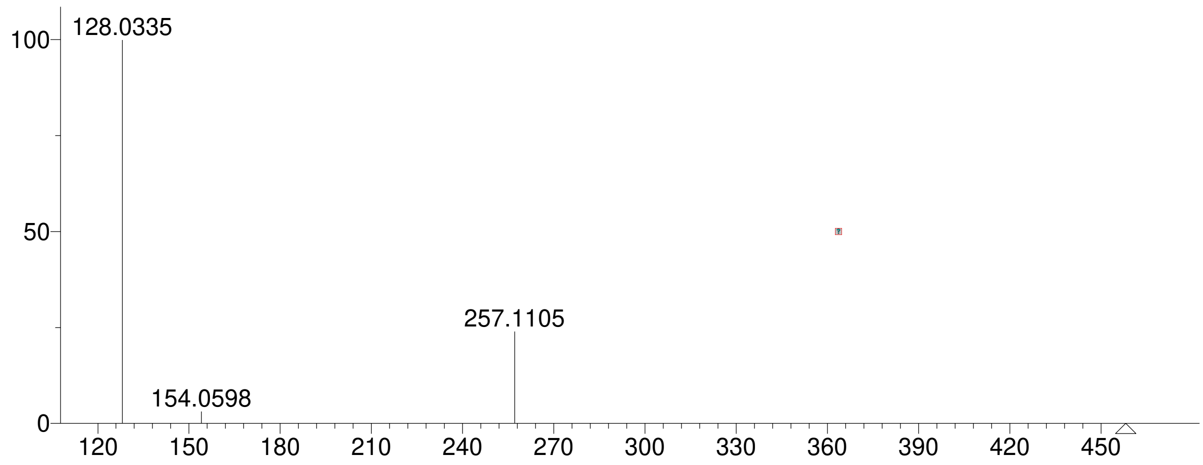

** Search Report Page 1 of 1 **

Unknown: Unknown (4.23_453.1474m/z)
Compound in Library Factor = 0

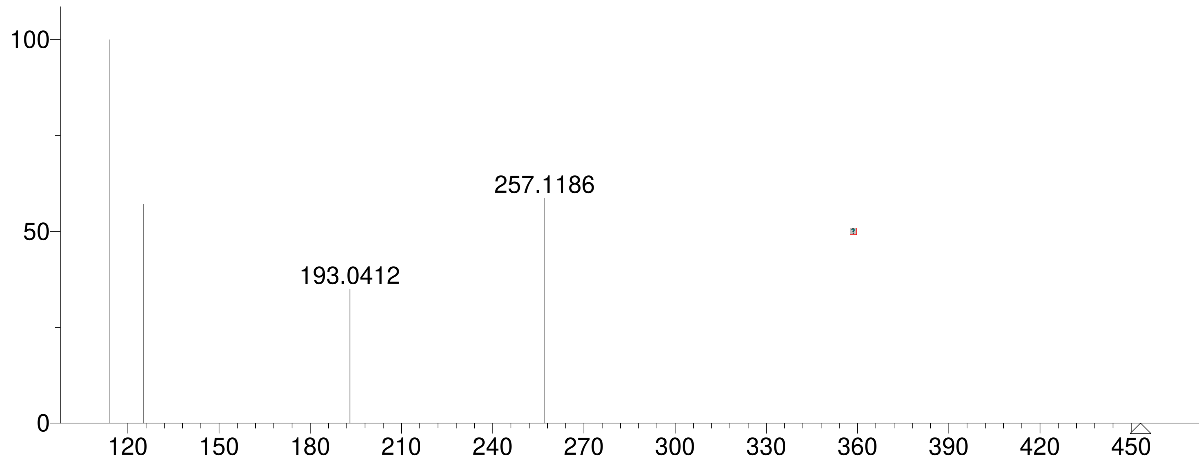

** Search Report Page 1 of 1 **

Unknown: Unknown (4.24_341.1436m/z)
Compound in Library Factor = 0

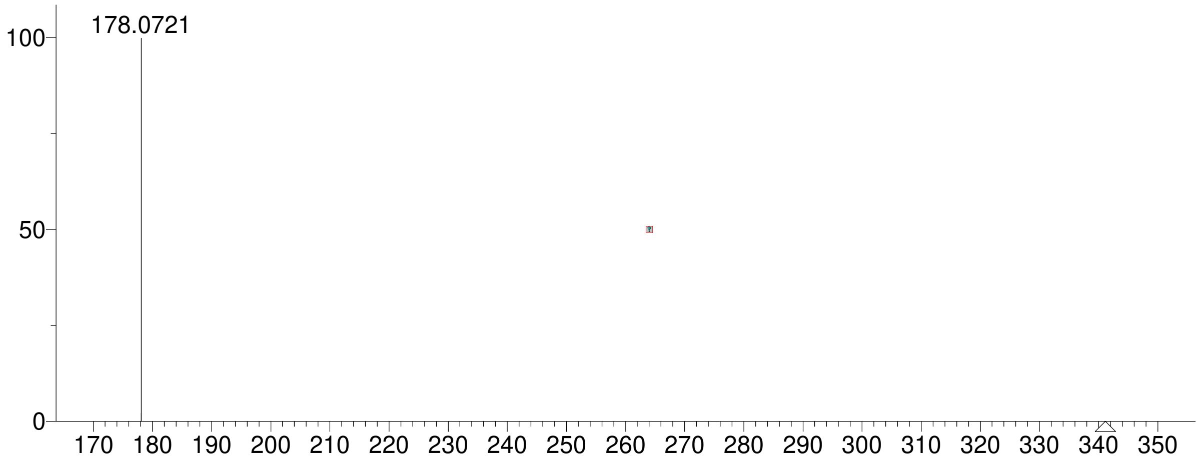

Unknown: Unknown (4.24_354.1044m/z)
Compound in Library Factor = -434

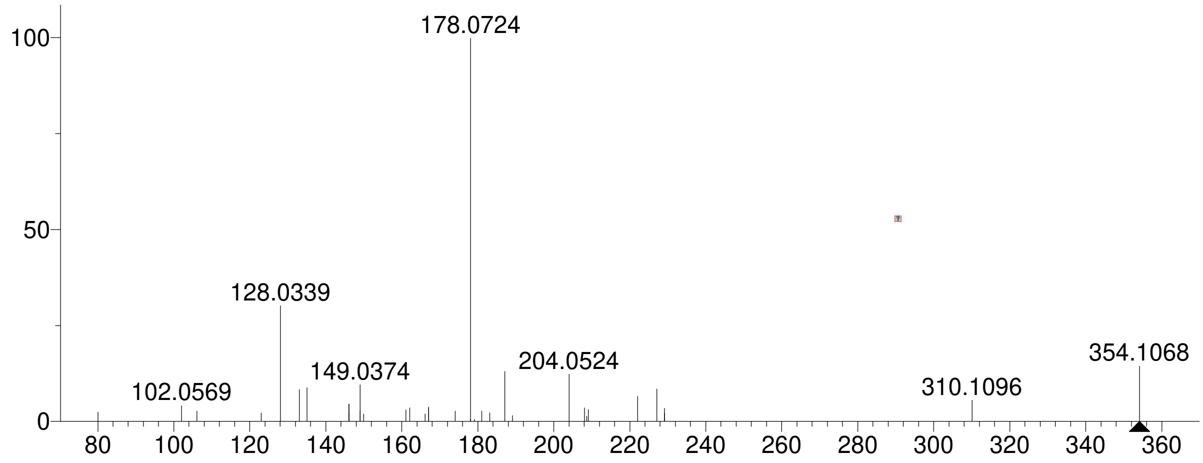

Hit 1 : Nizatidine [M+Na]+ HCD 10V P=354.1
C12H21N5O2S2; MF: 174; RMF: 820; Prob 94.3%; CAS: 76963-41-2; Lib: nist_msms; ID: 152083.

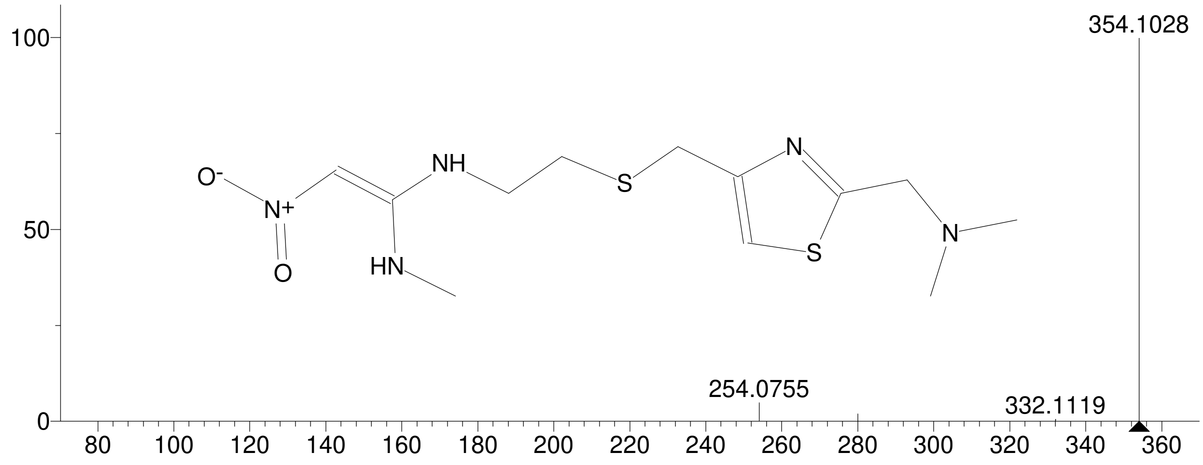

Hit 2 : 5'-O-Acetyladenosine [M+HCO2]- HCD 5V P=354.1
C12H15N5O5; MF: 15; RMF: 224; Prob 2.37%; CAS: 2140-25-2; Lib: nist_msms; ID: 228060.

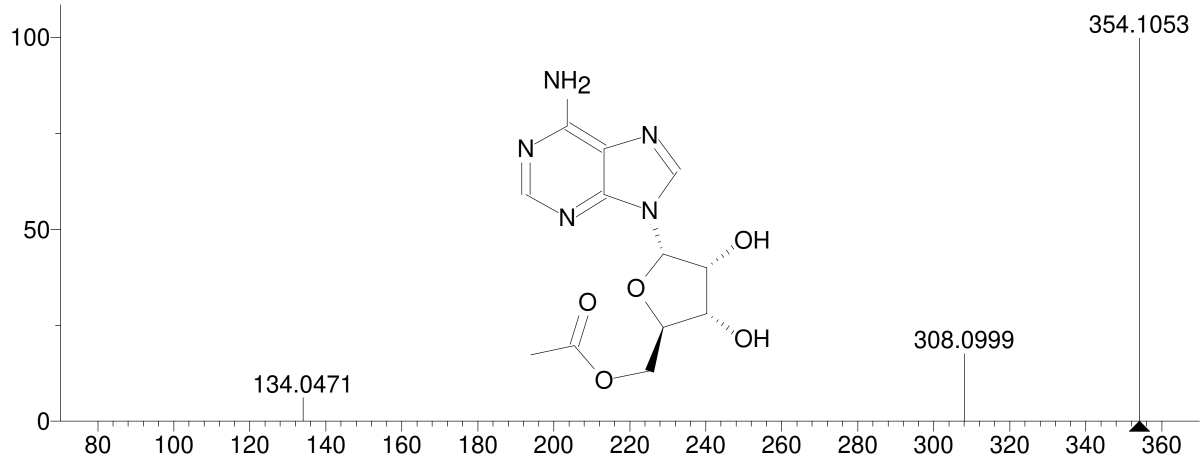

Hit 3 : rac-4-Sulfoxypropranolol [M-H]- HCD 17V P=354.1
C16H21NO6S; MF: 9; RMF: 212; Prob 1.86%; CAS: 87075-33-0; Lib: nist_msms; ID: 443588.

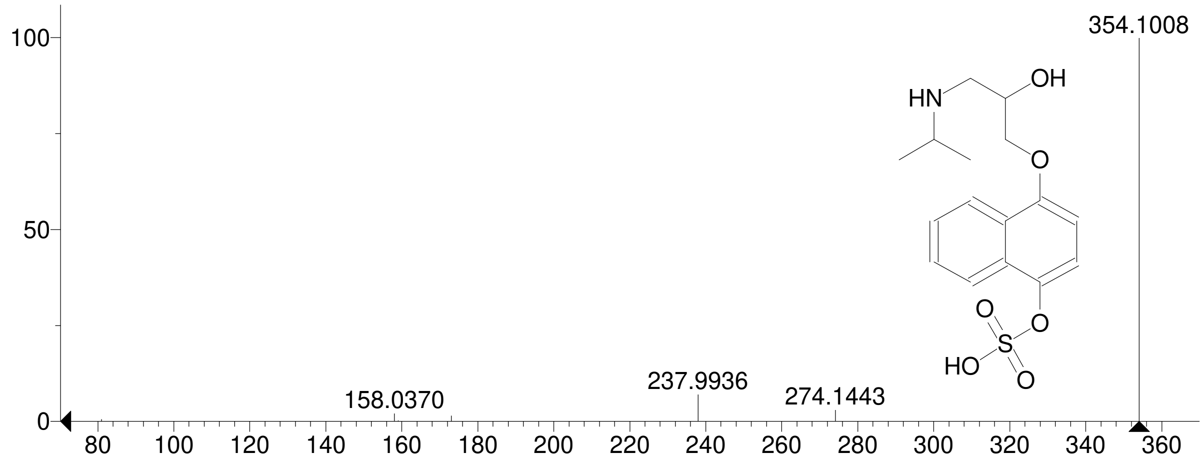

Hit 4 : 1H-Indole-2-propanoic acid, 1-[(4-chlorophenyl)methyl]-5-fluoro-α,α,3-trimethyl- [M-H-H2O]- HCD 31V P
C21H21ClFNO2; MF: 4; RMF: 167; Prob 1.50%; CAS: 103253-15-2; Lib: nist_msms; ID: 430036.

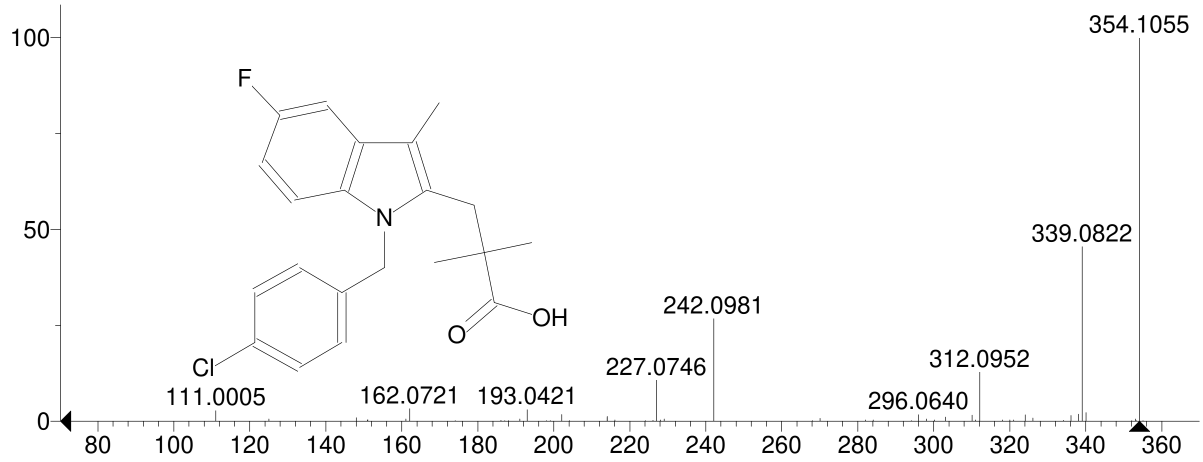

Unknown: Unknown (4.24_374.1548m/z)
Compound in Library Factor = 0

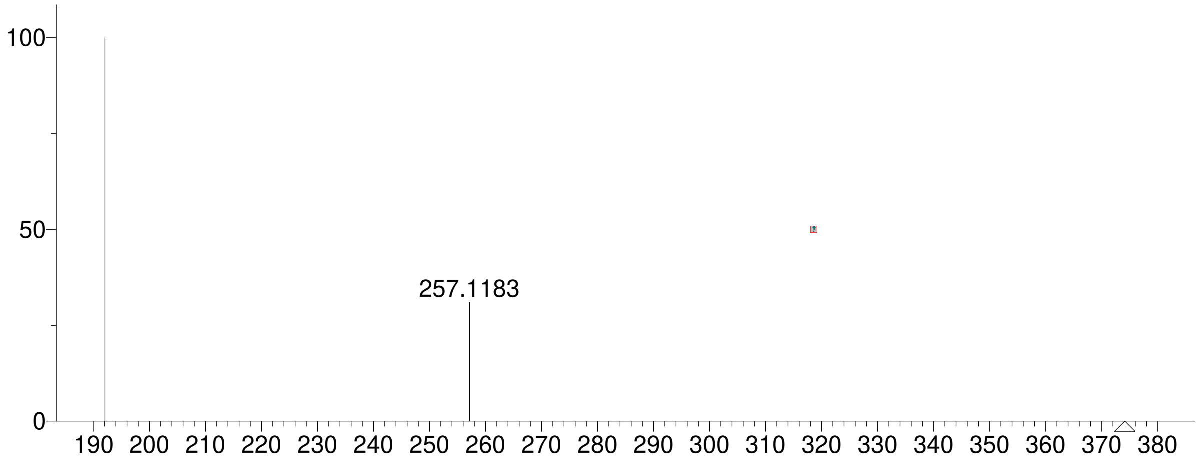

Hit 1 : Gatifloxacin [M-H]- HCD 82V P=374.2
C19H22FN3O4; MF: 0; RMF: 143; Prob 100.0%; CAS: 112811-59-3; Lib: nist_msms; ID: 357665.

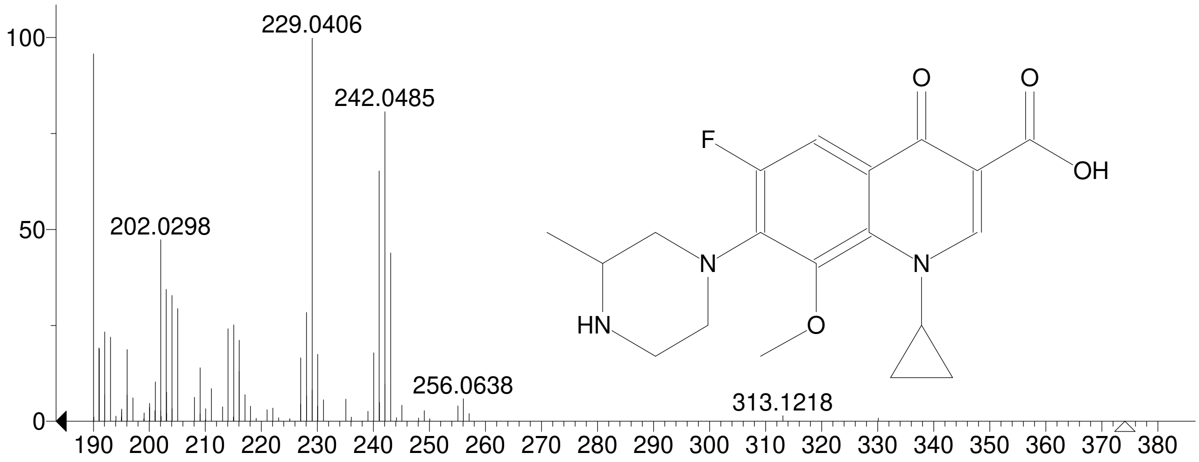

Unknown: Unknown (4.25_420.1492m/z)
Compound in Library Factor = 0

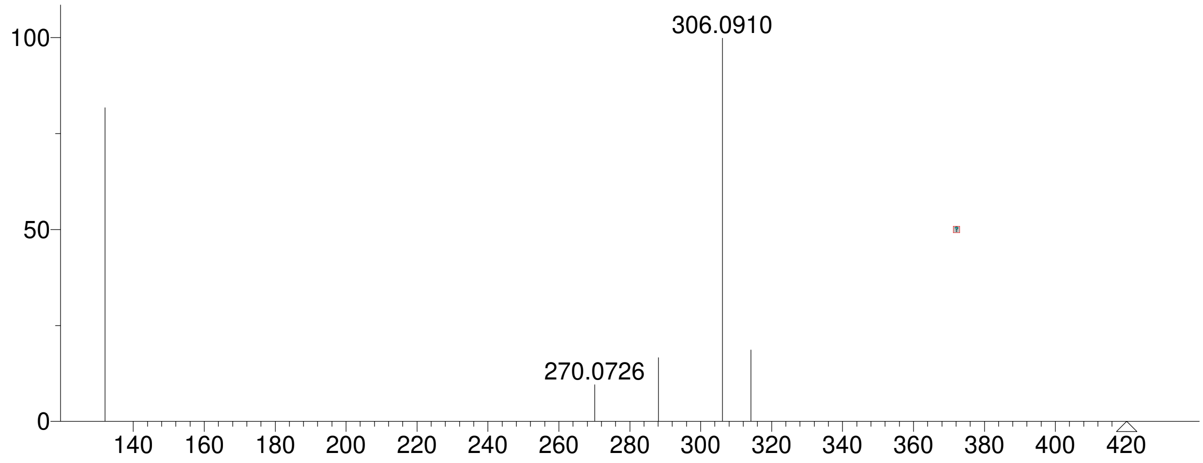

** Search Report Page 1 of 1 **

Unknown: Unknown (4.25_476.1507m/z)
Compound in Library Factor = 0

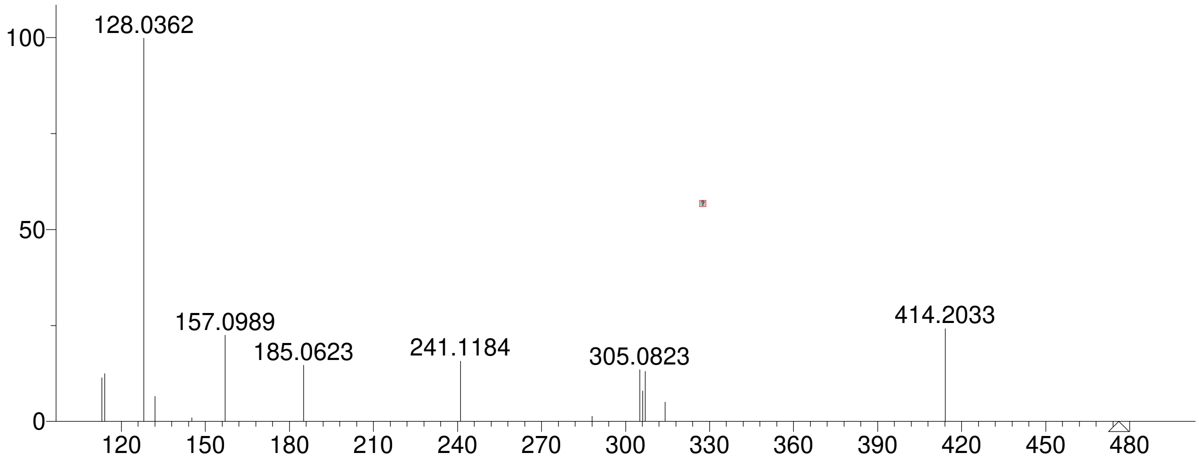

** Search Report Page 1 of 1 **

Unknown: Unknown (4.26_502.2020m/z)
Compound in Library Factor = 0

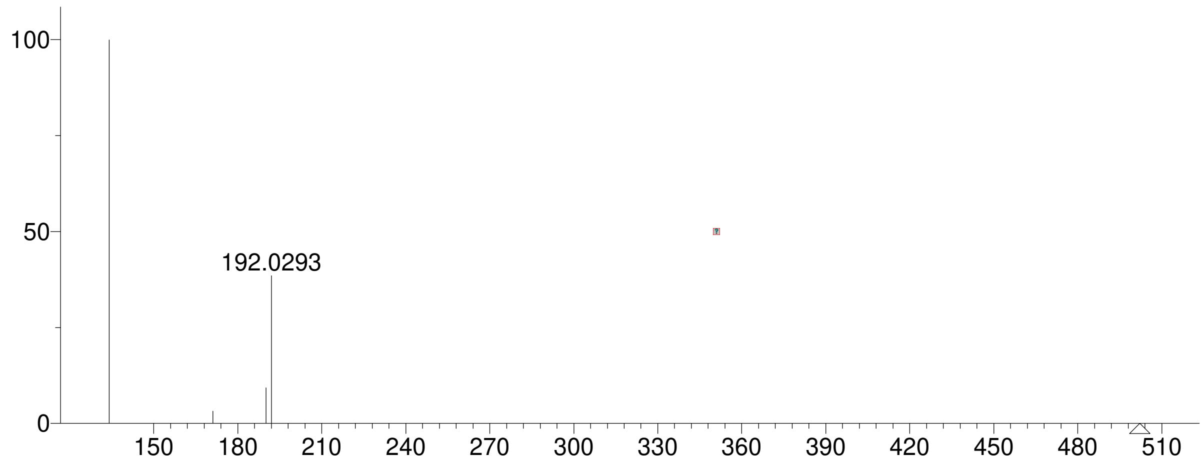

** Search Report Page 1 of 1 **

Unknown: Unknown (4.26_586.1295m/z)
Compound in Library Factor = 0

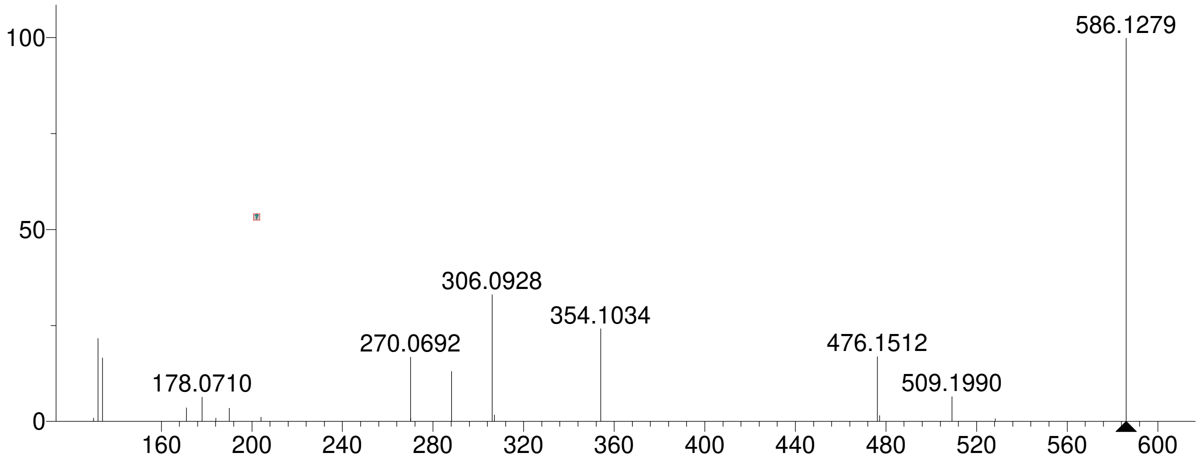

** Search Report Page 1 of 1 **

Unknown: Unknown (4.26_646.2093m/z)
Compound in Library Factor = 0

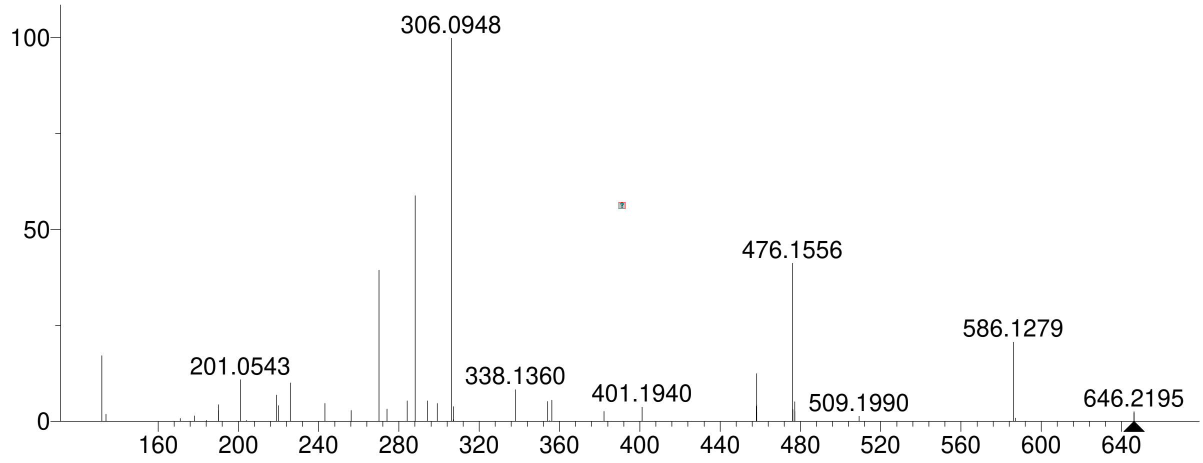

Unknown: Unknown (4.26_647.2143m/z)
Compound in Library Factor = 0

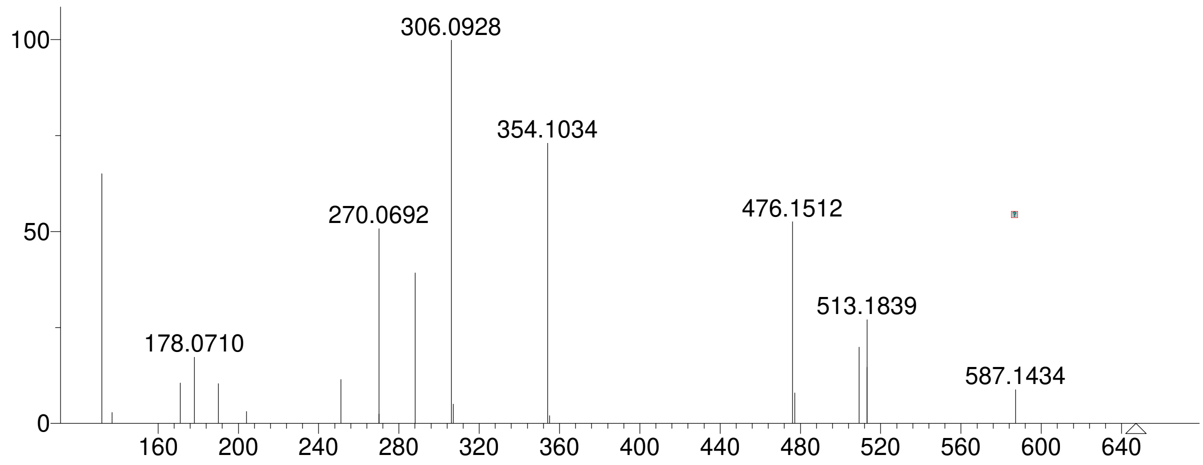

Hit 1 : NCGC00384546-01!7-[(2S,3R,4S,5S,6R)-4,5-dihydroxy-3-[(2R,3R,4R,5R,6S)-3,4,5-trihydroxy-6-methyloxa
C28H38O17; MF: 0; RMF: 12; Prob 50.0%; Lib: mona-export-gnps; ID: 6655.

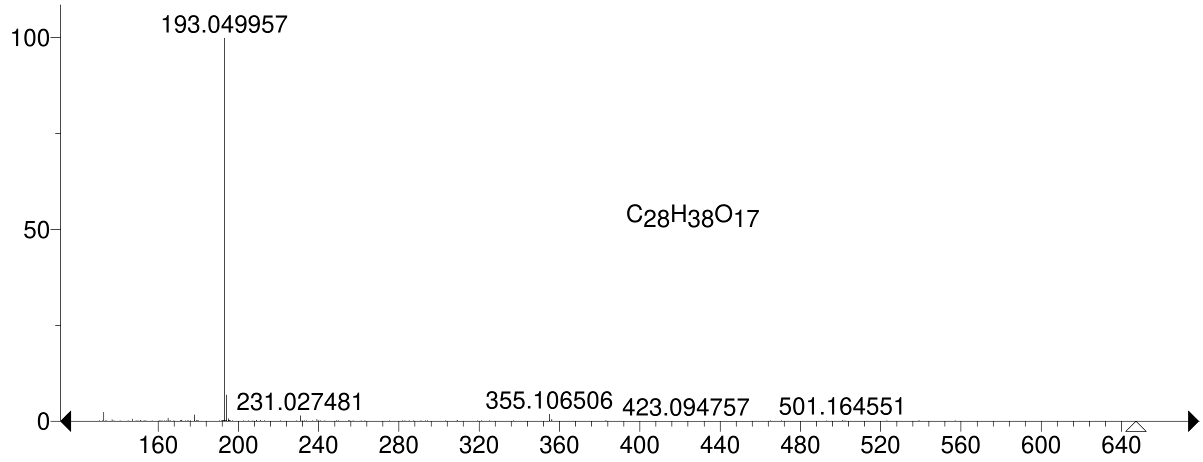

Hit 2 : 7-[(2S,3R,4S,5S,6R)-4,5-dihydroxy-3-[(2R,3R,4R,5R,6S)-3,4,5-trihydroxy-6-methyloxan-2-yl]oxy-6-[[(2R,3F
C28H38O17; MF: 0; RMF: 2; Prob 50.0%; Lib: anthocyanins mona; ID: 7149.

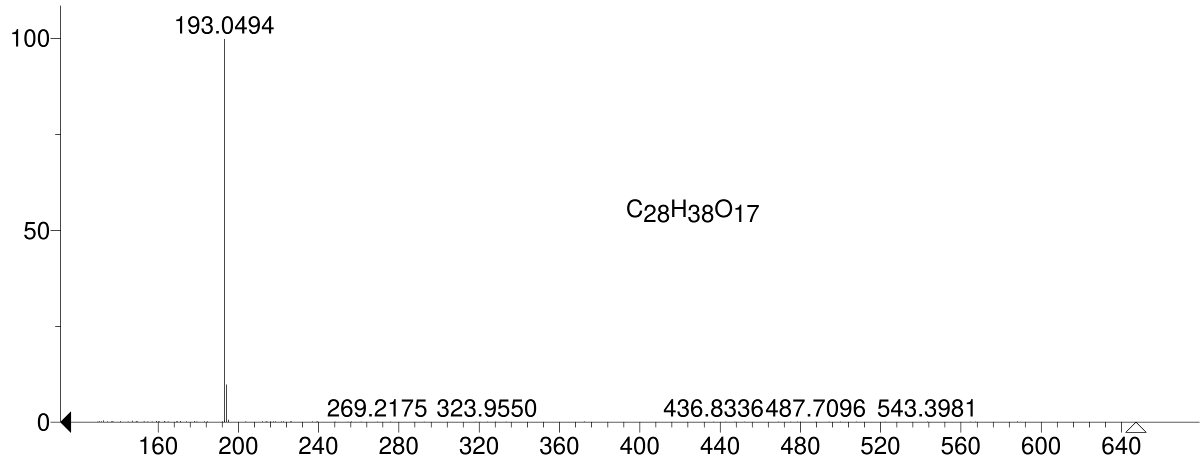

Unknown: Unknown (4.26_699.1242m/z)
Compound in Library Factor = 0

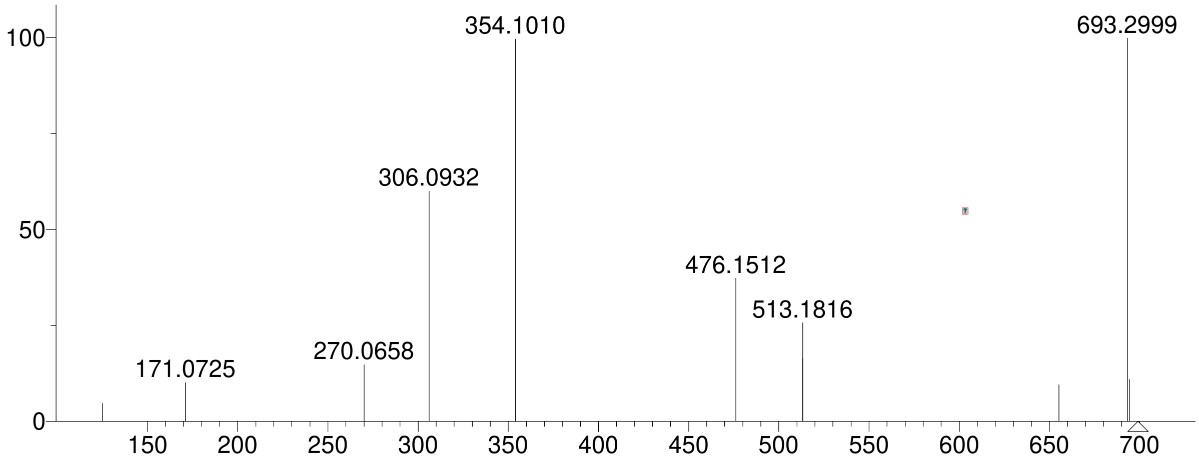

** Search Report Page 1 of 1 **

Unknown: Unknown (4.28_683.2515m/z)
Compound in Library Factor = 0

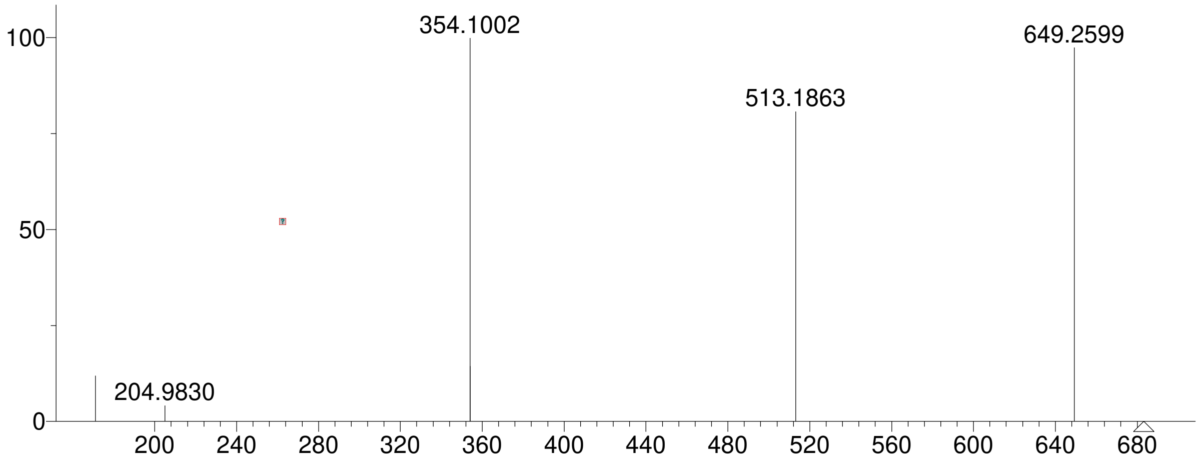

Unknown: Unknown (4.29_204.9803m/z)
Compound in Library Factor = 0

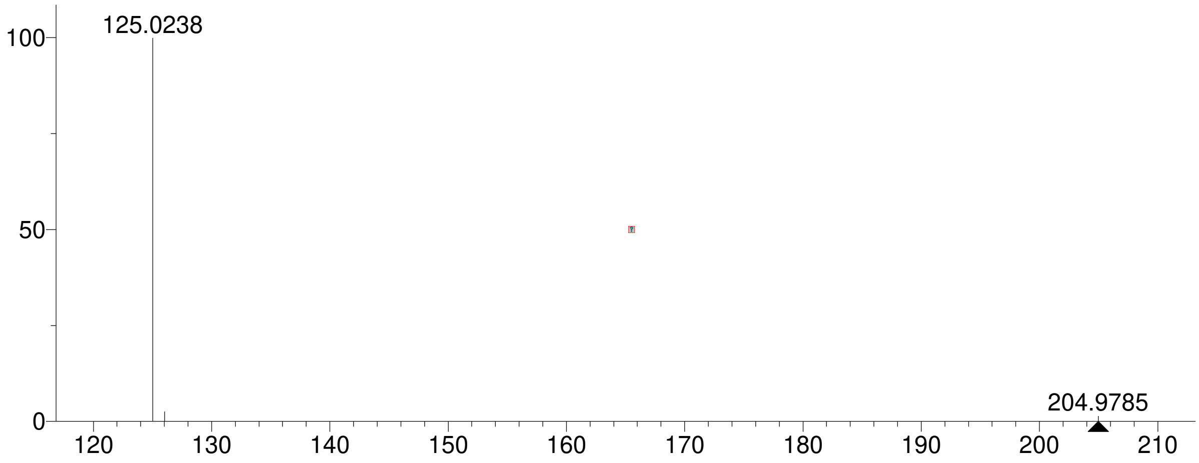

Unknown: Unknown (4.29_693.2963m/z)
Compound in Library Factor = -733

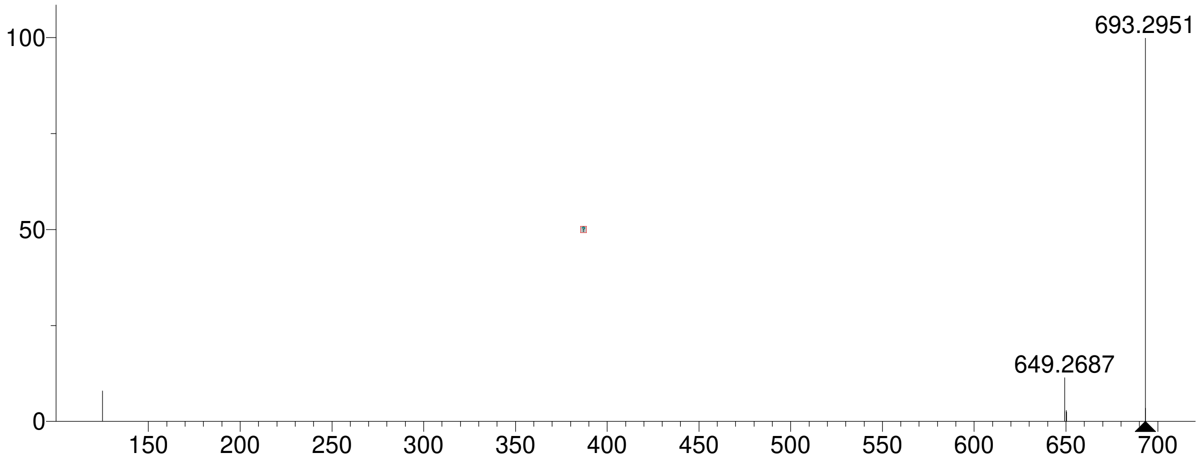

Hit 1 : Strictosidine hexoside ([H][C@@]1(C[C@]2([H])C(=CO[C@@]([H])(O[C@]3([H])O[C@]([H])(CO[C@]4([H])
C33H44N2O14; MF: 10; RMF: 618; Prob 100.0%; Lib: alkaloids; ID: 27.

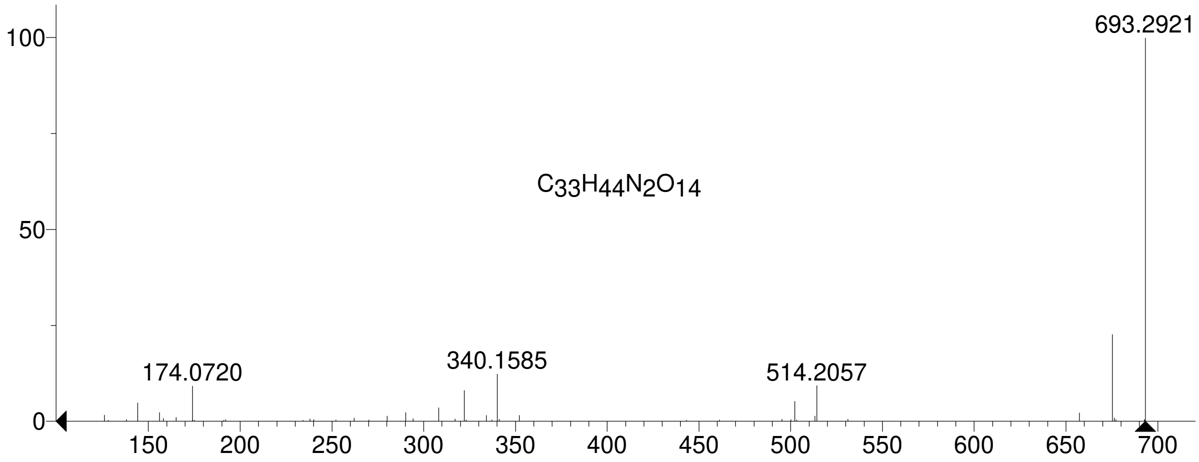

Unknown: Unknown (4.30_481.0932m/z)
Compound in Library Factor = 0

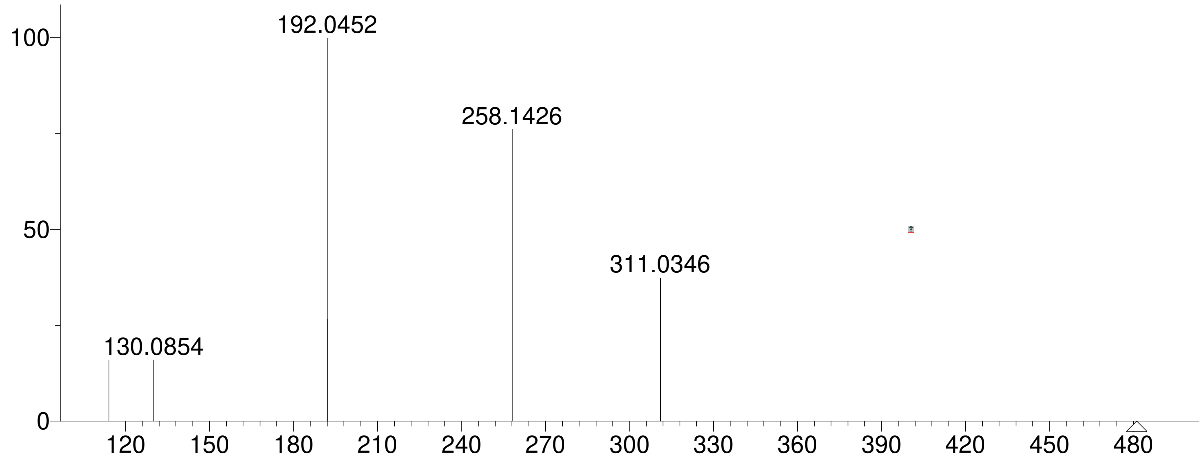

Hit 1 : Gossypin [M+H]+ HCD 95V P=481.1
C21H20O13; MF: 0; RMF: 16; Prob 50.0%; CAS: 652-78-8; Lib: nist_msms; ID: 111983.

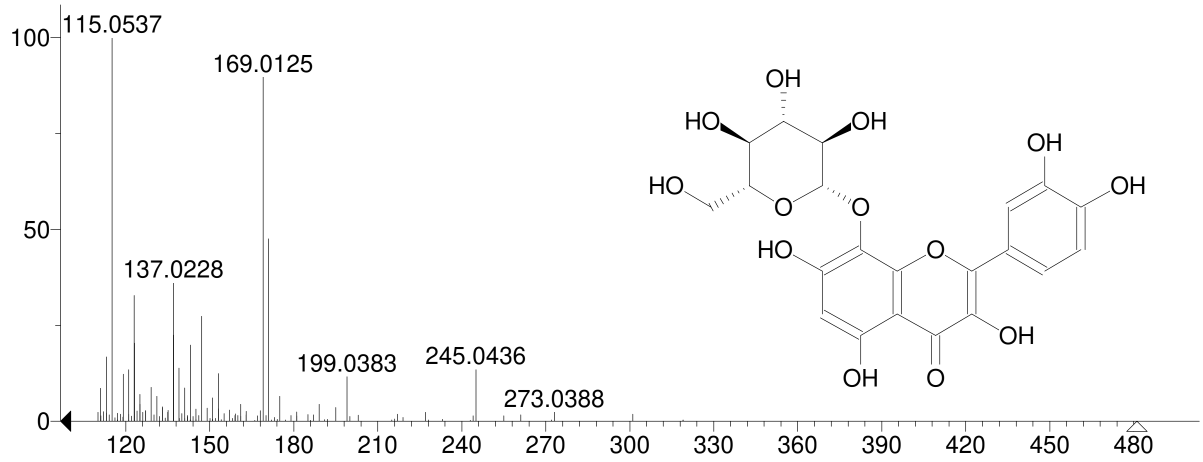

Hit 2 : Villol [M+K]+ 65HCD P=481.1
C23H22O9; MF: 0; RMF: 13; Prob 50.0%; Lib: mona-export-vf-npl_qexactive; ID: 8049.

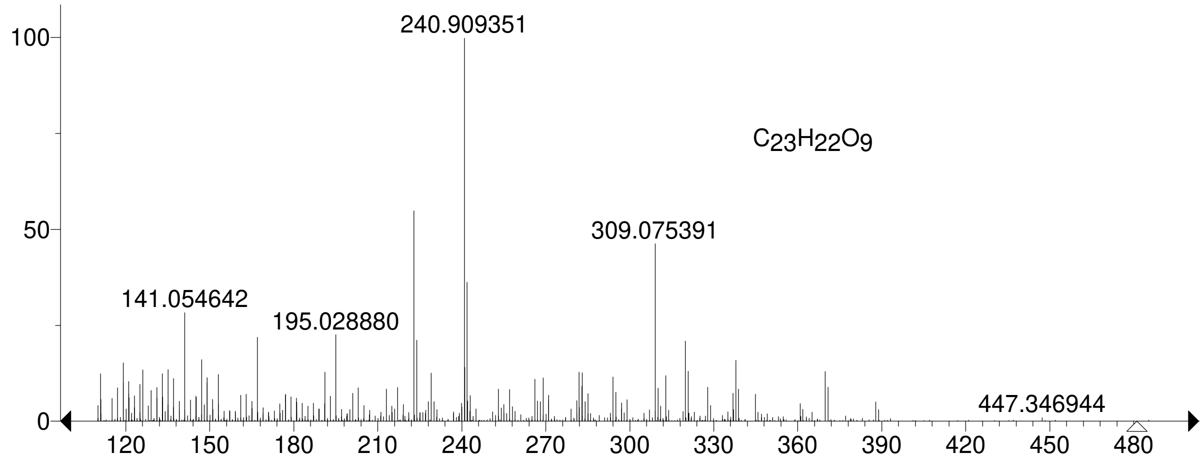

Unknown: Unknown (4.32_364.1387m/z)
Compound in Library Factor = 0

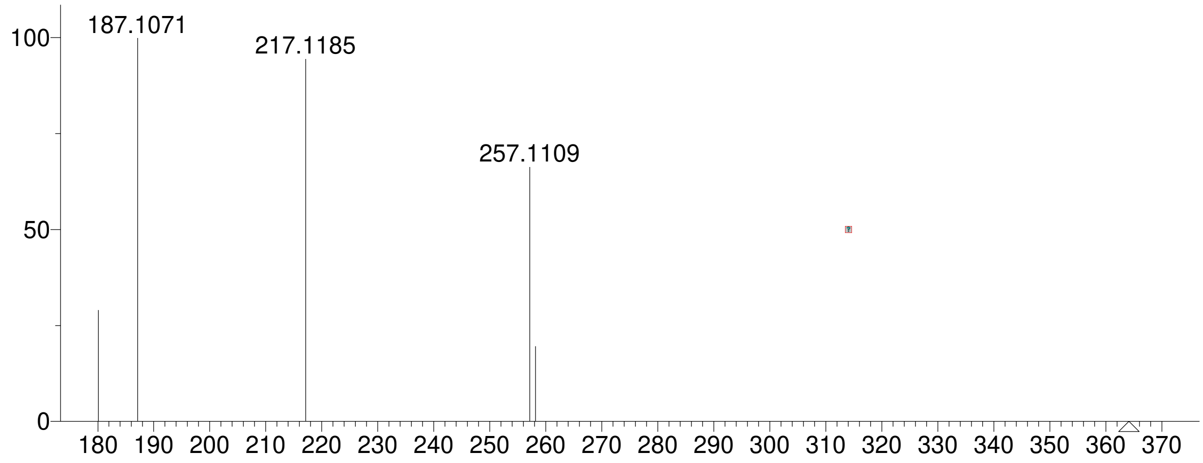

Hit 1 : methyl 2-(2,6-dihydroxy-4-methylbenzoyl)-3,5-dimethoxybenzoate [M+NH4]+ 35HCD P=364.1
C18H18O7; MF: 0; RMF: 18; Prob 100.0%; Lib: mona-export-vf-npl_qexactive; ID: 9811.

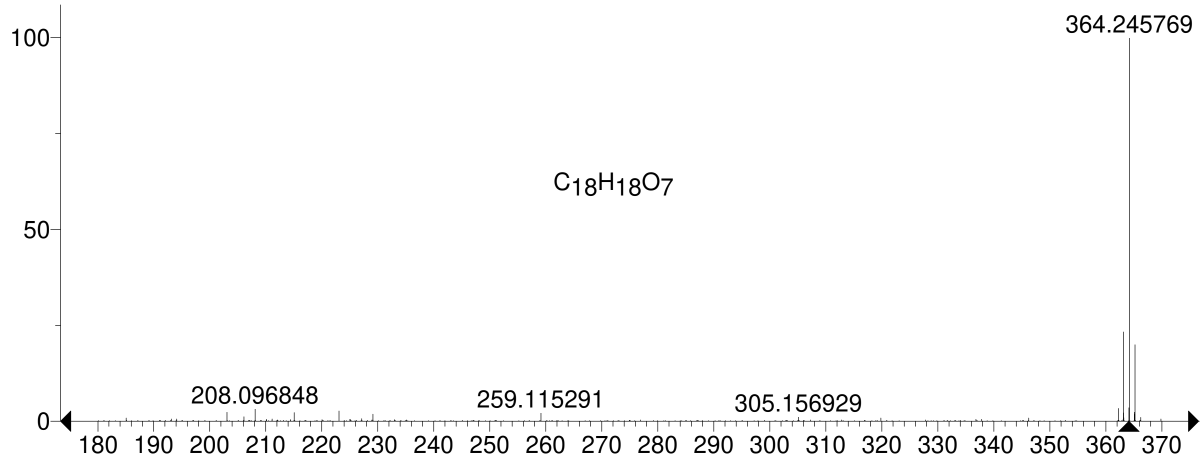

Unknown: Unknown (4.32_441.1862m/z)
Compound in Library Factor = 0

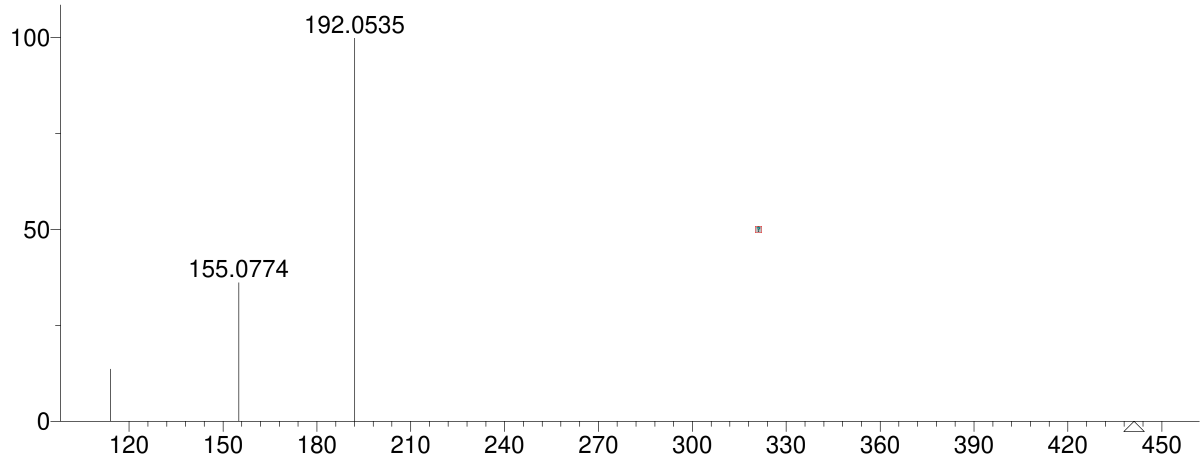

Hit 1 : NCGC00380134-01_C25H30O8_3-Furancarboxylic acid, (3R,5S,5aS,6S,9aR)-octahydro-9-hydroxy-2,2,5a
C25H30O8; MF: 0; RMF: 63; Prob 100.0%; Lib: mona-export-gnps; ID: 11250.

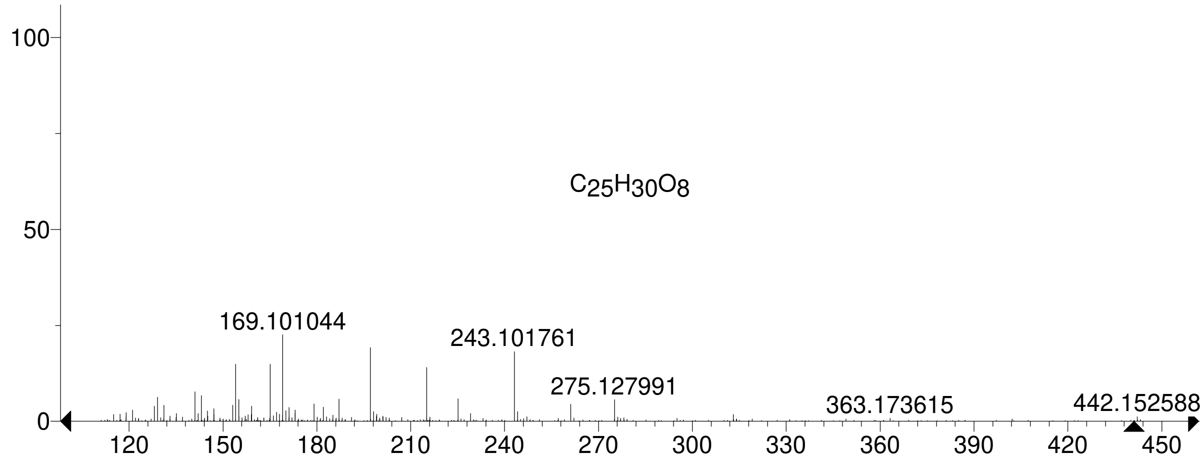

** Search Report Page 1 of 1 **

Unknown: Unknown (4.32_563.1022m/z)
Compound in Library Factor = 0

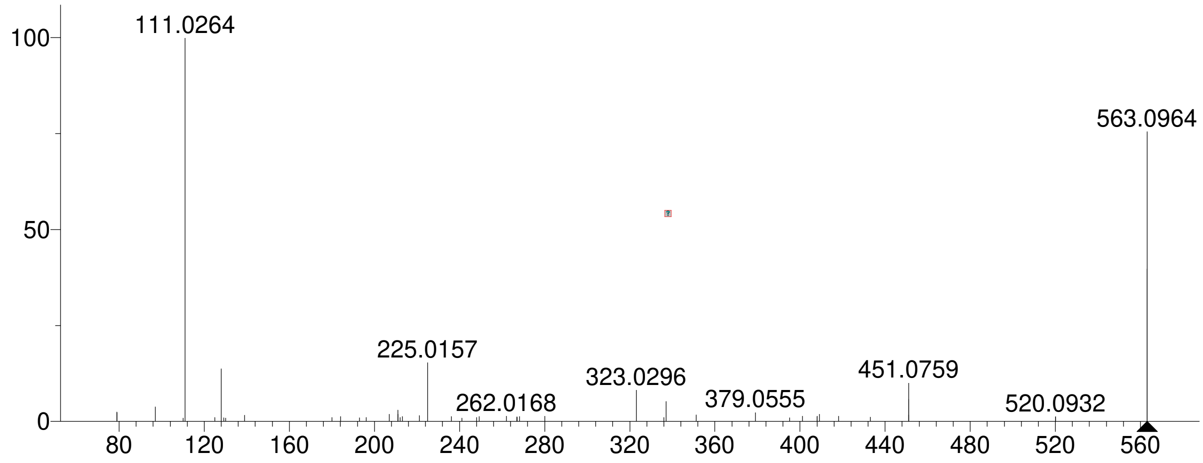

Hit 1 : Theaflavin 3-gallate [M-H]-=>563.1 IT 35% P=563.1
C36H28O16; MF: 0; RMF: 9; Prob 100.0%; CAS: 30462-34-1; Lib: nist_msms; ID: 394304.

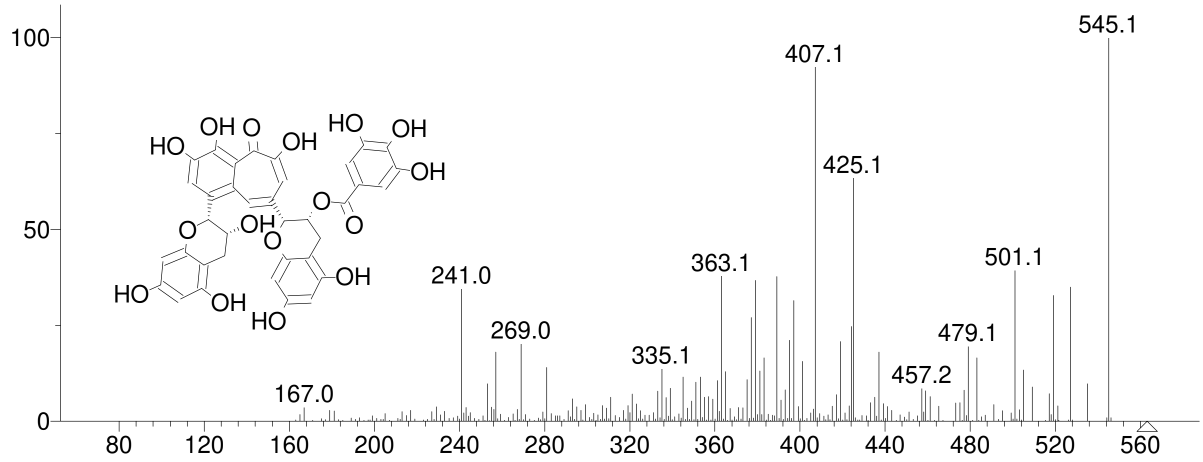

Unknown: Unknown (4.33_284.1238m/z)
Compound in Library Factor = -3273

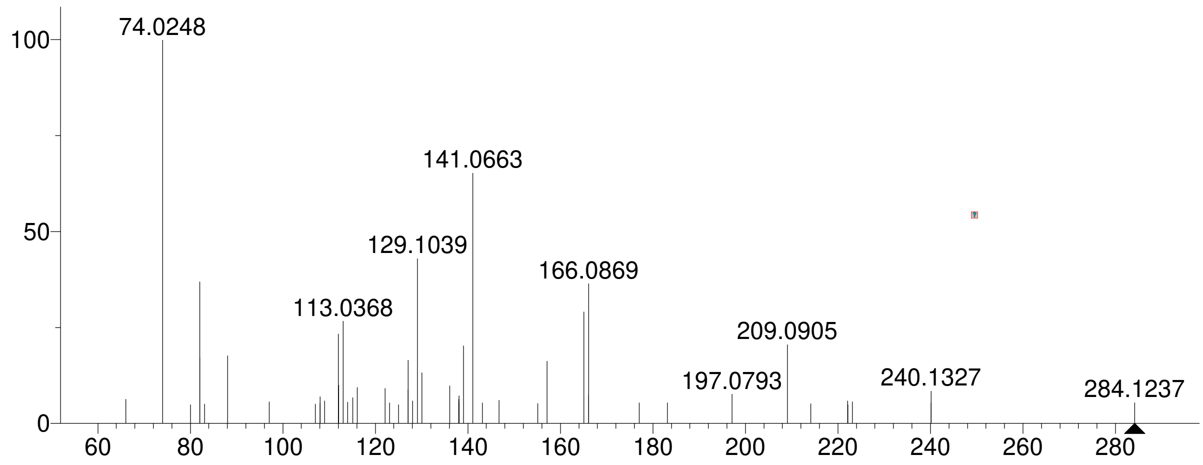

Hit 1 : O-(4-Hydroxybenzoyl)tropine [M+Na]+ HCD 5V P=284.1
C15H19NO3; MF: 3; RMF: 105; Prob 51.0%; CAS: 198898-04-3; Lib: nist_msms; ID: 552421.

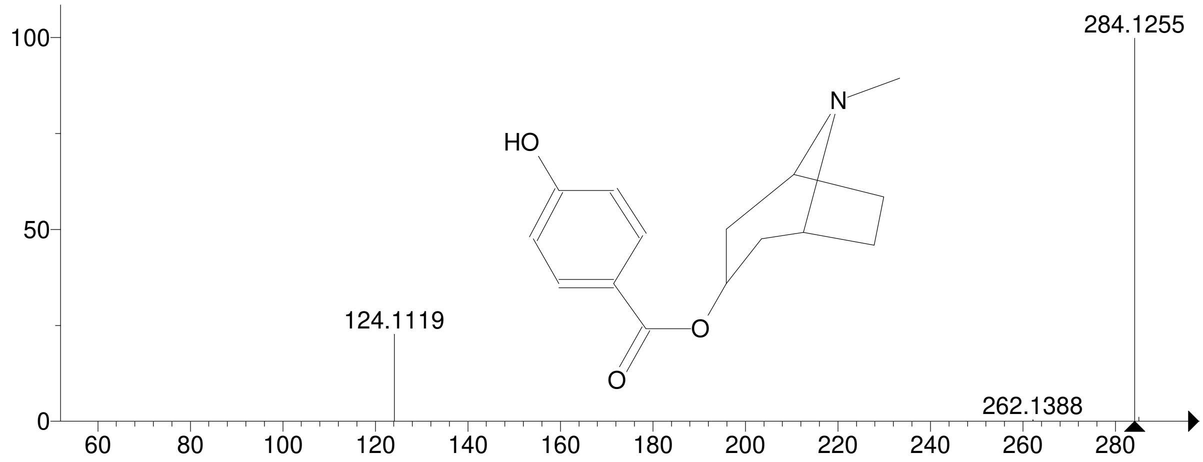

Hit 2 : Unknown (carbon number 12); PlaSMA ID-512 [M-H]- P=284.1
C12H19N3O5; MF: 2; RMF: 99; Prob 49.0%; Lib: biomsms-neg-plasma; ID: 1158.

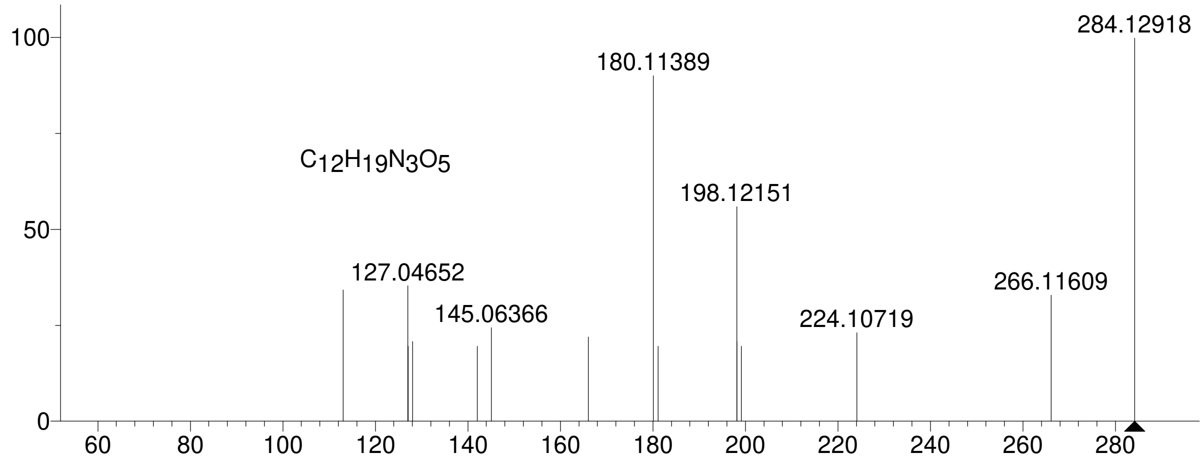

Unknown: Unknown (4.34_330.2021m/z)
Compound in Library Factor = -3273

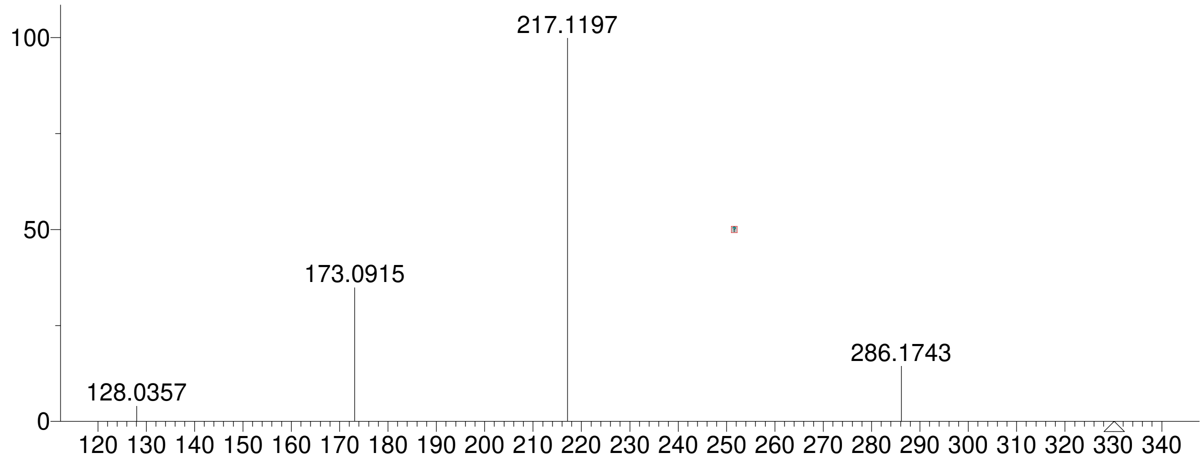

Hit 1 : Thr-Val-Leu [M-H]- IT-FT 35% P=330.2
C15H29N3O5; MF: 6; RMF: 254; Prob 28.0%; Lib: nist_msms; ID: 568882.

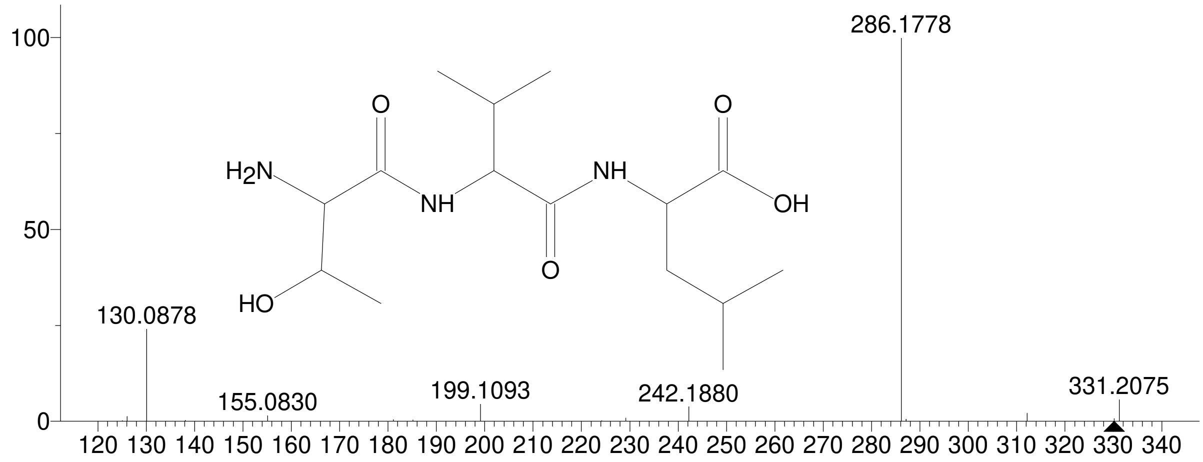

Hit 2 : Thr-Val-Leu [M-H]- IT-FT 35% P=330.2
C15H29N3O5; MF: 6; RMF: 254; Prob 28.0%; Lib: nist_msms2; ID: 72867.

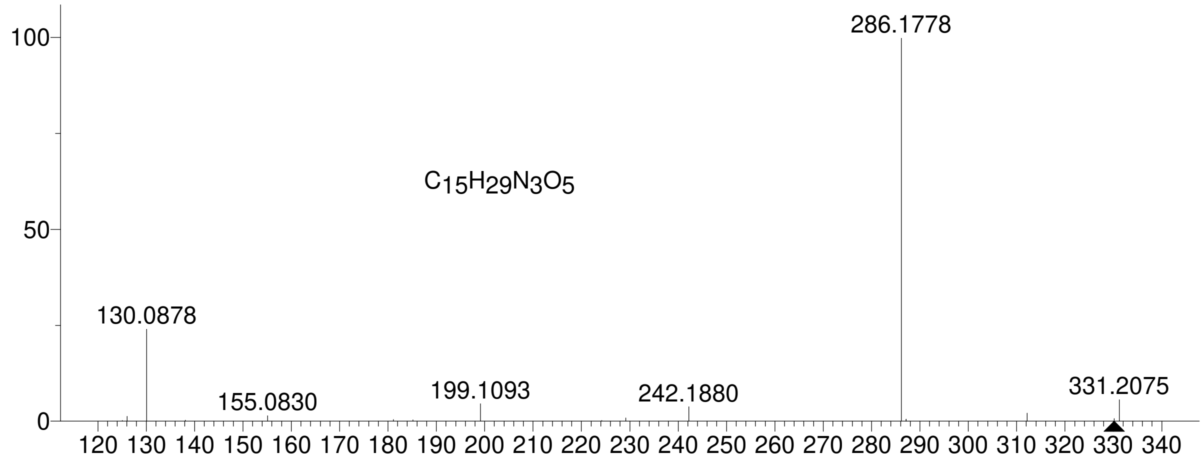

Hit 3 : β-Alanine, N-[2-(2-pyridinyl)-6-(1,2,4,5-tetrahydro-3H-3-benzazepin-3-yl)-4-pyrimidinyl]-, ethyl ester [M+H]-
C24H27N5O2; MF: 0; RMF: 24; Prob 22.0%; CAS: 1373423-53-0; Lib: nist_msms; ID: 274165.

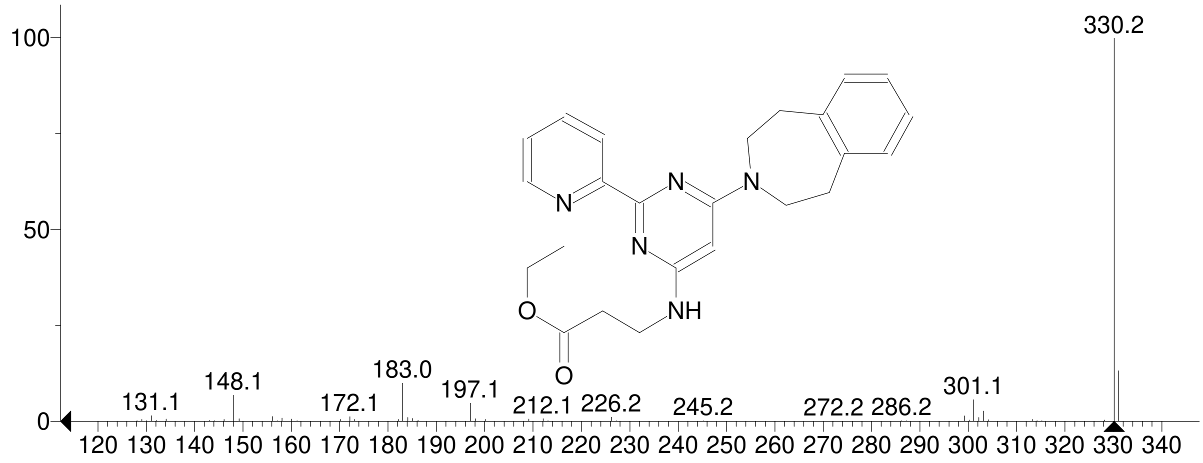

Hit 4 : β-Alanine, N-[2-(2-pyridinyl)-6-(1,2,4,5-tetrahydro-3H-3-benzazepin-3-yl)-4-pyrimidinyl]- [M+H-H2O]+=>33
C22H23N5O2; MF: 0; RMF: 22; Prob 22.0%; CAS: 1373422-53-7; Lib: nist_msms; ID: 273703.

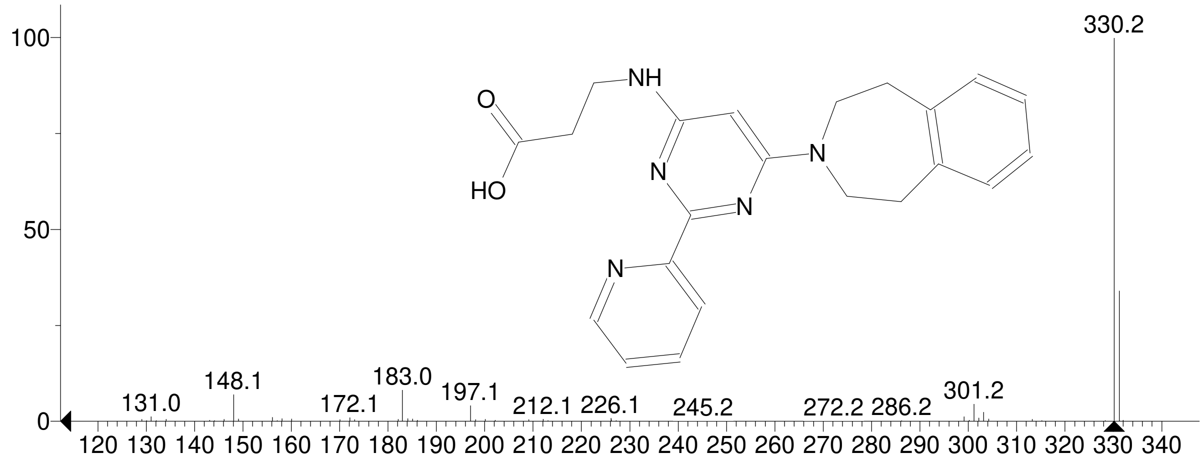

Unknown: Unknown (4.34_433.1815m/z)
Compound in Library Factor = -3273

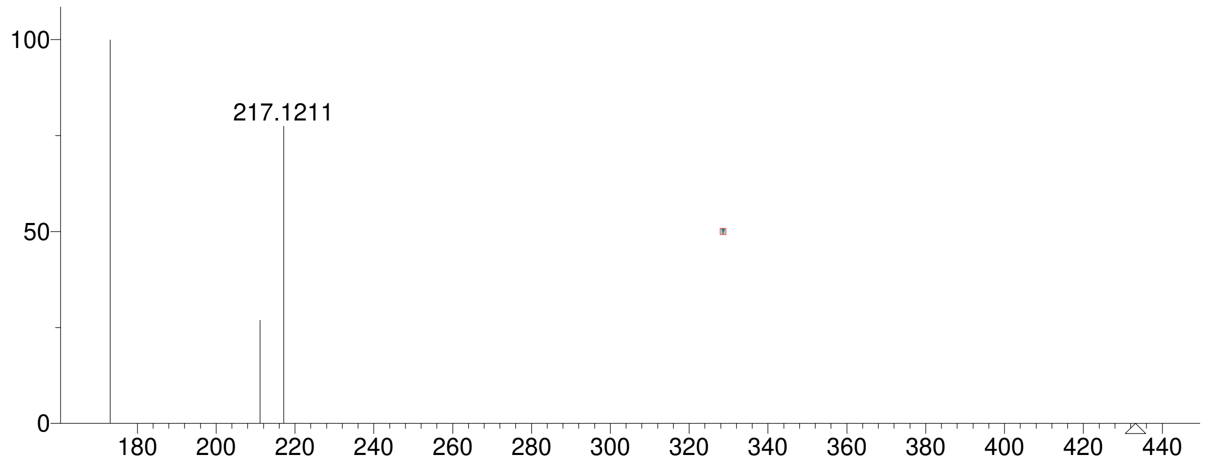

Hit 1 : Salvinorin A [M+H]+ 45HCD P=433.2
C23H28O8; MF: 1; RMF: 175; Prob 33.8%; Lib: mona-export-vf-npl_qexactive; ID: 13790.

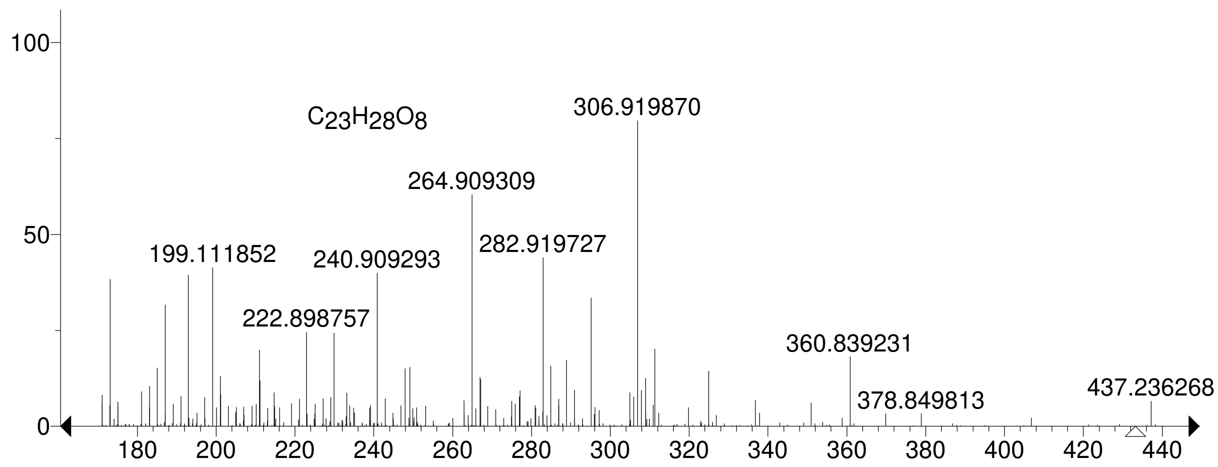

Hit 2 : Bixin [M+K]+ 45HCD P=433.2
C25H30O4; MF: 1; RMF: 153; Prob 33.8%; Lib: mona-export-vf-npl_qexactive; ID: 26504.

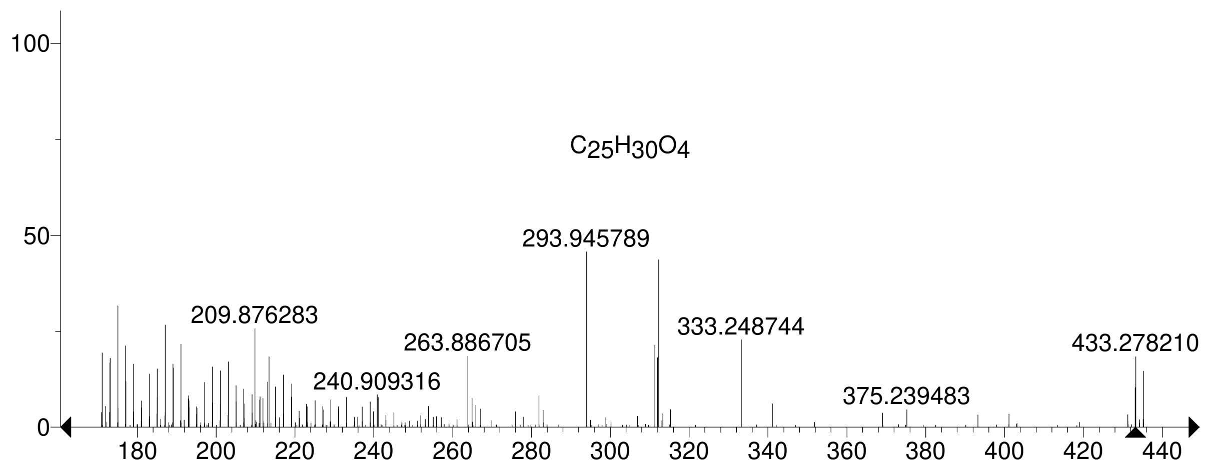

Hit 3 : 15β-Hydroxycyproterone acetate [M+H]+ HCD 30V P=433.2
C24H29ClO5; MF: 0; RMF: 74; Prob 32.5%; CAS: 65423-26-9; Lib: nist_msms; ID: 440396.

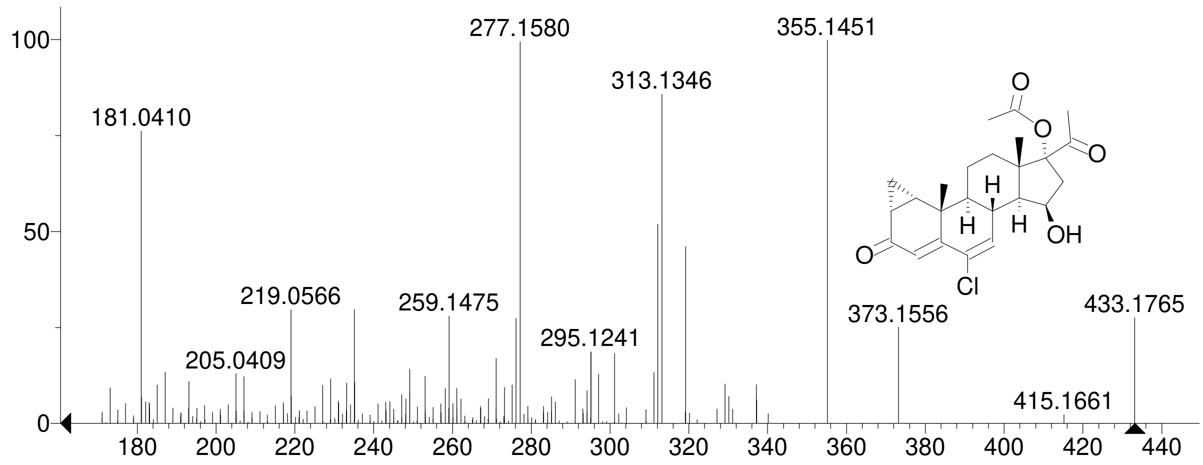

Unknown: Unknown (4.35_305.0689m/z)
Compound in Library Factor = -3273

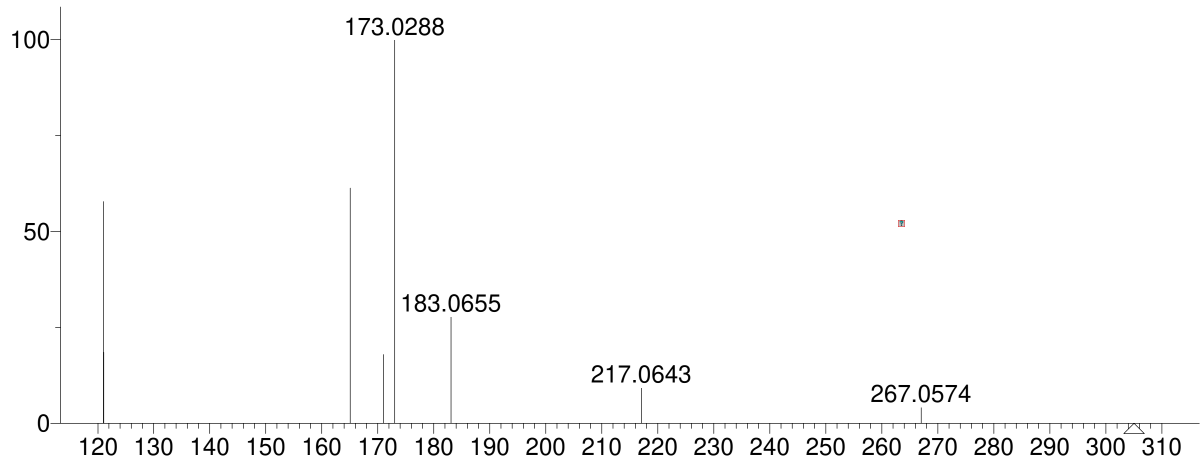

Hit 1 : Gallocatechin [M-H]- 45eV P=305.1
C15H14O7; MF: 1; RMF: 89; Prob 12.9%; Lib: msms-neg-metabobase; ID: 808.

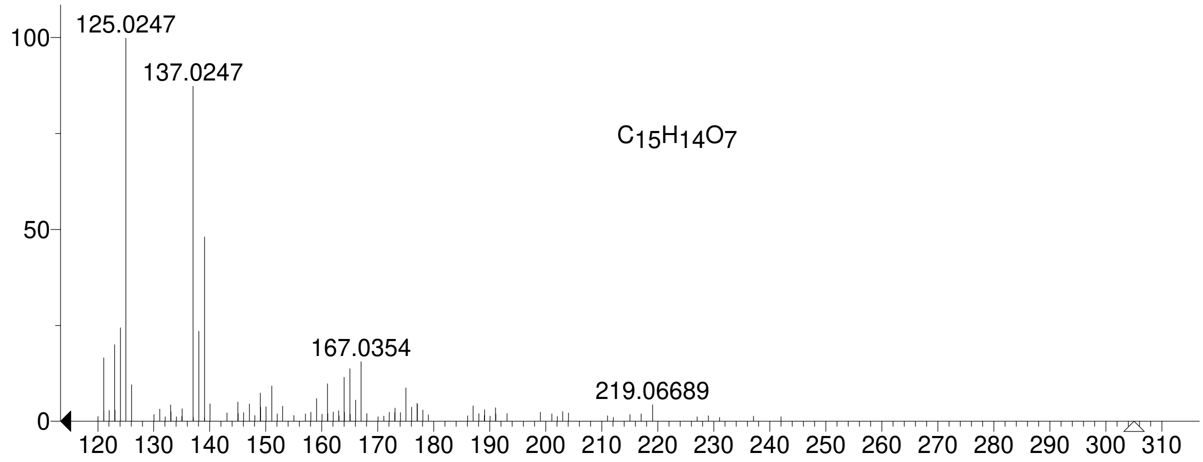

Hit 2 : Epigallocatechin [M-H]- 30 P=305.1
C15H14O7; MF: 0; RMF: 70; Prob 12.4%; Lib: mona-export-lc-ms-ms_negative_mode; ID: 12420.

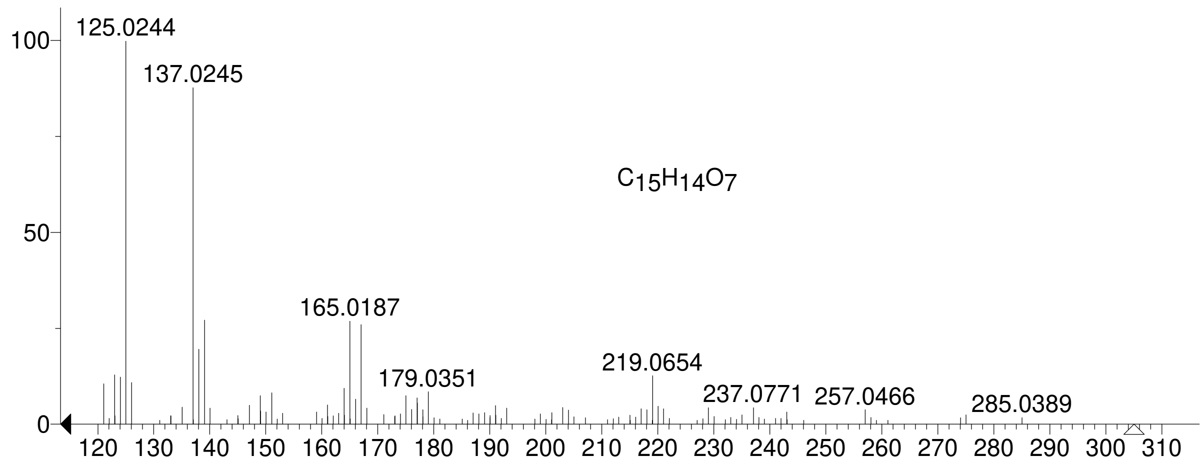

Hit 3 : (2S,3S)-2-(3,4,5-trihydroxyphenyl)-3,4-dihydro-2H-chromene-3,5,7-triol [M-H]- 65HCD P=305.1
C15H14O7; MF: 0; RMF: 43; Prob 12.4%; Lib: mona-export-lc-ms-ms_negative_mode; ID: 20161.

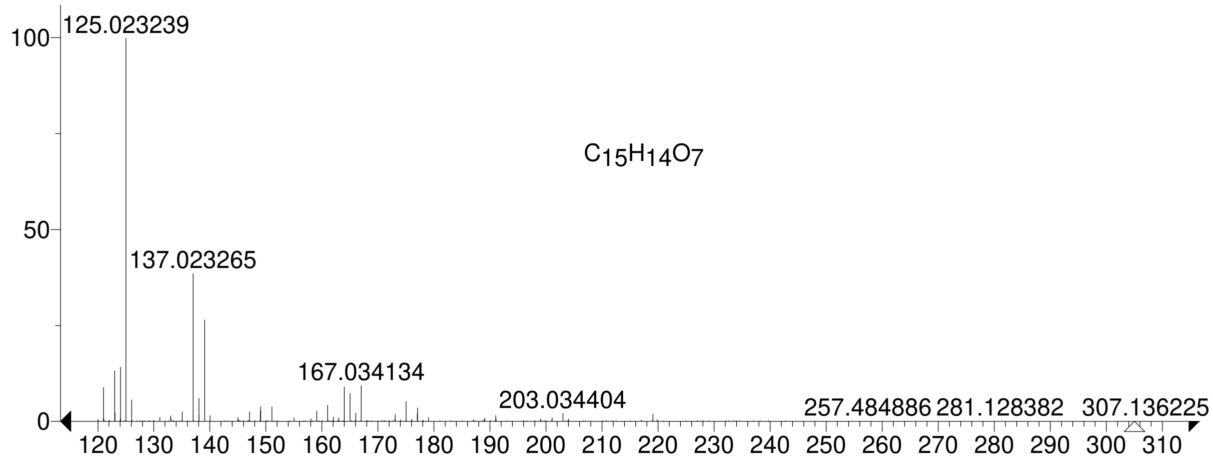

Hit 4 : 2,3-Trans-3,4-cis-Leucocyanidin [M-H]- 45eV P=305.1
C15H14O7; MF: 0; RMF: 27; Prob 12.4%; Lib: msms-neg-metabobase; ID: 828.

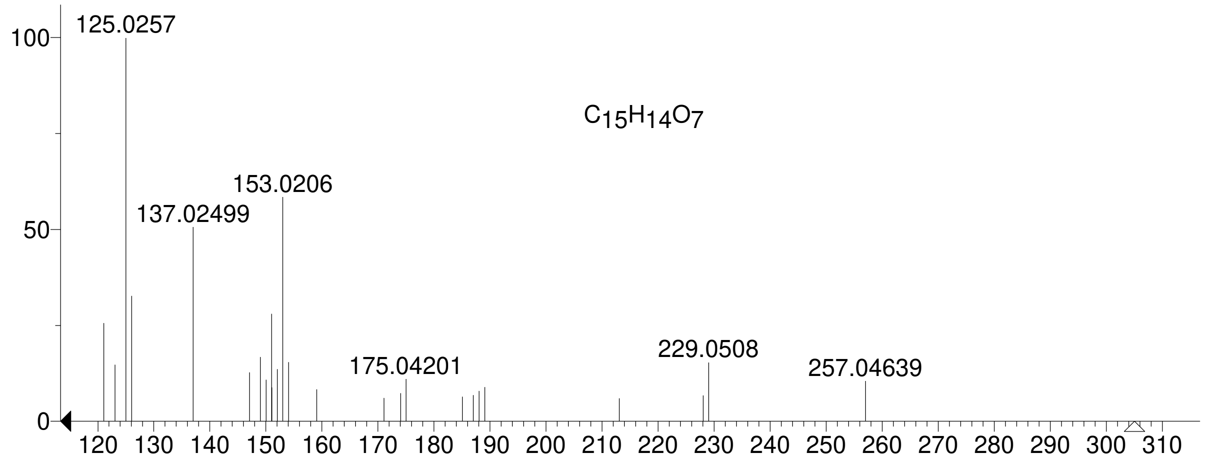

Hit 5 : (-)-Epigallocatechin [M-H]- HCD 48V P=305.1
C15H14O7; MF: 0; RMF: 24; Prob 12.4%; CAS: 970-74-1; Lib: nist_msms; ID: 235105.

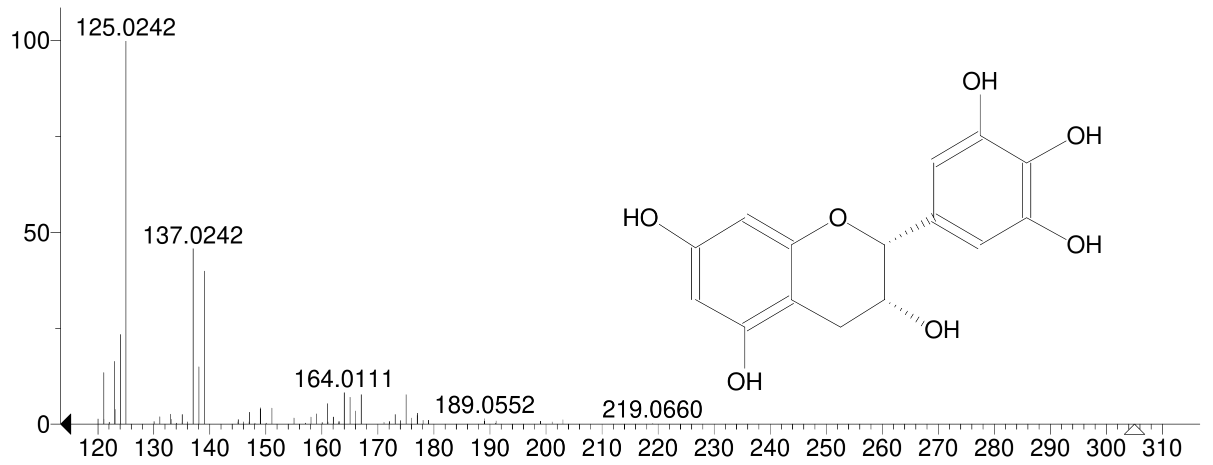

** Search Report Page 1 of 1 **

Unknown: Unknown (4.36_611.1256m/z)
Compound in Library Factor = 0

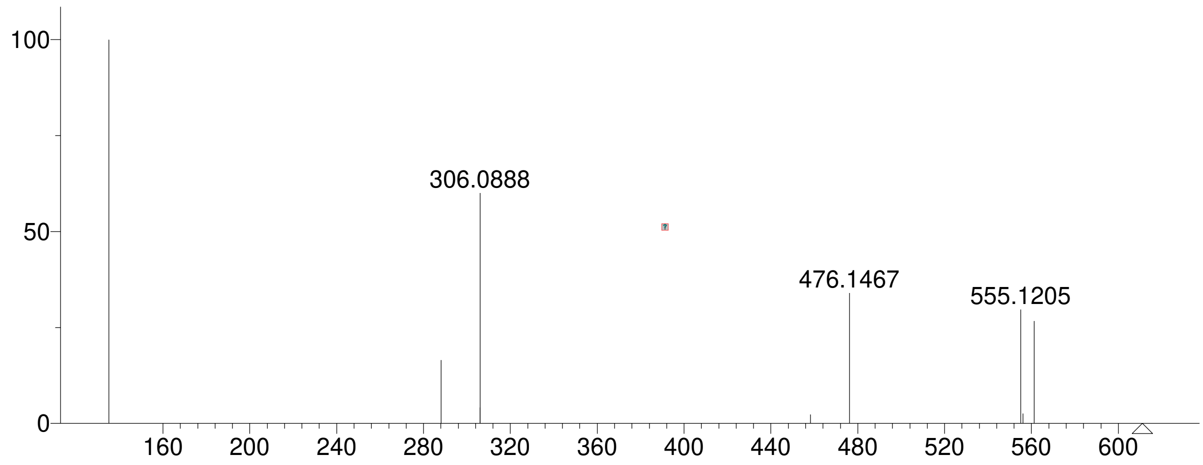

Unknown: Unknown (4.36_698.2907m/z)
Compound in Library Factor = 0

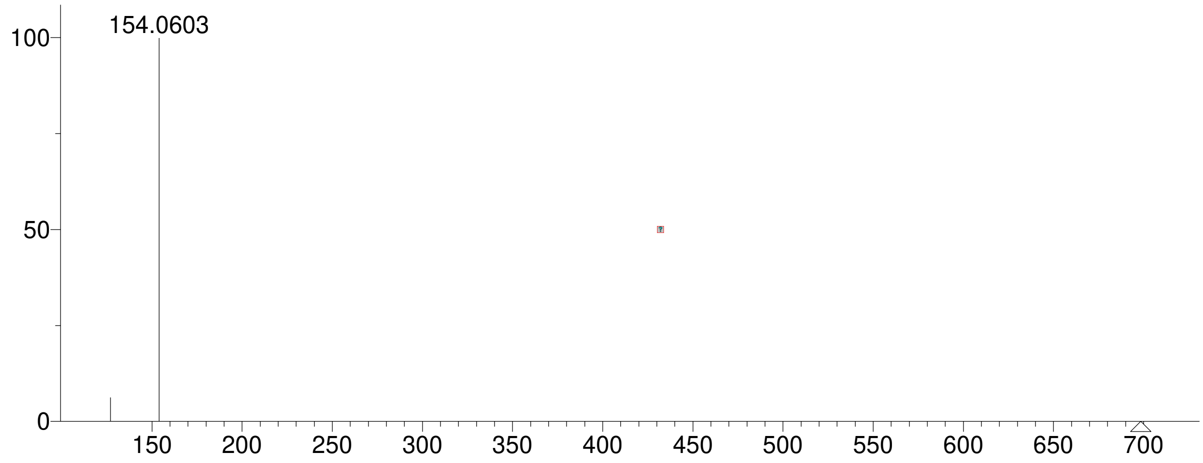

Unknown: Unknown (4.37_326.1339m/z)
Compound in Library Factor = -3273

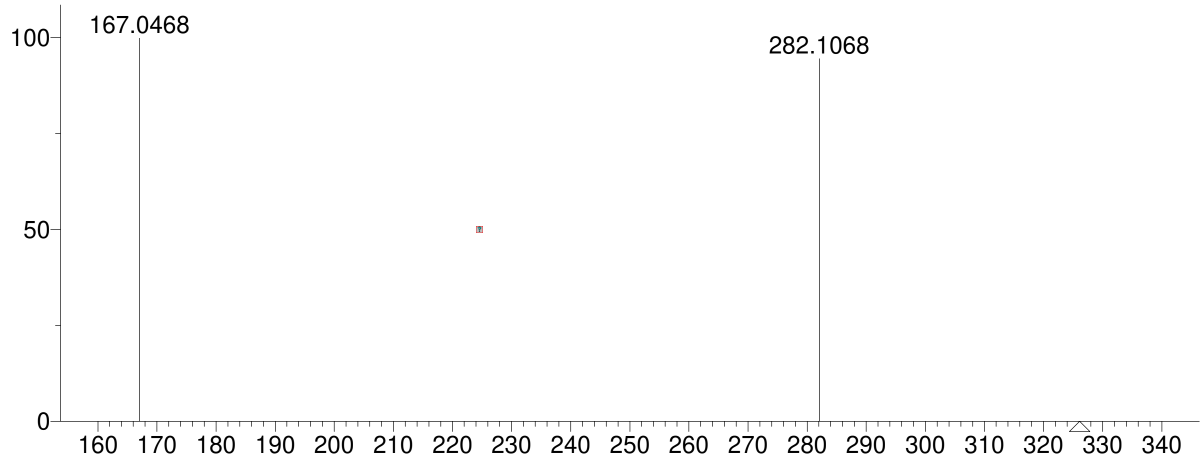

Hit 1 : Glycine, 1,1'-(1,8-dioxo-1,8-octanediyl)bis[glycyl- [M-H-C2H5NO2]- IT 35% P=326.1
C16H26N4O8; MF: 2; RMF: 307; Prob 35.1%; CAS: 143673-93-2; Lib: nist_msms; ID: 423624.

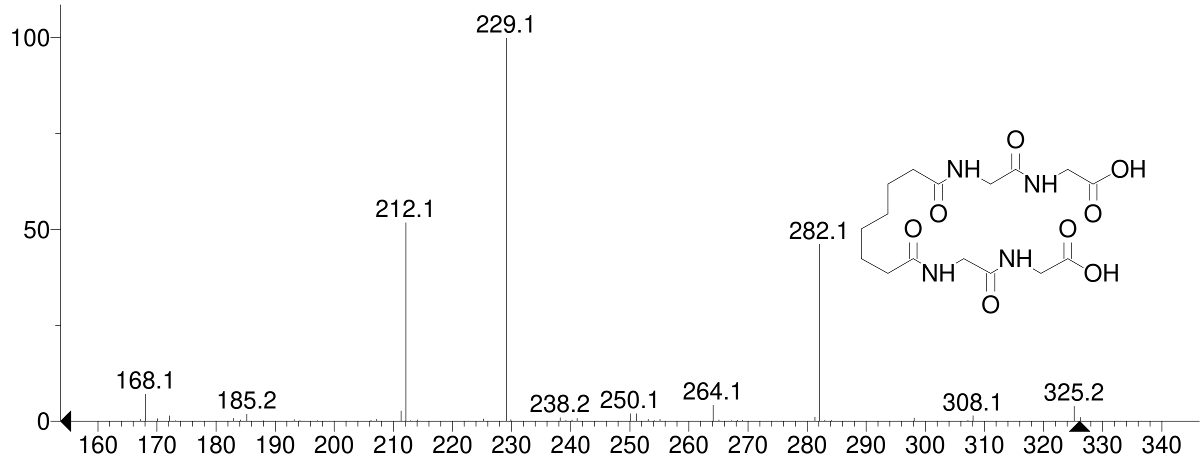

Hit 2 : Anhydrohaloxindole A [M+H]+ P=326.1
C21H21ClN2O; MF: 0; RMF: 21; Prob 32.4%; Lib: mona-export-gnps; ID: 4776.

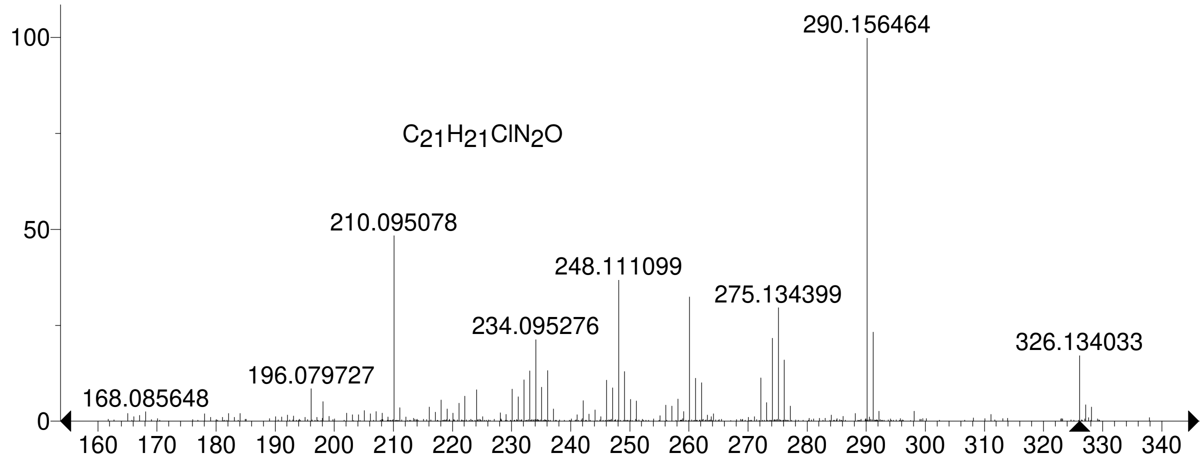

Hit 3 : (4-oxido-2,3,5,6,7,8-hexahydro-1H-pyrrolizin-4-ium-1-yl)methyl 2,3-dihydroxy-3-methylpentanoate [M+K]+
C14H25NO5; MF: 0; RMF: 11; Prob 32.4%; Lib: mona-export-vf-npl_qexactive; ID: 21020.

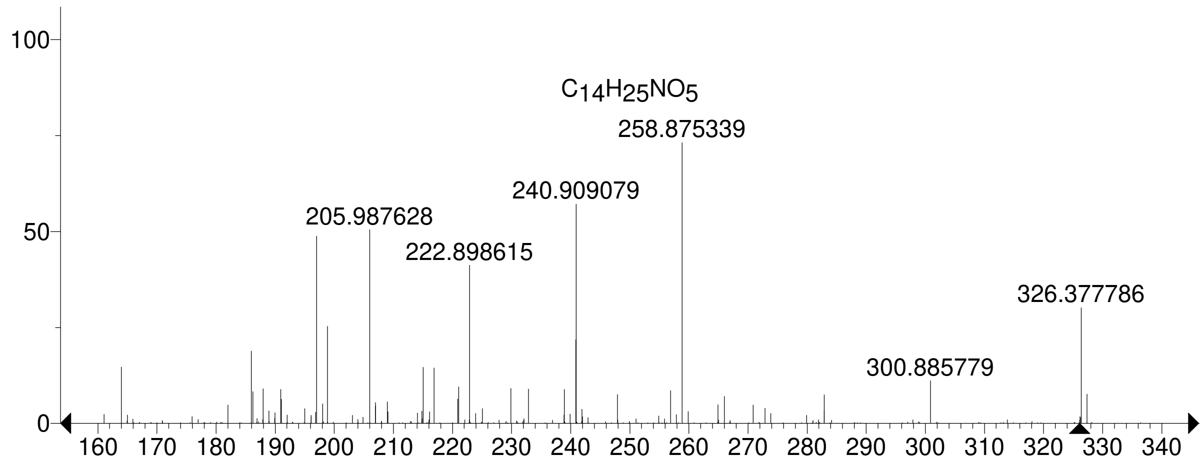

Unknown: Unknown (4.37_378.1771m/z)
Compound in Library Factor = -183

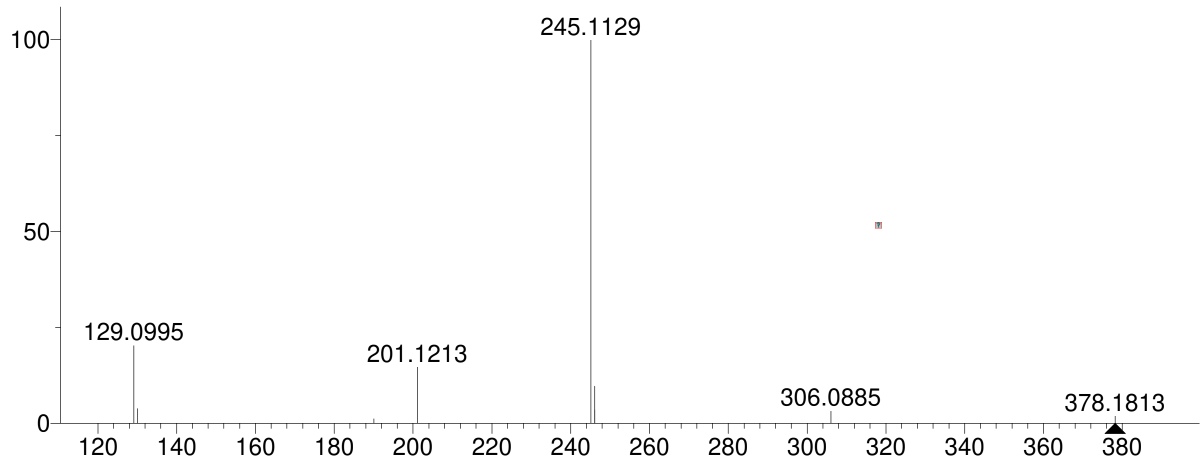

Hit 1 : 2-Oxo-3-hydroxylysergic acid diethylamide [M+Na]+ HCD 11V P=378.2
C20H25N3O3; MF: 322; RMF: 965; Prob 96.7%; CAS: 111295-09-1; Lib: nist_msms; ID: 326233.

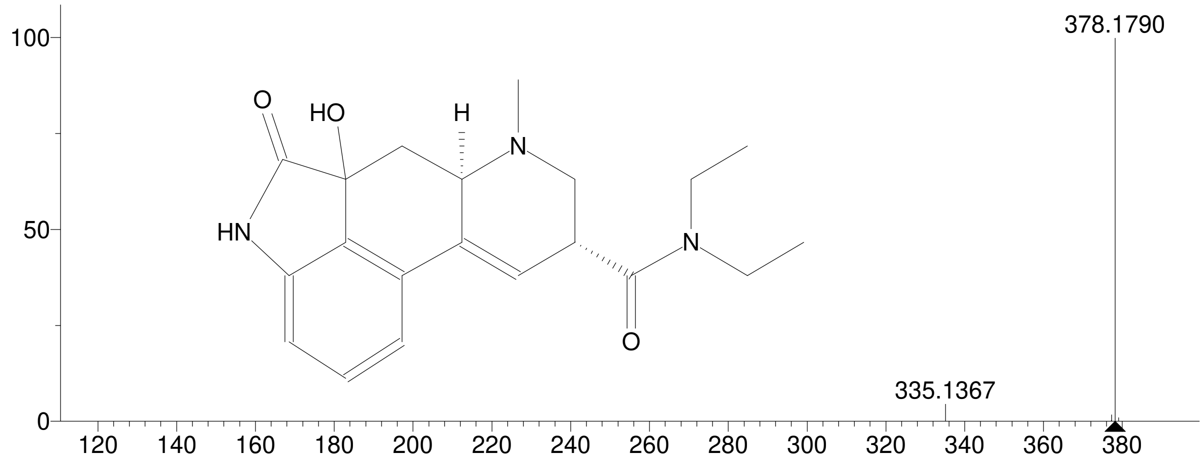

Hit 2 : Cys-Gln-Lys [M+H]+ QTOF 40V P=378.2
C14H27N5O5S; MF: 11; RMF: 264; Prob 0.96%; Lib: nist_msms; ID: 49701.

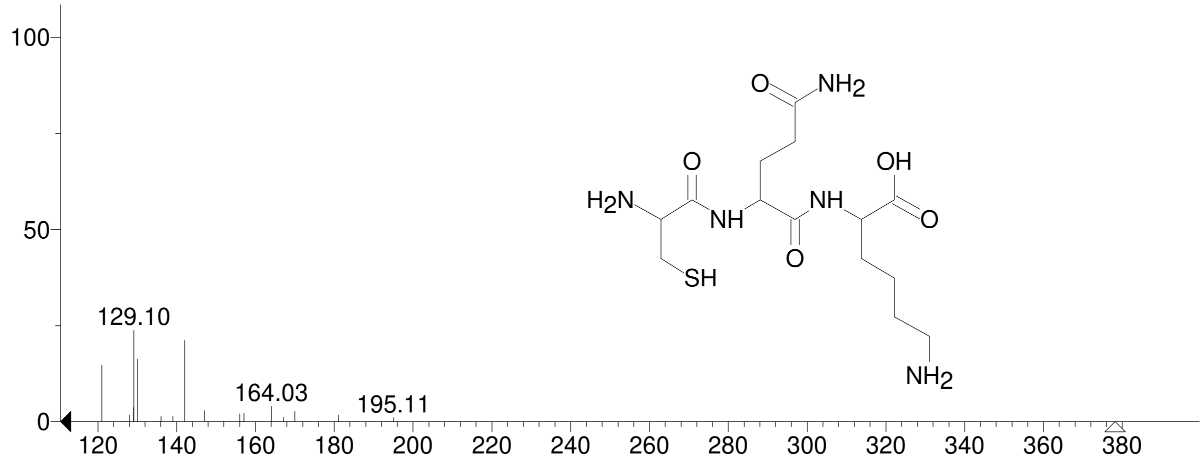

Hit 3 : Gln-Cys-Lys [M+H]+ QTOF 40V P=378.2
C14H27N5O5S; MF: 11; RMF: 225; Prob 0.96%; Lib: nist_msms; ID: 53021.

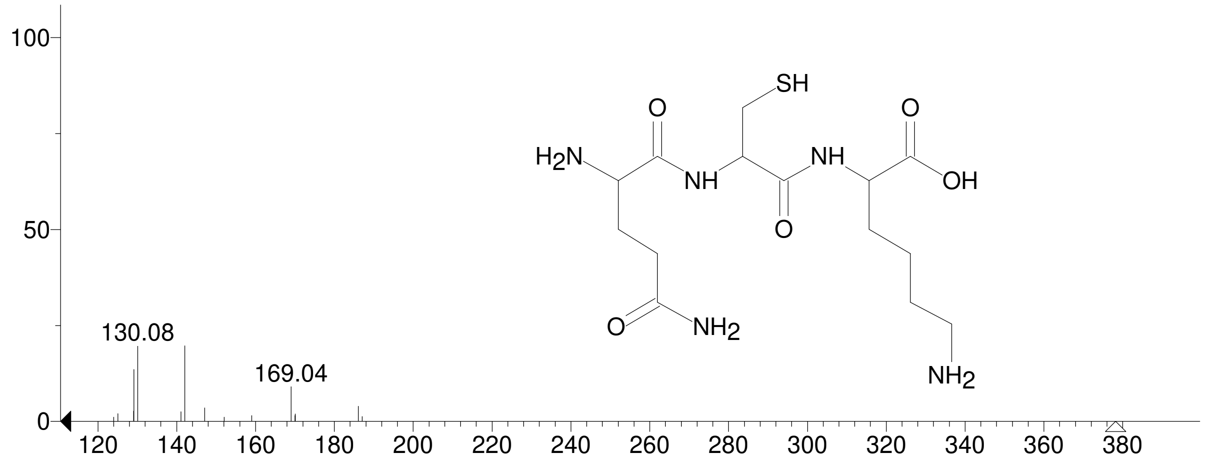

Hit 4 : Latrunculin B [M+H-H2O]+ HCD 10V P=378.2
C20H29NO5S; MF: 3; RMF: 99; Prob 0.72%; CAS: 76343-94-7; Lib: nist_msms; ID: 86160.

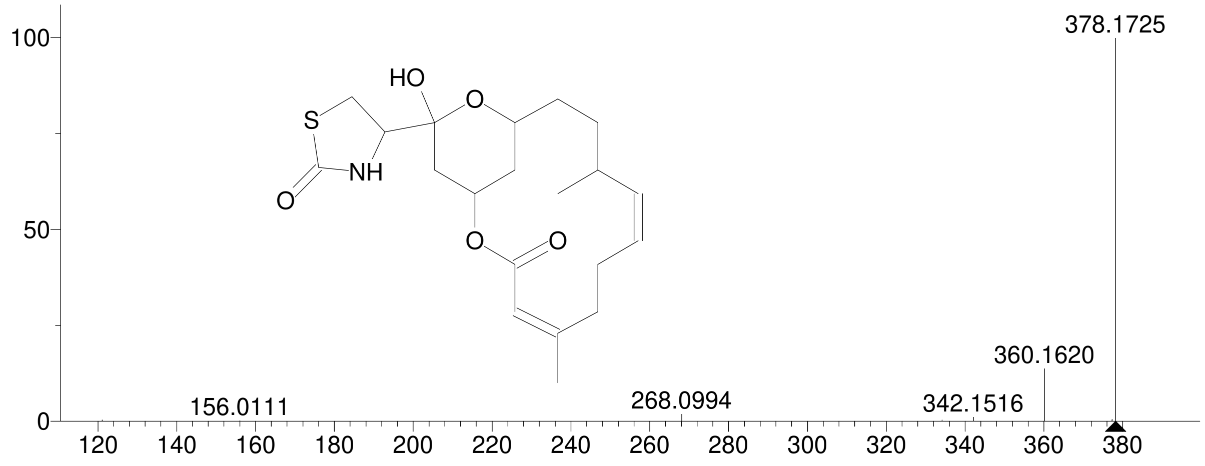

Hit 5 : (2R,3R,4S,5S,6R)-2-[3-hydroxy-2-(4-hydroxy-3-methoxyphenyl)propoxy]-6-(hydroxymethyl)oxane-3,4,5-tric
C16H24O9; MF: 0; RMF: 1; Prob 0.63%; Lib: mona-export-vf-npl_qexactive; ID: 10762.

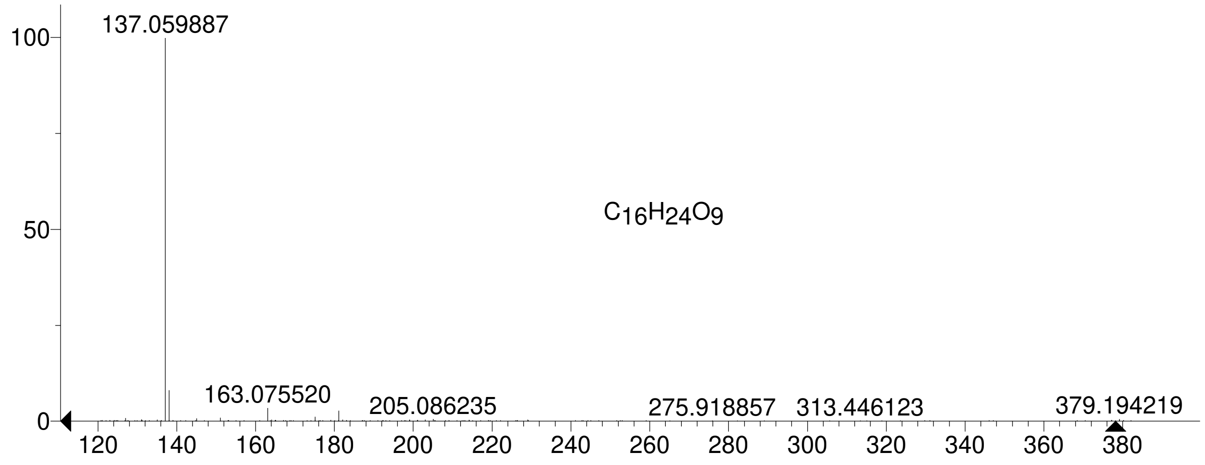

** Search Report Page 1 of 1 **

Unknown: Unknown (4.37_555.1236m/z)
Compound in Library Factor = 0

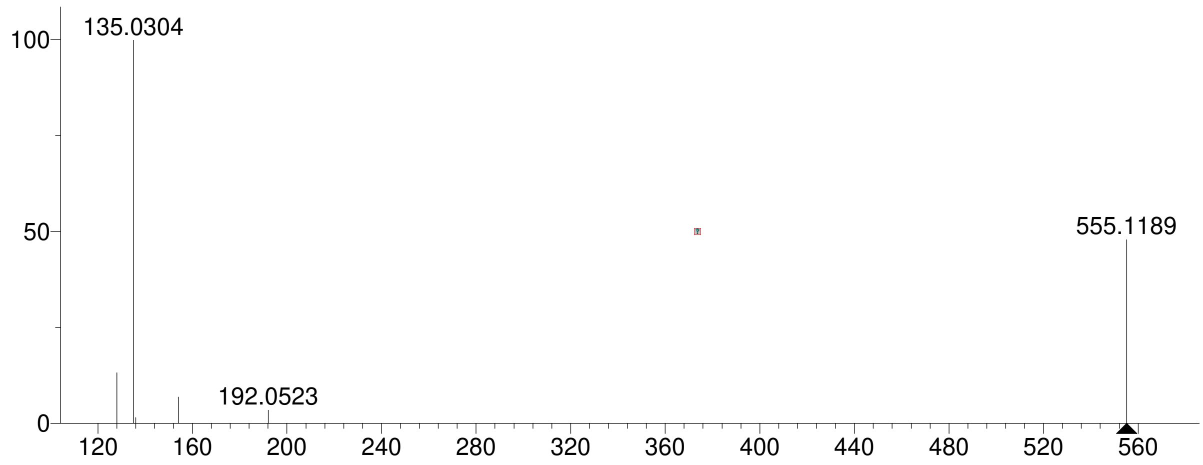

Hit 1 : Laccaic Acid A [M+NH4]+ 35HCD P=555.1
C26H19NO12; MF: 0; RMF: 5; Prob 100.0%; Lib: mona-export-vf-npl_qexactive; ID: 27181.

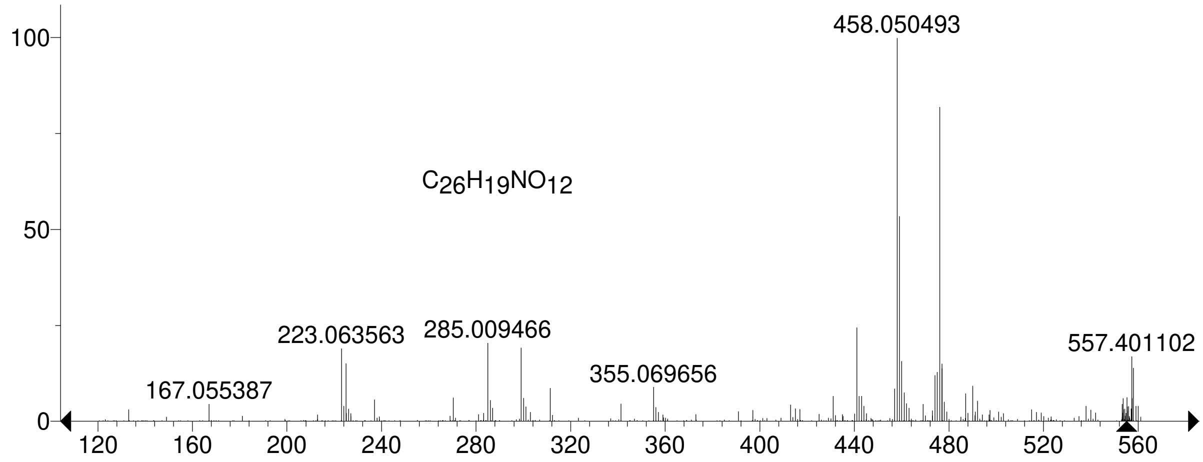

Unknown: Unknown (4.38_358.1968m/z)
Compound in Library Factor = -3273

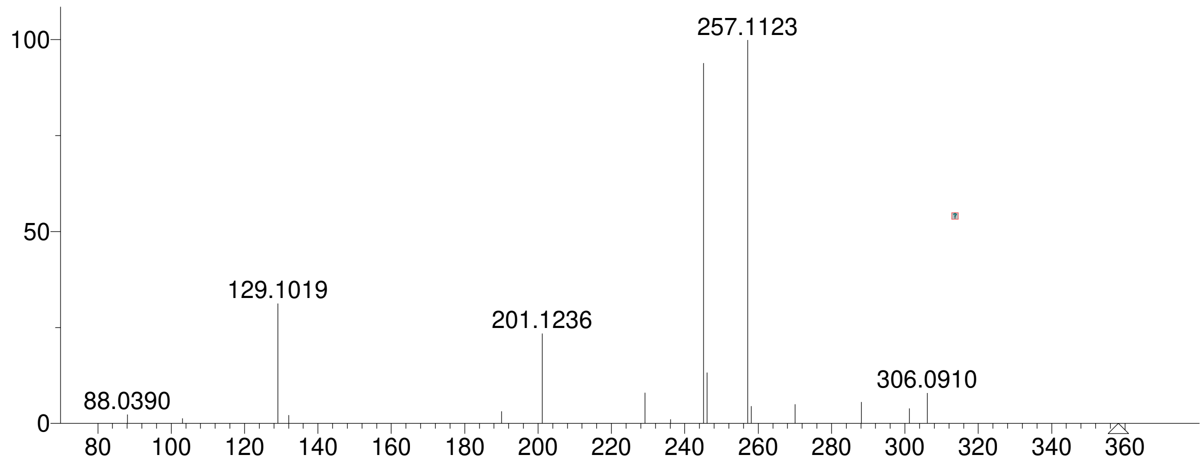

Hit 1 : Calpain Inhibitor II [M-H]-=>358.2 IT 35% P=358.2
C19H35N3O4S; MF: 1; RMF: 150; Prob 12.9%; CAS: 145757-50-2; Lib: nist_msms; ID: 81729.

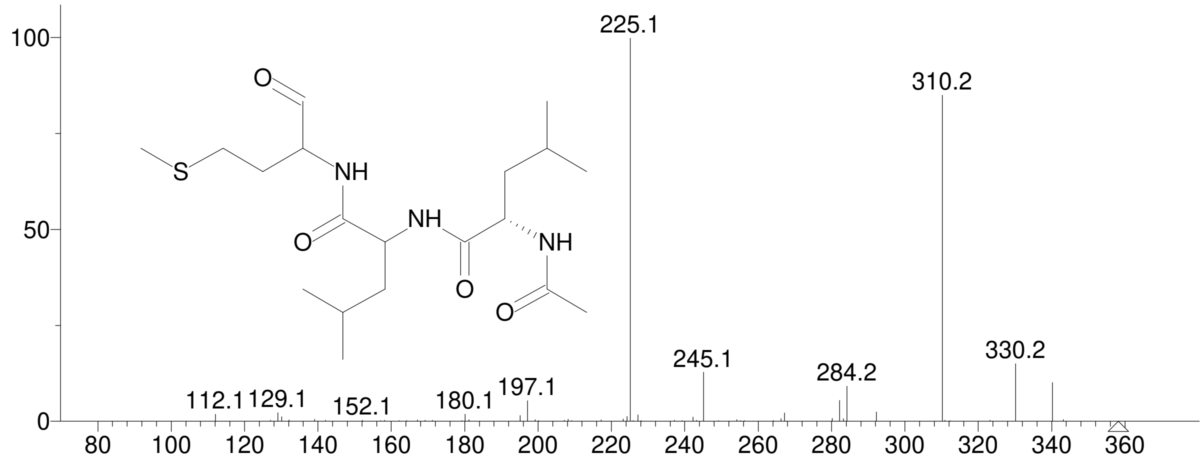

Hit 2 : Leukotriene D4 methyl ester [M+H-H2O]+=>390.2=>358.2 IT 35% P=358.2
C26H42N2O6S; MF: 0; RMF: 46; Prob 12.4%; Lib: nist_msms; ID: 92133.

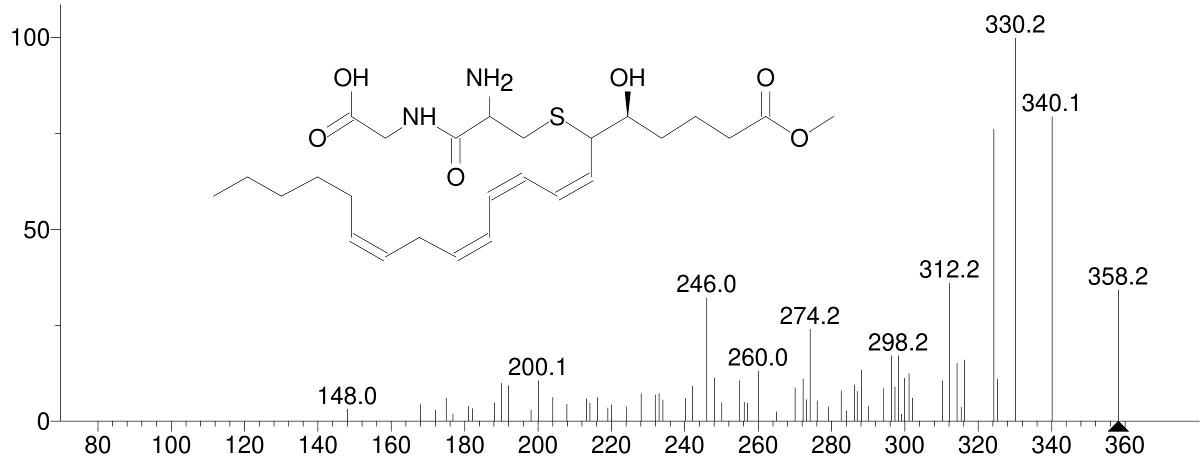

Hit 3 : Calpain Inhibitor II [M+Na]+=>377.2=>358.2 IT 35% P=358.2
C19H35N3O4S; MF: 0; RMF: 41; Prob 0.00%; CAS: 145757-50-2; Lib: nist_msms; ID: 81709.

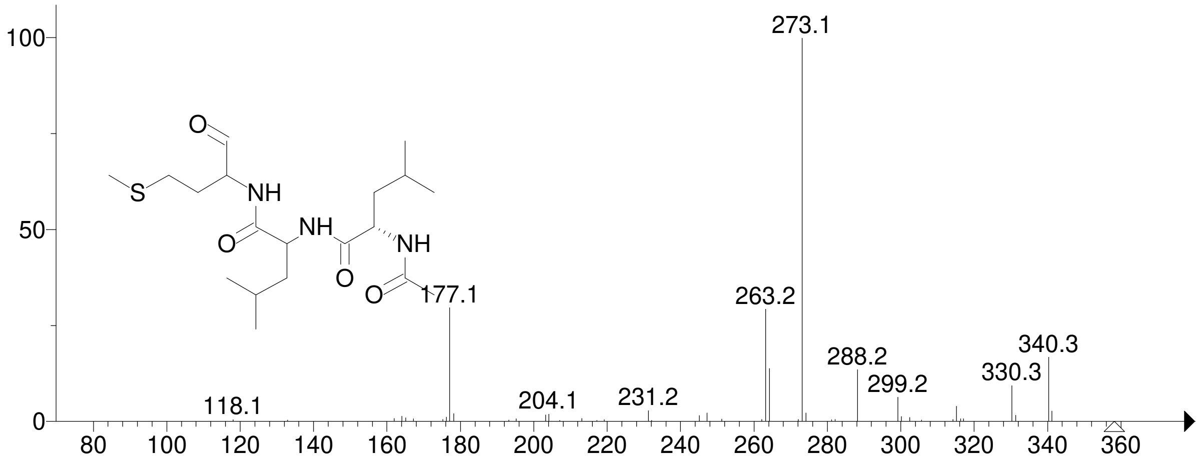

Hit 4 : cis-Moxifloxacin acyl-β-D-glucuronide [M+H-C6H8O6]+=>358.2 IT 35% P=358.2
C27H32FN3O10; MF: 0; RMF: 28; Prob 12.4%; CAS: 733002-61-4; Lib: nist_msms; ID: 463735.

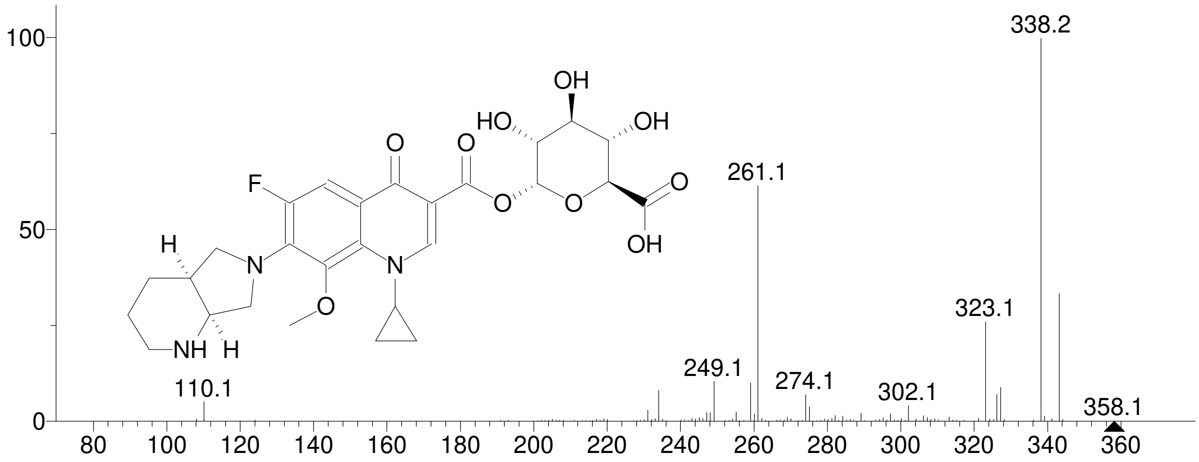

Hit 5 : Cyphenothrin [M+H]+=>358.2 IT 35% P=358.2
C24H25NO3; MF: 0; RMF: 28; Prob 12.4%; CAS: 39515-40-7; Lib: nist_msms; ID: 475533.

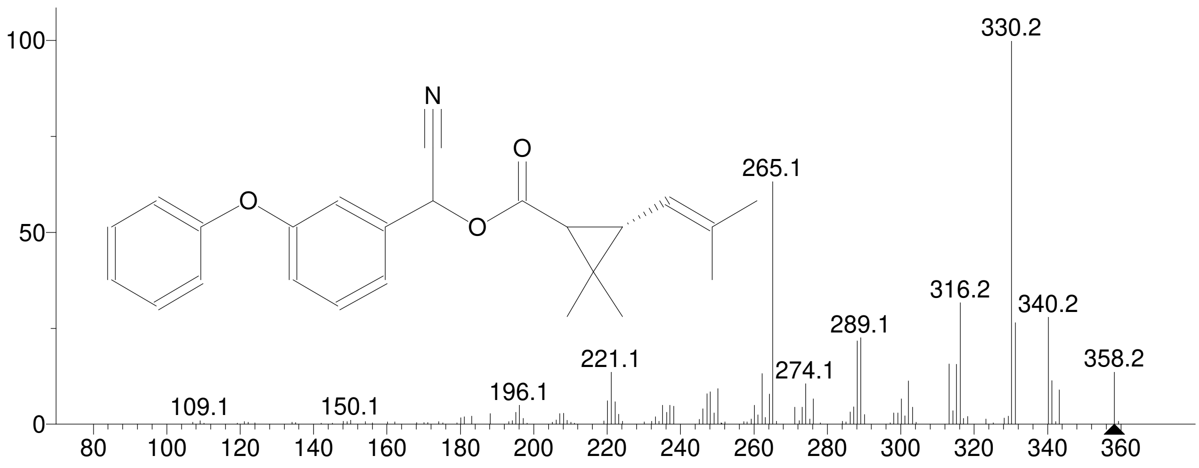

** Search Report Page 1 of 1 **

Unknown: Unknown (4.38_698.2844m/z)
Compound in Library Factor = 0

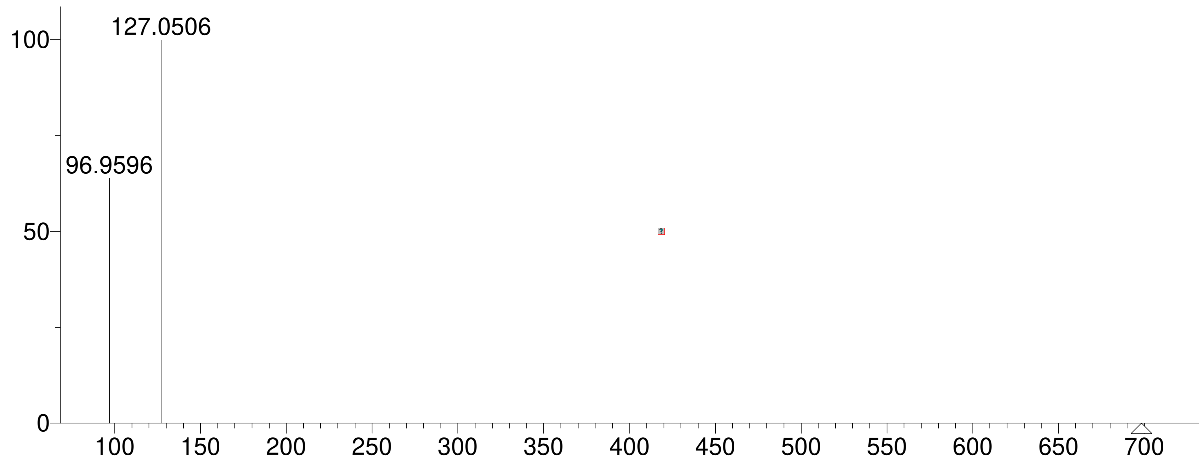

Unknown: Unknown (4.39_386.1435m/z)
Compound in Library Factor = 0

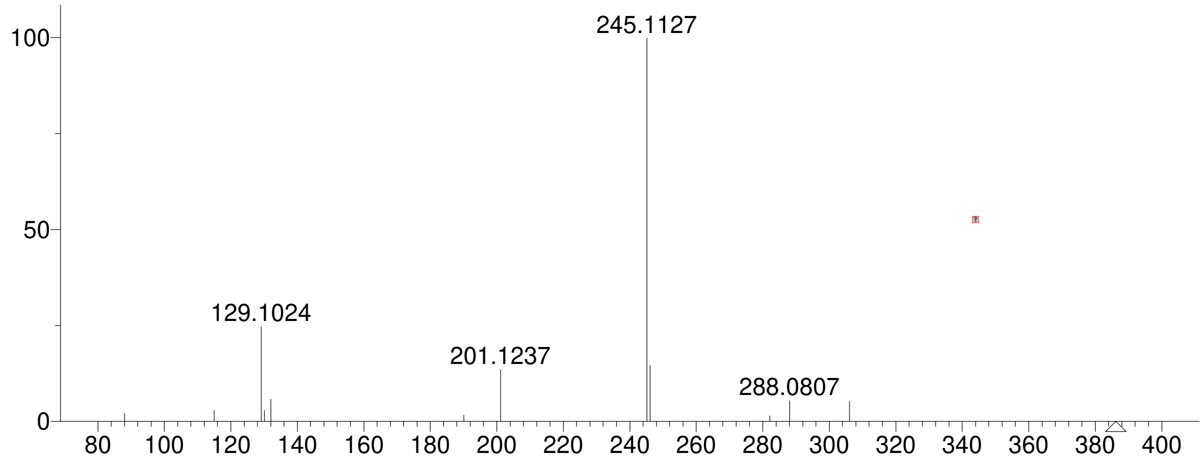

Hit 1 : 3-O-Feruloylquinic acid [M+NH4]+ 45HCD P=386.1
C17H20O9; MF: 0; RMF: 6; Prob 25.0%; Lib: mona-export-vf-npl_qexactive; ID: 6254.

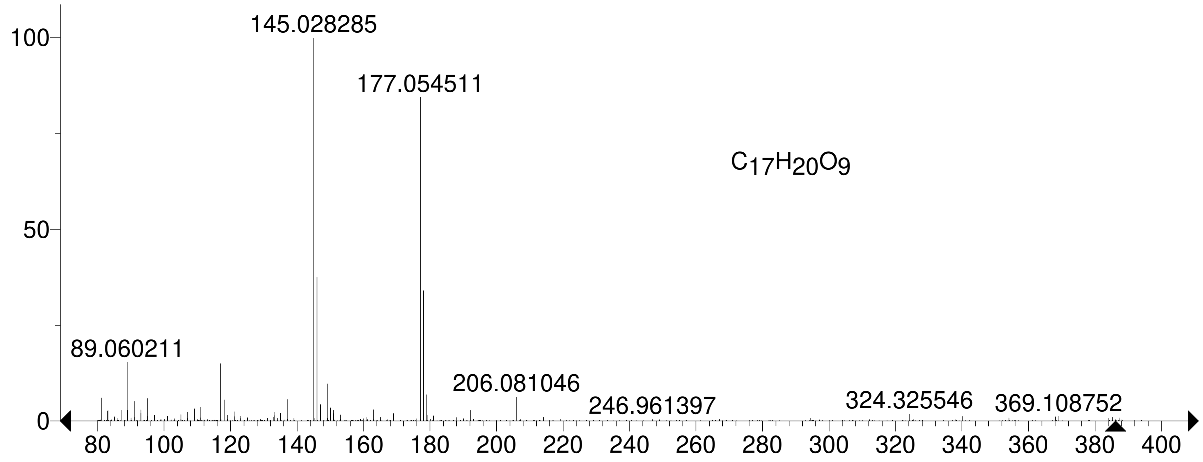

Hit 2 : methyl chlorogenate [M+NH4]+ 35HCD P=386.1
C17H20O9; MF: 0; RMF: 5; Prob 25.0%; Lib: mona-export-vf-npl_qexactive; ID: 5491.

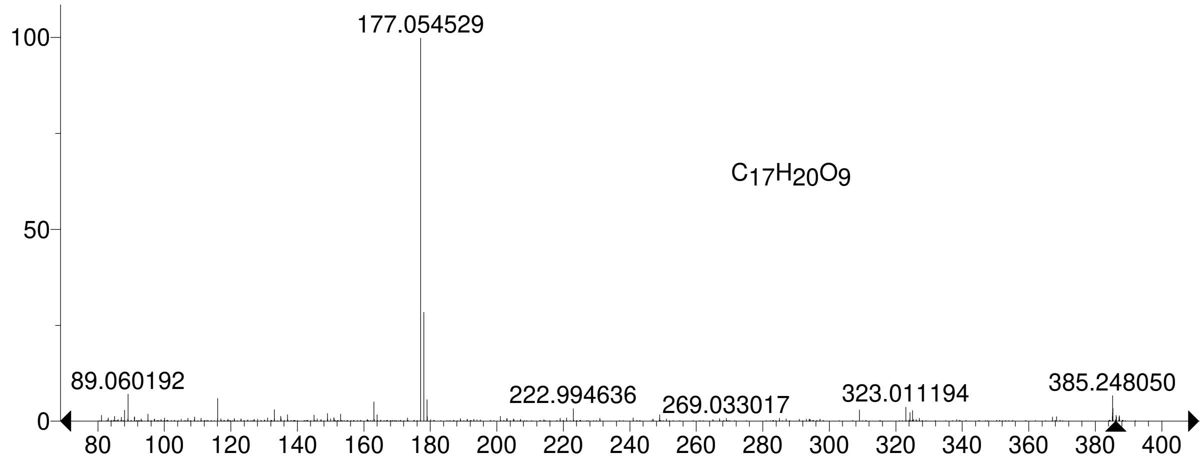

Hit 3 : (1R,3R,4S,5R)-1,3,4-trihydroxy-5-[(E)-3-(4-hydroxy-3-methoxyphenyl)prop-2-enoyl]oxycyclohexane-1-carb
C17H20O9; MF: 0; RMF: 3; Prob 25.0%; Lib: mona-export-vf-npl_qexactive; ID: 7930.

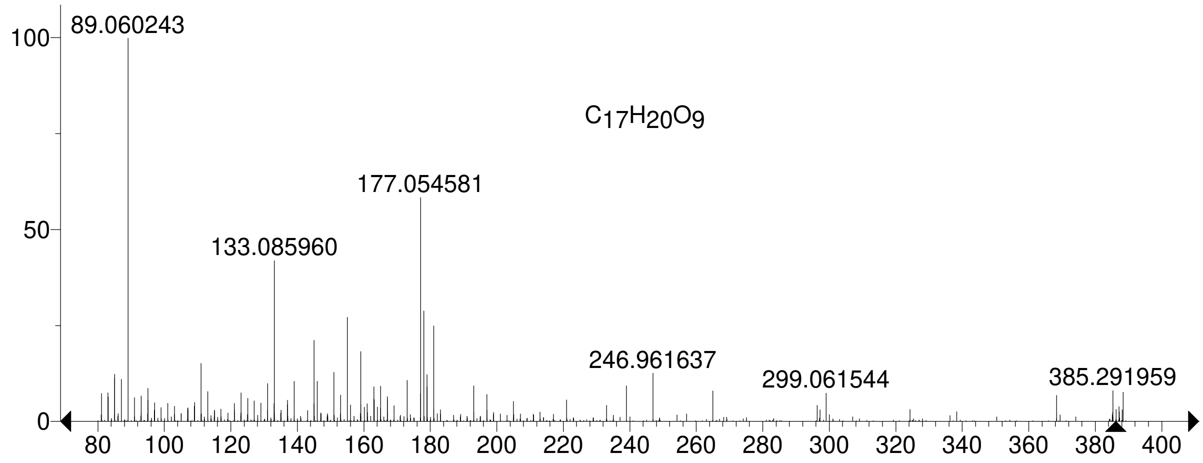

Hit 4 : [4-acetyloxy-2,5-dihydroxy-6-(hydroxymethyl)oxan-3-yl] (E)-3-(4-hydroxyphenyl)prop-2-enoate [M+NH4]+
C17H20O9; MF: 0; RMF: 2; Prob 25.0%; Lib: mona-export-vf-npl_qexactive; ID: 20331.

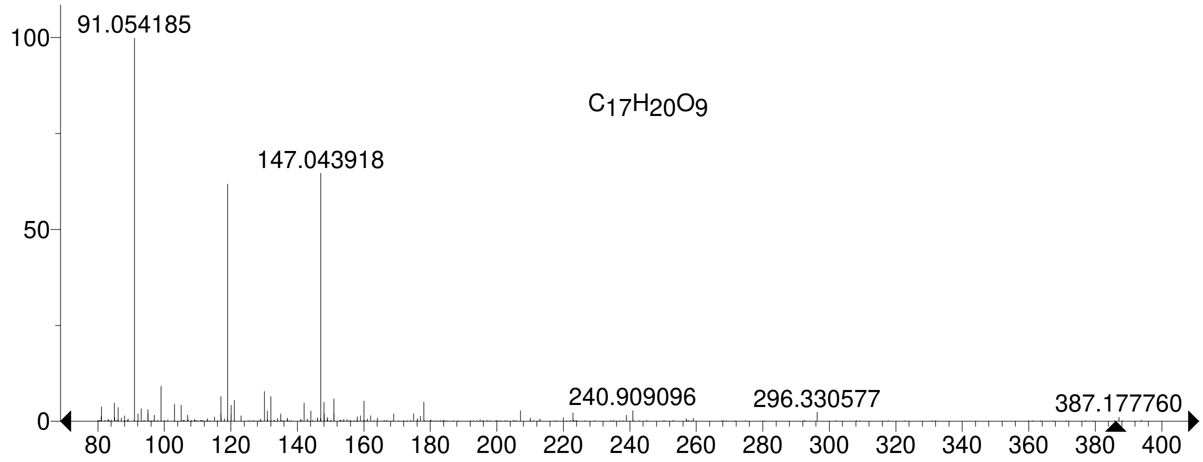

Unknown: Unknown (4.39_415.1710m/z)
Compound in Library Factor = -3273

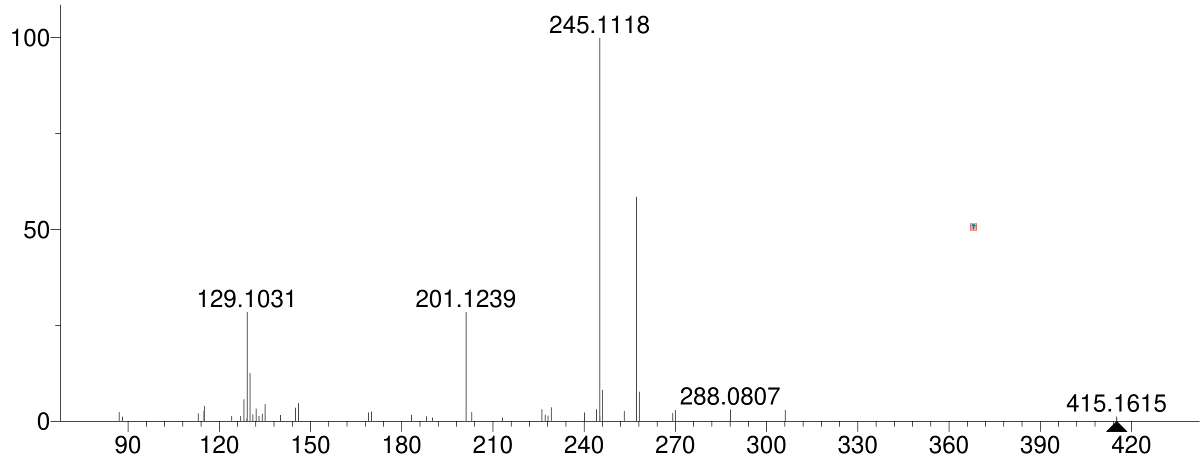

Hit 1 : Diltiazem [M+H]+ QTOF 3V P=415.2
C22H26N2O4S; MF: 6; RMF: 68; Prob 17.2%; CAS: 42399-41-7; Lib: nist_msms; ID: 20519.

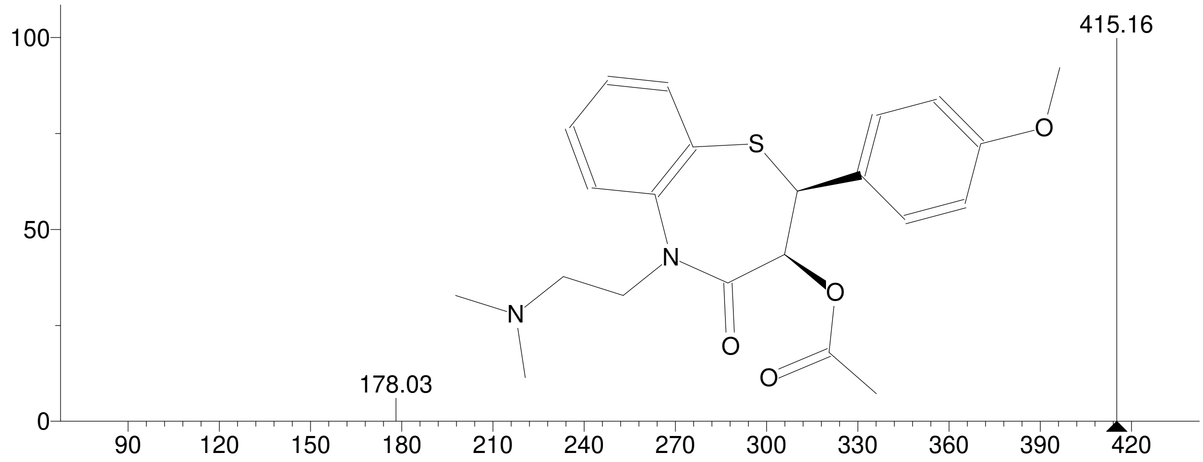

Hit 2 : Garcinone C [M+H]+ HCD 8V P=415.2
C23H26O7; MF: 2; RMF: 94; Prob 14.5%; CAS: 76996-27-5; Lib: nist_msms; ID: 389279.

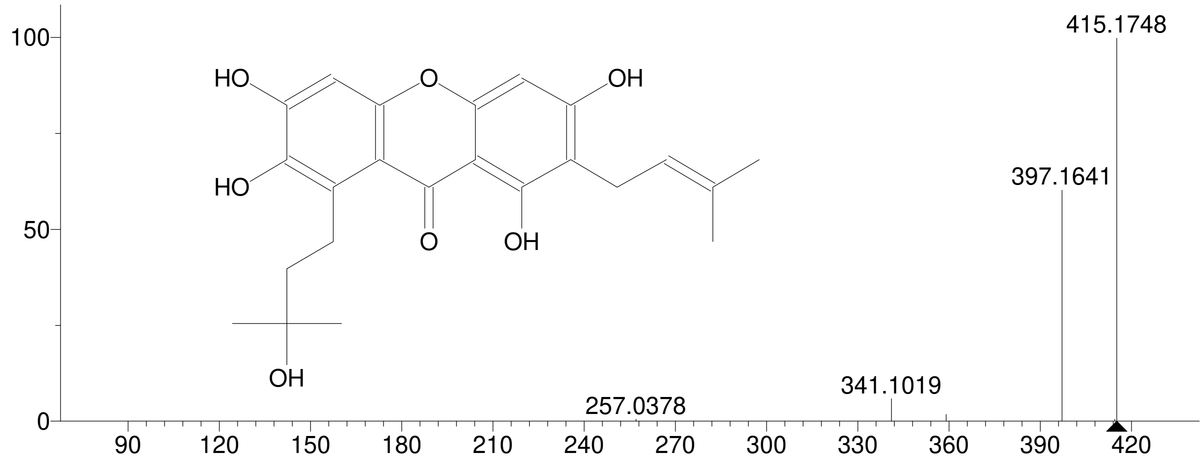

Hit 3 : 6-formyl-10-(hydroxymethyl)-5-methoxy-3-methylidene-2-oxo-2H,3H,3aH,4H,5H,8H,9H,11aH-cyclodeca[b]f
C21H28O7; MF: 2; RMF: 78; Prob 14.5%; Lib: mona-export-vf-npl_qexactive; ID: 16435.

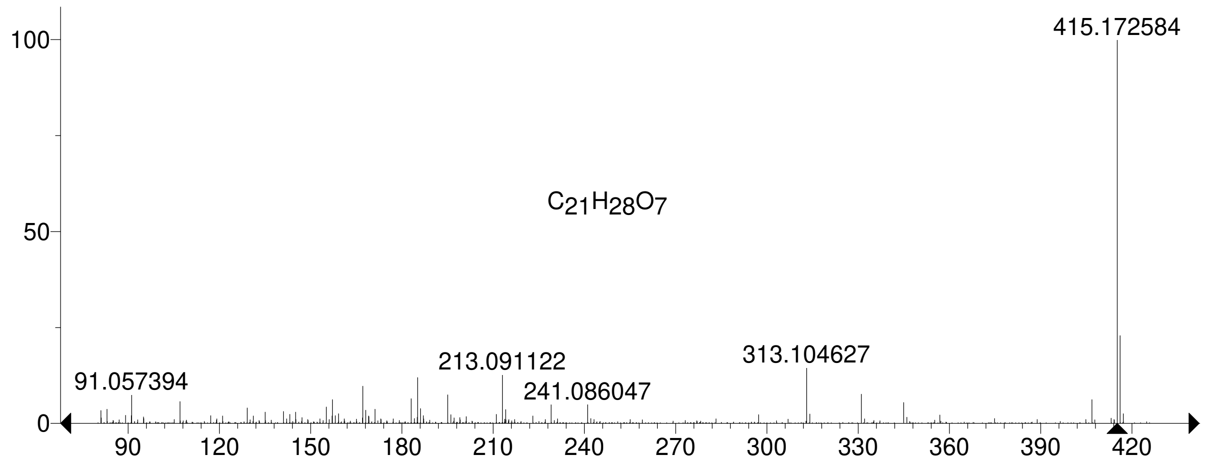

Hit 4 : Diltiazem HCl (Tiazac) [M+H]+ P=415.2
C22H27ClN2O4S; MF: 0; RMF: 25; Prob 13.4%; Lib: mona-export-gnps; ID: 648.

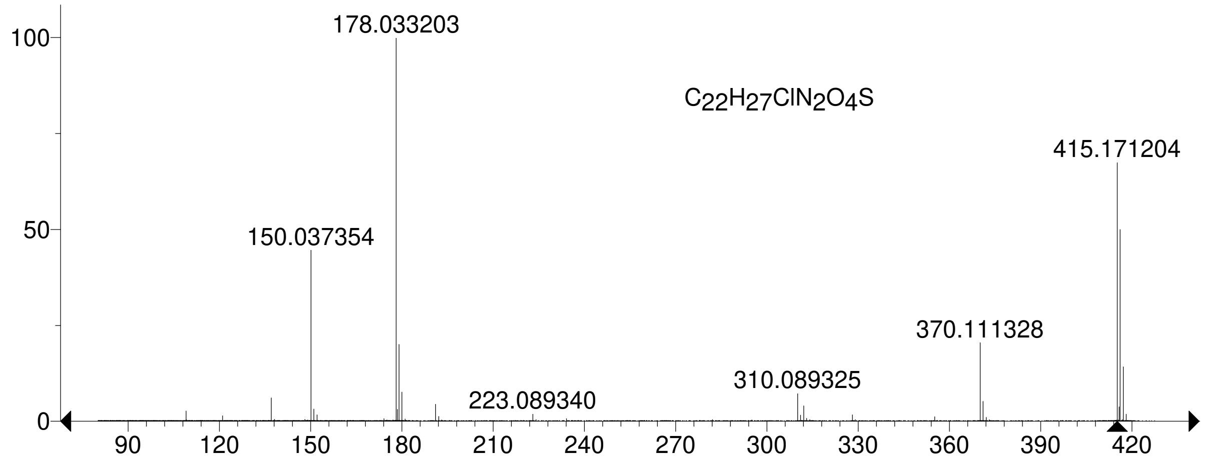

Hit 5 : NCGC00347525-02!2-amino-5-[2-[[2,3-dihydroxy-2-(1-hydroxyethyl)butanoyl]oxymethyl]-4-hydroxyanilino]-
C18H26N2O9; MF: 0; RMF: 23; Prob 13.4%; Lib: mona-export-gnps; ID: 6270.

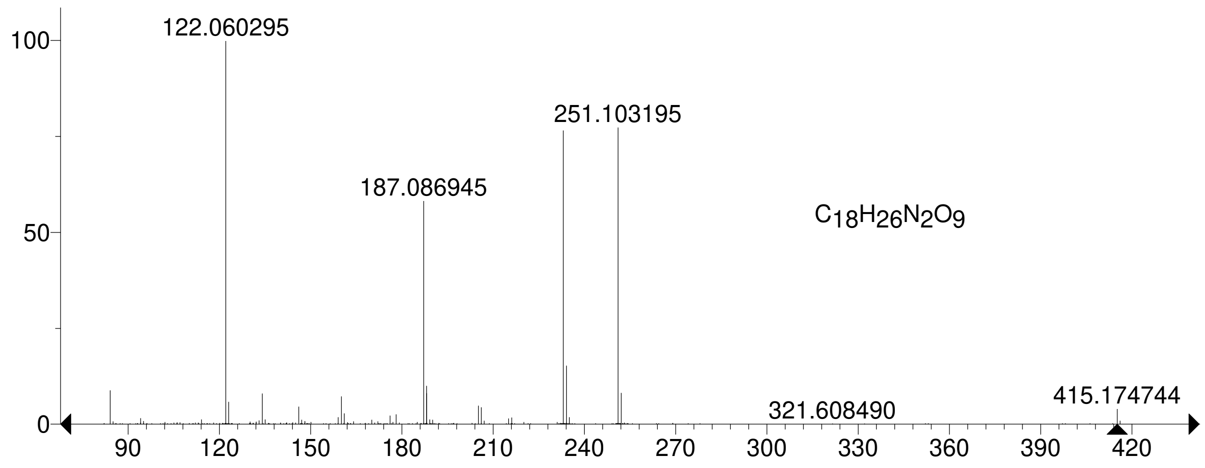

Unknown: Unknown (4.40_476.1510m/z)
Compound in Library Factor = 0

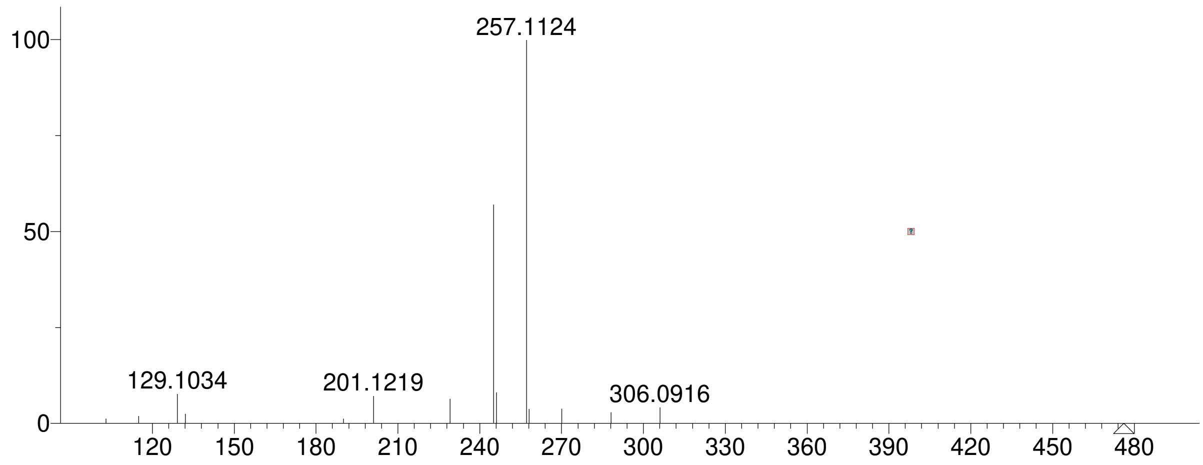

** Search Report Page 1 of 1 **

Unknown: Unknown (4.40_646.2090m/z)
Compound in Library Factor = 0

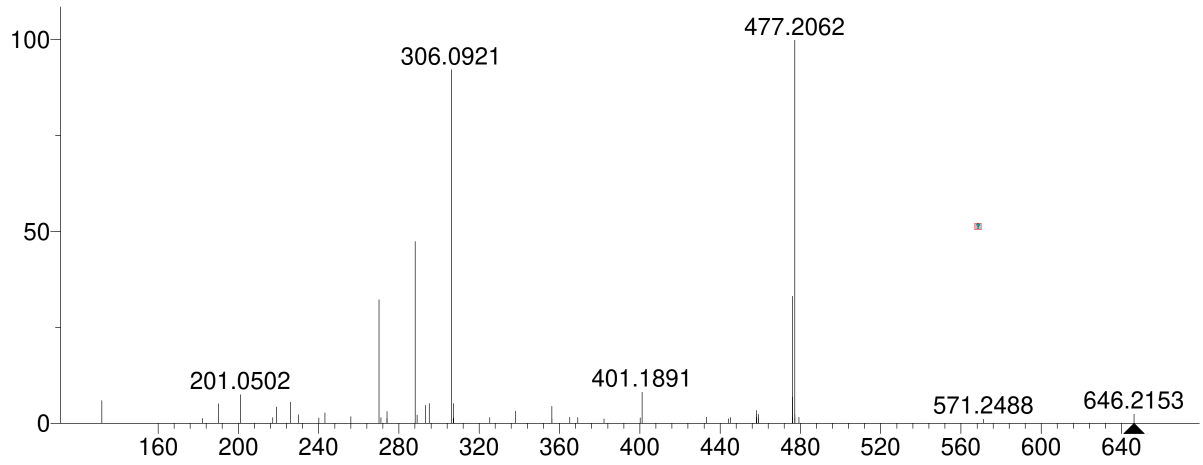

Unknown: Unknown (4.41_257.1133m/z)
Compound in Library Factor = -287

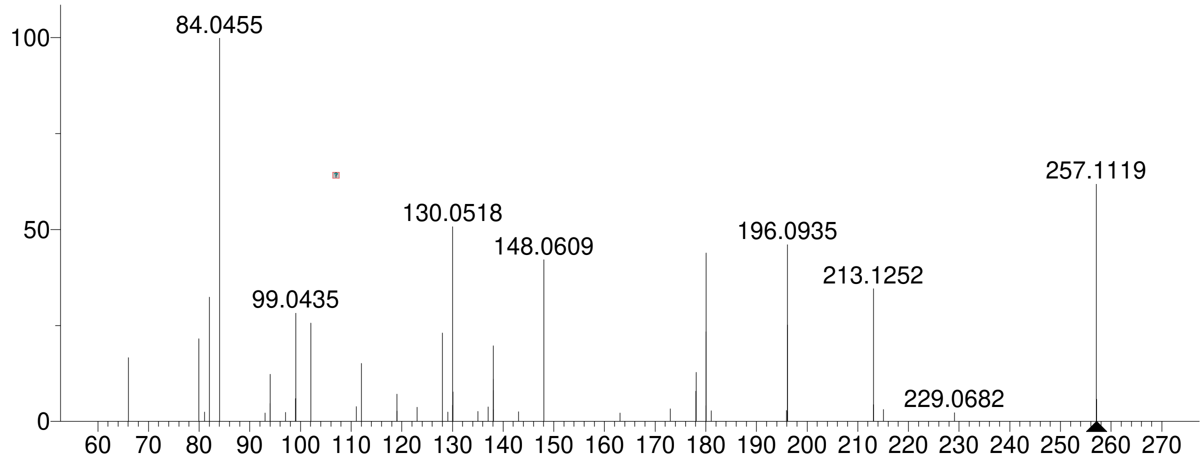

Hit 1 : L-Saccharopine [M-H-H2O]- HCD 24V P=257.1
C11H20N2O6; MF: 285; RMF: 540; Prob 98.8%; CAS: 997-68-2; Lib: nist_msms; ID: 125637.

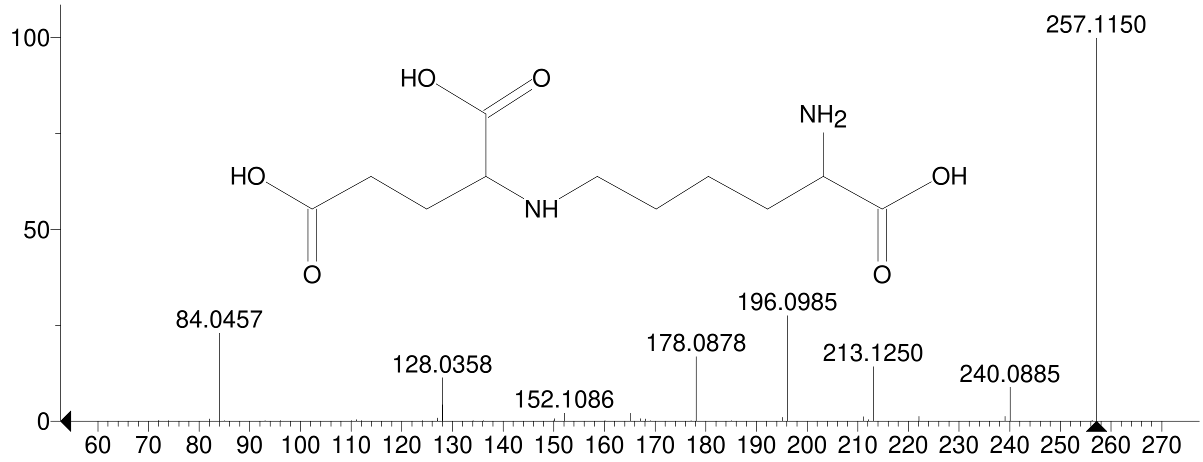

Hit 2 : α-Cyclohexylmandelic acid [M+Na]+ HCD 1V P=257.1
C14H18O3; MF: 59; RMF: 534; Prob 0.98%; CAS: 4335-77-7; Lib: nist_msms; ID: 492969.

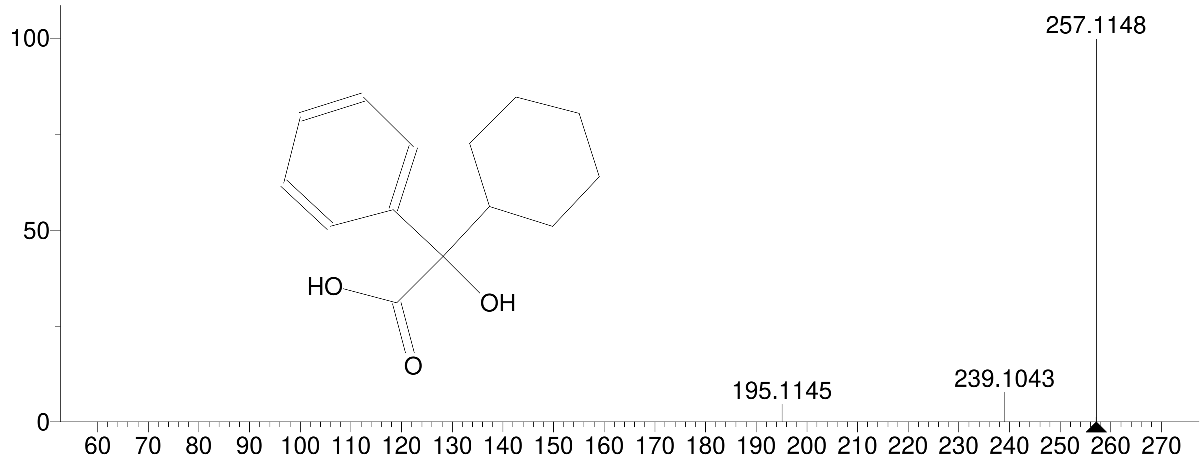

Hit 3 : 5'-O-Methylthymidine [M+H]+ 35eV P=257.1
C11H16N2O5; MF: 0; RMF: 32; Prob 0.17%; Lib: mona-export-fiehn_hilic; ID: 1695.

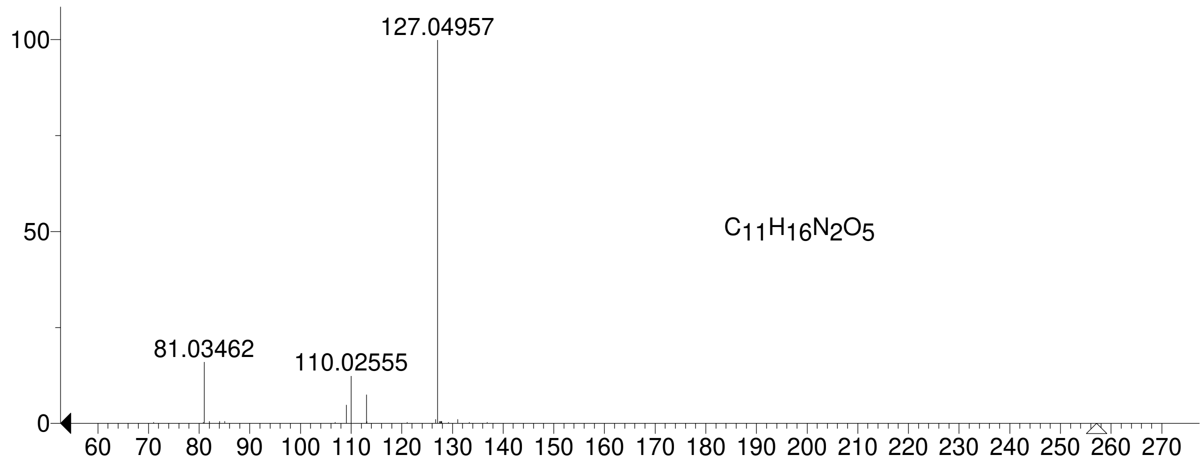

Unknown: Unknown (4.41_427.1709m/z)
Compound in Library Factor = -3273

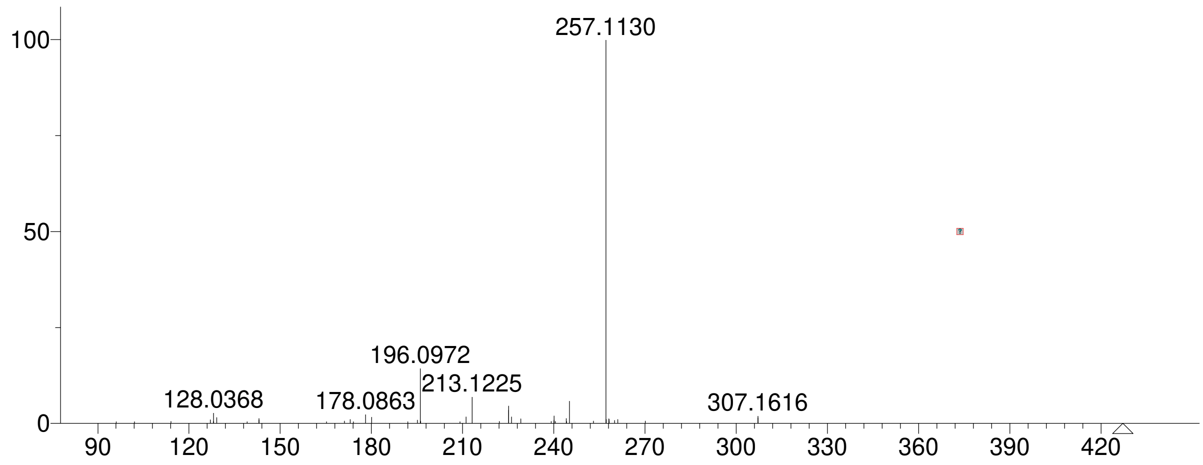

Hit 1 : [(6E,10Z)-10-(acetyloxymethyl)-6-formyl-3-methylidene-2-oxo-3a,4,5,8,9,11a-hexahydrocyclodeca[b]furan-
C22H28O7; MF: 1; RMF: 80; Prob 34.2%; Lib: mona-export-vf-npl_qexactive; ID: 23323.

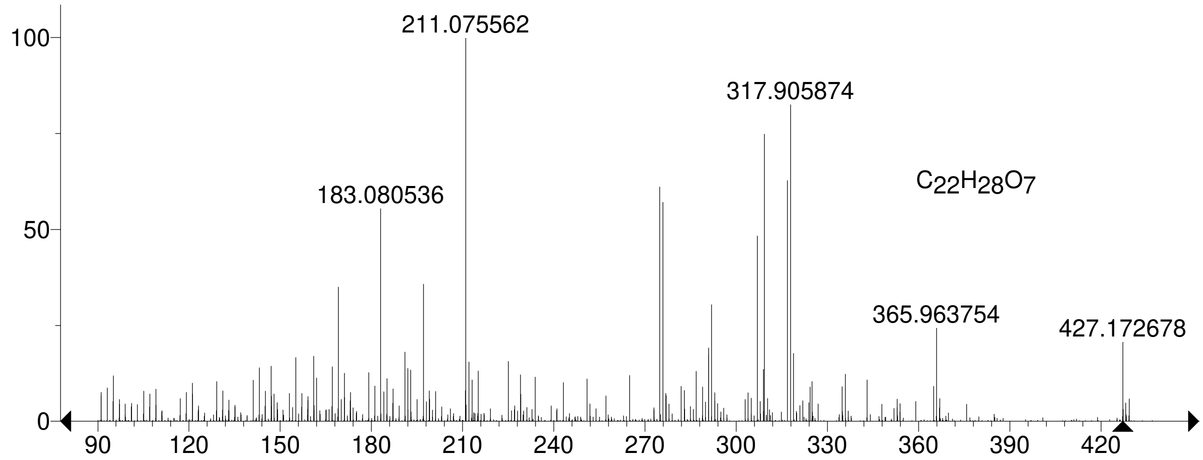

Hit 2 : Archangelicine [M+H]+ 35HCD P=427.2
C24H26O7; MF: 0; RMF: 26; Prob 32.9%; Lib: mona-export-vf-npl_qexactive; ID: 5194.

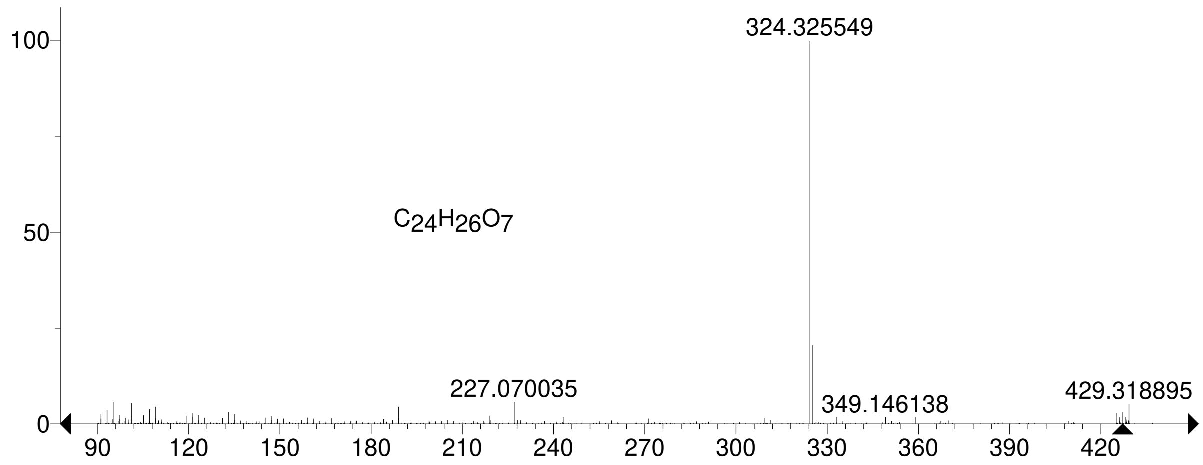

Hit 3 : [8,8-dimethyl-9-(3-methylbut-2-enoyloxy)-2-oxo-9,10-dihydropyrano[2,3-f]chromen-10-yl] 3-methylbut-2-en
C24H26O7; MF: 0; RMF: 19; Prob 32.9%; Lib: mona-export-vf-npl_qexactive; ID: 4551.

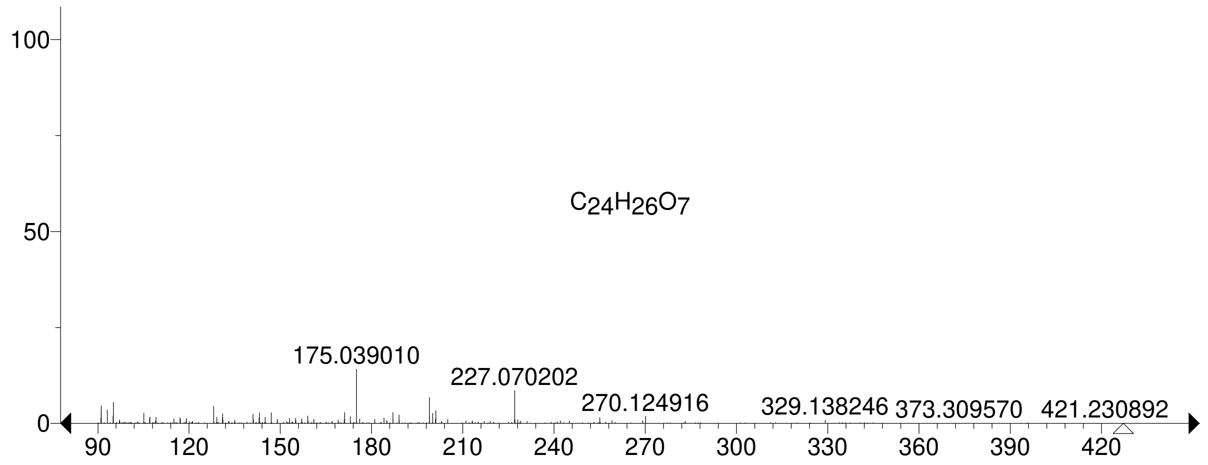

** Search Report Page 1 of 1 **

Unknown: Unknown (4.41_660.2226m/z)
Compound in Library Factor = 0

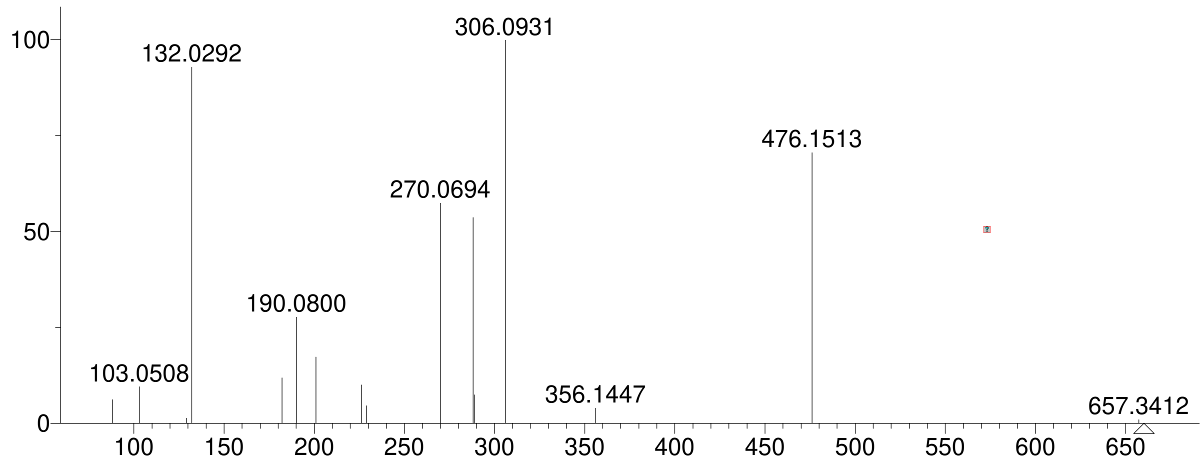

Unknown: Unknown (4.42_432.2329m/z)
Compound in Library Factor = -2176

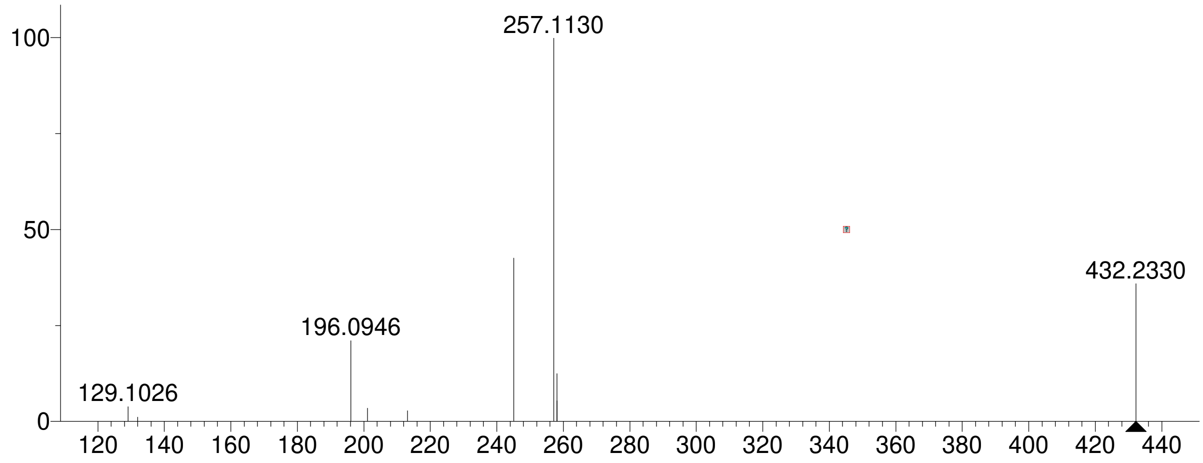

Hit 1 : Trp-Ala-Arg [M+H]+ QTOF 10V P=432.2
C20H29N7O4; MF: 44; RMF: 379; Prob 56.0%; Lib: nist_msms; ID: 54458.

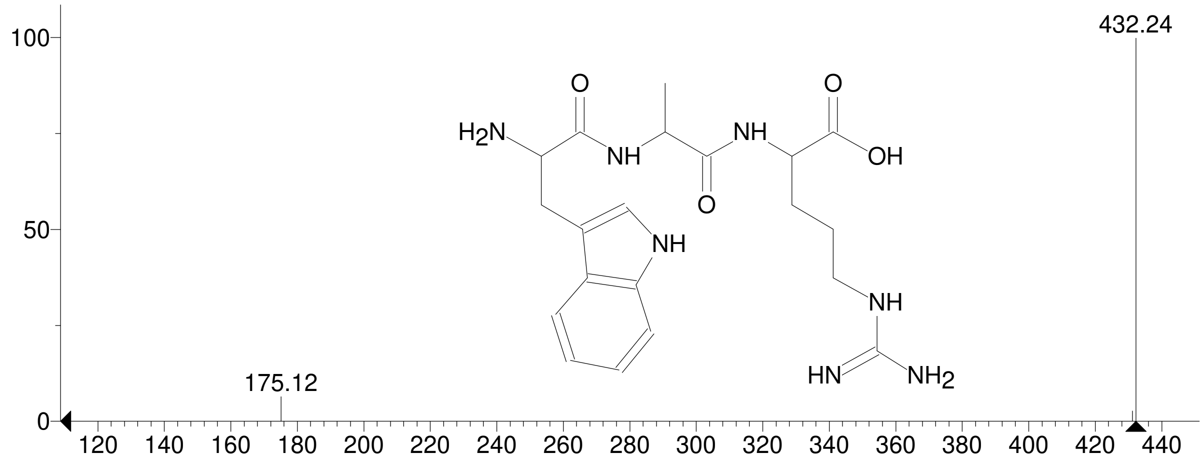

Hit 2 : Ala-Trp-Arg [M+H]+ QTOF 5V P=432.2
C20H29N7O4; MF: 22; RMF: 376; Prob 22.1%; Lib: nist_msms; ID: 49475.

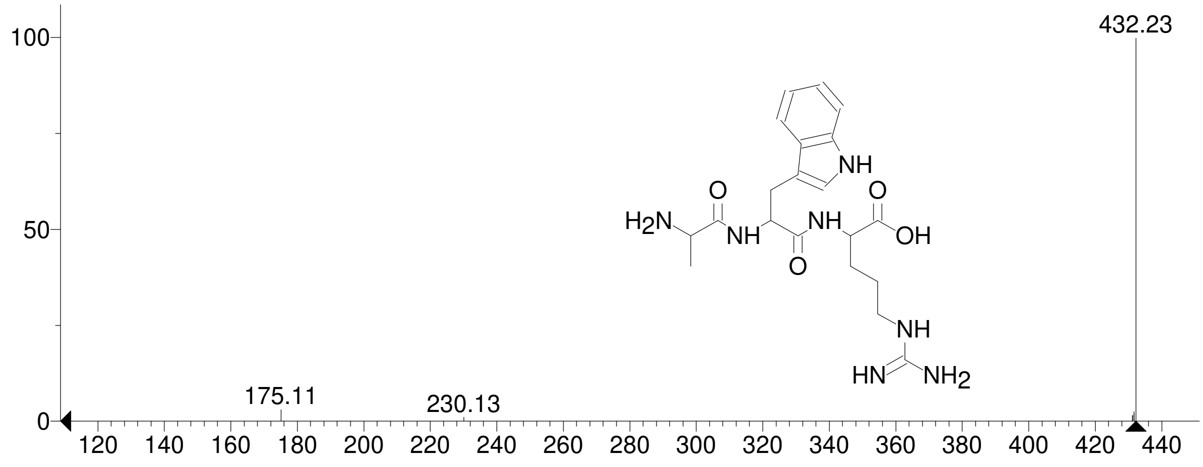

Hit 3 : NCGC00380807-01_C26H31N3O3_Pyrrolo[1,2-a]pyrazine-1,4-dione, 3-[[7-(1,1-dimethyl-2-propen-1-yl)-2,
C26H31N3O3; MF: 5; RMF: 249; Prob 12.1%; Lib: msms-neg-gnps; ID: 1604.

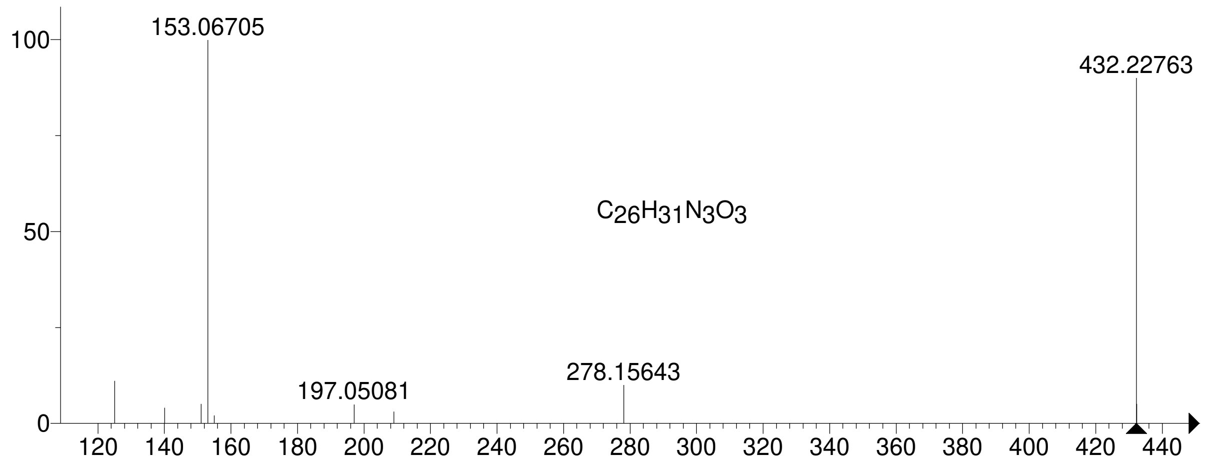

Hit 4 : Stephacidin A_130074 [M+H]+ P=432.2
C26H29N3O3; MF: 0; RMF: 186; Prob 9.74%; Lib: mona-export-gnps; ID: 5040.

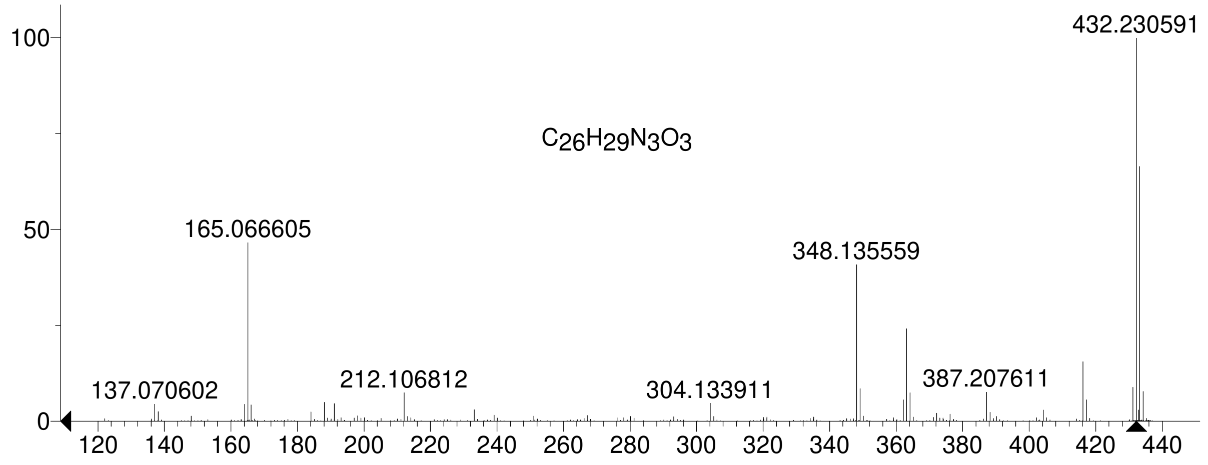

Unknown: Unknown (4.42_699.1211m/z)
Compound in Library Factor = 0

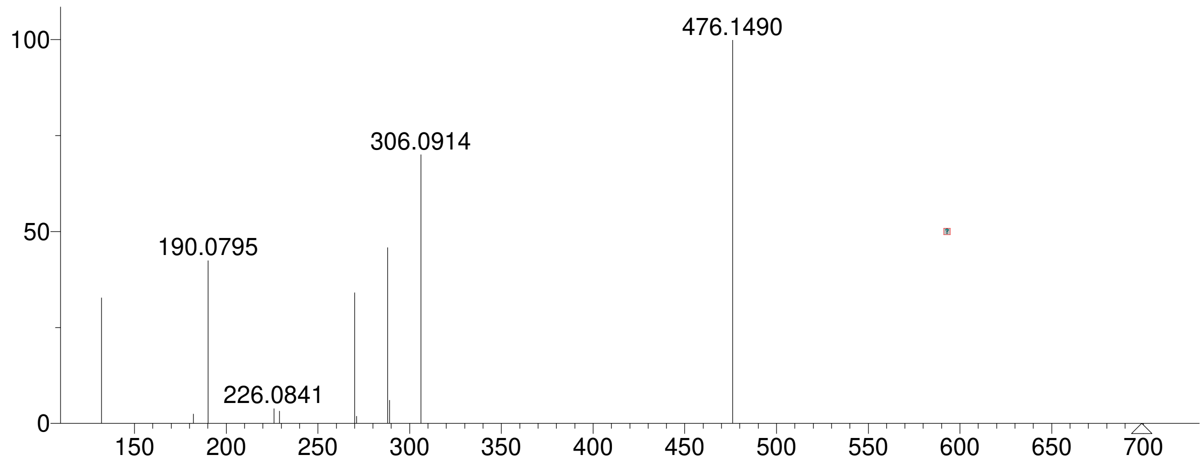

Unknown: Unknown (4.42_699.1219m/z)
Compound in Library Factor = 0

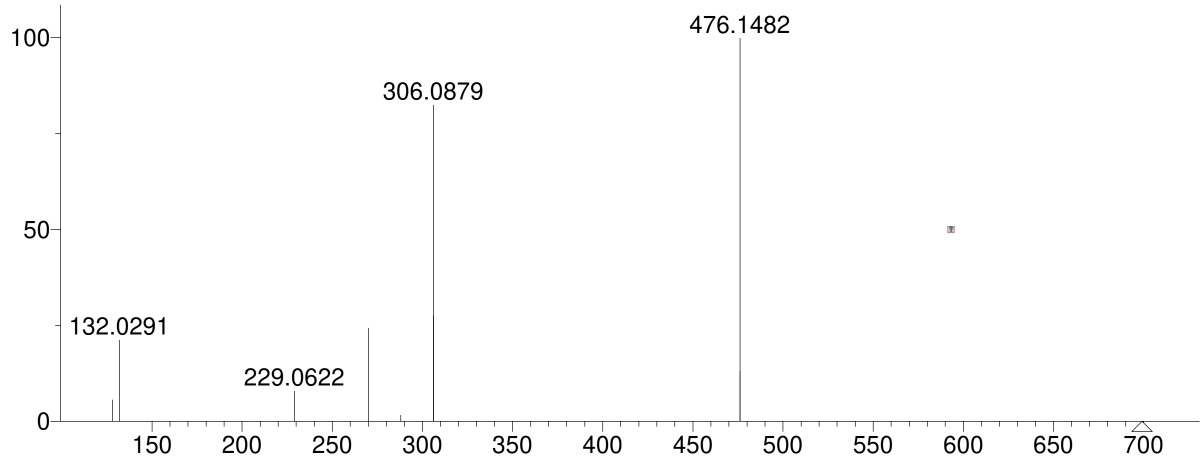

Unknown: Unknown (4.43_465.1492m/z)
Compound in Library Factor = 0

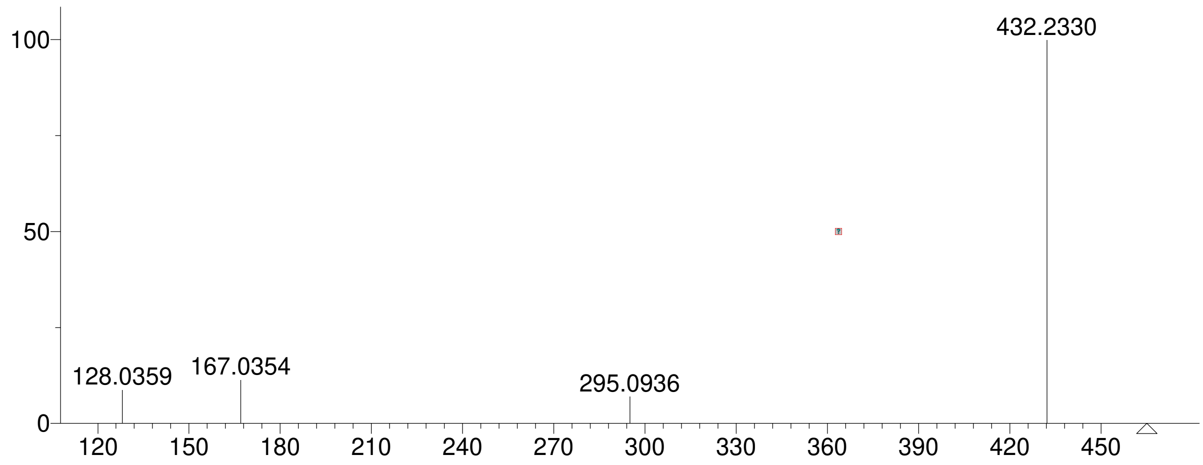

Hit 1 : [5-formyl-4,8-dihydroxy-3-(3-methoxy-3-oxoprop-1-en-2-yl)-8a-methyl-2,3,4,4a,5,6,7,8-octahydro-1H-napht
C21H30O9; MF: 0; RMF: 2; Prob 100.0%; Lib: mona-export-vf-npl_qexactive; ID: 10594.

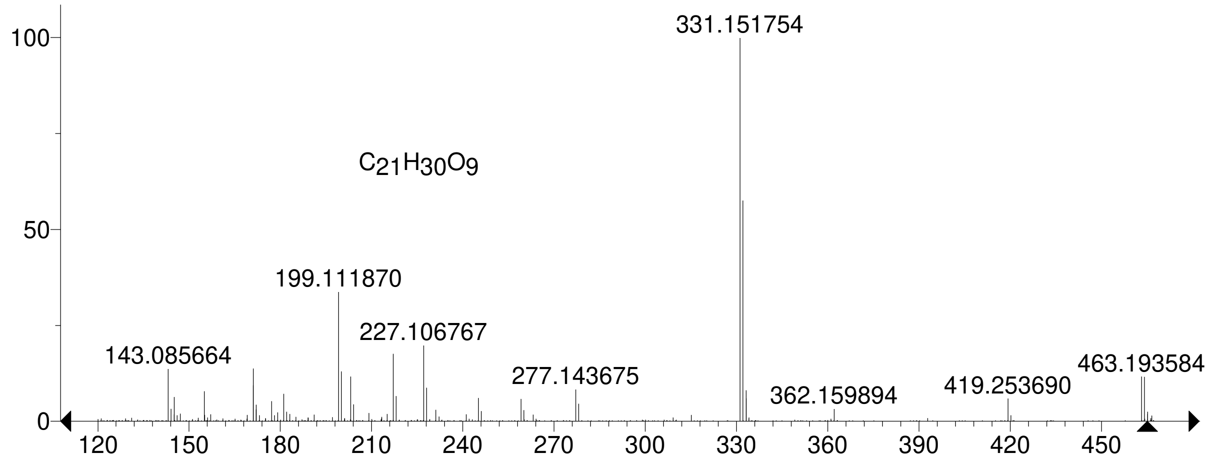

Unknown: Unknown (4.44_373.1713m/z)
Compound in Library Factor = 0

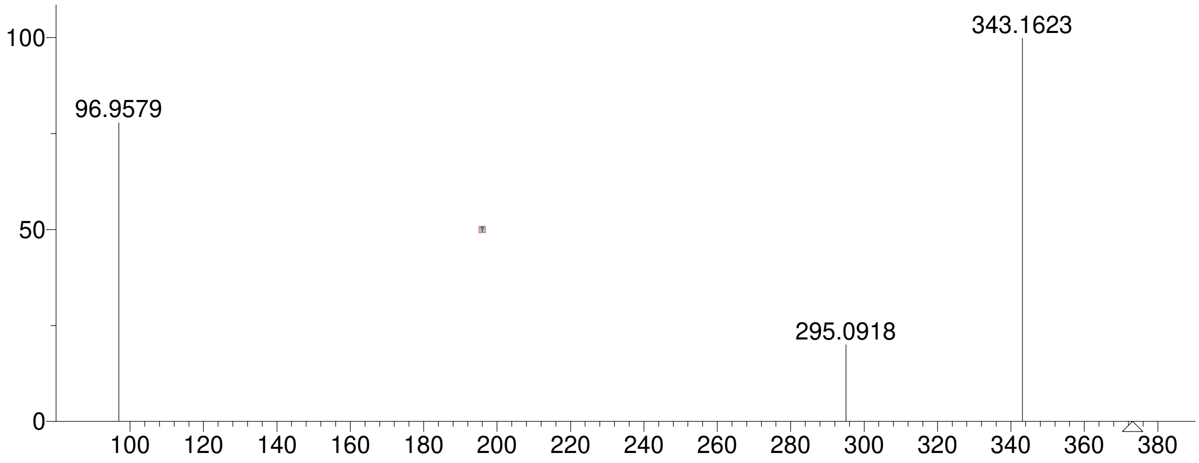

Unknown: Unknown (4.44_399.1492m/z)
Compound in Library Factor = 0

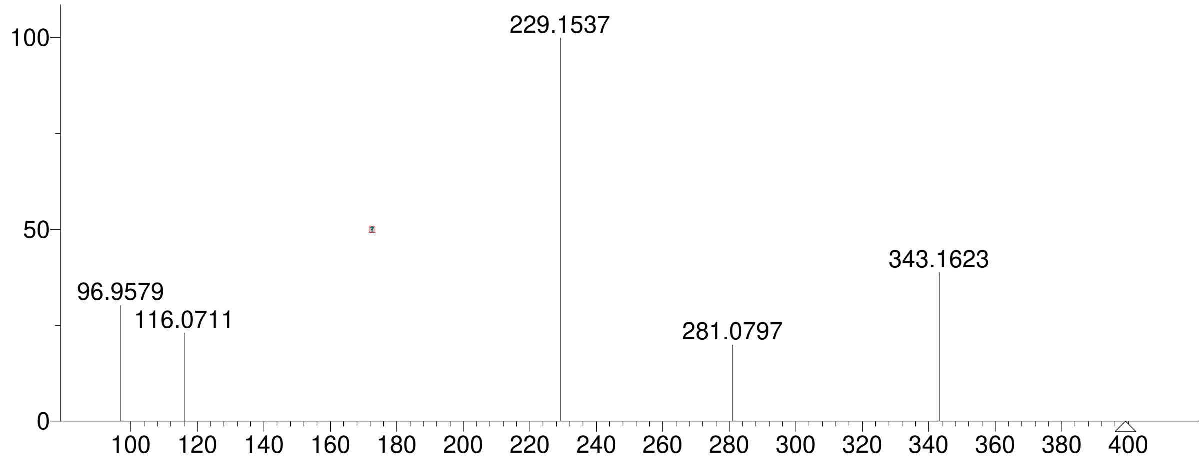

Unknown: Unknown (4.45_305.0692m/z)
Compound in Library Factor = -2387

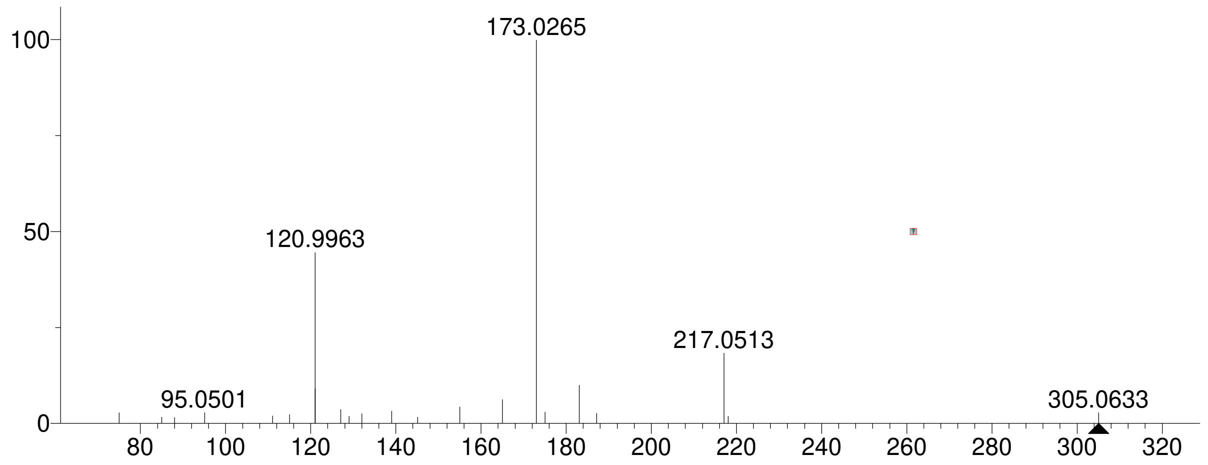

Hit 1 : Gallocatechin [M-H]- 45eV P=305.1
$C_{15}H_{14}O_7$; MF: 23; RMF: 625; Prob 15.4%; Lib: msms-neg-metabobase; ID: 810.

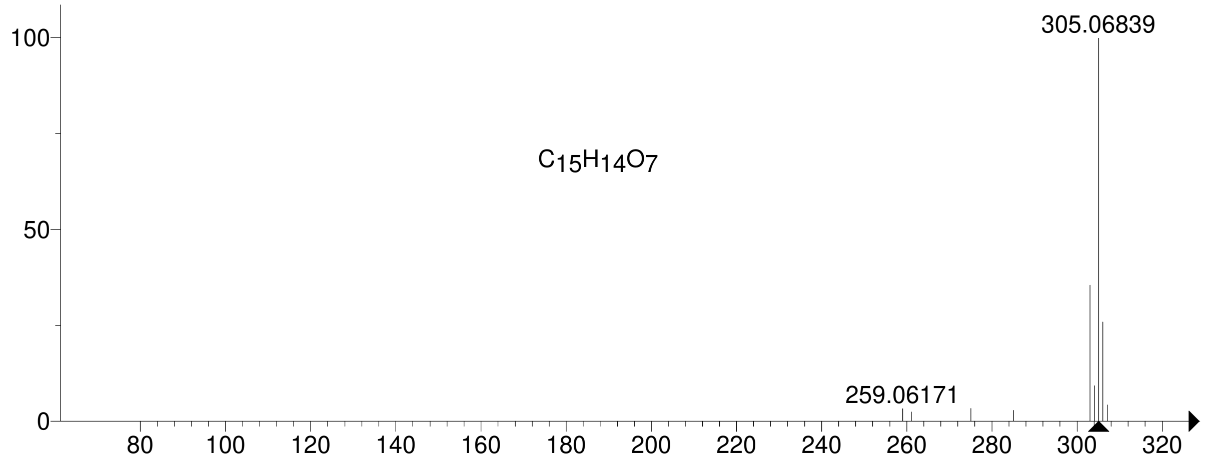

Hit 2 : 2,3-Trans-3,4-cis-Leucocyanidin [M-H]- 45eV P=305.1
$C_{15}H_{14}O_7$; MF: 13; RMF: 384; Prob 10.8%; Lib: msms-neg-metabobase; ID: 827.

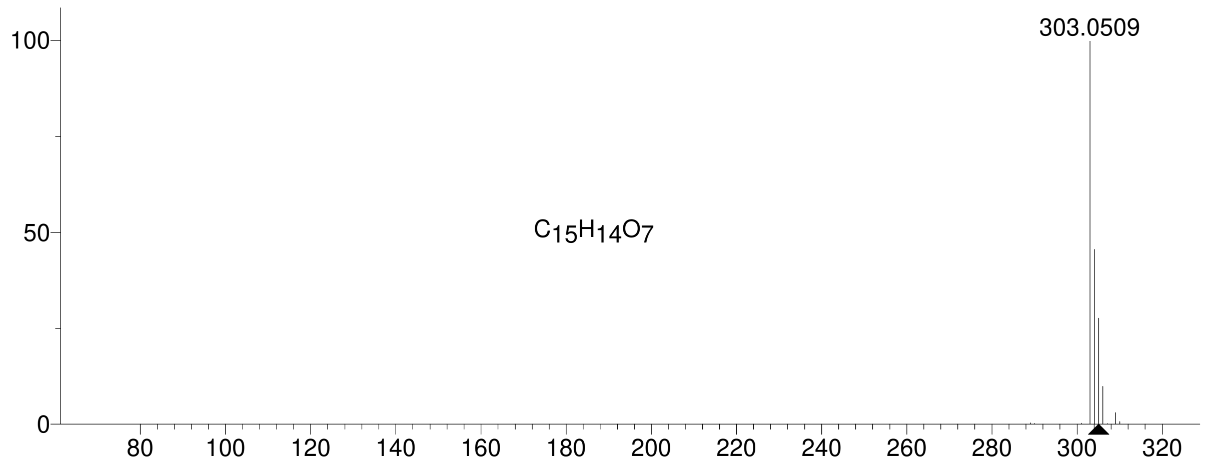

Hit 3 : Epigallocatechin [M-H]- 45eV P=305.1
C15H14O7; MF: 11; RMF: 547; Prob 10.0%; Lib: msms-neg-metabobase; ID: 857.

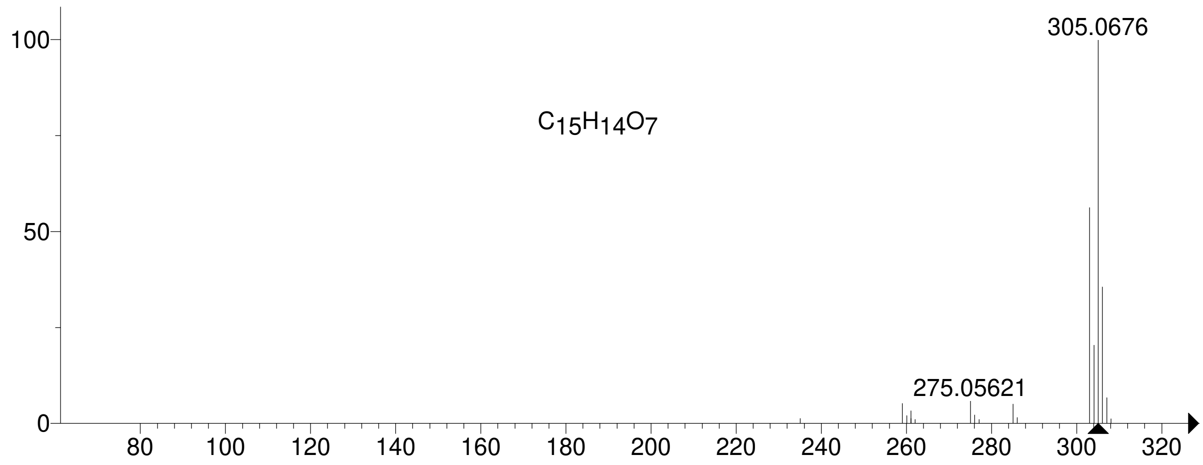

Hit 4 : (-)-Epigallocatechin [M-H]- HCD 13V P=305.1
C15H14O7; MF: 4; RMF: 134; Prob 7.66%; CAS: 970-74-1; Lib: nist_msms; ID: 235098.

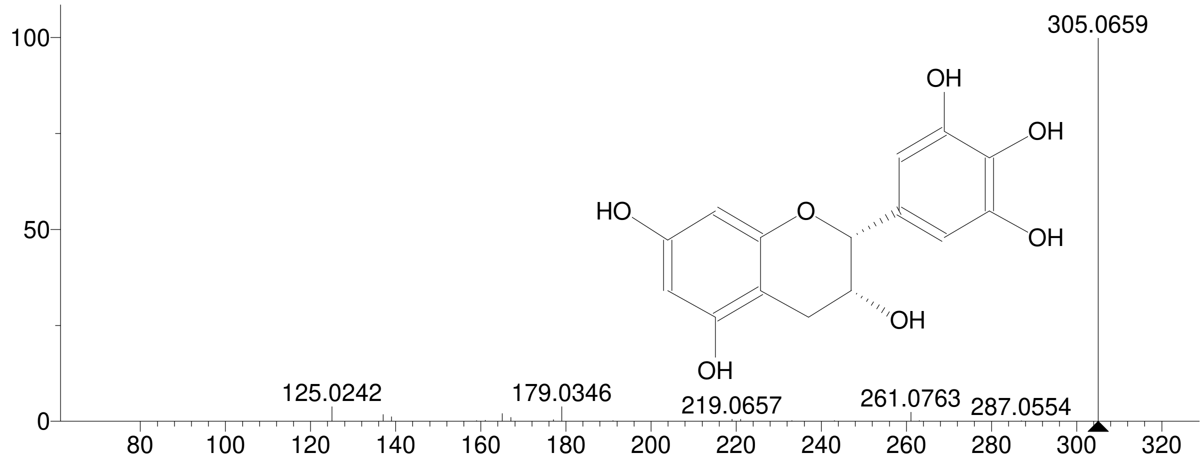

Hit 5 : Glutathione, oxidized [M-2H]2- HCD 8V P=305.1
C20H32N6O12S2; MF: 3; RMF: 122; Prob 7.36%; CAS: 27025-41-8; Lib: nist_msms; ID: 185529.

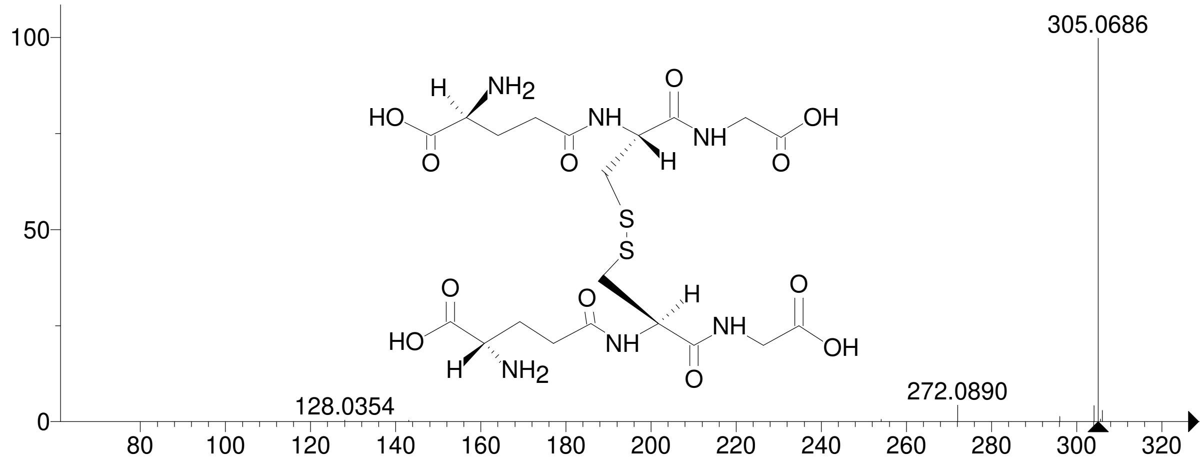

Unknown: Unknown (4.46_278.1133m/z)
Compound in Library Factor = 0

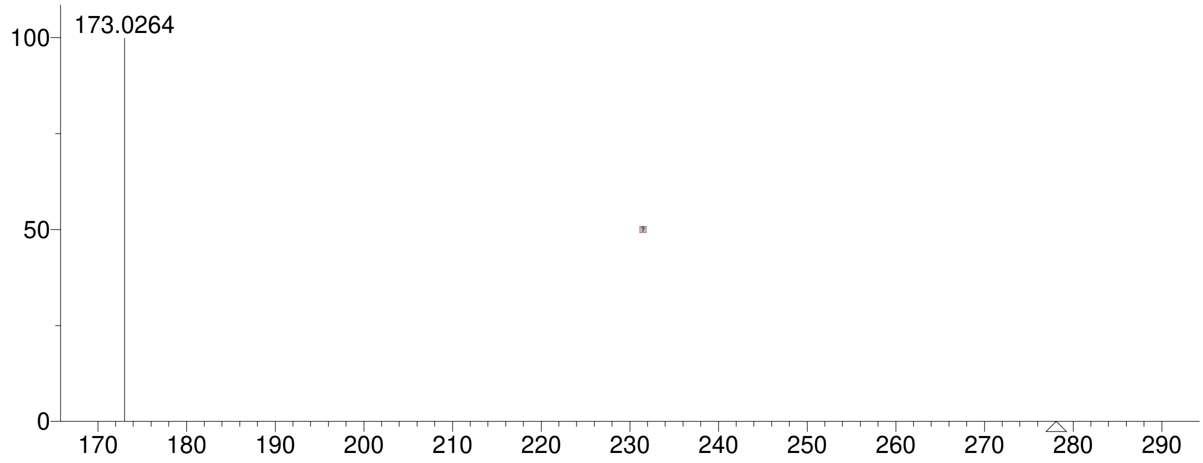

** Search Report Page 1 of 1 **

Unknown: Unknown (4.46_391.2067m/z)
Compound in Library Factor = 0

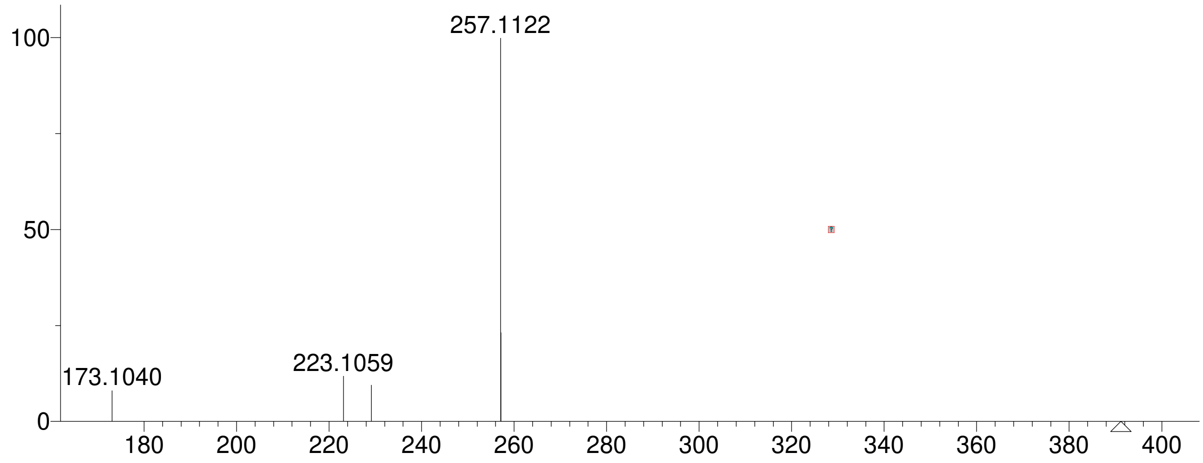

Hit 1 : Thromboxane B3 [M+Na]+ IT-FT 35% P=391.2
C20H32O6; MF: 0; RMF: 39; Prob 100.0%; CAS: 71953-80-5; Lib: nist_msms; ID: 85864.

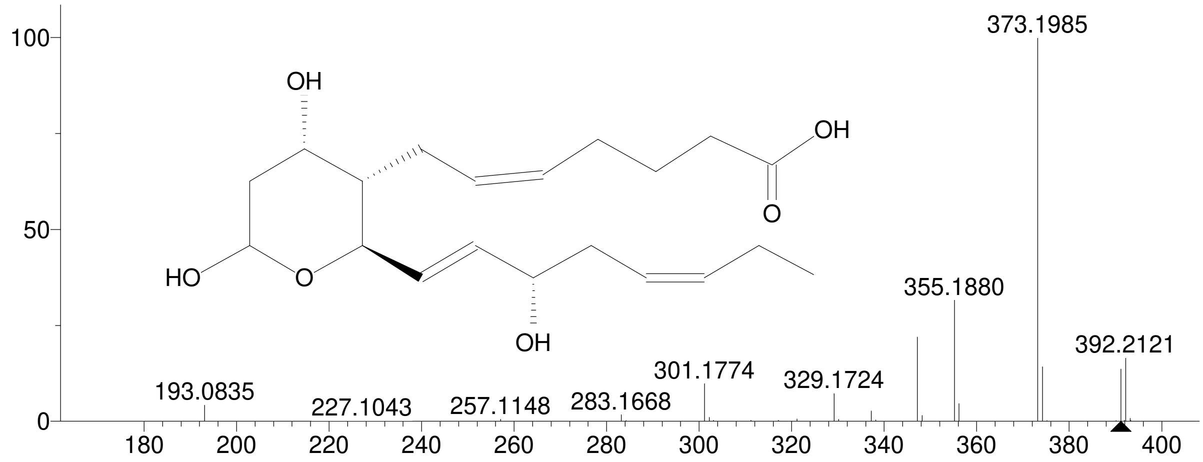

Unknown: Unknown (4.46_397.2197m/z)
Compound in Library Factor = -2176

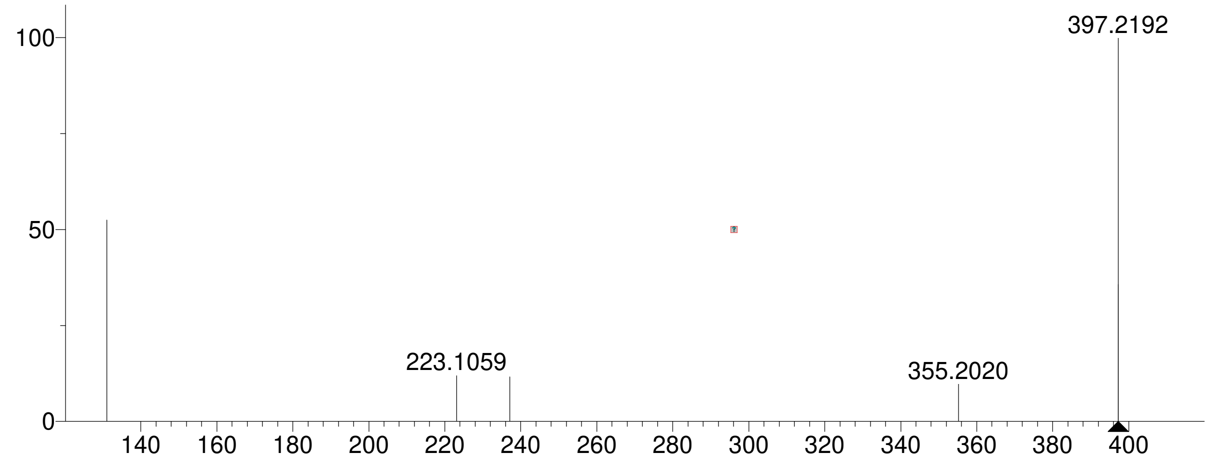

Hit 1 : NCGC00385079-01_C19H34O7_4-(4-Hydroxy-2,6,6-trimethyl-1-cyclohexen-1-yl)-2-butanyl beta-D-glucopy
C19H34O7; MF: 21; RMF: 593; Prob 43.9%; Lib: mona-export-gnps; ID: 14159.

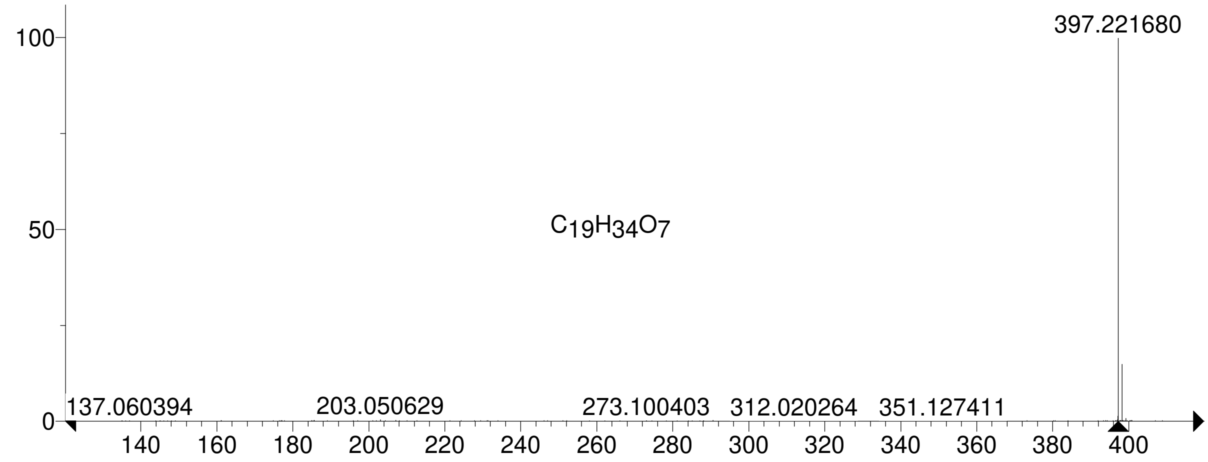

Hit 2 : (2R,3S,4S,5R,6R)-2-(hydroxymethyl)-6-[4-(4-hydroxy-2,6,6-trimethylcyclohexen-1-yl)butan-2-yloxy]oxane-3
C19H34O7; MF: 0; RMF: 516; Prob 18.7%; Lib: anthocyanins mona; ID: 7638.

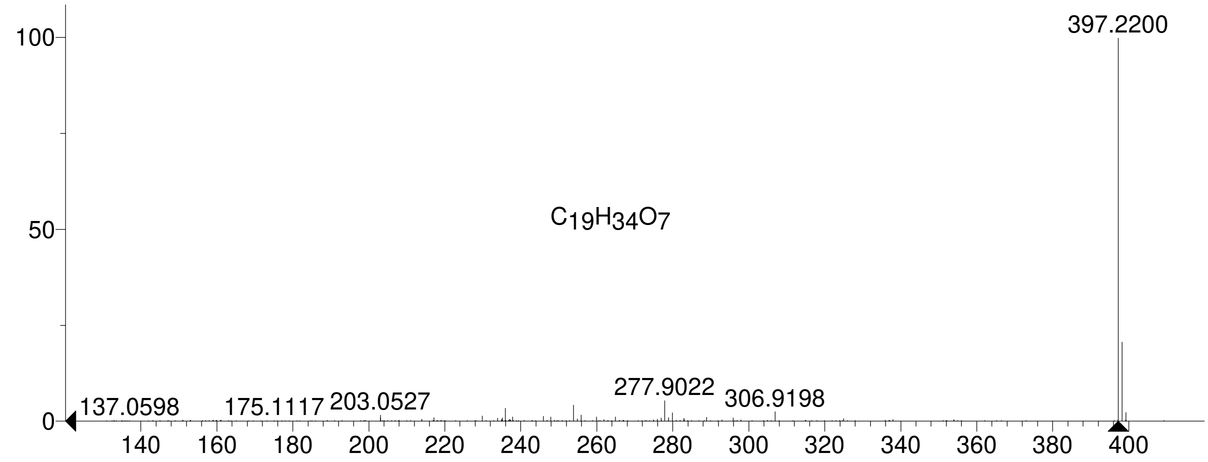

Hit 3 : O-acetylstemmadenine ([H]\C(C)=C1/CN2CC[C@]1([H])[C@](COC(C)=O)(C(=O)OC)C1=C(CC2)C2=CC=(
C23H28N2O4; MF: 0; RMF: 408; Prob 18.7%; Lib: alkaloids; ID: 12.

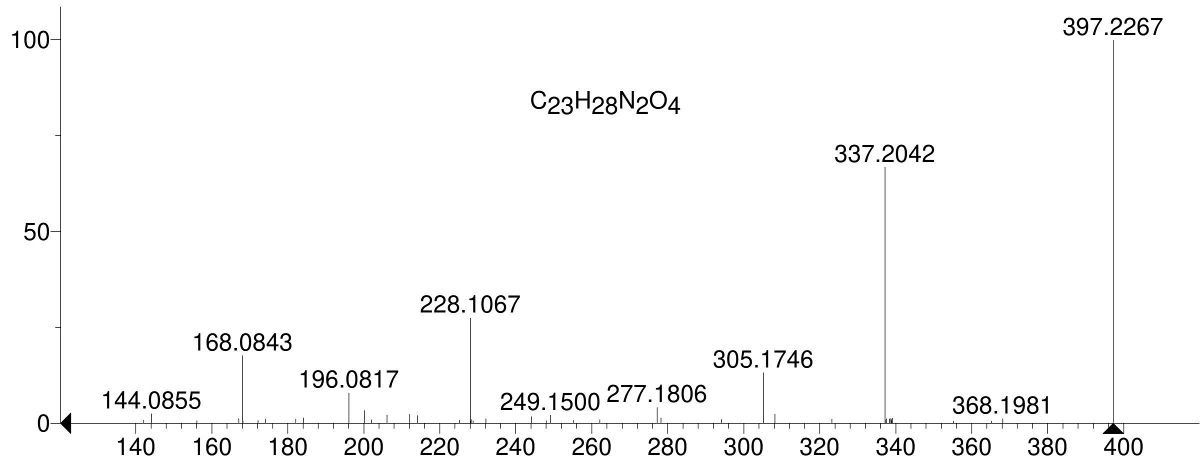

Hit 4 : 2,4-dihydroxy-5-methoxy-1'',6'',8''-trimethyldispiro[bis(oxolane)-3,2':5',7''-[3]oxatricyclo[6.3.1.0a^'',A^'1A^'2]c
C21H32O7; MF: 0; RMF: 115; Prob 18.7%; Lib: mona-export-vf-npl_qexactive; ID: 10632.

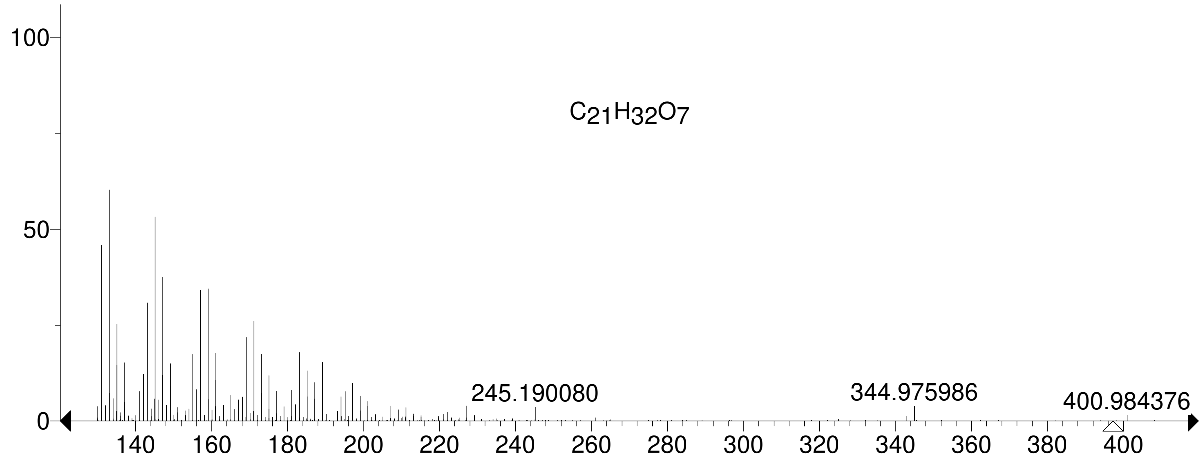

** Search Report Page 1 of 1 **

Unknown: Unknown (4.46_415.1701m/z)
Compound in Library Factor = 0

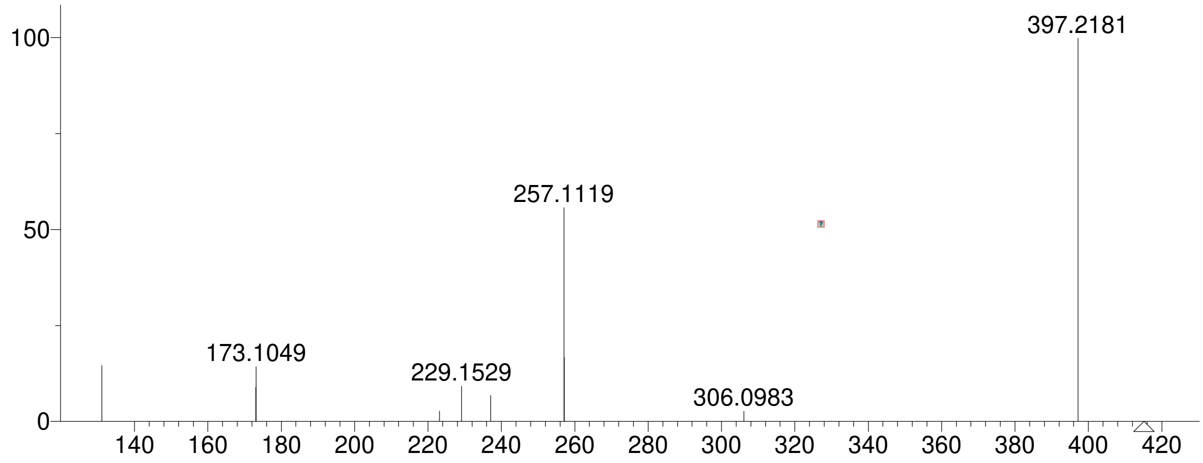

Hit 1 : 6-formyl-10-(hydroxymethyl)-5-methoxy-3-methylidene-2-oxo-2H,3H,3aH,4H,5H,8H,9H,11aH-cyclodeca[b]f
C21H28O7; MF: 0; RMF: 46; Prob 50.0%; Lib: mona-export-vf-npl_qexactive; ID: 16436.

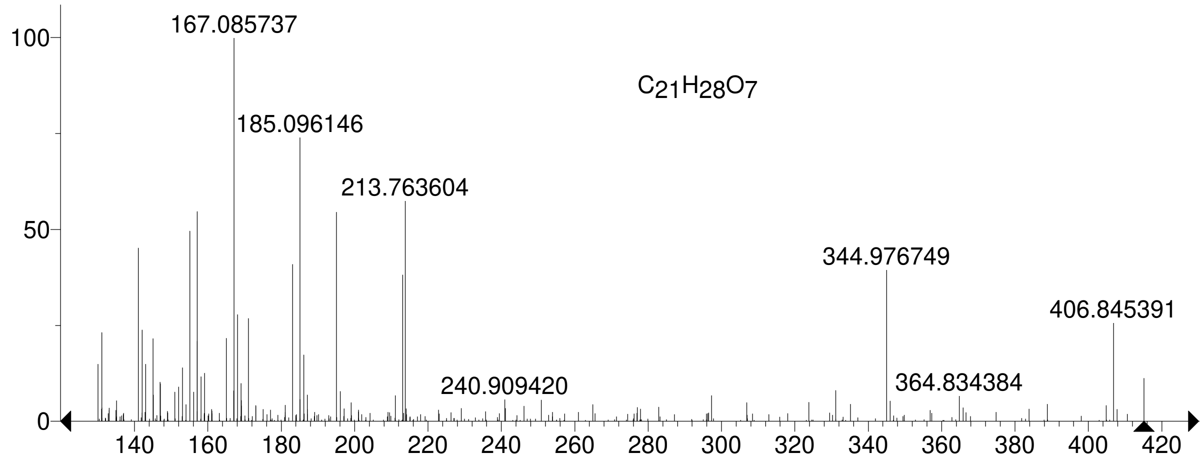

Hit 2 : Diltiazem [M+H]+ IT 35% P=415.2
C22H26N2O4S; MF: 0; RMF: 39; Prob 50.0%; CAS: 42399-41-7; Lib: nist_msms; ID: 281174.

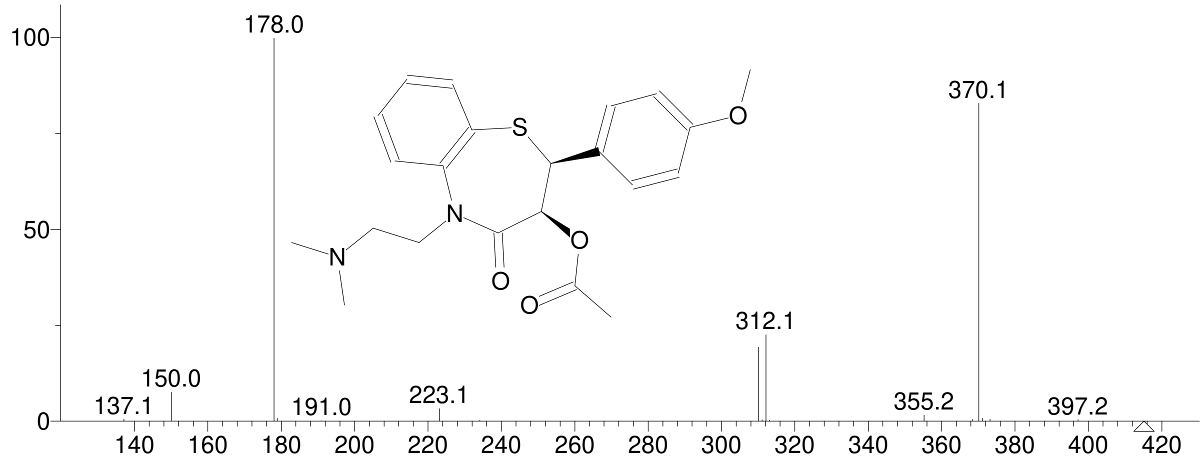

Unknown: Unknown (4.46_451.1374m/z)
Compound in Library Factor = -1309

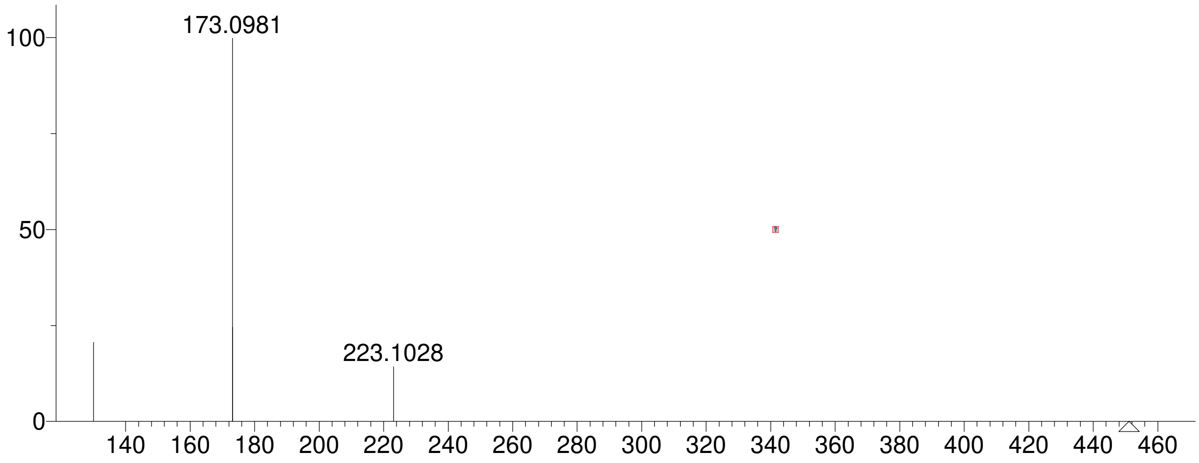

Hit 1 : 3-(N-Morpholino)-2-hydroxypropanesulfonic acid [2M+H]+ HCD 107V P=451.1
C7H15NO5S; MF: 59; RMF: 350; Prob 64.8%; CAS: 68399-77-9; Lib: nist_msms; ID: 58588.

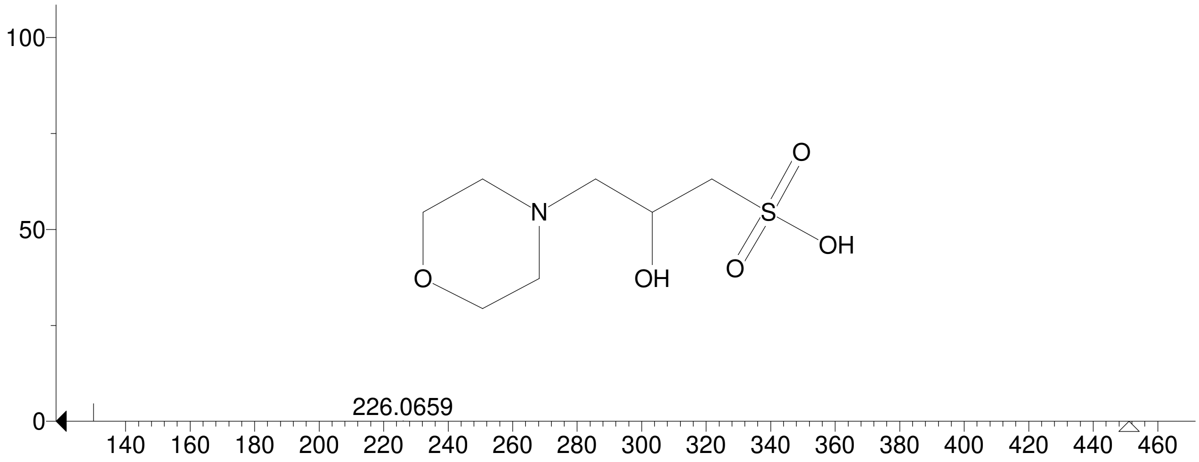

Hit 2 : [4,8-dihydroxy-3-(3-methoxy-3-oxoprop-1-en-2-yl)-8a-methyl-5-oxo-1,2,3,4,4a,6,7,8-octahydronaphthalen-2
C20H28O9; MF: 0; RMF: 78; Prob 11.7%; Lib: mona-export-vf-npl_qexactive; ID: 10661.

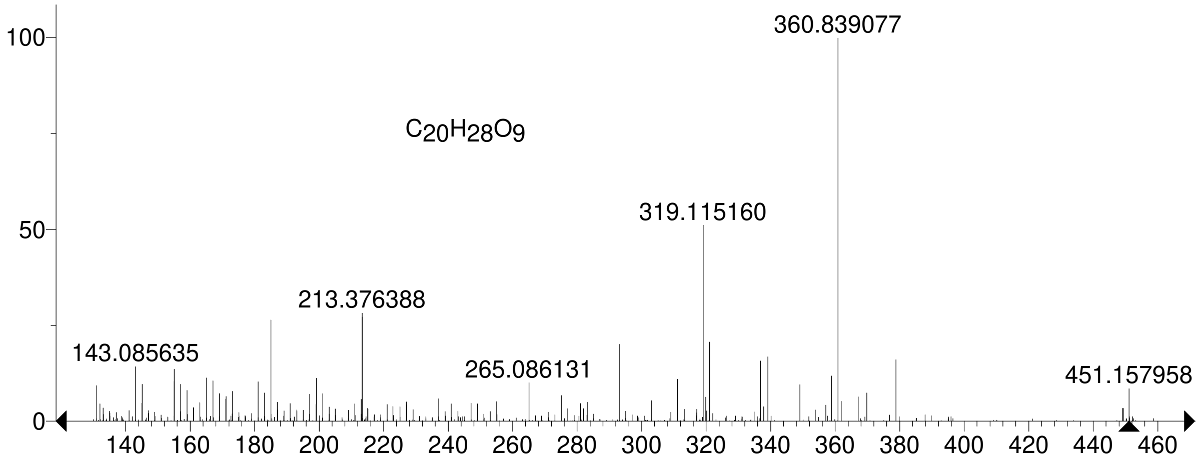

Hit 3 : 9,11,12a-trihydroxy-2,3-dimethoxy-8-(3-methylbut-2-enyl)-6,6a-dihydrochromeno[3,4-b]chromen-12-one [M
C23H24O8; MF: 0; RMF: 28; Prob 11.7%; Lib: mona-export-vf-npl_qexactive; ID: 18387.

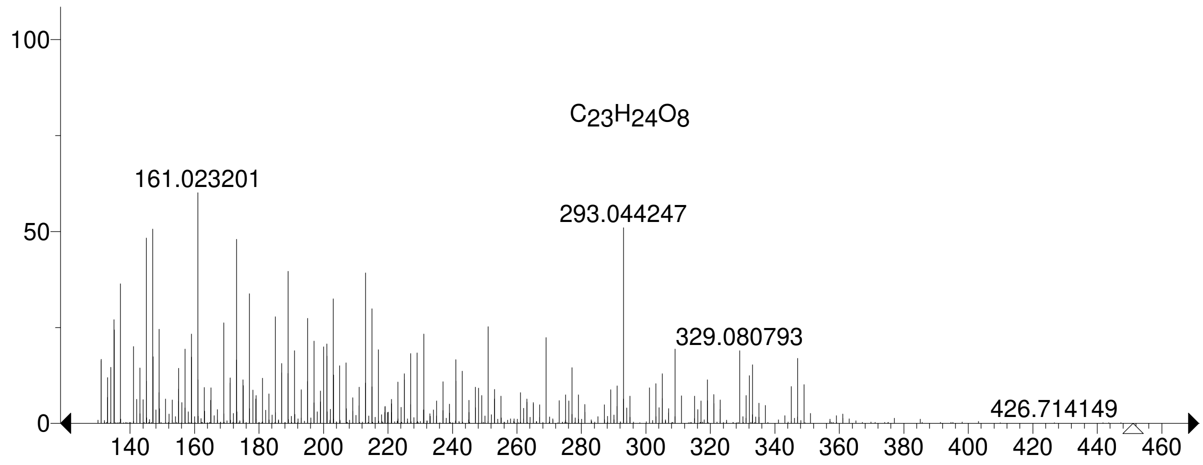

Hit 4 : 1',2'-dihydro-2',6-dihydroxyrotenone [M+Na]+ 45HCD P=451.1
C23H24O8; MF: 0; RMF: 25; Prob 11.7%; Lib: mona-export-vf-npl_qexactive; ID: 4145.

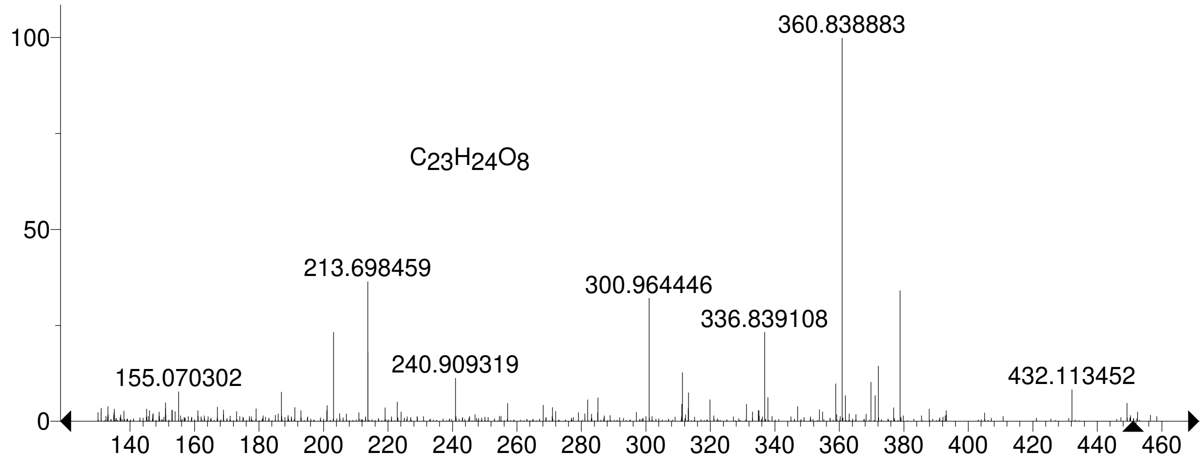

Unknown: Unknown (4.46_646.2109m/z)
Compound in Library Factor = 0

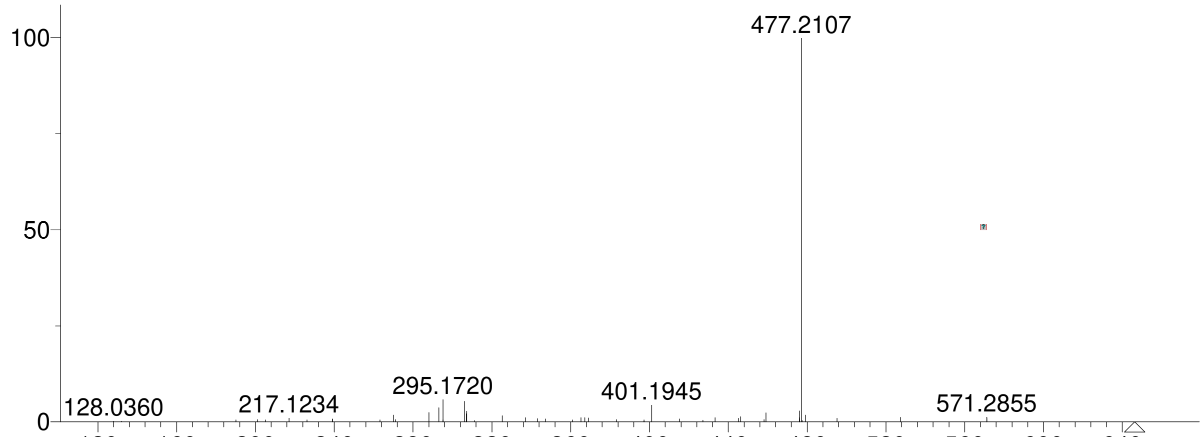

Unknown: Unknown (4.47_336.1434m/z)
Compound in Library Factor = 0

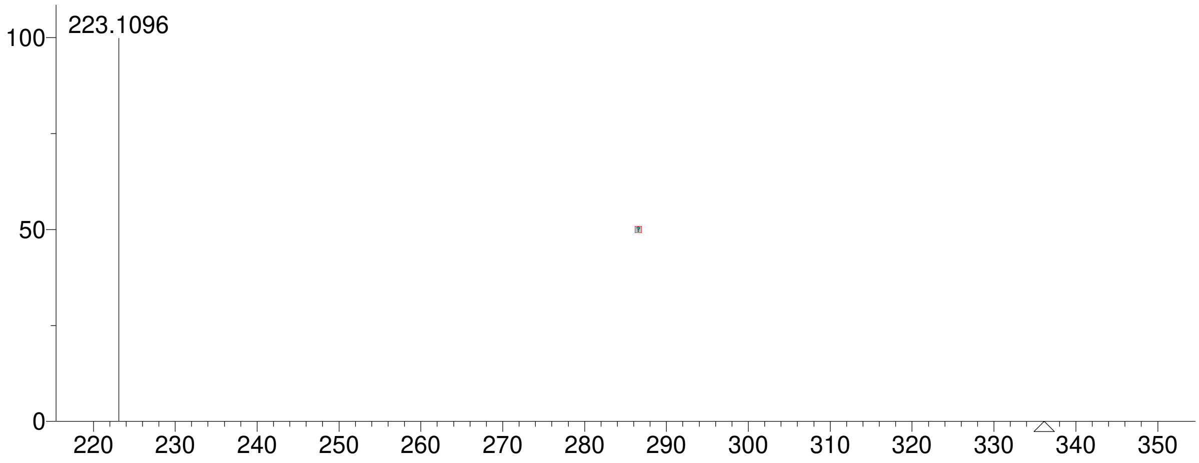

Unknown: Unknown (4.48_328.1495m/z)
Compound in Library Factor = 0

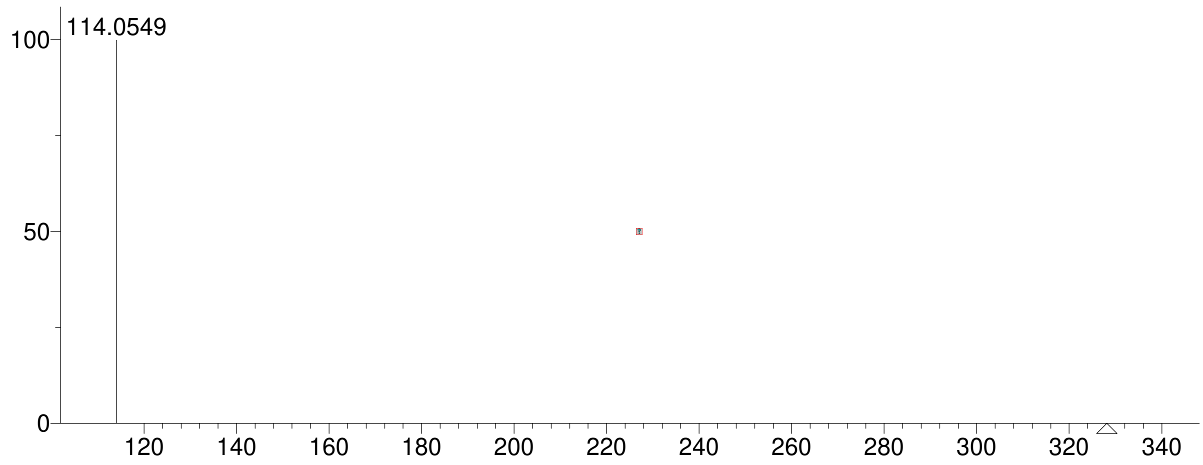

Hit 1 : Ac-Val-Ala-Asp-CHO [M-H]- QTOF 17V P=328.2
C14H23N3O6; MF: 0; RMF: 55; Prob 100.0%; Lib: nist_msms2; ID: 39356.

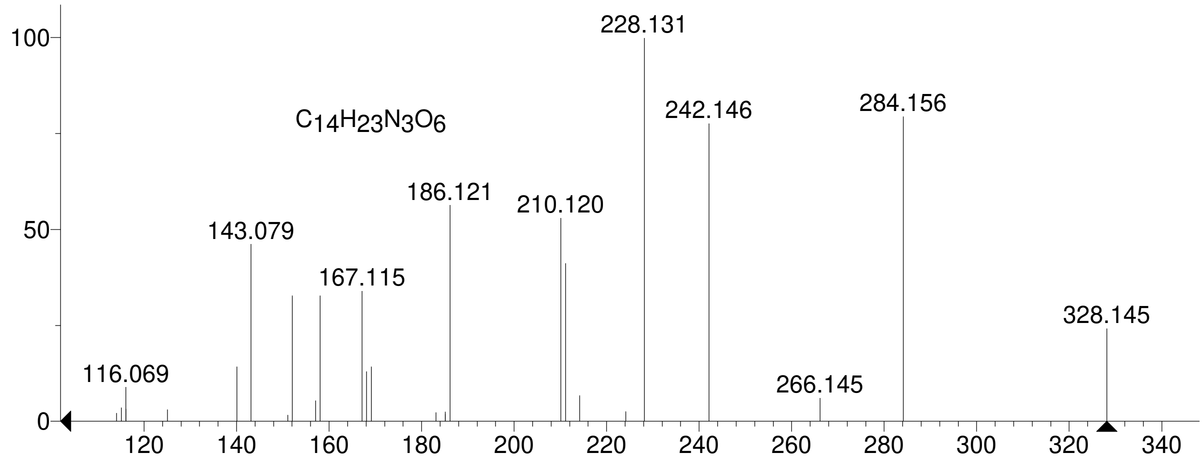

** Search Report Page 1 of 1 **

Unknown: Unknown (4.48_587.1132m/z)
Compound in Library Factor = 0

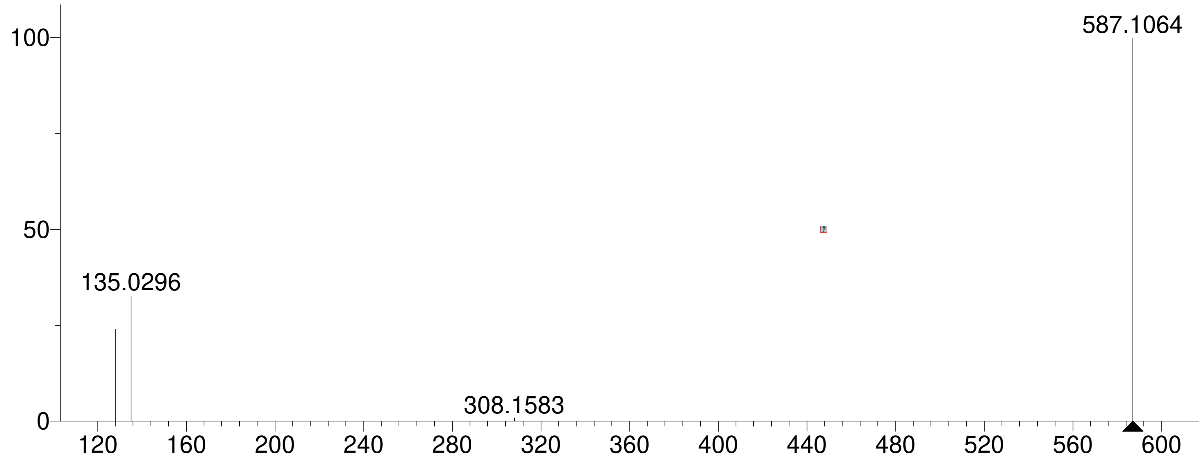

Hit 1 : 5,7-dihydroxy-2-phenyl-6-[3,4,5-trihydroxy-6-(hydroxymethyl)oxan-2-yl]-8-(3,4,5-trihydroxyoxan-2-yl)chrome
C26H28O13; MF: 0; RMF: 14; Prob 50.0%; Lib: anthocyanins mona; ID: 8504.

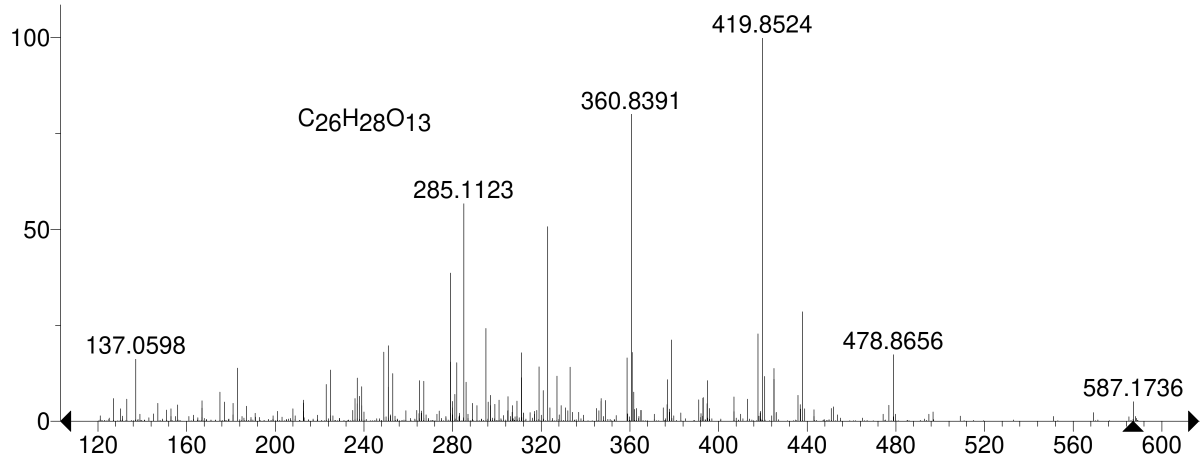

Hit 2 : Theaflavin [M+Na]+ 35HCD P=587.1
C29H24O12; MF: 0; RMF: 8; Prob 50.0%; Lib: mona-export-vf-npl_qexactive; ID: 2698.

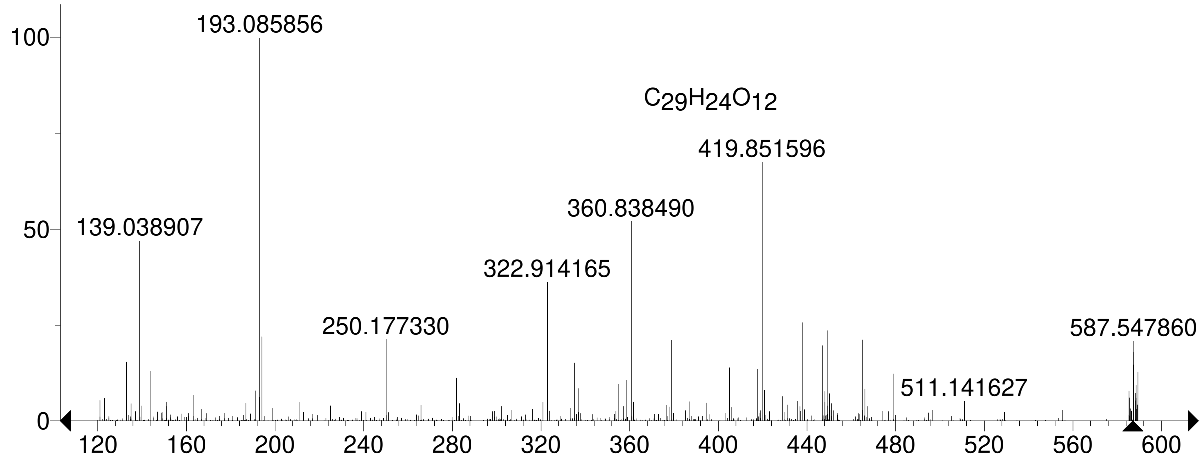

Unknown: Unknown (4.49_611.2305m/z)
Compound in Library Factor = -517

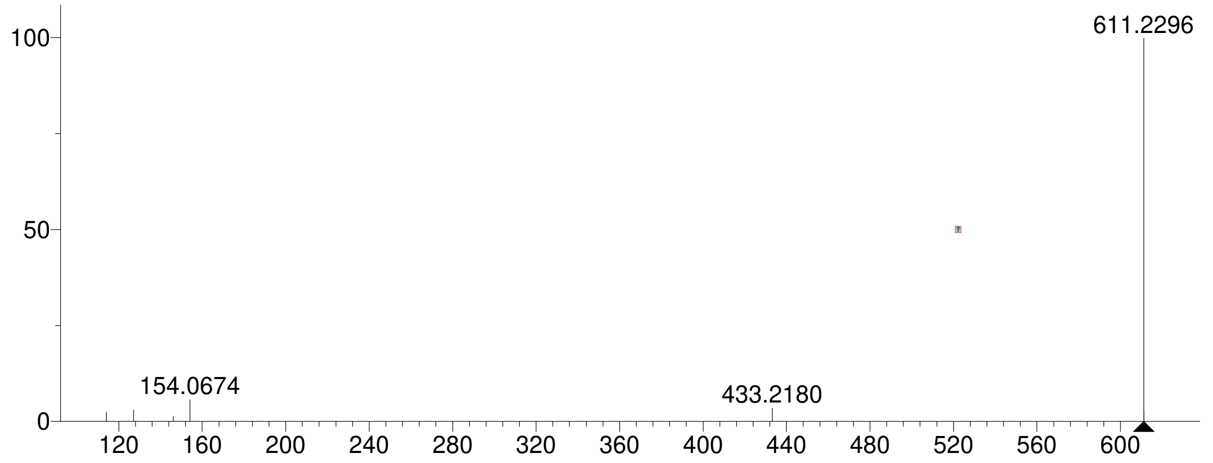

Hit 1 : NovobiocinA^ [M-H]- 10eV P=611.2
C31H36N2O11; MF: 204; RMF: 845; Prob 99.0%; Lib: mona-export-lc-ms-ms_negative_mode; ID: 23.

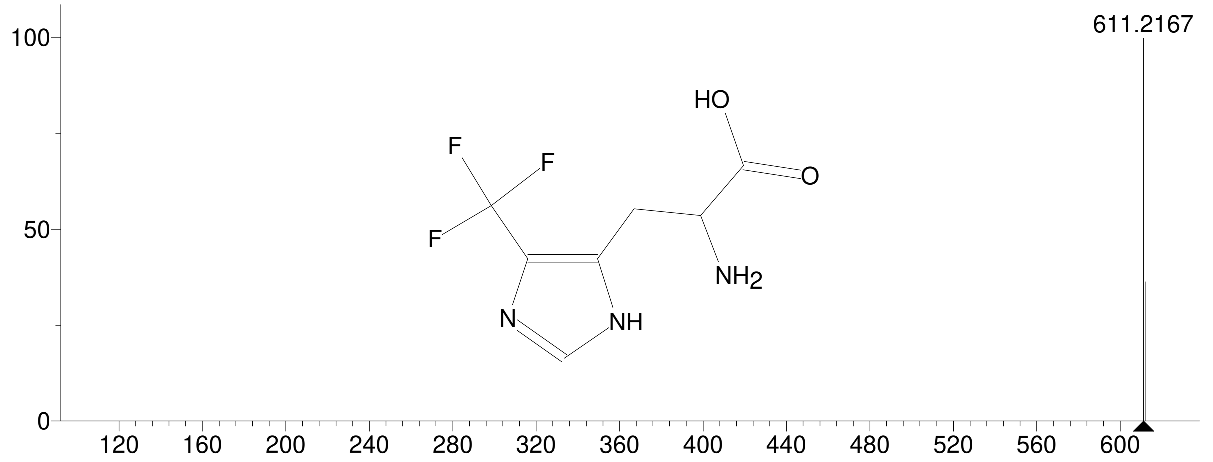

Hit 2 : 6-(furan-3-yl)-12,16,21-trihydroxy-7,15-dimethyl-9-oxo-3,17,19-trioxaheptacyclo[9.9.3.0A^'1,A^'1a^'o.0A^'2,
C31H40O10; MF: 0; RMF: 8; Prob 0.99%; Lib: mona-export-vf-npl_qexactive; ID: 8395.

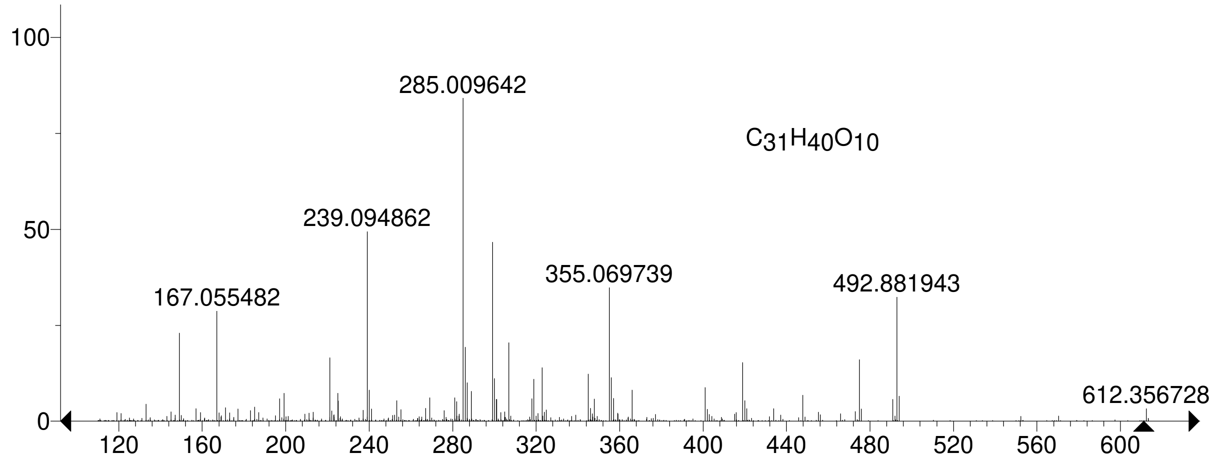

** Search Report Page 1 of 1 **

Unknown: Unknown (4.51_526.2026m/z)
Compound in Library Factor = 0

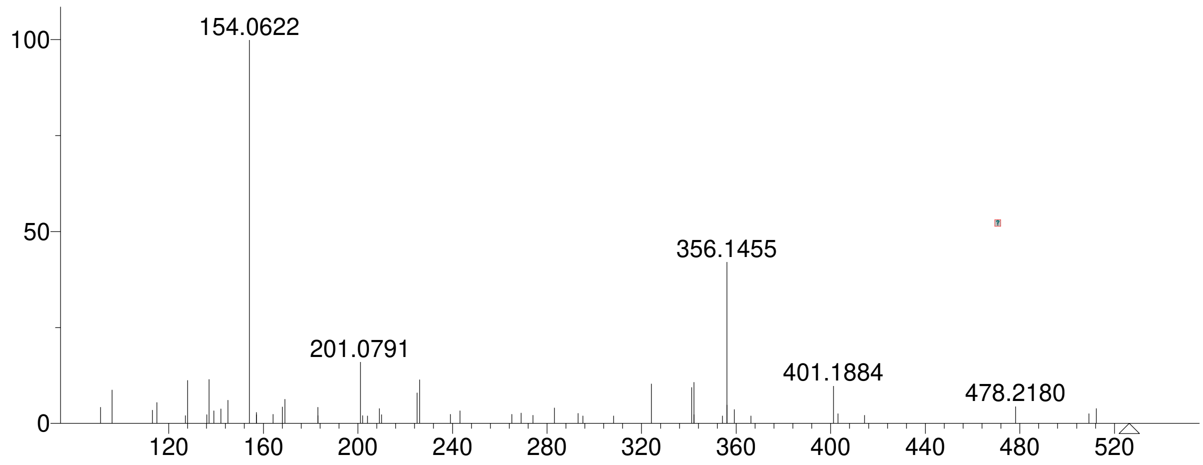

** Search Report Page 1 of 1 **

Unknown: Unknown (4.51_647.2658m/z)
Compound in Library Factor = 0

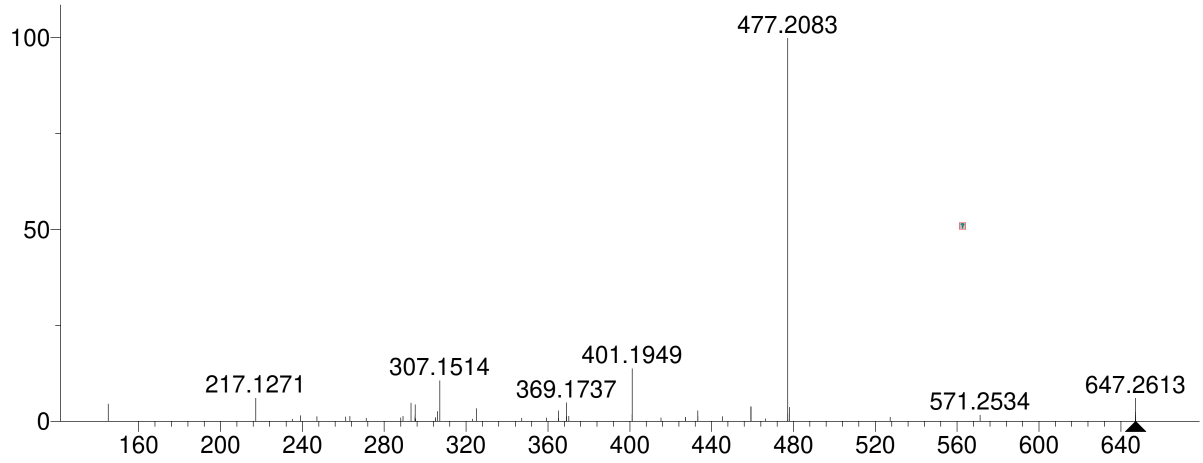

Hit 1 : Bavachin [2M-H]- HCD 9V P=647.3
C20H20O4; MF: 0; RMF: 36; Prob 100.0%; CAS: 19879-32-4; Lib: nist_msms; ID: 389268.

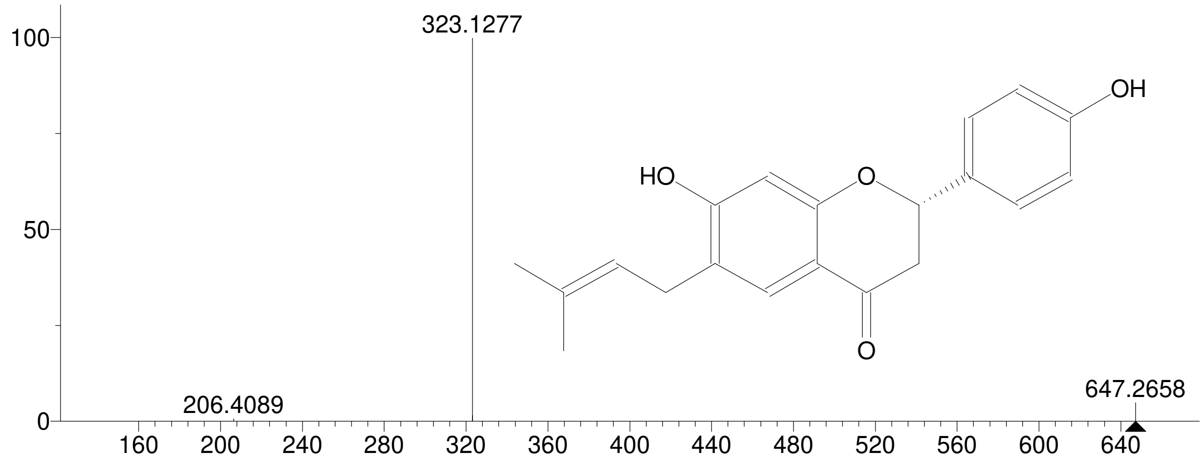

** Search Report Page 1 of 1 **

Unknown: Unknown (4.52_354.1279m/z)
Compound in Library Factor = 0

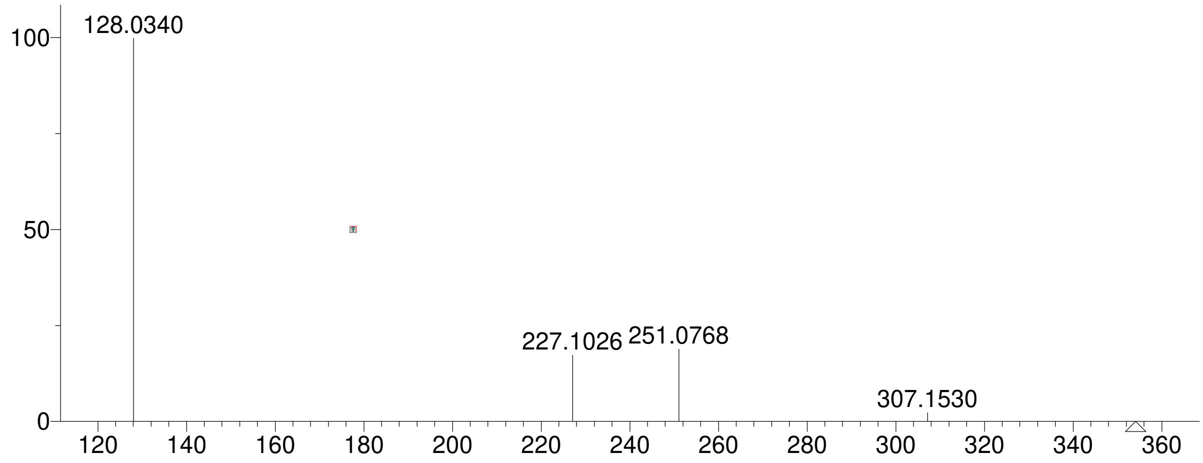

Unknown: Unknown (4.52_356.1452m/z)
Compound in Library Factor = -2387

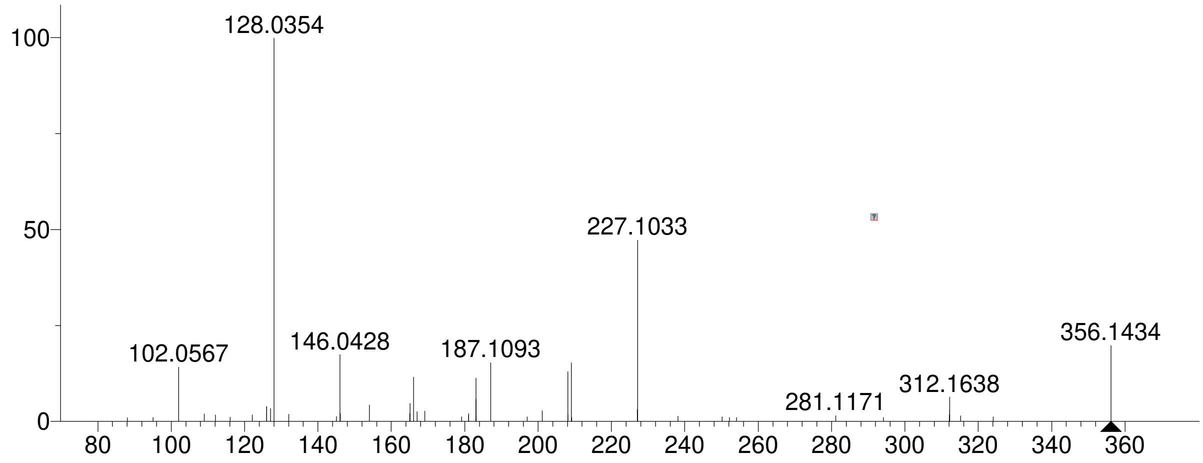

Hit 1 : Benzenesulfonamide, 4-(1,1-dimethylethyl)-N-[4-(1H-imidazol-1-yl)phenyl]- [M+H]+ HCD 21V P=356.1
C19H21N3O2S; MF: 25; RMF: 304; Prob 49.3%; CAS: 945526-43-2; Lib: nist_msms; ID: 355342.

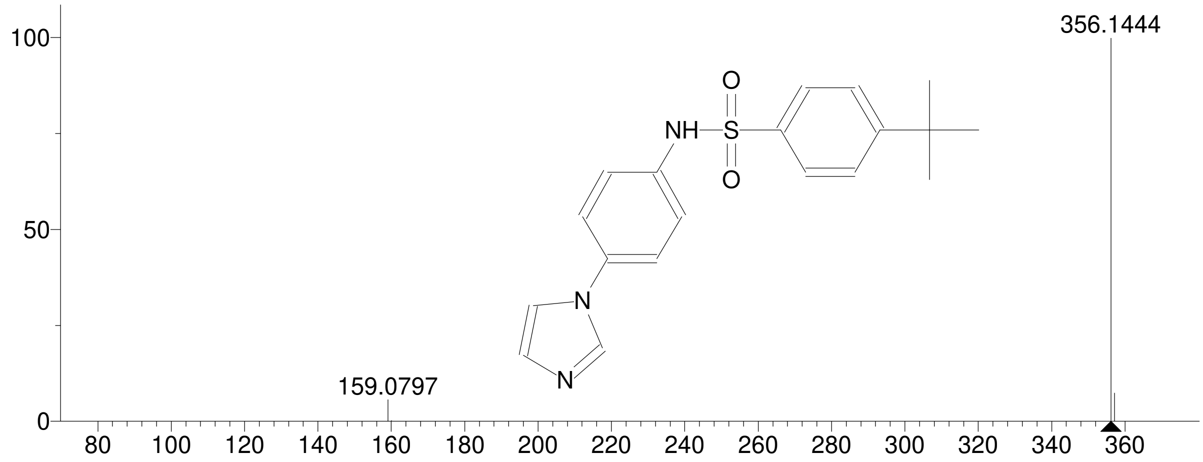

Hit 2 : Benzoic acid, 4-[[(2,4-diamino-7,8-dihydro-7-oxo-6-pteridinyl)methyl]methylamino]-, methyl ester [M+H]+ I
C16H17N7O3; MF: 8; RMF: 311; Prob 26.9%; CAS: 1330264-75-9; Lib: nist_msms; ID: 432535.

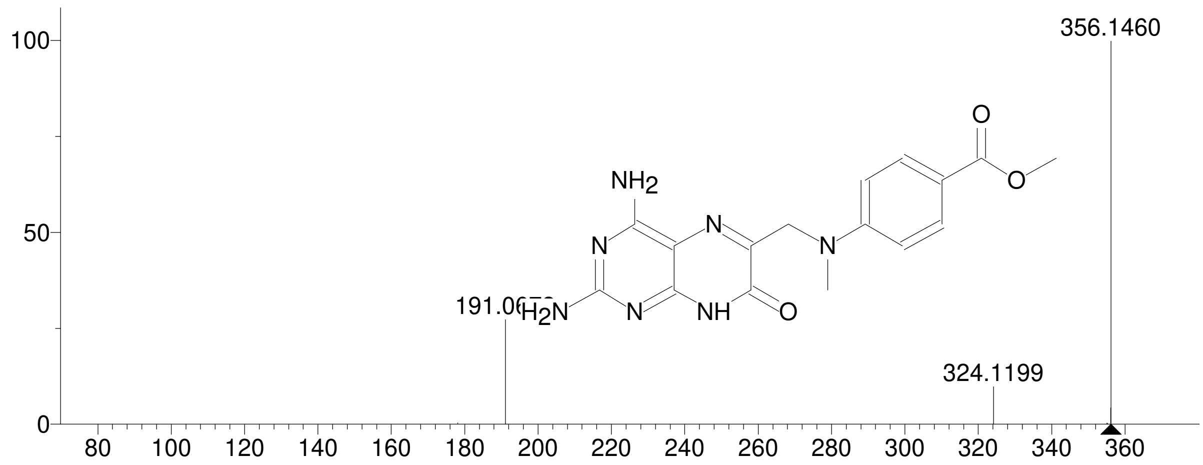

Hit 3 : Seneciphyllin [M+Na]+ HCD 17V P=356.1
C18H23NO5; MF: 5; RMF: 209; Prob 23.8%; CAS: 480-81-9; Lib: nist_msms; ID: 219524.

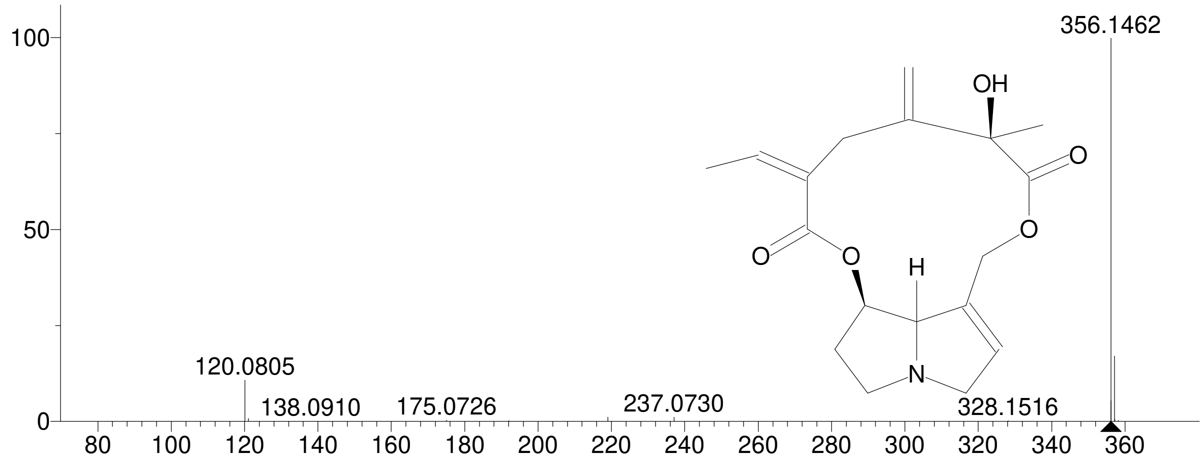

Unknown: Unknown (4.52_413.1645m/z)
Compound in Library Factor = 0

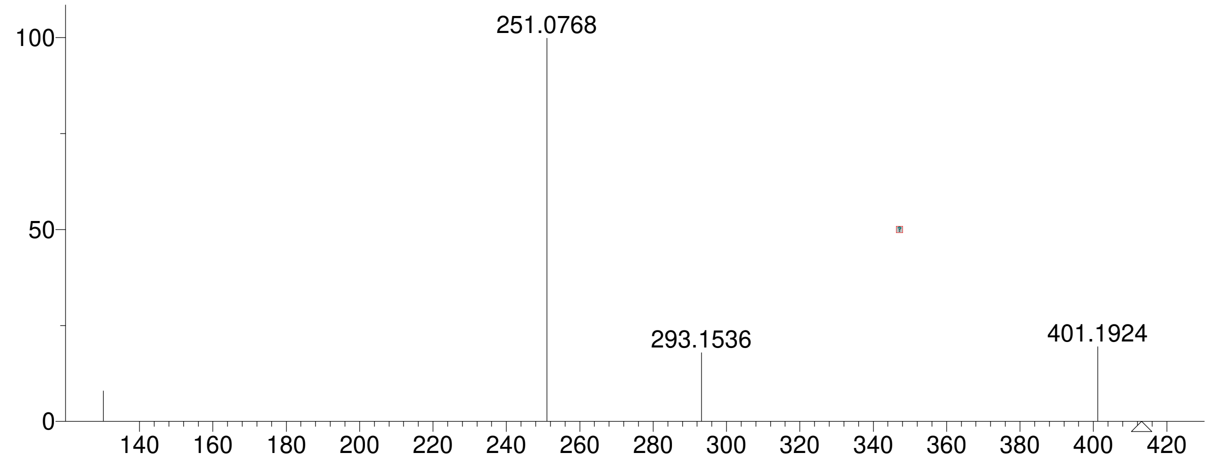

Hit 1 : purpactin A [M-H]- 45HCD P=413.2
C23H26O7; MF: 0; RMF: 52; Prob 20.0%; Lib: mona-export-lc-ms-ms_negative_mode; ID: 17700.

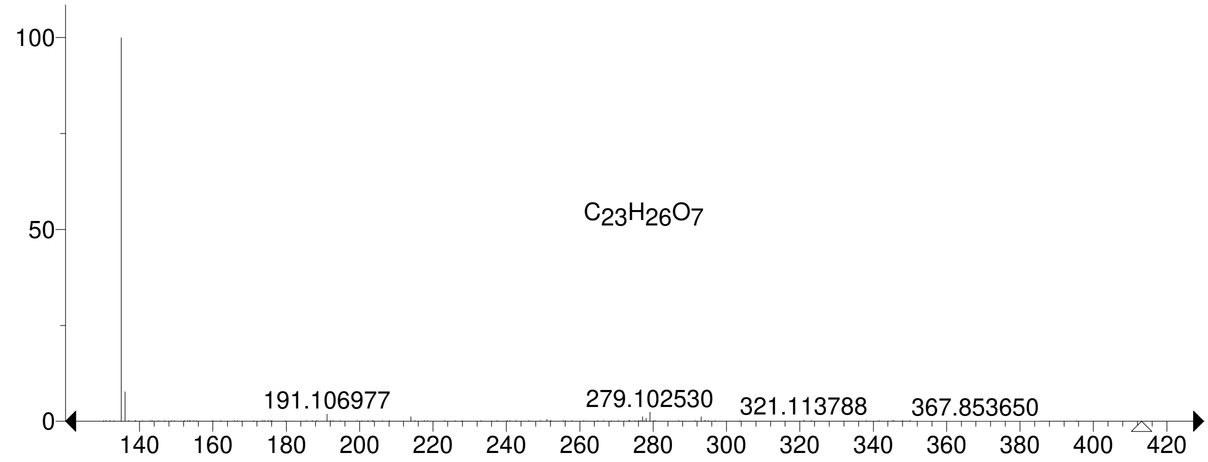

Hit 2 : 2-Hydroxymethylloratadine [M+H]+ HCD 132V P=413.2
C23H25ClN2O3; MF: 0; RMF: 44; Prob 20.0%; CAS: 609806-39-5; Lib: nist_msms; ID: 442227.

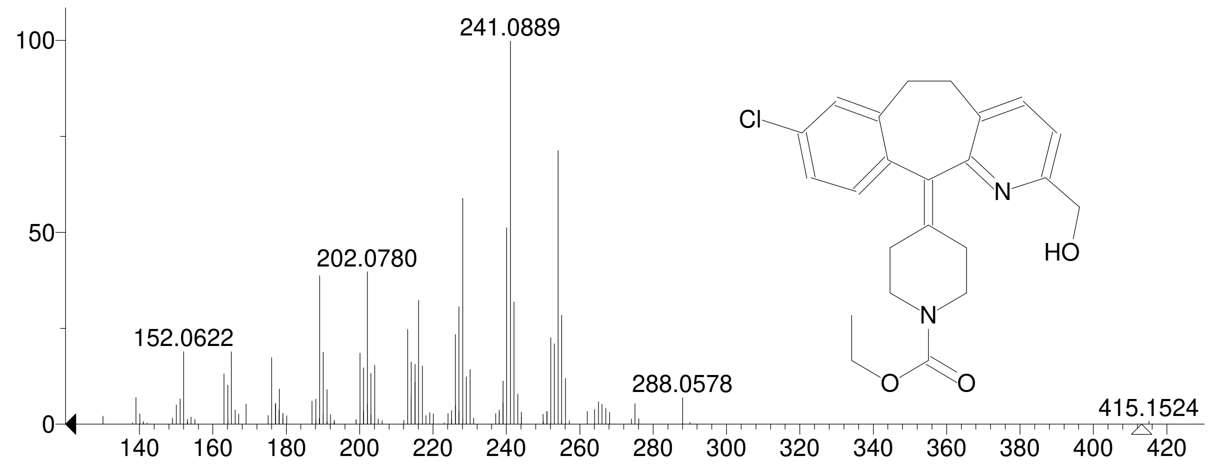

Hit 3 : (2S,3S)-6'-methyl-3-phenylspiro[oxirane-2,7'-quinazolino[3,2-a][1,4]benzodiazepine]-5',13'-dione [M+NH4]
C24H17N3O3; MF: 0; RMF: 42; Prob 20.0%; Lib: mona-export-vf-npl_qexactive; ID: 17221.

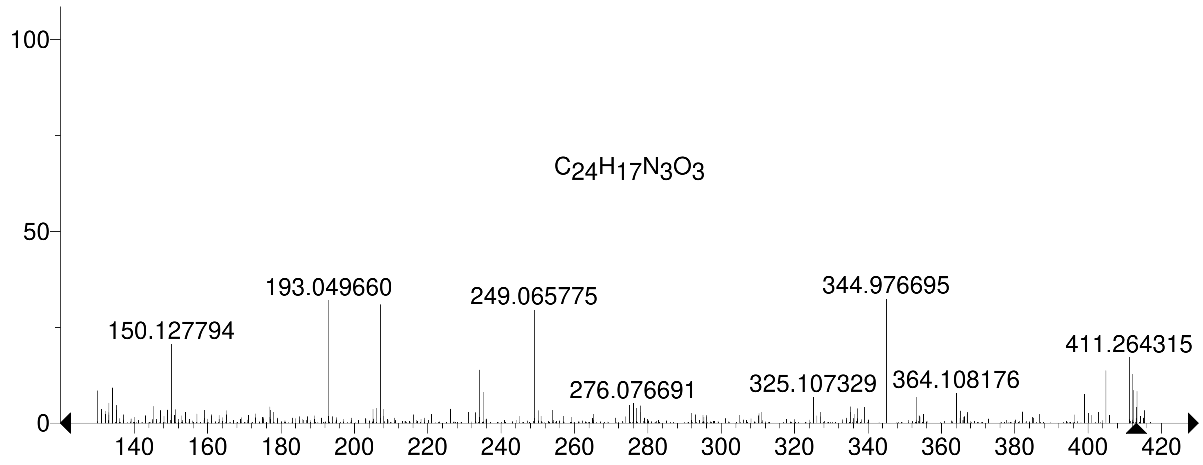

Hit 4 : Garcinone C [M-H]- HCD 90V P=413.2
C23H26O7; MF: 0; RMF: 36; Prob 20.0%; CAS: 76996-27-5; Lib: nist_msms; ID: 389327.

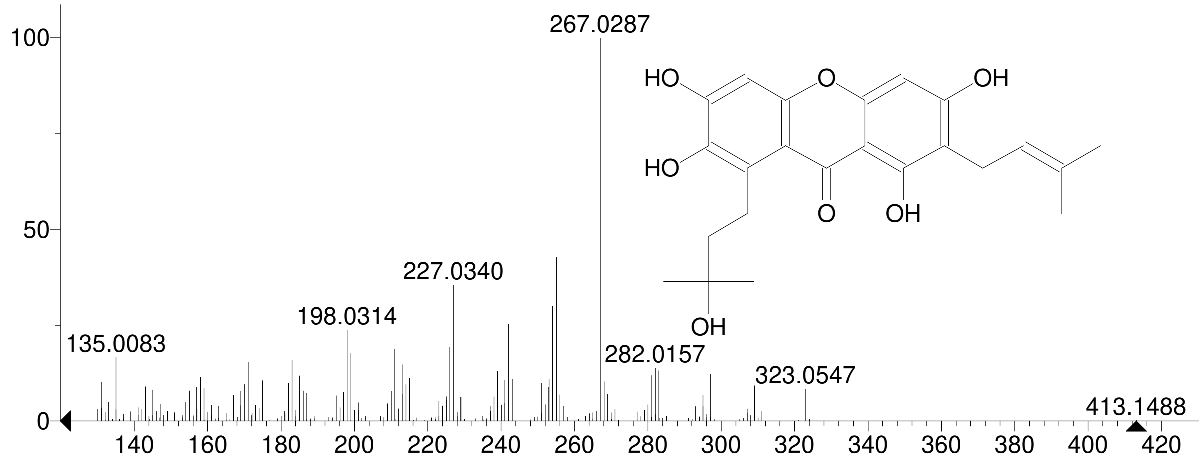

Hit 5 : 4-Hydroxymethylloratadine [M+H]+ HCD 132V P=413.2
C23H25ClN2O3; MF: 0; RMF: 24; Prob 20.0%; CAS: 609806-40-8; Lib: nist_msms; ID: 442293.

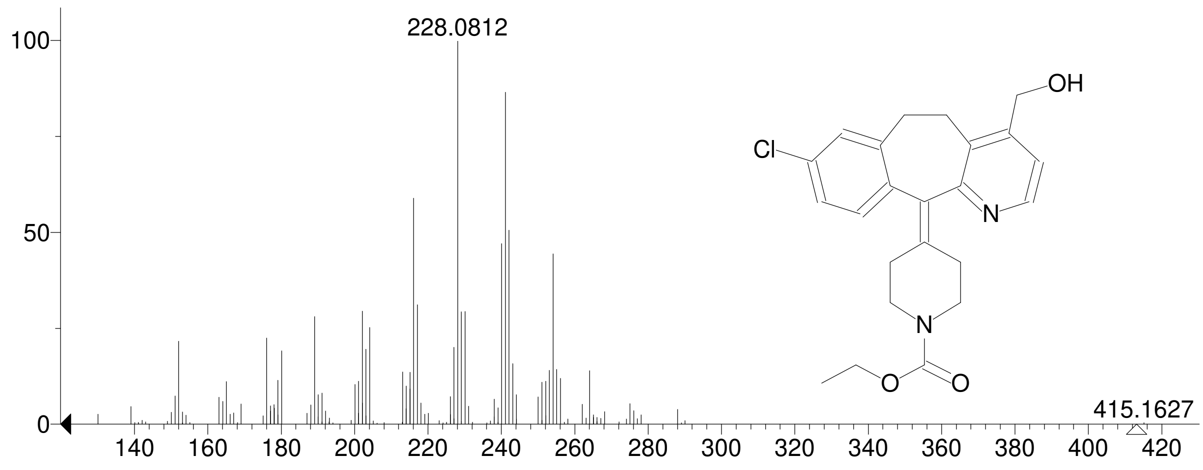

Unknown: Unknown (4.52_477.2073m/z)
Compound in Library Factor = -1309

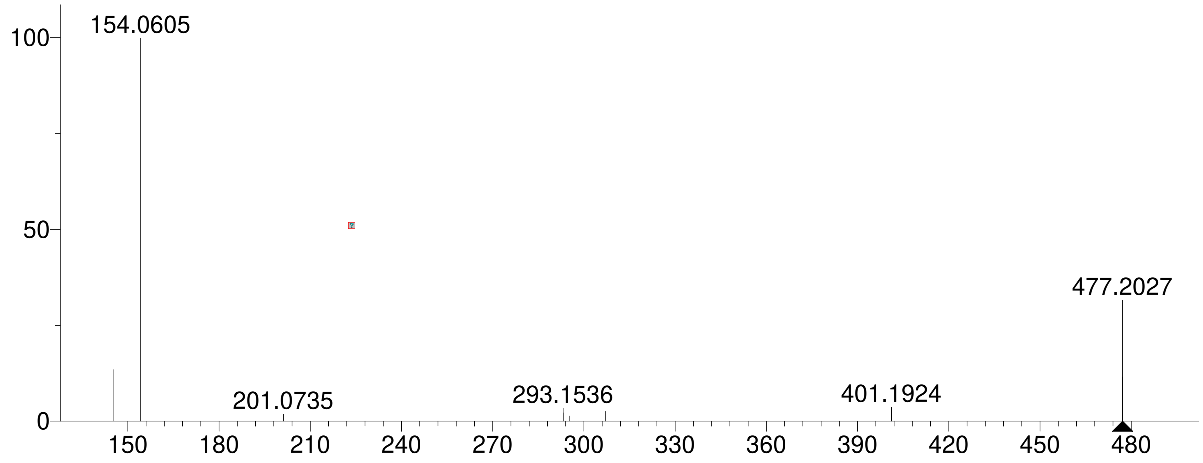

Hit 1 : 4-(2-Hydroxyethyl)piperazine-1-ethanesulfonic acid [2M+H]+ HCD 4V P=477.2
C8H18N2O4S; MF: 58; RMF: 625; Prob 73.1%; CAS: 7365-45-9; Lib: nist_msms; ID: 58512.

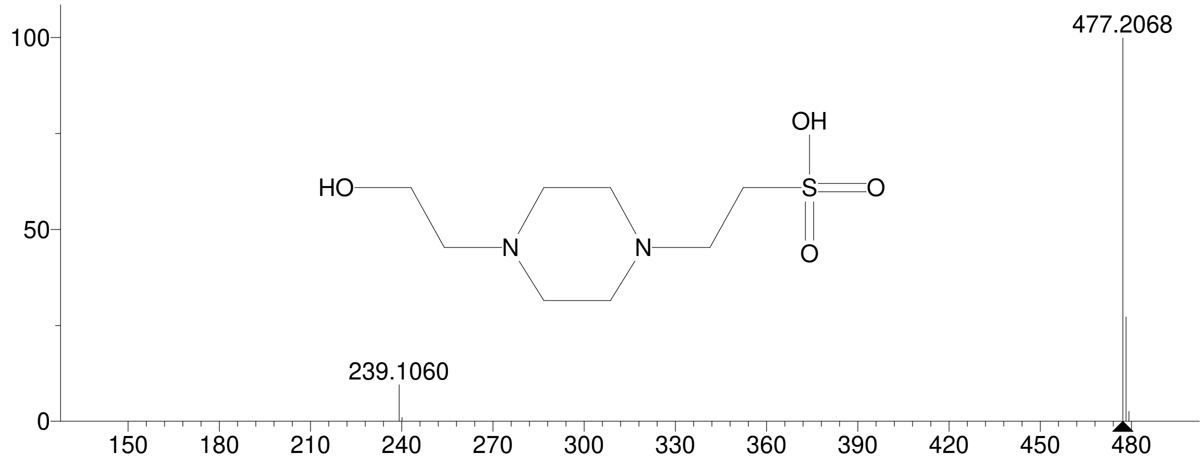

Hit 2 : 5-O-methyllicoricidin [M+K]+ 35HCD P=477.2
C27H34O5; MF: 0; RMF: 64; Prob 13.4%; Lib: mona-export-vf-npl_qexactive; ID: 964.

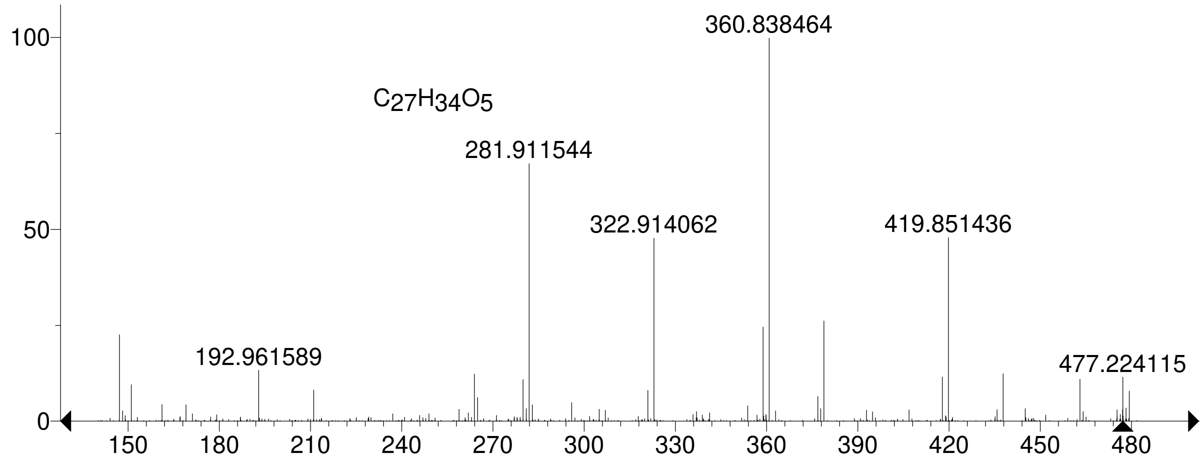

Hit 3 : 1,7-bis(4-hydroxyphenyl)-5-[(2R,3R,4S,5S,6R)-3,4,5-trihydroxy-6-(hydroxymethyl)oxan-2-yl]oxyheptan-3-or
C25H32O9; MF: 0; RMF: 3; Prob 13.4%; Lib: mona-export-vf-npl_qexactive; ID: 7567.

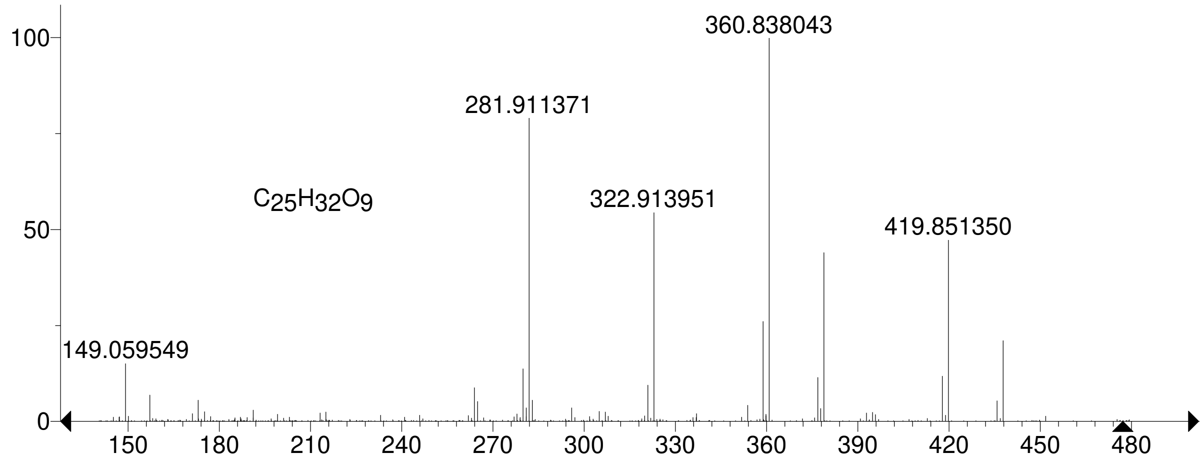

Unknown: Unknown (4.53_421.1338m/z)
Compound in Library Factor = 0

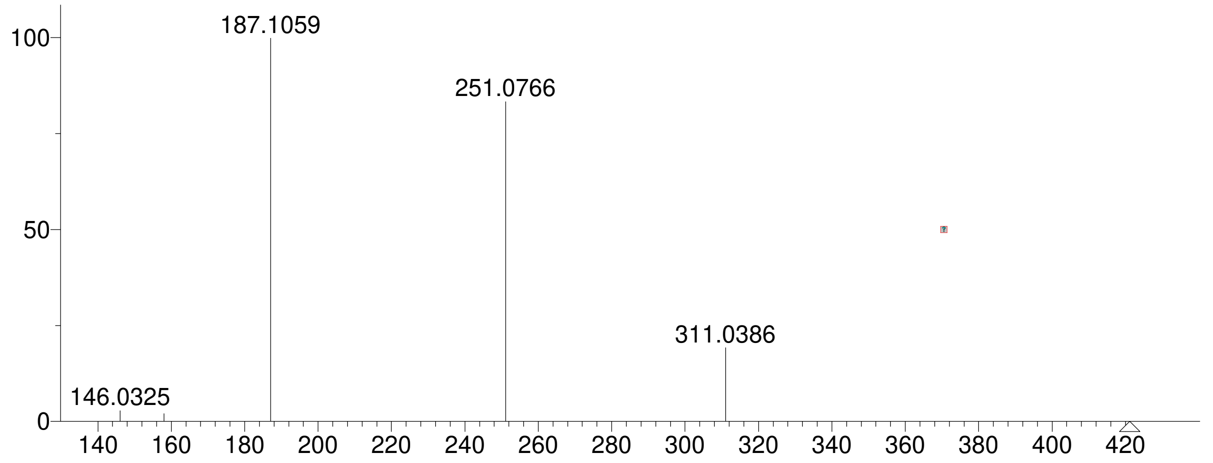

Hit 1 : lamalbid [M-H]- 35HCD P=421.1
C17H26O12; MF: 0; RMF: 62; Prob 33.3%; Lib: mona-export-lc-ms-ms_negative_mode; ID: 20249.

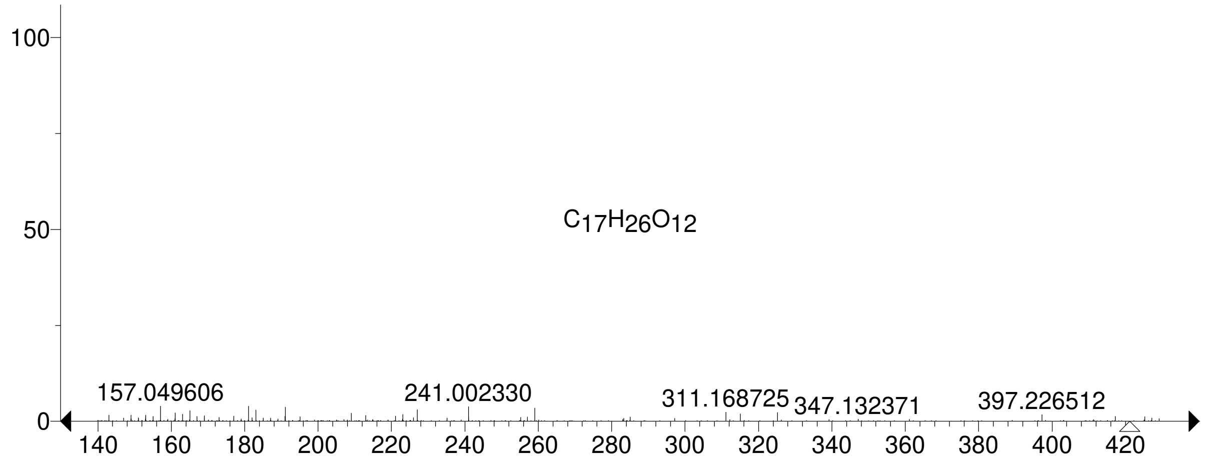

Hit 2 : Lamiide [M-H]- 65HCD P=421.1
C17H26O12; MF: 0; RMF: 14; Prob 33.3%; Lib: anthocyanins mona; ID: 7928.

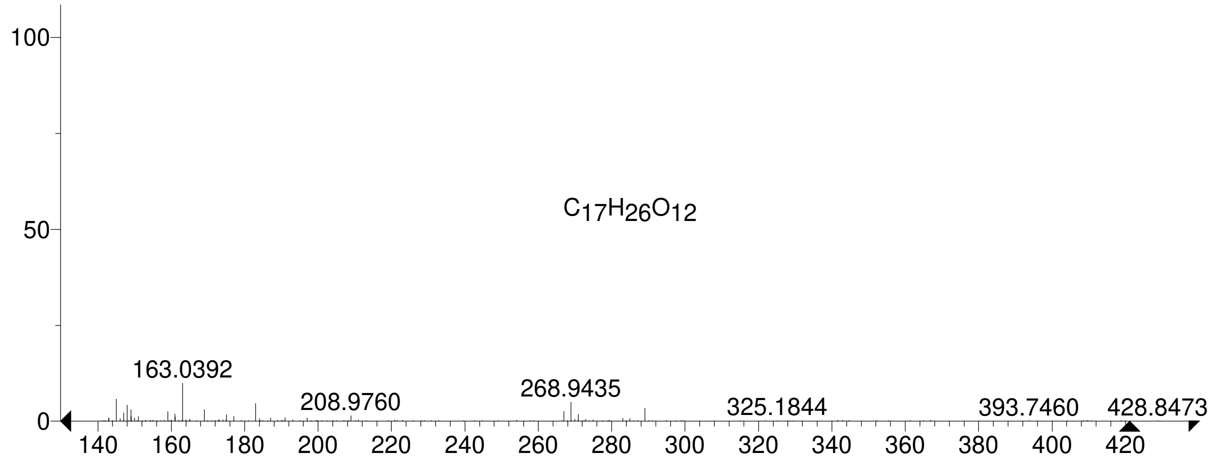

Hit 3 : methyl (1S,4aS,6R,7S,7aS)-5,6-dihydroxy-7-(hydroxymethyl)-1-[(2S,3R,4S,5S,6R)-3,4,5-trihydroxy-6-(hydr
C17H26O12; MF: 0; RMF: 7; Prob 33.3%; Lib: mona-export-lc-ms-ms_negative_mode; ID: 26931.

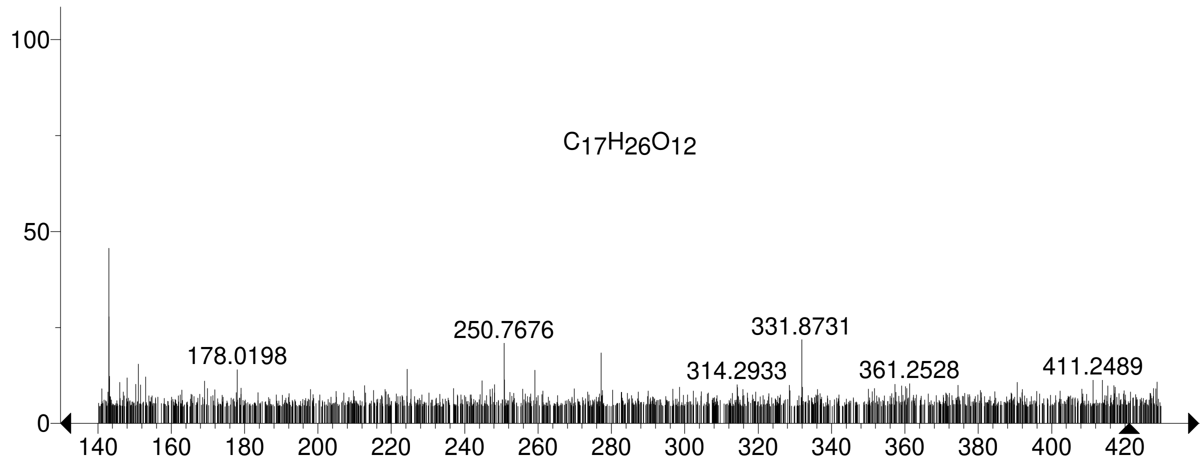

** Search Report Page 1 of 1 **

Unknown: Unknown (4.54_180.0653m/z)
Compound in Library Factor = 0

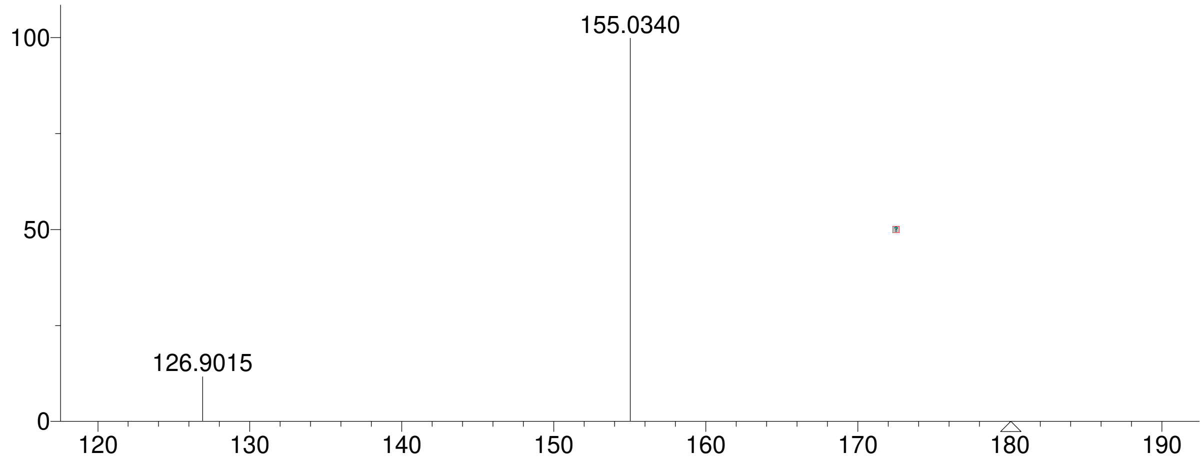

Unknown: Unknown (4.54_234.9906m/z)
Compound in Library Factor = -414

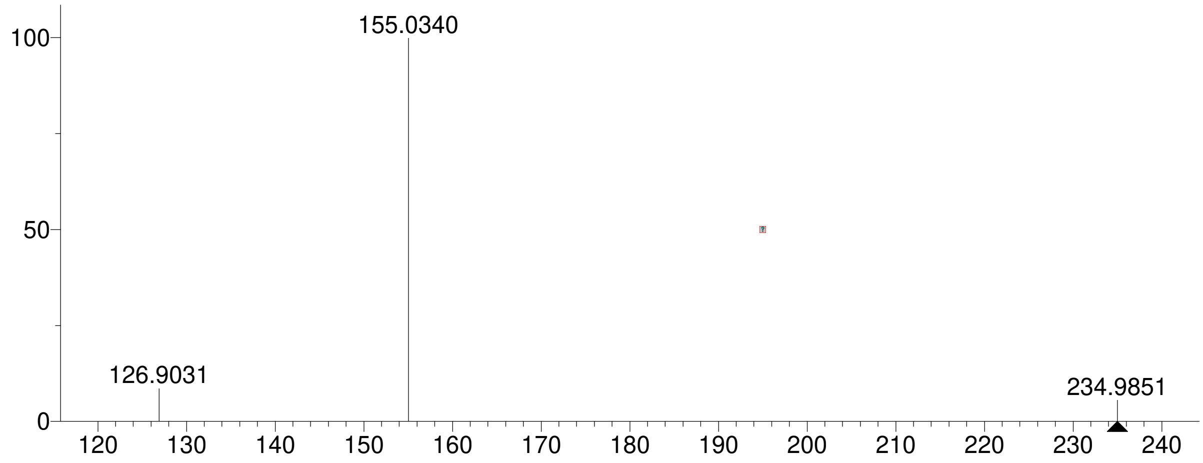

Hit 1 : (2,6-Dichlorophenoxy)acetic acid, methyl ester [M+H]+ HCD 2V P=235
C9H8Cl2O3; MF: 217; RMF: 909; Prob 56.0%; CAS: 1928-49-0; Lib: nist_msms; ID: 475304.

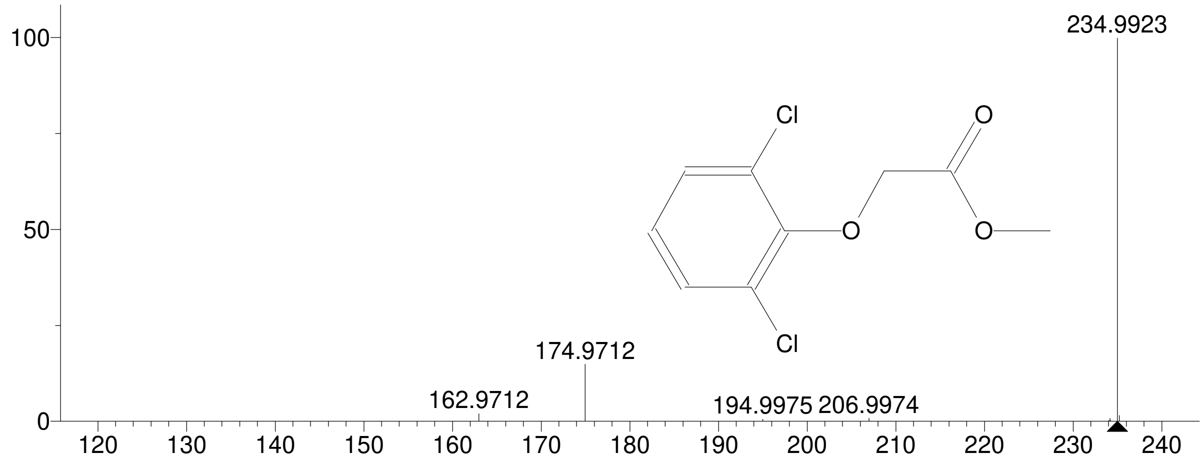

Hit 2 : Acetic acid, 2-(2,4-dichlorophenoxy)-, methyl ester [M+H]+ HCD 2V P=235
C9H8Cl2O3; MF: 210; RMF: 893; Prob 42.9%; CAS: 1928-38-7; Lib: nist_msms; ID: 484355.

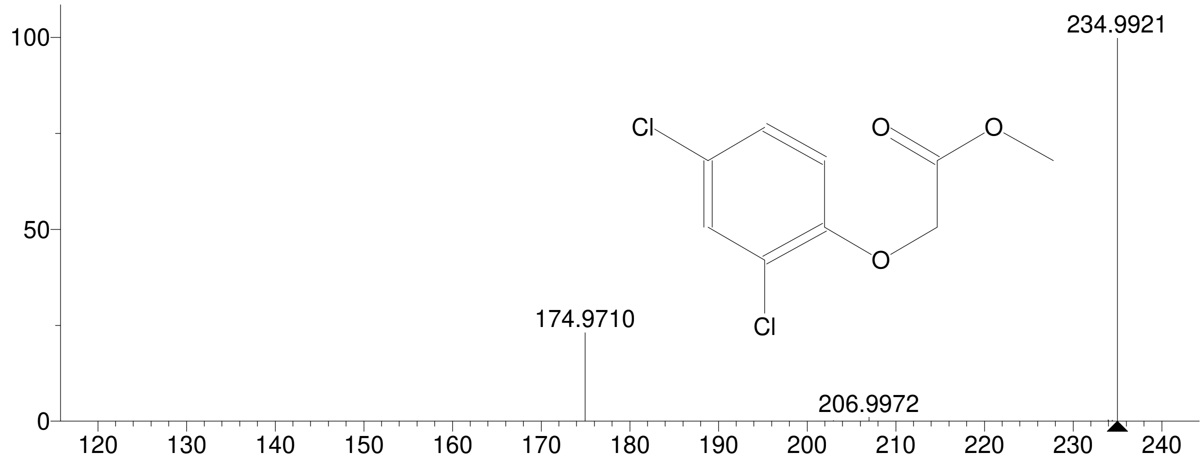

** Search Report Page 2 of 2 **

Hit 3 : Dicamba methyl ester [M+H]+ HCD 0V P=235
C9H8Cl2O3; MF: 55; RMF: 700; Prob 1.11%; CAS: 6597-78-0; Lib: nist_msms; ID: 484749.

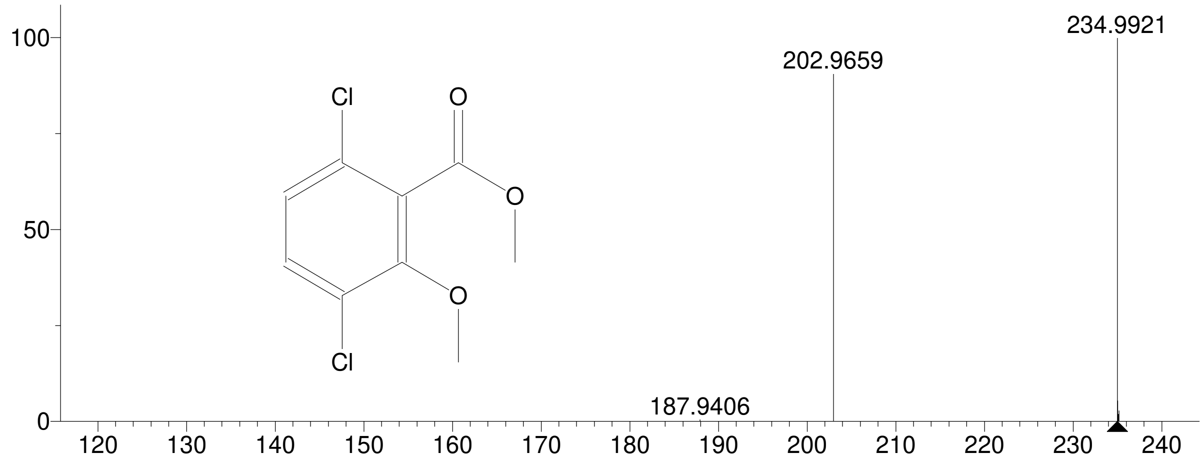

** Search Report Page 1 of 1 **

Unknown: Unknown (4.54_314.1232m/z)
Compound in Library Factor = 0

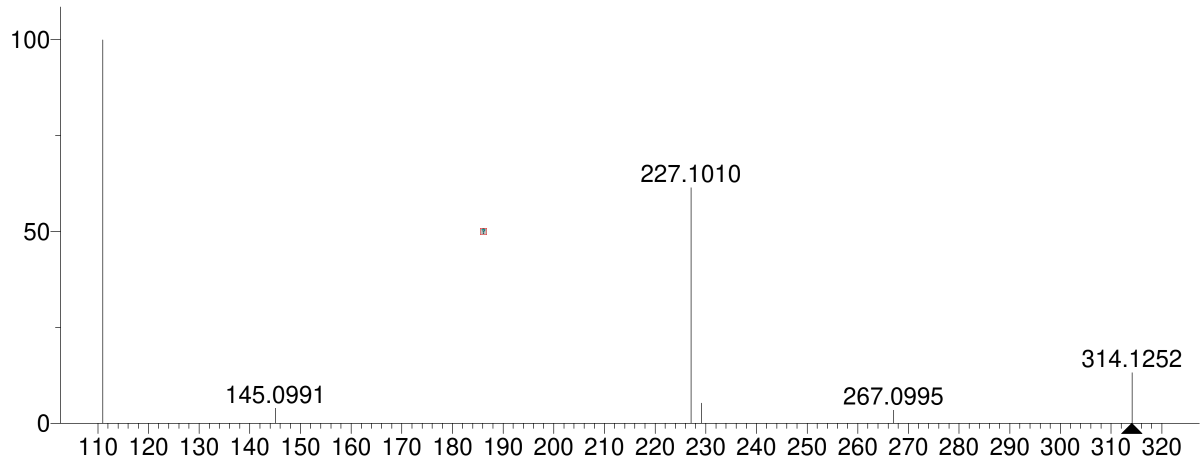

Unknown: Unknown (4.54_318.0955m/z)
Compound in Library Factor = 0

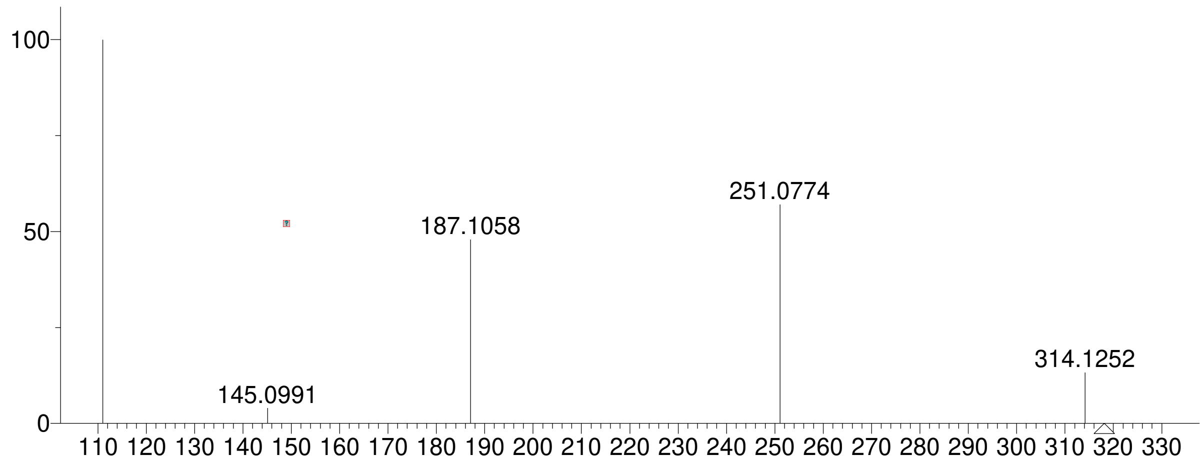

Hit 1 : (2Z)-4,6-dihydroxy-2-[(3-hydroxy-4-methoxyphenyl)methylidene]-1-benzofuran-3-one [M+NH4]+ 65HCD I
C16H12O6; MF: 0; RMF: 51; Prob 12.5%; Lib: mona-export-vf-npl_qexactive; ID: 5508.

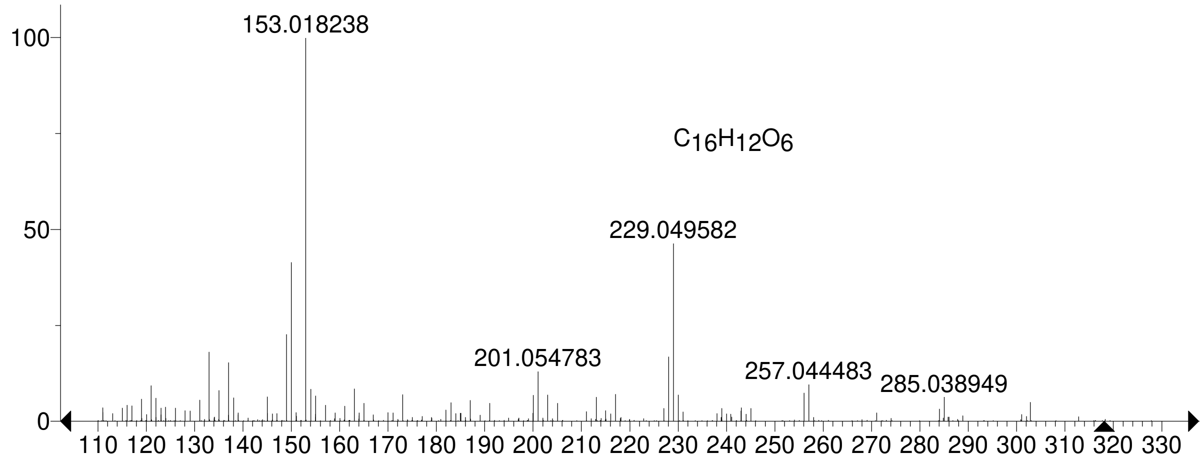

Hit 2 : Indican [M+Na]+ IT 35% P=318.1
C14H17NO6; MF: 0; RMF: 28; Prob 12.5%; CAS: 487-60-5; Lib: nist_msms; ID: 123247.

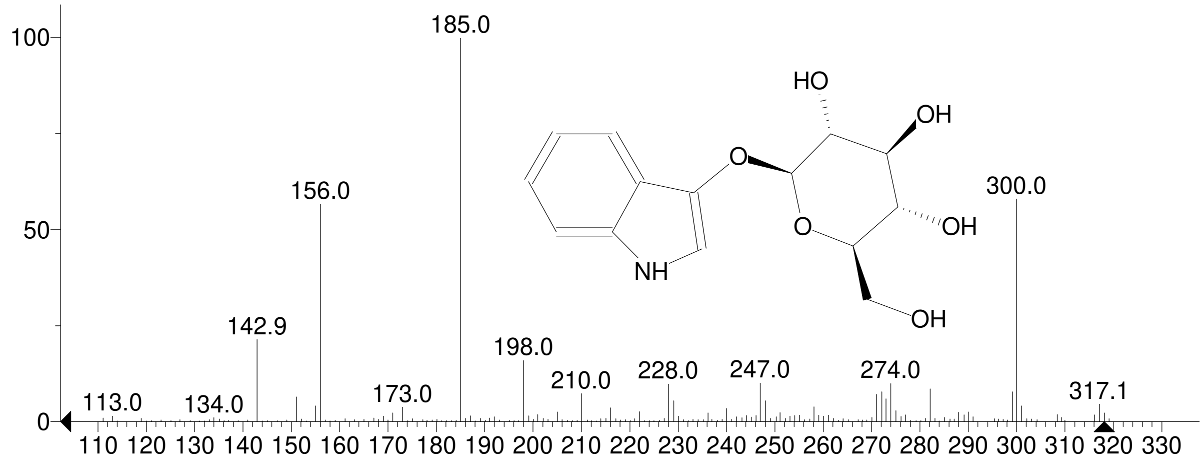

Hit 3 : carviolin [M+NH4]+ 45HCD P=318.1
C16H12O6; MF: 0; RMF: 17; Prob 12.5%; Lib: mona-export-vf-npl_qexactive; ID: 6509.

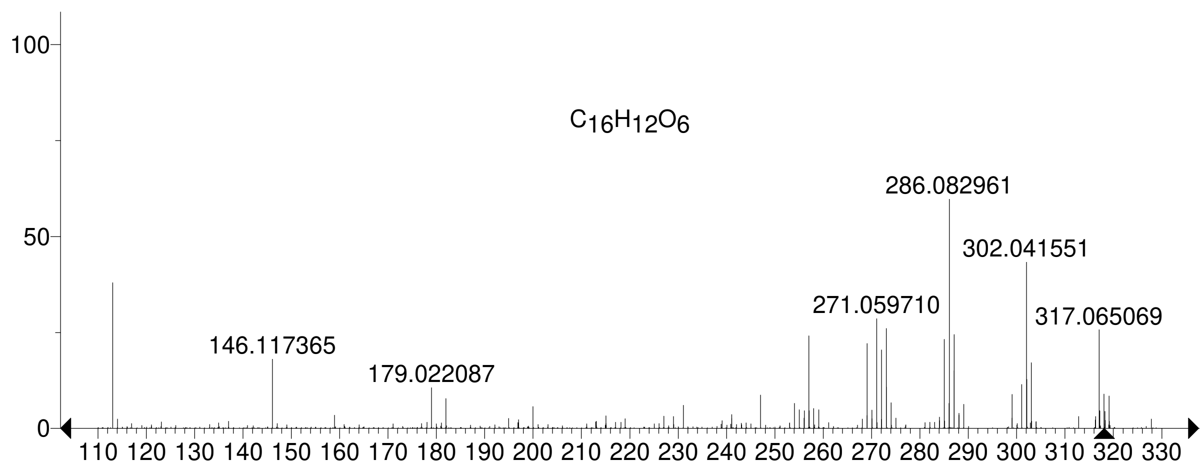

Hit 4 : Fallacinol [M+NH4]+ 65HCD P=318.1
C16H12O6; MF: 0; RMF: 15; Prob 12.5%; Lib: mona-export-vf-npl_qexactive; ID: 4923.

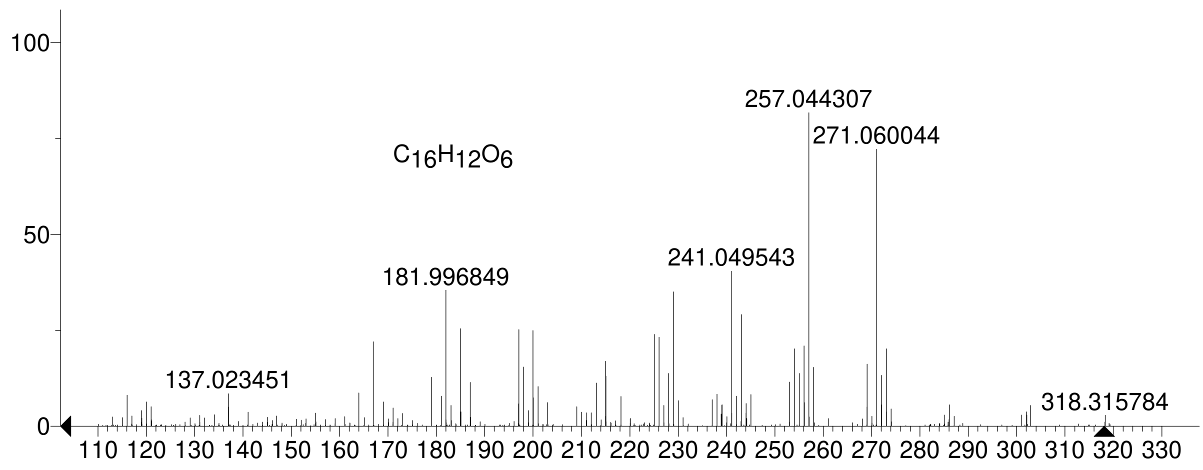

Hit 5 : (R)-Prunasin [M+Na]+ IT 35% P=318.1
C14H17NO6; MF: 0; RMF: 6; Prob 12.5%; CAS: 99-18-3; Lib: nist_msms; ID: 356333.

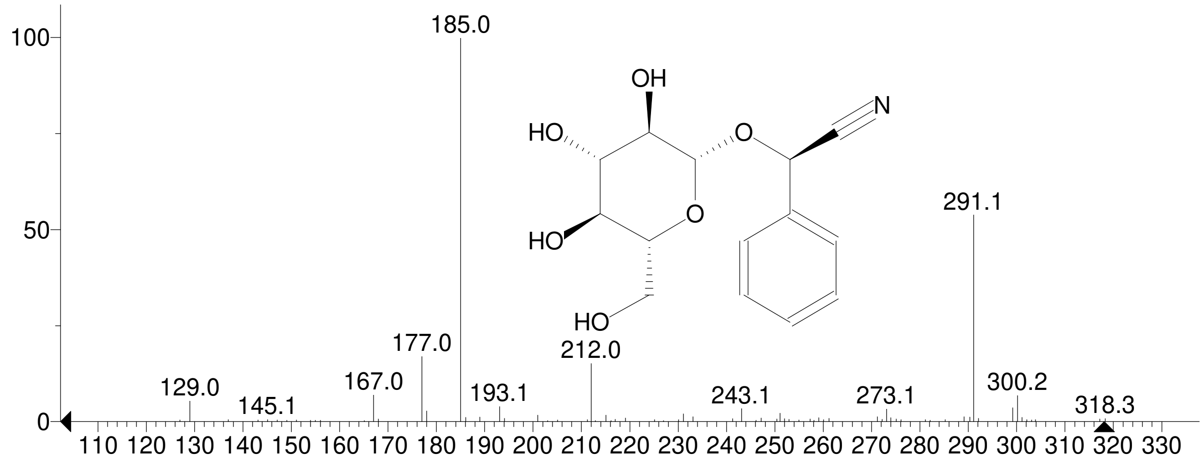

** Search Report Page 1 of 1 **

Unknown: Unknown (4.54_357.1620m/z)
Compound in Library Factor = 0

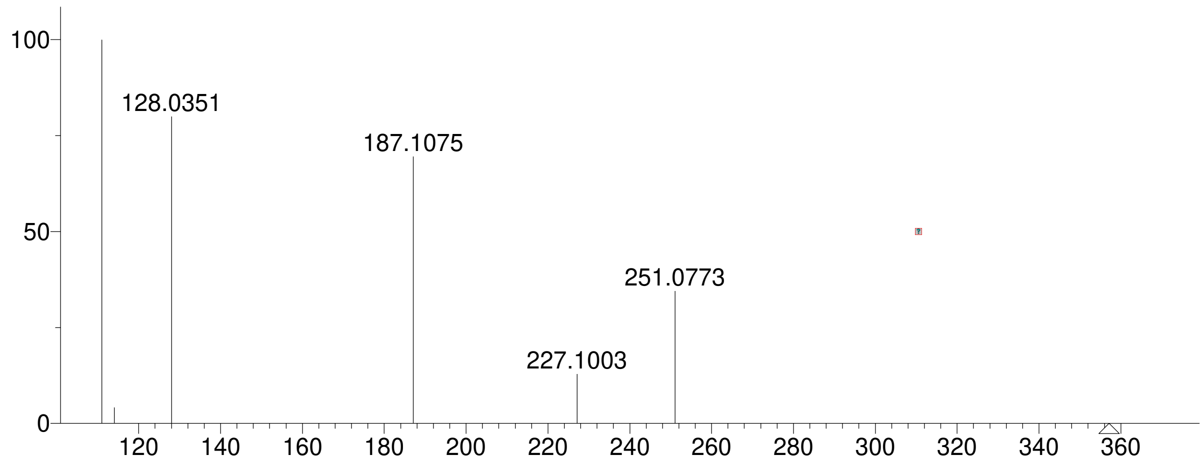

Hit 1 : NCGC00381117-01!2-amino-4-hydroxy-5-[[1-(4-methoxy-6-oxopyran-2-yl)-3-methylbutyl]amino]-5-oxopent
C16H24N2O7; MF: 0; RMF: 18; Prob 100.0%; Lib: mona-export-gnps; ID: 8549.

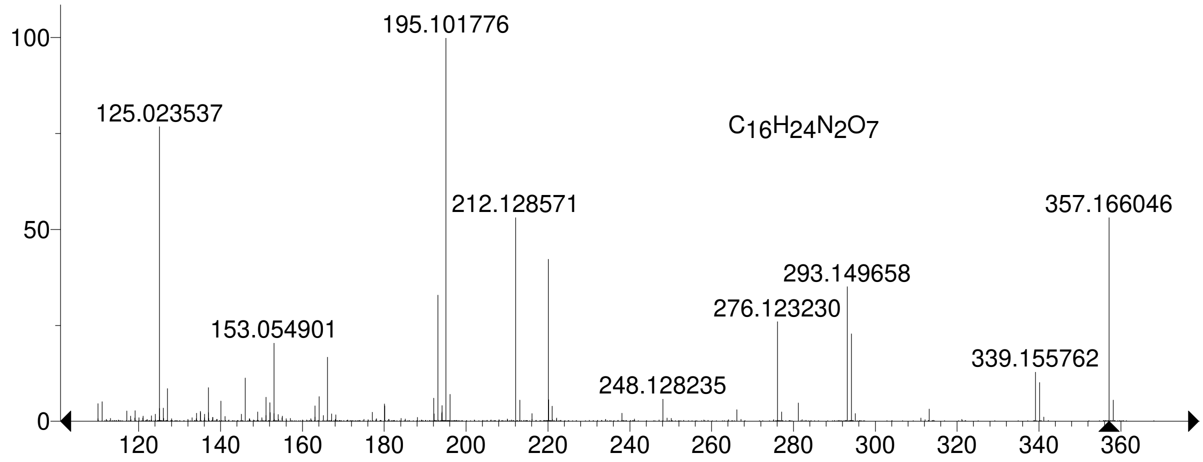

Unknown: Unknown (4.54_481.0940m/z)
Compound in Library Factor = 0

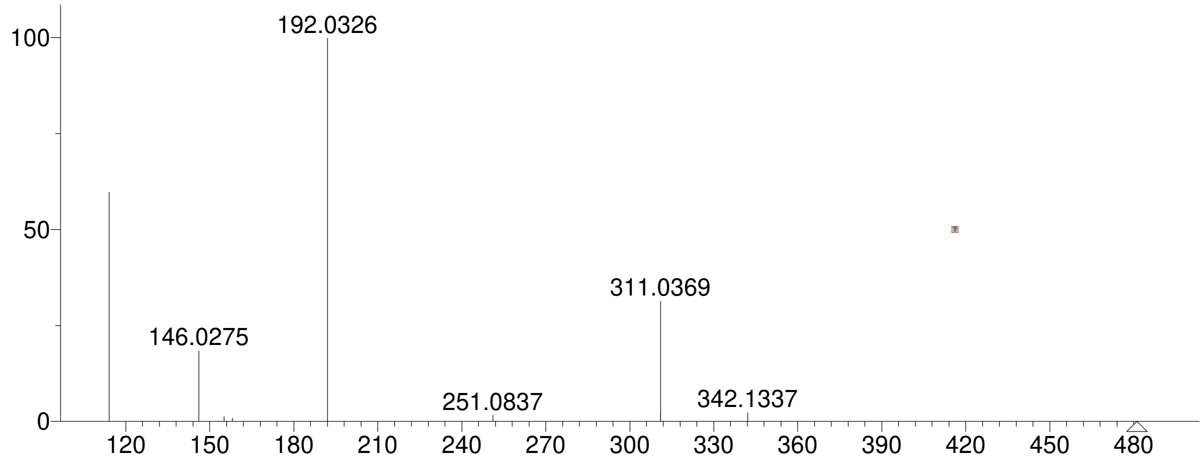

Hit 1 : Villol [M+K]+ 65HCD P=481.1
C23H22O9; MF: 0; RMF: 20; Prob 50.0%; Lib: mona-export-vf-npl_qexactive; ID: 8049.

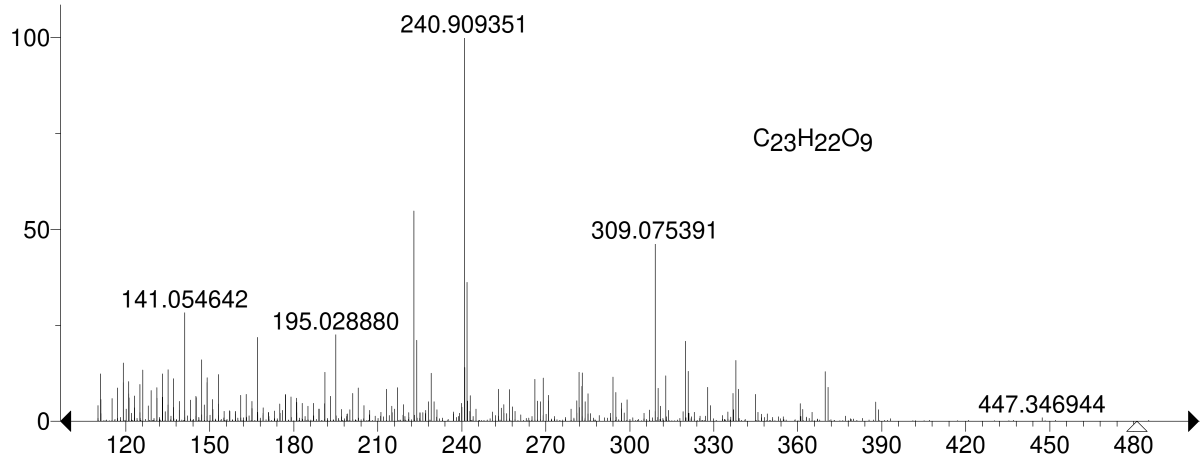

Hit 2 : Gossypin [M+H]+ HCD 95V P=481.1
C21H20O13; MF: 0; RMF: 18; Prob 50.0%; CAS: 652-78-8; Lib: nist_msms; ID: 111983.

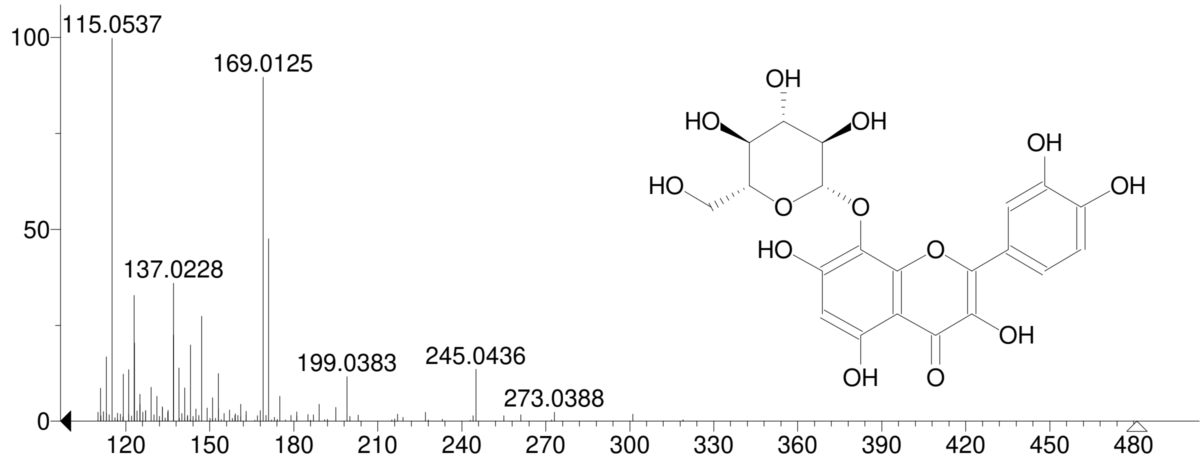

** Search Report Page 1 of 1 **

Unknown: Unknown (4.55_375.0919m/z)
Compound in Library Factor = 0

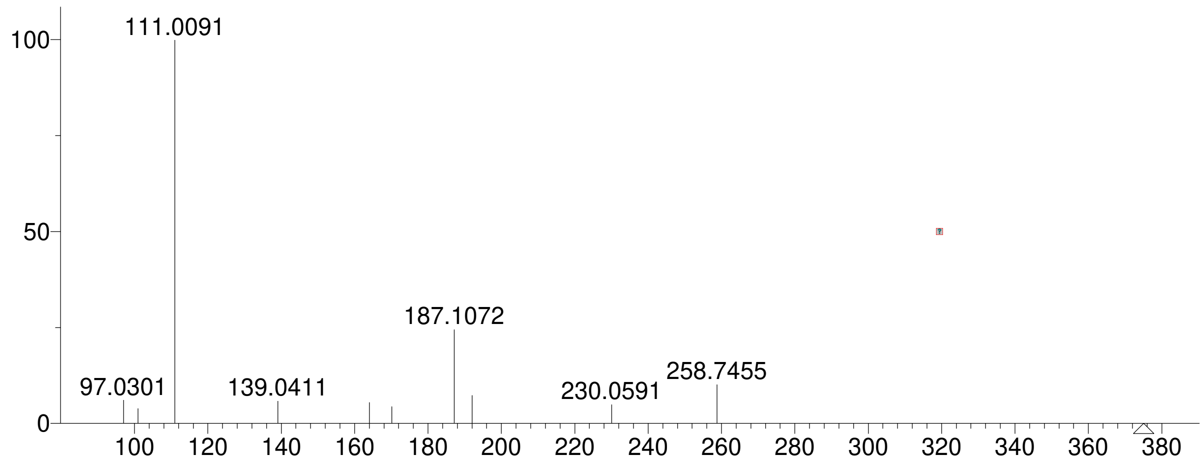

Unknown: Unknown (4.55_451.1364m/z)
Compound in Library Factor = 0

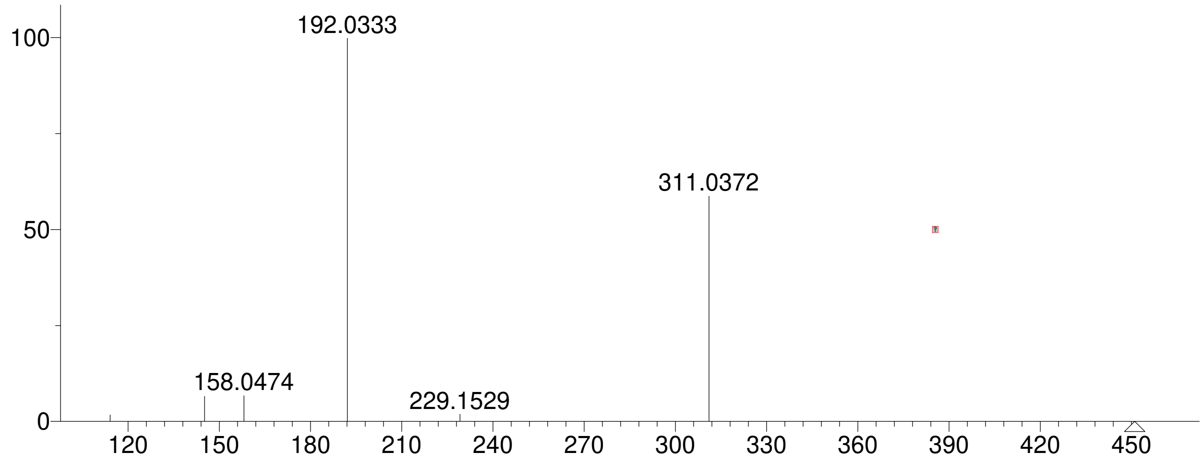

Hit 1 : NCGC00347590-02!9,11,12a-trihydroxy-2,3-dimethoxy-8-(3-methylbut-2-enyl)-6,6a-dihydrochromeno[3,4-b
C23H24O8; MF: 0; RMF: 36; Prob 16.7%; Lib: mona-export-gnps; ID: 8469.

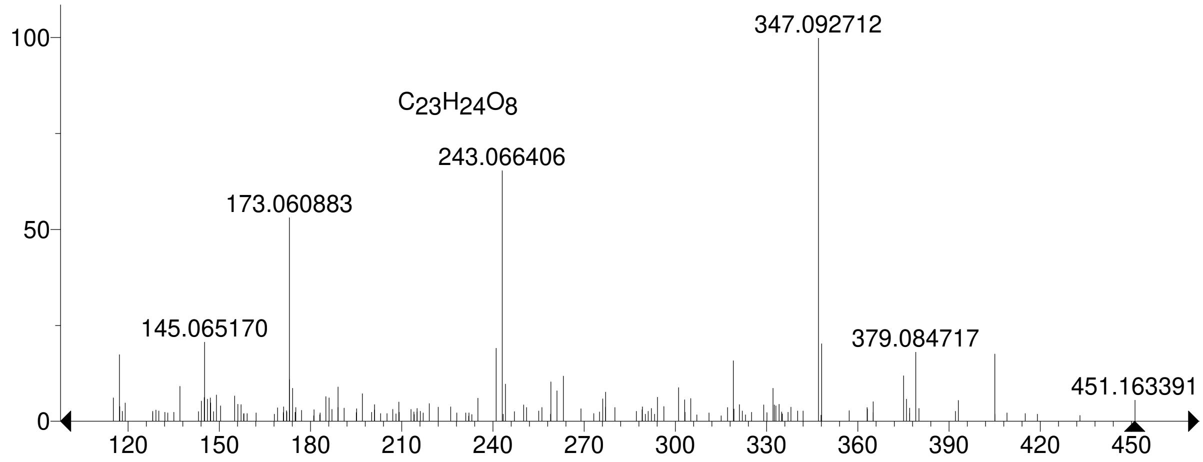

Hit 2 : [4,8-dihydroxy-3-(3-methoxy-3-oxoprop-1-en-2-yl)-8a-methyl-5-oxo-1,2,3,4,4a,6,7,8-octahydronaphthalen-2
C20H28O9; MF: 0; RMF: 19; Prob 16.7%; Lib: mona-export-vf-npl_qexactive; ID: 10662.

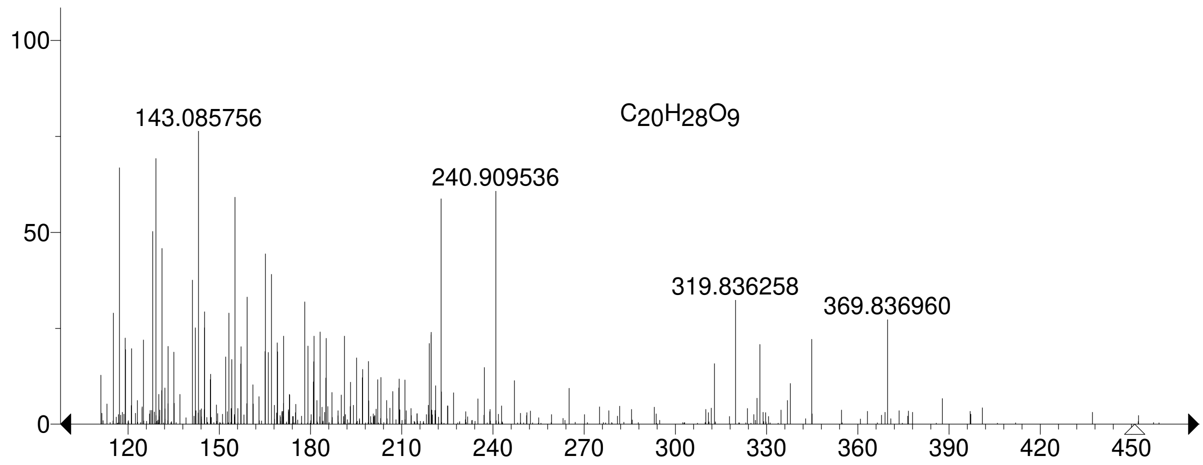

Hit 3 : 1',2'-dihydro-2',6-dihydroxyrotenone [M+Na]+ 45HCD P=451.1
C23H24O8; MF: 0; RMF: 15; Prob 16.7%; Lib: mona-export-vf-npl_qexactive; ID: 4145.

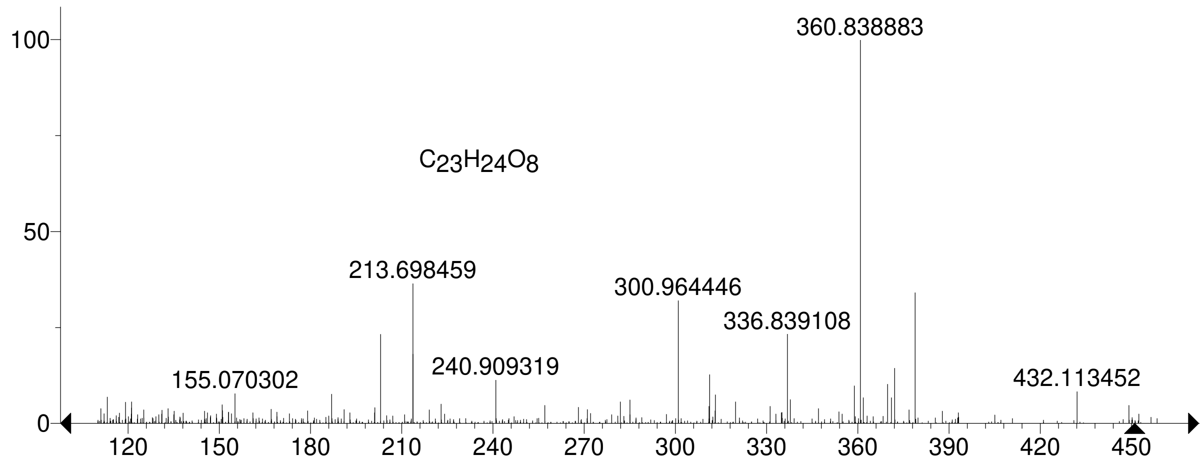

Hit 4 : 9,11,12a-trihydroxy-2,3-dimethoxy-8-(3-methylbut-2-enyl)-6,6a-dihydrochromeno[3,4-b]chromen-12-one [M
C23H24O8; MF: 0; RMF: 11; Prob 16.7%; Lib: mona-export-vf-npl_qexactive; ID: 18387.

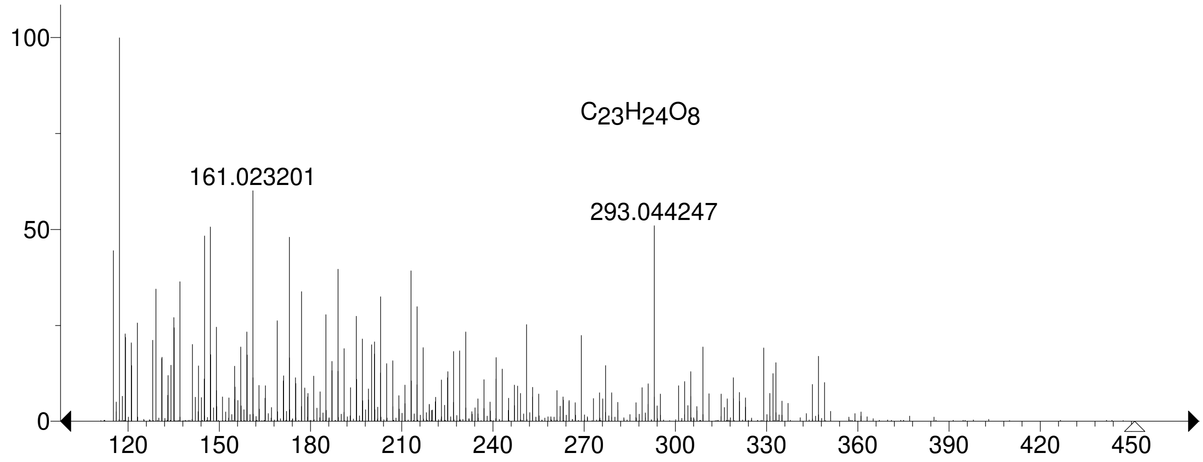

Hit 5 : 4-[(2R,3R,4S,5S,6R)-3,4,5-trihydroxy-6-(hydroxymethyl)oxan-2-yl]oxypentan-2-yl (E)-3-(4-hydroxyphenyl)p
C20H28O9; MF: 0; RMF: 5; Prob 16.7%; Lib: anthocyanins mona; ID: 6818.

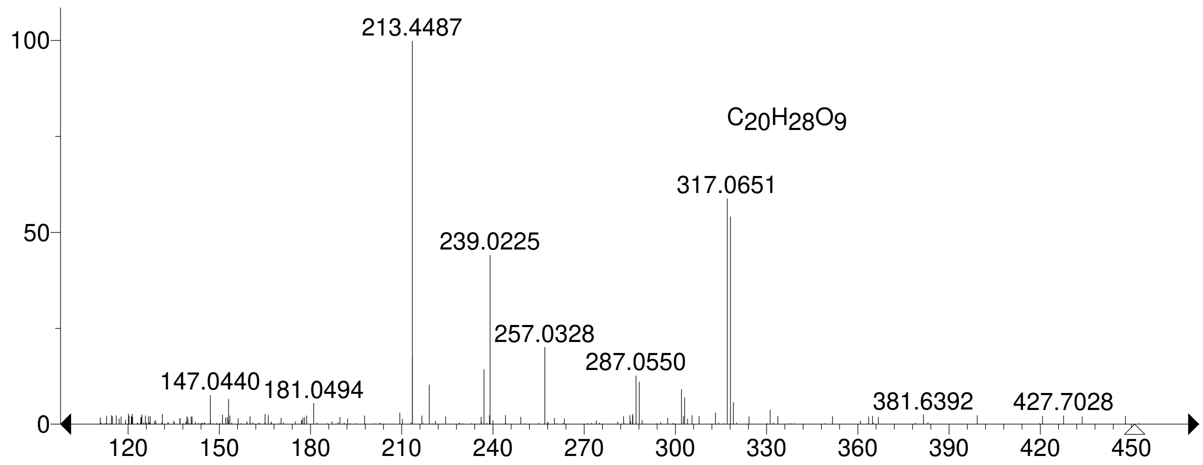

Unknown: Unknown (4.55_538.1160m/z)
Compound in Library Factor = 0

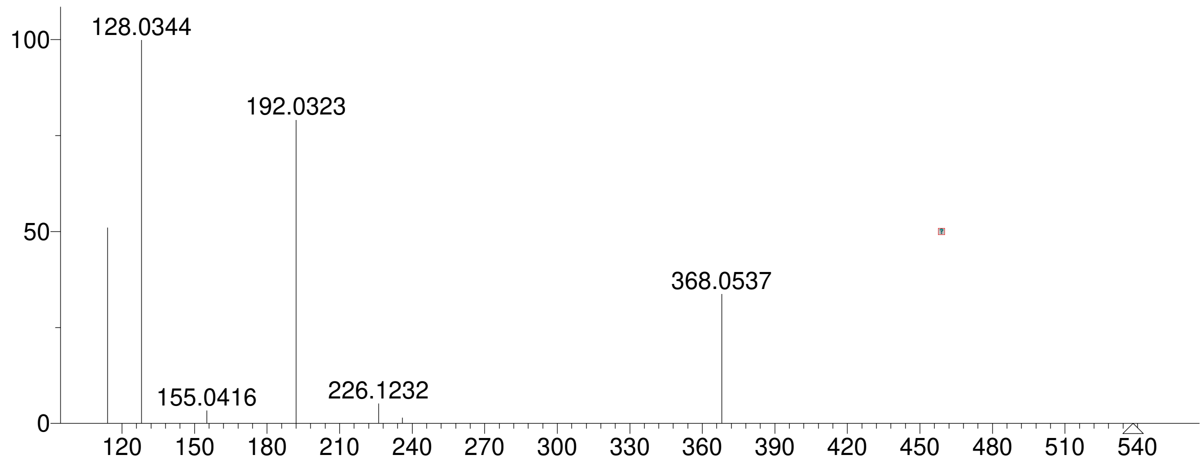

** Search Report Page 1 of 1 **

Unknown: Unknown (4.55_754.1744m/z)
Compound in Library Factor = 0

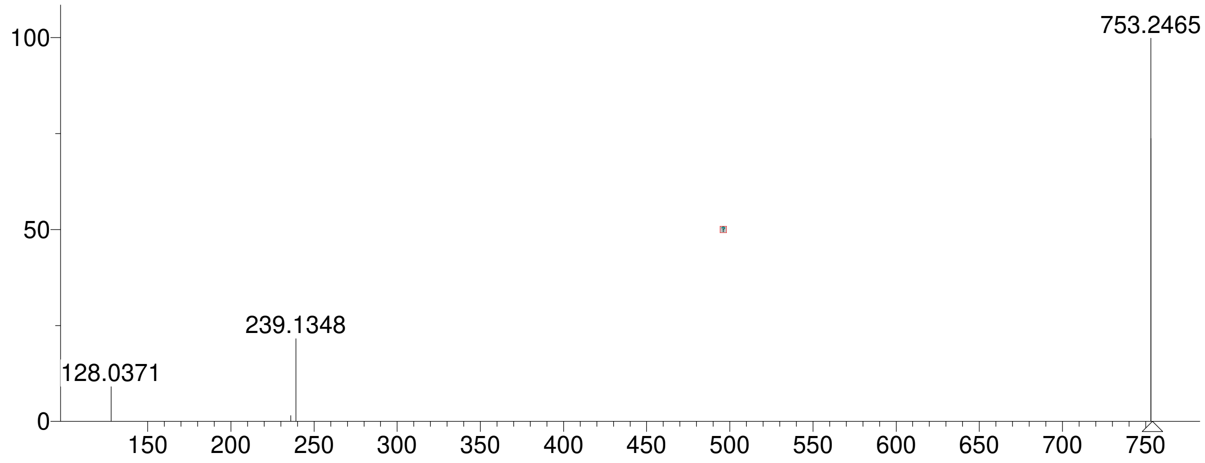

Unknown: Unknown (4.57_228.0870m/z)
Compound in Library Factor = 0

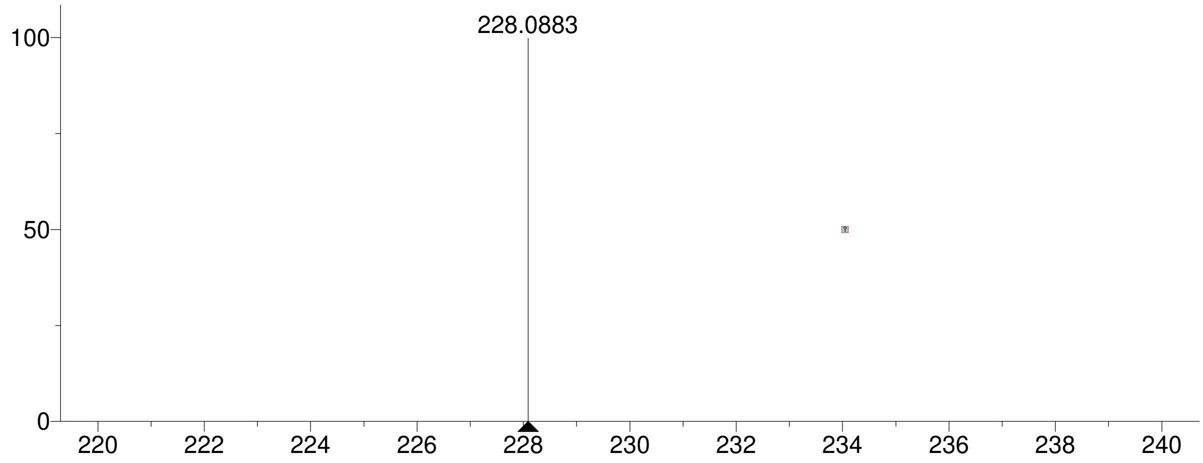

Unknown: Unknown (4.57_229.1544m/z)
Compound in Library Factor = -862

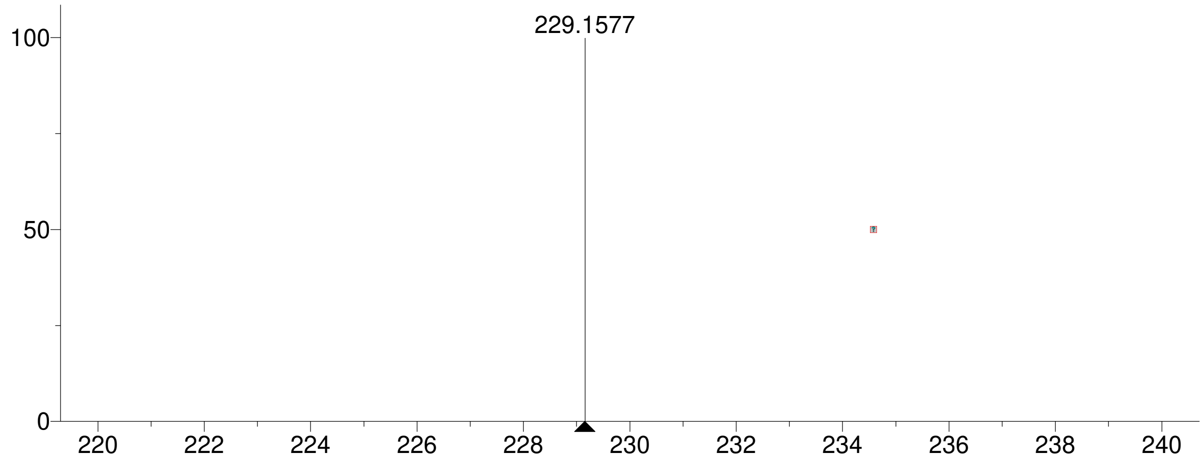

Hit 1 : Pro-Ile [M+H]+ QTOF 20V P=229.2
C11H20N2O3; MF: 364; RMF: 999; Prob 8.22%; Lib: nist_msms; ID: 48704.

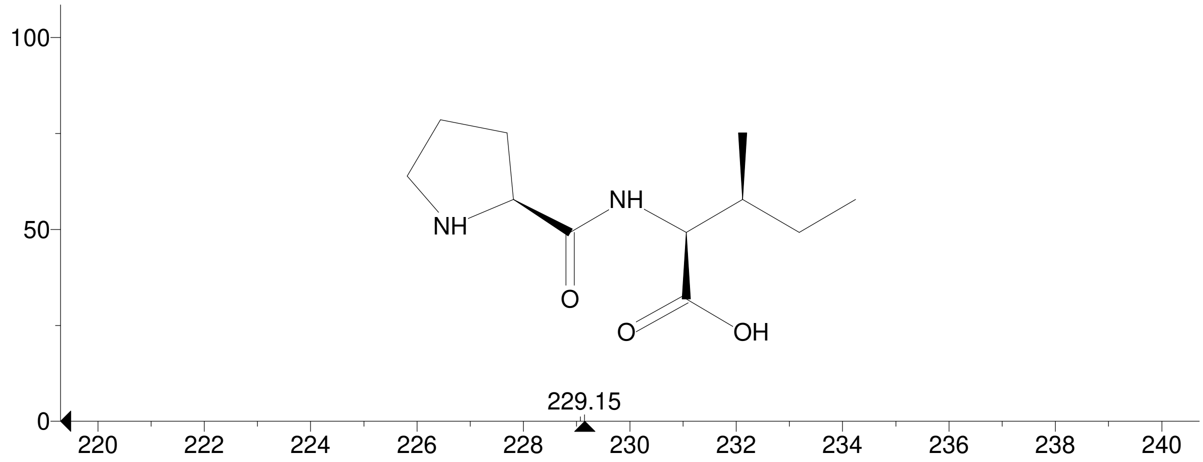

Hit 2 : Pro-Leu [M+H]+ QTOF 20V P=229.2
C11H20N2O3; MF: 364; RMF: 999; Prob 8.22%; Lib: nist_msms; ID: 48709.

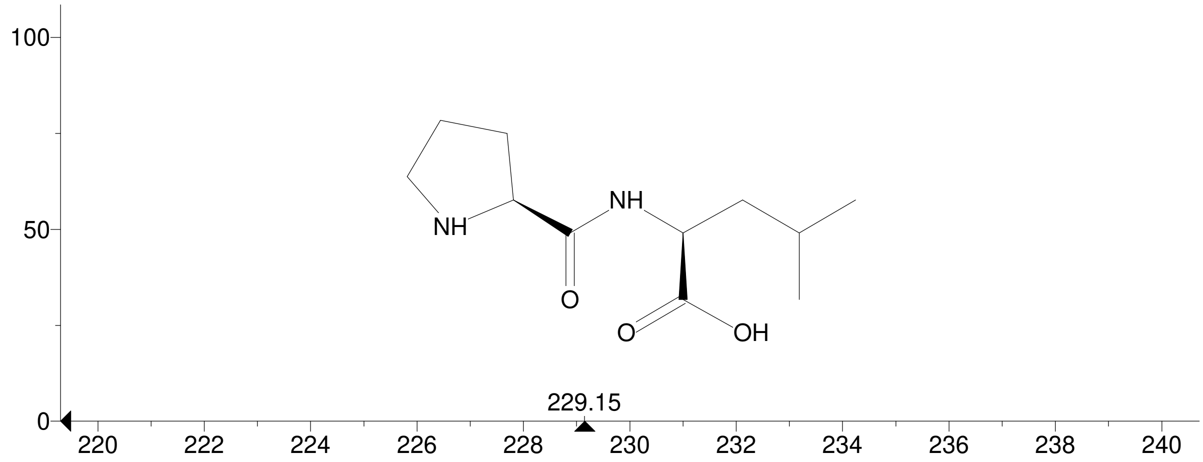

Hit 3 : Ile-Pro [M+H]+ QTOF 10V P=229.2
C11H20N2O3; MF: 364; RMF: 999; Prob 8.22%; Lib: nist_msms; ID: 55446.

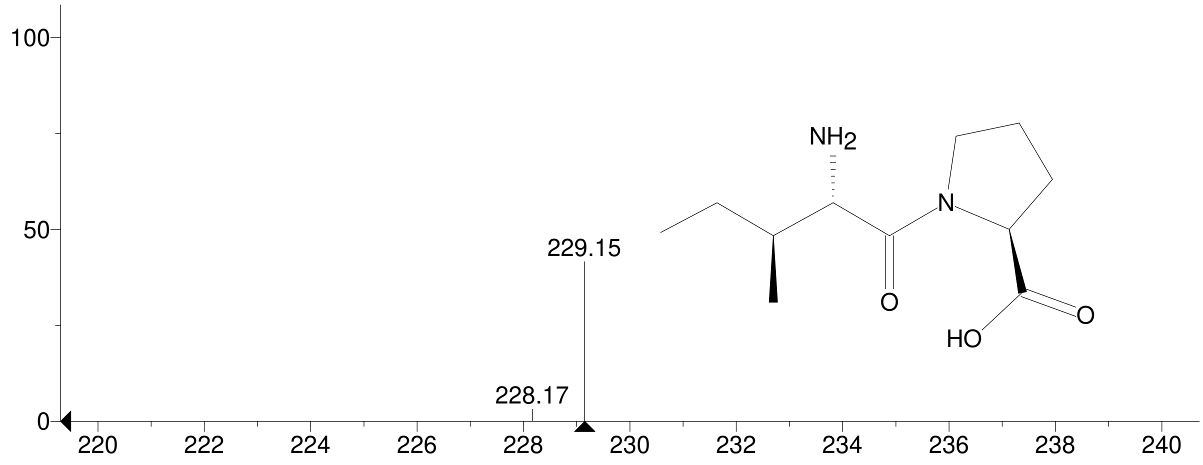

Hit 4 : DL-Leu-DL-Val [M-H]- IT-FT 35% P=229.2
C11H22N2O3; MF: 364; RMF: 999; Prob 8.22%; CAS: 35436-83-0; Lib: nist_msms; ID: 201269.

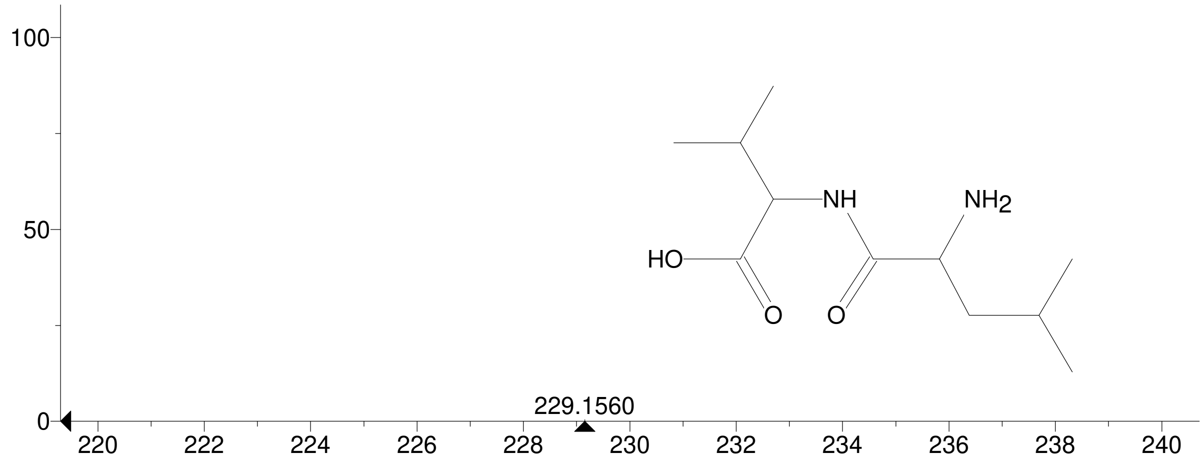

Hit 5 : Ile-Val [M-H]- HCD 25eV P=229.2
C11H22N2O3; MF: 364; RMF: 999; Prob 8.22%; CAS: 41017-96-3; Lib: nist_msms; ID: 567493.

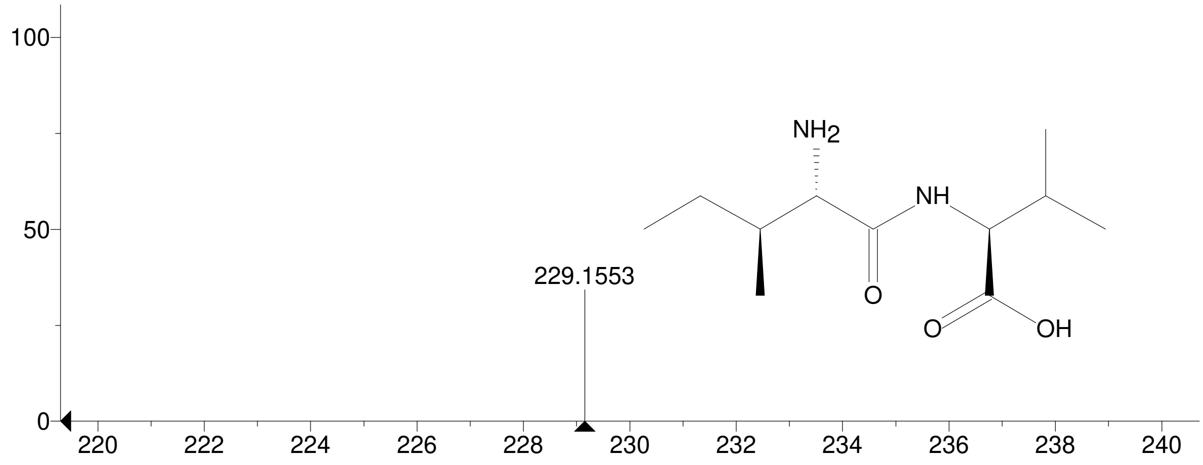

Unknown: Unknown (4.57_296.1229m/z)
Compound in Library Factor = 0

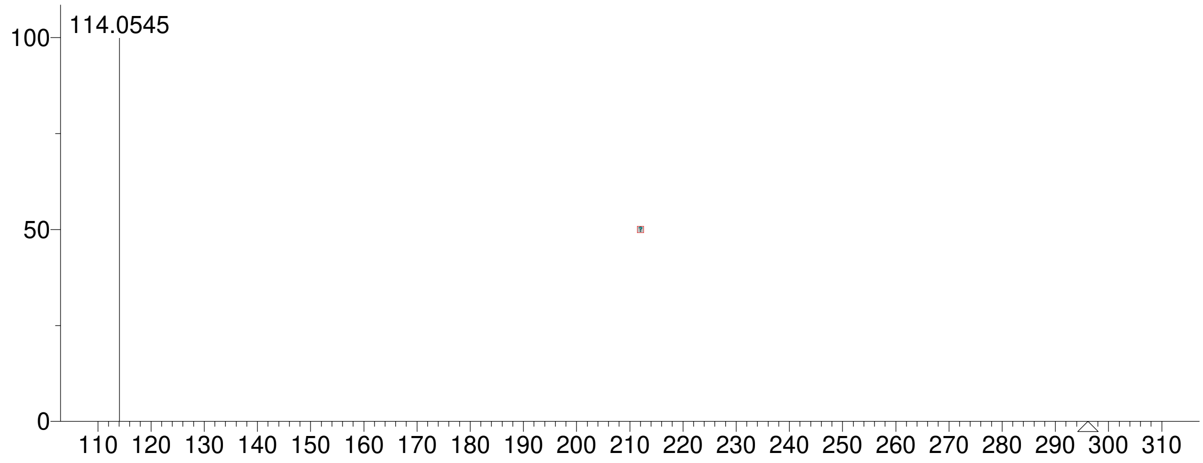

Unknown: Unknown (4.58_753.2379m/z)
Compound in Library Factor = 0

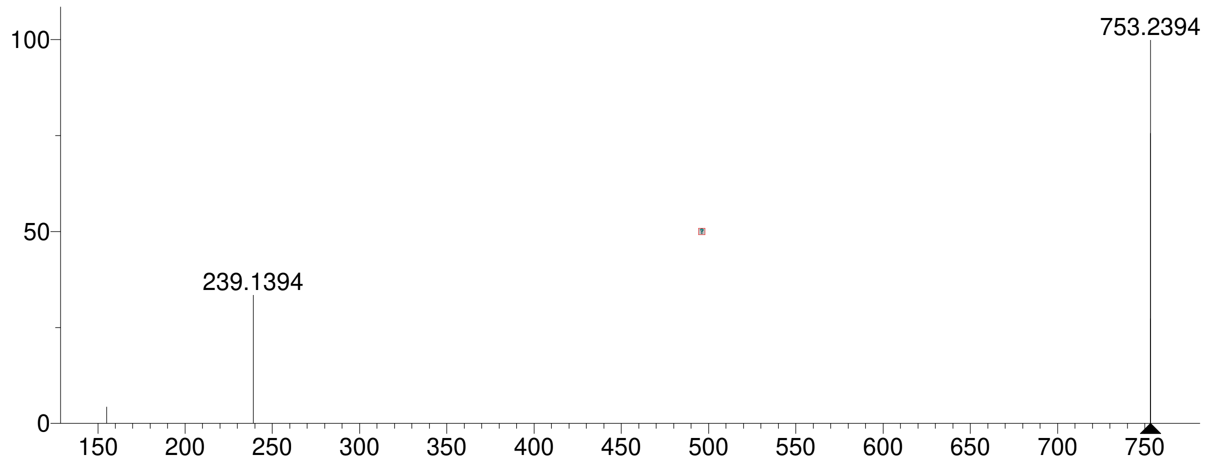

Unknown: Unknown (4.59_257.1108m/z)
Compound in Library Factor = 0

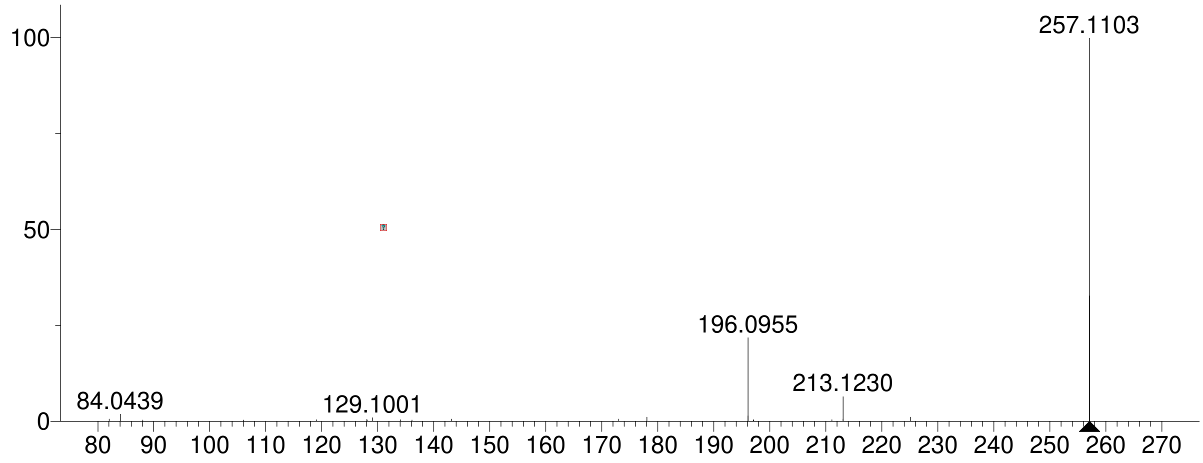

Hit 1 : 5'-O-Methylthymidine [M+H]+ 35eV P=257.1
C11H16N2O5; MF: 0; RMF: 9; Prob 100.0%; Lib: mona-export-fiehn_hilic; ID: 1695.

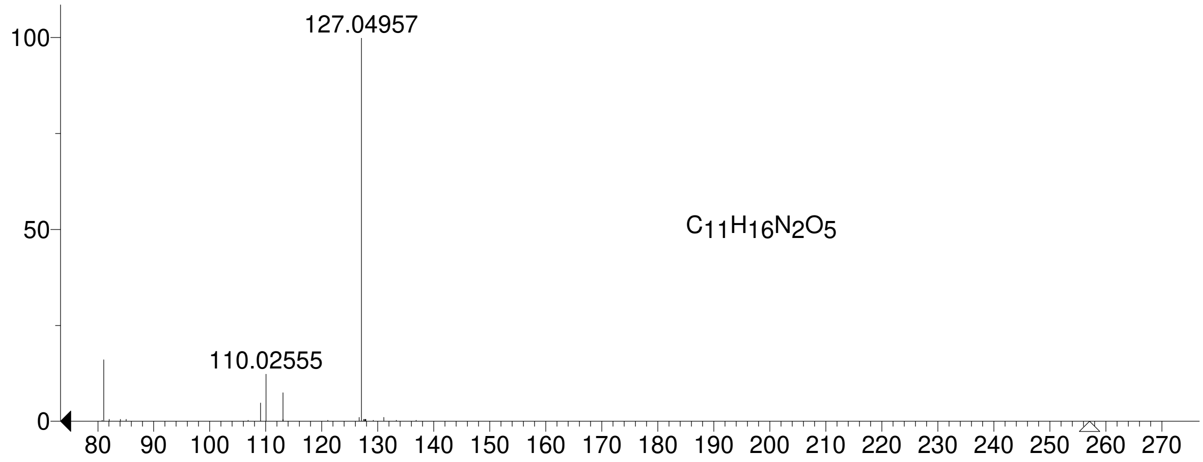

Unknown: Unknown (4.59_754.1649m/z)
Compound in Library Factor = 0

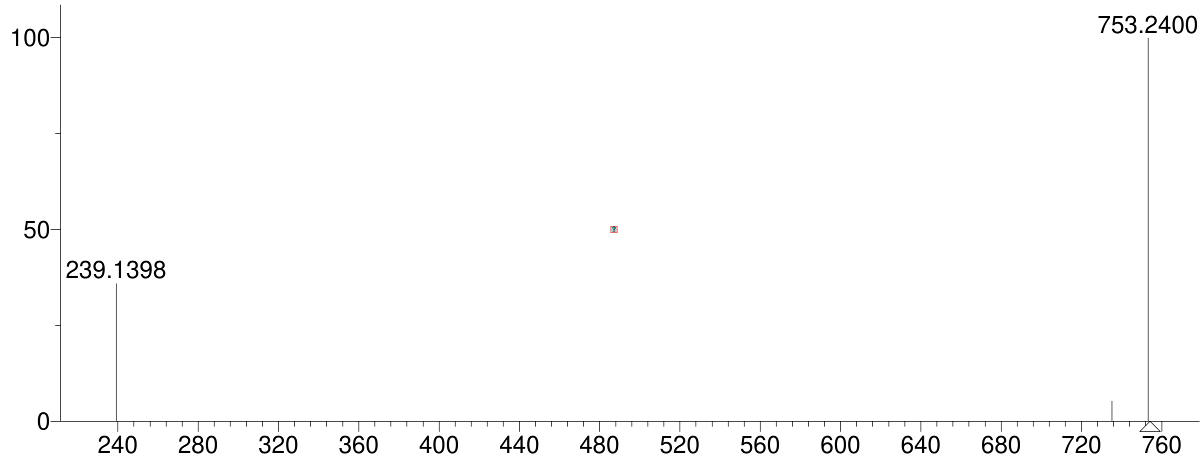

Unknown: Unknown (4.60_257.1133m/z)
Compound in Library Factor = -107

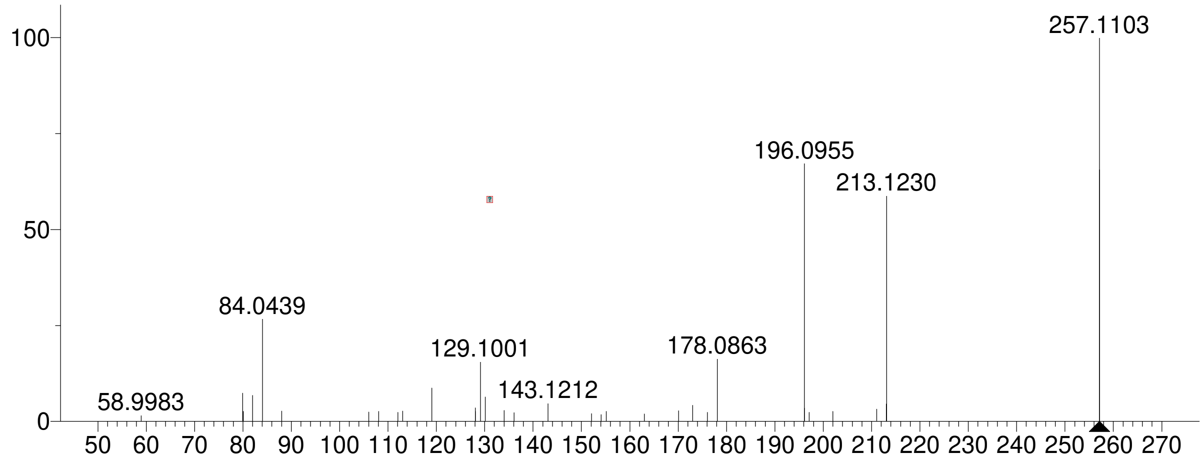

Hit 1 : L-Saccharopine [M-H-H2O]- HCD 24V P=257.1
C11H20N2O6; MF: 461; RMF: 720; Prob 98.8%; CAS: 997-68-2; Lib: nist_msms; ID: 125637.

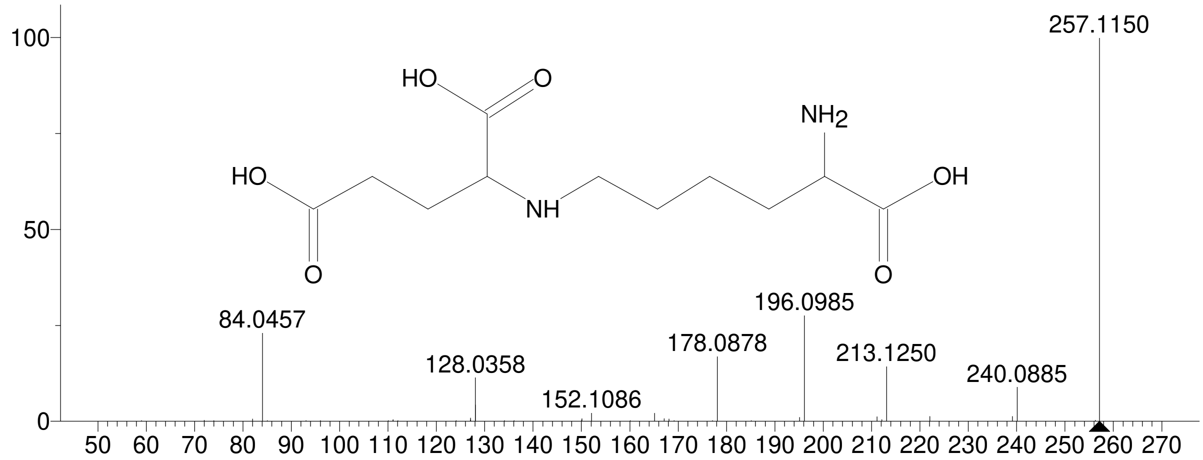

Hit 2 : α-Cyclohexylmandelic acid [M+Na]+ HCD 1V P=257.1
C14H18O3; MF: 60; RMF: 534; Prob 0.98%; CAS: 4335-77-7; Lib: nist_msms; ID: 492969.

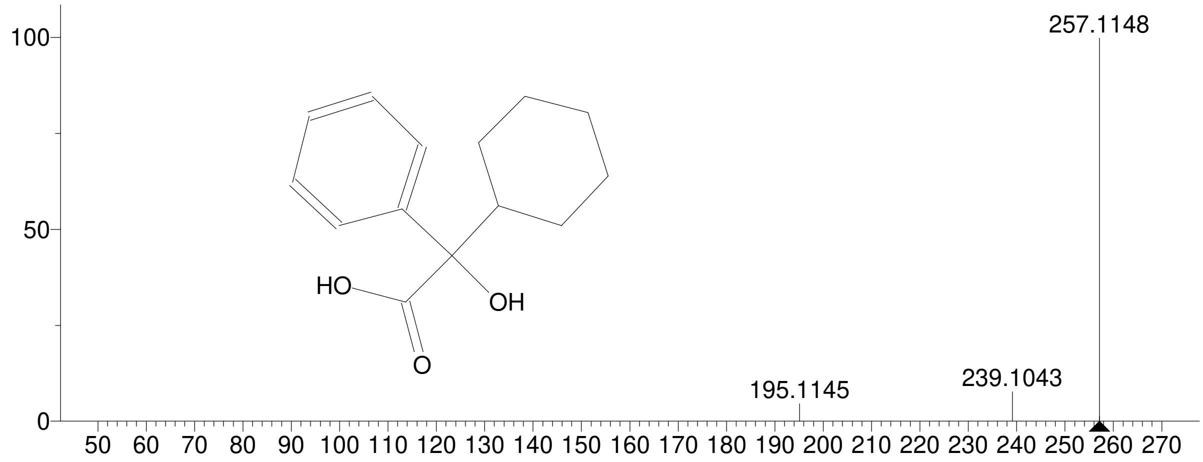

Hit 3 : 5'-O-Methylthymidine [M+H]+ 35eV P=257.1
C11H16N2O5; MF: 0; RMF: 21; Prob 0.17%; Lib: mona-export-fiehn_hilic; ID: 1695.

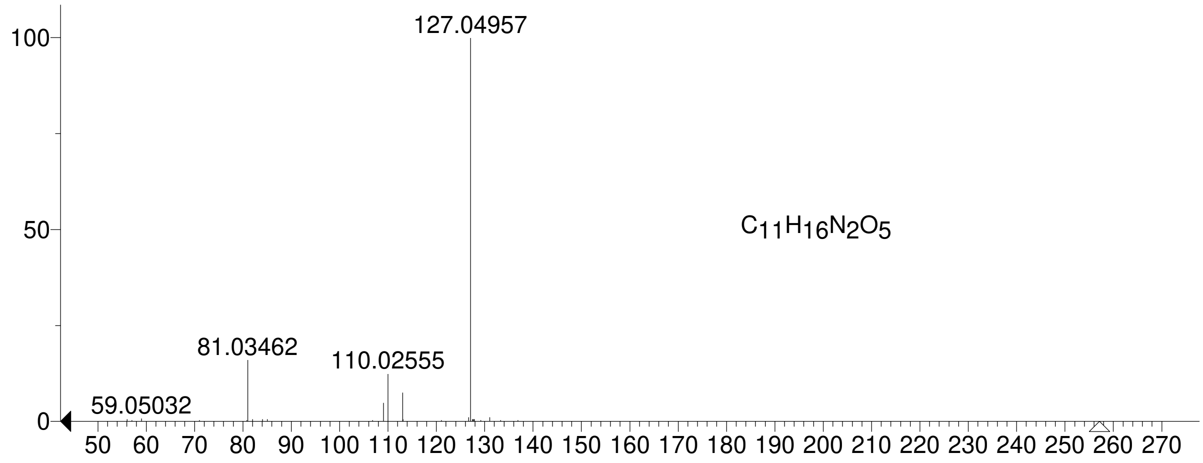

Unknown: Unknown (4.60_427.1711m/z)
Compound in Library Factor = -3273

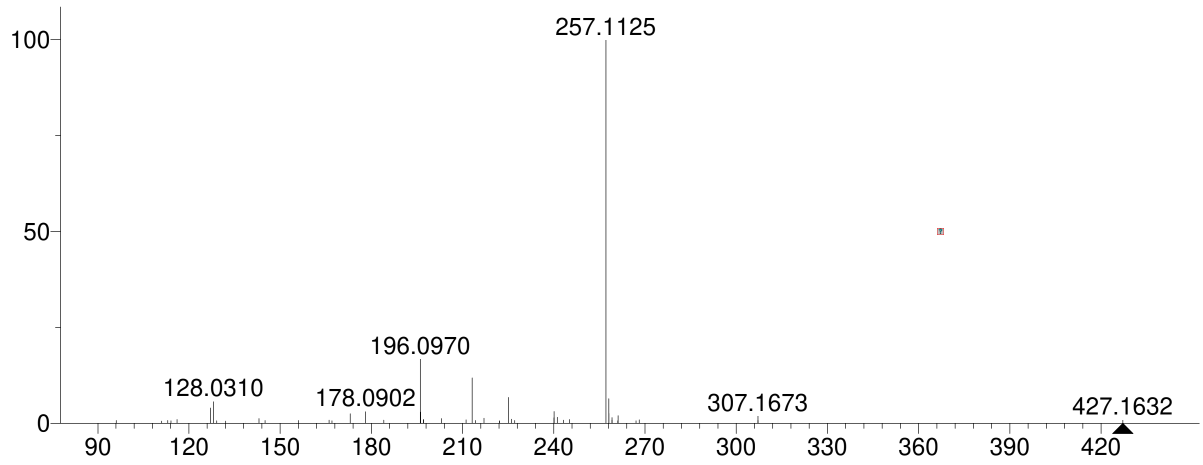

Hit 1 : [(6E,10Z)-10-(acetyloxymethyl)-6-formyl-3-methylidene-2-oxo-3a,4,5,8,9,11a-hexahydrocyclodeca[b]furan-
C22H28O7; MF: 2; RMF: 96; Prob 15.2%; Lib: mona-export-vf-npl_qexactive; ID: 23323.

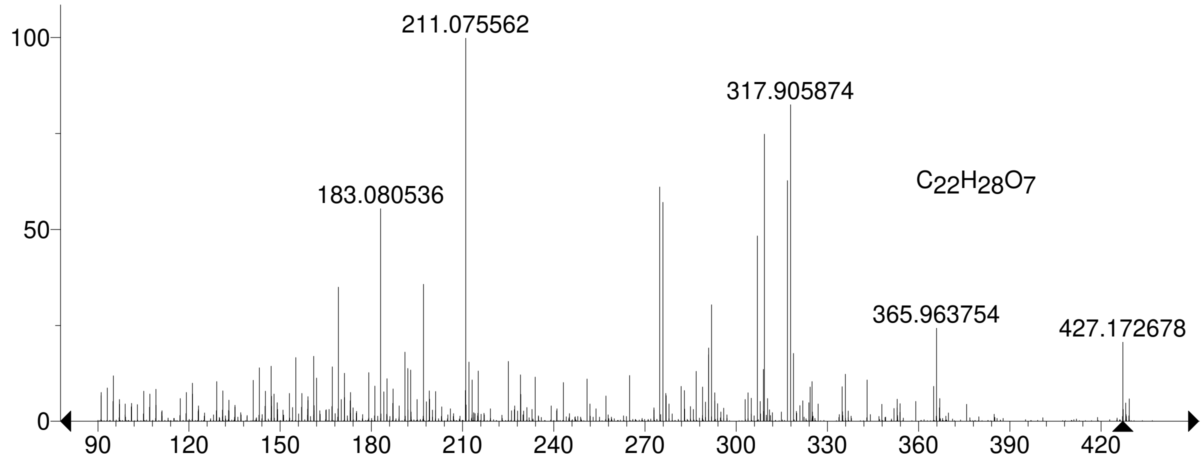

Hit 2 : Archangelicine [M+H]+ 35HCD P=427.2
C24H26O7; MF: 1; RMF: 42; Prob 14.6%; Lib: mona-export-vf-npl_qexactive; ID: 5194.

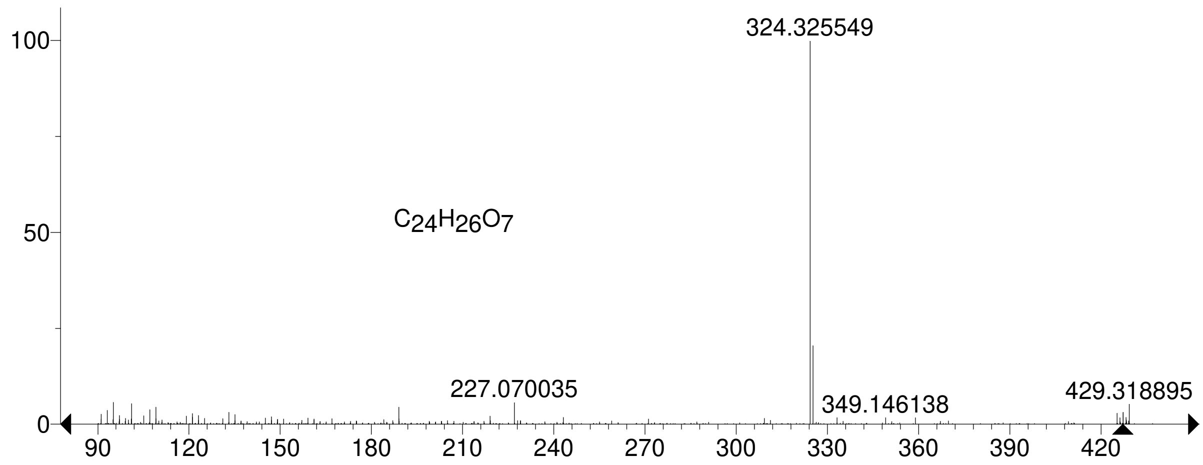

Hit 3 : [8,8-dimethyl-9-(3-methylbut-2-enoyloxy)-2-oxo-9,10-dihydropyrano[2,3-f]chromen-10-yl] 3-methylbut-2-en
C24H26O7; MF: 0; RMF: 34; Prob 14.0%; Lib: mona-export-vf-npl_qexactive; ID: 4551.

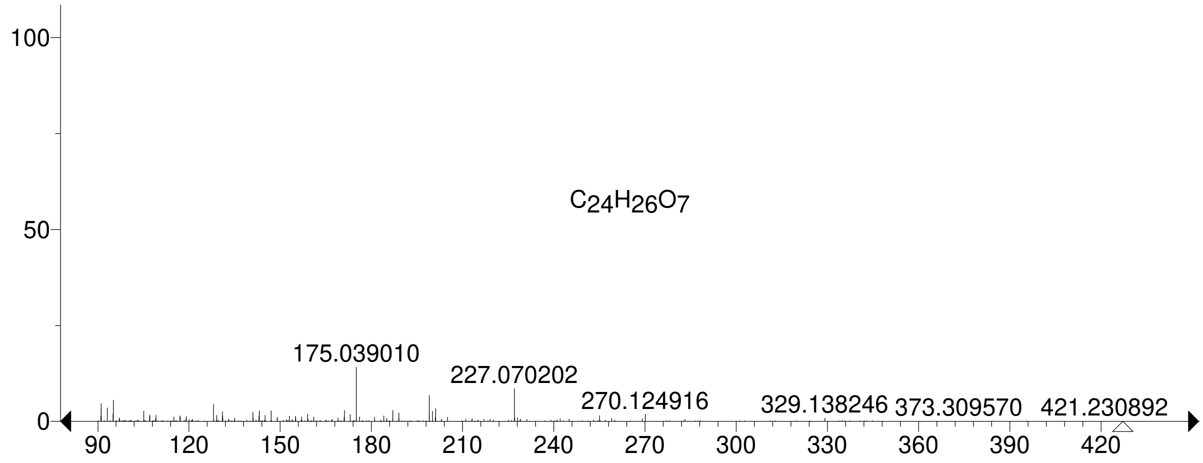

Hit 4 : PG 11:0; PG 2:0-9:0; [M-H]- [M-H]- P=427.2
C17H33O10P; MF: 0; RMF: 34; Prob 14.0%; Lib: msdial-insilicomsms-lipids-neg; ID: 324573.

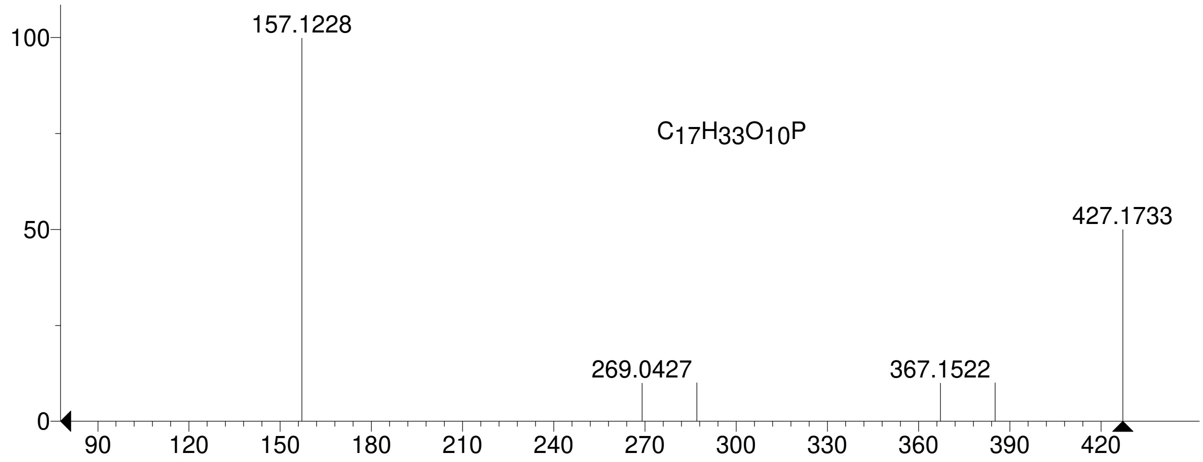

Hit 5 : PG 11:0; PG 3:0-8:0; [M-H]- [M-H]- P=427.2
C17H33O10P; MF: 0; RMF: 34; Prob 14.0%; Lib: msdial-insilicomsms-lipids-neg; ID: 324632.

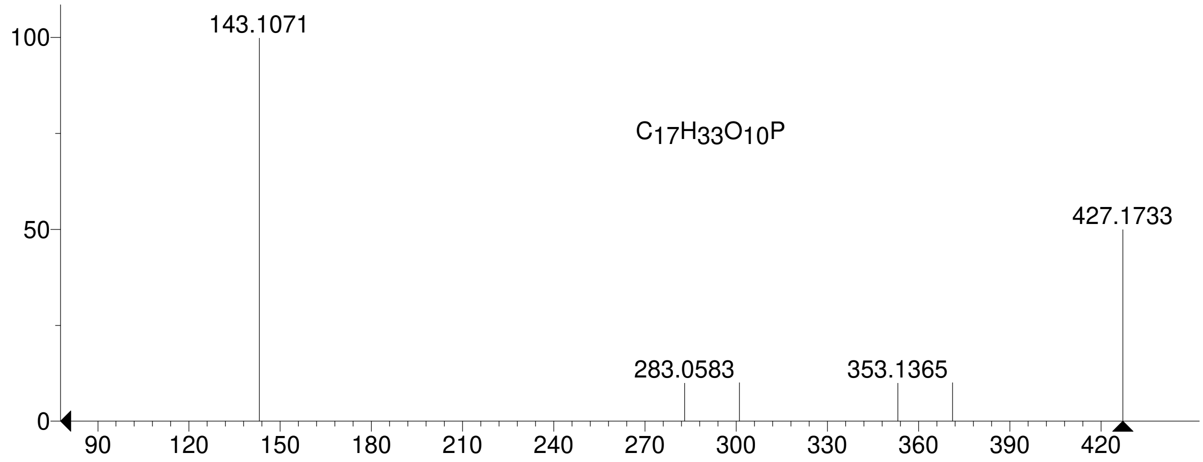

Unknown: Unknown (4.61_274.1073m/z)
Compound in Library Factor = -3273

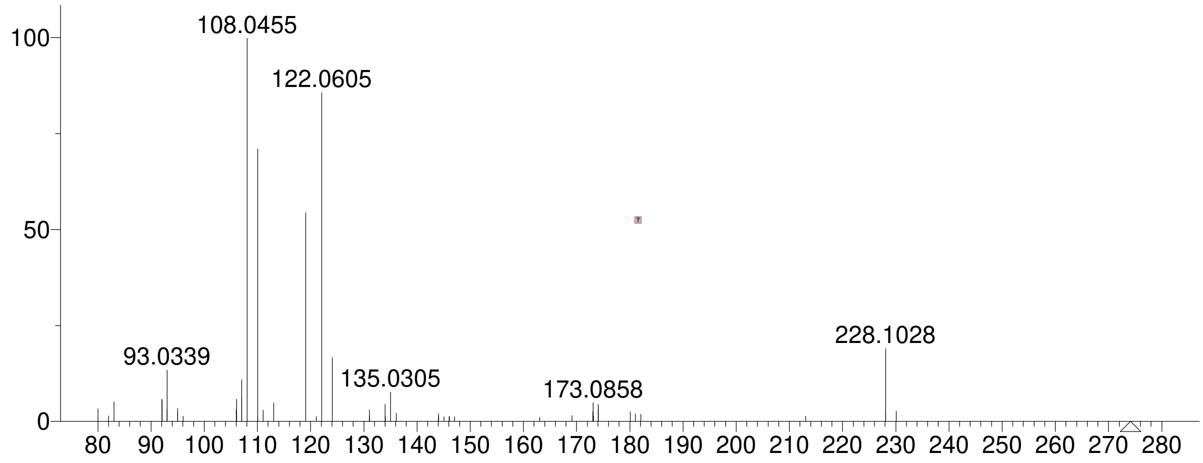

Hit 1 : L-Thyronine [M+H]+ IT-FT 35% P=274.1
C15H15NO4; MF: 12; RMF: 269; Prob 13.0%; CAS: 1596-67-4; Lib: nist_msms; ID: 277888.

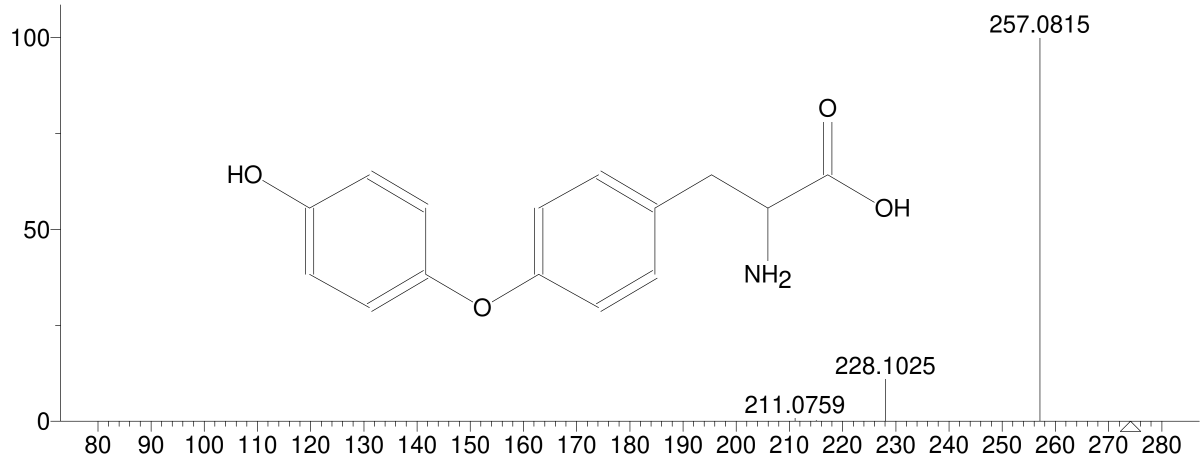

Hit 2 : Benzoic acid, 4-[[tetrahydro-2-(3-hydroxy-1-butenyl)-5-oxo-3-furanyl]amino]- [M+H-H2O]+ HCD 49V P=2
C15H17NO5; MF: 8; RMF: 132; Prob 11.0%; CAS: 144397-99-9; Lib: nist_msms; ID: 351123.

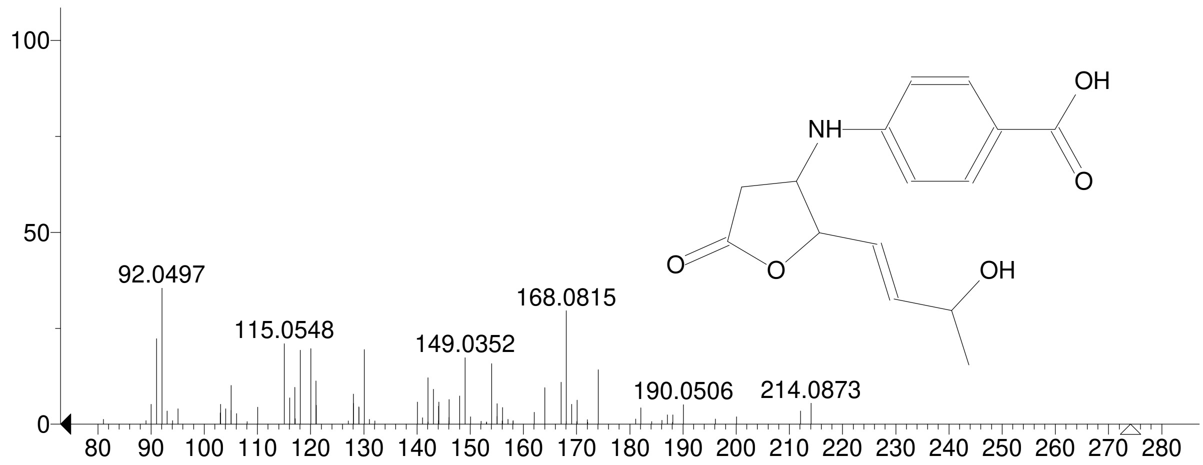

Hit 3 : NCGC00381099-01_C15H17NO5_Benzoic acid, 4-[[tetrahydro-2-[(2E)-1-hydroxy-2-buten-1-yl]-5-oxo-3-fur
C15H17NO5; MF: 4; RMF: 89; Prob 9.29%; Lib: mona-export-gnps; ID: 13377.

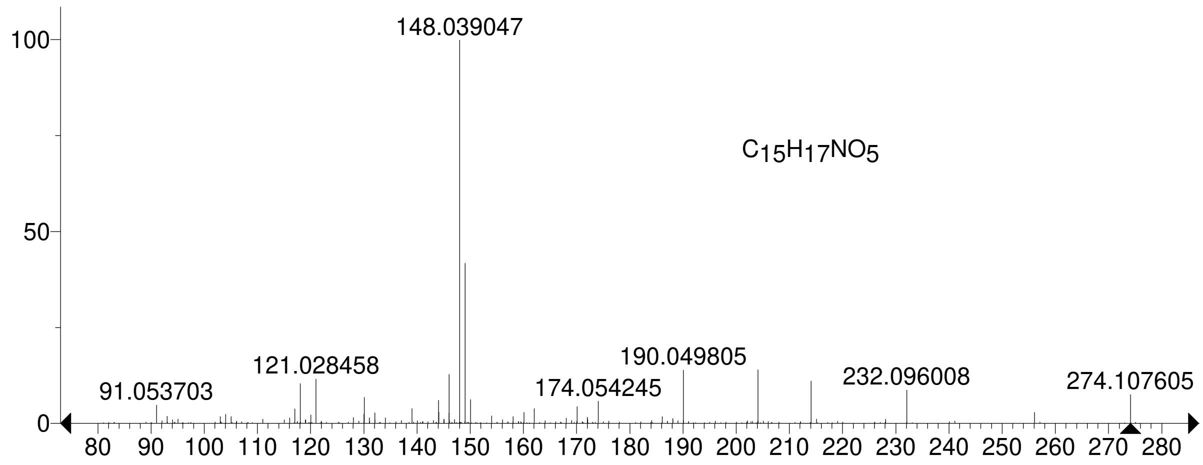

Hit 4 : NCGC00385442-01_C13H12O4_3-Hydroxy-2-phenyl-2,3,3a,7a-tetrahydro-5H-furo[3,2-b]pyran-5-one [M+
C13H12O4; MF: 3; RMF: 135; Prob 8.92%; Lib: mona-export-gnps; ID: 13007.

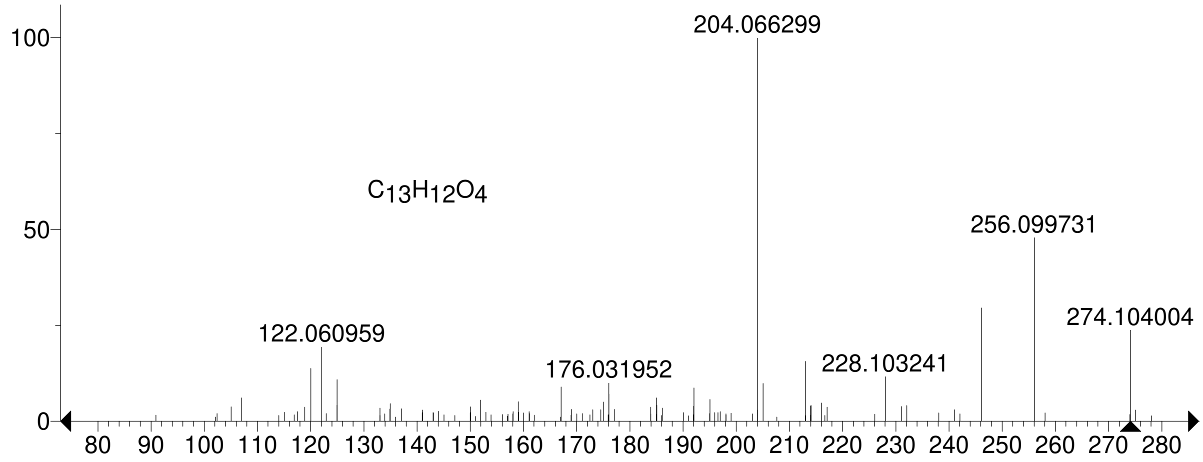

Hit 5 : NCGC00380910-01_C15H17NO5_2,6-Piperidinedione, 4-[2-[2-hydroxy-3-(hydroxymethyl)-5-methylphenyl
C15H17NO5; MF: 3; RMF: 112; Prob 8.92%; Lib: mona-export-gnps; ID: 12850.

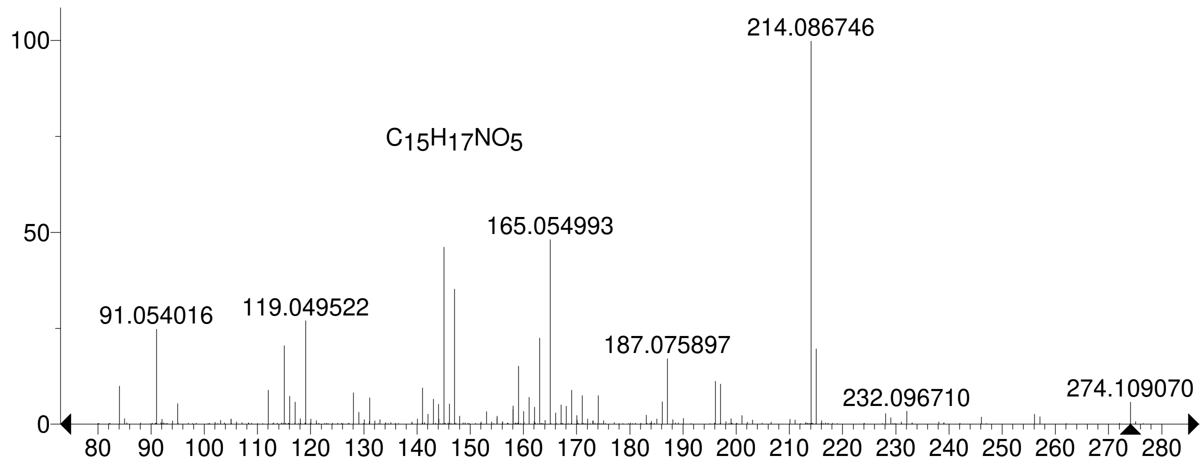

** Search Report Page 1 of 1 **

Unknown: Unknown (4.62_470.1761m/z)
Compound in Library Factor = 0

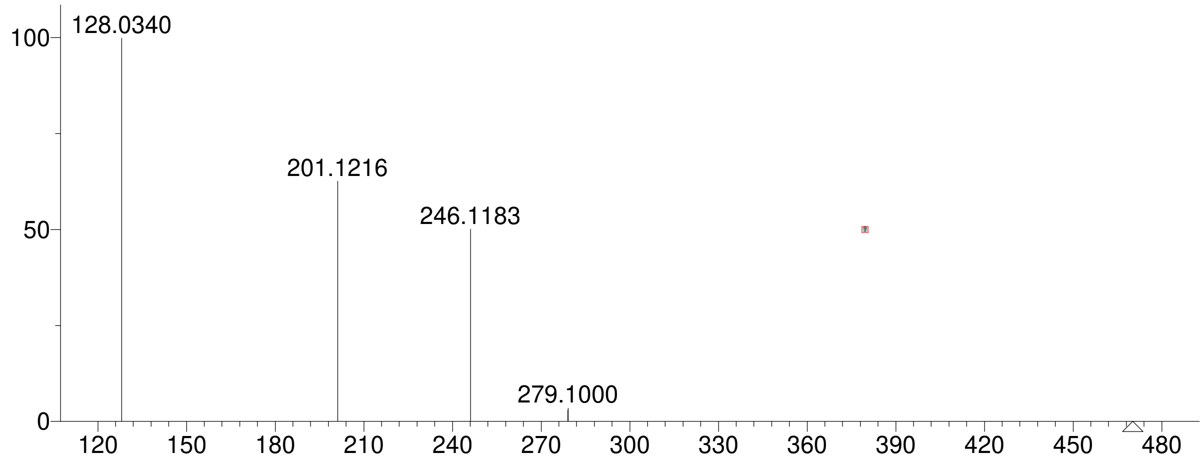

Unknown: Unknown (4.62_636.1522m/z)
Compound in Library Factor = 0

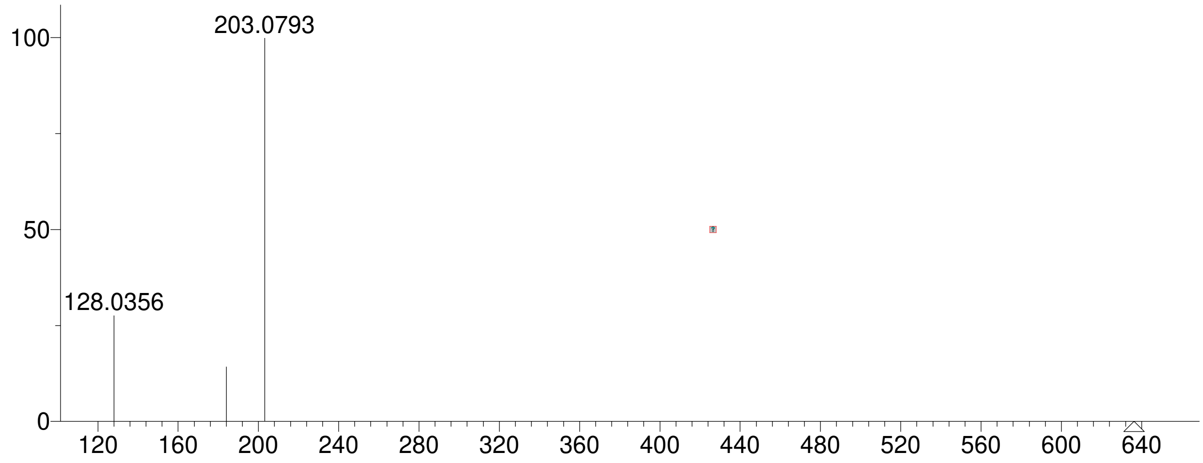

Unknown: Unknown (4.64_245.1126m/z)
Compound in Library Factor = -1090

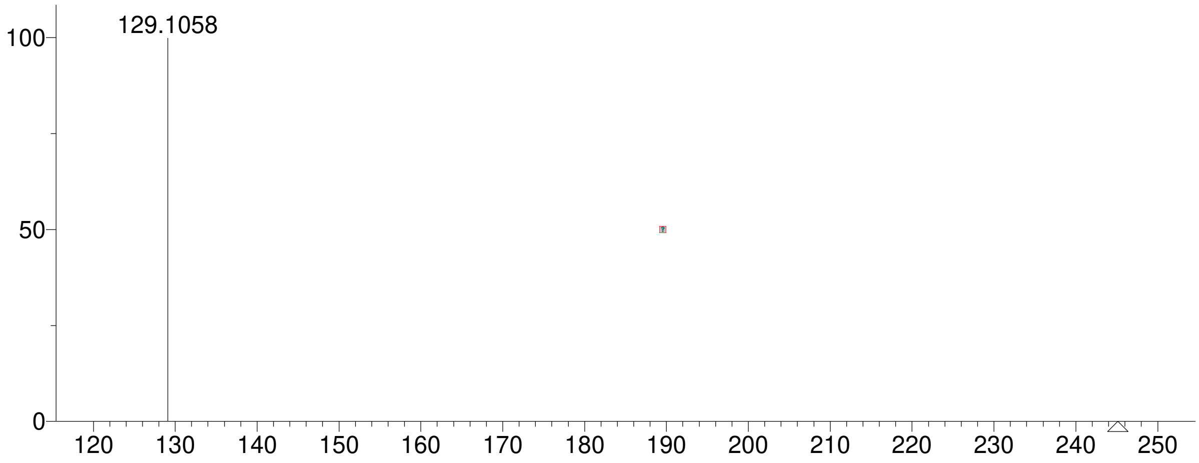

Hit 1 : Ile-Asp [M-H]- HCD 31eV P=245.1
C10H18N2O5; MF: 204; RMF: 911; Prob 33.6%; Lib: nist_msms; ID: 570706.

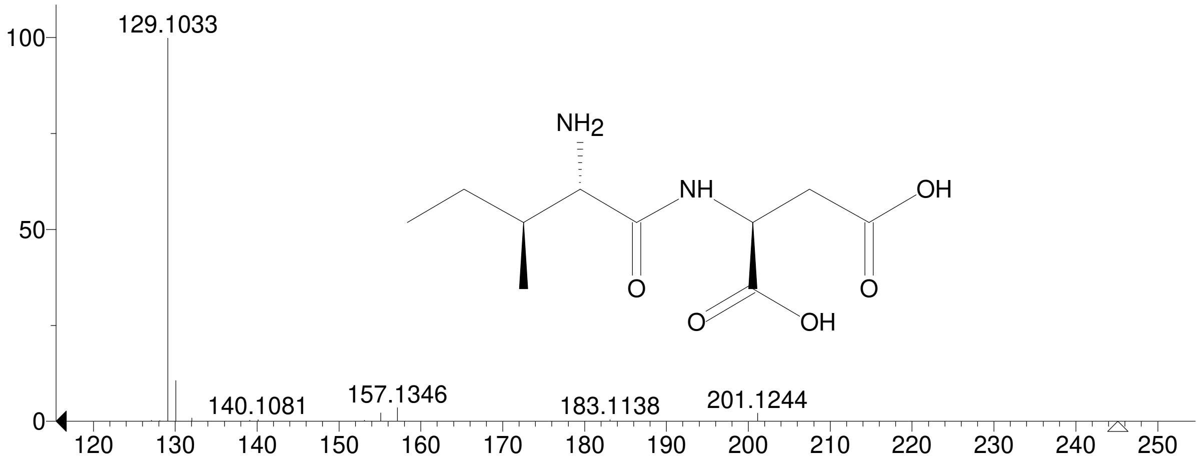

Hit 2 : Ile-Asp [M-H]- HCD 31eV P=245.1
C10H18N2O5; MF: 204; RMF: 911; Prob 33.6%; CAS: 54532-76-2; Lib: nist_msms2; ID: 84439.

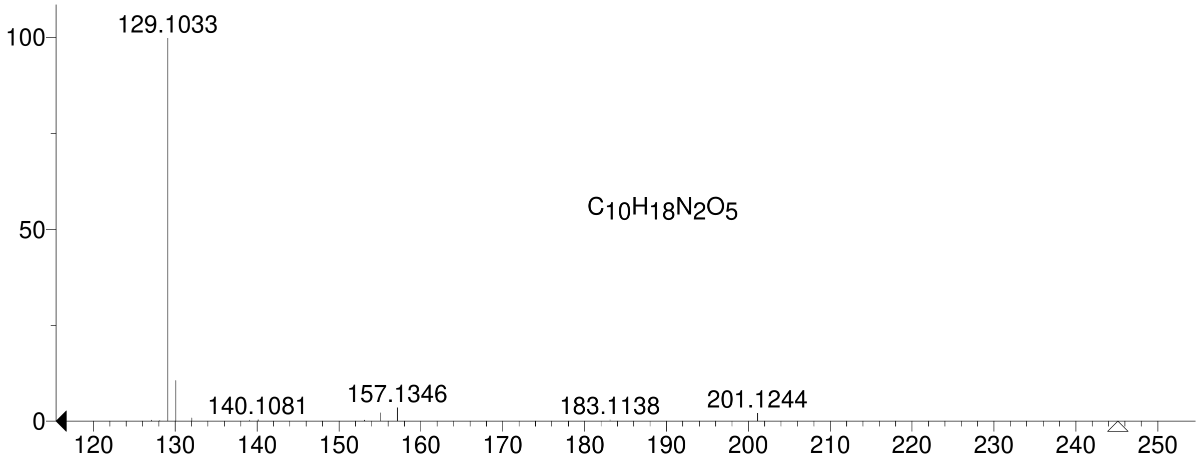

Hit 3 : Leu-Asp [M-H]- HCD 39eV P=245.1
C10H18N2O5; MF: 184; RMF: 892; Prob 15.3%; Lib: nist_msms; ID: 570776.

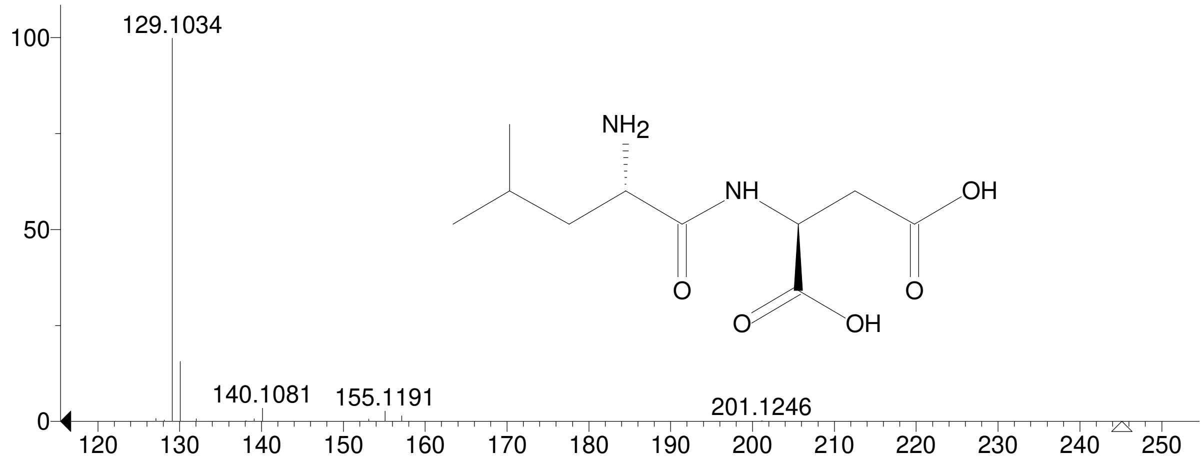

Hit 4 : Leu-Asp [M-H]- HCD 39eV P=245.1
C10H18N2O5; MF: 184; RMF: 892; Prob 15.3%; CAS: 32949-40-9; Lib: nist_msms2; ID: 84509.

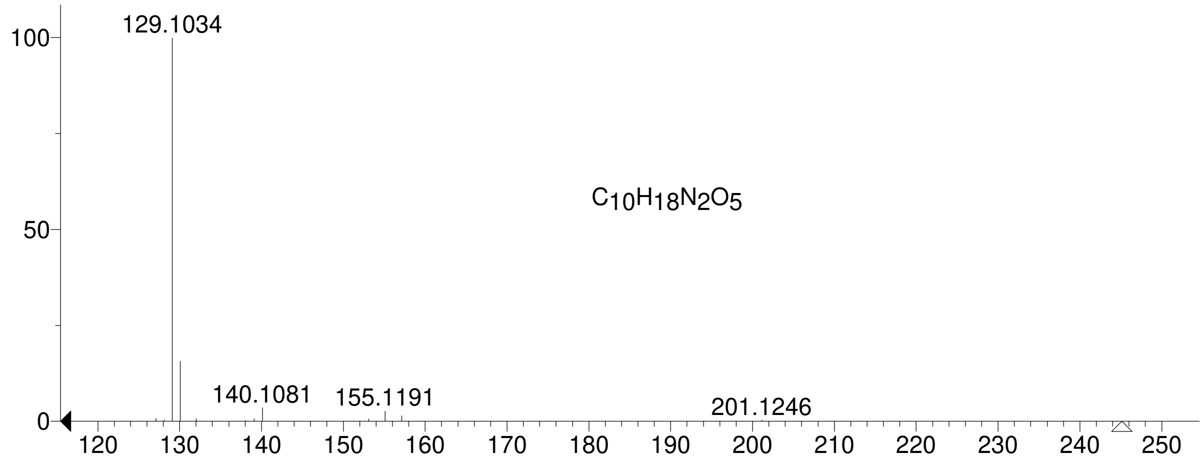

Hit 5 : Unknown (carbon number 10); PlaSMA ID-385 [M-H]- P=245.1
C10H18N2O5; MF: 14; RMF: 370; Prob 0.33%; Lib: biomsms-neg-plasma; ID: 1106.

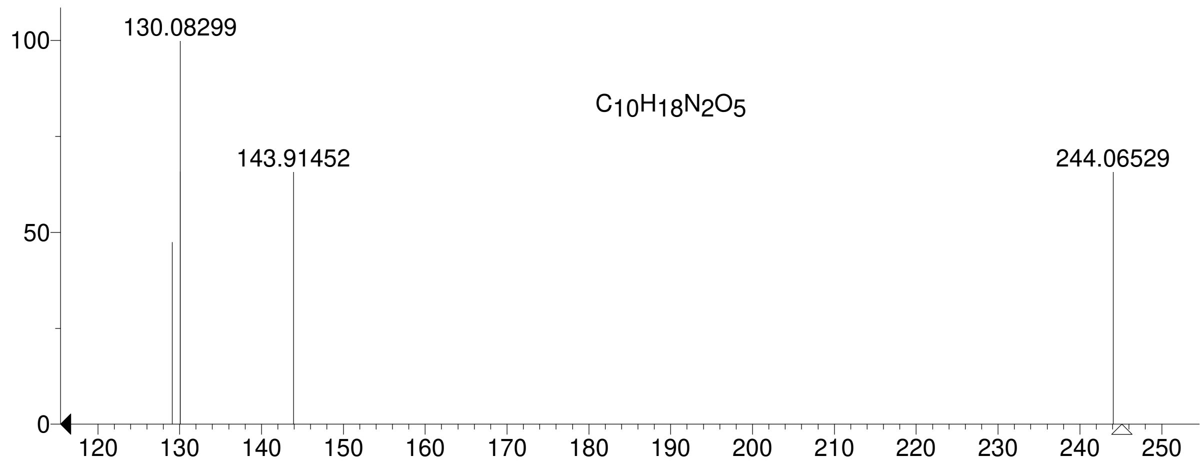

Unknown: Unknown (4.64_415.1711m/z)
Compound in Library Factor = -2387

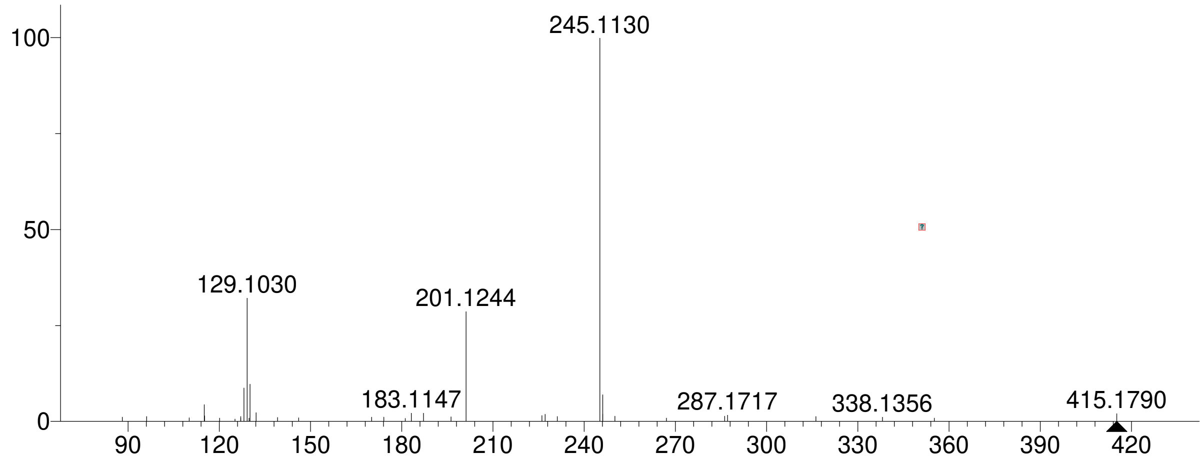

Hit 1 : Garcinone C [M+H]+ HCD 8V P=415.2
C23H26O7; MF: 14; RMF: 366; Prob 20.1%; CAS: 76996-27-5; Lib: nist_msms; ID: 389279.

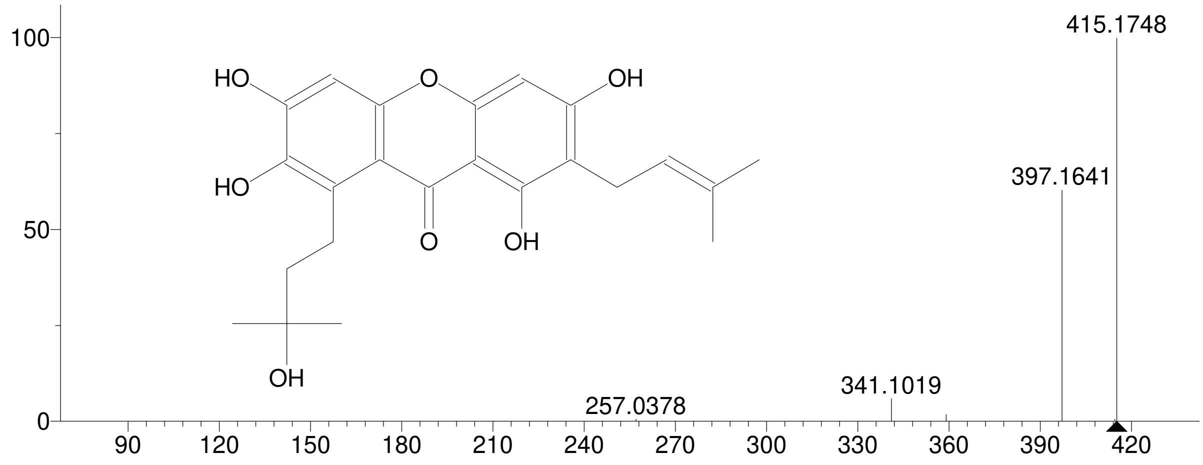

Hit 2 : Diltiazem [M+H]+ QTOF 3V P=415.2
C22H26N2O4S; MF: 12; RMF: 110; Prob 18.5%; CAS: 42399-41-7; Lib: nist_msms; ID: 20519.

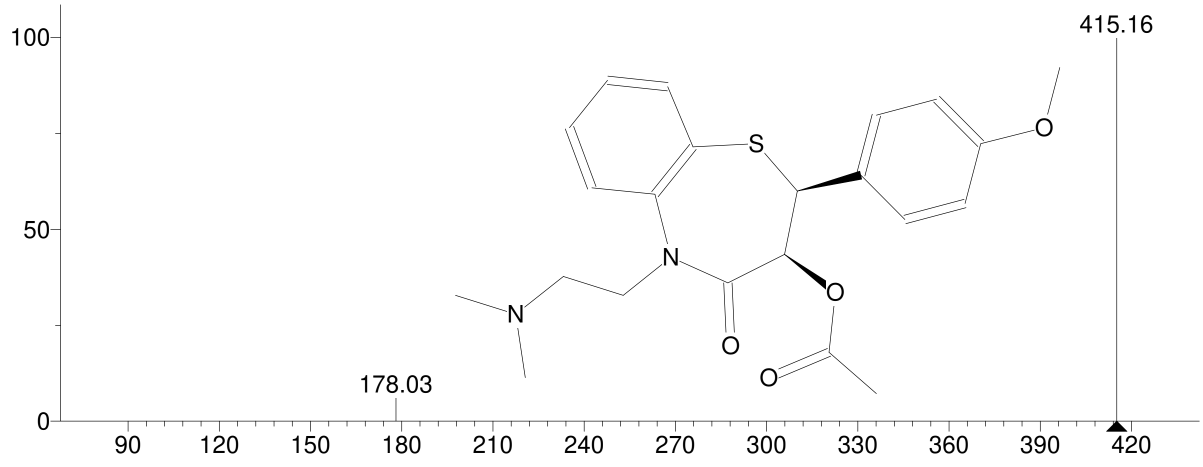

Hit 3 : 6-formyl-10-(hydroxymethyl)-5-methoxy-3-methylidene-2-oxo-2H,3H,3aH,4H,5H,8H,9H,11aH-cyclodeca[b]f
C21H28O7; MF: 2; RMF: 88; Prob 13.1%; Lib: mona-export-vf-npl_qexactive; ID: 16435.

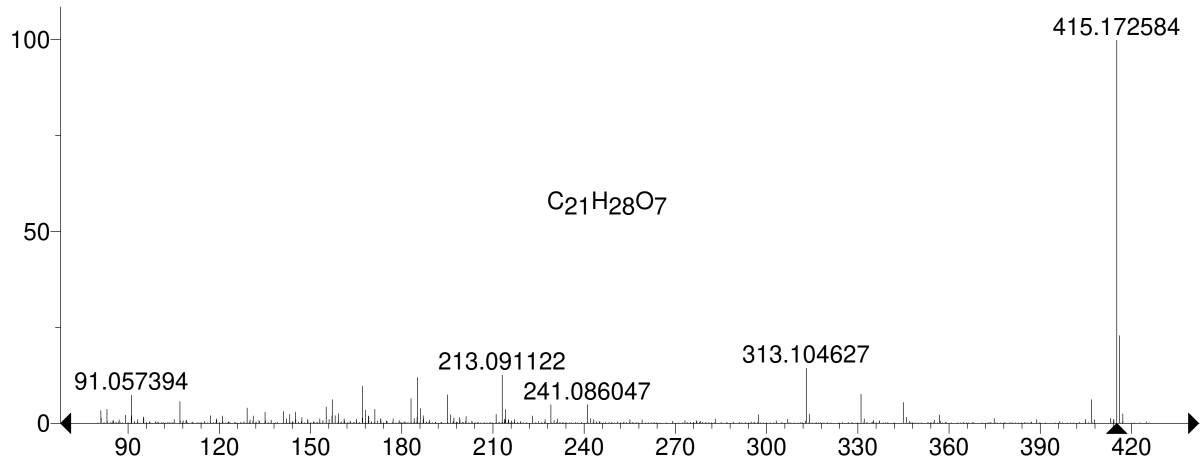

Hit 4 : Diltiazem HCl (Tiazac) [M+H]+ P=415.2
C22H27ClN2O4S; MF: 0; RMF: 35; Prob 12.1%; Lib: mona-export-gnps; ID: 648.

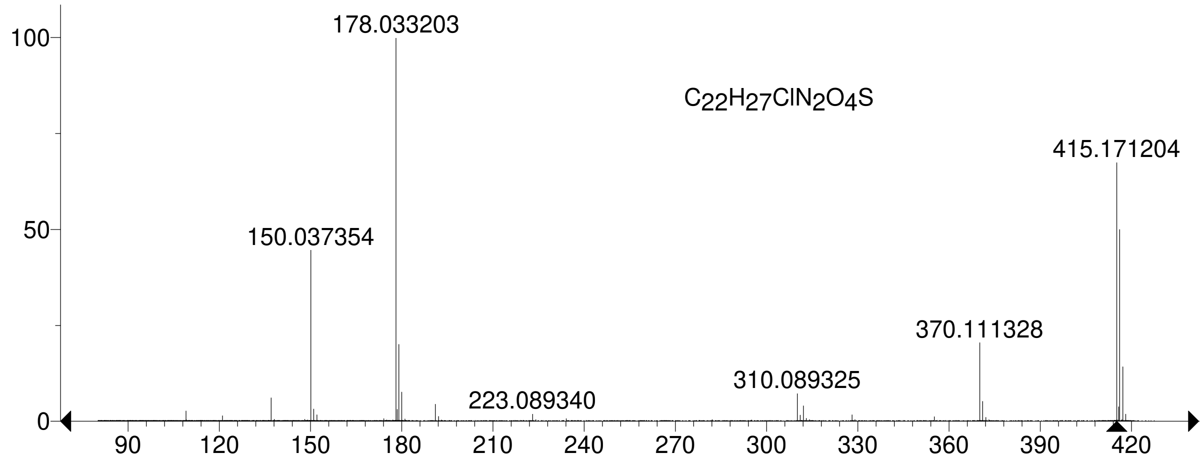

Hit 5 : NCGC00347525-03!2-amino-5-[2-[[2,3-dihydroxy-2-(1-hydroxyethyl)butanoyl]oxymethyl]-4-hydroxyanilino]-
C18H26N2O9; MF: 0; RMF: 21; Prob 12.1%; Lib: mona-export-gnps; ID: 9213.

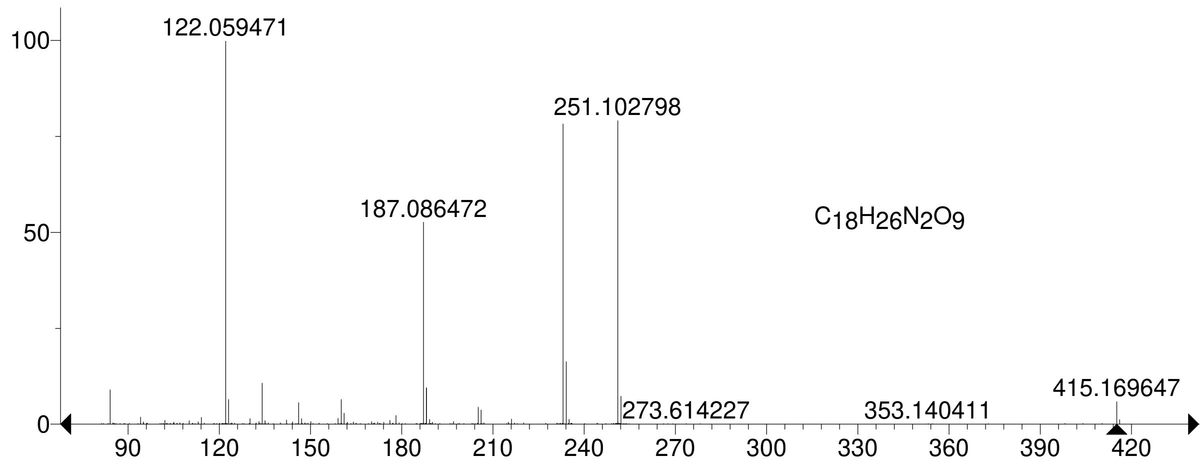

Unknown: Unknown (4.65_428.1787m/z)
Compound in Library Factor = 0

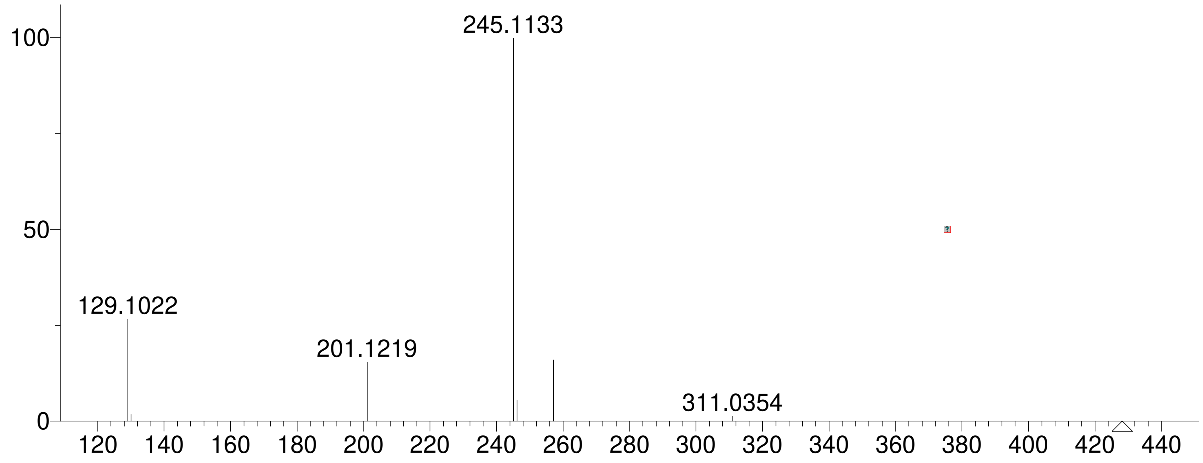

Unknown: Unknown (4.67_530.1961m/z)
Compound in Library Factor = 0

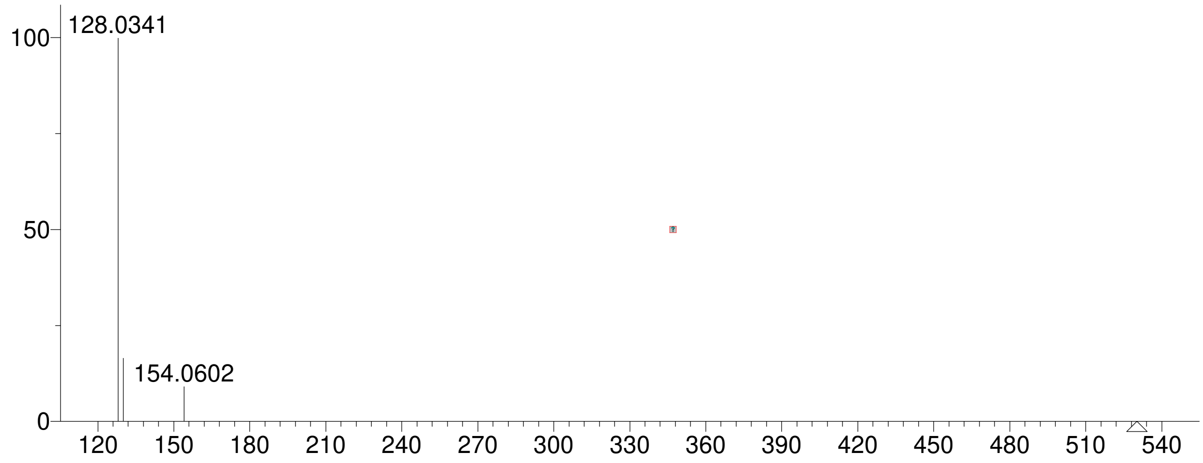

Hit 1 : Nilotinib [M+H]+ HCD 164V P=530.2
C28H22F3N7O; MF: 0; RMF: 29; Prob 100.0%; CAS: 641571-10-0; Lib: nist_msms; ID: 165144.

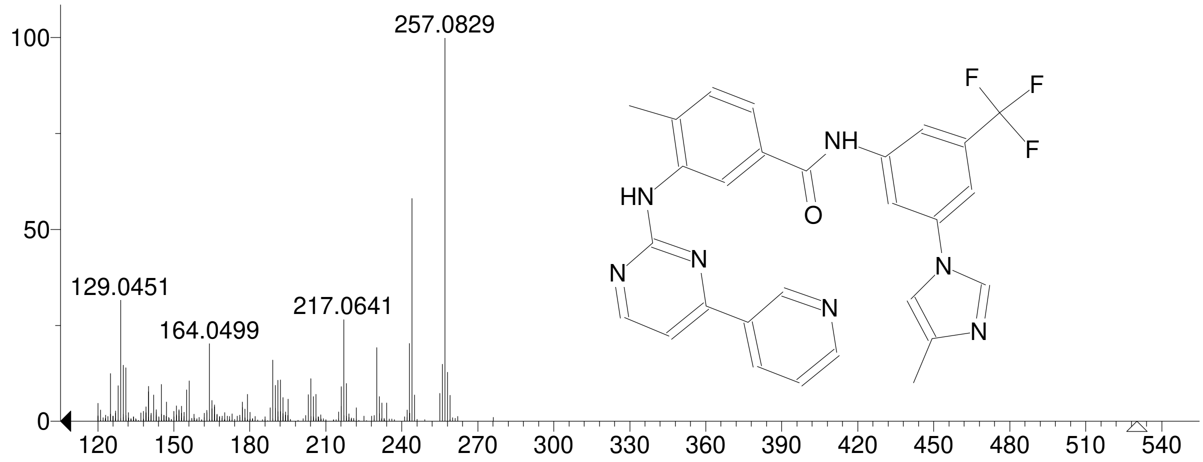

Unknown: Unknown (4.68_481.0940m/z)
Compound in Library Factor = -3273

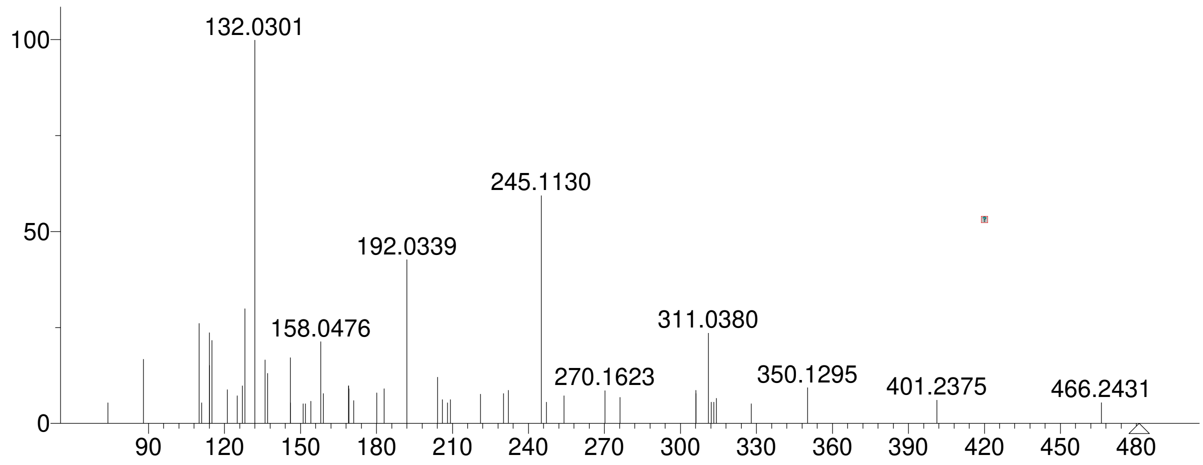

Hit 1 : Villol [M+K]+ 65HCD P=481.1
C23H22O9; MF: 1; RMF: 56; Prob 12.9%; Lib: mona-export-vf-npl_qexactive; ID: 8049.

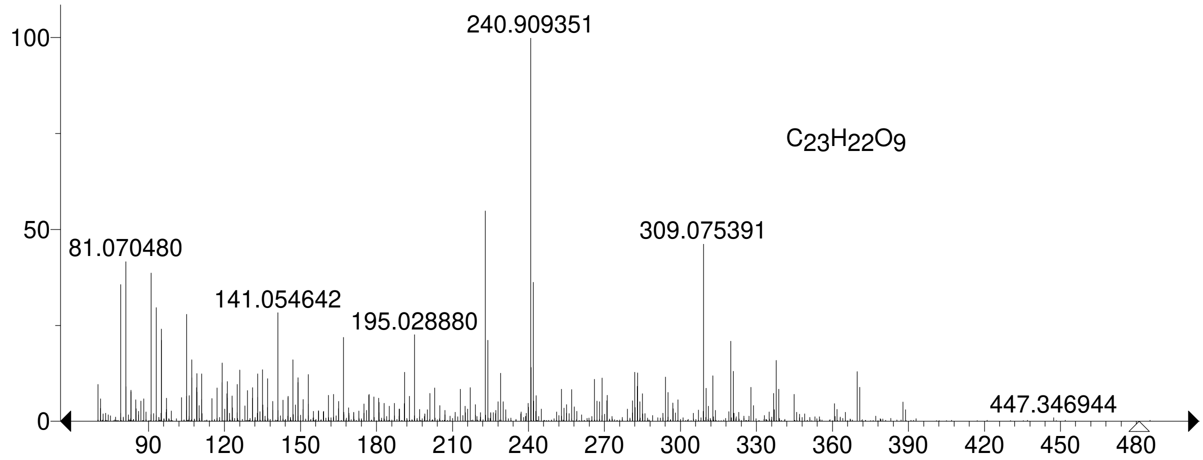

Hit 2 : Gossypin [M+H]+ HCD 77V P=481.1
C21H20O13; MF: 1; RMF: 38; Prob 12.9%; CAS: 652-78-8; Lib: nist_msms; ID: 111982.

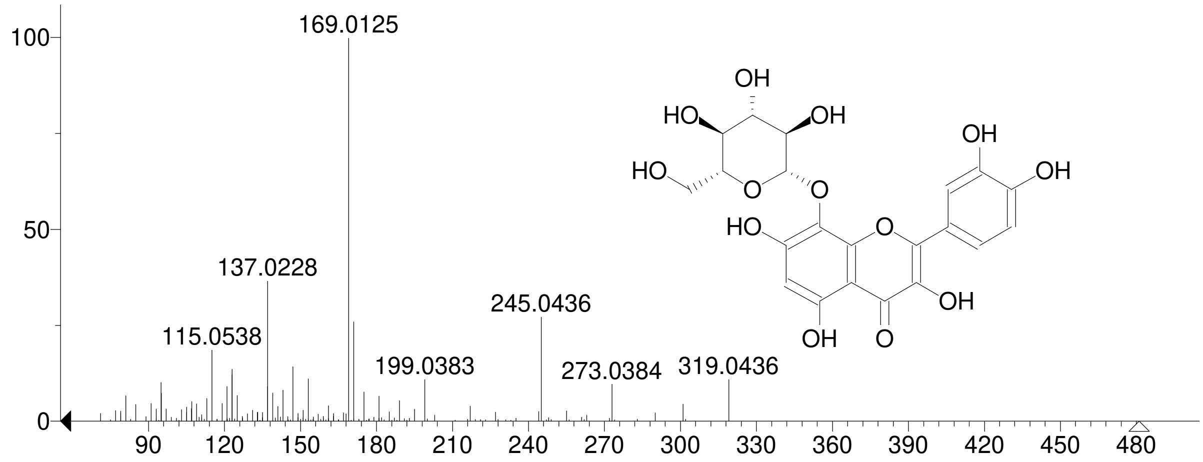

Hit 3 : Delphinidin 3-galactoside cation [Cat-H+OH]- HCD 72V P=481.1
C21H21O12; MF: 0; RMF: 45; Prob 12.4%; CAS: 197250-28-5; Lib: nist_msms; ID: 394731.

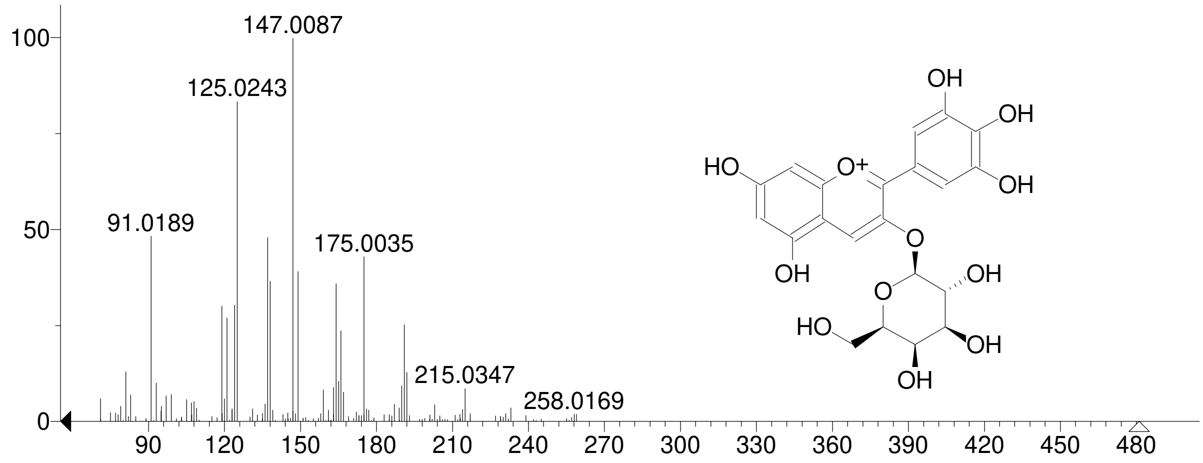

Hit 4 : Quercetagetin-7-O-glucoside [M+H]+ HCD 86V P=481.1
C21H20O13; MF: 0; RMF: 21; Prob 12.4%; CAS: 548-75-4; Lib: nist_msms; ID: 108302.

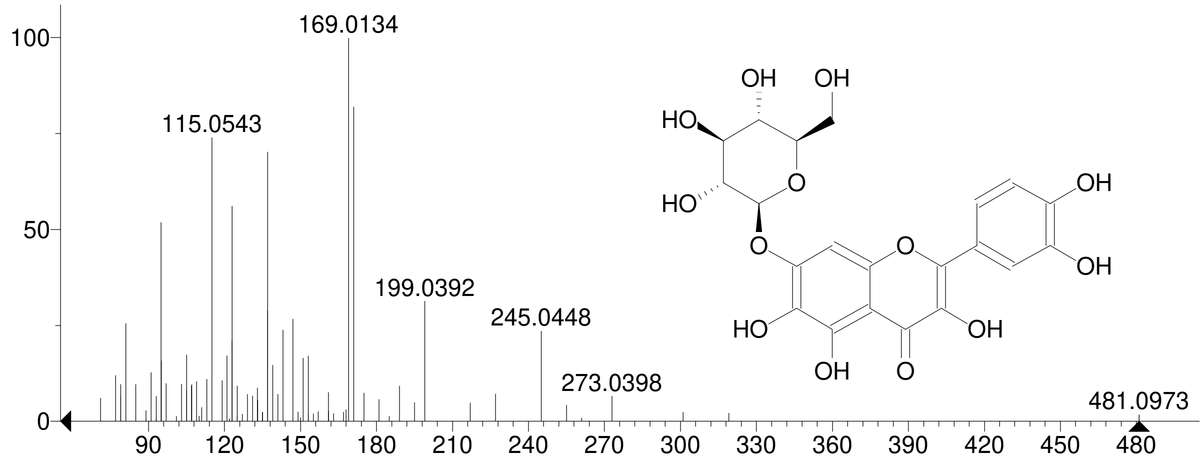

Hit 5 : Gossypetin-8-C-glucoside [M+H]+ 30eV P=481.1
C21H20O13; MF: 0; RMF: 17; Prob 12.4%; Lib: anthocyanins mona; ID: 673.

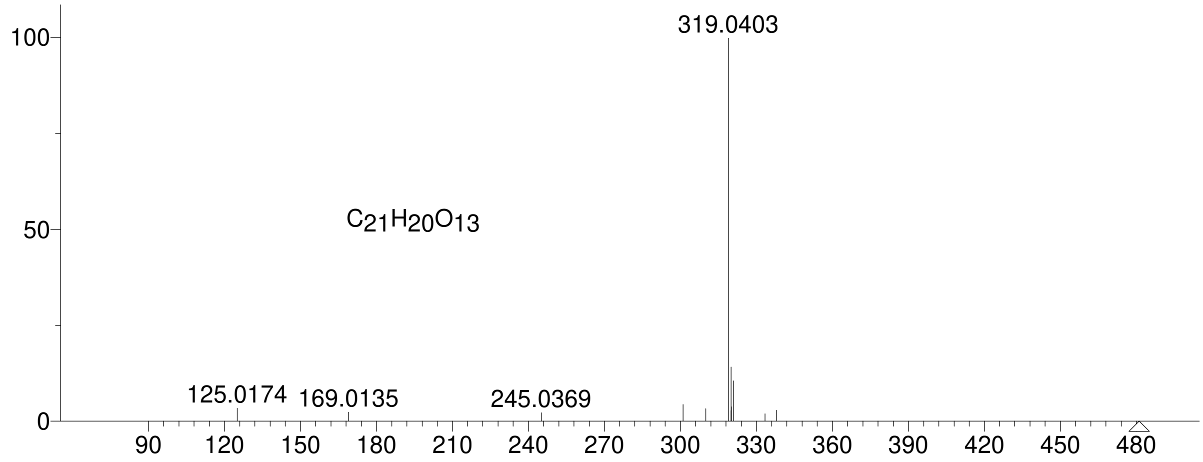

** Search Report Page 1 of 1 **

Unknown: Unknown (4.68_527.1880m/z)
Compound in Library Factor = 0

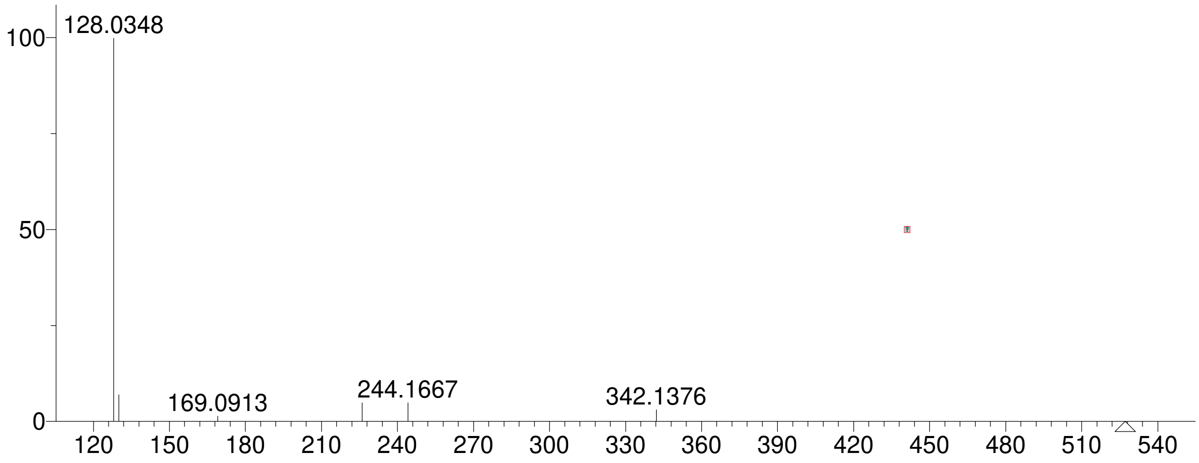

Unknown: Unknown (4.69_492.2552m/z)
Compound in Library Factor = 0

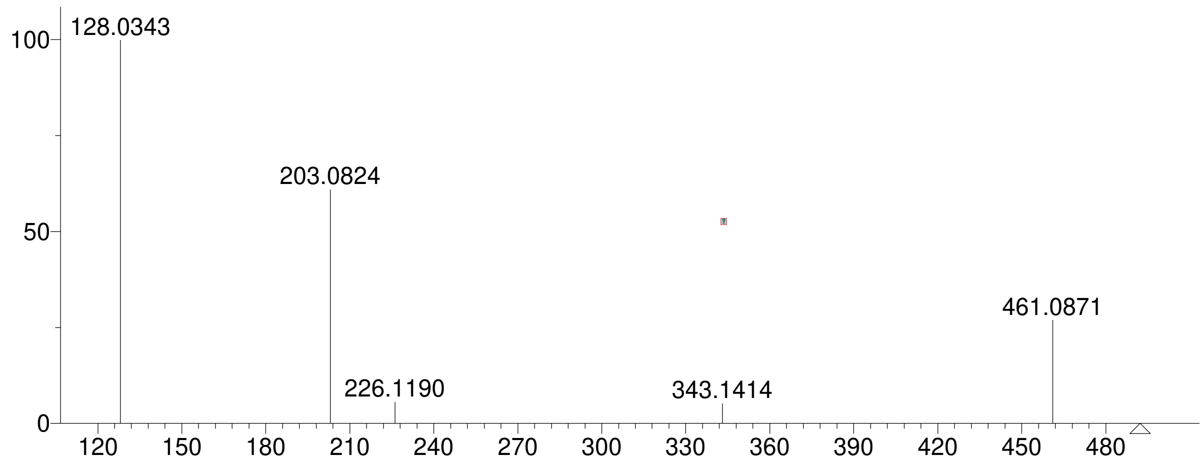

Hit 1 : Imatinib [M-H]- 120(no P=492.3
C29H31N7O; MF: 0; RMF: 119; Prob 100.0%; Lib: mona-export-lc-ms-ms_negative_mode; ID: 4786.

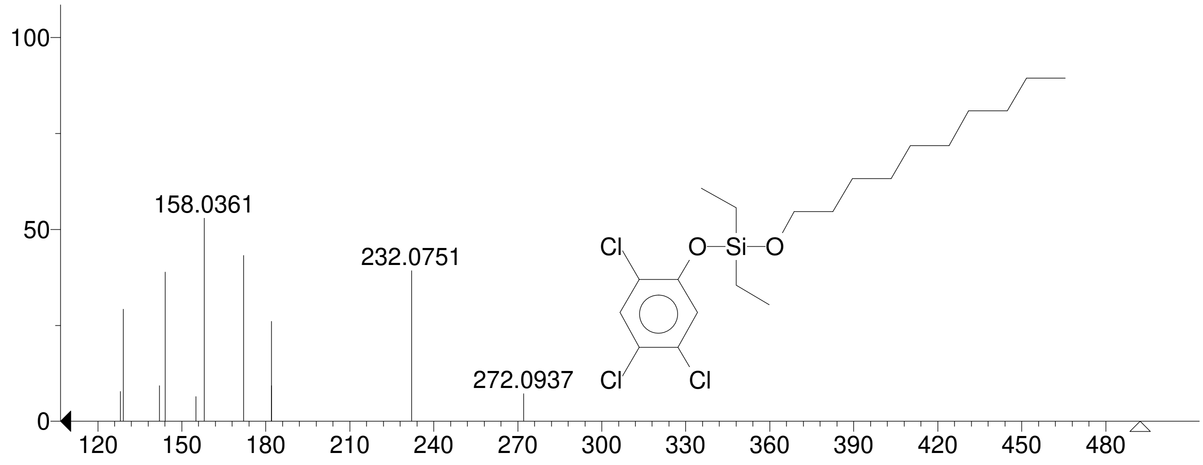

Unknown: Unknown (4.69_594.1938m/z)
Compound in Library Factor = 0

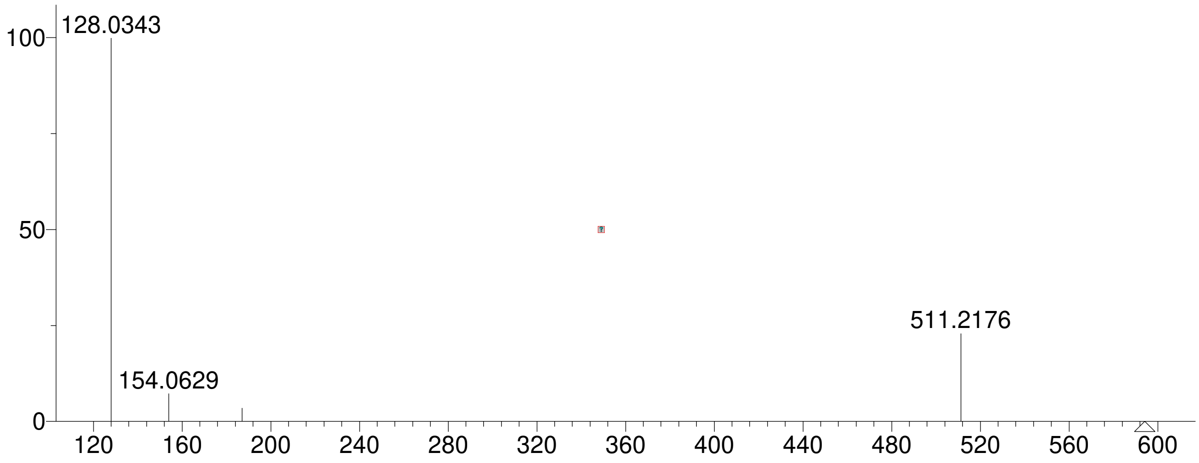

Unknown: Unknown (4.70_387.1743m/z)
Compound in Library Factor = -300

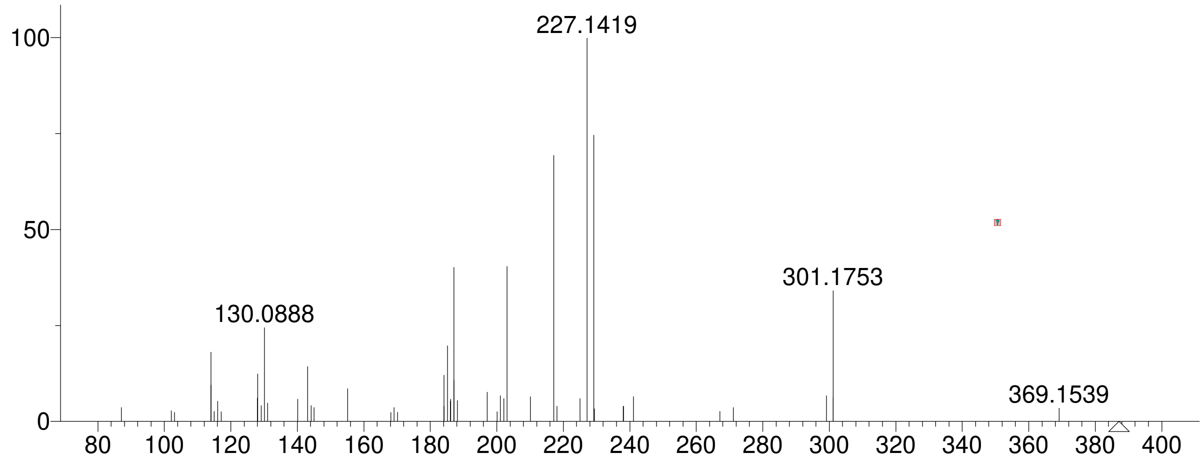

Hit 1 : Oridonin [M+Na]+ IT-FT 35% P=387.2
C20H28O6; MF: 243; RMF: 929; Prob 65.1%; CAS: 28957-04-2; Lib: nist_msms; ID: 238379.

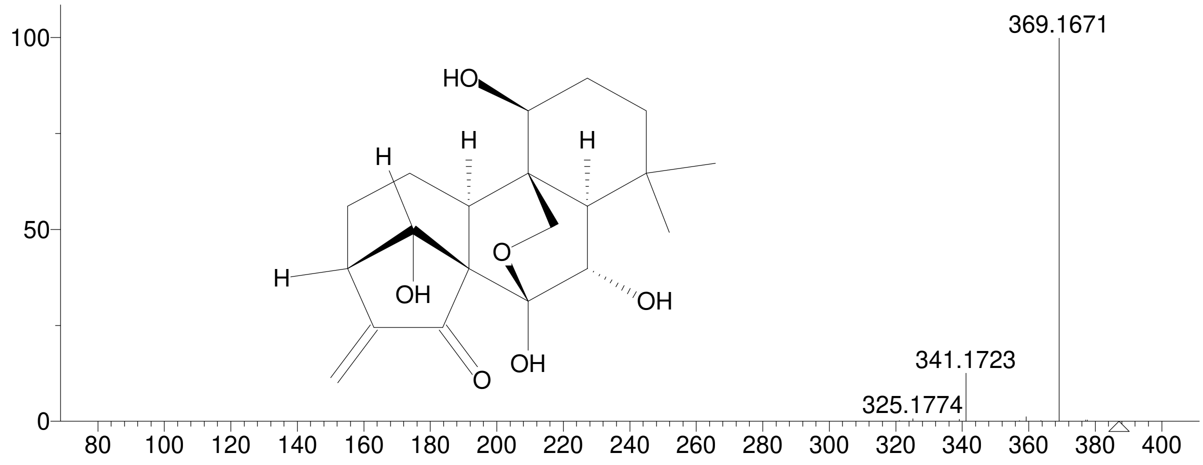

Hit 2 : Rubescensin A [M+Na]+ IT-FT 35% P=387.2
C20H28O6; MF: 225; RMF: 924; Prob 33.6%; CAS: 28957-10-0; Lib: nist_msms; ID: 401360.

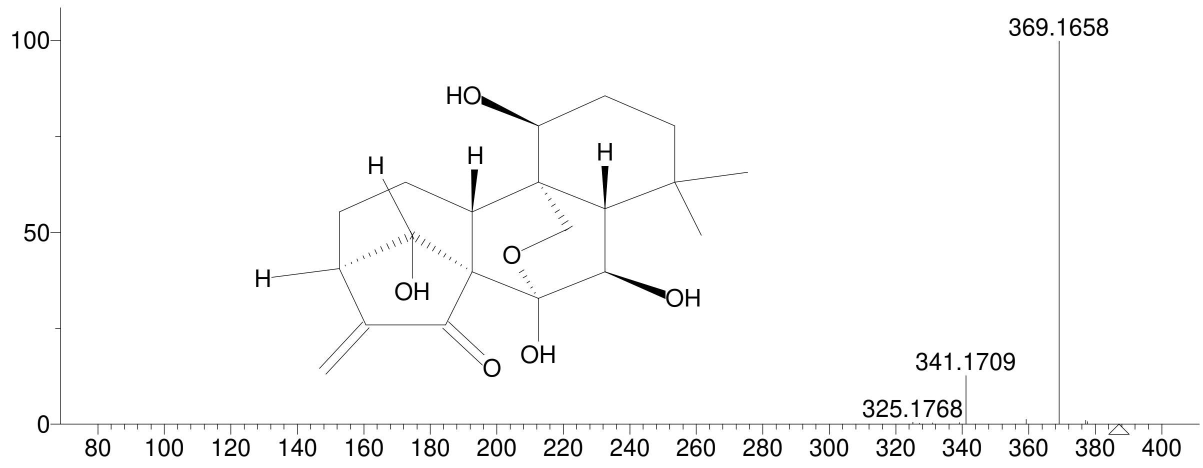

Hit 3 : Neurotensin (1-6) [M-2H]2- HCD 45eV P=387.2
C35H52N8O12; MF: 2; RMF: 54; Prob 0.33%; Lib: nist_msms2; ID: 74179.

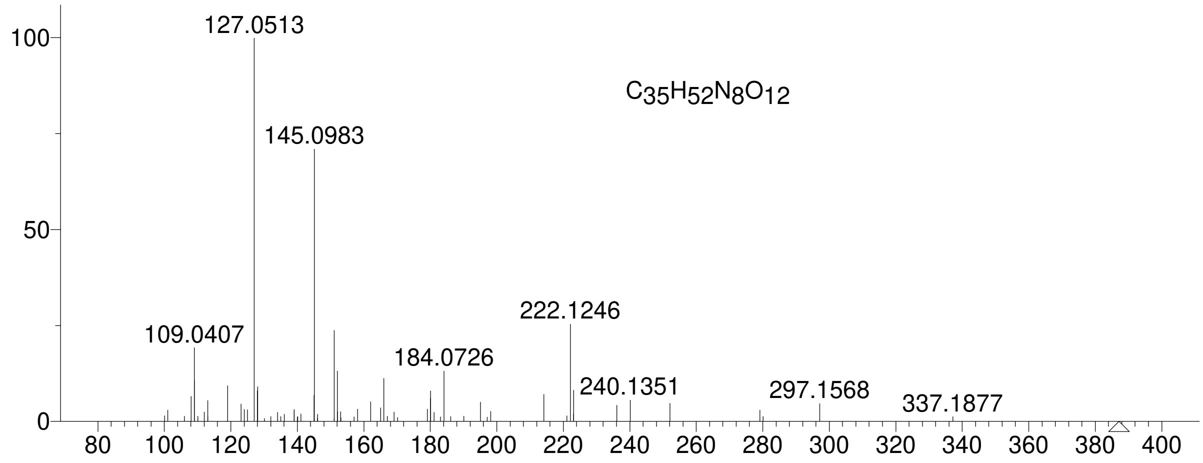

Hit 4 : N6-2-(4-Aminophenyl)ethyladenosine [M+H]+ HCD 85V P=387.2
C18H22N6O4; MF: 0; RMF: 26; Prob 0.30%; CAS: 89705-21-5; Lib: nist_msms; ID: 298733.

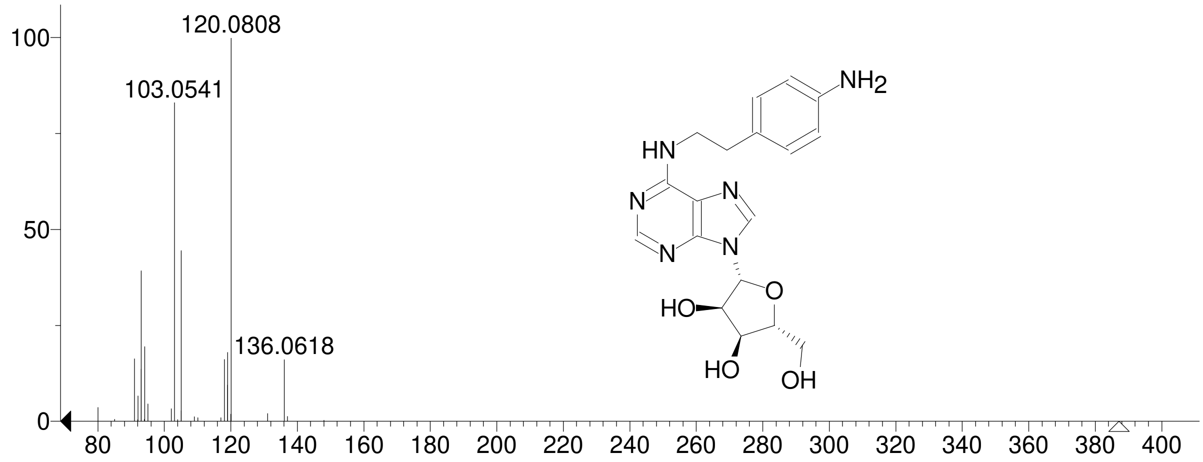

Hit 5 : NCGC00347508-02!4-[2-[[2,3-dihydroxy-2-(1-hydroxyethyl)butanoyl]oxymethyl]anilino]-4-oxobutanoic acid
C17H23NO8; MF: 0; RMF: 16; Prob 0.30%; Lib: mona-export-gnps; ID: 6723.

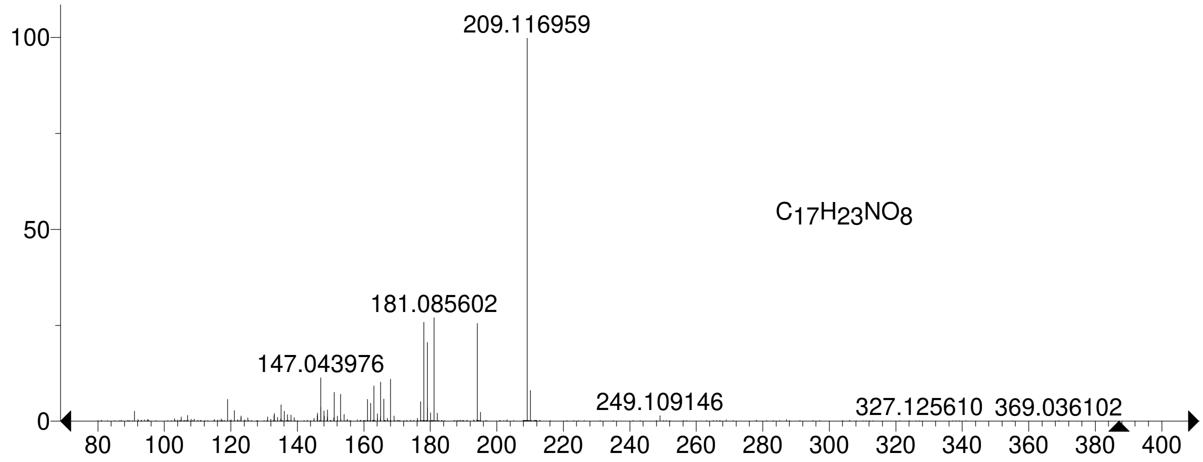

Unknown: Unknown (4.70_483.1620m/z)
Compound in Library Factor = 0

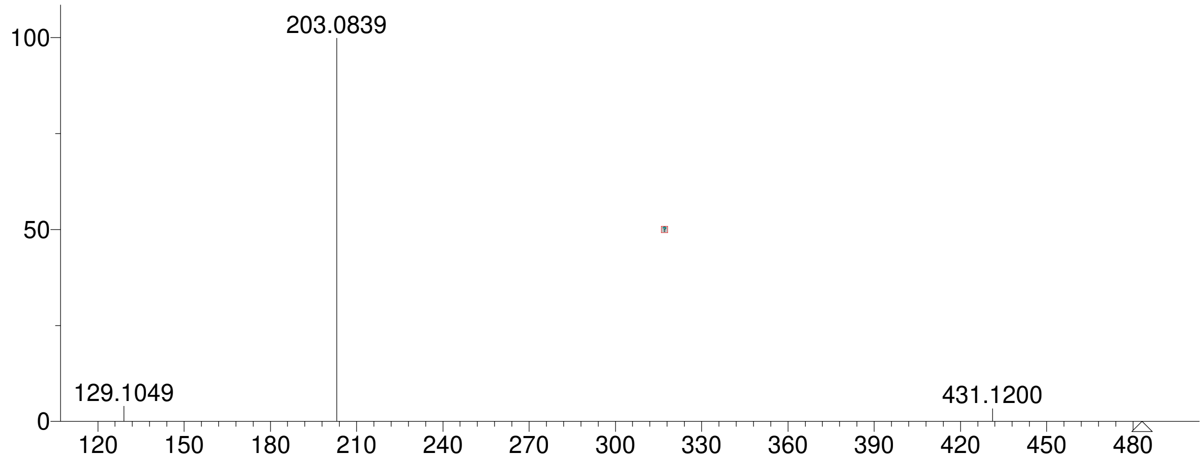

Hit 1 : [4,5,8-trihydroxy-5-(hydroxymethyl)-3-(3-methoxy-3-oxoprop-1-en-2-yl)-8a-methyl-1,2,3,4,4a,6,7,8-octahyd
C21H32O10; MF: 0; RMF: 37; Prob 100.0%; Lib: mona-export-vf-npl_qexactive; ID: 22398.

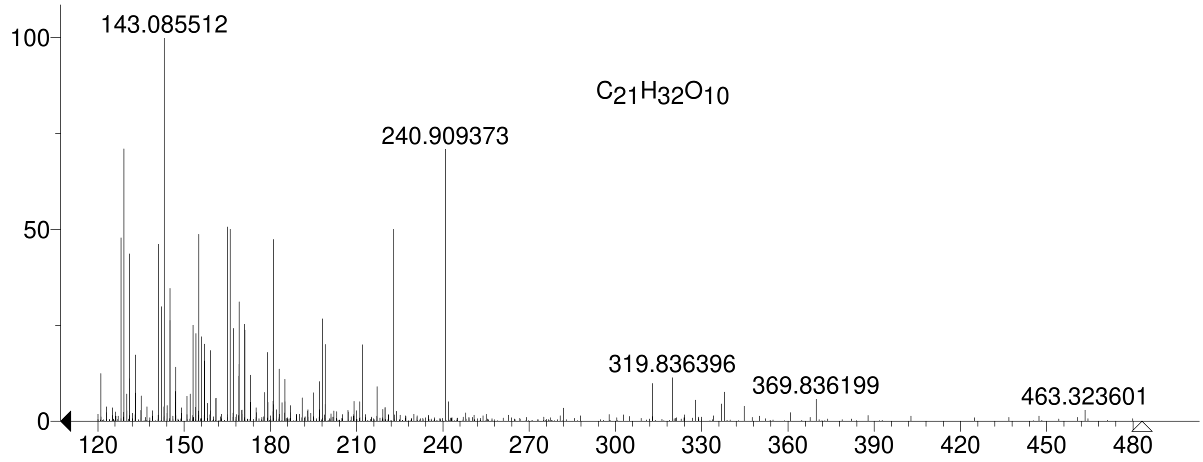

Unknown: Unknown (4.71_402.1165m/z)
Compound in Library Factor = 0

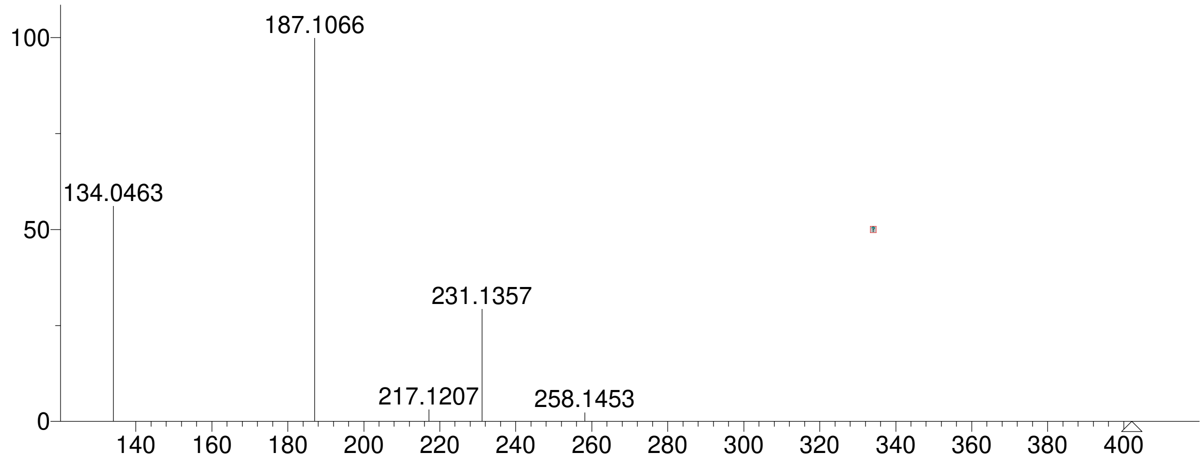

Unknown: Unknown (4.71_429.1528m/z)
Compound in Library Factor = 0

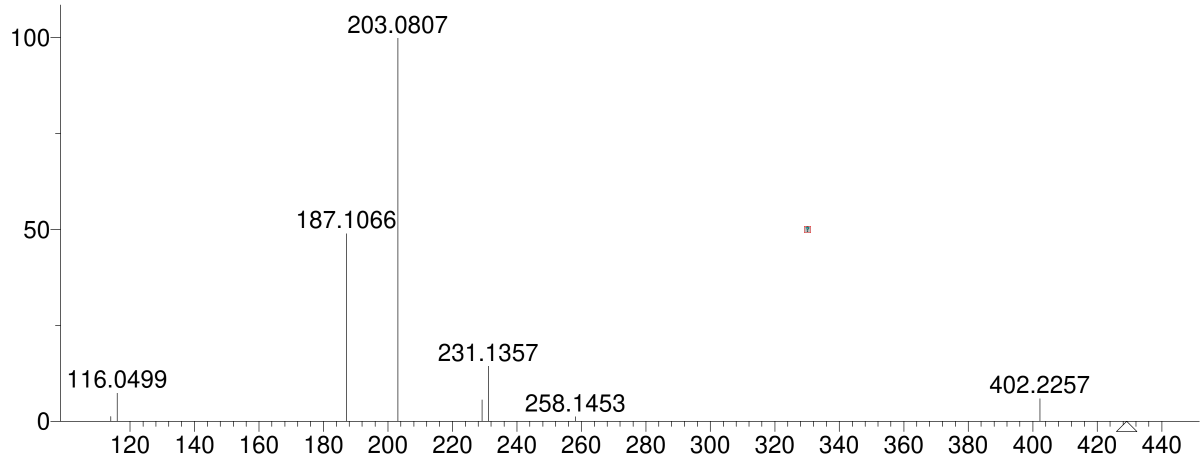

Hit 1 : 1',2'-dihydro-2',6-dihydroxyrotenone [M+H]+ 45HCD P=429.2
C23H24O8; MF: 0; RMF: 159; Prob 25.0%; Lib: mona-export-vf-npl_qexactive; ID: 4154.

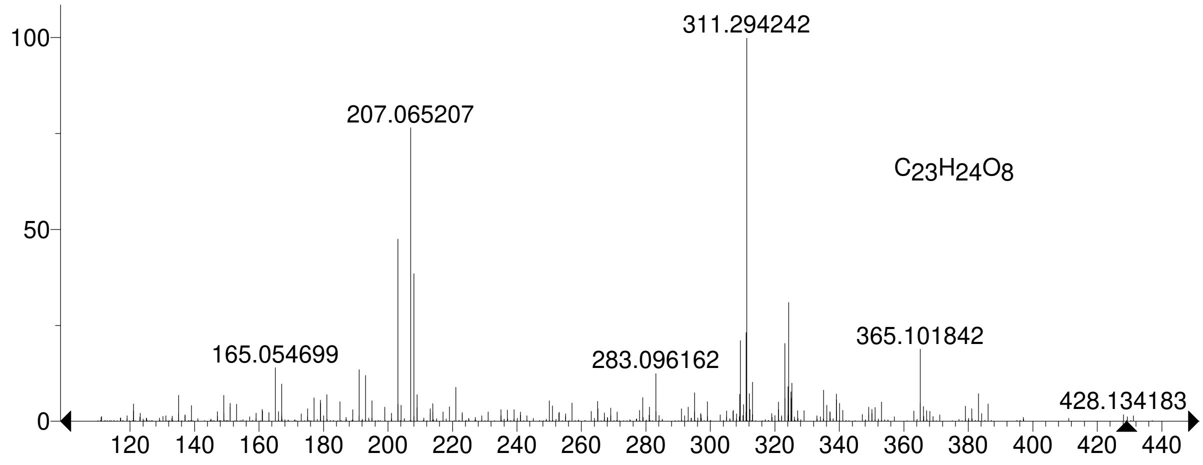

Hit 2 : Wortmannin [M+H]+ HCD 64V P=429.2
C23H24O8; MF: 0; RMF: 66; Prob 25.0%; CAS: 19545-26-7; Lib: nist_msms; ID: 168348.

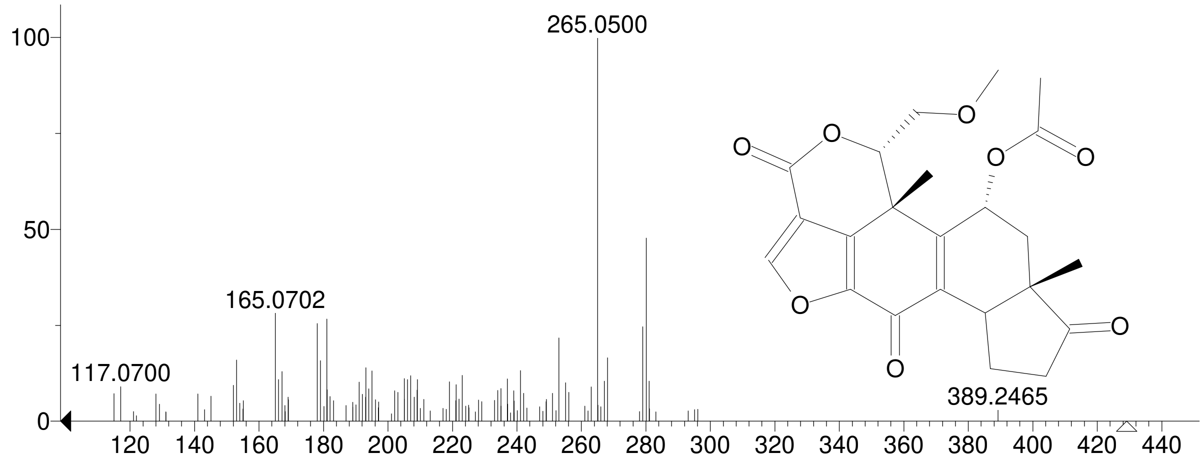

Hit 3 : 2,3-Dinorfluprostenol [M-H]- IT 35% P=429.2
C21H25F3O6; MF: 0; RMF: 52; Prob 25.0%; Lib: nist_msms; ID: 88297.

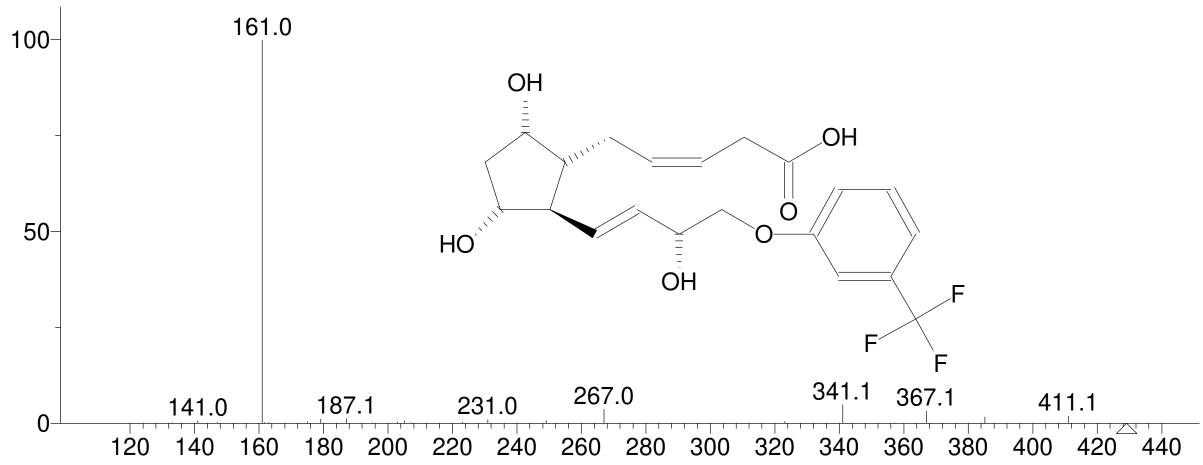

Hit 4 : 9,11,12a-trihydroxy-2,3-dimethoxy-8-(3-methylbut-2-enyl)-6,6a-dihydrochromeno[3,4-b]chromen-12-one [M
C23H24O8; MF: 0; RMF: 39; Prob 25.0%; Lib: mona-export-vf-npl_qexactive; ID: 18380.

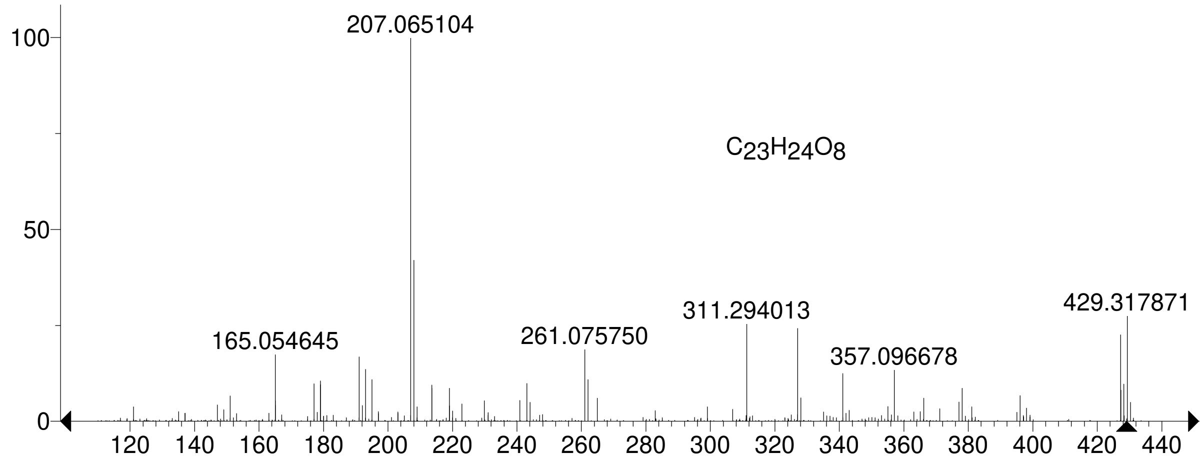

Unknown: Unknown (4.72_203.0817m/z)
Compound in Library Factor = -1323

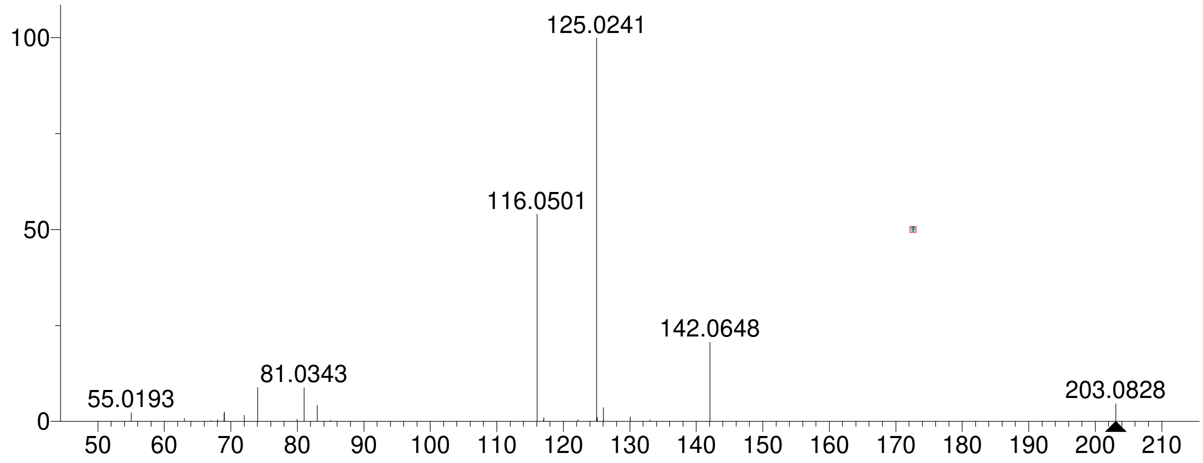

Hit 1 : D-Tryptophan [M-H]- 30 P=203.1
C11H12N2O2; MF: 478; RMF: 627; Prob 43.2%; Lib: msms neg mona_ipb_gnps_plasma; ID: 909.

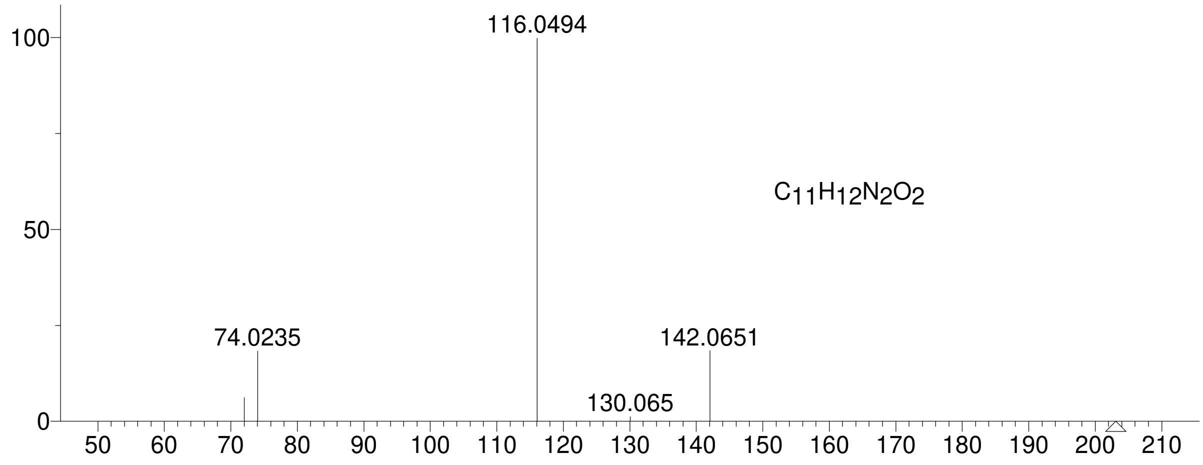

Hit 2 : L-Tryptophan [M-H]- 30 P=203.1
C11H12N2O2; MF: 477; RMF: 625; Prob 41.6%; Lib: msms neg mona_ipb_gnps_plasma; ID: 908.

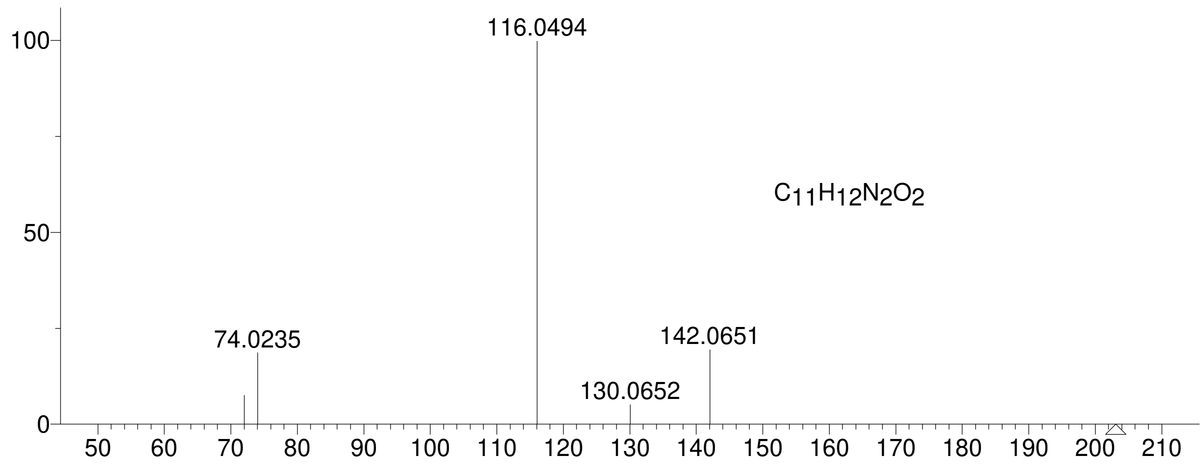

Hit 3 : L-TRYPTOPHAN
C11H12N2O2; MF: 424; RMF: 617; Prob 8.22%; Lib: embl neg mcg; ID: 600.

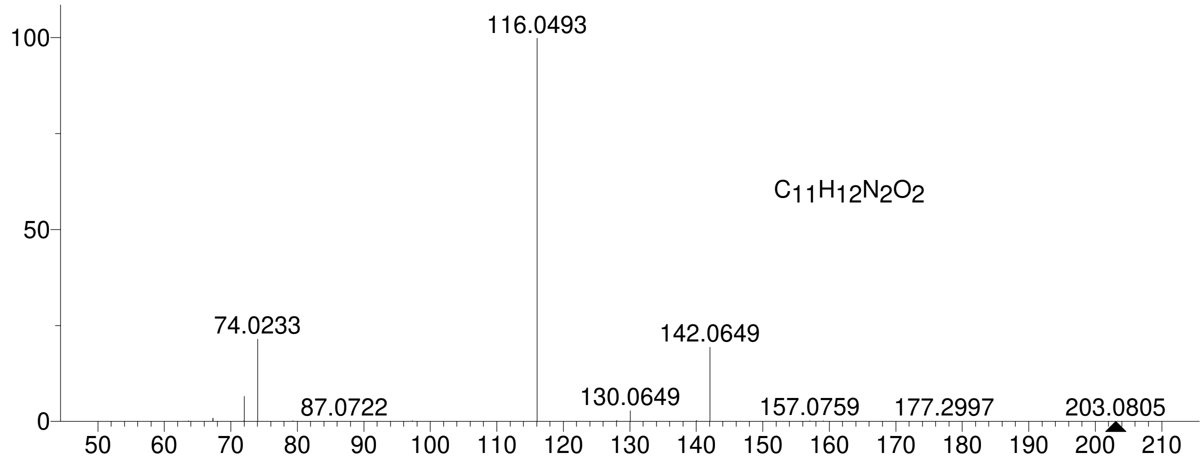

Hit 4 : Tryptophan [M-H]- 95eV P=203.1
C11H12N2O2; MF: 396; RMF: 625; Prob 2.37%; Lib: mona-export-lc-ms-ms_negative_mode; ID: 7650.

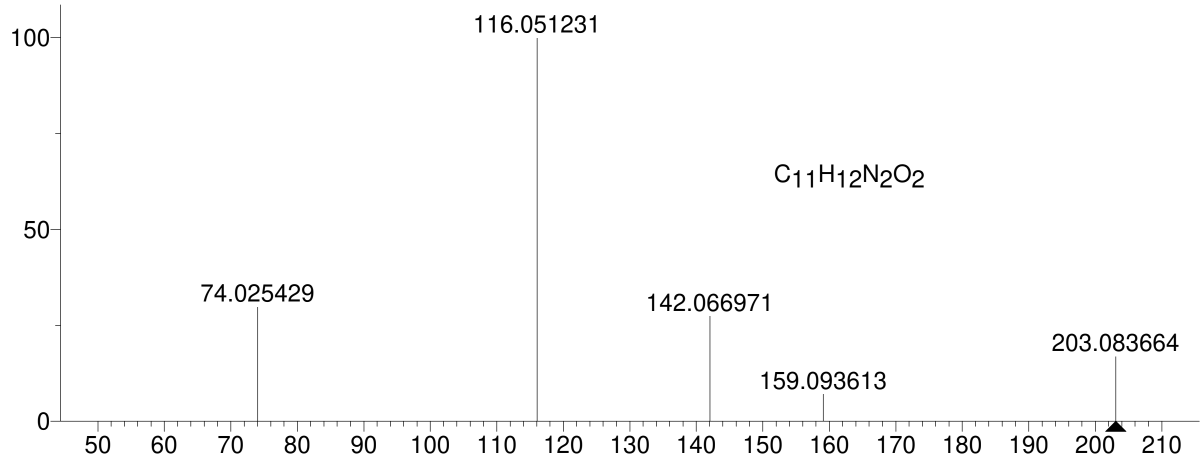

Hit 5 : Tryptophan; LC-ESI-ITFT; MS2; HCD; CE 95.0 eV; [M-H]- [M-H]- 95 P=203.1
C11H12N2O2; MF: 396; RMF: 625; Prob 2.37%; Lib: msms-neg-massbank; ID: 3682.

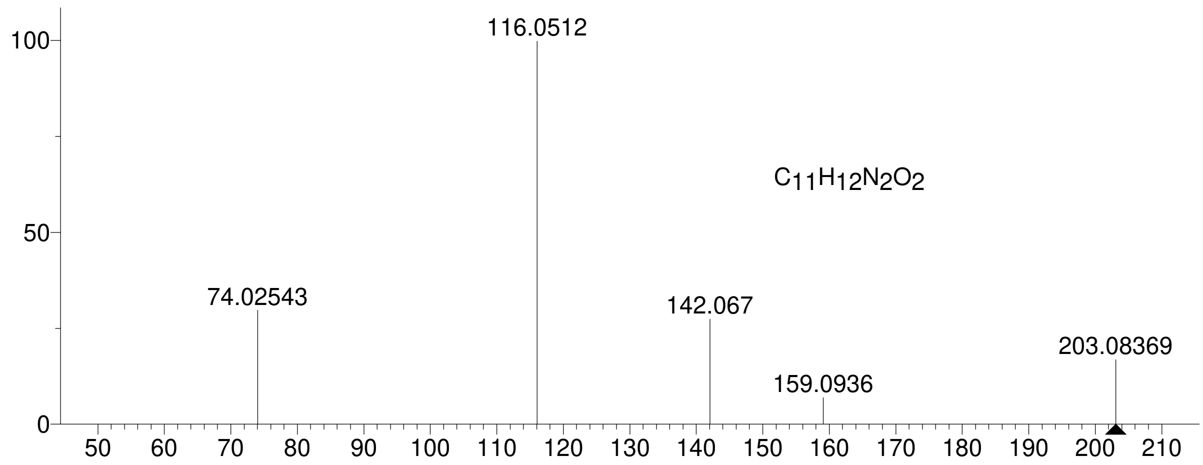

Unknown: Unknown (4.72_401.1912m/z)
Compound in Library Factor = 0

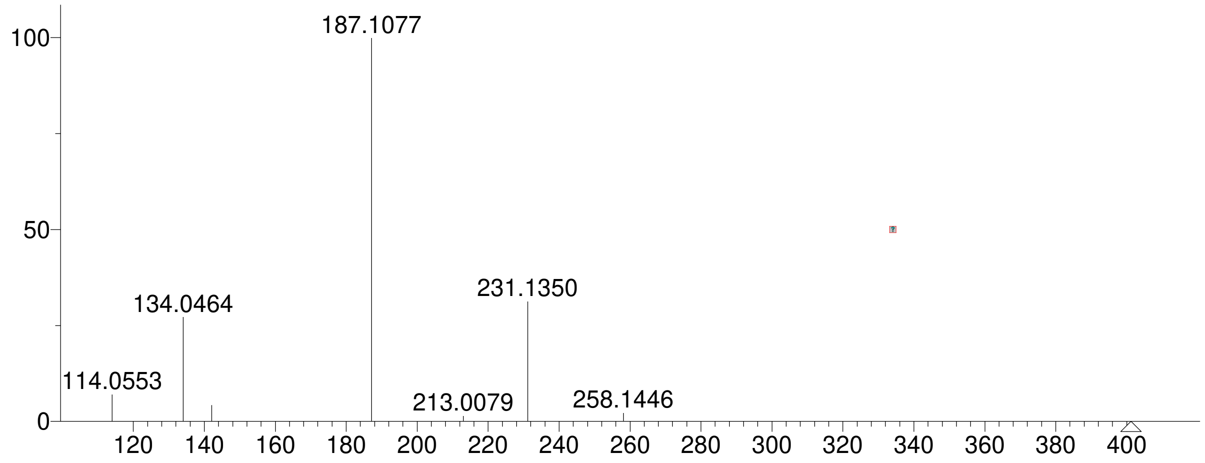

Hit 1 : NCGC00380382-01_C23H30O7_1-Phenanthrenecarboxylic acid, 10-(acetyloxy)-1,2,3,4,4a,9,10,10a-octal
C23H30O7; MF: 0; RMF: 23; Prob 50.0%; Lib: mona-export-gnps; ID: 13719.

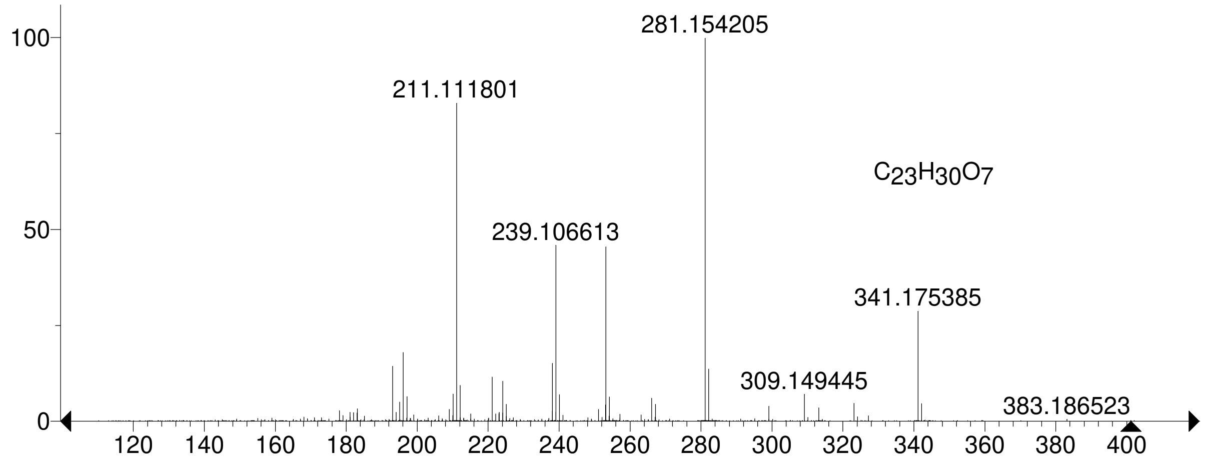

Hit 2 : NCGC00380669-01!2-(2,6-dimethoxy-4-prop-2-enylphenoxy)-1-(3,4,5-trimethoxyphenyl)propan-1-ol [M-H2
C23H30O7; MF: 0; RMF: 10; Prob 50.0%; Lib: mona-export-gnps; ID: 7642.

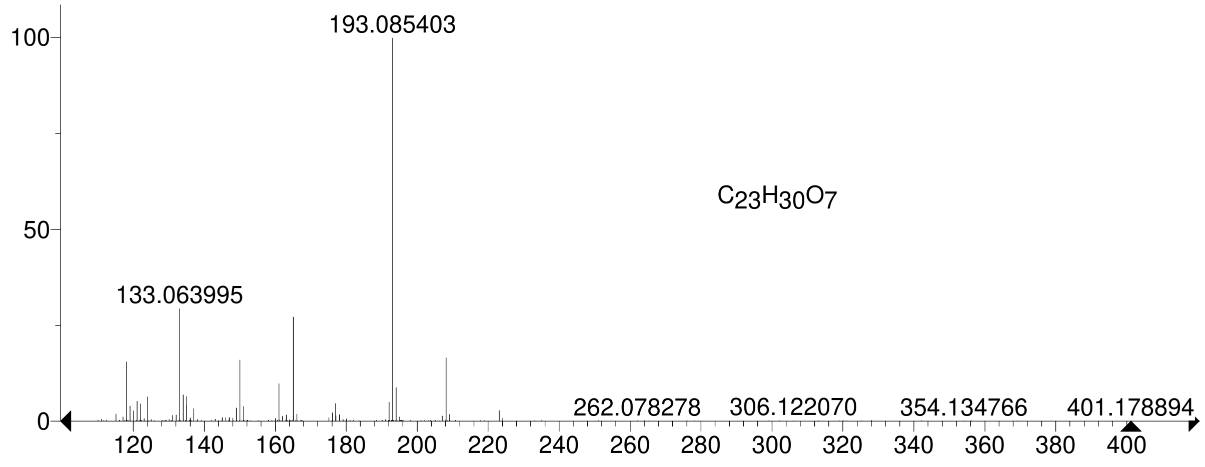

Unknown: Unknown (4.72_428.1992m/z)
Compound in Library Factor = 0

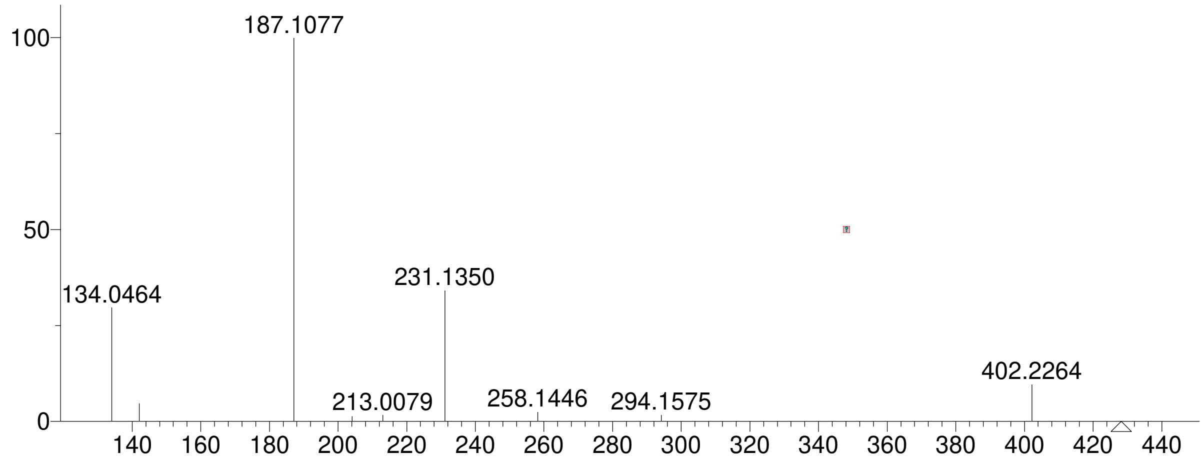

Unknown: Unknown (4.72_438.0635m/z)
Compound in Library Factor = 0

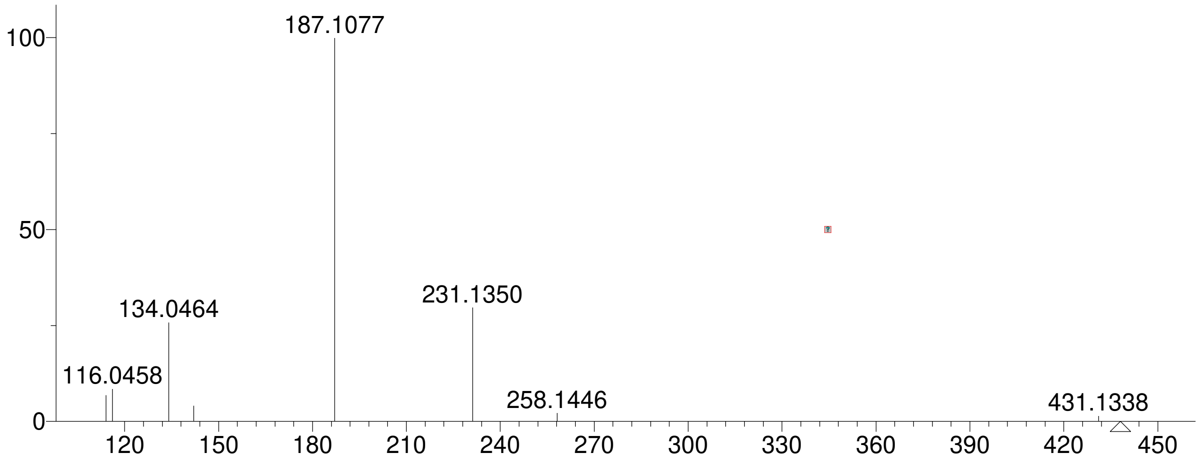

Unknown: Unknown (4.74_516.2068m/z)
Compound in Library Factor = 0

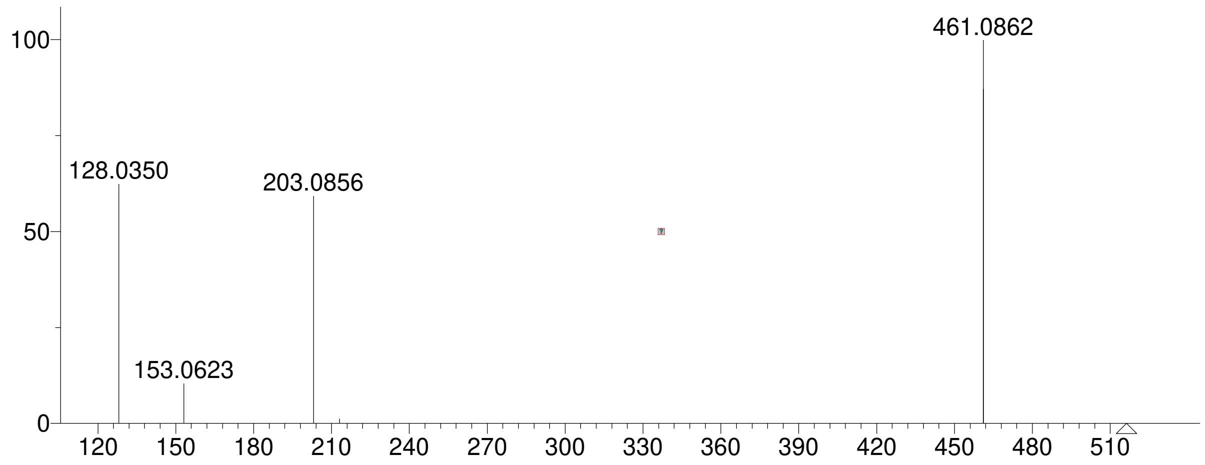

Hit 1 : 3,5-dihydroxy-4-[3,4,5-trihydroxy-6-[[4-(2-hydroxypropan-2-yl)cyclohexene-1-carbonyl]oxymethyl]oxan-2-yl]
C23H30O12; MF: 0; RMF: 5; Prob 50.0%; Lib: mona-export-vf-npl_qexactive; ID: 19776.

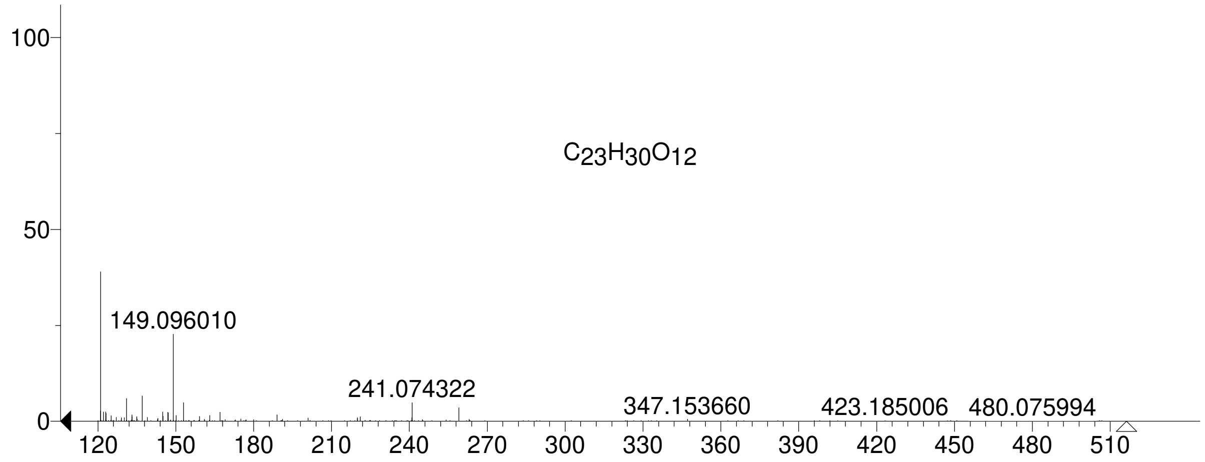

Hit 2 : 3,4-dihydroxy-5-[(2S,3R,4S,5S,6R)-3,4,5-trihydroxy-6-[[4-(2-hydroxypropan-2-yl)cyclohexene-1-carbonyl]ox
C23H30O12; MF: 0; RMF: 2; Prob 50.0%; Lib: mona-export-vf-npl_qexactive; ID: 24841.

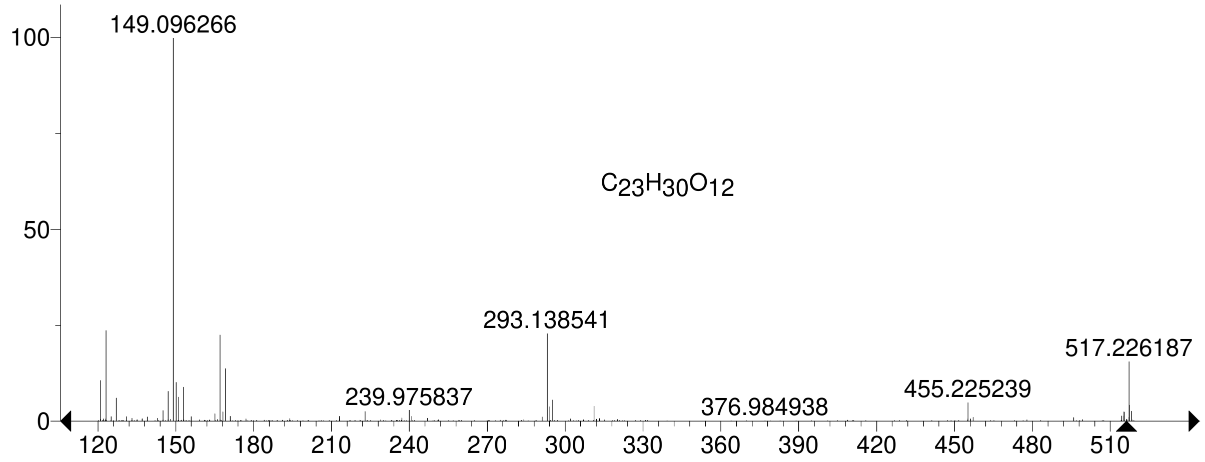

** Search Report Page 1 of 1 **

Unknown: Unknown (4.76_391.2063m/z)
Compound in Library Factor = 0

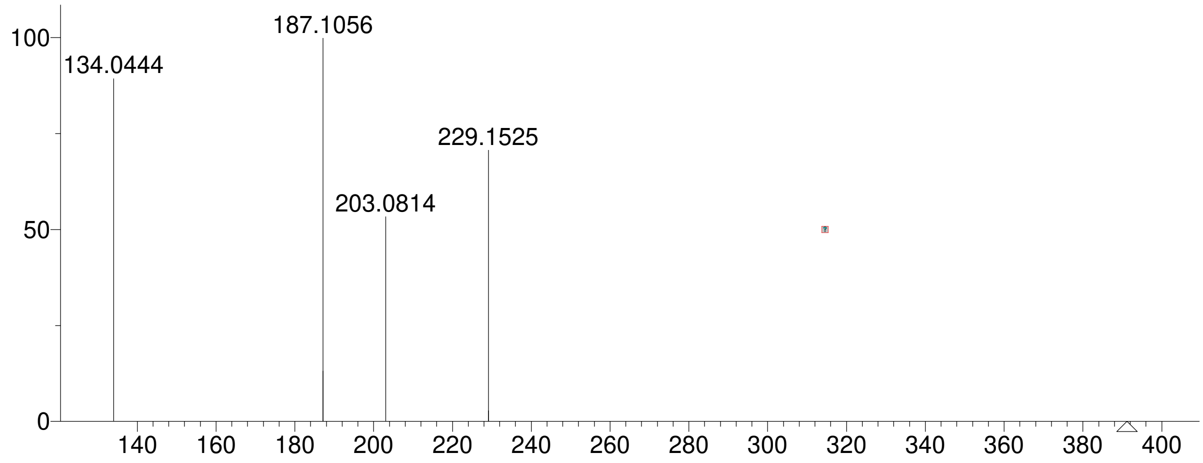

Hit 1 : Prostaglandin G2 [M+Na]+ HCD 39V P=391.2
C20H32O6; MF: 0; RMF: 19; Prob 100.0%; CAS: 51982-36-6; Lib: nist_msms; ID: 88305.

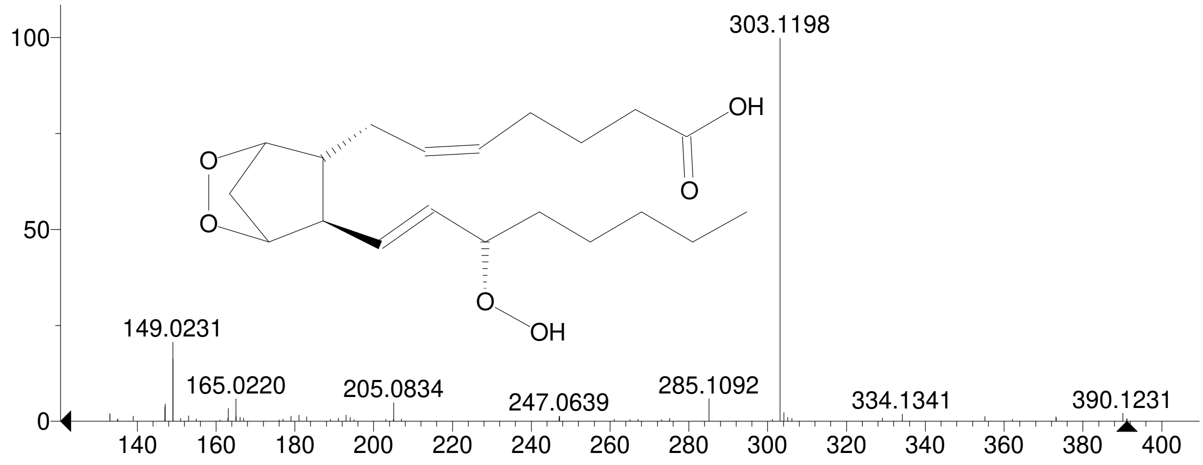

Unknown: Unknown (4.76_455.2017m/z)
Compound in Library Factor = 0

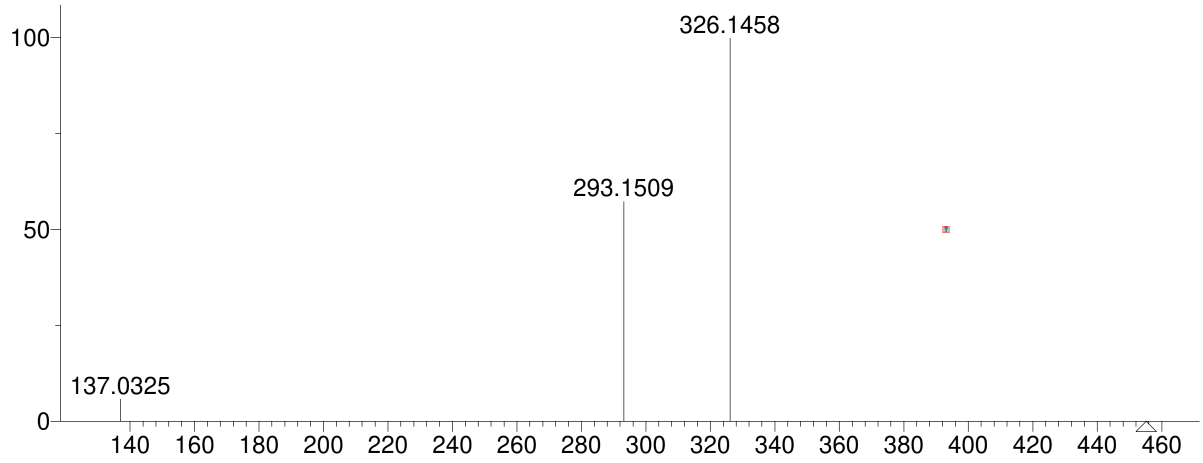

Hit 1 : 17-Trifluoromethylphenyltrinorprostaglandin F2α [M-H]- HCD 43V P=455.2
C24H31F3O5; MF: 0; RMF: 12; Prob 33.3%; CAS: 221246-34-0; Lib: nist_msms; ID: 93944.

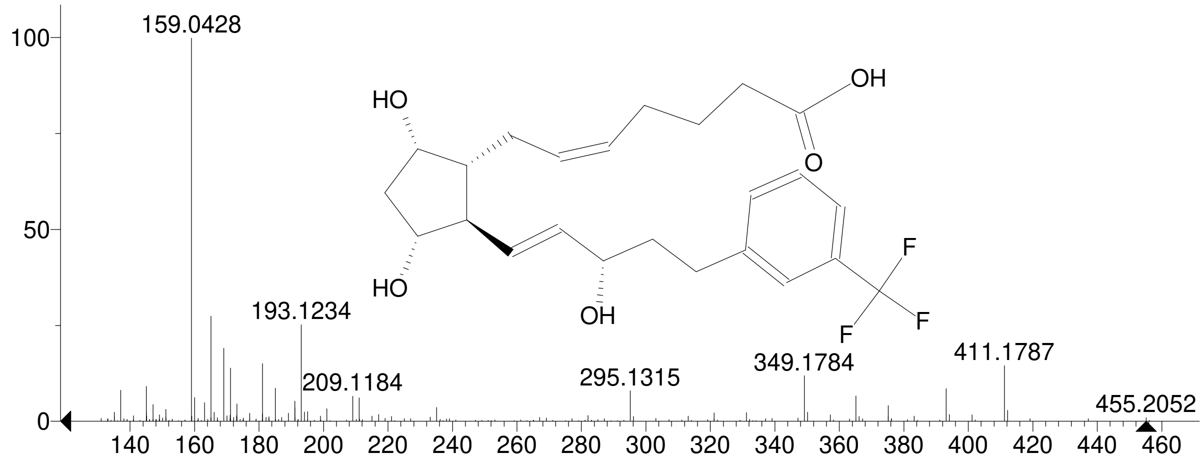

Hit 2 : Halcinonide [M+H]+ IT-FT 35% P=455.2
C24H32ClFO5; MF: 0; RMF: 8; Prob 33.3%; CAS: 3093-35-4; Lib: nist_msms; ID: 214403.

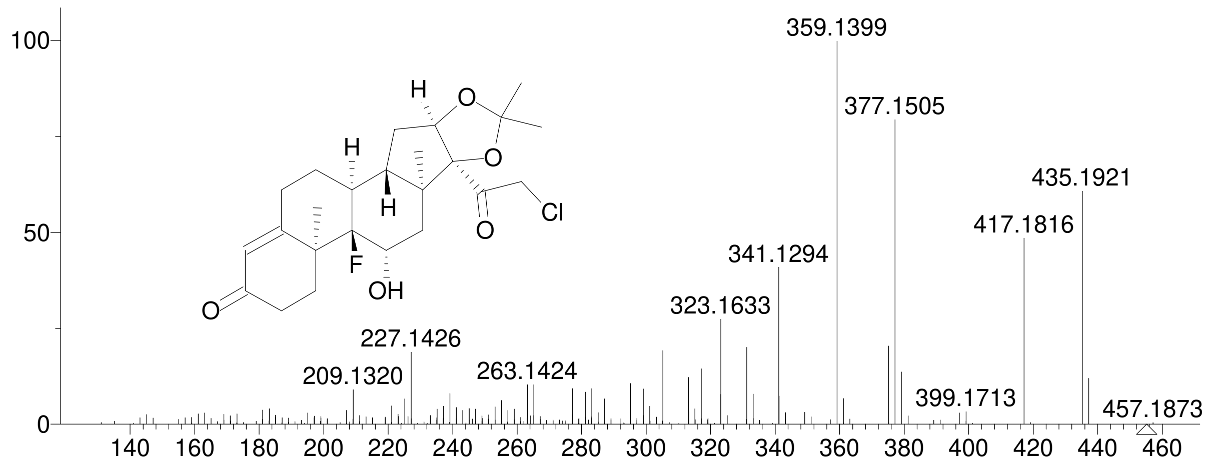

Hit 3 : 5-hydroxy-2,2,6,6-tetramethyl-4-[2-methyl-1-[2,4,6-trihydroxy-3-(2-methylpropanoyl)phenyl]propyl]cyclohex
C24H32O7; MF: 0; RMF: 3; Prob 33.3%; Lib: mona-export-vf-npl_qexactive; ID: 19755.

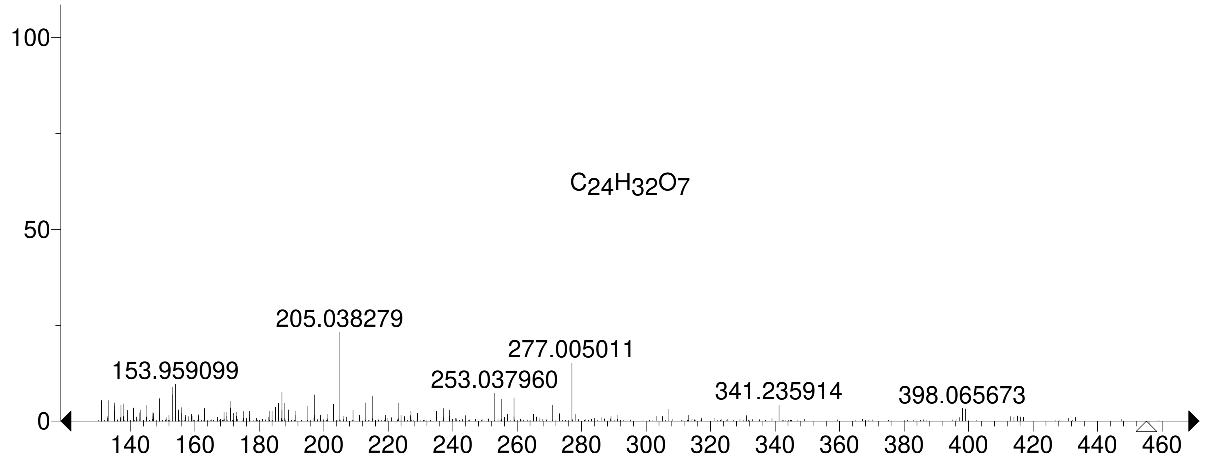

Unknown: Unknown (4.77_496.2037m/z)
Compound in Library Factor = -3273

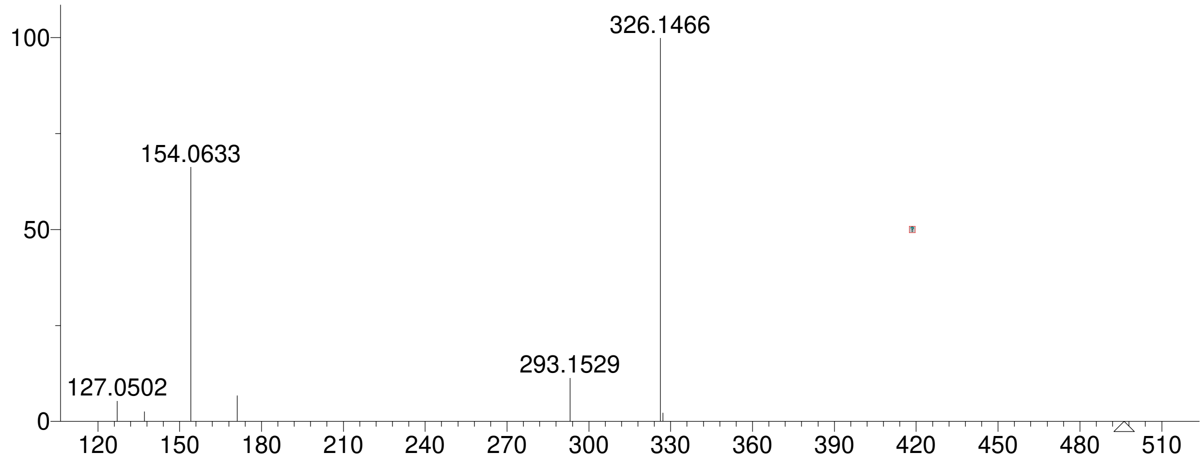

Hit 1 : (2R,3S,4S,5R,6S)-2-[(3,4,5-trihydroxyoxan-2-yl)oxymethyl]-6-(3,4,5-trimethoxyphenoxy)oxane-3,4,5-triol [M
C20H30O13; MF: 1; RMF: 259; Prob 25.5%; Lib: mona-export-vf-npl_qexactive; ID: 9258.

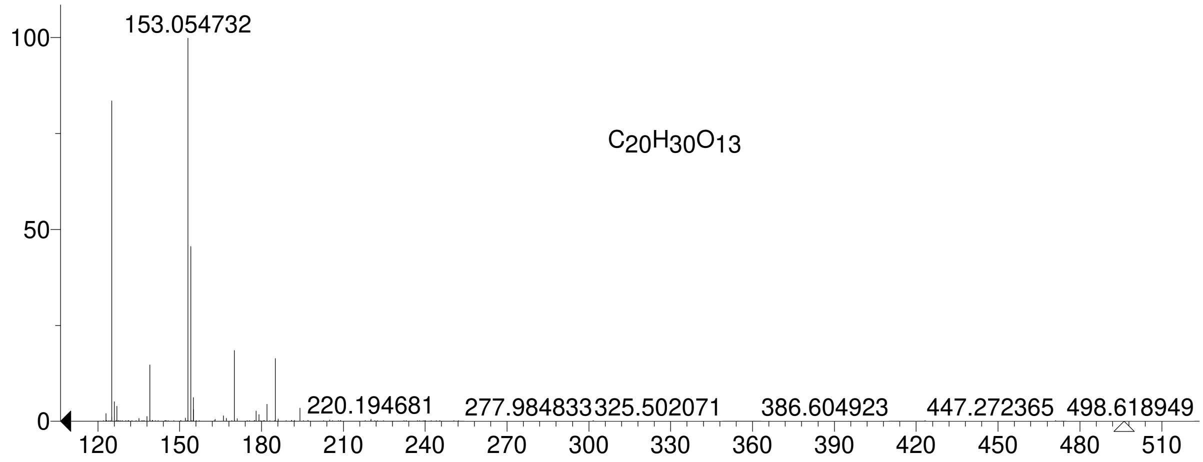

Hit 2 : (2R,3S,4S,5R,6S)-2-[[(2S,3R,4R)-3,4-dihydroxy-4-(hydroxymethyl)oxolan-2-yl]oxymethyl]-6-(3,4,5-trimetho
C20H30O13; MF: 1; RMF: 236; Prob 25.5%; Lib: mona-export-vf-npl_qexactive; ID: 9783.

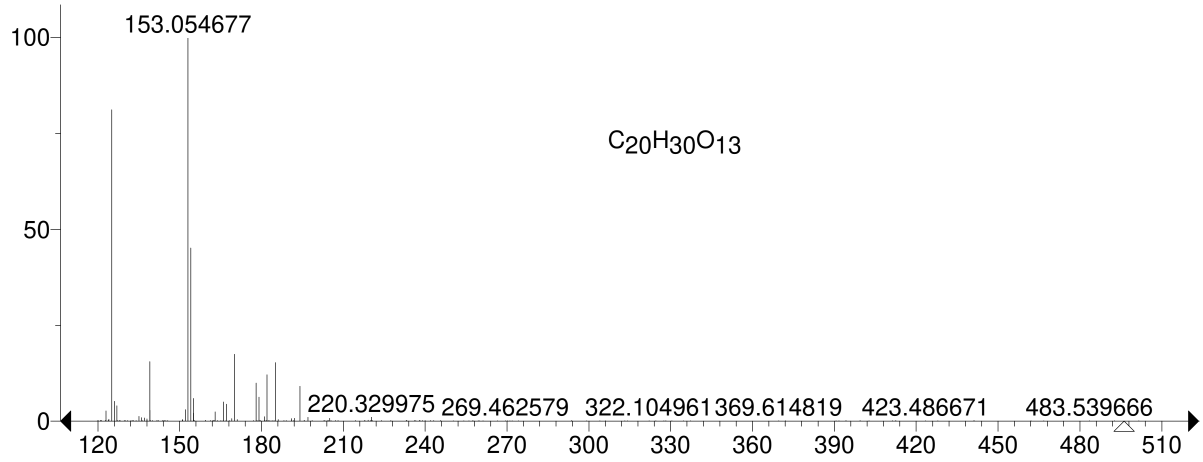

Hit 3 : NCGC00180025-03!(2R,3S,4S,5R,6S)-2-[[(2S,3R,4R)-3,4-dihydroxy-4-(hydroxymethyl)oxolan-2-yl]oxymetl
C20H30O13; MF: 0; RMF: 156; Prob 24.5%; Lib: mona-export-gnps; ID: 8565.

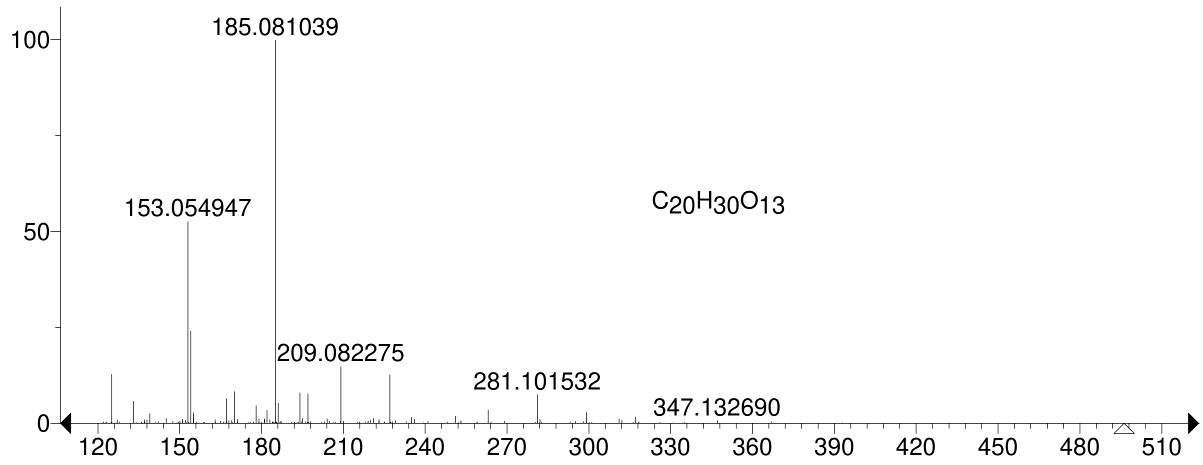

Hit 4 : NCGC00180666-02!(2R,3S,4S,5R,6S)-2-[(3,4,5-trihydroxyoxan-2-yl)oxymethyl]-6-(3,4,5-trimethoxyphenox
C20H30O13; MF: 0; RMF: 153; Prob 24.5%; Lib: mona-export-gnps; ID: 8997.

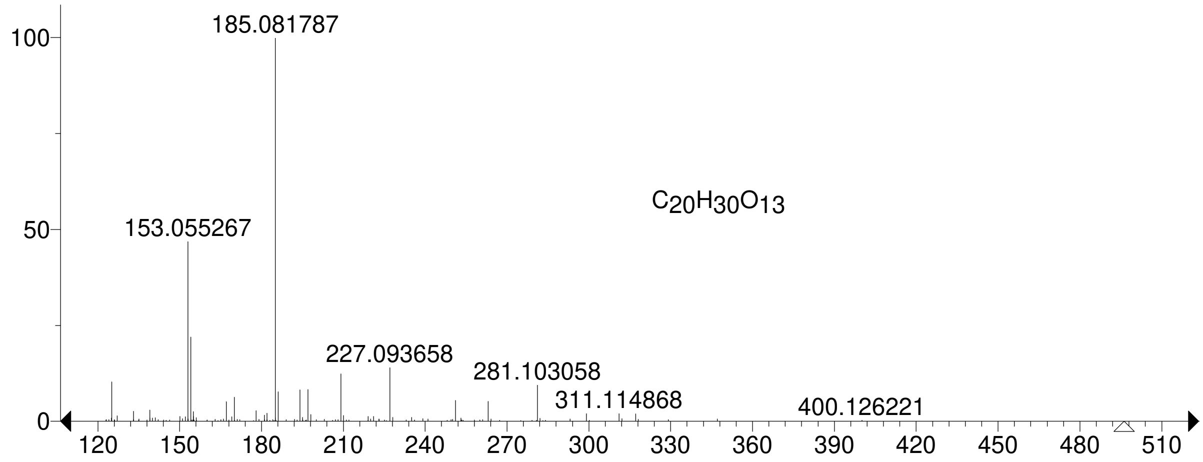

Unknown: Unknown (4.78_293.1493m/z)
Compound in Library Factor = -1453

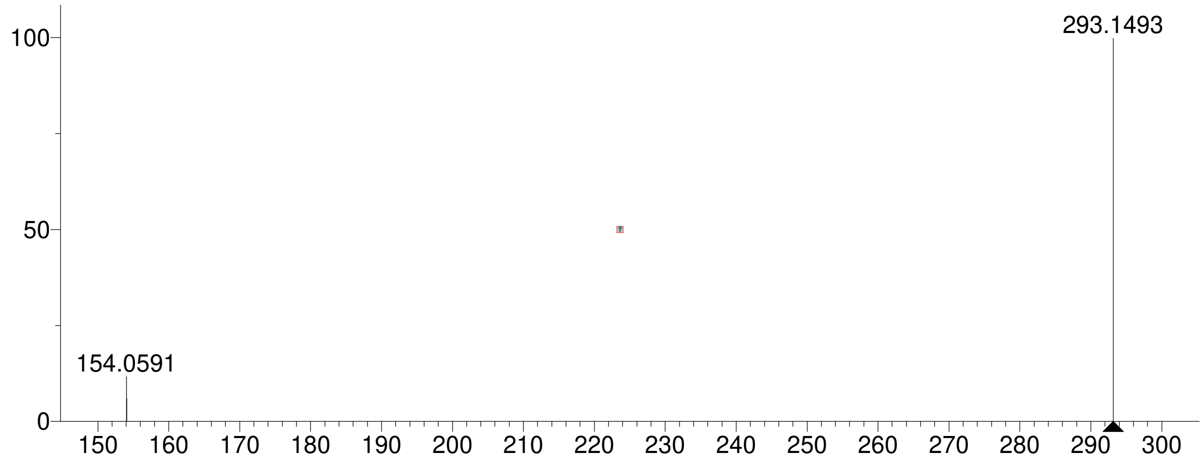

Hit 1 : Leu-Tyr [M-H]- HCD 14eV P=293.2
C15H22N2O4; MF: 238; RMF: 901; Prob 23.8%; Lib: nist_msms; ID: 567554.

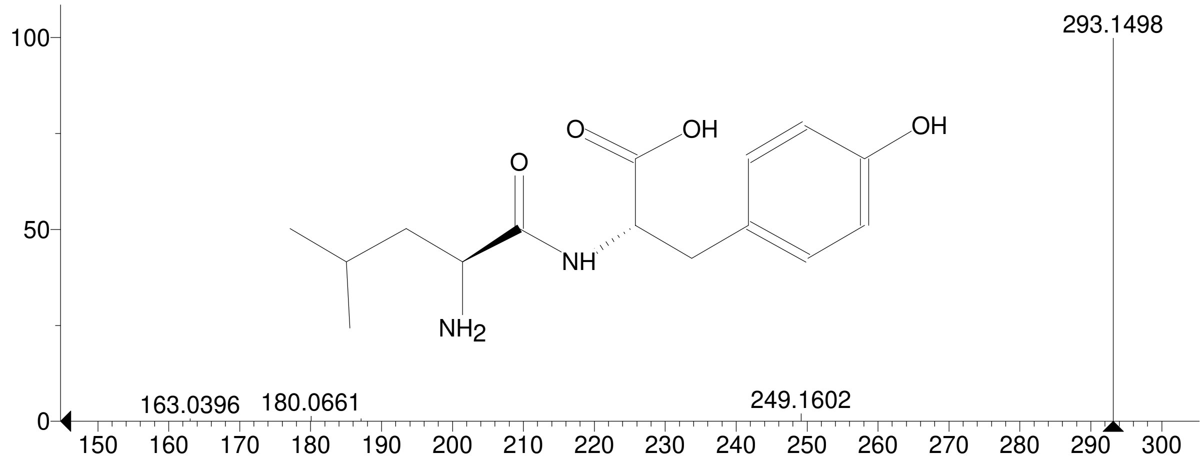

Hit 2 : Leu-Tyr [M-H]- HCD 14eV P=293.2
C15H22N2O4; MF: 238; RMF: 901; Prob 23.8%; CAS: 968-21-8; Lib: nist_msms2; ID: 70411.

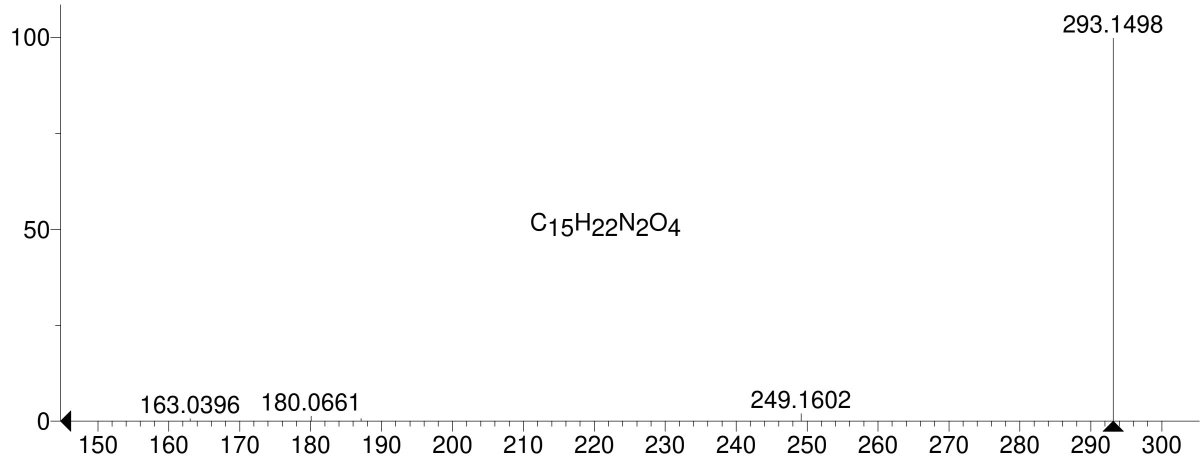

Hit 3 : Ile-Tyr [M-H]- HCD 14eV P=293.2
C15H22N2O4; MF: 235; RMF: 899; Prob 21.0%; Lib: nist_msms; ID: 567507.

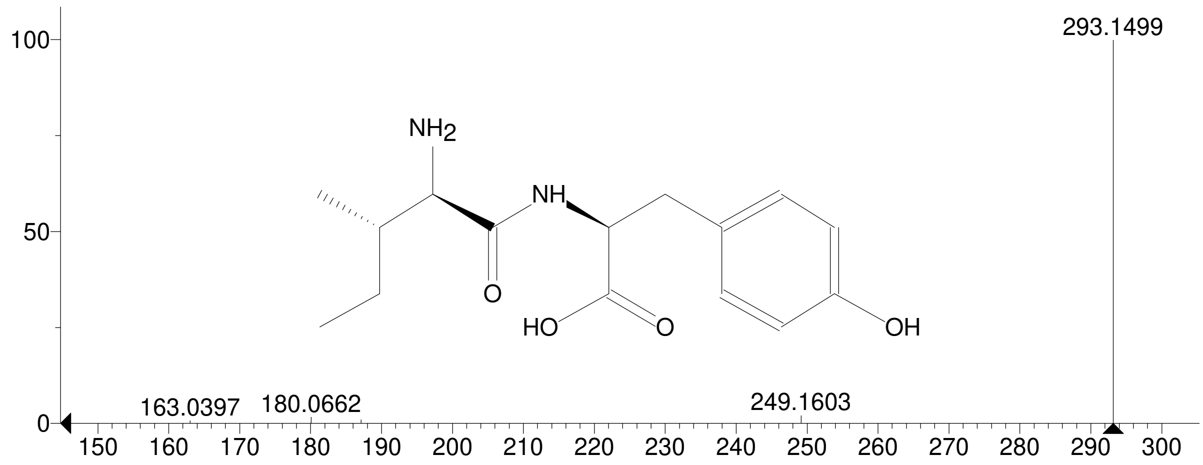

Hit 4 : Ile-Tyr [M-H]- HCD 14eV P=293.2
C15H22N2O4; MF: 235; RMF: 899; Prob 21.0%; CAS: 38579-21-4; Lib: nist_msms2; ID: 70364.

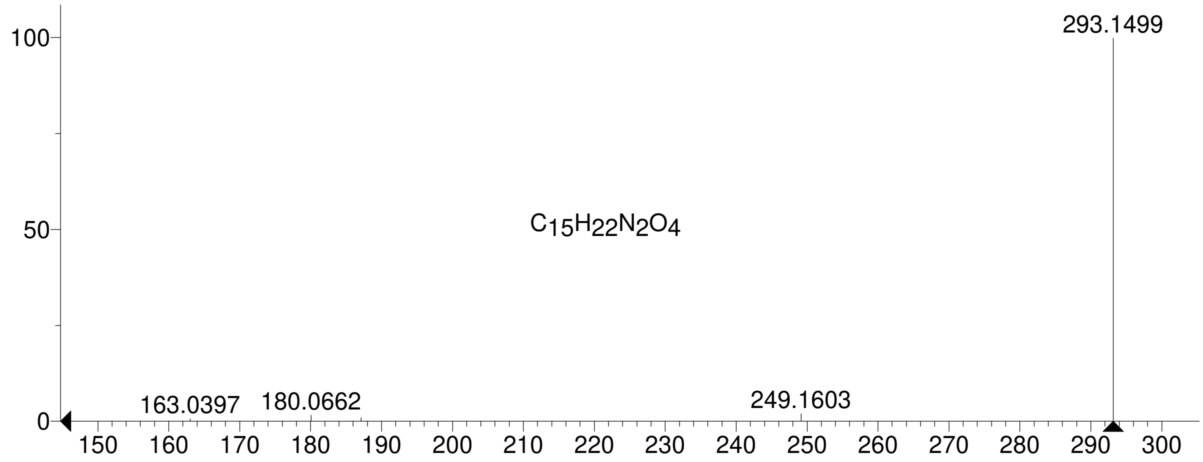

Hit 5 : Dicumyl peroxide [M+Na]+ HCD 2V P=293.2
C18H22O2; MF: 208; RMF: 888; Prob 6.17%; CAS: 80-43-3; Lib: nist_msms; ID: 153950.

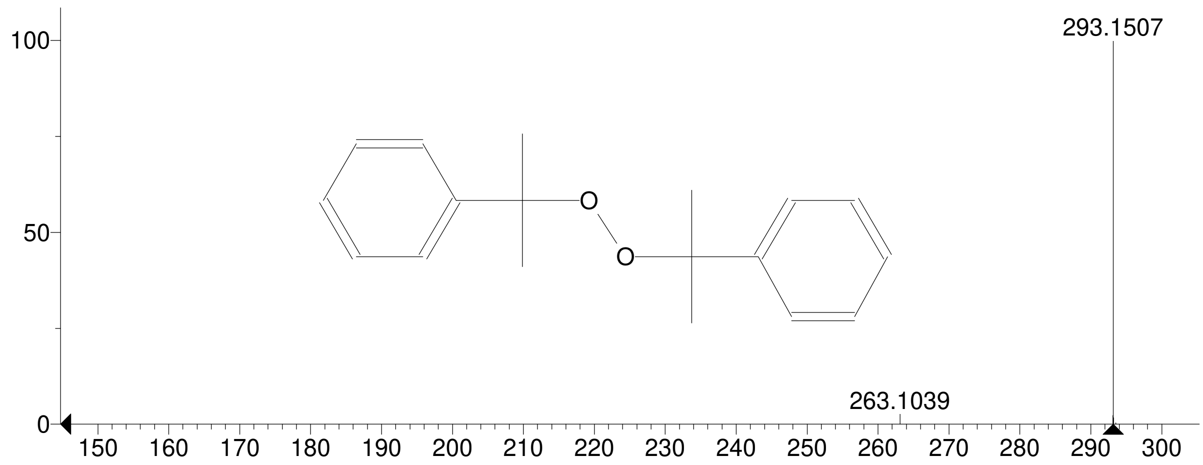

Unknown: Unknown (4.78_305.0681m/z)
Compound in Library Factor = -3273

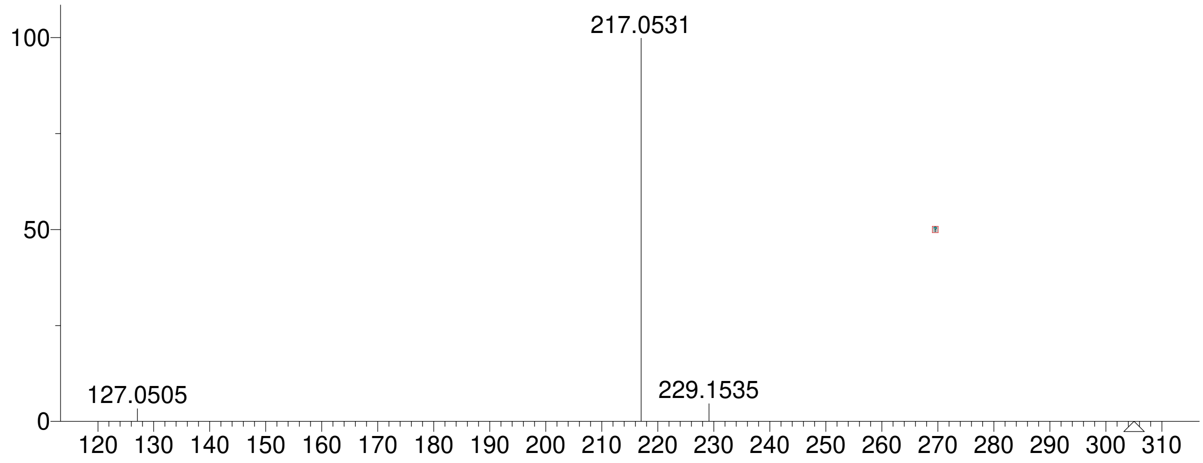

Hit 1 : 2,3-Trans-3,4-cis-Leucocyanidin [M-H]- 45eV P=305.1
C15H14O7; MF: 1; RMF: 199; Prob 9.35%; Lib: msms-neg-metabobase; ID: 829.

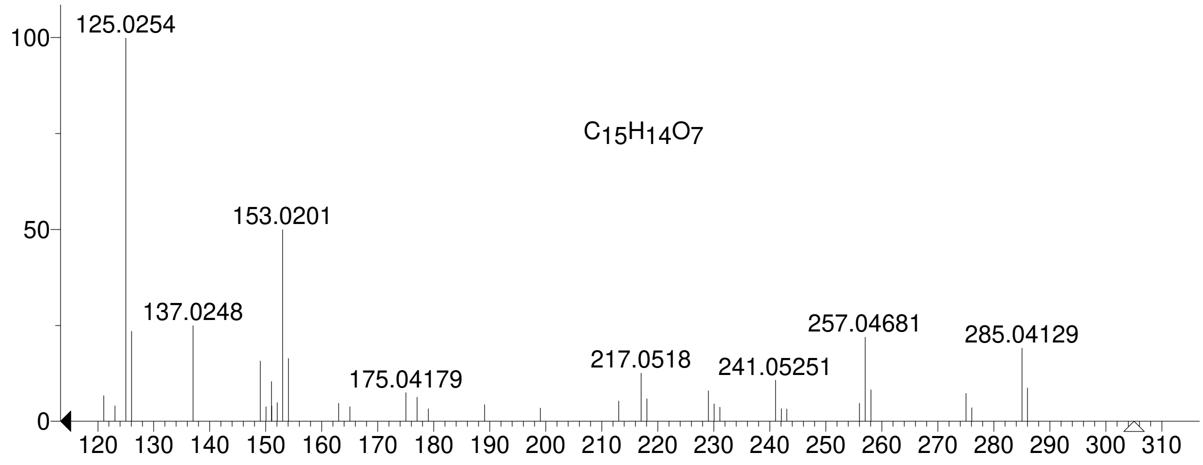

Hit 2 : 2,3-Trans-3,4-trans-Leucocyanidin [M-H]- 45eV P=305.1
C15H14O7; MF: 1; RMF: 183; Prob 9.35%; Lib: msms-neg-metabobase; ID: 823.

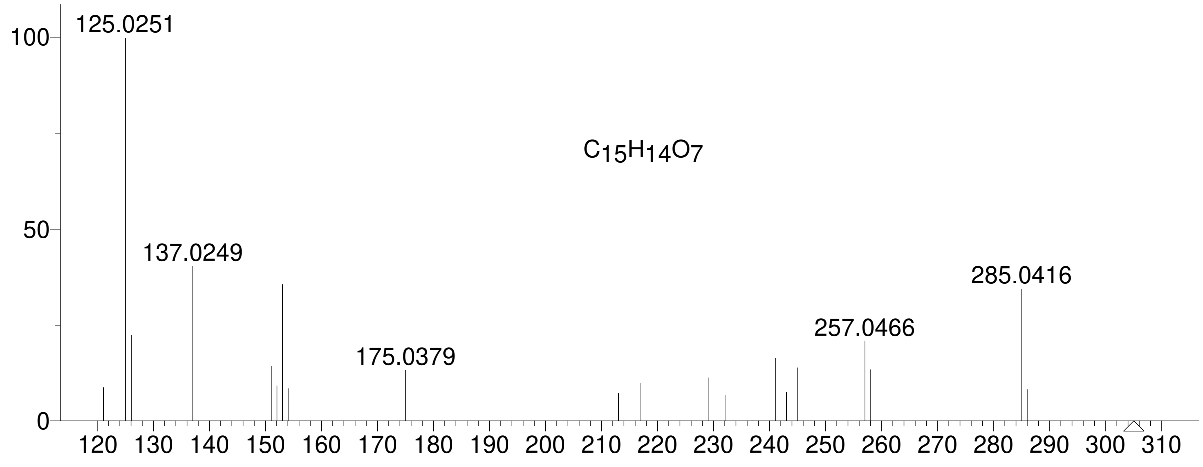

Hit 3 : 3,5,7,3',4'-Pentahydroxyflavanone [M+H]+ HCD 91V P=305.1
C15H12O7; MF: 1; RMF: 55; Prob 9.35%; CAS: 480-18-2; Lib: nist_msms; ID: 136825.

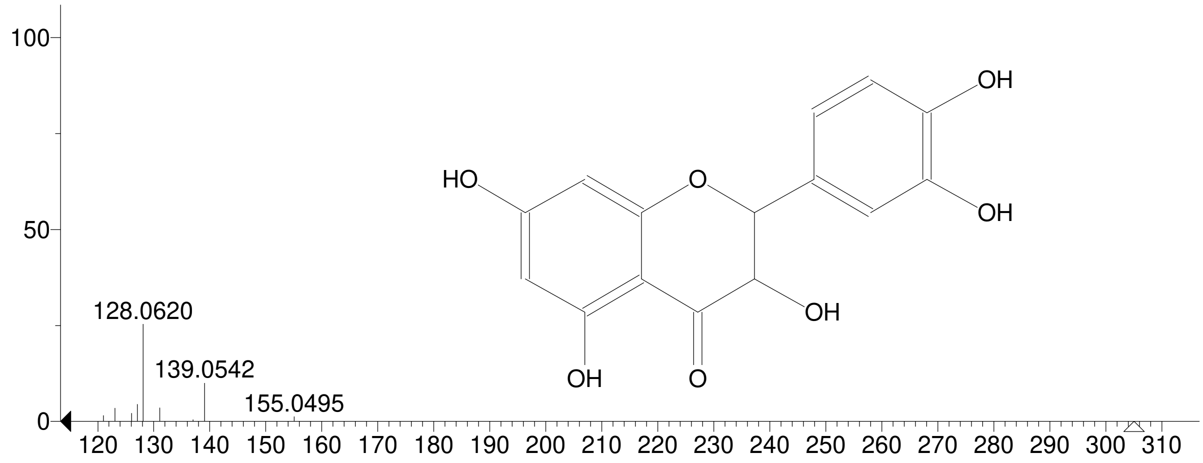

Hit 4 : Gallocatechin [M-H]- 20 P=305.1
C15H14O7; MF: 0; RMF: 105; Prob 8.99%; Lib: mona-export-lc-ms-ms_negative_mode; ID: 12384.

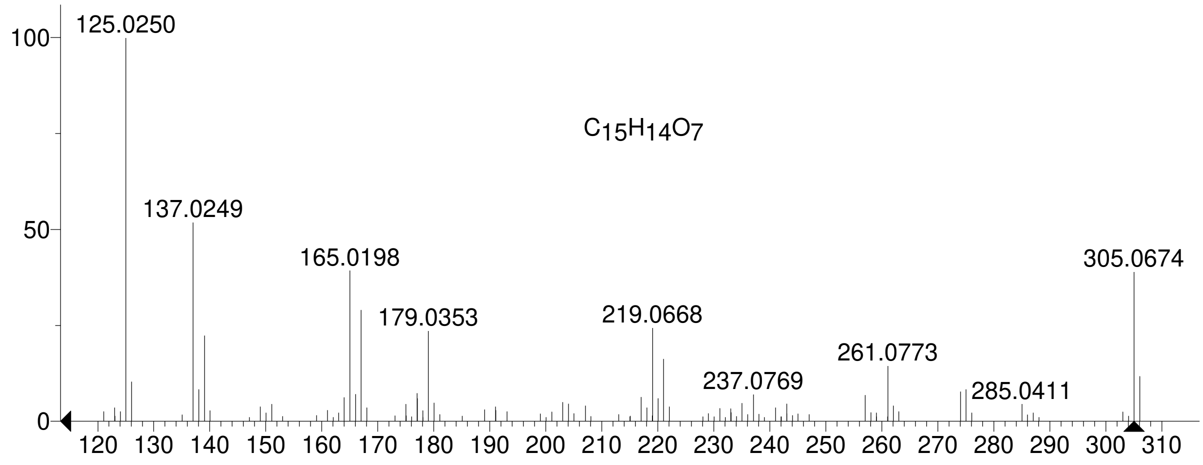

Hit 5 : Epigallocatechin [M-H]- 20 P=305.1
C15H14O7; MF: 0; RMF: 105; Prob 8.99%; Lib: mona-export-lc-ms-ms_negative_mode; ID: 12421.

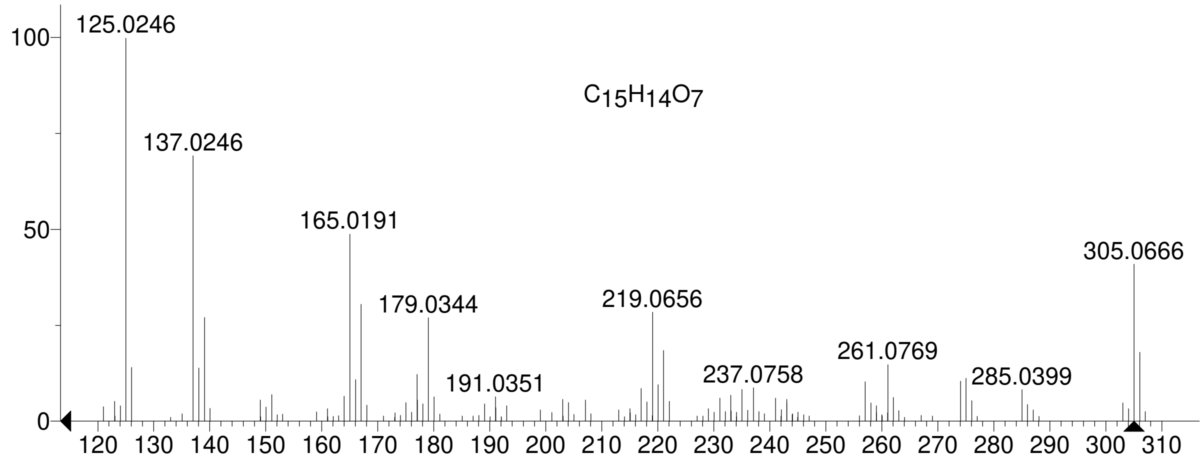

Unknown: Unknown (4.78_348.1187m/z)
Compound in Library Factor = 0

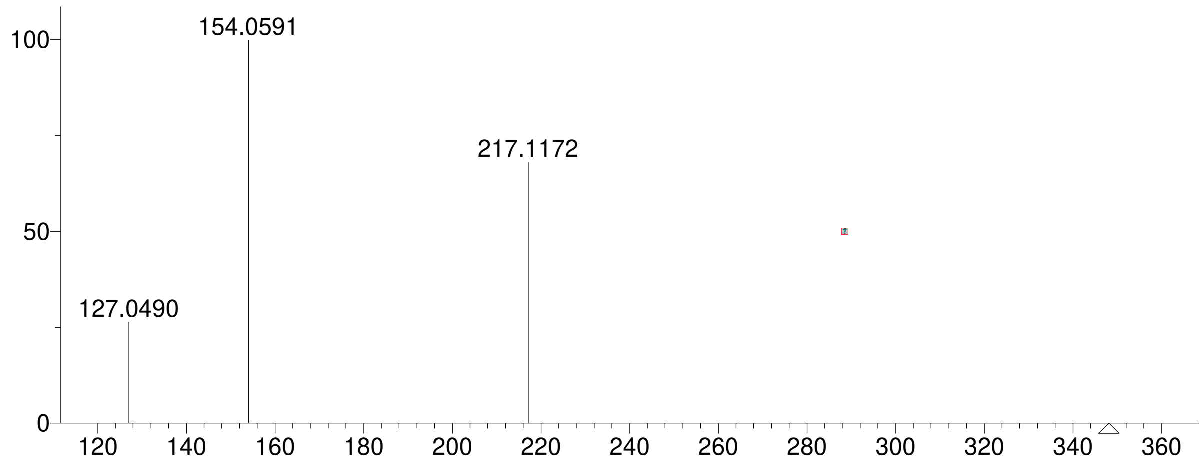

** Search Report Page 1 of 1 **

Unknown: Unknown (4.78_666.2621m/z)
Compound in Library Factor = 0

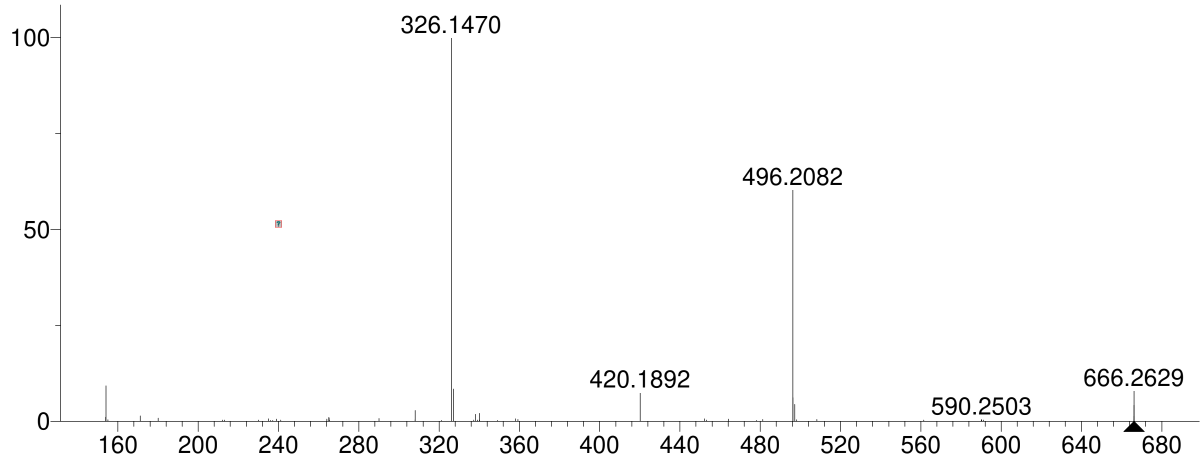

Unknown: Unknown (4.79_456.1625m/z)
Compound in Library Factor = 0

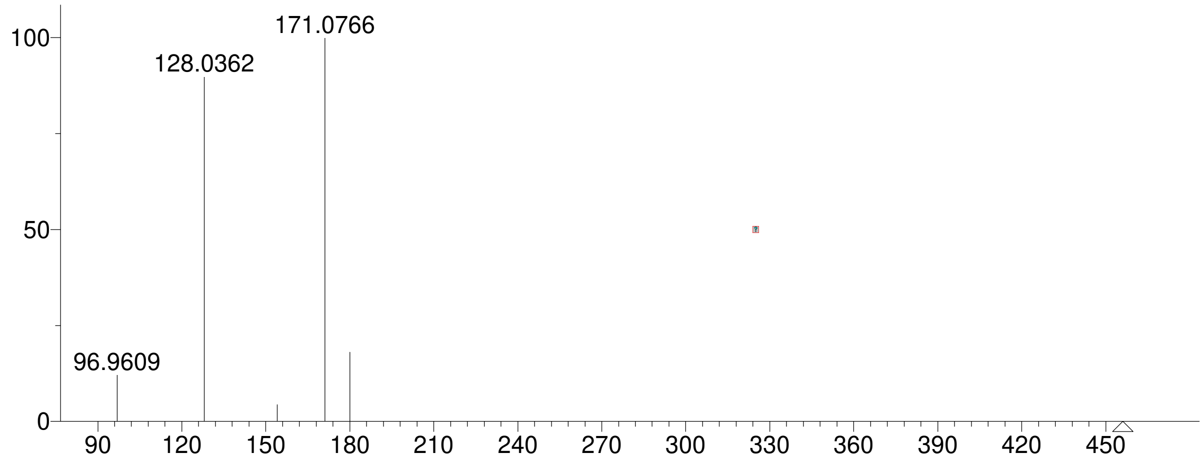

Hit 1 : Folinic acid [M+H-H2O]+ HCD 118V P=456.2
C20H23N7O7; MF: 0; RMF: 34; Prob 33.3%; CAS: 58-05-9; Lib: nist_msms; ID: 138163.

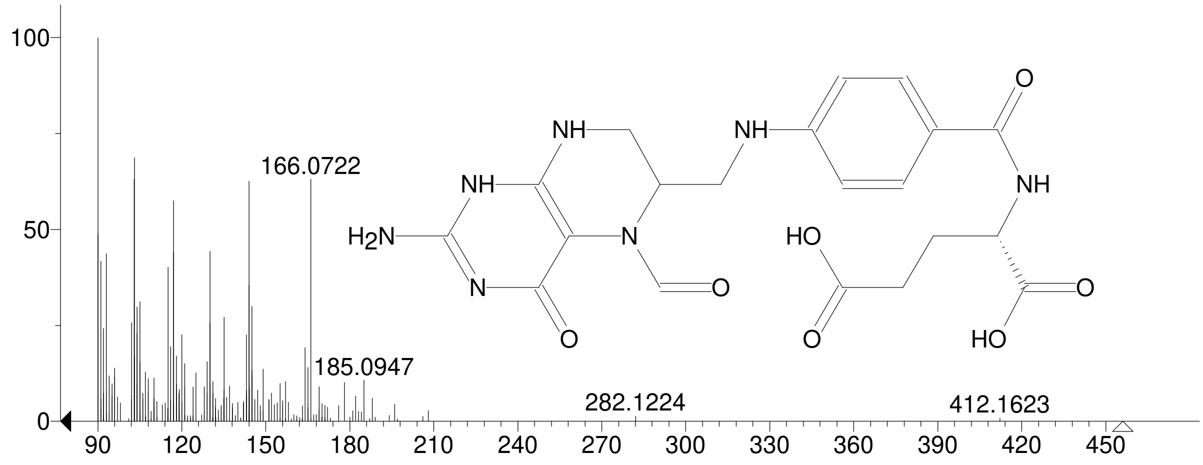

Hit 2 : Codeine-6-β-D-glucuronide [M-H-H2O]- HCD 72V P=456.2
C24H29NO9; MF: 0; RMF: 29; Prob 33.3%; CAS: 20736-11-2; Lib: nist_msms; ID: 322871.

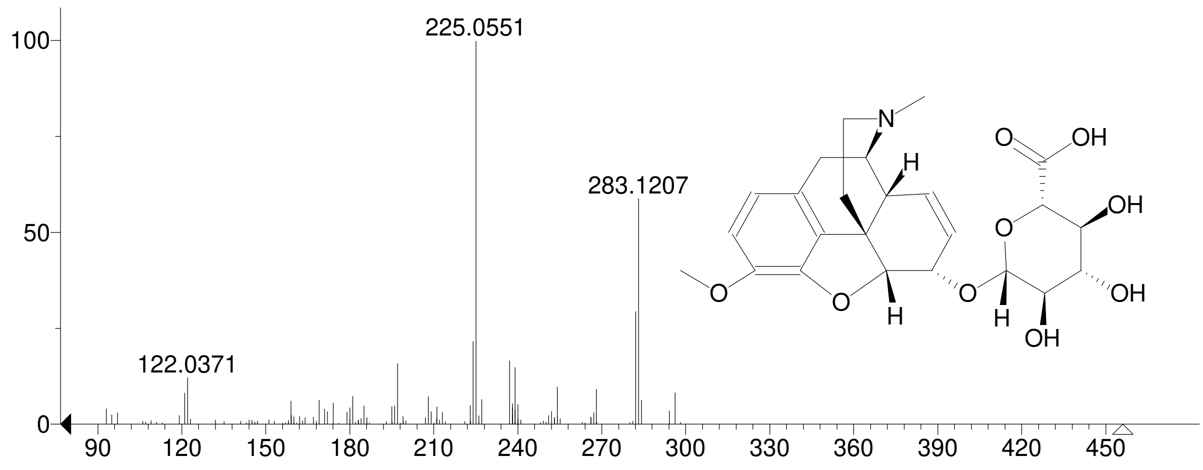

Hit 3 : Methopterine [M+H]+ HCD 82V P=456.2
C20H21N7O6; MF: 0; RMF: 8; Prob 33.3%; CAS: 2410-93-7; Lib: nist_msms; ID: 206196.

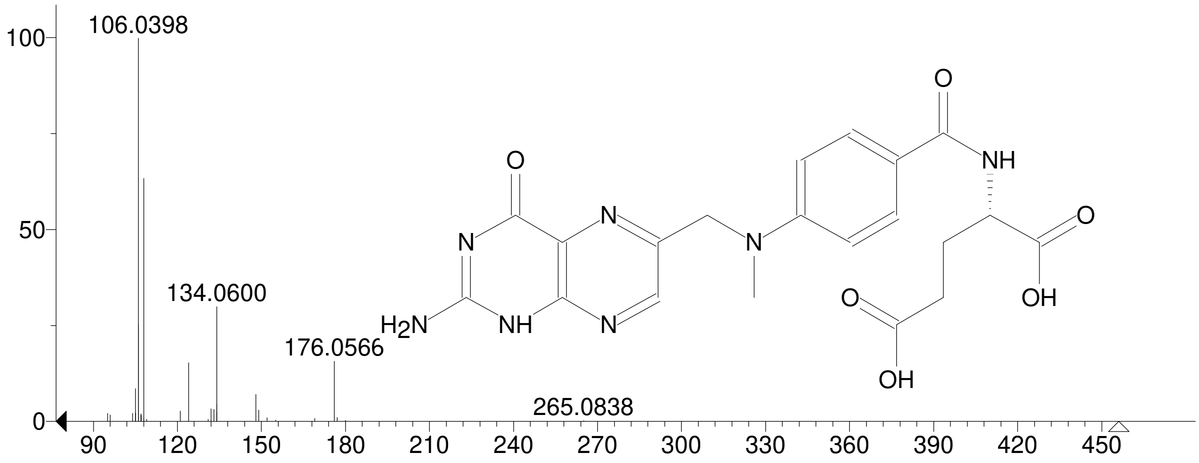

Unknown: Unknown (4.80_639.1685m/z)
Compound in Library Factor = -1072

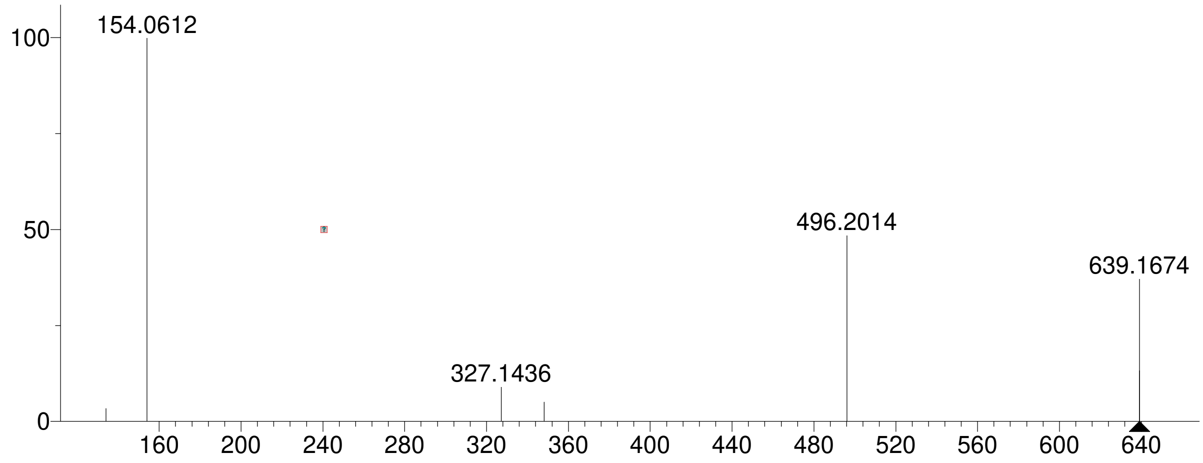

Hit 1 : 1-Naphthol β-D-glucuronide [2M-H]- HCD 3V P=639.2
C16H16O7; MF: 84; RMF: 548; Prob 51.2%; CAS: 17238-47-0; Lib: nist_msms; ID: 464073.

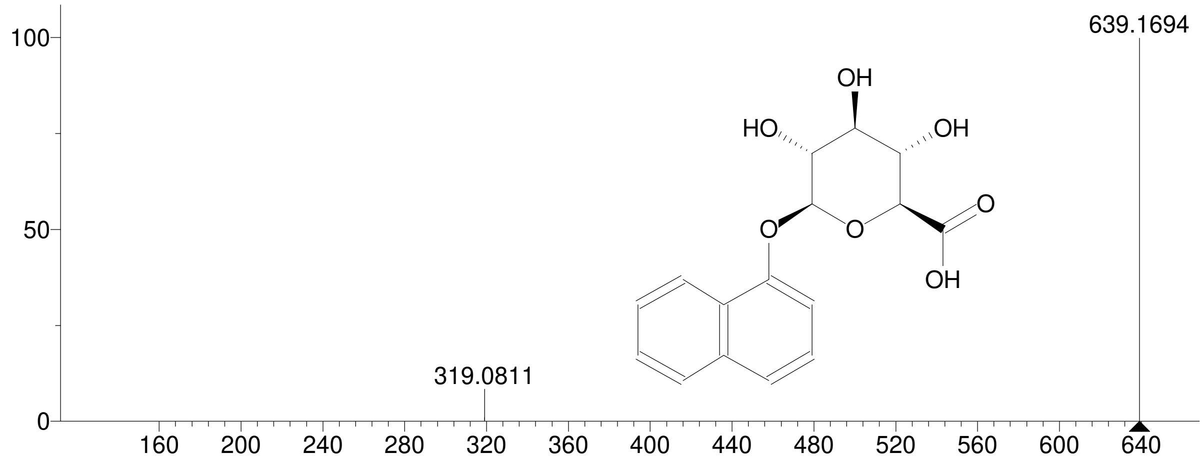

Hit 2 : NCGC00384507-01! [M-H]- P=639.2
C32H32O14; MF: 6; RMF: 355; Prob 6.04%; Lib: msms-neg-gnps; ID: 525.

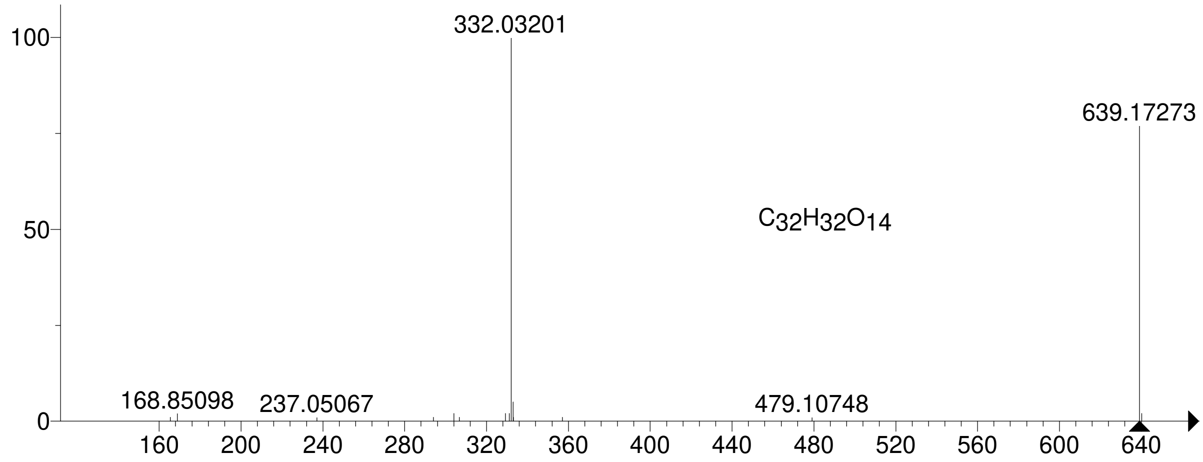

Hit 3 : _130087 [M+H]+ P=639.2
C32H30O14; MF: 0; RMF: 195; Prob 4.74%; Lib: mona-export-gnps; ID: 5046.

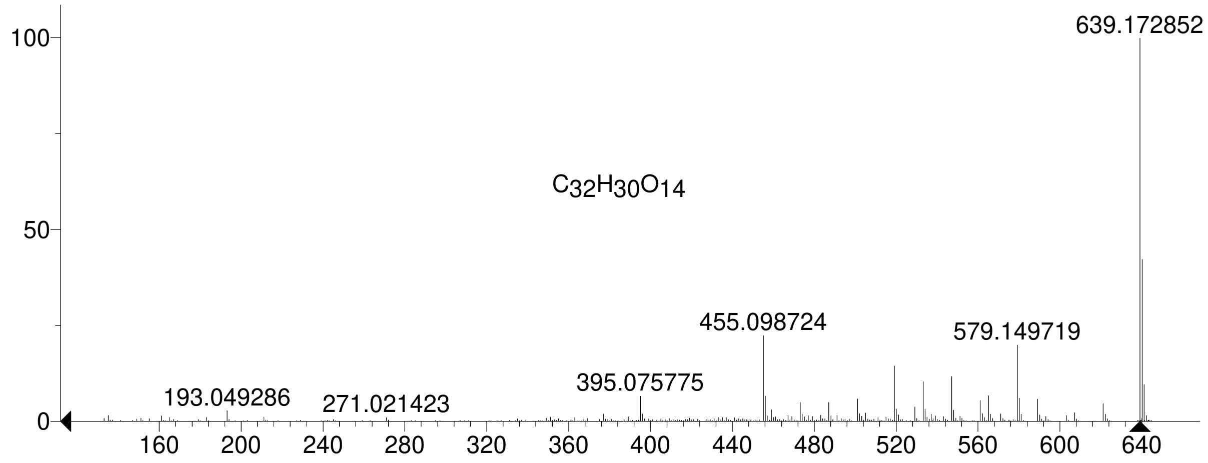

Hit 4 : NCGC00169779-03_C30H32O14_Spiro[cyclopenta[c]pyran-7(1H),2'(5'H)-furan]-4-carboxylic acid, 1-(beta-
C30H32O14; MF: 0; RMF: 194; Prob 4.74%; Lib: mona-export-gnps; ID: 13784.

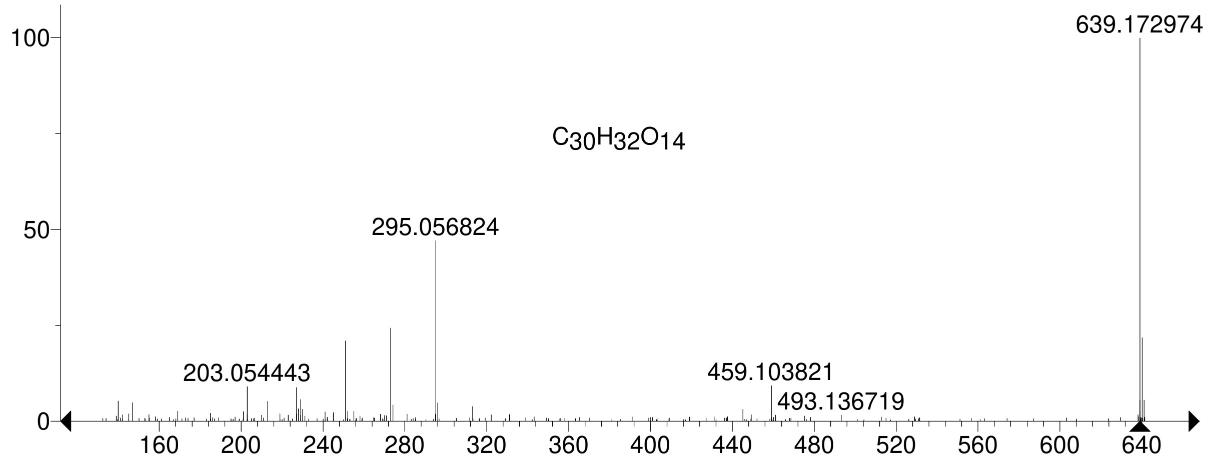

Hit 5 : _130088 [M+H]+ P=639.2
C32H30O14; MF: 0; RMF: 191; Prob 4.74%; Lib: mona-export-gnps; ID: 5047.

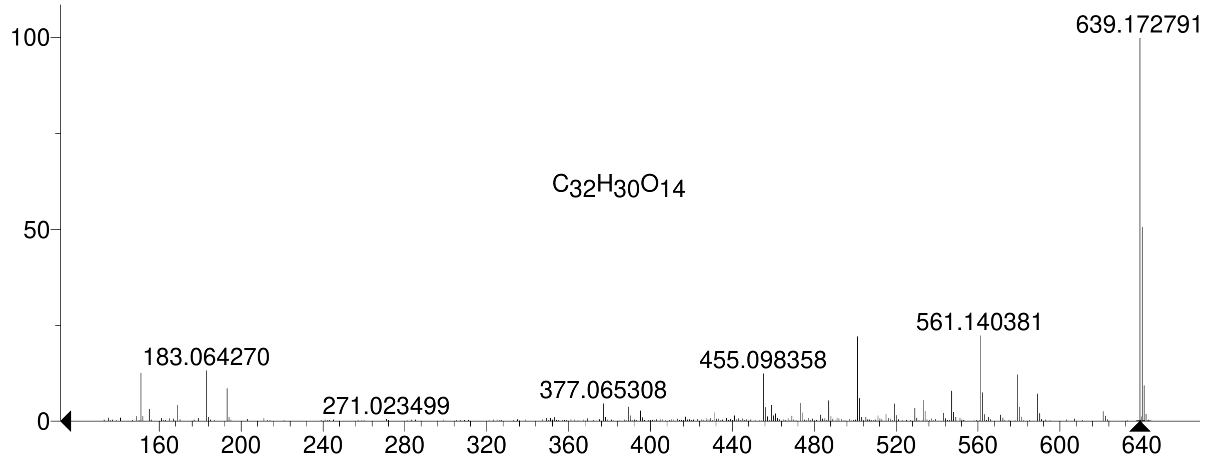

Unknown: Unknown (4.83_479.1630m/z)
Compound in Library Factor = 0

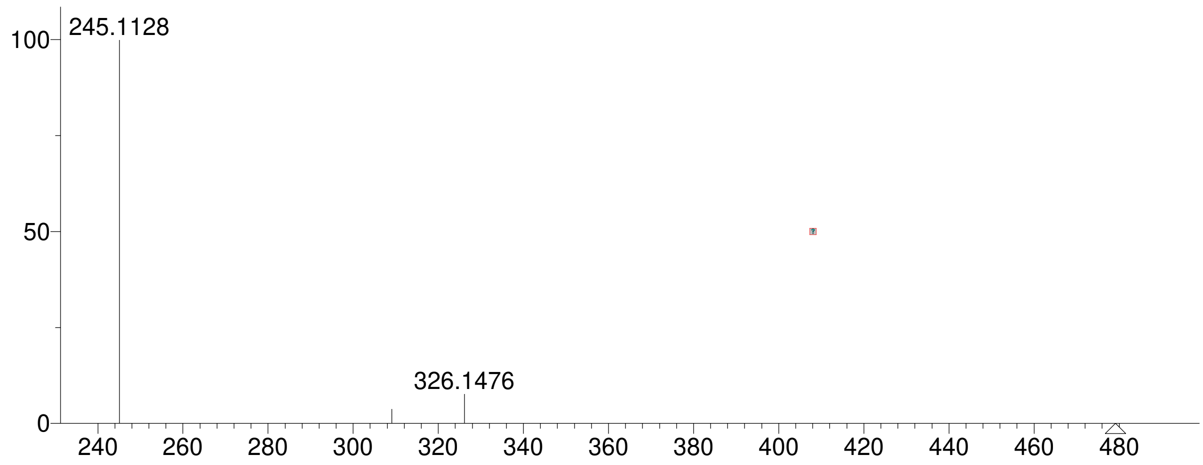

Hit 1 : 2-[3,4-dihydroxy-2,2-dimethyl-8-(3-methylbut-2-enoxy)-3,4-dihydrochromen-6-yl]-5,7-dihydroxy-2,3-dihydro
C25H28O8; MF: 0; RMF: 23; Prob 50.0%; Lib: mona-export-vf-npl_qexactive; ID: 16870.

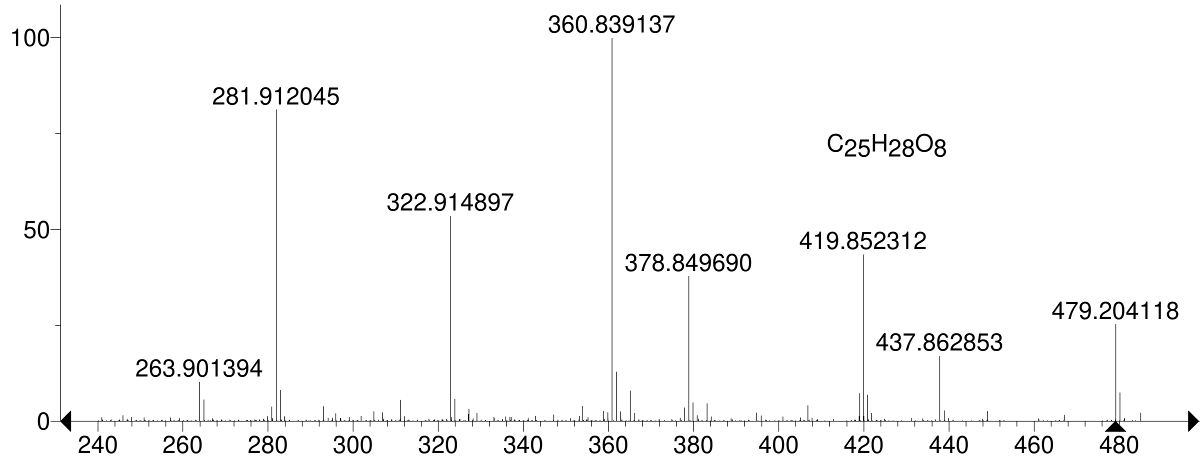

Hit 2 : 7-hydroxy-2,6,9,12,17,17-hexamethyl-22-methylidene-5,16,19,21-tetraoxaheptacyclo[10.8.1.1A^'3,a^'1.0A'
C25H28O8; MF: 0; RMF: 5; Prob 50.0%; Lib: mona-export-vf-npl_qexactive; ID: 20713.

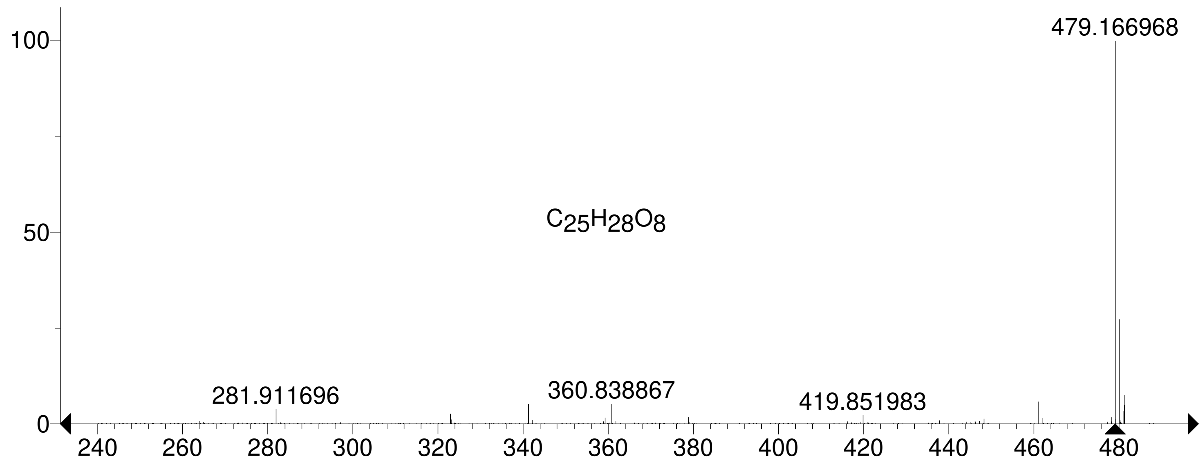

** Search Report Page 1 of 1 **

Unknown: Unknown (4.83_616.1396m/z)
Compound in Library Factor = 0

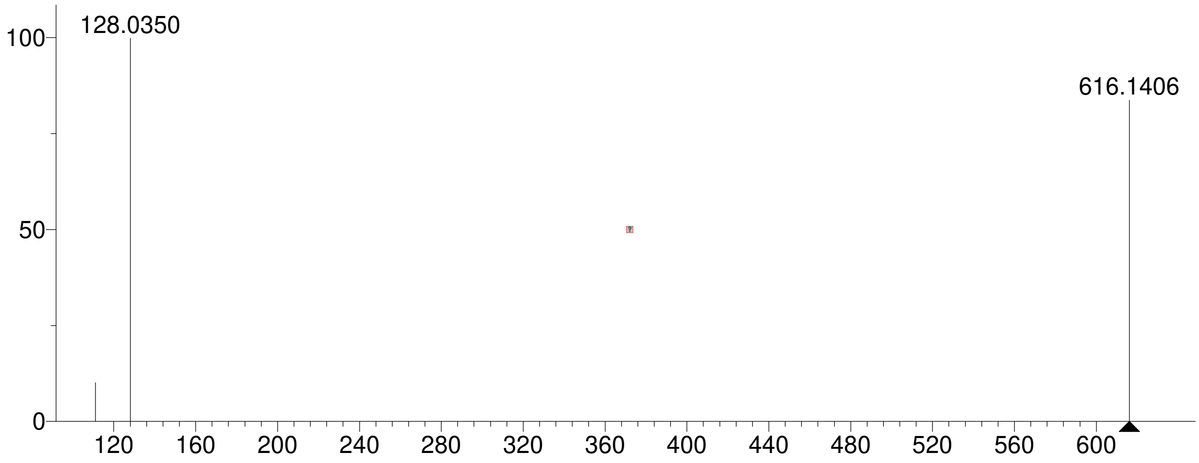

** Search Report Page 1 of 1 **

Unknown: Unknown (4.84_387.1863m/z)
Compound in Library Factor = 0

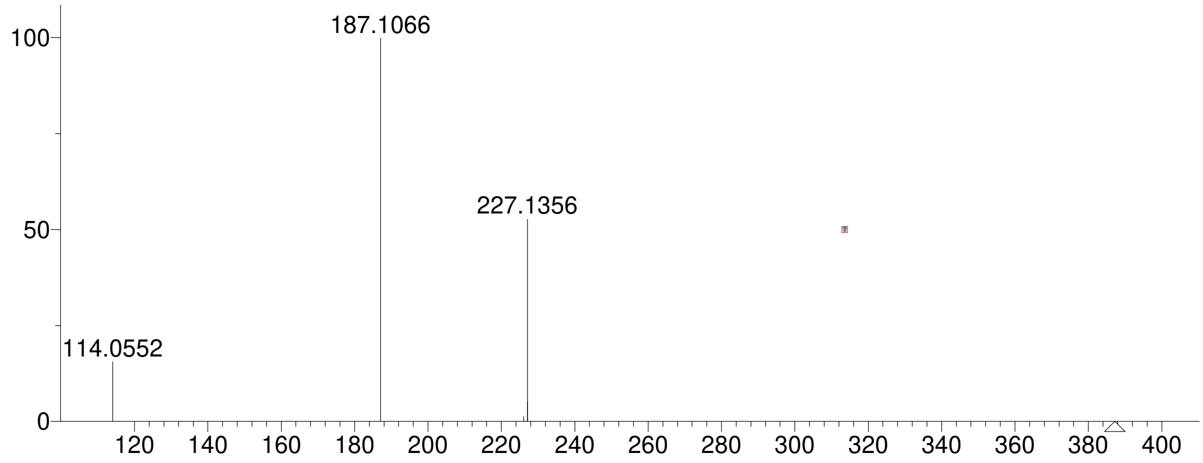

Unknown: Unknown (4.85_298.1392m/z)
Compound in Library Factor = 0

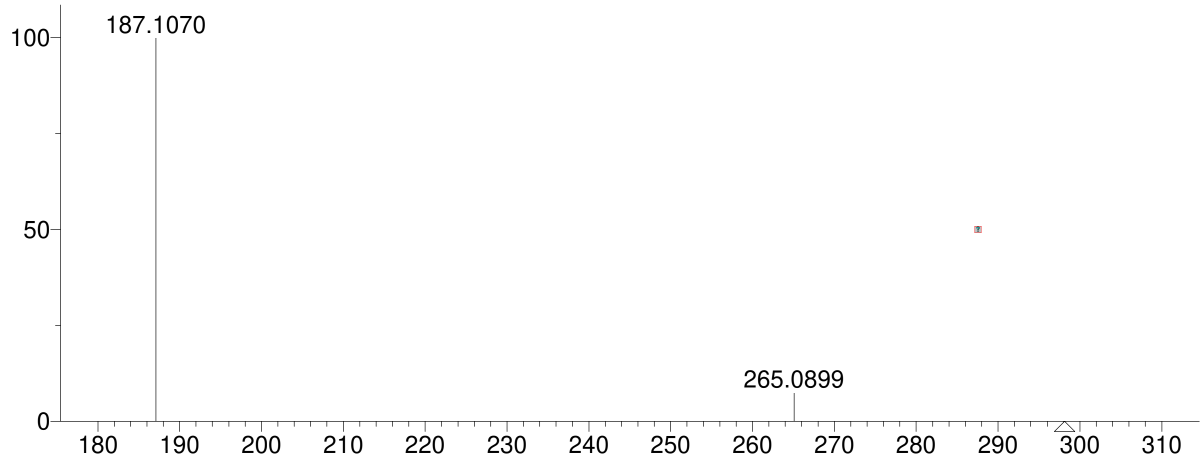

Unknown: Unknown (4.86_239.0547m/z)
Compound in Library Factor = 0

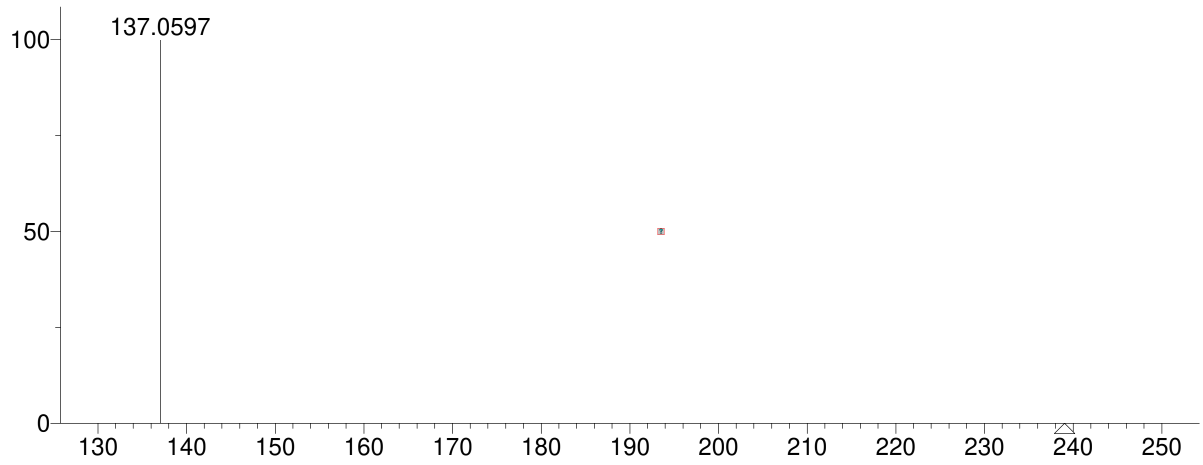

Hit 1 : NCGC00381344-01!8-hydroxy-6-methoxy-1-oxo-3,4-dihydroisochromene-7-carboxylic acid [M+H]+ P=23[illegible]
C11H10O6; MF: 0; RMF: 30; Prob 100.0%; Lib: mona-export-gnps; ID: 7519.

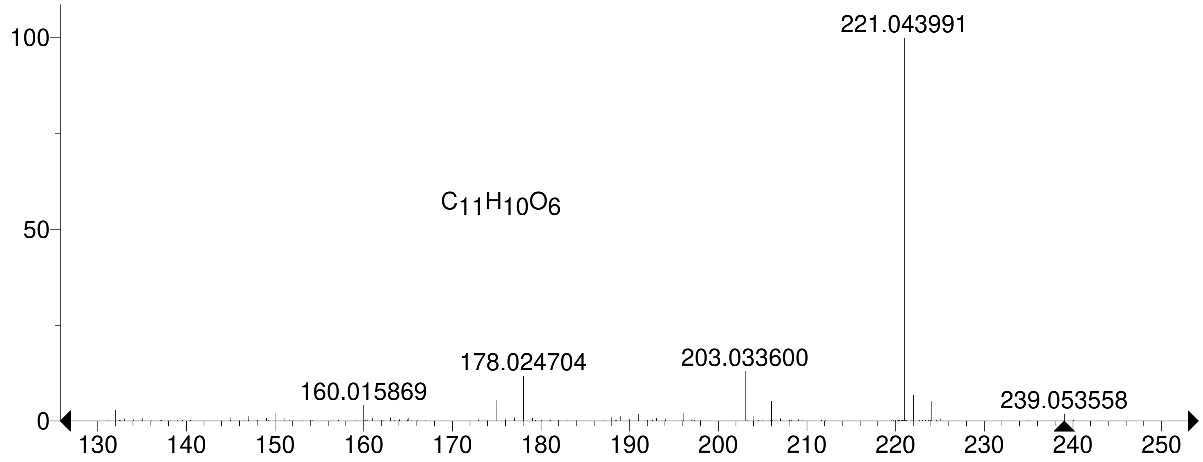

** Search Report Page 1 of 1 **

Unknown: Unknown (4.86_346.1594m/z)
Compound in Library Factor = 0

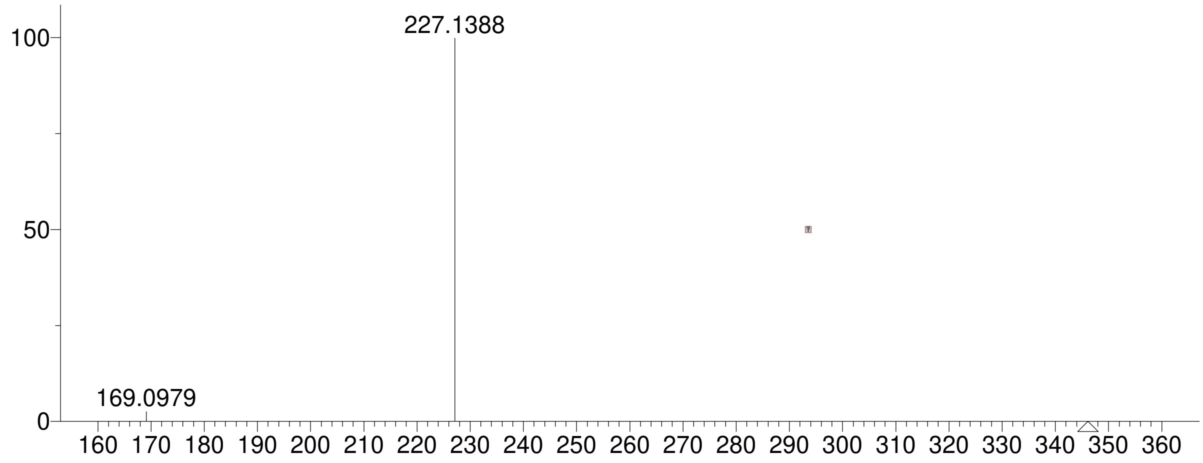

Unknown: Unknown (4.86_357.1651m/z)
Compound in Library Factor = 0

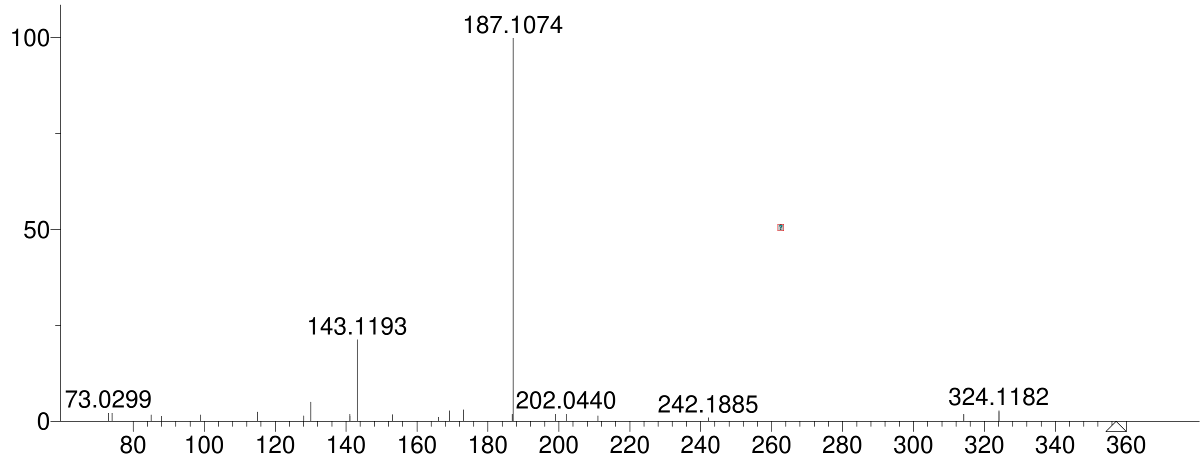

Hit 1 : [(1S,3aR,5R,5aR,8aR,9S,9aR)-1,5,8a-trimethyl-2,8-dioxo-3a,4,5,5a,9,9a-hexahydro-1H-azuleno[6,5-b]fura
C19H26O5; MF: 0; RMF: 19; Prob 50.0%; Lib: mona-export-vf-npl_qexactive; ID: 7288.

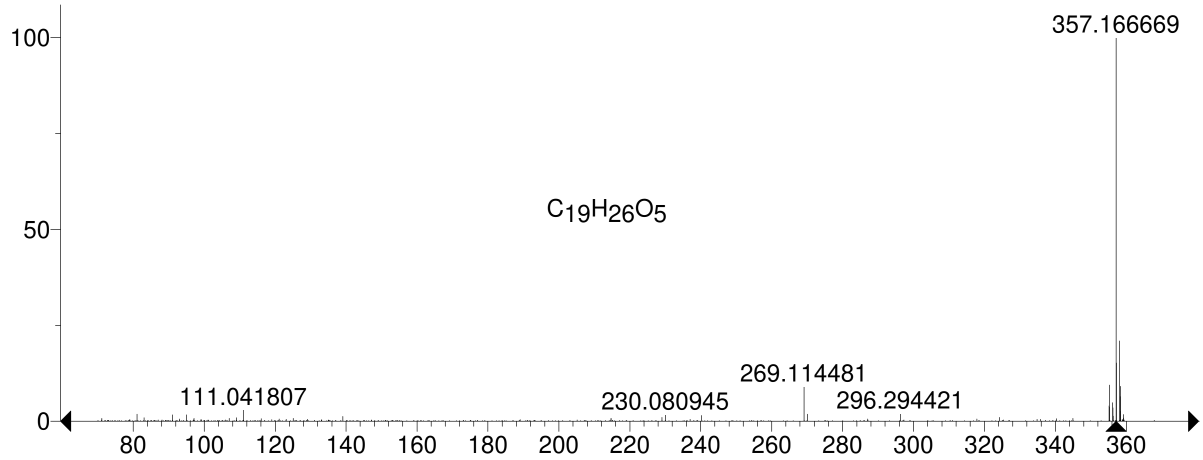

Hit 2 : NCGC00381117-01!2-amino-4-hydroxy-5-[[1-(4-methoxy-6-oxopyran-2-yl)-3-methylbutyl]amino]-5-oxopent
C16H24N2O7; MF: 0; RMF: 13; Prob 50.0%; Lib: mona-export-gnps; ID: 8549.

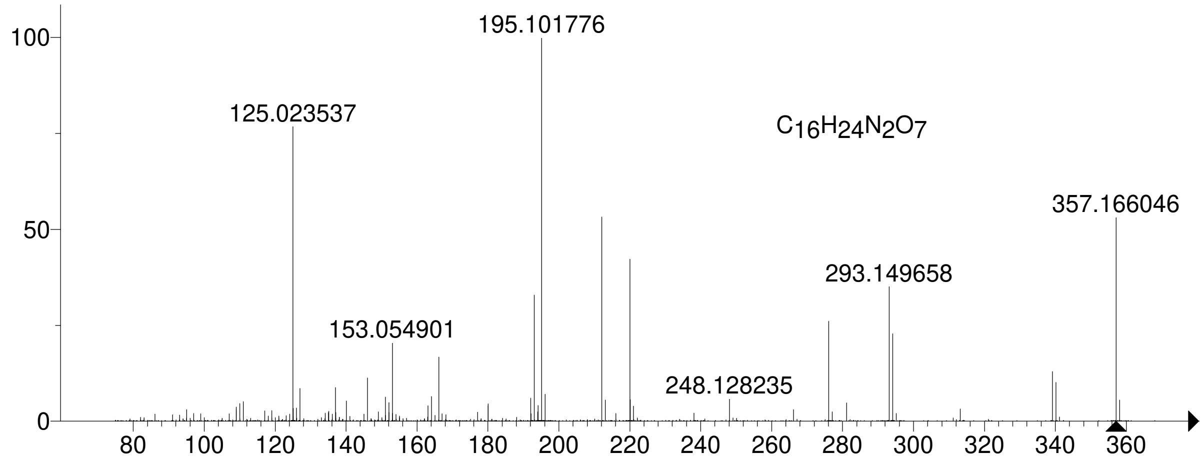

Unknown: Unknown (4.86_499.2510m/z)
Compound in Library Factor = -301

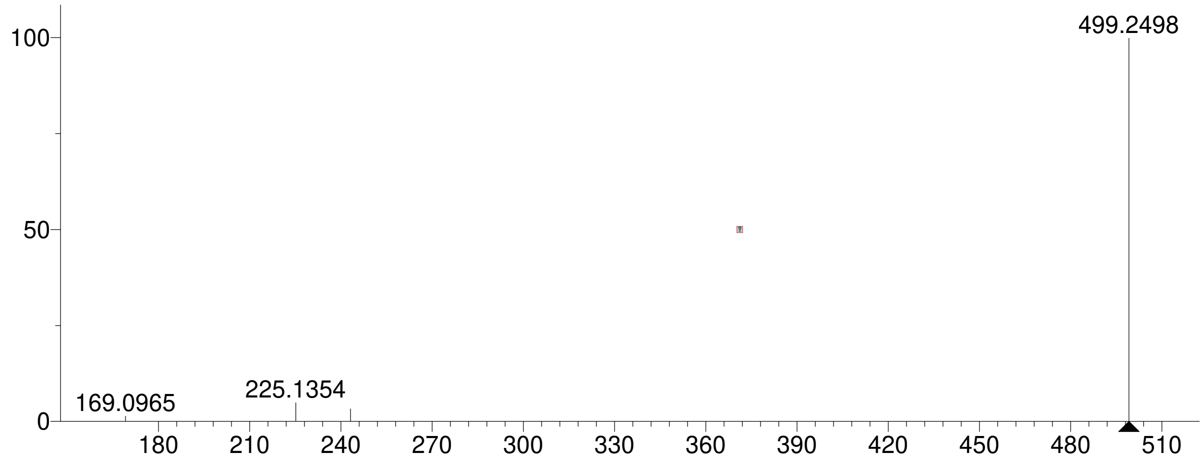

Hit 1 : Lactalbumin B (50-53) Alpha [Lactorphin Alpha], bovine [M+H]+ HCD 9eV P=499.3
C26H34N4O6; MF: 290; RMF: 937; Prob 81.3%; Lib: nist_msms2; ID: 47148.

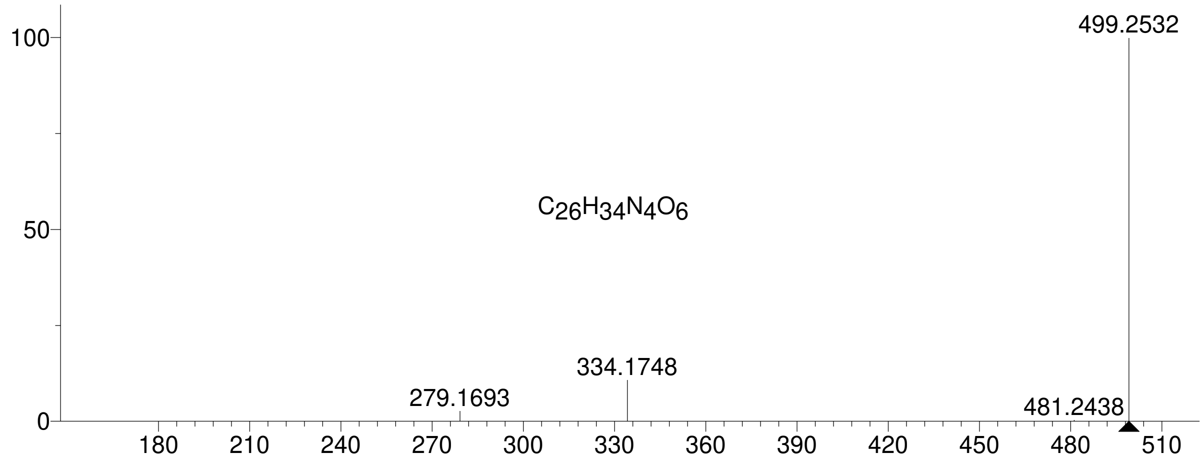

Hit 2 : Betamethasone 17-valerate [M+Na]+ HCD 14V P=499.2
C27H37FO6; MF: 247; RMF: 889; Prob 17.9%; CAS: 2152-44-5; Lib: nist_msms; ID: 309914.

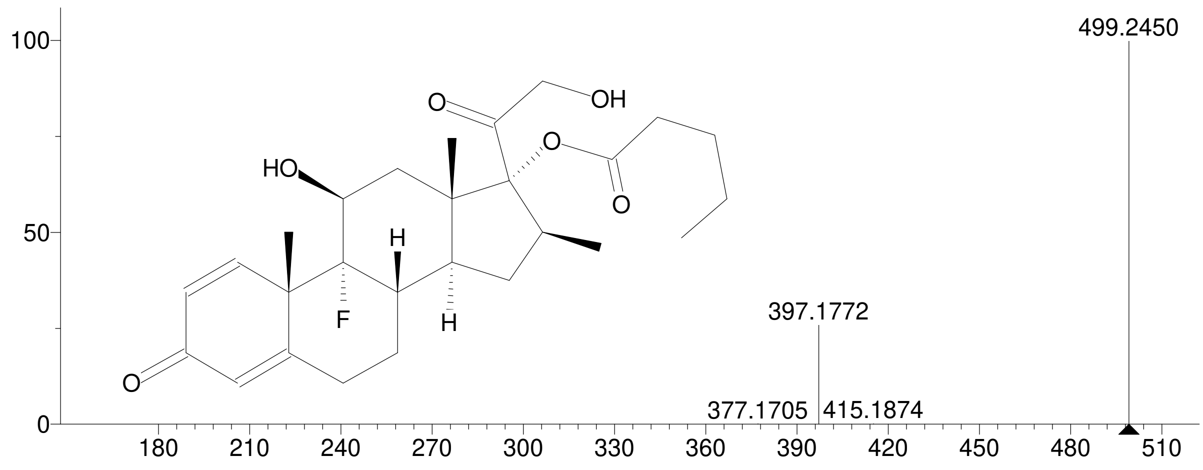

Hit 3 : PA 22:4; PA 2:0-20:4; [M-H]- [M-H]- P=499.2
C25H41O8P; MF: 20; RMF: 450; Prob 0.17%; Lib: msdial-insilicomsms-lipids-neg; ID: 315714.

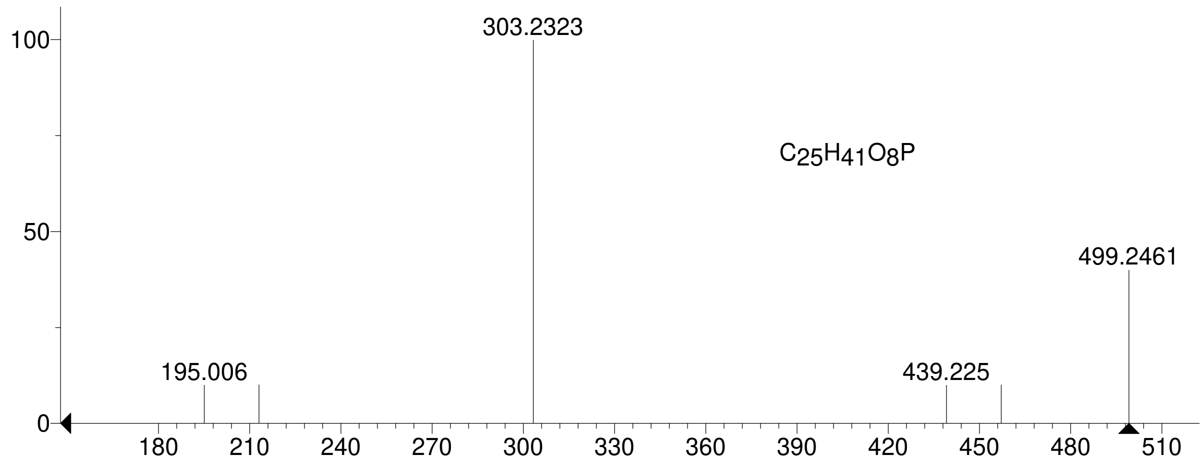

Hit 4 : PA 22:4; PA 4:0-18:4; [M-H]- [M-H]- P=499.2
C25H41O8P; MF: 20; RMF: 450; Prob 0.17%; Lib: msdial-insilicomsms-lipids-neg; ID: 315824.

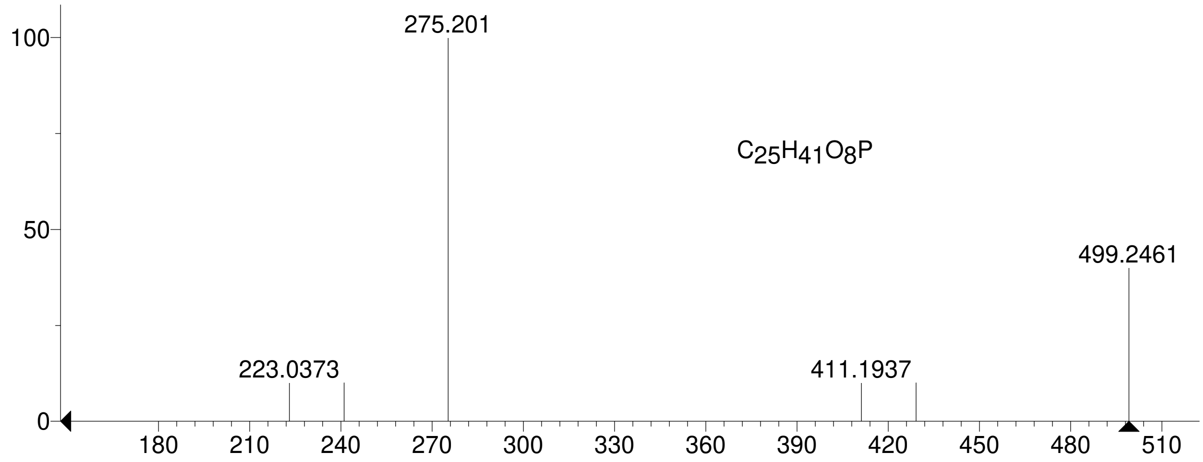

Hit 5 : PA 22:4; PA 6:0-16:4; [M-H]- [M-H]- P=499.2
C25H41O8P; MF: 20; RMF: 450; Prob 0.17%; Lib: msdial-insilicomsms-lipids-neg; ID: 315931.

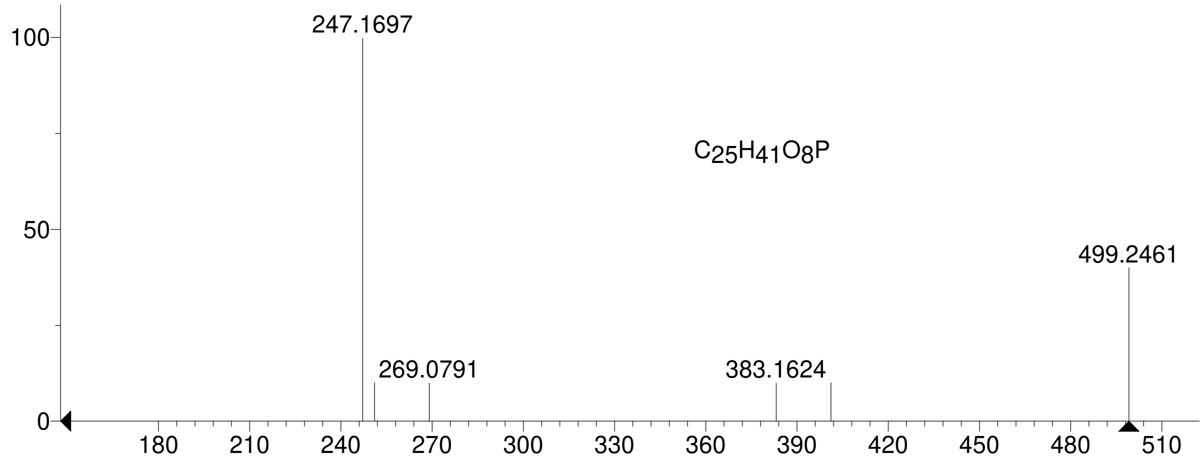

Unknown: Unknown (4.89_259.1286m/z)
Compound in Library Factor = -684

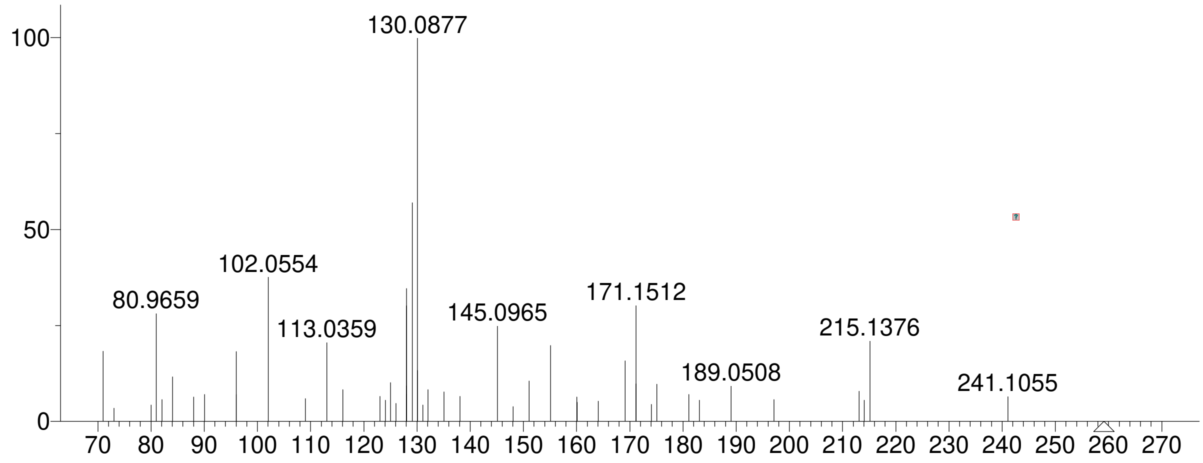

Hit 1 : Ile-Glu [M-H]- HCD 28eV P=259.1
C11H20N2O5; MF: 383; RMF: 639; Prob 38.6%; Lib: nist_msms; ID: 570717.

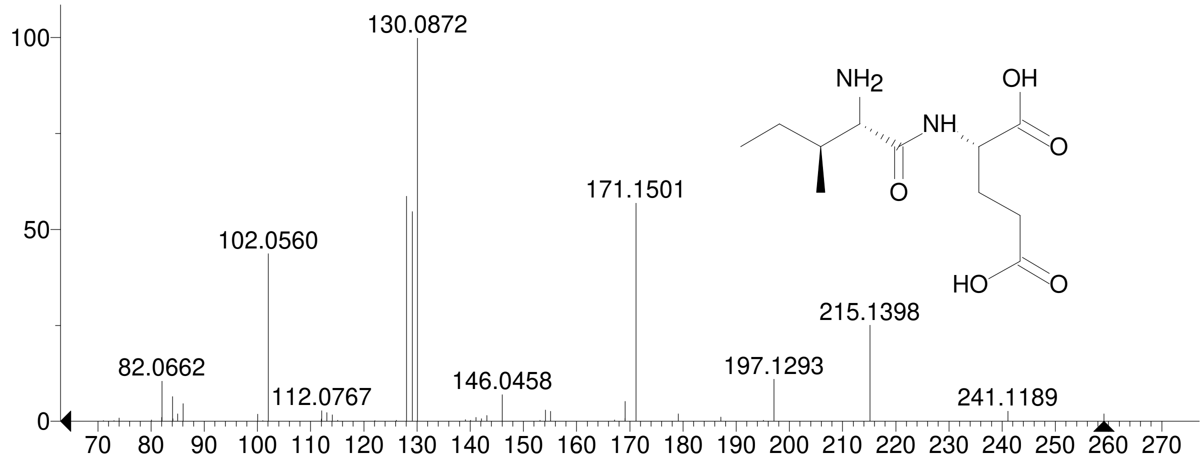

Hit 2 : Ile-Glu [M-H]- HCD 28eV P=259.1
C11H20N2O5; MF: 383; RMF: 639; Prob 38.6%; CAS: 42516-53-0; Lib: nist_msms2; ID: 84450.

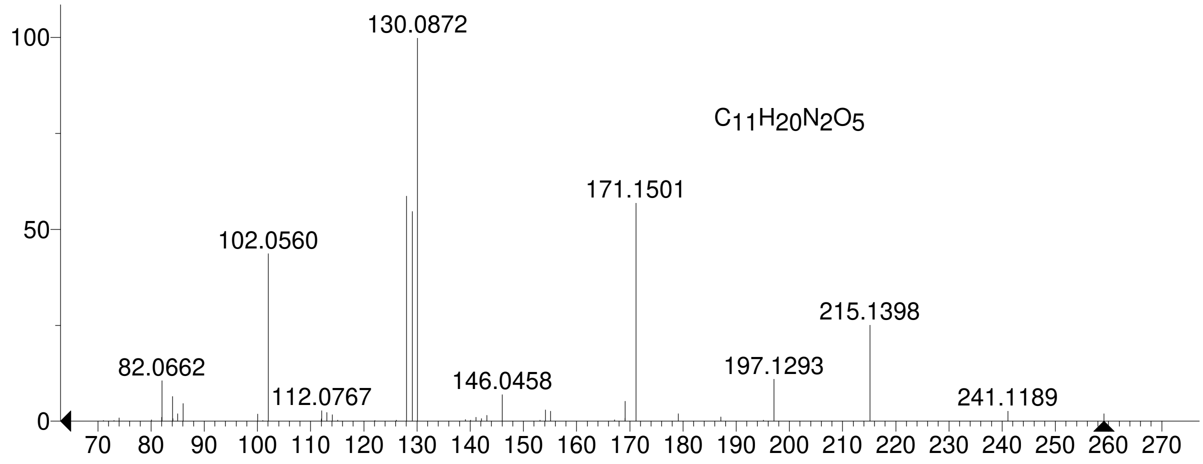

Hit 3 : Leu-Glu [M-H]- HCD 28eV P=259.1
C11H20N2O5; MF: 355; RMF: 625; Prob 11.1%; Lib: nist_msms; ID: 570785.

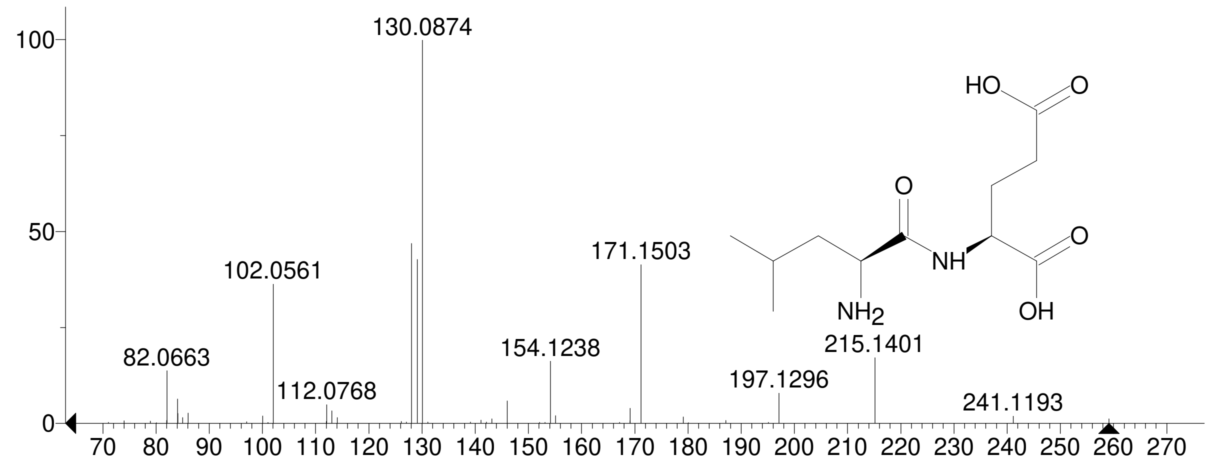

Hit 4 : Leu-Glu [M-H]- HCD 28eV P=259.1
C11H20N2O5; MF: 355; RMF: 625; Prob 11.1%; CAS: 16364-31-1; Lib: nist_msms2; ID: 84518.

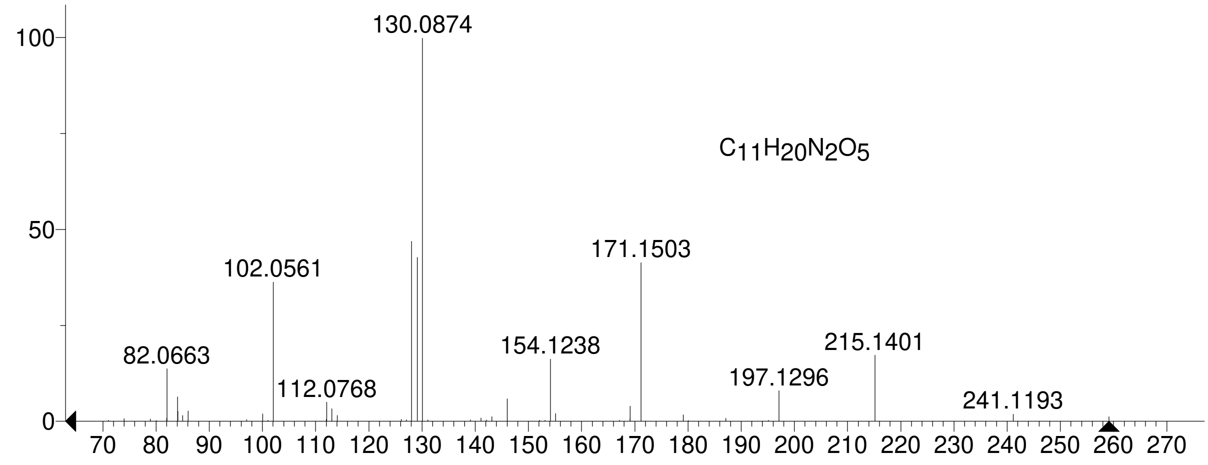

Hit 5 : Glu-Leu [M-H]- HCD 23eV P=259.1
C11H20N2O5; MF: 190; RMF: 492; Prob 0.26%; Lib: nist_msms; ID: 570062.

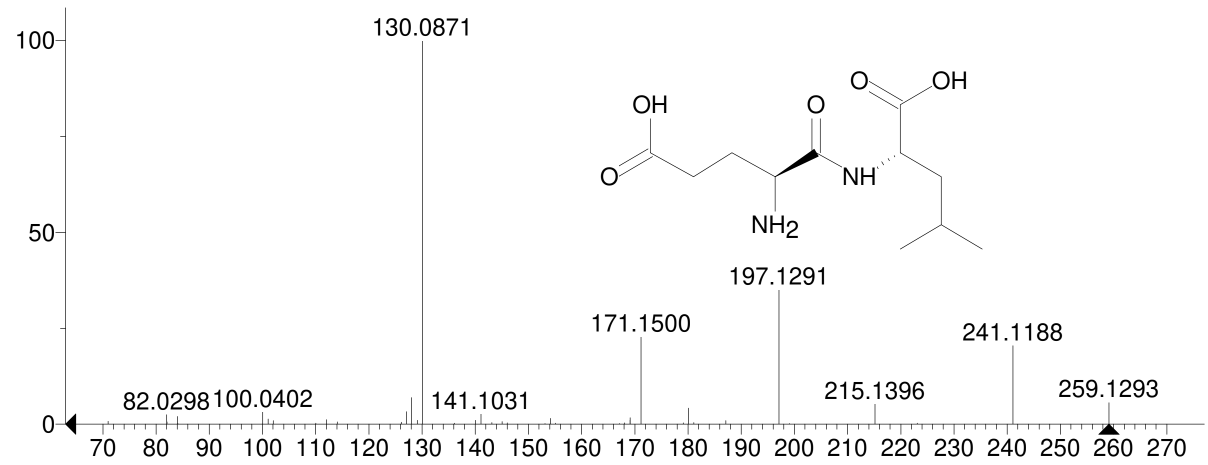

Unknown: Unknown (4.89_429.1869m/z)
Compound in Library Factor = -1309

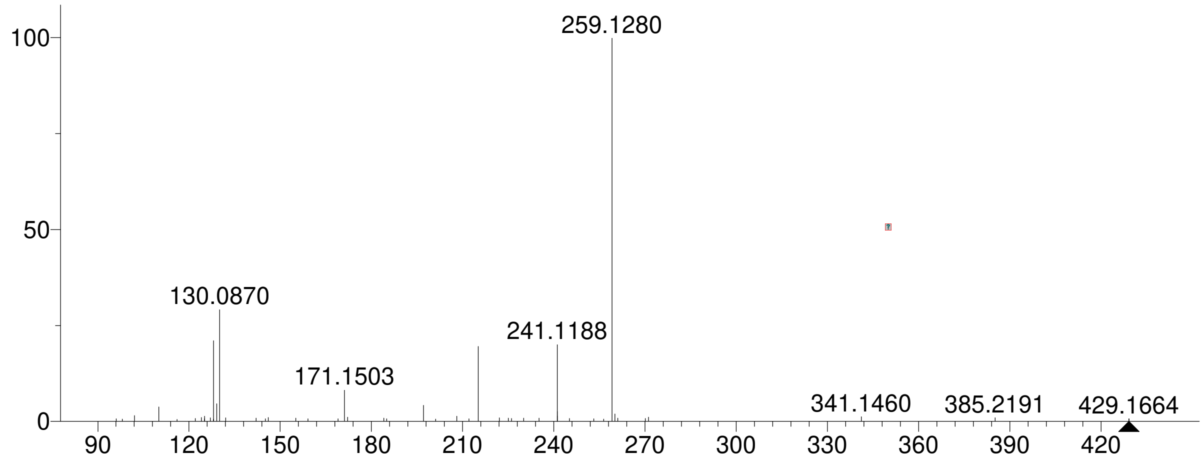

Hit 1 : Garcinone D [M+H]+ HCD 4V P=429.2
C24H28O7; MF: 57; RMF: 488; Prob 48.8%; CAS: 107390-08-9; Lib: nist_msms; ID: 258128.

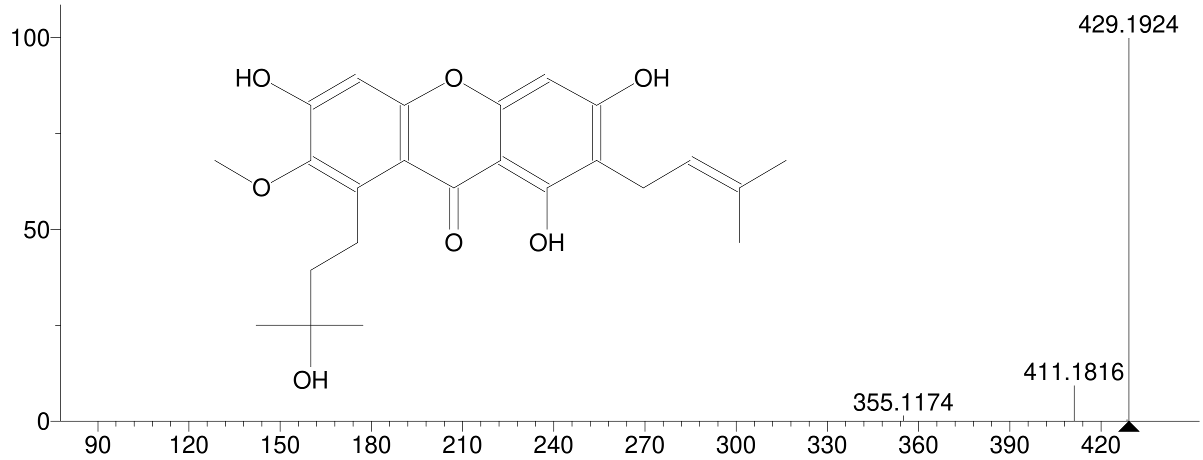

Hit 2 : 21-Carboxylic acid triamcinolone acetonide [M+H-HF]+ HCD 34V P=429.2
C24H29FO7; MF: 5; RMF: 102; Prob 9.73%; CAS: 53962-41-7; Lib: nist_msms; ID: 449217.

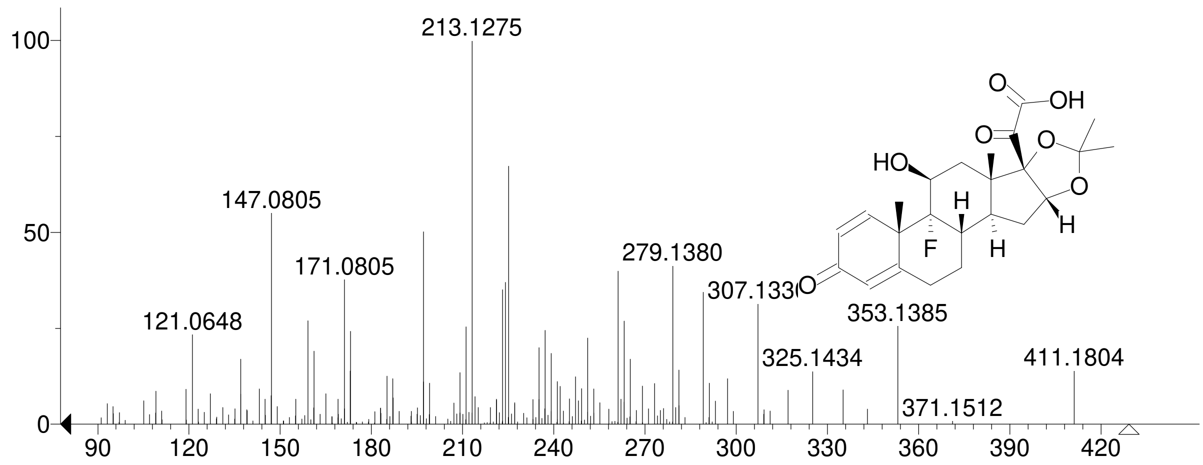

Hit 3 : Terretonin [M+H-C2H4O2]+ HCD 38V P=429.2
C26H32O9; MF: 4; RMF: 72; Prob 9.36%; CAS: 71911-90-5; Lib: nist_msms; ID: 460560.

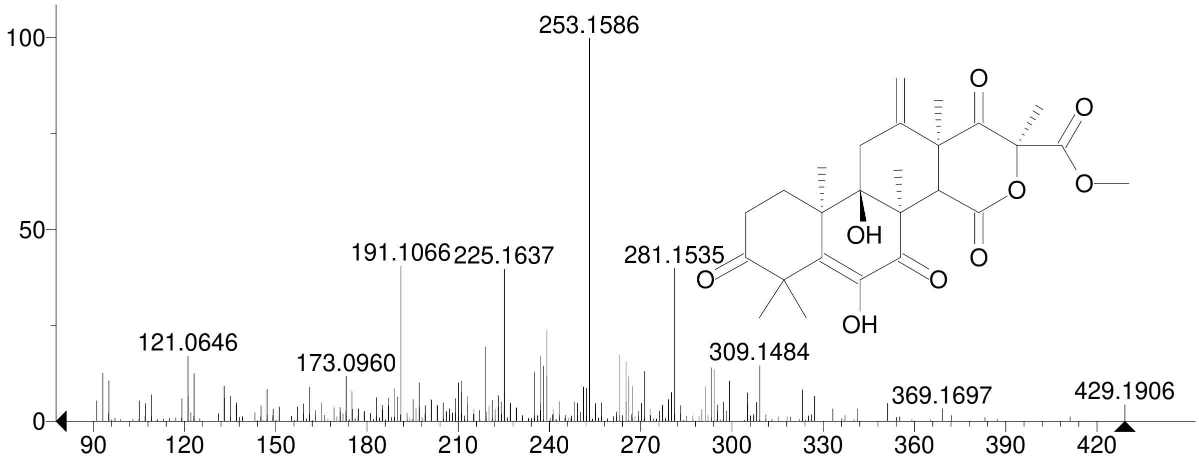

Hit 4 : (2R,3R,4S,5S,6R)-2-[6-hydroxy-3-[(E)-3-hydroxybut-1-enyl]-2,4,4-trimethylcyclohexyl]oxy-6-(hydroxymethy
C19H34O8; MF: 1; RMF: 51; Prob 8.27%; Lib: anthocyanins mona; ID: 9985.

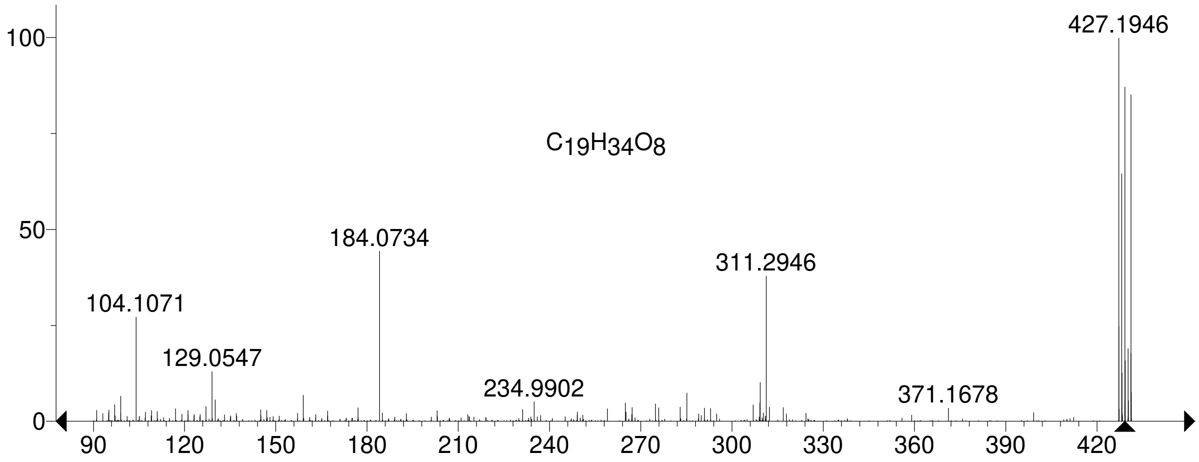

Hit 5 : [8-[2-(3-methylbutanoyloxy)propan-2-yl]-2-oxo-8,9-dihydrofuro[2,3-h]chromen-9-yl] (Z)-2-methylbut-2-enoa
C24H28O7; MF: 0; RMF: 16; Prob 7.95%; Lib: mona-export-vf-npl_qexactive; ID: 20018.

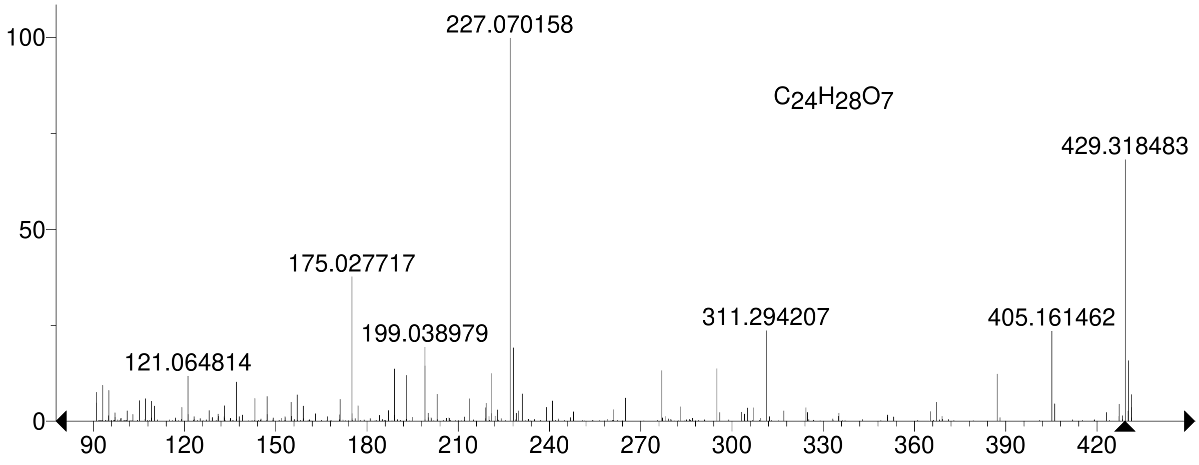

Unknown: Unknown (4.90_605.2552m/z)
Compound in Library Factor = 0

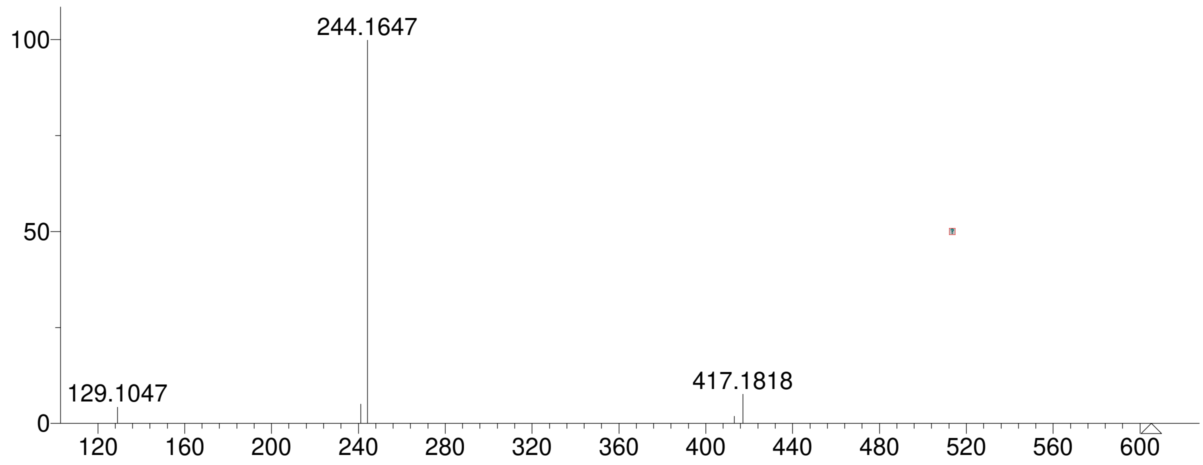

Hit 1 : 6-(furan-3-yl)-6,8,12,16,21-pentahydroxy-7,15-dimethyl-9-oxo-3,17,19-trioxaheptacyclo[9.9.3.0A^'1,A^'1a^'
C31H40O12; MF: 0; RMF: 12; Prob 33.3%; Lib: mona-export-vf-npl_qexactive; ID: 8383.

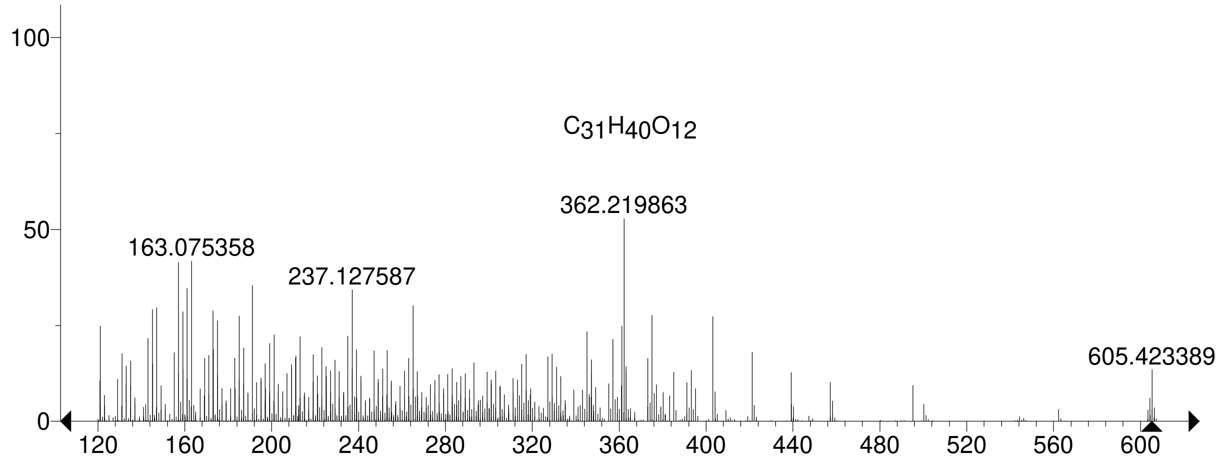

Hit 2 : [Trp7,Leu8] LH-RH, free acid [M-2H]2- HCD 70eV P=605.3
C60H72N14O14; MF: 0; RMF: 10; Prob 33.3%; Lib: nist_msms2; ID: 79092.

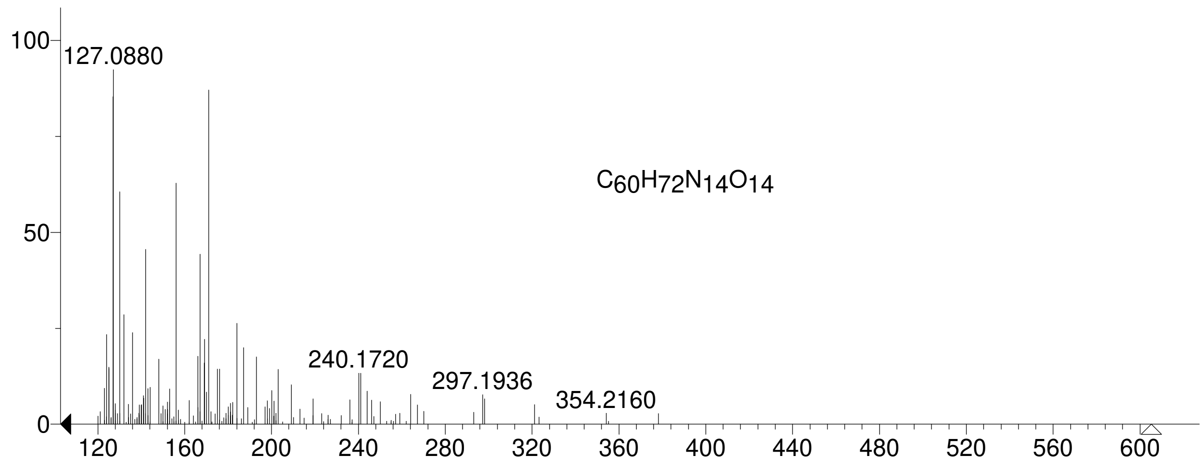

Hit 3 : NCGC00347665-02_C31H40O12_Butanoic acid, 2-methyl-, 9-(3-furanyl)decahydro-2,6,8,9,14-pentahydro
C31H40O12; MF: 0; RMF: 8; Prob 33.3%; Lib: mona-export-gnps; ID: 13856.

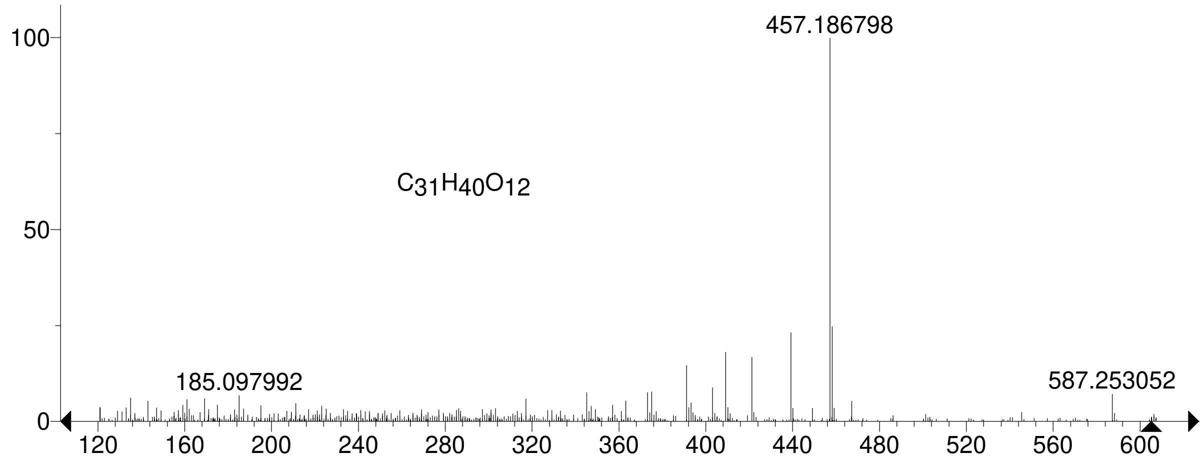

Unknown: Unknown (4.90_680.2744m/z)
Compound in Library Factor = 0

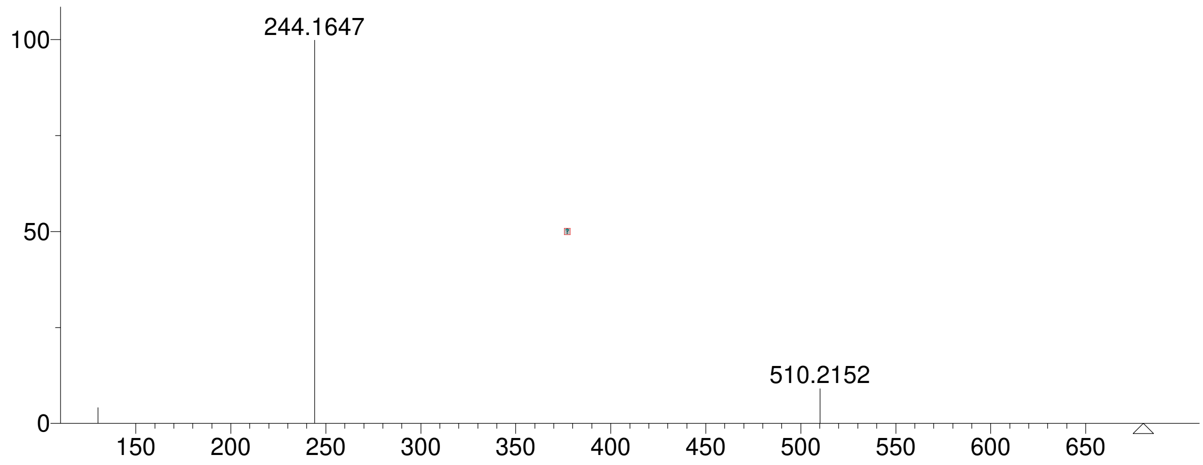

Unknown: Unknown (4.91_427.1782m/z)
Compound in Library Factor = -359

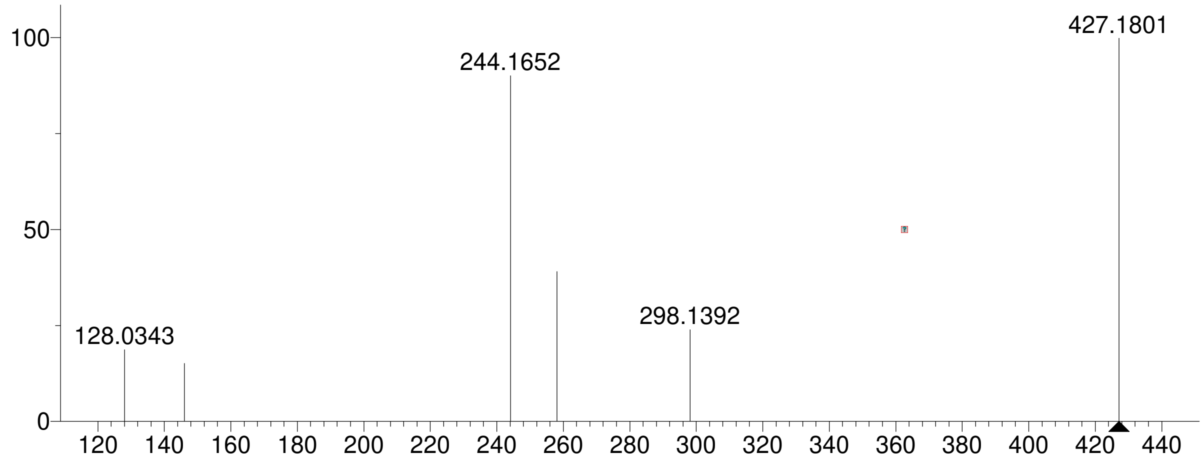

Hit 1 : 3-Isomangostin [M+OH]- HCD 21V P=427.2
C24H26O6; MF: 333; RMF: 976; Prob 92.8%; CAS: 19275-46-8; Lib: nist_msms; ID: 227726.

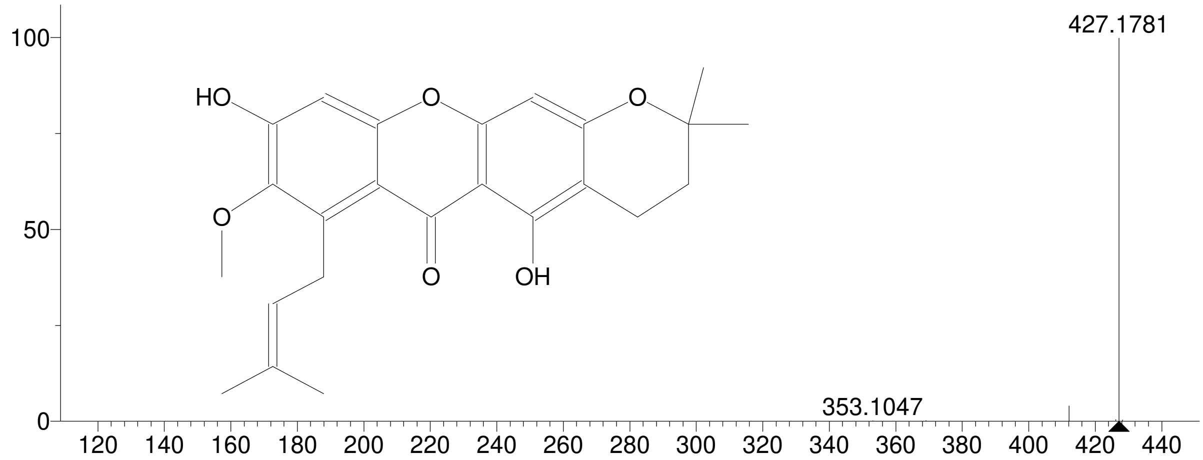

Hit 2 : Garcinone D [M-H]- HCD 25V P=427.2
C24H28O7; MF: 233; RMF: 926; Prob 6.91%; CAS: 107390-08-9; Lib: nist_msms; ID: 258174.

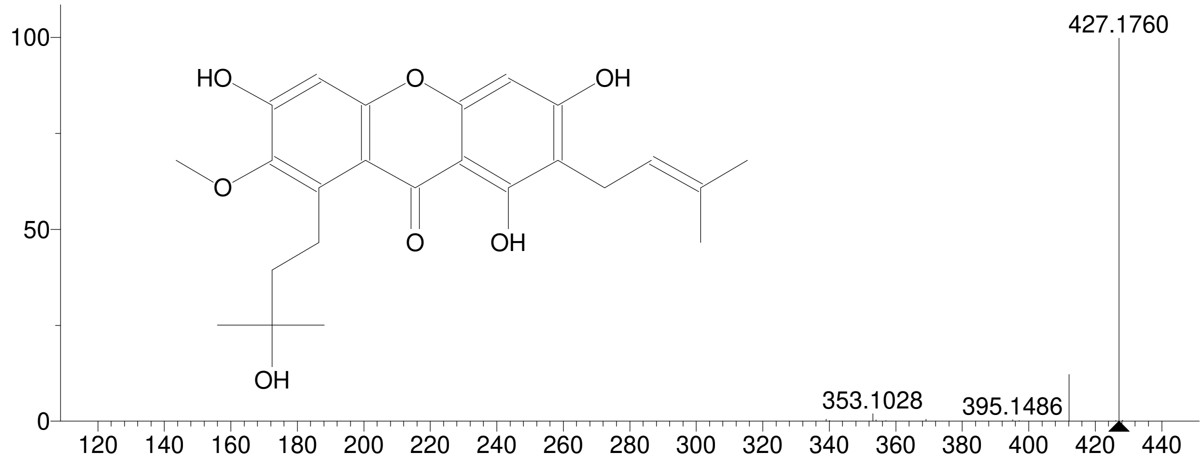

Hit 3 : Estrone glucuronide [M-H-H2O]- HCD 14V P=427.2
C24H30O8; MF: 68; RMF: 558; Prob 0.16%; CAS: 2479-90-5; Lib: nist_msms; ID: 144996.

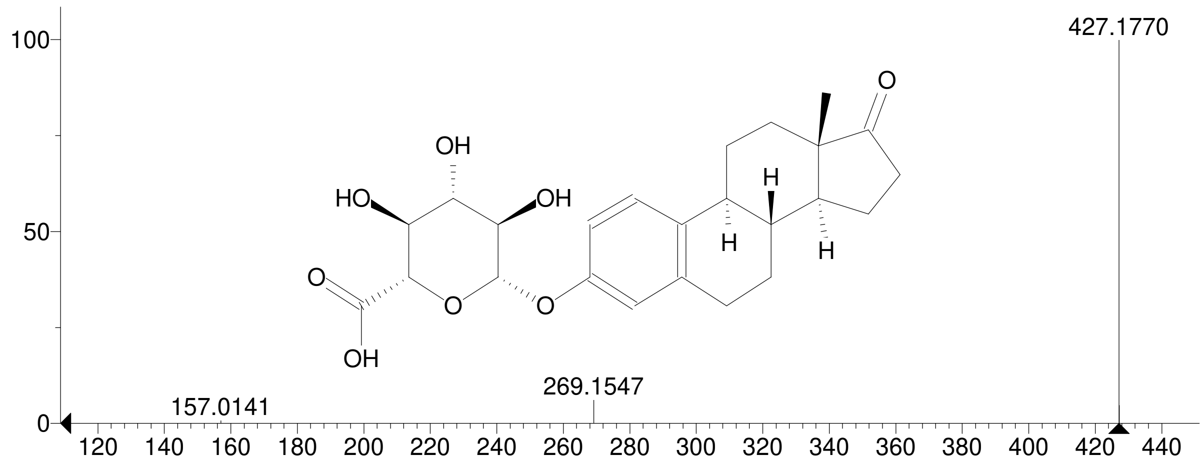

Hit 4 : [8,8-dimethyl-10-(3-methylbut-2-enoyloxy)-2-oxo-9,10-dihydropyrano[2,3-f]chromen-9-yl] 3-methylbutanoat
C24H28O7; MF: 0; RMF: 116; Prob 0.02%; Lib: mona-export-lc-ms-ms_negative_mode; ID: 22316.

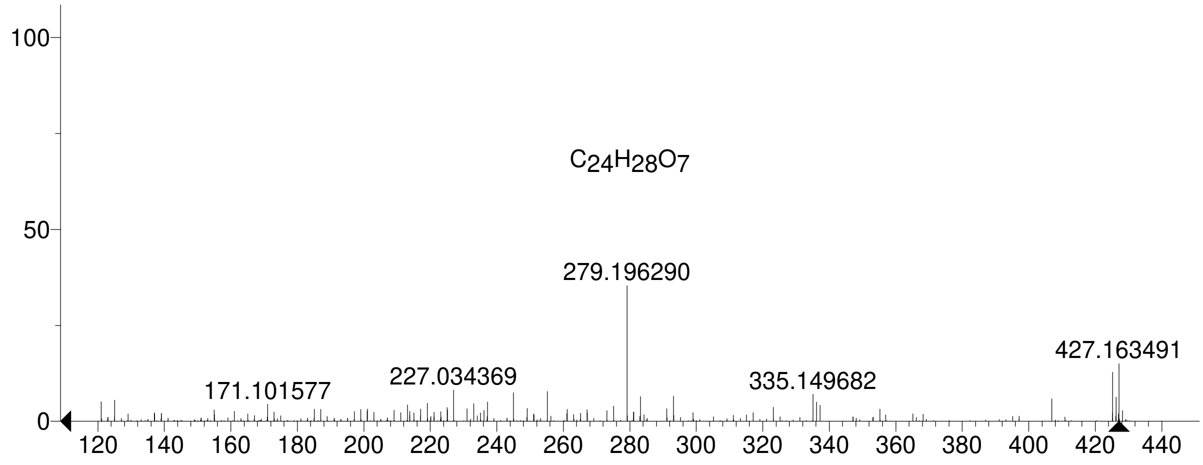

Hit 5 : NCGC00380647-01_C17H30O12_alpha-D-Glucopyranoside, alpha-D-glucopyranosyl, 3-(2-methylbutanoa
C17H30O12; MF: 0; RMF: 57; Prob 0.02%; Lib: mona-export-gnps; ID: 10315.

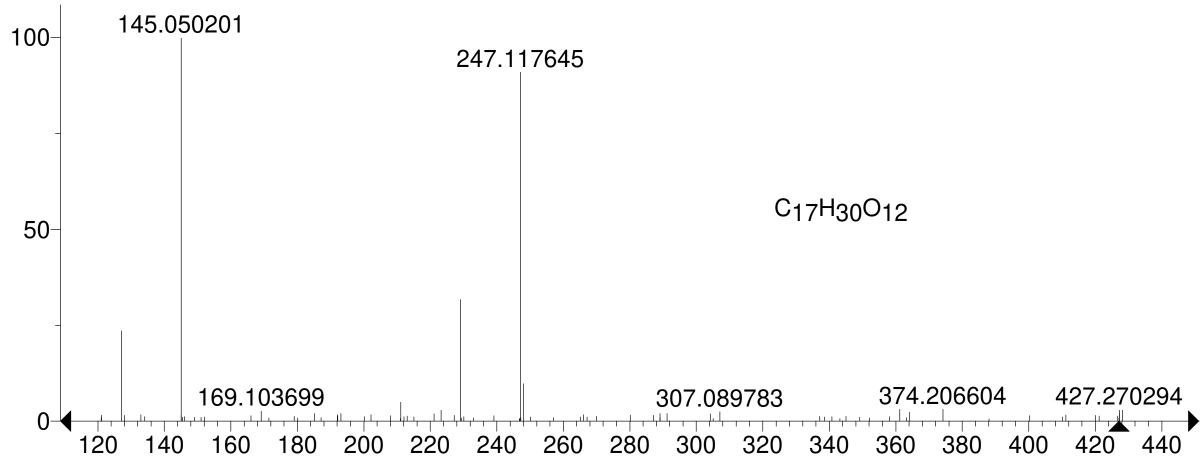

Unknown: Unknown (4.91_544.2129m/z)
Compound in Library Factor = 0

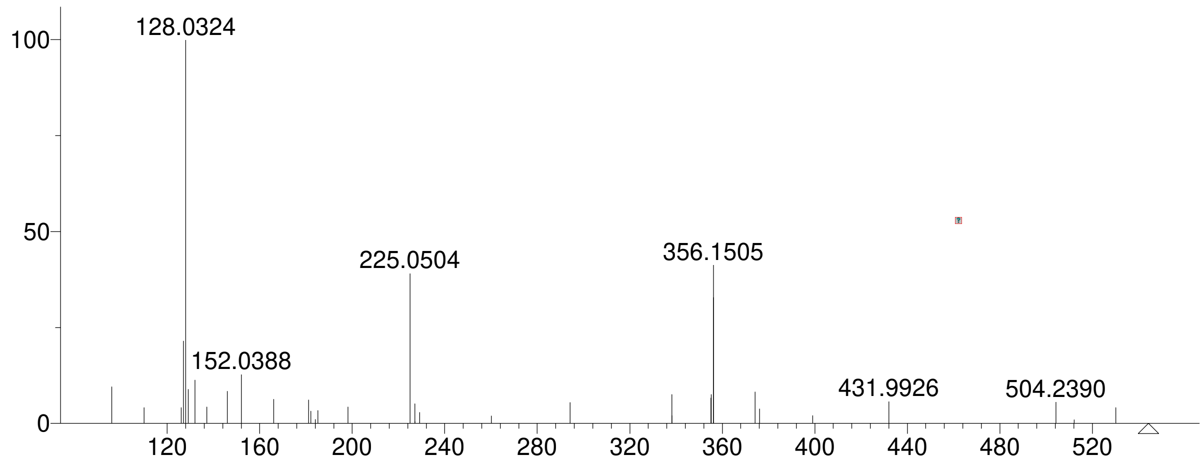

Unknown: Unknown (4.92_365.1342m/z)
Compound in Library Factor = 0

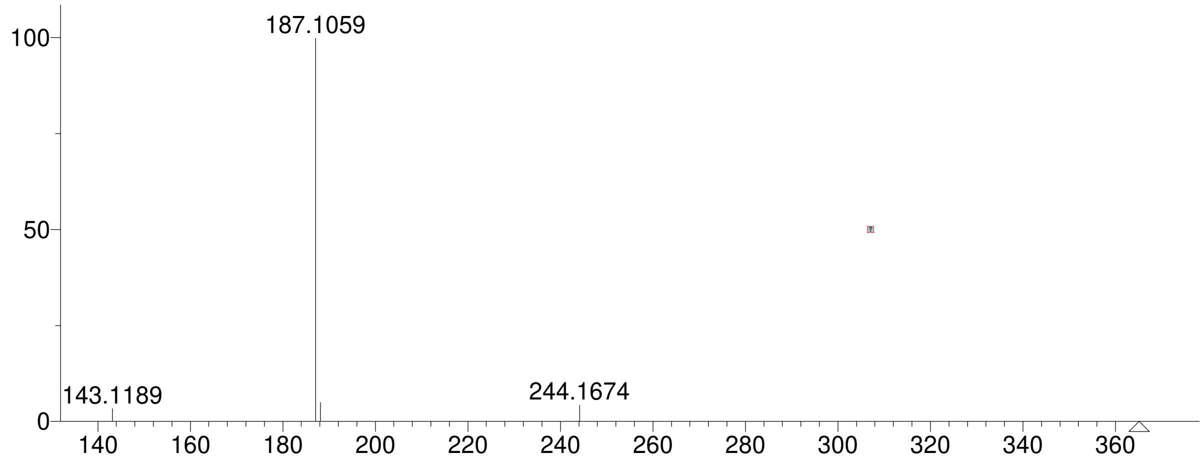

Hit 1 : [5-acetyloxy-3-(hydroxymethyl)-2-oxo-6-propan-2-ylcyclohex-3-en-1-yl] 3-methylbutanoate [M+K]+ 45HCD
C17H26O6; MF: 0; RMF: 25; Prob 100.0%; Lib: mona-export-vf-npl_qexactive; ID: 8246.

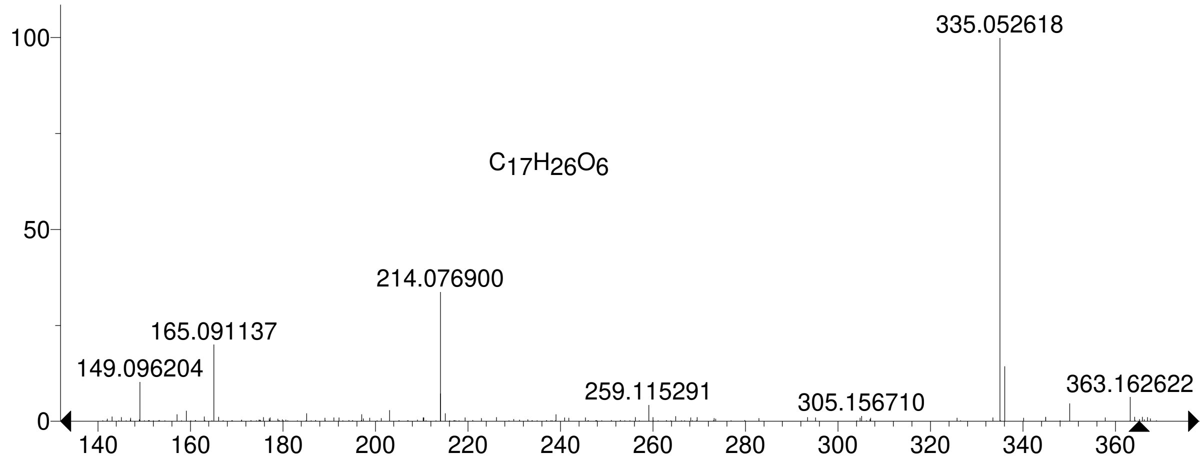

** Search Report Page 1 of 1 **

Unknown: Unknown (4.92_701.3099m/z)
Compound in Library Factor = 0

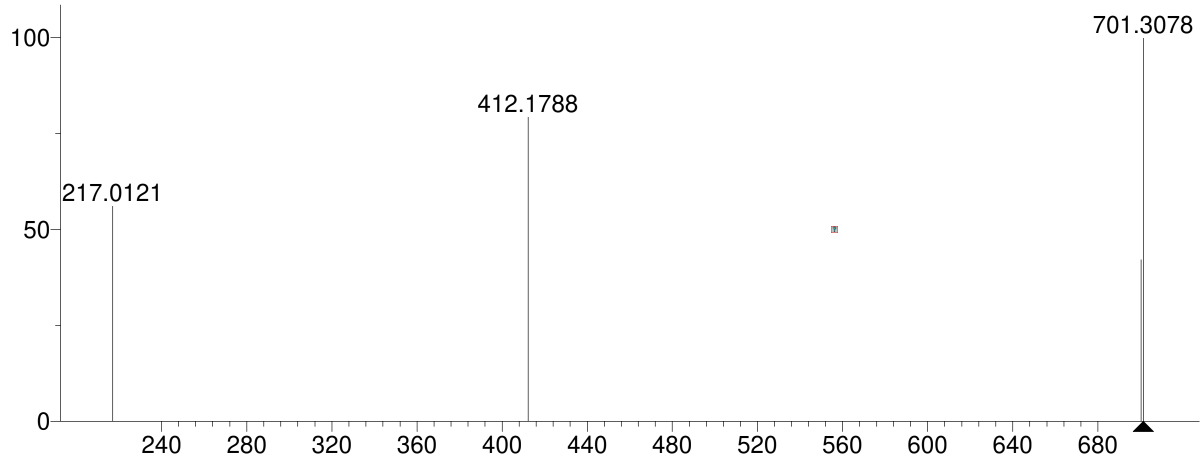

Unknown: Unknown (4.93_357.1652m/z)
Compound in Library Factor = 0

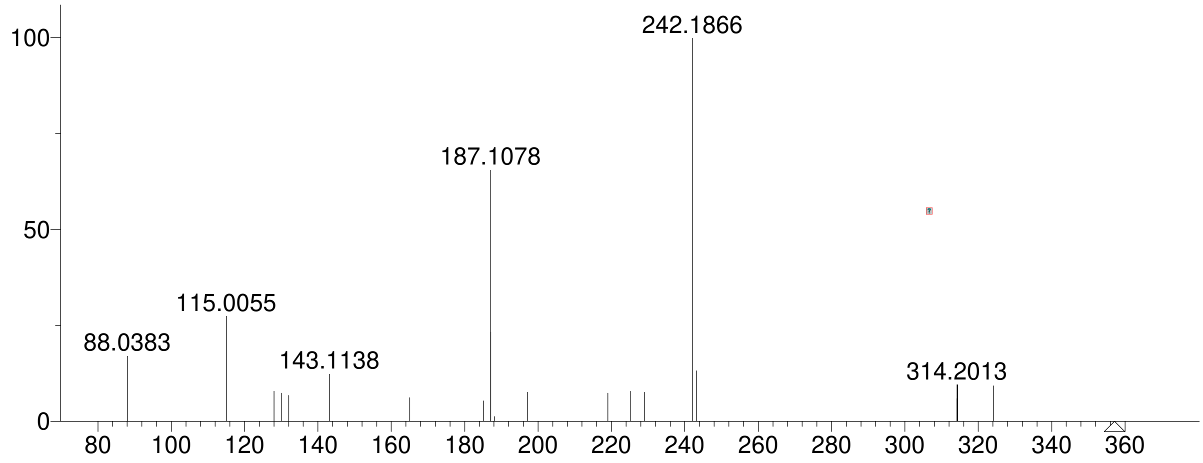

Hit 1 : [(1S,3aR,5R,5aR,8aR,9S,9aR)-1,5,8a-trimethyl-2,8-dioxo-3a,4,5,5a,9,9a-hexahydro-1H-azuleno[6,5-b]fura
C19H26O5; MF: 0; RMF: 10; Prob 50.0%; Lib: mona-export-vf-npl_qexactive; ID: 7288.

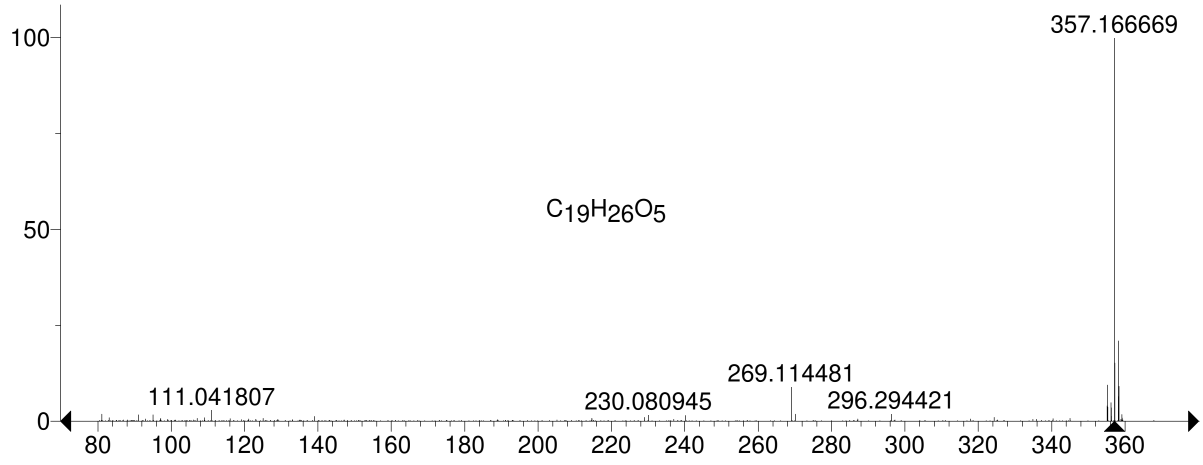

Hit 2 : NCGC00381117-01!2-amino-4-hydroxy-5-[[1-(4-methoxy-6-oxopyran-2-yl)-3-methylbutyl]amino]-5-oxopent
C16H24N2O7; MF: 0; RMF: 8; Prob 50.0%; Lib: mona-export-gnps; ID: 8549.

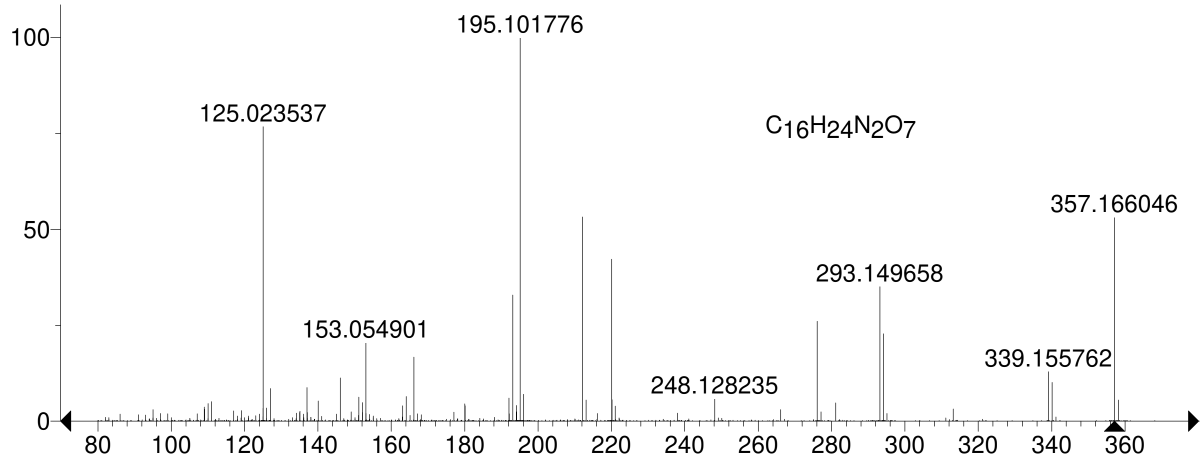

Unknown: Unknown (4.93_832.2981m/z)
Compound in Library Factor = 0

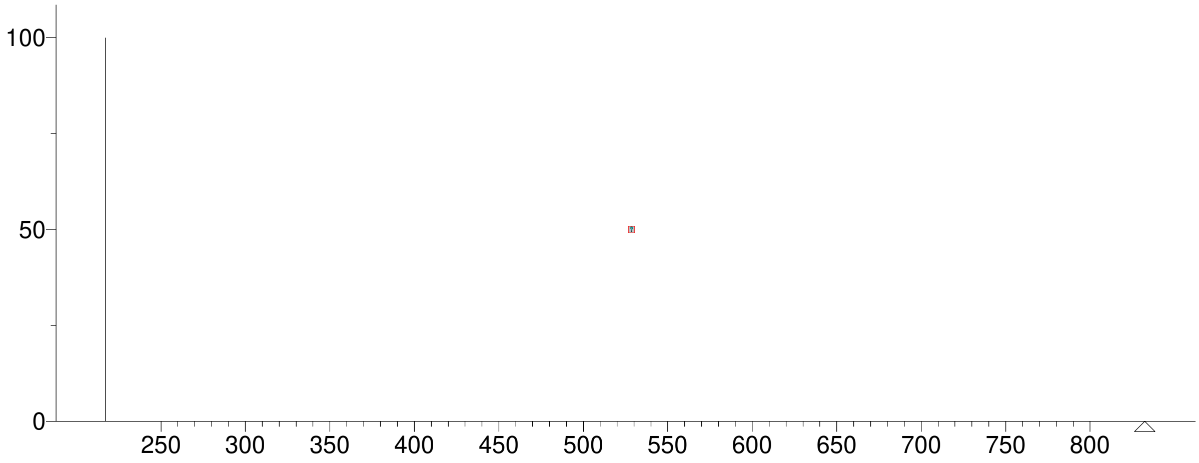

Unknown: Unknown (4.94_472.1933m/z)
Compound in Library Factor = 0

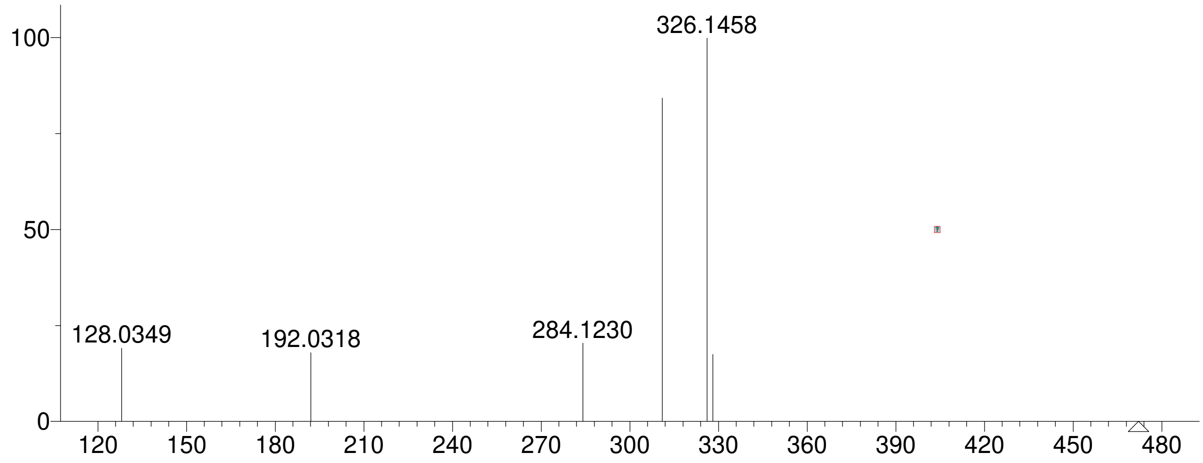

Hit 1 : methyl 3-(3,4-dihydroxyphenyl)-2-[[4-hydroxy-3-(3-methylbut-2-enyl)phenyl]methyl]-4-methoxy-5-oxofuran-2
C25H26O8; MF: 0; RMF: 12; Prob 100.0%; Lib: mona-export-vf-npl_qexactive; ID: 9391.

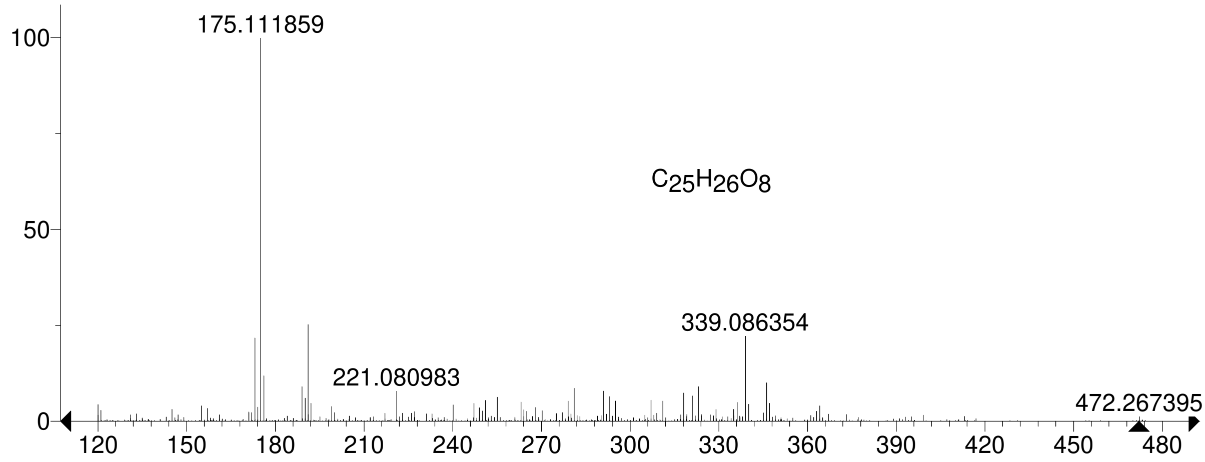

Unknown: Unknown (4.94_832.2663m/z)
Compound in Library Factor = 0

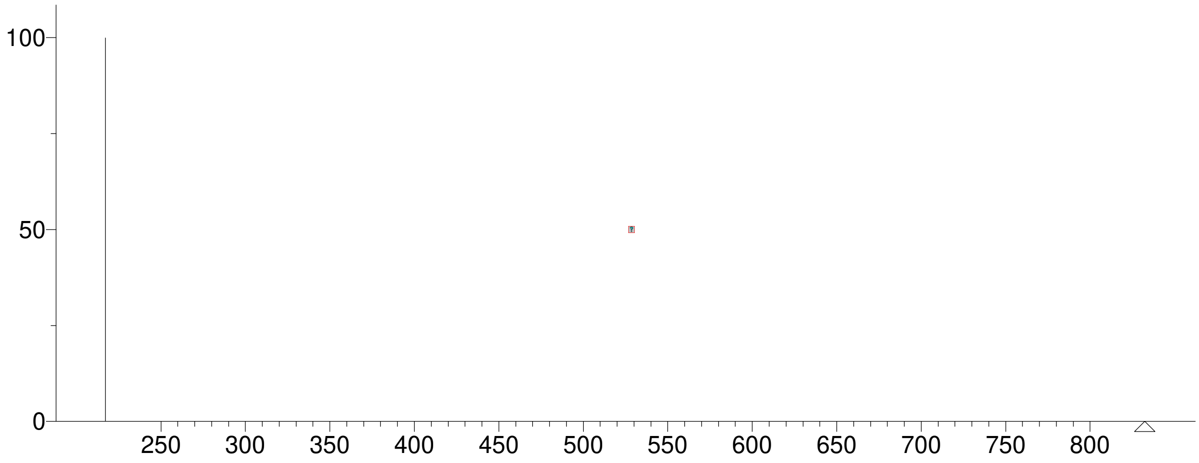

Unknown: Unknown (4.96_481.0946m/z)
Compound in Library Factor = -3273

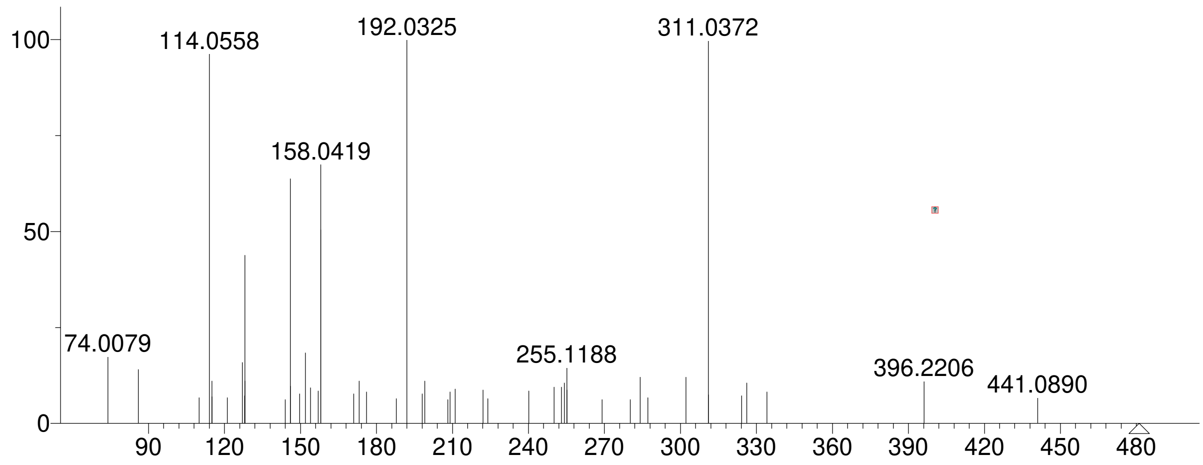

Hit 1 : Gossypin [M+H]+ HCD 95V P=481.1
C21H20O13; MF: 1; RMF: 66; Prob 17.1%; CAS: 652-78-8; Lib: nist_msms; ID: 111983.

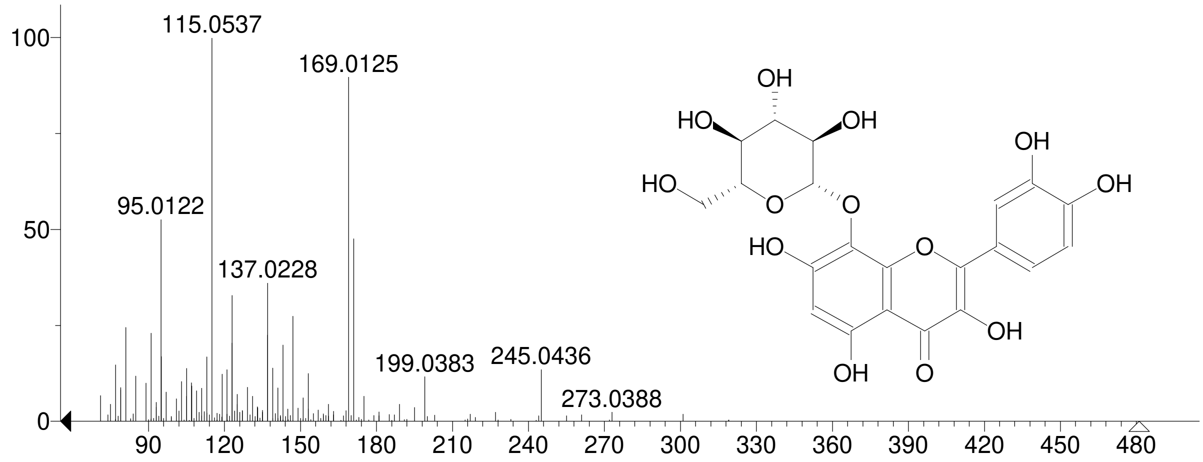

Hit 2 : Delphinidin 3-galactoside cation [Cat-H+OH]- HCD 91V P=481.1
C21H21O12; MF: 1; RMF: 37; Prob 17.1%; CAS: 197250-28-5; Lib: nist_msms; ID: 394733.

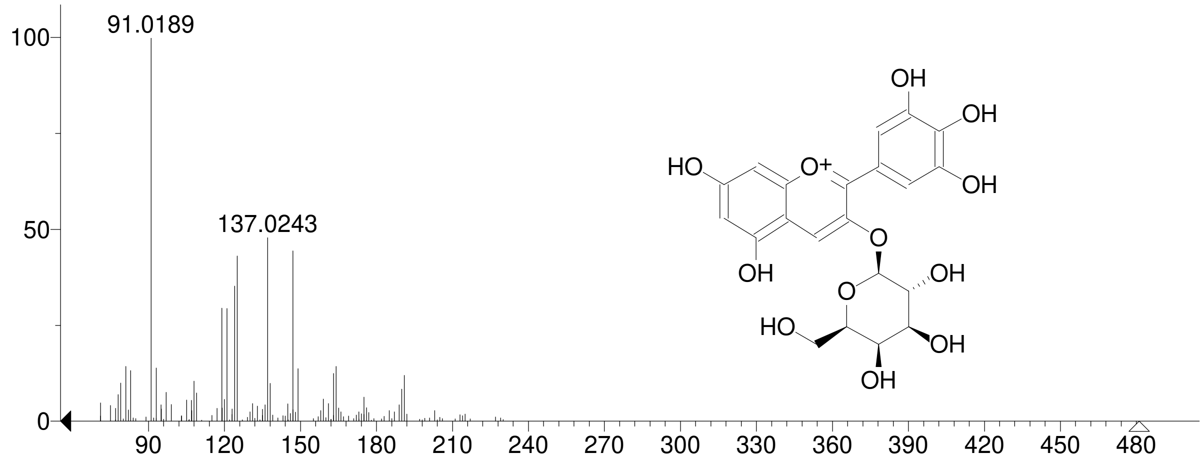

Hit 3 : Quercetagetin-7-O-glucoside [M+H]+ HCD 125V P=481.1
C21H20O13; MF: 0; RMF: 53; Prob 16.4%; CAS: 548-75-4; Lib: nist_msms; ID: 108305.

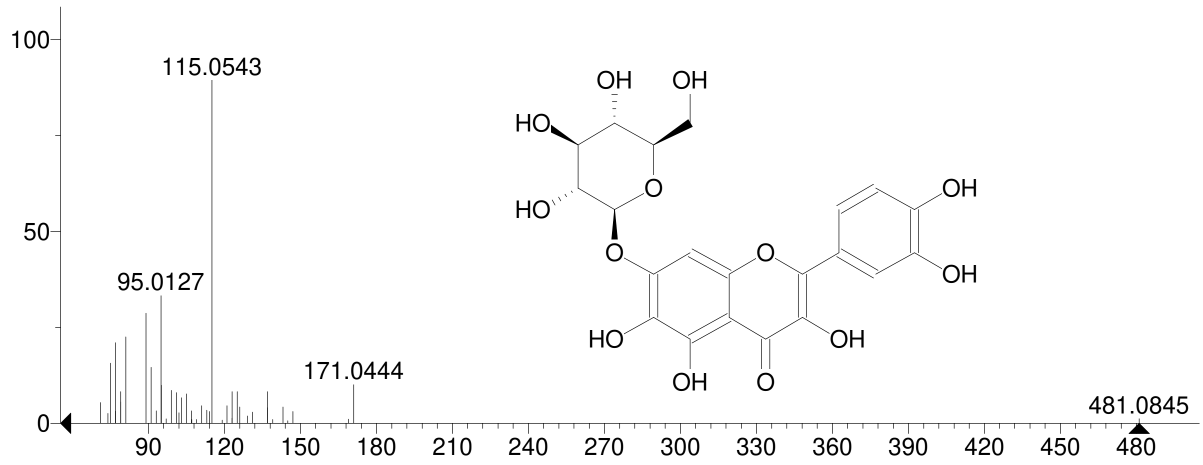

Hit 4 : Gossypetin-8-C-glucoside [M+H]+ 50eV P=481.1
C21H20O13; MF: 0; RMF: 17; Prob 16.4%; Lib: anthocyanins mona; ID: 680.

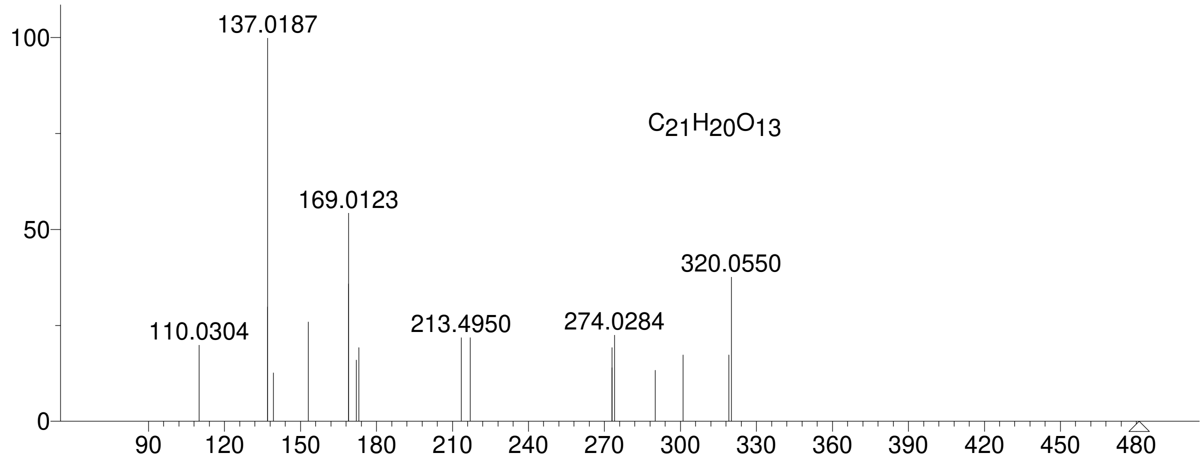

Hit 5 : NCGC00180655-02!5,7-dihydroxy-3-[(2S,3R,4S,5R,6R)-3,4,5-trihydroxy-6-(hydroxymethyl)oxan-2-yl]oxy-2
C21H20O13; MF: 0; RMF: 8; Prob 16.4%; Lib: mona-export-gnps; ID: 9816.

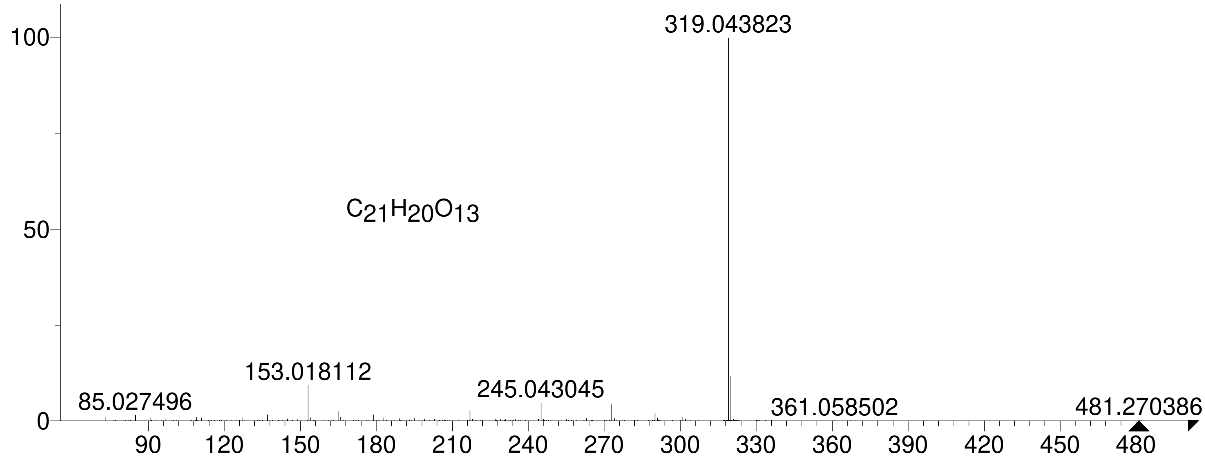

Unknown: Unknown (4.96_496.2031m/z)
Compound in Library Factor = -3273

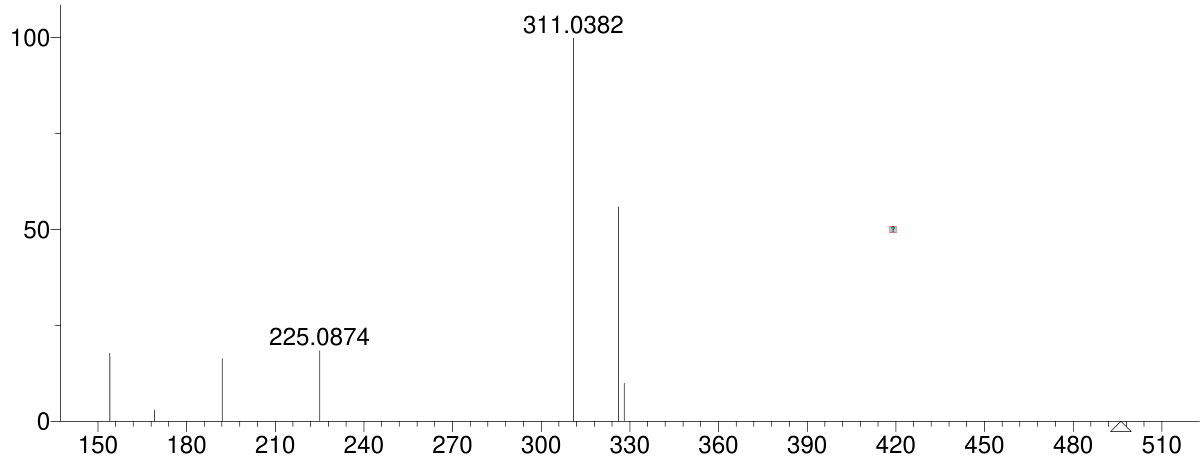

Hit 1 : (2R,3S,4S,5R,6S)-2-[(3,4,5-trihydroxyoxan-2-yl)oxymethyl]-6-(3,4,5-trimethoxyphenoxy)oxane-3,4,5-triol [M
C20H30O13; MF: 1; RMF: 174; Prob 25.7%; Lib: mona-export-vf-npl_qexactive; ID: 9258.

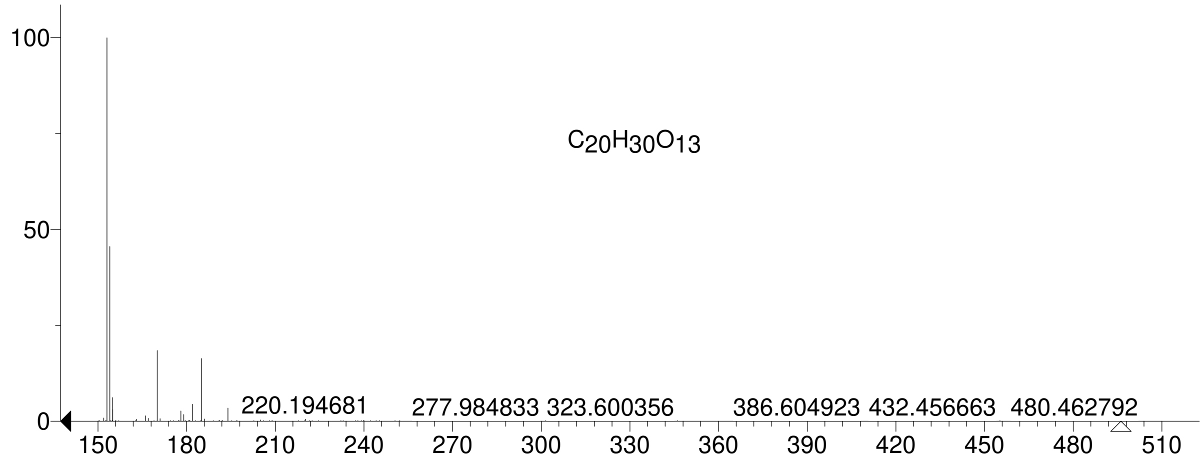

Hit 2 : (2R,3S,4S,5R,6S)-2-[[(2S,3R,4R)-3,4-dihydroxy-4-(hydroxymethyl)oxolan-2-yl]oxymethyl]-6-(3,4,5-trimetho
C20H30O13; MF: 0; RMF: 150; Prob 24.8%; Lib: mona-export-vf-npl_qexactive; ID: 9783.

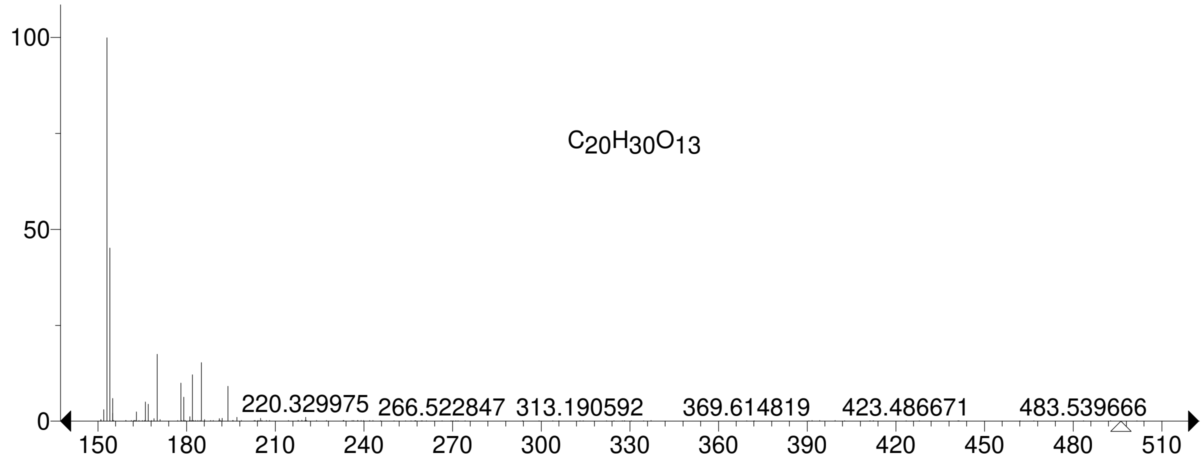

Hit 3 : NCGC00180025-03!(2R,3S,4S,5R,6S)-2-[[(2S,3R,4R)-3,4-dihydroxy-4-(hydroxymethyl)oxolan-2-yl]oxymetl
C20H30O13; MF: 0; RMF: 82; Prob 24.8%; Lib: mona-export-gnps; ID: 8565.

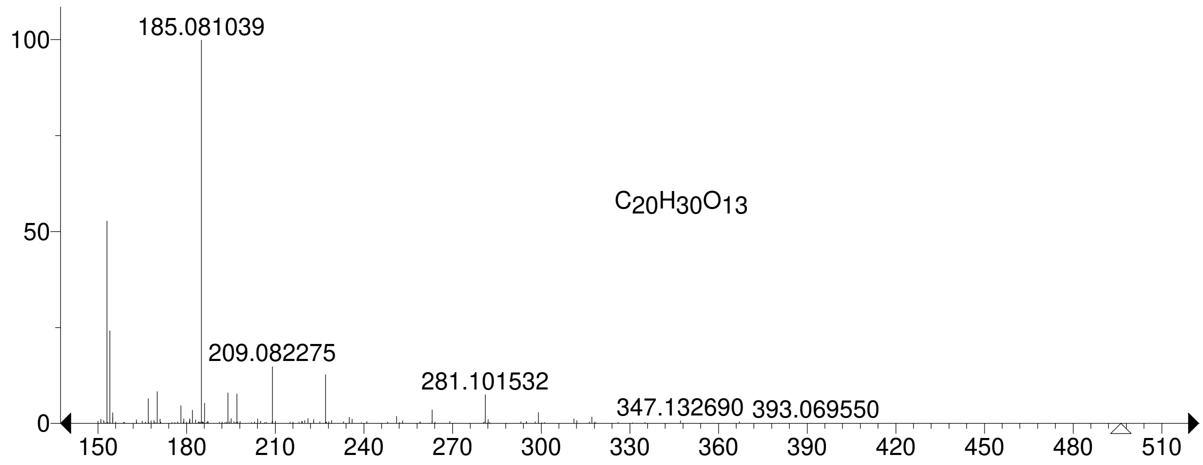

Hit 4 : NCGC00180666-02!(2R,3S,4S,5R,6S)-2-[(3,4,5-trihydroxyoxan-2-yl)oxymethyl]-6-(3,4,5-trimethoxyphenox
C20H30O13; MF: 0; RMF: 79; Prob 24.8%; Lib: mona-export-gnps; ID: 8997.

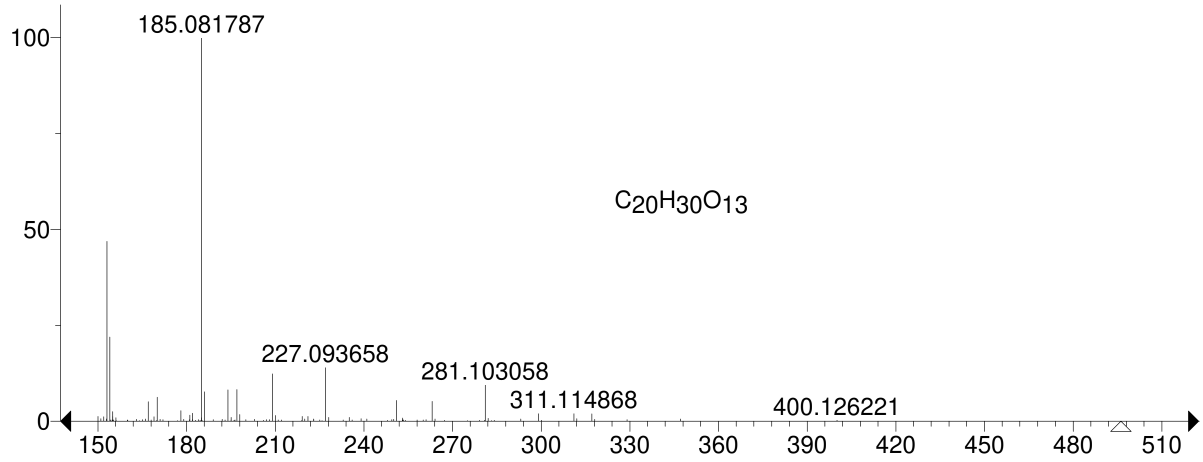

Unknown: Unknown (4.96_516.2160m/z)
Compound in Library Factor = 0

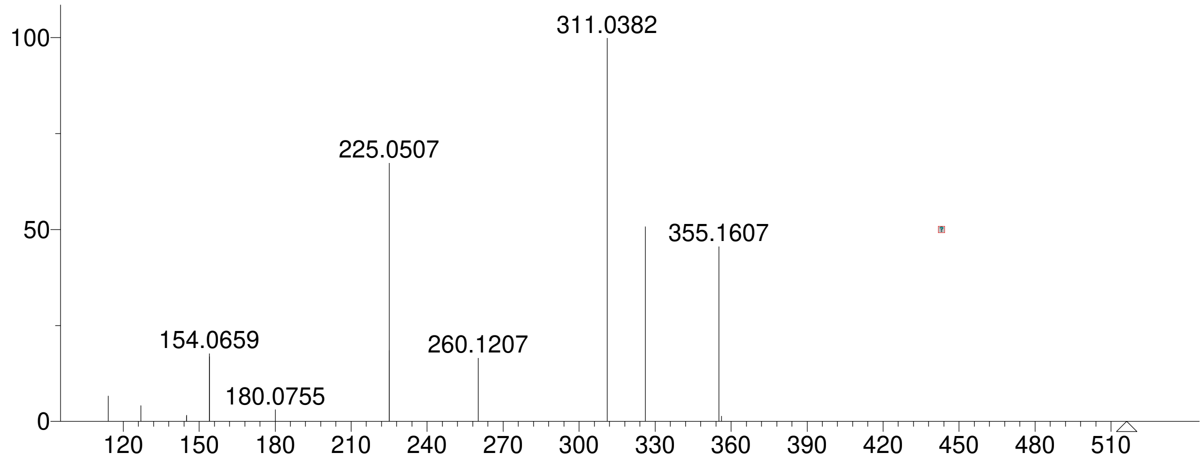

Hit 1 : Asp371, Tyrosinase (369-377), human [M+2H]2+ HCD 41eV P=516.2
C42H66N10O16S2; MF: 0; RMF: 23; Prob 100.0%; Lib: nist_msms2; ID: 47697.

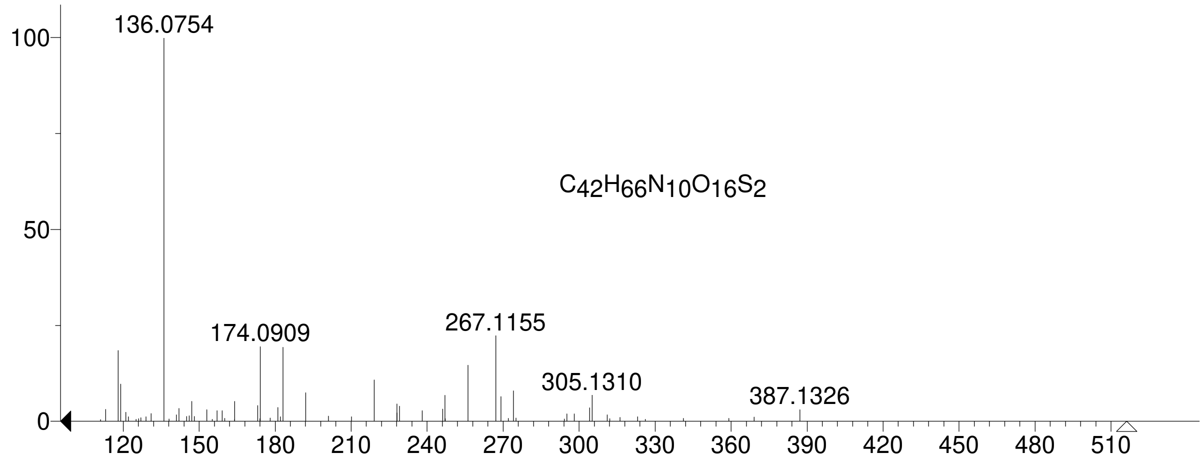

** Search Report Page 1 of 1 **

Unknown: Unknown (4.96_543.2289m/z)
Compound in Library Factor = 0

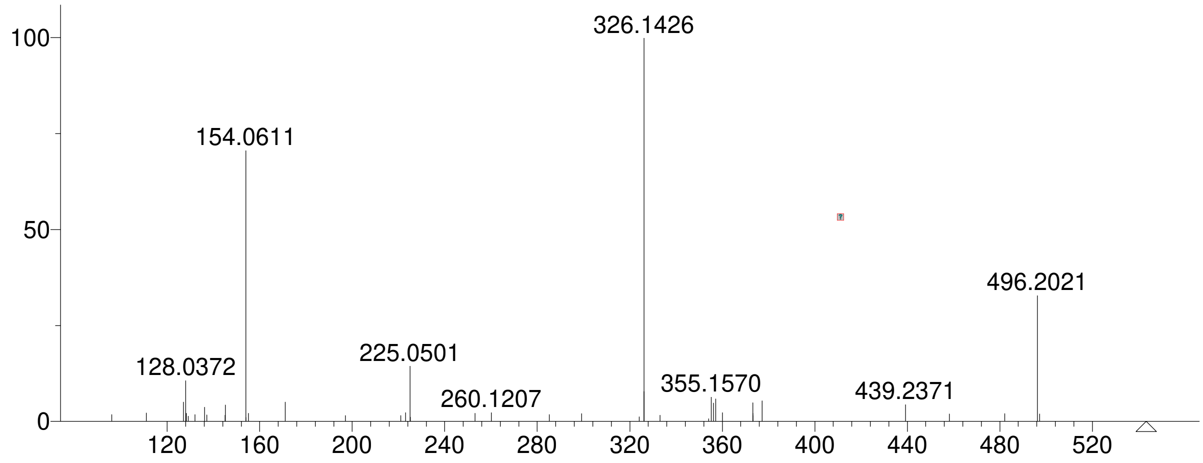

Hit 1 : methyl (2E)-3-[18-(acetyloxy)-7-(furan-3-yl)-17-hydroxy-1,8,15,15-tetramethyl-5-oxo-3,6,14-trioxapentacycl
C29H36O10; MF: 0; RMF: 34; Prob 100.0%; Lib: mona-export-lc-ms-ms_negative_mode; ID: 22886.

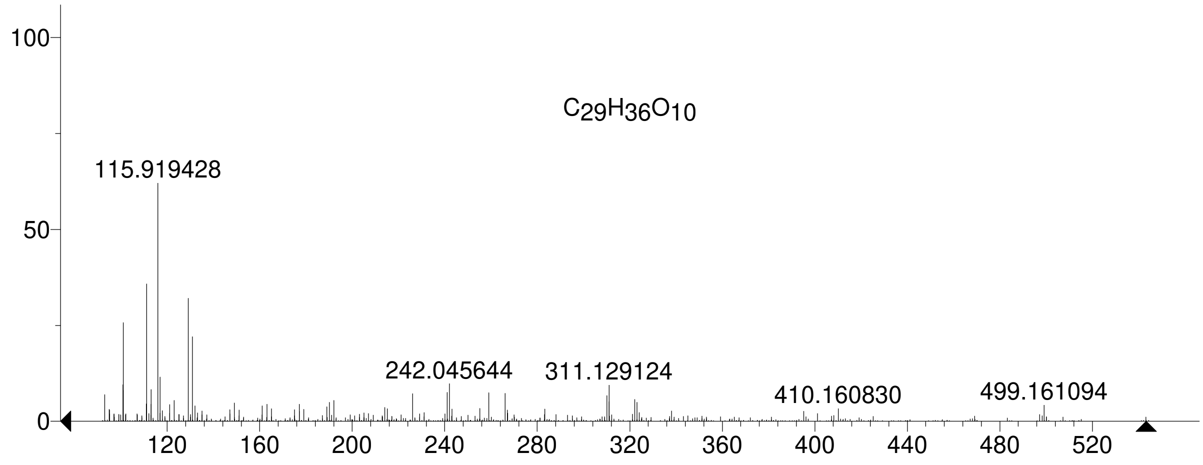

Unknown: Unknown (4.96_600.2412m/z)
Compound in Library Factor = 0

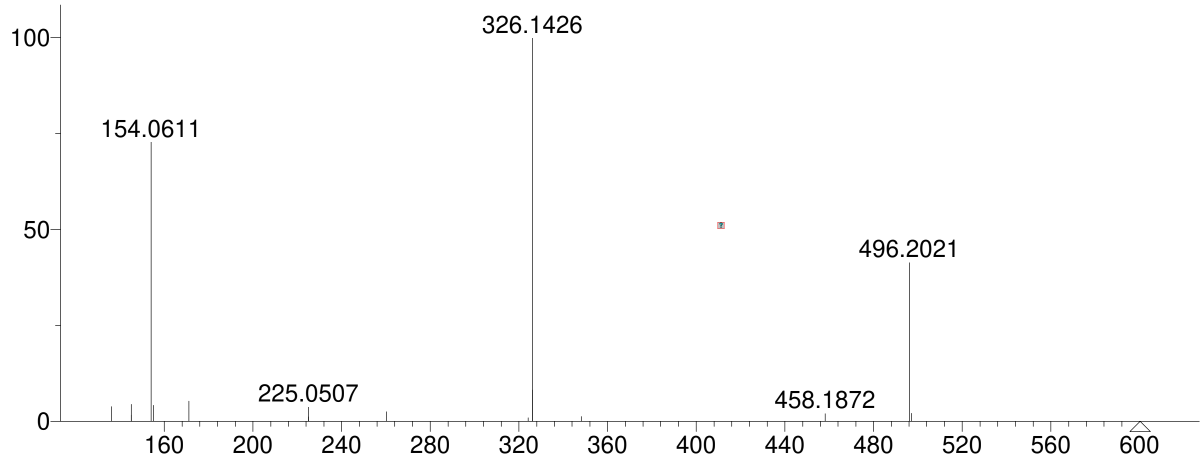

Unknown: Unknown (4.96_646.2562m/z)
Compound in Library Factor = 0

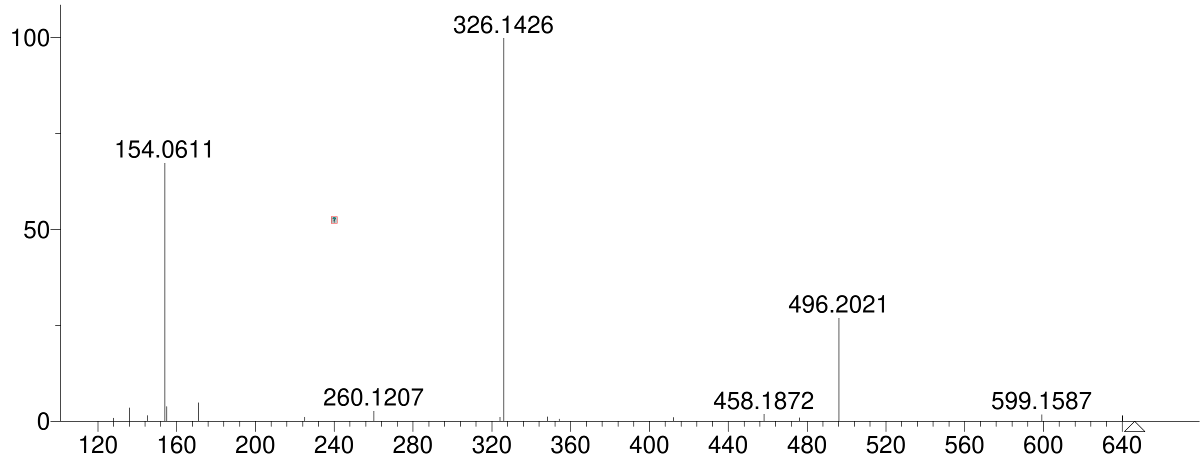

Unknown: Unknown (4.96_666.2618m/z)
Compound in Library Factor = 0

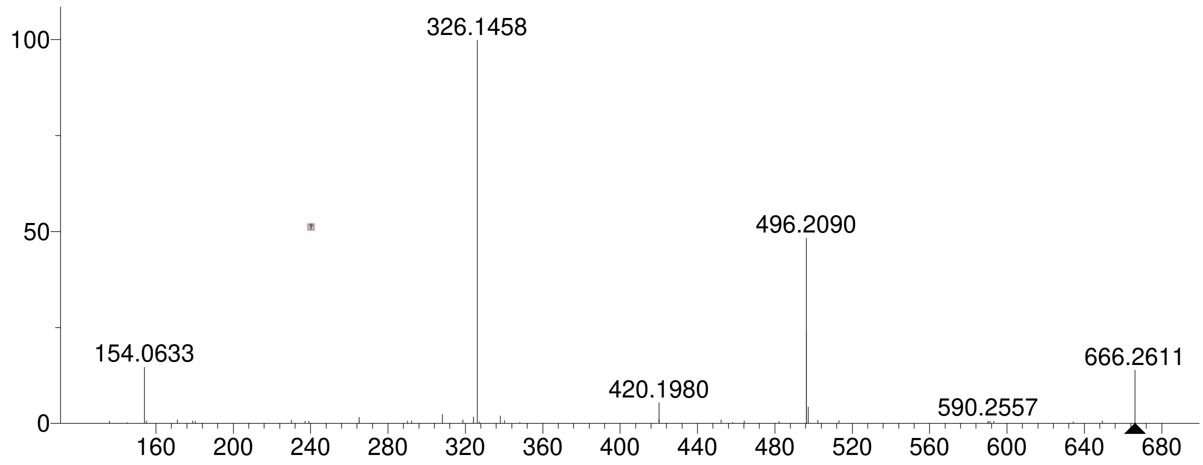

** Search Report Page 1 of 1 **

Unknown: Unknown (4.98_336.1427m/z)
Compound in Library Factor = 0

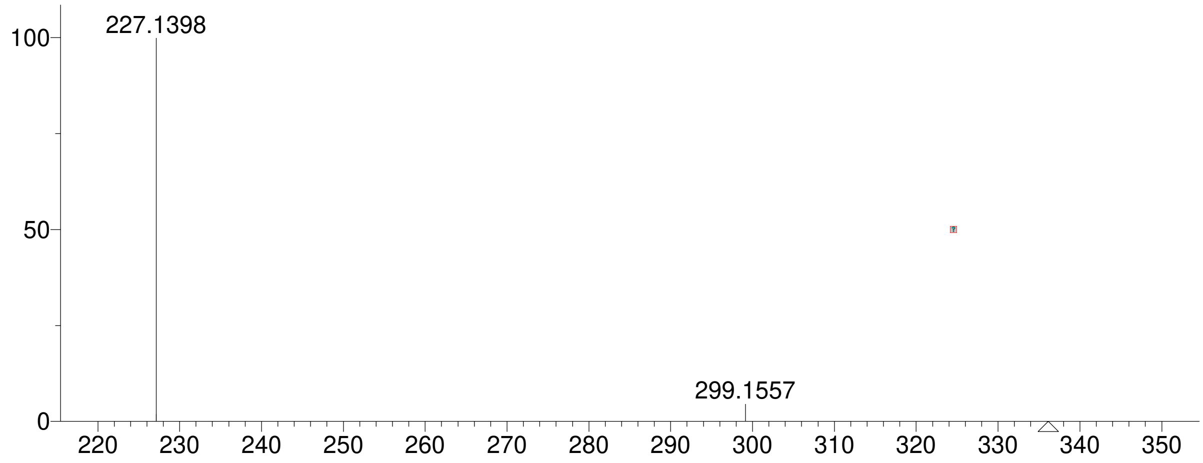

Unknown: Unknown (4.98_404.1530m/z)
Compound in Library Factor = 0

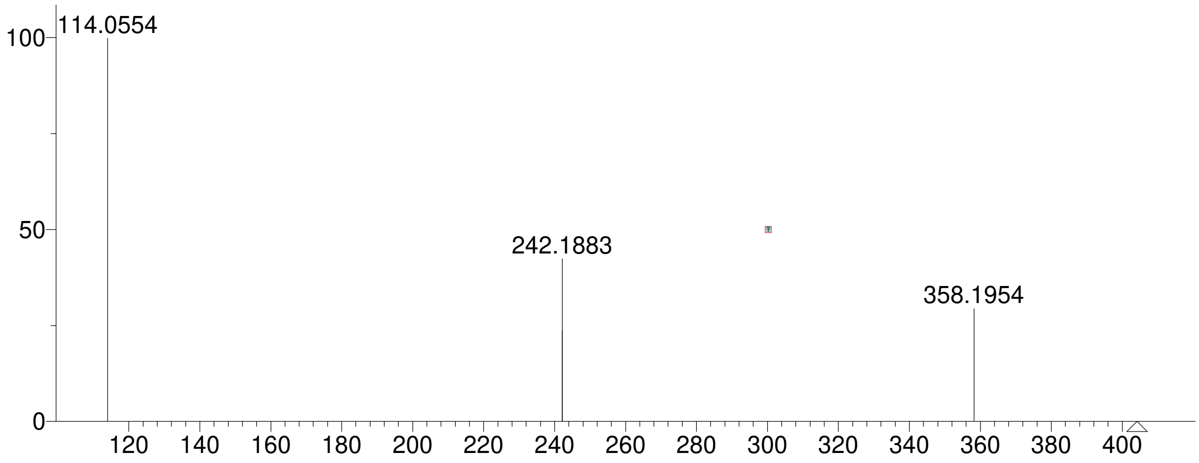

** Search Report Page 1 of 1 **

Unknown: Unknown (4.98_688.2476m/z)
Compound in Library Factor = 0

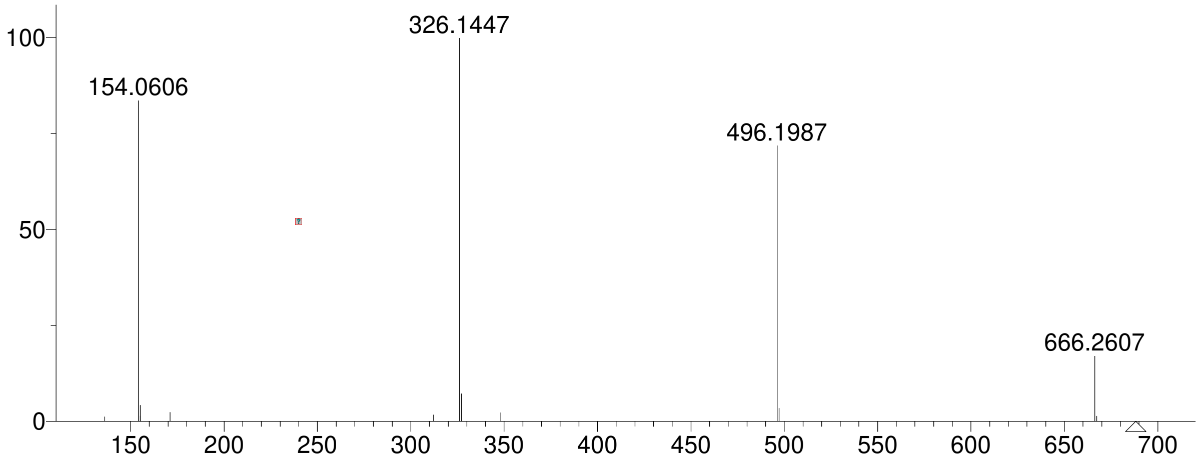

Unknown: Unknown (5.00_600.1442m/z)
Compound in Library Factor = 0

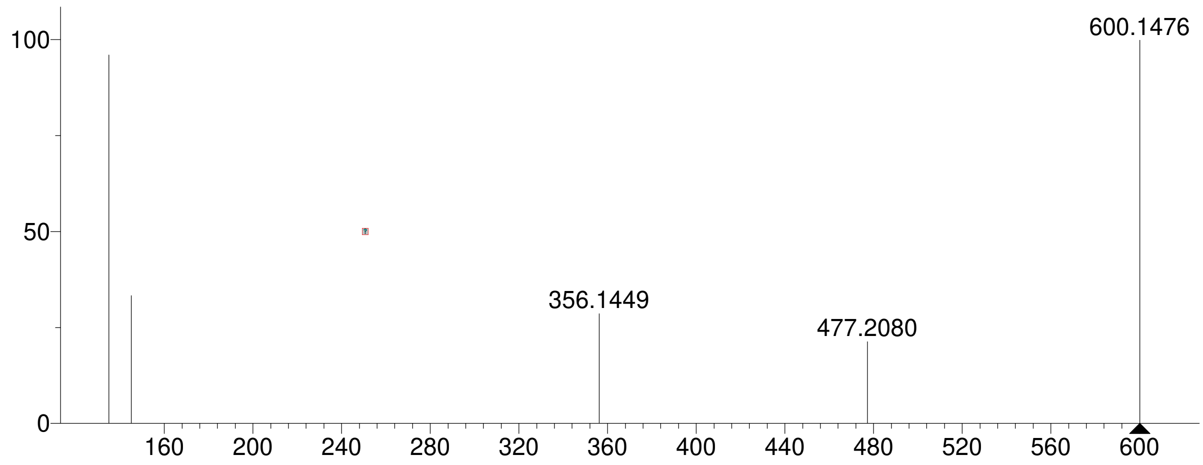

** Search Report Page 1 of 1 **

Unknown: Unknown (5.00_809.3218m/z)
Compound in Library Factor = 0

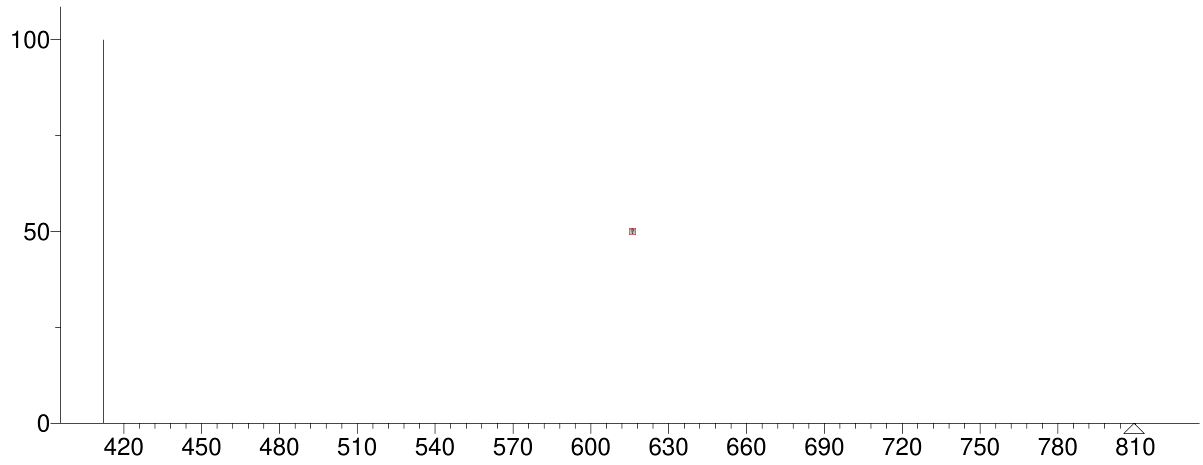

Unknown: Unknown (5.02_314.1601m/z)
Compound in Library Factor = 0

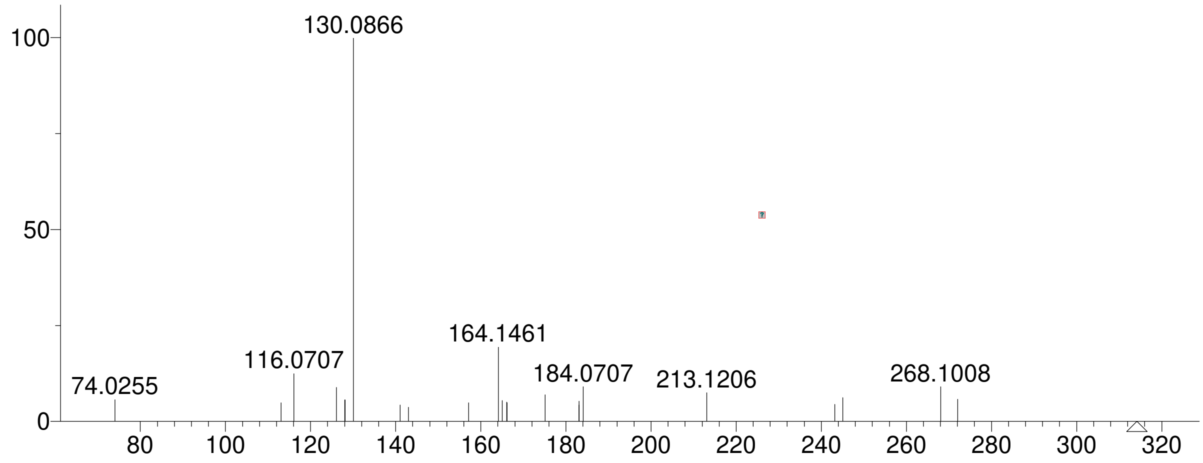

Hit 1 : [(2S,3S)-2-[(E,3R,4S)-3,4-dihydroxypent-1-enyl]-6-oxo-2,3-dihydropyran-3-yl] (E)-2-methylbut-2-enoate [M
C15H20O6; MF: 0; RMF: 7; Prob 100.0%; Lib: mona-export-vf-npl_qexactive; ID: 17659.

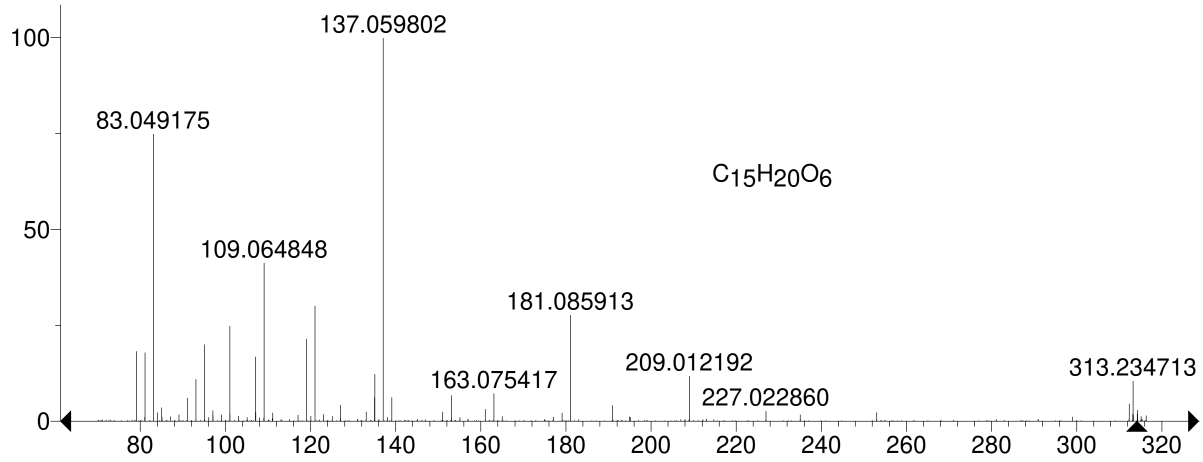

Unknown: Unknown (5.02_358.1972m/z)
Compound in Library Factor = -1309

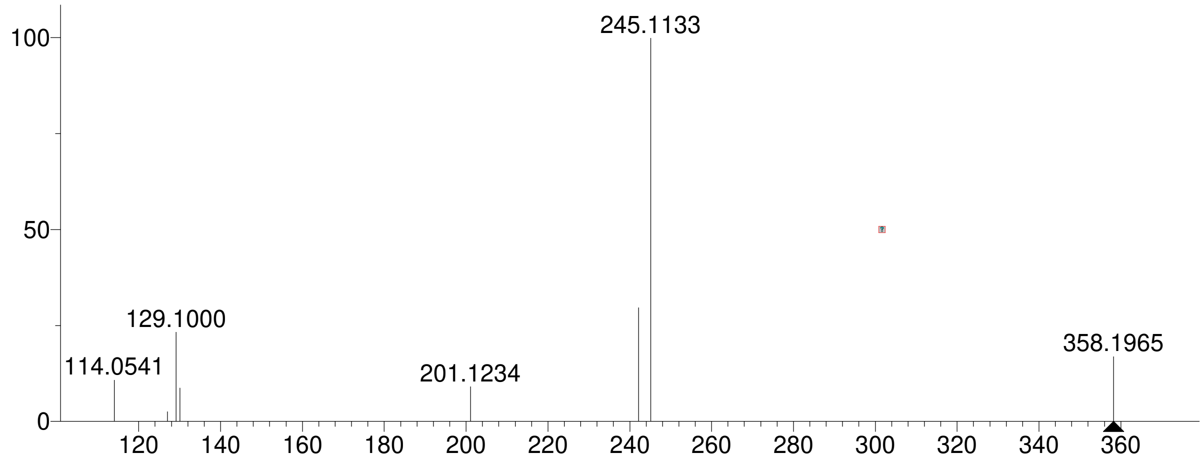

Hit 1 : 4-Hydroxyatorvastatin lactone [M-H-H2O]-=>493.2=>358.2 IT 35% P=358.2
C33H33FN2O5; MF: 58; RMF: 579; Prob 27.2%; CAS: 163217-70-7; Lib: nist_msms; ID: 440854.

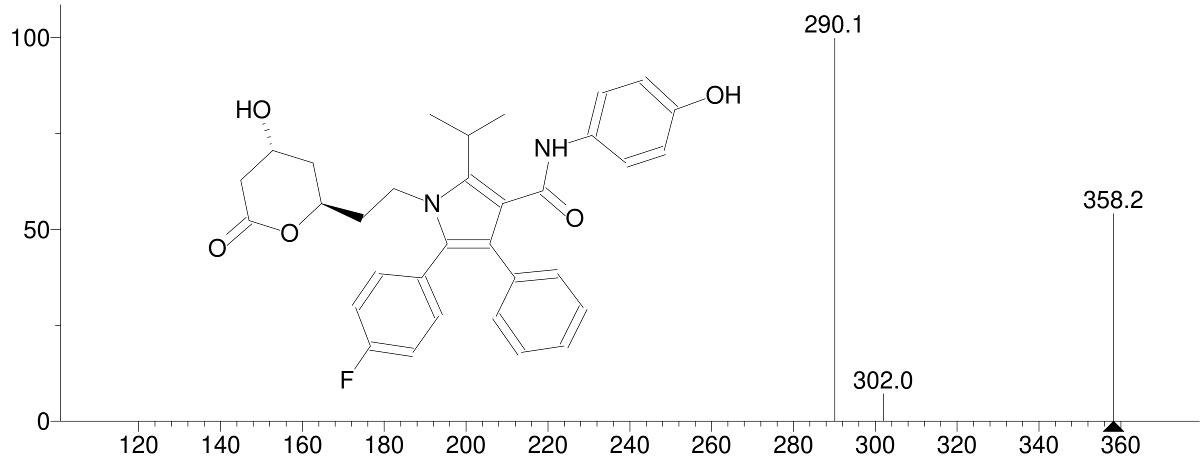

Hit 2 : 1H-Indole-3,5-dicarboxylic acid, 1-[3-[4-(decyloxy)phenoxy]-2-oxopropyl]-, 3-methyl ester [M-H]-=>358.2 I
C30H37NO7; MF: 2; RMF: 201; Prob 5.18%; CAS: 888320-29-4; Lib: nist_msms; ID: 374612.

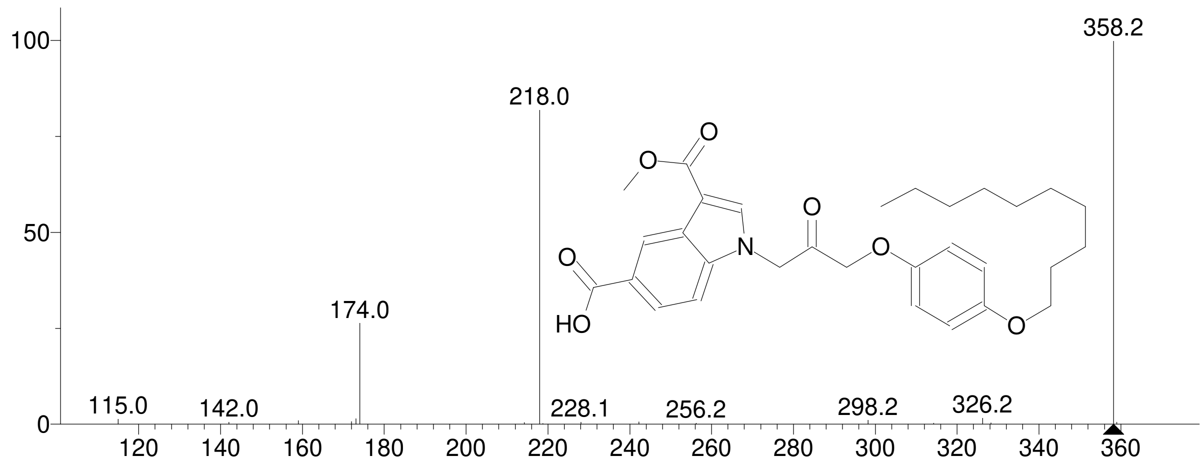

Hit 3 : Nalbuphine [M+H]+ 20-30-40%) P=358.2
C21H27NO4; MF: 1; RMF: 189; Prob 4.98%; Lib: mona-export-fiehn_hilic; ID: 599.

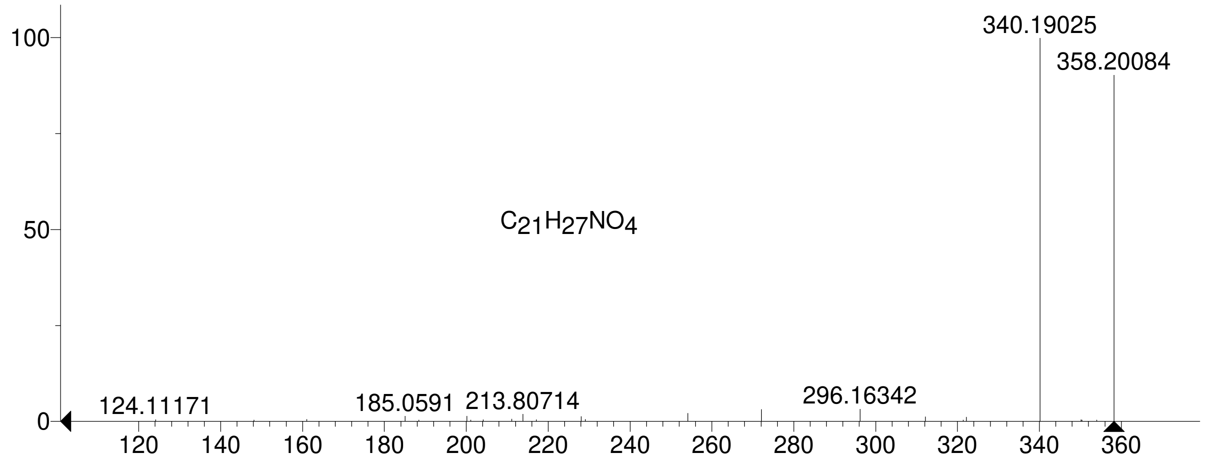

Hit 4 : Calpain Inhibitor II [M-H]-=>358.2 IT 35% P=358.2
C19H35N3O4S; MF: 1; RMF: 185; Prob 4.98%; CAS: 145757-50-2; Lib: nist_msms; ID: 81729.

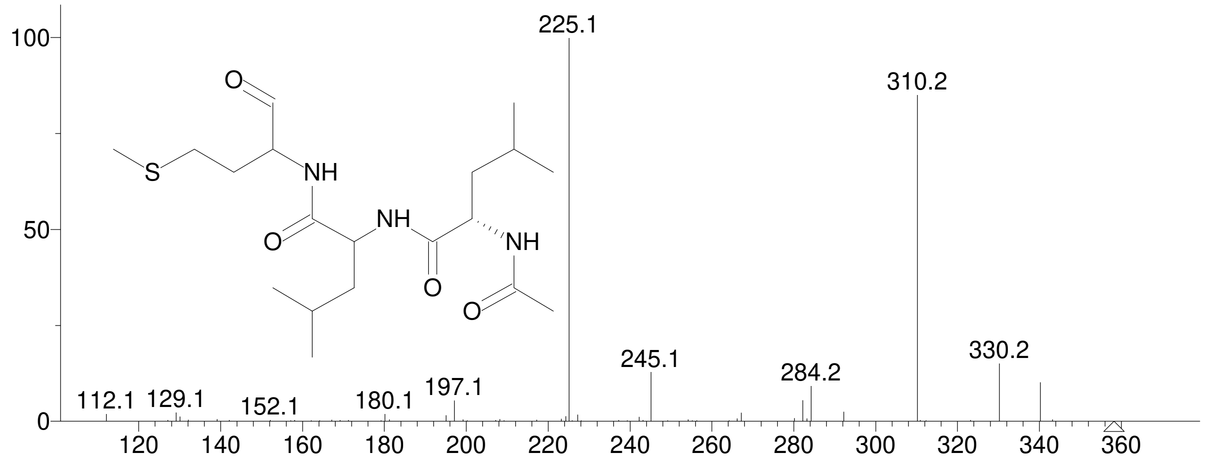

Hit 5 : 1H-Indole-3-carboxamide, 1-(5-fluoropentyl)-N-8-quinolinyl- [M+H]+=>358.2 IT 35% P=358.2
C23H22FN3O; MF: 1; RMF: 168; Prob 4.98%; Lib: nist_msms; ID: 344362.

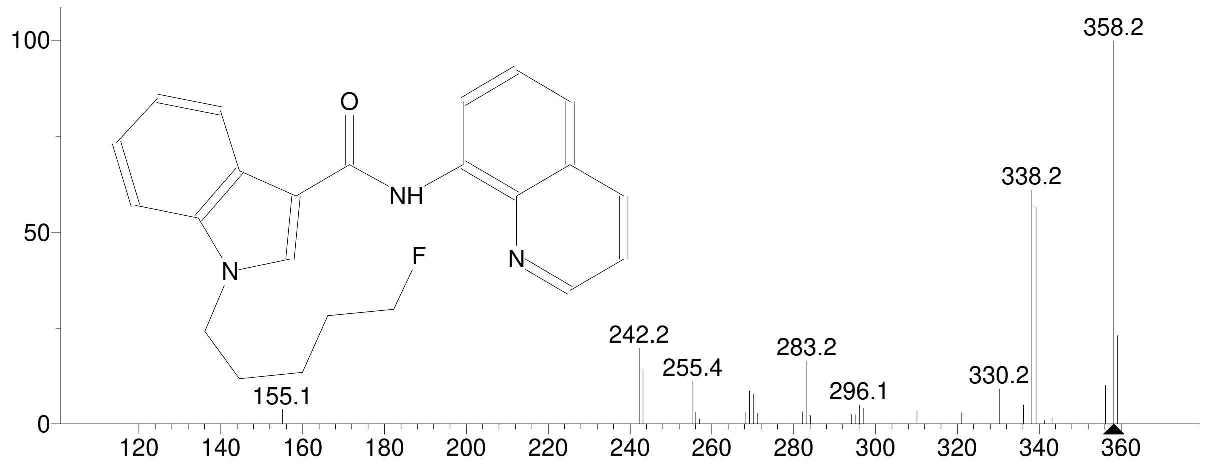

Unknown: Unknown (5.02_544.2163m/z)
Compound in Library Factor = 0

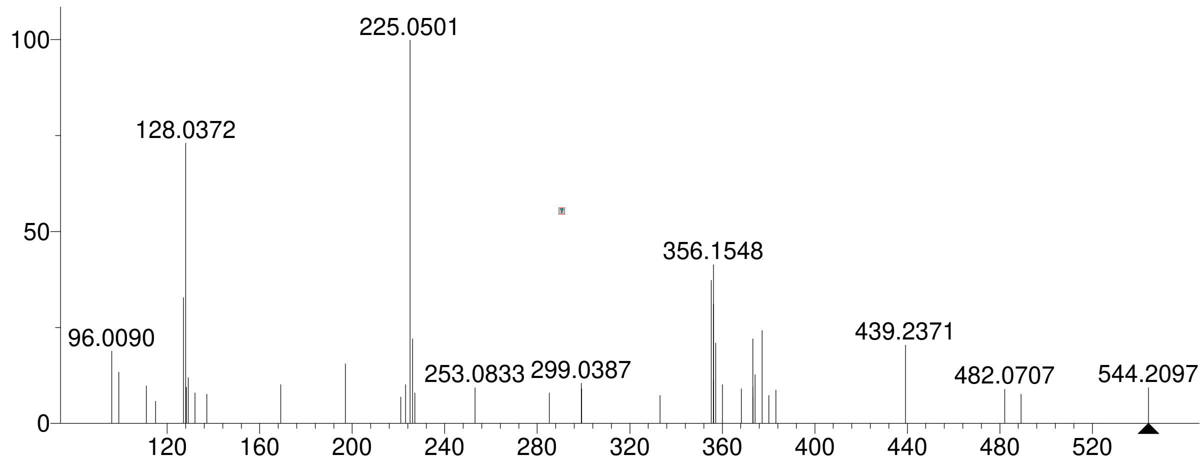

Unknown: Unknown (5.03_415.1707m/z)
Compound in Library Factor = 0

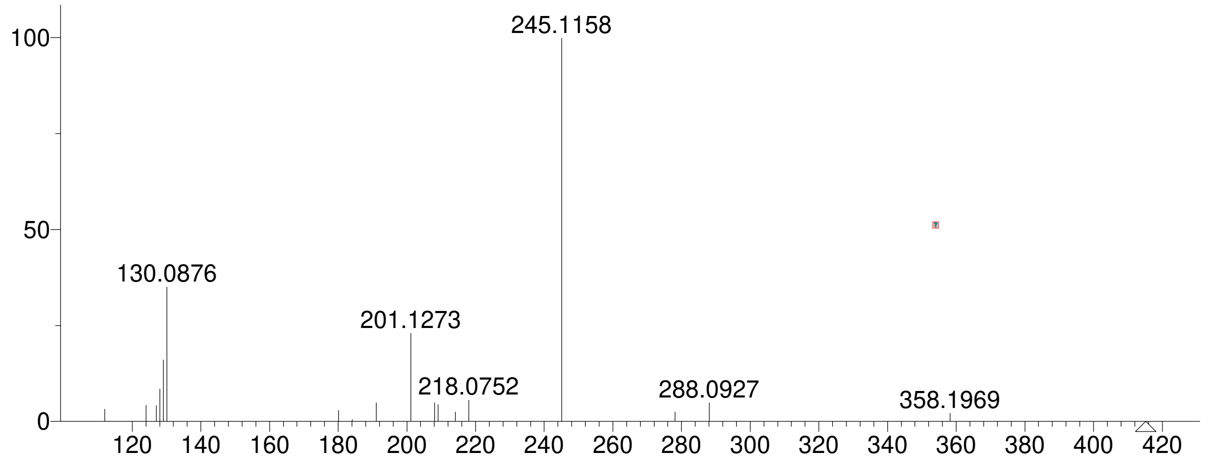

Hit 1 : 6-formyl-10-(hydroxymethyl)-5-methoxy-3-methylidene-2-oxo-2H,3H,3aH,4H,5H,8H,9H,11aH-cyclodeca[b]f
C21H28O7; MF: 0; RMF: 31; Prob 20.0%; Lib: mona-export-vf-npl_qexactive; ID: 16435.

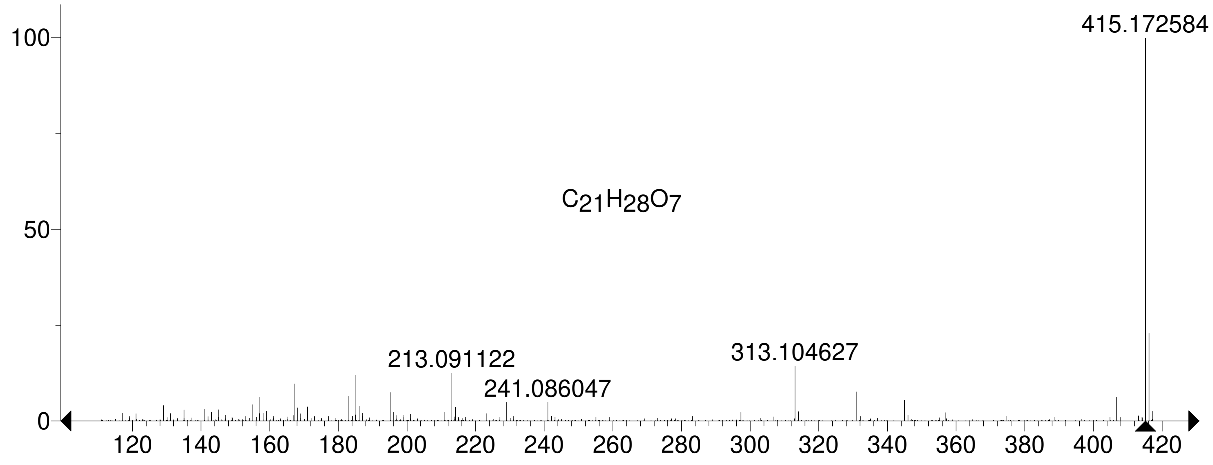

Hit 2 : NCGC00347525-03!2-amino-5-[2-[[2,3-dihydroxy-2-(1-hydroxyethyl)butanoyl]oxymethyl]-4-hydroxyanilino]-
C18H26N2O9; MF: 0; RMF: 10; Prob 20.0%; Lib: mona-export-gnps; ID: 9213.

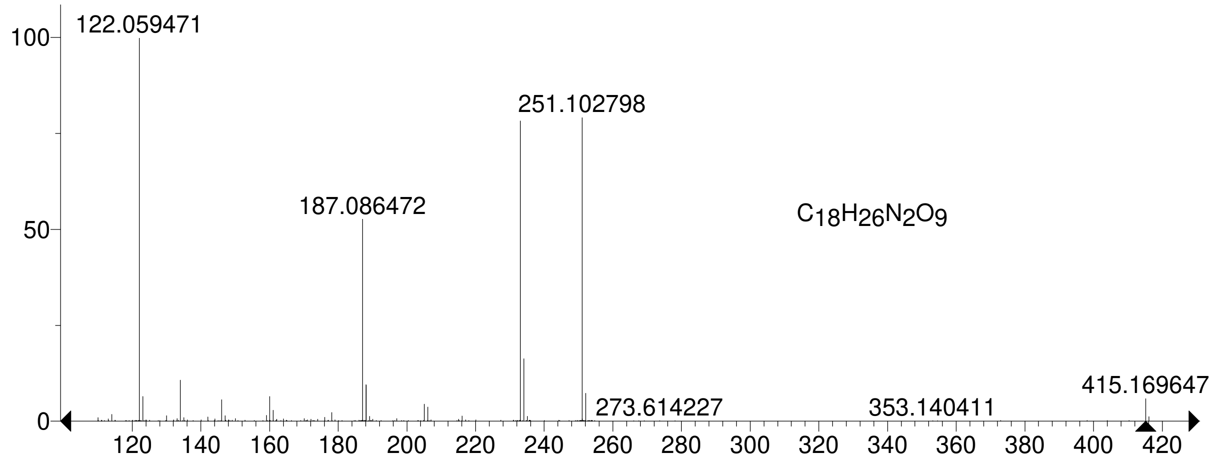

Hit 3 : Diltiazem HCl (Tiazac) [M+H]+ P=415.2
C22H27ClN2O4S; MF: 0; RMF: 3; Prob 20.0%; Lib: mona-export-gnps; ID: 648.

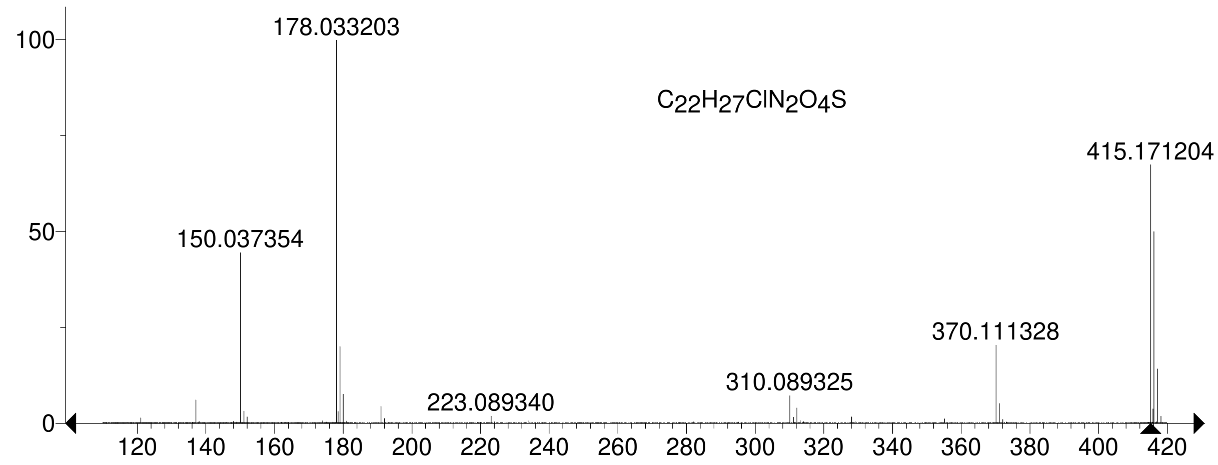

Hit 4 : NCGC00347525-02!2-amino-5-[2-[[2,3-dihydroxy-2-(1-hydroxyethyl)butanoyl]oxymethyl]-4-hydroxyanilino]-
C18H26N2O9; MF: 0; RMF: 3; Prob 20.0%; Lib: mona-export-gnps; ID: 6270.

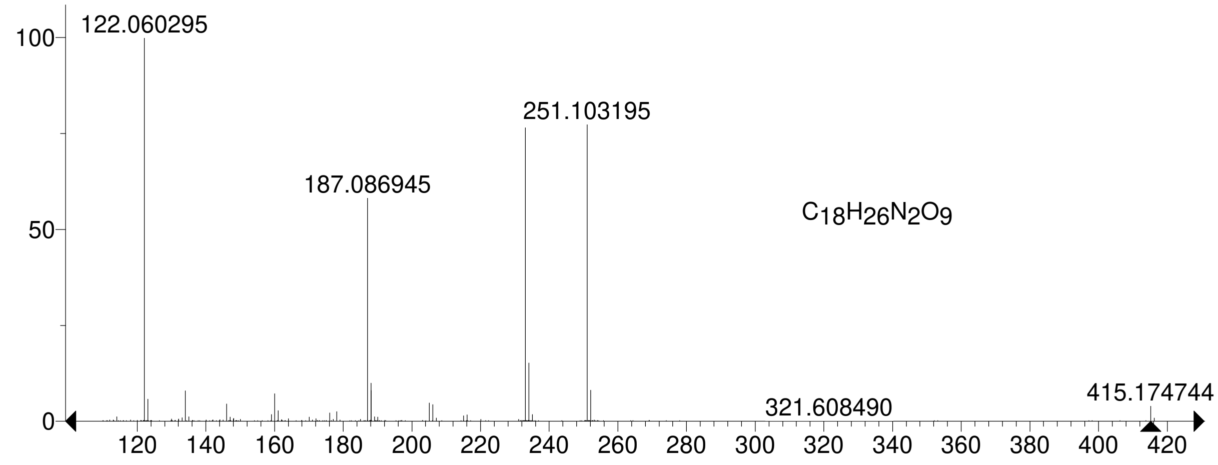

Hit 5 : 2-amino-5-[2-[[2,3-dihydroxy-2-(1-hydroxyethyl)butanoyl]oxymethyl]-4-hydroxyanilino]-5-oxopentanoic acid
C18H26N2O9; MF: 0; RMF: 2; Prob 20.0%; Lib: mona-export-vf-npl_qexactive; ID: 23034.

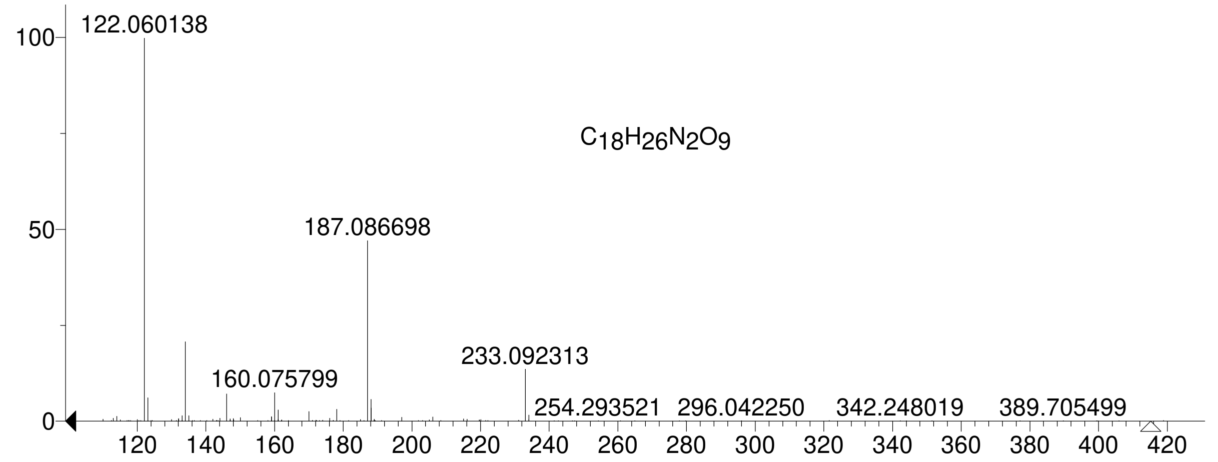

Unknown: Unknown (5.04_356.1808m/z)
Compound in Library Factor = 0

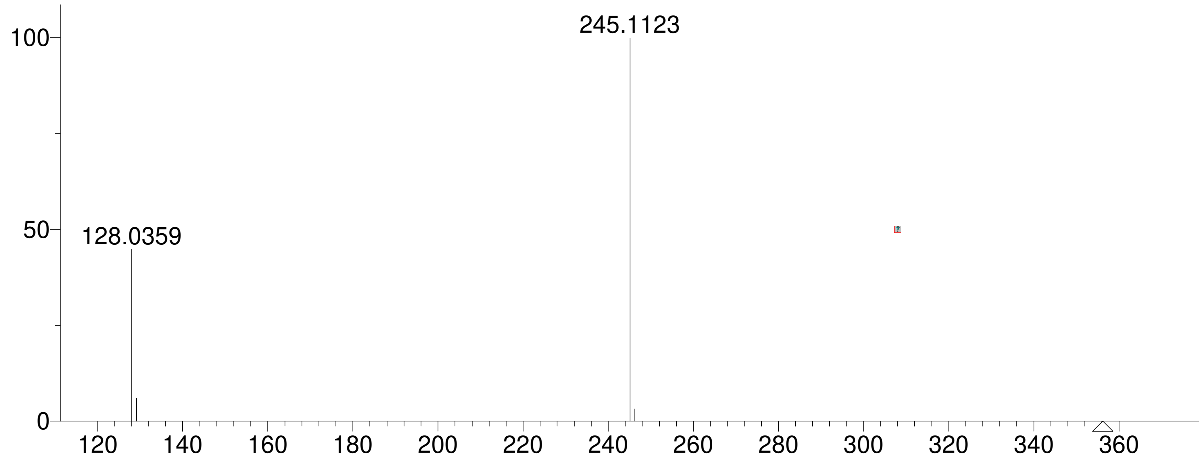

Unknown: Unknown (5.06_600.2406m/z)
Compound in Library Factor = 0

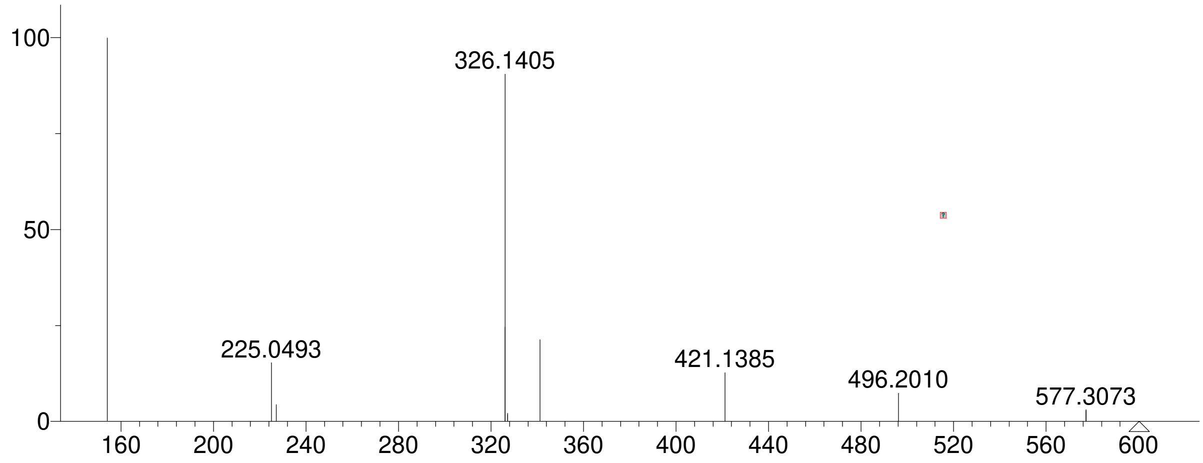

** Search Report Page 1 of 1 **

Unknown: Unknown (5.06_660.2276m/z)
Compound in Library Factor = 0

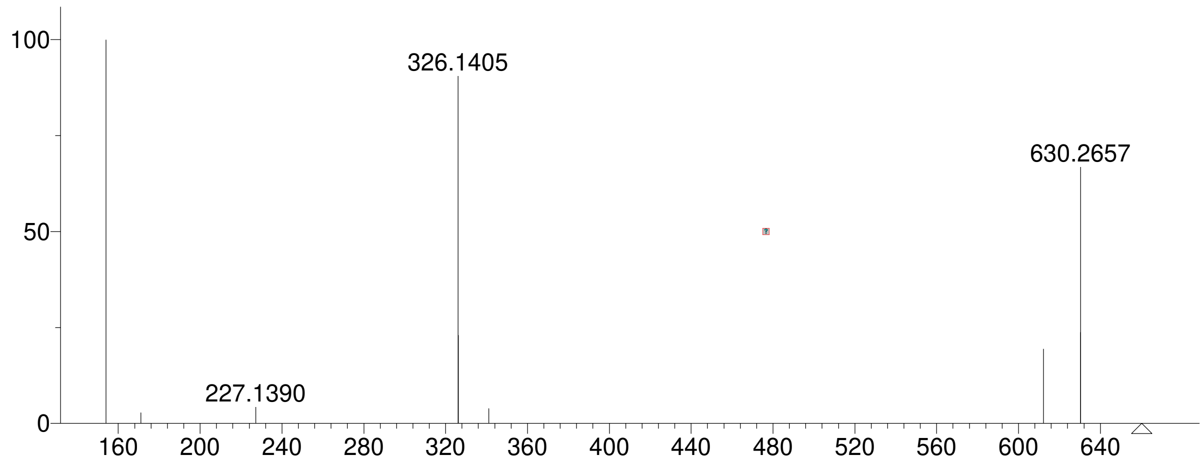

Unknown: Unknown (5.07_516.2152m/z)
Compound in Library Factor = 0

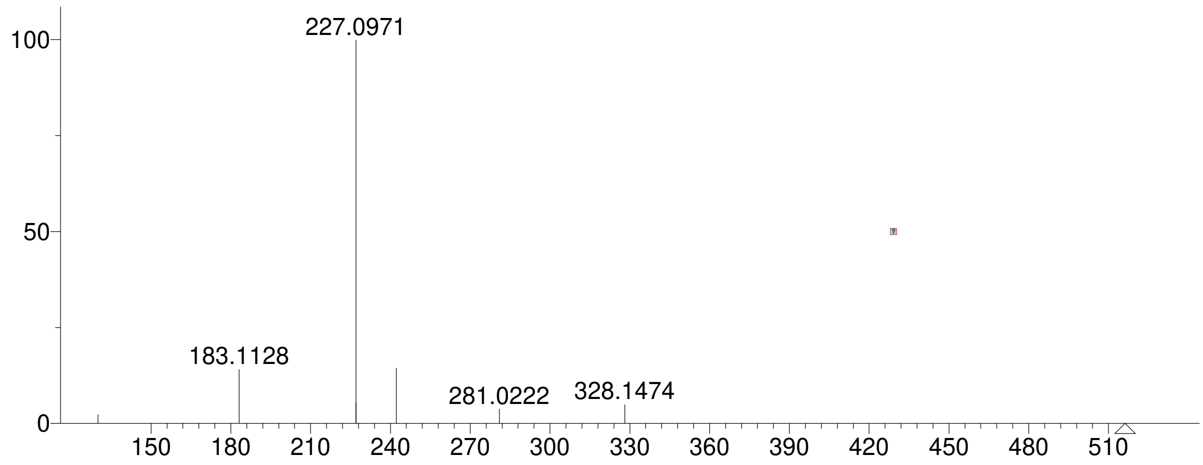

Hit 1 : Asp371, Tyrosinase (369-377), human [M+2H]2+ HCD 37eV P=516.2
C42H66N10O16S2; MF: 0; RMF: 78; Prob 100.0%; Lib: nist_msms2; ID: 47696.

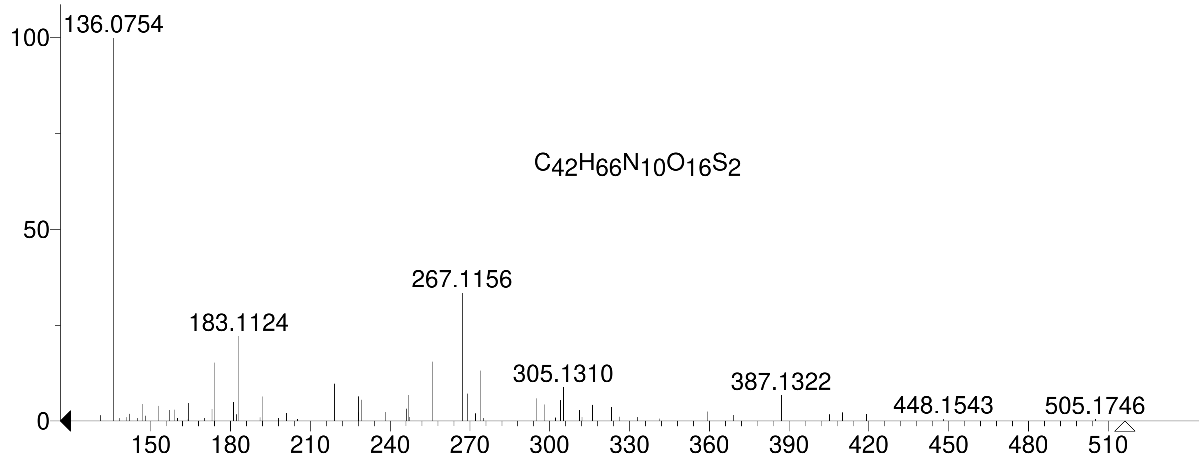

Unknown: Unknown (5.07_680.2778m/z)
Compound in Library Factor = 0

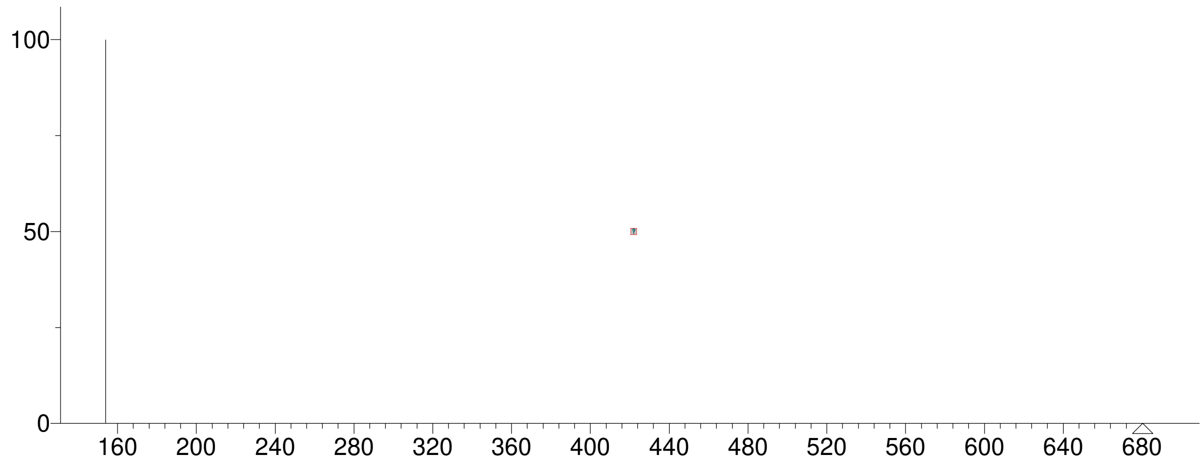

** Search Report Page 1 of 2 **

Unknown: Unknown (5.08_227.1028m/z)
Compound in Library Factor = -462

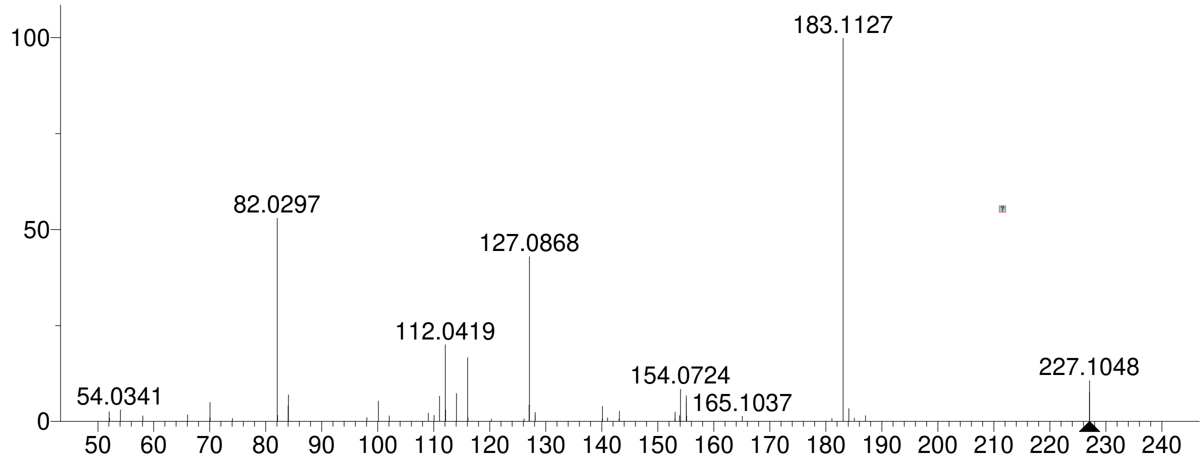

Hit 1 : Proline-hydroxyproline [M-H]- 20-30-40%) P=227.1
C10H16N2O4; MF: 185; RMF: 380; Prob 93.2%; Lib: mona-export-fiehn_hilic; ID: 1464.

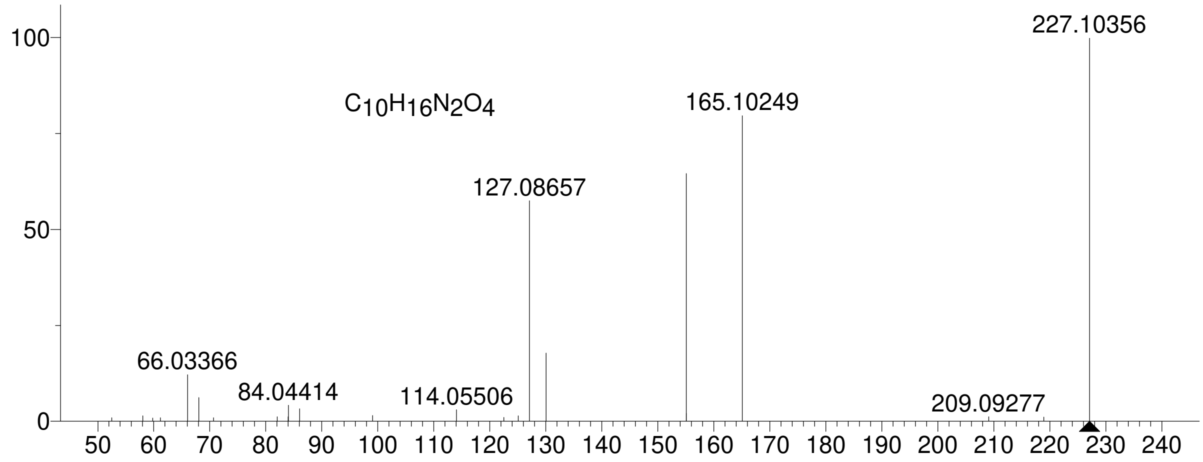

Hit 2 : PyroGlu-Pro [M+H]+ QTOF 6V P=227.1
C10H14N2O4; MF: 44; RMF: 238; Prob 3.09%; Lib: nist_msms; ID: 47866.

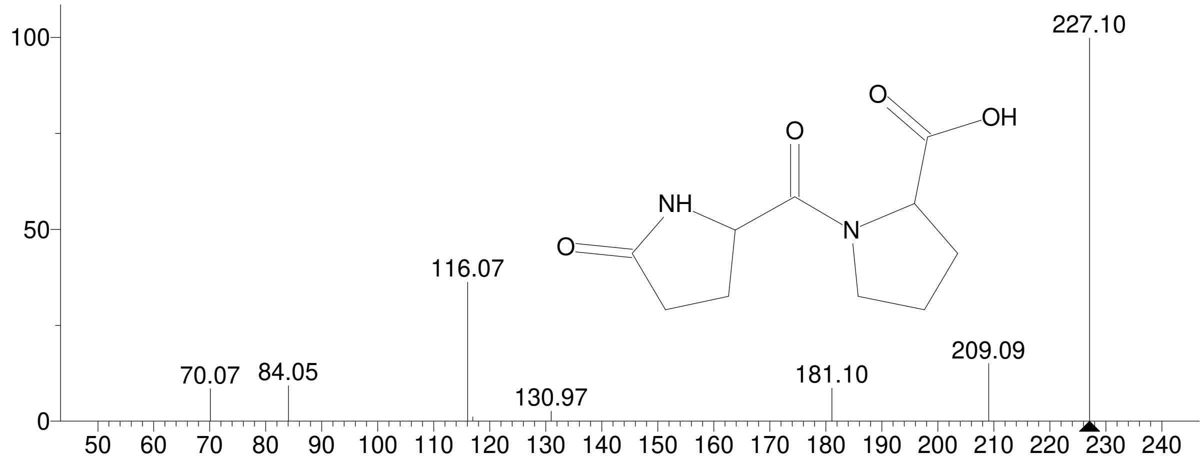

Hit 3 : Carbidopa [M+H]+ QTOF 4V P=227.1
C10H14N2O4; MF: 13; RMF: 166; Prob 0.84%; CAS: 28860-95-9; Lib: nist_msms; ID: 69025.

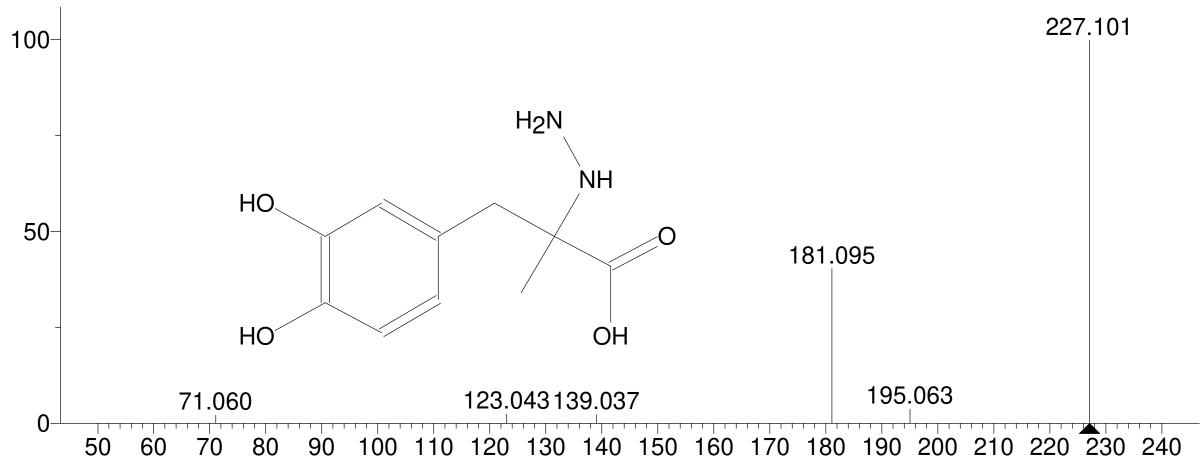

Hit 4 : (S)-Dibutyl 3-hydroxybutyl phosphate [M+H-C4H8]+ HCD 4V P=227.1
C12H27O5P; MF: 4; RMF: 148; Prob 0.61%; CAS: 89197-69-3; Lib: nist_msms; ID: 419318.

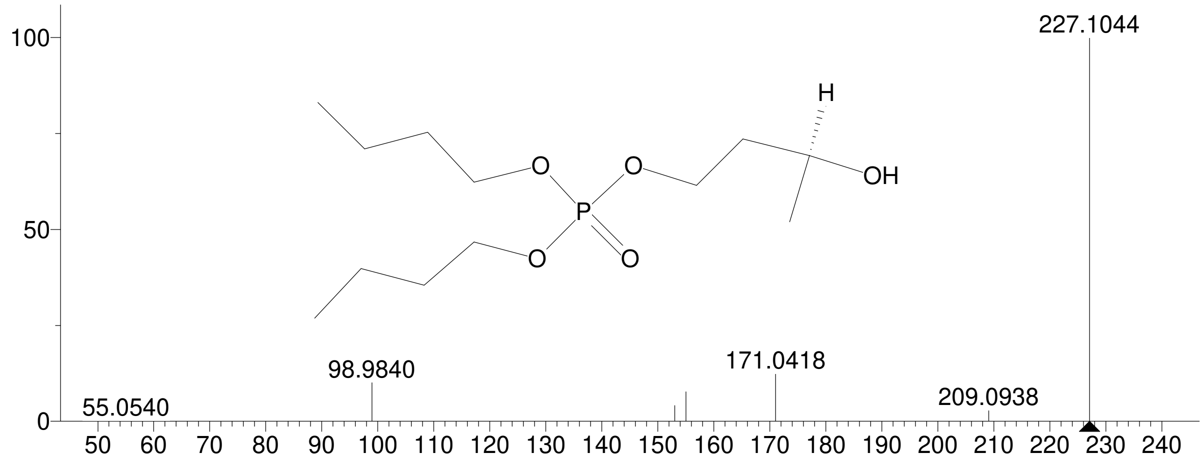

Hit 5 : Pro-Hyp [M-H]- 35eV P=227.1
C10H16N2O4; MF: 4; RMF: 99; Prob 0.61%; Lib: mona-export-fiehn_hilic; ID: 2943.

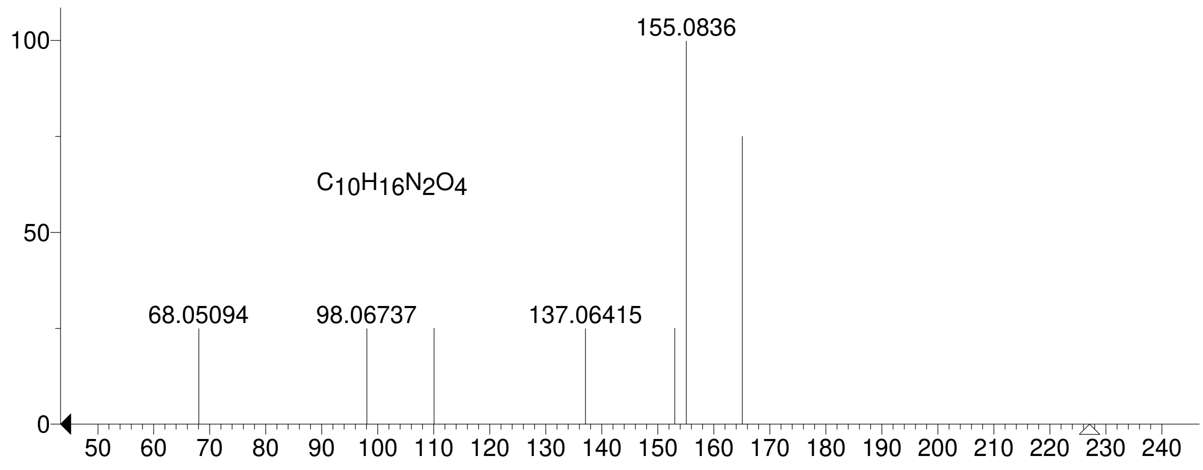

Unknown: Unknown (5.08_374.1567m/z)
Compound in Library Factor = 0

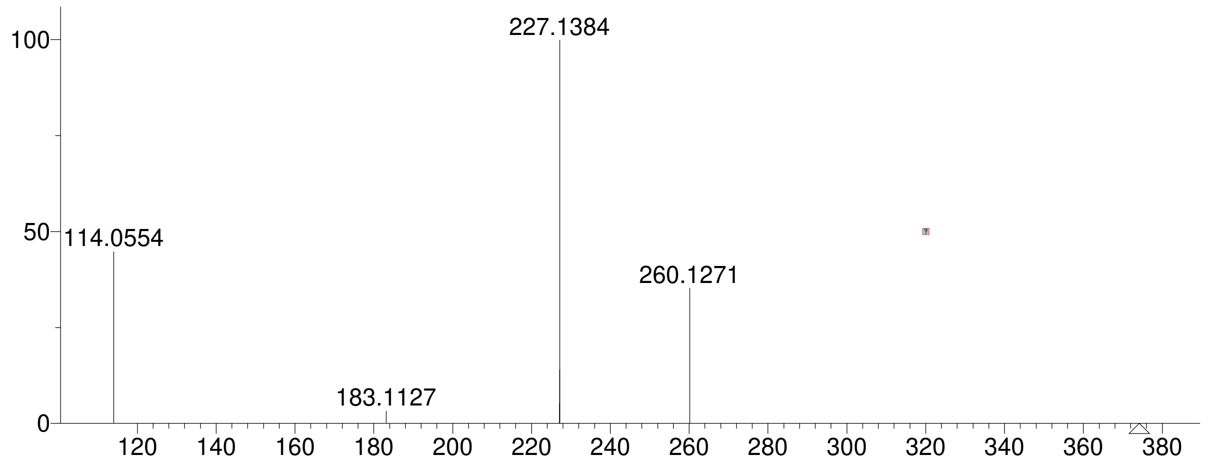

Hit 1 : (2S,3S)-3,5,7-trihydroxy-2-(4-hydroxyphenyl)-8-(3-methylbut-2-enyl)-2,3-dihydrochromen-4-one [M+NH4]+
C20H20O6; MF: 0; RMF: 55; Prob 33.3%; Lib: mona-export-vf-npl_qexactive; ID: 23299.

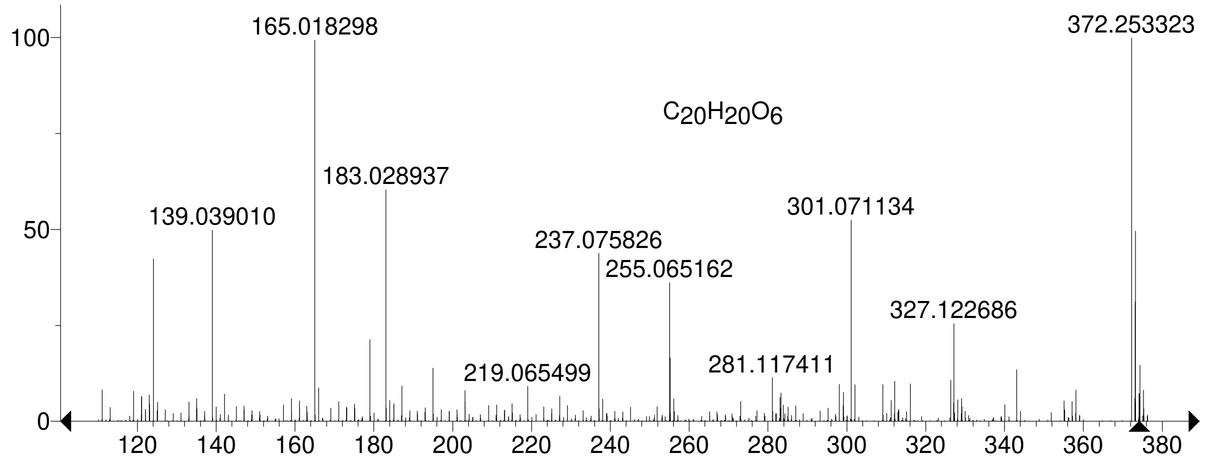

Hit 2 : dimethyl 2,4-bis(4-hydroxyphenyl)cyclobutane-1,3-dicarboxylate [M+NH4]+ 35HCD P=374.2
C20H20O6; MF: 0; RMF: 40; Prob 33.3%; Lib: mona-export-vf-npl_qexactive; ID: 23731.

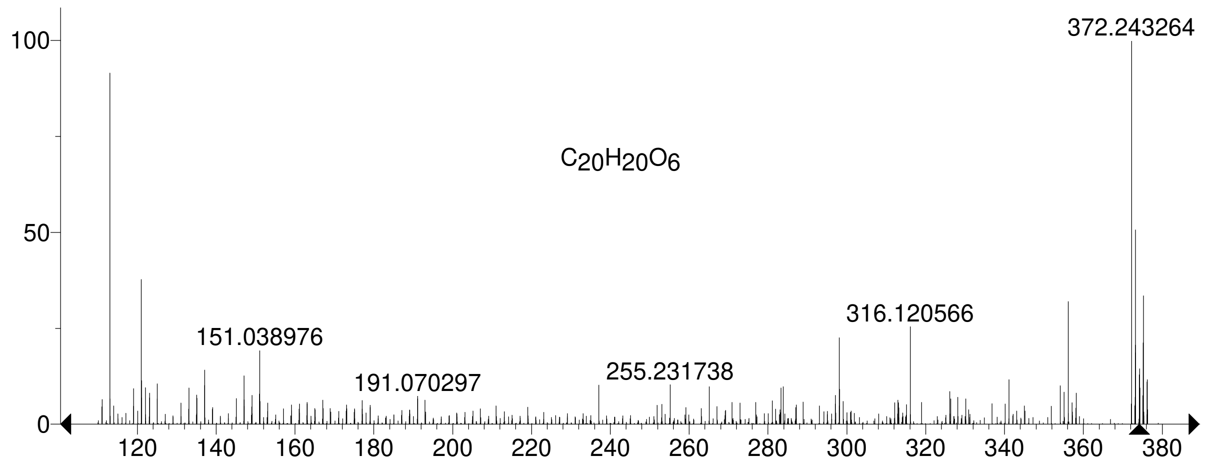

Hit 3 : (E)-1,4-bis(4-hydroxy-3-methoxyphenyl)-2,3-dimethylbut-2-ene-1,4-dione [M+NH4]+ 65HCD P=374.2
C20H20O6; MF: 0; RMF: 11; Prob 33.3%; Lib: mona-export-vf-npl_qexactive; ID: 19611.

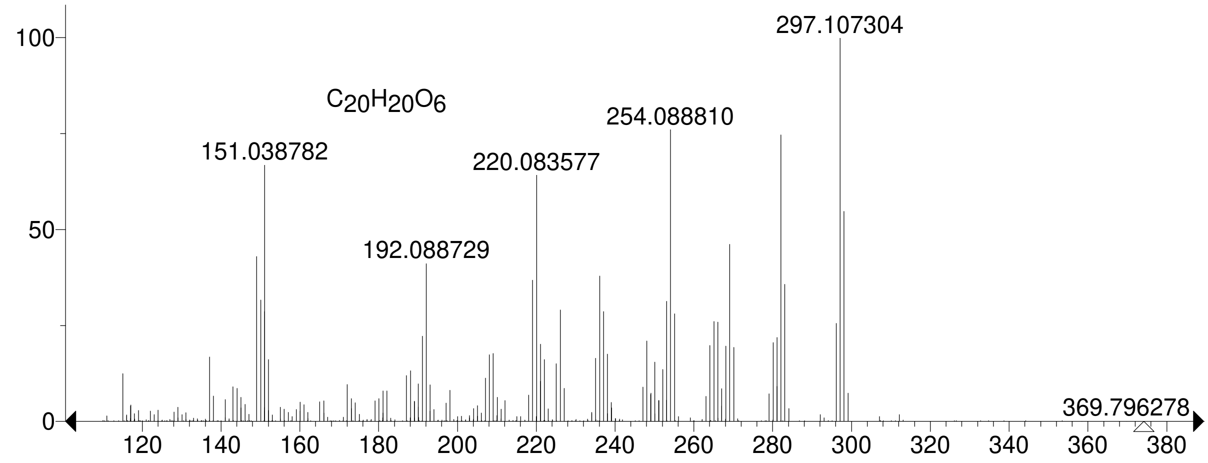

Unknown: Unknown (5.08_451.1708m/z)
Compound in Library Factor = 0

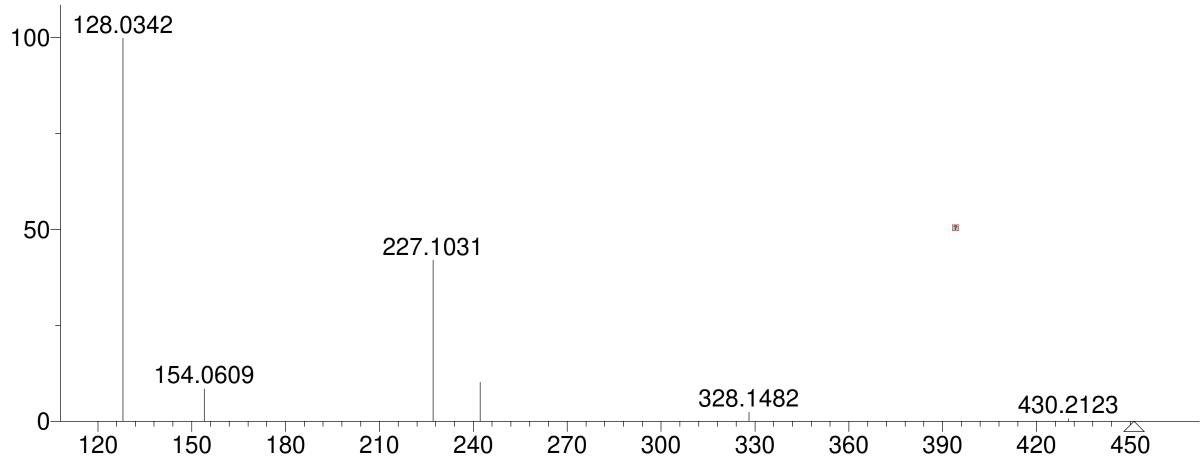

Hit 1 : 2H-Pyrido[3,2-b]-1,4-oxazin-3(4H)-one, 6-[[5-fluoro-2-[(3,4,5-trimethoxyphenyl)amino]-4-pyrimidinyl]amino]
C22H23FN6O5; MF: 0; RMF: 57; Prob 50.0%; CAS: 841290-80-0; Lib: nist_msms; ID: 251193.

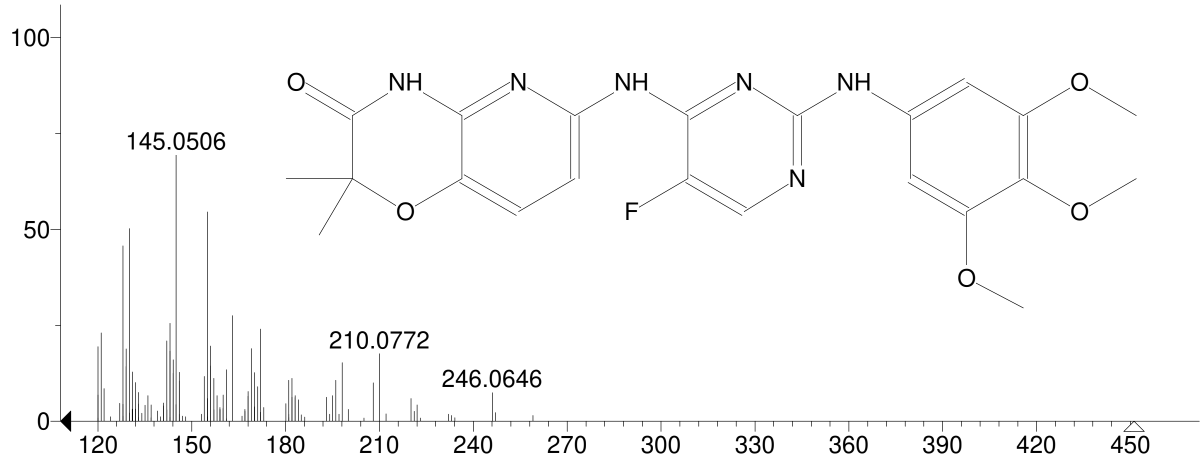

Hit 2 : α-Conotoxin IMI [M+3H]3+ IT 35% P=451.2
C52H78N20O15S4; MF: 0; RMF: 19; Prob 50.0%; Lib: nist_msms2; ID: 60571.

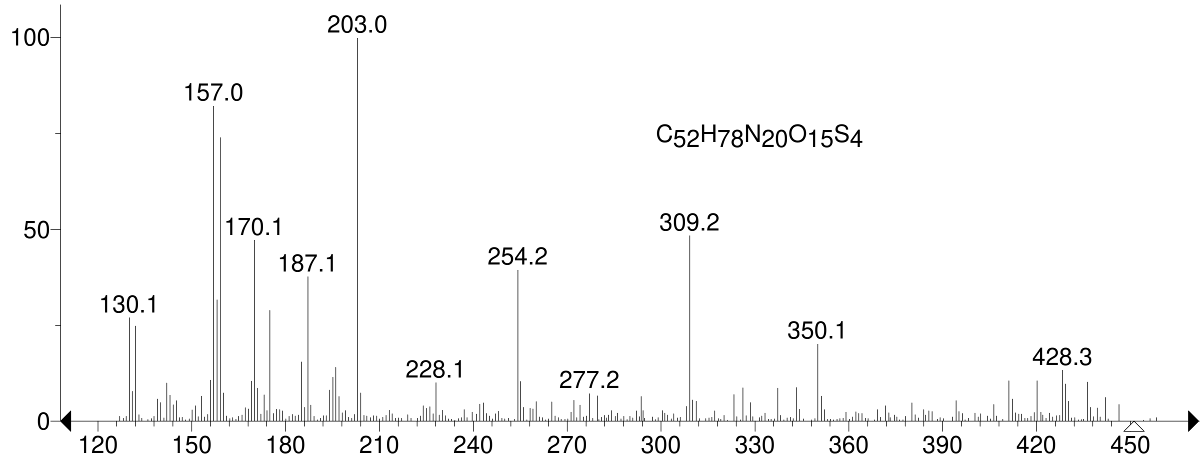

Unknown: Unknown (5.09_183.1128m/z)
Compound in Library Factor = -216

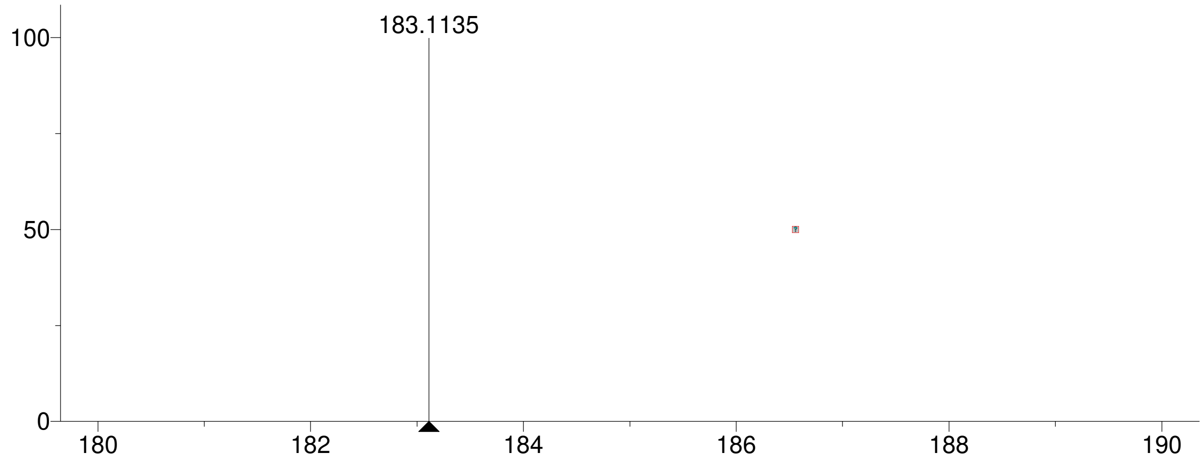

Hit 1 : 1-Trifluoromethoxyphenyl-3-(1-propionylpiperidin-4-yl)urea [M+H-C7H6F3NO]+ HCD 16V P=183.1
C16H20F3N3O3; MF: 364; RMF: 999; Prob 50.0%; CAS: 1222780-33-7; Lib: nist_msms; ID: 253650.

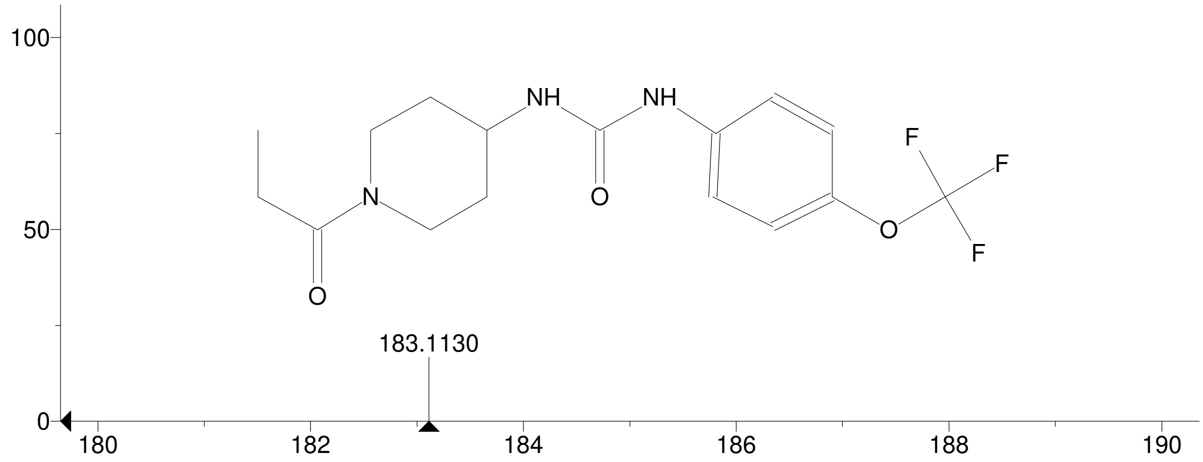

Hit 2 : Phe-Ala-Lys [M+2H]2+ QTOF 15V P=183.1
C18H28N4O4; MF: 364; RMF: 999; Prob 50.0%; Lib: nist_msms; ID: 50307.

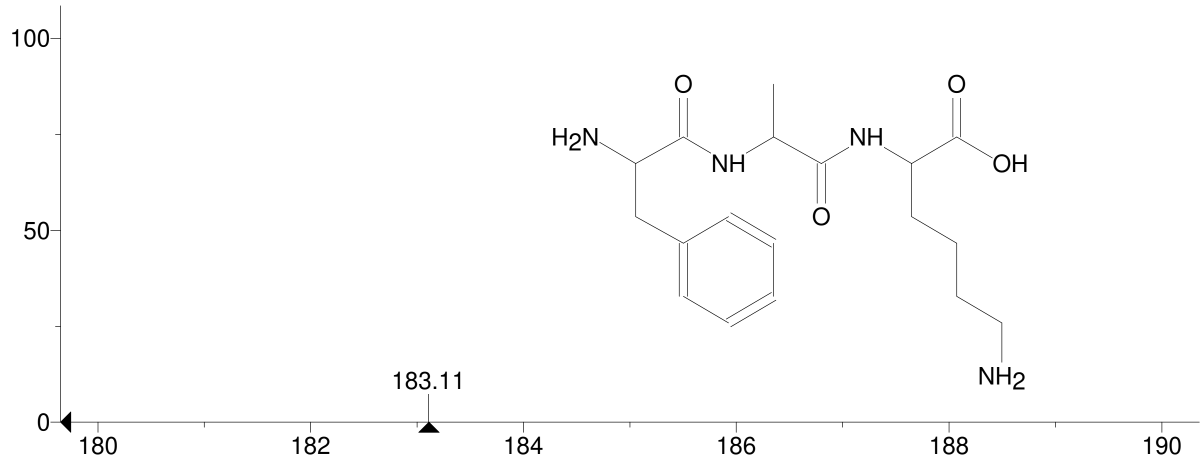

Unknown: Unknown (5.09_293.1127m/z)
Compound in Library Factor = 0

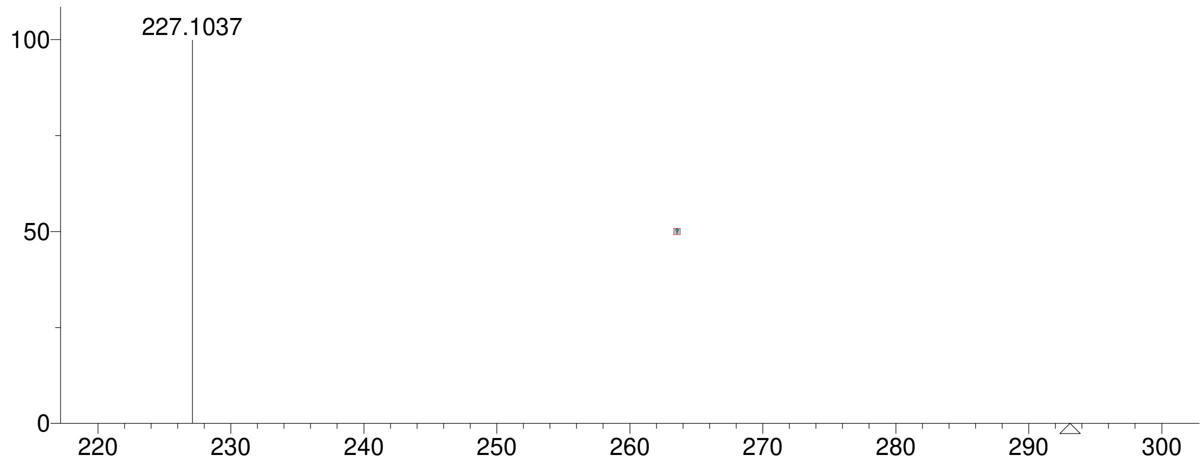

Hit 1 : (E)-2-(hydroxymethyl)-3-[3-(hydroxymethyl)-6-propan-2-ylcyclohex-2-en-1-yl]prop-2-enoic acid [M+K]+ 35
C14H22O4; MF: 0; RMF: 54; Prob 50.0%; Lib: mona-export-vf-npl_qexactive; ID: 17503.

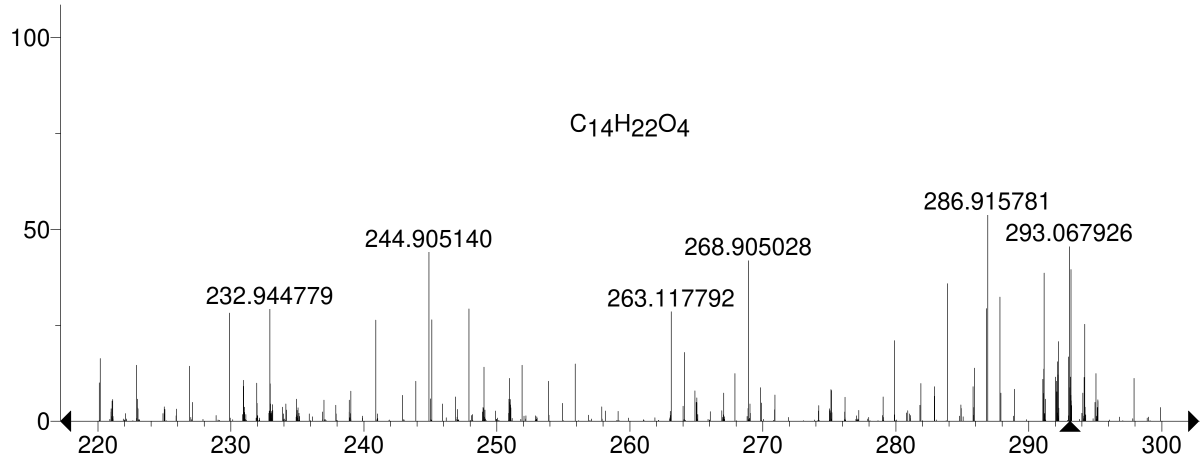

Hit 2 : 3-hydroxy-1a,5-bis(hydroxymethyl)-5,6b-dimethyl-1,3,3a,4,6,6a-hexahydrocyclopropa[e]inden-2-one [M+K
C14H22O4; MF: 0; RMF: 51; Prob 50.0%; Lib: mona-export-vf-npl_qexactive; ID: 8815.

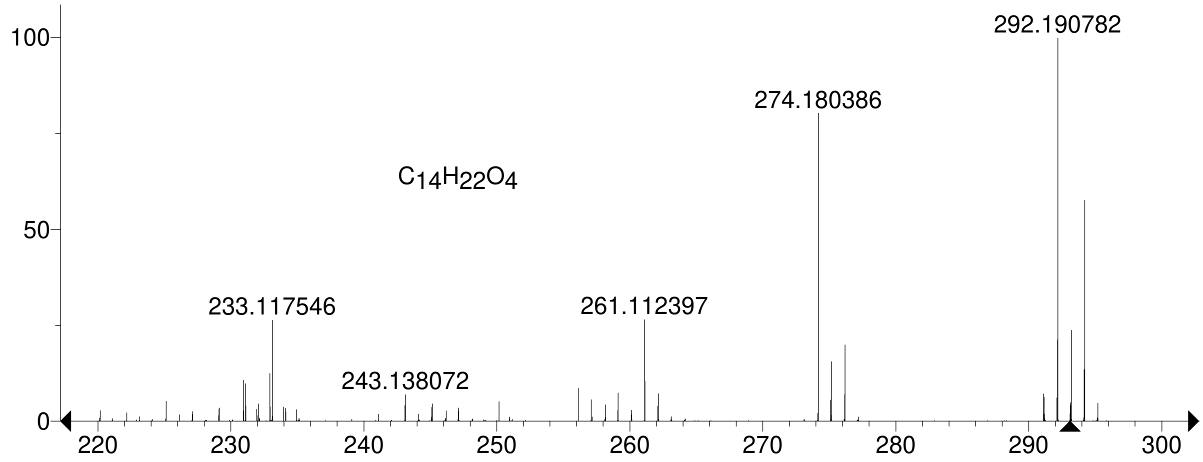

Unknown: Unknown (5.12_259.1289m/z)
Compound in Library Factor = 155

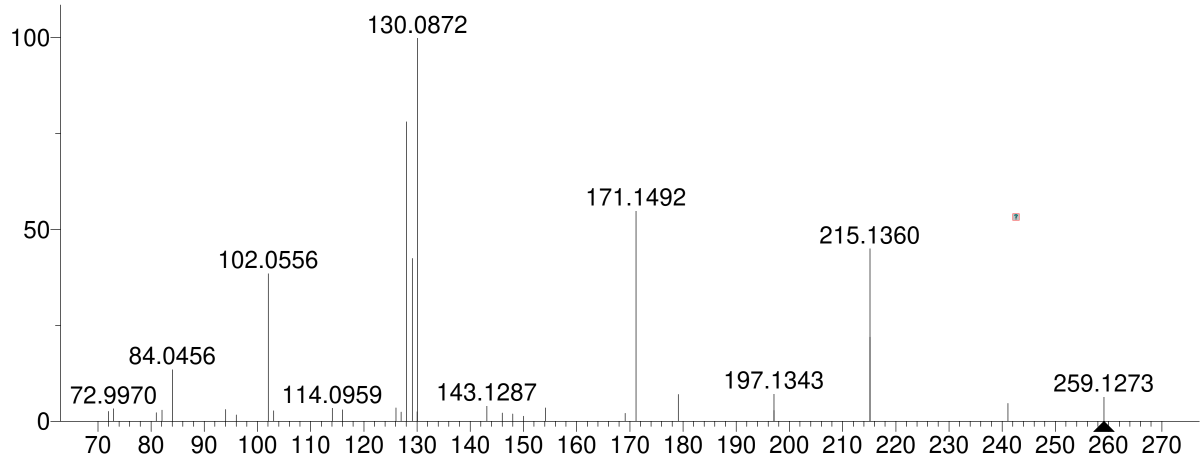

Hit 1 : Ile-Glu [M-H]- HCD 23eV P=259.1
C11H20N2O5; MF: 845; RMF: 901; Prob 31.0%; Lib: nist_msms; ID: 570716.

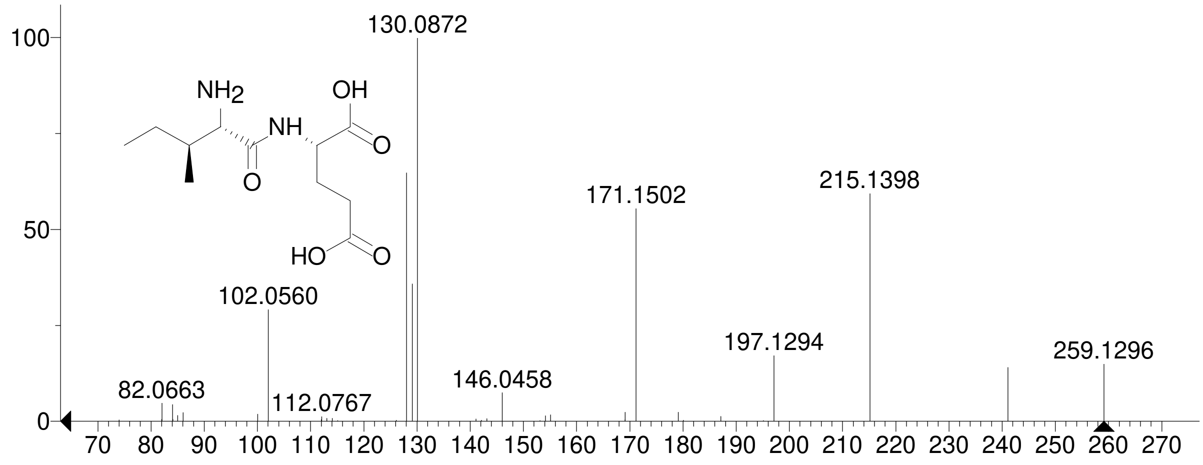

Hit 2 : Ile-Glu [M-H]- HCD 23eV P=259.1
C11H20N2O5; MF: 845; RMF: 901; Prob 31.0%; CAS: 42516-53-0; Lib: nist_msms2; ID: 84449.

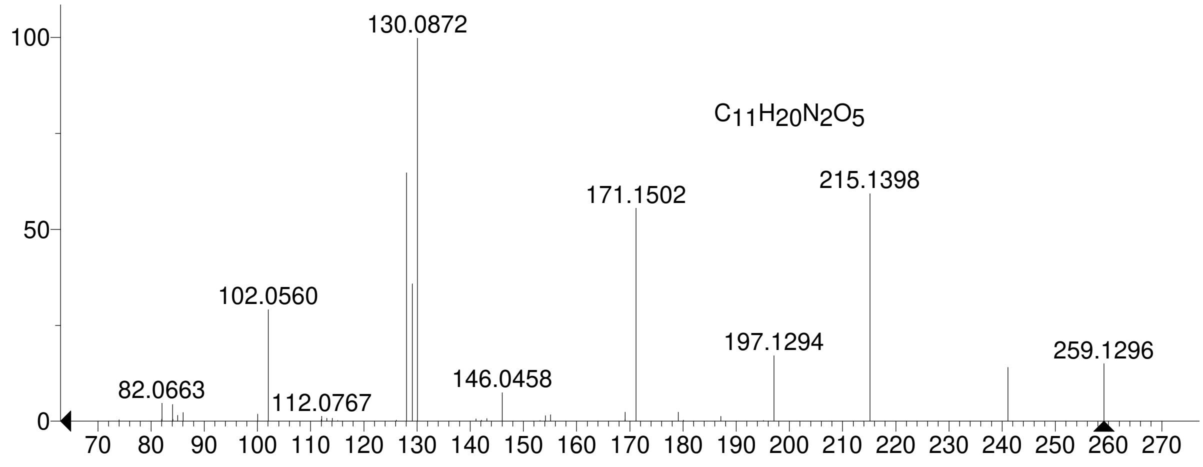

Hit 3 : Leu-Glu [M-H]- HCD 23eV P=259.1
C11H20N2O5; MF: 830; RMF: 898; Prob 18.8%; Lib: nist_msms; ID: 570784.

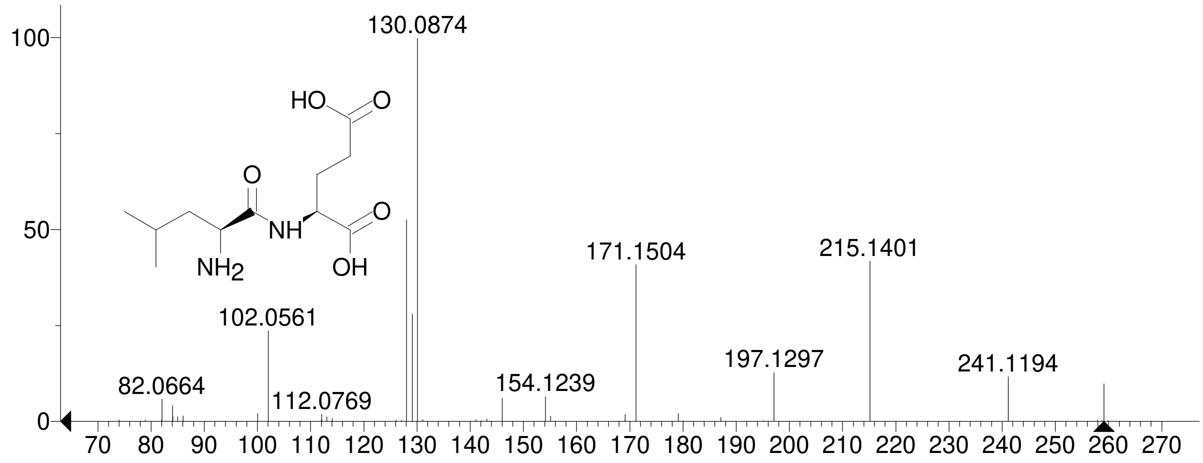

Hit 4 : Leu-Glu [M-H]- HCD 23eV P=259.1
C11H20N2O5; MF: 830; RMF: 898; Prob 18.8%; CAS: 16364-31-1; Lib: nist_msms2; ID: 84517.

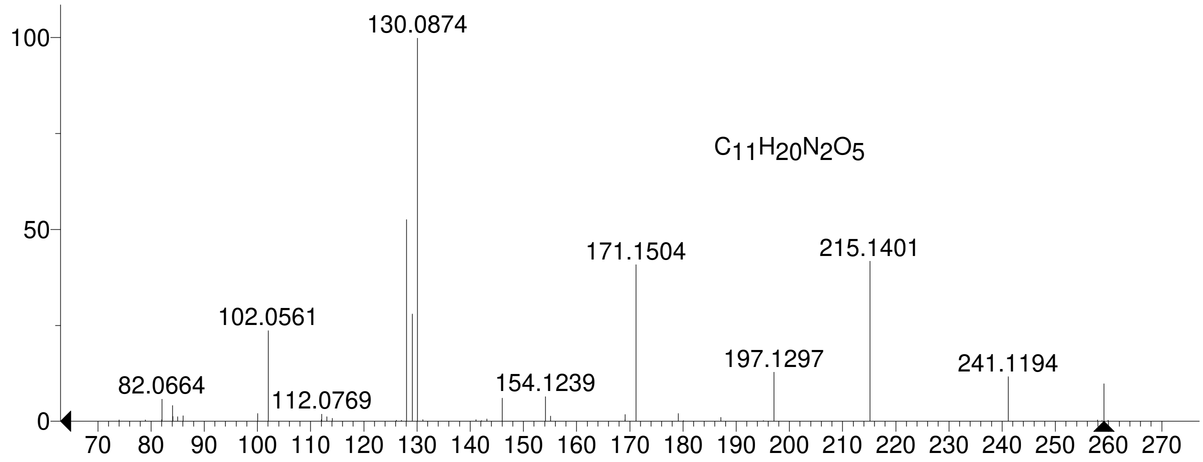

Hit 5 : Glu-Leu [M-H]- HCD 19eV P=259.1
C11H20N2O5; MF: 571; RMF: 674; Prob 0.18%; Lib: nist_msms; ID: 570061.

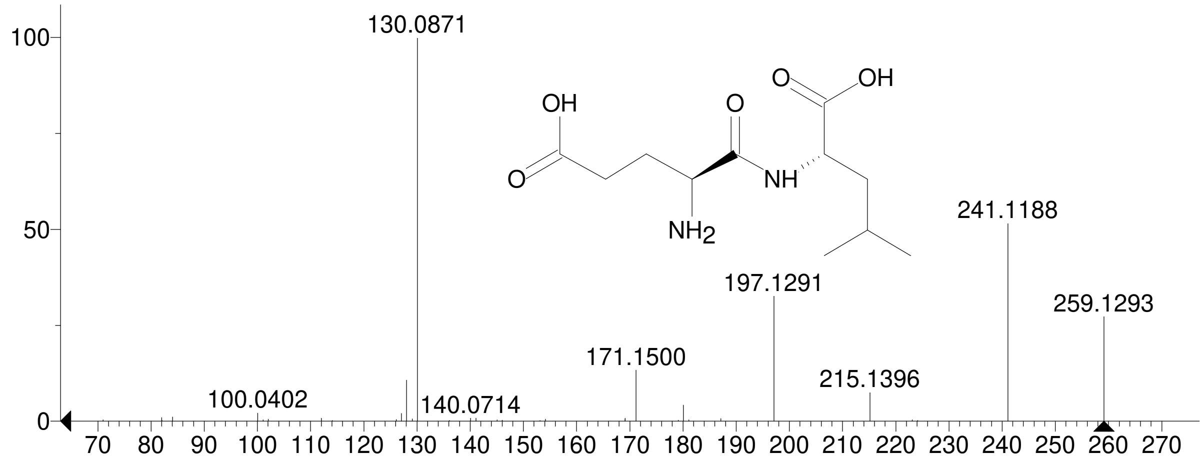

Unknown: Unknown (5.12_405.1395m/z)
Compound in Library Factor = 0

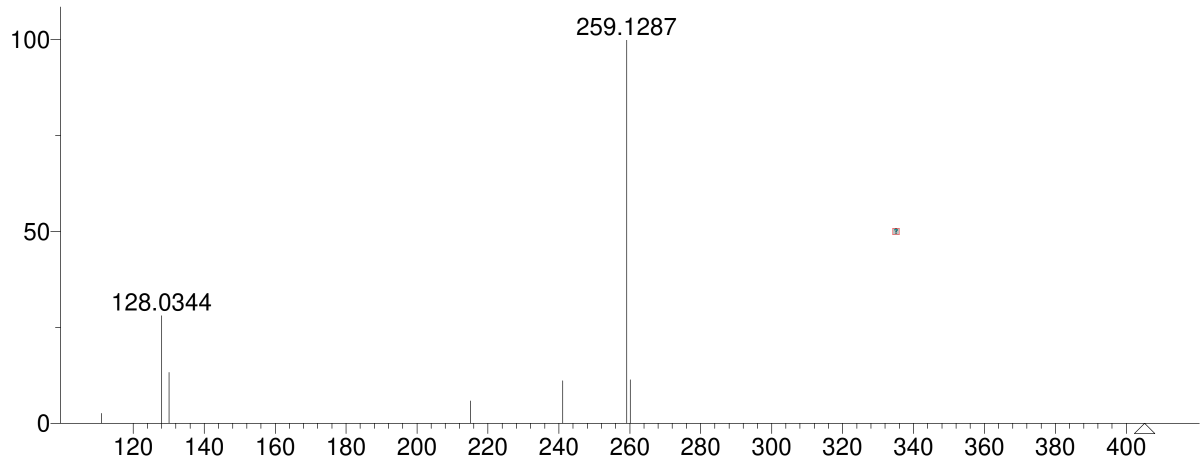

Hit 1 : Shanzhiside methyl ester [M-H]- 65HCD P=405.1
C17H26O11; MF: 0; RMF: 53; Prob 25.0%; Lib: anthocyanins mona; ID: 8593.

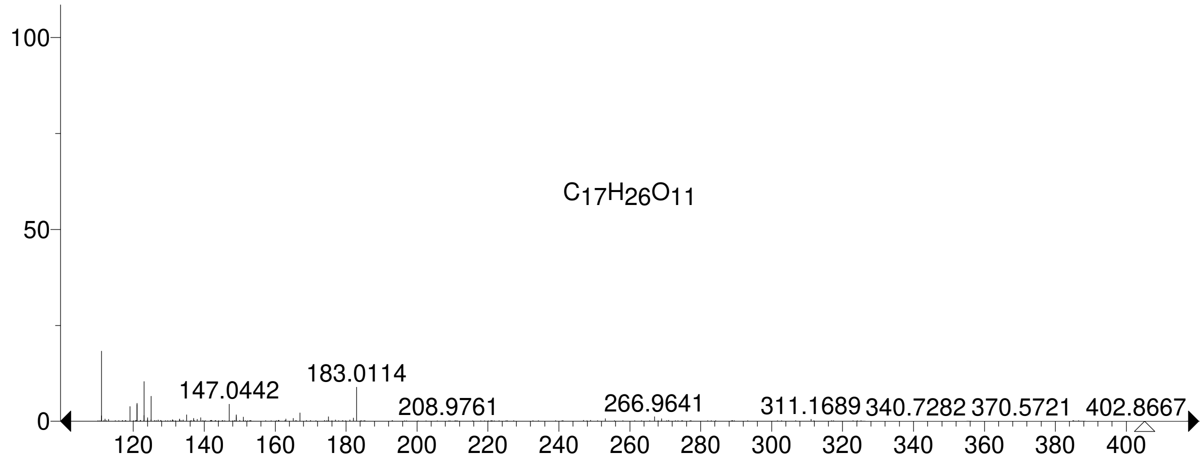

Hit 2 : methyl (1S,4aS,6R,7R,7aR)-5,6-dihydroxy-7-methyl-1-[(2S,3R,4S,5S,6R)-3,4,5-trihydroxy-6-(hydroxymethy
C17H26O11; MF: 0; RMF: 38; Prob 25.0%; Lib: mona-export-lc-ms-ms_negative_mode; ID: 20385.

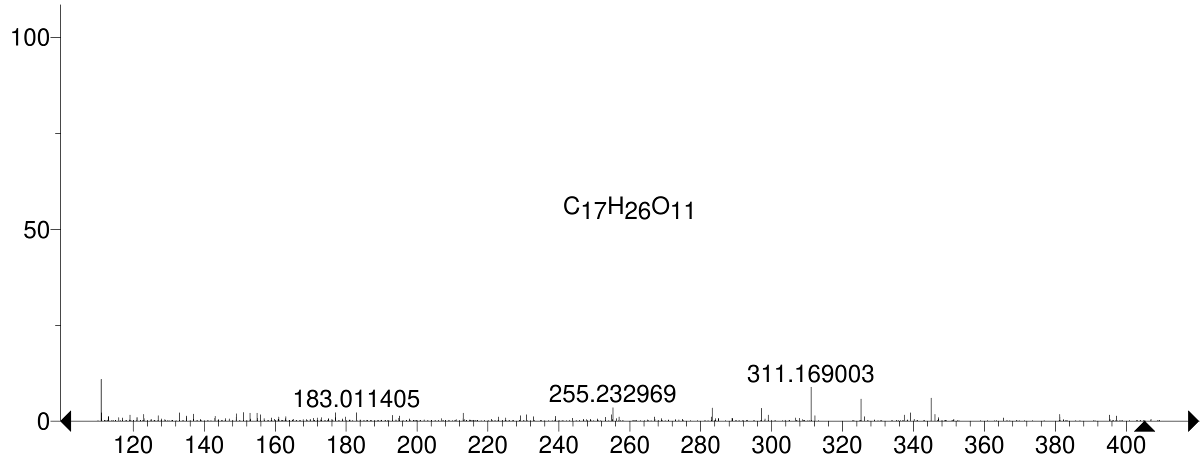

Hit 3 : methyl (1S,4aR,7aR)-4a-hydroxy-7-(hydroxymethyl)-1-[(2S,3R,4S,5S,6R)-3,4,5-trihydroxy-6-(hydroxymethy
C17H24O11; MF: 0; RMF: 7; Prob 25.0%; Lib: mona-export-vf-npl_qexactive; ID: 3655.

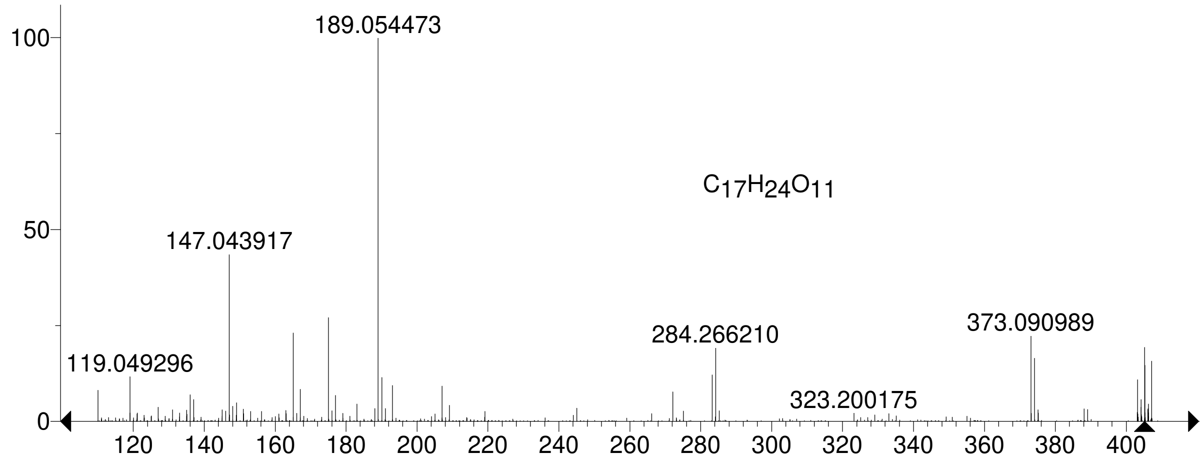

Hit 4 : gardenoside [M+H]+ 65HCD P=405.1
C17H24O11; MF: 0; RMF: 1; Prob 25.0%; Lib: anthocyanins mona; ID: 9043.

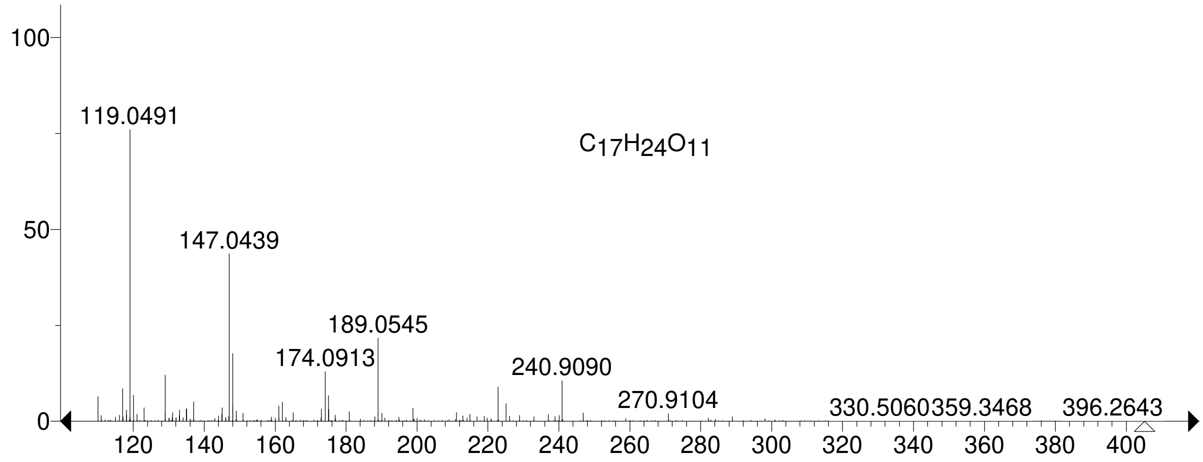

** Search Report Page 1 of 1 **

Unknown: Unknown (5.12_427.1346m/z)
Compound in Library Factor = 0

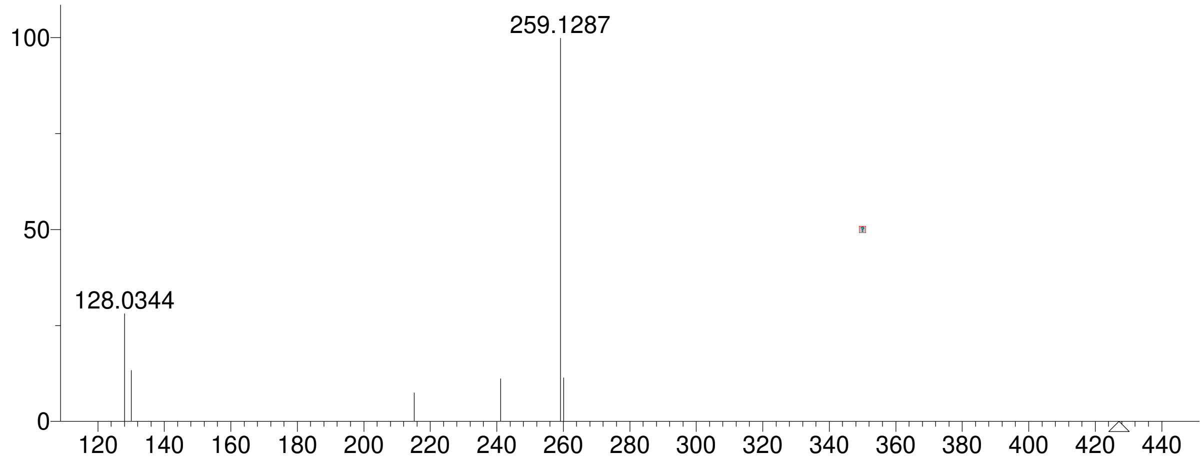

Unknown: Unknown (5.12_429.1868m/z)
Compound in Library Factor = -1142

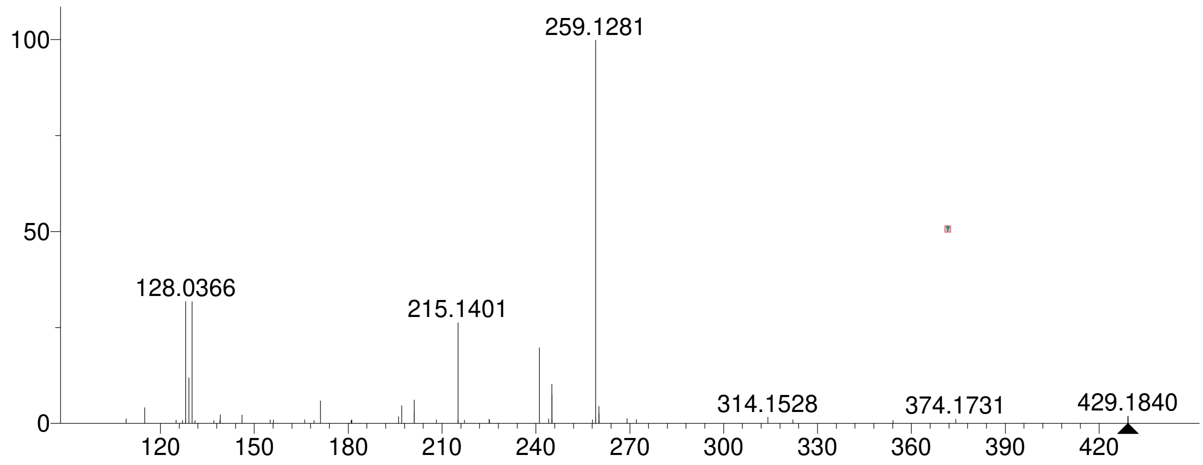

Hit 1 : Garcinone D [M+H]+ HCD 4V P=429.2
C24H28O7; MF: 71; RMF: 556; Prob 54.3%; CAS: 107390-08-9; Lib: nist_msms; ID: 258128.

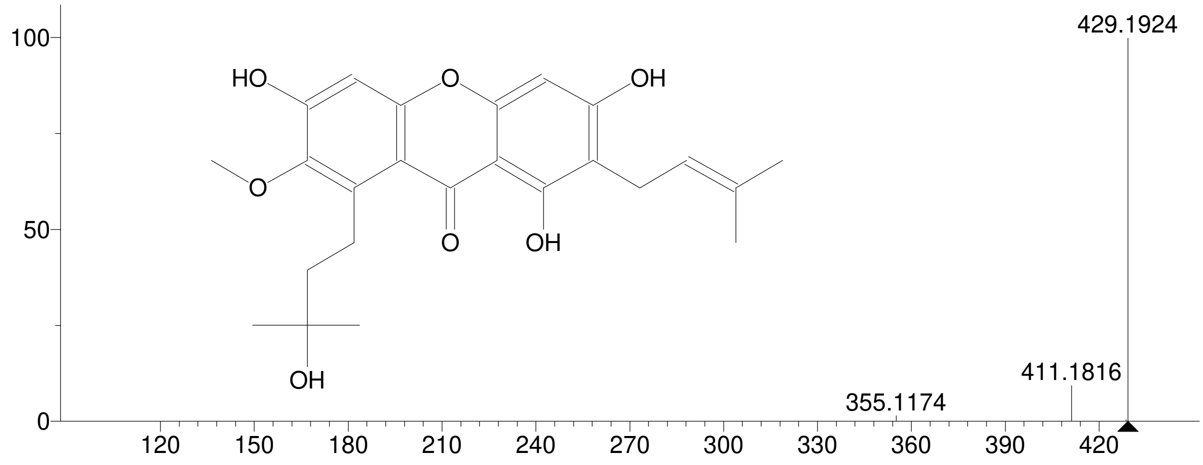

Hit 2 : 21-Carboxylic acid triamcinolone acetonide [M+H-HF]+ HCD 30V P=429.2
C24H29FO7; MF: 3; RMF: 86; Prob 8.23%; CAS: 53962-41-7; Lib: nist_msms; ID: 449216.

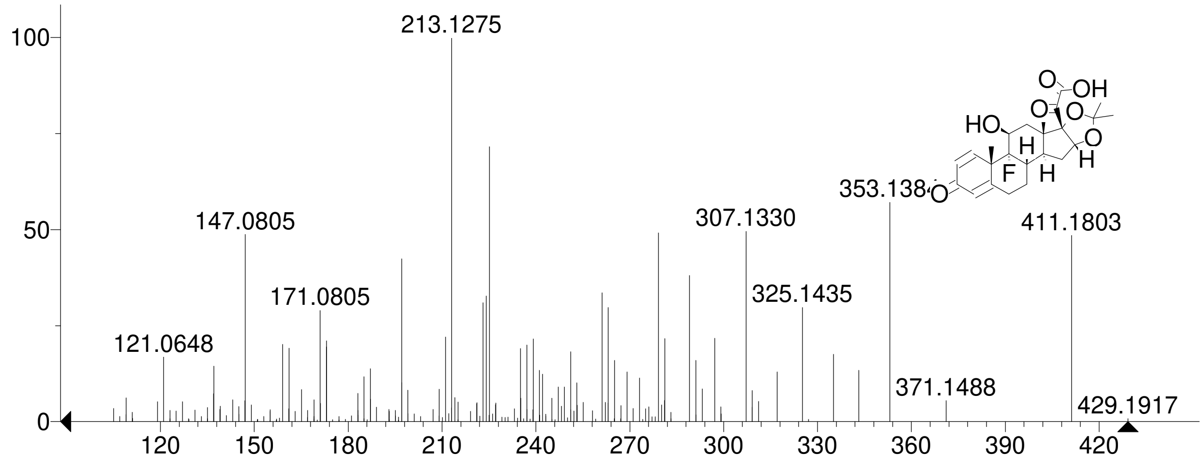

Hit 3 : Terretonin [M+H-C2H4O2]+ HCD 38V P=429.2
C26H32O9; MF: 2; RMF: 68; Prob 7.91%; CAS: 71911-90-5; Lib: nist_msms; ID: 460560.

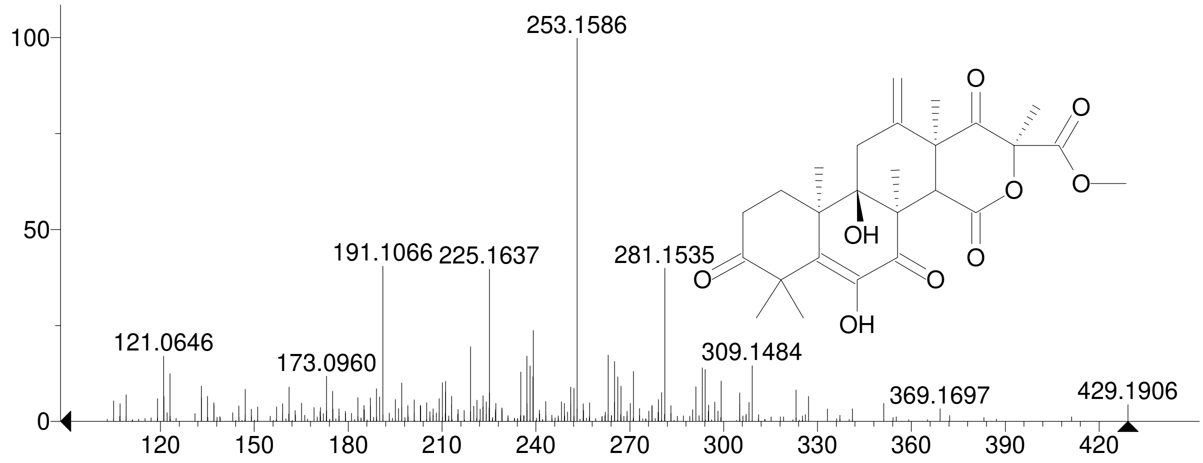

Hit 4 : (2R,3R,4S,5S,6R)-2-[6-hydroxy-3-[(E)-3-hydroxybut-1-enyl]-2,4,4-trimethylcyclohexyl]oxy-6-(hydroxymethy
C19H34O8; MF: 1; RMF: 57; Prob 7.60%; Lib: anthocyanins mona; ID: 9985.

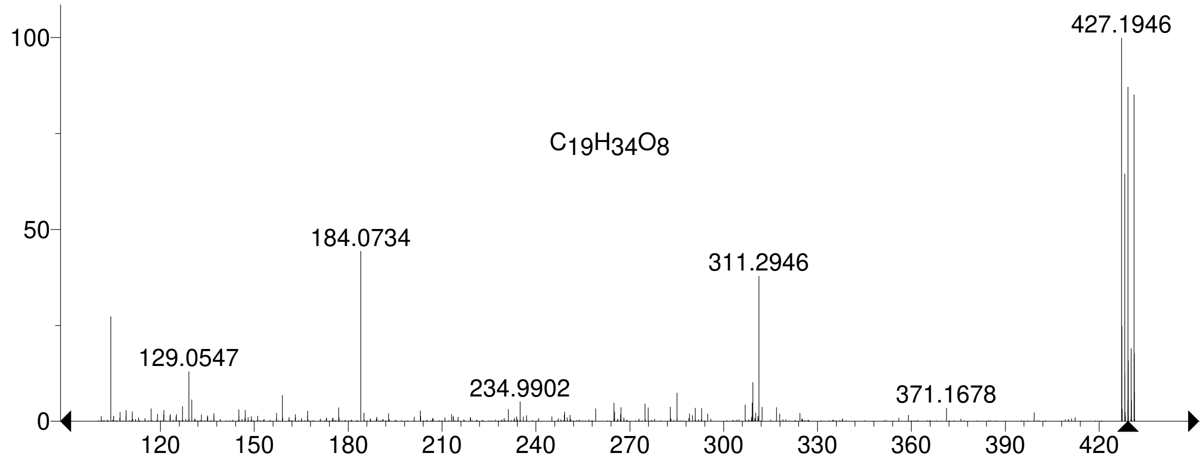

Hit 5 : [8,8-dimethyl-10-(3-methylbut-2-enoyloxy)-2-oxo-9,10-dihydropyrano[2,3-f]chromen-9-yl] 3-methylbutanoat
C24H28O7; MF: 0; RMF: 25; Prob 7.31%; Lib: mona-export-vf-npl_qexactive; ID: 19732.

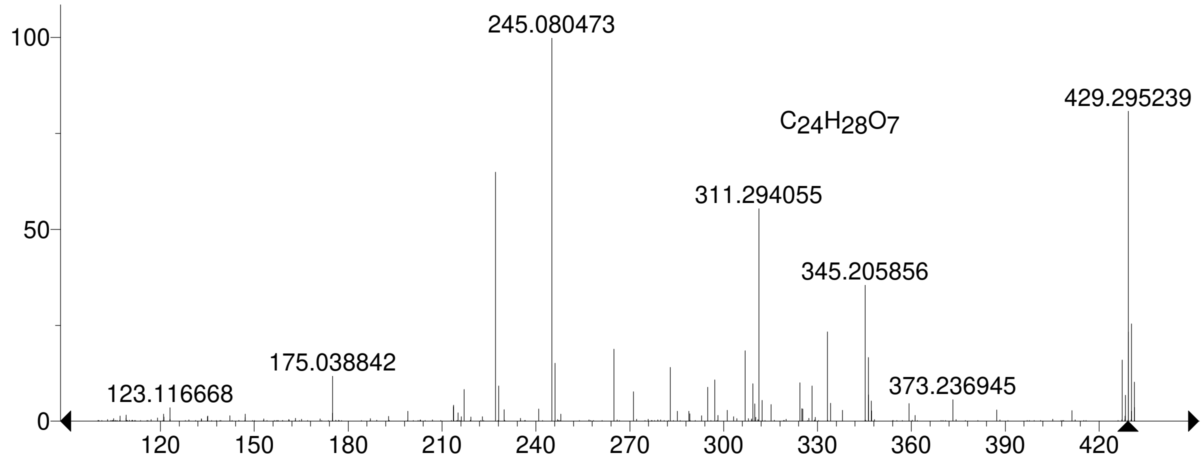

Unknown: Unknown (5.12_500.1857m/z)
Compound in Library Factor = 0

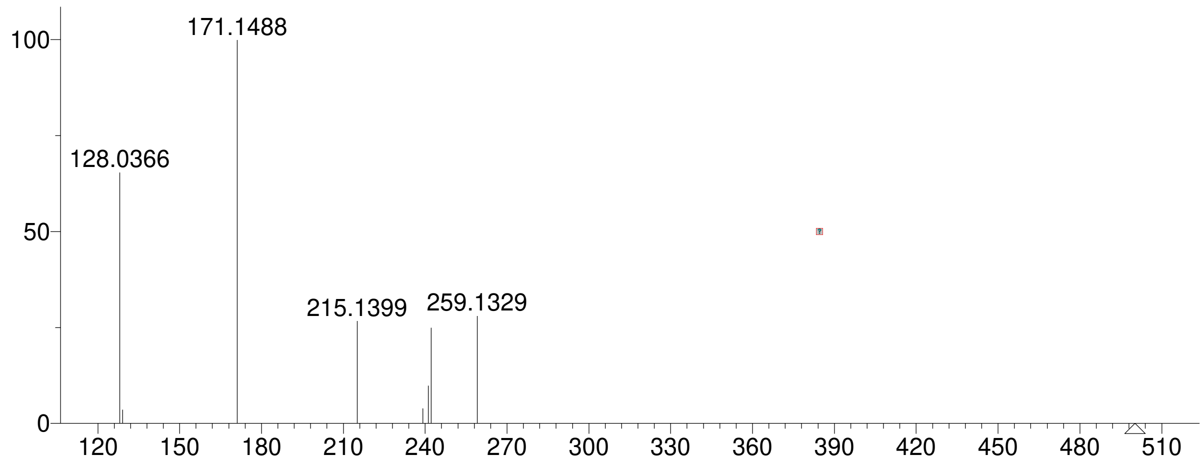

Unknown: Unknown (5.12_520.2500m/z)
Compound in Library Factor = 0

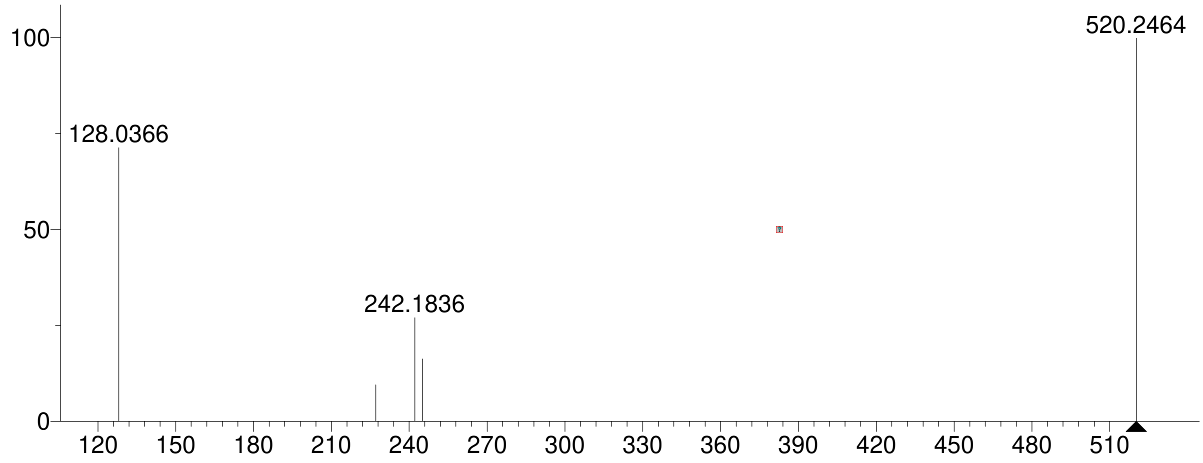

Hit 1 : NCGC00385440-01_C27H34O9_2-Butenoic acid, 2-(hydroxymethyl)-, (2E)-2-[[[(3aR,4R,6E,9S,10Z,11aS)-C27H34O9; MF: 0; RMF: 7; Prob 100.0%; Lib: mona-export-gnps; ID: 10916.

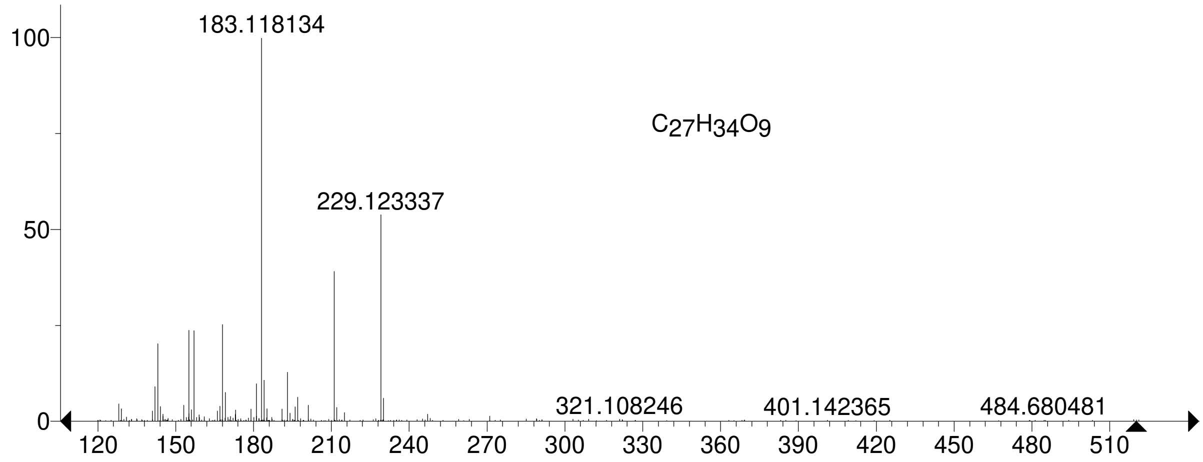

** Search Report Page 1 of 1 **

Unknown: Unknown (5.13_374.1539m/z)
Compound in Library Factor = 0

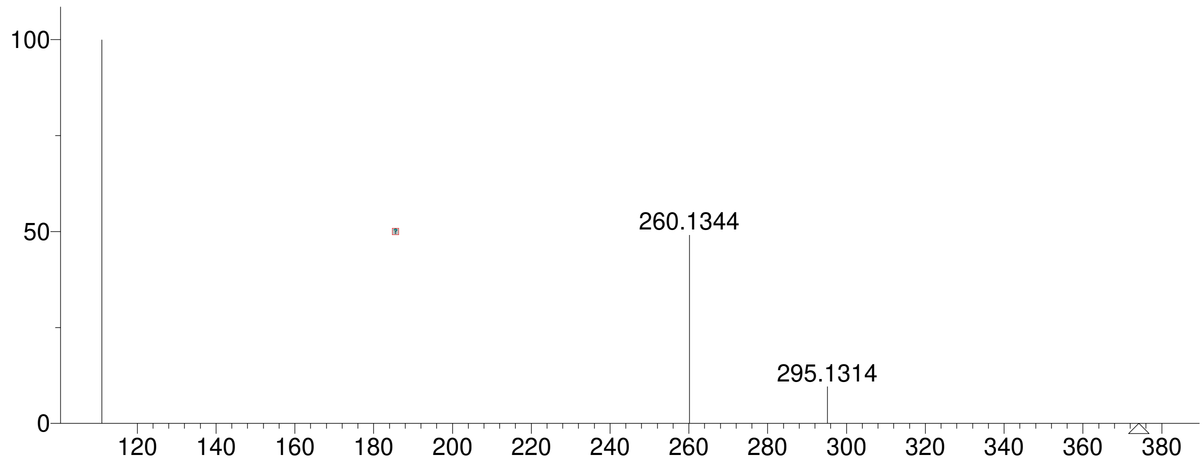

Hit 1 : Senecionine N-oxide [M+Na]+ HCD 29V P=374.2
C18H25NO6; MF: 0; RMF: 52; Prob 100.0%; CAS: 13268-67-2; Lib: nist_msms; ID: 257184.

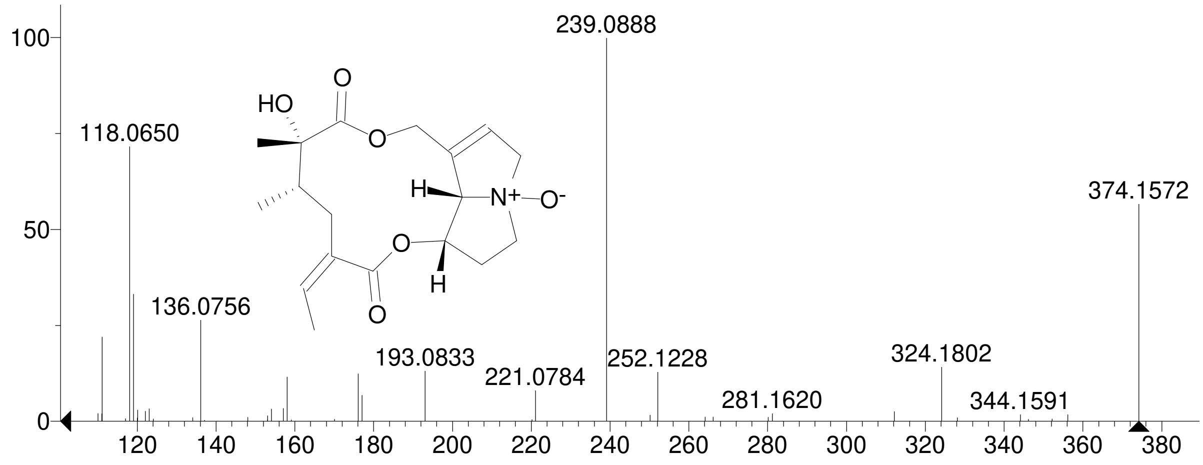

Unknown: Unknown (5.14_913.3931m/z)
Compound in Library Factor = 0

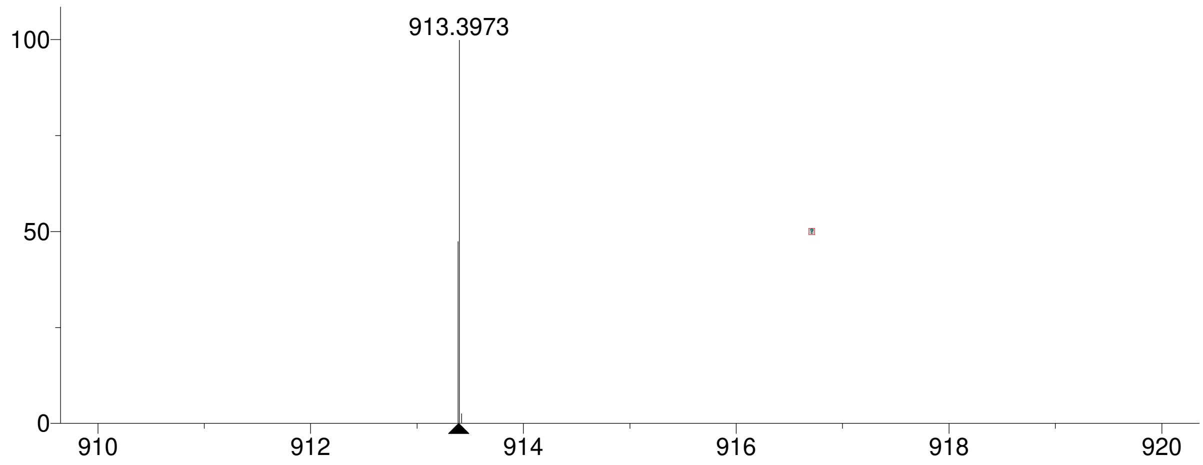

Unknown: Unknown (5.15_376.0985m/z)
Compound in Library Factor = 0

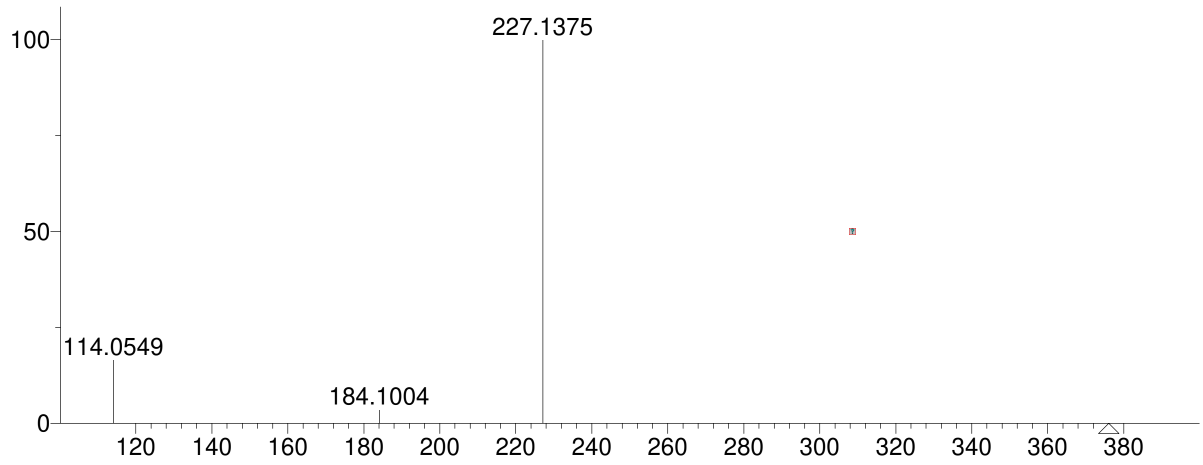

Hit 1 : 2-Pyridinecarboxylic acid, 6-[2-[5-chloro-2-[(2,4-difluorophenyl)methoxy]phenyl]-1-cyclopenten-1-yl]- [M-H]
C24H18ClF2NO3; MF: 0; RMF: 5; Prob 100.0%; CAS: 612831-24-0; Lib: nist_msms; ID: 371350.

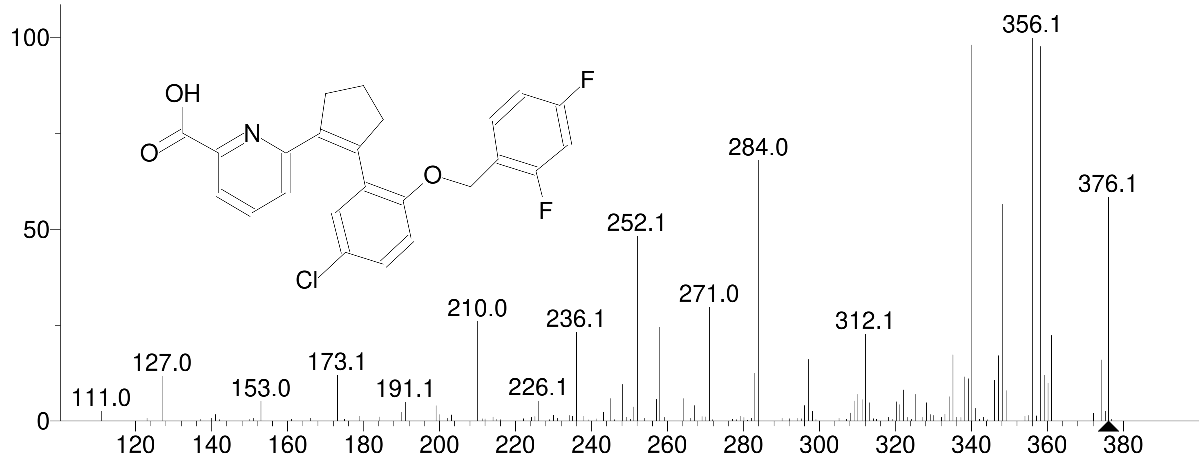

Unknown: Unknown (5.15_518.1860m/z)
Compound in Library Factor = 0

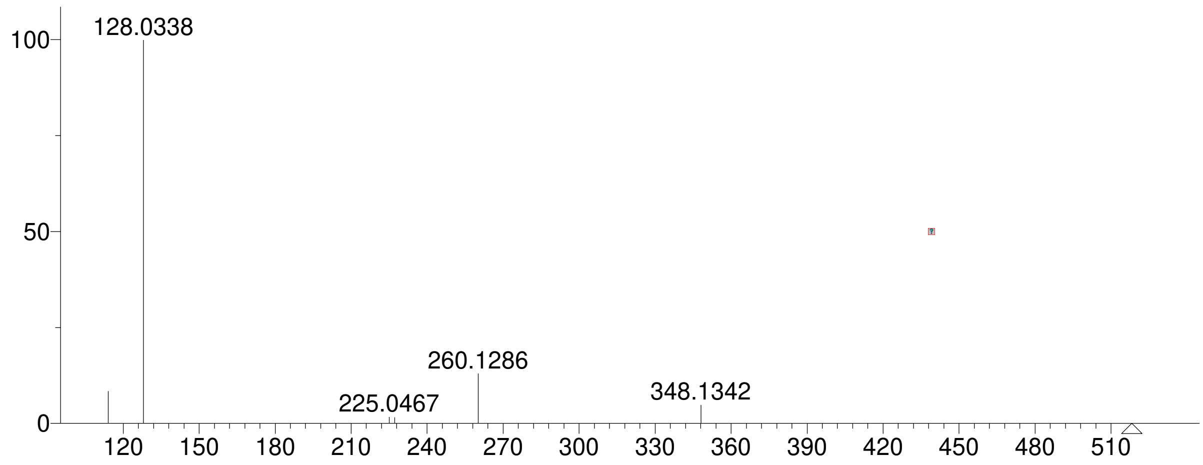

Hit 1 : 6-methoxy-7-[3,4,5-trihydroxy-6-[(3,4,5-trihydroxy-6-methyloxan-2-yl)oxymethyl]oxan-2-yl]oxychromen-2-or
C22H28O13; MF: 0; RMF: 3; Prob 100.0%; Lib: anthocyanins mona; ID: 5871.

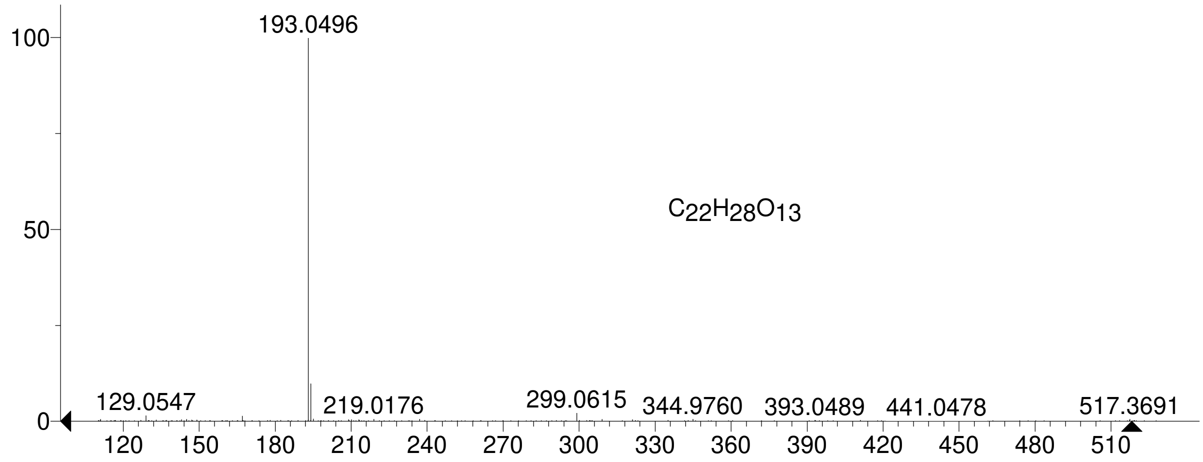

** Search Report Page 1 of 1 **

Unknown: Unknown (5.15_600.2419m/z)
Compound in Library Factor = 0

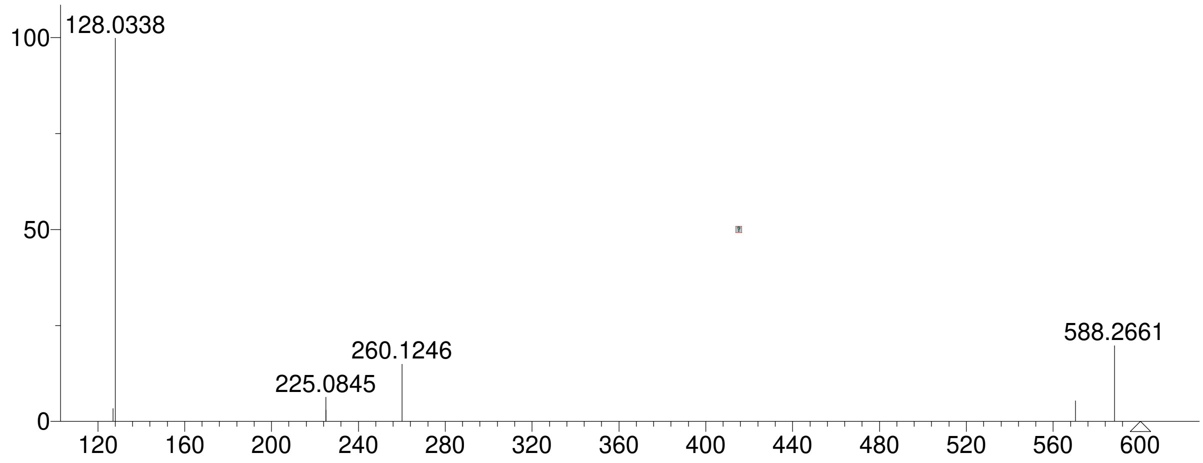

Unknown: Unknown (5.16_409.1959m/z)
Compound in Library Factor = 0

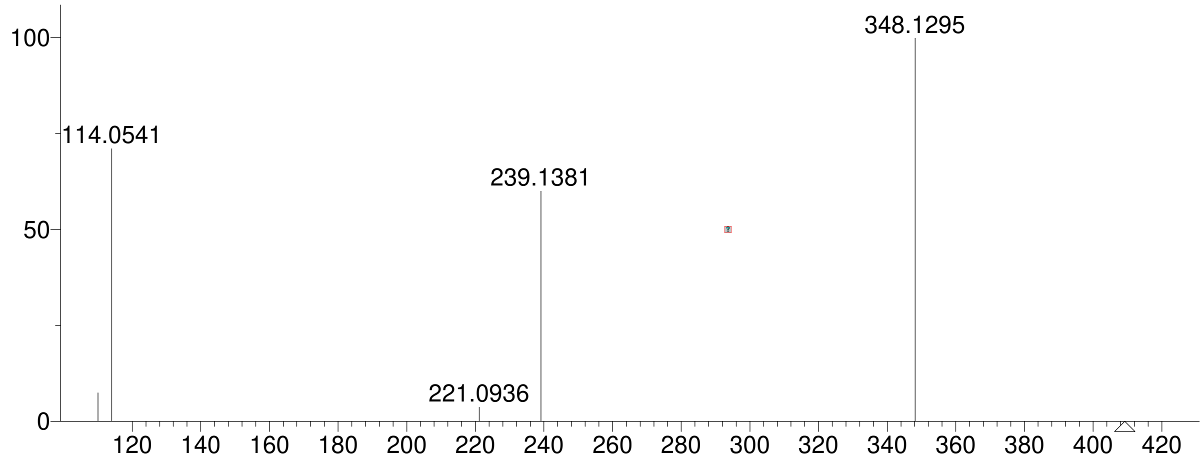

Unknown: Unknown (5.18_420.1402m/z)
Compound in Library Factor = 0

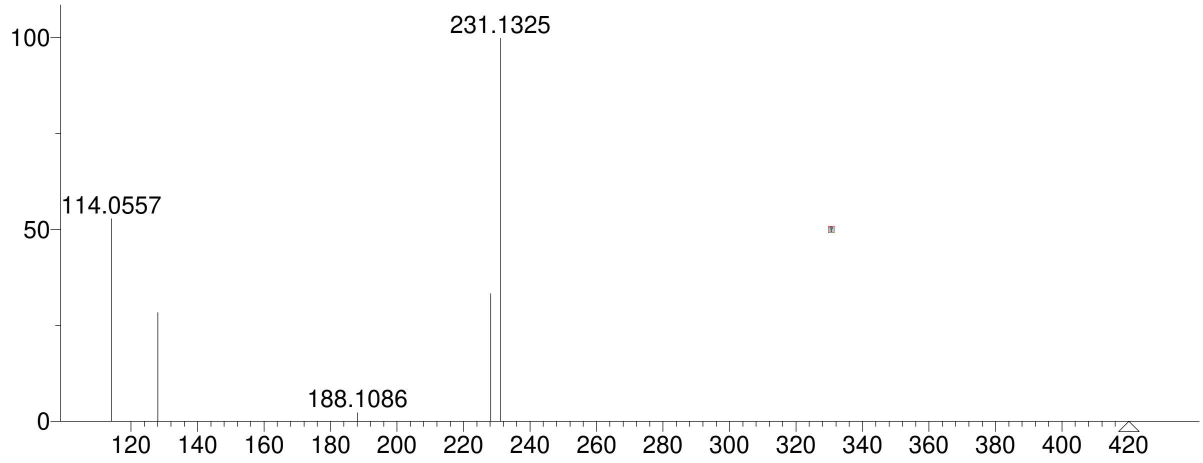

Unknown: Unknown (5.19_456.1611m/z)
Compound in Library Factor = 0

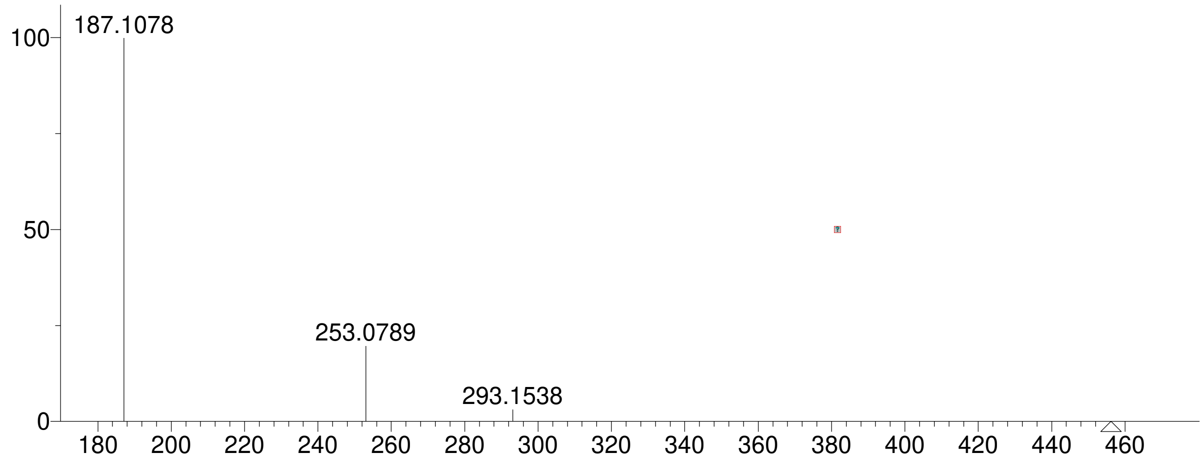

Hit 1 : Folinic acid [M+H-H2O]+ HCD 72V P=456.2
C20H23N7O7; MF: 0; RMF: 33; Prob 100.0%; CAS: 58-05-9; Lib: nist_msms; ID: 138160.

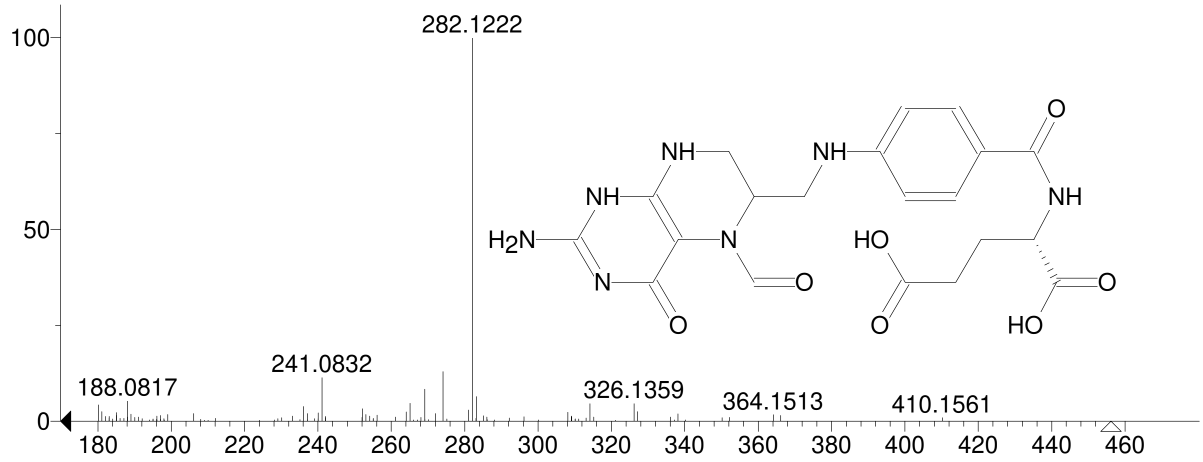

Unknown: Unknown (5.19_584.3038m/z)
Compound in Library Factor = -3273

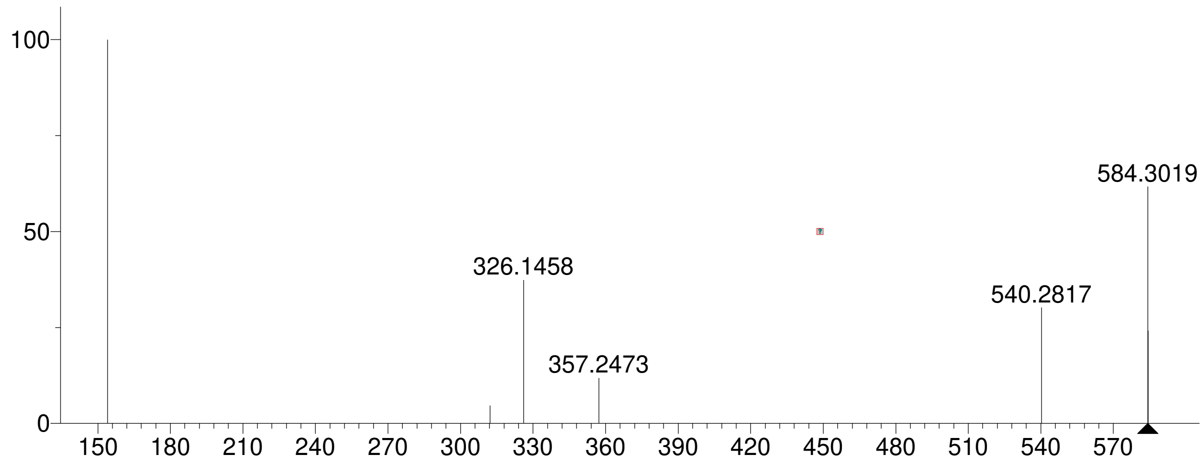

Hit 1 : [DAla6,Des-Gly10] LH-RH, ethyl amide [M+2H]2+ QTOF 4V P=584.3
C56H78N16O12; MF: 14; RMF: 371; Prob 61.5%; Lib: nist_msms2; ID: 38383.

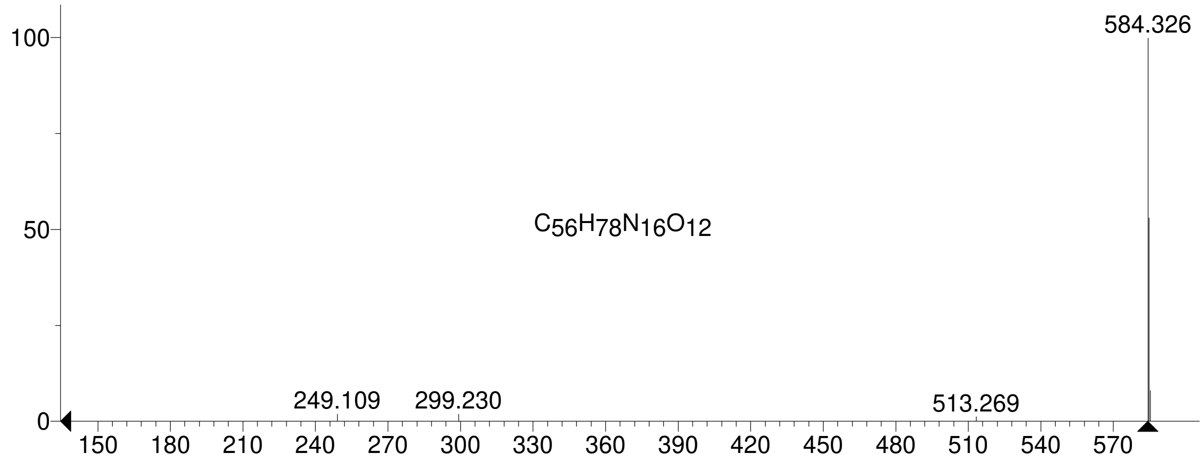

Hit 2 : NCGC00380379-01_C29H42O11_ [M+NH4]+ P=584.3
C29H42O11; MF: 0; RMF: 39; Prob 38.5%; Lib: mona-export-gnps; ID: 12061.

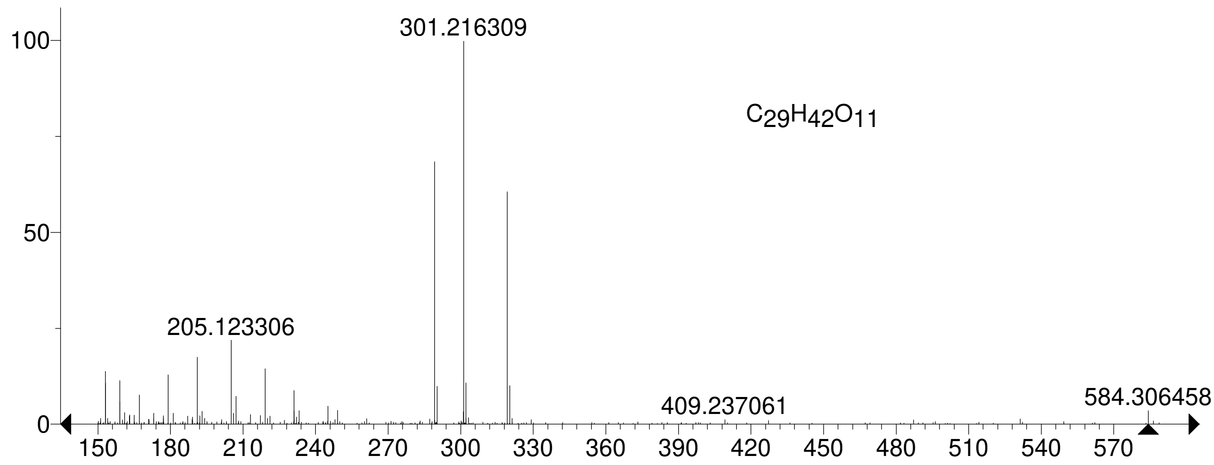

Unknown: Unknown (5.20_401.1916m/z)
Compound in Library Factor = 0

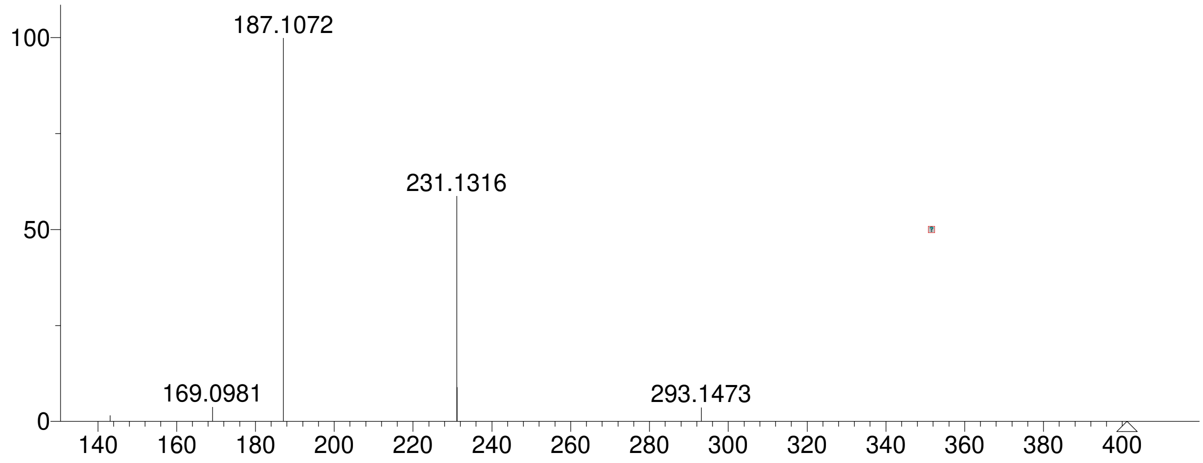

Hit 1 : NCGC00380382-01_C23H30O7_1-Phenanthrenecarboxylic acid, 10-(acetyloxy)-1,2,3,4,4a,9,10,10a-octah
C23H30O7; MF: 0; RMF: 26; Prob 100.0%; Lib: mona-export-gnps; ID: 13719.

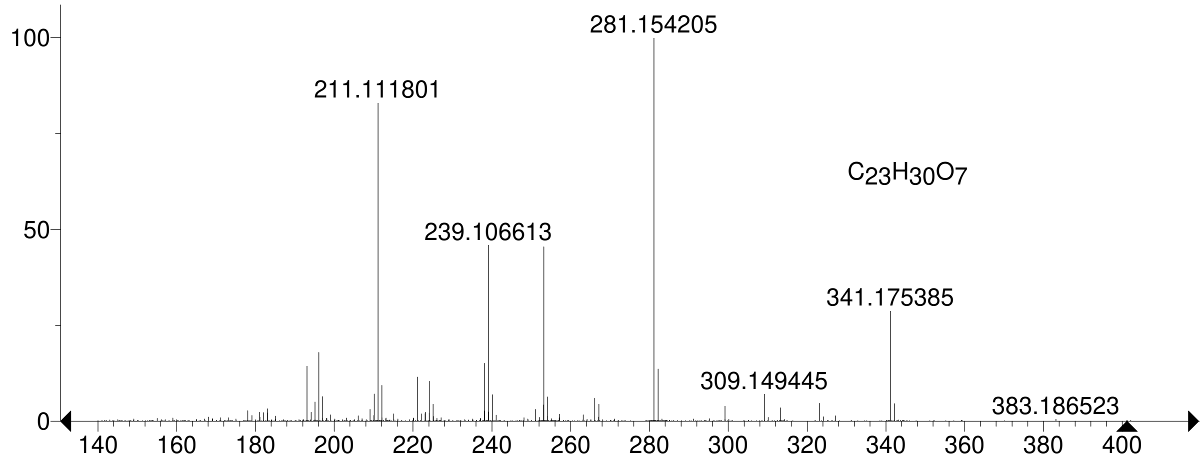

Unknown: Unknown (5.21_355.1607m/z)
Compound in Library Factor = 0

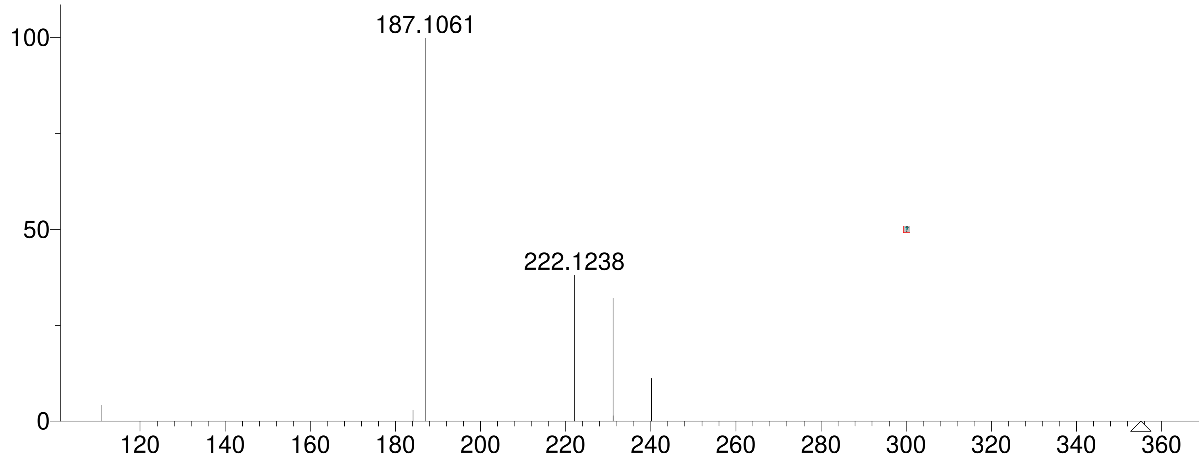

Unknown: Unknown (5.23_291.0977m/z)
Compound in Library Factor = -791

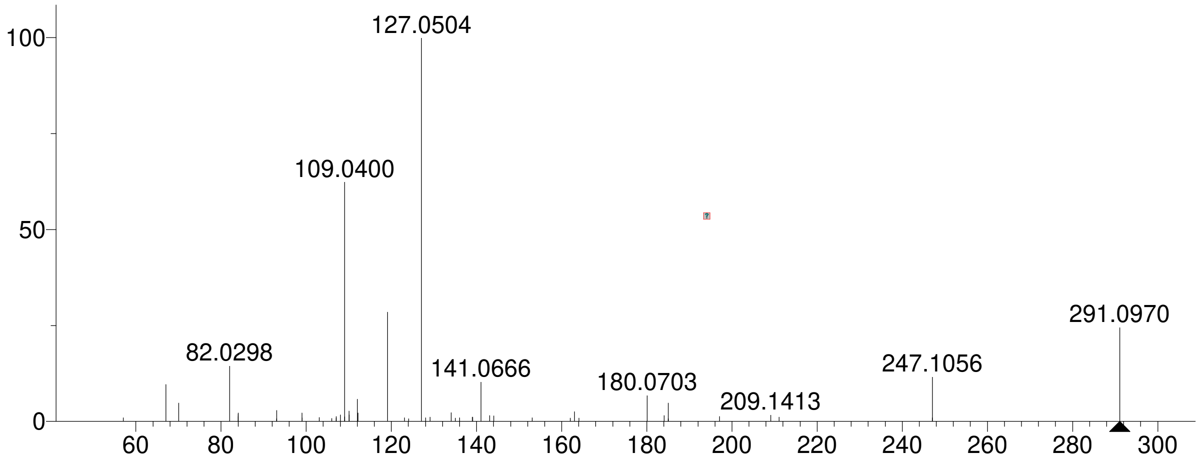

Hit 1 : N,N'-Dicarbobenzyloxy-L-ornithine [M-H-C7H8O]- HCD 0V P=291.1
C21H24N2O6; MF: 119; RMF: 658; Prob 20.6%; CAS: 2274-58-0; Lib: nist_msms; ID: 305180.

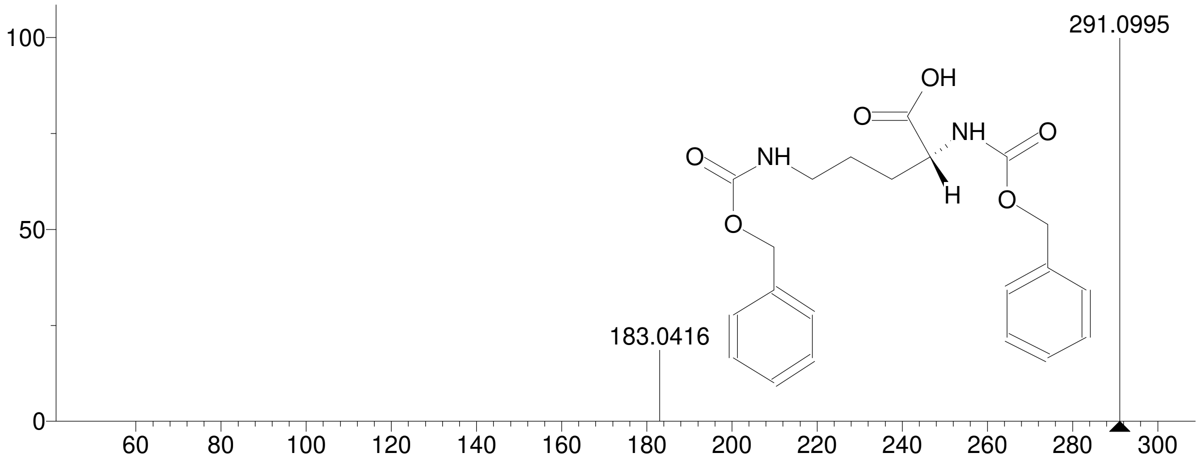

Hit 2 : 5a,6-Anhydrotetracycline [M-H-NH3]-=>319.1=>291.1 IT 35% P=291.1
C22H22N2O7; MF: 20; RMF: 325; Prob 1.56%; CAS: 1665-56-1; Lib: nist_msms; ID: 309452.

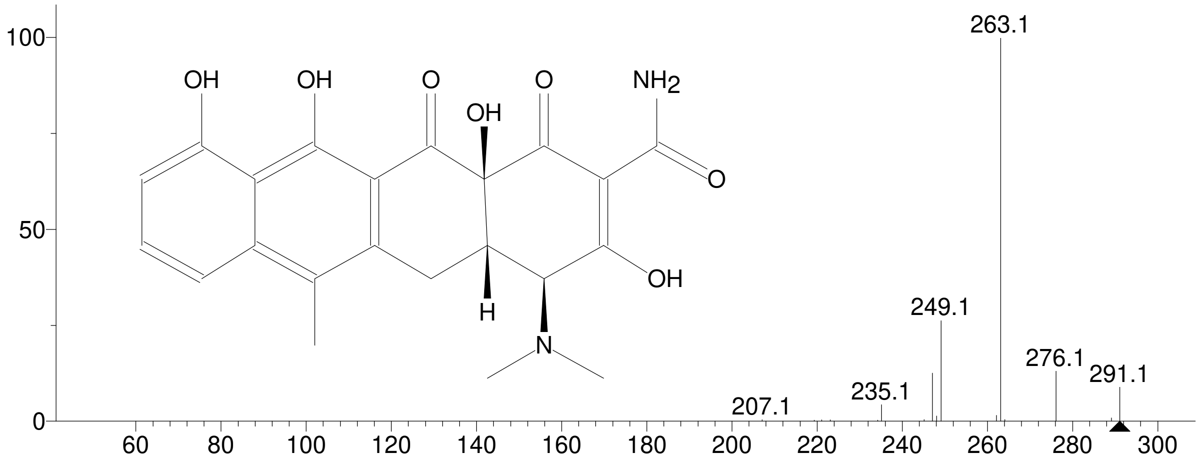

Hit 3 : Oxytetracycline [M-H-CHNO]-=>309.1=>291.1 IT 35% P=291.1
C22H24N2O9; MF: 11; RMF: 339; Prob 1.13%; CAS: 79-57-2; Lib: nist_msms; ID: 481729.

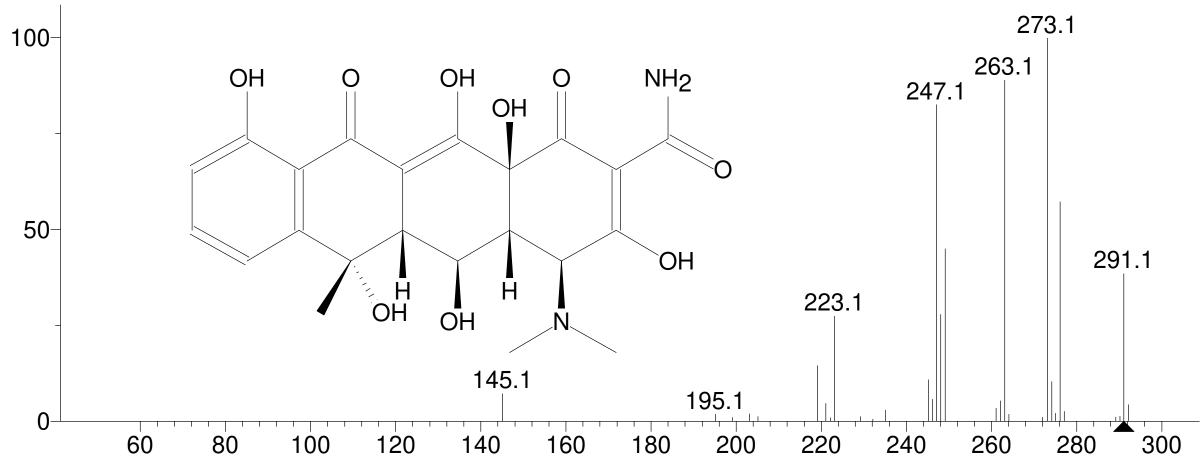

Hit 4 : Uncarine C [M+H-CH4O]+=>309.2=>291.1 IT 35% P=291.1
C21H24N2O4; MF: 3; RMF: 198; Prob 0.84%; CAS: 5629-60-7; Lib: nist_msms; ID: 227867.

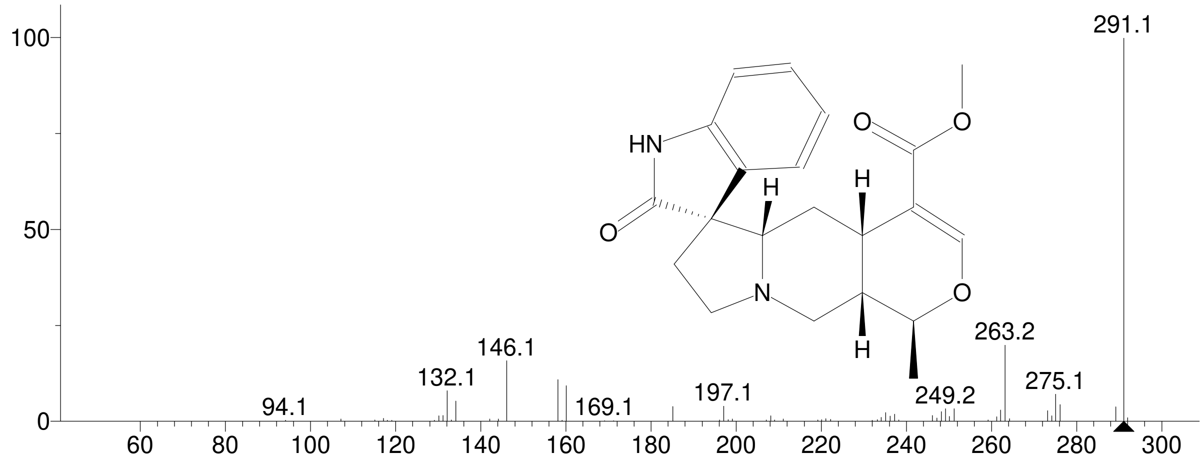

Hit 5 : 2',3'-Dihydrocephalomannine [M+H-C25H33NO10]+=>309.1=>291.1 IT 35% P=291.1
C45H55NO14; MF: 3; RMF: 167; Prob 0.84%; CAS: 159001-25-9; Lib: nist_msms; ID: 458904.

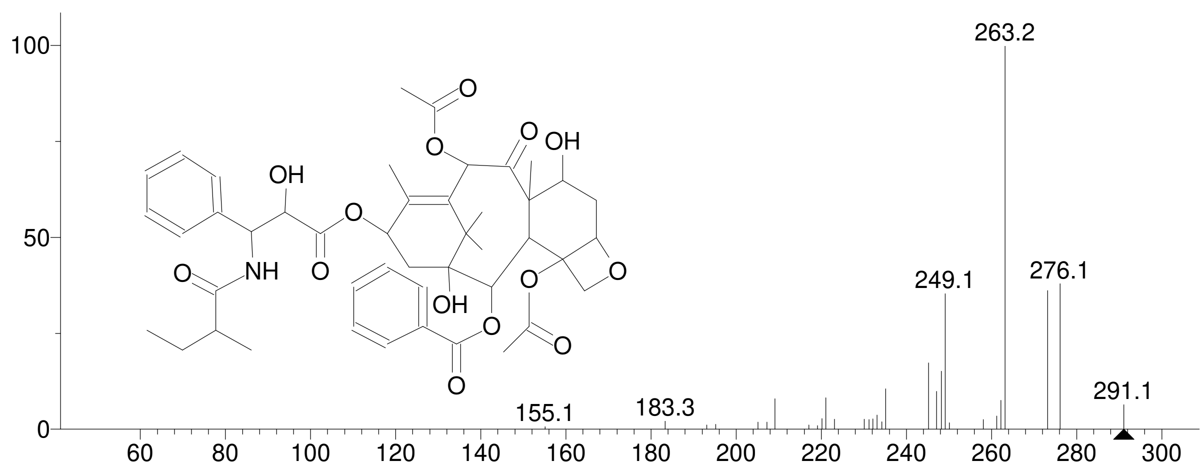

Unknown: Unknown (5.23_348.1206m/z)
Compound in Library Factor = 0

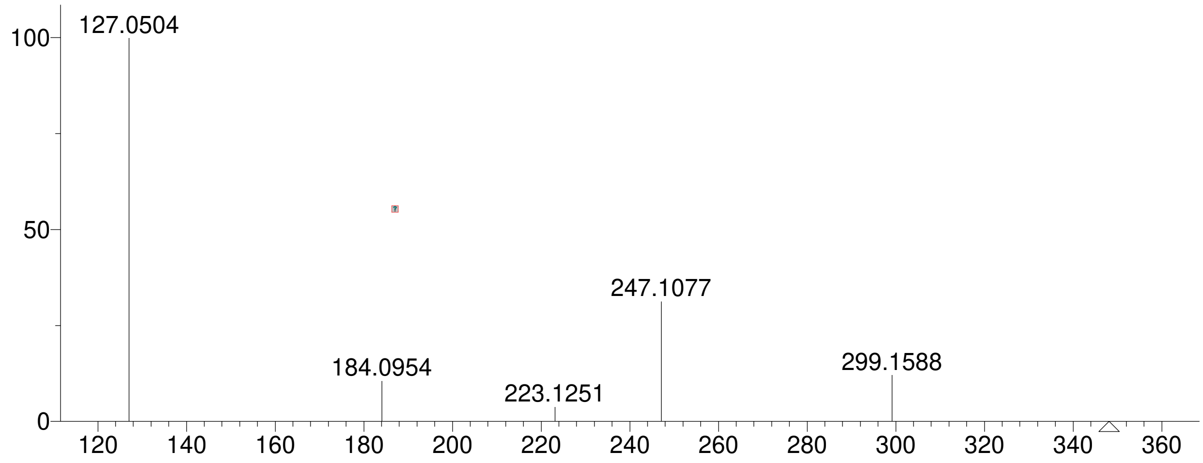

Hit 1 : Nitidine cation [Cat]+ HCD 62V P=348.1
C21H18NO4; MF: 0; RMF: 101; Prob 33.3%; CAS: 6872-57-7; Lib: nist_msms; ID: 218853.

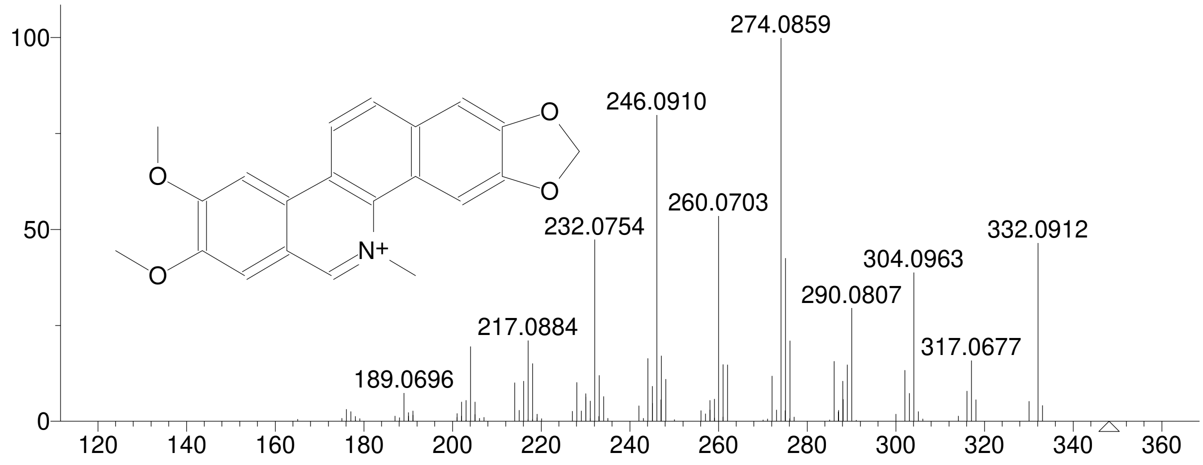

Hit 2 : (-)-Coralyne cation [Cat-CH4]+ HCD 76V P=348.1
C22H22NO4; MF: 0; RMF: 84; Prob 33.3%; CAS: 6872-73-7; Lib: nist_msms; ID: 217308.

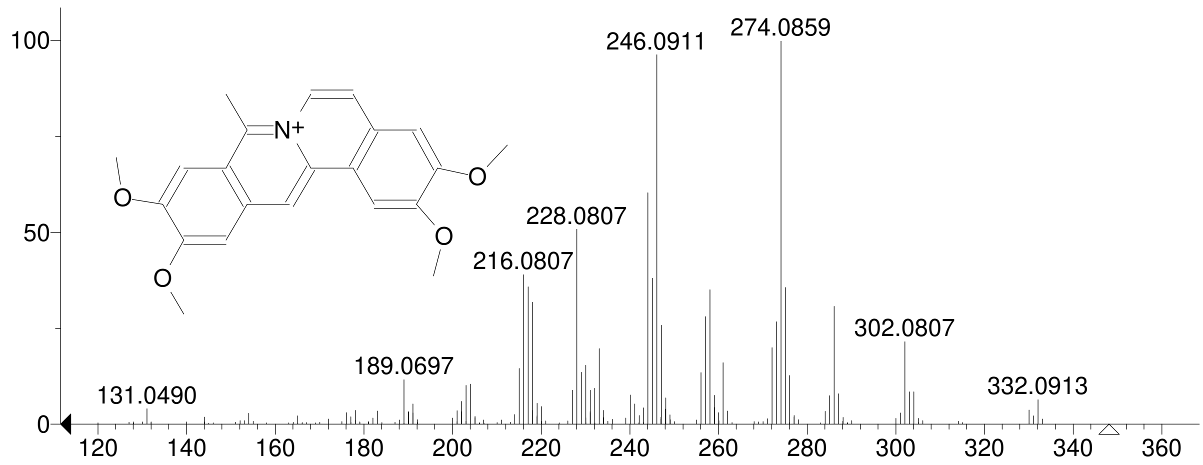

Hit 3 : Chelerythrine cation [Cat]+ QTOF 45V P=348.1
C21H18NO4; MF: 0; RMF: 67; Prob 33.3%; CAS: 34316-15-9; Lib: nist_msms; ID: 66141.

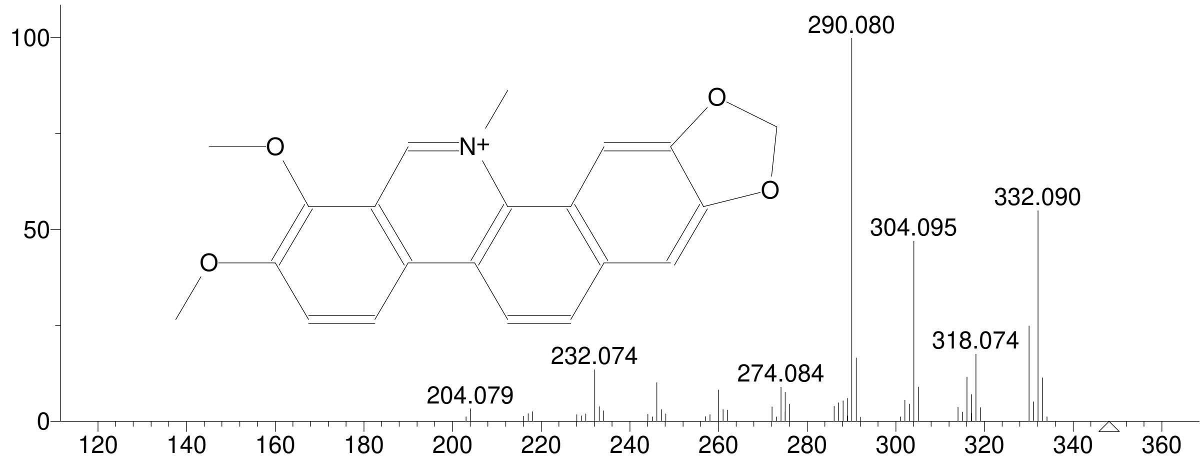

Unknown: Unknown (5.23_372.2125m/z)
Compound in Library Factor = 0

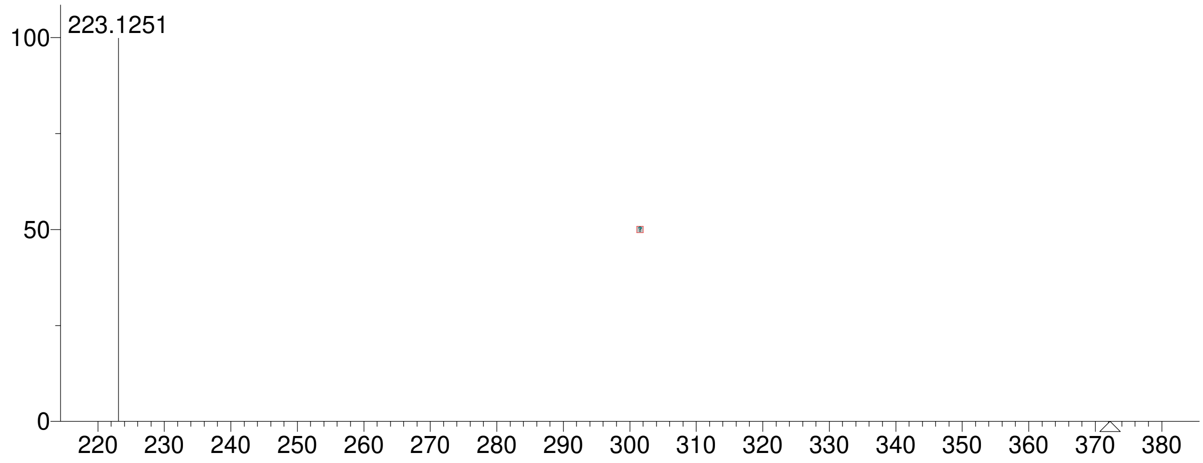

Unknown: Unknown (5.23_680.2787m/z)
Compound in Library Factor = 0

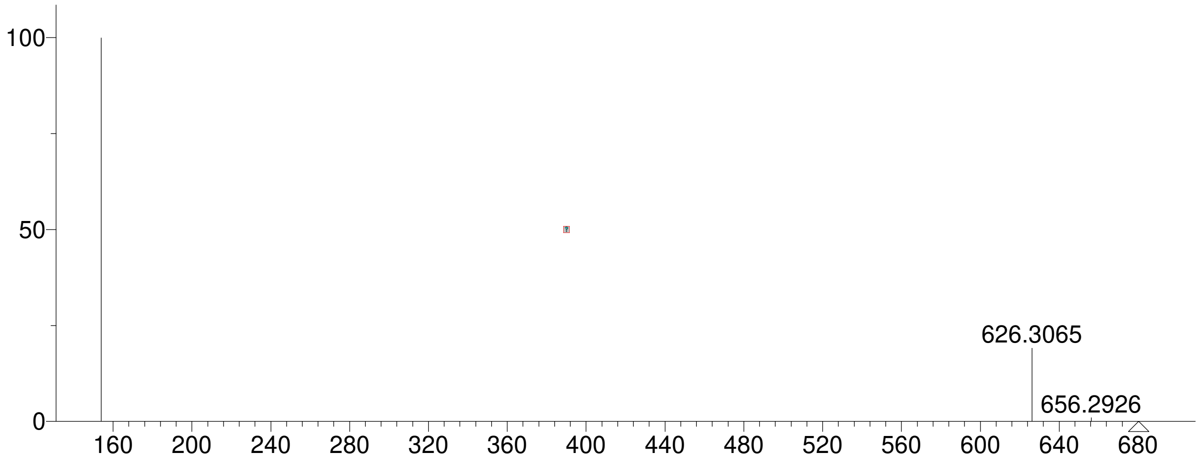

Unknown: Unknown (5.26_178.1225m/z)
Compound in Library Factor = -379

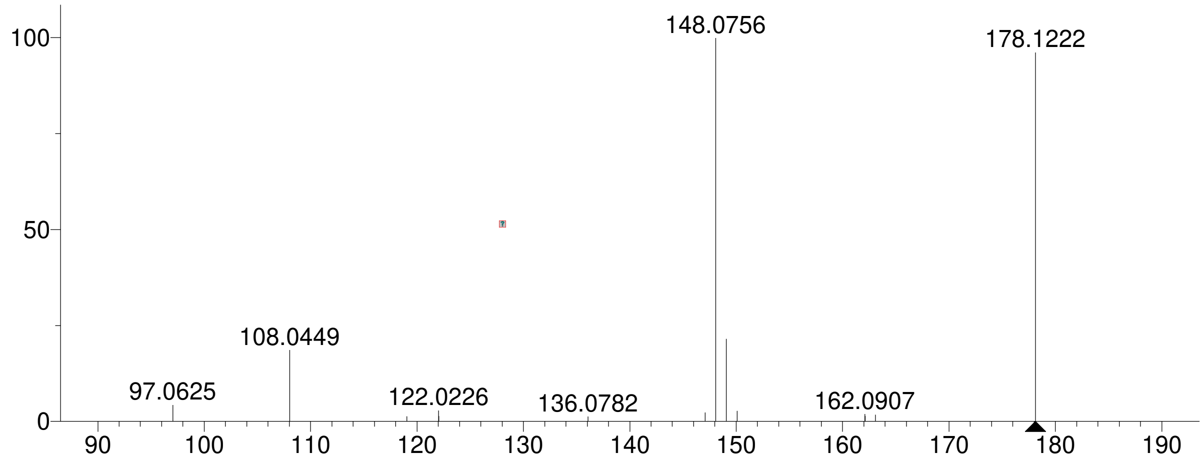

Hit 1 : 1H-Inden-2-amine, 2,3-dihydro-5-methoxy-6-methyl- [M+H]+ HCD 3V P=178.1
C11H15NO; MF: 297; RMF: 921; Prob 92.5%; CAS: 136468-19-4; Lib: nist_msms; ID: 364124.

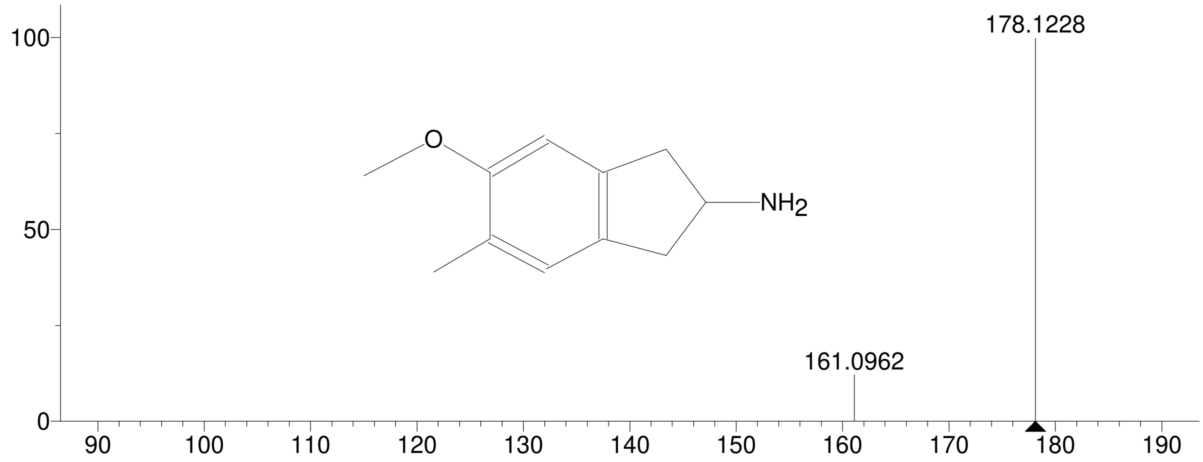

Hit 2 : 4-Benzofuranethanamine, 2,3-dihydro-α-methyl- [M+H]+ HCD 0V P=178.1
C11H15NO; MF: 138; RMF: 638; Prob 2.33%; CAS: 1203430-05-0; Lib: nist_msms; ID: 426816.

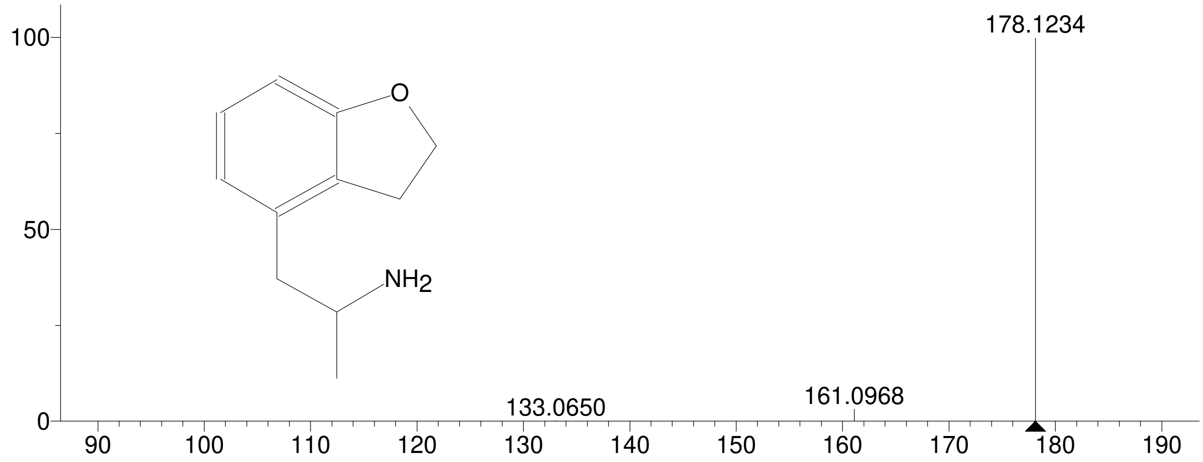

Hit 3 : 2-Methylmethcathinone [M+H]+ HCD 3V P=178.1
C11H15NO; MF: 124; RMF: 637; Prob 1.45%; CAS: 1246911-71-6; Lib: nist_msms; ID: 249570.

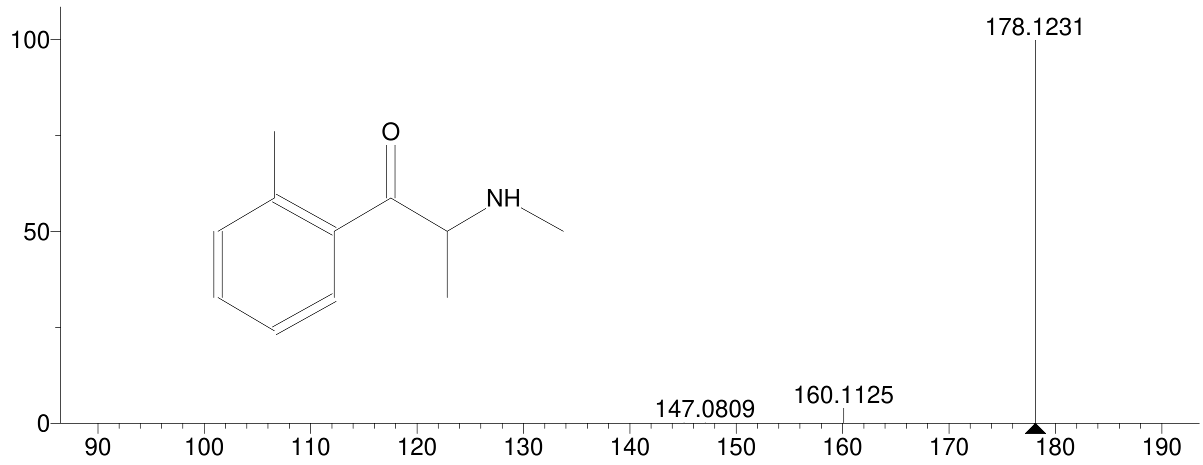

Hit 4 : 4-Methylmethcathinone [M+H]+ HCD 3V P=178.1
C11H15NO; MF: 119; RMF: 636; Prob 1.17%; CAS: 1189805-46-6; Lib: nist_msms; ID: 325130.

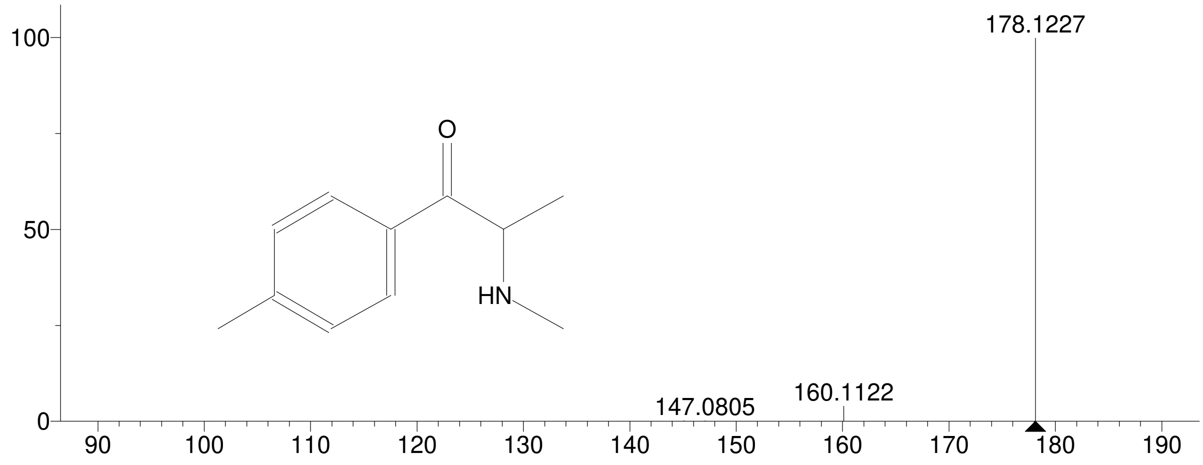

Hit 5 : 6-(2-Aminopropyl)-2,3-dihydrobenzofuran [M+H]+ HCD 1V P=178.1
C11H15NO; MF: 96; RMF: 612; Prob 0.42%; CAS: 152623-93-3; Lib: nist_msms; ID: 253794.

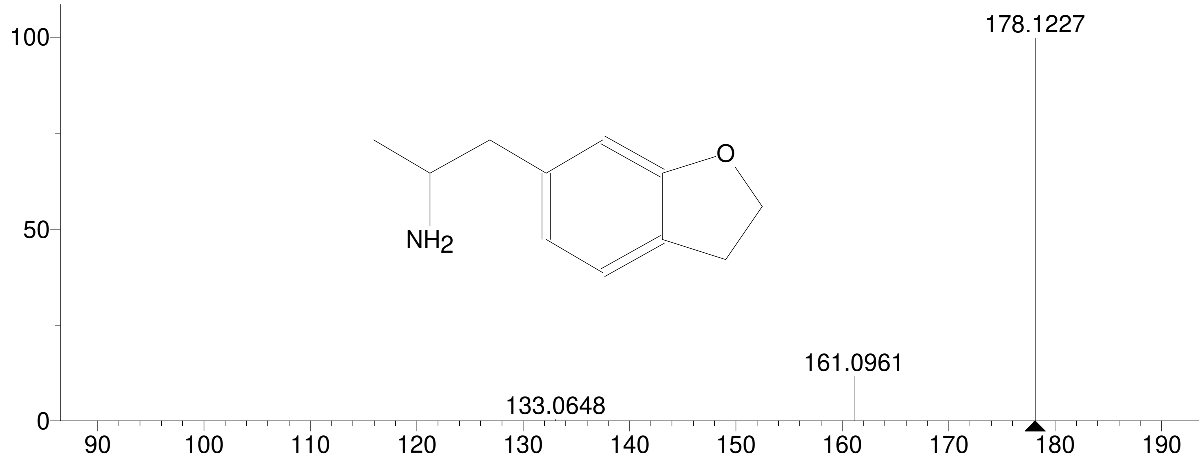

Unknown: Unknown (5.26_222.0760m/z)
Compound in Library Factor = -642

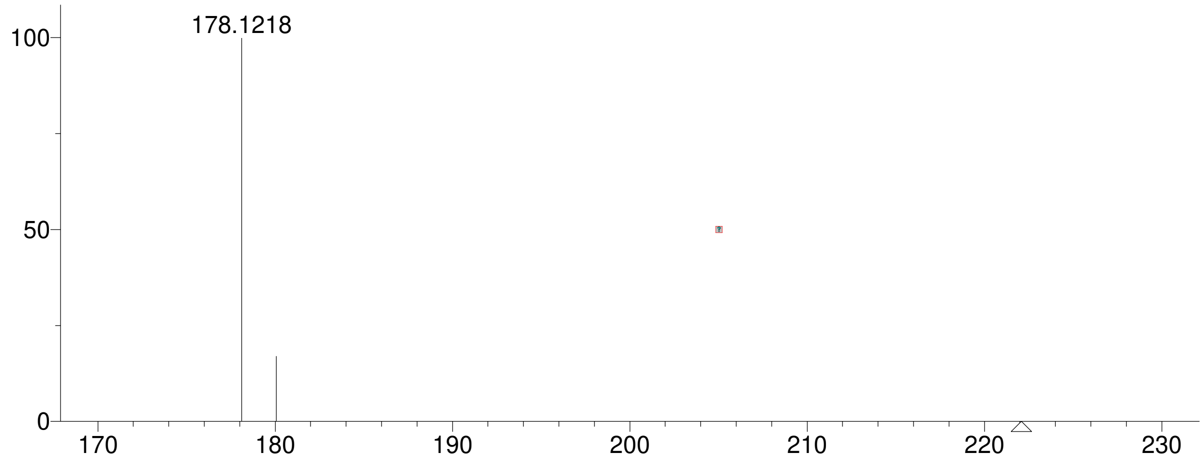

Hit 1 : N-Acetyl-L-tyrosine [M-H]- HCD 28V P=222.1
C11H13NO4; MF: 112; RMF: 380; Prob 94.0%; CAS: 537-55-3; Lib: nist_msms; ID: 280195.

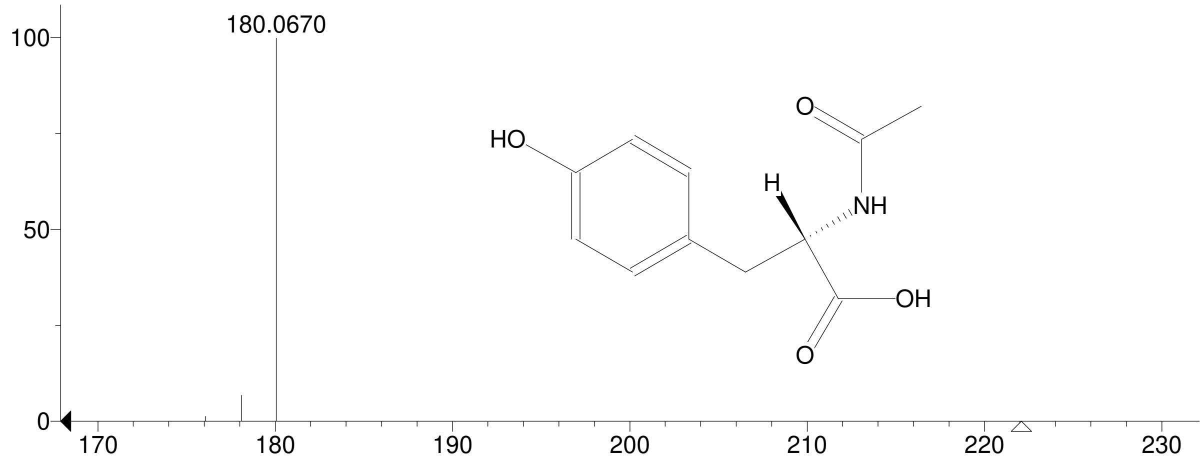

Hit 2 : NCGC00381349-01_C11H13NO5_1H-Pyrrole-3-propanoic acid, 5-acetyl-4-(carboxymethyl)- [M-H2O+H]+
C11H13NO5; MF: 1; RMF: 122; Prob 5.94%; Lib: mona-export-gnps; ID: 11085.

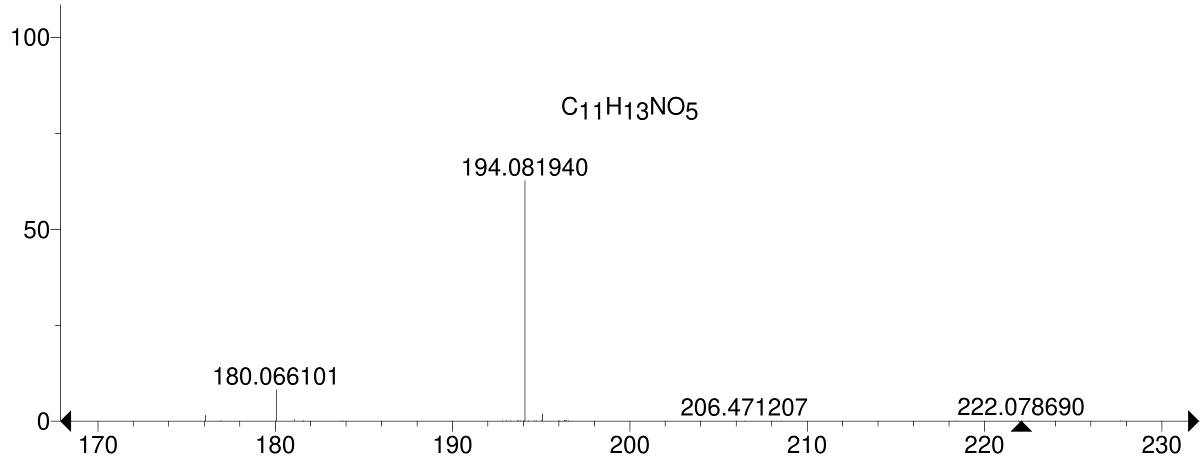

Unknown: Unknown (5.26_224.1278m/z)
Compound in Library Factor = -3273

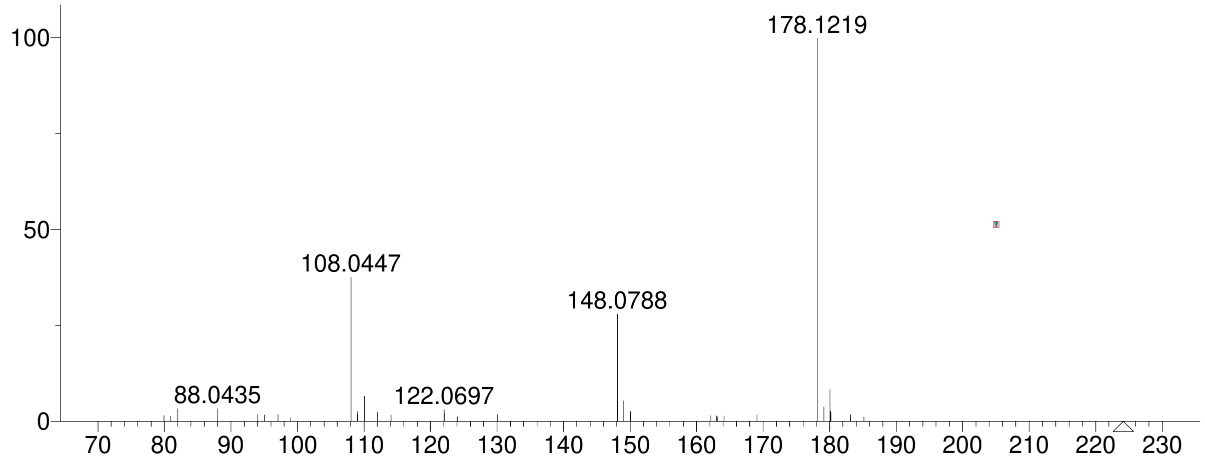

Hit 1 : 3,4-Dimethoxymethcathinone [M+H]+ HCD 40V P=224.1
C12H17NO3; MF: 2; RMF: 143; Prob 20.5%; CAS: 802301-79-7; Lib: nist_msms; ID: 547282.

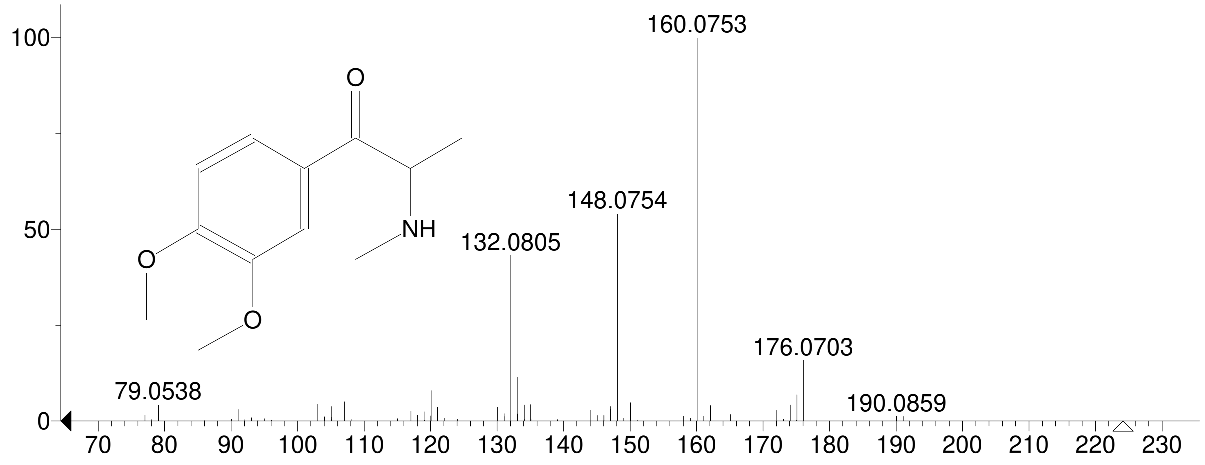

Hit 2 : Bufexamac [M+H]+ IT 35% P=224.1
C12H17NO3; MF: 2; RMF: 66; Prob 20.5%; CAS: 2438-72-4; Lib: nist_msms; ID: 492682.

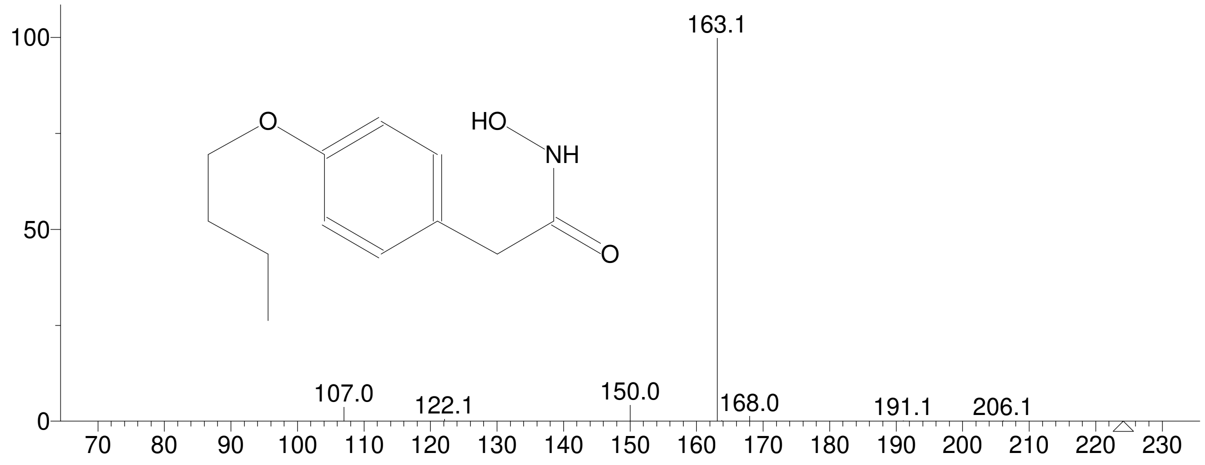

Hit 3 : N-(3-Oxooctanoyl)-L-homoserine lactone [M+H-H2O]+ HCD 20V P=224.1
C12H19NO4; MF: 2; RMF: 60; Prob 20.5%; CAS: 147795-39-9; Lib: nist_msms; ID: 315228.

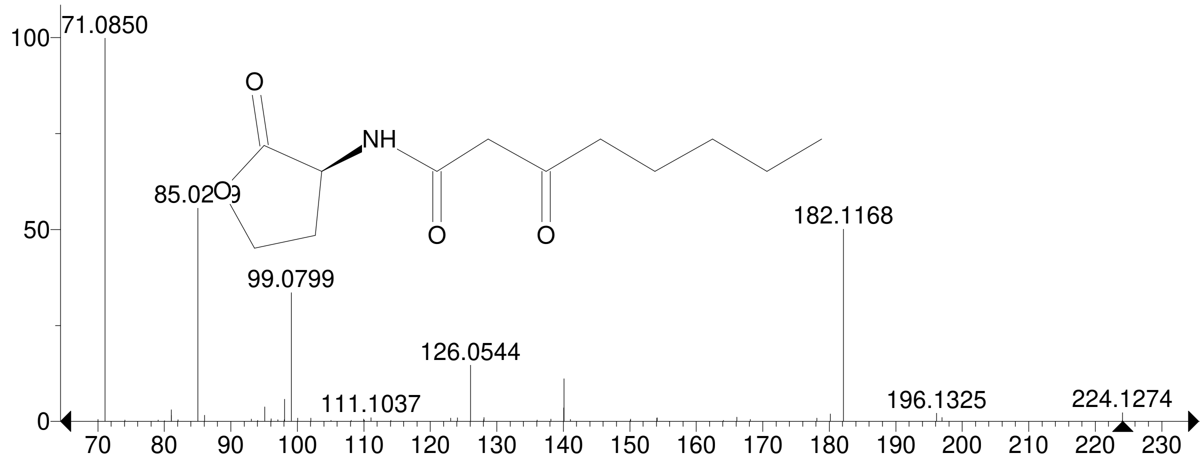

Hit 4 : 1-(3,5-Dihydroxyphenyl)-2-[(1,1-dimethylethyl)amino]ethanone [M+H]+ HCD 33V P=224.1
C12H17NO3; MF: 1; RMF: 53; Prob 19.7%; CAS: 139508-58-0; Lib: nist_msms; ID: 437127.

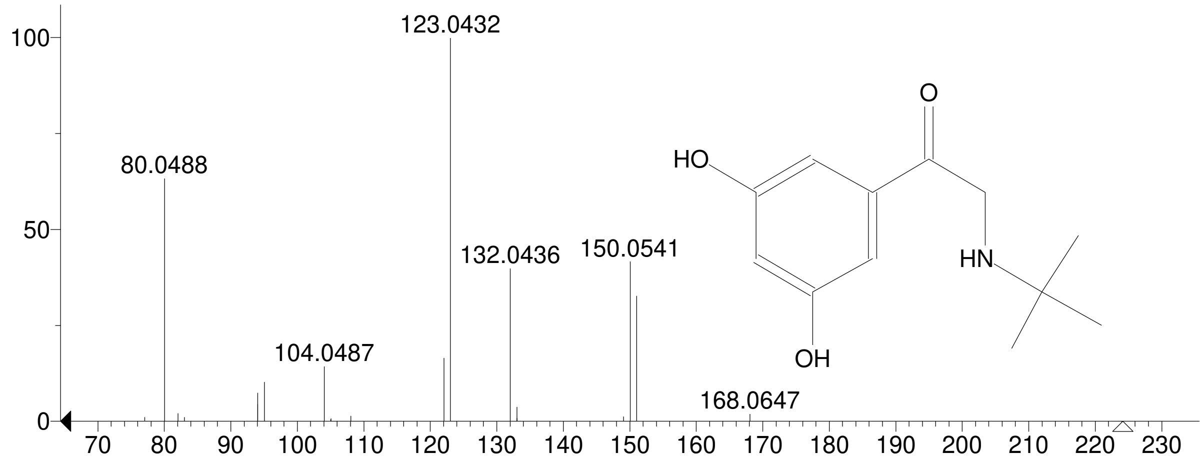

Hit 5 : Cerulenin [M+H]+ IT-FT 35% P=224.1
C12H17NO3; MF: 0; RMF: 131; Prob 18.9%; CAS: 17397-89-6; Lib: nist_msms; ID: 538608.

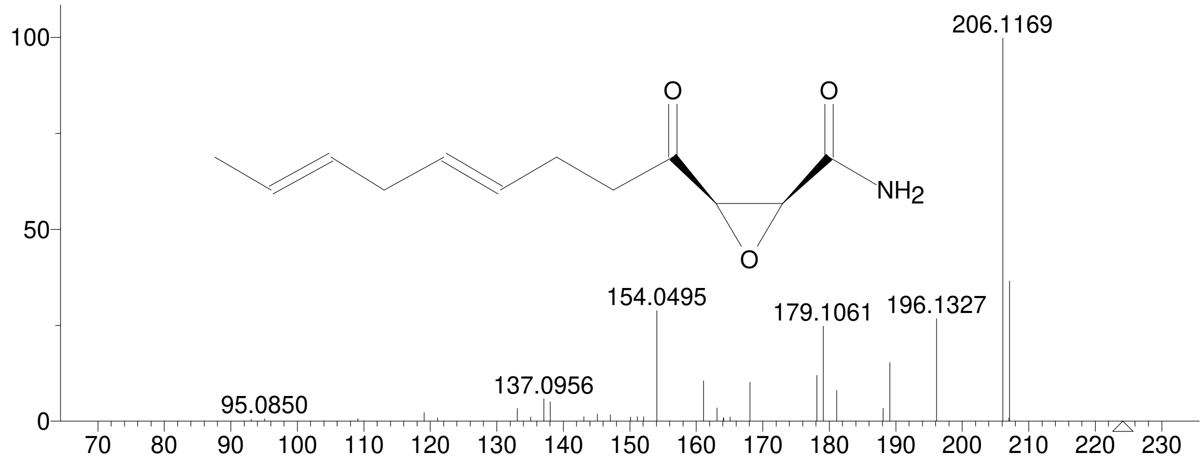

** Search Report Page 1 of 1 **

Unknown: Unknown (5.26_366.1544m/z)
Compound in Library Factor = -3273

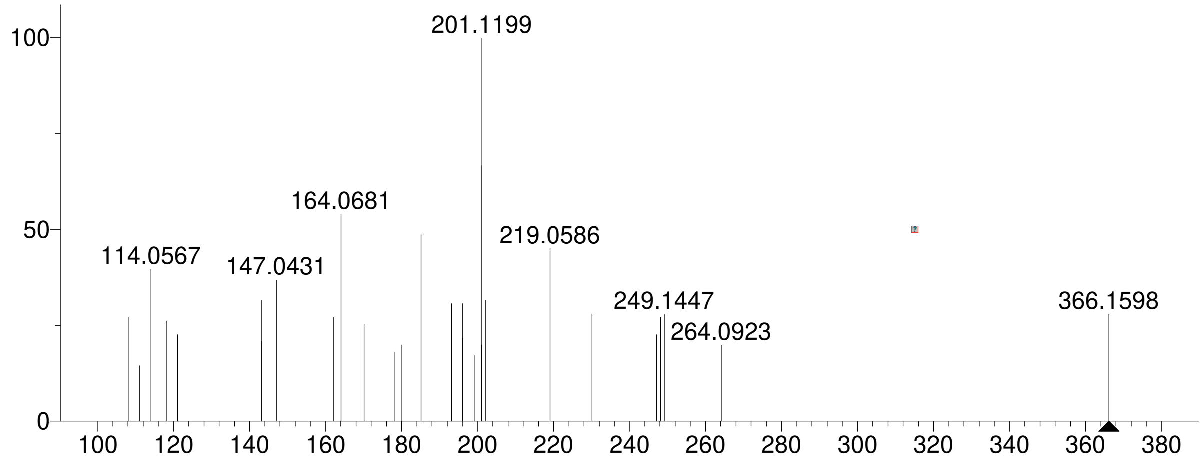

Hit 1 : Prazosin [M+H-H2O]+ HCD 32V P=366.2
C19H21N5O4; MF: 2; RMF: 117; Prob 100.0%; CAS: 19216-56-9; Lib: nist_msms; ID: 135102.

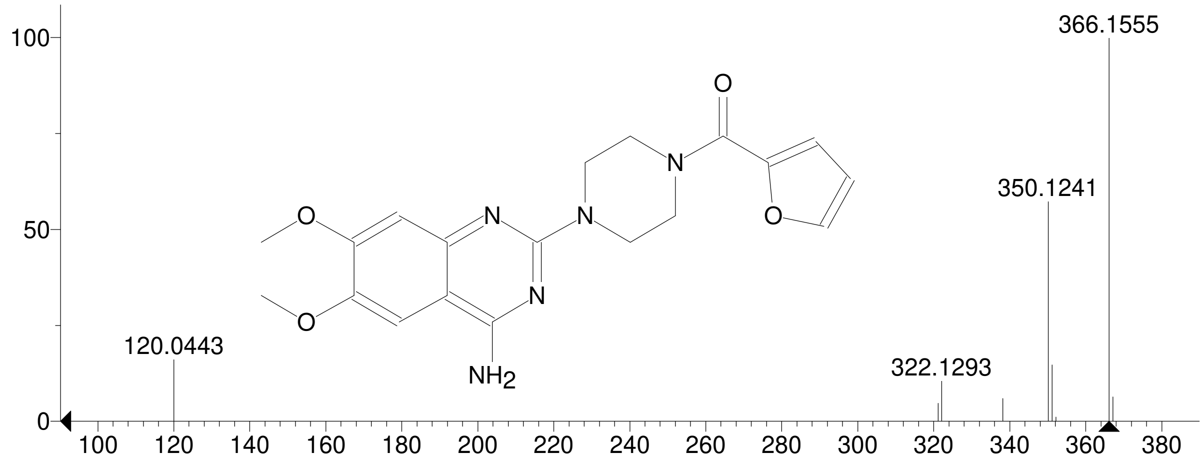

Unknown: Unknown (5.26_654.2082m/z)
Compound in Library Factor = 0

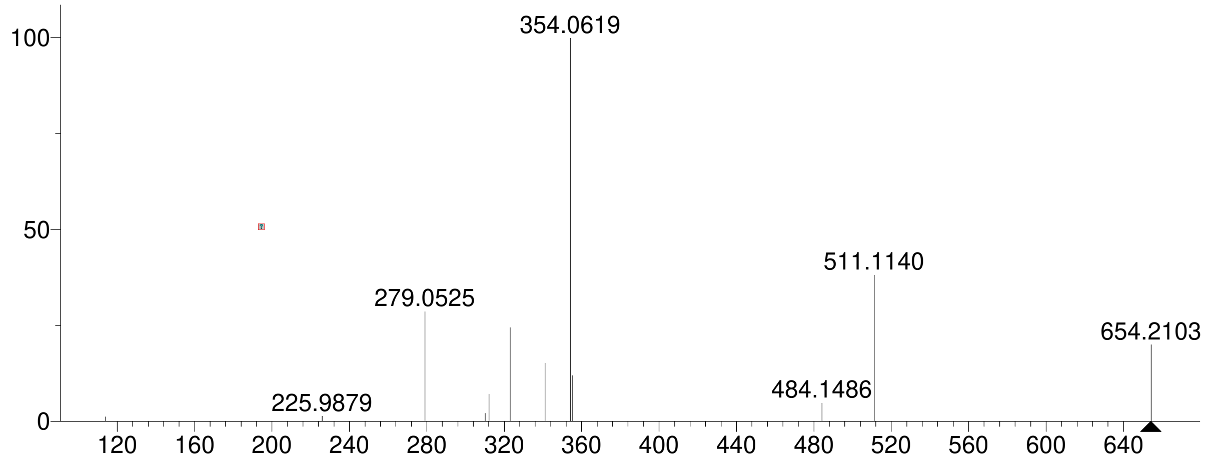

Unknown: Unknown (5.27_625.2542m/z)
Compound in Library Factor = -3273

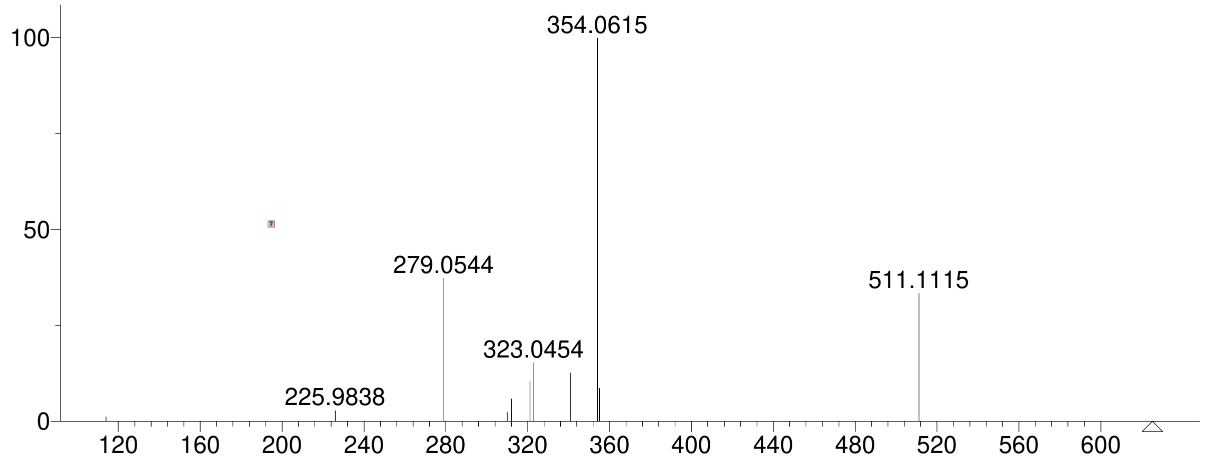

Hit 1 : N-Caffeoyl-O-methyltyramine [2M-H]- IT 35% P=625.3
C18H19NO4; MF: 3; RMF: 146; Prob 100.0%; CAS: 189307-47-9; Lib: nist_msms; ID: 221344.

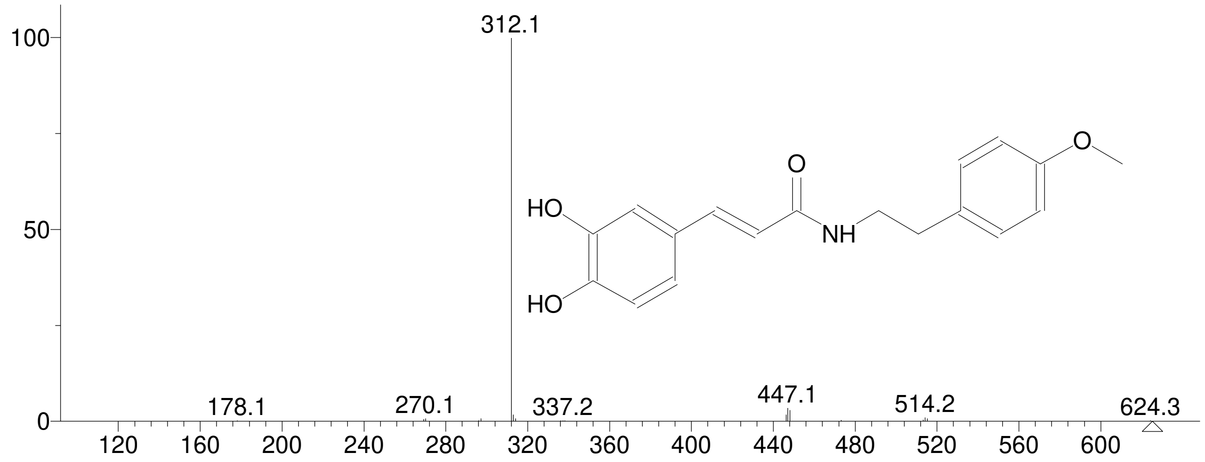

Unknown: Unknown (5.28_545.1283m/z)
Compound in Library Factor = -1523

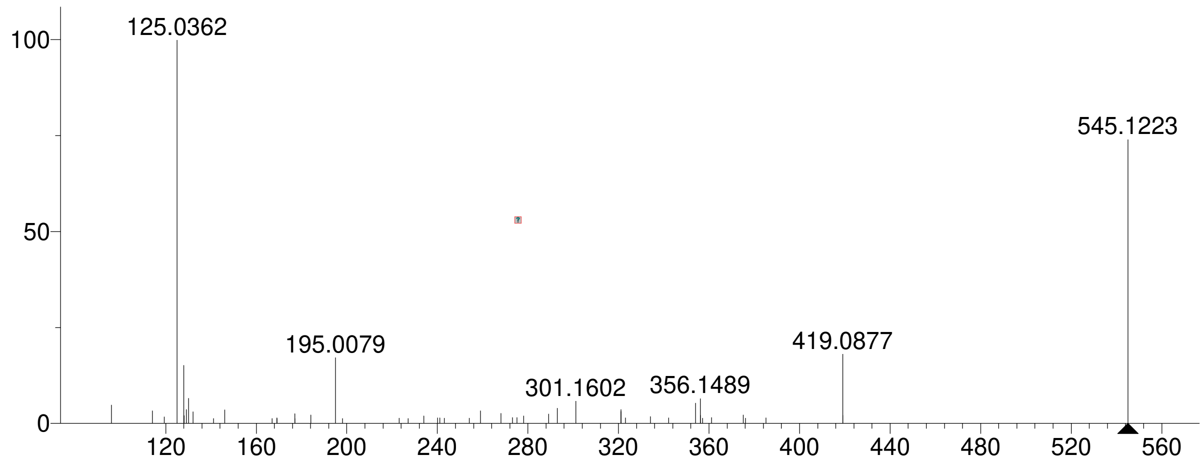

Hit 1 : Iridin [M+Na]+ HCD 21V P=545.1
C24H26O13; MF: 43; RMF: 577; Prob 47.5%; CAS: 491-74-7; Lib: nist_msms; ID: 256973.

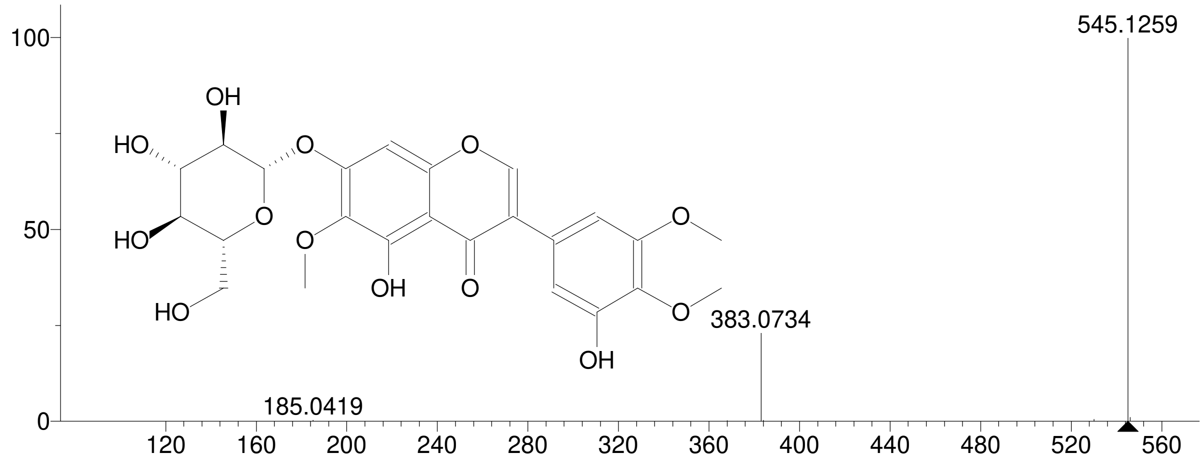

Hit 2 : (3S,5R,8S)-2-[(benzoyloxy)methyl]-8-methyl-3-{[(2S,3R,4S,5S,6R)-3,4,5-trihydroxy-6-(hydroxymethyl)oxan
C23H28O13S; MF: 0; RMF: 51; Prob 10.5%; Lib: mona-export-vf-npl_qexactive; ID: 21547.

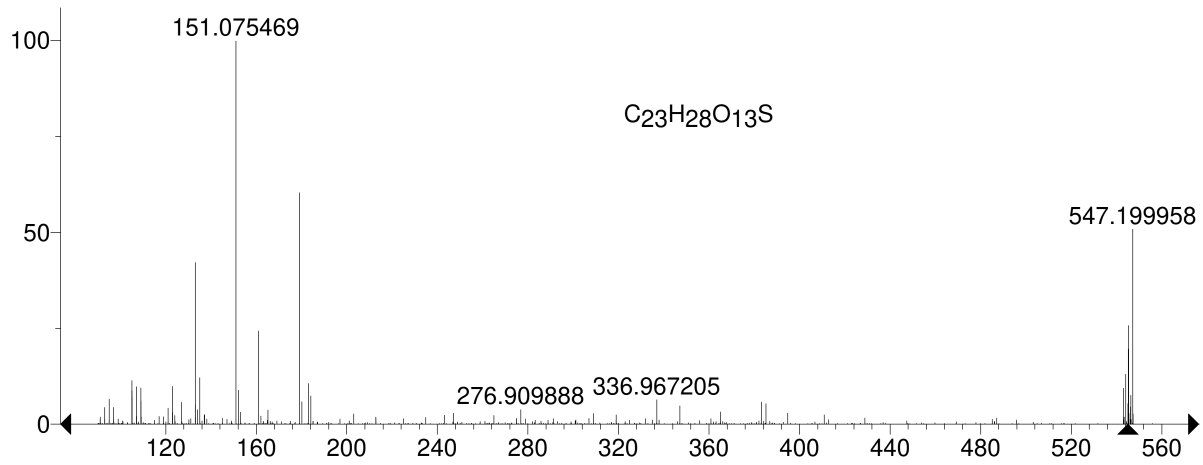

Hit 3 : salviaflaside [M+Na]+ 35HCD P=545.1
C24H26O13; MF: 0; RMF: 39; Prob 10.5%; Lib: mona-export-vf-npl_qexactive; ID: 20881.

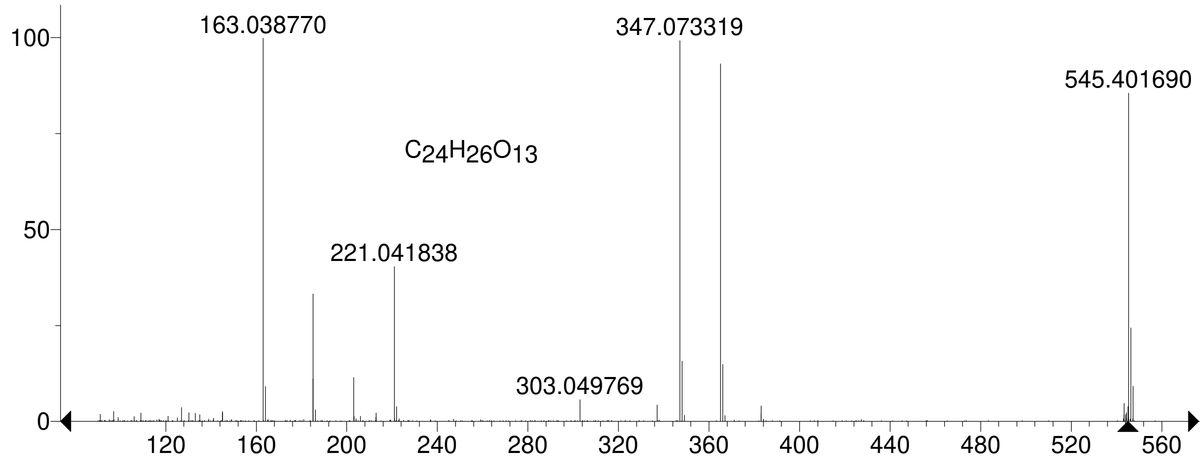

Hit 4 : Perseitol Heptaacetate [M+K]+ 45HCD P=545.1
C21H30O14; MF: 0; RMF: 25; Prob 10.5%; Lib: mona-export-vf-npl_qexactive; ID: 26654.

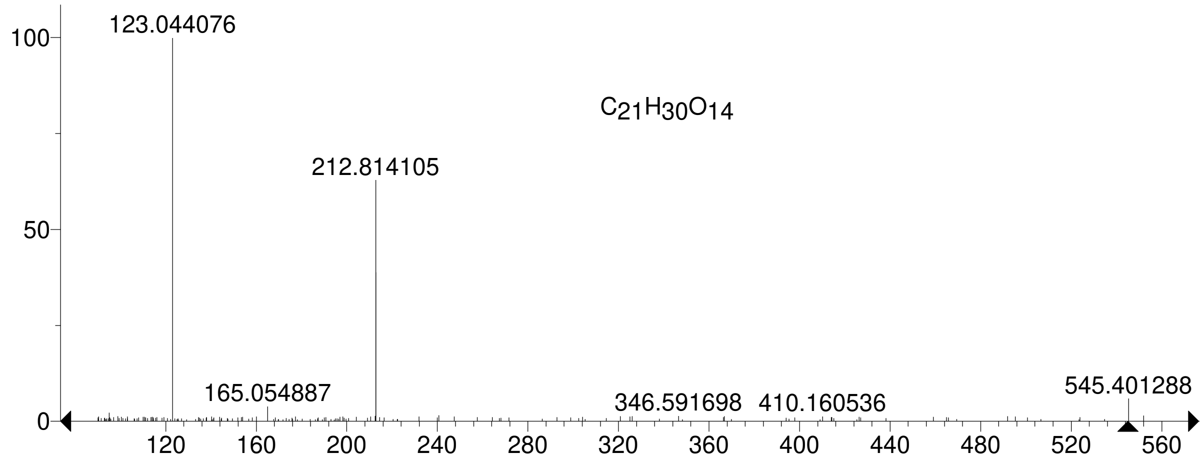

Hit 5 : 5-hydroxy-3-(5-hydroxy-2,4-dimethoxyphenyl)-6-methoxy-7-[3,4,5-trihydroxy-6-(hydroxymethyl)oxan-2-yl]ox
C24H26O13; MF: 0; RMF: 13; Prob 10.5%; Lib: anthocyanins mona; ID: 9886.

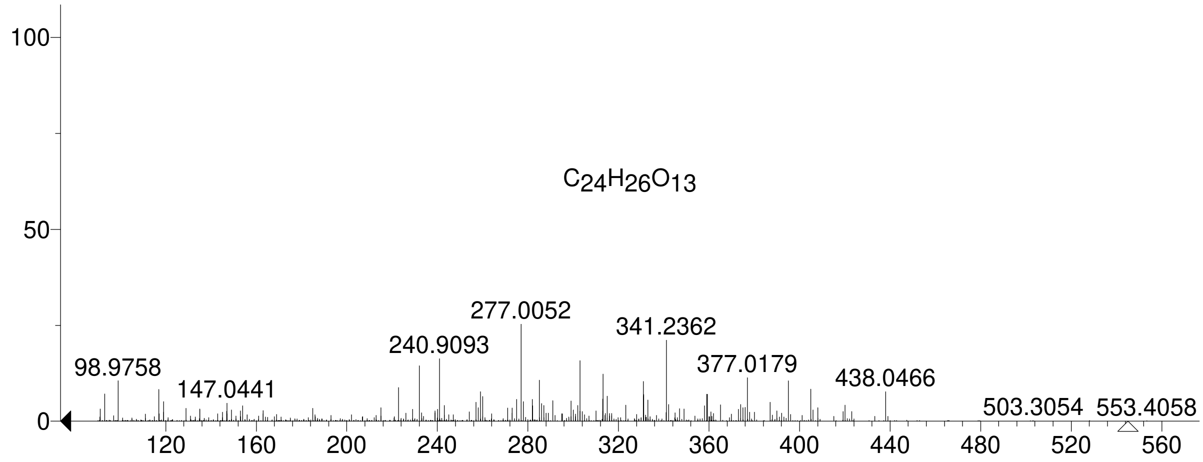

Unknown: Unknown (5.31_378.1245m/z)
Compound in Library Factor = 0

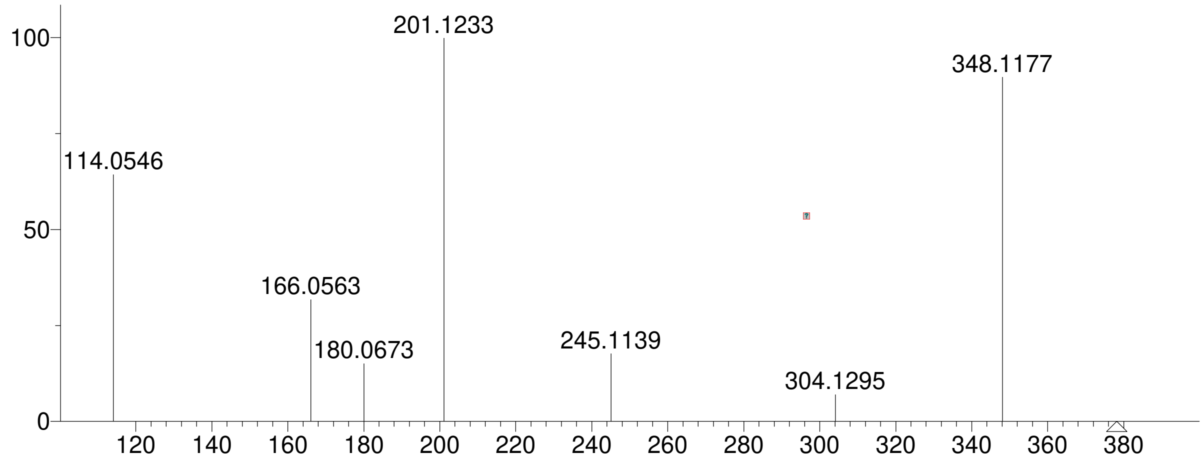

Hit 1 : Benzenesulfonamide, 4-(4,5-dihydro-3,5-diphenyl-1H-pyrazol-1-yl)- [M+H]+ HCD 121V P=378.1
C21H19N3O2S; MF: 0; RMF: 62; Prob 100.0%; CAS: 10179-57-4; Lib: nist_msms; ID: 420765.

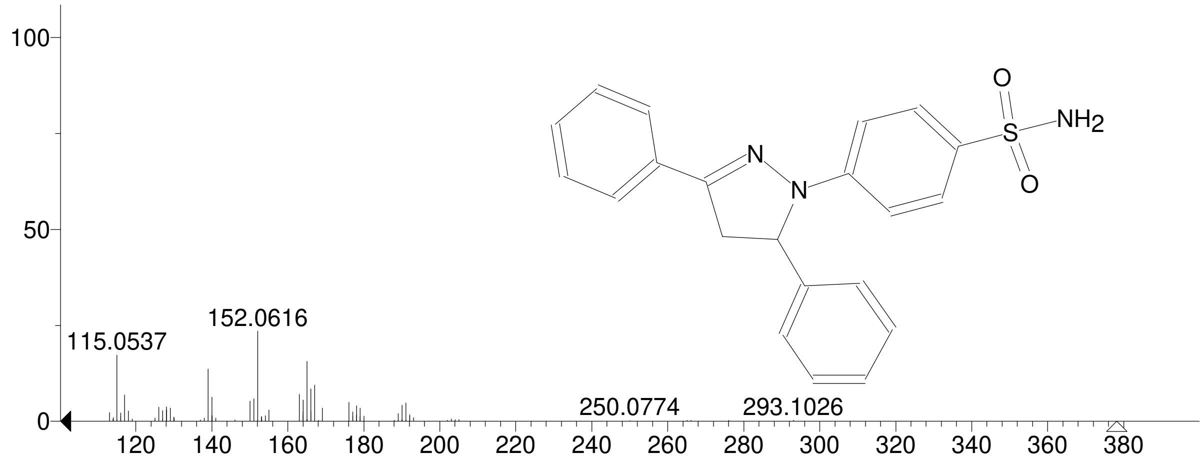

Unknown: Unknown (5.31_389.1918m/z)
Compound in Library Factor = 0

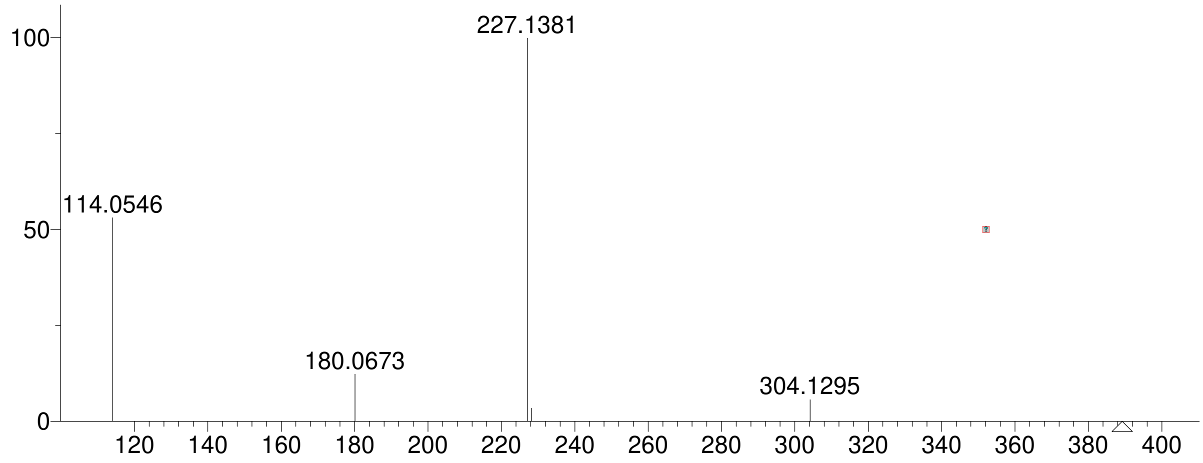

Hit 1 : 3-[2-(6,7-dihydroxy-1,2,4a-trimethylspiro[3,4,6,7,8,8a-hexahydro-2H-naphthalene-5,2'-oxirane]-1-yl)ethyl]-2
C20H30O6; MF: 0; RMF: 20; Prob 50.0%; Lib: mona-export-vf-npl_qexactive; ID: 9850.

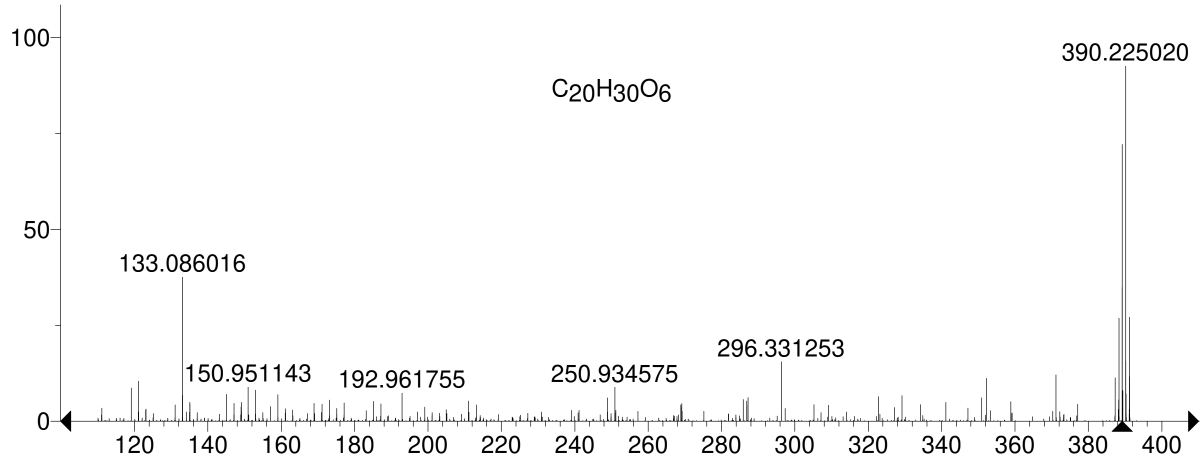

Hit 2 : 20-Trifluoroleukotriene B4 [M-H]- HCD 31V P=389.2
C20H29F3O4; MF: 0; RMF: 7; Prob 50.0%; CAS: 115178-97-7; Lib: nist_msms; ID: 88774.

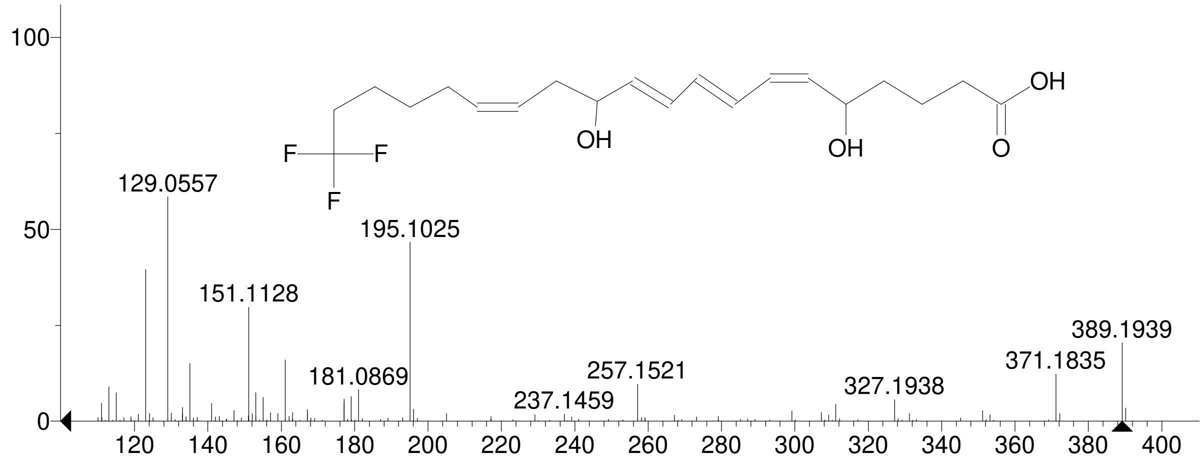

** Search Report Page 1 of 1 **

Unknown: Unknown (5.32_419.1554m/z)
Compound in Library Factor = -3273

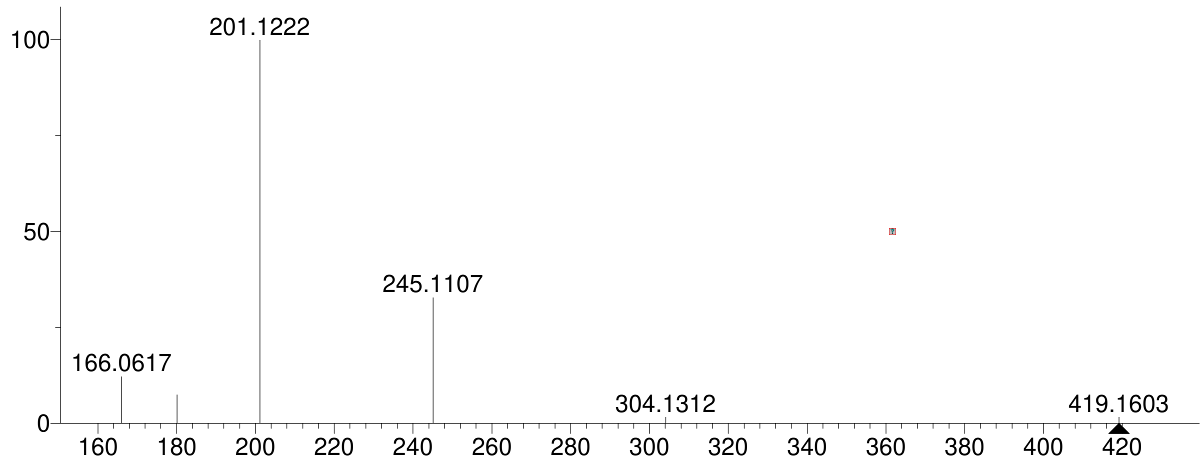

Hit 1 : 4-Morpholinopropanesulfonic acid [2M+H]+ HCD 99V P=419.2
C7H15NO4S; MF: 13; RMF: 215; Prob 100.0%; CAS: 1132-61-2; Lib: nist_msms; ID: 58672.

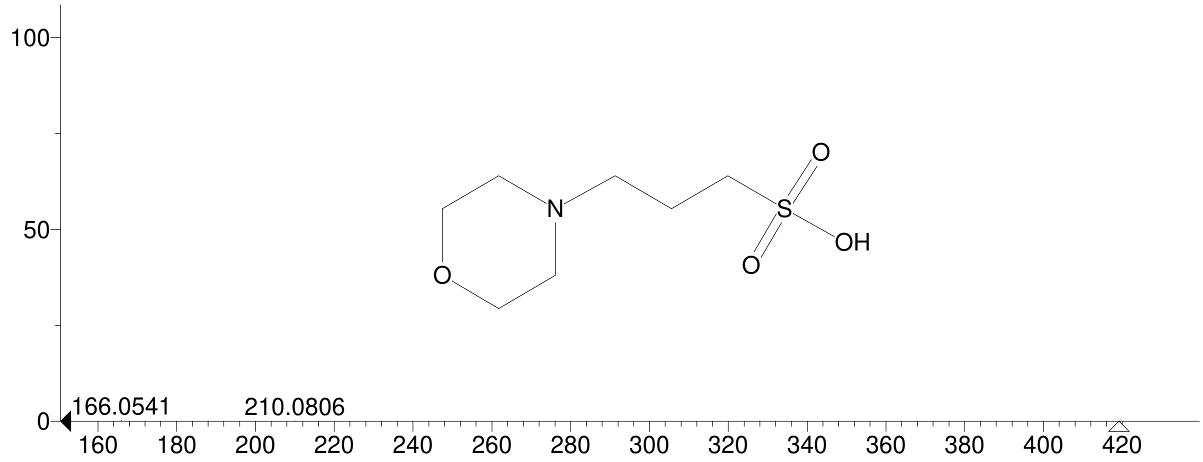

** Search Report Page 1 of 1 **

Unknown: Unknown (5.33_371.1799m/z)
Compound in Library Factor = 0

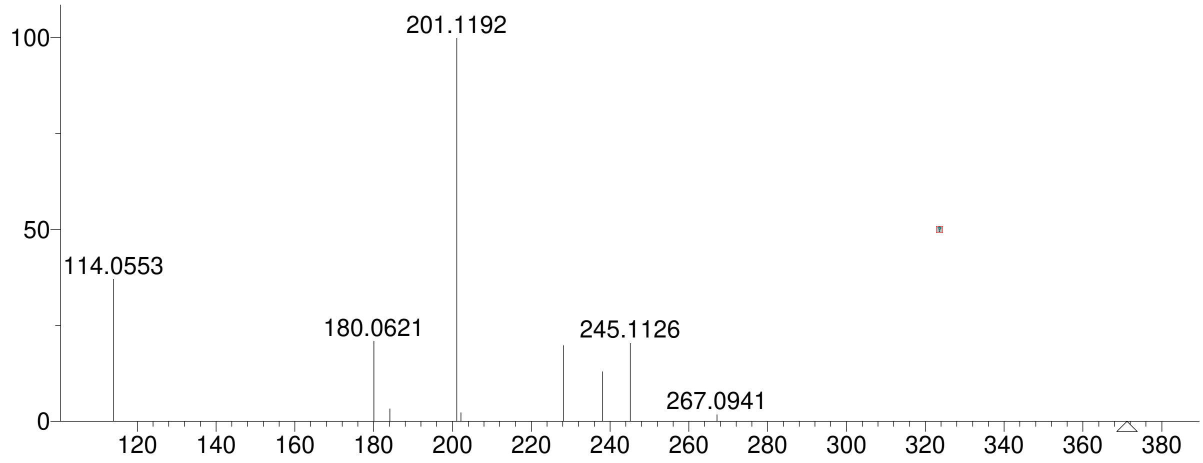

Hit 1 : 4-[2-[(2-ethyl-2,3-dihydroxybutanoyl)oxymethyl]anilino]-4-oxobutanoic acid [M+NH4]+ 35HCD P=371.2
C17H23NO7; MF: 0; RMF: 6; Prob 50.0%; Lib: mona-export-vf-npl_qexactive; ID: 23566.

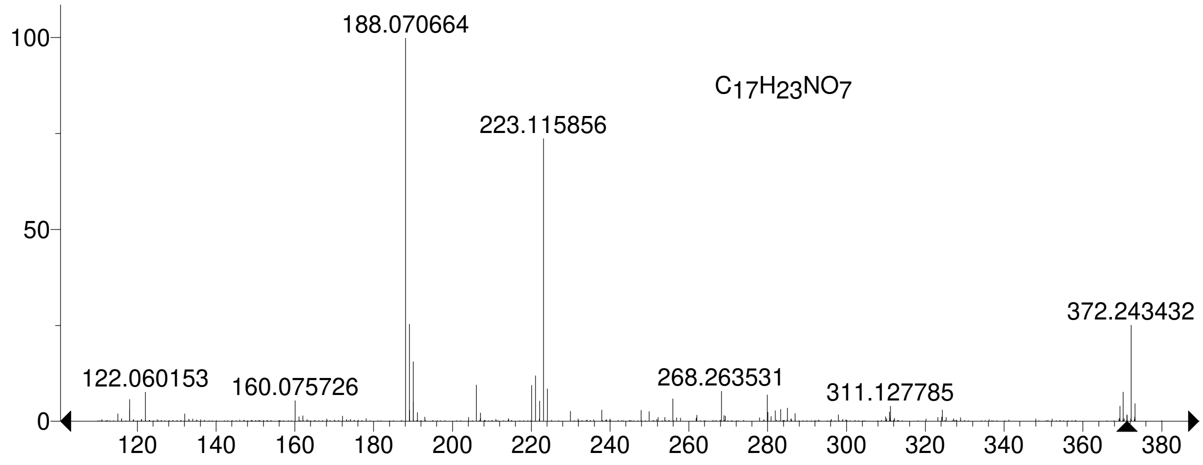

Hit 2 : Ingenol [M+Na]+ IT-FT 35% P=371.2
C20H28O5; MF: 0; RMF: 4; Prob 50.0%; CAS: 30220-46-3; Lib: nist_msms; ID: 364298.

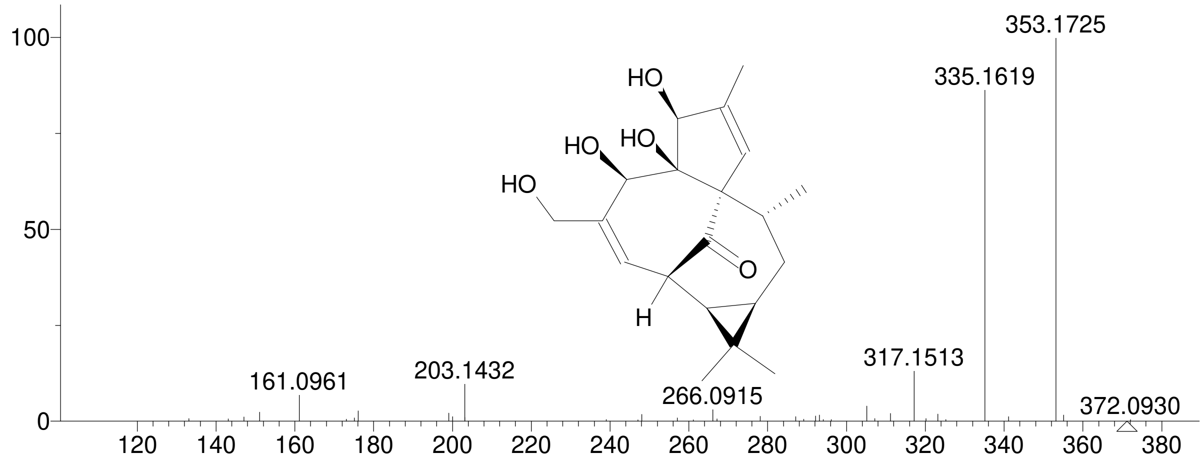

** Search Report Page 1 of 1 **

Unknown: Unknown (5.33_543.2294m/z)
Compound in Library Factor = 0

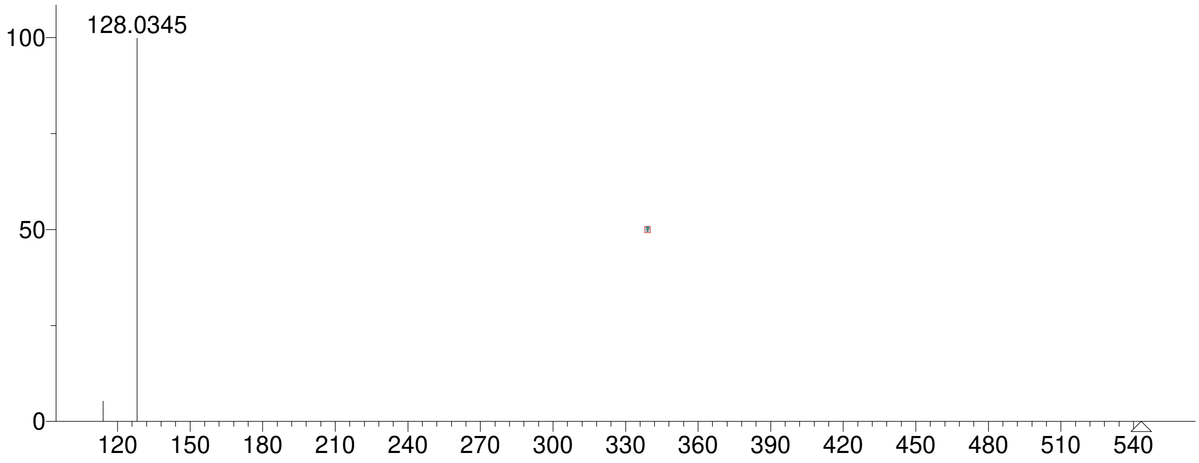

Unknown: Unknown (5.34_229.1545m/z)
Compound in Library Factor = -3273

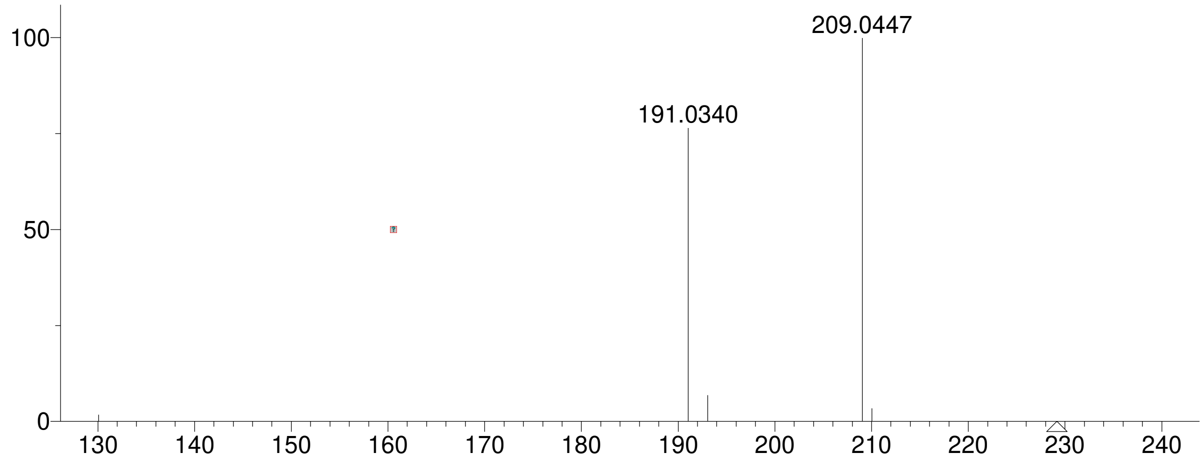

Hit 1 : Val-Leu [M-H]- HCD 36eV P=229.2
C11H22N2O3; MF: 2; RMF: 85; Prob 50.0%; CAS: 3989-97-7; Lib: nist_msms; ID: 568172.

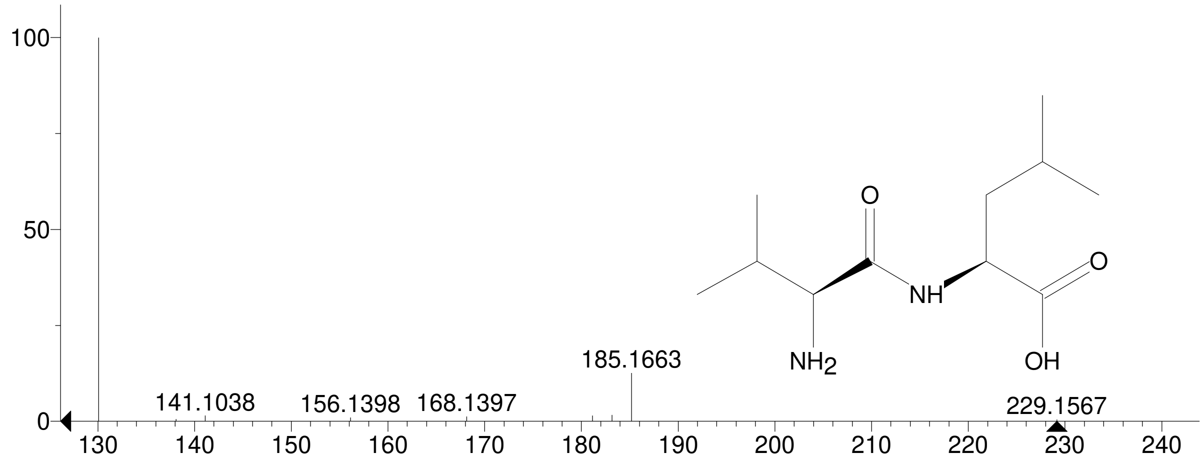

Hit 2 : Val-Ile [M-H]- HCD 36eV P=229.2
C11H22N2O3; MF: 2; RMF: 85; Prob 50.0%; CAS: 20556-14-3; Lib: nist_msms; ID: 571092.

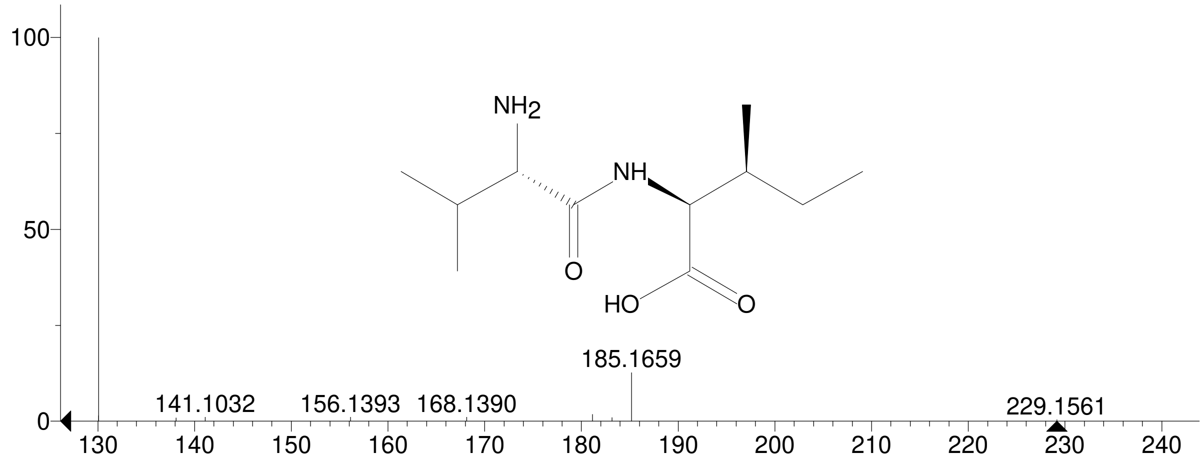

Unknown: Unknown (5.34_289.0013m/z)
Compound in Library Factor = -1309

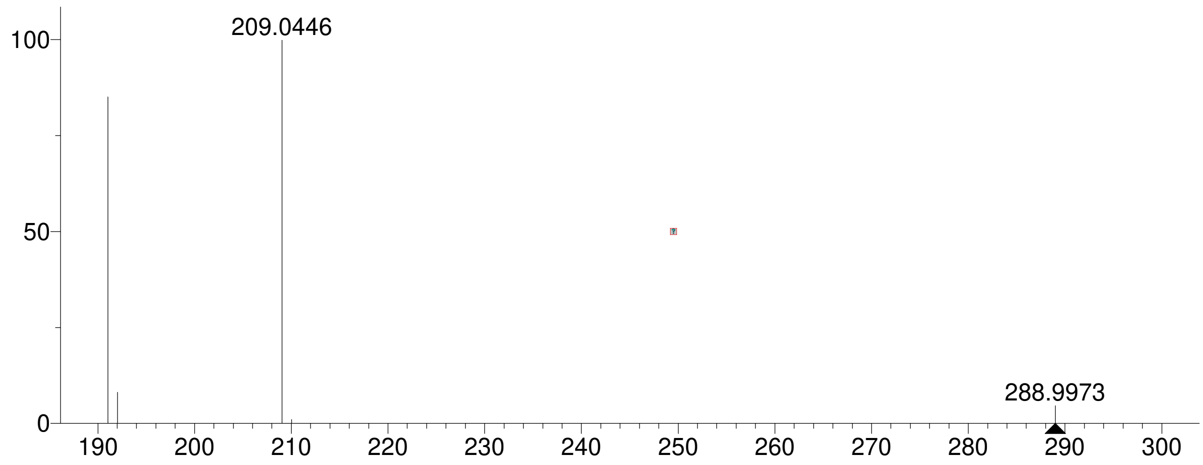

Hit 1 : Catechin [M-H]- QQQ 20V P=289
C15H14O6; MF: 59; RMF: 534; Prob 30.4%; Lib: mona-export-lc-ms-ms_negative_mode; ID: 14518.

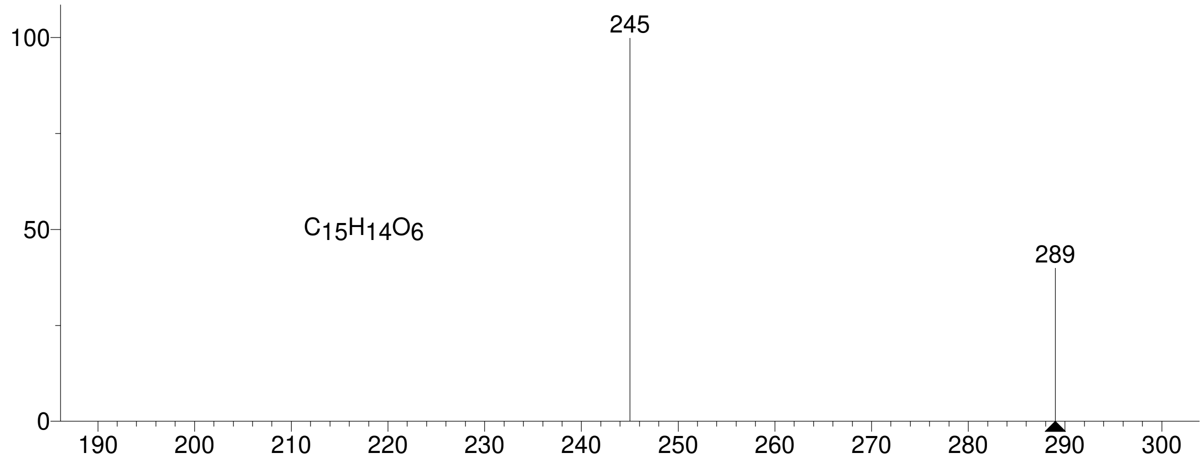

Hit 2 : Epicatechin [M-H]- QQQ 20V P=289
C15H14O6; MF: 59; RMF: 534; Prob 30.4%; Lib: mona-export-lc-ms-ms_negative_mode; ID: 14624.

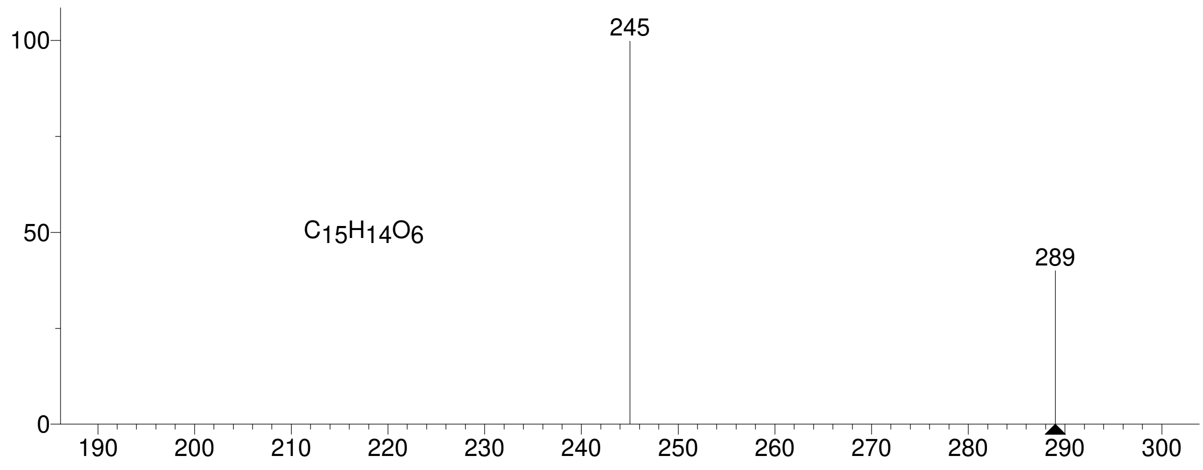

Hit 3 : Lormetazepam [M+H]+=>289.0 IT 35% P=289
C16H12Cl2N2O2; MF: 6; RMF: 141; Prob 6.01%; CAS: 848-75-9; Lib: nist_msms; ID: 186240.

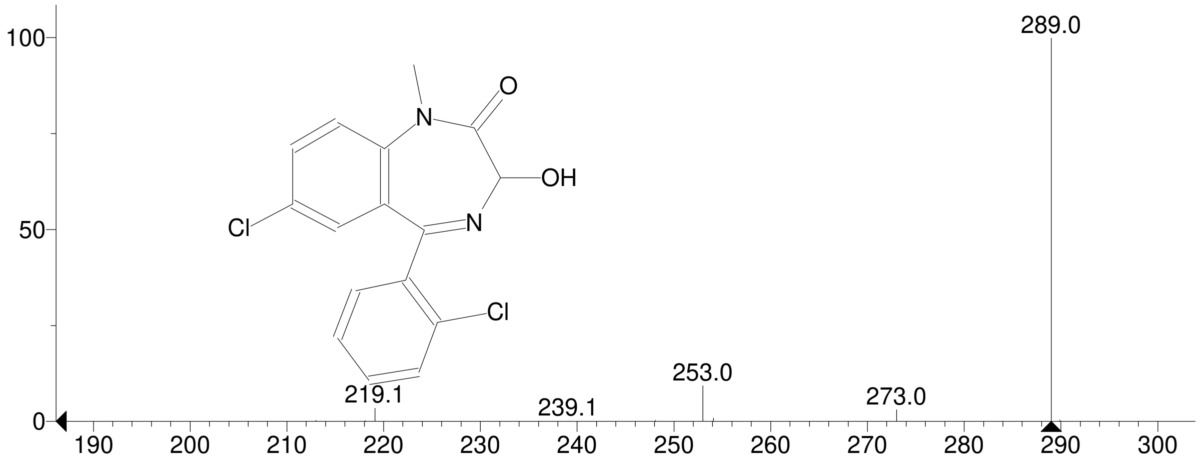

Hit 4 : Benzoic acid, 2-[(2-bromophenyl)methoxy]- [M+H]+=>289.0 IT 35% P=289
C14H11BrO3; MF: 2; RMF: 107; Prob 5.07%; CAS: 743456-83-9; Lib: nist_msms; ID: 420103.

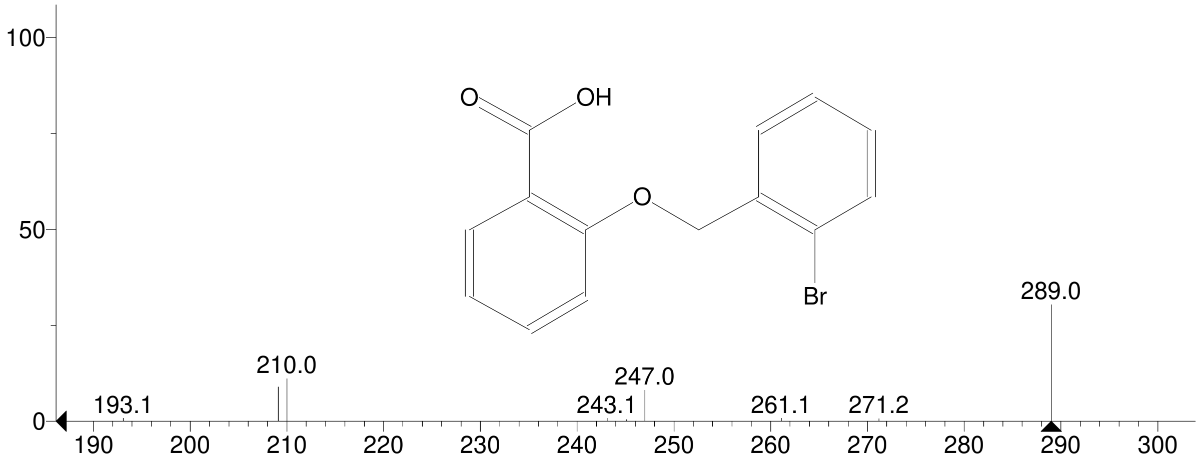

Hit 5 : Permethrin [M+H-H2O]+=>317.0=>289.0 IT 35% P=289
C21H20Cl2O3; MF: 0; RMF: 81; Prob 4.68%; CAS: 52645-53-1; Lib: nist_msms; ID: 284116.

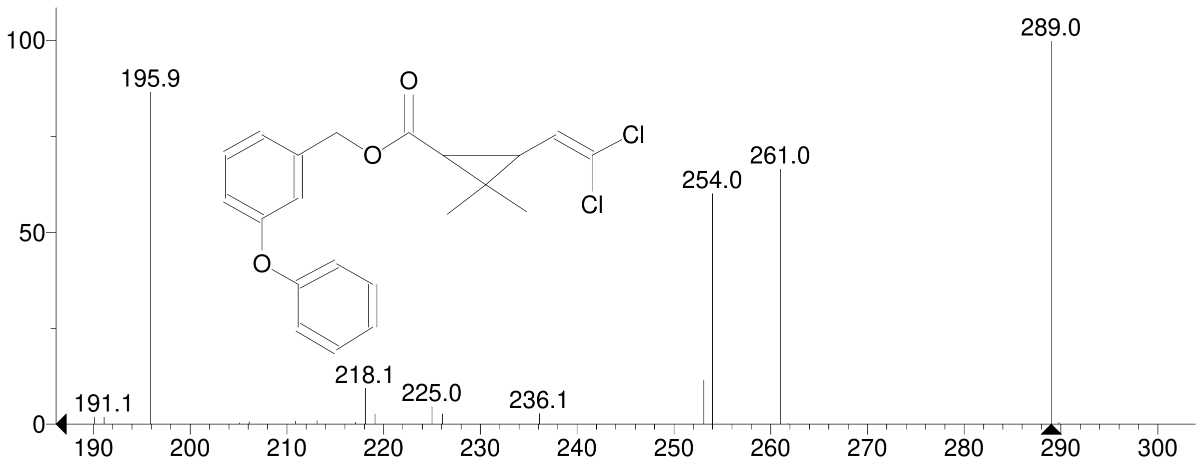

Unknown: Unknown (5.34_599.2685m/z)
Compound in Library Factor = 0

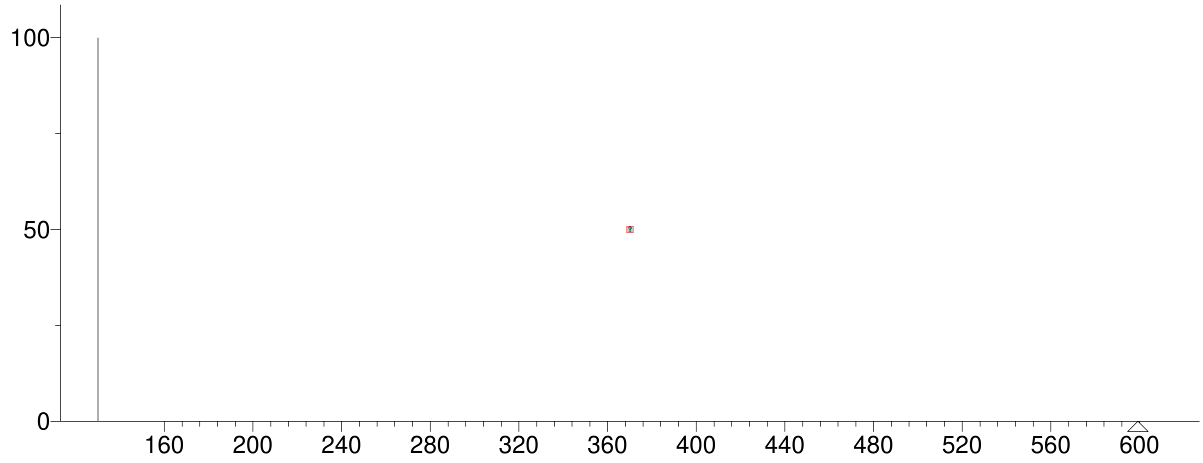

Unknown: Unknown (5.36_265.0925m/z)
Compound in Library Factor = -3273

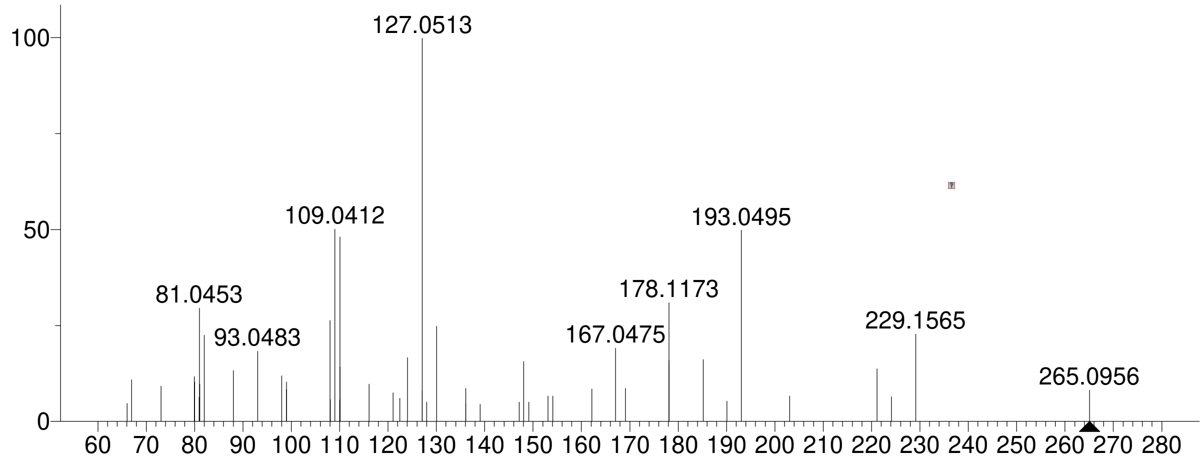

Hit 1 : Molsidomine [M+Na]+ HCD 7V P=265.1
C9H14N4O4; MF: 2; RMF: 93; Prob 35.1%; CAS: 25717-80-0; Lib: nist_msms; ID: 189037.

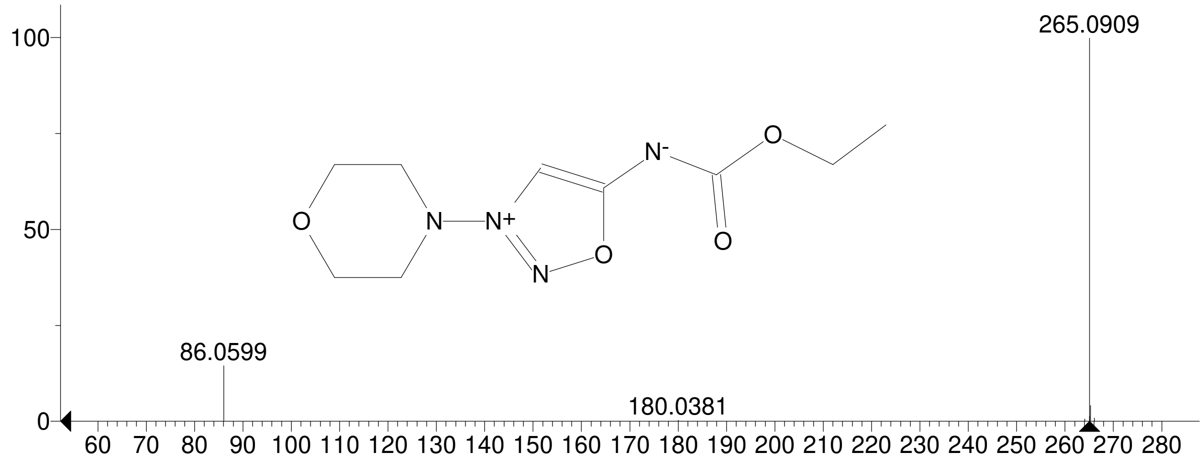

Hit 2 : Unknown (carbon number 10); PlaSMA ID-439 [M-H]- P=265.1
C10H18O8; MF: 0; RMF: 55; Prob 32.4%; Lib: biomsms-neg-plasma; ID: 1132.

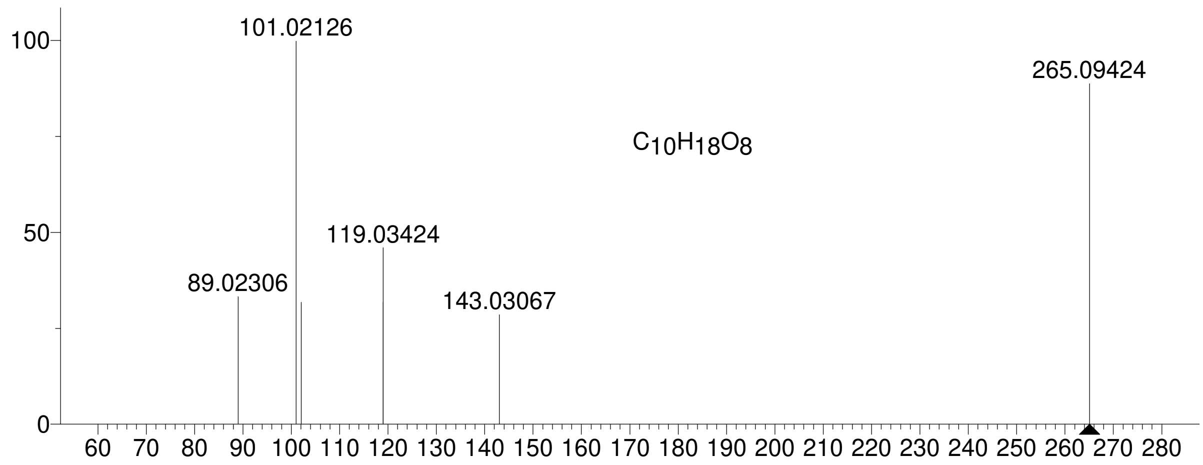

Hit 3 : 1,4-D-Xylobiose [M+H-H2O]+ IT 35% P=265.1
C10H18O9; MF: 0; RMF: 31; Prob 32.4%; CAS: 6860-47-5; Lib: nist_msms; ID: 530813.

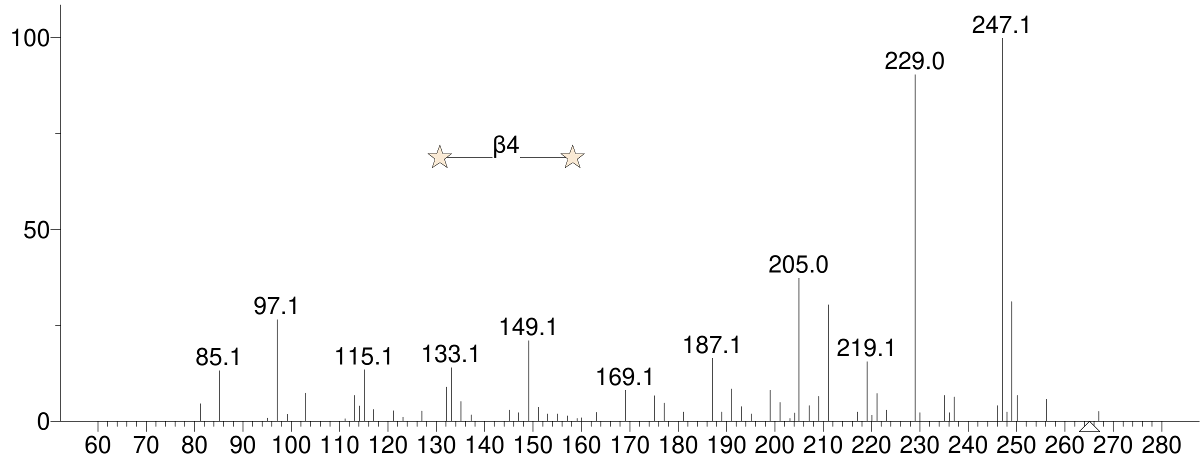

Unknown: Unknown (5.36_273.0052m/z)
Compound in Library Factor = -2387

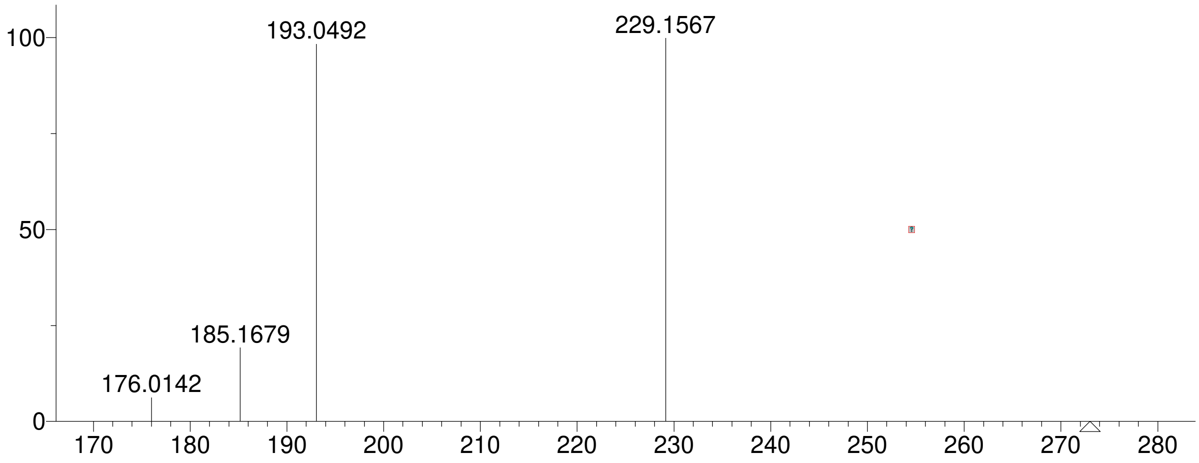

Hit 1 : ferulic acid + sulfate (CCR vessel) [M-H]- P=273
C10H10O7S; MF: 12; RMF: 524; Prob 100.0%; Lib: phytofrag2018_final_060219; ID: 938.

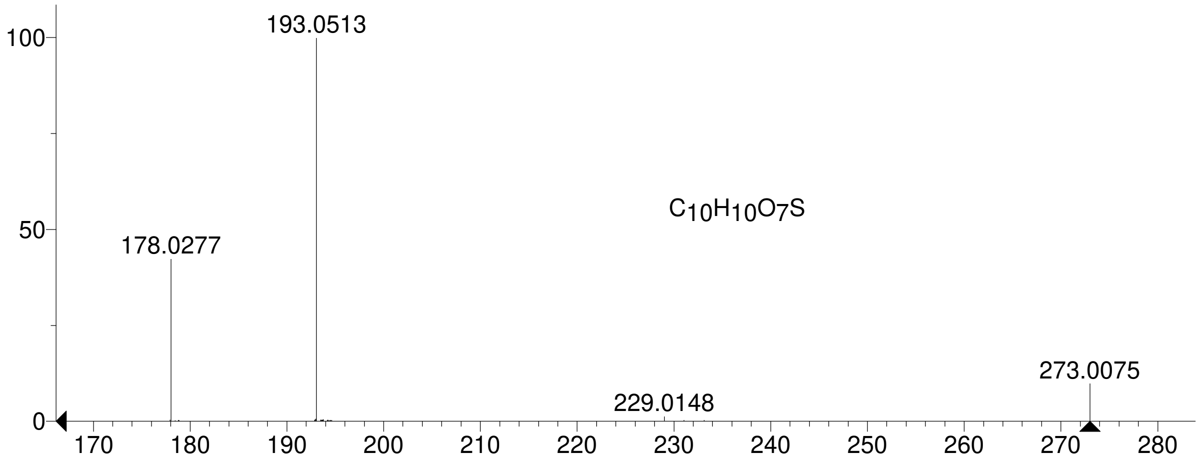

Unknown: Unknown (5.36_429.1864m/z)
Compound in Library Factor = -3273

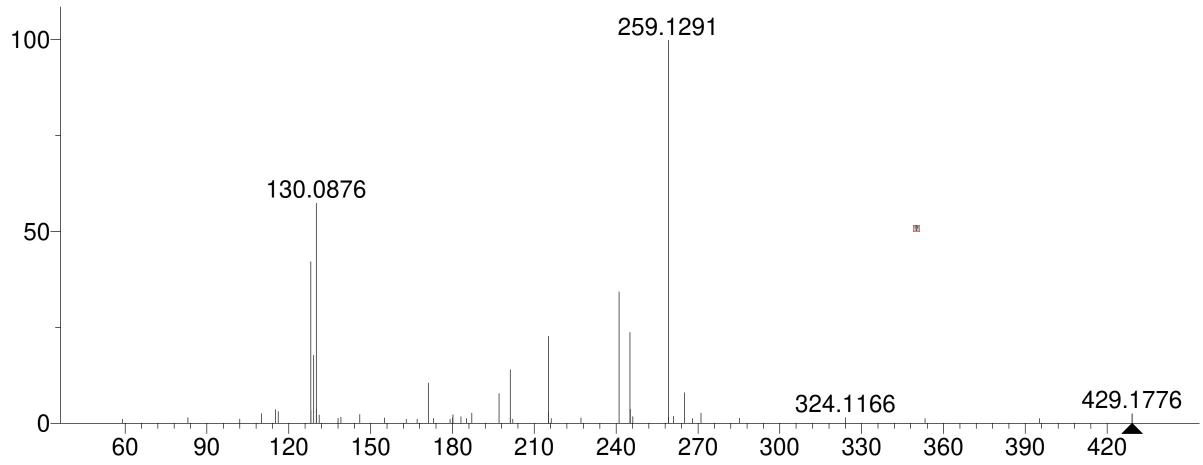

Hit 1 : (2R,3R,4S,5S,6R)-2-[6-hydroxy-3-[(E)-3-hydroxybut-1-enyl]-2,4,4-trimethylcyclohexyl]oxy-6-(hydroxymethy
C19H34O8; MF: 1; RMF: 57; Prob 51.0%; Lib: anthocyanins mona; ID: 9985.

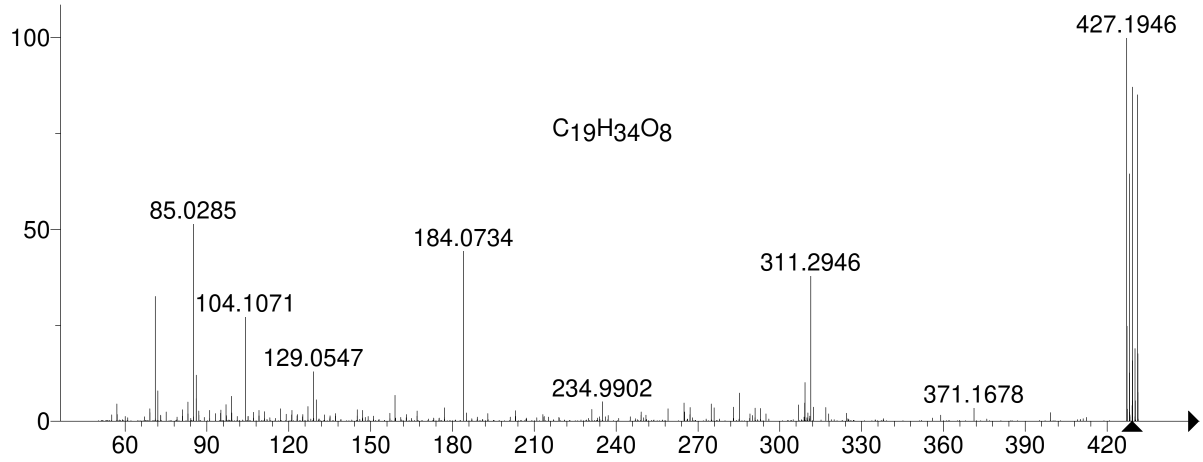

Hit 2 : NCGC00169368-02!3,6-bis(3,4,5-trimethoxyphenyl)-1,3,3a,4,6,6a-hexahydrofuro[3,4-c]furan [M-H2O+H]+
C24H30O8; MF: 0; RMF: 14; Prob 49.0%; Lib: mona-export-gnps; ID: 9133.

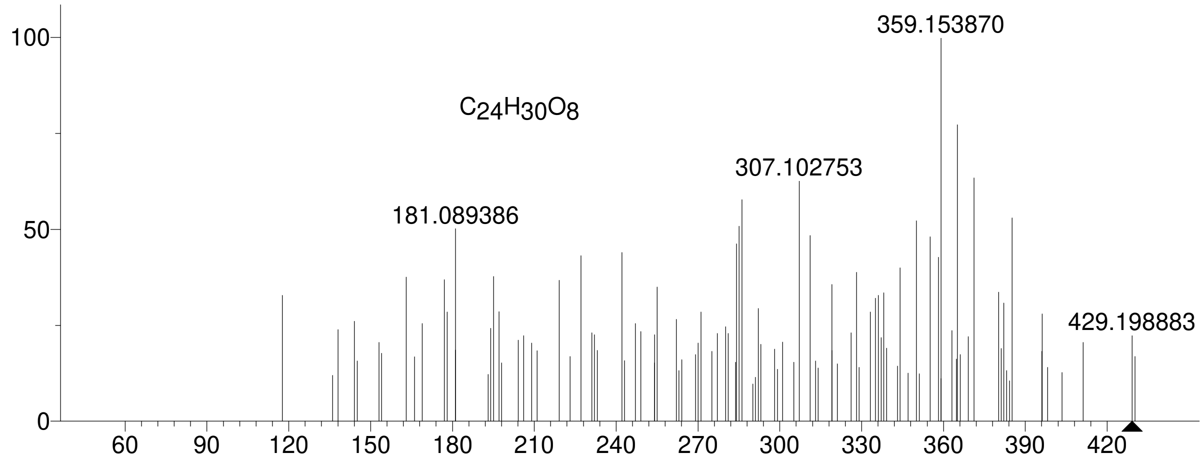

** Search Report Page 1 of 1 **

Unknown: Unknown (5.37_435.1500m/z)
Compound in Library Factor = -3273

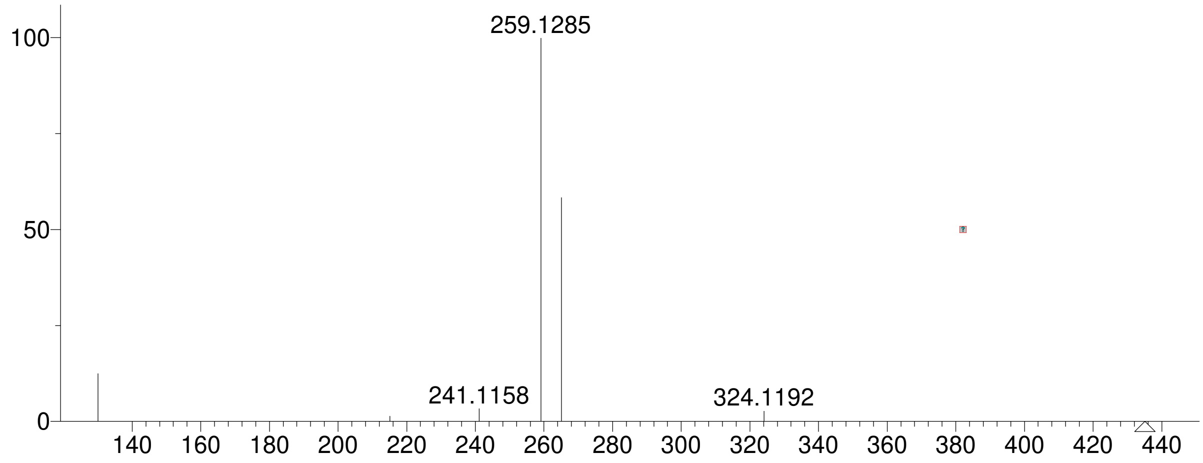

Hit 1 : Methotrexate [M-H-H2O]- HCD 13V P=435.2
C20H22N8O5; MF: 3; RMF: 110; Prob 100.0%; CAS: 59-05-2; Lib: nist_msms; ID: 82226.

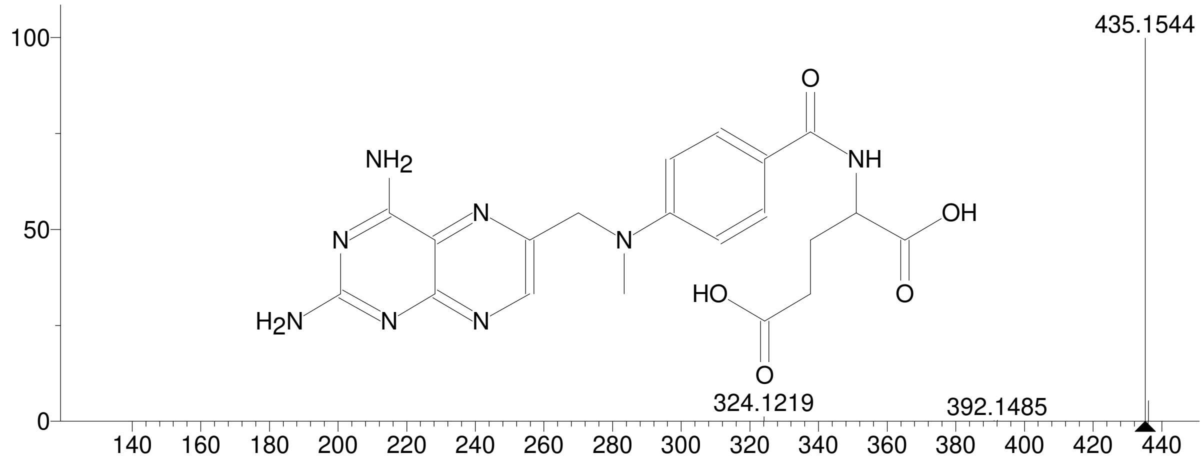

Unknown: Unknown (5.37_647.2652m/z)
Compound in Library Factor = -3273

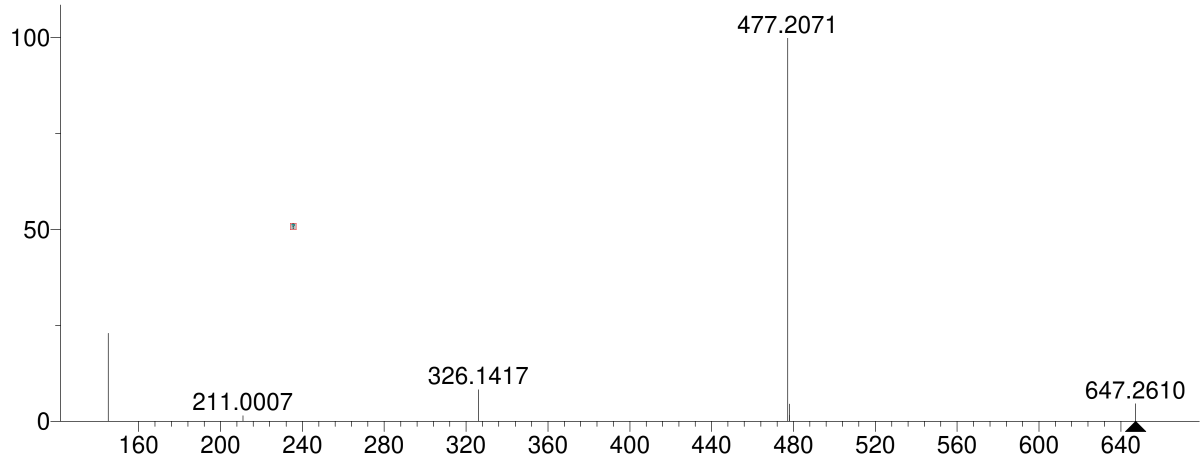

Hit 1 : Bavachin [2M-H]- HCD 9V P=647.3
C20H20O4; MF: 1; RMF: 41; Prob 100.0%; CAS: 19879-32-4; Lib: nist_msms; ID: 389268.

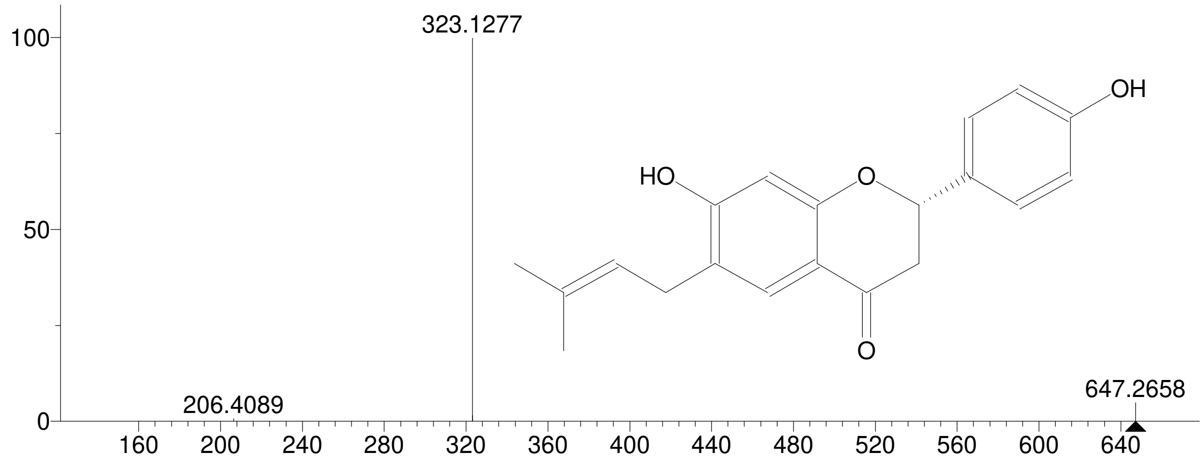

Unknown: Unknown (5.38_193.0491m/z)
Compound in Library Factor = -1389

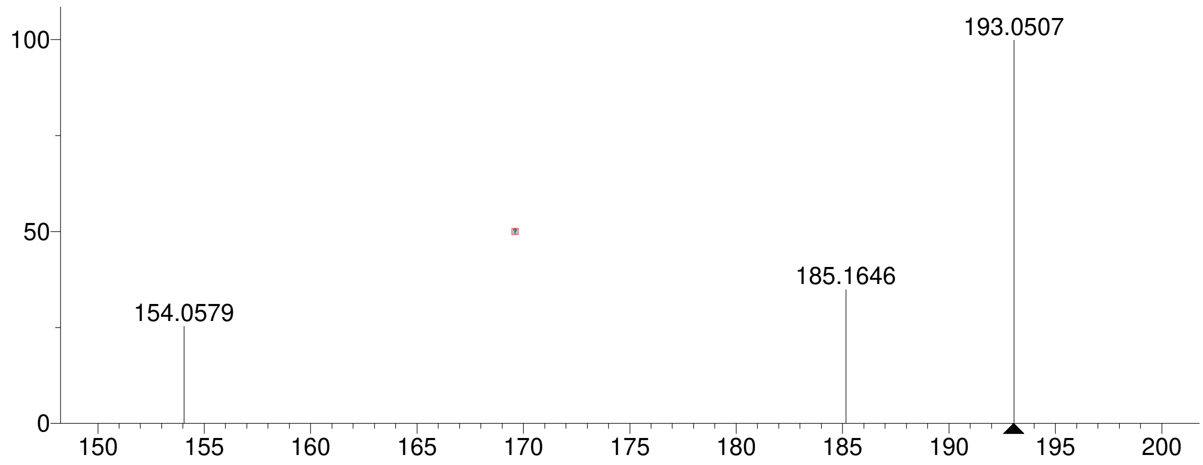

Hit 1 : 6,7-Dihydroxy-4-methylcoumarin [M+H]+ HCD 42V P=193
C10H8O4; MF: 268; RMF: 789; Prob 15.3%; CAS: 529-84-0; Lib: nist_msms; ID: 111709.

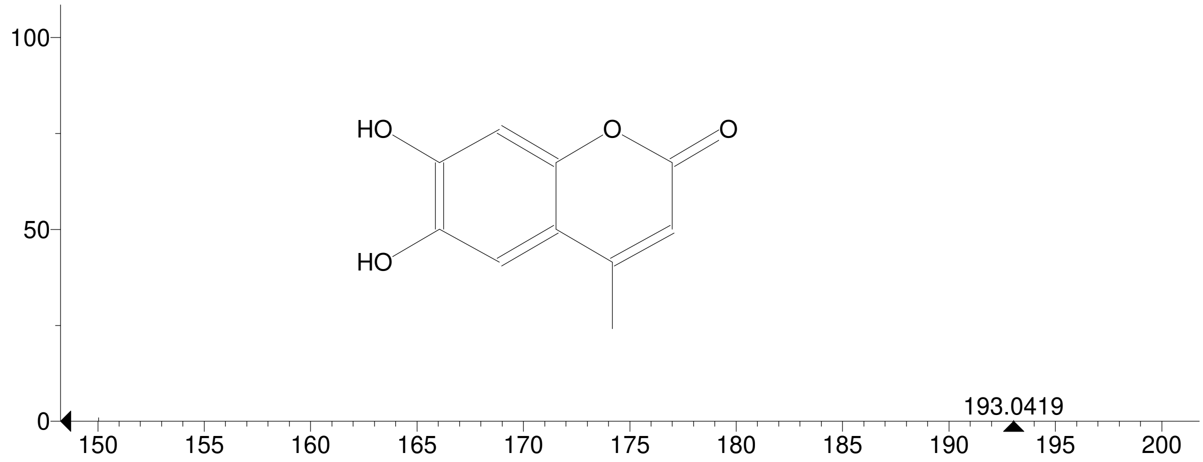

Hit 2 : 5,7-Dihydroxy-4-methylcoumarin [M+H]+ HCD 42V P=193
C10H8O4; MF: 268; RMF: 789; Prob 15.3%; CAS: 2107-76-8; Lib: nist_msms; ID: 111824.

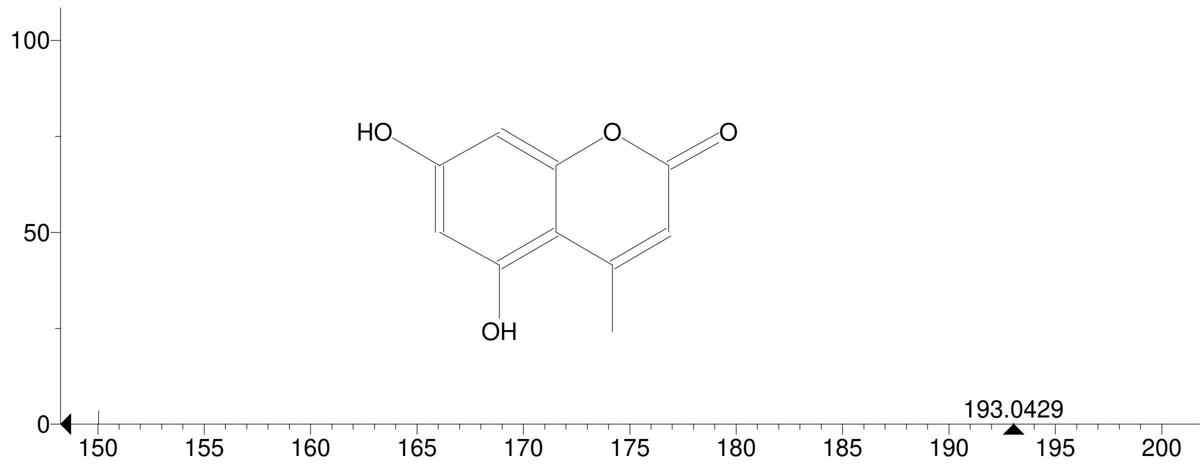

Hit 3 : 7,8-Dihydroxy-4-methylcoumarin [M+H]+ HCD 42V P=193
$C_{10}H_8O_4$; MF: 268; RMF: 789; Prob 15.3%; CAS: 2107-77-9; Lib: nist_msms; ID: 111845.

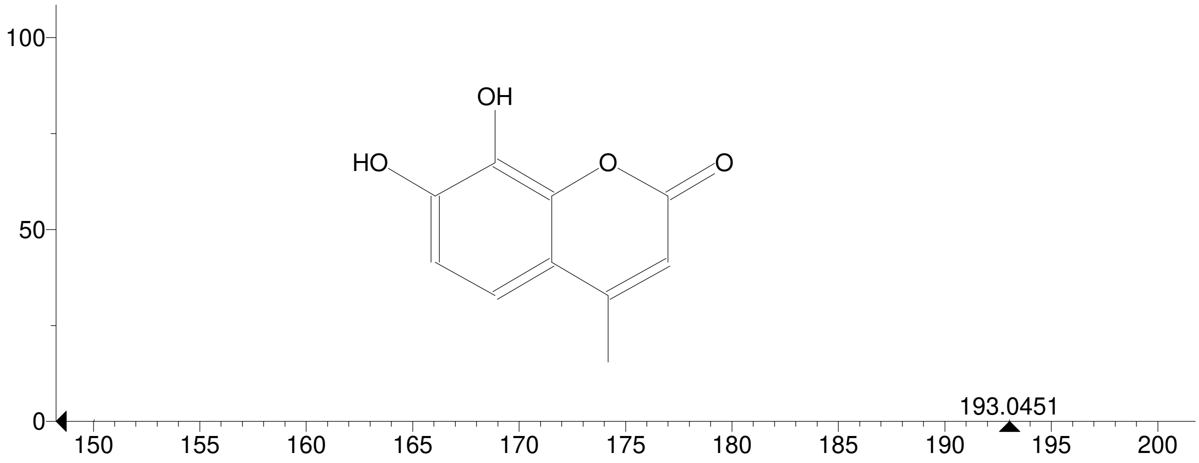

Hit 4 : 4-Hydroxy-7-methoxycoumarin [M+H]+ HCD 52V P=193
$C_{10}H_8O_4$; MF: 268; RMF: 789; Prob 15.3%; CAS: 17575-15-4; Lib: nist_msms; ID: 177257.

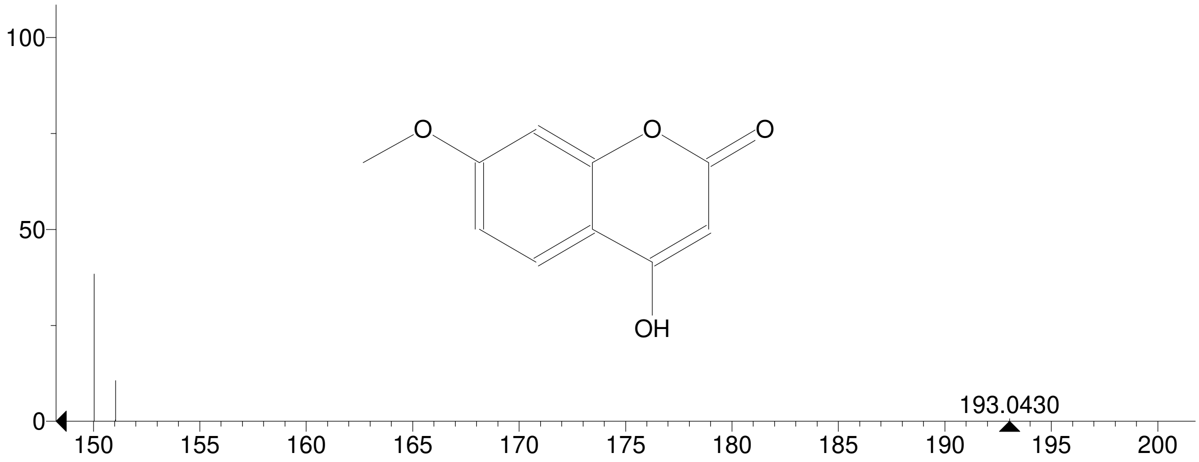

Hit 5 : 4-Hydroxy-6-methoxycoumarin [M+H]+ HCD 9V P=193
$C_{10}H_8O_4$; MF: 268; RMF: 789; Prob 15.3%; CAS: 13252-84-1; Lib: nist_msms; ID: 178730.

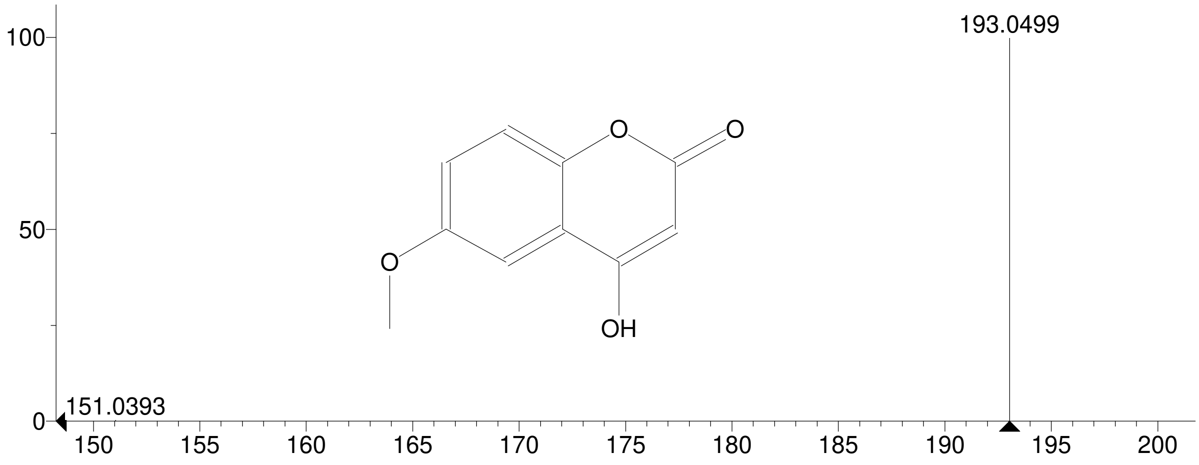

Unknown: Unknown (5.38_446.1650m/z)
Compound in Library Factor = 0

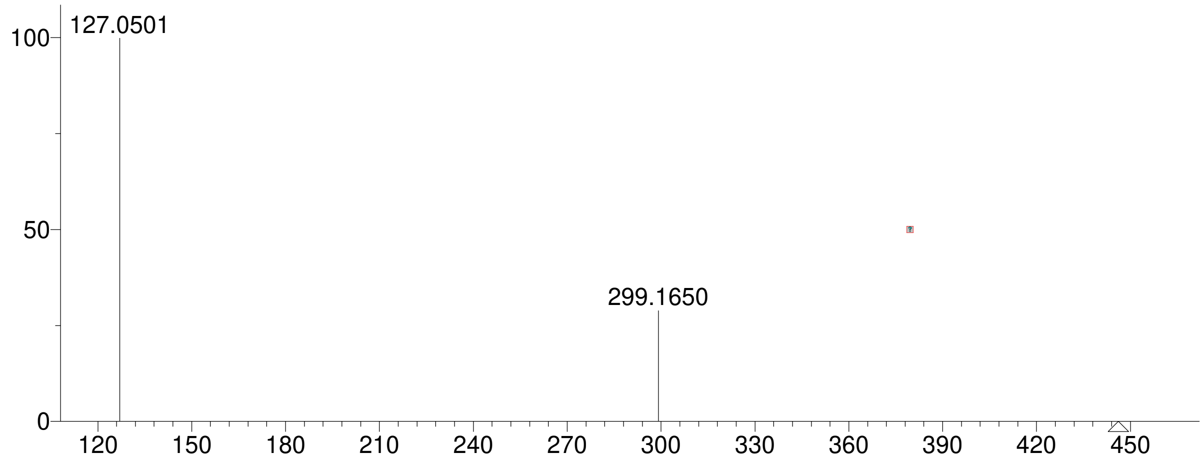

Unknown: Unknown (5.39_529.1679m/z)
Compound in Library Factor = 0

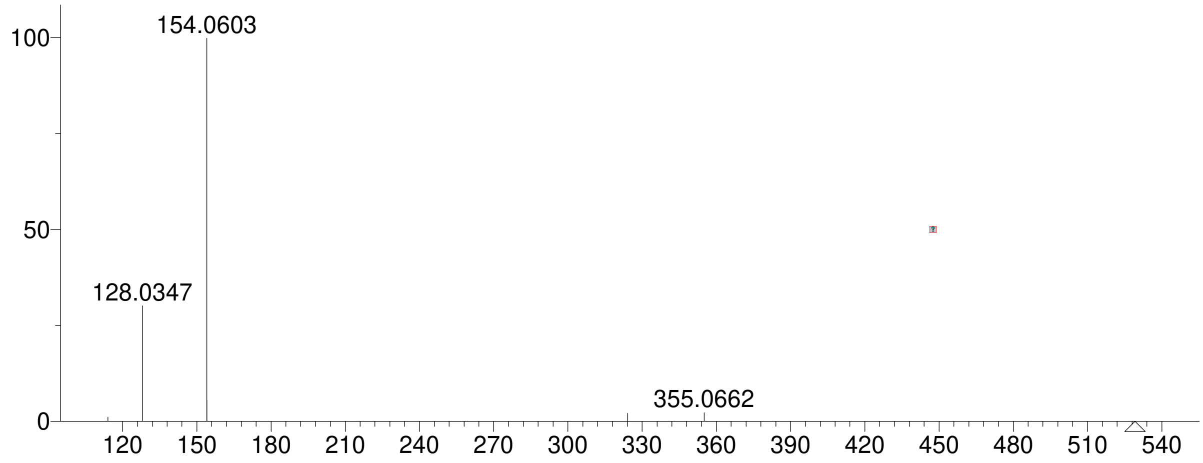

** Search Report Page 1 of 1 **

Unknown: Unknown (5.39_654.2090m/z)
Compound in Library Factor = 0

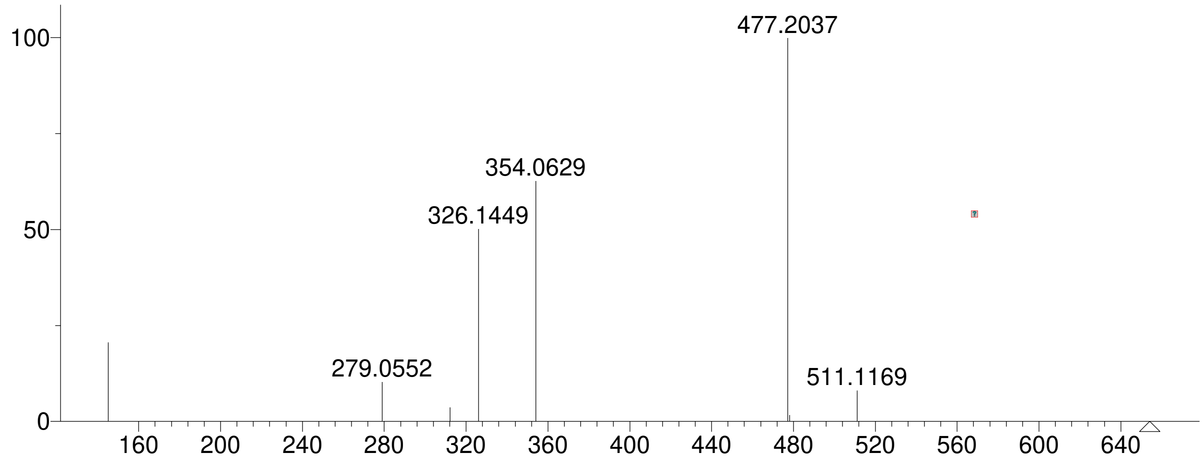

Unknown: Unknown (5.41_178.1227m/z)
Compound in Library Factor = -1275

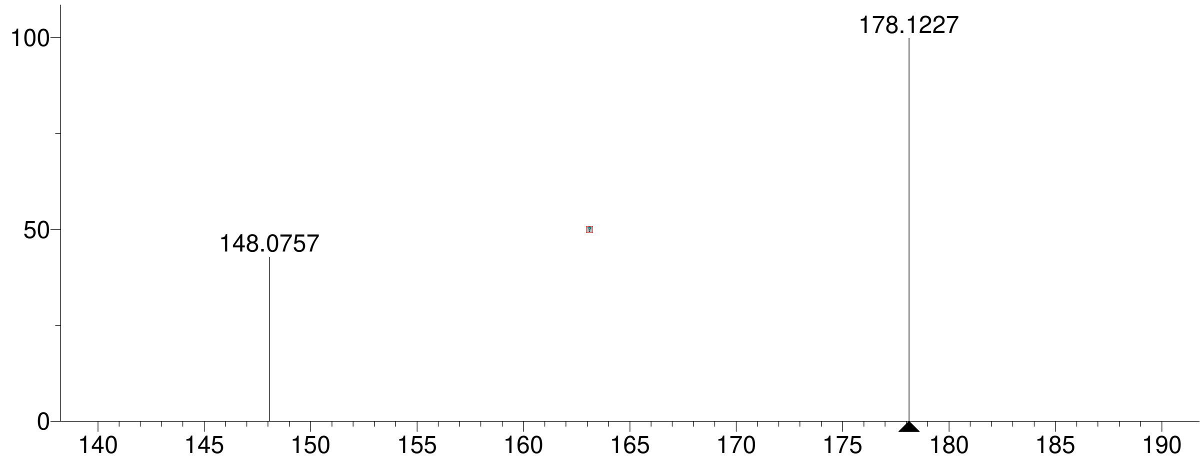

Hit 1 : 1H-Inden-2-amine, 2,3-dihydro-5-methoxy-6-methyl- [M+H]+ HCD 3V P=178.1
C11H15NO; MF: 322; RMF: 944; Prob 47.1%; CAS: 136468-19-4; Lib: nist_msms; ID: 364124.

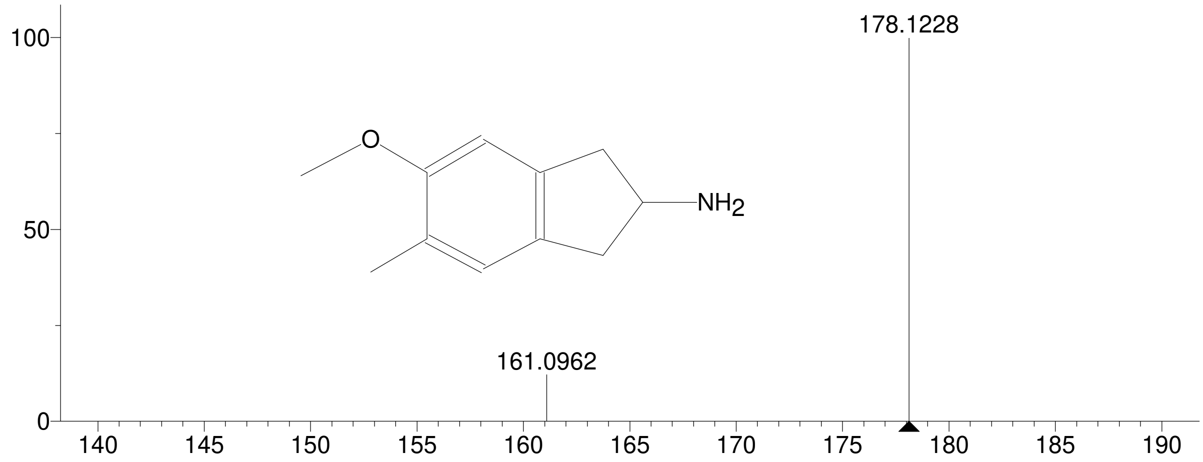

Hit 2 : Rebemide [M+H]+ 30eV P=178.1
C11H15NO; MF: 292; RMF: 836; Prob 13.1%; Lib: mona-export-massbank; ID: 26955.

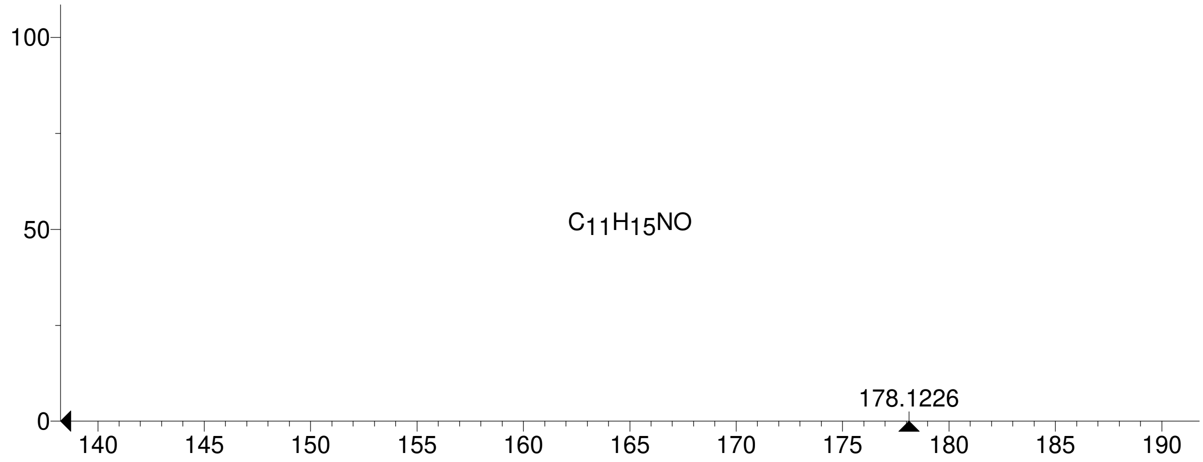

Hit 3 : N-tert-Butyl-α-phenylnitrone [M+H]+ QTOF 3V P=178.1
C11H15NO; MF: 292; RMF: 836; Prob 13.1%; CAS: 3376-24-7; Lib: nist_msms; ID: 67834.

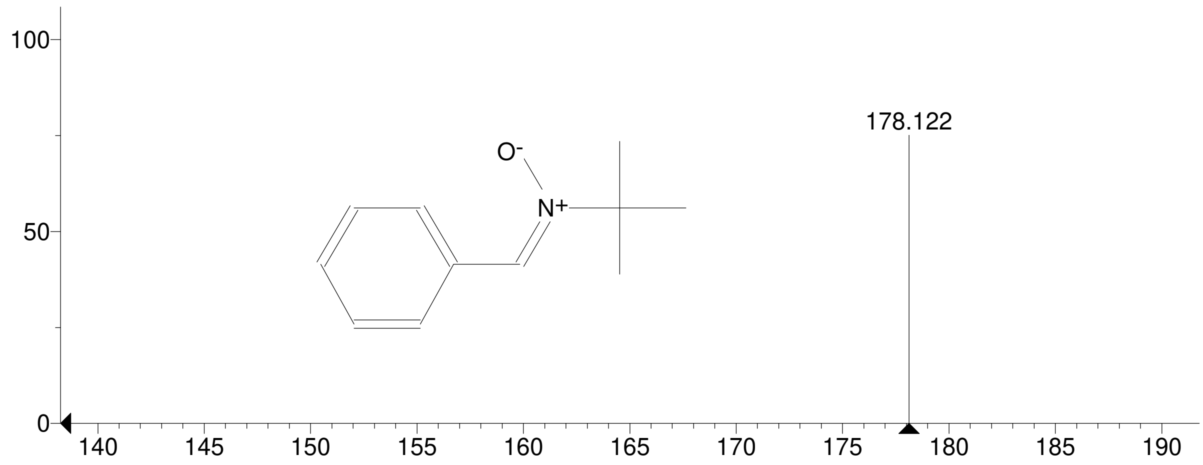

Hit 4 : N-Acetylamphetamine [M+H]+ HCD 5V P=178.1
C11H15NO; MF: 292; RMF: 836; Prob 13.1%; CAS: 14383-60-9; Lib: nist_msms; ID: 243432.

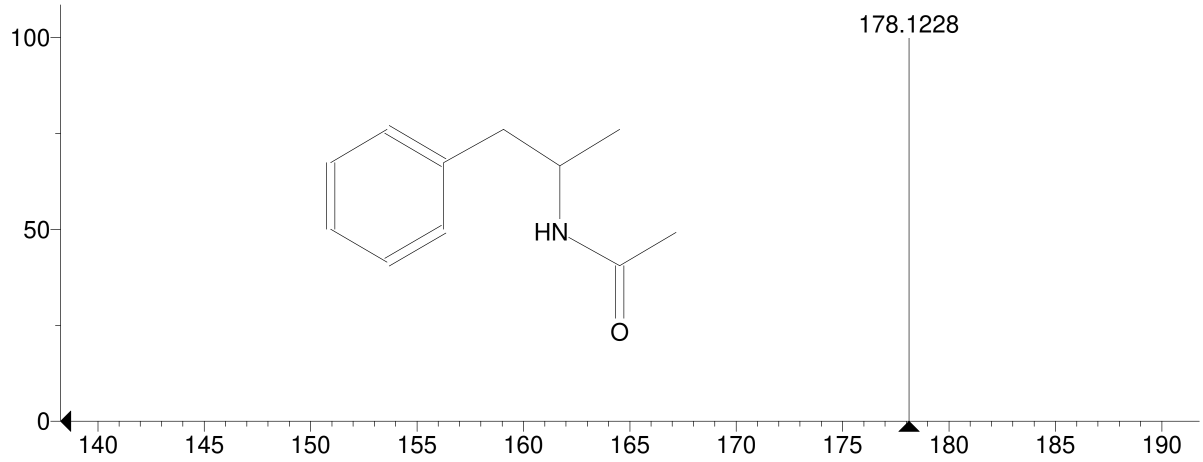

Hit 5 : 4-Benzofuranethanamine, 2,3-dihydro-α-methyl- [M+H]+ HCD 0V P=178.1
C11H15NO; MF: 245; RMF: 823; Prob 2.73%; CAS: 1203430-05-0; Lib: nist_msms; ID: 426816.

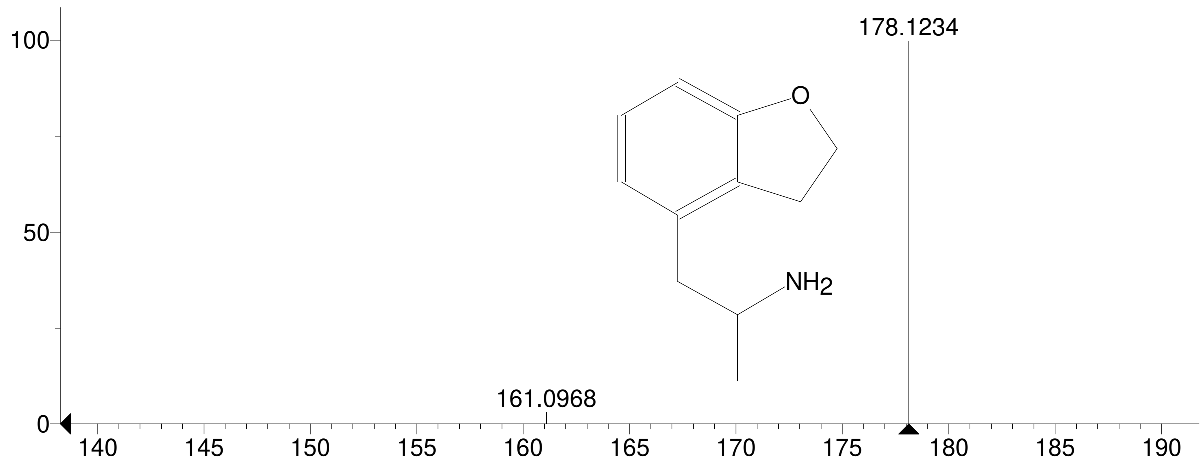

Unknown: Unknown (5.41_494.1765m/z)
Compound in Library Factor = 0

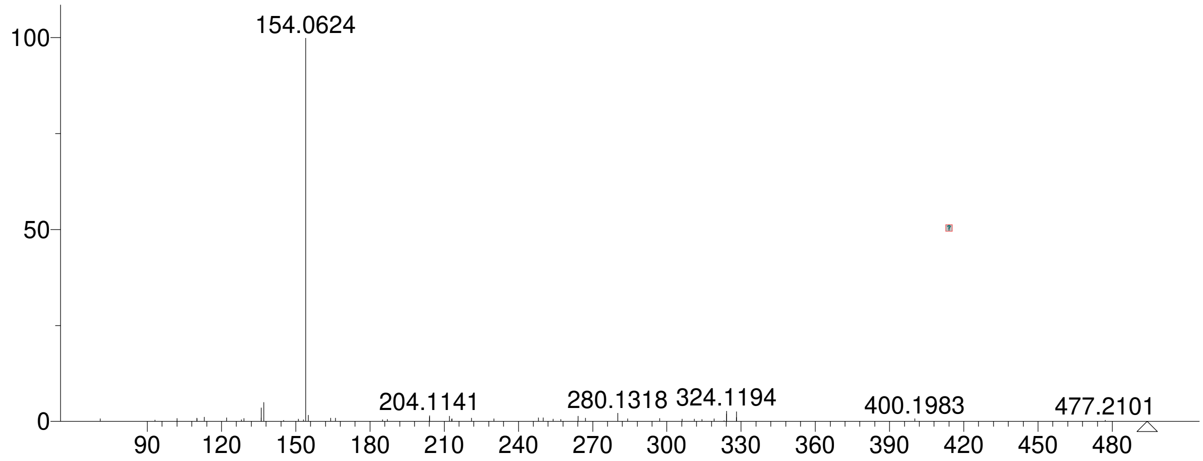

Unknown: Unknown (5.41_625.2560m/z)
Compound in Library Factor = -3273

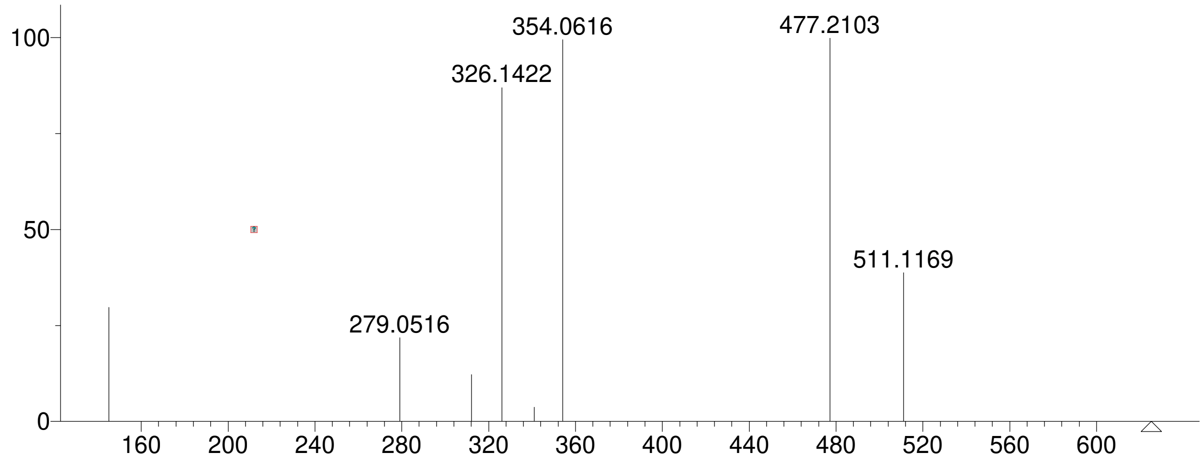

Hit 1 : N-Caffeoyl-O-methyltyramine [2M-H]- IT 35% P=625.3
C18H19NO4; MF: 4; RMF: 170; Prob 54.2%; CAS: 189307-47-9; Lib: nist_msms; ID: 221344.

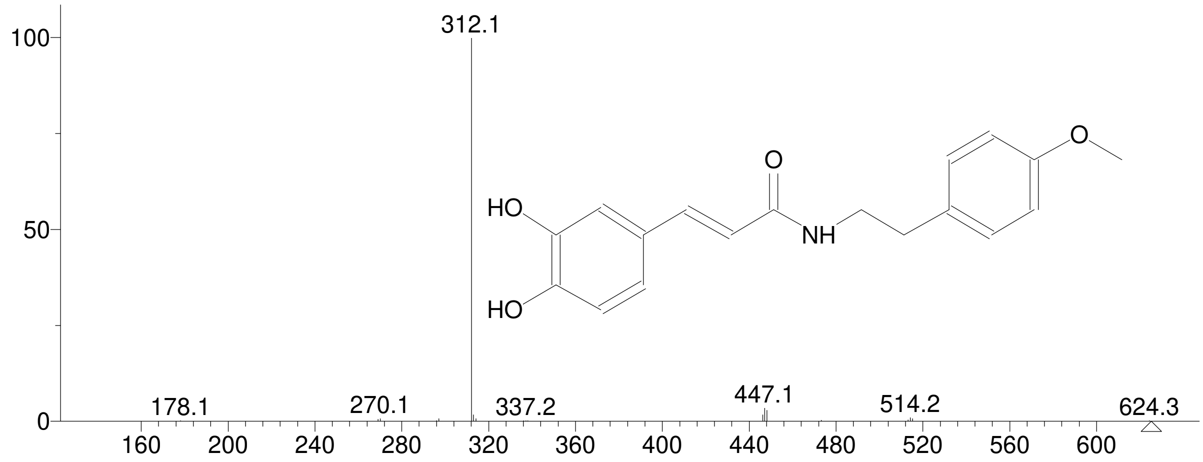

Hit 2 : Collagen Binding Fragment [M+2H]2+ QQQ 22.5V P=625.3
C52H76N14O20S; MF: 0; RMF: 4; Prob 45.8%; CAS: 94040-53-6; Lib: nist_msms2; ID: 3608.

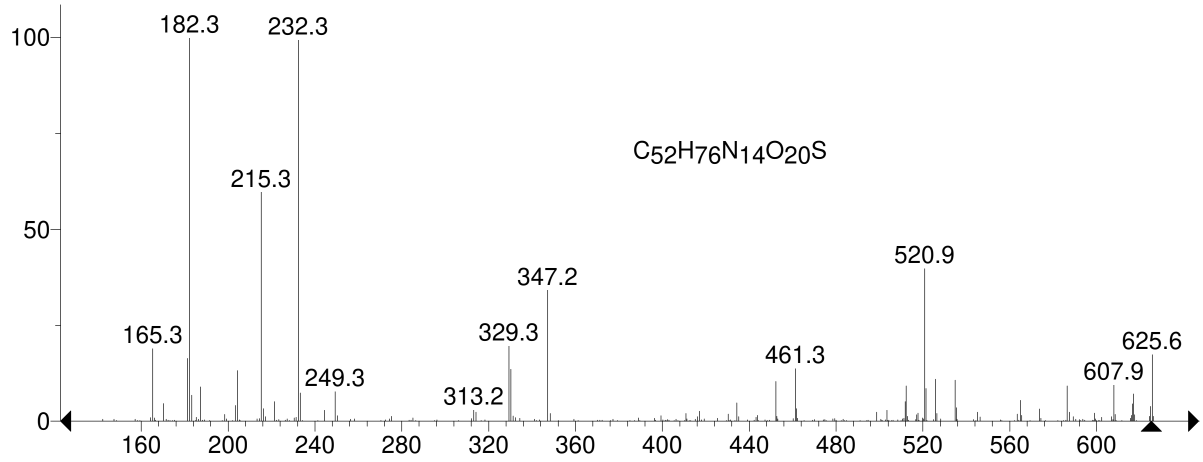

Unknown: Unknown (5.44_376.1028m/z)
Compound in Library Factor = 0

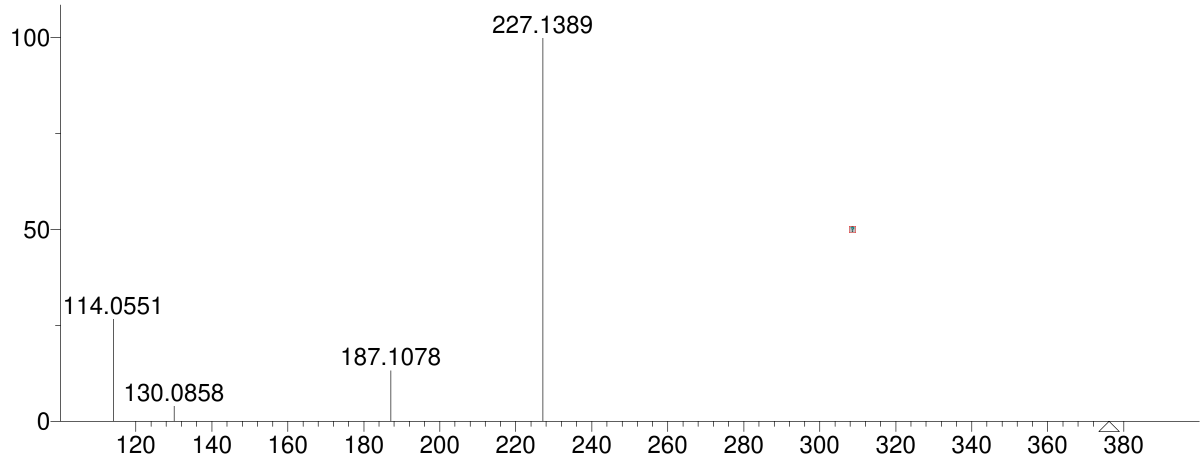

Hit 1 : 1-(3,4-dihydroxyphenyl)-6,7-dihydroxy-1,2-dihydronaphthalene-2,3-dicarboxylic acid [M+NH4]+ 45HCD F
C18H14O8; MF: 0; RMF: 5; Prob 100.0%; Lib: mona-export-vf-npl_qexactive; ID: 24902.

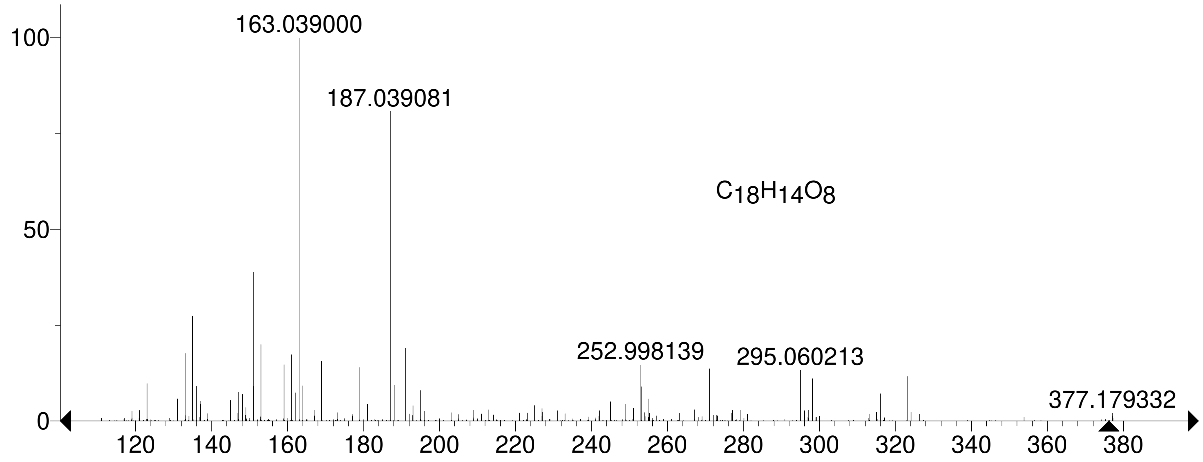

Unknown: Unknown (5.44_415.1740m/z)
Compound in Library Factor = 0

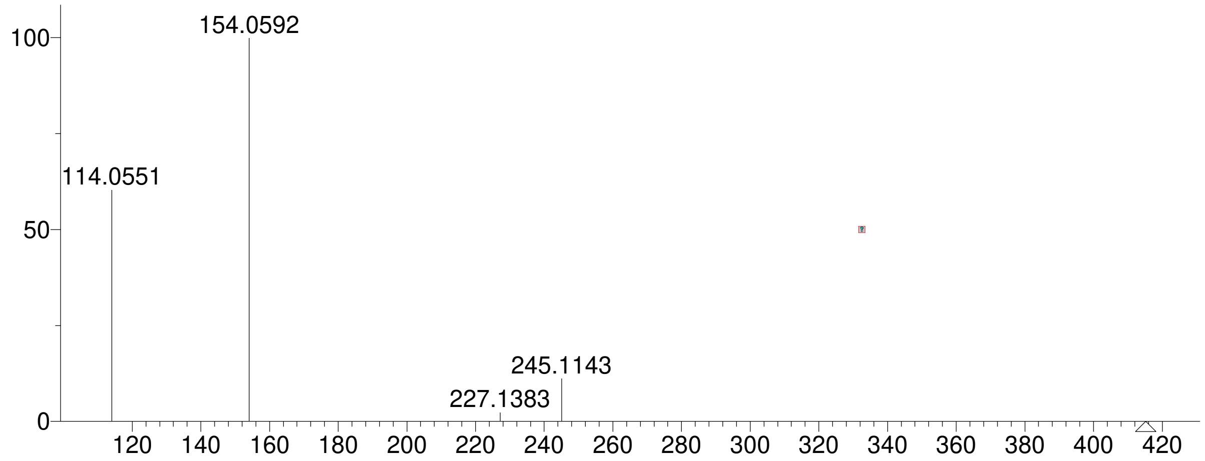

Hit 1 : 2-amino-5-[2-[[2,3-dihydroxy-2-(1-hydroxyethyl)butanoyl]oxymethyl]-4-hydroxyanilino]-5-oxopentanoic acid
C18H26N2O9; MF: 0; RMF: 55; Prob 25.0%; Lib: mona-export-vf-npl_qexactive; ID: 23033.

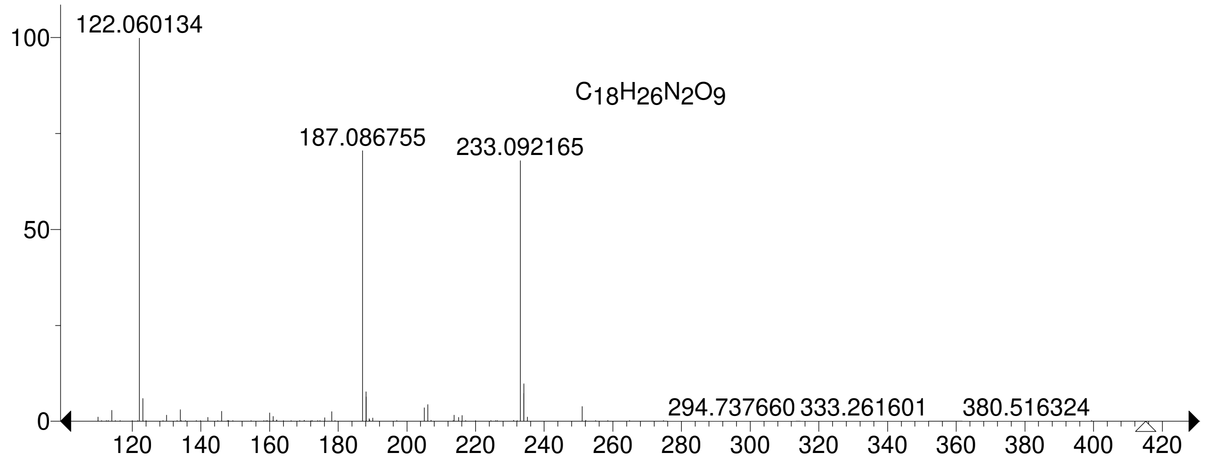

Hit 2 : NCGC00347525-03!2-amino-5-[2-[[2,3-dihydroxy-2-(1-hydroxyethyl)butanoyl]oxymethyl]-4-hydroxyanilino]-
C18H26N2O9; MF: 0; RMF: 36; Prob 25.0%; Lib: mona-export-gnps; ID: 9213.

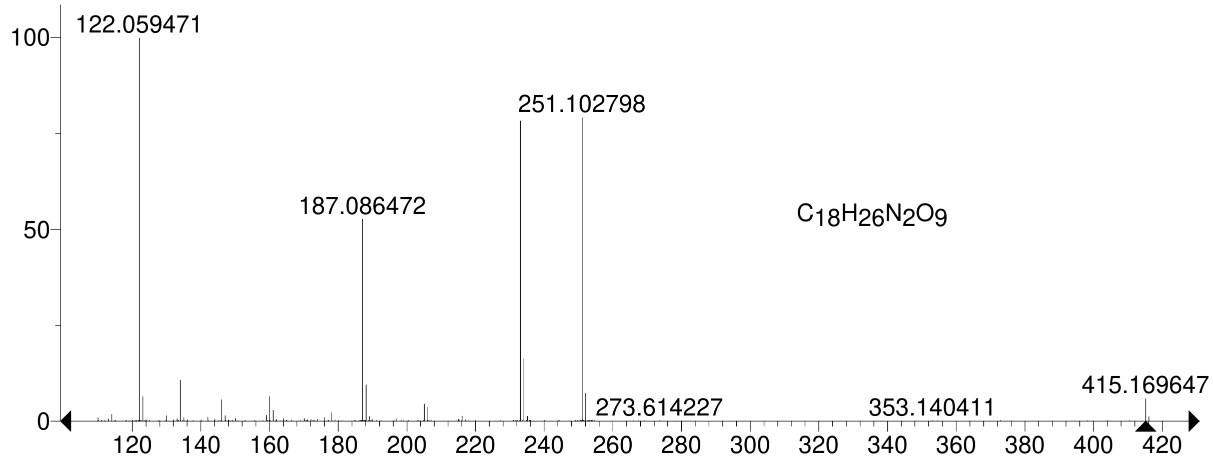

Hit 3 : NCGC00347525-02!2-amino-5-[2-[[2,3-dihydroxy-2-(1-hydroxyethyl)butanoyl]oxymethyl]-4-hydroxyanilino]-
C18H26N2O9; MF: 0; RMF: 29; Prob 25.0%; Lib: mona-export-gnps; ID: 6270.

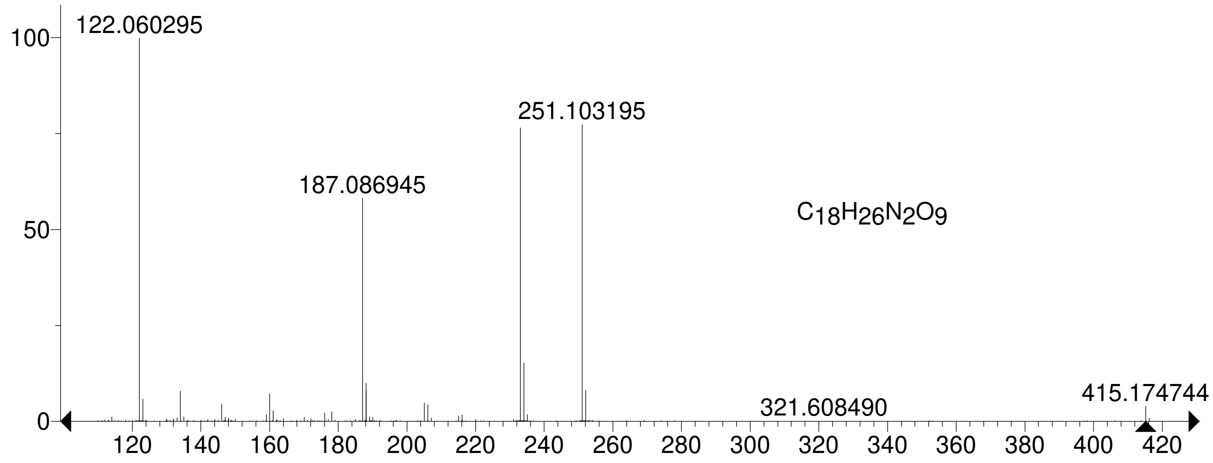

Hit 4 : 6-formyl-10-(hydroxymethyl)-5-methoxy-3-methylidene-2-oxo-2H,3H,3aH,4H,5H,8H,9H,11aH-cyclodeca[b]f
C21H28O7; MF: 0; RMF: 8; Prob 25.0%; Lib: mona-export-vf-npl_qexactive; ID: 16435.

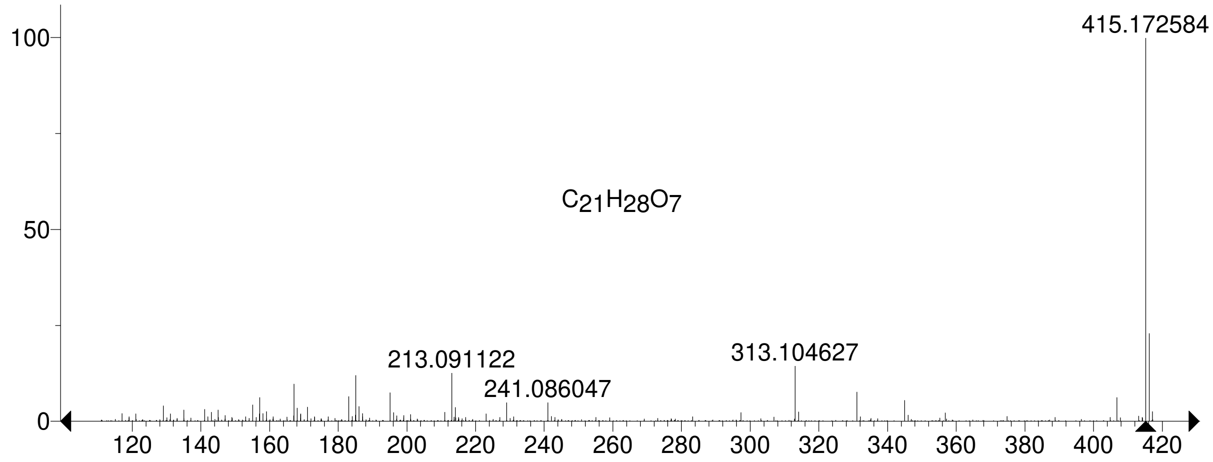

** Search Report Page 1 of 1 **

Unknown: Unknown (5.45_486.2050m/z)
Compound in Library Factor = 0

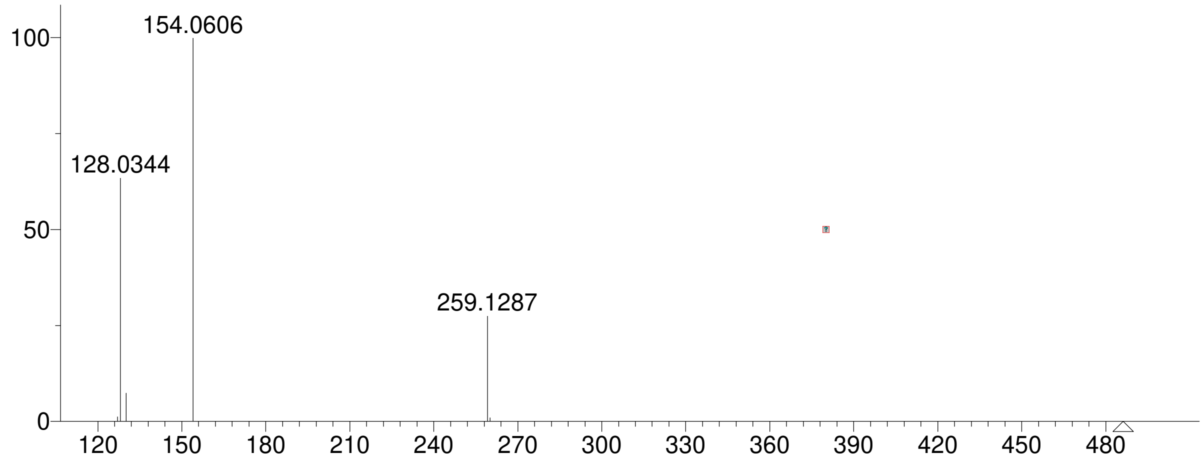

Hit 1 : rac-N-Allyloxycarbonyl-cis-moxifloxacin acyl-β-D-glucuronide allyl ester [M+H-C9H12O6]+ HCD 126V P=
C34H40FN3O12; MF: 0; RMF: 93; Prob 100.0%; Lib: nist_msms; ID: 431491.

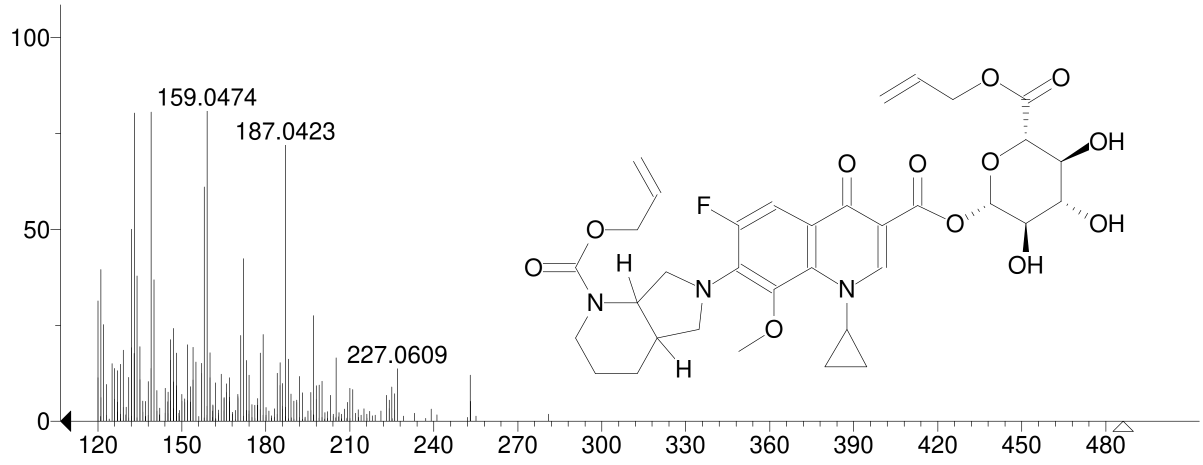

Unknown: Unknown (5.46_429.1863m/z)
Compound in Library Factor = 0

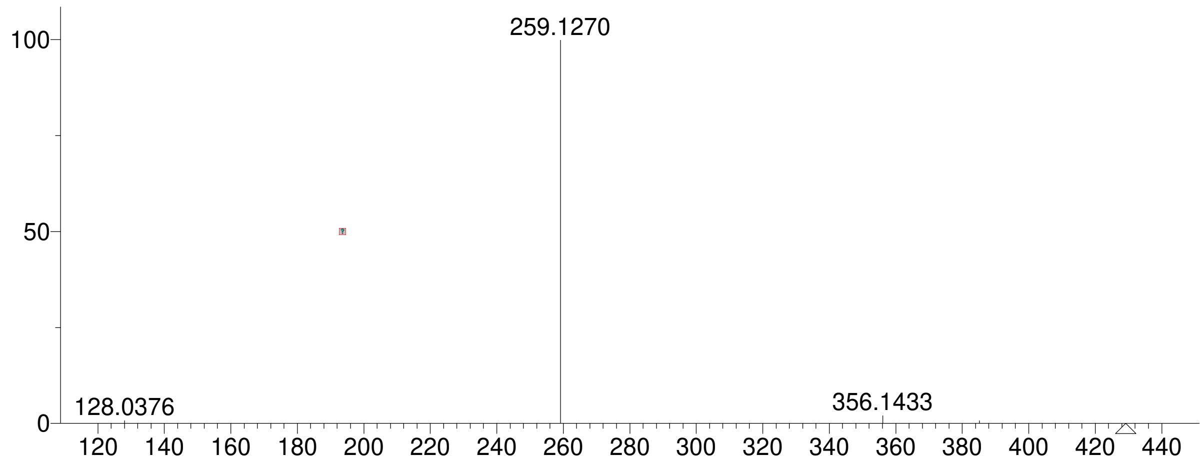

Hit 1 : (2R,3R,4S,5S,6R)-2-[6-hydroxy-3-[(E)-3-hydroxybut-1-enyl]-2,4,4-trimethylcyclohexyl]oxy-6-(hydroxymethy
C19H34O8; MF: 0; RMF: 15; Prob 50.0%; Lib: anthocyanins mona; ID: 9988.

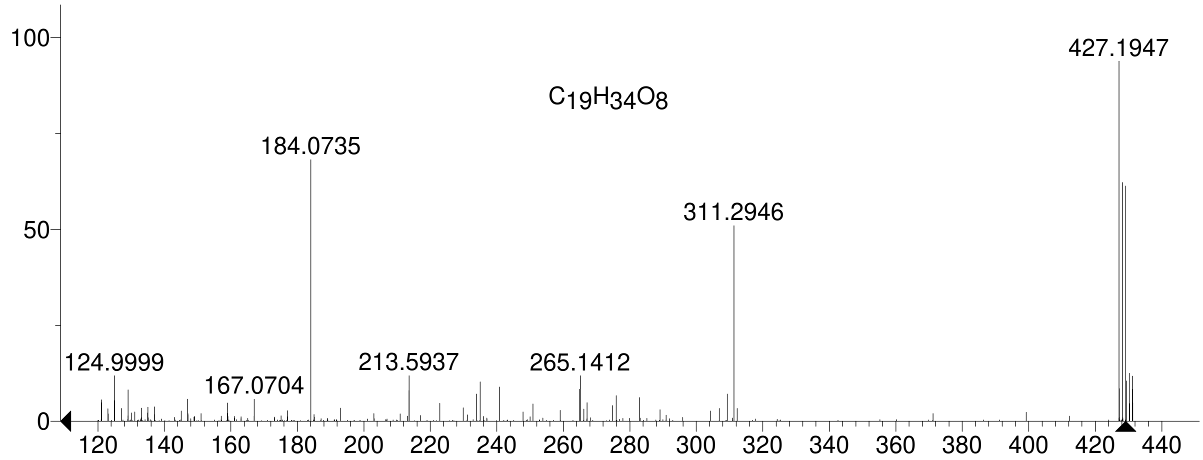

Hit 2 : NCGC00169368-02!3,6-bis(3,4,5-trimethoxyphenyl)-1,3,3a,4,6,6a-hexahydrofuro[3,4-c]furan [M-H2O+H]+
C24H30O8; MF: 0; RMF: 10; Prob 50.0%; Lib: mona-export-gnps; ID: 9133.

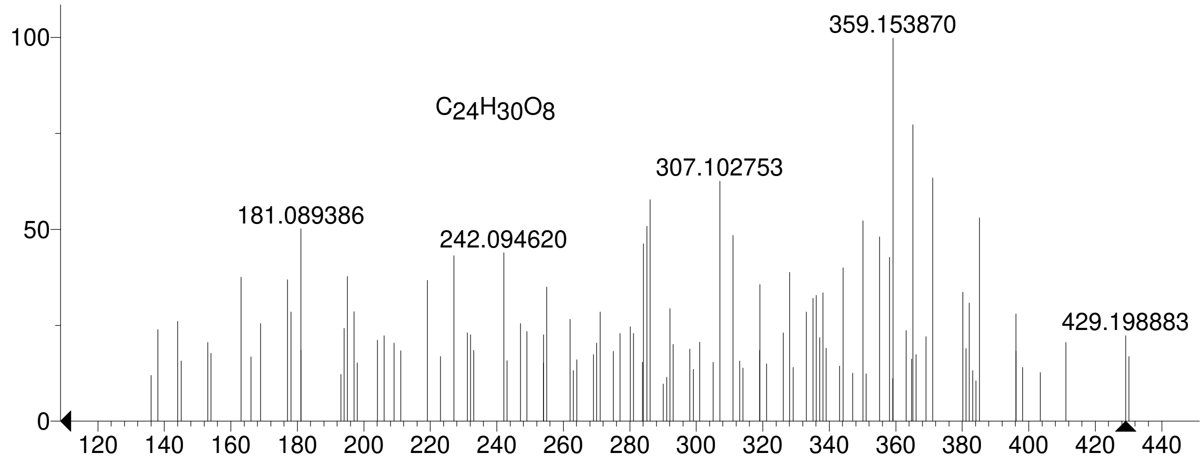

** Search Report Page 1 of 1 **

Unknown: Unknown (5.46_544.2146m/z)
Compound in Library Factor = 0

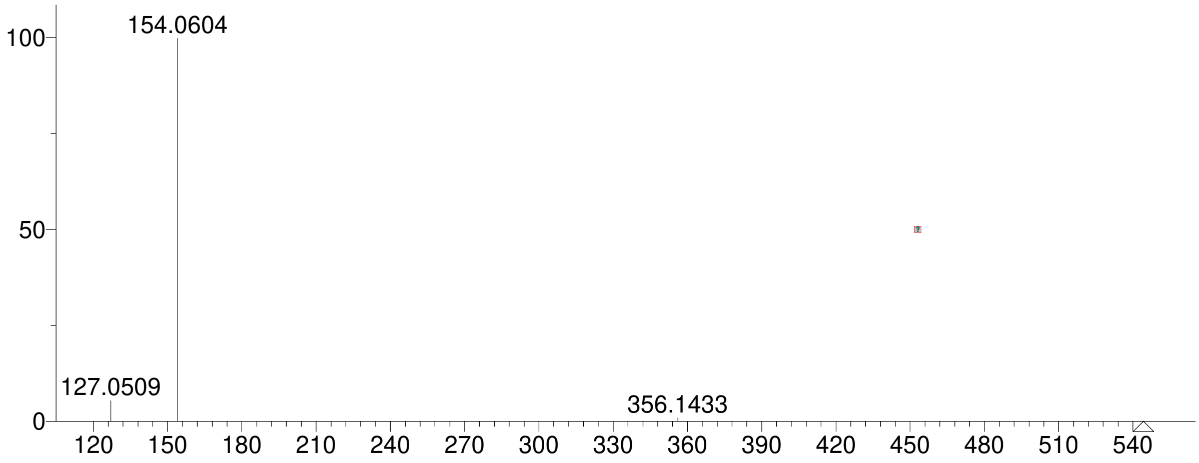

Unknown: Unknown (5.46_750.2982m/z)
Compound in Library Factor = 0

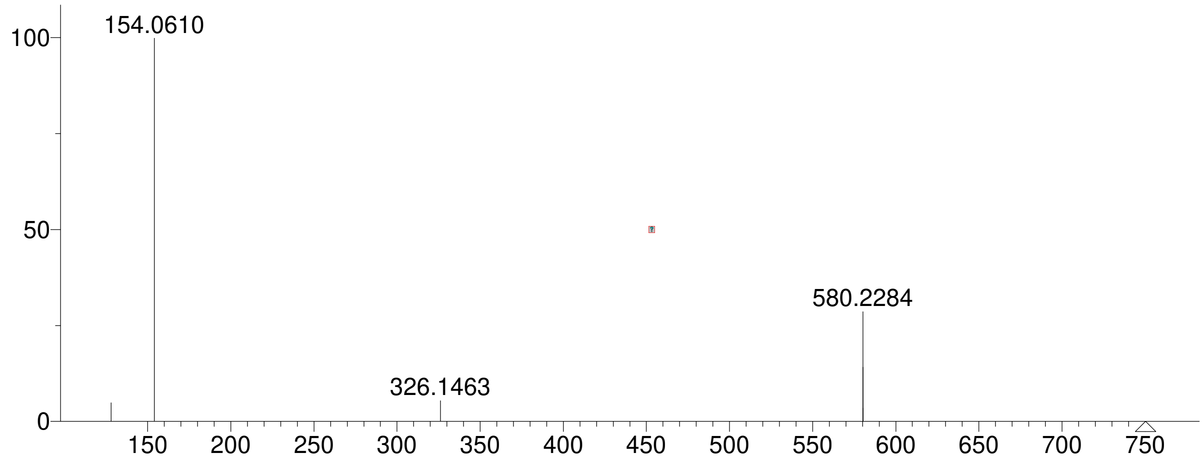

Unknown: Unknown (5.48_314.1598m/z)
Compound in Library Factor = -3273

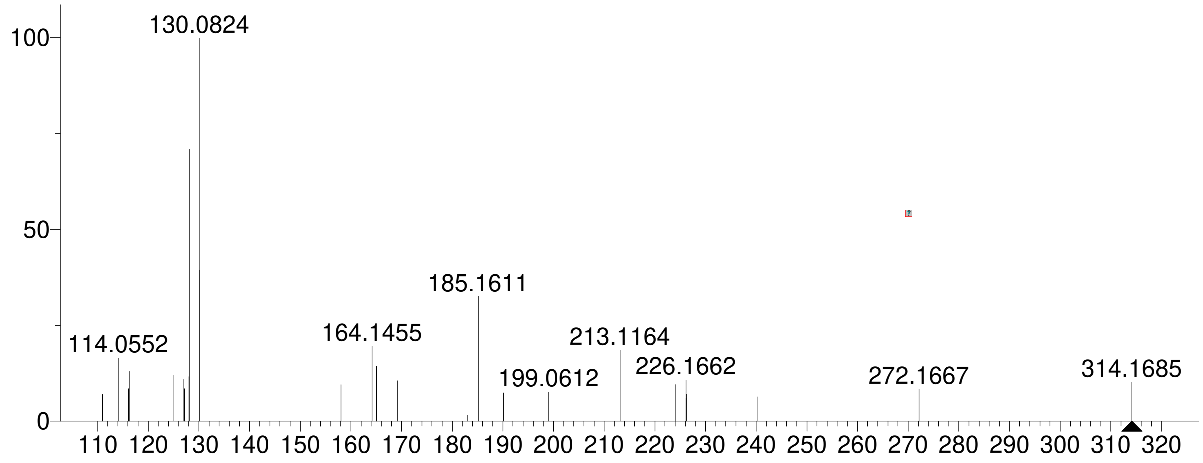

Hit 1 : MMV675993 [M+H]+ 20-30-40%) P=314.2
C16H19N5O2; MF: 1; RMF: 110; Prob 51.0%; Lib: mona-export-pathogen_box; ID: 219.

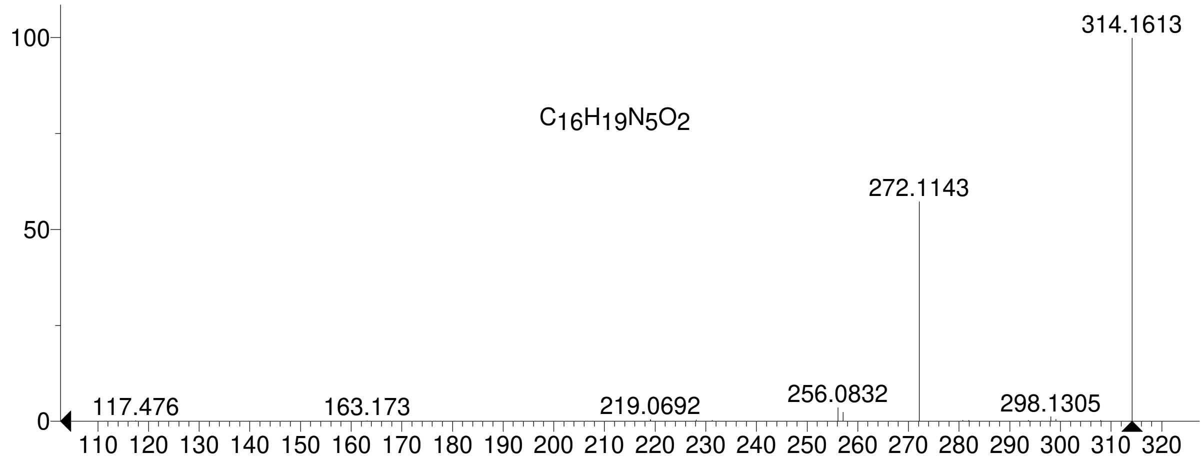

Hit 2 : [(2S,3S)-2-[(E,3R,4S)-3,4-dihydroxypent-1-enyl]-6-oxo-2,3-dihydropyran-3-yl] (E)-2-methylbut-2-enoate [M
C15H20O6; MF: 0; RMF: 21; Prob 49.0%; Lib: mona-export-vf-npl_qexactive; ID: 17659.

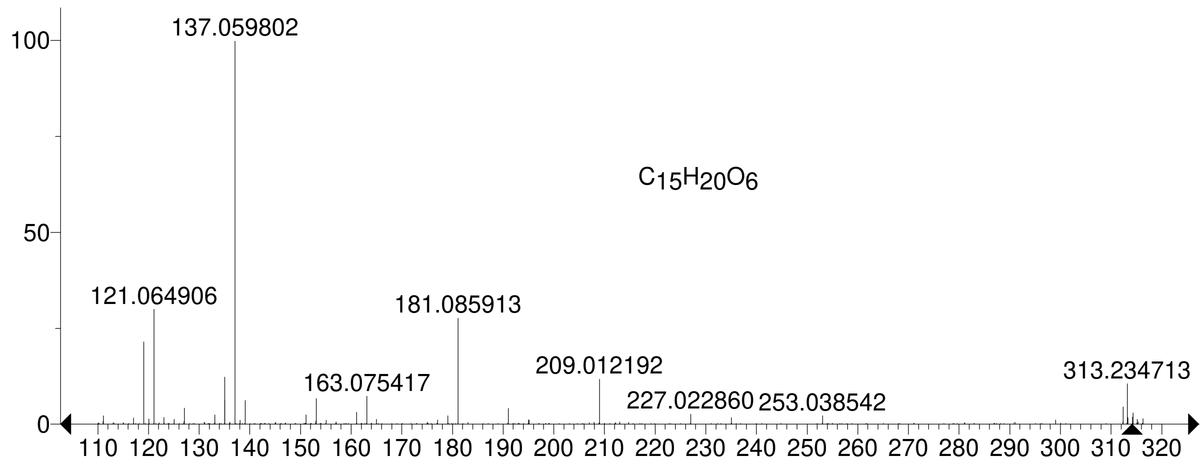

** Search Report Page 1 of 1 **

Unknown: Unknown (5.48_891.4320m/z)
Compound in Library Factor = -3273

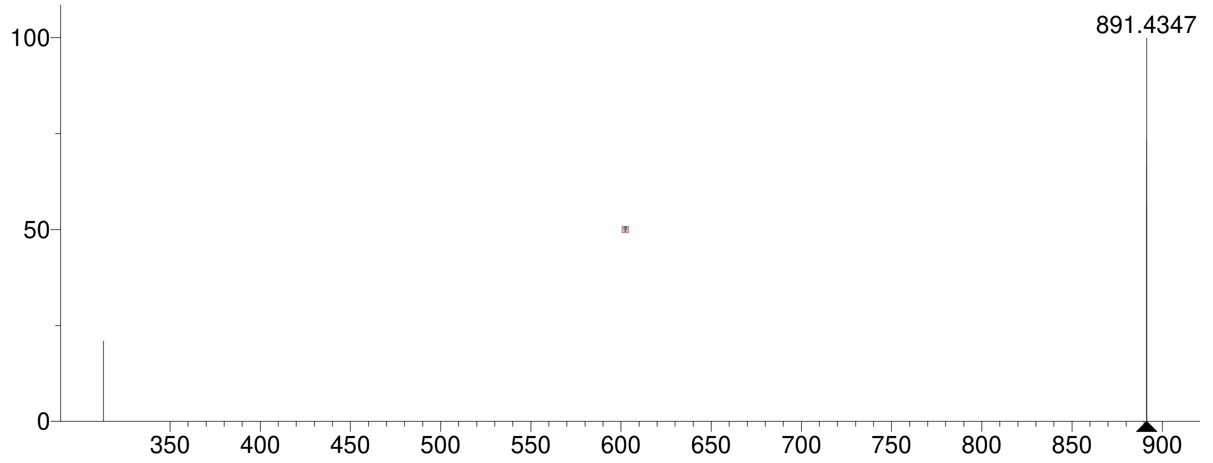

Hit 1 : Ruscin [M+Na]+ HCD 64V P=891.4
C44H68O17; MF: 5; RMF: 342; Prob 52.0%; CAS: 39491-41-3; Lib: nist_msms; ID: 227132.

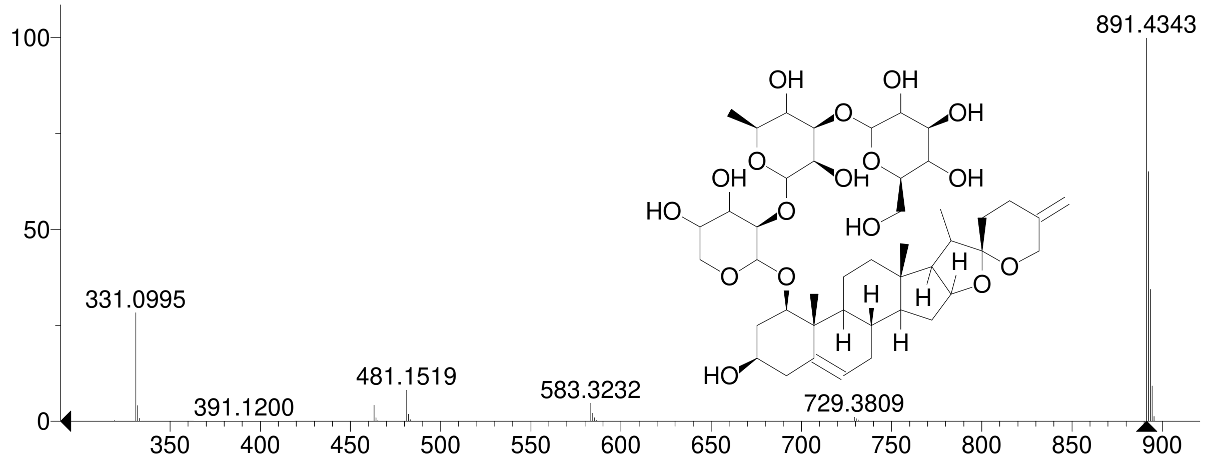

Hit 2 : NCGC00347384-02_C44H68O17_(1beta,3alpha,9xi,14xi)-1-Hydroxyspirosta-5,25(27)-dien-3-yl beta-D-glu
C44H68O17; MF: 3; RMF: 401; Prob 48.0%; Lib: mona-export-gnps; ID: 10545.

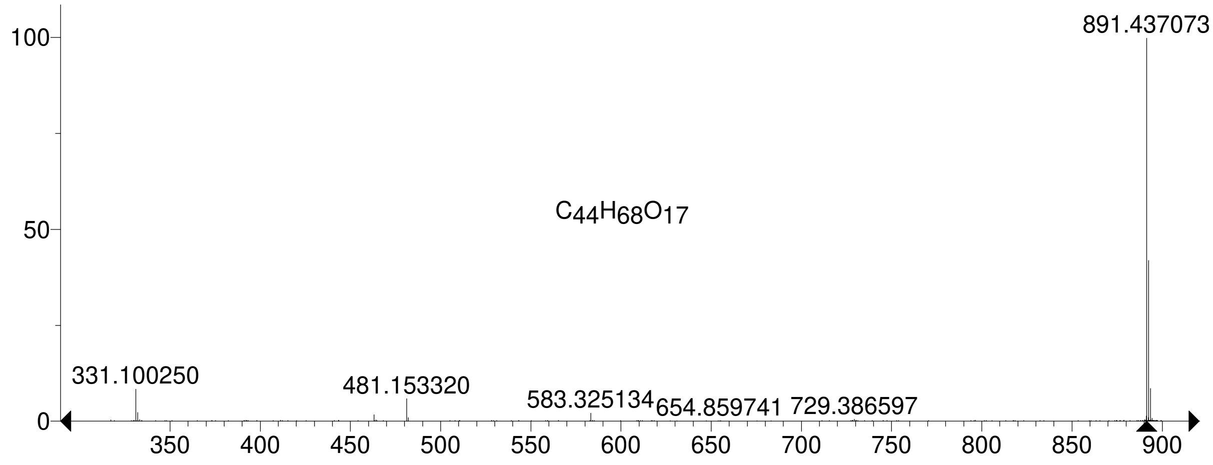

Unknown: Unknown (5.50_401.1916m/z)
Compound in Library Factor = 0

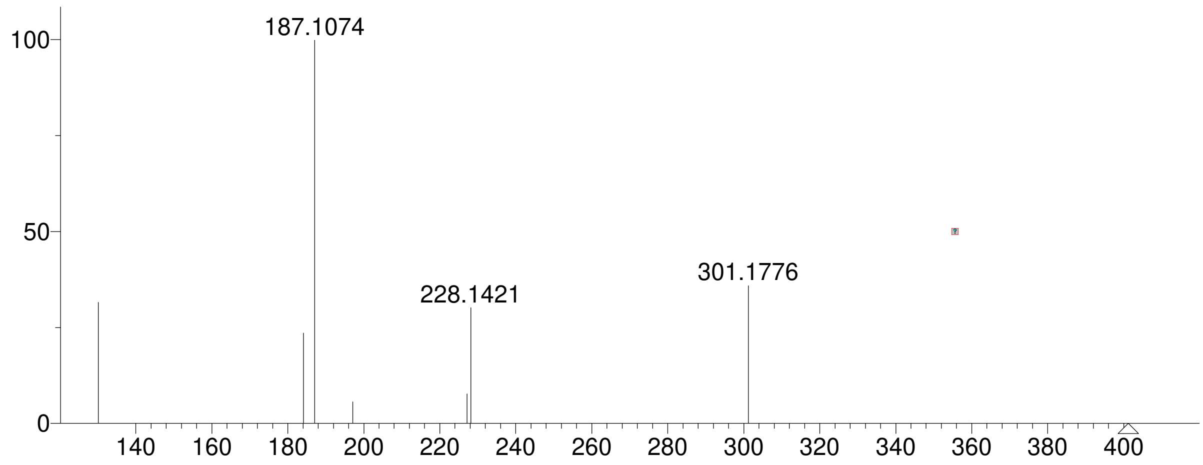

Hit 1 : NCGC00380382-01_C23H30O7_1-Phenanthrenecarboxylic acid, 10-(acetyloxy)-1,2,3,4,4a,9,10,10a-octah
C23H30O7; MF: 0; RMF: 14; Prob 100.0%; Lib: mona-export-gnps; ID: 13719.

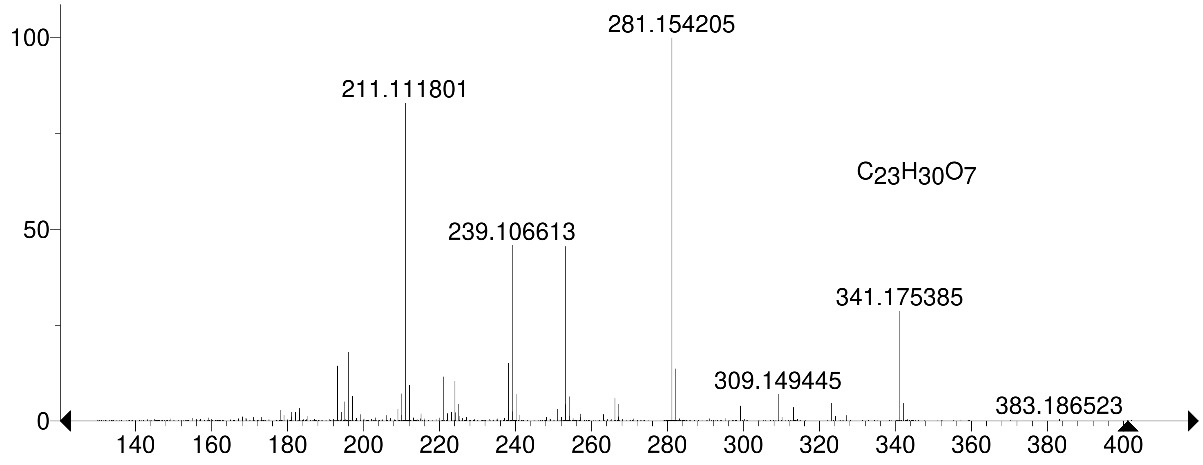

** Search Report Page 1 of 1 **

Unknown: Unknown (5.51_431.1774m/z)
Compound in Library Factor = 0

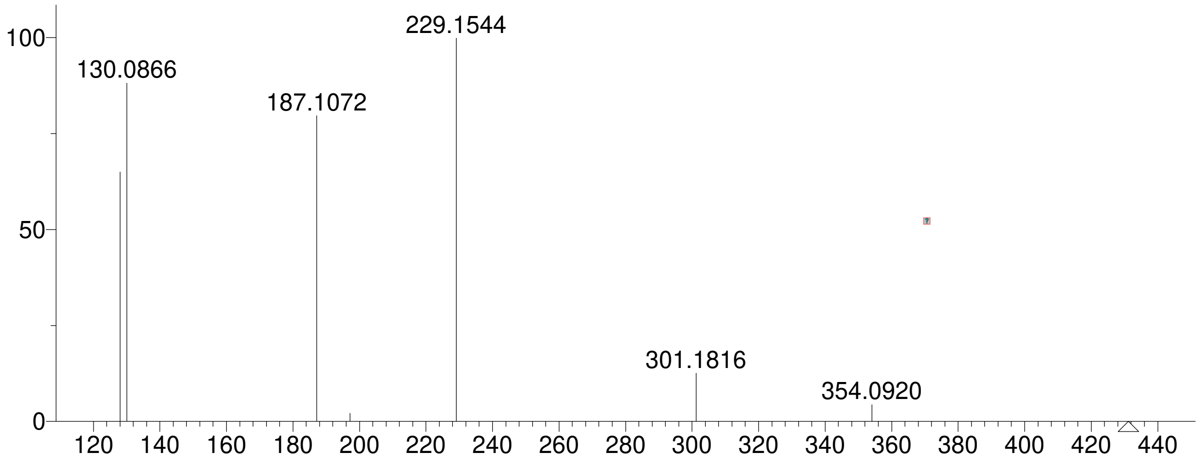

** Search Report Page 1 of 1 **

Unknown: Unknown (5.51_516.2136m/z)
Compound in Library Factor = 0

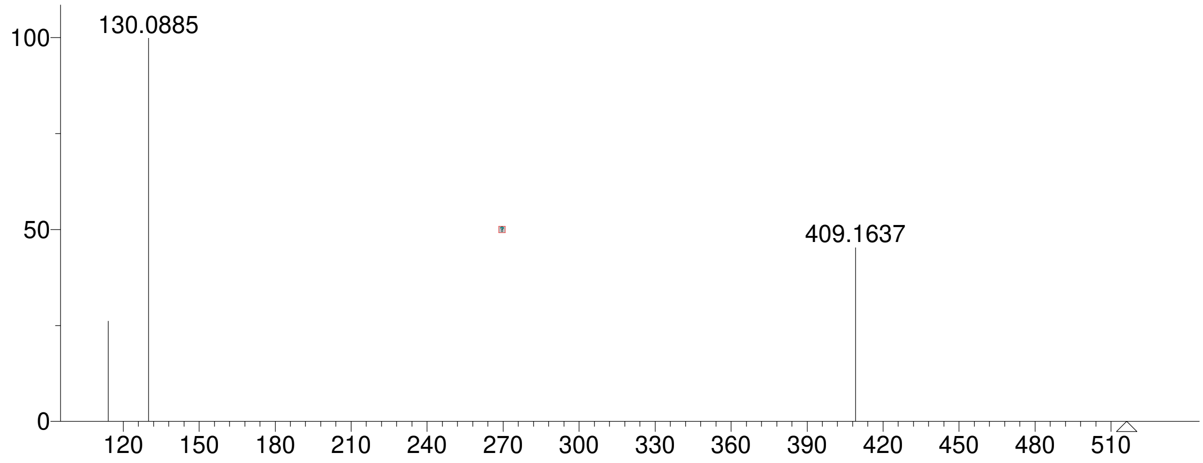

** Search Report Page 1 of 1 **

Unknown: Unknown (5.53_371.1805m/z)
Compound in Library Factor = 0

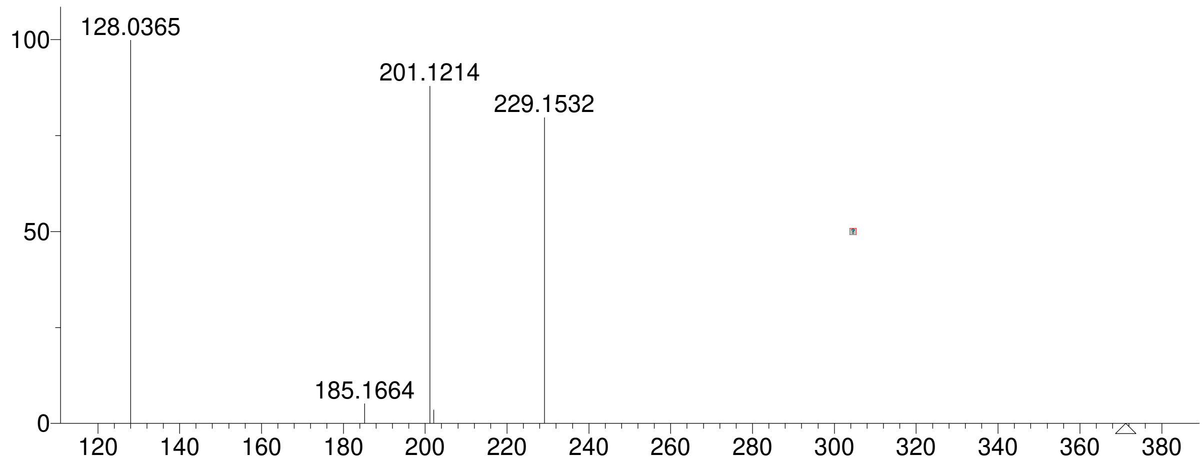

Unknown: Unknown (5.53_433.1926m/z)
Compound in Library Factor = 0

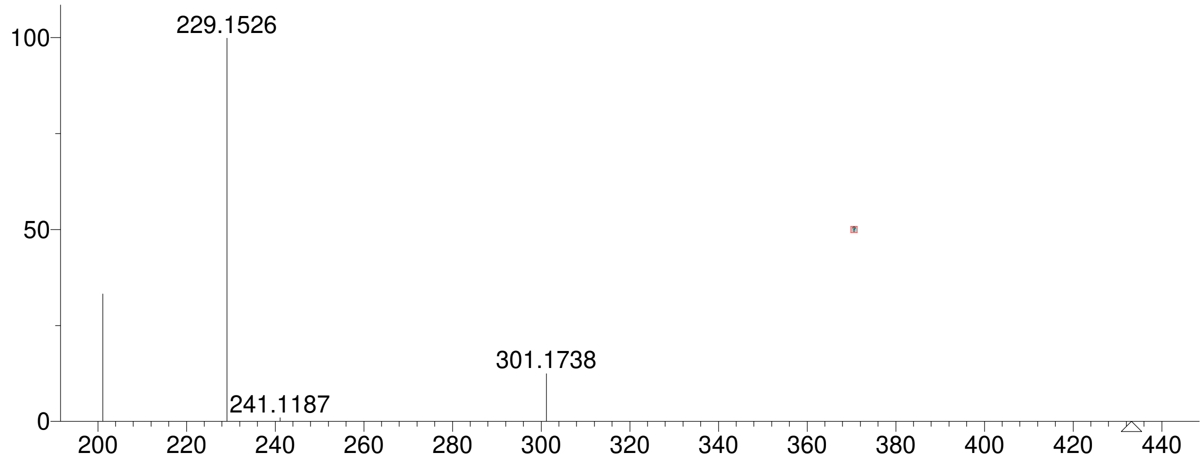

Unknown: Unknown (5.53_440.1892m/z)
Compound in Library Factor = 0

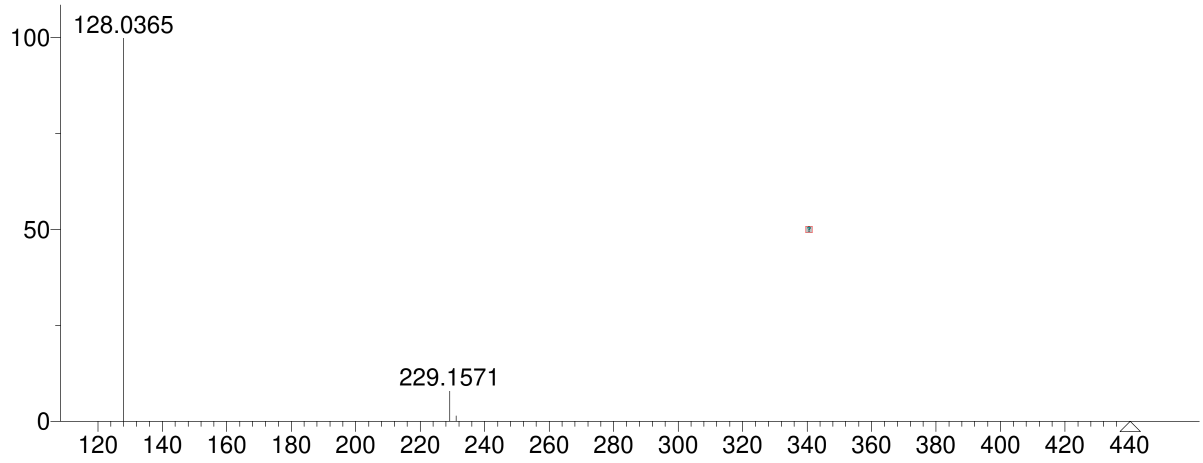

Unknown: Unknown (5.53_527.2092m/z)
Compound in Library Factor = -3273

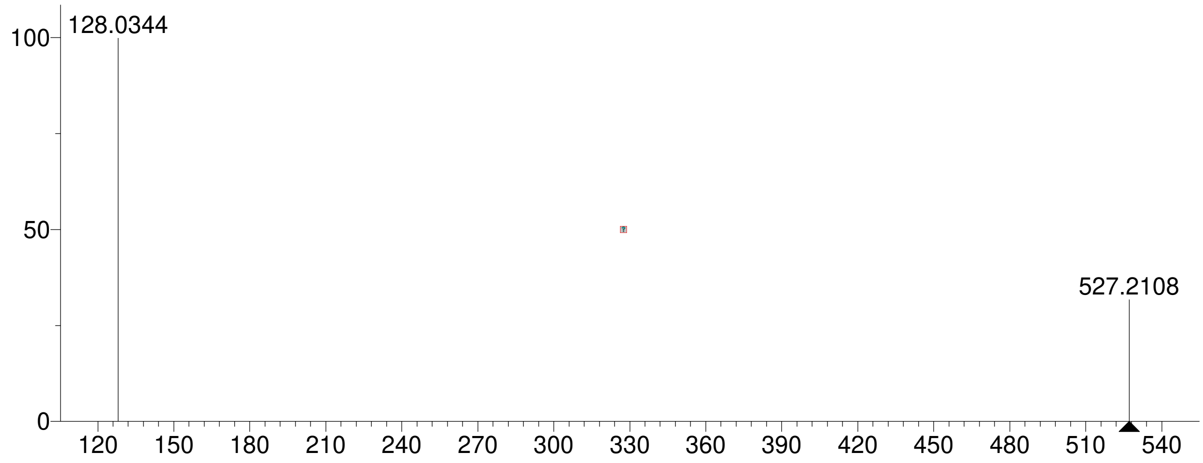

Hit 1 : NCGC00380661-01_C23H36O12_ [M+Na]+ P=527.2
C23H36O12; MF: 1; RMF: 310; Prob 34.2%; Lib: mona-export-gnps; ID: 13348.

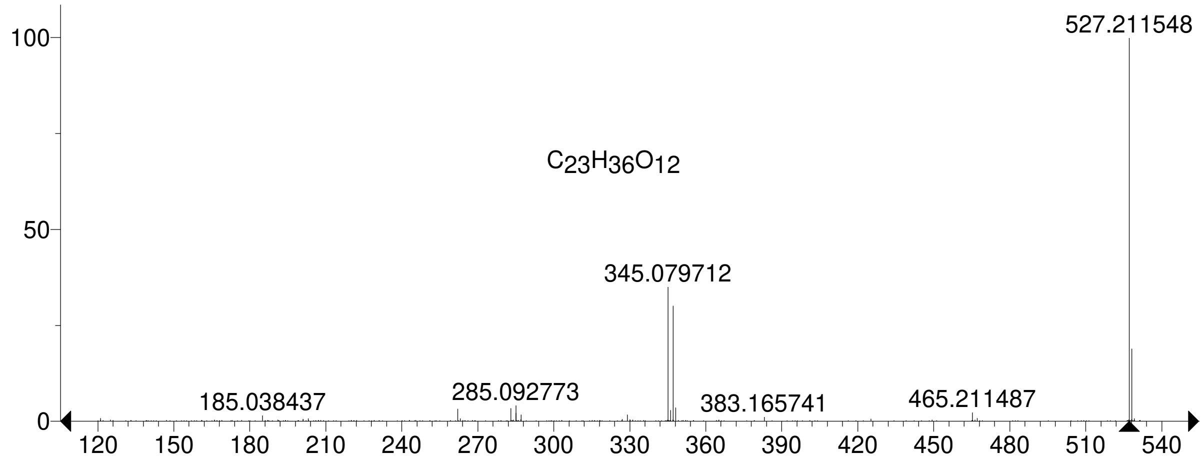

Hit 2 : 13-(acetyloxy)-1,6-dihydroxy-8-(hydroxymethyl)-4,12,12,15-tetramethyl-5-oxotetracyclo[8.5.0.0A^'2,a^!P.0A
C27H36O8; MF: 0; RMF: 64; Prob 32.9%; Lib: mona-export-vf-npl_qexactive; ID: 20116.

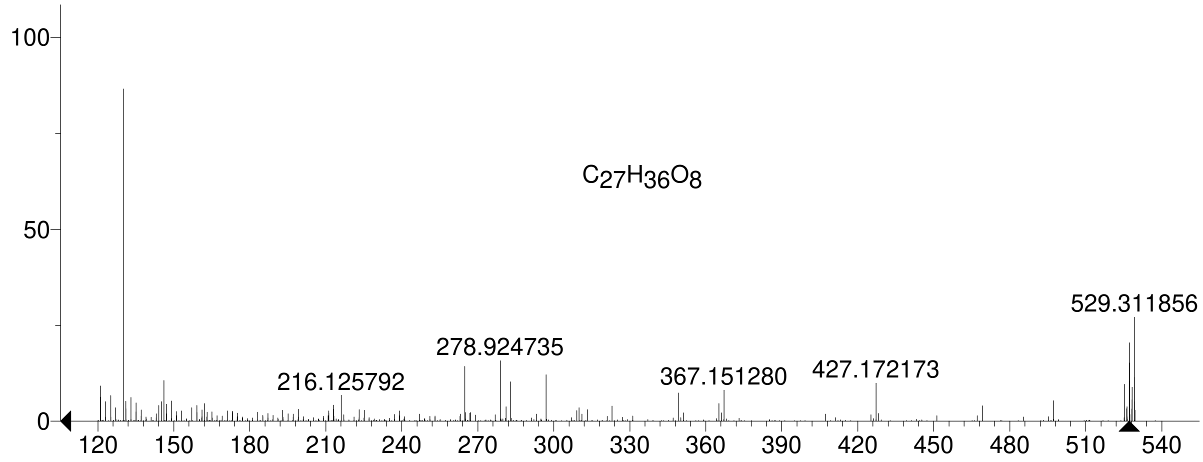

Hit 3 : [(1aR,1bS,2S,5aS,6R,6aS)-5a-hydroxy-1a-methyl-2-[(2S,3R,4S,5S,6R)-3,4,5-trihydroxy-6-(hydroxymethyl)
C25H36O12; MF: 0; RMF: 16; Prob 32.9%; Lib: mona-export-lc-ms-ms_negative_mode; ID: 27028.

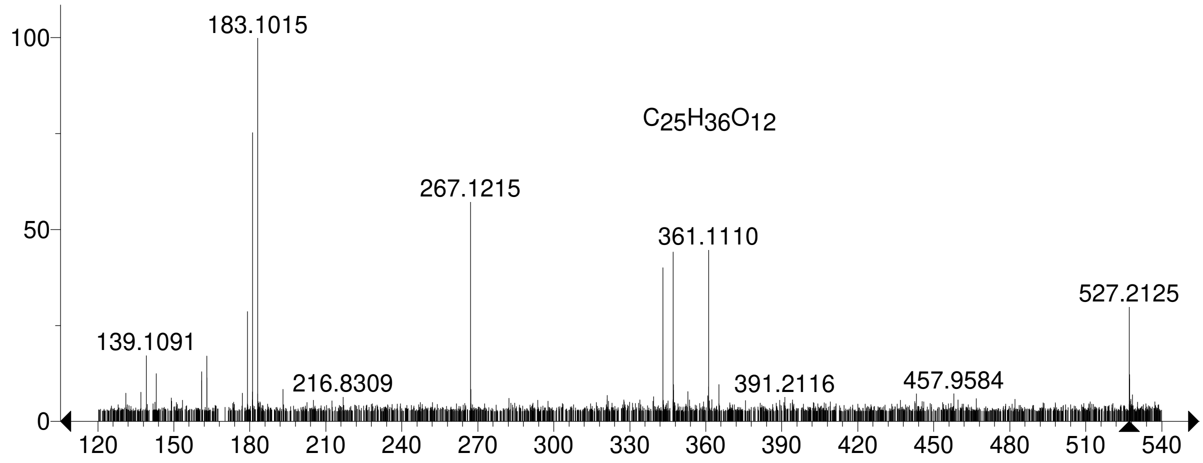

Unknown: Unknown (5.55_659.2521m/z)
Compound in Library Factor = -3273

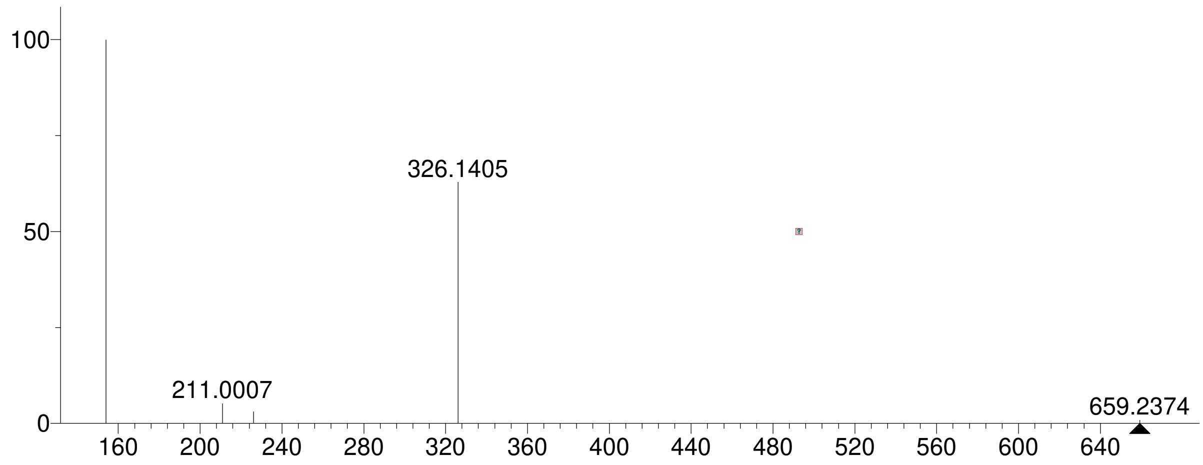

Hit 1 : Extracellular Death Factor [M-H]- HCD 26eV P=659.3
C27H36N10O10; MF: 1; RMF: 61; Prob 51.0%; CAS: 960129-66-2; Lib: nist_msms2; ID: 88872.

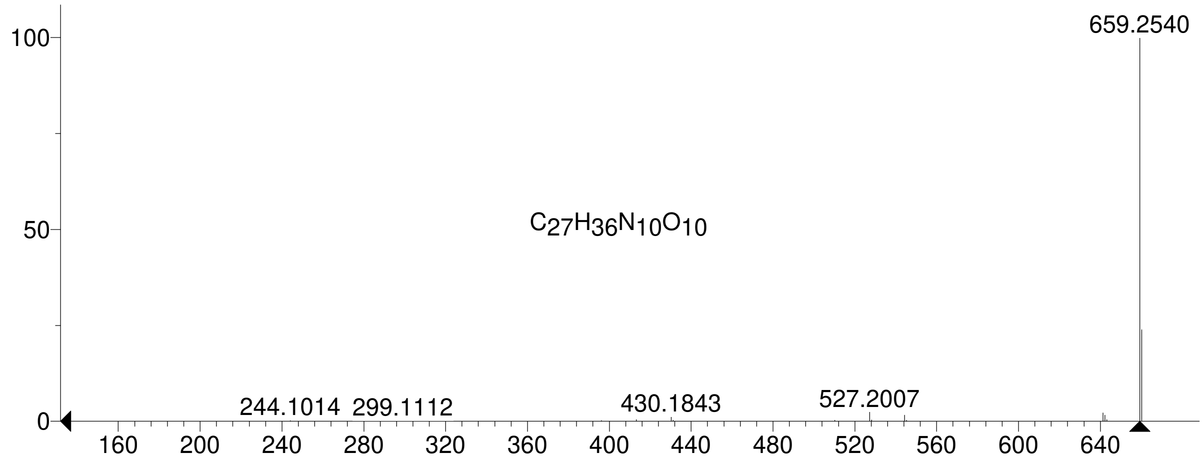

Hit 2 : G0 [M+2H]2+ IT 35% P=659.3
C50H84N4O36; MF: 0; RMF: 13; Prob 49.0%; CAS: 84808-02-6; Lib: nist_msms; ID: 523891.

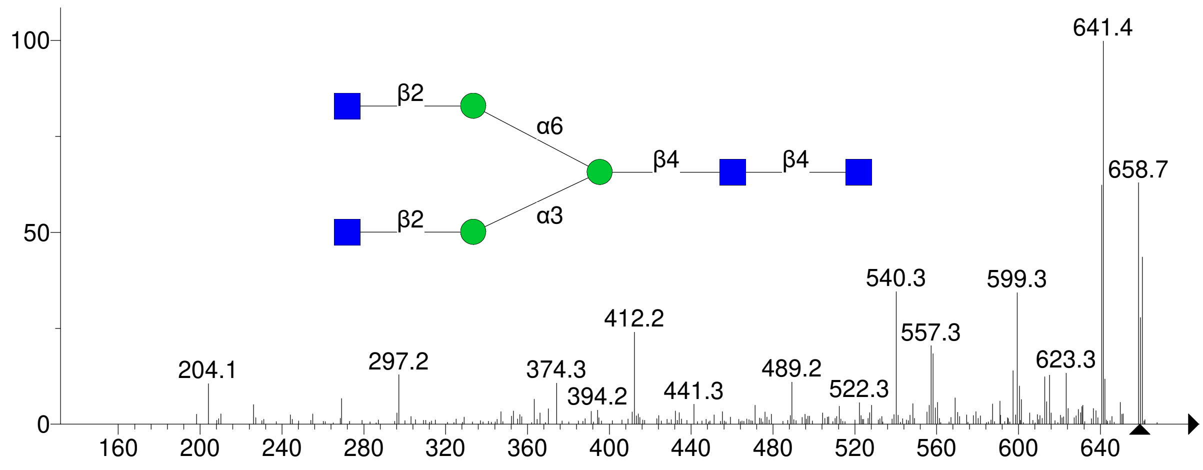

Unknown: Unknown (5.55_718.3115m/z)
Compound in Library Factor = 0

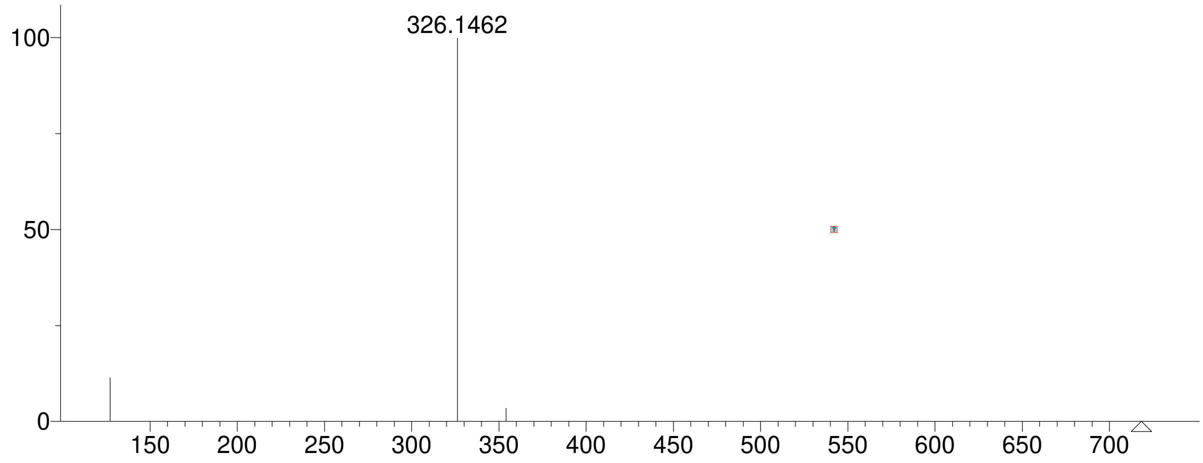

Unknown: Unknown (5.56_391.2062m/z)
Compound in Library Factor = 0

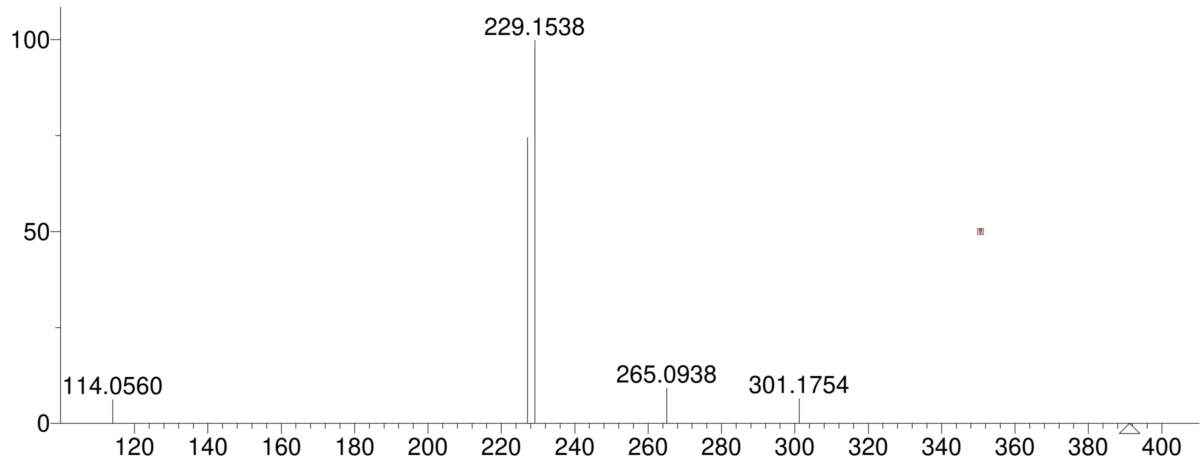

Hit 1 : Thromboxane B3 [M+Na]+ HCD 31V P=391.2
C20H32O6; MF: 0; RMF: 53; Prob 50.0%; CAS: 71953-80-5; Lib: nist_msms; ID: 85863.

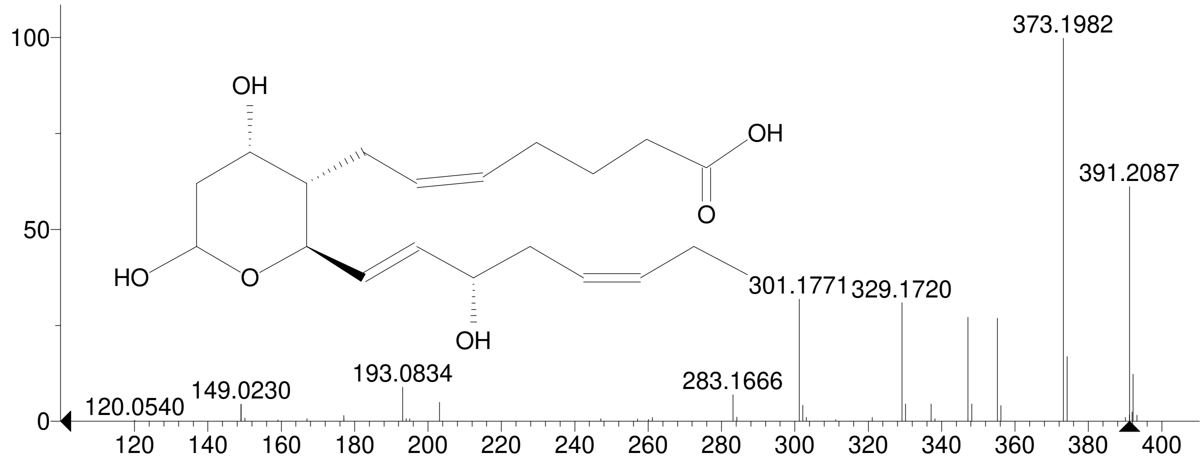

Hit 2 : Prostaglandin G2 [M+Na]+ HCD 23V P=391.2
C20H32O6; MF: 0; RMF: 12; Prob 50.0%; CAS: 51982-36-6; Lib: nist_msms; ID: 88303.

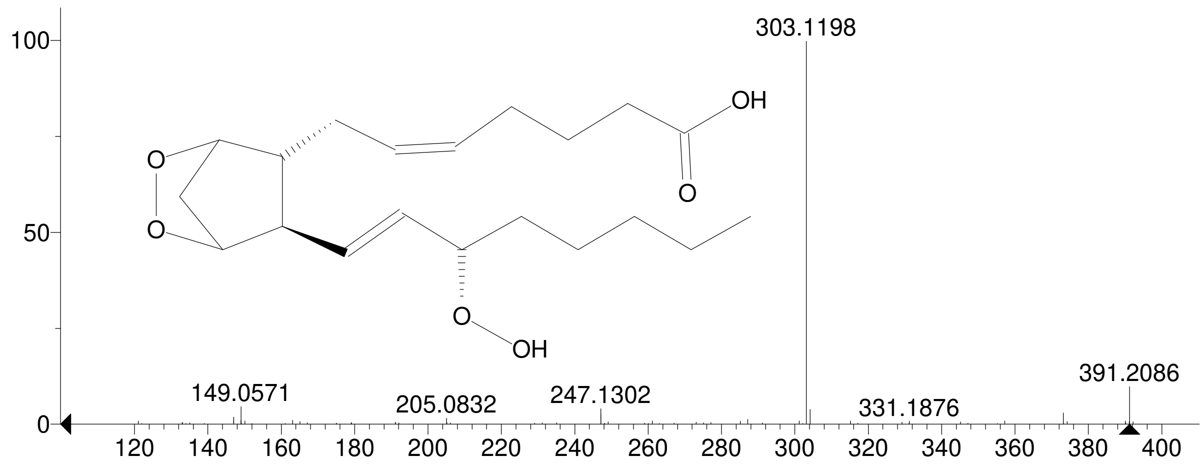

Unknown: Unknown (5.57_582.1900m/z)
Compound in Library Factor = 0

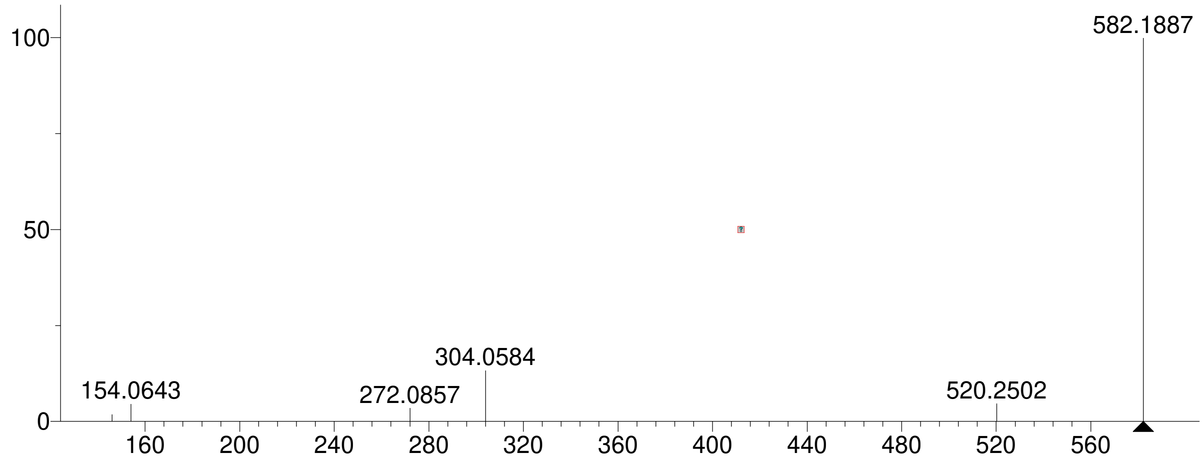

** Search Report Page 1 of 1 **

Unknown: Unknown (5.57_680.2785m/z)
Compound in Library Factor = 0

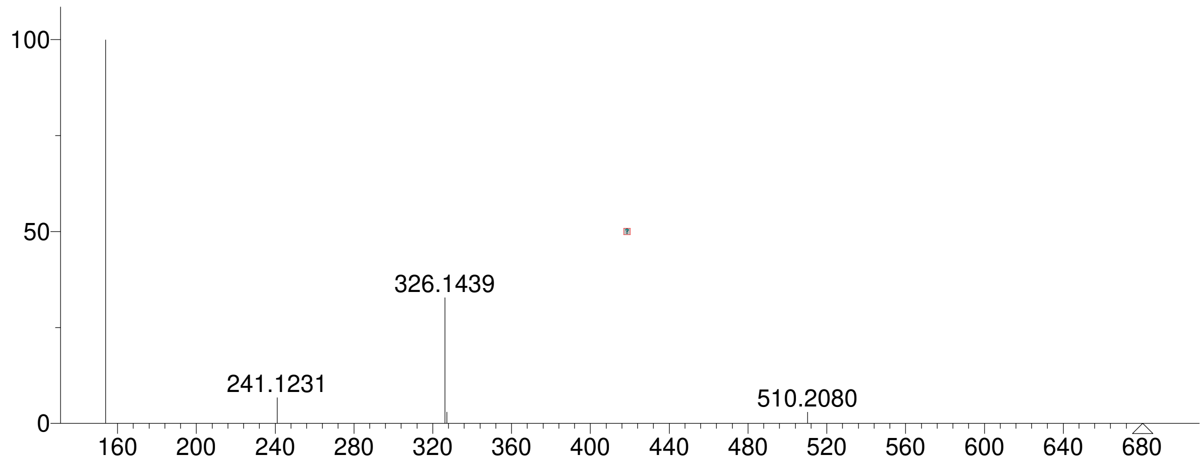

Unknown: Unknown (5.60_328.1504m/z)
Compound in Library Factor = -3273

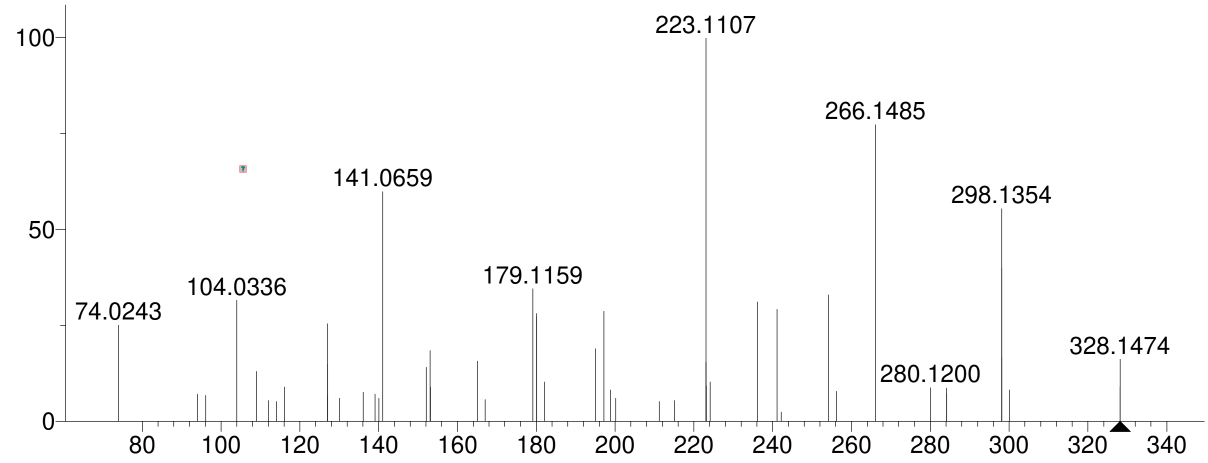

Hit 1 : Ac-Val-Ala-Asp-CHO [M-H]- HCD 11eV P=328.2
C14H23N3O6; MF: 23; RMF: 147; Prob 53.8%; Lib: nist_msms2; ID: 75914.

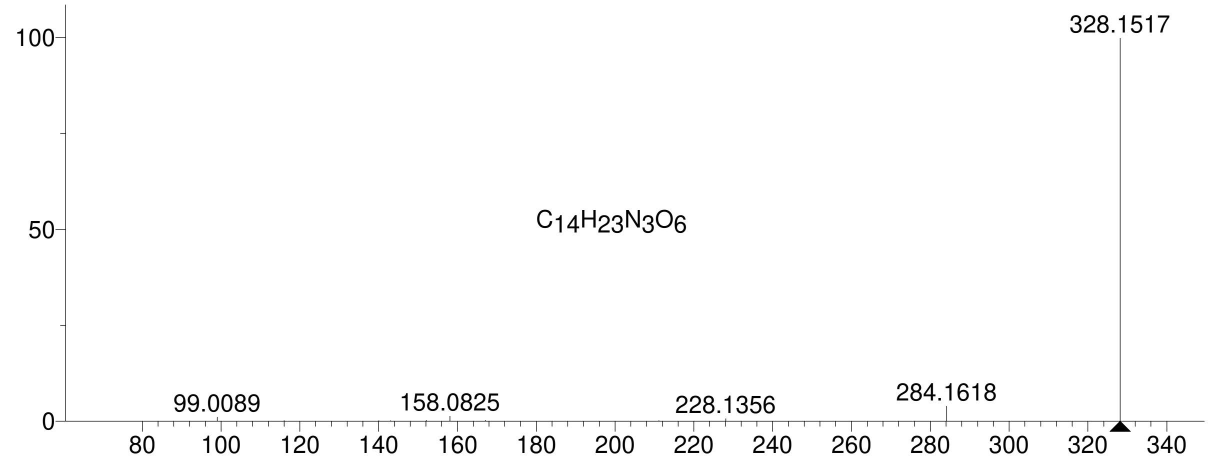

Hit 2 : Glycine, 1,1'-(1,8-dioxo-1,8-octanediyl)bis[glycyl- [M+H-C2H5NO2]+ HCD 26V P=328.2
C16H26N4O8; MF: 3; RMF: 48; Prob 24.5%; CAS: 143673-93-2; Lib: nist_msms; ID: 423558.

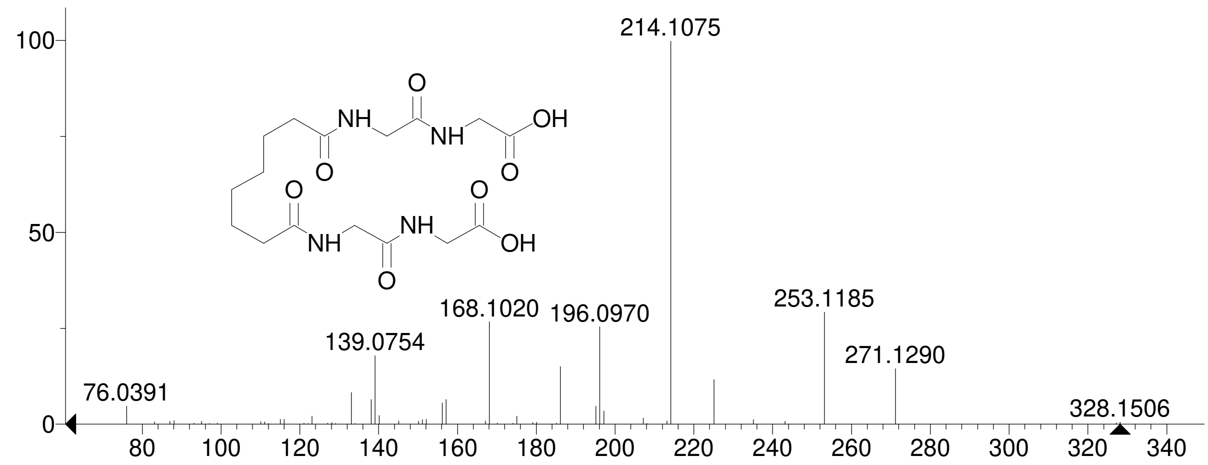

Hit 3 : MMV1088520 [M+H]+ 20-30-40%) P=328.1
C18H21N3OS; MF: 0; RMF: 78; Prob 21.7%; Lib: mona-export-pathogen_box; ID: 224.

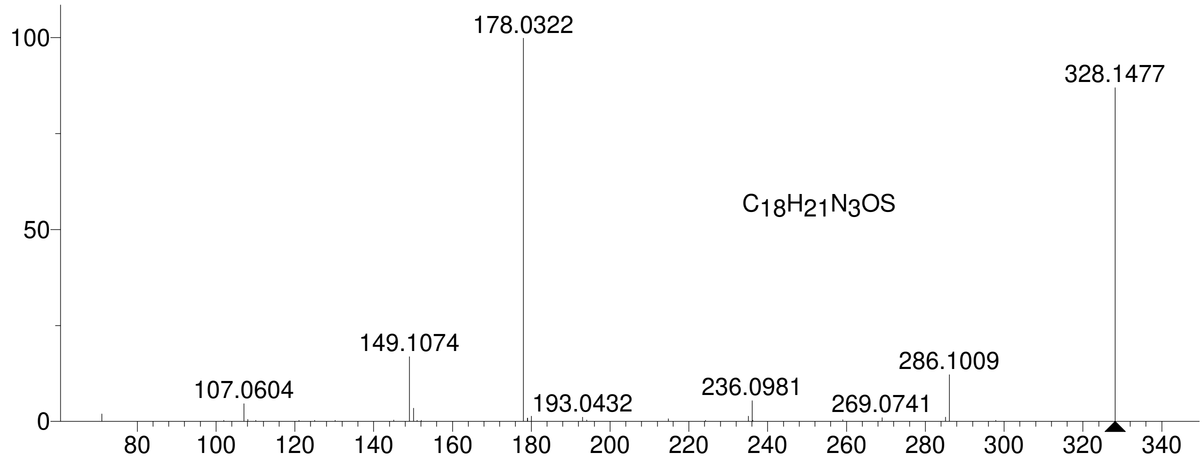

Unknown: Unknown (5.60_352.1431m/z)
Compound in Library Factor = 0

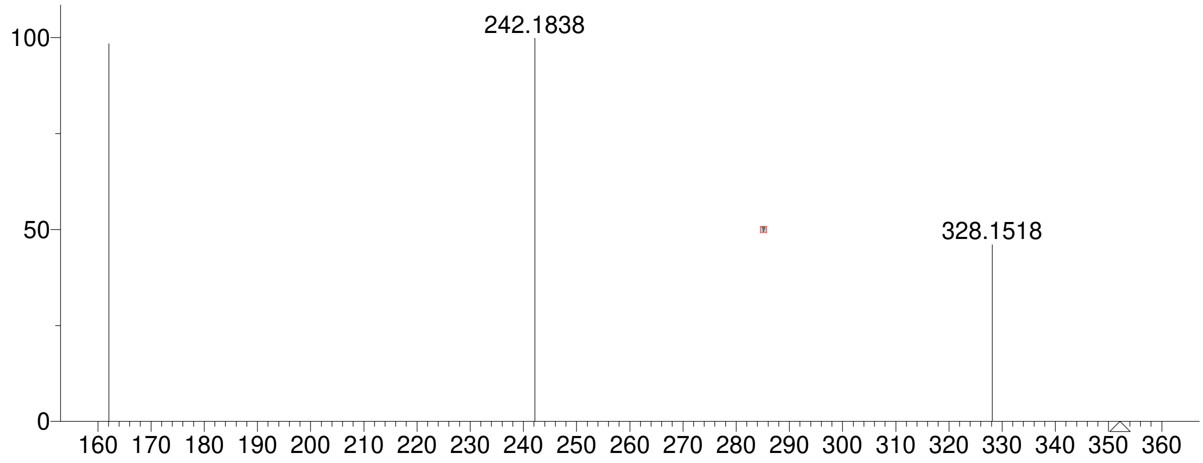

Hit 1 : Bisphenol BP [M+H-H]+ HCD 112V P=352.1
C25H20O2; MF: 0; RMF: 33; Prob 100.0%; CAS: 1844-01-5; Lib: nist_msms; ID: 403492.

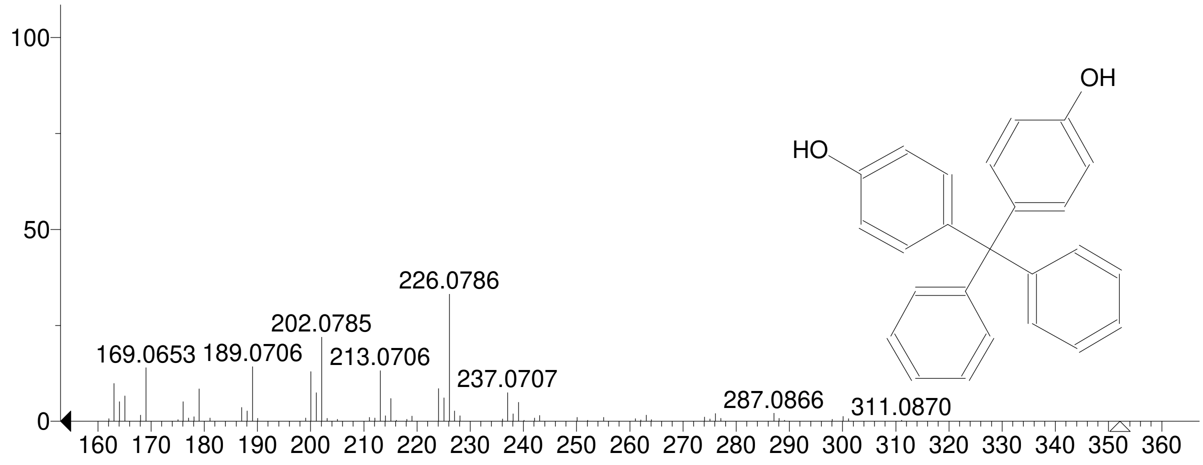

** Search Report Page 1 of 1 **

Unknown: Unknown (5.60_514.1995m/z)
Compound in Library Factor = 0

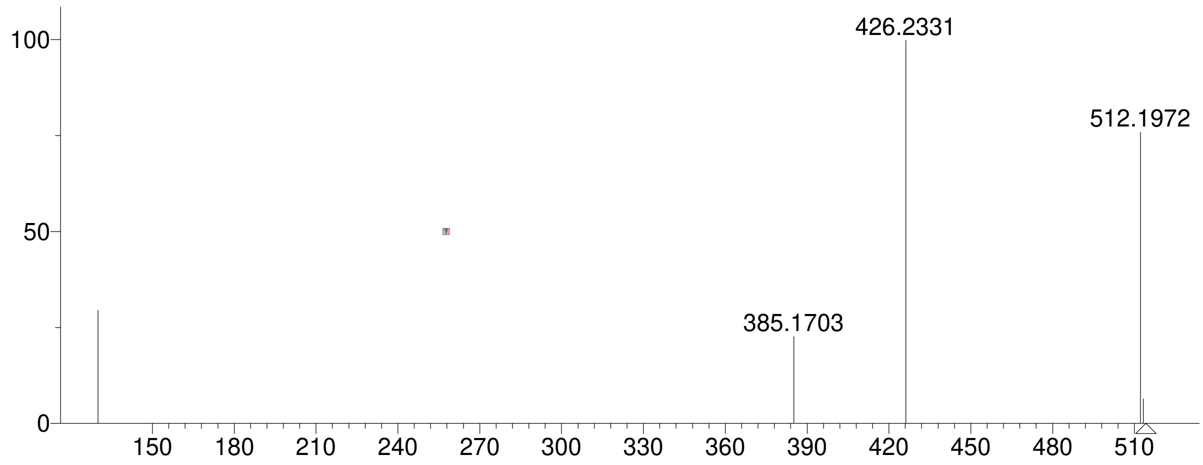

Unknown: Unknown (5.60_520.2512m/z)
Compound in Library Factor = 0

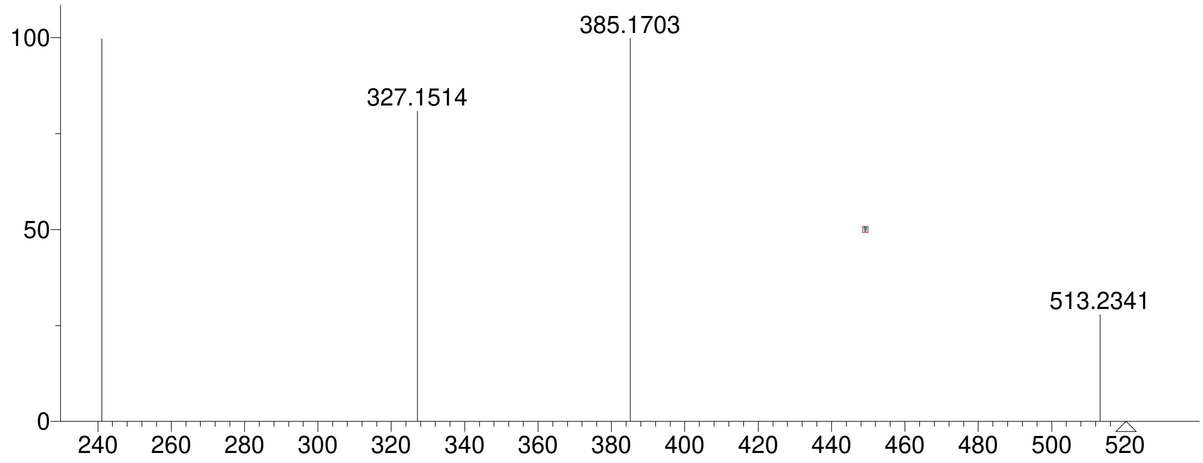

Hit 1 : NCGC00385440-01_C27H34O9_2-Butenoic acid, 2-(hydroxymethyl)-, (2E)-2-[[[(3aR,4R,6E,9S,10Z,11aS)-
C27H34O9; MF: 0; RMF: 39; Prob 100.0%; Lib: mona-export-gnps; ID: 10916.

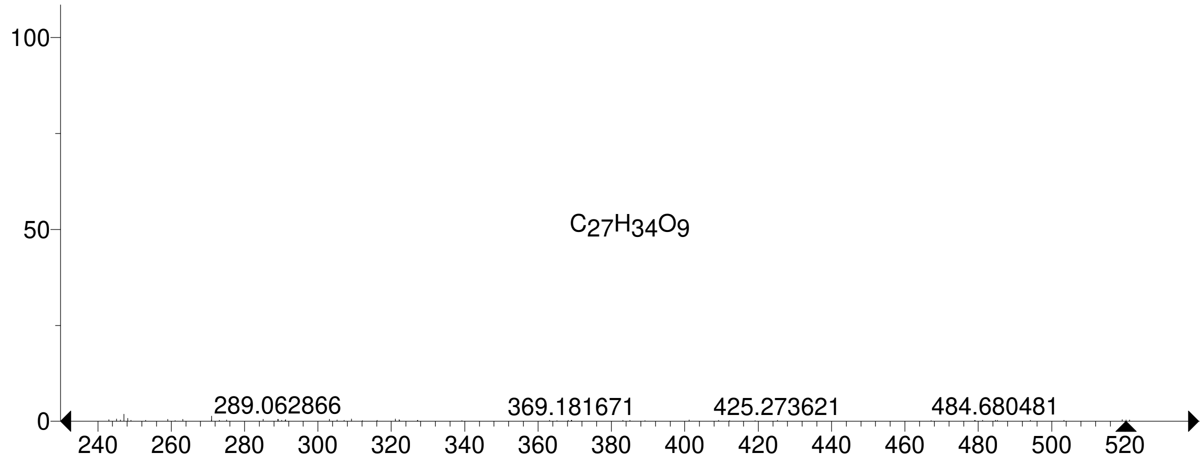

** Search Report Page 1 of 1 **

Unknown: Unknown (5.61_350.1470m/z)
Compound in Library Factor = 0

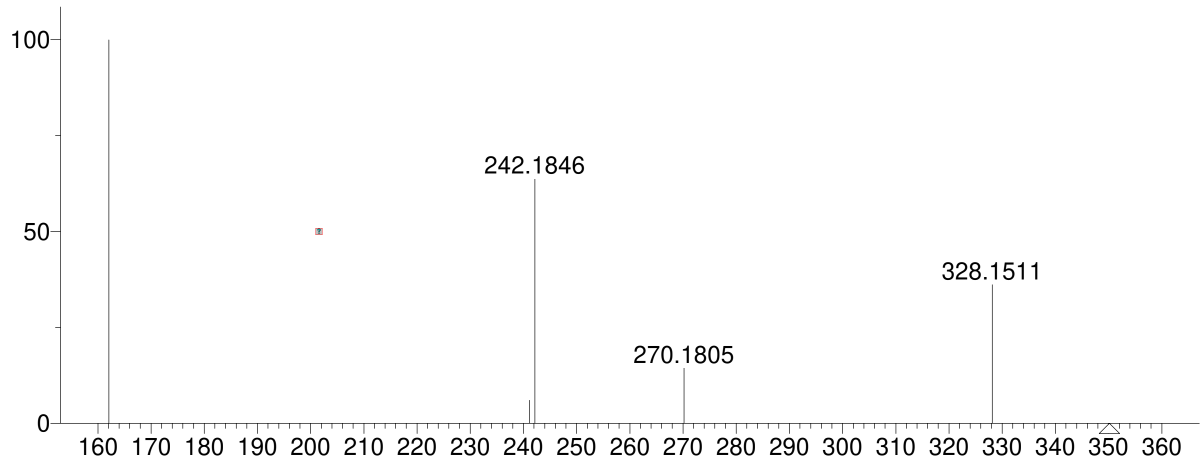

Hit 1 : 4-Methylerlotinib [M+H-C3H6O]+ HCD 112V P=350.1
C23H25N3O4; MF: 0; RMF: 57; Prob 100.0%; CAS: 1346601-52-2; Lib: nist_msms; ID: 423719.

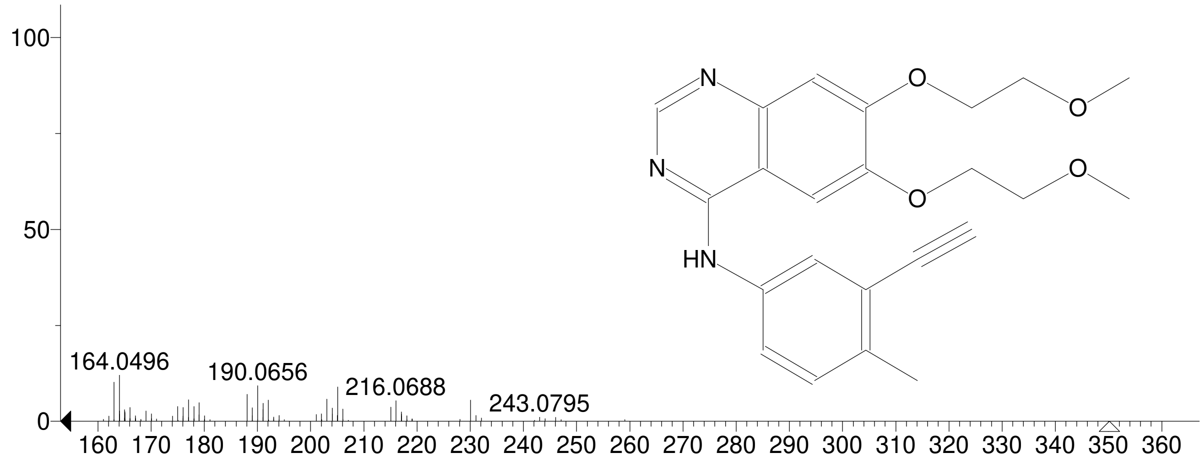

** Search Report Page 1 of 1 **

Unknown: Unknown (5.61_426.2336m/z)
Compound in Library Factor = 0

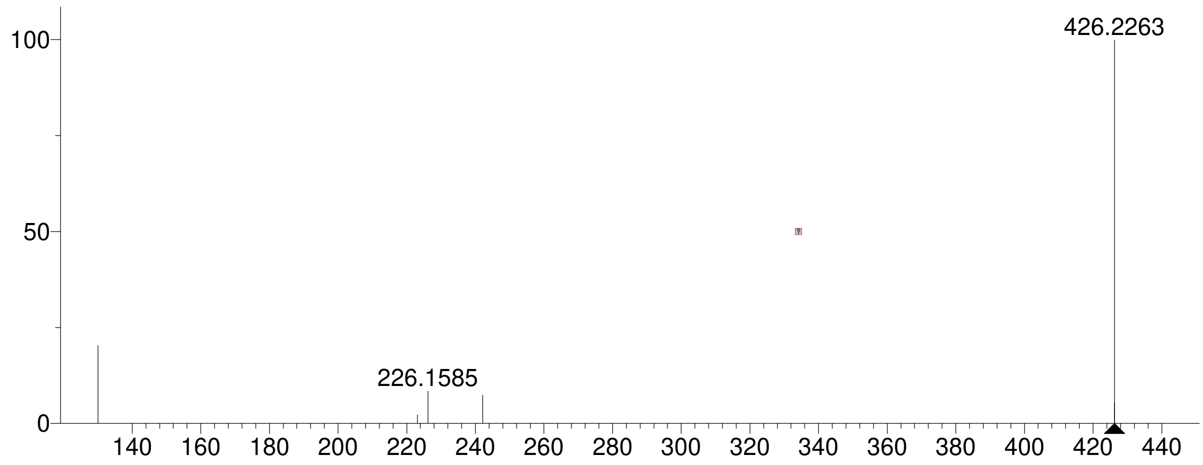

Unknown: Unknown (5.61_512.1998m/z)
Compound in Library Factor = -1142

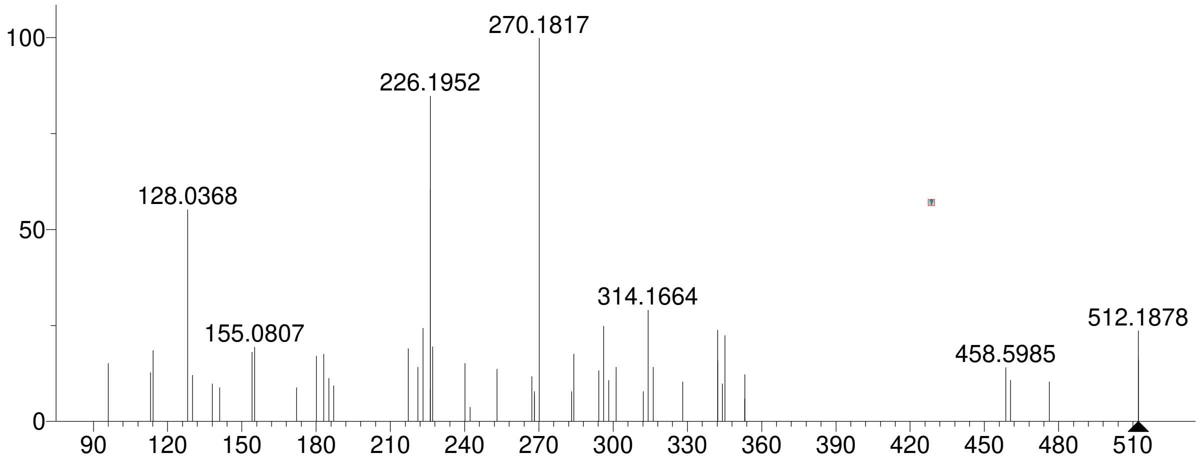

Hit 1 : Lewis X trisaccharide [M+H-H2O]+ HCD 5V P=512.2
C20H35NO15; MF: 124; RMF: 562; Prob 79.9%; CAS: 71208-06-5; Lib: nist_msms; ID: 184475.

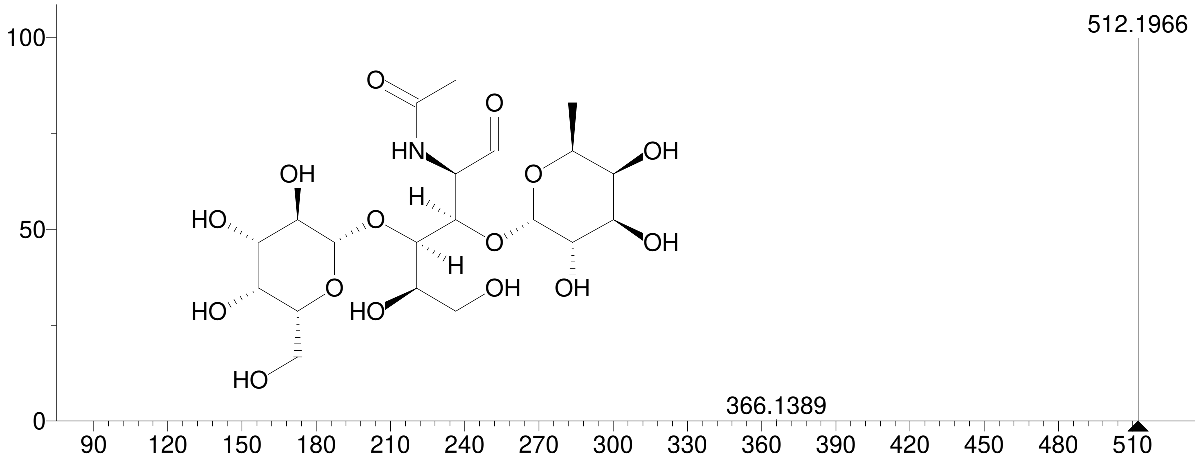

Hit 2 : Octanediamide, N1-hydroxy-N8-[4-[4-[(1-oxo-5-hexyn-1-yl)amino]benzoyl]phenyl]- [M+Cl]- HCD 17V P=[illegible]
C27H31N3O5; MF: 57; RMF: 274; Prob 12.4%; CAS: 930772-88-6; Lib: nist_msms; ID: 248435.

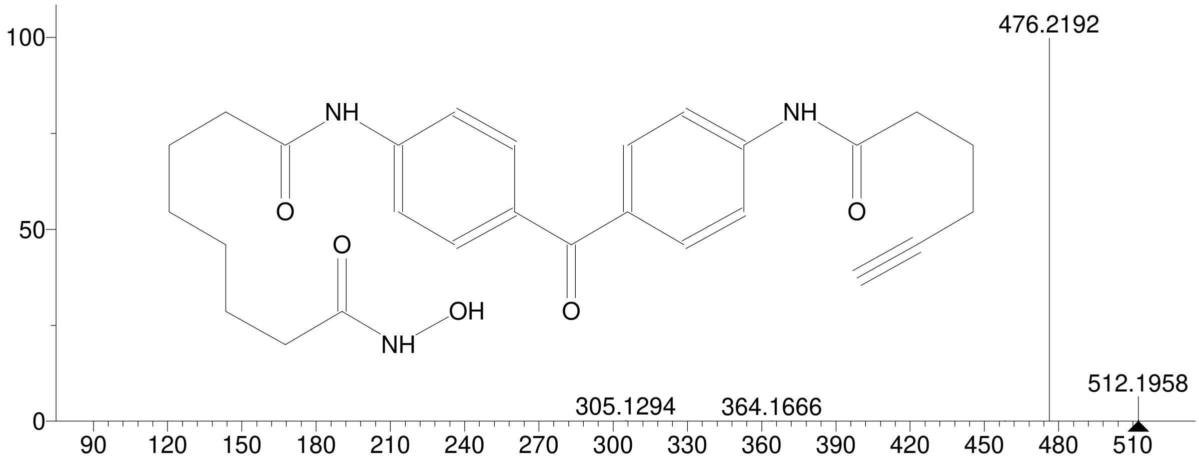

Hit 3 : Lewis A trisaccharide [M+H-H2O]+ HCD 5V P=512.2
C20H35NO15; MF: 29; RMF: 181; Prob 3.57%; CAS: 56570-03-7; Lib: nist_msms; ID: 183642.

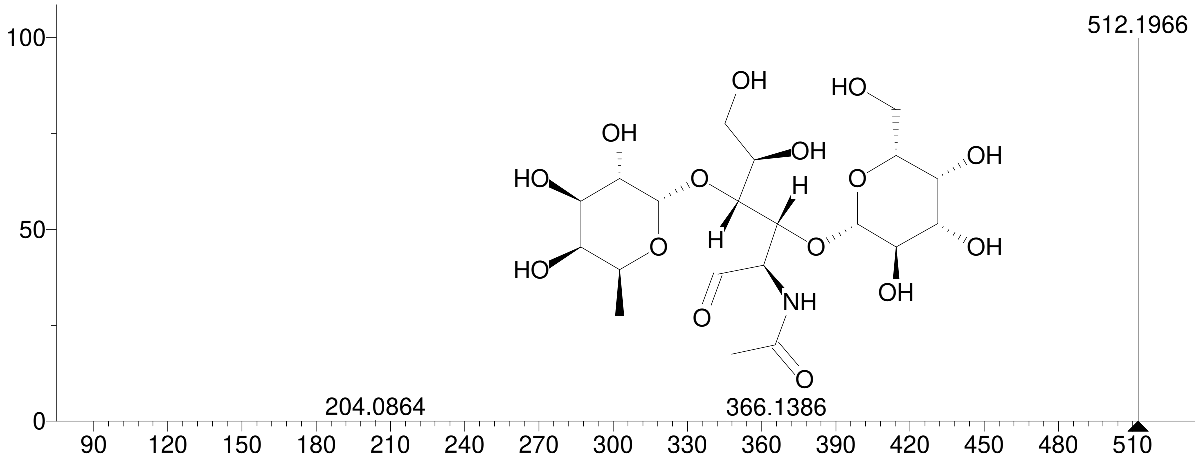

Hit 4 : Blood Group A Trisaccharide [M+H-H2O]+ HCD 5V P=512.2
C20H35NO15; MF: 9; RMF: 173; Prob 1.62%; CAS: 49777-13-1; Lib: nist_msms; ID: 184976.

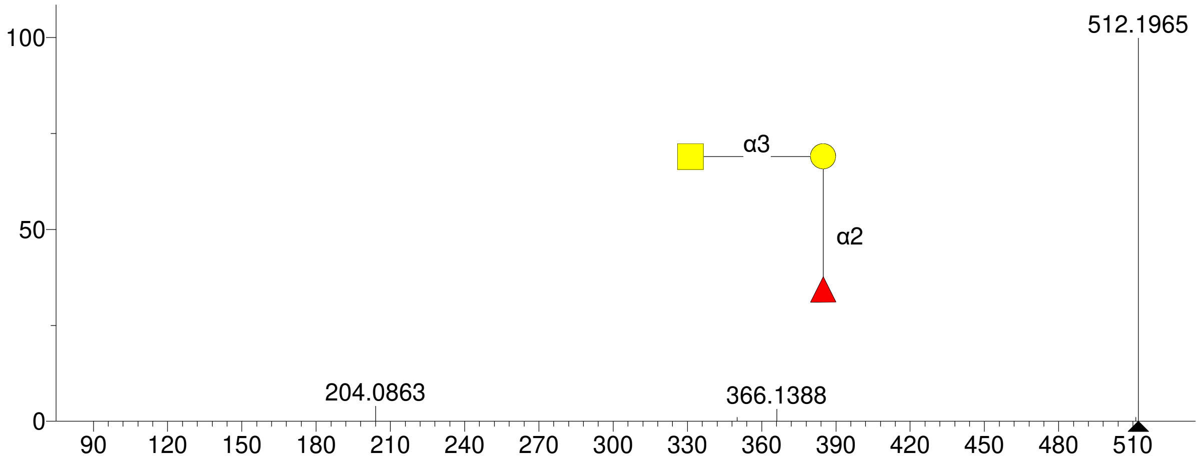

Hit 5 : Blood Group H Trisaccharide [M+H-H2O]+ HCD 10V P=512.2
C20H35NO15; MF: 6; RMF: 153; Prob 1.43%; CAS: 60797-31-1; Lib: nist_msms; ID: 182491.

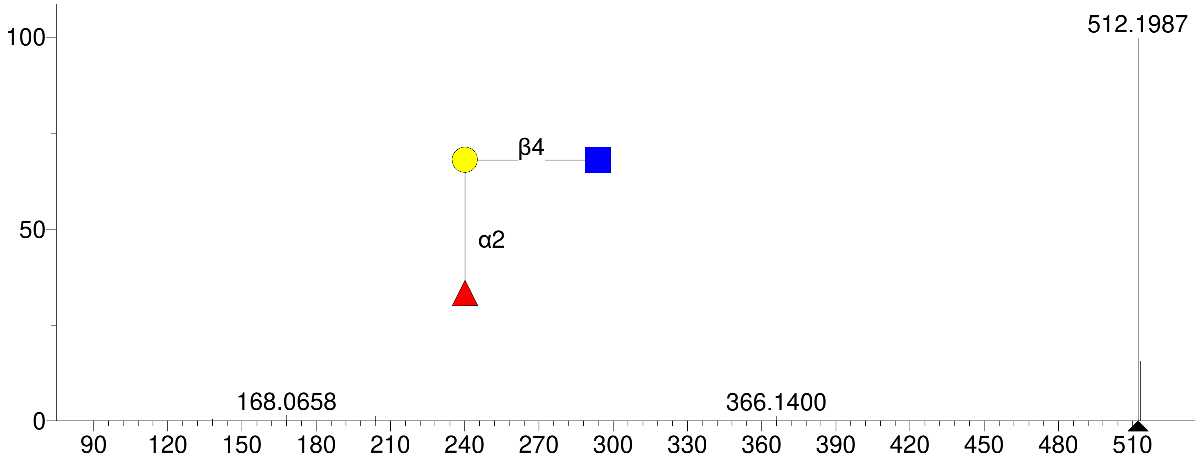

** Search Report Page 1 of 1 **

Unknown: Unknown (5.62_500.1931m/z)
Compound in Library Factor = 0

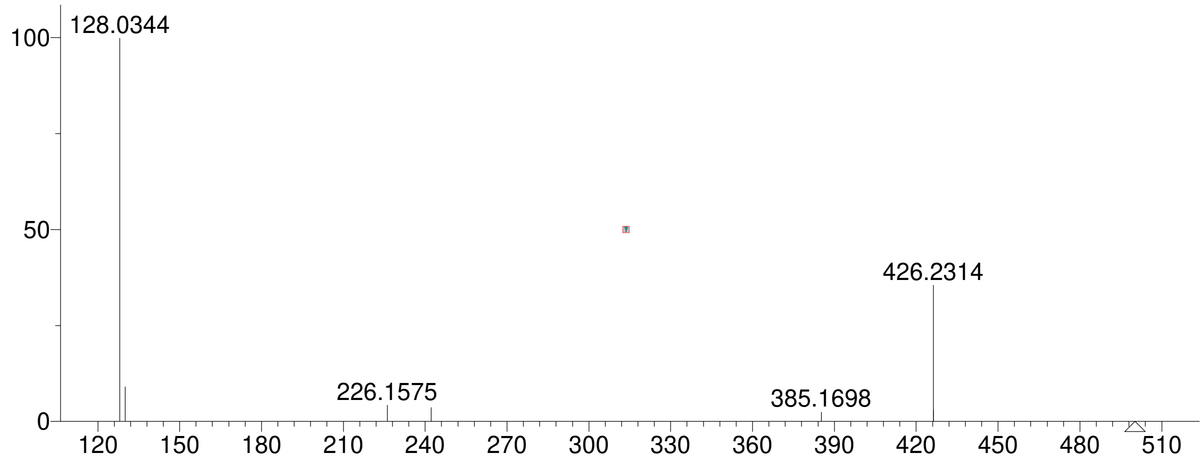

Hit 1 : N-[4,5-dihydroxy-2-[[4-[hydroxy(phenyl)methyl]-5-oxo-3-phenyl-2H-furan-2-yl]oxy]-6-(methoxymethyl)oxan-3
C26H29NO9; MF: 0; RMF: 26; Prob 50.0%; Lib: mona-export-vf-npl_qexactive; ID: 17390.

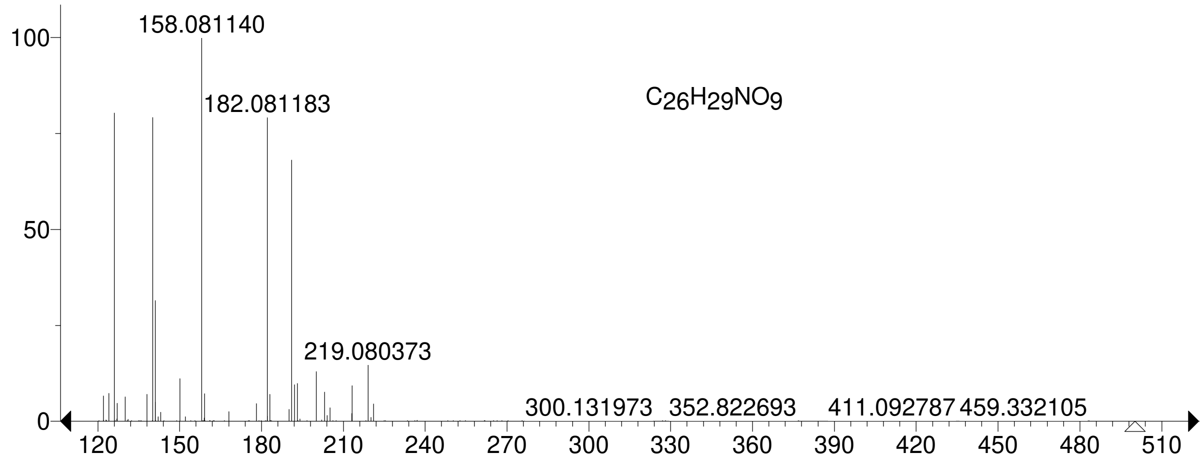

Hit 2 : NCGC00347672-02!N-[4,5-dihydroxy-2-[[4-[hydroxy(phenyl)methyl]-5-oxo-3-phenyl-2H-furan-2-yl]oxy]-6-(m
C26H29NO9; MF: 0; RMF: 19; Prob 50.0%; Lib: mona-export-gnps; ID: 9789.

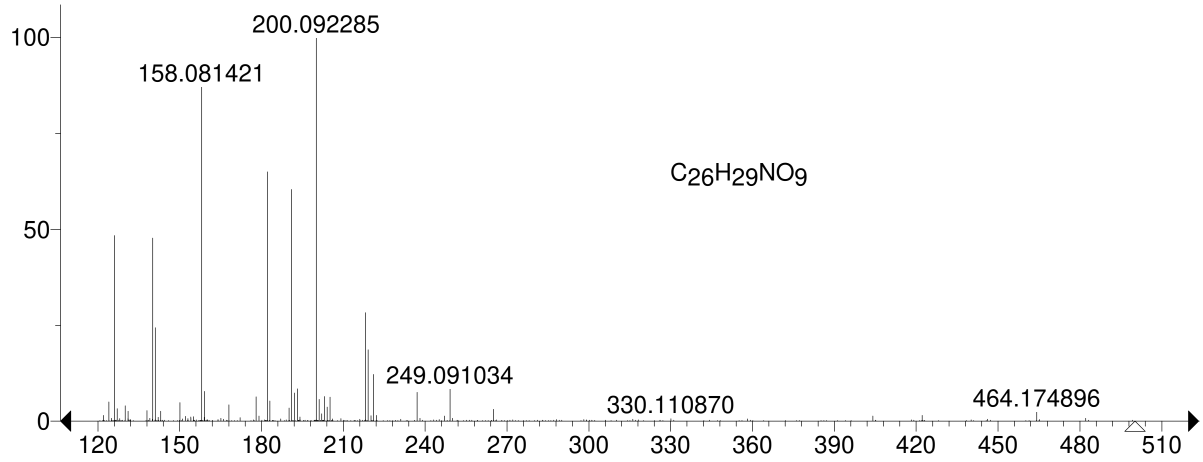

Unknown: Unknown (5.62_541.2962m/z)
Compound in Library Factor = -372

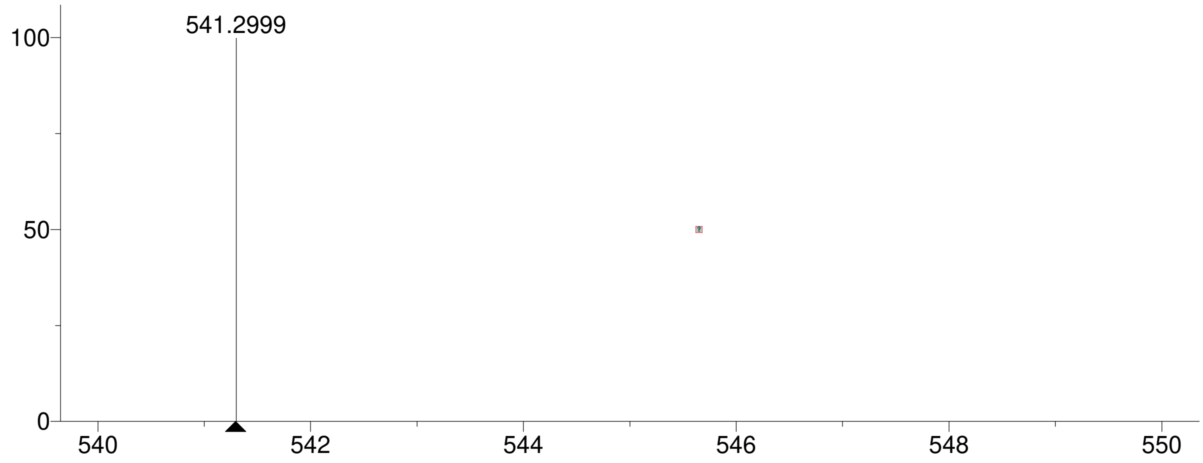

Hit 1 : Fulvestrant 9-sulfone [M-H]-=>541.3 IT 35% P=541.3
C32H47F5O4S; MF: 364; RMF: 999; Prob 16.8%; CAS: 98008-06-1; Lib: nist_msms; ID: 439666.

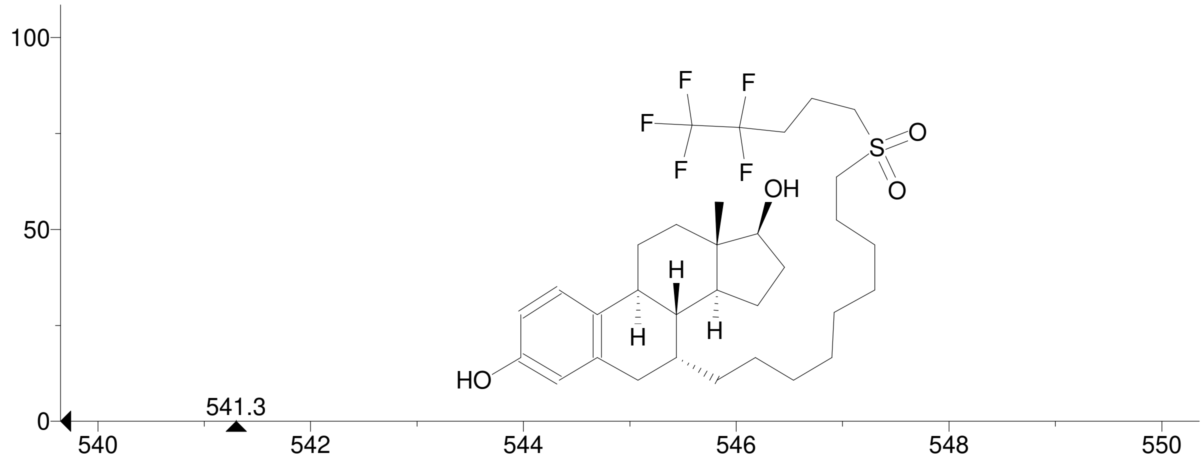

Hit 2 : PA 25:4; PA 3:0-22:4; [M-H]- [M-H]- P=541.3
C28H47O8P; MF: 364; RMF: 999; Prob 16.8%; Lib: msdial-insilicomsms-lipids-neg; ID: 315783.

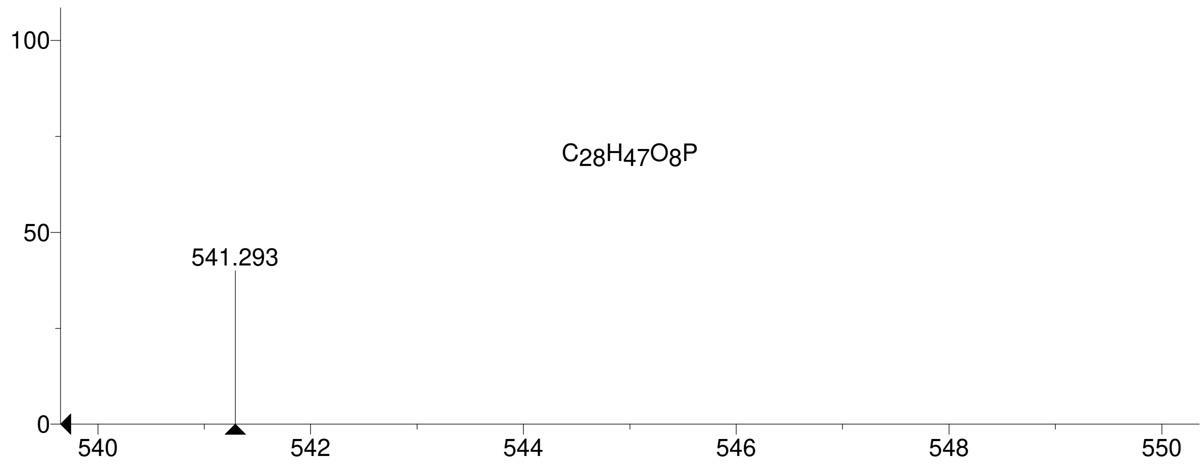

Hit 3 : PA 25:4; PA 5:0-20:4; [M-H]- [M-H]- P=541.3
C28H47O8P; MF: 364; RMF: 999; Prob 16.8%; Lib: msdial-insilicomsms-lipids-neg; ID: 315891.

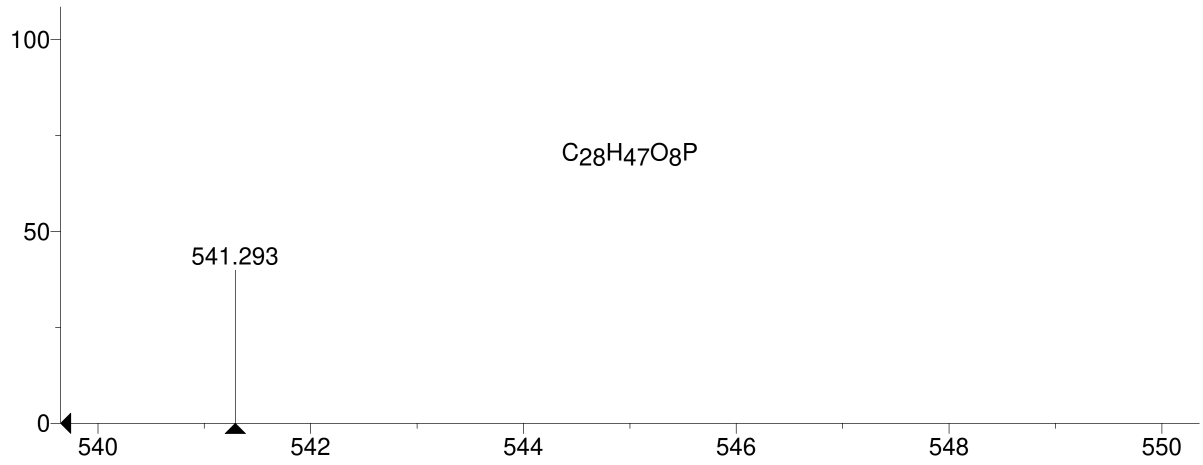

Hit 4 : PA 25:4; PA 7:0-18:4; [M-H]- [M-H]- P=541.3
C28H47O8P; MF: 364; RMF: 999; Prob 16.8%; Lib: msdial-insilicomsms-lipids-neg; ID: 315995.

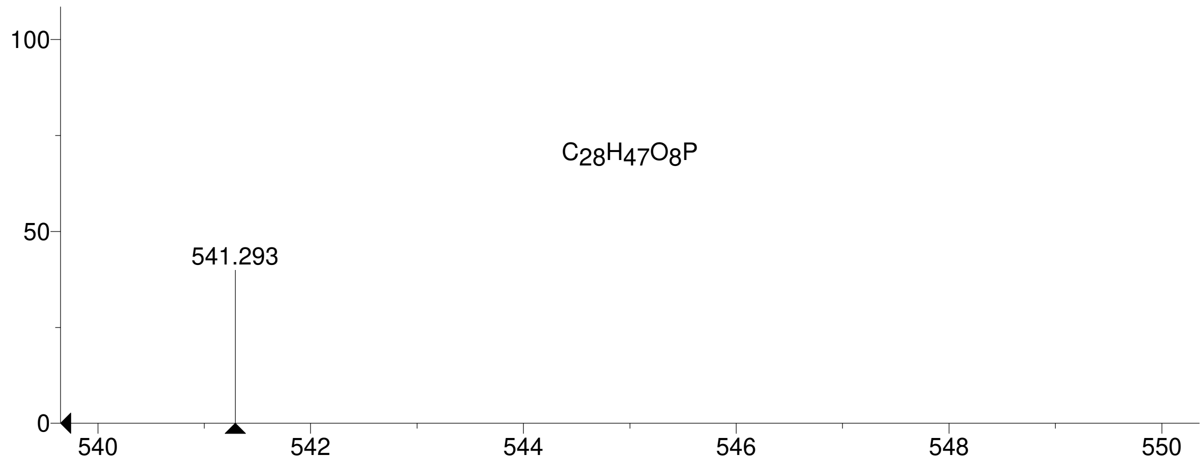

Hit 5 : PA 25:4; PA 9:0-16:4; [M-H]- [M-H]- P=541.3
C28H47O8P; MF: 364; RMF: 999; Prob 16.8%; Lib: msdial-insilicomsms-lipids-neg; ID: 316096.

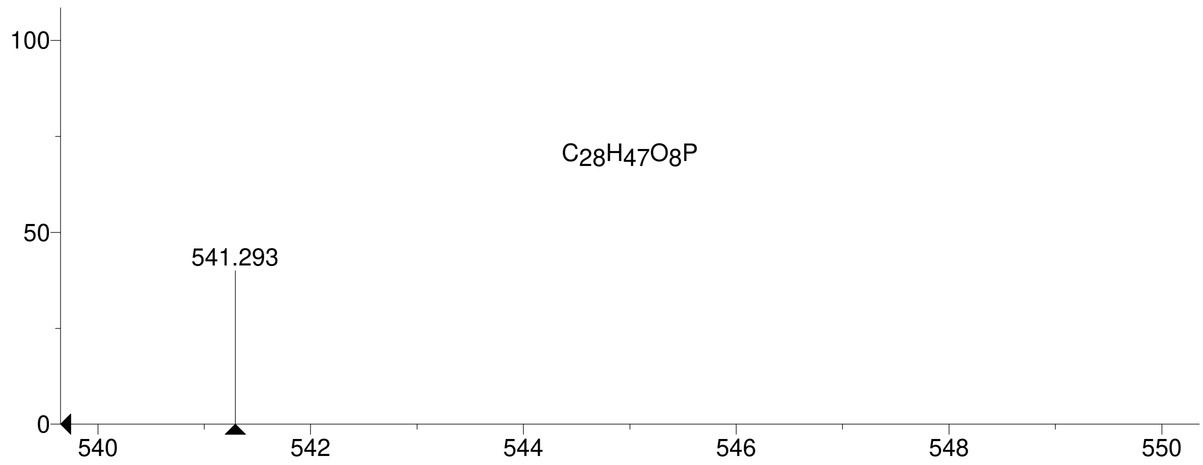

Unknown: Unknown (5.63_490.2380m/z)
Compound in Library Factor = 0

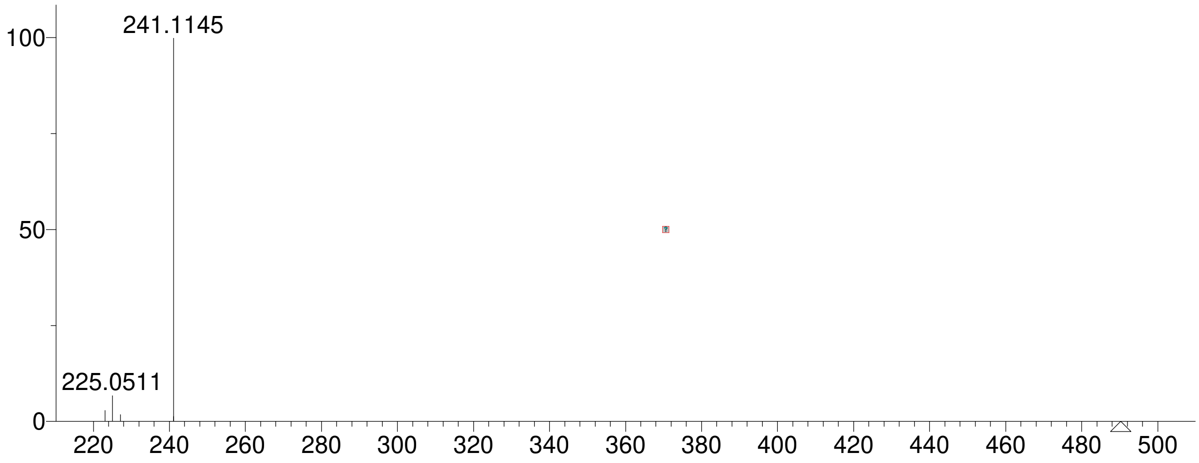

Unknown: Unknown (5.64_369.1765m/z)
Compound in Library Factor = -1090

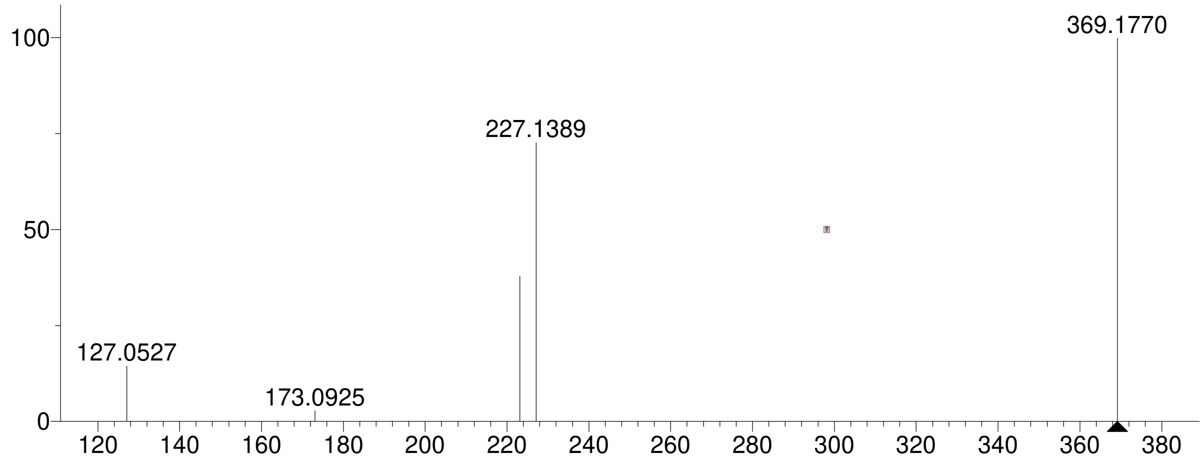

Hit 1 : 5β-Androstan-3α-ol-17-one sulfate [M-H]- HCD 33V P=369.2
C19H30O5S; MF: 209; RMF: 662; Prob 40.8%; CAS: 977-36-6; Lib: nist_msms; ID: 231644.

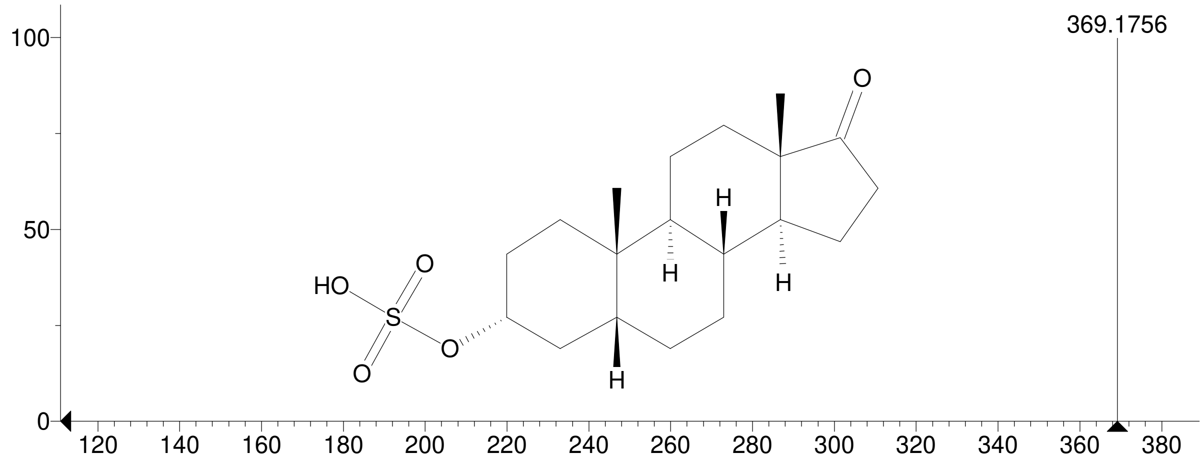

Hit 2 : 5-Androsten-3β,17β-diol-3-sulfate [M-H]- HCD 27V P=369.2
C19H30O5S; MF: 209; RMF: 662; Prob 40.8%; CAS: 977-33-3; Lib: nist_msms; ID: 231655.

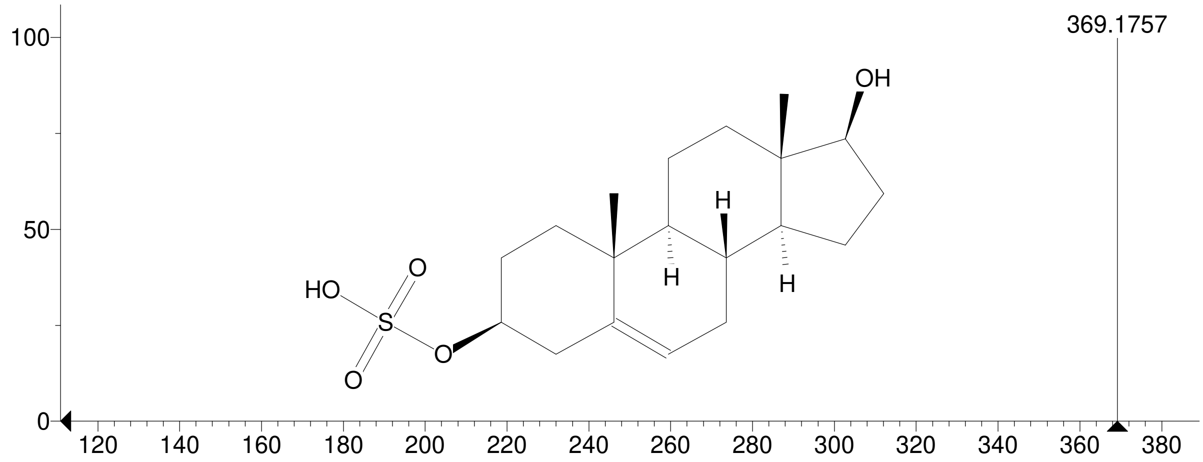

Hit 3 : 5α-Androstan-3β-ol-17-one sulfate [M-H]- HCD 40V P=369.2
C19H30O5S; MF: 172; RMF: 661; Prob 9.92%; CAS: 977-35-5; Lib: nist_msms; ID: 231625.

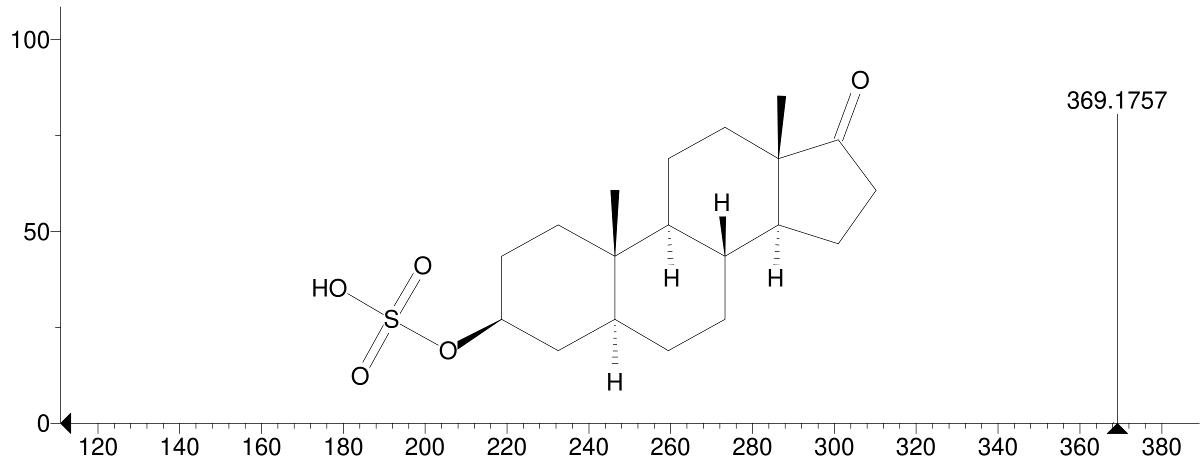

Hit 4 : 5α-Androstan-17β-ol-3-one sulfate [M-H]- HCD 40V P=369.2
C19H30O5S; MF: 160; RMF: 658; Prob 6.60%; CAS: 2641-48-7; Lib: nist_msms; ID: 231920.

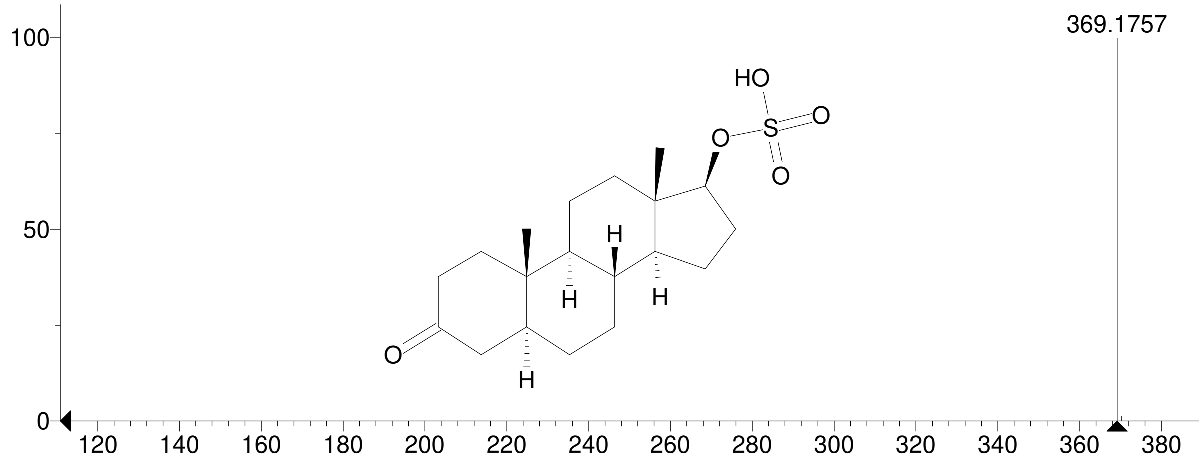

Hit 5 : 4-Androsten-17β-ol-3-one sulfate [M+H]+ HCD 14V P=369.2
C19H28O5S; MF: 100; RMF: 653; Prob 1.17%; CAS: 651-45-6; Lib: nist_msms; ID: 231503.

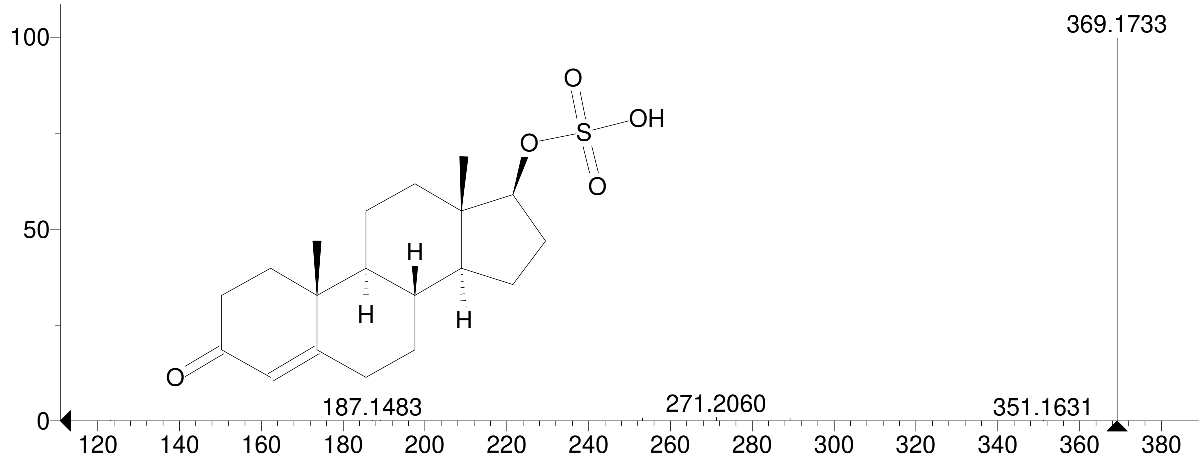

Unknown: Unknown (5.66_443.1945m/z)
Compound in Library Factor = -2387

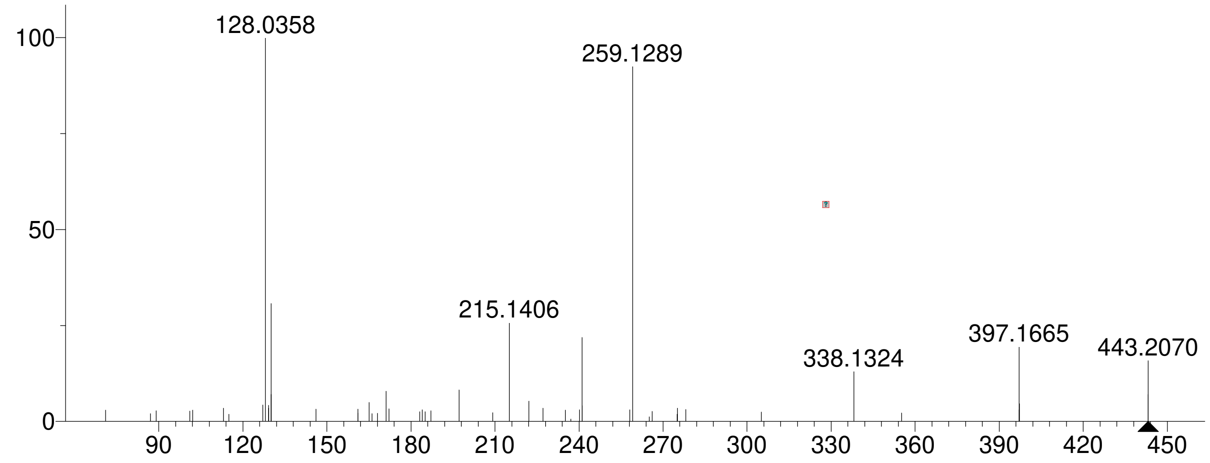

Hit 1 : Mitoxantrone [M-H]- HCD 17V P=443.2
C22H28N4O6; MF: 28; RMF: 504; Prob 27.0%; CAS: 65271-80-9; Lib: nist_msms; ID: 343812.

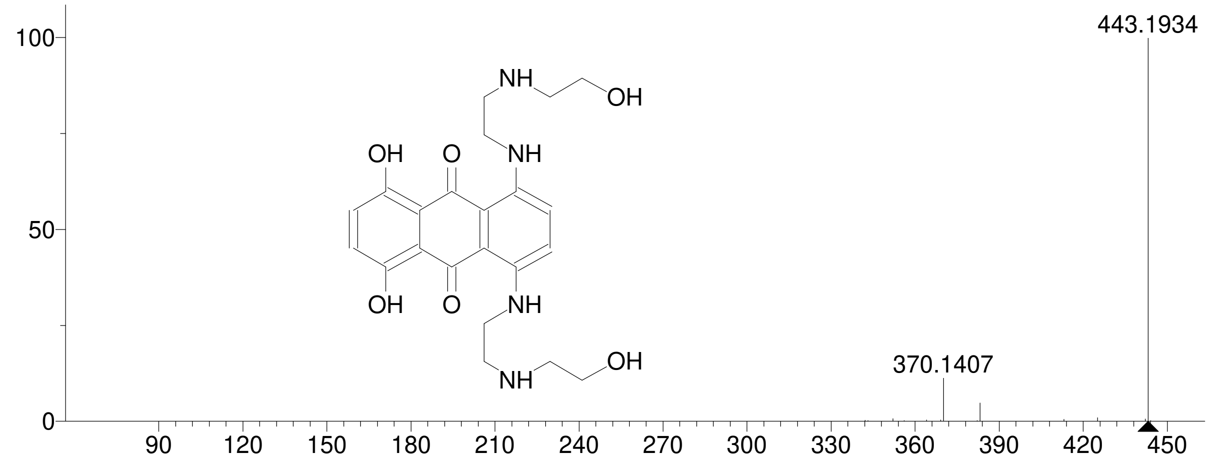

Hit 2 : C21H32O10; PlaSMA ID-1175 [M-H]- P=443.2
C21H32O10; MF: 16; RMF: 175; Prob 18.0%; Lib: biomsms-neg-plasma; ID: 867.

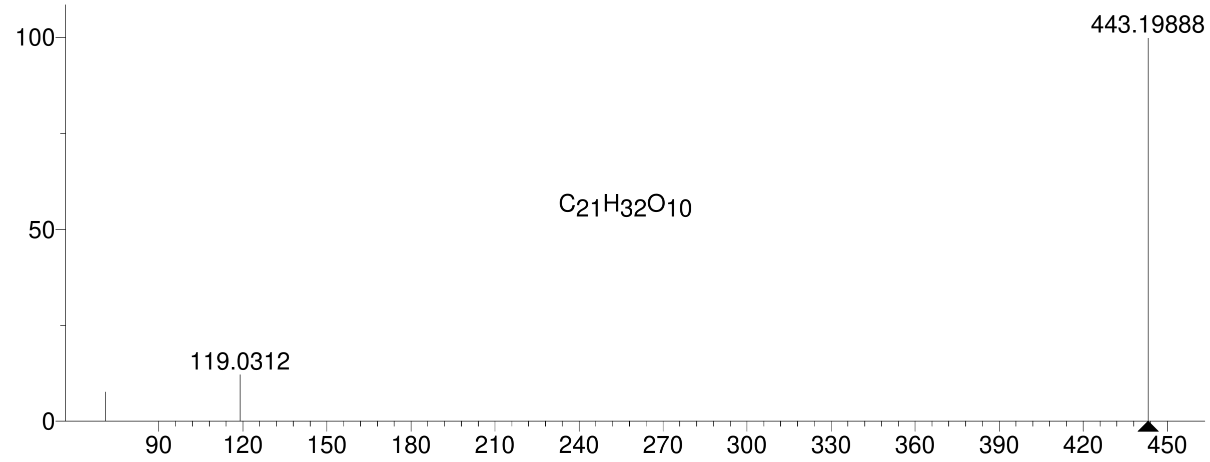

Hit 3 : N(6)-((Aminoethylamino)carbonyl)methylphenyladenosine [M-H-C5H8O4]- HCD 22V P=443.2
C28H32N8O6; MF: 5; RMF: 201; Prob 12.3%; CAS: 96760-69-9; Lib: nist_msms; ID: 298641.

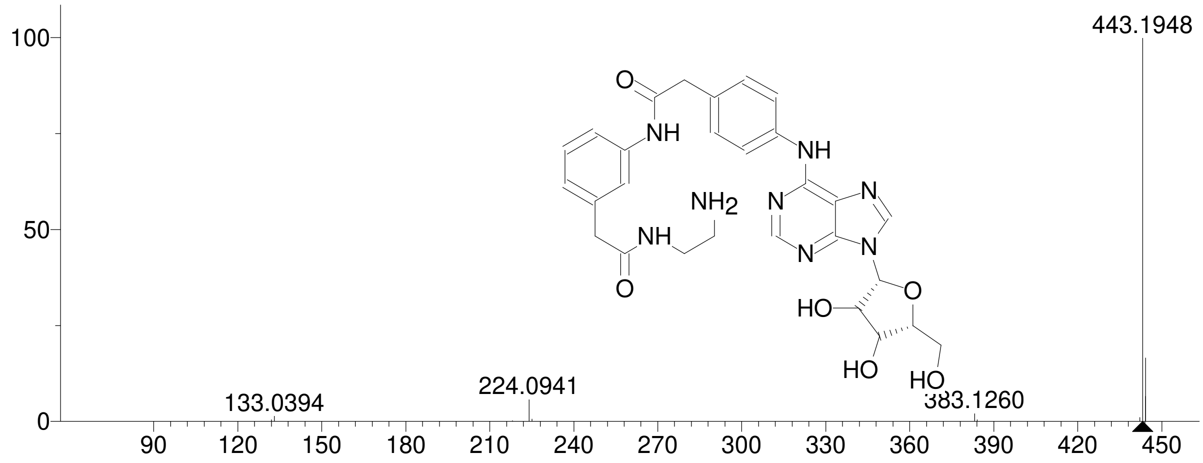

Hit 4 : [4,5,8-trihydroxy-5-(hydroxymethyl)-3-(3-methoxy-3-oxoprop-1-en-2-yl)-8a-methyl-1,2,3,4,4a,6,7,8-octahyd
C21H32O10; MF: 5; RMF: 78; Prob 12.3%; Lib: mona-export-lc-ms-ms_negative_mode; ID: 22850.

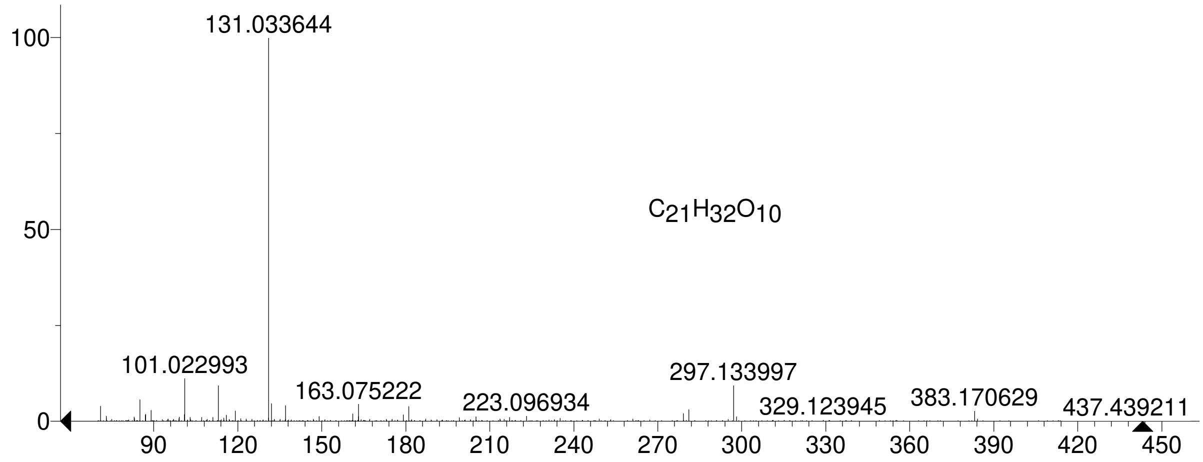

Hit 5 : β-Lipotropin (61-64) [M+H]+ IT 35% P=443.2
C22H26N4O6; MF: 1; RMF: 83; Prob 10.4%; Lib: nist_msms2; ID: 1128.

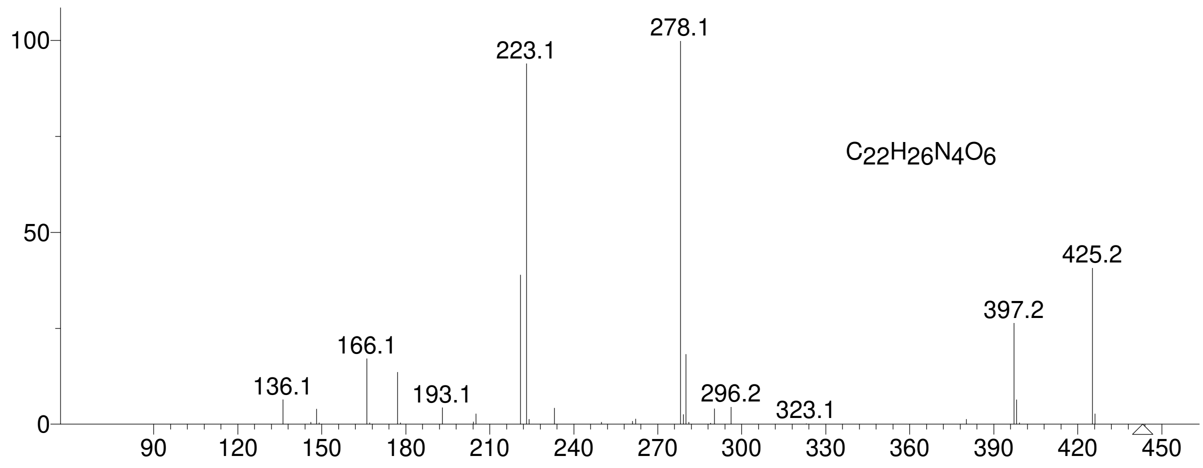

Unknown: Unknown (5.67_344.1802m/z)
Compound in Library Factor = 0

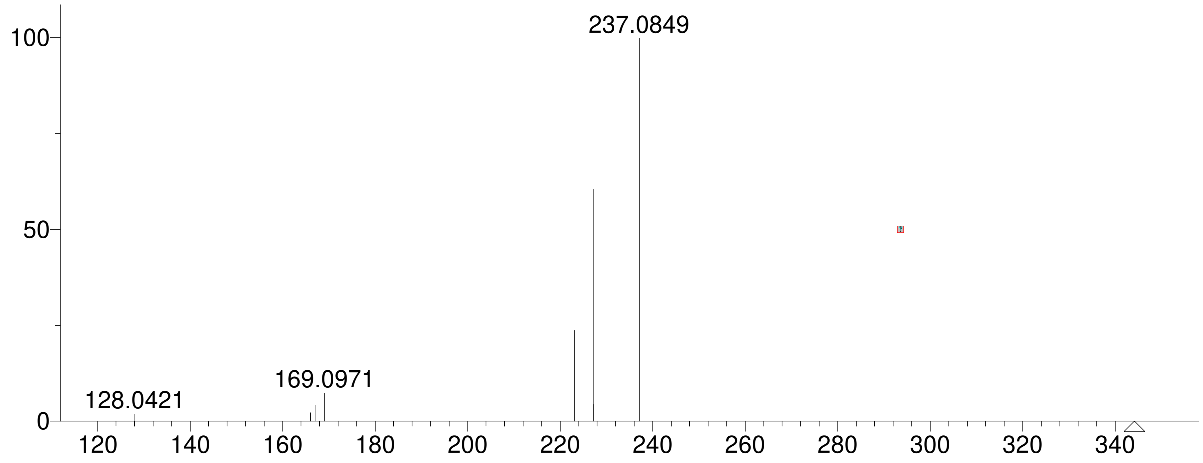

Hit 1 : Clemastine Fumarate [M+H]+ P=344.2
C25H30ClNO5; MF: 0; RMF: 6; Prob 100.0%; Lib: mona-export-gnps; ID: 639.

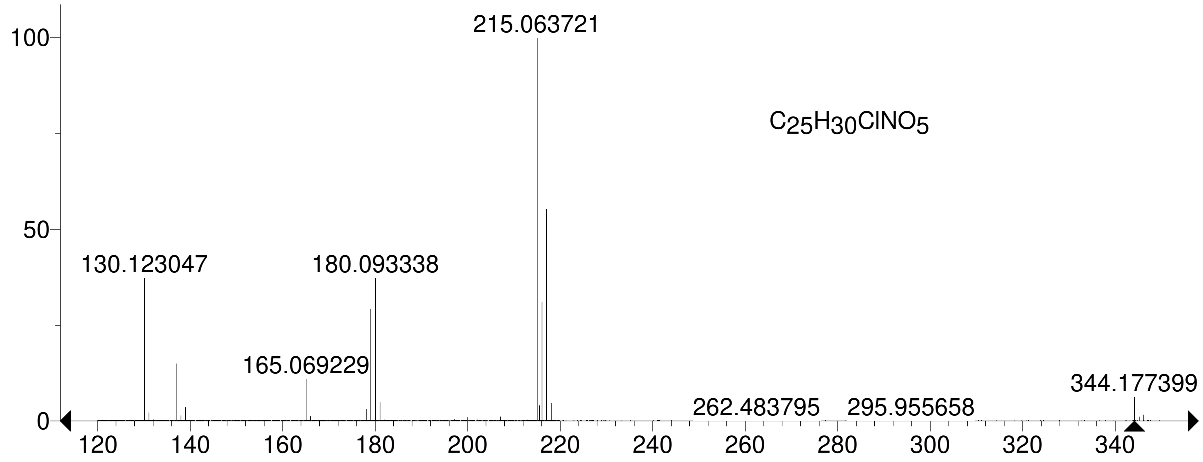

Unknown: Unknown (5.67_421.1592m/z)
Compound in Library Factor = 0

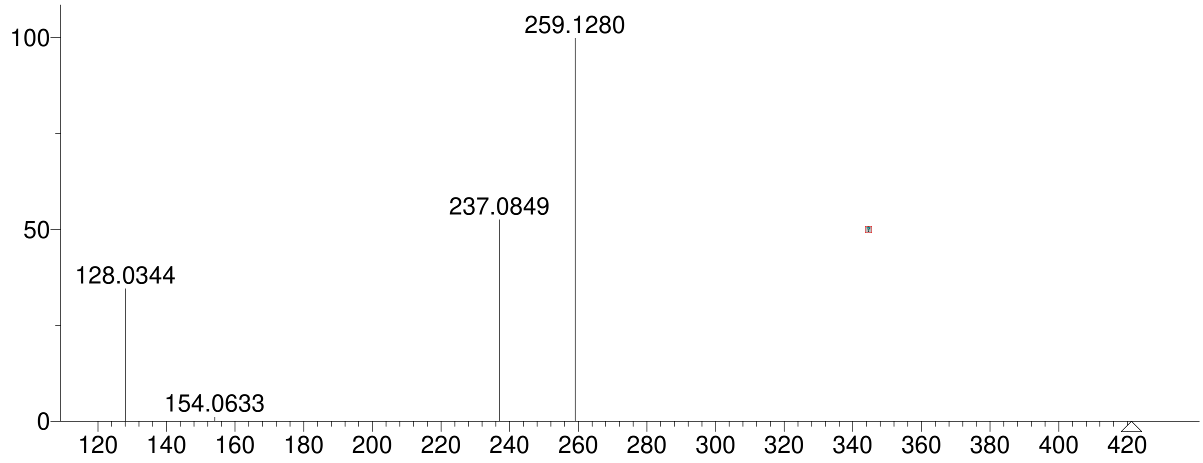

Hit 1 : 13(R),14(R)-Epoxyfluprostenol isopropyl ester [M+H-C3H12O3]+ IT-FT 35% P=421.2
C26H35F3O7; MF: 0; RMF: 200; Prob 33.3%; Lib: nist_msms; ID: 254119.

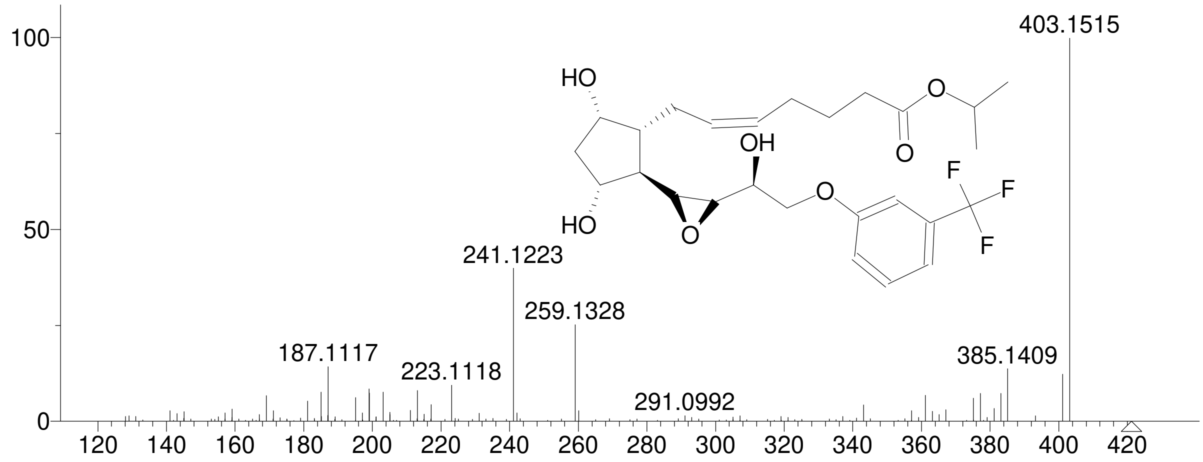

Hit 2 : 2-[[(2R,3S)-2-(3,4-dihydroxyphenyl)-3,5-dihydroxy-3,4-dihydro-2H-chromen-7-yl]oxy]oxane-3,4,5-triol [M-H
C20H22O10; MF: 0; RMF: 65; Prob 33.3%; Lib: mona-export-itree; ID: 2026.

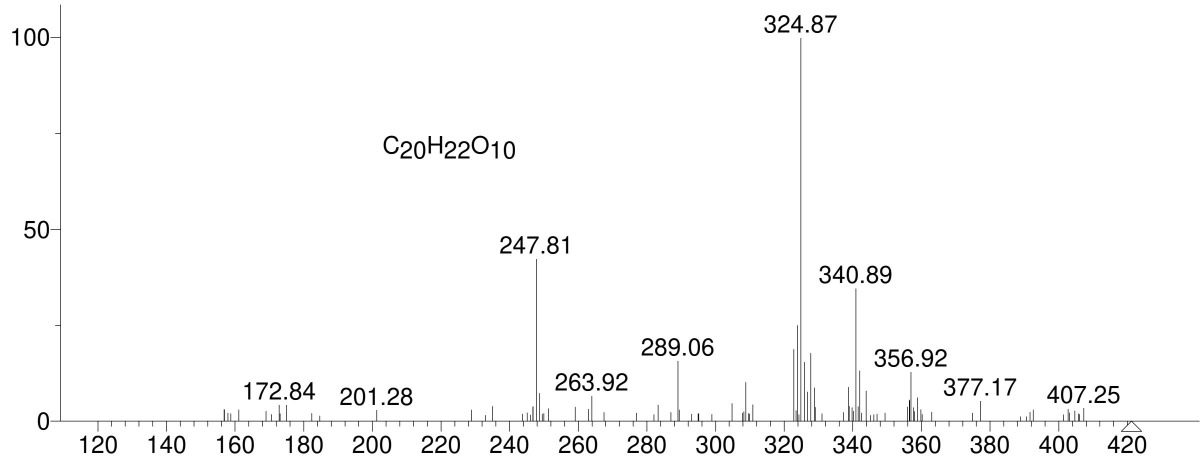

Hit 3 : 2-amino-5-[2-[[(3S)-2,3-dihydroxy-2-[(1S)-1-hydroxyethyl]butanoyl]oxymethyl]anilino]-5-oxopentanoic acid
C18H26N2O8; MF: 0; RMF: 5; Prob 33.3%; Lib: mona-export-vf-npl_qexactive; ID: 16602.

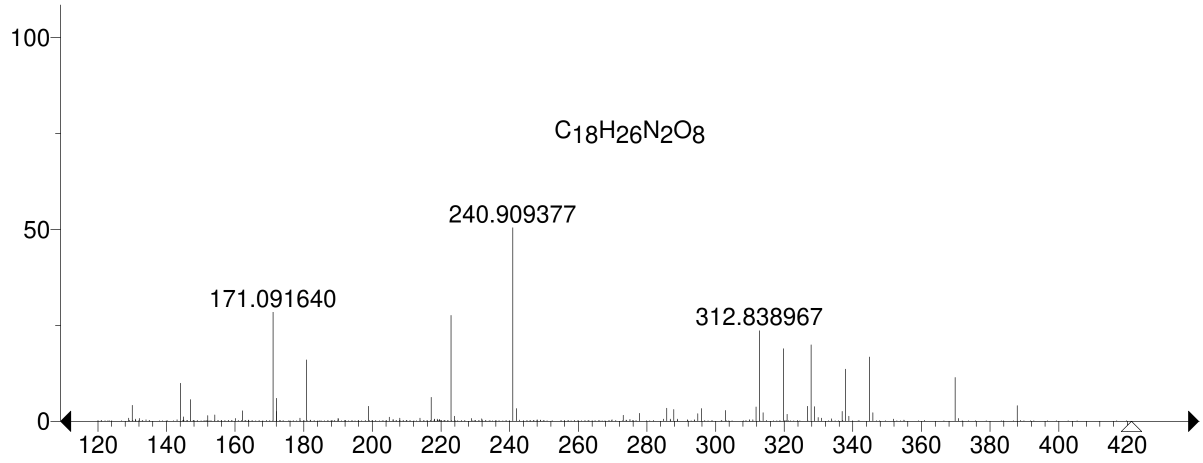

** Search Report Page 1 of 1 **

Unknown: Unknown (5.68_376.1046m/z)
Compound in Library Factor = -3273

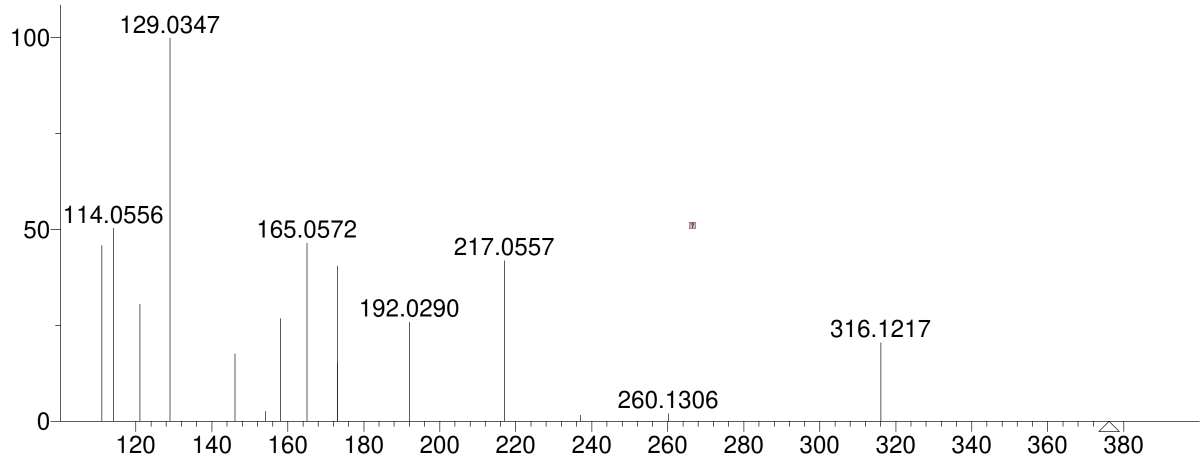

Hit 1 : 1-(3,4-dihydroxyphenyl)-6,7-dihydroxy-1,2-dihydronaphthalene-2,3-dicarboxylic acid [M+NH4]+ 45HCD F
C18H14O8; MF: 1; RMF: 65; Prob 100.0%; Lib: mona-export-vf-npl_qexactive; ID: 24902.

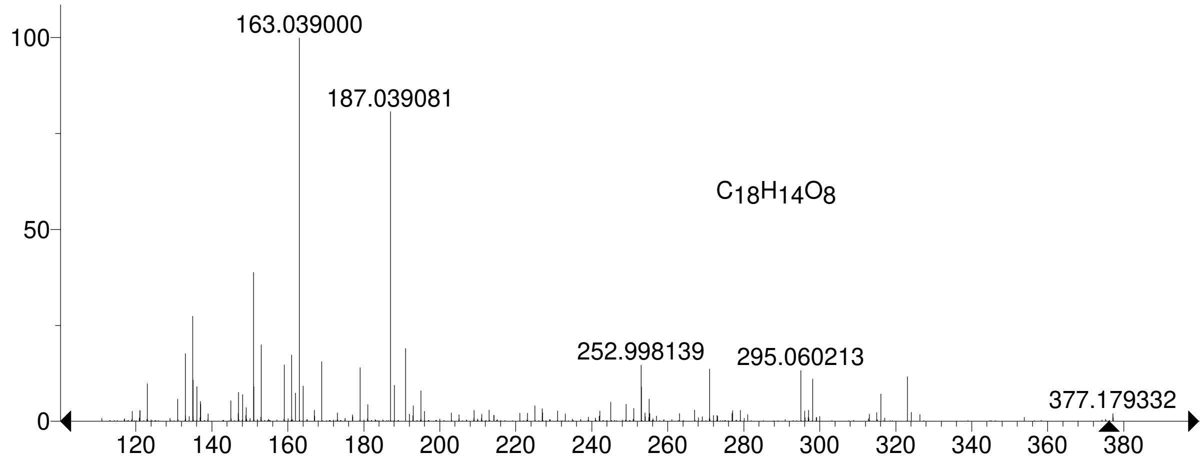

Unknown: Unknown (5.68_380.0386m/z)
Compound in Library Factor = 0

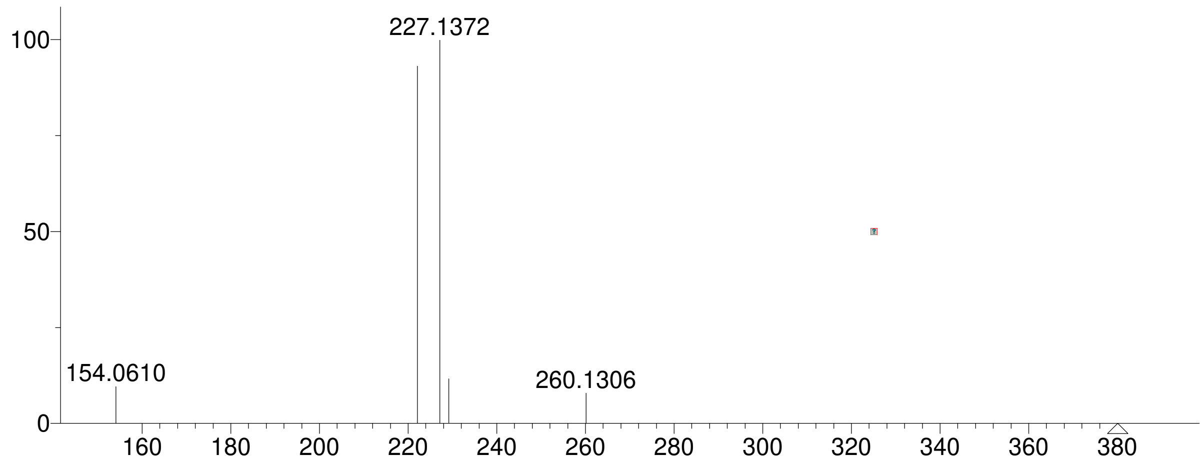

Unknown: Unknown (5.68_397.1697m/z)
Compound in Library Factor = -2387

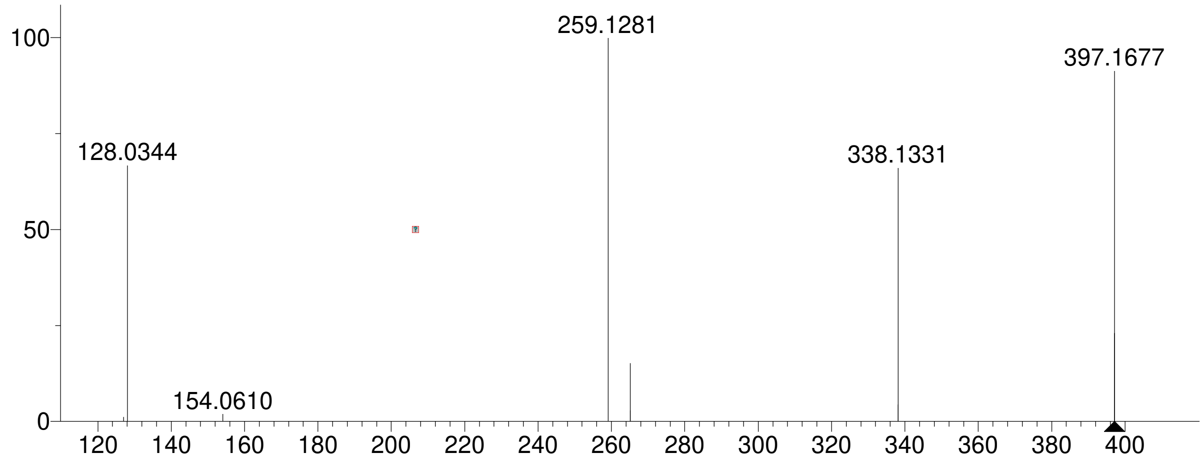

Hit 1 : Benzoic acid, 2-(diphenylphosphino)-4-[21-[(3aS,4S,6aR)-hexahydro-2-oxo-1H-thieno[3,4-d]imidazol-4-yl]-
C41H53N4O8PS; MF: 40; RMF: 578; Prob 31.5%; CAS: 608514-42-7; Lib: nist_msms; ID: 376808.

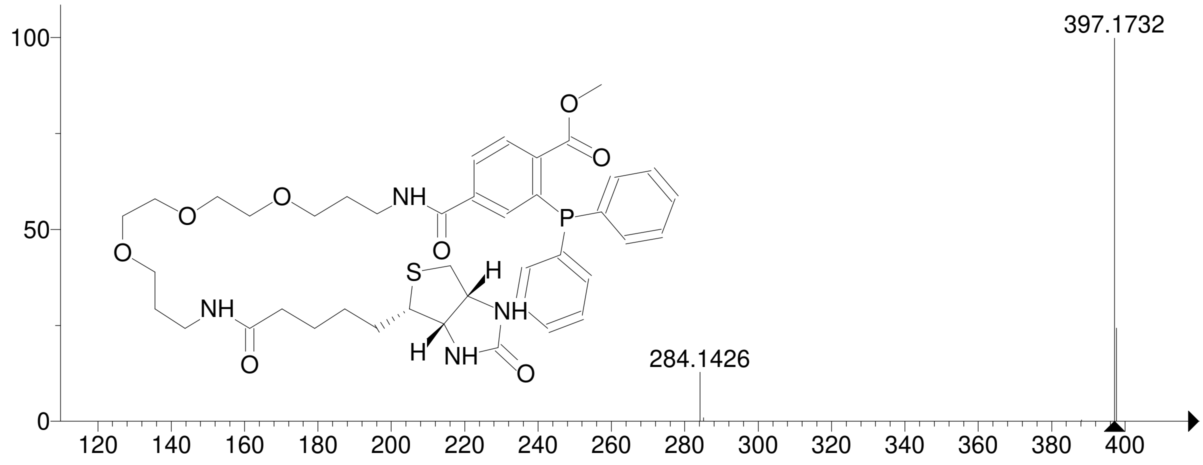

Hit 2 : 3-[1-(3-Aminopropyl)-1H-indol-3-yl]-4-(1-methyl-1H-indol-3-yl)-1H-pyrrole-2,5-dione [M-H]- HCD 29V P=
C24H22N4O2; MF: 28; RMF: 470; Prob 20.9%; CAS: 138516-31-1; Lib: nist_msms; ID: 321330.

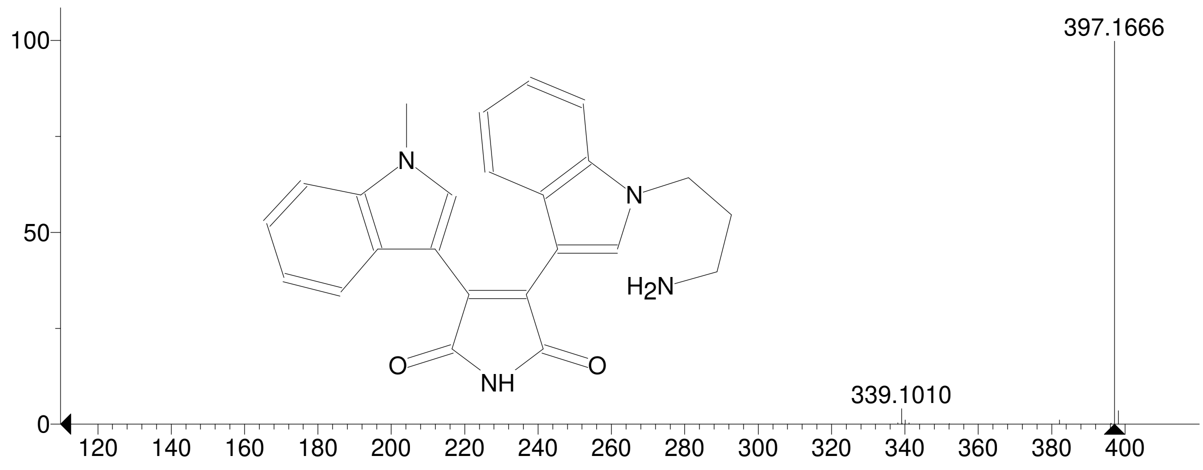

Hit 3 : 1-Naphthalenesulfonamide, N-(10-aminodecyl)-5-chloro- [M+H]+ HCD 23V P=397.2
C20H29ClN2O2S; MF: 22; RMF: 458; Prob 16.5%; CAS: 80467-76-1; Lib: nist_msms; ID: 382032.

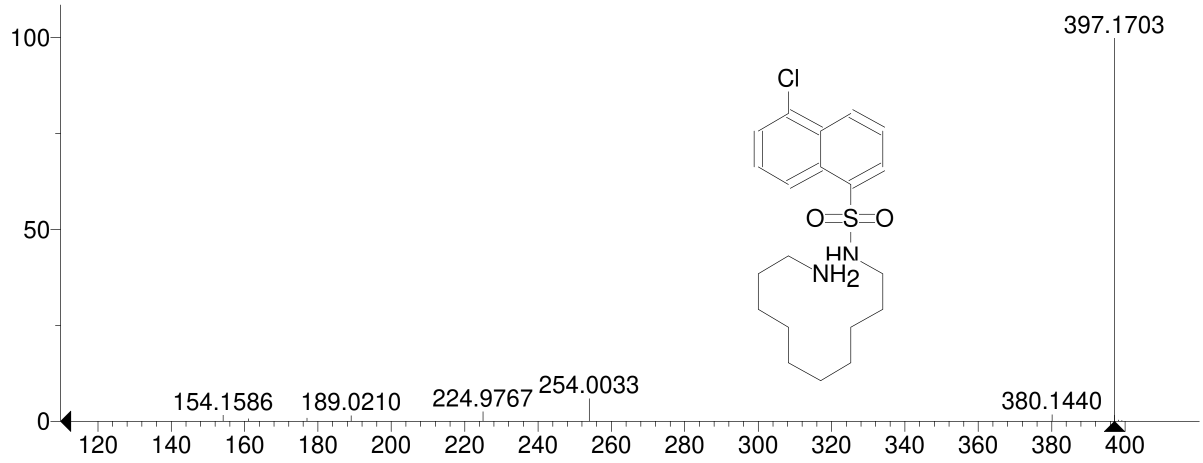

Hit 4 : Unknown (carbon number 12); PlaSMA ID-998 [M-H]- P=397.2
C12H36N2O6P2S; MF: 13; RMF: 369; Prob 11.9%; Lib: biomsms-neg-plasma; ID: 1342.

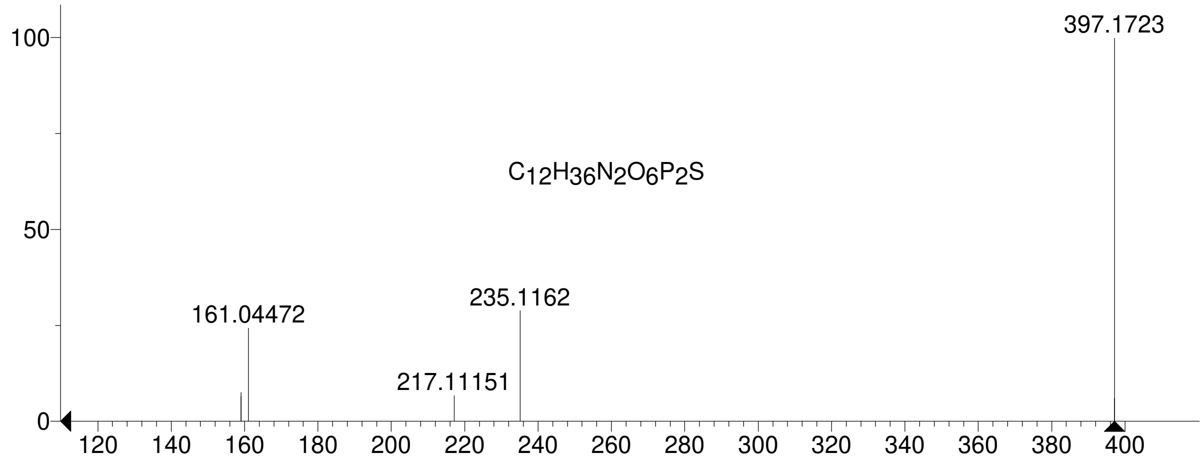

Hit 5 : Atorvastatin [M-H-C7H12O4]- HCD 17V P=397.2
C33H35FN2O5; MF: 12; RMF: 411; Prob 11.5%; CAS: 134523-00-5; Lib: nist_msms; ID: 322266.

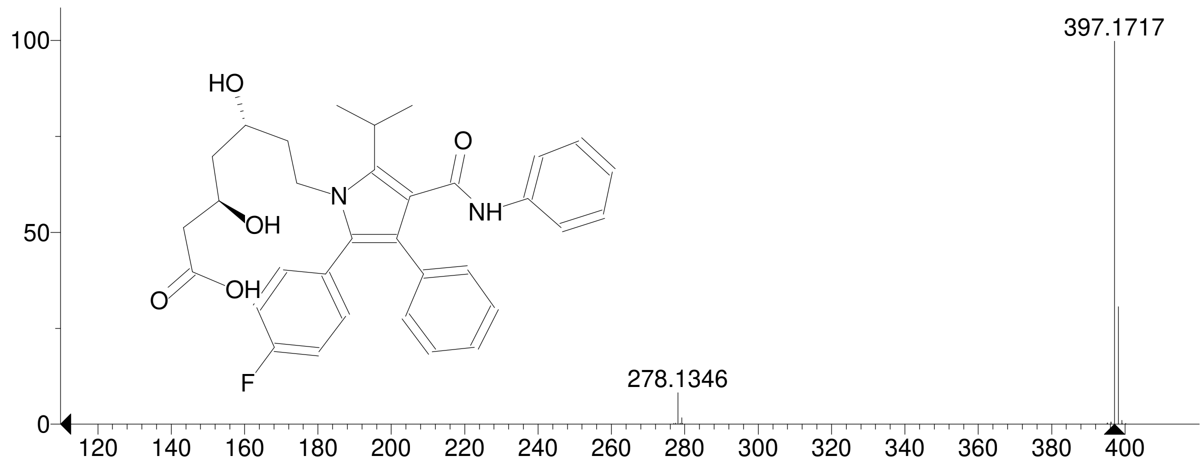

Unknown: Unknown (5.68_443.1839m/z)
Compound in Library Factor = 0

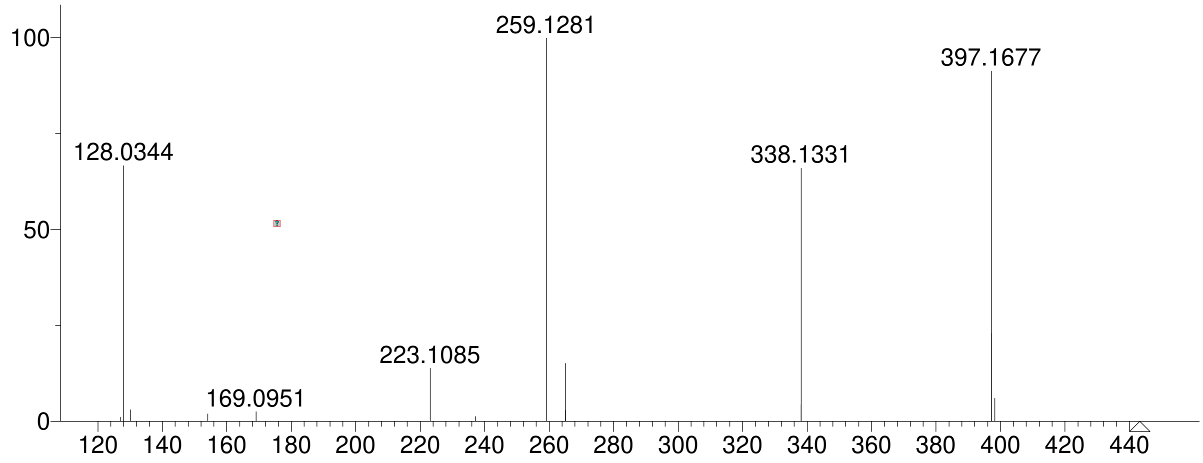

Hit 1 : Strophanthidin [M+K]+ 65HCD P=443.2
C23H32O6; MF: 0; RMF: 8; Prob 100.0%; Lib: mona-export-vf-npl_qexactive; ID: 3261.

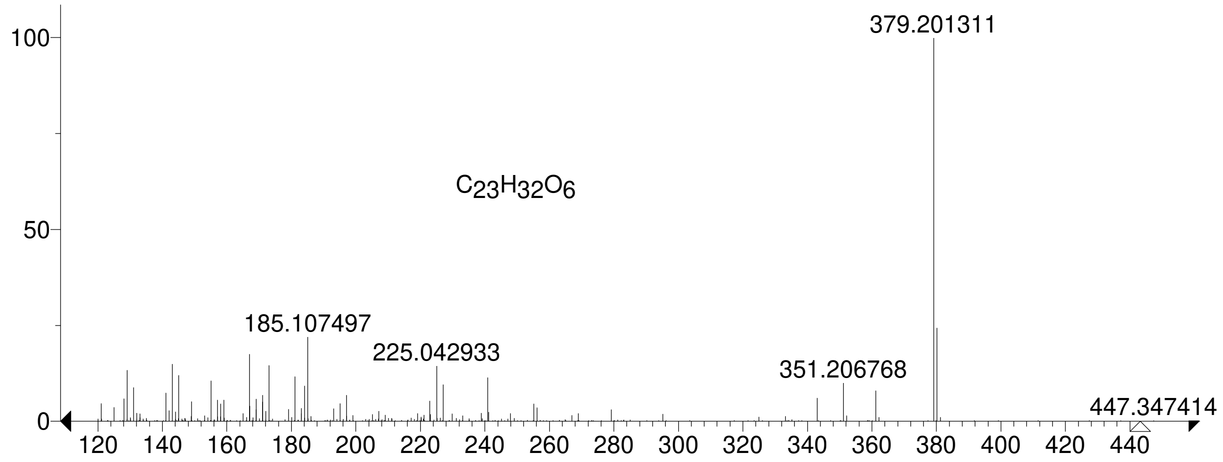

Unknown: Unknown (5.68_484.2035m/z)
Compound in Library Factor = -3273

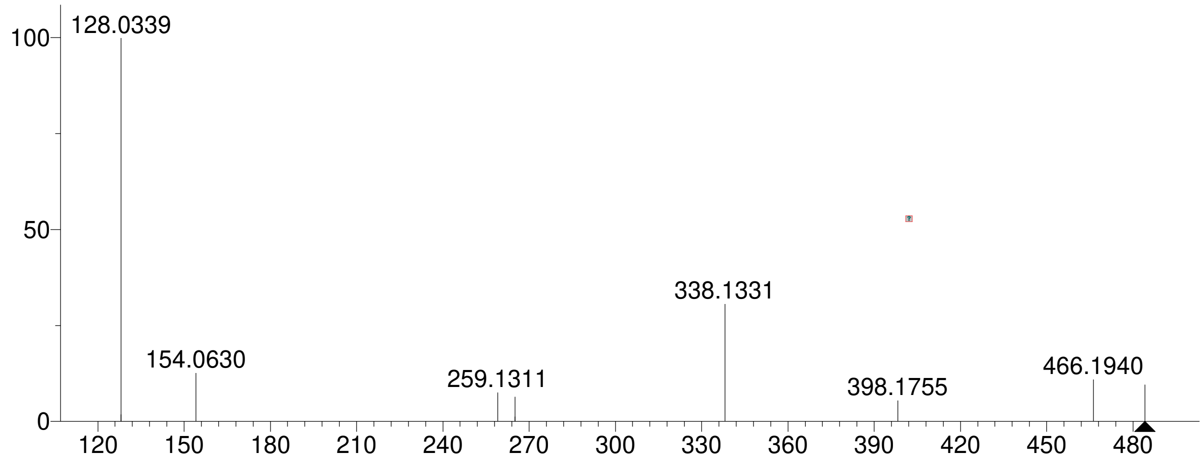

Hit 1 : Fexofenadine [M+H]+=>484.2 IT 35% P=484.2
C32H39NO4; MF: 8; RMF: 364; Prob 30.6%; CAS: 83799-24-0; Lib: nist_msms; ID: 123829.

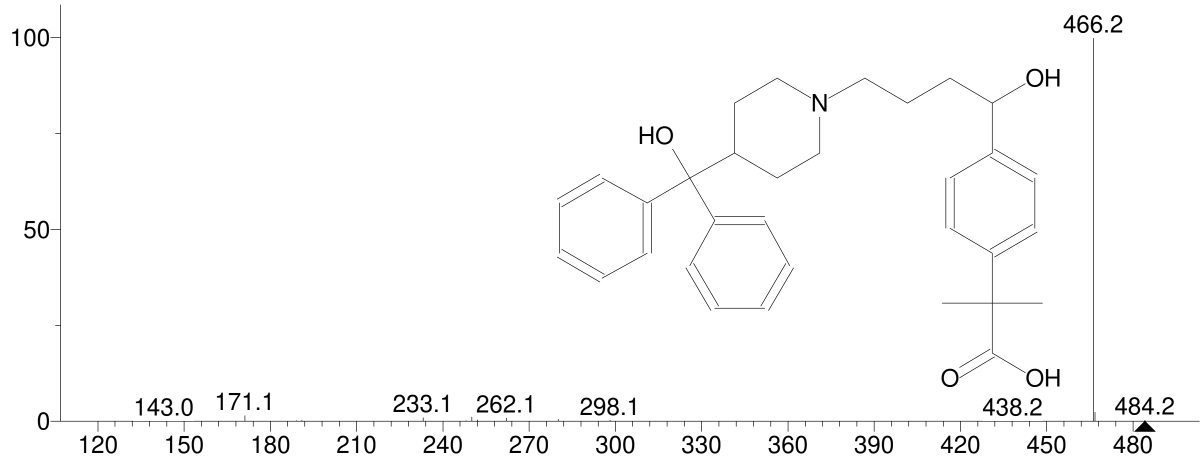

Hit 2 : [1,1'-Biphenyl]-2-carboxylic acid, 4'-[[2,3-dimethyl-5-[[[(1S)-1-(4-nitrophenyl)ethyl]amino]carbonyl]-1H-indol·
C33H29N3O5; MF: 1; RMF: 161; Prob 23.4%; CAS: 1338259-05-4; Lib: nist_msms; ID: 253488.

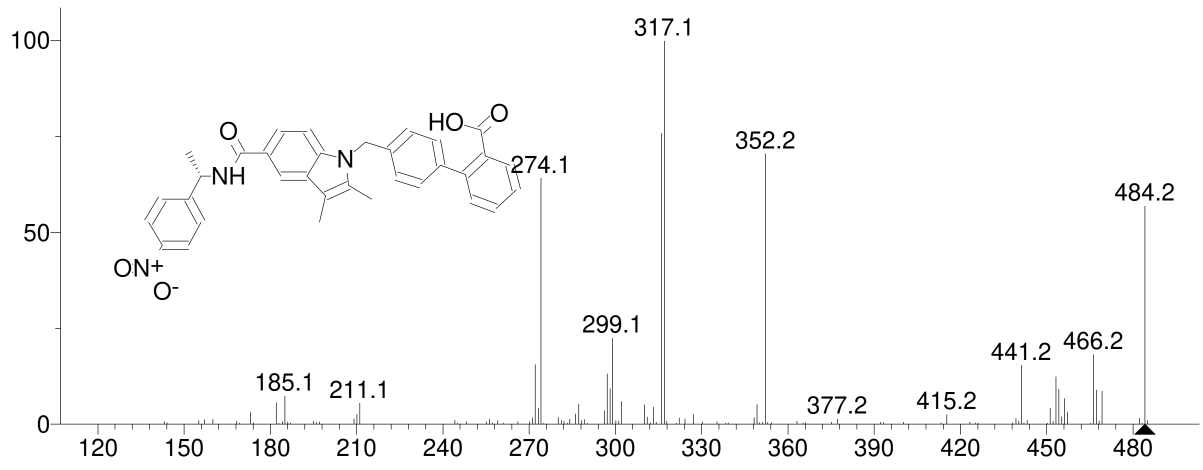

Hit 3 : NCGC00381190-01_C27H29N5O5_ [M-H2O-H]- P=484.2
C27H29N5O5; MF: 1; RMF: 124; Prob 23.4%; Lib: msms-neg-gnps; ID: 1973.

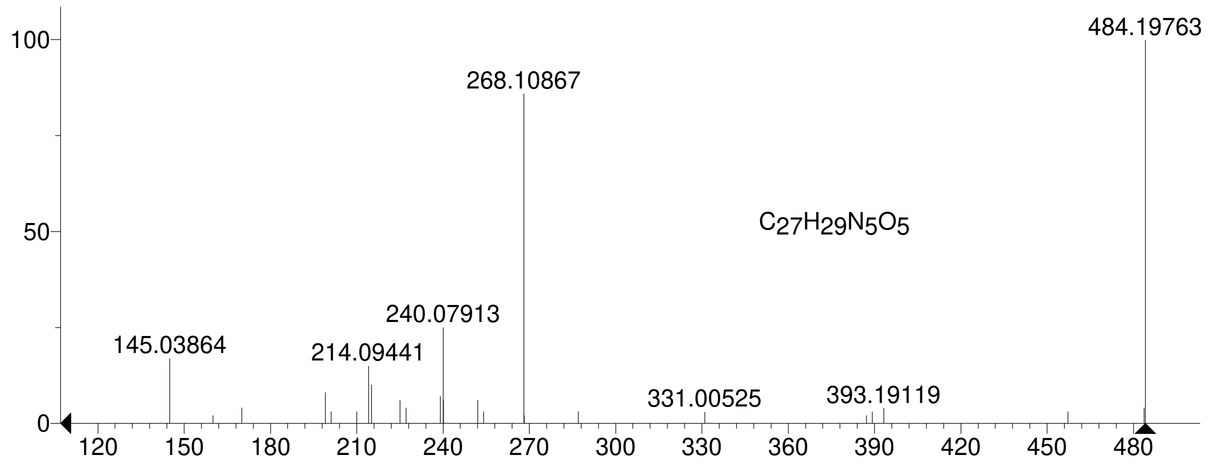

Hit 4 : Halofantrine [M+H+2i]+=>484.2 IT 35% P=484.2
C26H30Cl2F3NO; MF: 0; RMF: 42; Prob 22.5%; CAS: 69756-53-2; Lib: nist_msms; ID: 522837.

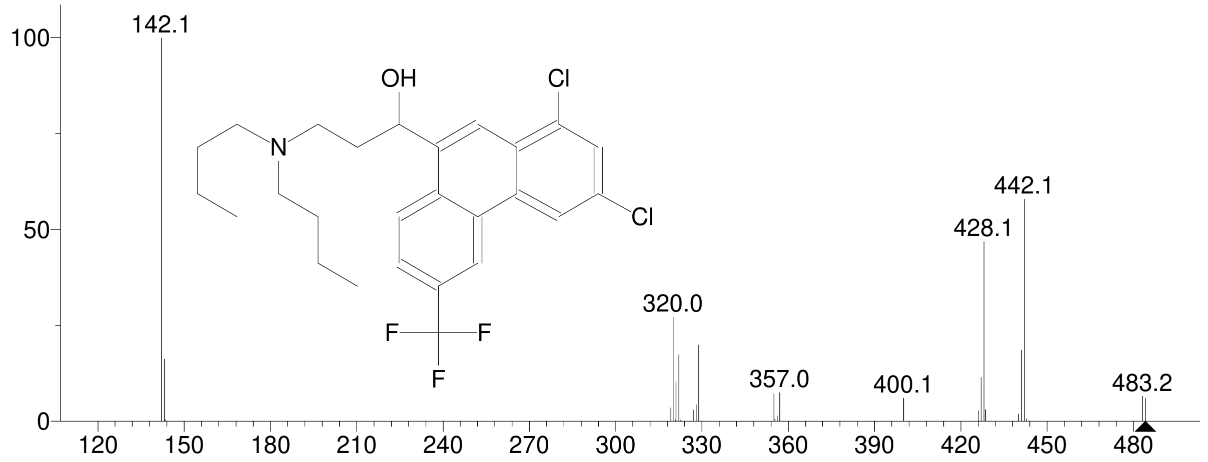

Unknown: Unknown (5.70_355.1617m/z)
Compound in Library Factor = 0

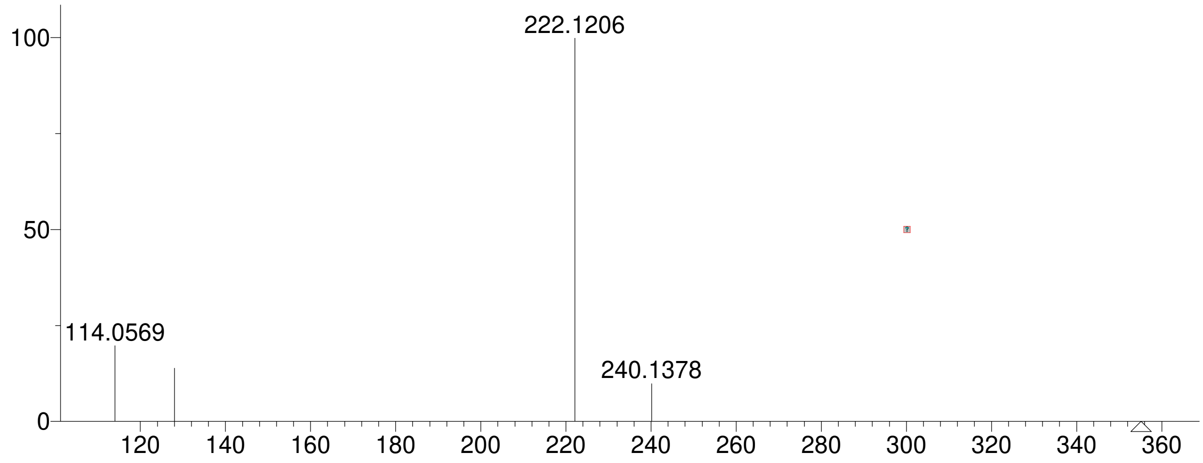

Unknown: Unknown (5.71_707.1927m/z)
Compound in Library Factor = 0

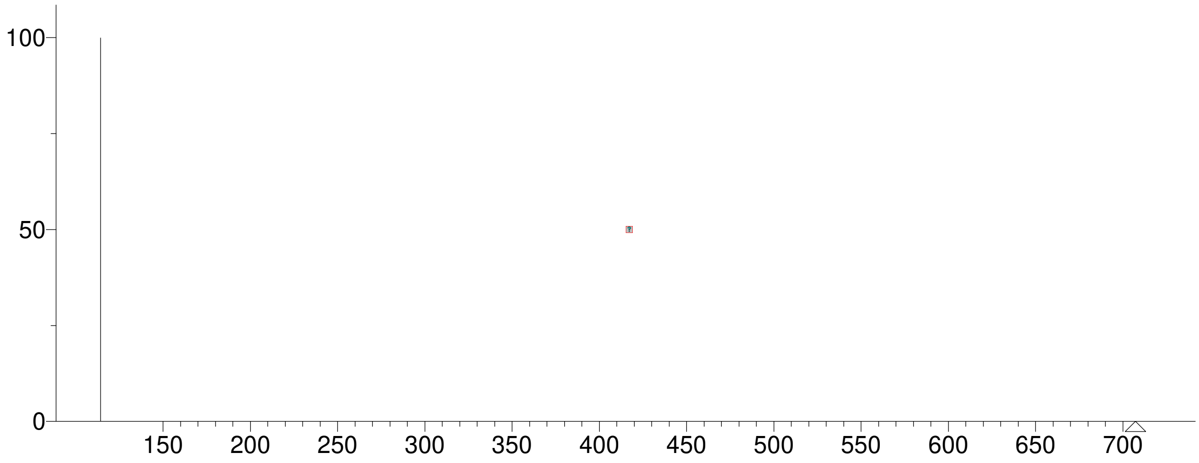

Unknown: Unknown (5.74_227.1023m/z)
Compound in Library Factor = -178

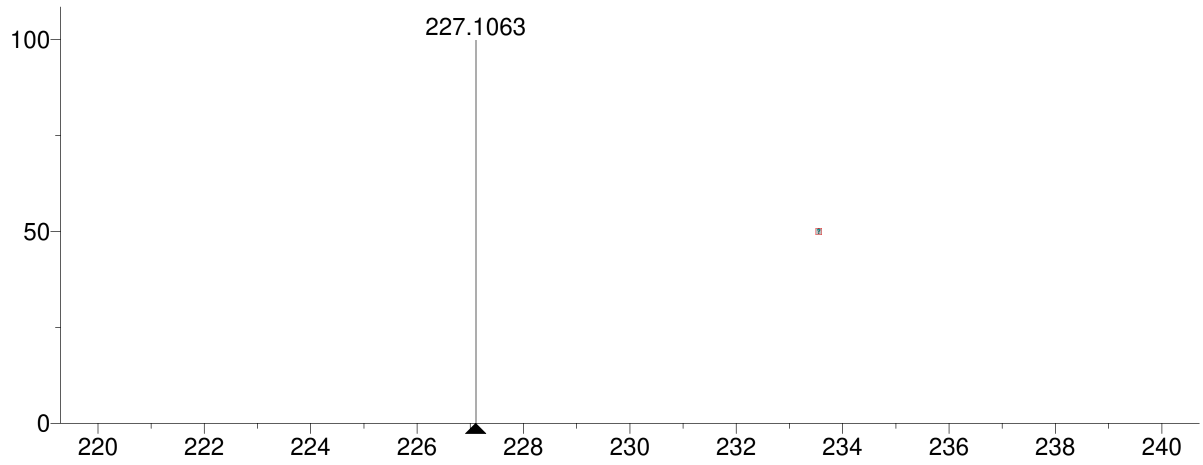

Hit 1 : PyroGlu-Pro [M+H]+ QTOF 10V P=227.1
C10H14N2O4; MF: 364; RMF: 999; Prob 16.6%; Lib: nist_msms; ID: 47868.

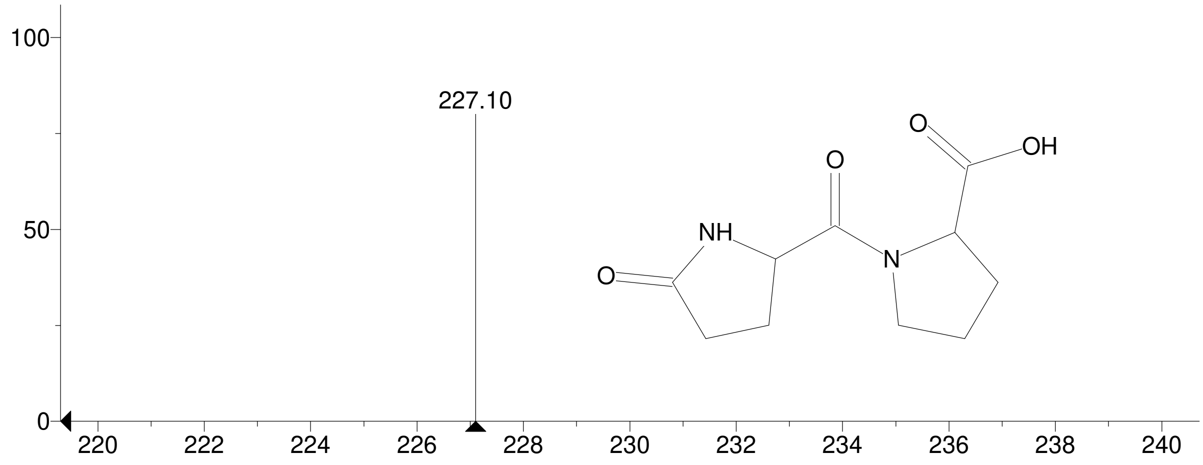

Hit 2 : Carbidopa [M+H]+ QTOF 17V P=227.1
C10H14N2O4; MF: 364; RMF: 999; Prob 16.6%; CAS: 28860-95-9; Lib: nist_msms; ID: 69030.

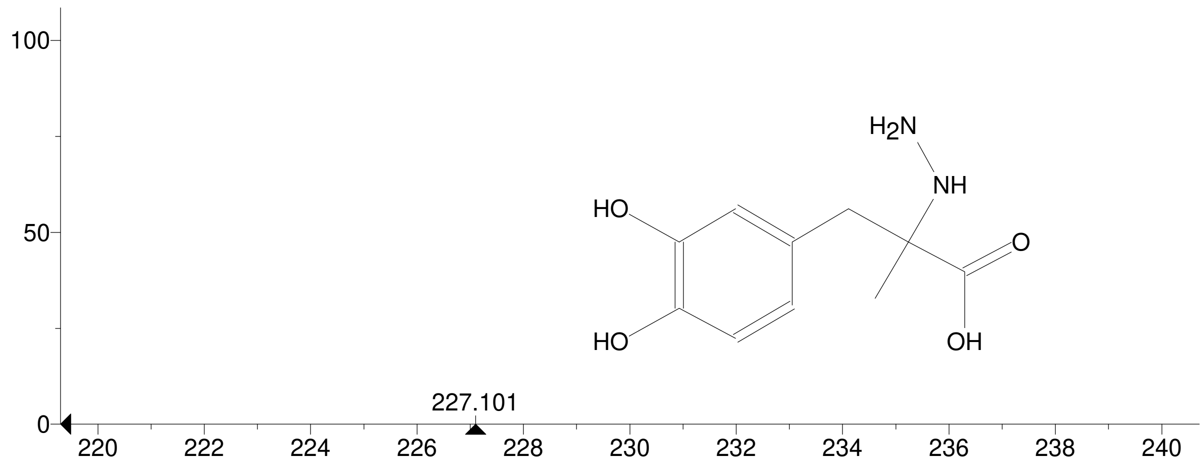

Hit 3 : 2,5-Dimethoxy-4-nitrophenethylamine [M+H]+ HCD 9V P=227.1
C10H14N2O4; MF: 364; RMF: 999; Prob 16.6%; CAS: 261789-00-8; Lib: nist_msms; ID: 355204.

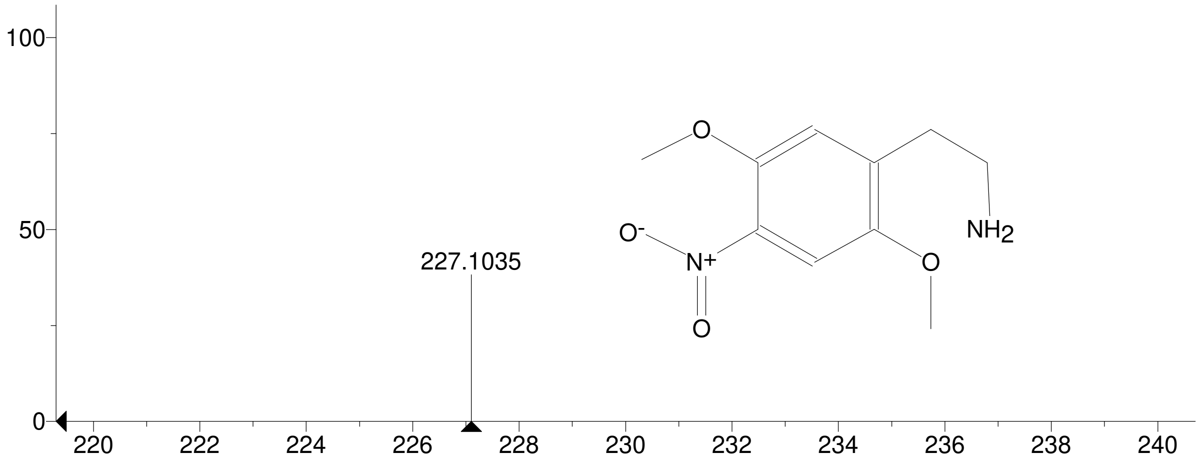

Hit 4 : (S)-Dibutyl 3-hydroxybutyl phosphate [M+H-C4H8]+ HCD 9V P=227.1
C12H27O5P; MF: 364; RMF: 999; Prob 16.6%; CAS: 89197-69-3; Lib: nist_msms; ID: 419320.

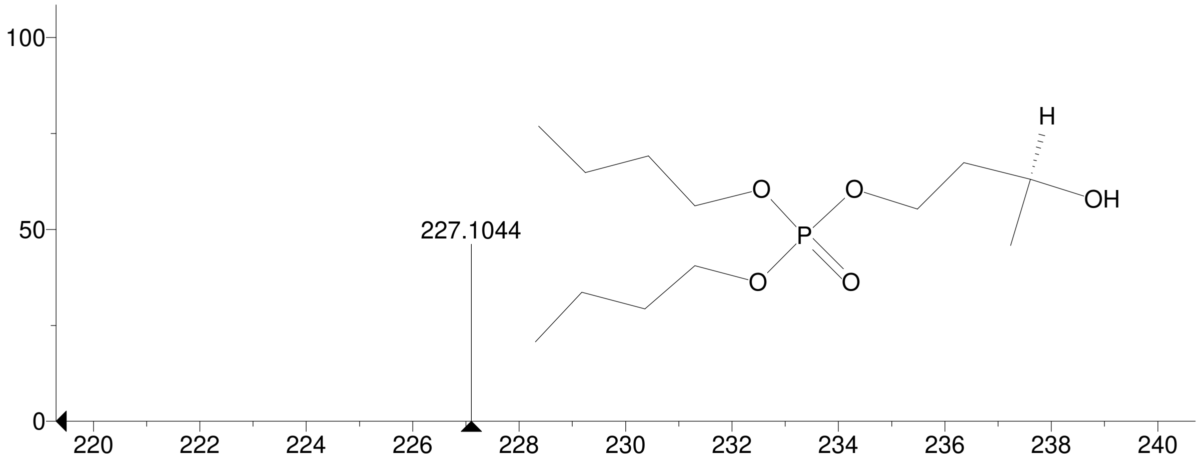

Hit 5 : Proline-hydroxyproline [M-H]- 20-30-40%) P=227.1
C10H16N2O4; MF: 364; RMF: 999; Prob 16.6%; Lib: mona-export-fiehn_hilic; ID: 1464.

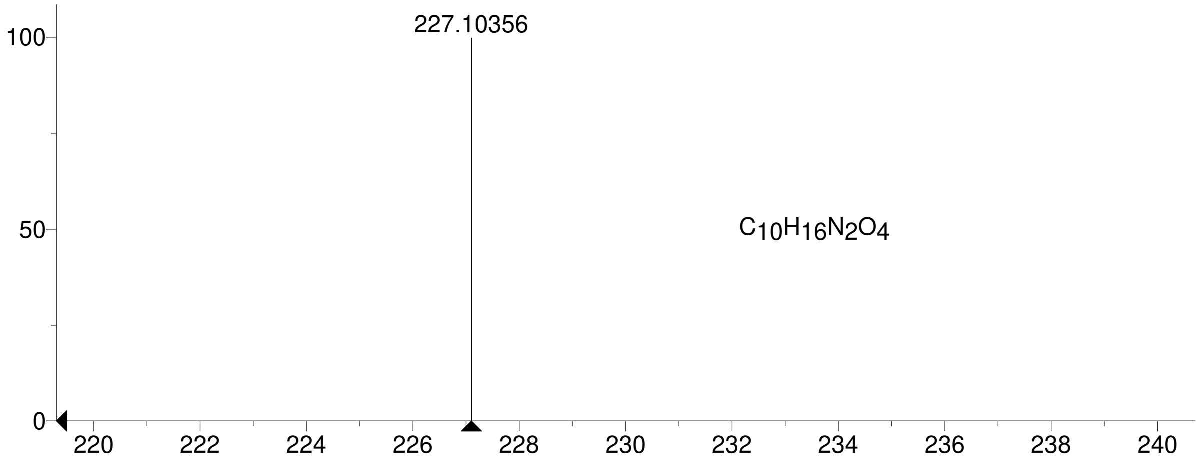

Unknown: Unknown (5.74_393.1654m/z)
Compound in Library Factor = -3273

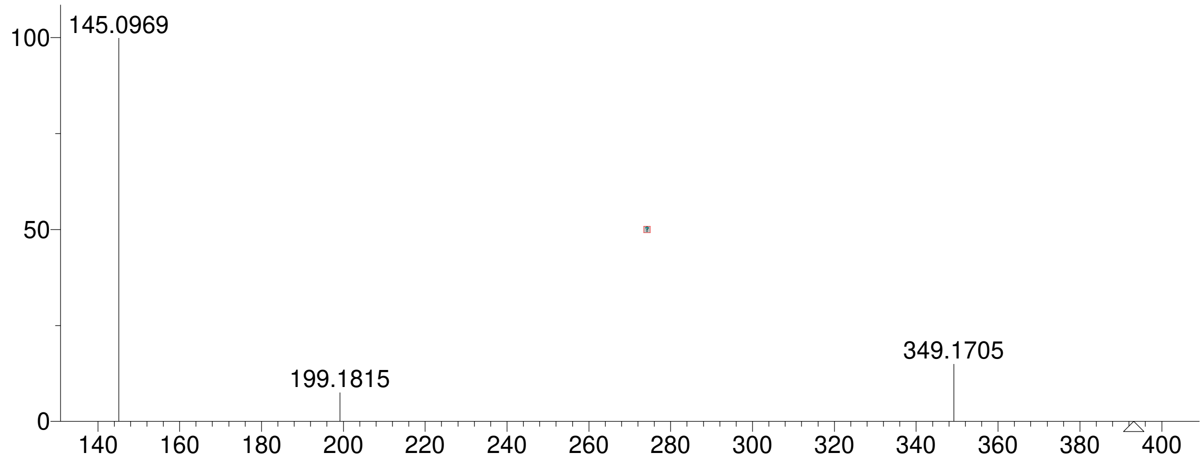

Hit 1 : Sparfloxacin [M+H]+ P=393.2
C19H22F2N4O3; MF: 4; RMF: 423; Prob 54.2%; Lib: mona-export-gnps; ID: 3028.

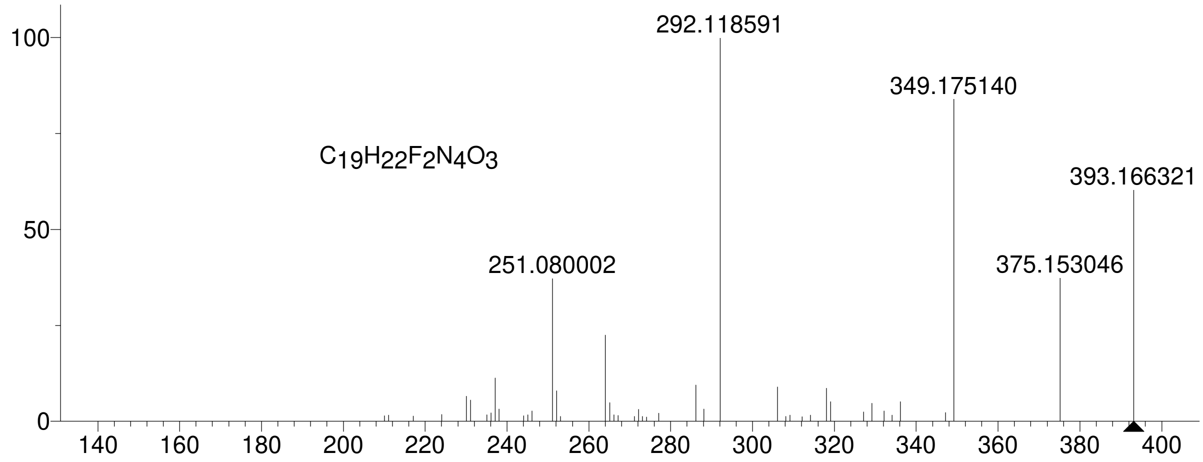

Hit 2 : (E)-3-(4-acetyloxy-2,3-dihydroxy-2,5,5,8a-tetramethyl-3,4,4a,6,7,8-hexahydro-1H-naphthalen-1-yl)prop-2-e
C19H30O6; MF: 0; RMF: 325; Prob 45.8%; Lib: mona-export-vf-npl_qexactive; ID: 20133.

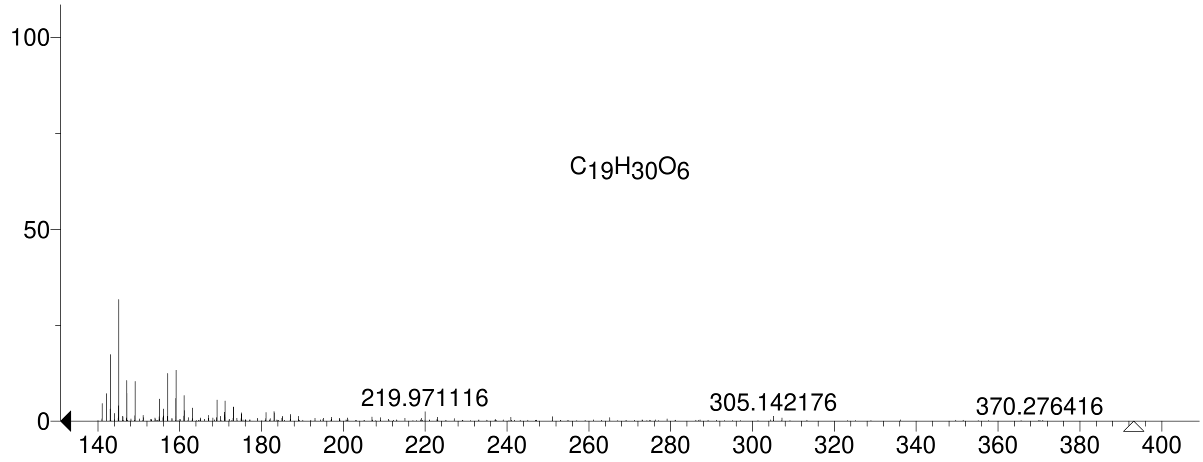

Unknown: Unknown (5.75_349.1754m/z)
Compound in Library Factor = -313

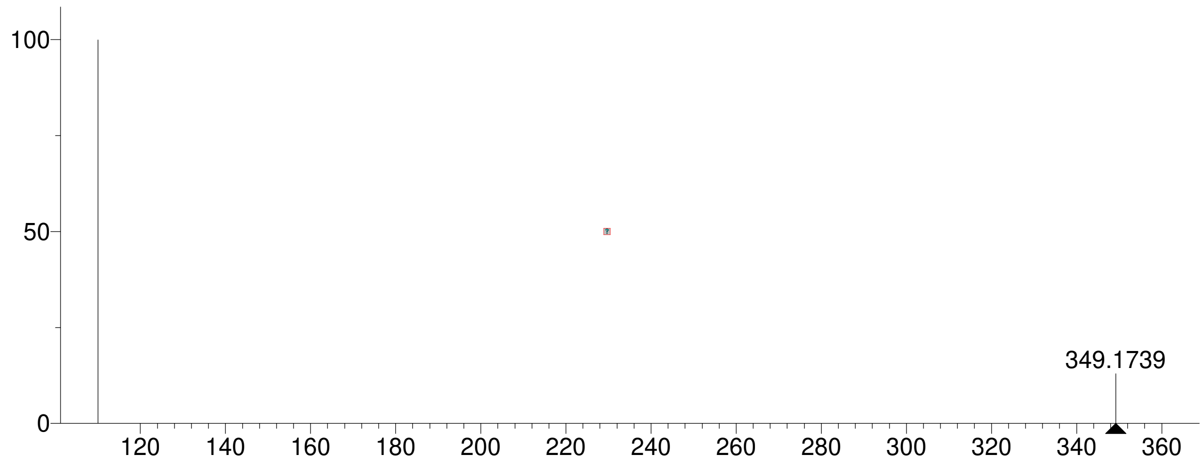

Hit 1 : Enalaprilat tert-butyl ester [M+H-C4H8]+ HCD 10V P=349.2
C22H32N2O5; MF: 311; RMF: 956; Prob 77.9%; CAS: 674796-29-3; Lib: nist_msms; ID: 437459.

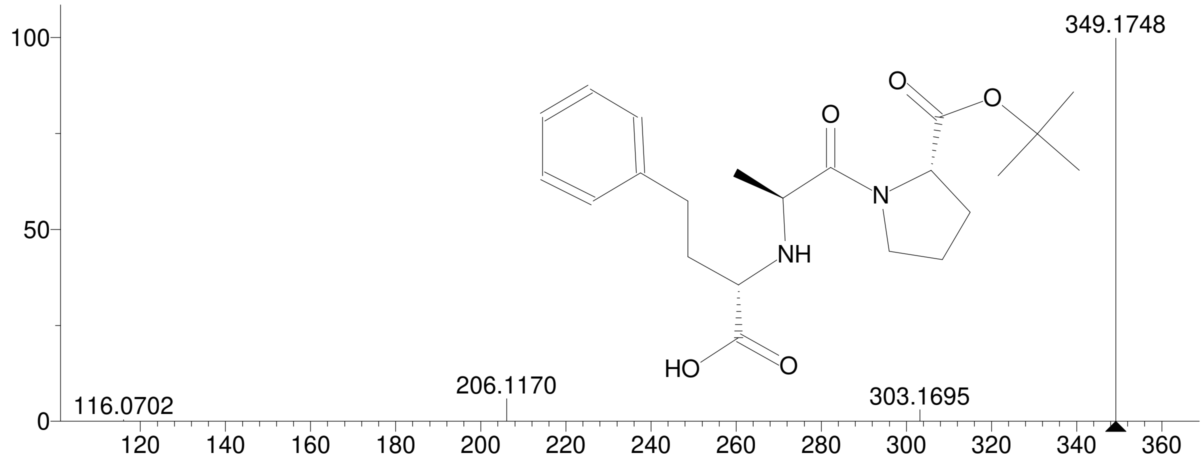

Hit 2 : Imidocarb [M+H]+ 10eV P=349.2
C19H20N6O; MF: 280; RMF: 908; Prob 21.2%; Lib: mona-export-massbank; ID: 356.

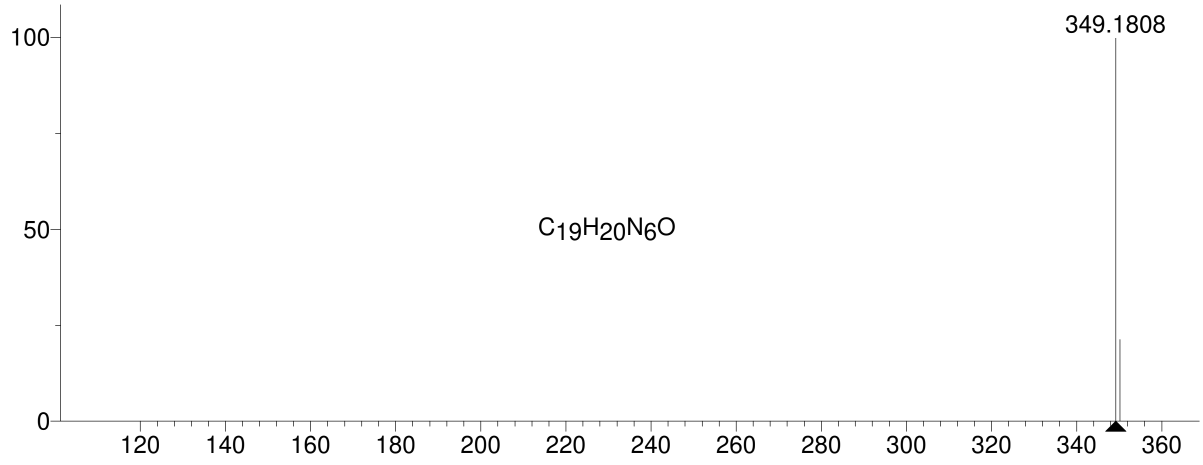

Hit 3 : MMV085071 [M+H]+ 20-30-40%) P=349.2
C19H20N6O; MF: 16; RMF: 301; Prob 0.21%; Lib: mona-export-pathogen_box; ID: 278.

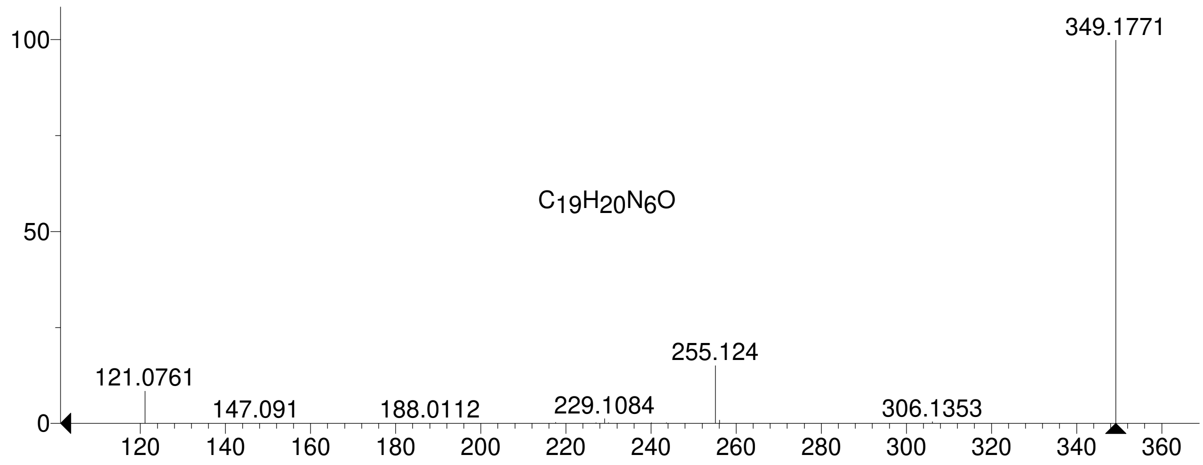

Hit 4 : Enalaprilat [M+H]+ HCD 13V P=349.2
C18H24N2O5; MF: 14; RMF: 304; Prob 0.19%; CAS: 76420-72-9; Lib: nist_msms; ID: 120441.

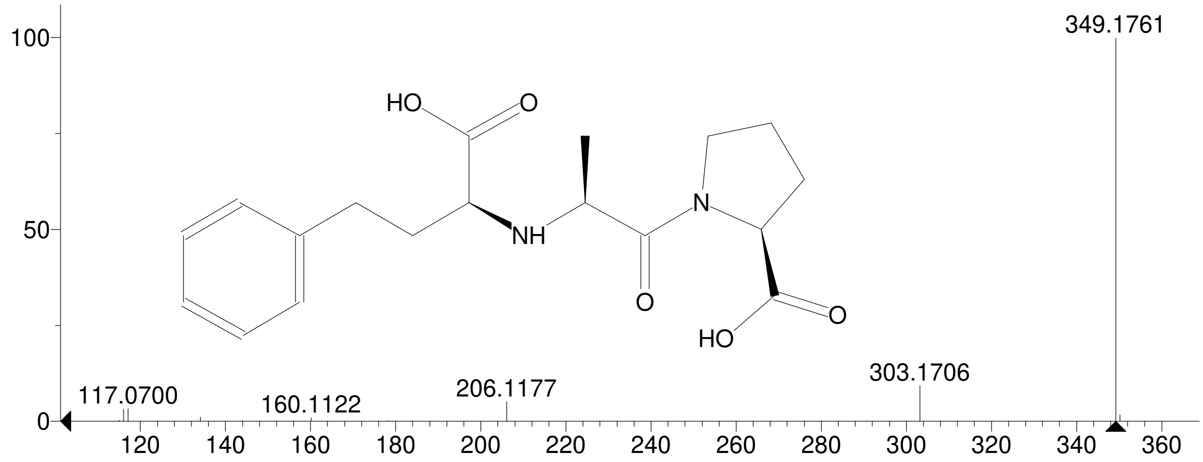

Hit 5 : methyl (2E,4E,8E)-7,13-dihydroxy-4,8,12-trimethyltetradeca-2,4,8-trienoate [M+K]+ 35HCD P=349.2
C18H30O4; MF: 1; RMF: 244; Prob 0.12%; Lib: mona-export-vf-npl_qexactive; ID: 25033.

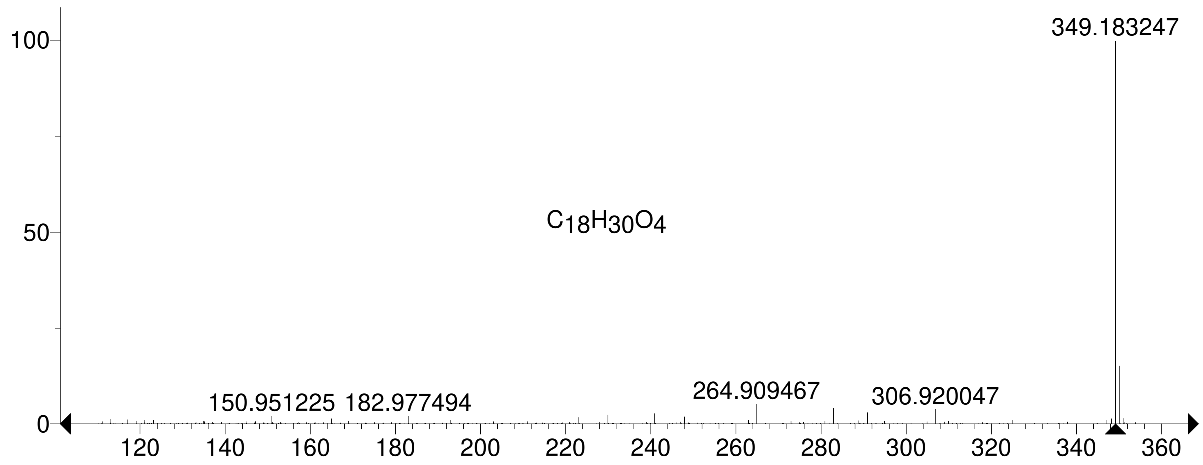

Unknown: Unknown (5.76_255.1337m/z)
Compound in Library Factor = -3273

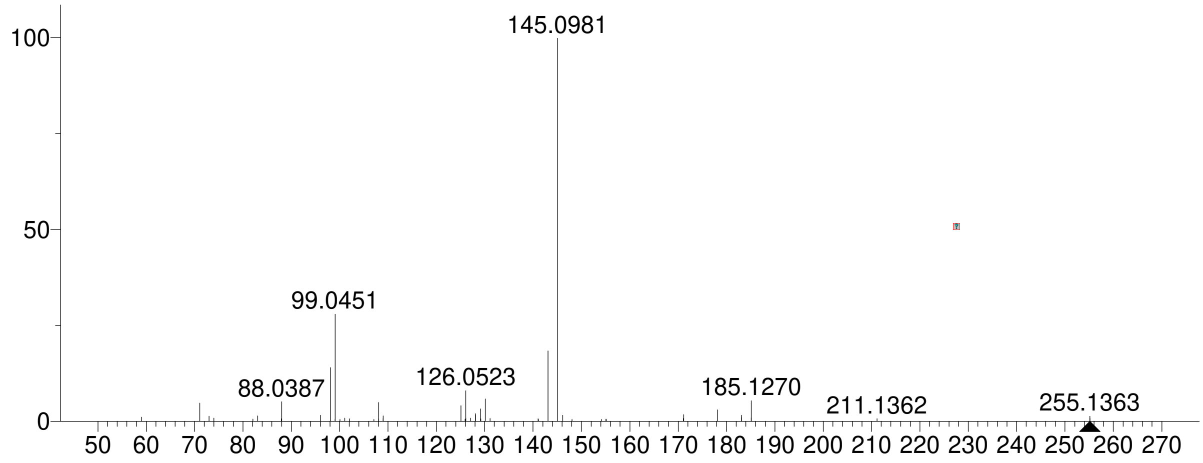

Hit 1 : N6-2-(4-Aminophenyl)ethyladenosine [M+H-C5H8O4]+ HCD 10V P=255.1
C18H22N6O4; MF: 3; RMF: 75; Prob 27.4%; CAS: 89705-21-5; Lib: nist_msms; ID: 298742.

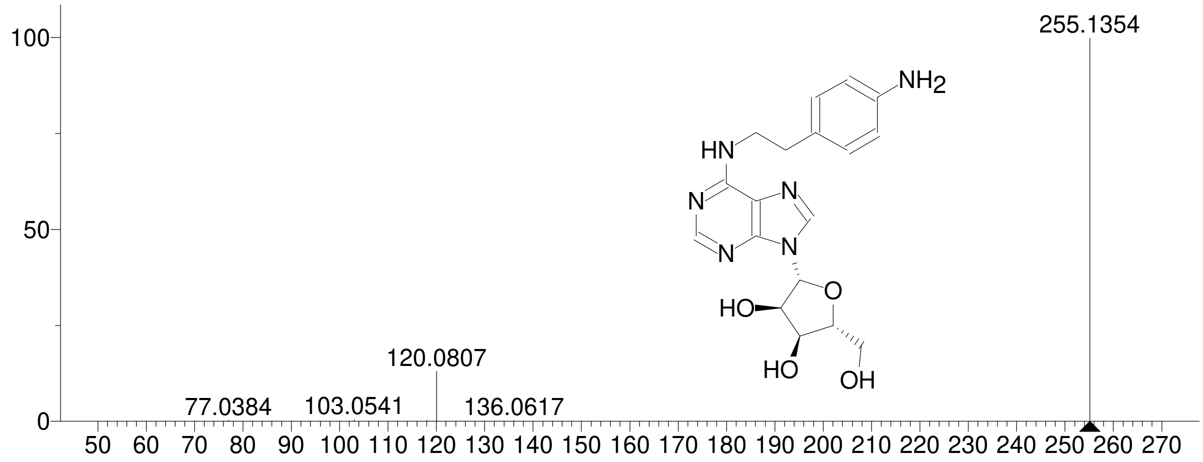

Hit 2 : Secobarbital [M+OH]- IT 35% P=255.1
C12H18N2O3; MF: 0; RMF: 73; Prob 24.2%; CAS: 76-73-3; Lib: nist_msms; ID: 14139.

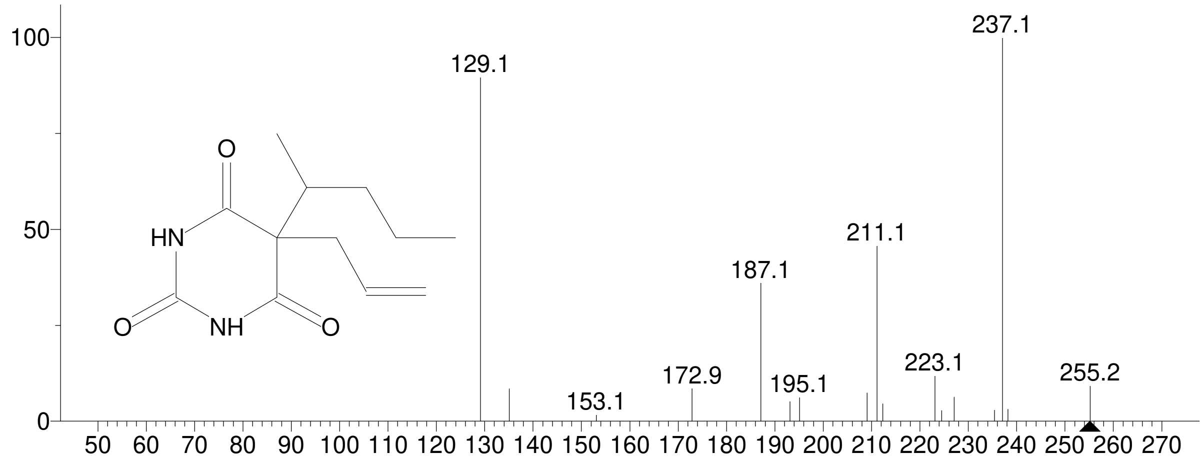

Hit 3 : Helenine [M+Na]+ 35HCD P=255.1
C15H20O2; MF: 0; RMF: 26; Prob 24.2%; Lib: mona-export-vf-npl_qexactive; ID: 2647.

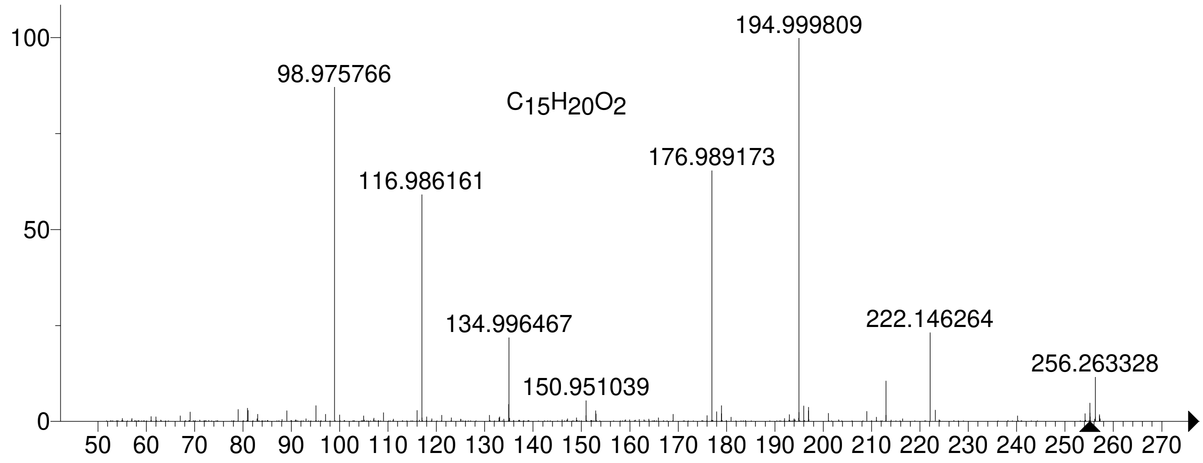

Hit 4 : Hexobarbital [M+H+H2O]+ IT 35% P=255.1
C12H16N2O3; MF: 0; RMF: 11; Prob 24.2%; CAS: 56-29-1; Lib: nist_msms; ID: 14133.

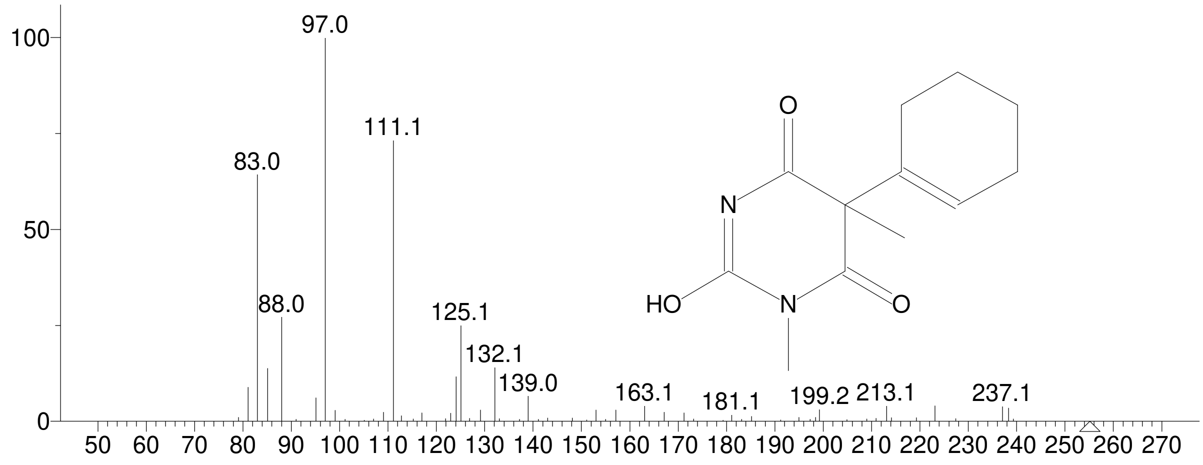

** Search Report Page 1 of 1 **

Unknown: Unknown (5.76_367.1602m/z)
Compound in Library Factor = 0

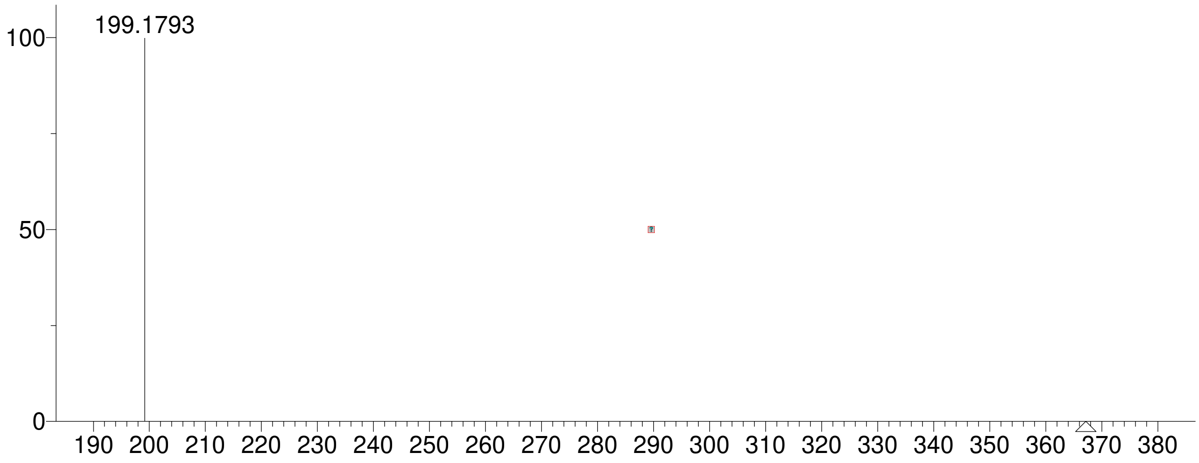

Unknown: Unknown (5.76_743.1542m/z)
Compound in Library Factor = 0

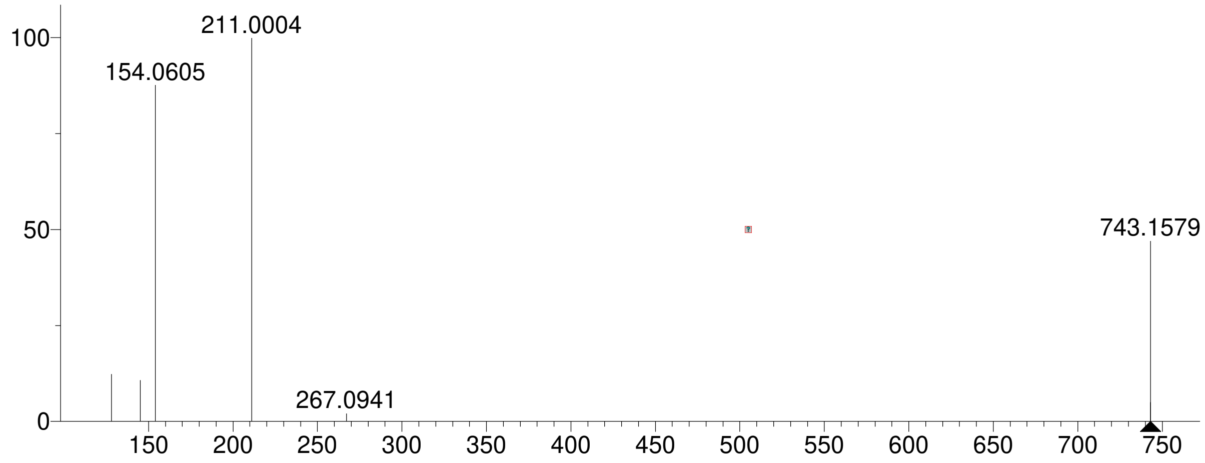

Hit 1 : [4,5-diacetyloxy-6-[7-hydroxy-5-methoxy-4-oxo-2-[4-[3,4,5-trihydroxy-6-(hydroxymethyl)oxan-2-yl]oxypheny
C33H36O17; MF: 0; RMF: 13; Prob 100.0%; Lib: mona anthocyanidin-o-glycosides; ID: 7016.

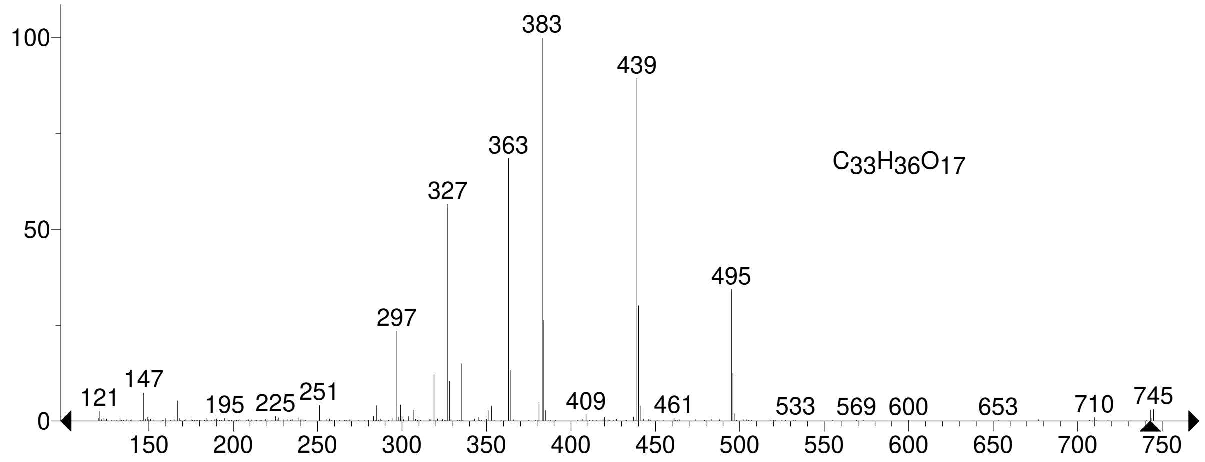

** Search Report Page 1 of 1 **

Unknown: Unknown (5.77_385.1692m/z)
Compound in Library Factor = 0

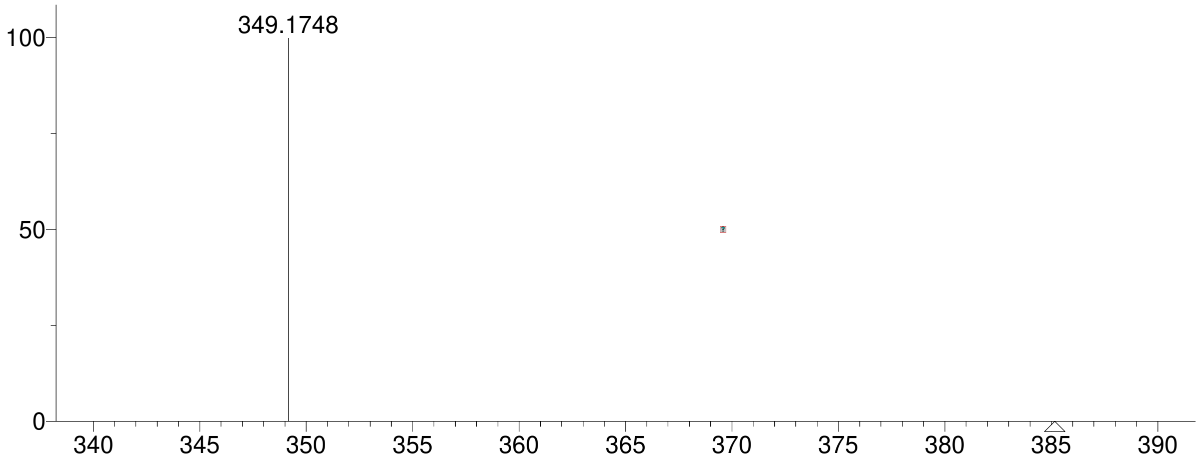

Unknown: Unknown (5.78_372.2128m/z)
Compound in Library Factor = 0

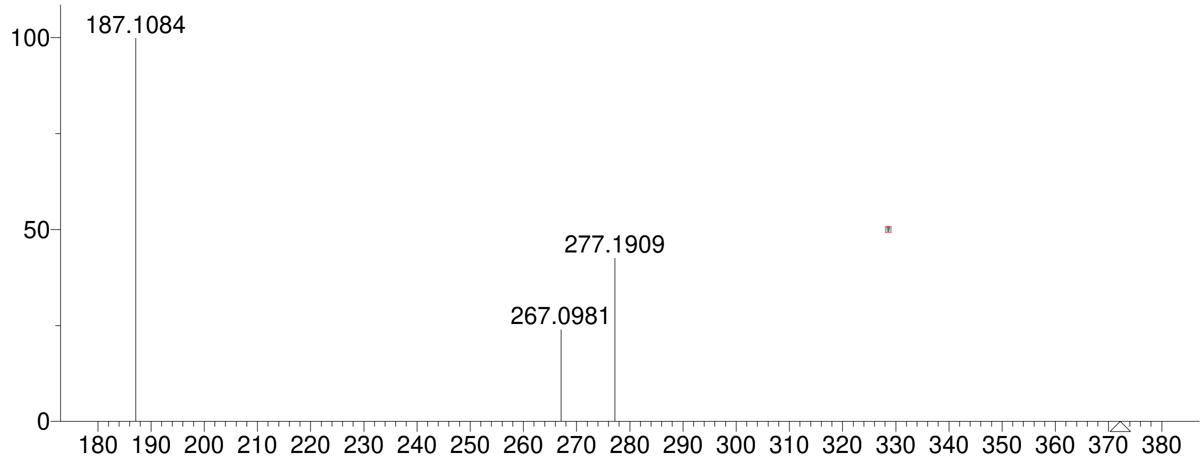

Unknown: Unknown (5.80_298.0922m/z)
Compound in Library Factor = 0

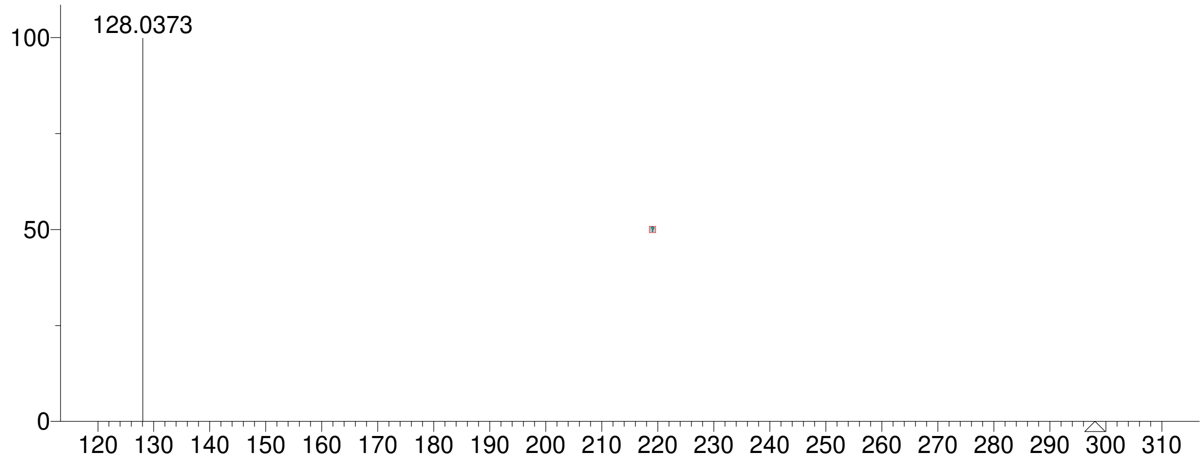

** Search Report Page 1 of 1 **

Unknown: Unknown (5.80_420.1380m/z)
Compound in Library Factor = 0

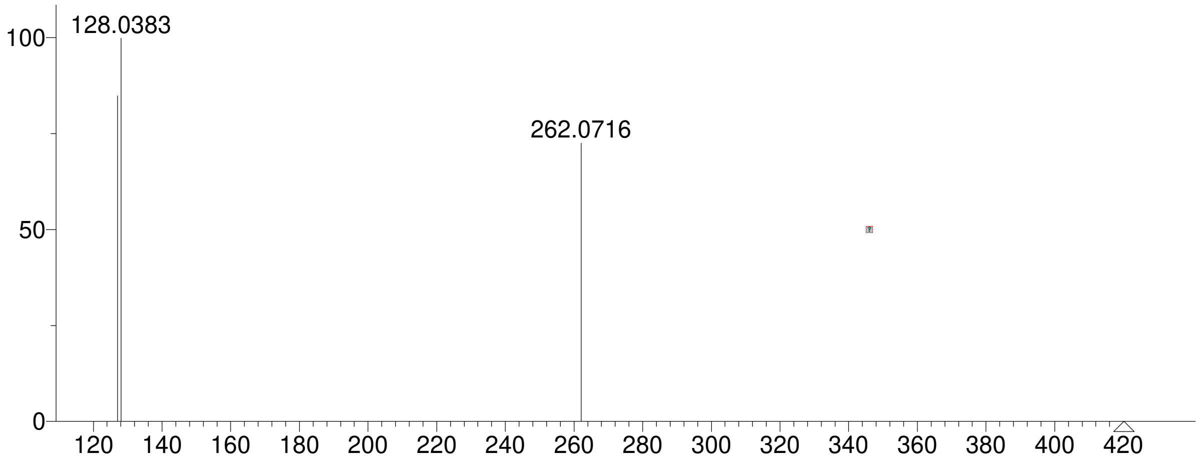

** Search Report Page 1 of 1 **

Unknown: Unknown (5.80_510.1826m/z)
Compound in Library Factor = 0

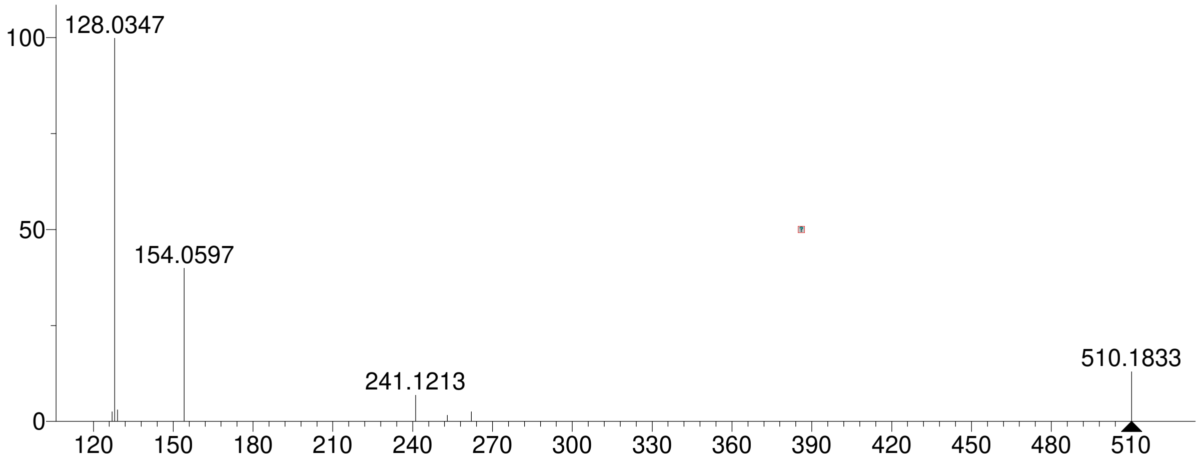

Unknown: Unknown (5.80_680.2400m/z)
Compound in Library Factor = 0

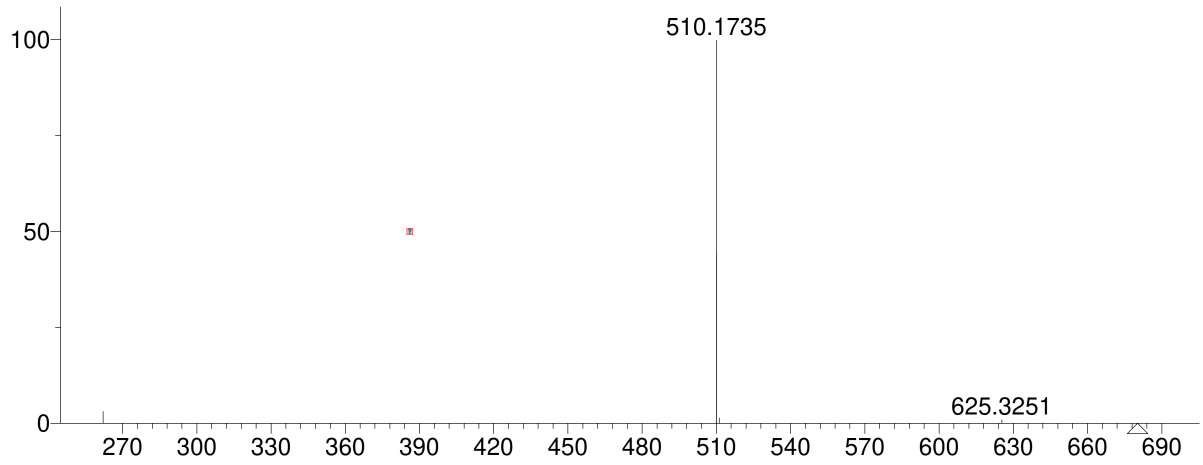

Unknown: Unknown (5.82_227.1026m/z)
Compound in Library Factor = -3273

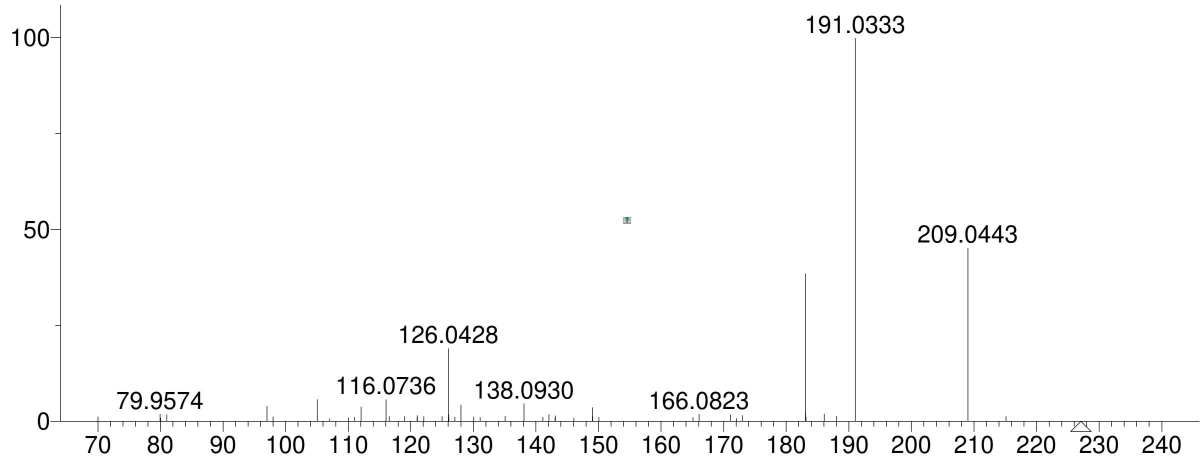

Hit 1 : 2,5-Dimethoxy-4-nitrophenethylamine [M+H]+ HCD 34V P=227.1
C10H14N2O4; MF: 2; RMF: 50; Prob 17.4%; CAS: 261789-00-8; Lib: nist_msms; ID: 355212.

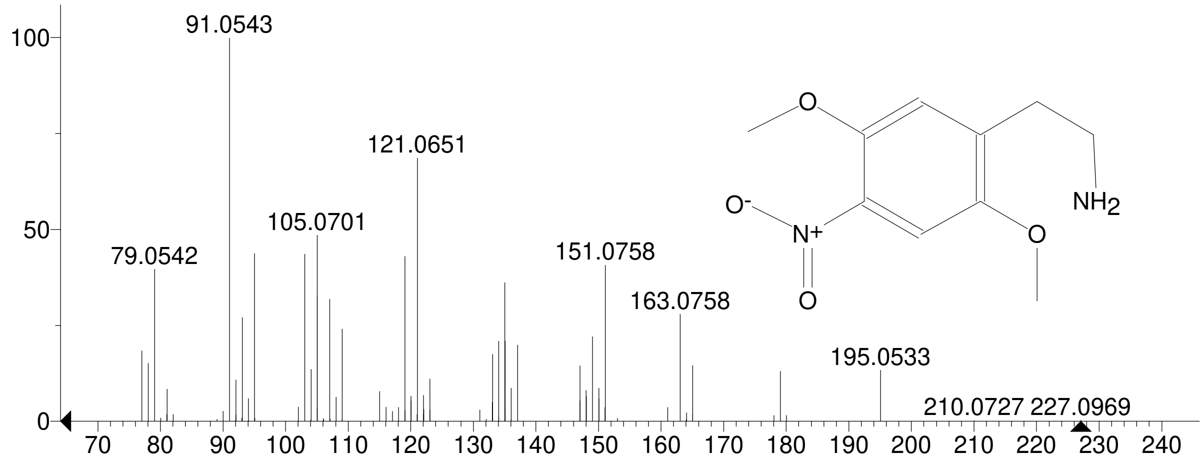

Hit 2 : Pro-Hyp [M-H]- 35eV P=227.1
C10H16N2O4; MF: 2; RMF: 46; Prob 17.4%; Lib: mona-export-fiehn_hilic; ID: 2943.

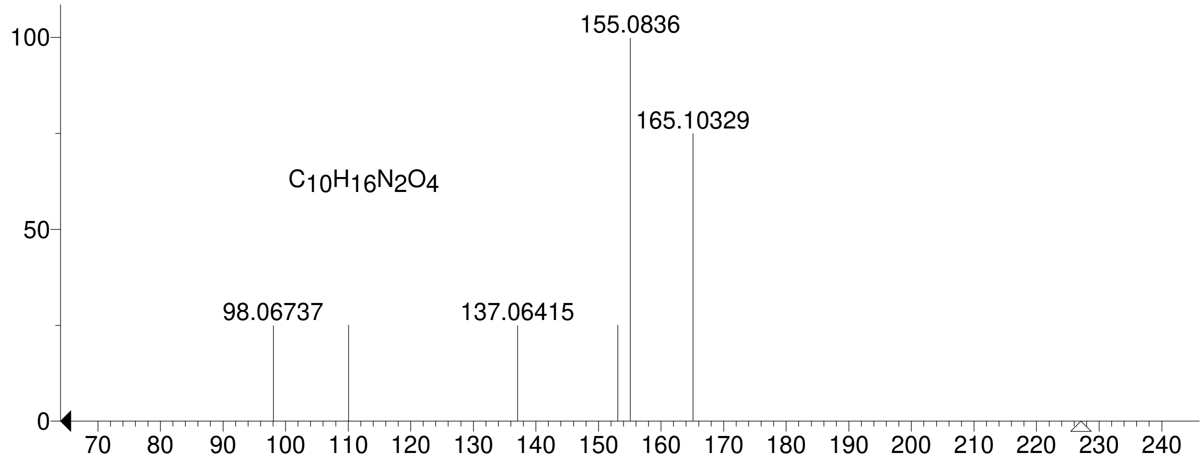

Hit 3 : PyroGlu-Pro [M+H]+ QTOF 12V P=227.1
C10H14N2O4; MF: 1; RMF: 85; Prob 16.8%; Lib: nist_msms; ID: 47869.

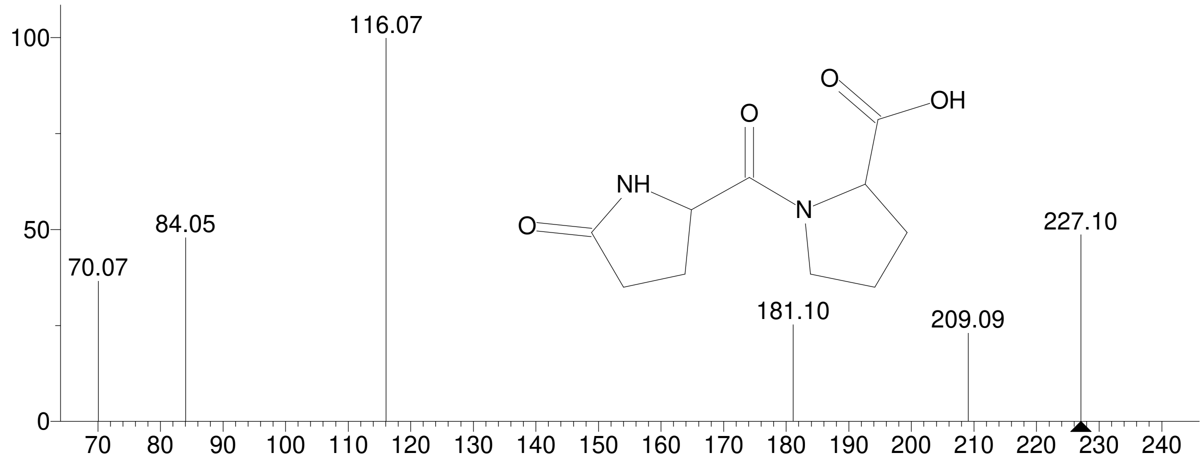

Hit 4 : Carbidopa [M+H]+ HCD 40V P=227.1
C10H14N2O4; MF: 0; RMF: 39; Prob 16.1%; CAS: 28860-95-9; Lib: nist_msms; ID: 313736.

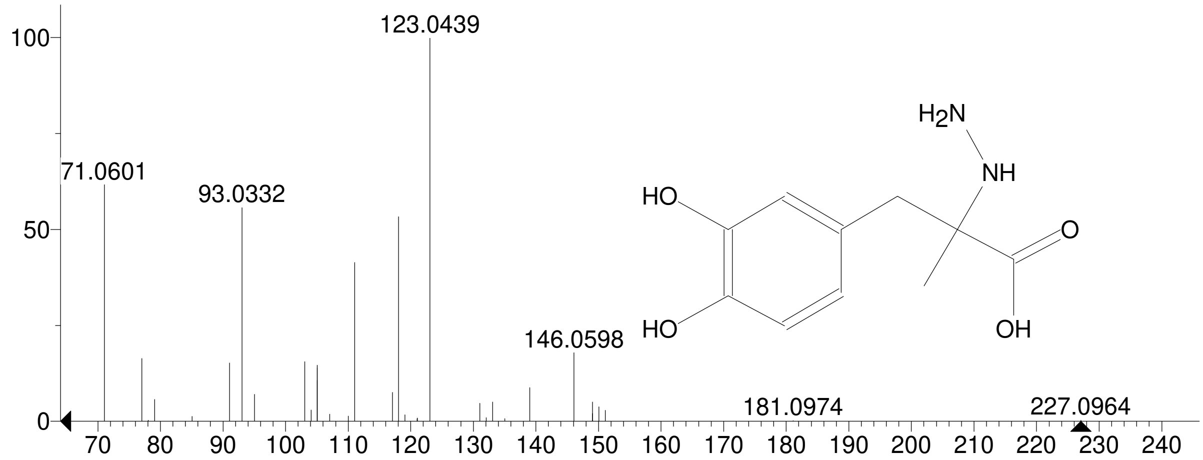

Hit 5 : Proline-hydroxyproline [M-H]- 20-30-40%) P=227.1
C10H16N2O4; MF: 0; RMF: 27; Prob 16.1%; Lib: mona-export-fiehn_hilic; ID: 1464.

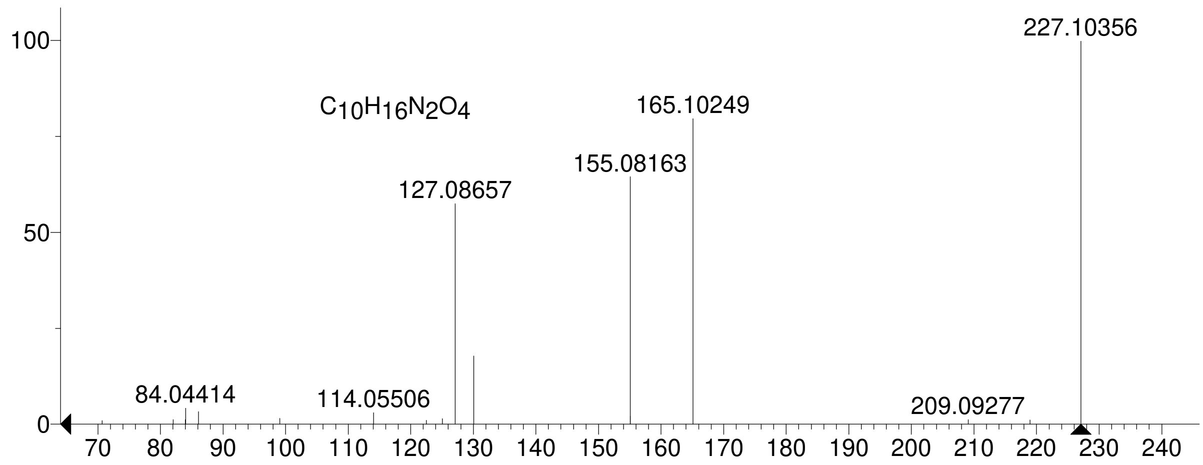

** Search Report Page 1 of 1 **

Unknown: Unknown (5.82_470.2595m/z)
Compound in Library Factor = -2387

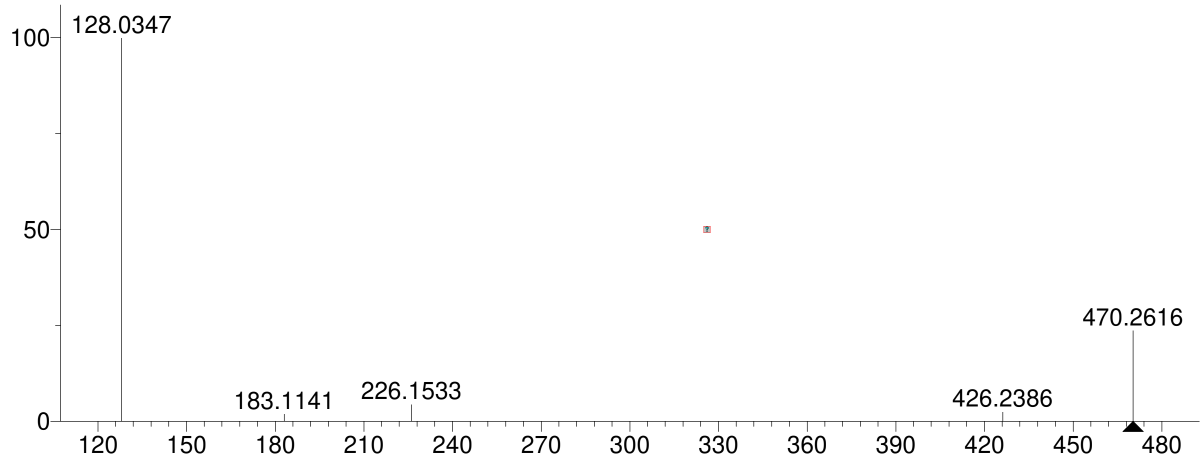

Hit 1 : Angiogenin (108-123) [M+4H]4+ HCD 7eV P=470.3
C83H132N26O24; MF: 11; RMF: 536; Prob 100.0%; Lib: nist_msms2; ID: 51723.

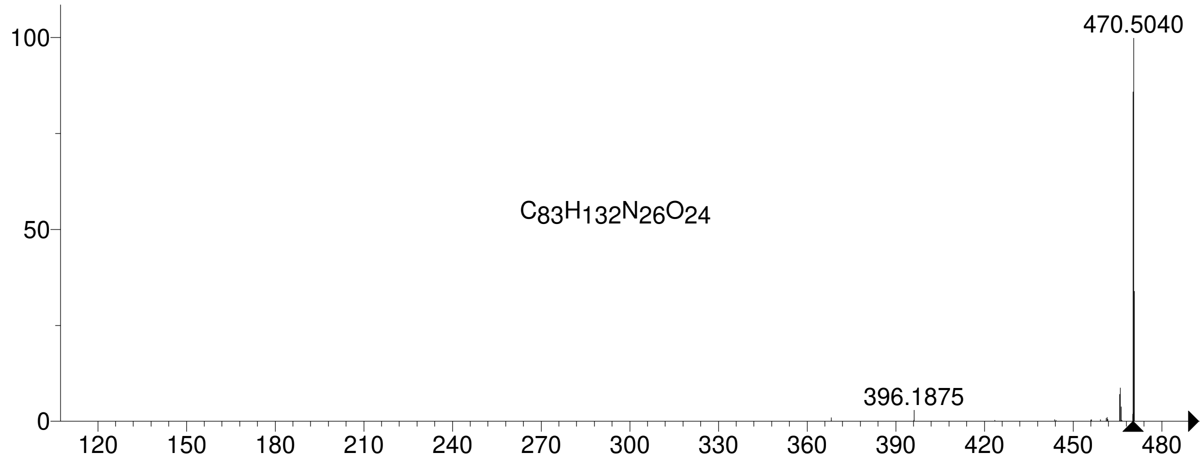

Unknown: Unknown (5.83_289.0007m/z)
Compound in Library Factor = 0

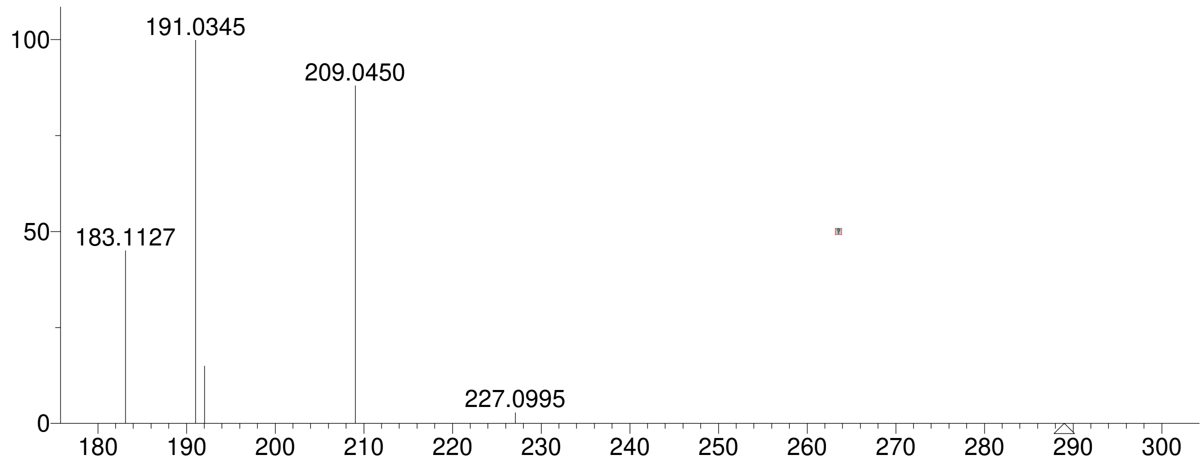

Hit 1 : Boscalid [M+H-HCl]+=>289.0 IT 35% P=289
C18H12Cl2N2O; MF: 0; RMF: 73; Prob 14.3%; CAS: 188425-85-6; Lib: nist_msms; ID: 472656.

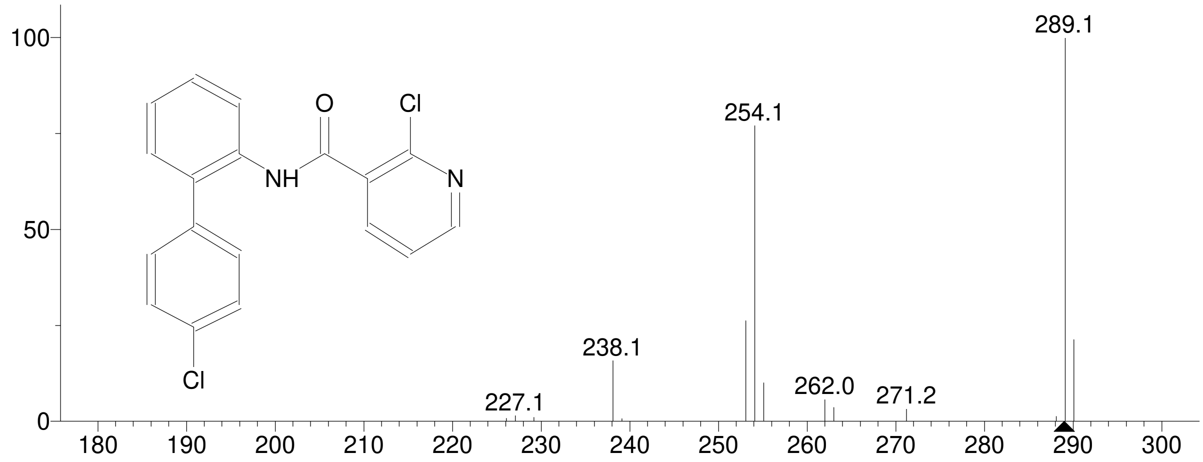

Hit 2 : Argininosuccinate [M-H]- 30V P=289
C10H18N4O6; MF: 0; RMF: 32; Prob 14.3%; Lib: mona-export-lc-ms-ms_negative_mode; ID: 5510.

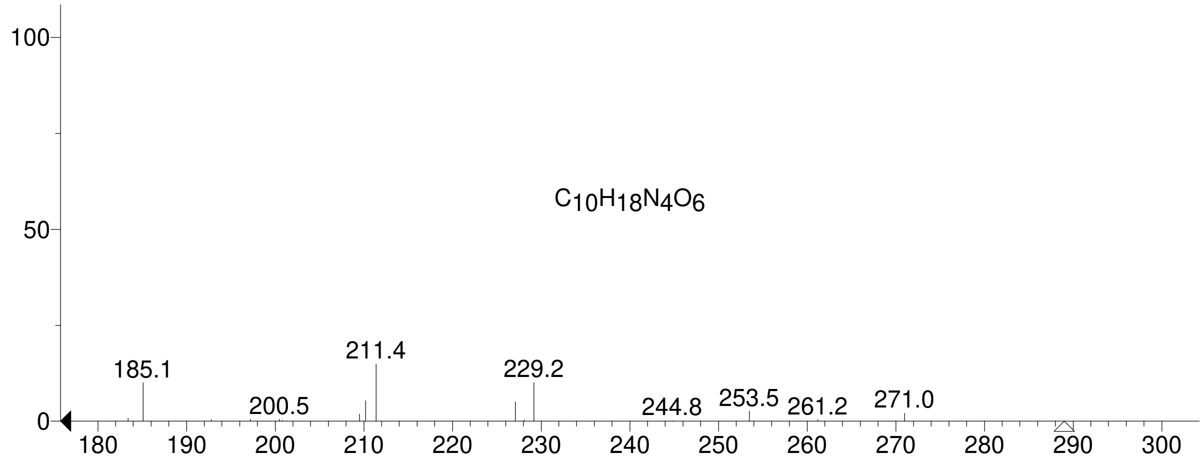

Hit 3 : Glycyrrhizic acid [M-H-C30H46O4]-=>289.0 IT 35% P=289
C42H62O16; MF: 0; RMF: 19; Prob 14.3%; CAS: 1405-86-3; Lib: nist_msms; ID: 233516.

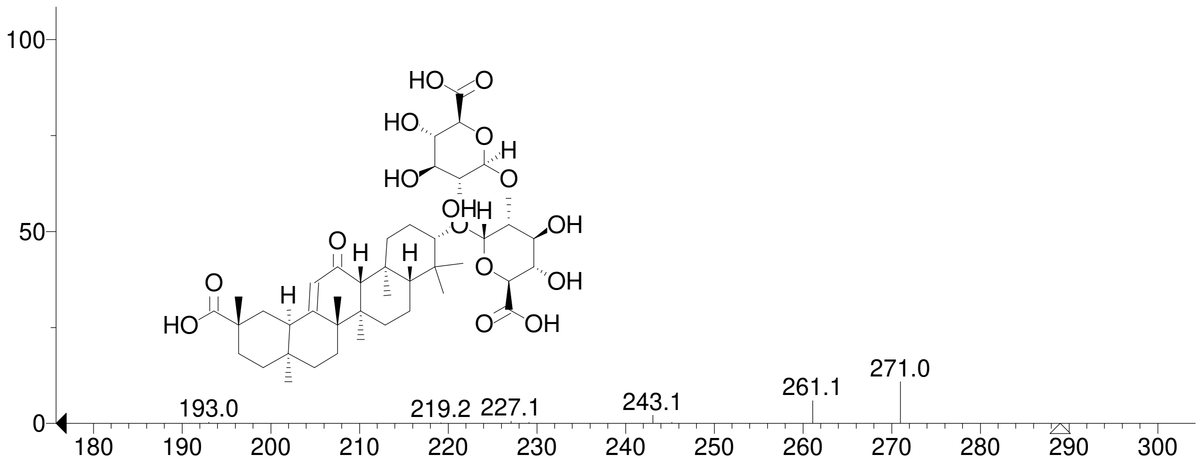

Hit 4 : (-)-Epicatechin gallate [M-H]-=>289.0 IT 35% P=289
C22H18O10; MF: 0; RMF: 11; Prob 14.3%; CAS: 1257-08-5; Lib: nist_msms; ID: 233423.

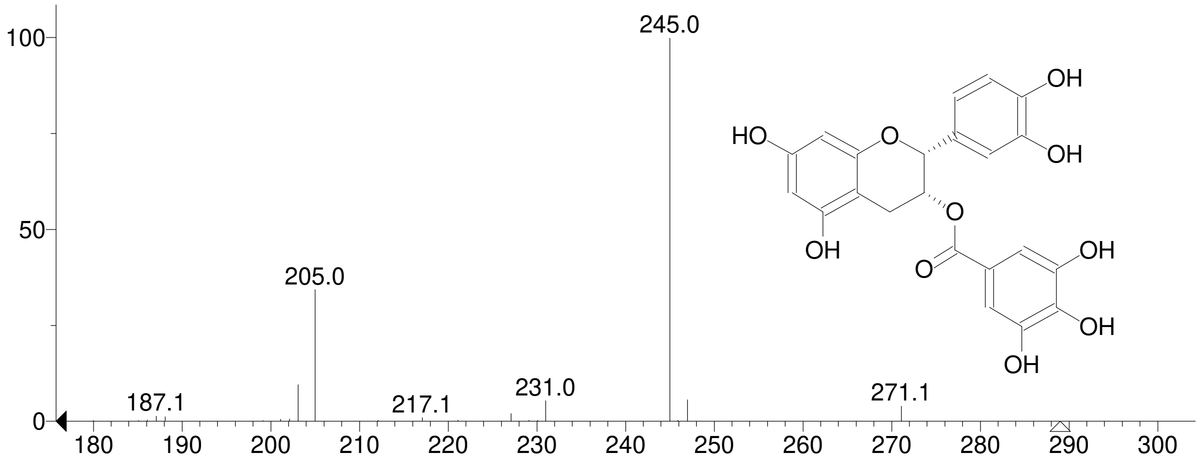

Hit 5 : D-Glucose, 2-deoxy-2-[(7-nitro-2,1,3-benzoxadiazol-4-yl)amino]- [M+H-2H2O]+=>289.0 IT 35% P=289
C12H14N4O8; MF: 0; RMF: 8; Prob 14.3%; CAS: 186689-07-6; Lib: nist_msms; ID: 253354.

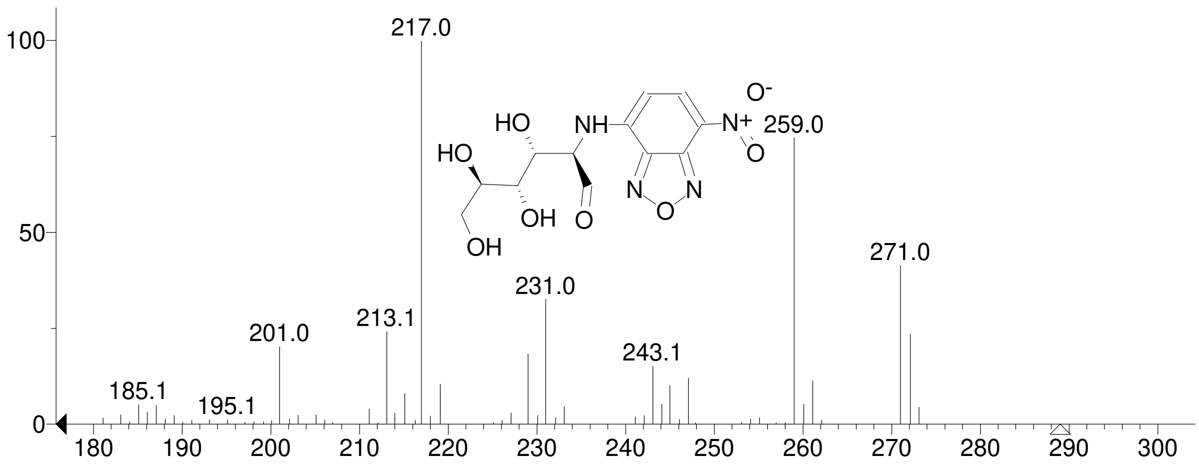

Unknown: Unknown (5.84_490.2388m/z)
Compound in Library Factor = 0

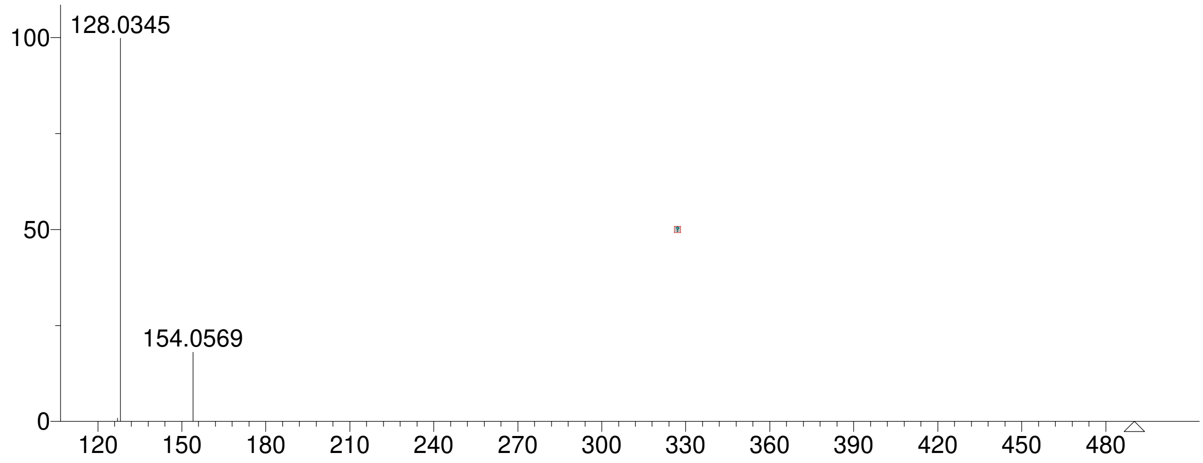

Hit 1 : Trp-Glu-Arg [M+H]+ QTOF 40V P=490.2
C22H31N7O6; MF: 0; RMF: 53; Prob 33.3%; Lib: nist_msms; ID: 54512.

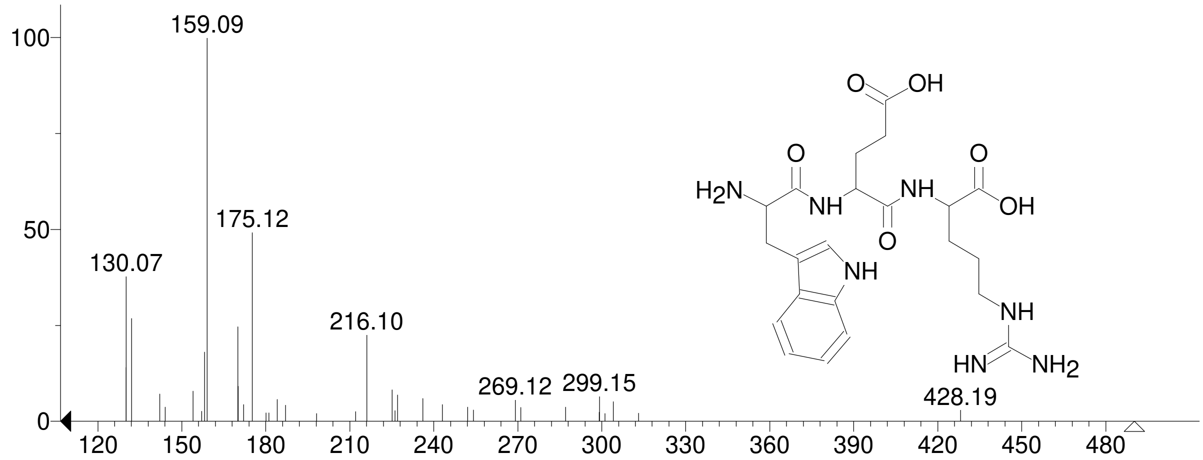

Hit 2 : Ethynylestradiol 3-β-D-glucuronide [M+NH4]+ HCD 156V P=490.2
C26H32O8; MF: 0; RMF: 24; Prob 33.3%; CAS: 60134-76-1; Lib: nist_msms; ID: 438271.

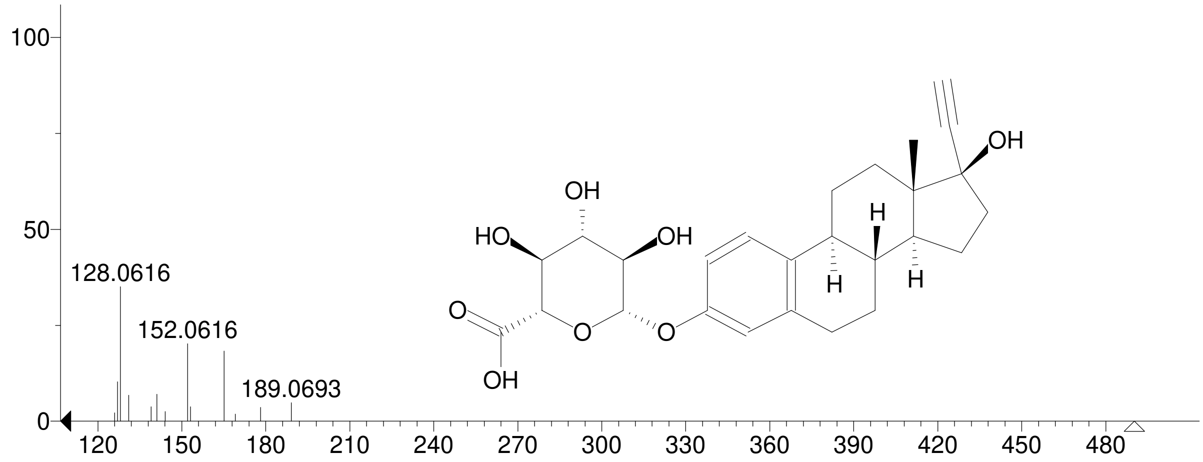

Hit 3 : Ethynylestradiol 17-β-D-glucuronide [M+NH4]+ HCD 156V P=490.2
C26H32O8; MF: 0; RMF: 22; Prob 33.3%; CAS: 75803-39-3; Lib: nist_msms; ID: 438418.

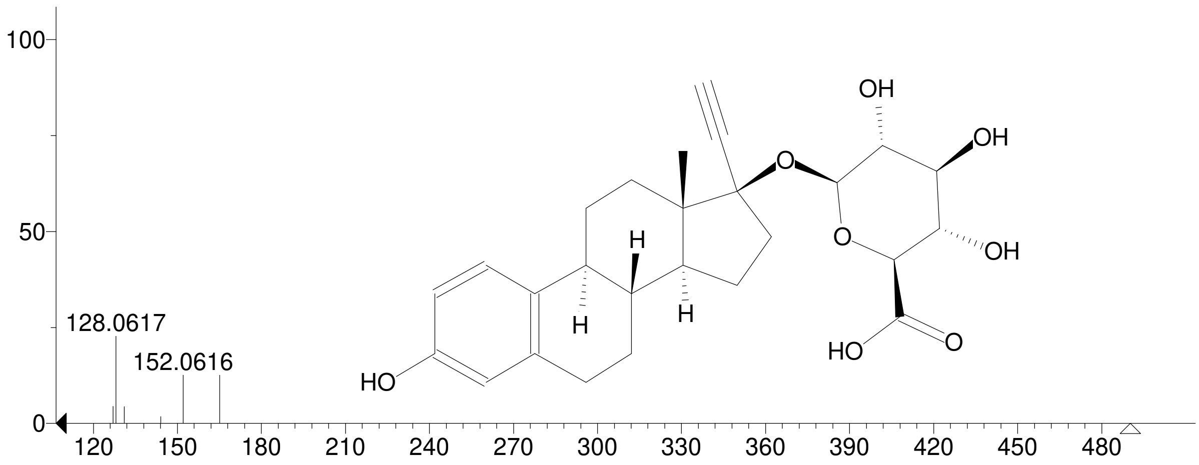

** Search Report Page 1 of 1 **

Unknown: Unknown (5.86_356.1459m/z)
Compound in Library Factor = 0

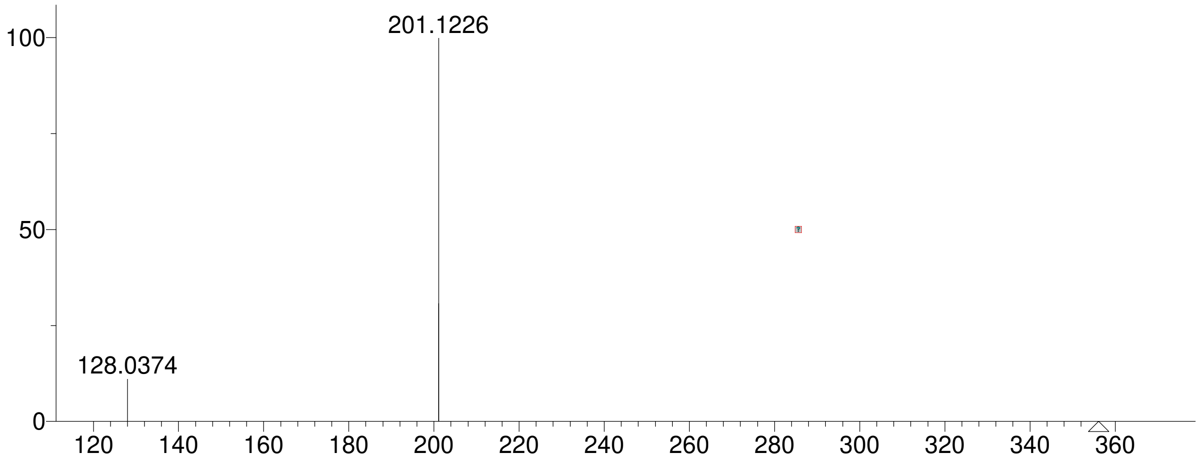

Unknown: Unknown (5.86_516.2167m/z)
Compound in Library Factor = 0

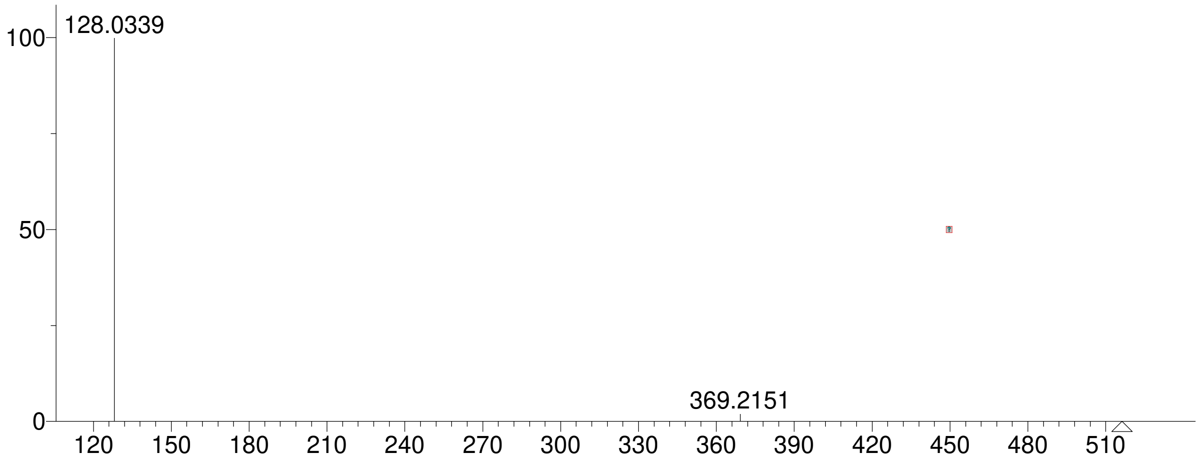

Unknown: Unknown (5.88_413.1651m/z)
Compound in Library Factor = 0

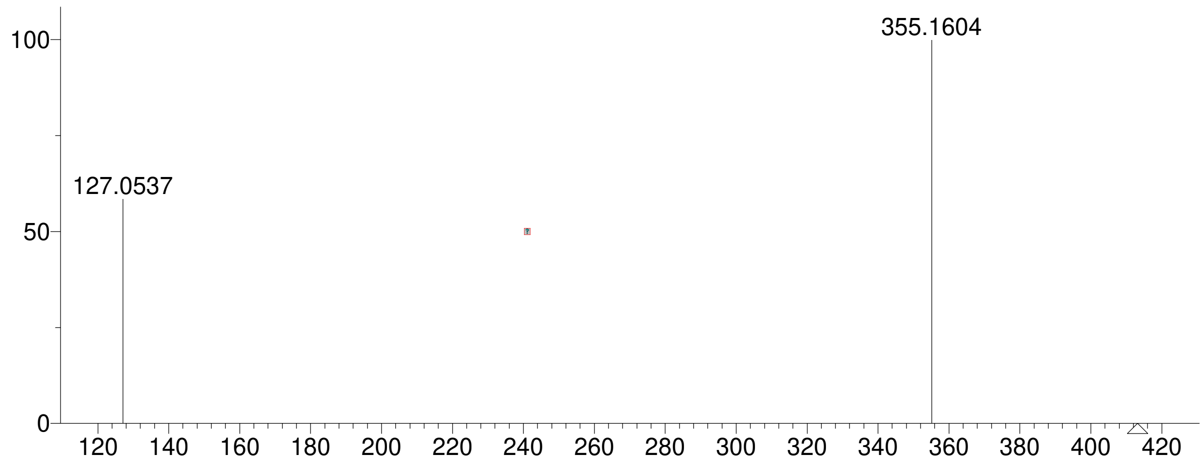

Hit 1 : 2-Hydroxymethylloratadine [M+H]+ HCD 132V P=413.2
C23H25ClN2O3; MF: 0; RMF: 27; Prob 50.0%; CAS: 609806-39-5; Lib: nist_msms; ID: 442227.

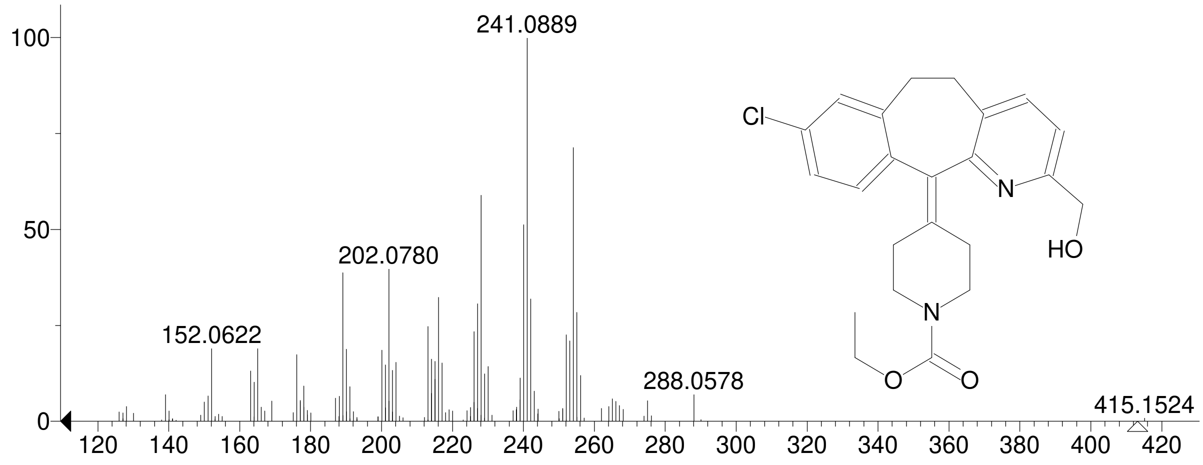

Hit 2 : 4-Hydroxymethylloratadine [M+H]+ HCD 132V P=413.2
C23H25ClN2O3; MF: 0; RMF: 23; Prob 50.0%; CAS: 609806-40-8; Lib: nist_msms; ID: 442293.

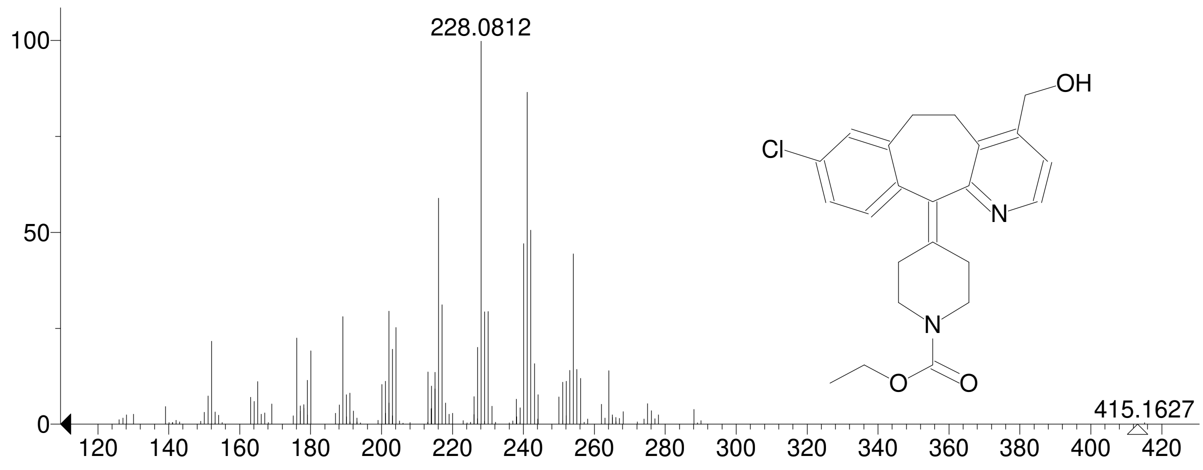

** Search Report Page 1 of 1 **

Unknown: Unknown (5.88_486.2067m/z)
Compound in Library Factor = 0

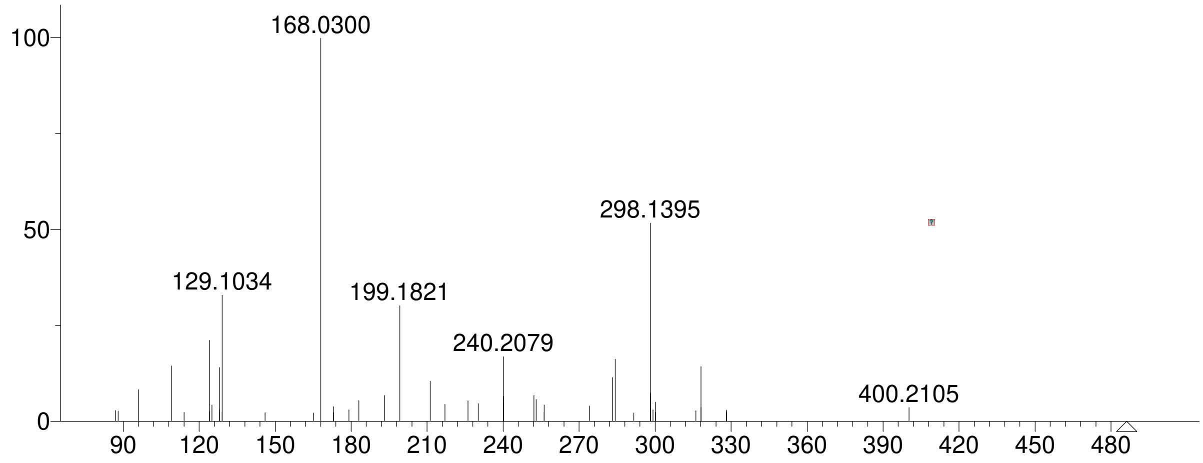

Hit 1 : rac-N-Allyloxycarbonyl-cis-moxifloxacin acyl-β-D-glucuronide allyl ester [M+H-C9H12O6]+ HCD 126V P=
C34H40FN3O12; MF: 0; RMF: 27; Prob 100.0%; Lib: nist_msms; ID: 431491.

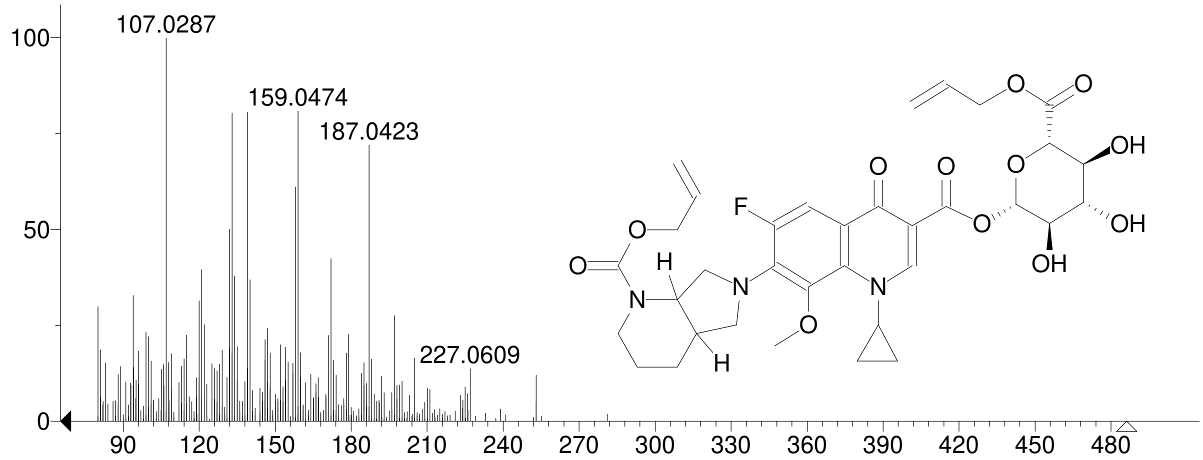

** Search Report Page 1 of 1 **

Unknown: Unknown (5.94_246.9906m/z)
Compound in Library Factor = 0

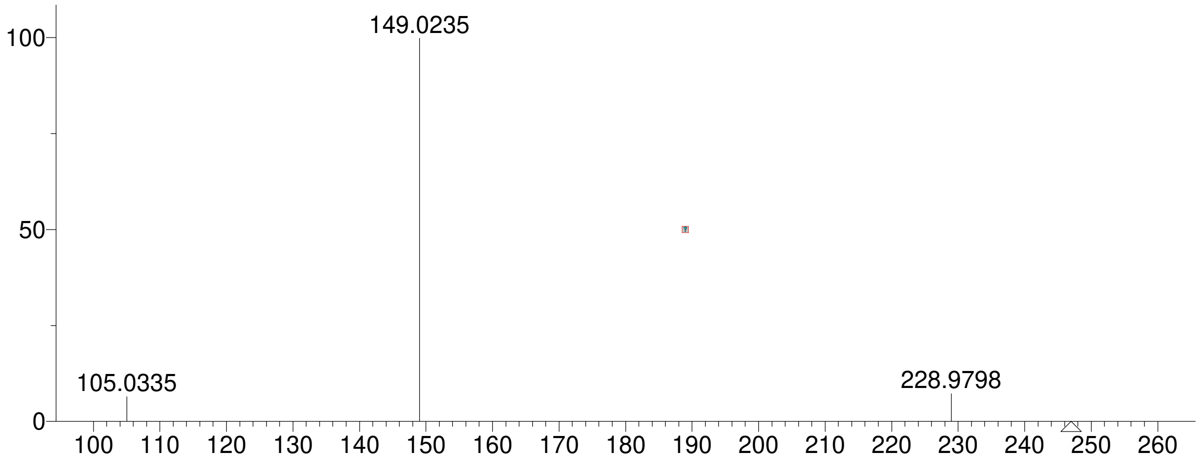

Unknown: Unknown (5.94_472.2409m/z)
Compound in Library Factor = -3273

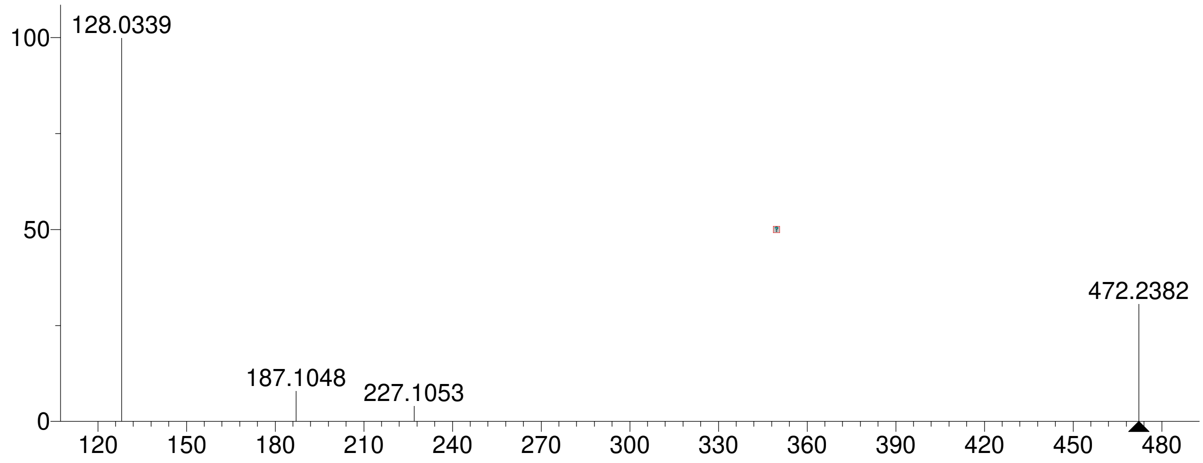

Hit 1 : lysoPE 16:2 [M+Na]+ 25eV P=472.2
C21H40NO7P; MF: 2; RMF: 98; Prob 100.0%; Lib: mona-export-in-silico_spectra; ID: 35970.

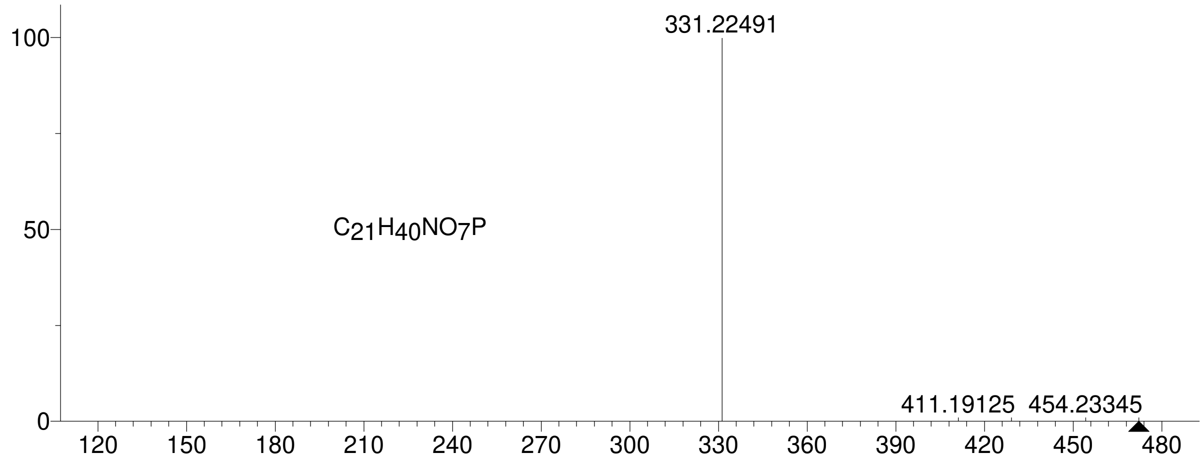

Unknown: Unknown (5.94_545.1998m/z)
Compound in Library Factor = -3273

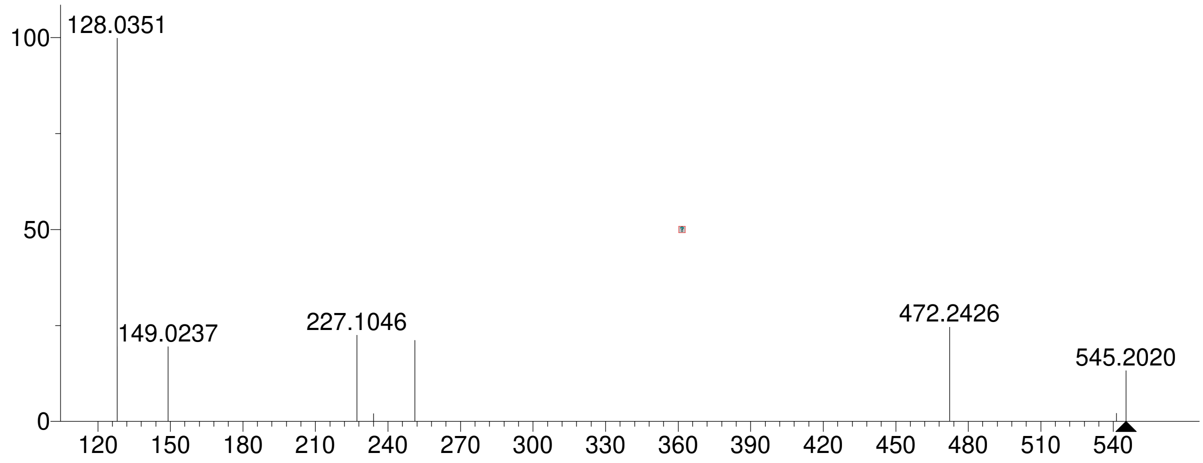

Hit 1 : 2-(hydroxymethyl)-6-[5-[3-(hydroxymethyl)-5-(3-hydroxypropyl)-7-methoxy-2,3-dihydro-1-benzofuran-2-yl]-2
C26H34O11; MF: 2; RMF: 179; Prob 10.7%; Lib: mona-export-vf-npl_qexactive; ID: 2287.

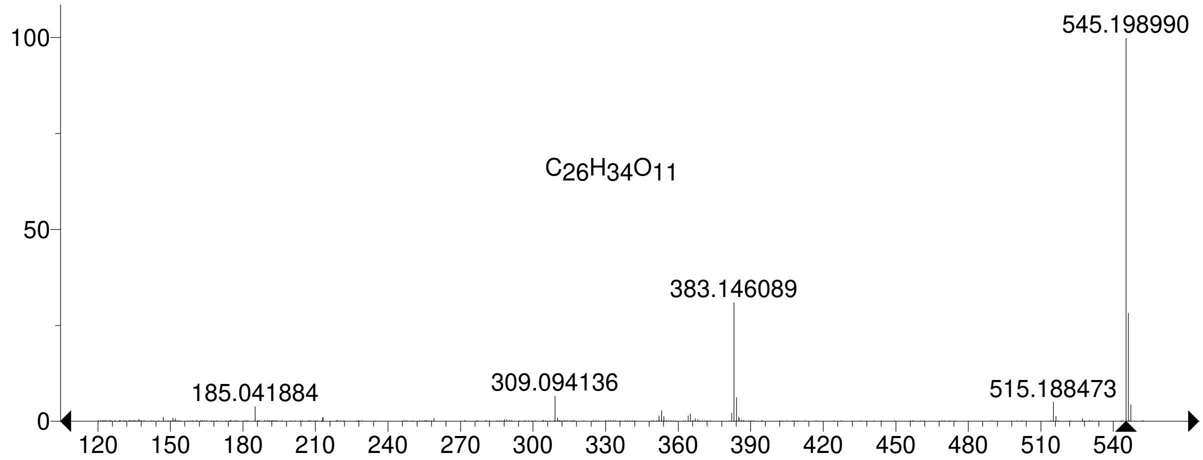

Hit 2 : (7R,8S)-dihydrodehydrodiconiferyl alcohol 9-O-beta-D-glucopyranoside [M+H]+ P=545.2
C26H34O11; MF: 1; RMF: 196; Prob 10.3%; Lib: mona-export-gnps; ID: 5804.

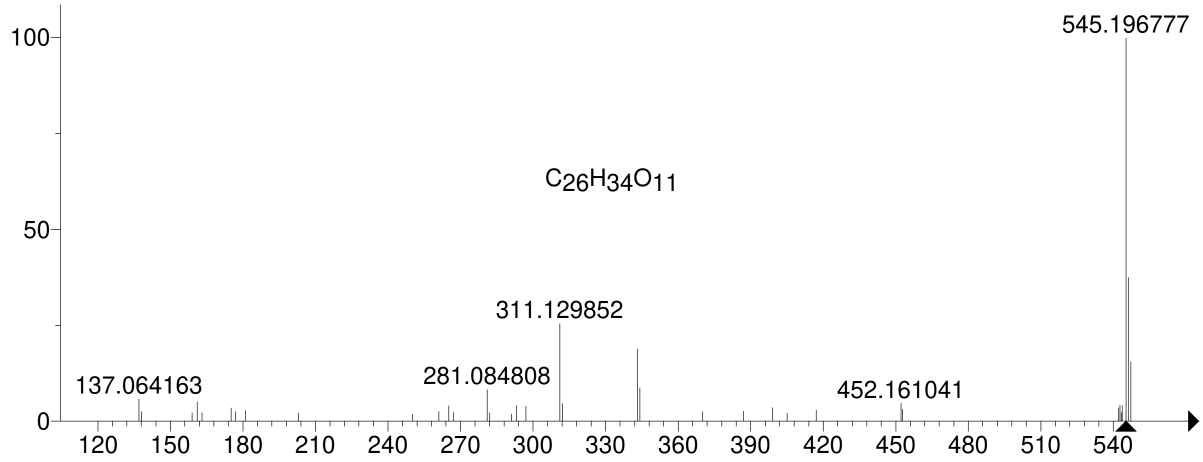

Hit 3 : NCGC00384838-01_C26H34O11_3-[2-(4-Hydroxy-3-methoxyphenyl)-3-(hydroxymethyl)-7-methoxy-2,3-dil
C26H34O11; MF: 0; RMF: 205; Prob 9.87%; Lib: mona-export-gnps; ID: 10977.

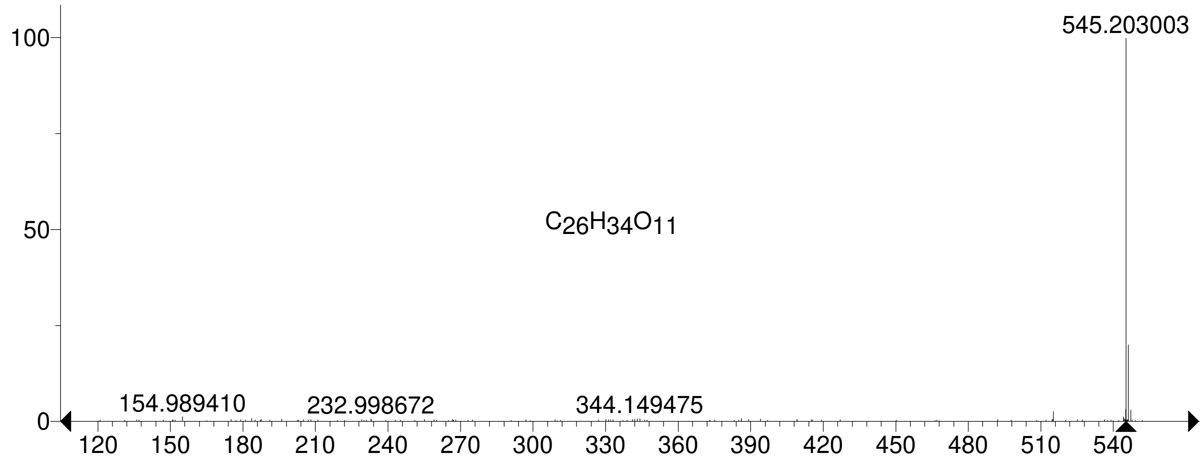

Hit 4 : (2R,3R,4S,5S,6R)-2-[3-[2-(4-hydroxy-3-methoxyphenyl)-3-(hydroxymethyl)-7-methoxy-2,3-dihydro-1-benzo
C26H34O11; MF: 0; RMF: 184; Prob 9.87%; Lib: anthocyanins mona; ID: 7235.

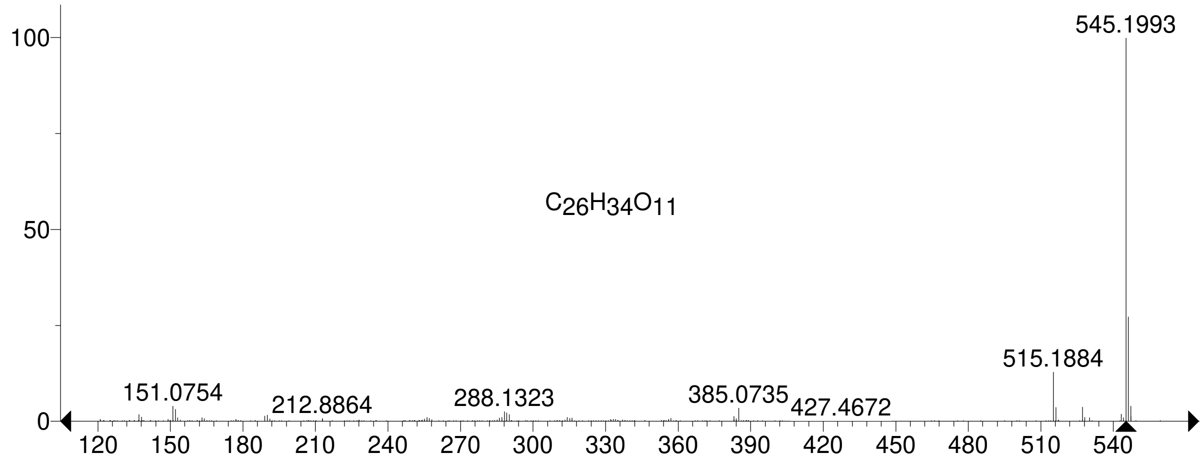

Hit 5 : NCGC00385233-01_C26H34O11_2-{2-Hydroxy-5-[(1E)-3-hydroxy-1-propen-1-yl]-3-methoxyphenyl}-3-(4-h
C26H34O11; MF: 0; RMF: 184; Prob 9.87%; Lib: mona-export-gnps; ID: 12015.

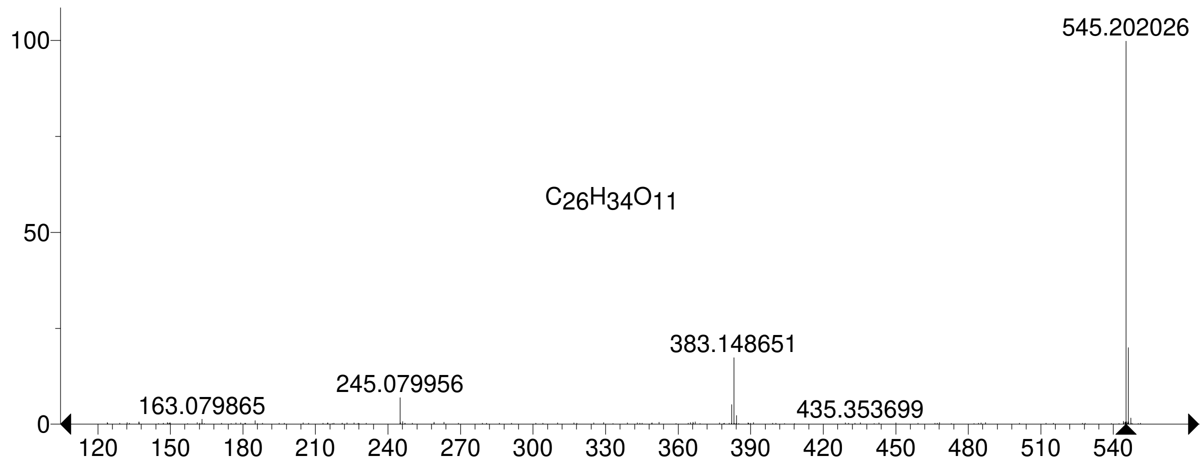

Unknown: Unknown (5.94_609.2043m/z)
Compound in Library Factor = 0

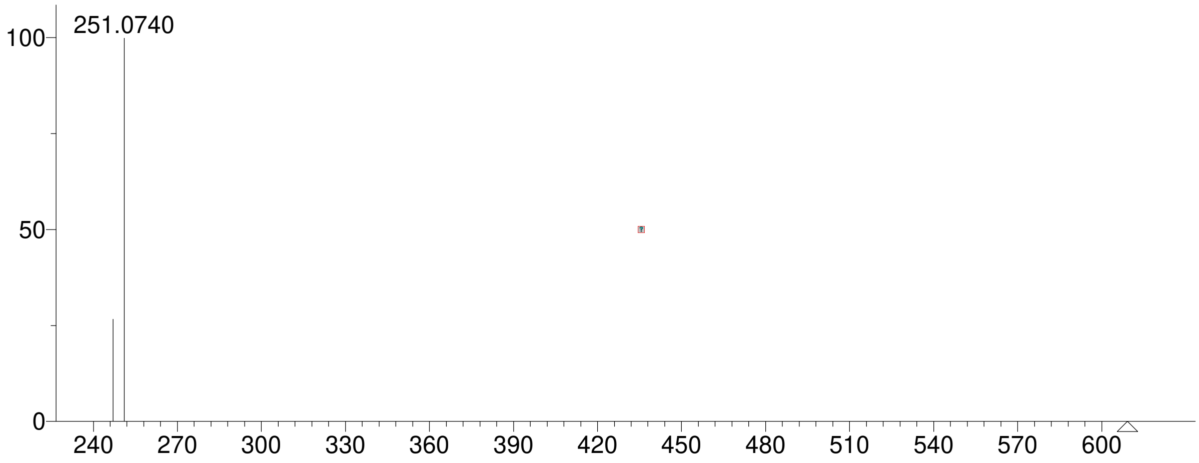

Unknown: Unknown (5.96_328.1871m/z)
Compound in Library Factor = 0

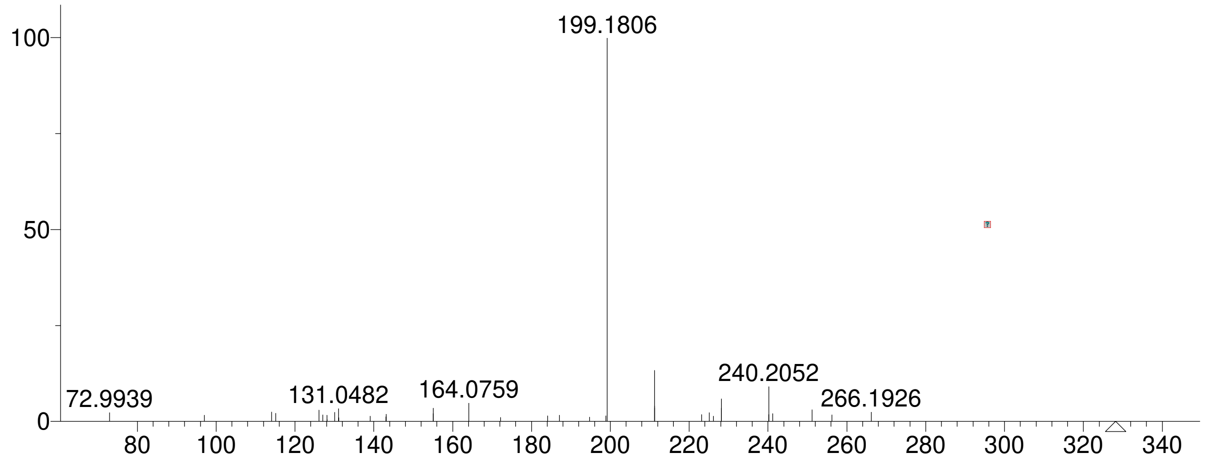

Hit 1 : CAPSAICIN [M+Na]+ 65HCD P=328.2
C18H27NO3; MF: 0; RMF: 4; Prob 33.3%; Lib: mona-export-vf-npl_qexactive; ID: 2967.

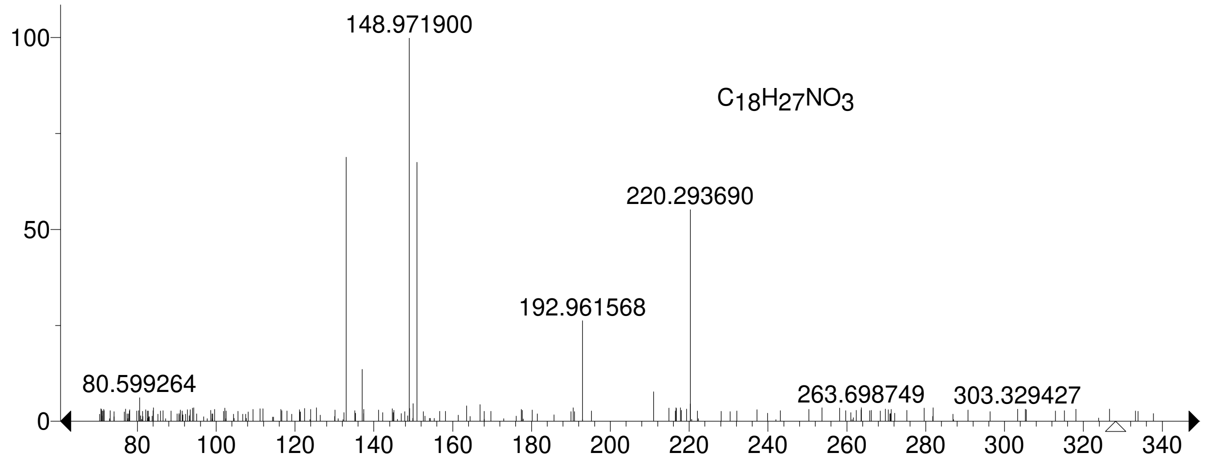

Hit 2 : Capsaicin [M+Na]+ 35HCD P=328.2
C18H27NO3; MF: 0; RMF: 2; Prob 33.3%; Lib: mona-export-vf-npl_qexactive; ID: 647.

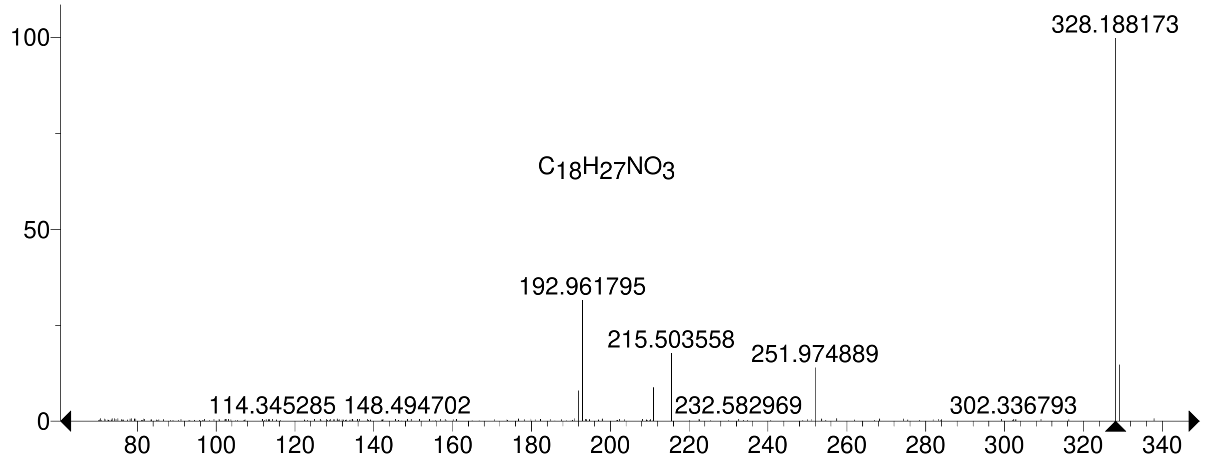

Hit 3 : Benactyzine [M+H]+ 20-30-40%) P=328.2
C20H25NO3; MF: 0; RMF: 1; Prob 33.3%; Lib: mona-export-fiehn_hilic; ID: 218.

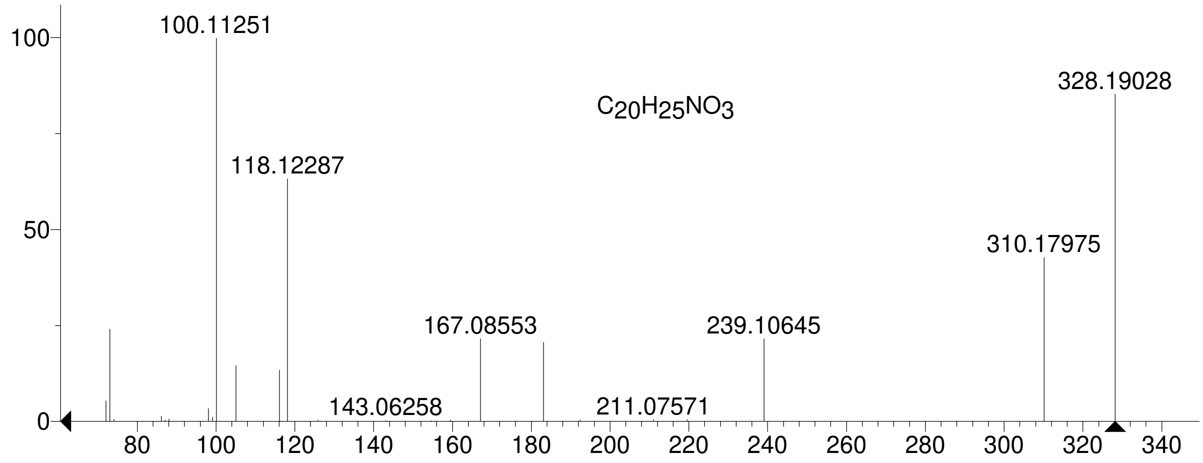

** Search Report Page 1 of 1 **

Unknown: Unknown (5.96_368.1362m/z)
Compound in Library Factor = 0

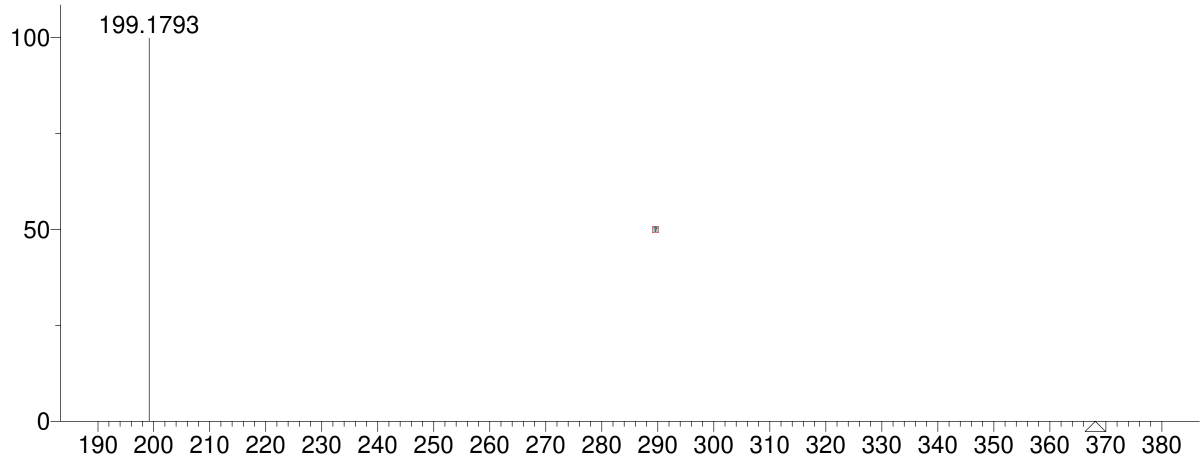

** Search Report Page 1 of 1 **

Unknown: Unknown (5.96_527.2093m/z)
Compound in Library Factor = 0

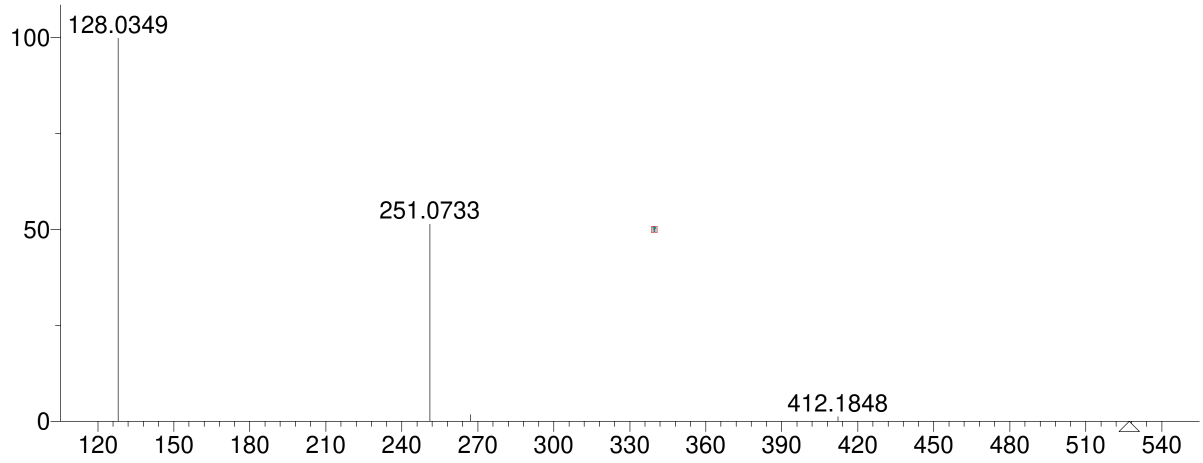

Hit 1 : [(1aR,1bS,2S,5aS,6R,6aS)-5a-hydroxy-1a-methyl-2-[(2S,3R,4S,5S,6R)-3,4,5-trihydroxy-6-(hydroxymethyl)
C25H36O12; MF: 0; RMF: 12; Prob 50.0%; Lib: mona-export-lc-ms-ms_negative_mode; ID: 20478.

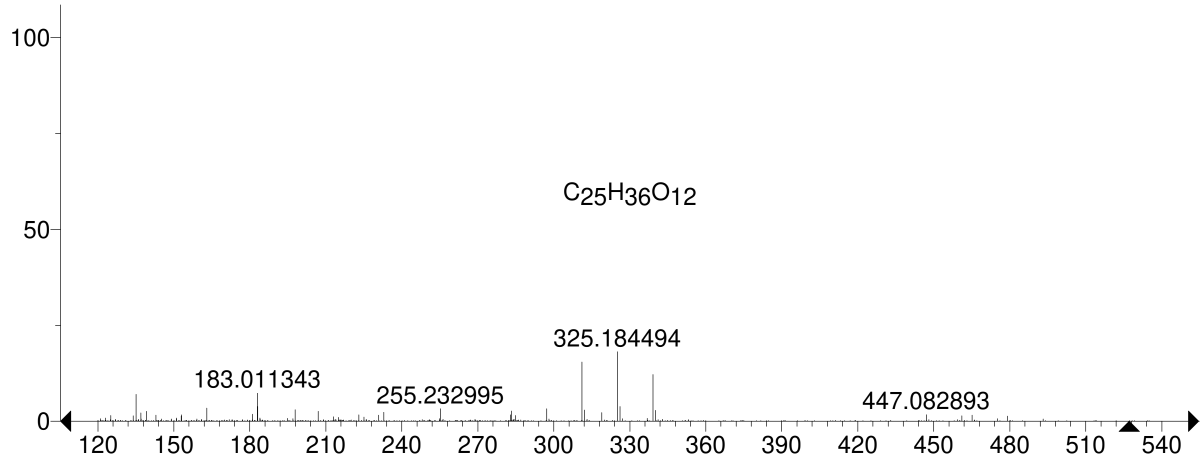

Hit 2 : 13-(acetyloxy)-1,6-dihydroxy-8-(hydroxymethyl)-4,12,12,15-tetramethyl-5-oxotetracyclo[8.5.0.0A^'2,a^!P.0A
C27H36O8; MF: 0; RMF: 2; Prob 50.0%; Lib: mona-export-vf-npl_qexactive; ID: 20116.

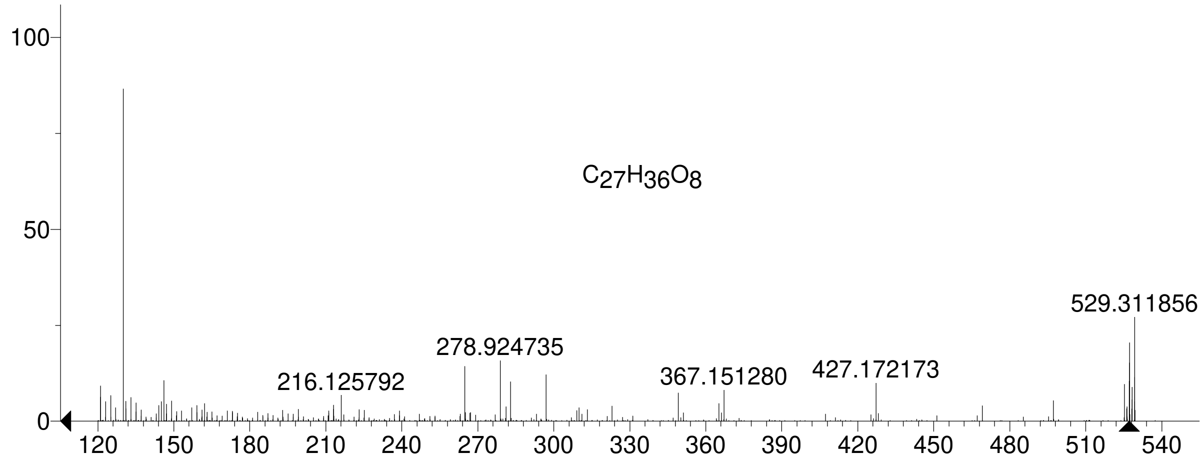

Unknown: Unknown (5.97_367.1603m/z)
Compound in Library Factor = 0

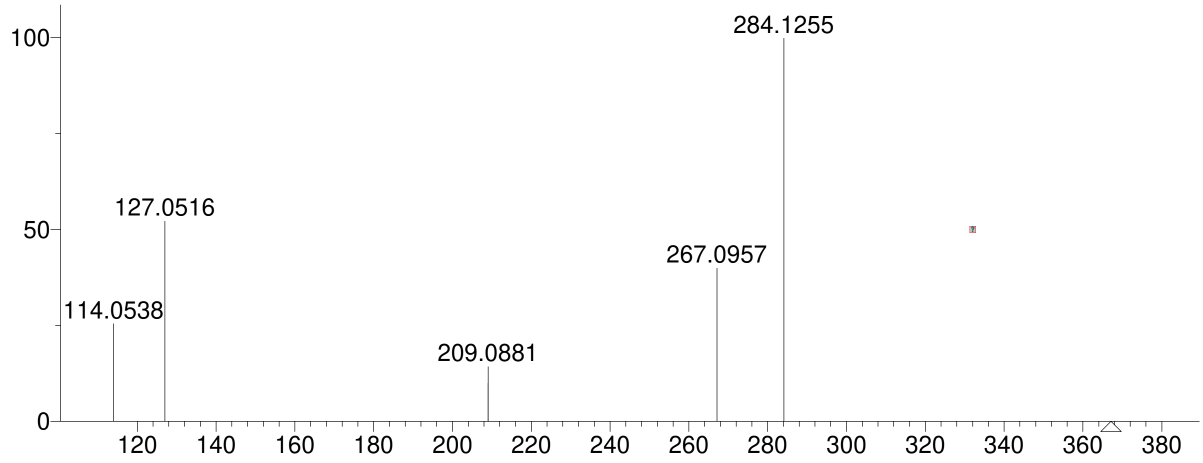

Hit 1 : Boldenone sulfate [M+H]+ HCD 117V P=367.2
C19H26O5S; MF: 0; RMF: 78; Prob 100.0%; CAS: 87331-43-9; Lib: nist_msms; ID: 383658.

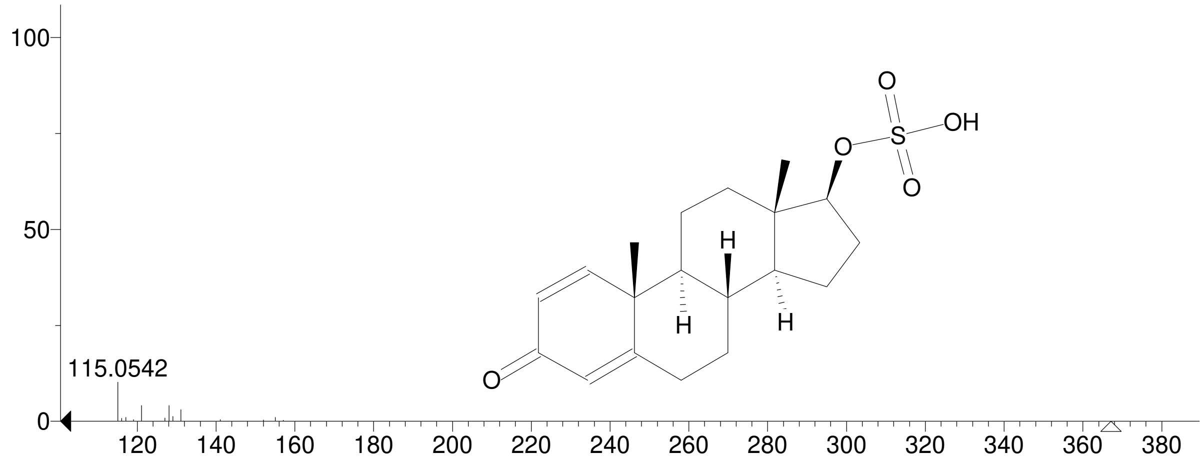

** Search Report Page 1 of 1 **

Unknown: Unknown (5.98_412.1823m/z)
Compound in Library Factor = 0

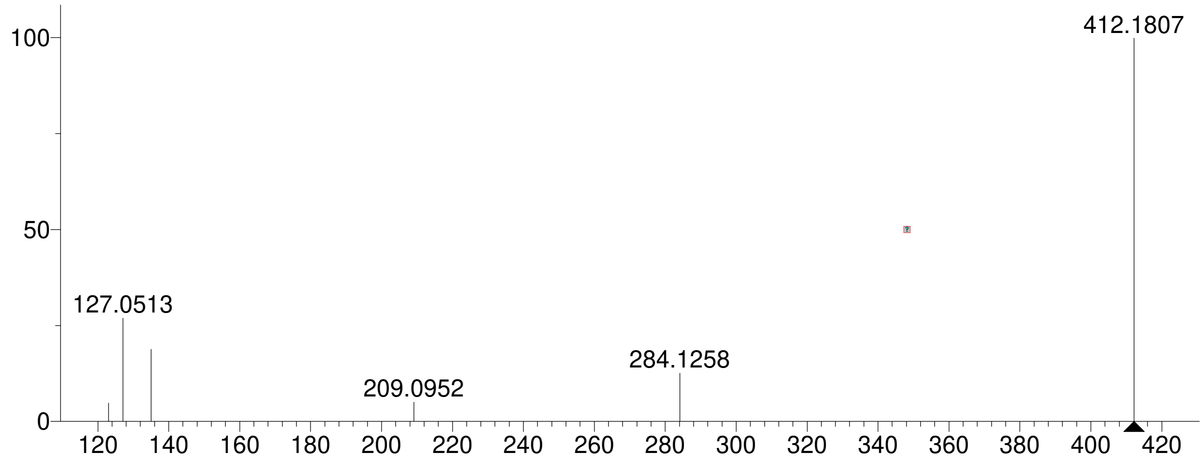

Unknown: Unknown (6.00_178.1230m/z)
Compound in Library Factor = -1144

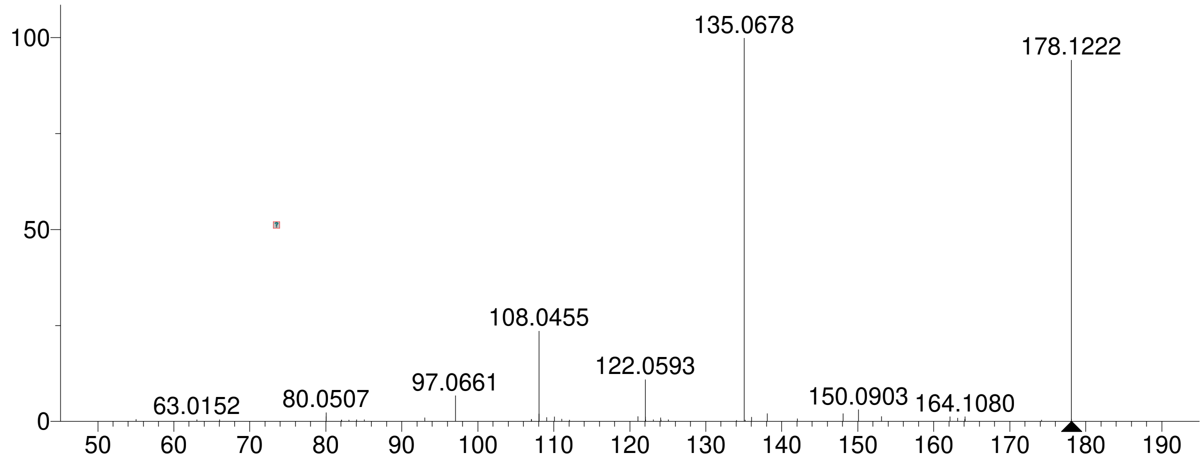

Hit 1 : 1H-Inden-2-amine, 2,3-dihydro-5-methoxy-6-methyl- [M+H]+ HCD 3V P=178.1
C11H15NO; MF: 292; RMF: 920; Prob 34.8%; CAS: 136468-19-4; Lib: nist_msms; ID: 364124.

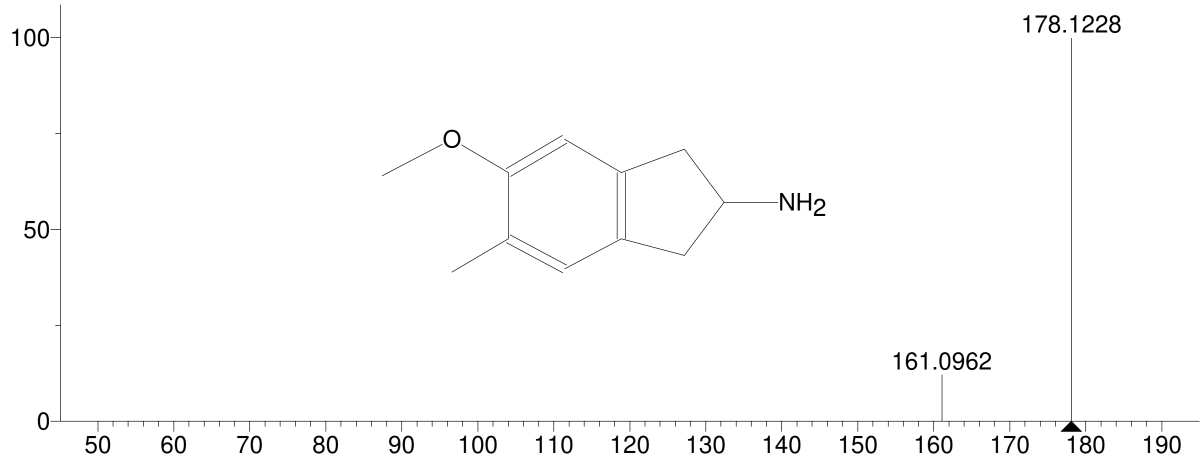

Hit 2 : 2-Methylmethcathinone [M+H]+ HCD 3V P=178.1
C11H15NO; MF: 289; RMF: 926; Prob 30.7%; CAS: 1246911-71-6; Lib: nist_msms; ID: 249570.

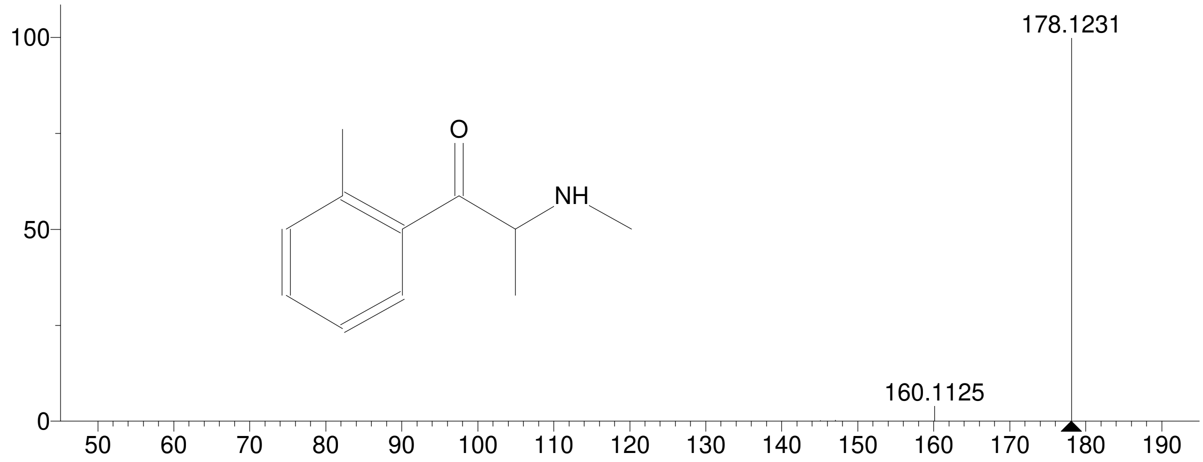

Hit 3 : 4-Methylmethcathinone [M+H]+ HCD 3V P=178.1
C11H15NO; MF: 286; RMF: 925; Prob 27.2%; CAS: 1189805-46-6; Lib: nist_msms; ID: 325130.

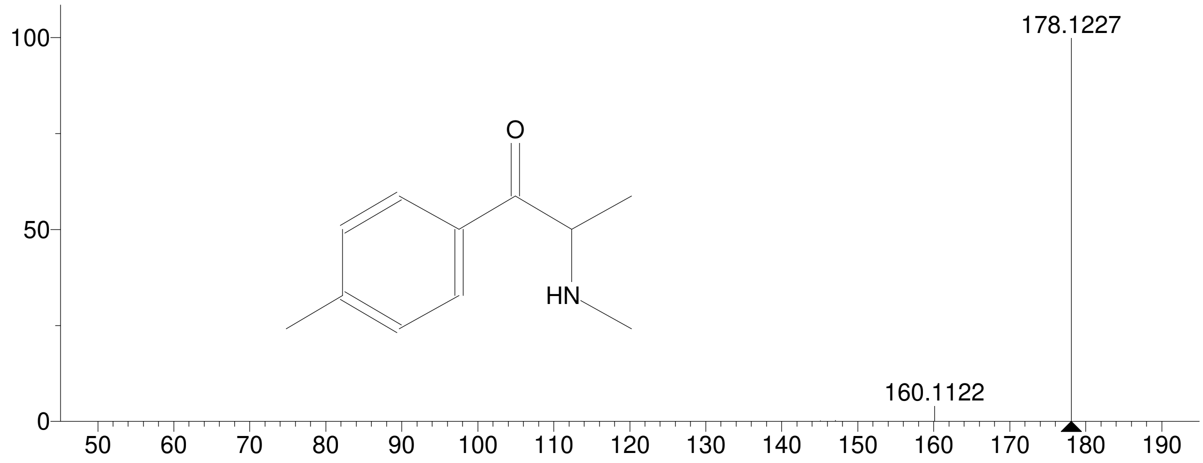

Hit 4 : 3-Methylmethcathinone [M+H]+ HCD 5V P=178.1
C11H15NO; MF: 238; RMF: 899; Prob 5.63%; CAS: 1246911-86-3; Lib: nist_msms; ID: 249602.

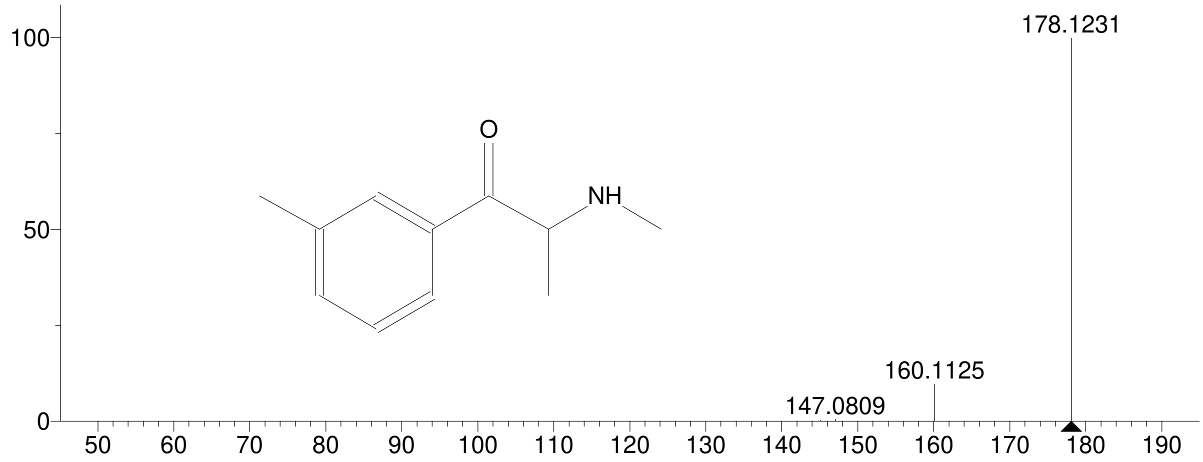

Hit 5 : N-tert-Butyl-α-phenylnitrone [M+H]+ HCD 1V P=178.1
C11H15NO; MF: 183; RMF: 644; Prob 1.09%; CAS: 3376-24-7; Lib: nist_msms; ID: 313590.

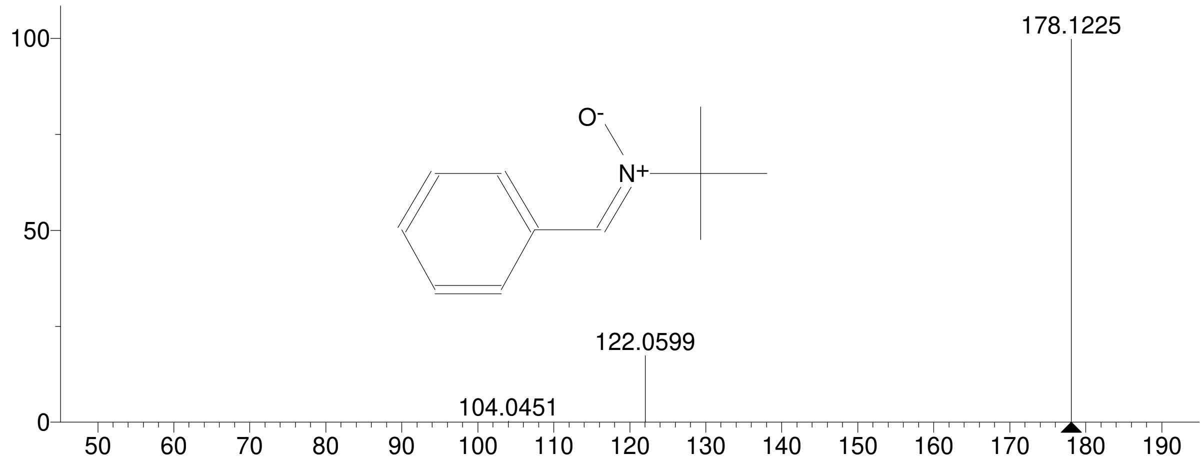

Unknown: Unknown (6.00_224.1283m/z)
Compound in Library Factor = -3273

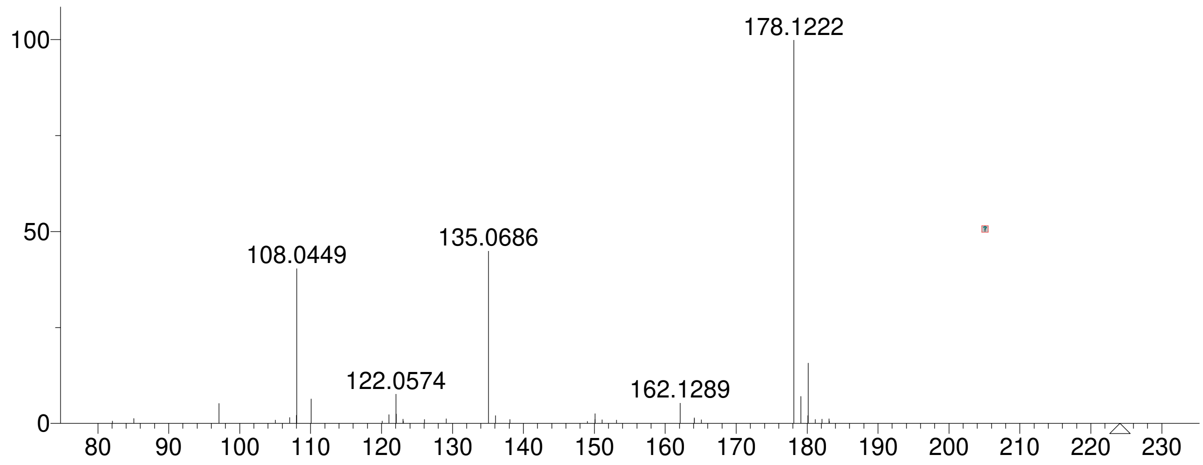

Hit 1 : 1-(3,5-Dihydroxyphenyl)-2-[(1,1-dimethylethyl)amino]ethanone [M+H]+ HCD 33V P=224.1
C12H17NO3; MF: 12; RMF: 110; Prob 22.6%; CAS: 139508-58-0; Lib: nist_msms; ID: 437127.

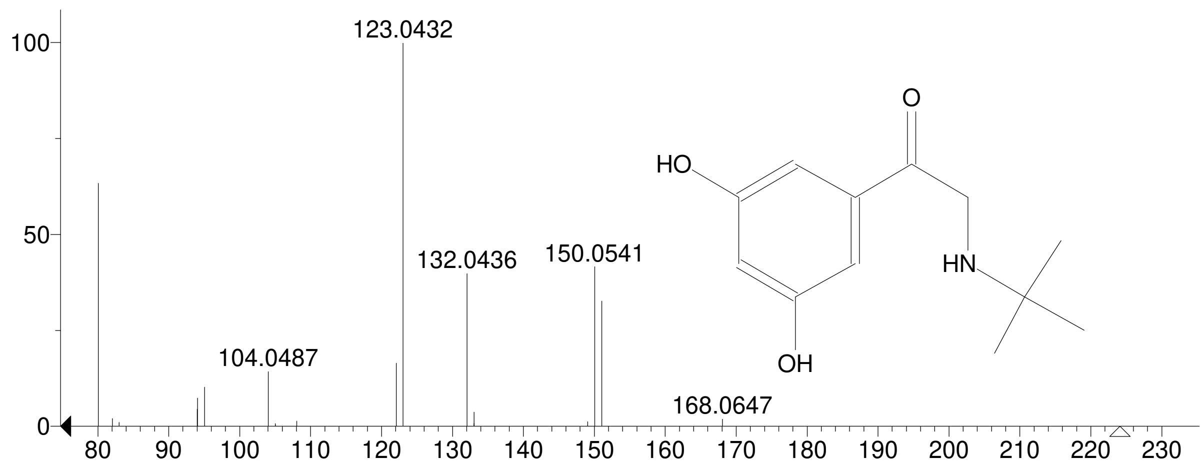

Hit 2 : N-(3-Oxooctanoyl)-L-homoserine lactone [M+H-H2O]+ HCD 20V P=224.1
C12H19NO4; MF: 9; RMF: 106; Prob 19.9%; CAS: 147795-39-9; Lib: nist_msms; ID: 315228.

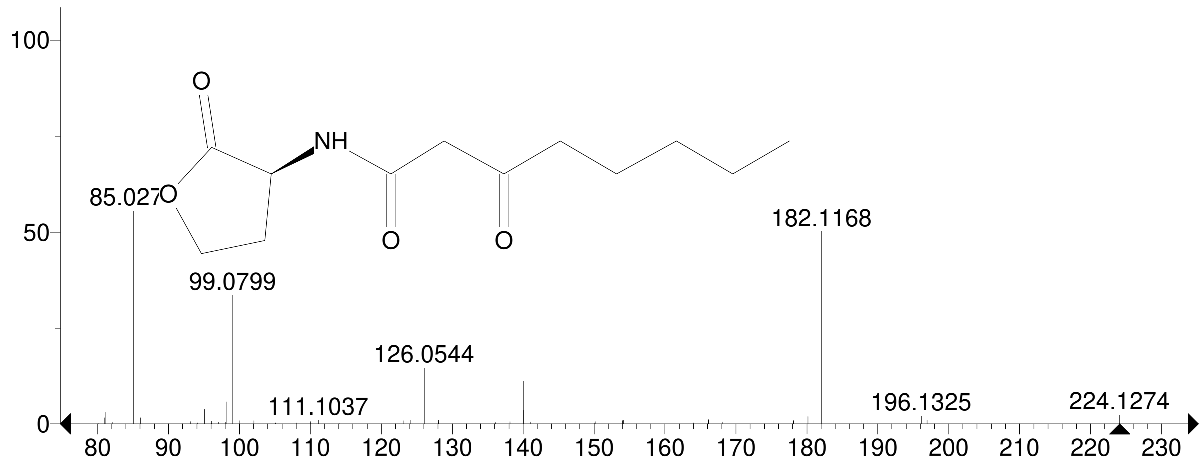

Hit 3 : Bufexamac [M+H]+ 45(no P=224.1
C12H17NO3; MF: 2; RMF: 72; Prob 15.3%; Lib: mona-export-massbank; ID: 12554.

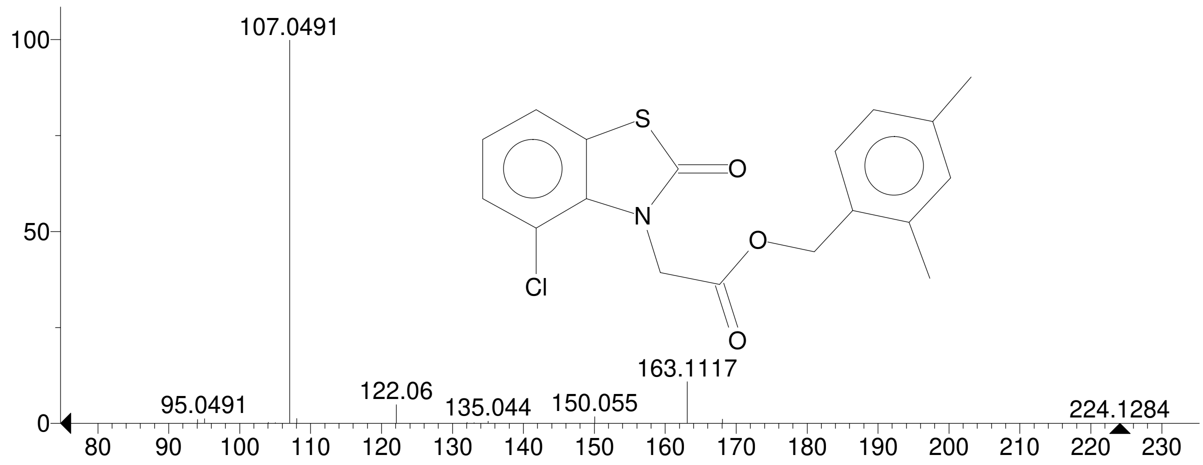

Hit 4 : Cerulenin [M+H]+ IT-FT 35% P=224.1
C12H17NO3; MF: 0; RMF: 124; Prob 14.1%; CAS: 17397-89-6; Lib: nist_msms; ID: 538608.

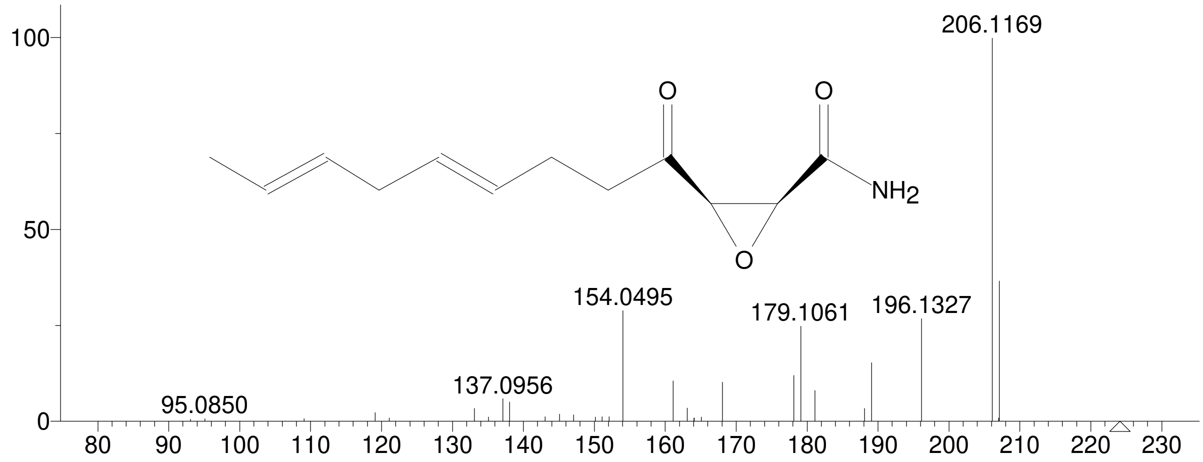

Hit 5 : Vanillic acid diethylamide [M+H]+ HCD 80V P=224.1
C12H17NO3; MF: 0; RMF: 19; Prob 14.1%; CAS: 304-84-7; Lib: nist_msms; ID: 133849.

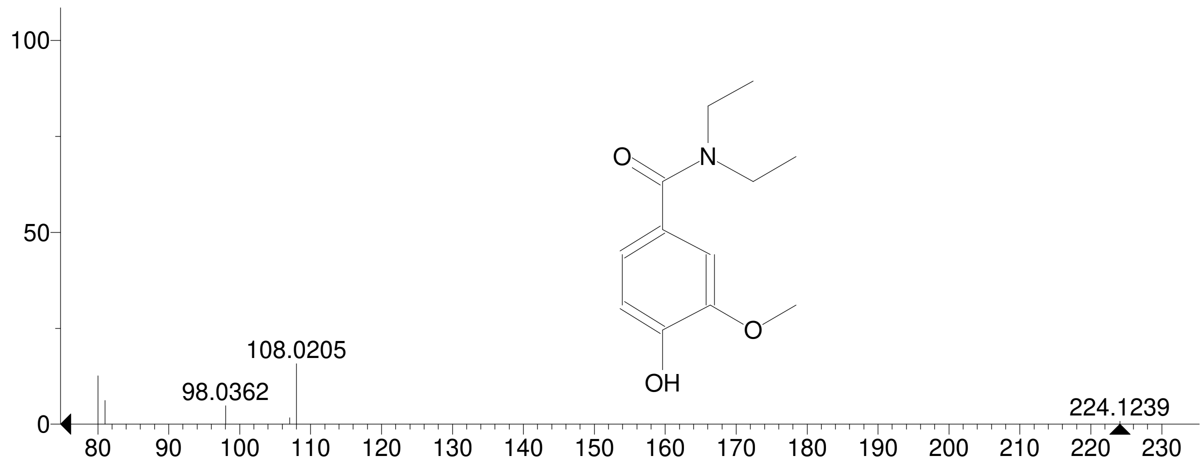

Unknown: Unknown (6.00_625.2534m/z)
Compound in Library Factor = -3273

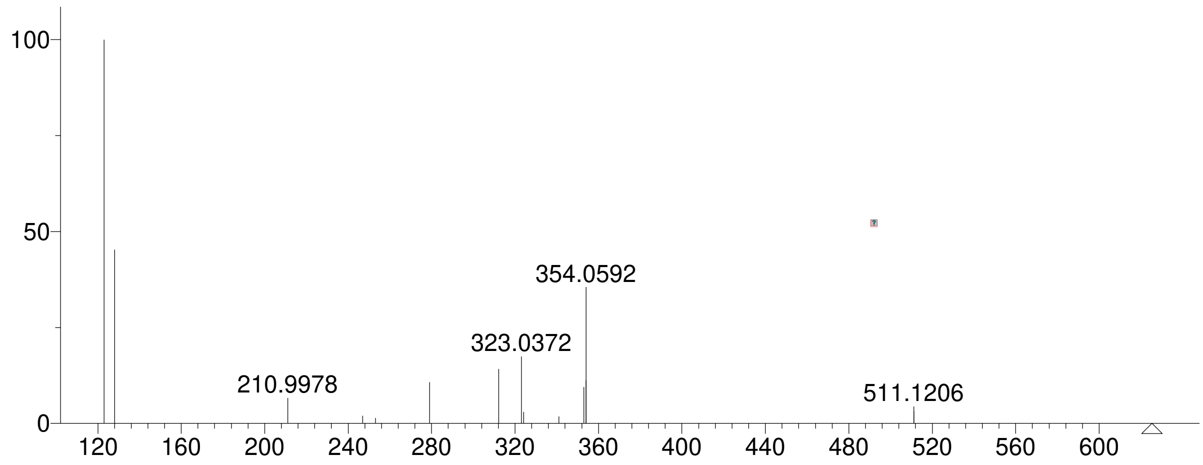

Hit 1 : N-Caffeoyl-O-methyltyramine [2M-H]- IT 35% P=625.3
C18H19NO4; MF: 9; RMF: 319; Prob 57.9%; CAS: 189307-47-9; Lib: nist_msms; ID: 221344.

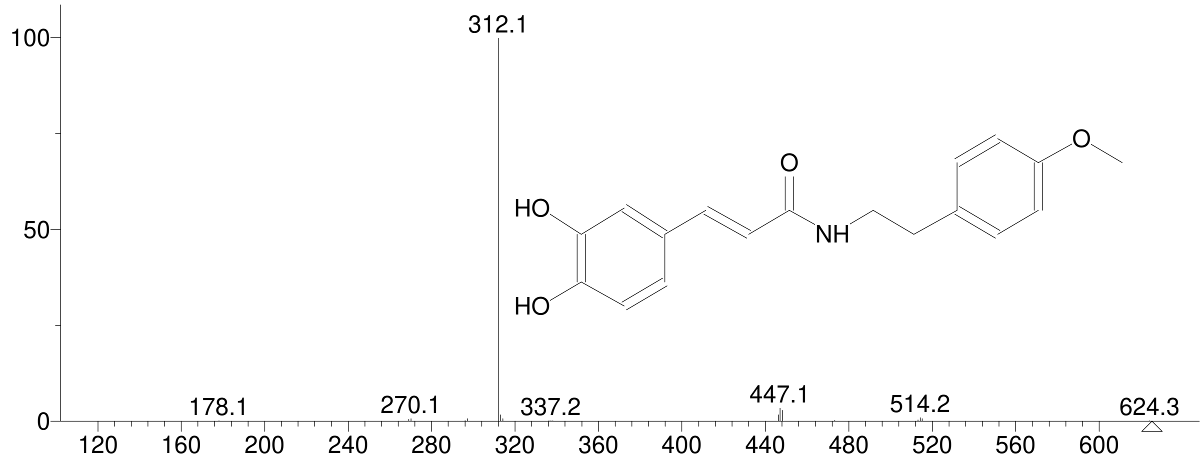

Hit 2 : NCGC00385791-01! [M+FA-H]- P=625.3
C29H40O12; MF: 0; RMF: 43; Prob 42.1%; Lib: mona-export-gnps; ID: 6389.

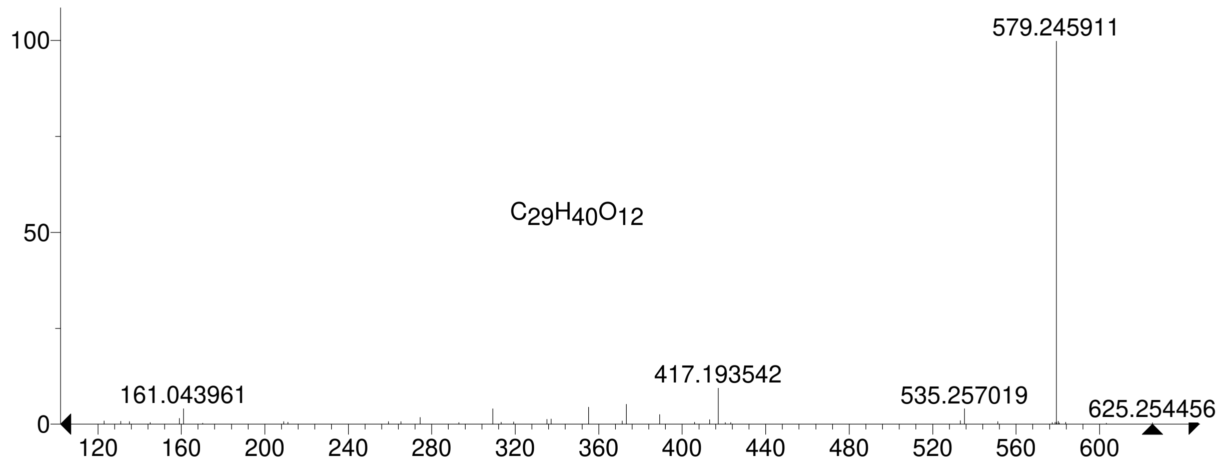

Unknown: Unknown (6.02_654.2085m/z)
Compound in Library Factor = 0

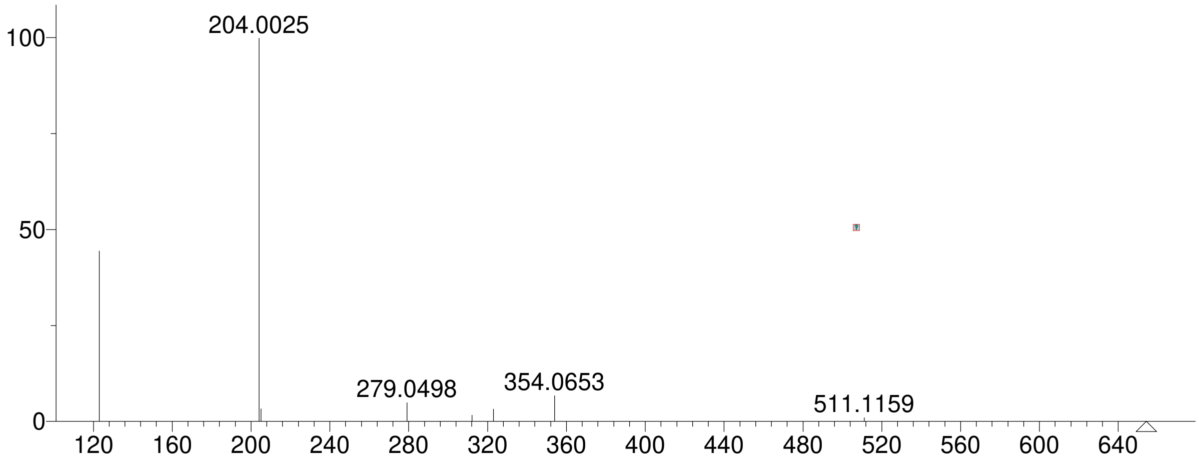

Unknown: Unknown (6.03_356.1447m/z)
Compound in Library Factor = -3273

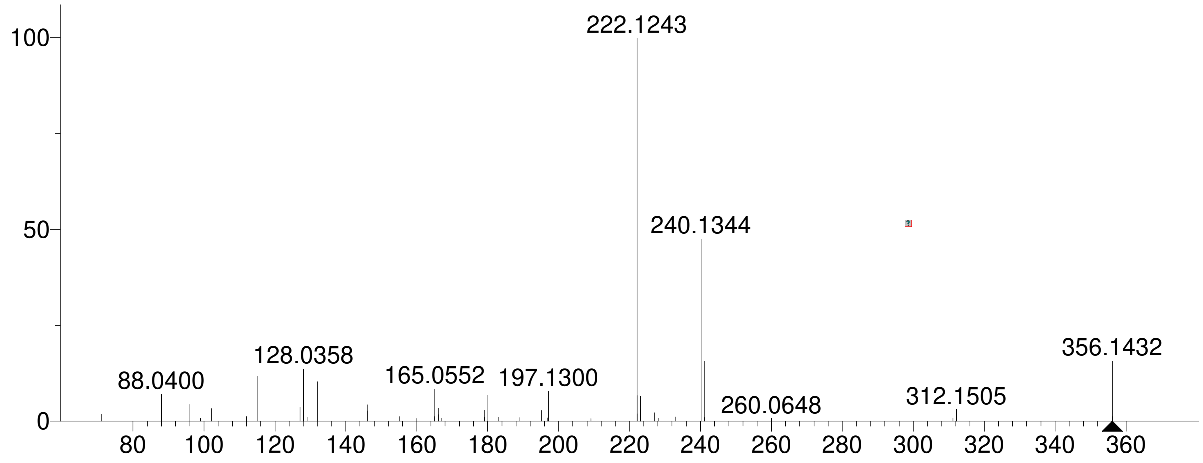

Hit 1 : Benzenesulfonamide, 4-(1,1-dimethylethyl)-N-[4-(1H-imidazol-1-yl)phenyl]- [M+H]+ HCD 21V P=356.1
C19H21N3O2S; MF: 18; RMF: 235; Prob 26.5%; CAS: 945526-43-2; Lib: nist_msms; ID: 355342.

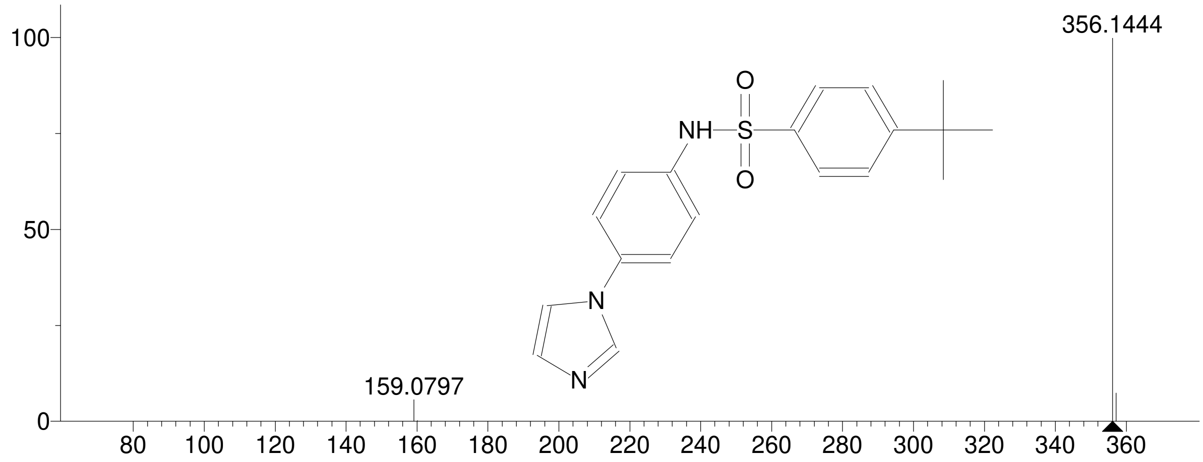

Hit 2 : Ethanone, 2-(2-chlorophenyl)-1-[1-(5-hydroxypentyl)-1H-indol-3-yl]- [M+H]+ HCD 12V P=356.1
C21H22ClNO2; MF: 12; RMF: 221; Prob 20.8%; CAS: 1449690-09-8; Lib: nist_msms; ID: 269487.

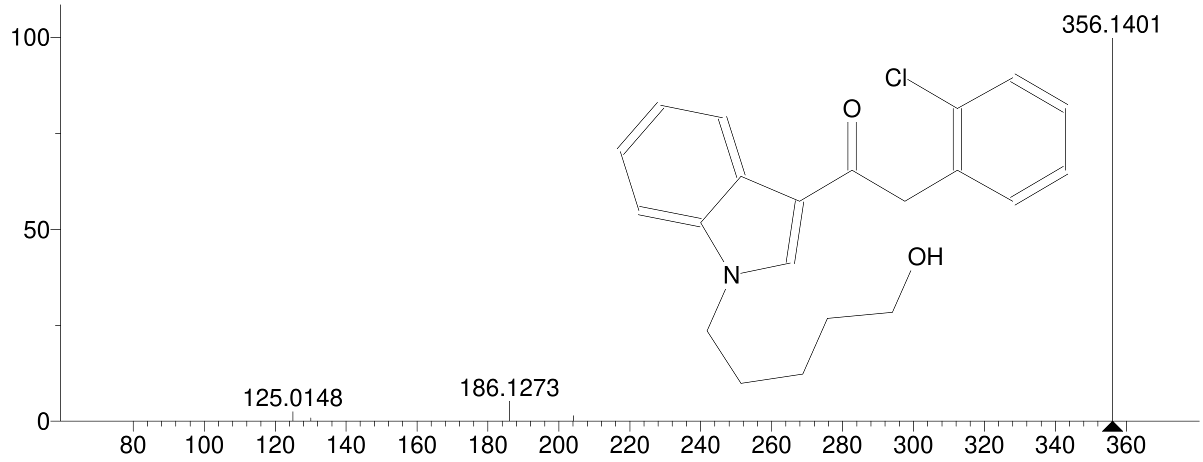

Hit 3 : Ethanone, 2-(2-chlorophenyl)-1-[1-(4-hydroxypentyl)-1H-indol-3-yl]- [M+H]+ HCD 10V P=356.1
C21H22ClNO2; MF: 8; RMF: 229; Prob 17.6%; Lib: nist_msms; ID: 347686.

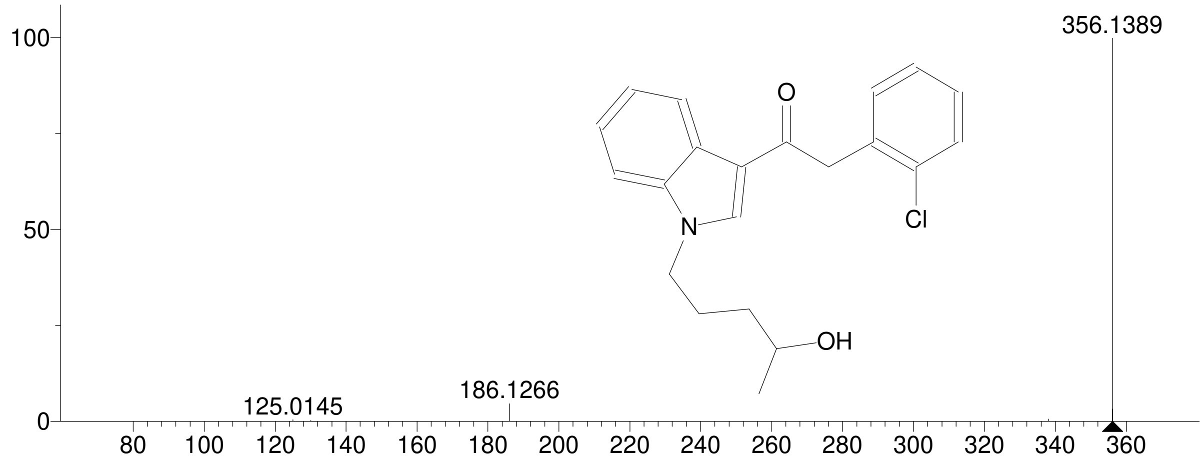

Hit 4 : Benzoic acid, 4-[[(2,4-diamino-7,8-dihydro-7-oxo-6-pteridinyl)methyl]methylamino]-, methyl ester [M+H]+
C16H17N7O3; MF: 8; RMF: 217; Prob 17.6%; CAS: 1330264-75-9; Lib: nist_msms; ID: 432535.

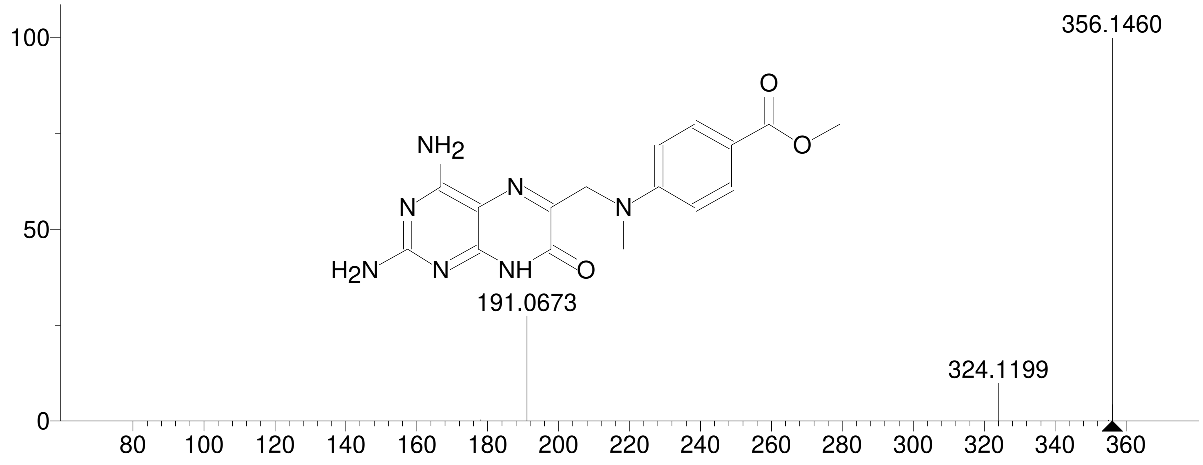

Hit 5 : Seneciphyllin [M+Na]+ HCD 21V P=356.1
C18H23NO5; MF: 8; RMF: 182; Prob 17.6%; CAS: 480-81-9; Lib: nist_msms; ID: 219526.

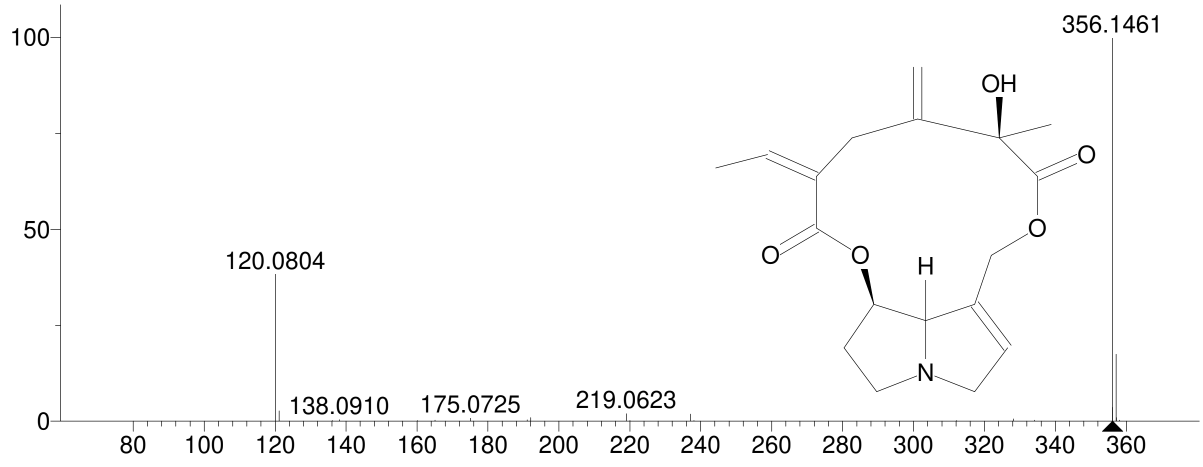

Unknown: Unknown (6.04_793.3395m/z)
Compound in Library Factor = 0

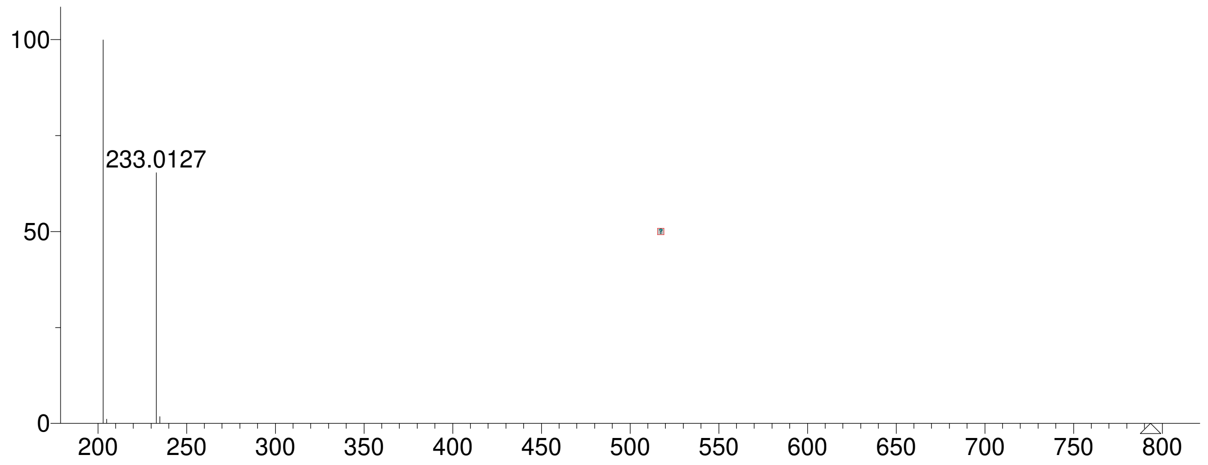

Hit 1 : [(1R,3R,10S,13R,14S,17R)-3-[(2R)-5-(3,5-dihydroxy-4-methoxy-6-methyloxan-2-yl)oxy-3,4-dihydroxy-6-(hy
C38H58O15; MF: 0; RMF: 56; Prob 100.0%; Lib: mona-export-vf-npl_qexactive; ID: 14720.

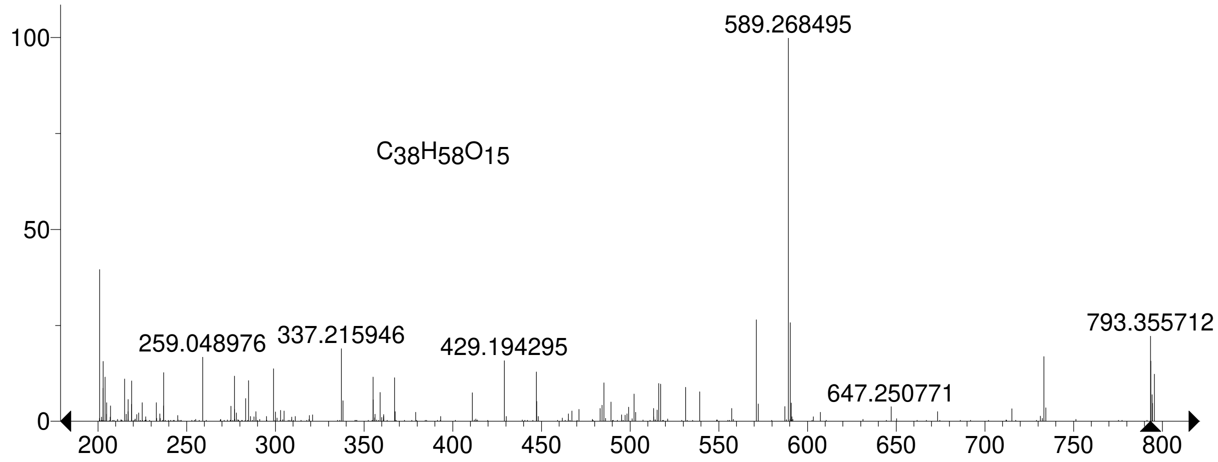

Unknown: Unknown (6.05_328.1499m/z)
Compound in Library Factor = 0

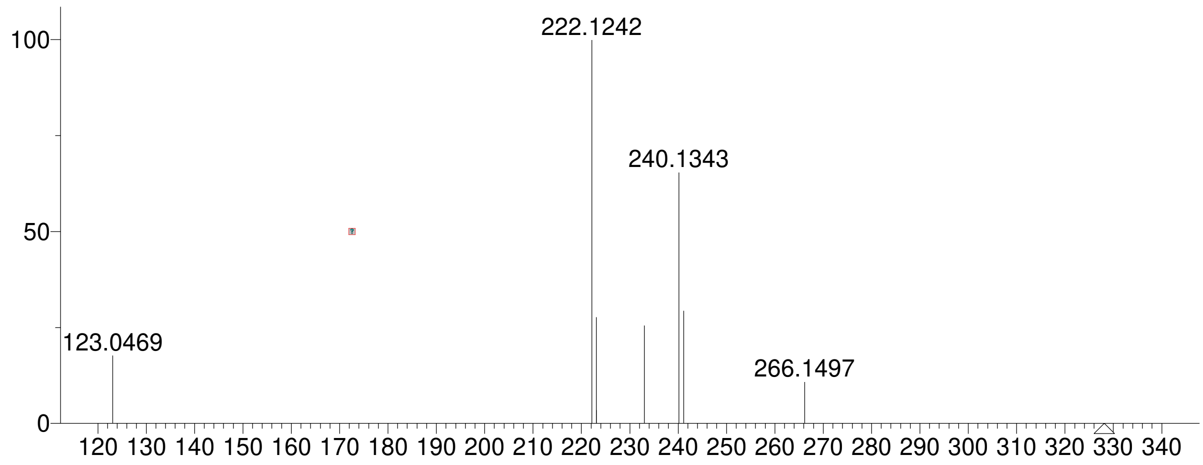

Hit 1 : Ac-Val-Ala-Asp-CHO [M-H]- QTOF 17V P=328.2
C14H23N3O6; MF: 0; RMF: 19; Prob 100.0%; Lib: nist_msms2; ID: 39356.

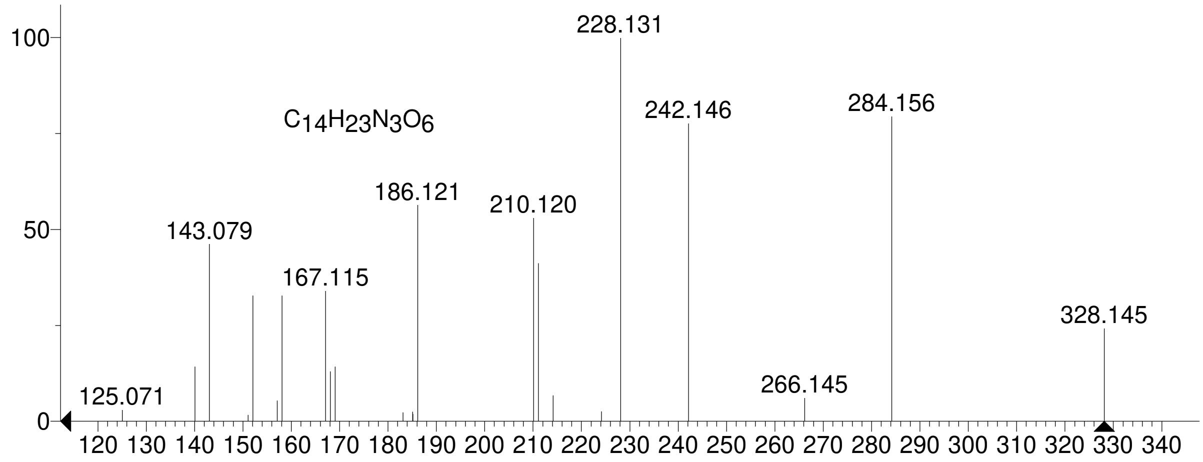

Unknown: Unknown (6.06_233.0118m/z)
Compound in Library Factor = -3273

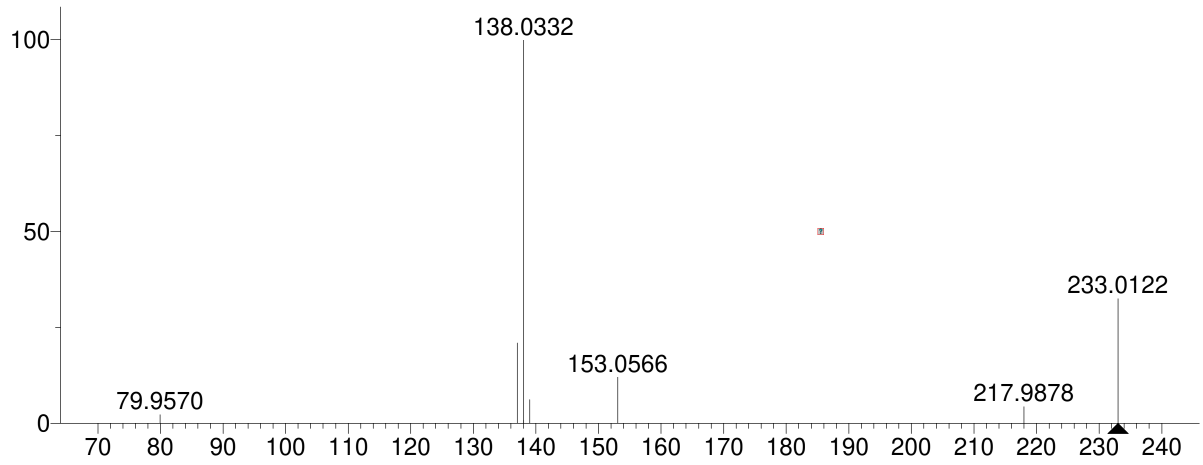

Hit 1 : Phaclofen [M+H-NH3]+ HCD 9V P=233
C9H13ClNO3P; MF: 7; RMF: 262; Prob 100.0%; CAS: 114012-12-3; Lib: nist_msms; ID: 119883.

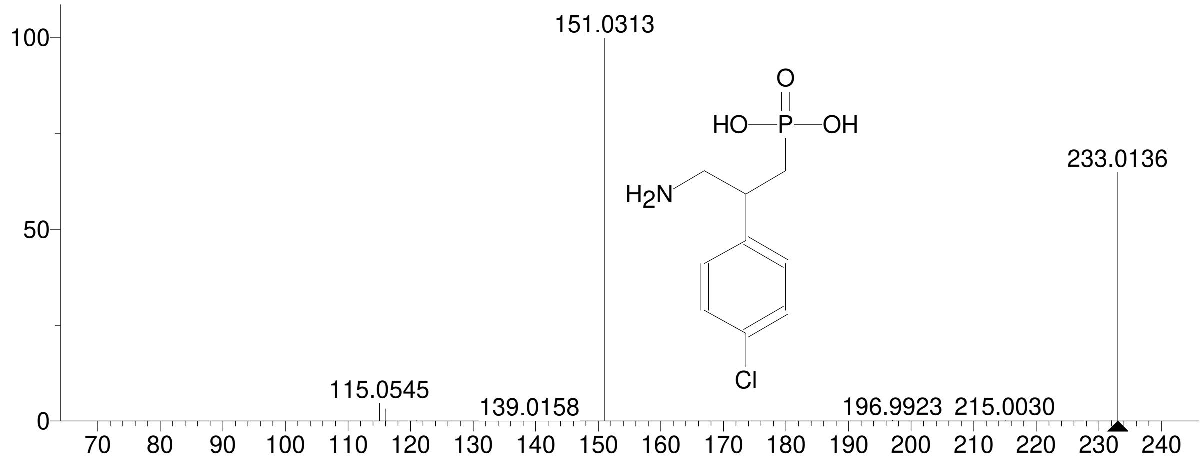

** Search Report Page 1 of 1 **

Unknown: Unknown (6.06_459.0010m/z)
Compound in Library Factor = 0

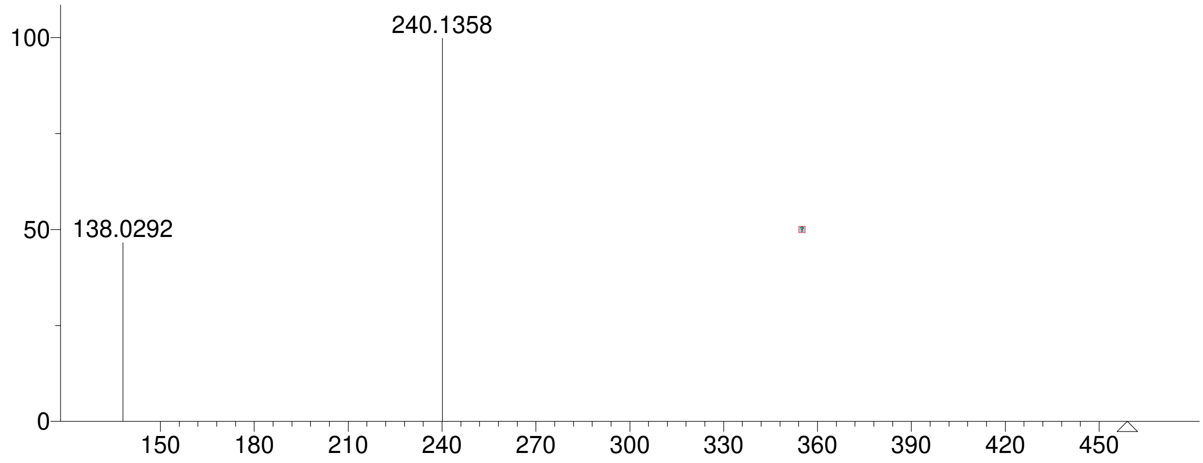

Hit 1 : Selumetinib [M+H+2i]+ HCD 146V P=459
C17H15BrClFN4O3; MF: 0; RMF: 105; Prob 100.0%; CAS: 606143-52-6; Lib: nist_msms; ID: 520928.

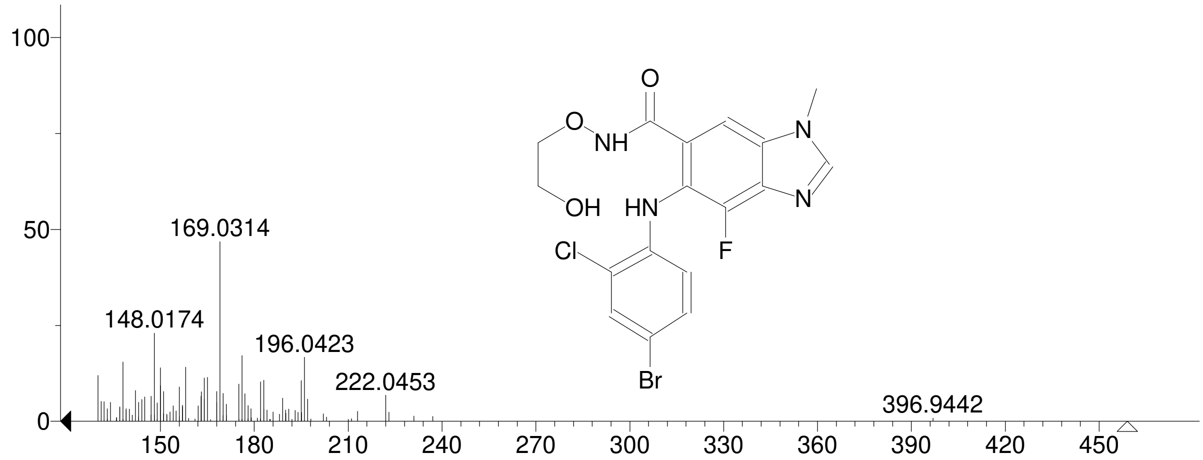

Unknown: Unknown (6.07_233.1552m/z)
Compound in Library Factor = 0

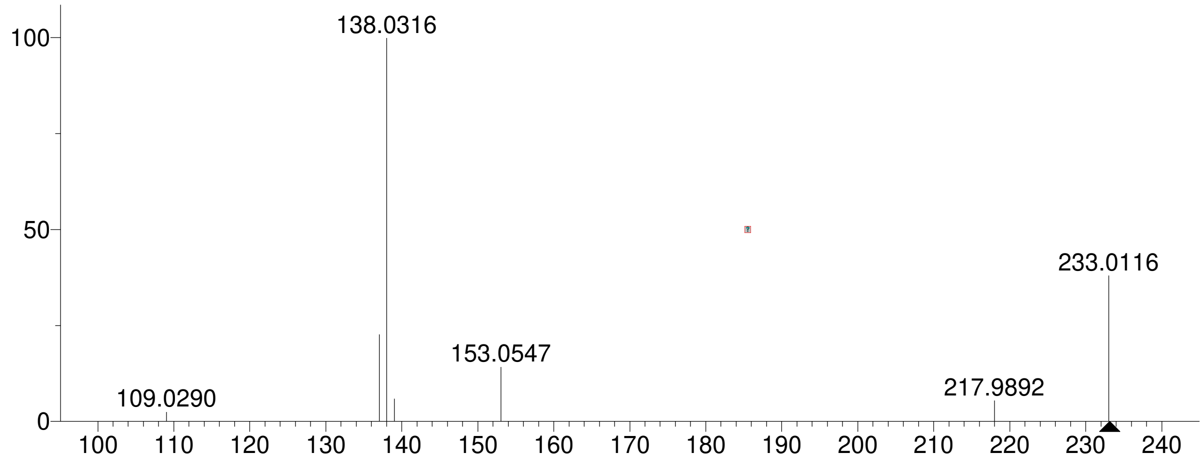

Hit 1 : COSTUNOLIDE [M+H]+ 10 P=233.2
C15H20O2; MF: 0; RMF: 77; Prob 14.3%; Lib: mona-export-vf-npl_qtof; ID: 2204.

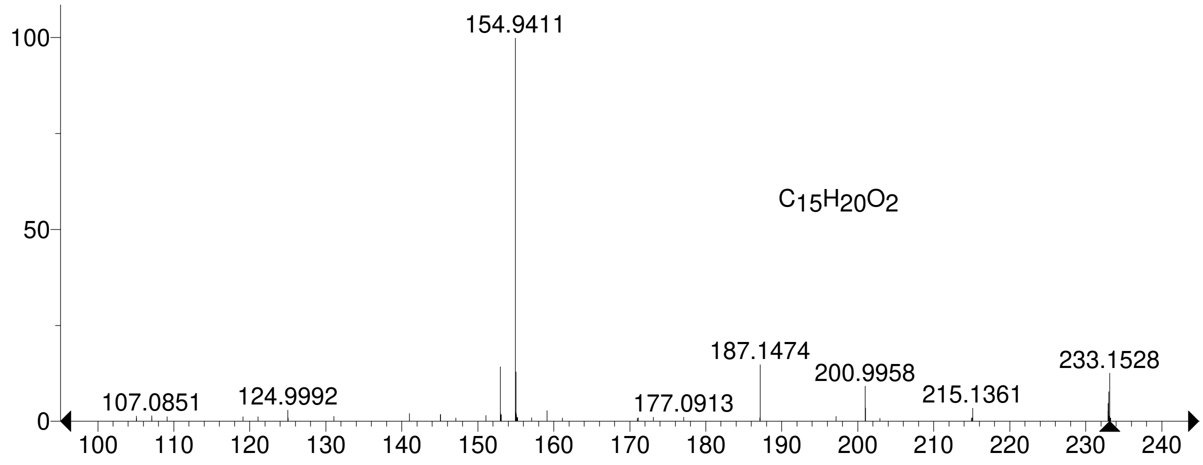

Hit 2 : NCGC00384653-01_C15H22O3_2-[(1S,2S,4aR,8aS)-1-Hydroxy-4a-methyl-8-methylenedecahydro-2-naph
C15H22O3; MF: 0; RMF: 13; Prob 14.3%; Lib: mona-export-gnps; ID: 13411.

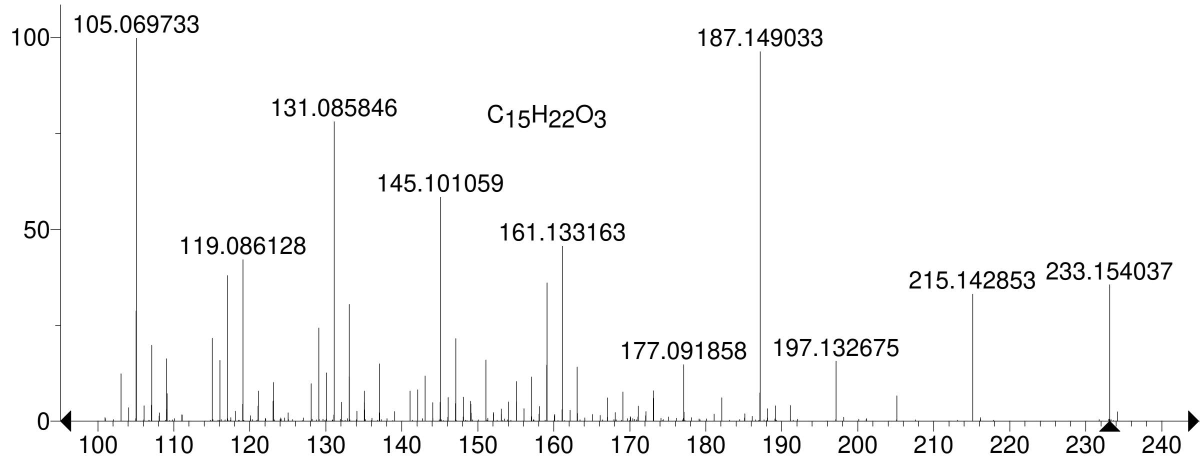

Hit 3 : NCGC00385912-01_C15H22O3_3-Cycloheptene-1-acetic acid, 5-methyl-alpha-methylene-4-(3-oxobutyl)-,
C15H22O3; MF: 0; RMF: 11; Prob 14.3%; Lib: mona-export-gnps; ID: 14383.

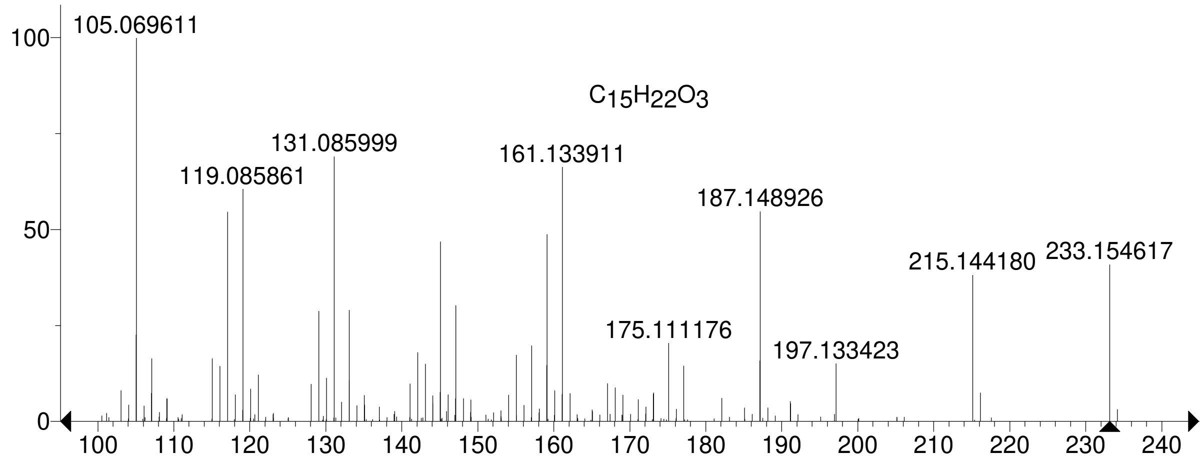

Hit 4 : (4S,5Z,6S)-4-(2-methoxy-2-oxoethyl)-5-[2-[(E)-3-phenylprop-2-enoyl]oxyethylidene]-6-[(2S,3R,4S,5S,6R)-3
C15H22O2; MF: 0; RMF: 8; Prob 14.3%; Lib: mona-export-lc-ms-ms_negative_mode; ID: 19611.

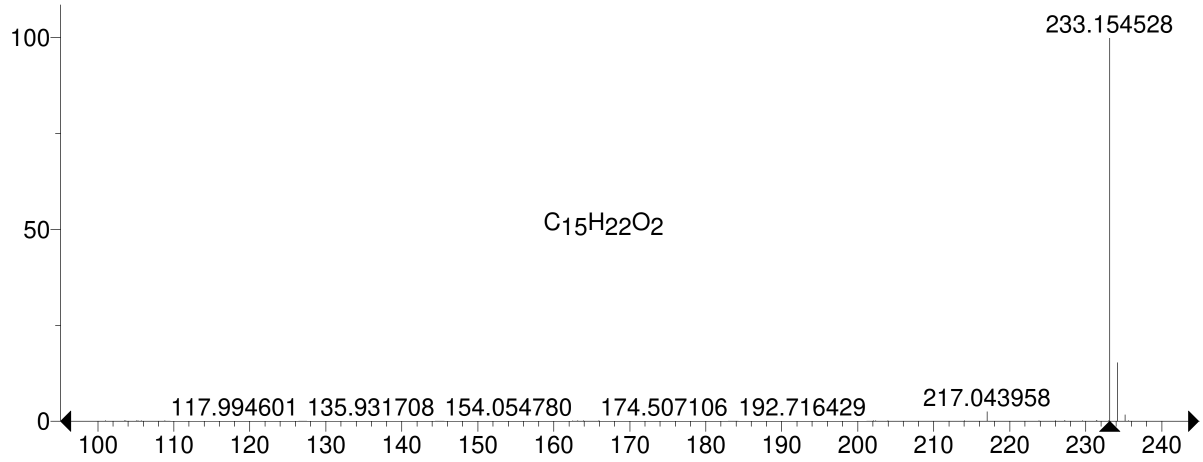

Hit 5 : 5,8,11,14-Eicosatetraenoic acid, 16-hydroxy-, (5Z,8Z,11Z,14Z,16R)- [M-H-C5H10O]- IT 35% P=233.2
C20H32O3; MF: 0; RMF: 4; Prob 14.3%; CAS: 183509-22-0; Lib: nist_msms; ID: 193173.

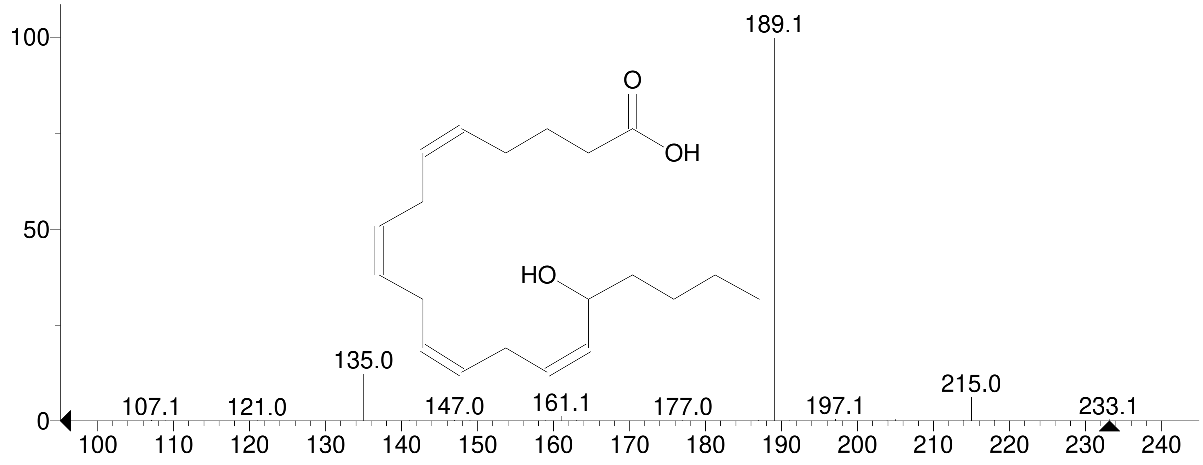

Unknown: Unknown (6.07_489.0120m/z)
Compound in Library Factor = 0

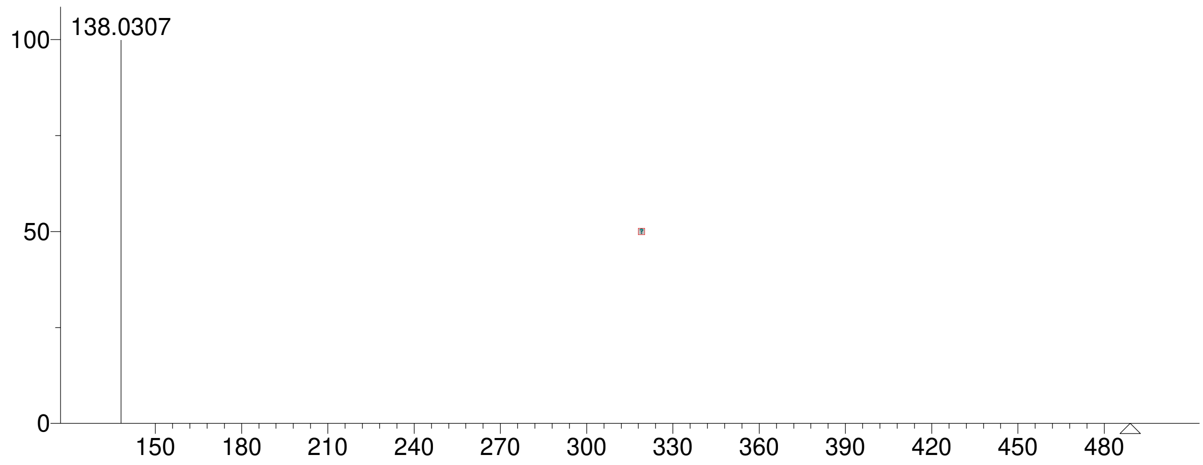

Unknown: Unknown (6.14_390.1330m/z)
Compound in Library Factor = 0

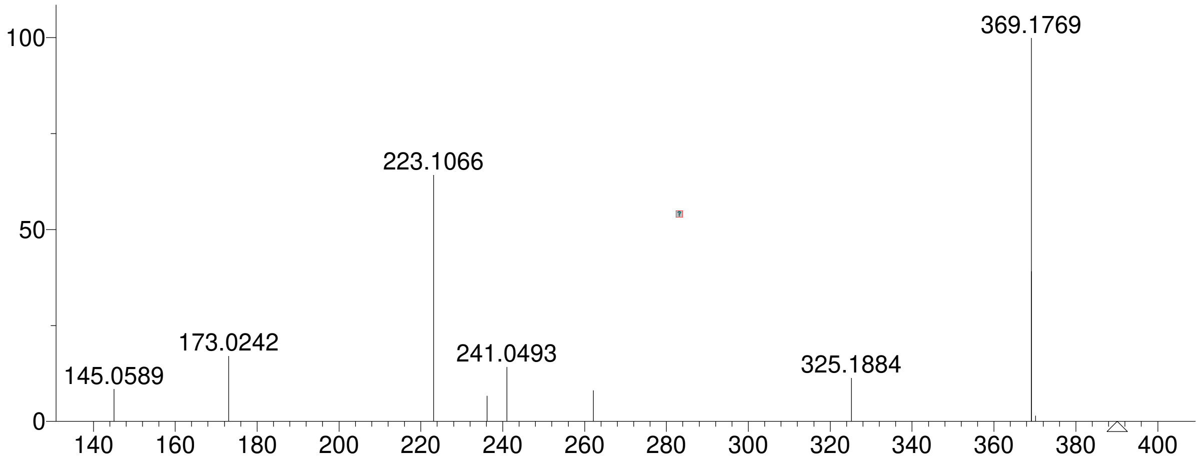

Unknown: Unknown (6.14_565.2231m/z)
Compound in Library Factor = 0

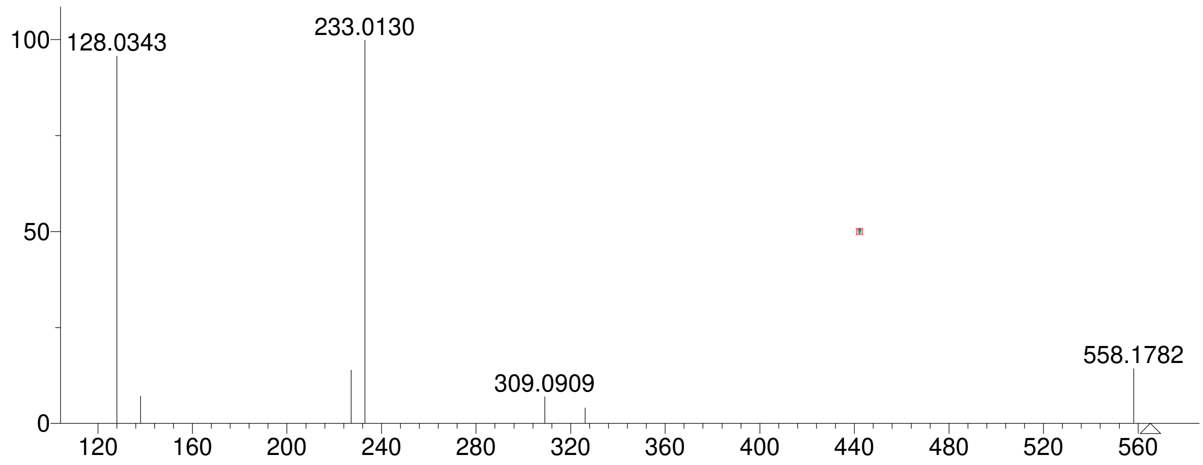

Hit 1 : Theaflavin [M+H]+ 35% P=565.2
C29H24O12; MF: 0; RMF: 13; Prob 50.0%; Lib: mona-export-itree; ID: 673.

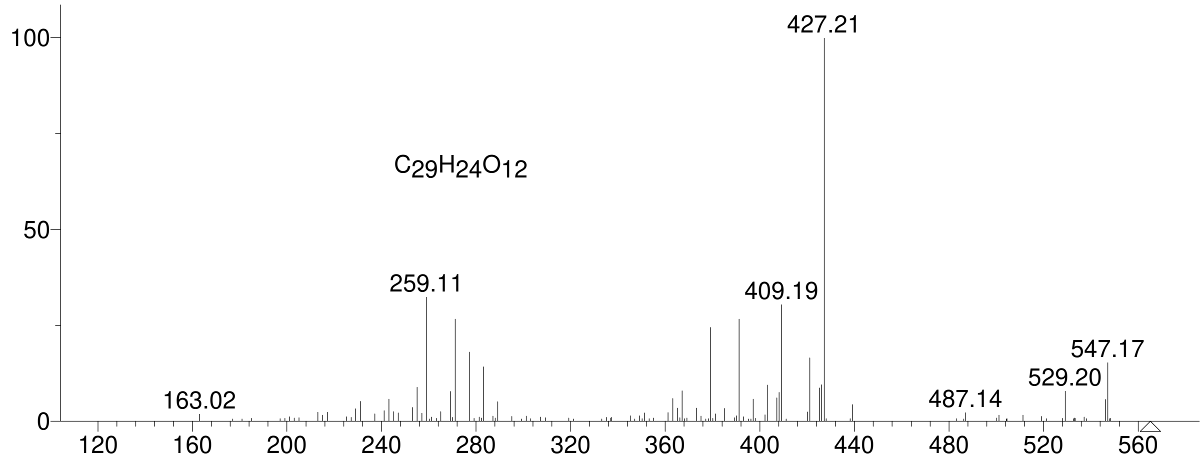

Hit 2 : (1R,7S,8E,9S,14S,15S,17R)-8-ethylidene-15-(1-hydroxypropan-2-yl)-17-methyl-7-{[(2S,3R,4S,5S,6R)-3,4,5
C26H38O12; MF: 0; RMF: 1; Prob 50.0%; Lib: mona-export-vf-npl_qexactive; ID: 12805.

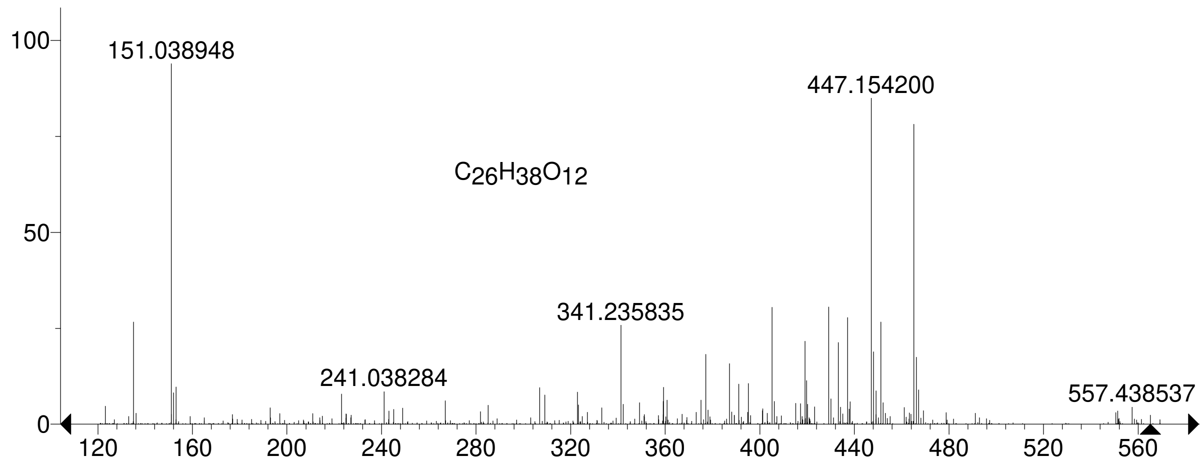

Unknown: Unknown (6.15_369.1772m/z)
Compound in Library Factor = -791

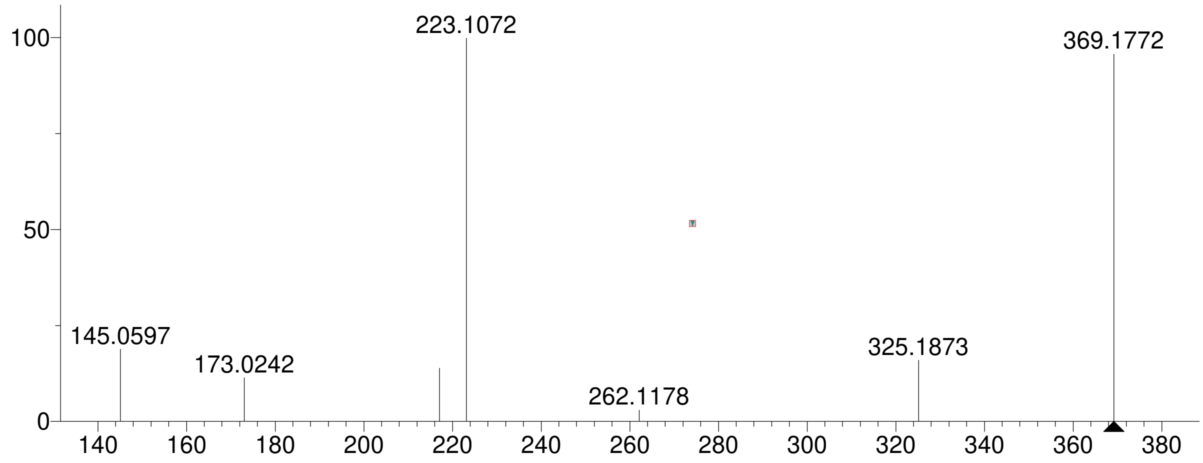

Hit 1 : 5β-Androstan-3α-ol-17-one sulfate [M-H]- HCD 33V P=369.2
C19H30O5S; MF: 185; RMF: 608; Prob 41.1%; CAS: 977-36-6; Lib: nist_msms; ID: 231644.

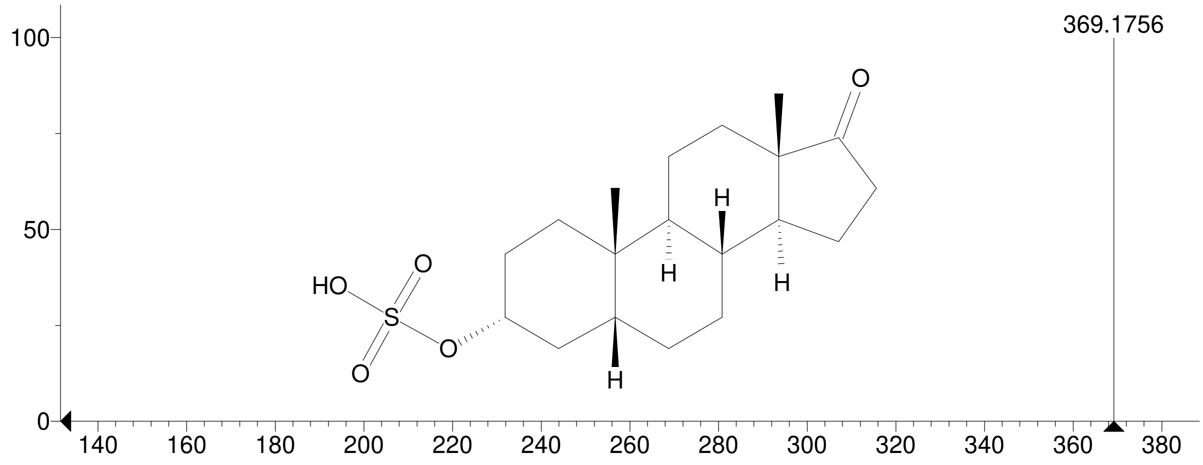

Hit 2 : 5-Androsten-3β,17β-diol-3-sulfate [M-H]- HCD 27V P=369.2
C19H30O5S; MF: 185; RMF: 608; Prob 41.1%; CAS: 977-33-3; Lib: nist_msms; ID: 231655.

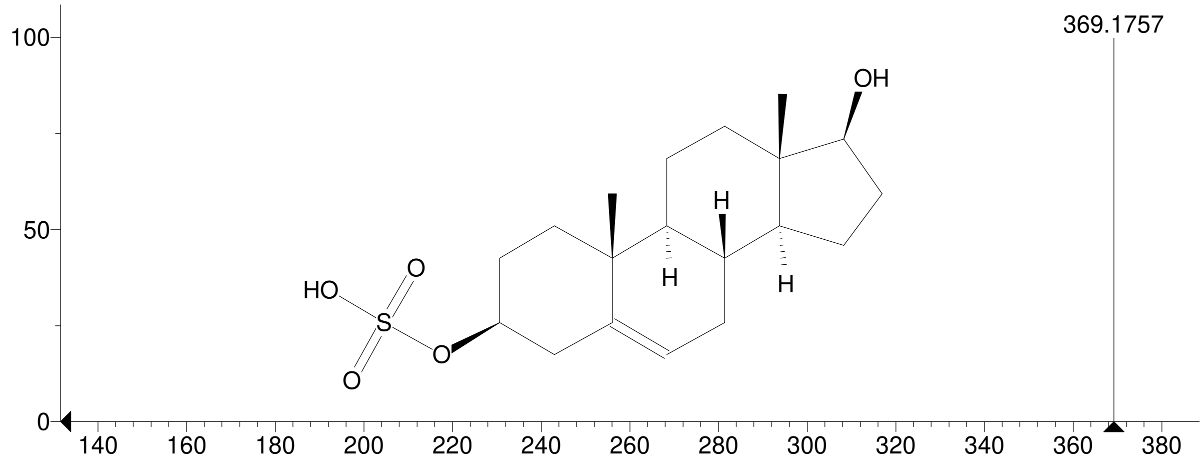

Hit 3 : 5α-Androstan-3β-ol-17-one sulfate [M-H]- HCD 40V P=369.2
C19H30O5S; MF: 148; RMF: 607; Prob 10.0%; CAS: 977-35-5; Lib: nist_msms; ID: 231625.

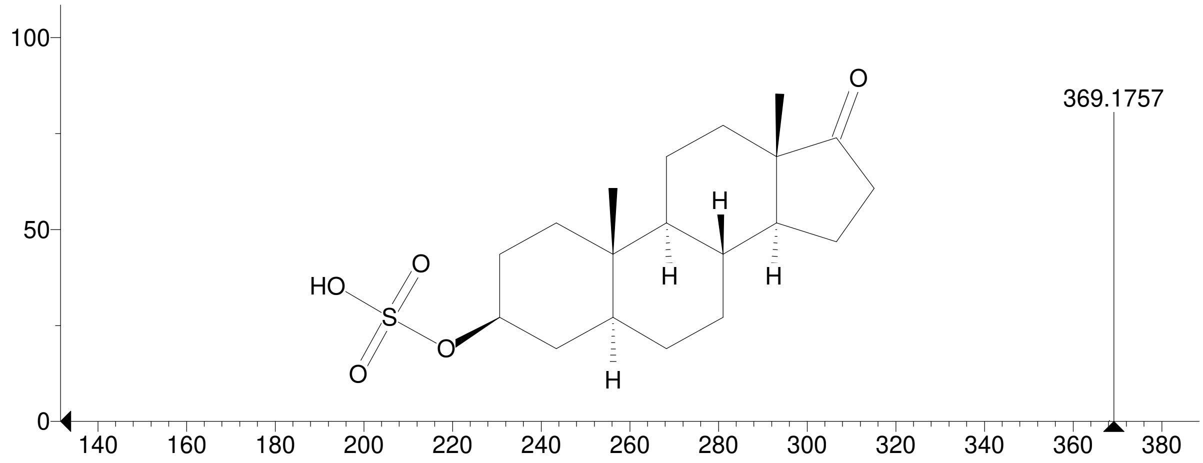

Hit 4 : 5α-Androstan-17β-ol-3-one sulfate [M-H]- HCD 40V P=369.2
C19H30O5S; MF: 137; RMF: 604; Prob 6.87%; CAS: 2641-48-7; Lib: nist_msms; ID: 231920.

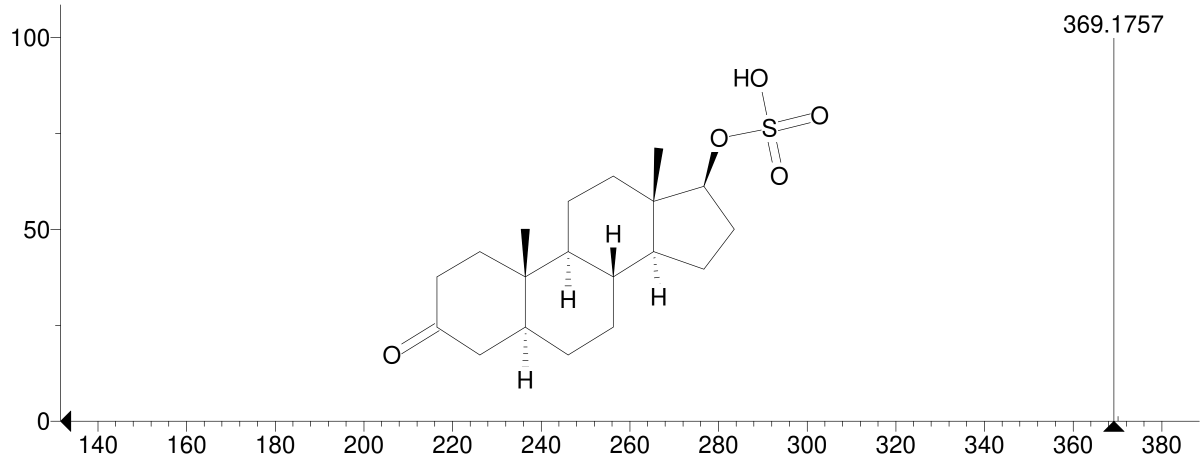

Hit 5 : Androsterone sulfate [M-H]- HCD 35V P=369.2
C19H30O5S; MF: 41; RMF: 547; Prob 0.54%; CAS: 2479-86-9; Lib: nist_msms; ID: 117993.

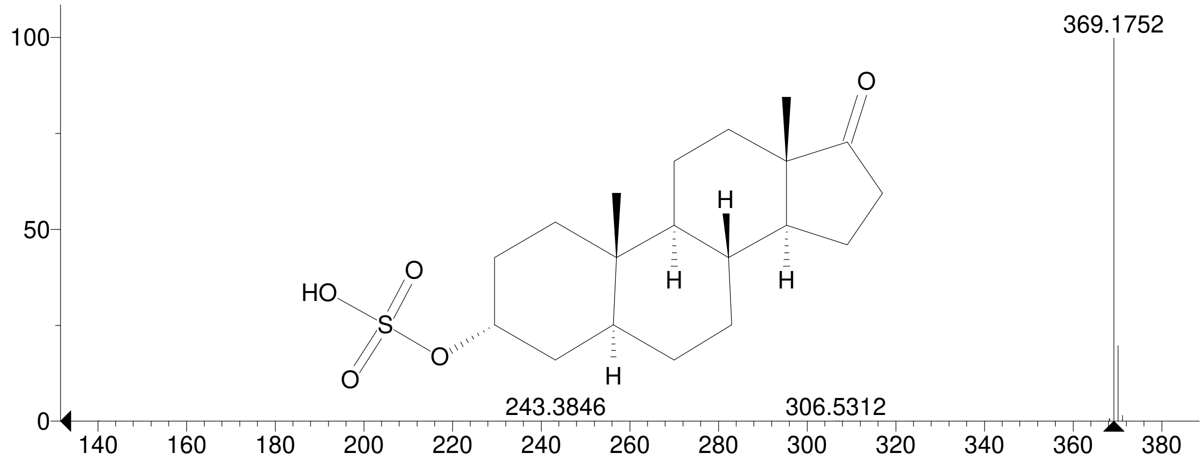

Unknown: Unknown (6.15_488.1581m/z)
Compound in Library Factor = 0

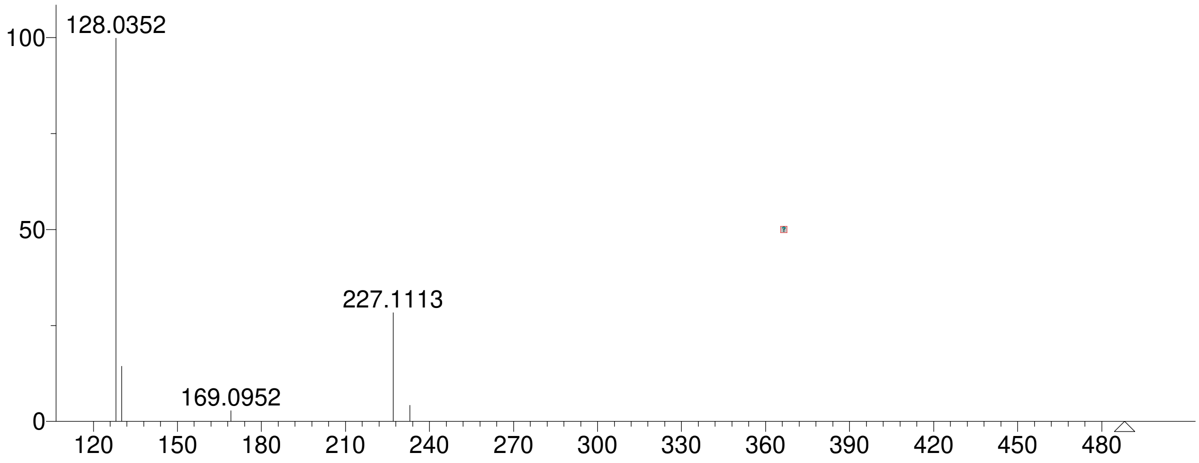

** Search Report Page 1 of 1 **

Unknown: Unknown (6.16_609.2057m/z)
Compound in Library Factor = 0

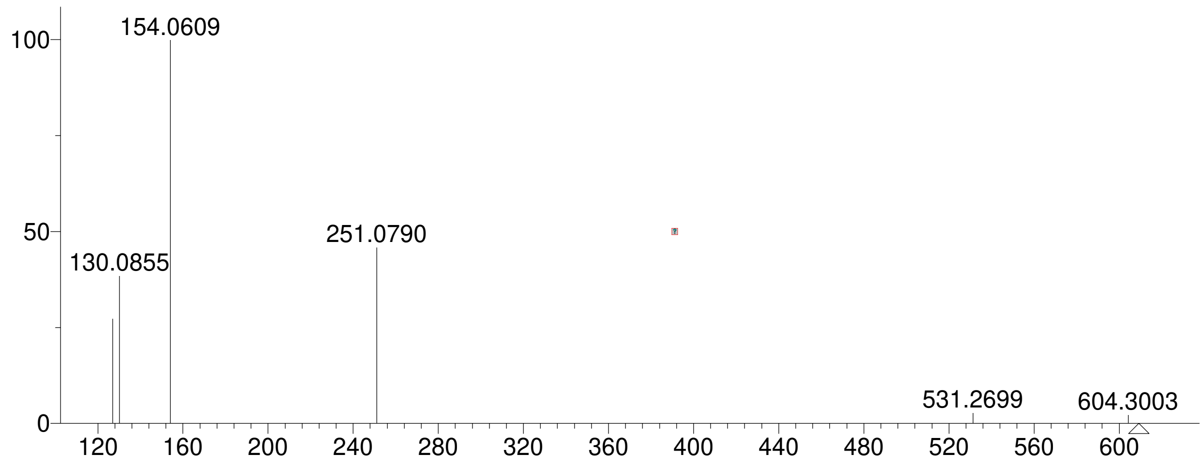

Unknown: Unknown (6.18_531.2658m/z)
Compound in Library Factor = 0

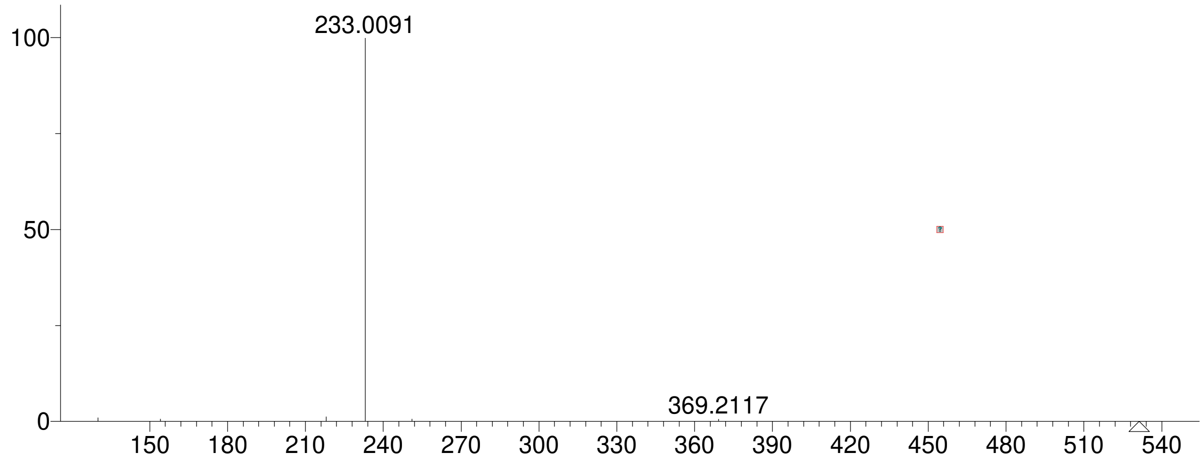

Unknown: Unknown (6.20_243.1703m/z)
Compound in Library Factor = -2176

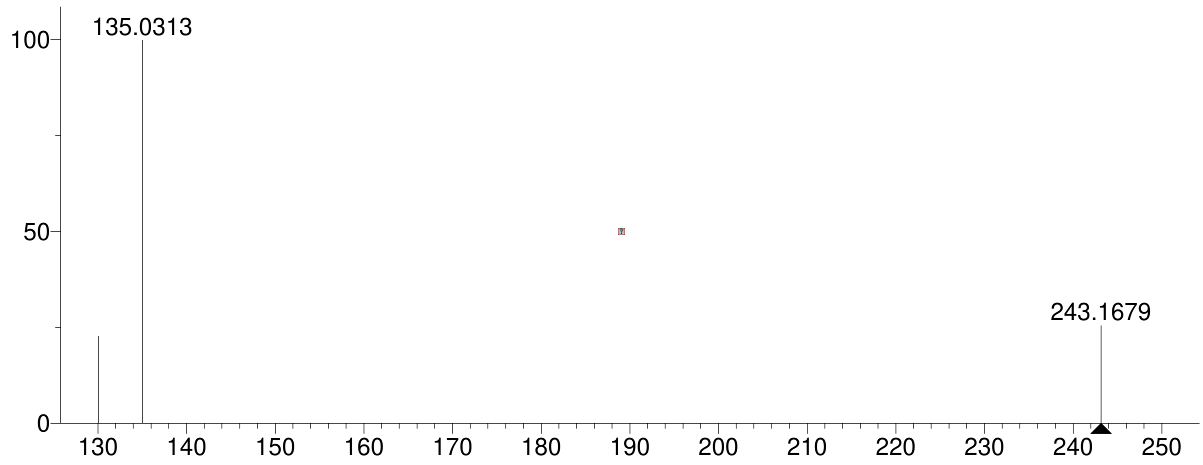

Hit 1 : Leu-Leu [M-H]- HCD 14eV P=243.2
C12H24N2O3; MF: 144; RMF: 487; Prob 31.0%; CAS: 3303-31-9; Lib: nist_msms; ID: 568071.

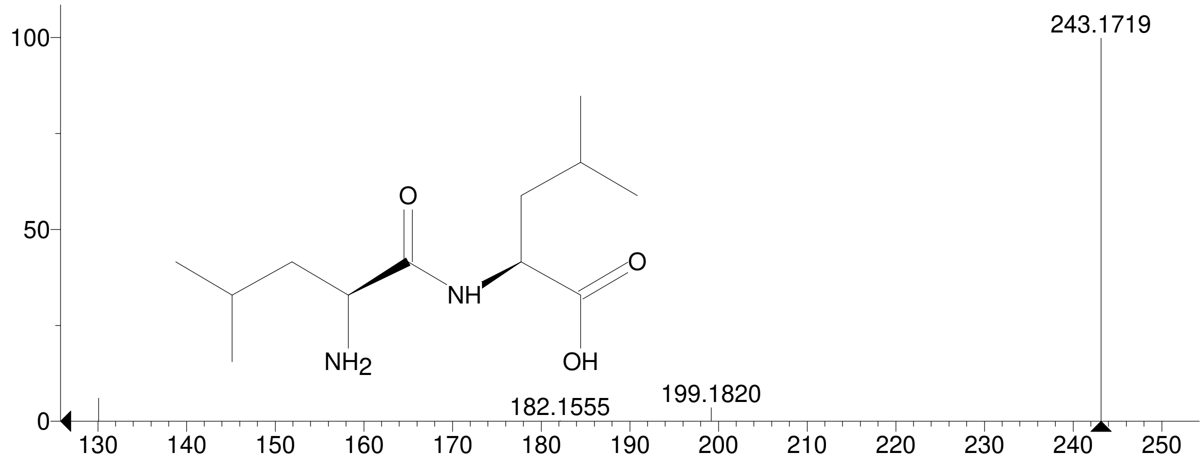

Hit 2 : Ile-Leu [M-H]- HCD 14eV P=243.2
C12H24N2O3; MF: 134; RMF: 479; Prob 21.9%; Lib: nist_msms; ID: 570160.

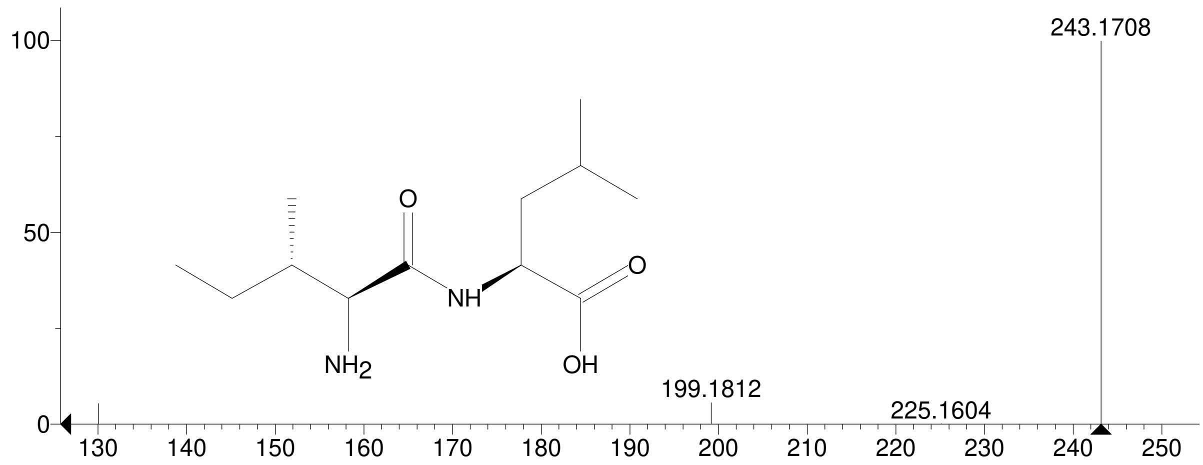

Hit 3 : Ile-Leu [M-H]- HCD 14eV P=243.2
C12H24N2O3; MF: 134; RMF: 479; Prob 21.9%; CAS: 26462-22-6; Lib: nist_msms2; ID: 83886.

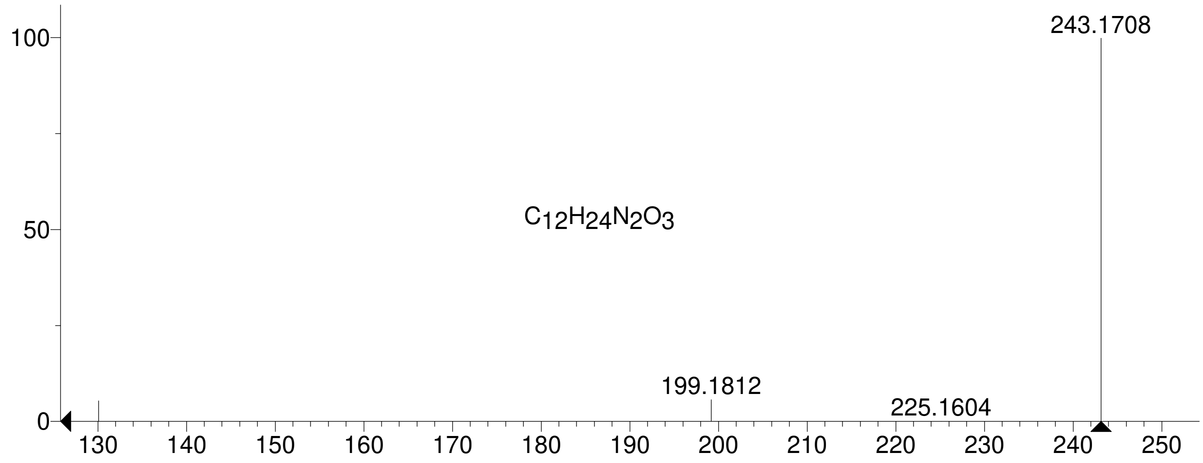

Hit 4 : Leu-Ile [M-H]- HCD 14eV P=243.2
C12H24N2O3; MF: 105; RMF: 474; Prob 6.20%; Lib: nist_msms; ID: 571156.

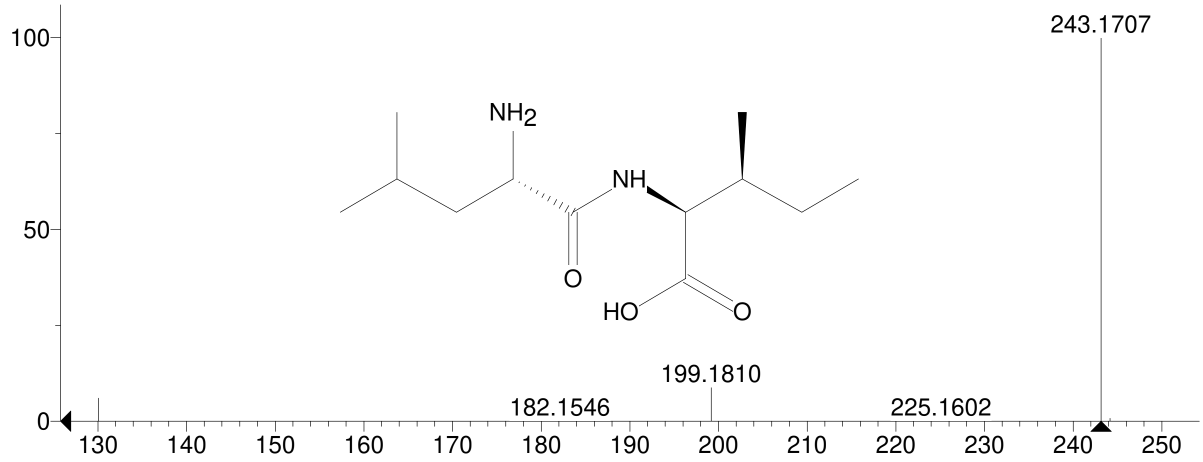

Hit 5 : Leu-Ile [M-H]- HCD 14eV P=243.2
C12H24N2O3; MF: 105; RMF: 474; Prob 6.20%; CAS: 36077-41-5; Lib: nist_msms2; ID: 84889.

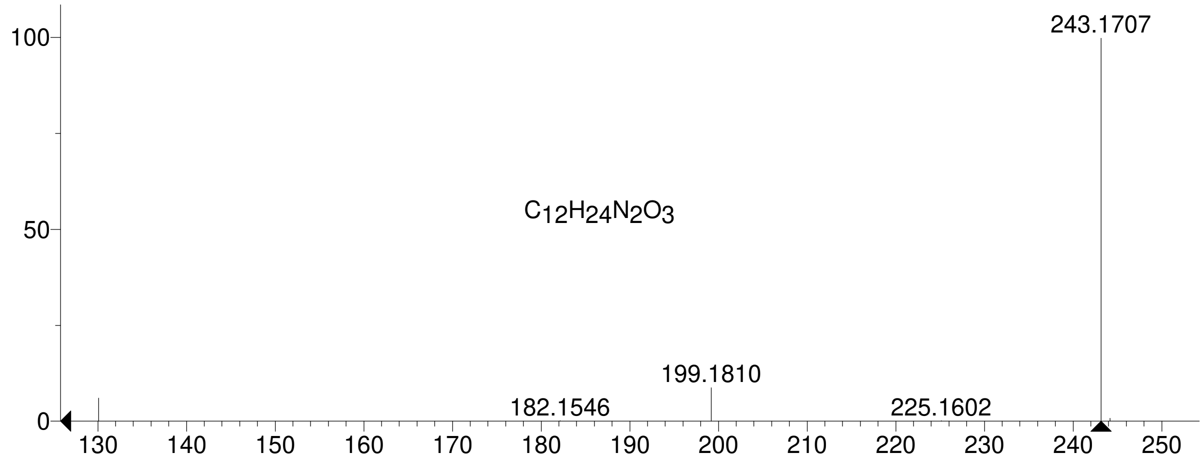

Unknown: Unknown (6.20_369.2134m/z)
Compound in Library Factor = -3273

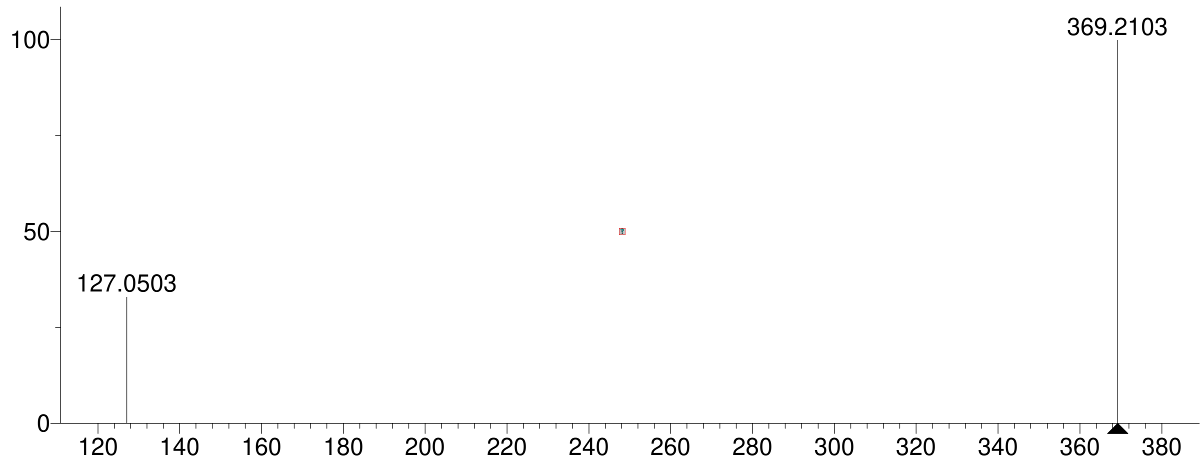

Hit 1 : Neuropeptide S, rat [M+6H]6+ QTOF 2V P=369.2
C95H160N34O27; MF: 7; RMF: 434; Prob 24.7%; Lib: nist_msms2; ID: 35976.

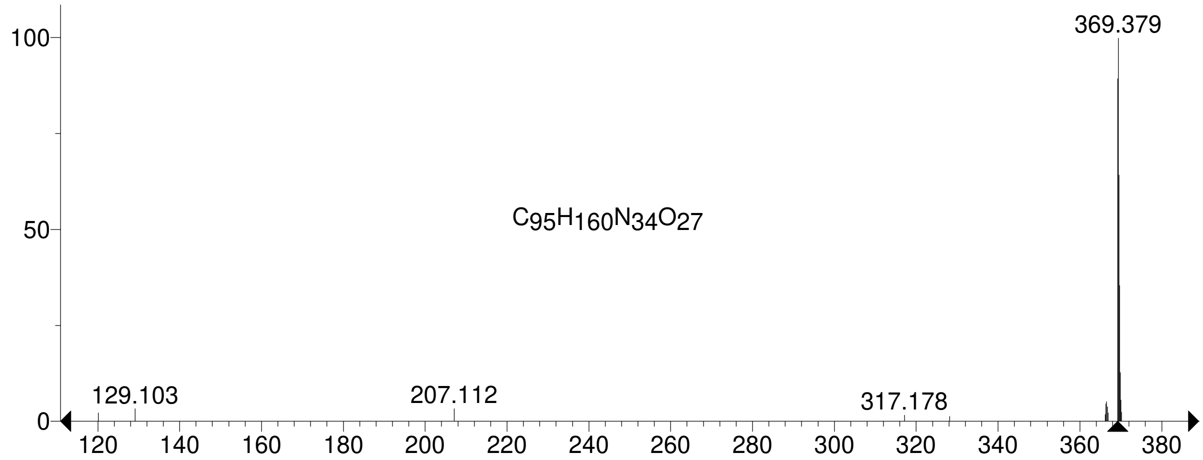

Hit 2 : Corynantheidine [M+H]+ P=369.2
C22H28N2O3; MF: 1; RMF: 168; Prob 19.4%; Lib: mona-export-gnps; ID: 15019.

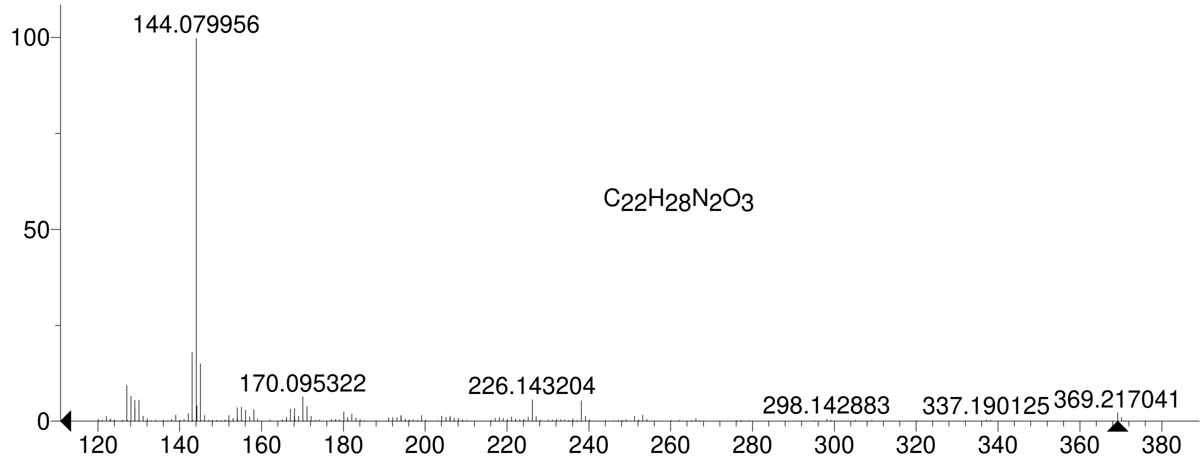

Hit 3 : Voacangine [M+H]+ P=369.2
C22H28N2O3; MF: 0; RMF: 303; Prob 18.6%; Lib: mona-export-gnps; ID: 15078.

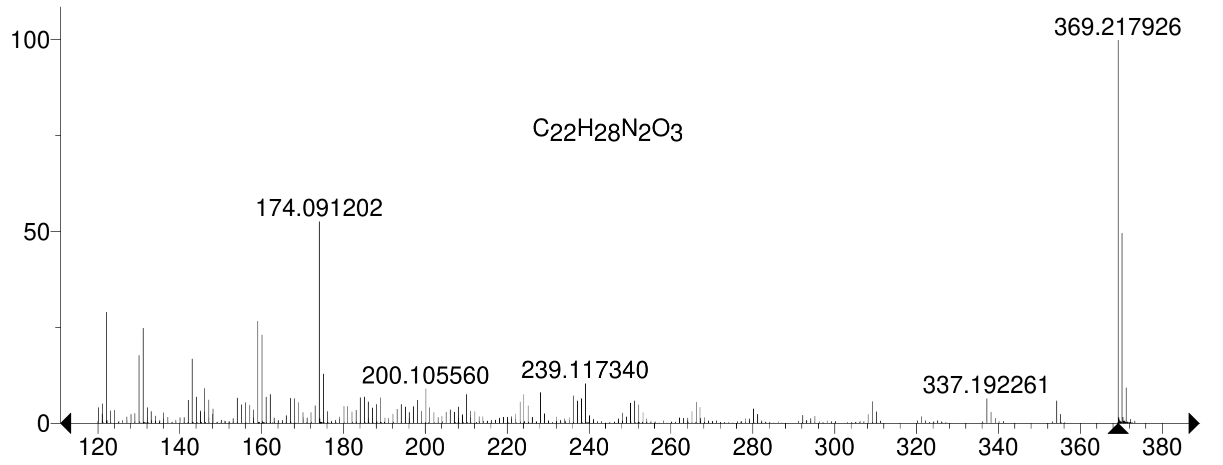

Hit 4 : Alpha-methylyohimbine [M+H]+ P=369.2
C22H28N2O3; MF: 0; RMF: 246; Prob 18.6%; Lib: mona-export-gnps; ID: 14961.

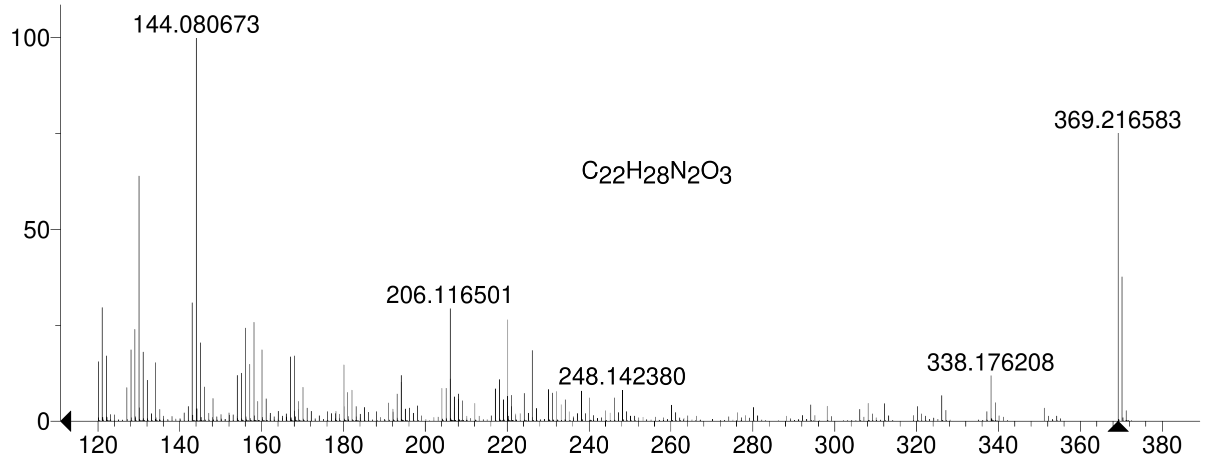

Hit 5 : Pesticide4_Boscalid_C18H12Cl2N2O_3-Pyridinecarboxamide, 2-chloro-N-(4'-chloro[1,1'-biphenyl]-2-yl)- [
C18H12Cl2N2O; MF: 0; RMF: 3; Prob 18.6%; Lib: mona-export-gnps; ID: 10170.

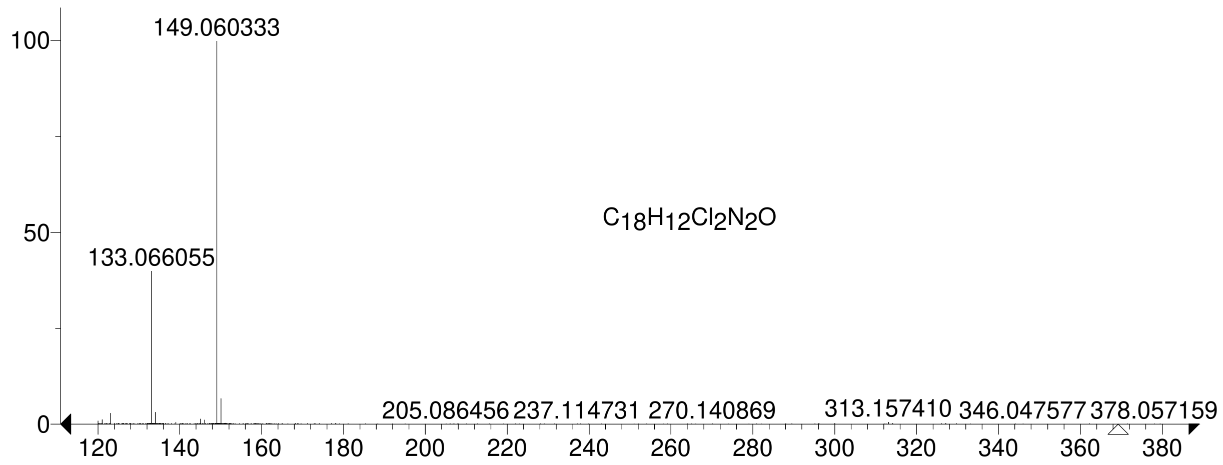

Unknown: Unknown (6.20_386.1909m/z)
Compound in Library Factor = -287

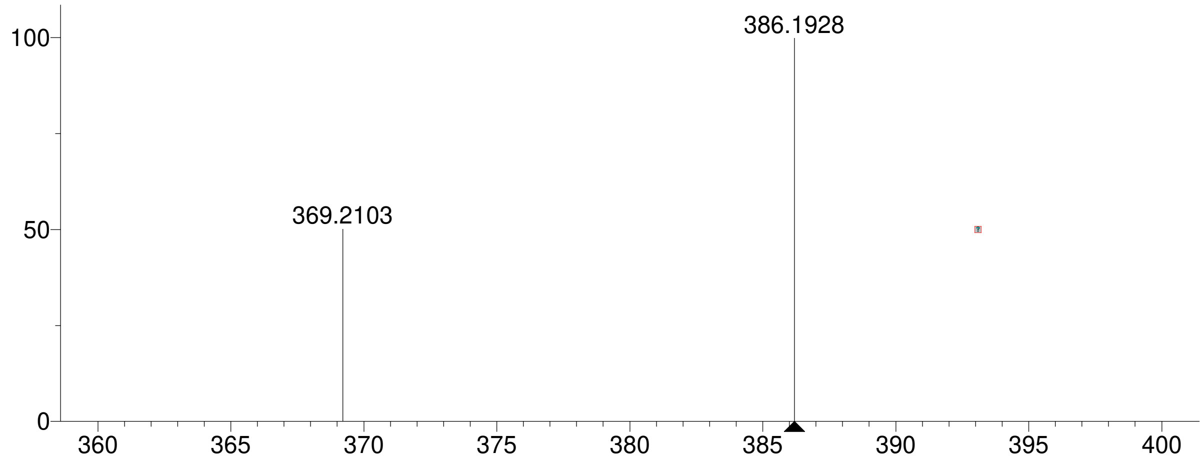

Hit 1 : Methanone, [5-(2-fluorophenyl)-1-pentyl-1H-pyrrol-3-yl]-1-naphthalenyl- [M+H]+ HCD 11V P=386.2
C26H24FNO; MF: 282; RMF: 815; Prob 49.8%; CAS: 914458-26-7; Lib: nist_msms; ID: 248825.

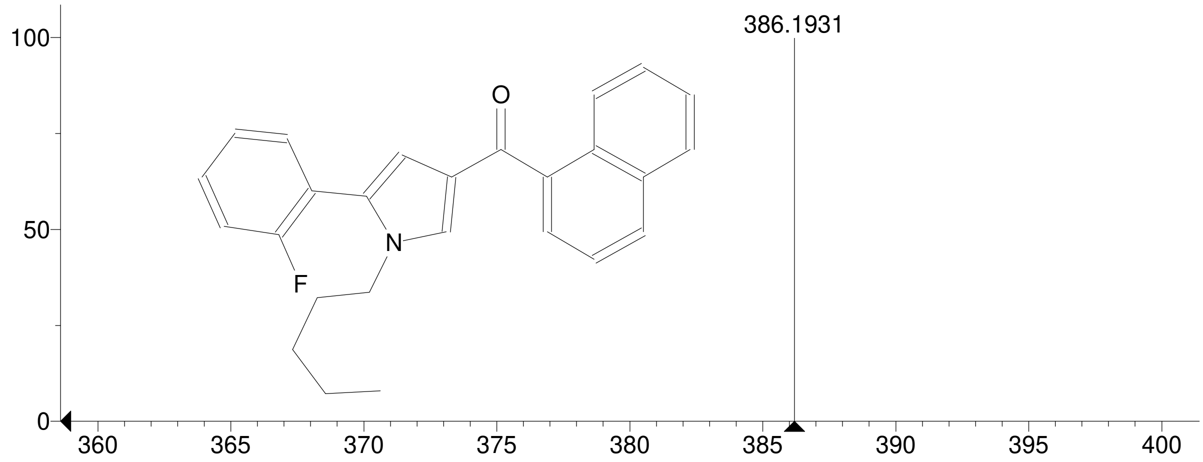

Hit 2 : Methanone, [5-(3-fluorophenyl)-1-pentyl-1H-pyrrol-3-yl]-1-naphthalenyl- [M+H]+ HCD 11V P=386.2
C26H24FNO; MF: 282; RMF: 815; Prob 49.8%; CAS: 914458-31-4; Lib: nist_msms; ID: 248873.

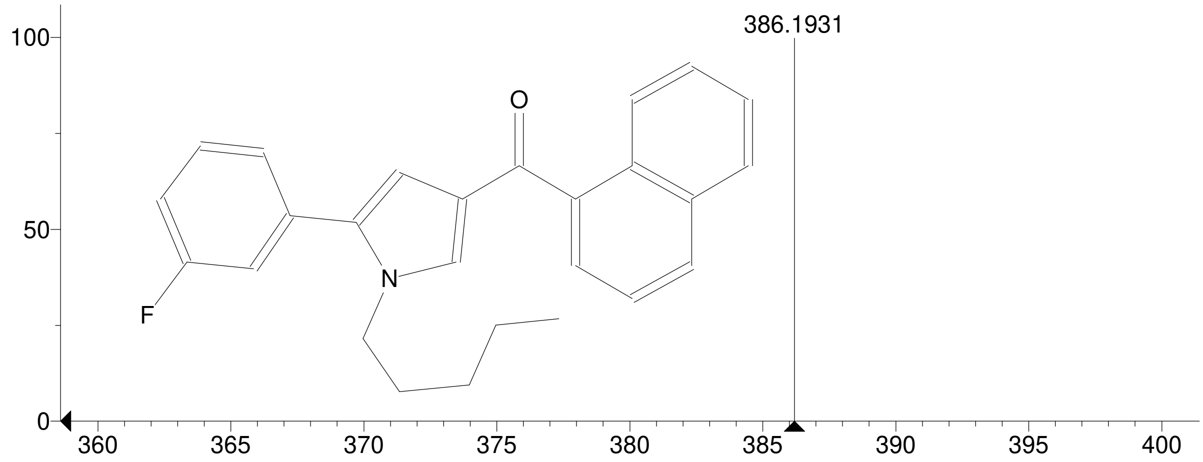

Hit 3 : Pro-His-Cys-Lys-Arg-Met [M+2H]2+ HCD 13eV P=386.2
C31H54N12O7S2; MF: 60; RMF: 707; Prob 0.49%; Lib: nist_msms2; ID: 44917.

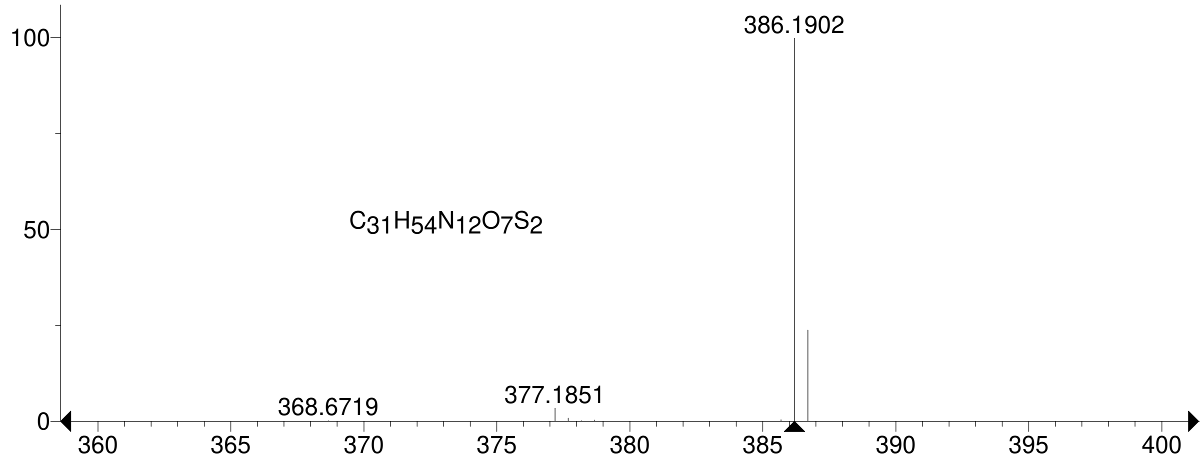

Unknown: Unknown (6.21_293.1130m/z)
Compound in Library Factor = -3273

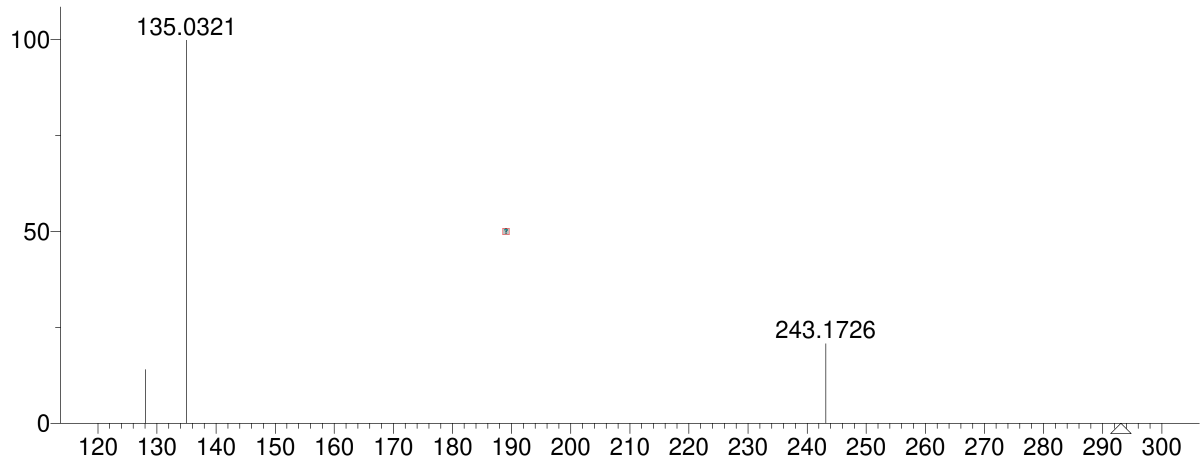

Hit 1 : Phe-Glu [M-H]- HCD 46eV P=293.1
C14H18N2O5; MF: 1; RMF: 166; Prob 16.9%; Lib: nist_msms; ID: 570598.

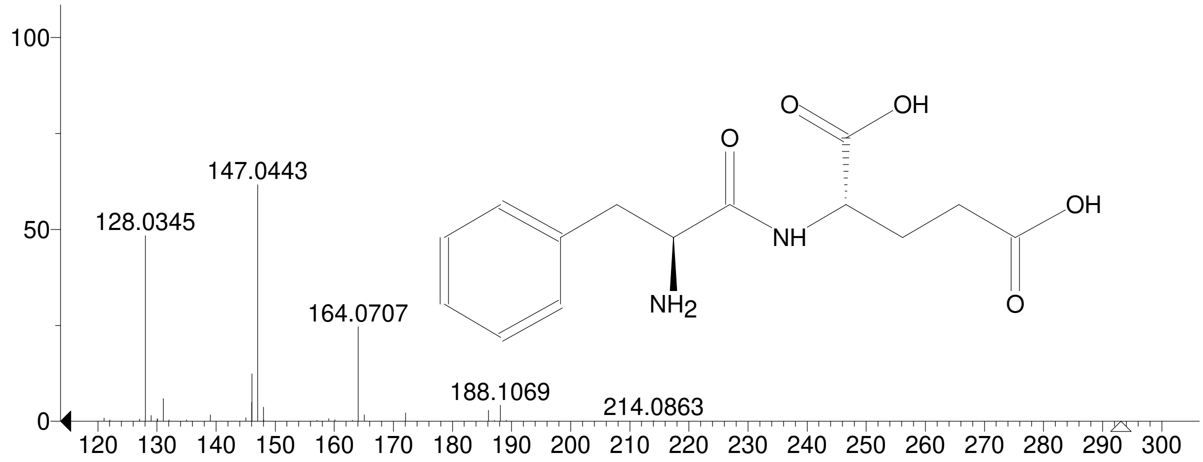

Hit 2 : Phe-Glu [M-H]- HCD 46eV P=293.1
C14H18N2O5; MF: 1; RMF: 166; Prob 16.9%; CAS: 3617-45-6; Lib: nist_msms2; ID: 84331.

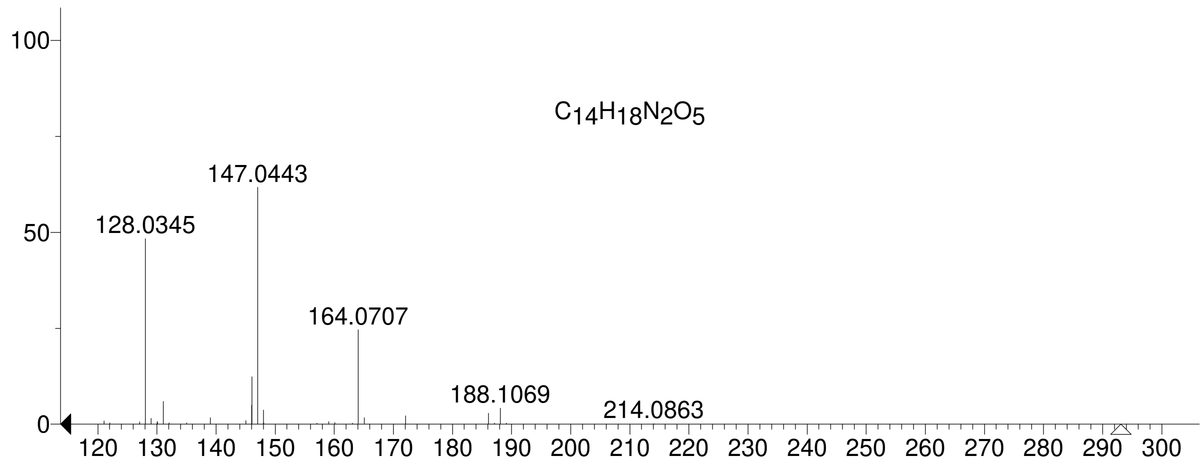

Hit 3 : Glu-Phe [M-H]- IT-FT 35% P=293.1
C14H18N2O5; MF: 1; RMF: 60; Prob 16.9%; Lib: nist_msms; ID: 570035.

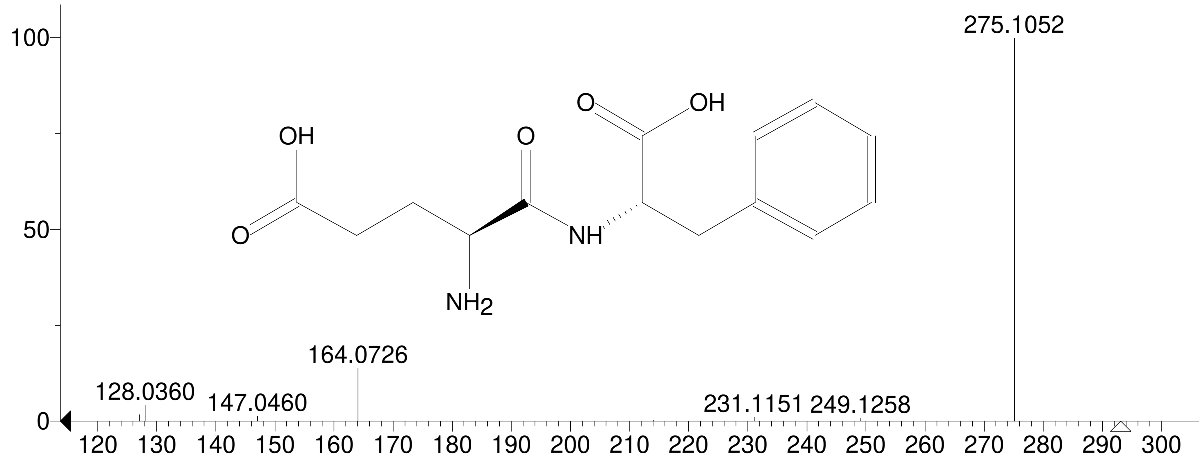

Hit 4 : Glu-Phe [M-H]- IT-FT 35% P=293.1
C14H18N2O5; MF: 1; RMF: 60; Prob 16.9%; CAS: 20556-22-3; Lib: nist_msms2; ID: 83761.

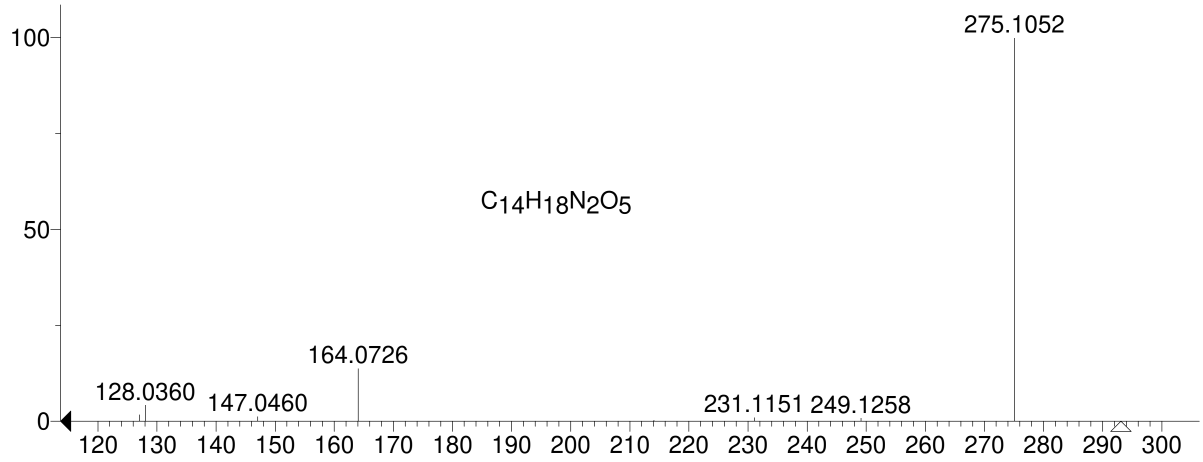

Hit 5 : Unknown (carbon number 14); PlaSMA ID-549 [M-H]- P=293.1
C14H18N2O5; MF: 0; RMF: 72; Prob 16.2%; Lib: biomsms-neg-plasma; ID: 1167.

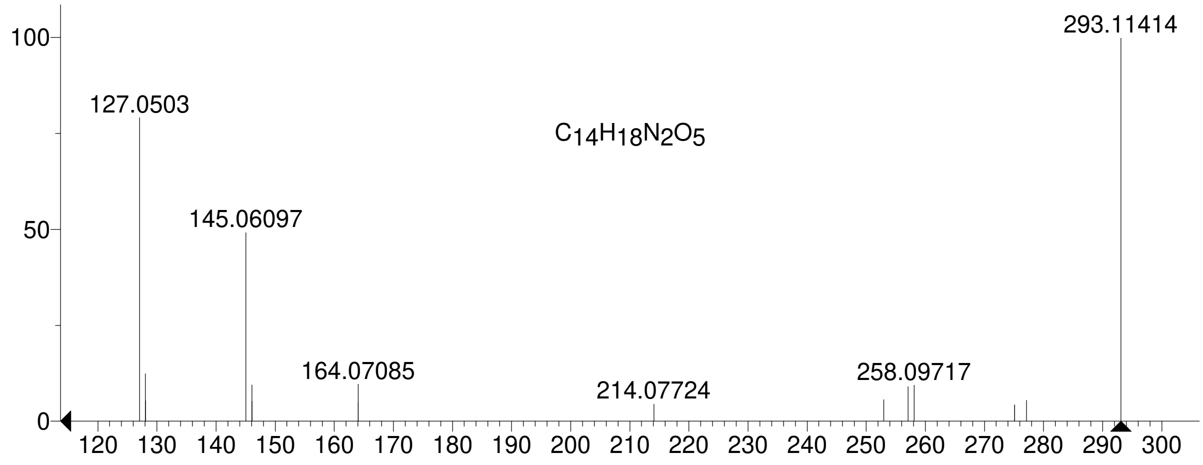

Unknown: Unknown (6.23_557.2081m/z)
Compound in Library Factor = 0

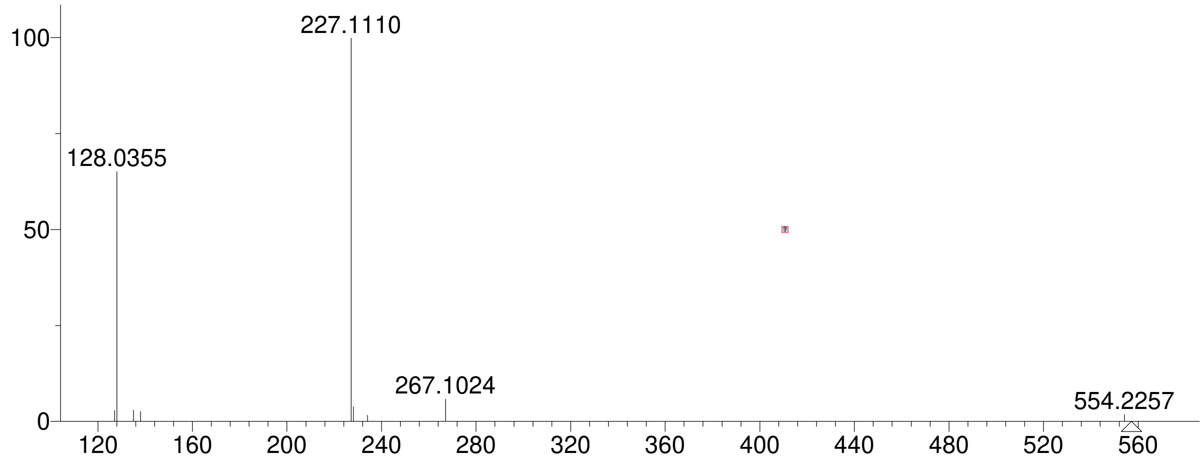

** Search Report Page 1 of 1 **

Unknown: Unknown (6.24_567.2285m/z)
Compound in Library Factor = 0

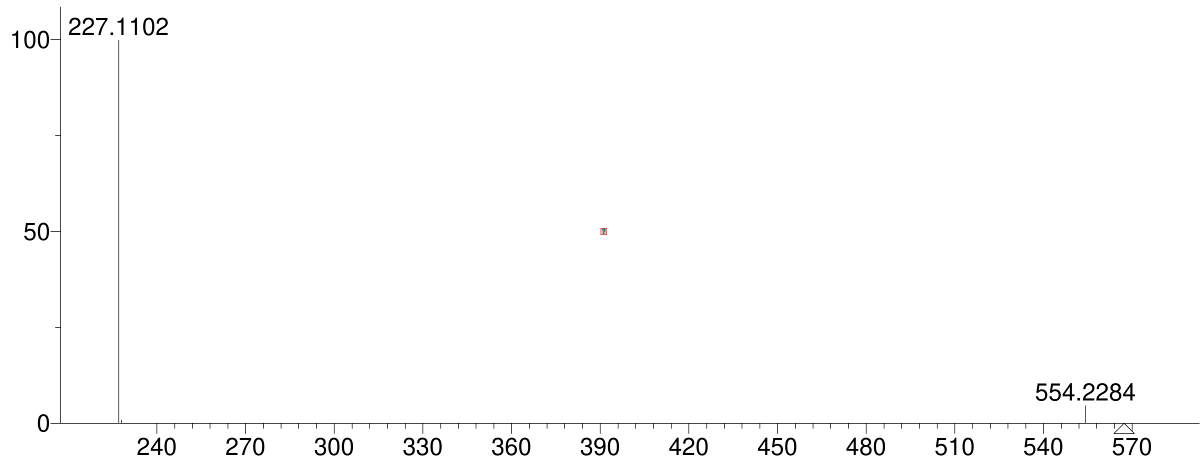

Hit 1 : chaetoglobosins [M+K]+ 35HCD P=567.2
C32H36N2O5; MF: 0; RMF: 10; Prob 100.0%; Lib: mona-export-vf-npl_qexactive; ID: 23206.

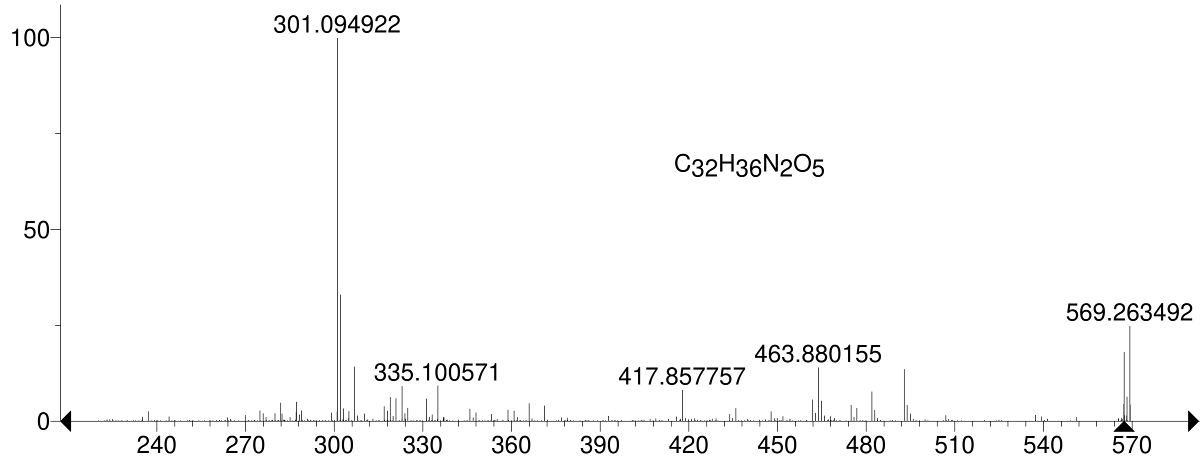

Unknown: Unknown (6.26_178.1227m/z)
Compound in Library Factor = -862

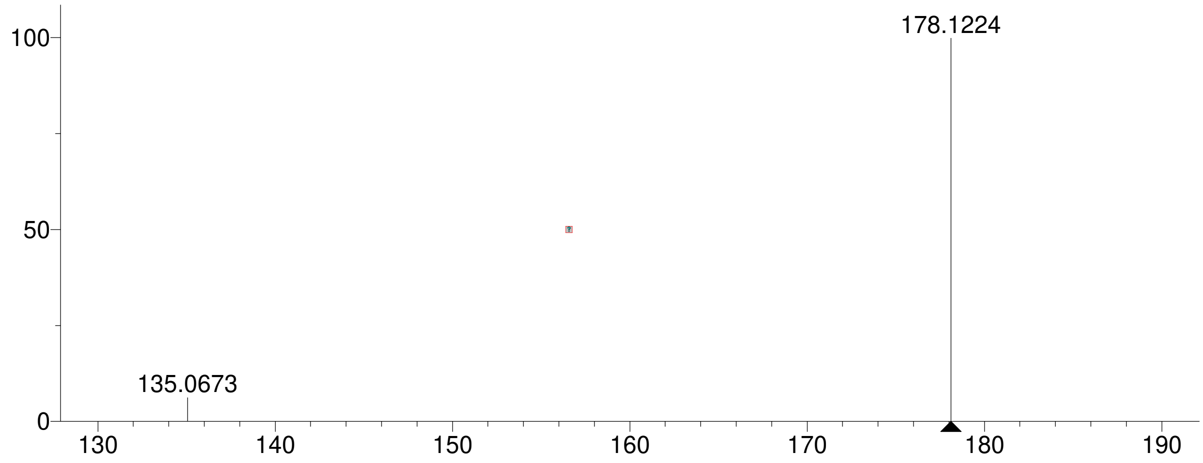

Hit 1 : Rebemide [M+H]+ 30eV P=178.1
C11H15NO; MF: 352; RMF: 970; Prob 15.3%; Lib: mona-export-massbank; ID: 26955.

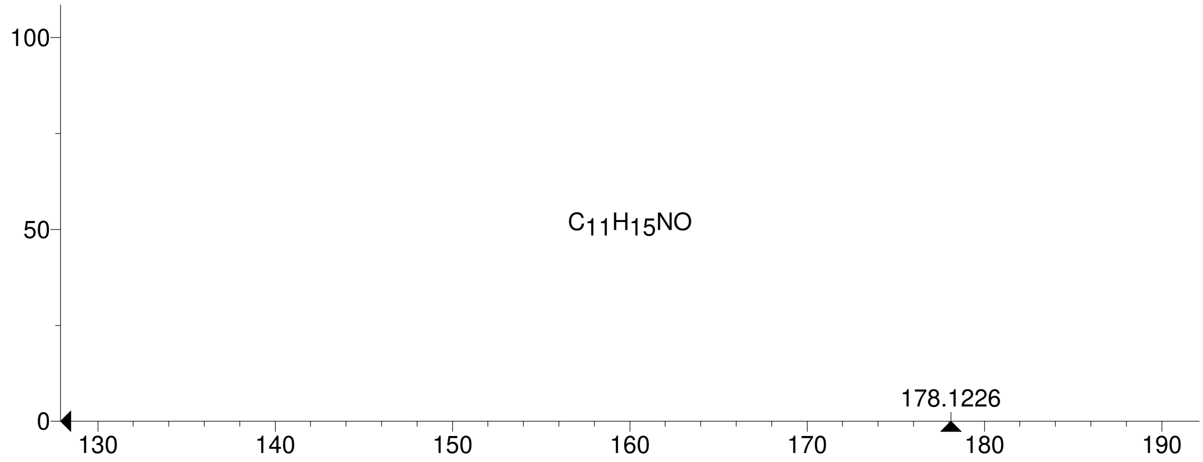

Hit 2 : N-tert-Butyl-α-phenylnitrone [M+H]+ QTOF 3V P=178.1
C11H15NO; MF: 352; RMF: 970; Prob 15.3%; CAS: 3376-24-7; Lib: nist_msms; ID: 67834.

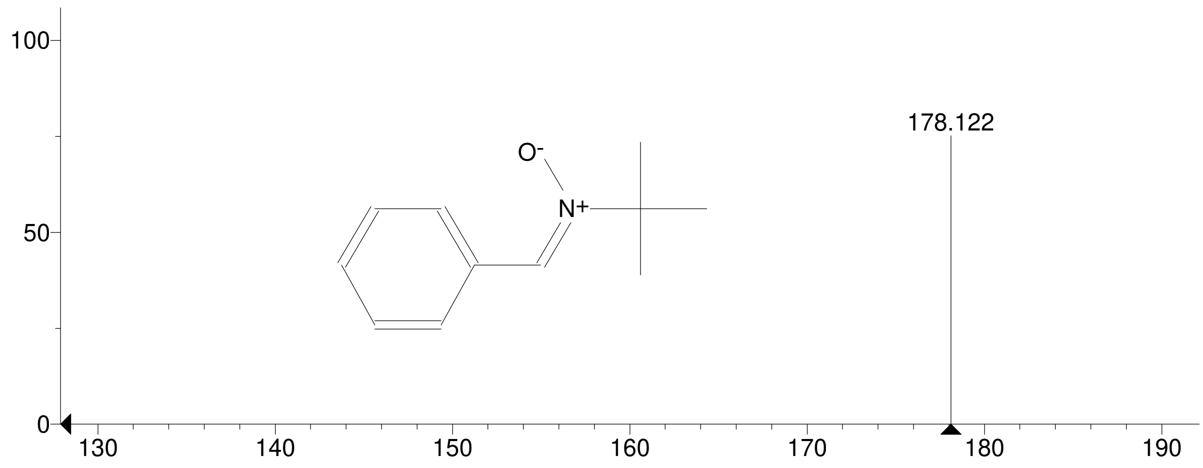

Hit 3 : N-Acetylamphetamine [M+H]+ HCD 46V P=178.1
C11H15NO; MF: 352; RMF: 970; Prob 15.3%; CAS: 14383-60-9; Lib: nist_msms; ID: 243444.

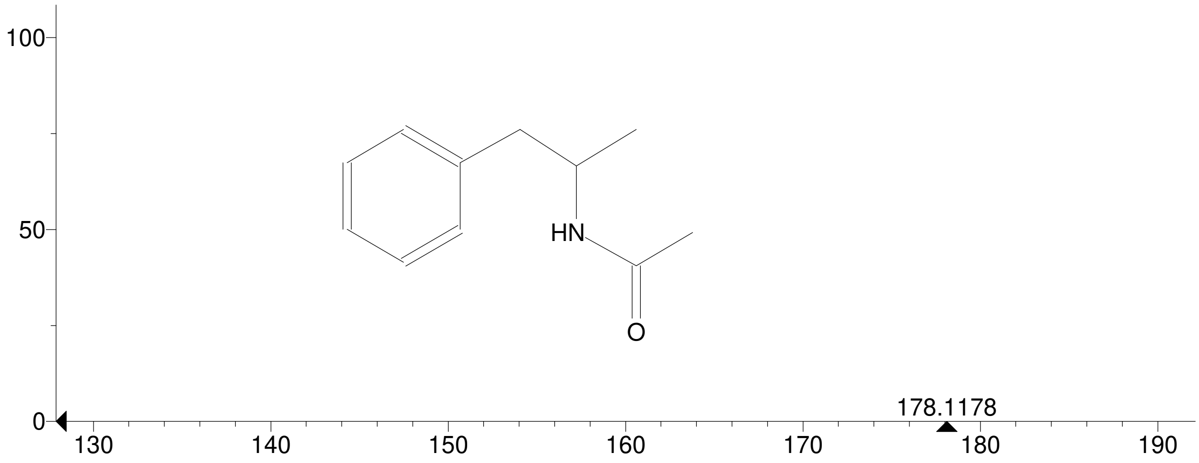

Hit 4 : 2-Methylmethcathinone [M+H]+ HCD 3V P=178.1
C11H15NO; MF: 346; RMF: 978; Prob 12.0%; CAS: 1246911-71-6; Lib: nist_msms; ID: 249570.

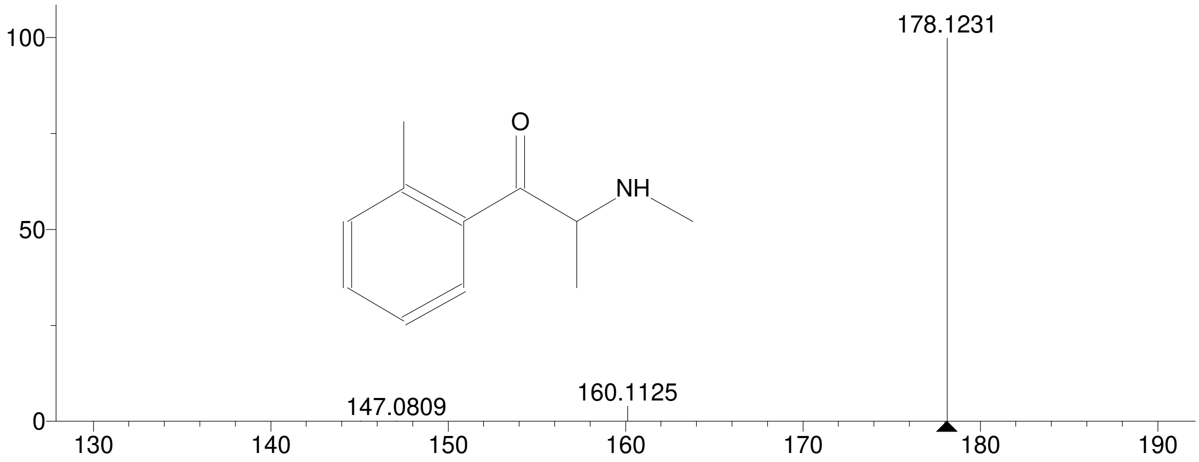

Hit 5 : 4-Methylmethcathinone [M+H]+ HCD 3V P=178.1
C11H15NO; MF: 345; RMF: 978; Prob 11.5%; CAS: 1189805-46-6; Lib: nist_msms; ID: 325130.

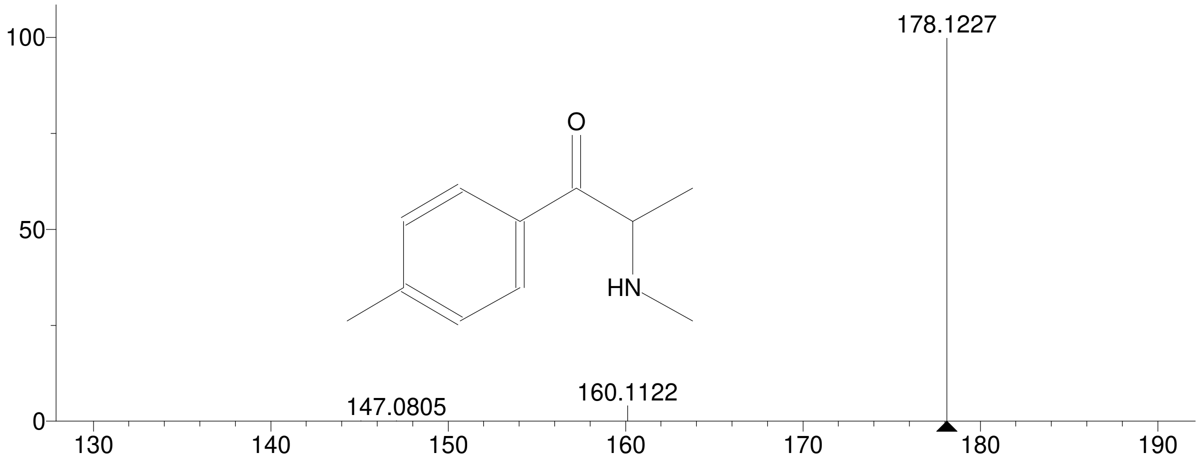

Unknown: Unknown (6.26_180.1379m/z)
Compound in Library Factor = -1848

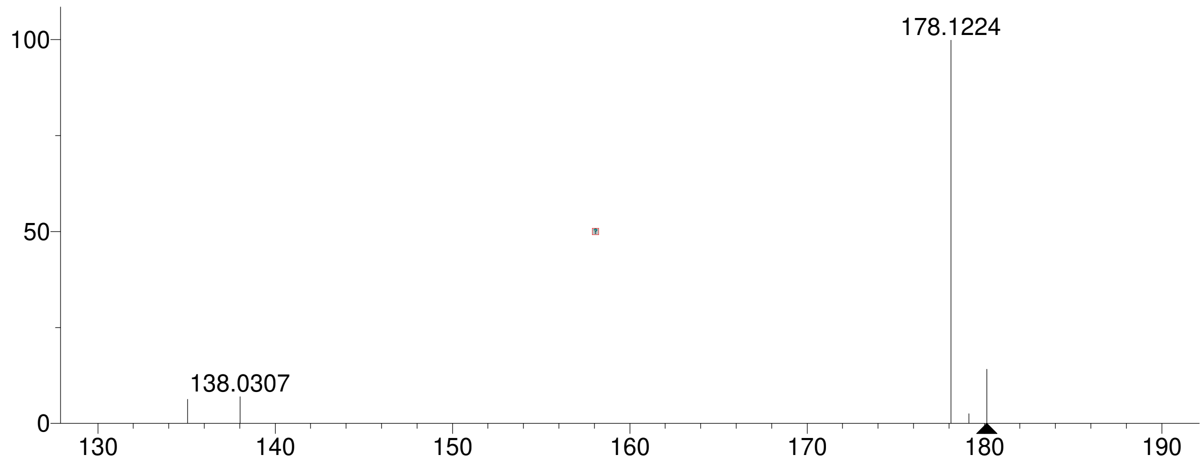

Hit 1 : Mexiletine [M+H]+ HCD 1V P=180.1
C11H17NO; MF: 88; RMF: 329; Prob 17.9%; CAS: 31828-71-4; Lib: nist_msms; ID: 134450.

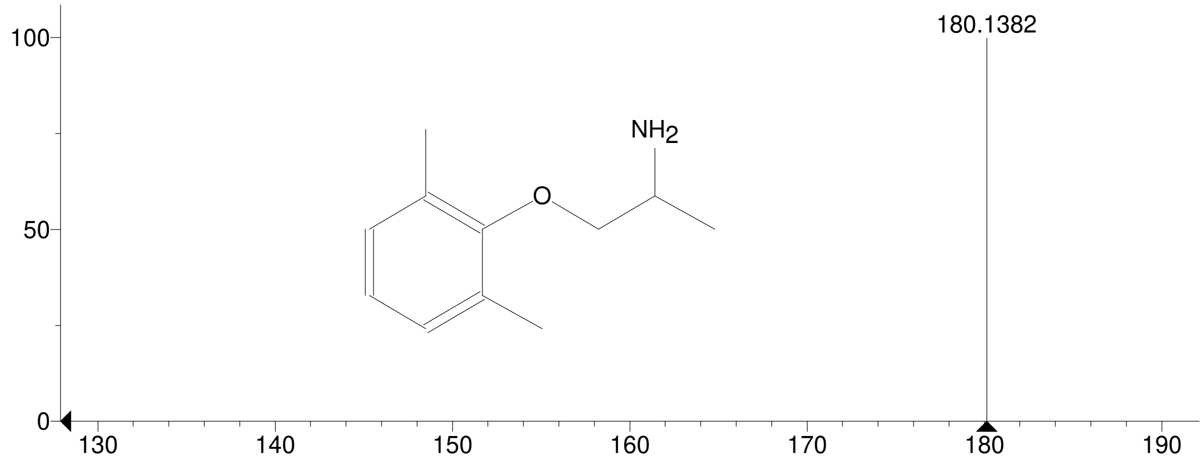

Hit 2 : p-(Isoamyloxy)aniline [M+H]+ HCD 10V P=180.1
C11H17NO; MF: 88; RMF: 329; Prob 17.9%; CAS: 5198-05-0; Lib: nist_msms; ID: 190379.

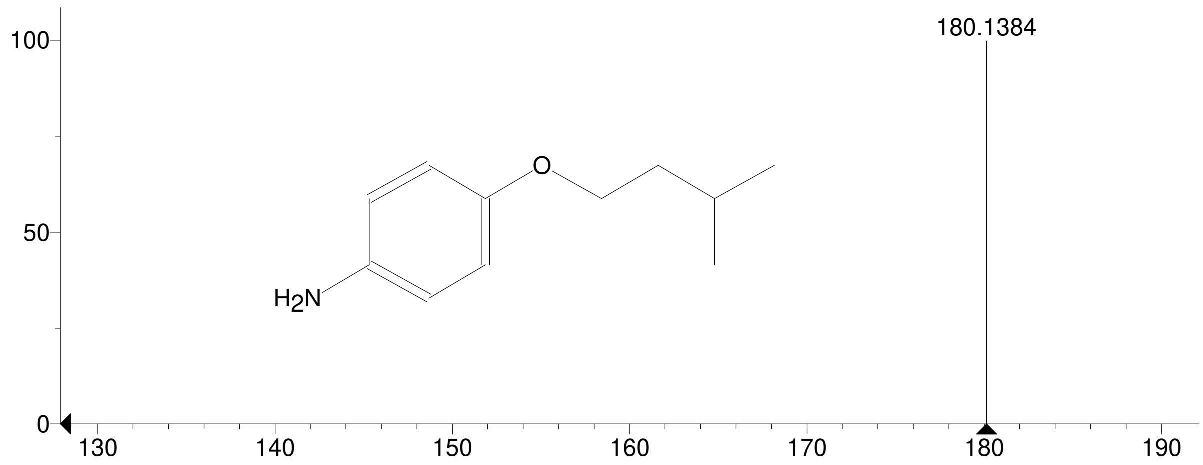

Hit 3 : Thiocarlide [M+H-C12H15SON]+ HCD 12V P=180.1
C23H32N2O2S; MF: 88; RMF: 329; Prob 17.9%; CAS: 910-86-1; Lib: nist_msms; ID: 333192.

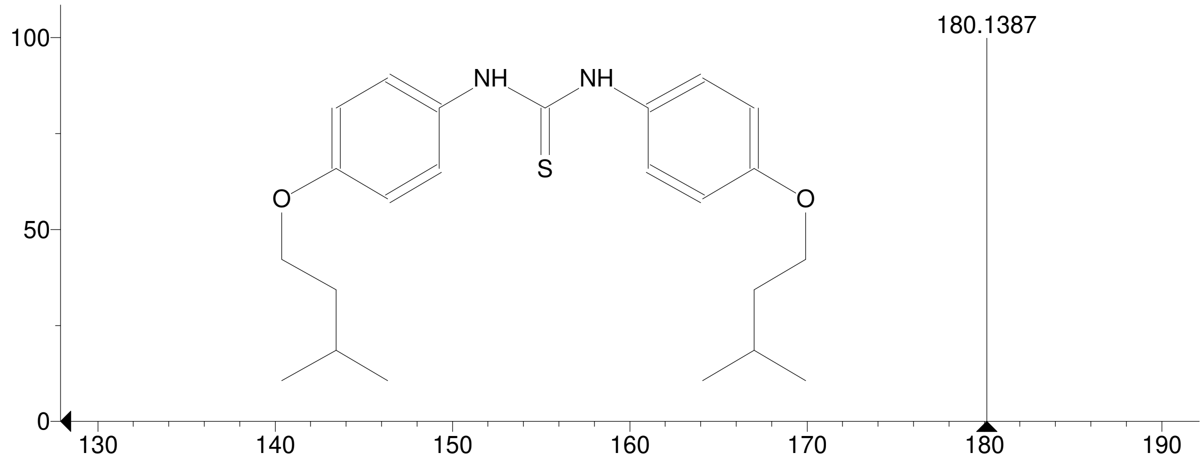

Hit 4 : 1-(Cyclopentylcarbonyl)-4-piperidinamine [M+H-NH3]+ HCD 27V P=180.1
C11H20N2O; MF: 88; RMF: 329; Prob 17.9%; CAS: 926234-06-2; Lib: nist_msms; ID: 560807.

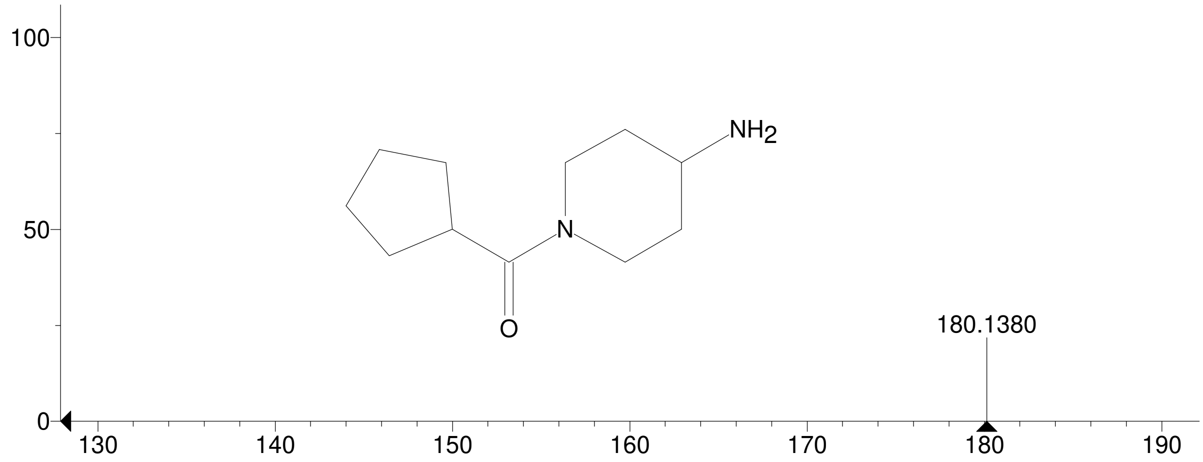

Hit 5 : 4-Methylephedrine [M+H]+ HCD 1V P=180.1
C11H17NO; MF: 58; RMF: 338; Prob 4.97%; CAS: 27465-54-9; Lib: nist_msms; ID: 196793.

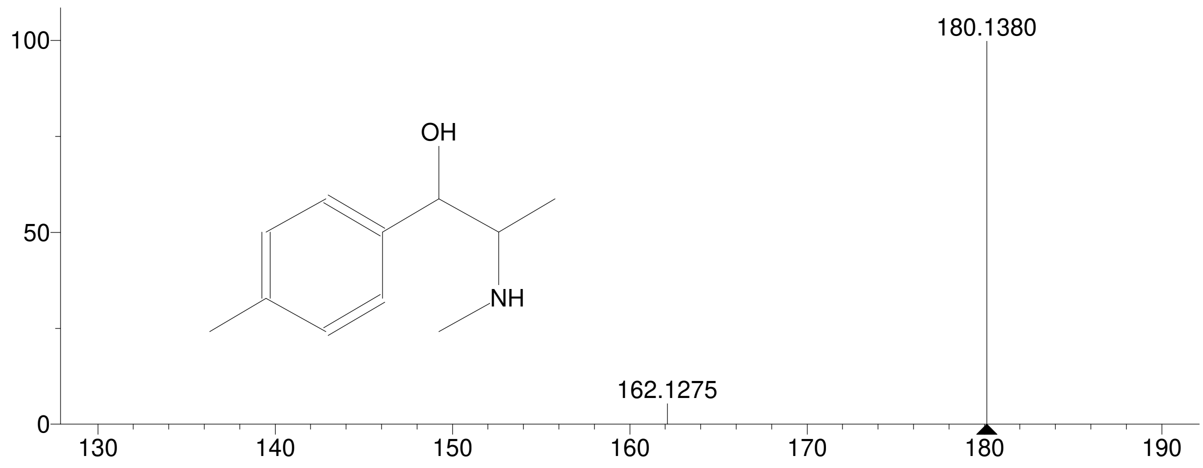

** Search Report Page 1 of 1 **

Unknown: Unknown (6.26_224.1280m/z)
Compound in Library Factor = -3273

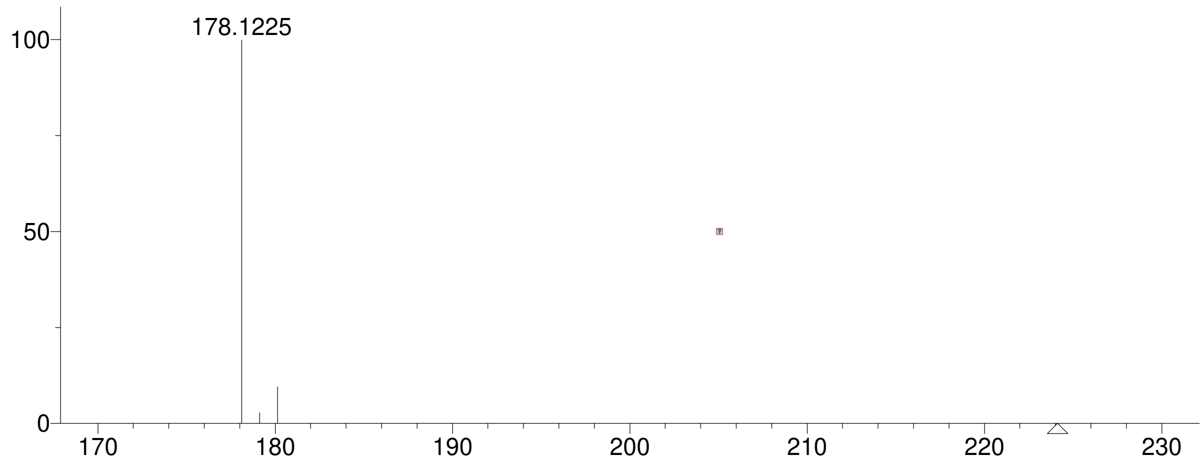

Hit 1 : N-(3-Oxooctanoyl)-L-homoserine lactone [M+H-H2O]+ IT-FT 35% P=224.1
C12H19NO4; MF: 10; RMF: 140; Prob 56.6%; CAS: 147795-39-9; Lib: nist_msms; ID: 315231.

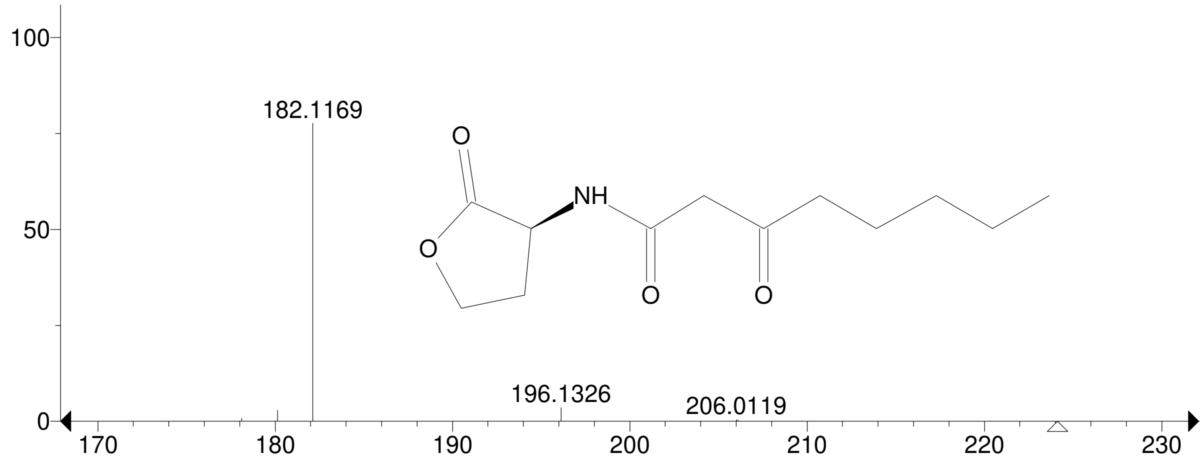

Hit 2 : Cerulenin [M+H]+ IT-FT 35% P=224.1
C12H17NO3; MF: 3; RMF: 216; Prob 43.4%; CAS: 17397-89-6; Lib: nist_msms; ID: 538608.

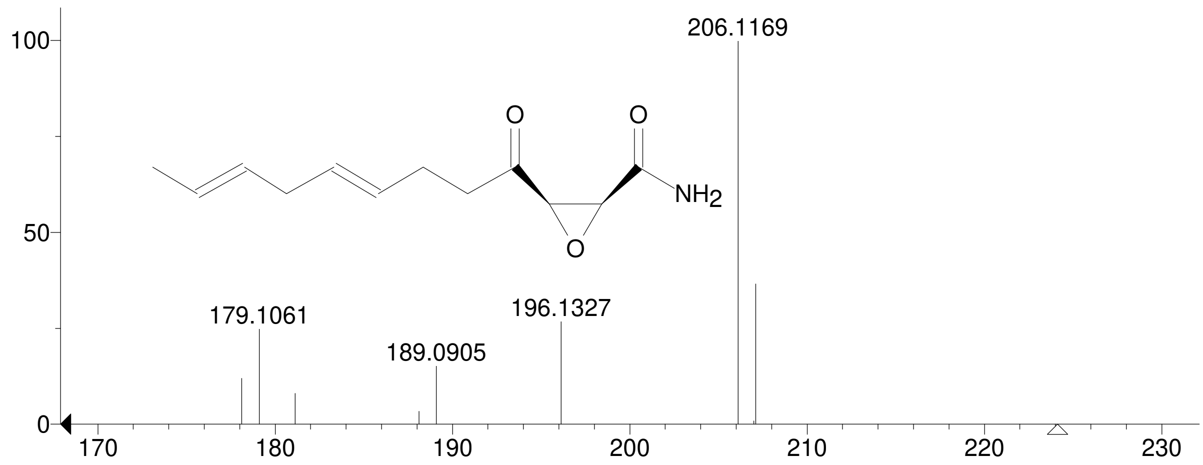

Unknown: Unknown (6.26_449.1544m/z)
Compound in Library Factor = 0

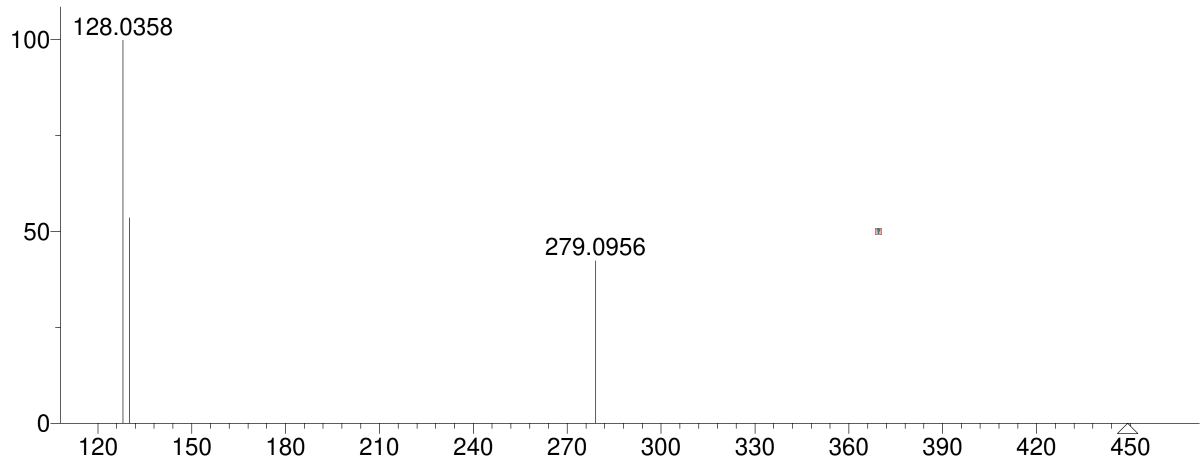

Hit 1 : NCGC00385098-01![8,8-dimethyl-9-(3-methylbut-2-enoyloxy)-2-oxo-9,10-dihydropyrano[2,3-f]chromen-10-
C24H26O7; MF: 0; RMF: 54; Prob 100.0%; Lib: mona-export-gnps; ID: 7369.

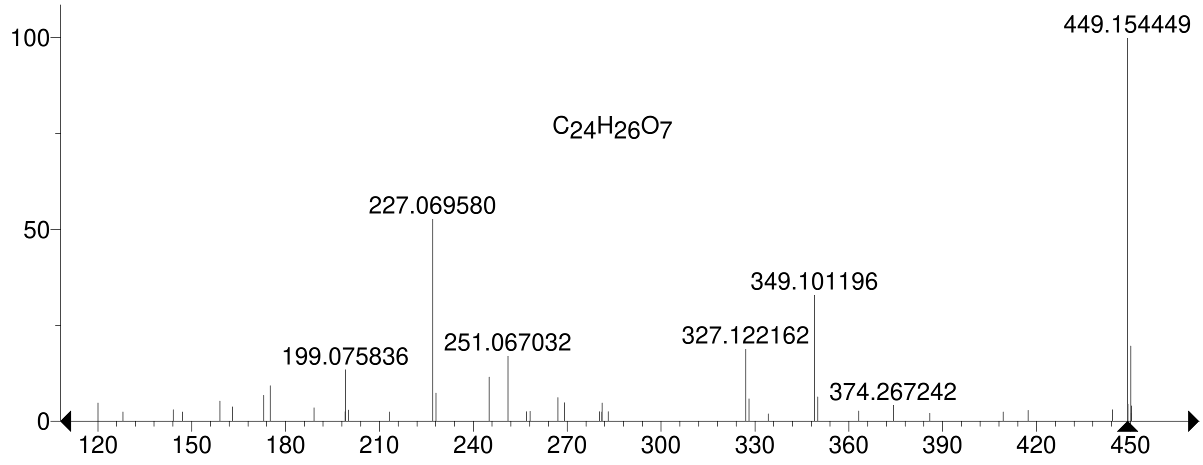

Unknown: Unknown (6.26_625.2551m/z)
Compound in Library Factor = -3273

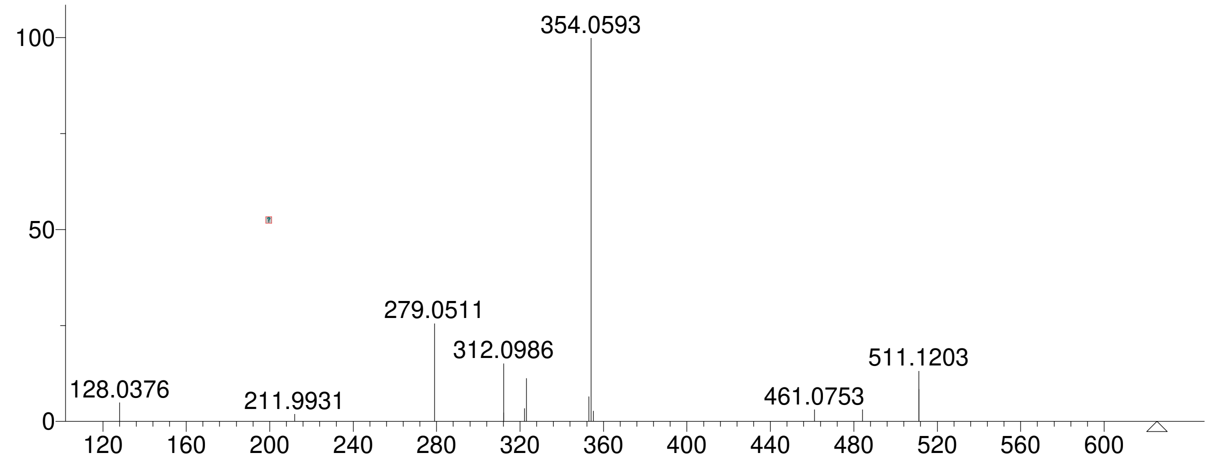

Hit 1 : N-Caffeoyl-O-methyltyramine [2M-H]- IT 35% P=625.3
C18H19NO4; MF: 6; RMF: 259; Prob 56.0%; CAS: 189307-47-9; Lib: nist_msms; ID: 221344.

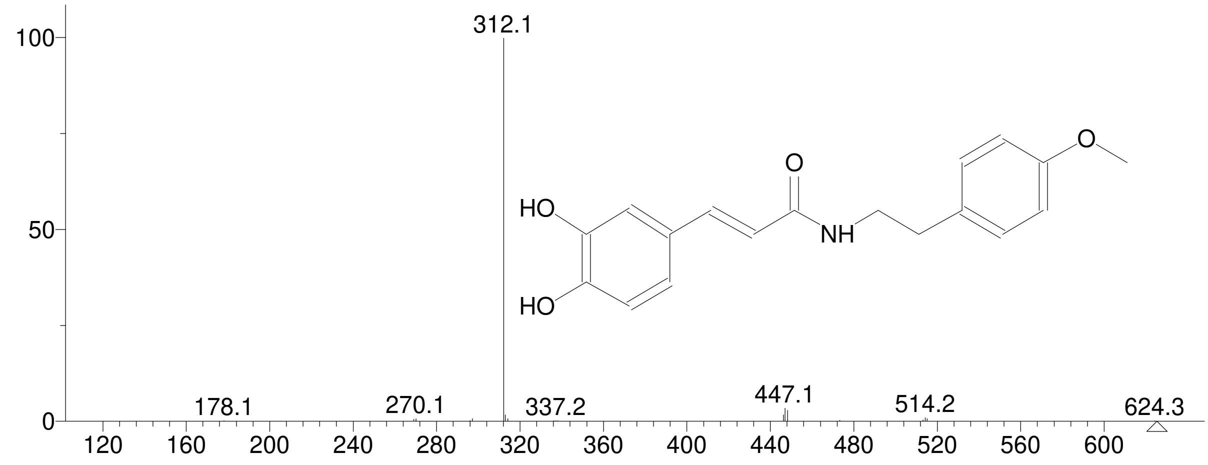

Hit 2 : Collagen Binding Fragment [M+2H]2+ QQQ 22.5V P=625.3
C52H76N14O20S; MF: 0; RMF: 6; Prob 44.0%; CAS: 94040-53-6; Lib: nist_msms2; ID: 3608.

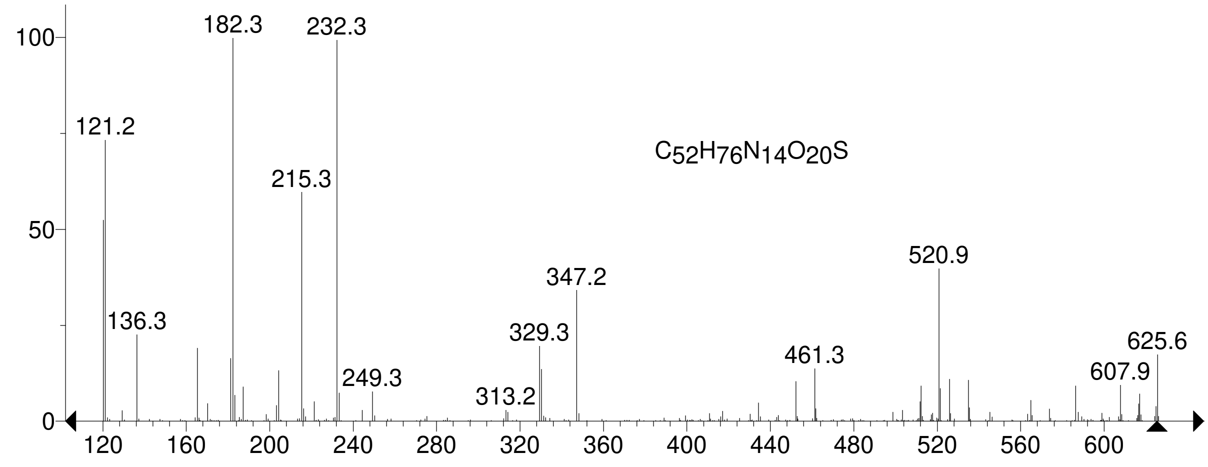

** Search Report Page 1 of 1 **

Unknown: Unknown (6.26_654.2087m/z)
Compound in Library Factor = 0

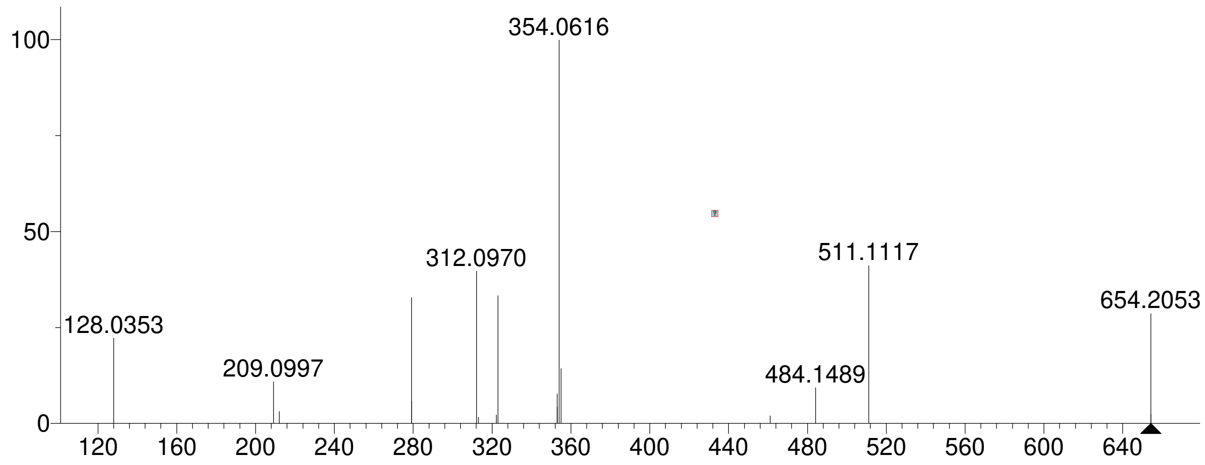

Unknown: Unknown (6.27_405.2218m/z)
Compound in Library Factor = 0

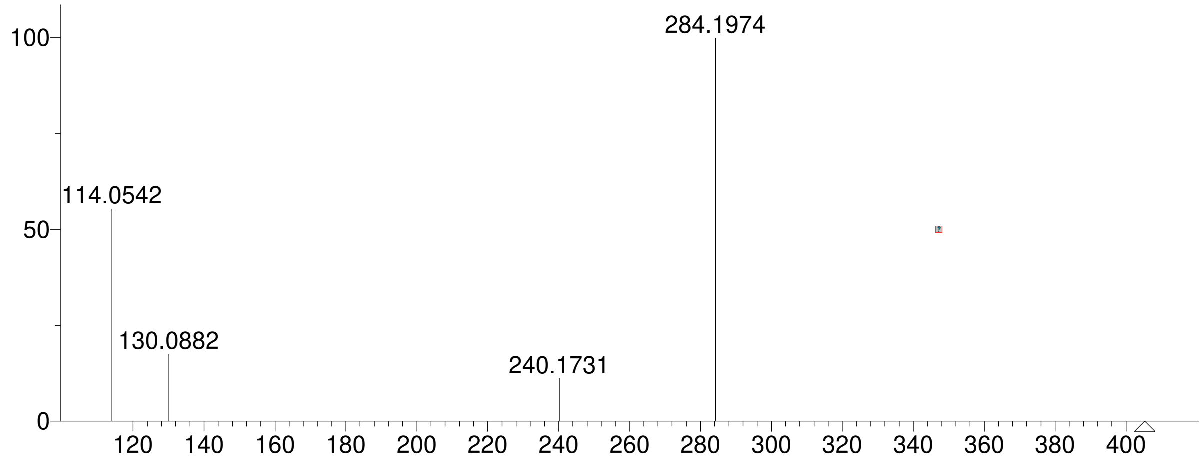

Hit 1 : MLS001076491-01!Cortell [M+H]+ P=405.2
C23H32O6; MF: 0; RMF: 12; Prob 100.0%; Lib: mona-export-gnps; ID: 4335.

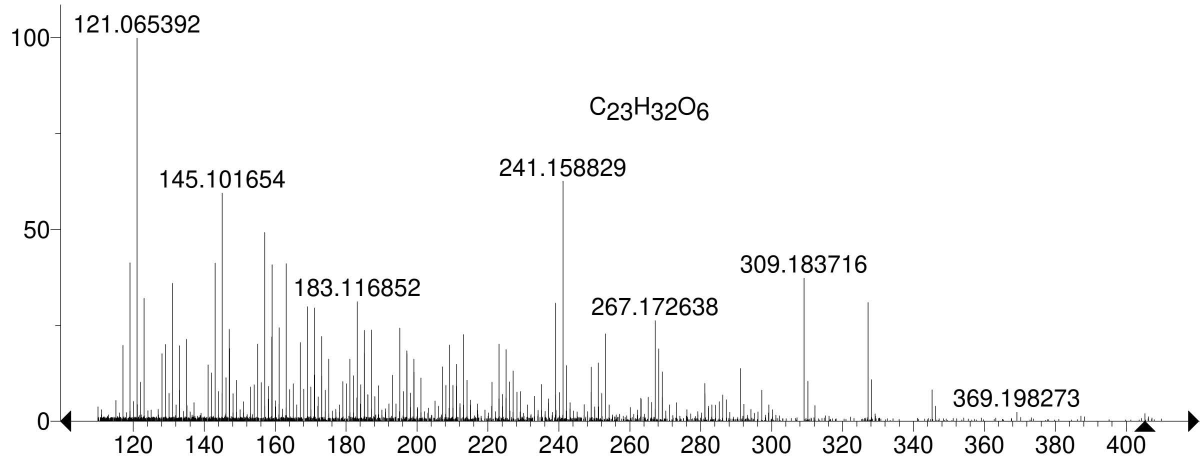

Unknown: Unknown (6.29_528.2129m/z)
Compound in Library Factor = 0

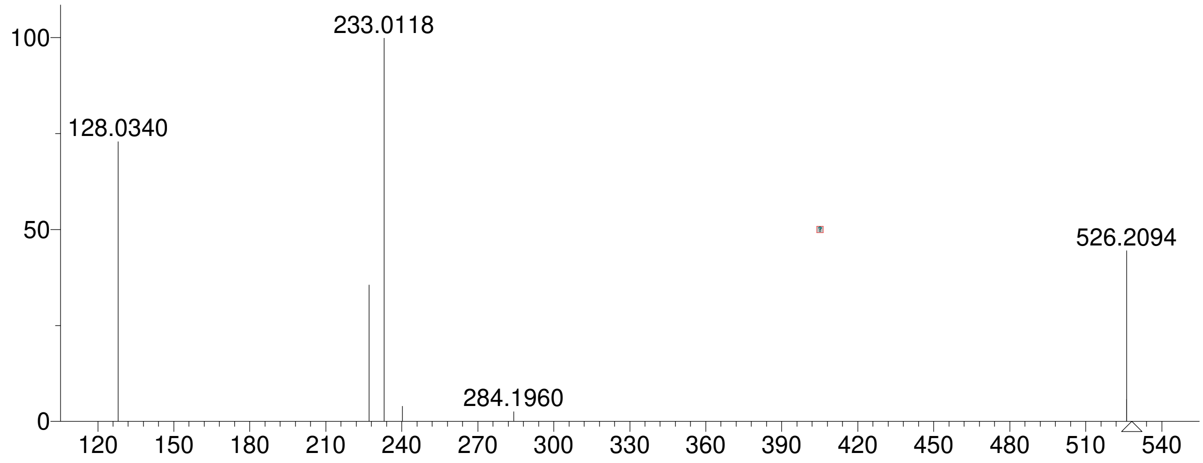

** Search Report Page 1 of 1 **

Unknown: Unknown (6.30_517.1680m/z)
Compound in Library Factor = 0

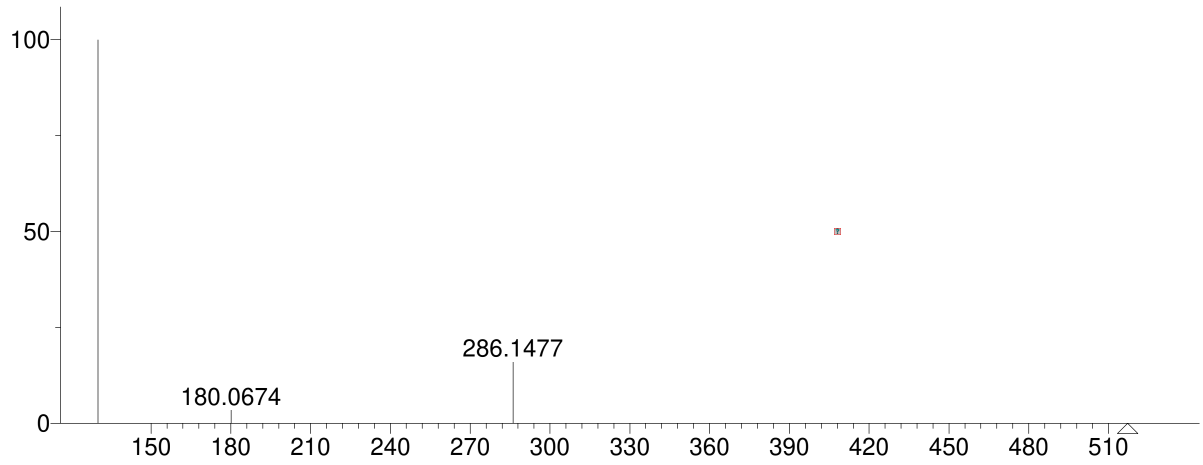

Unknown: Unknown (6.31_267.0719m/z)
Compound in Library Factor = -862

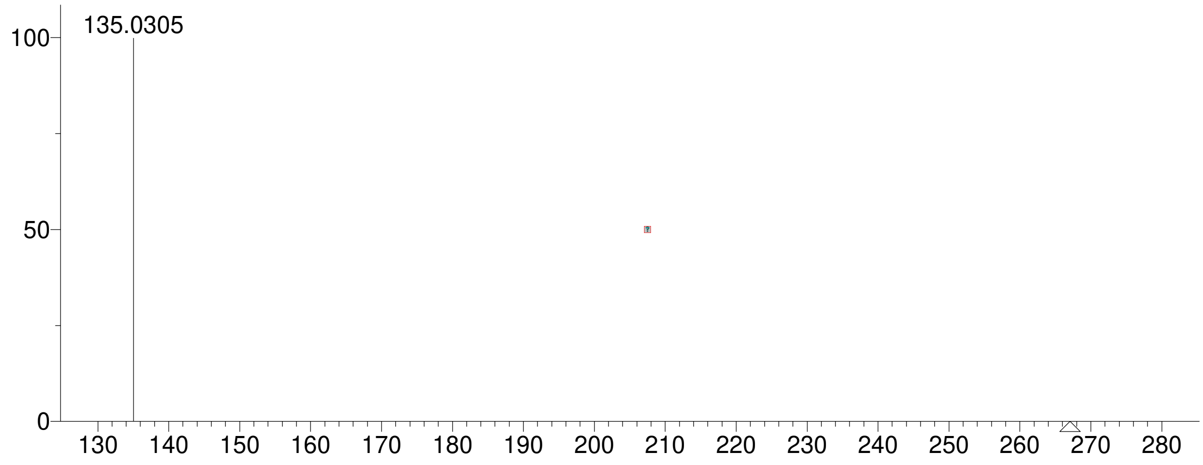

Hit 1 : Inosine [M-H]- 60 P=267.1
C10H12N4O5; MF: 364; RMF: 999; Prob 58.5%; Lib: msms neg mona_ipb_gnps_plasma; ID: 950.

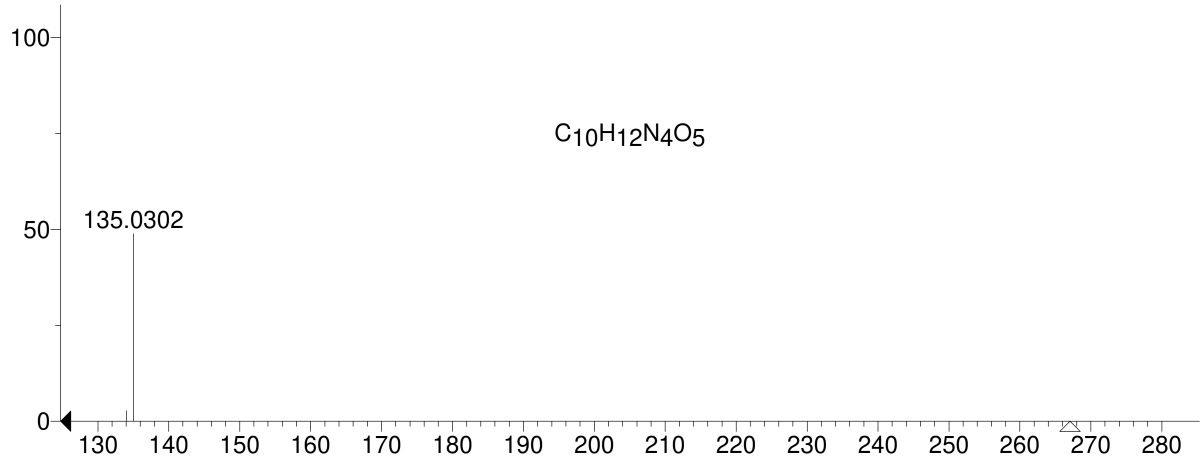

Hit 2 : Ribavirin [M+Na]+ HCD 24V P=267.1
C8H12N4O5; MF: 353; RMF: 989; Prob 40.1%; CAS: 36791-04-5; Lib: nist_msms; ID: 489043.

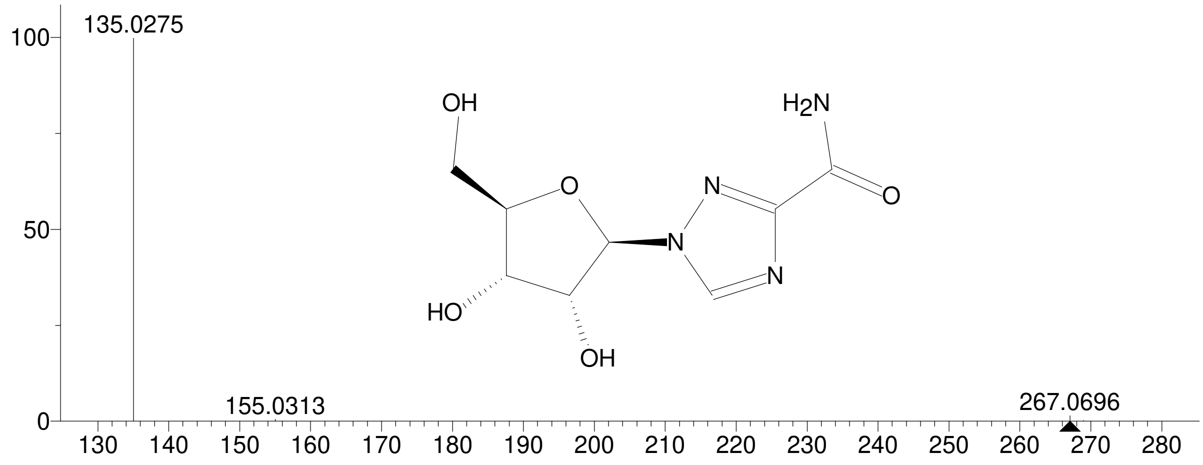

Hit 3 : Inosine; LC-ESI-QTOF; MS2; CE [M-H]- P=267.1
C10H12N4O5; MF: 209; RMF: 849; Prob 1.23%; Lib: msms-neg-massbank; ID: 4345.

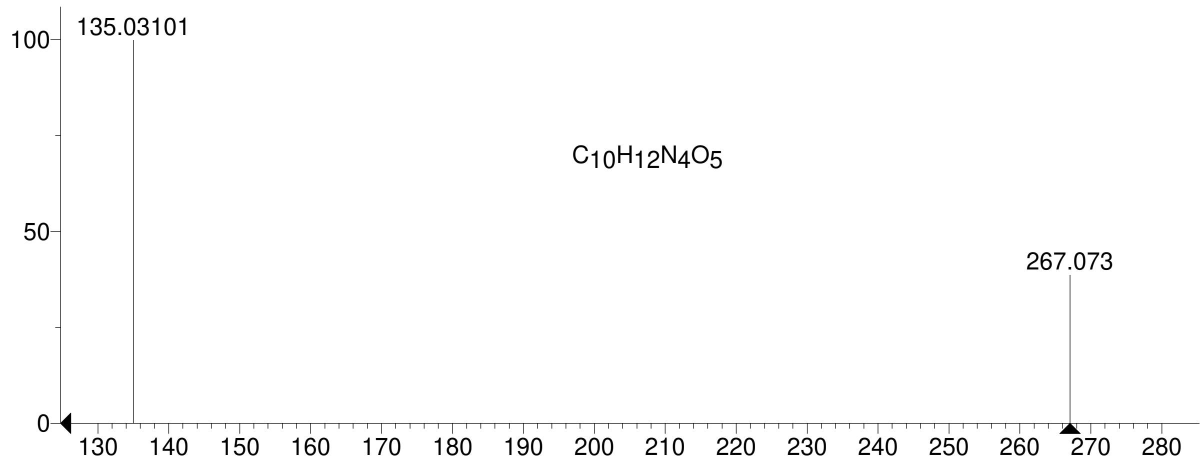

Hit 4 : INOSINE [M-H]- P=267.1
C10H12N4O5; MF: 86; RMF: 708; Prob 0.05%; Lib: msms-neg-gnps; ID: 167.

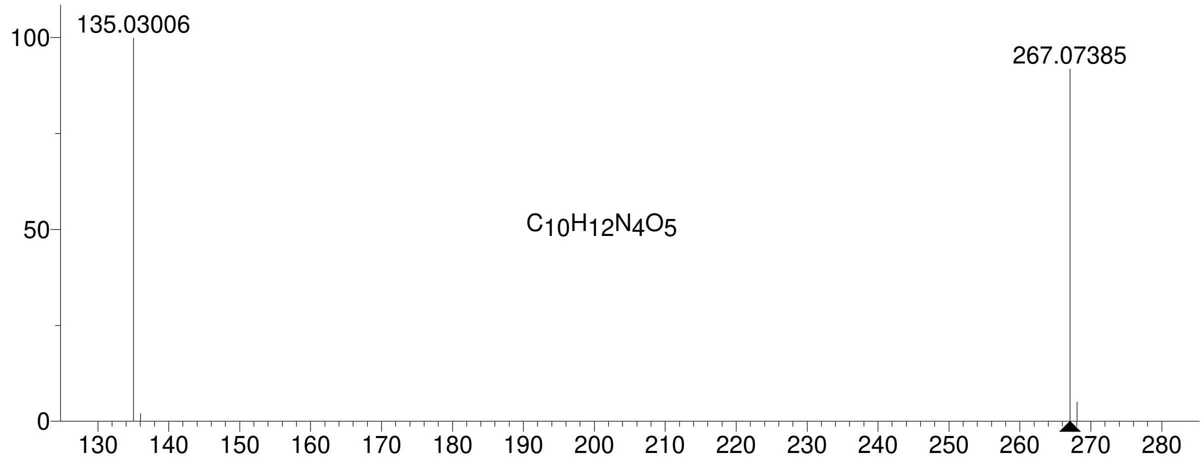

Hit 5 : INOSINE
C10H12N4O5; MF: 71; RMF: 707; Prob 0.03%; Lib: embl neg mcg; ID: 113.

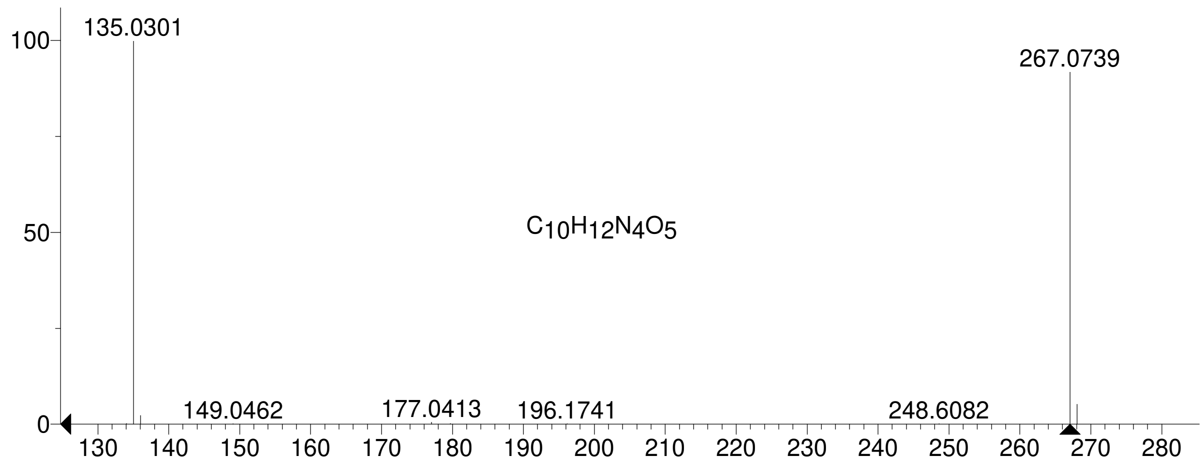

Unknown: Unknown (6.31_328.1852m/z)
Compound in Library Factor = 0

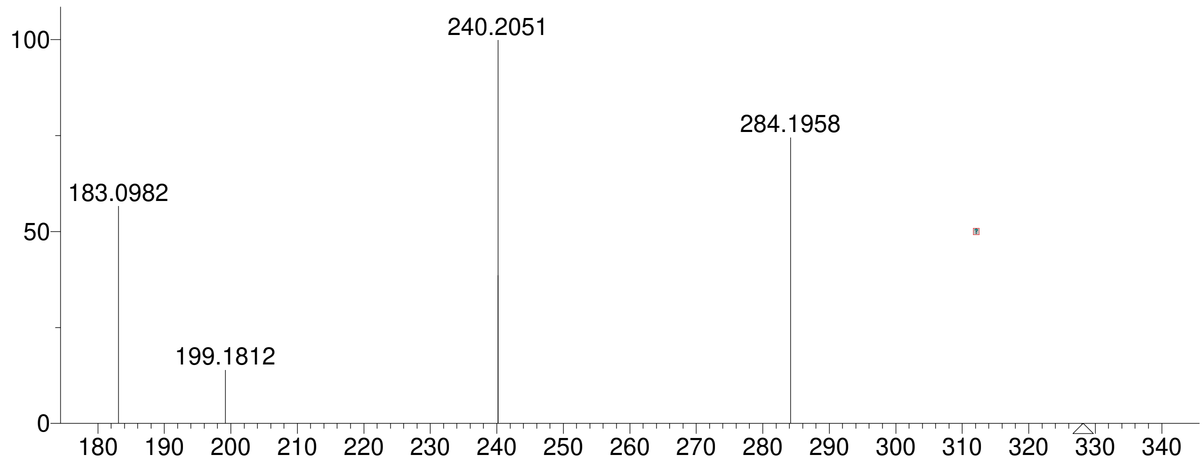

Hit 1 : Methanone, [1-(2-chloropentyl)-1H-indol-3-yl](2,2,3,3-tetramethylcyclopropyl)- [M+H-H2O]+ IT 35% P=3:
C21H28ClNO; MF: 0; RMF: 29; Prob 20.0%; Lib: nist_msms; ID: 260480.

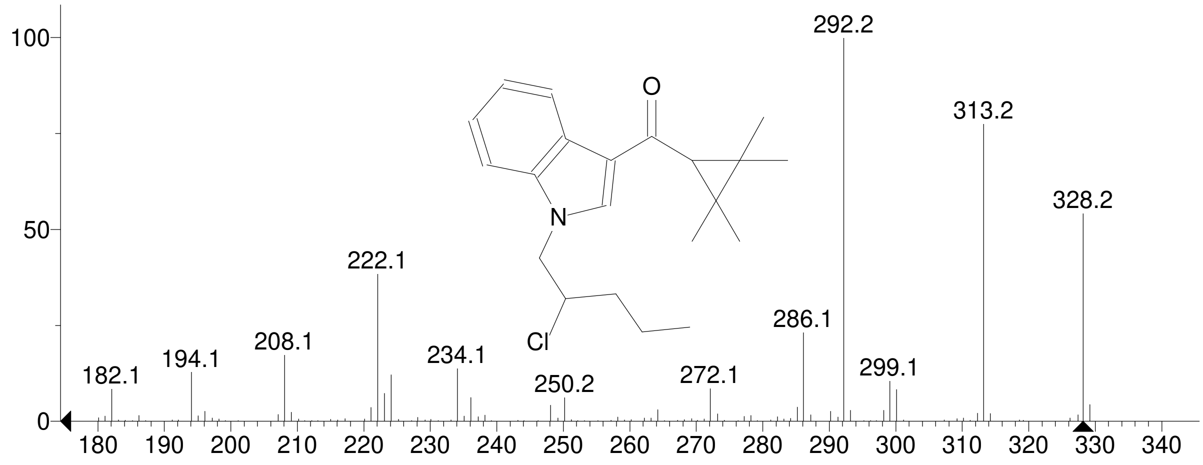

Hit 2 : Methanone, [1-(3-chloropentyl)-1H-indol-3-yl](2,2,3,3-tetramethylcyclopropyl)- [M+H-H2O]+ HCD 59V P=
C21H28ClNO; MF: 0; RMF: 22; Prob 20.0%; Lib: nist_msms; ID: 260534.

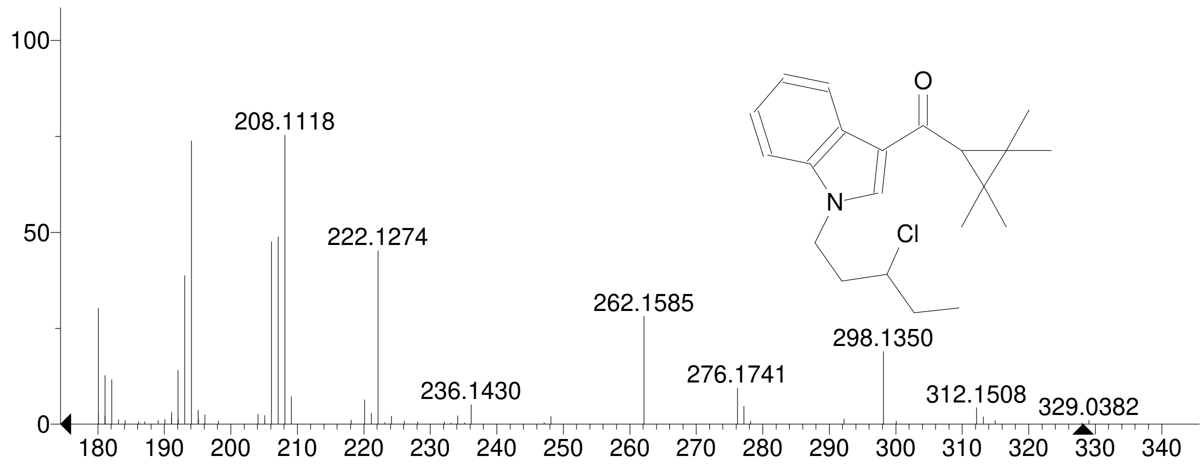

Hit 3 : Methanone, [1-(5-chloropentyl)-1H-indol-3-yl](2,2,3,3-tetramethylcyclopropyl)- [M+H-H2O]+ HCD 49V P=
C21H28ClNO; MF: 0; RMF: 20; Prob 20.0%; CAS: 1445577-42-3; Lib: nist_msms; ID: 271990.

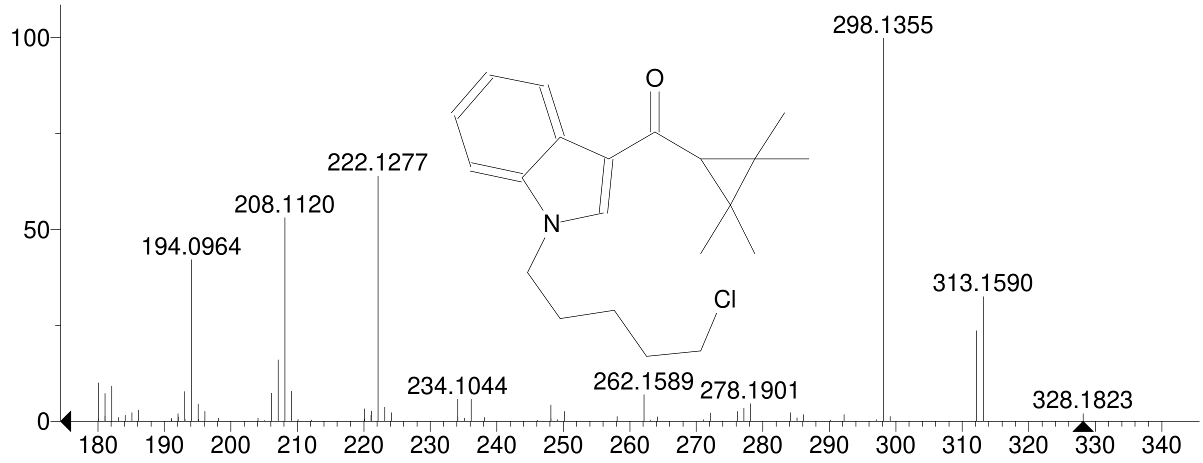

Hit 4 : Methanone, [1-(4-chloropentyl)-1H-indol-3-yl](2,2,3,3-tetramethylcyclopropyl)- [M+H-H2O]+ IT 35% P=3
C21H28ClNO; MF: 0; RMF: 15; Prob 20.0%; Lib: nist_msms; ID: 260614.

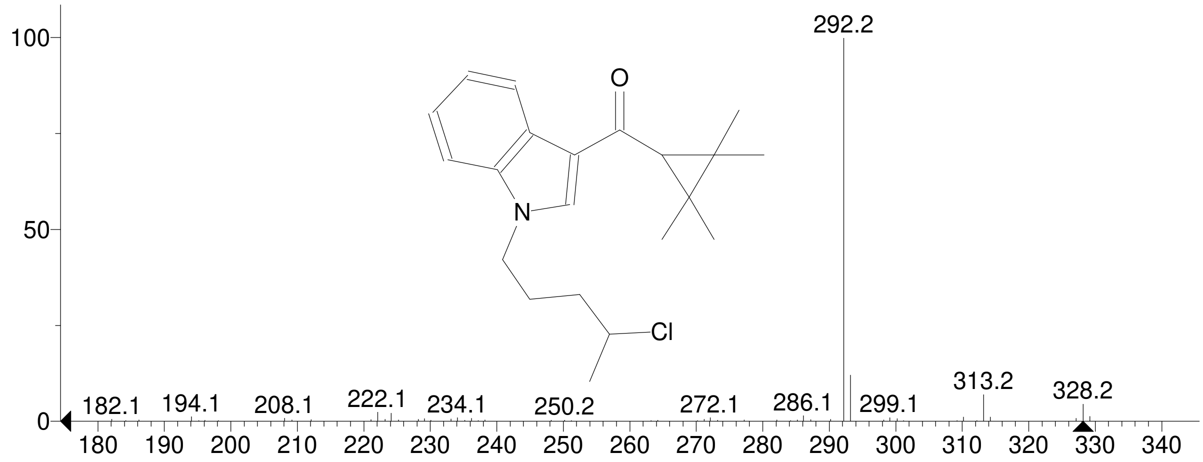

Hit 5 : CAPSAICIN [M+Na]+ 35HCD P=328.2
C18H27NO3; MF: 0; RMF: 3; Prob 20.0%; Lib: mona-export-vf-npl_qexactive; ID: 2965.

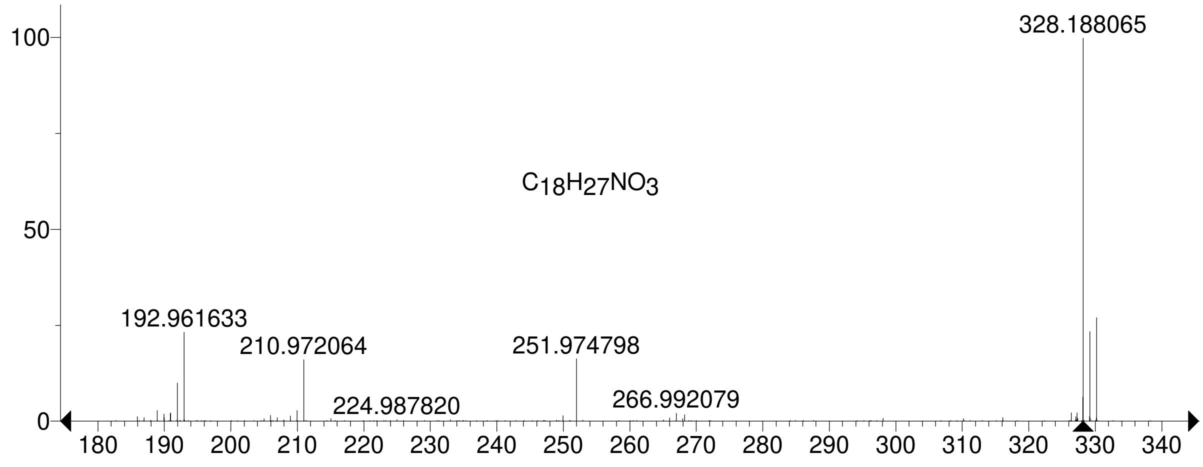

Unknown: Unknown (6.31_364.1632m/z)
Compound in Library Factor = 0

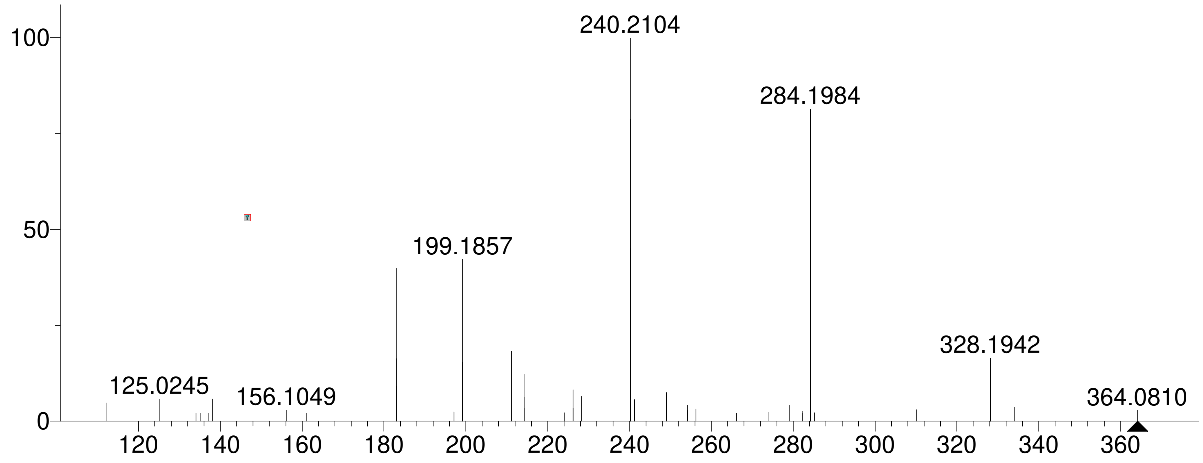

Hit 1 : Cys-Asn-Lys [M+H]+ QQQ 20V P=364.2
C13H25N5O5S; MF: 0; RMF: 44; Prob 12.5%; Lib: nist_msms; ID: 42766.

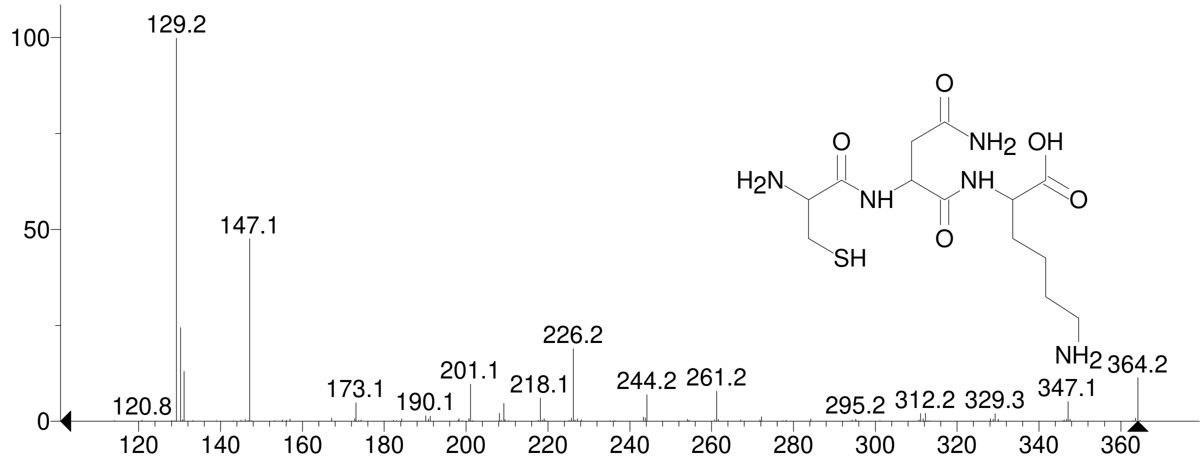

Hit 2 : (2R,3S,4S,5R,6S)-2-(hydroxymethyl)-6-(3,4,5-trimethoxyphenoxy)oxane-3,4,5-triol [M+NH4]+ 35HCD P=
C15H22O9; MF: 0; RMF: 26; Prob 12.5%; Lib: mona-export-vf-npl_qexactive; ID: 9871.

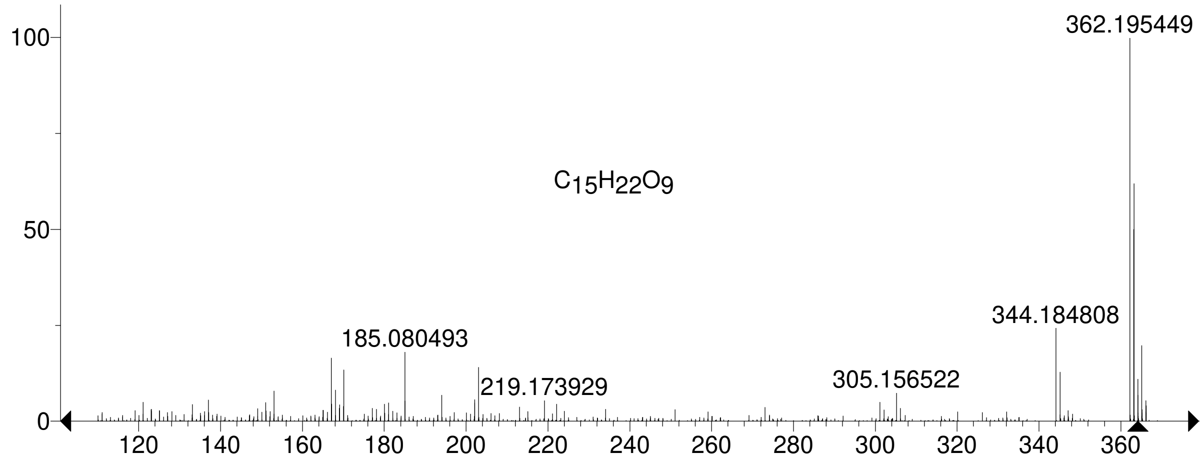

Hit 3 : aucubin [M+NH4]+ 65HCD P=364.2
C15H22O9; MF: 0; RMF: 24; Prob 12.5%; Lib: mona-export-vf-npl_qexactive; ID: 3774.

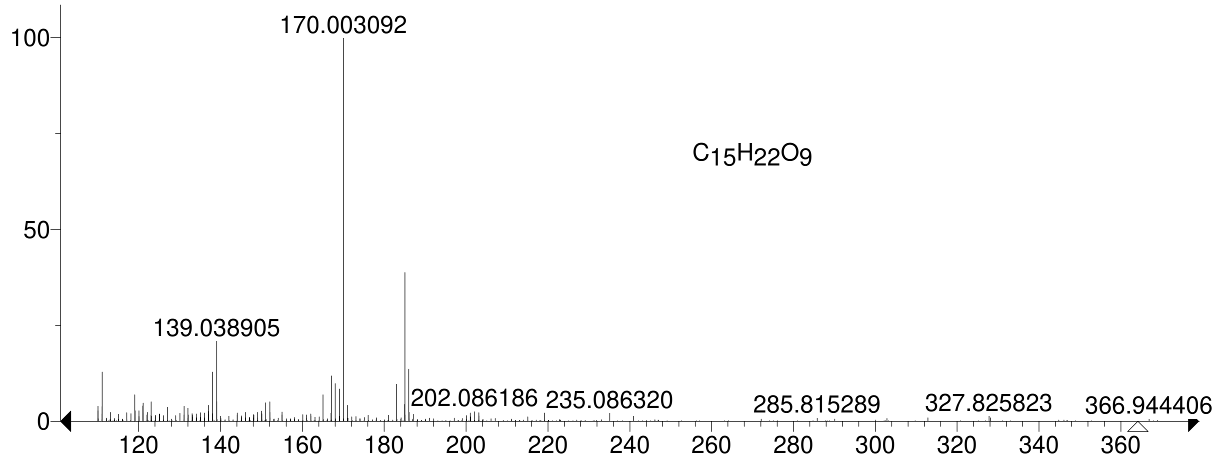

Hit 4 : Asn-Cys-Lys [M+H]+ IT 35% P=364.2
C13H25N5O5S; MF: 0; RMF: 23; Prob 12.5%; Lib: nist_msms; ID: 46916.

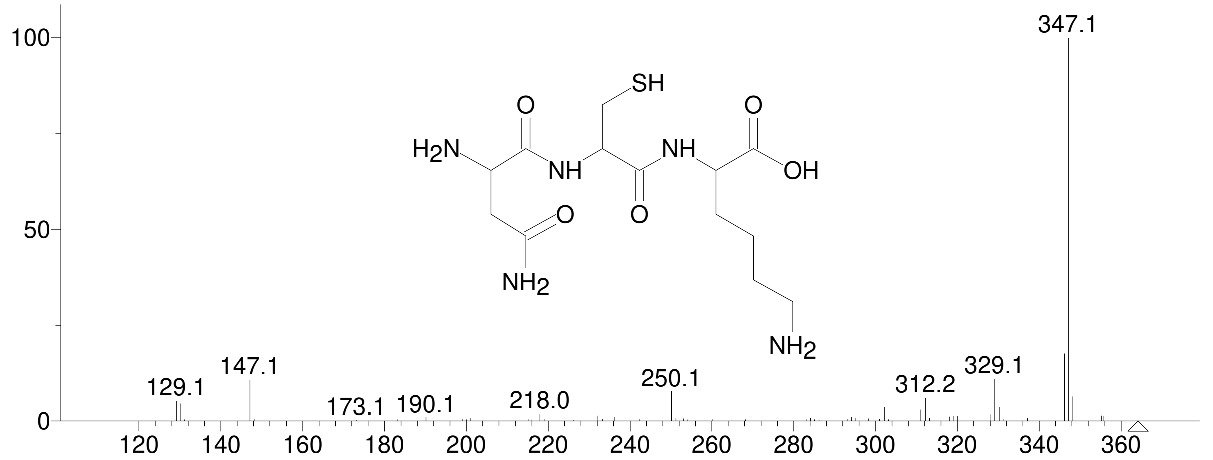

Hit 5 : NCGC00380729-01_C21H21N3O3_8',8'-Dimethyl-5a',6'-dihydro-3'H,5'H,8'H-spiro[indole-2,7'-pyrrolo[1',2':
C21H21N3O3; MF: 0; RMF: 14; Prob 12.5%; Lib: mona-export-gnps; ID: 11547.

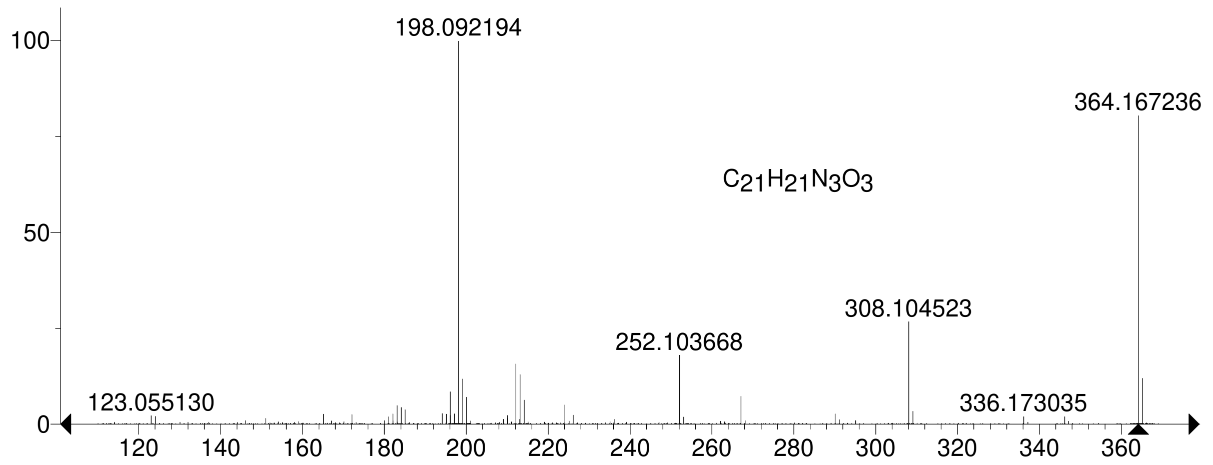

** Search Report Page 1 of 1 **

Unknown: Unknown (6.31_558.1803m/z)
Compound in Library Factor = -3273

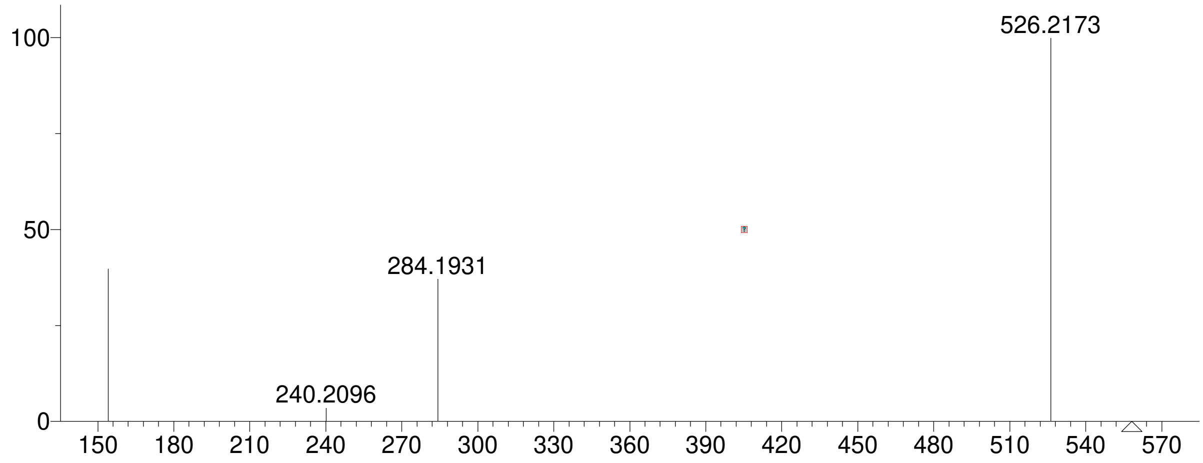

Hit 1 : LNH [M-H+HCO2]2- IT 35% P=558.2
C40H68N2O31; MF: 2; RMF: 266; Prob 100.0%; CAS: 64003-51-6; Lib: nist_msms; ID: 532404.

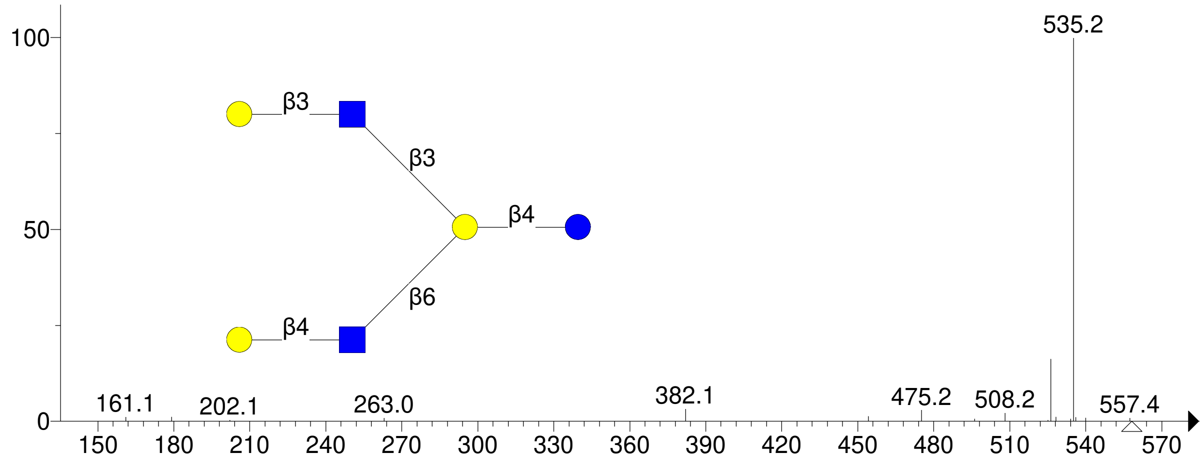

Unknown: Unknown (6.32_378.1765m/z)
Compound in Library Factor = 0

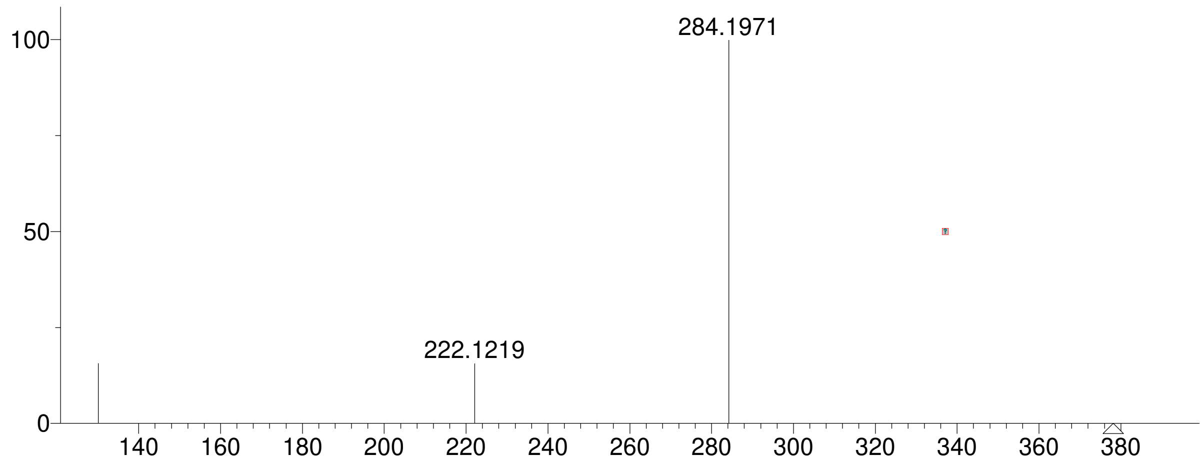

Hit 1 : (2R,3R,4S,5S,6R)-2-[3-hydroxy-2-(4-hydroxy-3-methoxyphenyl)propoxy]-6-(hydroxymethyl)oxane-3,4,5-tric
C16H24O9; MF: 0; RMF: 2; Prob 100.0%; Lib: mona-export-vf-npl_qexactive; ID: 10762.

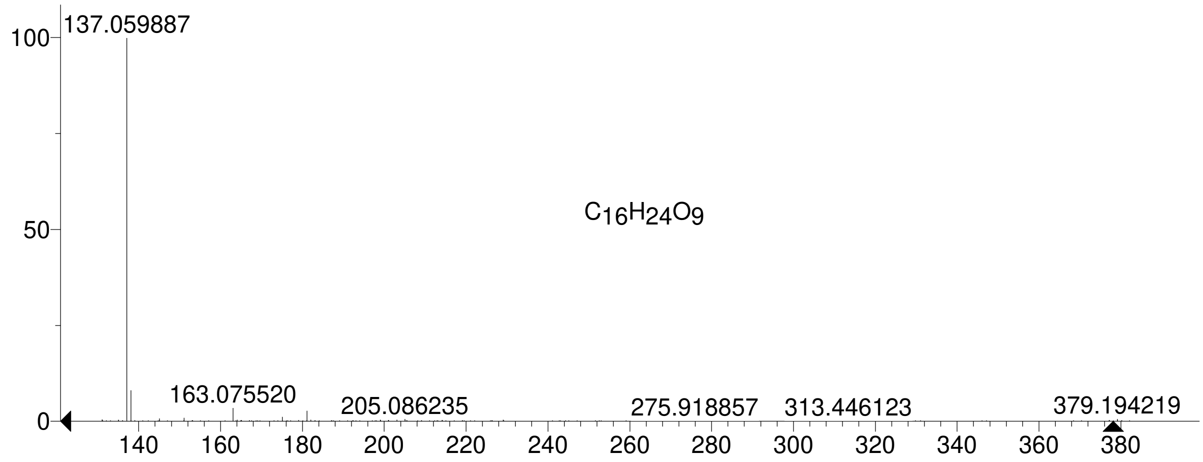

Unknown: Unknown (6.32_516.1858m/z)
Compound in Library Factor = 0

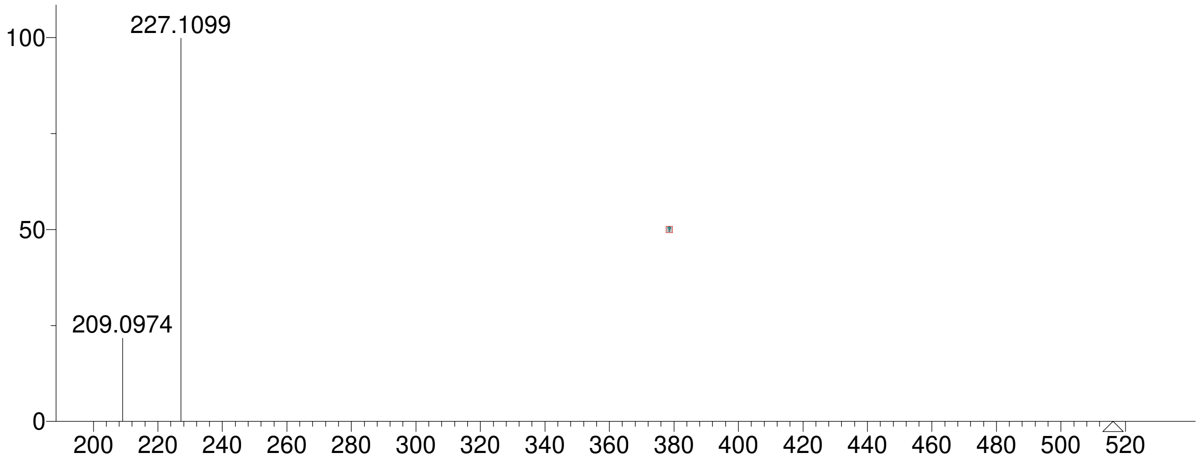

** Search Report Page 1 of 1 **

Unknown: Unknown (6.33_526.2159m/z)
Compound in Library Factor = 0

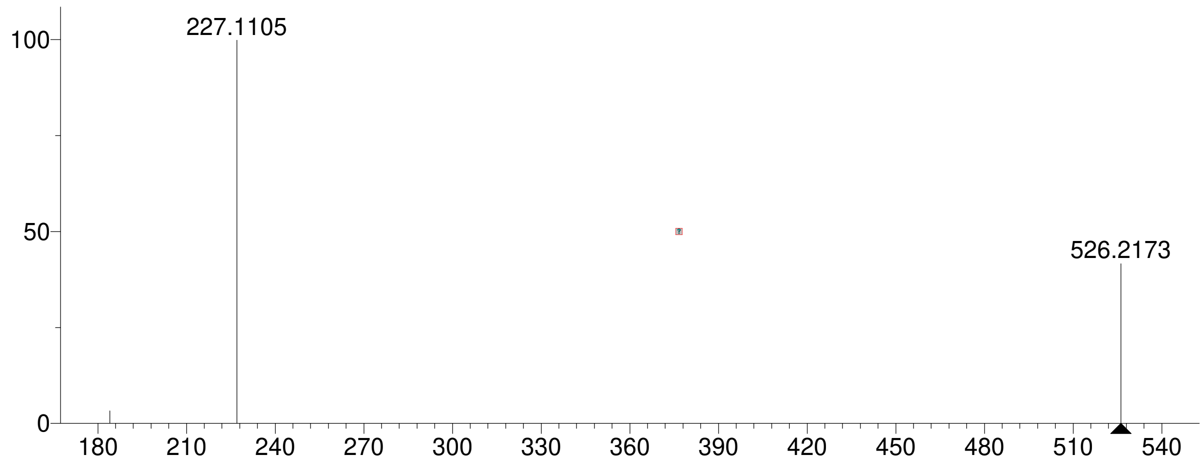

Unknown: Unknown (6.33_567.2282m/z)
Compound in Library Factor = 0

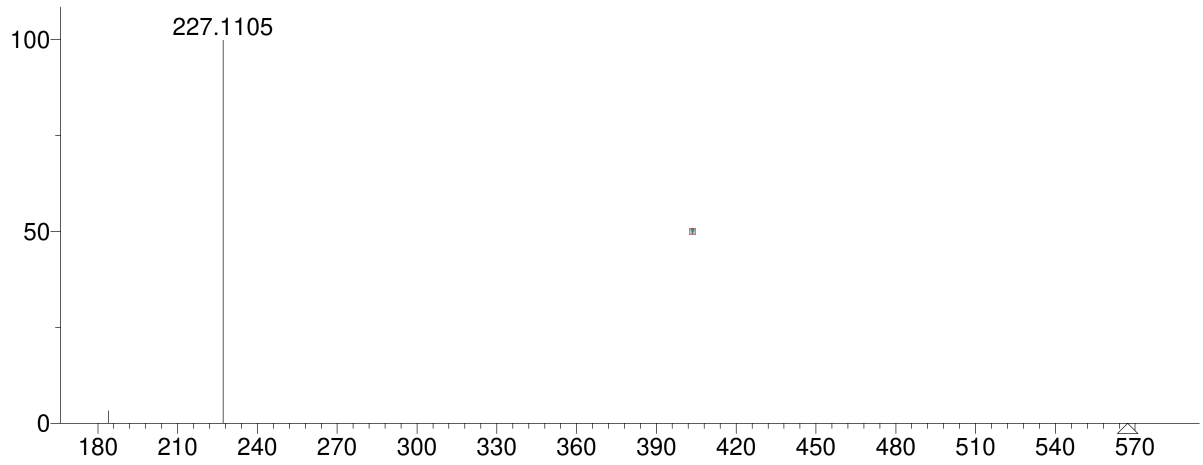

Hit 1 : chaetoglobosins [M+K]+ 35HCD P=567.2
C32H36N2O5; MF: 0; RMF: 11; Prob 100.0%; Lib: mona-export-vf-npl_qexactive; ID: 23206.

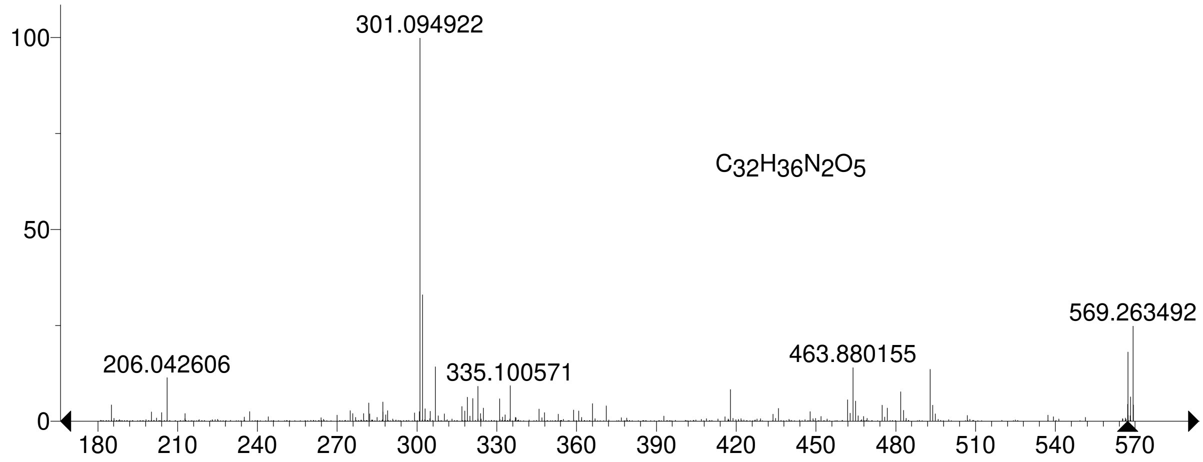

Unknown: Unknown (6.35_403.1695m/z)
Compound in Library Factor = -3273

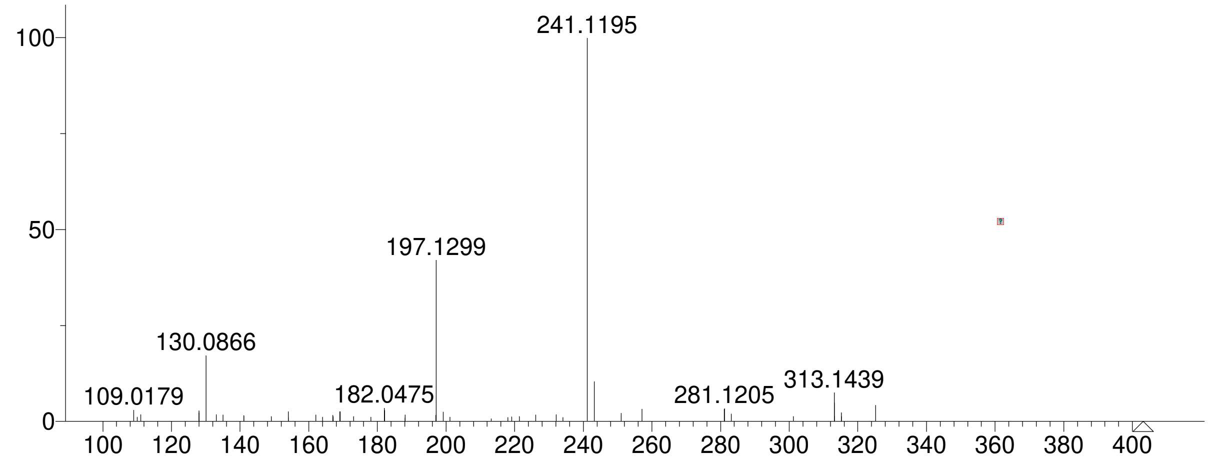

Hit 1 : (+)-Cloprostenol methyl ester [M+H-2H2O]+ HCD 32V P=403.2
C23H31ClO6; MF: 4; RMF: 89; Prob 28.3%; CAS: 56687-85-5; Lib: nist_msms; ID: 544277.

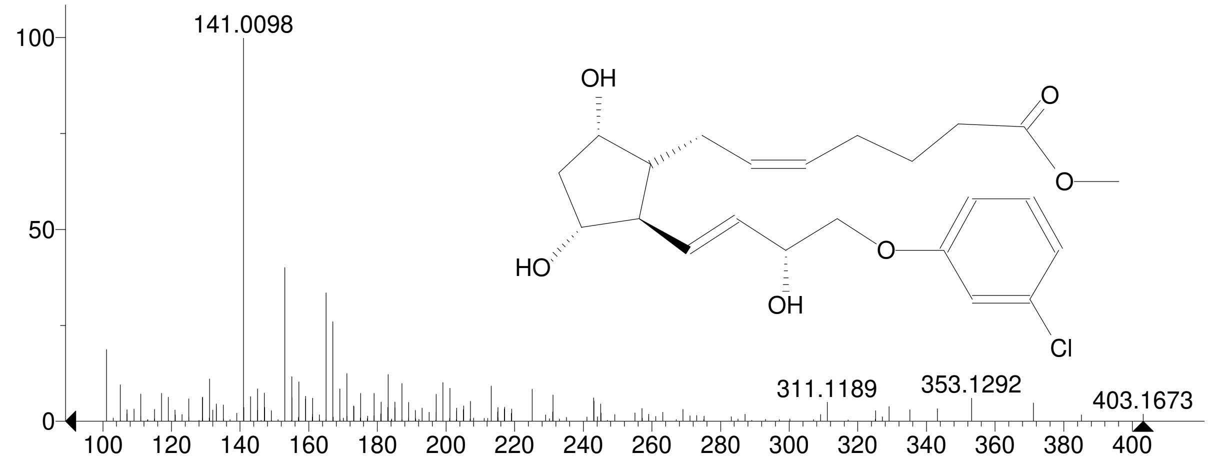

Hit 2 : NCGC00380324-01_C23H29ClO5_Benzaldehyde, 3-chloro-4,6-dihydroxy-2-methyl-5-[(2E,6E)-3-methyl-7-
C23H29ClO5; MF: 0; RMF: 24; Prob 23.9%; Lib: mona-export-gnps; ID: 13042.

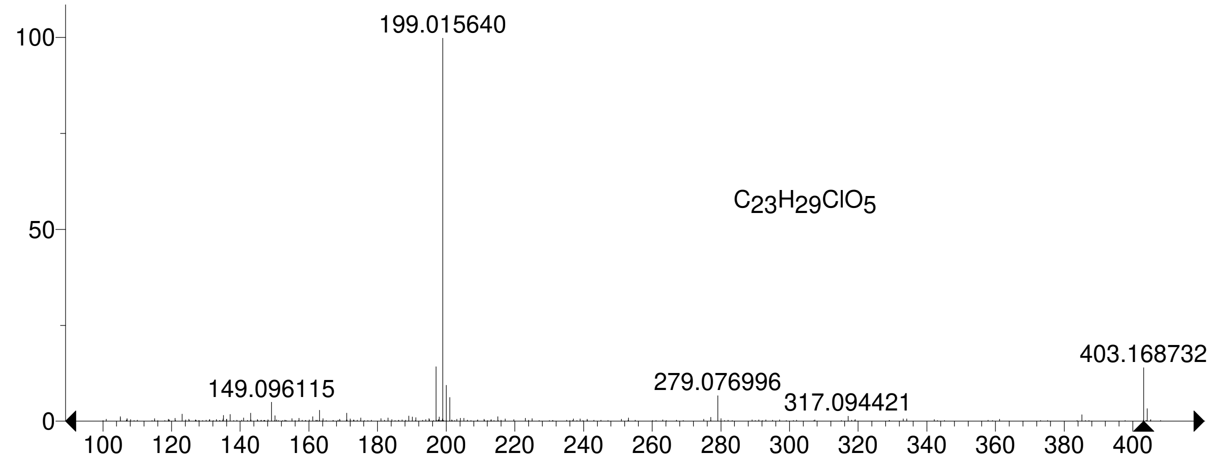

Hit 3 : [(1R,5S,6S)-3-(hydroxymethyl)-5-[(Z)-2-methylbut-2-enoyl]oxy-2-oxo-6-propan-2-ylcyclohex-3-en-1-yl] (E)-4
C20H28O7; MF: 0; RMF: 24; Prob 23.9%; Lib: mona-export-vf-npl_qexactive; ID: 1510.

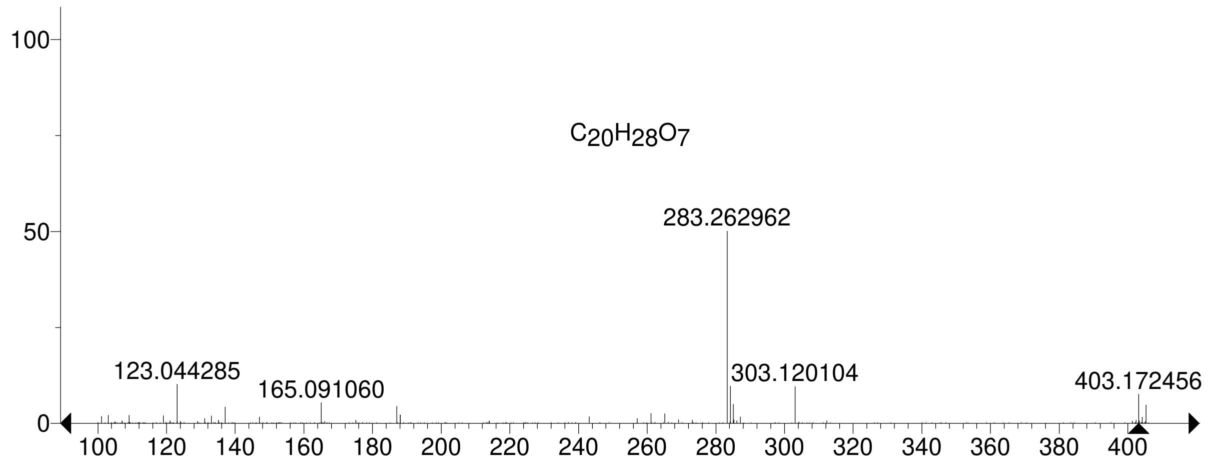

Hit 4 : 17-Phenyltrinorprostaglandin A2 [M+Cl]- HCD 30V P=403.2
C23H28O4; MF: 0; RMF: 4; Prob 23.9%; CAS: 38315-51-4; Lib: nist_msms; ID: 319742.

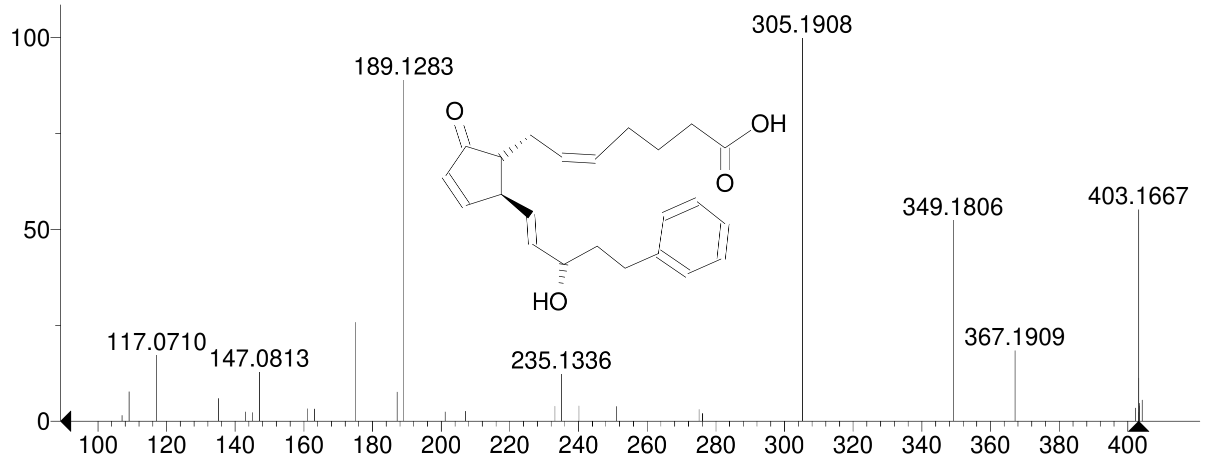

** Search Report Page 1 of 1 **

Unknown: Unknown (6.37_355.1605m/z)
Compound in Library Factor = -2632

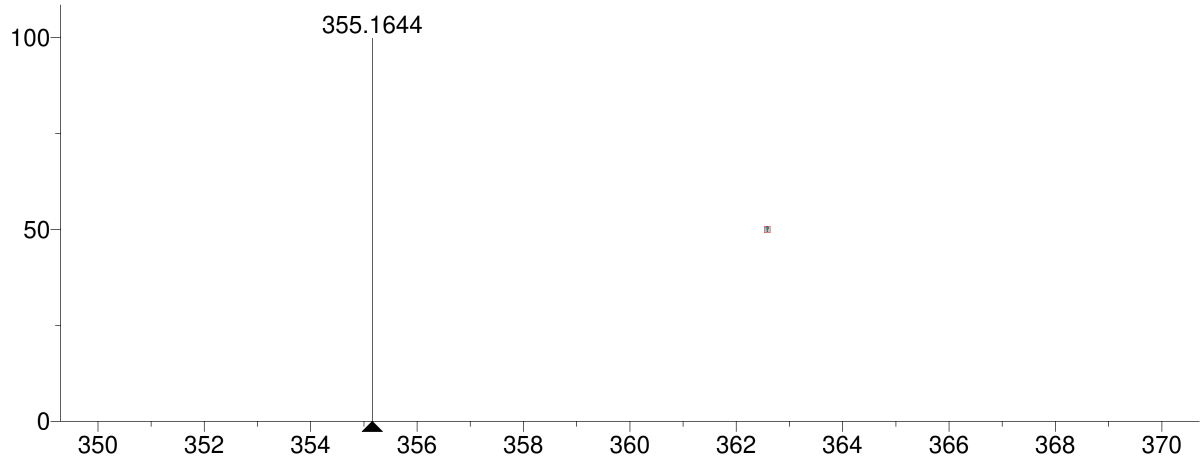

Hit 1 : Isoxaben [M+Na]+ HCD 17V P=355.2
C18H24N2O4; MF: 364; RMF: 999; Prob 50.0%; CAS: 82558-50-7; Lib: nist_msms; ID: 397190.

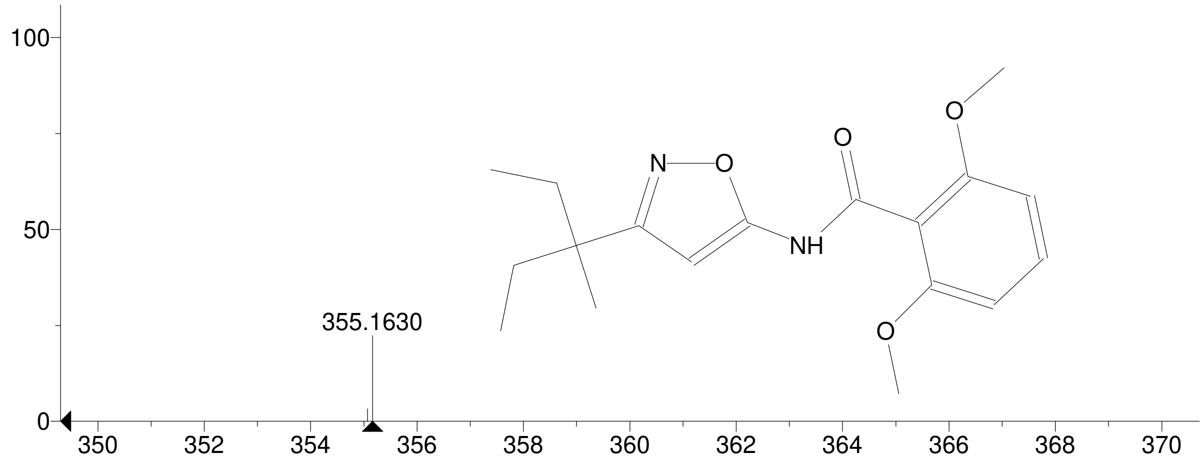

Hit 2 : pGlu-Glu-Pro-amide [M+H]+ HCD 7eV P=355.2
C15H22N4O6; MF: 364; RMF: 999; Prob 50.0%; CAS: 85541-78-2; Lib: nist_msms; ID: 564355.

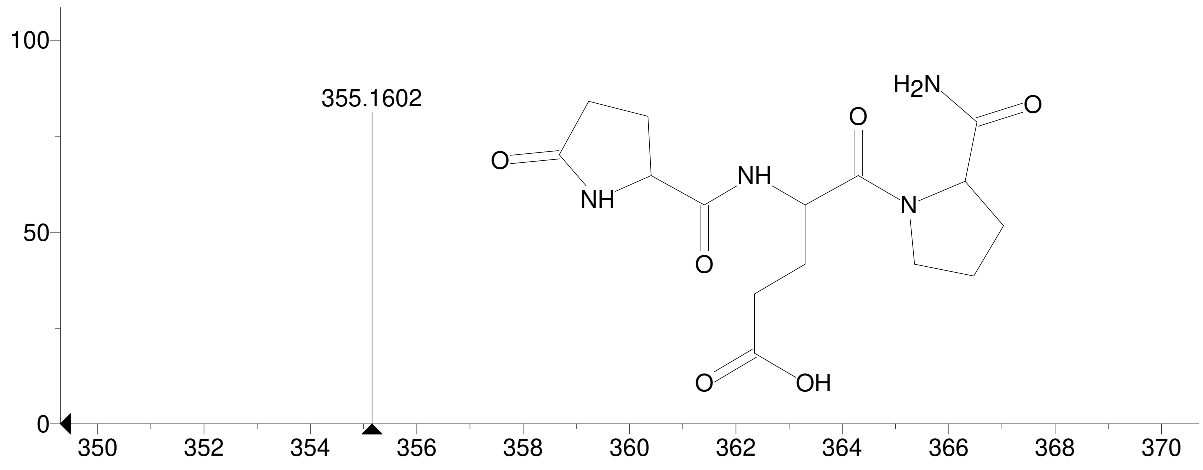

Unknown: Unknown (6.37_427.1787m/z)
Compound in Library Factor = 0

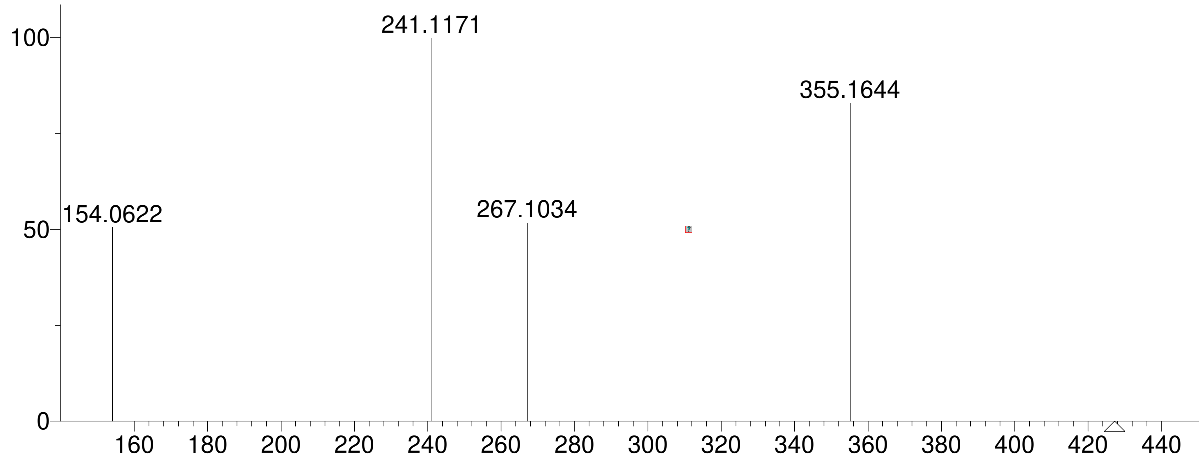

Hit 1 : [8,8-dimethyl-9-(3-methylbut-2-enoyloxy)-2-oxo-9,10-dihydropyrano[2,3-f]chromen-10-yl] 3-methylbut-2-en
C24H26O7; MF: 0; RMF: 20; Prob 33.3%; Lib: mona-export-vf-npl_qexactive; ID: 4551.

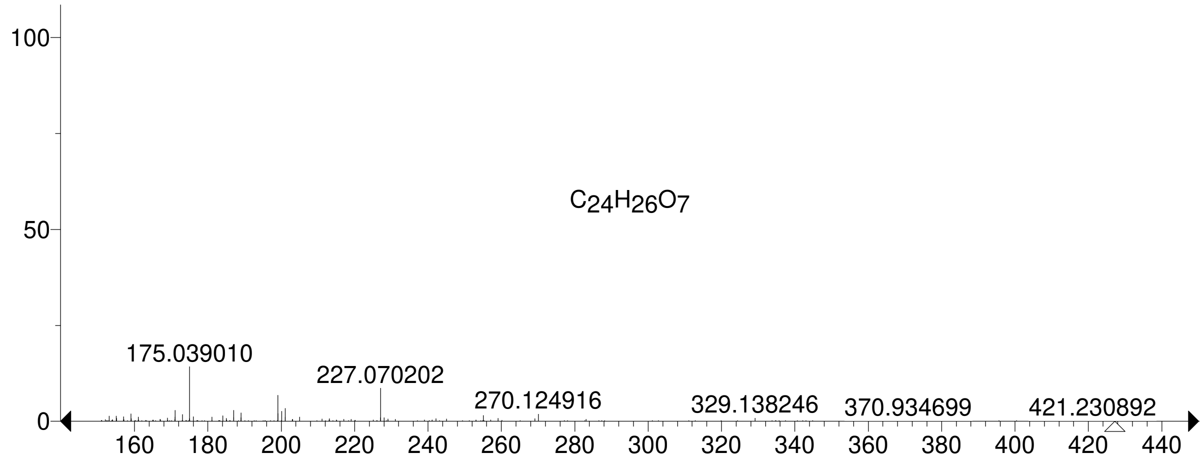

Hit 2 : Garcinone D [M-H]- HCD 55V P=427.2
C24H28O7; MF: 0; RMF: 9; Prob 33.3%; CAS: 107390-08-9; Lib: nist_msms; ID: 258178.

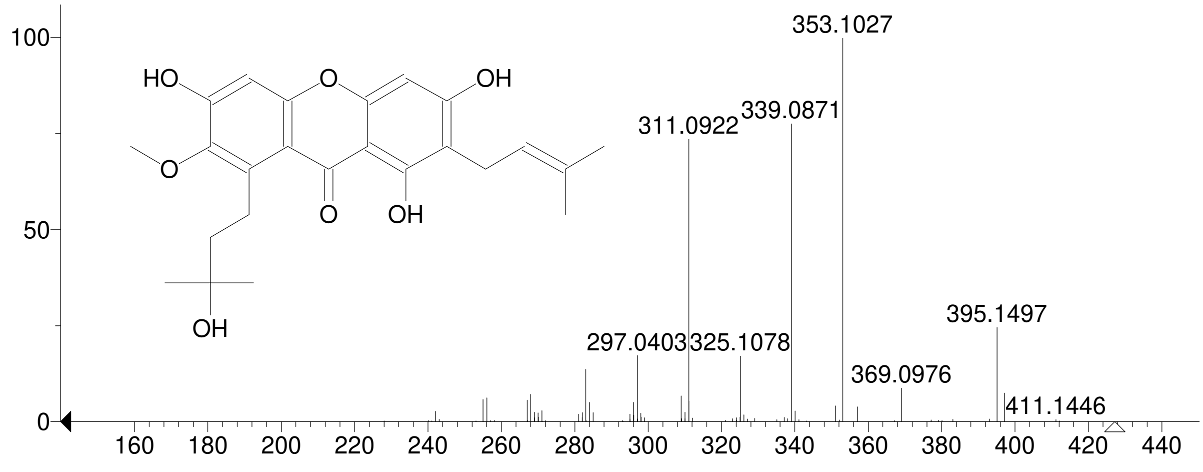

Hit 3 : Archangelicine [M+H]+ 35HCD P=427.2
C24H26O7; MF: 0; RMF: 3; Prob 33.3%; Lib: mona-export-vf-npl_qexactive; ID: 5194.

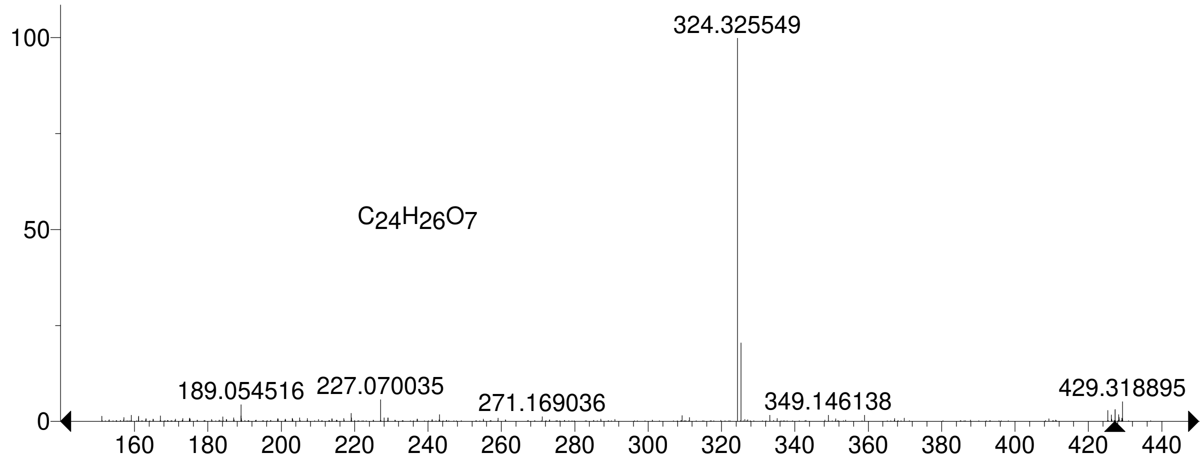

Unknown: Unknown (6.37_484.2041m/z)
Compound in Library Factor = 0

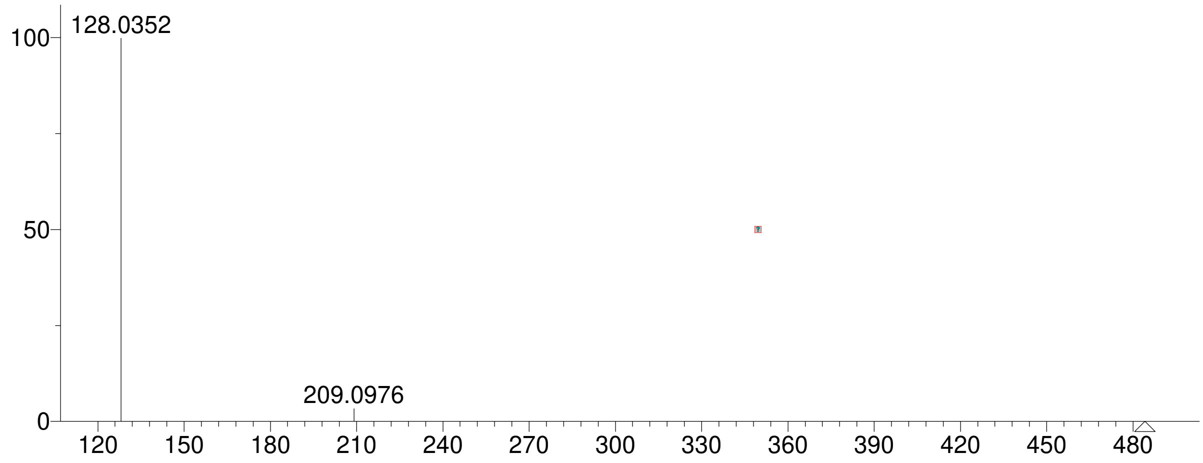

Hit 1 : [1,1'-Biphenyl]-2-carboxylic acid, 4'-[[2,3-dimethyl-5-[[[(1S)-1-(4-nitrophenyl)ethyl]amino]carbonyl]-1H-indol·
C33H29N3O5; MF: 0; RMF: 46; Prob 100.0%; CAS: 1338259-05-4; Lib: nist_msms; ID: 253488.

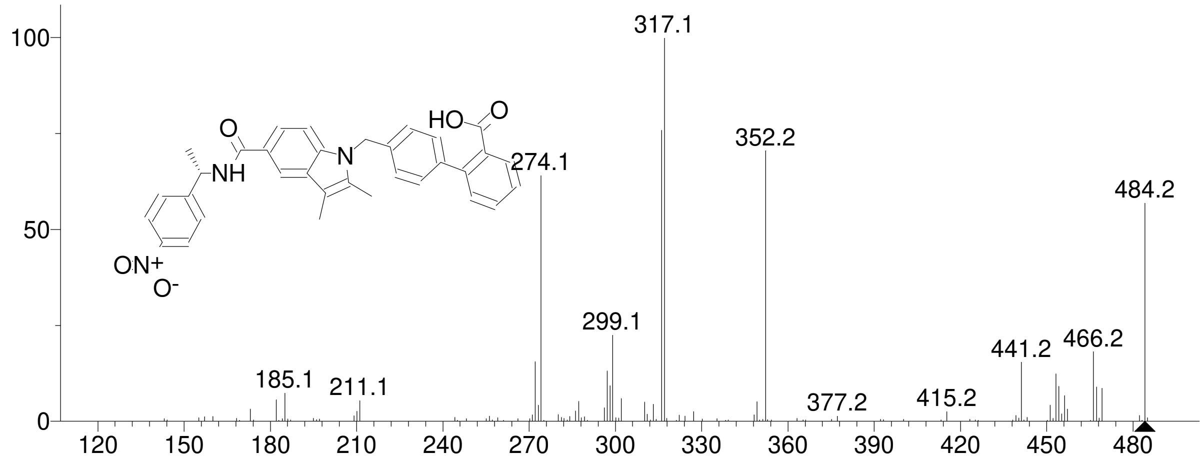

Unknown: Unknown (6.37_486.2075m/z)
Compound in Library Factor = 0

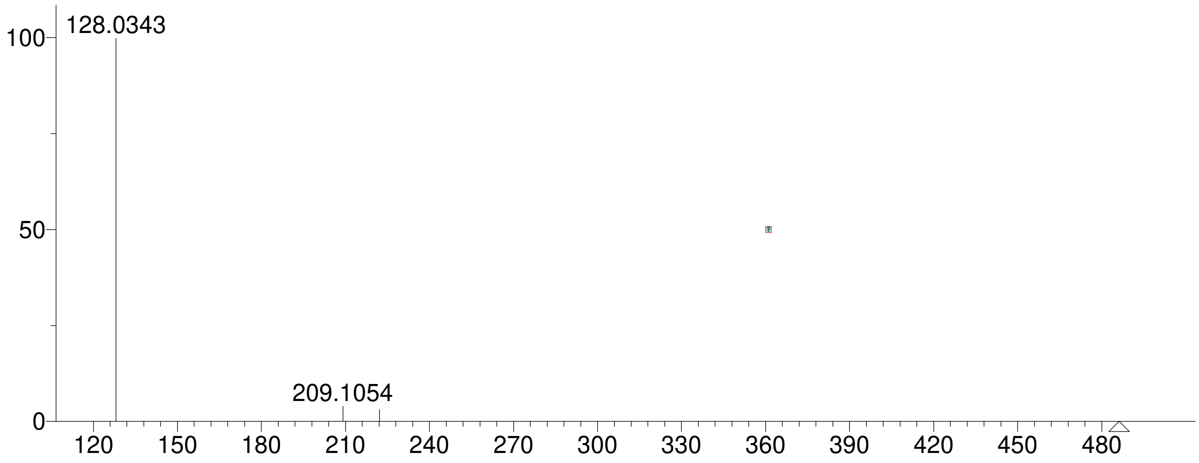

Hit 1 : rac-N-Allyloxycarbonyl-cis-moxifloxacin acyl-β-D-glucuronide allyl ester [M+H-C9H12O6]+ HCD 126V P=
C34H40FN3O12; MF: 0; RMF: 20; Prob 50.0%; Lib: nist_msms; ID: 431491.

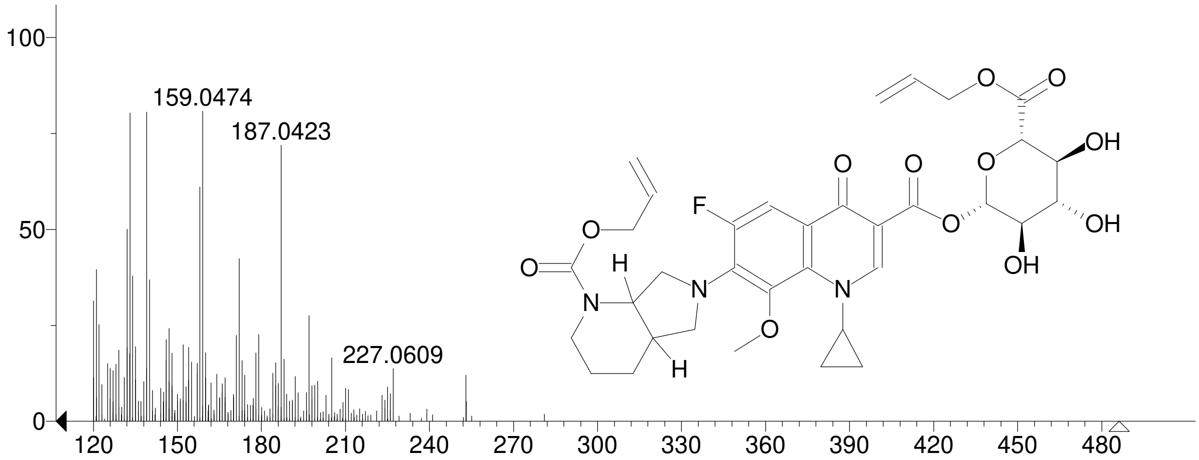

Hit 2 : 6-(furan-3-yl)-13-hydroxy-5,12,16,16-tetramethyl-7,10,17-trioxahexacyclo[13.3.3.0A^'1,A^'1a^*u.0A^'2,A^'1.
C26H28O8; MF: 0; RMF: 19; Prob 50.0%; Lib: mona-export-vf-npl_qexactive; ID: 18767.

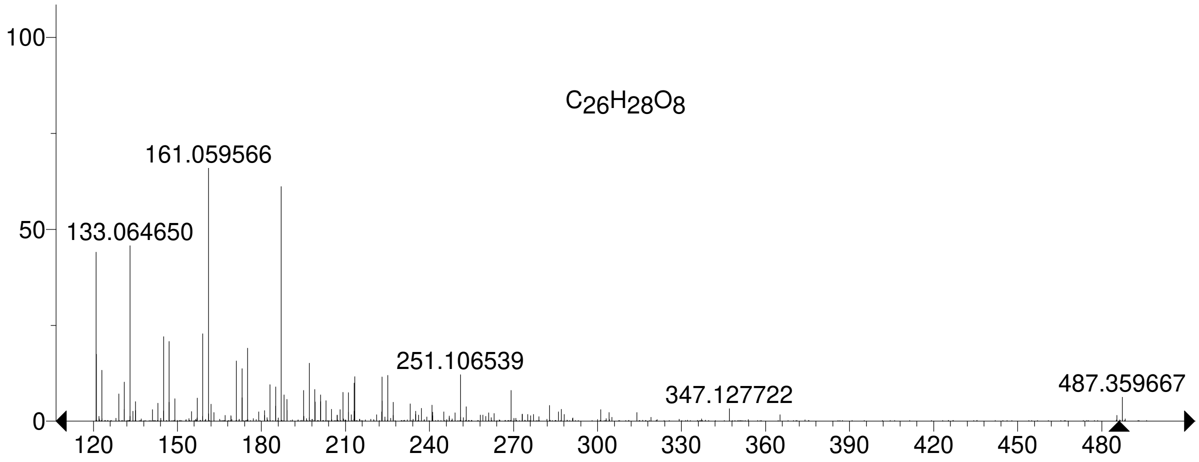

Unknown: Unknown (6.38_282.0969m/z)
Compound in Library Factor = -3273

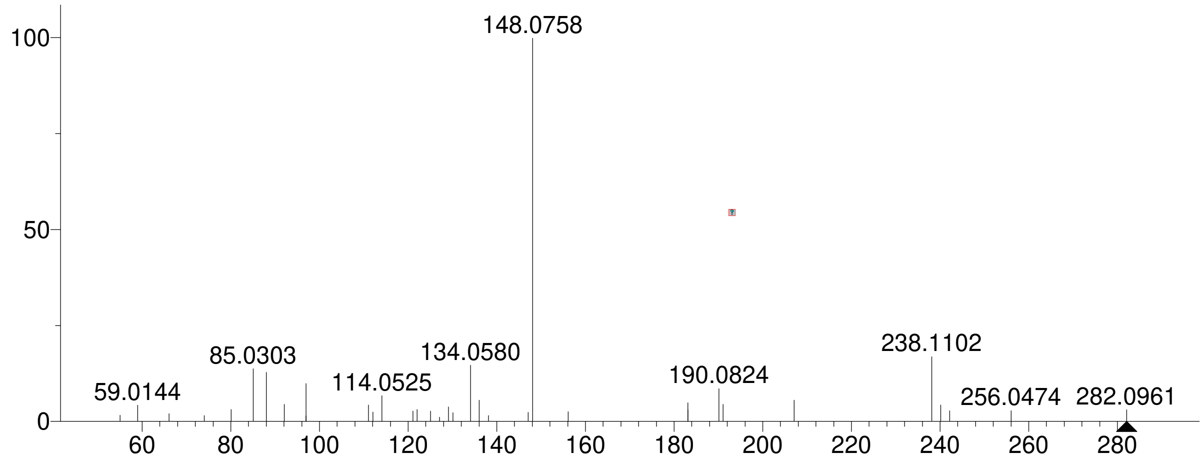

Hit 1 : (Z)-2-methyl-4-[(2R,3R,4S,5S,6R)-3,4,5-trihydroxy-6-(hydroxymethyl)oxan-2-yl]oxybut-2-enenitrile [M+Na]-
C11H17NO6; MF: 2; RMF: 99; Prob 52.0%; Lib: anthocyanins mona; ID: 4576.

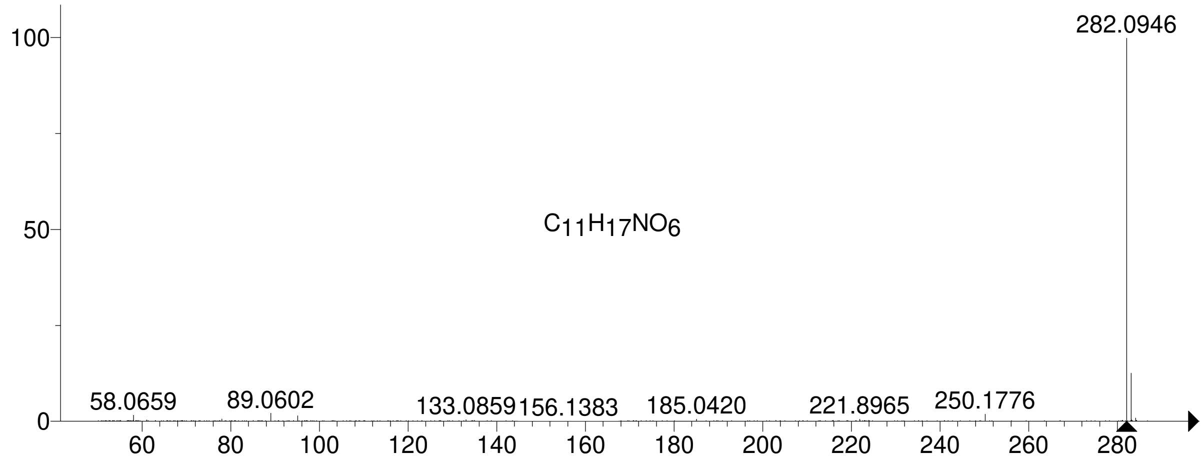

Hit 2 : 4-hydroxy-3-[(4-hydroxy-6-methyl-2-oxopyran-3-yl)methyl]-6-methylpyran-2-one [M+NH4]+ 65HCD P=28
C13H12O6; MF: 0; RMF: 65; Prob 48.0%; Lib: mona-export-vf-npl_qexactive; ID: 21516.

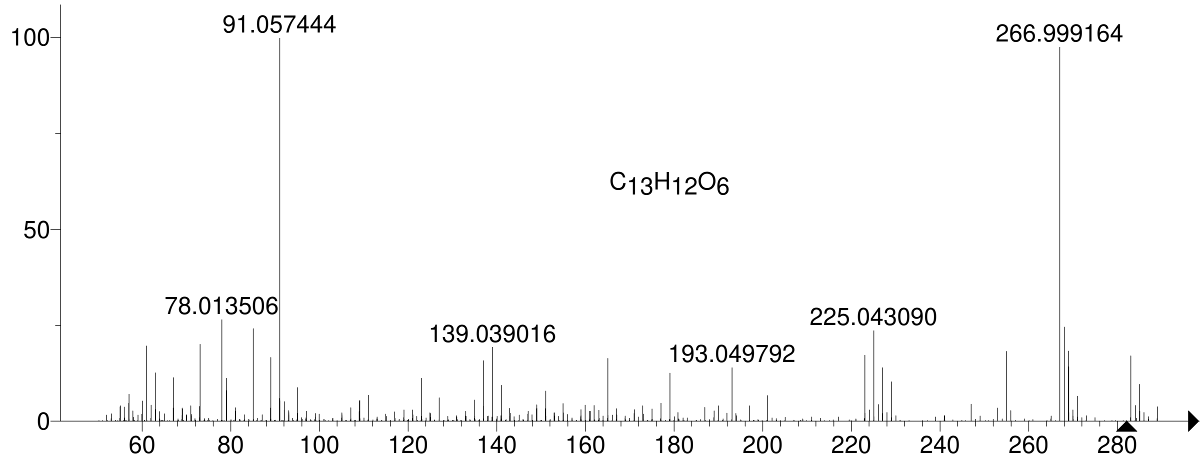

Unknown: Unknown (6.39_417.0935m/z)
Compound in Library Factor = 0

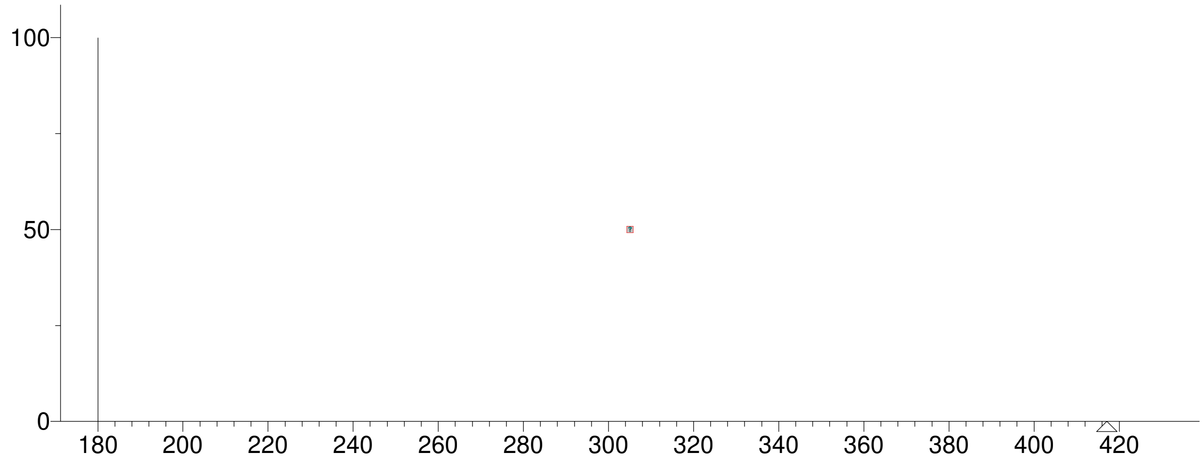

Unknown: Unknown (6.43_284.1952m/z)
Compound in Library Factor = 0

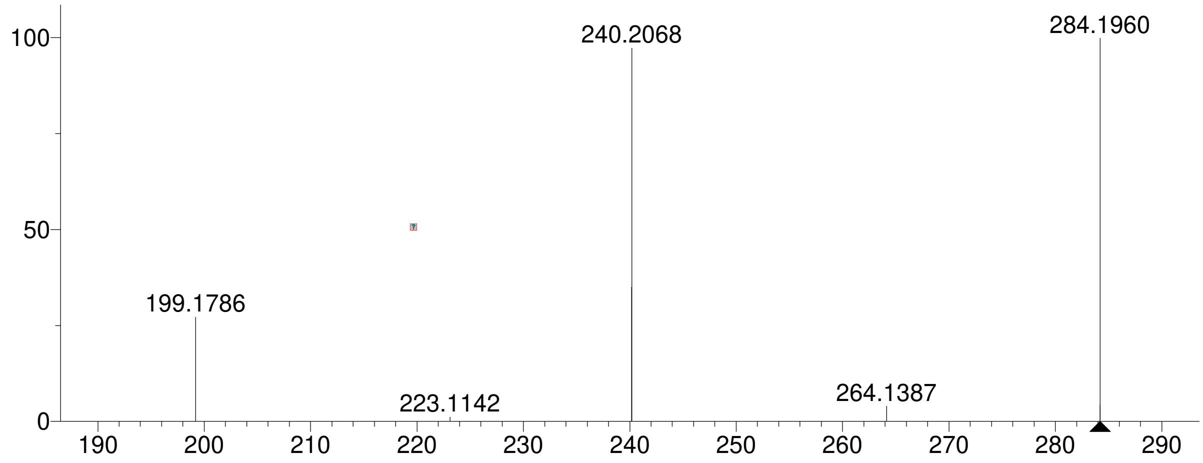

Unknown: Unknown (6.43_298.1398m/z)
Compound in Library Factor = -3273

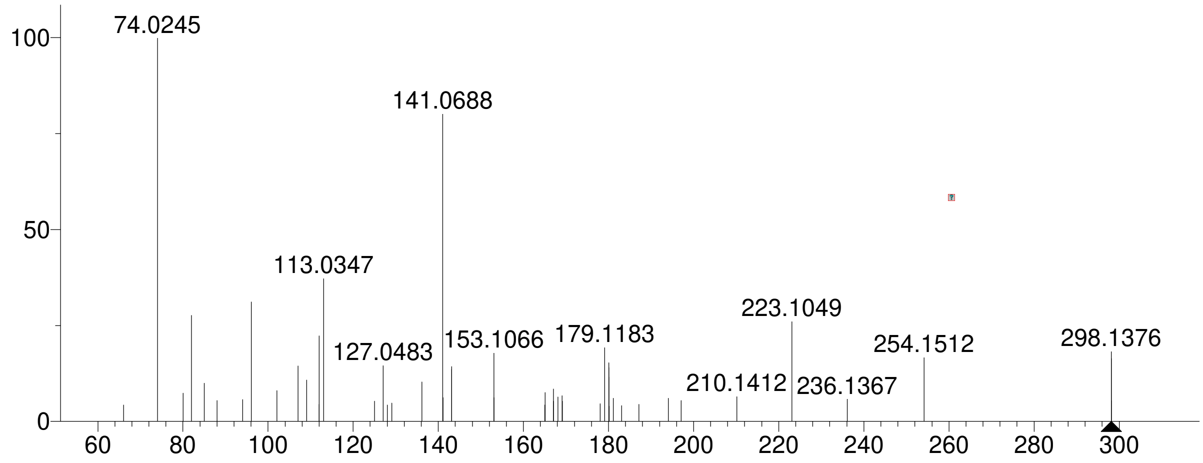

Hit 1 : Noratropine [M+Na]+ HCD 8V P=298.1
C16H21NO3; MF: 5; RMF: 183; Prob 100.0%; CAS: 16839-98-8; Lib: nist_msms; ID: 197150.

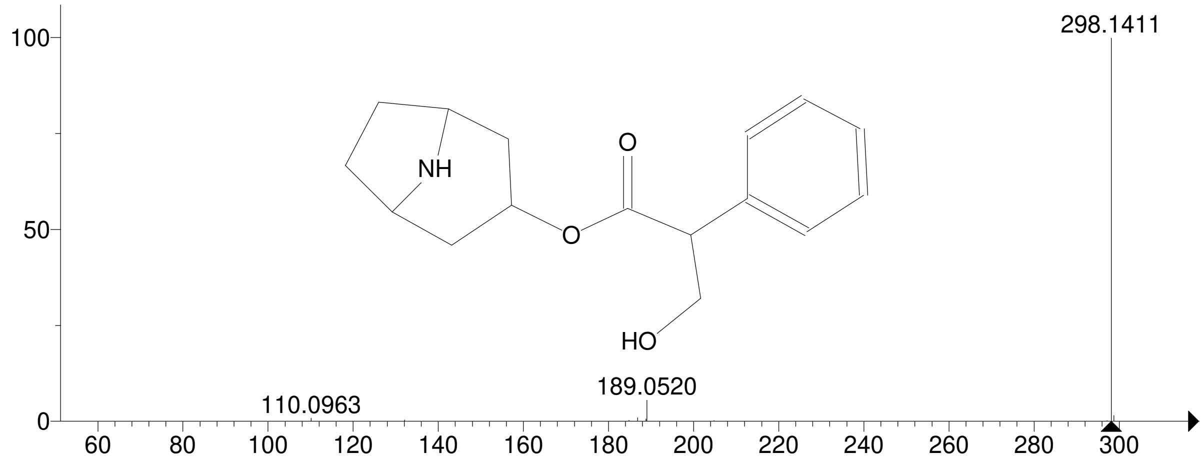

** Search Report Page 1 of 1 **

Unknown: Unknown (6.44_328.1862m/z)
Compound in Library Factor = 0

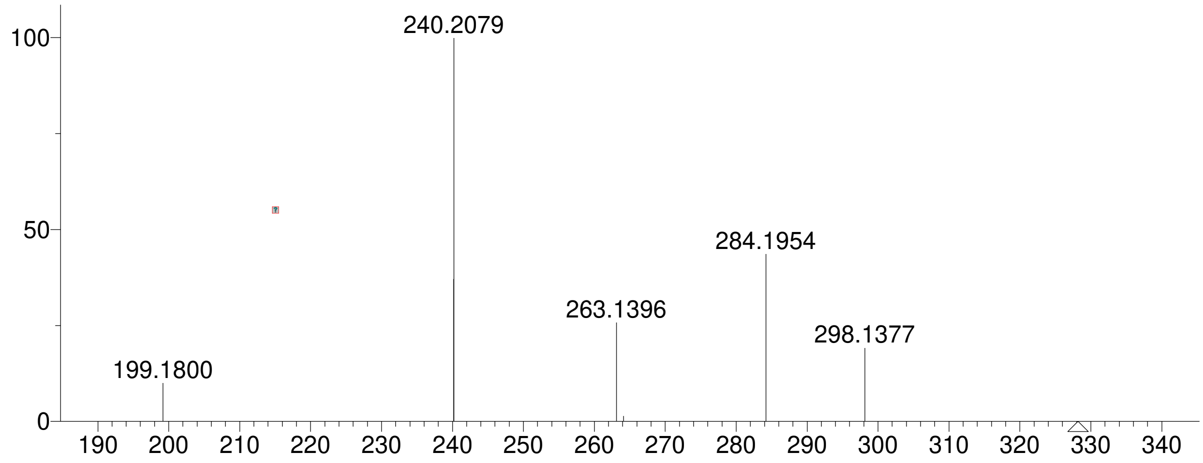

Hit 1 : CAPSAICIN [M+Na]+ 35HCD P=328.2
C18H27NO3; MF: 0; RMF: 4; Prob 100.0%; Lib: mona-export-vf-npl_qexactive; ID: 2965.

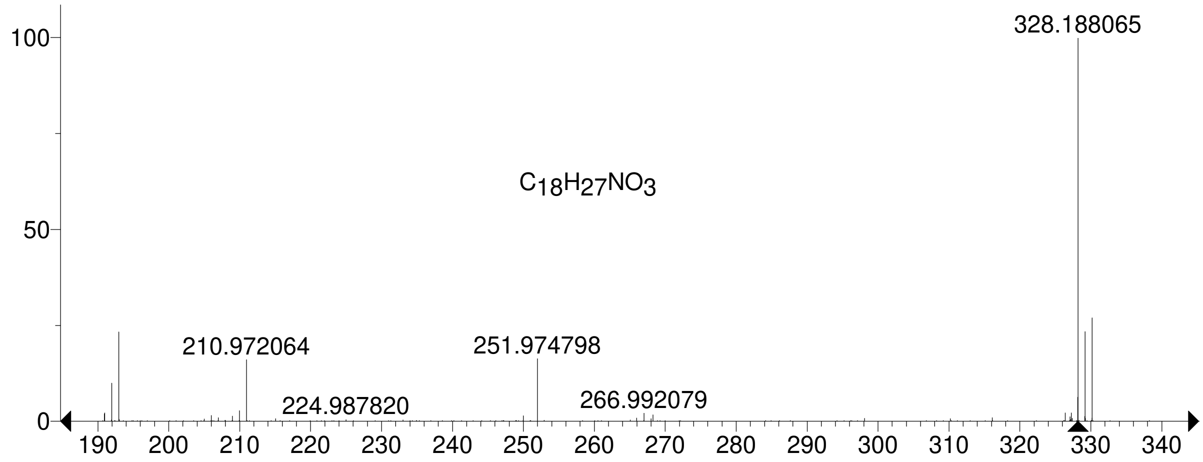

Unknown: Unknown (6.44_366.1603m/z)
Compound in Library Factor = 0

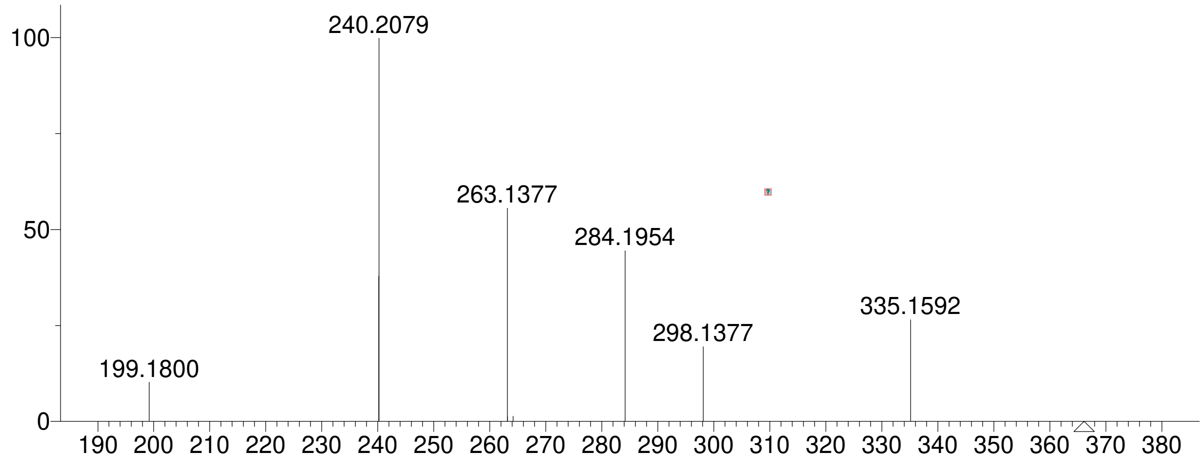

Hit 1 : Amyloid β-Protein (1-15) [M+5H]5+ HCD 24eV P=366.2
C78H107N25O27; MF: 0; RMF: 53; Prob 100.0%; CAS: 183745-81-5; Lib: nist_msms2; ID: 57159.

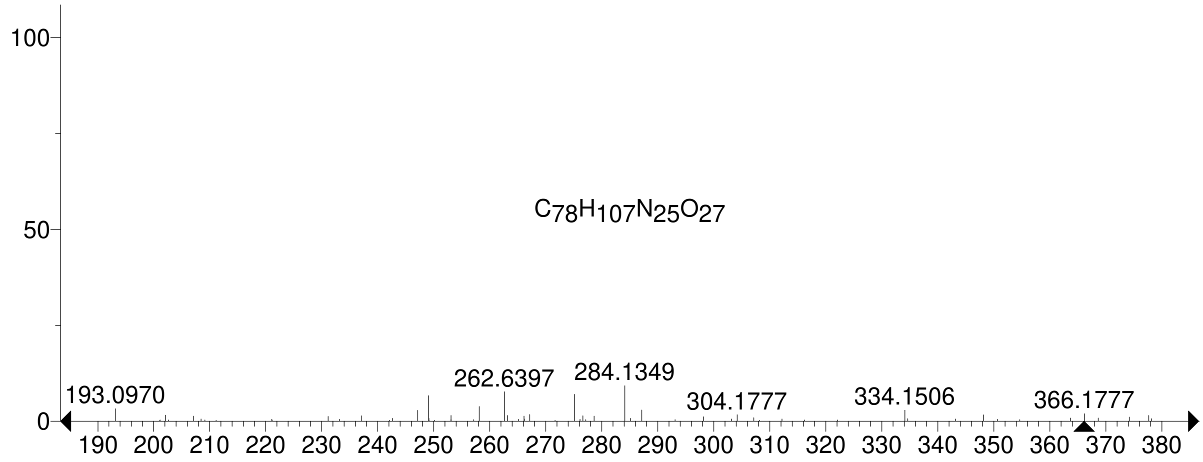

** Search Report Page 1 of 1 **

Unknown: Unknown (6.45_342.1657m/z)
Compound in Library Factor = 0

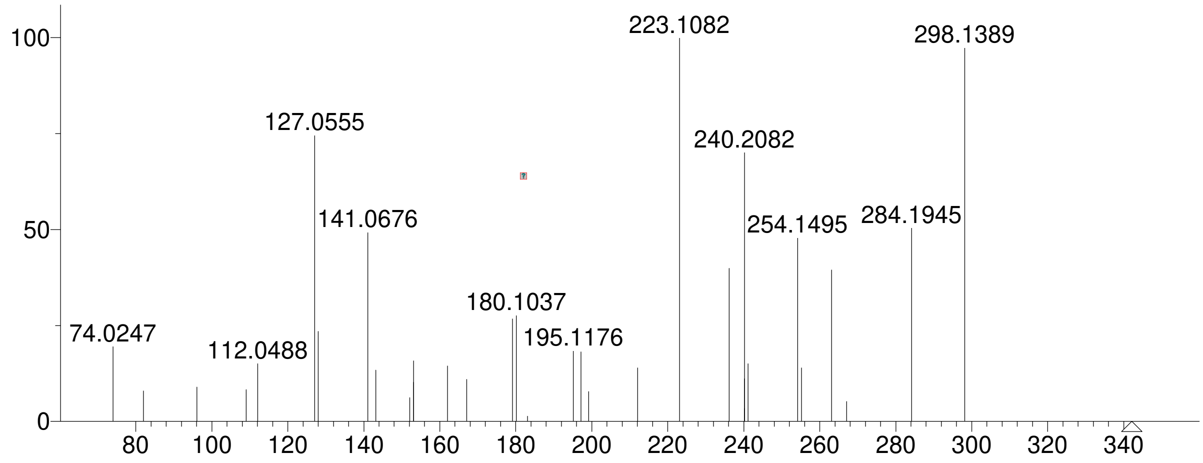

Unknown: Unknown (6.45_364.1632m/z)
Compound in Library Factor = 0

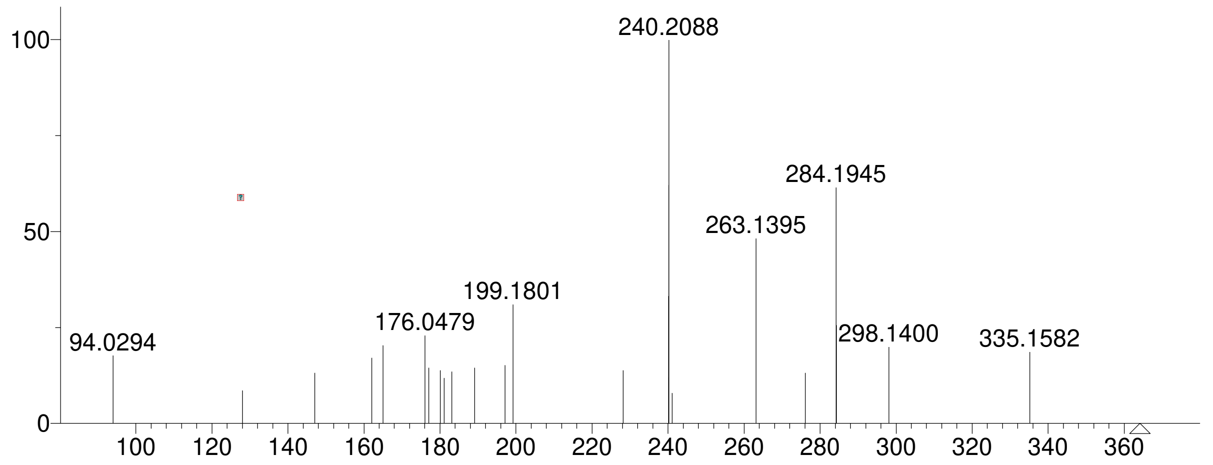

Hit 1 : (2R,3S,4S,5R,6S)-2-(hydroxymethyl)-6-(3,4,5-trimethoxyphenoxy)oxane-3,4,5-triol [M+NH4]+ 65HCD P=
C15H22O9; MF: 0; RMF: 46; Prob 12.5%; Lib: mona-export-vf-npl_qexactive; ID: 9873.

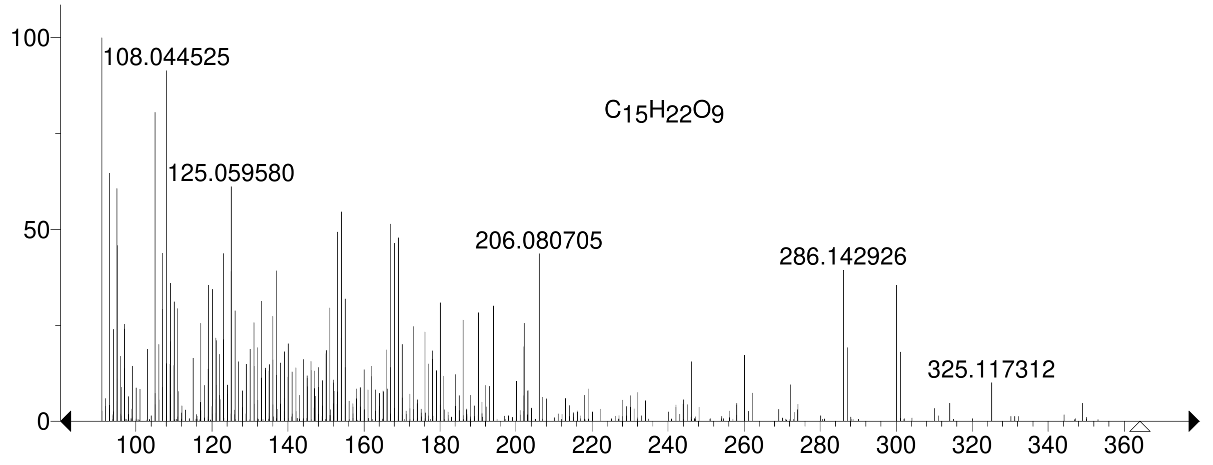

Hit 2 : aucubin [M+NH4]+ 65HCD P=364.2
C15H22O9; MF: 0; RMF: 31; Prob 12.5%; Lib: mona-export-vf-npl_qexactive; ID: 3774.

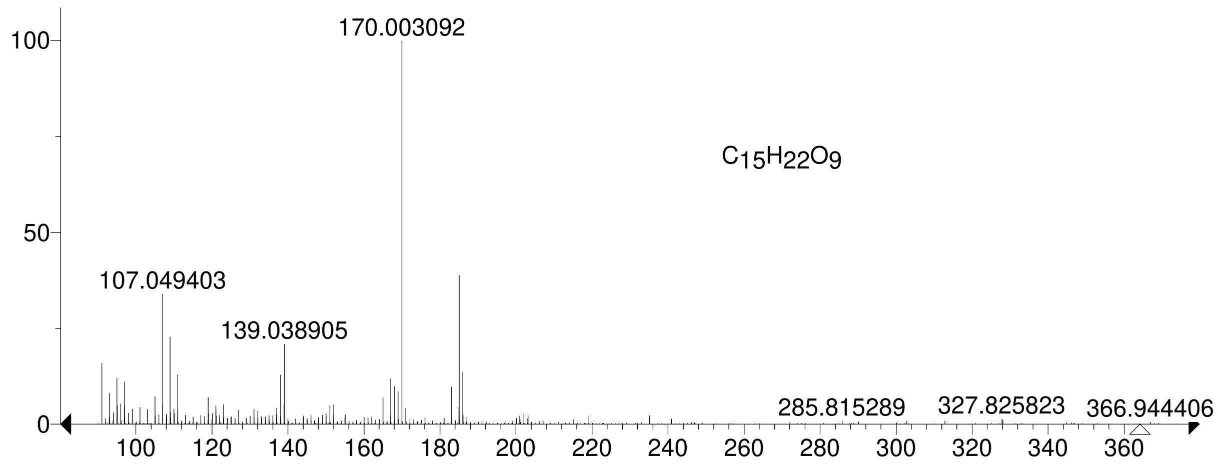

Hit 3 : NCGC00380729-01_C21H21N3O3_8',8'-Dimethyl-5a',6'-dihydro-3'H,5'H,8'H-spiro[indole-2,7'-pyrrolo[1',2':
C21H21N3O3; MF: 0; RMF: 20; Prob 12.5%; Lib: mona-export-gnps; ID: 11547.

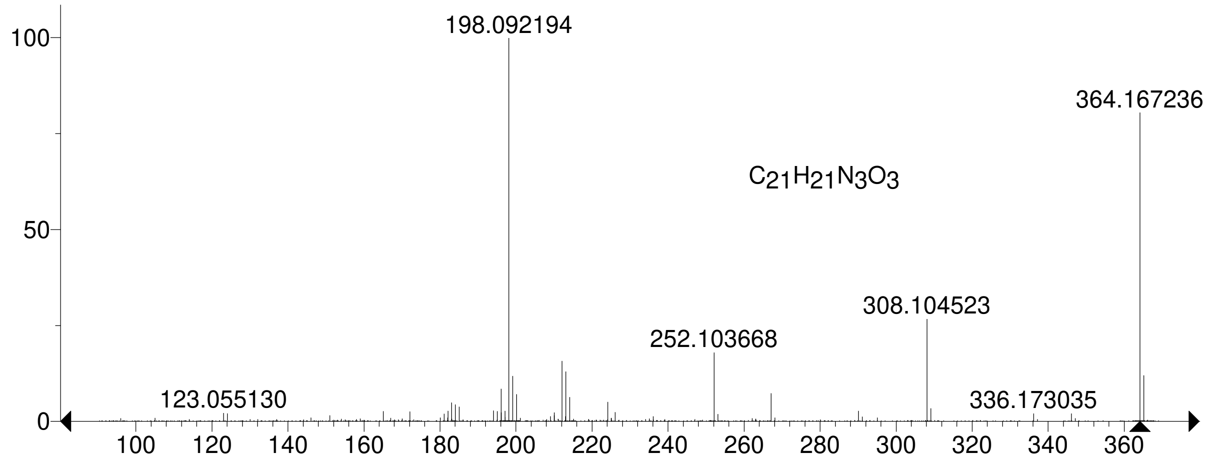

Hit 4 : Cys-Asn-Lys [M+H]+ QQQ 20V P=364.2
C13H25N5O5S; MF: 0; RMF: 18; Prob 12.5%; Lib: nist_msms; ID: 42766.

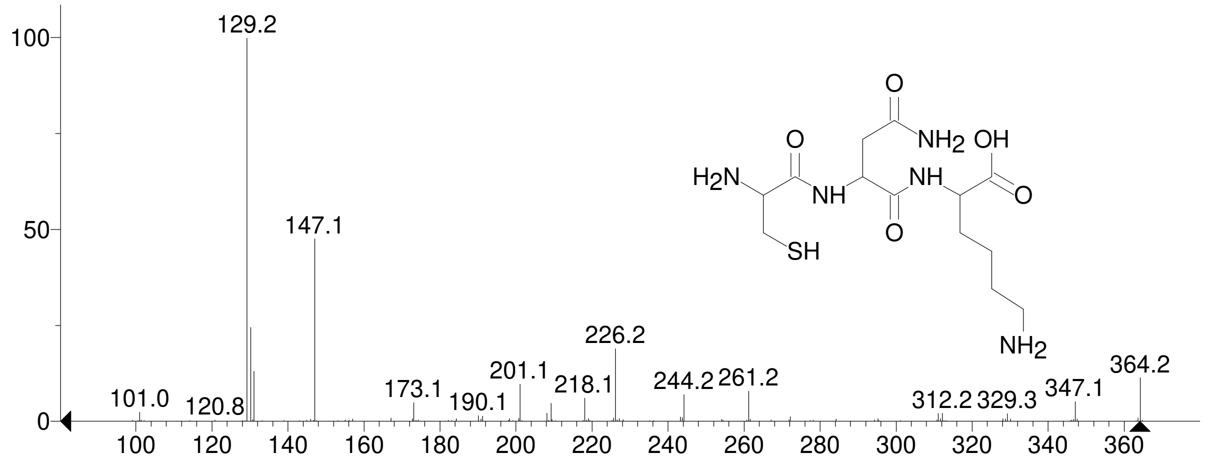

Hit 5 : 2-[(4-hydroxy-3,5-dimethoxyphenyl)methoxy]-6-(hydroxymethyl)oxane-3,4,5-triol [M+NH4]+ 35HCD P=36
C15H22O9; MF: 0; RMF: 13; Prob 12.5%; Lib: mona-export-vf-npl_qexactive; ID: 20389.

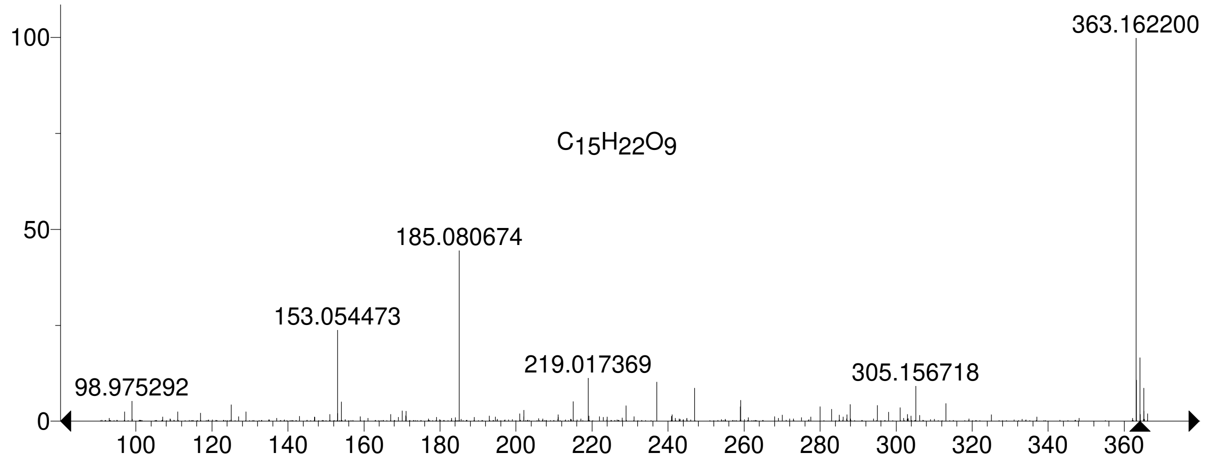

Unknown: Unknown (6.45_425.1913m/z)
Compound in Library Factor = -3273

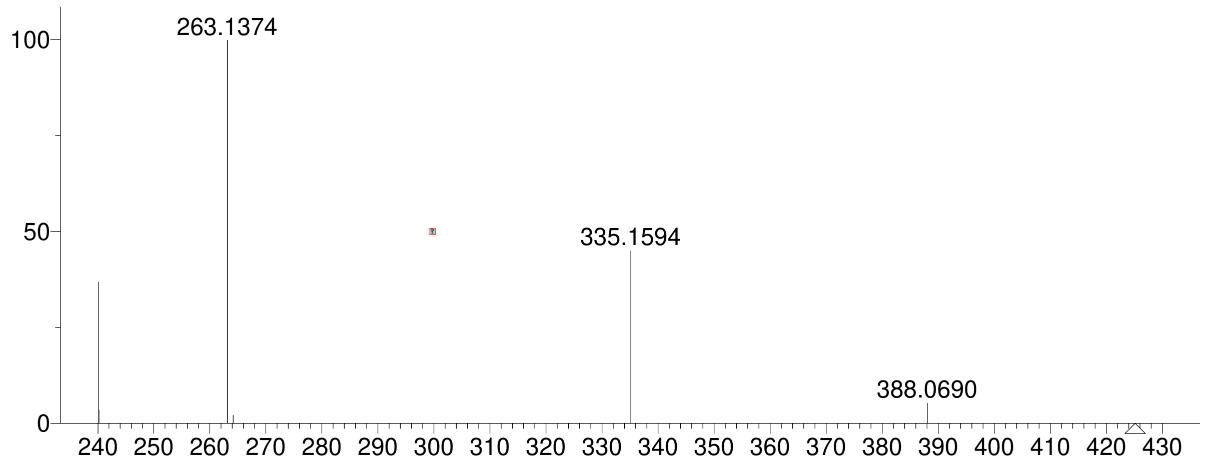

Hit 1 : Clindamycin [M+H]+ 45% (P=425.2
C18H33ClN2O5S; MF: 11; RMF: 271; Prob 42.1%; Lib: mona-export-massbank; ID: 7097.

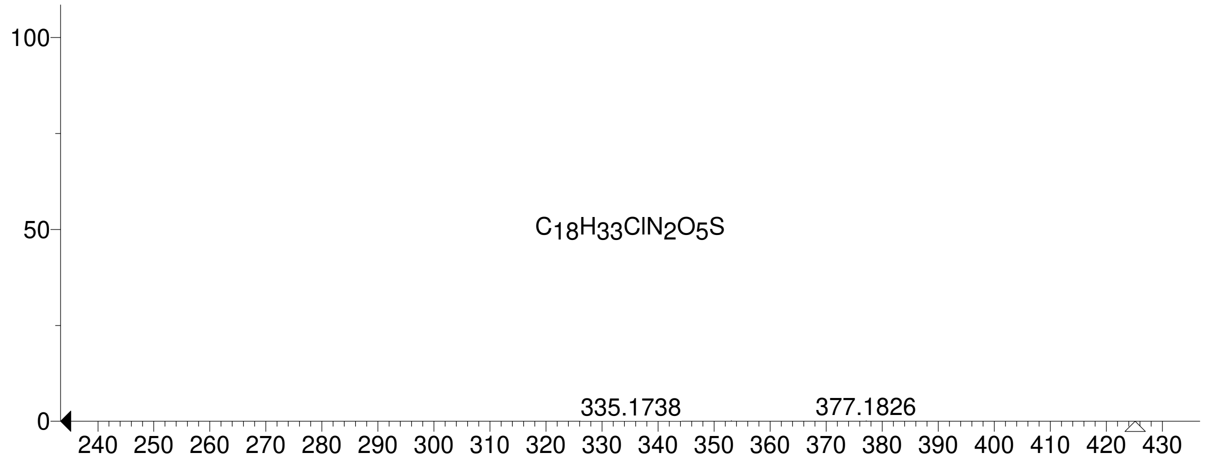

Hit 2 : MLS001304044-01! [M+H]+ P=425.2
C18H36Cl2N2O6S; MF: 0; RMF: 30; Prob 28.9%; Lib: mona-export-gnps; ID: 3214.

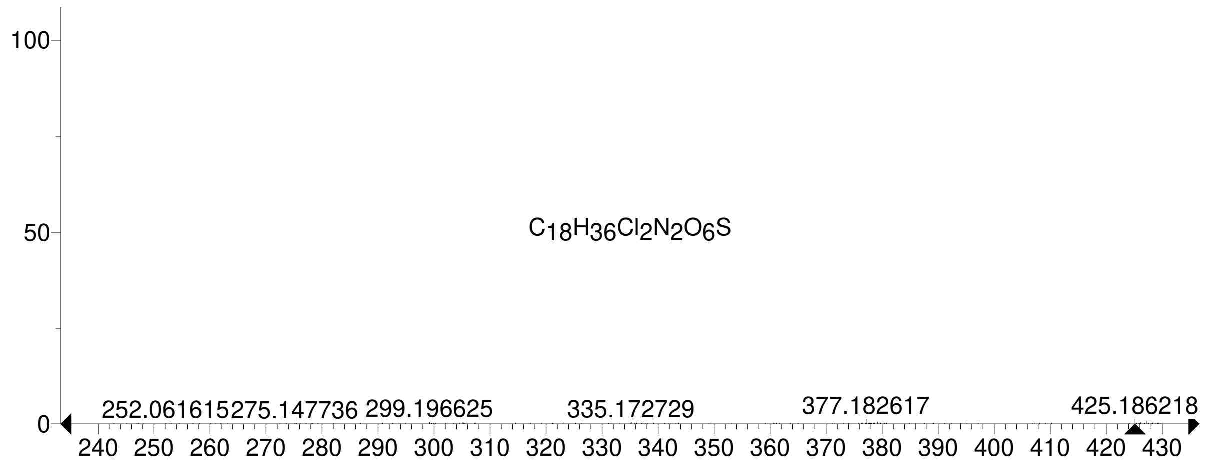

Hit 3 : MLS001183714-01!Clindamycin [M+H]+ P=425.2
C18H33ClN2O5S; MF: 0; RMF: 29; Prob 28.9%; Lib: mona-export-gnps; ID: 3814.

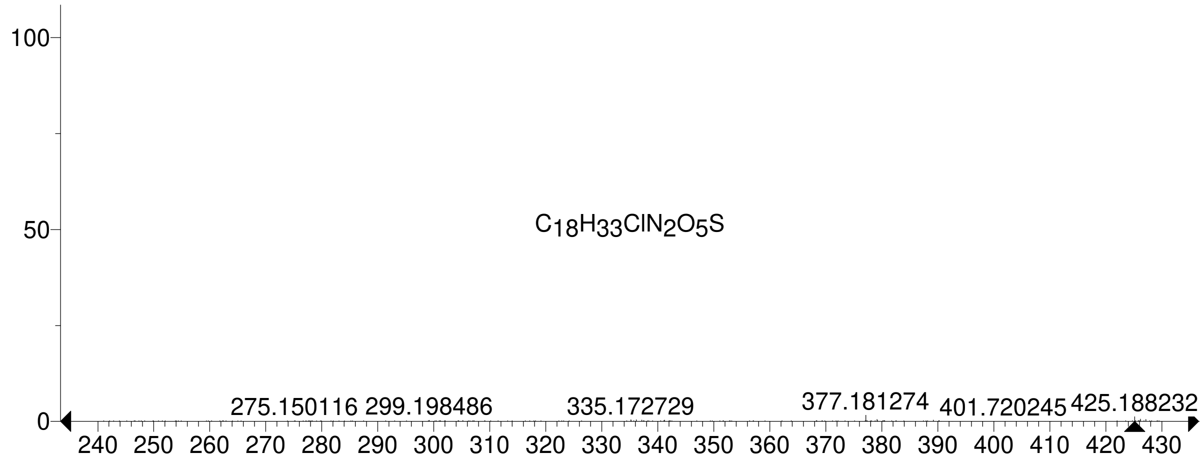

Unknown: Unknown (6.45_464.0891m/z)
Compound in Library Factor = 0

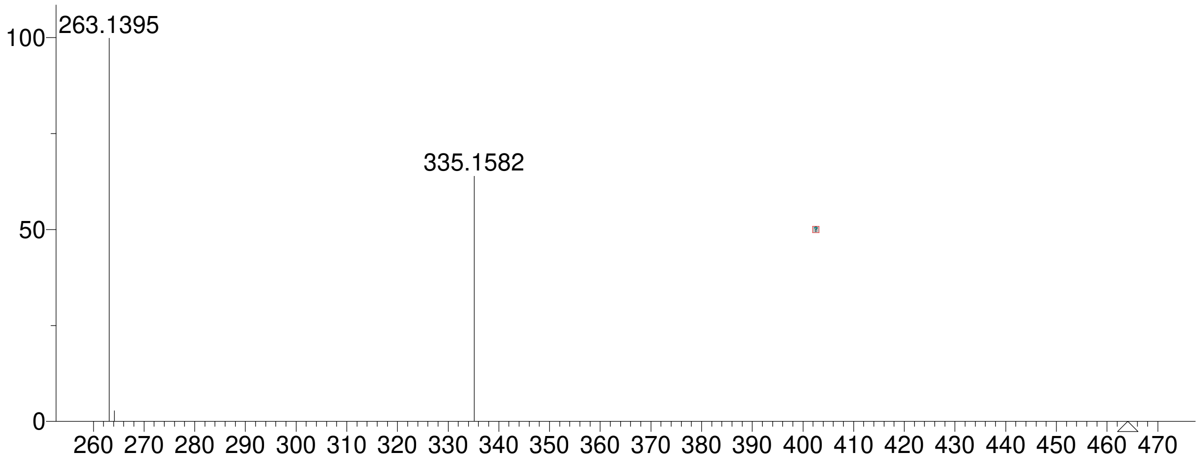

Unknown: Unknown (6.47_293.1490m/z)
Compound in Library Factor = -349

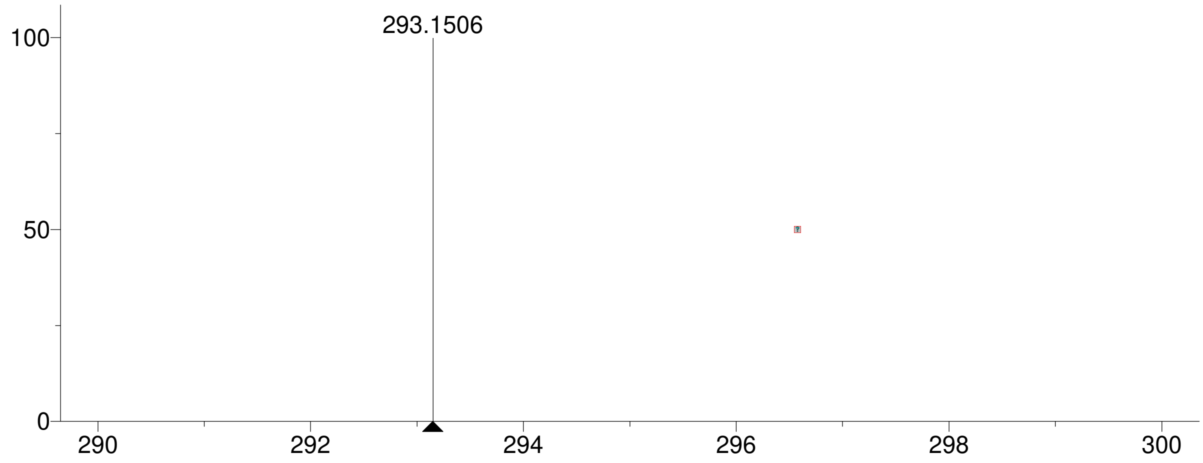

Hit 1 : Dicumyl peroxide [M+Na]+ HCD 26V P=293.2
C18H22O2; MF: 364; RMF: 999; Prob 11.1%; CAS: 80-43-3; Lib: nist_msms; ID: 153958.

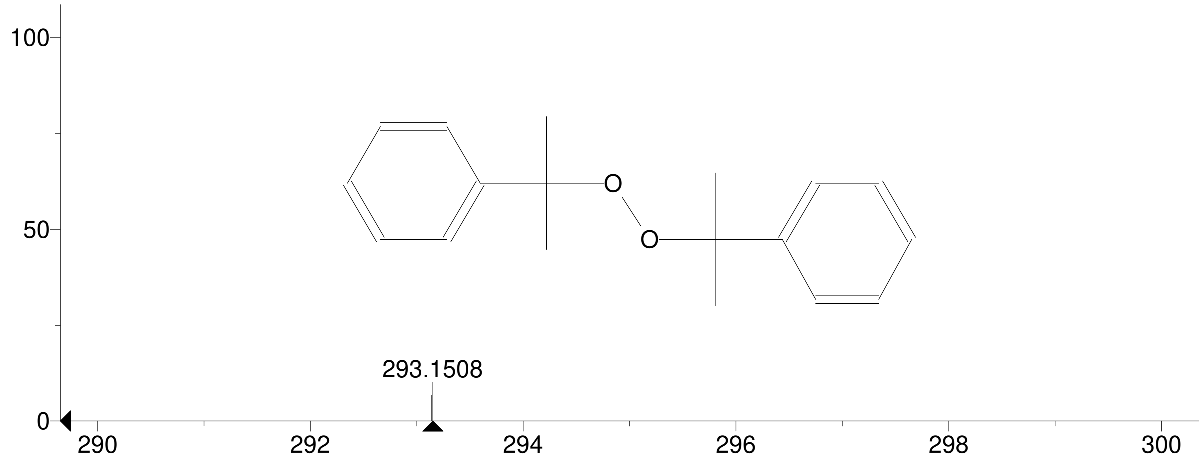

Hit 2 : Ile-Tyr [M-H]- HCD 29eV P=293.2
C15H22N2O4; MF: 364; RMF: 999; Prob 11.1%; Lib: nist_msms; ID: 567514.

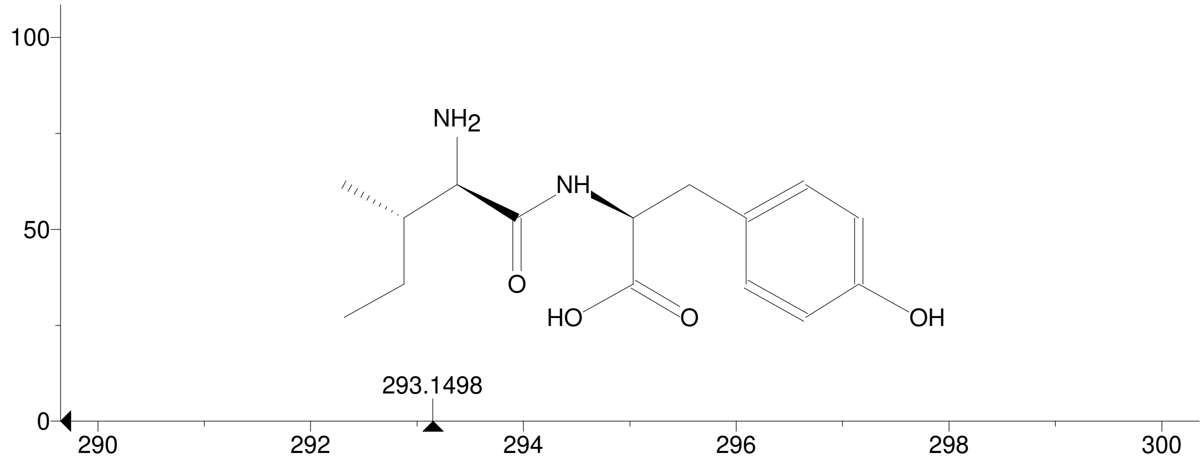

Hit 3 : Ile-Tyr [M-H]- HCD 29eV P=293.2
C15H22N2O4; MF: 364; RMF: 999; Prob 11.1%; CAS: 38579-21-4; Lib: nist_msms2; ID: 70371.

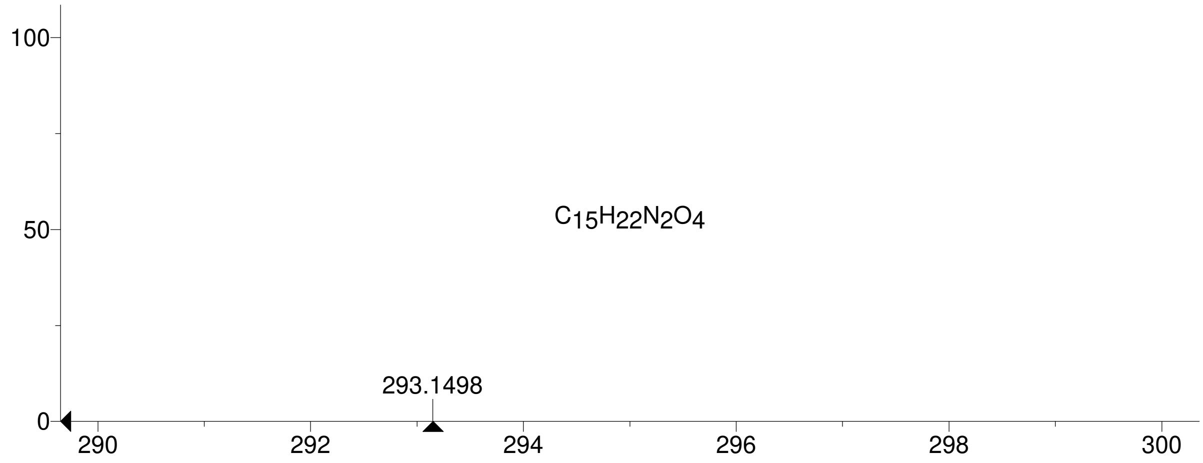

Hit 4 : Leu-Tyr [M-H]- HCD 14eV P=293.2
C15H22N2O4; MF: 364; RMF: 999; Prob 11.1%; Lib: nist_msms; ID: 567554.

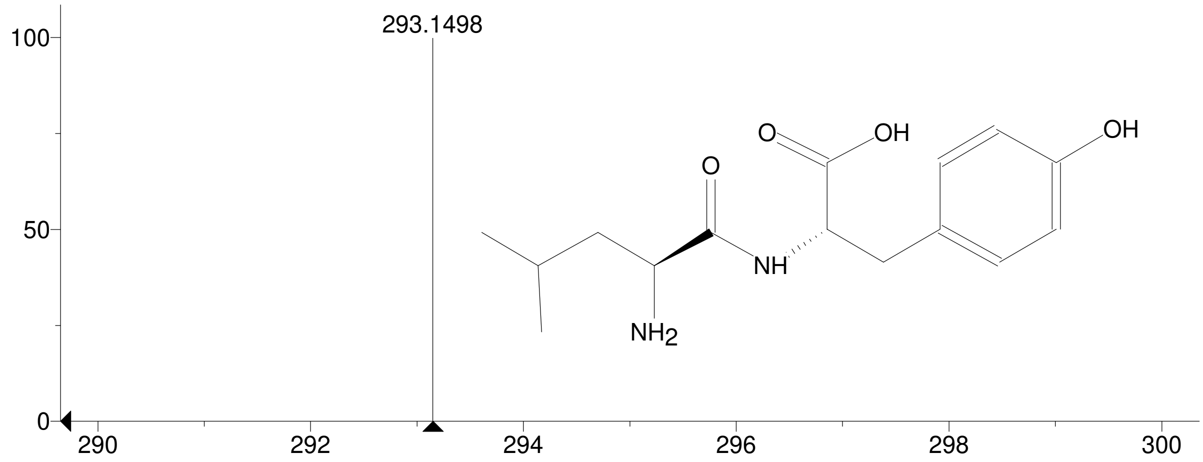

Hit 5 : Tyr-Leu [M-H]- HCD 38eV P=293.2
C15H22N2O4; MF: 364; RMF: 999; Prob 11.1%; Lib: nist_msms; ID: 570430.

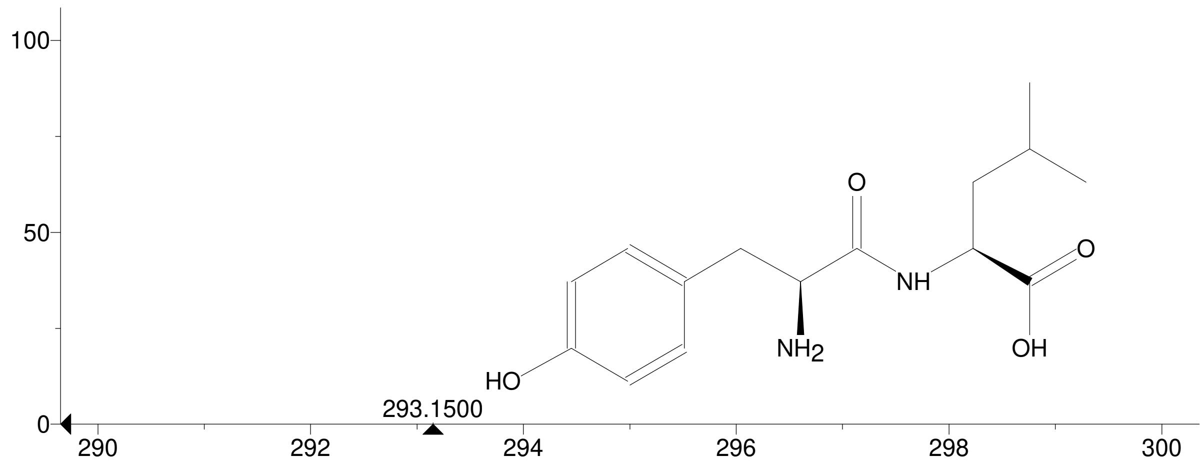

Unknown: Unknown (6.47_415.1799m/z)
Compound in Library Factor = 0

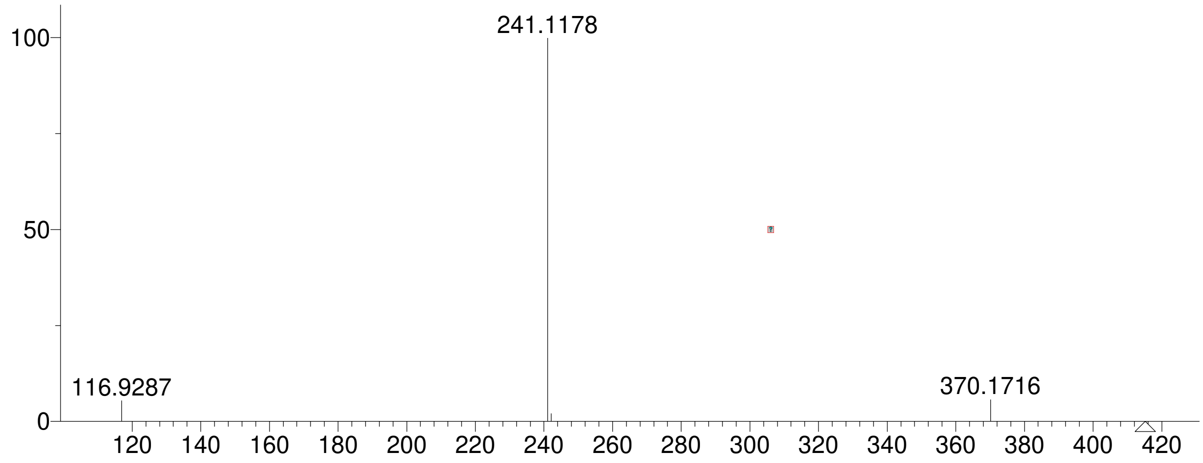

Hit 1 : methyl (1R,4aS,9S,10S)-10-acetyloxy-5,9-dihydroxy-1,4a-dimethyl-2-oxo-7-propan-2-yl-10,10a-dihydro-9H
C23H28O7; MF: 0; RMF: 119; Prob 50.0%; Lib: mona-export-lc-ms-ms_negative_mode; ID: 22118.

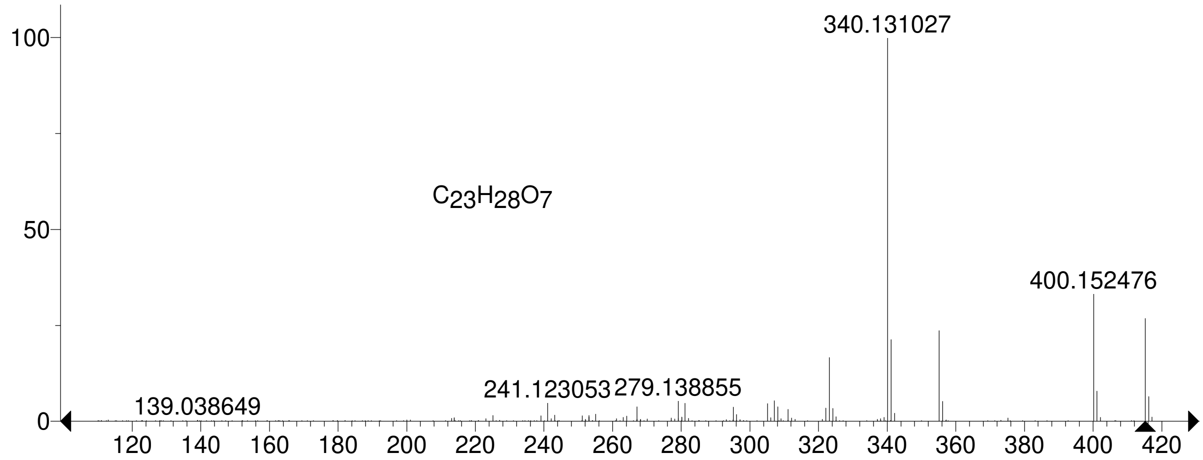

Hit 2 : Dehydrorotenone [M+Na]+ 35% P=415.2
C23H20O6; MF: 0; RMF: 15; Prob 50.0%; Lib: mona-export-itree; ID: 708.

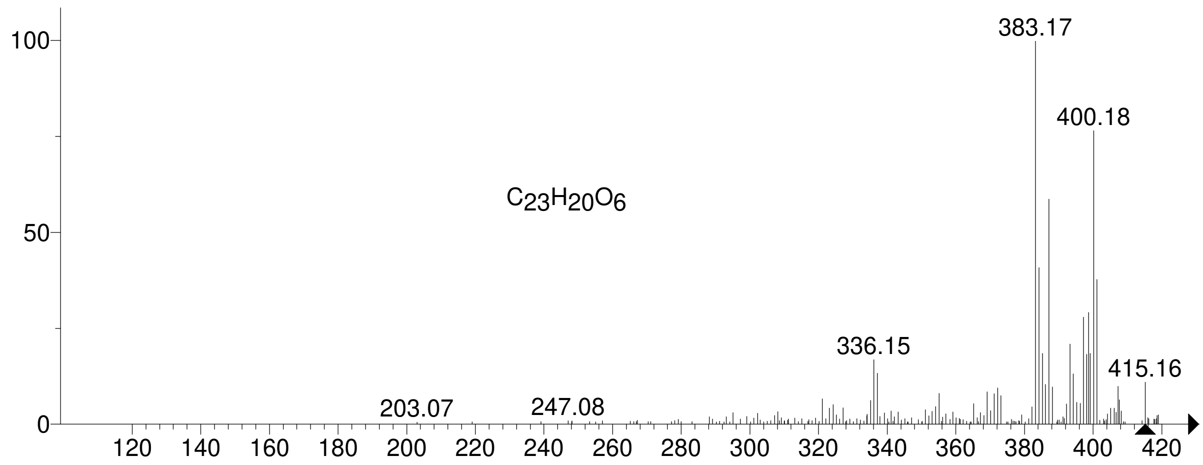

** Search Report Page 1 of 1 **

Unknown: Unknown (6.48_642.2137m/z)
Compound in Library Factor = 0

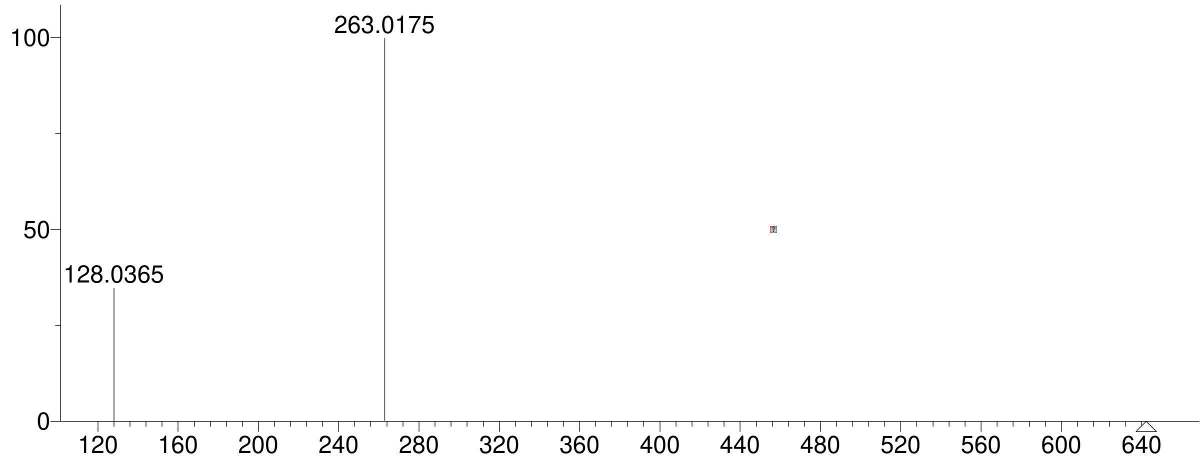

** Search Report Page 1 of 1 **

Unknown: Unknown (6.49_370.1609m/z)
Compound in Library Factor = -2387

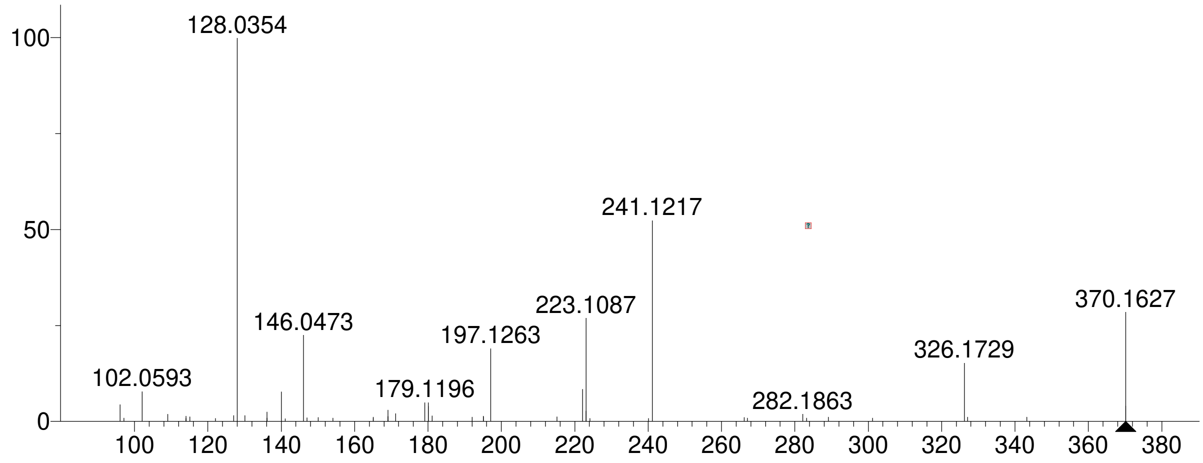

Hit 1 : Tenovin-1 [M+H]+ HCD 11V P=370.2
C20H23N3O2S; MF: 19; RMF: 343; Prob 100.0%; CAS: 380315-80-0; Lib: nist_msms; ID: 334898.

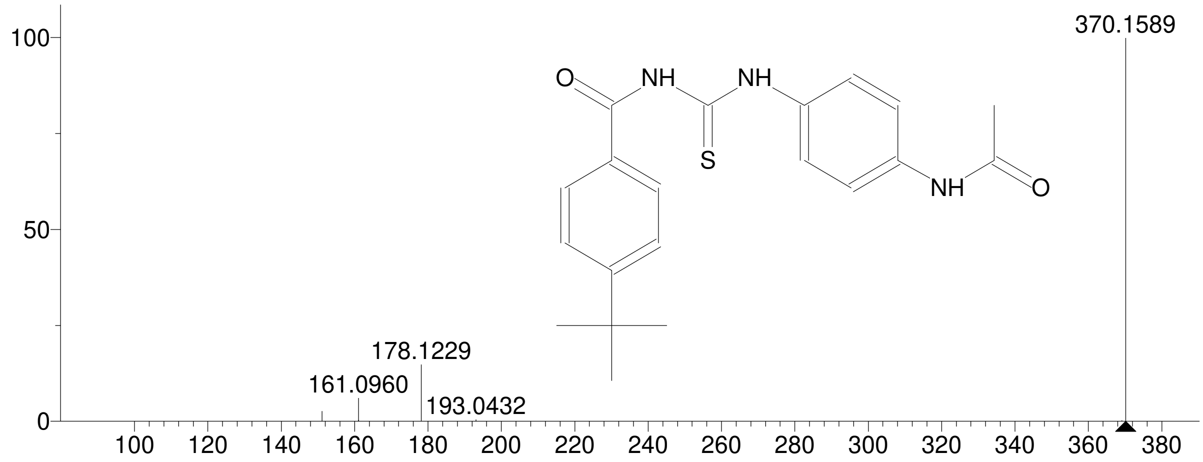

Unknown: Unknown (6.49_397.1686m/z)
Compound in Library Factor = 0

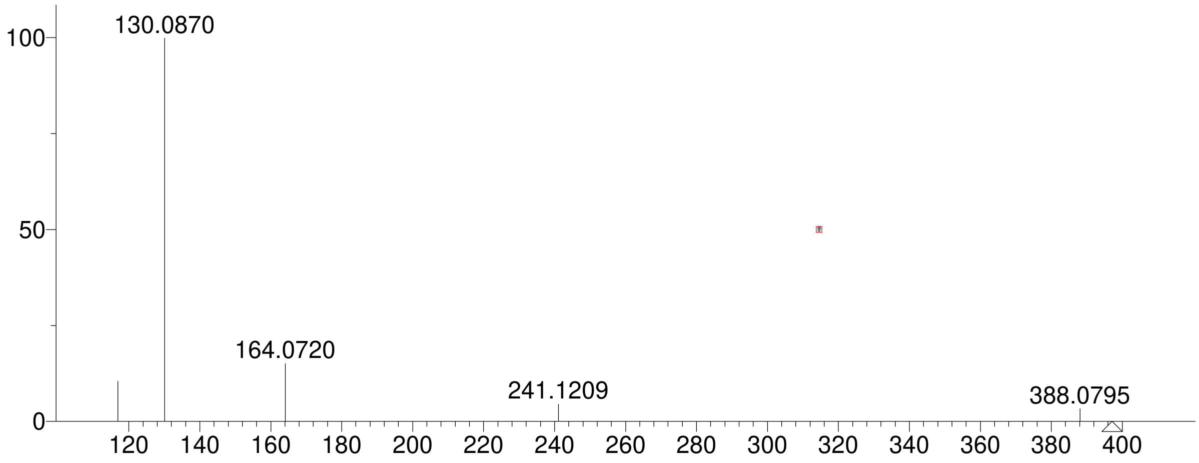

Unknown: Unknown (6.49_799.2444m/z)
Compound in Library Factor = -2387

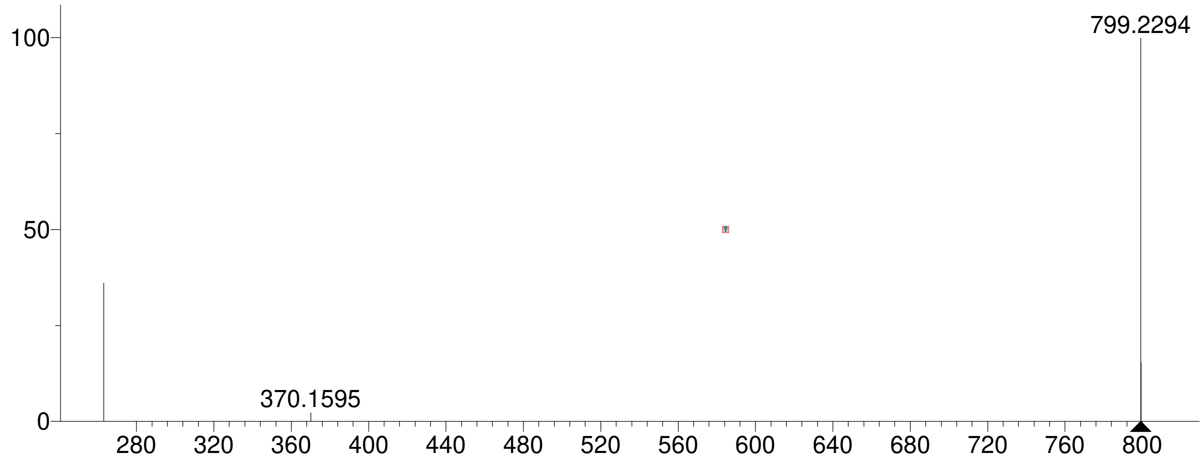

Hit 1 : NCGC00380574-01_C39H44O18_beta-D-Glucopyranoside, (1aS,1bS,2S,5aR,6S,6aS)-6-[[6-deoxy-3,4-bis
C39H44O18; MF: 18; RMF: 522; Prob 64.7%; Lib: msms-neg-gnps; ID: 1536.

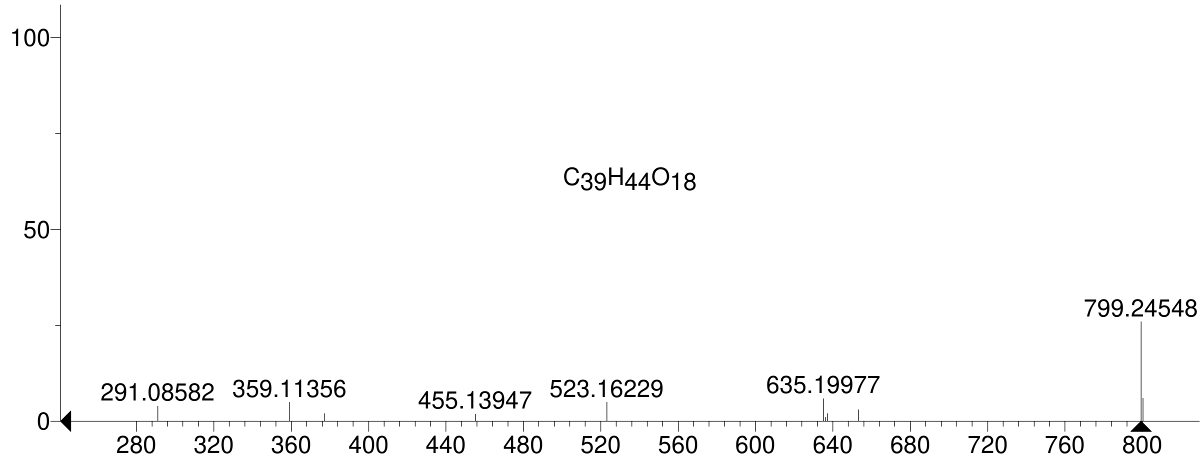

Hit 2 : Corticotropin Releasing Factor, porcine [M+6H]6+ QTOF 5V P=799.2
C209H337N61O64S2; MF: 1; RMF: 178; Prob 35.3%; Lib: nist_msms2; ID: 33744.

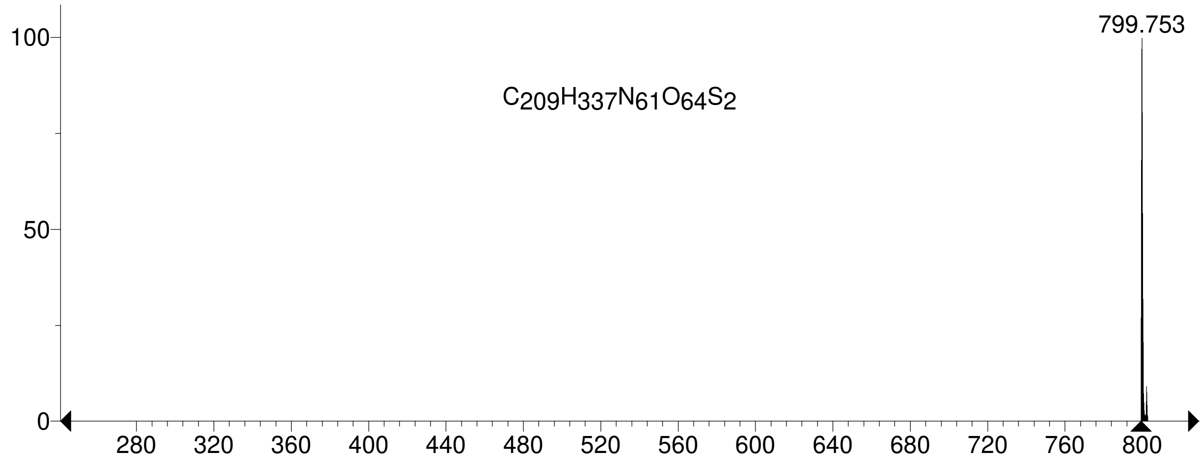

Unknown: Unknown (6.51_301.0912m/z)
Compound in Library Factor = 0

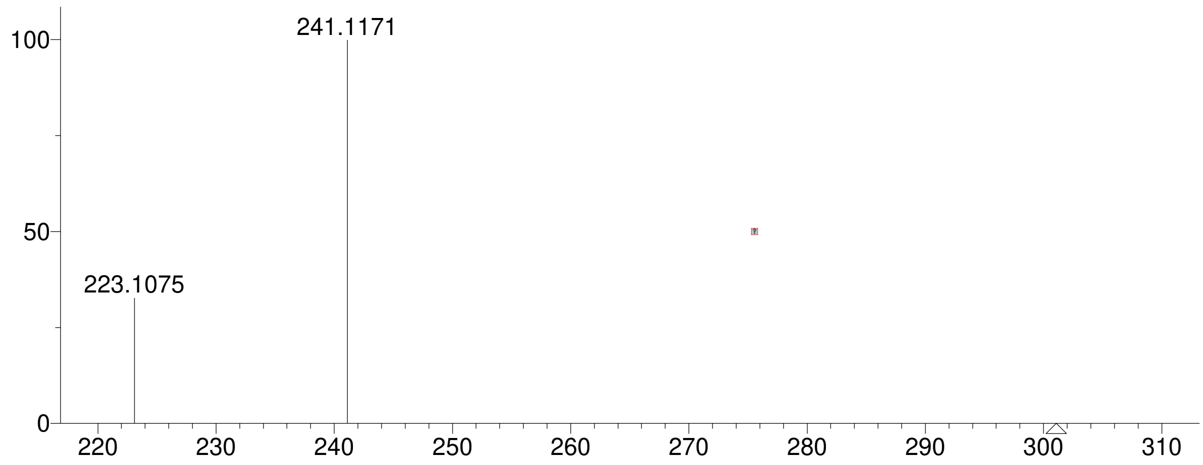

Unknown: Unknown (6.51_327.1331m/z)
Compound in Library Factor = -3273

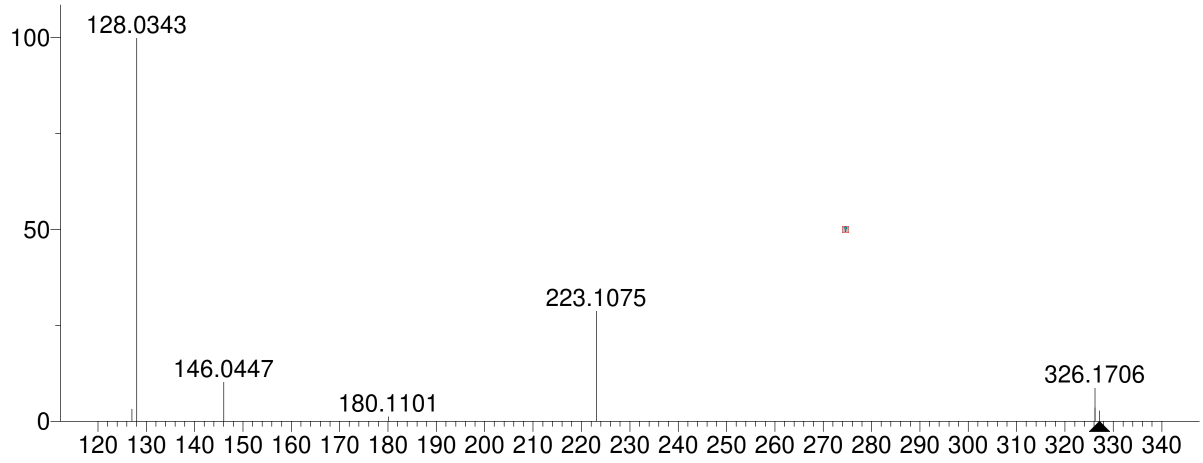

Hit 1 : Coumarin-suberoylanilide hydroxamic acid [M-H-H2O]- HCD 9V P=327.1
C18H22N2O5; MF: 22; RMF: 143; Prob 25.6%; Lib: nist_msms; ID: 154743.

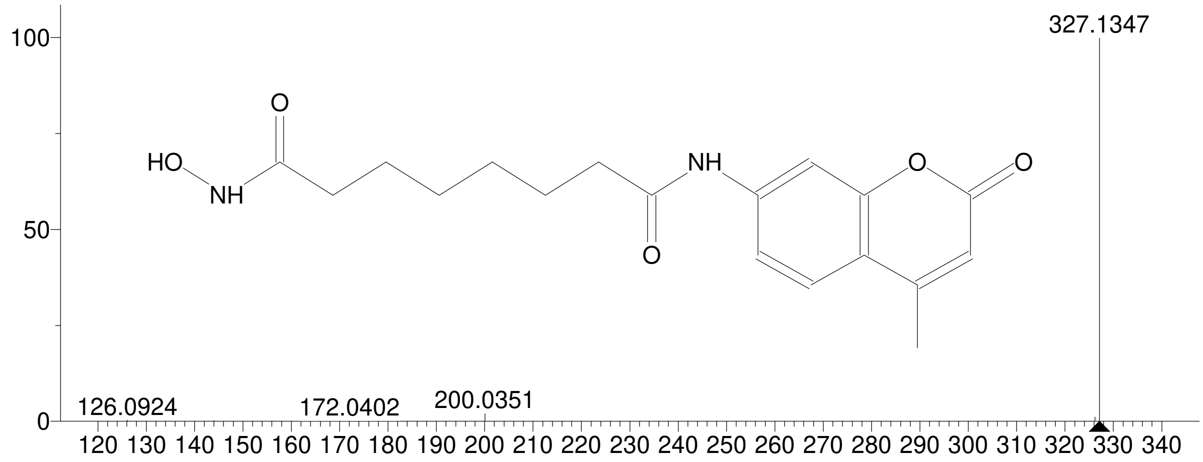

Hit 2 : Scriptaid [M+H]+ HCD 6V P=327.1
C18H18N2O4; MF: 19; RMF: 228; Prob 22.6%; CAS: 287383-59-9; Lib: nist_msms; ID: 319992.

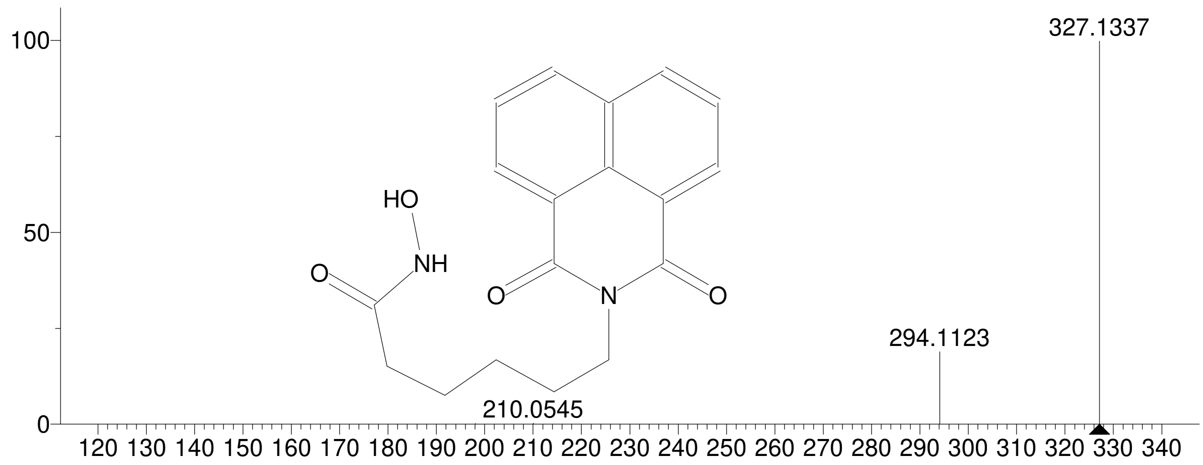

Hit 3 : 5-Hydroxy-3,4-dihydro-2(1H)-quinolinone [2M+H]+ HCD 1V P=327.1
C9H9NO2; MF: 6; RMF: 168; Prob 14.6%; CAS: 30389-33-4; Lib: nist_msms; ID: 441569.

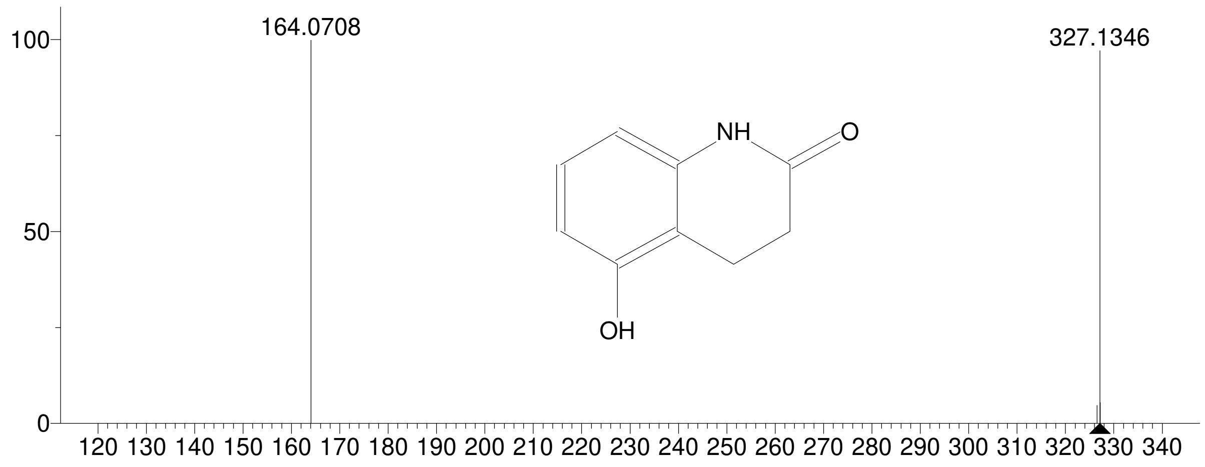

Hit 4 : Phe-Tyr [M-H]- HCD 16eV P=327.1
C18H20N2O4; MF: 3; RMF: 121; Prob 12.9%; Lib: nist_msms; ID: 567427.

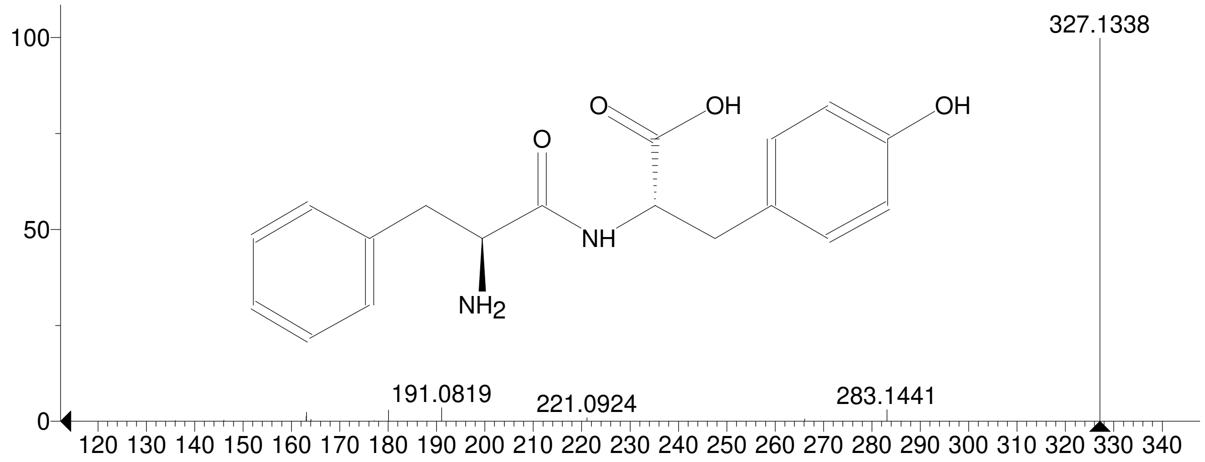

Hit 5 : Phe-Tyr [M-H]- HCD 16eV P=327.1
C18H20N2O4; MF: 3; RMF: 121; Prob 12.9%; CAS: 17355-18-9; Lib: nist_msms2; ID: 70284.

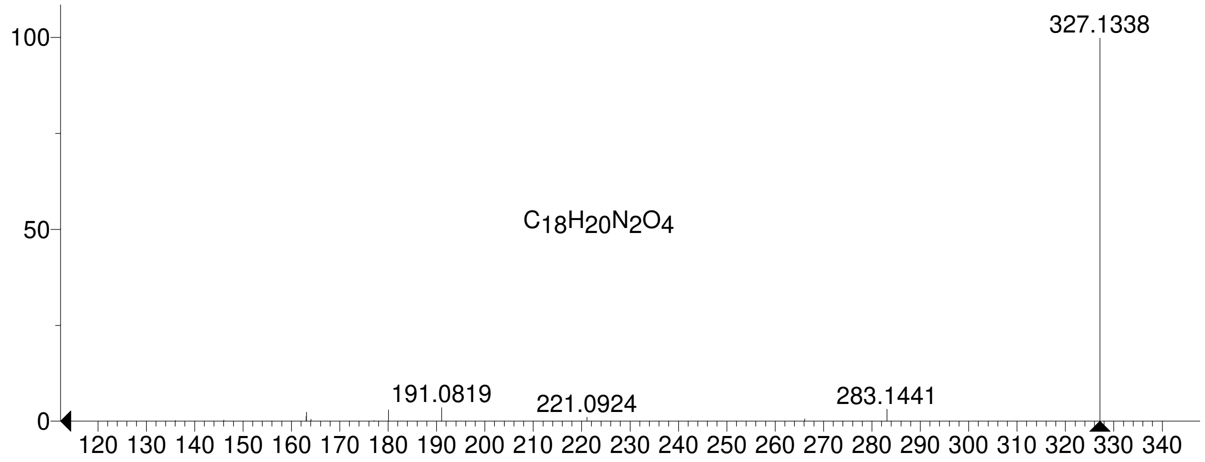

Unknown: Unknown (6.51_397.2199m/z)
Compound in Library Factor = -2387

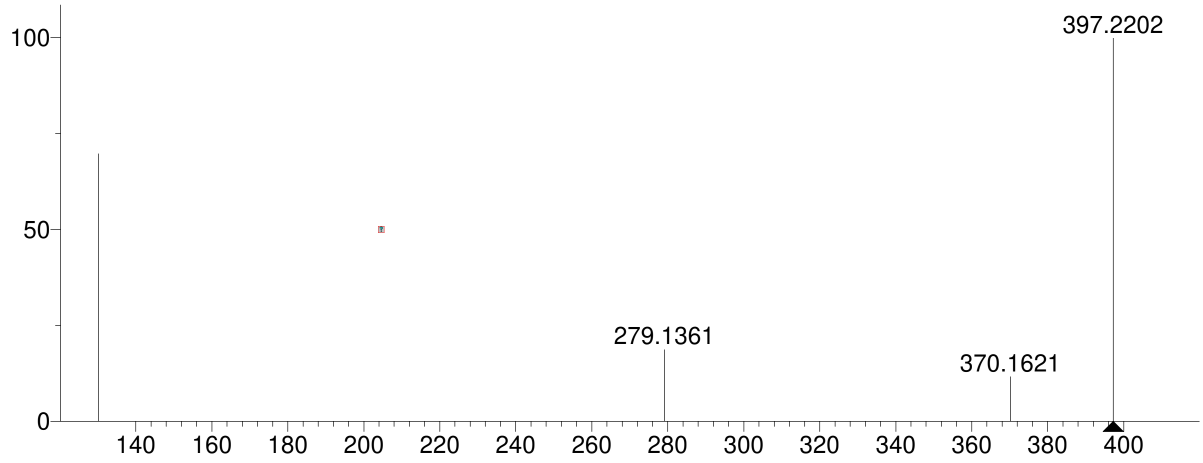

Hit 1 : NCGC00385079-01_C19H34O7_4-(4-Hydroxy-2,6,6-trimethyl-1-cyclohexen-1-yl)-2-butanyl beta-D-glucopy
C19H34O7; MF: 24; RMF: 623; Prob 41.8%; Lib: mona-export-gnps; ID: 14159.

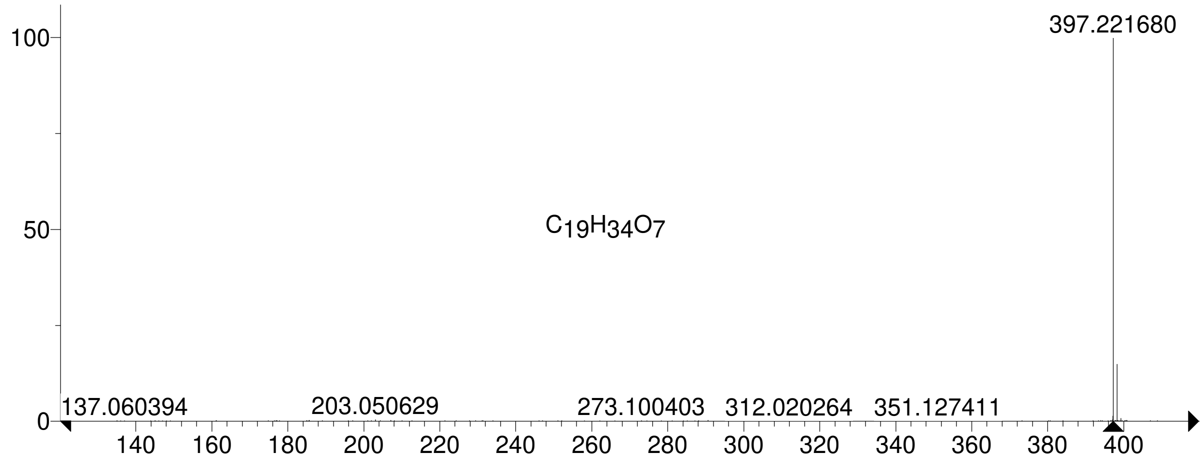

Hit 2 : (2R,3S,4S,5R,6R)-2-(hydroxymethyl)-6-[4-(4-hydroxy-2,6,6-trimethylcyclohexen-1-yl)butan-2-yloxy]oxane-3
C19H34O7; MF: 6; RMF: 537; Prob 21.5%; Lib: anthocyanins mona; ID: 7638.

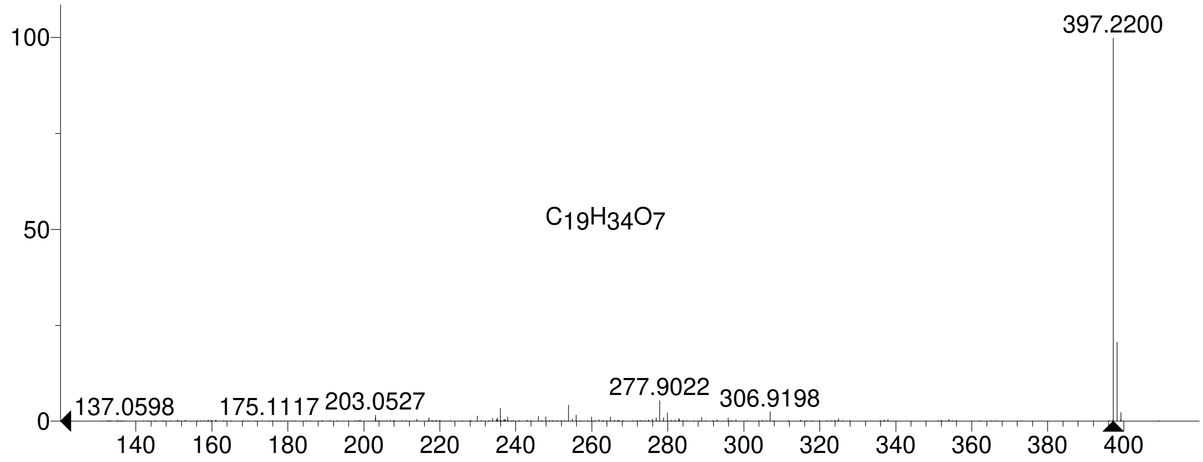

Hit 3 : O-acetylstemmadenine ([H]\C(C)=C1/CN2CC[C@]1([H])[C@](COC(C)=O)(C(=O)OC)C1=C(CC2)C2=CC=(
C23H28N2O4; MF: 4; RMF: 419; Prob 19.9%; Lib: alkaloids; ID: 12.

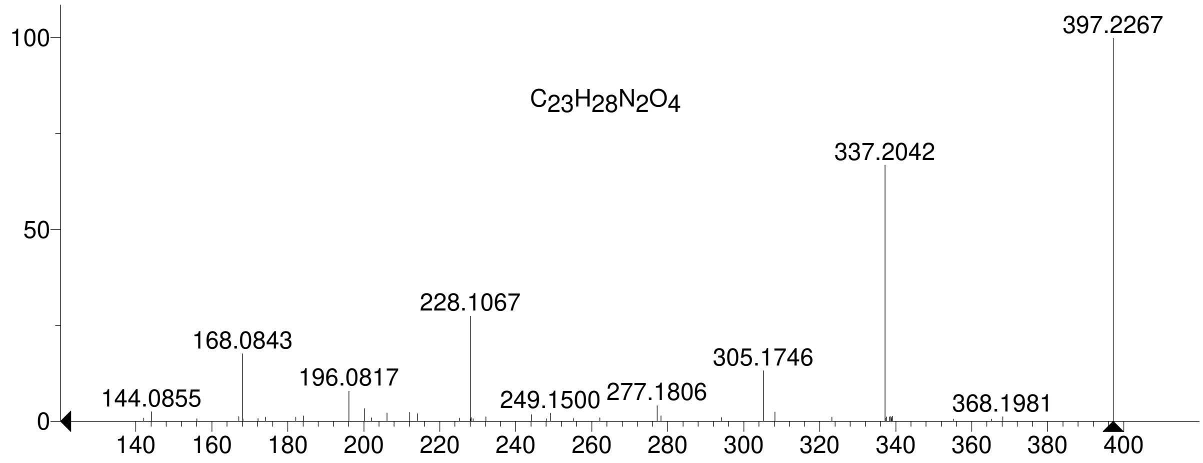

Hit 4 : 2,4-dihydroxy-5-methoxy-1'',6'',8''-trimethyldispiro[bis(oxolane)-3,2':5',7''-[3]oxatricyclo[6.3.1.0a^'',A^'1A^'2]c
C21H32O7; MF: 0; RMF: 75; Prob 16.8%; Lib: mona-export-vf-npl_qexactive; ID: 10630.

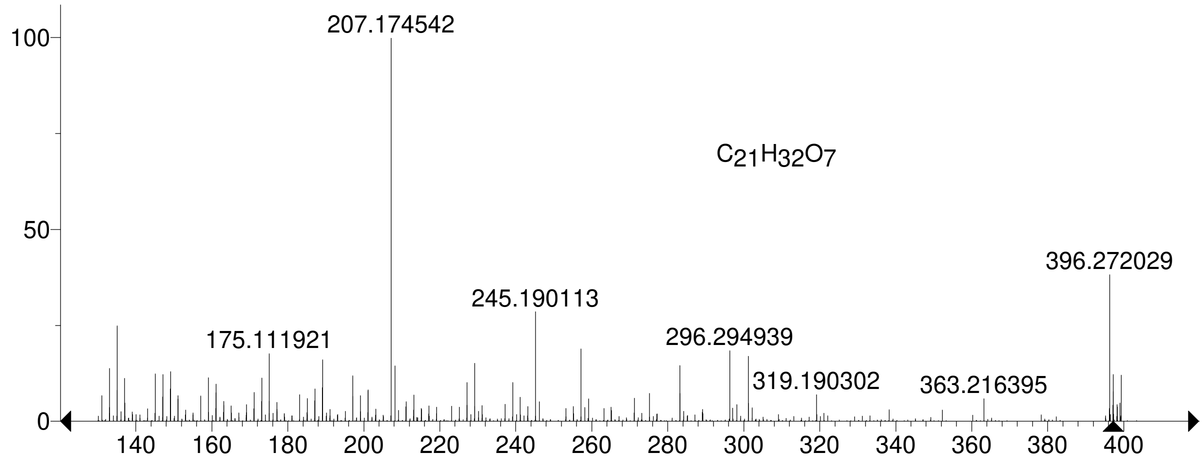

Unknown: Unknown (6.54_590.2881m/z)
Compound in Library Factor = 0

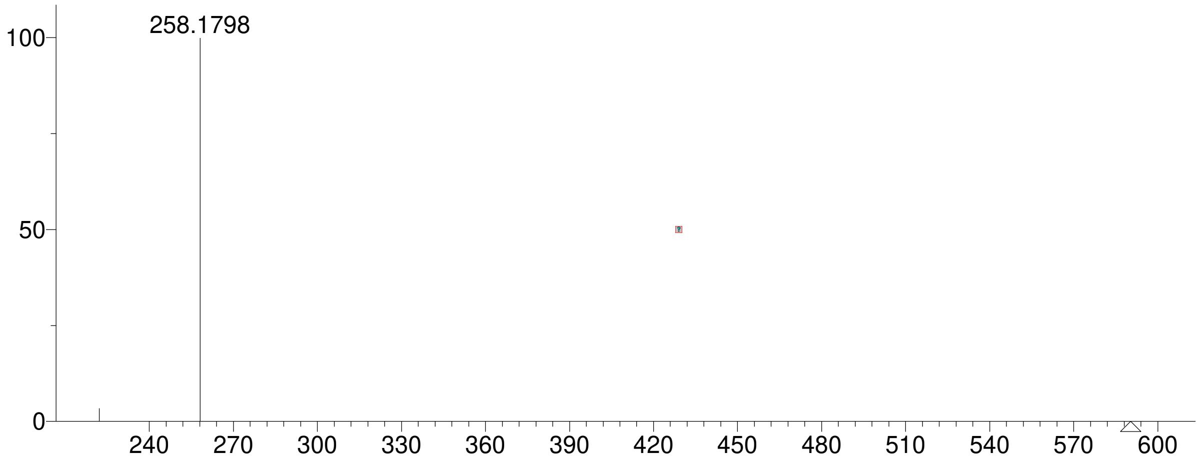

Unknown: Unknown (6.55_356.1455m/z)
Compound in Library Factor = -3273

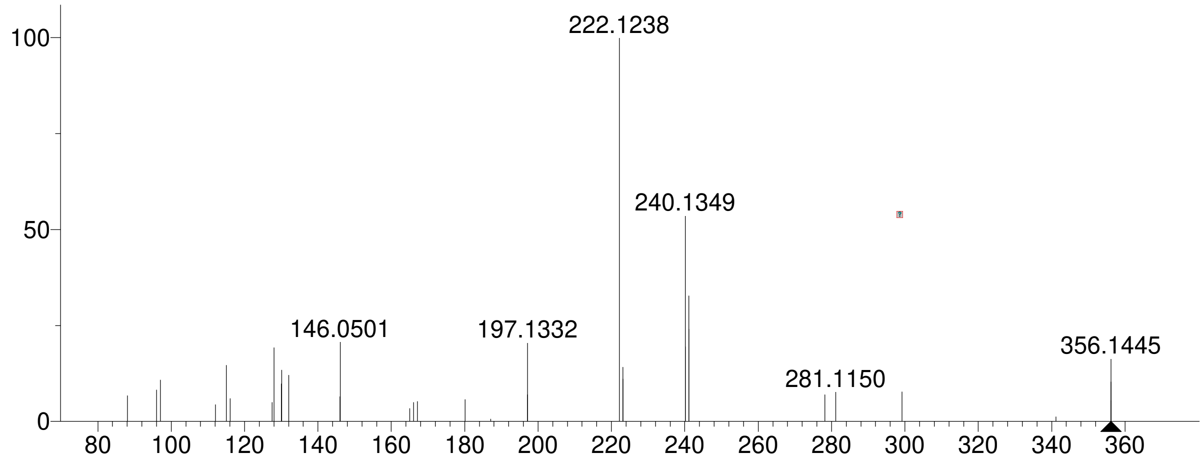

Hit 1 : Benzenesulfonamide, 4-(1,1-dimethylethyl)-N-[4-(1H-imidazol-1-yl)phenyl]- [M+H]+ HCD 21V P=356.1
C19H21N3O2S; MF: 15; RMF: 201; Prob 42.2%; CAS: 945526-43-2; Lib: nist_msms; ID: 355342.

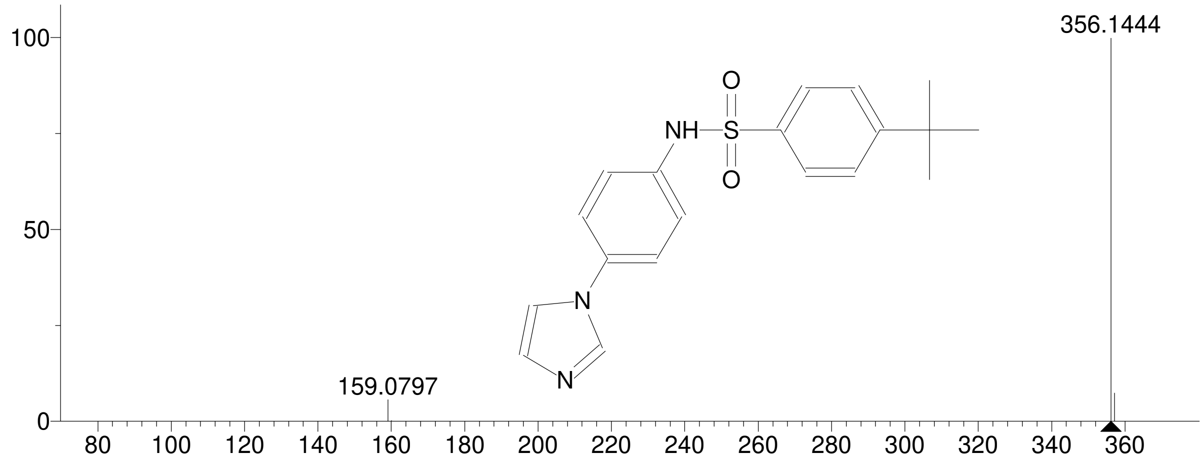

Hit 2 : Benzoic acid, 4-[[(2,4-diamino-7,8-dihydro-7-oxo-6-pteridinyl)methyl]methylamino]-, methyl ester [M+H]+ I
C16H17N7O3; MF: 6; RMF: 182; Prob 30.7%; CAS: 1330264-75-9; Lib: nist_msms; ID: 432535.

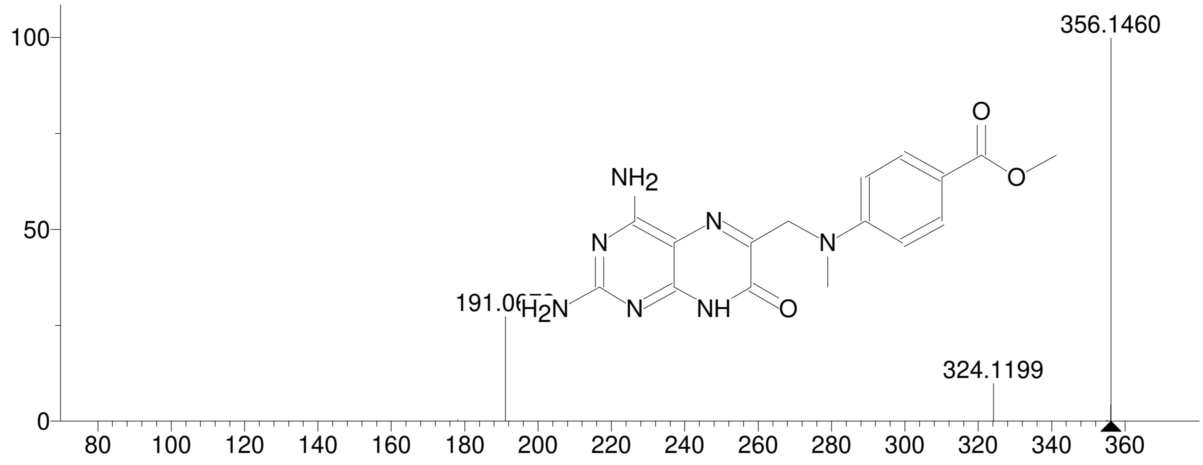

Hit 3 : Seneciphyllin [M+Na]+ HCD 17V P=356.1
C18H23NO5; MF: 3; RMF: 163; Prob 27.1%; CAS: 480-81-9; Lib: nist_msms; ID: 219524.

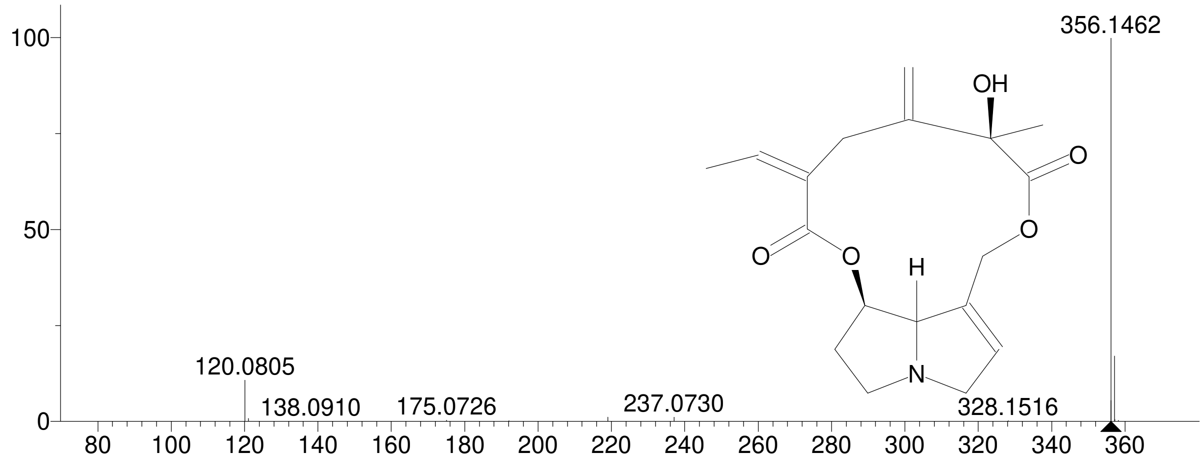

Unknown: Unknown (6.55_395.1500m/z)
Compound in Library Factor = 0

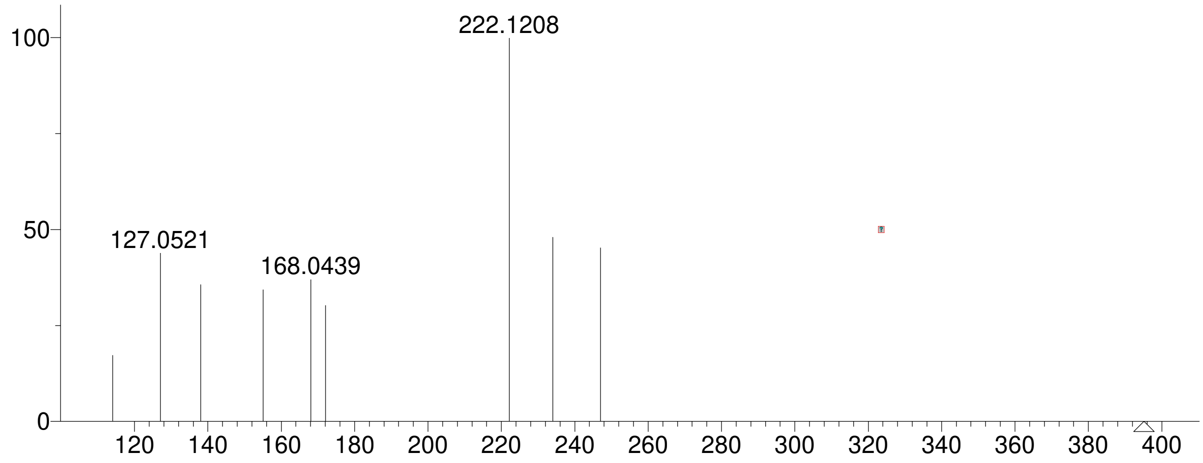

Hit 1 : Rotenone [M+H]+ HCD 126V P=395.1
C23H22O6; MF: 0; RMF: 47; Prob 10.0%; CAS: 83-79-4; Lib: nist_msms; ID: 491890.

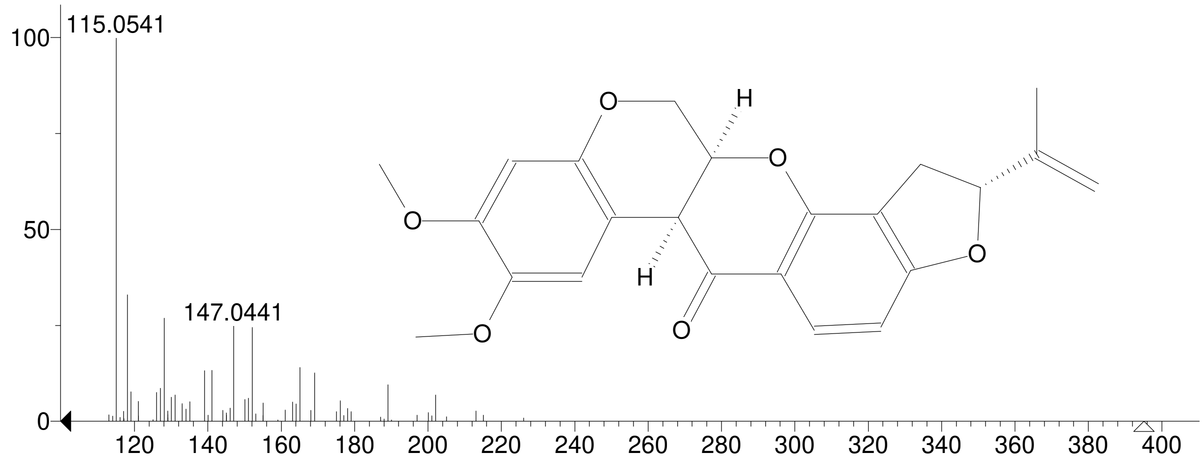

Hit 2 : (-)-Deguelin [M+H]+ HCD 154V P=395.1
C23H22O6; MF: 0; RMF: 46; Prob 10.0%; CAS: 522-17-8; Lib: nist_msms; ID: 165224.

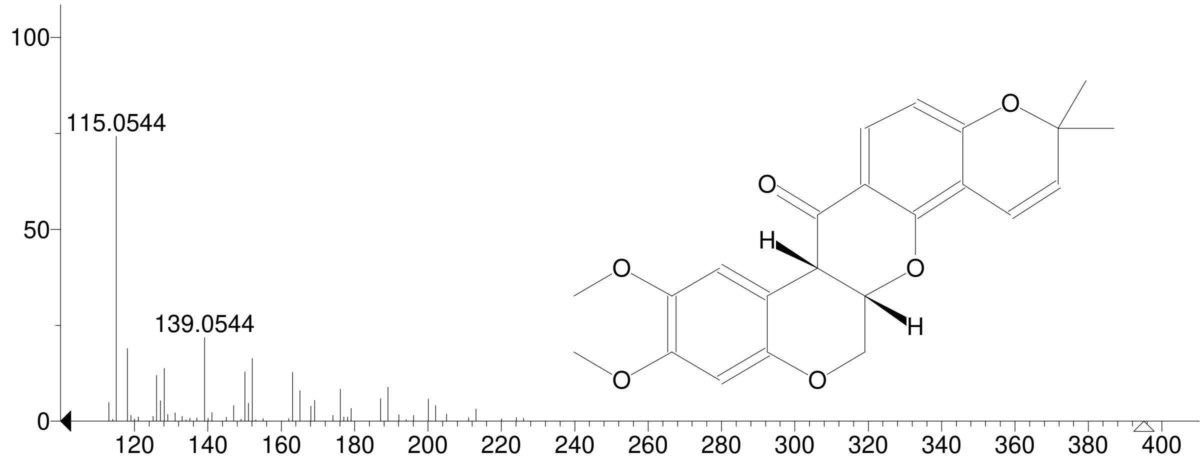

Hit 3 : Garcinone C [M-H-H2O]- HCD 126V P=395.2
C23H26O7; MF: 0; RMF: 28; Prob 10.0%; CAS: 76996-27-5; Lib: nist_msms; ID: 389342.

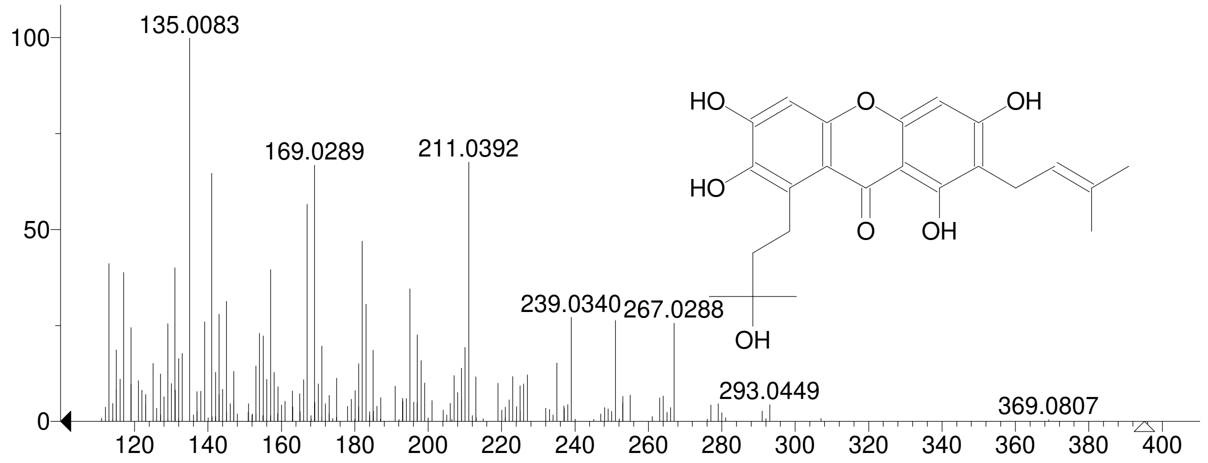

Hit 4 : NCGC00160168-01!MACLUROXANTHONE [M+H]+ P=395.1
C23H22O6; MF: 0; RMF: 23; Prob 10.0%; Lib: mona-export-gnps; ID: 1120.

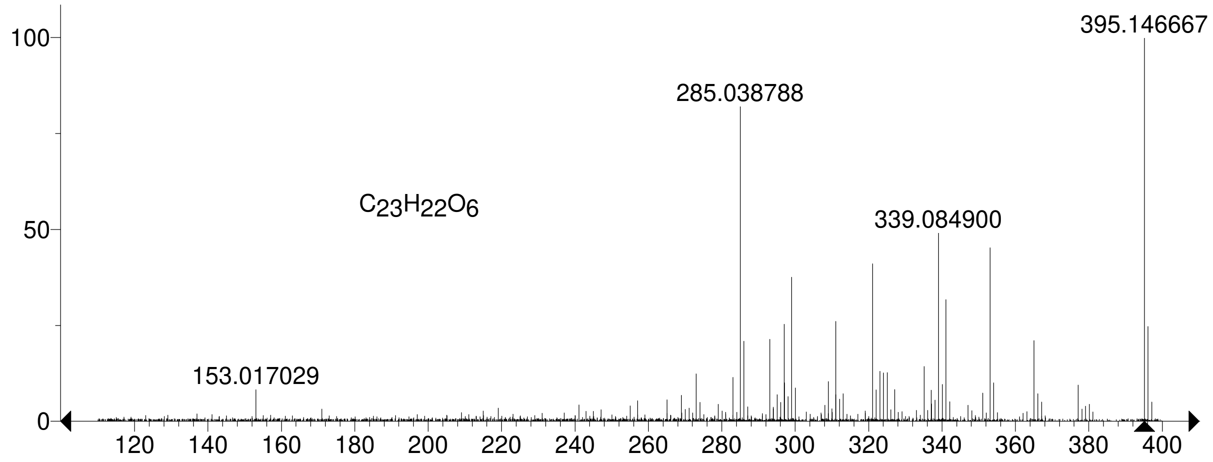

Hit 5 : NCGC00386021-01!8,8-dimethyl-3-(2,4,5-trimethoxyphenyl)pyrano[2,3-f]chromen-4-one [M+H]+ P=395.1
C23H22O6; MF: 0; RMF: 23; Prob 10.0%; Lib: mona-export-gnps; ID: 9396.

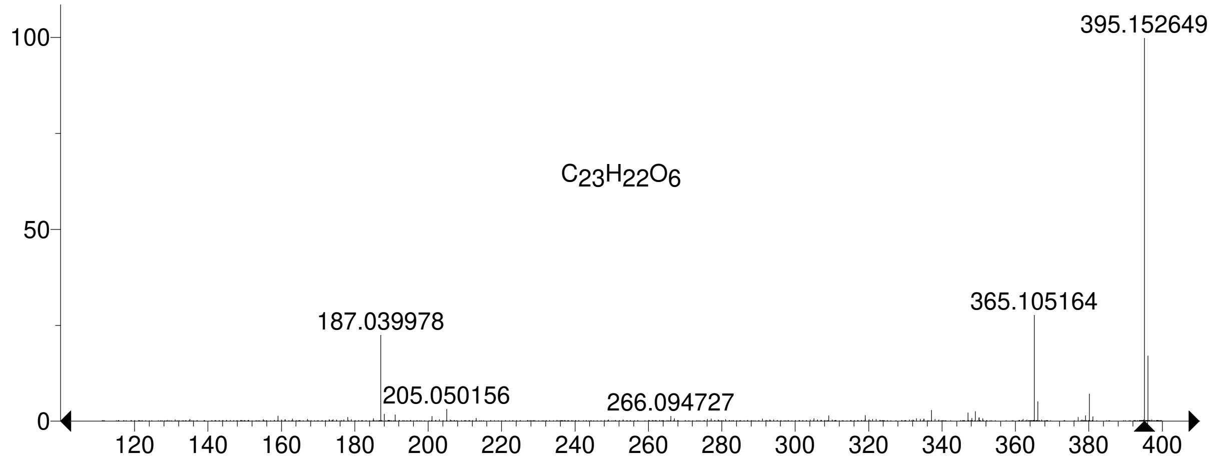

** Search Report Page 1 of 1 **

Unknown: Unknown (6.58_564.2410m/z)
Compound in Library Factor = -3273

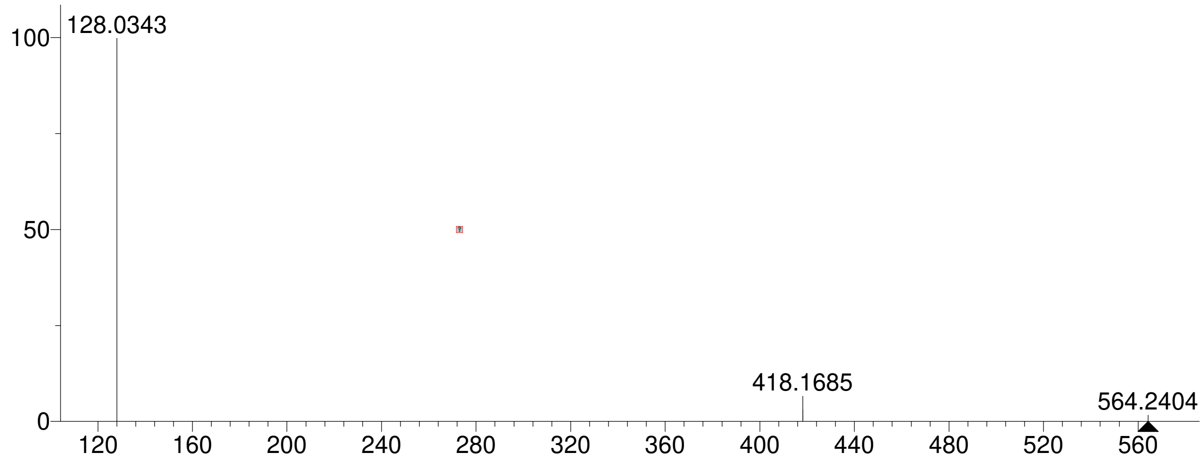

Hit 1 : Curine [M+H-CH5N]+ HCD 33V P=564.2
C36H38N2O6; MF: 5; RMF: 278; Prob 55.4%; CAS: 436-05-5; Lib: nist_msms; ID: 385681.

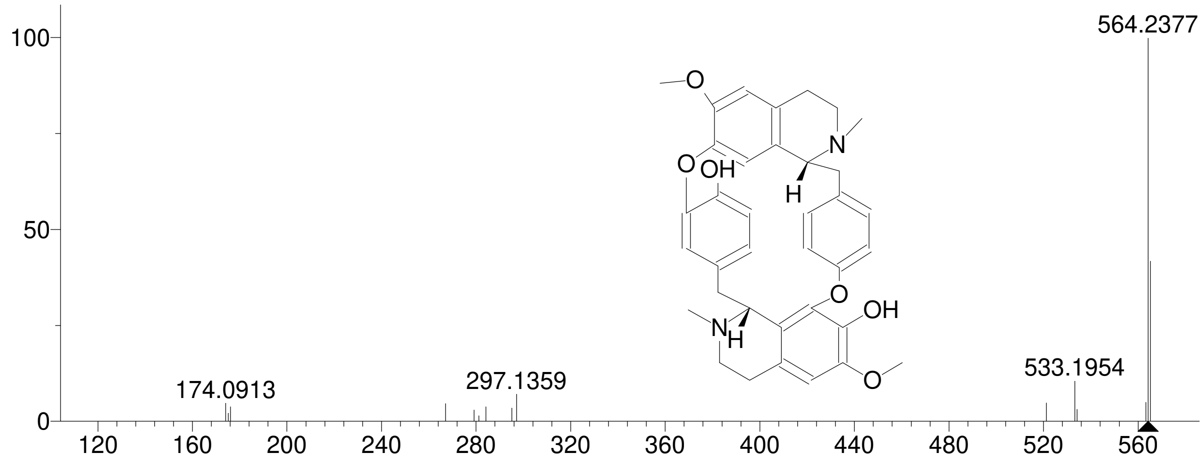

Hit 2 : Cepharanthine [M+H-C2H5N]+ HCD 33V P=564.2
C37H38N2O6; MF: 0; RMF: 72; Prob 44.6%; CAS: 481-49-2; Lib: nist_msms; ID: 217080.

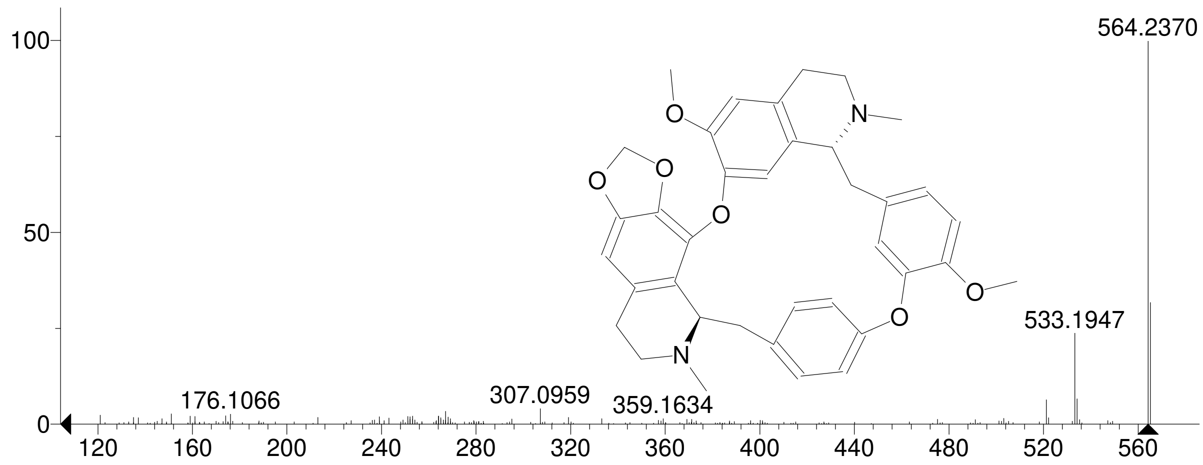

** Search Report Page 1 of 1 **

Unknown: Unknown (6.59_530.1985m/z)
Compound in Library Factor = 0

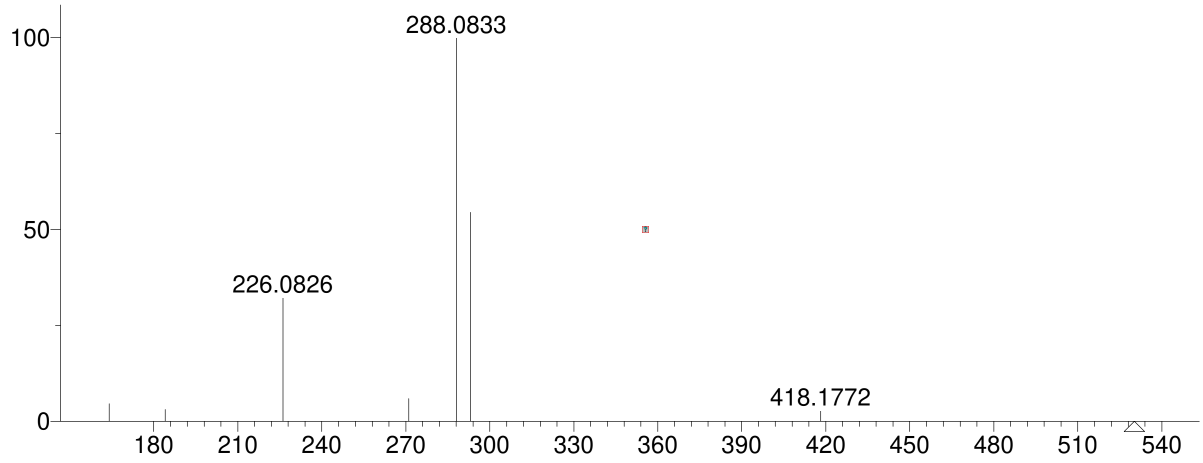

Unknown: Unknown (6.60_630.2065m/z)
Compound in Library Factor = 0

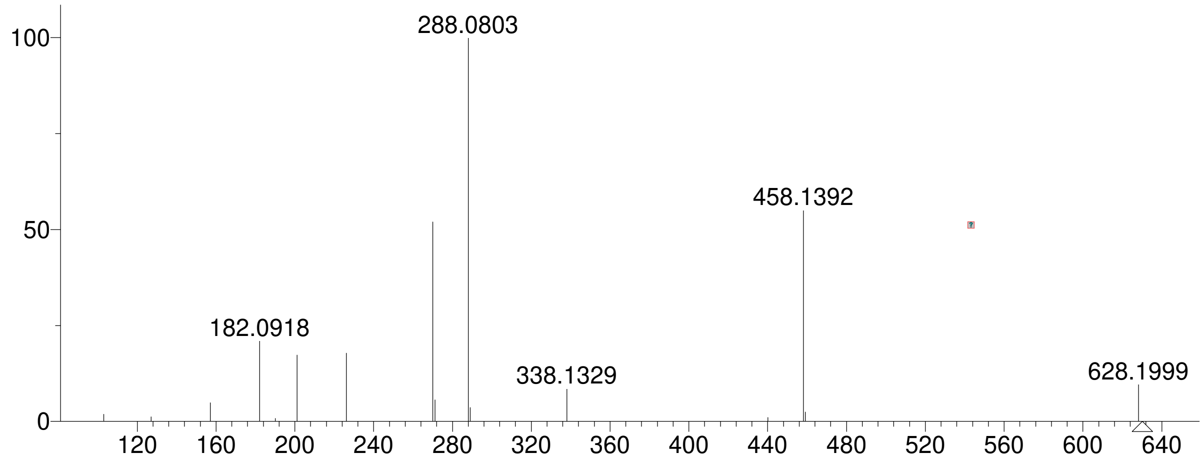

Hit 1 : NCGC00384724-01_C27H32O16_beta-D-Glucopyranoside, 4-carboxy-2-methoxyphenyl 2-O-[(2S,3R,4S)-C27H32O16; MF: 0; RMF: 16; Prob 100.0%; Lib: mona-export-gnps; ID: 11374.

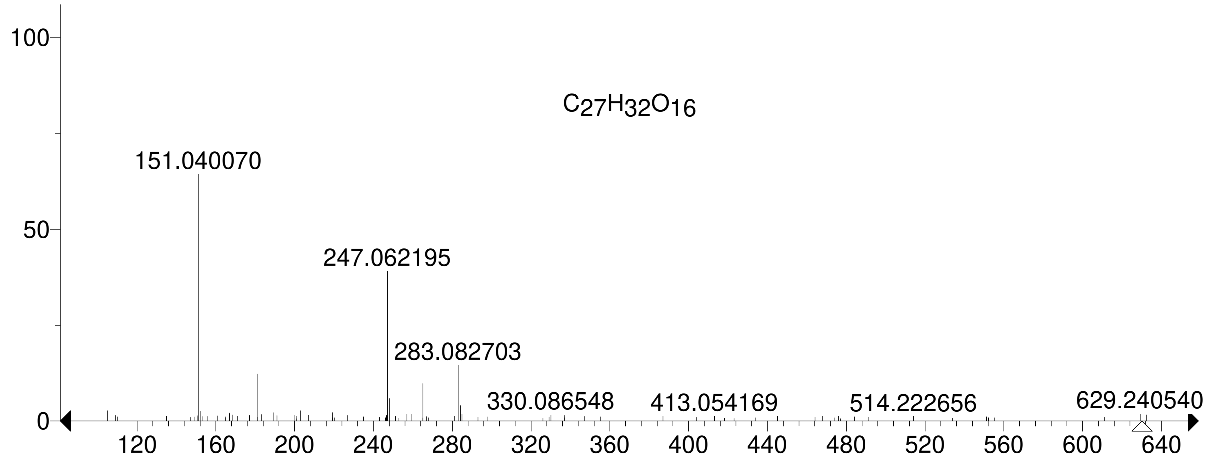

** Search Report Page 1 of 1 **

Unknown: Unknown (6.61_458.1396m/z)
Compound in Library Factor = 0

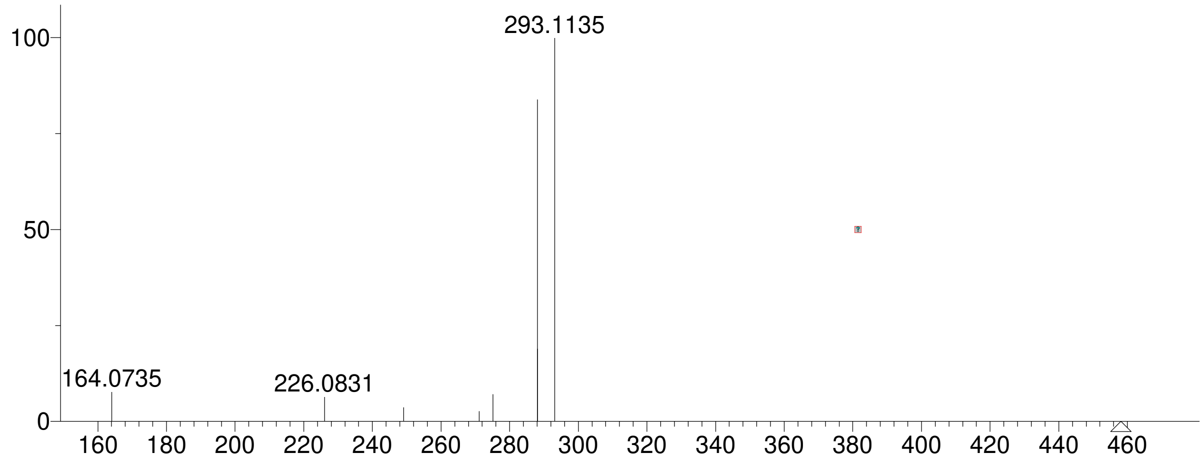

Unknown: Unknown (6.61_463.1711m/z)
Compound in Library Factor = 0

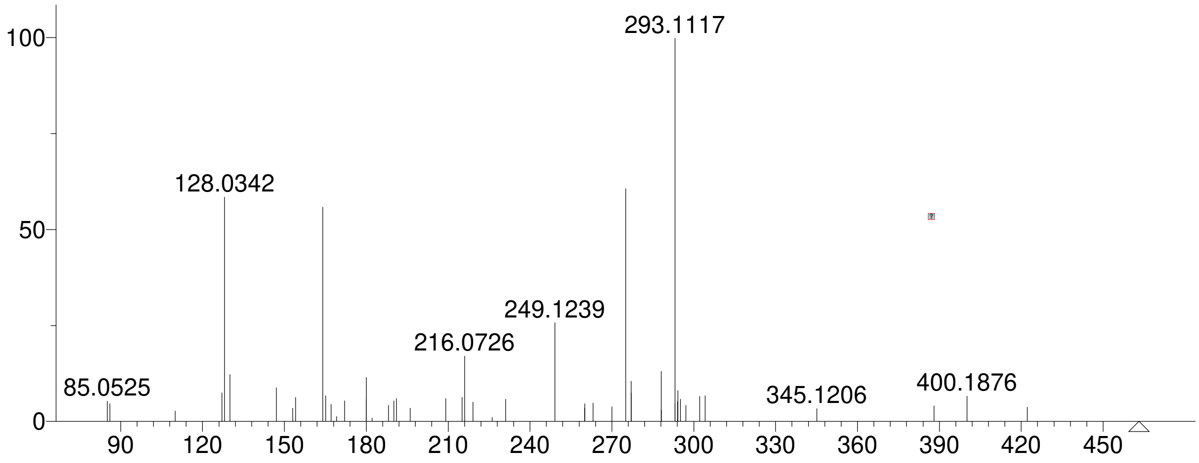

Hit 1 : [(1S,6S,7R)-6-acetyloxy-1-(3-methylbutanoyloxy)spiro[4a,5,6,7a-tetrahydro-1H-cyclopenta[c]pyran-7,2'-oxi
C22H32O8; MF: 0; RMF: 31; Prob 100.0%; Lib: mona-export-vf-npl_qexactive; ID: 1819.

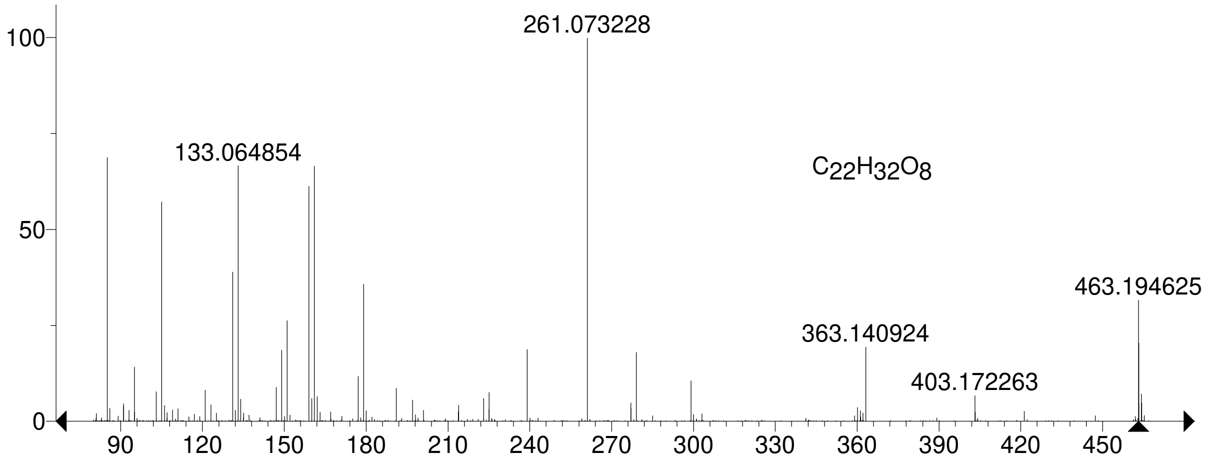

** Search Report Page 1 of 1 **

Unknown: Unknown (6.61_628.1987m/z)
Compound in Library Factor = 0

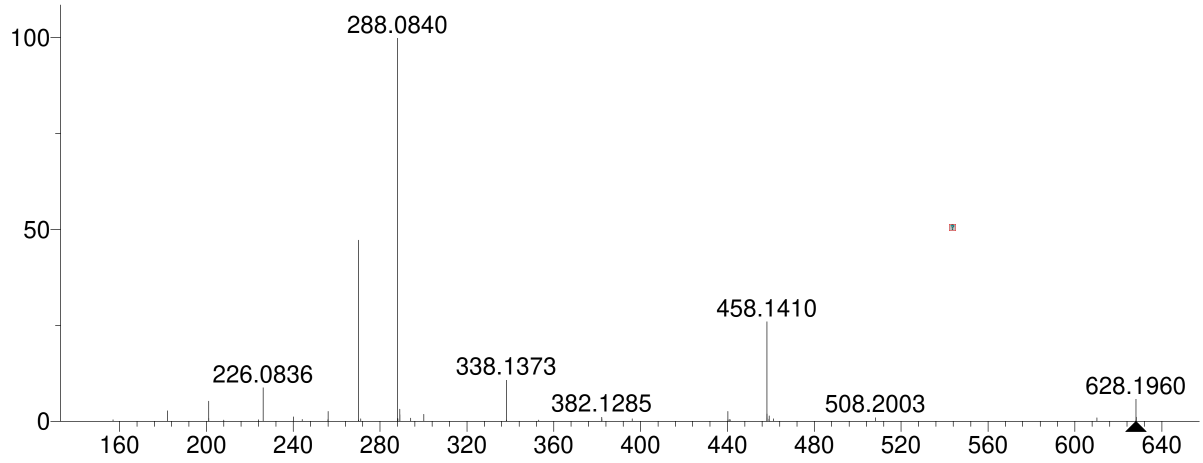

Unknown: Unknown (6.62_453.1499m/z)
Compound in Library Factor = 0

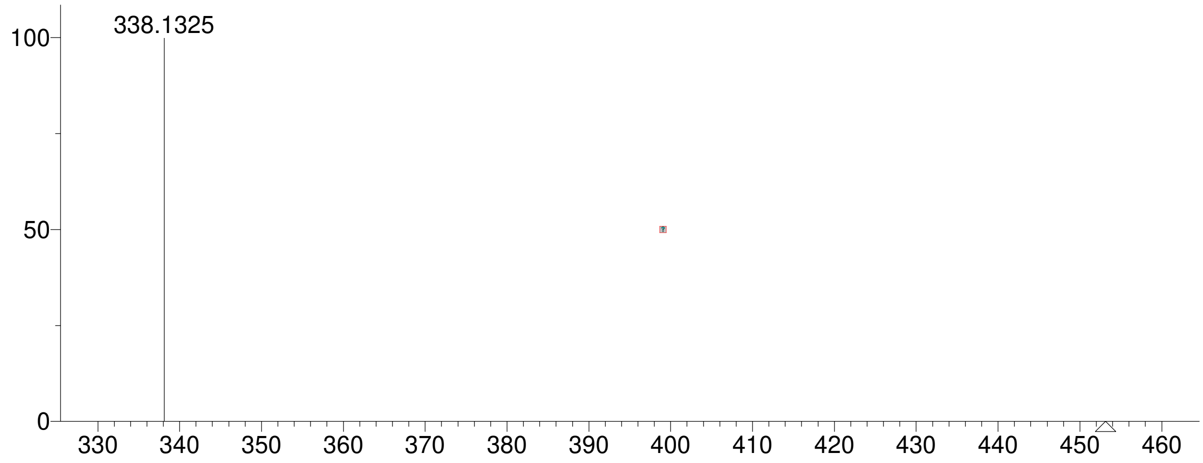

Unknown: Unknown (6.63_854.4749m/z)
Compound in Library Factor = -2860

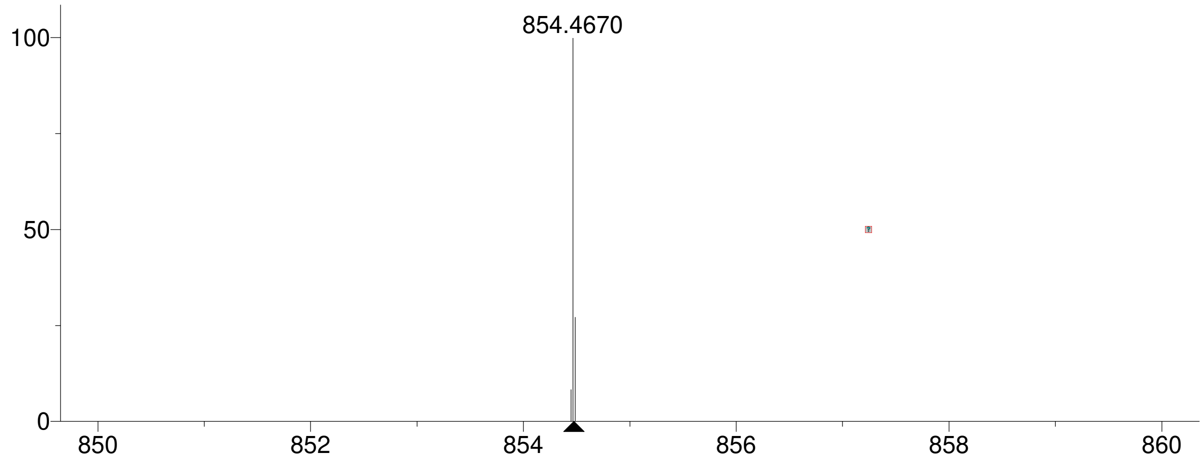

Hit 1 : OxPS 38:6+3O; OxPS 16:0-22:6+3O; [M-H]- [M-H]- P=854.5
C44H74NO13P; MF: 302; RMF: 859; Prob 33.3%; Lib: msdial-insilicomsms-lipids-neg; ID: 315604.

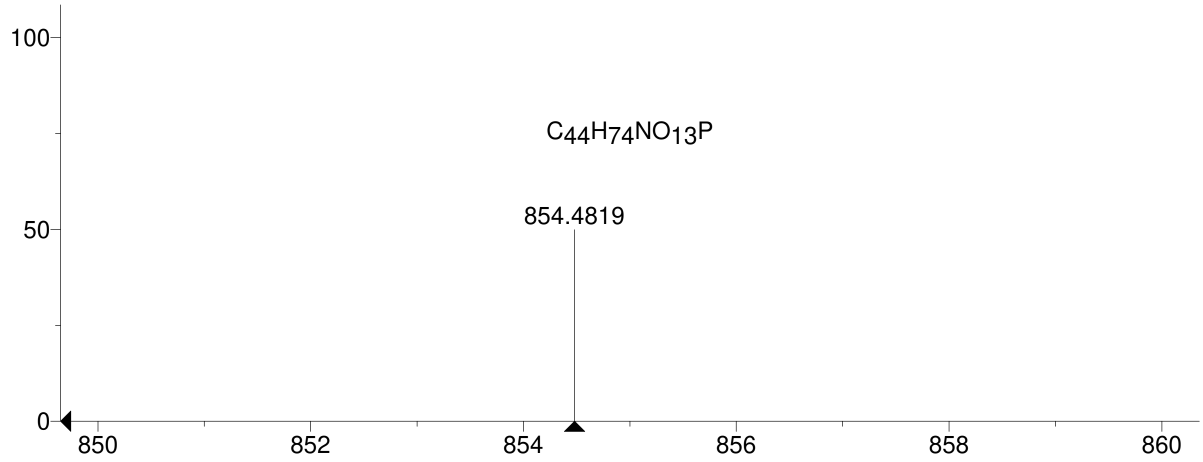

Hit 2 : OxPS 38:6+3O; OxPS 18:1-20:5+3O; [M-H]- [M-H]- P=854.5
C44H74NO13P; MF: 302; RMF: 859; Prob 33.3%; Lib: msdial-insilicomsms-lipids-neg; ID: 315667.

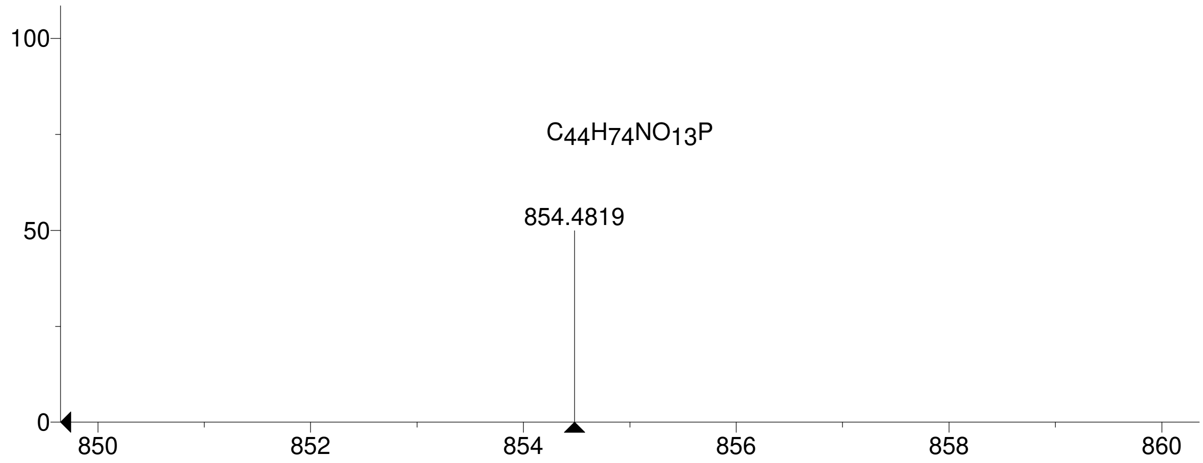

Hit 3 : OxPS 38:6+3O(2Cyc); OxPS 18:1-20:5+3O(2Cyc); [M-H]- [M-H]- P=854.5
C44H74NO13P; MF: 302; RMF: 859; Prob 33.3%; Lib: msdial-insilicomsms-lipids-neg; ID: 315668.

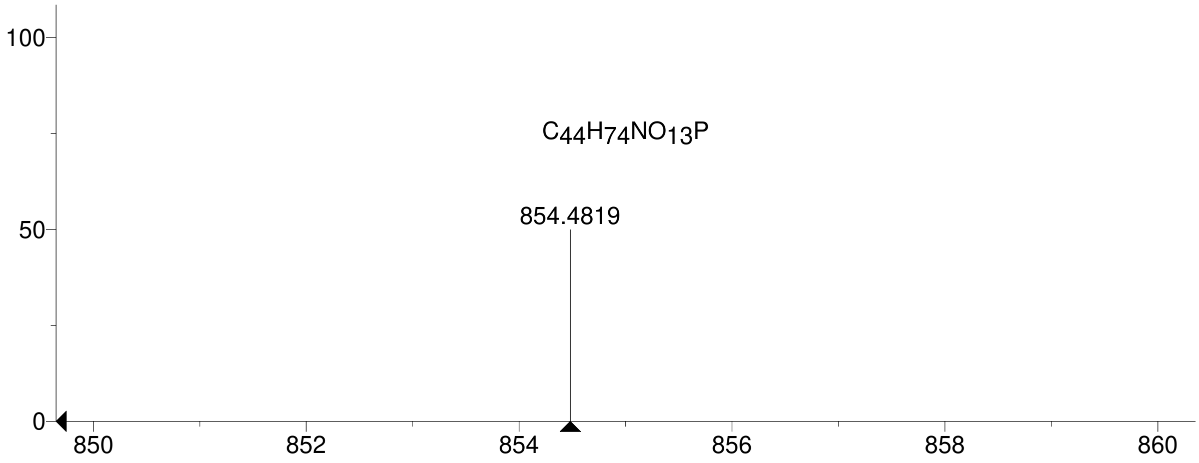

Unknown: Unknown (6.66_546.2404m/z)
Compound in Library Factor = 0

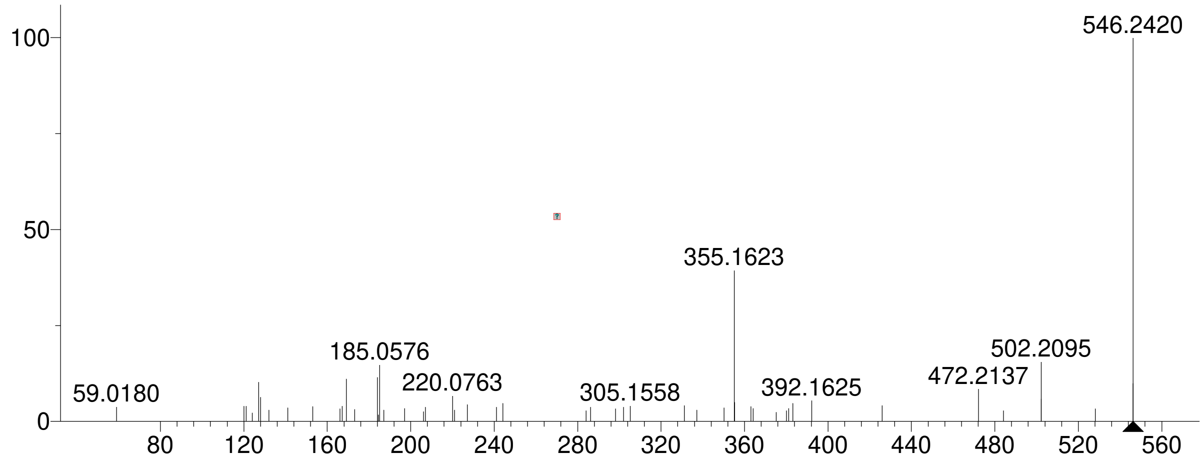

Unknown: Unknown (6.67_479.1688m/z)
Compound in Library Factor = -2387

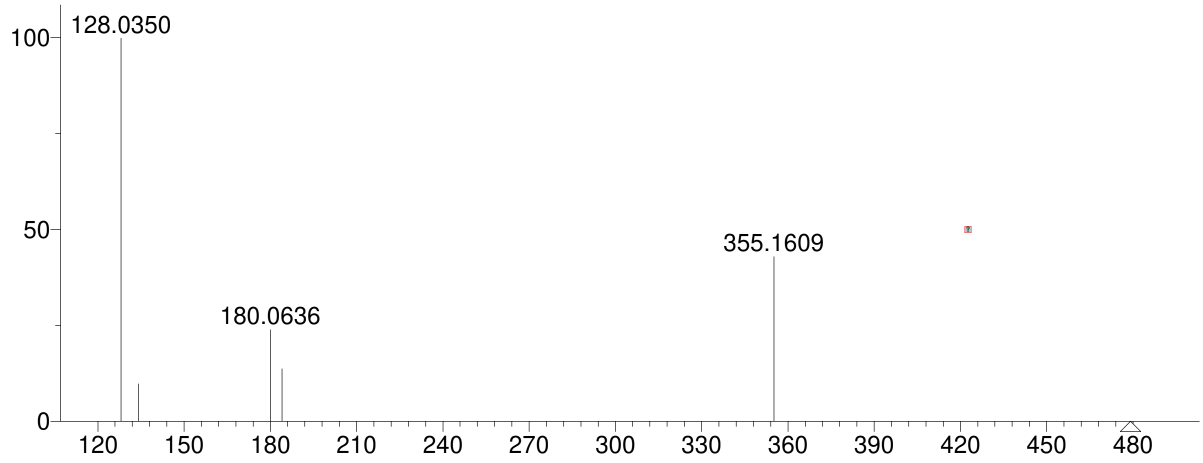

Hit 1 : β-Carboline-1-propionic acid [2M-H]- HCD 62V P=479.2
C14H12N2O2; MF: 10; RMF: 266; Prob 32.1%; CAS: 89915-39-9; Lib: nist_msms; ID: 217004.

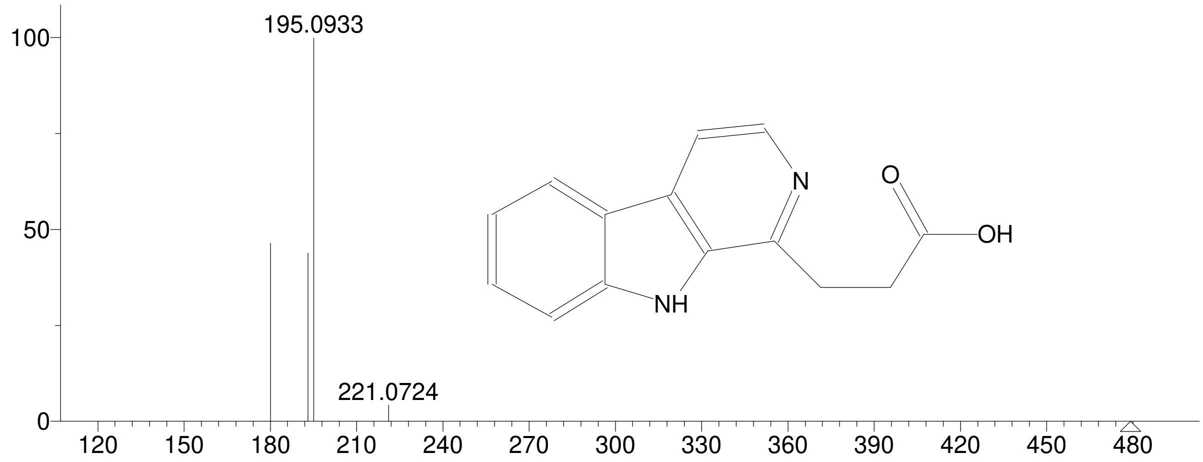

Hit 2 : 2-[3,4-dihydroxy-2,2-dimethyl-8-(3-methylbut-2-enoxy)-3,4-dihydrochromen-6-yl]-5,7-dihydroxy-2,3-dihydro
C25H28O8; MF: 0; RMF: 16; Prob 22.6%; Lib: mona-export-vf-npl_qexactive; ID: 16870.

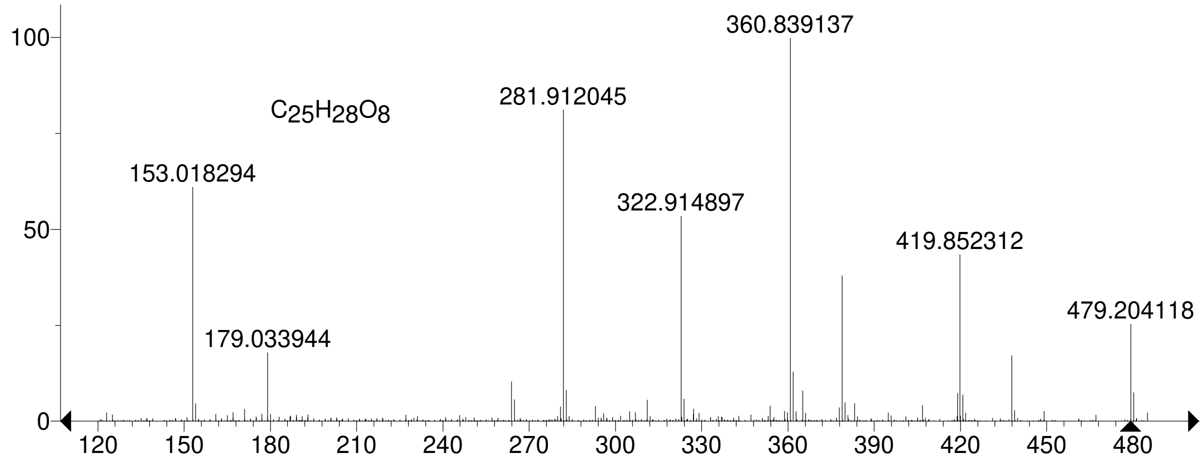

Hit 3 : 7-hydroxy-2,6,9,12,17,17-hexamethyl-22-methylidene-5,16,19,21-tetraoxaheptacyclo[10.8.1.1A^'3,a^'1.0A'
C25H28O8; MF: 0; RMF: 10; Prob 22.6%; Lib: mona-export-vf-npl_qexactive; ID: 20713.

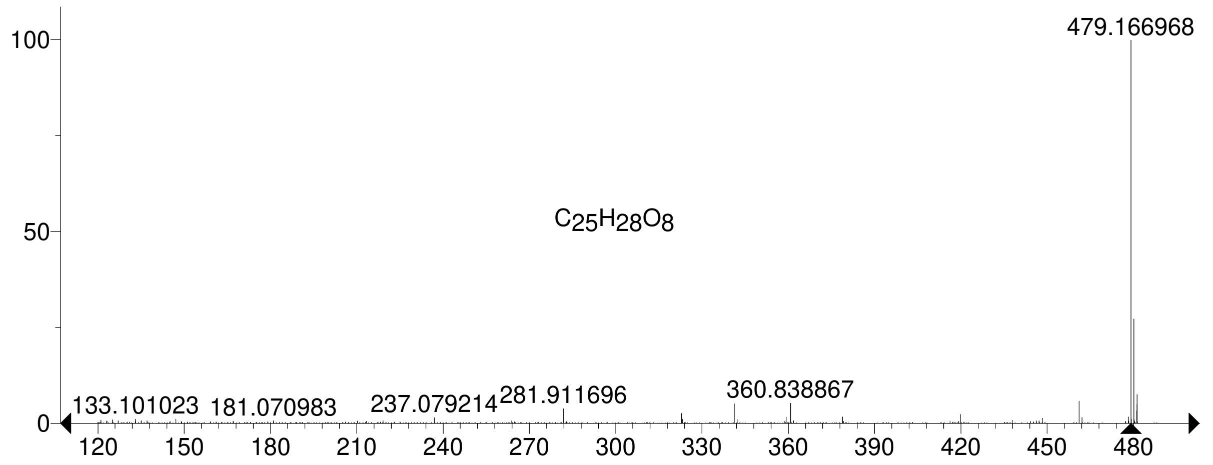

Hit 4 : Chrysomycin B [M+H-H2O]+ HCD 124V P=479.2
C27H28O9; MF: 0; RMF: 10; Prob 22.6%; CAS: 83852-56-6; Lib: nist_msms; ID: 379206.

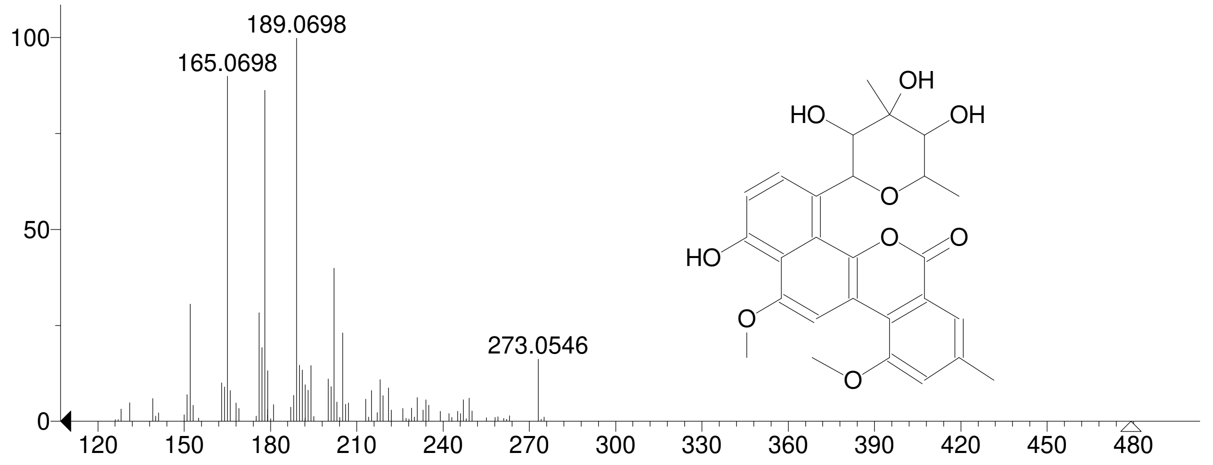

Unknown: Unknown (6.67_498.2194m/z)
Compound in Library Factor = 0

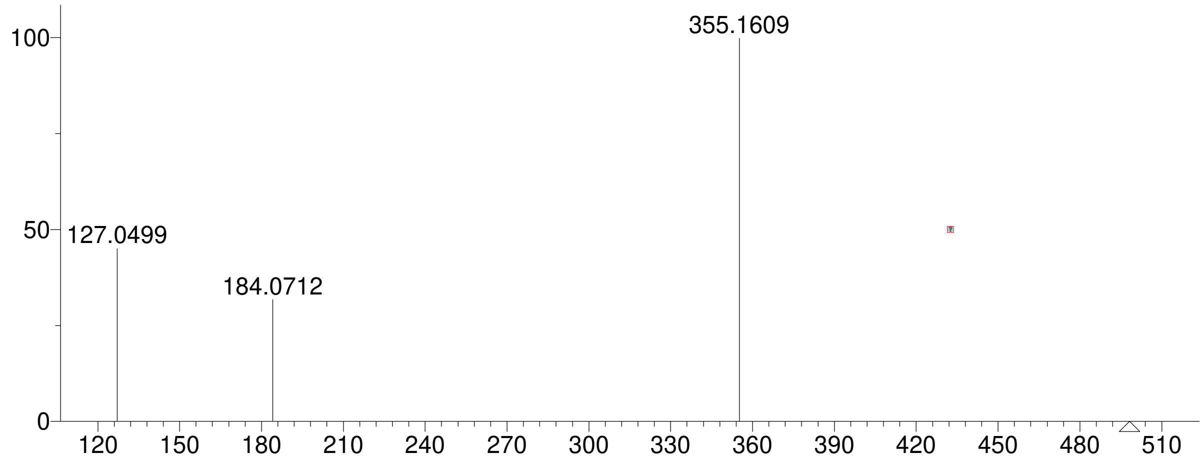

Unknown: Unknown (6.69_402.1175m/z)
Compound in Library Factor = 0

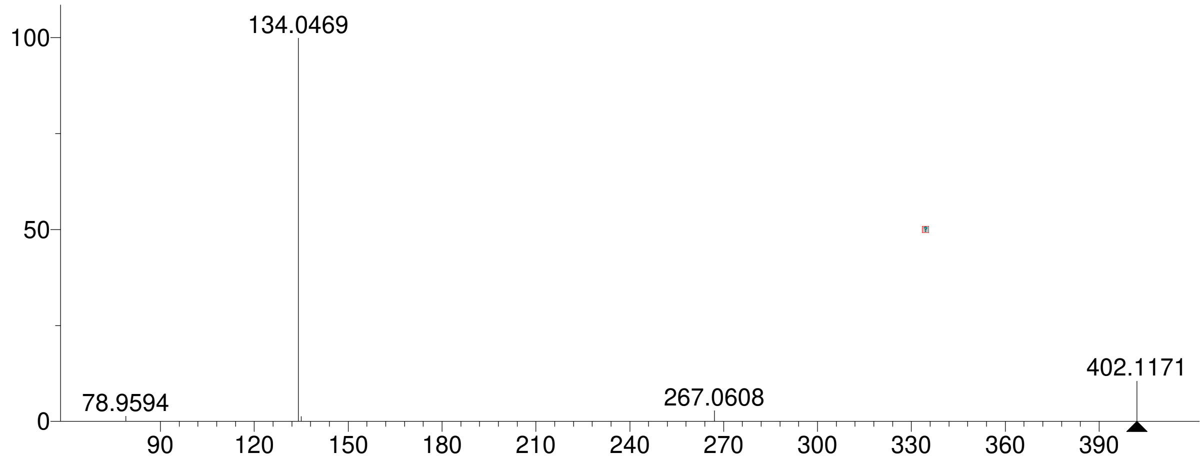

Unknown: Unknown (6.69_402.1756m/z)
Compound in Library Factor = 0

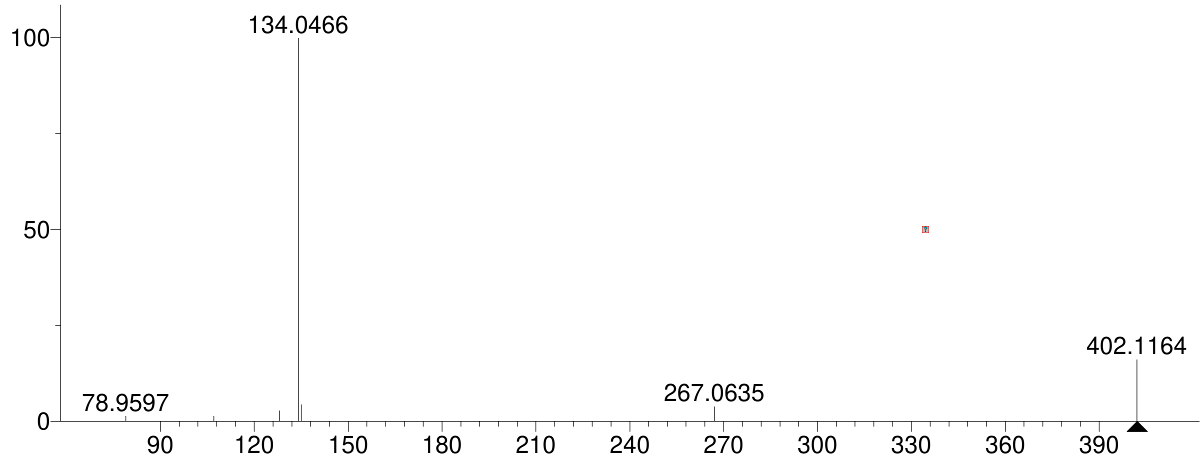

Hit 1 : Fumitremorgin C [M+Na]+ IT 35% P=402.2
C22H25N3O3; MF: 0; RMF: 68; Prob 100.0%; CAS: 118974-02-0; Lib: nist_msms; ID: 362872.

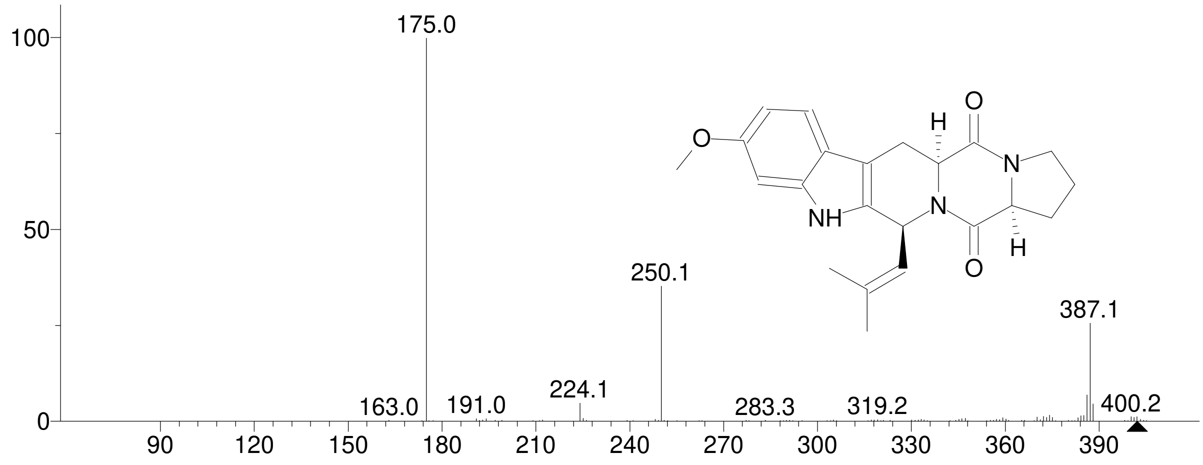

Unknown: Unknown (6.71_242.9953m/z)
Compound in Library Factor = 0

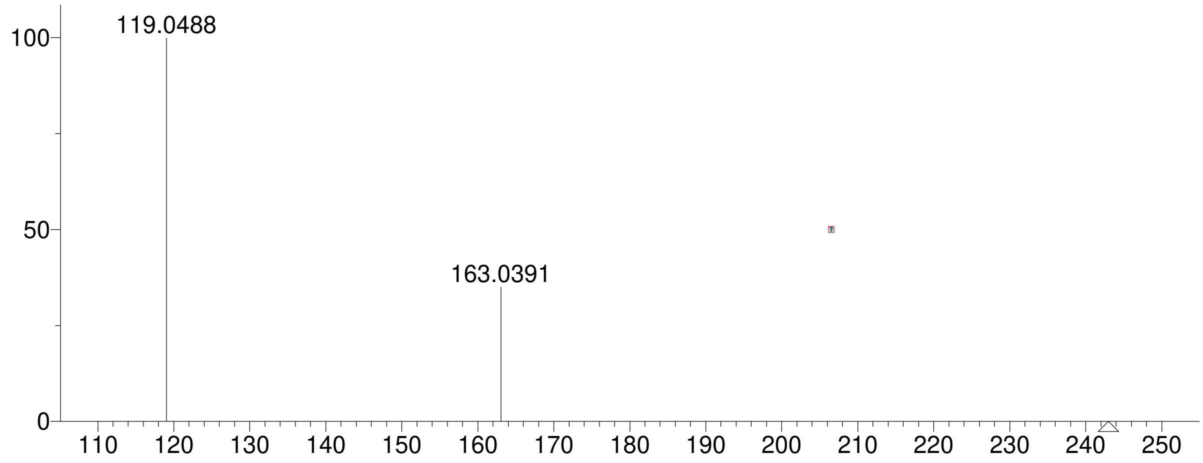

** Search Report Page 1 of 1 **

Unknown: Unknown (6.71_842.4260m/z)
Compound in Library Factor = -3273

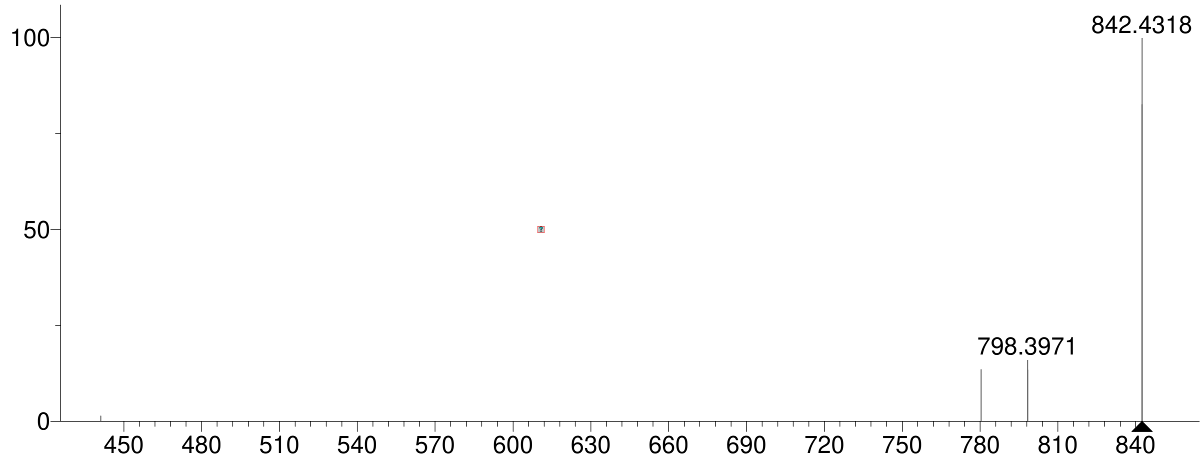

Hit 1 : L-Glutamic acid, L-methionyl-L-alanylglycyl-L-prolyl-L-histidyl-L-prolyl-L-valyl-L-isoleucyl-L-valyl-L-isoleucyl
C75H118N20O22S; MF: 7; RMF: 437; Prob 100.0%; CAS: 249537-73-3; Lib: nist_msms; ID: 265777.

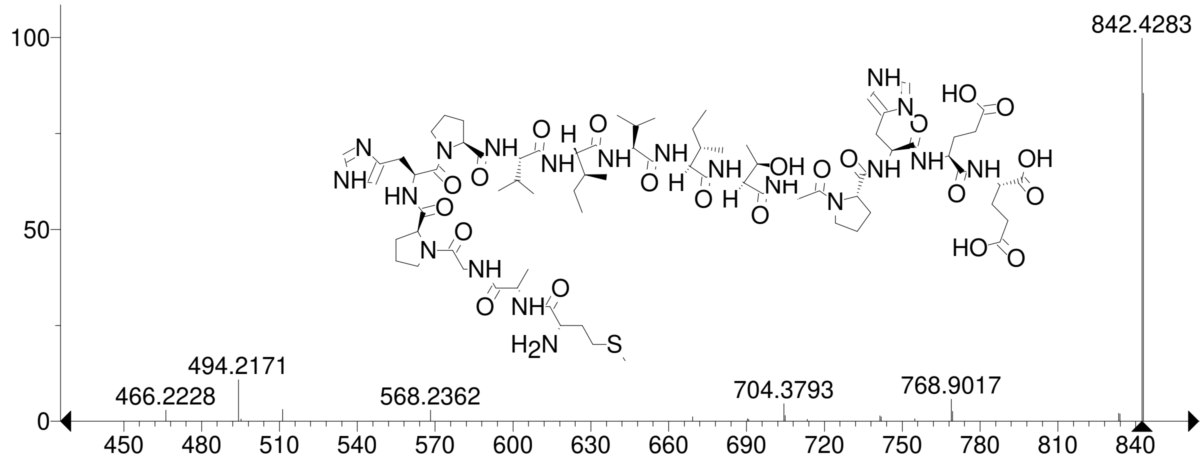

Unknown: Unknown (6.73_329.0861m/z)
Compound in Library Factor = 0

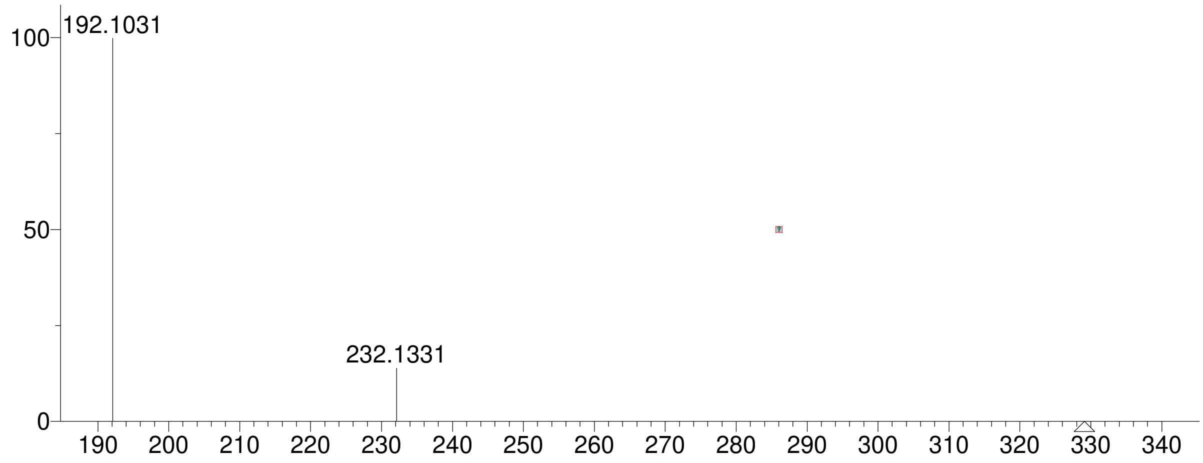

Unknown: Unknown (6.73_338.1236m/z)
Compound in Library Factor = 0

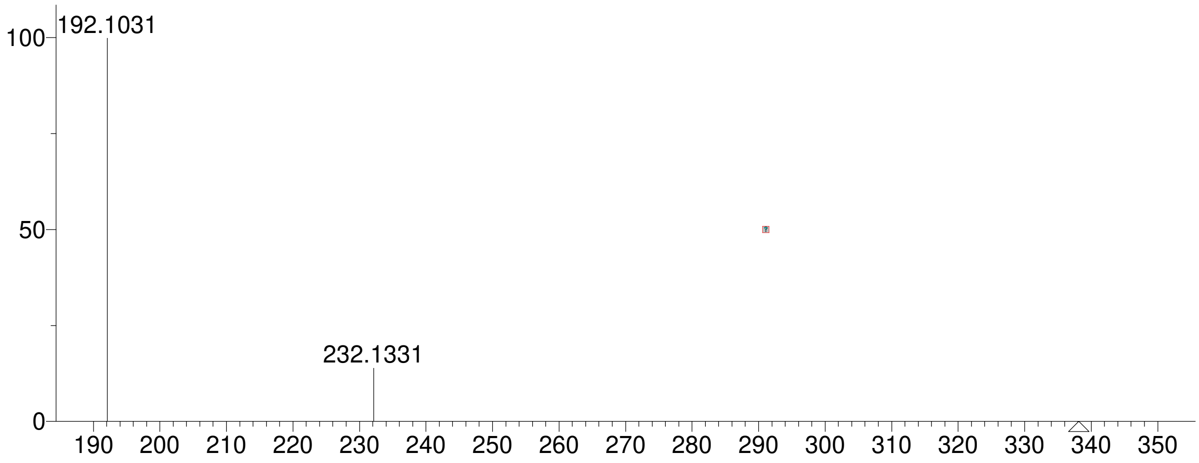

Hit 1 : barceloneic acid A [M+NH4]+ 35HCD P=338.1
C16H16O7; MF: 0; RMF: 20; Prob 50.0%; Lib: mona-export-vf-npl_qexactive; ID: 6133.

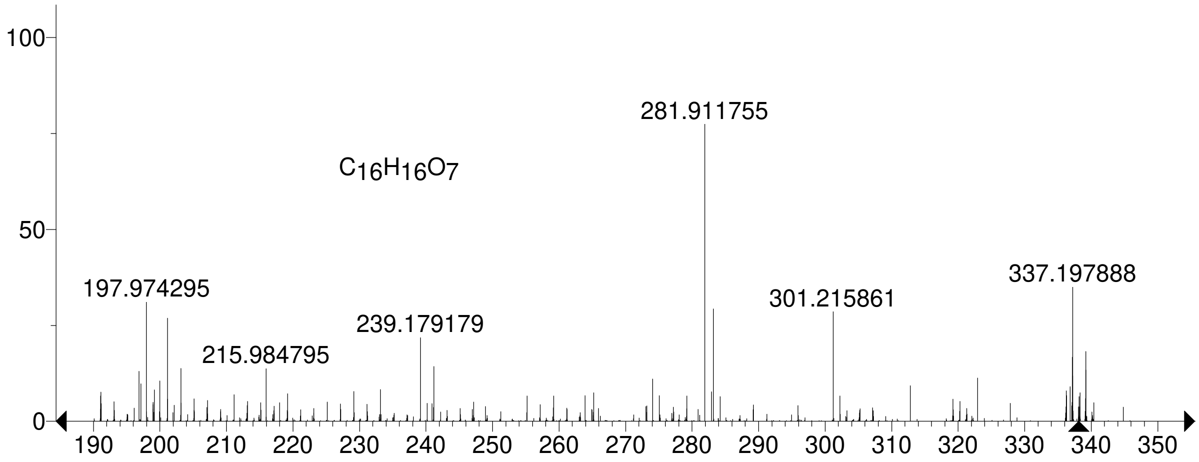

Hit 2 : Met-Gly-Met [M+H]+ QQQ 14V P=338.1
C12H23N3O4S2; MF: 0; RMF: 9; Prob 50.0%; Lib: nist_msms; ID: 9505.

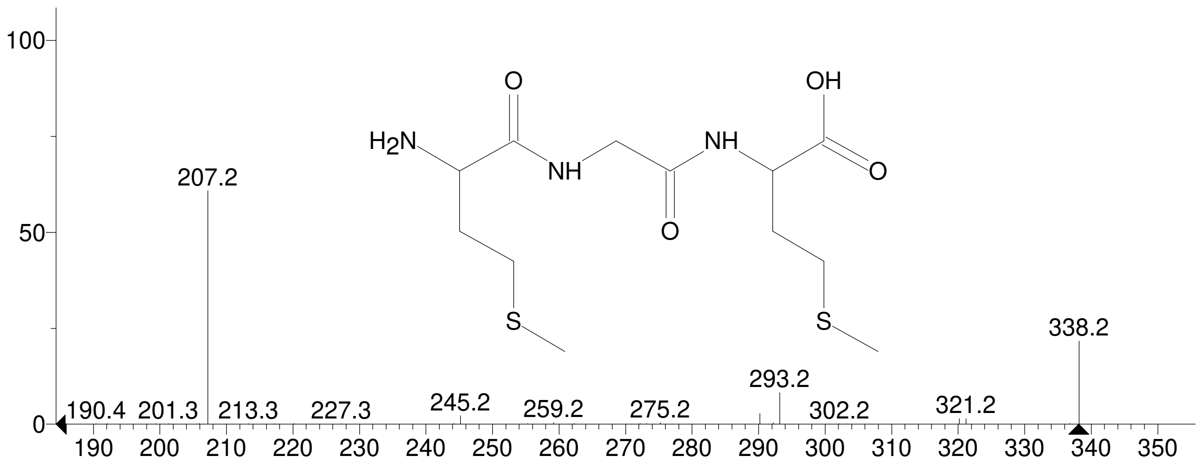

** Search Report Page 1 of 1 **

Unknown: Unknown (6.73_341.1229m/z)
Compound in Library Factor = 0

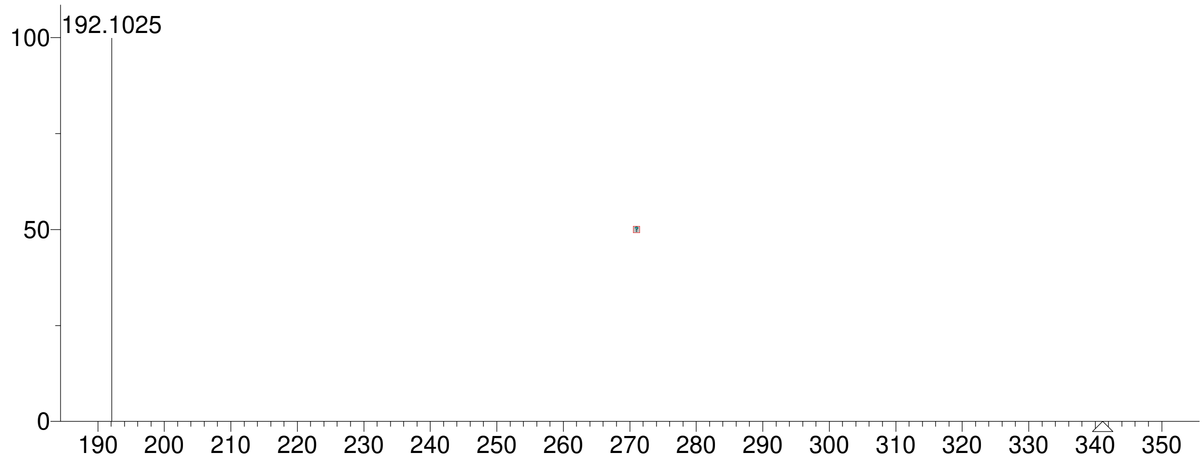

Unknown: Unknown (6.75_263.1391m/z)
Compound in Library Factor = -201

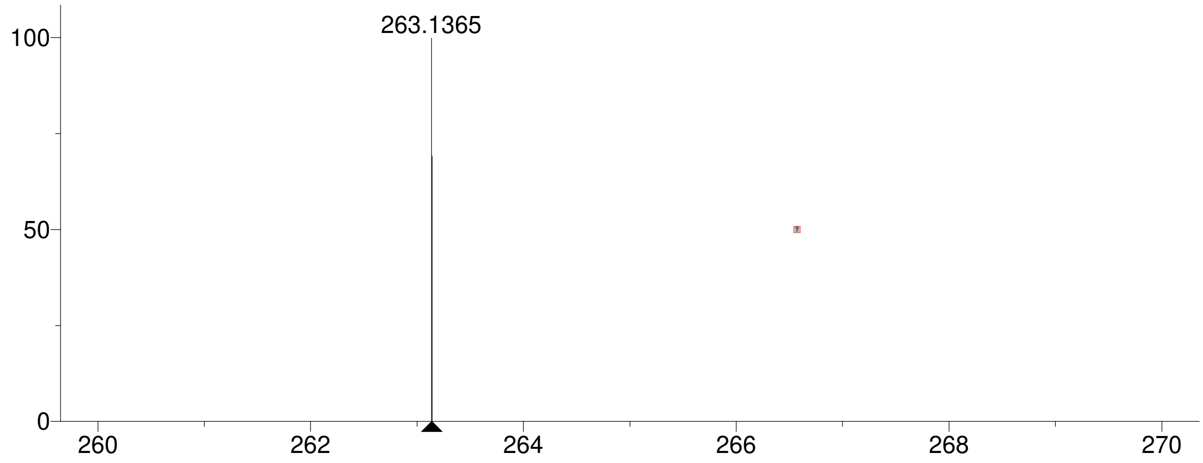

Hit 1 : Unknown (carbon number 14); PlaSMA ID-436 [M-H]- P=263.1
C14H20N2O3; MF: 470; RMF: 872; Prob 90.9%; Lib: biomsms-neg-plasma; ID: 1129.

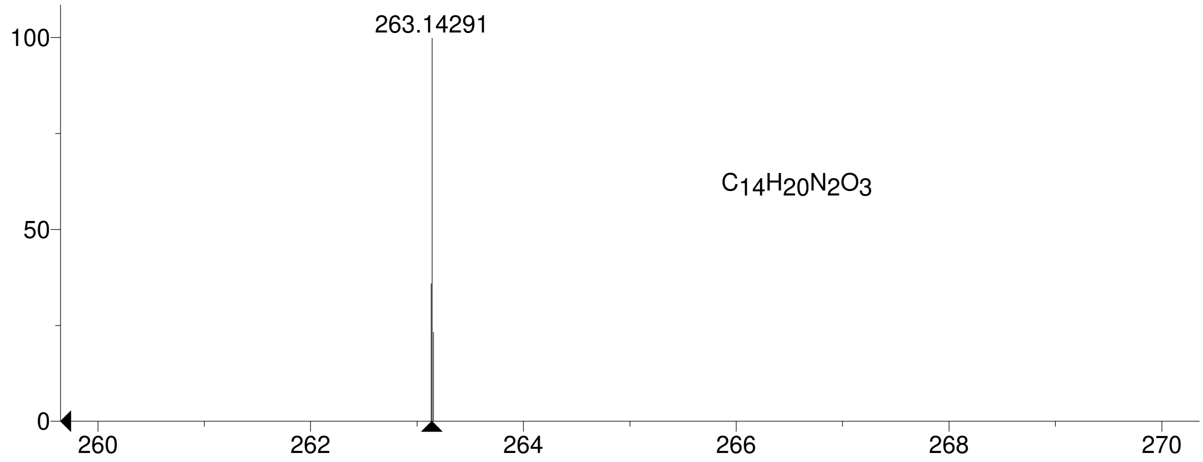

Hit 2 : Methohexital [M+H]+ 20-30-40%) P=263.1
C14H18N2O3; MF: 262; RMF: 768; Prob 0.90%; Lib: mona-export-fiehn_hilic; ID: 520.

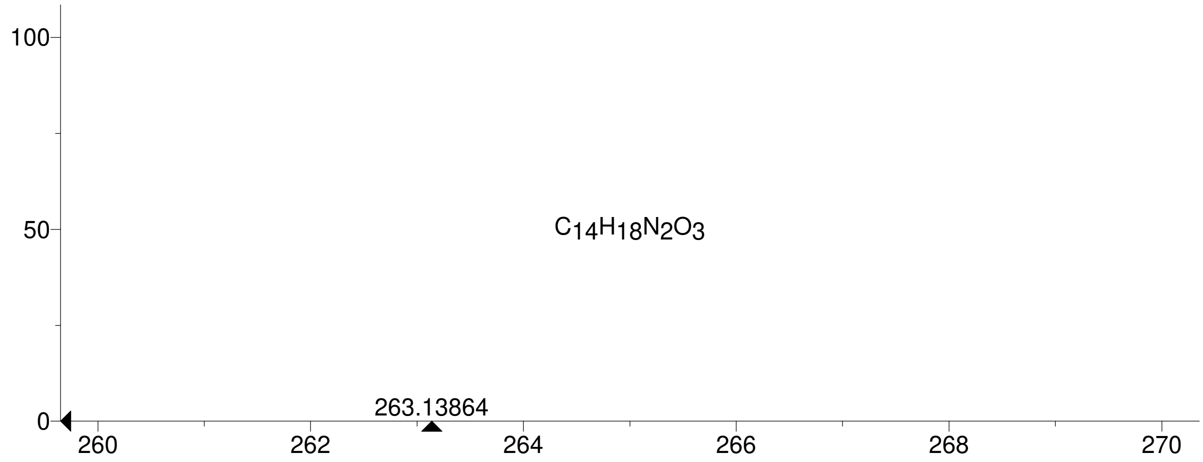

Hit 3 : Phe-Pro [M+H]+ QTOF 4V P=263.1
C14H18N2O3; MF: 262; RMF: 768; Prob 0.90%; Lib: nist_msms; ID: 47950.

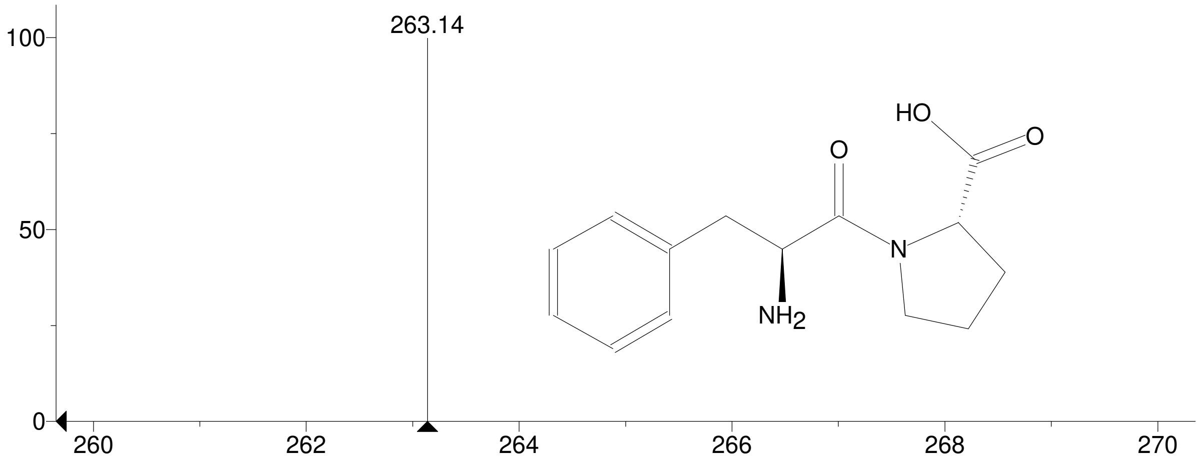

Hit 4 : Pro-Phe [M+H]+ QTOF 4V P=263.1
C14H18N2O3; MF: 262; RMF: 768; Prob 0.90%; Lib: nist_msms; ID: 48686.

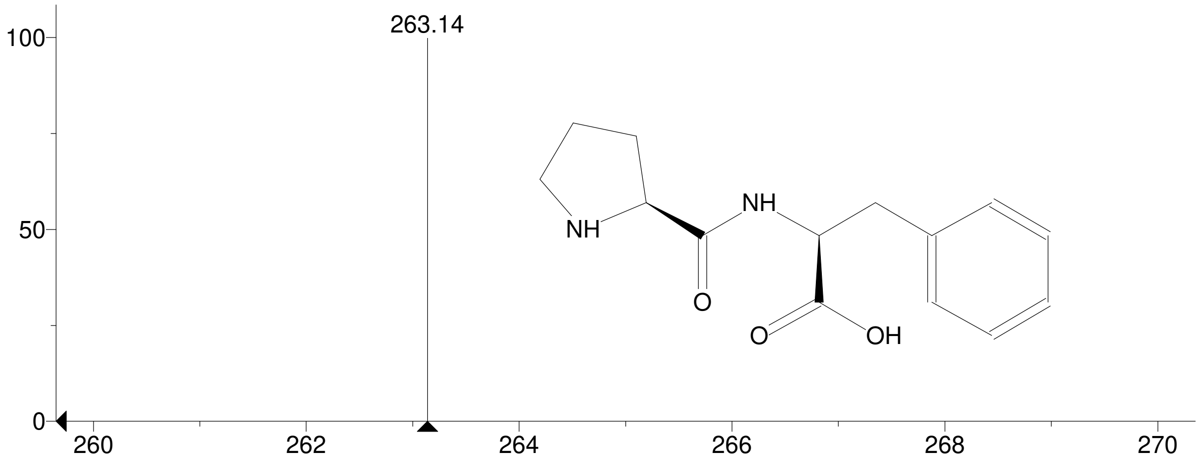

Hit 5 : Suberoylanilide hydroxamic acid [M-H]- HCD 10V P=263.1
C14H20N2O3; MF: 262; RMF: 768; Prob 0.90%; CAS: 149647-78-9; Lib: nist_msms; ID: 159154.

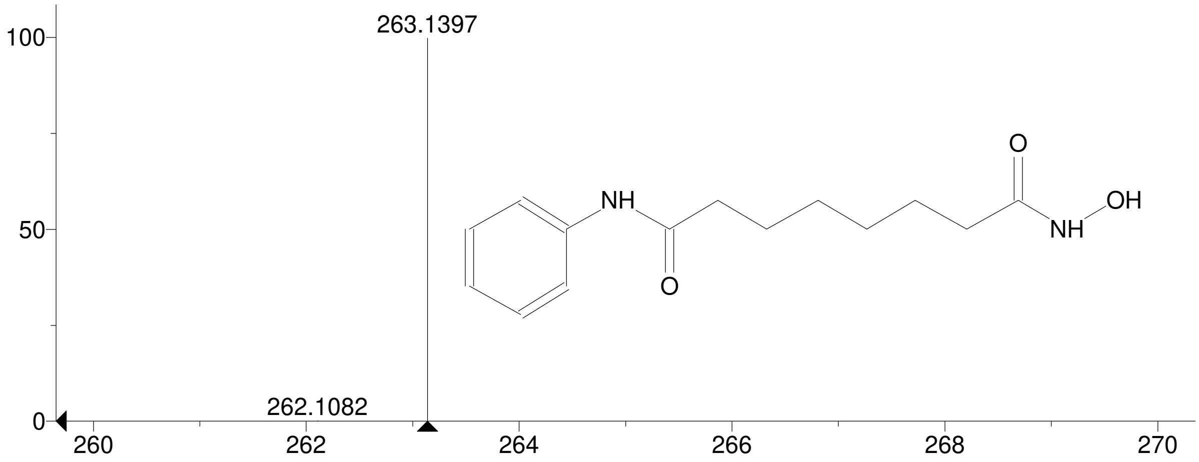

Unknown: Unknown (6.75_589.2713m/z)
Compound in Library Factor = 0

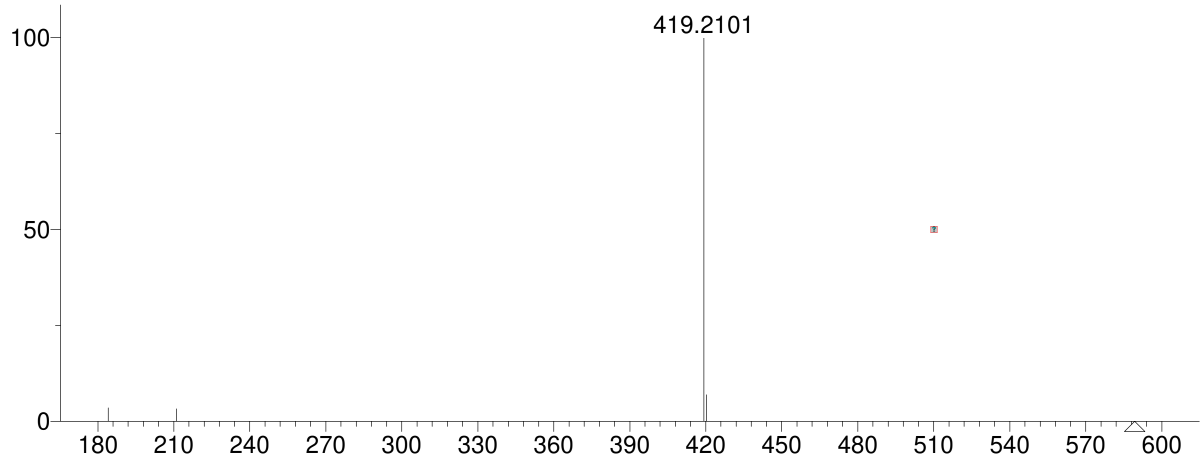

Unknown: Unknown (6.77_462.1622m/z)
Compound in Library Factor = 0

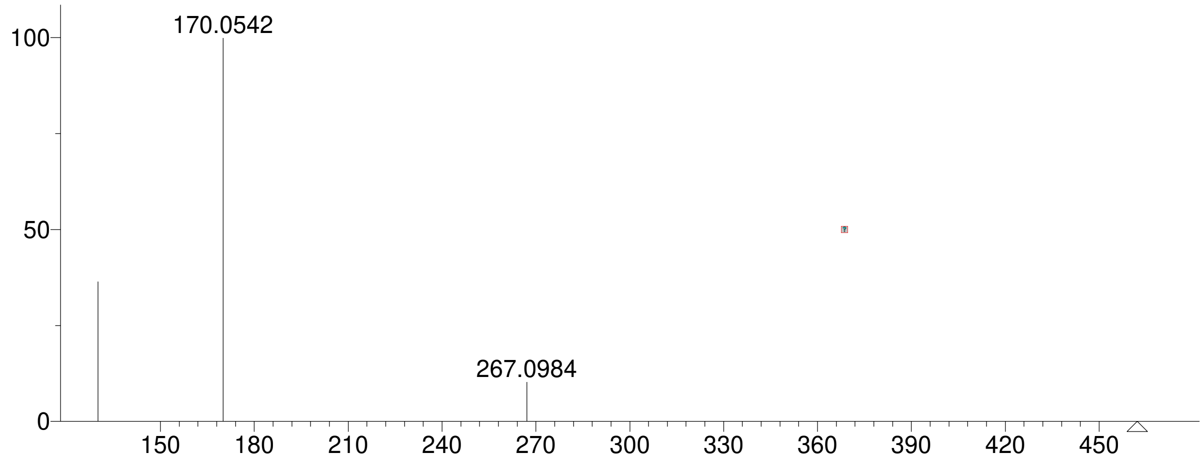

Hit 1 : 4-(2-Nitroethyl)phenyl 6-O-I^'2-D-xylopyranosyl-I^'2-D-glucopyranoside [M+H]+ 35HCD P=462.2
C19H27NO12; MF: 0; RMF: 1; Prob 100.0%; Lib: mona-export-vf-npl_qexactive; ID: 21502.

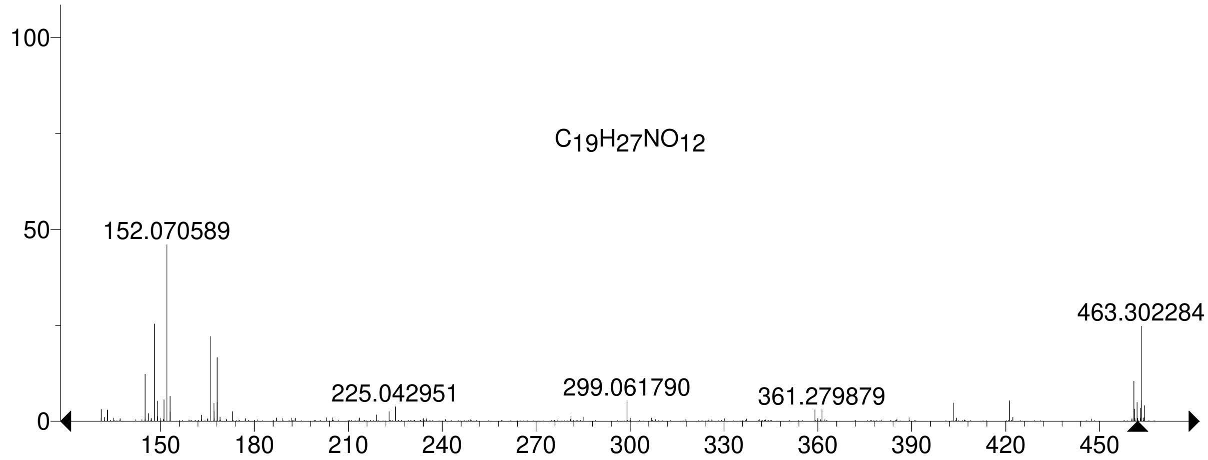

Unknown: Unknown (6.80_241.1183m/z)
Compound in Library Factor = -3273

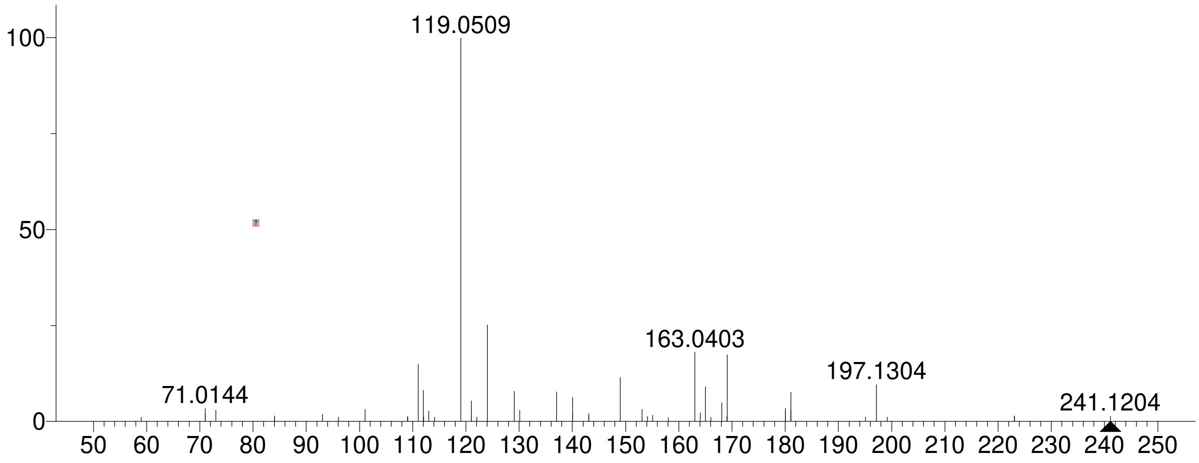

Hit 1 : Pyroglutamyl-Isoleucine; PlaSMA ID-369 [M-H]- P=241.1
C11H18N2O4; MF: 8; RMF: 118; Prob 30.9%; Lib: biomsms-neg-plasma; ID: 400.

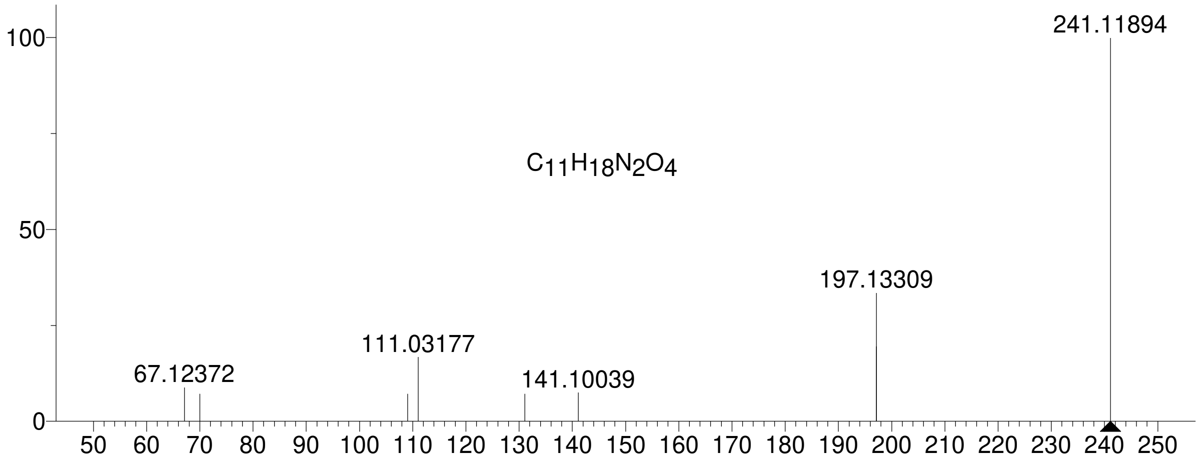

Hit 2 : Meprobamate [M+Na]+ HCD 19V P=241.1
C9H18N2O4; MF: 3; RMF: 66; Prob 24.9%; CAS: 57-53-4; Lib: nist_msms; ID: 196308.

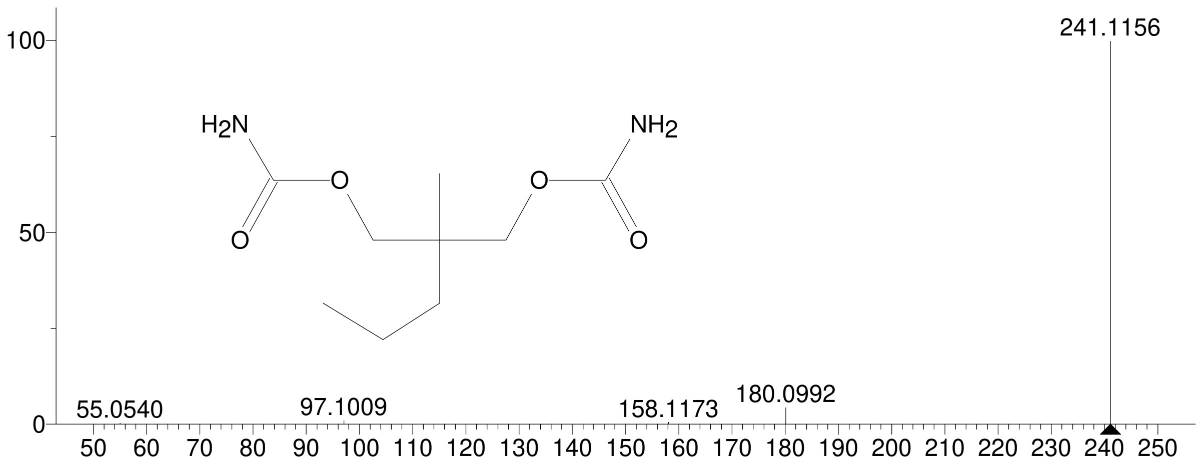

Hit 3 : Dictazoline E [M+H]+ P=241.1
C27H26N8O2; MF: 0; RMF: 37; Prob 22.0%; Lib: mona-export-gnps; ID: 4845.

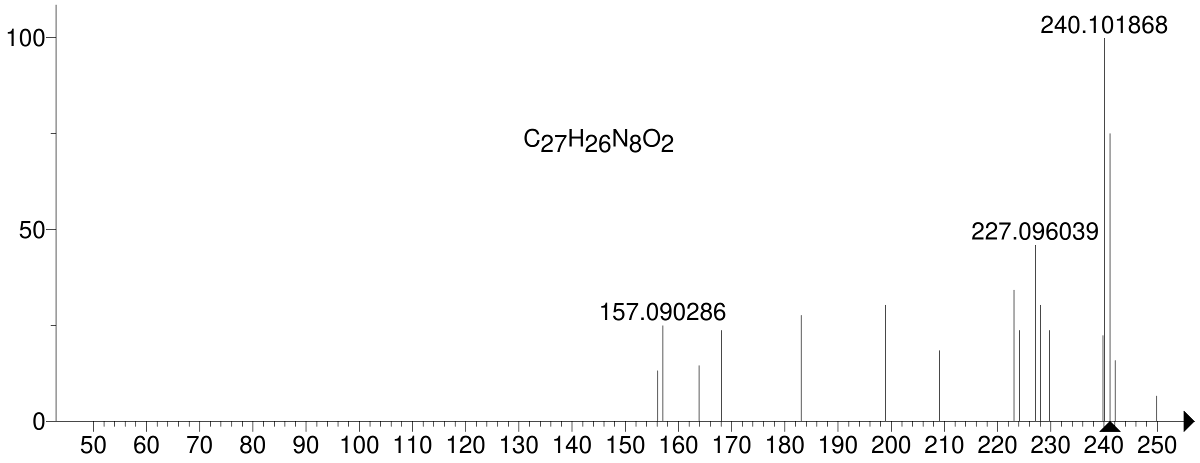

Hit 4 : Ser-Leu [M+Na]+ IT 35% P=241.1
C9H18N2O4; MF: 0; RMF: 2; Prob 22.0%; CAS: 6665-16-3; Lib: nist_msms; ID: 45588.

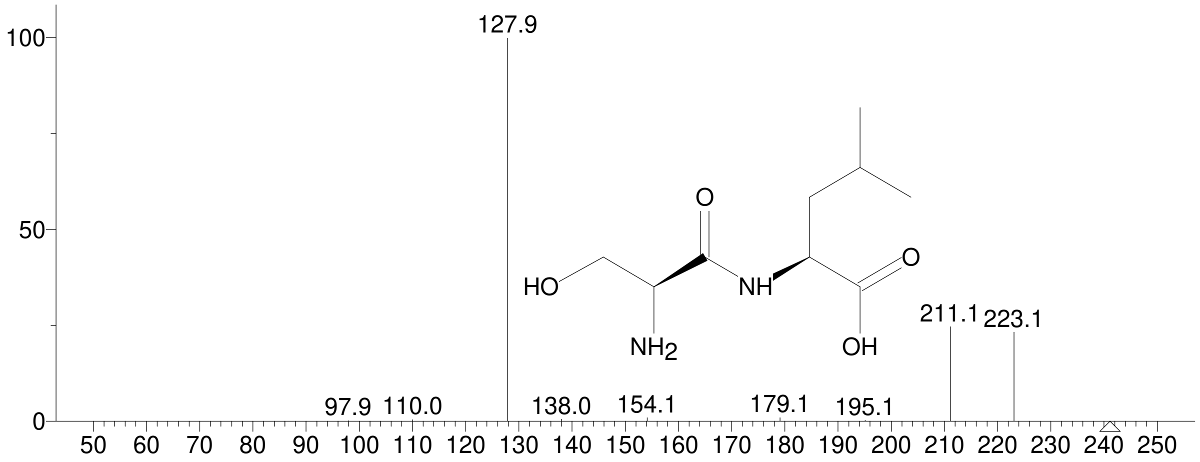

** Search Report Page 1 of 1 **

Unknown: Unknown (6.83_298.1394m/z)
Compound in Library Factor = 0

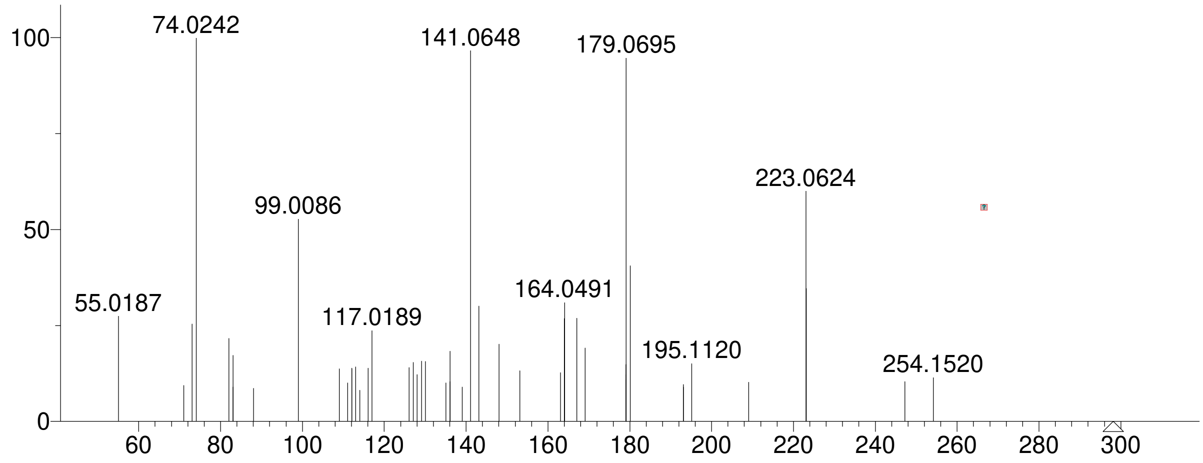

Hit 1 : Noratropine [M+Na]+ IT 35% P=298.1
C16H21NO3; MF: 0; RMF: 7; Prob 100.0%; CAS: 16839-98-8; Lib: nist_msms; ID: 197153.

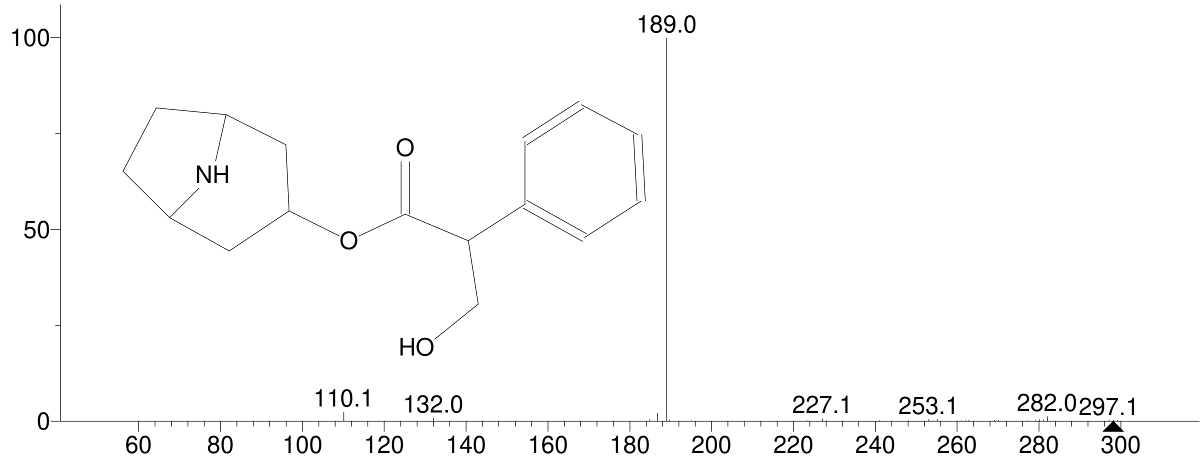

Unknown: Unknown (6.83_756.1864m/z)
Compound in Library Factor = 0

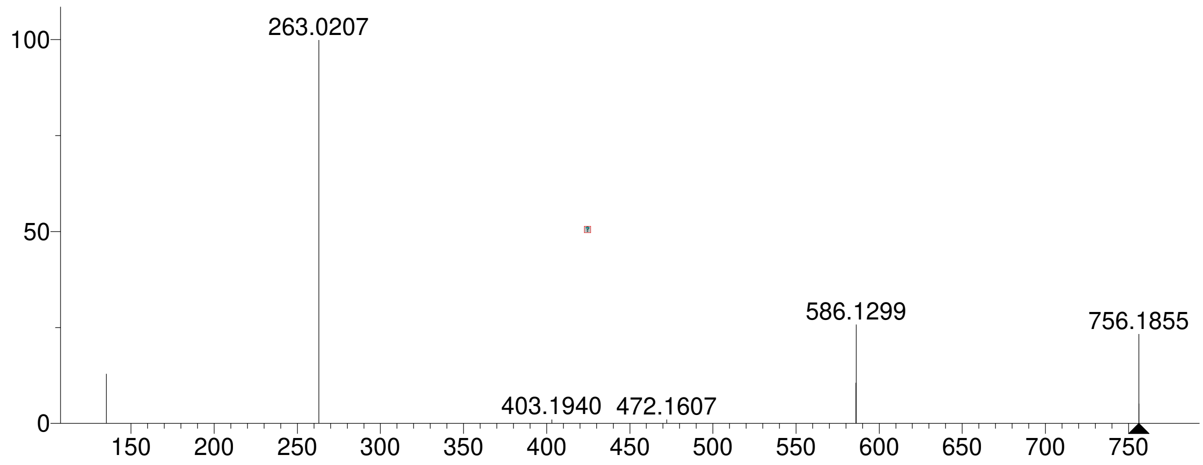

Unknown: Unknown (6.84_263.0221m/z)
Compound in Library Factor = 0

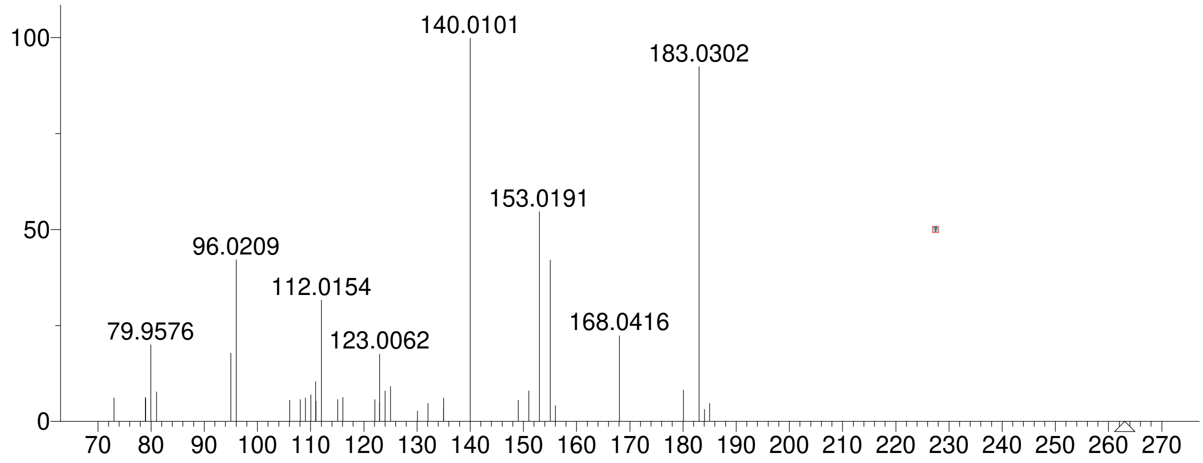

Hit 1 : 3-Methoxy-4-hydroxyphenylglycol sulfate [M-H]- QTOF 20V P=263
C9H12O7S; MF: 0; RMF: 44; Prob 100.0%; CAS: 3415-67-6; Lib: nist_msms; ID: 29981.

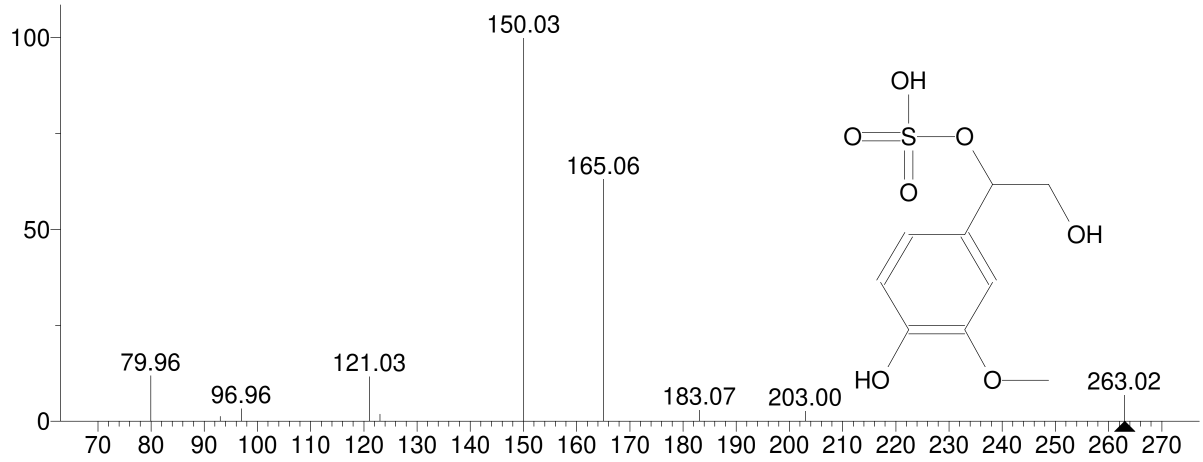

** Search Report Page 1 of 1 **

Unknown: Unknown (6.84_453.1503m/z)
Compound in Library Factor = 0

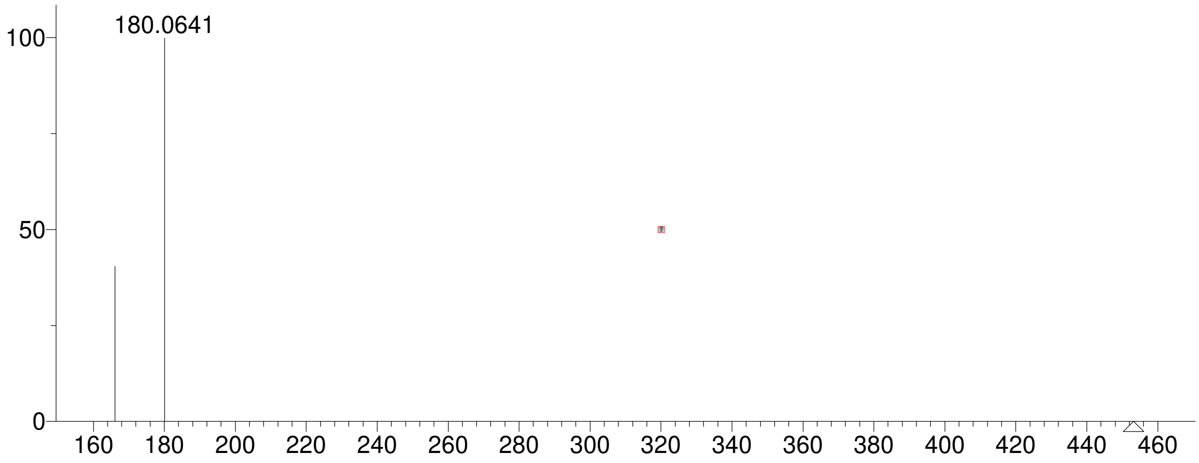

Unknown: Unknown (6.84_642.2164m/z)
Compound in Library Factor = 0

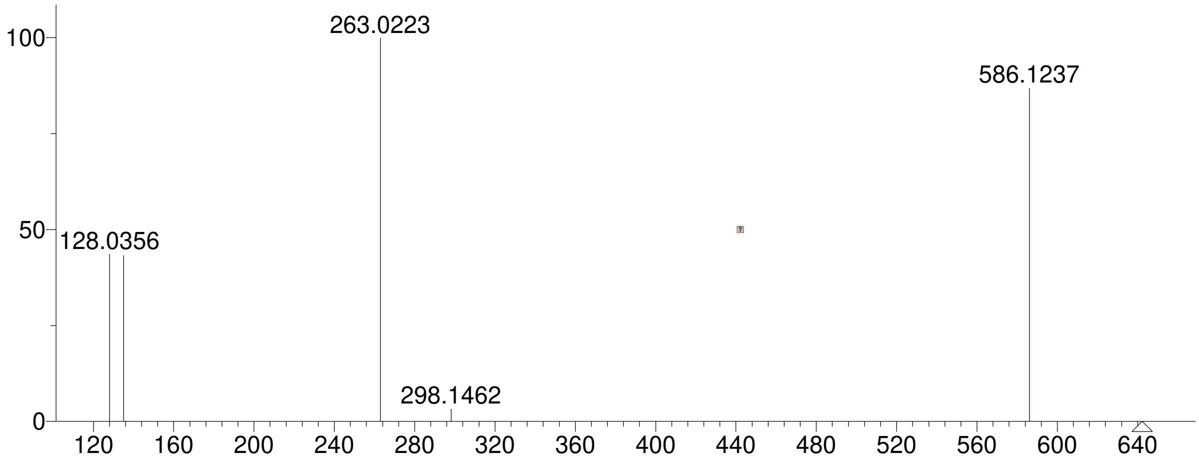

** Search Report Page 1 of 1 **

Unknown: Unknown (6.85_408.1762m/z)
Compound in Library Factor = 0

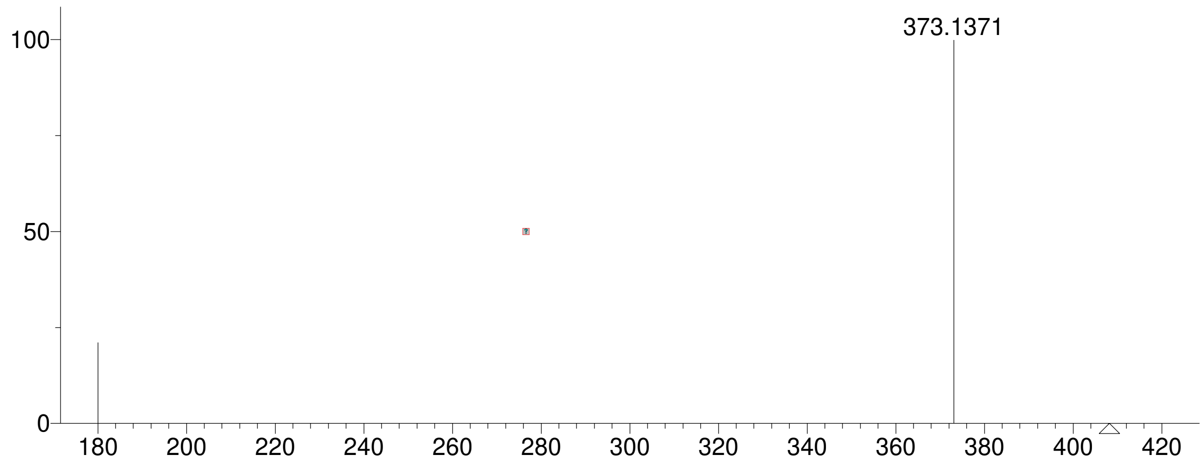

Hit 1 : Trifluoperazine [M+H]+ 35eV P=408.2
C21H24F3N3S; MF: 0; RMF: 33; Prob 100.0%; Lib: mona-export-fiehn_hilic; ID: 2383.

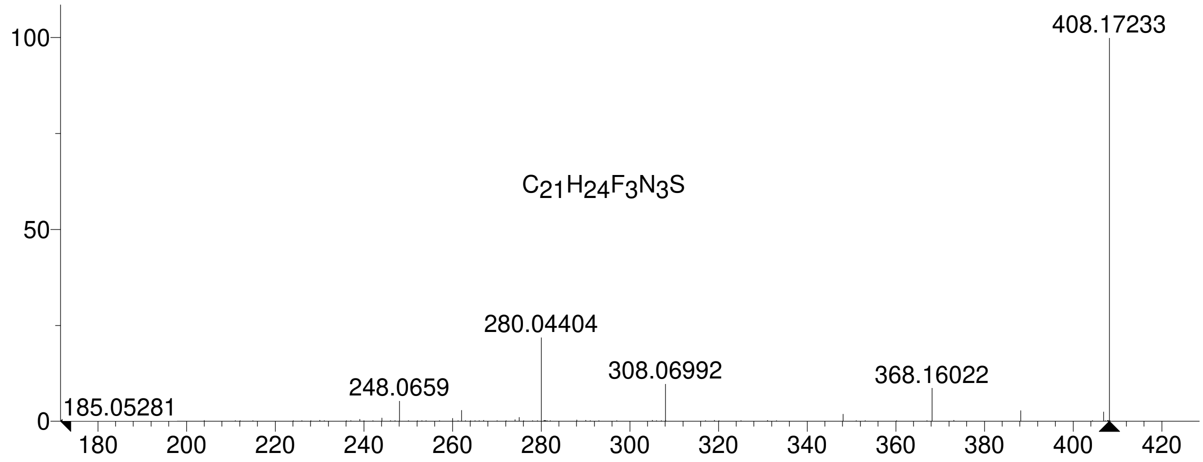

Unknown: Unknown (6.86_358.1972m/z)
Compound in Library Factor = -1523

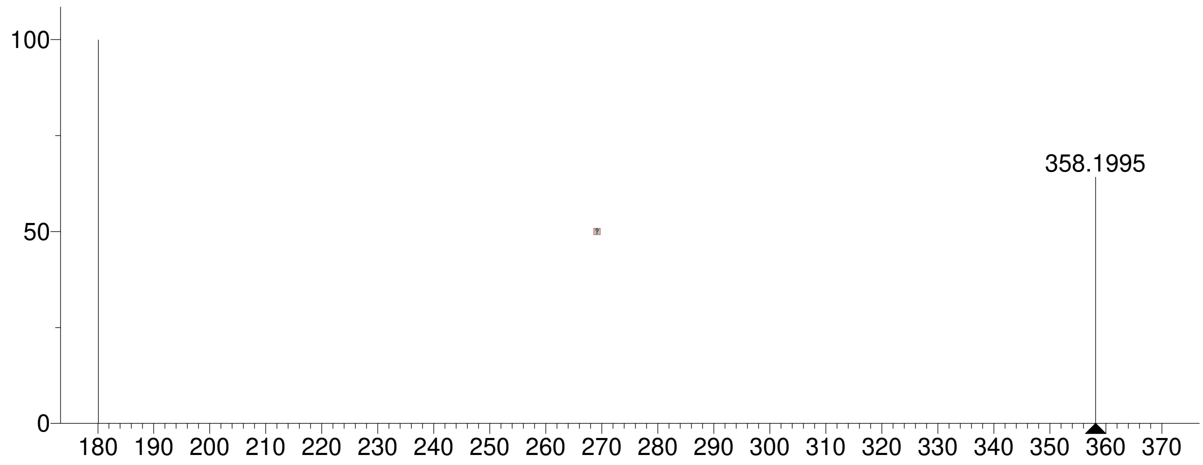

Hit 1 : 4-Hydroxyatorvastatin lactone [M-H-H2O]-=>493.2=>358.2 IT 35% P=358.2
C33H33FN2O5; MF: 58; RMF: 579; Prob 39.4%; CAS: 163217-70-7; Lib: nist_msms; ID: 440854.

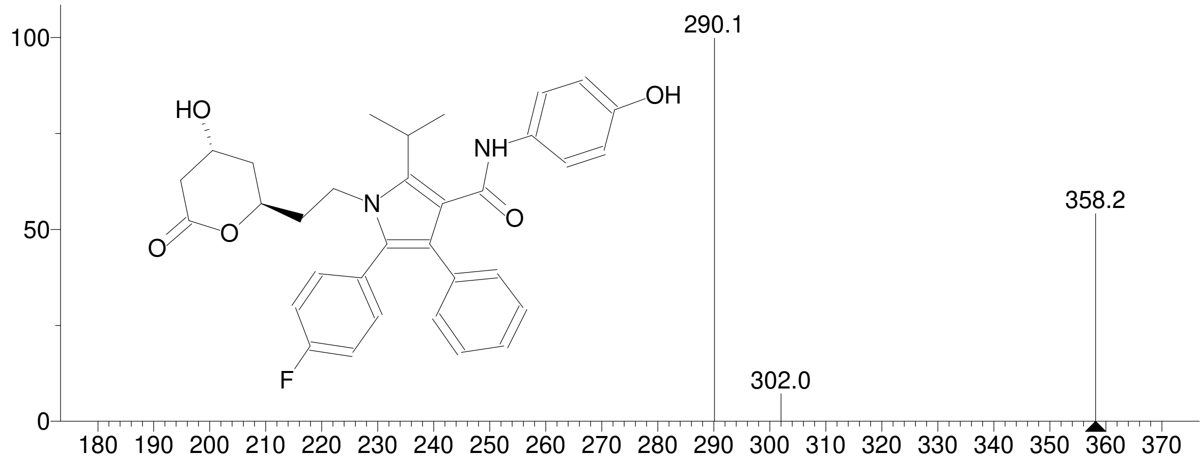

Hit 2 : 1H-Indole-3,5-dicarboxylic acid, 1-[3-[4-(decyloxy)phenoxy]-2-oxopropyl]-, 3-methyl ester [M-H]-=>358.2 I
C30H37NO7; MF: 15; RMF: 456; Prob 8.69%; CAS: 888320-29-4; Lib: nist_msms; ID: 374612.

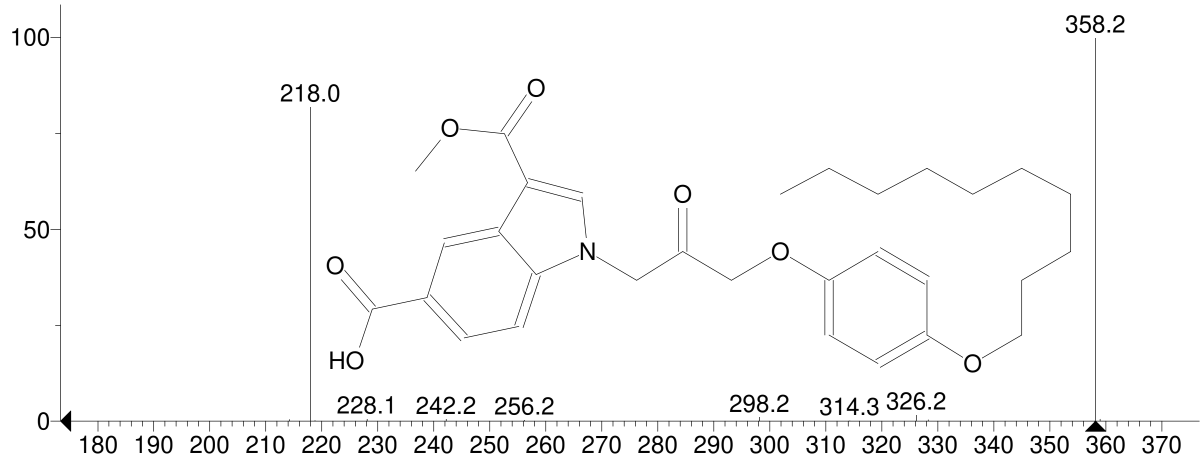

Hit 3 : Nalbuphine [M+H]+ 20-30-40%) P=358.2
C21H27NO4; MF: 6; RMF: 410; Prob 6.31%; Lib: mona-export-fiehn_hilic; ID: 599.

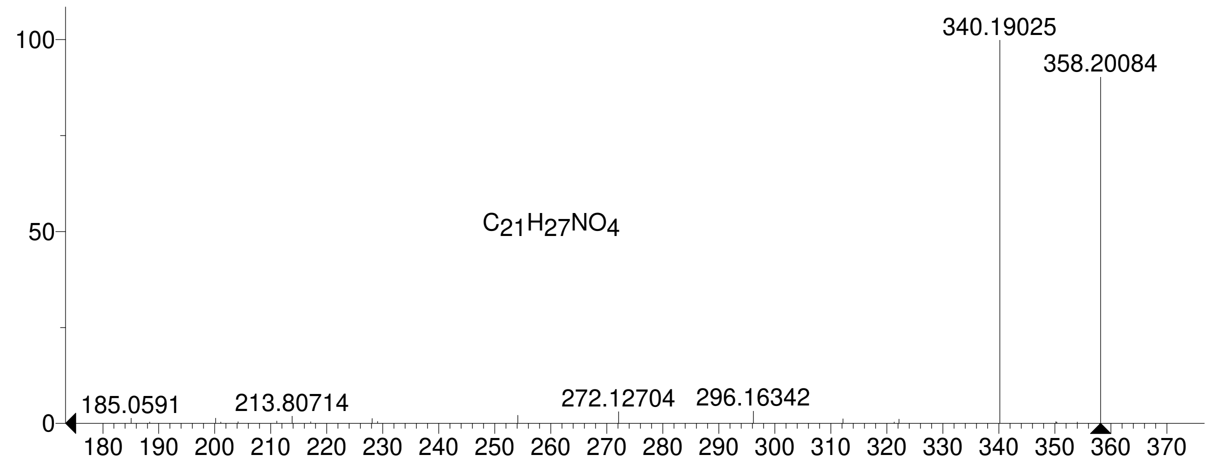

Hit 4 : 1H-Indole-3-carboxamide, 1-(5-fluoropentyl)-N-8-quinolinyl- [M+H]+=>358.2 IT 35% P=358.2
C23H22FN3O; MF: 4; RMF: 320; Prob 5.82%; Lib: nist_msms; ID: 344362.

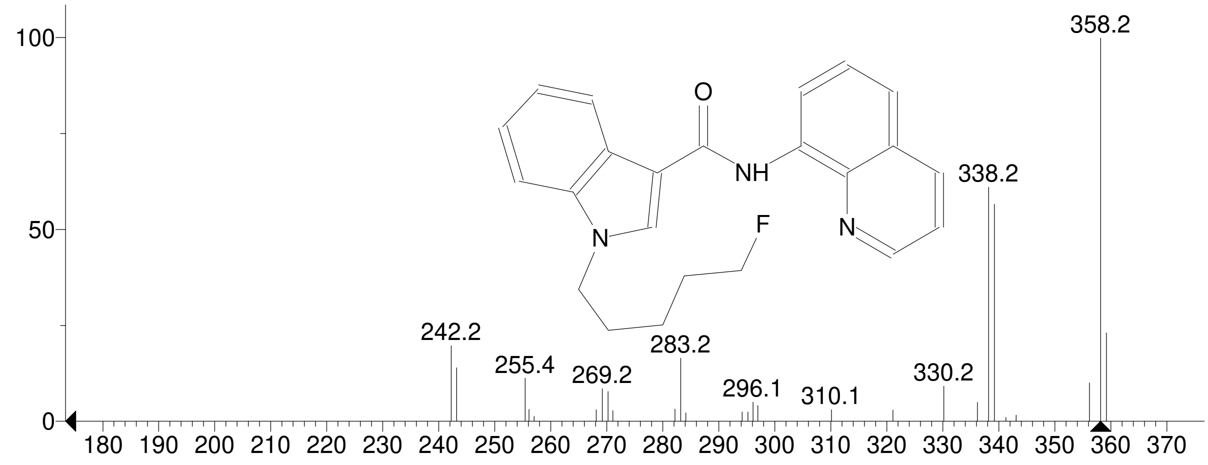

Hit 5 : Propanoic acid, 2-[[4-[2-[[(cyclohexylamino)carbonyl](4-cyclohexylbutyl)amino]ethyl]phenyl]thio]-2-methyl-
C29H46N2O3S; MF: 1; RMF: 241; Prob 5.14%; CAS: 265129-71-3; Lib: nist_msms; ID: 374428.

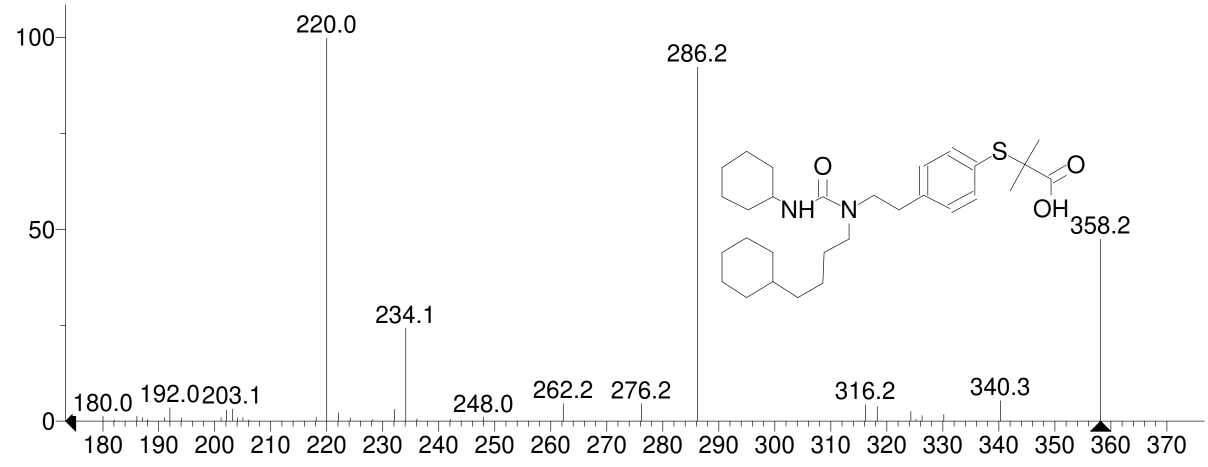

Unknown: Unknown (6.86_405.1643m/z)
Compound in Library Factor = 0

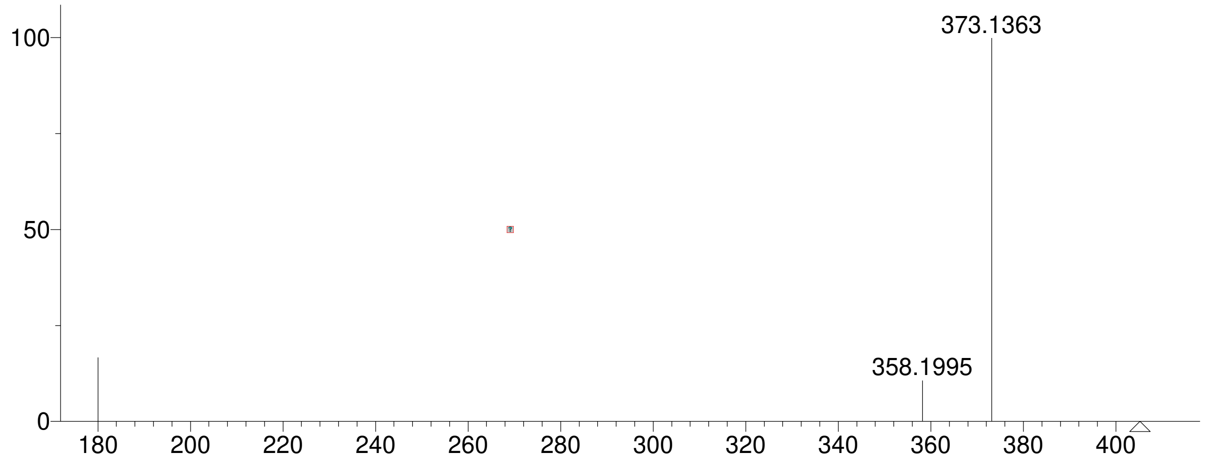

Hit 1 : NCGC00169052-03_C23H26O5_6H-Furo[2,3-h]-2-benzopyran-6,8(6aH)-dione, 9-acetyl-3-[(1E,3E,5S)-3,5
C23H26O5; MF: 0; RMF: 41; Prob 33.3%; Lib: mona-export-gnps; ID: 14072.

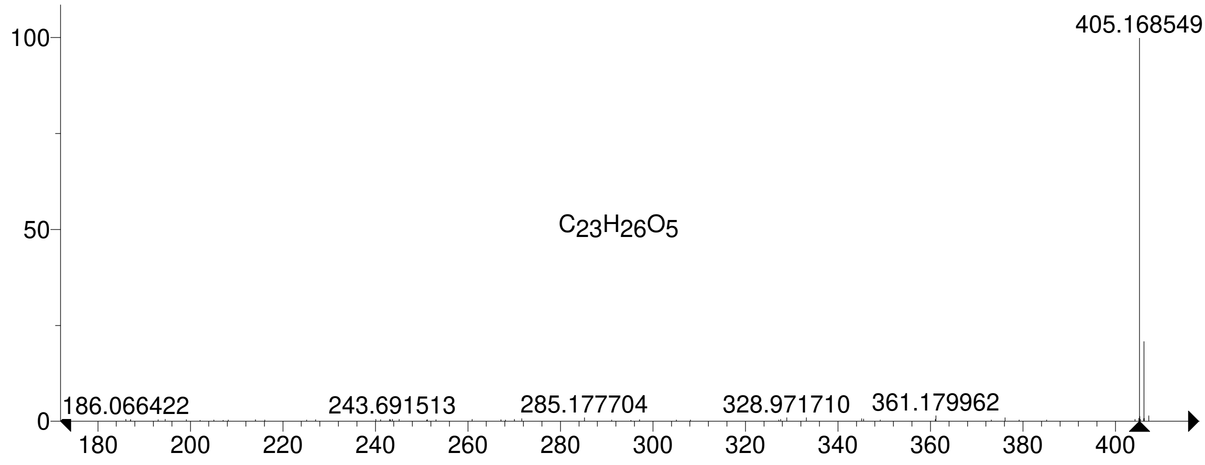

Hit 2 : Methanone, [1-(5-fluoropentyl)-6-nitro-1H-indol-3-yl]-1-naphthalenyl- [M+H]+ IT 35% P=405.2
C24H21FN2O3; MF: 0; RMF: 13; Prob 33.3%; CAS: 335161-27-8; Lib: nist_msms; ID: 422537.

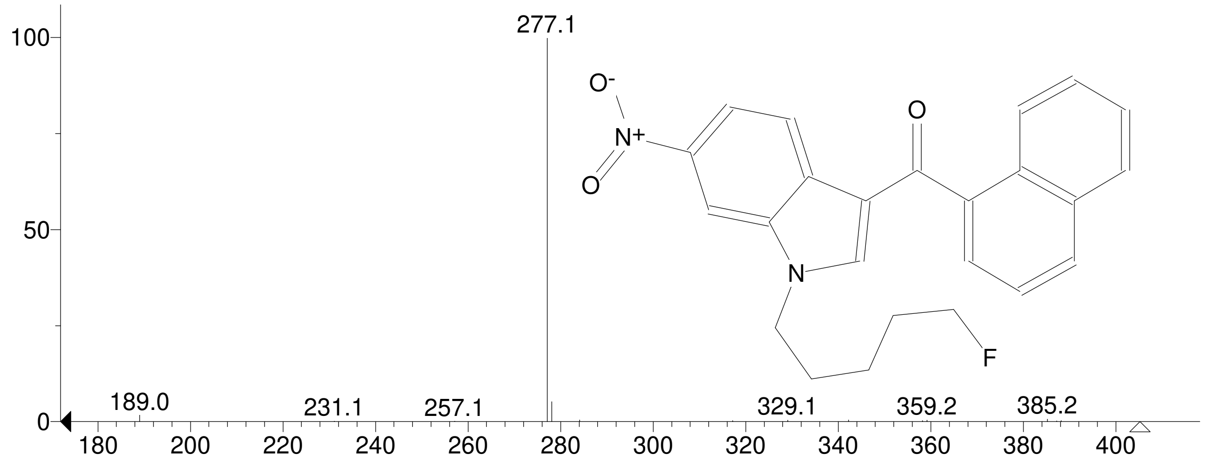

Hit 3 : Ochrephilone [M+Na]+ 35HCD P=405.2
C23H26O5; MF: 0; RMF: 5; Prob 33.3%; Lib: mona-export-vf-npl_qexactive; ID: 21688.

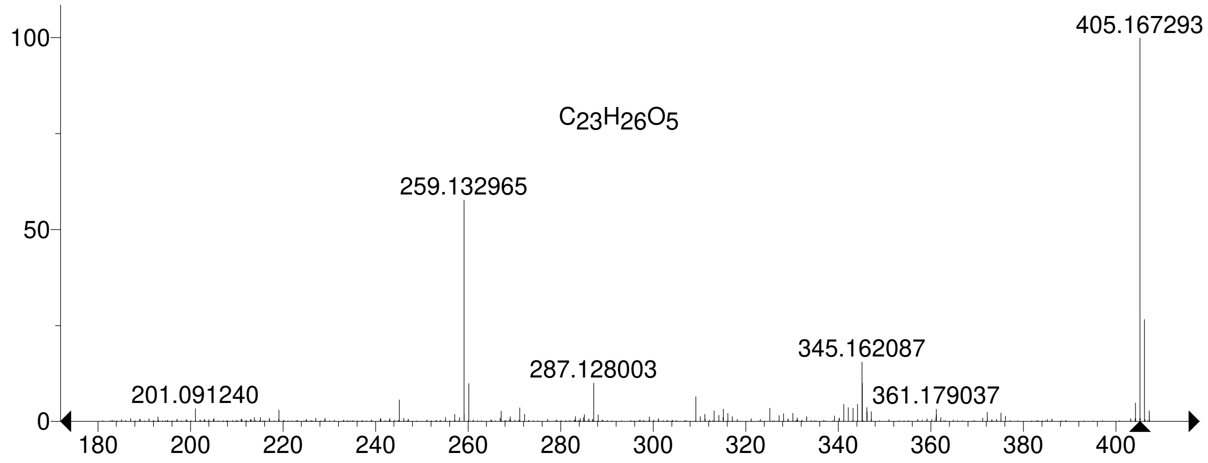

Unknown: Unknown (6.87_169.0130m/z)
Compound in Library Factor = -349

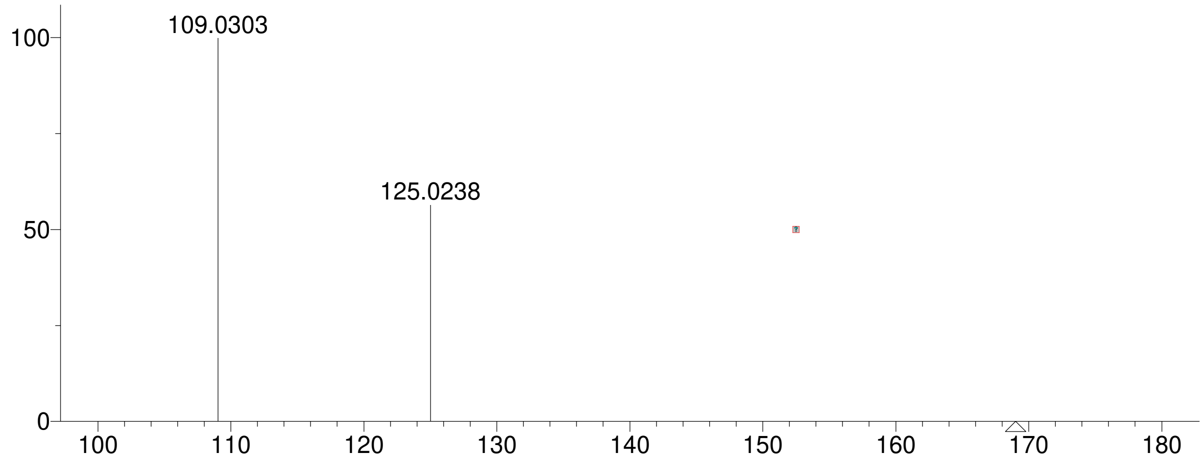

Hit 1 : (-)-Gallocatechin 3-gallate [M-H-C15H12O6]- IT-FT 35% P=169
C22H18O11; MF: 364; RMF: 999; Prob 20.9%; CAS: 4233-96-9; Lib: nist_msms; ID: 390513.

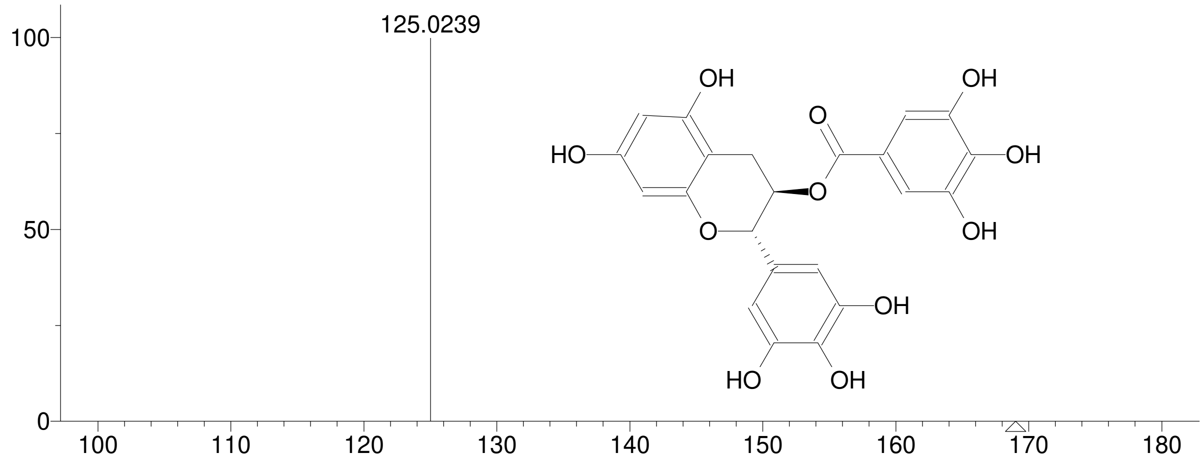

Hit 2 : (-)-Epicatechin gallate [M-H-C15H12O5]- IT-FT 35% P=169
C22H18O10; MF: 361; RMF: 995; Prob 18.5%; CAS: 1257-08-5; Lib: nist_msms; ID: 459026.

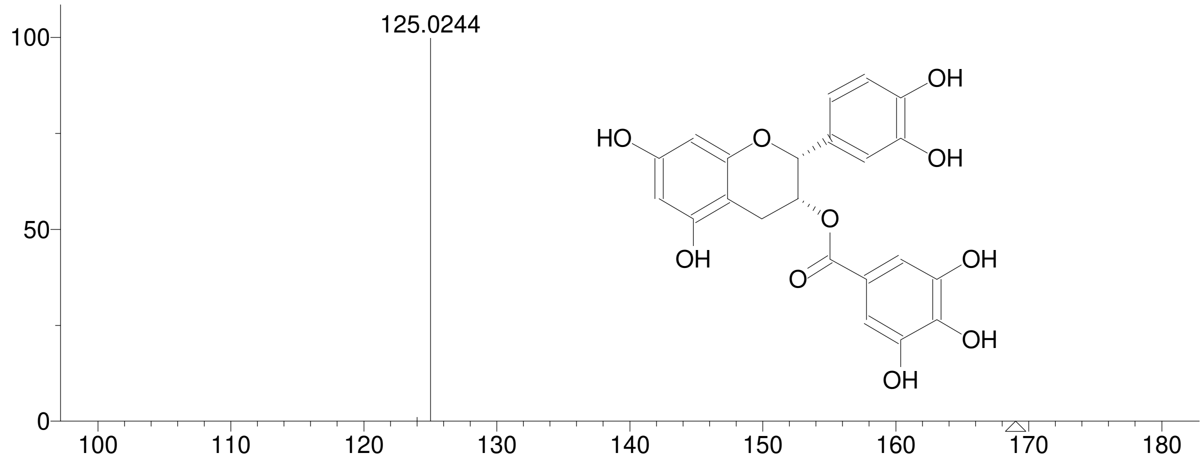

Hit 3 : (-)-Catechin gallate [M-H-C15H12O5]- IT-FT 35% P=169
C22H18O10; MF: 360; RMF: 993; Prob 17.8%; CAS: 130405-40-2; Lib: nist_msms; ID: 552347.

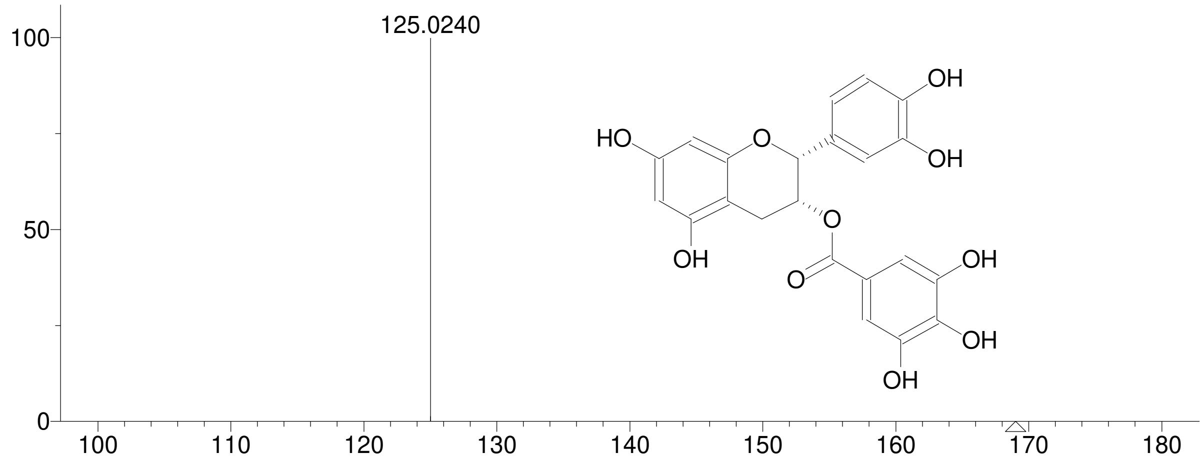

Hit 4 : Epigallocatechin gallate [M-H-C15H12O6]- IT-FT 35% P=169
C22H18O11; MF: 359; RMF: 992; Prob 17.1%; CAS: 989-51-5; Lib: nist_msms; ID: 336528.

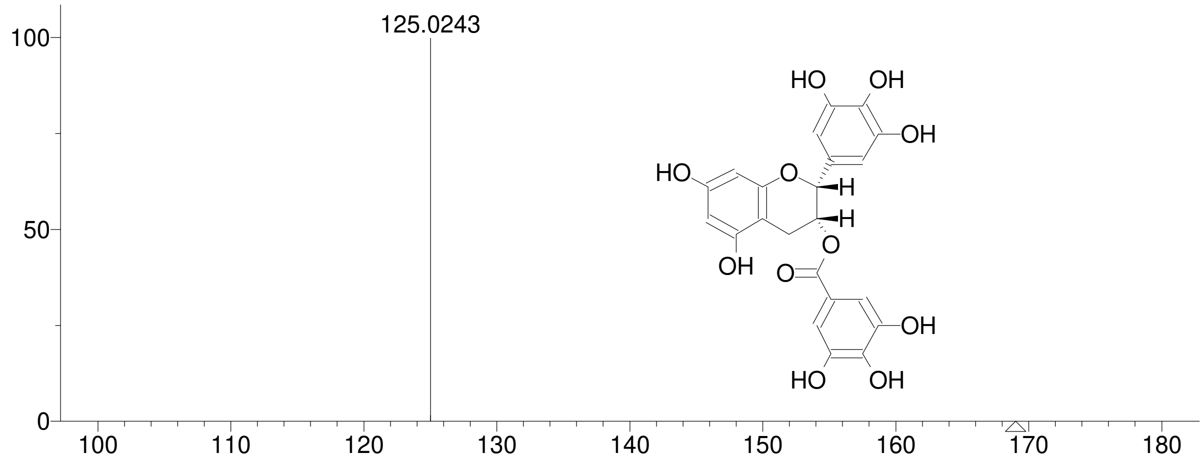

Hit 5 : Gallic acid [M-H]- IT-FT 35% P=169
C7H6O5; MF: 354; RMF: 986; Prob 13.8%; CAS: 149-91-7; Lib: nist_msms; ID: 285660.

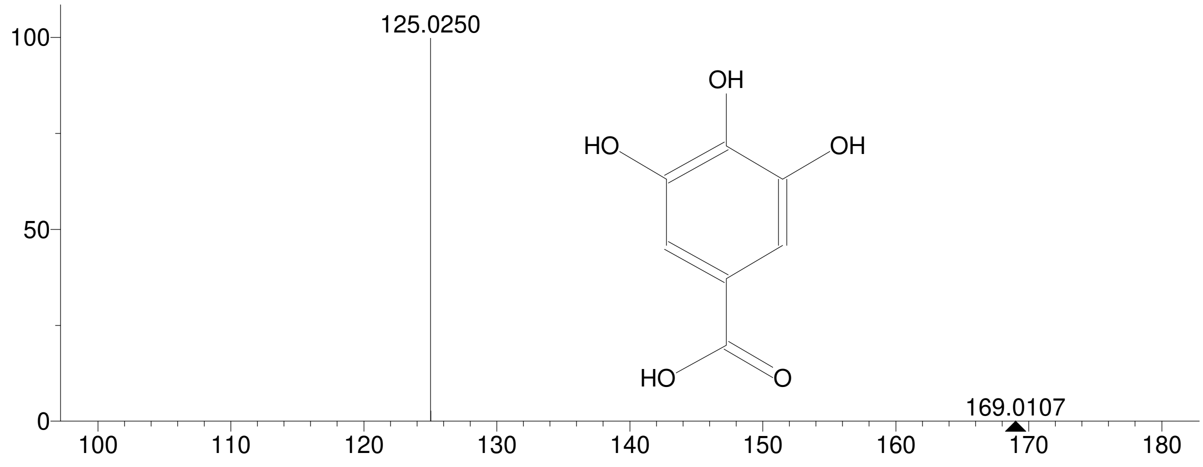

Unknown: Unknown (6.89_418.1118m/z)
Compound in Library Factor = -1309

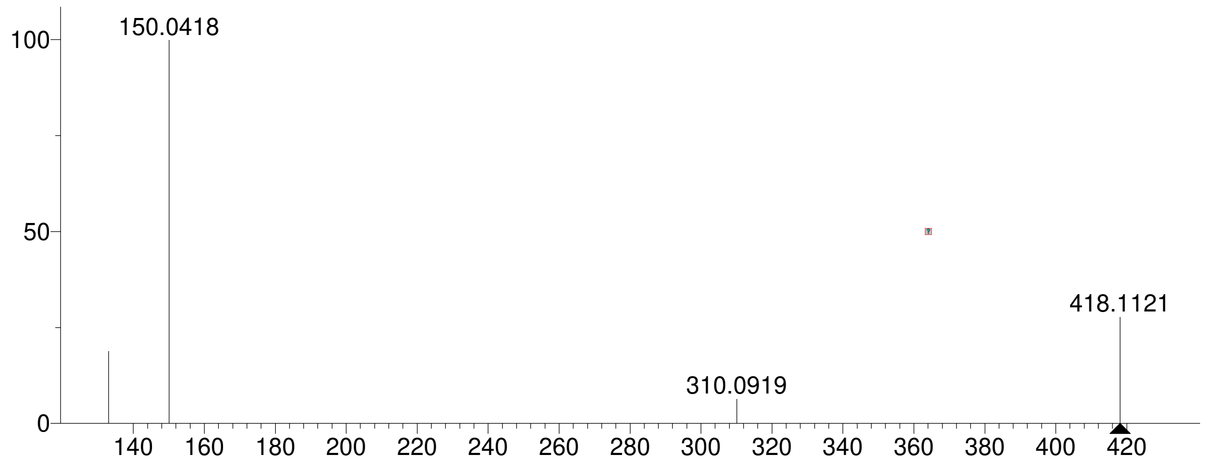

Hit 1 : 1H- Pyrazole-4-carboxylic acid, 1-(2,4-difluorophenyl)-5-[[2-[[(1,1-dimethylethyl)amino] thioxomethyl]hydraz
C17H19F2N5O2S; MF: 124; RMF: 785; Prob 76.7%; CAS: 264233-05-8; Lib: nist_msms; ID: 273564.

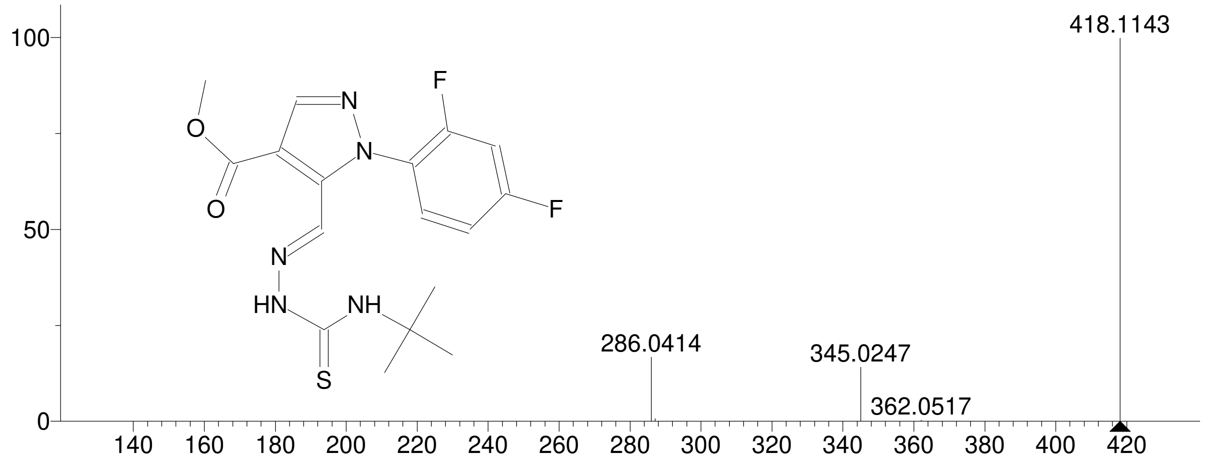

Hit 2 : Galacto-N-biose [M+Cl]- HCD 8V P=418.1
C14H25NO11; MF: 68; RMF: 451; Prob 14.6%; CAS: 20972-29-6; Lib: nist_msms; ID: 531643.

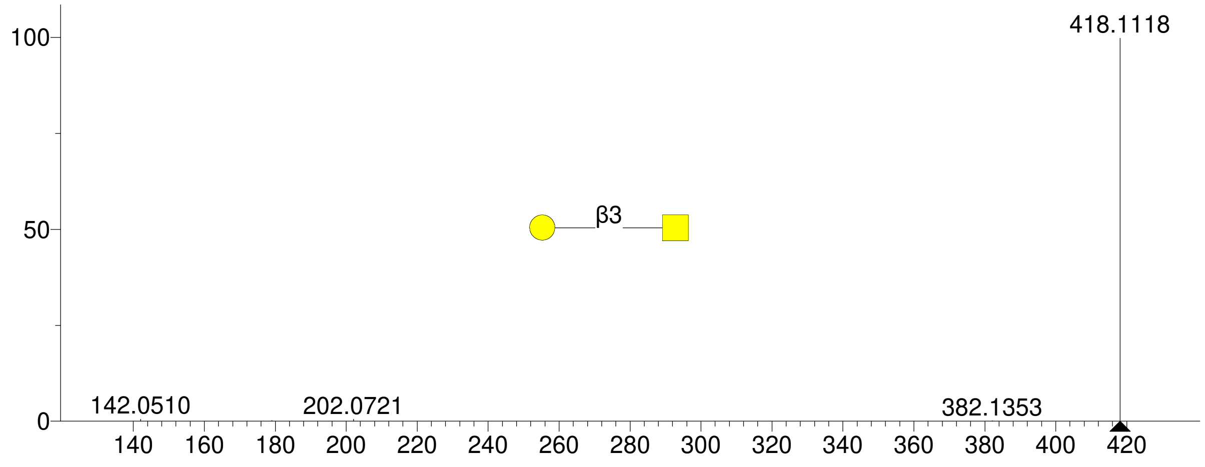

Hit 3 : 2-Acetamido-2-deoxy-3-O-(β-D-galactopyranosyl)-D-glucopyranose [M+Cl]- HCD 8V P=418.1
C14H25NO11; MF: 47; RMF: 448; Prob 6.22%; CAS: 489-52-1; Lib: nist_msms; ID: 534172.

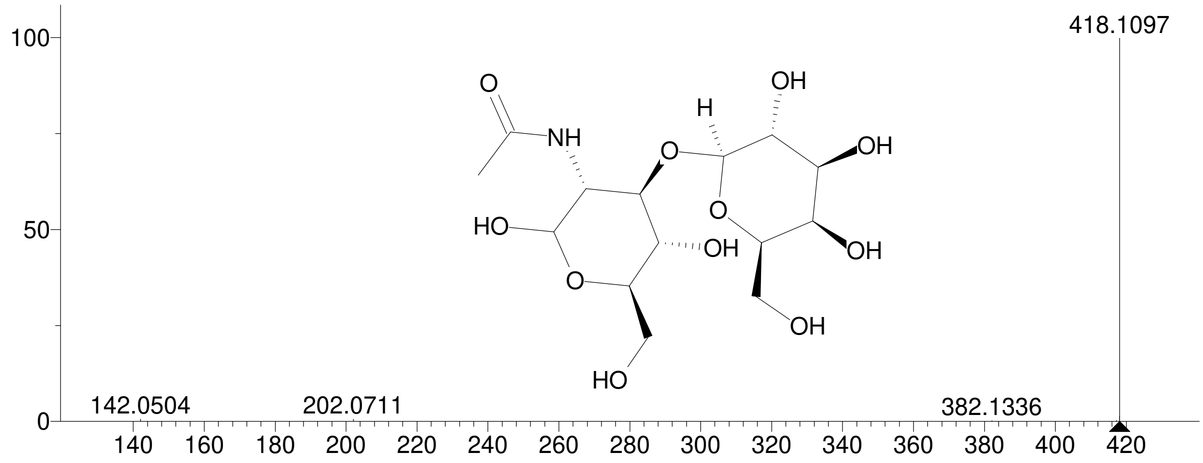

Hit 4 : N-Acetyl-D-lactosamine [M+Cl]- HCD 12V P=418.1
C14H25NO11; MF: 25; RMF: 400; Prob 2.45%; CAS: 32181-59-2; Lib: nist_msms; ID: 478644.

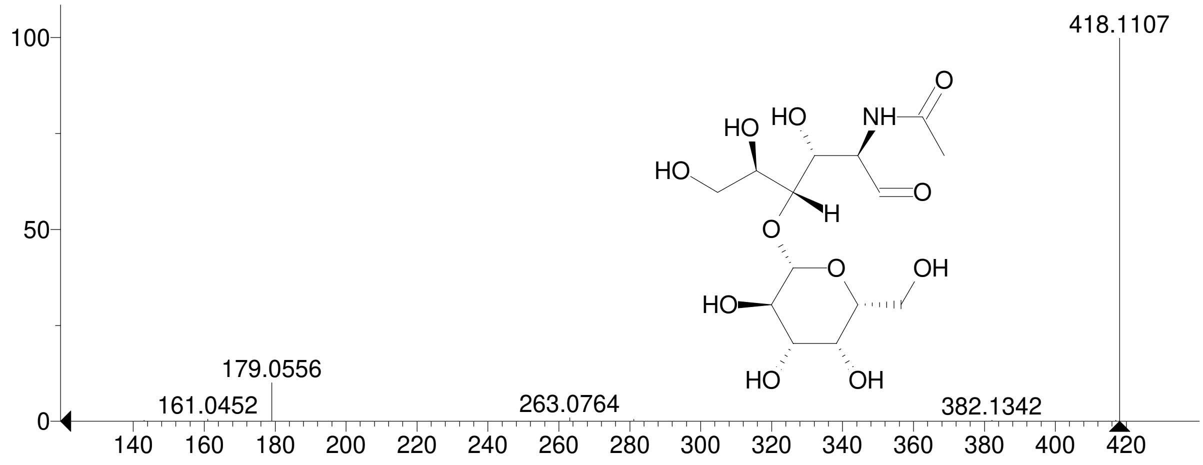

Unknown: Unknown (6.91_427.1725m/z)
Compound in Library Factor = 0

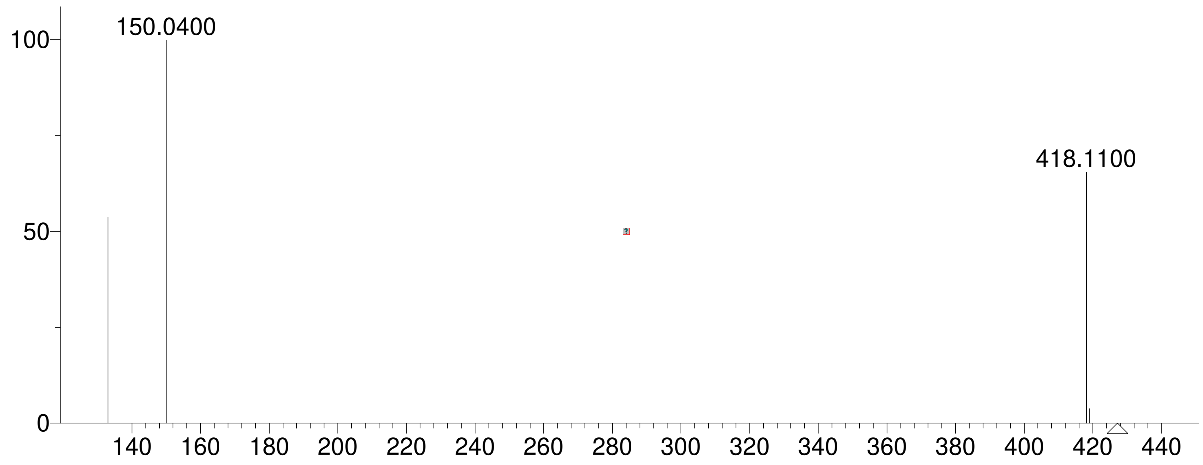

Hit 1 : [(6E,10Z)-10-(acetyloxymethyl)-6-formyl-3-methylidene-2-oxo-3a,4,5,8,9,11a-hexahydrocyclodeca[b]furan-
C22H28O7; MF: 0; RMF: 6; Prob 100.0%; Lib: mona-export-vf-npl_qexactive; ID: 23325.

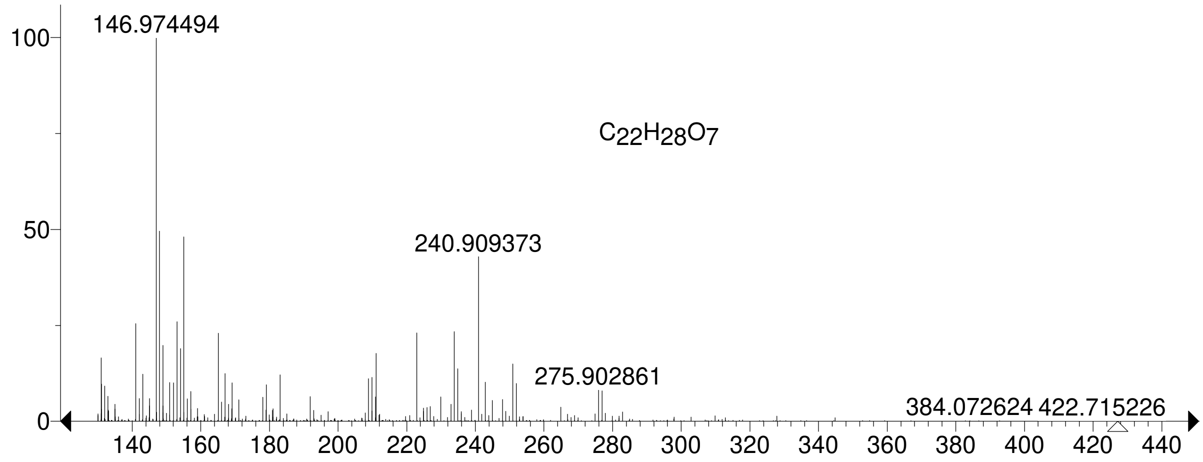

Unknown: Unknown (6.92_312.1554m/z)
Compound in Library Factor = -2176

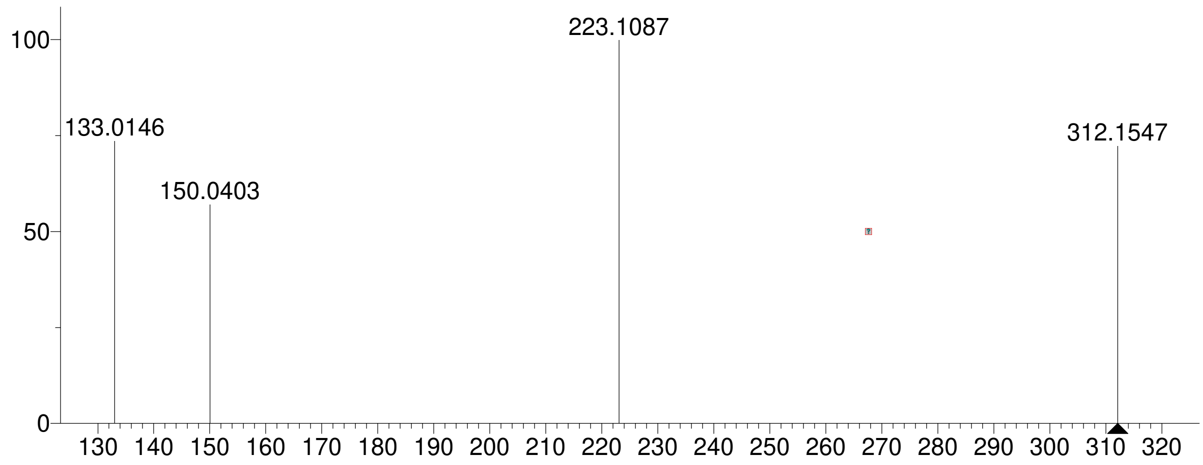

Hit 1 : (-)-Atropine [M+Na]+ HCD 16V P=312.2
C17H23NO3; MF: 86; RMF: 471; Prob 52.4%; CAS: 101-31-5; Lib: nist_msms; ID: 385892.

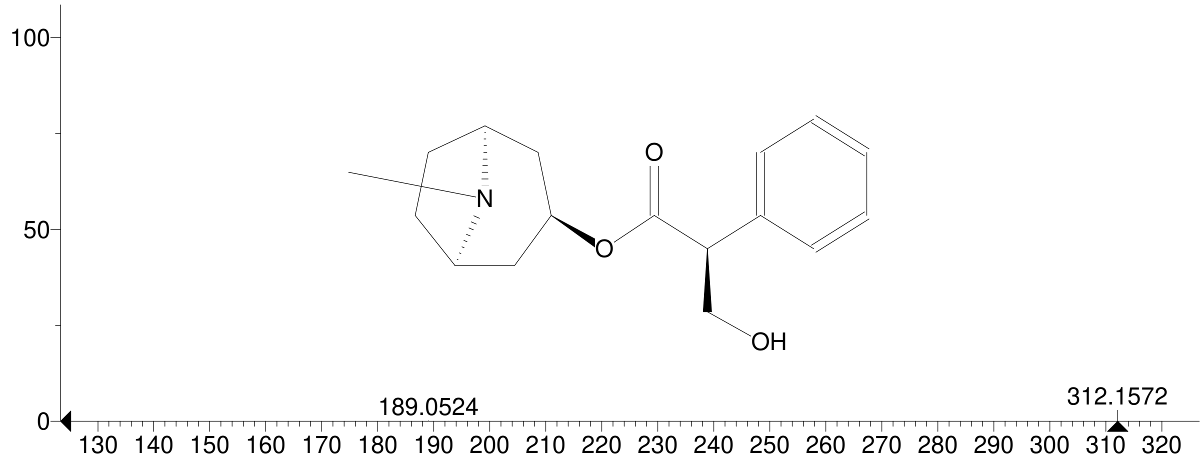

Hit 2 : Atropine [M+Na]+ HCD 6V P=312.2
C17H23NO3; MF: 60; RMF: 473; Prob 15.7%; CAS: 51-55-8; Lib: nist_msms; ID: 385579.

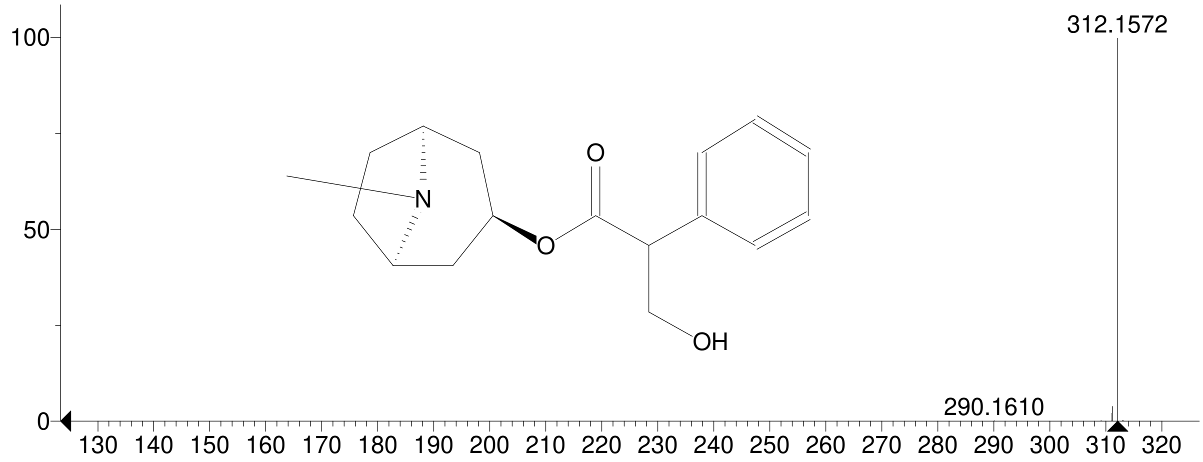

Hit 3 : Leonurine [M+H]+ HCD 15V P=312.2
C14H21N3O5; MF: 49; RMF: 462; Prob 10.8%; CAS: 24697-74-3; Lib: nist_msms; ID: 234001.

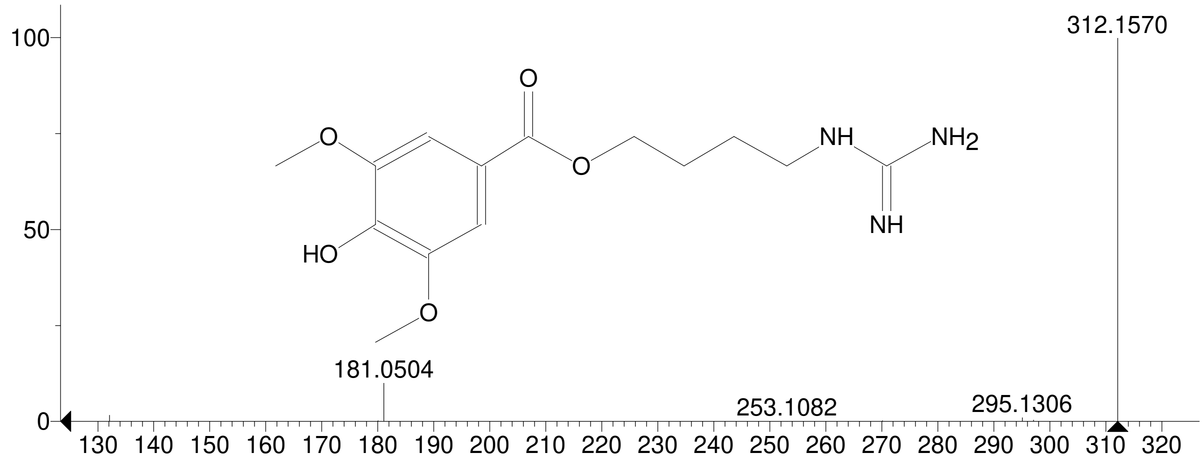

Hit 4 : Ac-Pro-Gly-Pro (PGP) [M+H]+ HCD 6eV P=312.2
C14H21N3O5; MF: 42; RMF: 450; Prob 8.25%; CAS: 292171-04-1; Lib: nist_msms; ID: 564205.

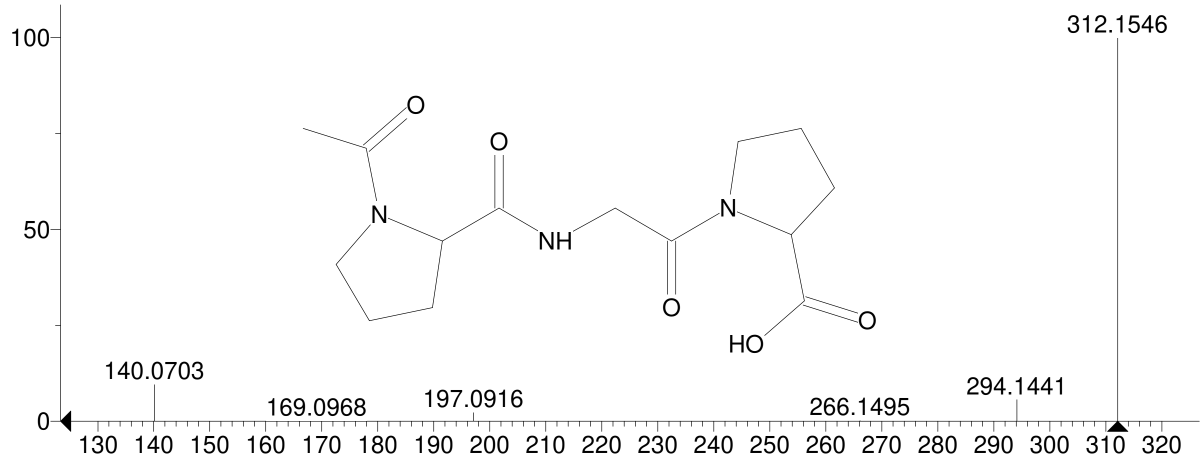

Hit 5 : Vardenafil [M+H-C6H13SO2N2]+ HCD 21V P=312.2
C23H32N6O4S; MF: 35; RMF: 557; Prob 6.32%; CAS: 224785-90-4; Lib: nist_msms; ID: 329143.

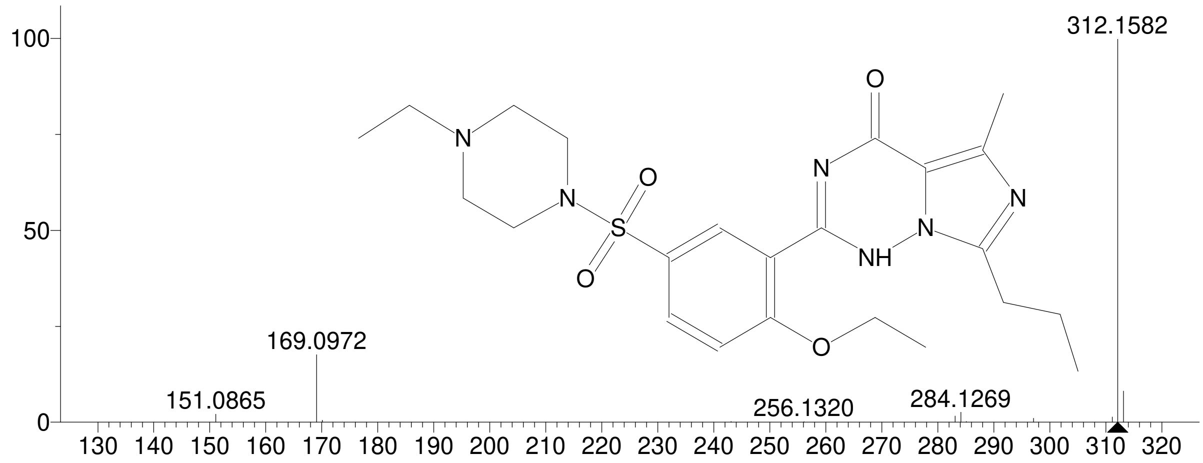

Unknown: Unknown (6.93_343.1963m/z)
Compound in Library Factor = 0

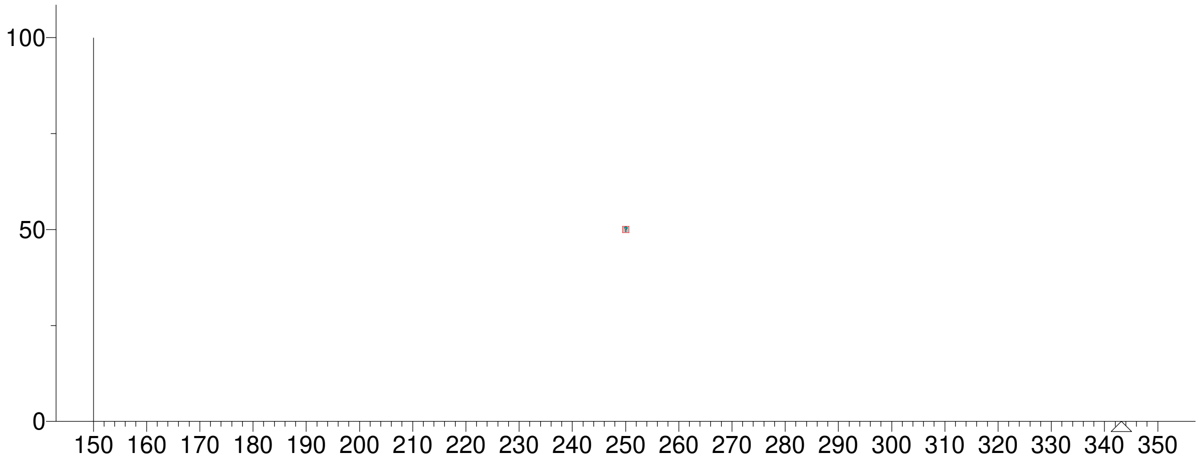

Unknown: Unknown (6.94_366.1543m/z)
Compound in Library Factor = 0

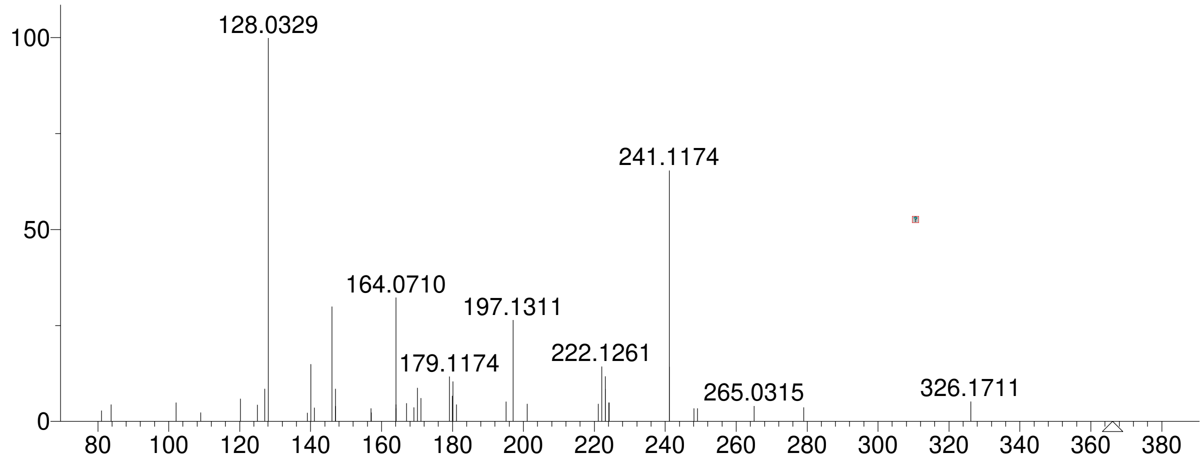

Hit 1 : Prazosin [M+H-H2O]+ HCD 62V P=366.2
C19H21N5O4; MF: 0; RMF: 16; Prob 100.0%; CAS: 19216-56-9; Lib: nist_msms; ID: 135105.

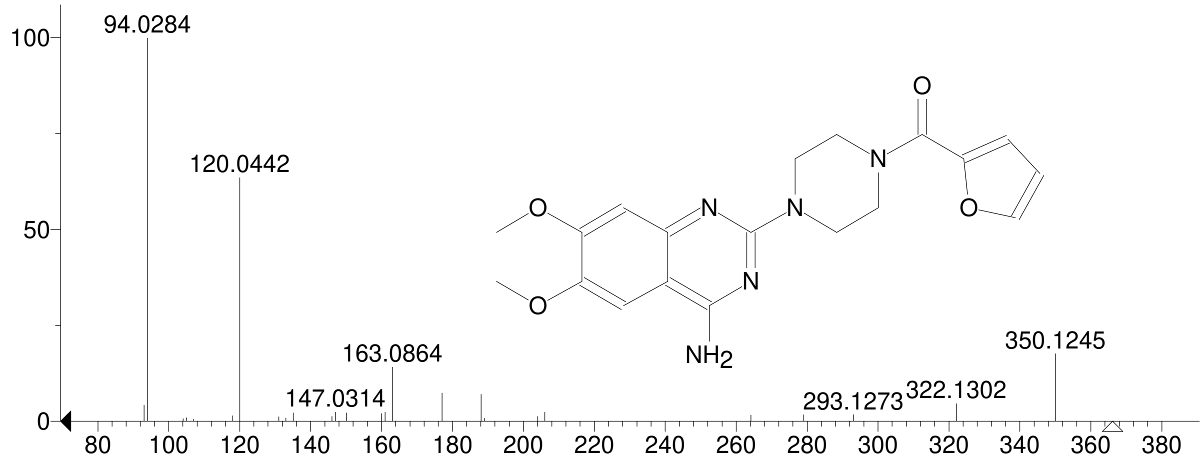

** Search Report Page 1 of 1 **

Unknown: Unknown (6.94_373.1585m/z)
Compound in Library Factor = 0

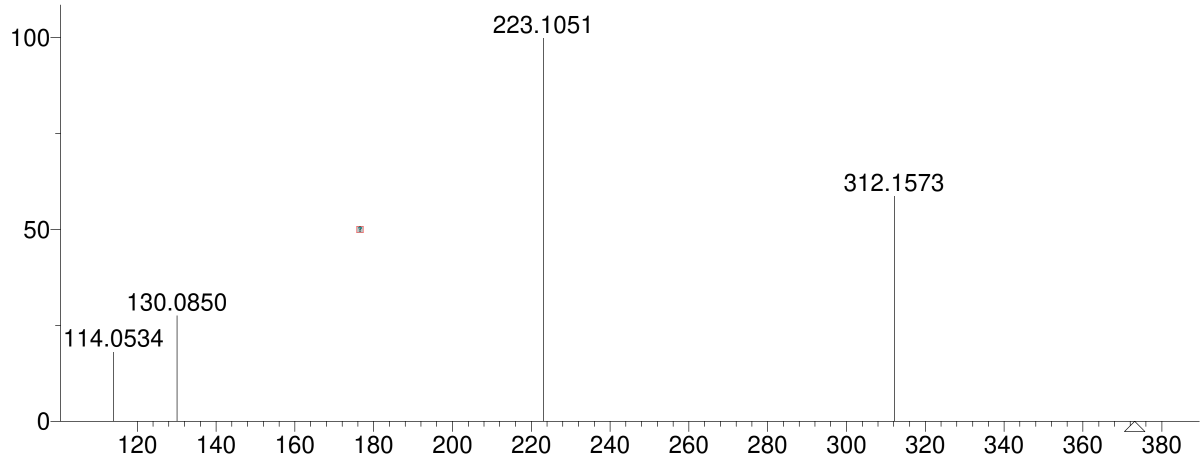

Hit 1 : 15β-Hydroxycyproterone acetate [M+H-C2H4O2]+ HCD 33V P=373.2
C24H29ClO5; MF: 0; RMF: 59; Prob 100.0%; CAS: 65423-26-9; Lib: nist_msms; ID: 440422.

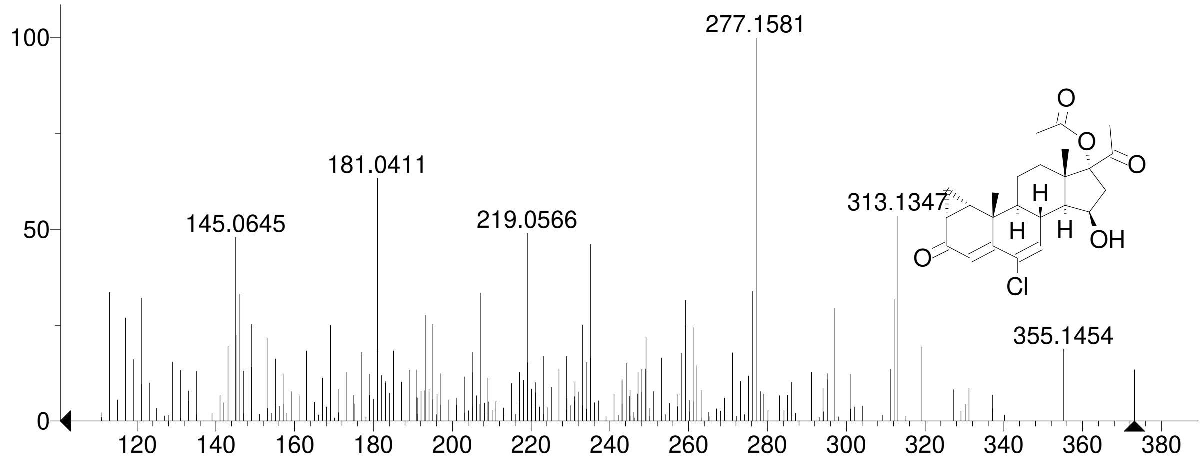

Unknown: Unknown (6.97_642.2129m/z)
Compound in Library Factor = 0

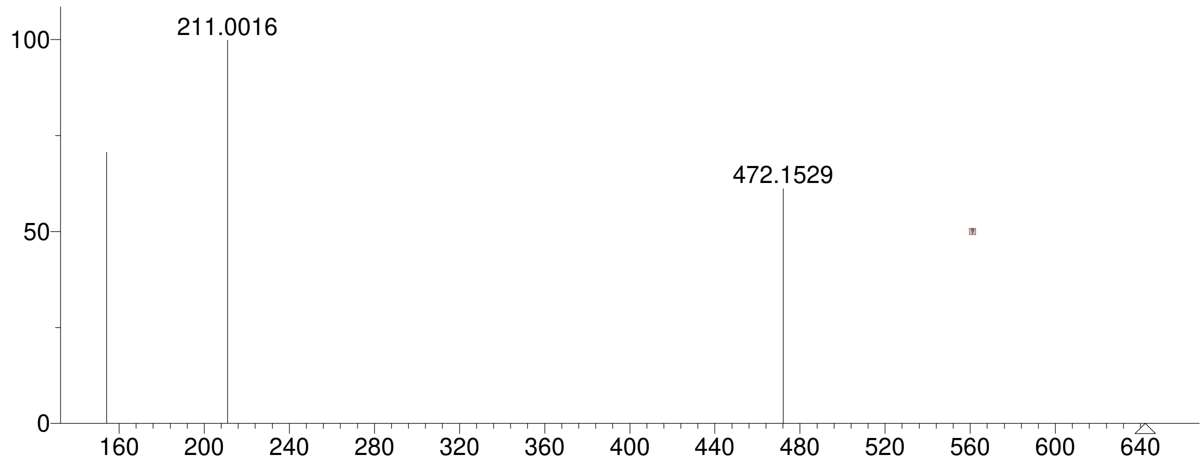

Unknown: Unknown (7.01_344.1814m/z)
Compound in Library Factor = -3273

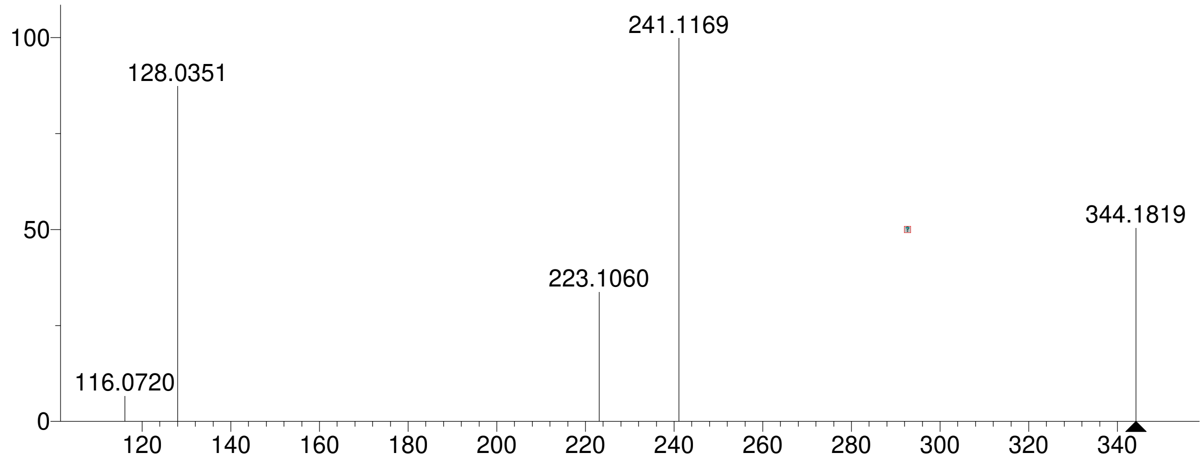

Hit 1 : NCGC00347618-02!3-hydroxy-N-(1-hydroxy-4-methylpentan-2-yl)-5-oxo-6-phenylhexanamide [M+Na]+ P
C18H27NO4; MF: 8; RMF: 341; Prob 40.1%; Lib: mona-export-gnps; ID: 9879.

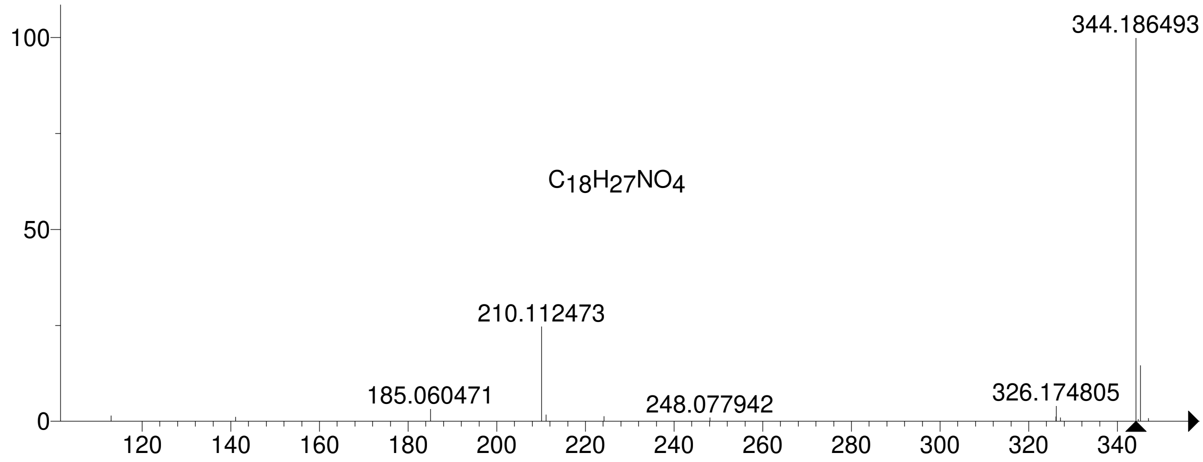

Hit 2 : Clemastine Fumarate [M+H]+ P=344.2
C25H30ClNO5; MF: 0; RMF: 48; Prob 29.9%; Lib: mona-export-gnps; ID: 639.

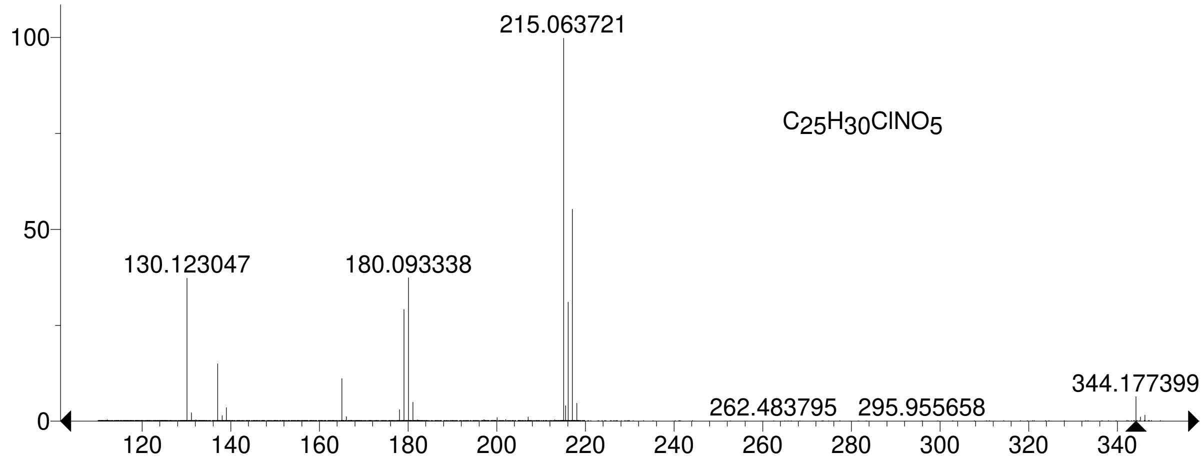

Hit 3 : 4-amino-2-hydroxy-5-[[1-hydroxy-1-(5-oxo-6-bicyclo[4.1.0]hept-3-enyl)propan-2-yl]amino]-5-oxopentanoic a
C15H22N2O6; MF: 0; RMF: 16; Prob 29.9%; Lib: mona-export-vf-npl_qexactive; ID: 16561.

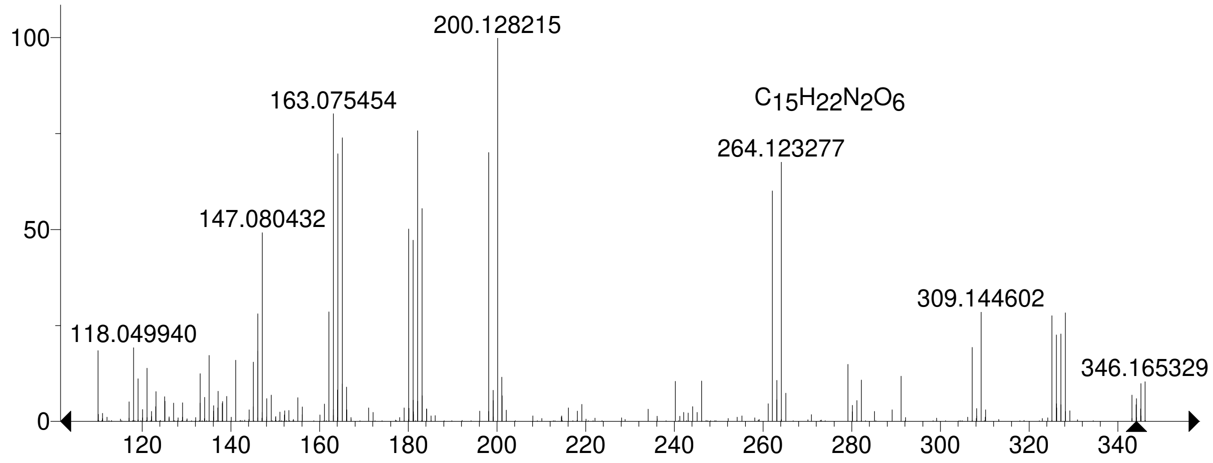

** Search Report Page 1 of 1 **

Unknown: Unknown (7.01_370.1610m/z)
Compound in Library Factor = -1523

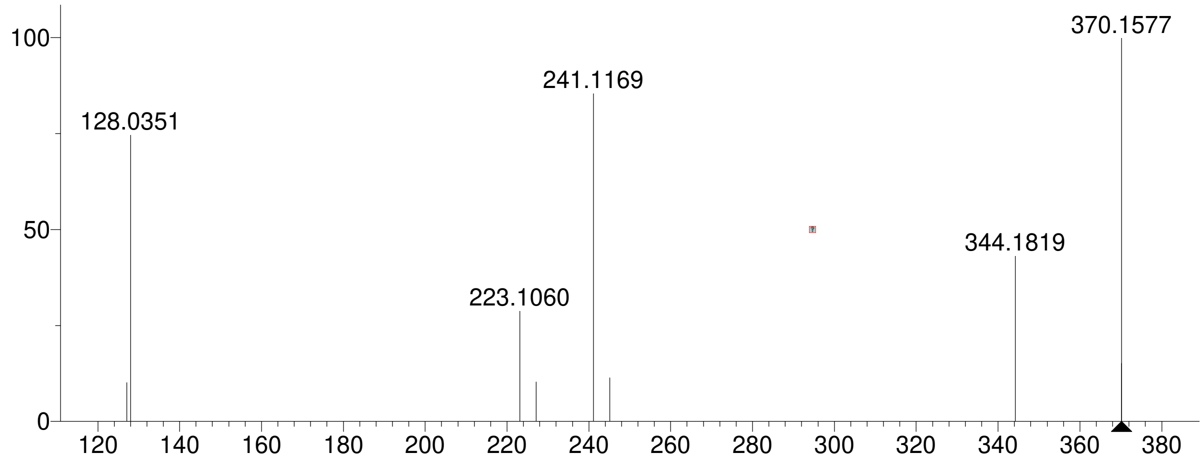

Hit 1 : Tenovin-1 [M+H]+ HCD 11V P=370.2
C20H23N3O2S; MF: 44; RMF: 522; Prob 100.0%; CAS: 380315-80-0; Lib: nist_msms; ID: 334898.

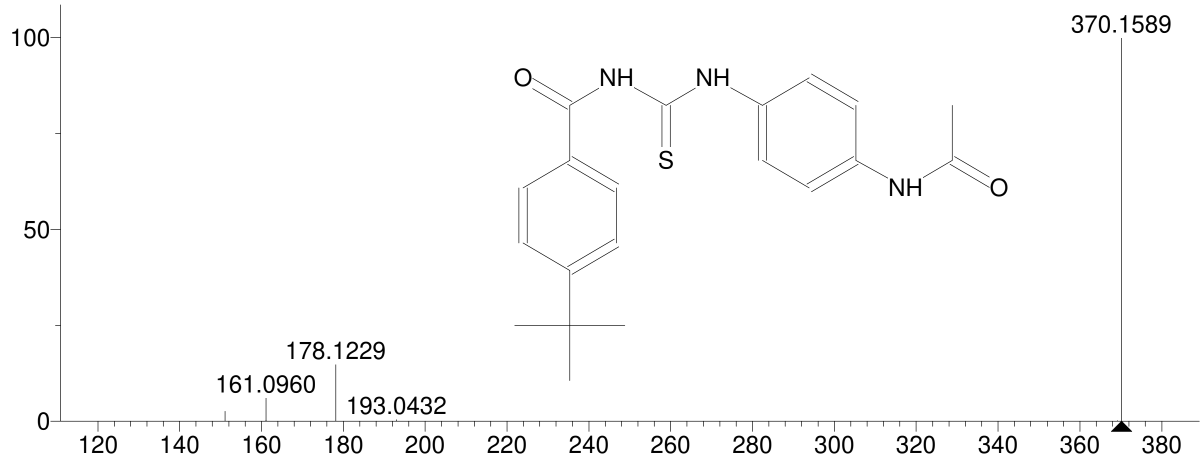

Unknown: Unknown (7.01_429.1857m/z)
Compound in Library Factor = 0

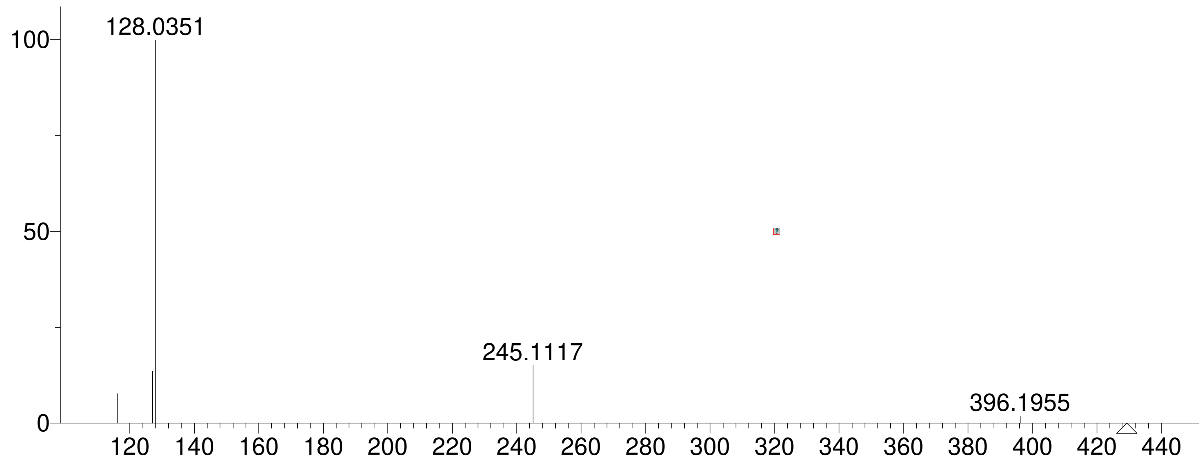

Hit 1 : (2R,3R,4S,5S,6R)-2-[6-hydroxy-3-[(E)-3-hydroxybut-1-enyl]-2,4,4-trimethylcyclohexyl]oxy-6-(hydroxymethy
C19H34O8; MF: 0; RMF: 3; Prob 100.0%; Lib: anthocyanins mona; ID: 9985.

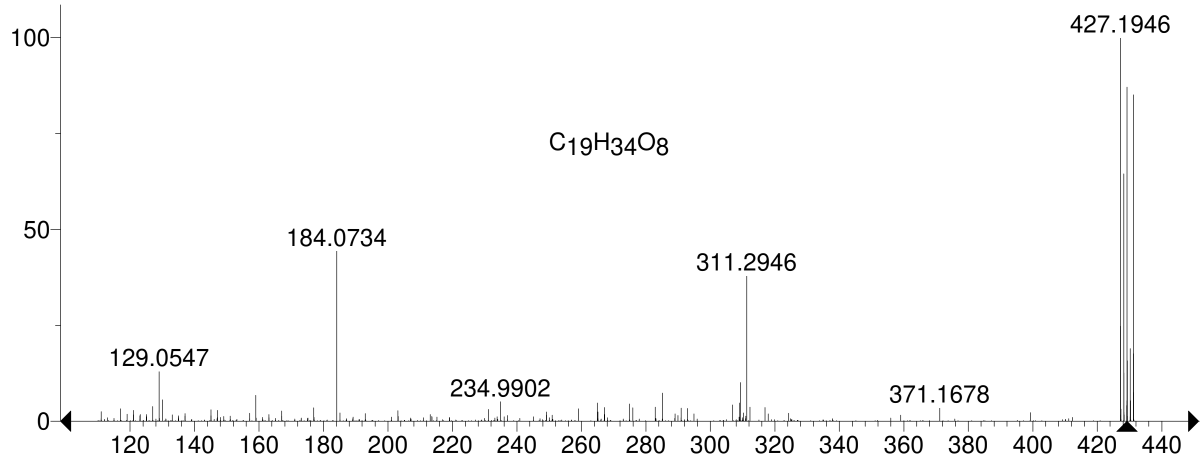

Unknown: Unknown (7.01_783.3779m/z)
Compound in Library Factor = 0

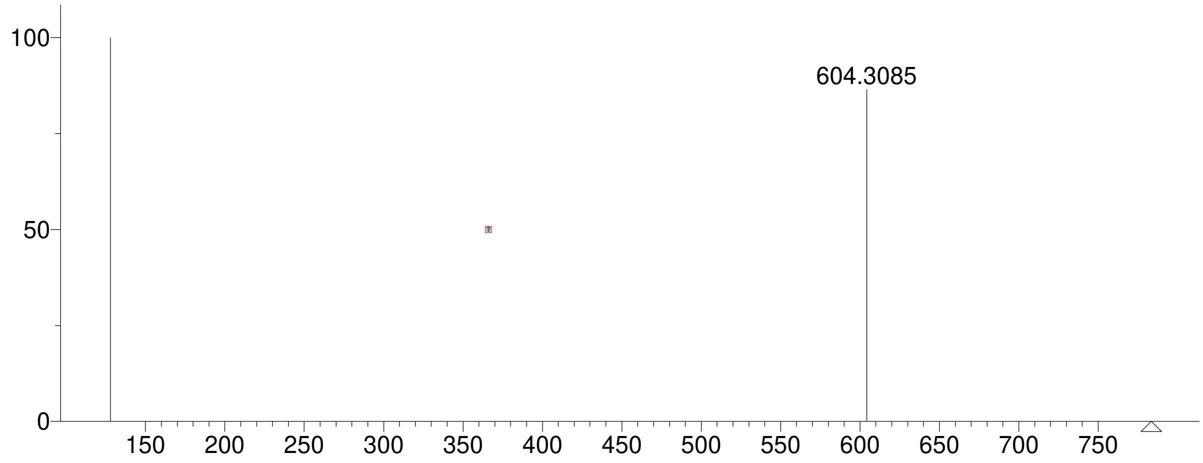

Unknown: Unknown (7.02_425.2029m/z)
Compound in Library Factor = -404

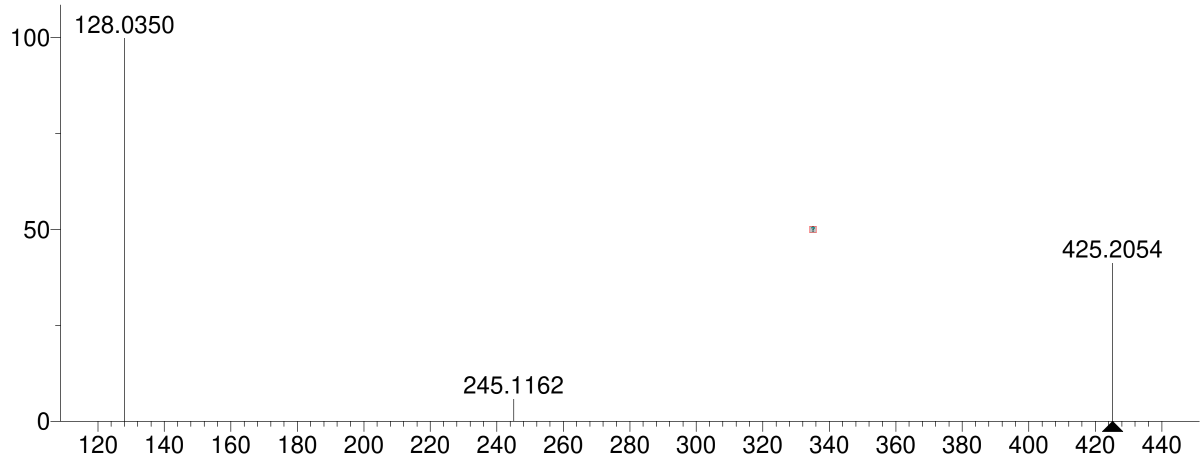

Hit 1 : Benazepril [M+H]+ HCD 13V P=425.2
C24H28N2O5; MF: 287; RMF: 917; Prob 94.6%; CAS: 86541-75-5; Lib: nist_msms; ID: 119286.

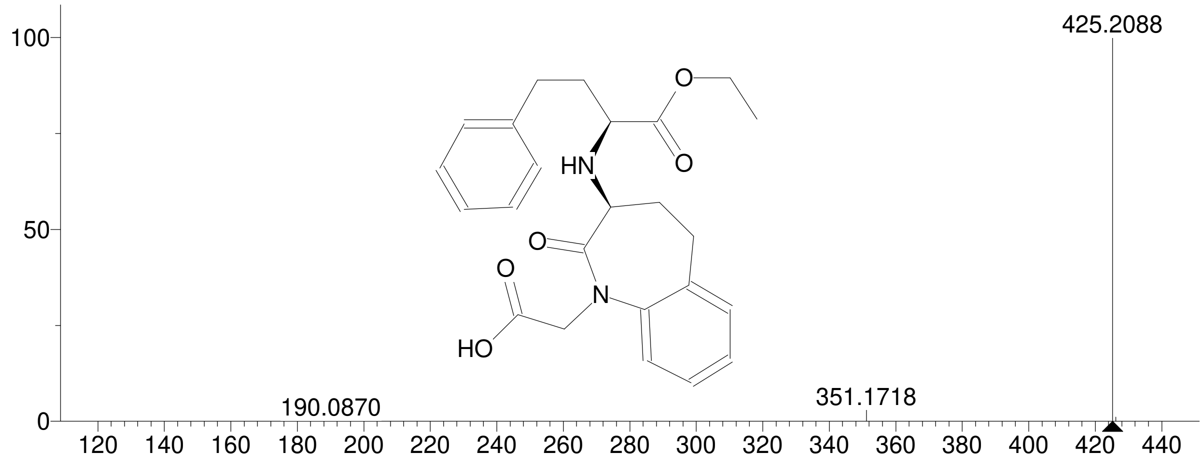

Hit 2 : Telmisartan [M-H-CO2]-=>440.3=>425.2 IT 35% P=425.2
C33H30N4O2; MF: 139; RMF: 840; Prob 2.72%; CAS: 144701-48-4; Lib: nist_msms; ID: 255935.

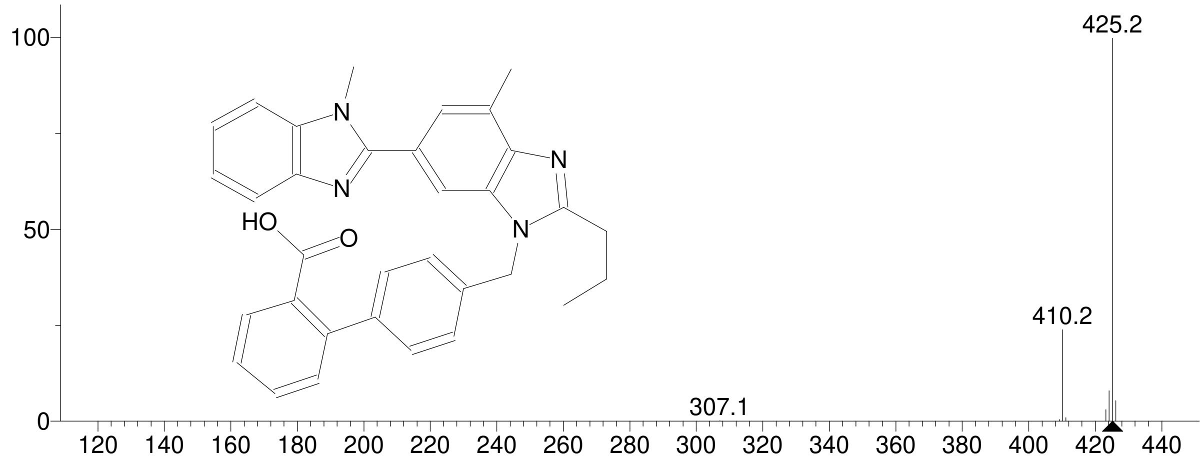

Hit 3 : Trandolaprilat [M+Na]+ HCD 15V P=425.2
C22H30N2O5; MF: 134; RMF: 811; Prob 2.19%; CAS: 87679-71-8; Lib: nist_msms; ID: 434140.

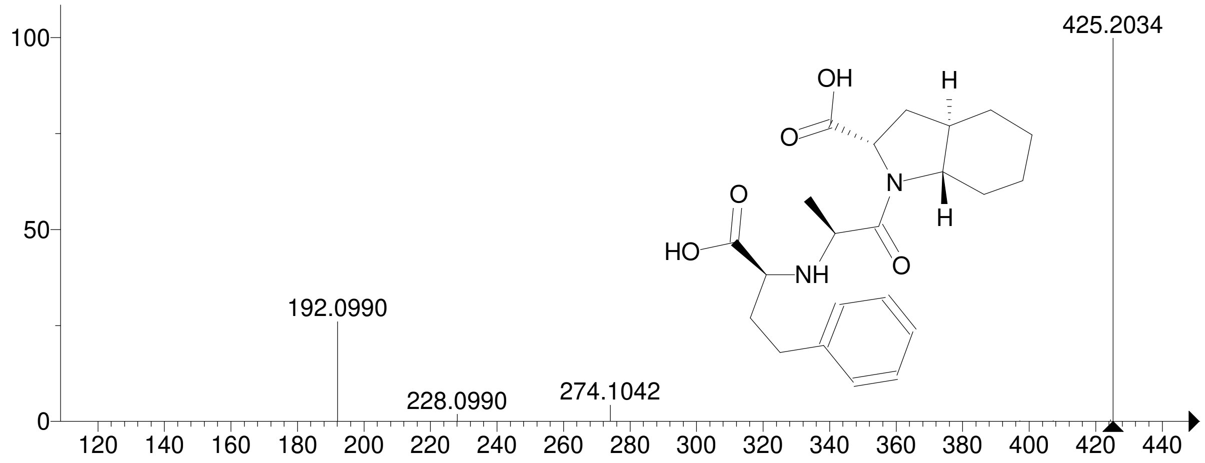

Hit 4 : Tyr-Asp-Lys [M+H]+ QQQ 14V P=425.2
C19H28N4O7; MF: 16; RMF: 460; Prob 0.12%; Lib: nist_msms; ID: 42317.

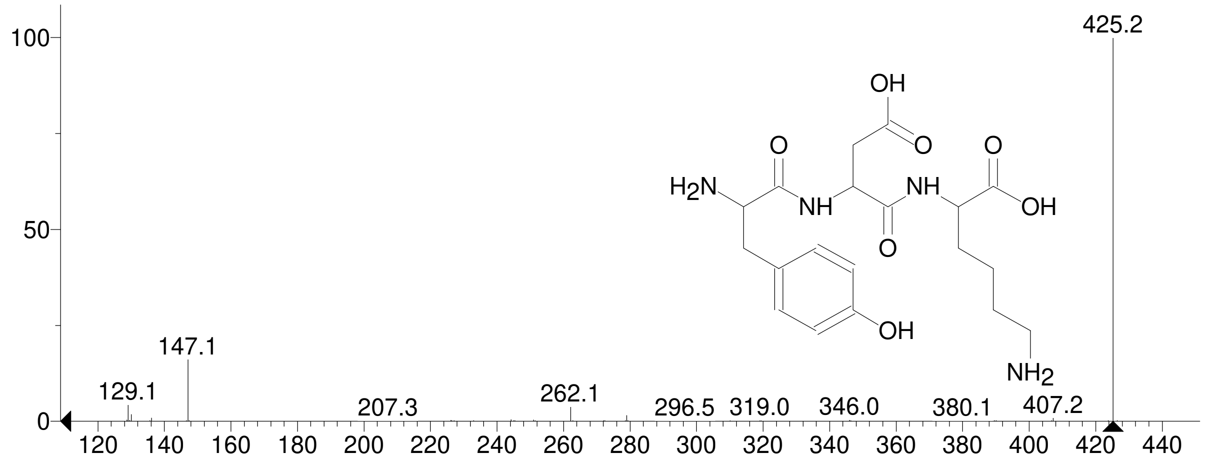

Hit 5 : Asp-Tyr-Lys [M+H]+ QTOF 10V P=425.2
C19H28N4O7; MF: 6; RMF: 339; Prob 0.08%; Lib: nist_msms; ID: 50040.

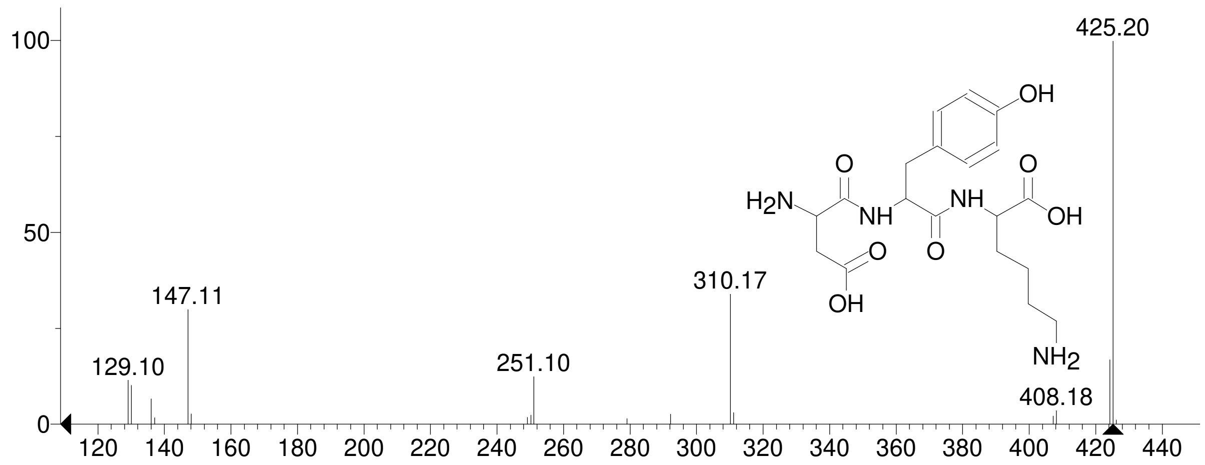

Unknown: Unknown (7.02_467.1660m/z)
Compound in Library Factor = 0

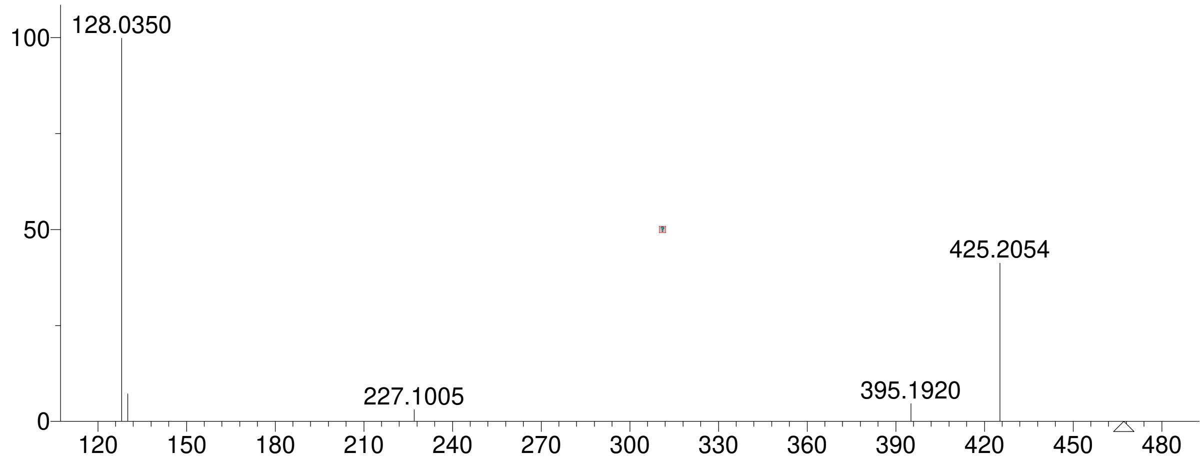

Hit 1 : MMV689243 [M+H]+ 20-30-40%) P=467.2
C23H20F6N4; MF: 0; RMF: 89; Prob 16.7%; Lib: mona-export-pathogen_box; ID: 361.

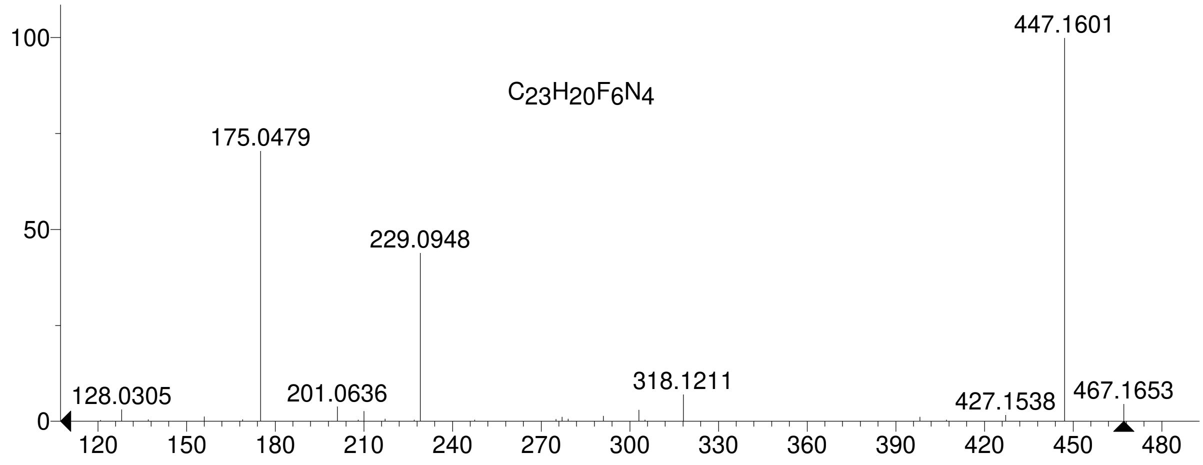

Hit 2 : 11-(hydroxymethyl)-2,6,6,14,19-pentamethyl-7,16,18-trioxapentacyclo[12.6.1.0A^'2,A^'1A^'2.0a^*u,A^'1a^'c
C24H28O8; MF: 0; RMF: 51; Prob 16.7%; Lib: mona-export-vf-npl_qexactive; ID: 21163.

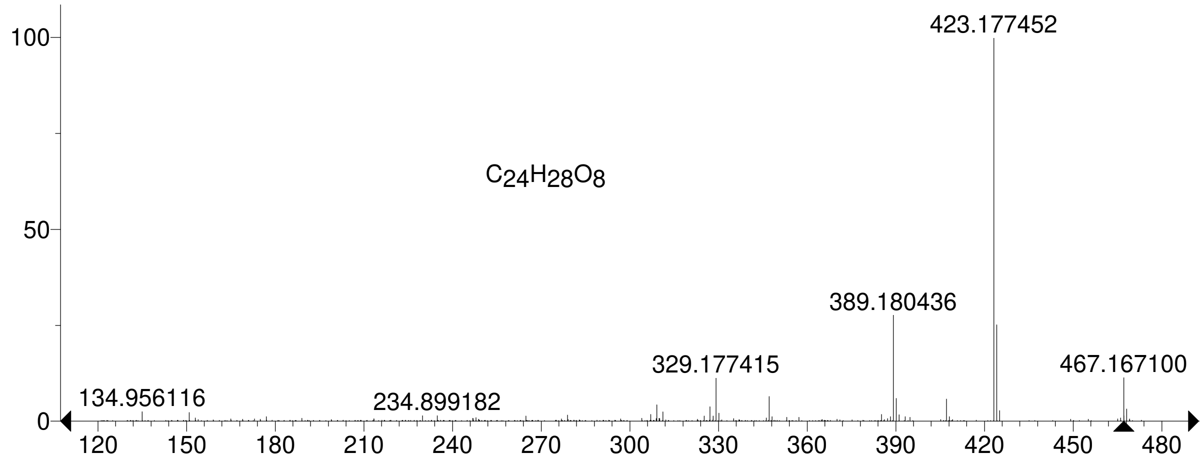

Hit 3 : 11,17-Difuroatemometasone [M+H-C5H5ClO3]+ IT 35% P=467.2
C32H32Cl2O8; MF: 0; RMF: 48; Prob 16.7%; CAS: 1370190-33-2; Lib: nist_msms; ID: 436547.

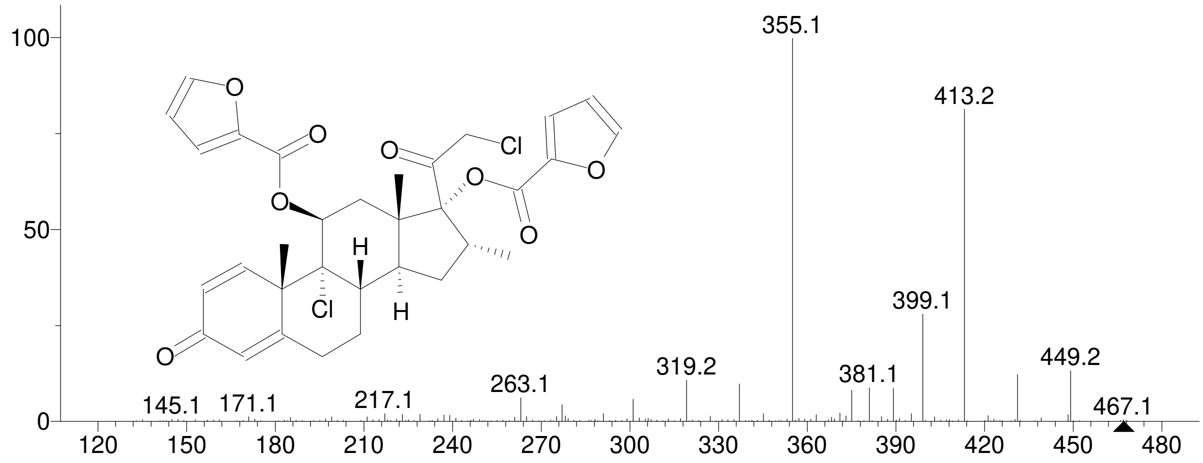

Hit 4 : Azadirachtin B [M+H-C7H16O6]+ IT 35% P=467.2
C33H42O14; MF: 0; RMF: 36; Prob 16.7%; CAS: 95507-03-2; Lib: nist_msms; ID: 223464.

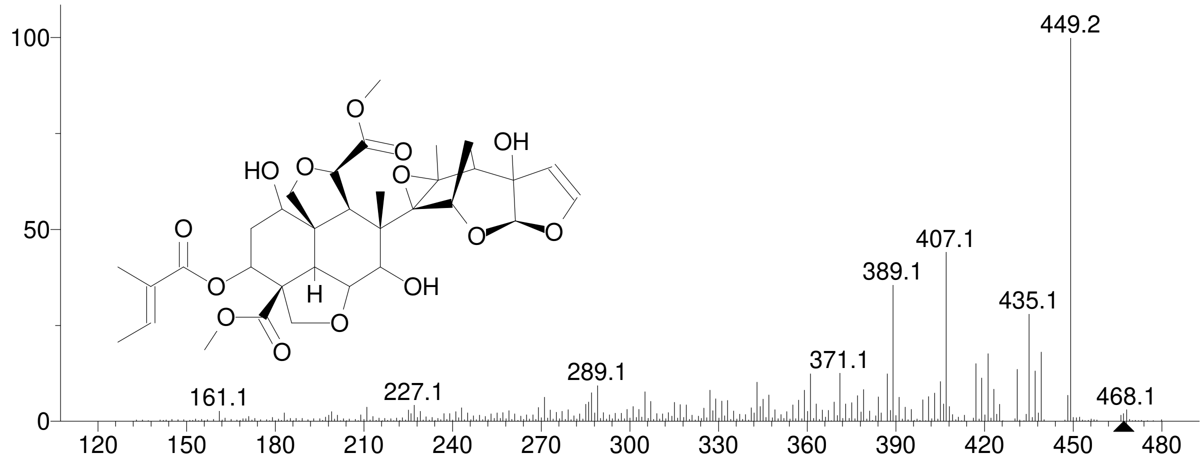

Hit 5 : Man3 b [M+H+Na]2+ IT 35% P=467.2
C34H58N2O26; MF: 0; RMF: 19; Prob 16.7%; Lib: nist_msms; ID: 184041.

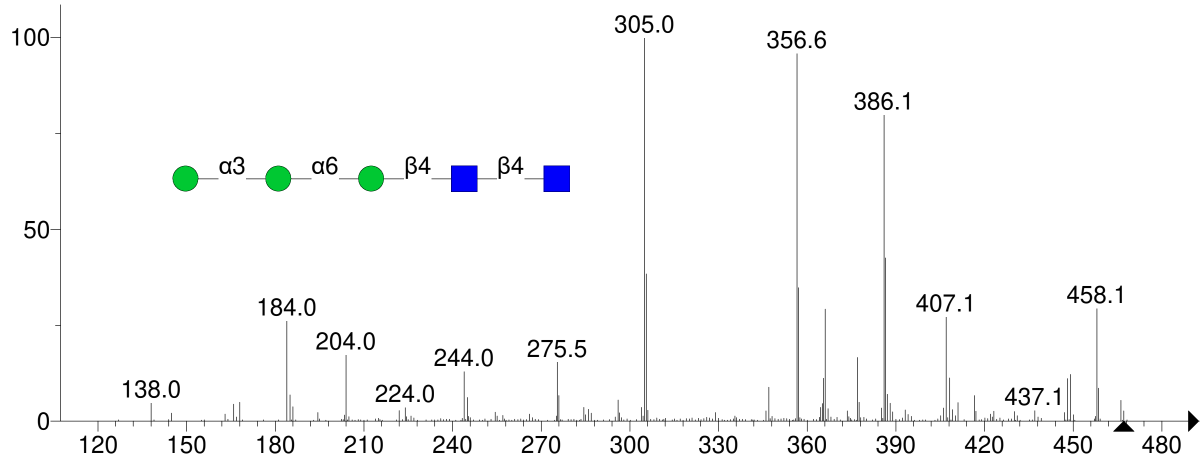

** Search Report Page 1 of 1 **

Unknown: Unknown (7.03_283.1532m/z)
Compound in Library Factor = 0

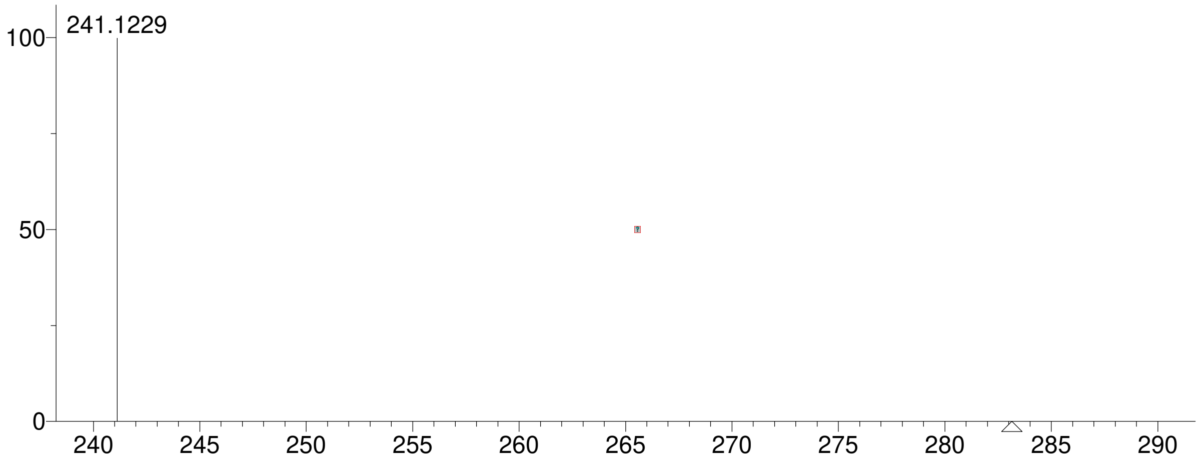

Unknown: Unknown (7.03_415.1695m/z)
Compound in Library Factor = -3273

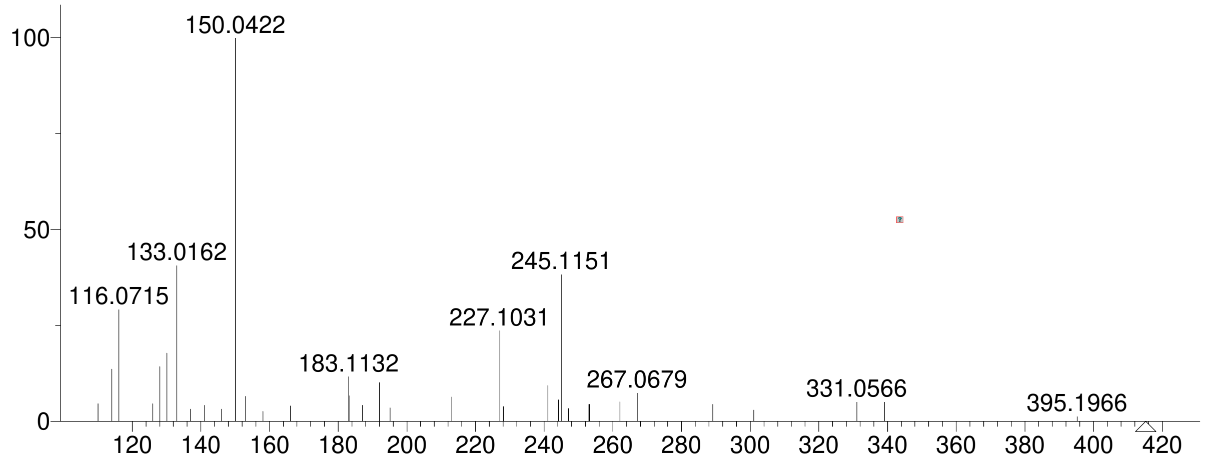

Hit 1 : Diltiazem [M+H]+ HCD 74V P=415.2
C22H26N2O4S; MF: 10; RMF: 417; Prob 22.1%; CAS: 42399-41-7; Lib: nist_msms; ID: 281169.

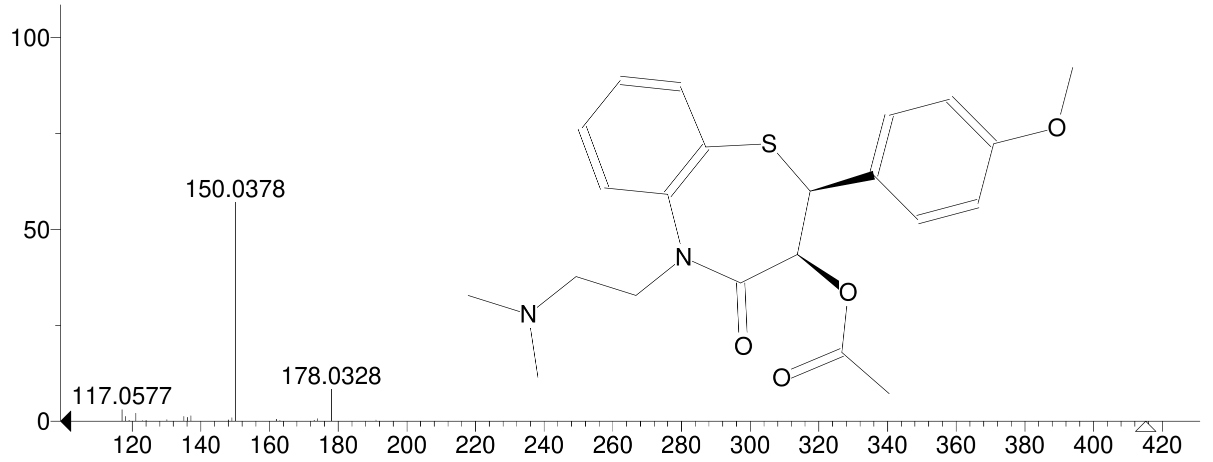

Hit 2 : 6-formyl-10-(hydroxymethyl)-5-methoxy-3-methylidene-2-oxo-2H,3H,3aH,4H,5H,8H,9H,11aH-cyclodeca[b]f
C21H28O7; MF: 1; RMF: 54; Prob 16.1%; Lib: mona-export-vf-npl_qexactive; ID: 16435.

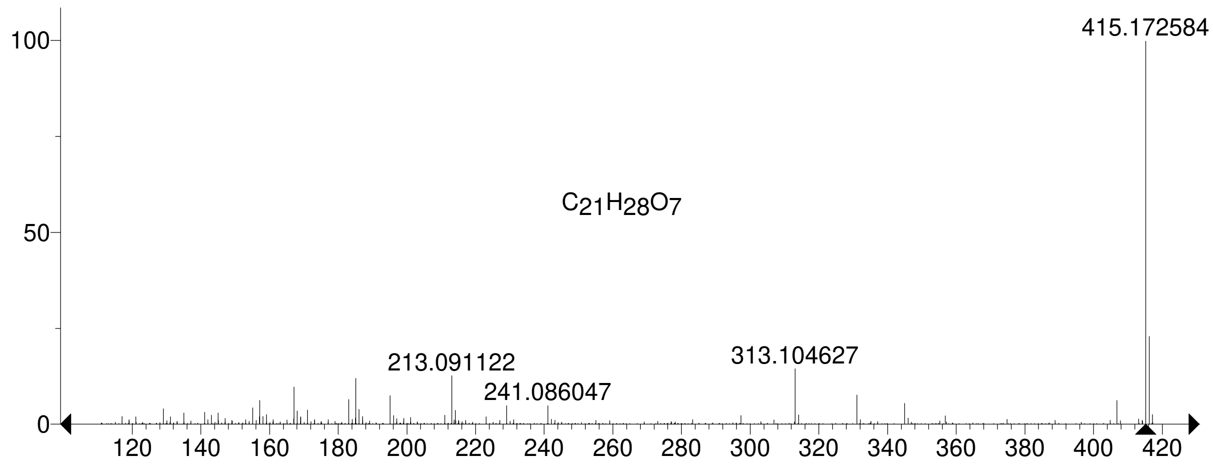

Hit 3 : Diltiazem HCl (Tiazac) [M+H]+ P=415.2
C22H27ClN2O4S; MF: 0; RMF: 155; Prob 15.4%; Lib: mona-export-gnps; ID: 648.

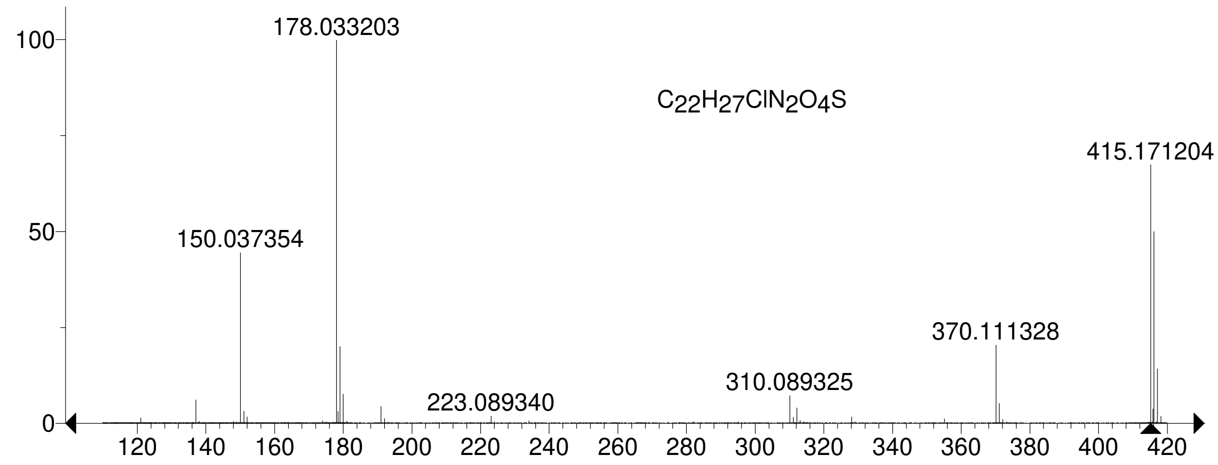

Hit 4 : 2-amino-5-[2-[[2,3-dihydroxy-2-(1-hydroxyethyl)butanoyl]oxymethyl]-4-hydroxyanilino]-5-oxopentanoic acid
C18H26N2O9; MF: 0; RMF: 16; Prob 15.4%; Lib: mona-export-vf-npl_qexactive; ID: 23033.

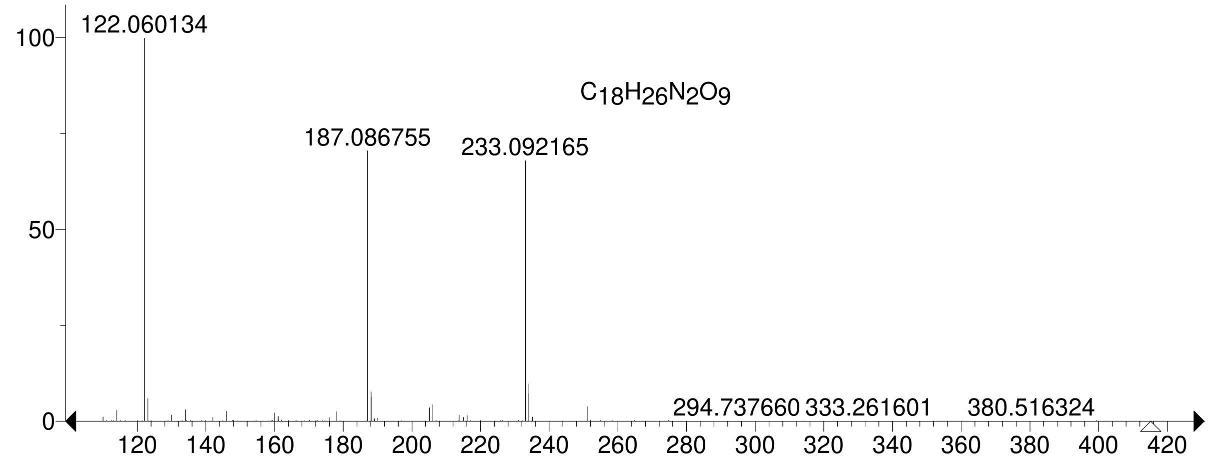

Hit 5 : NCGC00347525-03!2-amino-5-[2-[[2,3-dihydroxy-2-(1-hydroxyethyl)butanoyl]oxymethyl]-4-hydroxyanilino]-
C18H26N2O9; MF: 0; RMF: 15; Prob 15.4%; Lib: mona-export-gnps; ID: 9213.

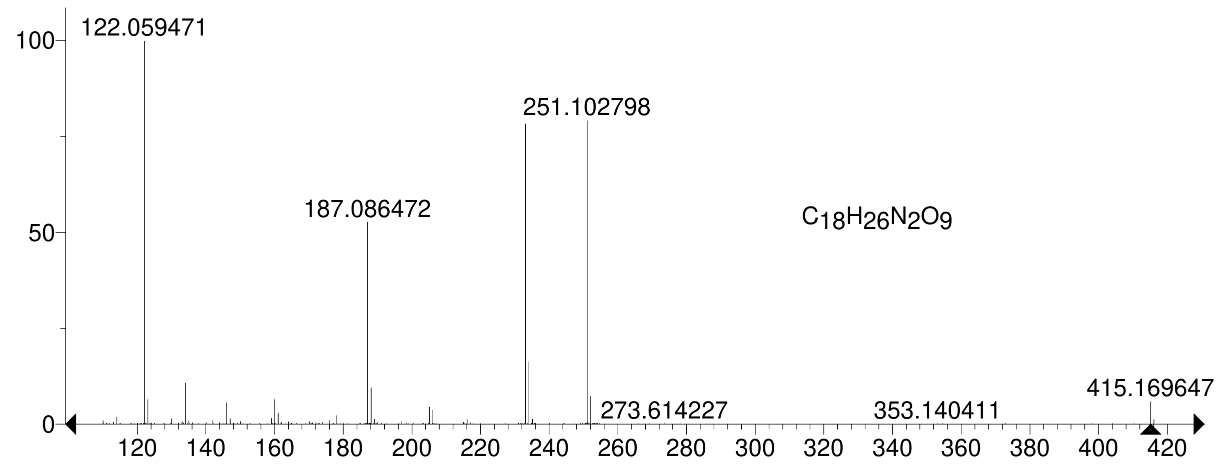

** Search Report Page 1 of 1 **

Unknown: Unknown (7.04_241.1184m/z)
Compound in Library Factor = -3273

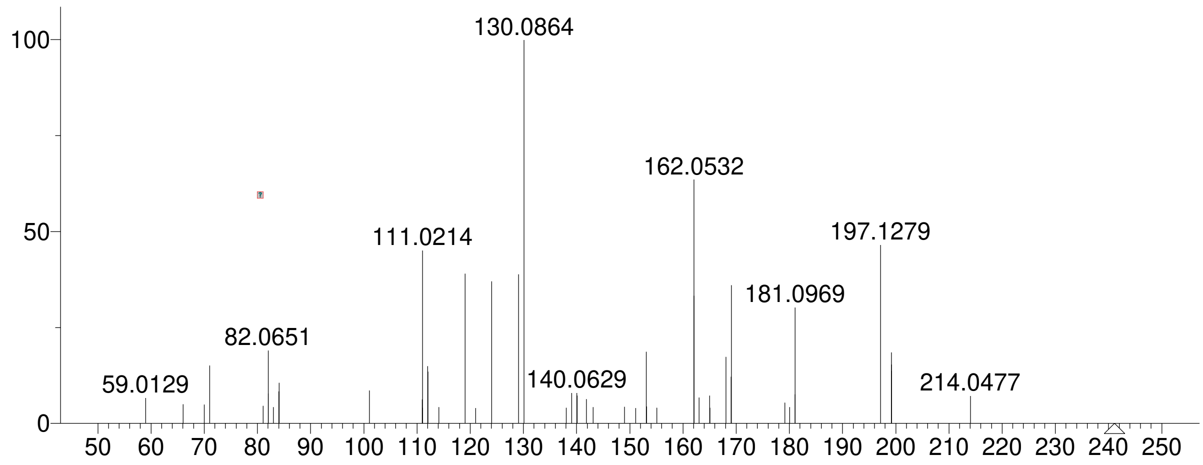

Hit 1 : Pyroglutamyl-Isoleucine; PlaSMA ID-369 [M-H]- P=241.1
C11H18N2O4; MF: 1; RMF: 112; Prob 100.0%; Lib: biomsms-neg-plasma; ID: 400.

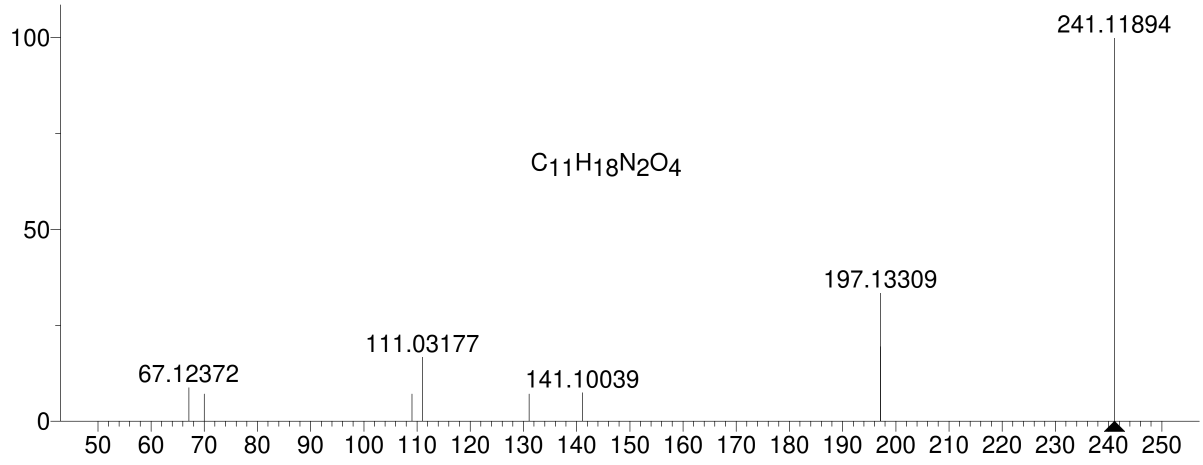

Unknown: Unknown (7.04_559.2717m/z)
Compound in Library Factor = -2387

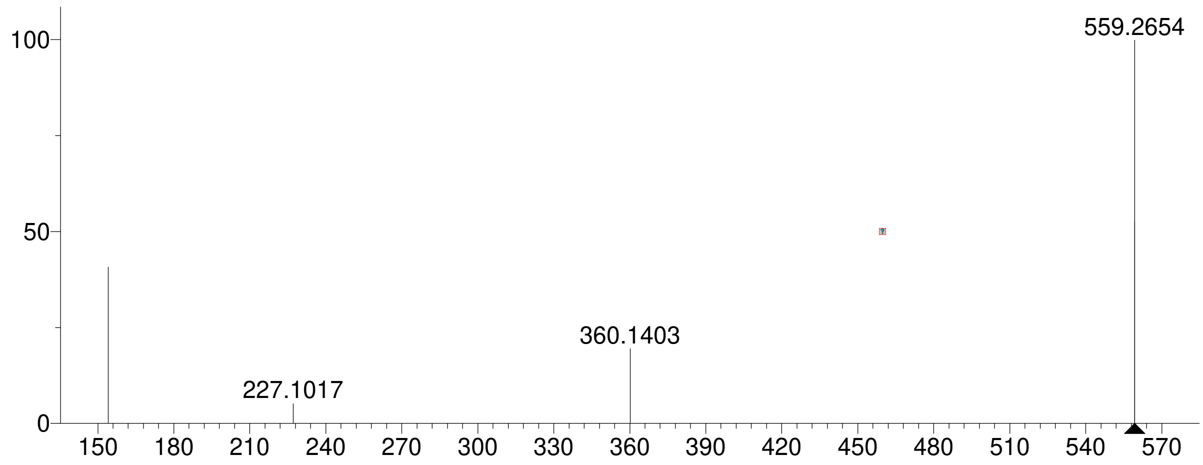

Hit 1 : PG 21:4; PG 3:0-18:4; [M-H]- [M-H]- P=559.3
C27H45O10P; MF: 12; RMF: 347; Prob 37.5%; Lib: msdial-insilicomsms-lipids-neg; ID: 324655.

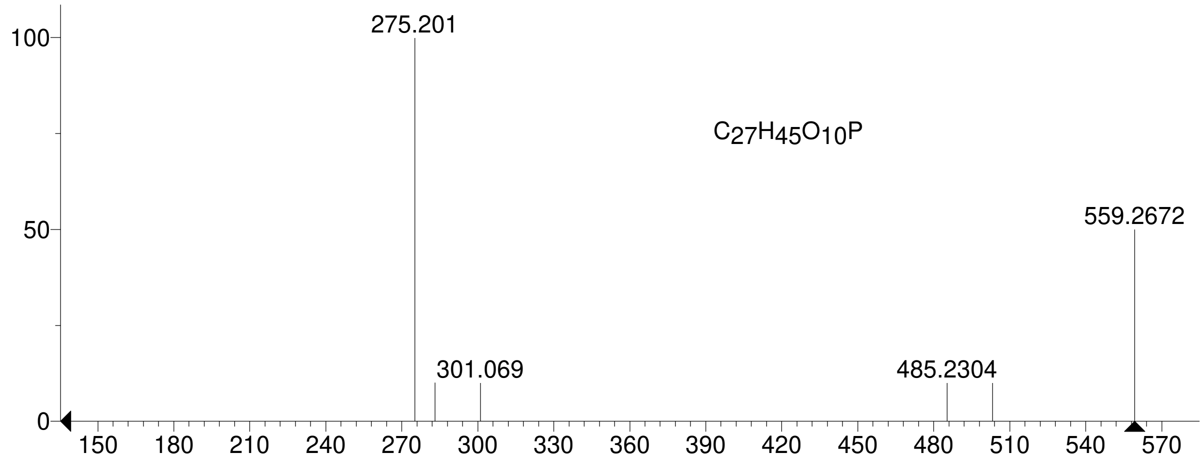

Hit 2 : PG 21:4; PG 5:0-16:4; [M-H]- [M-H]- P=559.3
C27H45O10P; MF: 12; RMF: 347; Prob 37.5%; Lib: msdial-insilicomsms-lipids-neg; ID: 324764.

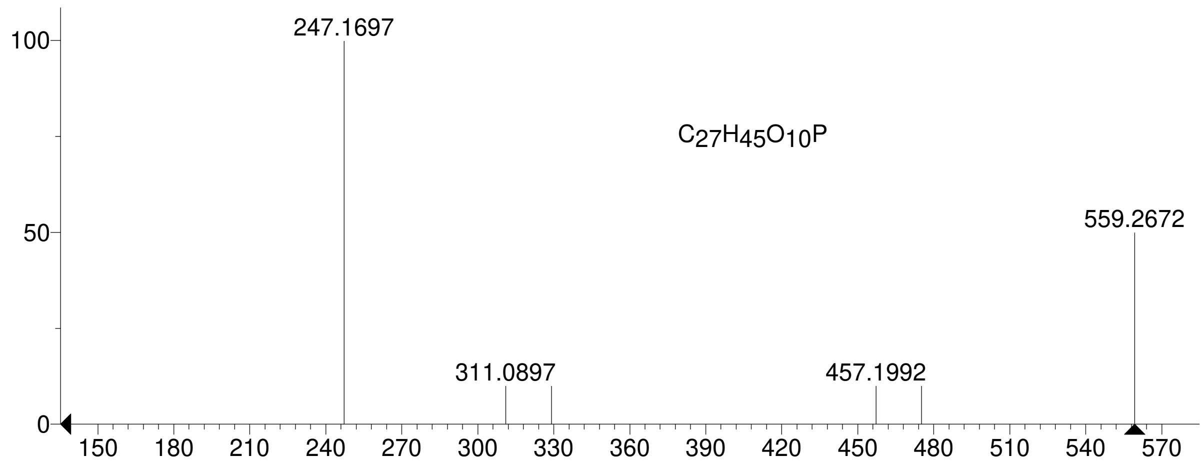

Hit 3 : (3S,4S,5R)-4-[(2R,3R)-2,3-dihydroxy-3-[(2S,3R,5R,10R,13R,14S,17S)-2,3,14-trihydroxy-10,13-dimethyl-6-
C29H44O8; MF: 0; RMF: 334; Prob 25.0%; Lib: mona-export-vf-npl_qexactive; ID: 9538.

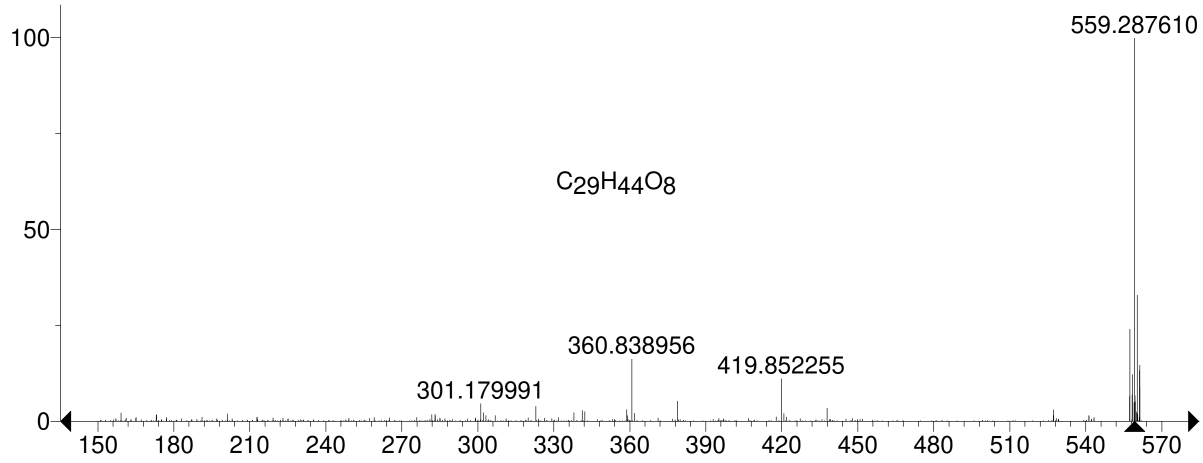

Unknown: Unknown (7.05_530.1961m/z)
Compound in Library Factor = 0

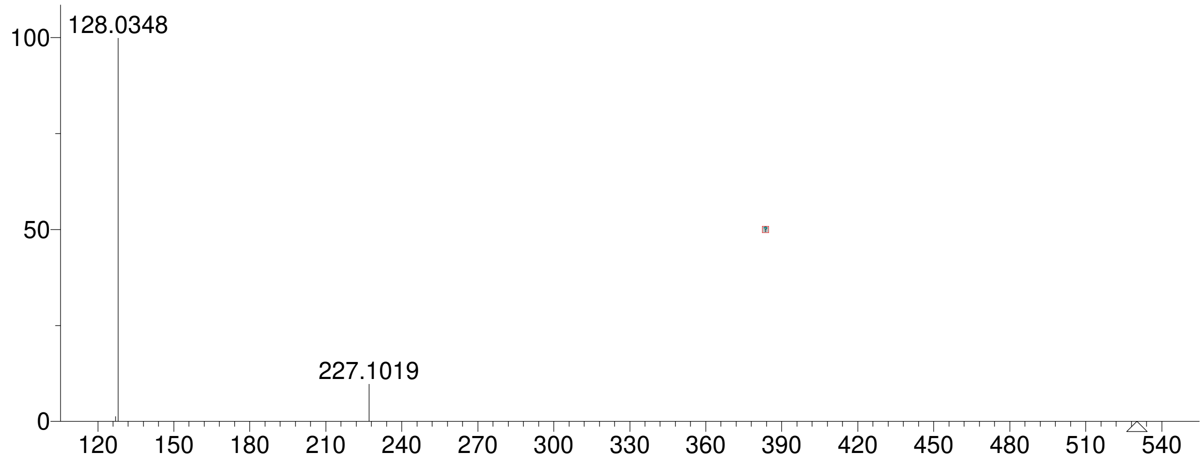

Hit 1 : Nilotinib [M+H]+ HCD 196V P=530.2
C28H22F3N7O; MF: 0; RMF: 25; Prob 50.0%; CAS: 641571-10-0; Lib: nist_msms; ID: 165147.

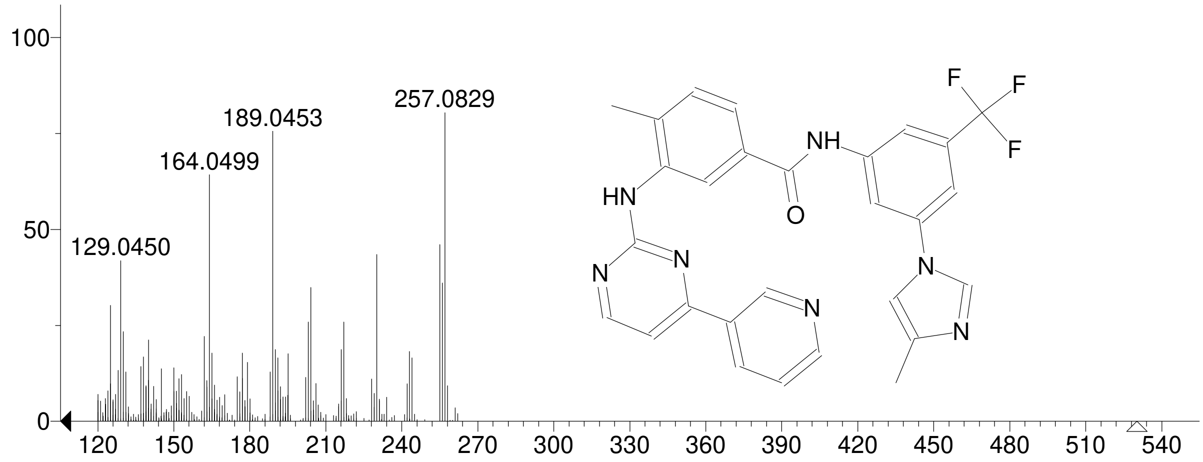

Hit 2 : Nilotinib (AMN-107) [M+H]+ P=530.2
C28H22F3N7O; MF: 0; RMF: 1; Prob 50.0%; Lib: mona-export-gnps; ID: 336.

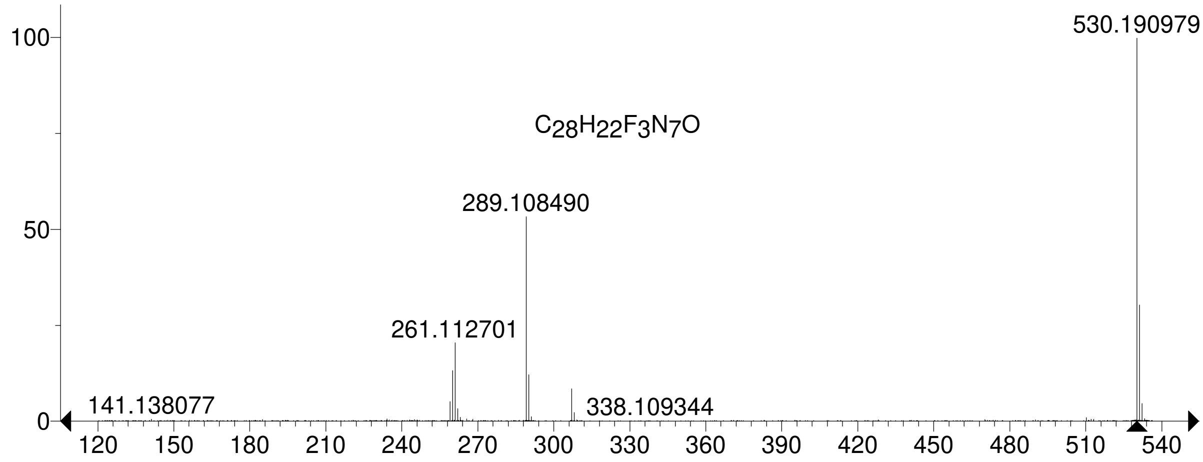

Unknown: Unknown (7.06_787.3338m/z)
Compound in Library Factor = 0

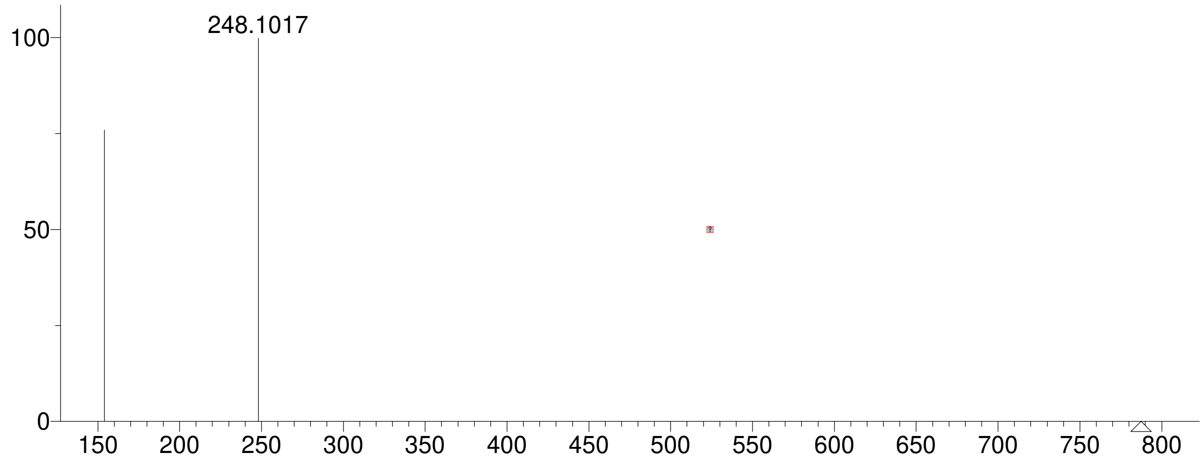

Unknown: Unknown (7.07_403.1006m/z)
Compound in Library Factor = 0

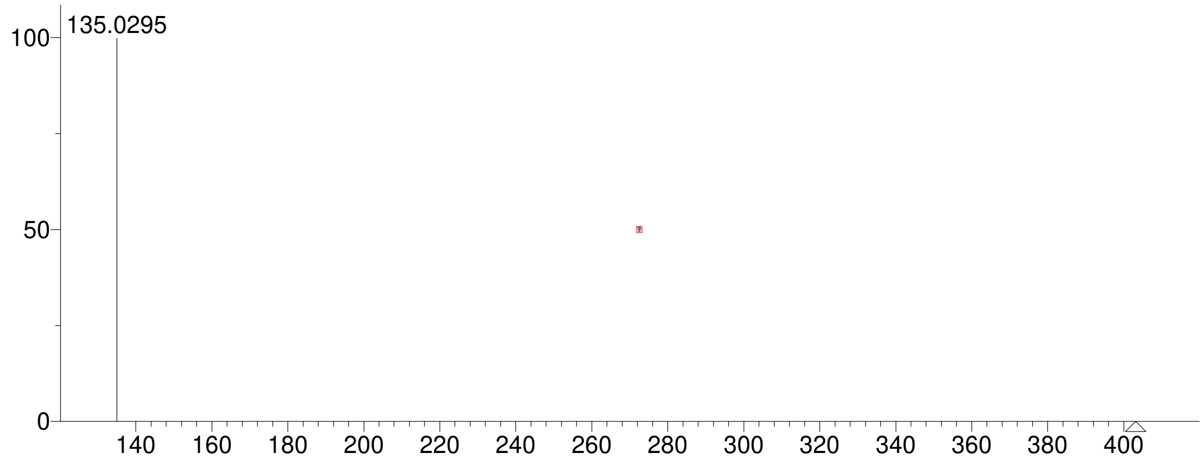

Unknown: Unknown (7.08_386.1047m/z)
Compound in Library Factor = 0

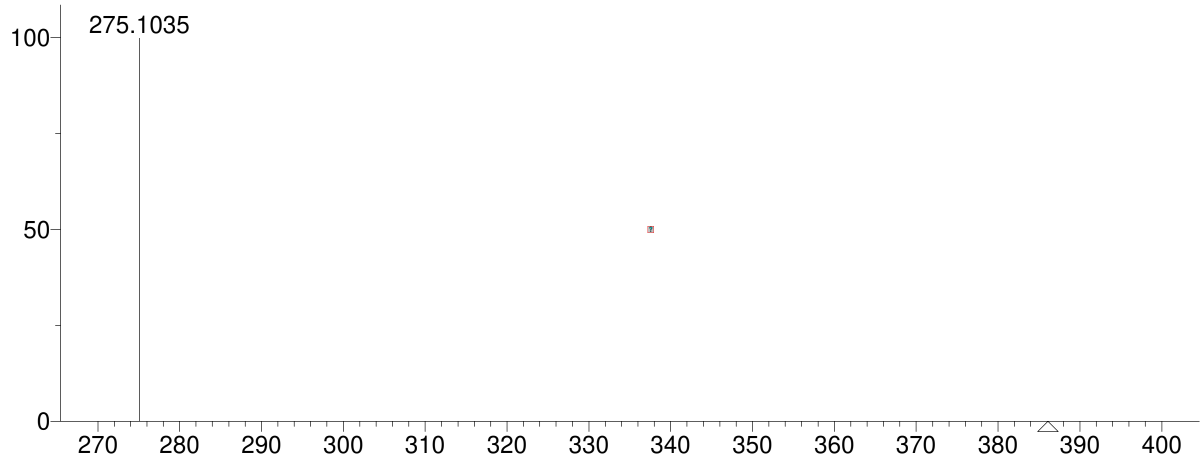

Unknown: Unknown (7.08_424.1203m/z)
Compound in Library Factor = -472

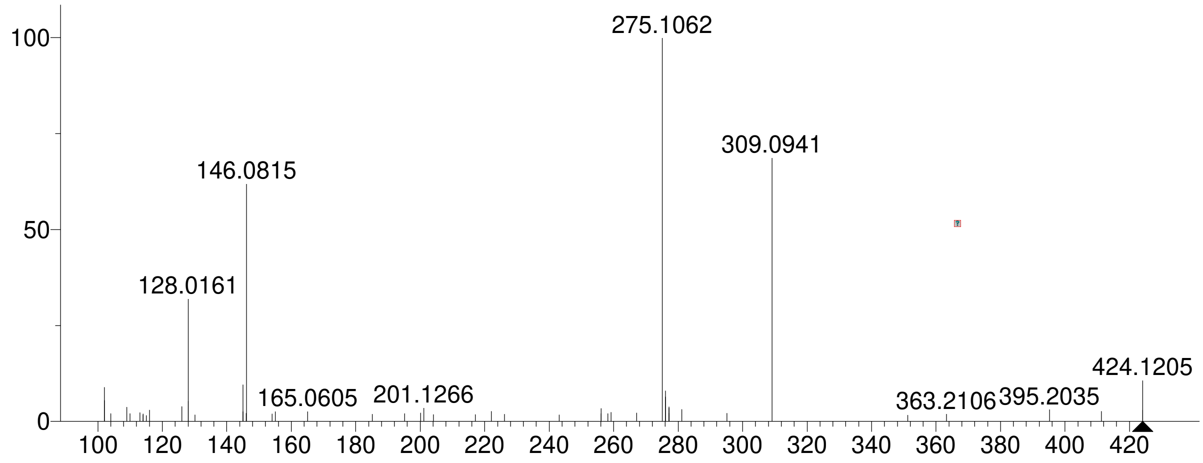

Hit 1 : Benzenesulfonamide, N-[[(1,1-dimethylethyl)amino]carbonyl]-2-(3-methoxyphenoxy)-5-nitro- [M+H]+ HCD
C18H21N3O7S; MF: 153; RMF: 698; Prob 94.1%; CAS: 945716-28-9; Lib: nist_msms; ID: 371131.

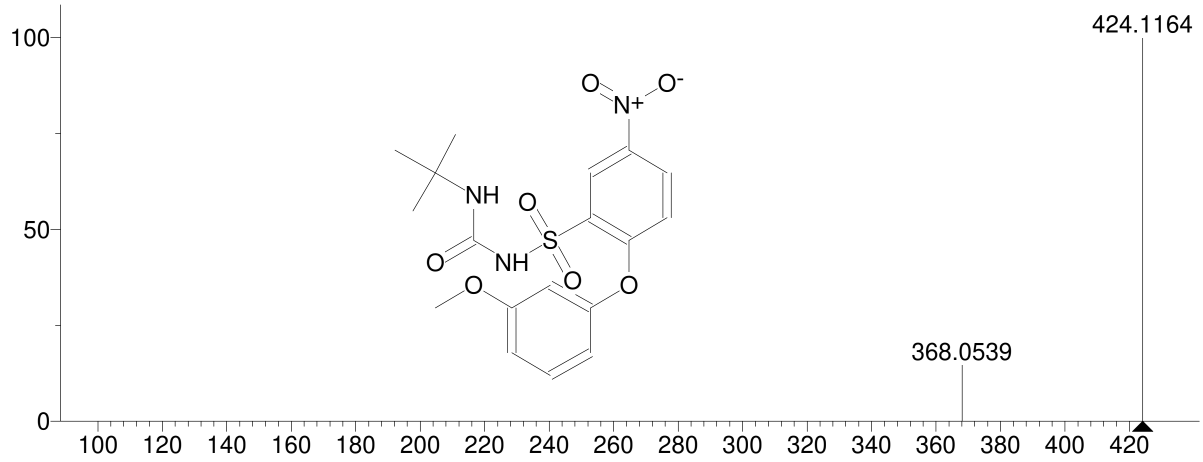

Hit 2 : N6-(4-Methoxybenzoyl)adenosine [M+Na]+ HCD 12V P=424.1
C18H19N5O6; MF: 14; RMF: 208; Prob 3.27%; CAS: 56883-05-7; Lib: nist_msms; ID: 298845.

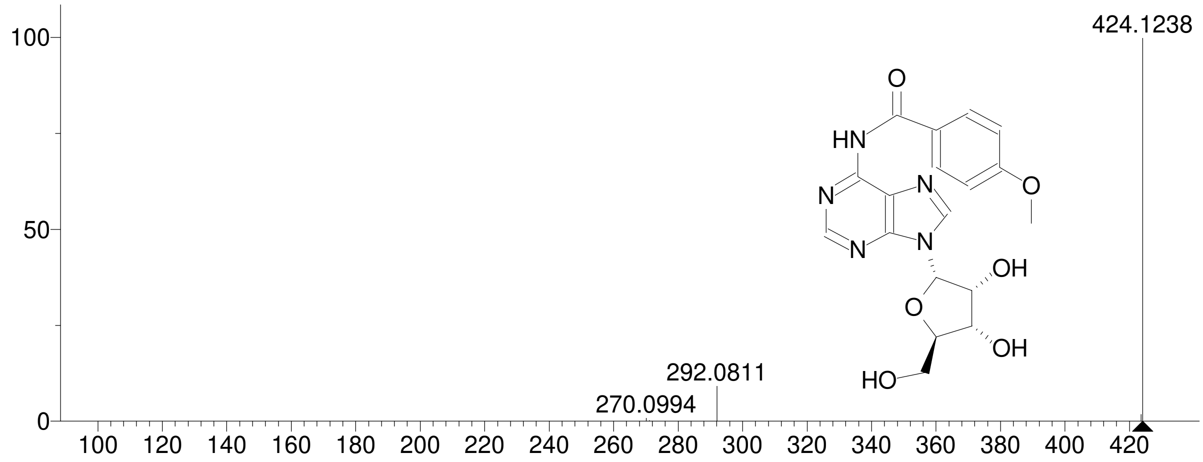

Hit 3 : Spiro[isobenzofuran-1(3H),9'-[9H]xanthen]-3-one, 3'-(4-aminophenoxy)-6'-hydroxy- [M+H]+ IT-FT 35% P
C26H17NO5; MF: 8; RMF: 289; Prob 2.57%; CAS: 359010-70-1; Lib: nist_msms; ID: 376892.

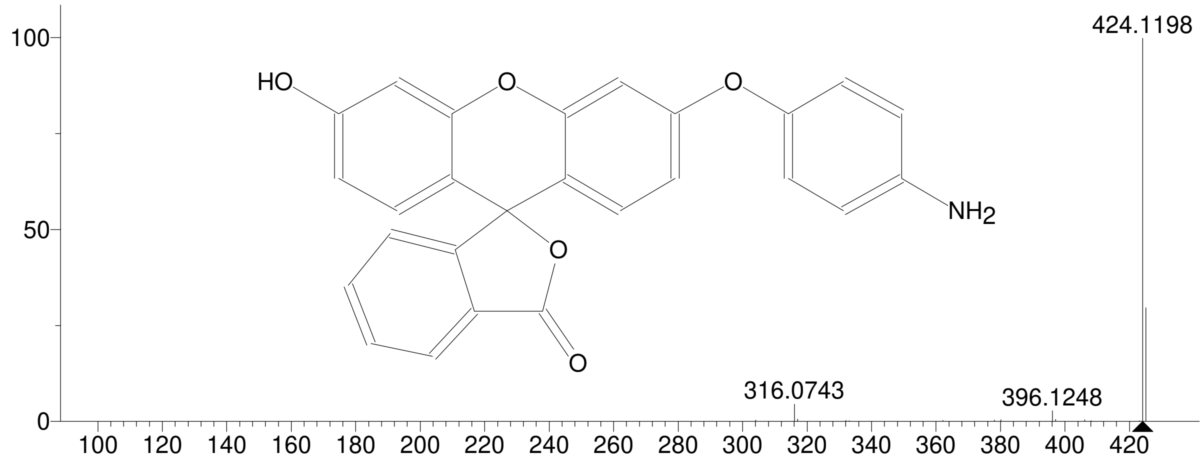

Unknown: Unknown (7.10_431.2032m/z)
Compound in Library Factor = -1523

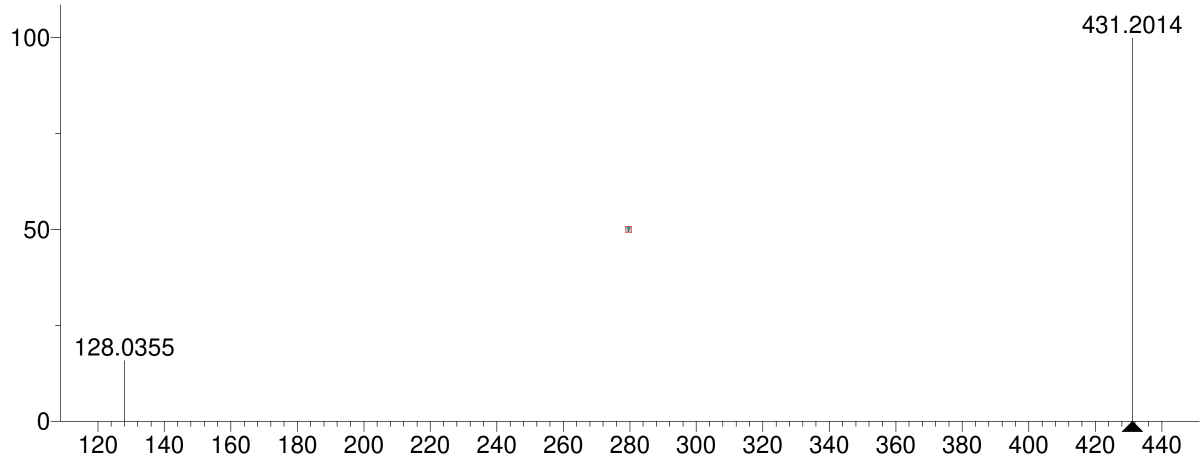

Hit 1 : Eplerenone hydroxy acid [M-H]- HCD 19V P=431.2
C24H32O7; MF: 136; RMF: 868; Prob 65.9%; CAS: 579484-30-3; Lib: nist_msms; ID: 437832.

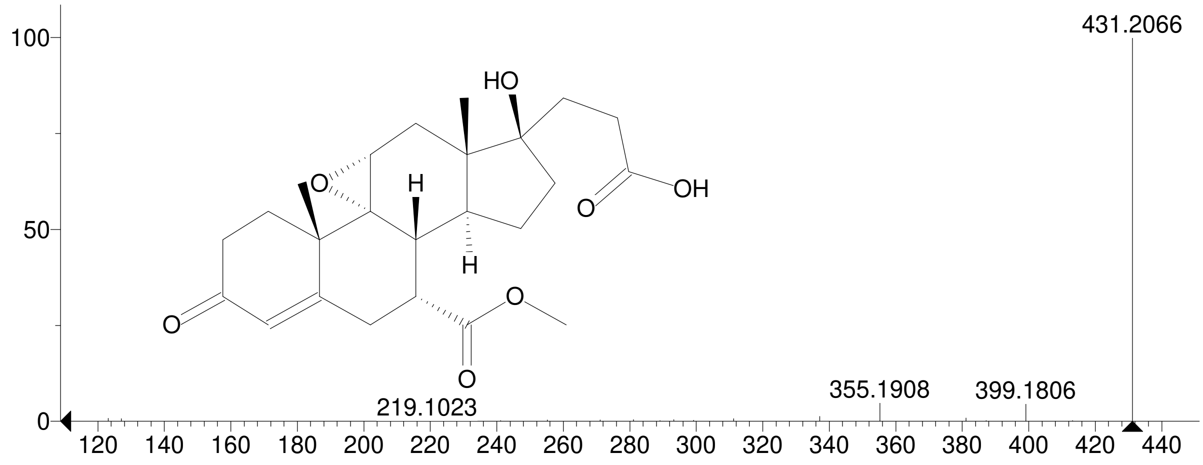

Hit 2 : 6β-Hydroxyeplerenone [M+H]+ HCD 12V P=431.2
C24H30O7; MF: 90; RMF: 852; Prob 13.9%; CAS: 209253-80-5; Lib: nist_msms; ID: 441588.

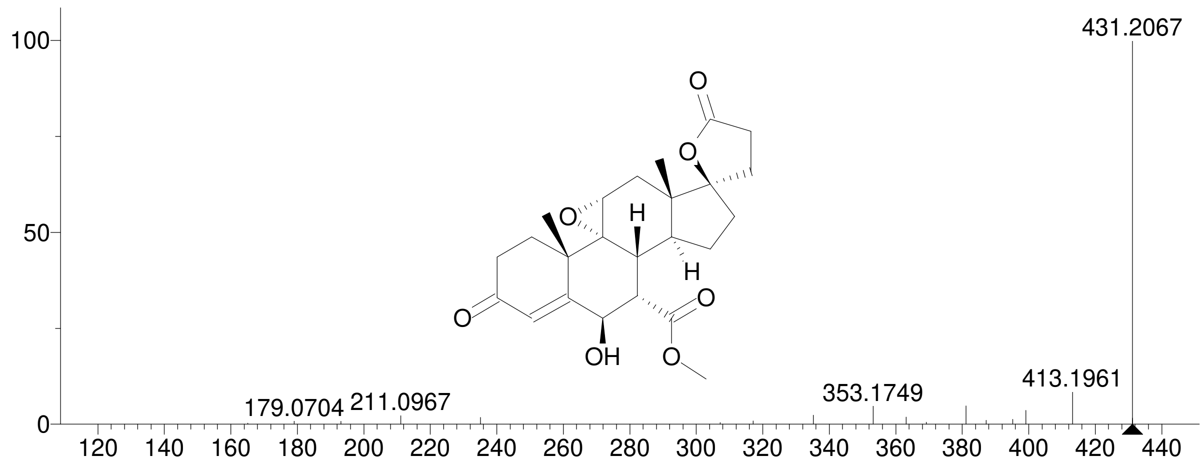

Hit 3 : NCGC00381060-01_C24H32O7_Benzoic acid, 4-hydroxy-, (1aS,2aR,3S,5R,6S,7aR)-3-(acetyloxy)decahy
C24H32O7; MF: 79; RMF: 771; Prob 9.54%; Lib: msms-neg-gnps; ID: 1864.

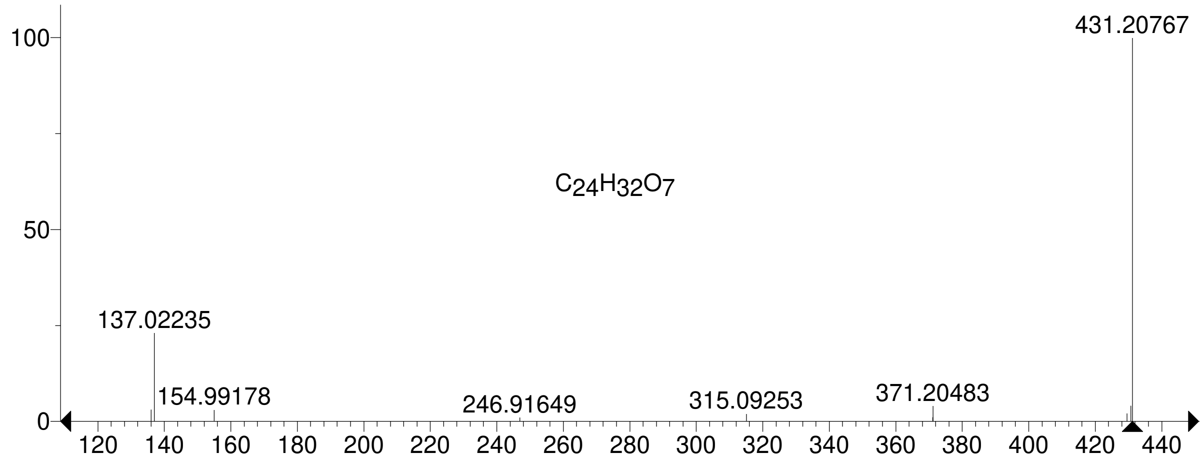

Hit 4 : NCGC00380477-01_C24H32O7_Benzoic acid, 4-hydroxy-, (3R,4S,8aS)-2-(acetyloxy)-1,2,3,3a,4,7,8,8a-oc
C24H32O7; MF: 59; RMF: 663; Prob 4.35%; Lib: msms-neg-gnps; ID: 1602.

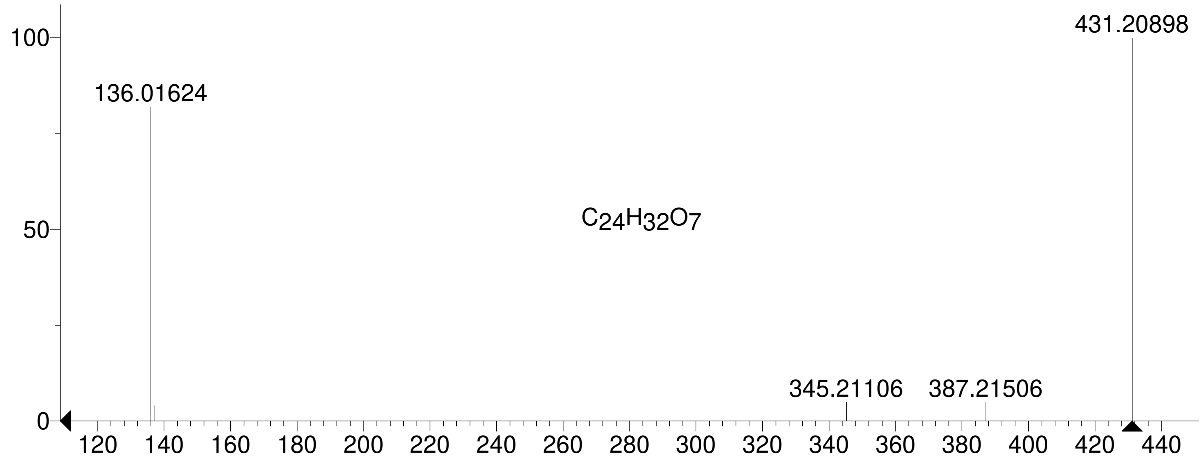

Hit 5 : NCGC00347874-02!5-hydroxy-2,2,6,6-tetramethyl-4-[2-methyl-1-[2,4,6-trihydroxy-3-(2-methylpropanoyl)ph
C24H32O7; MF: 13; RMF: 378; Prob 0.91%; Lib: msms-neg-gnps; ID: 896.

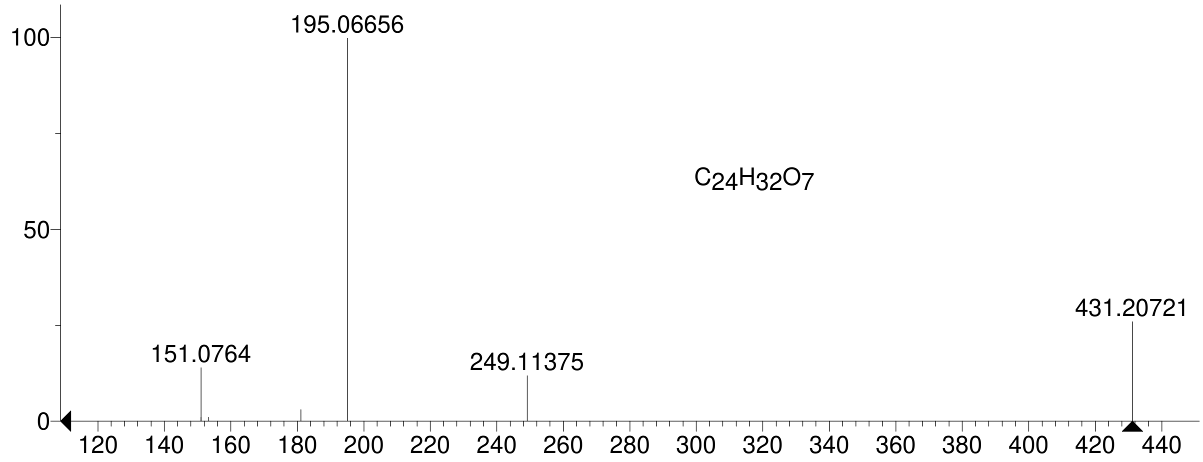

Unknown: Unknown (7.11_419.1560m/z)
Compound in Library Factor = 0

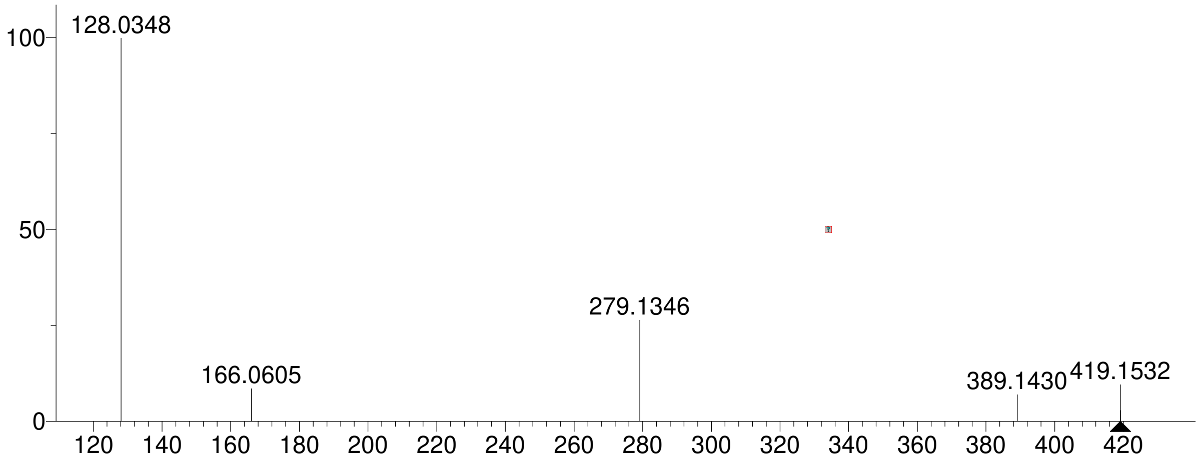

Unknown: Unknown (7.12_355.1611m/z)
Compound in Library Factor = 0

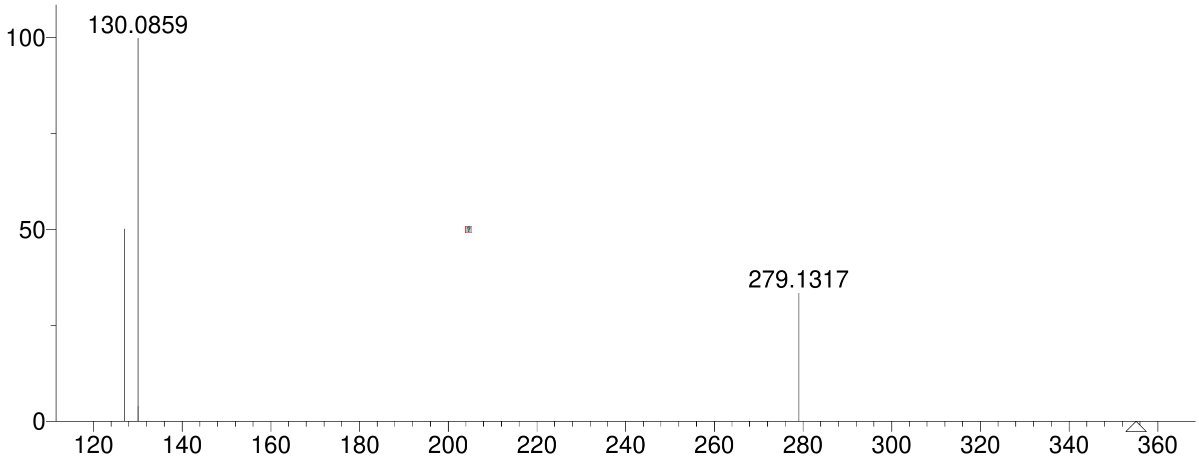

Unknown: Unknown (7.12_787.3384m/z)
Compound in Library Factor = 0

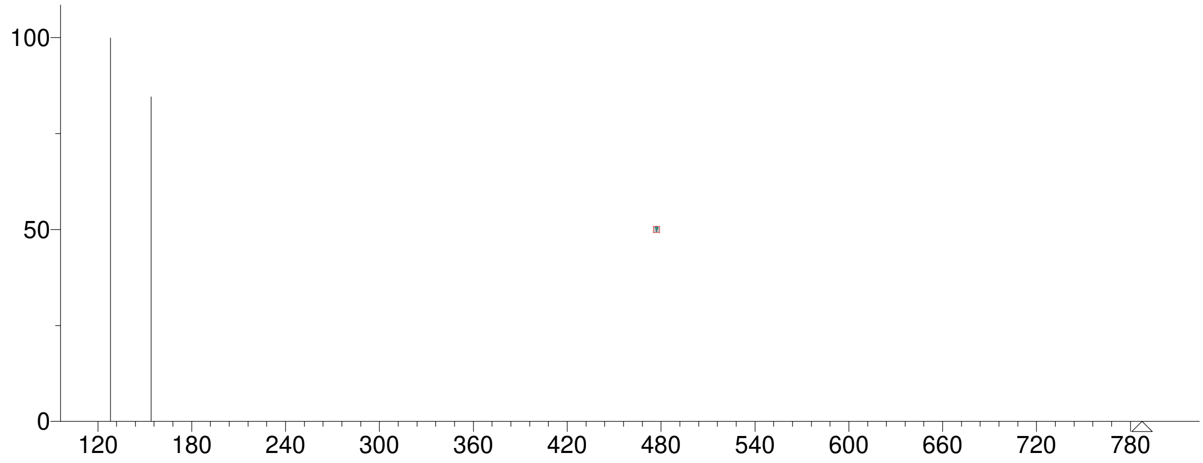

Unknown: Unknown (7.13_338.1245m/z)
Compound in Library Factor = 0

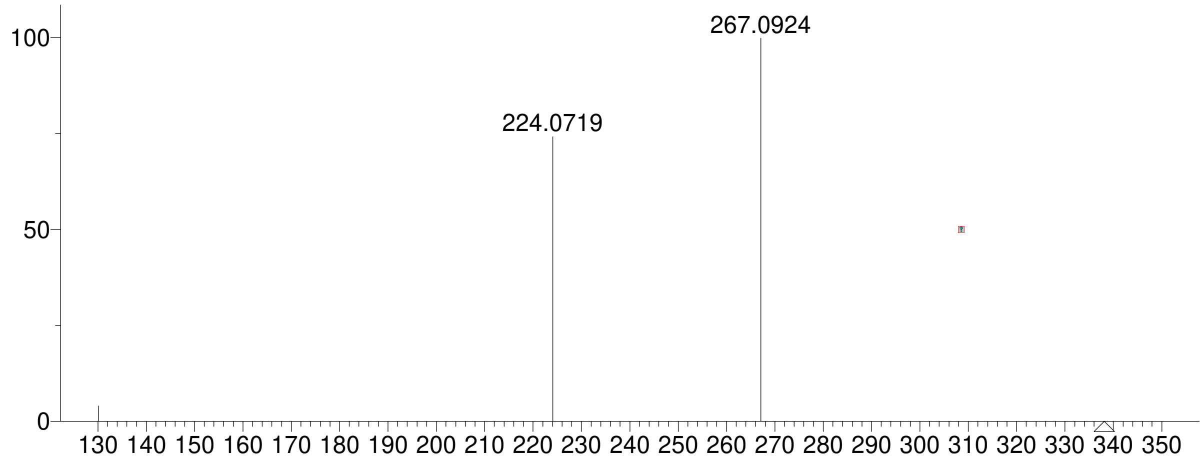

Hit 1 : 1-Naphthol β-D-glucuronide [M+NH4]+ IT 35% P=338.1
C16H16O7; MF: 0; RMF: 45; Prob 50.0%; CAS: 17238-47-0; Lib: nist_msms; ID: 464034.

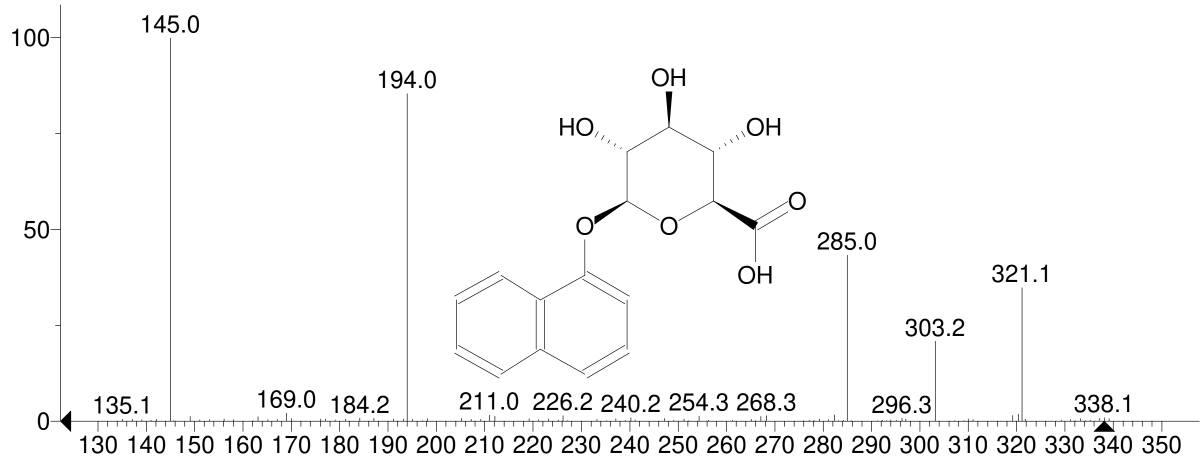

Hit 2 : barceloneic acid A [M+NH4]+ 35HCD P=338.1
C16H16O7; MF: 0; RMF: 6; Prob 50.0%; Lib: mona-export-vf-npl_qexactive; ID: 6133.

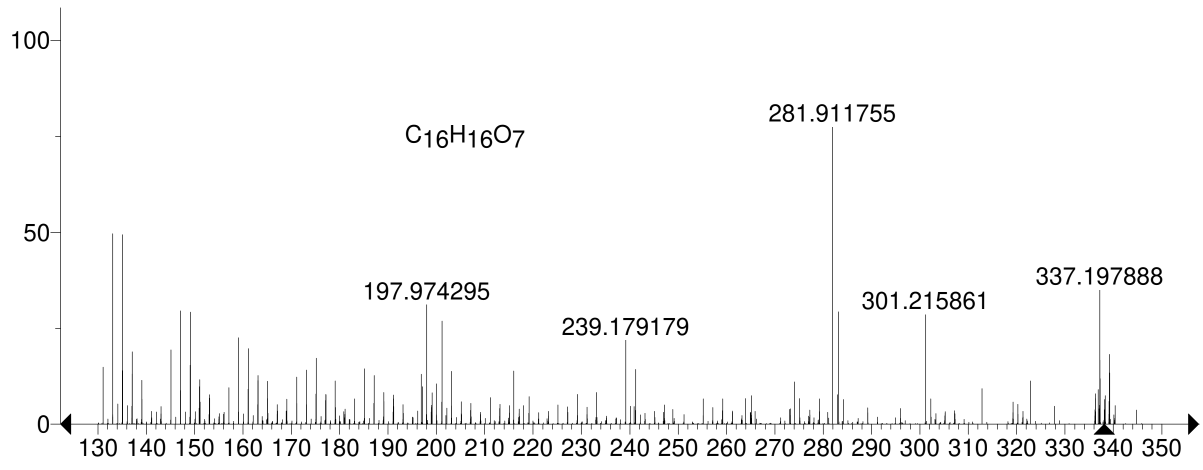

Unknown: Unknown (7.13_426.1964m/z)
Compound in Library Factor = 0

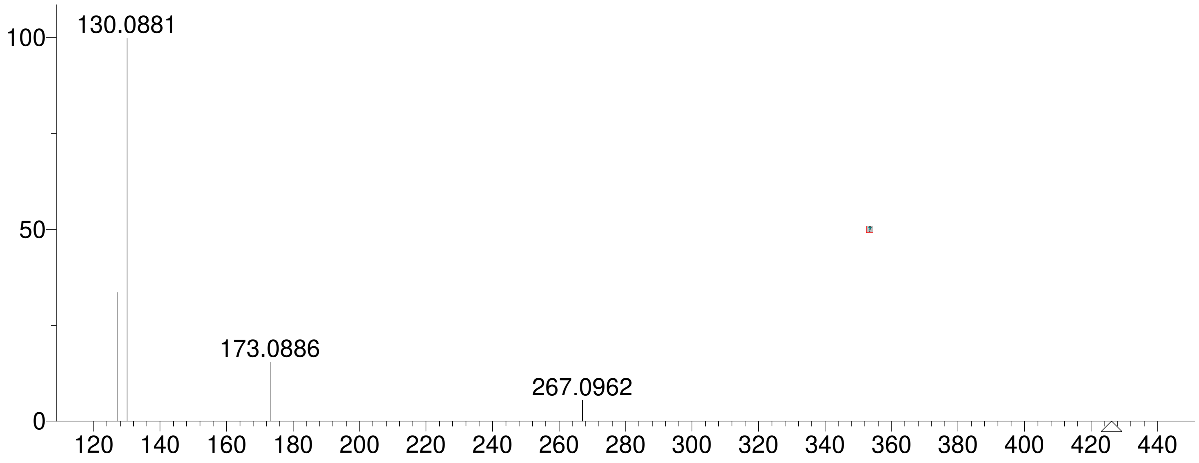

Unknown: Unknown (7.14_387.1755m/z)
Compound in Library Factor = 0

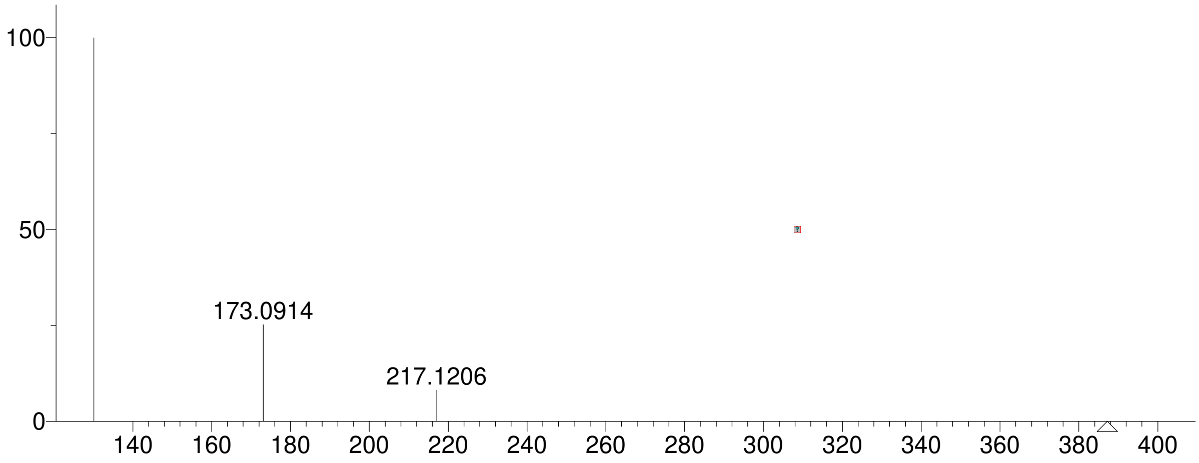

** Search Report Page 1 of 1 **

Unknown: Unknown (7.15_338.1215m/z)
Compound in Library Factor = 0

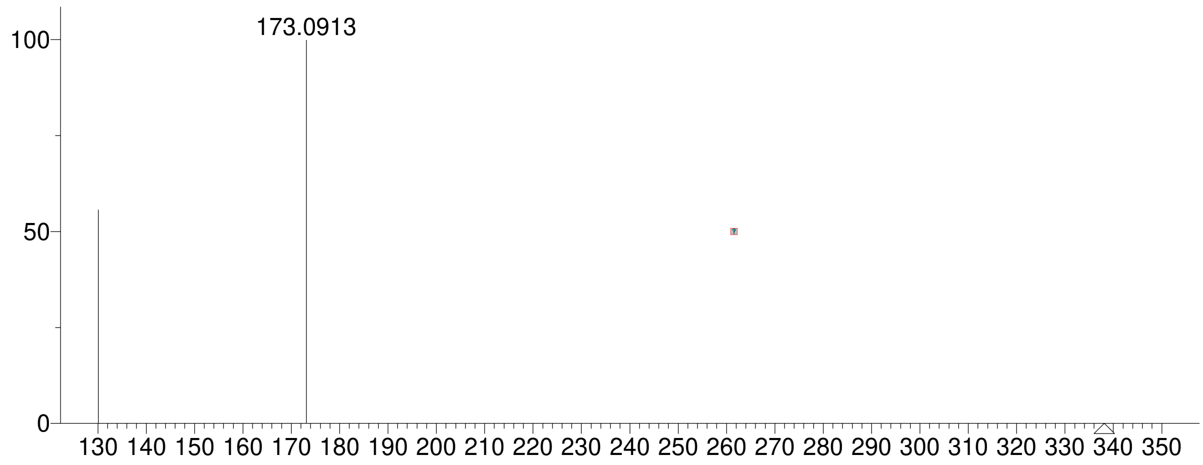

Hit 1 : barceloneic acid A [M+NH4]+ 45HCD P=338.1
C16H16O7; MF: 0; RMF: 59; Prob 100.0%; Lib: mona-export-vf-npl_qexactive; ID: 6134.

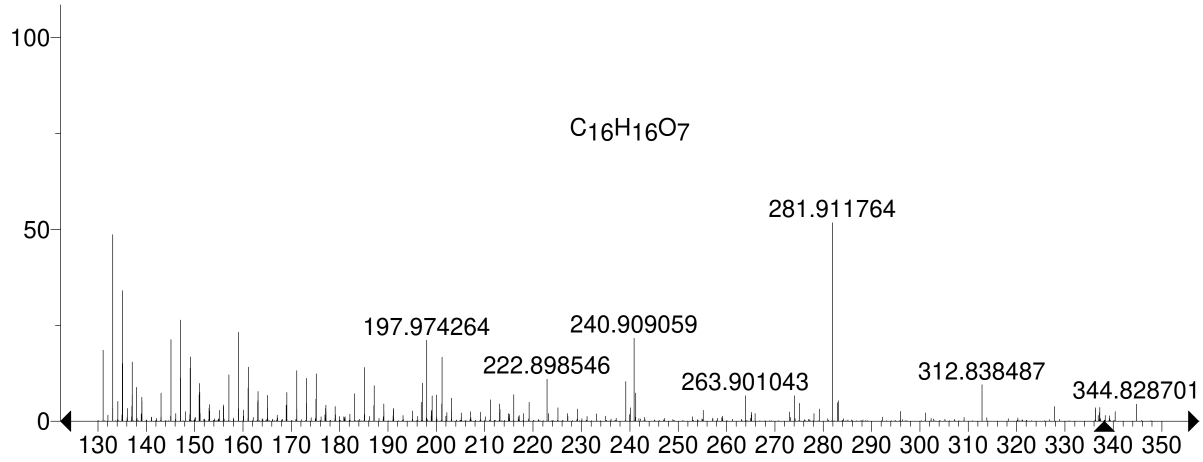

Unknown: Unknown (7.16_396.1241m/z)
Compound in Library Factor = -3273

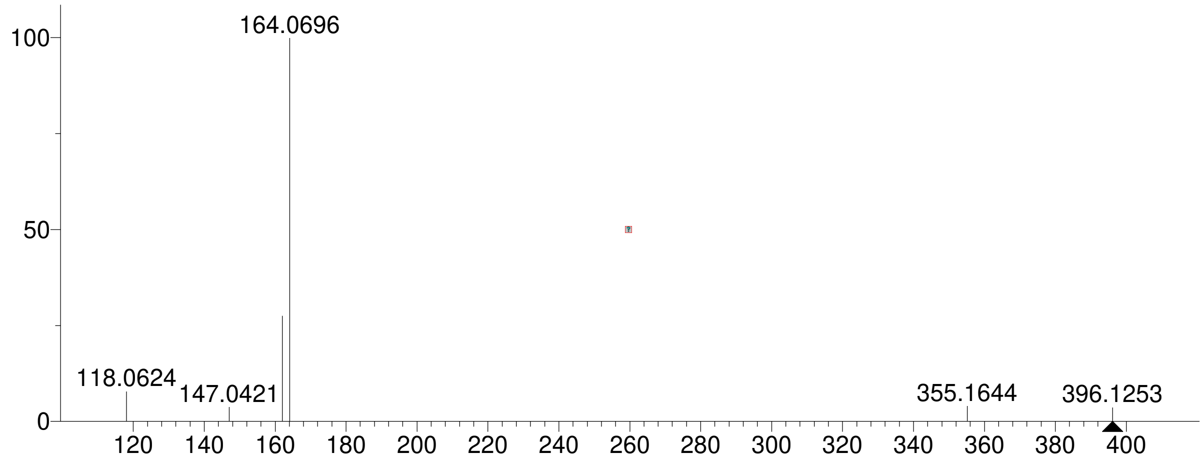

Hit 1 : 2-Oxiraneacetic acid, 3-phenyl-, cyano(6-methoxy-2-naphthalenyl)methyl ester [M+Na]+ HCD 7V P=396
C23H19NO4; MF: 75; RMF: 650; Prob 100.0%; CAS: 1028430-42-3; Lib: nist_msms; ID: 375384.

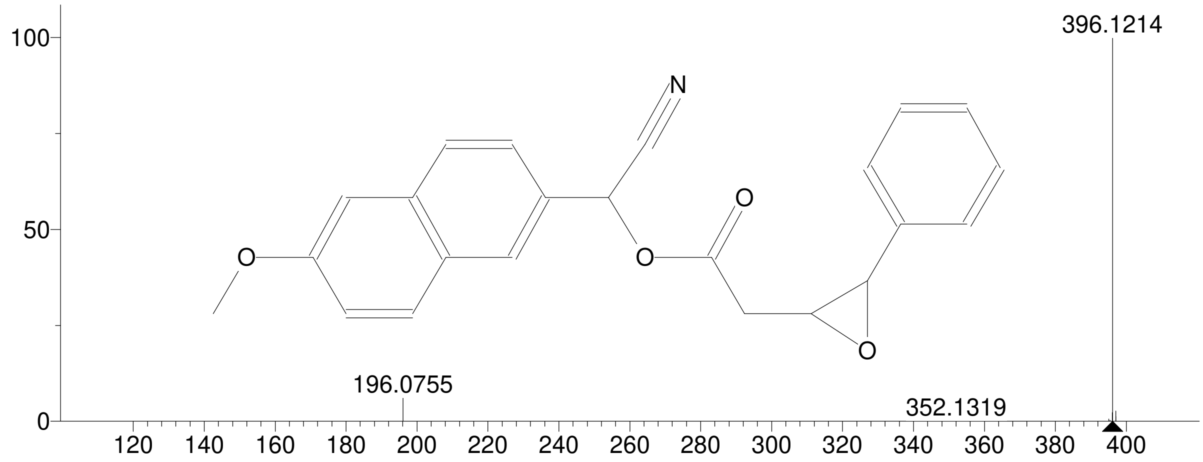

Unknown: Unknown (7.16_566.1841m/z)
Compound in Library Factor = 0

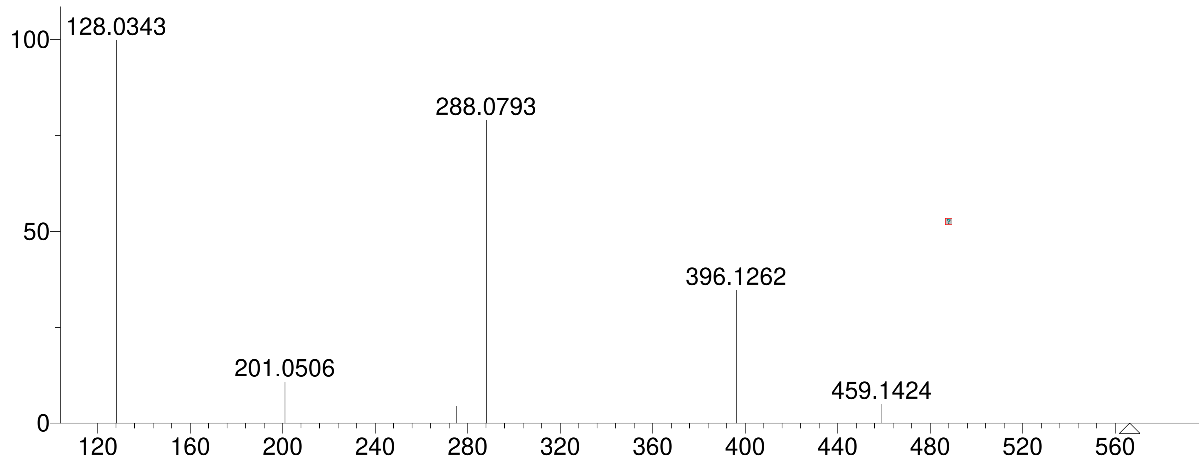

Hit 1 : 5,7-dihydroxy-2-phenyl-6-[3,4,5-trihydroxy-6-(hydroxymethyl)oxan-2-yl]-8-(3,4,5-trihydroxyoxan-2-yl)chrome
C26H28O13; MF: 0; RMF: 15; Prob 100.0%; Lib: anthocyanins mona; ID: 8496.

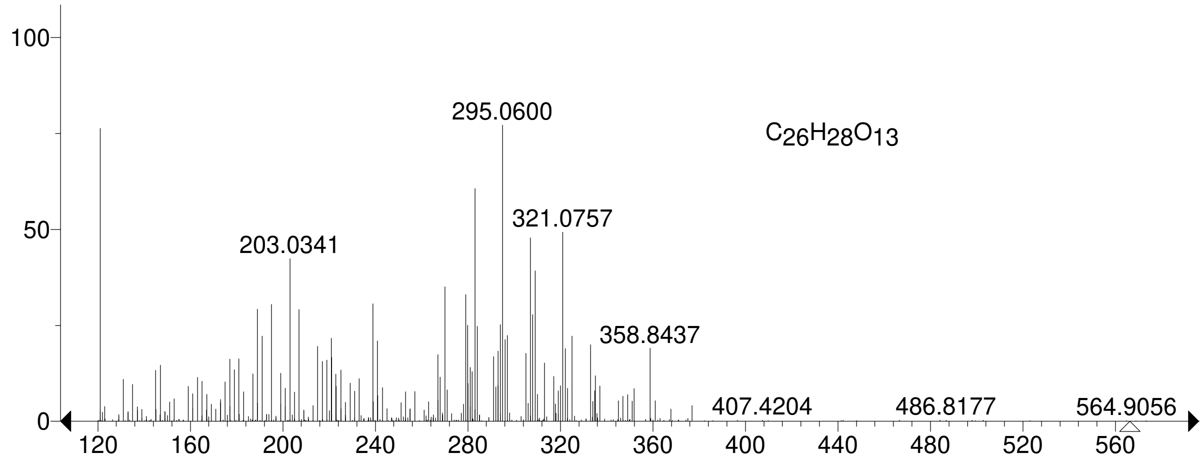

Unknown: Unknown (7.17_348.1441m/z)
Compound in Library Factor = -1072

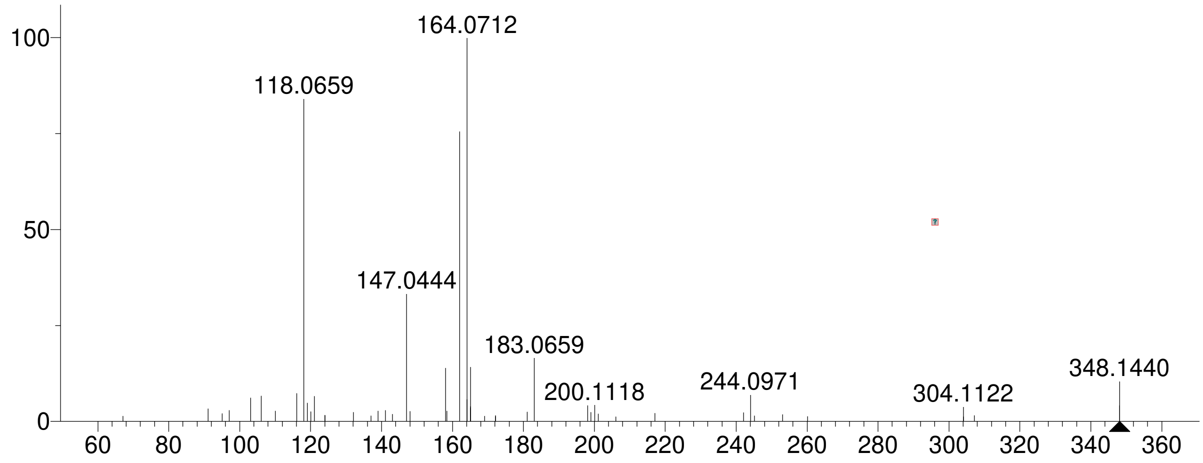

Hit 1 : 1H-Indazole-3-carboxamide, N-[(1S)-1-(aminocarbonyl)-2-methylpropyl]-1-(5-chloropentyl)- [M+H-NH3]+ I
C18H25ClN4O2; MF: 108; RMF: 649; Prob 69.2%; CAS: 1801552-02-2; Lib: nist_msms; ID: 426676.

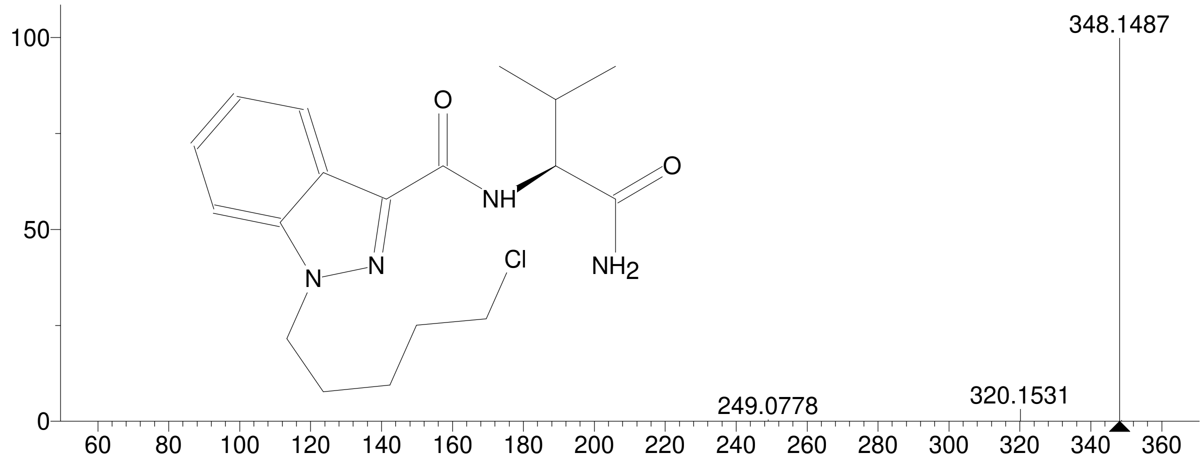

Hit 2 : Monocrotaline [M+Na]+ HCD 41V P=348.1
C16H23NO6; MF: 38; RMF: 140; Prob 10.1%; CAS: 315-22-0; Lib: nist_msms; ID: 187311.

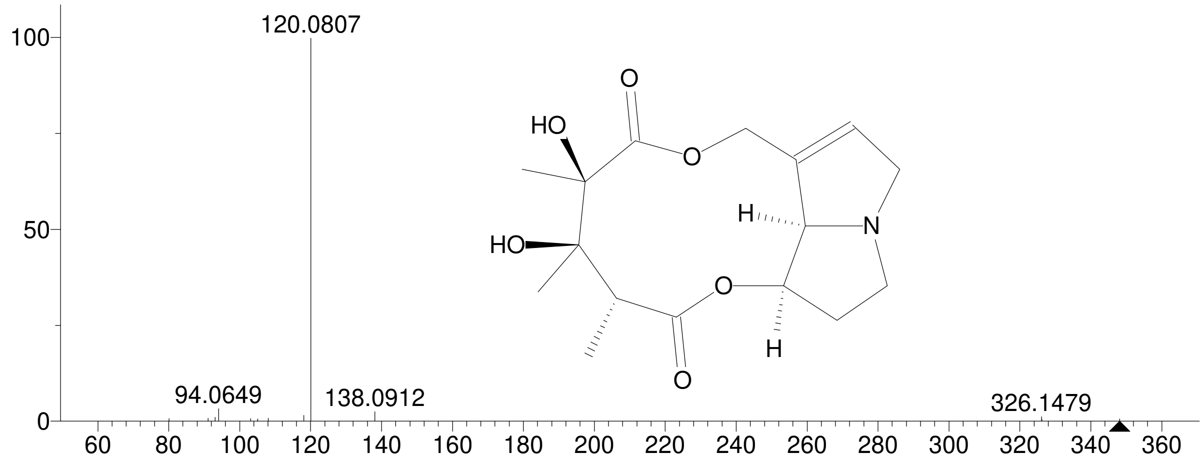

Hit 3 : 3-Pyridinecarboxamide, 6-amino-N-[3-[4-(4-morpholinyl)pyrido[3',2':4,5]furo[3,2-d]pyrimidin-2-yl]phenyl]- [[illegible]
C25H21N7O3; MF: 17; RMF: 177; Prob 4.28%; CAS: 371942-69-7; Lib: nist_msms; ID: 262100.

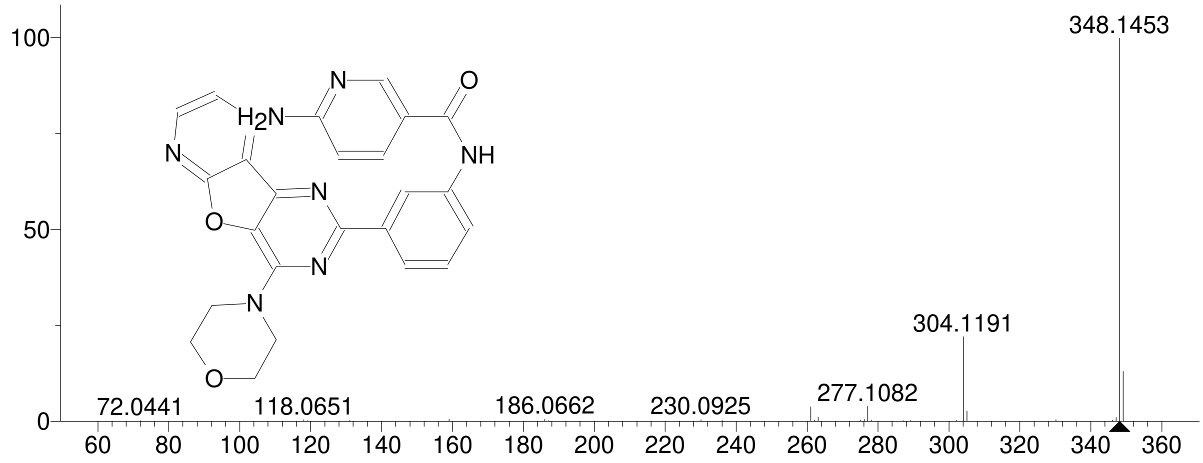

Hit 4 : His-Gly-His [M-H]- HCD 13eV P=348.1
C14H19N7O4; MF: 7; RMF: 181; Prob 3.02%; Lib: nist_msms; ID: 574325.

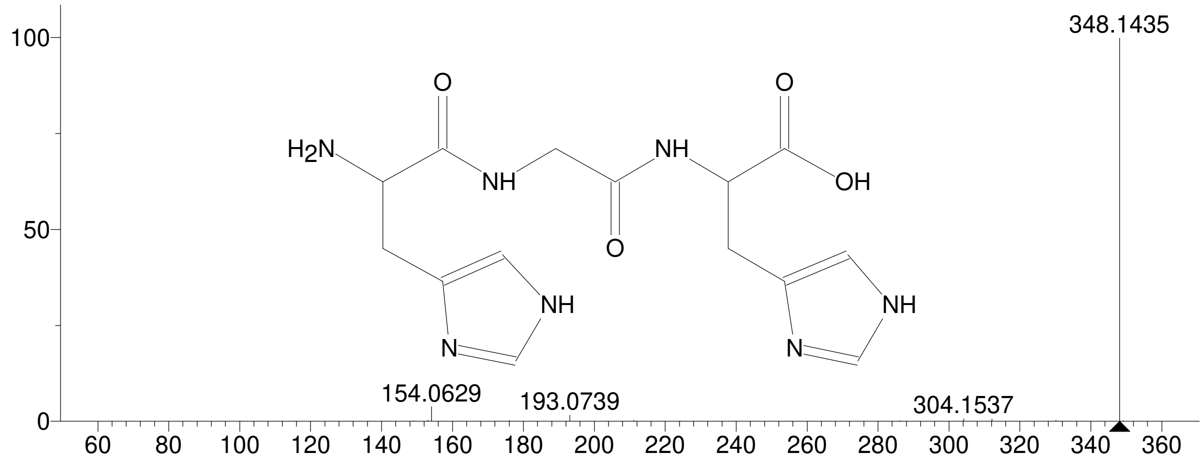

Hit 5 : His-Gly-His [M-H]- HCD 13eV P=348.1
C14H19N7O4; MF: 7; RMF: 181; Prob 3.02%; CAS: 38689-38-2; Lib: nist_msms2; ID: 89451.

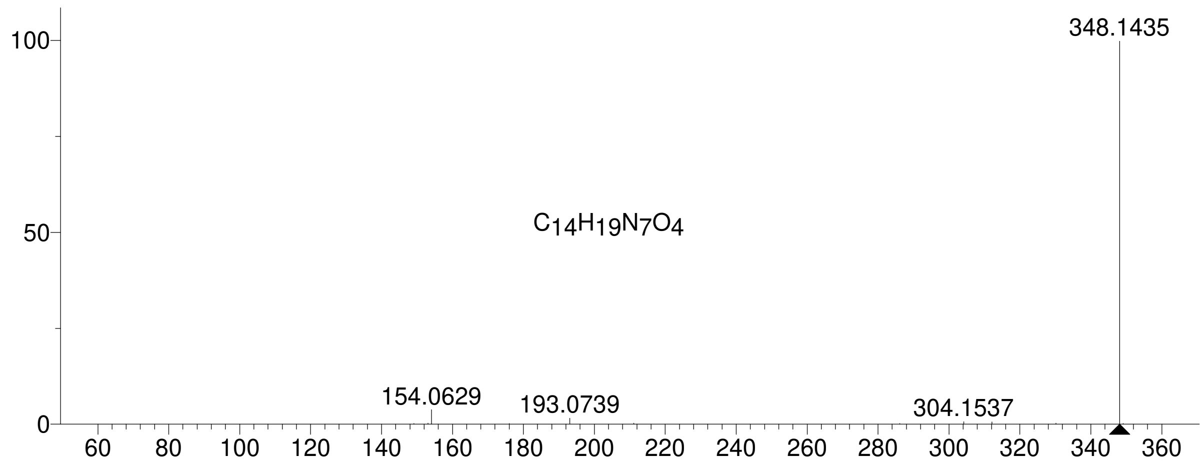

** Search Report Page 1 of 1 **

Unknown: Unknown (7.18_787.3366m/z)
Compound in Library Factor = 0

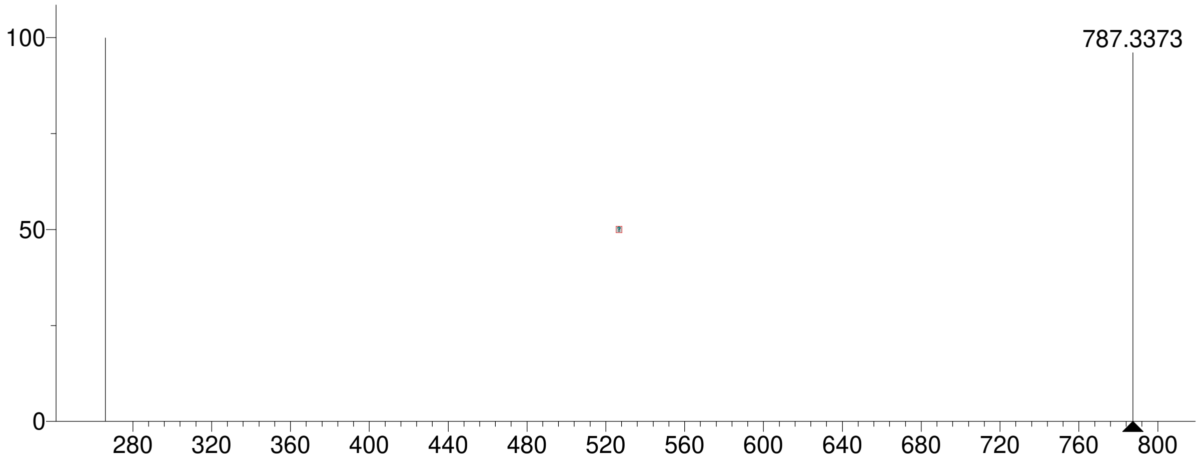

Unknown: Unknown (7.19_458.1409m/z)
Compound in Library Factor = 0

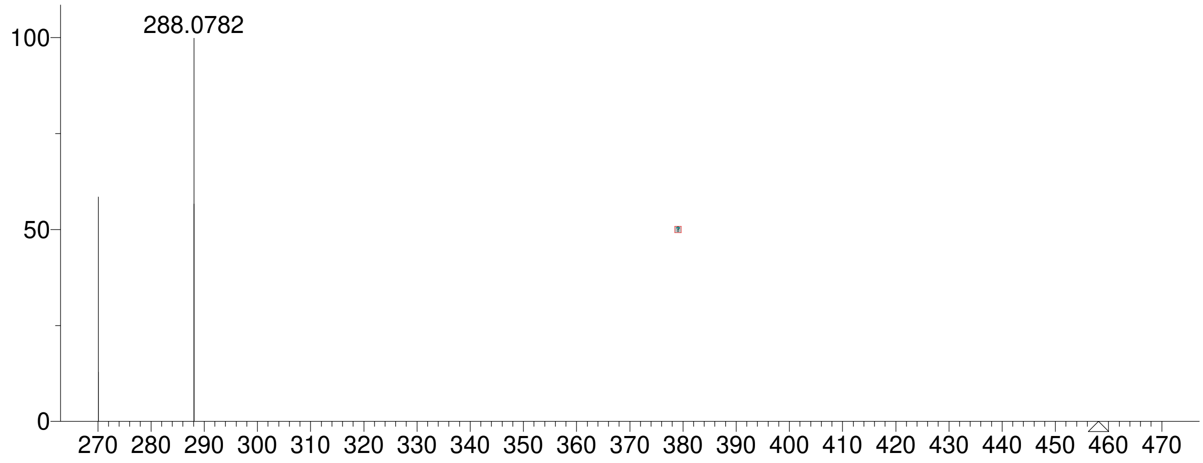

** Search Report Page 1 of 1 **

Unknown: Unknown (7.19_628.1990m/z)
Compound in Library Factor = 0

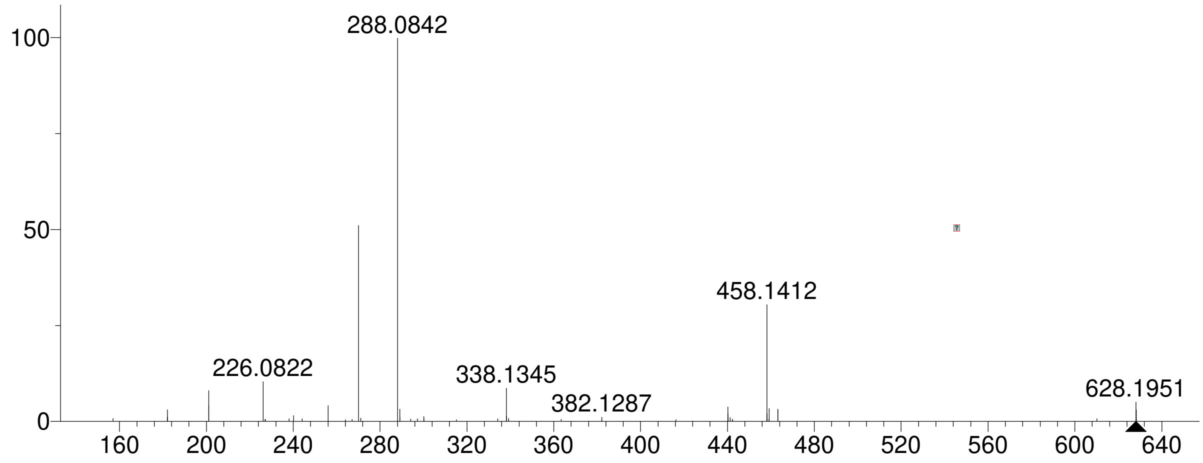

Unknown: Unknown (7.20_243.1706m/z)
Compound in Library Factor = -526

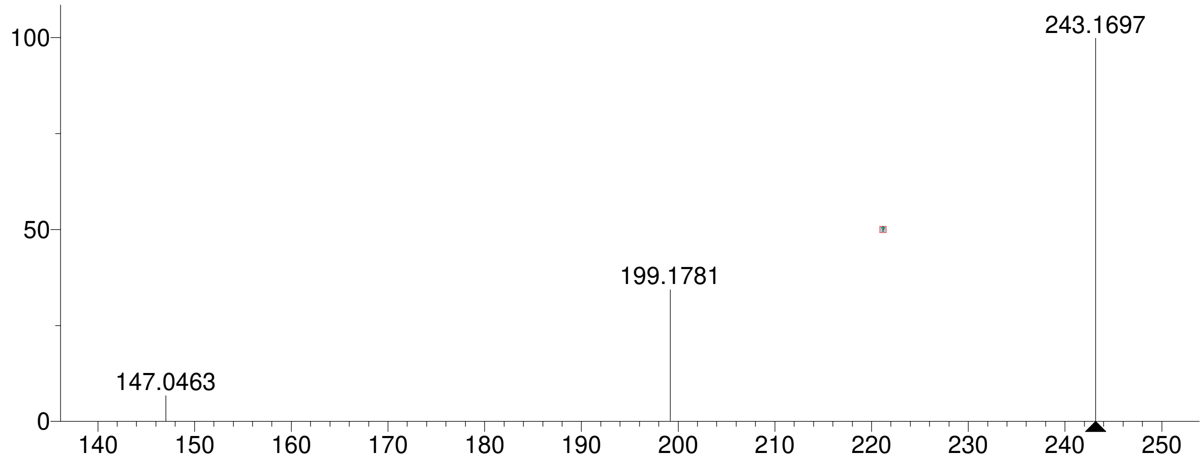

Hit 1 : Ile-Leu [M-H]- HCD 18eV P=243.2
C12H24N2O3; MF: 569; RMF: 971; Prob 29.5%; Lib: nist_msms; ID: 570161.

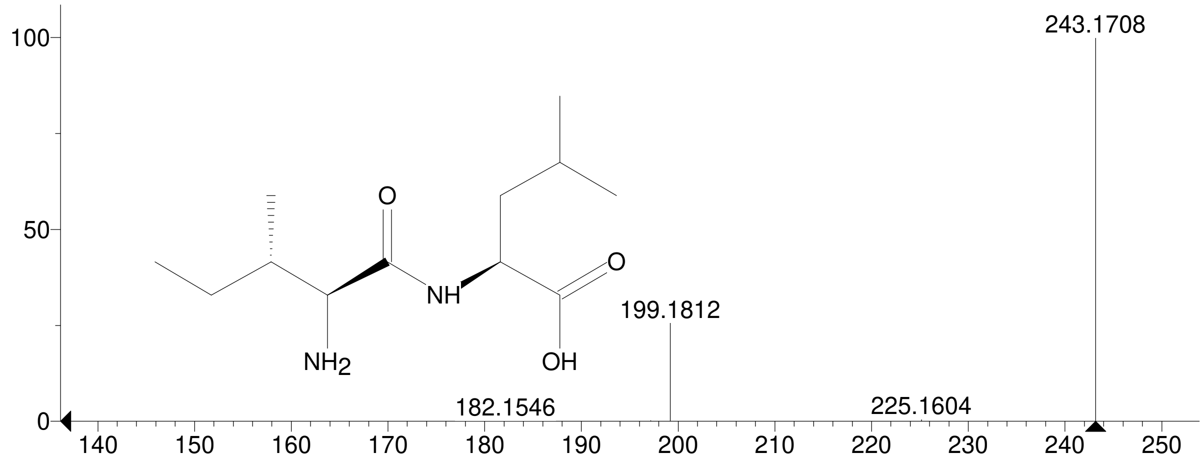

Hit 2 : Ile-Leu [M-H]- HCD 18eV P=243.2
C12H24N2O3; MF: 569; RMF: 971; Prob 29.5%; CAS: 26462-22-6; Lib: nist_msms2; ID: 83887.

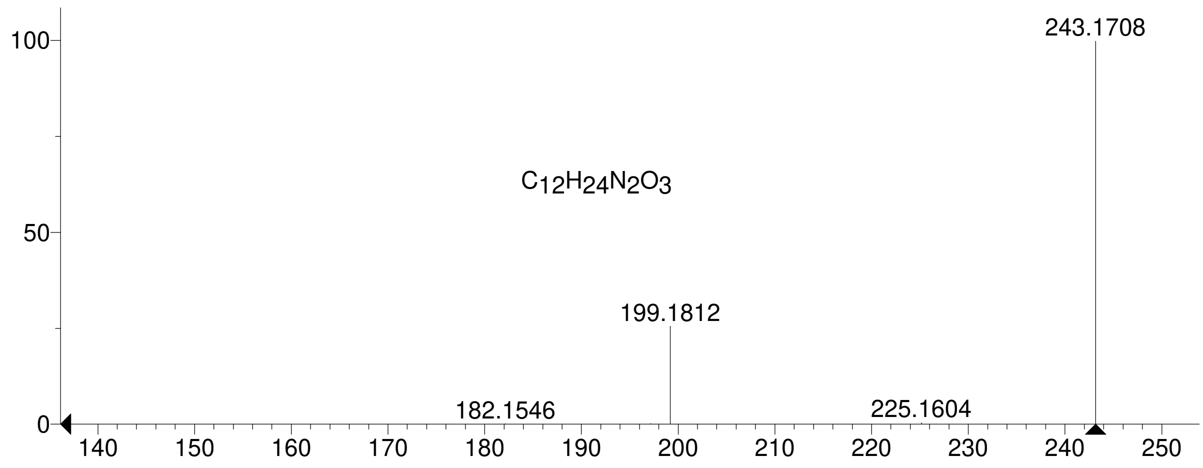

Hit 3 : Leu-Ile [M-H]- HCD 18eV P=243.2
C12H24N2O3; MF: 540; RMF: 965; Prob 8.36%; Lib: nist_msms; ID: 571157.

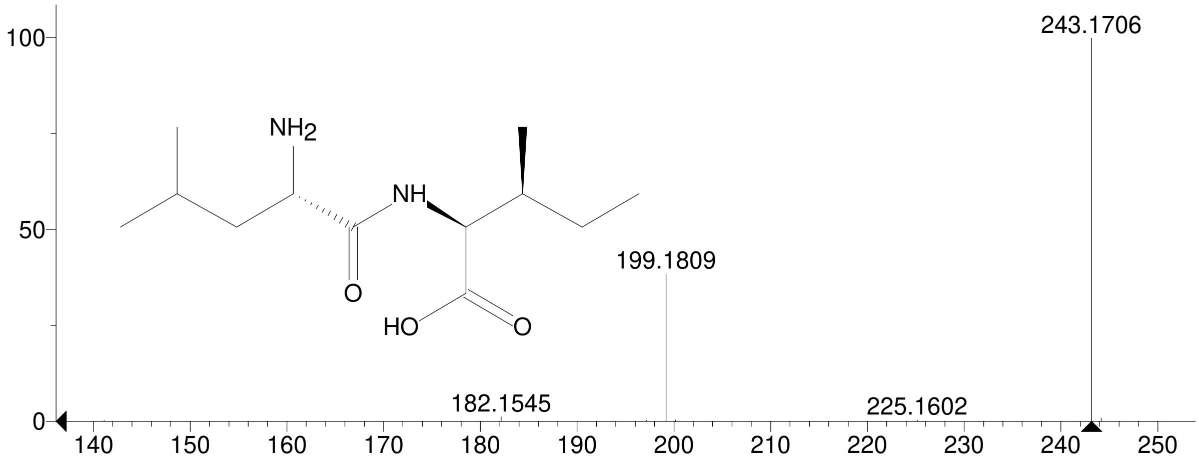

Hit 4 : Leu-Ile [M-H]- HCD 18eV P=243.2
C12H24N2O3; MF: 540; RMF: 965; Prob 8.36%; CAS: 36077-41-5; Lib: nist_msms2; ID: 84890.

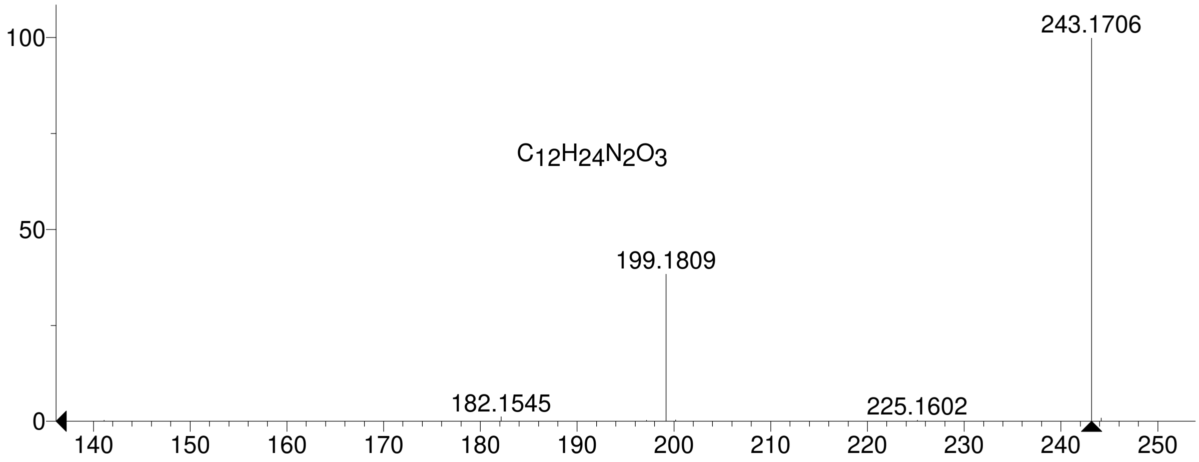

Hit 5 : Leu-Leu [M-H]- HCD 18eV P=243.2
C12H24N2O3; MF: 539; RMF: 958; Prob 8.04%; CAS: 3303-31-9; Lib: nist_msms; ID: 568072.

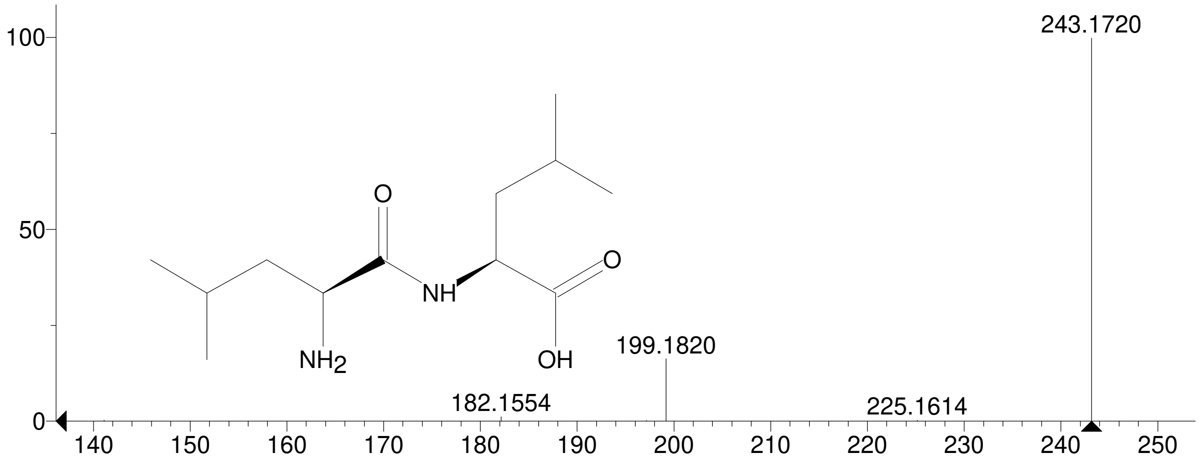

Unknown: Unknown (7.25_415.1709m/z)
Compound in Library Factor = -3273

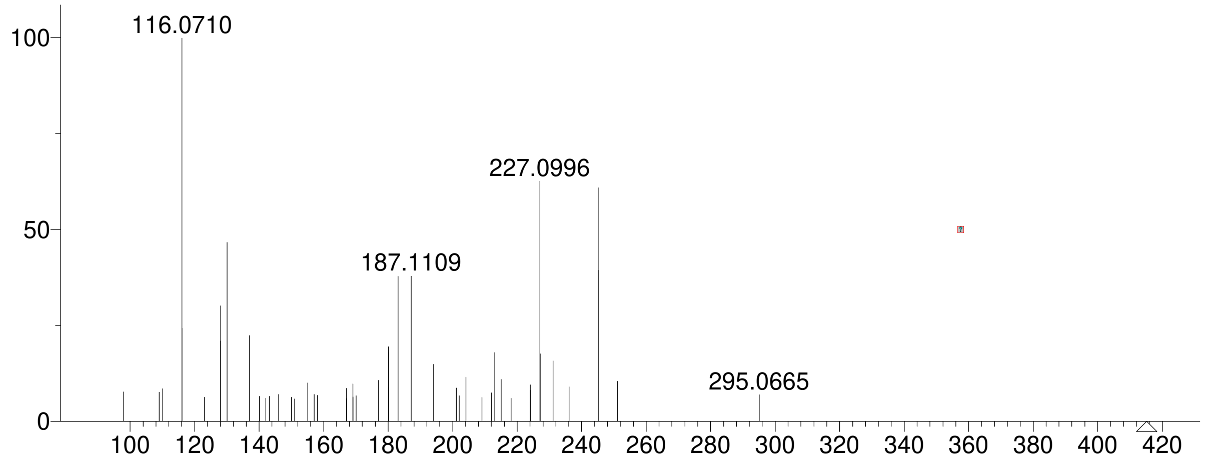

Hit 1 : 6-formyl-10-(hydroxymethyl)-5-methoxy-3-methylidene-2-oxo-2H,3H,3aH,4H,5H,8H,9H,11aH-cyclodeca[b]f
C21H28O7; MF: 7; RMF: 111; Prob 20.6%; Lib: mona-export-vf-npl_qexactive; ID: 16435.

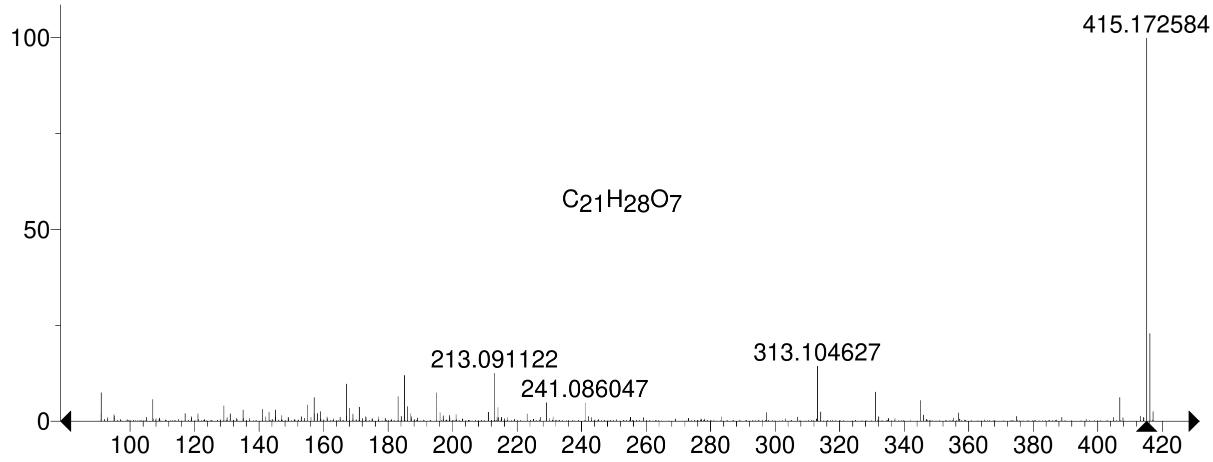

Hit 2 : NCGC00347525-03!2-amino-5-[2-[[2,3-dihydroxy-2-(1-hydroxyethyl)butanoyl]oxymethyl]-4-hydroxyanilino]-
C18H26N2O9; MF: 2; RMF: 65; Prob 16.6%; Lib: mona-export-gnps; ID: 9213.

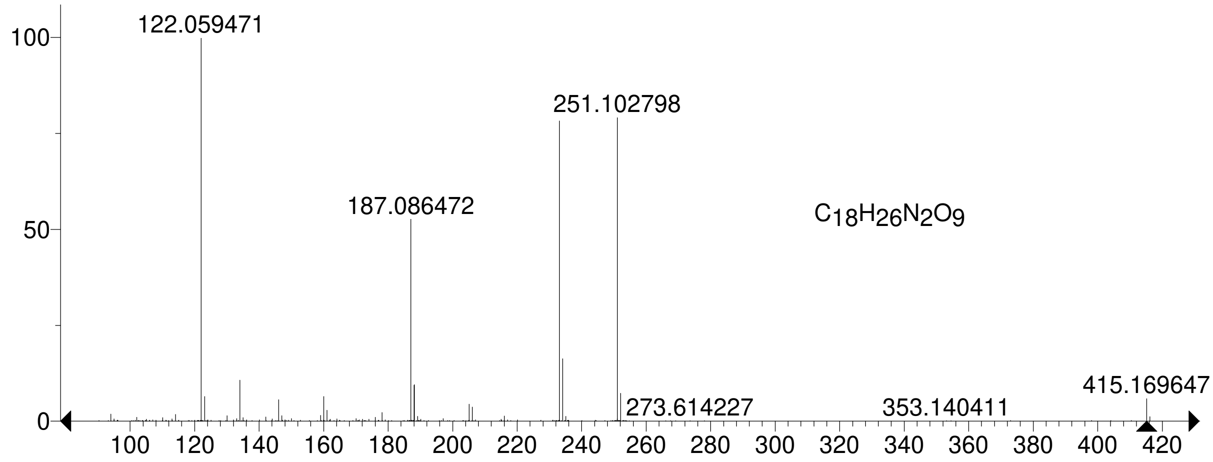

Hit 3 : NCGC00347525-02!2-amino-5-[2-[[2,3-dihydroxy-2-(1-hydroxyethyl)butanoyl]oxymethyl]-4-hydroxyanilino]-
C18H26N2O9; MF: 1; RMF: 63; Prob 16.0%; Lib: mona-export-gnps; ID: 6270.

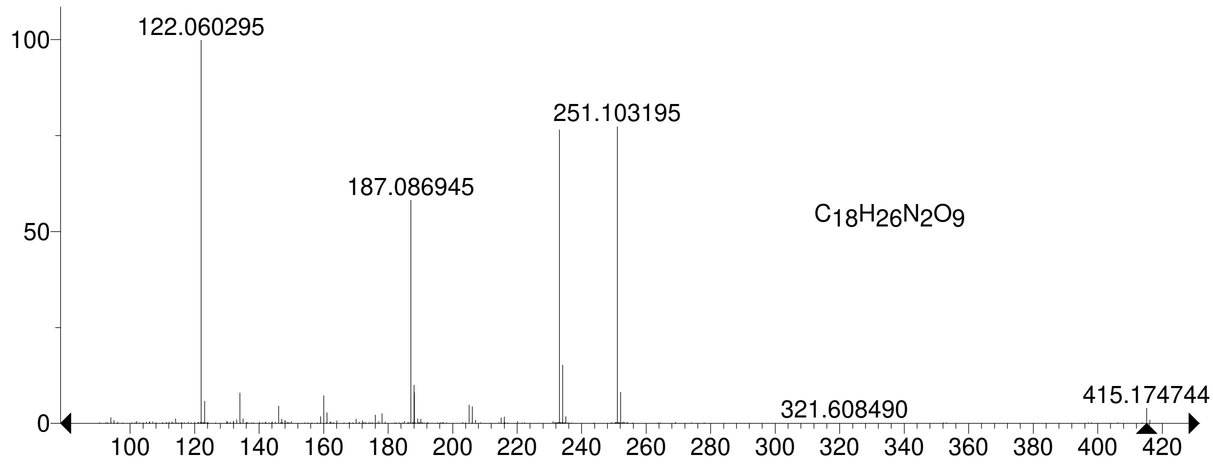

Hit 4 : 2-amino-5-[2-[[2,3-dihydroxy-2-(1-hydroxyethyl)butanoyl]oxymethyl]-4-hydroxyanilino]-5-oxopentanoic acid
C18H26N2O9; MF: 1; RMF: 53; Prob 16.0%; Lib: mona-export-vf-npl_qexactive; ID: 23032.

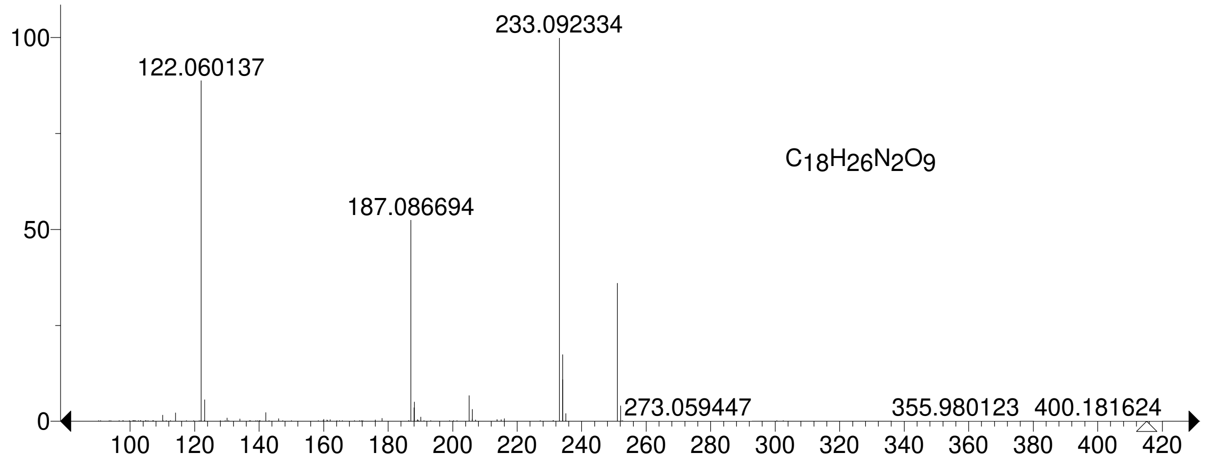

Hit 5 : Diltiazem HCl (Tiazac) [M+H]+ P=415.2
C22H27ClN2O4S; MF: 0; RMF: 19; Prob 15.4%; Lib: mona-export-gnps; ID: 648.

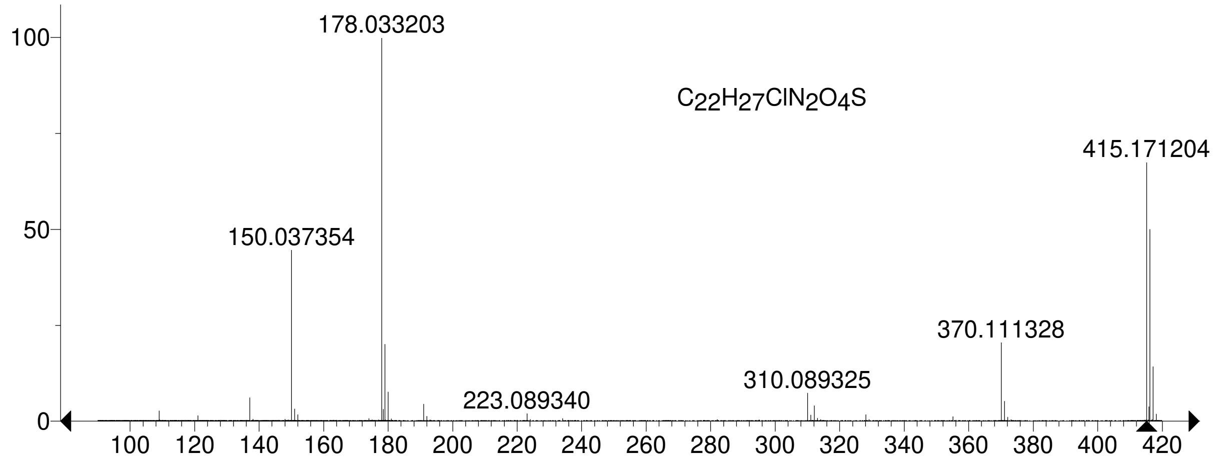

Unknown: Unknown (7.27_444.1866m/z)
Compound in Library Factor = 0

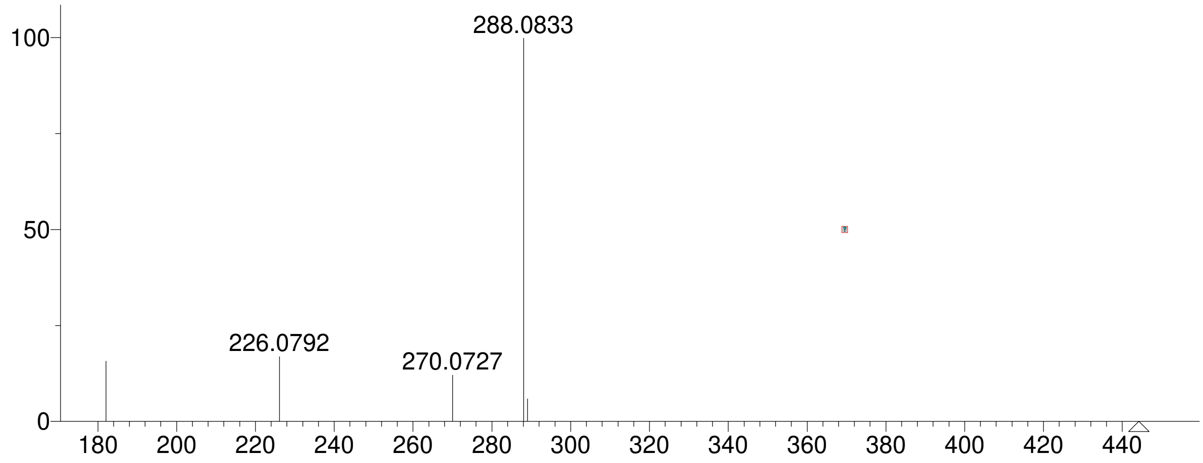

Unknown: Unknown (7.27_628.1985m/z)
Compound in Library Factor = 0

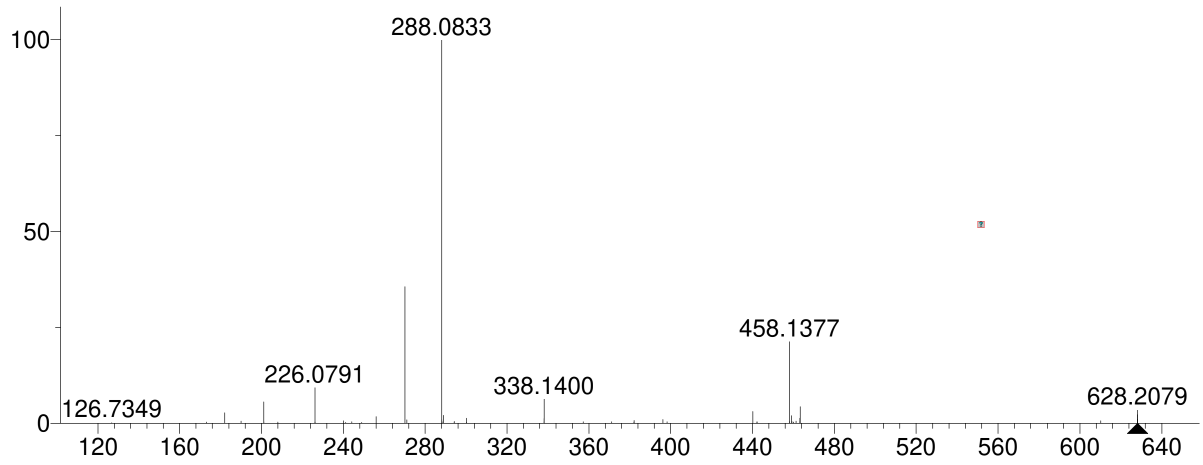

Unknown: Unknown (7.28_458.1397m/z)
Compound in Library Factor = 0

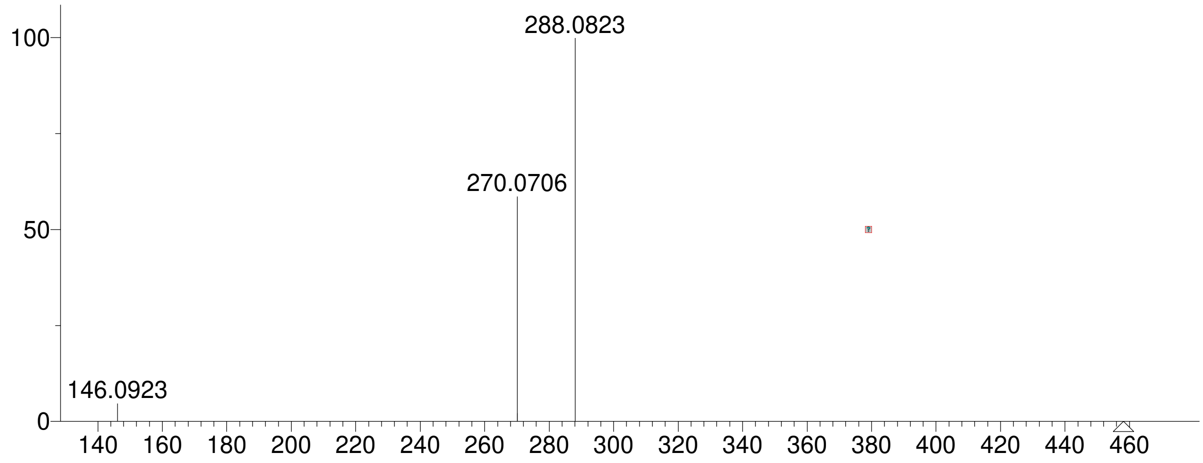

Unknown: Unknown (7.32_275.1024m/z)
Compound in Library Factor = 0

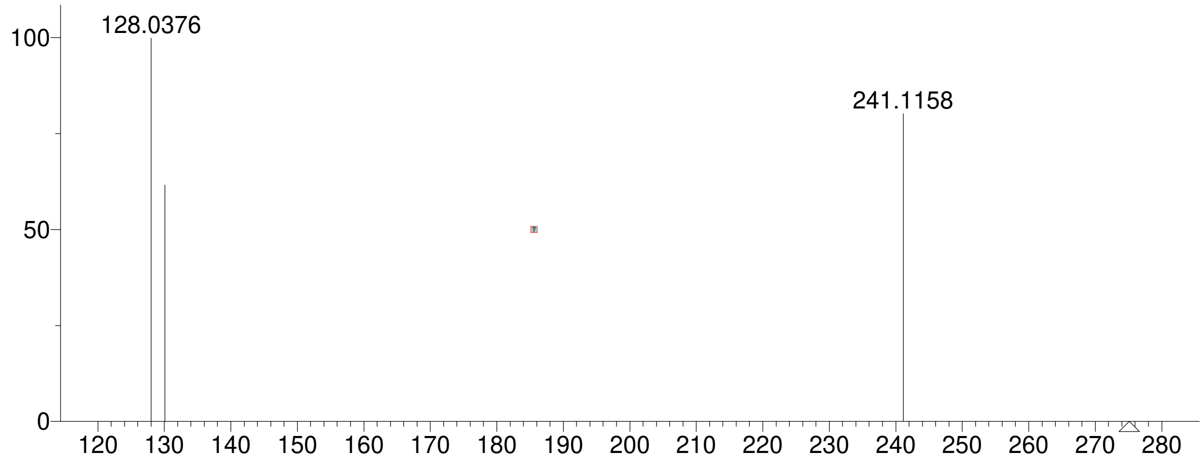

Unknown: Unknown (7.32_484.2071m/z)
Compound in Library Factor = 0

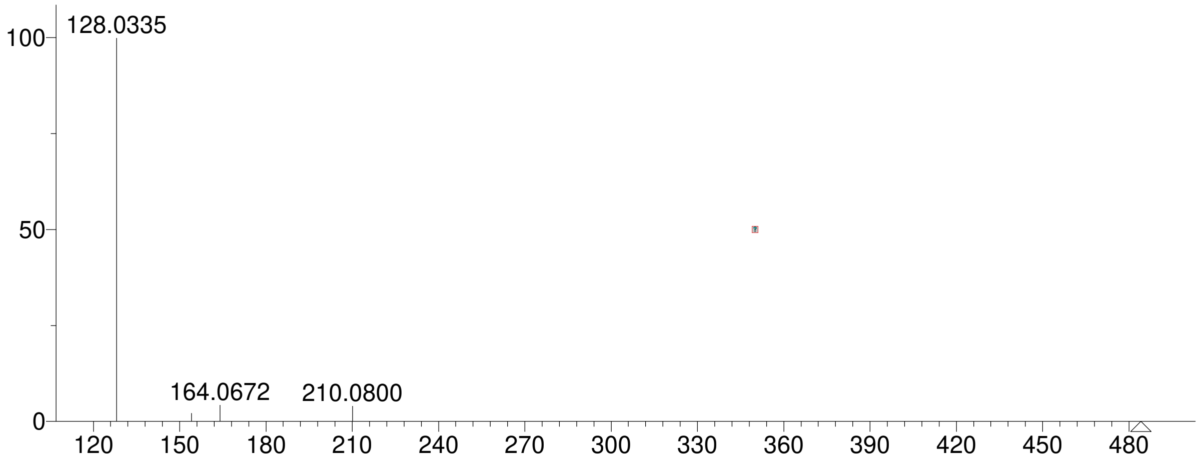

Unknown: Unknown (7.33_437.0864m/z)
Compound in Library Factor = 0

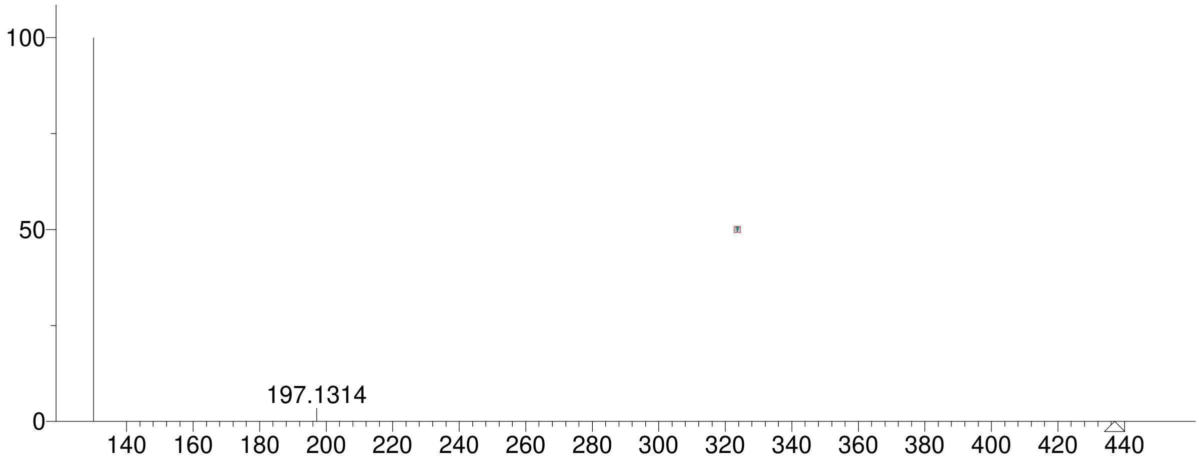

Unknown: Unknown (7.33_612.3720m/z)
Compound in Library Factor = -2632

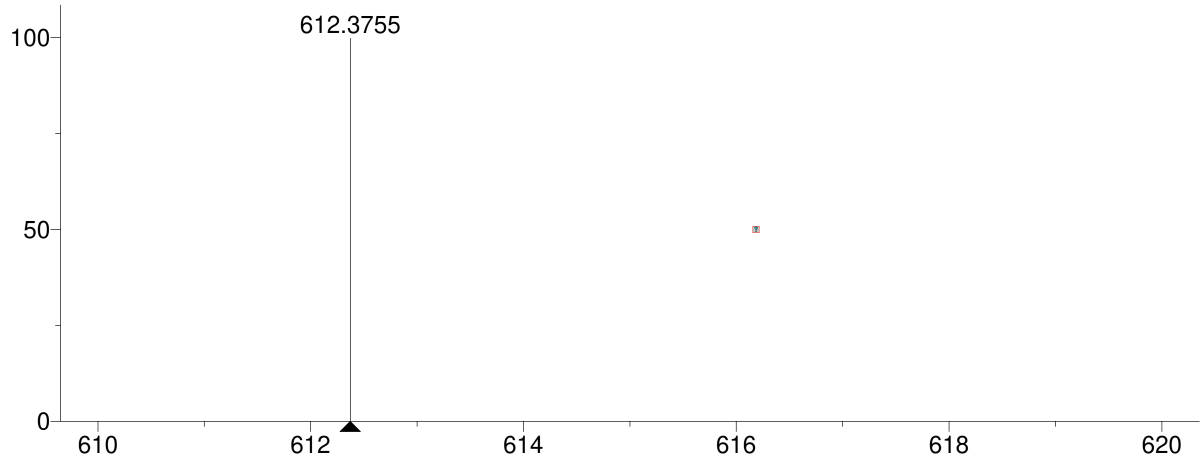

Hit 1 : PE 27:4 [M-H]- 40eV P=612.4
C32H56NO8P; MF: 364; RMF: 999; Prob 14.3%; Lib: mona-export-in-silico_spectra; ID: 82189.

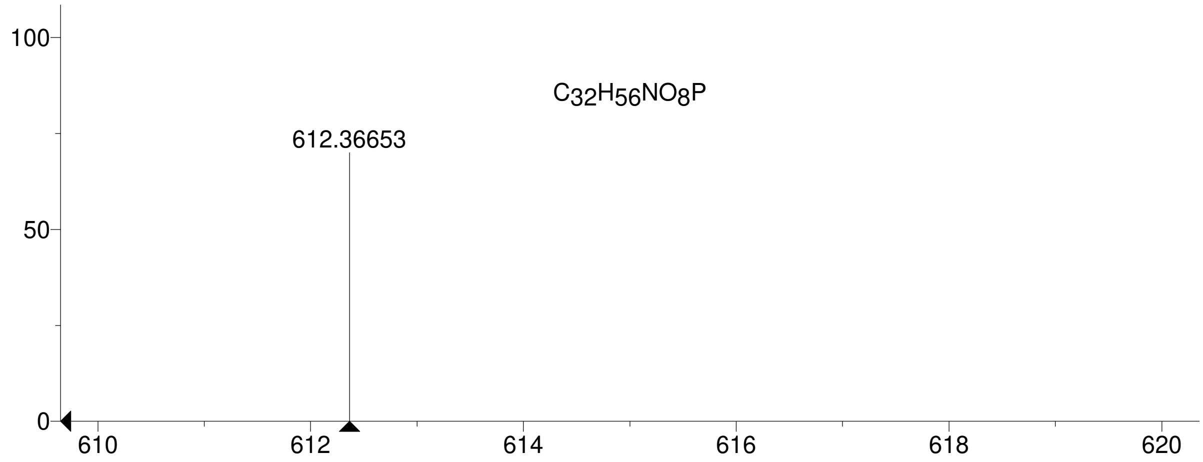

Hit 2 : PE 27:5 [M+H]+ 25eV P=612.4
C32H54NO8P; MF: 364; RMF: 999; Prob 14.3%; Lib: mona-export-in-silico_spectra; ID: 86869.

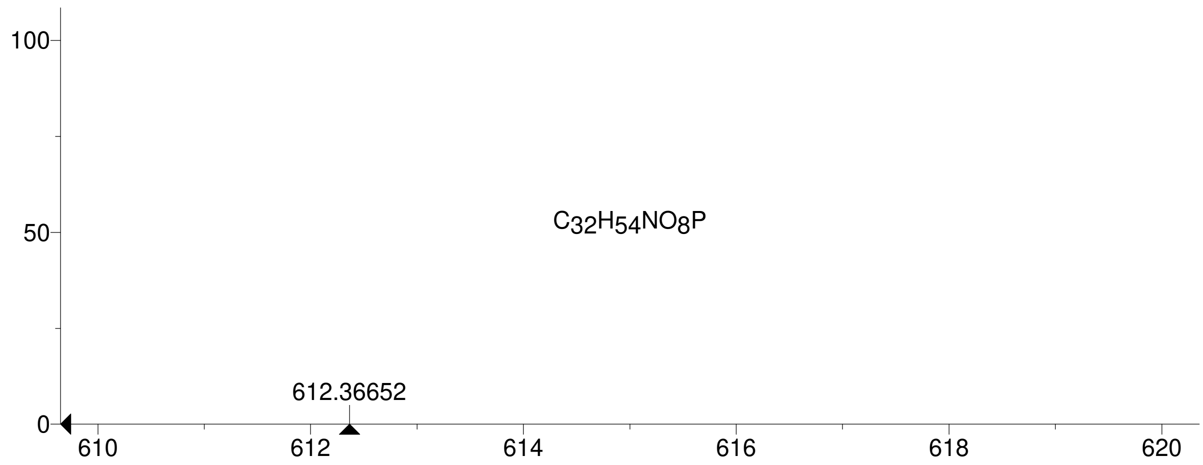

Hit 3 : PE 27:4; PE 3:0-24:4; [M-H]- [M-H]- P=612.4
C32H56NO8P; MF: 364; RMF: 999; Prob 14.3%; Lib: msdial-insilicomsms-lipids-neg; ID: 322789.

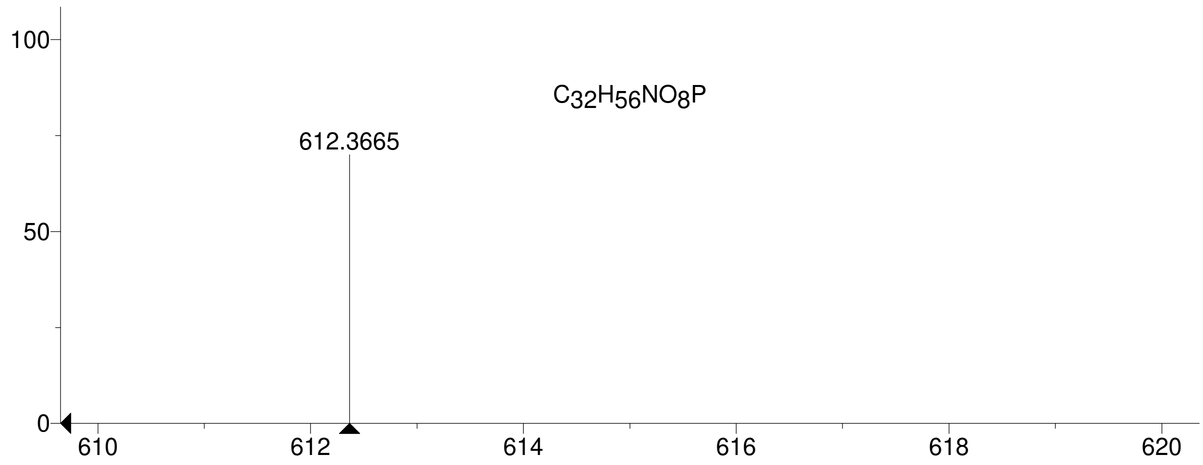

Hit 4 : PE 27:4; PE 5:0-22:4; [M-H]- [M-H]- P=612.4
C32H56NO8P; MF: 364; RMF: 999; Prob 14.3%; Lib: msdial-insilicomsms-lipids-neg; ID: 322899.

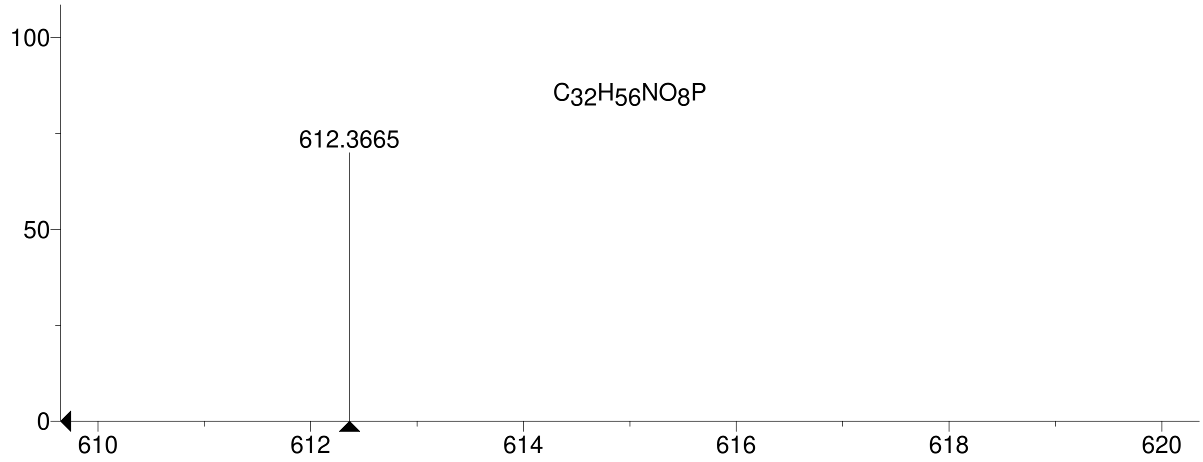

Hit 5 : PE 27:4; PE 7:0-20:4; [M-H]- [M-H]- P=612.4
C32H56NO8P; MF: 364; RMF: 999; Prob 14.3%; Lib: msdial-insilicomsms-lipids-neg; ID: 323003.

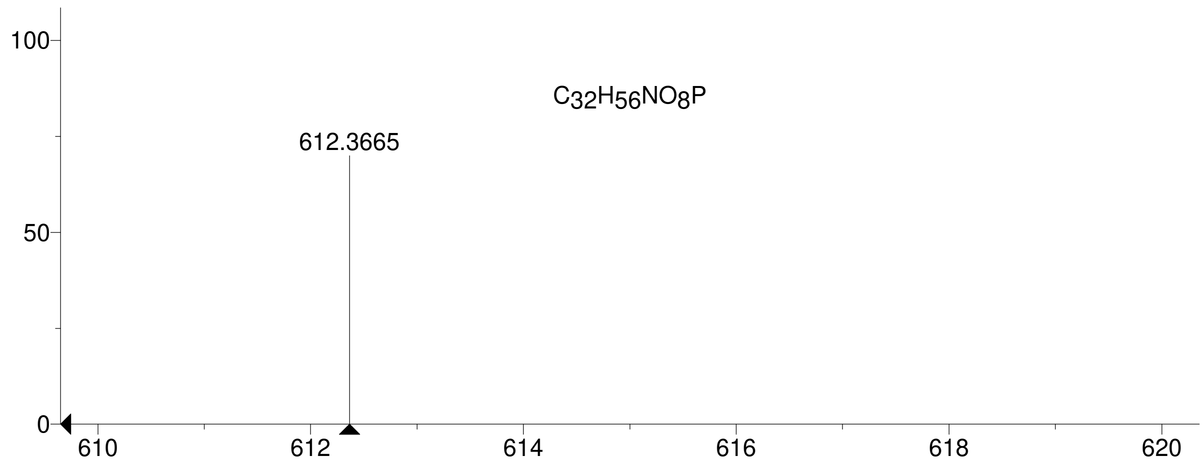

Unknown: Unknown (7.34_388.1714m/z)
Compound in Library Factor = 0

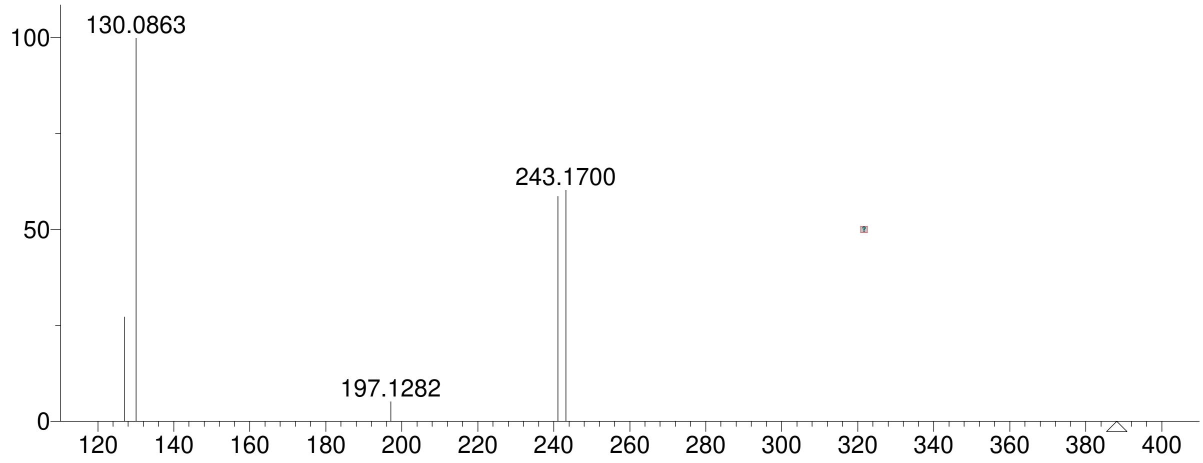

Unknown: Unknown (7.34_549.2259m/z)
Compound in Library Factor = 0

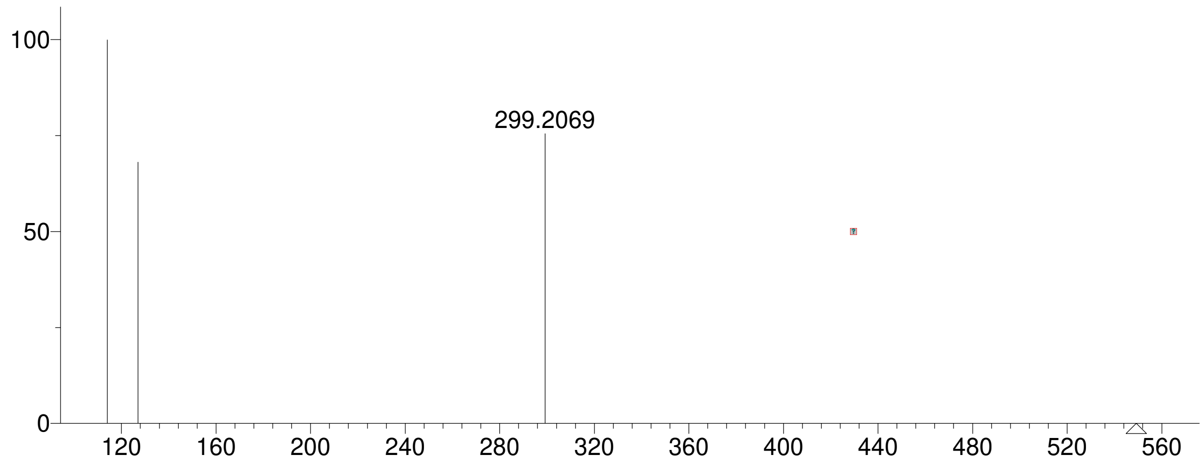

** Search Report Page 1 of 1 **

Unknown: Unknown (7.34_634.2461m/z)
Compound in Library Factor = 0

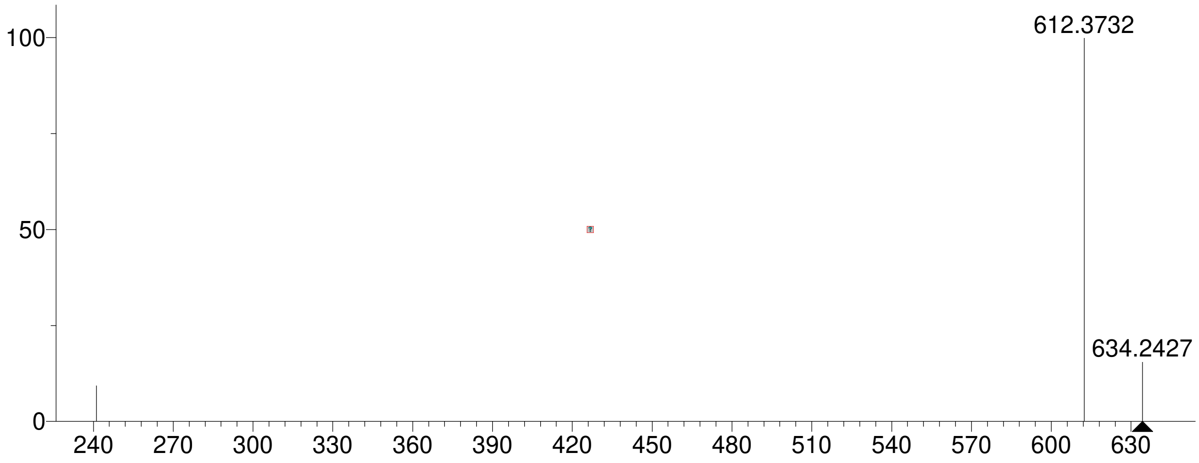

** Search Report Page 1 of 1 **

Unknown: Unknown (7.34_656.2787m/z)
Compound in Library Factor = 0

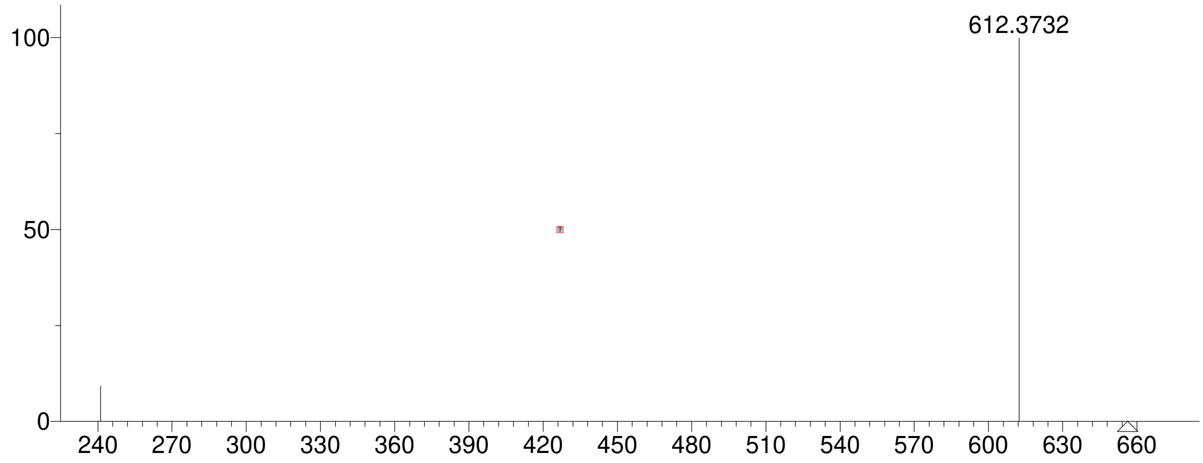

Unknown: Unknown (7.36_405.2229m/z)
Compound in Library Factor = 0

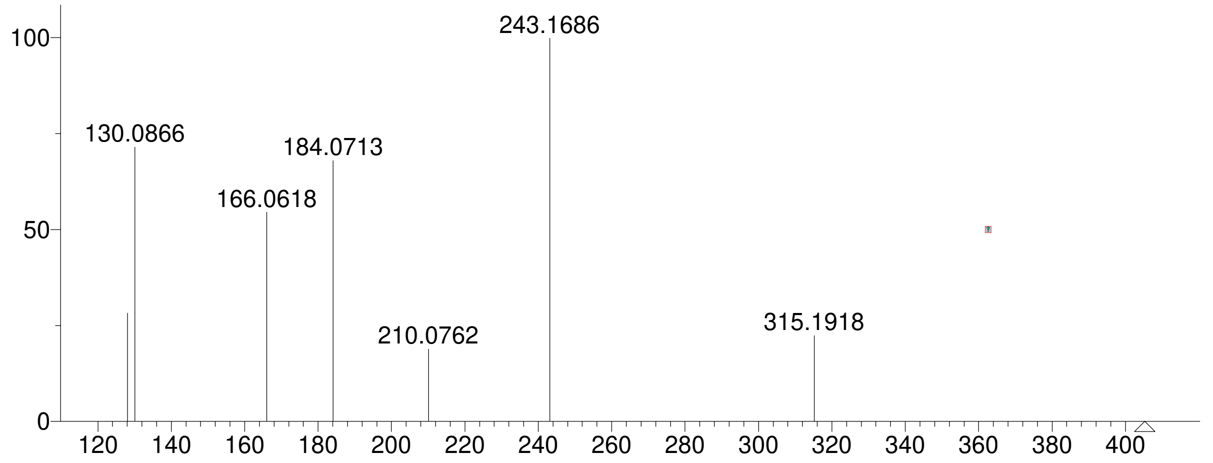

Hit 1 : MLS002637530-01!Cortell50-03-3 [M+H]+ P=405.2
C23H32O6; MF: 0; RMF: 31; Prob 50.0%; Lib: mona-export-gnps; ID: 3679.

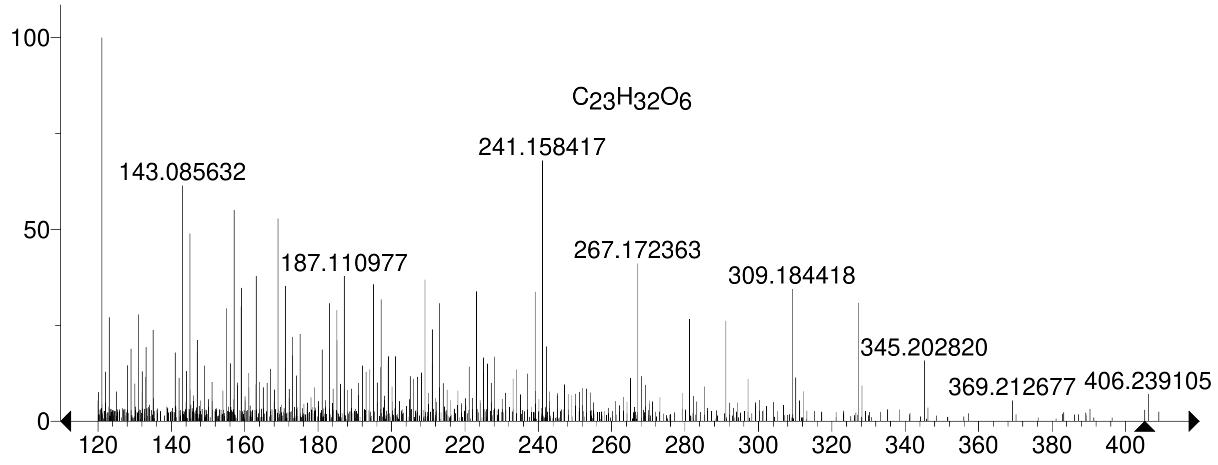

Hit 2 : MLS001076491-01!Cortell [M+H]+ P=405.2
C23H32O6; MF: 0; RMF: 27; Prob 50.0%; Lib: mona-export-gnps; ID: 4335.

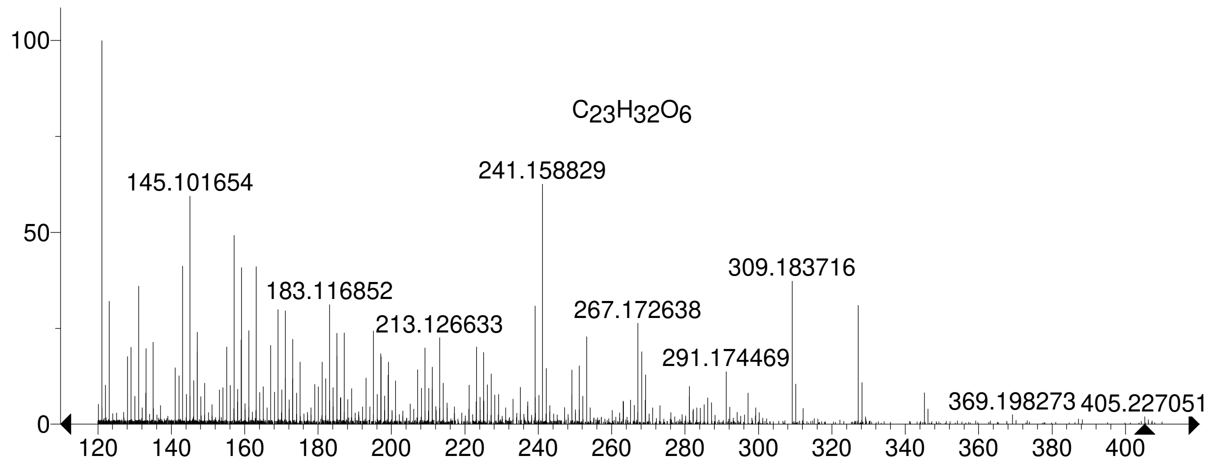

** Search Report Page 1 of 1 **

Unknown: Unknown (7.37_465.1983m/z)
Compound in Library Factor = 0

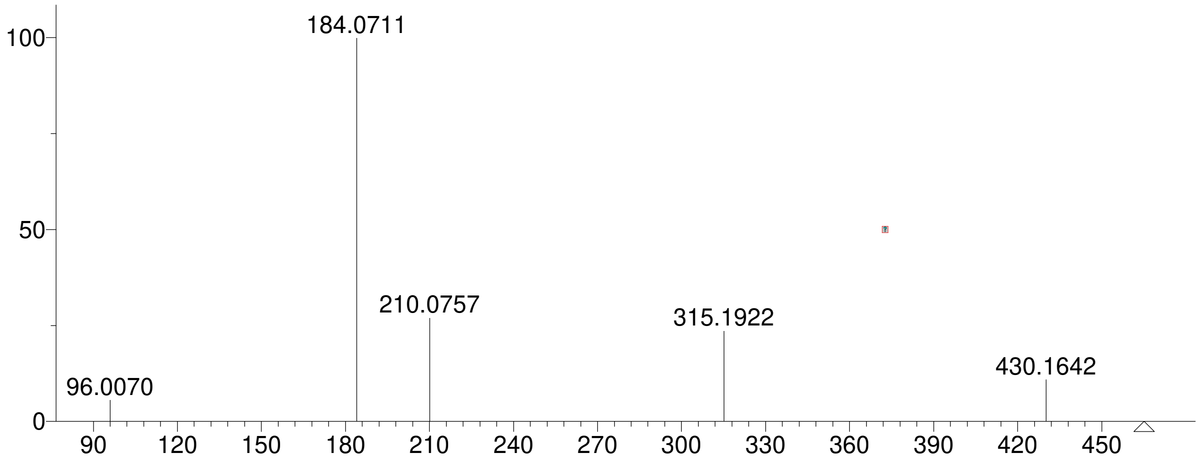

Hit 1 : Salannin [M+H]+=>565.3=>465.2 IT 35% P=465.2
C34H44O9; MF: 0; RMF: 45; Prob 50.0%; CAS: 992-20-1; Lib: nist_msms; ID: 223511.

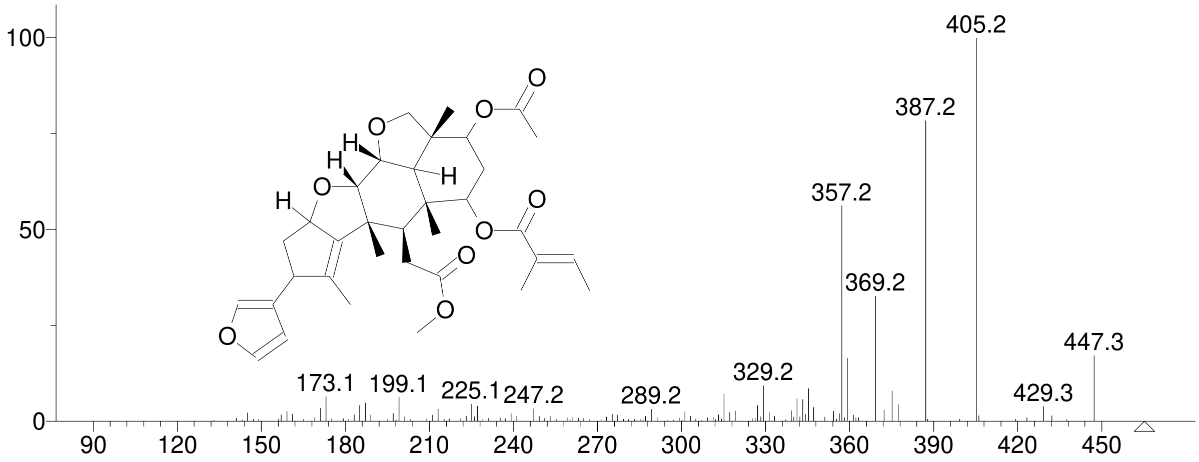

Hit 2 : Azadirachtin A [M+H-C7H16O6]+=>465.2 IT 35% P=465.2
C35H44O16; MF: 0; RMF: 33; Prob 50.0%; CAS: 11141-17-6; Lib: nist_msms; ID: 223393.

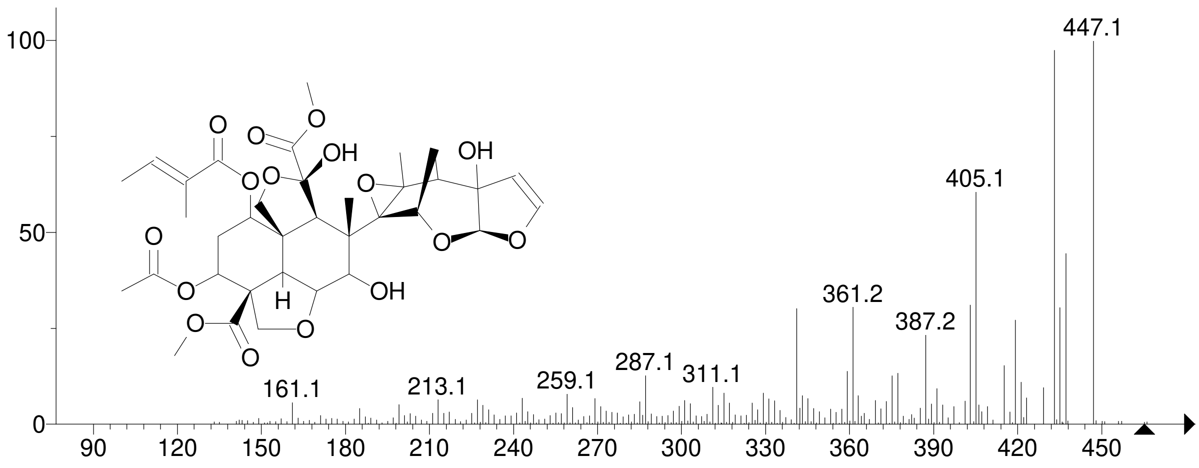

Unknown: Unknown (7.37_476.1674m/z)
Compound in Library Factor = 0

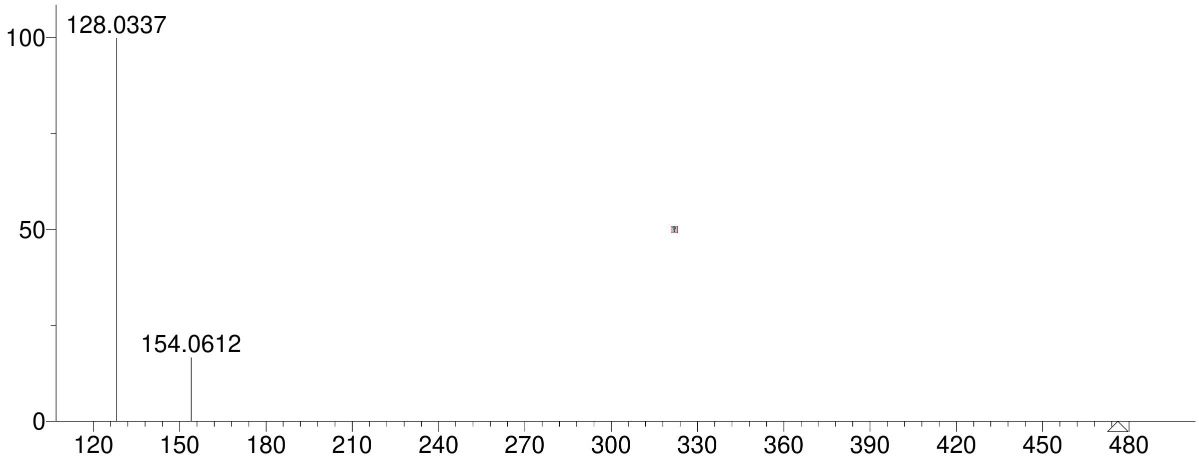

Unknown: Unknown (7.38_381.1756m/z)
Compound in Library Factor = 0

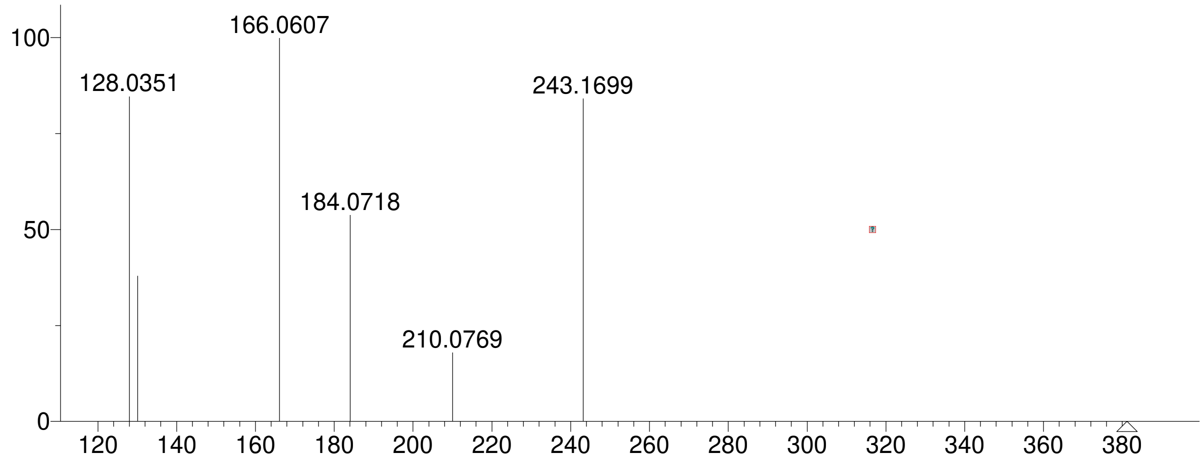

Unknown: Unknown (7.38_413.1665m/z)
Compound in Library Factor = -3273

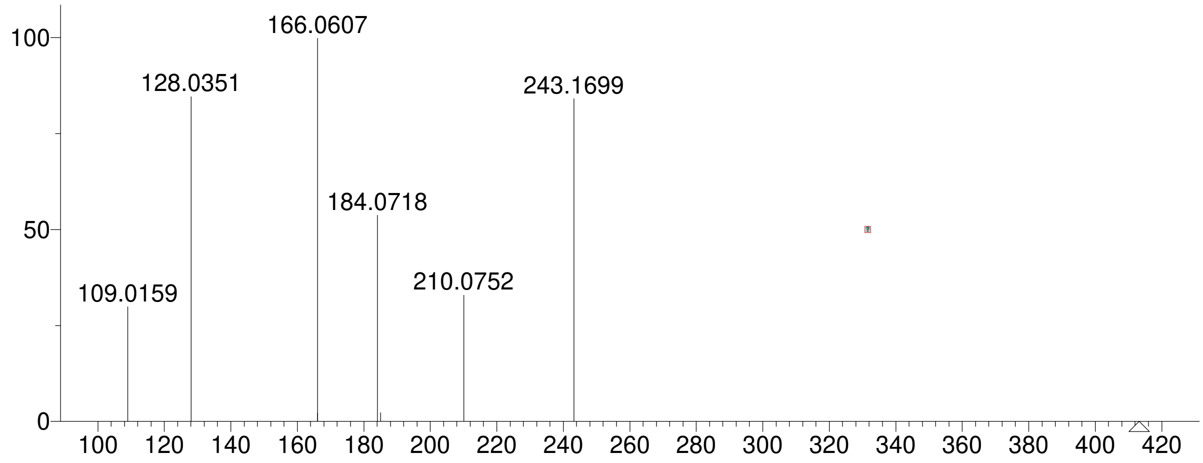

Hit 1 : N-(sec-Butyl)-N-(4-(sec-butyl(trifluoroacetyl)amino)phenyl-2,2,2-trifluoroacetamide [M+H]+ HCD 107V P
C18H22F6N2O2; MF: 1; RMF: 166; Prob 25.7%; CAS: 883829-91-2; Lib: nist_msms; ID: 199644.

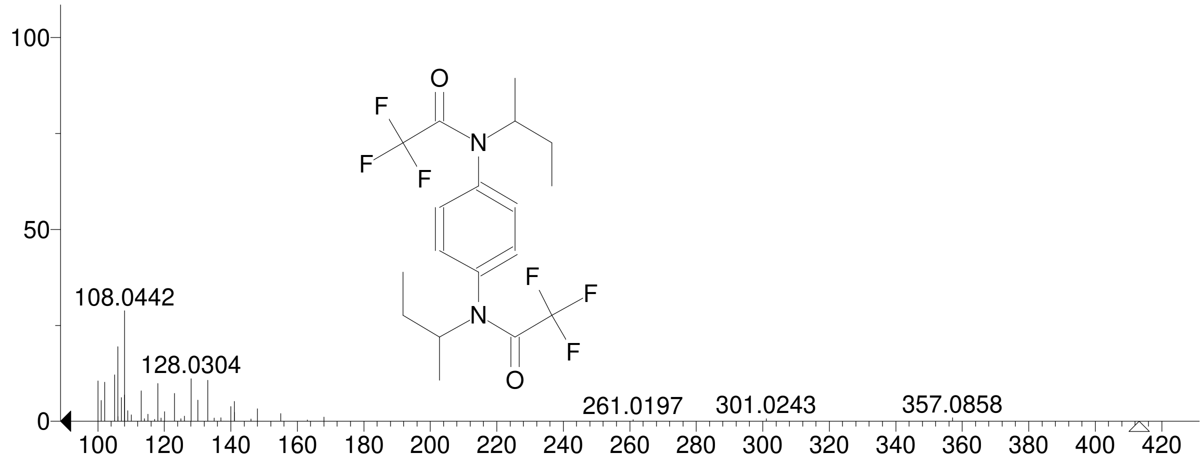

Hit 2 : 2-Hydroxymethylloratadine [M+H]+ HCD 132V P=413.2
C23H25ClN2O3; MF: 0; RMF: 28; Prob 24.8%; CAS: 609806-39-5; Lib: nist_msms; ID: 442227.

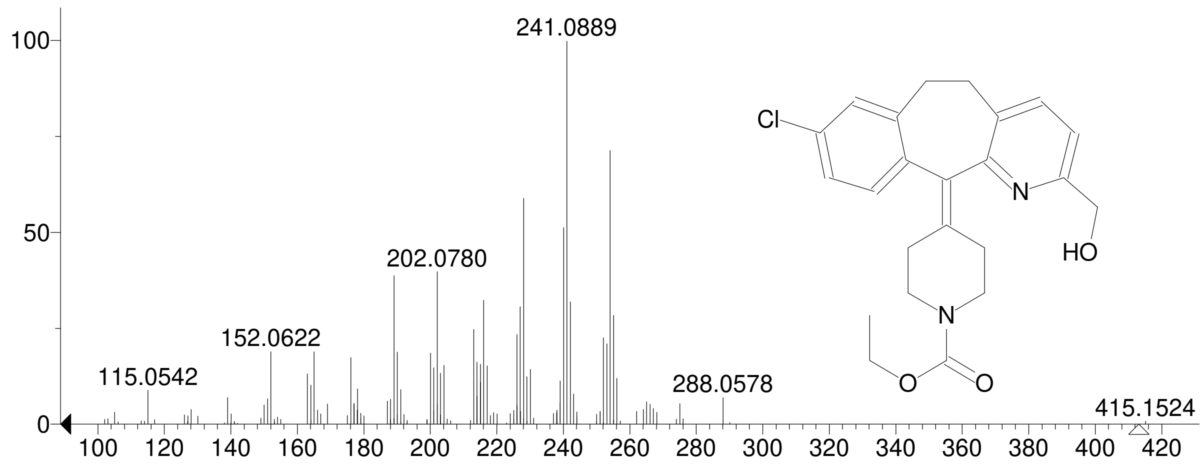

Hit 3 : 4-Hydroxymethylloratadine [M+H]+ HCD 132V P=413.2
C23H25ClN2O3; MF: 0; RMF: 24; Prob 24.8%; CAS: 609806-40-8; Lib: nist_msms; ID: 442293.

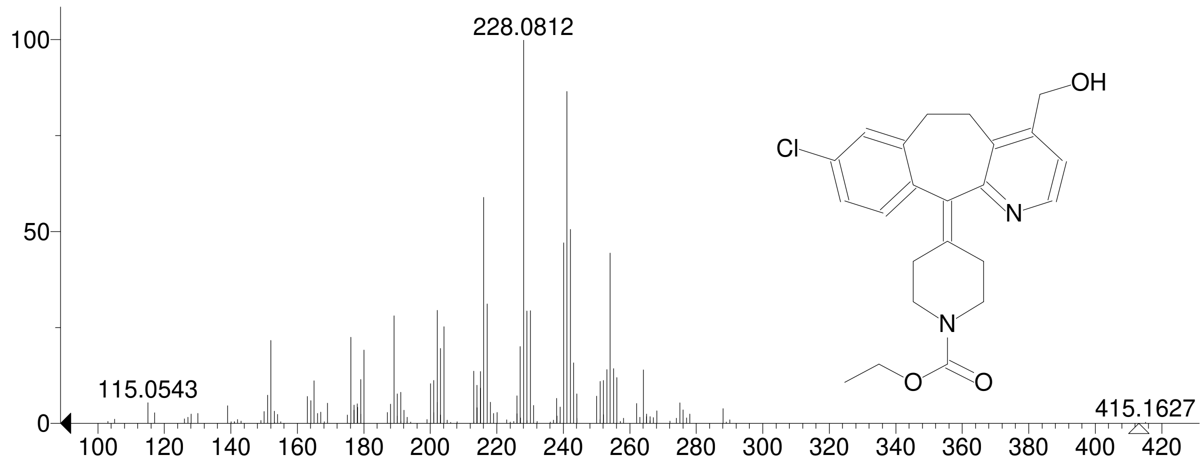

Hit 4 : NCGC00380162-01_C16H28O12_alpha-D-Glucopyranoside, alpha-D-glucopyranosyl, 3-(2-methylpropanc
C16H28O12; MF: 0; RMF: 7; Prob 24.8%; Lib: mona-export-gnps; ID: 12080.

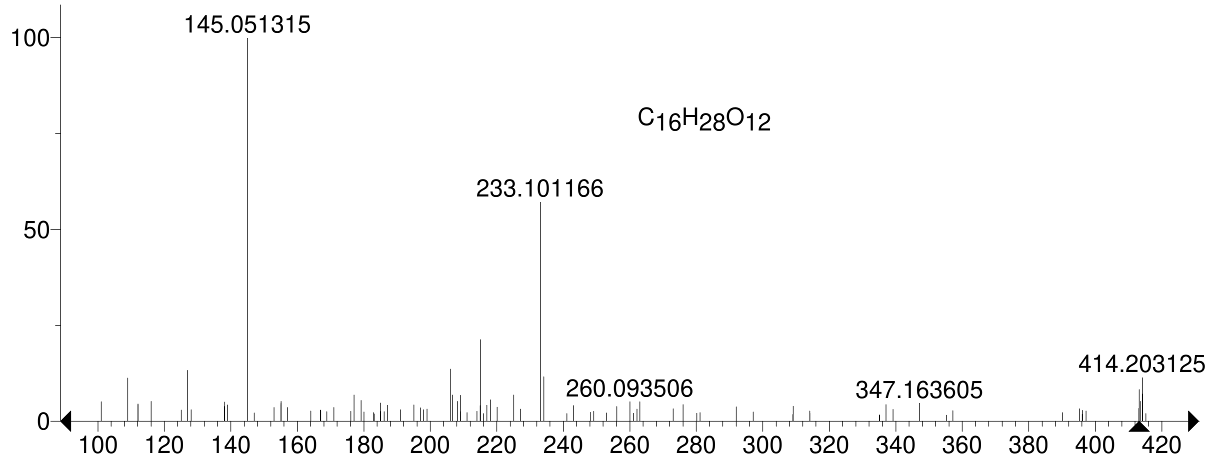

Unknown: Unknown (7.38_467.1654m/z)
Compound in Library Factor = -3273

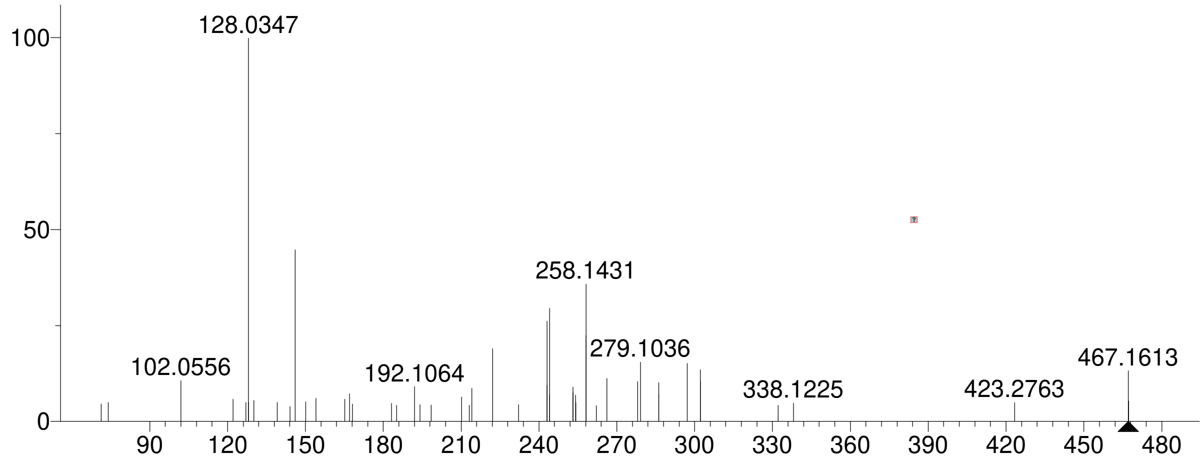

Hit 1 : Man3 a [M+H+Na]2+ HCD 8V P=467.2
C34H58N2O26; MF: 2; RMF: 162; Prob 17.2%; CAS: 70858-45-6; Lib: nist_msms; ID: 184753.

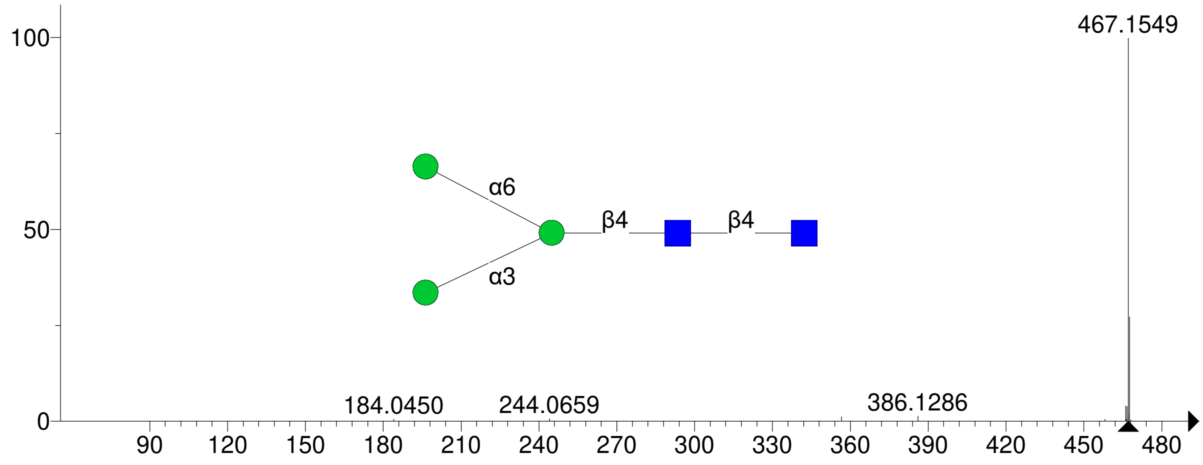

Hit 2 : 11,17-Difuroatemometasone [M+H-C5H5ClO3]+ HCD 9V P=467.2
C32H32Cl2O8; MF: 2; RMF: 139; Prob 17.2%; CAS: 1370190-33-2; Lib: nist_msms; ID: 436531.

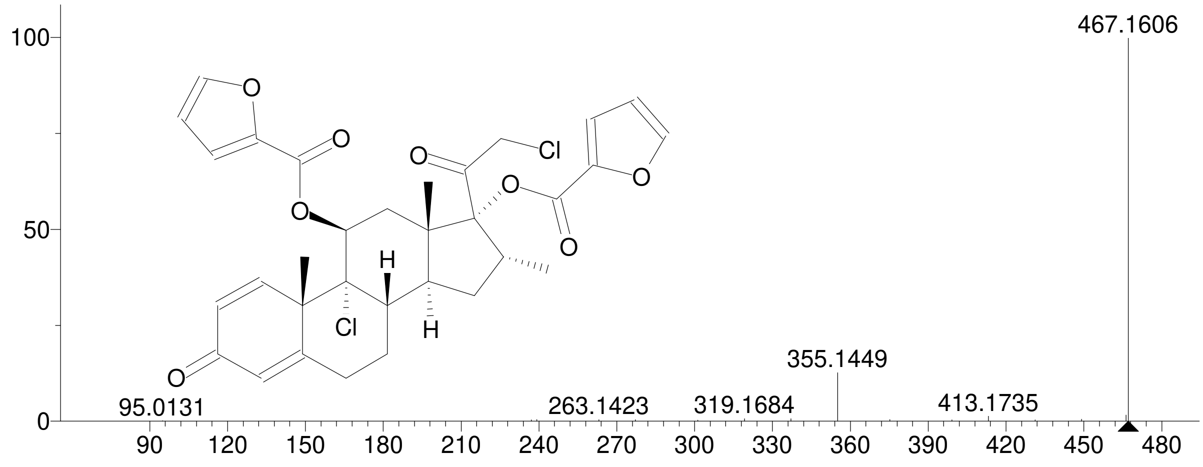

Hit 3 : MMV689243 [M+H]+ 20-30-40%) P=467.2
C23H20F6N4; MF: 2; RMF: 74; Prob 17.2%; Lib: mona-export-pathogen_box; ID: 361.

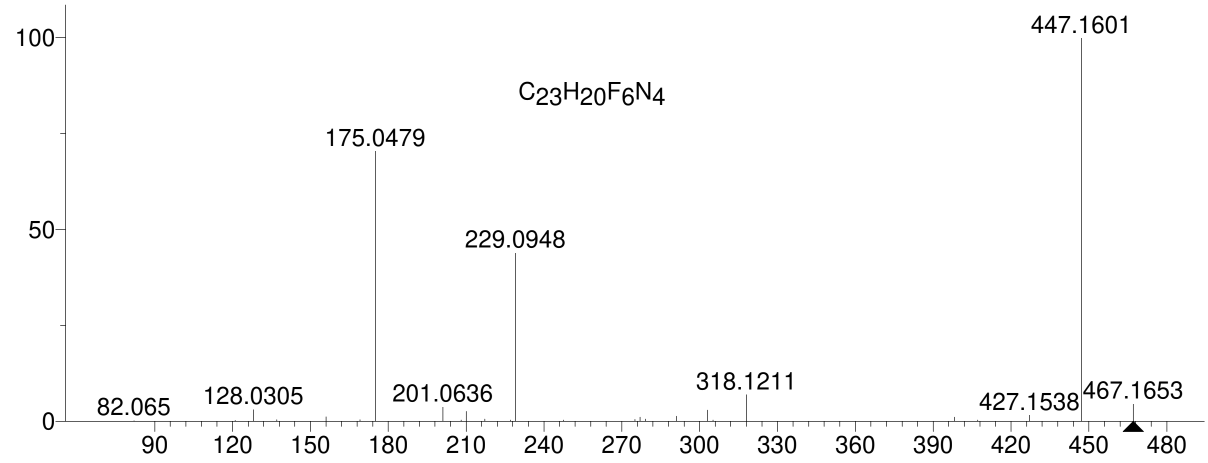

Hit 4 : Man3 b [M+H+Na]2+ HCD 8V P=467.2
C34H58N2O26; MF: 1; RMF: 153; Prob 16.5%; Lib: nist_msms; ID: 184028.

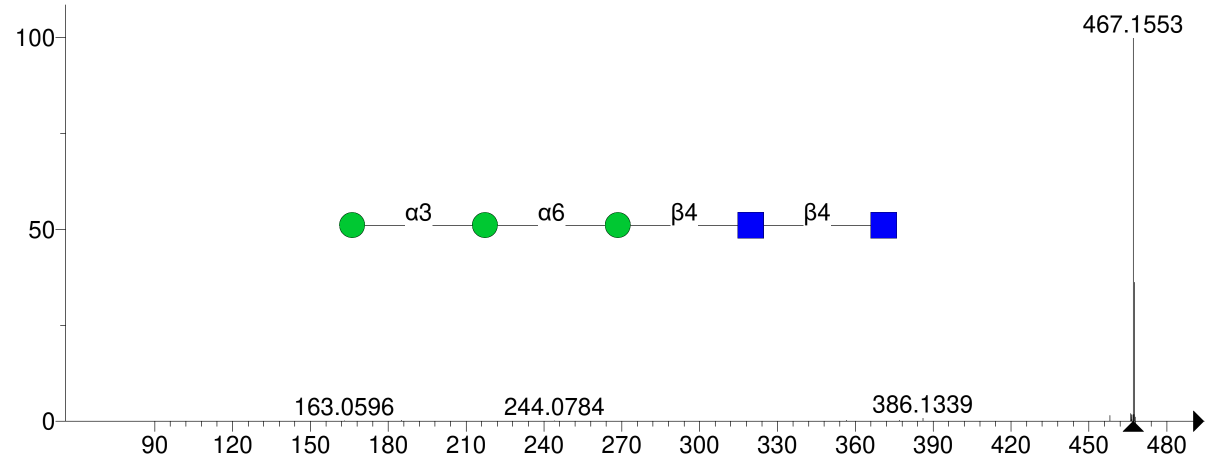

Hit 5 : Azadirachtin B [M+H-C7H16O6]+ IT-FT 35% P=467.2
C33H42O14; MF: 0; RMF: 43; Prob 15.9%; CAS: 95507-03-2; Lib: nist_msms; ID: 223463.

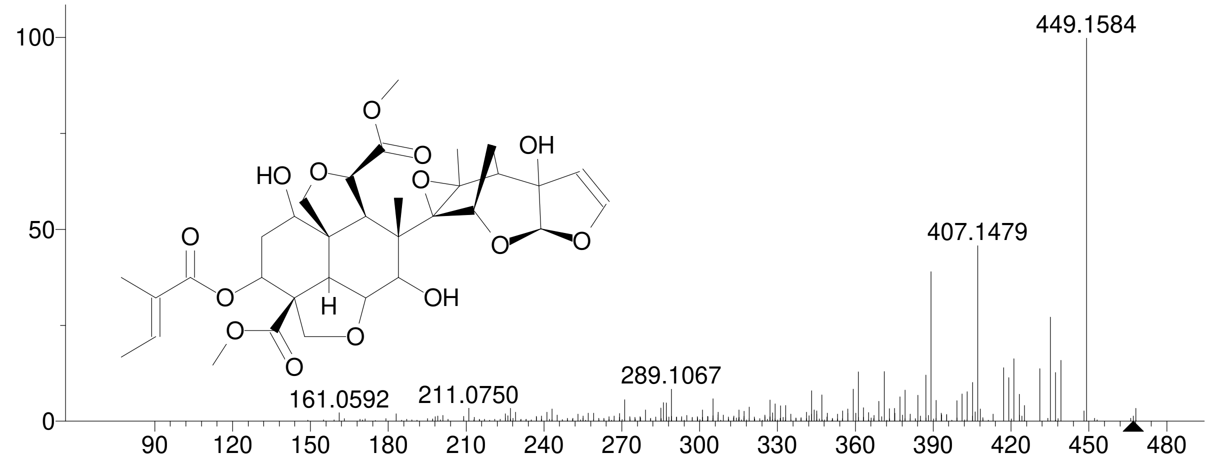

Unknown: Unknown (7.38_502.2038m/z)
Compound in Library Factor = 0

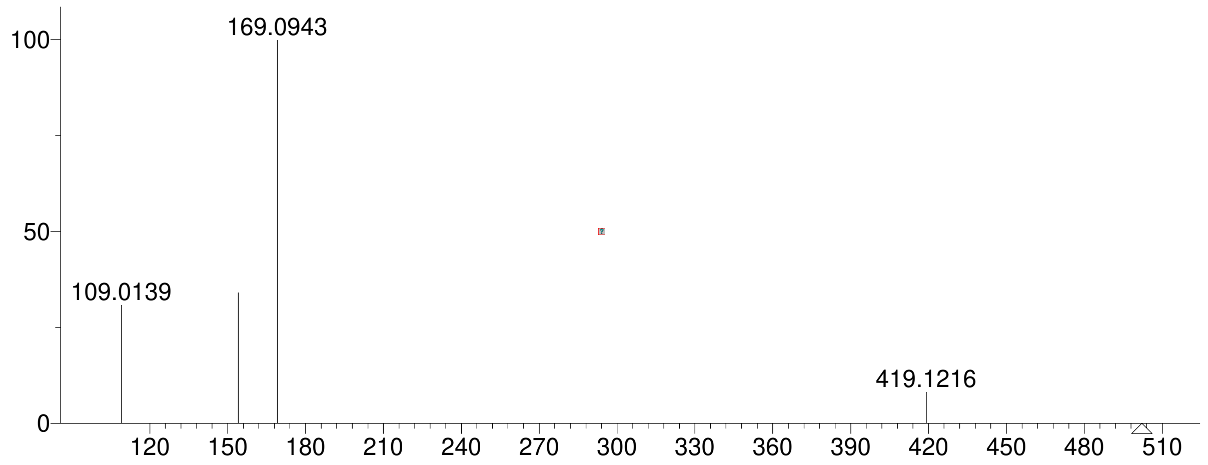

Hit 1 : (2R,7S,13R,14R,16S,19R,20S)-19-(furan-3-yl)-11-hydroxy-9,9,13,20-tetramethyl-4,8,15,18-tetraoxahexacy
C26H28O9; MF: 0; RMF: 88; Prob 100.0%; Lib: mona-export-vf-npl_qexactive; ID: 9558.

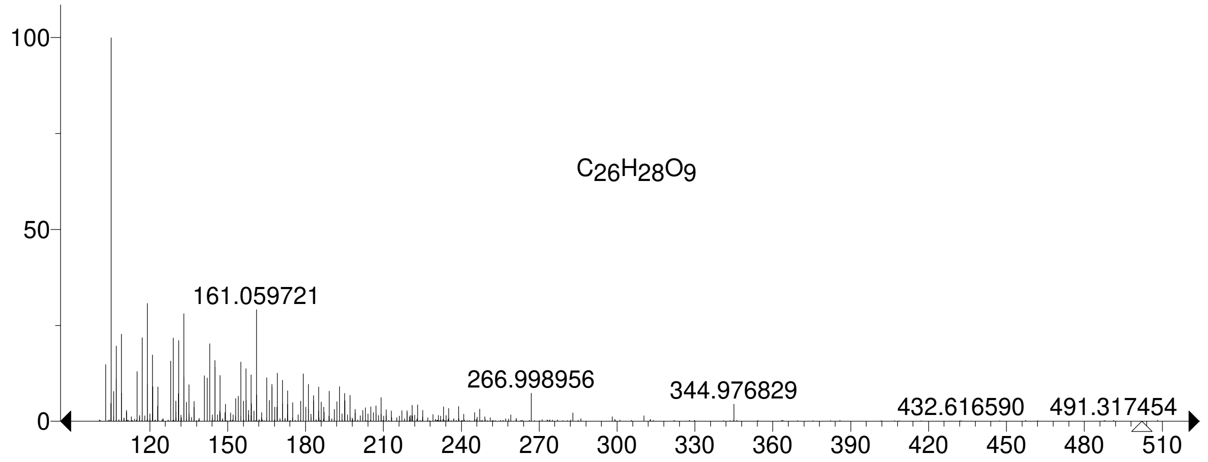

Unknown: Unknown (7.39_389.1454m/z)
Compound in Library Factor = 0

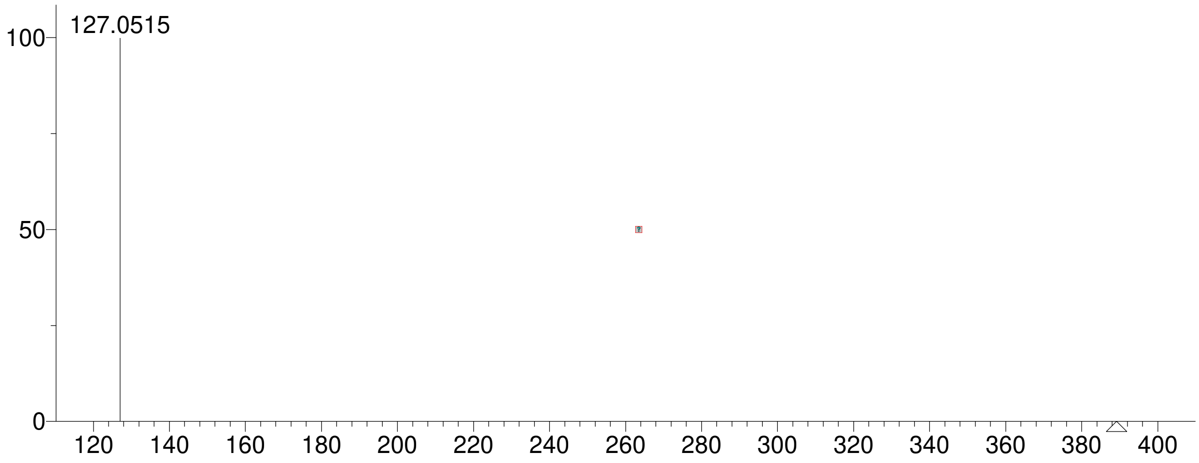

Unknown: Unknown (7.43_413.2016m/z)
Compound in Library Factor = 0

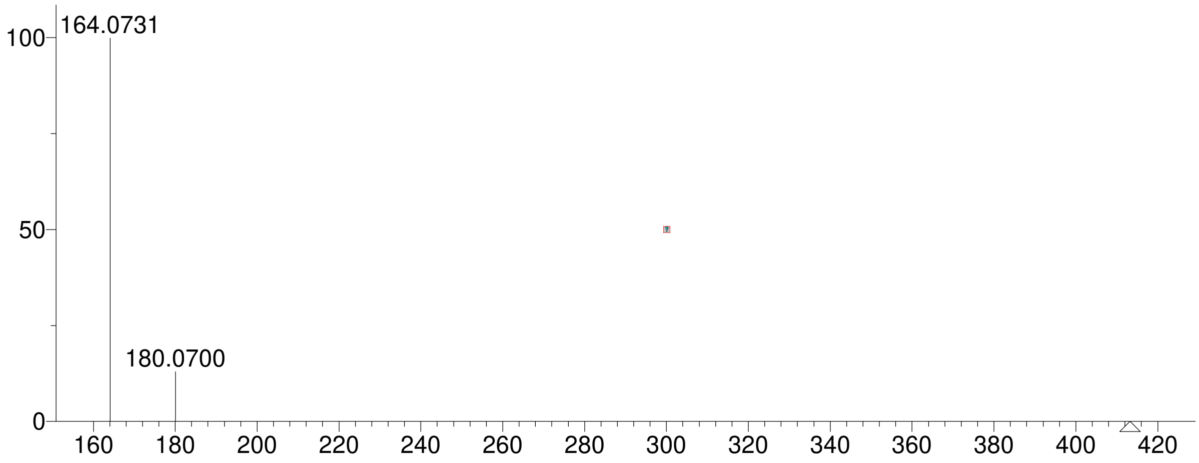

Unknown: Unknown (7.44_526.2869m/z)
Compound in Library Factor = 0

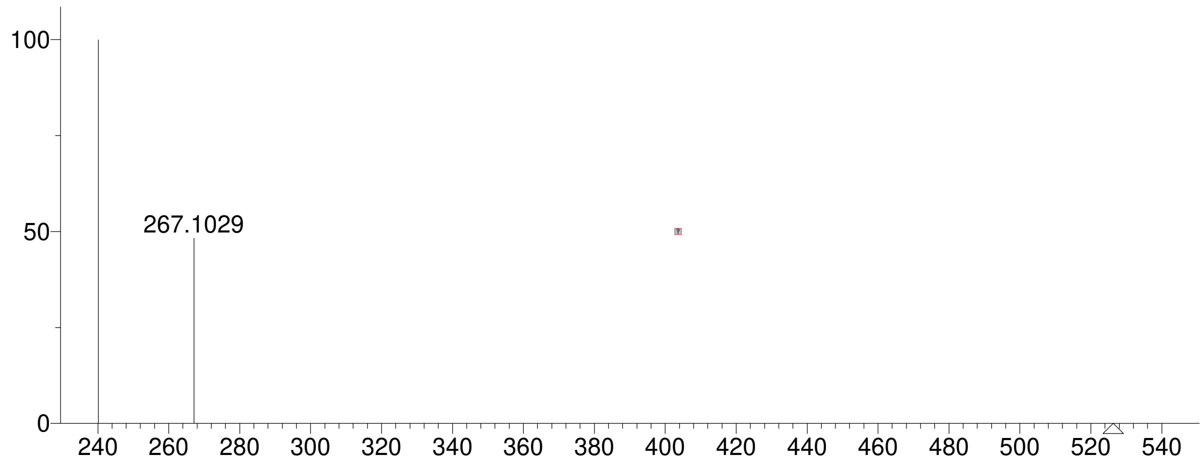

Unknown: Unknown (7.45_344.1810m/z)
Compound in Library Factor = 0

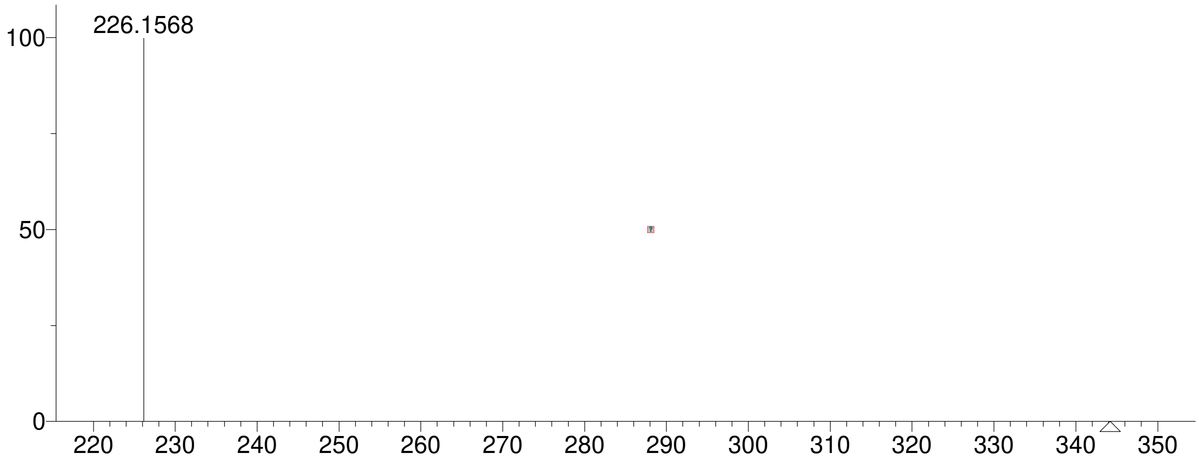

Unknown: Unknown (7.45_441.1862m/z)
Compound in Library Factor = 0

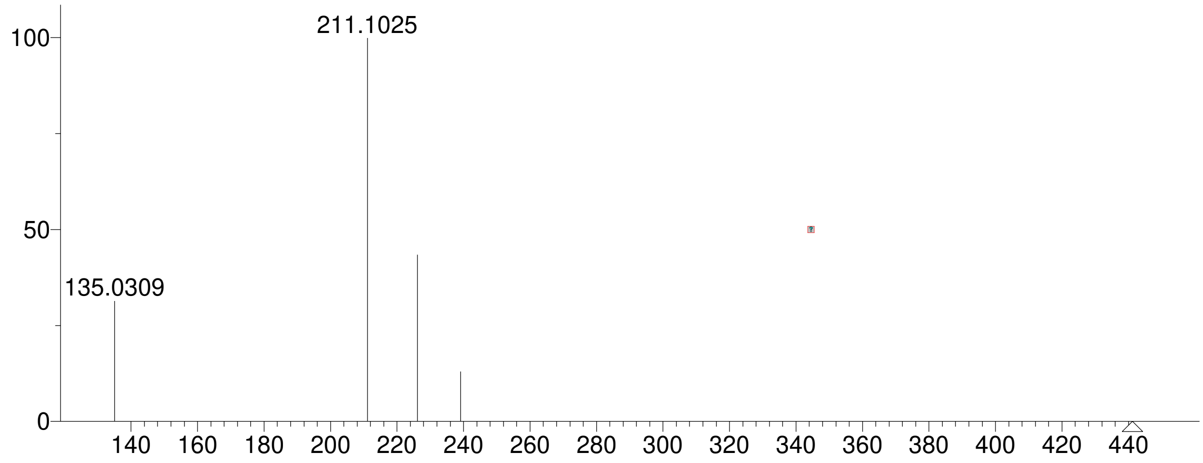

** Search Report Page 1 of 1 **

Unknown: Unknown (7.45_722.1774m/z)
Compound in Library Factor = 0

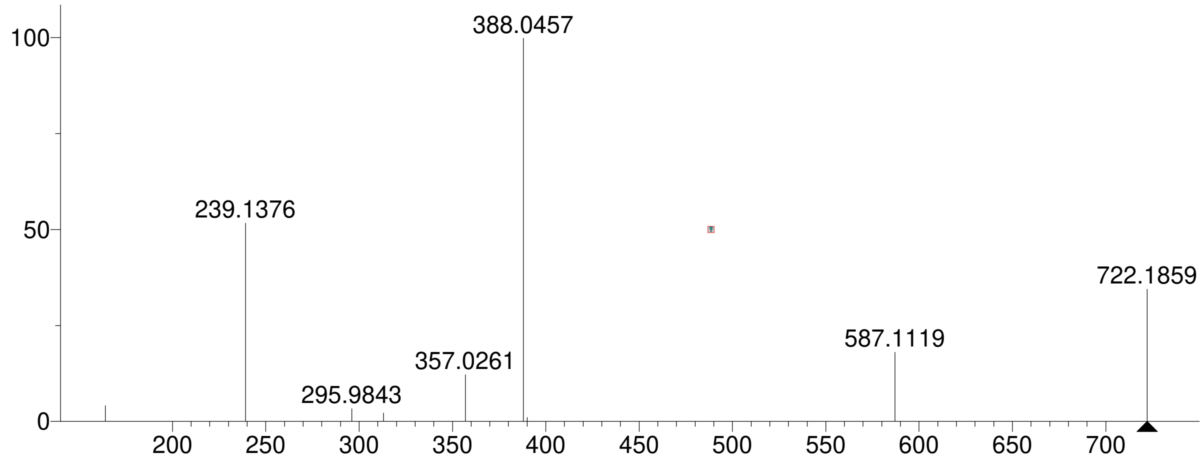

Unknown: Unknown (7.46_757.1732m/z)
Compound in Library Factor = 0

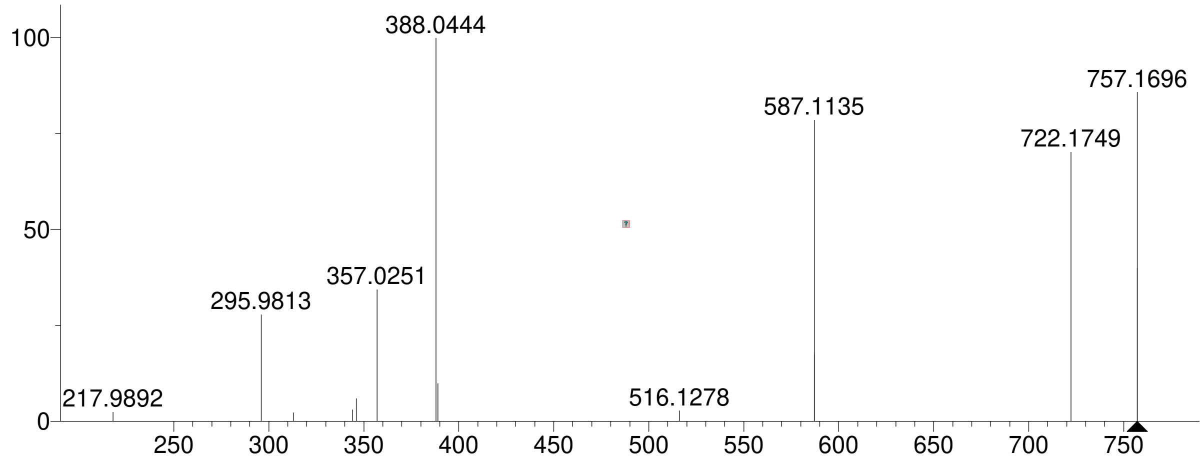

Unknown: Unknown (7.47_258.1126m/z)
Compound in Library Factor = -1309

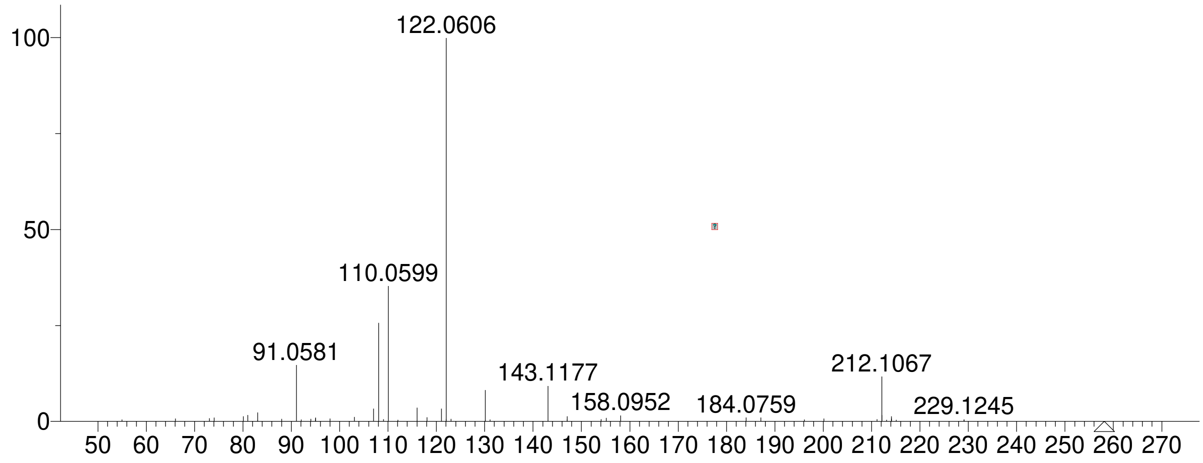

Hit 1 : Tolmetin [M+H]+ HCD 49V P=258.1
C15H15NO3; MF: 59; RMF: 261; Prob 42.4%; CAS: 26171-23-3; Lib: nist_msms; ID: 135560.

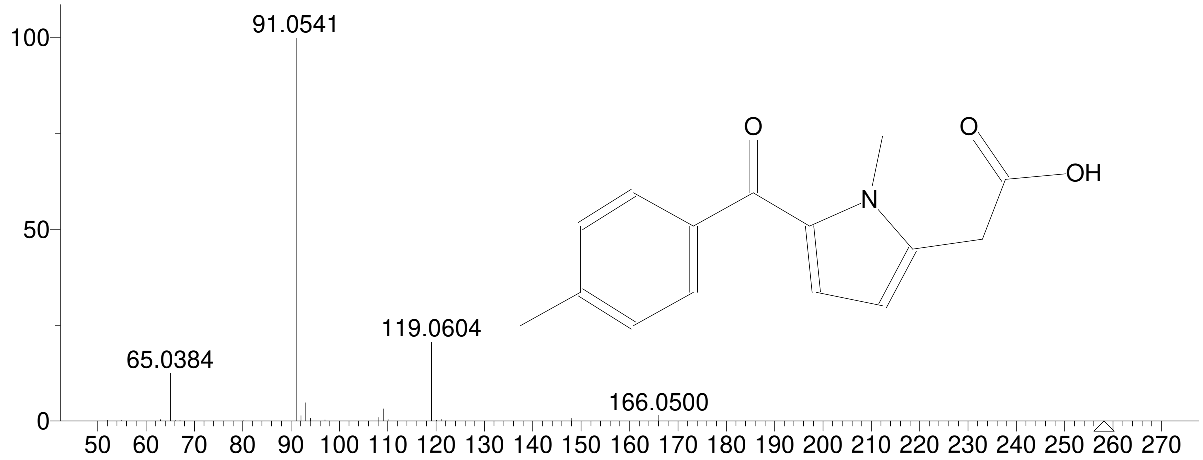

Hit 2 : NCGC00017285-06_C15H15NO3_9-Methoxy-2,2-dimethyl-2,6-dihydro-5H-pyrano[3,2-c]quinolin-5-one [M
C15H15NO3; MF: 5; RMF: 186; Prob 8.30%; Lib: mona-export-gnps; ID: 11787.

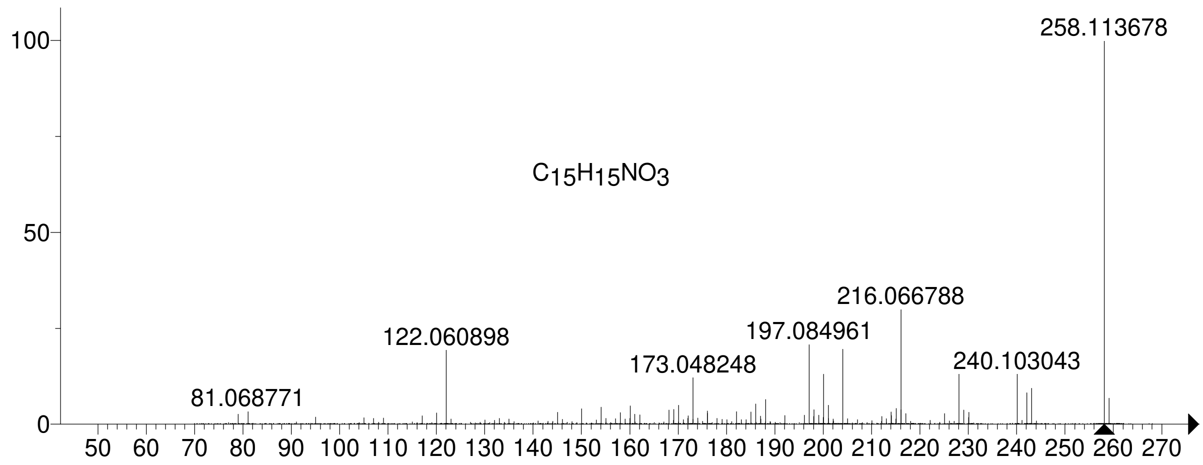

Hit 3 : 9-Methoxy-2,2-dimethyl-2,6-dihydro-pyrano[3,2-c]quinolin-5-one [M+H]+ 65HCD P=258.1
C15H15NO3; MF: 5; RMF: 156; Prob 8.30%; Lib: mona-export-vf-npl_qexactive; ID: 17121.

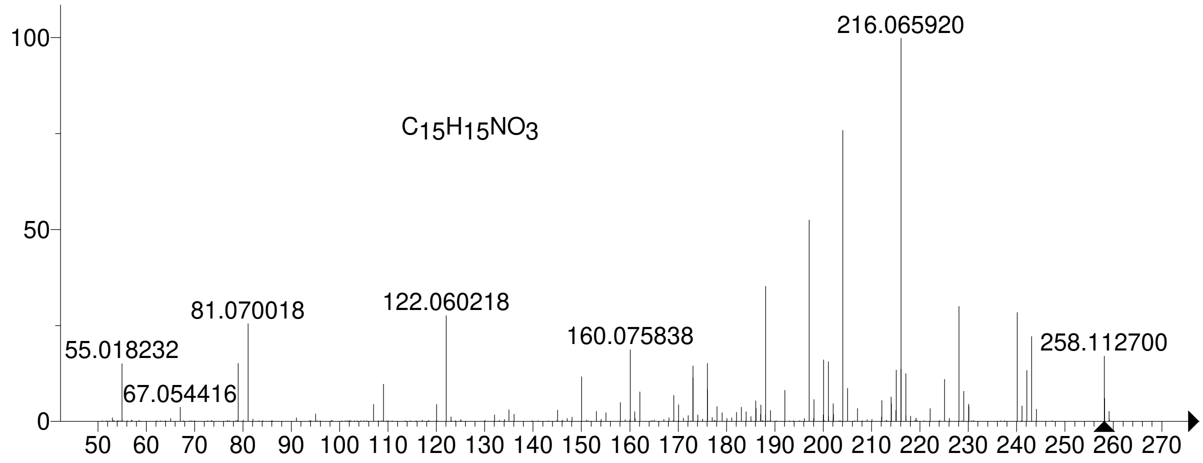

Hit 4 : NCGC00017285-04!HAPLAMINE [M+H]+ P=258.1
C15H15NO3; MF: 1; RMF: 140; Prob 7.01%; Lib: mona-export-gnps; ID: 1103.

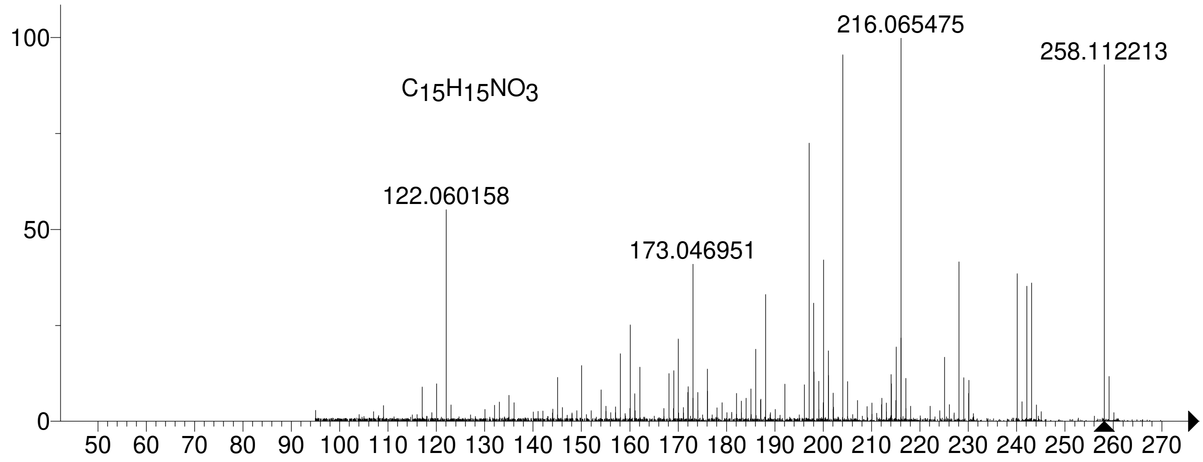

Hit 5 : NCGC00244969-01!4-[[2-[(E)-3-hydroxybut-1-enyl]-5-oxooxolan-3-yl]amino]benzaldehyde [M-H2O+H]+ P
C15H17NO4; MF: 1; RMF: 72; Prob 7.01%; Lib: mona-export-gnps; ID: 8338.

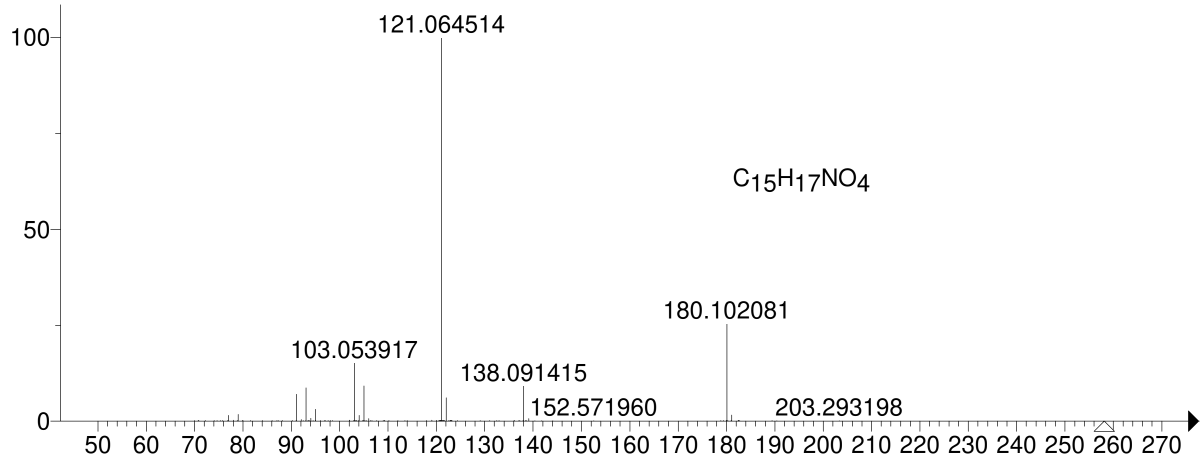

Unknown: Unknown (7.48_445.1912m/z)
Compound in Library Factor = 0

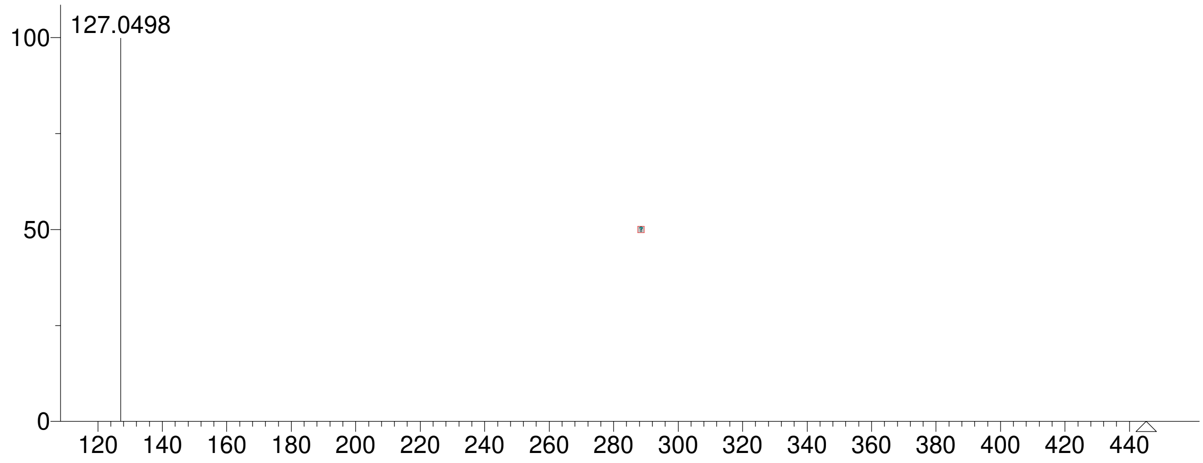

Hit 1 : epiyangambin [M-H]- 45HCD P=445.2
C24H30O8; MF: 0; RMF: 33; Prob 100.0%; Lib: mona-export-lc-ms-ms_negative_mode; ID: 18558.

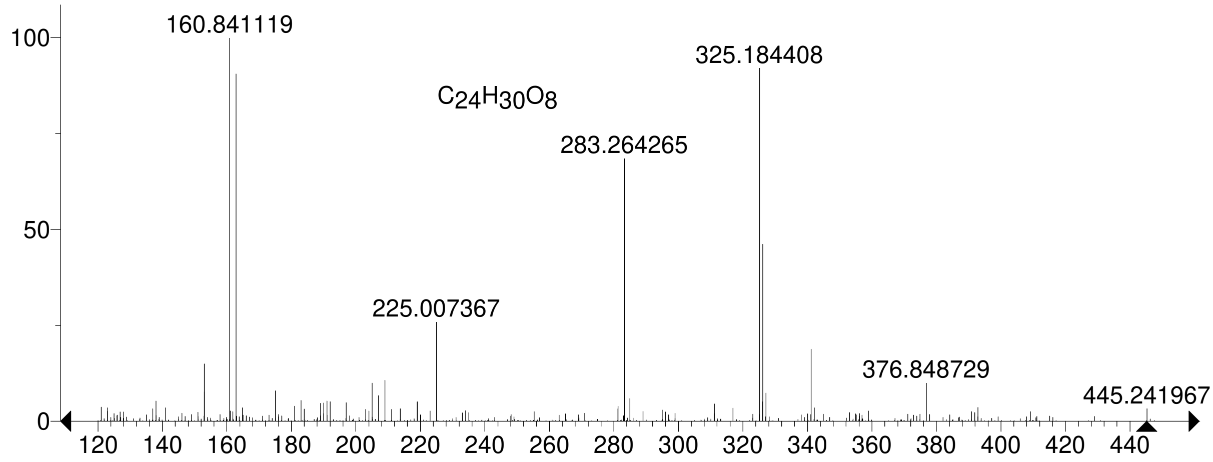

Unknown: Unknown (7.48_679.1020m/z)
Compound in Library Factor = 0

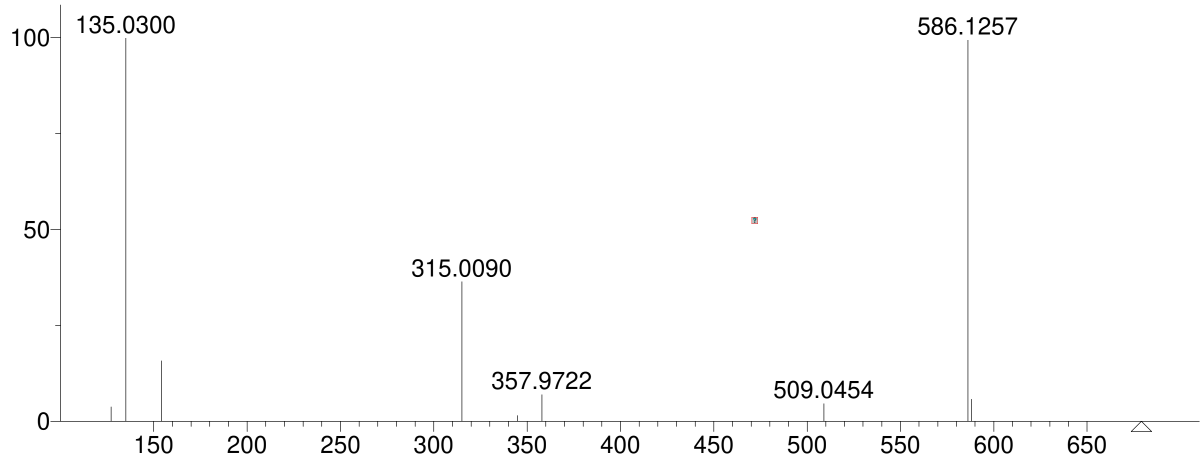

Unknown: Unknown (7.48_695.3736m/z)
Compound in Library Factor = -3273

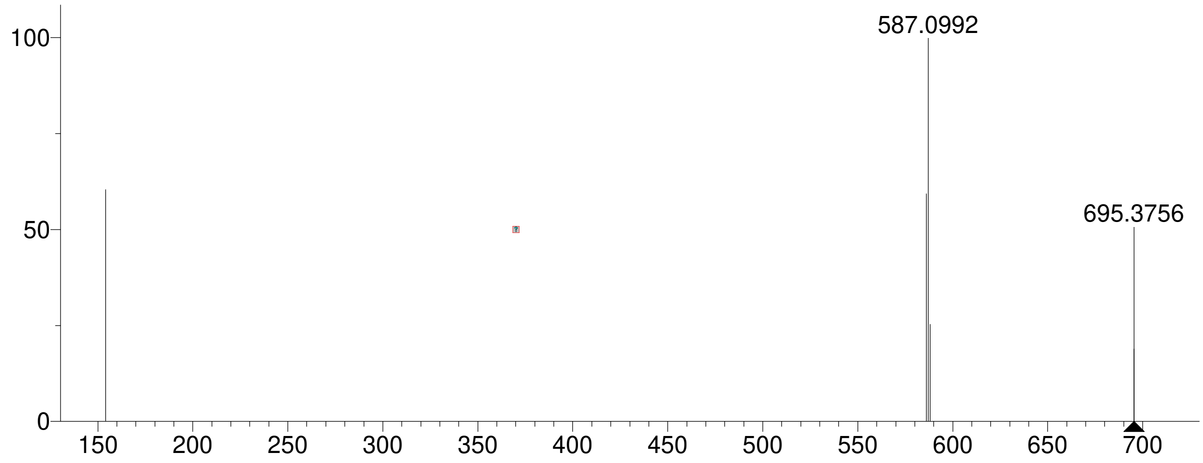

Hit 1 : PI 24:1 [M-H]- 40eV P=695.4
C33H61O13P; MF: 8; RMF: 301; Prob 9.09%; Lib: mona-export-in-silico_spectra; ID: 96230.

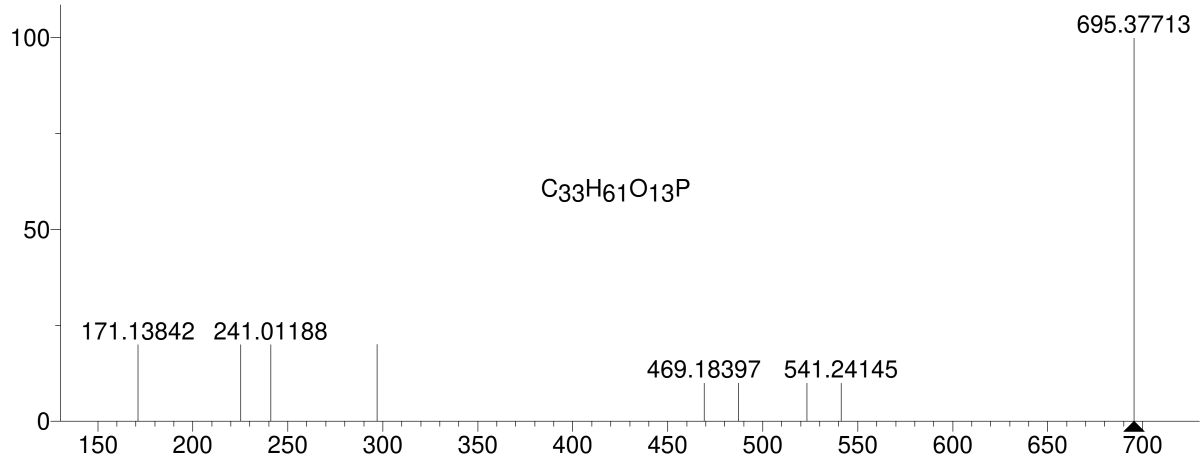

Hit 2 : PI 24:1; PI 9:0-15:1; [M-H]- [M-H]- P=695.4
C33H61O13P; MF: 8; RMF: 301; Prob 9.09%; Lib: msdial-insilicomsms-lipids-neg; ID: 326872.

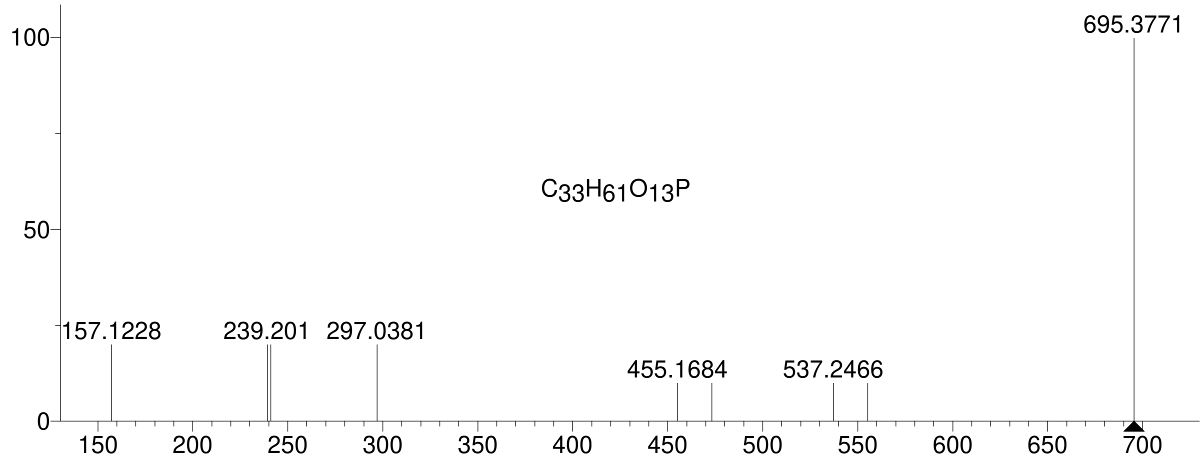

Hit 3 : PI 24:1; PI 10:0-14:1; [M-H]- [M-H]- P=695.4
C33H61O13P; MF: 8; RMF: 301; Prob 9.09%; Lib: msdial-insilicomsms-lipids-neg; ID: 326923.

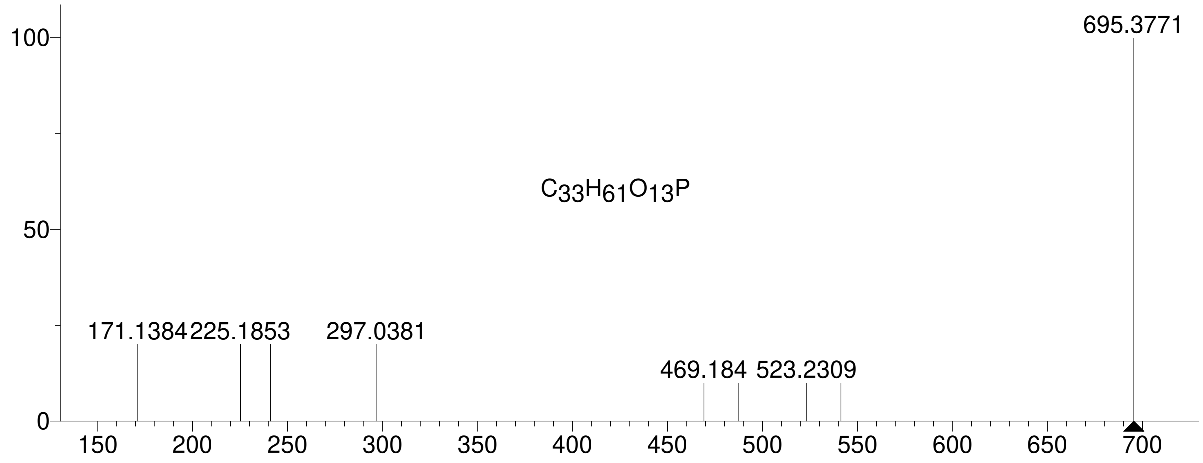

Hit 4 : PI 24:1; PI 11:0-13:1; [M-H]- [M-H]- P=695.4
C33H61O13P; MF: 8; RMF: 301; Prob 9.09%; Lib: msdial-insilicomsms-lipids-neg; ID: 326973.

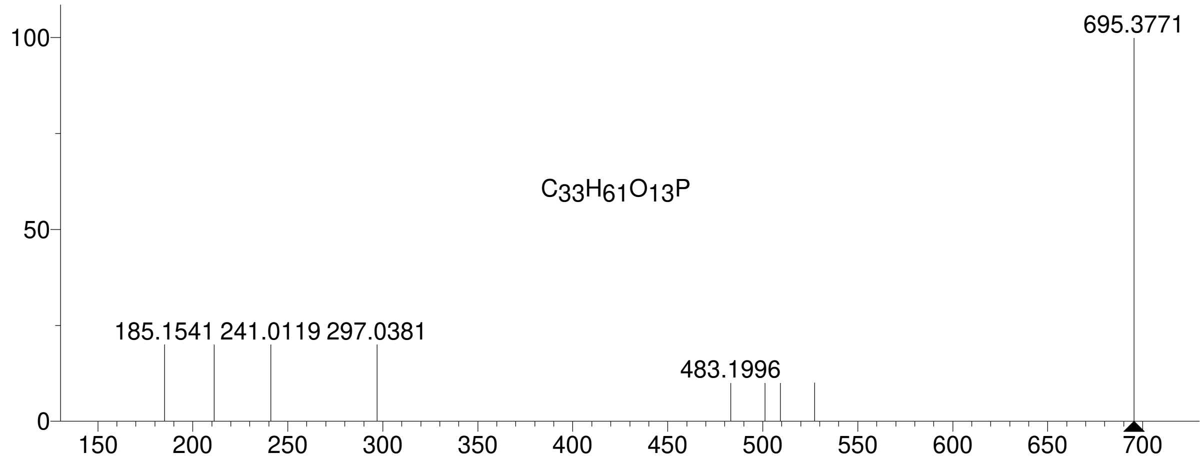

Hit 5 : PI 24:1; PI 2:0-22:1; [M-H]- [M-H]- P=695.4
C33H61O13P; MF: 8; RMF: 283; Prob 9.09%; Lib: msdial-insilicomsms-lipids-neg; ID: 326501.

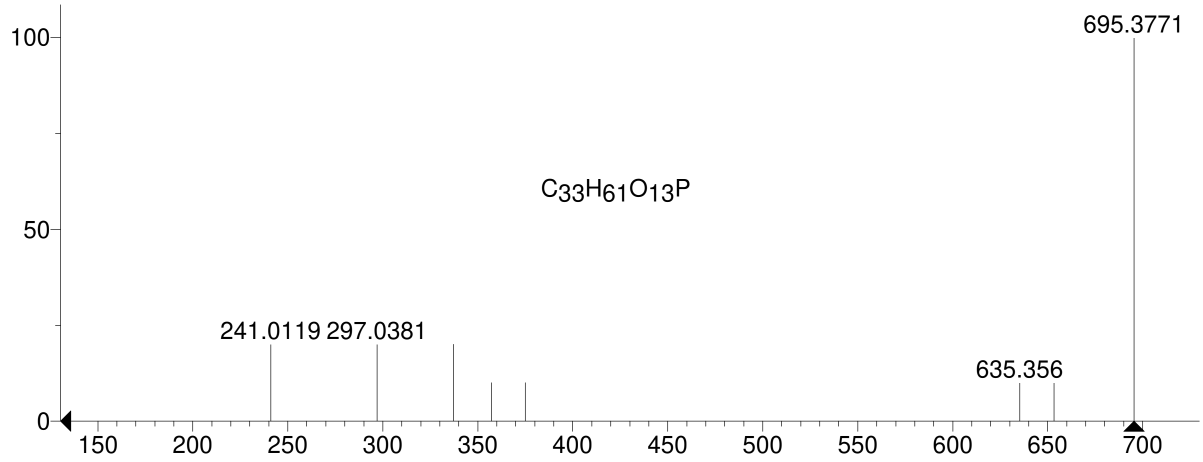

Unknown: Unknown (7.48_723.1822m/z)
Compound in Library Factor = 0

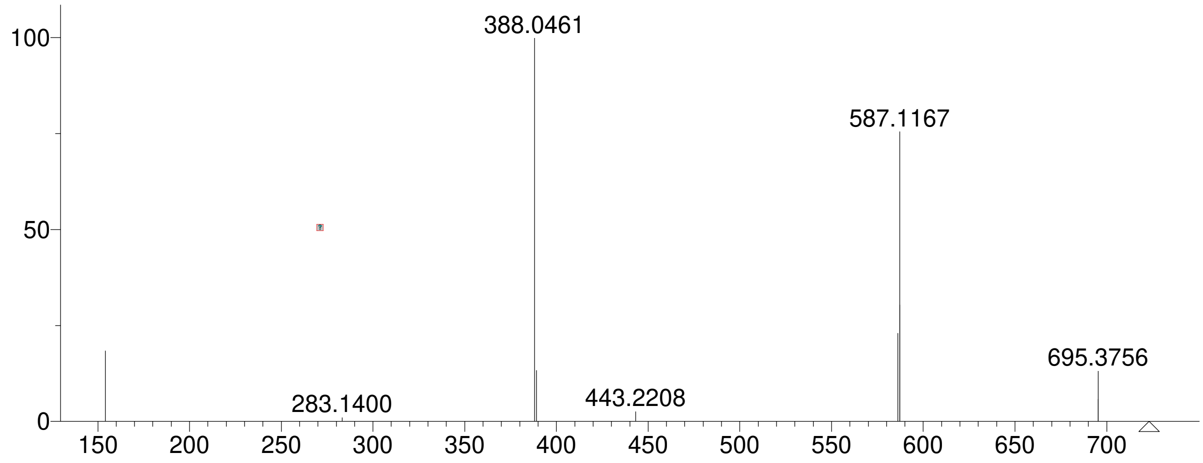

Unknown: Unknown (7.49_477.1831m/z)
Compound in Library Factor = 0

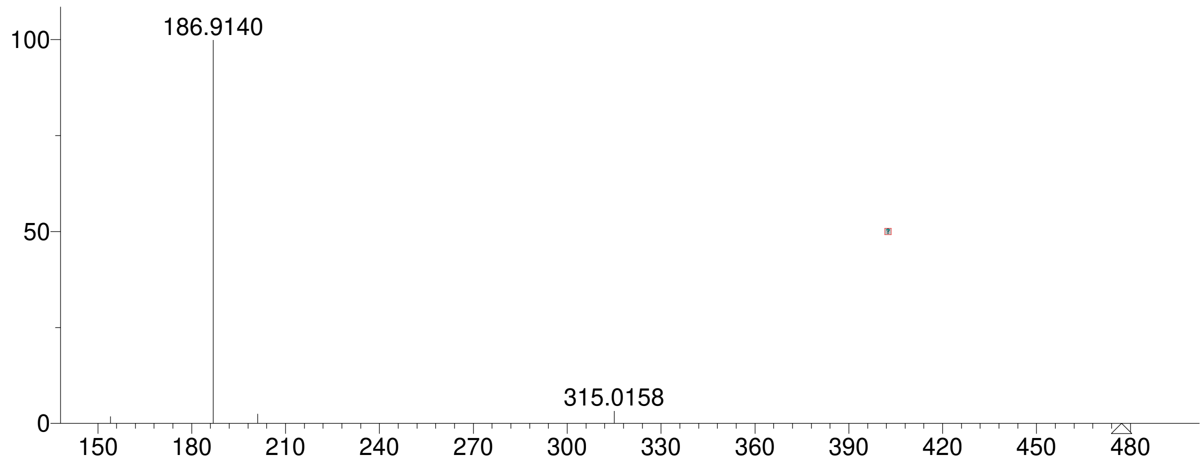

Unknown: Unknown (7.49_648.0272m/z)
Compound in Library Factor = 0

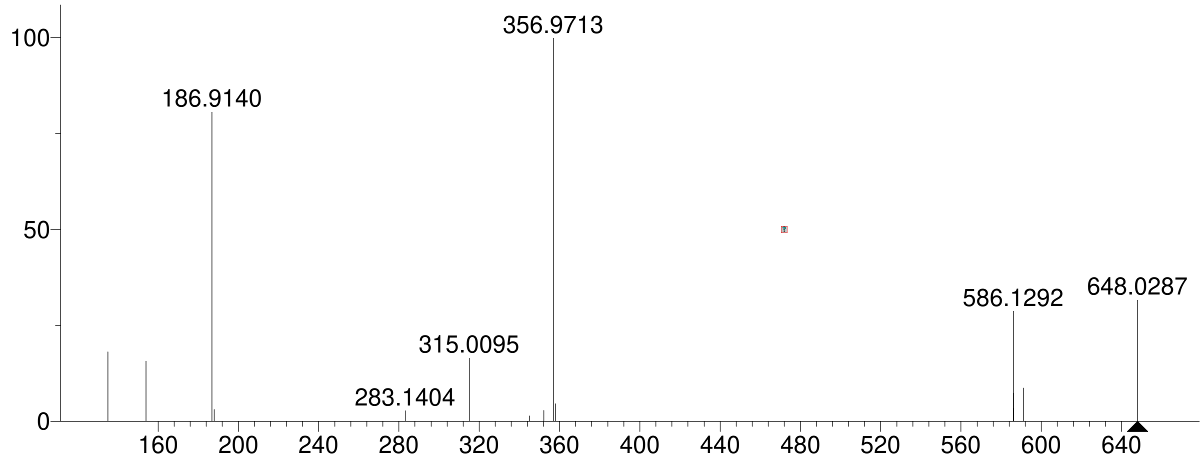

Unknown: Unknown (7.51_559.2237m/z)
Compound in Library Factor = 0

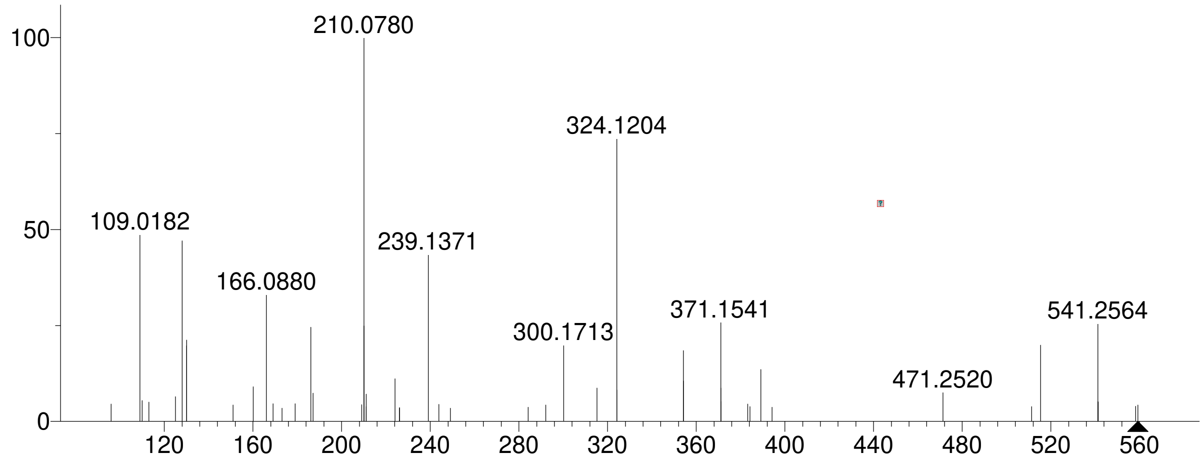

Hit 1 : methyl (2R)-2-[(1S,2R,4S,9R,10R,14S,15S,17S)-17-(acetyloxy)-9-(furan-3-yl)-1-hydroxy-10,14,16,16-tetrai
C29H36O11; MF: 0; RMF: 41; Prob 100.0%; Lib: mona-export-lc-ms-ms_negative_mode; ID: 20348.

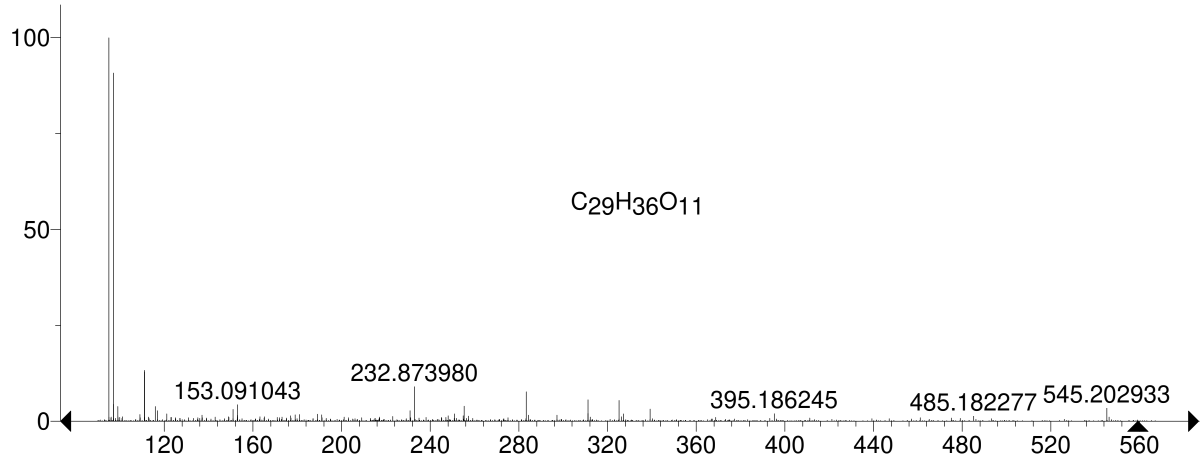

Unknown: Unknown (7.52_369.1772m/z)
Compound in Library Factor = -885

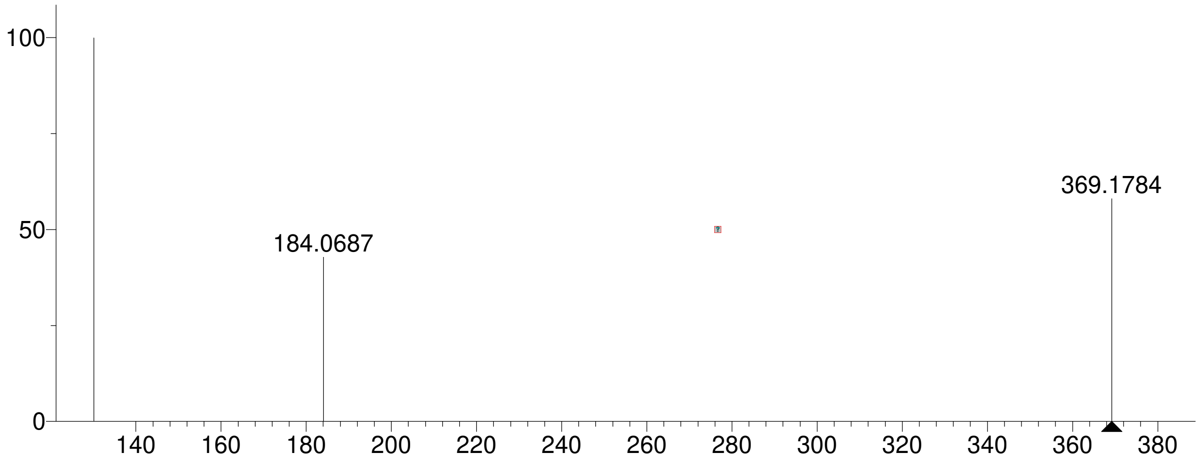

Hit 1 : 5β-Androstan-3α-ol-17-one sulfate [M-H]- HCD 33V P=369.2
C19H30O5S; MF: 165; RMF: 537; Prob 41.0%; CAS: 977-36-6; Lib: nist_msms; ID: 231644.

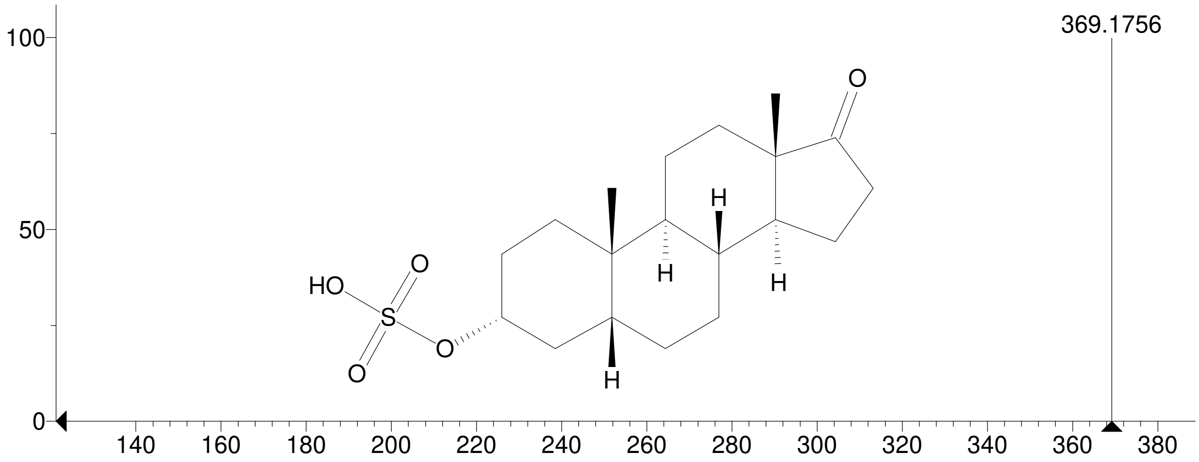

Hit 2 : 5-Androsten-3β,17β-diol-3-sulfate [M-H]- HCD 27V P=369.2
C19H30O5S; MF: 165; RMF: 537; Prob 41.0%; CAS: 977-33-3; Lib: nist_msms; ID: 231655.

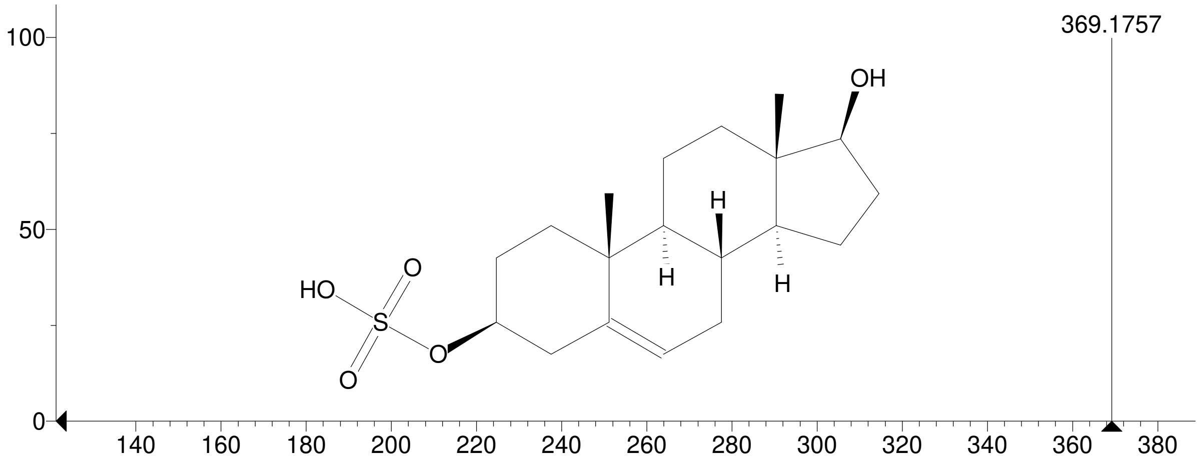

Hit 3 : 5α-Androstan-3β-ol-17-one sulfate [M-H]- HCD 40V P=369.2
C19H30O5S; MF: 129; RMF: 537; Prob 10.1%; CAS: 977-35-5; Lib: nist_msms; ID: 231625.

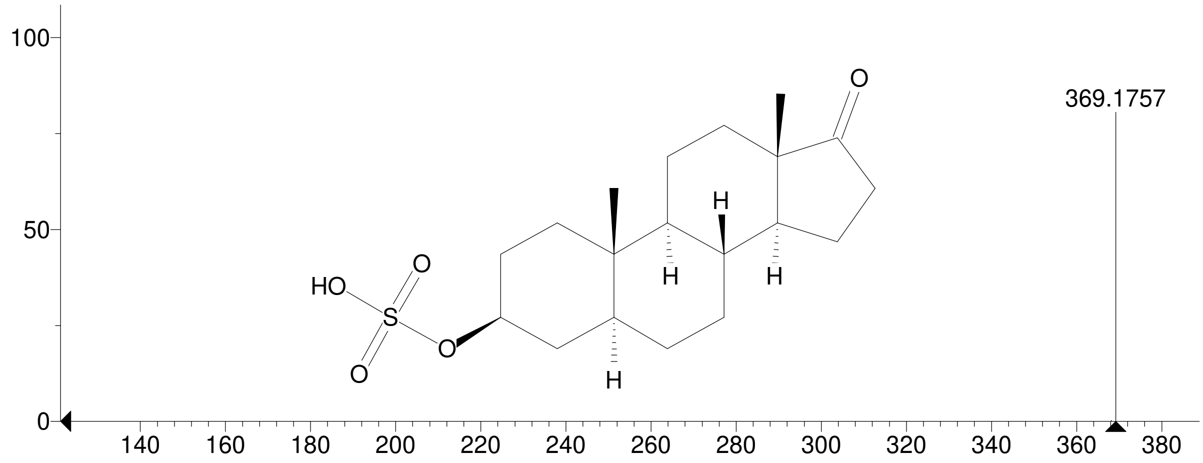

Hit 4 : 5α-Androstan-17β-ol-3-one sulfate [M-H]- HCD 40V P=369.2
C19H30O5S; MF: 118; RMF: 534; Prob 6.95%; CAS: 2641-48-7; Lib: nist_msms; ID: 231920.

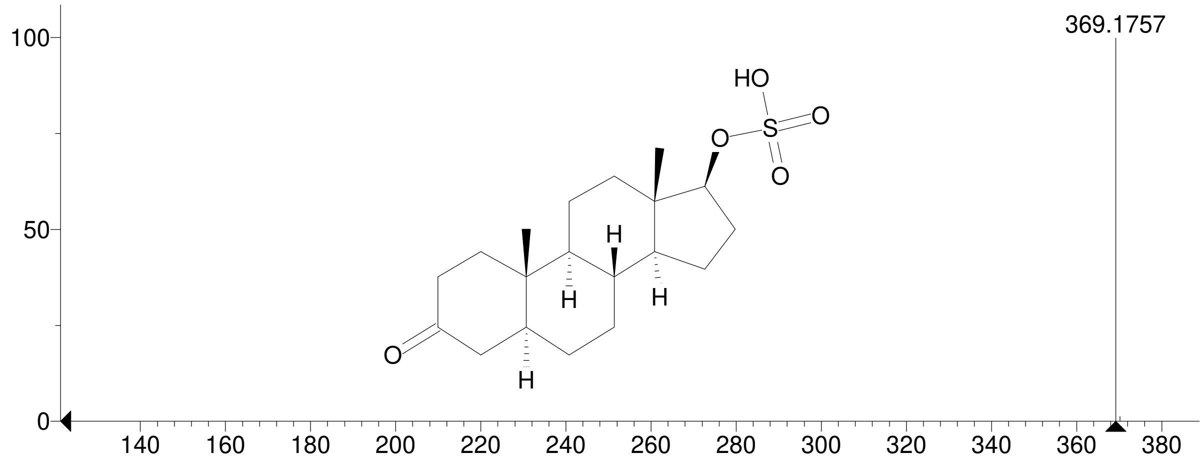

Hit 5 : Androsterone sulfate [M-H]- HCD 35V P=369.2
C19H30O5S; MF: 34; RMF: 484; Prob 0.65%; CAS: 2479-86-9; Lib: nist_msms; ID: 117993.

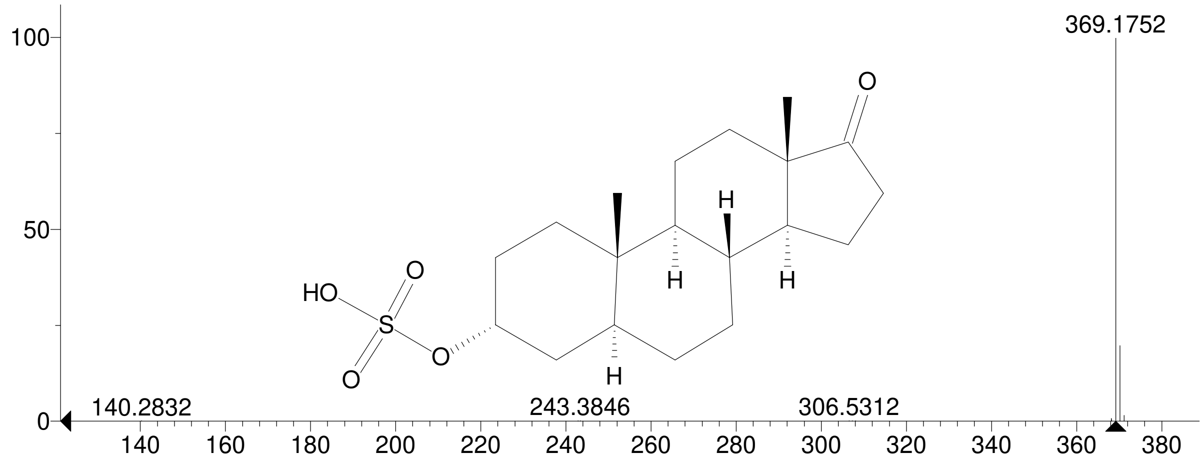

Unknown: Unknown (7.53_226.1548m/z)
Compound in Library Factor = 0

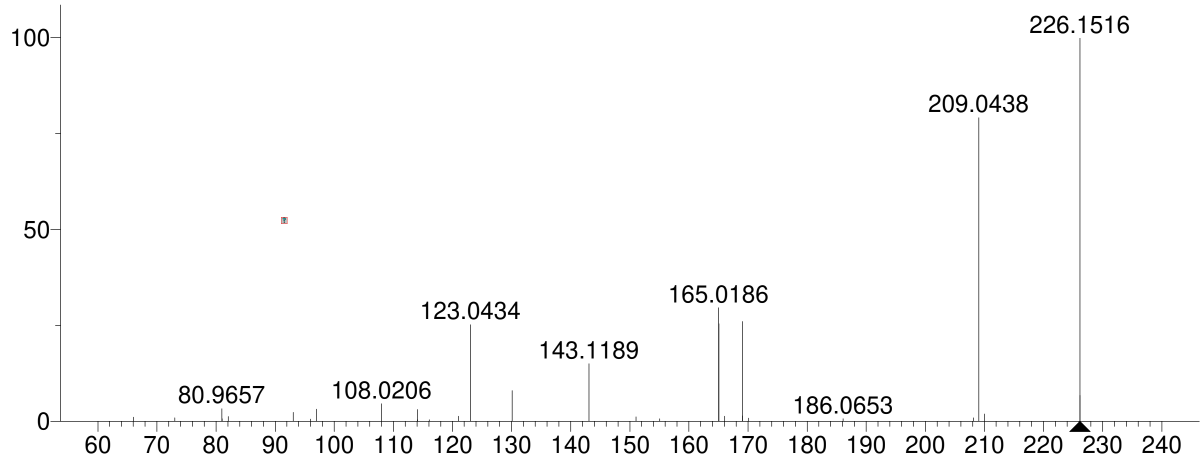

Unknown: Unknown (7.53_653.3601m/z)
Compound in Library Factor = -3273

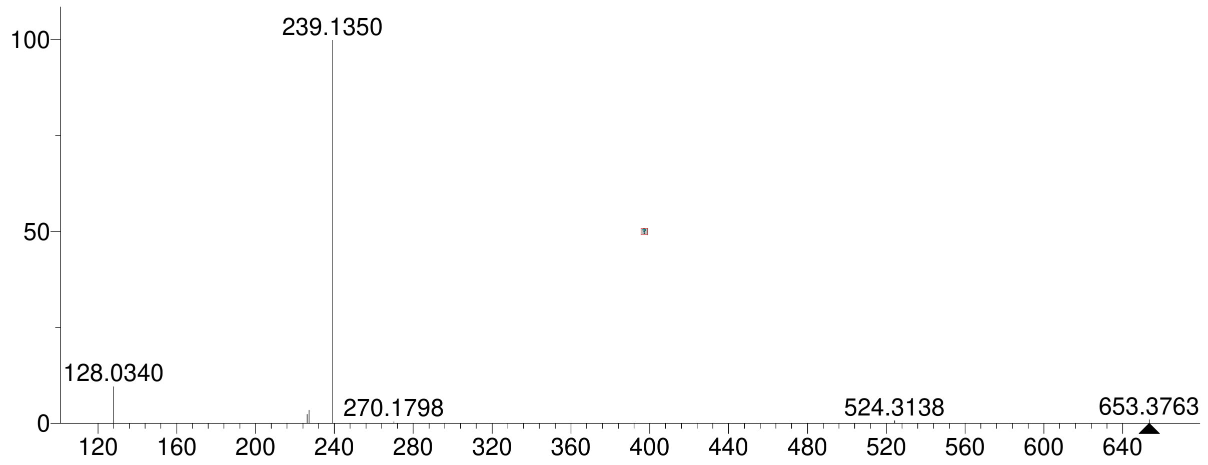

Hit 1 : SQDG 22:0 [M-H]- 40eV P=653.4
C31H58O12S; MF: 3; RMF: 81; Prob 18.4%; Lib: mona-export-in-silico_spectra; ID: 119065.

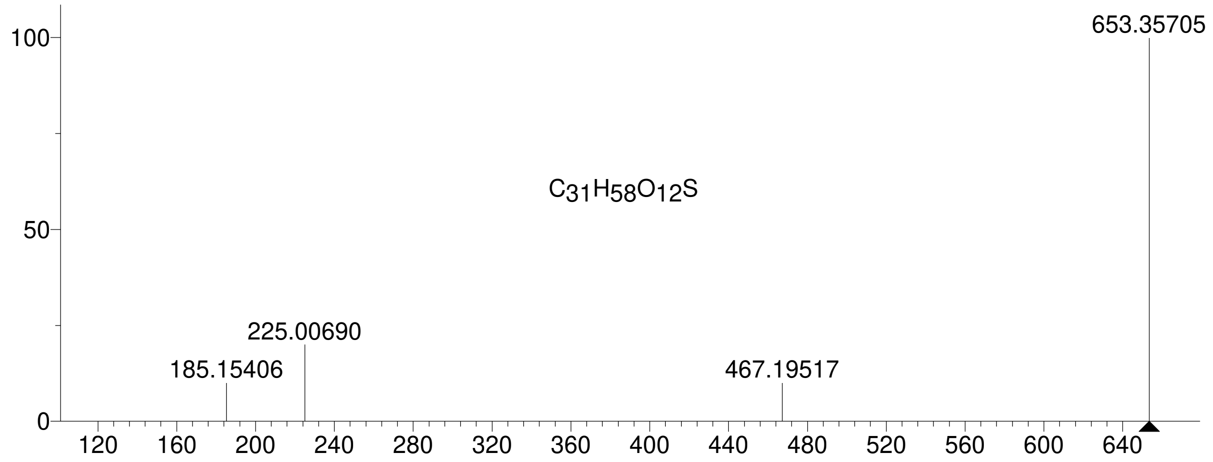

Hit 2 : MGDG 24:5; MGDG 2:0-22:5; [M+FA-H]- [M+FA-H]- P=653.4
C33H52O10; MF: 0; RMF: 40; Prob 16.3%; Lib: msdial-insilicomsms-lipids-neg; ID: 311312.

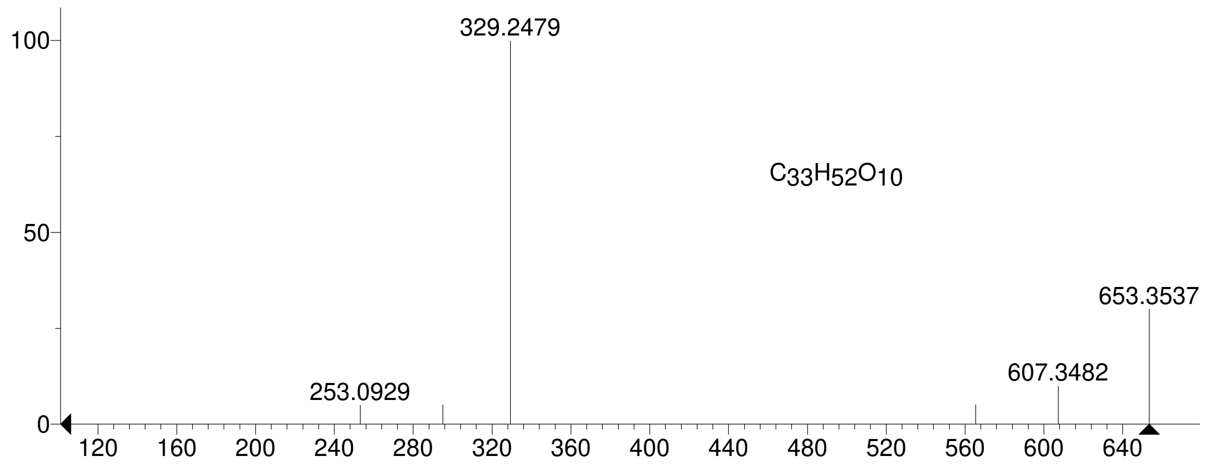

Hit 3 : MGDG 24:5; MGDG 4:0-20:5; [M+FA-H]- [M+FA-H]- P=653.4
C33H52O10; MF: 0; RMF: 40; Prob 16.3%; Lib: msdial-insilicomsms-lipids-neg; ID: 311422.

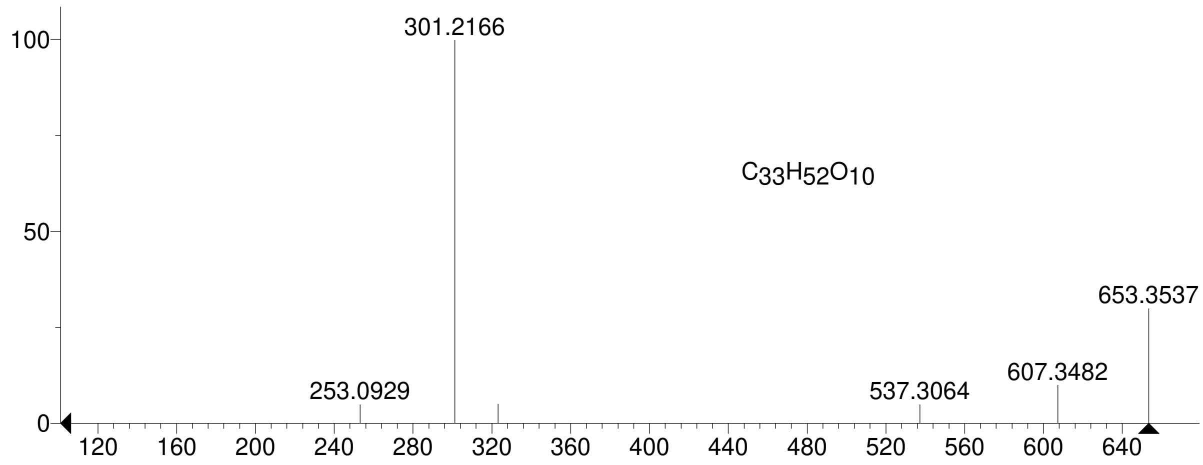

Hit 4 : MGDG 24:5; MGDG 6:0-18:5; [M+FA-H]- [M+FA-H]- P=653.4
C33H52O10; MF: 0; RMF: 40; Prob 16.3%; Lib: msdial-insilicomsms-lipids-neg; ID: 311528.

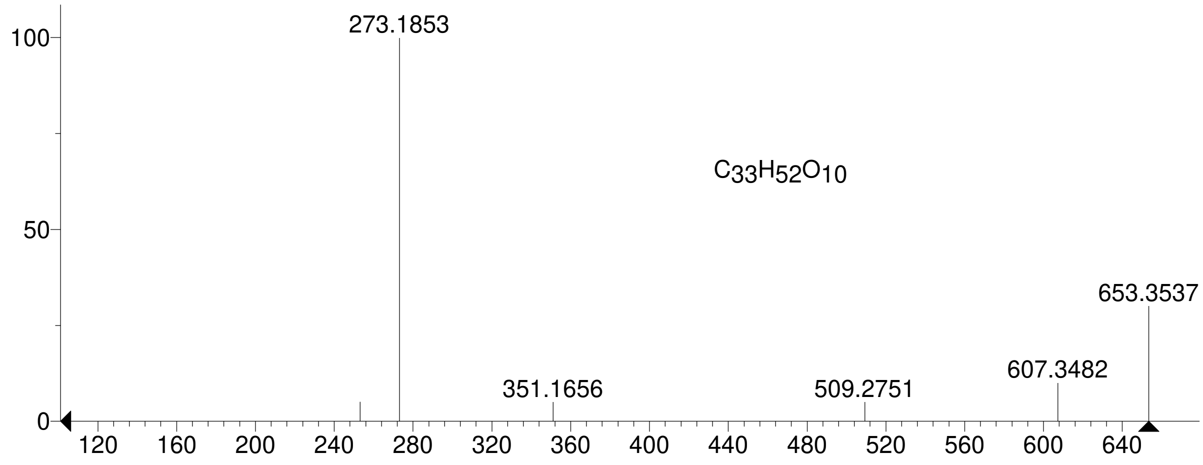

Hit 5 : MGDG 23:5; MGDG 3:0-20:5; [M+Hac-H]- [M+Hac-H]- P=653.4
C32H50O10; MF: 0; RMF: 40; Prob 16.3%; Lib: msdial-insilicomsms-lipids-neg; ID: 313254.

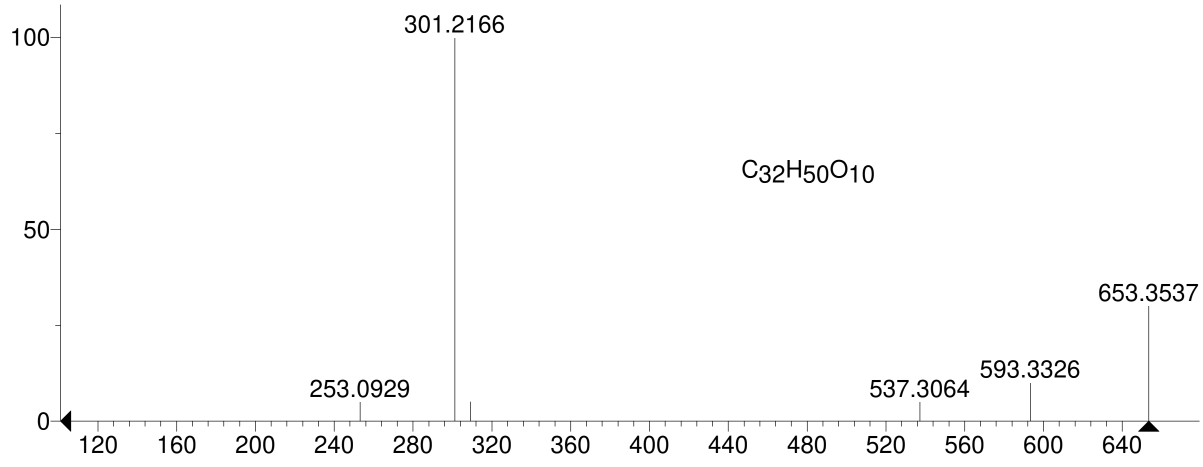

Unknown: Unknown (7.54_289.0014m/z)
Compound in Library Factor = -1523

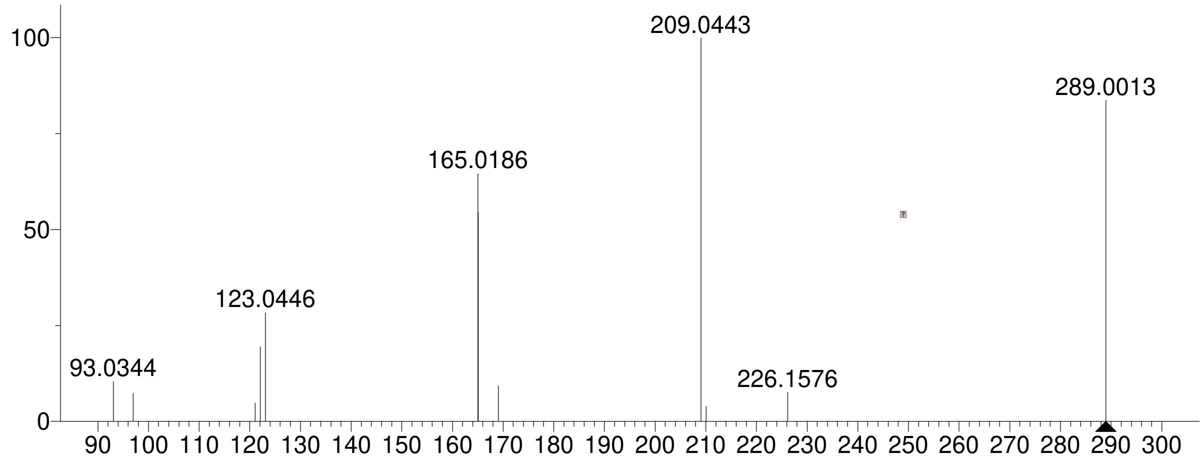

Hit 1 : Catechin [M-H]- QQQ 20V P=289
C15H14O6; MF: 59; RMF: 534; Prob 32.0%; Lib: mona-export-lc-ms-ms_negative_mode; ID: 14518.

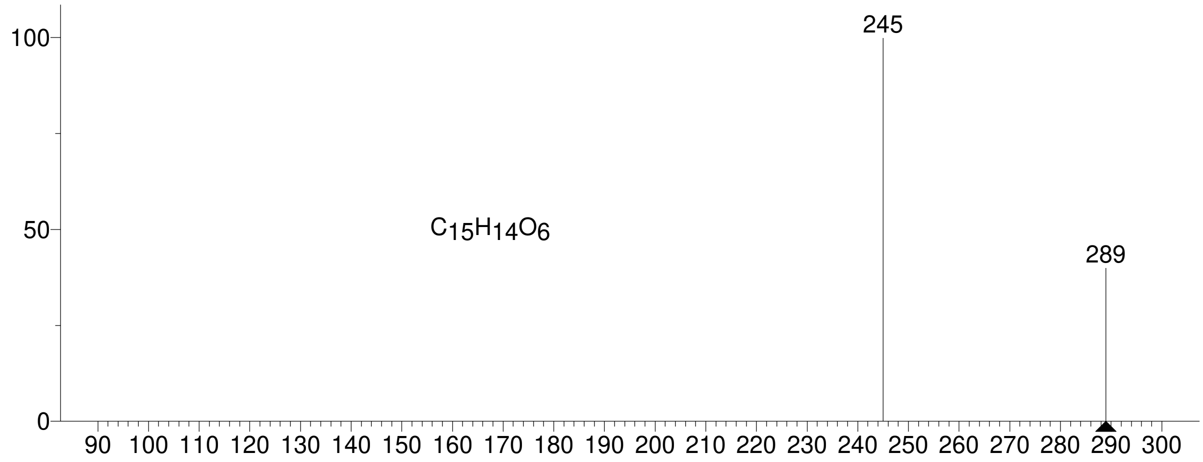

Hit 2 : Epicatechin [M-H]- QQQ 20V P=289
C15H14O6; MF: 59; RMF: 534; Prob 32.0%; Lib: mona-export-lc-ms-ms_negative_mode; ID: 14624.

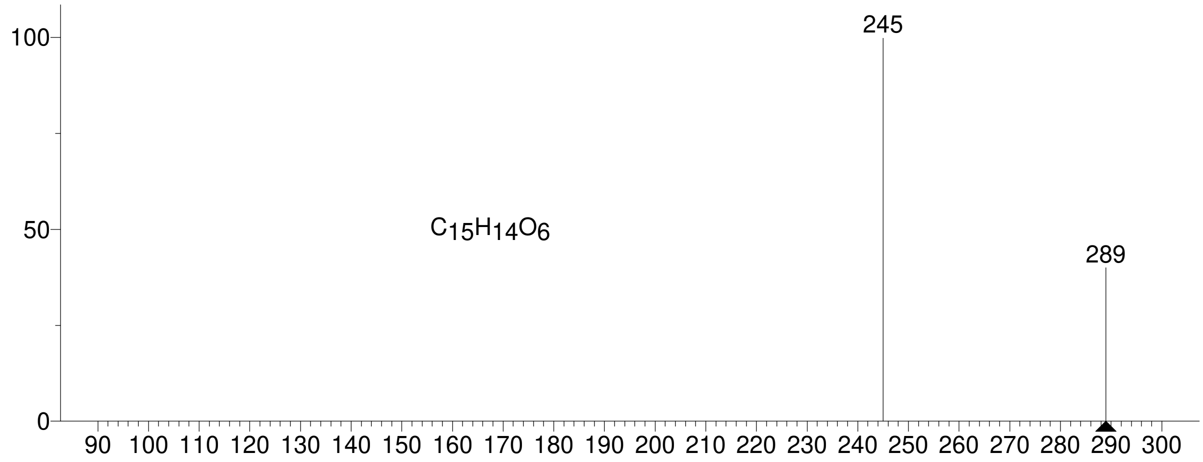

Hit 3 : Lormetazepam [M+H]+=>289.0 IT 35% P=289
C16H12Cl2N2O2; MF: 17; RMF: 416; Prob 7.18%; CAS: 848-75-9; Lib: nist_msms; ID: 186240.

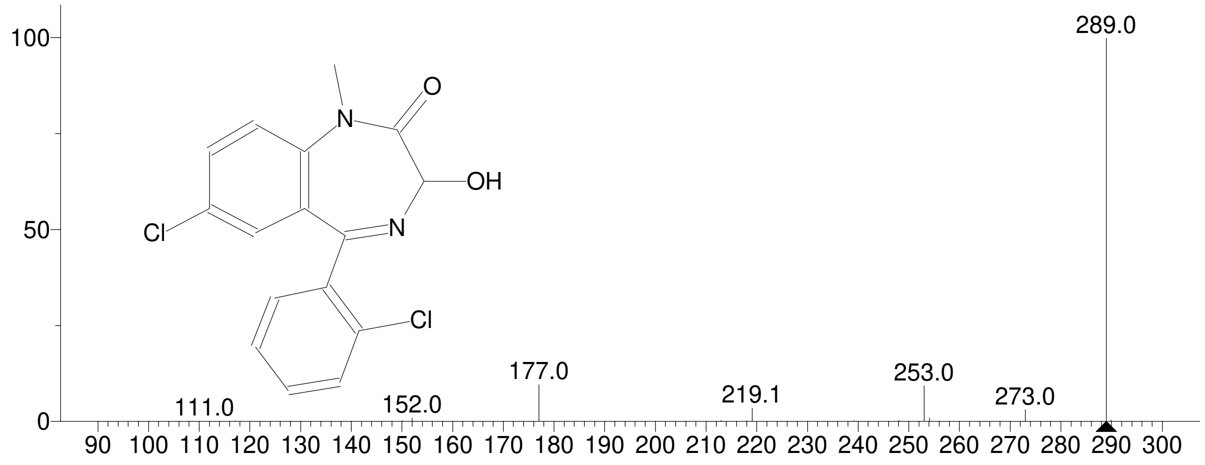

Hit 4 : Permethrin [M+H-H2O]+=>317.0=>289.0 IT 35% P=289
C21H20Cl2O3; MF: 4; RMF: 344; Prob 4.64%; CAS: 52645-53-1; Lib: nist_msms; ID: 284116.

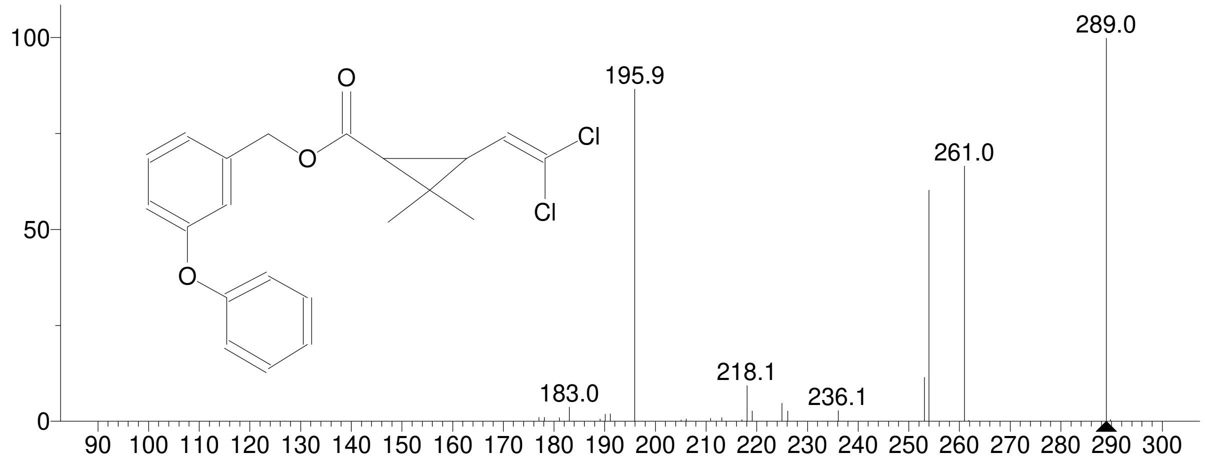

Hit 5 : Lignan J1 [M+H-CH2O]+=>317.0=>289.0 IT 35% P=289
C21H14O7; MF: 1; RMF: 153; Prob 4.10%; CAS: 27041-98-1; Lib: nist_msms; ID: 400690.

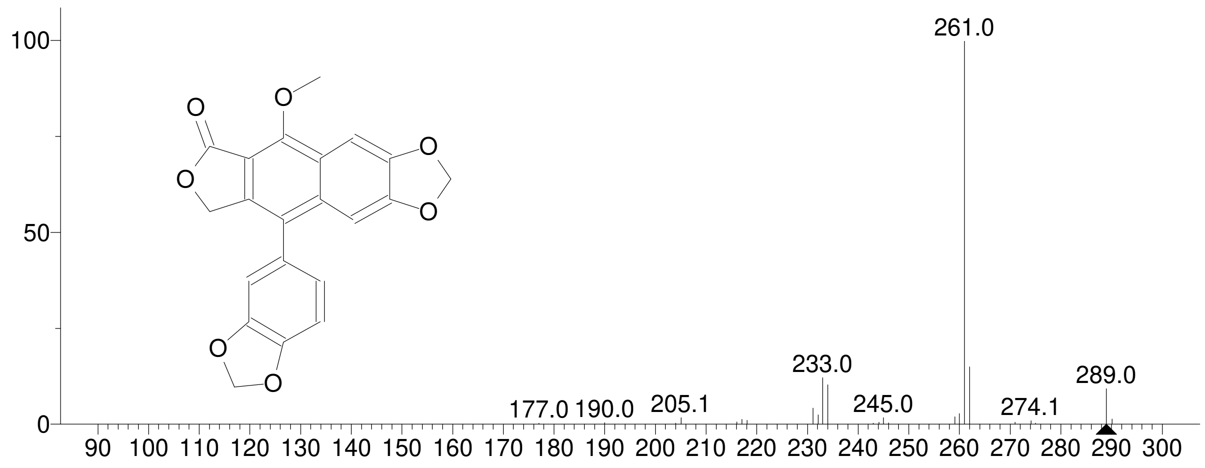

Unknown: Unknown (7.54_524.1543m/z)
Compound in Library Factor = 0

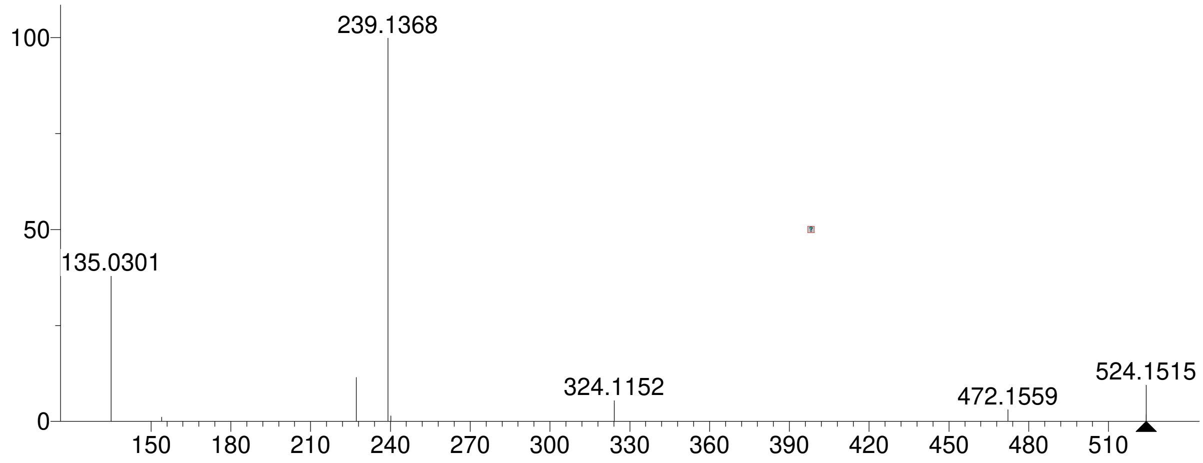

Unknown: Unknown (7.54_623.2424m/z)
Compound in Library Factor = 0

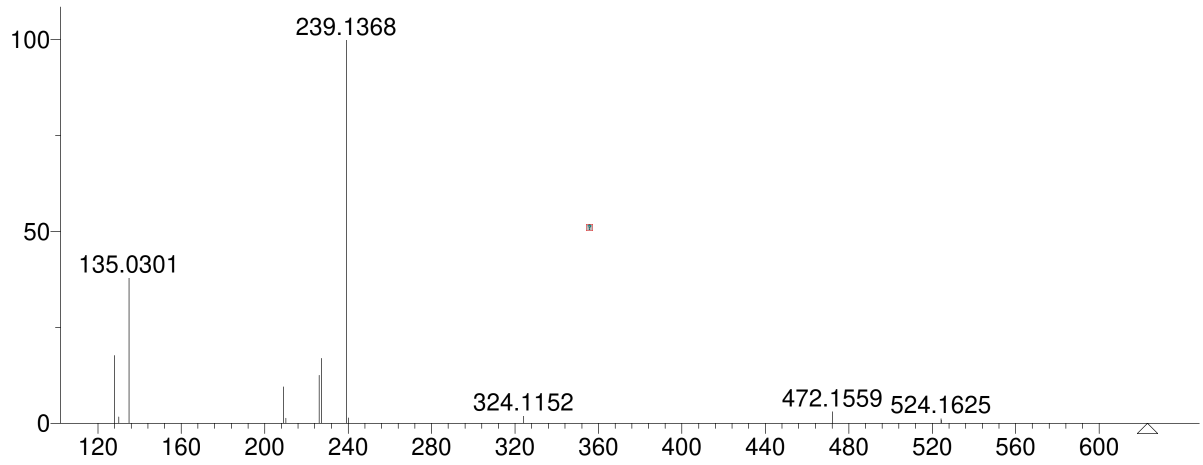

Hit 1 : Grossamide or its isomer (Not validated) ; PlaSMA ID-1722 [M-H]- P=623.2
C36H36N2O8; MF: 0; RMF: 8; Prob 100.0%; Lib: biomsms-neg-plasma; ID: 728.

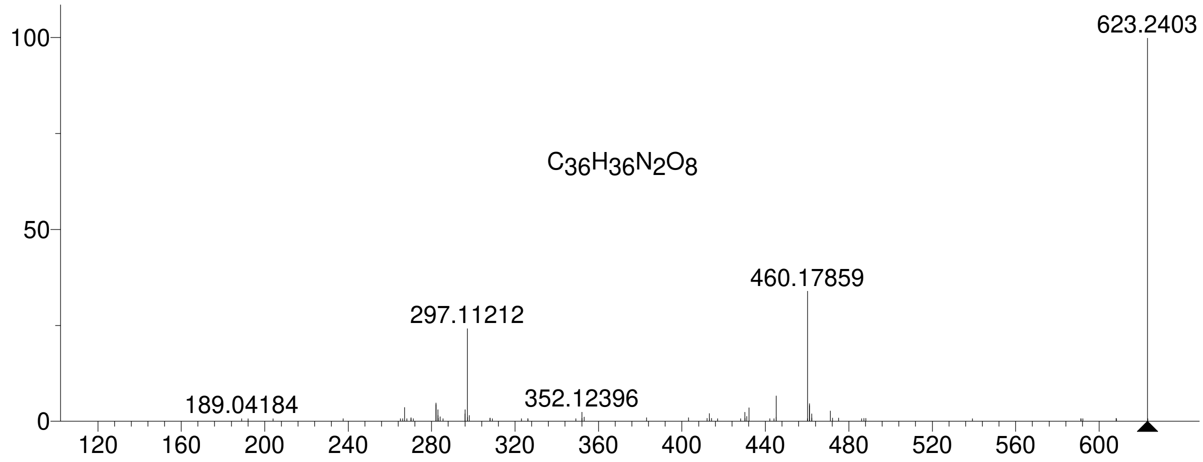

Unknown: Unknown (7.54_642.2151m/z)
Compound in Library Factor = 0

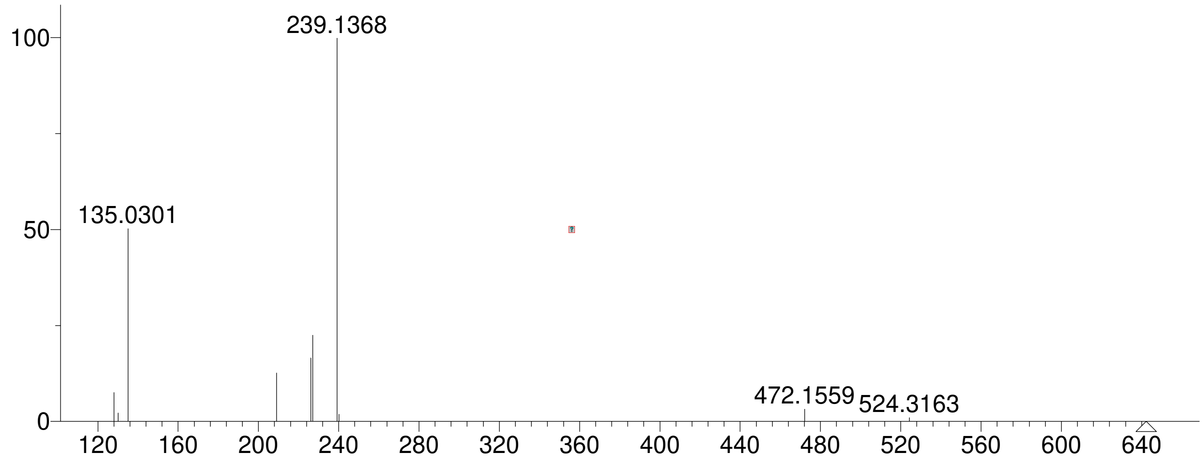

Unknown: Unknown (7.54_725.1844m/z)
Compound in Library Factor = 0

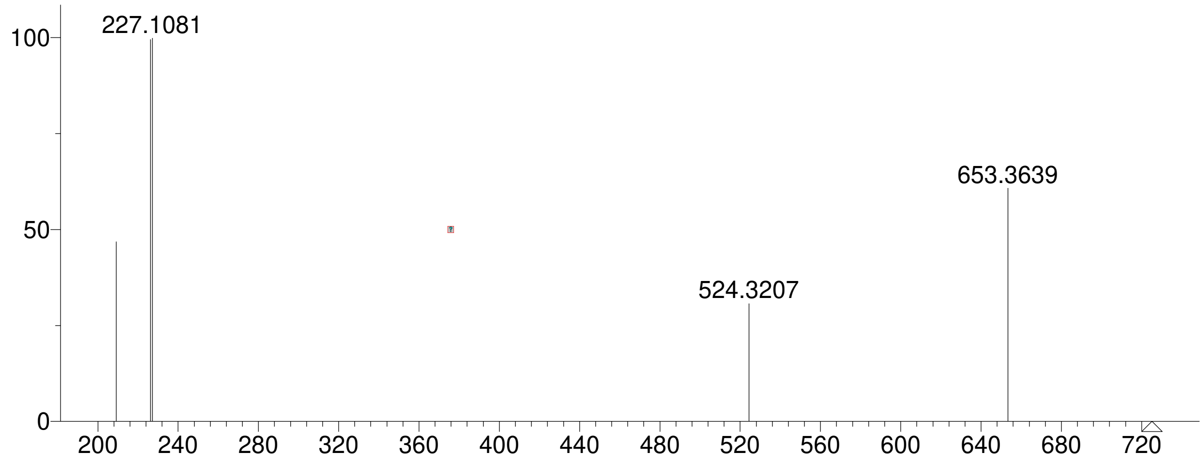

Unknown: Unknown (7.56_559.2716m/z)
Compound in Library Factor = 0

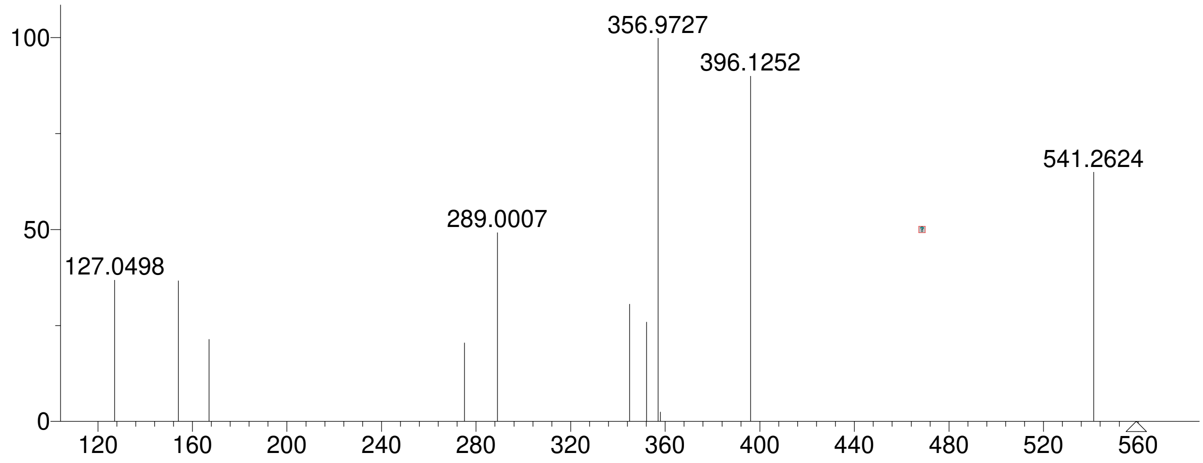

Hit 1 : (3S,4S,5R)-4-[(2R,3R)-2,3-dihydroxy-3-[(2S,3R,5R,10R,13R,14S,17S)-2,3,14-trihydroxy-10,13-dimethyl-6-
C29H44O8; MF: 0; RMF: 39; Prob 100.0%; Lib: mona-export-vf-npl_qexactive; ID: 9539.

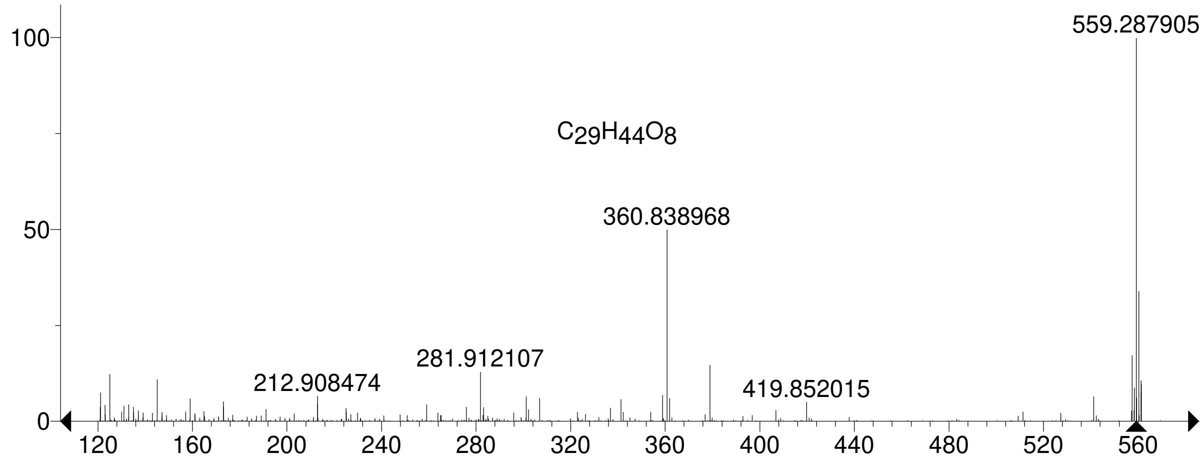

Unknown: Unknown (7.57_289.0014m/z)
Compound in Library Factor = -1523

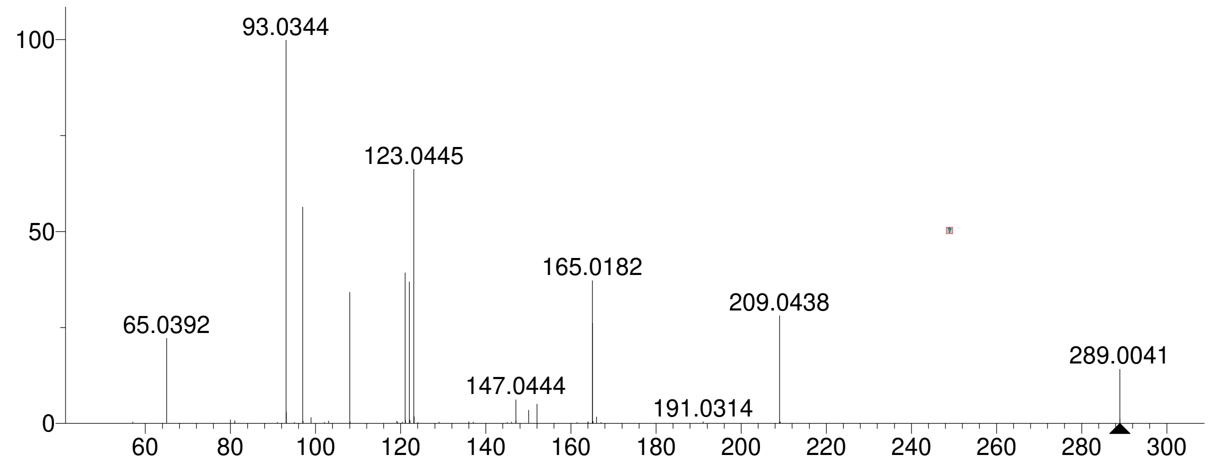

Hit 1 : Catechin [M-H]- QQQ 20V P=289
C15H14O6; MF: 51; RMF: 515; Prob 28.9%; Lib: mona-export-lc-ms-ms_negative_mode; ID: 14518.

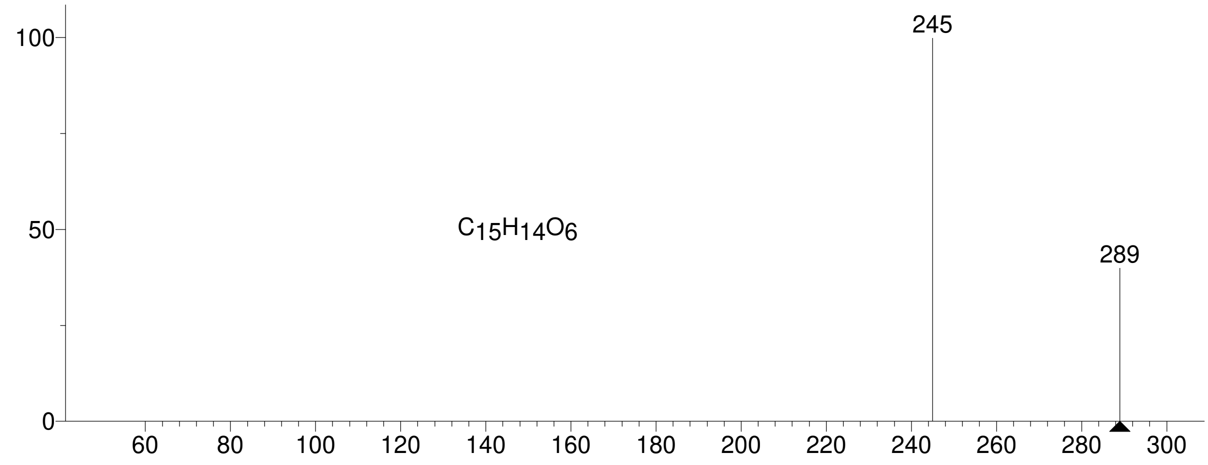

Hit 2 : Epicatechin [M-H]- QQQ 20V P=289
C15H14O6; MF: 51; RMF: 515; Prob 28.9%; Lib: mona-export-lc-ms-ms_negative_mode; ID: 14624.

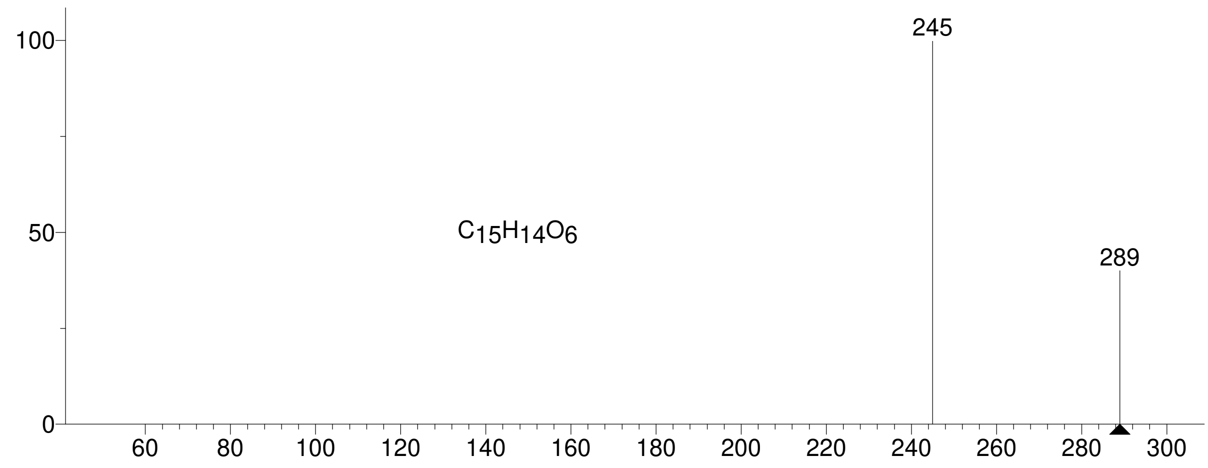

Hit 3 : Lormetazepam [M+H]+=>289.0 IT 35% P=289
C16H12Cl2N2O2; MF: 5; RMF: 200; Prob 6.10%; CAS: 848-75-9; Lib: nist_msms; ID: 186240.

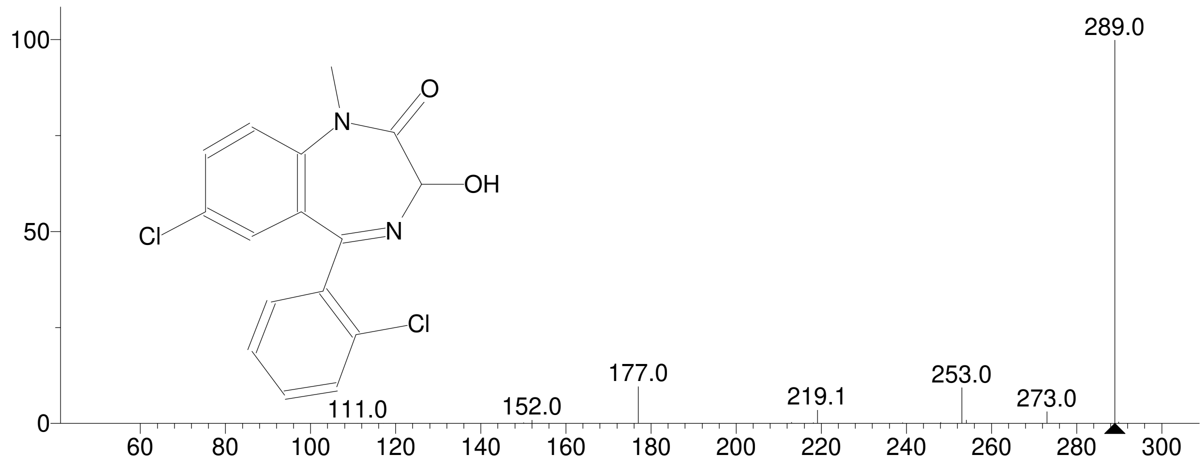

Hit 4 : Permethrin [M+H-H2O]+=>317.0=>289.0 IT 35% P=289
C21H20Cl2O3; MF: 3; RMF: 297; Prob 5.63%; CAS: 52645-53-1; Lib: nist_msms; ID: 284116.

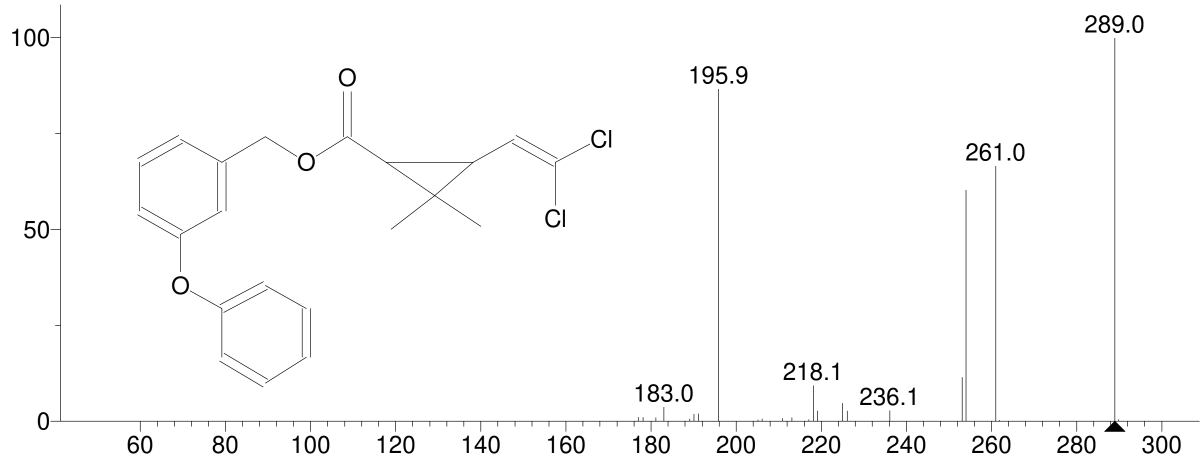

Hit 5 : Lignan J1 [M+H-CH2O]+=>317.0=>289.0 IT 35% P=289
C21H14O7; MF: 1; RMF: 132; Prob 5.19%; CAS: 27041-98-1; Lib: nist_msms; ID: 400690.

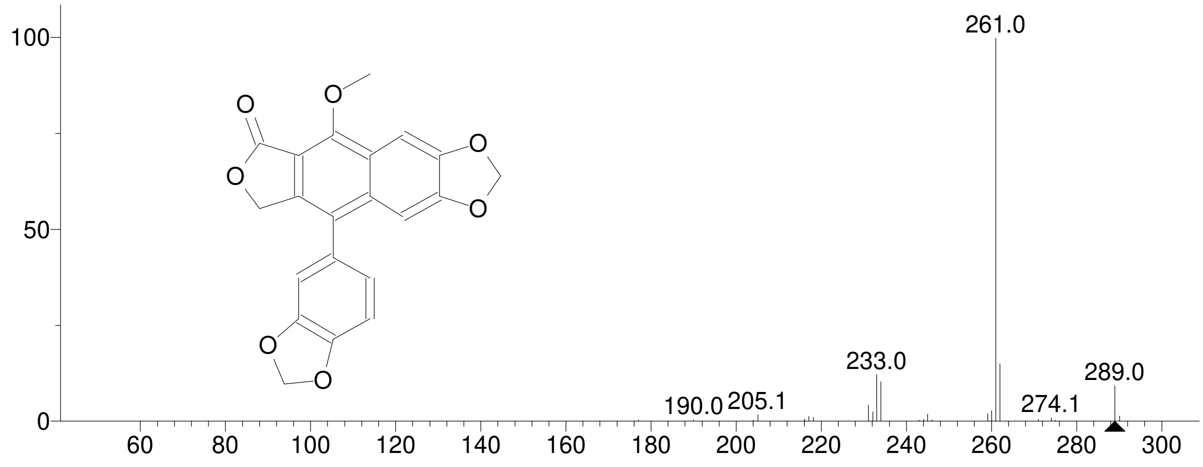

Unknown: Unknown (7.58_624.2501m/z)
Compound in Library Factor = -3273

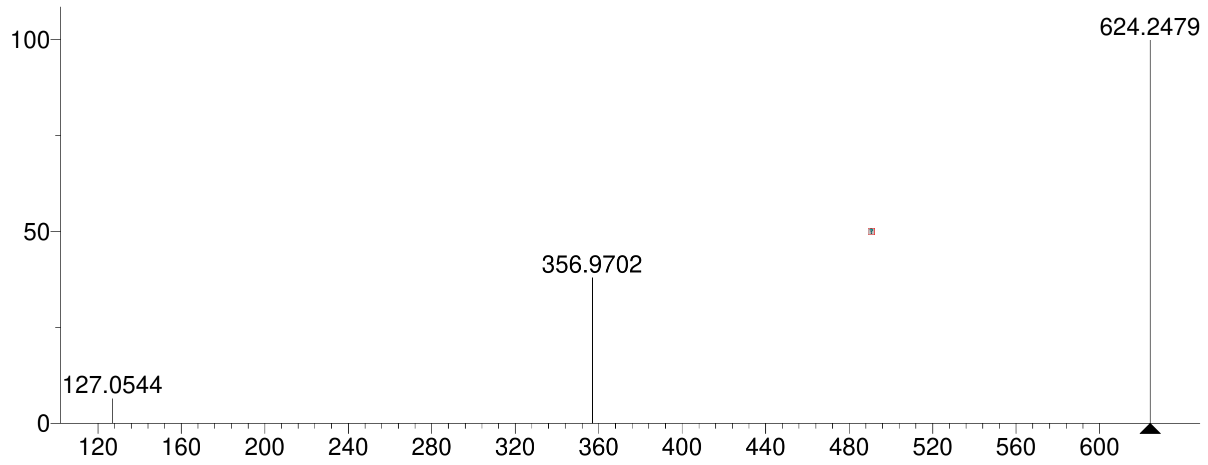

Hit 1 : (Thr28,Nle31)-Cholecystokinin-33 (25-33) (sulfated) [M-2H]2- HCD 25eV P=624.2
C55H74N14O18S; MF: 10; RMF: 541; Prob 100.0%; Lib: nist_msms2; ID: 80226.

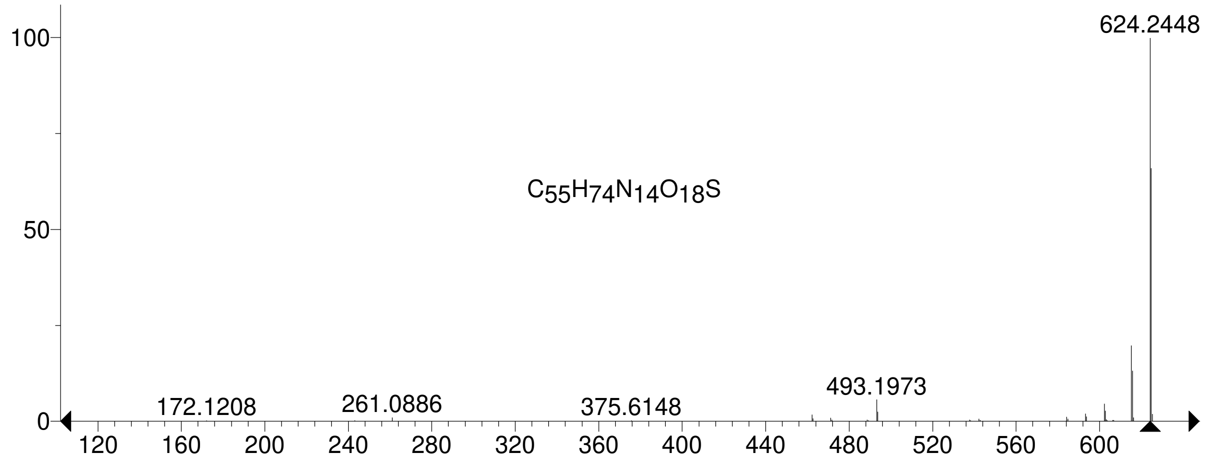

Unknown: Unknown (7.58_649.0267m/z)
Compound in Library Factor = 0

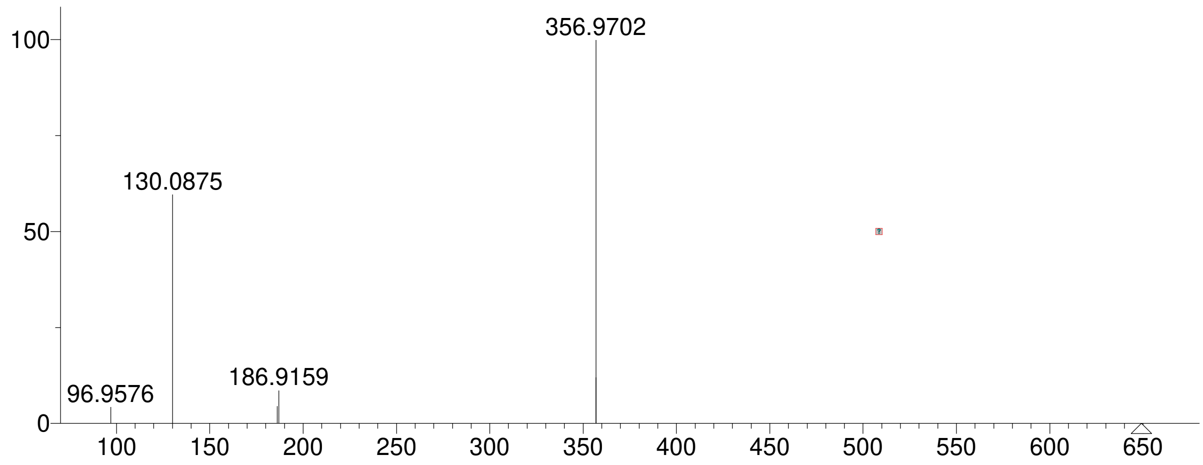

Unknown: Unknown (7.59_468.1469m/z)
Compound in Library Factor = -3273

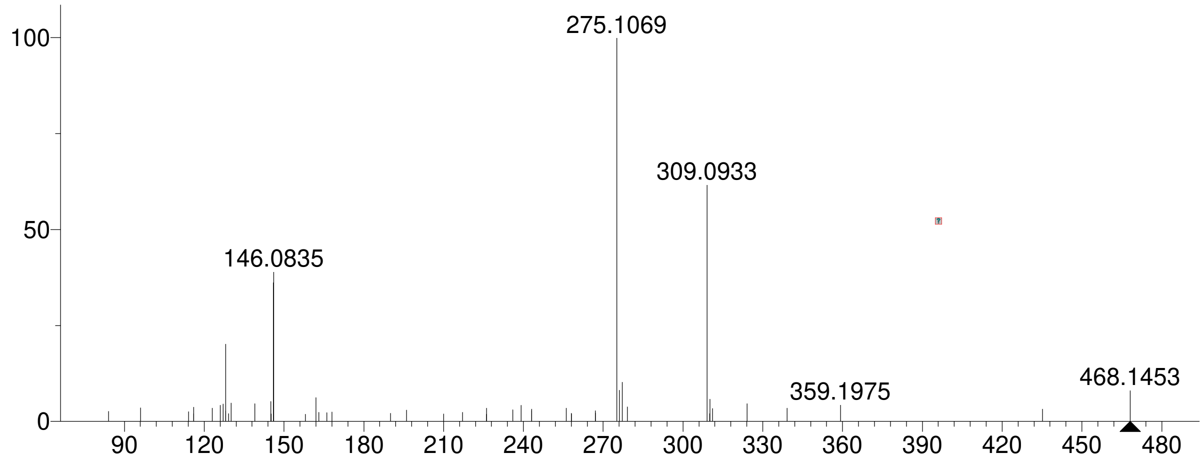

Hit 1 : NCGC00380387-01_C21H27NO11_ [M-H]- P=468.2
C21H27NO11; MF: 1; RMF: 106; Prob 25.7%; Lib: msms-neg-gnps; ID: 1703.

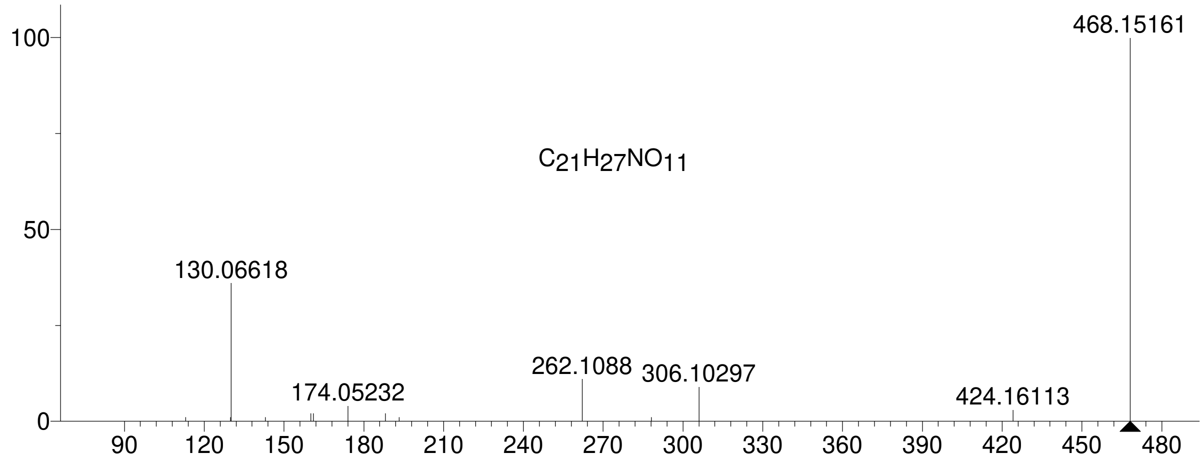

Hit 2 : astilbin [M+NH4]+ 45HCD P=468.2
C21H22O11; MF: 0; RMF: 20; Prob 24.8%; Lib: anthocyanins mona; ID: 5106.

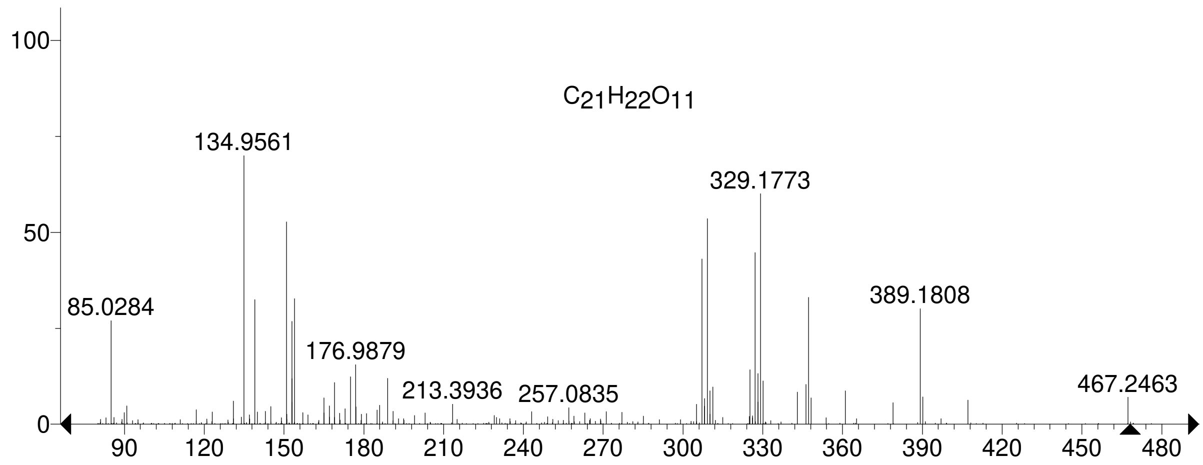

Hit 3 : 2-[1-[4,5-dihydroxy-3-[(2S,3R,4S,5S,6R)-3,4,5-trihydroxy-6-(hydroxymethyl)oxan-2-yl]oxyoxan-2-yl]indol-3-y
C21H27NO11; MF: 0; RMF: 11; Prob 24.8%; Lib: mona-export-lc-ms-ms_negative_mode; ID: 22556.

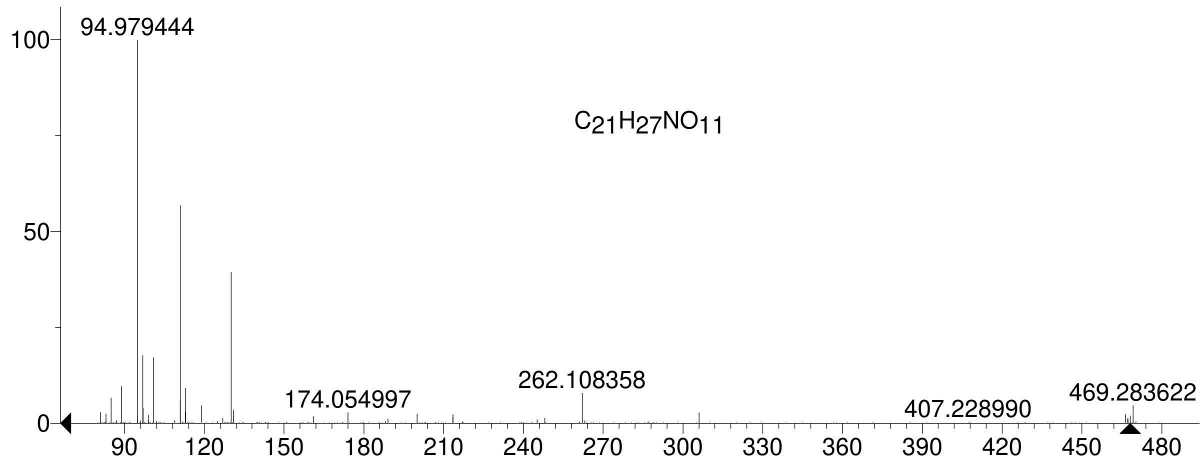

Hit 4 : Rhizocarpic Acid [M-H]- 65HCD P=468.1
C28H23NO6; MF: 0; RMF: 1; Prob 24.8%; Lib: mona-export-lc-ms-ms_negative_mode; ID: 23806.

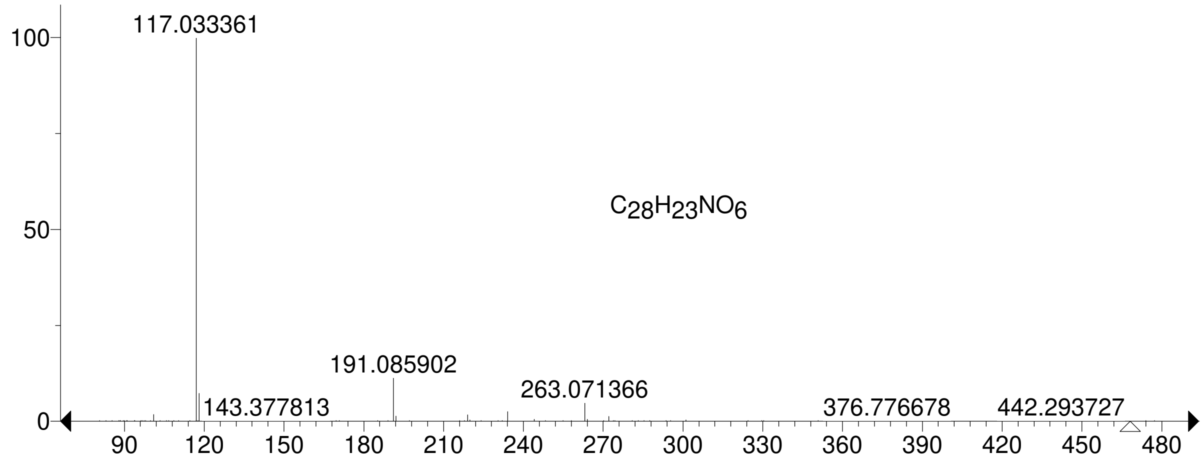

Unknown: Unknown (7.59_725.1823m/z)
Compound in Library Factor = 0

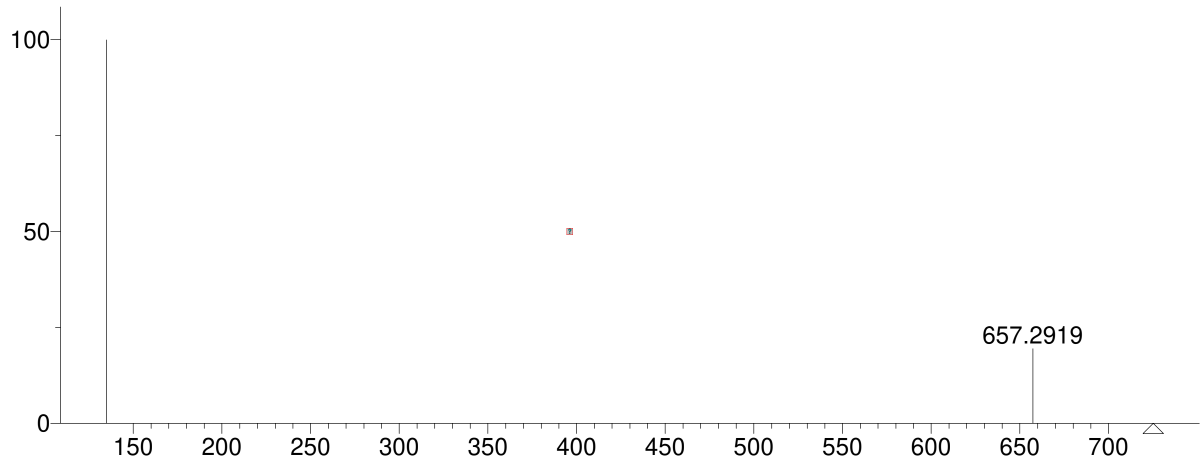

Unknown: Unknown (7.60_433.1707m/z)
Compound in Library Factor = 0

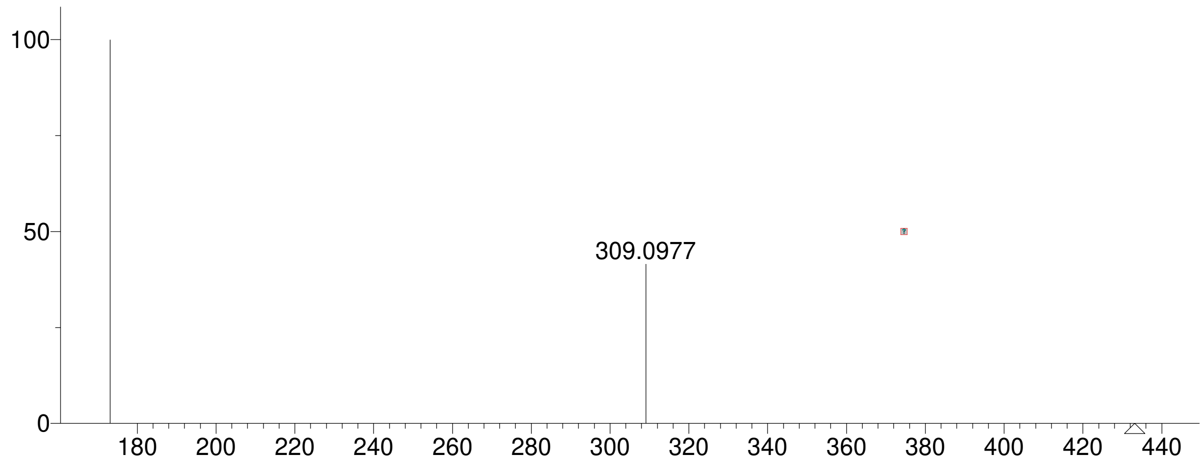

Hit 1 : osmanthuside H [M+H]+ 65HCD P=433.2
C19H28O11; MF: 0; RMF: 153; Prob 100.0%; Lib: mona-export-vf-npl_qexactive; ID: 4341.

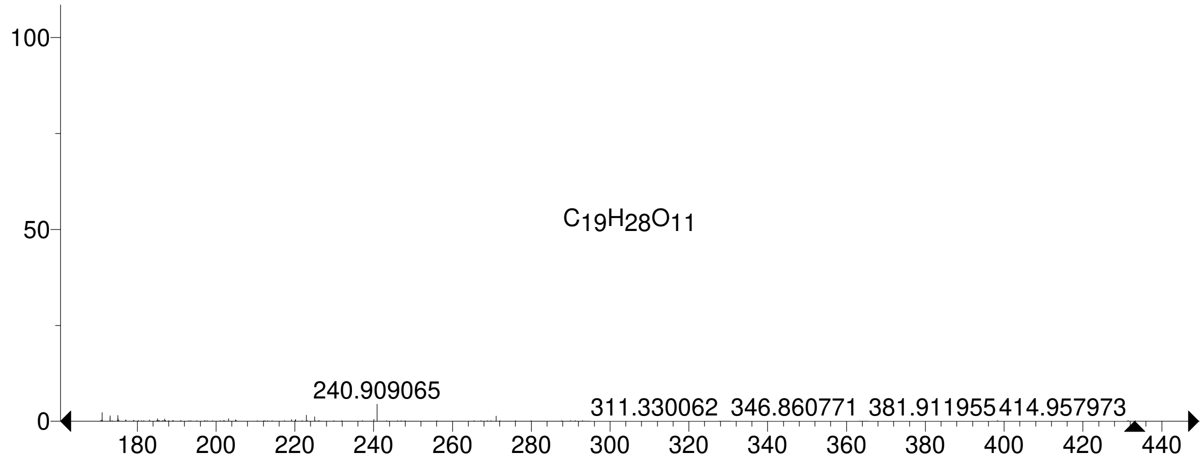

Unknown: Unknown (7.60_656.2881m/z)
Compound in Library Factor = 0

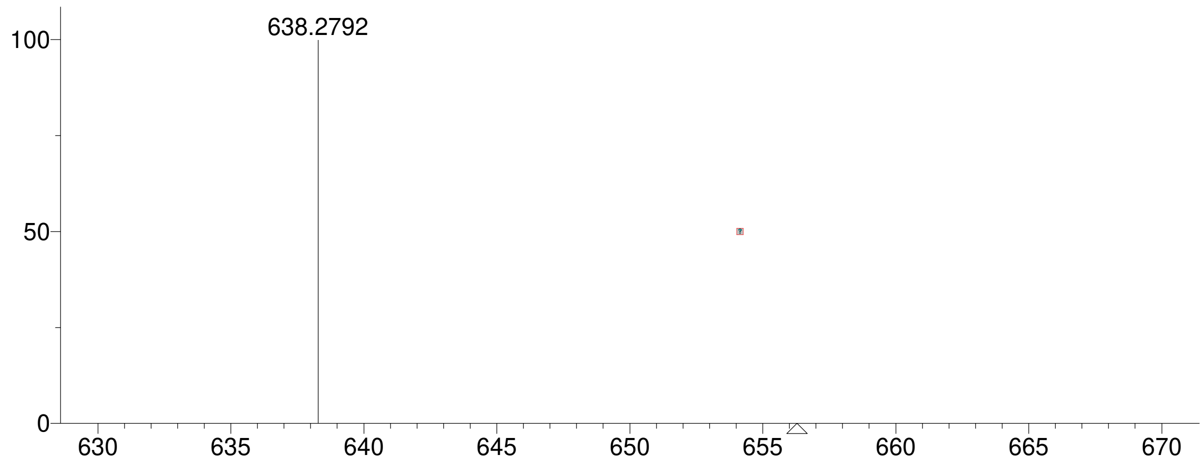

Unknown: Unknown (7.61_385.1949m/z)
Compound in Library Factor = 0

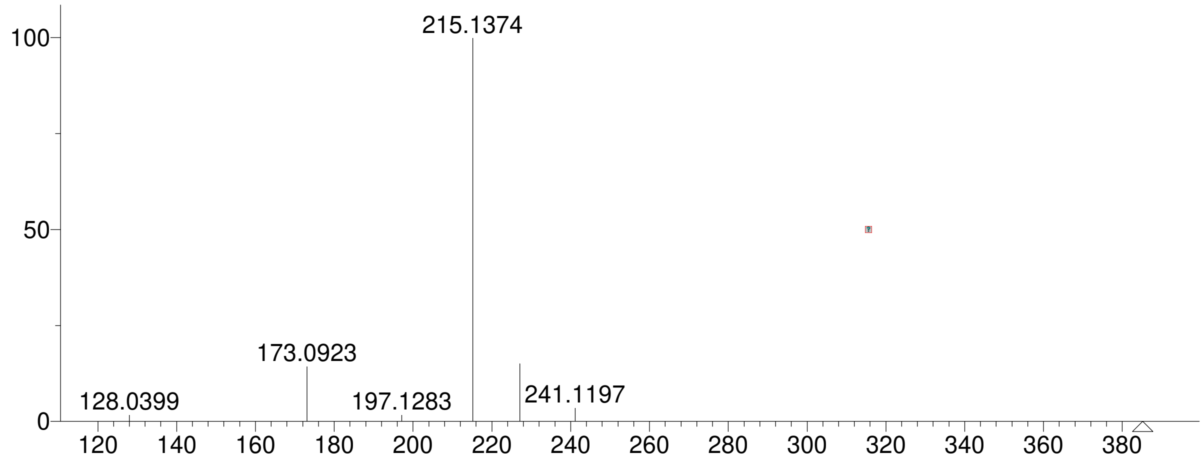

Unknown: Unknown (7.62_197.1282m/z)
Compound in Library Factor = -1072

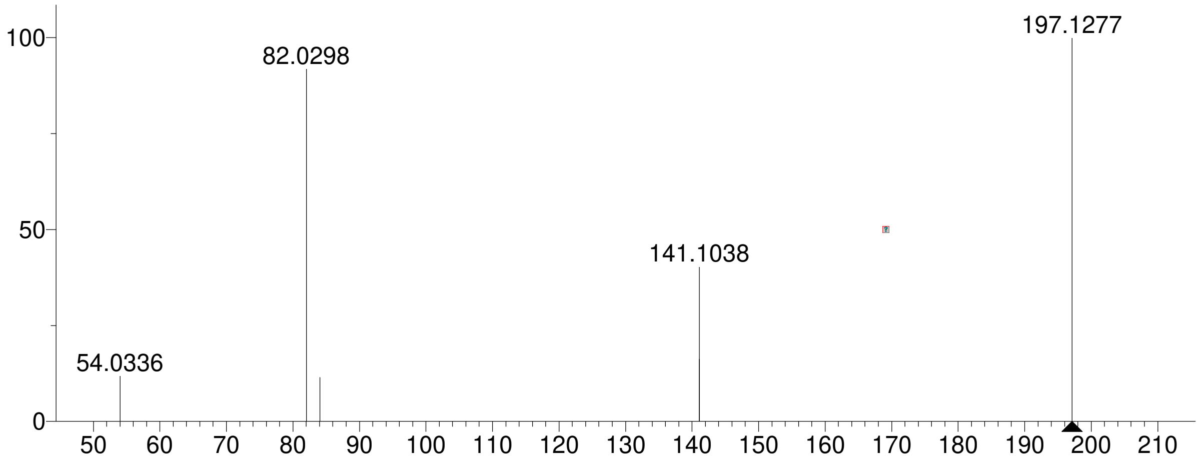

Hit 1 : L-N5-(1-Imino-3-pentenyl)ornithine [M+H-NH3]+ HCD 9V P=197.1
C10H19N3O2; MF: 88; RMF: 609; Prob 71.7%; CAS: 751479-44-4; Lib: nist_msms; ID: 165493.

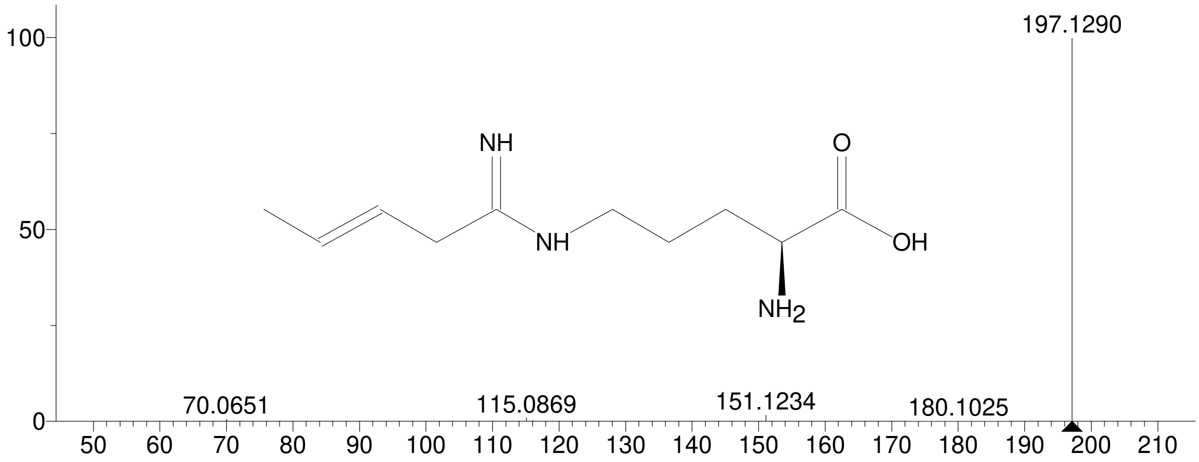

Hit 2 : D-Desthiobiotin [M+H-H2O]+ HCD 15V P=197.1
C10H18N2O3; MF: 18; RMF: 544; Prob 10.4%; CAS: 533-48-2; Lib: nist_msms; ID: 489080.

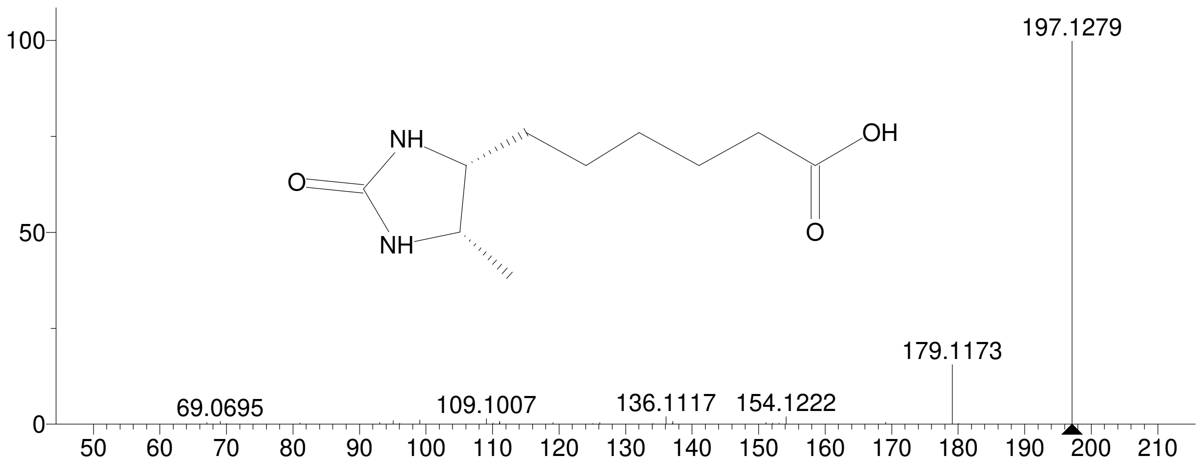

Hit 3 : NCGC00381364-01!3-propan-2-yl-2,3,6,7,8,8a-hexahydropyrrolo[1,2-a]pyrazine-1,4-dione [M+H]+ P=197
C10H16N2O2; MF: 5; RMF: 378; Prob 6.73%; Lib: mona-export-gnps; ID: 6310.

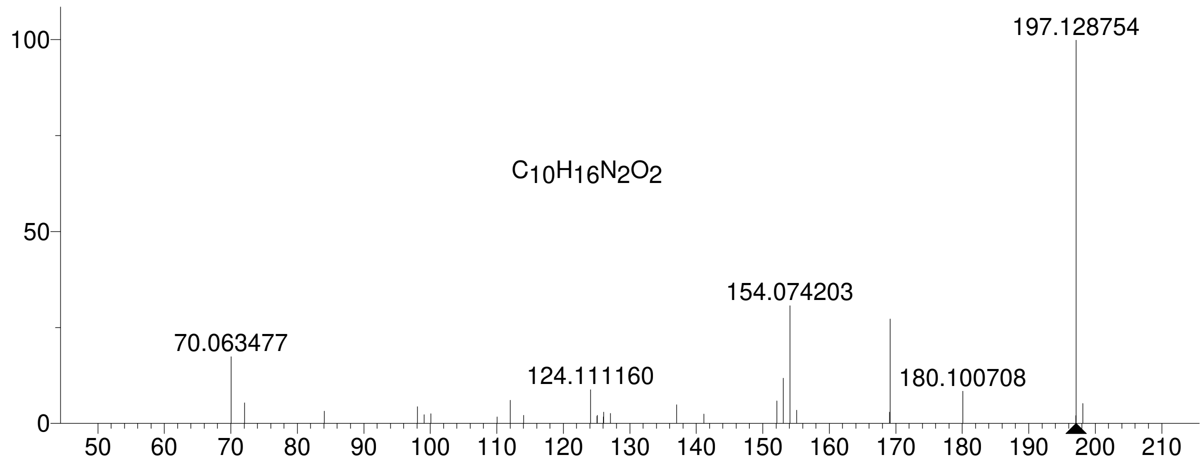

Hit 4 : Val-Phe-Lys [M+2H]2+ QTOF 5V P=197.1
C20H32N4O4; MF: 1; RMF: 232; Prob 5.69%; Lib: nist_msms; ID: 54272.

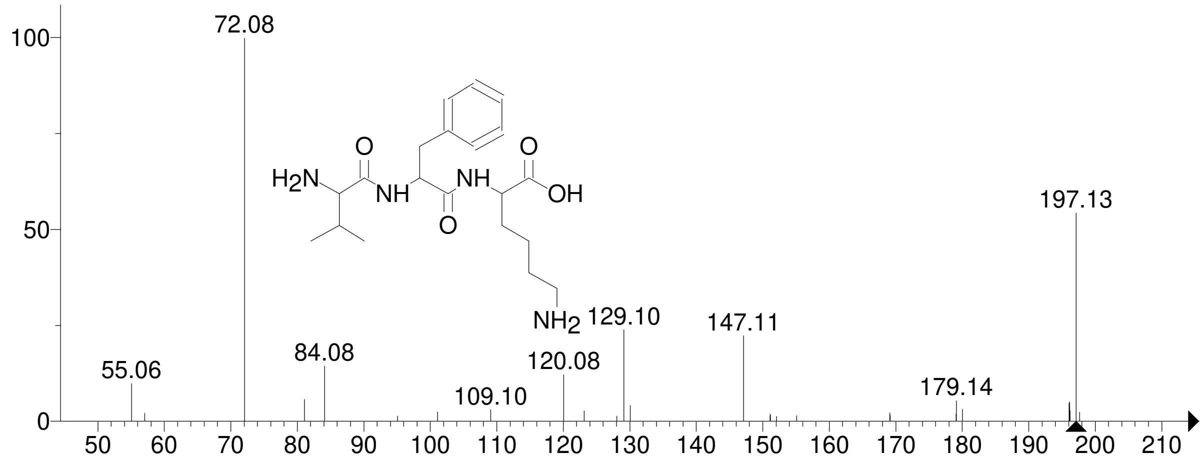

Hit 5 : NCGC00381364-02_C10H16N2O2_Pyrrolo[1,2-a]pyrazine-1,4-dione, hexahydro-3-(1-methylethyl)- [M+H]
C10H16N2O2; MF: 0; RMF: 255; Prob 5.47%; Lib: mona-export-gnps; ID: 10512.

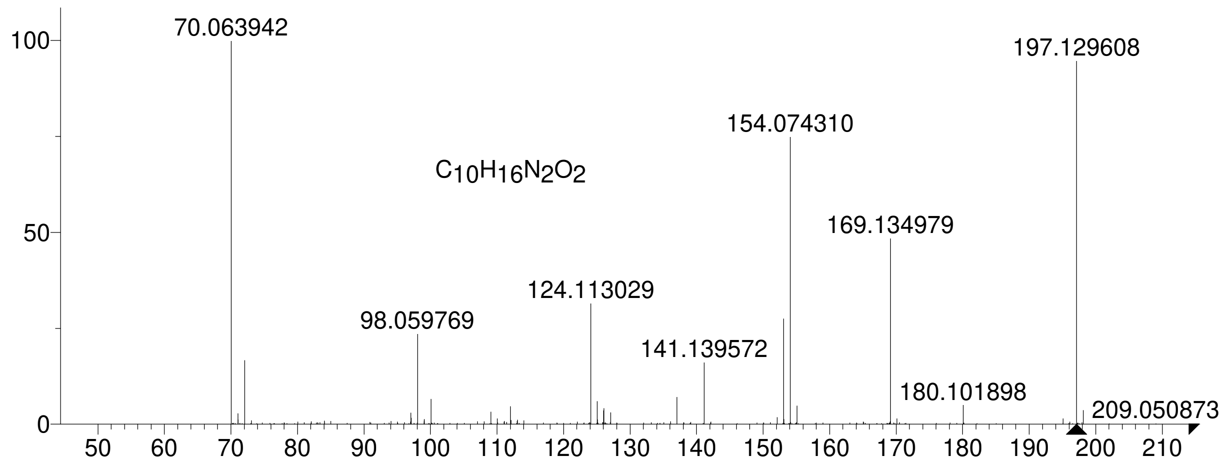

Unknown: Unknown (7.62_387.1743m/z)
Compound in Library Factor = -3273

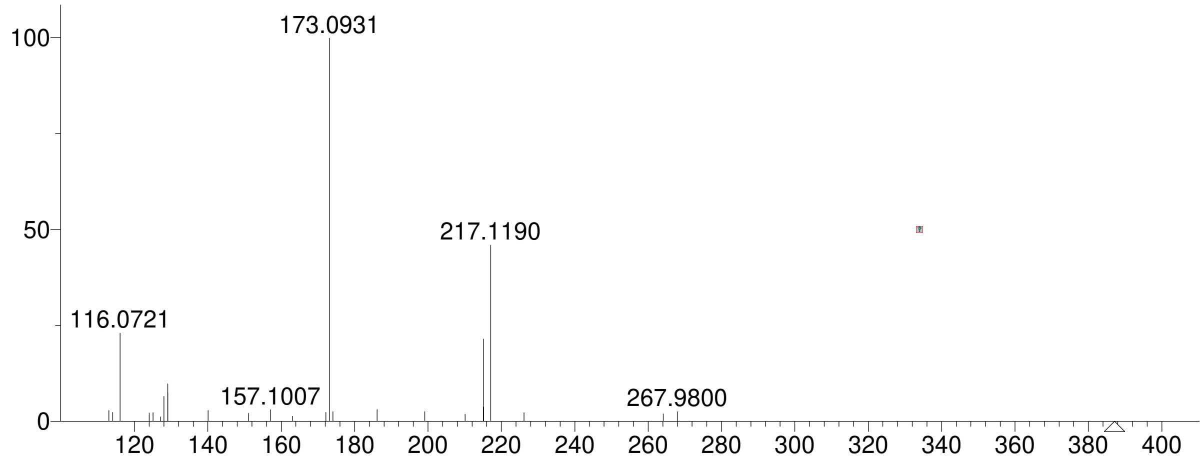

Hit 1 : Neurotensin (1-6) [M-2H]2- HCD 55eV P=387.2
C35H52N8O12; MF: 13; RMF: 117; Prob 60.8%; Lib: nist_msms2; ID: 74180.

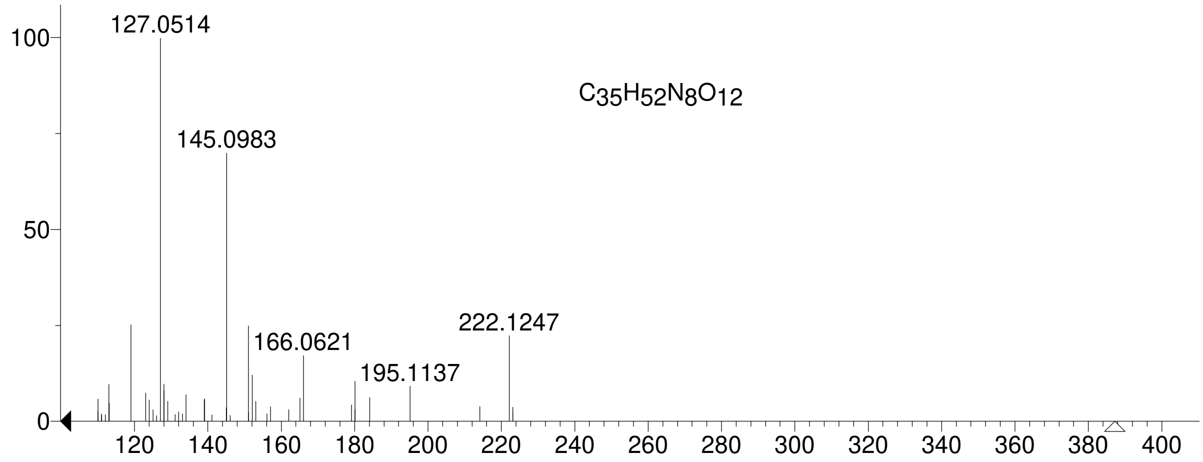

Hit 2 : NCGC00347508-02!4-[2-[[2,3-dihydroxy-2-(1-hydroxyethyl)butanoyl]oxymethyl]anilino]-4-oxobutanoic acid
C17H23NO8; MF: 0; RMF: 4; Prob 39.2%; Lib: mona-export-gnps; ID: 6723.

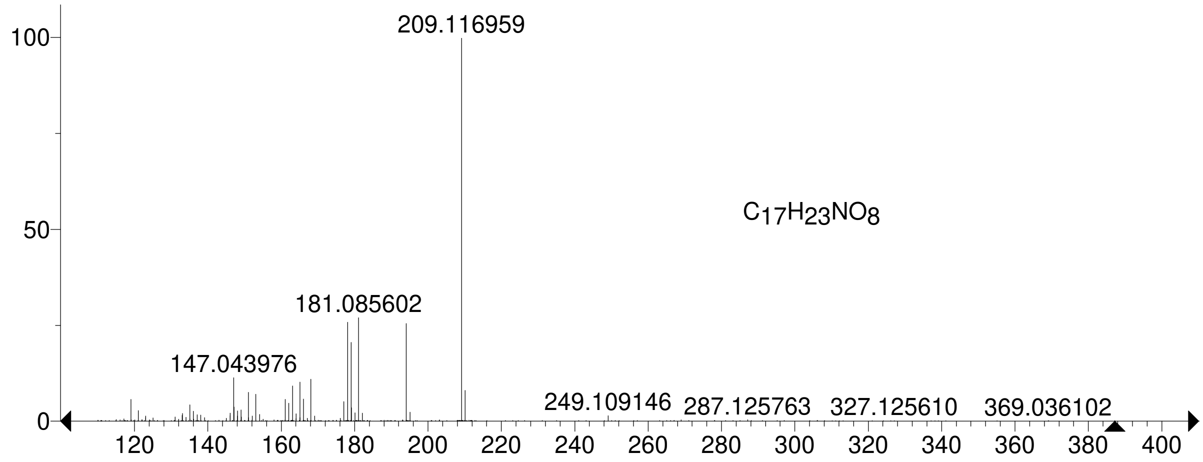

Unknown: Unknown (7.63_241.1186m/z)
Compound in Library Factor = -566

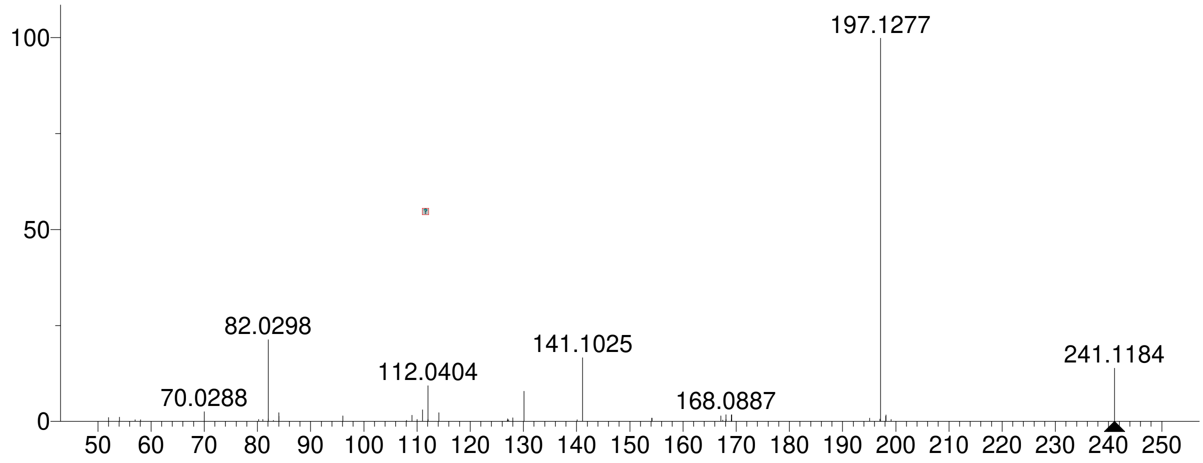

Hit 1 : Pyroglutamyl-Isoleucine; PlaSMA ID-369 [M-H]- P=241.1
C11H18N2O4; MF: 401; RMF: 590; Prob 100.0%; Lib: biomsms-neg-plasma; ID: 400.

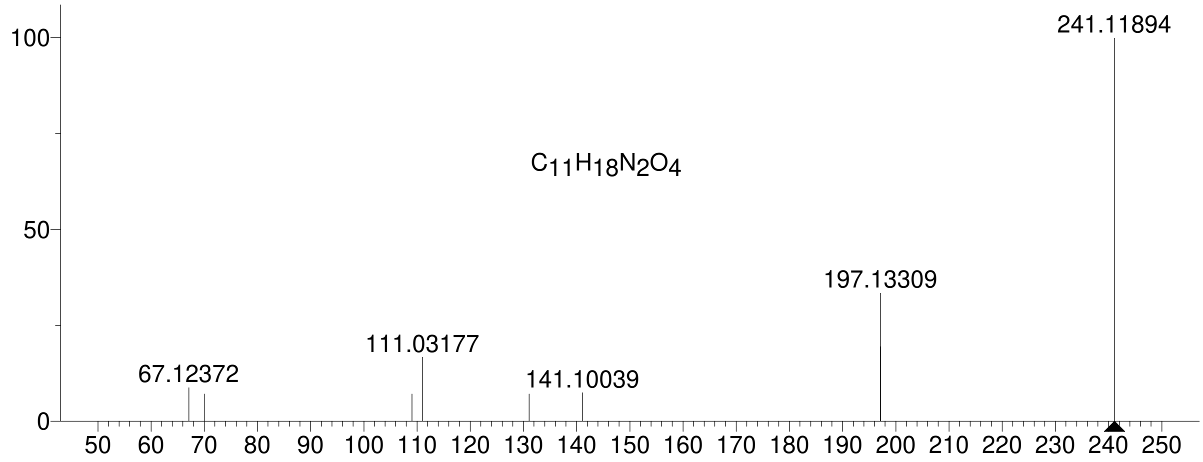

Unknown: Unknown (7.64_371.1229m/z)
Compound in Library Factor = 0

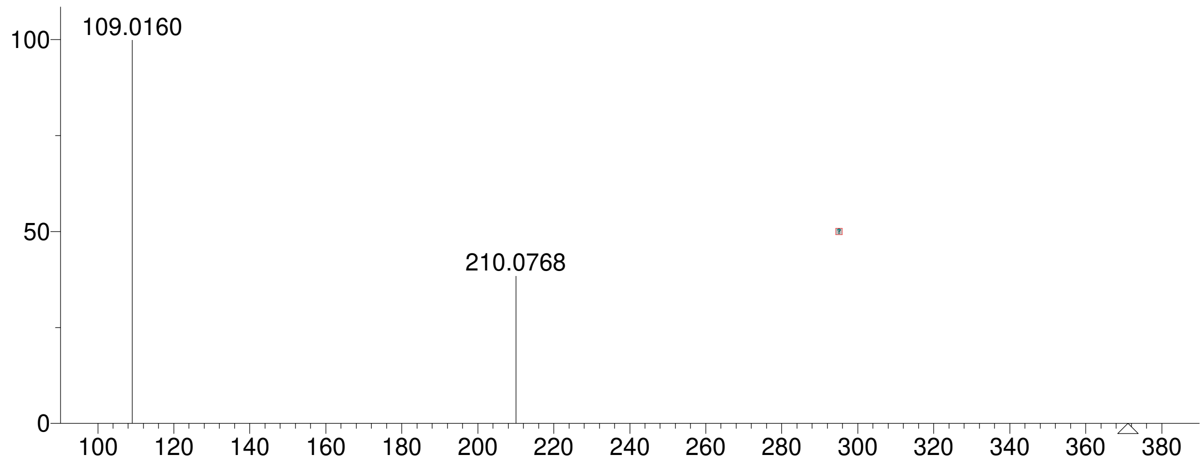

Hit 1 : 7,8-dihydroxy-3-[(1E,3E)-7-hydroxy-3,5-dimethylhepta-1,3-dienyl]-7-methyl-8H-isochromen-6-one [M+K]+
C19H24O5; MF: 0; RMF: 29; Prob 50.0%; Lib: mona-export-vf-npl_qexactive; ID: 16500.

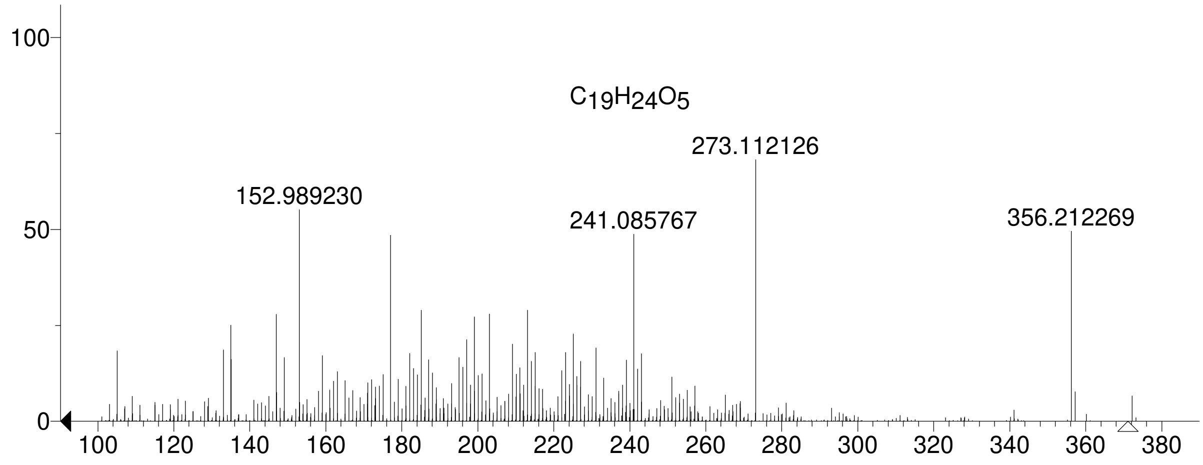

Hit 2 : 1-[2-hydroxy-4-(3-hydroxy-5-methylphenoxy)-6-methylphenyl]-3-methylbutane-2,3-diol [M+K]+ 65HCD P=
C19H24O5; MF: 0; RMF: 8; Prob 50.0%; Lib: mona-export-vf-npl_qexactive; ID: 22293.

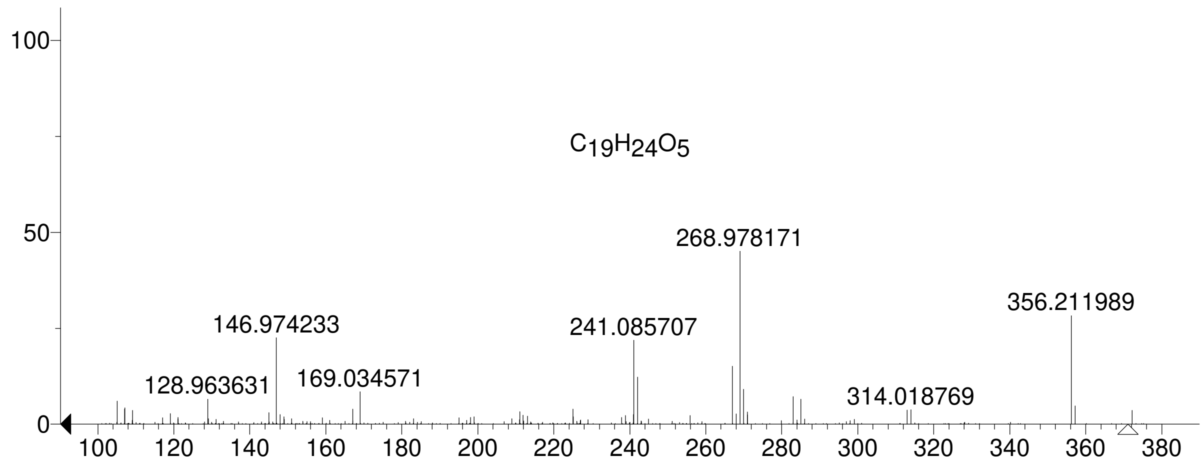

** Search Report Page 1 of 1 **

Unknown: Unknown (7.64_726.3414m/z)
Compound in Library Factor = 0

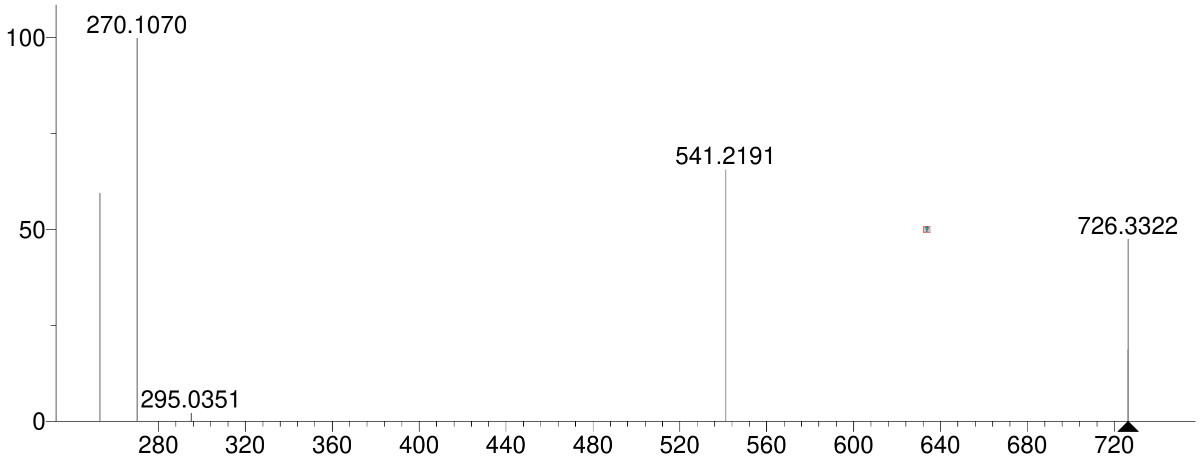

Unknown: Unknown (7.65_302.1490m/z)
Compound in Library Factor = 0

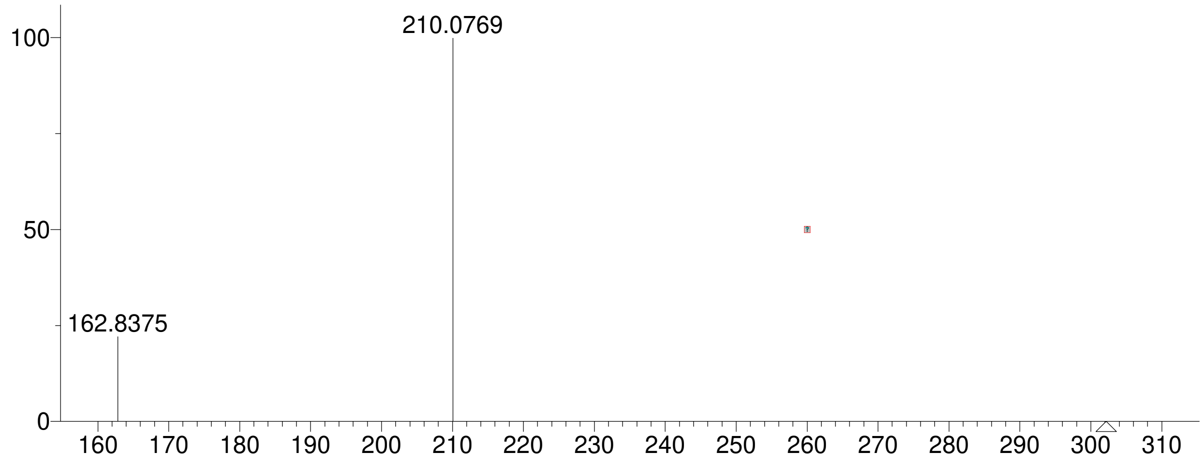

** Search Report Page 1 of 1 **

Unknown: Unknown (7.66_338.1323m/z)
Compound in Library Factor = 0

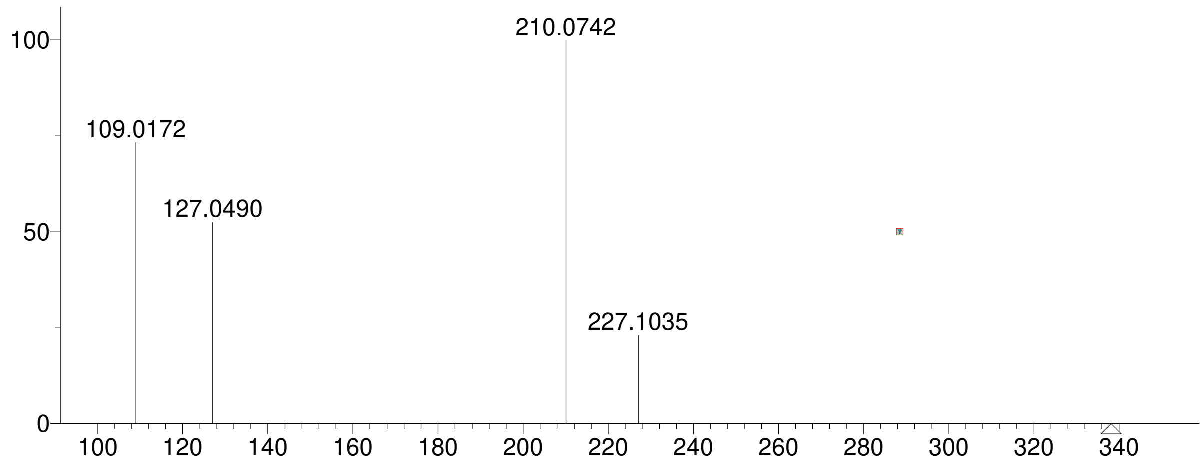

Hit 1 : Ethanone, 2-(2-chlorophenyl)-1-[1-(4-hydroxypentyl)-1H-indol-3-yl]- [M+H-H2O]+ HCD 108V P=338.1
C21H22ClNO2; MF: 0; RMF: 48; Prob 50.0%; Lib: nist_msms; ID: 347725.

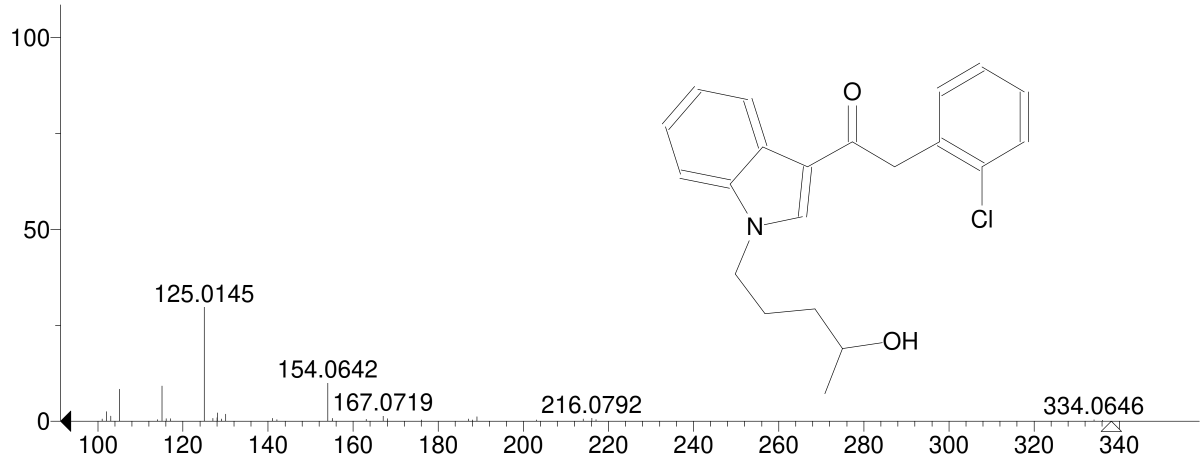

Hit 2 : Ethanone, 2-(2-chlorophenyl)-1-[1-(5-hydroxypentyl)-1H-indol-3-yl]- [M+H-H2O]+ HCD 108V P=338.1
C21H22ClNO2; MF: 0; RMF: 43; Prob 50.0%; CAS: 1449690-09-8; Lib: nist_msms; ID: 269519.

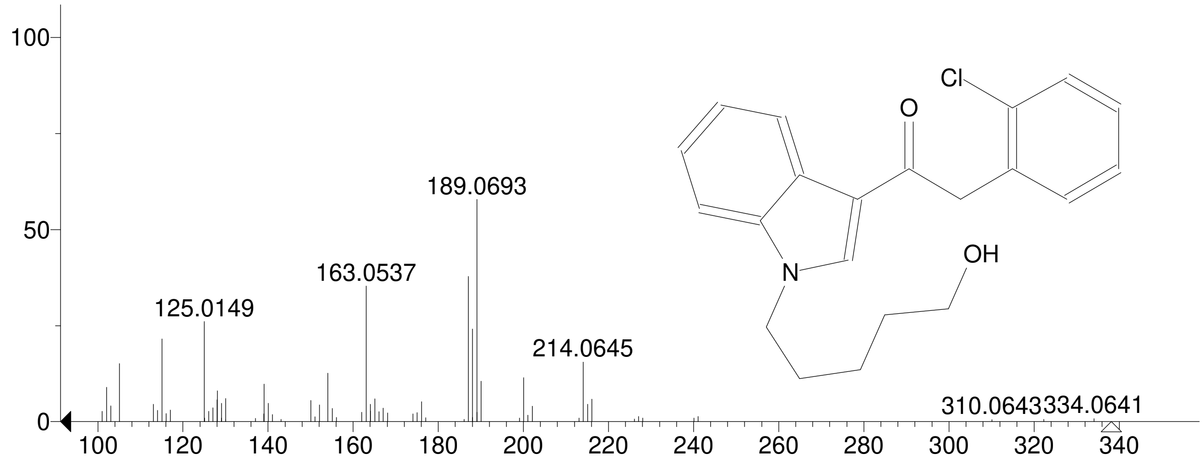

Unknown: Unknown (7.66_356.1453m/z)
Compound in Library Factor = 0

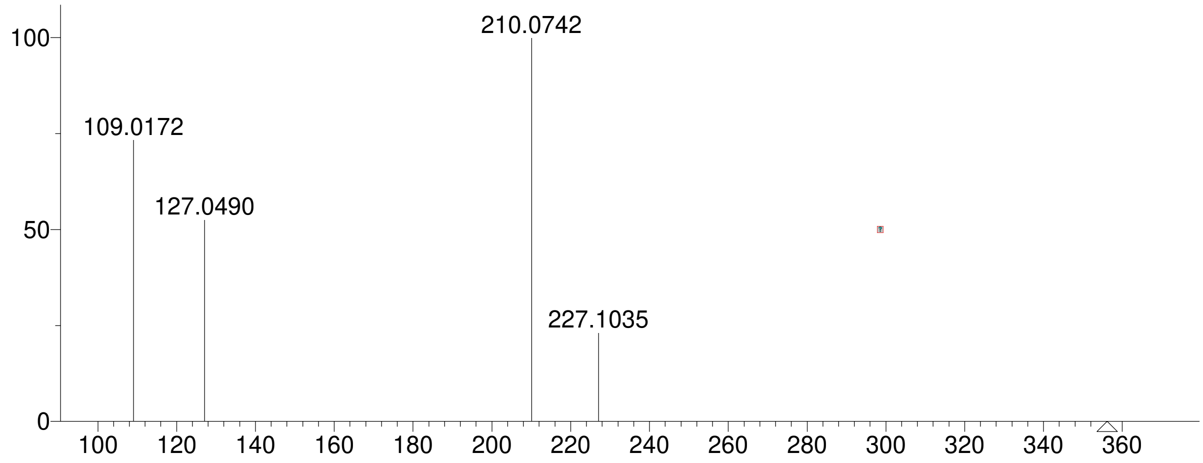

Hit 1 : Benzoic acid, 4-[[(2,4-diamino-7,8-dihydro-7-oxo-6-pteridinyl)methyl]methylamino]-, methyl ester [M+H]+
C16H17N7O3; MF: 0; RMF: 61; Prob 100.0%; CAS: 1330264-75-9; Lib: nist_msms; ID: 432548.

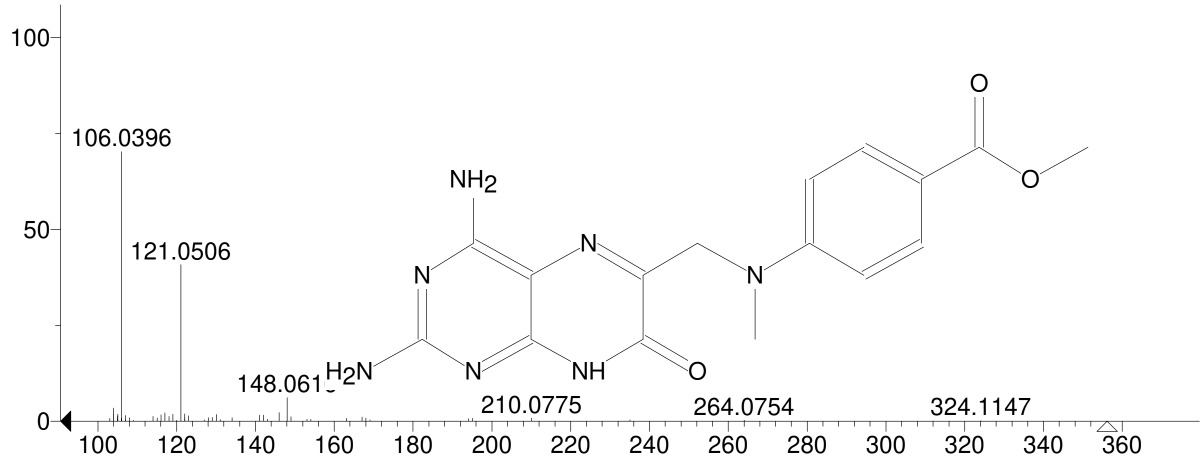

Unknown: Unknown (7.67_553.2609m/z)
Compound in Library Factor = -791

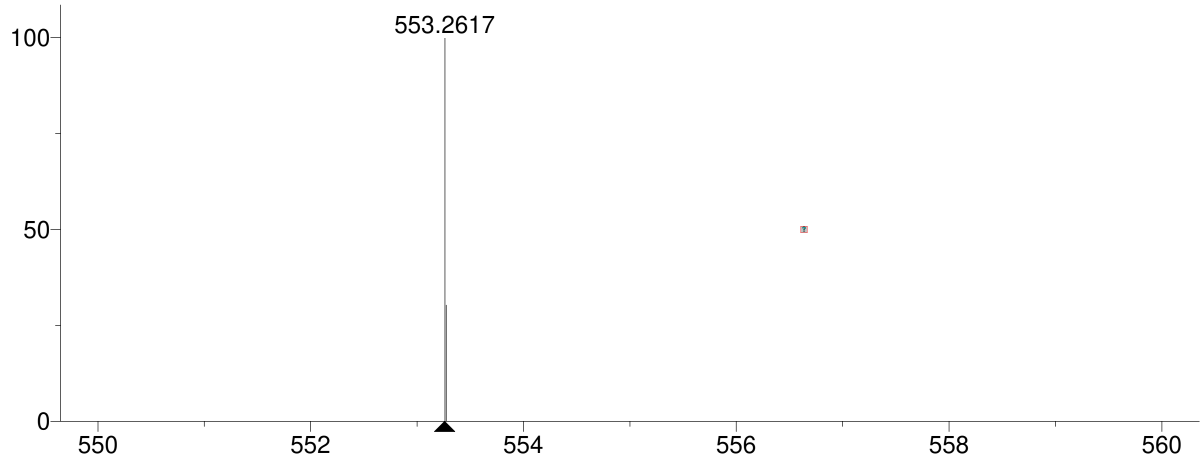

Hit 1 : (2S,3R,5S,9R)-2,3-dihydroxy-14-(hydroxymethyl)-5,9-dimethyl-14-{[(2S,3R,4S,5S,6R)-3,4,5-trihydroxy-6-(h
C26H42O11; MF: 142; RMF: 769; Prob 45.2%; Lib: anthocyanins mona; ID: 5886.

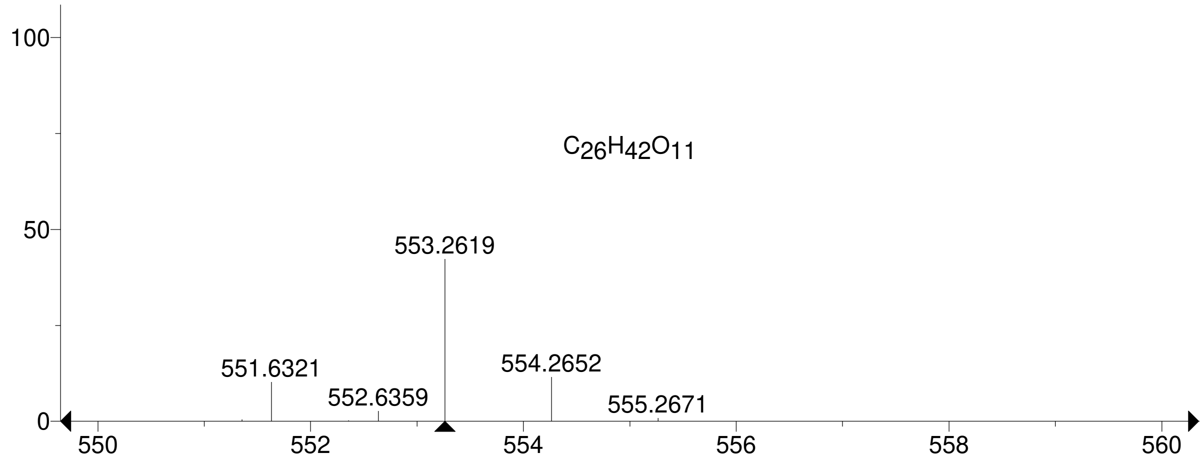

Hit 2 : (2S,3R,5S,9R)-2,3-dihydroxy-14-(hydroxymethyl)-5,9-dimethyl-14-{[(2S,3R,4S,5S,6R)-3,4,5-trihydroxy-6-(h
C26H42O11; MF: 142; RMF: 769; Prob 45.2%; Lib: mona-export-vf-npl_qexactive; ID: 17888.

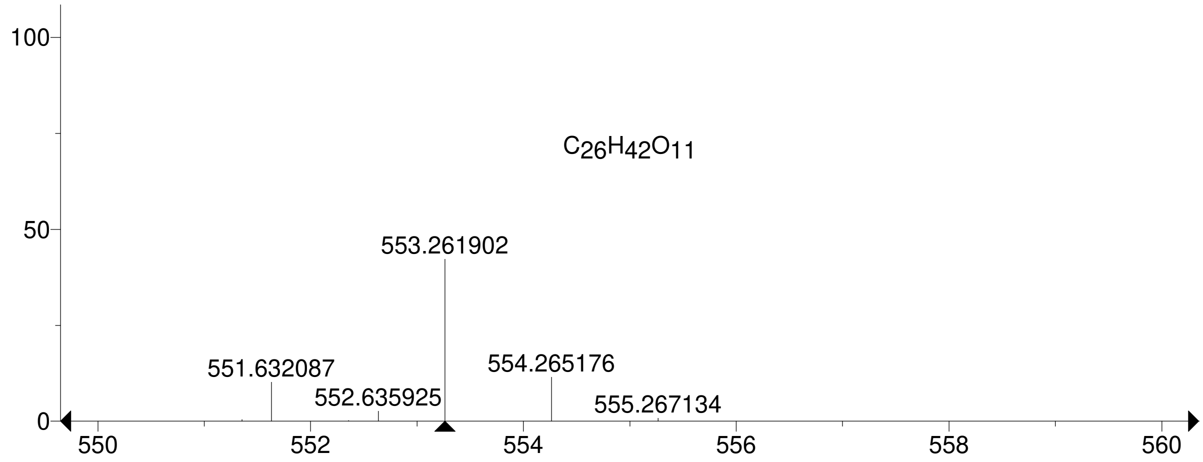

Hit 3 : NCGC00380707-01_C26H42O11_(5xi,6alpha,7alpha,9xi,16xi)-16-(beta-D-Glucopyranosyloxy)-6,7,17-trihy
C26H42O11; MF: 96; RMF: 786; Prob 9.54%; Lib: mona-export-gnps; ID: 13741.

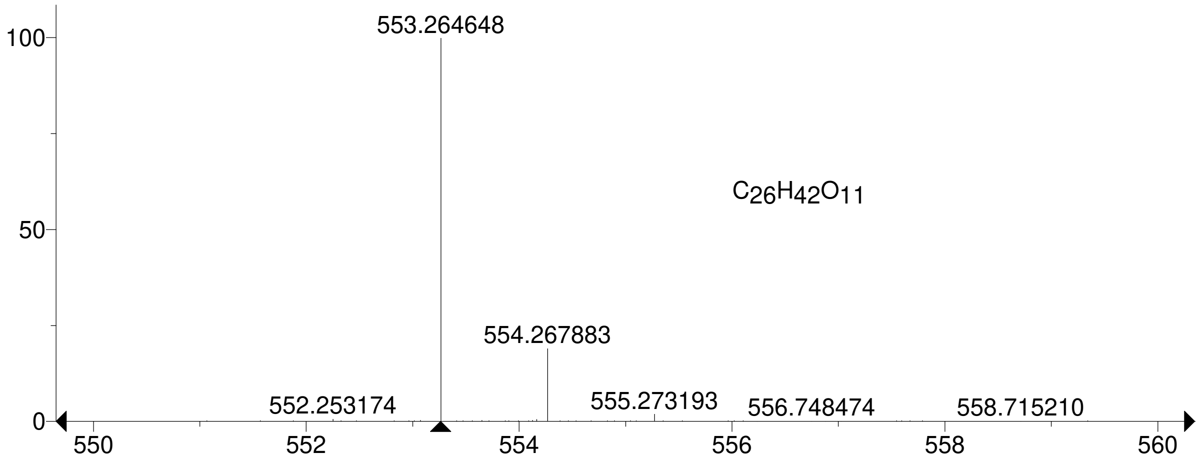

Unknown: Unknown (7.68_401.1531m/z)
Compound in Library Factor = 0

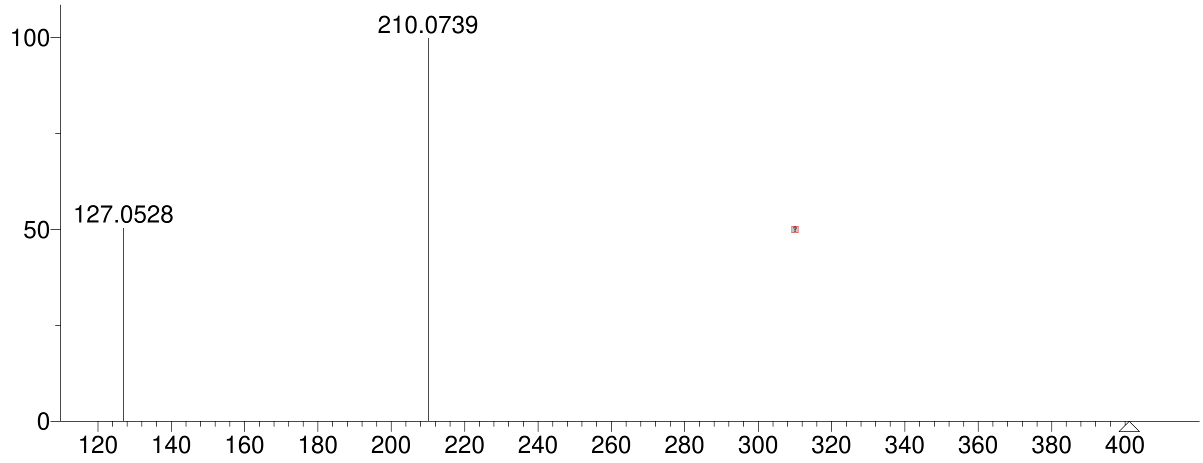

Hit 1 : Cnicin [M+Na]+ HCD 104V P=401.2
C20H26O7; MF: 0; RMF: 109; Prob 50.0%; CAS: 24394-09-0; Lib: nist_msms; ID: 236009.

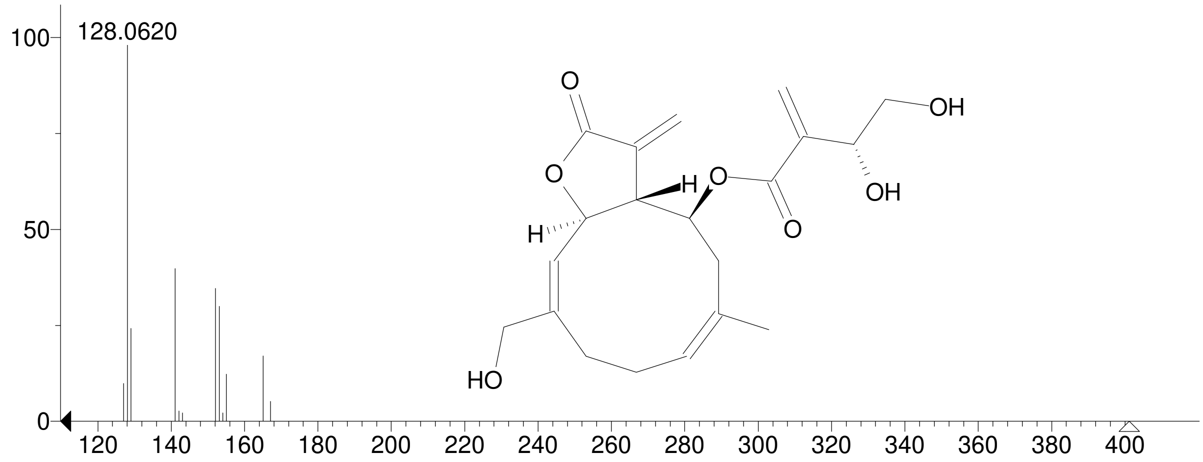

Hit 2 : (2aR,4S,6R,7R,9R,9aR)-5'-(furan-3-yl)-4,9-dihydroxy-2a-(hydroxymethyl)-7-methylspiro[1,3,4,5,5a,7,8,9-oc
C20H26O7; MF: 0; RMF: 7; Prob 50.0%; Lib: mona-export-vf-npl_qexactive; ID: 23517.

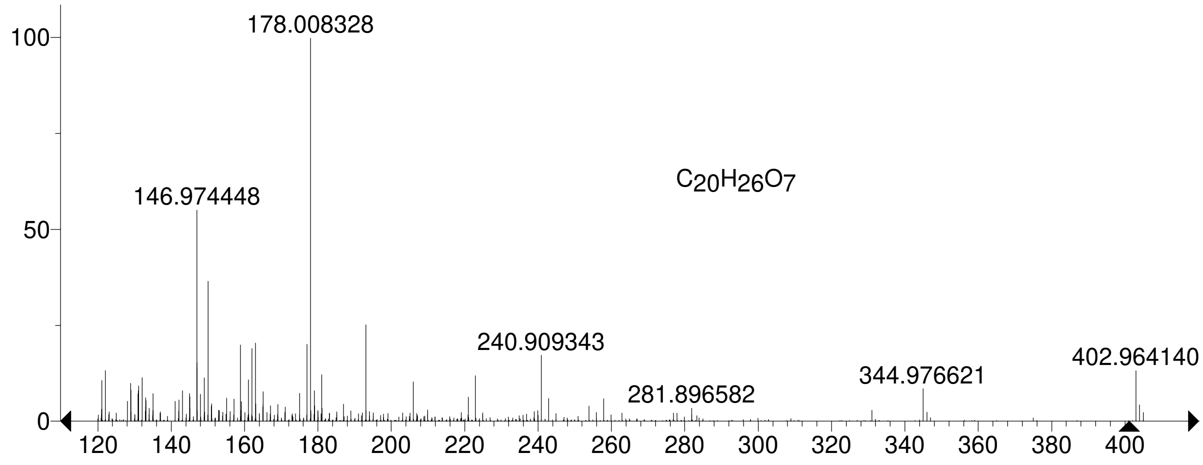

Unknown: Unknown (7.69_344.1809m/z)
Compound in Library Factor = 0

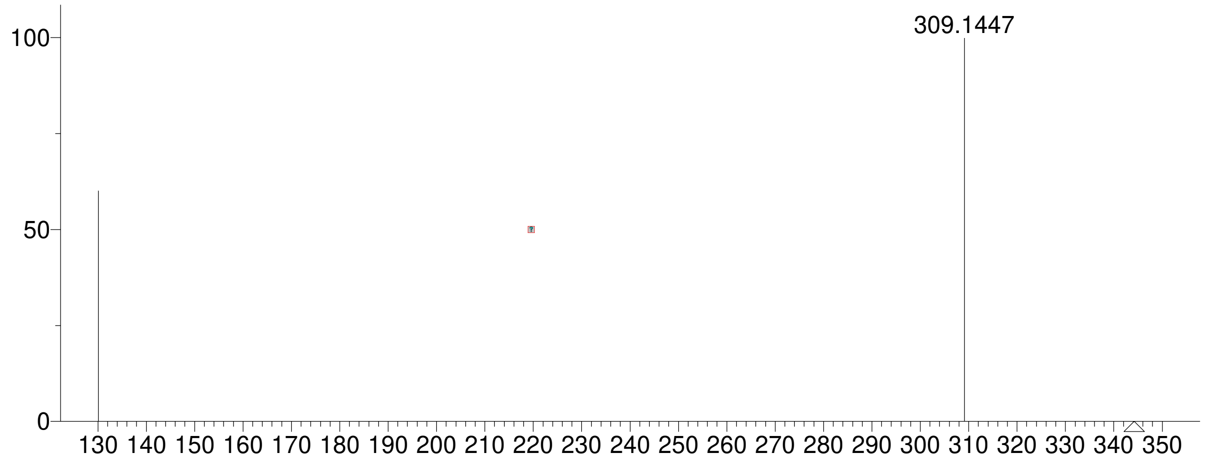

Hit 1 : 4-amino-2-hydroxy-5-[[1-hydroxy-1-(5-oxo-6-bicyclo[4.1.0]hept-3-enyl)propan-2-yl]amino]-5-oxopentanoic a
C15H22N2O6; MF: 0; RMF: 107; Prob 100.0%; Lib: mona-export-vf-npl_qexactive; ID: 16561.

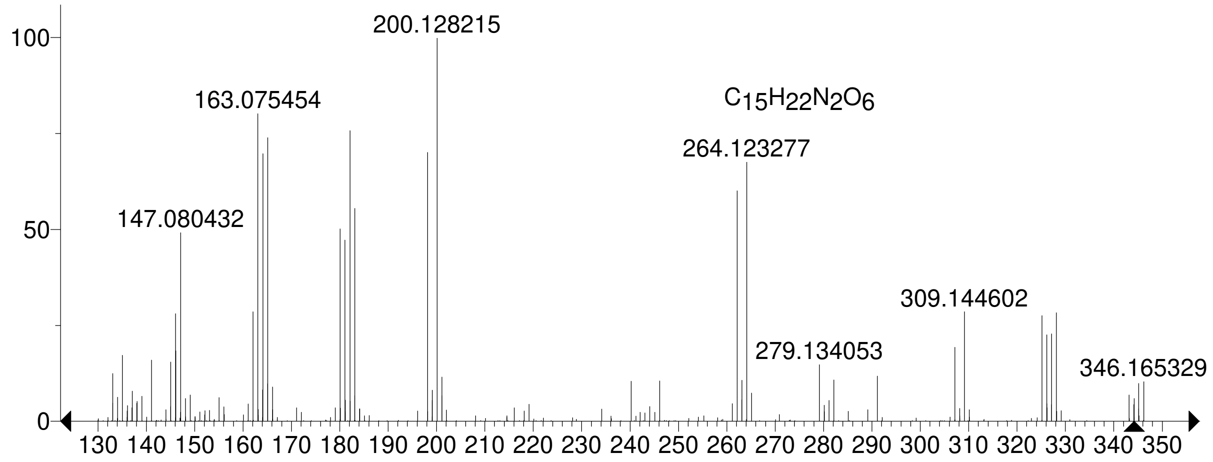

Unknown: Unknown (7.72_281.0868m/z)
Compound in Library Factor = -441

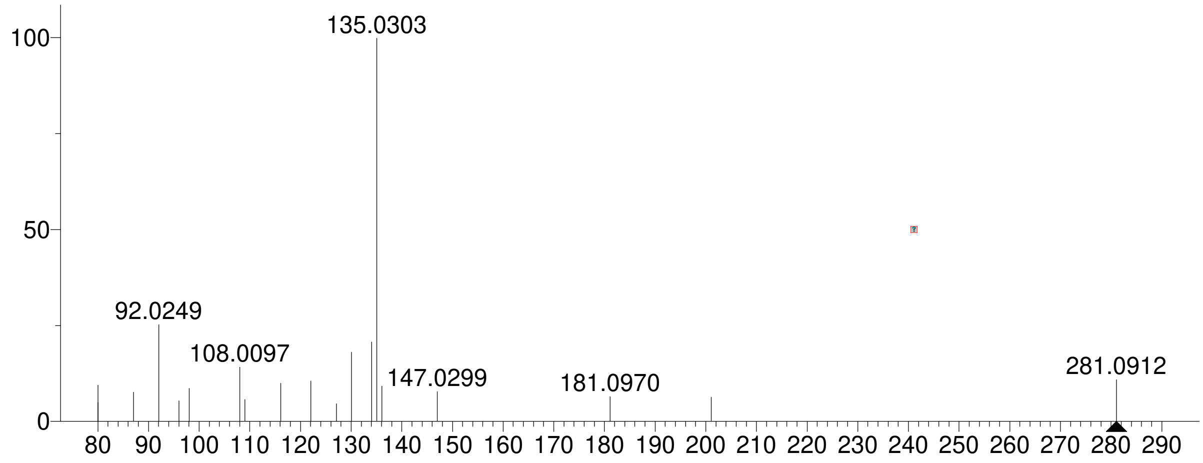

Hit 1 : 1H- Pyrazole-4-carboxylic acid, 1-(2,4-difluorophenyl)-5-[[2-[[(1,1-dimethylethyl)amino] thioxomethyl]hydraz
C17H19F2N5O2S; MF: 210; RMF: 870; Prob 38.5%; CAS: 264233-05-8; Lib: nist_msms; ID: 273544.

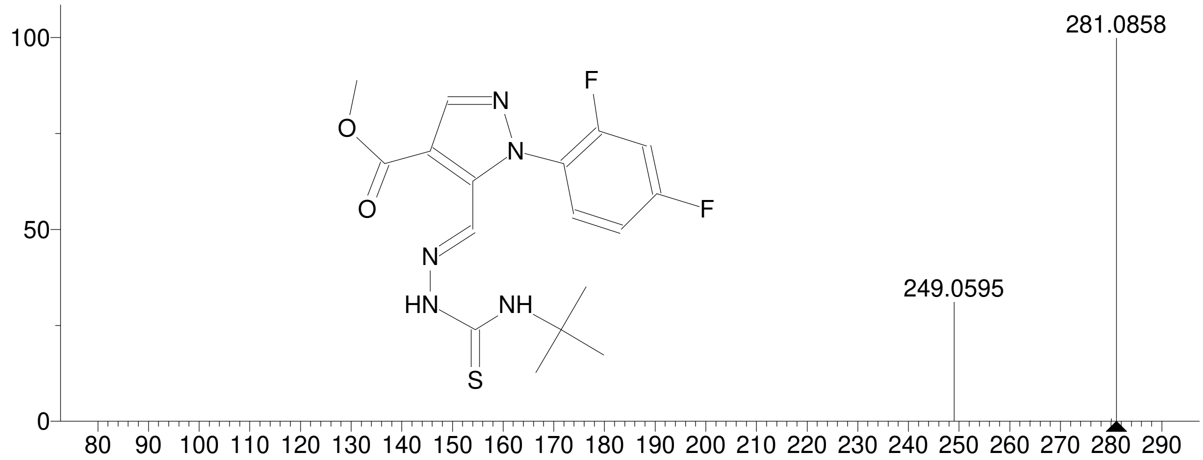

Hit 2 : 2'-O-Methylinosine [M-H]- 20-30-40%) P=281.1
C11H14N4O5; MF: 210; RMF: 575; Prob 38.5%; Lib: msms-neg-fiehnhilic; ID: 31.

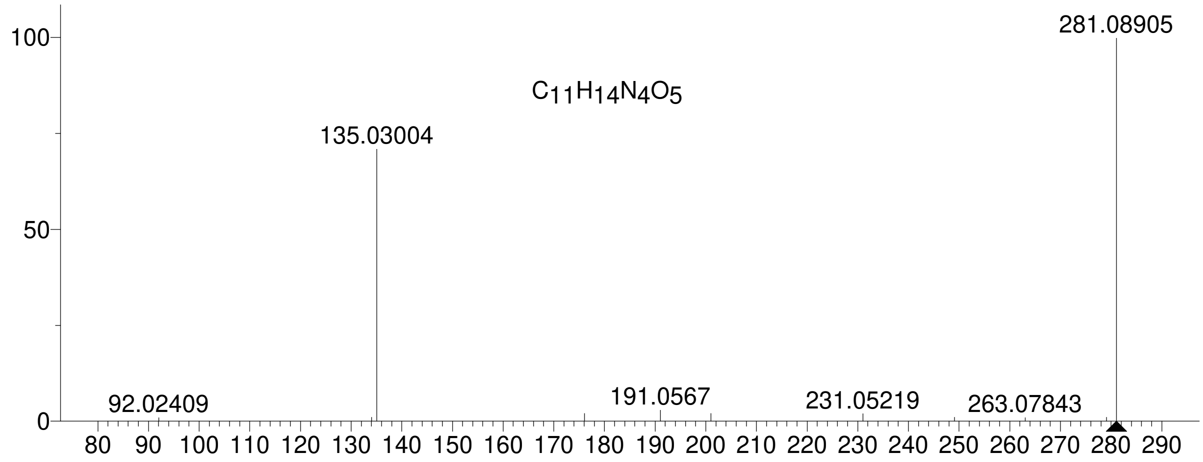

Hit 3 : 3'-O-Methylinosine [M-H]- 20-30-40%) P=281.1
C11H14N4O5; MF: 182; RMF: 614; Prob 11.1%; Lib: mona-export-fiehn_hilic; ID: 938.

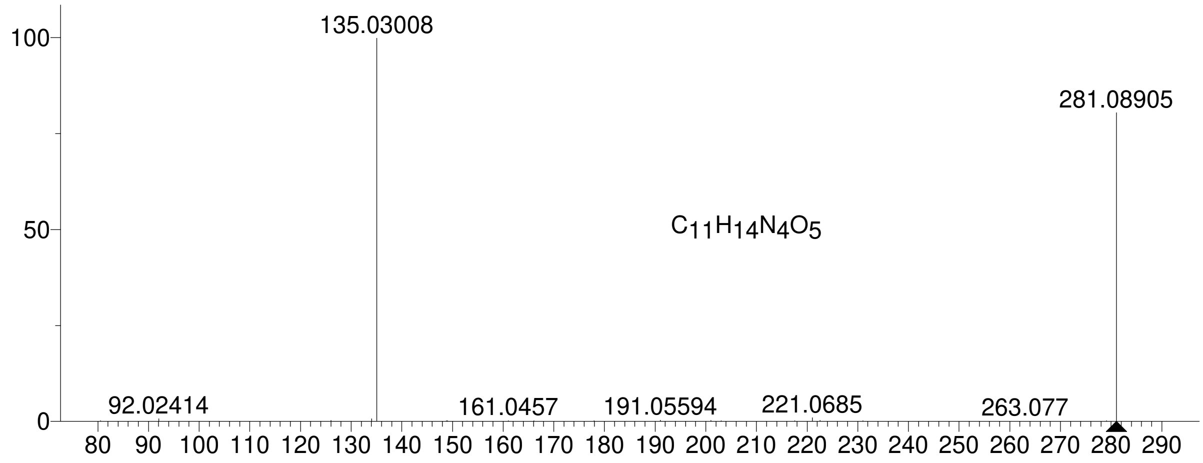

Hit 4 : 1,4-D-Xylobiose [M-H]- HCD 4V P=281.1
C10H18O9; MF: 169; RMF: 674; Prob 7.18%; CAS: 6860-47-5; Lib: nist_msms; ID: 530861.

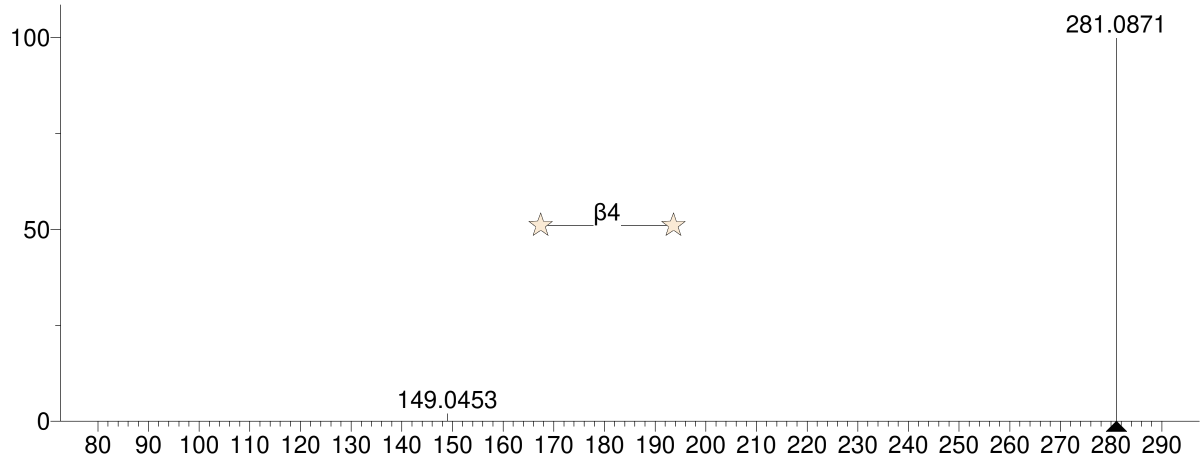

Hit 5 : NCGC00380761-01_C11H14N4O5_6H-Purin-6-one, 1,9-dihydro-9-(3-O-methylpentofuranosyl)- [M-H]- P=
C11H14N4O5; MF: 155; RMF: 575; Prob 4.49%; Lib: mona-export-gnps; ID: 13281.

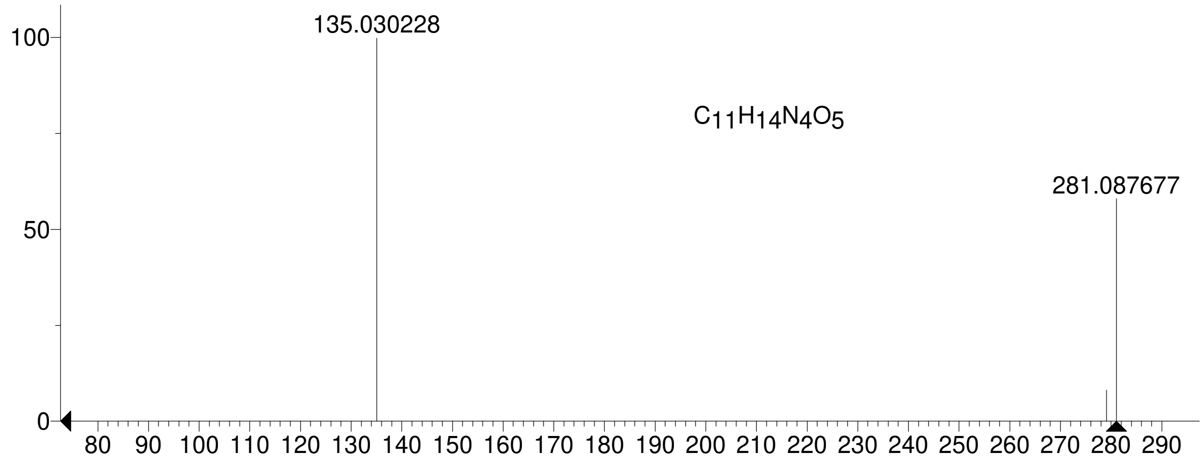

** Search Report Page 1 of 1 **

Unknown: Unknown (7.73_400.1704m/z)
Compound in Library Factor = 0

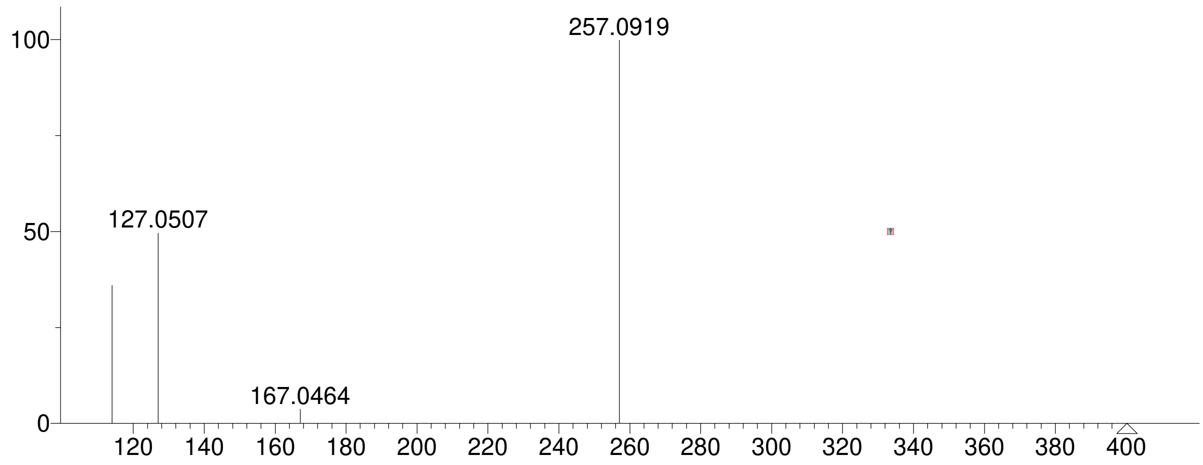

Hit 1 : 7-Hydroxyquetiapine [M+H]+ HCD 128V P=400.2
C21H25N3O3S; MF: 0; RMF: 106; Prob 50.0%; CAS: 139079-39-3; Lib: nist_msms; ID: 324168.

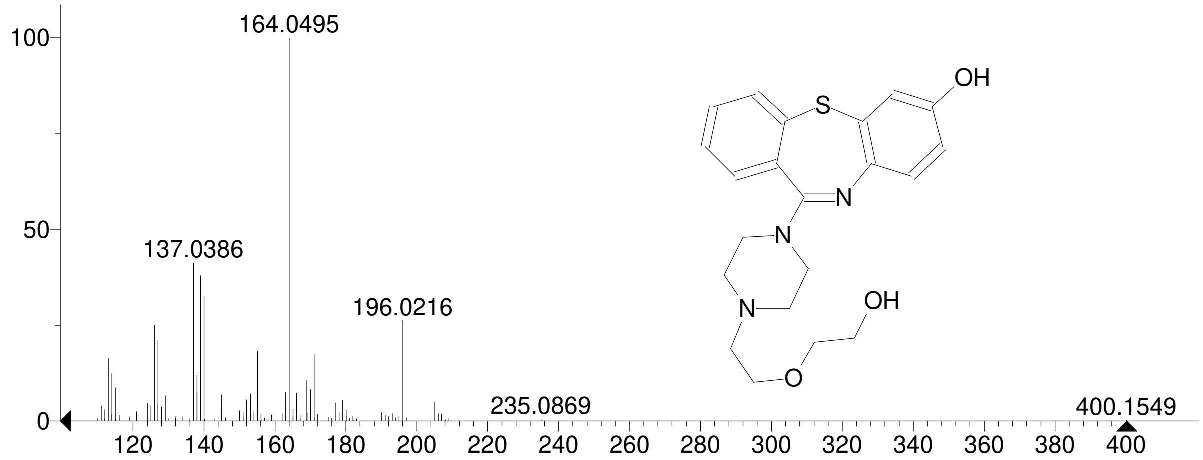

Hit 2 : Quetiapine sulfoxide [M+H]+ HCD 128V P=400.2
C21H25N3O3S; MF: 0; RMF: 61; Prob 50.0%; CAS: 329216-63-9; Lib: nist_msms; ID: 452545.

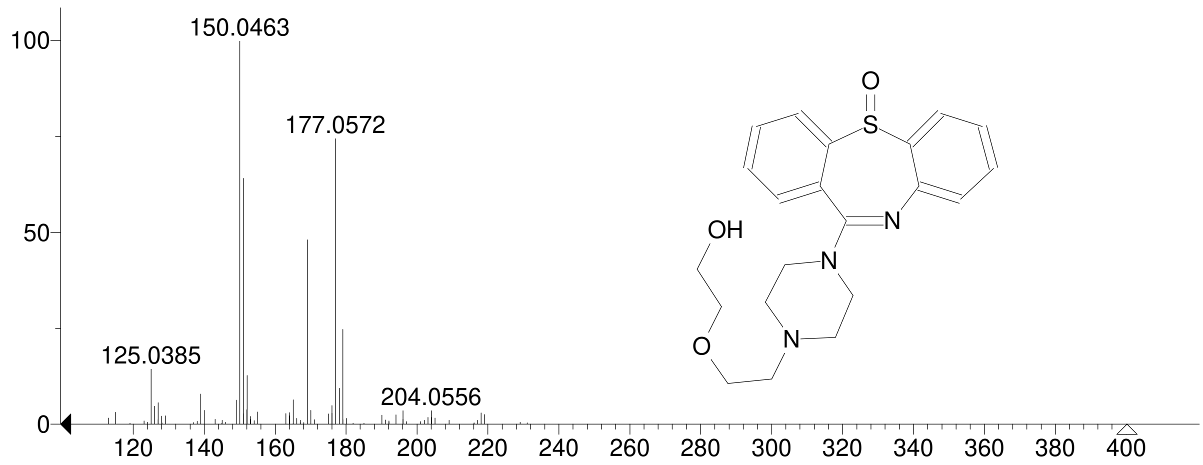

Unknown: Unknown (7.73_419.0634m/z)
Compound in Library Factor = 0

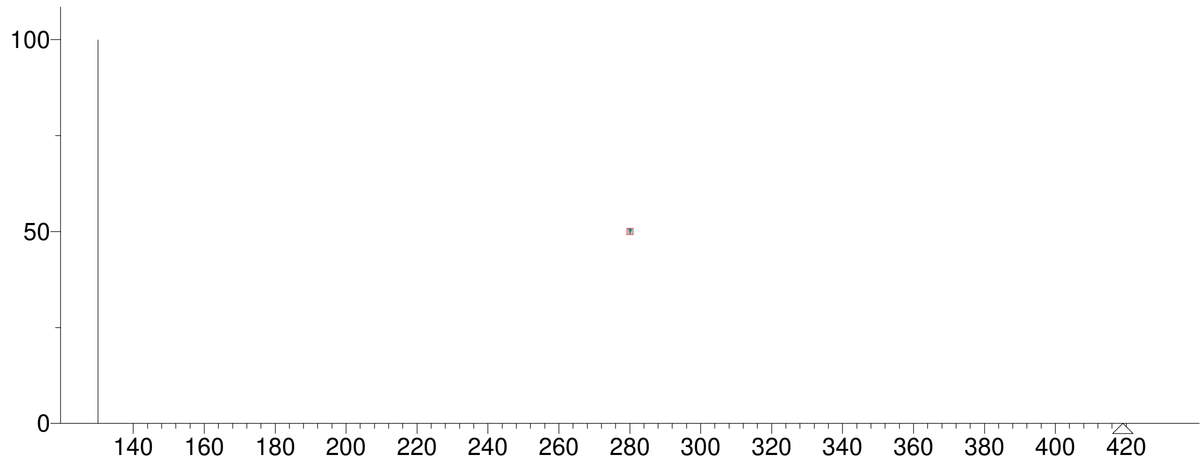

** Search Report Page 1 of 1 **

Unknown: Unknown (7.75_327.1327m/z)
Compound in Library Factor = 0

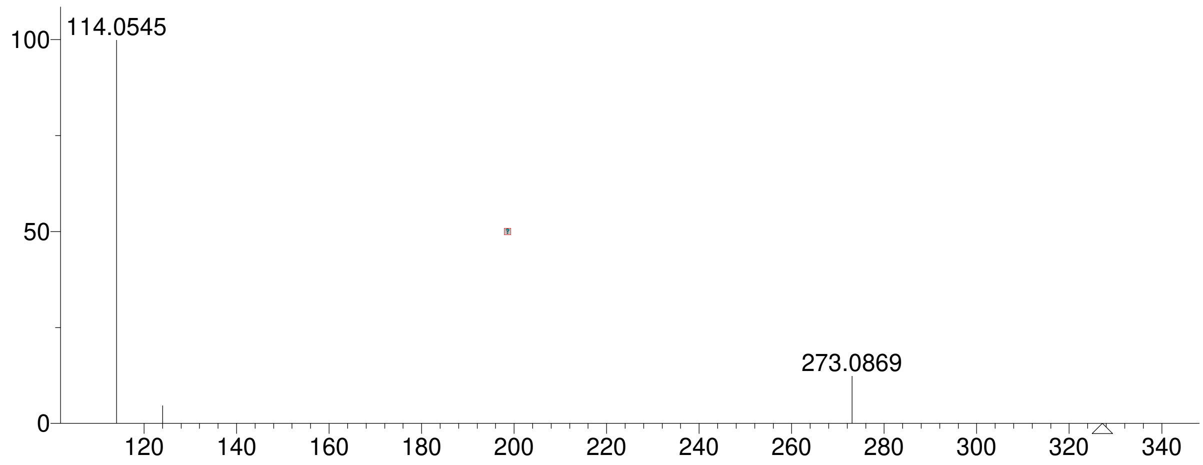

Unknown: Unknown (7.75_403.1614m/z)
Compound in Library Factor = -2387

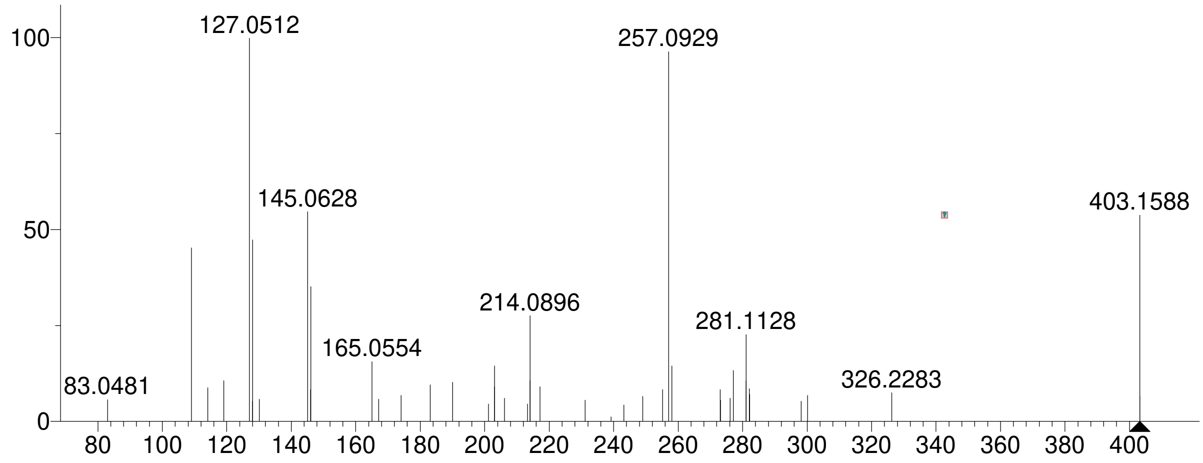

Hit 1 : C18H28O10; PlaSMA ID-1025 [M-H]- P=403.2
C18H28O10; MF: 15; RMF: 247; Prob 44.1%; Lib: biomsms-neg-plasma; ID: 839.

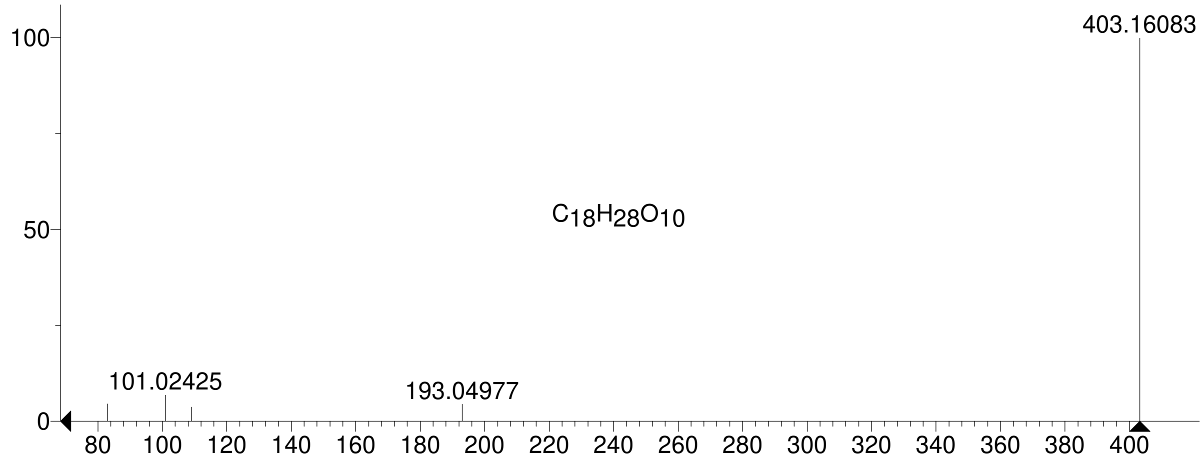

Hit 2 : (Z)-3-(4-hydroxyphenyl)-N-[4-[[(E)-3-(4-hydroxyphenyl)prop-2-enoyl]amino]butyl]prop-2-enamide [M+Na]+
C22H24N2O4; MF: 2; RMF: 225; Prob 28.5%; Lib: mona-export-vf-npl_qexactive; ID: 17140.

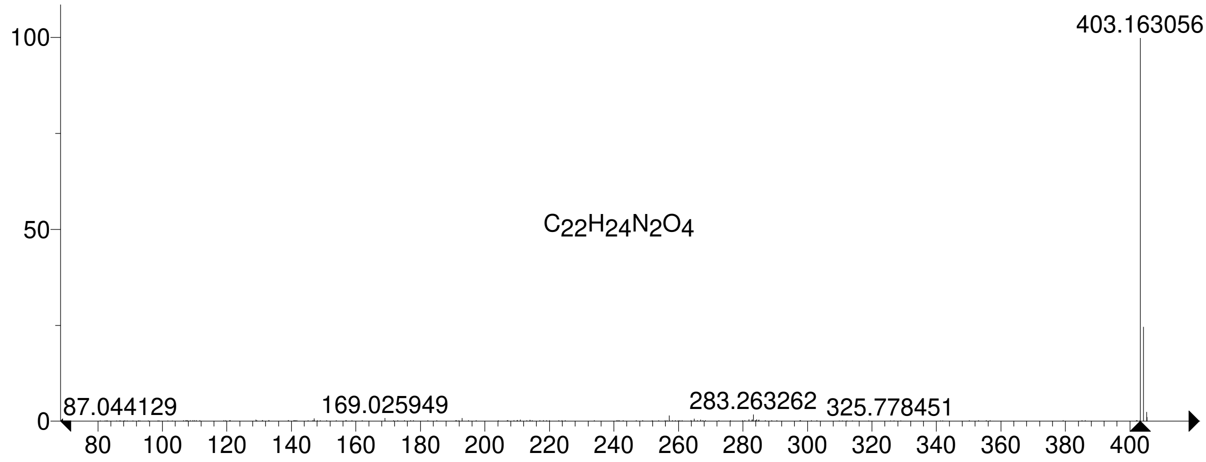

Hit 3 : 12:4+4O fatty acyl hexoside; PlaSMA ID-1026 [M-H]- P=403.2
C18H28O10; MF: 1; RMF: 204; Prob 27.4%; Lib: biomsms-neg-plasma; ID: 674.

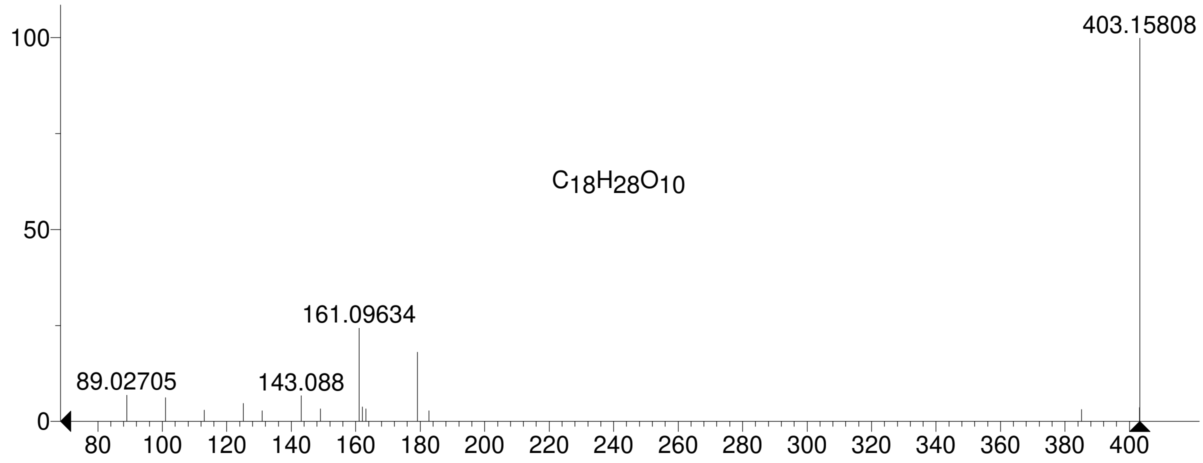

Unknown: Unknown (7.76_579.2575m/z)
Compound in Library Factor = 0

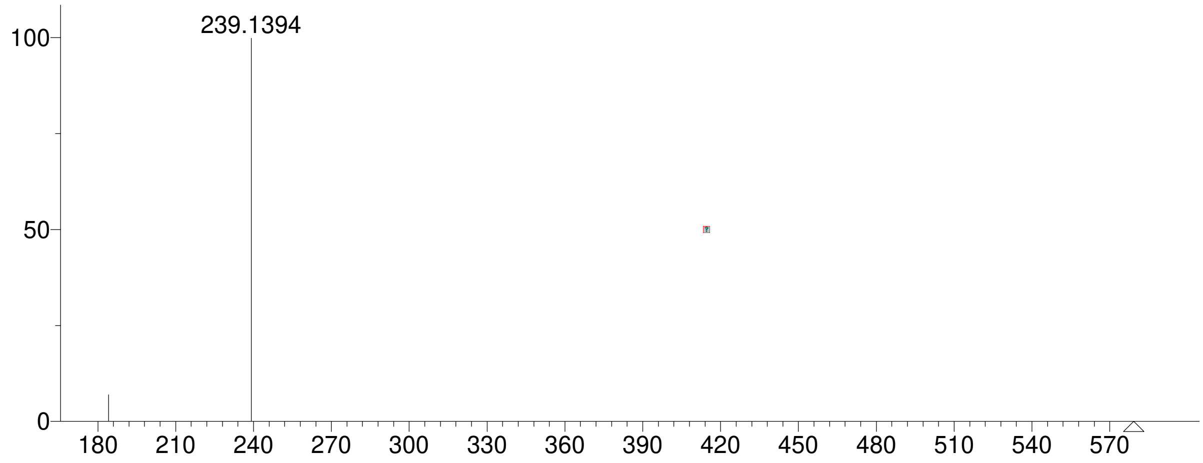

Unknown: Unknown (7.76_990.5013m/z)
Compound in Library Factor = 0

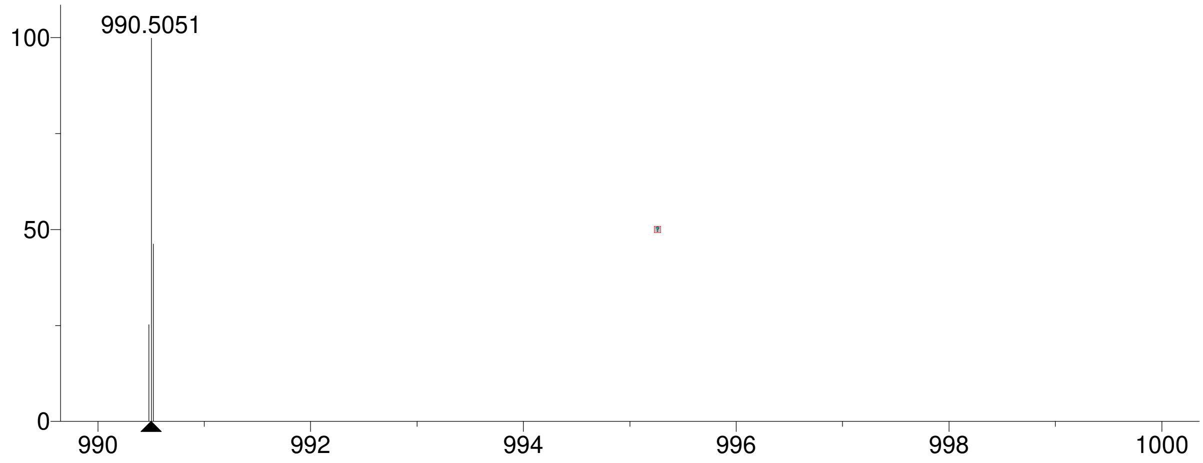

Unknown: Unknown (7.77_298.1396m/z)
Compound in Library Factor = -3273

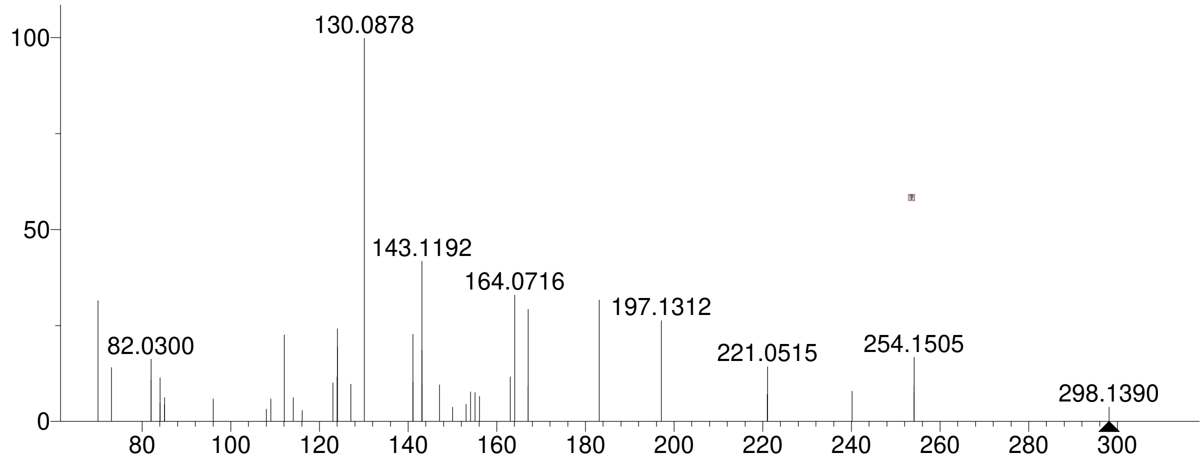

Hit 1 : Noratropine [M+Na]+ HCD 8V P=298.1
C16H21NO3; MF: 1; RMF: 77; Prob 100.0%; CAS: 16839-98-8; Lib: nist_msms; ID: 197150.

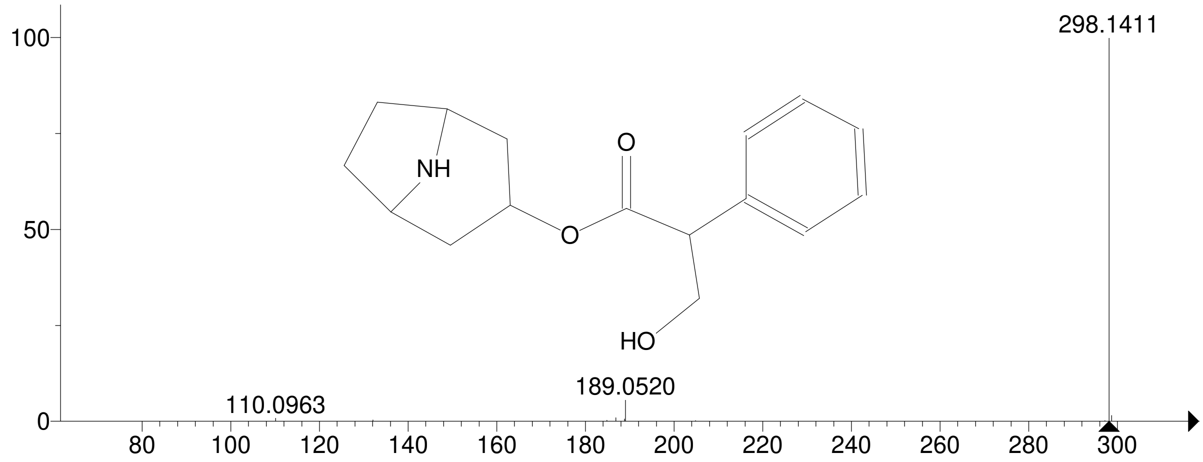

Unknown: Unknown (7.77_388.1502m/z)
Compound in Library Factor = 0

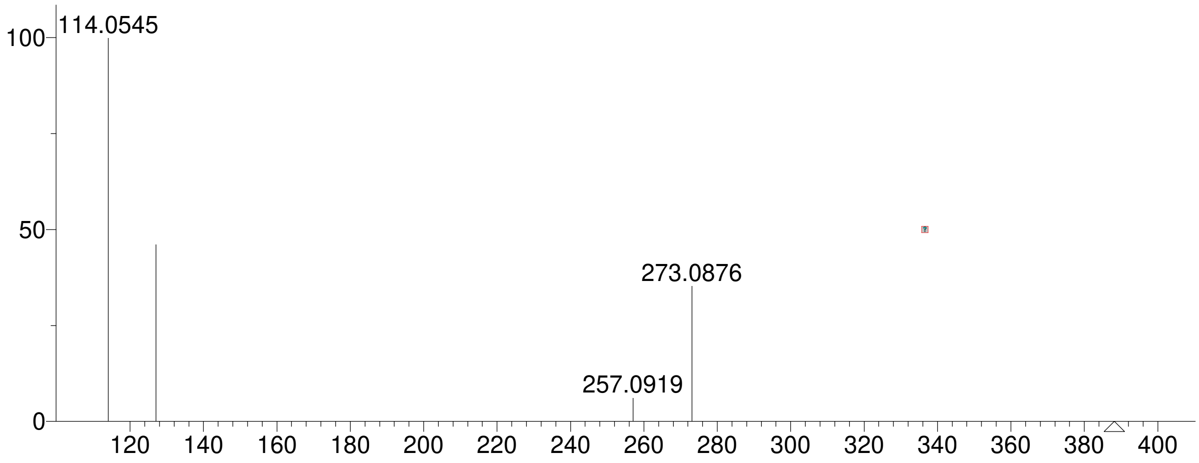

Unknown: Unknown (7.77_542.2090m/z)
Compound in Library Factor = 0

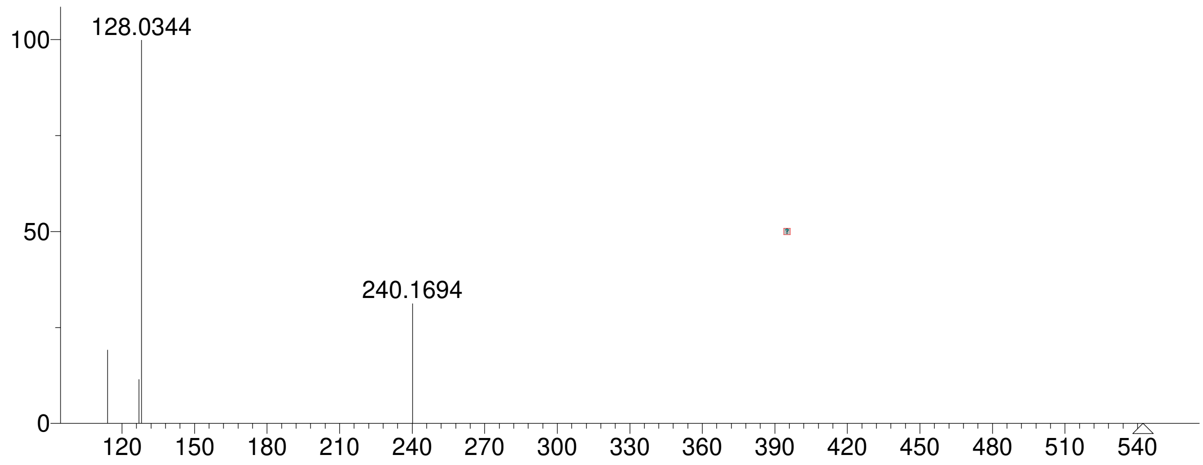

Unknown: Unknown (7.78_348.1441m/z)
Compound in Library Factor = 0

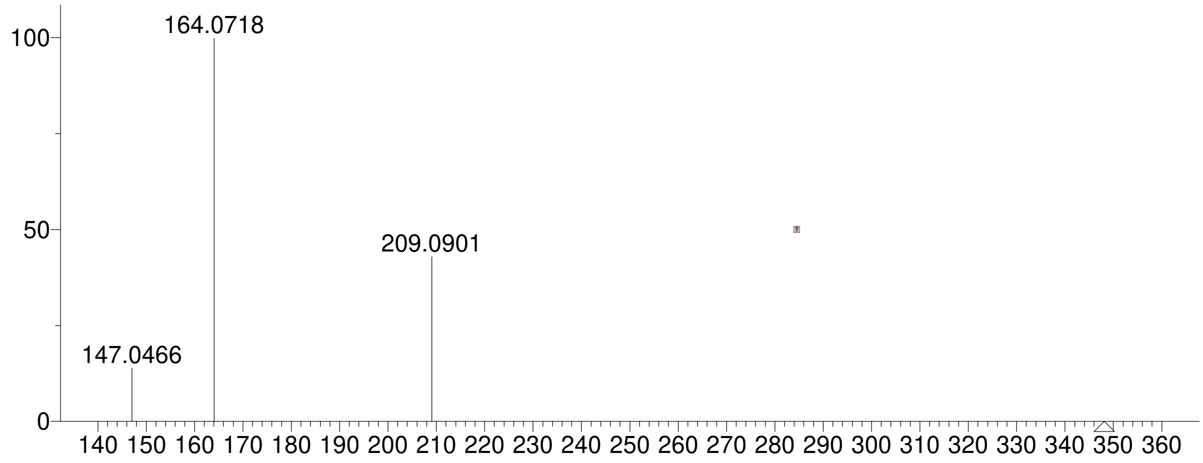

Hit 1 : 4,5-dihydroxy-4,5,6-trimethyl-2,8-dioxa-13-azatricyclo[8.5.1.0A^'1A^'3,A^'1a^!P]hexadec-10-ene-3,7-dione
C16H23NO6; MF: 0; RMF: 76; Prob 50.0%; Lib: mona-export-vf-npl_qexactive; ID: 24612.

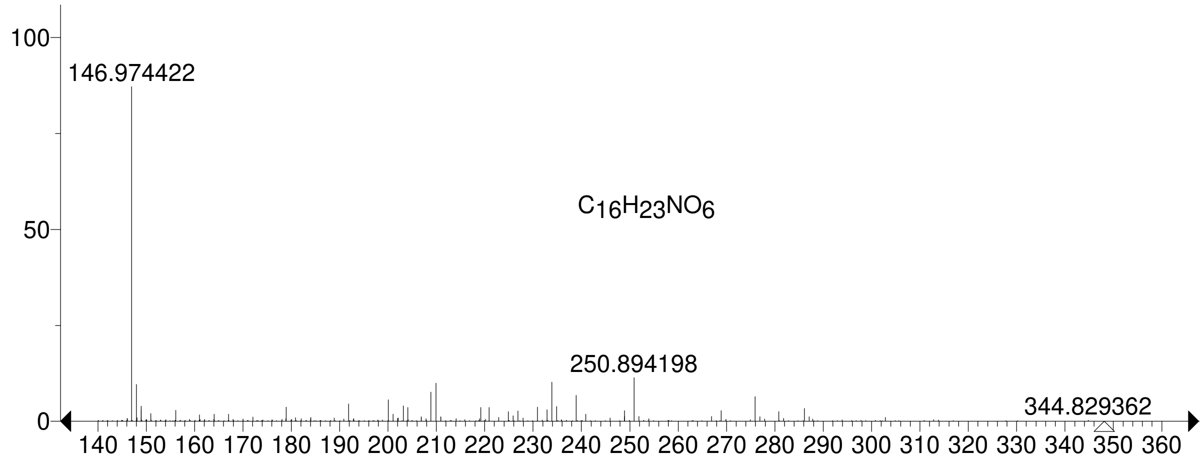

Hit 2 : Nafamostat mesylate [M+H]+ P=348.1
C21H25N5O8S2; MF: 0; RMF: 43; Prob 50.0%; Lib: mona-export-gnps; ID: 457.

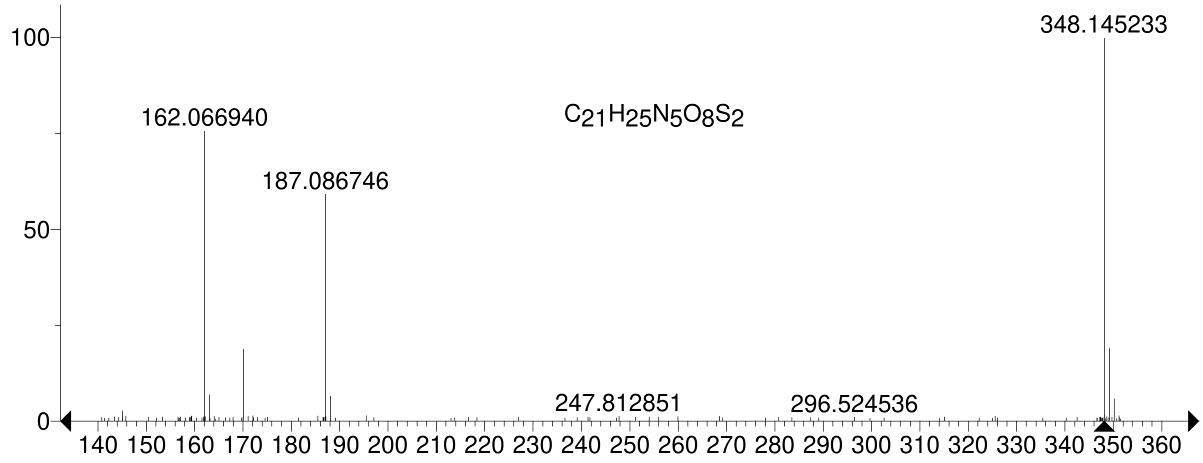

Unknown: Unknown (7.79_364.1623m/z)
Compound in Library Factor = -3273

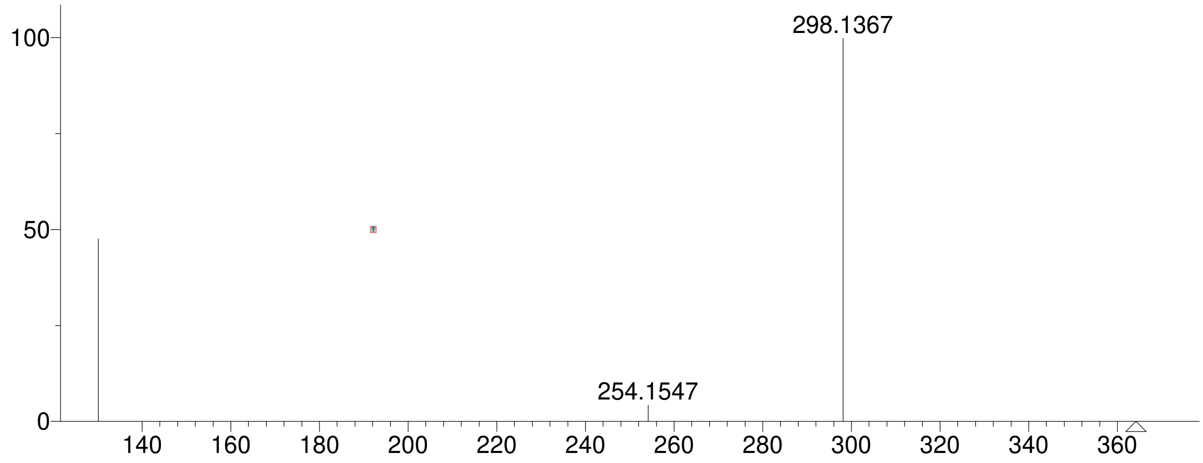

Hit 1 : Asn-Cys-Lys [M+H]+ QTOF 40V P=364.2
$C_{13}H_{25}N_5O_5S$; MF: 13; RMF: 382; Prob 33.4%; Lib: nist_msms; ID: 52440.

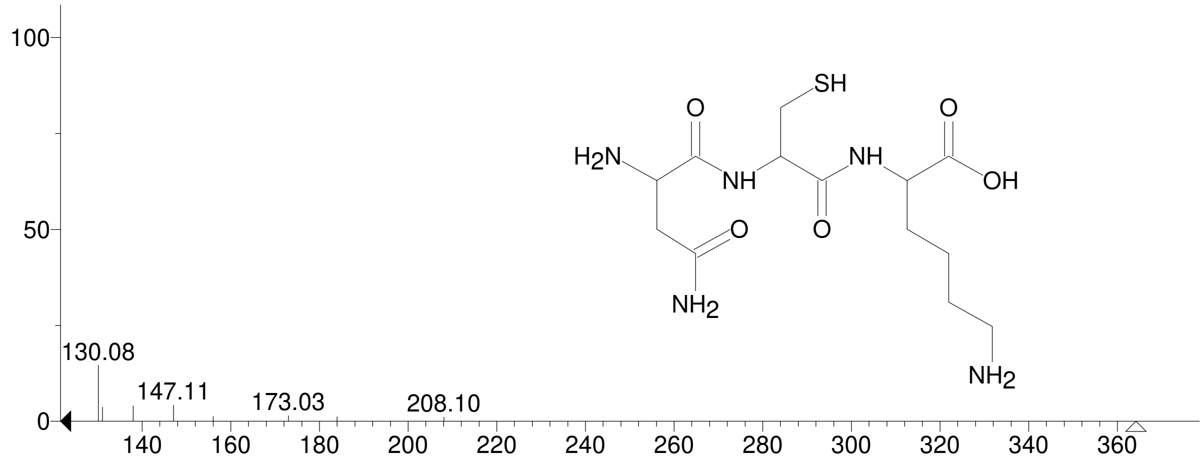

Hit 2 : Cys-Asn-Lys [M+H]+ QTOF 40V P=364.2
$C_{13}H_{25}N_5O_5S$; MF: 7; RMF: 259; Prob 26.3%; Lib: nist_msms; ID: 49672.

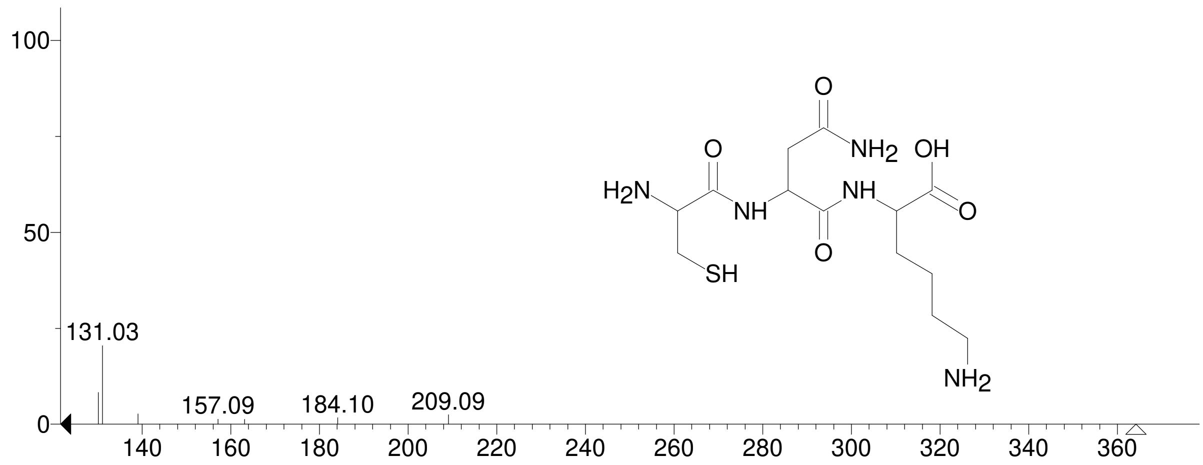

Hit 3 : aucubin [M+NH4]+ 45HCD P=364.2
C15H22O9; MF: 0; RMF: 19; Prob 20.1%; Lib: mona-export-vf-npl_qexactive; ID: 3773.

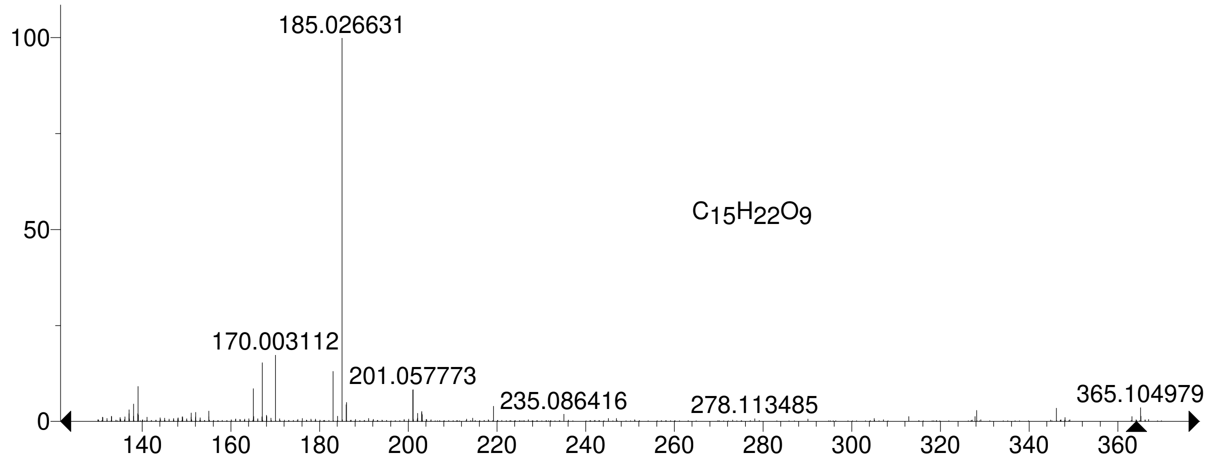

Hit 4 : (2R,3S,4S,5R,6S)-2-(hydroxymethyl)-6-(3,4,5-trimethoxyphenoxy)oxane-3,4,5-triol [M+NH4]+ 45HCD P=
C15H22O9; MF: 0; RMF: 12; Prob 20.1%; Lib: mona-export-vf-npl_qexactive; ID: 9872.

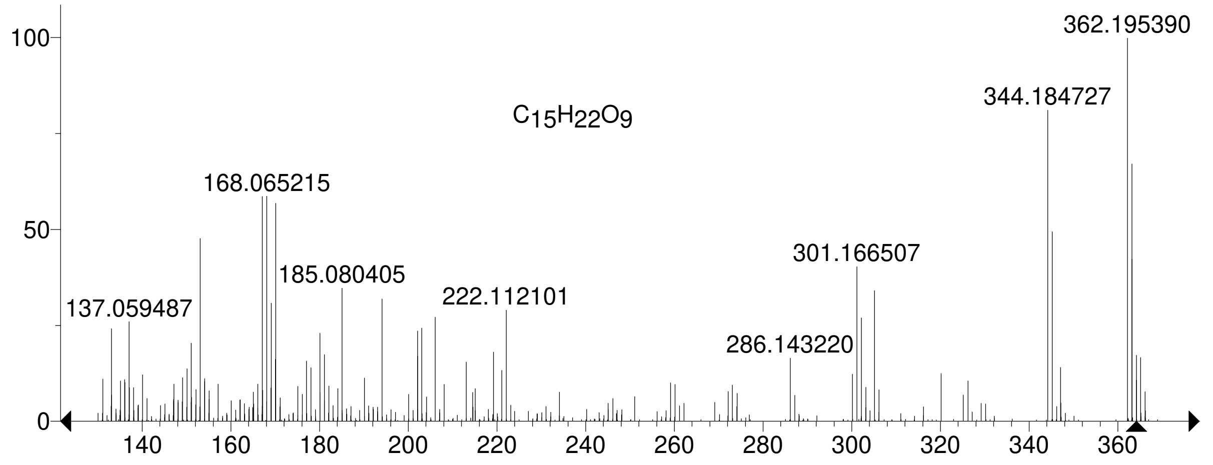

** Search Report Page 1 of 1 **

Unknown: Unknown (7.80_240.1710m/z)
Compound in Library Factor = 0

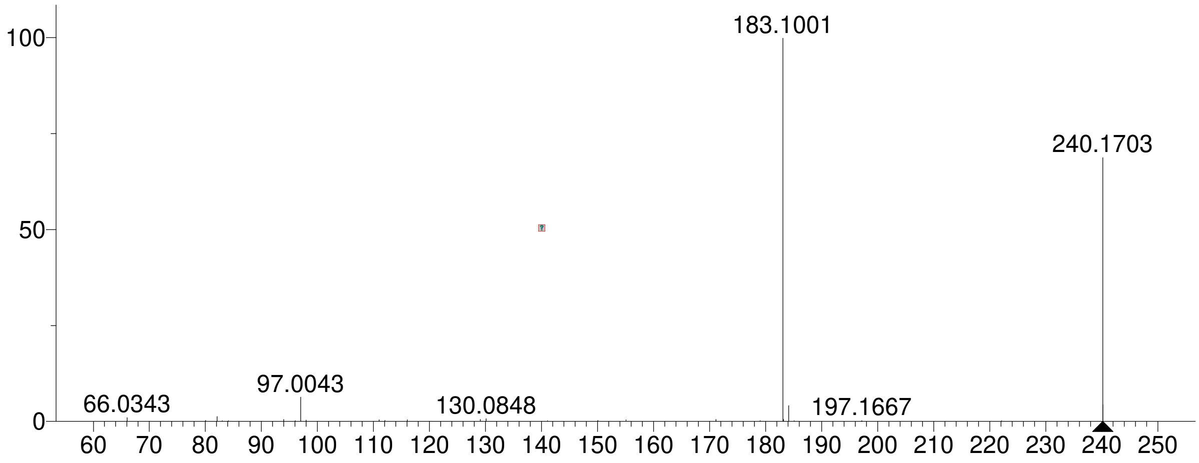

Unknown: Unknown (7.81_284.1970m/z)
Compound in Library Factor = 0

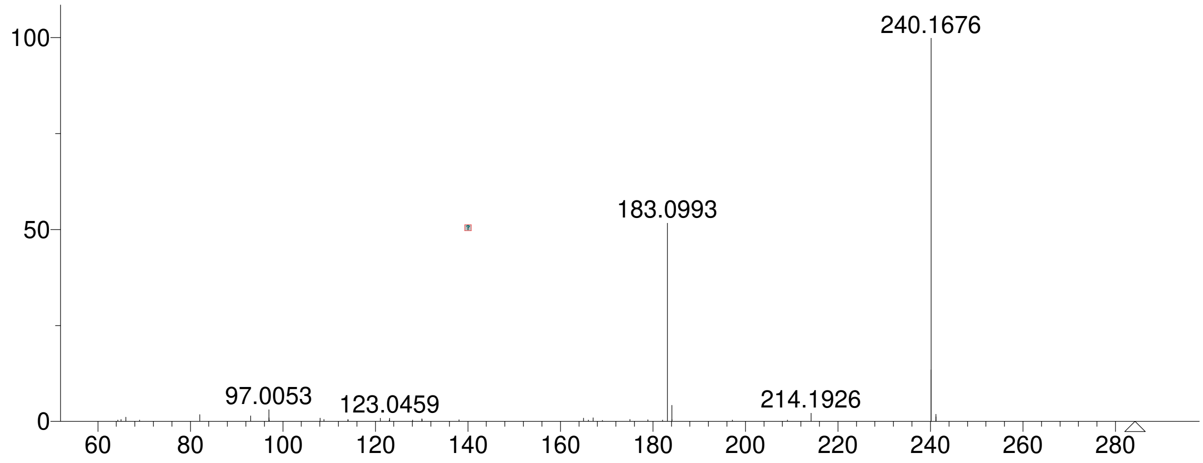

Hit 1 : Dodecyl phosphate [M+NH4]+ QQQ 7V P=284.2
C12H27O4P; MF: 0; RMF: 2; Prob 100.0%; CAS: 2627-35-2; Lib: nist_msms; ID: 180585.

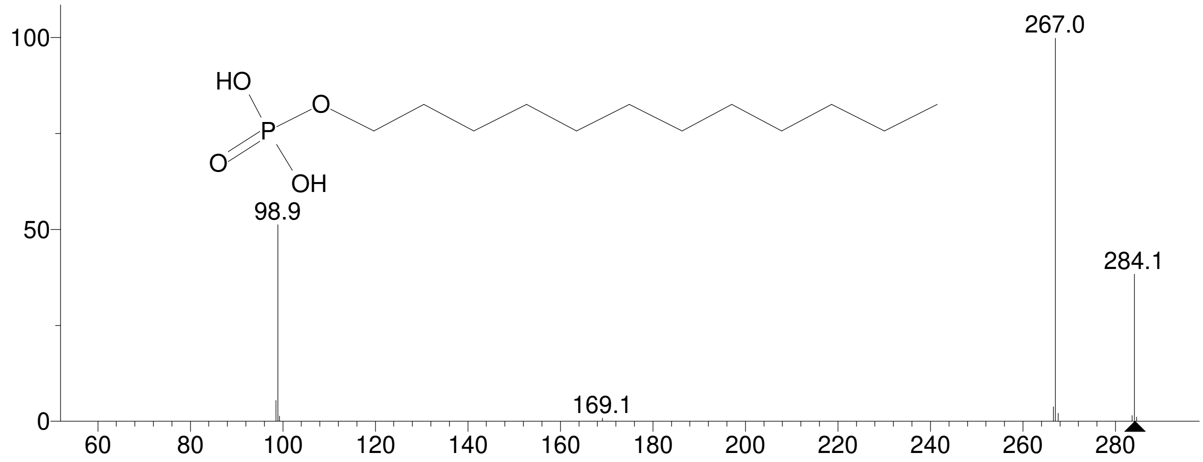

Unknown: Unknown (7.81_328.1502m/z)
Compound in Library Factor = 0

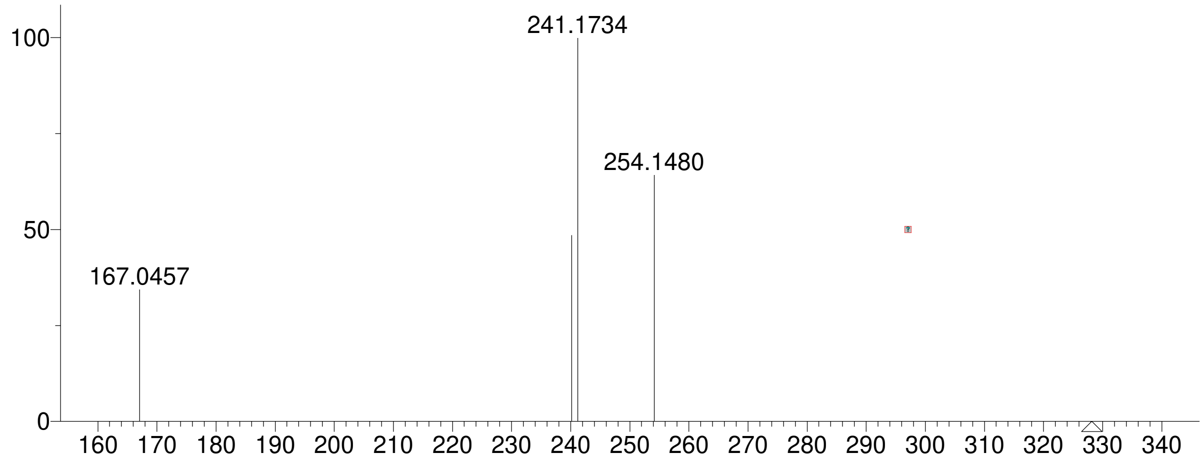

** Search Report Page 1 of 1 **

Unknown: Unknown (7.81_410.1447m/z)
Compound in Library Factor = 0

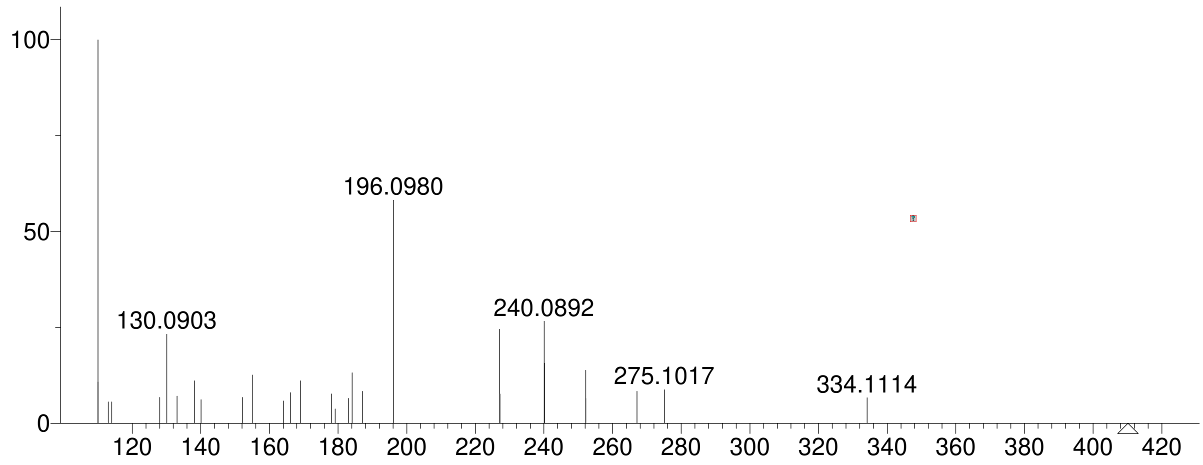

Hit 1 : NCGC00179757-02![(2R,3S,4S,5R,6S)-3,4,5-trihydroxy-6-(4-hydroxyphenoxy)oxan-2-yl]methyl 4-hydroxyb
C19H20O9; MF: 0; RMF: 15; Prob 50.0%; Lib: mona-export-gnps; ID: 9157.

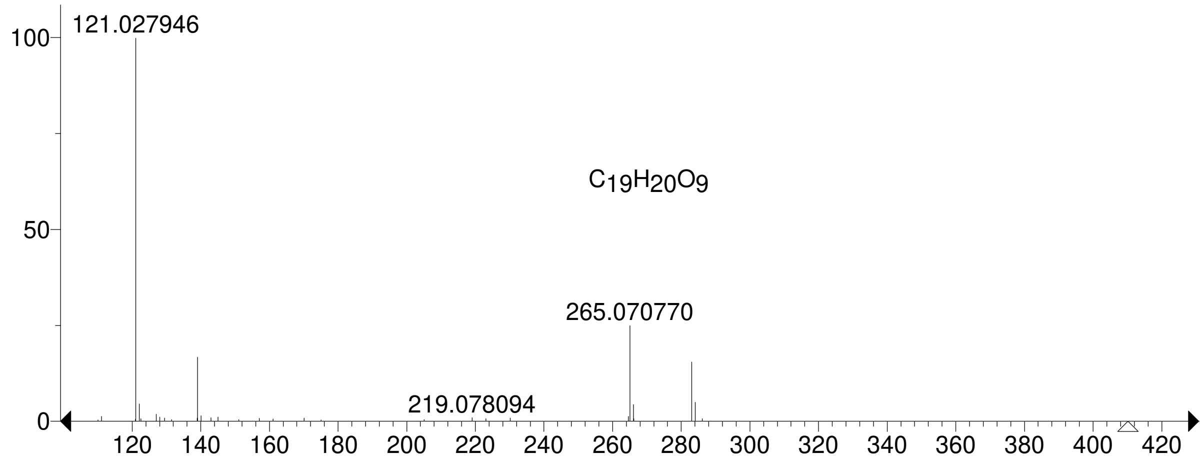

Hit 2 : Pemetrexed [M+H-H2O]+ HCD 49V P=410.1
C20H21N5O6; MF: 0; RMF: 3; Prob 50.0%; CAS: 137281-23-3; Lib: nist_msms; ID: 349927.

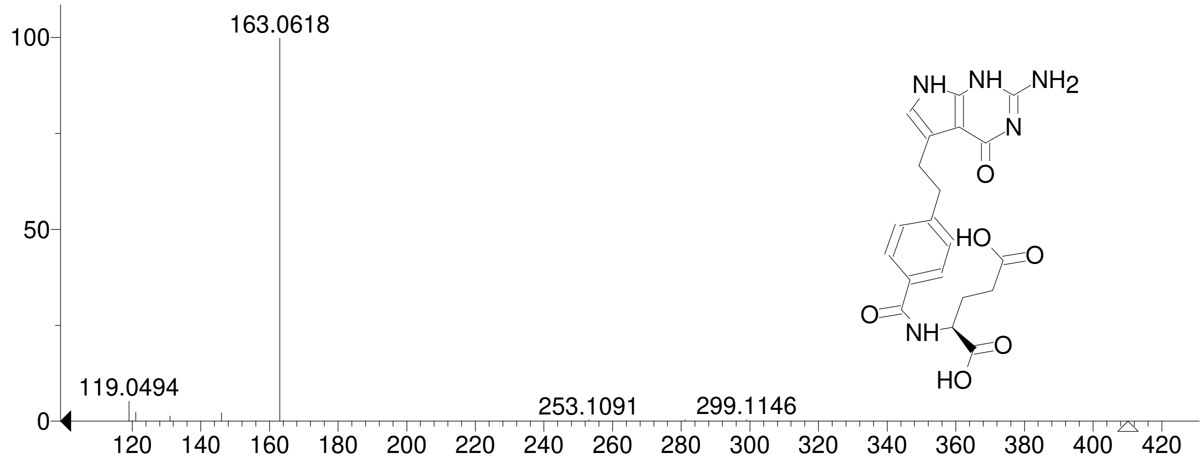

Unknown: Unknown (7.81_606.2376m/z)
Compound in Library Factor = 0

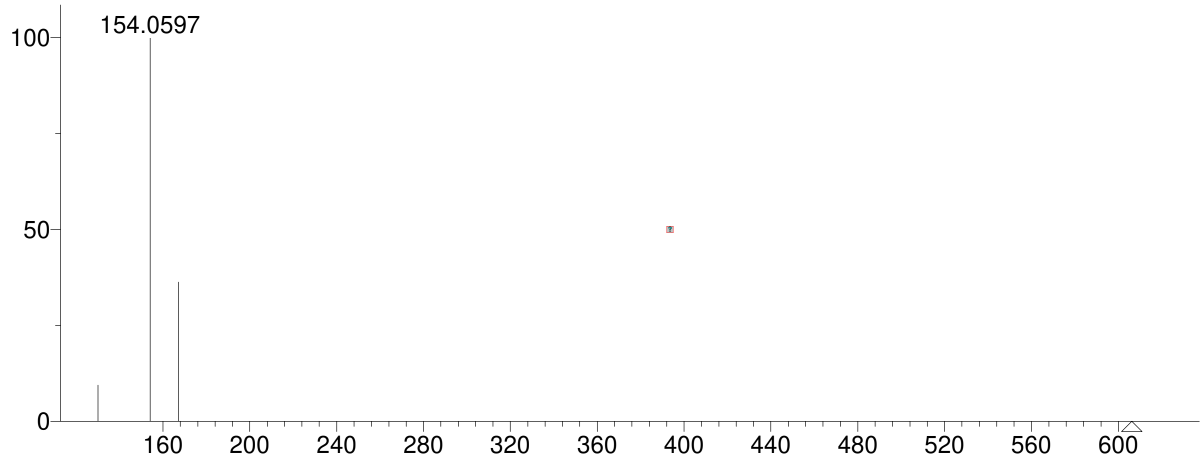

Unknown: Unknown (7.82_628.3305m/z)
Compound in Library Factor = -3273

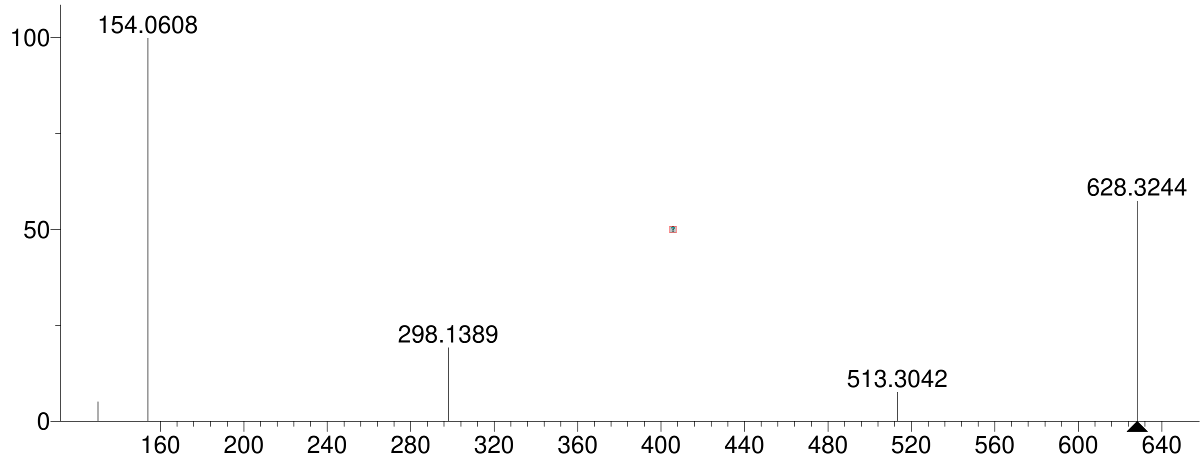

Hit 1 : PS 25:4; PS 9:0-16:4; [M-H]- [M-H]- P=628.3
C31H52NO10P; MF: 6; RMF: 295; Prob 15.6%; Lib: msdial-insilicomsms-lipids-neg; ID: 330095.

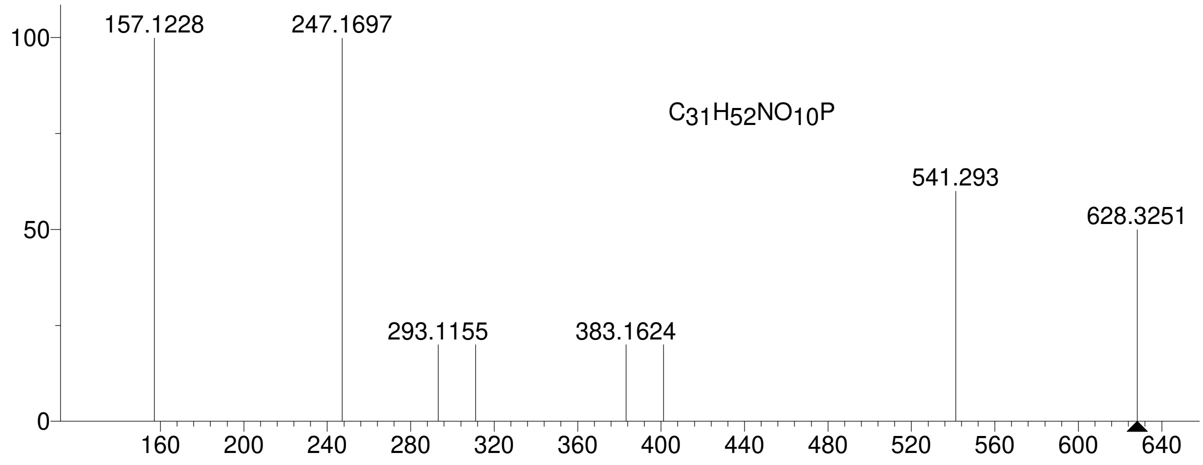

Hit 2 : PS 25:4; PS 3:0-22:4; [M-H]- [M-H]- P=628.3
C31H52NO10P; MF: 4; RMF: 228; Prob 14.4%; Lib: msdial-insilicomsms-lipids-neg; ID: 329782.

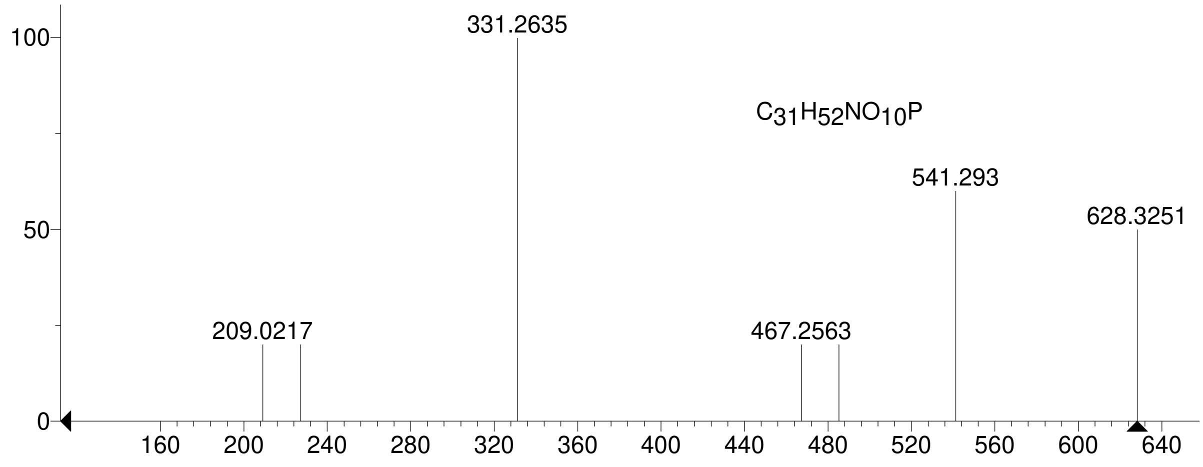

Hit 3 : PS 25:4; PS 5:0-20:4; [M-H]- [M-H]- P=628.3
C31H52NO10P; MF: 4; RMF: 228; Prob 14.4%; Lib: msdial-insilicomsms-lipids-neg; ID: 329890.

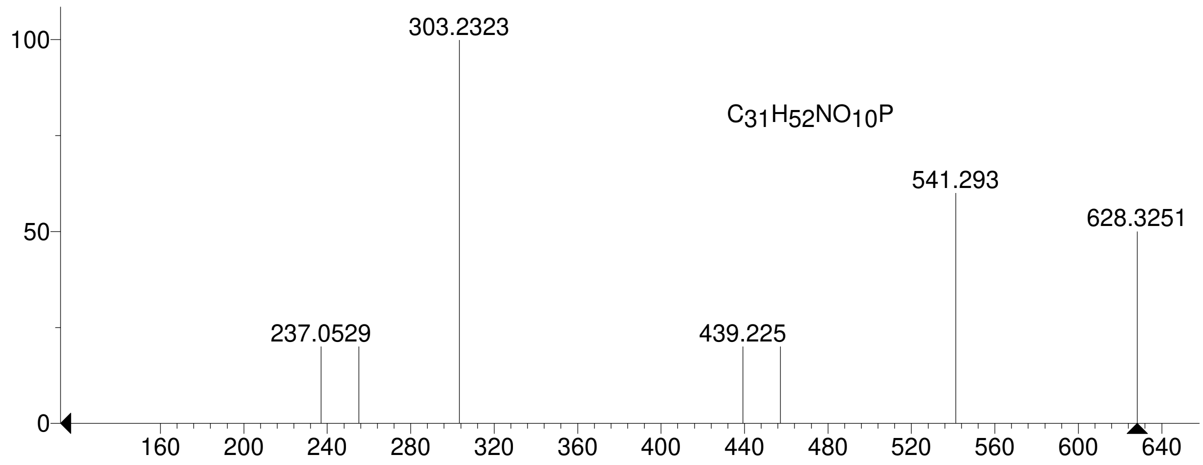

Hit 4 : PS 25:4; PS 7:0-18:4; [M-H]- [M-H]- P=628.3
C31H52NO10P; MF: 4; RMF: 228; Prob 14.4%; Lib: msdial-insilicomsms-lipids-neg; ID: 329994.

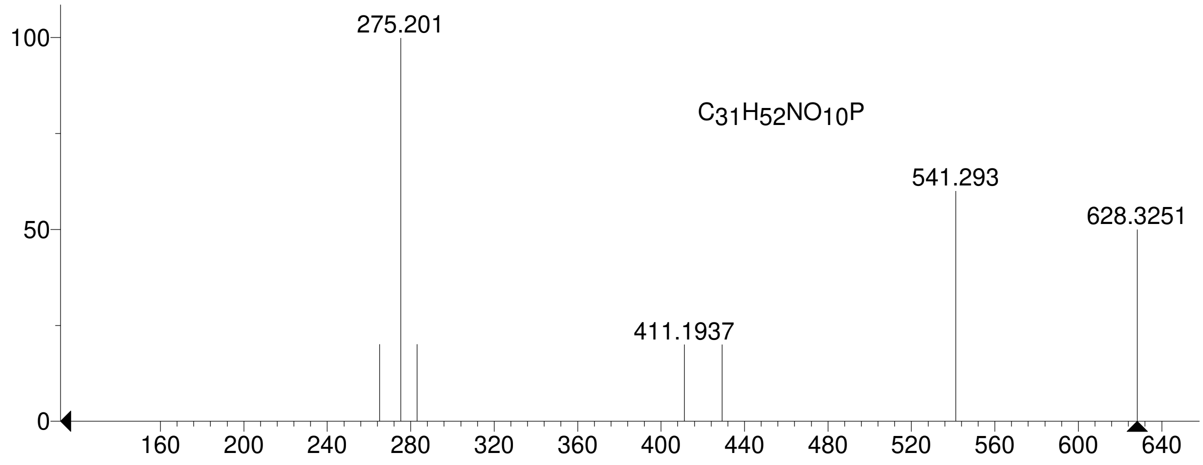

Hit 5 : PC 22:5; PC 2:0-20:5; [M+FA-H]- [M+FA-H]- P=628.3
C30H50NO8P; MF: 3; RMF: 170; Prob 13.8%; Lib: msdial-insilicomsms-lipids-neg; ID: 317606.

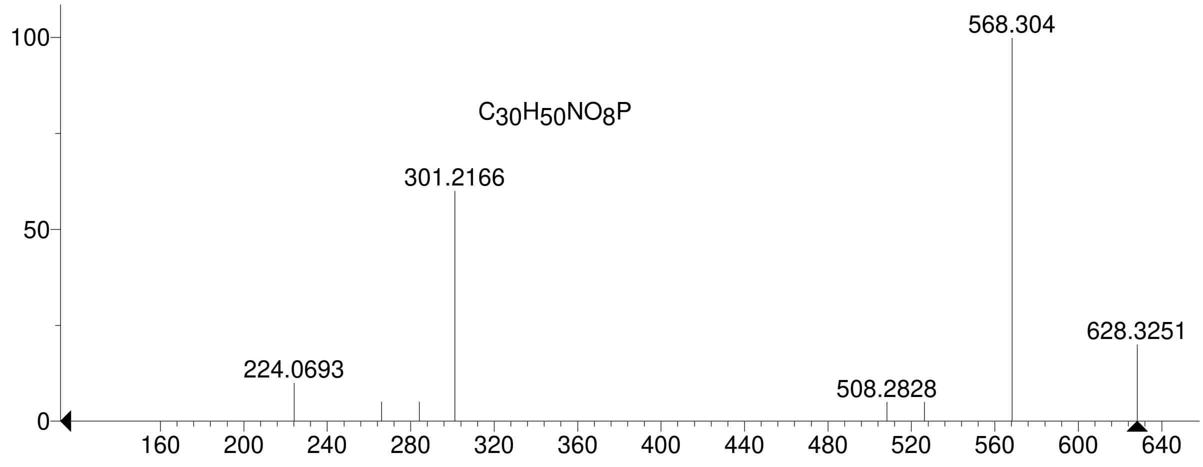

Unknown: Unknown (7.84_279.0169m/z)
Compound in Library Factor = 0

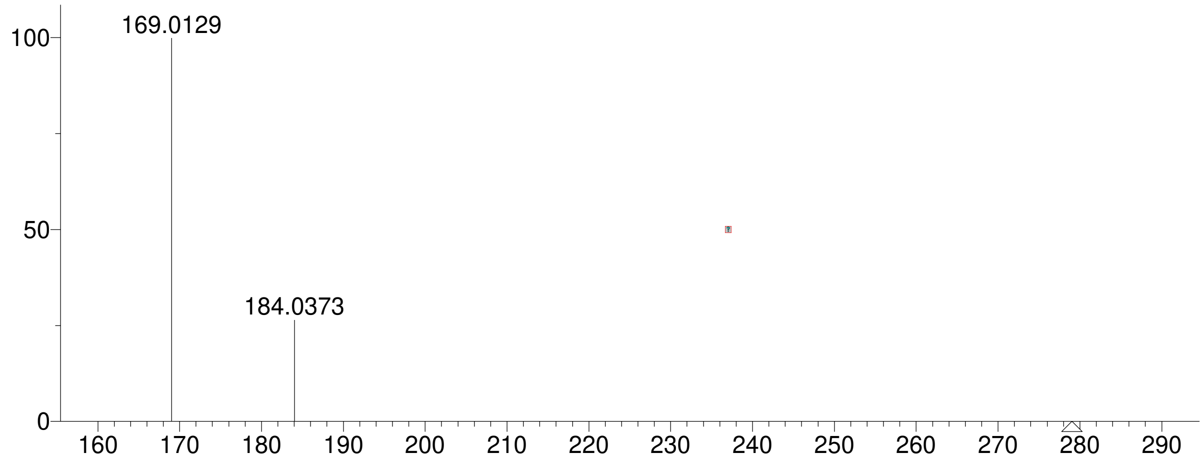

Unknown: Unknown (7.85_324.1538m/z)
Compound in Library Factor = 0

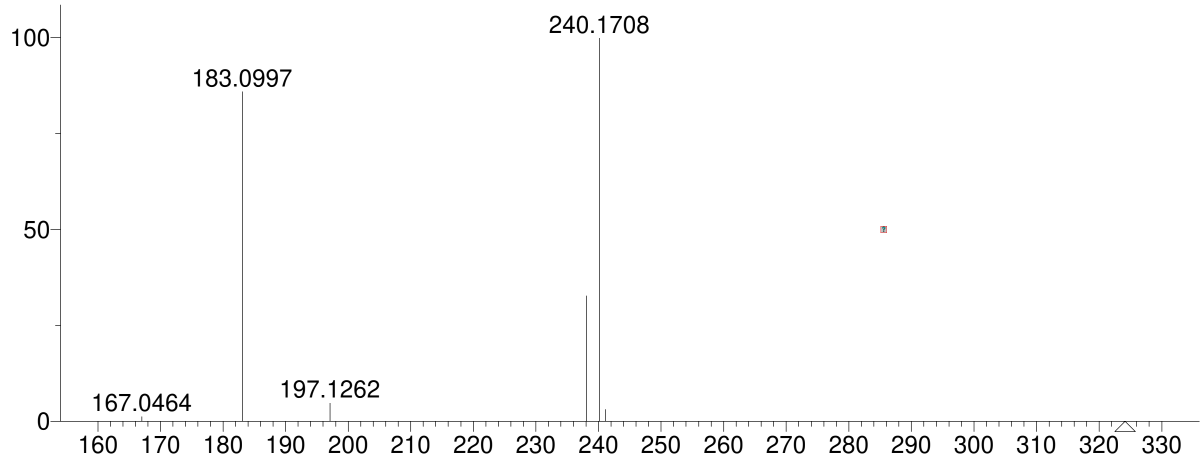

** Search Report Page 1 of 1 **

Unknown: Unknown (7.85_357.1631m/z)
Compound in Library Factor = 0

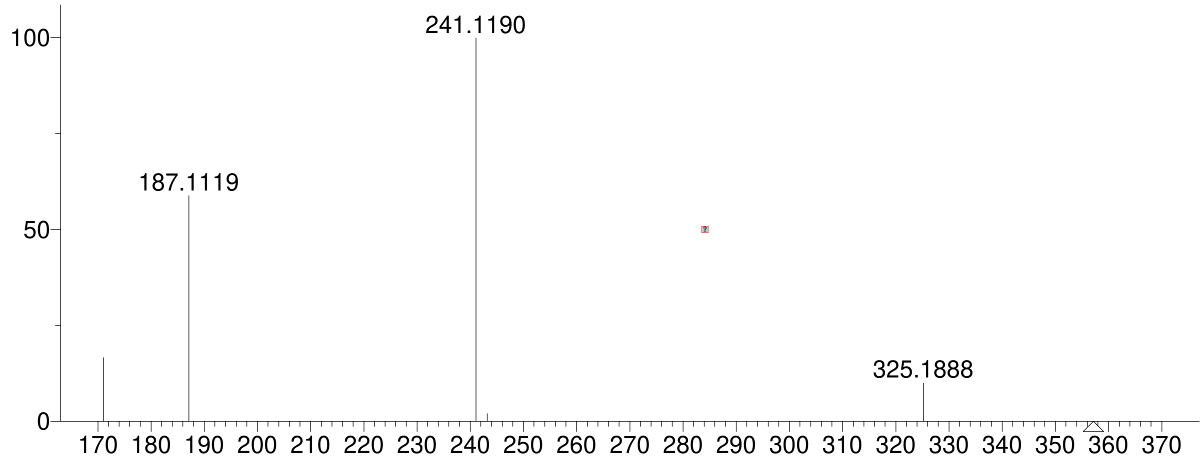

Hit 1 : NCGC00381117-01!2-amino-4-hydroxy-5-[[1-(4-methoxy-6-oxopyran-2-yl)-3-methylbutyl]amino]-5-oxopenta
C16H24N2O7; MF: 0; RMF: 28; Prob 100.0%; Lib: mona-export-gnps; ID: 8549.

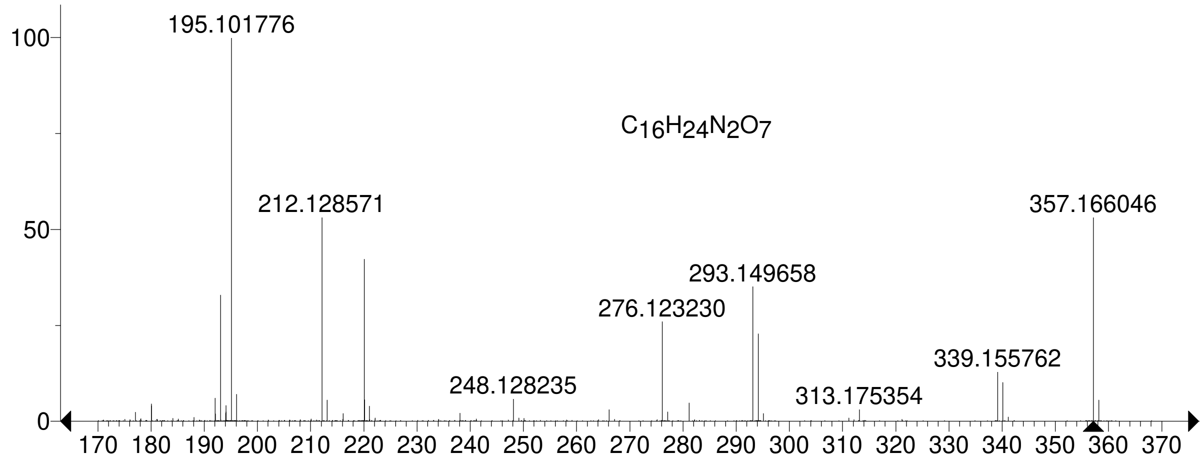

Unknown: Unknown (7.85_369.1771m/z)
Compound in Library Factor = -2387

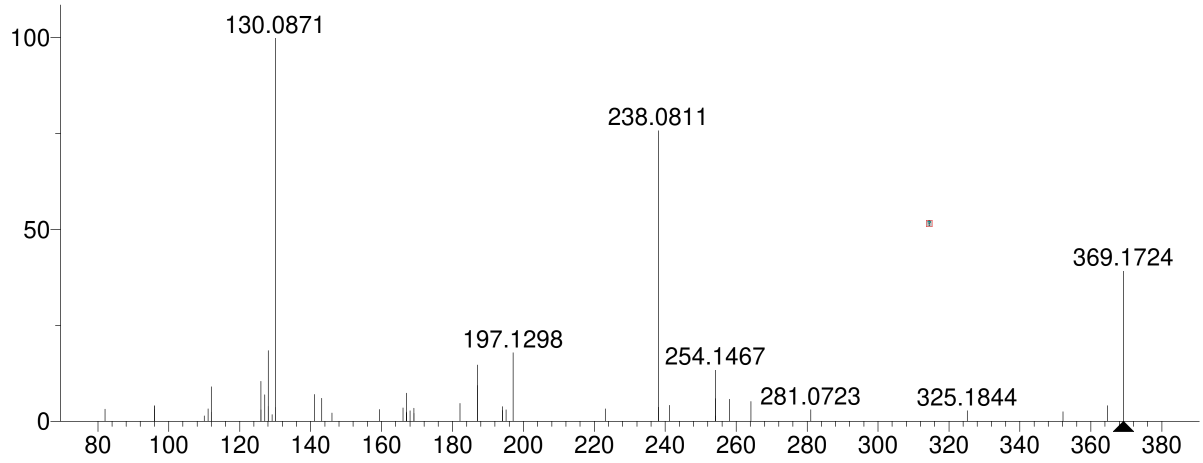

Hit 1 : 5-Androsten-3β,17β-diol-3-sulfate [M-H]- HCD 27V P=369.2
C19H30O5S; MF: 24; RMF: 274; Prob 19.5%; CAS: 977-33-3; Lib: nist_msms; ID: 231655.

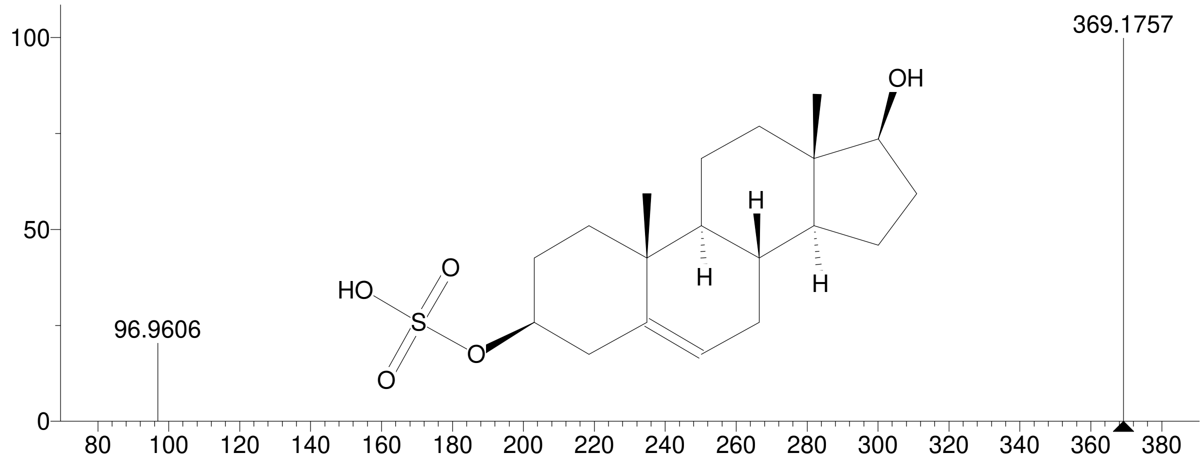

Hit 2 : 5β-Androstan-3α-ol-17-one sulfate [M-H]- HCD 33V P=369.2
C19H30O5S; MF: 19; RMF: 266; Prob 15.7%; CAS: 977-36-6; Lib: nist_msms; ID: 231644.

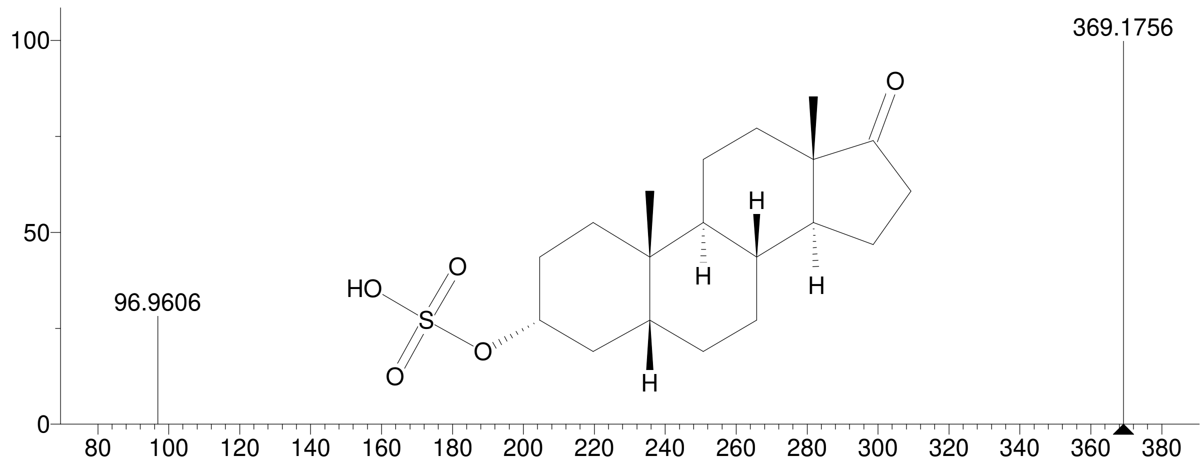

Hit 3 : 5α-Androstan-3β-ol-17-one sulfate [M-H]- HCD 33V P=369.2
C19H30O5S; MF: 18; RMF: 276; Prob 15.1%; CAS: 977-35-5; Lib: nist_msms; ID: 231624.

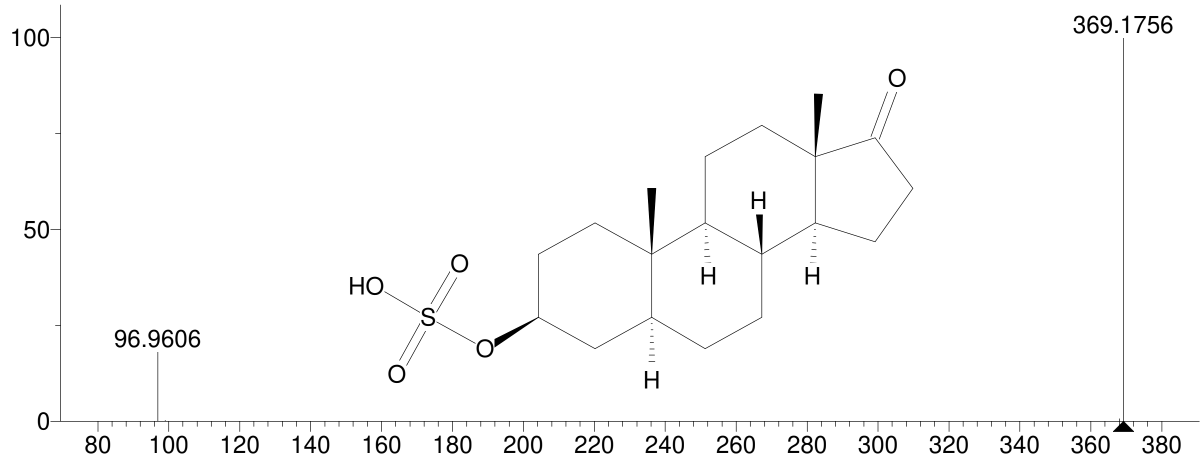

Hit 4 : 5α-Androstan-17β-ol-3-one sulfate [M-H]- HCD 40V P=369.2
C19H30O5S; MF: 18; RMF: 269; Prob 15.1%; CAS: 2641-48-7; Lib: nist_msms; ID: 231920.

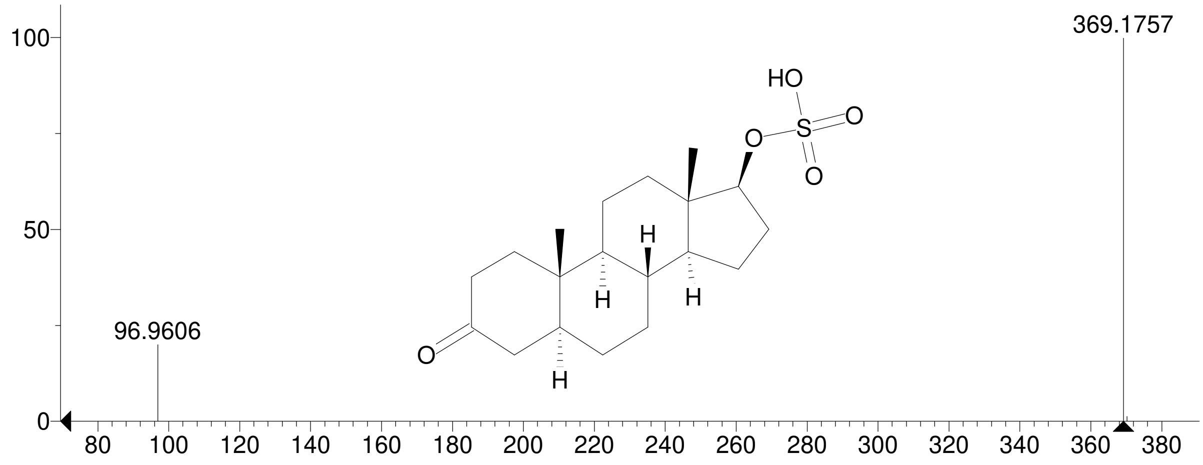

Hit 5 : Androsterone sulfate [M-H]- HCD 29V P=369.2
C19H30O5S; MF: 5; RMF: 250; Prob 9.75%; CAS: 2479-86-9; Lib: nist_msms; ID: 117992.

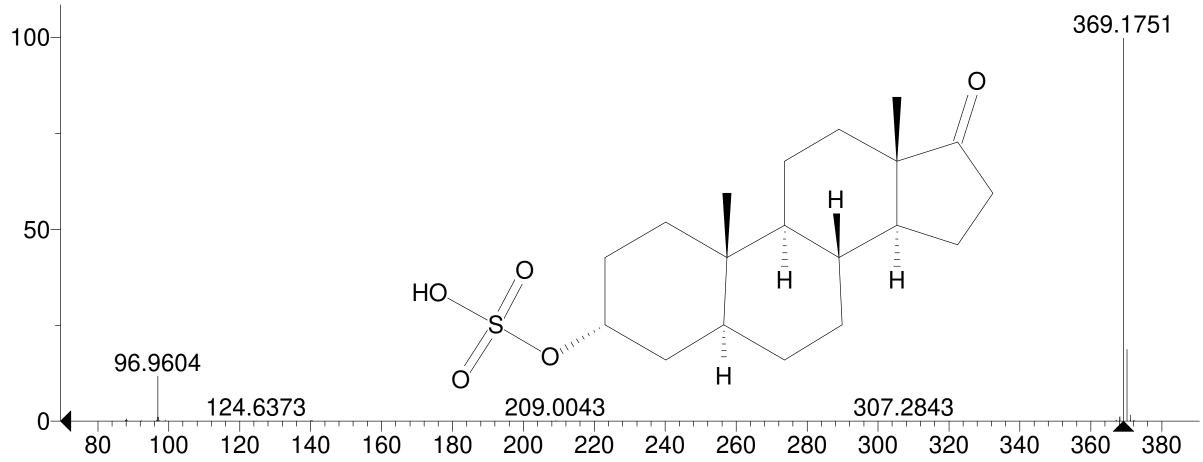

Unknown: Unknown (7.85_472.1921m/z)
Compound in Library Factor = 0

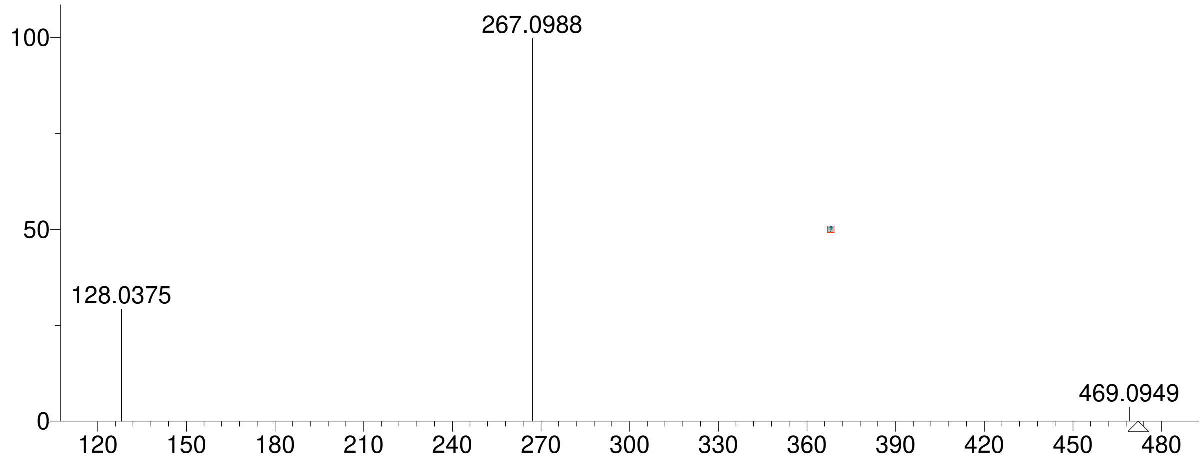

Hit 1 : methyl 3-(3,4-dihydroxyphenyl)-2-[[4-hydroxy-3-(3-methylbut-2-enyl)phenyl]methyl]-4-methoxy-5-oxofuran-
C25H26O8; MF: 0; RMF: 38; Prob 100.0%; Lib: mona-export-vf-npl_qexactive; ID: 9392.

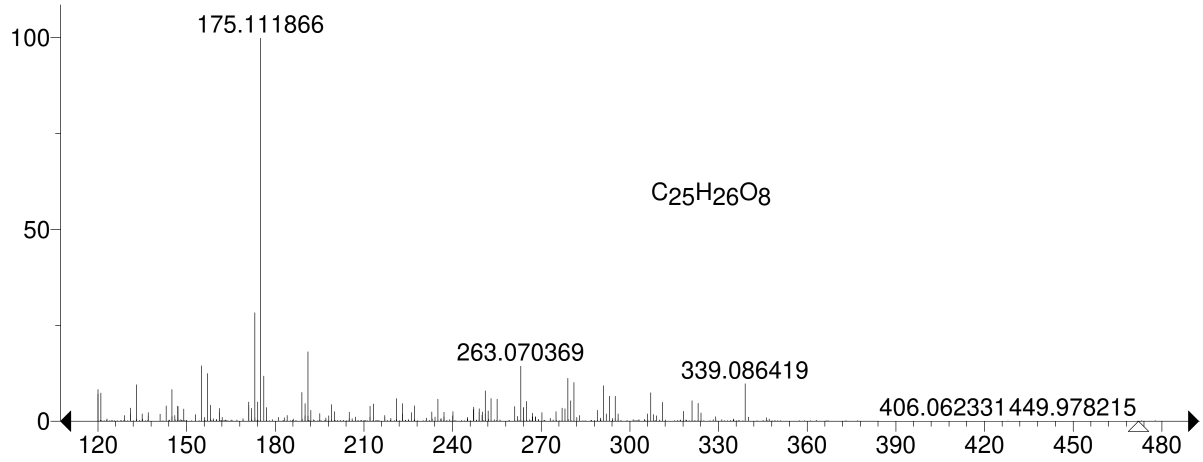

Unknown: Unknown (7.87_243.1703m/z)
Compound in Library Factor = 0

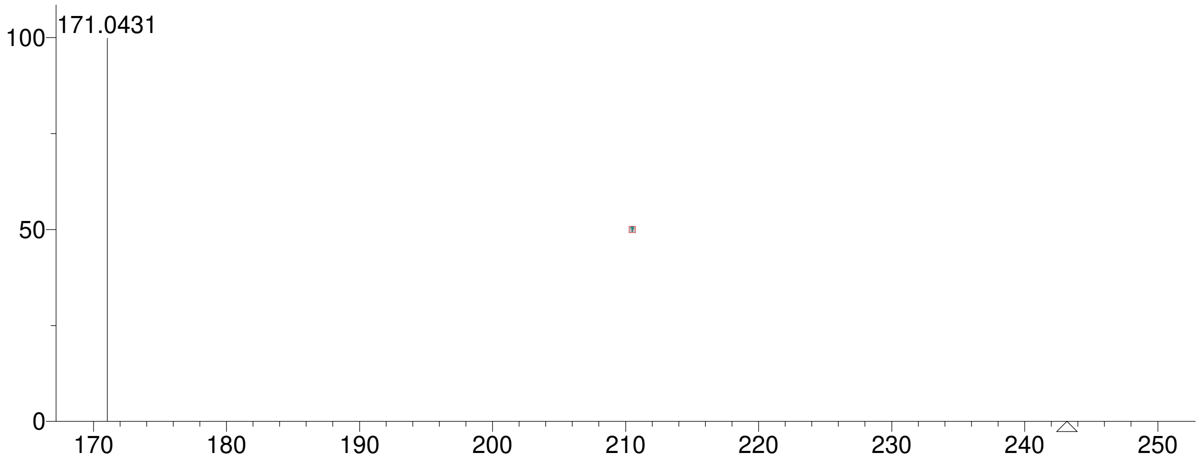

Unknown: Unknown (7.88_387.0857m/z)
Compound in Library Factor = 0

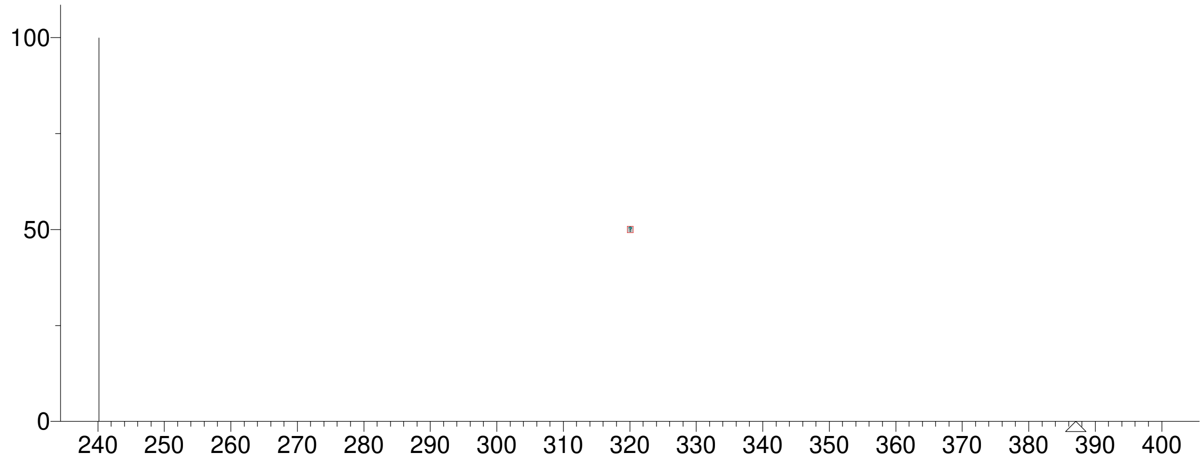

Unknown: Unknown (7.88_429.1856m/z)
Compound in Library Factor = 0

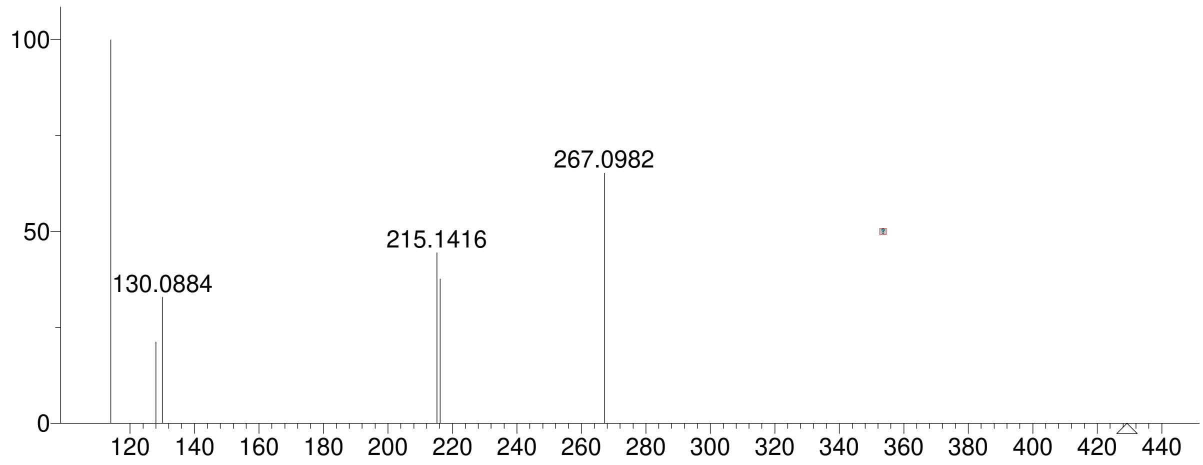

Hit 1 : (2R,3R,4S,5S,6R)-2-[6-hydroxy-3-[(E)-3-hydroxybut-1-enyl]-2,4,4-trimethylcyclohexyl]oxy-6-(hydroxymethy
C19H34O8; MF: 0; RMF: 41; Prob 100.0%; Lib: anthocyanins mona; ID: 9985.

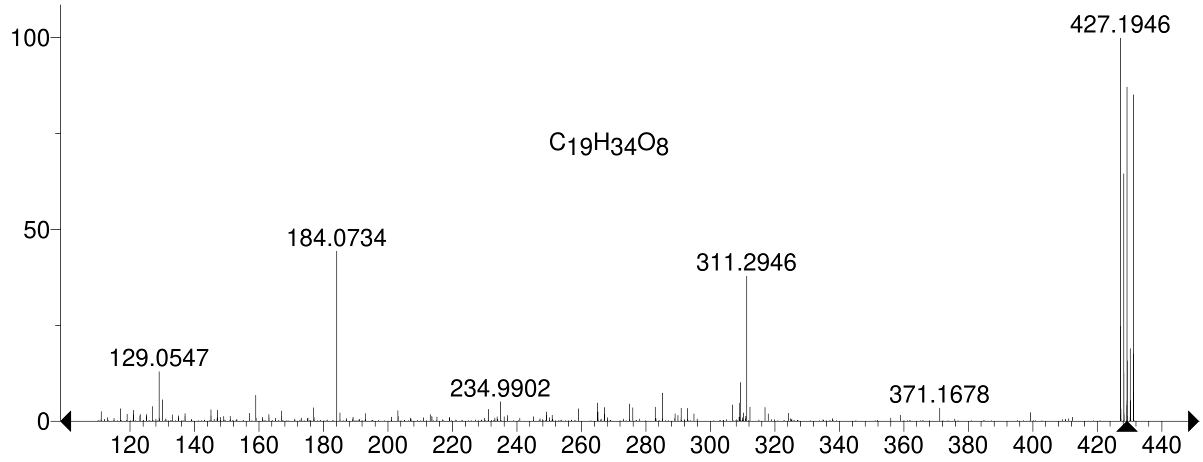

** Search Report Page 1 of 1 **

Unknown: Unknown (7.88_579.2579m/z)
Compound in Library Factor = 0

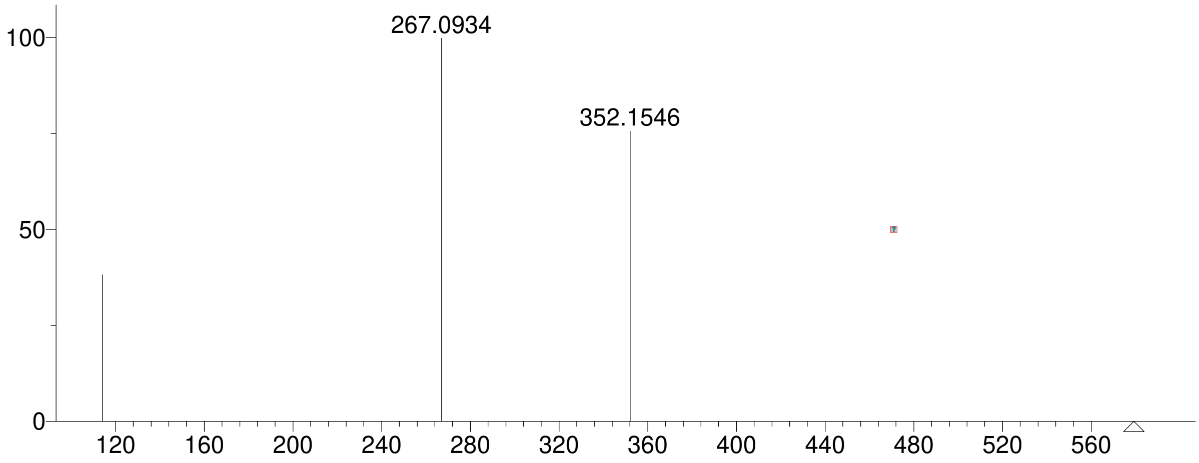

Hit 1 : Hematoporphyrin [M-H-H2O]- HCD 75V P=579.3
C34H38N4O6; MF: 0; RMF: 15; Prob 100.0%; CAS: 14459-29-1; Lib: nist_msms; ID: 289029.

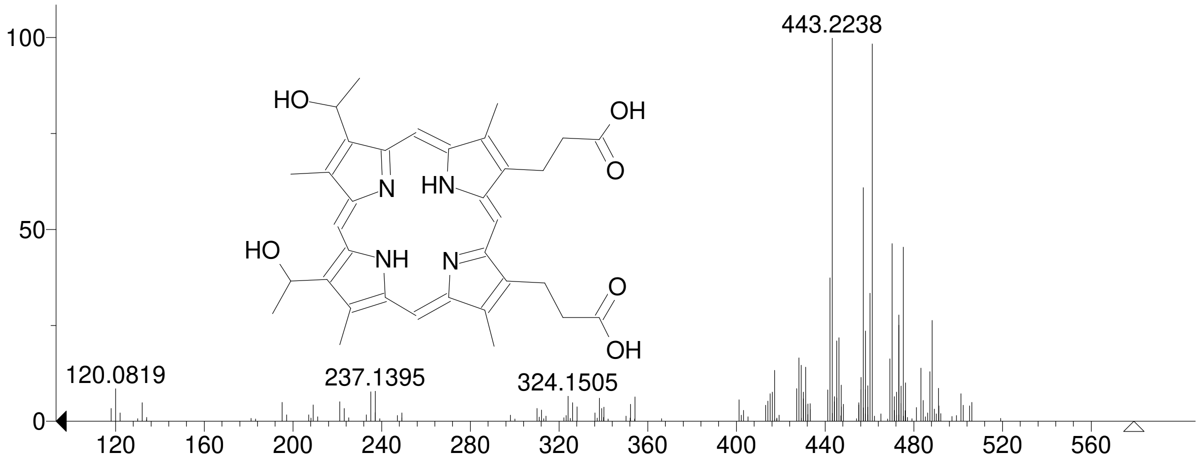

** Search Report Page 1 of 1 **

Unknown: Unknown (7.89_240.1708m/z)
Compound in Library Factor = 0

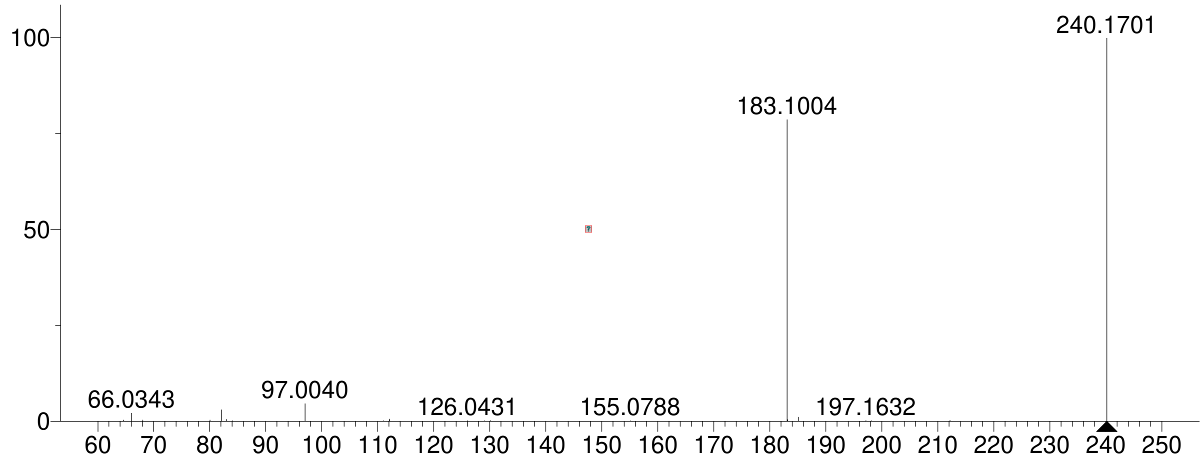

Unknown: Unknown (7.89_284.1970m/z)
Compound in Library Factor = 0

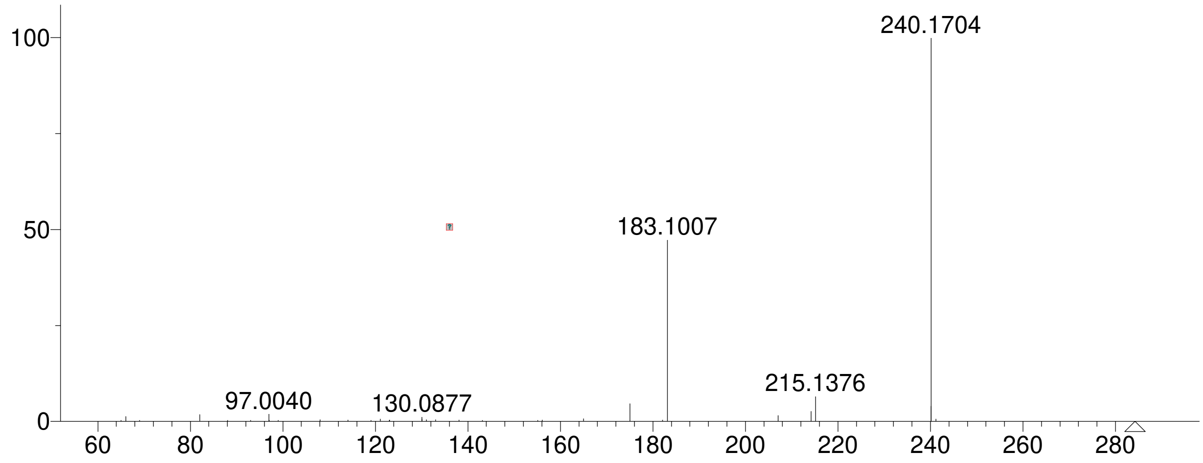

** Search Report Page 1 of 1 **

Unknown: Unknown (7.89_500.2347m/z)
Compound in Library Factor = 0

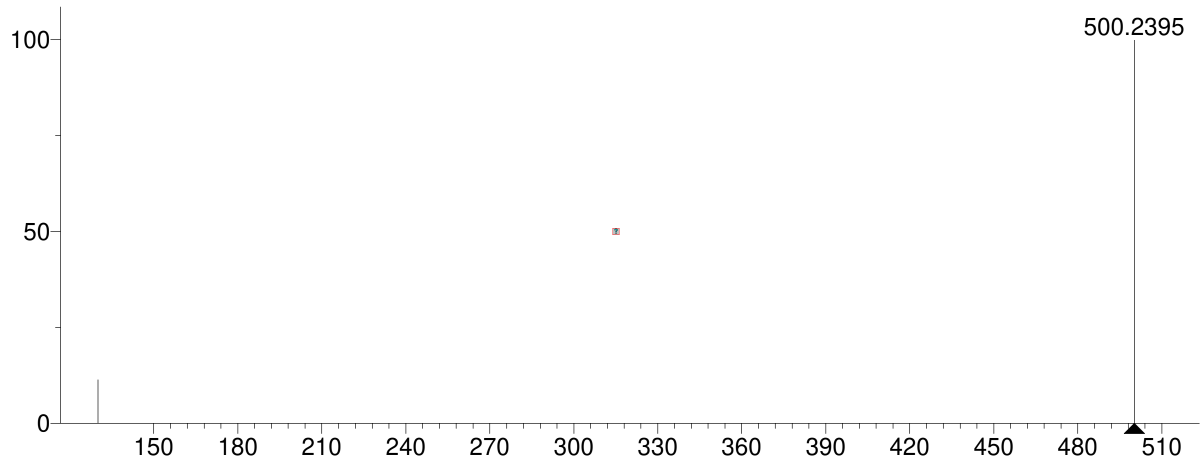

Unknown: Unknown (7.89_770.2049m/z)
Compound in Library Factor = 0

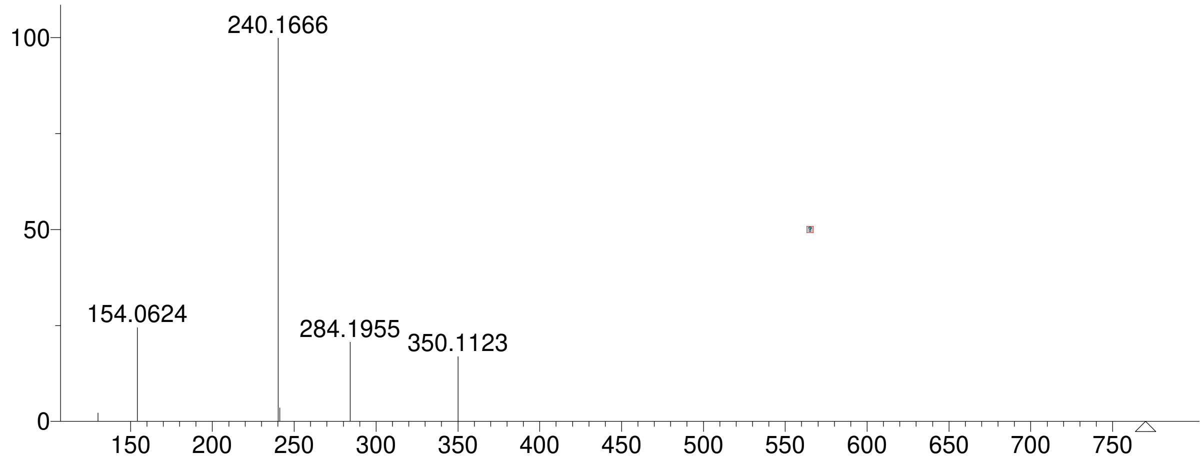

Unknown: Unknown (7.90_385.1962m/z)
Compound in Library Factor = 0

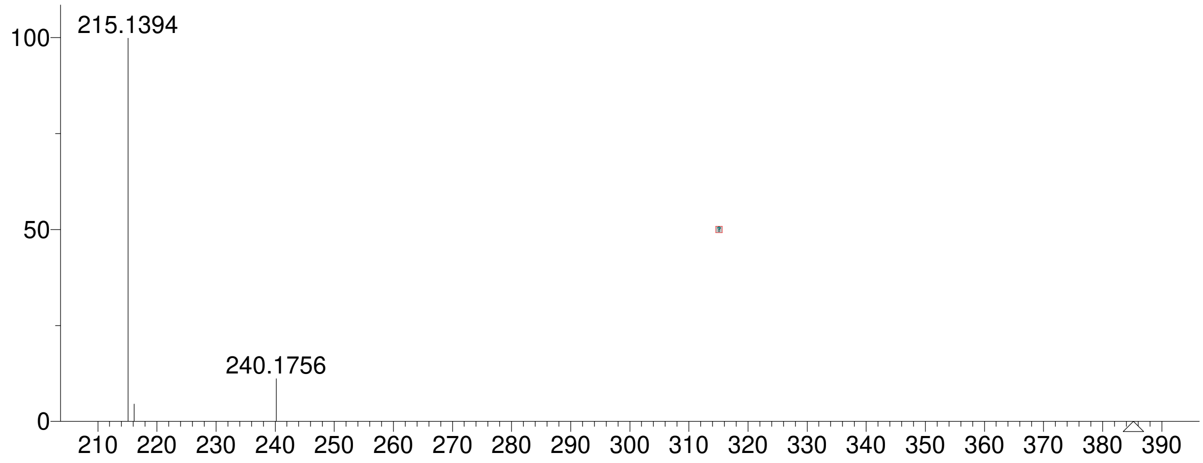

Unknown: Unknown (7.92_514.2128m/z)
Compound in Library Factor = 0

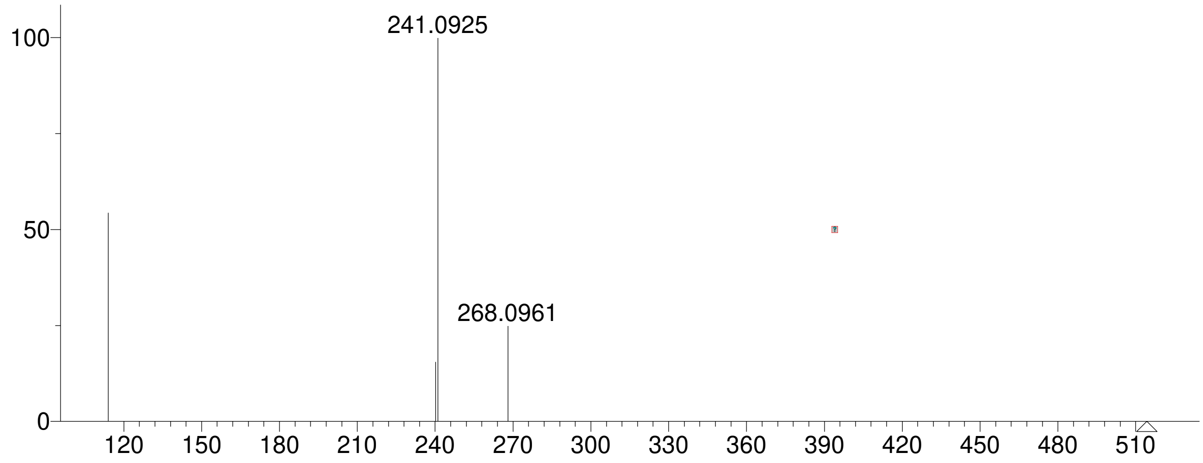

Unknown: Unknown (7.94_324.1461m/z)
Compound in Library Factor = 0

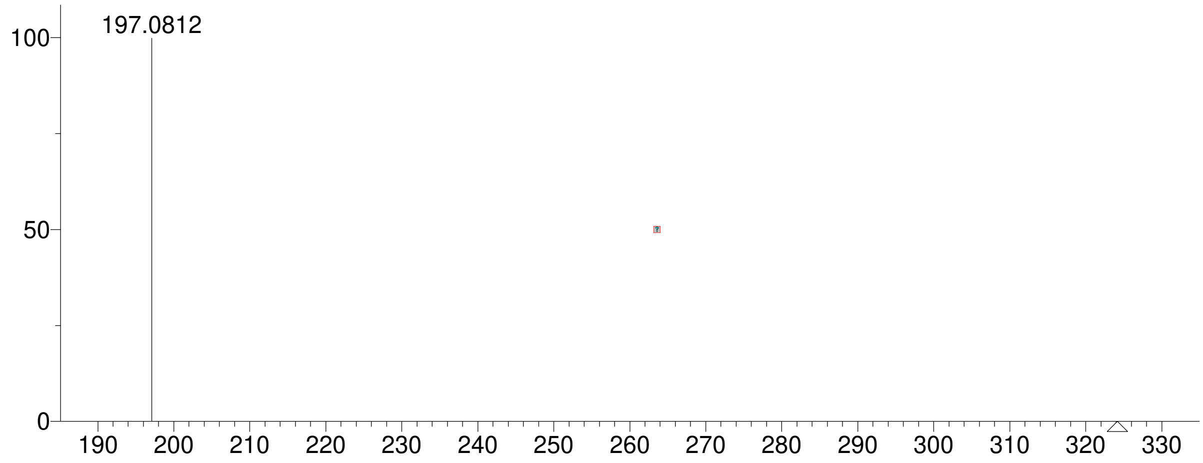

Hit 1 : NCGC00169062-03! [M+NH4]+ P=324.1
C16H18O6; MF: 0; RMF: 59; Prob 33.3%; Lib: mona-export-gnps; ID: 6069.

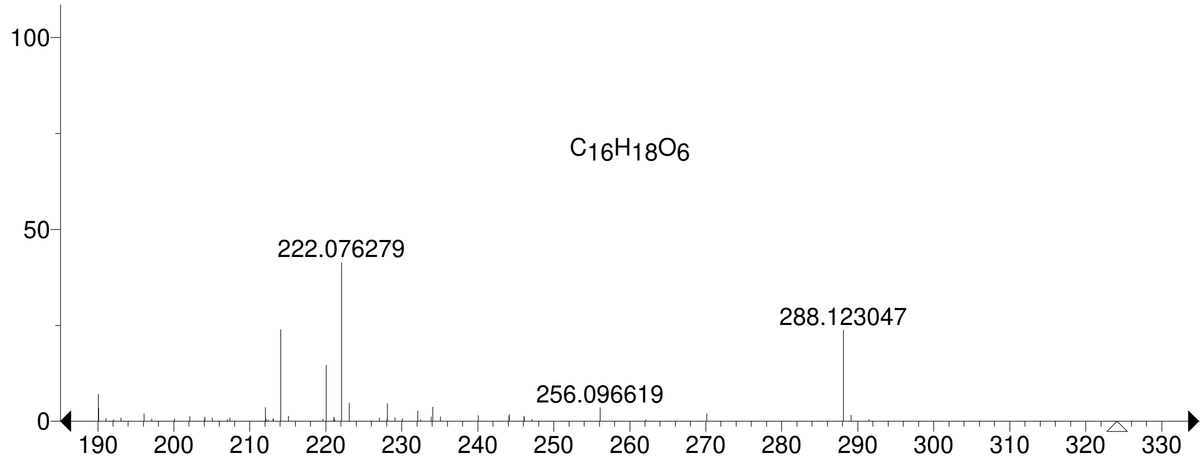

Hit 2 : NCGC00385408-01_C17H17N5O2_3-(1H-Imidazol-4-ylmethyl)-6-(1H-indol-3-ylmethyl)-2,5-piperazinedion
C17H17N5O2; MF: 0; RMF: 41; Prob 33.3%; Lib: mona-export-gnps; ID: 10874.

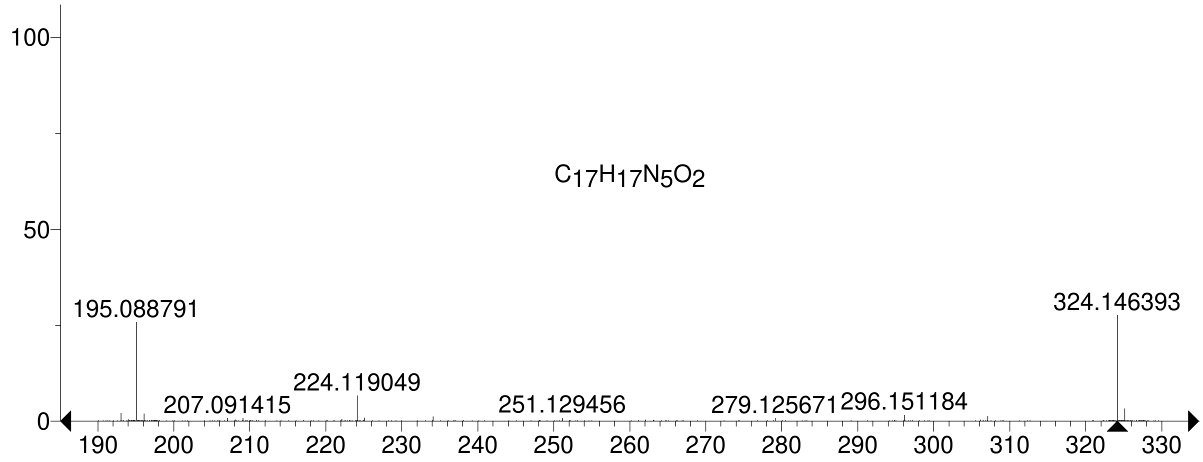

Hit 3 : 15,17-dihydroxy-9-methyl-4,10-dioxatricyclo[11.4.0.0A^'3,a^*u]heptadeca-1(17),13,15-triene-2,11-dione [M
C16H18O6; MF: 0; RMF: 36; Prob 33.3%; Lib: mona-export-vf-npl_qexactive; ID: 1491.

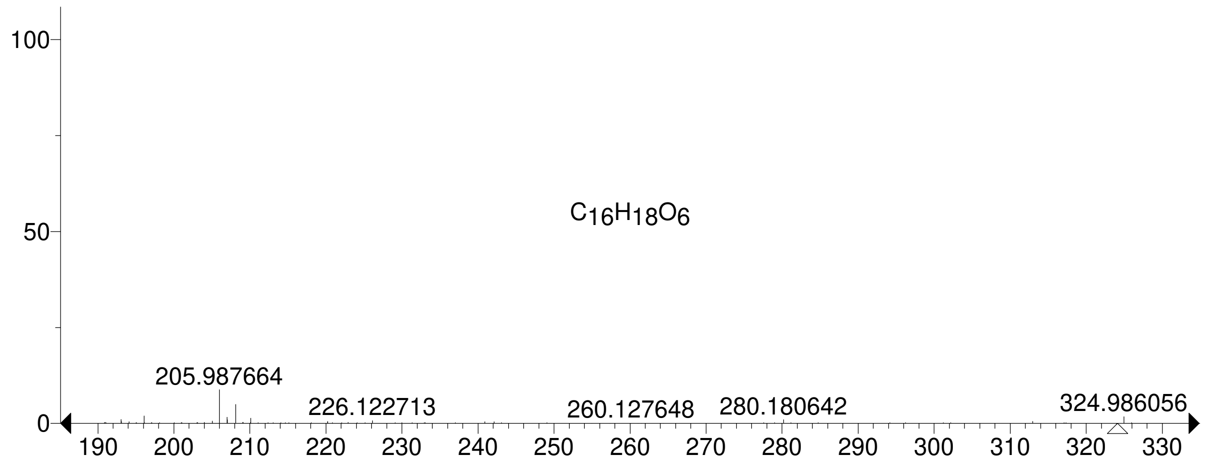

Unknown: Unknown (7.94_406.1831m/z)
Compound in Library Factor = 0

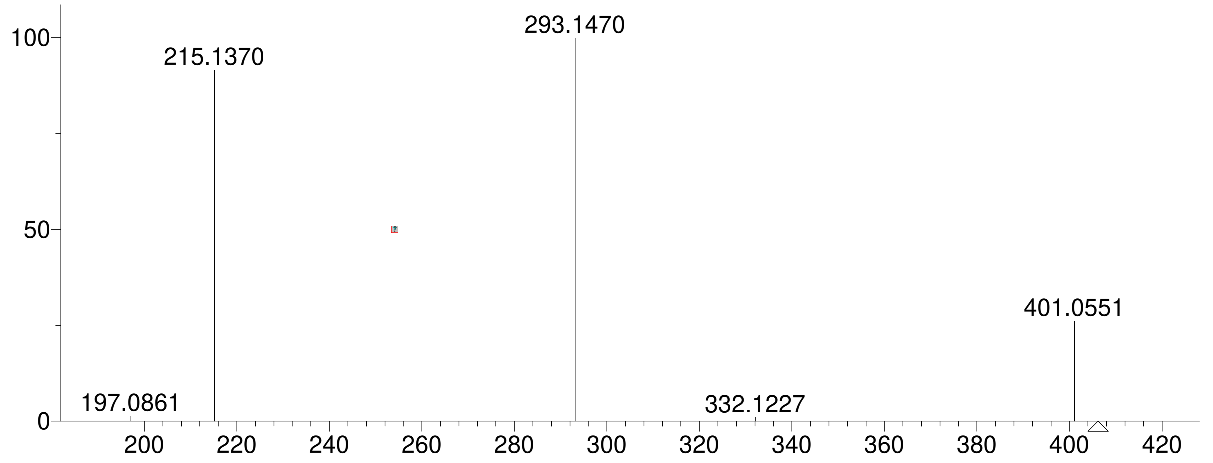

Hit 1 : 2-[(8S,9R)-9-acetyloxy-2-oxo-8,9-dihydrofuro[2,3-h]chromen-8-yl]propan-2-yl 3-methylbutanoate [M+NH4]
C21H24O7; MF: 0; RMF: 3; Prob 33.3%; Lib: mona-export-vf-npl_qexactive; ID: 9901.

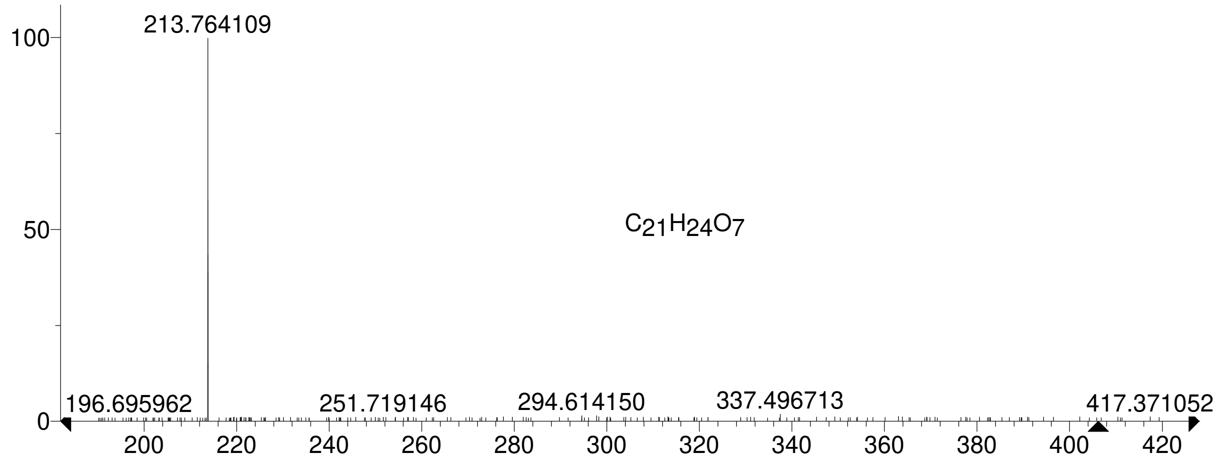

Hit 2 : NCGC00384593-01![3-hydroxy-3-methyl-1-(7-oxofuro[3,2-g]chromen-9-yl)oxybutan-2-yl] 3-methylbutanoat
C21H24O7; MF: 0; RMF: 2; Prob 33.3%; Lib: mona-export-gnps; ID: 9744.

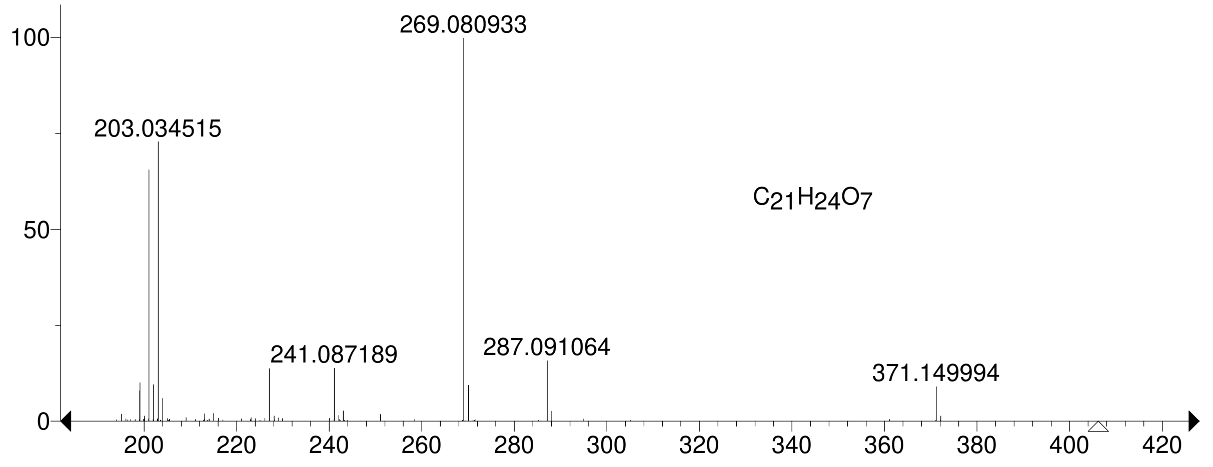

Hit 3 : Nebivolol (Bystolic) [M+H]+ P=406.2
C22H26ClF2NO4; MF: 0; RMF: 1; Prob 33.3%; Lib: mona-export-gnps; ID: 512.

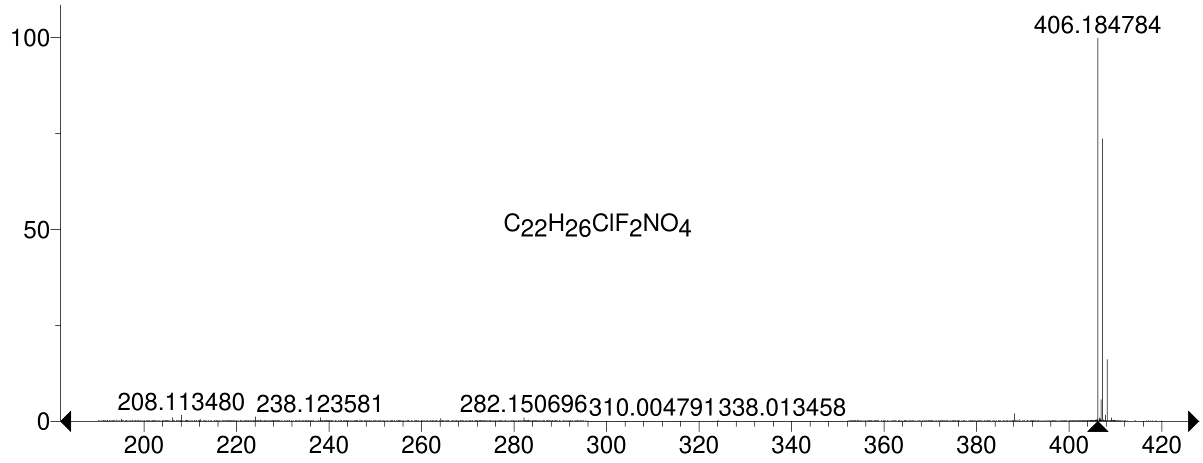

Unknown: Unknown (7.94_463.2065m/z)
Compound in Library Factor = 0

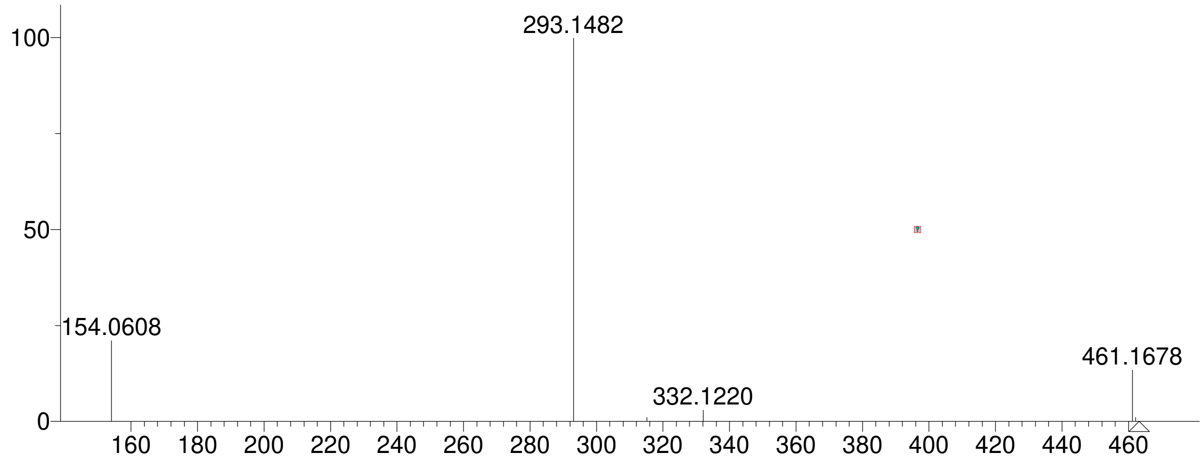

Unknown: Unknown (7.95_289.0371m/z)
Compound in Library Factor = -2176

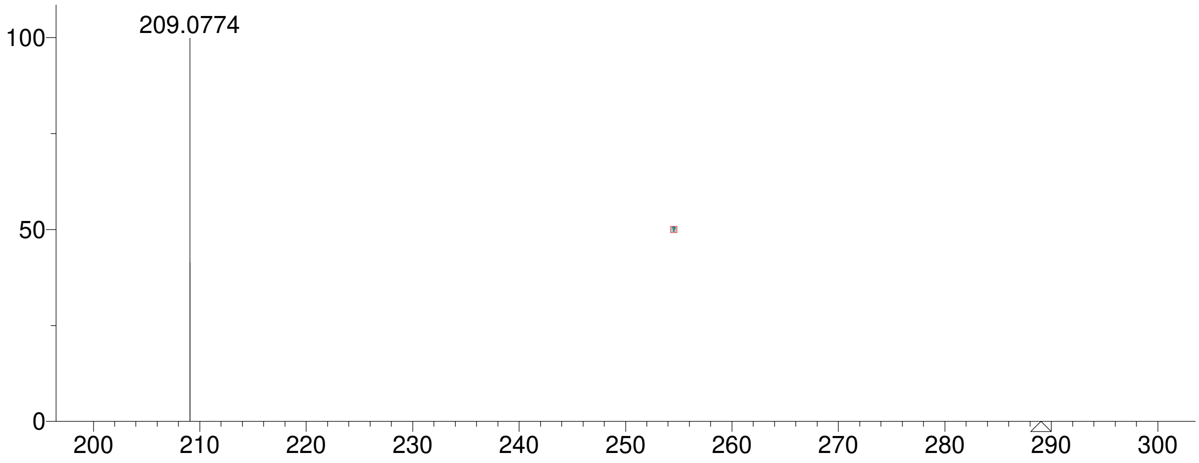

Hit 1 : Unknown (carbon number 11); PlaSMA ID-529 [M-H]- P=289
C11H14O7S; MF: 27; RMF: 445; Prob 77.3%; Lib: biomsms-neg-plasma; ID: 1163.

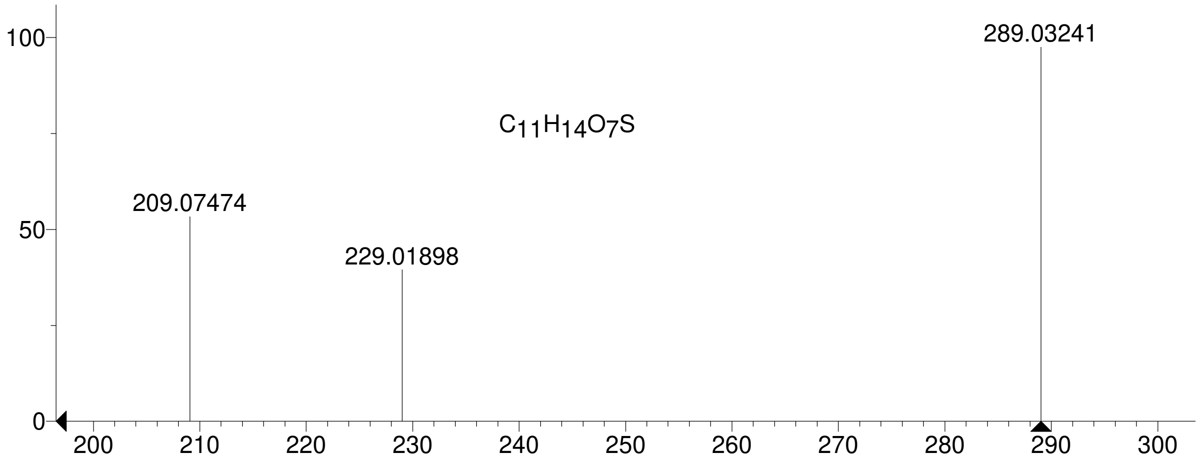

Hit 2 : 13-methoxy-1,6-diazatetracyclo[7.6.1.0a^*u,A^'1a^!P.0A^'1a^'o,A^'1a^*u]hexadeca-3,5,7,9(16),10,12,14-he
C15H10N2O2; MF: 0; RMF: 15; Prob 22.7%; Lib: mona-export-vf-npl_qexactive; ID: 23654.

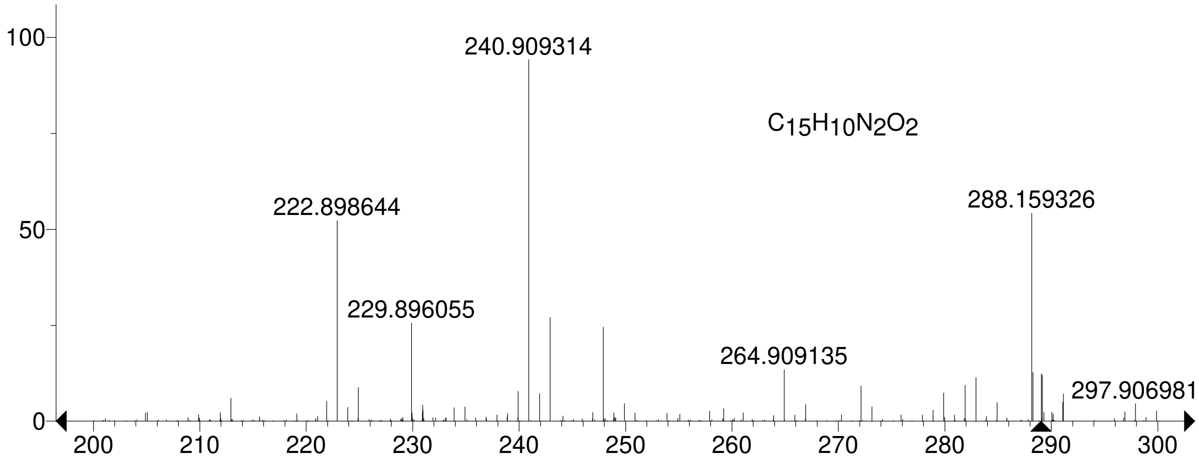

Unknown: Unknown (7.95_401.0522m/z)
Compound in Library Factor = 0

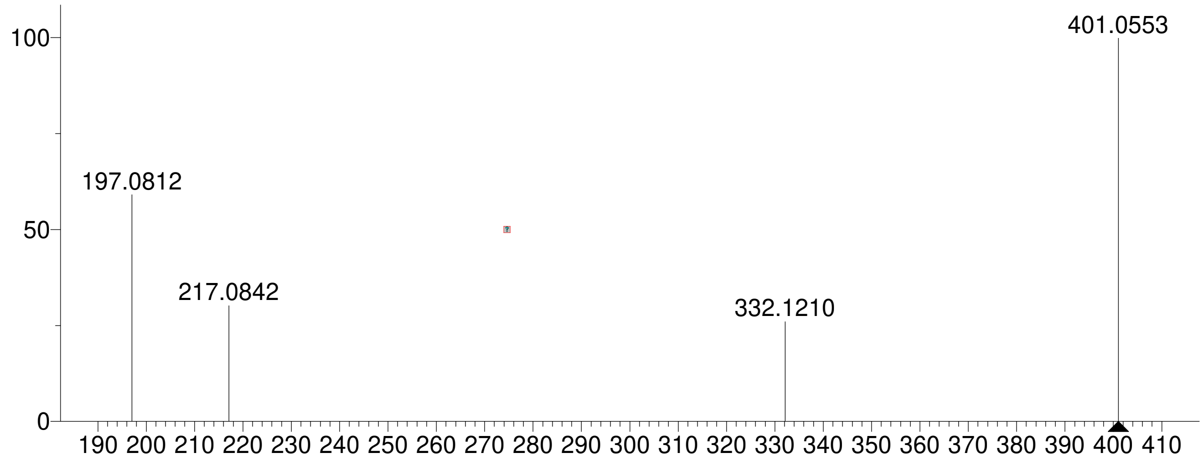

Unknown: Unknown (7.97_423.1748m/z)
Compound in Library Factor = 0

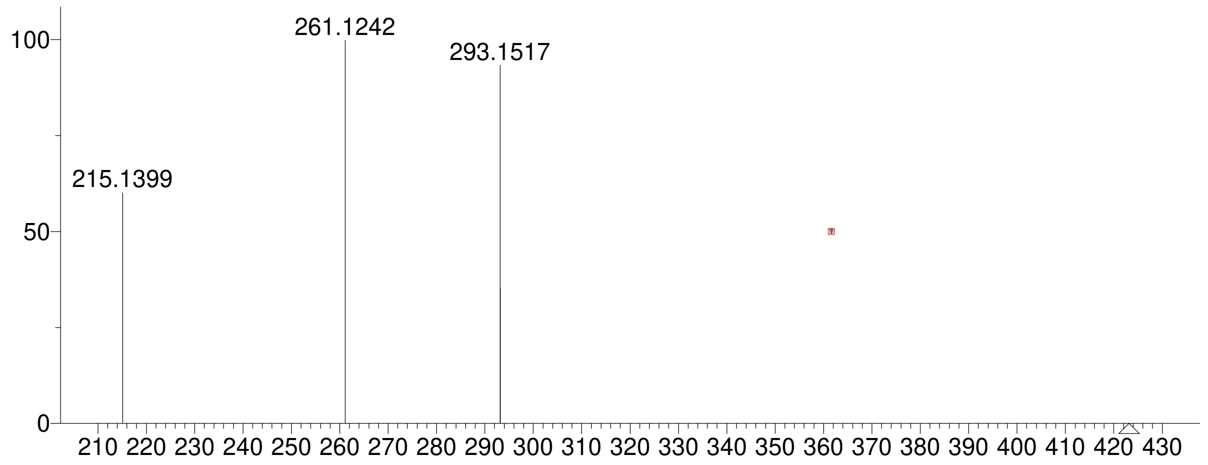

Unknown: Unknown (7.98_468.1470m/z)
Compound in Library Factor = -3273

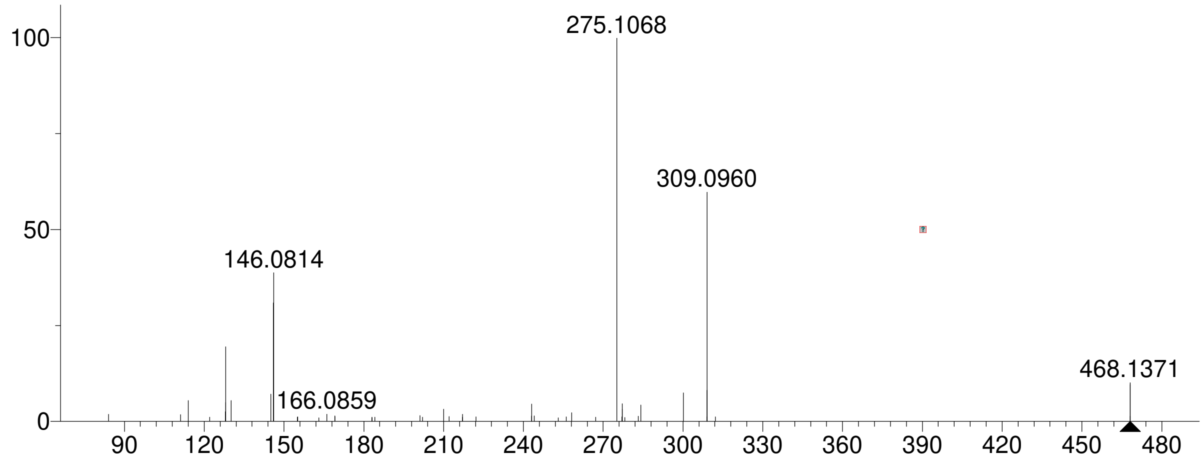

Hit 1 : NCGC00380387-01_C21H27NO11_ [M-H]- P=468.2
C21H27NO11; MF: 1; RMF: 126; Prob 25.7%; Lib: msms-neg-gnps; ID: 1703.

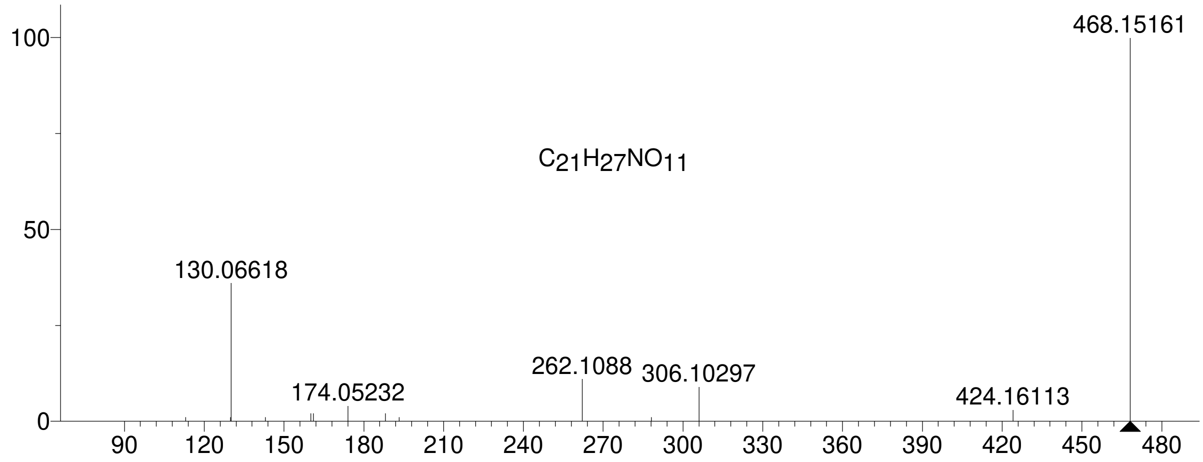

Hit 2 : 5'-O-b-D dihydroascorbigen (CCR vessel) [M-H]- P=468.2
C21H27NO11; MF: 0; RMF: 77; Prob 24.8%; Lib: phytofrag2018_final_060219; ID: 941.

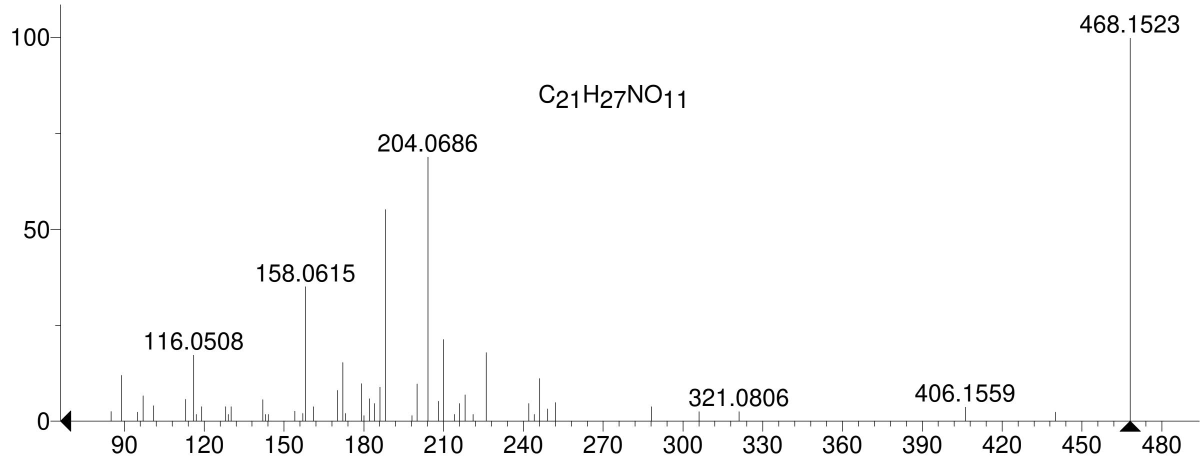

Hit 3 : 2-[1-[4,5-dihydroxy-3-[(2S,3R,4S,5S,6R)-3,4,5-trihydroxy-6-(hydroxymethyl)oxan-2-yl]oxyoxan-2-yl]indol-3-y
C21H27NO11; MF: 0; RMF: 13; Prob 24.8%; Lib: mona-export-lc-ms-ms_negative_mode; ID: 22556.

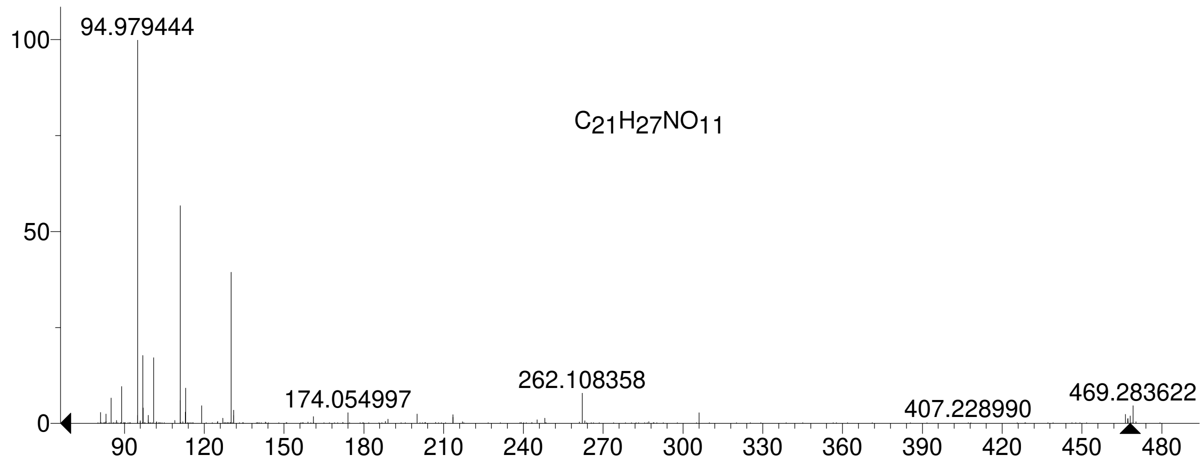

Hit 4 : astilbin [M+NH4]+ 45HCD P=468.2
C21H22O11; MF: 0; RMF: 7; Prob 24.8%; Lib: anthocyanins mona; ID: 5106.

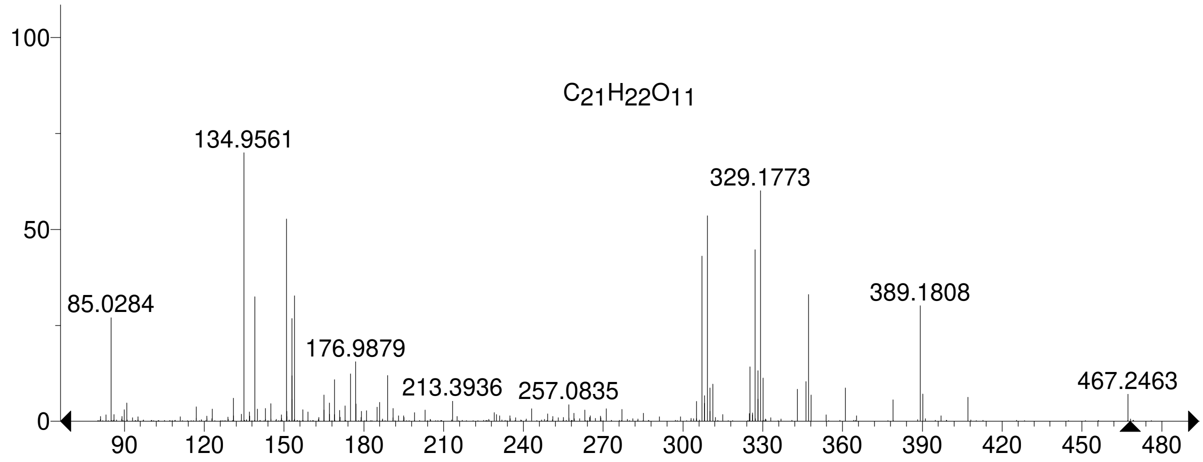

Unknown: Unknown (7.98_714.3311m/z)
Compound in Library Factor = 0

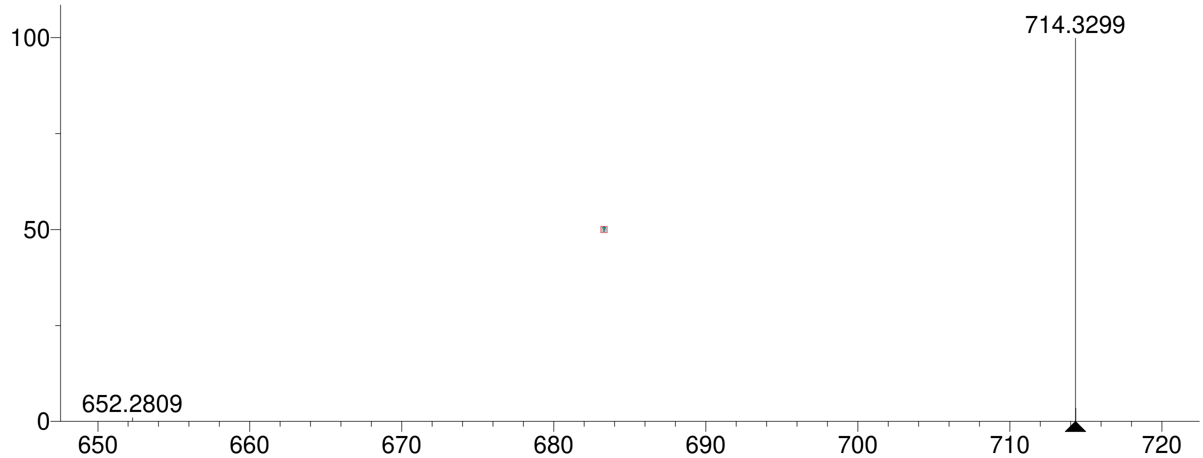

Unknown: Unknown (7.99_387.1576m/z)
Compound in Library Factor = 0

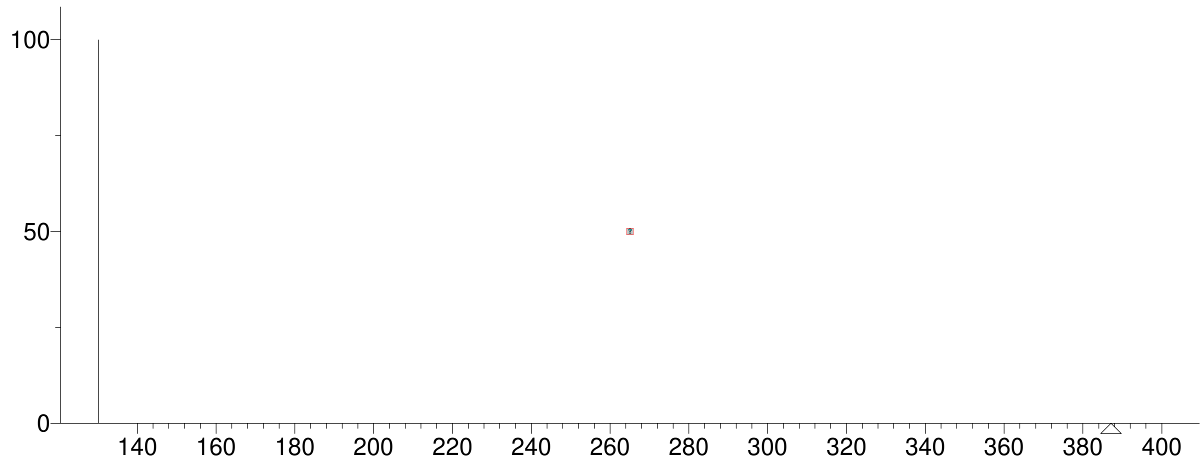

Unknown: Unknown (8.02_405.2221m/z)
Compound in Library Factor = 0

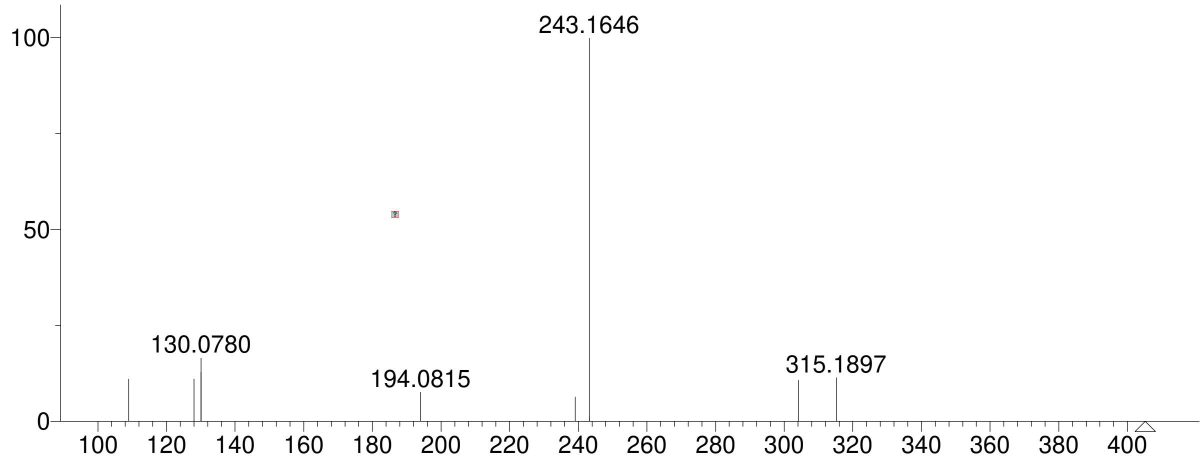

Hit 1 : MLS002637530-01!Cortell50-03-3 [M+H]+ P=405.2
C23H32O6; MF: 0; RMF: 57; Prob 50.0%; Lib: mona-export-gnps; ID: 3679.

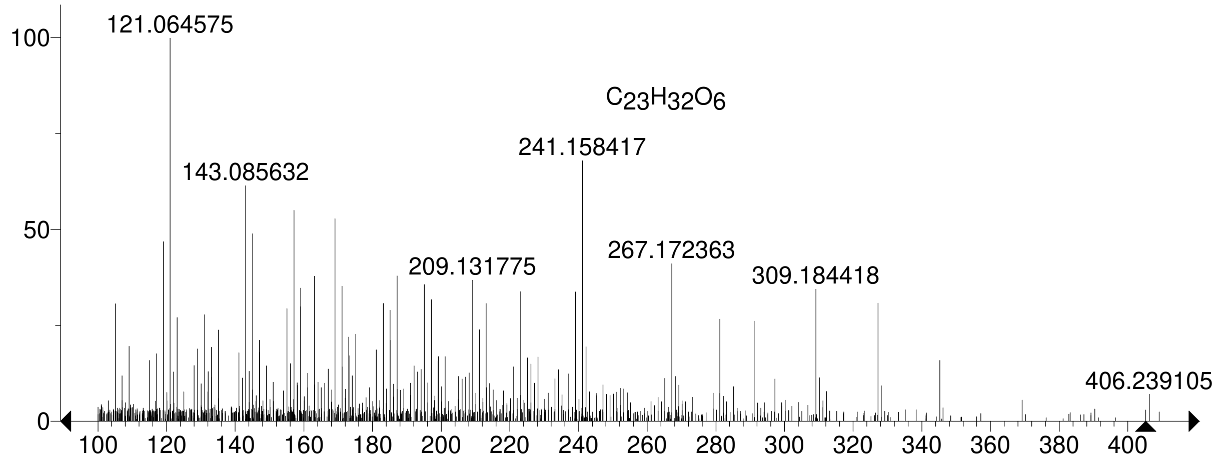

Hit 2 : MLS001076491-01!Cortell [M+H]+ P=405.2
C23H32O6; MF: 0; RMF: 48; Prob 50.0%; Lib: mona-export-gnps; ID: 4335.

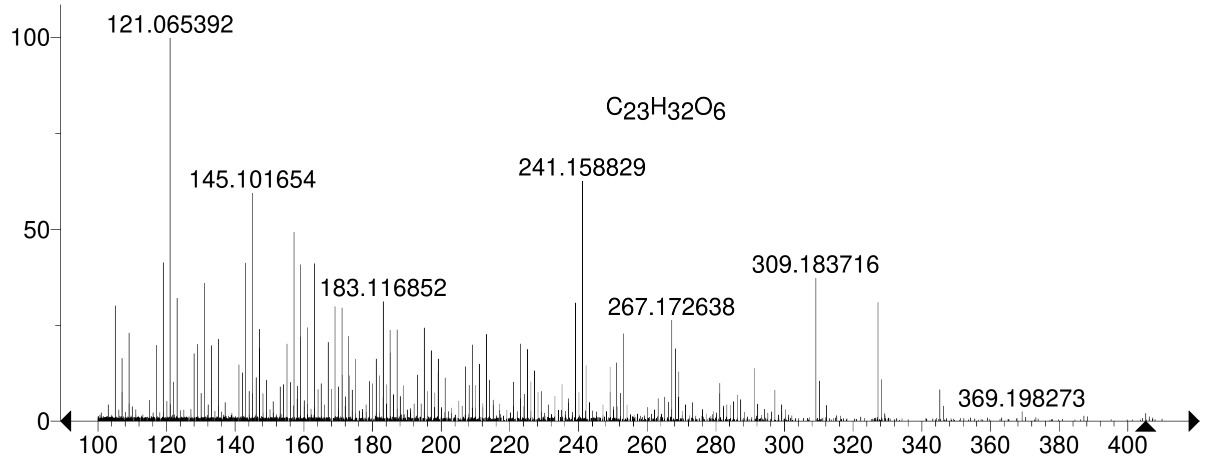

Unknown: Unknown (8.04_549.2256m/z)
Compound in Library Factor = 0

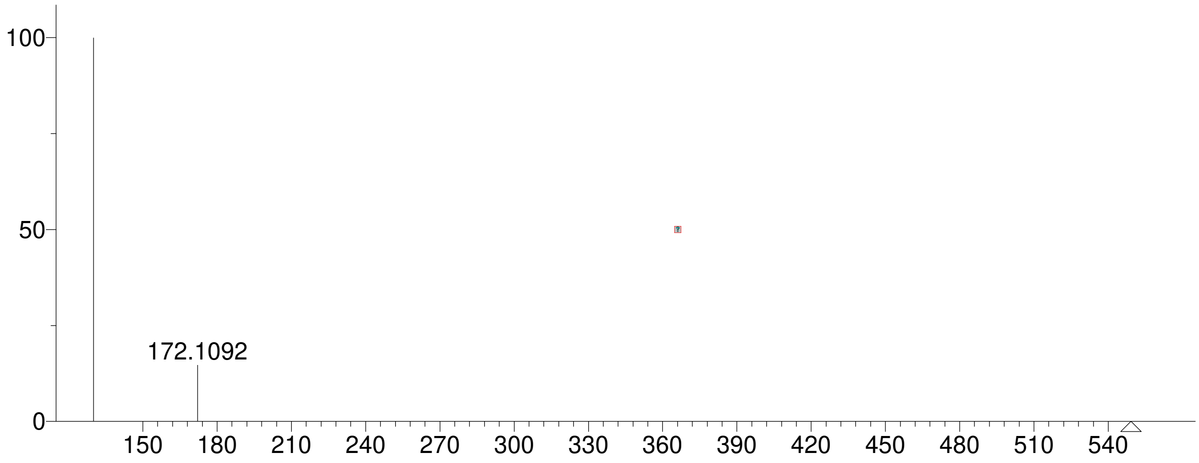

** Search Report Page 1 of 1 **

Unknown: Unknown (8.05_656.2835m/z)
Compound in Library Factor = 0

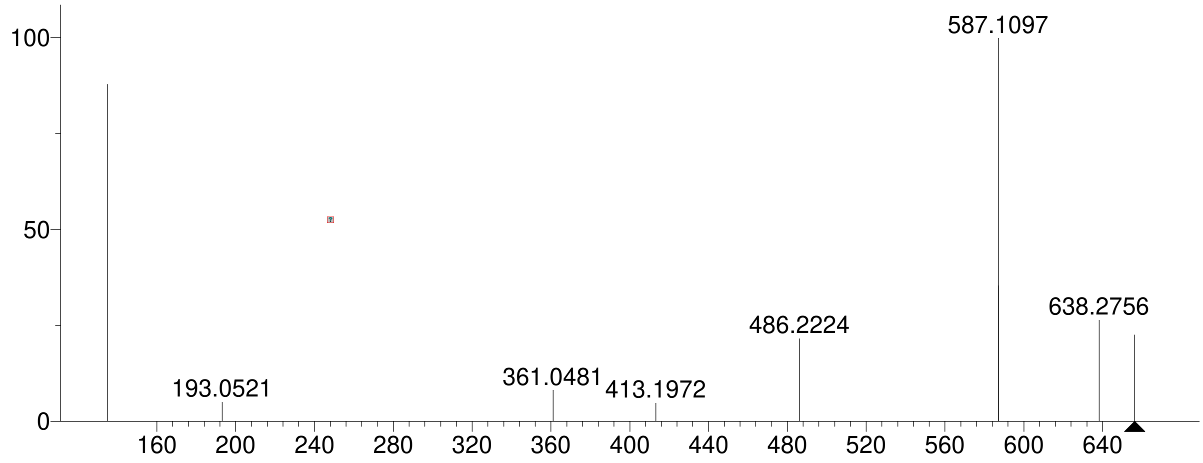

** Search Report Page 1 of 1 **

Unknown: Unknown (8.05_757.1726m/z)
Compound in Library Factor = 0

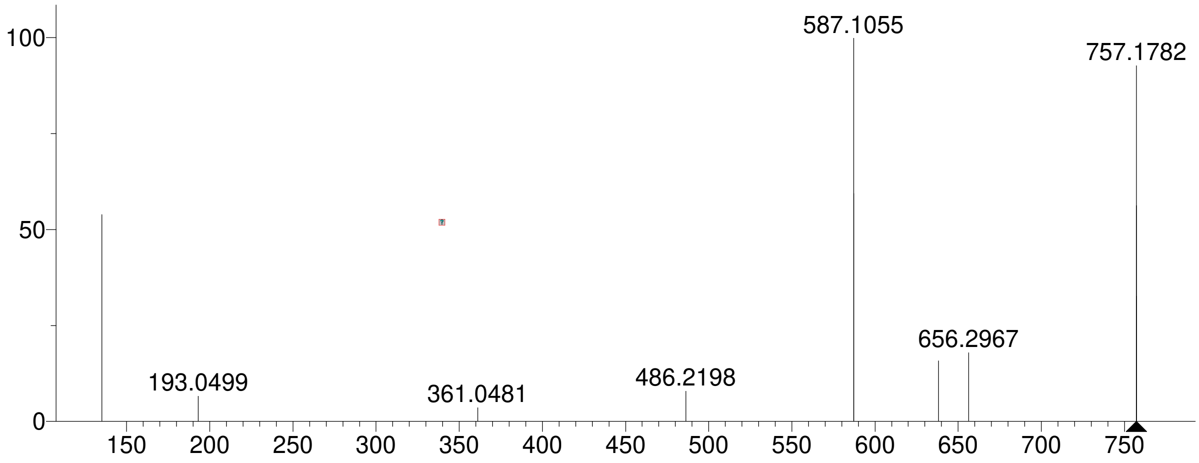

** Search Report Page 1 of 1 **

Unknown: Unknown (8.06_387.0845m/z)
Compound in Library Factor = -3273

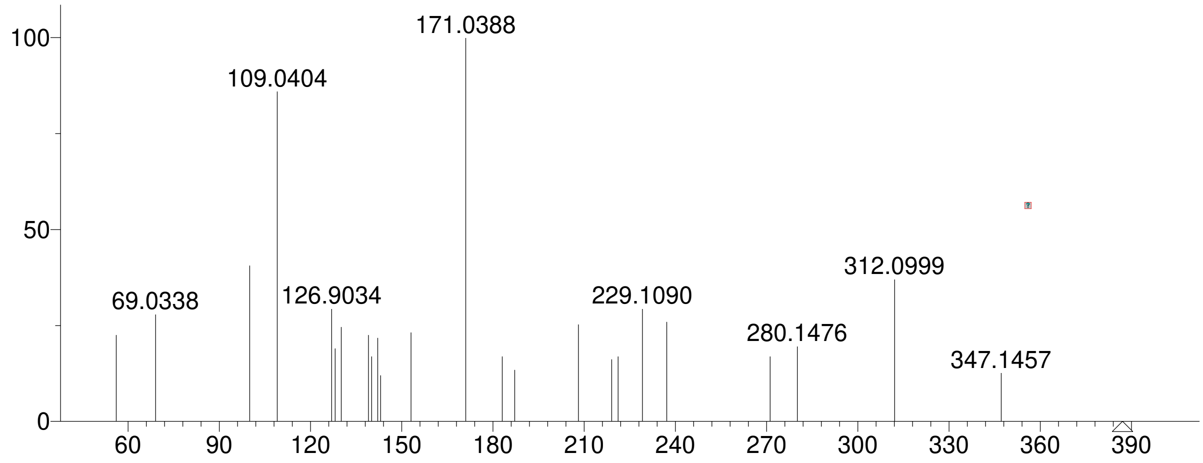

Hit 1 : Pamoic acid [M-H]- HCD 61V P=387.1
C23H16O6; MF: 12; RMF: 151; Prob 100.0%; CAS: 130-85-8; Lib: nist_msms; ID: 152472.

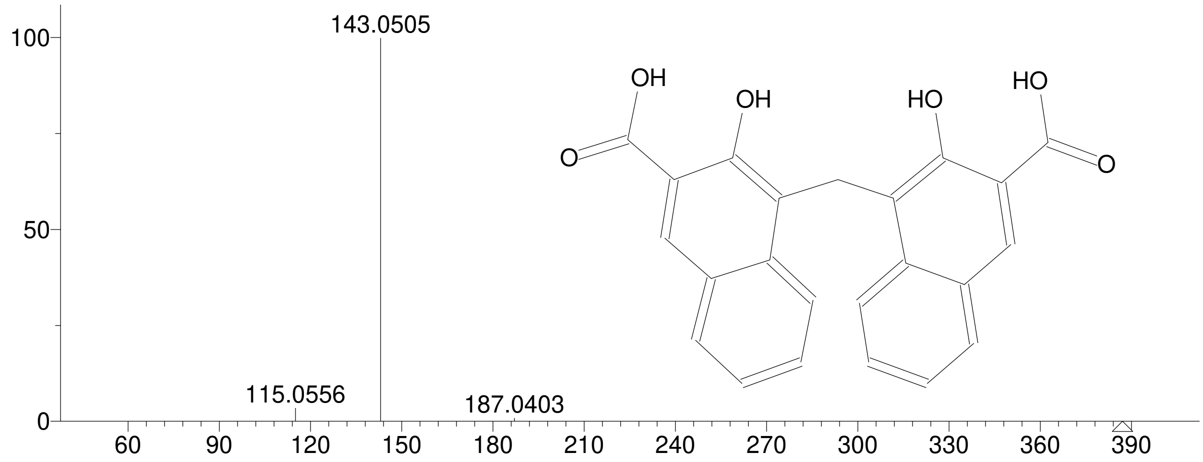

Unknown: Unknown (8.07_253.1184m/z)
Compound in Library Factor = -3273

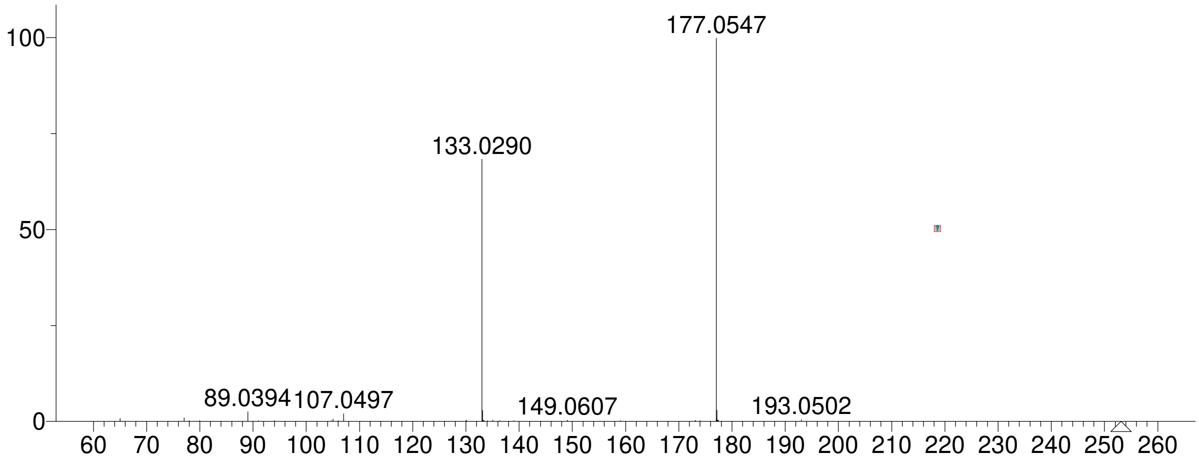

Hit 1 : Tyr-Ala [M+H]+ HCD 80eV P=253.1
C12H16N2O4; MF: 12; RMF: 71; Prob 16.5%; CAS: 730-08-5; Lib: nist_msms; ID: 564073.

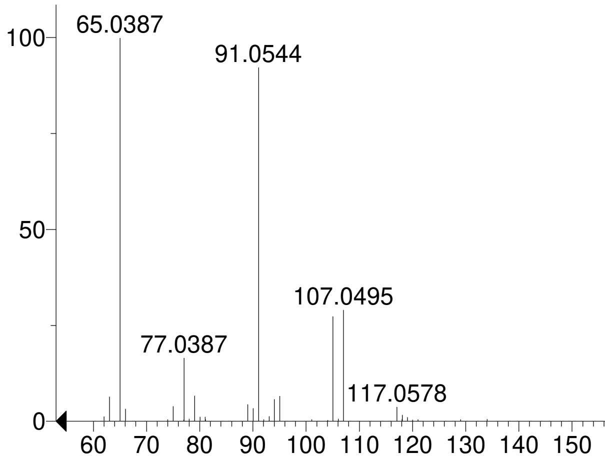

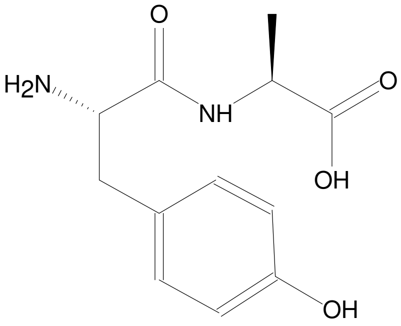

Hit 2 : Ala-Tyr [M+H]+ HCD 80eV P=253.1
C12H16N2O4; MF: 11; RMF: 67; Prob 15.8%; CAS: 3061-88-9; Lib: nist_msms; ID: 562749.

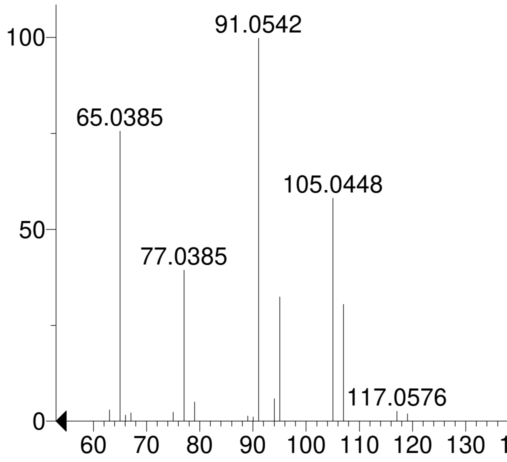

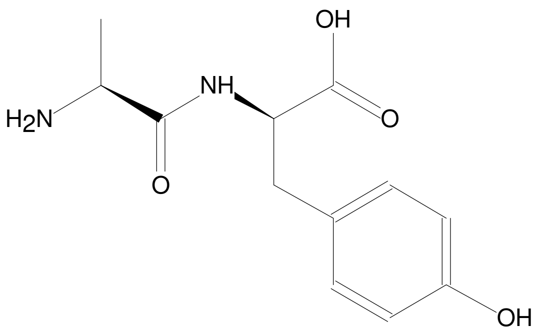

Hit 3 : Phe-Ser [M+H]+ HCD 80eV P=253.1
C12H16N2O4; MF: 9; RMF: 43; Prob 14.6%; Lib: nist_msms; ID: 562028.

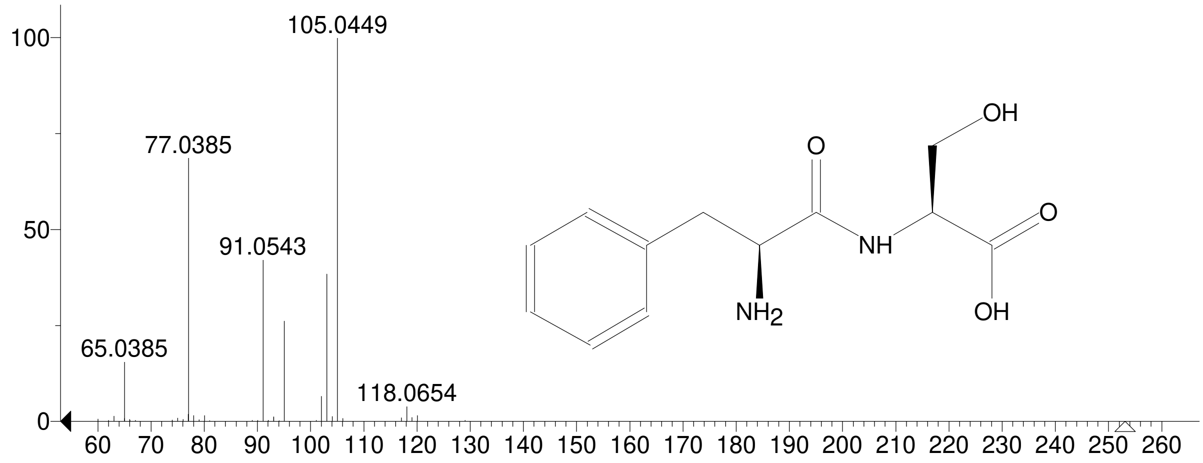

Hit 4 : Phe-Ser [M+H]+ HCD 80eV P=253.1
C12H16N2O4; MF: 9; RMF: 43; Prob 14.6%; CAS: 16053-39-7; Lib: nist_msms2; ID: 41828.

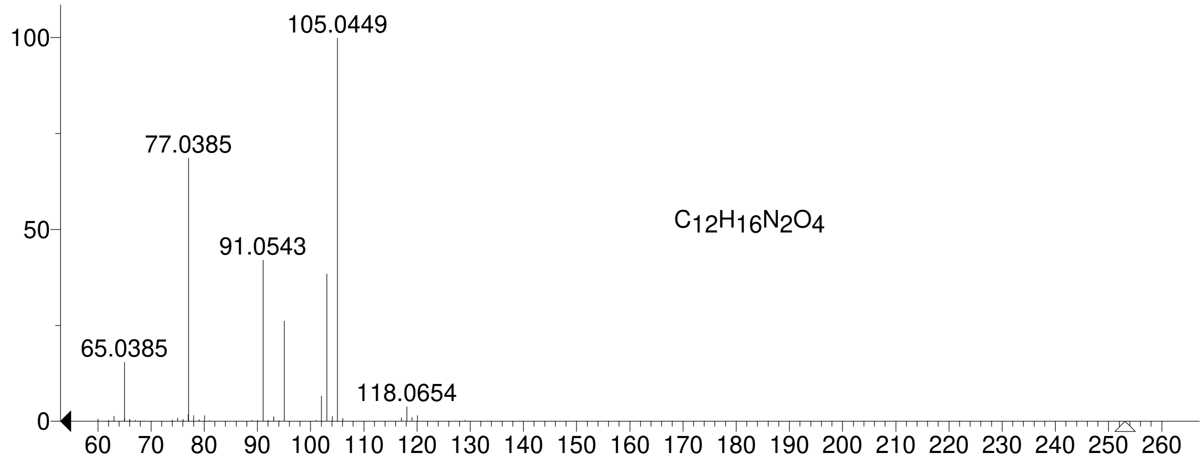

Hit 5 : Ser-Phe [M+H]+ HCD 80eV P=253.1
C12H16N2O4; MF: 8; RMF: 39; Prob 14.0%; Lib: nist_msms; ID: 571676.

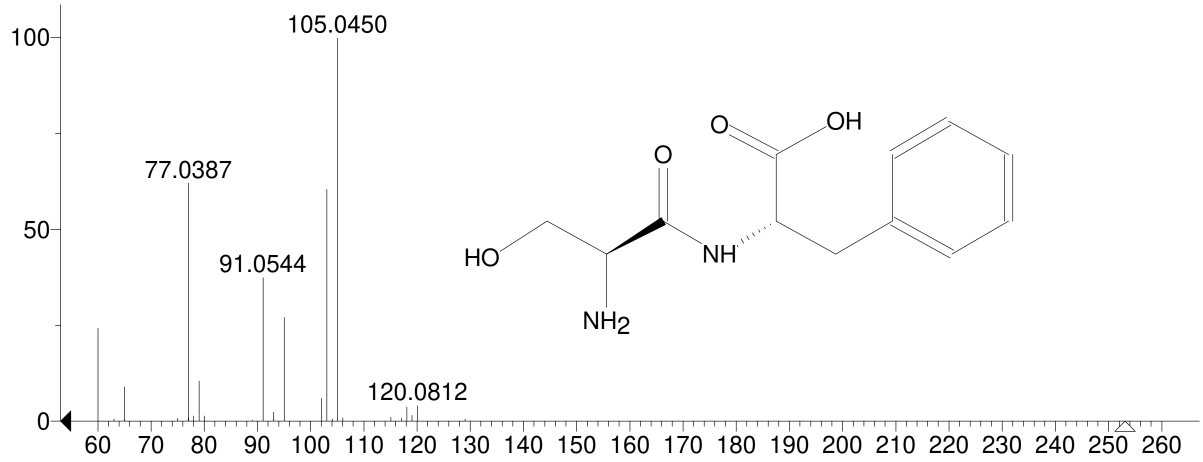

** Search Report Page 1 of 1 **

Unknown: Unknown (8.07_656.2897m/z)
Compound in Library Factor = 0

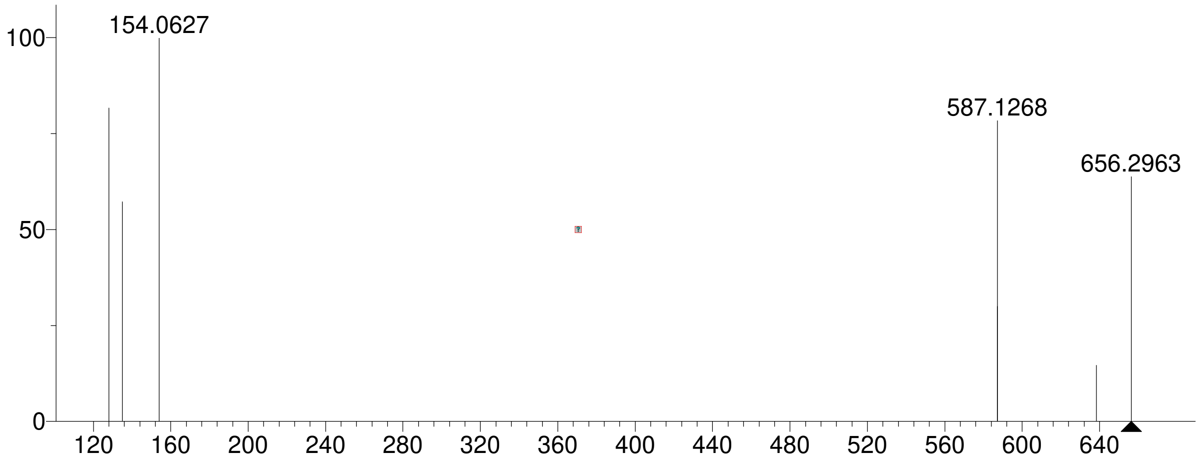

Unknown: Unknown (8.09_302.1024m/z)
Compound in Library Factor = 0

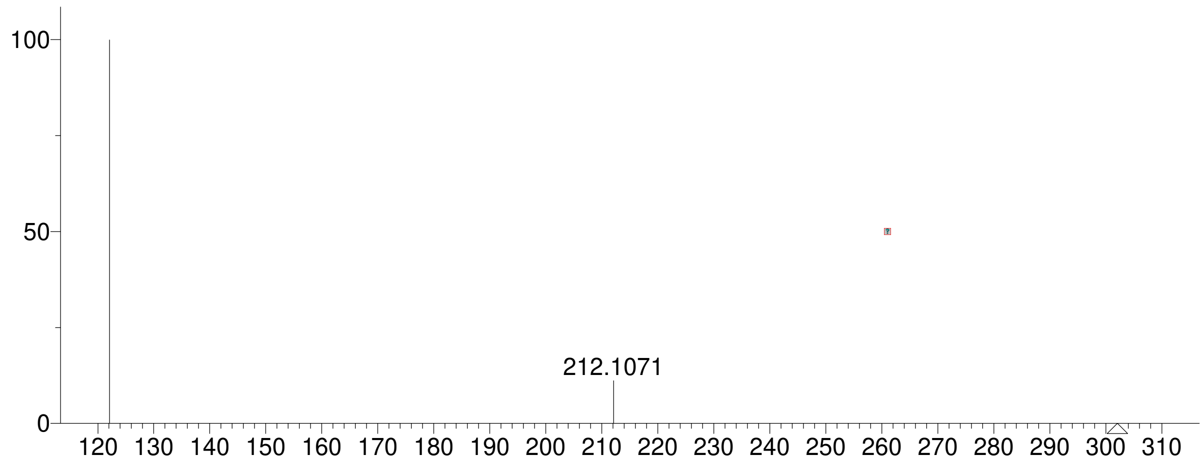

Hit 1 : Petunidin 3-glucoside cation [Cat]+=>317.1=>302.1 IT 35% P=302.1
C22H23O12; MF: 0; RMF: 67; Prob 8.33%; CAS: 71991-88-3; Lib: nist_msms; ID: 394992.

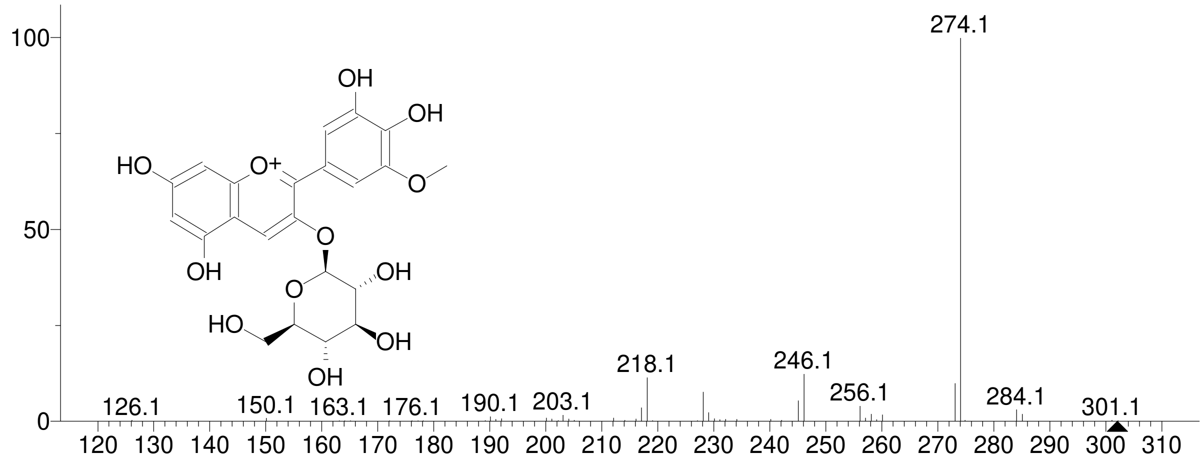

Hit 2 : Quinapril [M+Na]+=>302.1 IT 35% P=302.1
C25H30N2O5; MF: 0; RMF: 60; Prob 8.33%; CAS: 85441-61-8; Lib: nist_msms; ID: 127674.

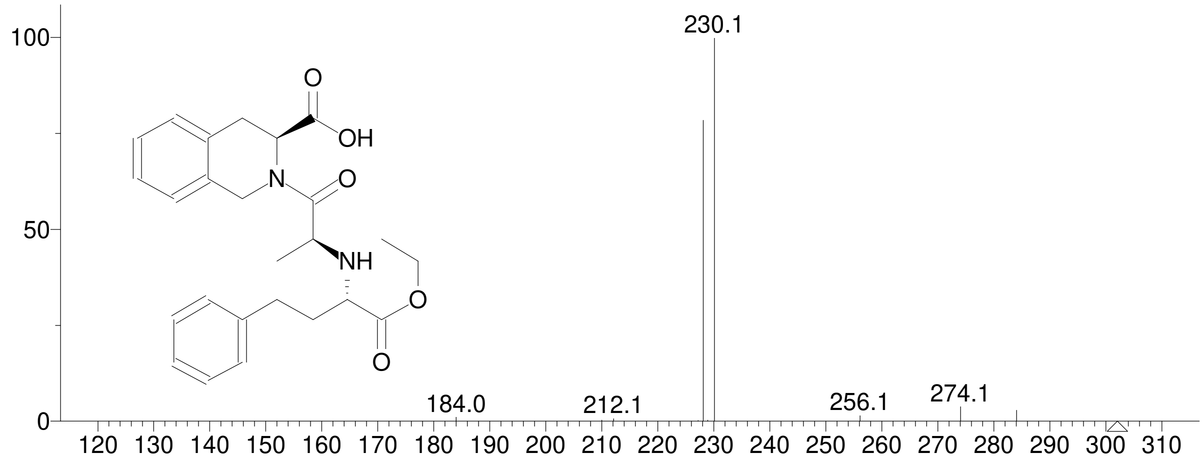

Hit 3 : Trandolapril [M+Na]+=>302.1 IT 35% P=302.1
C24H34N2O5; MF: 0; RMF: 50; Prob 8.33%; CAS: 87679-37-6; Lib: nist_msms; ID: 116294.

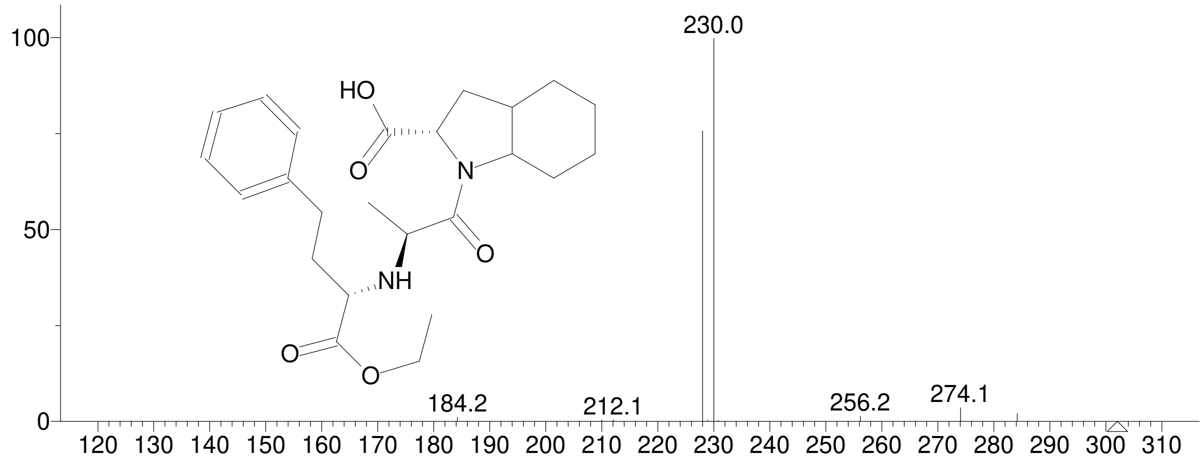

Hit 4 : Dipyridamole [M+H]+=>385.3=>302.1 IT 35% P=302.1
C24H40N8O4; MF: 0; RMF: 34; Prob 8.33%; CAS: 58-32-2; Lib: nist_msms; ID: 314331.

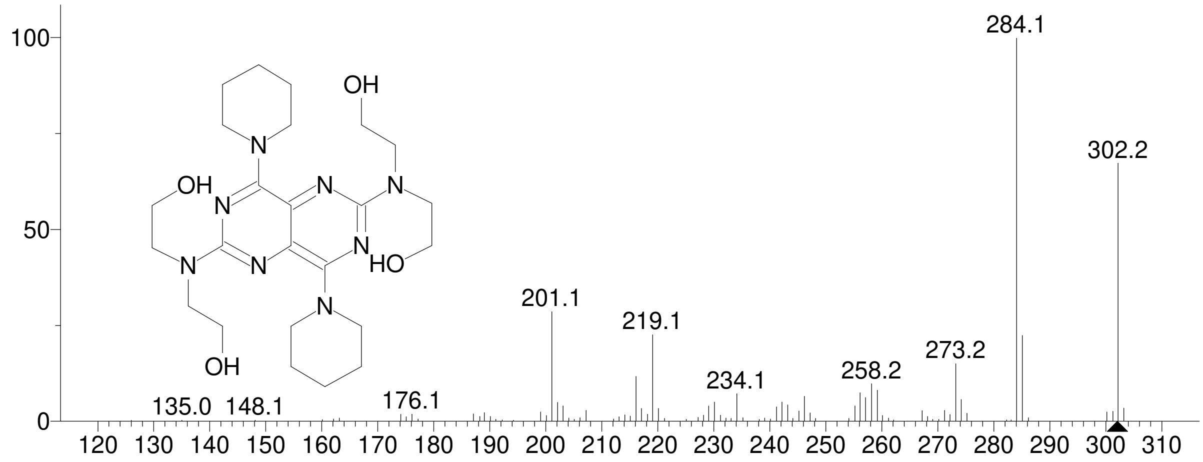

Hit 5 : Norfloxacin [M+H]+=>302.1 IT 35% P=302.1
C16H18FN3O3; MF: 0; RMF: 32; Prob 8.33%; CAS: 70458-96-7; Lib: nist_msms; ID: 282030.

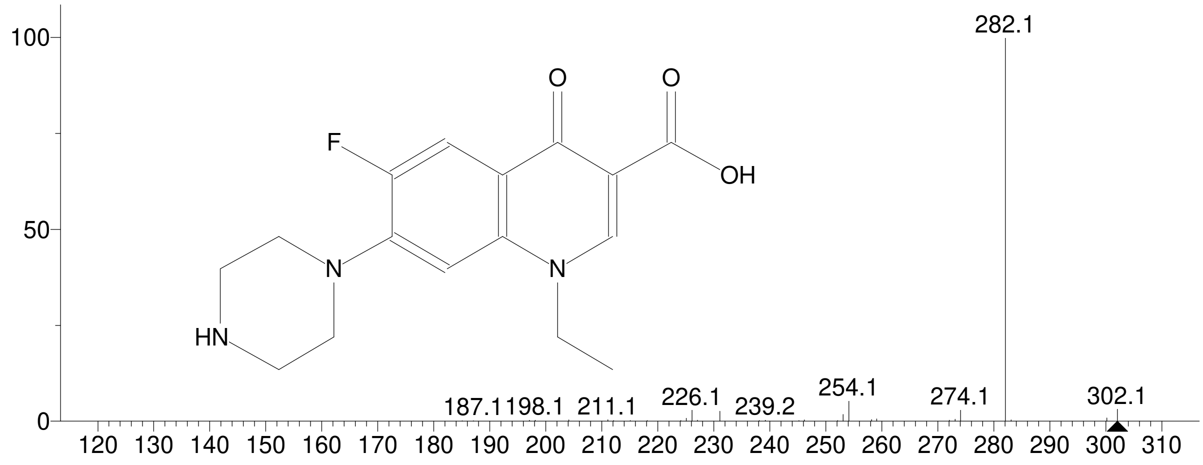

Unknown: Unknown (8.09_434.0519m/z)
Compound in Library Factor = 0

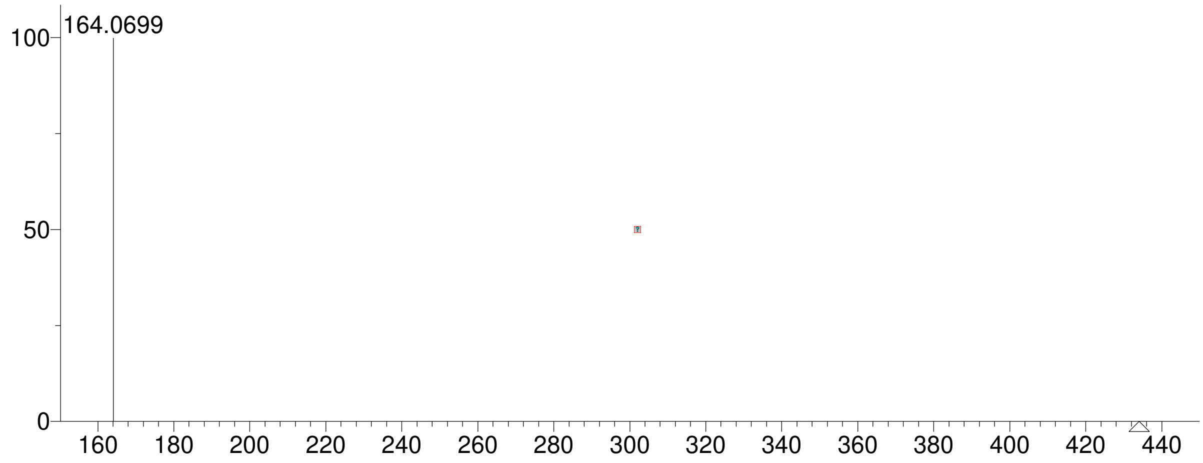

Unknown: Unknown (8.10_258.1124m/z)
Compound in Library Factor = -2387

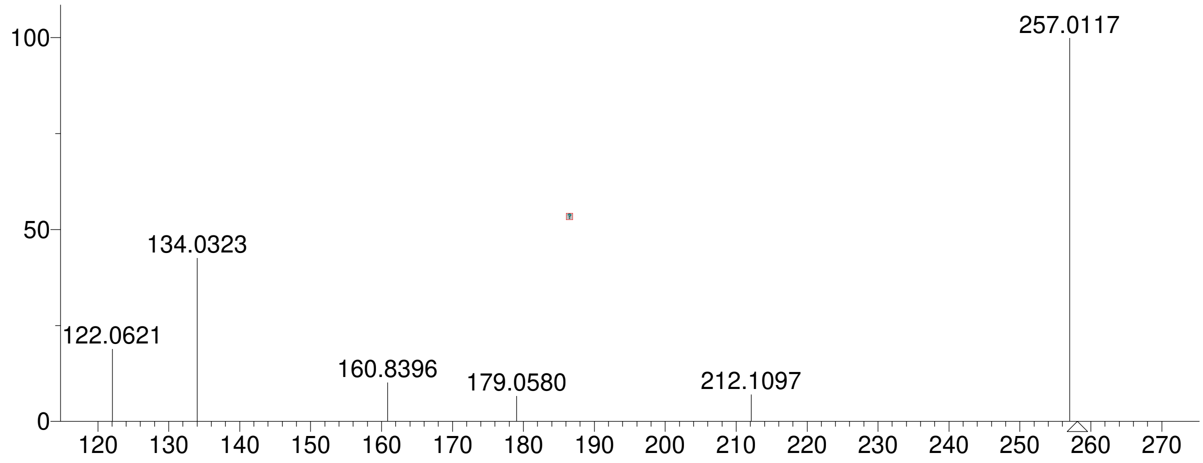

Hit 1 : Tolmetin [M+H]+ IT-FT 35% P=258.1
C15H15NO3; MF: 17; RMF: 261; Prob 23.4%; CAS: 26171-23-3; Lib: nist_msms; ID: 135565.

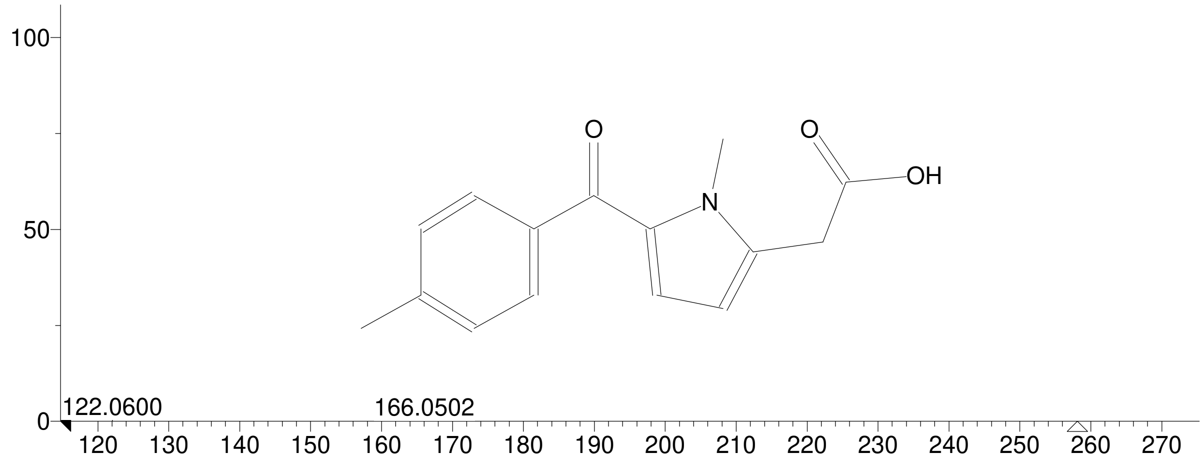

Hit 2 : NCGC00244969-01!4-[[2-[(E)-3-hydroxybut-1-enyl]-5-oxooxolan-3-yl]amino]benzaldehyde [M-H2O+H]+ P
C15H17NO4; MF: 0; RMF: 115; Prob 12.8%; Lib: mona-export-gnps; ID: 8338.

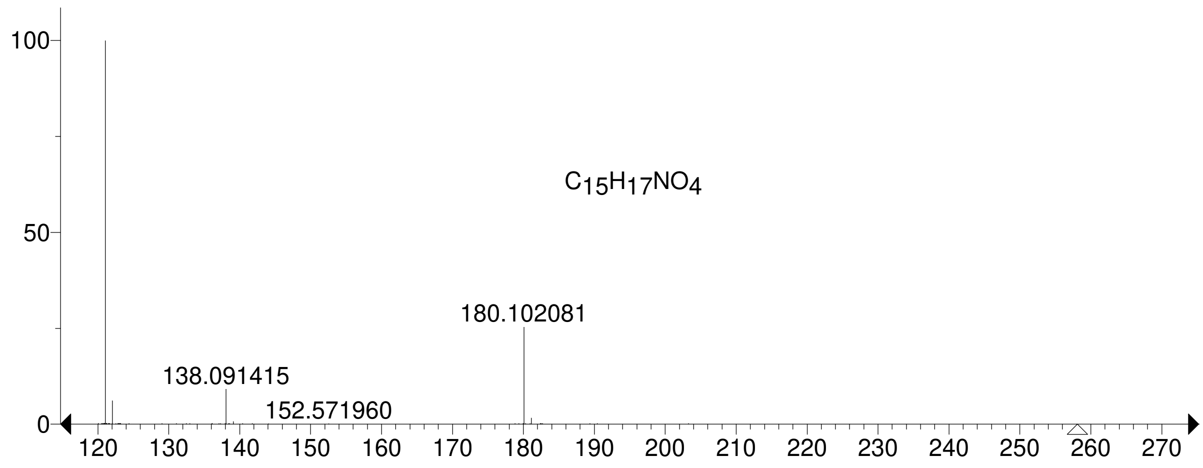

Hit 3 : NCGC00017285-06_C15H15NO3_9-Methoxy-2,2-dimethyl-2,6-dihydro-5H-pyrano[3,2-c]quinolin-5-one [M
C15H15NO3; MF: 0; RMF: 83; Prob 12.8%; Lib: mona-export-gnps; ID: 11787.

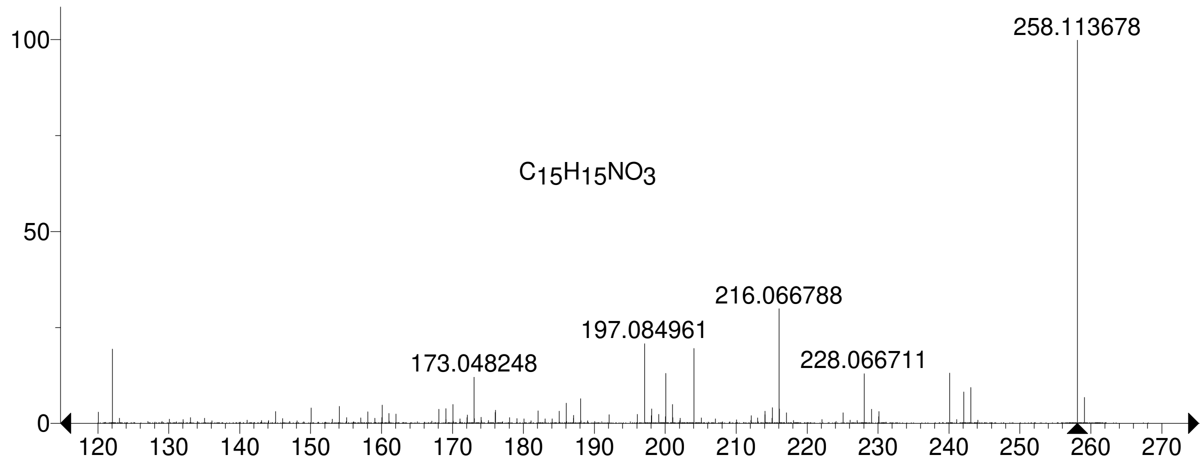

Hit 4 : 9-Methoxy-2,2-dimethyl-2,6-dihydro-pyrano[3,2-c]quinolin-5-one [M+H]+ 65HCD P=258.1
C15H15NO3; MF: 0; RMF: 77; Prob 12.8%; Lib: mona-export-vf-npl_qexactive; ID: 17121.

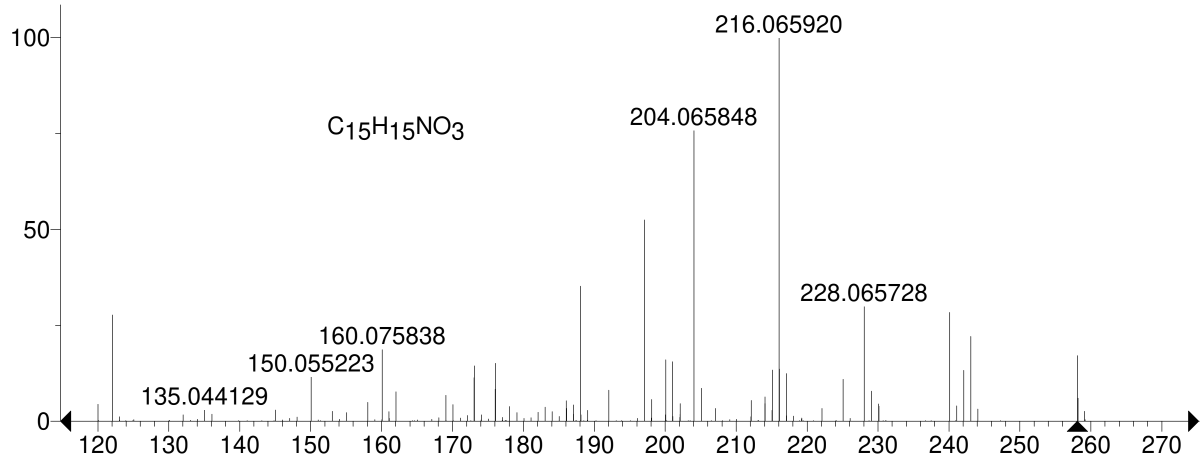

Hit 5 : NCGC00017285-04!HAPLAMINE [M+H]+ P=258.1
C15H15NO3; MF: 0; RMF: 60; Prob 12.8%; Lib: mona-export-gnps; ID: 1103.

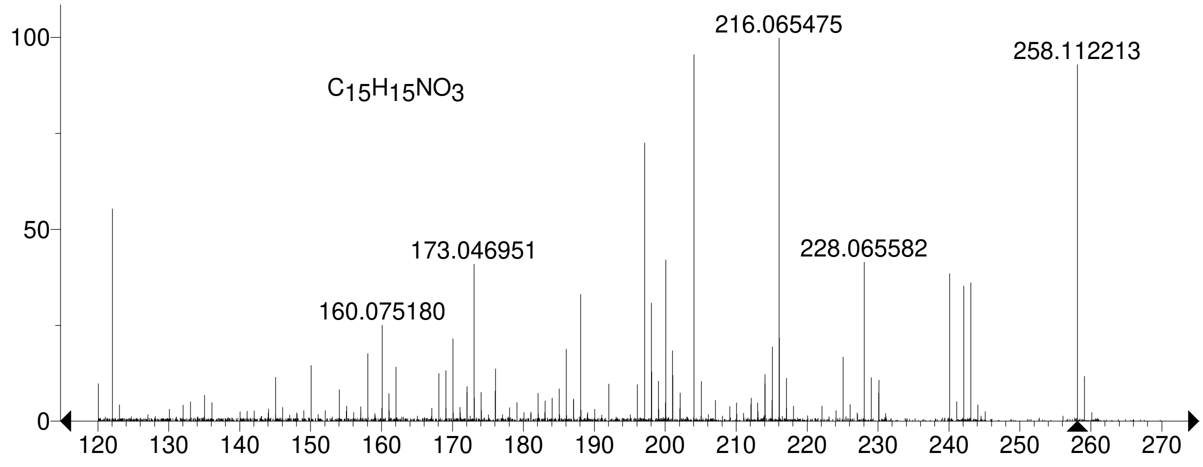

Unknown: Unknown (8.10_693.2246m/z)
Compound in Library Factor = 0

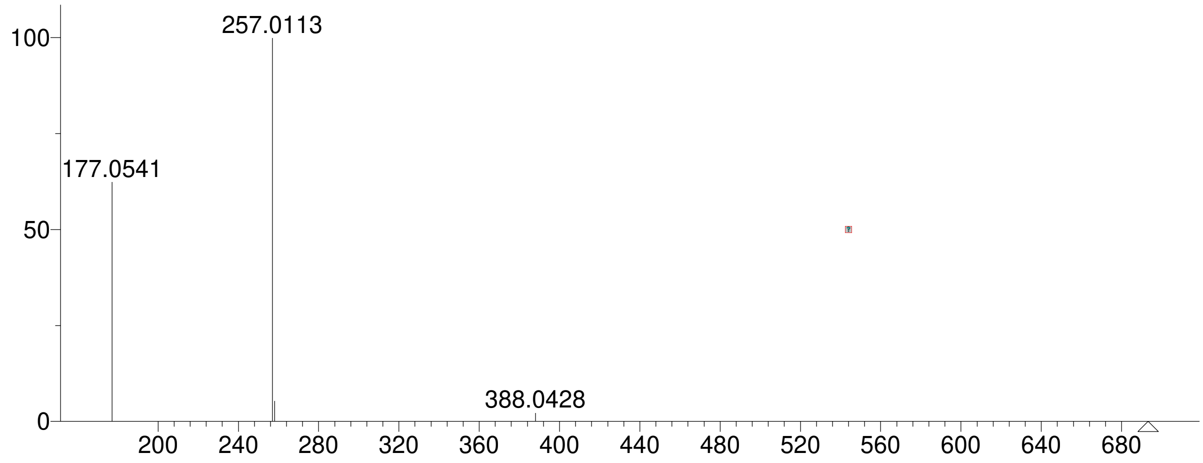

Unknown: Unknown (8.10_722.1788m/z)
Compound in Library Factor = 0

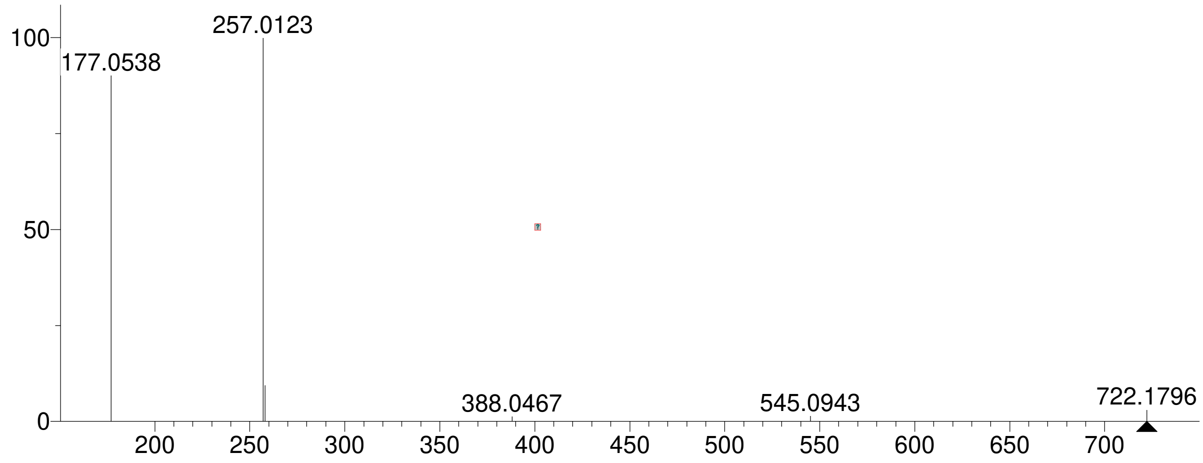

Unknown: Unknown (8.11_305.0689m/z)
Compound in Library Factor = -1142

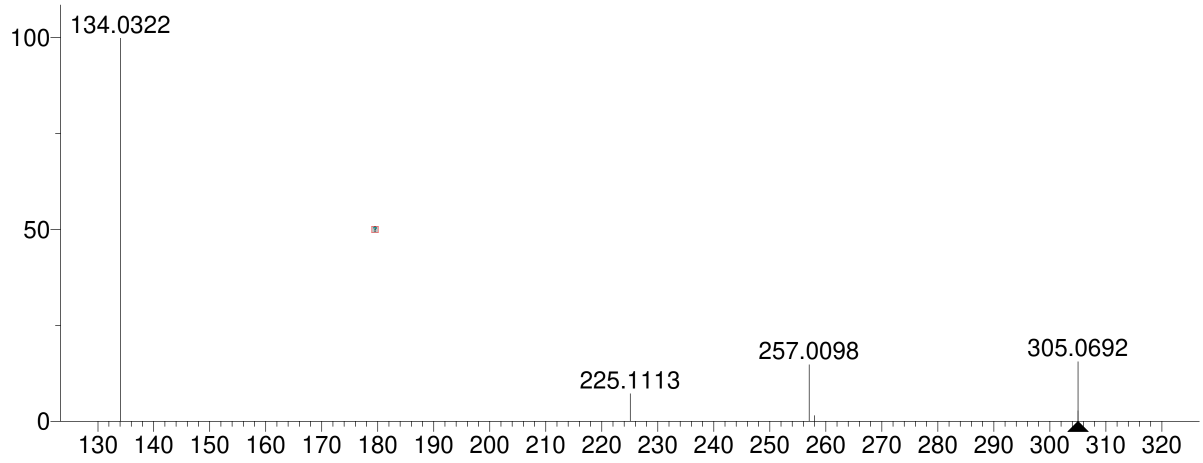

Hit 1 : 12-hydroxy Jasmonic acid sulfate () [M-H]- P=305.1
C12H18O7S; MF: 114; RMF: 429; Prob 65.2%; Lib: phytofrag2018_final_060219; ID: 8.

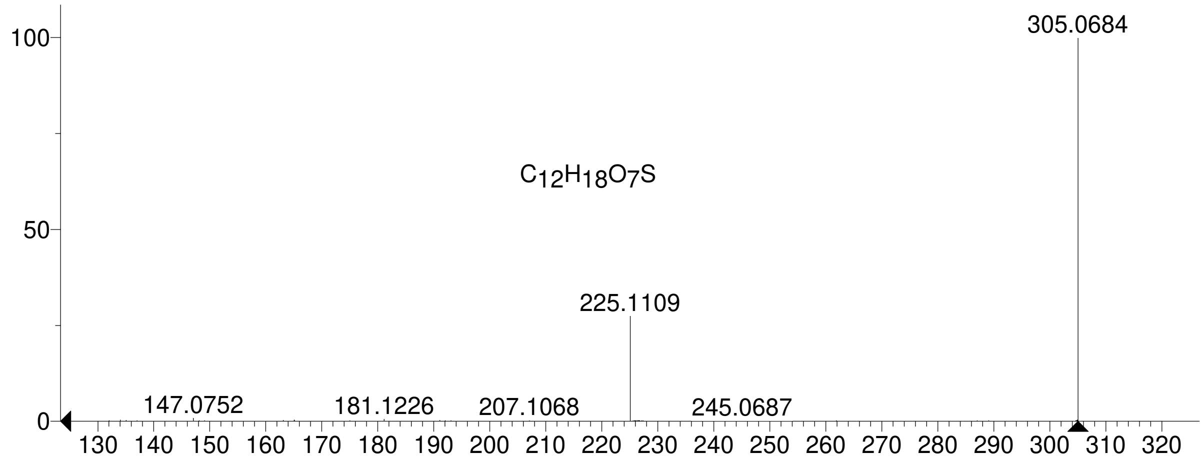

Hit 2 : Sulfo jasmonate; PlaSMA ID-611 [M-H]- P=305.1
C12H18O7S; MF: 77; RMF: 367; Prob 15.9%; Lib: biomsms-neg-plasma; ID: 293.

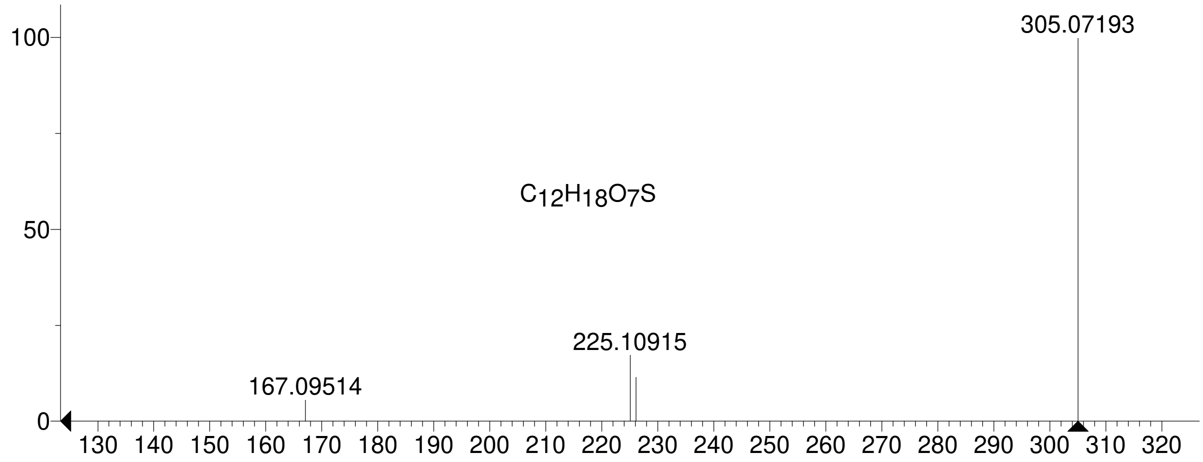

Hit 3 : Gallocatechin [M-H]- 45eV P=305.1
C15H14O7; MF: 17; RMF: 577; Prob 2.81%; Lib: msms-neg-metabobase; ID: 810.

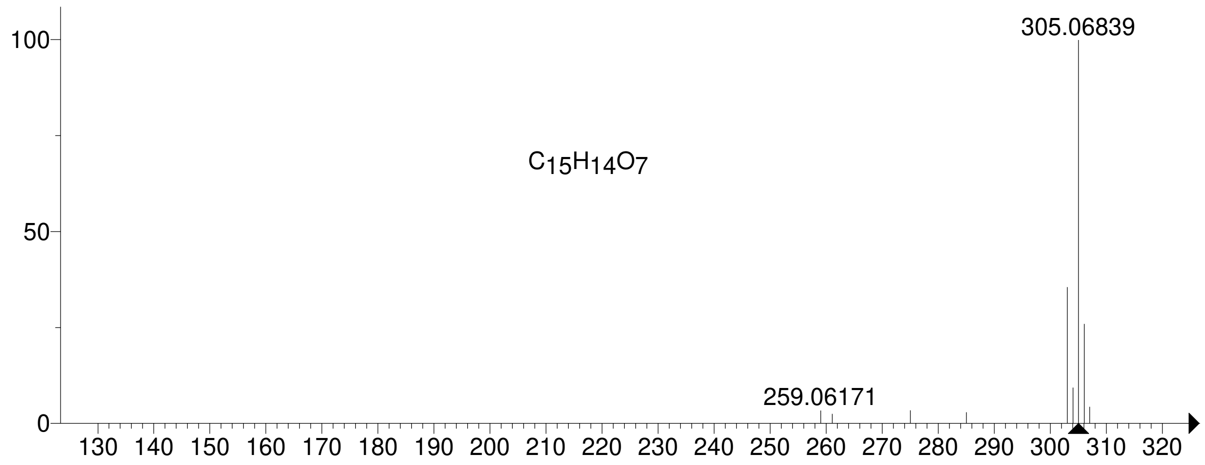

Hit 4 : Glutathione, oxidized [M-2H]2- HCD 8V P=305.1
C20H32N6O12S2; MF: 13; RMF: 307; Prob 2.38%; CAS: 27025-41-8; Lib: nist_msms; ID: 185529.

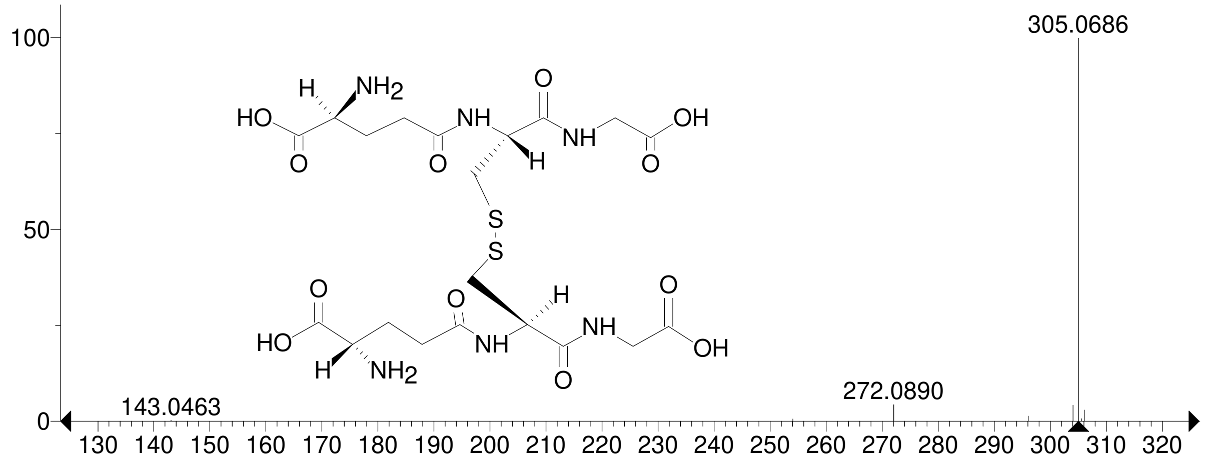

Hit 5 : (-)-Epigallocatechin [M-H]- HCD 13V P=305.1
C15H14O7; MF: 11; RMF: 310; Prob 2.19%; CAS: 970-74-1; Lib: nist_msms; ID: 235098.

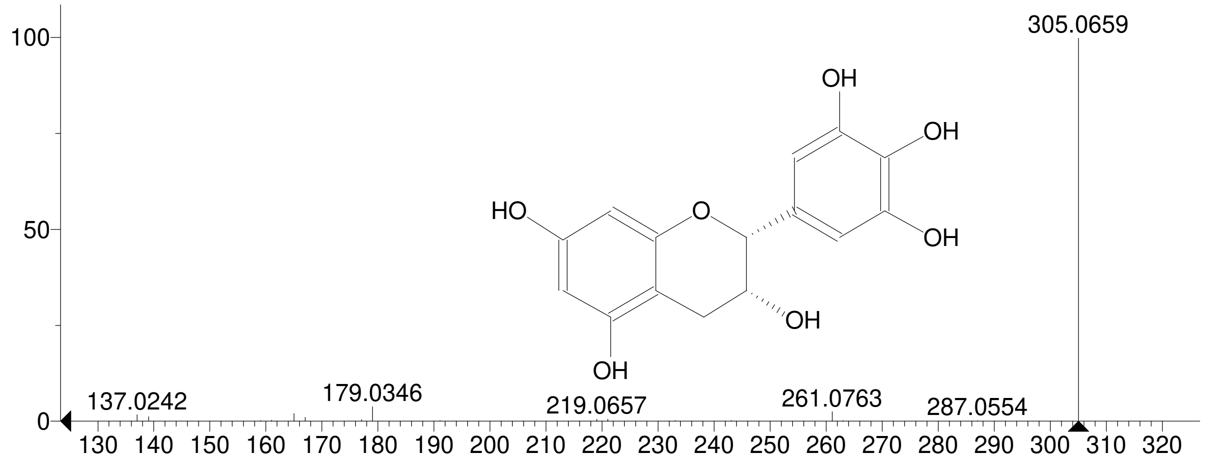

Unknown: Unknown (8.11_414.0856m/z)
Compound in Library Factor = 0

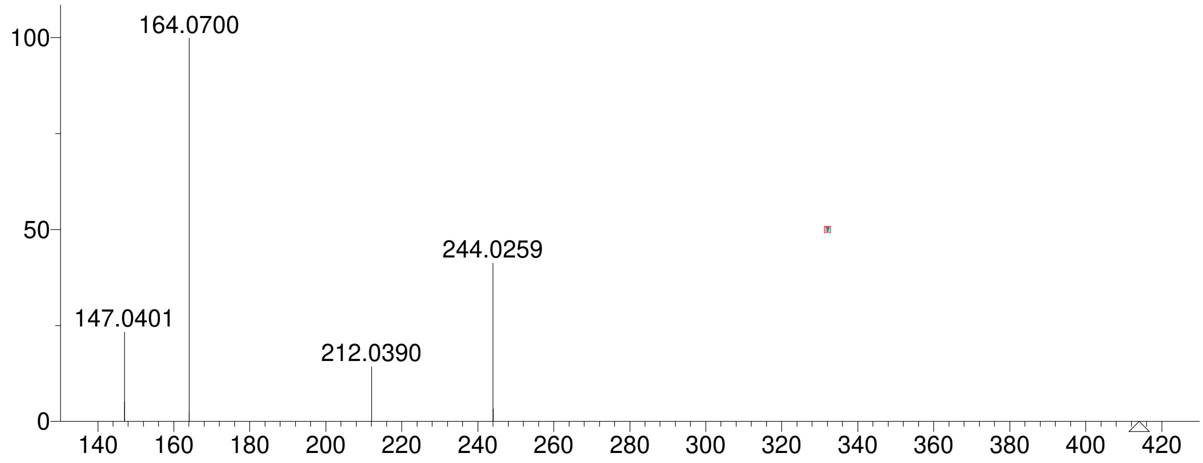

Hit 1 : 3H-Cyclopenta[c]quinoline, 4-(6-bromo-1,3-benzodioxol-5-yl)-3a,4,5,9b-tetrahydro-8-(1-methylethyl)-, (3aR
C22H22BrNO2; MF: 0; RMF: 89; Prob 100.0%; CAS: 1392487-51-2; Lib: nist_msms; ID: 509997.

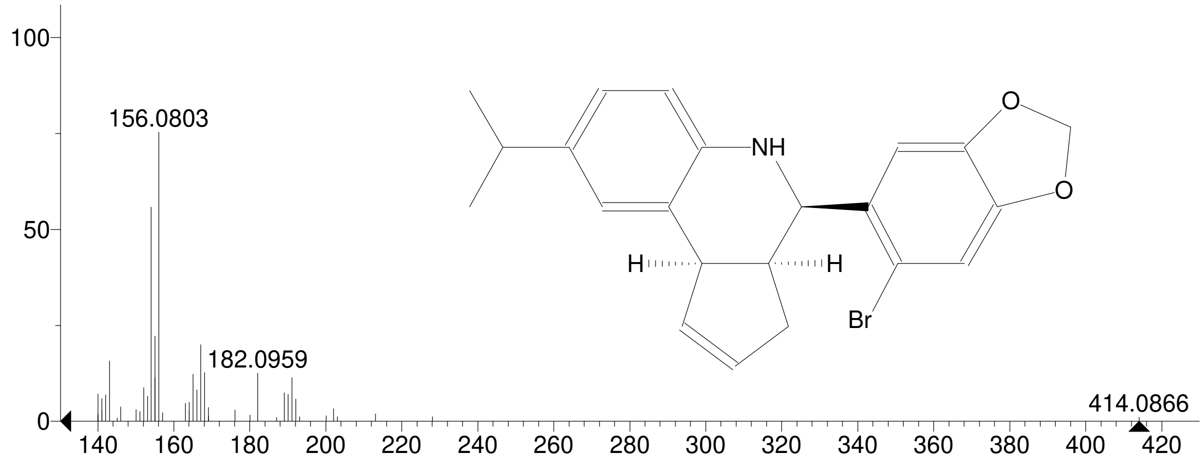

** Search Report Page 1 of 1 **

Unknown: Unknown (8.11_722.1756m/z)
Compound in Library Factor = 0

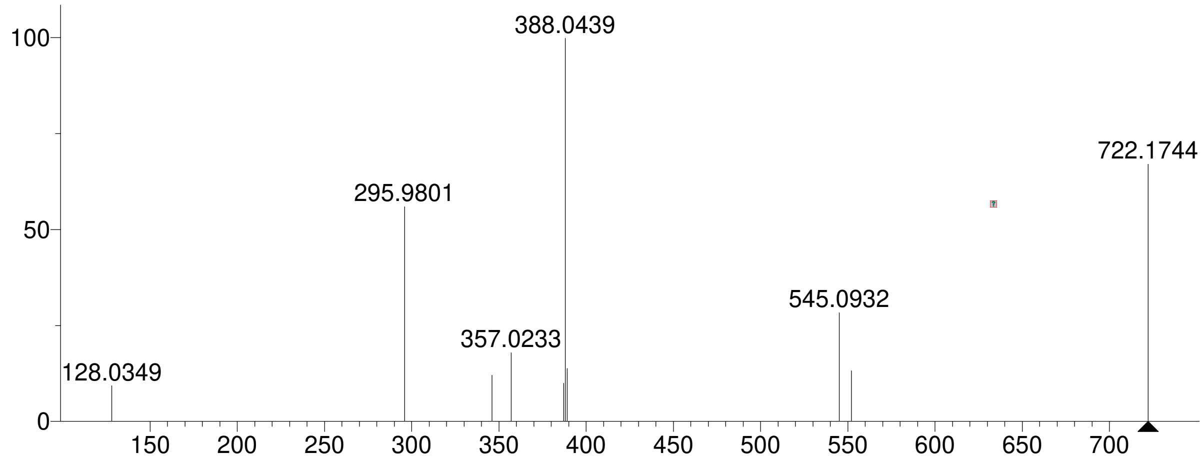

Unknown: Unknown (8.15_257.1622m/z)
Compound in Library Factor = 0

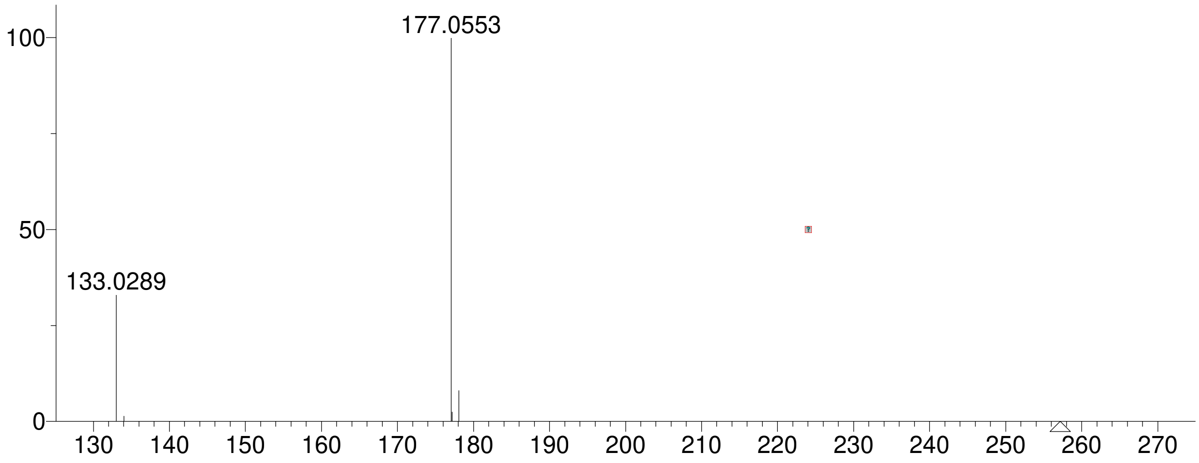

Unknown: Unknown (8.15_481.0820m/z)
Compound in Library Factor = 0

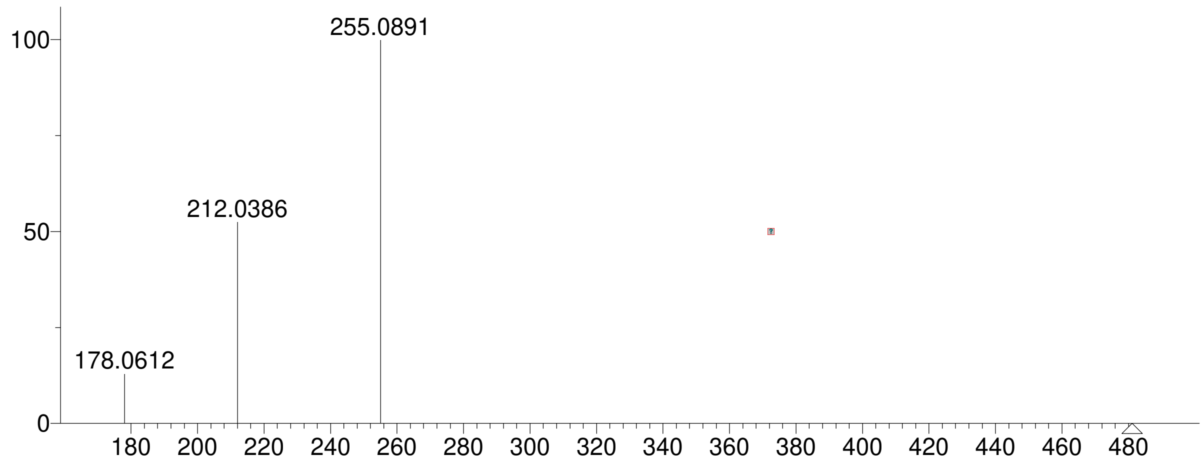

Unknown: Unknown (8.16_255.0874m/z)
Compound in Library Factor = 0

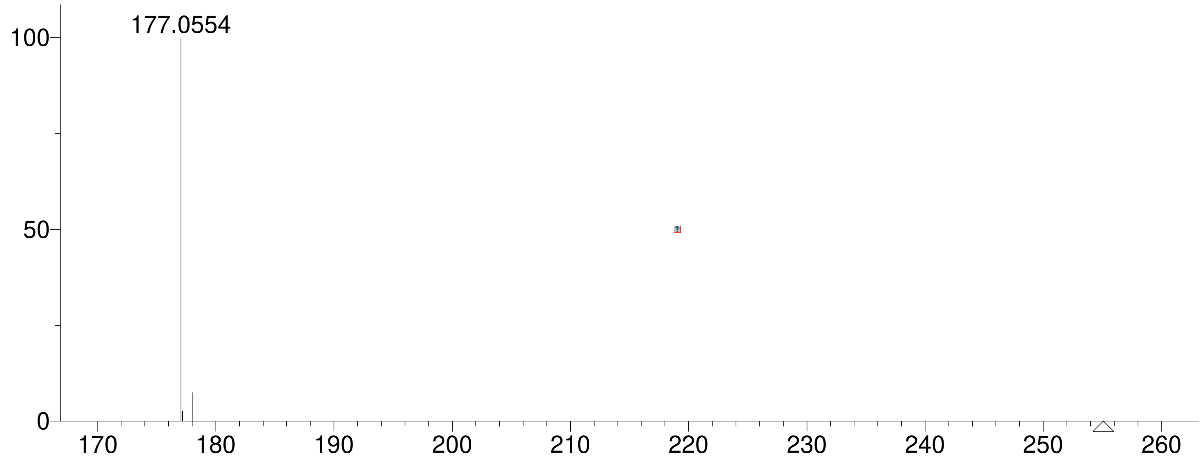

Hit 1 : phenylglucoside [M-H]- 65HCD P=255.1
C12H16O6; MF: 0; RMF: 41; Prob 100.0%; Lib: mona-export-lc-ms-ms_negative_mode; ID: 17536.

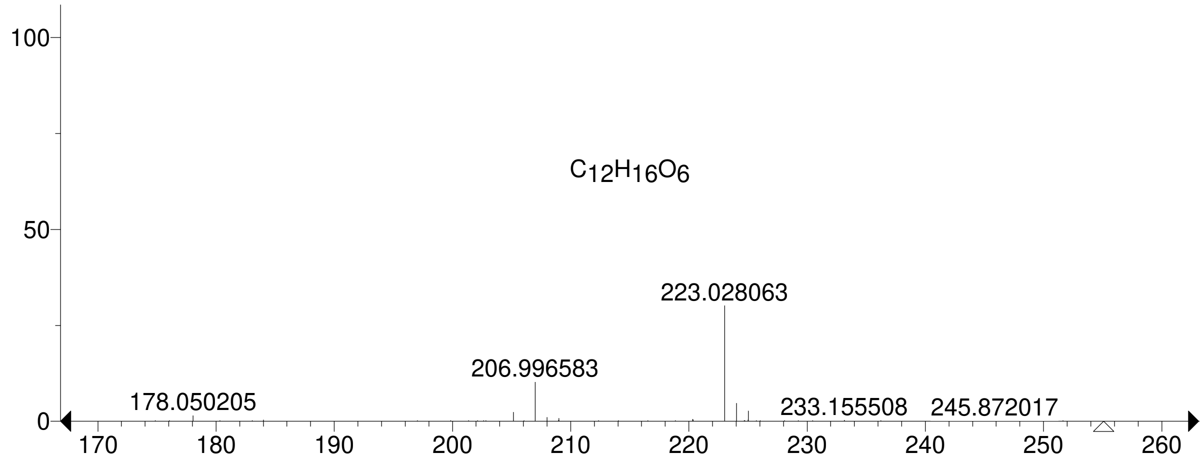

Unknown: Unknown (8.16_375.1300m/z)
Compound in Library Factor = -979

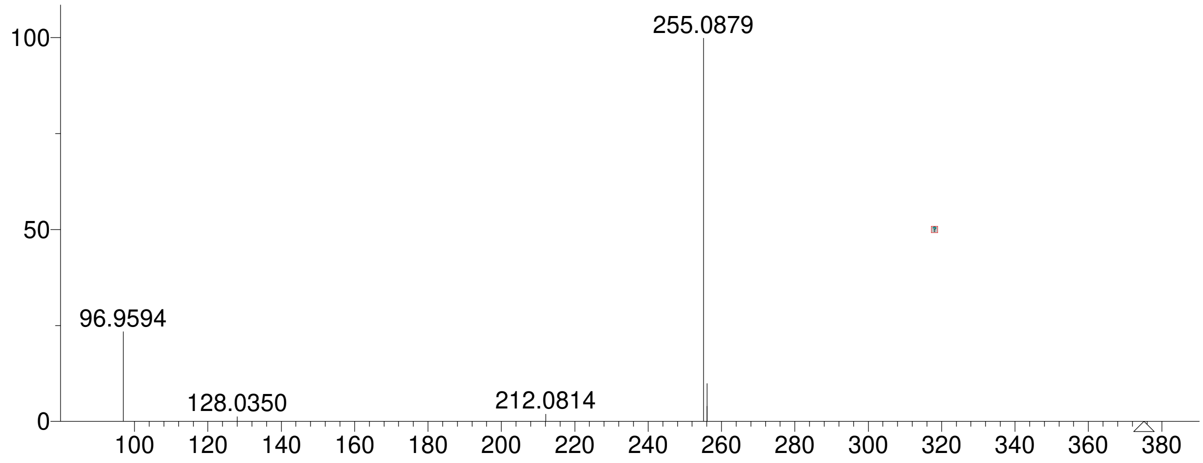

Hit 1 : (-)-Riboflavin [M-H]- IT 35% P=375.1
C17H20N4O6; MF: 278; RMF: 926; Prob 84.9%; CAS: 83-88-5; Lib: nist_msms; ID: 280474.

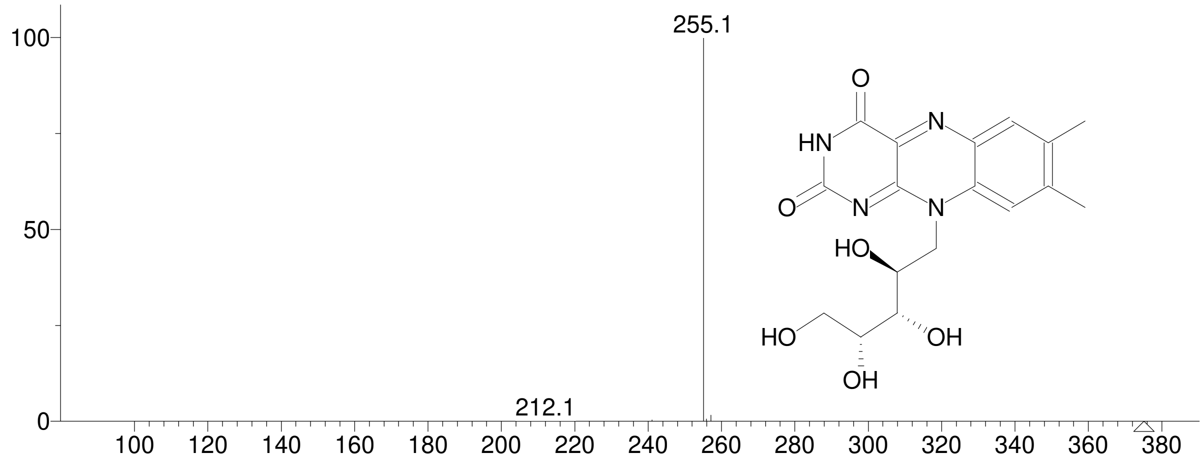

Hit 2 : RIBOFLAVIN [M-H]- P=375.1
C17H20N4O6; MF: 208; RMF: 804; Prob 12.4%; Lib: msms-neg-gnps; ID: 298.

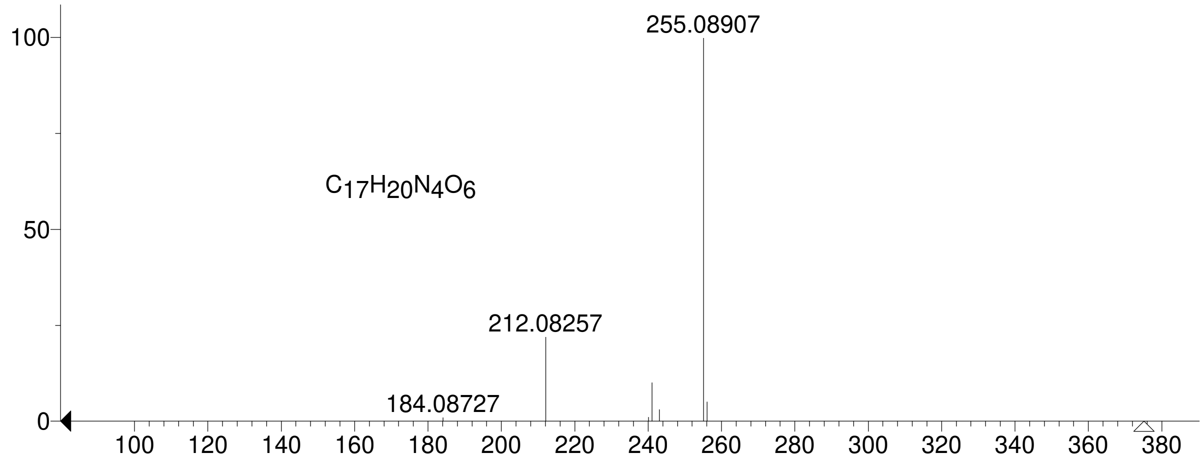

Hit 3 : RIBOFLAVIN
C17H20N4O6; MF: 94; RMF: 782; Prob 0.75%; Lib: embl neg mcg; ID: 324.

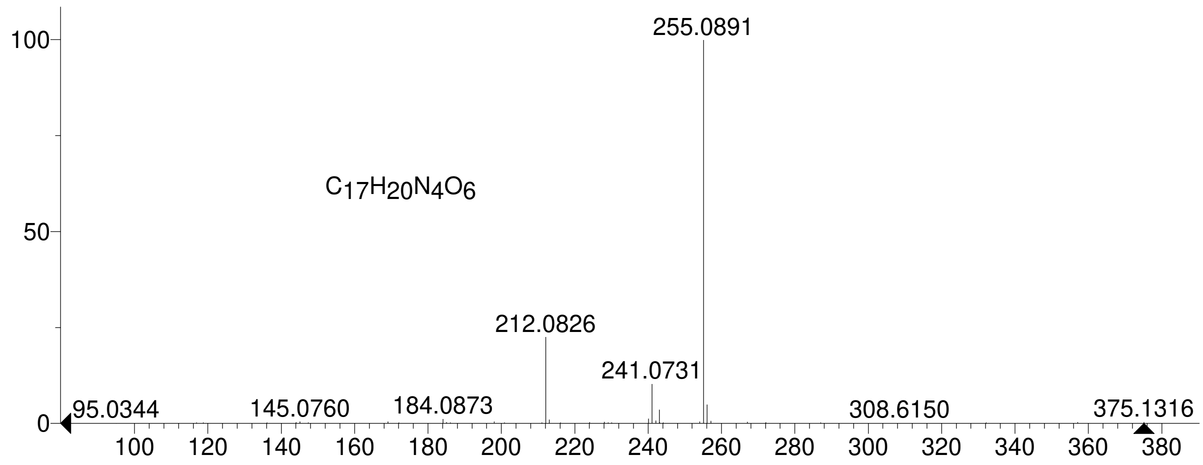

Hit 4 : Riboflavin [M-H]- 35eV P=375.1
C17H20N4O6; MF: 91; RMF: 664; Prob 0.66%; Lib: msms-neg-fiehnhilic; ID: 1245.

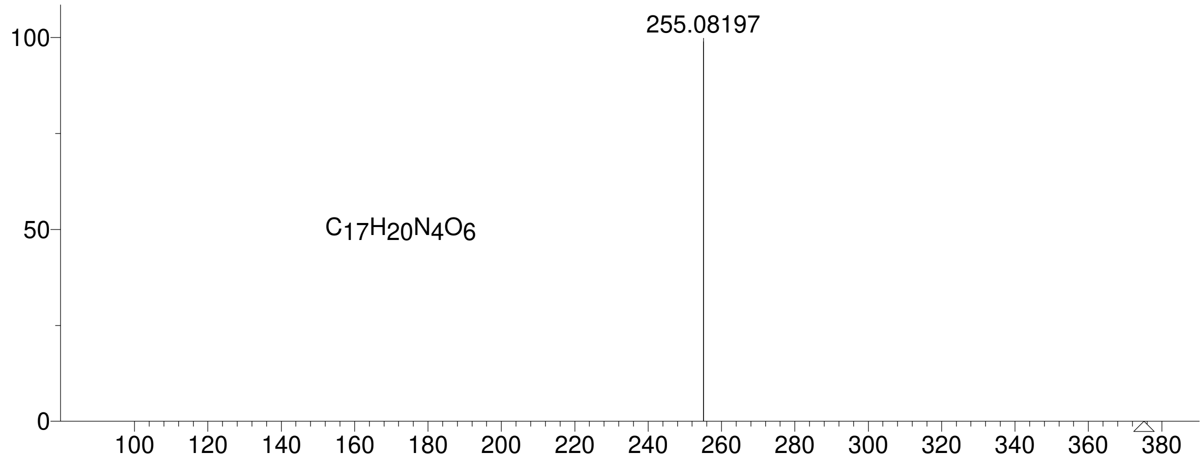

Hit 5 : (-)-Riboflavin; LC-ESI-QTOF; MS2; CE [M-H]- P=375.1
C17H20N4O6; MF: 85; RMF: 790; Prob 0.52%; Lib: msms-neg-massbank; ID: 4608.

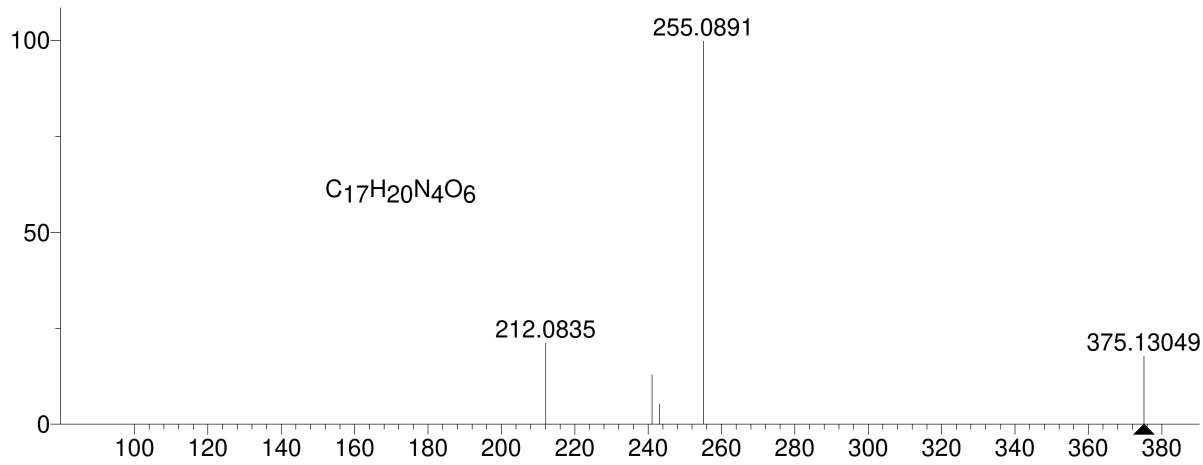

Unknown: Unknown (8.17_257.0120m/z)
Compound in Library Factor = 0

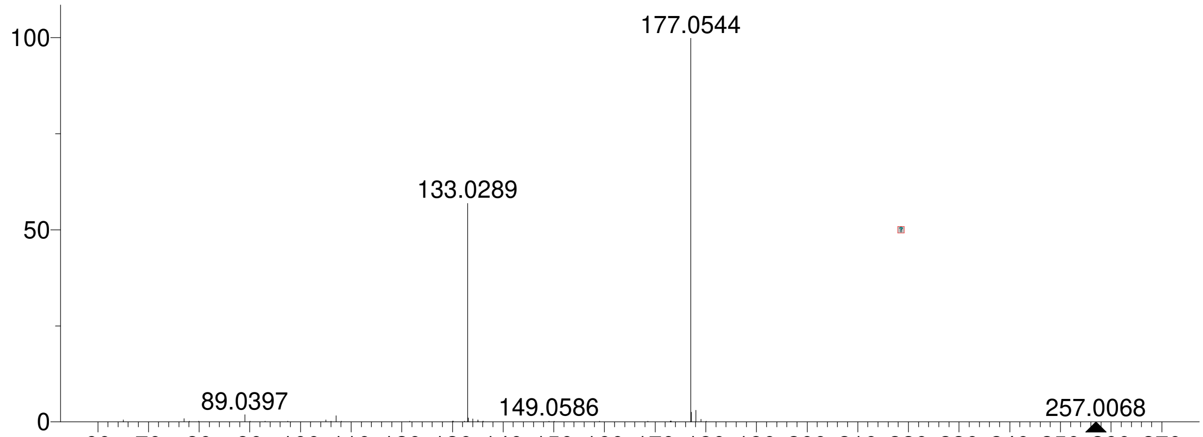

** Search Report Page 1 of 1 **

Unknown: Unknown (8.18_537.0116m/z)
Compound in Library Factor = 0

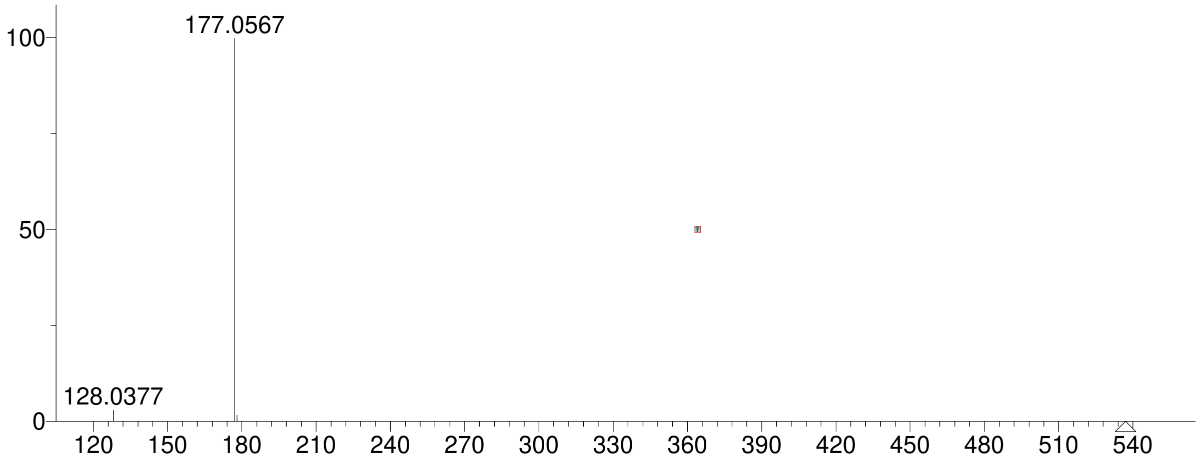

** Search Report Page 1 of 1 **

Unknown: Unknown (8.27_389.1458m/z)
Compound in Library Factor = 0

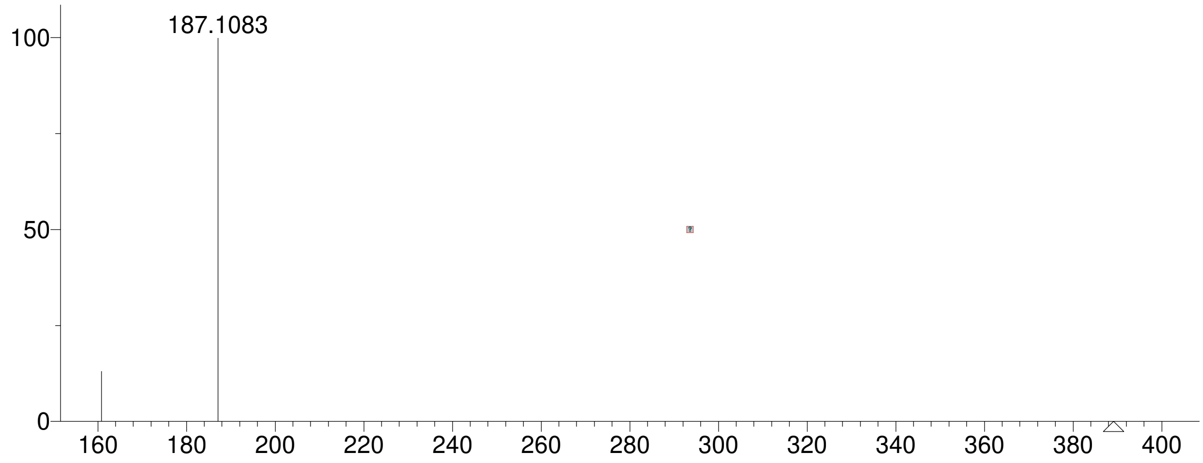

Hit 1 : geniposide [M+H]+ 40 P=389.1
; MF: 0; RMF: 9; Prob 50.0%; Lib: mona-export-vf-npl_qtof; ID: 1651.

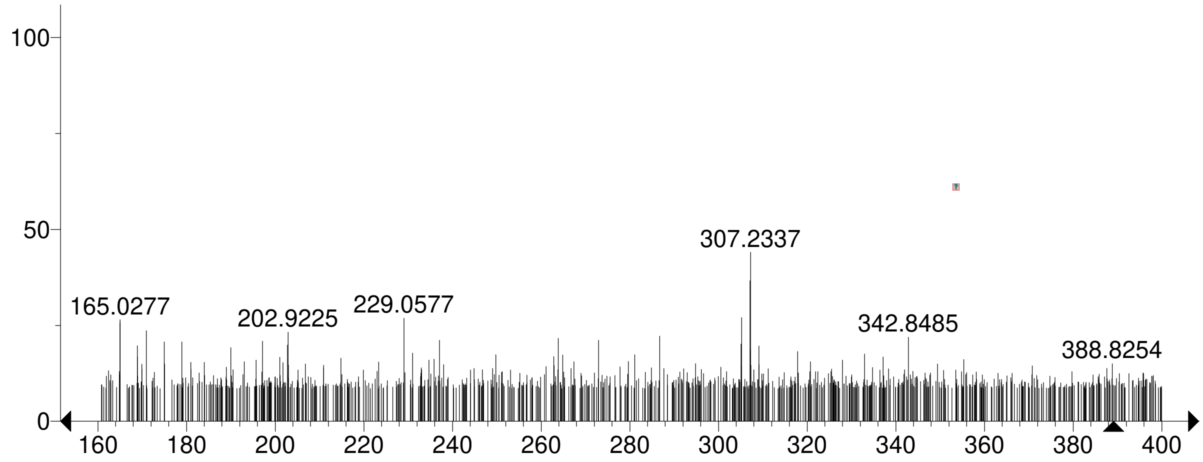

Hit 2 : methyl (1S)-7-hydroxy-7-methyl-1-[(2S,3R,4S,5S,6R)-3,4,5-trihydroxy-6-(hydroxymethyl)oxan-2-yl]oxy-4a,5
C17H26O10; MF: 0; RMF: 4; Prob 50.0%; Lib: mona-export-lc-ms-ms_negative_mode; ID: 20423.

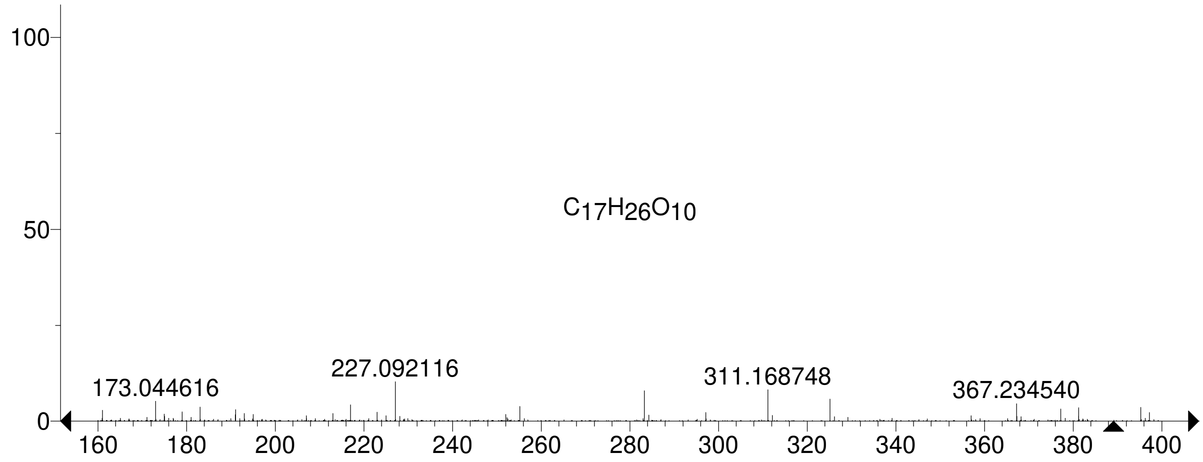

Unknown: Unknown (8.31_197.1282m/z)
Compound in Library Factor = -344

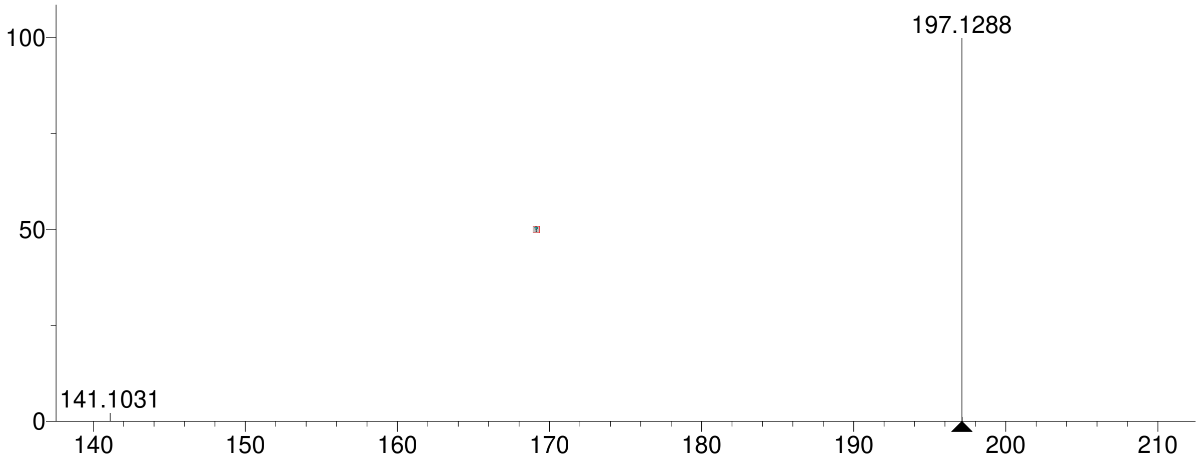

Hit 1 : L-N5-(1-Imino-3-pentenyl)ornithine [M+H-NH3]+ HCD 61V P=197.1
C10H19N3O2; MF: 357; RMF: 984; Prob 97.2%; CAS: 751479-44-4; Lib: nist_msms; ID: 165506.

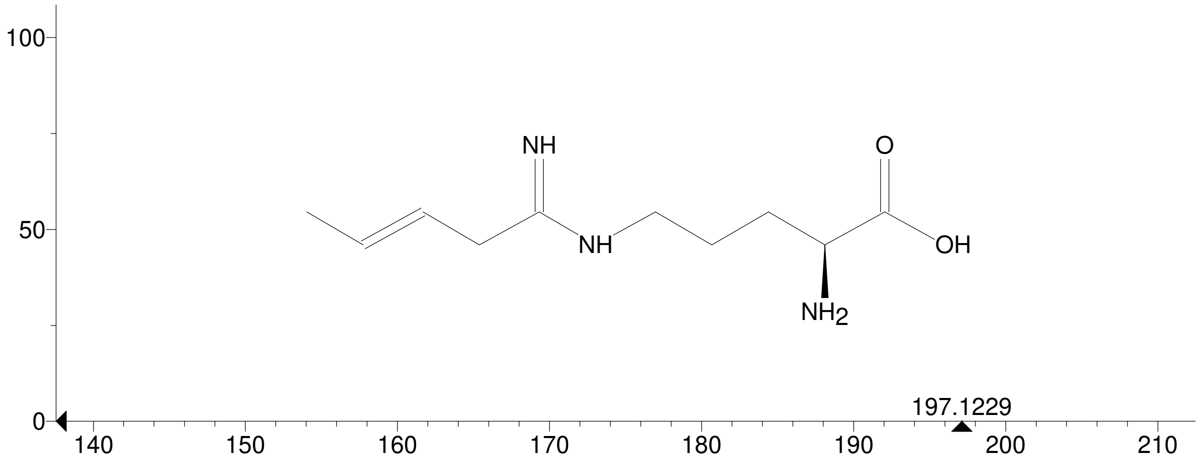

Hit 2 : D-Desthiobiotin [M+H-H2O]+ HCD 15V P=197.1
C10H18N2O3; MF: 207; RMF: 901; Prob 2.72%; CAS: 533-48-2; Lib: nist_msms; ID: 489080.

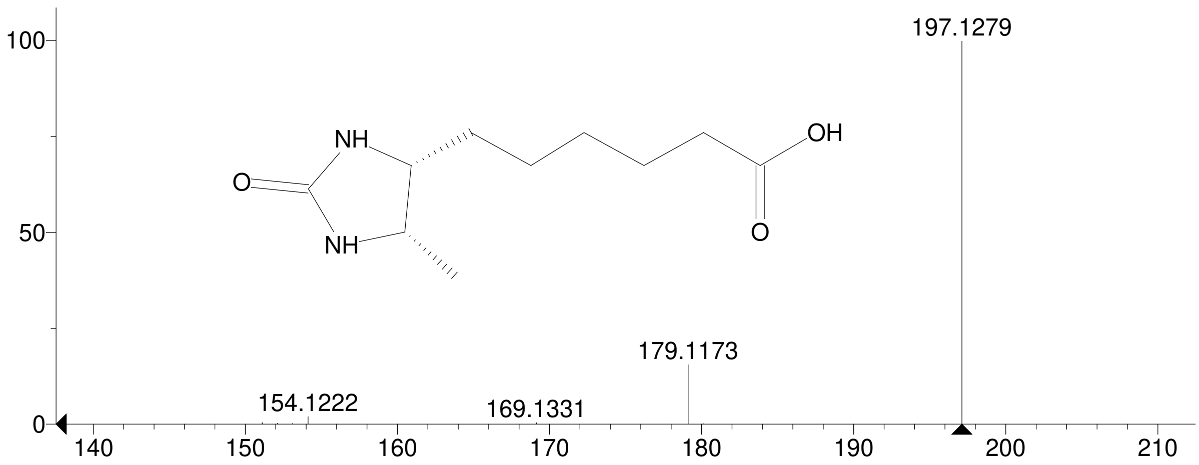

Hit 3 : NCGC00381364-01!3-propan-2-yl-2,3,6,7,8,8a-hexahydropyrrolo[1,2-a]pyrazine-1,4-dione [M+H]+ P=197
C10H16N2O2; MF: 43; RMF: 696; Prob 0.06%; Lib: mona-export-gnps; ID: 6310.

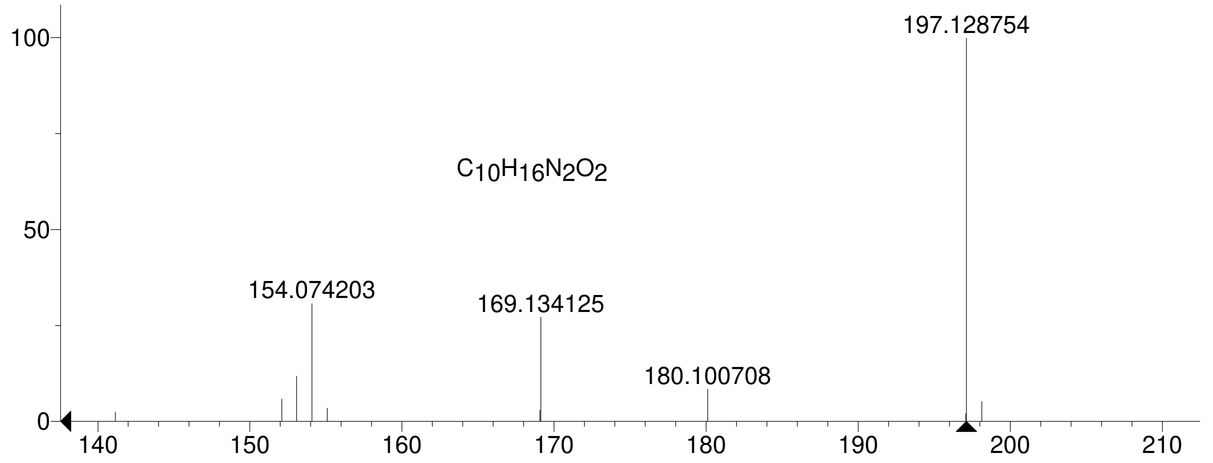

Hit 4 : Val-Phe-Lys [M+2H]2+ QTOF 20V P=197.1
C20H32N4O4; MF: 23; RMF: 406; Prob 0.02%; Lib: nist_msms; ID: 54274.

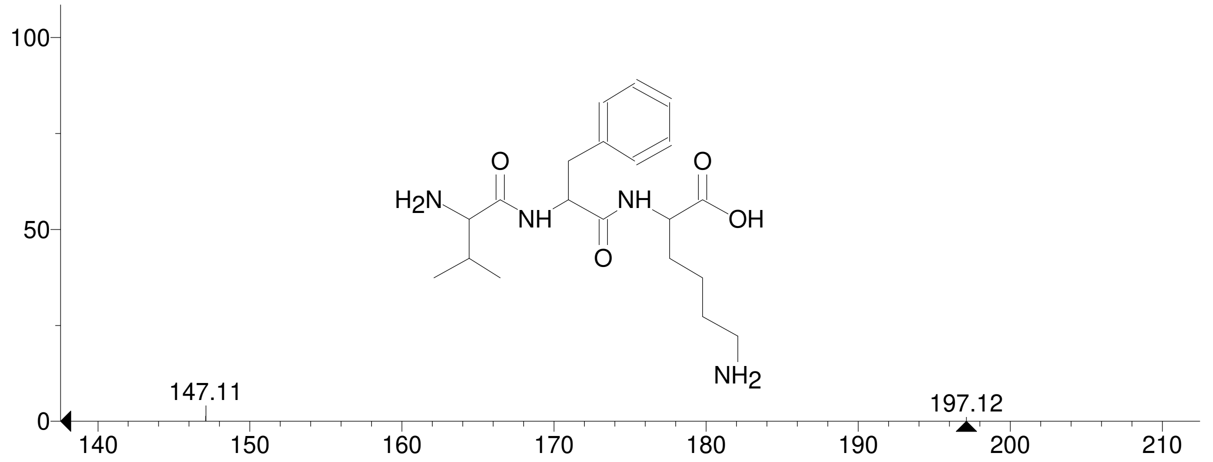

Hit 5 : NCGC00381364-02_C10H16N2O2_Pyrrolo[1,2-a]pyrazine-1,4-dione, hexahydro-3-(1-methylethyl)- [M+H]
C10H16N2O2; MF: 5; RMF: 551; Prob 0.01%; Lib: mona-export-gnps; ID: 10512.

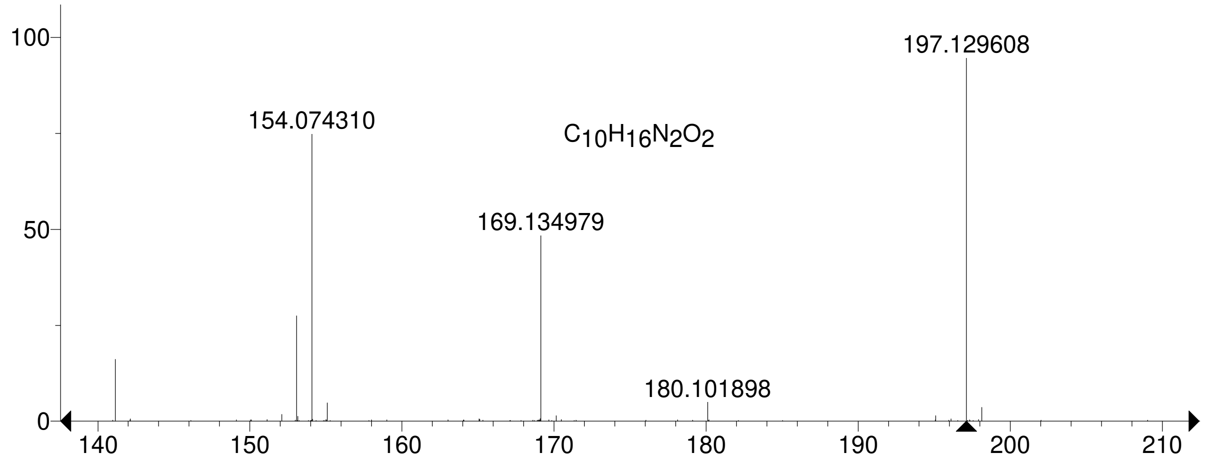

Unknown: Unknown (8.31_241.1188m/z)
Compound in Library Factor = -589

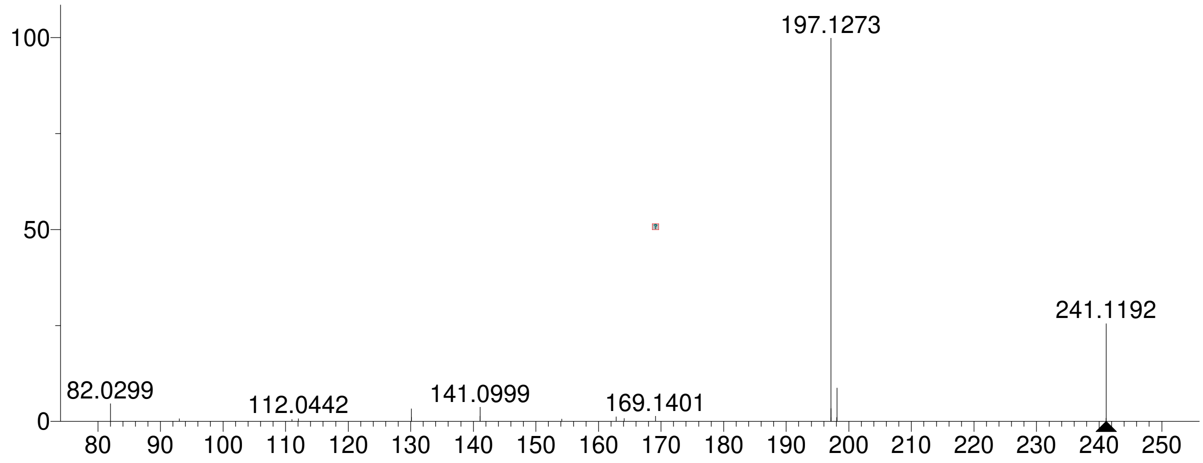

Hit 1 : Pyroglutamyl-Isoleucine; PlaSMA ID-369 [M-H]- P=241.1
C11H18N2O4; MF: 378; RMF: 665; Prob 100.0%; Lib: biomsms-neg-plasma; ID: 400.

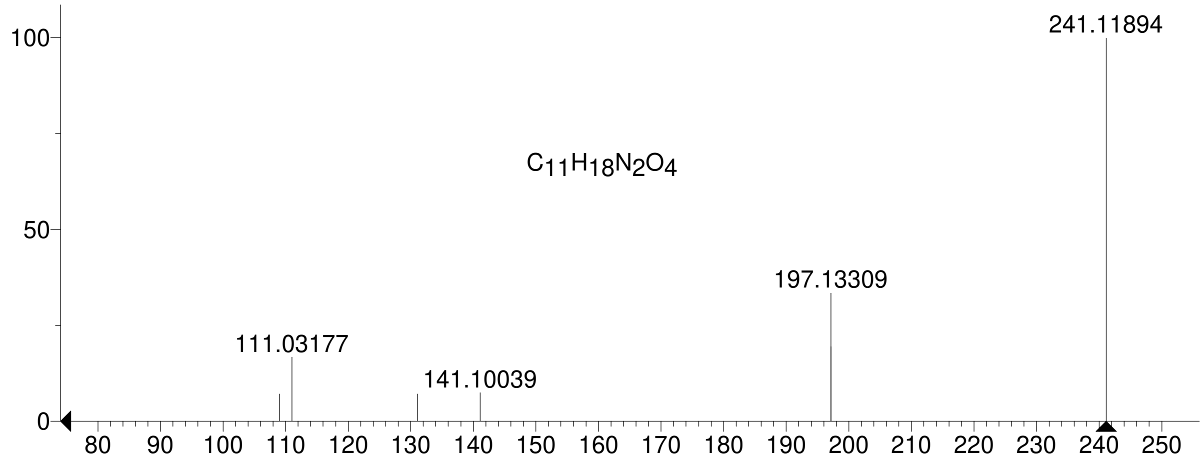

** Search Report Page 1 of 1 **

Unknown: Unknown (8.31_401.9606m/z)
Compound in Library Factor = 0

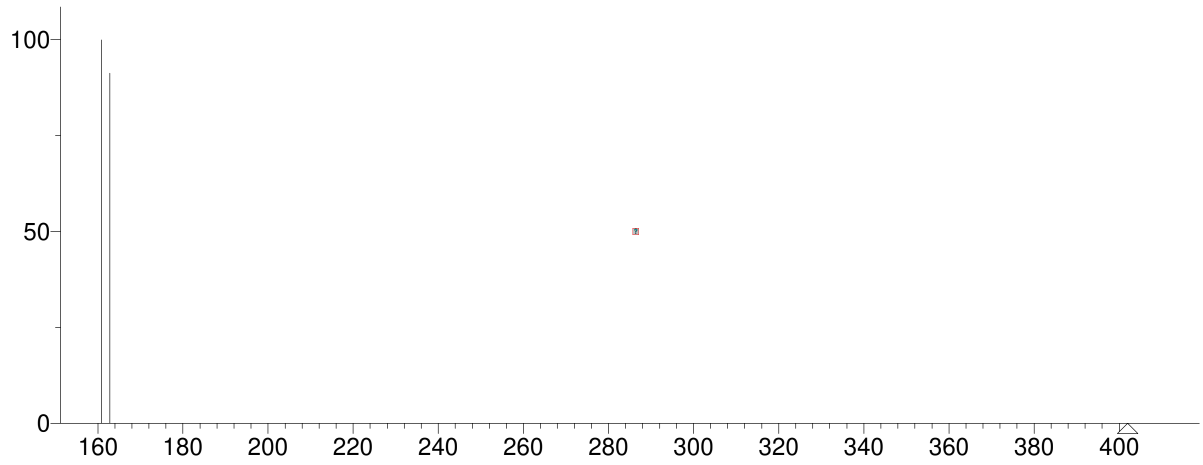

Unknown: Unknown (8.31_403.9564m/z)
Compound in Library Factor = 0

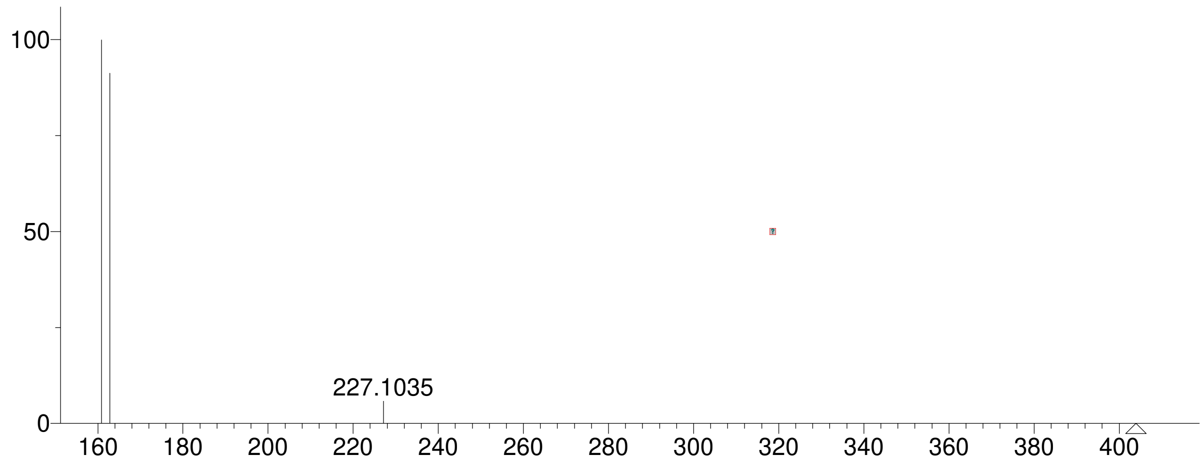

Unknown: Unknown (8.31_483.2463m/z)
Compound in Library Factor = -2176

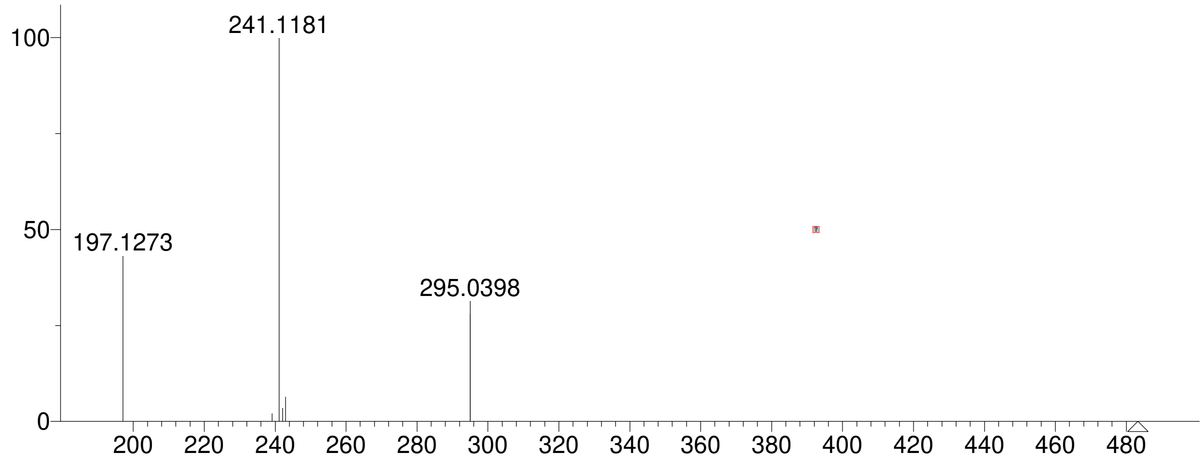

Hit 1 : Tetrahydro-L-biopterin [2M+H]+ HCD 14V P=483.2
C9H15N5O3; MF: 27; RMF: 125; Prob 51.9%; CAS: 62989-33-7; Lib: nist_msms; ID: 318091.

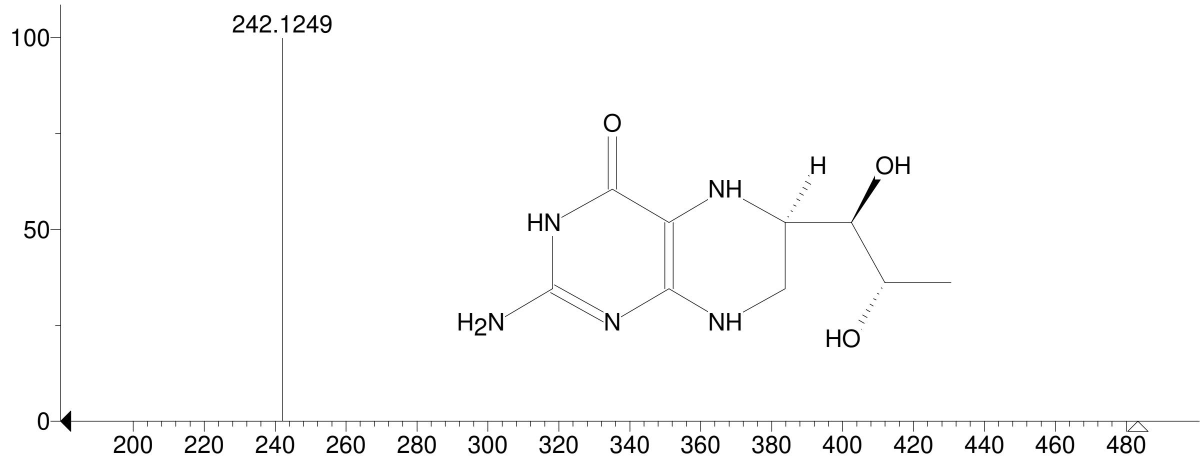

Hit 2 : 4-(Dimethylamino)azobenzene N-oxide [2M+H]+ HCD 15V P=483.3
C14H15N3O; MF: 3; RMF: 101; Prob 17.4%; CAS: 2747-31-1; Lib: nist_msms; ID: 119538.

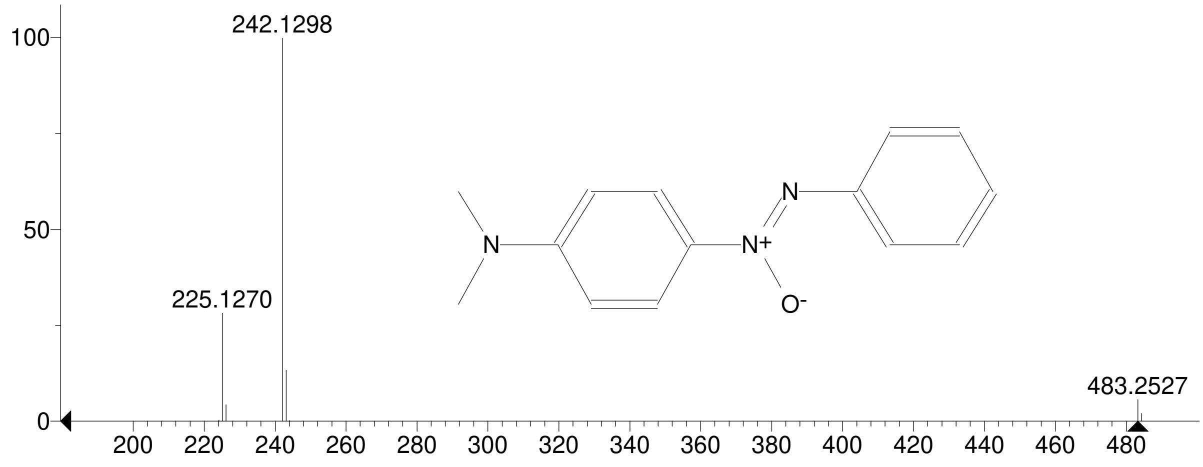

Hit 3 : 4-[5-[[4-[5-[acetyl(hydroxy)amino]pentylamino]-4-oxobutanoyl]-hydroxyamino]pentylamino]-4-oxobutanoic a
C20H36N4O8; MF: 0; RMF: 64; Prob 15.4%; Lib: mona-export-vf-npl_qexactive; ID: 19031.

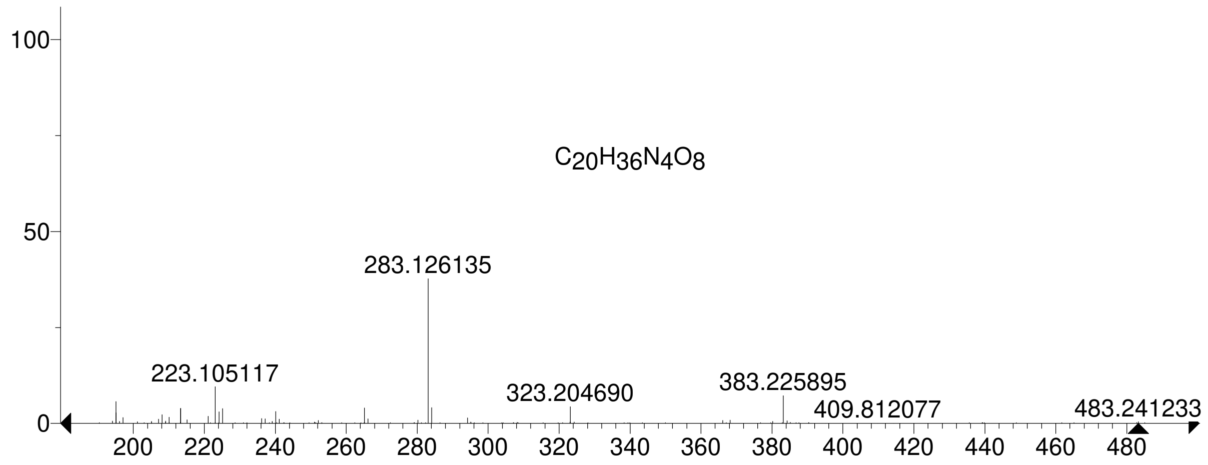

Hit 4 : NCGC00380283-01!4-[5-[[4-[5-[acetyl(hydroxy)amino]pentylamino]-4-oxobutanoyl]-hydroxyamino]pentylam
C20H36N4O8; MF: 0; RMF: 38; Prob 15.4%; Lib: mona-export-gnps; ID: 6572.

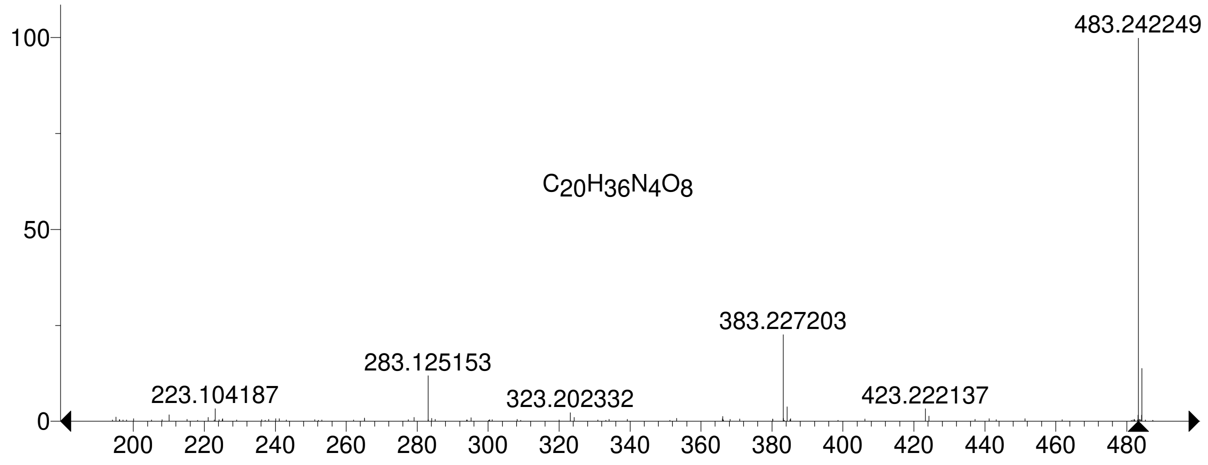

** Search Report Page 1 of 1 **

Unknown: Unknown (8.38_579.2537m/z)
Compound in Library Factor = 0

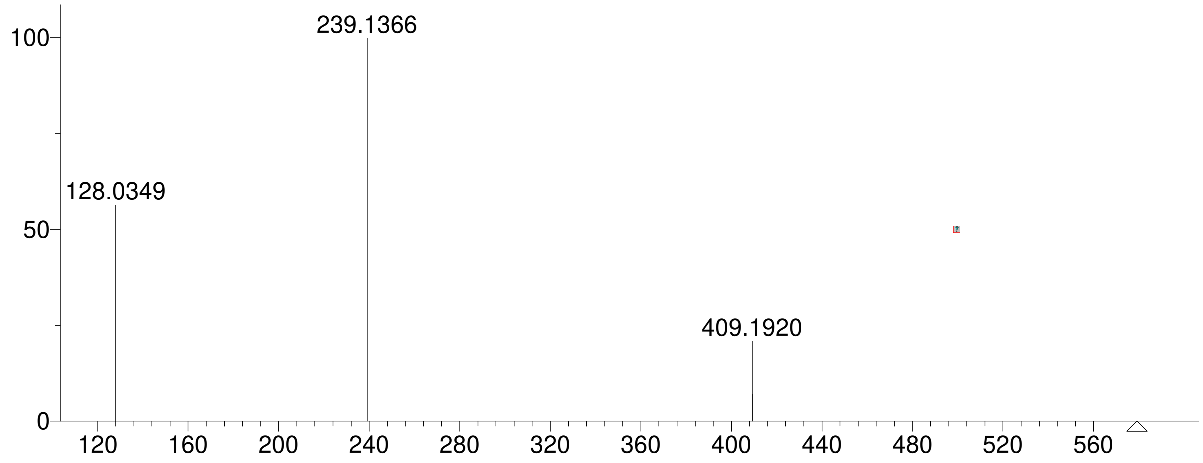

Unknown: Unknown (8.40_356.1277m/z)
Compound in Library Factor = -1072

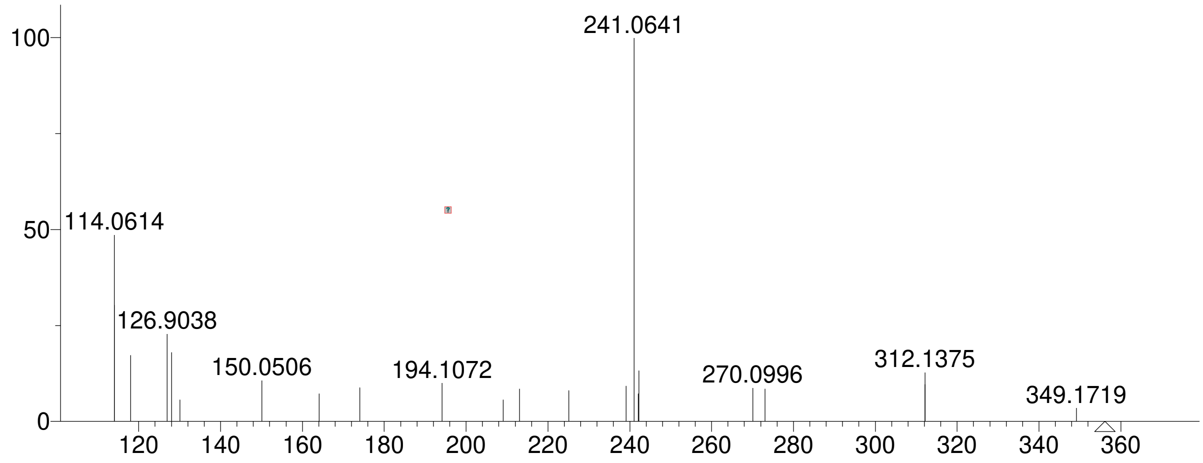

Hit 1 : 1H-Indole-1-butanoic acid, 3-(1-naphthalenylcarbonyl)- [M-H]- IT 35% P=356.1
C23H19NO3; MF: 76; RMF: 449; Prob 100.0%; CAS: 1307803-52-6; Lib: nist_msms; ID: 346136.

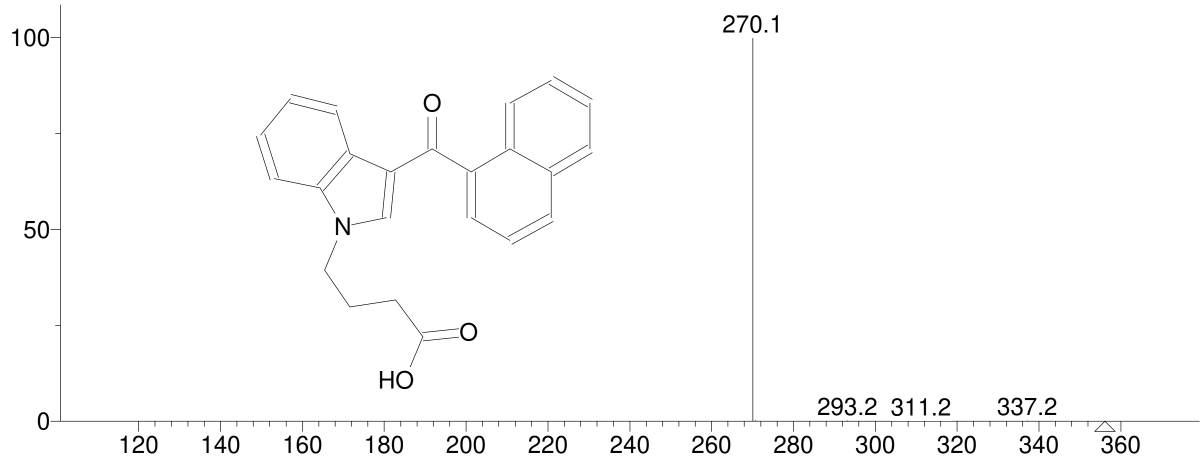

Unknown: Unknown (8.40_409.1966m/z)
Compound in Library Factor = 0

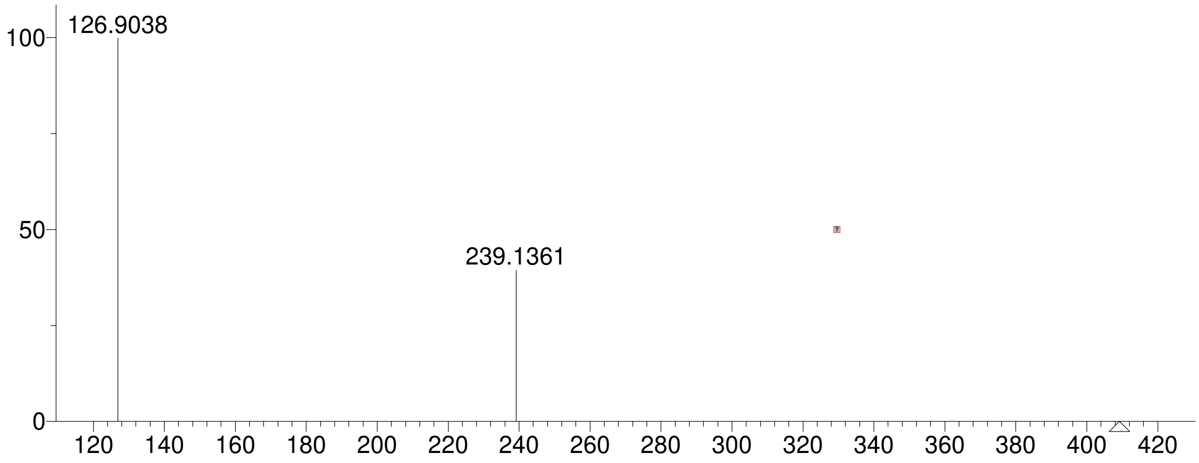

Hit 1 : Met-Met-Lys [M+H]+ QQQ 32V P=409.2
C16H32N4O4S2; MF: 0; RMF: 15; Prob 100.0%; Lib: nist_msms; ID: 40876.

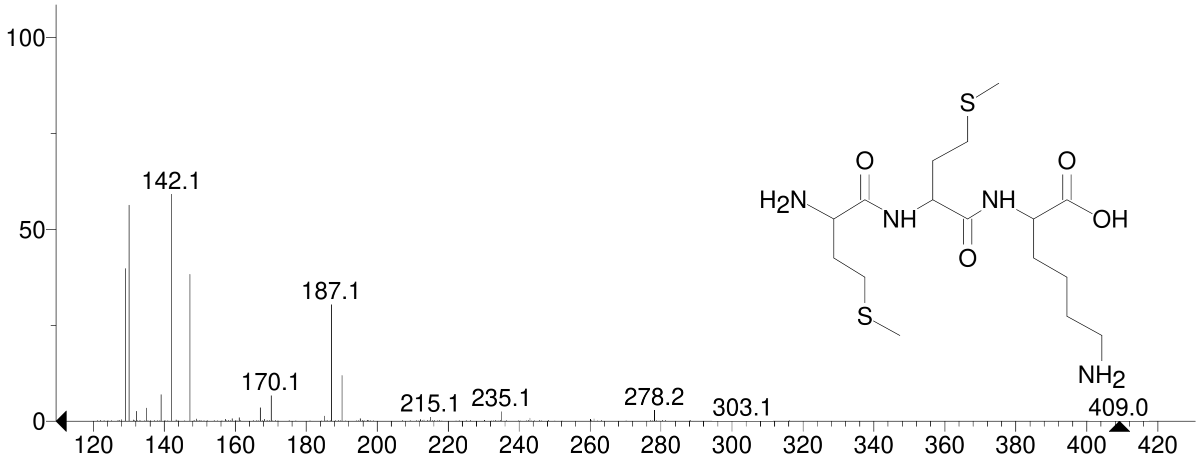

Unknown: Unknown (8.41_400.0035m/z)
Compound in Library Factor = 0

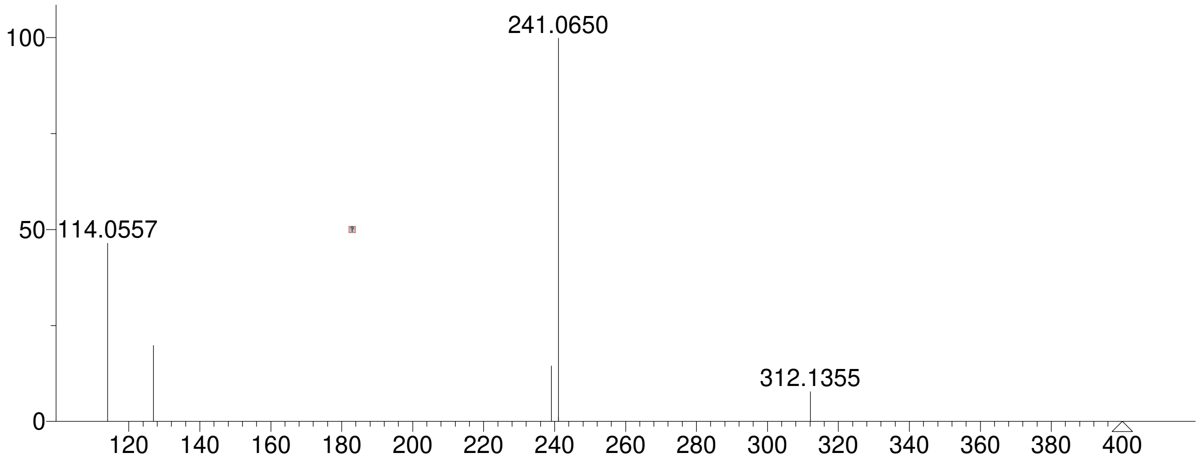

Unknown: Unknown (8.41_579.2468m/z)
Compound in Library Factor = 0

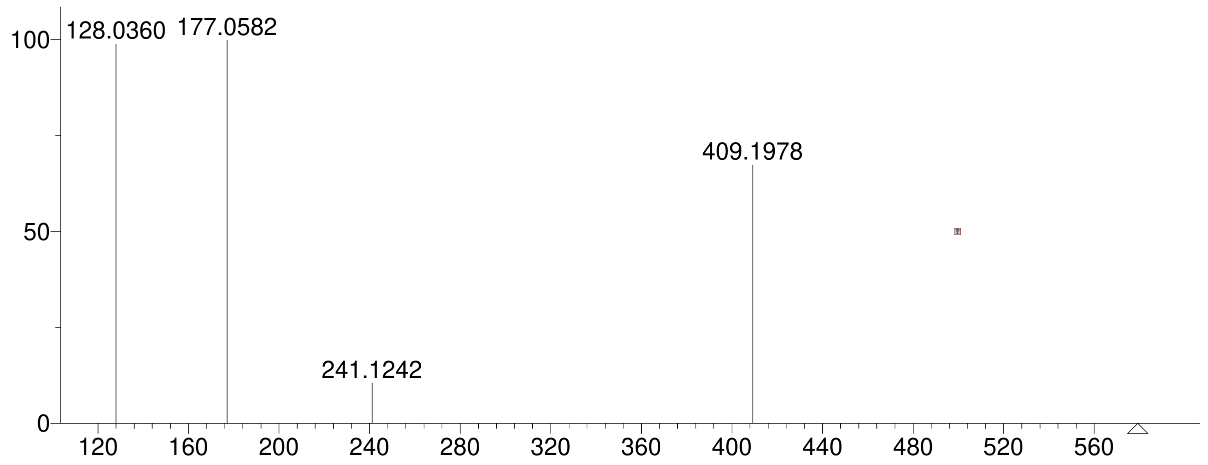

Hit 1 : HYDRANGENOSIDE C [M+H]+ 35HCD P=579.2
C29H38O12; MF: 0; RMF: 71; Prob 50.0%; Lib: mona-export-vf-npl_qexactive; ID: 1009.

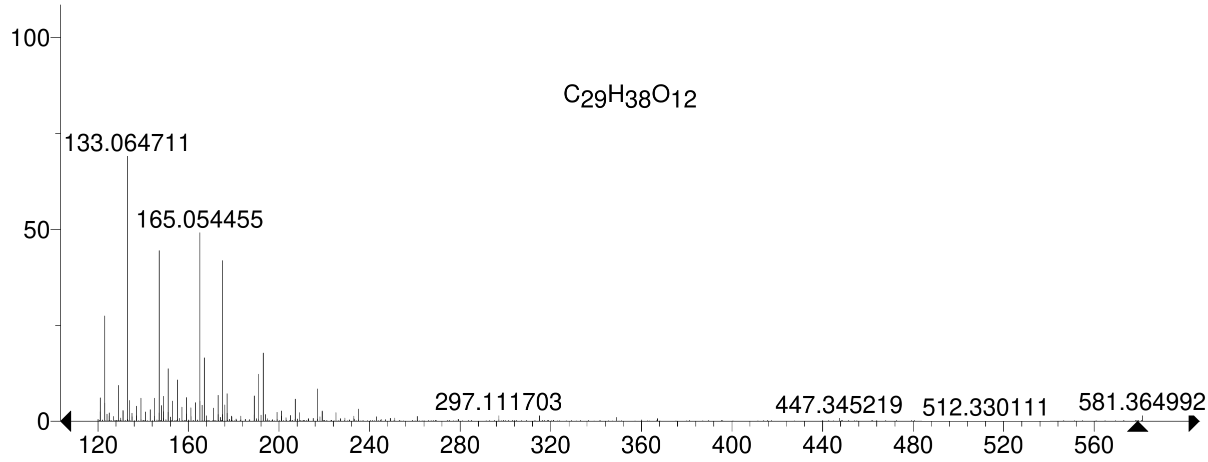

Hit 2 : NCGC00385509-01_C29H38O12_Methyl 2-(hexopyranosyloxy)-4-({6-[2-(4-hydroxyphenyl)ethyl]-4-oxotetra
C29H38O12; MF: 0; RMF: 49; Prob 50.0%; Lib: mona-export-gnps; ID: 11095.

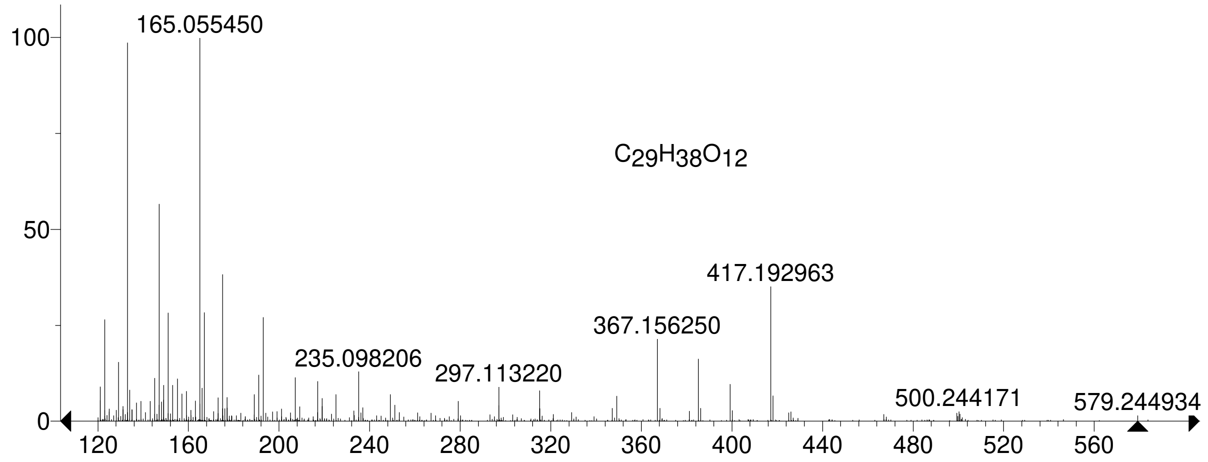

Unknown: Unknown (8.43_387.1541m/z)
Compound in Library Factor = -3273

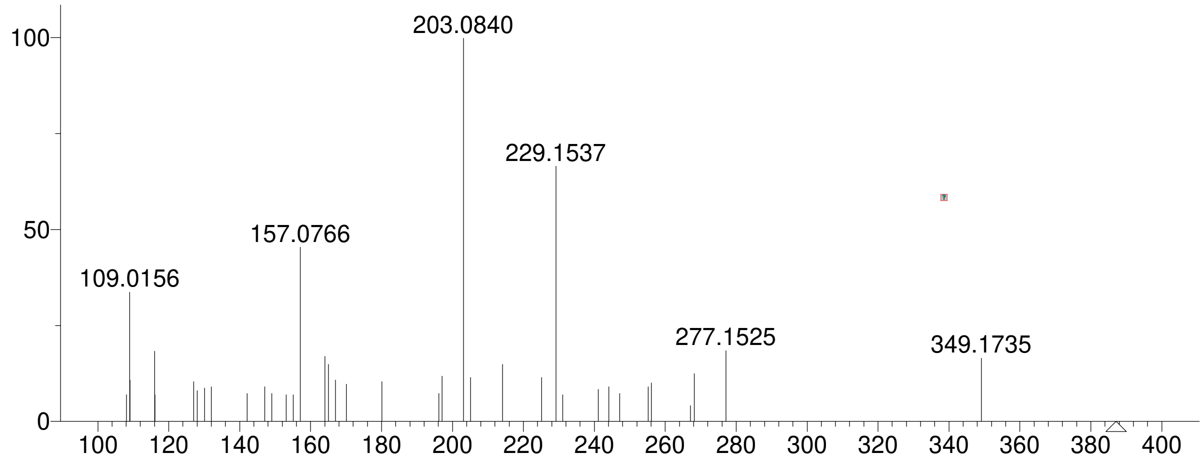

Hit 1 : NCGC00179845-03!5-chloro-3-[(E)-3,4-dihydroxy-3,5-dimethylhept-1-enyl]-7,8-dihydroxy-7-methyl-8,8a-dil
C19H27ClO6; MF: 1; RMF: 43; Prob 51.0%; Lib: mona-export-gnps; ID: 8510.

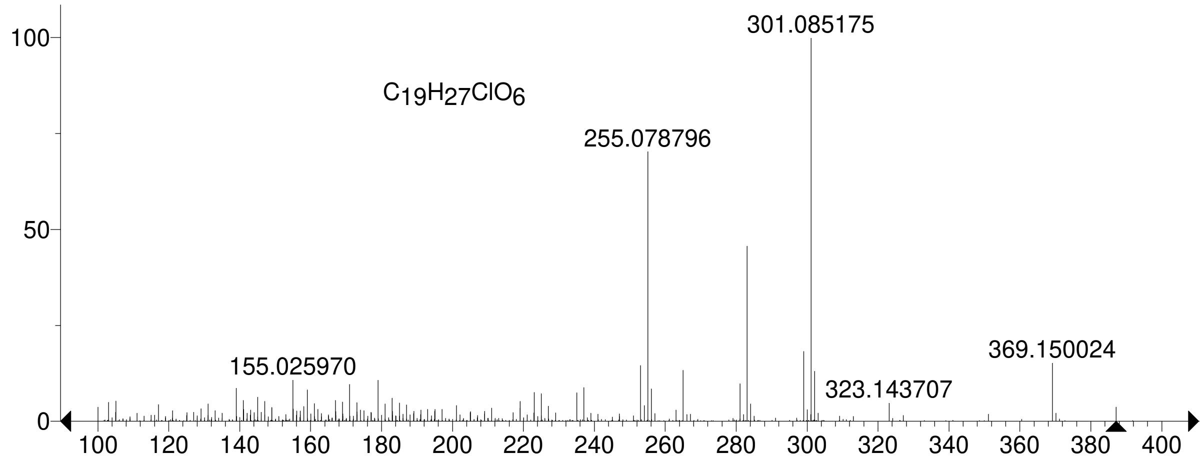

Hit 2 : 1H-Indazole-3-carboxamide, N-[(1S)-1-(aminocarbonyl)-2-methylpropyl]-1-(5-chloropentyl)- [M+Na]+ HCD
C18H25ClN4O2; MF: 0; RMF: 17; Prob 49.0%; CAS: 1801552-02-2; Lib: nist_msms; ID: 426703.

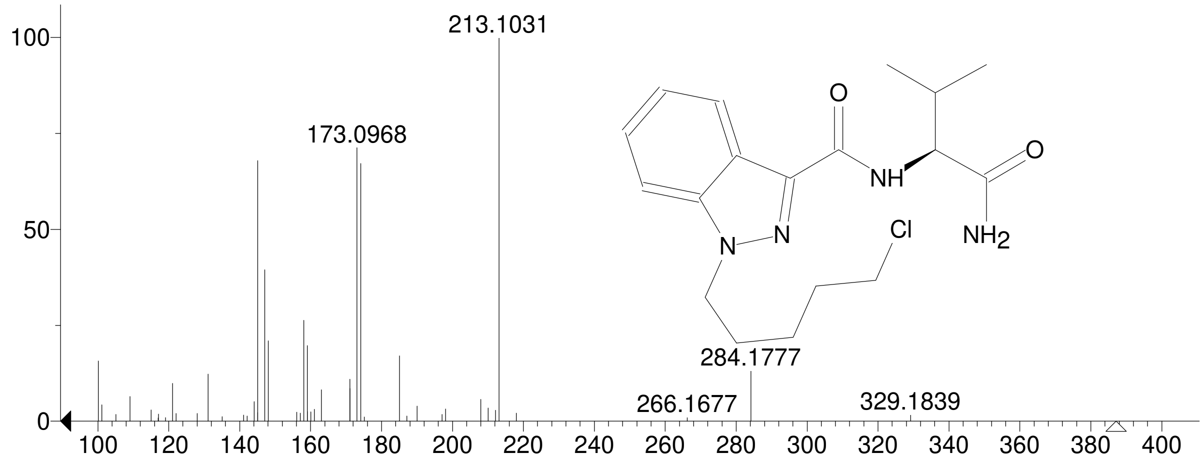

Unknown: Unknown (8.43_443.9918m/z)
Compound in Library Factor = 0

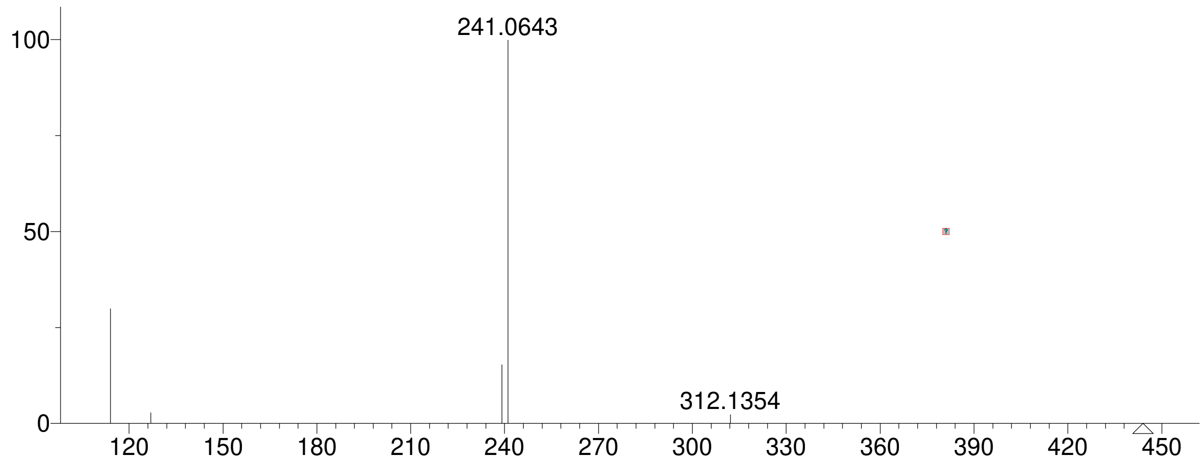

Unknown: Unknown (8.44_300.0170m/z)
Compound in Library Factor = 0

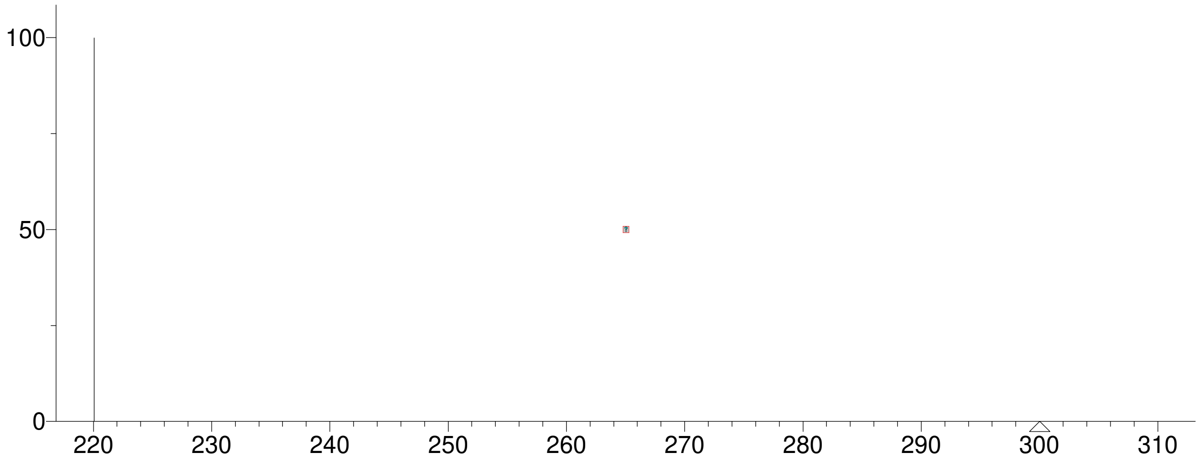

Unknown: Unknown (8.44_439.2073m/z)
Compound in Library Factor = -3273

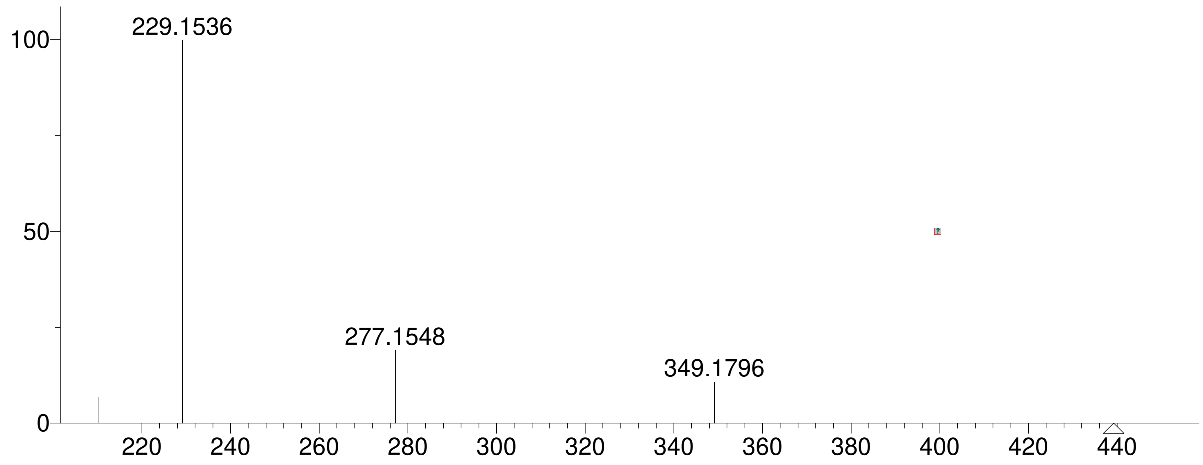

Hit 1 : Deacetoxy-7-Oxogedunin [M+H]+ 35HCD P=439.2
C26H30O6; MF: 1; RMF: 90; Prob 34.2%; Lib: mona-export-vf-npl_qexactive; ID: 25777.

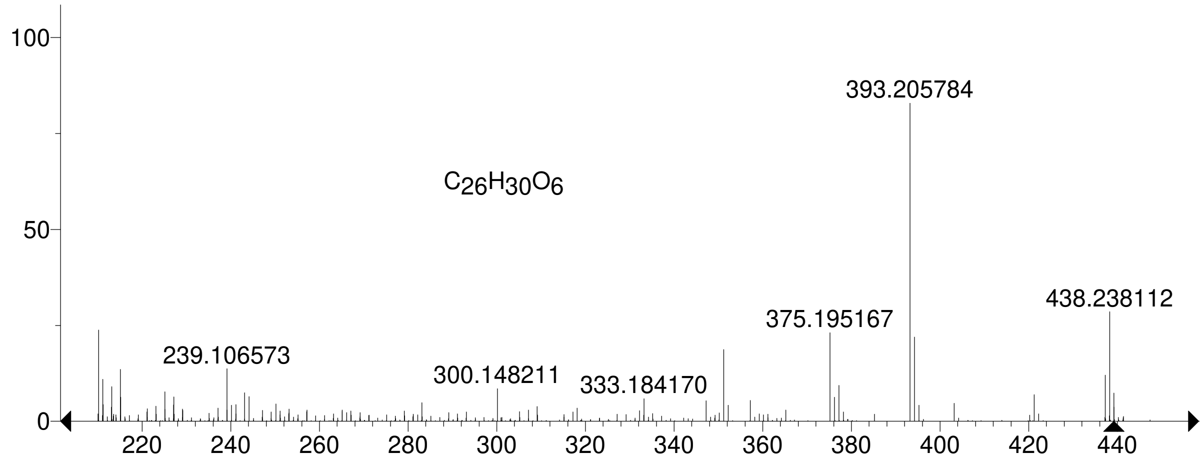

Hit 2 : 17-Trifluoromethylphenyl-13,14-dihydrotrinorprostaglandin F2α [M-H-H2O]- HCD 41V P=439.2
C24H33F3O5; MF: 0; RMF: 13; Prob 32.9%; CAS: 294856-01-2; Lib: nist_msms; ID: 93984.

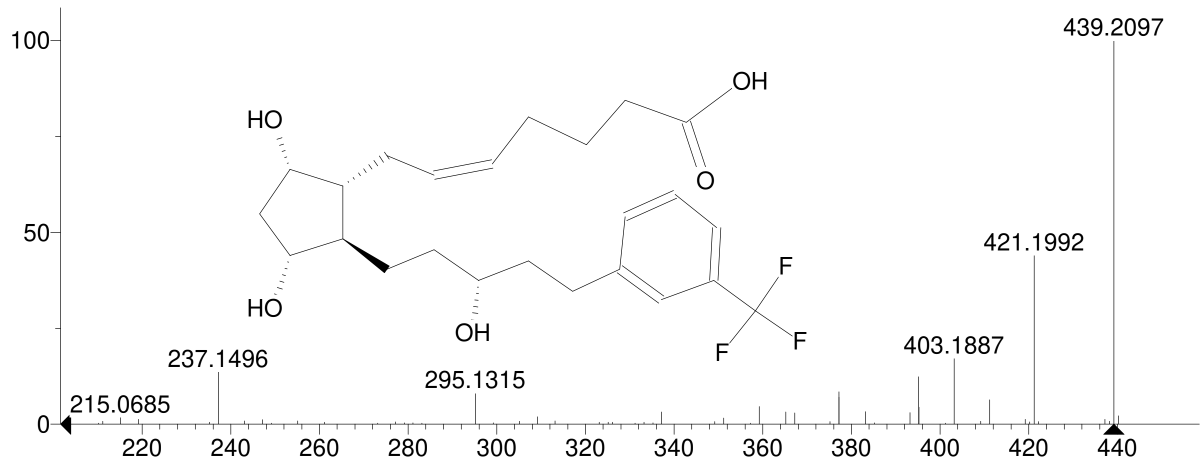

Hit 3 : NCGC00385780-01!5,7-dihydroxy-3-[4-hydroxy-2-methoxy-3-(3-methylbut-2-enyl)phenyl]-6-(3-methylbut-2
C26H30O6; MF: 0; RMF: 10; Prob 32.9%; Lib: mona-export-gnps; ID: 8555.

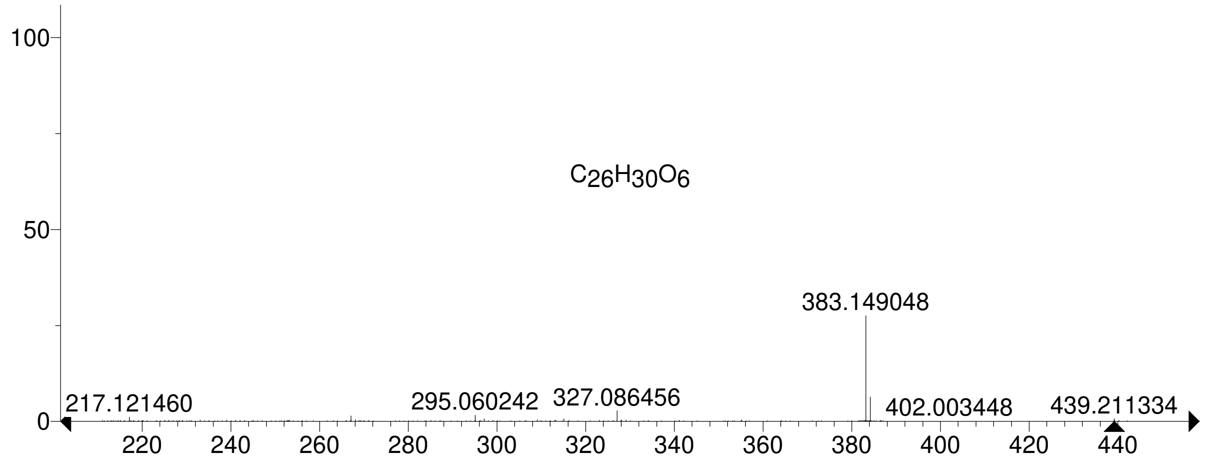

Unknown: Unknown (8.44_736.4349m/z)
Compound in Library Factor = -3273

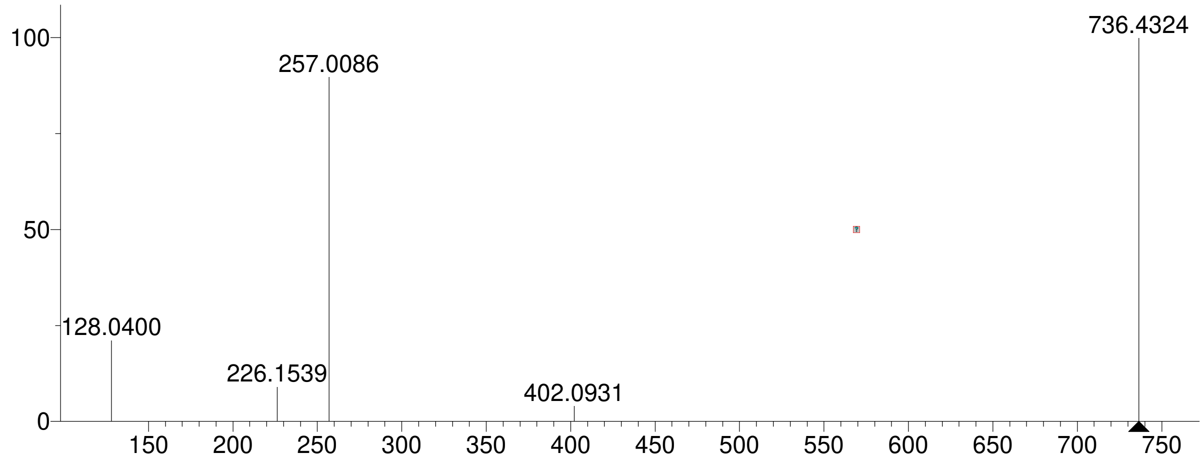

Hit 1 : Dynorphin A (1-12), porcine [M-2H]2- HCD 39eV P=736.4
C69H114N22O14; MF: 2; RMF: 423; Prob 52.0%; Lib: nist_msms2; ID: 77027.

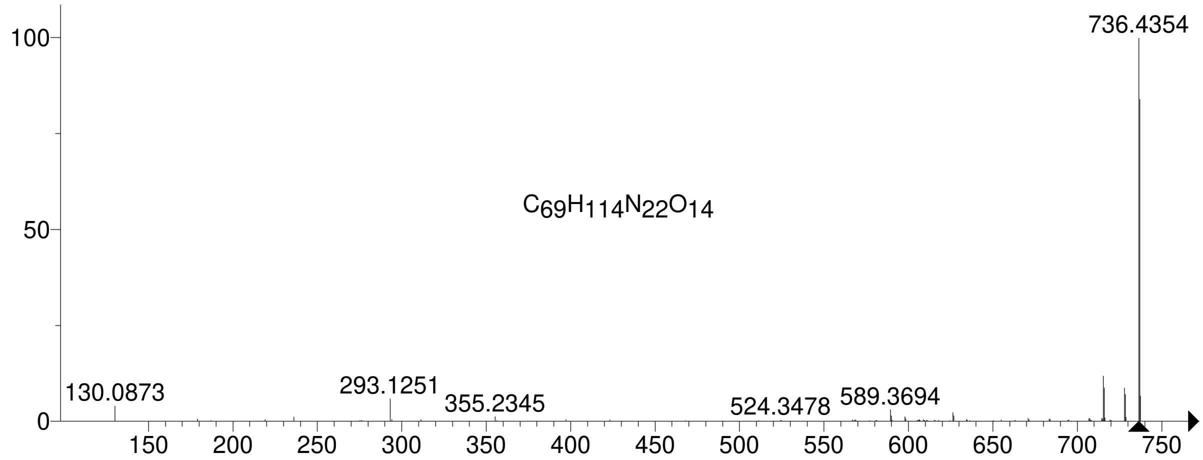

Hit 2 : 16-{[(2R,3R,4S,5S,6R)-4,5-dihydroxy-6-(hydroxymethyl)-3-{[(2S,3R,4R,5R,6S)-3,4,5-trihydroxy-6-methylox
C39H63NO12; MF: 0; RMF: 40; Prob 48.0%; Lib: mona-export-lc-ms-ms_negative_mode; ID: 22838.

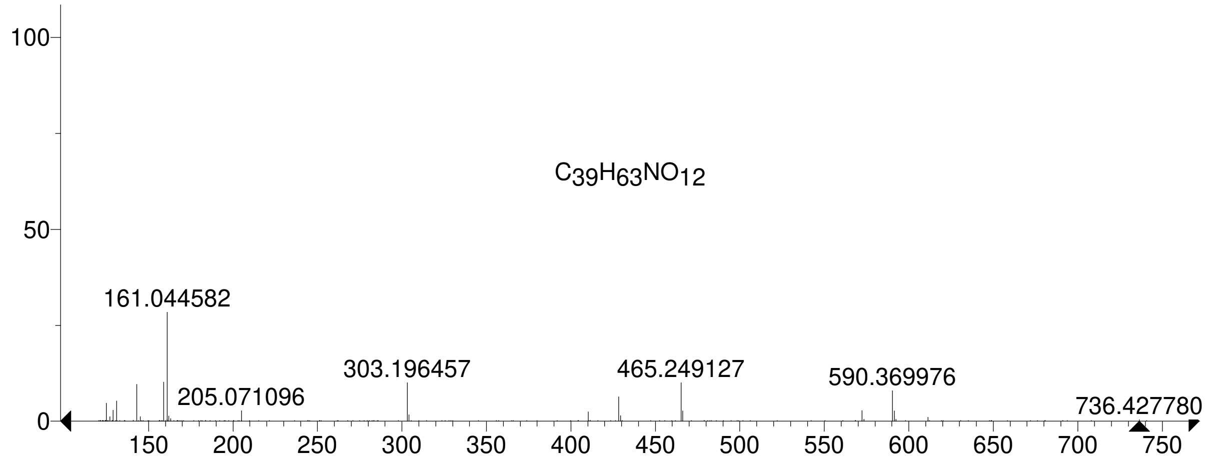

** Search Report Page 1 of 1 **

Unknown: Unknown (8.45_502.2016m/z)
Compound in Library Factor = 0

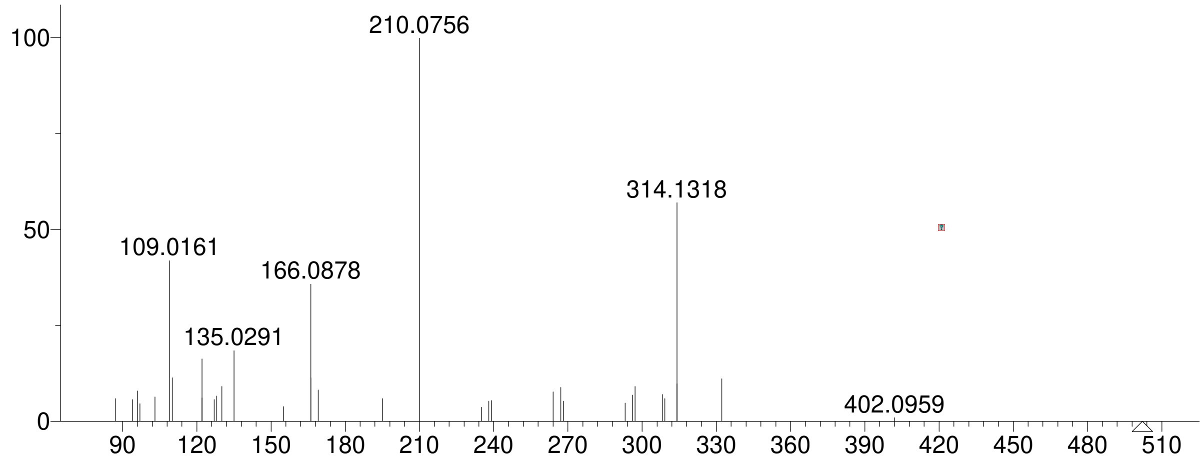

Hit 1 : Irinotecan [M+H-C5H11N]+ HCD 130V P=502.2
C33H38N4O6; MF: 0; RMF: 40; Prob 50.0%; CAS: 97682-44-5; Lib: nist_msms; ID: 218337.

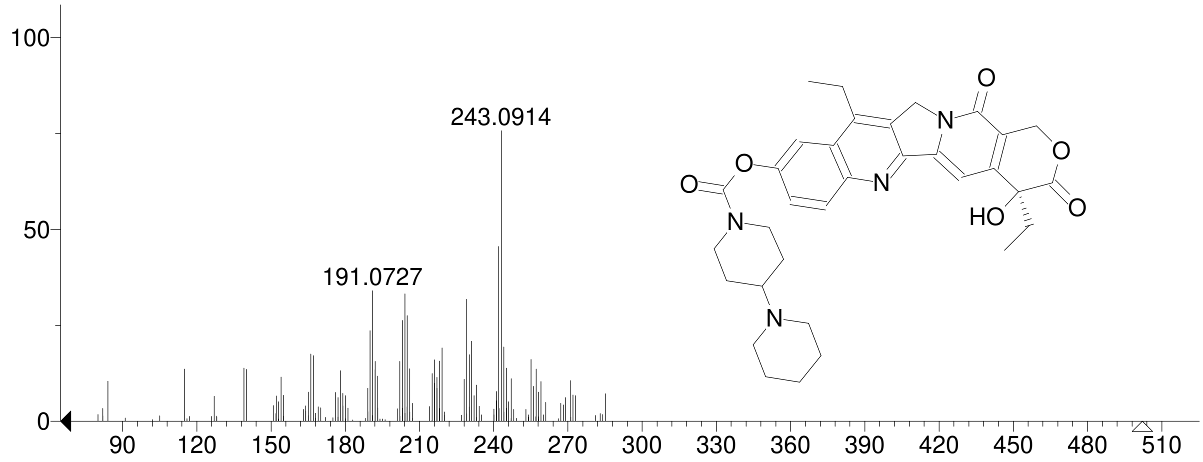

Hit 2 : "Adefovir Dipivoxil (Preveon, Hepsera)" [M+H]+ P=502.2
C20H32N5O8P; MF: 0; RMF: 13; Prob 50.0%; Lib: mona-export-gnps; ID: 580.

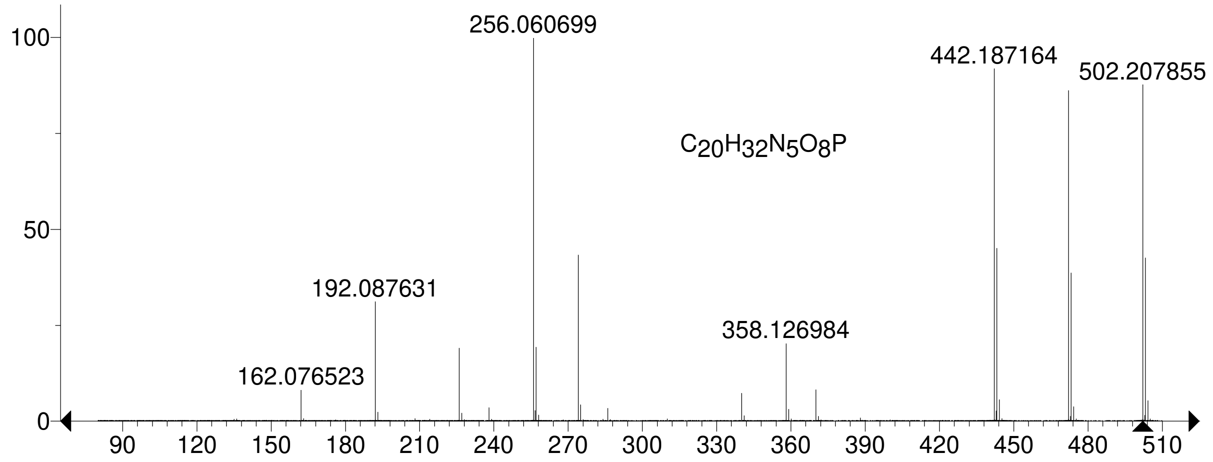

Unknown: Unknown (8.46_300.1330m/z)
Compound in Library Factor = 0

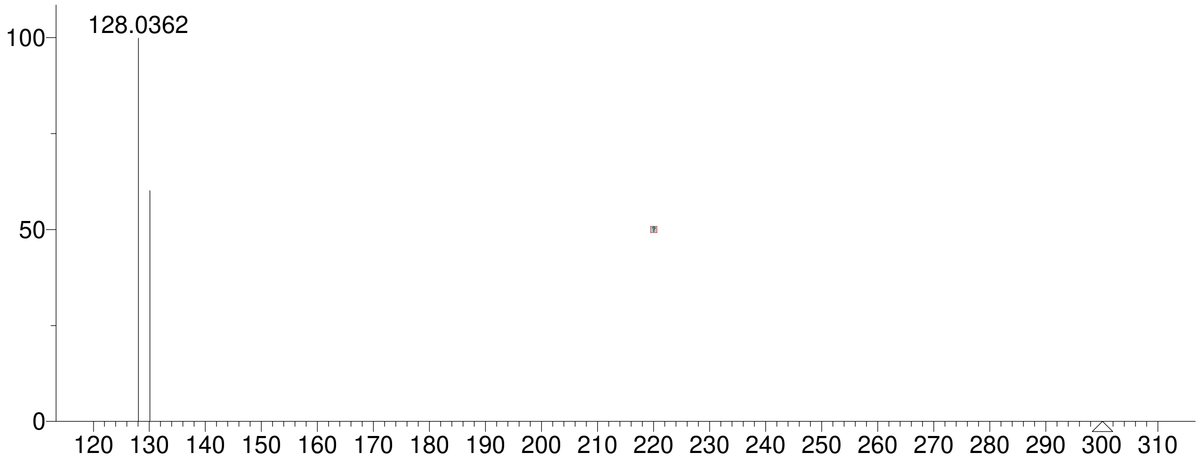

Unknown: Unknown (8.46_399.2118m/z)
Compound in Library Factor = -3273

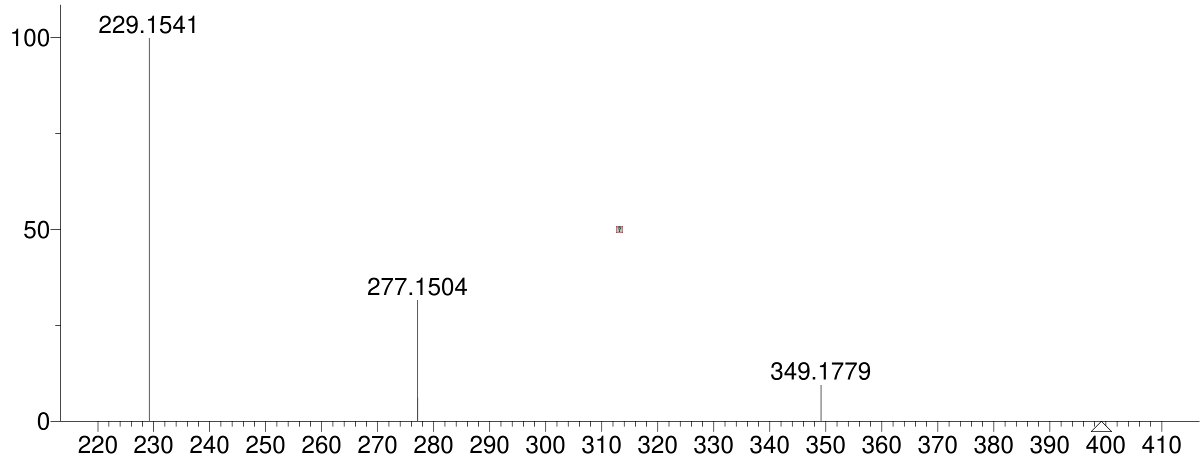

Hit 1 : Ser-His-Arg [M+H]+ QTOF 30V P=399.2
C15H26N8O5; MF: 1; RMF: 135; Prob 51.0%; Lib: nist_msms; ID: 53772.

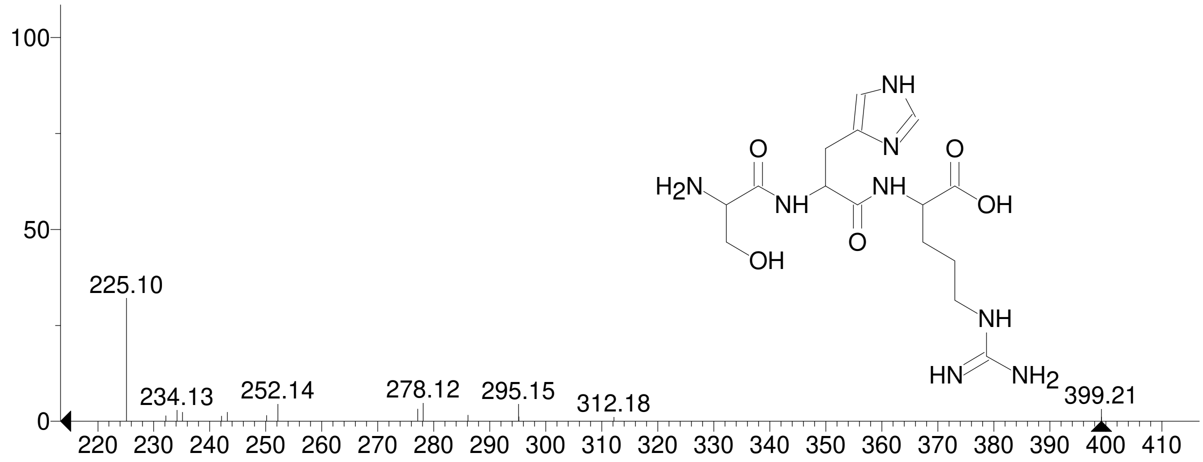

Hit 2 : N-Desmethylvinblastine [M+2H]2+ HCD 43V P=399.2
C45H56N4O9; MF: 0; RMF: 29; Prob 49.0%; CAS: 18172-50-4; Lib: nist_msms; ID: 435593.

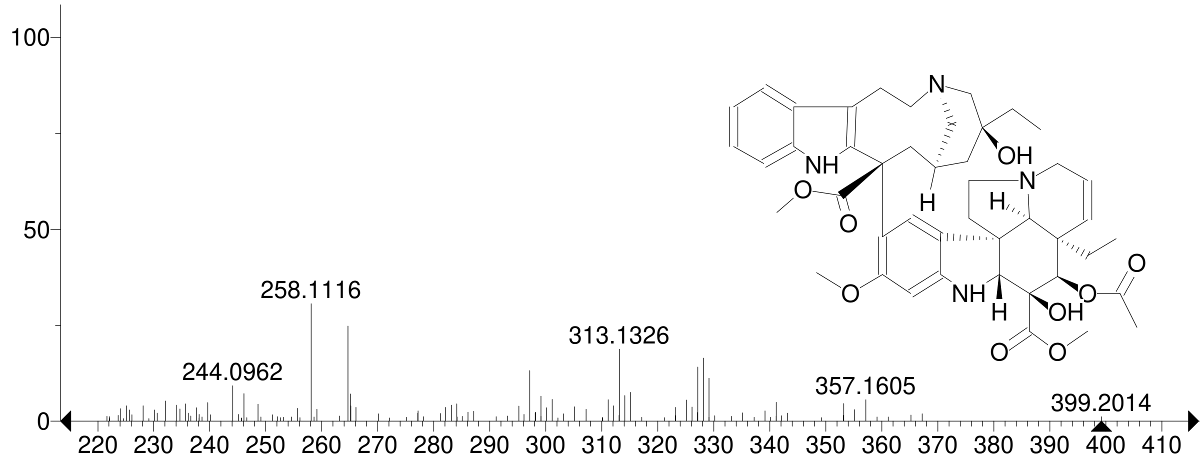

Unknown: Unknown (8.48_770.2048m/z)
Compound in Library Factor = 0

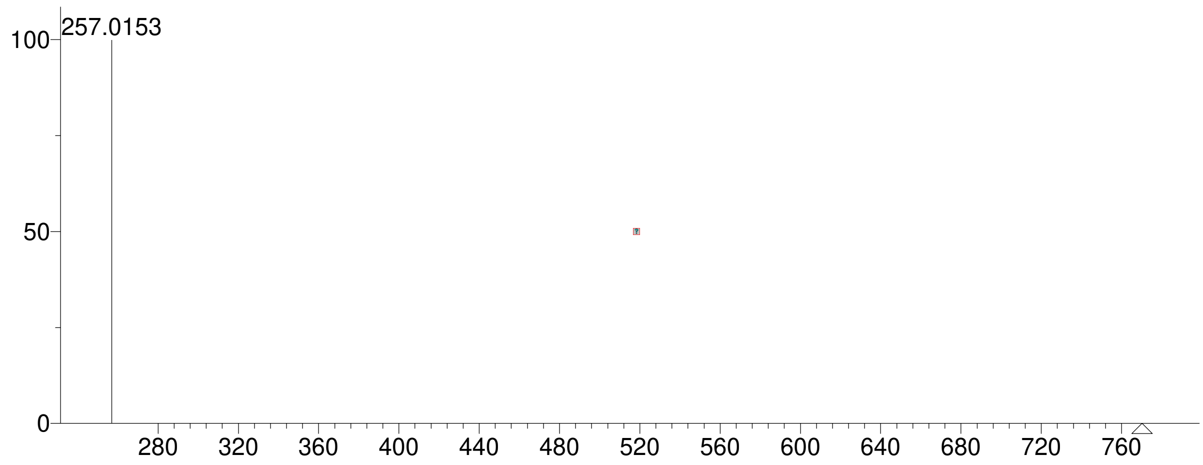

Unknown: Unknown (8.49_370.1608m/z)
Compound in Library Factor = 0

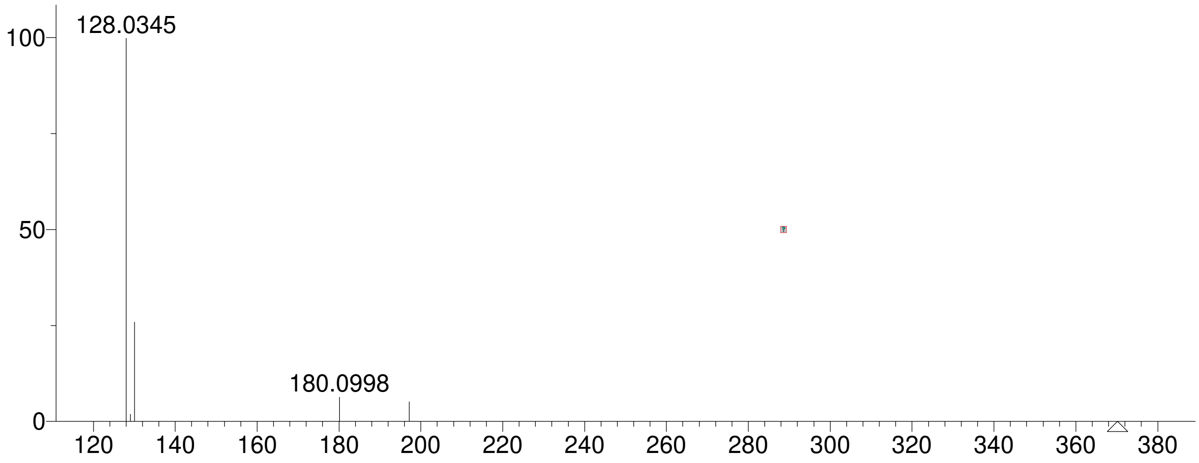

Unknown: Unknown (8.49_449.1546m/z)
Compound in Library Factor = 0

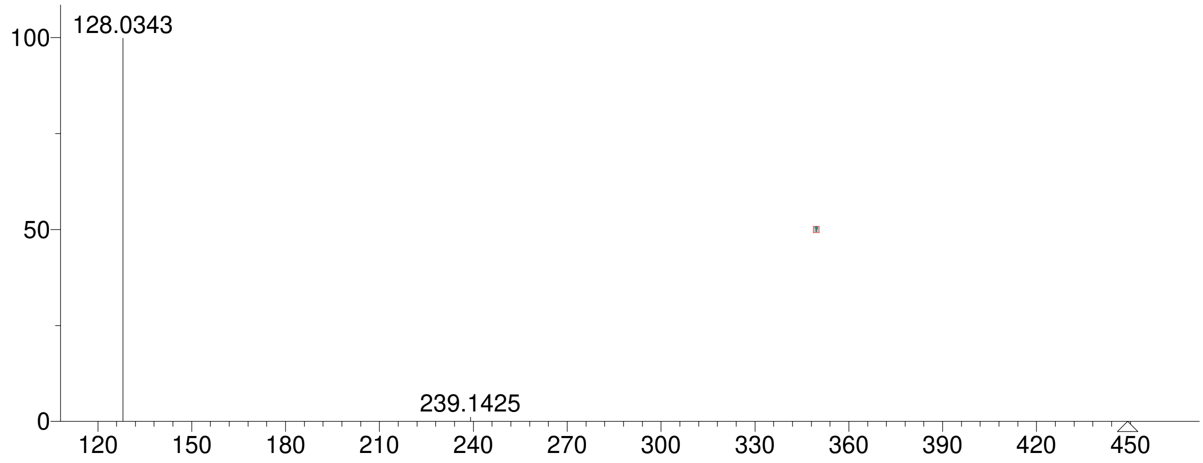

Hit 1 : NCGC00385098-01![8,8-dimethyl-9-(3-methylbut-2-enoyloxy)-2-oxo-9,10-dihydropyrano[2,3-f]chromen-10-
C24H26O7; MF: 0; RMF: 75; Prob 100.0%; Lib: mona-export-gnps; ID: 7369.

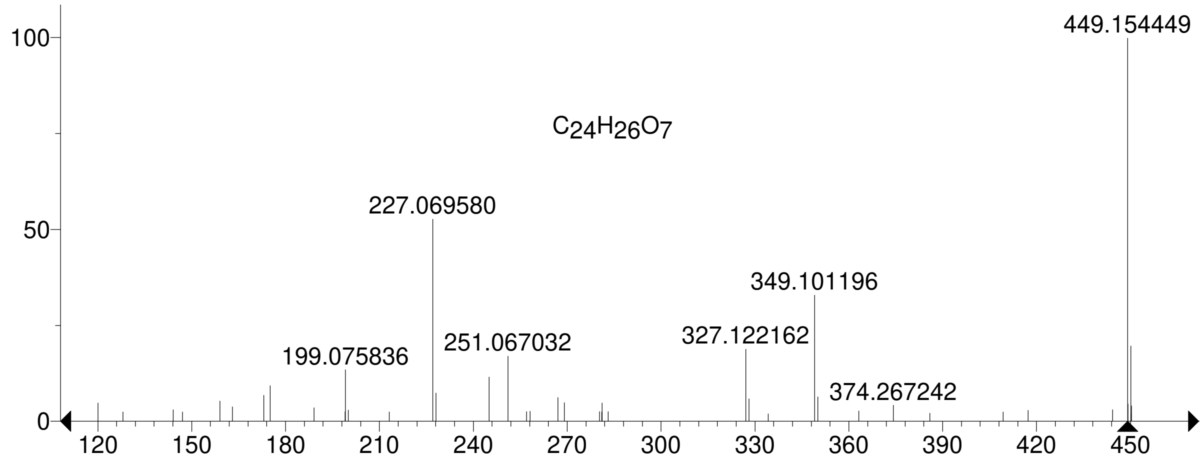

** Search Report Page 1 of 1 **

Unknown: Unknown (8.52_468.0824m/z)
Compound in Library Factor = 0

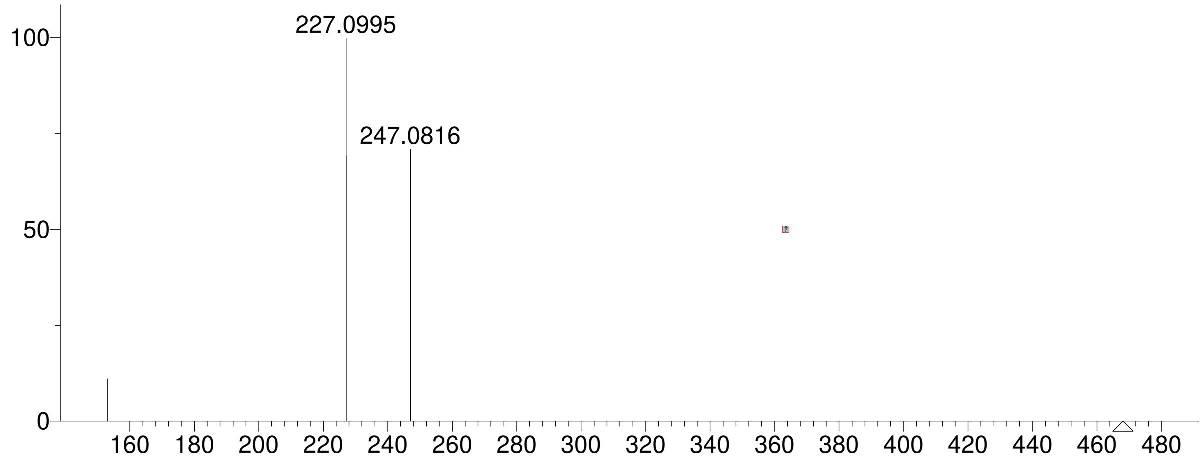

Hit 1 : 1H-Indole-5-sulfonamide, N-(3-chlorophenyl)-3-[[3,5-dimethyl-4-[(4-methyl-1-piperazinyl)carbonyl]-1H-pyrro
C28H30ClN5O4S; MF: 0; RMF: 17; Prob 100.0%; CAS: 658084-23-2; Lib: nist_msms; ID: 347792.

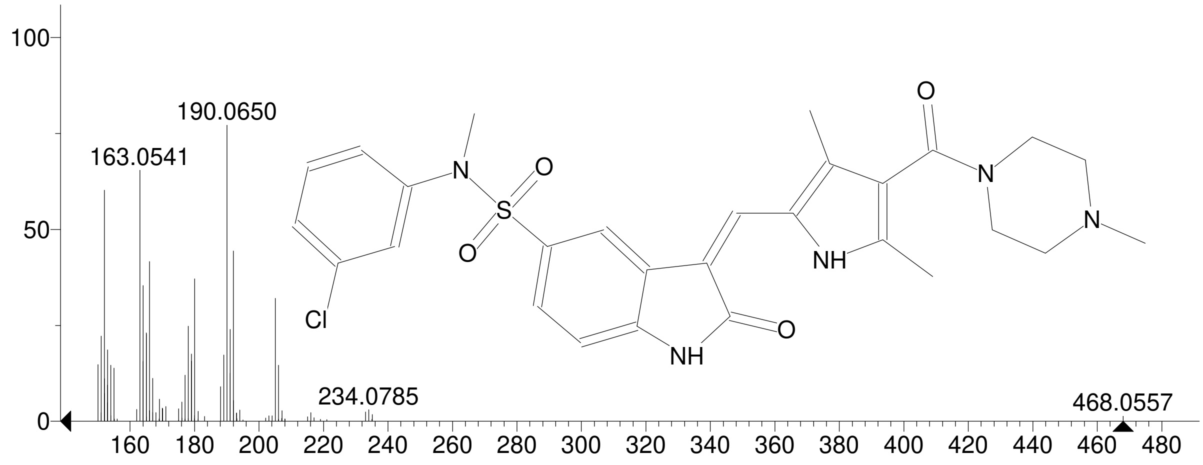

Unknown: Unknown (8.52_493.1565m/z)
Compound in Library Factor = 0

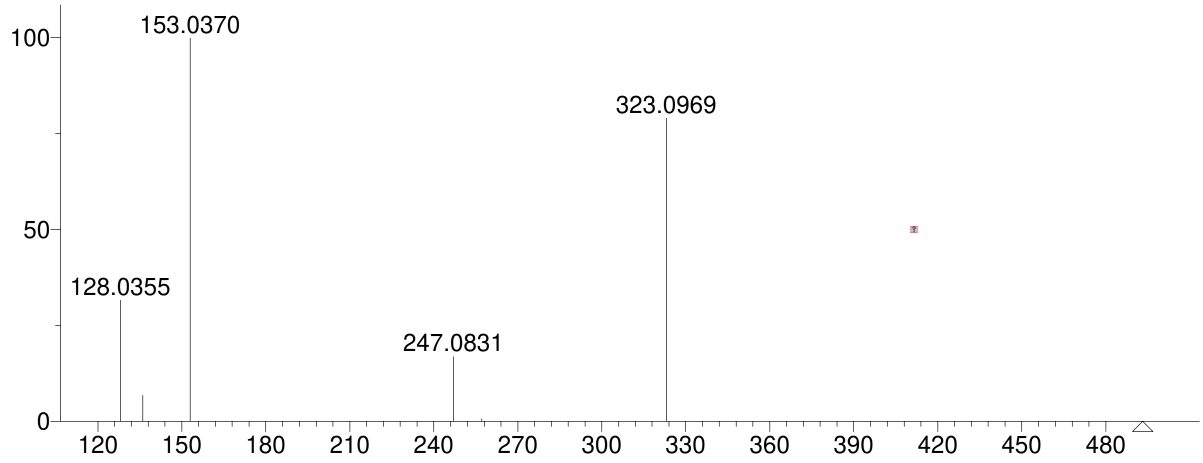

Hit 1 : Blood Group B Trisaccharide [M+Na-H2O]+ IT 35% P=493.2
C18H32O15; MF: 0; RMF: 2; Prob 100.0%; CAS: 49777-14-2; Lib: nist_msms; ID: 533822.

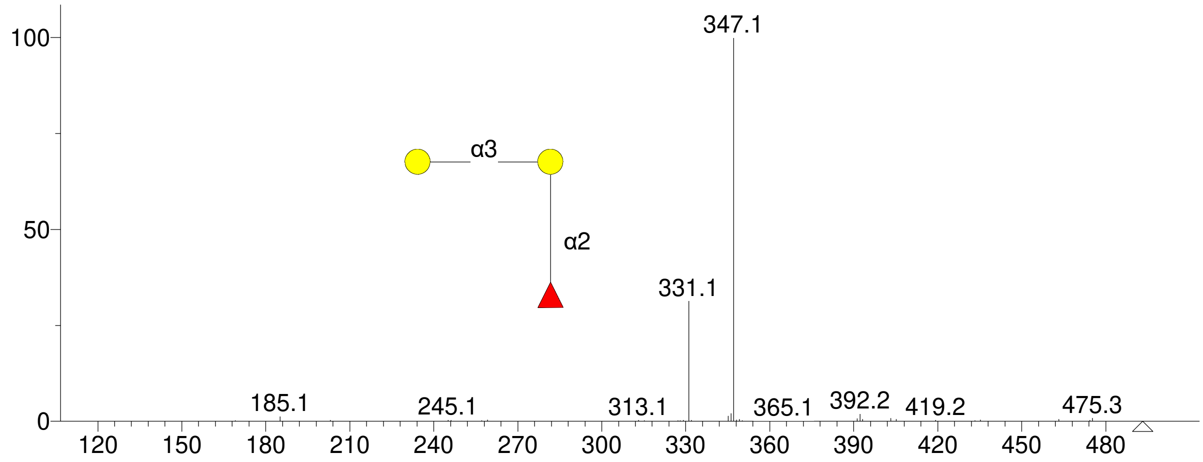

Unknown: Unknown (8.53_332.1244m/z)
Compound in Library Factor = -3273

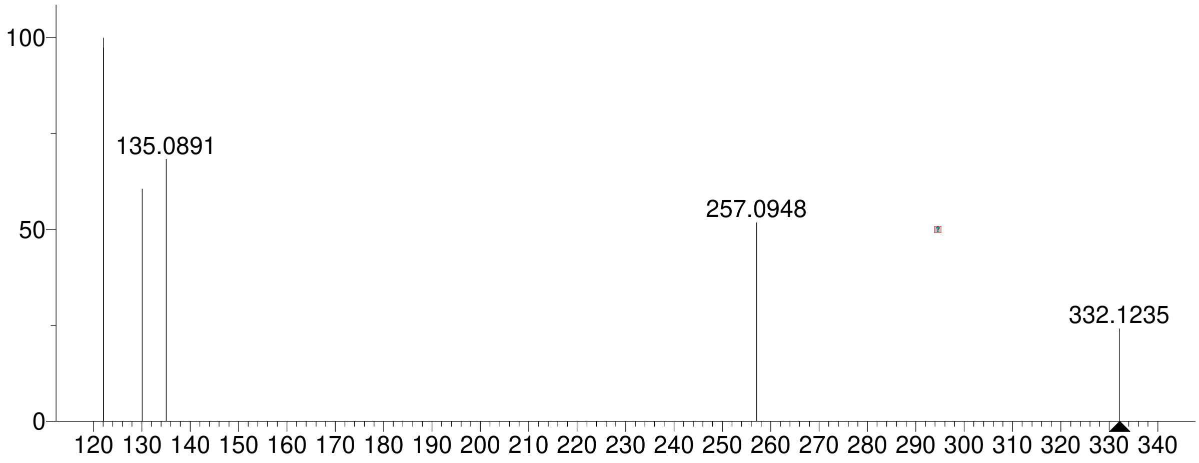

Hit 1 : Glu-Trp [M-H]- HCD 9eV P=332.1
C16H19N3O5; MF: 19; RMF: 237; Prob 23.0%; Lib: nist_msms; ID: 567396.

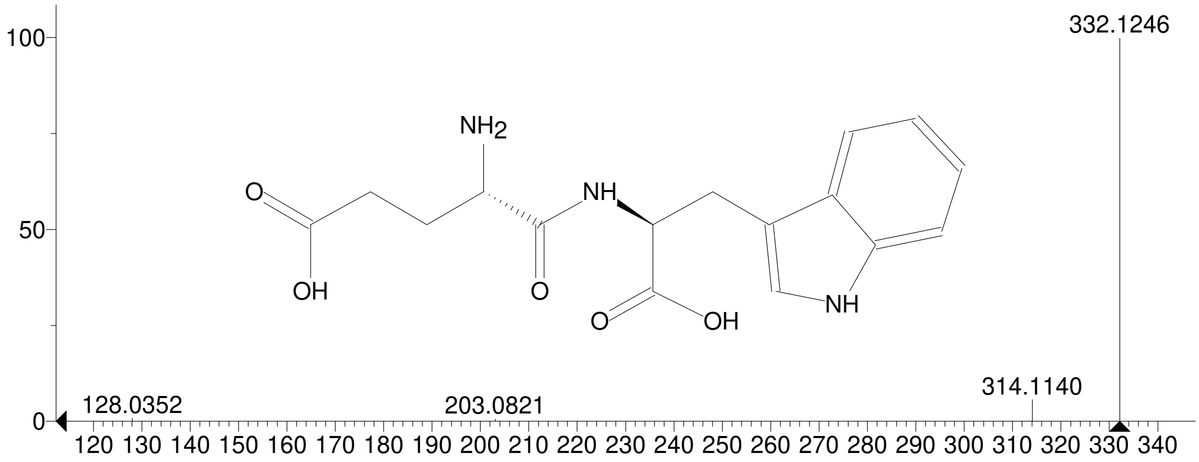

Hit 2 : Glu-Trp [M-H]- HCD 9eV P=332.1
C16H19N3O5; MF: 19; RMF: 237; Prob 23.0%; CAS: 38101-59-6; Lib: nist_msms2; ID: 70253.

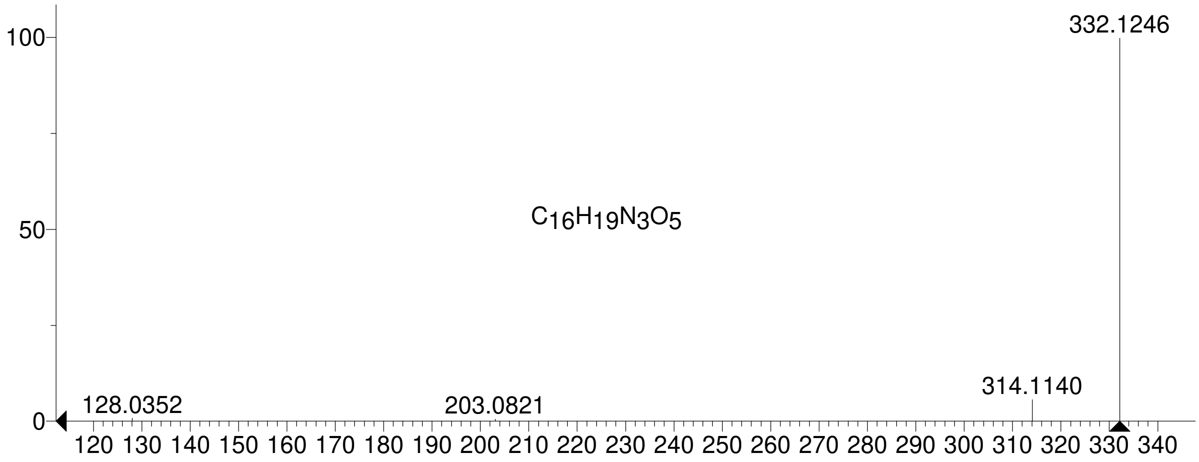

Hit 3 : Ciodrin [M+NH4]+ HCD 1V P=332.1
C14H19O6P; MF: 10; RMF: 279; Prob 16.7%; CAS: 7700-17-6; Lib: nist_msms; ID: 472079.

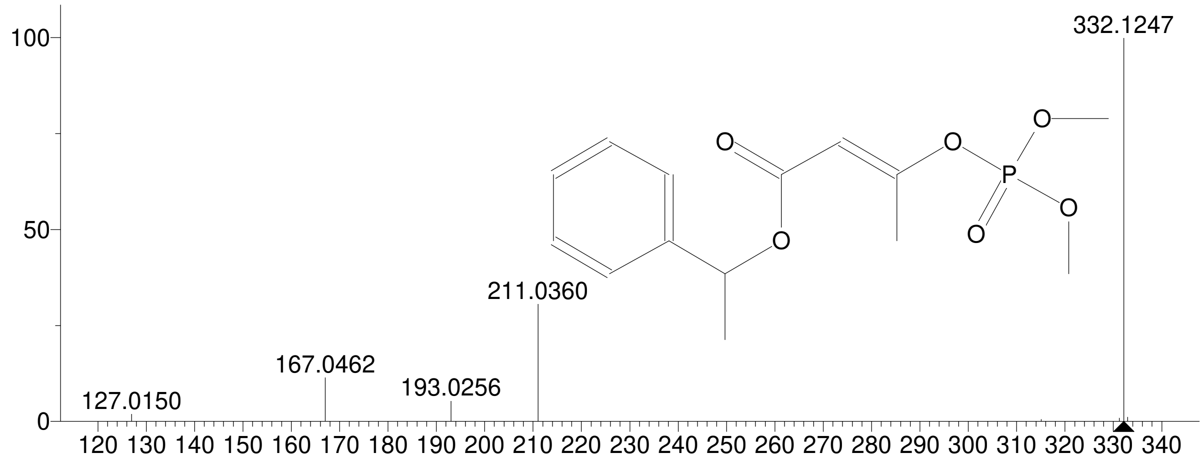

Hit 4 : Trp-Glu [M-H]- HCD 14eV P=332.1
C16H19N3O5; MF: 4; RMF: 210; Prob 13.1%; Lib: nist_msms; ID: 566618.

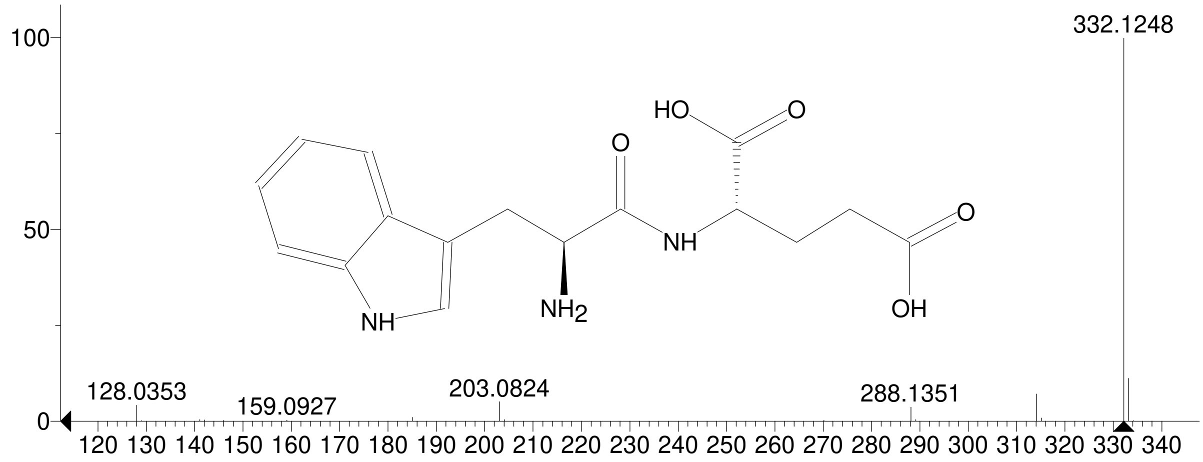

Hit 5 : Trp-Glu [M-H]- HCD 14eV P=332.1
C16H19N3O5; MF: 4; RMF: 210; Prob 13.1%; CAS: 36099-95-3; Lib: nist_msms2; ID: 69475.

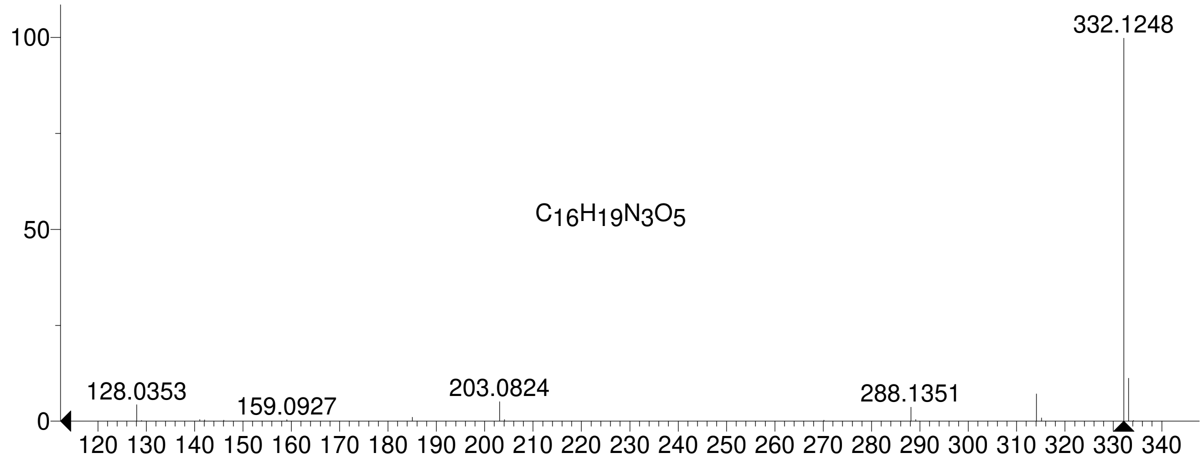

Unknown: Unknown (8.55_503.1763m/z)
Compound in Library Factor = -885

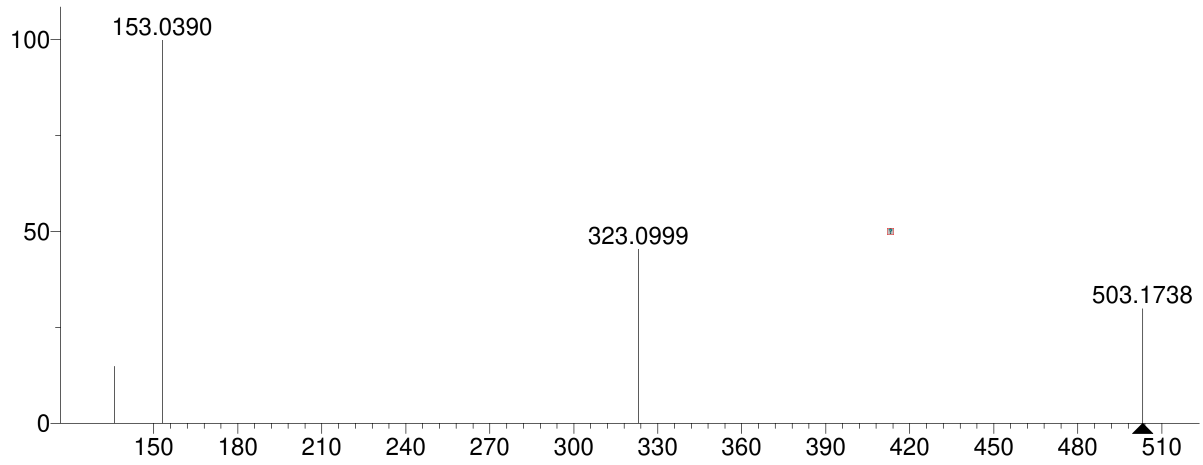

Hit 1 : Melezitose [M-H]- 35% P=503.2
C18H32O16; MF: 99; RMF: 707; Prob 87.1%; Lib: mona-export-itree; ID: 3188.

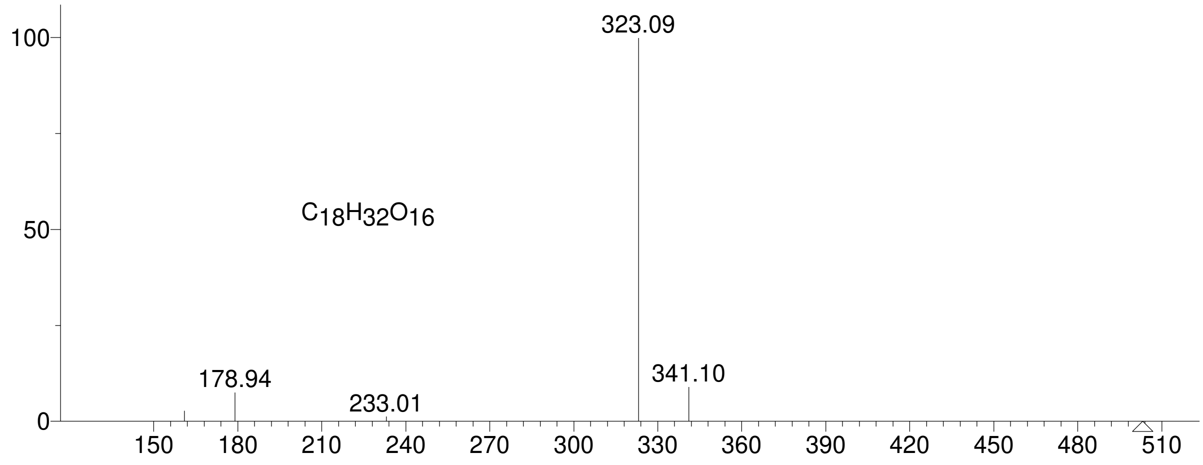

Hit 2 : NCGC00380568-01!2-(hydroxymethyl)-6-[[3,4,5-trihydroxy-6-(4-prop-2-enylphenoxy)oxan-2-yl]methoxy]oxa
C21H30O11; MF: 9; RMF: 344; Prob 7.45%; Lib: mona-export-gnps; ID: 8317.

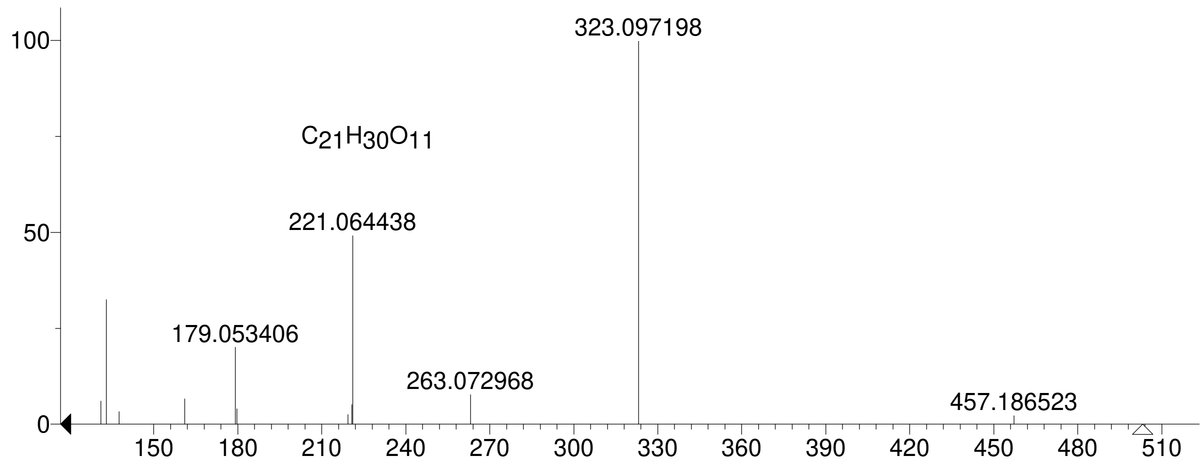

Hit 3 : [3-(13-methyltetradecyl)-5-sulfooxyphenyl] hydrogen sulfate [M+Na]+ 65HCD P=503.2
C21H36O8S2; MF: 0; RMF: 38; Prob 5.40%; Lib: mona-export-vf-npl_qexactive; ID: 18237.

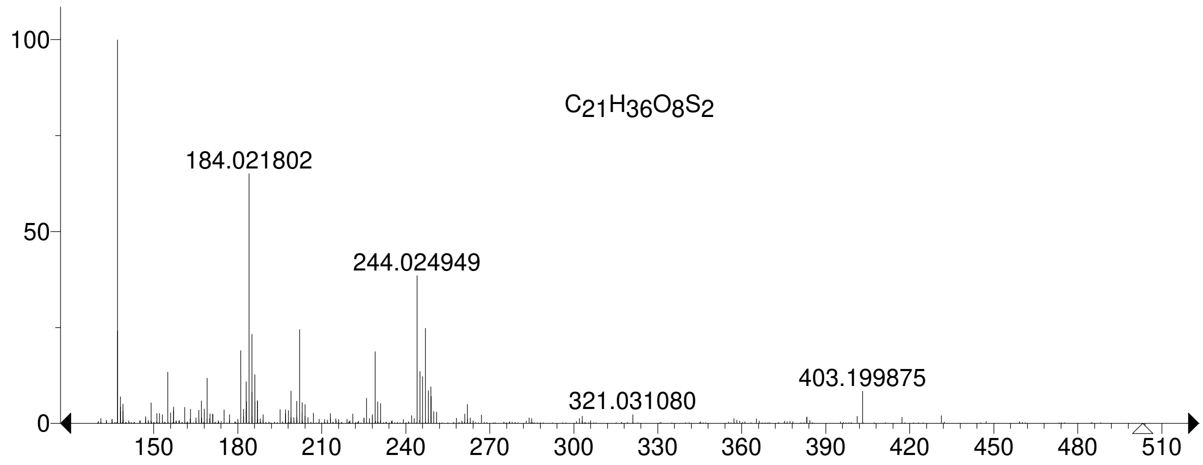

Unknown: Unknown (8.55_579.2545m/z)
Compound in Library Factor = 0

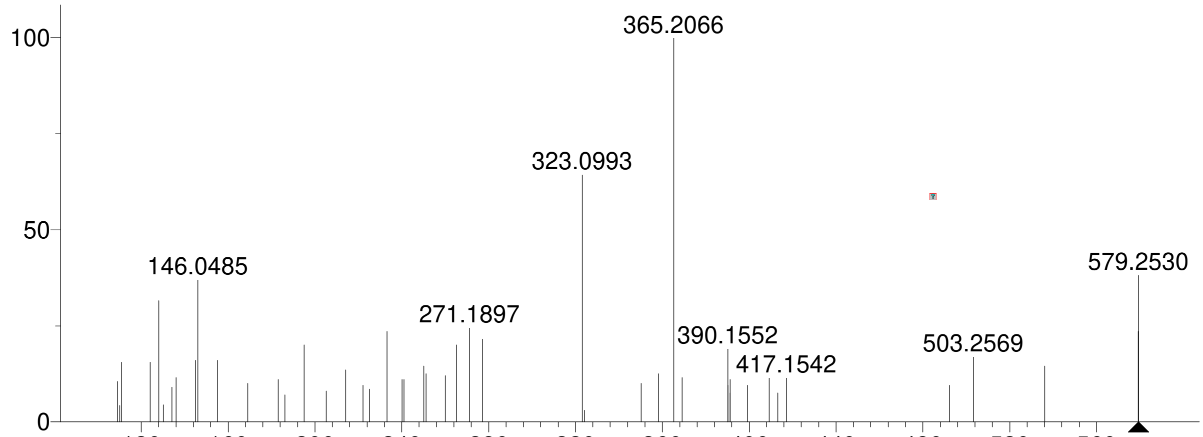

Hit 1 : Naringin M-H 35 P=579.2
C27H32O14; MF: 0; RMF: 21; Prob 100.0%; Lib: anthocyanins mona; ID: 1179.

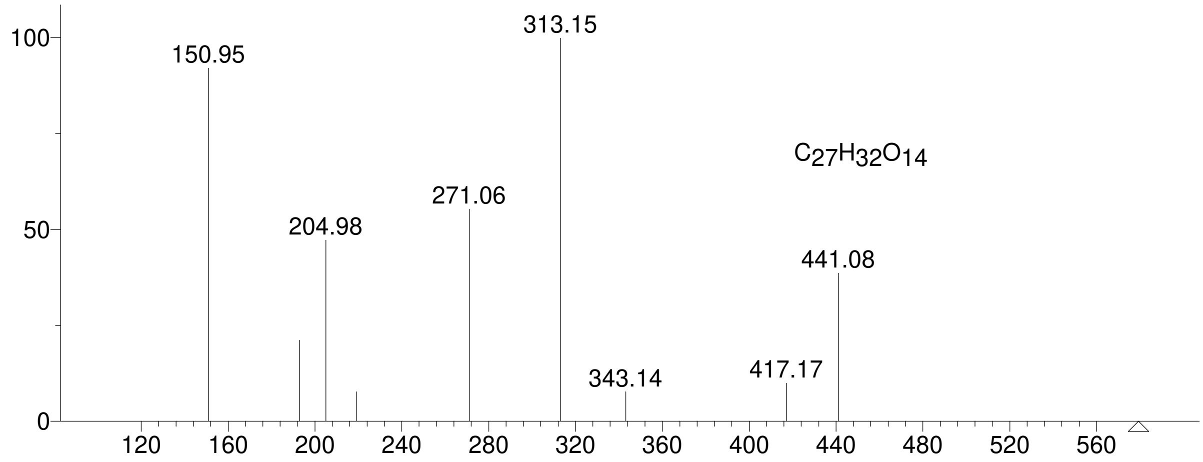

Unknown: Unknown (8.57_431.0641m/z)
Compound in Library Factor = -885

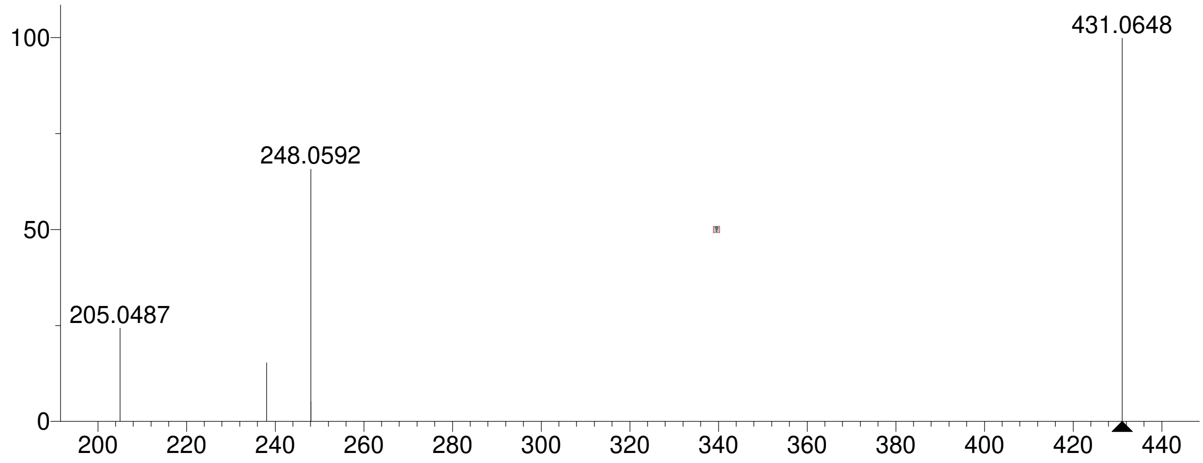

Hit 1 : Bicalutamide [M+H]+ HCD 7V P=431.1
C18H14F4N2O4S; MF: 112; RMF: 664; Prob 94.0%; CAS: 90357-06-5; Lib: nist_msms; ID: 116227.

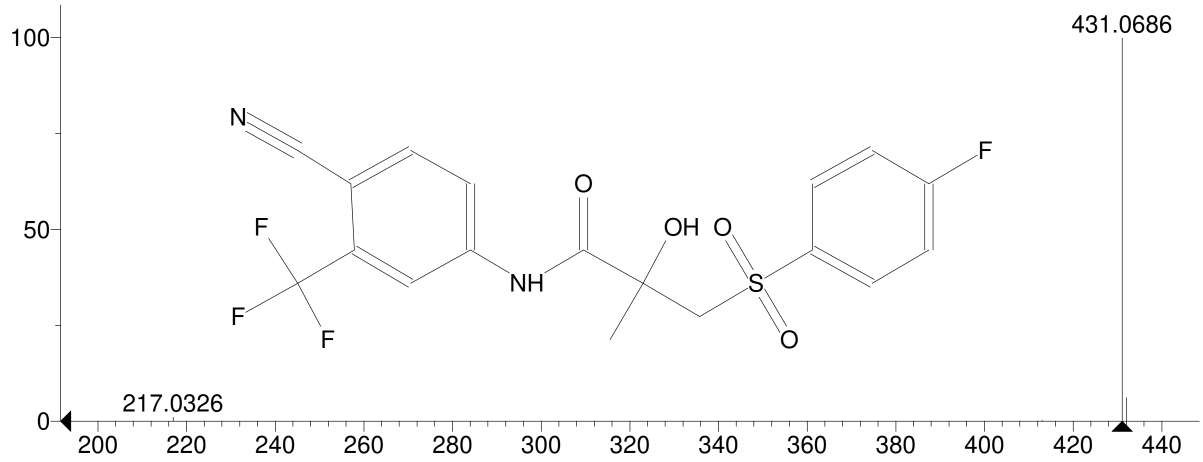

Hit 2 : Bicalutamide (Casodex) [M+H]+ P=431.1
C18H14F4N2O4S; MF: 1; RMF: 195; Prob 5.94%; Lib: mona-export-gnps; ID: 365.

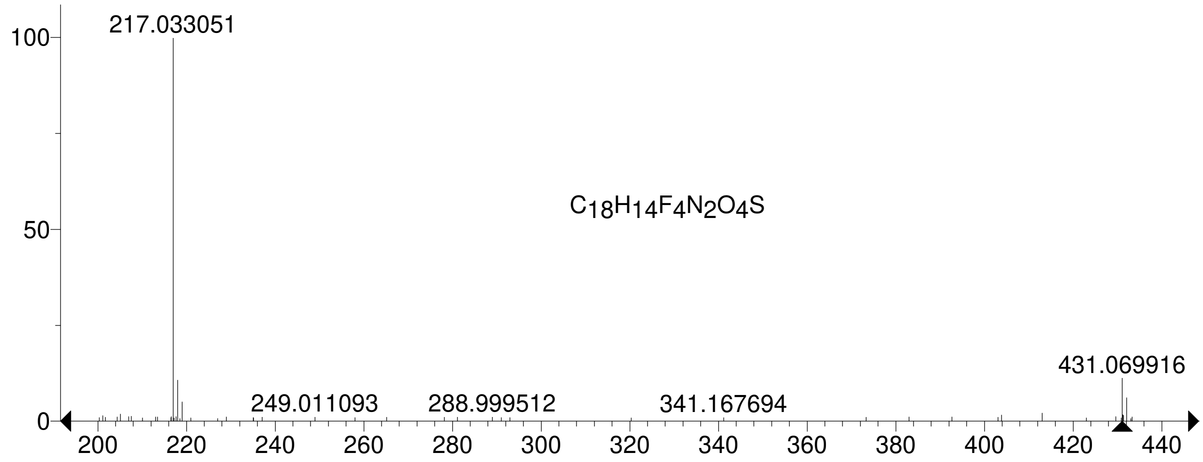

** Search Report Page 1 of 1 **

Unknown: Unknown (8.58_403.1608m/z)
Compound in Library Factor = 0

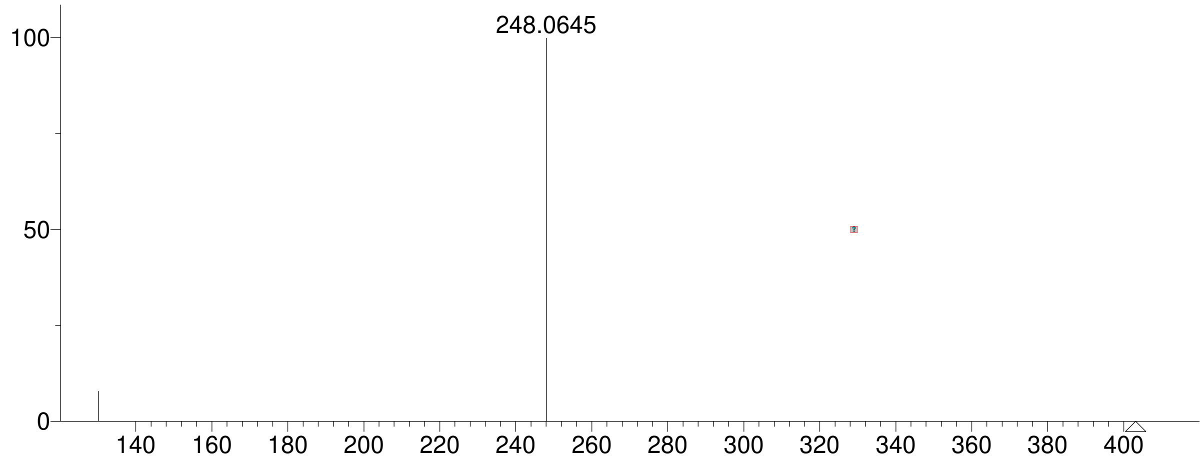

Unknown: Unknown (8.58_493.1560m/z)
Compound in Library Factor = 0

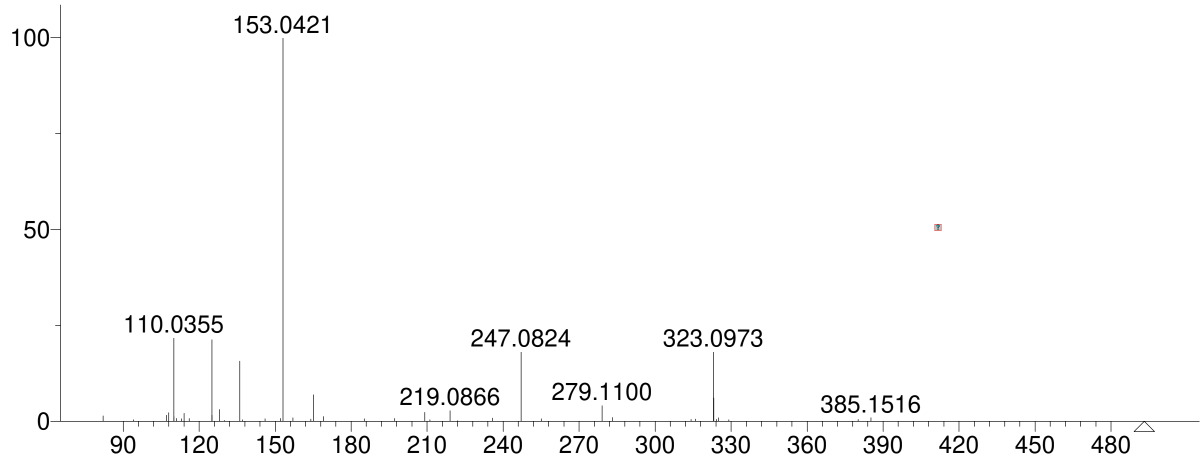

Hit 1 : Blood Group B Trisaccharide [M+Na-H2O]+ HCD 44V P=493.2
C18H32O15; MF: 0; RMF: 3; Prob 100.0%; CAS: 49777-14-2; Lib: nist_msms; ID: 533818.

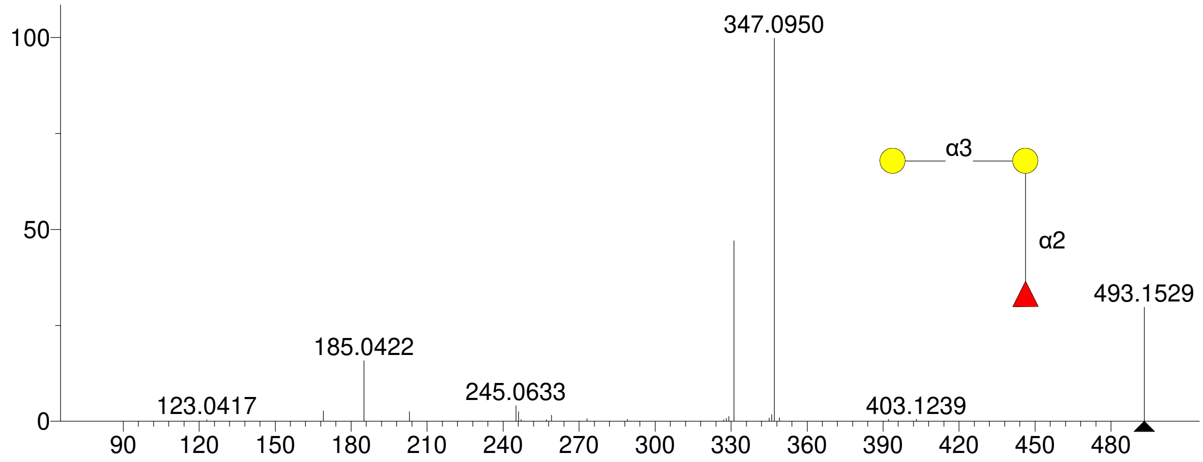

Unknown: Unknown (8.61_557.1987m/z)
Compound in Library Factor = 0

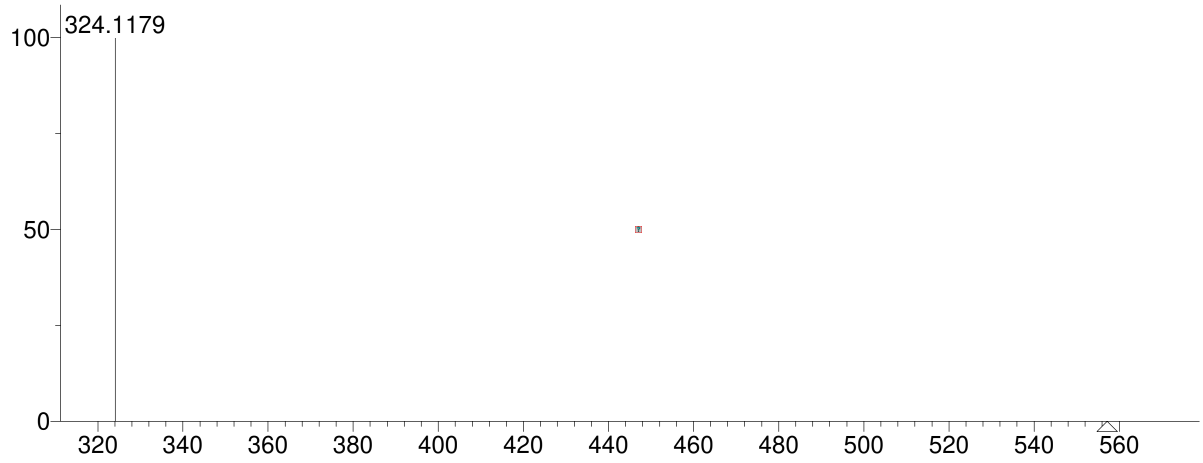

Hit 1 : NCGC00381419-01_C28H30N4O7_ [M+Na]+ P=557.2
C28H30N4O7; MF: 0; RMF: 178; Prob 33.3%; Lib: mona-export-gnps; ID: 12094.

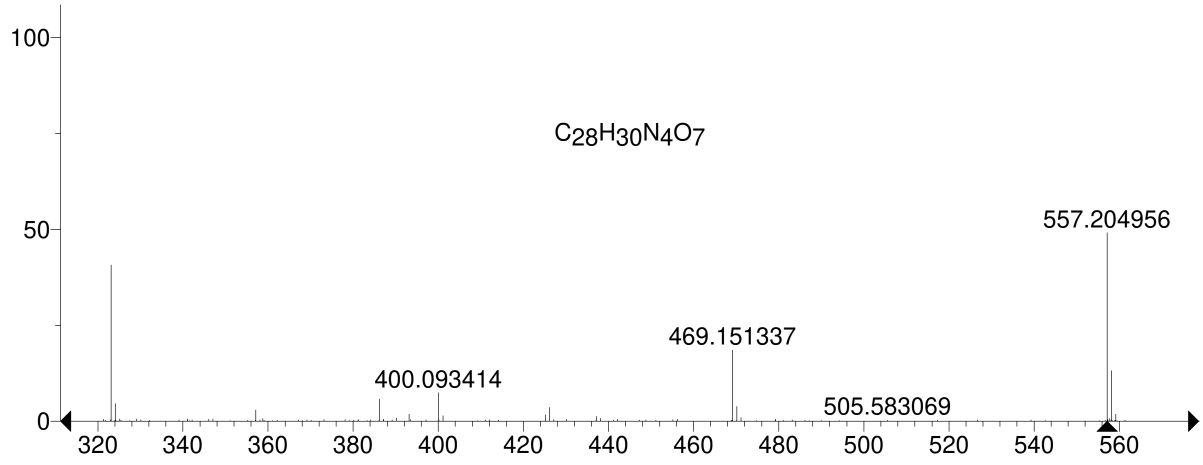

Hit 2 : 2-[4-[(3R,3aR,6S,6aR)-3-(3,4-dimethoxyphenyl)-1,3,3a,4,6,6a-hexahydrofuro[3,4-c]furan-6-yl]-2-methoxypl
C27H34O11; MF: 0; RMF: 61; Prob 33.3%; Lib: anthocyanins mona; ID: 3790.

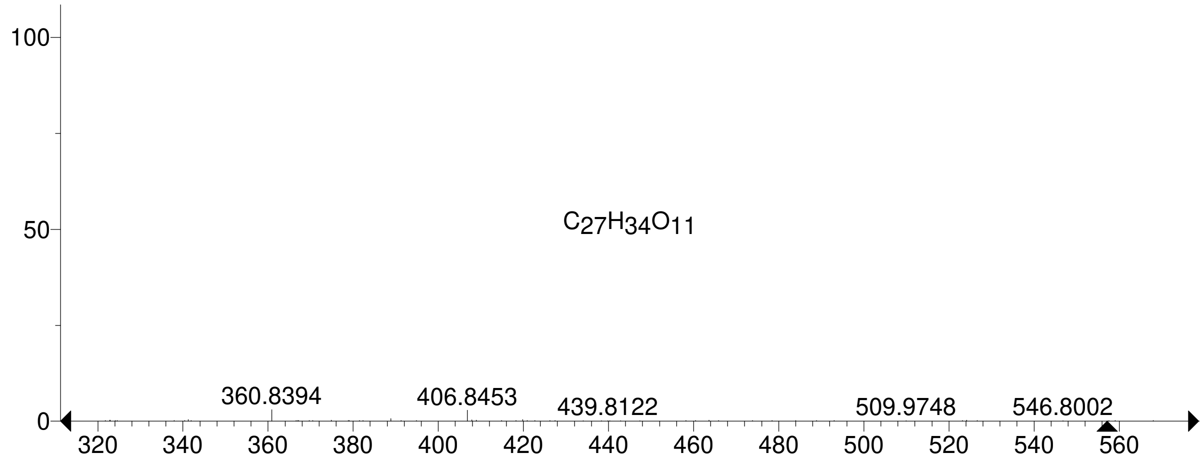

Hit 3 : arctiin [M+Na]+ 35HCD P=557.2
C27H34O11; MF: 0; RMF: 5; Prob 33.3%; Lib: anthocyanins mona; ID: 3554.

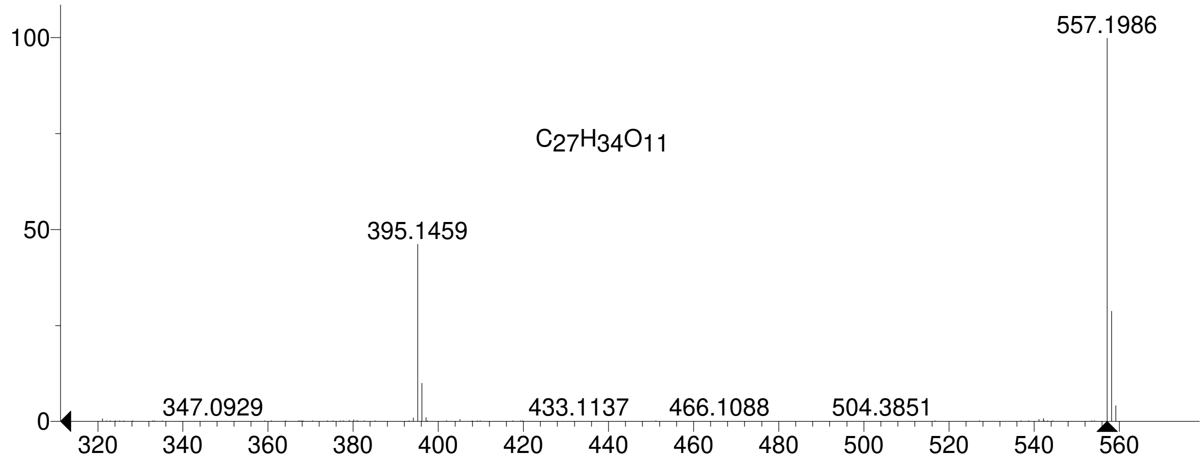

** Search Report Page 1 of 1 **

Unknown: Unknown (8.62_362.1341m/z)
Compound in Library Factor = 0

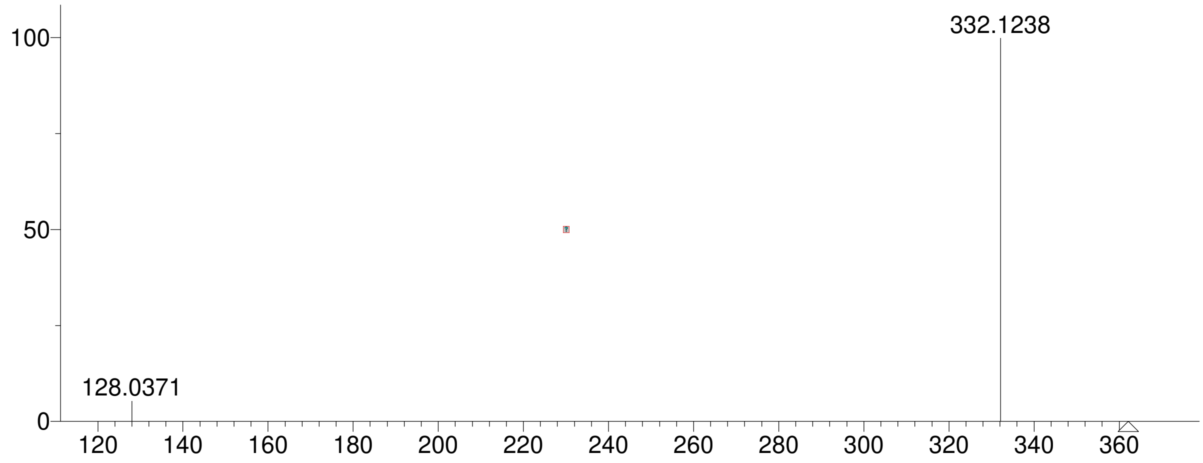

Hit 1 : (Z)-Pyriminobac-methyl [M+H]+ HCD 115V P=362.1
C17H19N3O6; MF: 0; RMF: 45; Prob 100.0%; CAS: 147411-70-9; Lib: nist_msms; ID: 469207.

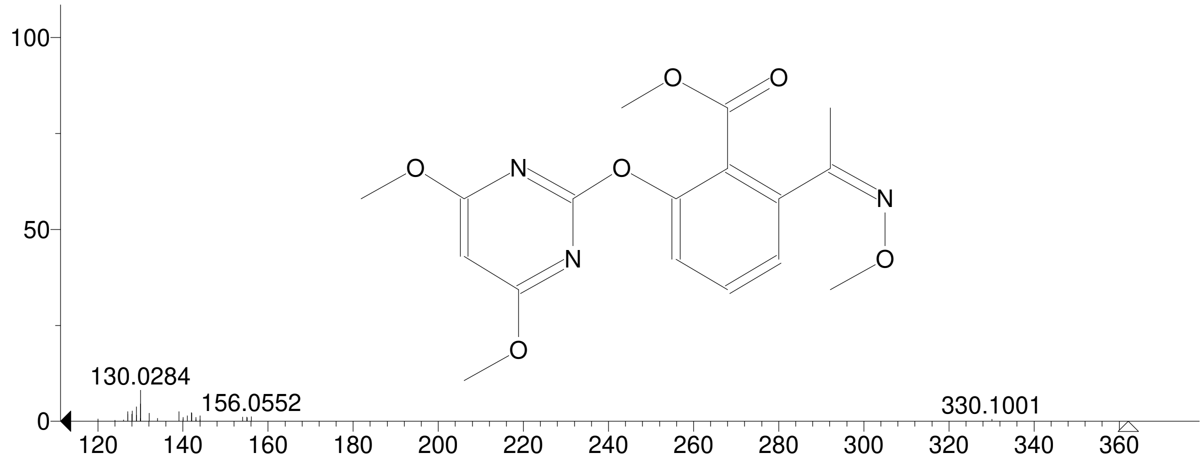

Unknown: Unknown (8.67_401.0537m/z)
Compound in Library Factor = 0

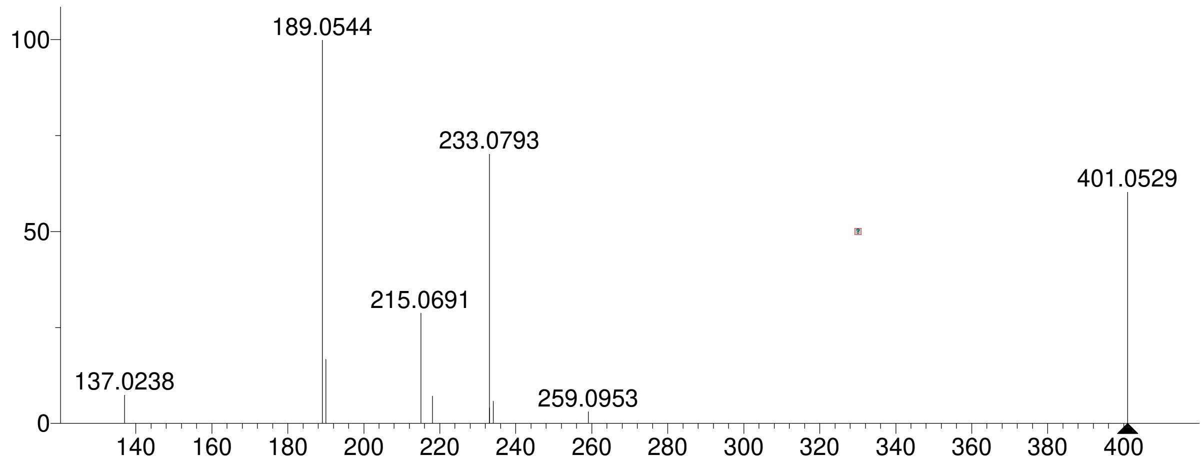

Unknown: Unknown (8.68_472.1934m/z)
Compound in Library Factor = 0

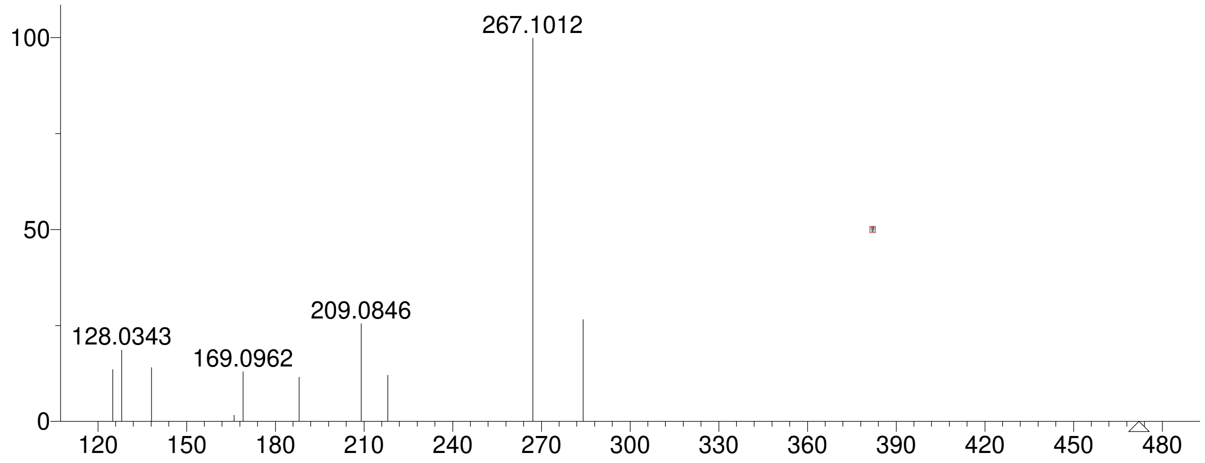

Hit 1 : methyl 3-(3,4-dihydroxyphenyl)-2-[[4-hydroxy-3-(3-methylbut-2-enyl)phenyl]methyl]-4-methoxy-5-oxofuran-2
C25H26O8; MF: 0; RMF: 39; Prob 100.0%; Lib: mona-export-vf-npl_qexactive; ID: 9391.

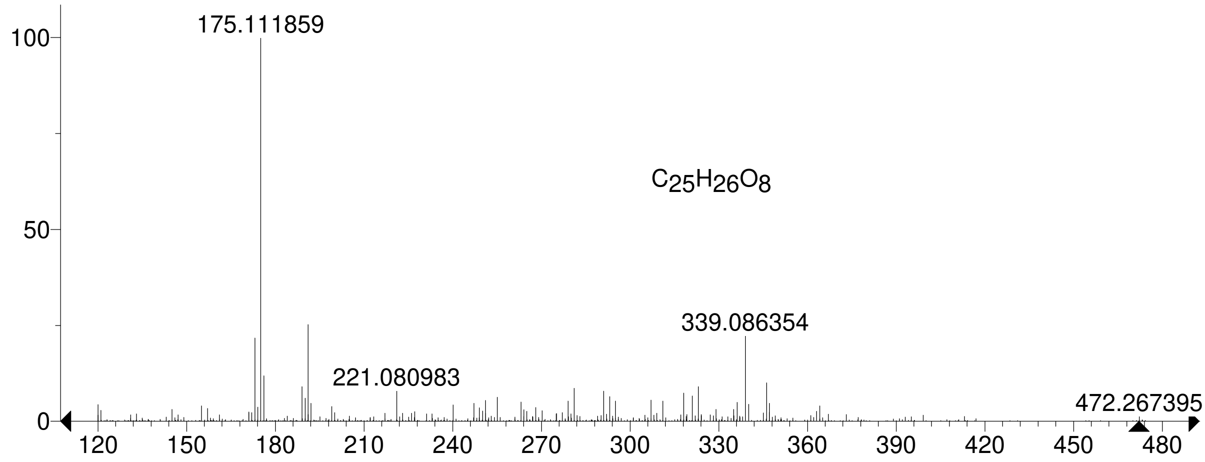

Unknown: Unknown (8.69_544.2140m/z)
Compound in Library Factor = 0

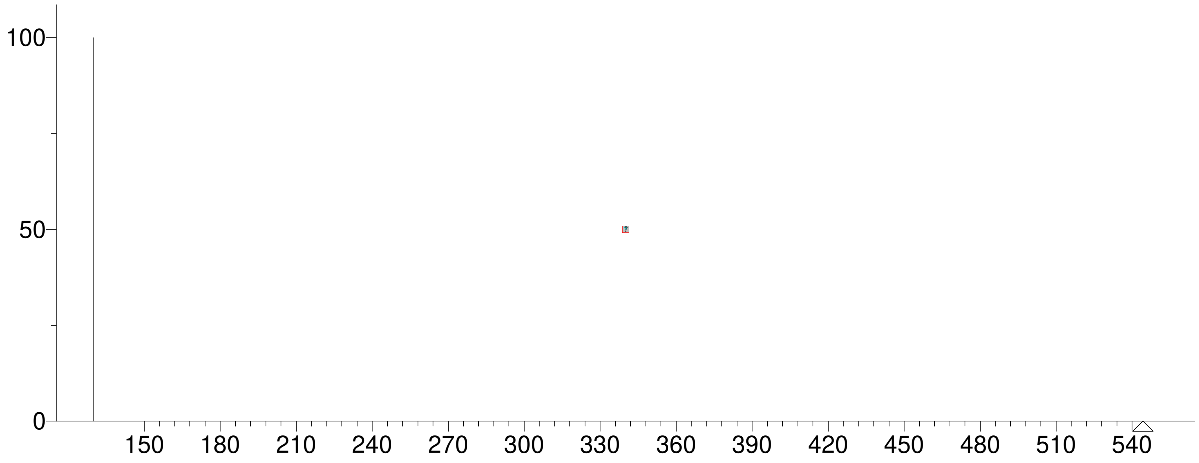

Unknown: Unknown (8.72_332.1241m/z)
Compound in Library Factor = -2387

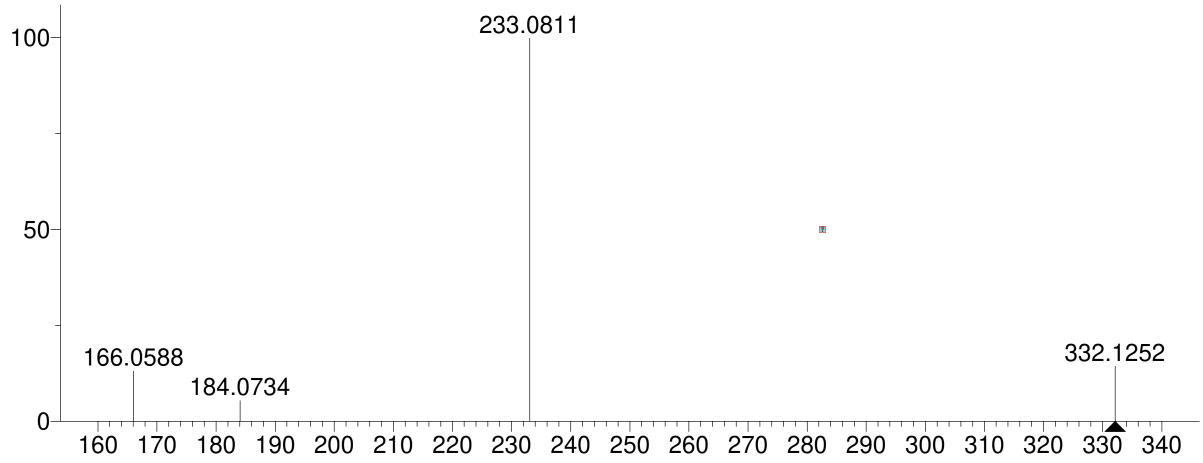

Hit 1 : Glu-Trp [M-H]- HCD 9eV P=332.1
C16H19N3O5; MF: 39; RMF: 319; Prob 32.1%; Lib: nist_msms; ID: 567396.

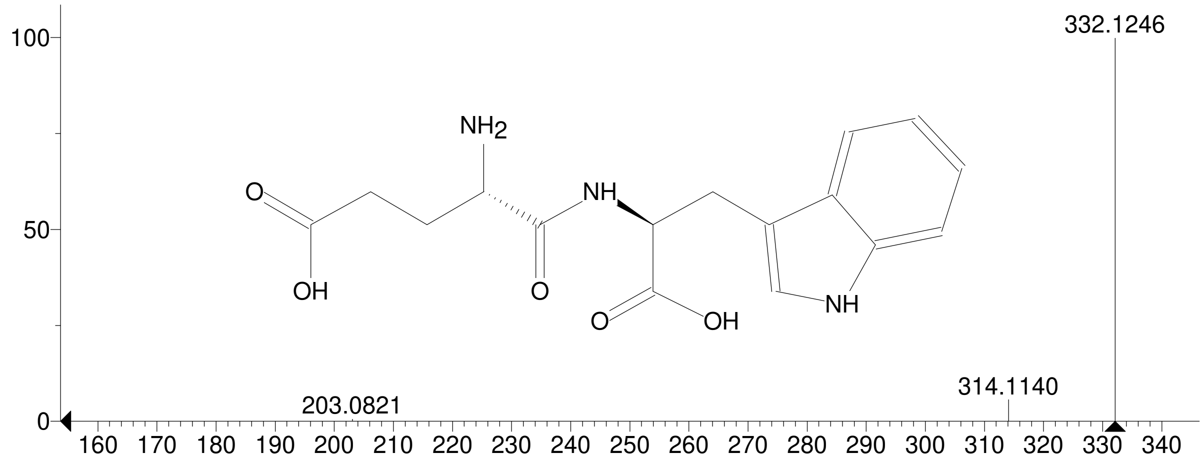

Hit 2 : Glu-Trp [M-H]- HCD 9eV P=332.1
C16H19N3O5; MF: 39; RMF: 319; Prob 32.1%; CAS: 38101-59-6; Lib: nist_msms2; ID: 70253.

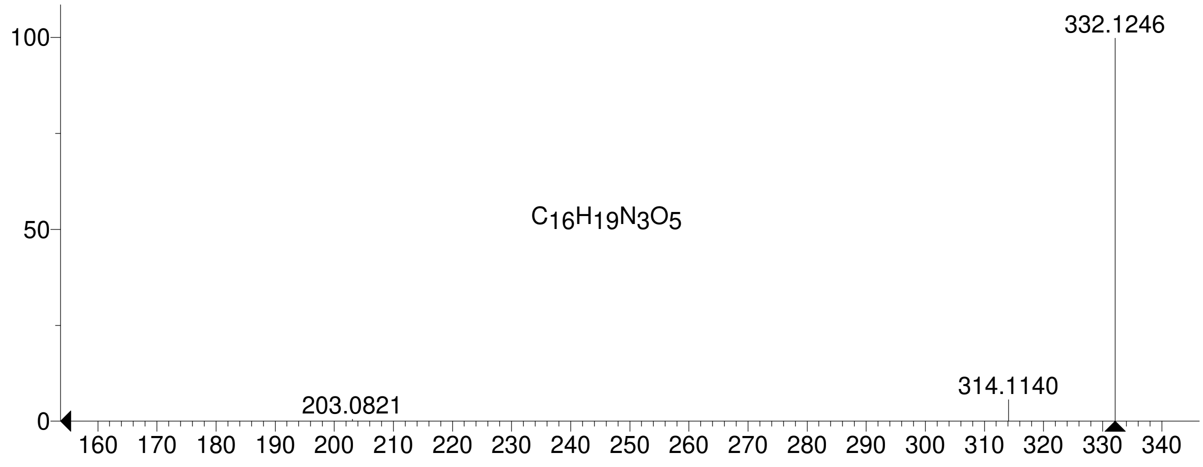

Hit 3 : Nizatidine [M+H]+ HCD 13V P=332.1
C12H21N5O2S2; MF: 13; RMF: 306; Prob 9.60%; CAS: 76963-41-2; Lib: nist_msms; ID: 152049.

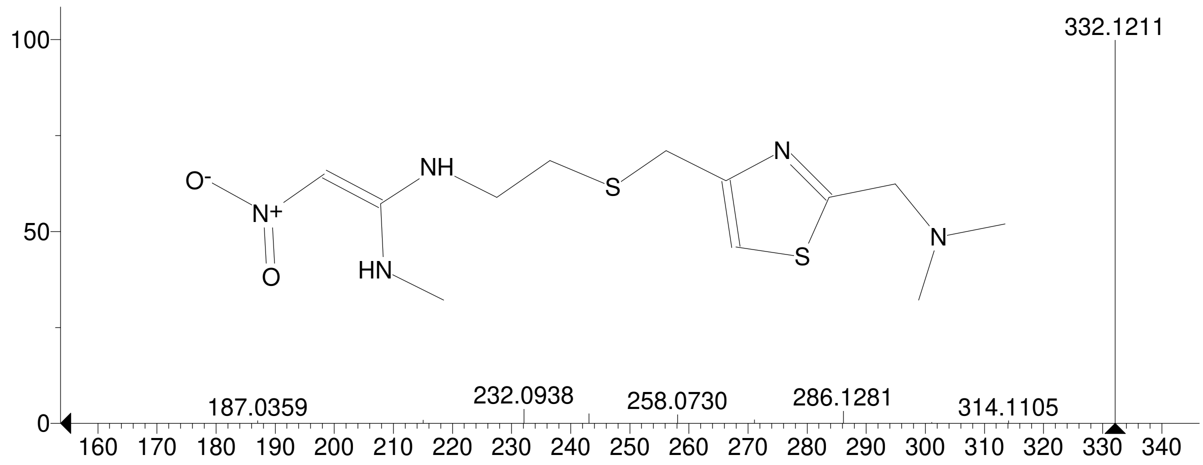

Hit 4 : Trp-Glu [M-H]- HCD 14eV P=332.1
C16H19N3O5; MF: 11; RMF: 288; Prob 8.86%; Lib: nist_msms; ID: 566618.

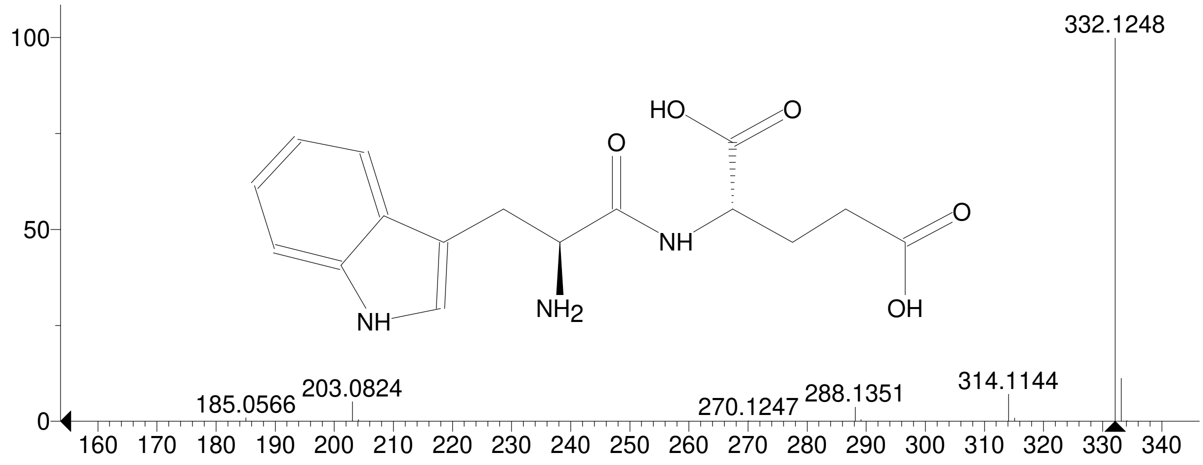

Hit 5 : Trp-Glu [M-H]- HCD 14eV P=332.1
C16H19N3O5; MF: 11; RMF: 288; Prob 8.86%; CAS: 36099-95-3; Lib: nist_msms2; ID: 69475.

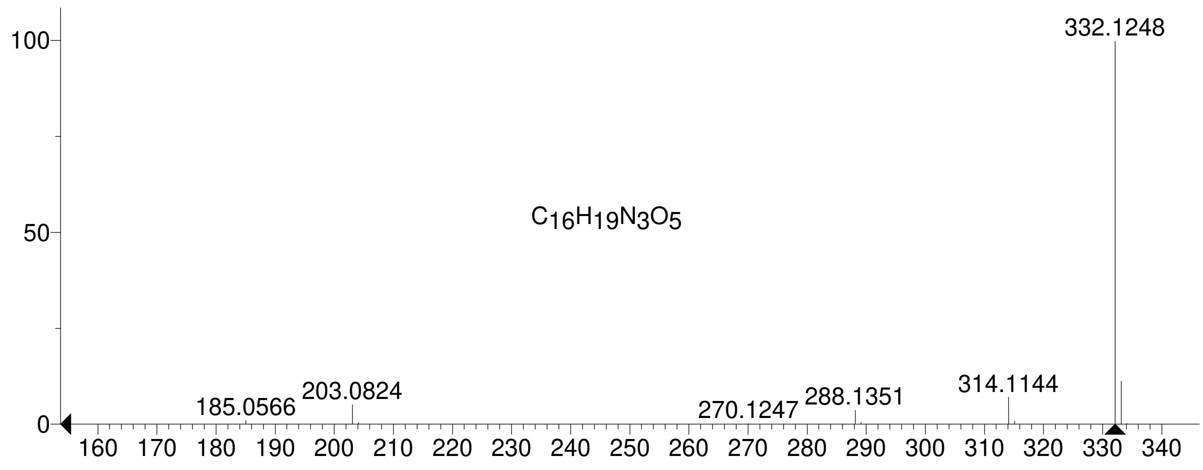

Unknown: Unknown (8.72_443.9930m/z)
Compound in Library Factor = 0

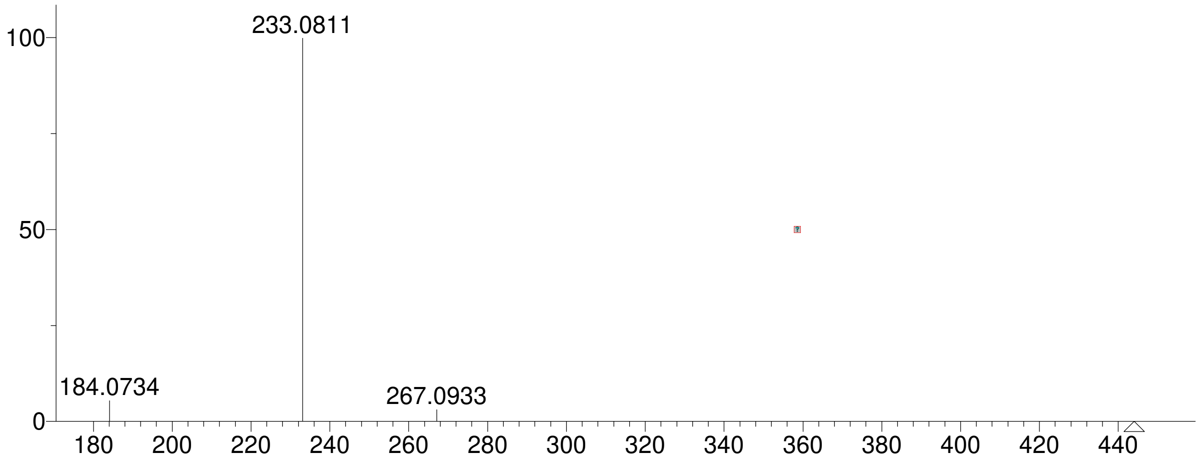

Unknown: Unknown (8.74_614.2961m/z)
Compound in Library Factor = -733

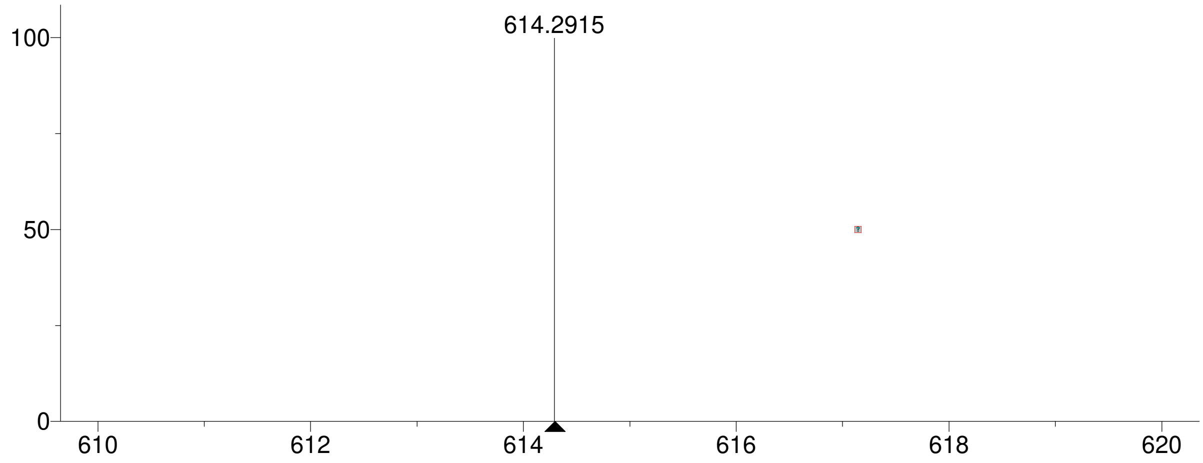

Hit 1 : NCGC00381019-01_C33H40O10_1H-Cyclopentacyclododecene-4,10(2H,5H)-dione, 3a,9,13-tris(acetylox
C33H40O10; MF: 5; RMF: 373; Prob 100.0%; Lib: mona-export-gnps; ID: 11327.

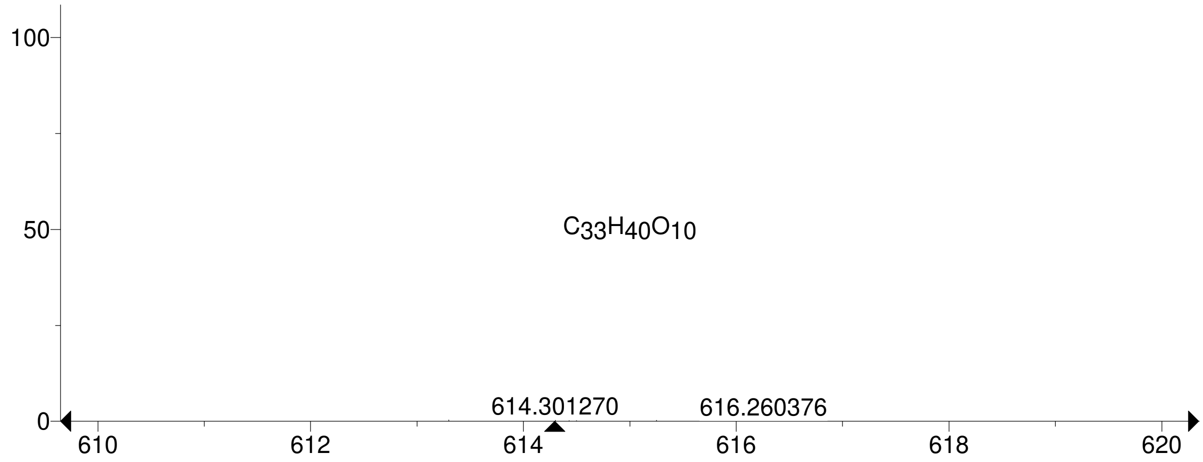

Unknown: Unknown (8.77_209.0443m/z)
Compound in Library Factor = -1309

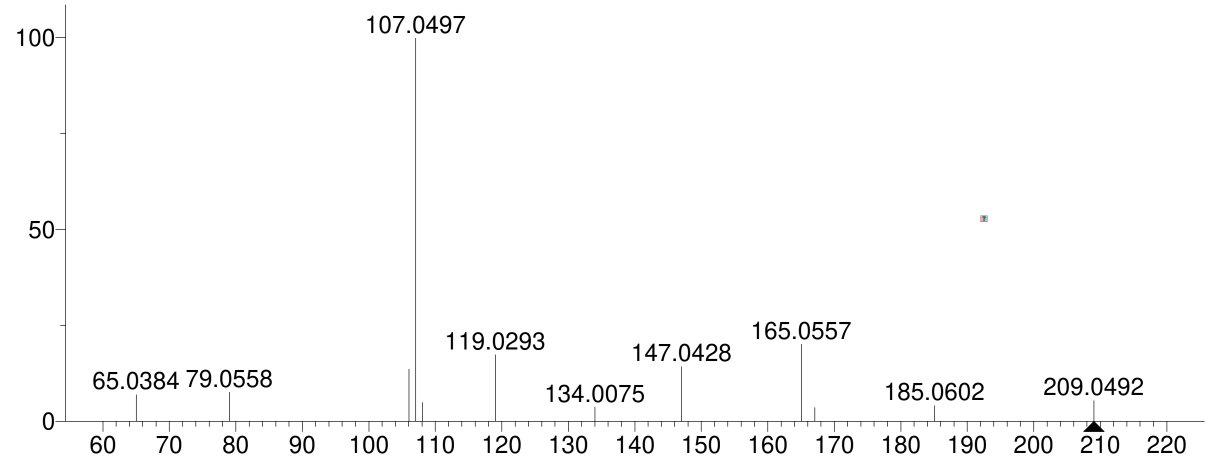

Hit 1 : Fraxetin [M+H]+ HCD 10V P=209
$C_{10}H_8O_5$; MF: 78; RMF: 280; Prob 37.5%; CAS: 574-84-5; Lib: nist_msms; ID: 106679.

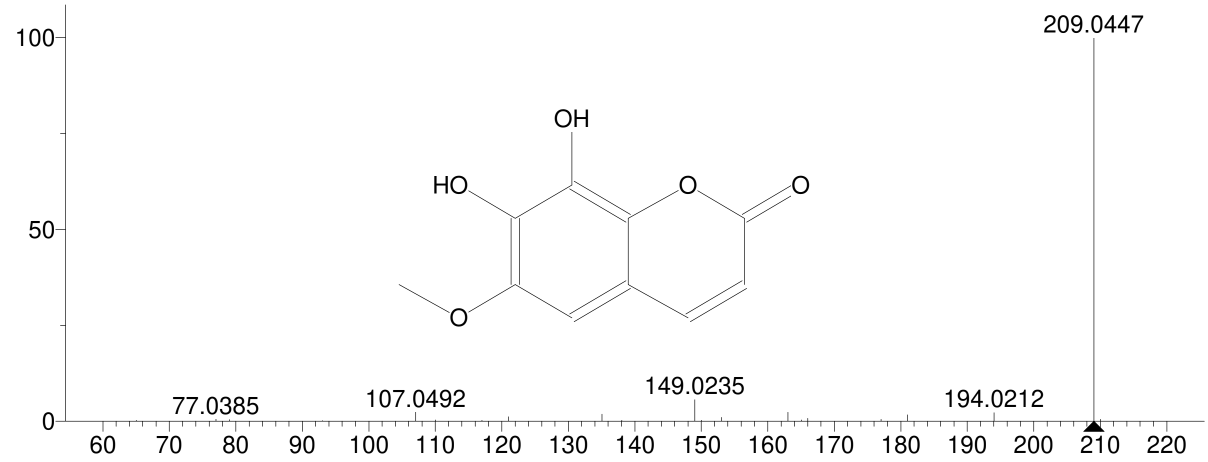

Hit 2 : Hydroxyferulic acid ; PlaSMA ID-282 [M-H]- P=209
$C_{10}H_{10}O_5$; MF: 71; RMF: 323; Prob 28.7%; Lib: biomsms-neg-plasma; ID: 535.

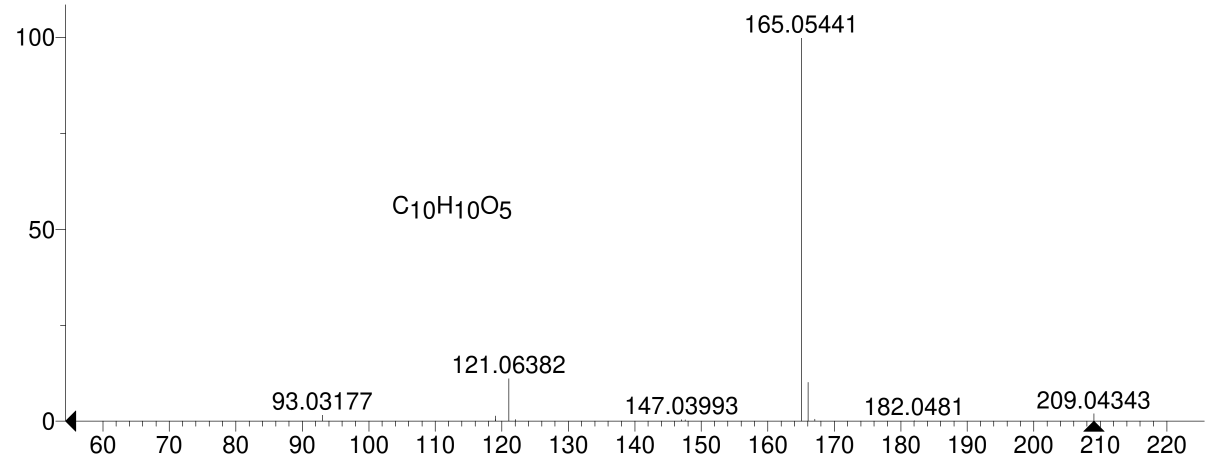

Hit 3 : Chorismic acid [M+H-H2O]+ HCD 20V P=209
C10H10O6; MF: 18; RMF: 215; Prob 5.67%; CAS: 617-12-9; Lib: nist_msms; ID: 242934.

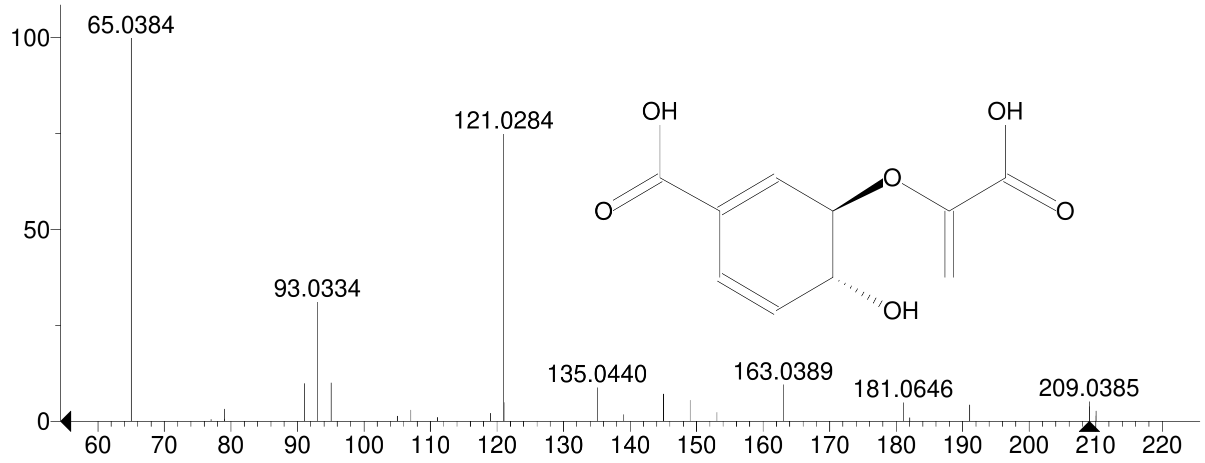

Hit 4 : NCGC00017270-07!7,8-dihydroxy-6-methoxychromen-2-one [M+H]+ P=209
C10H8O5; MF: 8; RMF: 200; Prob 4.00%; Lib: mona-export-gnps; ID: 6636.

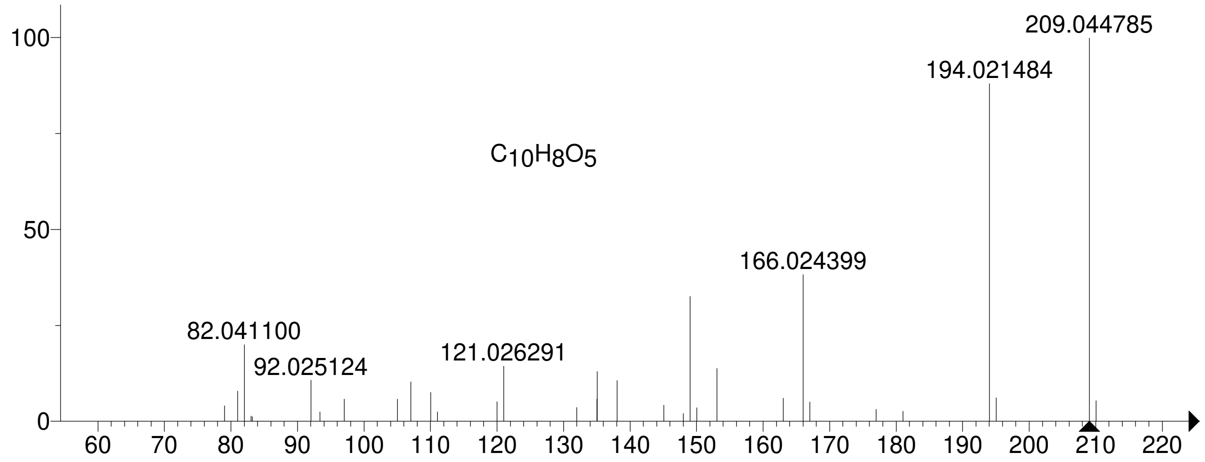

Hit 5 : 2,4-diacetylphloroglucinol [M-H]- 35HCD P=209
C10H10O5; MF: 5; RMF: 189; Prob 3.54%; Lib: mona-export-lc-ms-ms_negative_mode; ID: 18149.

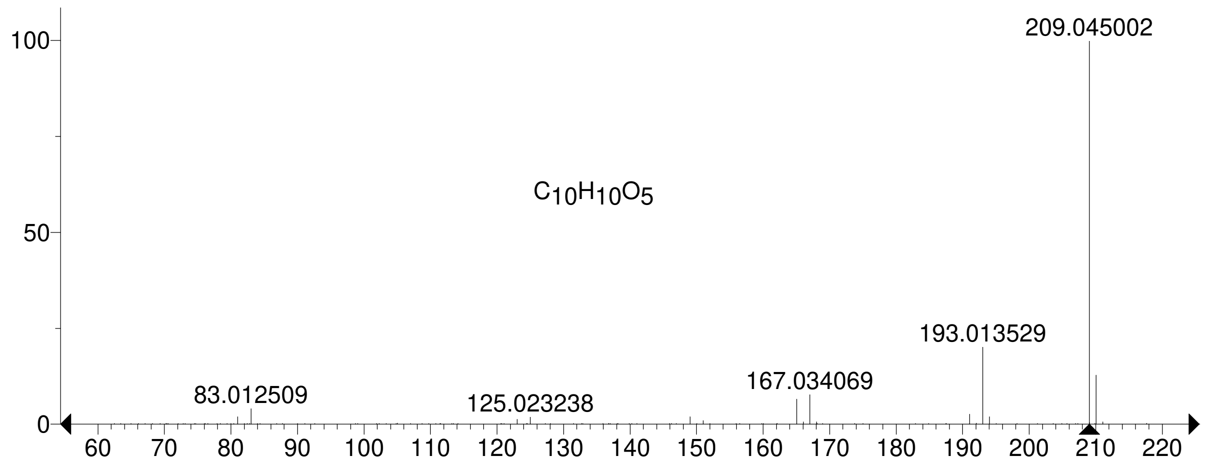

** Search Report Page 1 of 1 **

Unknown: Unknown (8.77_371.1331m/z)
Compound in Library Factor = 0

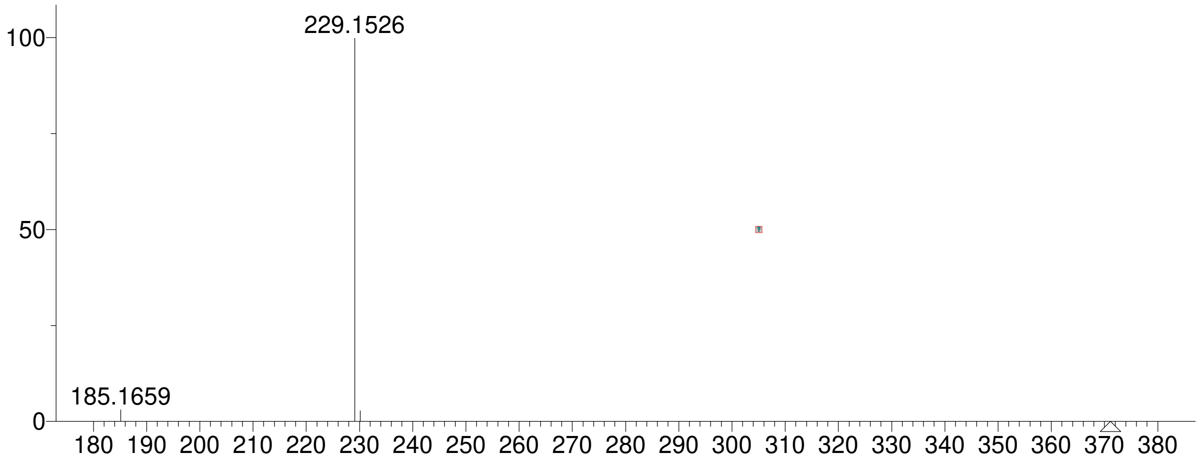

Unknown: Unknown (8.77_399.2115m/z)
Compound in Library Factor = -3273

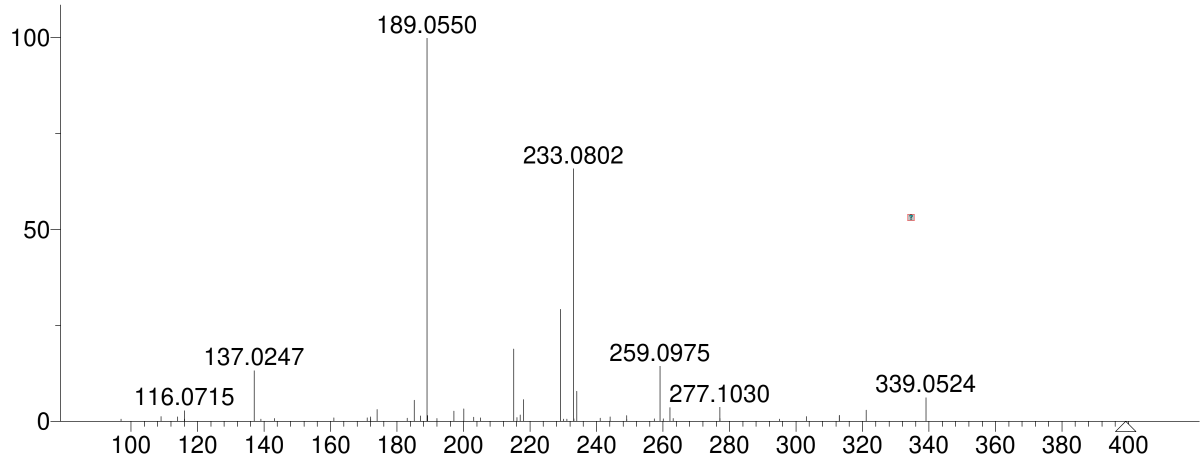

Hit 1 : N-Desmethylvinblastine [M+2H]2+ HCD 114V P=399.2
C45H56N4O9; MF: 1; RMF: 54; Prob 25.7%; CAS: 18172-50-4; Lib: nist_msms; ID: 435598.

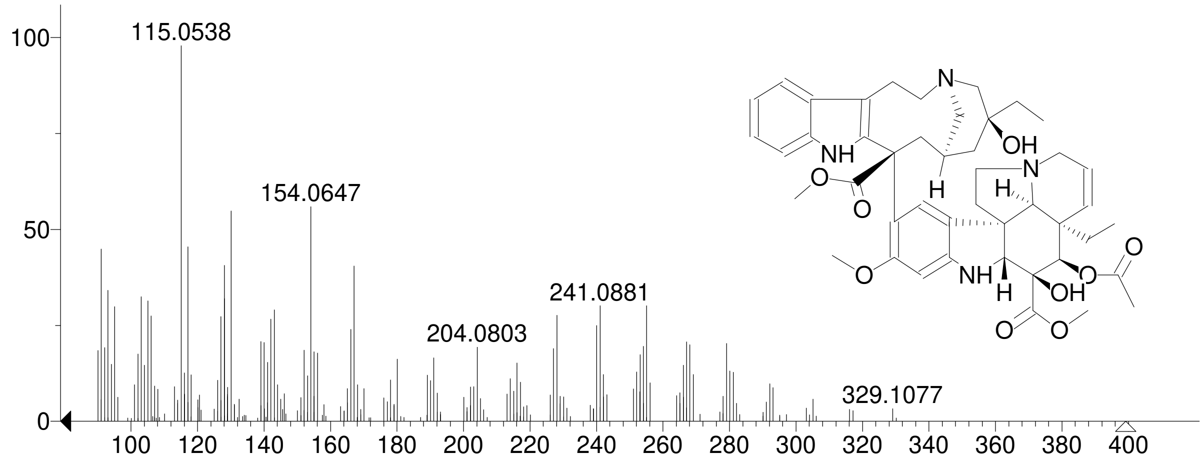

Hit 2 : His-Ser-Arg [M+H]+ QTOF 40V P=399.2
C15H26N8O5; MF: 0; RMF: 20; Prob 24.8%; Lib: nist_msms; ID: 51161.

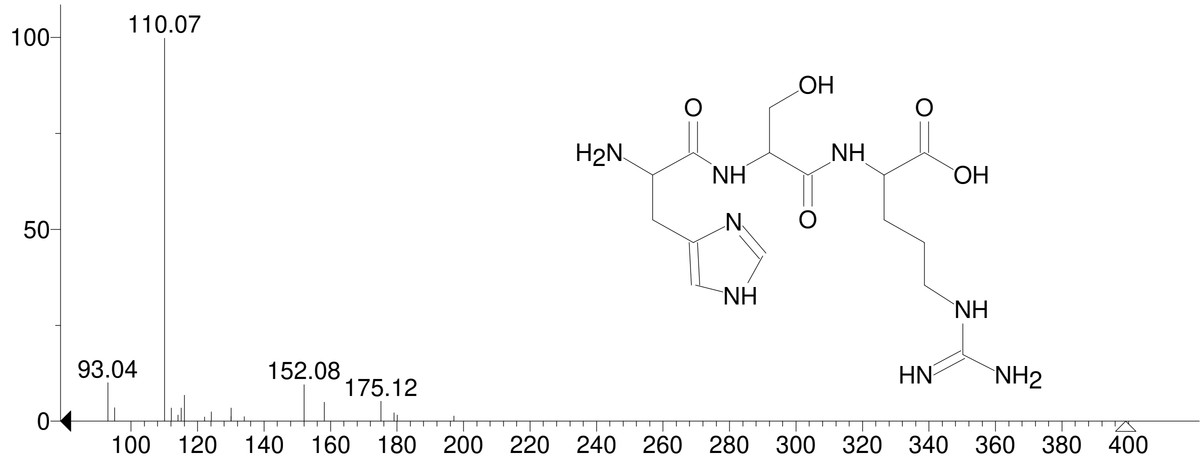

Hit 3 : Ser-His-Arg [M+H]+ QTOF 30V P=399.2
C15H26N8O5; MF: 0; RMF: 18; Prob 24.8%; Lib: nist_msms; ID: 53772.

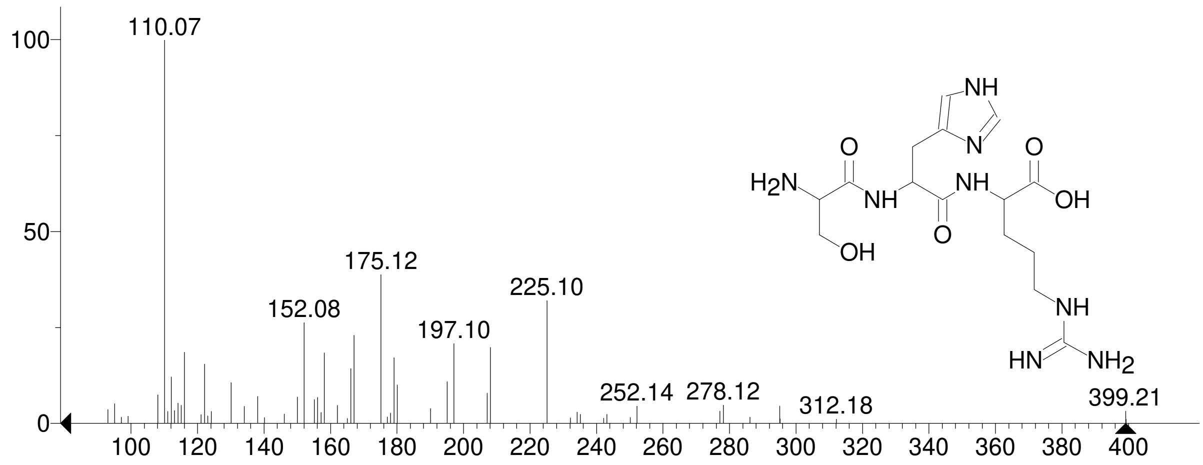

Hit 4 : NCGC00380576-01_C22H32O5_2-Pentenoic acid, 3-[(acetyloxy)methyl]-5-[(1S,4aR,8aR)-2-formyl-1,4,4a,
C22H32O5; MF: 0; RMF: 1; Prob 24.8%; Lib: mona-export-gnps; ID: 13905.

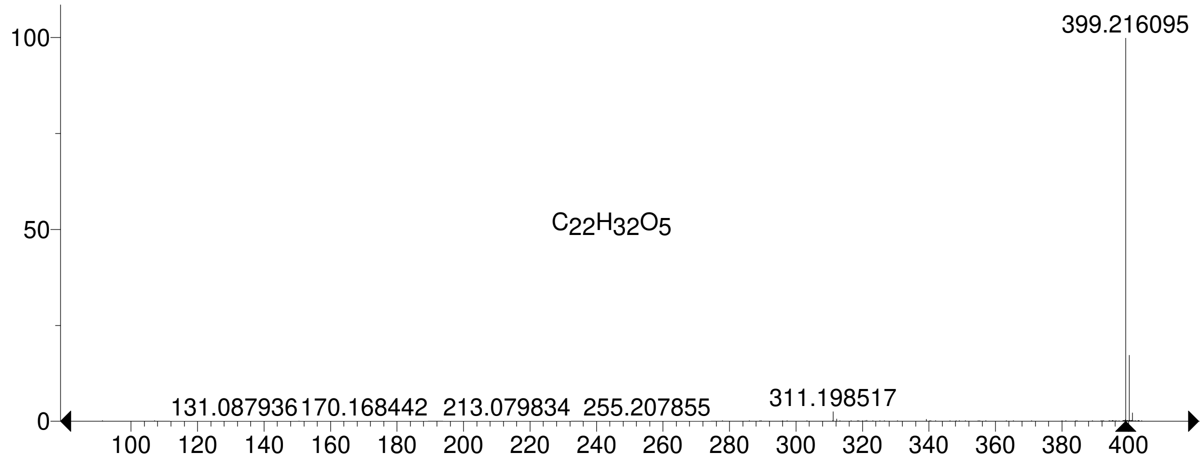

** Search Report Page 1 of 1 **

Unknown: Unknown (8.77_437.1022m/z)
Compound in Library Factor = 0

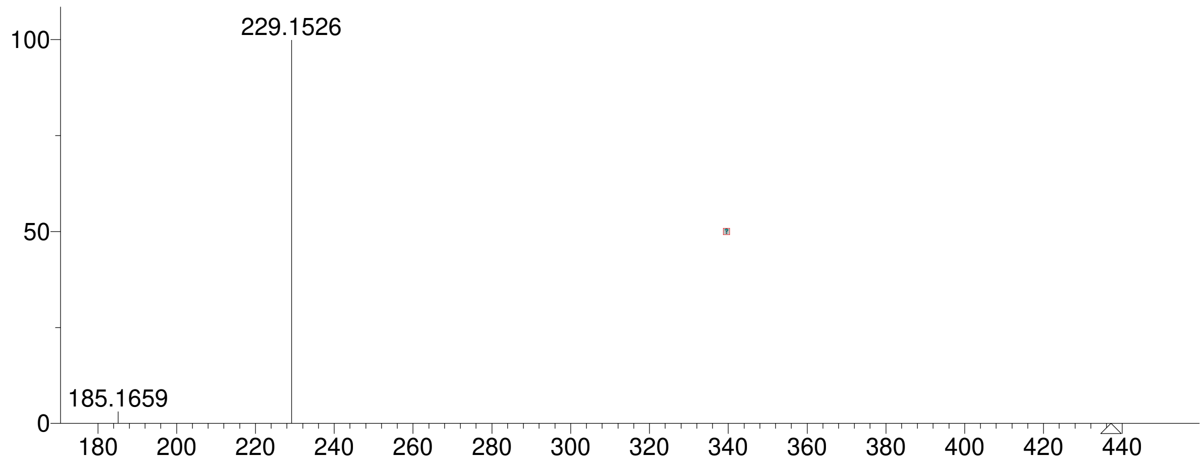

Hit 1 : (5-benzoyloxy-4,6-dihydroxy-3-methoxycyclohexen-1-yl)methyl benzoate [M+K]+ 45HCD P=437.1
C22H22O7; MF: 0; RMF: 15; Prob 100.0%; Lib: mona-export-vf-npl_qexactive; ID: 1880.

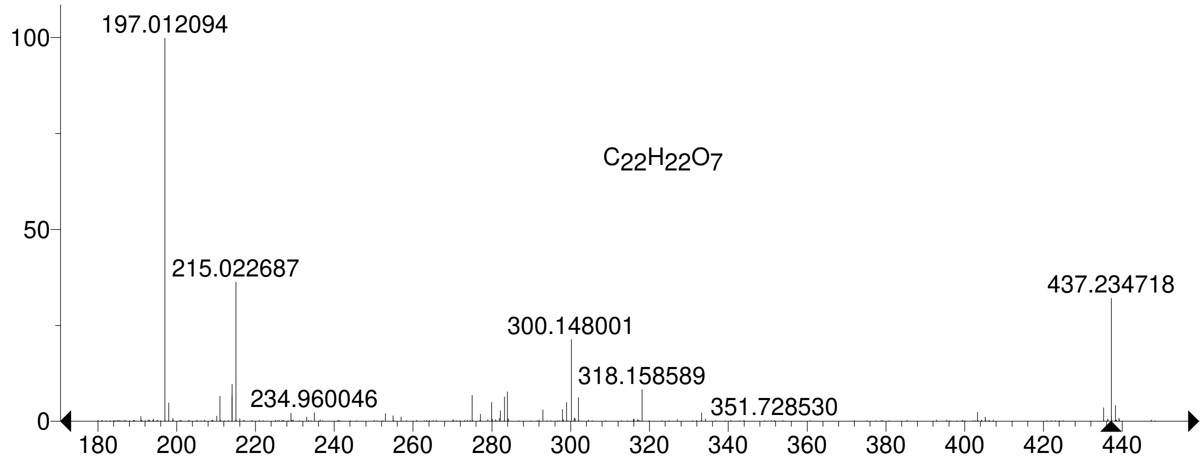

Unknown: Unknown (8.77_586.2720m/z)
Compound in Library Factor = 0

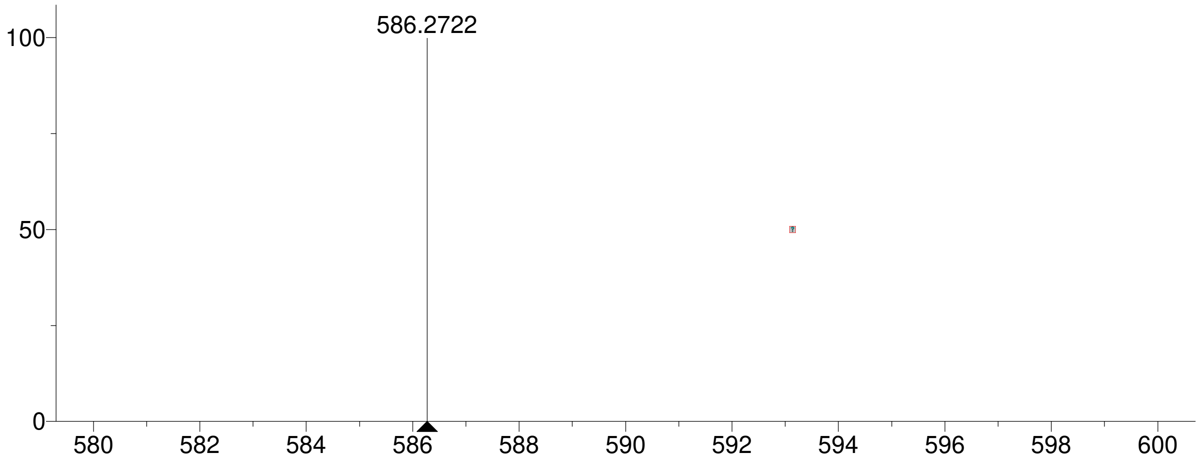

Unknown: Unknown (8.77_640.3310m/z)
Compound in Library Factor = -2632

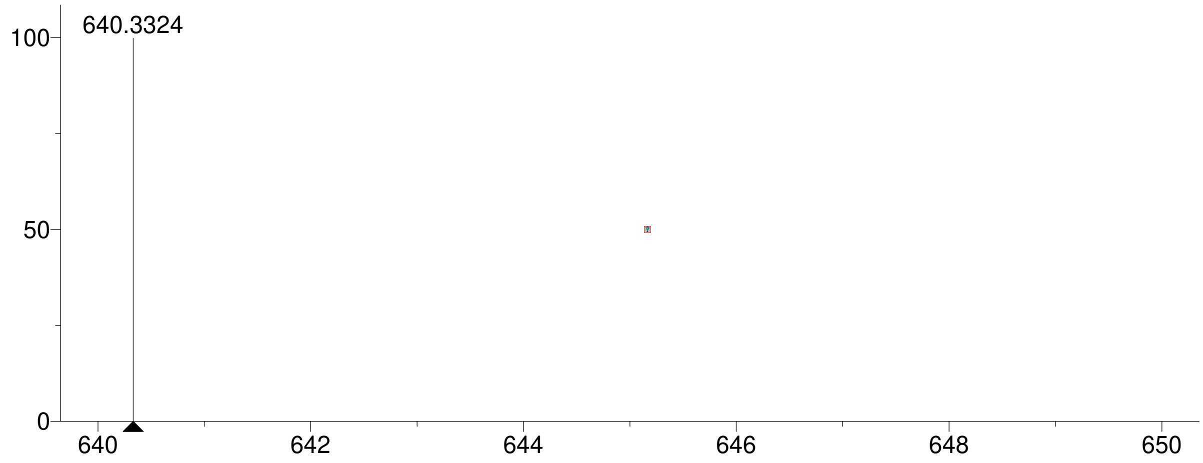

Hit 1 : PS 26:5 [M-H]- 40eV P=640.3
C32H52NO10P; MF: 364; RMF: 999; Prob 20.0%; Lib: mona-export-in-silico_spectra; ID: 106181.

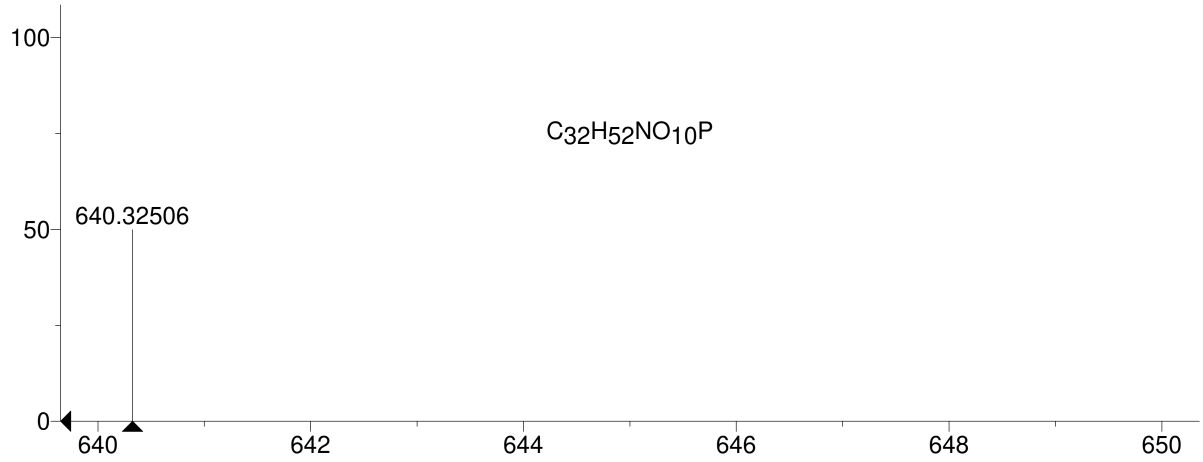

Hit 2 : PS 26:5; PS 4:0-22:5; [M-H]- [M-H]- P=640.3
C32H52NO10P; MF: 364; RMF: 999; Prob 20.0%; Lib: msdial-insilicomsms-lipids-neg; ID: 329842.

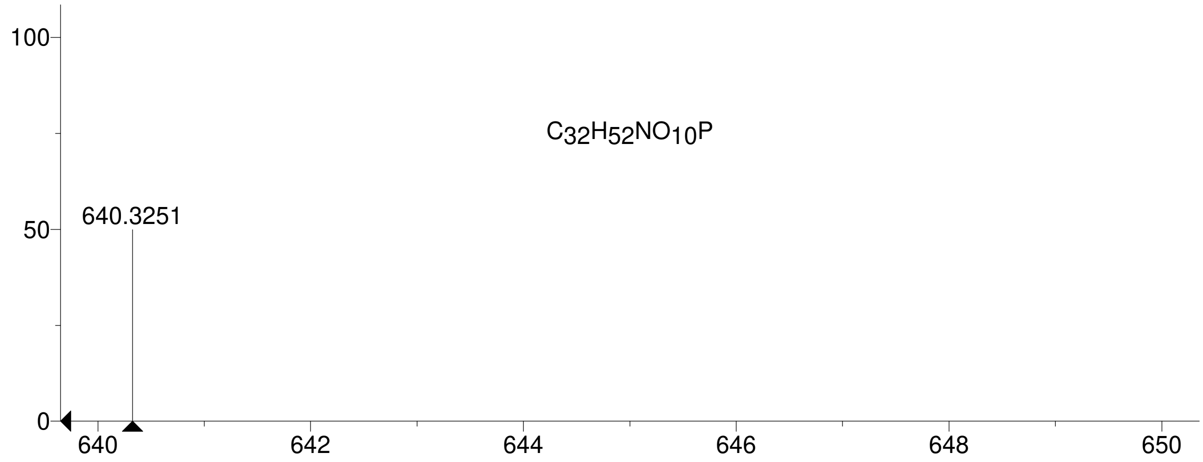

Hit 3 : PS 26:5; PS 6:0-20:5; [M-H]- [M-H]- P=640.3
C32H52NO10P; MF: 364; RMF: 999; Prob 20.0%; Lib: msdial-insilicomsms-lipids-neg; ID: 329948.

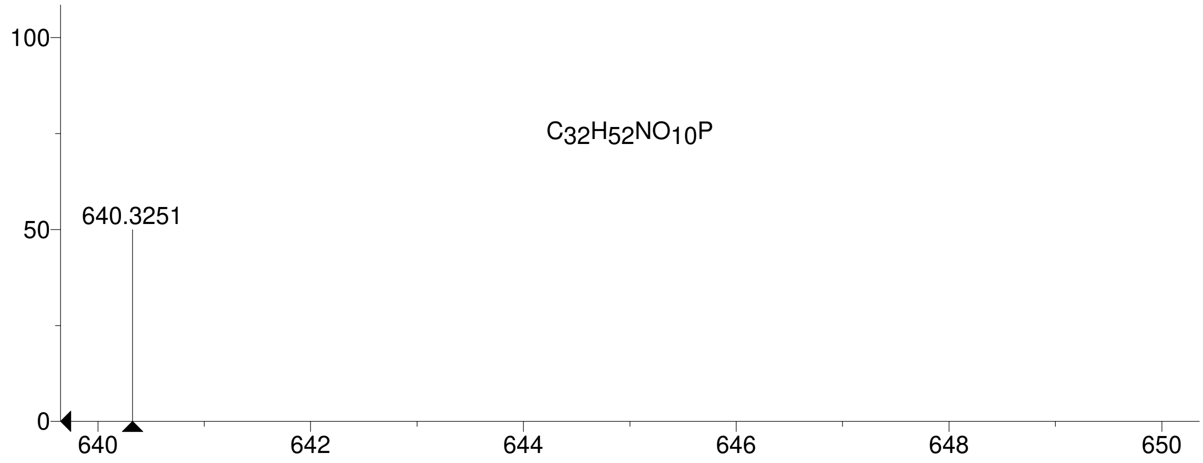

Hit 4 : PS 26:5; PS 8:0-18:5; [M-H]- [M-H]- P=640.3
C32H52NO10P; MF: 364; RMF: 999; Prob 20.0%; Lib: msdial-insilicomsms-lipids-neg; ID: 330050.

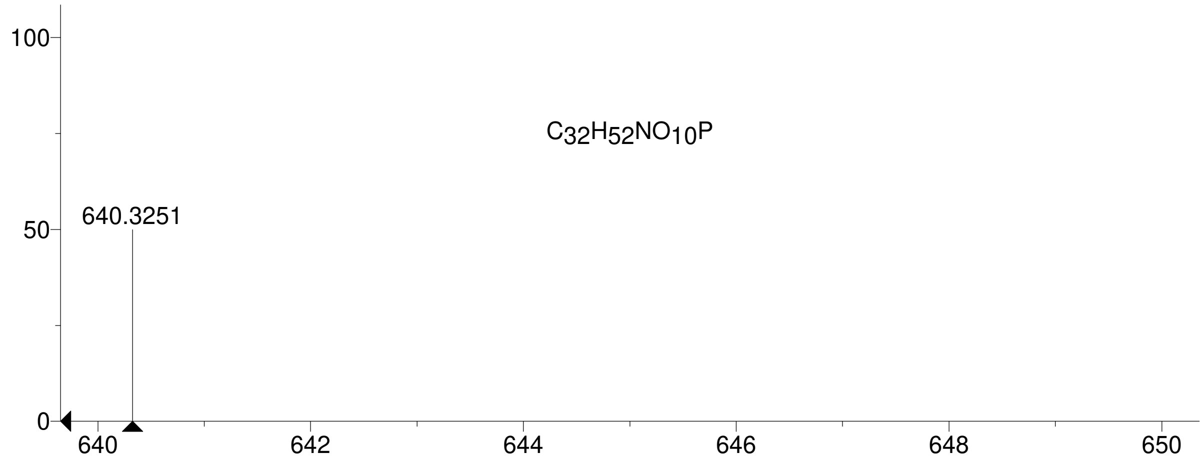

Hit 5 : PS 26:6 [M+H]+ 25eV P=640.3
C32H50NO10P; MF: 364; RMF: 999; Prob 20.0%; Lib: mona-export-in-silico_spectra; ID: 110545.

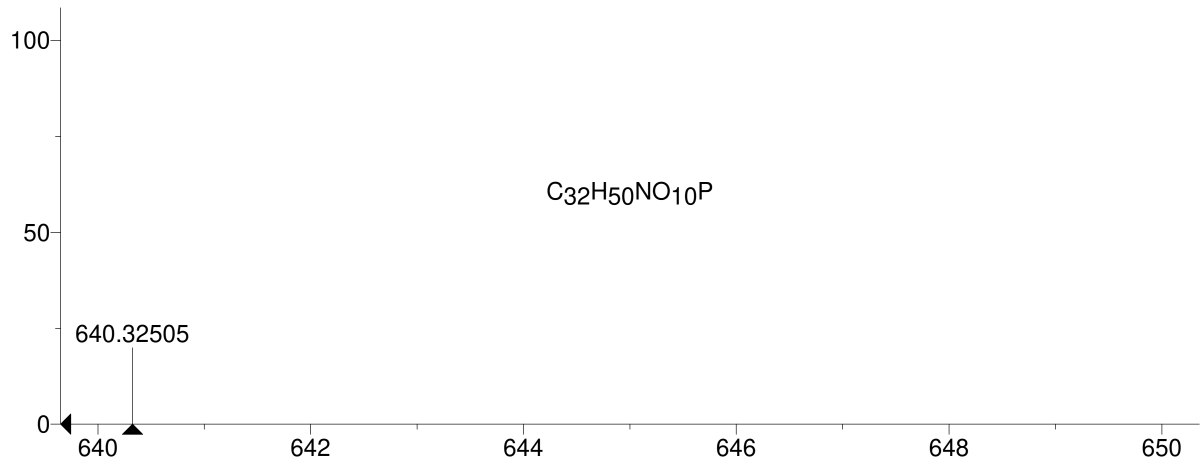

** Search Report Page 1 of 1 **

Unknown: Unknown (8.79_520.2040m/z)
Compound in Library Factor = -3273

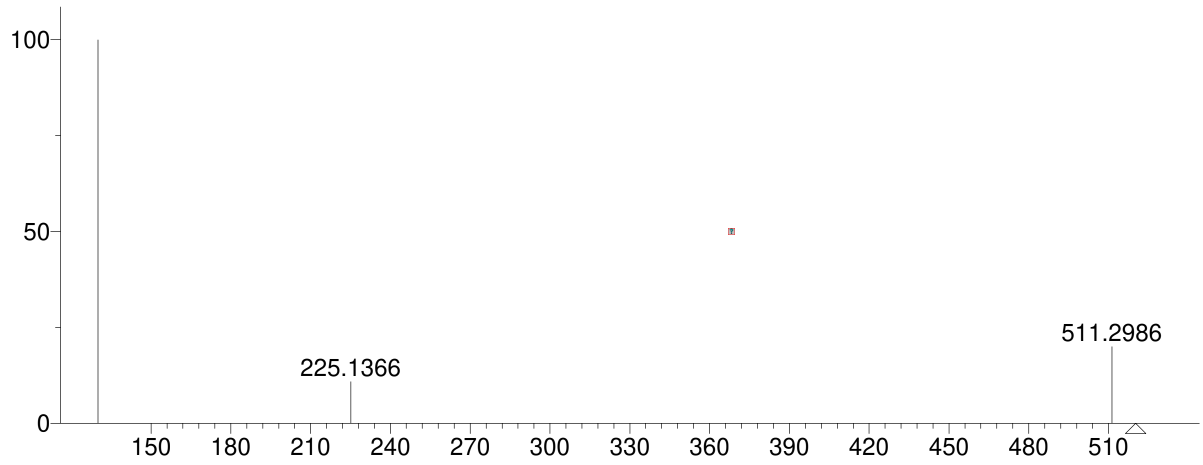

Hit 1 : [Lys8]-Vasopressin [M+2H]2+=>520.2 IT 35% P=520.2
C46H65N13O12S2; MF: 1; RMF: 369; Prob 100.0%; CAS: 50-57-7; Lib: nist_msms; ID: 113393.

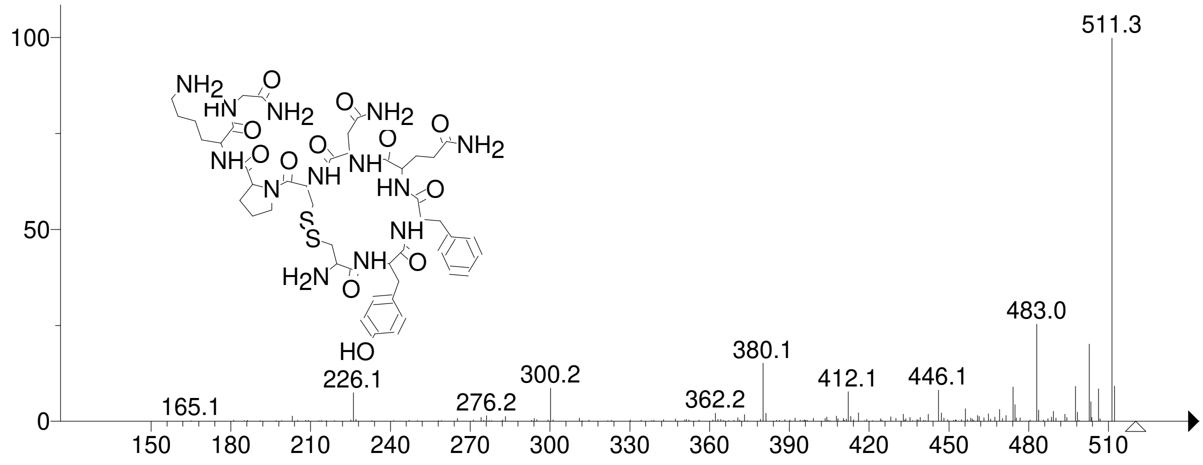

Unknown: Unknown (8.80_222.0754m/z)
Compound in Library Factor = -2387

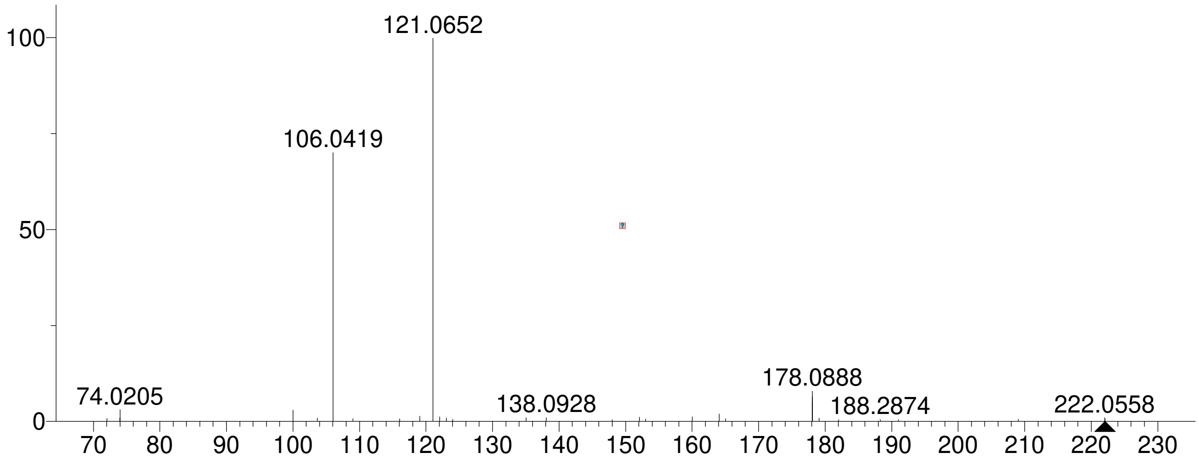

Hit 1 : N-Acetyl-L-tyrosine [M-H]- HCD 19V P=222.1
C11H13NO4; MF: 20; RMF: 172; Prob 51.2%; CAS: 537-55-3; Lib: nist_msms; ID: 280193.

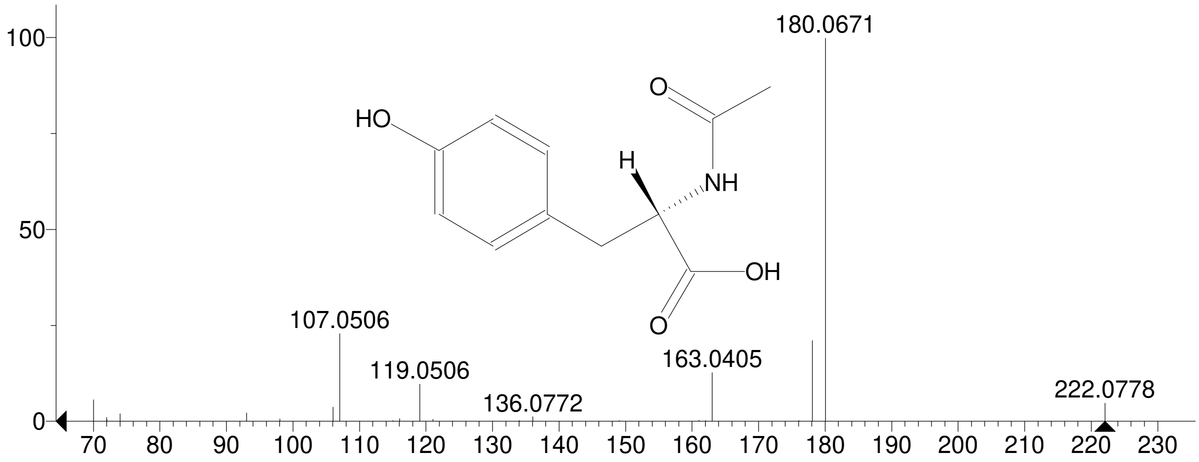

Hit 2 : N-Benzoylaminopurine [M+H-H2O]+ HCD 11V P=222.1
C12H9N5O; MF: 1; RMF: 44; Prob 24.9%; CAS: 4005-49-6; Lib: nist_msms; ID: 144557.

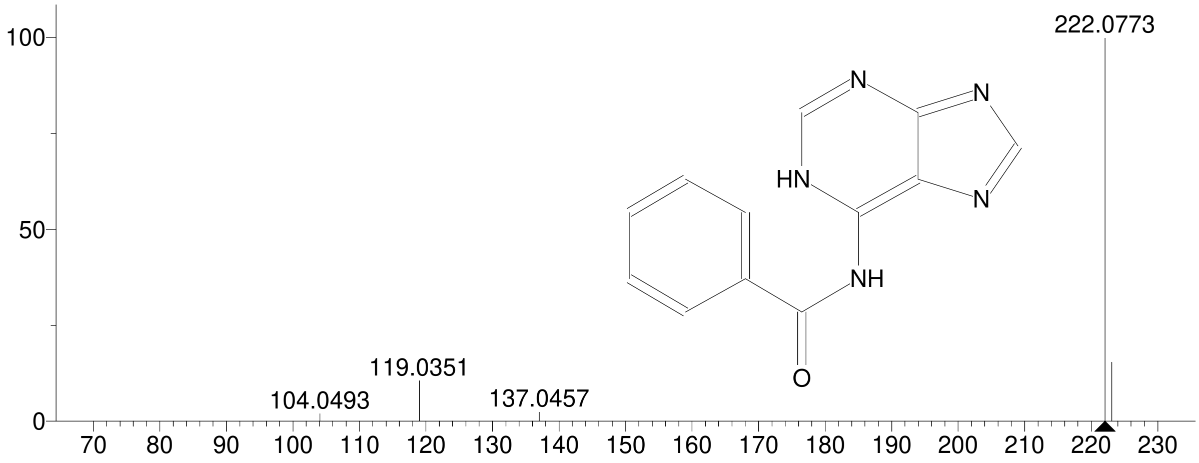

Hit 3 : NCGC00381349-01_C11H13NO5_1H-Pyrrole-3-propanoic acid, 5-acetyl-4-(carboxymethyl)- [M-H2O+H]+
C11H13NO5; MF: 0; RMF: 13; Prob 23.9%; Lib: mona-export-gnps; ID: 11085.

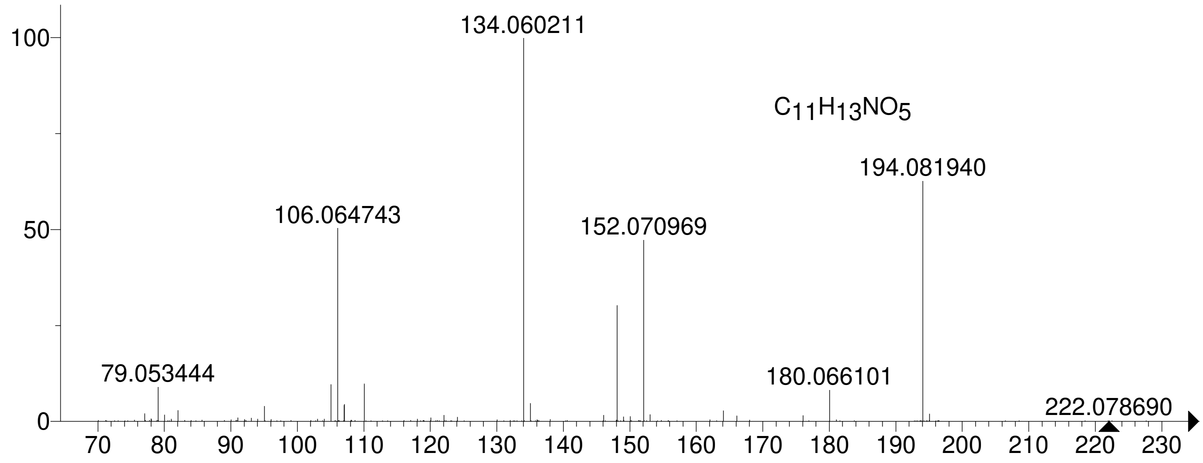

Unknown: Unknown (8.80_277.1549m/z)
Compound in Library Factor = -344

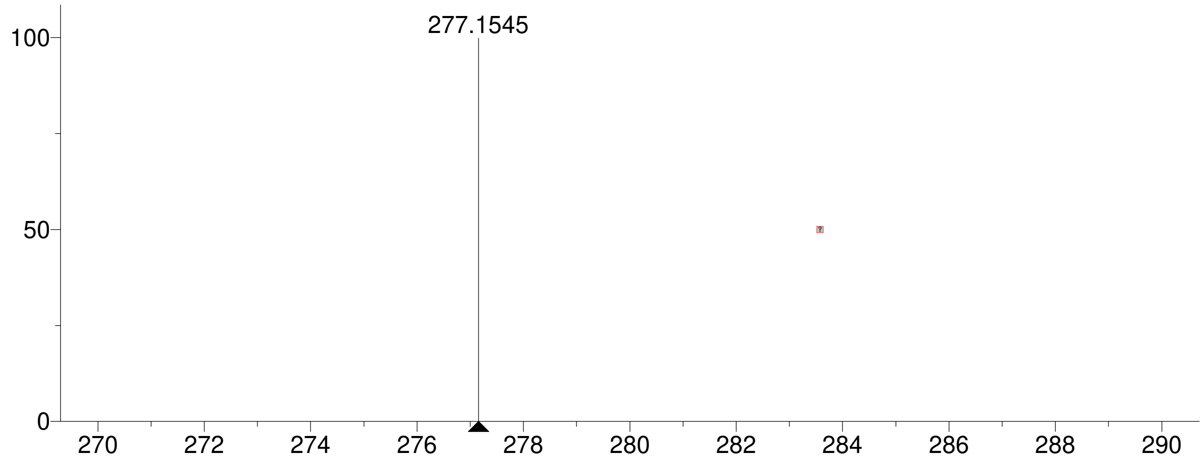

Hit 1 : Phe-Leu [M-H]- HCD 24eV P=277.2
C15H22N2O3; MF: 364; RMF: 999; Prob 12.4%; Lib: nist_msms; ID: 570091.

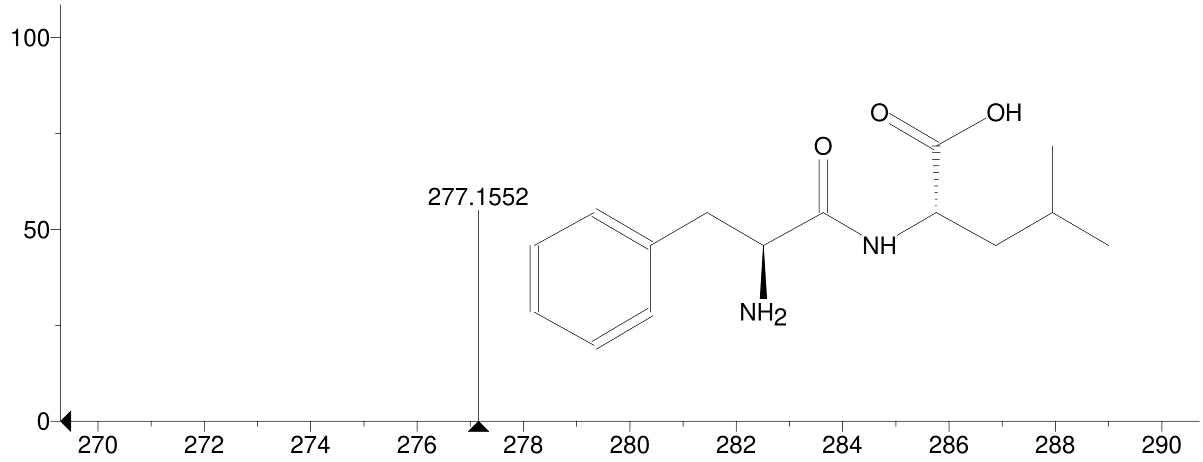

Hit 2 : Ile-Phe [M-H]- HCD 20eV P=277.2
C15H22N2O3; MF: 364; RMF: 999; Prob 12.4%; Lib: nist_msms; ID: 570144.

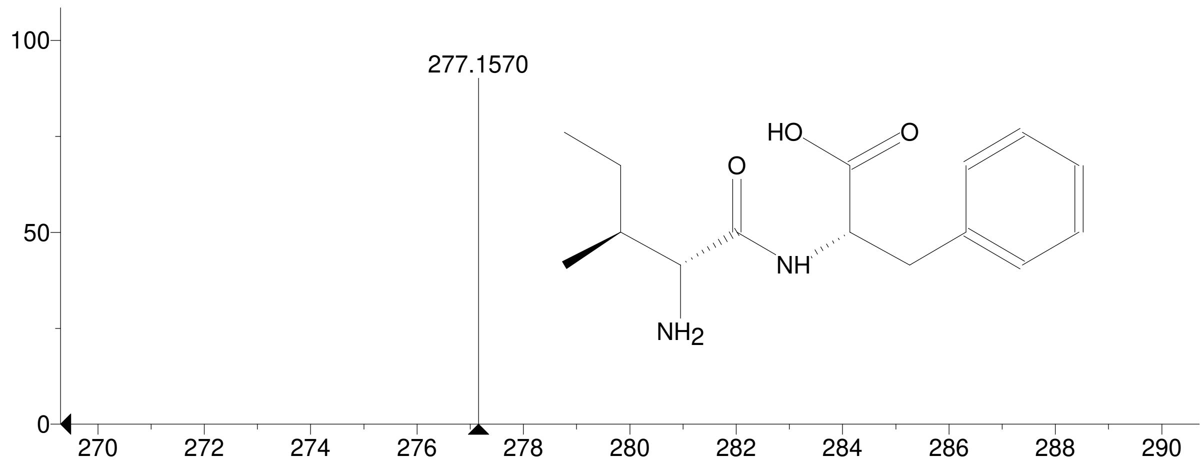

Hit 3 : Leu-Phe [M-H]- HCD 24eV P=277.2
C15H22N2O3; MF: 364; RMF: 999; Prob 12.4%; Lib: nist_msms; ID: 570205.

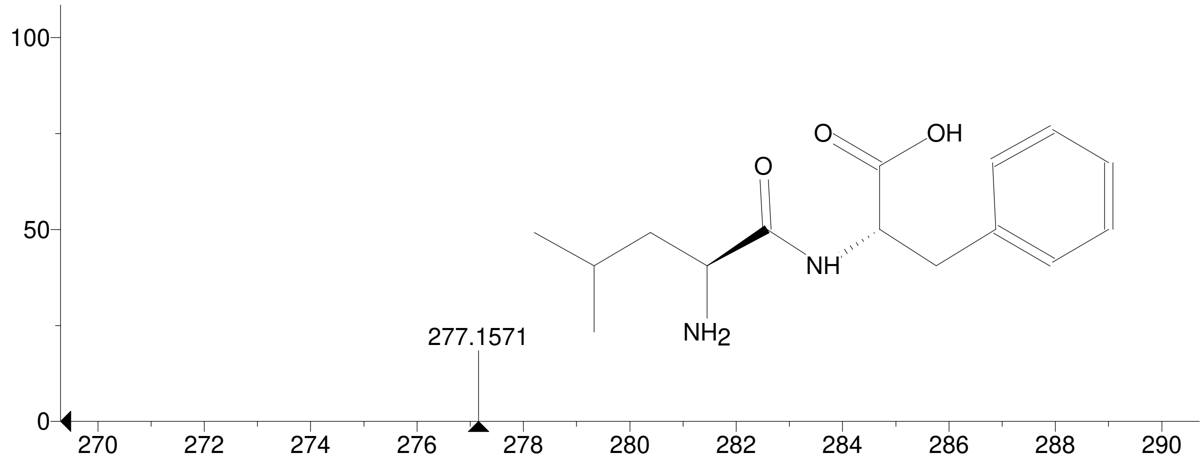

Hit 4 : Phe-Ile [M-H]- HCD 36eV P=277.2
C15H22N2O3; MF: 364; RMF: 999; Prob 12.4%; Lib: nist_msms; ID: 570606.

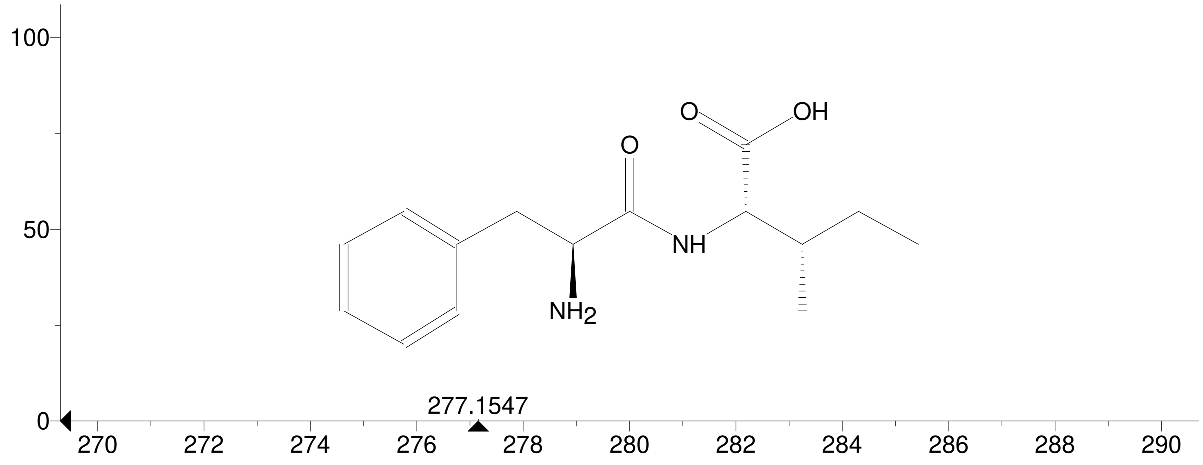

Hit 5 : Phe-Leu [M-H]- HCD 24eV P=277.2
C15H22N2O3; MF: 364; RMF: 999; Prob 12.4%; CAS: 3303-55-7; Lib: nist_msms2; ID: 83817.

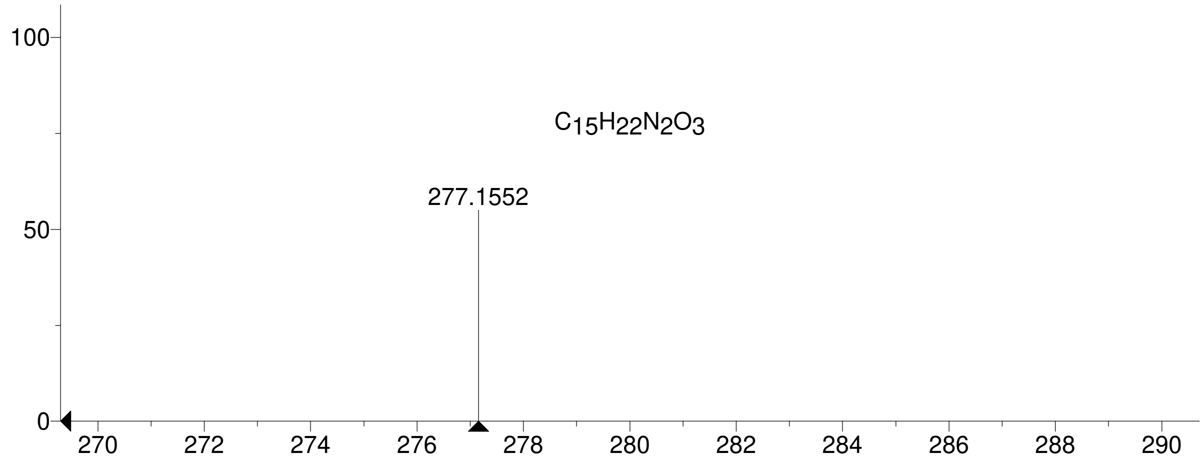

** Search Report Page 1 of 1 **

Unknown: Unknown (8.81_387.0854m/z)
Compound in Library Factor = -3273

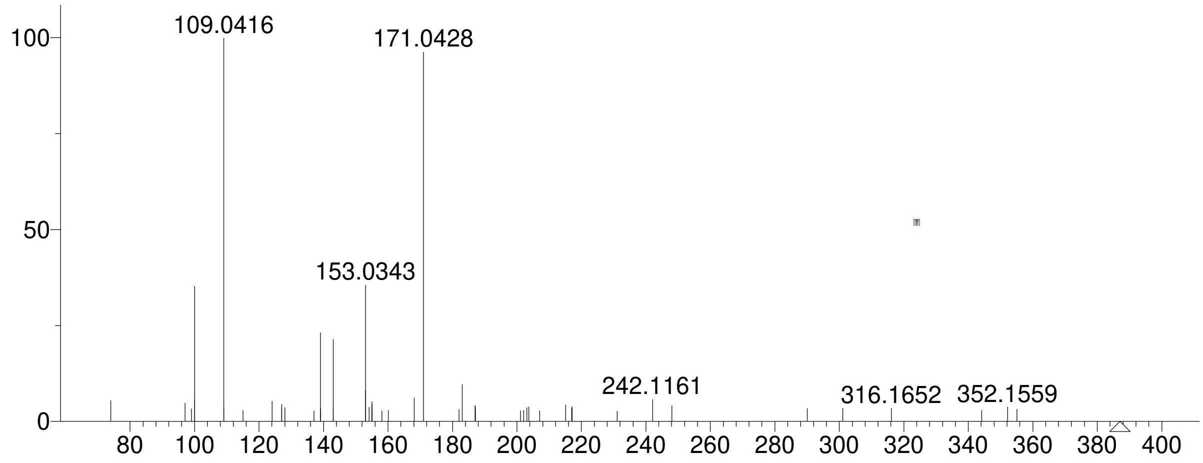

Hit 1 : Pamoic acid [M-H]- HCD 61V P=387.1
C23H16O6; MF: 22; RMF: 249; Prob 100.0%; CAS: 130-85-8; Lib: nist_msms; ID: 152472.

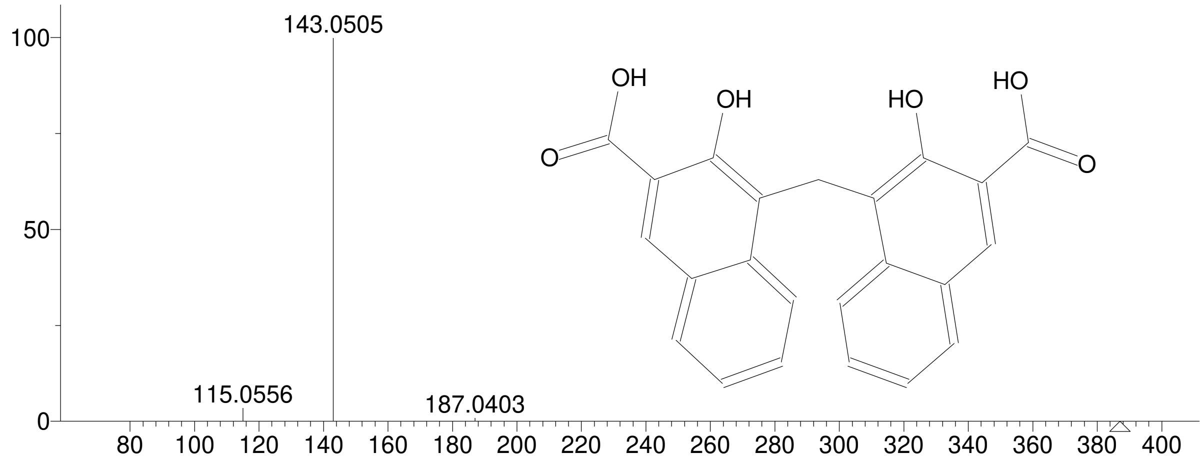

Unknown: Unknown (8.83_352.1399m/z)
Compound in Library Factor = 0

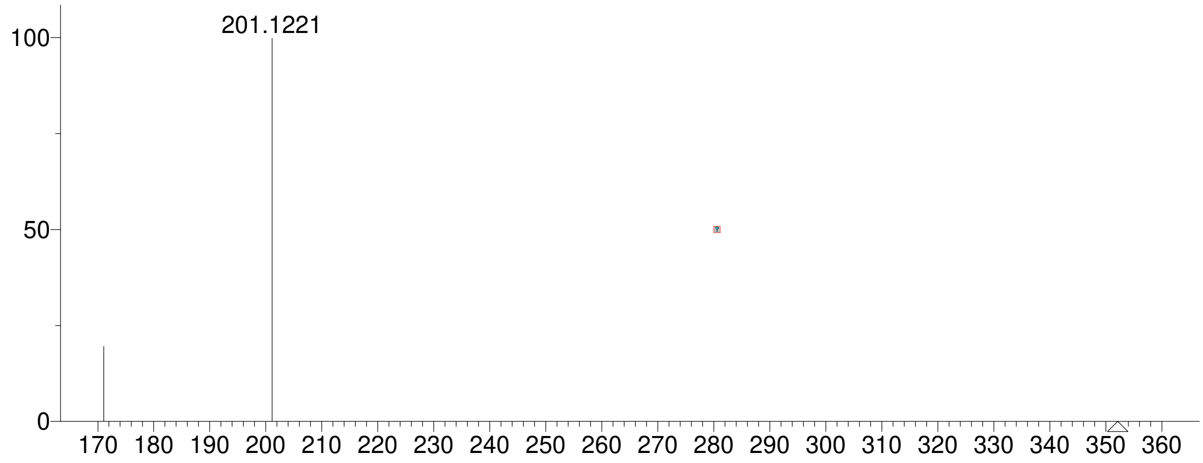

Unknown: Unknown (8.83_371.1804m/z)
Compound in Library Factor = 0

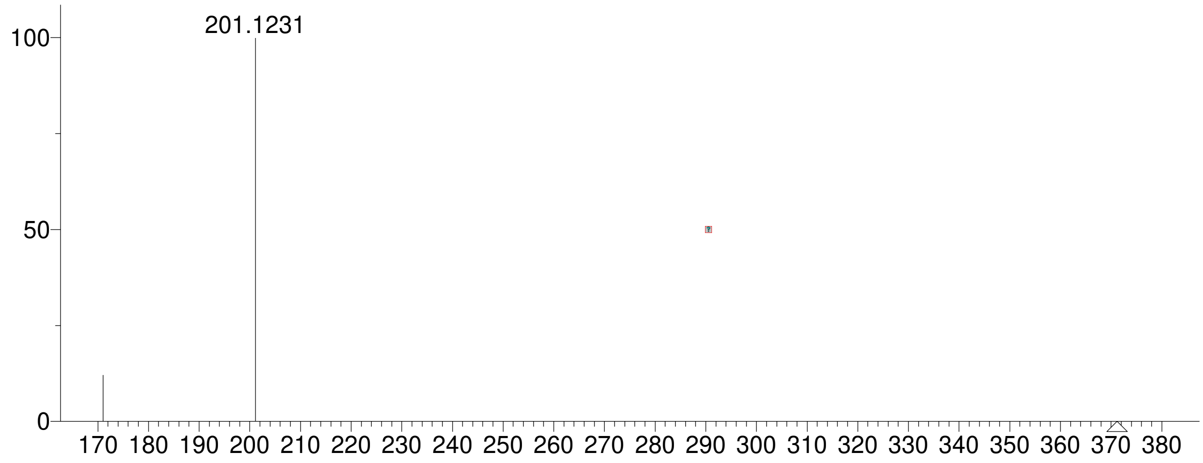

Unknown: Unknown (8.84_517.2286m/z)
Compound in Library Factor = -2176

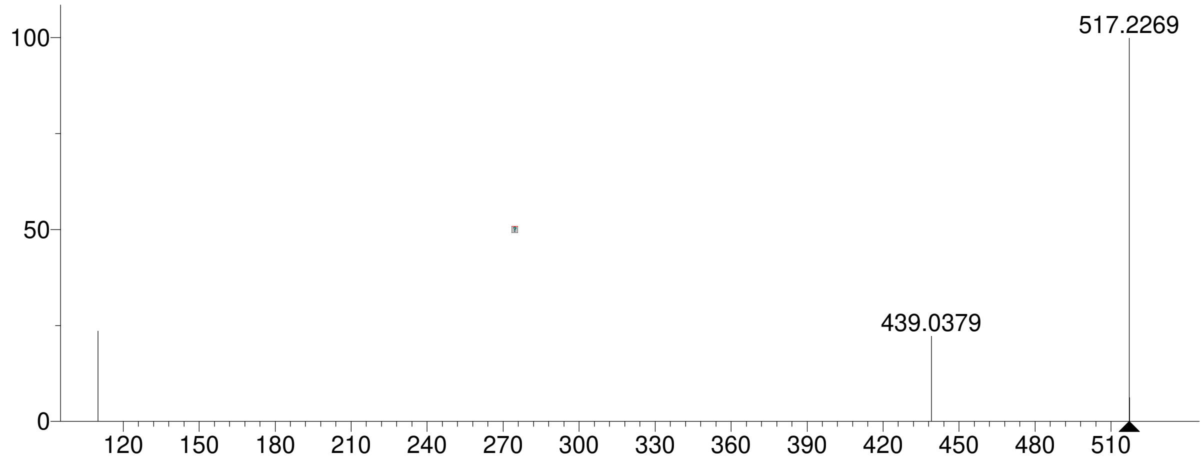

Hit 1 : Experimental Allergic Encephalitogenic Peptide [M-2H]2- HCD 20eV P=517.2
C46H64N14O14; MF: 39; RMF: 695; Prob 100.0%; Lib: nist_msms2; ID: 78587.

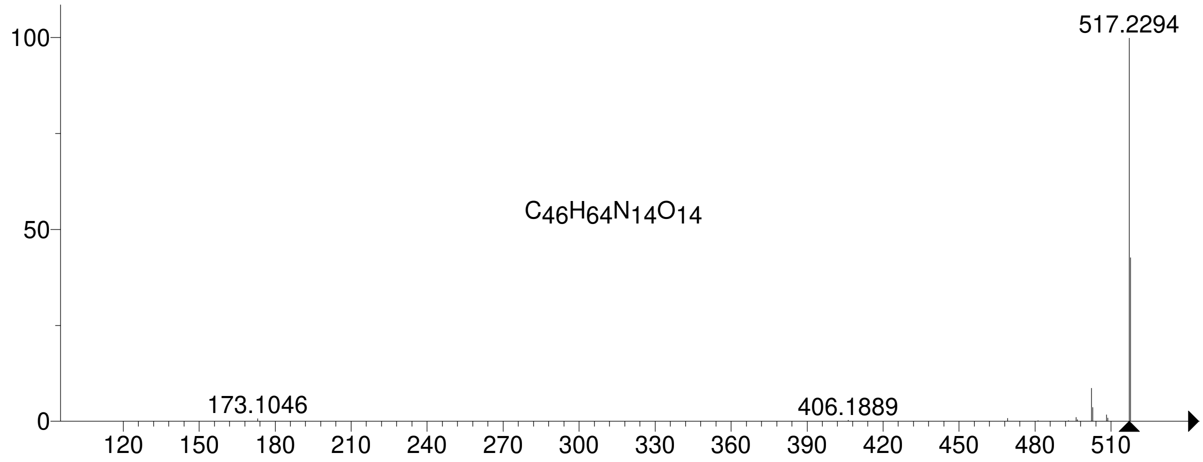

** Search Report Page 1 of 1 **

Unknown: Unknown (8.84_608.0820m/z)
Compound in Library Factor = 0

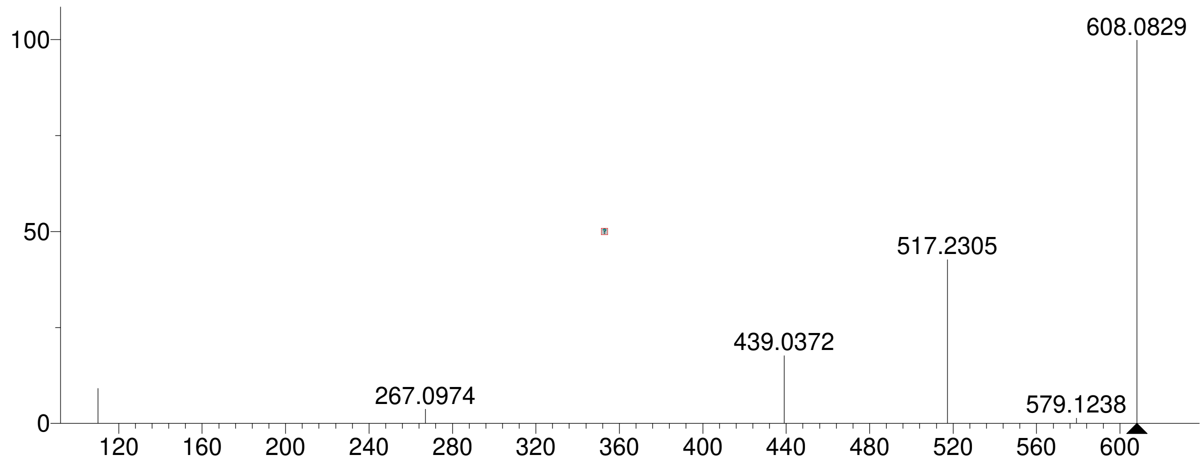

Unknown: Unknown (8.84_658.2354m/z)
Compound in Library Factor = 0

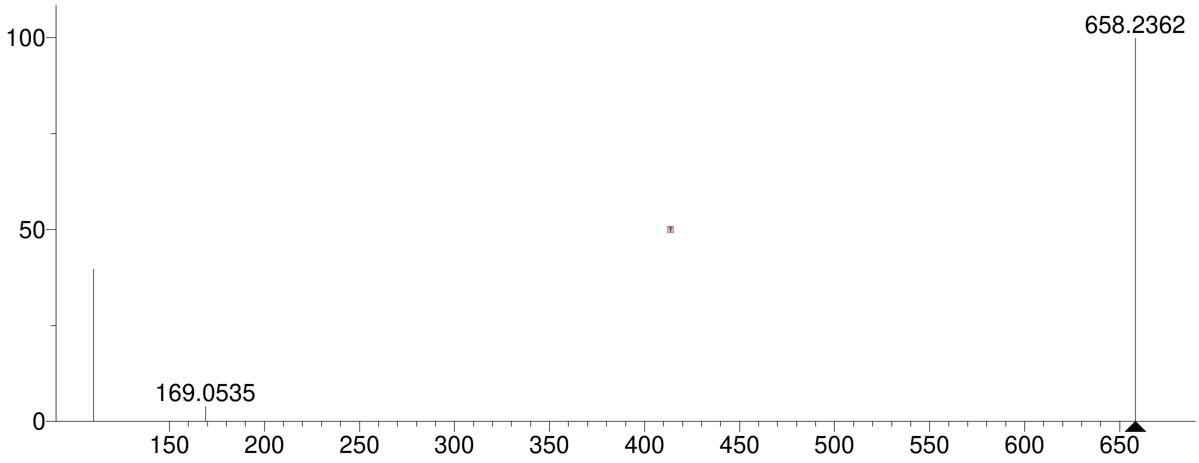

Unknown: Unknown (8.84_998.5149m/z)
Compound in Library Factor = 0

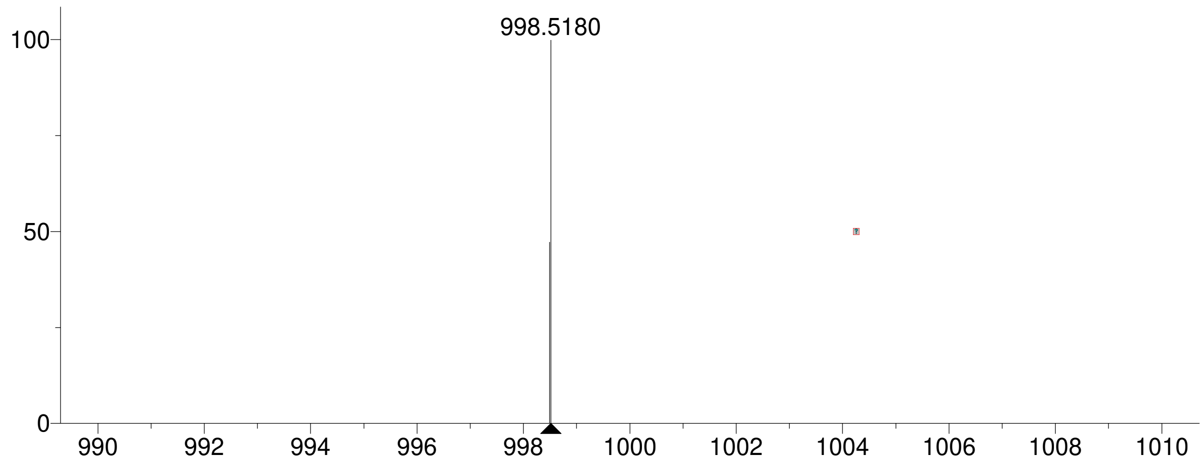

Unknown: Unknown (8.85_463.9725m/z)
Compound in Library Factor = 0

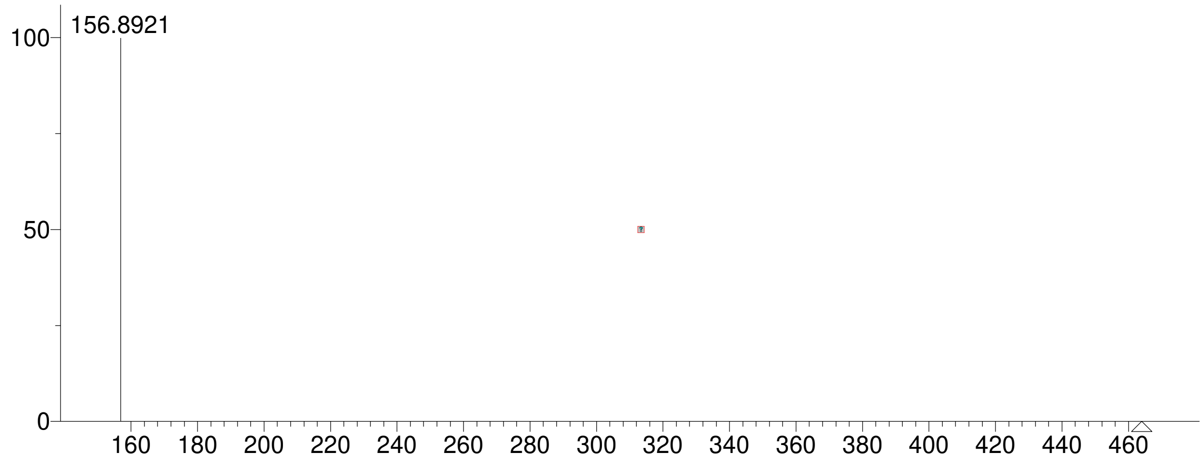

Unknown: Unknown (8.85_473.9997m/z)
Compound in Library Factor = 0

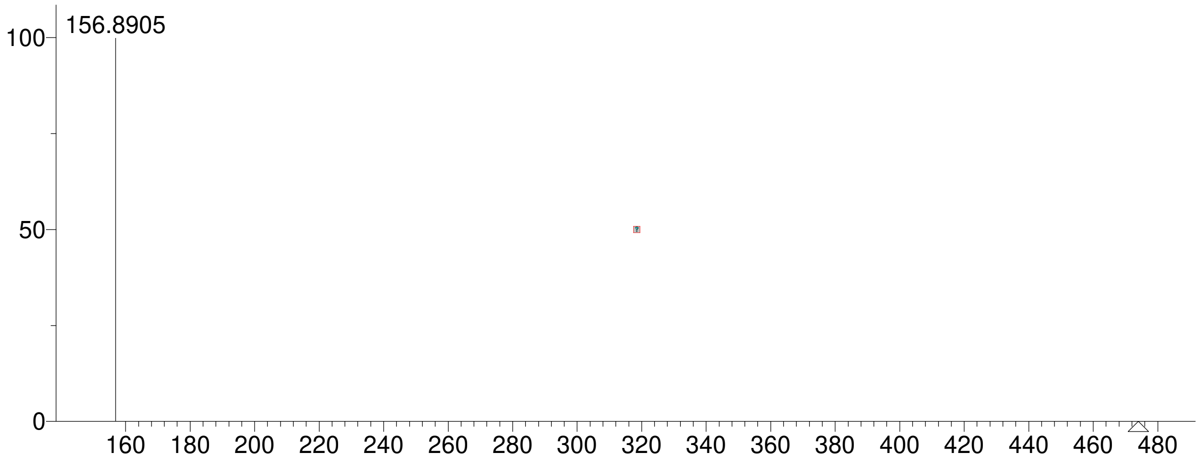

Unknown: Unknown (8.85_517.2247m/z)
Compound in Library Factor = -2176

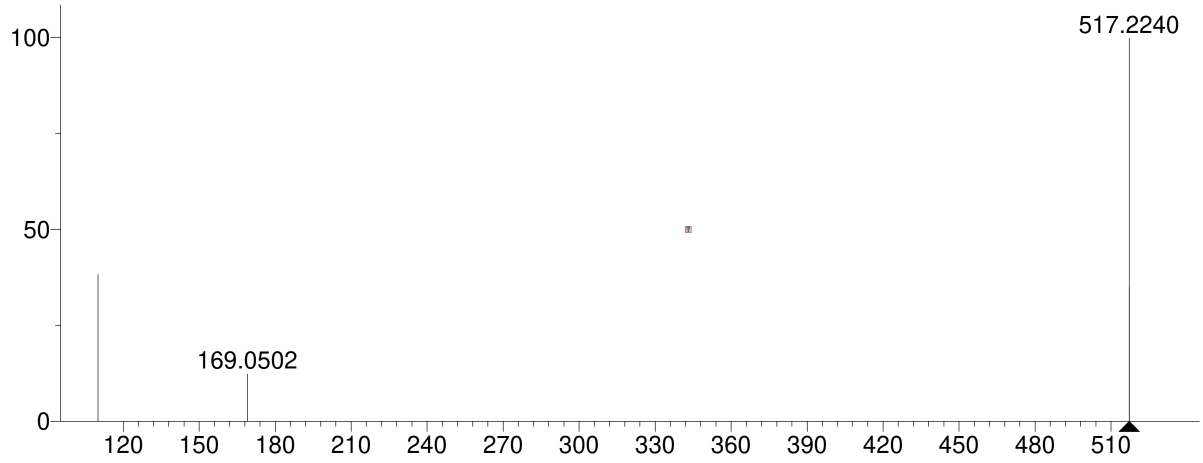

Hit 1 : Experimental Allergic Encephalitogenic Peptide [M-2H]2- HCD 20eV P=517.2
C46H64N14O14; MF: 36; RMF: 678; Prob 60.9%; Lib: nist_msms2; ID: 78587.

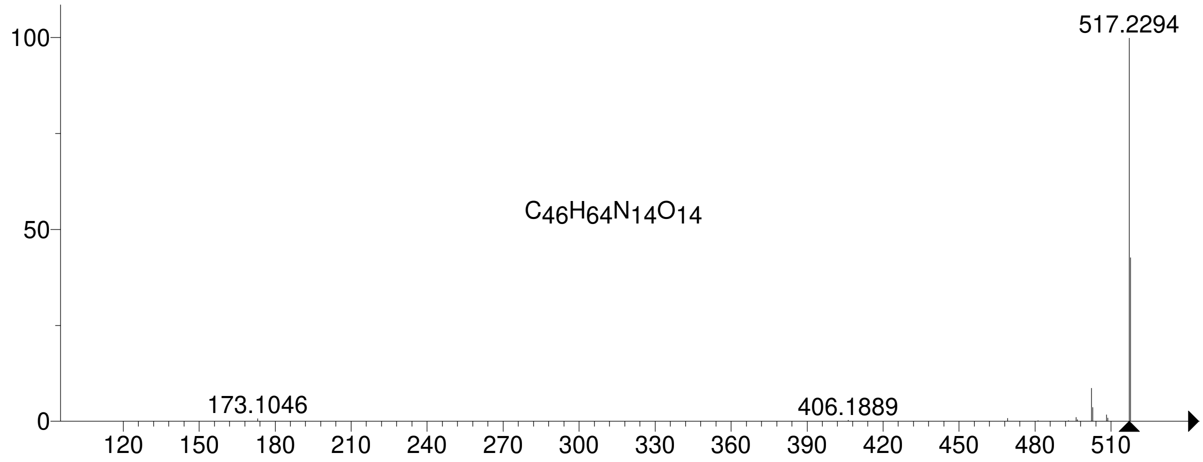

Hit 2 : PG 18:4; PG 2:0-16:4; [M-H]- [M-H]- P=517.2
C24H39O10P; MF: 14; RMF: 376; Prob 24.1%; Lib: msdial-insilicomsms-lipids-neg; ID: 324587.

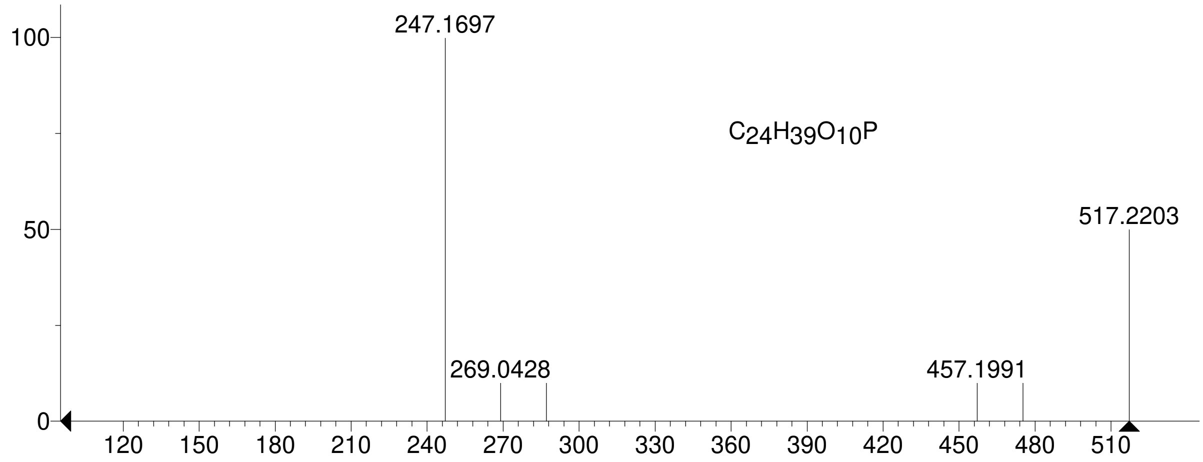

Hit 3 : (2R,4'aR,5'S,6'R,6'aS,10'aR,10'bR)-6'-(acetyloxy)-4'a,6'a,10'b-trimethyl-5''-oxo-decahydro-1'H-dispiro[oxira
C26H38O8; MF: 0; RMF: 219; Prob 15.1%; Lib: mona-export-vf-npl_qexactive; ID: 8713.

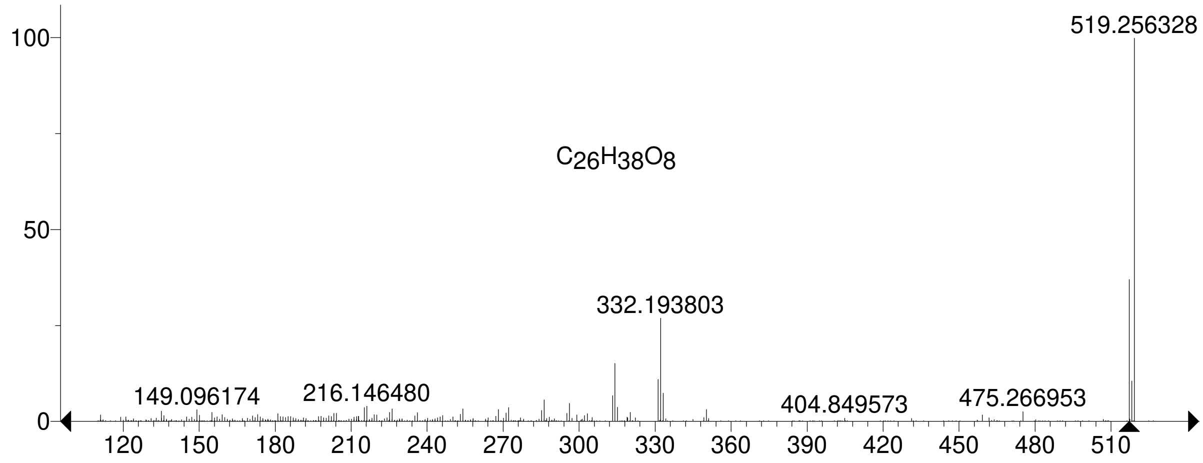

Unknown: Unknown (8.85_732.1352m/z)
Compound in Library Factor = 0

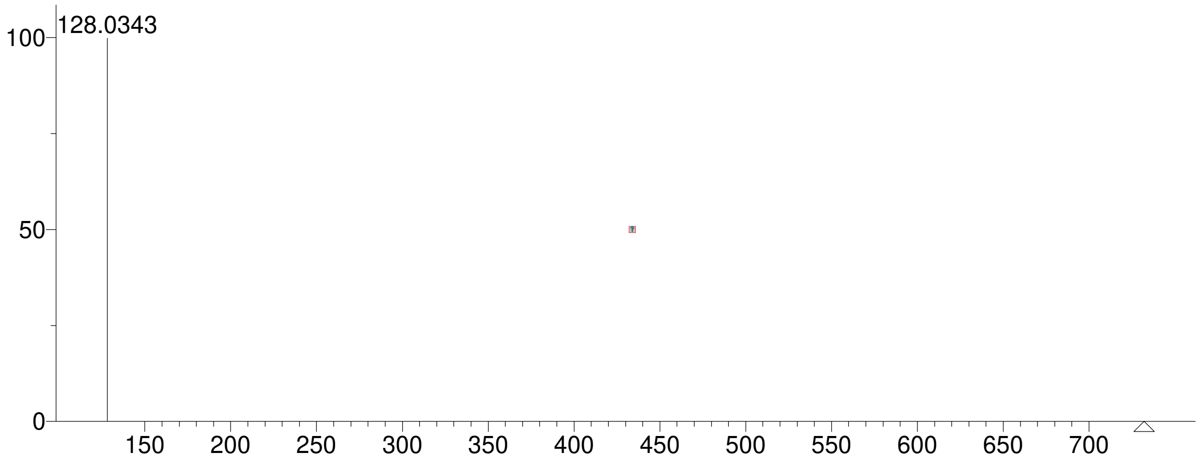

** Search Report Page 1 of 1 **

Unknown: Unknown (8.86_598.0523m/z)
Compound in Library Factor = 0

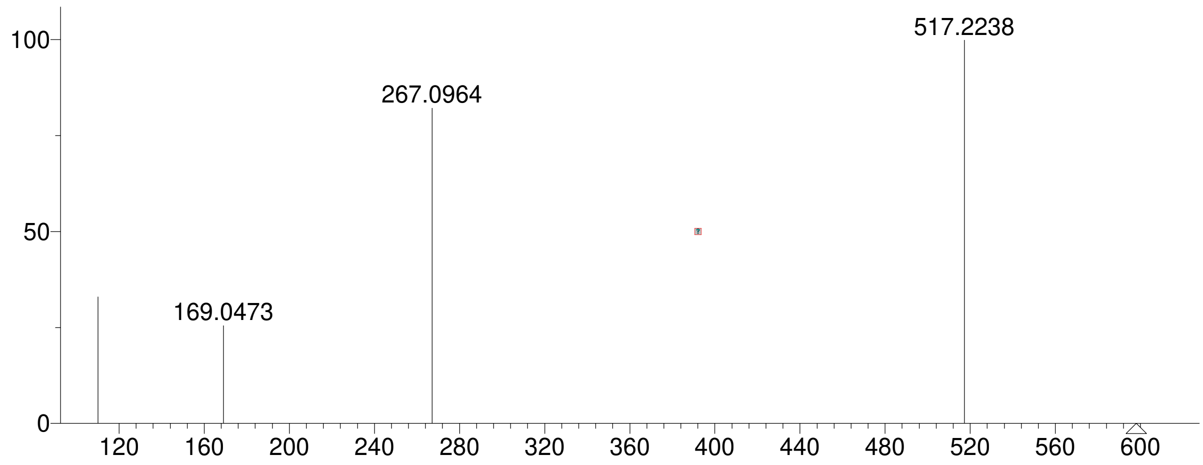

Unknown: Unknown (8.88_800.1985m/z)
Compound in Library Factor = 0

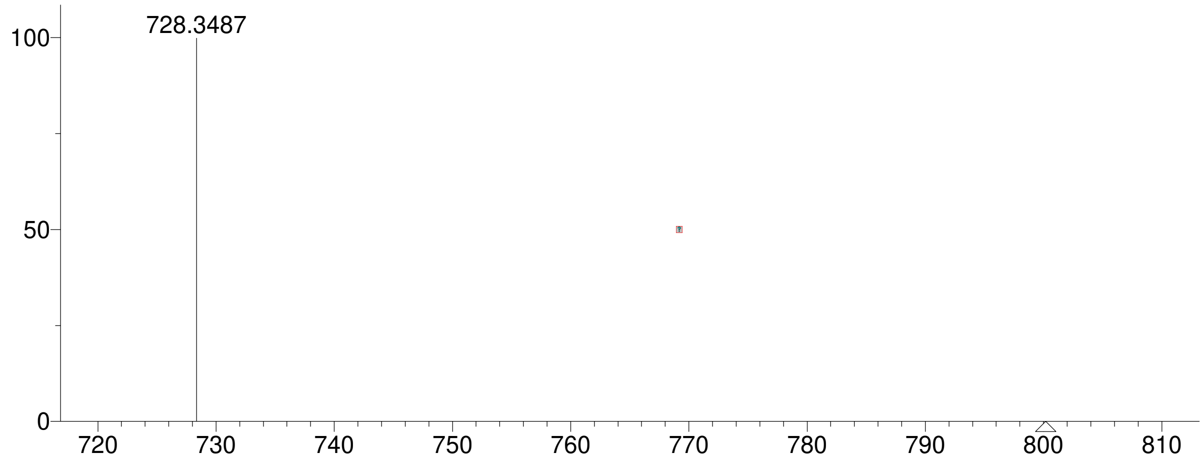

Unknown: Unknown (8.89_253.1330m/z)
Compound in Library Factor = -156

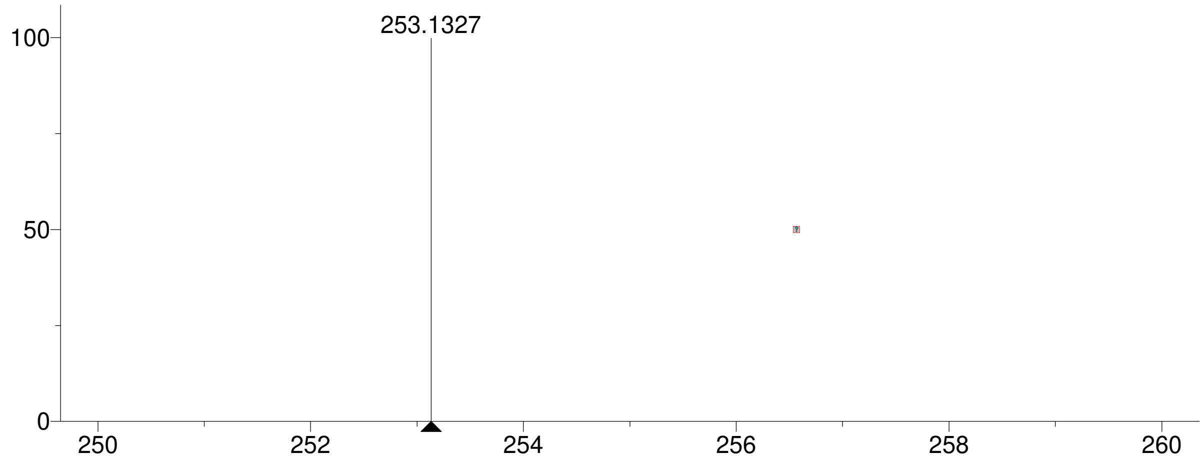

Hit 1 : His-Val [M-H]- HCD 32eV P=253.1
C11H18N4O3; MF: 364; RMF: 999; Prob 14.4%; Lib: nist_msms; ID: 567465.

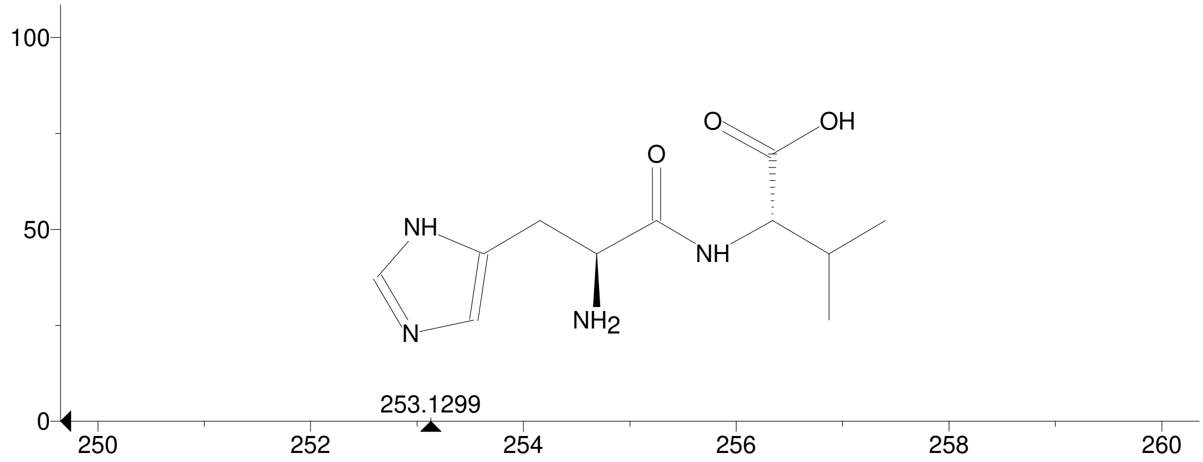

Hit 2 : Val-His [M-H]- HCD 22eV P=253.1
C11H18N4O3; MF: 364; RMF: 999; Prob 14.4%; Lib: nist_msms; ID: 568742.

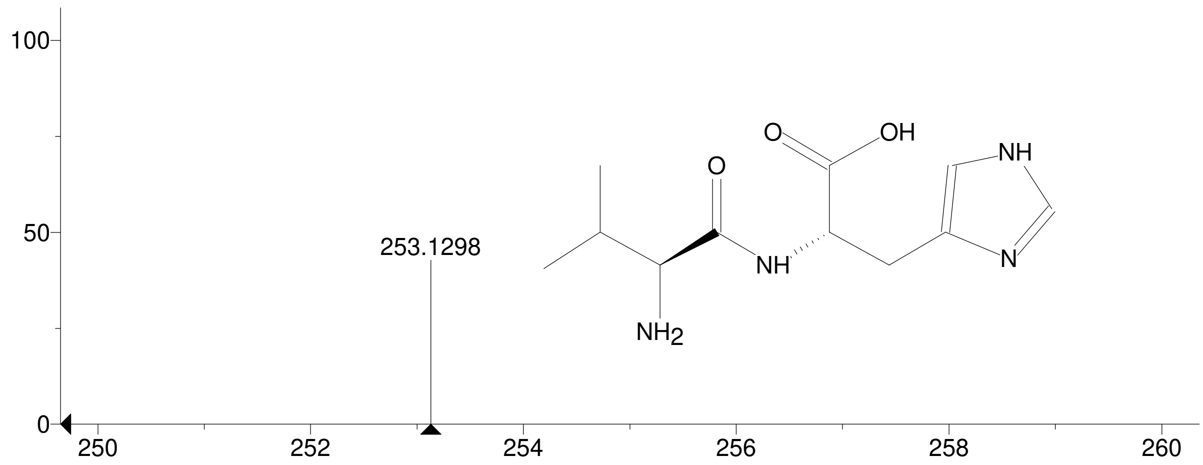

Hit 3 : His-Val [M-H]- HCD 32eV P=253.1
C11H18N4O3; MF: 364; RMF: 999; Prob 14.4%; CAS: 76019-15-3; Lib: nist_msms2; ID: 70322.

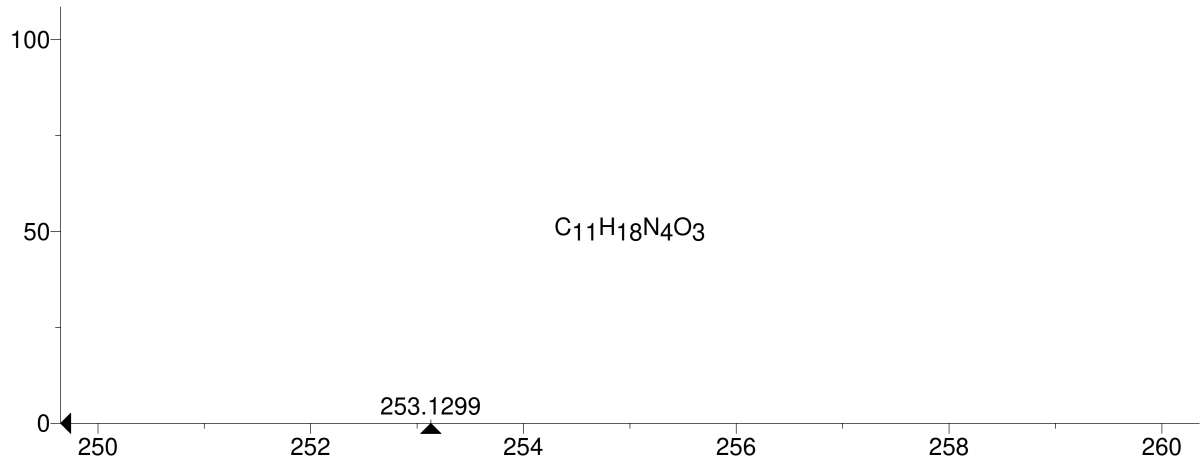

Hit 4 : Val-His [M-H]- HCD 22eV P=253.1
C11H18N4O3; MF: 364; RMF: 999; Prob 14.4%; Lib: nist_msms2; ID: 71676.

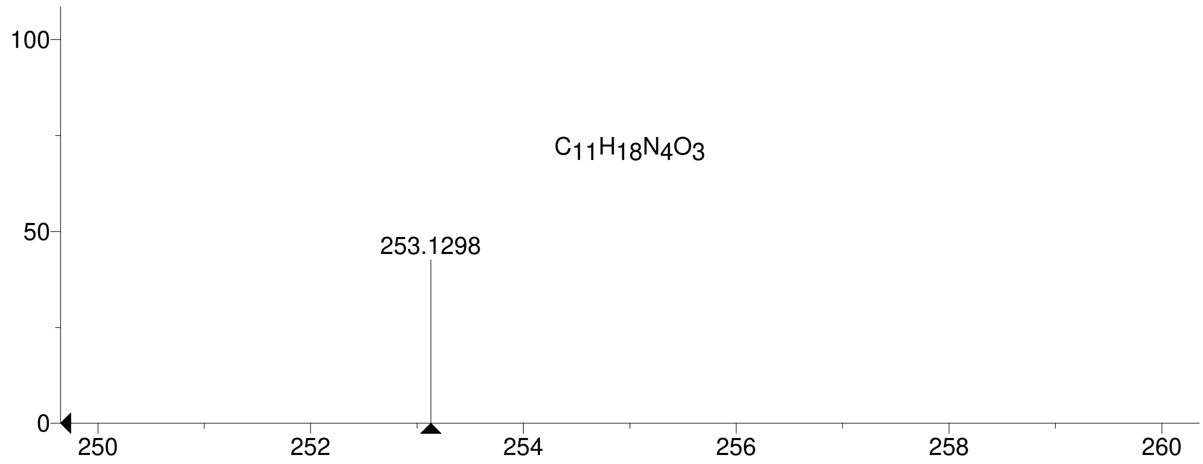

Hit 5 : Eremanthin [M+Na]+ P=253.1
C15H18O2; MF: 364; RMF: 999; Prob 14.4%; Lib: mona-export-gnps; ID: 2910.

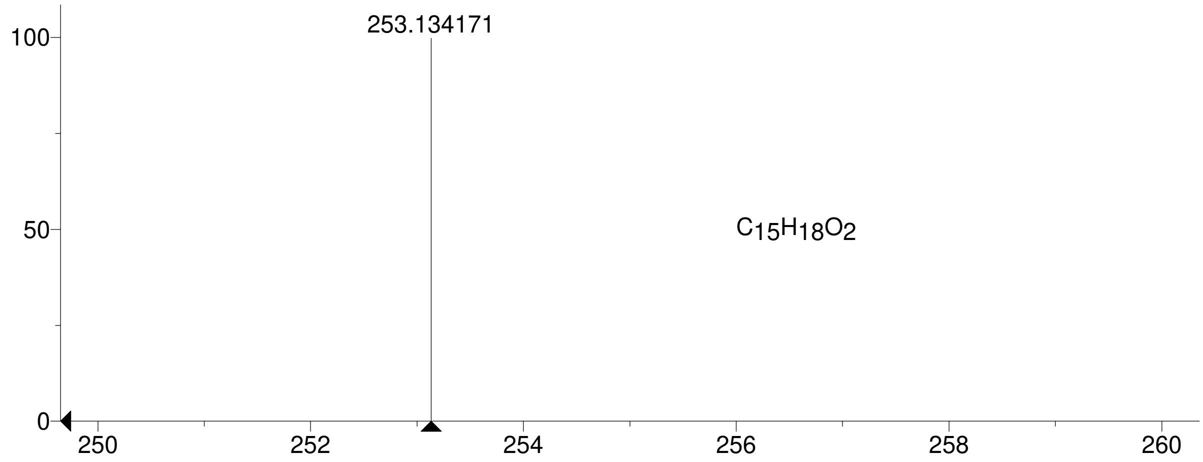

Unknown: Unknown (8.89_297.1235m/z)
Compound in Library Factor = 0

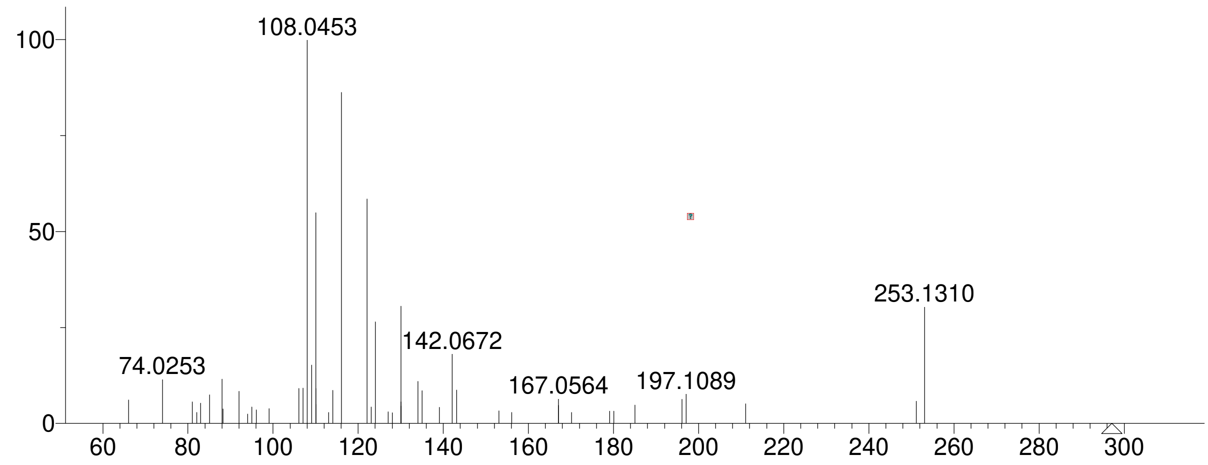

Hit 1 : 1H-Indole-6-carboxamide, N-hydroxy-1-[(4-methoxyphenyl)methyl]- [M+H]+ IT-FT 35% P=297.1
C17H16N2O3; MF: 0; RMF: 30; Prob 100.0%; CAS: 950762-95-5; Lib: nist_msms; ID: 254263.

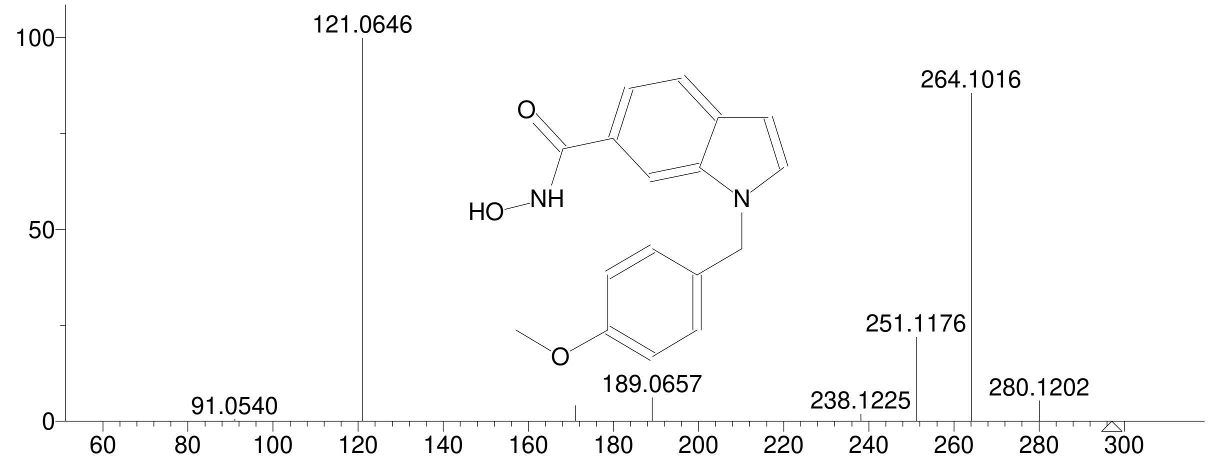

** Search Report Page 1 of 1 **

Unknown: Unknown (8.91_508.1815m/z)
Compound in Library Factor = 0

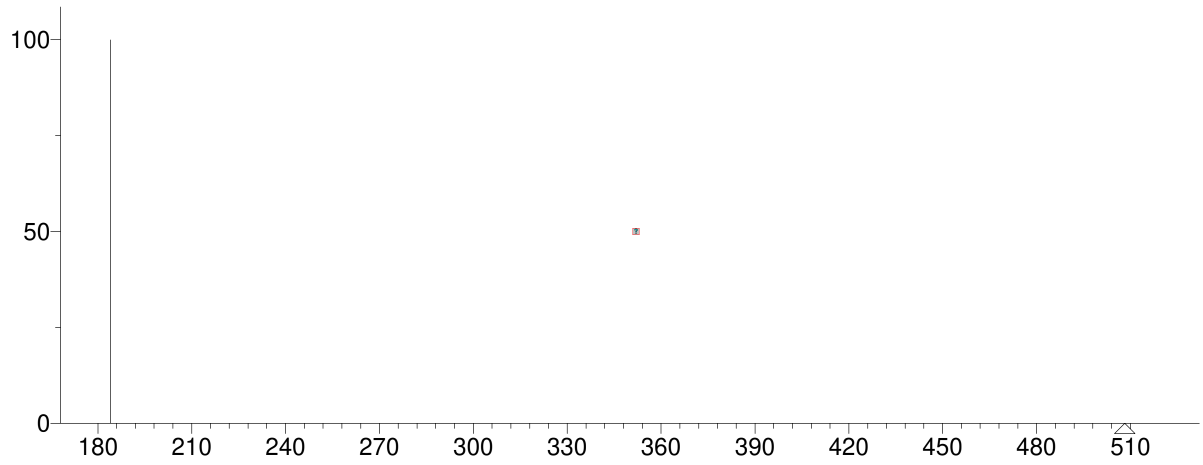

Unknown: Unknown (8.94_433.0803m/z)
Compound in Library Factor = -674

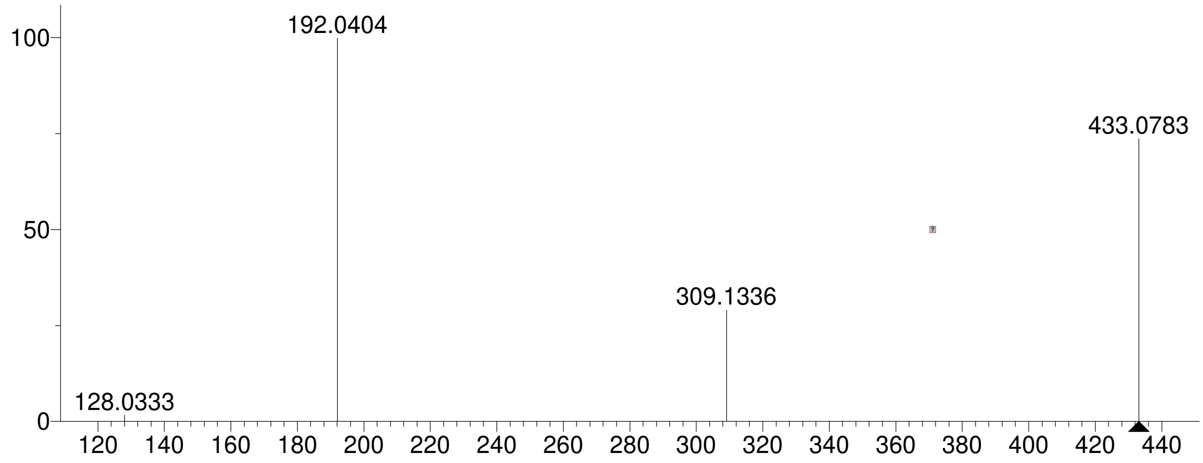

Hit 1 : Avicularin [M-H]- HCD 12V P=433.1
C20H18O11; MF: 175; RMF: 806; Prob 84.8%; CAS: 572-30-5; Lib: nist_msms; ID: 385062.

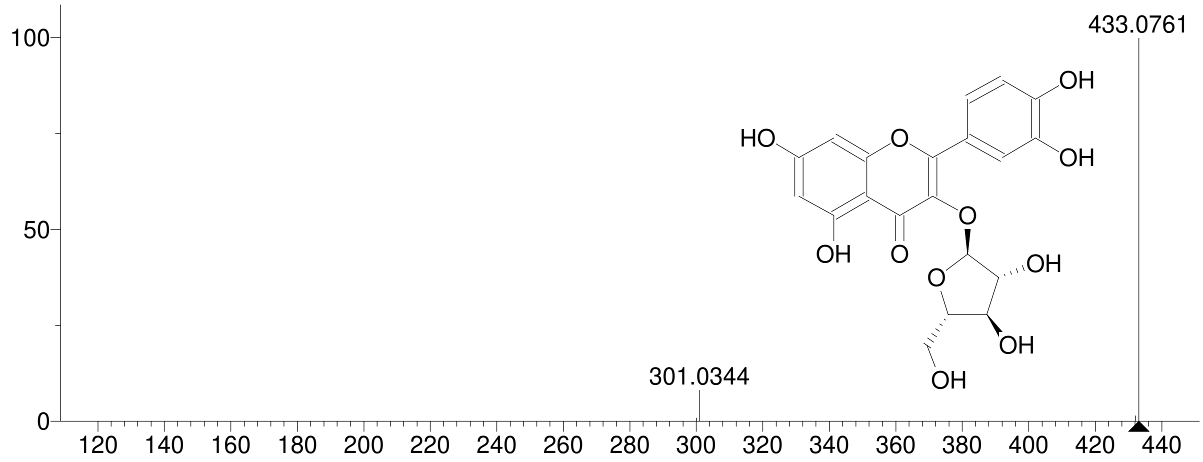

Hit 2 : Quercetin-3-O-xyloside [M-H]- 10eV P=433.1
C20H18O11; MF: 75; RMF: 709; Prob 6.31%; Lib: anthocyanins mona; ID: 2984.

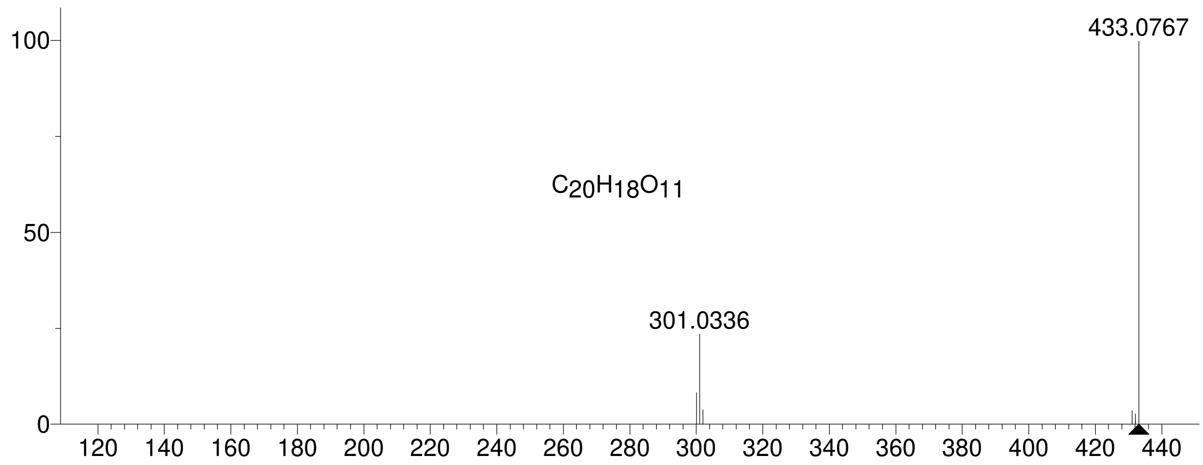

Hit 3 : Guaiaverin [M-H]- HCD 17V P=433.1
C20H18O11; MF: 55; RMF: 550; Prob 2.87%; CAS: 22255-13-6; Lib: nist_msms; ID: 389162.

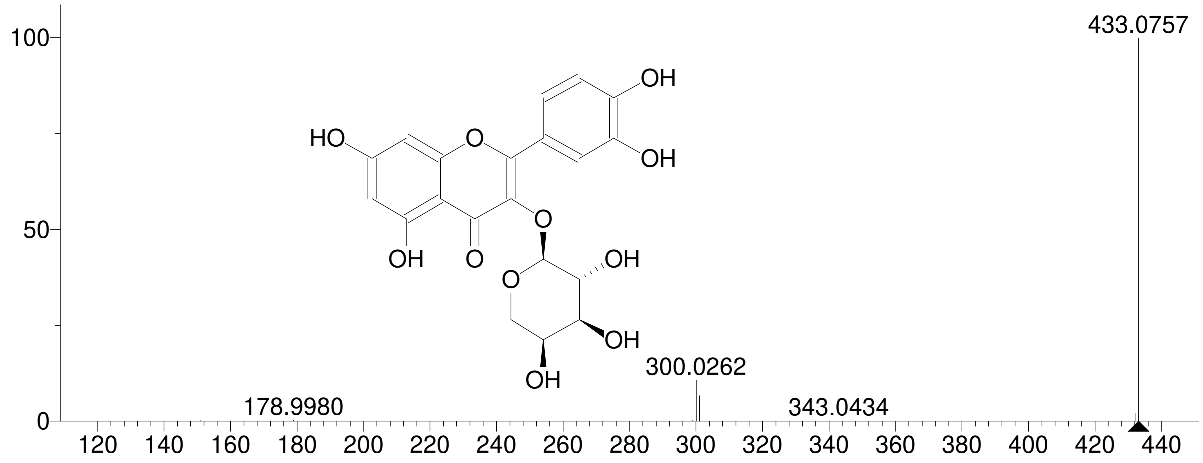

Hit 4 : Bensulfuron-methyl [M+Na]+ HCD 12V P=433.1
C16H18N4O7S; MF: 40; RMF: 513; Prob 1.74%; CAS: 83055-99-6; Lib: nist_msms; ID: 139117.

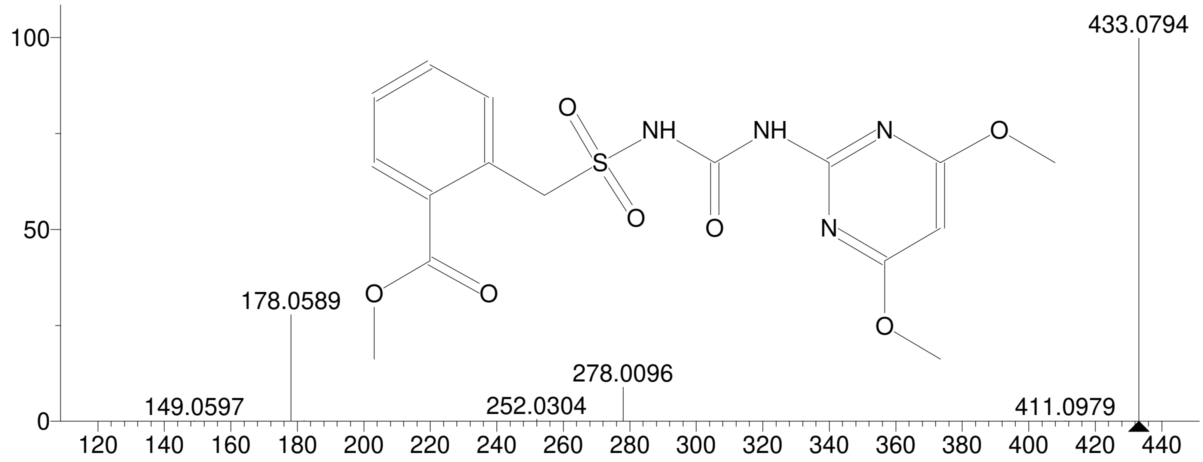

Hit 5 : quercetin-3-O-pentoside [M-H]- P=433.1
C20H18O11; MF: 10; RMF: 375; Prob 0.48%; Lib: msms-neg-gnps; ID: 2272.

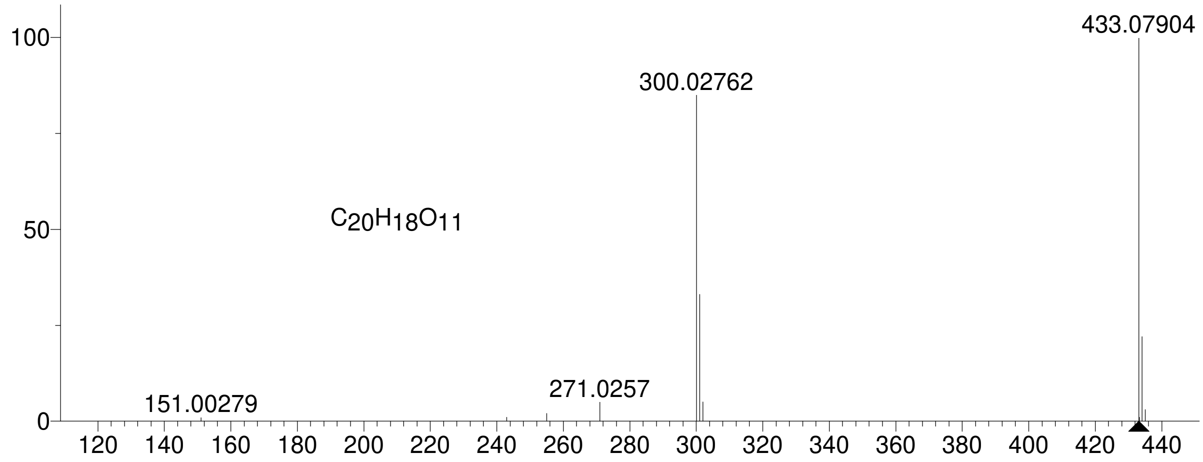

Unknown: Unknown (8.94_544.2115m/z)
Compound in Library Factor = 0

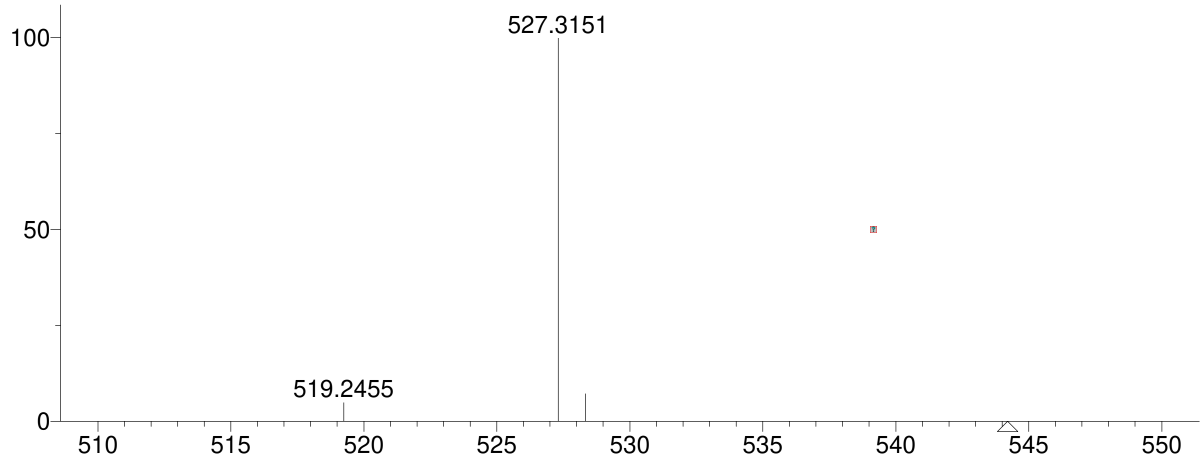

Unknown: Unknown (8.96_371.1799m/z)
Compound in Library Factor = 0

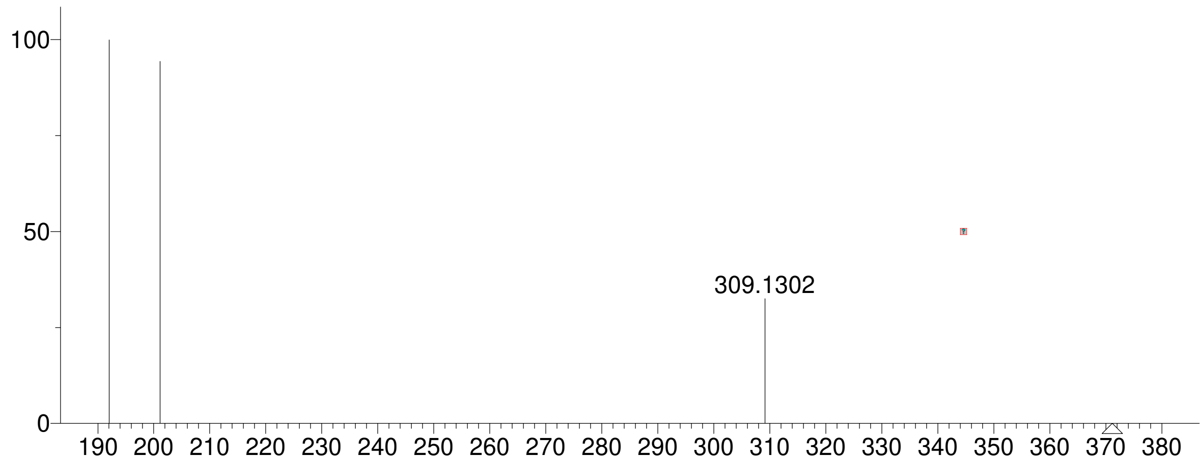

Unknown: Unknown (8.97_387.1762m/z)
Compound in Library Factor = 0

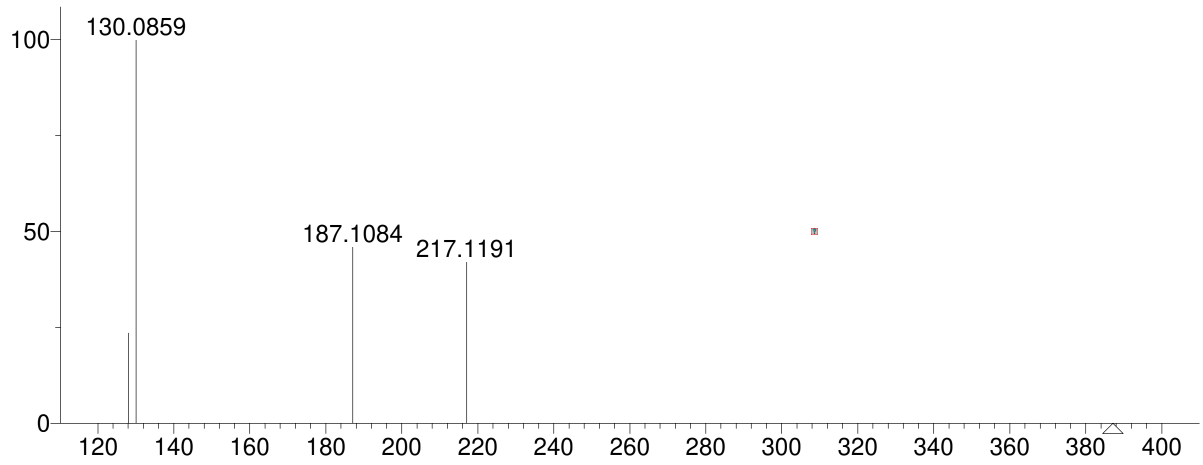

Hit 1 : Neurotensin (1-6) [M-2H]2- HCD 55eV P=387.2
C35H52N8O12; MF: 0; RMF: 63; Prob 50.0%; Lib: nist_msms2; ID: 74180.

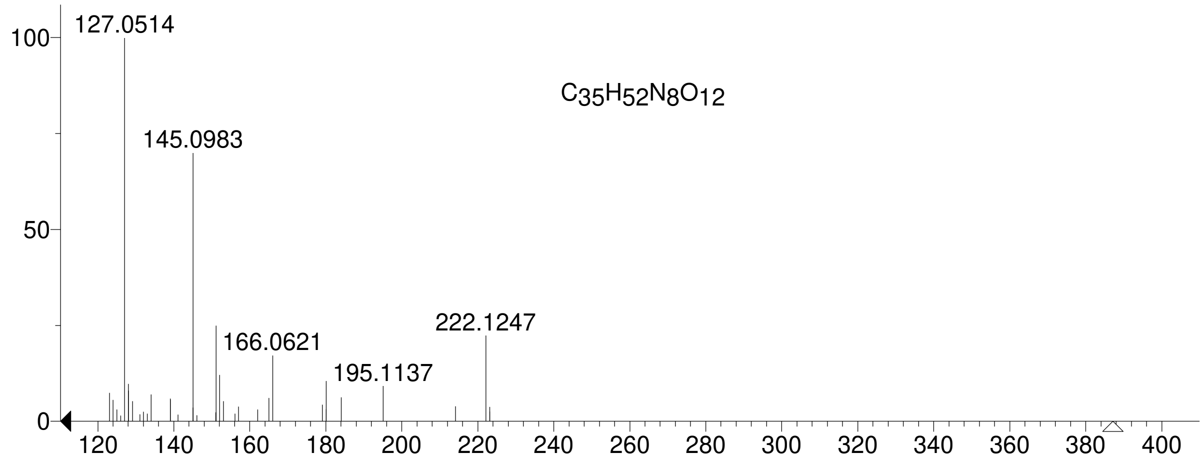

Hit 2 : NCGC00380363-01!8-(2,3-dihydroxy-3-methylbutyl)-5,7-dimethoxy-2-phenyl-2,3-dihydrochromen-4-one [M
C22H26O6; MF: 0; RMF: 11; Prob 50.0%; Lib: mona-export-gnps; ID: 9549.

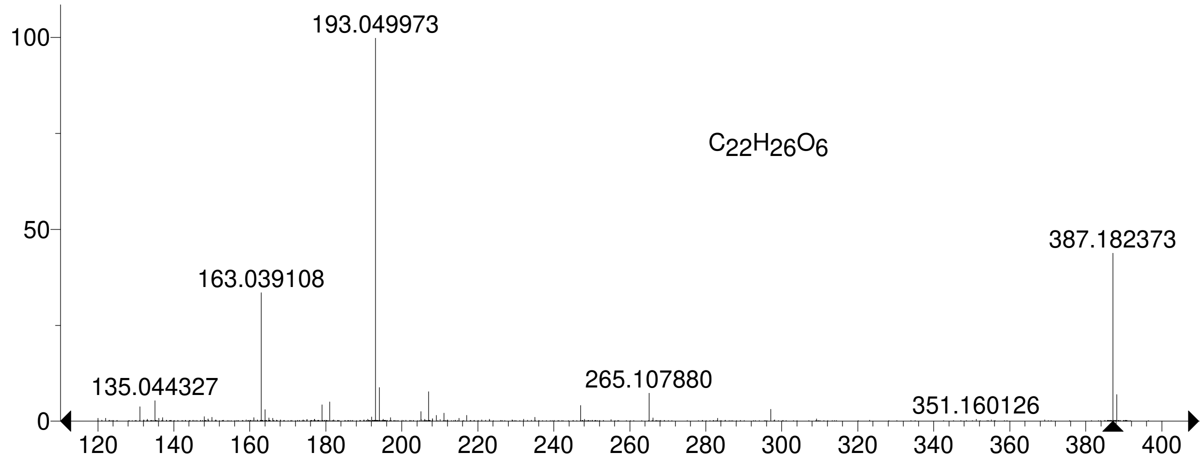

Unknown: Unknown (8.97_516.2188m/z)
Compound in Library Factor = 0

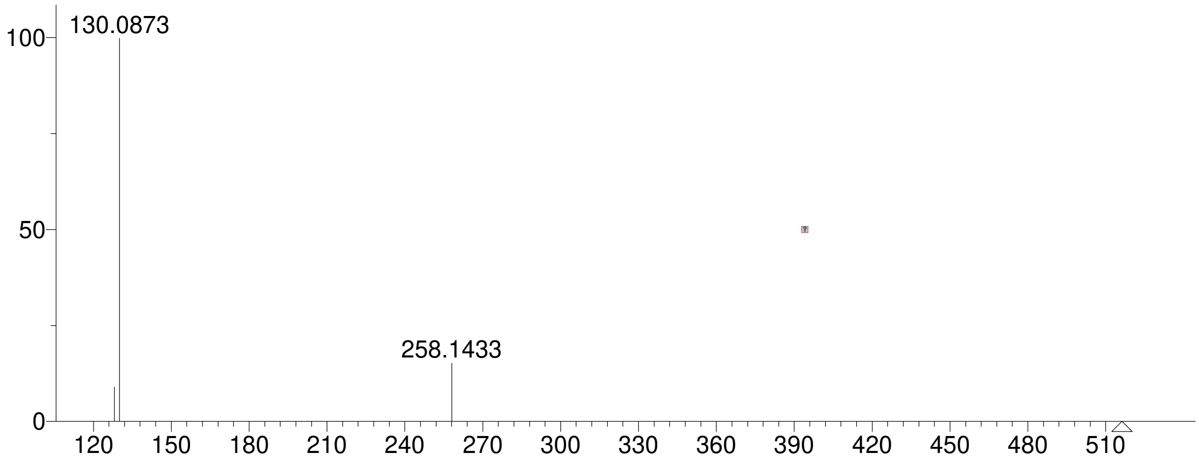

Unknown: Unknown (8.97_527.3187m/z)
Compound in Library Factor = 0

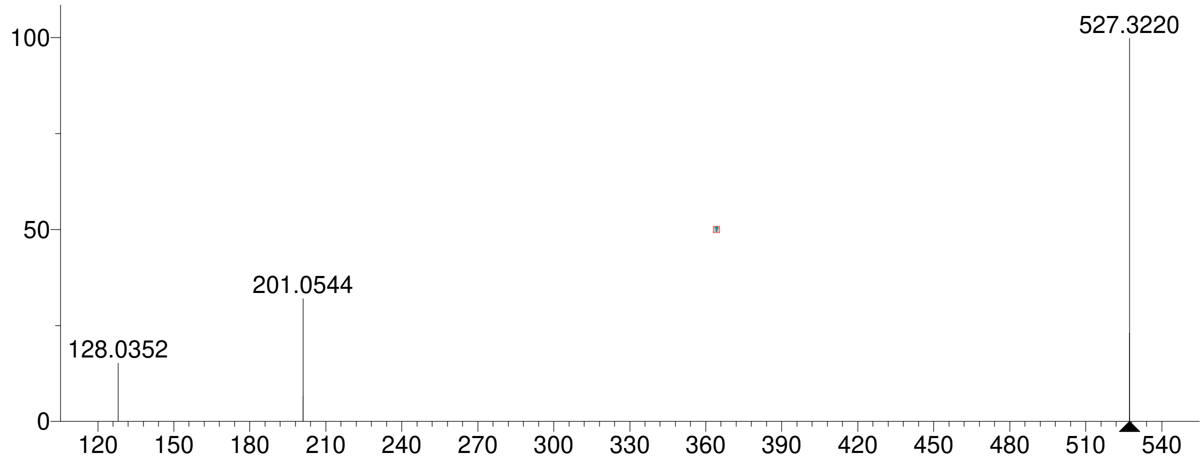

Hit 1 : [(1R)-1-[(2S,4aR,4bS,7S,8aS)-7-hydroxy-2,4b,8,8-tetramethyl-4,4a,5,6,7,8a,9,10-octahydro-3H-phenanthr
C28H46O9; MF: 0; RMF: 101; Prob 100.0%; Lib: mona-export-vf-npl_qtof; ID: 1224.

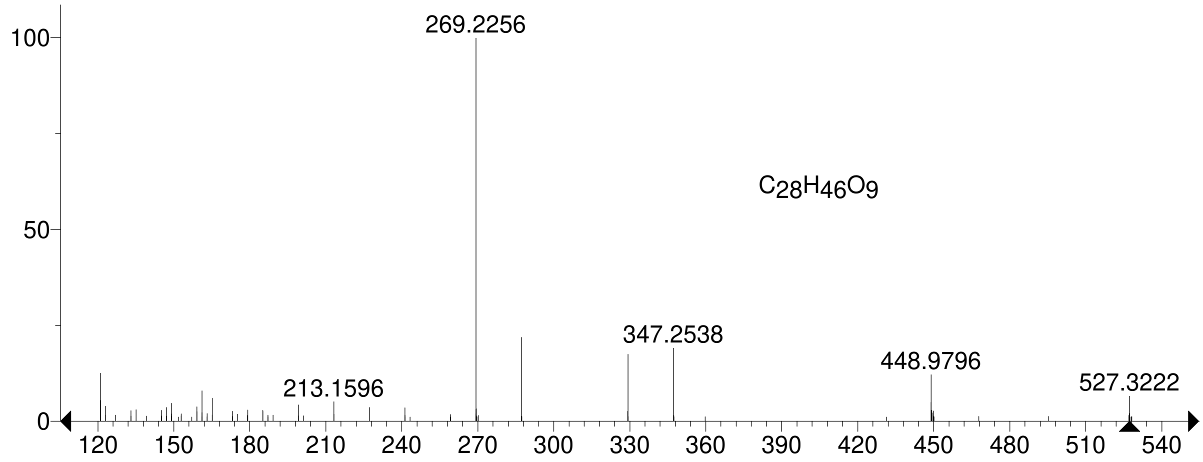

Unknown: Unknown (8.98_541.1685m/z)
Compound in Library Factor = 0

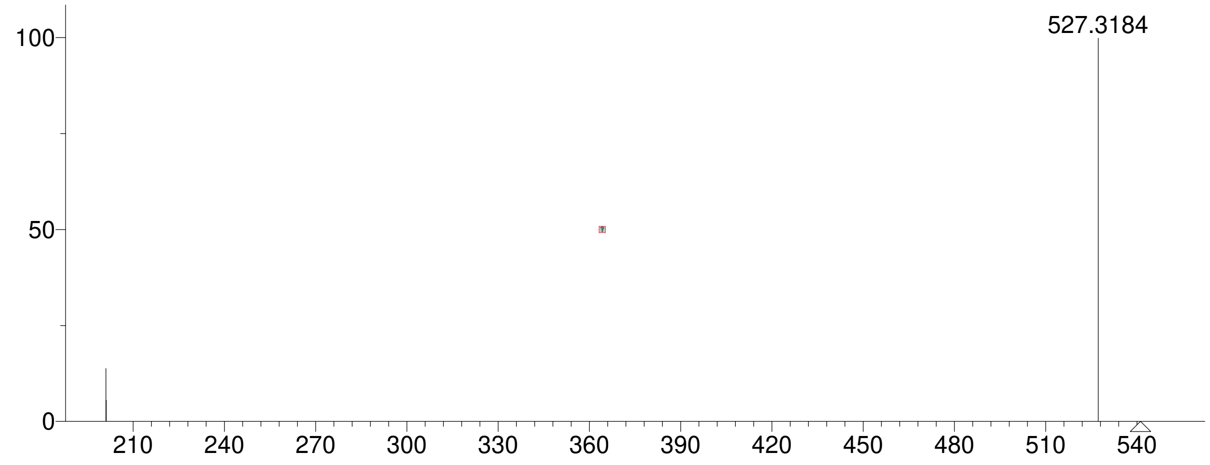

Hit 1 : 7-Benzyloxy-2-(2,2-diphenyl-1,3-benzodioxol-5-yl)-5-hydroxy-H-1-benzopyran-4-one [M+H]+ HCD 48V F
C35H24O6; MF: 0; RMF: 31; Prob 100.0%; CAS: 1391054-52-6; Lib: nist_msms; ID: 448012.

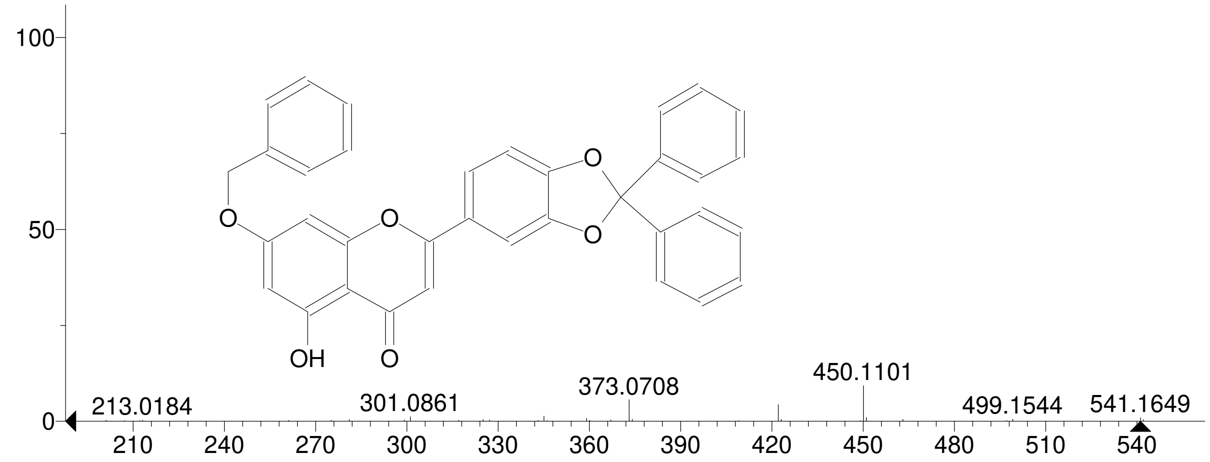

Unknown: Unknown (9.01_472.1918m/z)
Compound in Library Factor = 0

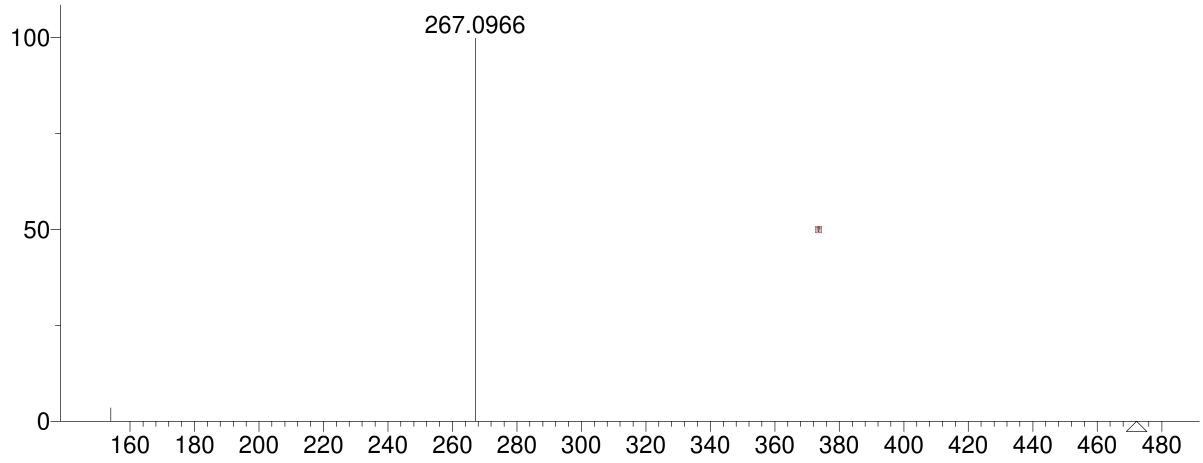

Unknown: Unknown (9.04_500.1774m/z)
Compound in Library Factor = 0

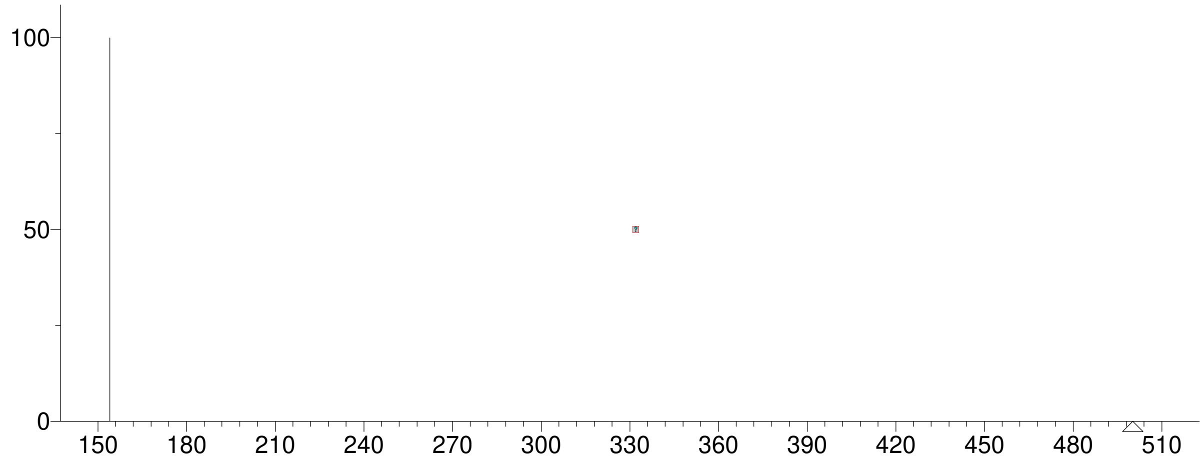

Unknown: Unknown (9.05_470.0149m/z)
Compound in Library Factor = 0

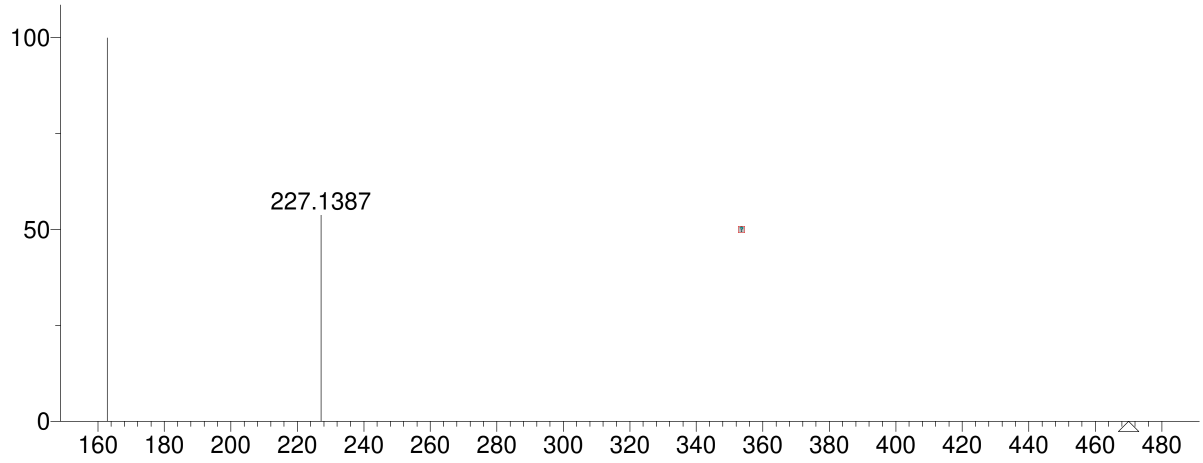

** Search Report Page 1 of 1 **

Unknown: Unknown (9.06_704.1041m/z)
Compound in Library Factor = 0

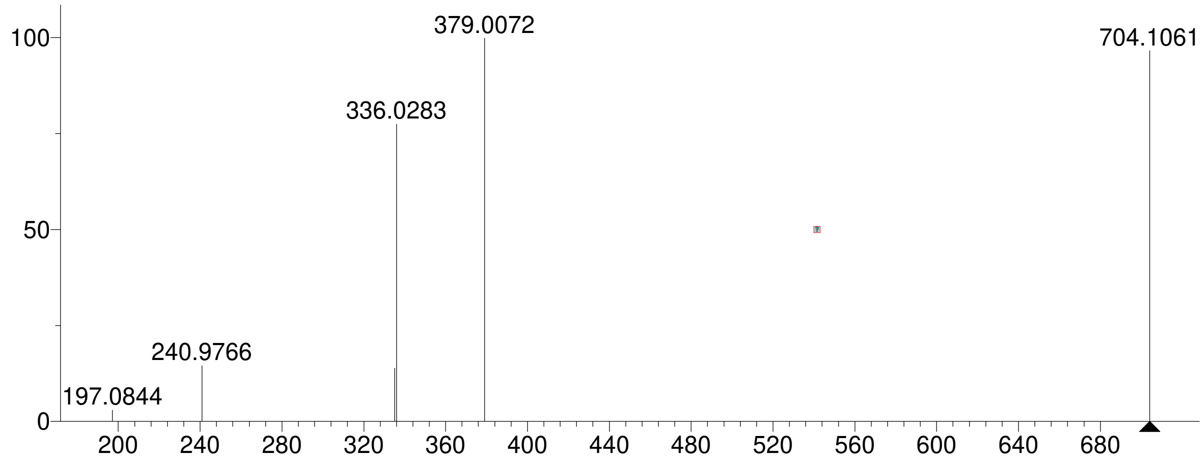

Unknown: Unknown (9.07_520.2290m/z)
Compound in Library Factor = -3273

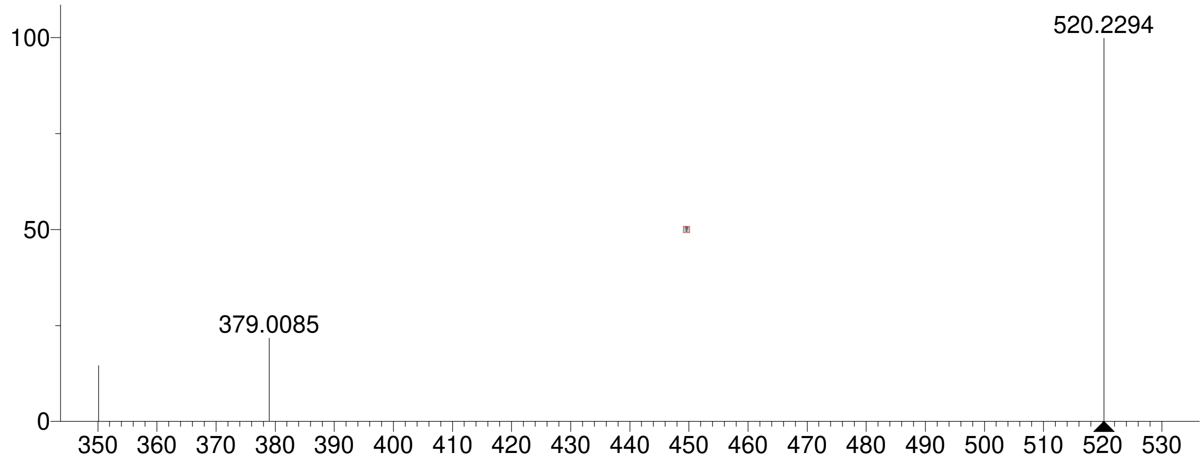

Hit 1 : NCGC00380810-01! [M+H]+ P=520.2
C30H33NO7; MF: 7; RMF: 620; Prob 56.0%; Lib: mona-export-gnps; ID: 6611.

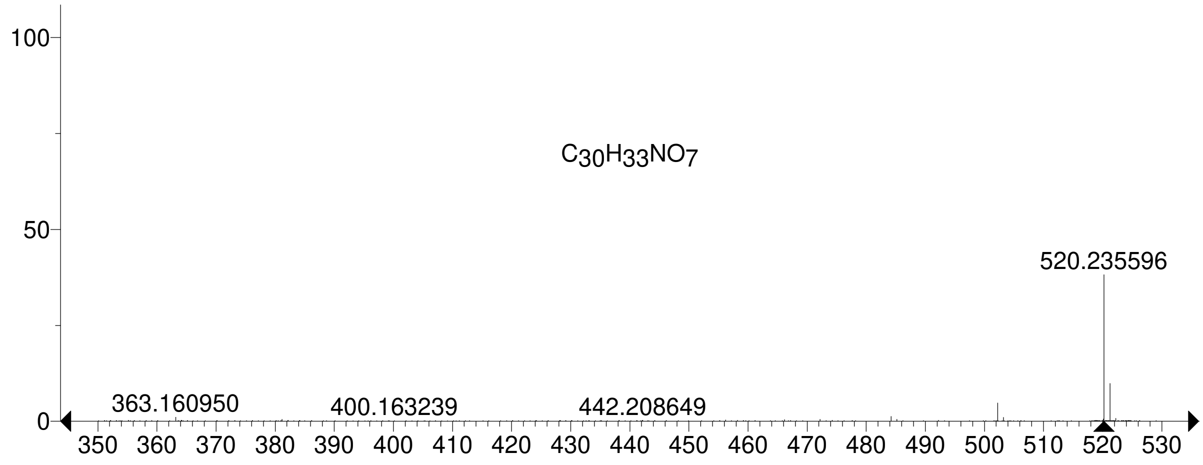

Hit 2 : _130047 [M+Na]+ P=520.2
C28H35NO7; MF: 1; RMF: 233; Prob 44.0%; Lib: mona-export-gnps; ID: 5021.

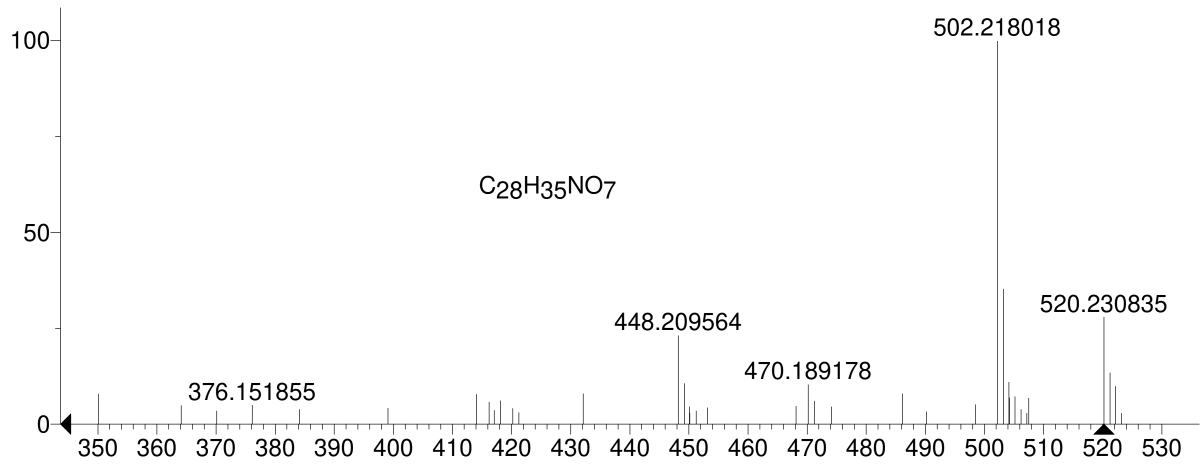

Unknown: Unknown (9.08_429.1860m/z)
Compound in Library Factor = 0

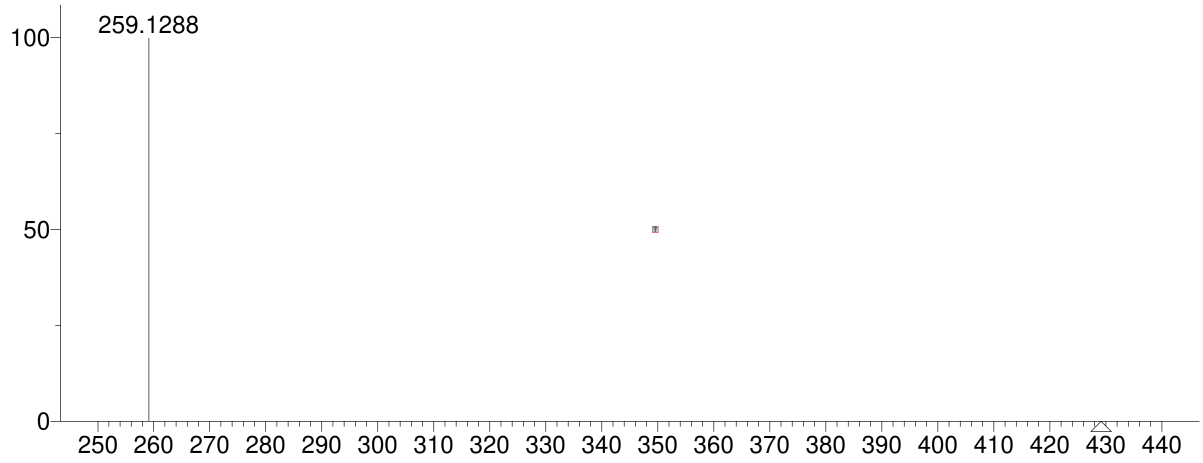

Hit 1 : (2R,3R,4S,5S,6R)-2-[6-hydroxy-3-[(E)-3-hydroxybut-1-enyl]-2,4,4-trimethylcyclohexyl]oxy-6-(hydroxymethy
C19H34O8; MF: 0; RMF: 19; Prob 100.0%; Lib: anthocyanins mona; ID: 9988.

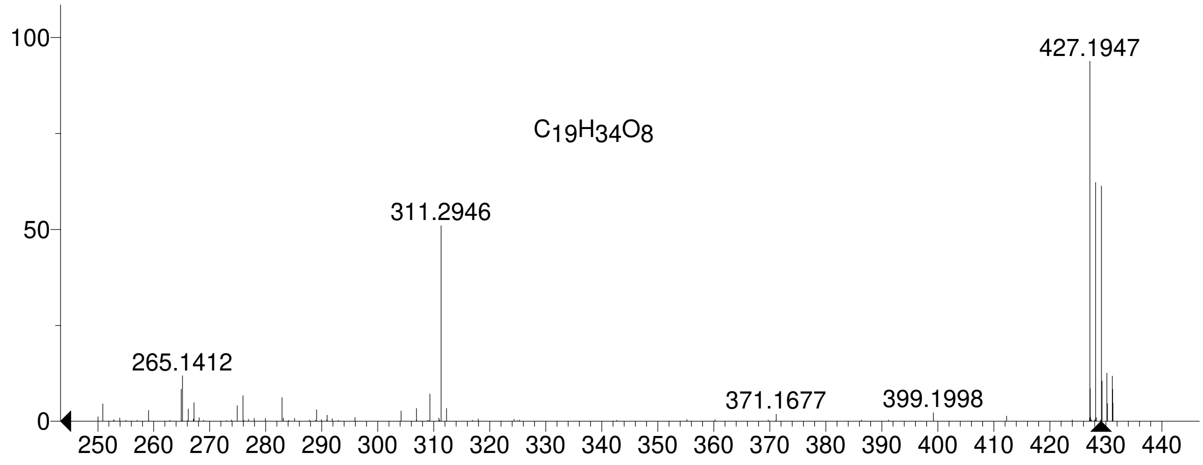

** Search Report Page 1 of 2 **

Unknown: Unknown (9.09_437.1028m/z)
Compound in Library Factor = 0

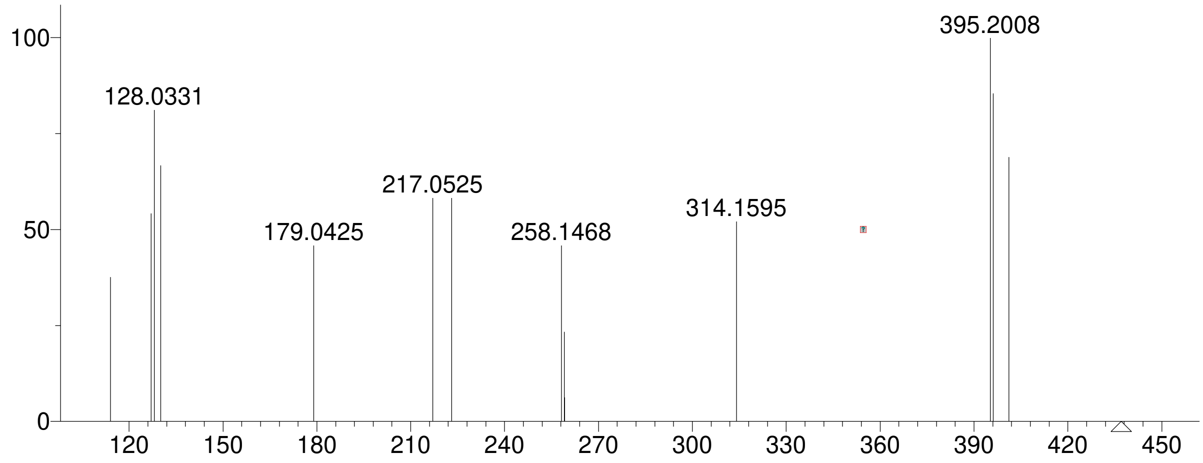

Hit 1 : asperuloside [M+Na]+ 35HCD P=437.1
C18H22O11; MF: 0; RMF: 18; Prob 20.0%; Lib: mona-export-vf-npl_qexactive; ID: 10939.

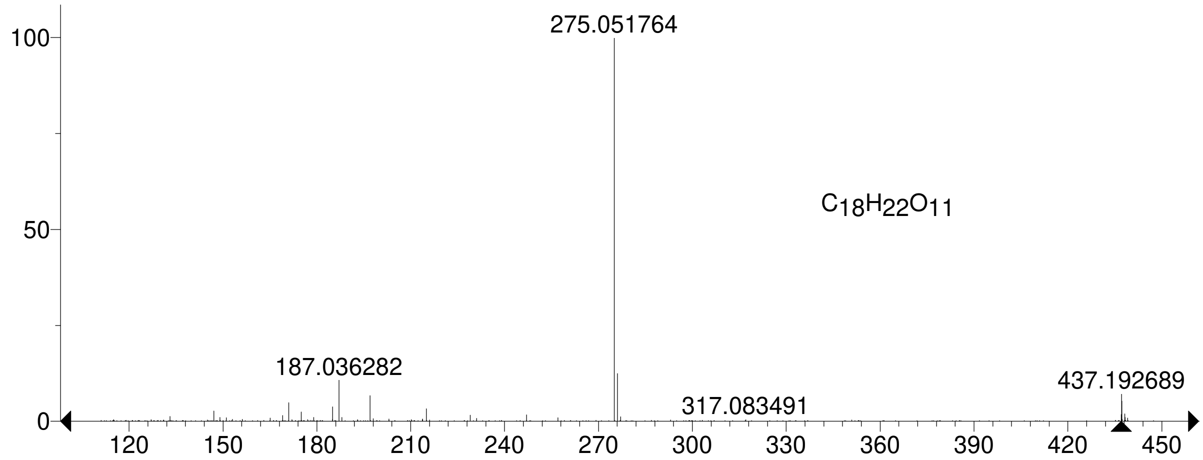

Hit 2 : Ouabain [M-H]-=>437.1 IT 35% P=437.1
C29H44O12; MF: 0; RMF: 10; Prob 20.0%; CAS: 630-60-4; Lib: nist_msms; ID: 153450.

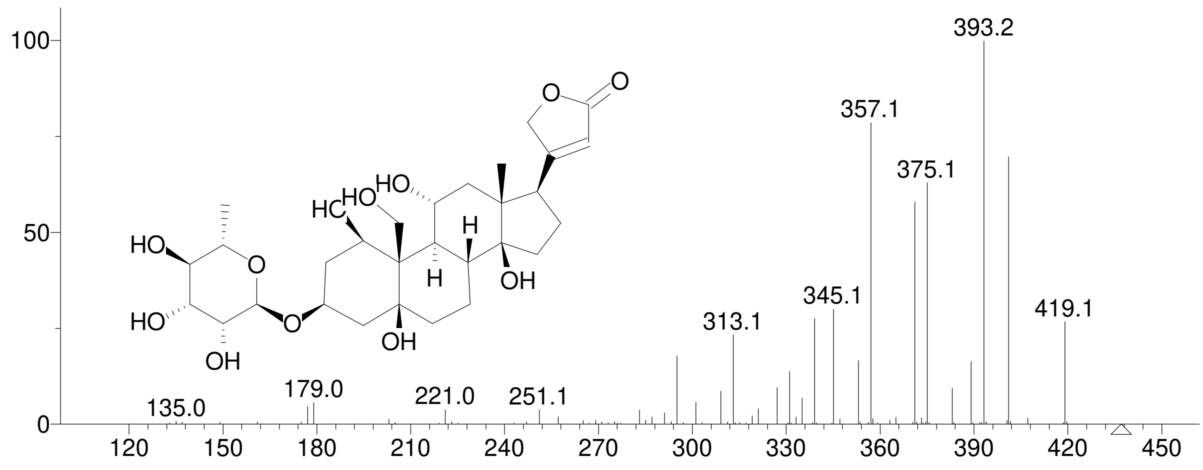

Hit 3 : (5-benzoyloxy-4,6-dihydroxy-3-methoxycyclohexen-1-yl)methyl benzoate [M+K]+ 35HCD P=437.1
C22H22O7; MF: 0; RMF: 7; Prob 20.0%; Lib: mona-export-vf-npl_qexactive; ID: 1879.

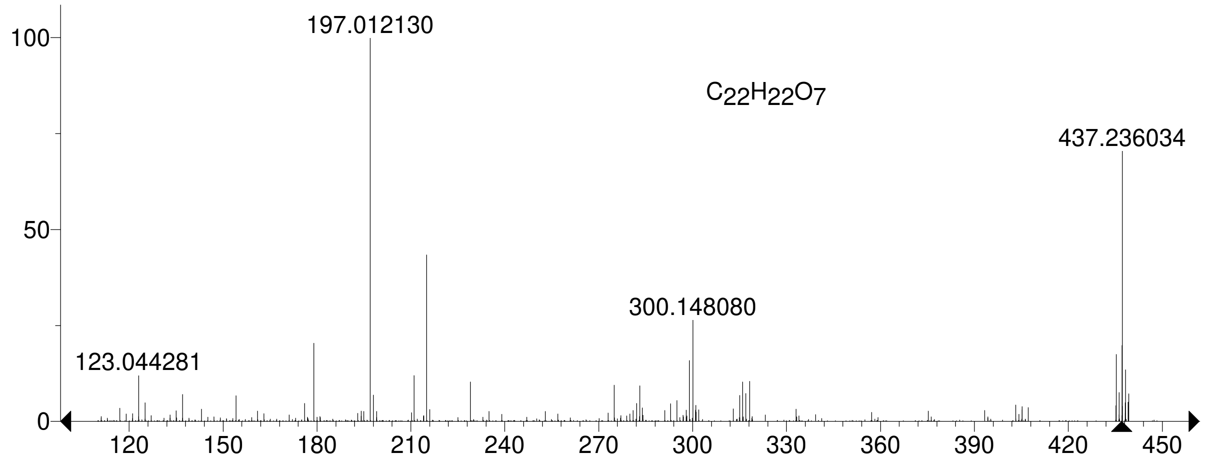

Hit 4 : Pranlukast [M+H-N2]+=>437.1 IT 35% P=437.1
C27H23N5O4; MF: 0; RMF: 7; Prob 20.0%; CAS: 103177-37-3; Lib: nist_msms; ID: 334232.

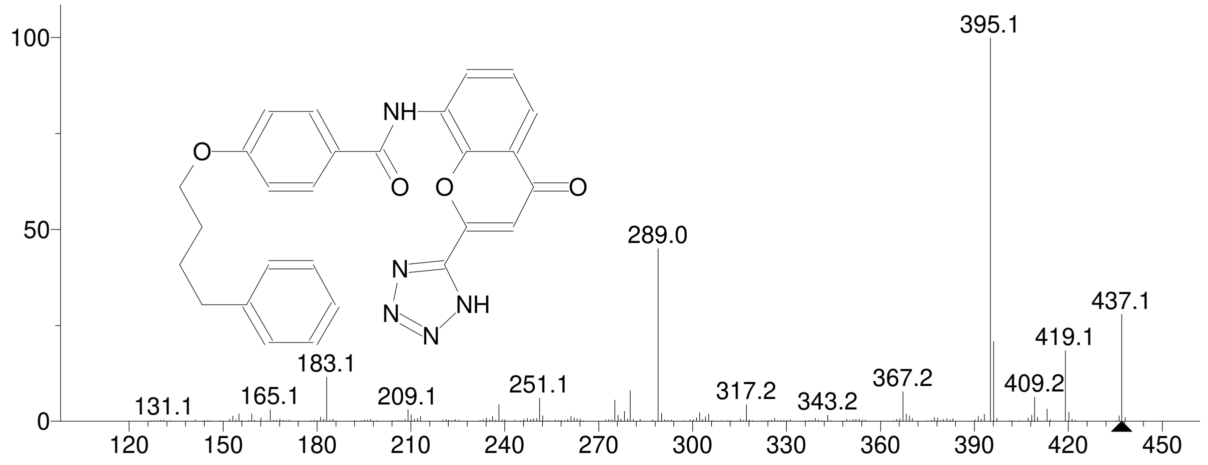

Hit 5 : 3-[3-[3,4-dihydroxy-5-(6-oxo-3H-purin-9-yl)oxolan-2-yl]propanoyl]benzoic acid [M+Na]+ 45HCD P=437.1
C19H18N4O7; MF: 0; RMF: 3; Prob 20.0%; Lib: mona-export-vf-npl_qexactive; ID: 17321.

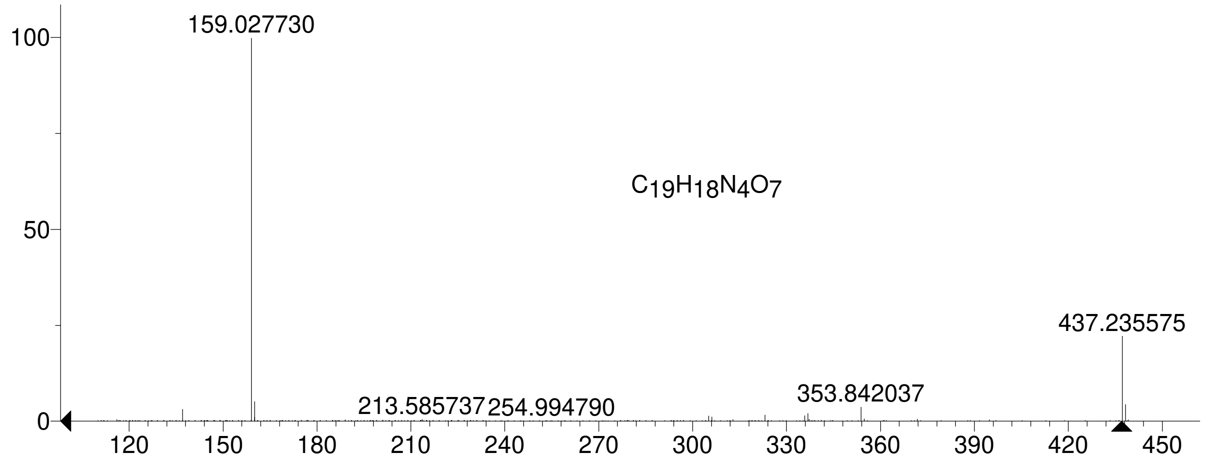

Unknown: Unknown (9.09_508.1820m/z)
Compound in Library Factor = 0

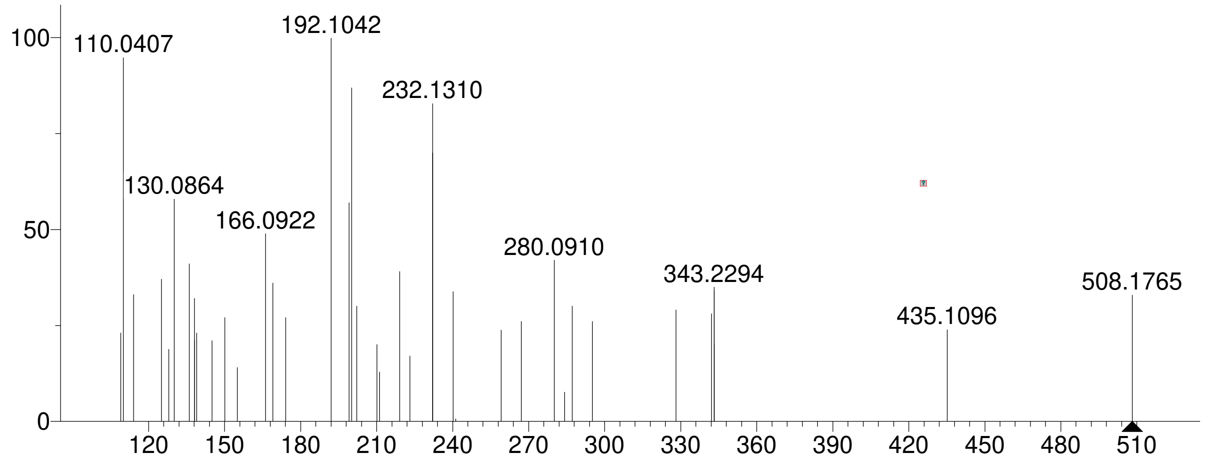

Unknown: Unknown (9.10_383.1330m/z)
Compound in Library Factor = 0

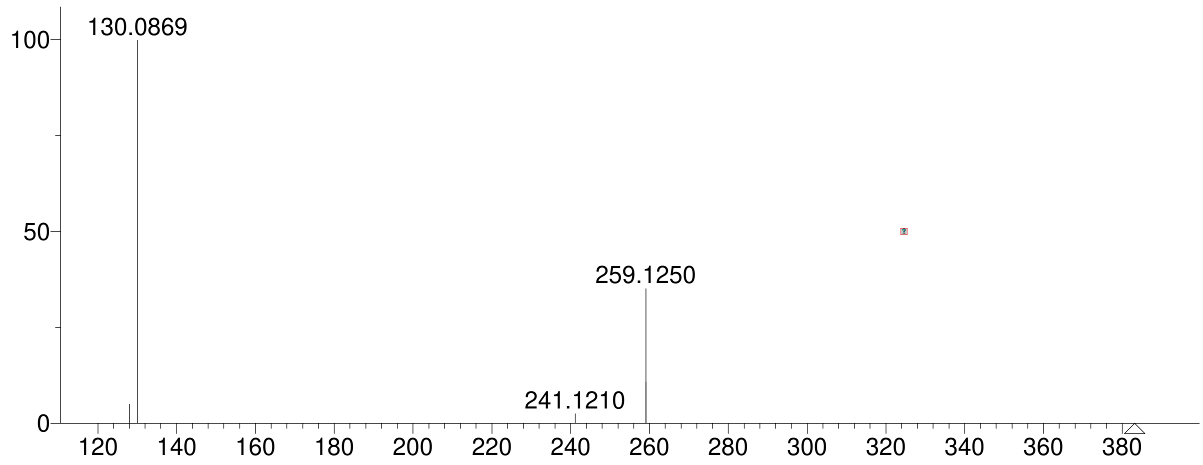

Hit 1 : dihydroferuloyl-beta-keto acid + deoxyhexose (curcumin) [M-H]- P=383.1
C18H24O9; MF: 0; RMF: 8; Prob 100.0%; Lib: phytofrag2018_final_060219; ID: 1018.

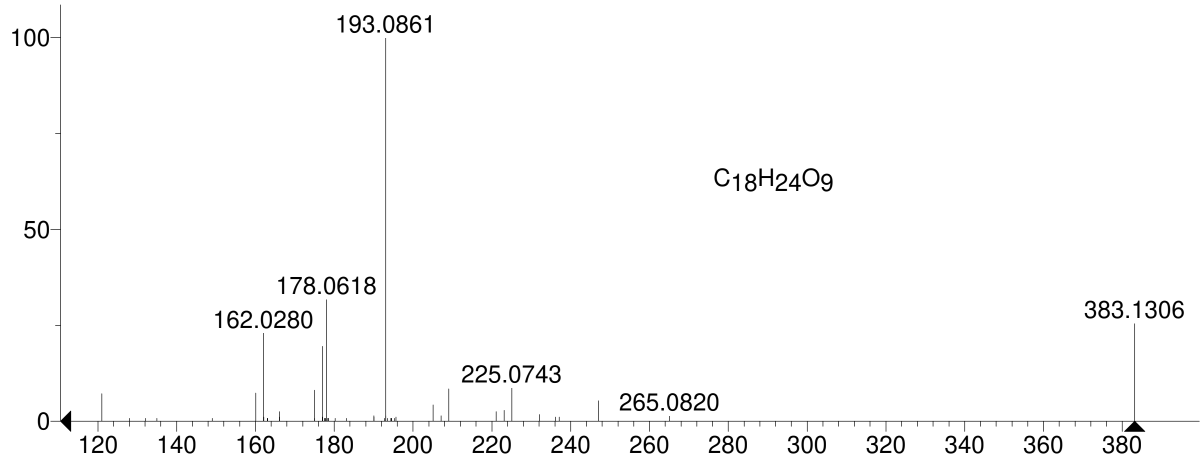

Unknown: Unknown (9.10_498.2445m/z)
Compound in Library Factor = 0

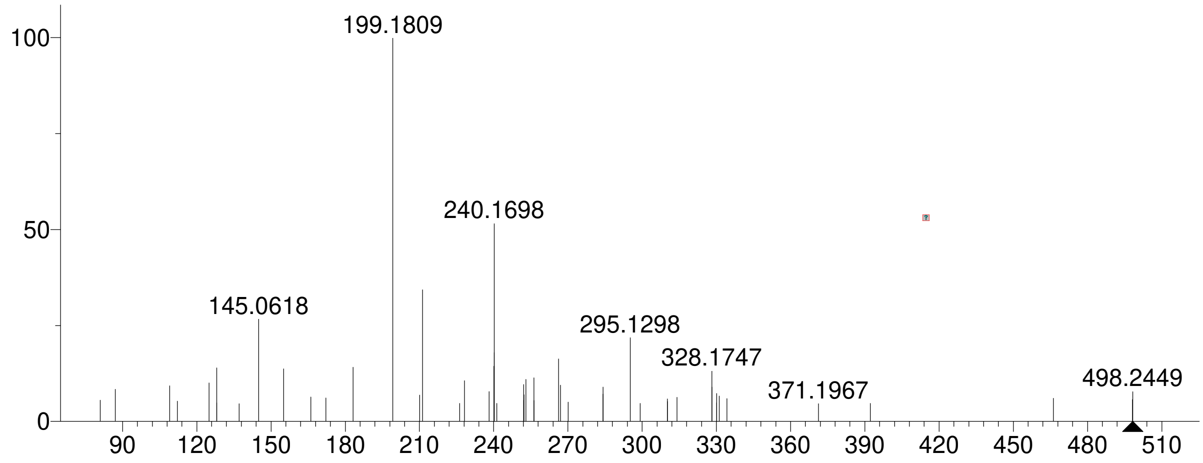

Hit 1 : NCGC00169355-02_C28H37NO8_Pentanedioic acid, 2-[(6'S,8a'S)-3',4',4'a,5',6,6',7',8,8',8'a-decahydro-4,[illegible]
C28H37NO8; MF: 0; RMF: 52; Prob 8.33%; Lib: mona-export-gnps; ID: 10695.

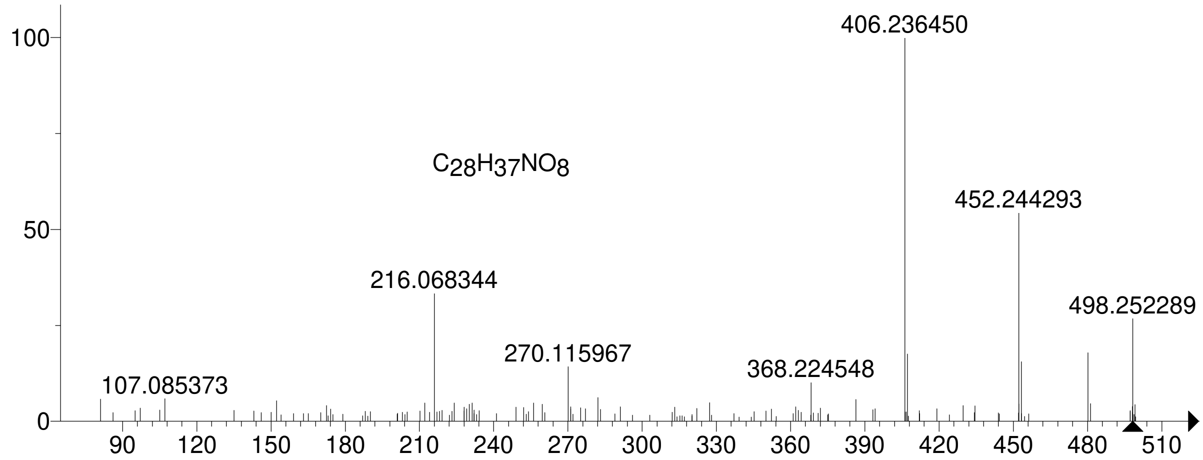

Hit 2 : PC 12:0; PC 6:0-6:0; [M+FA-H]- [M+FA-H]- P=498.2
C20H40NO8P; MF: 0; RMF: 37; Prob 8.33%; Lib: msdial-insilicomsms-lipids-neg; ID: 317805.

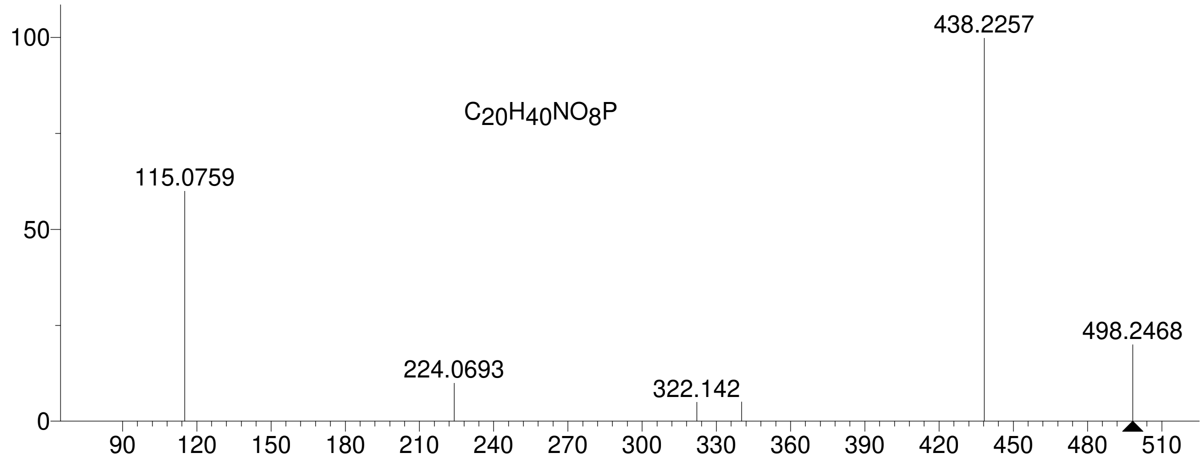

Hit 3 : PC 12:0; PC 2:0-10:0; [M+FA-H]- [M+FA-H]- P=498.2
C20H40NO8P; MF: 0; RMF: 35; Prob 8.33%; Lib: msdial-insilicomsms-lipids-neg; ID: 317575.

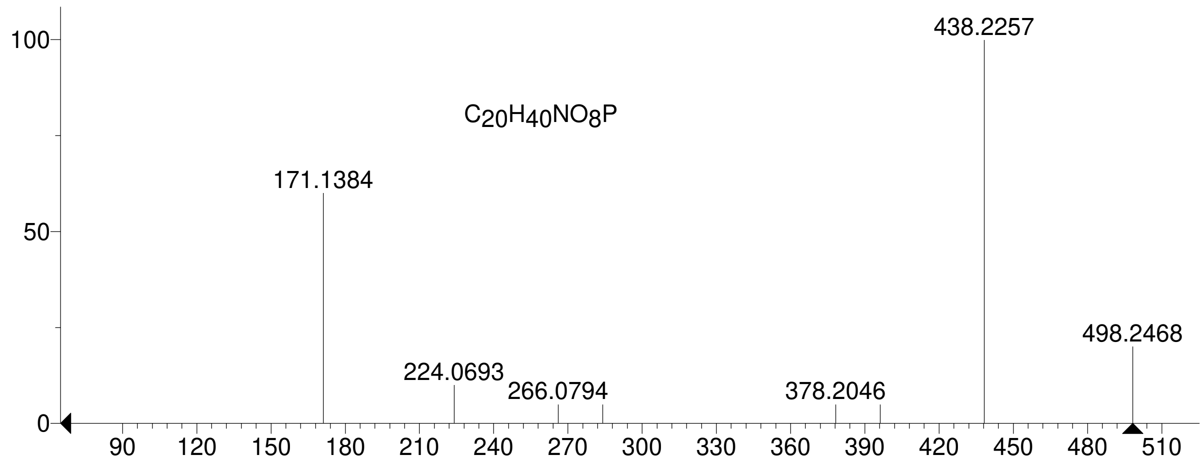

Hit 4 : PC 12:0; PC 3:0-9:0; [M+FA-H]- [M+FA-H]- P=498.2
C20H40NO8P; MF: 0; RMF: 35; Prob 8.33%; Lib: msdial-insilicomsms-lipids-neg; ID: 317634.

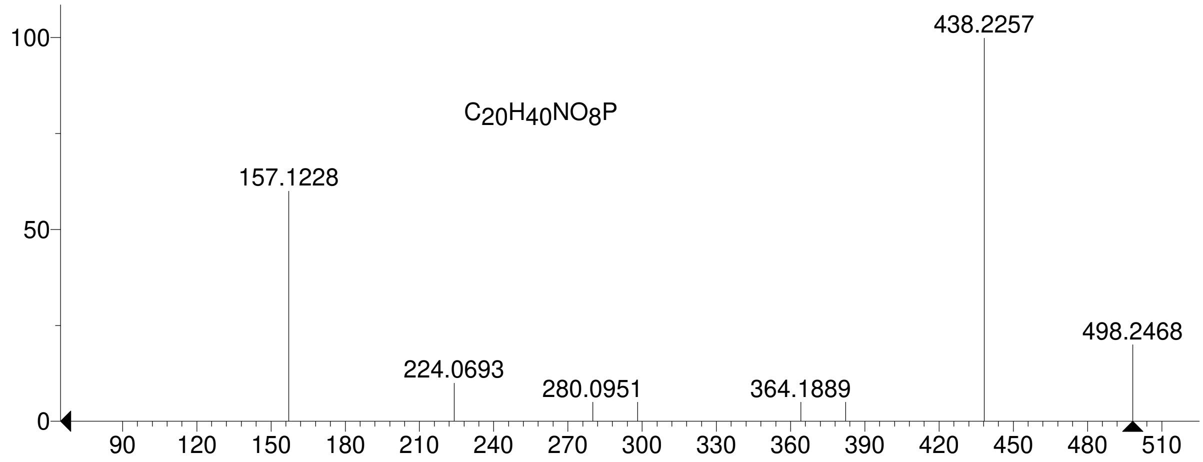

Hit 5 : PC 11:0; PC 2:0-9:0; [M+Hac-H]- [M+Hac-H]- P=498.2
C19H38NO8P; MF: 0; RMF: 35; Prob 8.33%; Lib: msdial-insilicomsms-lipids-neg; ID: 319465.

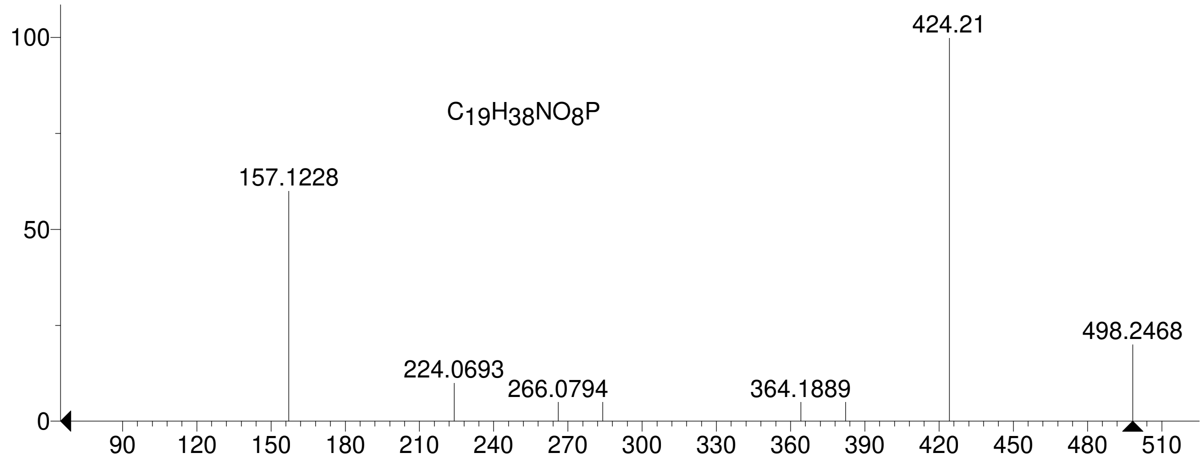

Unknown: Unknown (9.11_337.0917m/z)
Compound in Library Factor = -3273

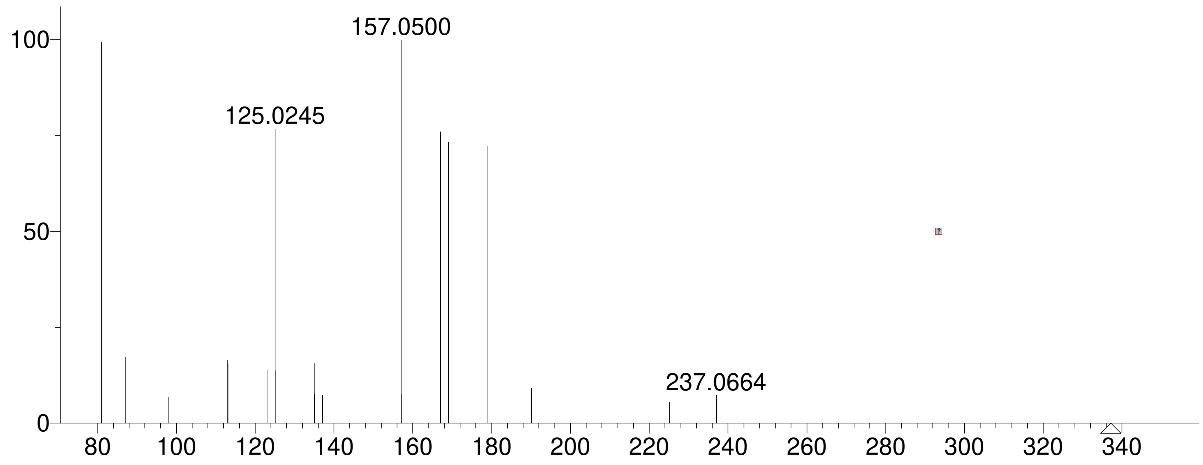

Hit 1 : Chlorogenic acid [M+H-H2O]+ HCD 87V P=337.1
C16H18O9; MF: 1; RMF: 36; Prob 10.4%; CAS: 327-97-9; Lib: nist_msms; ID: 100727.

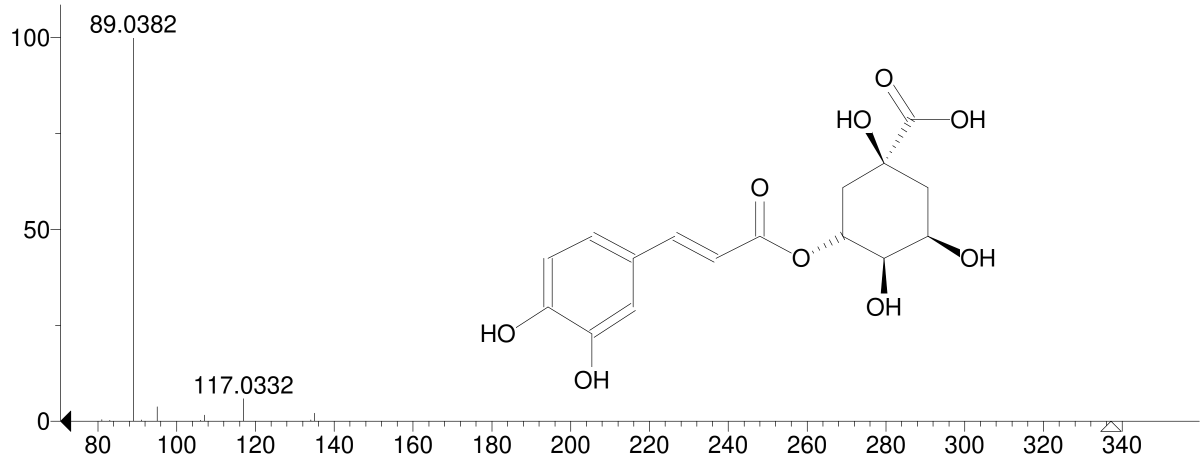

Hit 2 : Neochlorogenic acid [M+H-H2O]+ HCD 60V P=337.1
C16H18O9; MF: 0; RMF: 57; Prob 9.95%; CAS: 906-33-2; Lib: nist_msms; ID: 239448.

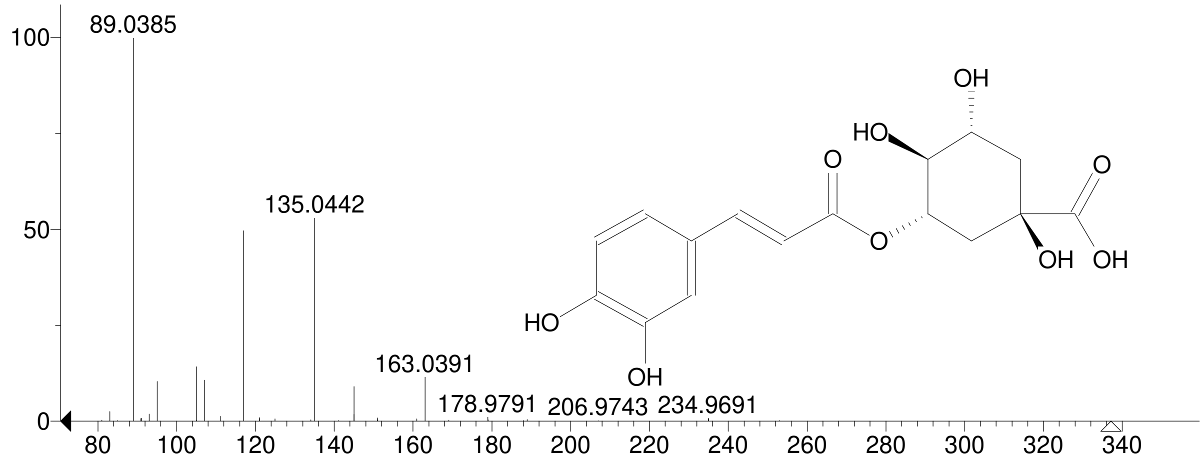

Hit 3 : NCGC00180823-02!(3R,4R,5R)-5-[(E)-3-(3,4-dihydroxyphenyl)prop-2-enoyl]oxy-3,4-dihydroxycyclohexene
C16H16O8; MF: 0; RMF: 51; Prob 9.95%; Lib: mona-export-gnps; ID: 9151.

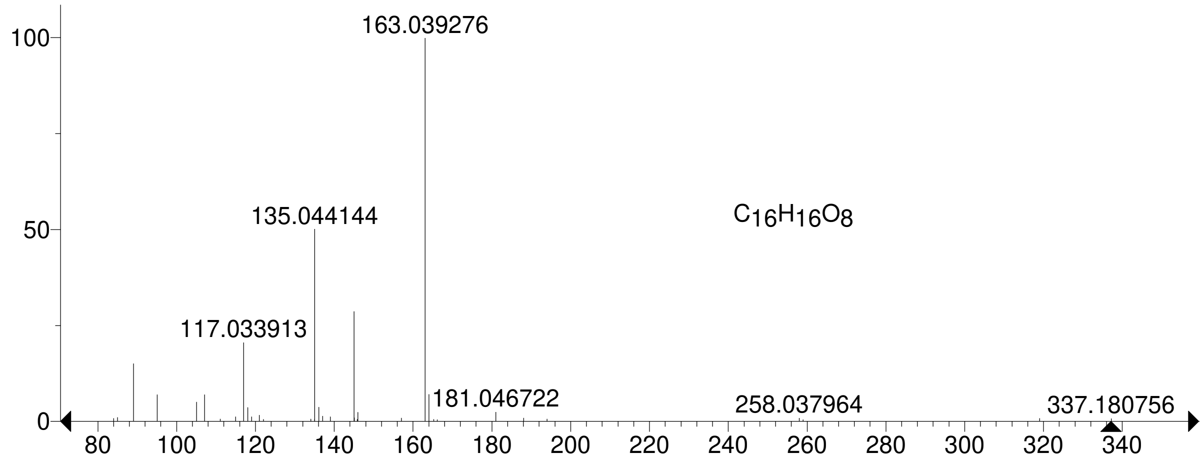

Hit 4 : NCGC00380360-01!7-methoxy-6-[(2S,3R,4S,5S,6R)-3,4,5-trihydroxy-6-(hydroxymethyl)oxan-2-yl]oxychron
C16H18O9; MF: 0; RMF: 46; Prob 9.95%; Lib: mona-export-gnps; ID: 9547.

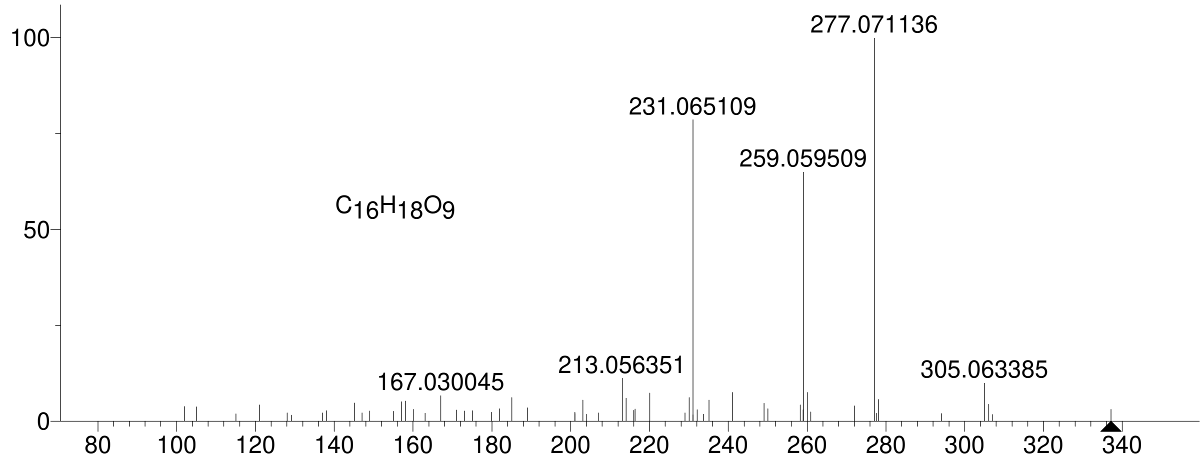

Hit 5 : (1R,3R,4S,5R)-1,3,4-trihydroxy-5-[(E)-3-(4-hydroxyphenyl)prop-2-enoyl]oxycyclohexane-1-carboxylic acid
C16H18O8; MF: 0; RMF: 39; Prob 9.95%; Lib: mona-export-lc-ms-ms_negative_mode; ID: 19000.

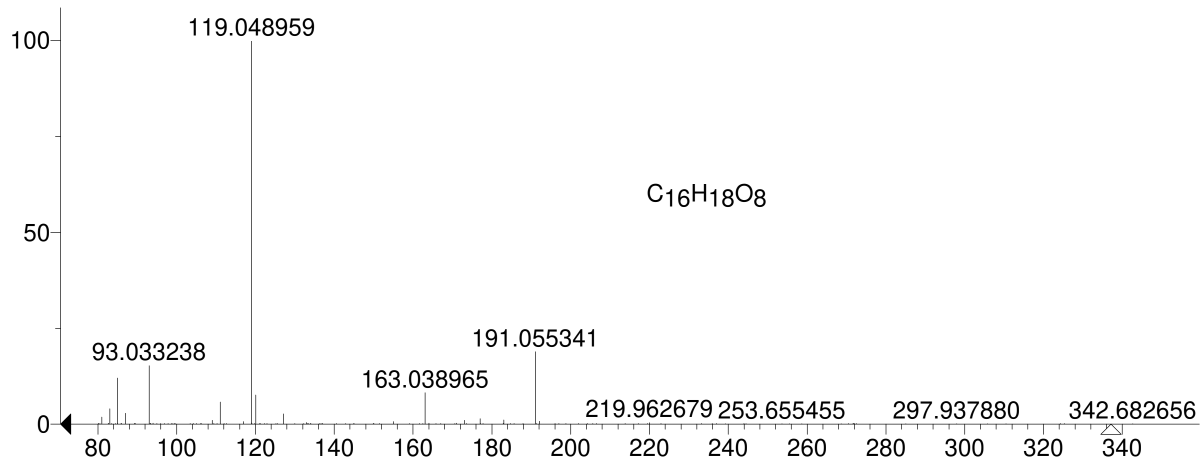

Unknown: Unknown (9.11_378.1655m/z)
Compound in Library Factor = 0

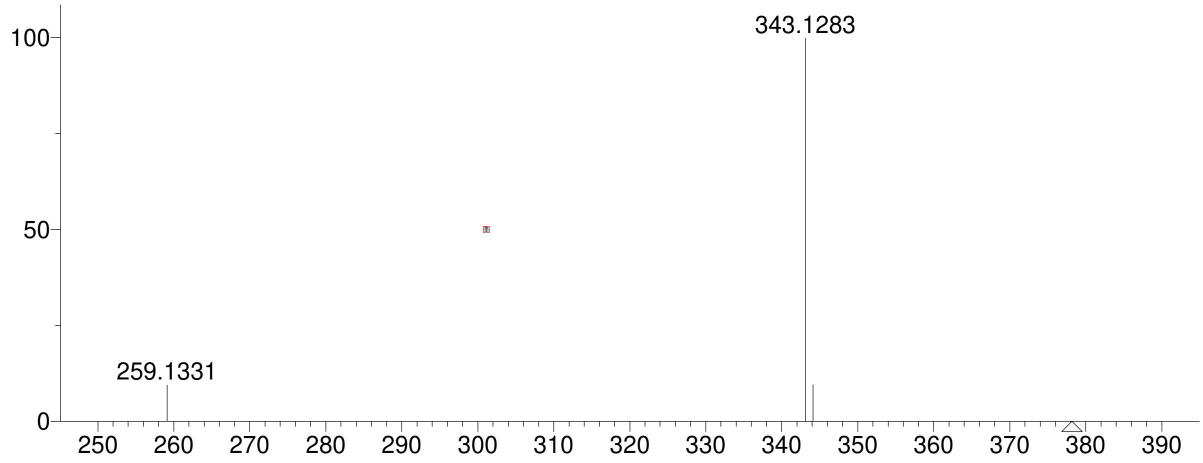

Unknown: Unknown (9.13_381.1178m/z)
Compound in Library Factor = 0

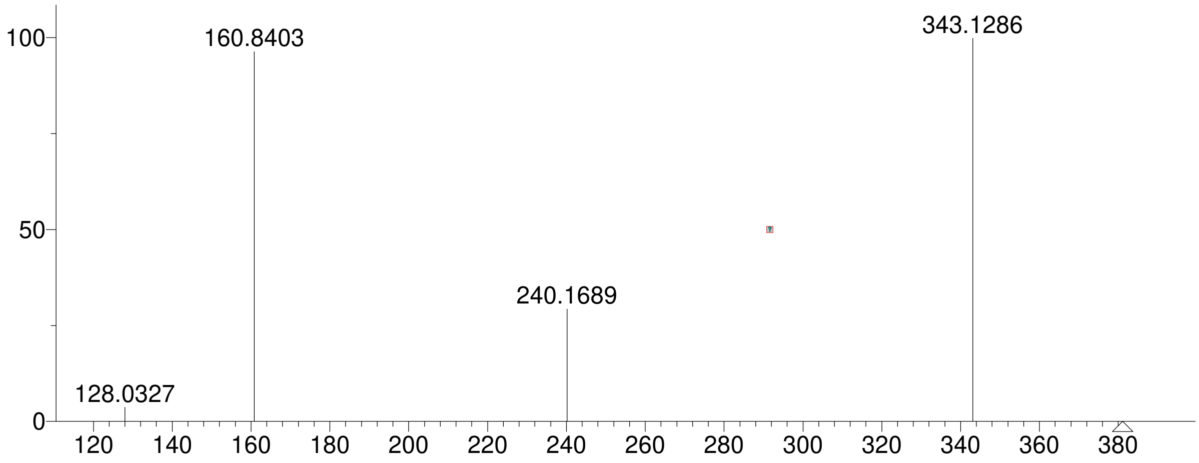

Unknown: Unknown (9.13_433.0800m/z)
Compound in Library Factor = -3273

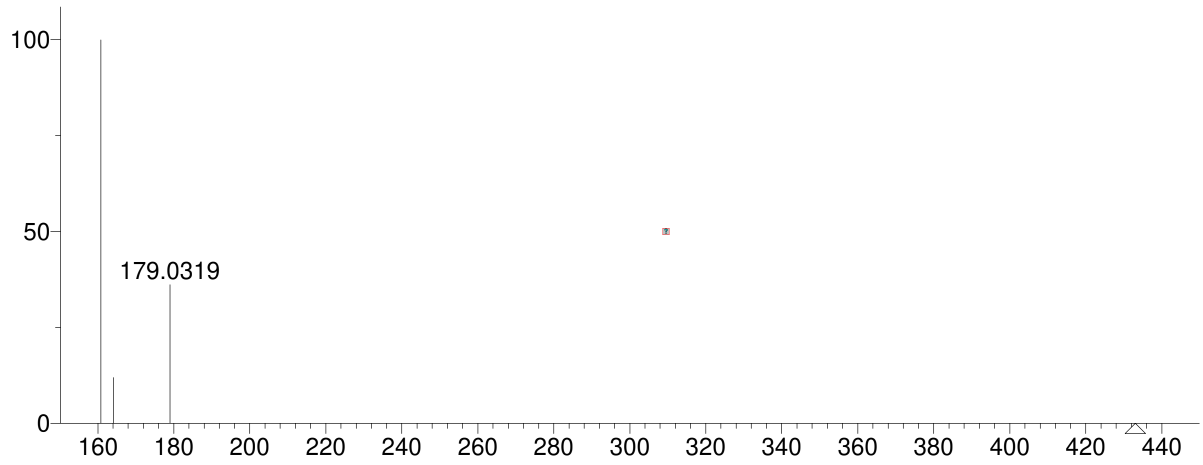

Hit 1 : NCGC00168846-02!(2R,3S)-2-[(3,4-dihydroxyphenyl)methyl]-3-[(E)-3-(3,4-dihydroxyphenyl)prop-2-enoyl]o
C20H18O11; MF: 4; RMF: 258; Prob 100.0%; Lib: mona-export-gnps; ID: 8149.

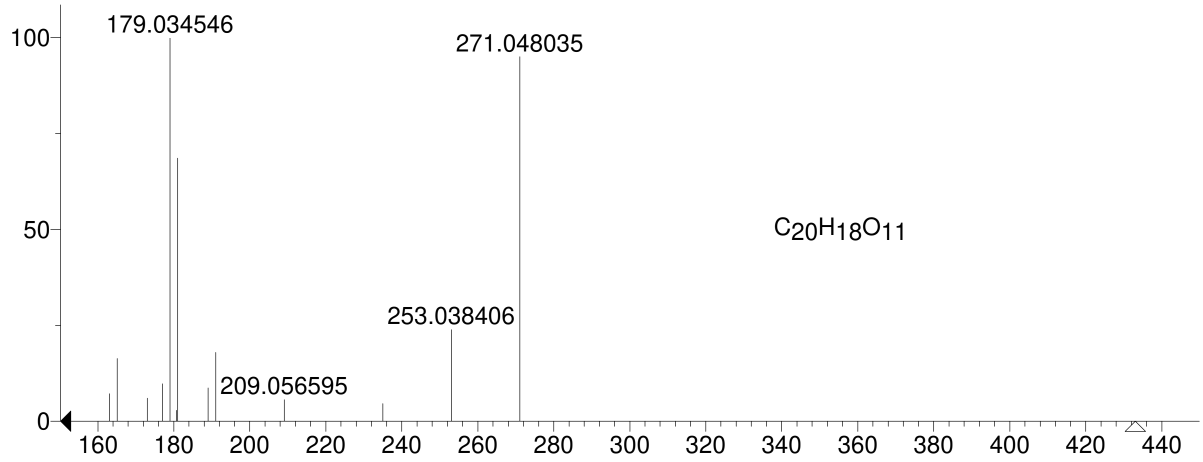

Unknown: Unknown (9.14_439.2188m/z)
Compound in Library Factor = 0

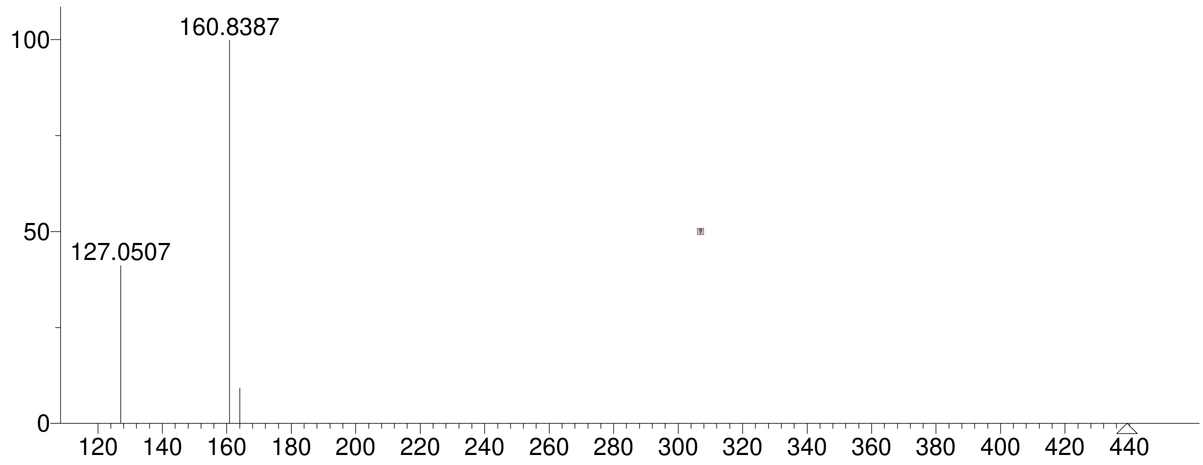

Unknown: Unknown (9.15_339.1073m/z)
Compound in Library Factor = 0

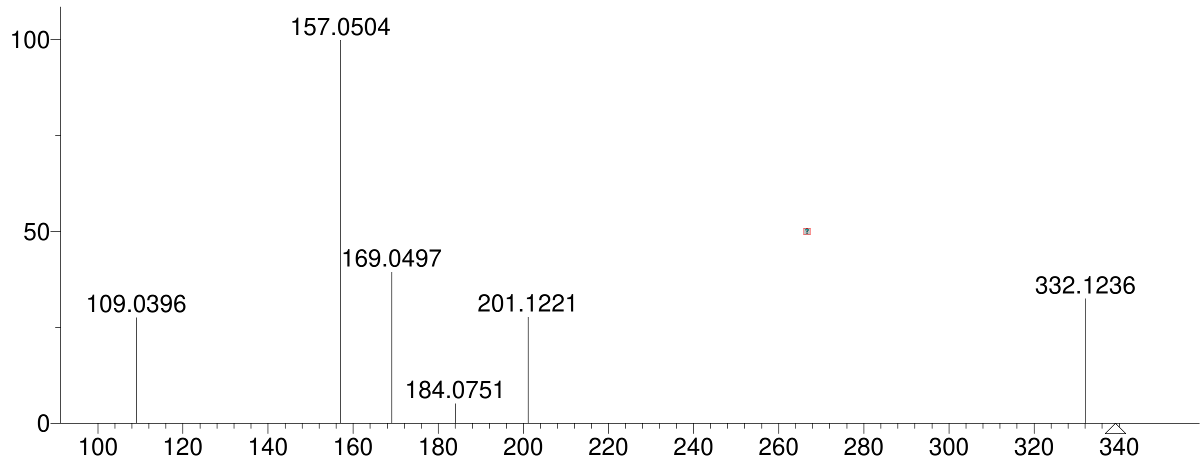

Hit 1 : (1R,3R,4S,5R)-1,3,4-trihydroxy-5-[(E)-3-(4-hydroxyphenyl)prop-2-enoyl]oxycyclohexane-1-carboxylic acid
C16H18O8; MF: 0; RMF: 12; Prob 100.0%; Lib: mona-export-vf-npl_qexactive; ID: 6316.

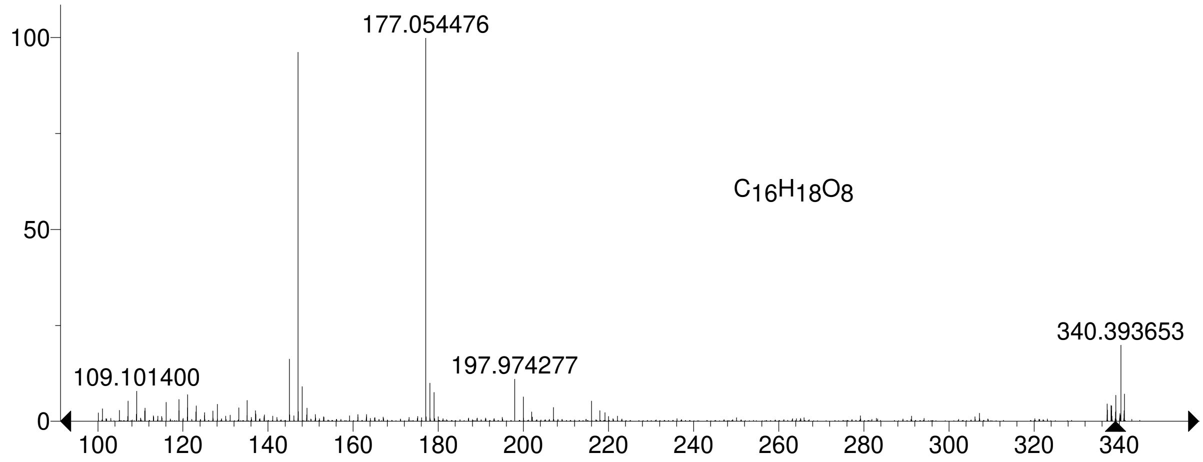

Unknown: Unknown (9.15_342.9132m/z)
Compound in Library Factor = 0

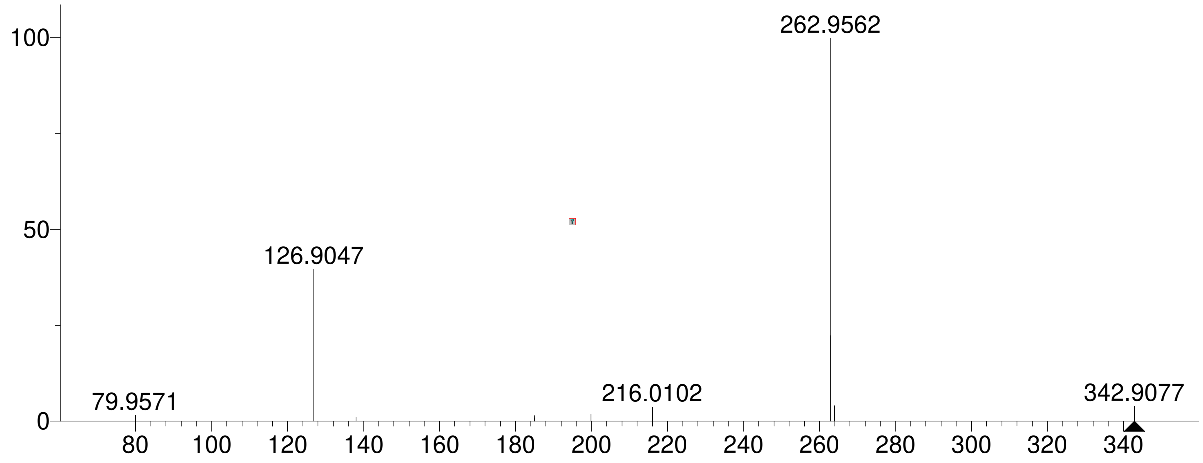

Unknown: Unknown (9.15_534.2217m/z)
Compound in Library Factor = 0

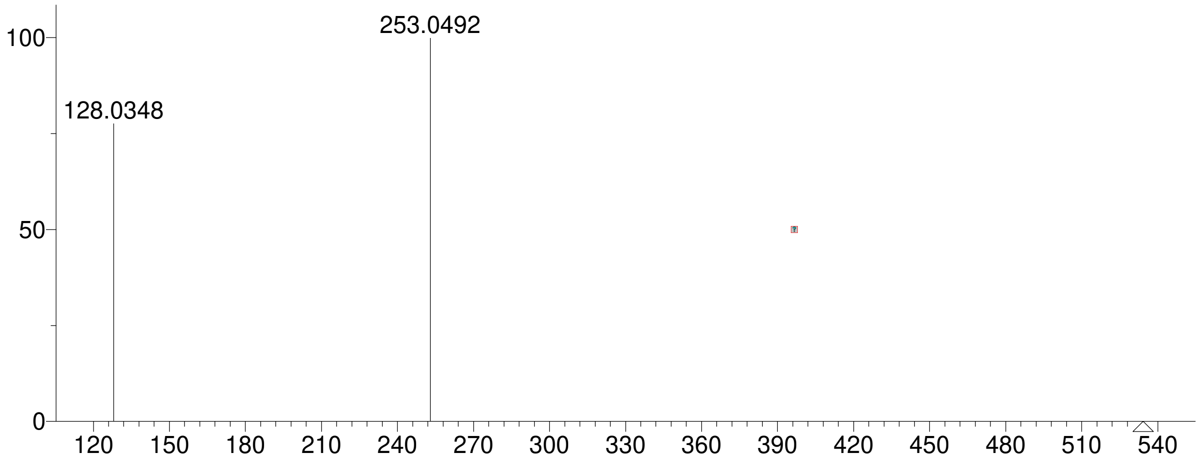

Unknown: Unknown (9.17_387.0861m/z)
Compound in Library Factor = 0

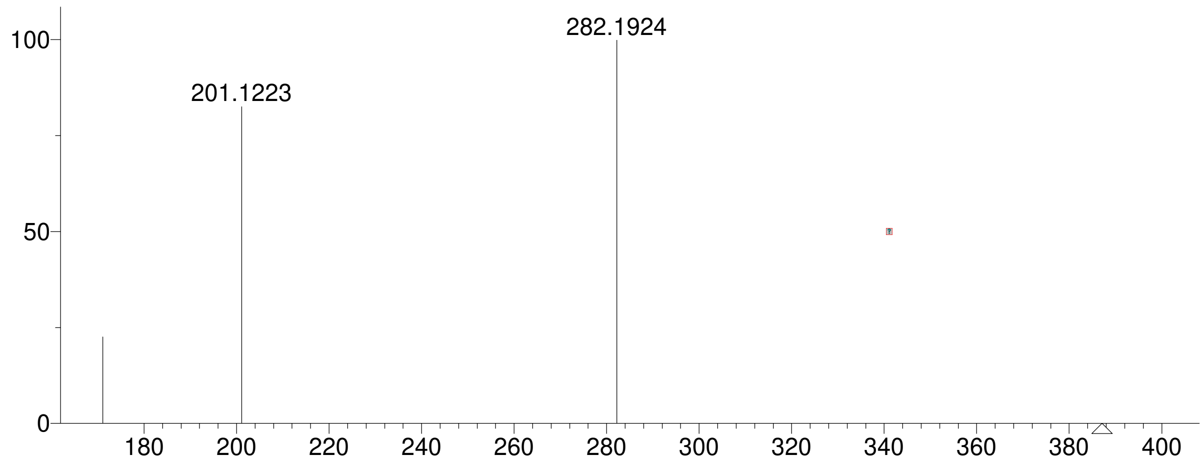

** Search Report Page 1 of 1 **

Unknown: Unknown (9.19_359.0327m/z)
Compound in Library Factor = -3273

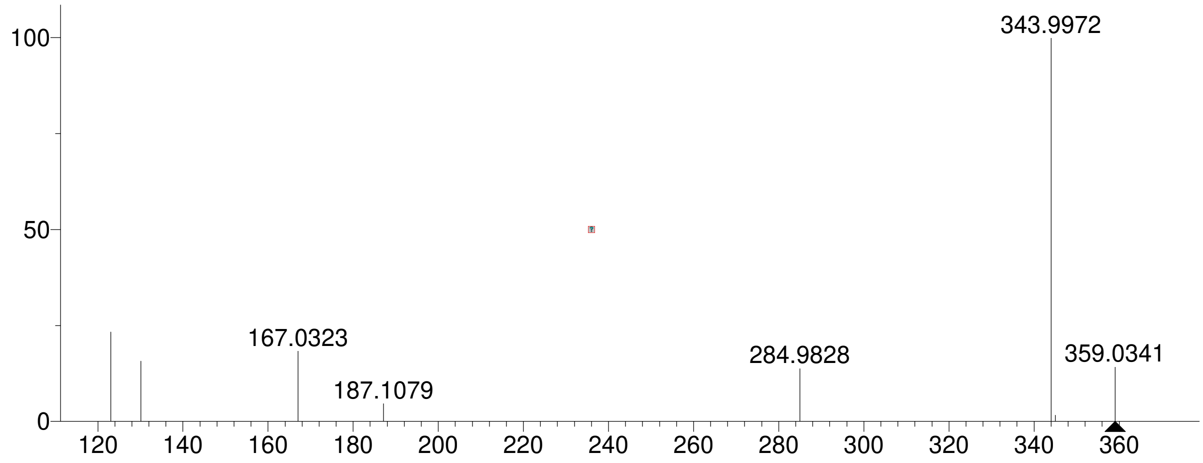

Hit 1 : 8-Chloroquinolin-2-ol [2M+H]+ HCD 1V P=359
C9H6ClNO; MF: 7; RMF: 177; Prob 54.2%; CAS: 23981-25-1; Lib: nist_msms; ID: 535966.

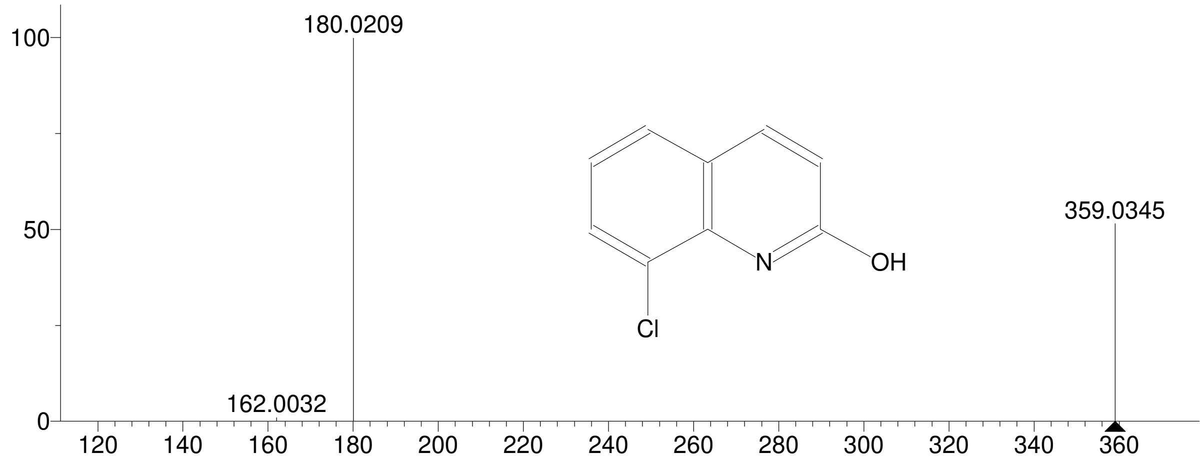

Hit 2 : 6-Chloroquinolin-2-ol [2M+H]+ HCD 1V P=359
C9H6ClNO; MF: 3; RMF: 138; Prob 45.8%; CAS: 1810-67-9; Lib: nist_msms; ID: 535872.

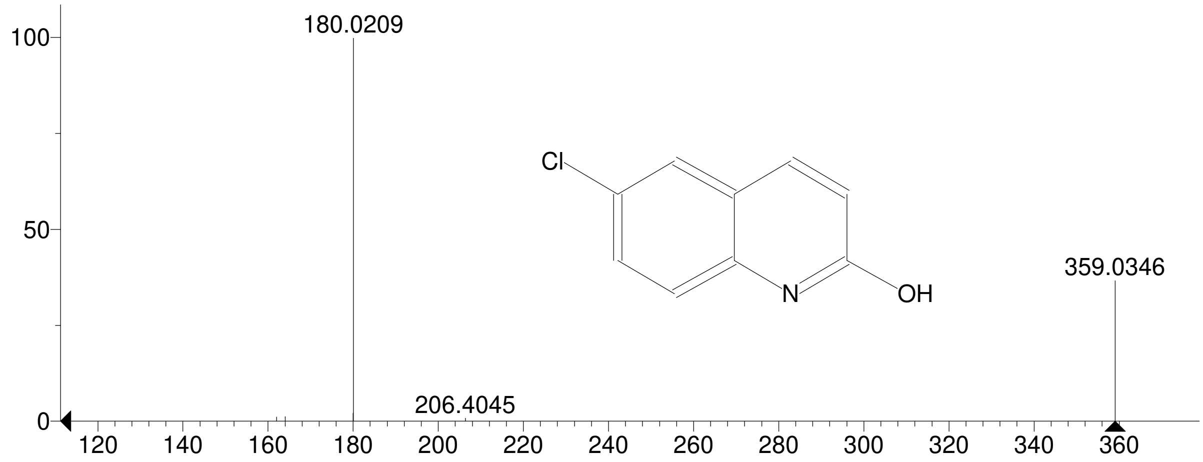

Unknown: Unknown (9.21_167.0343m/z)
Compound in Library Factor = 785

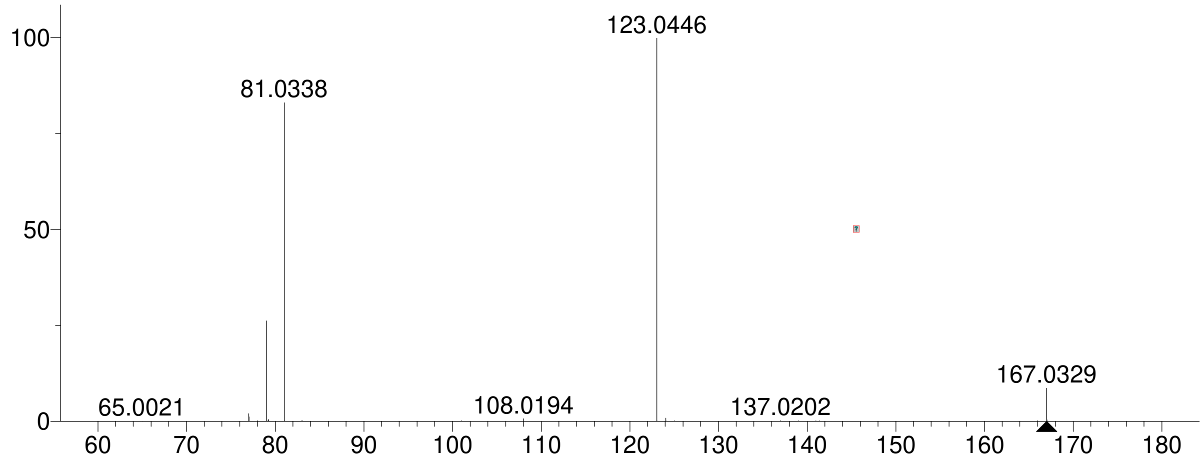

Hit 1 : Lecanoric acid [M-H-C8H6O3]- HCD 21V P=167
C16H14O7; MF: 967; RMF: 977; Prob 98.7%; CAS: 480-56-8; Lib: nist_msms; ID: 455654.

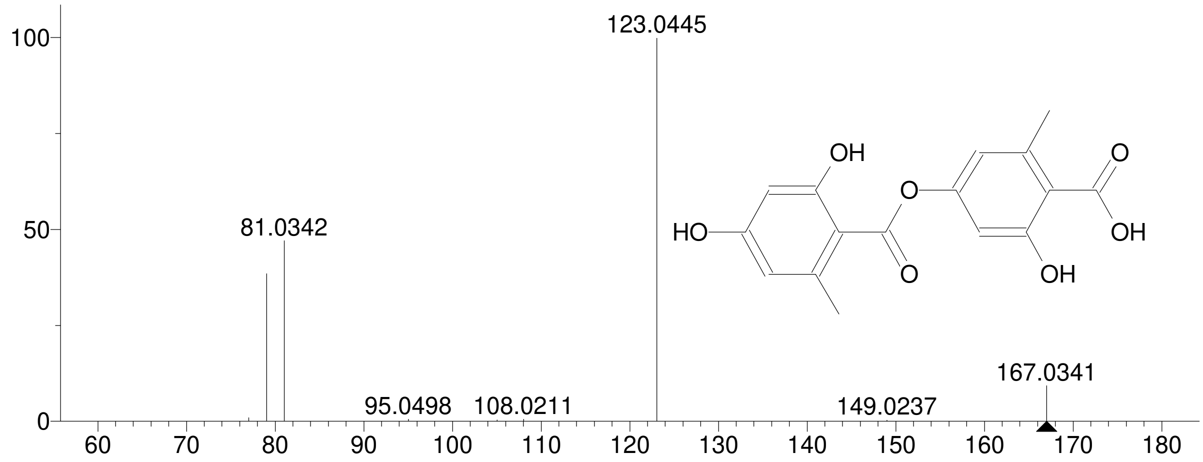

Hit 2 : Evernic acid [M-H-C9H8O3]- HCD 21V P=167
C17H16O7; MF: 725; RMF: 854; Prob 0.98%; CAS: 537-09-7; Lib: nist_msms; ID: 459272.

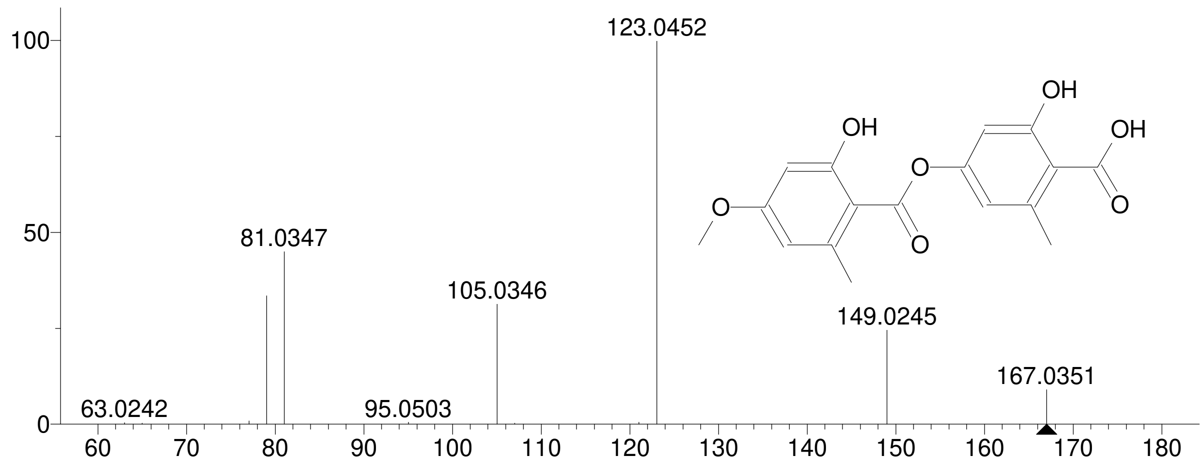

Hit 3 : Homogentisic acid [M-H]- HCD 6V P=167
C8H8O4; MF: 699; RMF: 871; Prob 0.29%; CAS: 451-13-8; Lib: nist_msms; ID: 545998.

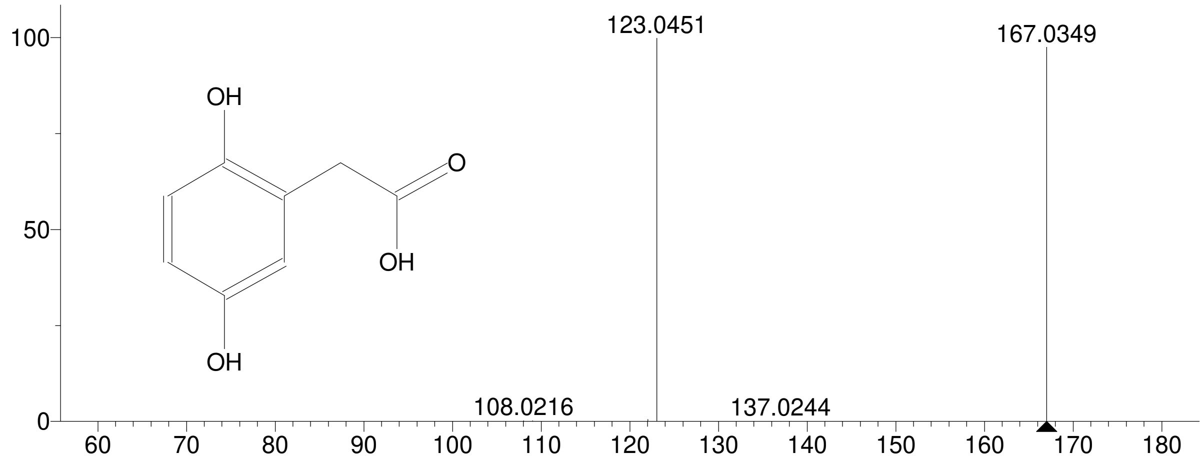

Hit 4 : 2,6-Dihydroxy-4-methylbenzoic acid [M-H]- HCD 25V P=167
C8H8O4; MF: 626; RMF: 792; Prob 0.04%; CAS: 480-67-1; Lib: nist_msms; ID: 534660.

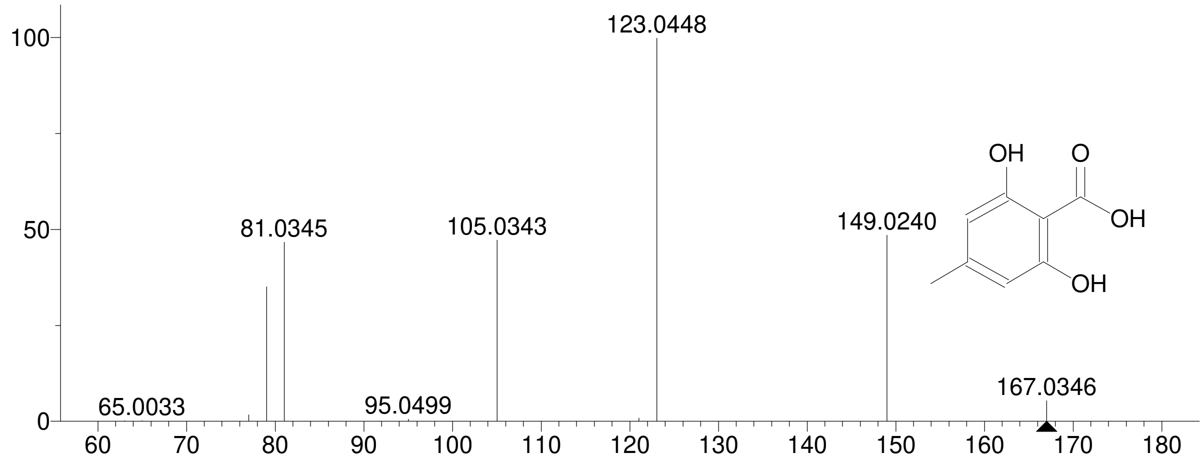

Hit 5 : 3,4-Dihydroxyphenylacetic acid [M-H]- HCD 3V P=167
C8H8O4; MF: 551; RMF: 941; Prob 0.00%; CAS: 102-32-9; Lib: nist_msms; ID: 286720.

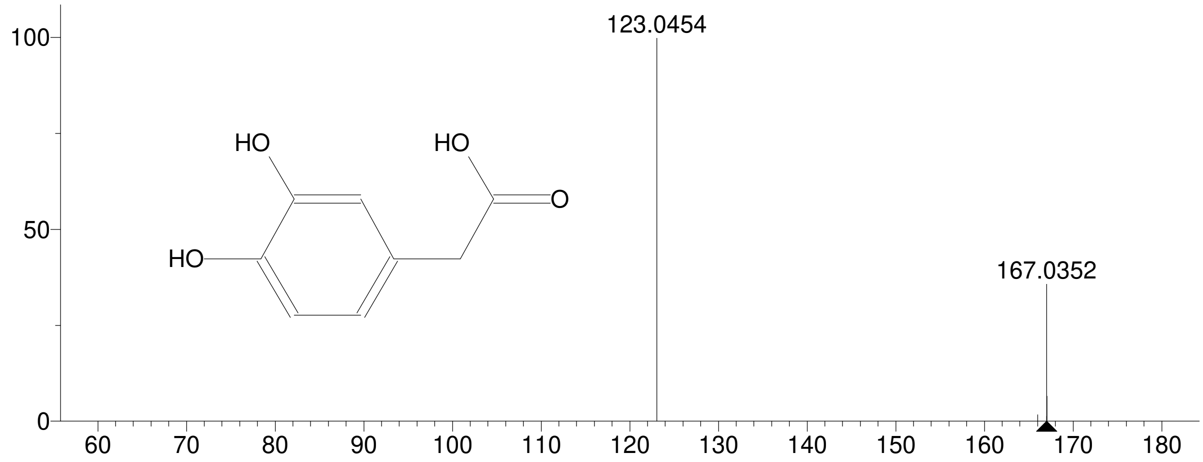

Unknown: Unknown (9.21_401.1908m/z)
Compound in Library Factor = 0

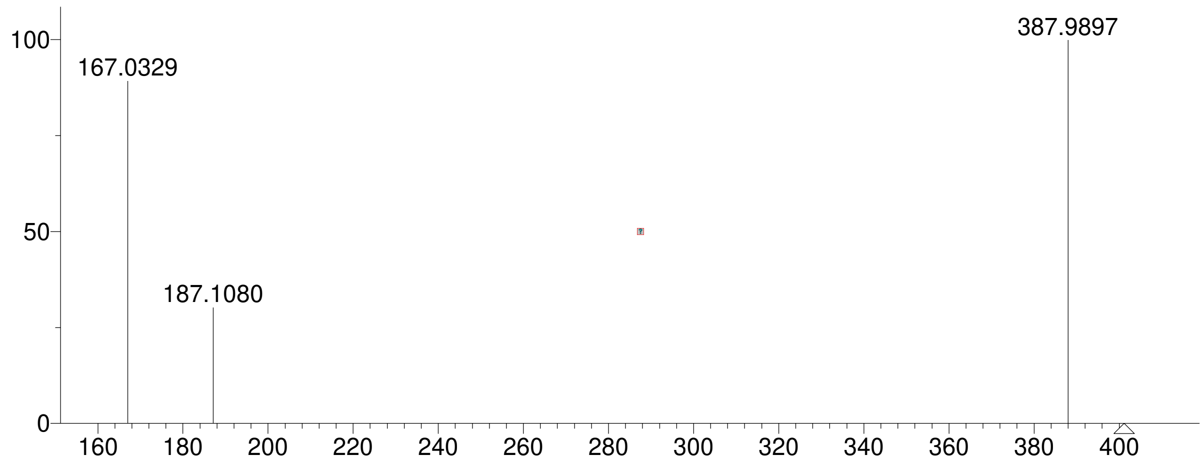

Unknown: Unknown (9.21_425.0169m/z)
Compound in Library Factor = 0

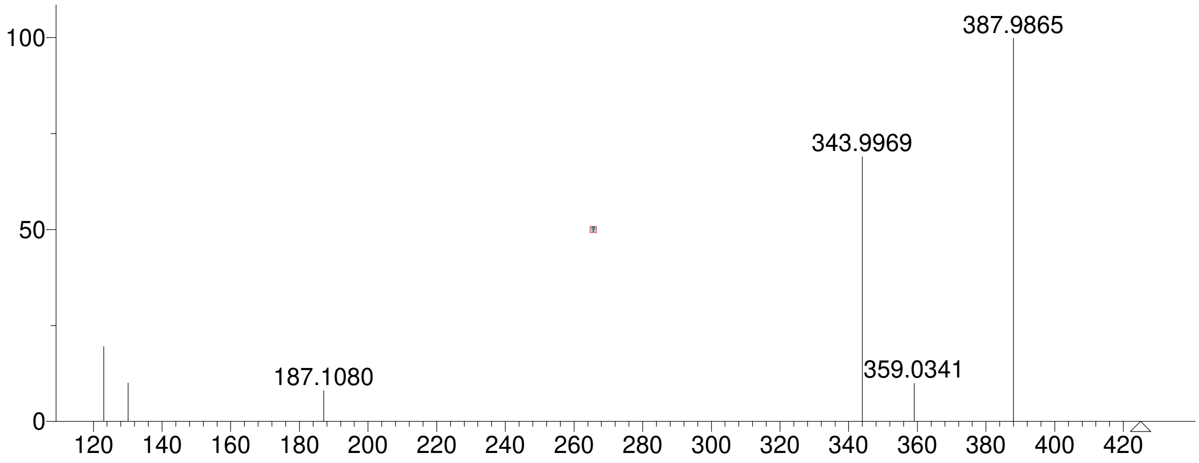

Unknown: Unknown (9.21_546.0459m/z)
Compound in Library Factor = 0

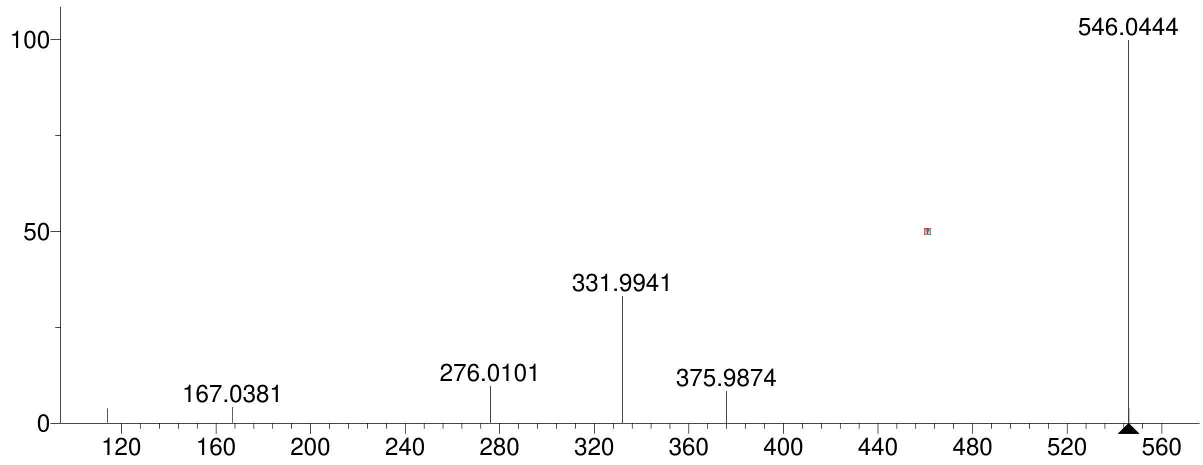

Unknown: Unknown (9.22_123.0445m/z)
Compound in Library Factor = -302

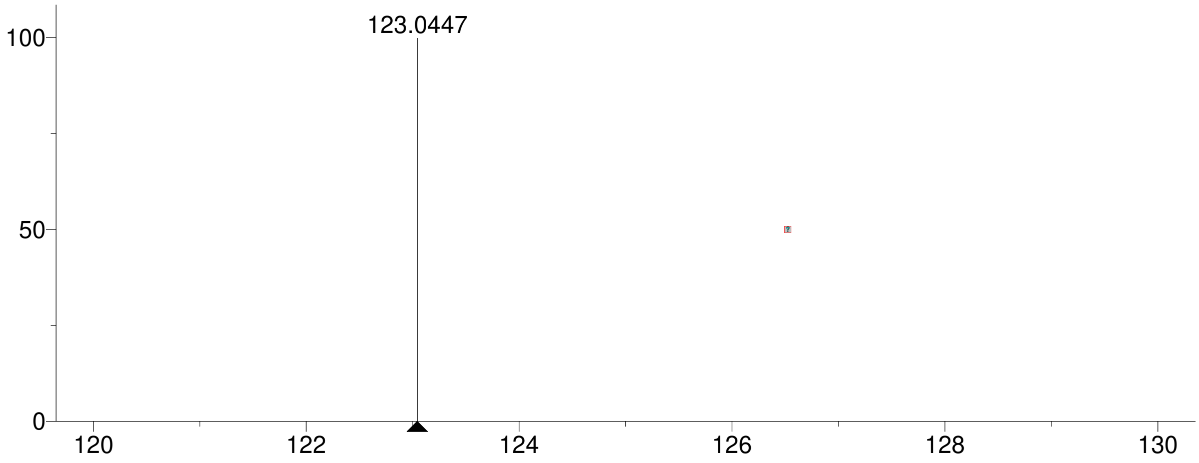

Hit 1 : 4-Hydroxybenzaldehyde [M+H]+ HCD 12V P=123
C7H6O2; MF: 364; RMF: 999; Prob 2.33%; CAS: 123-08-0; Lib: nist_msms; ID: 136890.

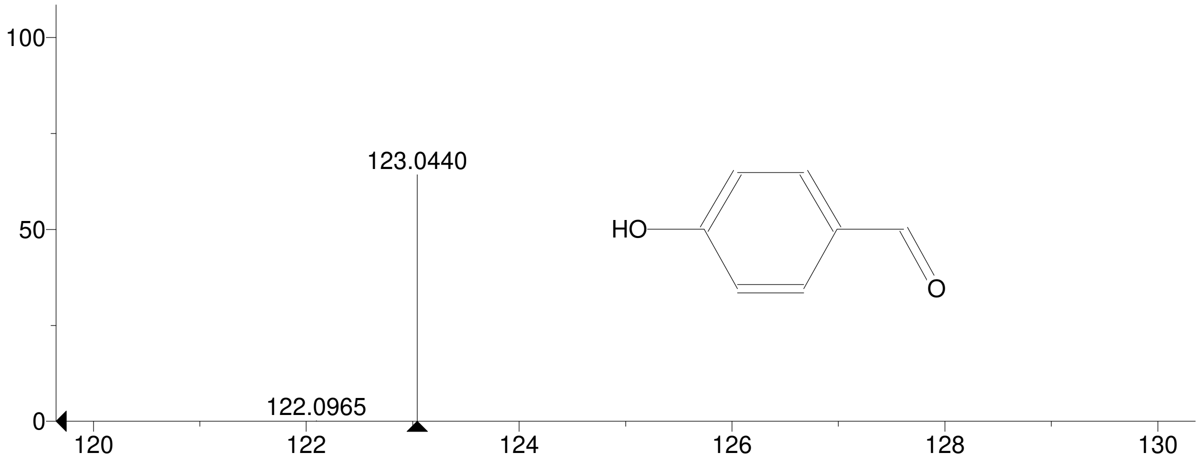

Hit 2 : Maackiaine [M+H-C9H6O3]+ HCD 22V P=123
C16H12O5; MF: 364; RMF: 999; Prob 2.33%; CAS: 2035-15-6; Lib: nist_msms; ID: 223828.

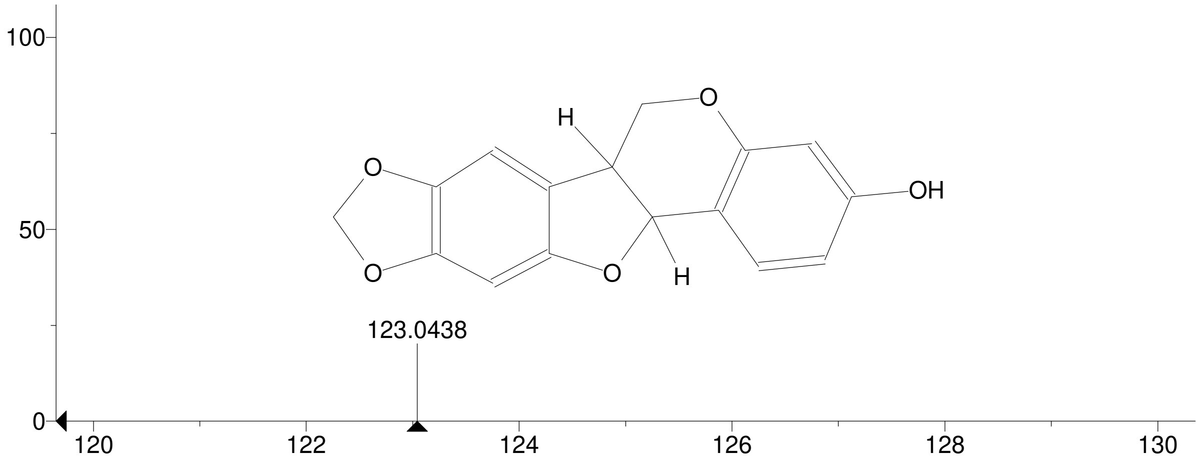

Hit 3 : Helicid [M+H-C6H10O5]+ HCD 14V P=123
C13H16O7; MF: 364; RMF: 999; Prob 2.33%; CAS: 80154-34-3; Lib: nist_msms; ID: 256928.

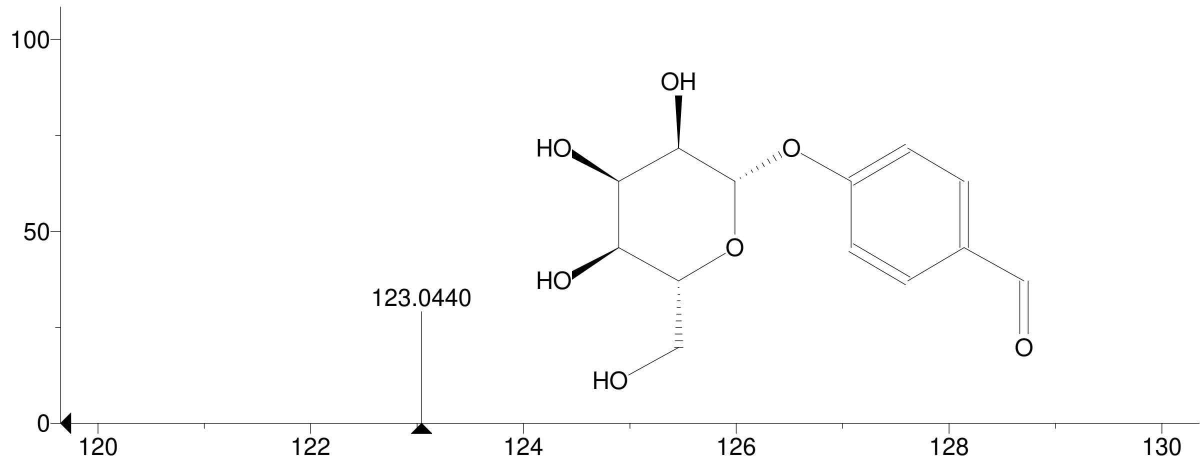

Hit 4 : Glabridin [M+H-C13H14O2]+ HCD 31V P=123
C20H20O4; MF: 364; RMF: 999; Prob 2.33%; CAS: 59870-68-7; Lib: nist_msms; ID: 257694.

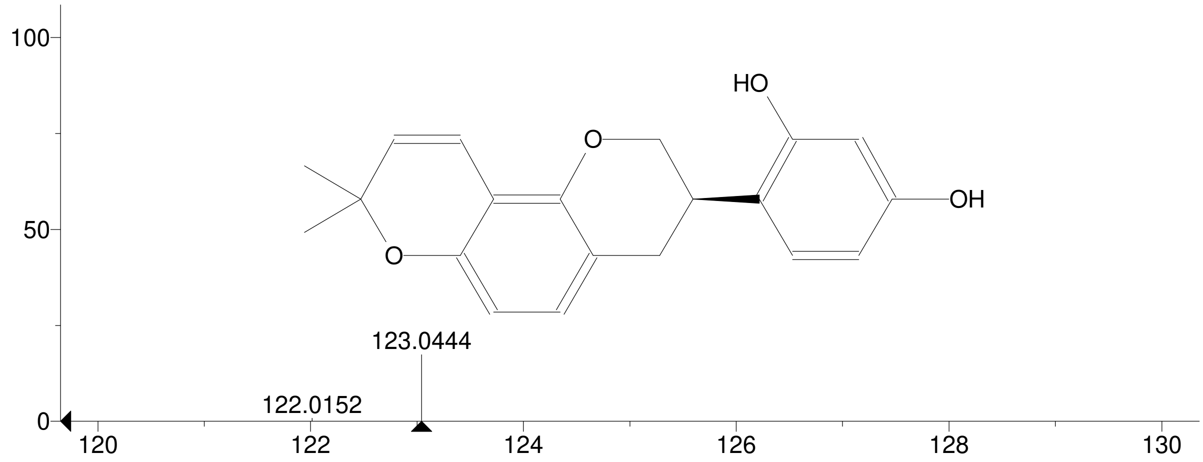

Hit 5 : 3-Hydroxybenzyl alcohol [M-H]- IT-FT 35% P=123
C7H8O2; MF: 364; RMF: 999; Prob 2.33%; CAS: 620-24-6; Lib: nist_msms; ID: 278139.

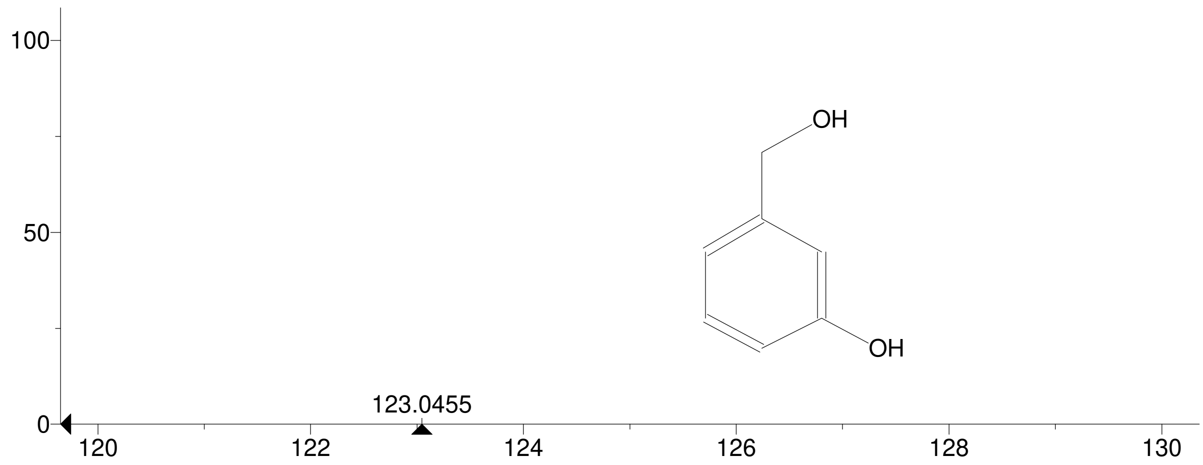

** Search Report Page 1 of 1 **

Unknown: Unknown (9.22_357.2118m/z)
Compound in Library Factor = 0

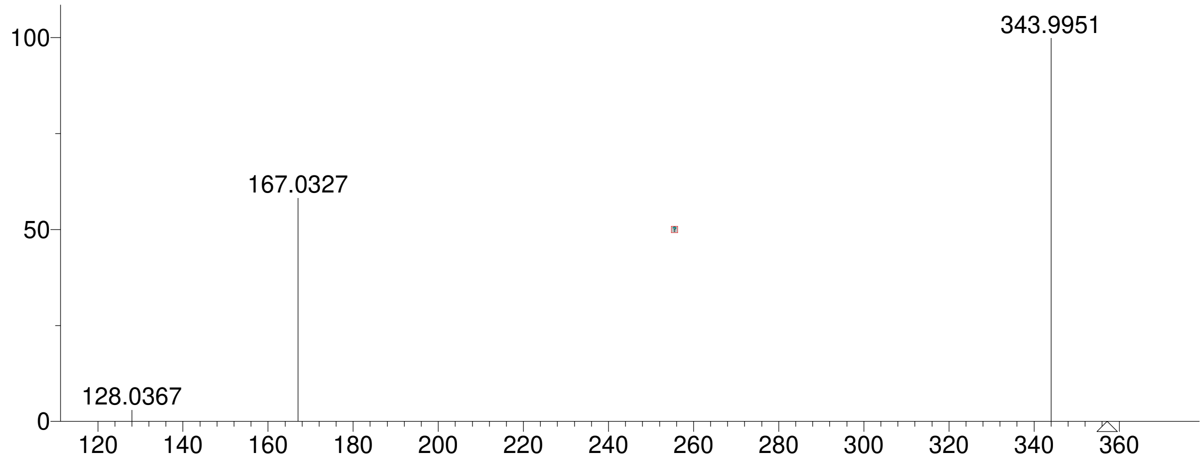

Unknown: Unknown (9.22_387.9876m/z)
Compound in Library Factor = 0

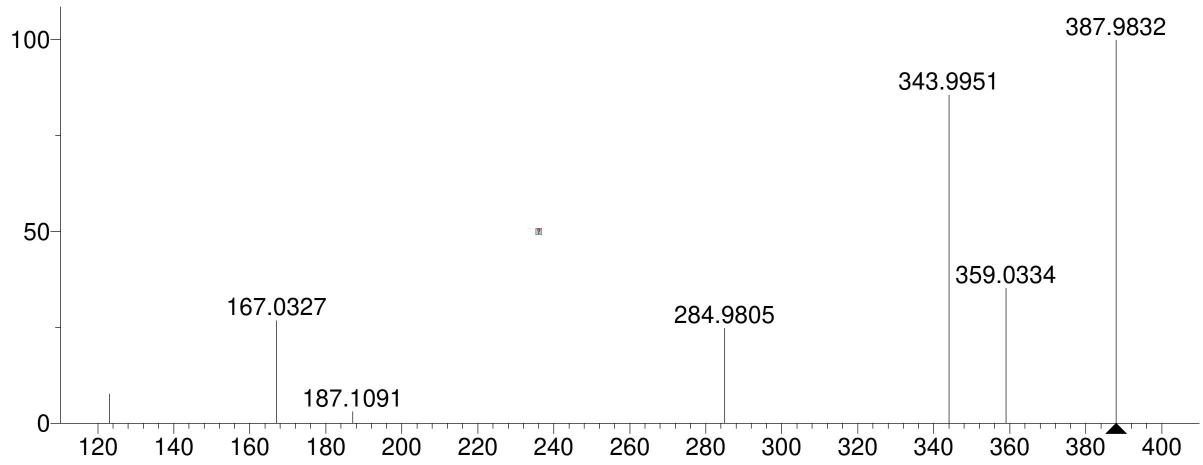

Unknown: Unknown (9.23_167.1560m/z)
Compound in Library Factor = 0

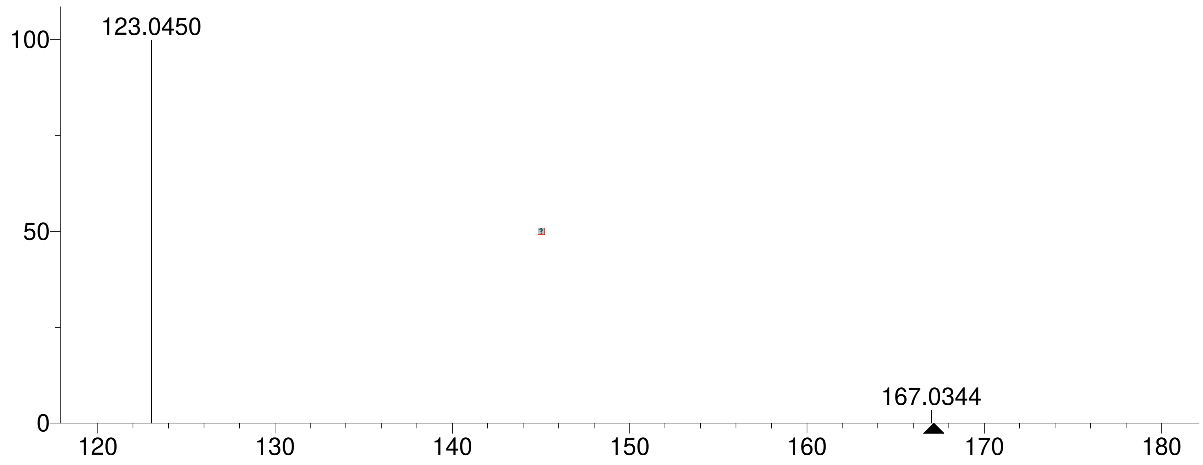

** Search Report Page 1 of 1 **

Unknown: Unknown (9.24_357.0560m/z)
Compound in Library Factor = 0

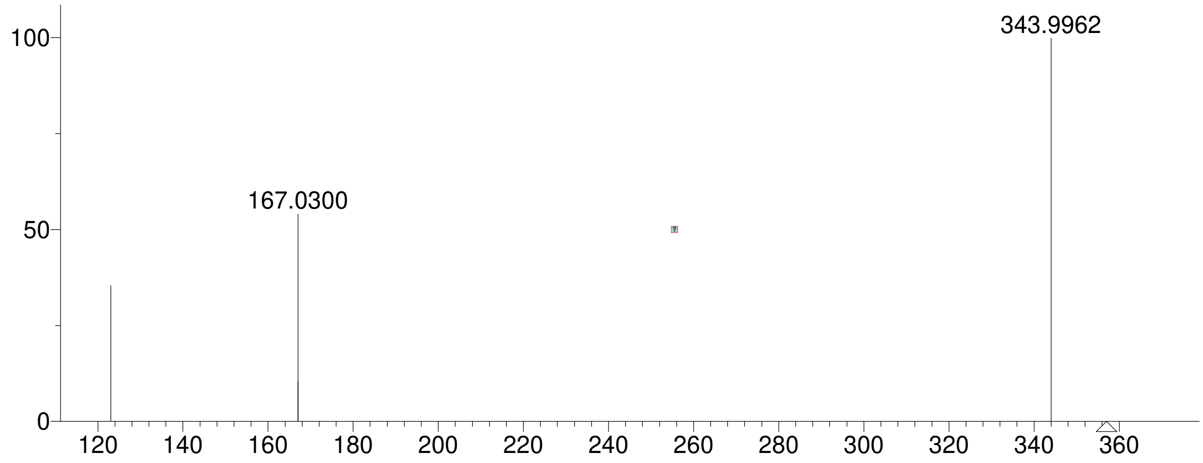

Unknown: Unknown (9.28_316.1654m/z)
Compound in Library Factor = -1523

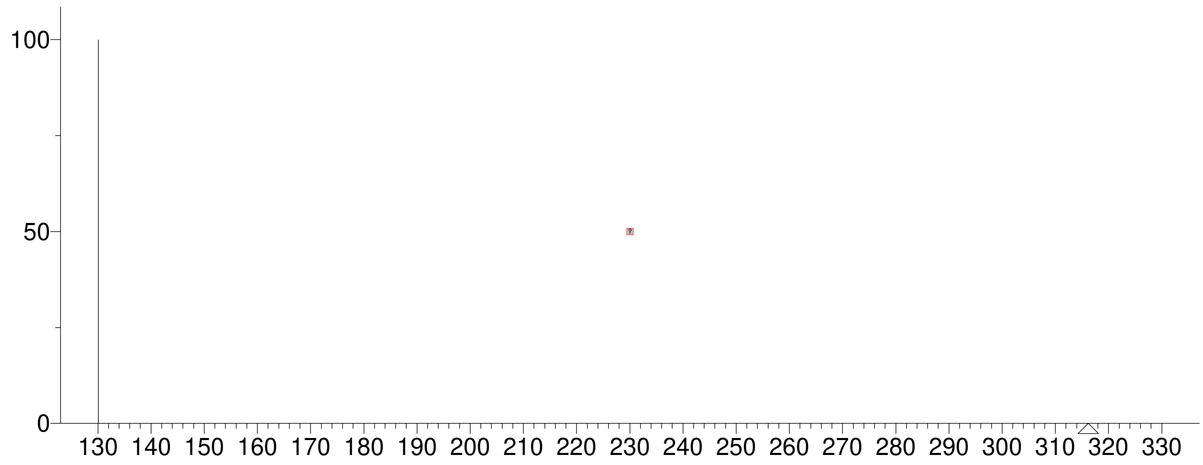

Hit 1 : Trp-Leu [M-H]- HCD 50eV P=316.2
C17H23N3O3; MF: 79; RMF: 827; Prob 62.2%; CAS: 13123-35-8; Lib: nist_msms; ID: 570423.

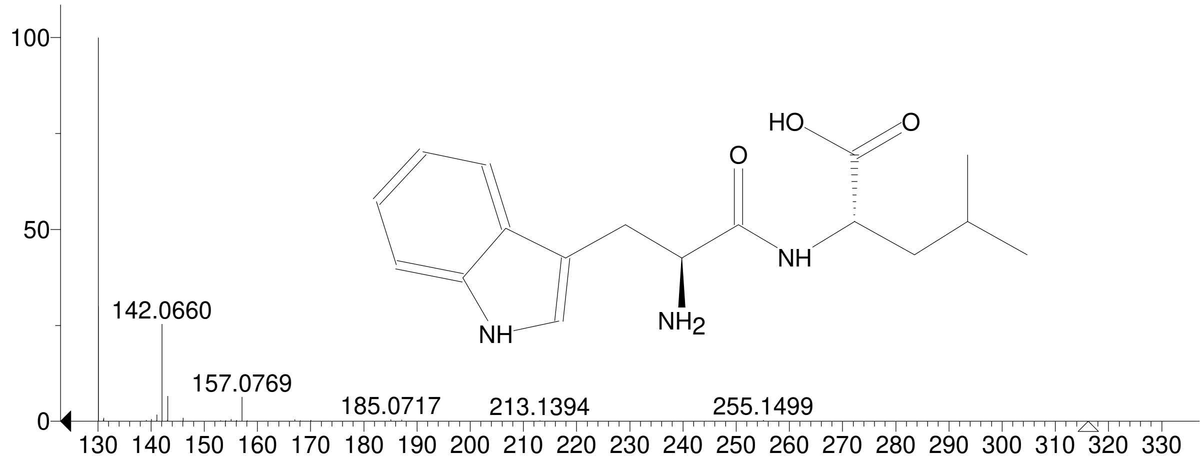

Hit 2 : Trp-Ile [M-H]- HCD 41eV P=316.2
C17H23N3O3; MF: 36; RMF: 757; Prob 13.7%; Lib: nist_msms; ID: 571121.

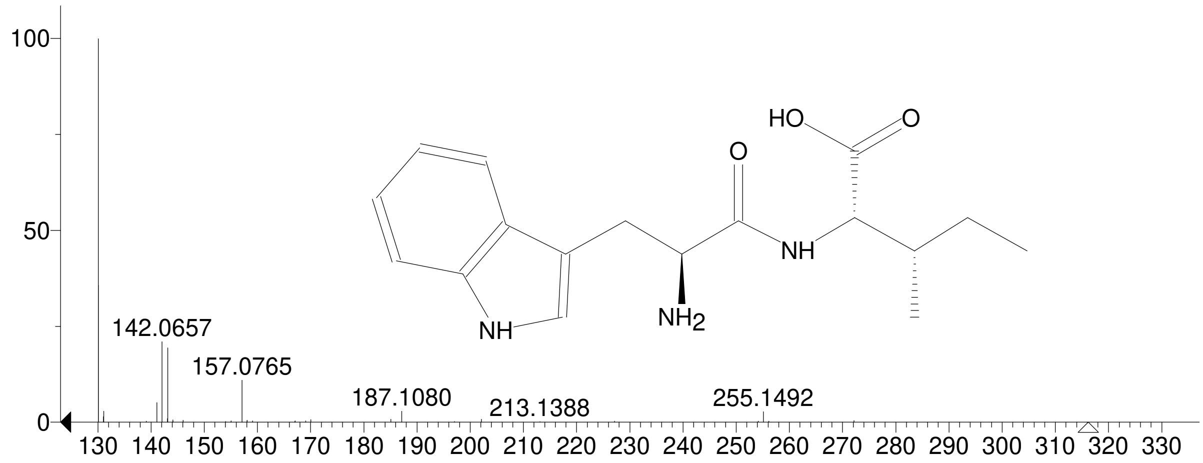

Hit 3 : Trp-Ile [M-H]- HCD 41eV P=316.2
C17H23N3O3; MF: 36; RMF: 757; Prob 13.7%; CAS: 64339-42-0; Lib: nist_msms2; ID: 84854.

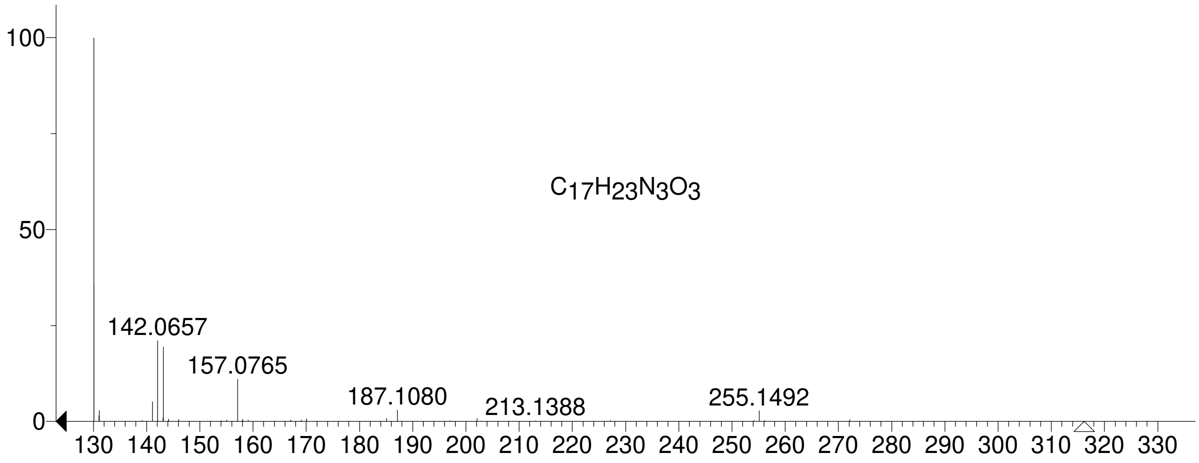

Hit 4 : Ile-Trp [M-H]- HCD 50eV P=316.2
C17H23N3O3; MF: 1; RMF: 125; Prob 3.44%; Lib: nist_msms; ID: 567504.

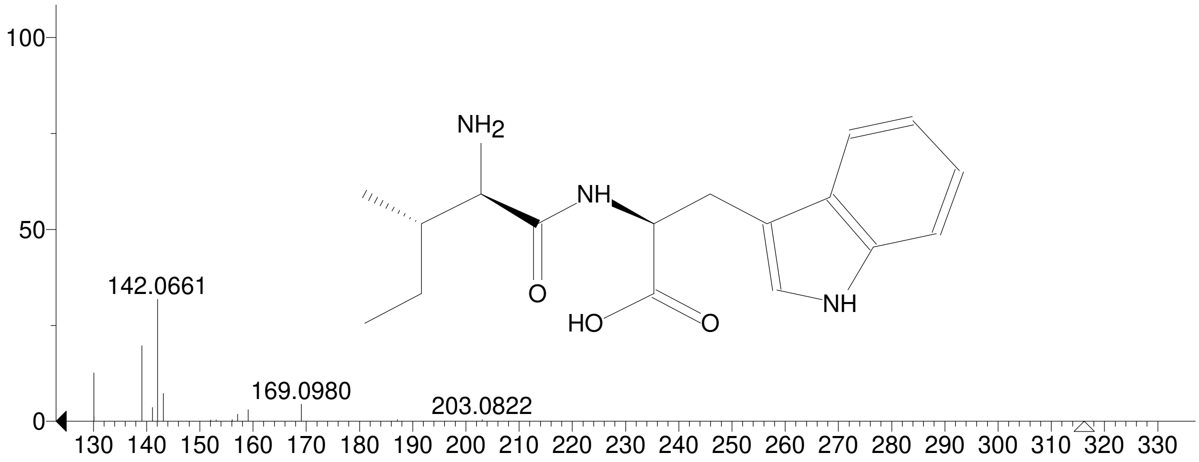

Hit 5 : Ile-Trp [M-H]- HCD 50eV P=316.2
C17H23N3O3; MF: 1; RMF: 125; Prob 3.44%; CAS: 13589-06-5; Lib: nist_msms2; ID: 70361.

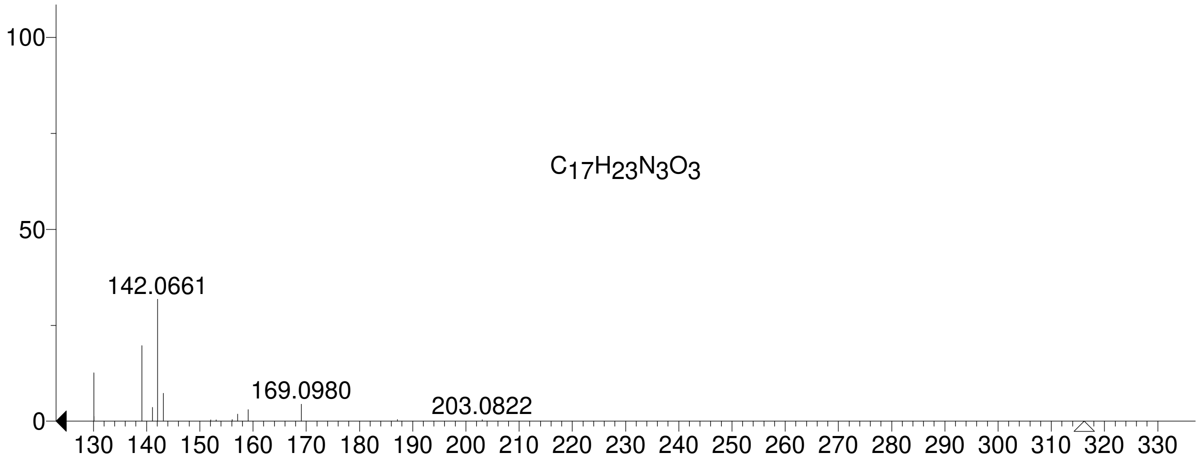

Unknown: Unknown (9.30_356.1807m/z)
Compound in Library Factor = 0

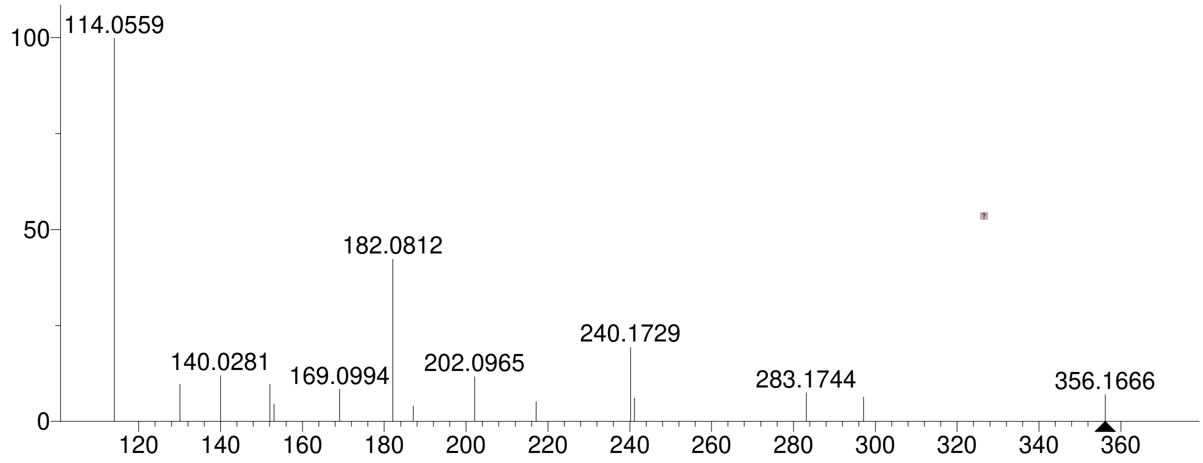

Unknown: Unknown (9.30_428.2011m/z)
Compound in Library Factor = 0

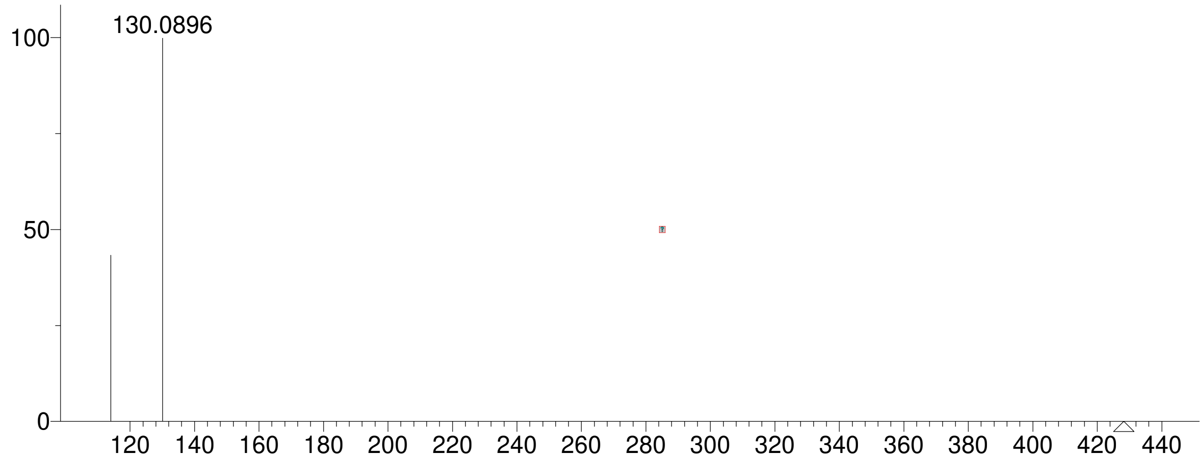

Unknown: Unknown (9.32_253.1318m/z)
Compound in Library Factor = -1309

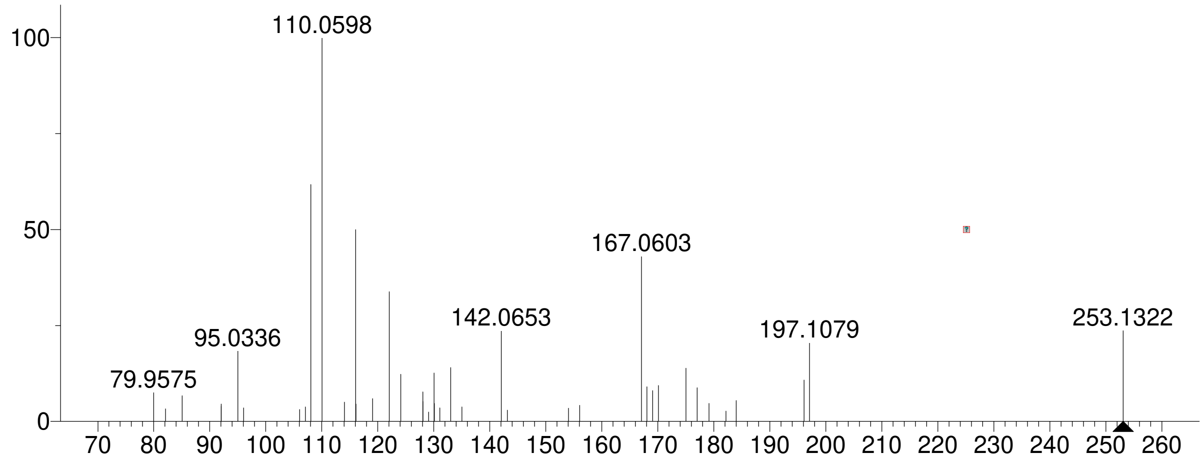

Hit 1 : 1-Piperidinecarboxamide, N-phenyl-4-(3-quinolinylmethyl)- [M+H-C6H7N]+ HCD 12V P=253.1
C22H23N3O; MF: 102; RMF: 455; Prob 45.3%; CAS: 959151-50-9; Lib: nist_msms; ID: 371887.

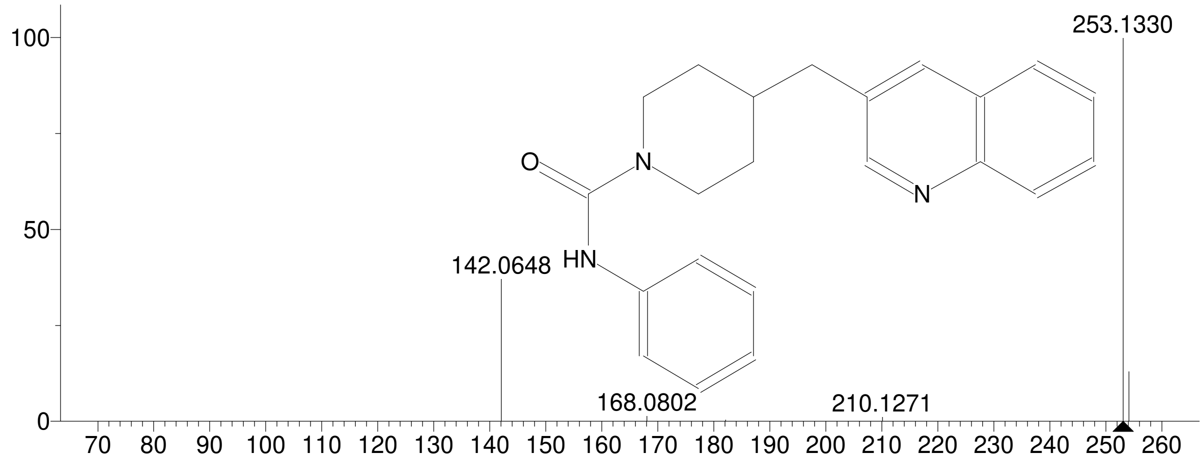

Hit 2 : His-Pro [M+H]+ HCD 5eV P=253.1
C11H16N4O3; MF: 50; RMF: 257; Prob 9.03%; Lib: nist_msms; ID: 561427.

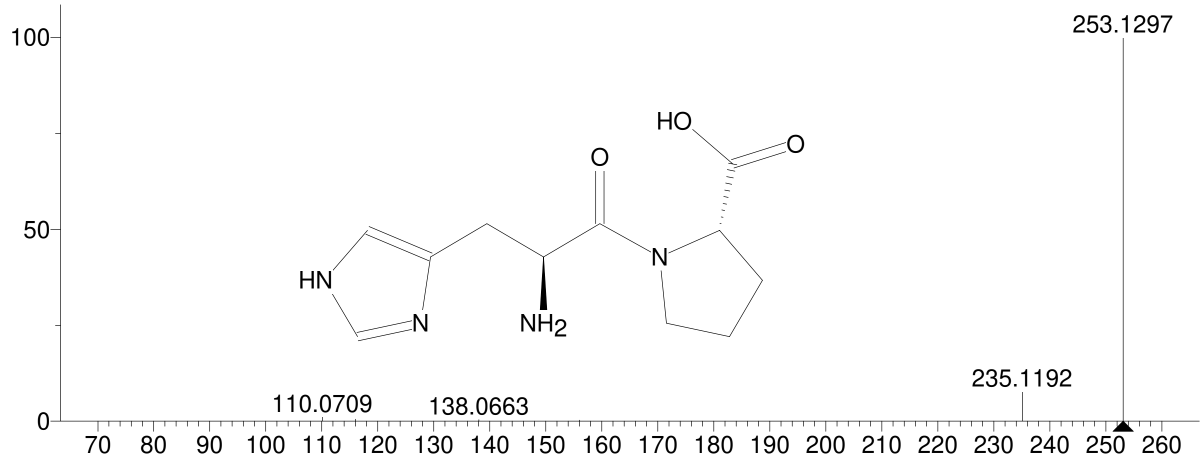

Hit 3 : His-Pro [M+H]+ HCD 5eV P=253.1
C11H16N4O3; MF: 50; RMF: 257; Prob 9.03%; CAS: 20930-58-9; Lib: nist_msms2; ID: 41227.

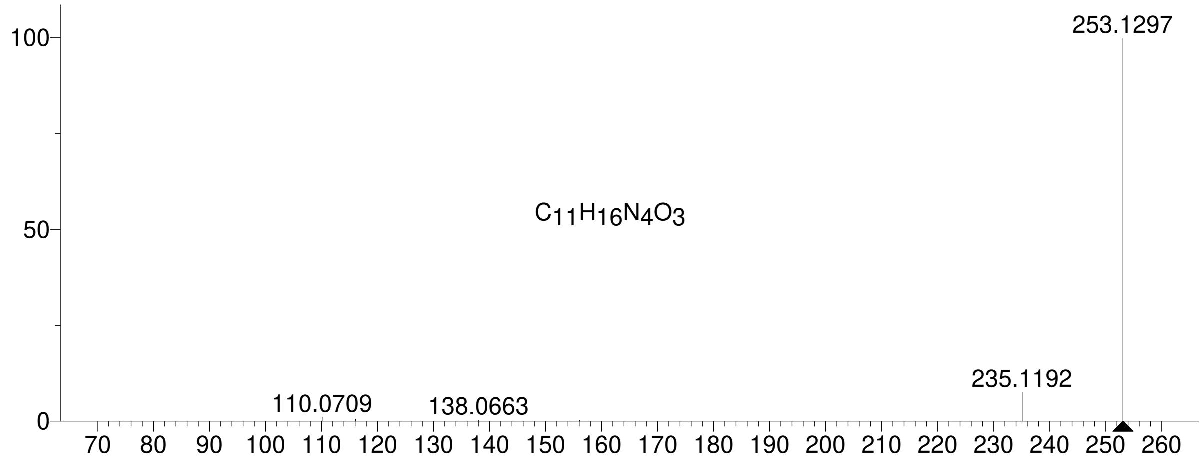

Hit 4 : Pro-His [M+H]+ HCD 6eV P=253.1
C11H16N4O3; MF: 40; RMF: 207; Prob 6.38%; Lib: nist_msms; ID: 565663.

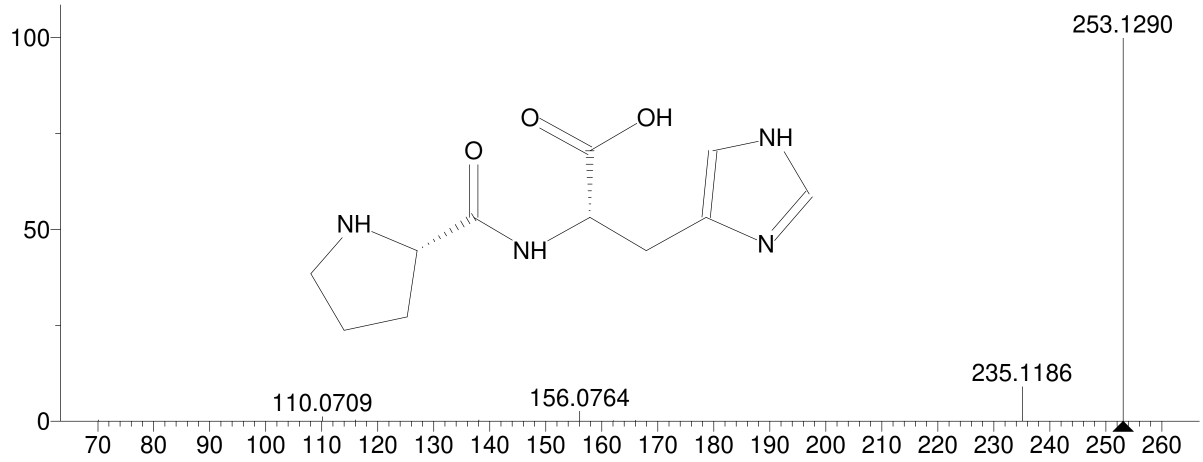

Hit 5 : Pro-His [M+H]+ HCD 6eV P=253.1
C11H16N4O3; MF: 40; RMF: 207; Prob 6.38%; Lib: nist_msms2; ID: 68520.

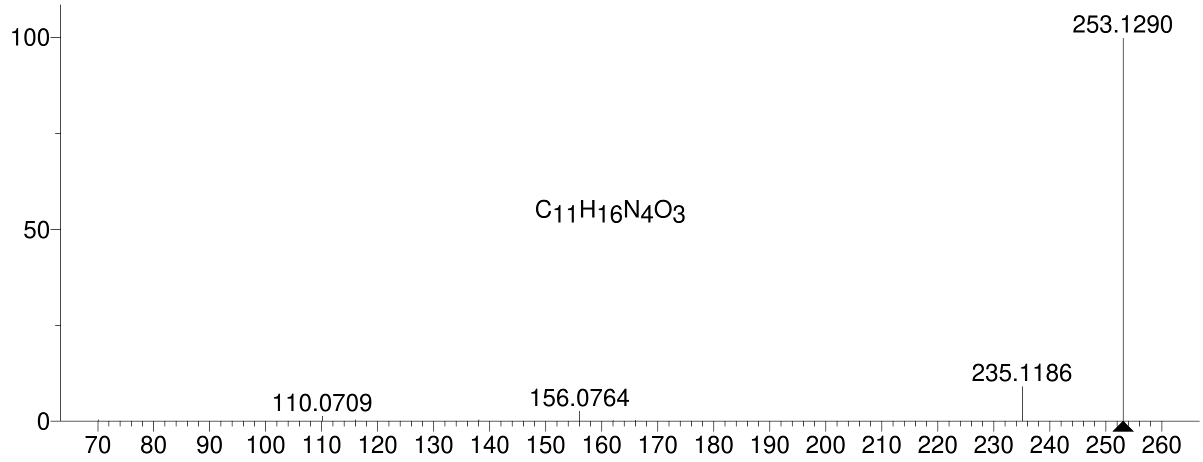

** Search Report Page 1 of 1 **

Unknown: Unknown (9.32_297.1237m/z)
Compound in Library Factor = -3273

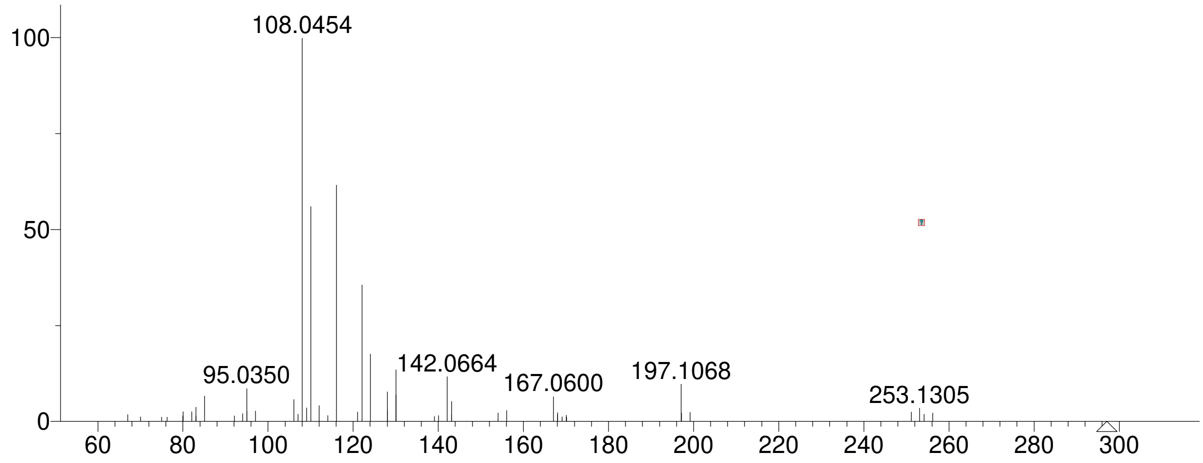

Hit 1 : 1H-Indole-6-carboxamide, N-hydroxy-1-[(4-methoxyphenyl)methyl]- [M+H]+ HCD 53V P=297.1
C17H16N2O3; MF: 2; RMF: 41; Prob 100.0%; CAS: 950762-95-5; Lib: nist_msms; ID: 254260.

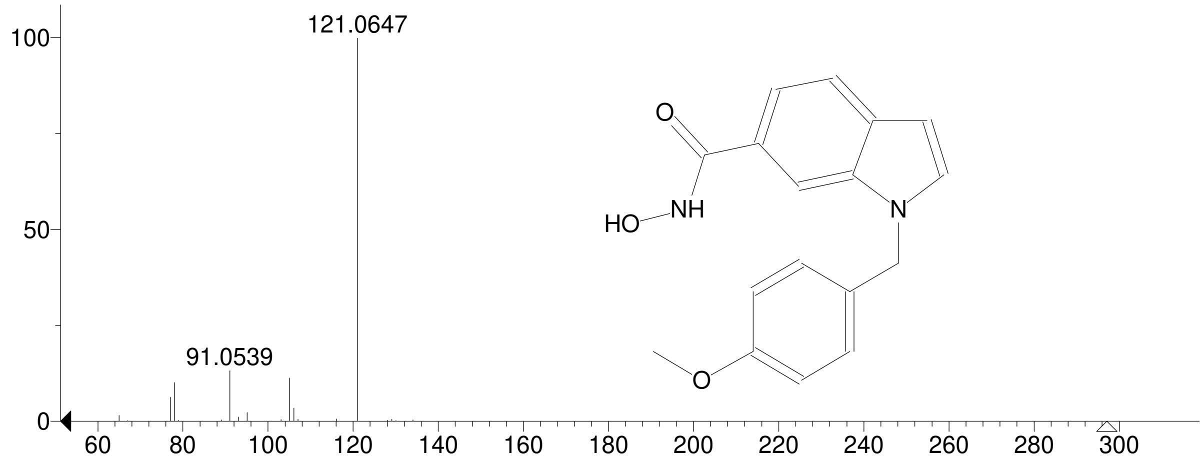

** Search Report Page 1 of 1 **

Unknown: Unknown (9.32_387.1756m/z)
Compound in Library Factor = 0

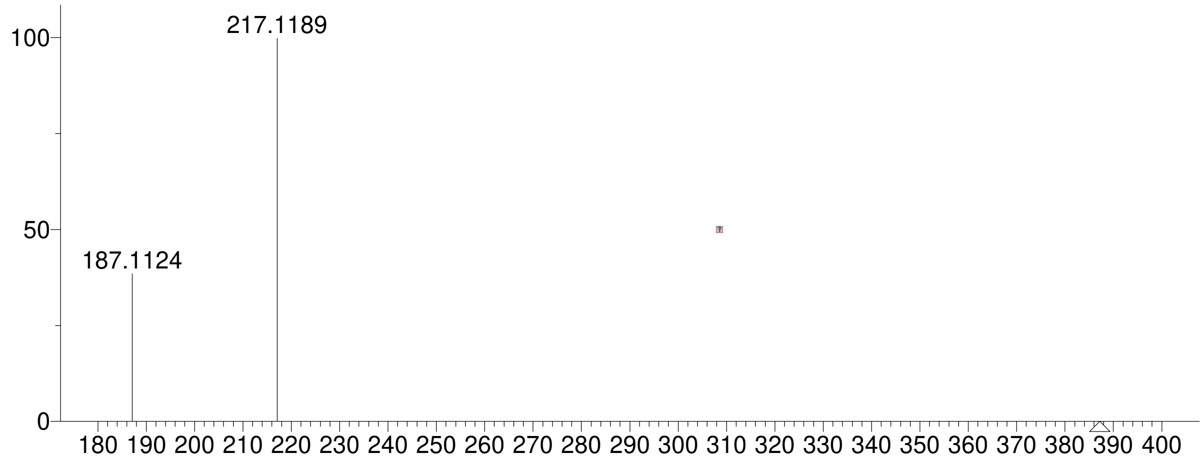

** Search Report Page 1 of 1 **

Unknown: Unknown (9.32_404.1449m/z)
Compound in Library Factor = 0

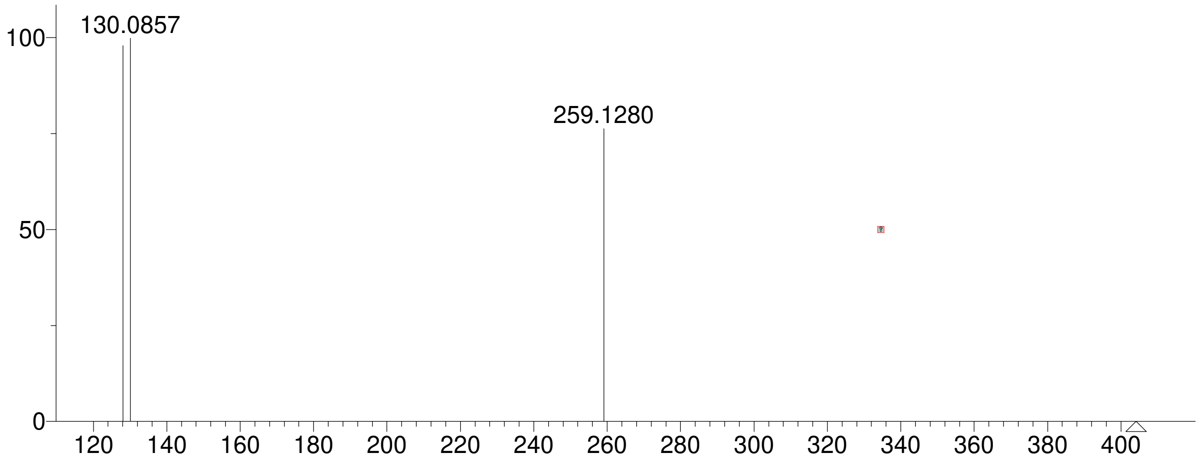

Unknown: Unknown (9.34_457.1839m/z)
Compound in Library Factor = -3273

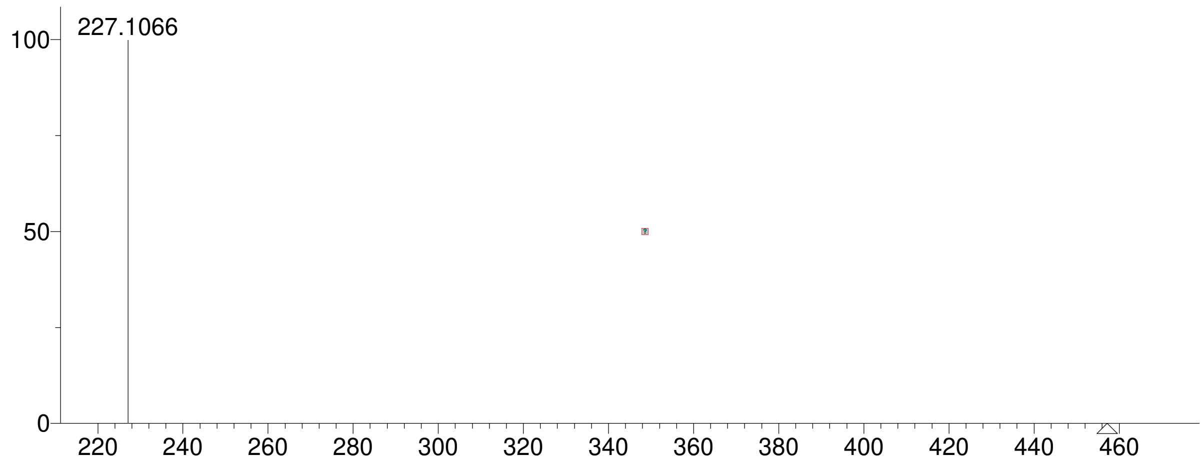

Hit 1 : NCGC00179819-03_C25H30O9_(4R,6aR,9S,9aR,9bR)-9-Methyl-3,6-bis(methylene)-2,8-dioxododecahydı
C25H30O9; MF: 2; RMF: 340; Prob 17.8%; Lib: mona-export-gnps; ID: 13119.

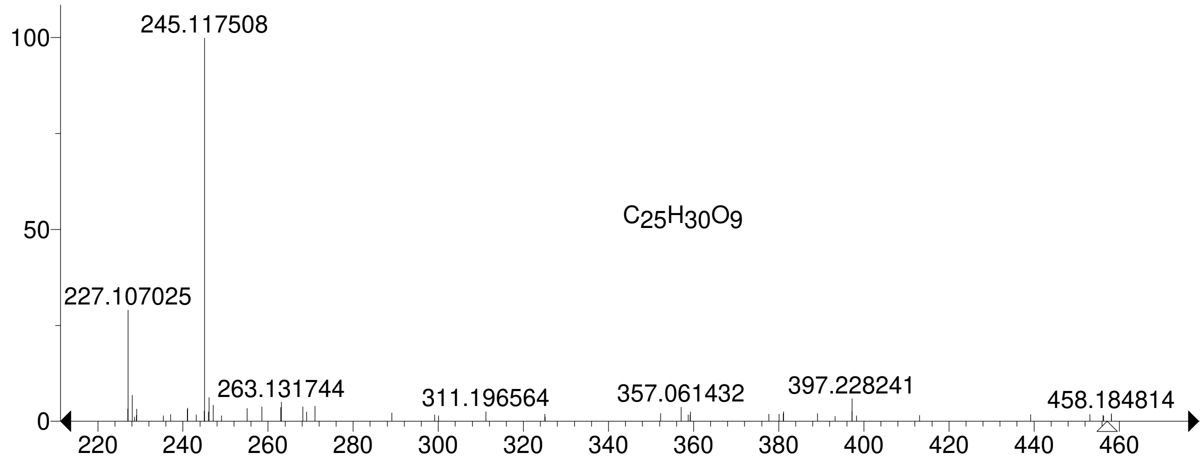

Hit 2 : 7-hydroxy-2,6,9,12,17,17-hexamethyl-22-methylidene-5,16,19,21-tetraoxaheptacyclo[10.8.1.1A^'3,a^'1.0A'
C25H28O8; MF: 0; RMF: 124; Prob 16.4%; Lib: mona-export-vf-npl_qexactive; ID: 20723.

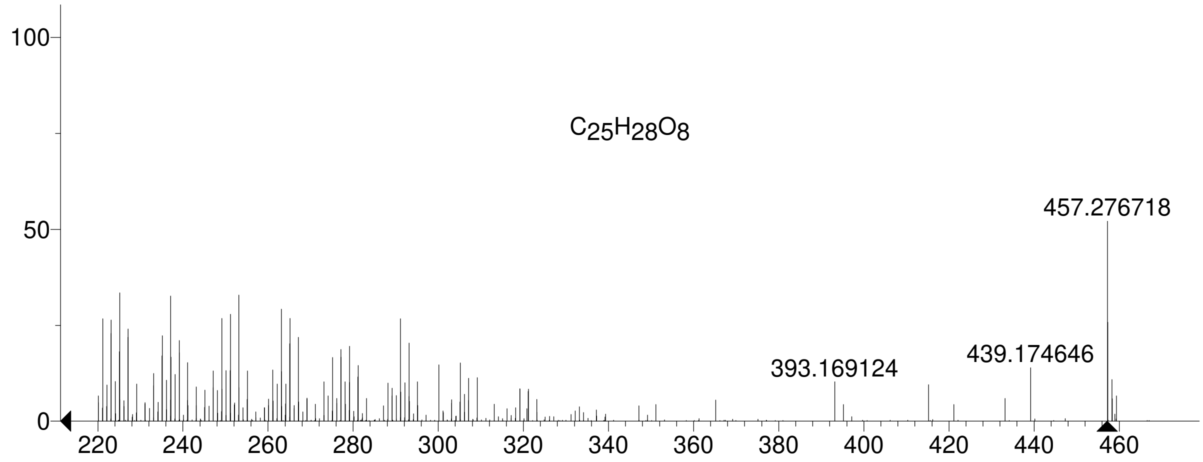

Hit 3 : NCGC00347648-02_C25H28O8_12H-6,13-Epoxy-8,12a-methano-2H,7H-furo[3',4':6,7]oxireno[3,4]heptale
C25H28O8; MF: 0; RMF: 87; Prob 16.4%; Lib: mona-export-gnps; ID: 10982.

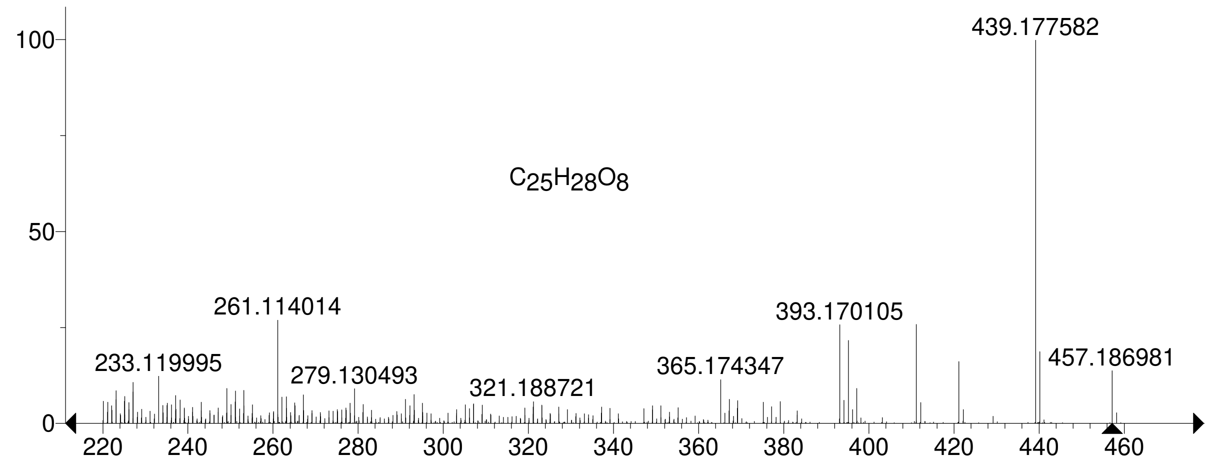

Hit 4 : 2-[3,4-dihydroxy-2,2-dimethyl-8-(3-methylbut-2-enoxy)-3,4-dihydrochromen-6-yl]-5,7-dihydroxy-2,3-dihydro
C25H28O8; MF: 0; RMF: 81; Prob 16.4%; Lib: mona-export-vf-npl_qexactive; ID: 16880.

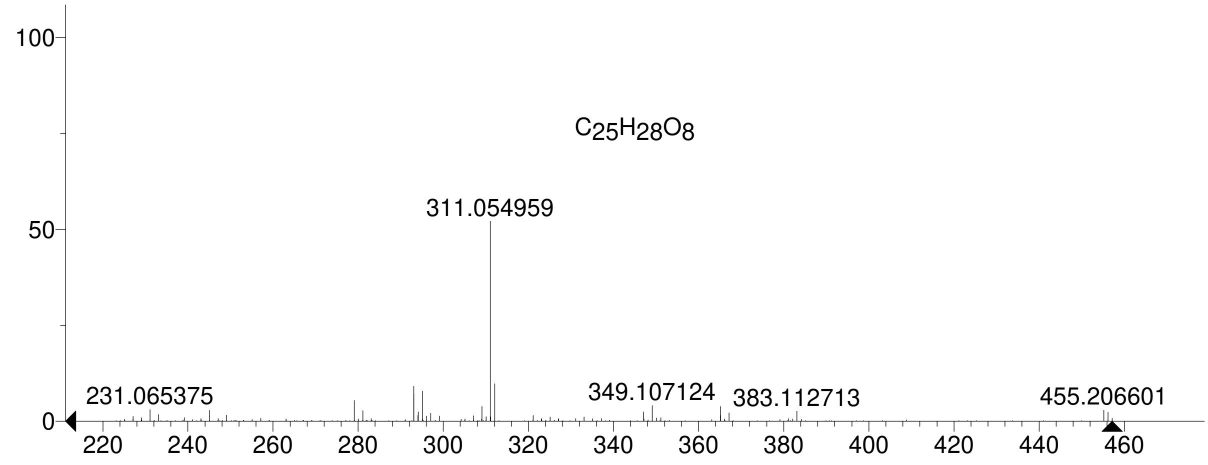

Hit 5 : Lobaric Acid [M+H]+ 65HCD P=457.2
C25H28O8; MF: 0; RMF: 42; Prob 16.4%; Lib: mona-export-vf-npl_qexactive; ID: 27036.

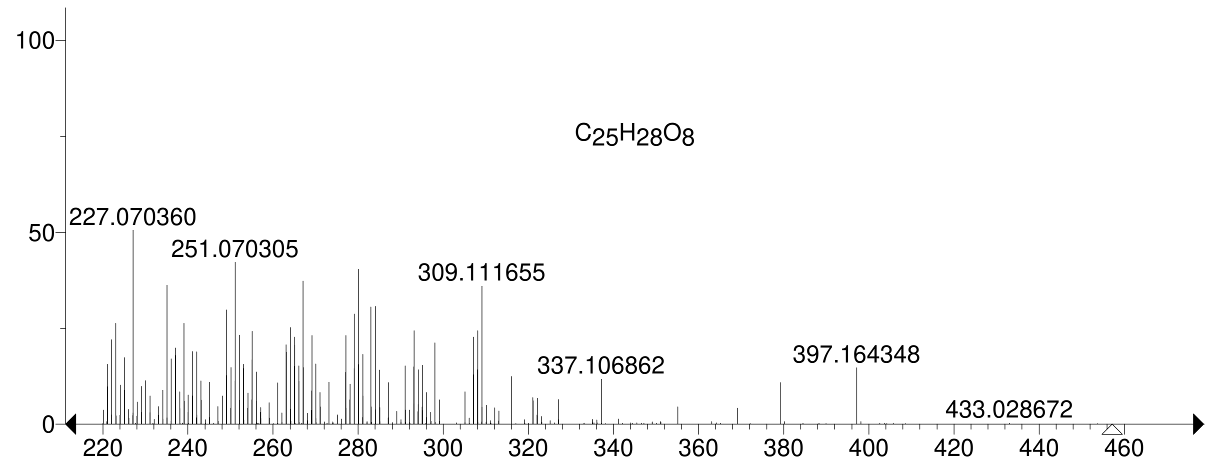

Unknown: Unknown (9.34_498.2437m/z)
Compound in Library Factor = 0

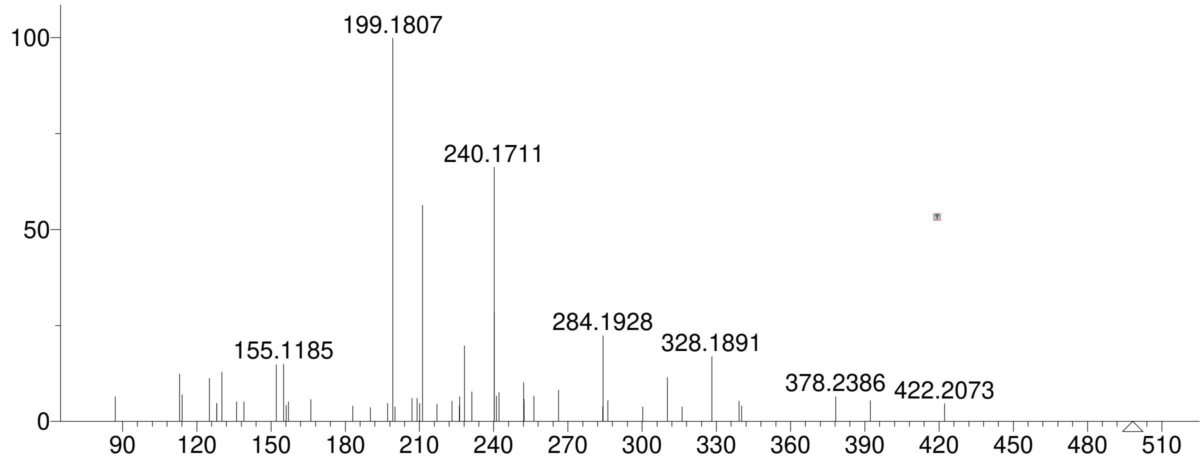

Hit 1 : _130048 [M+H]+ P=498.2
C28H35NO7; MF: 0; RMF: 32; Prob 33.3%; Lib: mona-export-gnps; ID: 5022.

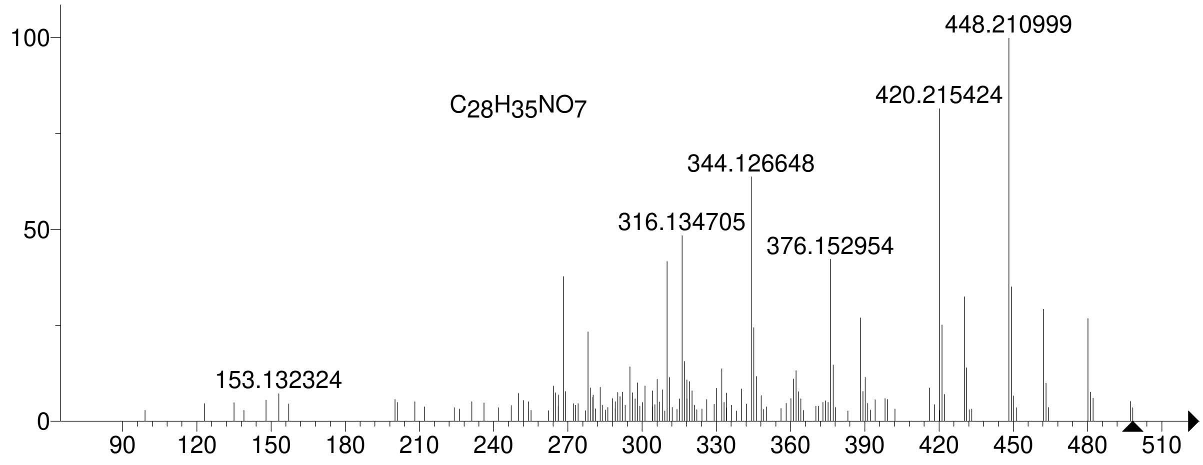

Hit 2 : _130047 [M+H]+ P=498.2
C28H35NO7; MF: 0; RMF: 26; Prob 33.3%; Lib: mona-export-gnps; ID: 5020.

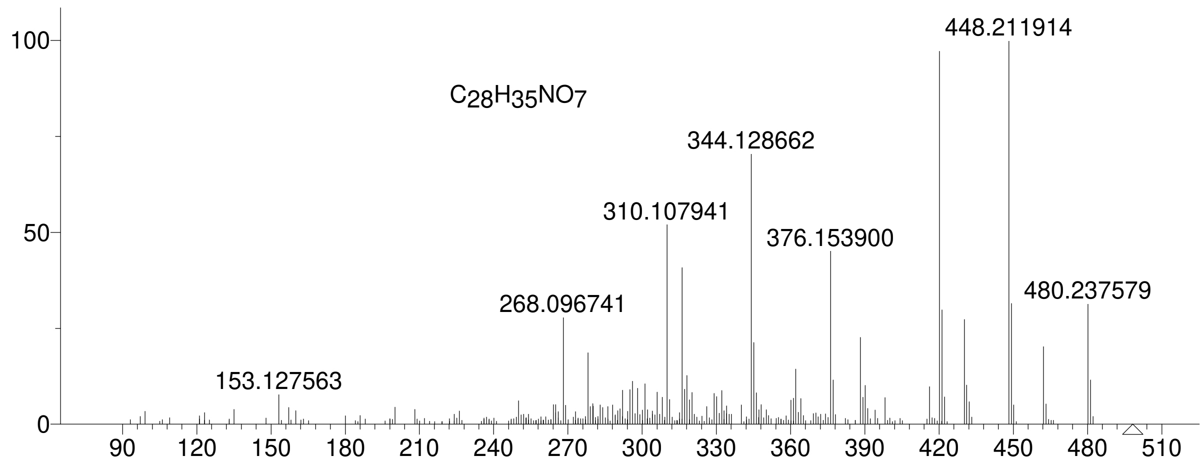

Hit 3 : NCGC00169355-02_C28H37NO8_Pentanedioic acid, 2-[(6'S,8a'S)-3',4',4'a,5',6,6',7',8,8',8'a-decahydro-4,(
C28H37NO8; MF: 0; RMF: 11; Prob 33.3%; Lib: mona-export-gnps; ID: 10695.

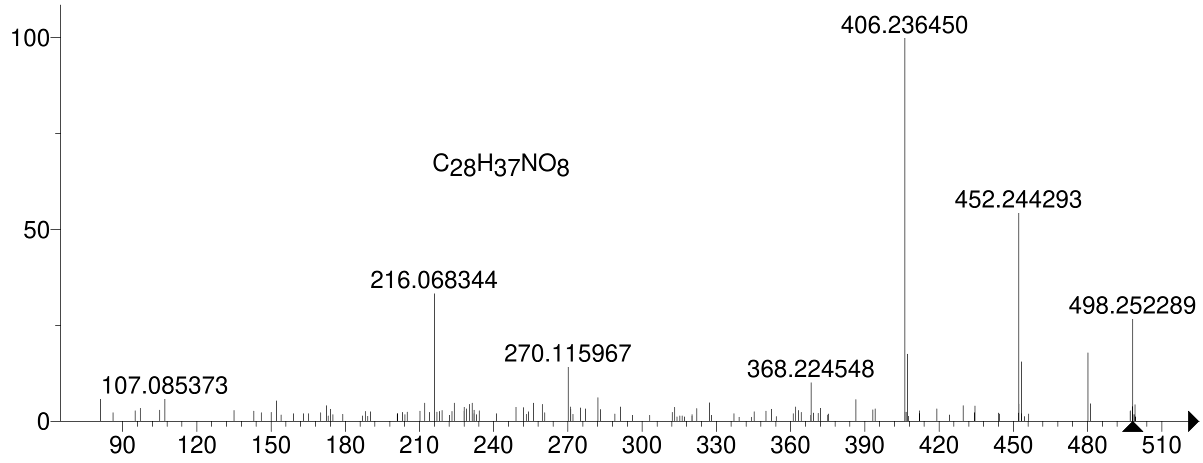

Unknown: Unknown (9.34_800.2006m/z)
Compound in Library Factor = 0

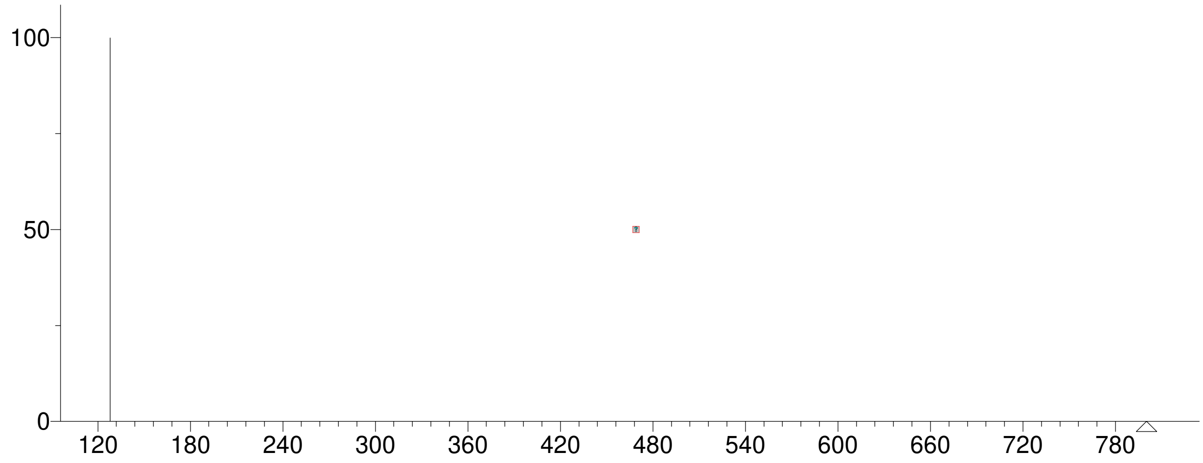

Unknown: Unknown (9.35_329.1054m/z)
Compound in Library Factor = -674

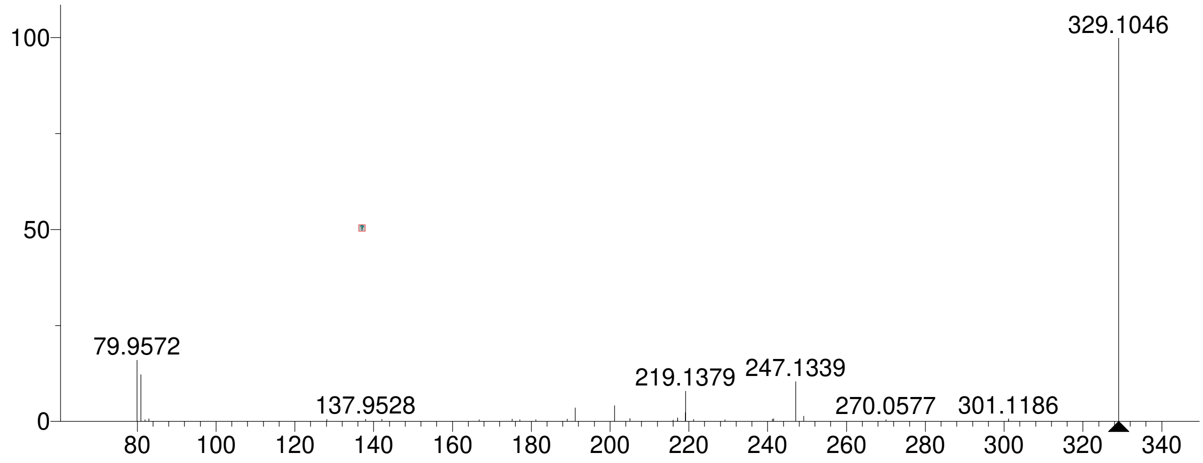

Hit 1 : 4,2'-Dihydroxy-3,4',6'-trimethoxychalcone [M-H]- HCD 11V P=329.1
C18H18O6; MF: 175; RMF: 817; Prob 81.3%; CAS: 1013916-01-2; Lib: nist_msms; ID: 145423.

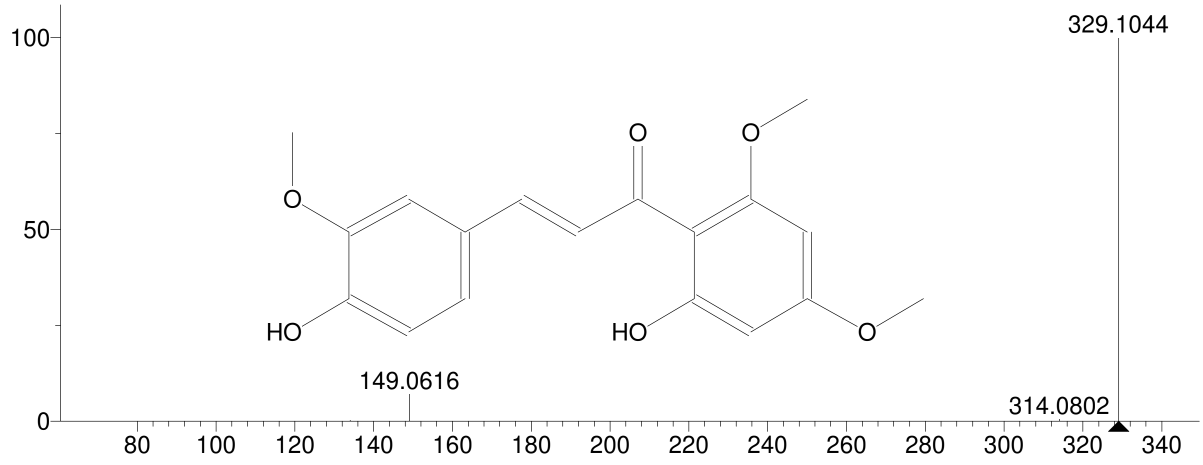

Hit 2 : 3,2'-Dihydroxy-4,4',6'-trimethoxychalcone [M-H]- HCD 9V P=329.1
C18H18O6; MF: 117; RMF: 776; Prob 15.0%; CAS: 38186-71-9; Lib: nist_msms; ID: 145561.

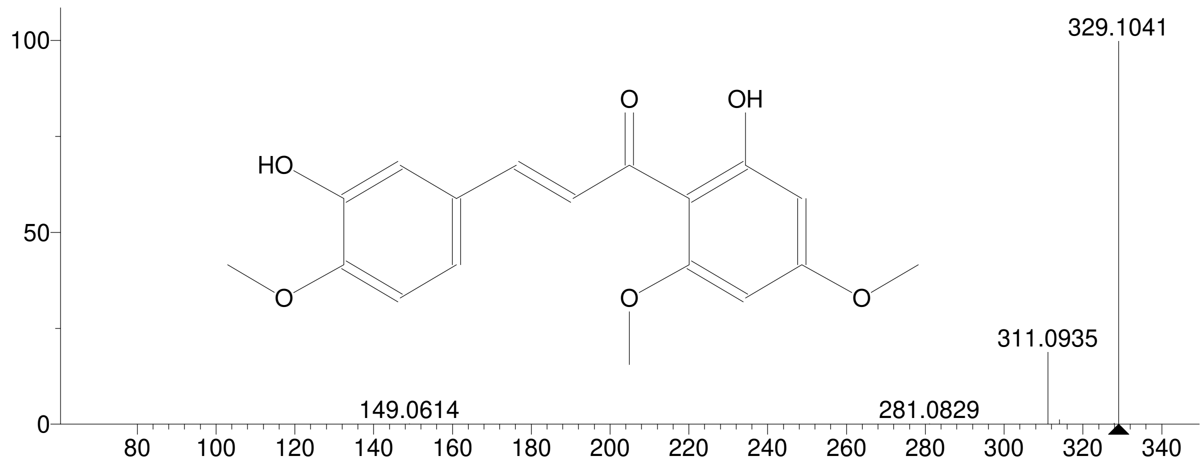

Hit 3 : Pesticide4_Halofenozide_C18H19ClN2O2_N'-Benzoyl-4-chloro-N'-(2-methyl-2-propanyl)benzohydrazide [
C18H19ClN2O2; MF: 10; RMF: 300; Prob 0.99%; Lib: msms-neg-gnps; ID: 1678.

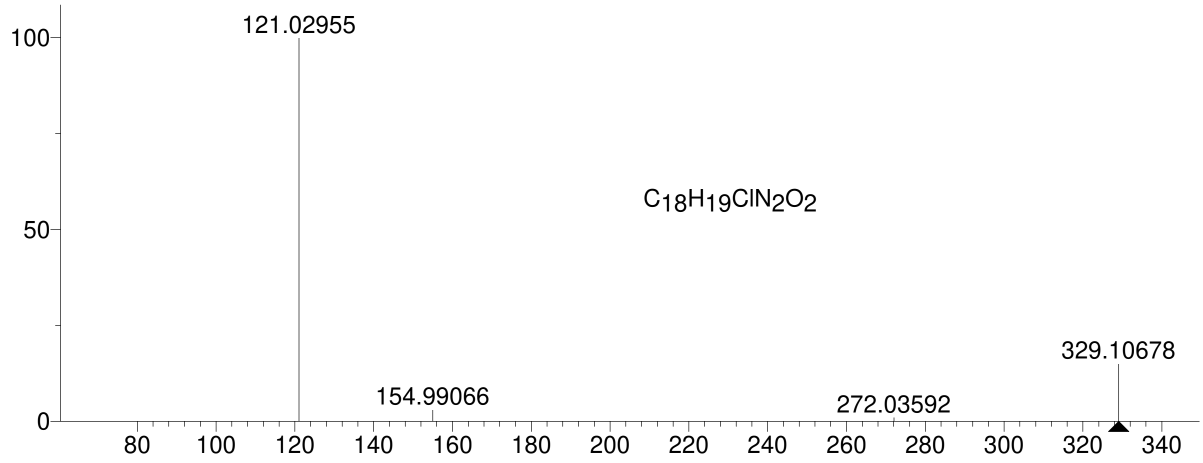

Hit 4 : 3OH-pCAlc(8-O-4)3OH-pCAlc2 (YT3-073) [M-H]- P=329.1
C18H18O6; MF: 4; RMF: 531; Prob 0.78%; Lib: phytofrag2018_final_060219; ID: 47.

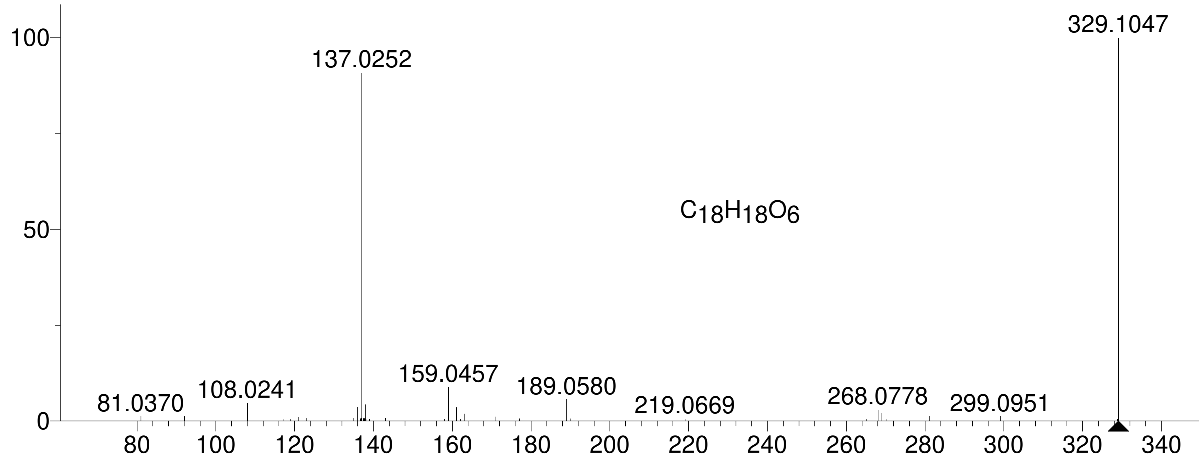

Hit 5 : "NCGC00160293-01!6-Methyl-5,7a,13,13b-tetraaza-pentaphene-8,14-dione" [M+H]+ P=329.1
C19H12N4O2; MF: 0; RMF: 350; Prob 0.65%; Lib: mona-export-gnps; ID: 1015.

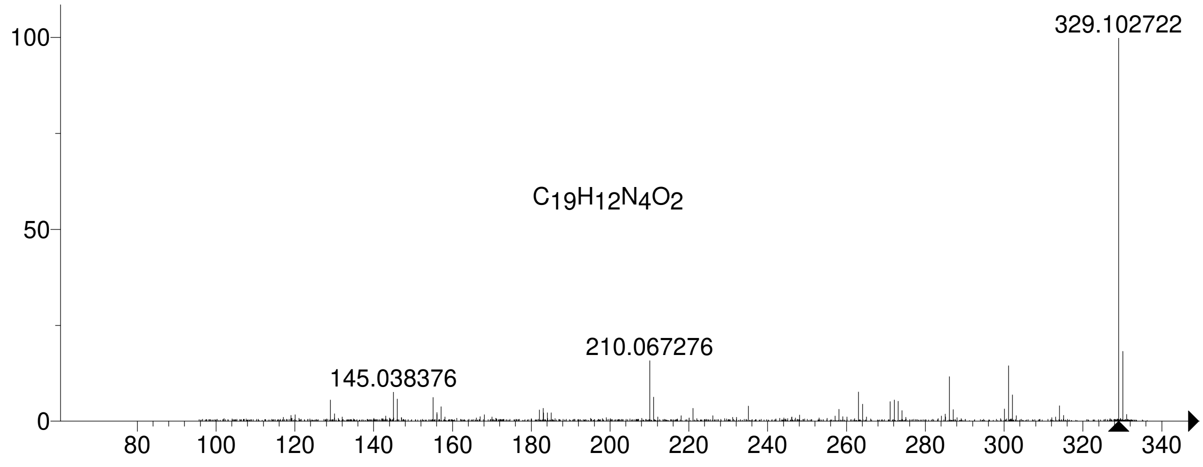

Unknown: Unknown (9.36_429.1862m/z)
Compound in Library Factor = 0

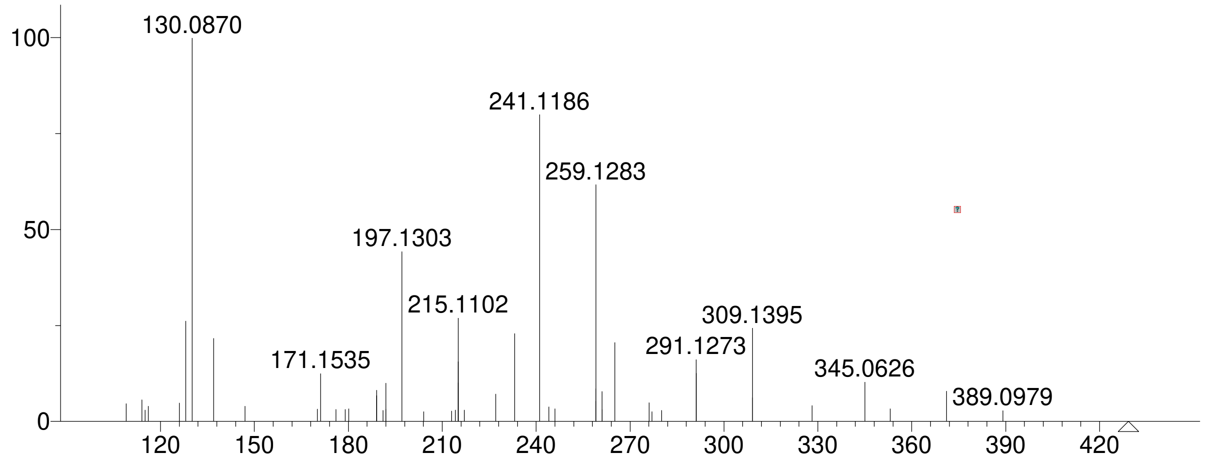

Hit 1 : (2R,3R,4S,5S,6R)-2-[6-hydroxy-3-[(E)-3-hydroxybut-1-enyl]-2,4,4-trimethylcyclohexyl]oxy-6-(hydroxymethy
C19H34O8; MF: 0; RMF: 25; Prob 50.0%; Lib: anthocyanins mona; ID: 9985.

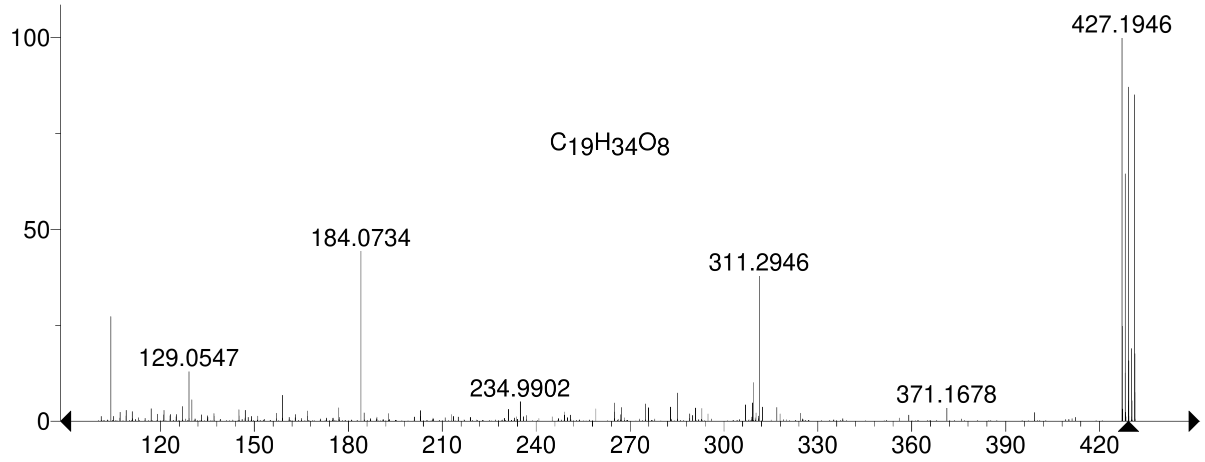

Hit 2 : NCGC00169368-02!3,6-bis(3,4,5-trimethoxyphenyl)-1,3,3a,4,6,6a-hexahydrofuro[3,4-c]furan [M-H2O+H]+
C24H30O8; MF: 0; RMF: 14; Prob 50.0%; Lib: mona-export-gnps; ID: 9133.

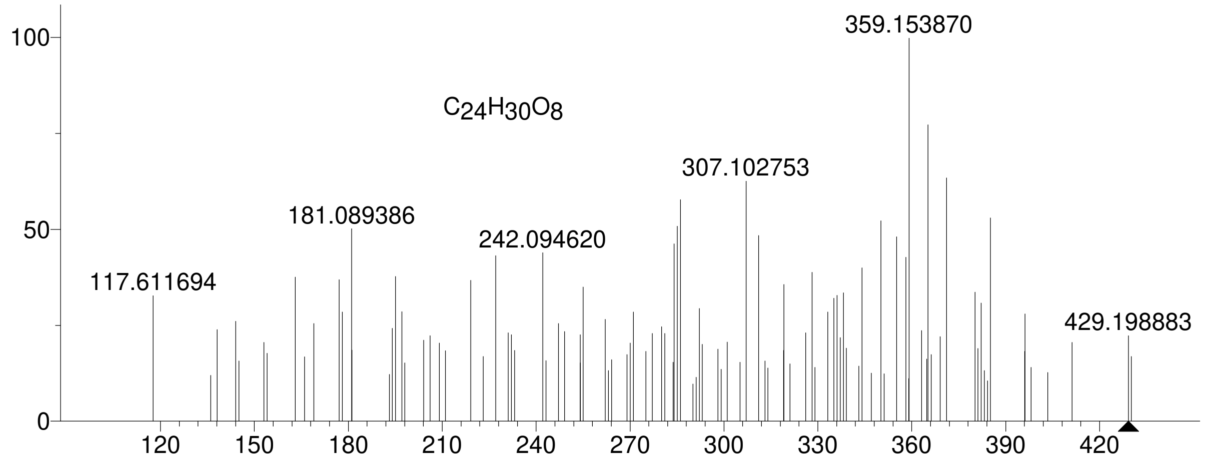

Unknown: Unknown (9.36_476.0702m/z)
Compound in Library Factor = 0

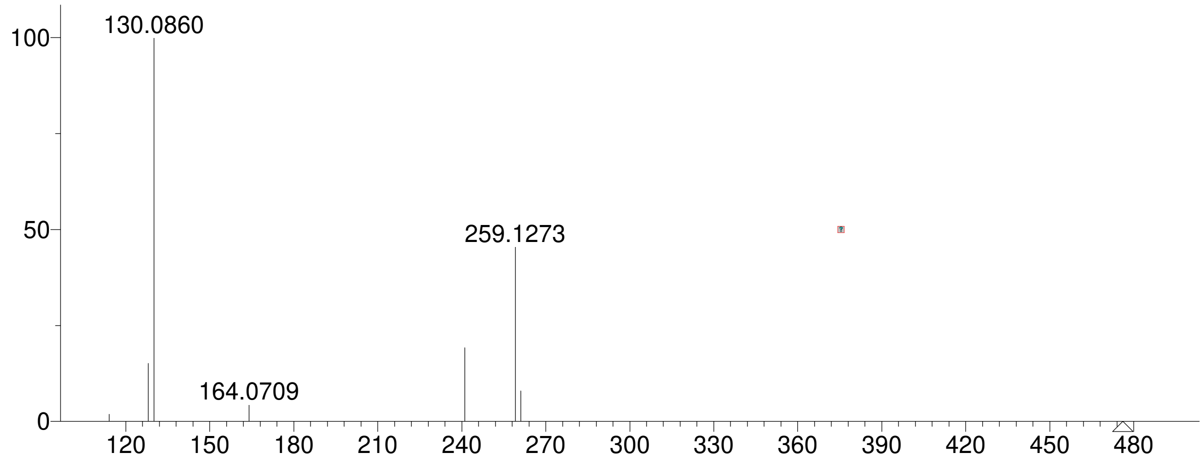

Unknown: Unknown (9.36_576.2302m/z)
Compound in Library Factor = 0

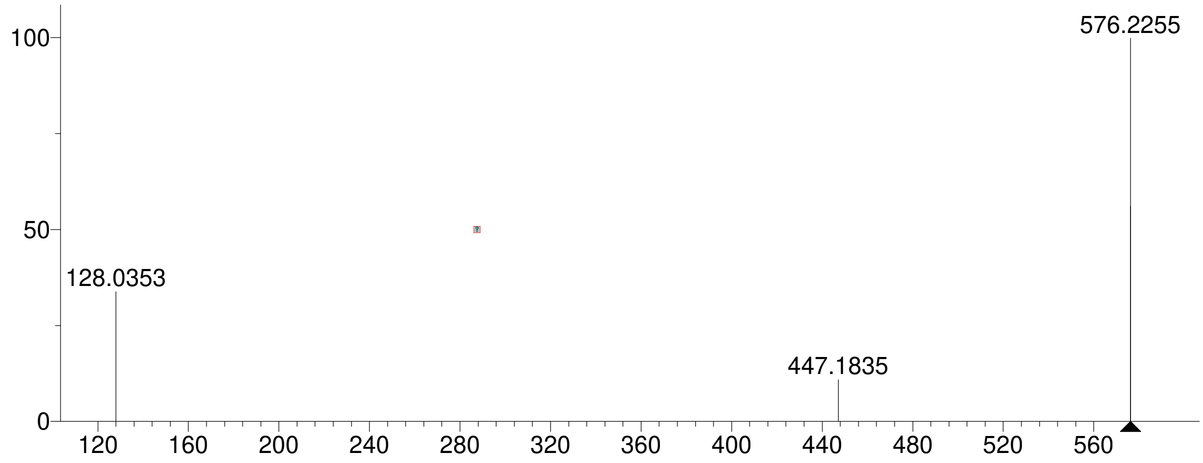

** Search Report Page 1 of 1 **

Unknown: Unknown (9.37_339.1073m/z)
Compound in Library Factor = 0

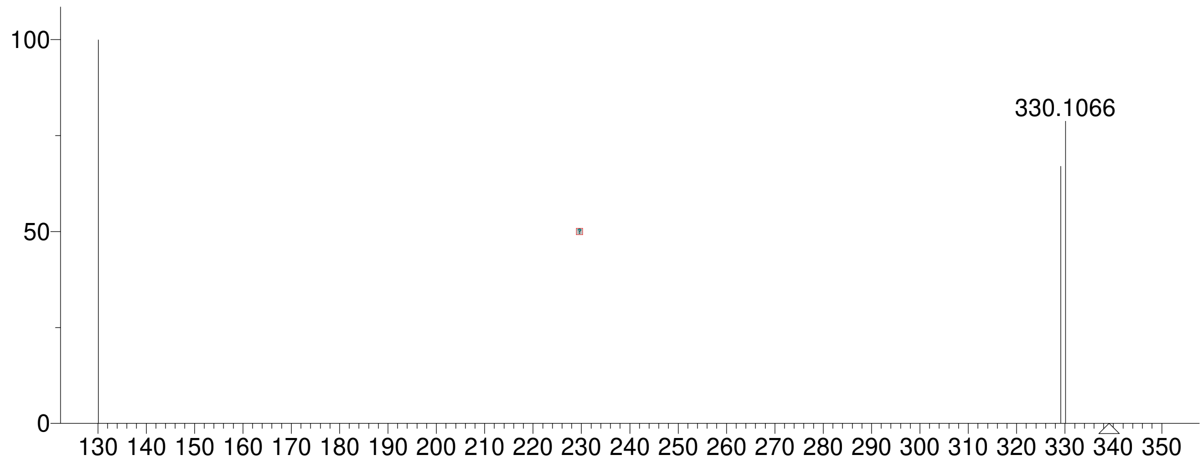

Hit 1 : (1R,3R,4S,5R)-1,3,4-trihydroxy-5-[(E)-3-(4-hydroxyphenyl)prop-2-enoyl]oxycyclohexane-1-carboxylic acid
C16H18O8; MF: 0; RMF: 19; Prob 50.0%; Lib: mona-export-vf-npl_qexactive; ID: 6316.

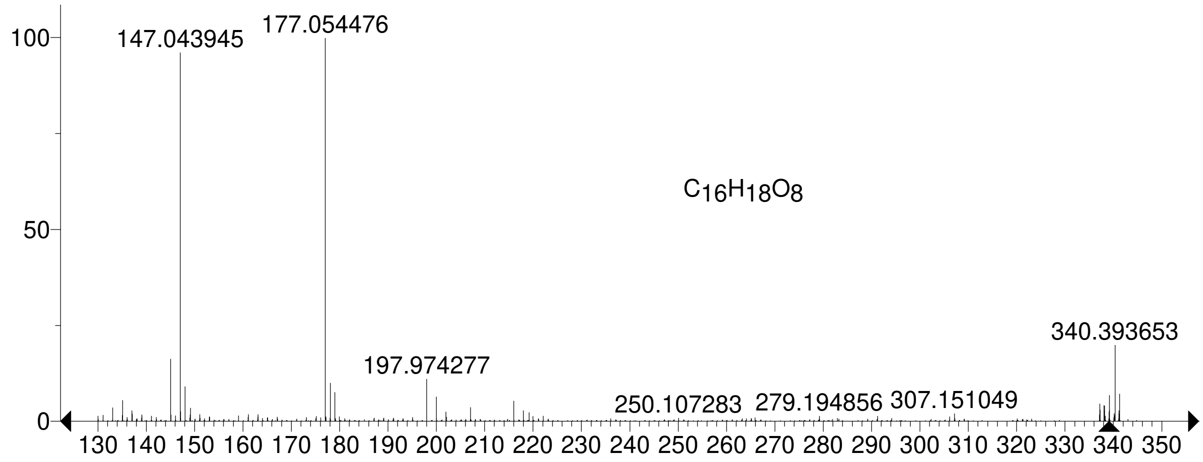

Hit 2 : NCGC00384821-01_C16H18O8_Cyclohexanecarboxylic acid, 1,3,4-trihydroxy-5-[[(2E)-3-(4-hydroxypheny
C16H18O8; MF: 0; RMF: 10; Prob 50.0%; Lib: mona-export-gnps; ID: 10980.

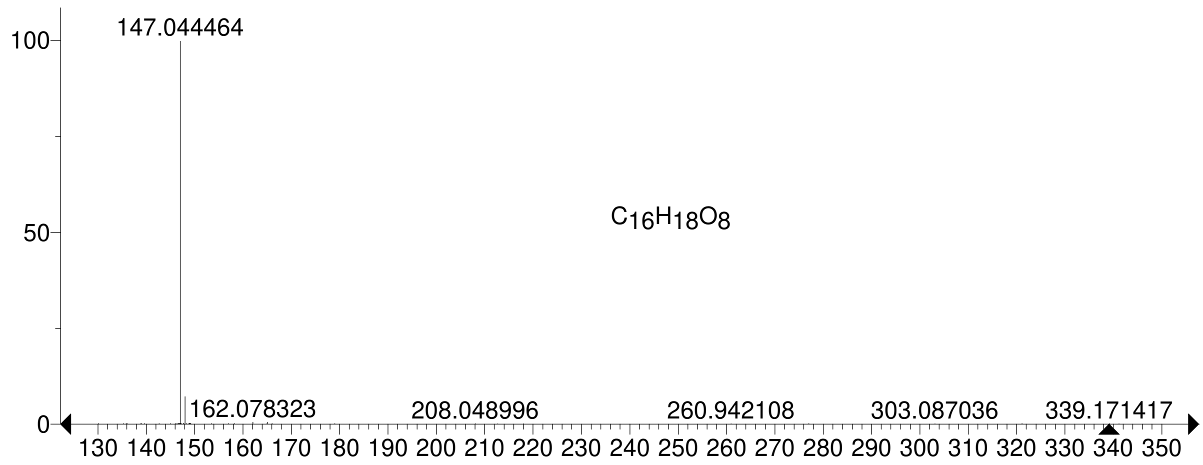

Unknown: Unknown (9.37_356.8917m/z)
Compound in Library Factor = 0

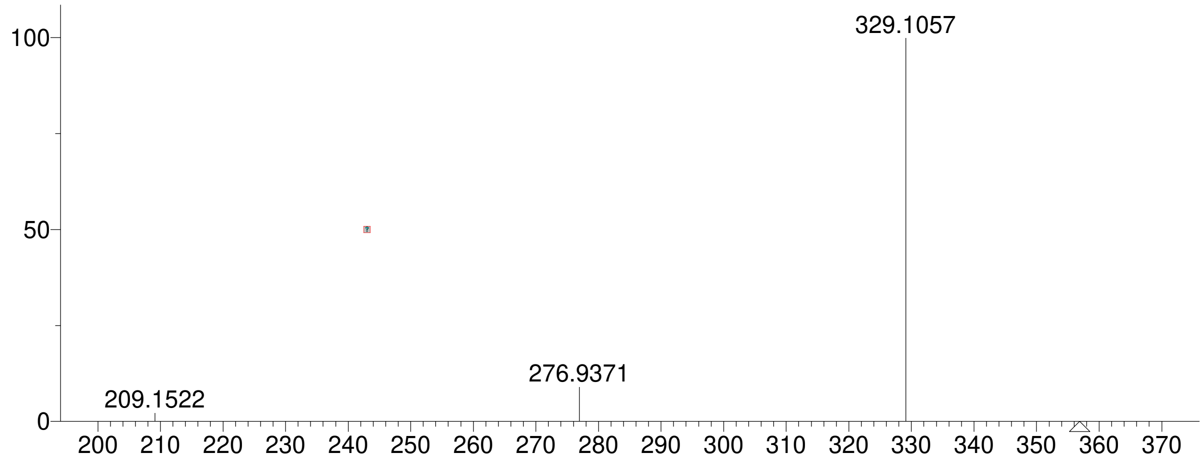

Unknown: Unknown (9.37_425.1442m/z)
Compound in Library Factor = 0

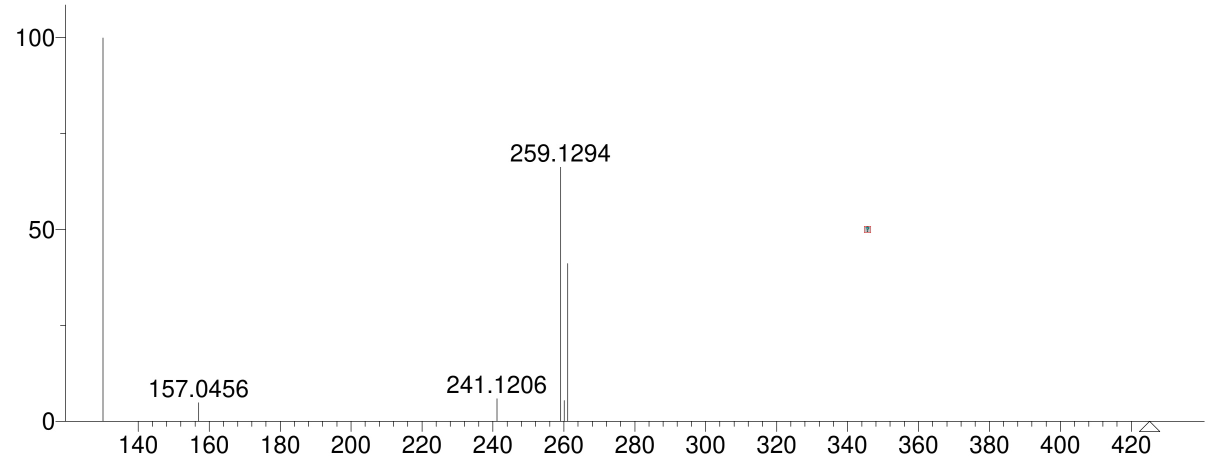

Hit 1 : Ginkgolide B [M+H]+ HCD 34V P=425.1
C20H24O10; MF: 0; RMF: 89; Prob 20.0%; CAS: 15291-77-7; Lib: nist_msms; ID: 352887.

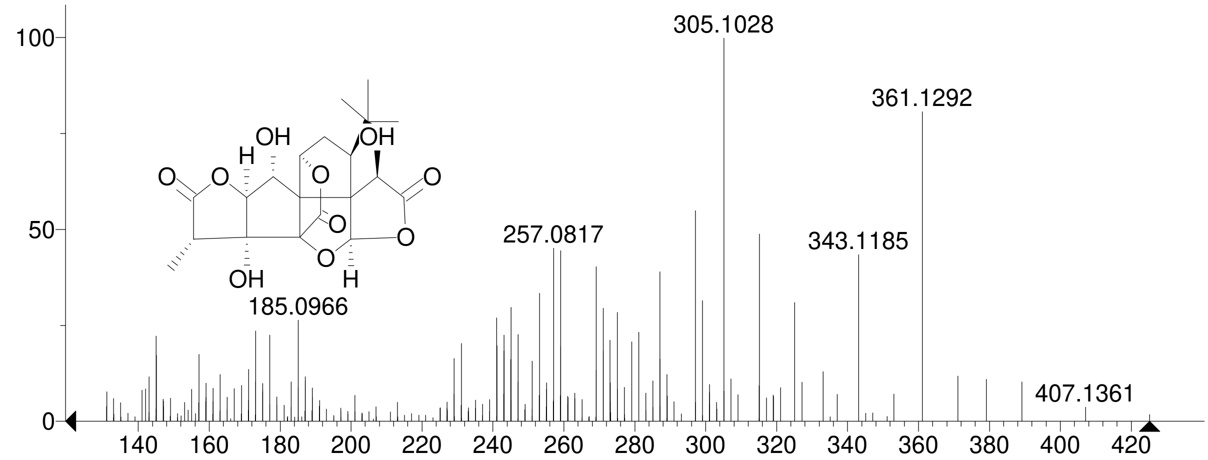

Hit 2 : NCGC00381128-01_C18H26O10_4-(beta-D-Glucopyranosyloxy)benzyl 2,3-dihydroxy-3-methylbutanoate
C18H26O10; MF: 0; RMF: 80; Prob 20.0%; Lib: mona-export-gnps; ID: 11687.

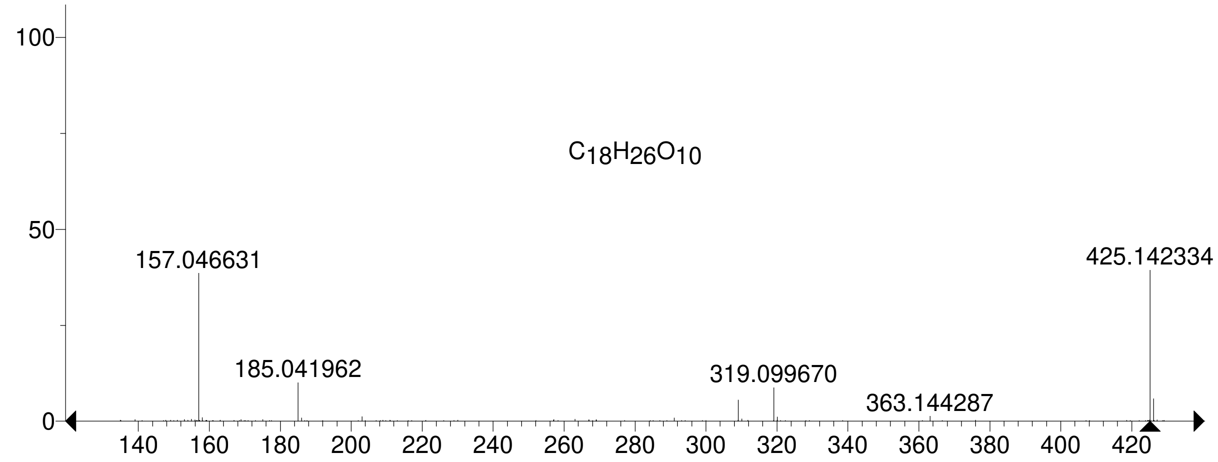

Hit 3 : ginkgolide B [M+H]+ 35HCD P=425.1
C20H24O10; MF: 0; RMF: 60; Prob 20.0%; Lib: mona-export-vf-npl_qexactive; ID: 5950.

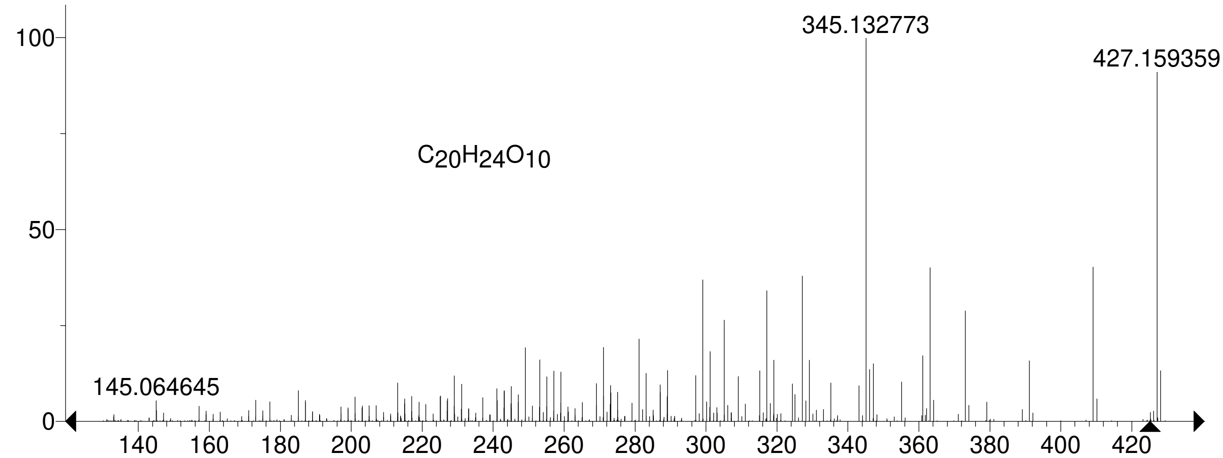

Hit 4 : [(2R,3S,4S,5R,6S)-3,4,5-trihydroxy-6-[(E)-3-phenylprop-2-enoyl]oxyoxan-2-yl]methyl 3,4-dihydroxy-2-meth
C20H24O10; MF: 0; RMF: 10; Prob 20.0%; Lib: mona-export-vf-npl_qexactive; ID: 9484.

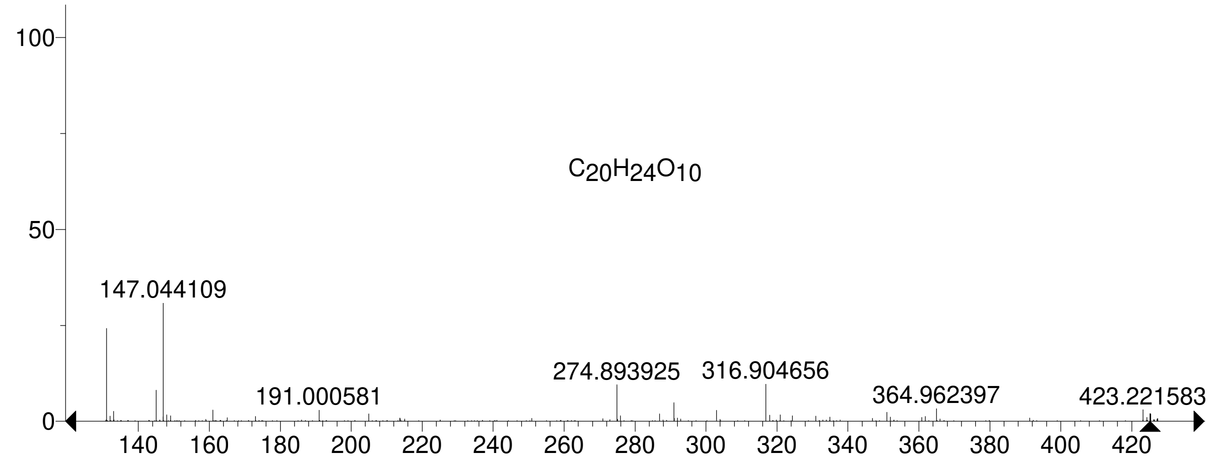

Hit 5 : NCGC00380741-01_C20H24O10_6-O-(3,4-Dihydroxy-2-methylenebutanoyl)-1-O-[(2E)-3-phenyl-2-propen
C20H24O10; MF: 0; RMF: 9; Prob 20.0%; Lib: mona-export-gnps; ID: 11636.

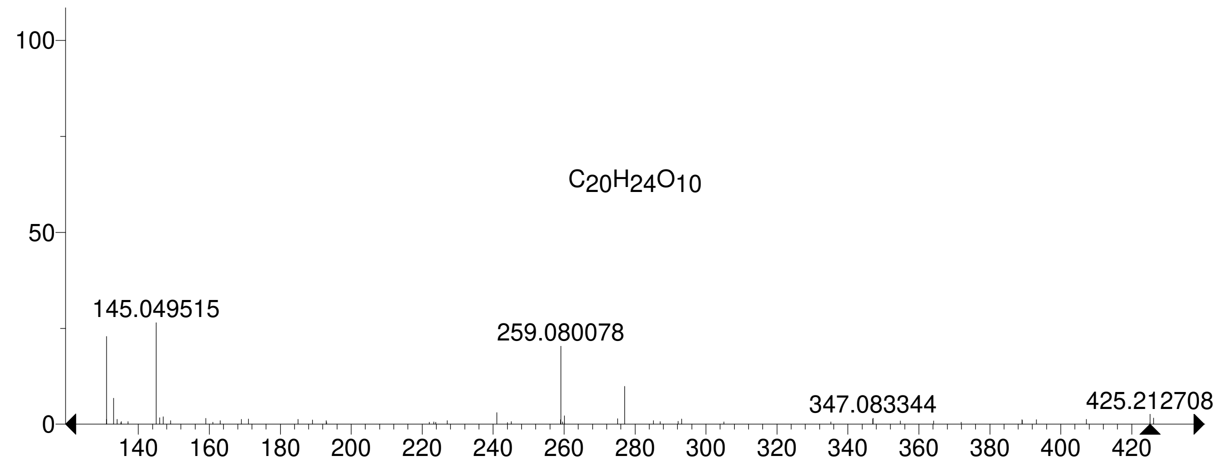

** Search Report Page 1 of 1 **

Unknown: Unknown (9.38_387.9947m/z)
Compound in Library Factor = 0

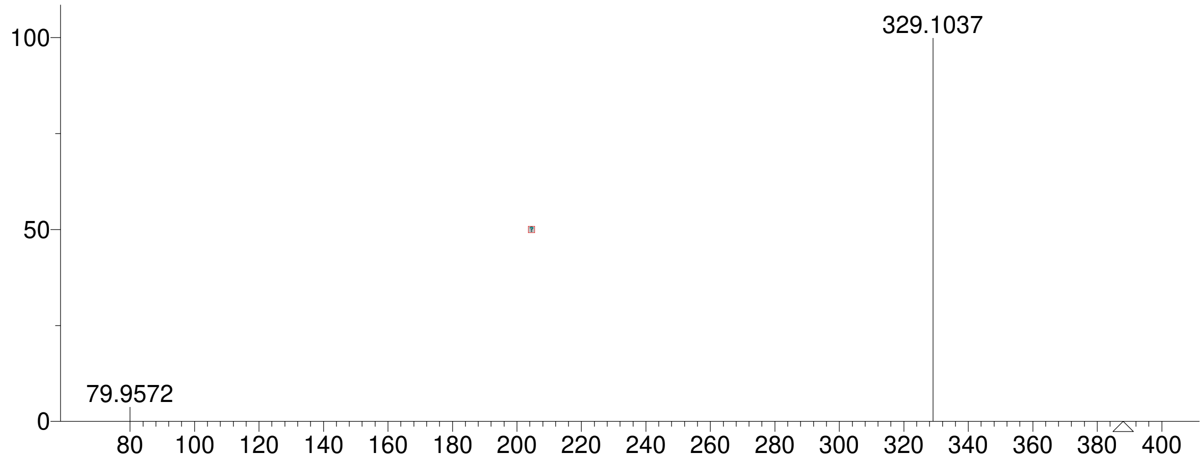

Unknown: Unknown (9.38_494.1639m/z)
Compound in Library Factor = 0

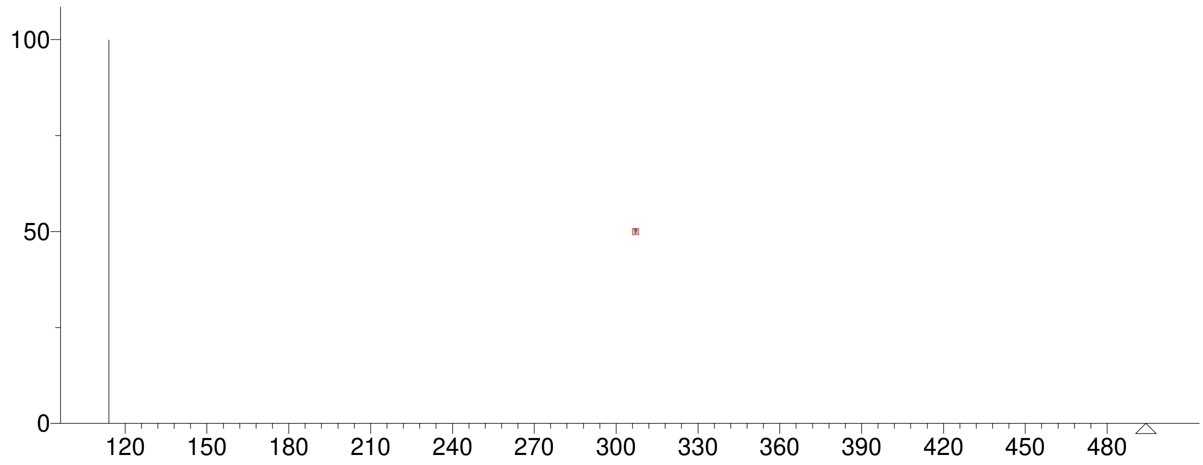

Unknown: Unknown (9.39_356.1807m/z)
Compound in Library Factor = 0

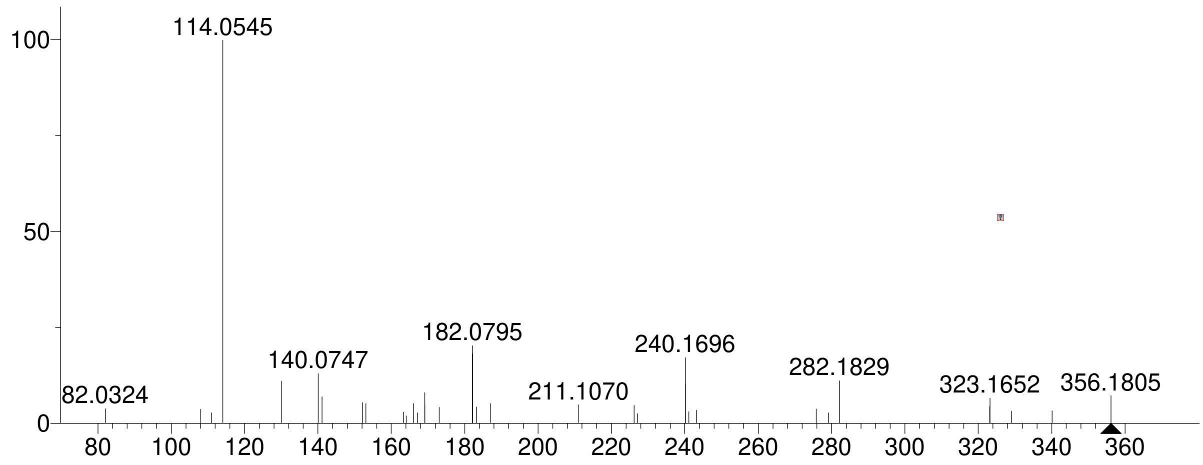

Unknown: Unknown (9.39_449.1526m/z)
Compound in Library Factor = 0

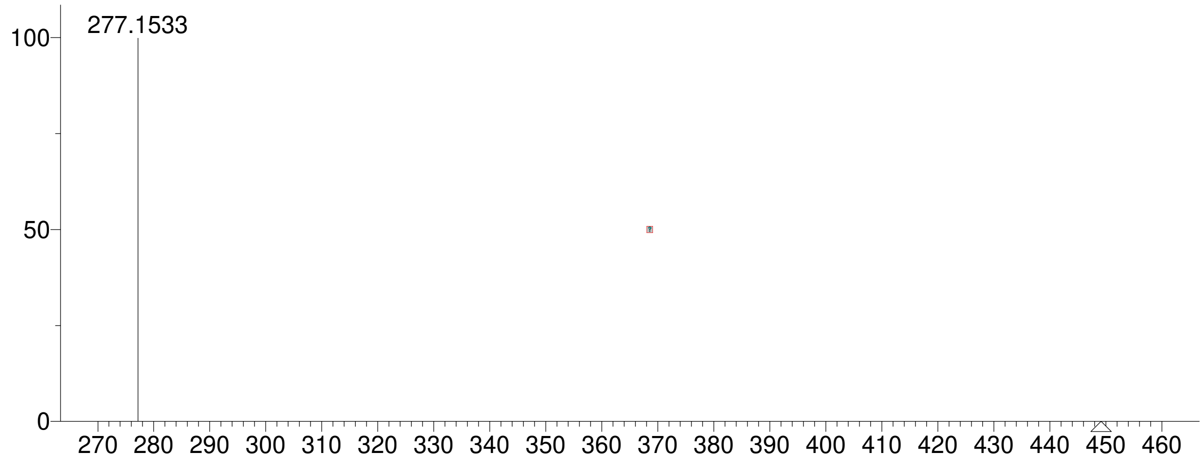

Unknown: Unknown (9.41_439.2073m/z)
Compound in Library Factor = 0

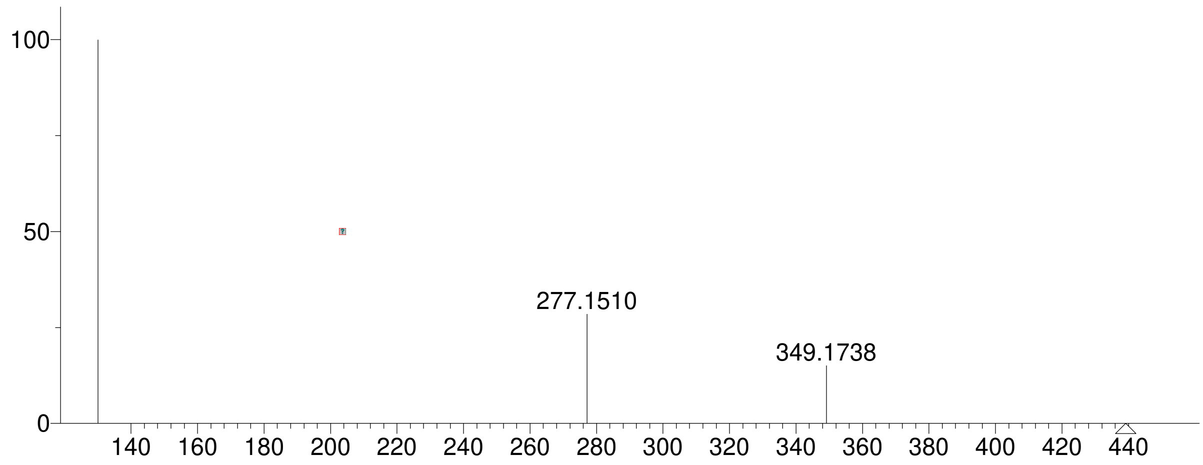

Hit 1 : Deacetoxy-7-Oxogedunin [M+H]+ 35HCD P=439.2
C26H30O6; MF: 0; RMF: 17; Prob 50.0%; Lib: mona-export-vf-npl_qexactive; ID: 25777.

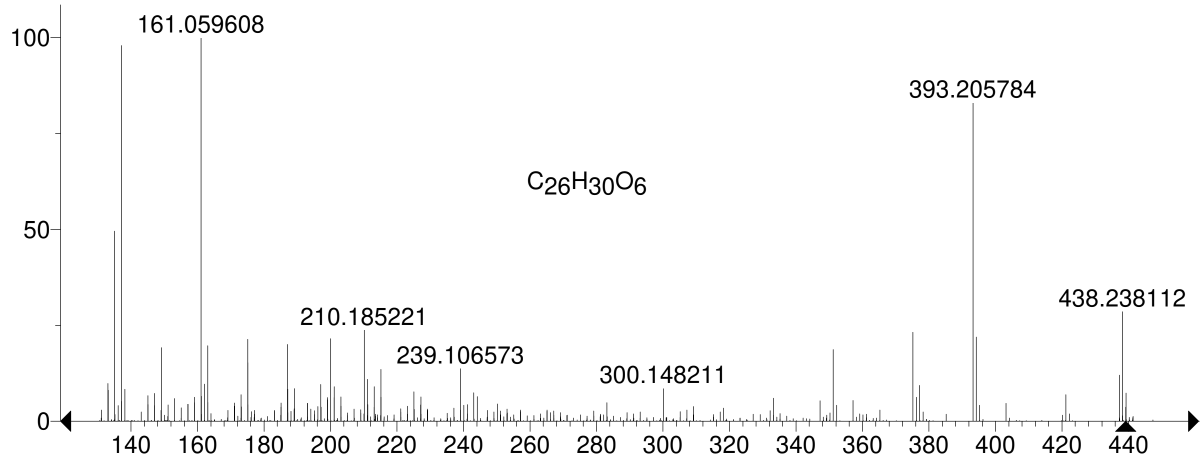

Hit 2 : 17-Trifluoromethylphenyl-13,14-dihydrotrinorprostaglandin F2α [M-H-H2O]- HCD 41V P=439.2
C24H33F3O5; MF: 0; RMF: 10; Prob 50.0%; CAS: 294856-01-2; Lib: nist_msms; ID: 93984.

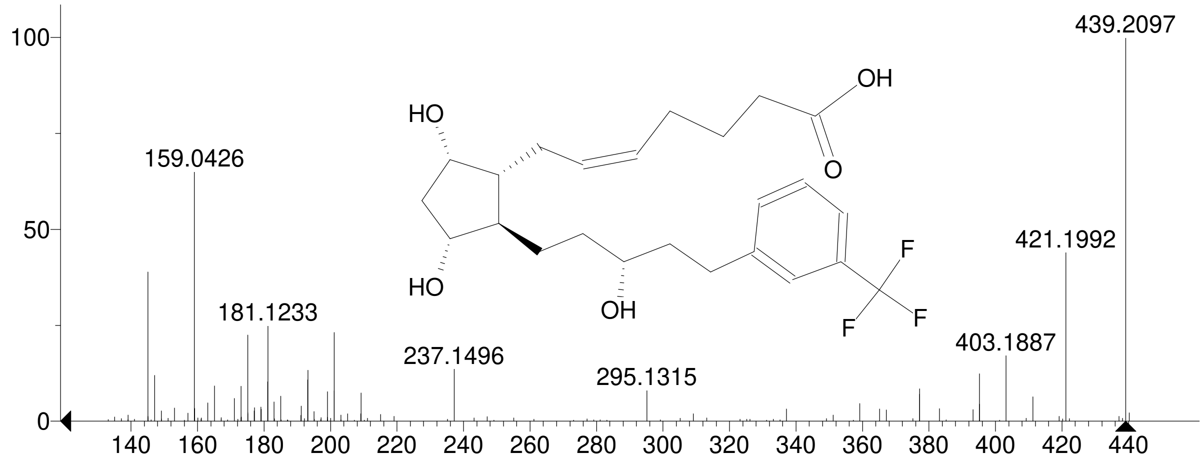

Unknown: Unknown (9.44_277.1549m/z)
Compound in Library Factor = -1453

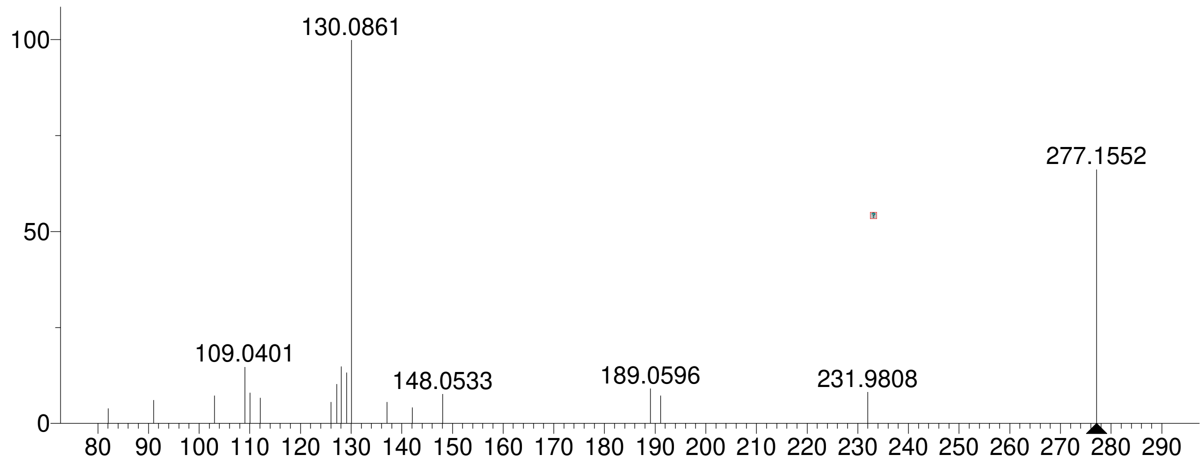

Hit 1 : Phe-Leu [M-H]- HCD 13eV P=277.2
C15H22N2O3; MF: 236; RMF: 564; Prob 43.1%; Lib: nist_msms; ID: 570088.

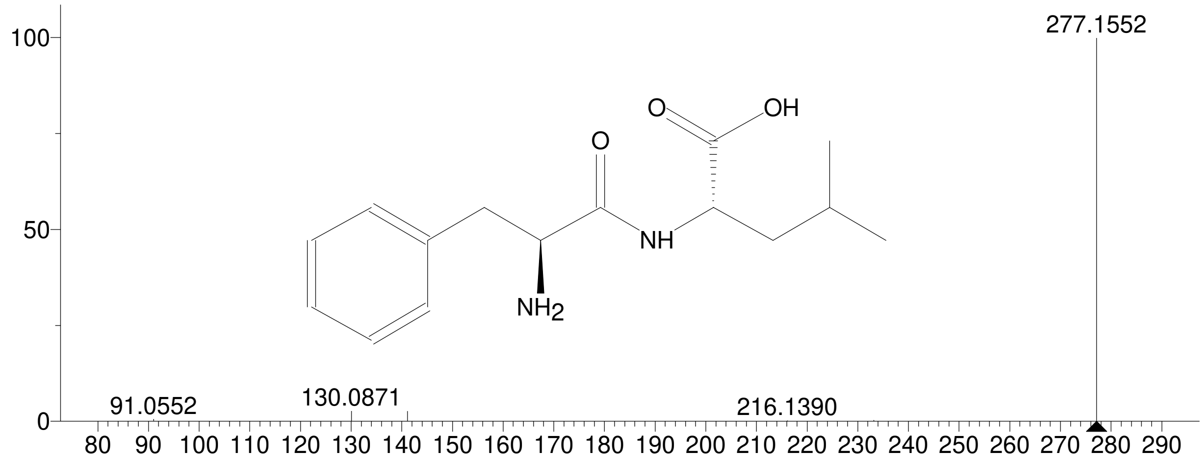

Hit 2 : Phe-Leu [M-H]- HCD 13eV P=277.2
C15H22N2O3; MF: 236; RMF: 564; Prob 43.1%; CAS: 3303-55-7; Lib: nist_msms2; ID: 83814.

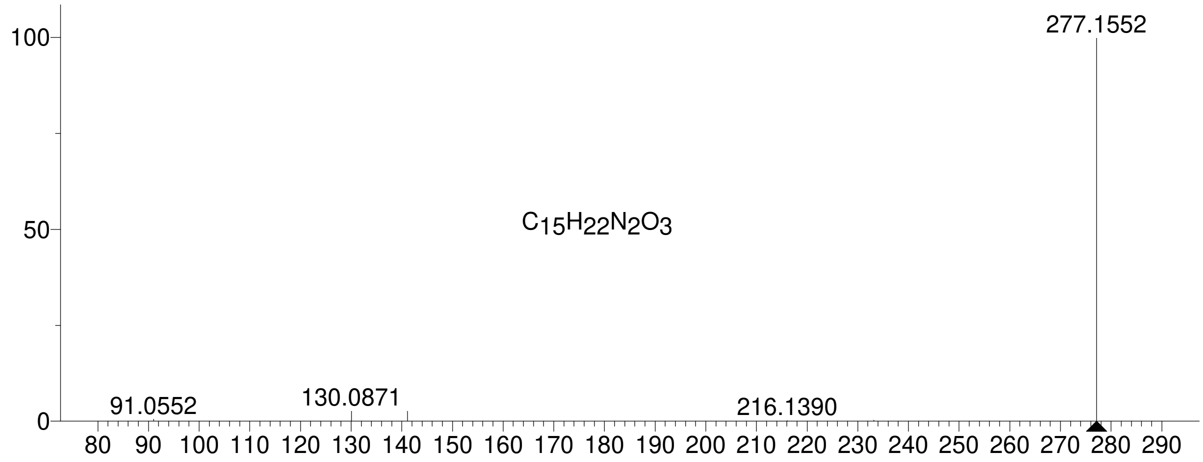

Hit 3 : Phe-Ile [M-H]- HCD 16eV P=277.2
C15H22N2O3; MF: 127; RMF: 582; Prob 2.79%; Lib: nist_msms; ID: 570602.

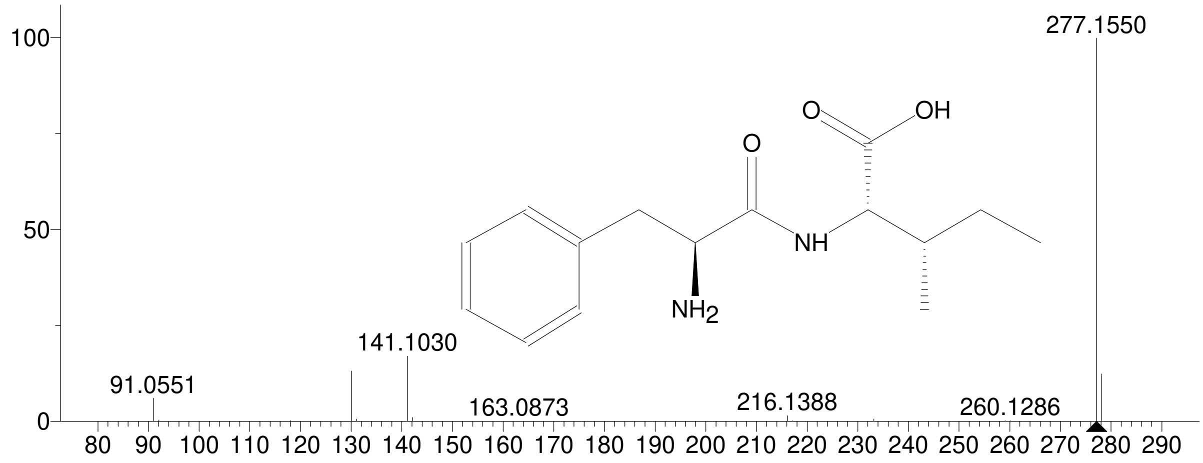

Hit 4 : Phe-Ile [M-H]- HCD 16eV P=277.2
C15H22N2O3; MF: 127; RMF: 582; Prob 2.79%; CAS: 22951-94-6; Lib: nist_msms2; ID: 84335.

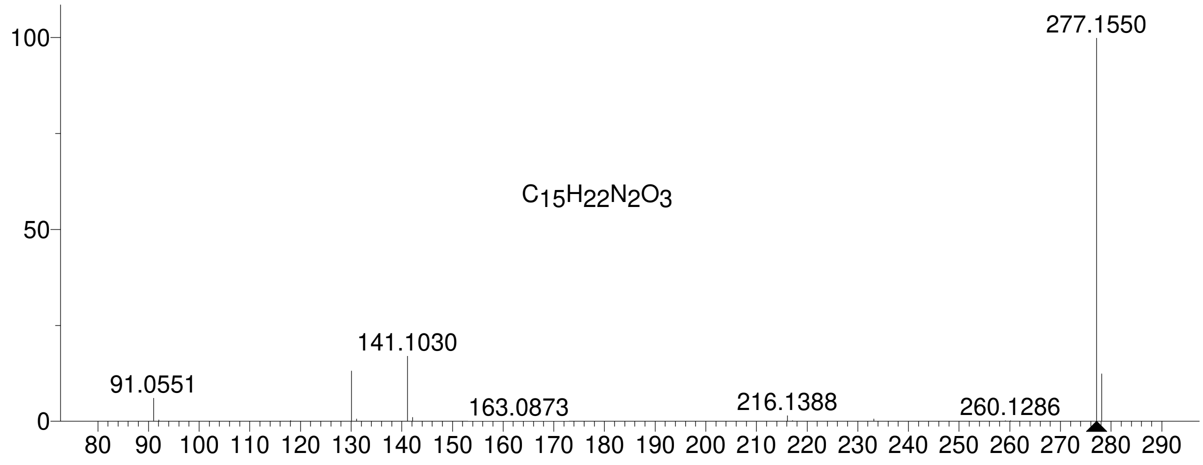

Hit 5 : Ile-Phe [M-H]- HCD 13eV P=277.2
C15H22N2O3; MF: 116; RMF: 502; Prob 1.91%; Lib: nist_msms; ID: 570142.

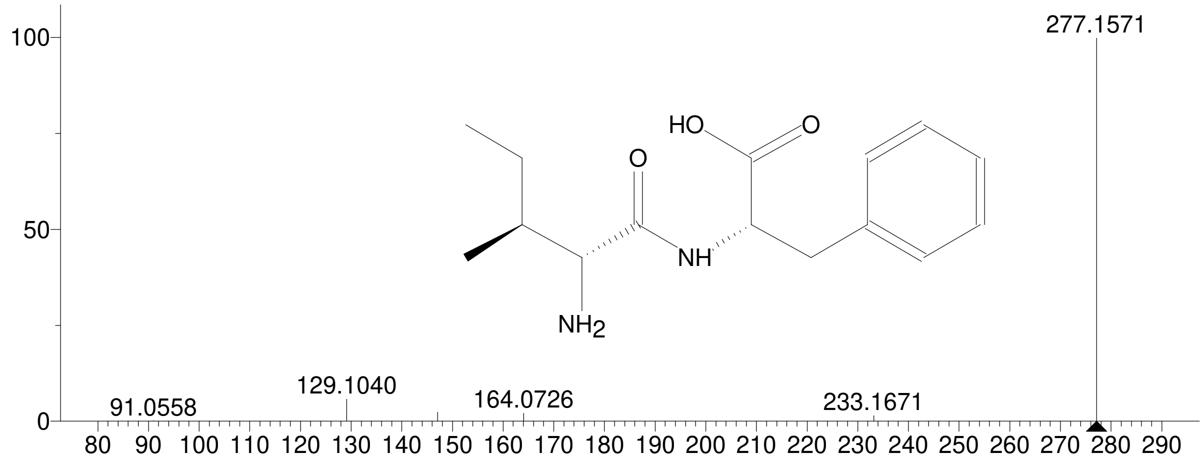

Unknown: Unknown (9.44_586.0112m/z)
Compound in Library Factor = 0

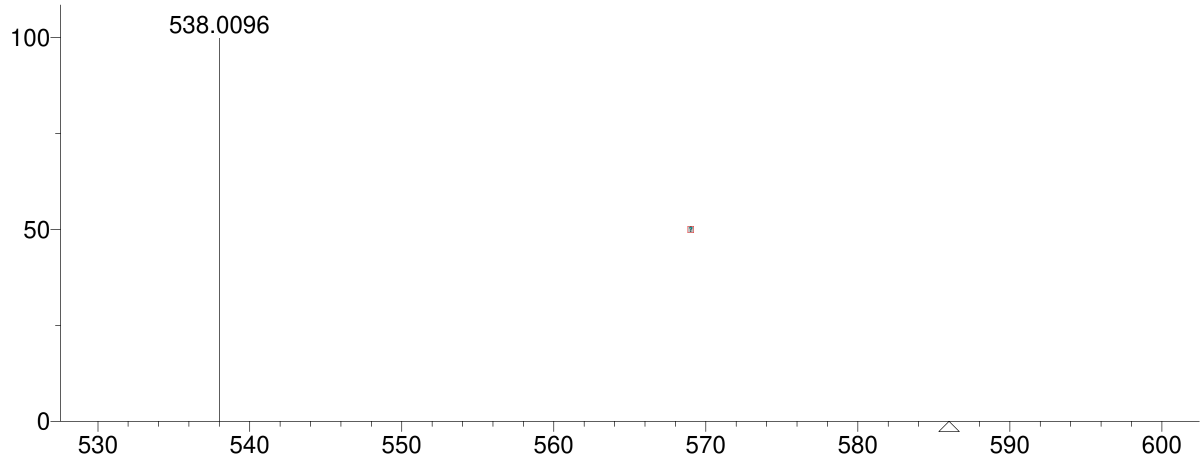

Unknown: Unknown (9.45_165.0548m/z)
Compound in Library Factor = -1224

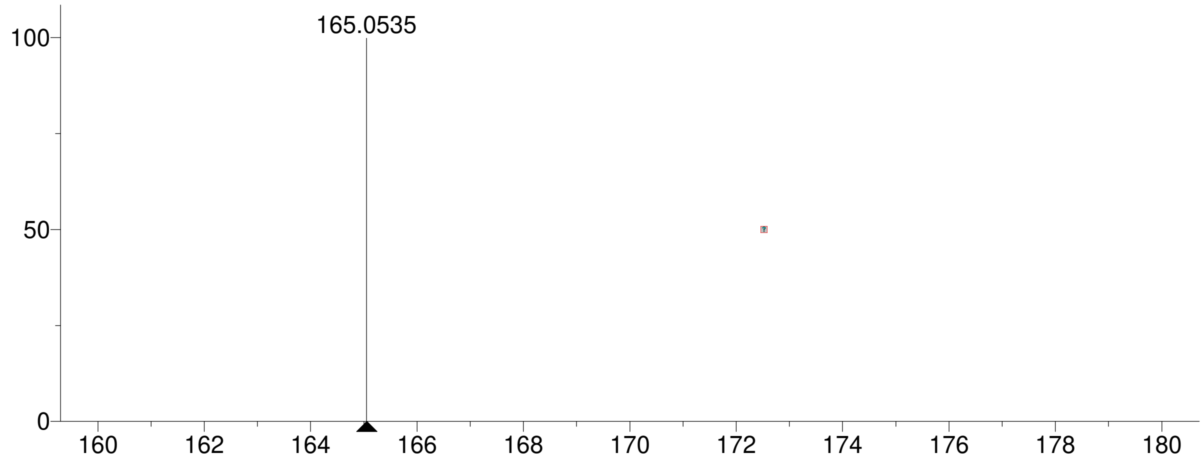

Hit 1 : Caffeyl alcohol; PlaSMA ID-148 [M-H]- P=165.1
C9H10O3; MF: 364; RMF: 999; Prob 2.07%; Lib: biomsms-neg-plasma; ID: 778.

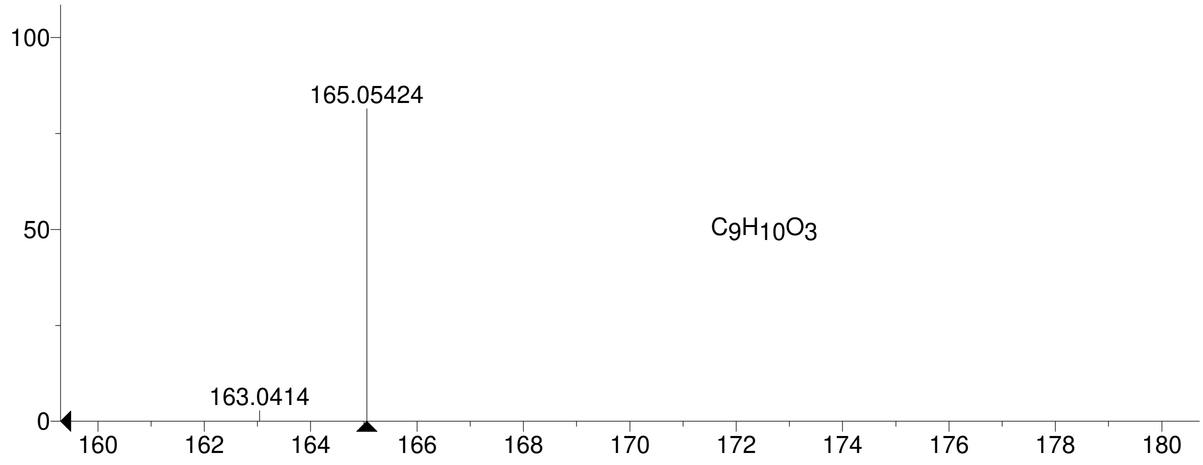

Hit 2 : 3-(3-Hydroxyphenyl)propionic acid [M-H]- 20-30-40%) P=165.1
C9H10O3; MF: 364; RMF: 999; Prob 2.07%; Lib: mona-export-fiehn_hilic; ID: 947.

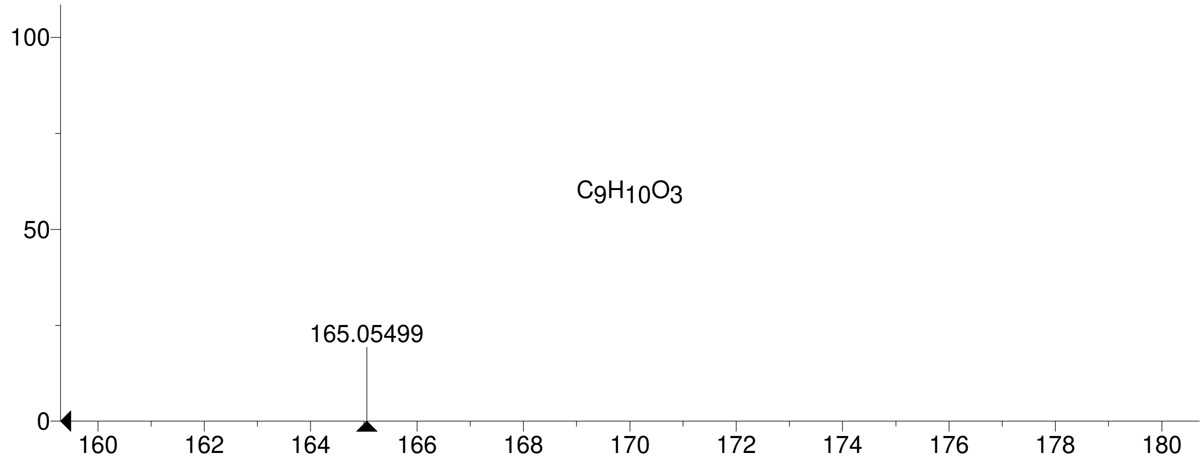

Hit 3 : 3-(2-Hydroxyphenyl)propanoic acid [M-H]- 35eV P=165.1
C9H10O3; MF: 364; RMF: 999; Prob 2.07%; Lib: mona-export-fiehn_hilic; ID: 2491.

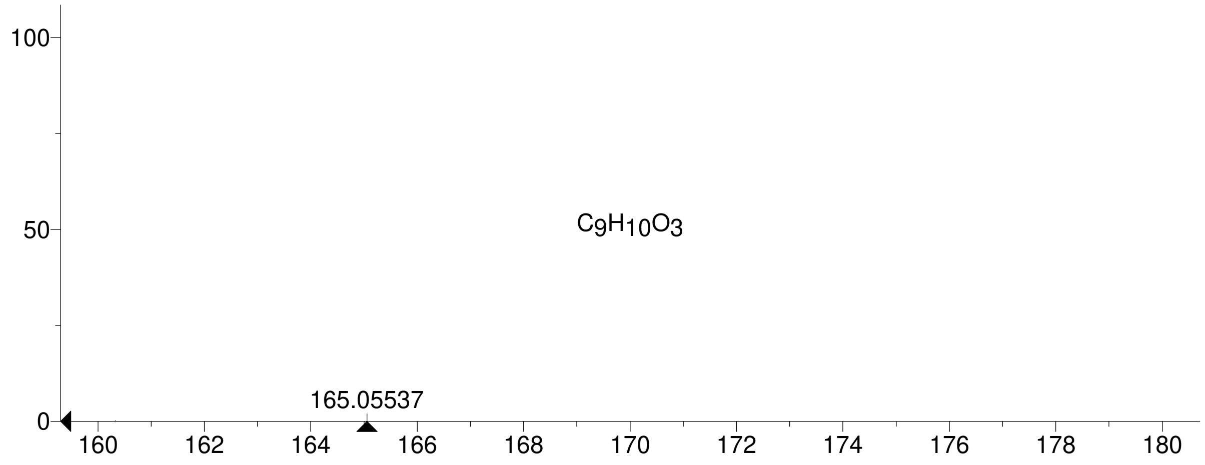

Hit 4 : 3-Phenyllactic acid [M-H]- 35eV P=165.1
C9H10O3; MF: 364; RMF: 999; Prob 2.07%; Lib: mona-export-fiehn_hilic; ID: 2516.

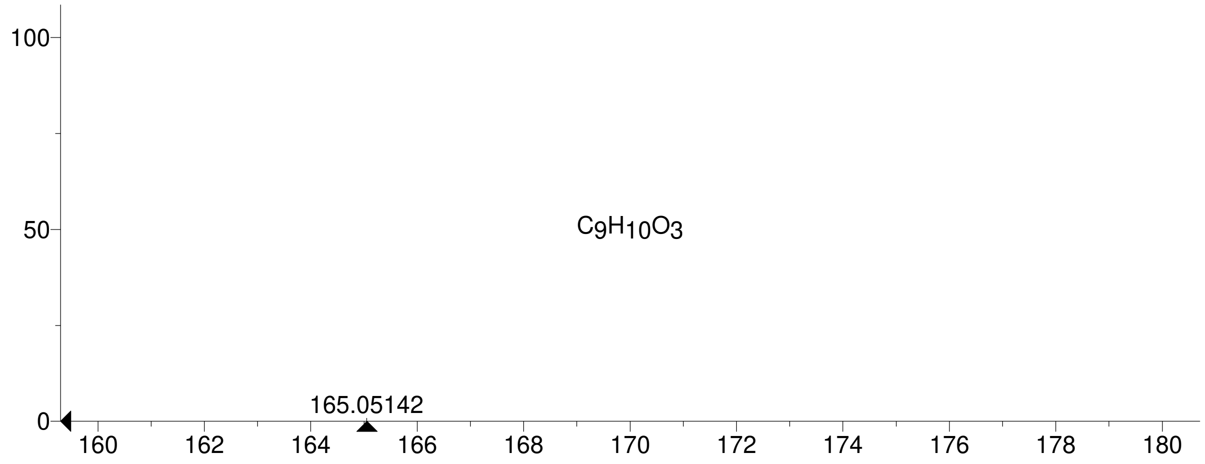

Hit 5 : p-coumaric acid [M+H]+ P=165.1
C9H8O3; MF: 364; RMF: 999; Prob 2.07%; Lib: mona-export-gnps; ID: 5826.

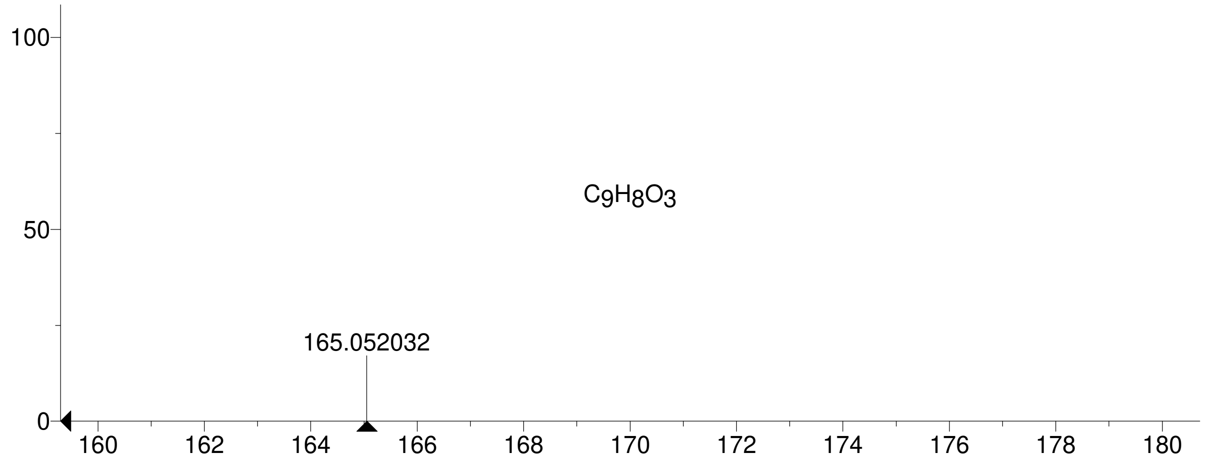

Unknown: Unknown (9.45_484.0206m/z)
Compound in Library Factor = 0

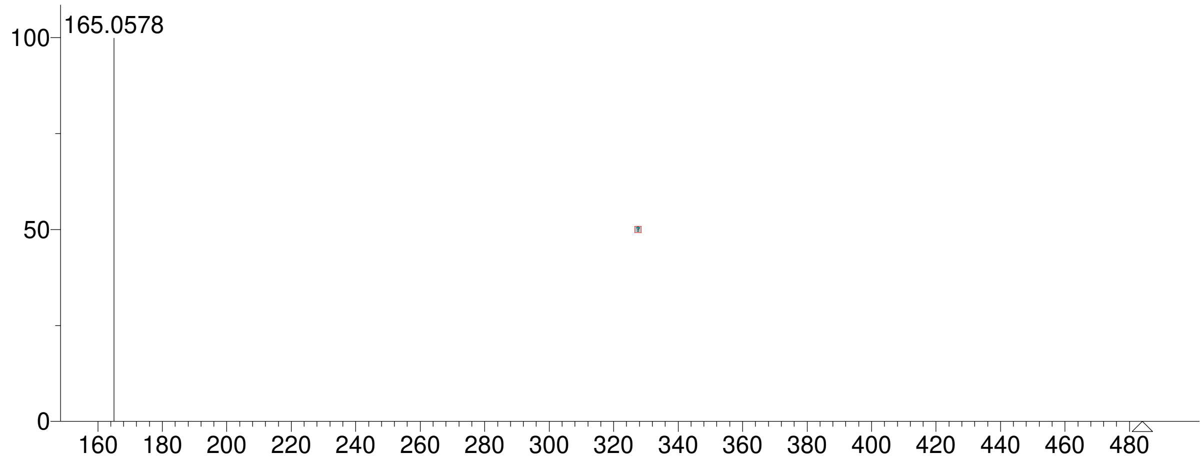

Unknown: Unknown (9.46_494.1676m/z)
Compound in Library Factor = 0

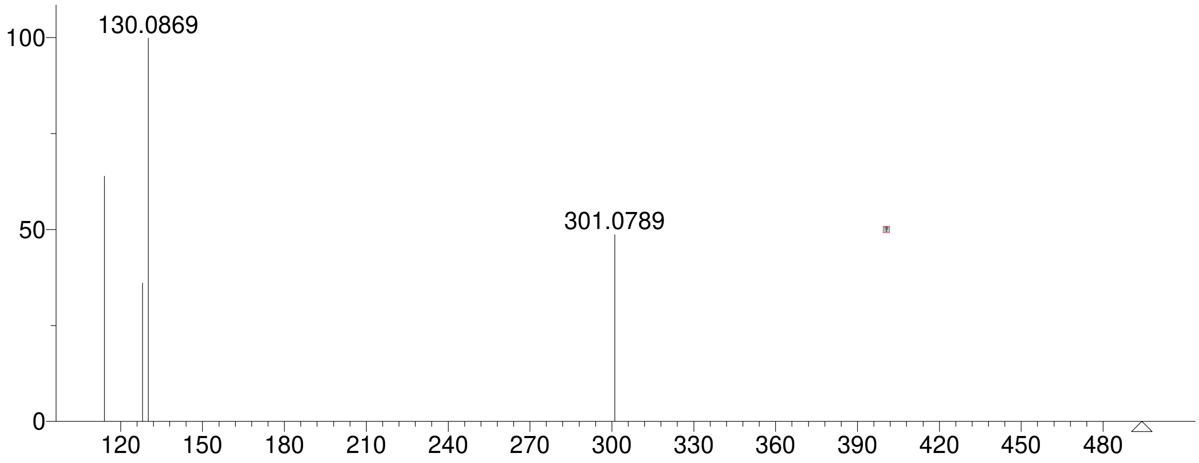

Unknown: Unknown (9.47_325.0910m/z)
Compound in Library Factor = 0

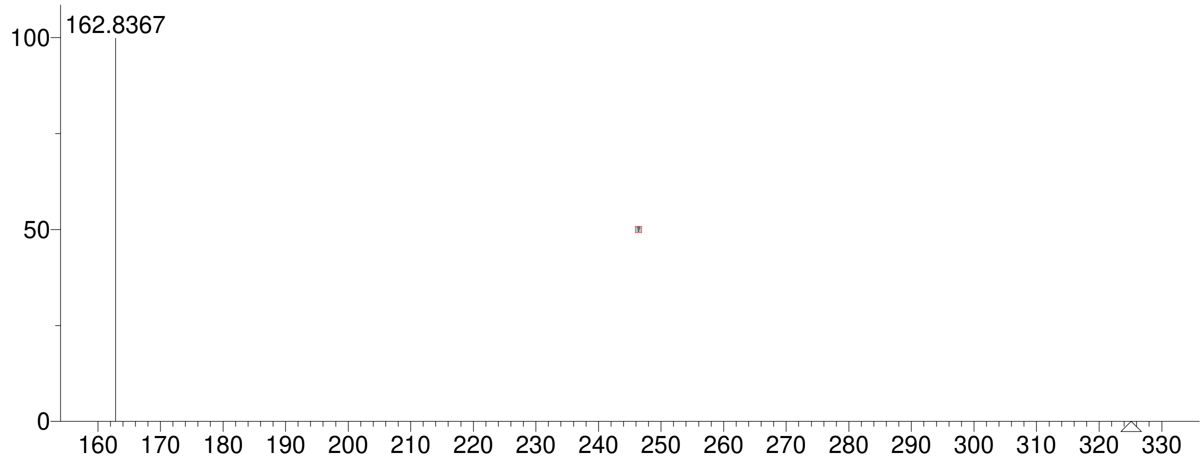

** Search Report Page 1 of 1 **

Unknown: Unknown (9.47_401.1908m/z)
Compound in Library Factor = 0

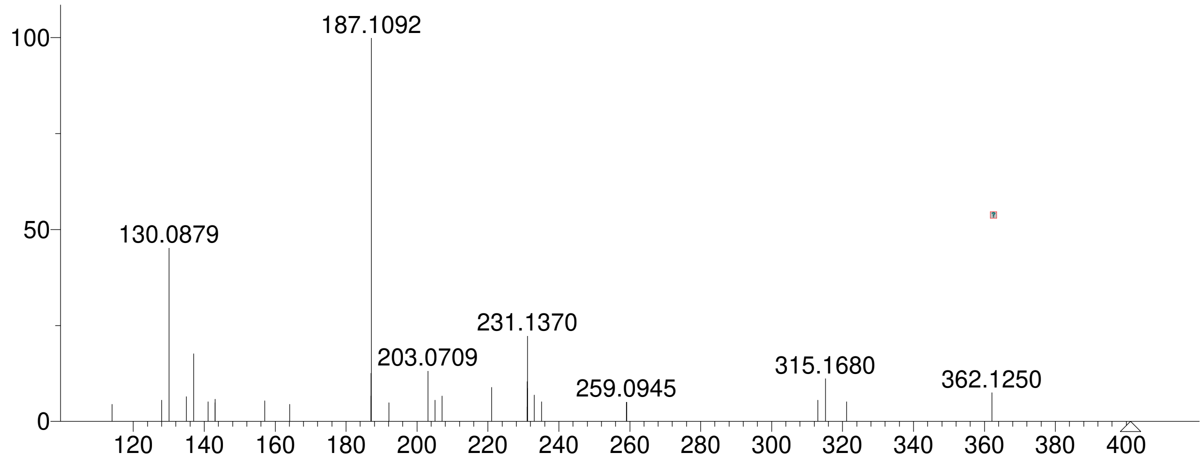

** Search Report Page 1 of 1 **

Unknown: Unknown (9.47_506.1770m/z)
Compound in Library Factor = 0

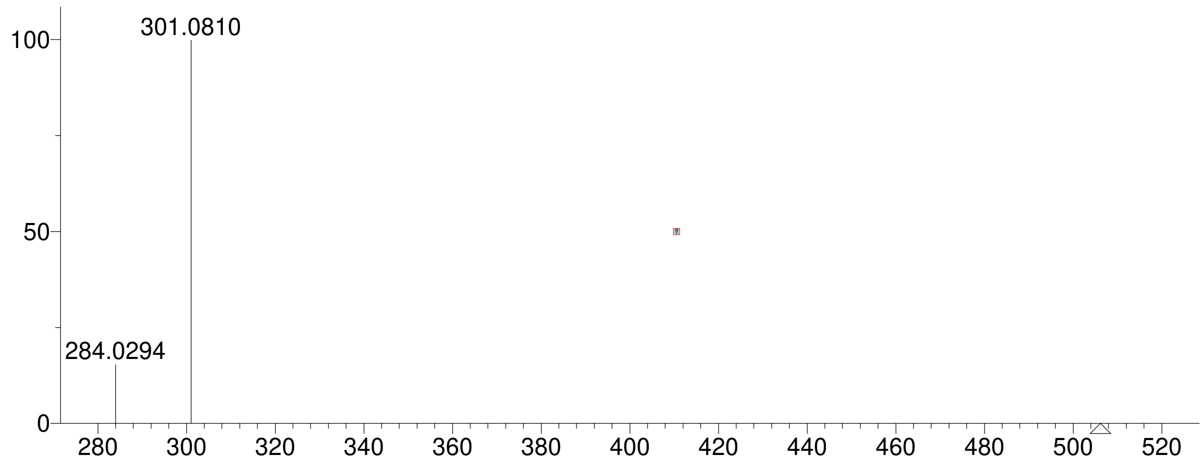

Unknown: Unknown (9.47_584.0141m/z)
Compound in Library Factor = 0

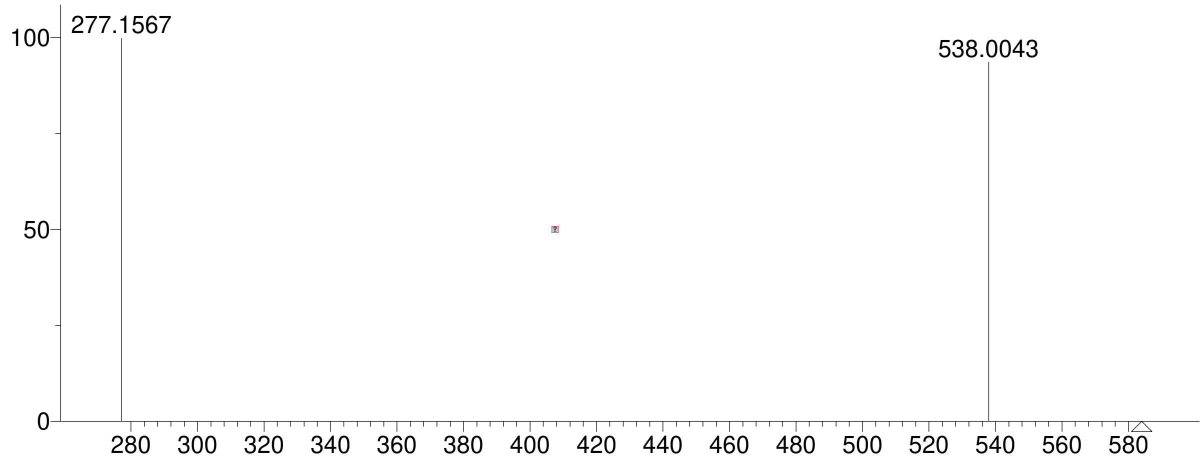

Unknown: Unknown (9.49_409.9693m/z)
Compound in Library Factor = 0

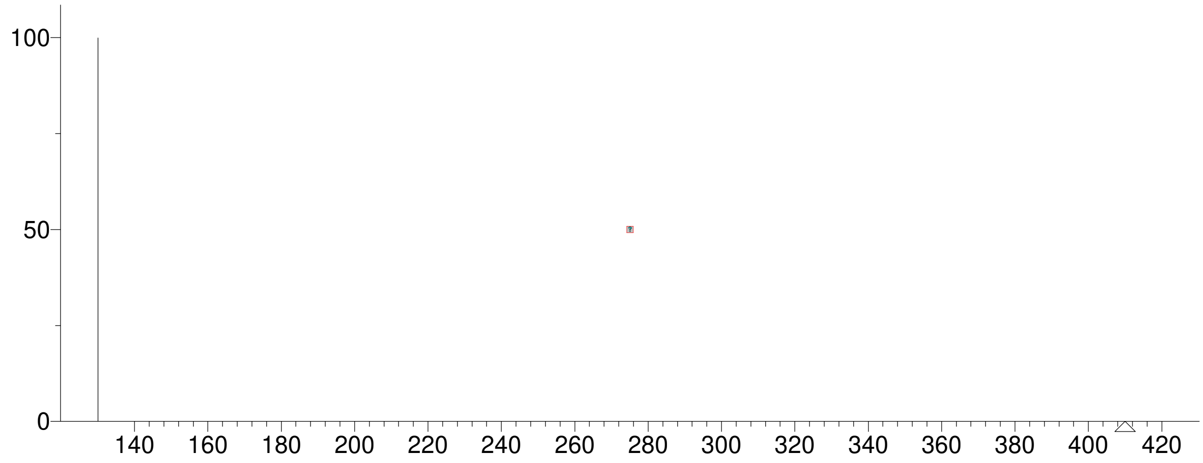

Unknown: Unknown (9.49_415.2064m/z)
Compound in Library Factor = 0

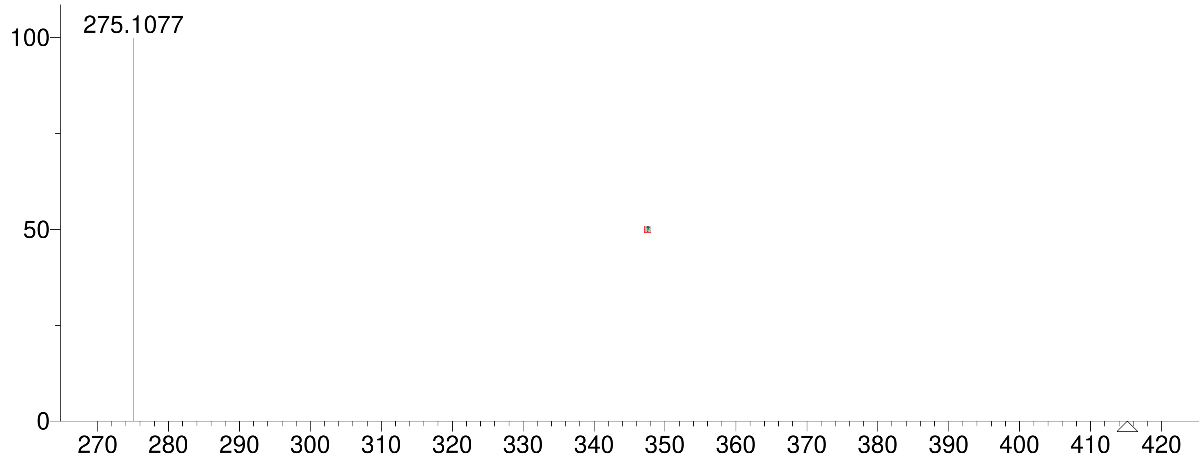

Unknown: Unknown (9.50_437.9407m/z)
Compound in Library Factor = 0

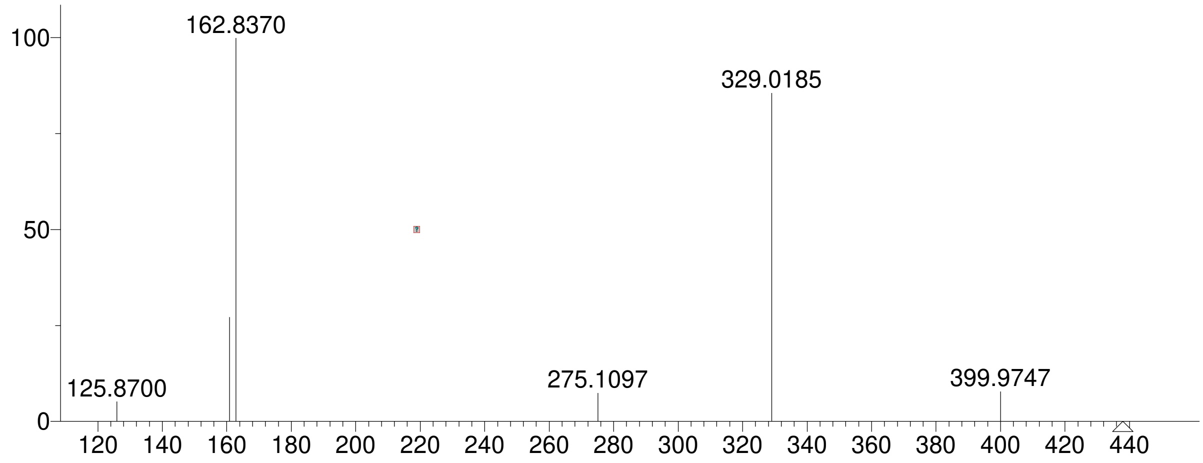

Unknown: Unknown (9.51_275.1030m/z)
Compound in Library Factor = 114

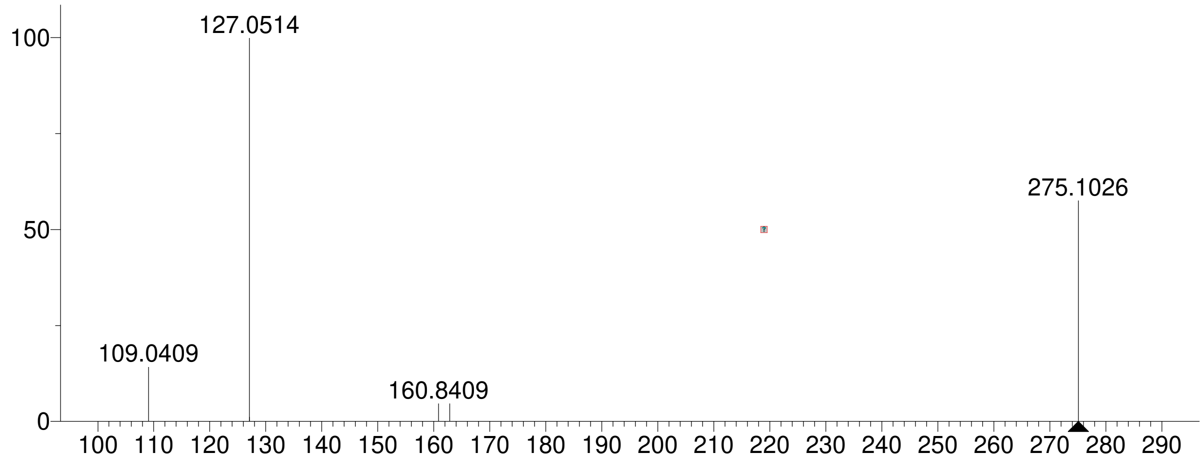

Hit 1 : Unknown (carbon number 14); PlaSMA ID-486 [M-H]- P=275.1
C14H16N2O4; MF: 696; RMF: 952; Prob 99.0%; Lib: biomsms-neg-plasma; ID: 1148.

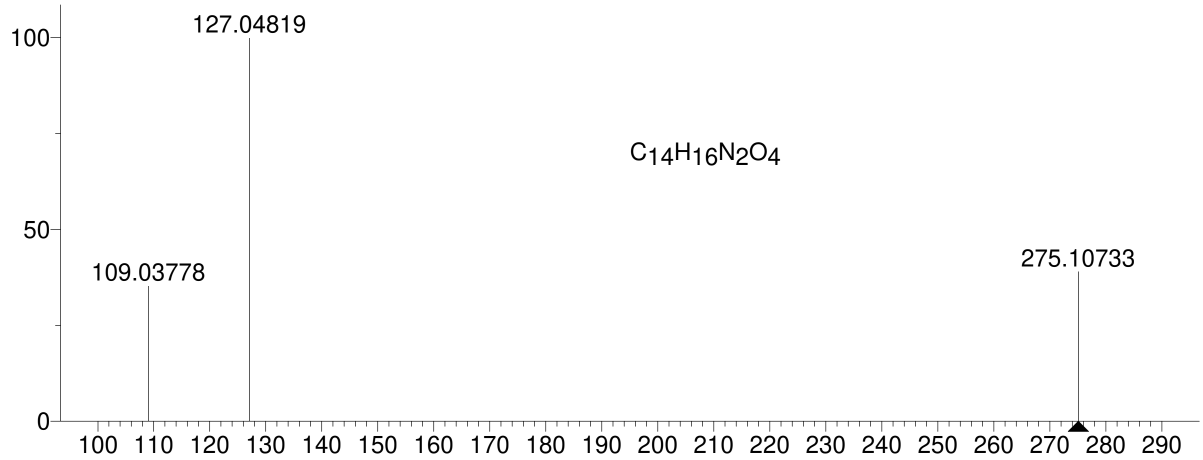

Hit 2 : Unknown (carbon number 14); PlaSMA ID-487 [M-H]- P=275.1
C14H16N2O4; MF: 399; RMF: 862; Prob 0.98%; Lib: biomsms-neg-plasma; ID: 1149.

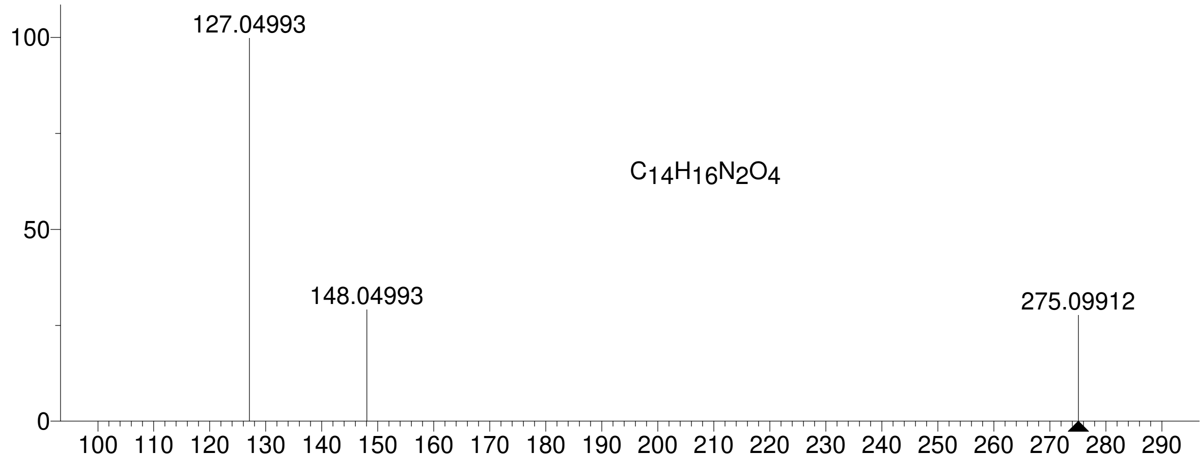

Hit 3 : PRI_275.1026_12.7 [M+H]+ 15(no P=275.1
; MF: 63; RMF: 535; Prob 0.00%; Lib: mona-export-massbank_eu; ID: 429.

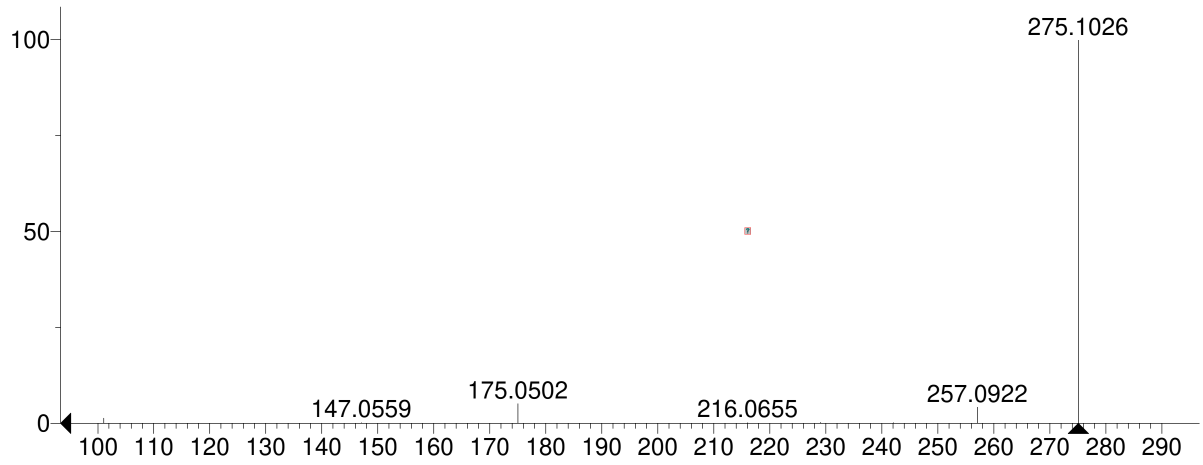

Hit 4 : Trigonelline [2M+H]+ HCD 1V P=275.1
C7H7NO2; MF: 43; RMF: 536; Prob 0.00%; CAS: 535-83-1; Lib: nist_msms; ID: 118719.

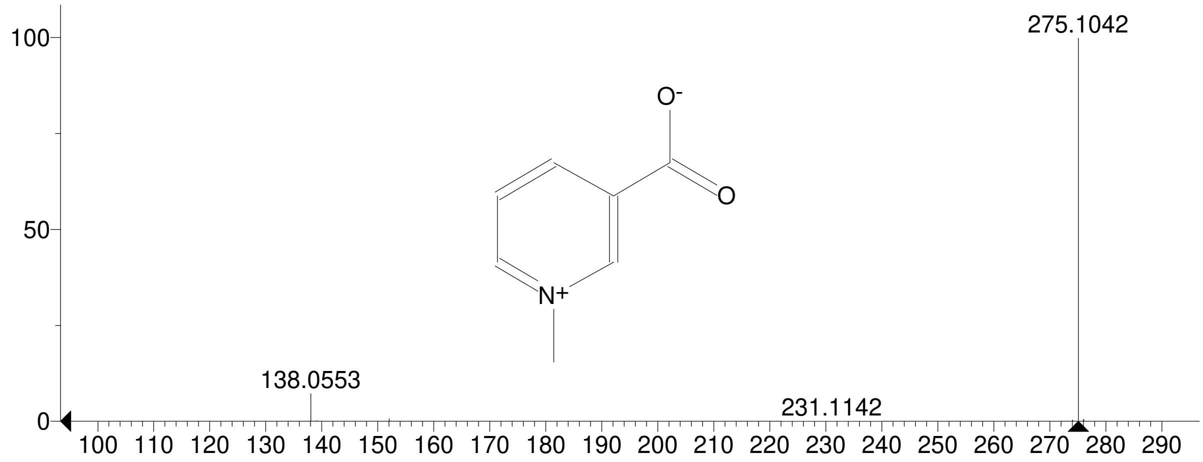

Hit 5 : NCGC00381382-01_C14H14N2O4_Cyclohepta[b]pyrrole-3-propanoic acid, alpha-(acetylamino)-1,4-dihyd
C14H14N2O4; MF: 0; RMF: 30; Prob 0.00%; Lib: mona-export-gnps; ID: 12062.

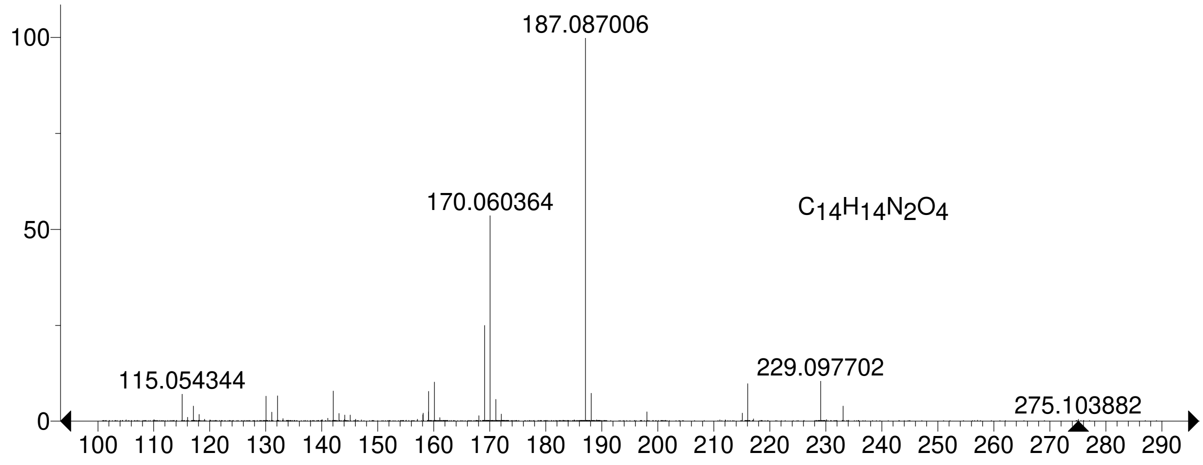

Unknown: Unknown (9.51_548.2359m/z)
Compound in Library Factor = -3273

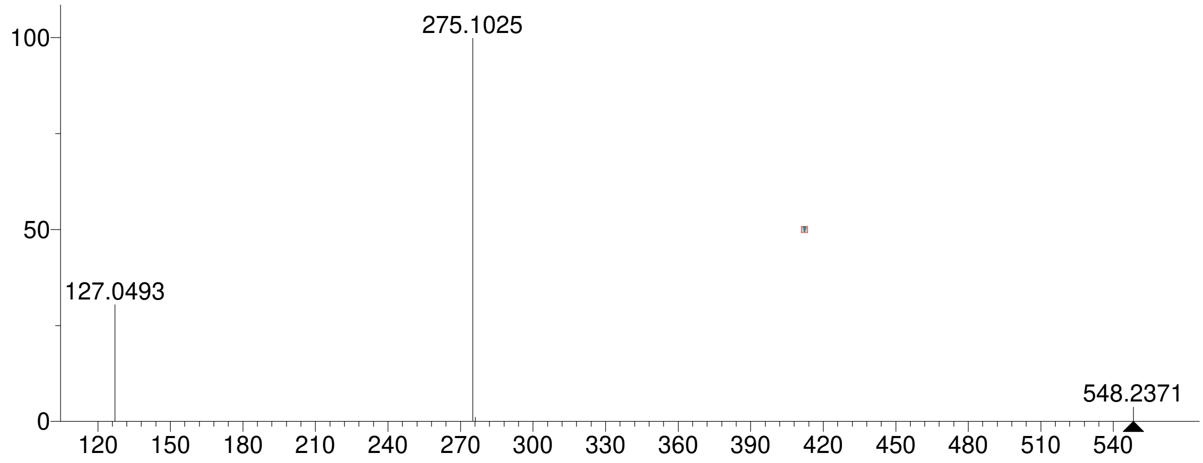

Hit 1 : Virginiamycin component b [M+Na]+ HCD 32V P=548.2
C28H35N3O7; MF: 5; RMF: 152; Prob 55.4%; Lib: nist_msms; ID: 353177.

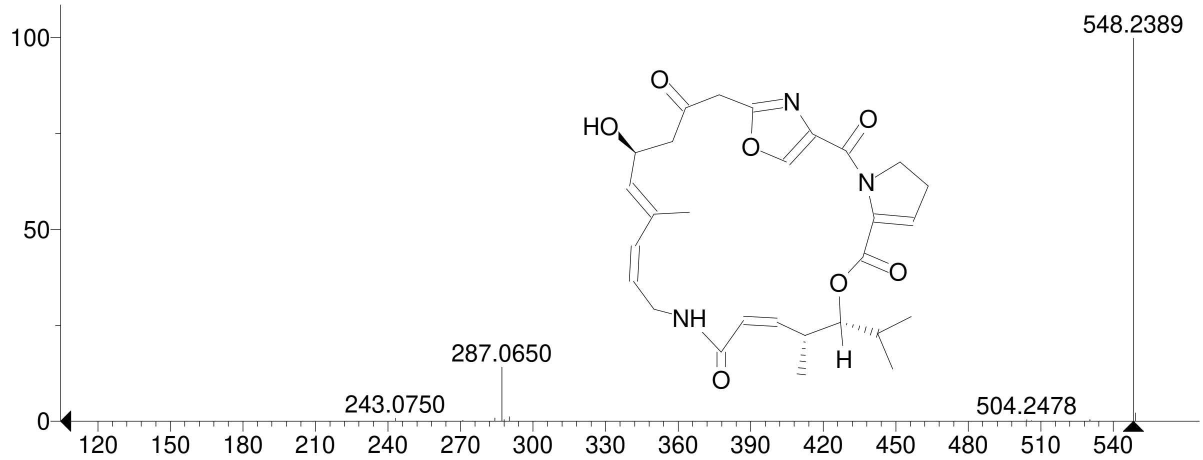

Hit 2 : Tobramycin, inactivated by 2'' phosphorylation [M+H]+ P=548.2
C18H38N5O12P; MF: 0; RMF: 12; Prob 44.6%; Lib: anthocyanins mona; ID: 9671.

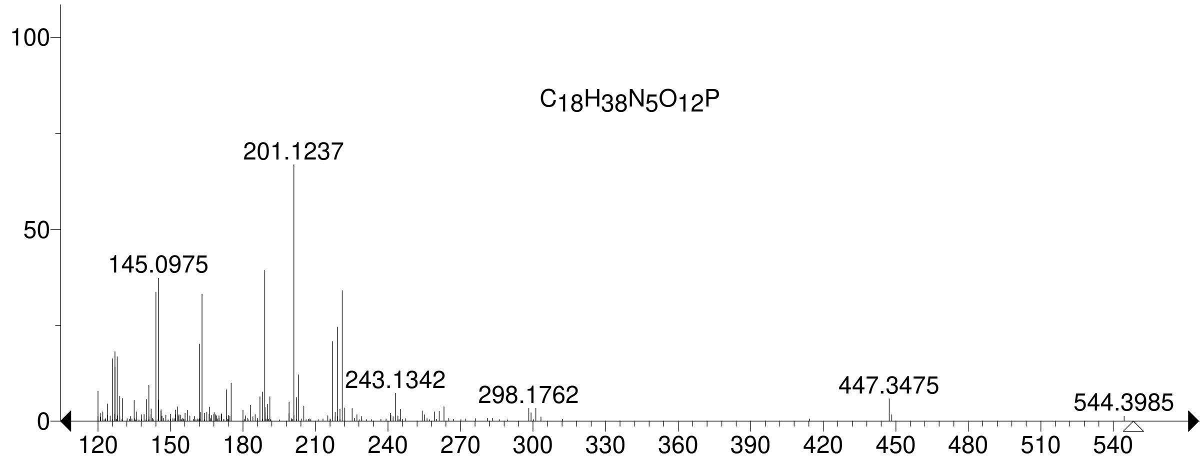

Unknown: Unknown (9.52_435.9433m/z)
Compound in Library Factor = 0

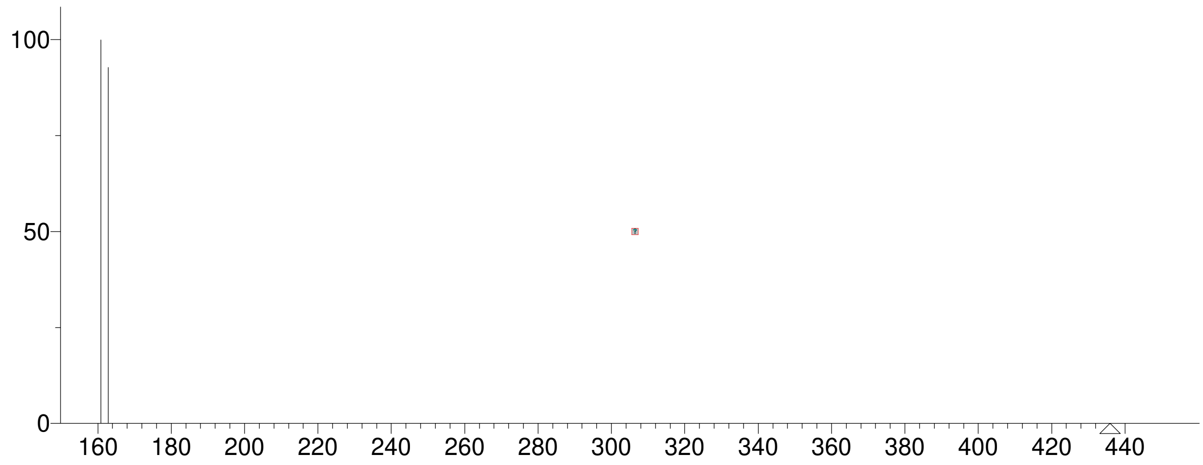

** Search Report Page 1 of 1 **

Unknown: Unknown (9.53_375.0270m/z)
Compound in Library Factor = 0

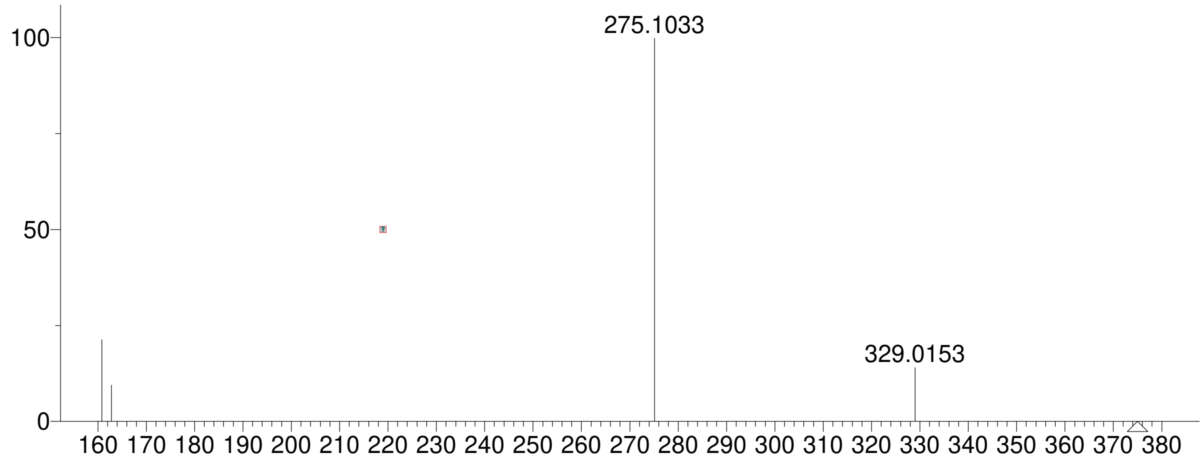

Unknown: Unknown (9.55_425.1442m/z)
Compound in Library Factor = -3273

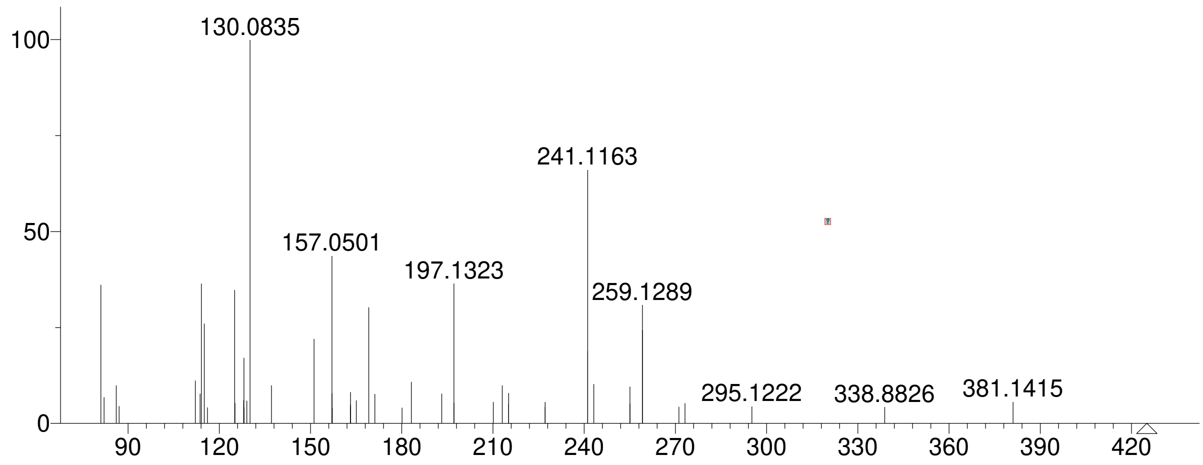

Hit 1 : Ginkgolide B [M+H]+ HCD 51V P=425.1
C20H24O10; MF: 8; RMF: 161; Prob 12.8%; CAS: 15291-77-7; Lib: nist_msms; ID: 352890.

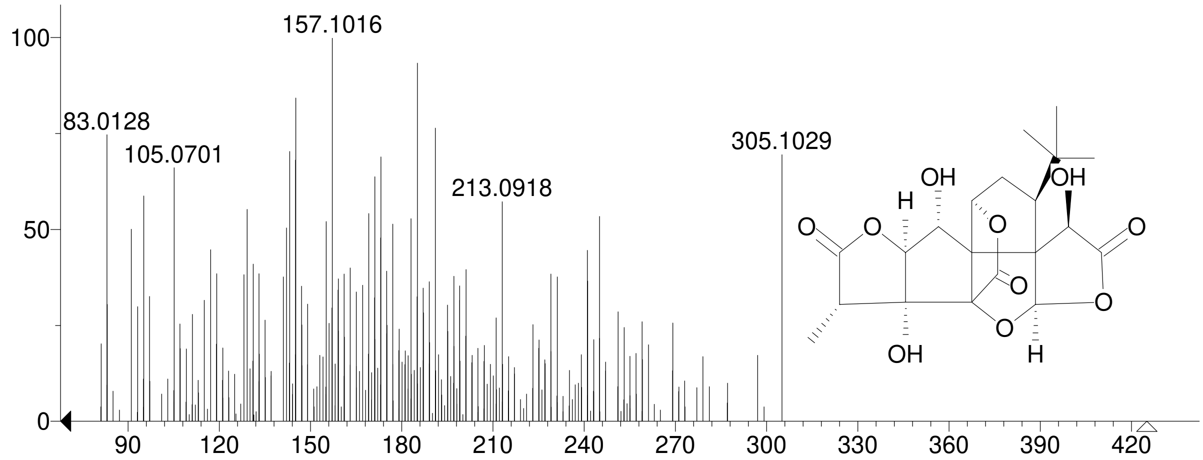

Hit 2 : ginkgolide B [M+H]+ 45HCD P=425.1
C20H24O10; MF: 7; RMF: 150; Prob 12.3%; Lib: mona-export-vf-npl_qexactive; ID: 5951.

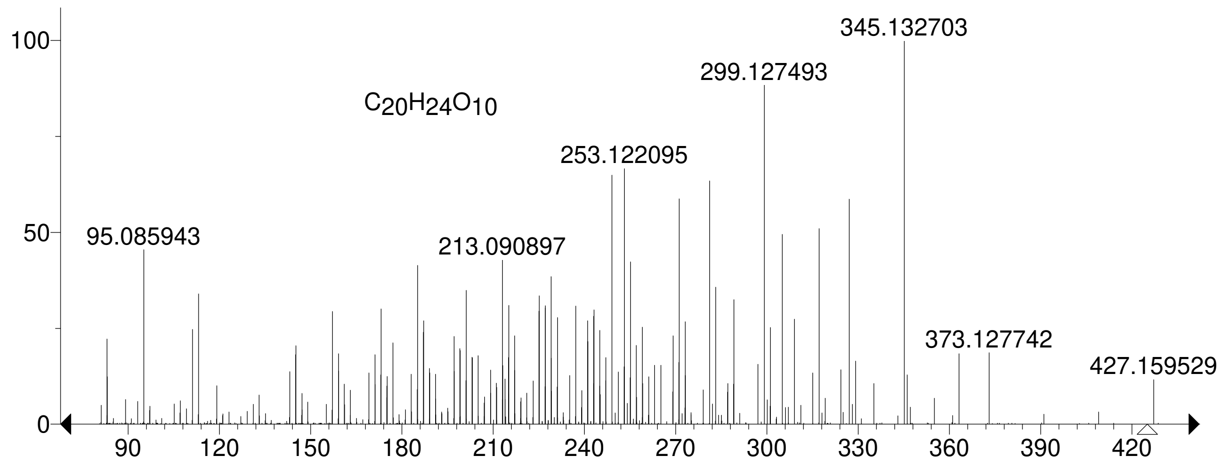

Hit 3 : [(2R,3S,4S,5R,6S)-3,4,5-trihydroxy-6-[(E)-3-phenylprop-2-enoyl]oxyoxan-2-yl]methyl 3,4-dihydroxy-2-methy
C20H24O10; MF: 1; RMF: 46; Prob 9.68%; Lib: mona-export-vf-npl_qexactive; ID: 9484.

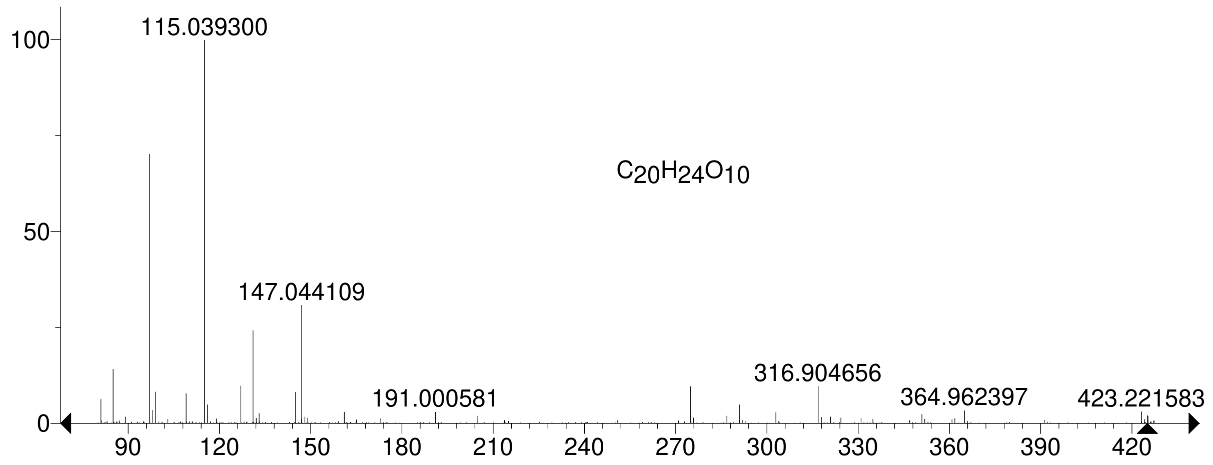

Hit 4 : NCGC00381128-01_C18H26O10_4-(beta-D-Glucopyranosyloxy)benzyl 2,3-dihydroxy-3-methylbutanoate
C18H26O10; MF: 0; RMF: 102; Prob 9.31%; Lib: mona-export-gnps; ID: 11687.

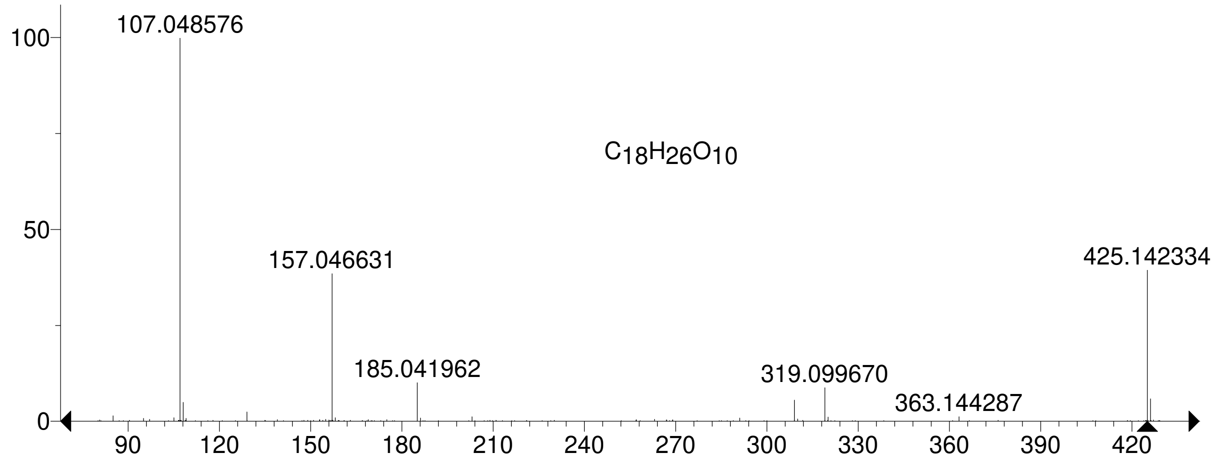

Hit 5 : NCGC00380741-01_C20H24O10_6-O-(3,4-Dihydroxy-2-methylenebutanoyl)-1-O-[(2E)-3-phenyl-2-propen
C20H24O10; MF: 0; RMF: 49; Prob 9.31%; Lib: mona-export-gnps; ID: 11636.

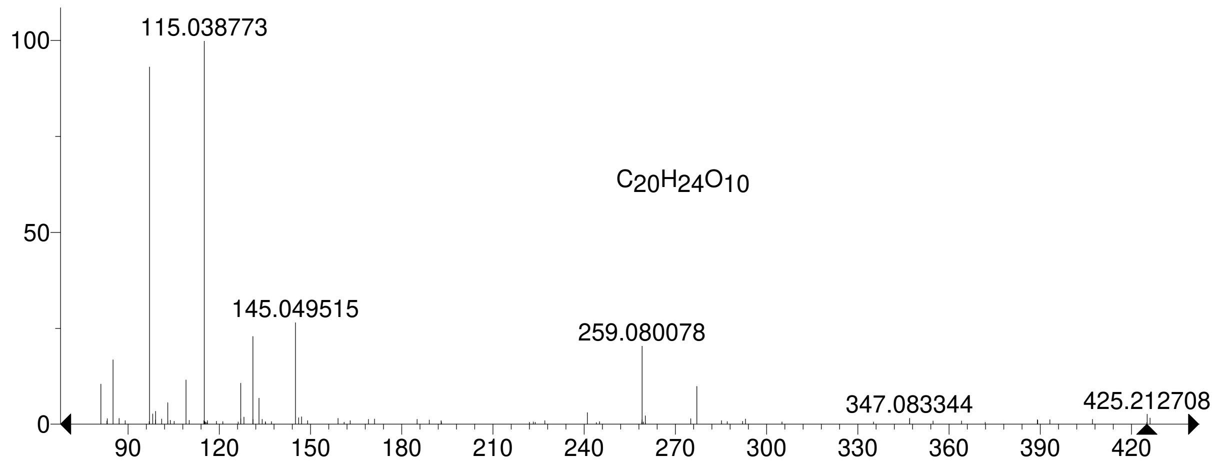

Unknown: Unknown (9.55_429.1868m/z)
Compound in Library Factor = 0

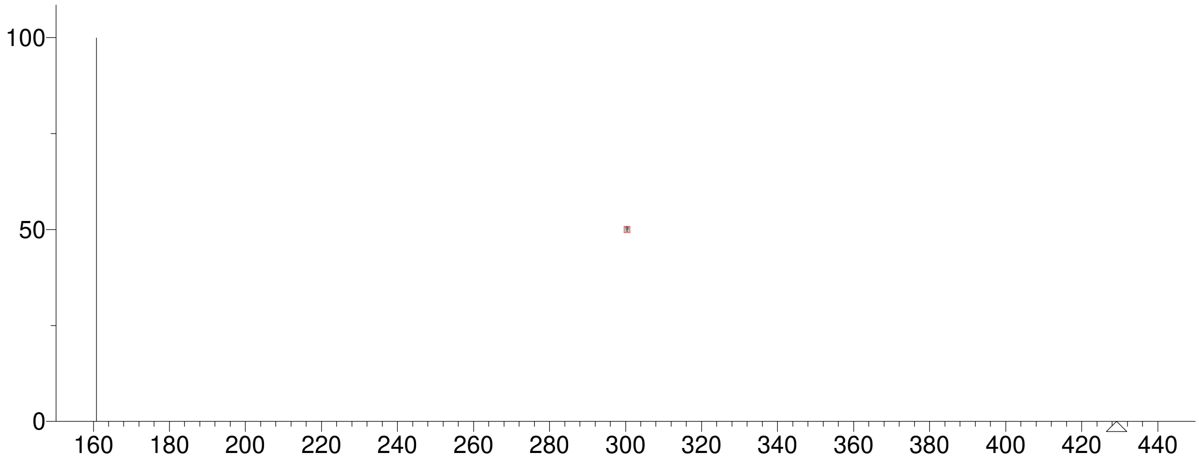

Unknown: Unknown (9.55_534.2213m/z)
Compound in Library Factor = 0

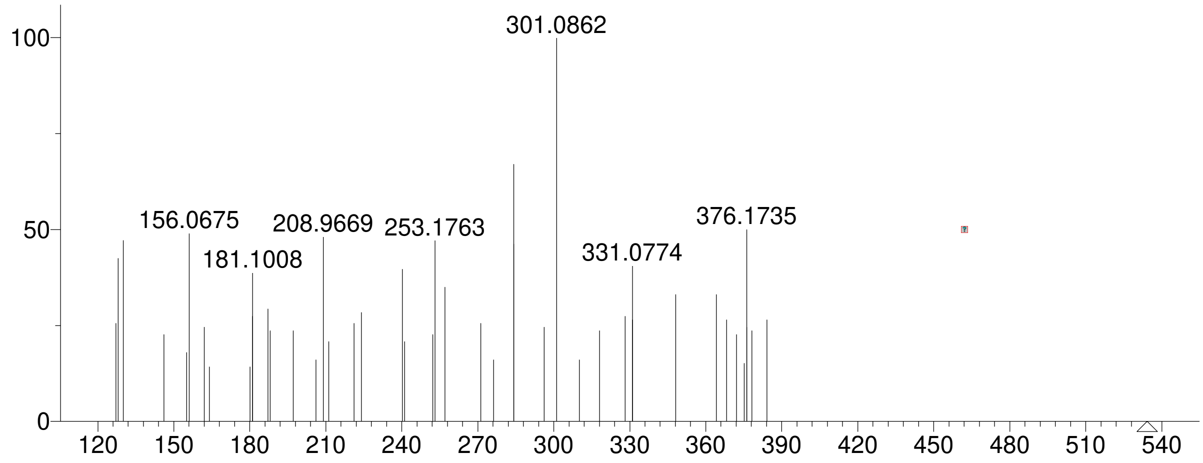

Unknown: Unknown (9.57_358.1957m/z)
Compound in Library Factor = 0

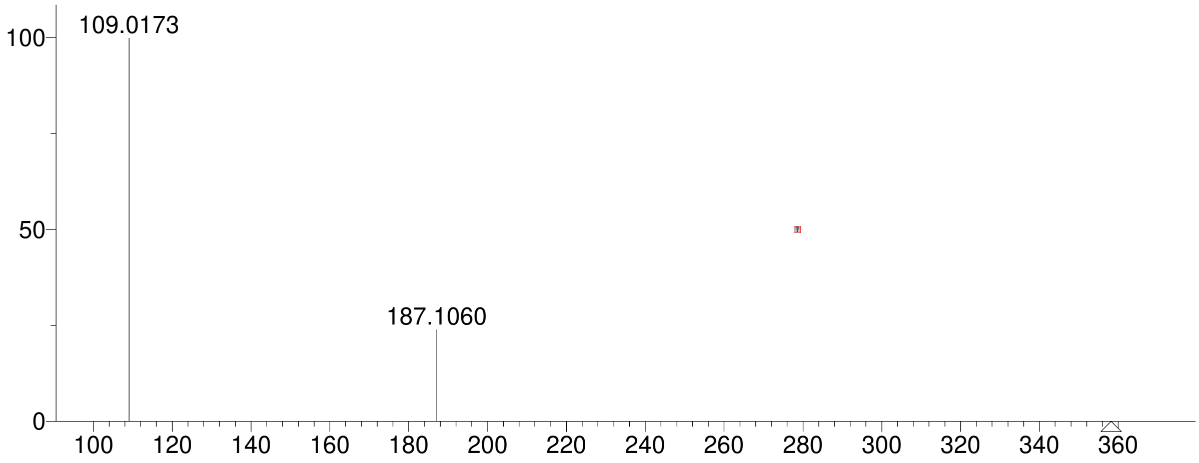

Unknown: Unknown (9.58_447.1865m/z)
Compound in Library Factor = -264

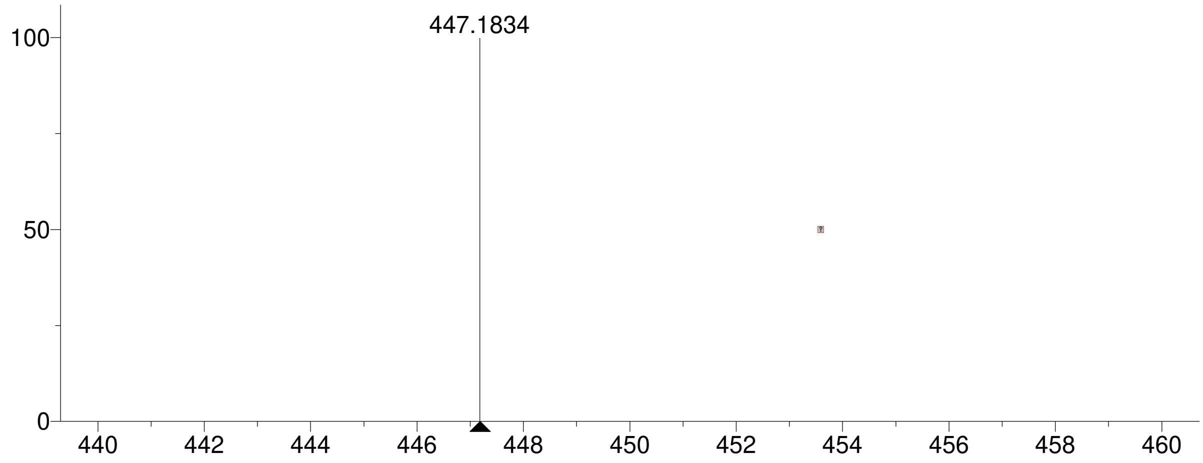

Hit 1 : 21-Carboxylic acid triamcinolone acetonide [M-H]- HCD 40V P=447.2
C24H29FO7; MF: 364; RMF: 999; Prob 92.2%; CAS: 53962-41-7; Lib: nist_msms; ID: 449262.

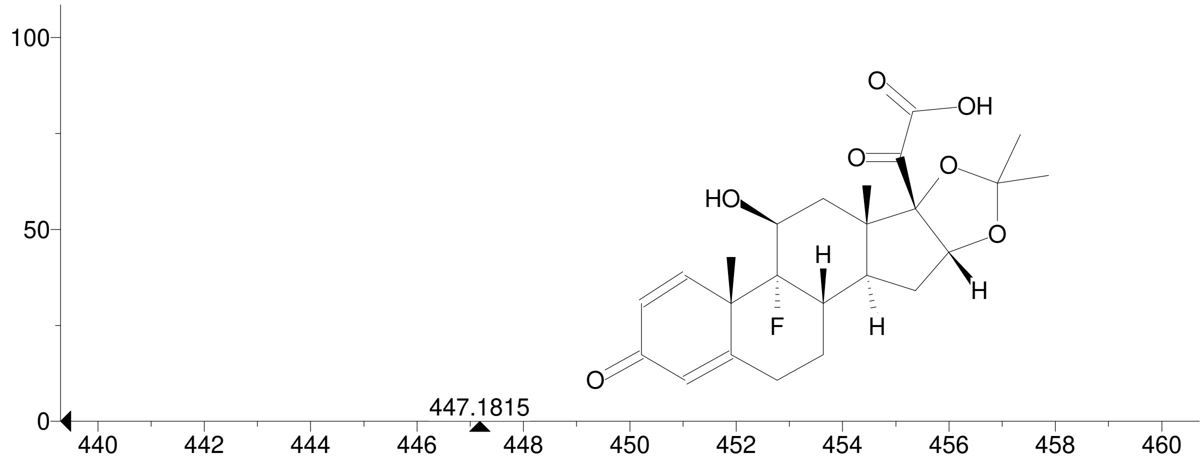

Hit 2 : [(2R,3R,4S,5S,6R)-2-(acetyloxymethyl)-3,5-dihydroxy-6-[(2S,3R)-2,3,4-trihydroxybutoxy]oxan-4-yl] hexanoa
C18H32O11; MF: 257; RMF: 906; Prob 6.12%; Lib: anthocyanins mona; ID: 5054.

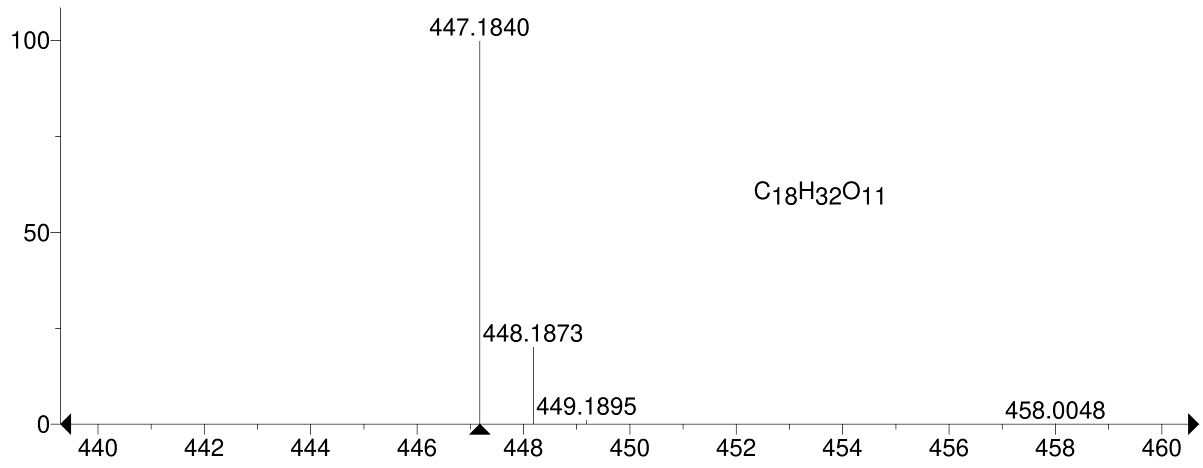

Hit 3 : NCGC00381210-01_C18H32O11_(2E)-4-Hydroxy-3,4-dimethyl-2-penten-1-yl 6-O-[(2S,3R,4R)-3,4-dihydro
C18H32O11; MF: 227; RMF: 925; Prob 1.70%; Lib: mona-export-gnps; ID: 12907.

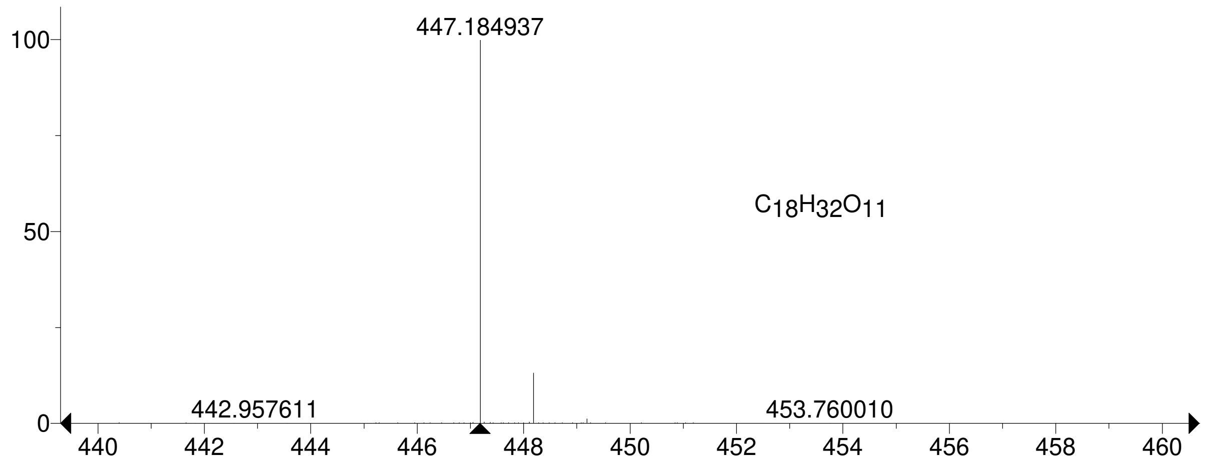

Unknown: Unknown (9.59_525.2660m/z)
Compound in Library Factor = -3273

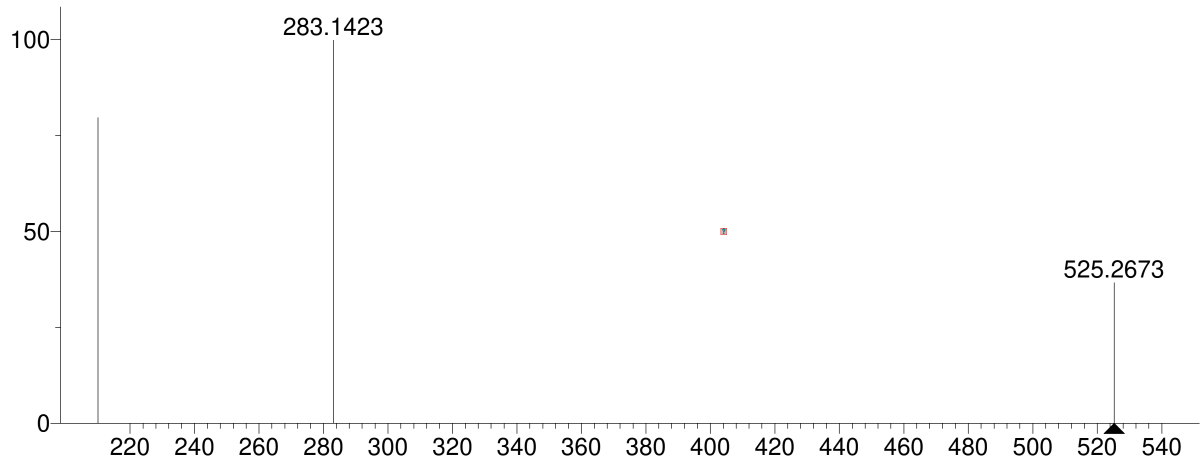

Hit 1 : PA 24:5; PA 2:0-22:5; [M-H]- [M-H]- P=525.3
C27H43O8P; MF: 6; RMF: 199; Prob 26.5%; Lib: msdial-insilicomsms-lipids-neg; ID: 315724.

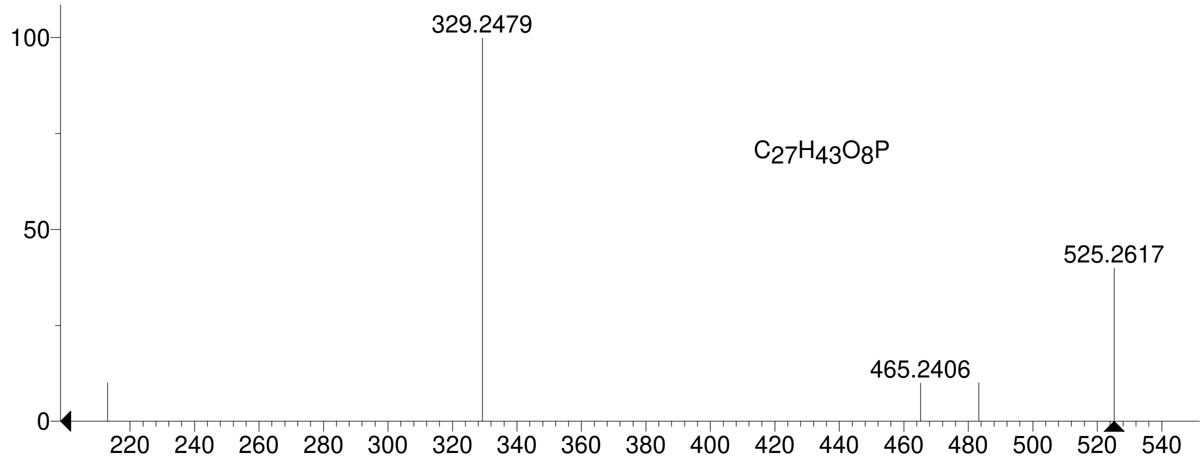

Hit 2 : PA 24:5; PA 4:0-20:5; [M-H]- [M-H]- P=525.3
C27H43O8P; MF: 5; RMF: 194; Prob 25.5%; Lib: msdial-insilicomsms-lipids-neg; ID: 315834.

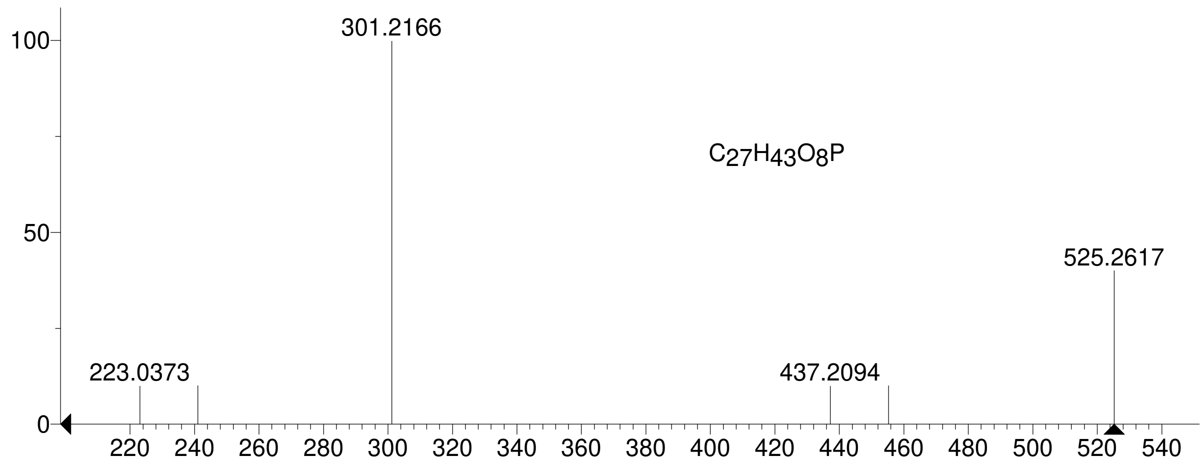

Hit 3 : PA 24:5; PA 6:0-18:5; [M-H]- [M-H]- P=525.3
C27H43O8P; MF: 5; RMF: 194; Prob 25.5%; Lib: msdial-insilicomsms-lipids-neg; ID: 315940.

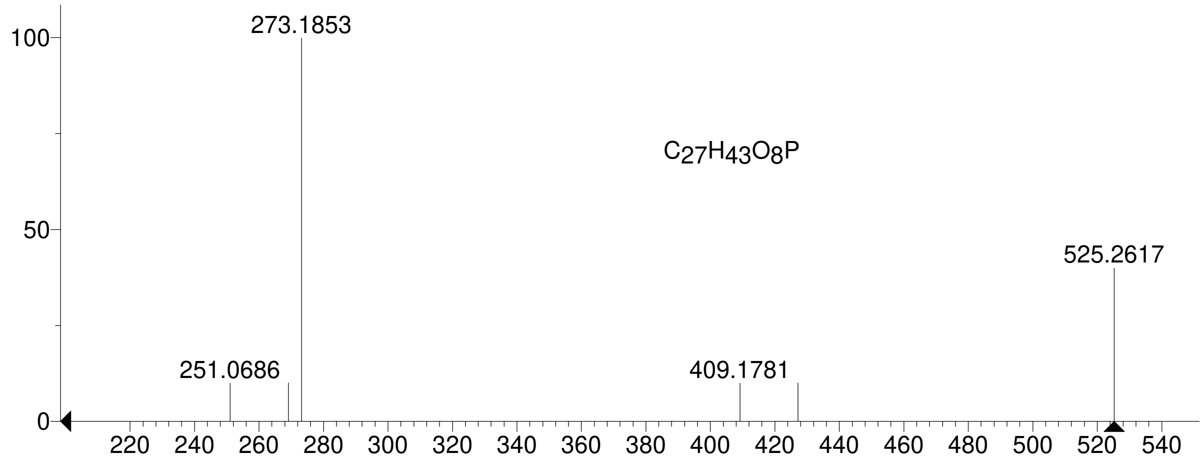

Hit 4 : FMRF Amide-Like Peptide I, lobster [M-2H]2- HCD 18eV P=525.3
C47H72N16O12; MF: 2; RMF: 284; Prob 22.5%; Lib: nist_msms2; ID: 79285.

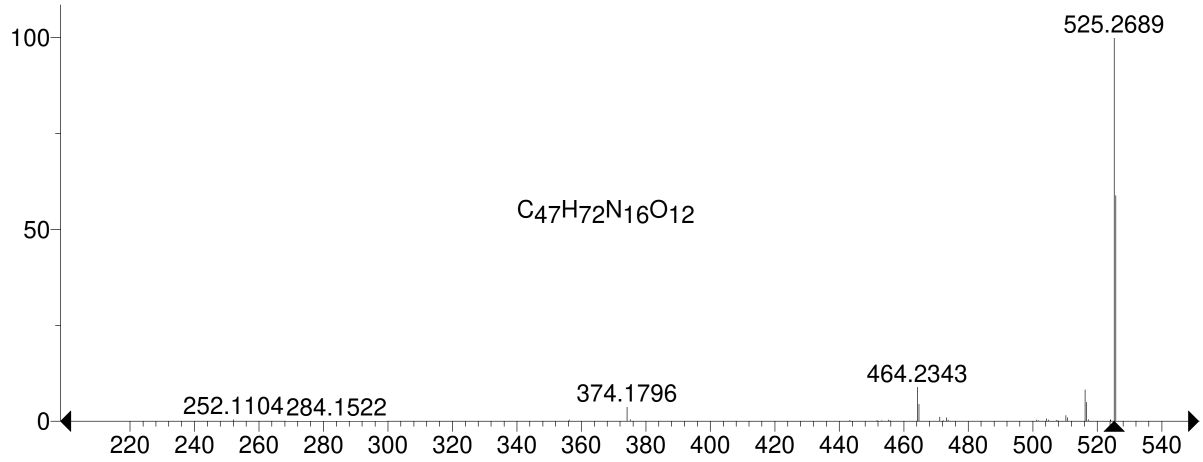

Unknown: Unknown (9.61_277.1547m/z)
Compound in Library Factor = -216

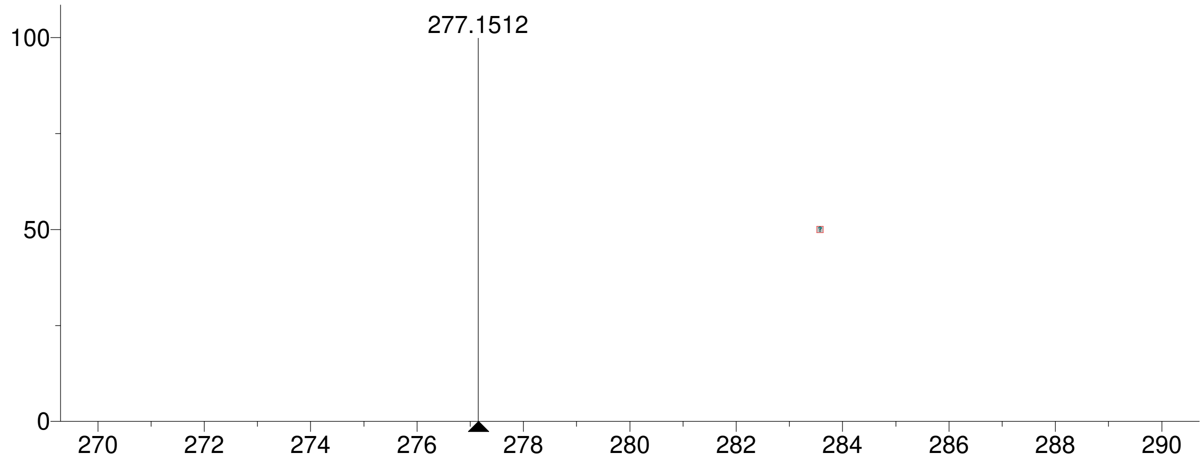

Hit 1 : Phe-Leu [M-H]- HCD 24eV P=277.2
C15H22N2O3; MF: 364; RMF: 999; Prob 12.5%; Lib: nist_msms; ID: 570091.

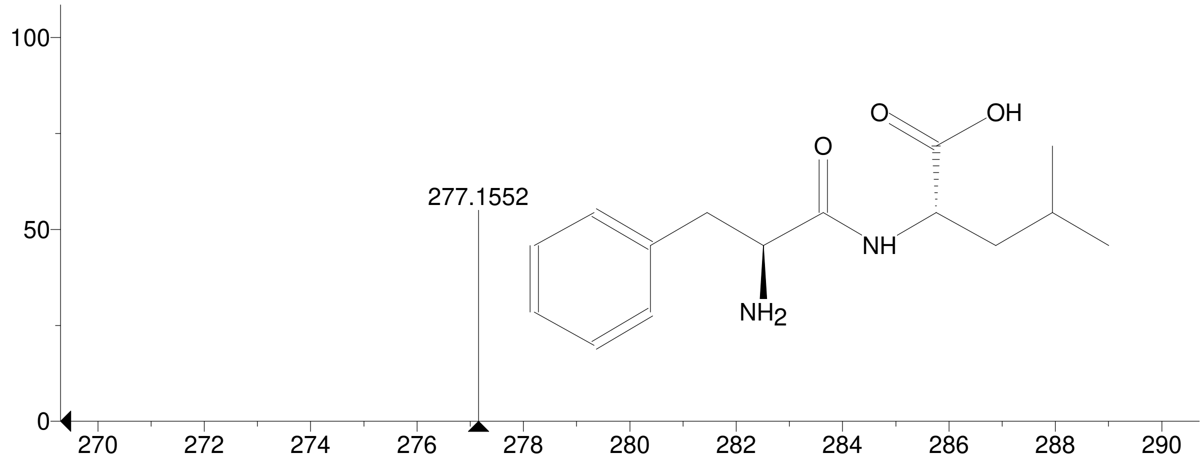

Hit 2 : Ile-Phe [M-H]- HCD 20eV P=277.2
C15H22N2O3; MF: 364; RMF: 999; Prob 12.5%; Lib: nist_msms; ID: 570144.

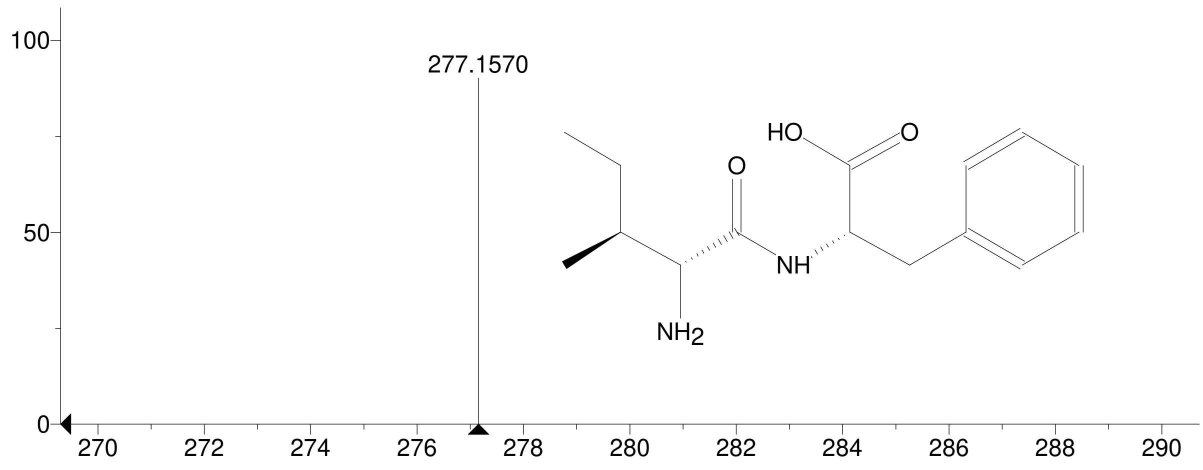

Hit 3 : Leu-Phe [M-H]- HCD 24eV P=277.2
C15H22N2O3; MF: 364; RMF: 999; Prob 12.5%; Lib: nist_msms; ID: 570205.

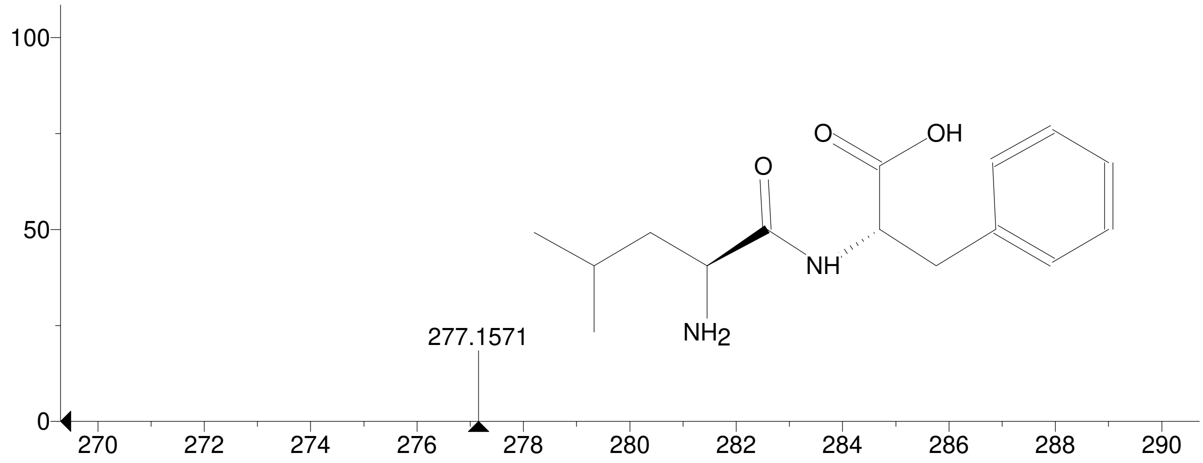

Hit 4 : Phe-Ile [M-H]- HCD 36eV P=277.2
C15H22N2O3; MF: 364; RMF: 999; Prob 12.5%; Lib: nist_msms; ID: 570606.

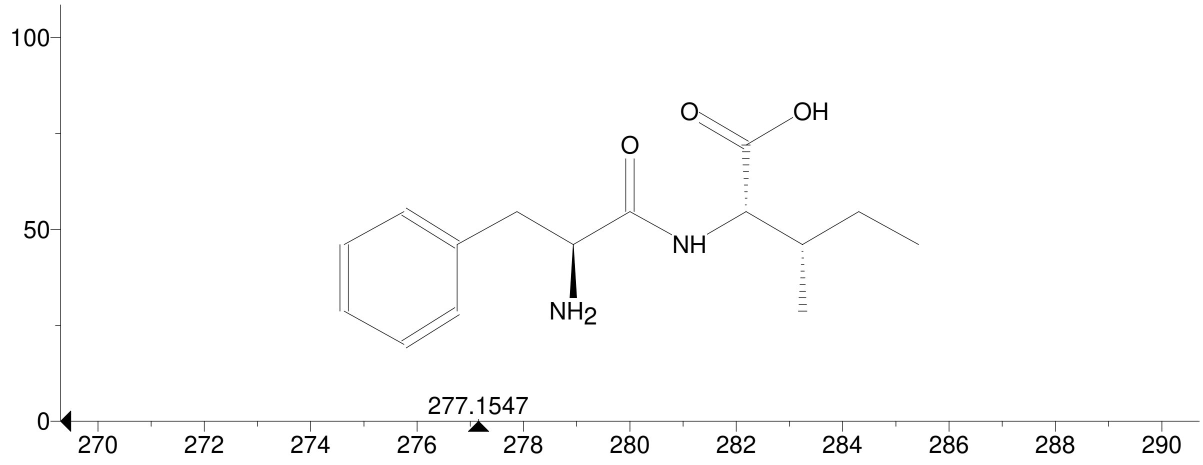

Hit 5 : Phe-Leu [M-H]- HCD 24eV P=277.2
C15H22N2O3; MF: 364; RMF: 999; Prob 12.5%; CAS: 3303-55-7; Lib: nist_msms2; ID: 83817.

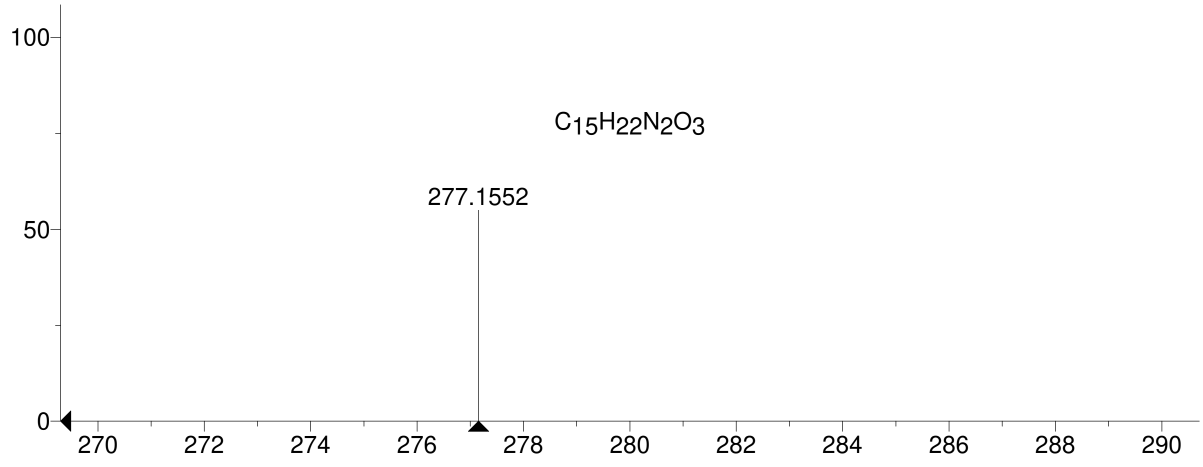

Unknown: Unknown (9.61_289.0009m/z)
Compound in Library Factor = 0

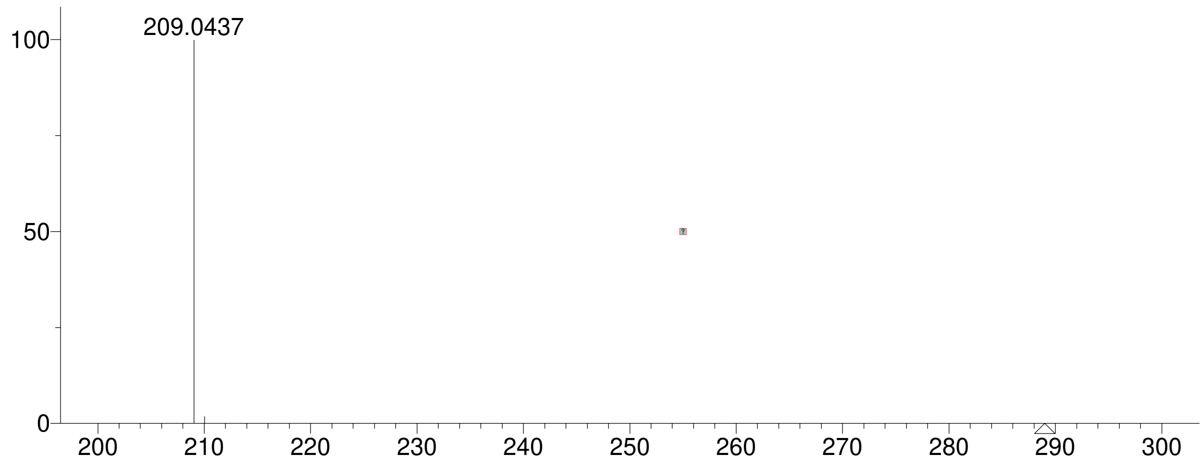

Unknown: Unknown (9.64_417.0841m/z)
Compound in Library Factor = 0

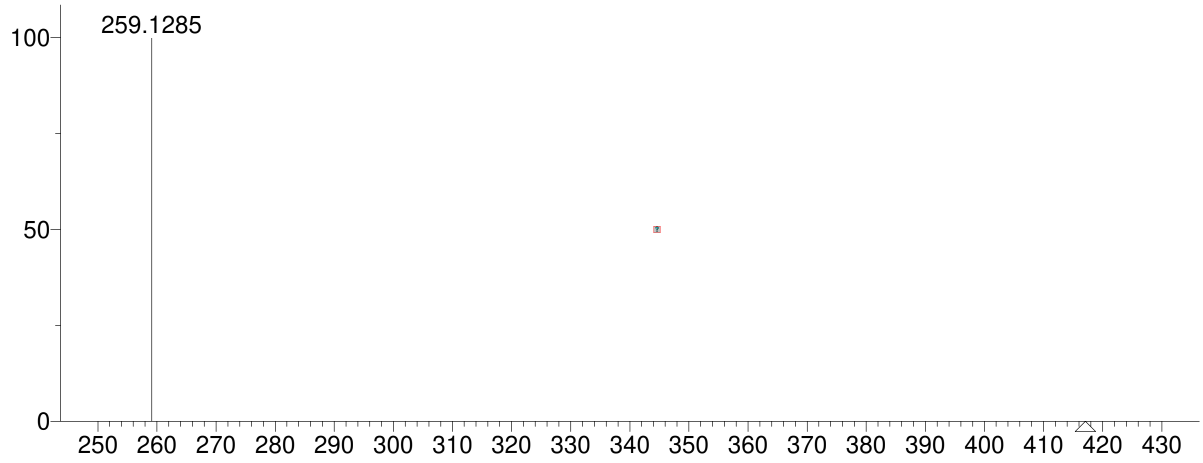

** Search Report Page 1 of 1 **

Unknown: Unknown (9.66_429.1860m/z)
Compound in Library Factor = 0

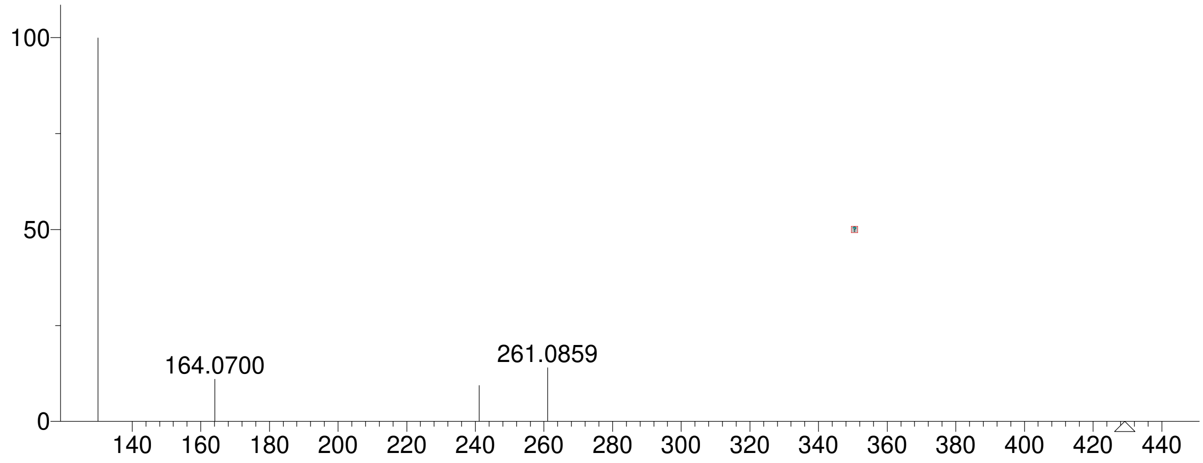

Hit 1 : (2R,3R,4S,5S,6R)-2-[6-hydroxy-3-[(E)-3-hydroxybut-1-enyl]-2,4,4-trimethylcyclohexyl]oxy-6-(hydroxymethy
C19H34O8; MF: 0; RMF: 7; Prob 100.0%; Lib: anthocyanins mona; ID: 9963.

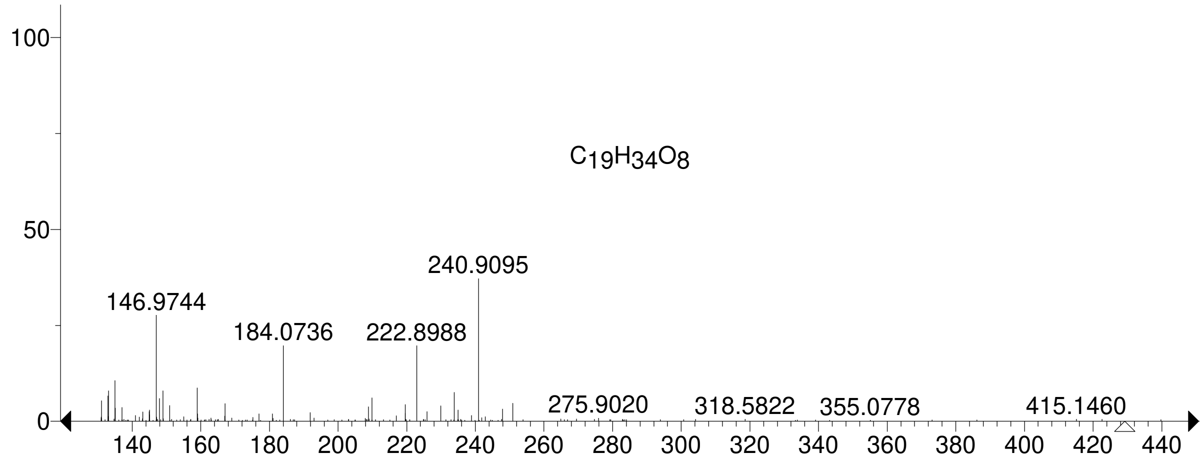

Unknown: Unknown (9.66_449.1548m/z)
Compound in Library Factor = 0

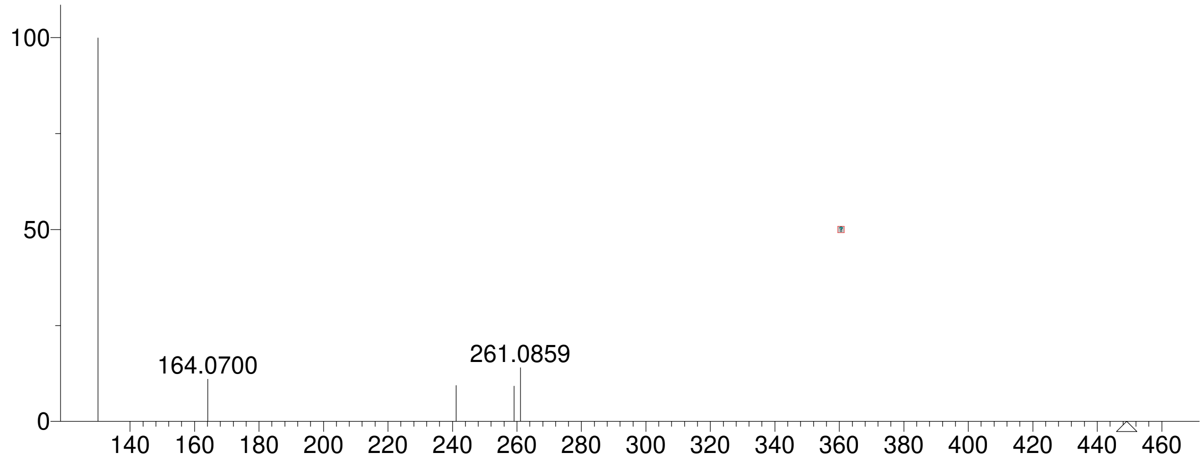

Hit 1 : Archangelicine [M+Na]+ 35HCD P=449.2
C24H26O7; MF: 0; RMF: 1; Prob 100.0%; Lib: mona-export-vf-npl_qexactive; ID: 5185.

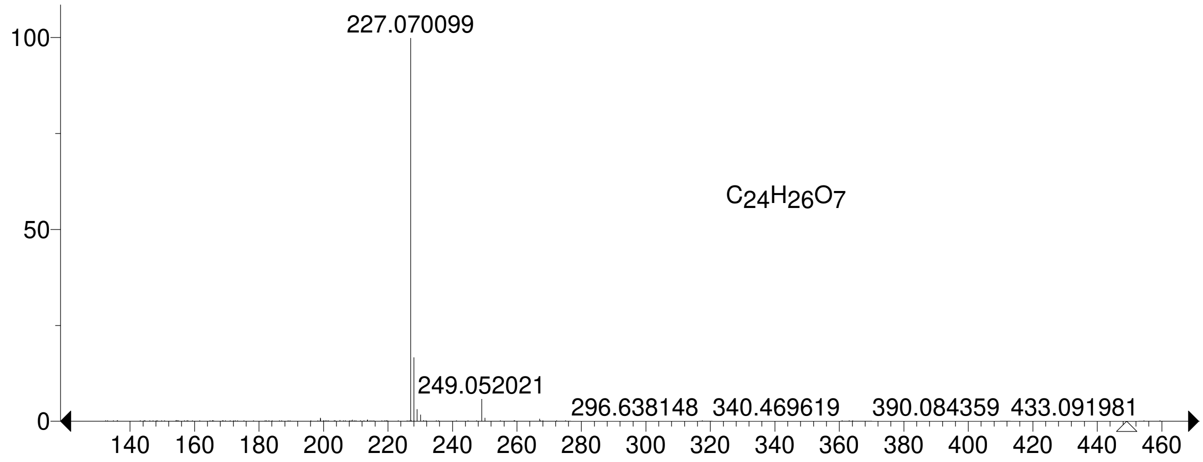

Unknown: Unknown (9.66_514.2395m/z)
Compound in Library Factor = 0

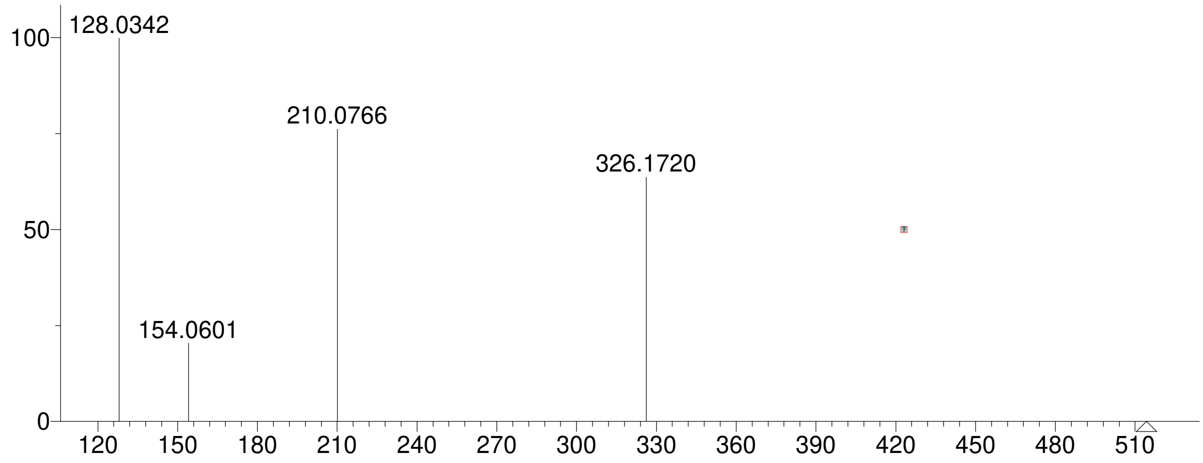

Hit 1 : Harringtonine [M+H-H2O]+ HCD 164V P=514.2
C28H37NO9; MF: 0; RMF: 66; Prob 16.7%; CAS: 26833-85-2; Lib: nist_msms; ID: 359996.

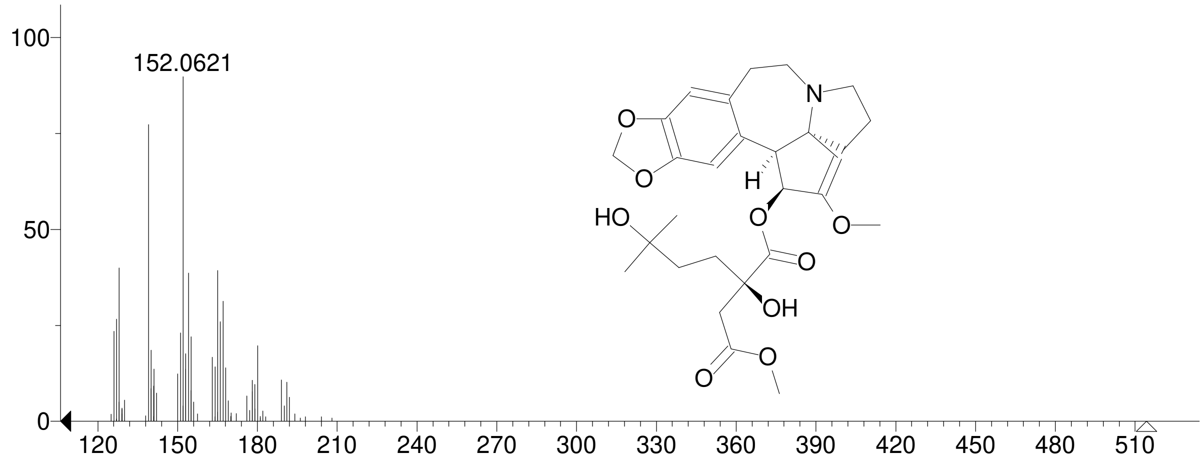

Hit 2 : (2S)-2-[(3R,7R,8R,8aS)-3,4'-dihydroxy-4,4,7,8a-tetramethyl-6'-oxospiro[2,3,4a,5,6,7-hexahydro-1H-naphth
C28H37NO8; MF: 0; RMF: 25; Prob 16.7%; Lib: mona-export-lc-ms-ms_negative_mode; ID: 19562.

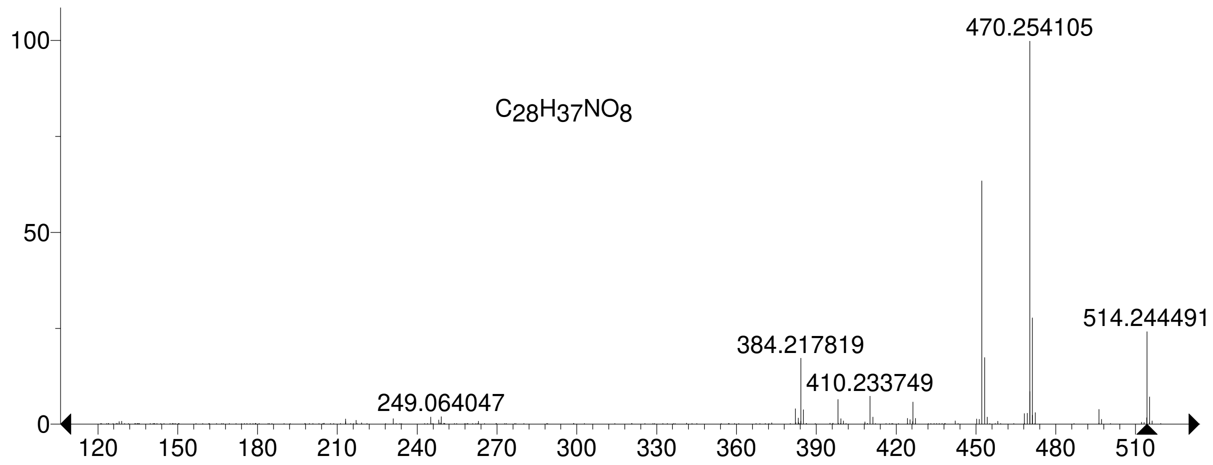

Hit 3 : 2-[(3R,8aS)-3,4'-dihydroxy-4,4,7,8a-tetramethyl-6'-oxospiro[2,3,4a,5,6,7-hexahydro-1H-naphthalene-8,2'-3,
C28H37NO8; MF: 0; RMF: 15; Prob 16.7%; Lib: mona-export-lc-ms-ms_negative_mode; ID: 19532.

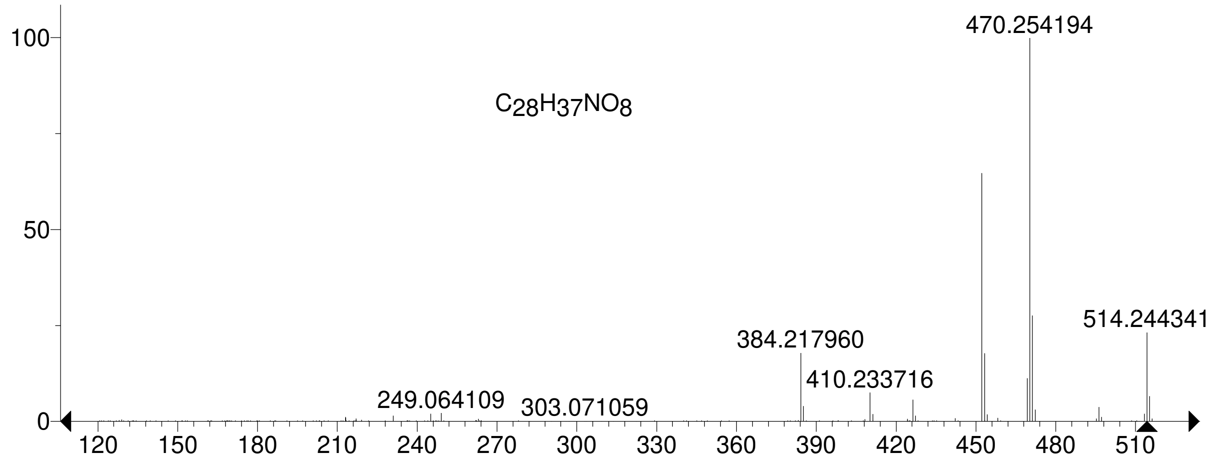

Hit 4 : (1R,4S,7R,8S)-7-[(2E,4E)-hexa-2,4-dienoyl]-3-hydroxy-8-[(2S)-3-hydroxy-2,4-dimethyl-5-oxo-2,5-dihydrofu
C28H32O8; MF: 0; RMF: 11; Prob 16.7%; Lib: mona-export-vf-npl_qexactive; ID: 21801.

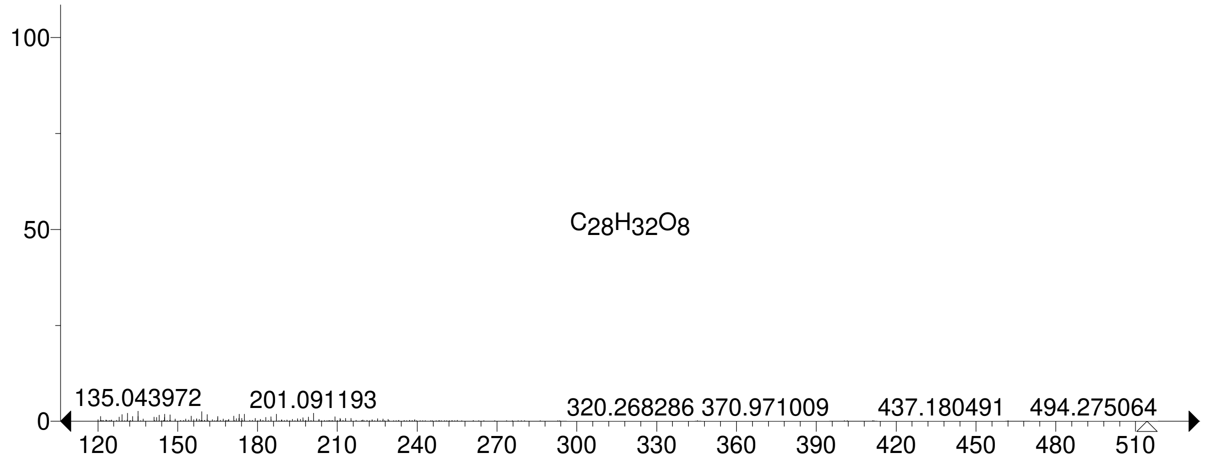

Hit 5 : (2E,4E)-1-[(1R,3S,8R,10S,14S)-12-[(2E,4E)-hexa-2,4-dienoyl]-1,6,8,13-tetrahydroxy-3,7,10,14-tetramethyl
C28H32O8; MF: 0; RMF: 9; Prob 16.7%; Lib: mona-export-vf-npl_qexactive; ID: 20659.

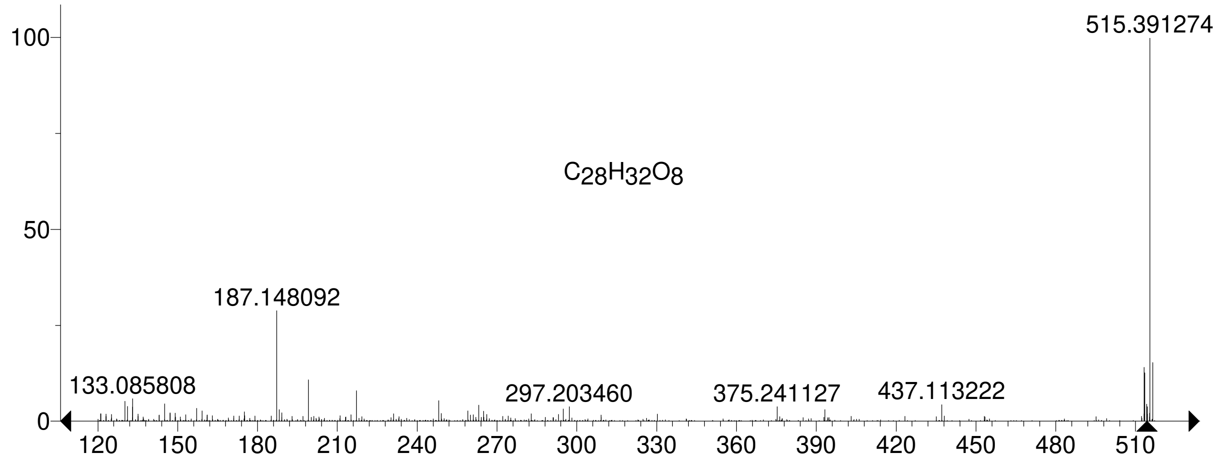

Unknown: Unknown (9.67_443.2028m/z)
Compound in Library Factor = -3273

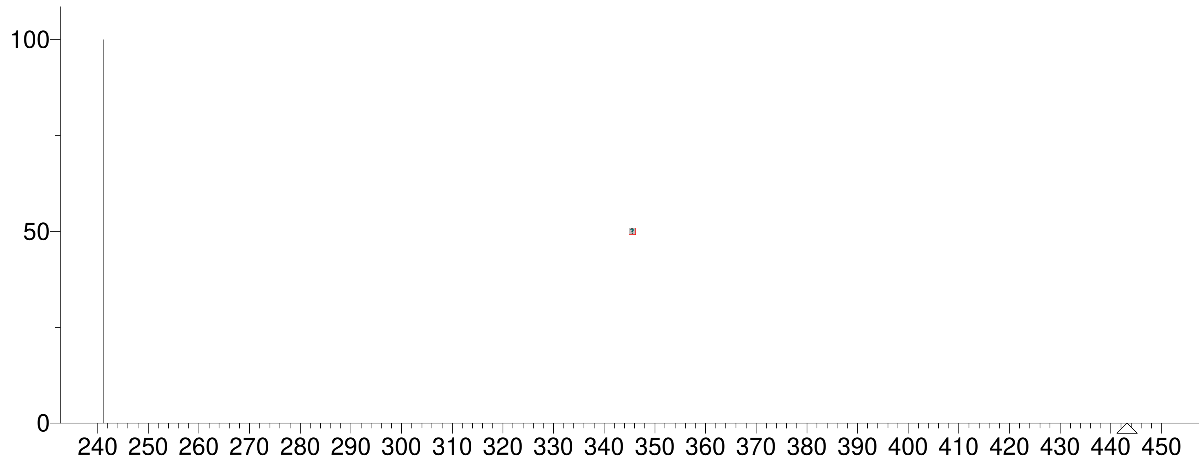

Hit 1 : methyl 10-acetyloxy-4,5,9-trihydroxy-1,4a-dimethyl-7-propan-2-yl-2,3,4,9,10,10a-hexahydrophenanthrene-
C23H32O7; MF: 2; RMF: 307; Prob 52.0%; Lib: mona-export-vf-npl_qexactive; ID: 22545.

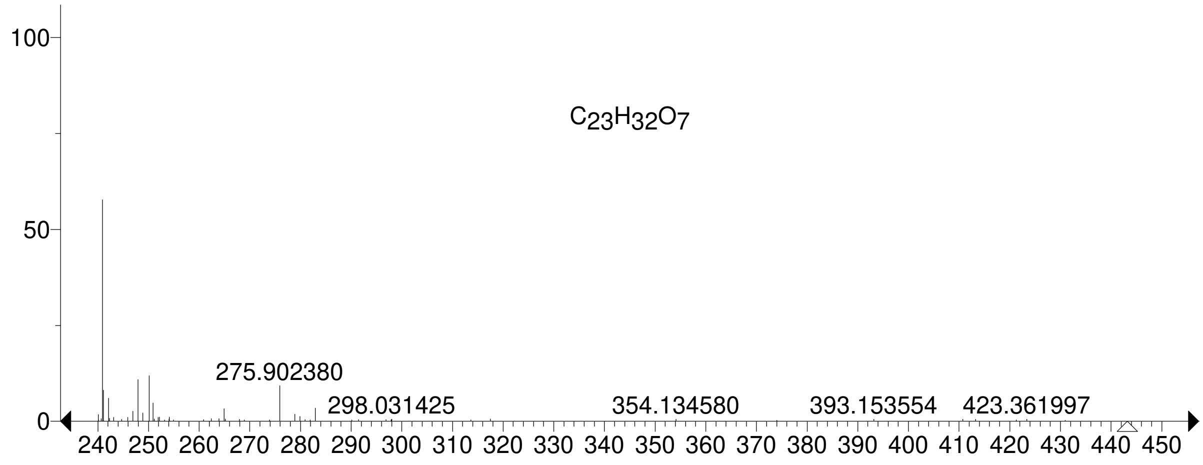

Hit 2 : NCGC00380442-01_C25H30O7_3H-5,13b-Methano-1H-furo[3',4':6,7]heptaleno[3,2-c]pyran-1,4,9(3aH,7H)
C25H30O7; MF: 0; RMF: 55; Prob 48.0%; Lib: mona-export-gnps; ID: 13756.

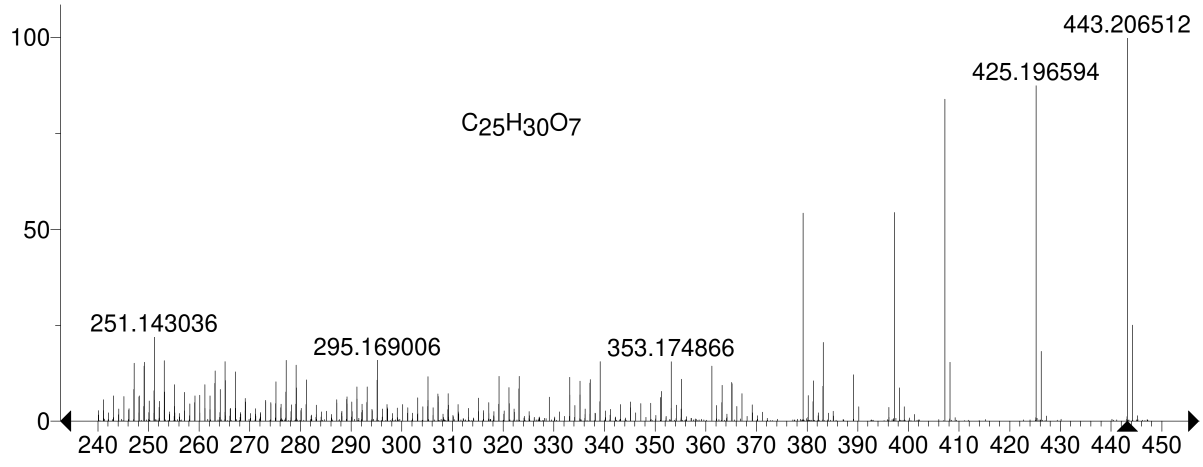

Unknown: Unknown (9.68_534.2214m/z)
Compound in Library Factor = 0

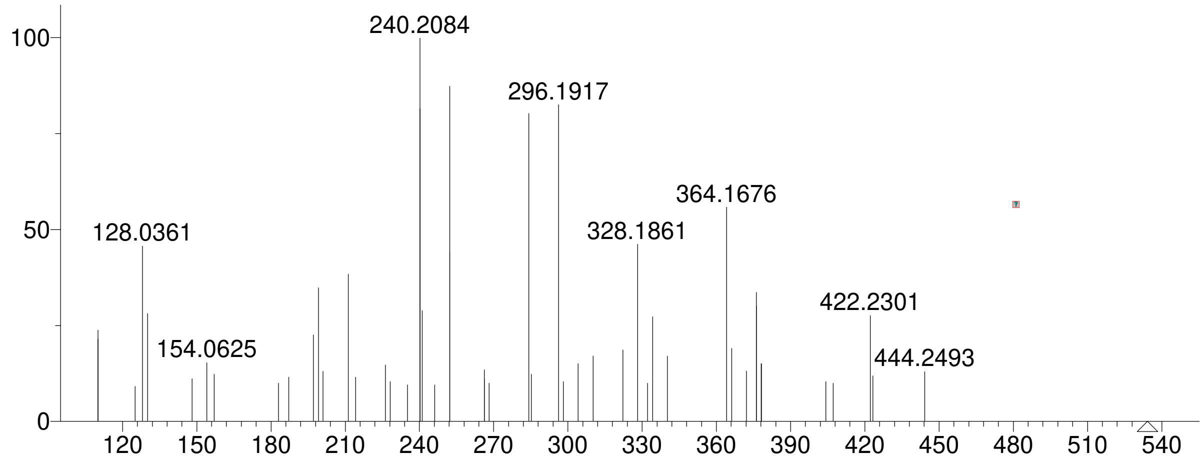

Unknown: Unknown (9.68_536.2153m/z)
Compound in Library Factor = 0

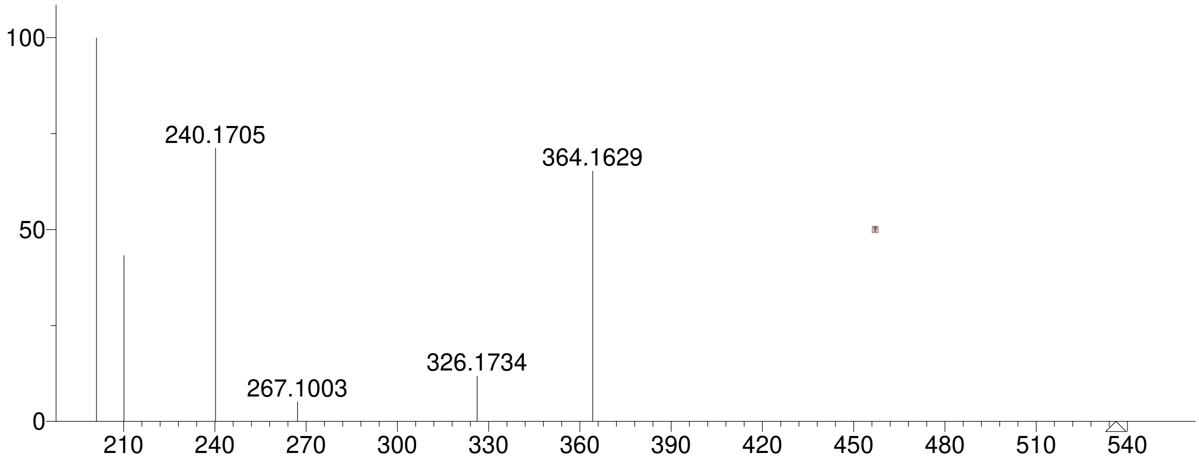

Unknown: Unknown (9.68_663.2492m/z)
Compound in Library Factor = 0

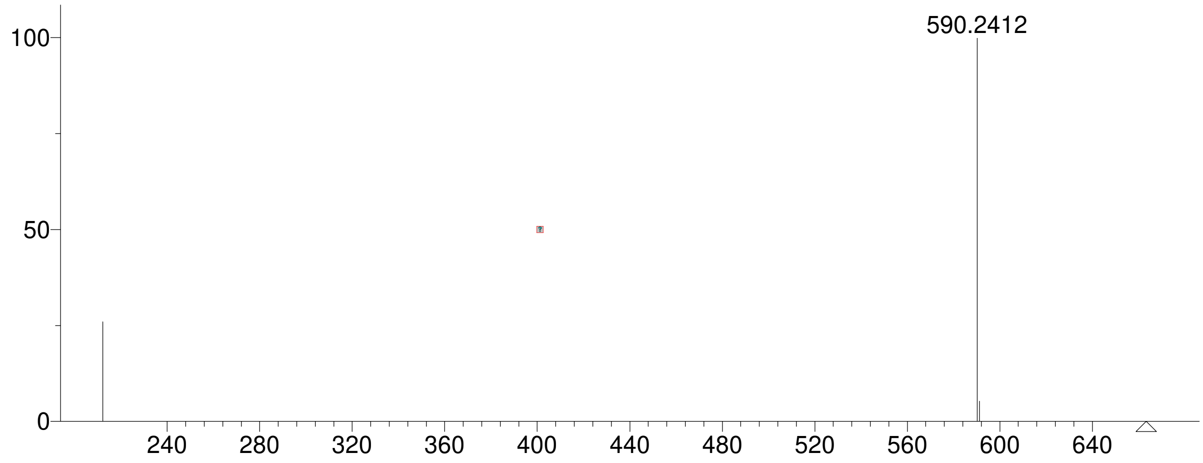

Unknown: Unknown (9.69_548.2381m/z)
Compound in Library Factor = -235

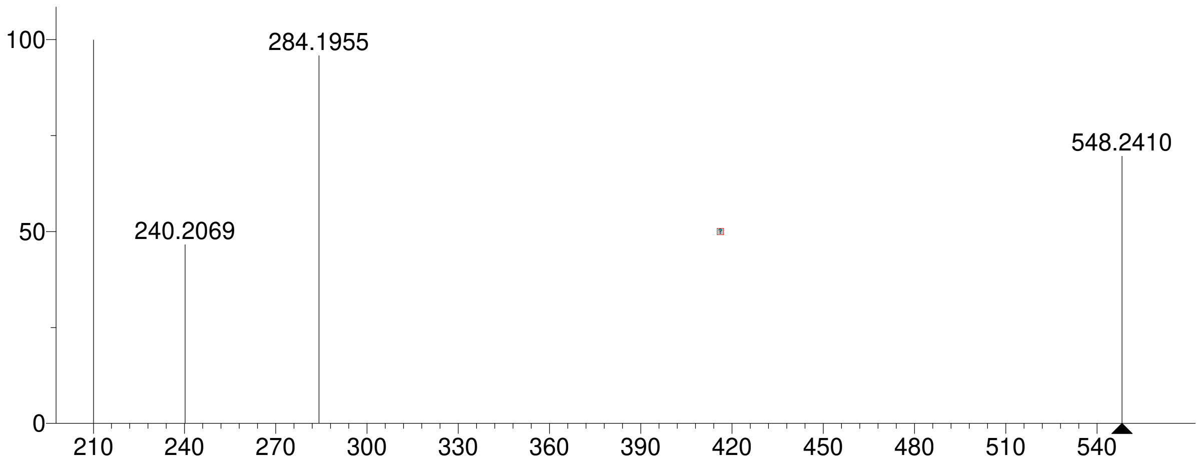

Hit 1 : Darunavir [M+H]+ HCD 5V P=548.2
C27H37N3O7S; MF: 288; RMF: 924; Prob 98.6%; CAS: 206361-99-1; Lib: nist_msms; ID: 355571.

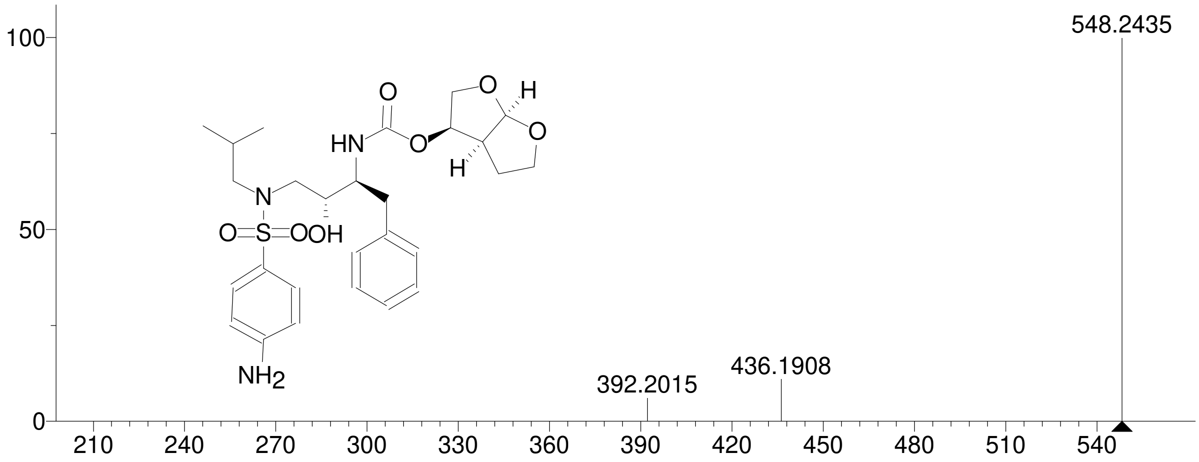

Hit 2 : Virginiamycin component b [M+Na]+ HCD 32V P=548.2
C28H35N3O7; MF: 21; RMF: 429; Prob 0.98%; Lib: nist_msms; ID: 353177.

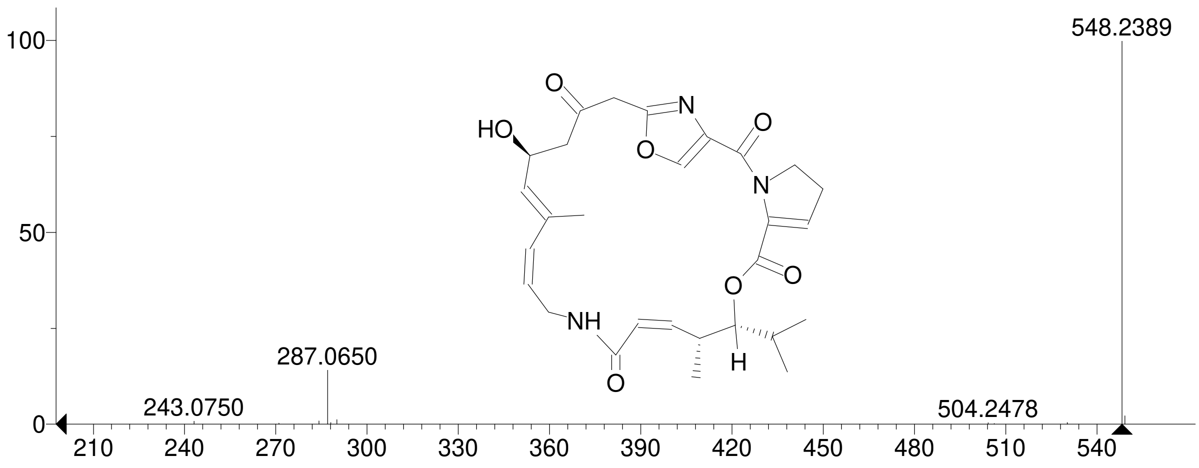

Hit 3 : Darunavir Ethanolate (Prezista) [M+H]+ P=548.2
C29H43N3O8S; MF: 0; RMF: 42; Prob 0.41%; Lib: mona-export-gnps; ID: 538.

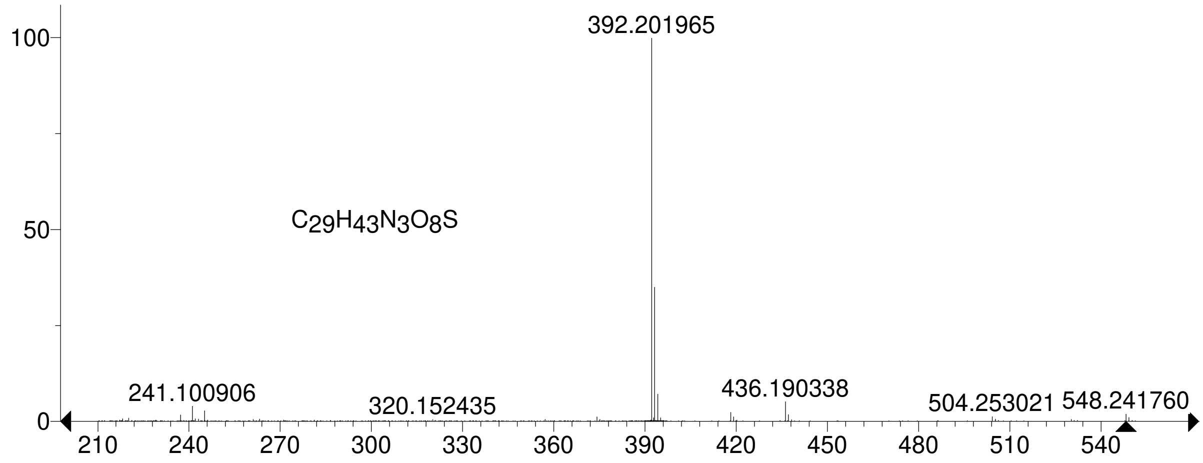

Unknown: Unknown (9.69_760.3070m/z)
Compound in Library Factor = 0

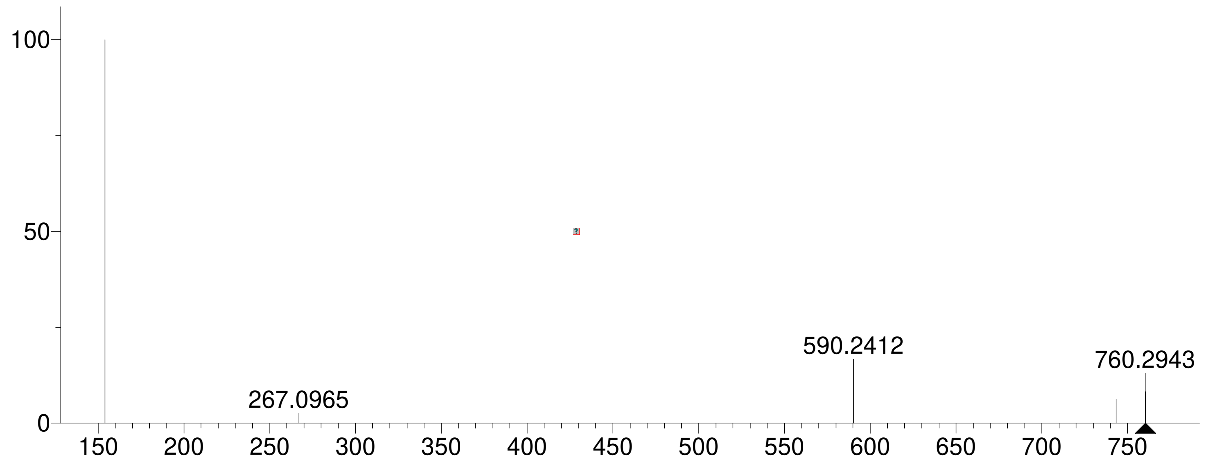

Hit 1 : NCGC00380723-01!(2S,3R,4S,5S,6R)-2-[4-[(3S,3aR,6S,6aR)-6-[3,5-dimethoxy-4-[(2S,3R,4S,5S,6R)-3,4,5
C34H46O18; MF: 0; RMF: 20; Prob 100.0%; Lib: mona-export-gnps; ID: 7024.

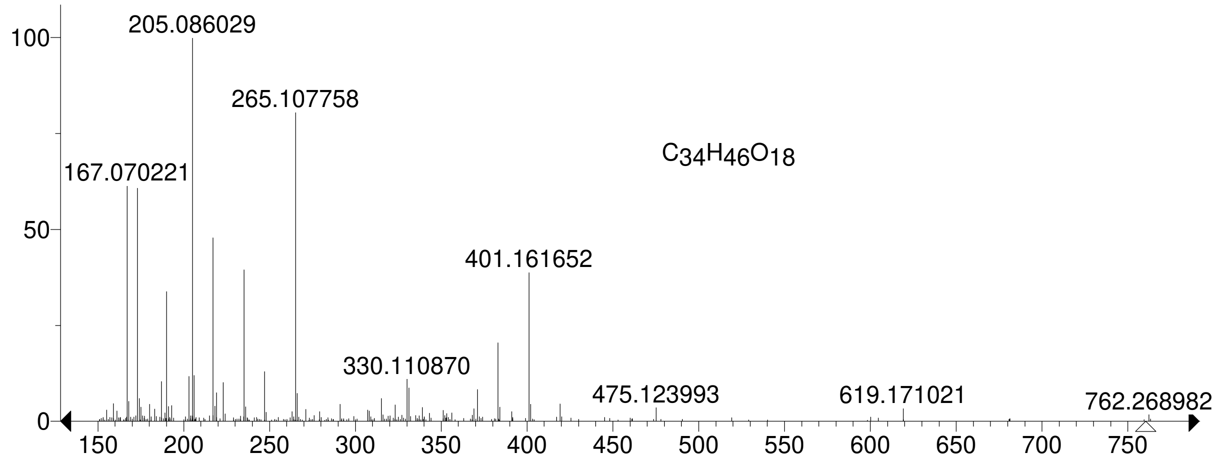

Unknown: Unknown (9.71_401.1911m/z)
Compound in Library Factor = 0

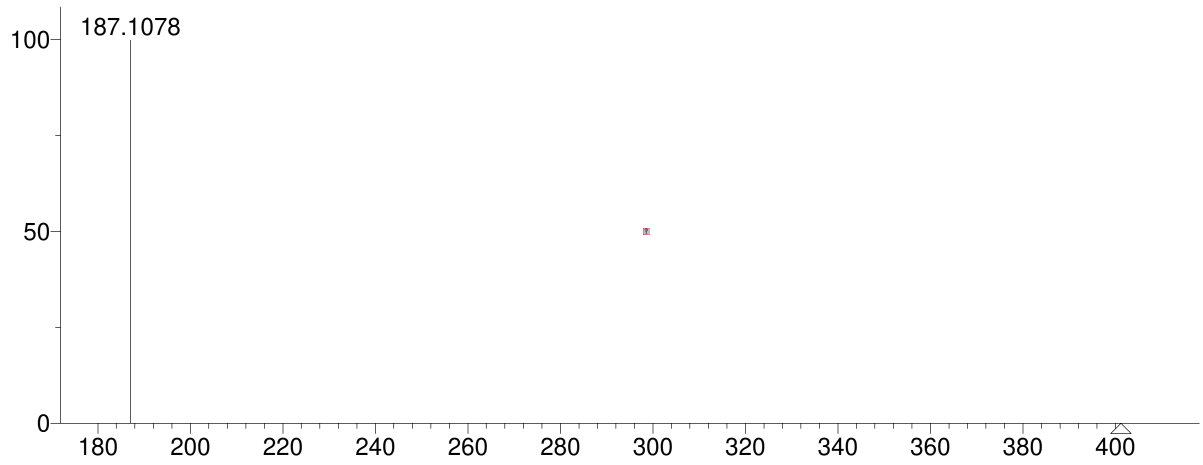

Hit 1 : NCGC00380382-01_C23H30O7_1-Phenanthrenecarboxylic acid, 10-(acetyloxy)-1,2,3,4,4a,9,10,10a-octah
C23H30O7; MF: 0; RMF: 17; Prob 100.0%; Lib: mona-export-gnps; ID: 13719.

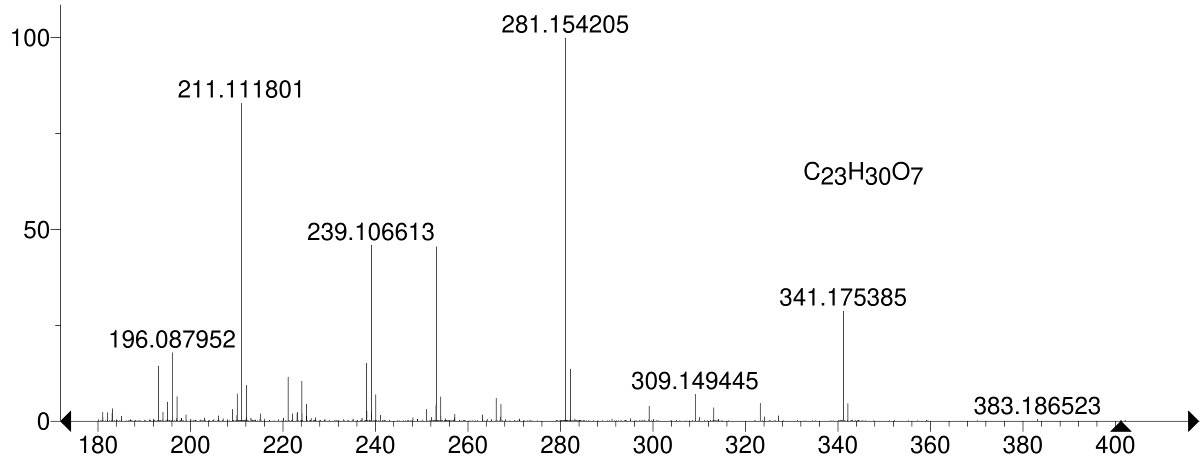

Unknown: Unknown (9.72_576.2297m/z)
Compound in Library Factor = 0

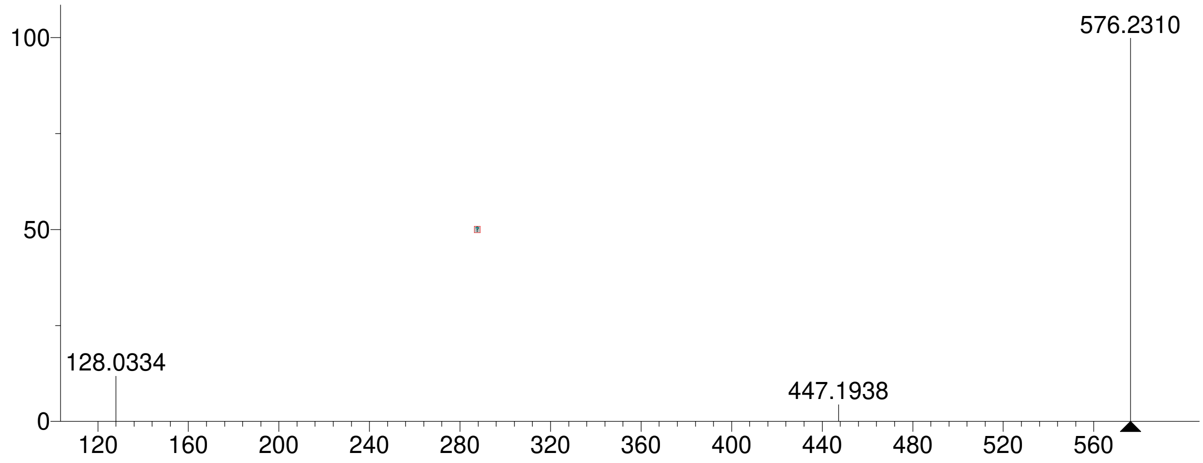

Unknown: Unknown (9.78_399.2107m/z)
Compound in Library Factor = 0

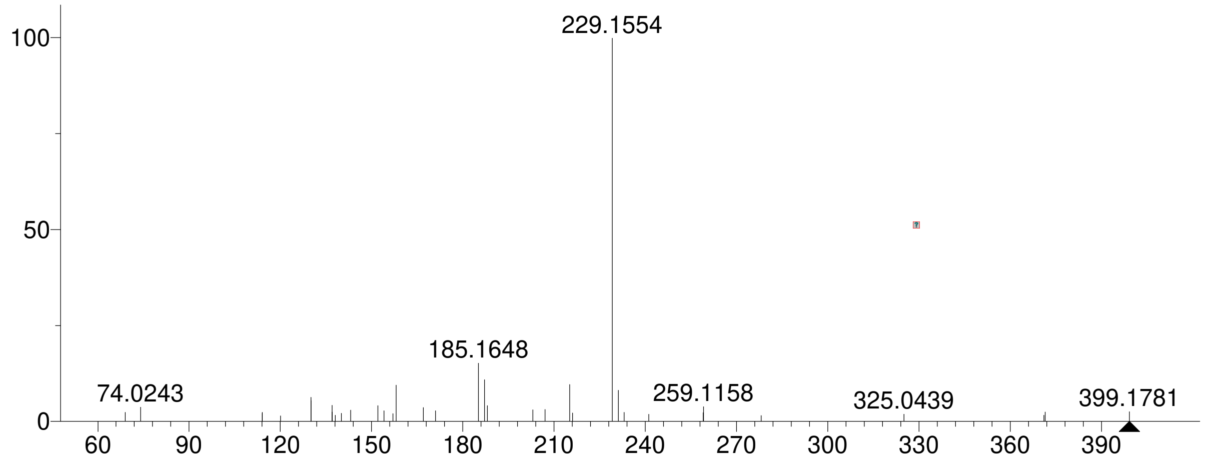

Hit 1 : N-Desmethylvinblastine [M+2H]2+ HCD 14V P=399.2
C45H56N4O9; MF: 0; RMF: 89; Prob 20.0%; CAS: 18172-50-4; Lib: nist_msms; ID: 435586.

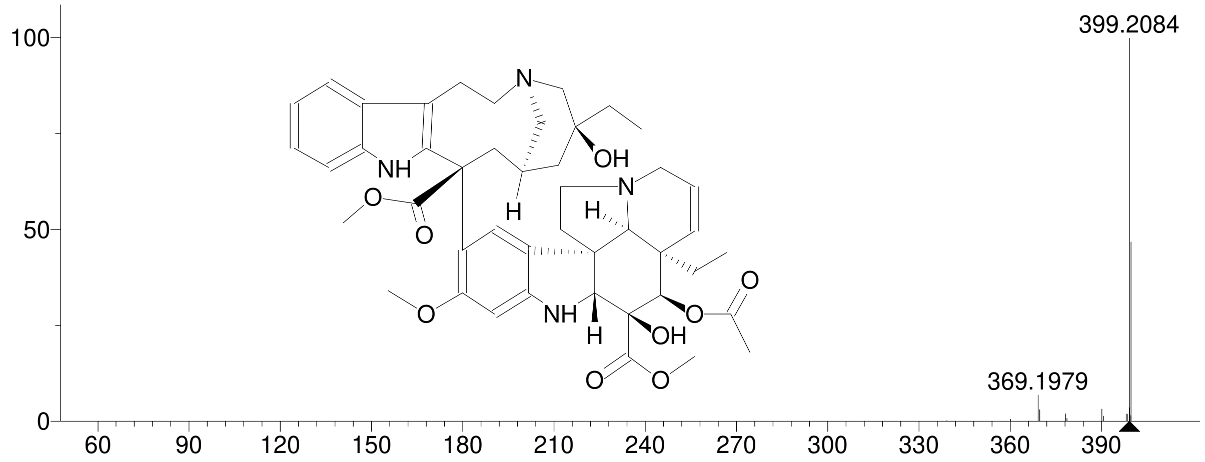

Hit 2 : Ser-His-Arg [M+H]+ QTOF 30V P=399.2
C15H26N8O5; MF: 0; RMF: 20; Prob 20.0%; Lib: nist_msms; ID: 53772.

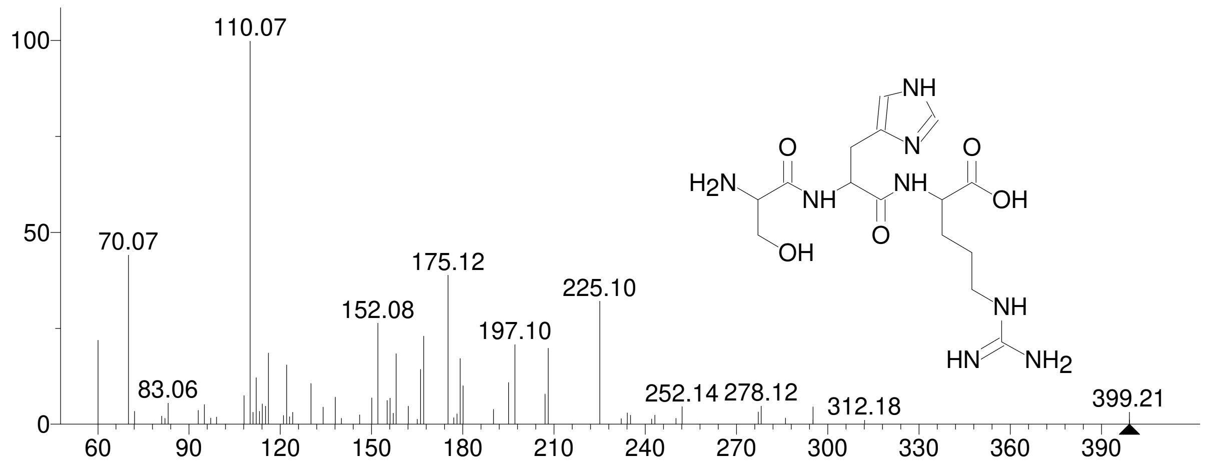

Hit 3 : His-Ser-Arg [M+H]+ QTOF 30V P=399.2
C15H26N8O5; MF: 0; RMF: 19; Prob 20.0%; Lib: nist_msms; ID: 51160.

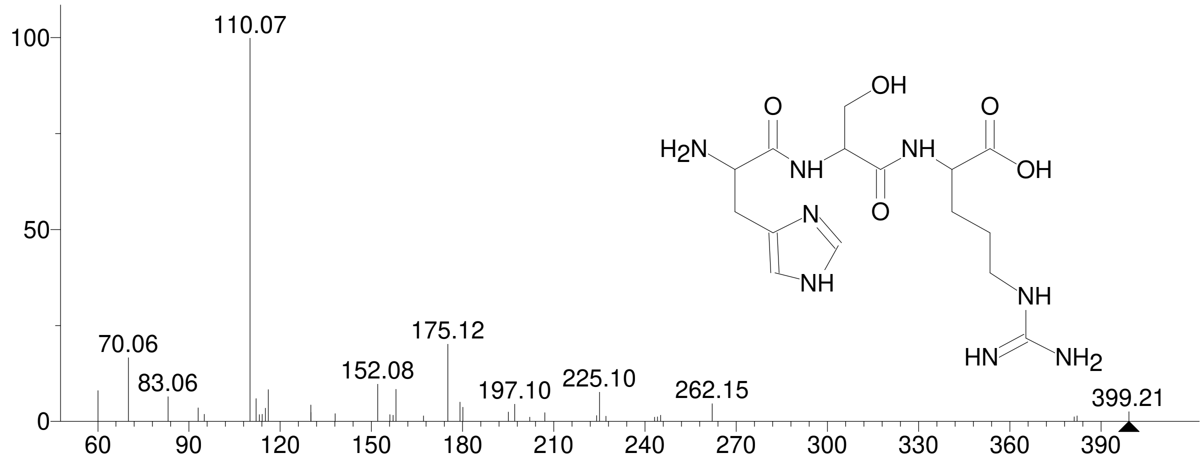

Hit 4 : NCGC00380576-01_C22H32O5_2-Pentenoic acid, 3-[(acetyloxy)methyl]-5-[(1S,4aR,8aR)-2-formyl-1,4,4a,
C22H32O5; MF: 0; RMF: 4; Prob 20.0%; Lib: mona-export-gnps; ID: 13905.

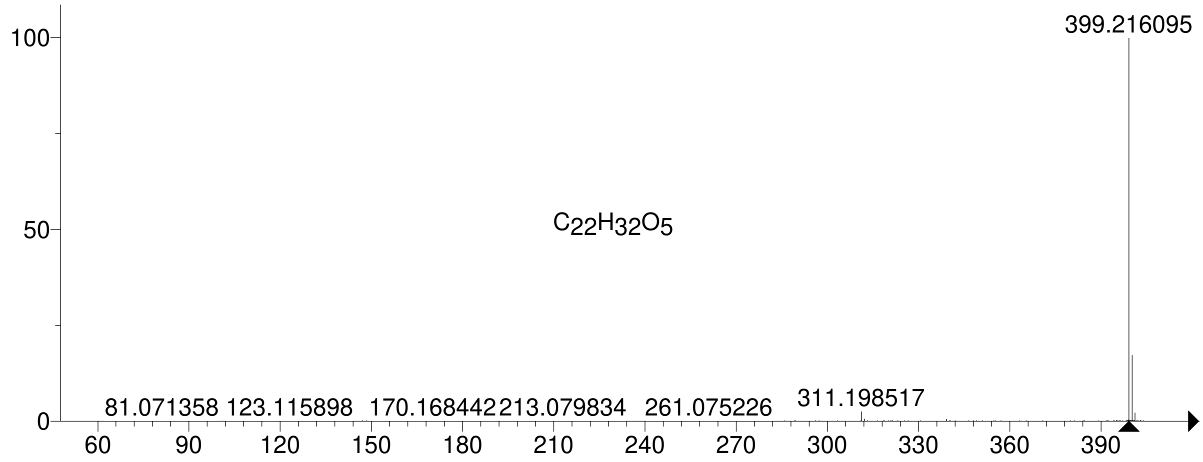

Hit 5 : 7S,8,17S-Trihydroxydocosa-4Z,9E,11E,13Z,15E,19Z-hexaenoic acid [M+Na]+ HCD 31V P=399.2
C22H32O5; MF: 0; RMF: 4; Prob 20.0%; CAS: 872993-05-0; Lib: nist_msms; ID: 118172.

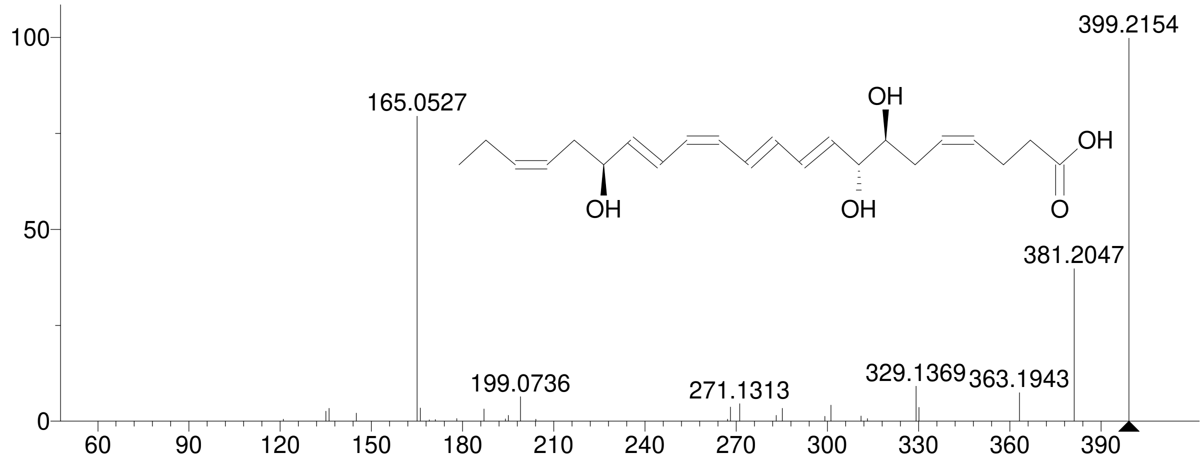

Unknown: Unknown (9.78_506.1743m/z)
Compound in Library Factor = 0

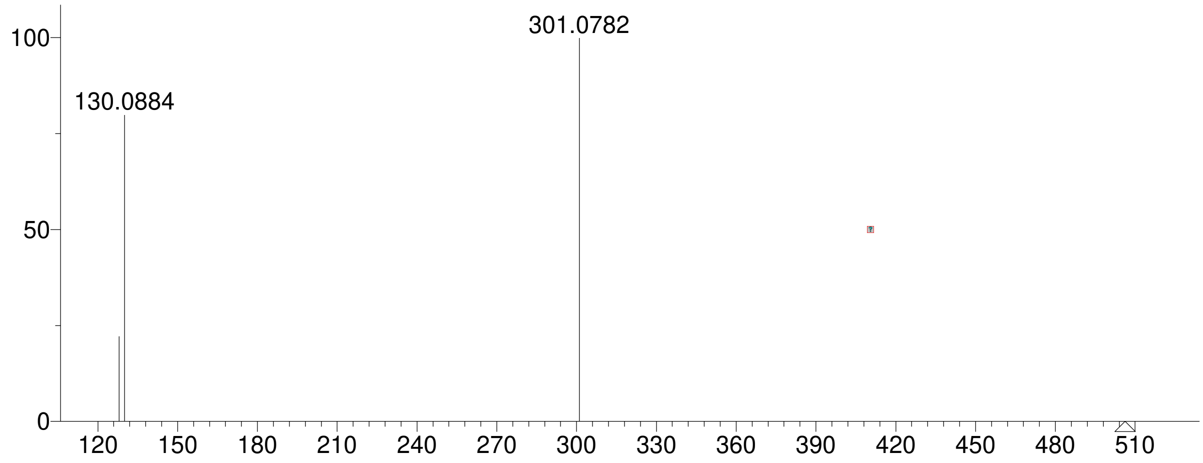

Unknown: Unknown (9.78_563.2440m/z)
Compound in Library Factor = 0

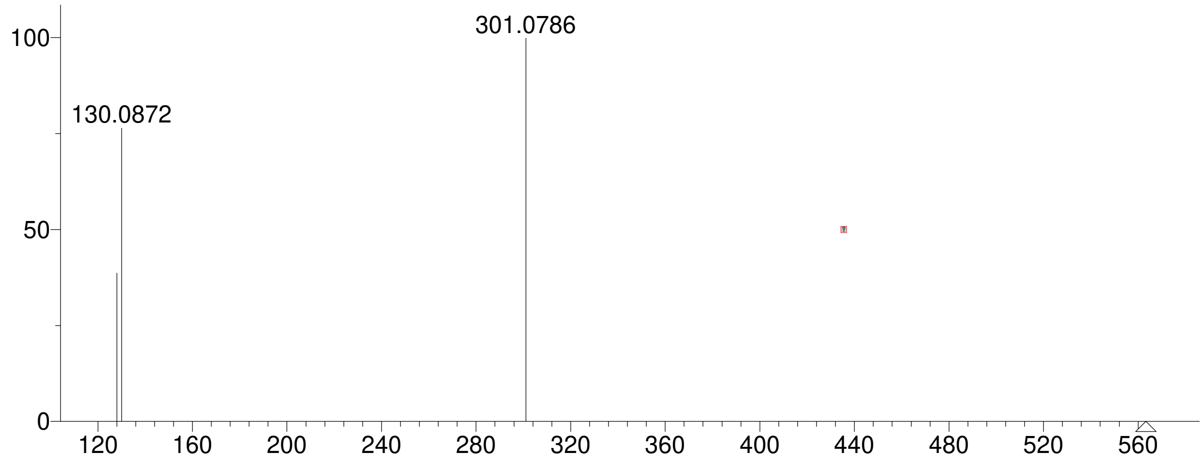

Unknown: Unknown (9.80_203.0006m/z)
Compound in Library Factor = 0

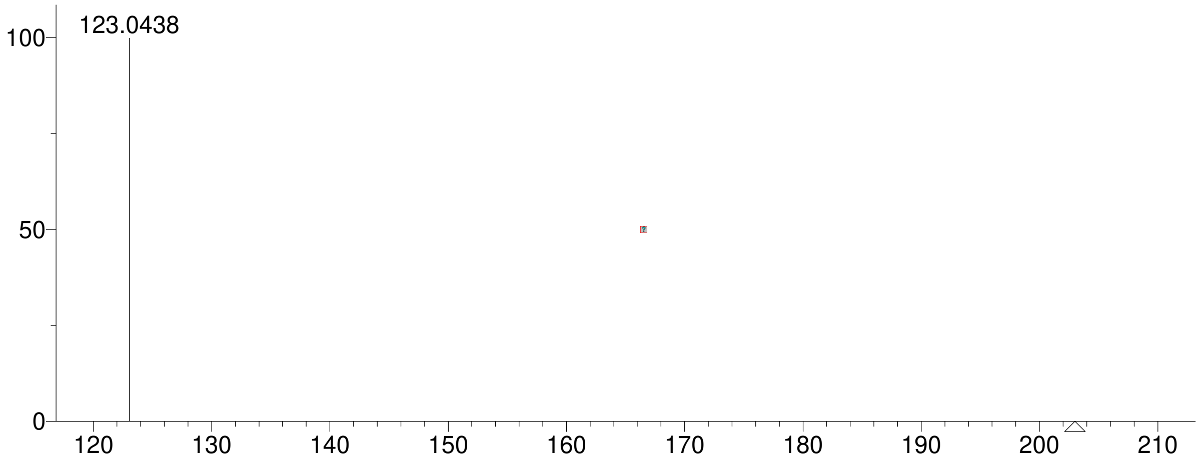

Unknown: Unknown (9.83_415.1697m/z)
Compound in Library Factor = 0

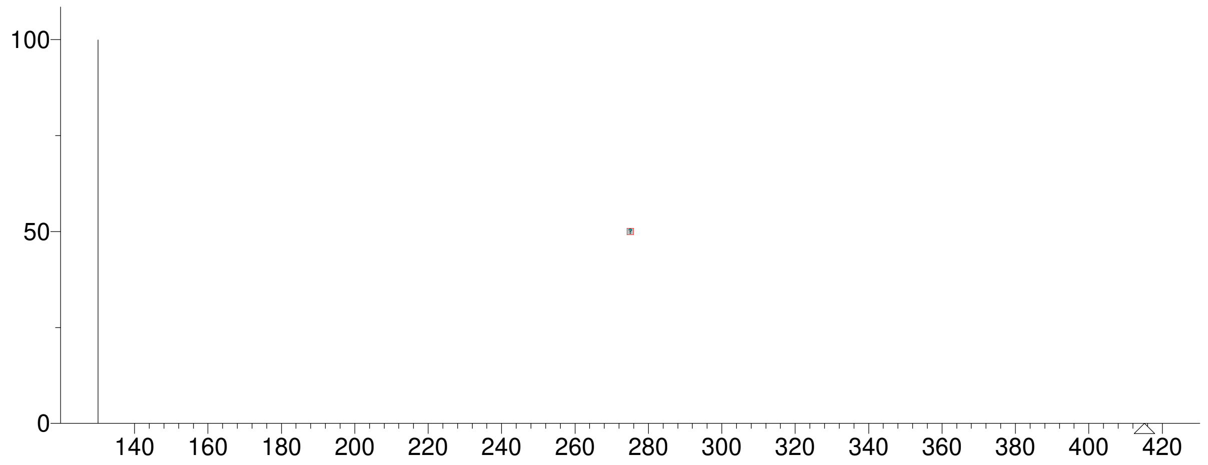

Hit 1 : NCGC00347525-02!2-amino-5-[2-[[2,3-dihydroxy-2-(1-hydroxyethyl)butanoyl]oxymethyl]-4-hydroxyanilino]-C18H26N2O9; MF: 0; RMF: 9; Prob 100.0%; Lib: mona-export-gnps; ID: 6270.

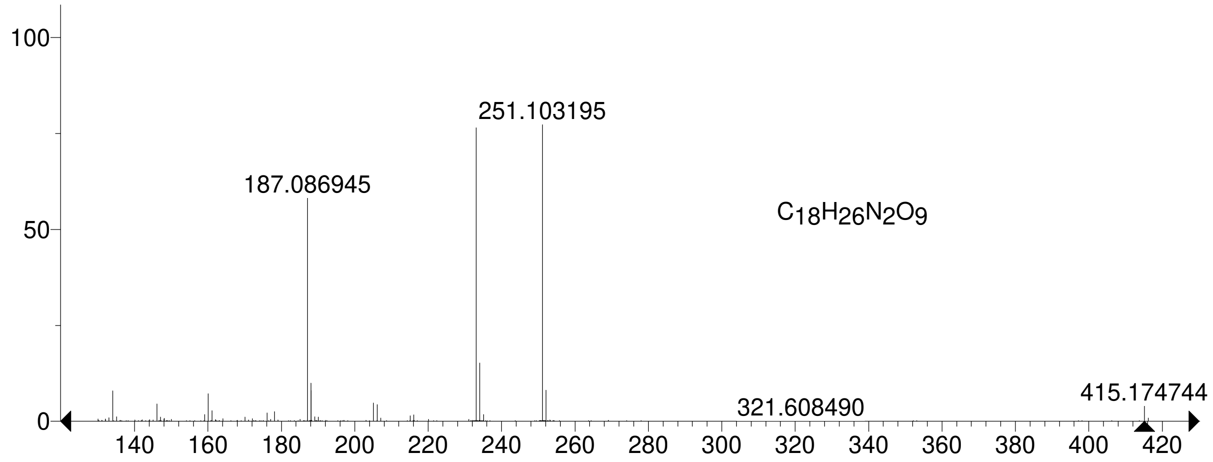

** Search Report Page 1 of 1 **

Unknown: Unknown (9.85_508.1820m/z)
Compound in Library Factor = 0

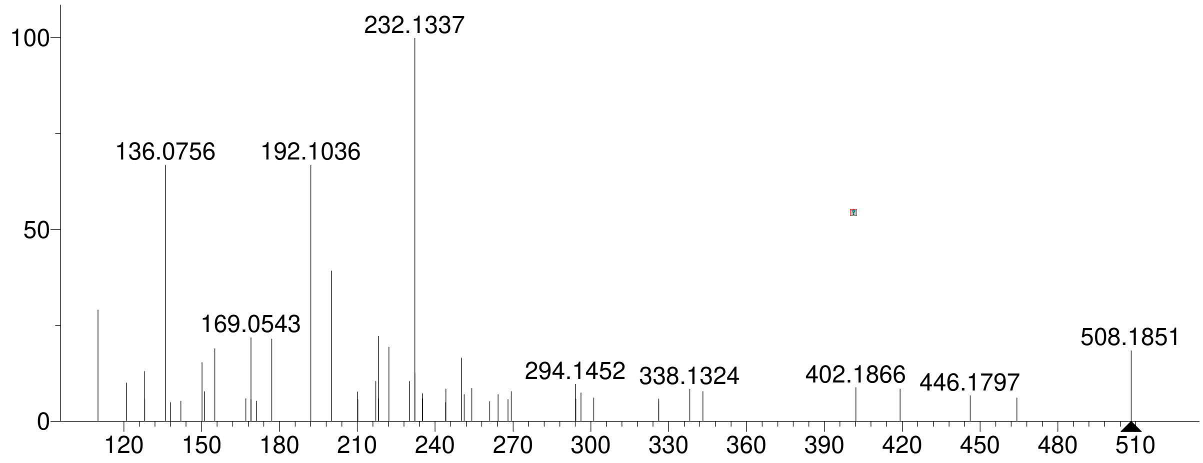

Unknown: Unknown (9.86_358.1962m/z)
Compound in Library Factor = 0

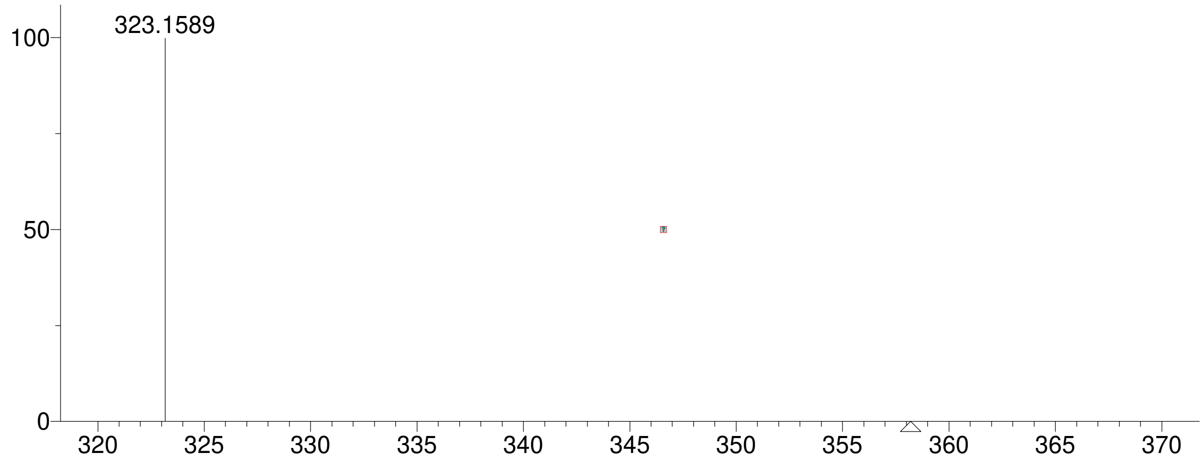

Unknown: Unknown (9.87_760.3046m/z)
Compound in Library Factor = 0

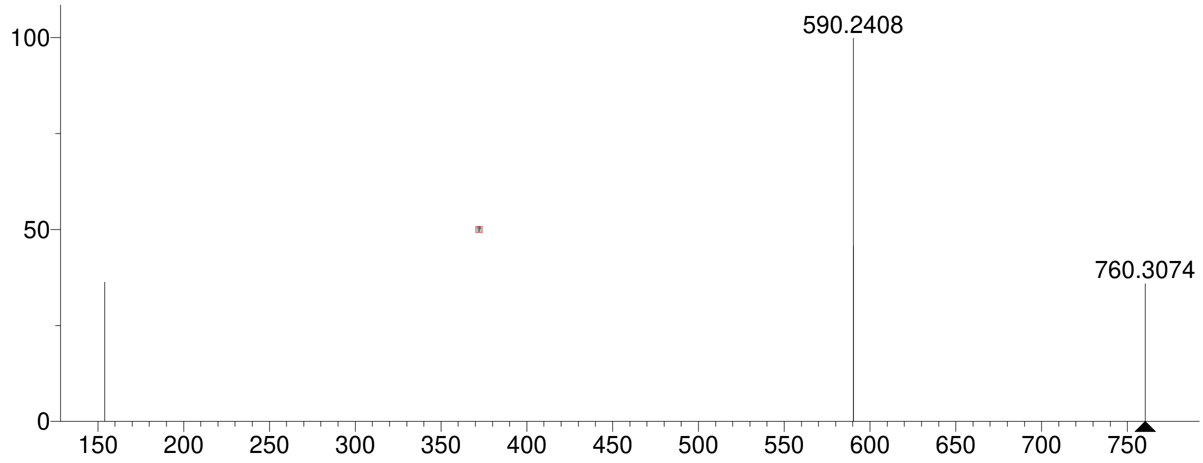

Unknown: Unknown (9.88_605.2459m/z)
Compound in Library Factor = 0

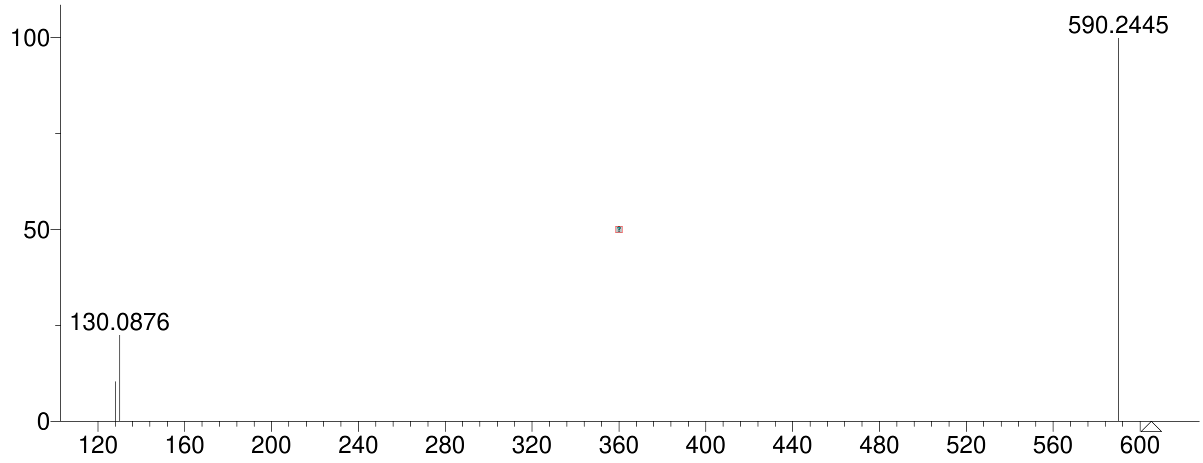

Unknown: Unknown (9.91_283.1537m/z)
Compound in Library Factor = -379

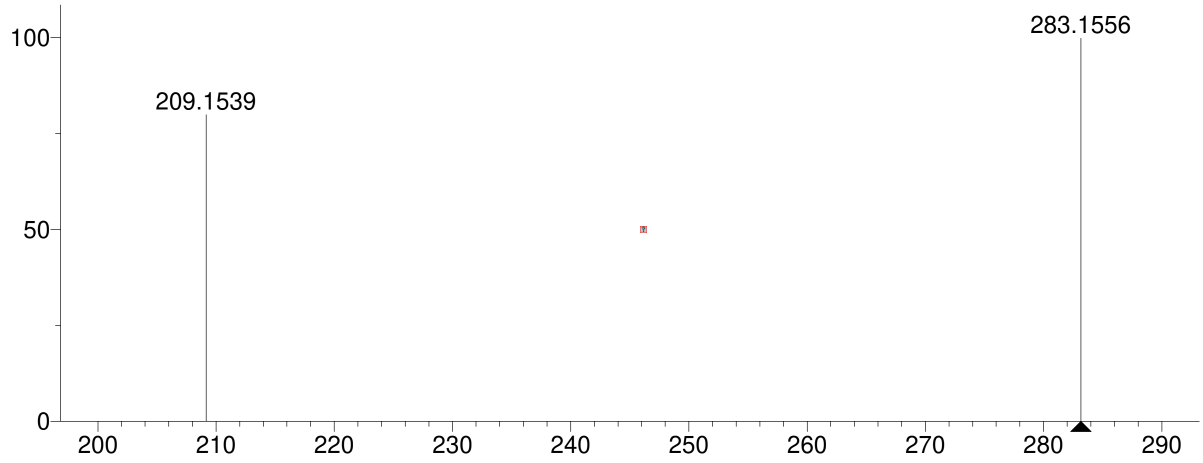

Hit 1 : Artemisinin [M+H]+ HCD 2V P=283.2
C15H22O5; MF: 302; RMF: 972; Prob 96.9%; CAS: 63968-64-9; Lib: nist_msms; ID: 118848.

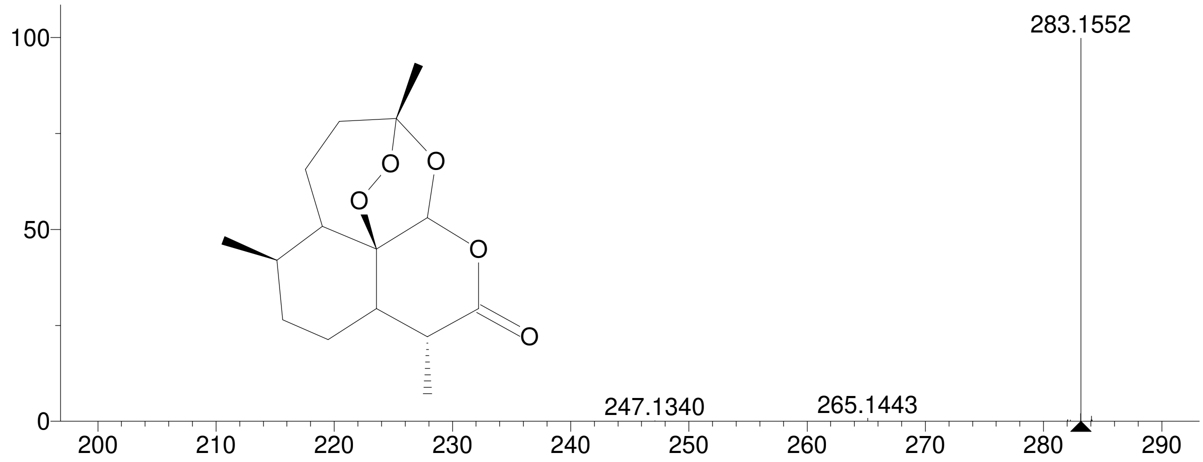

Hit 2 : Octyl gallate [M+H]+ HCD 1V P=283.2
C15H22O5; MF: 143; RMF: 705; Prob 2.44%; CAS: 1034-01-1; Lib: nist_msms; ID: 138058.

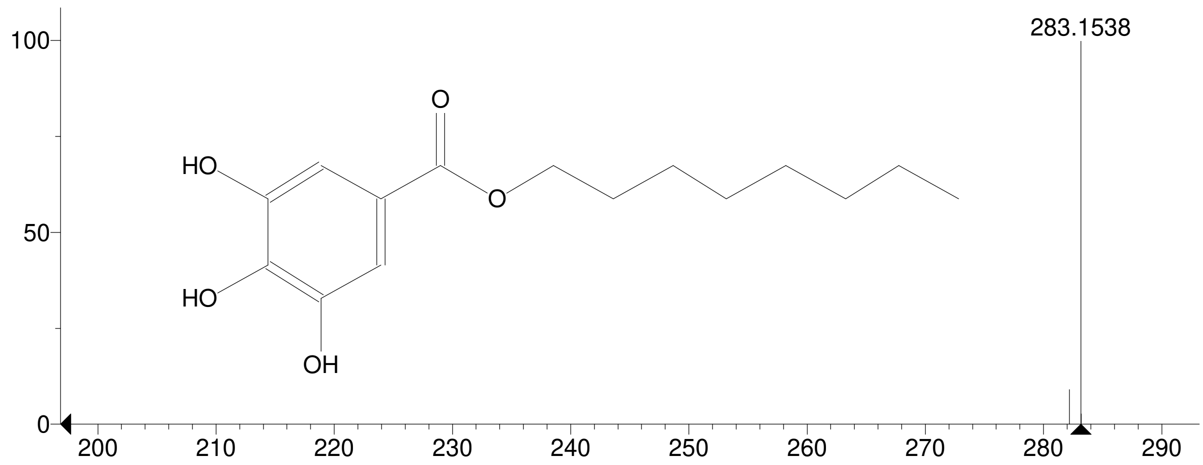

Hit 3 : NCGC00347773-02!1-[4,5-bis(hydroxymethyl)-3-methoxy-2-methylphenoxy]-3-methylbutane-2,3-diol [M-H
C15H24O6; MF: 51; RMF: 621; Prob 0.20%; Lib: mona-export-gnps; ID: 6416.

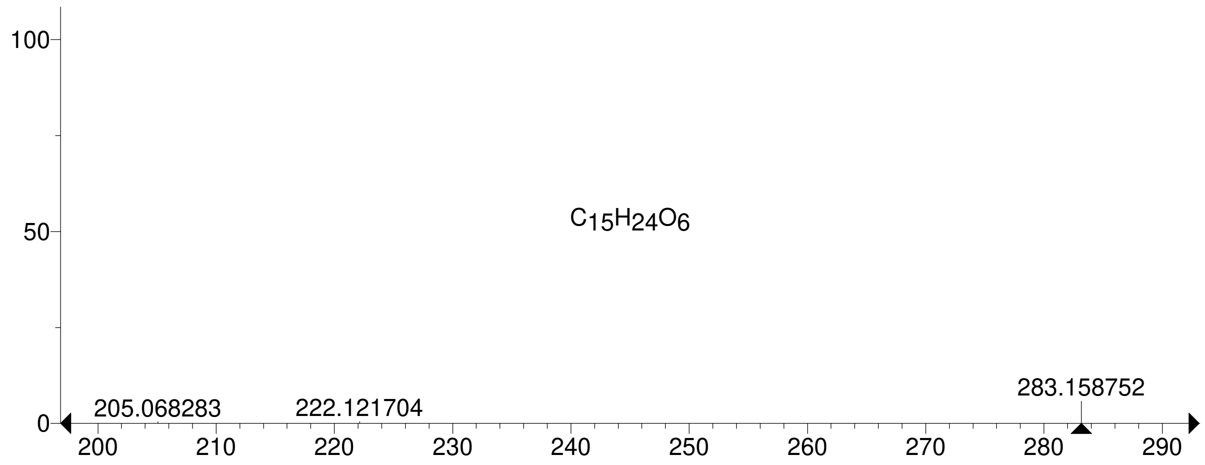

Hit 4 : isomer of dihydrophaseic acid [M+H]+ P=283.2
C15H22O5; MF: 41; RMF: 586; Prob 0.14%; Lib: mona-export-gnps; ID: 14793.

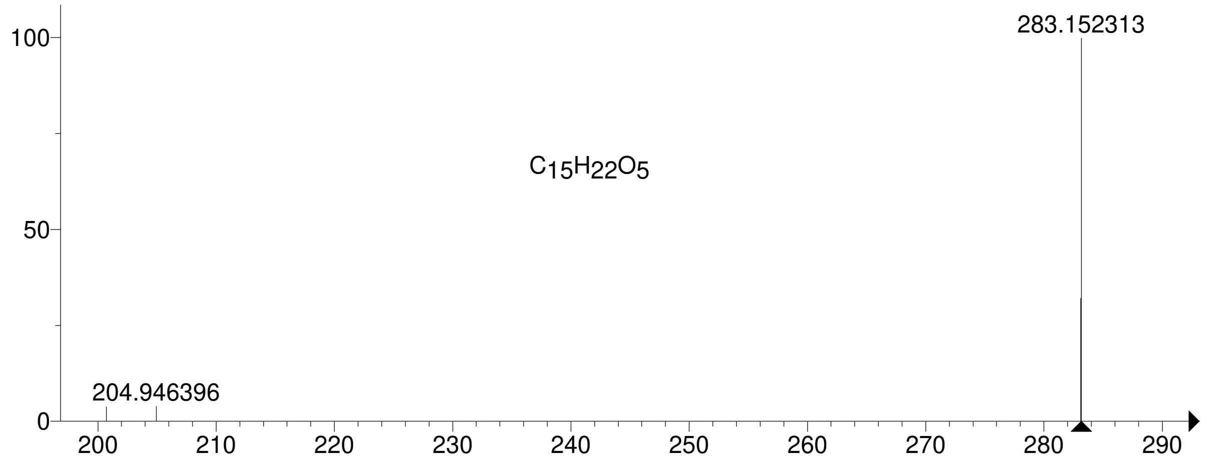

Hit 5 : Cyclopentanehexanoic acid, 2-[(2Z)-4-carboxy-2-buten-1-yl]-3,5-dihydroxy-γ-oxo-, (1R,2R,3S,5S)- [M-H-C
C16H24O7; MF: 9; RMF: 366; Prob 0.03%; CAS: 133161-96-3; Lib: nist_msms; ID: 264214.

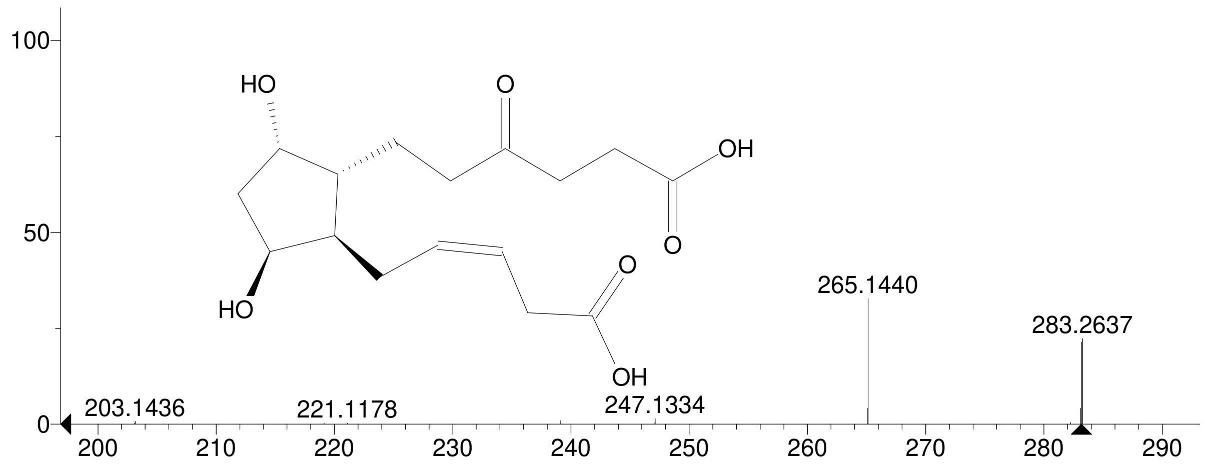

Unknown: Unknown (9.96_923.4119m/z)
Compound in Library Factor = 0

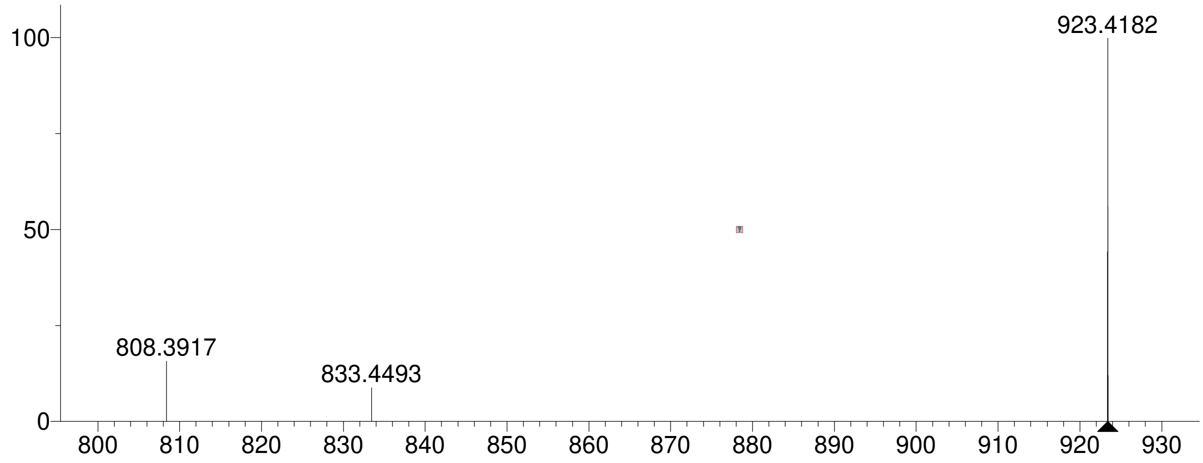

Unknown: Unknown (9.97_433.0796m/z)
Compound in Library Factor = 0

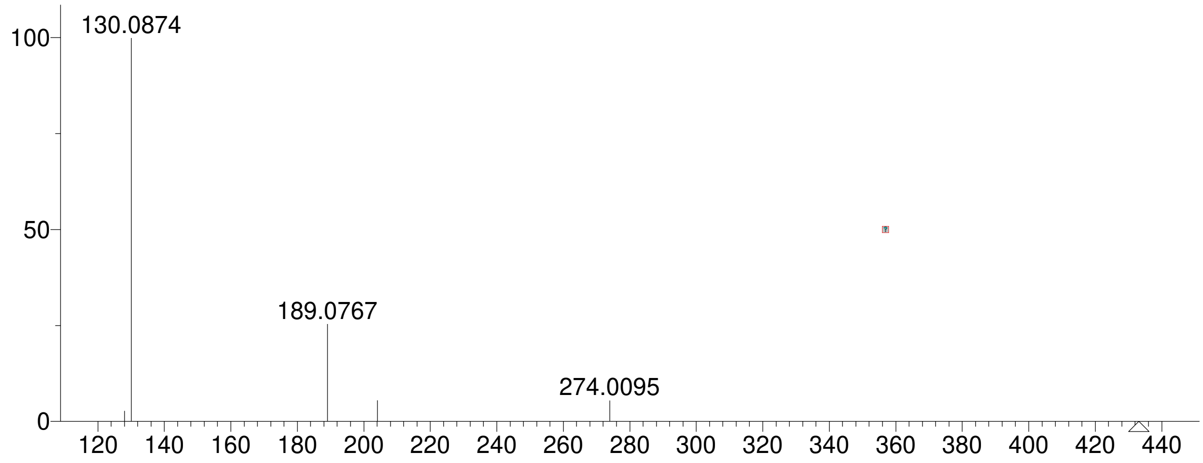

Hit 1 : Quercetin-3-O-xyloside [M-H]- 30eV P=433.1
C20H18O11; MF: 0; RMF: 10; Prob 33.3%; Lib: mona anthocyanidin-o-glycosides; ID: 2991.

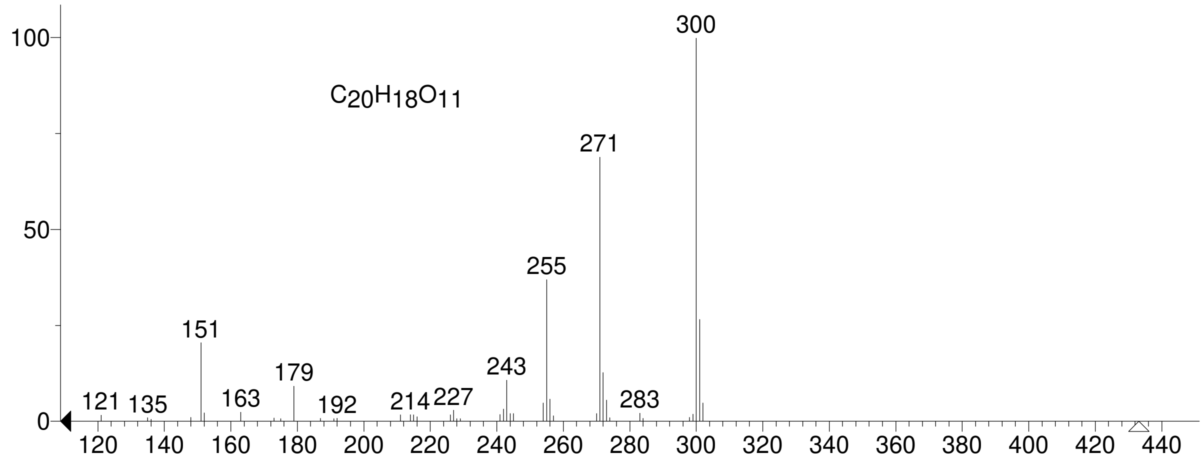

Hit 2 : guajavarin [M-H]- 25HCD P=433.1
C20H18O11; MF: 0; RMF: 4; Prob 33.3%; Lib: mona anthocyanidin-o-glycosides; ID: 5117.

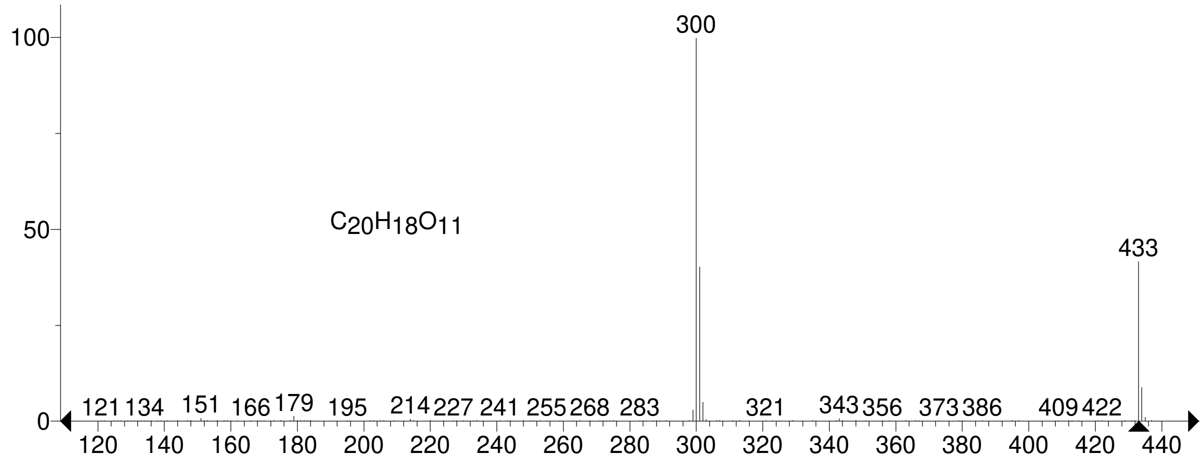

Hit 3 : avicularin [M-H]- 35HCD P=433.1
C20H18O11; MF: 0; RMF: 4; Prob 33.3%; Lib: mona anthocyanidin-o-glycosides; ID: 5329.

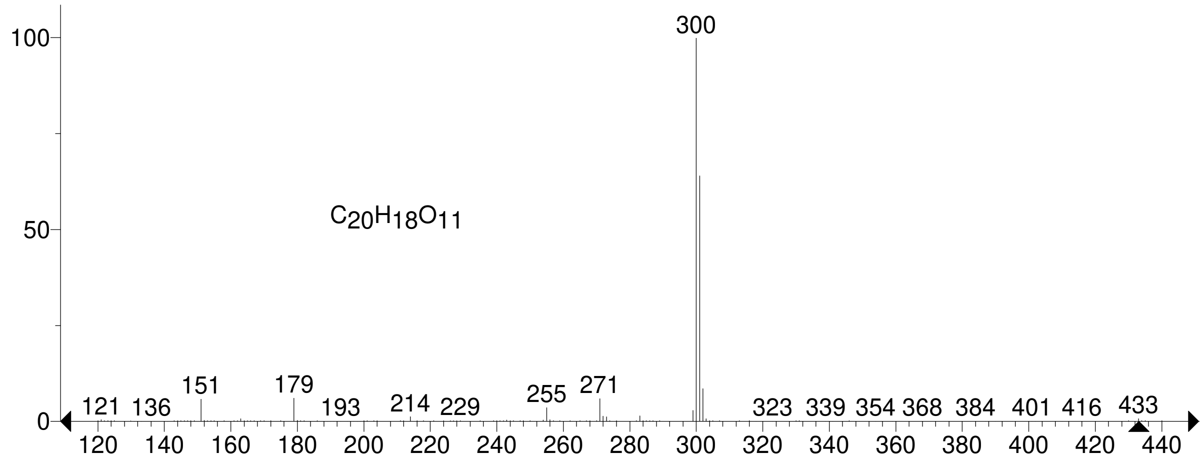

Unknown: Unknown (9.98_372.2121m/z)
Compound in Library Factor = -733

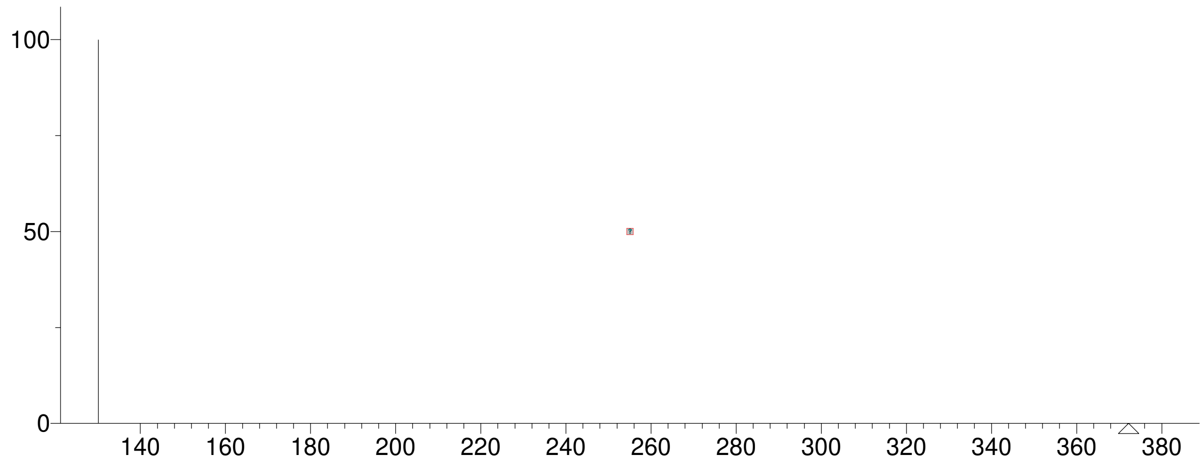

Hit 1 : Unknown (carbon number 17); PlaSMA ID-910 [M-H]- P=372.2
C17H31N3O6; MF: 6; RMF: 309; Prob 100.0%; Lib: biomsms-neg-plasma; ID: 1304.

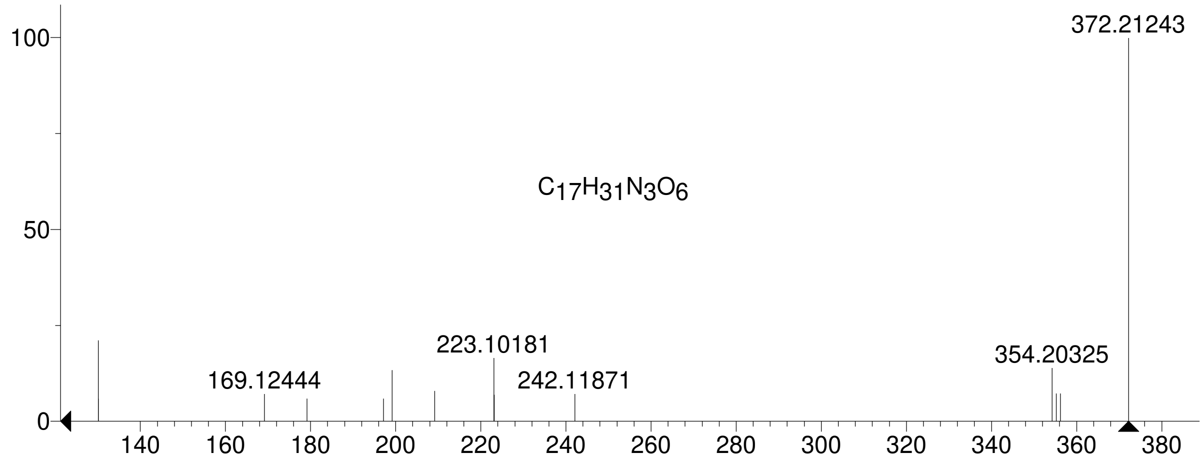

Unknown: Unknown (9.98_494.1655m/z)
Compound in Library Factor = 0

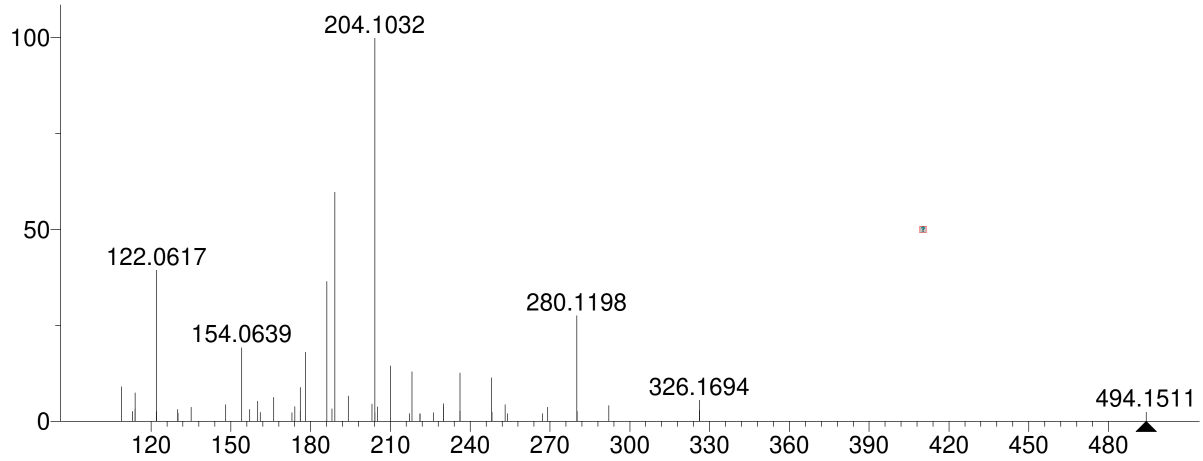

Supplement: Supplementary file 1 [file File S1 - All significants matched against MONA libraries based on precursor and MSMS.pdf › Supplementary Material/File S1 - All significants matched against MONA libraries based on precursor and MSMS.pdf]
